# Identification and Evolution of the Silkworm *Helitrons* and their Contribution to Transcripts

**DOI:** 10.1093/dnares/dst024

**Published:** 2013-06-14

**Authors:** Min-Jin Han, Yi-Hong Shen, Meng-Shu Xu, Hong-Yu Liang, Hua-Hao Zhang, Ze Zhang

**Affiliations:** 1 State Key Laboratory of Silkworm Genome Biology, The Key Sericultural Laboratory of Agricultural Ministry, Southwest University, Chongqing400715, China; 2 School of Life Sciences, Chongqing University, Chongqing400044, China

**Keywords:** *Helitron*, silkworm, gene fragment acquisition, evolution, transcript

## Abstract

In this study, we developed a structure-based approach to identify *Helitrons* in four lepidopterans and systematically analysed *Helitrons* in the silkworm genome. We found that the content of *Helitrons* varied greatly among genomes. The silkworm genome harboured 67 555 *Helitron*-related sequences that could be classified into 21 families and accounted for ∼4.23% of the genome. Thirteen of the families were new. Three families were putatively autonomous and included the replication initiator motif and helicase domain. The silkworm *Helitrons* were widely and randomly distributed in the genome. Most *Helitron* families radiated within the past 2 million years and experienced a single burst of expansion. These *Helitron* families captured 3724 gene fragments and contributed to at least 1.4% of the silkworm full-length cDNAs, suggesting important roles of *Helitrons* in the evolution of the silkworm genes. In addition, we found that some new *Helitrons* were generated by combinations of other *Helitrons*. Overall, the results presented in this study provided insights into the generation and evolution of *Helitron* transposons and their contribution to transcripts.

## 1. Introduction

*Helitrons*, originally discovered in the genomes of the model organisms *Arabidopsis*, rice, and *Caenorhabditis elegans*, are classified as DNA transposable elements (TEs).^1^ However, their sequence structures and mechanisms of transposition are completely different from those of other DNA transposons. They are thought to transpose via a rolling circle mechanism, because some intact *Helitrons* encode proteins that include the replication initiator (Rep) motif and helicase domain. These two genetic elements are necessary for bacterial IS91 (insertion sequence 91) transposition through a rolling circle mechanism.^2^ Although *Helitrons* do not have terminal inverted repeats or target site duplications, they preferentially insert into the dinucleotide AT and are characterized by a TC dinucleotide at the 5′-end, a CTRR motif at the 3′-end, and often a palindromic sequence near the 3′-terminus.

In recent years, *Helitrons* have been identified in almost all eukaryotic genomes. They constitute 0–5% of total genomic DNA in some model organisms. For example, they comprise >2% of the genome in *C. elegans*,^1^ >0.5% in frog,^3^ ∼3% in *Nematostella vectensis*,^4^ <0.1% in *Aspergillus nidulans*,^5^ ∼3% in bat,^6^ ∼2% in maize,^7,8^ and 1–5% in fruit flies.^9^ Furthermore, *Helitron* content is often highly variable among closely related species. For instance, they occupy 1–5% of genomic DNA in different fruit flies and 0.03–2.09% in different rice species.^9–11^

*Helitrons* vary greatly in sequence length, even within the same *Helitron* family, in part because different gene fragments are captured by these elements. More than half of the *Helitrons* in the maize B73 genome contained gene fragments whose lengths ranged from tens of base pairs (bp) to ten or more kilobase pairs (kbp).^7,8^ Furthermore, genes captured by *Helitrons* reshuffled the transcriptome of maize.^12^ Hence, *Helitrons*, creating the diversity of coding regions, can lead to the evolution of new functional genes.^12,13^

Although an increasing number of *Helitrons* are being identified in eukaryotic genomes, little is known about *Helitrons* in Lepidoptera. Recently, the genome sequences of three lepidopterans, *Heliconius melpomene*, *Danaus plexippus* (both Nymphalidae), and *Manduca sexta* (Sphingidae) were released, in addition to the previously available silkworm (*Bombyx mori*; Bombycidae) genome.^14–16^ Taken together, they provide an excellent resource for investigating *Helitrons* in Lepidoptera. The silkworm and *M. sexta* are moths, while *H. melpomene* and *D. plexippus* belong to butterfly. They diverged ∼100 million years ago (mya).^15–17^

The silkworm is a model insect for Lepidoptera and has important economic value for its silk and as a bioreactor. Approximately 40% of its genome consists of known TEs, with *Helitrons* comprising only 0.1%.^18^ In this study, we developed a structure-based approach to rescan the new silkworm genome assembly to identify *Helitrons*. We found that the silkworm genome harbours 21 *Helitron* families that occupy ∼4.23% of the genomic DNA. Thirteen of these families are new and three are putative autonomous elements. Estimates of insertion date and diversity for each *Helitron* family suggested that most *Helitron* families experienced a single rapid expansion within the past 2 million years (my). Strikingly, these *Helitron* families captured 3724 fragments from 268 genes and contributed to at least 1.4% of silkworm full-length cDNAs. A comparative analysis of *Helitrons* within Lepidoptera was also performed.

## 2. Materials and Methods

### 2.1. Identification and characterization of *Helitrons*

Genome sequences were downloaded for the following Lepidopterans as indicated: silkworm new assembly from SilkDB (http://silkworm.swu.edu.cn/silkdb); *H. melpomene* from the *Heliconius* genome project (http://butterflygenome.org/); *D. plexippus* V3 from MonarchBase (http://monarchbase.umassmed.edu/home.html), and *M. sexta* from NCBI (http://www.ncbi.nlm.nih.gov/nuccore/AIXA00000000).

**Figure 1.**
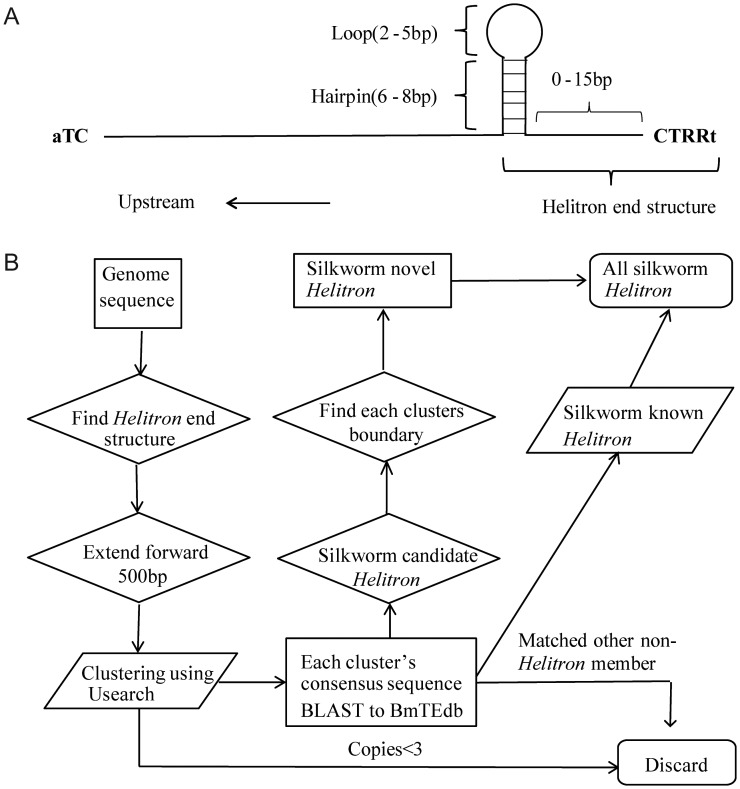
*Helitrons* in the silkworm. (A) *Helitron* structure. (B) Pipeline for *Helitron* identification.

To identify *Helitrons*, a series of Perl scripts were written to search for *Helitron* sequence characteristics, similar to ‘HelSearch’.^11^ Briefly, the method included four steps (Fig. 1): (I) a Perl script found *Helitron* end structures, includes hairpins, loops, and CTRRT motifs; (II) another Perl script scanned upstream from *Helitron* end structure; (III) all sequences were clustered using Usearch;^19^ (IV) the *Helitron* boundaries were sought. In the step IV, we extended the sequences of each cluster in both directions using a Perl script and aligned them using MUSCLE,^20^ then the *Helitron* boundaries were manually defined. Finally, we modified Yang and Bennetzen's method of classification.^8^ Sequences with identities >80% in the 30 bp of both their 5′- and 3′-ends were classified as members of the same family. Full-length sequences with identity >80% were classified in the same subfamily. Our programme and readme file are available upon request.

To estimate copy numbers, we generated a consensus sequence for each *Helitron* family using DAMBE.^21^ We used these consensus sequences as queries for BLASTN searches of the corresponding genome database. In this step, a *Helitron* family was defined by *E* < *e*^–6^, pair-wise length >80 bp, and a minimum nucleotide identity rate of >80%. Sequences with a TC dinucleotide at the 5′-end and a CTRR motif at the 3′-end were defined as intact *Helitrons*.

Copy numbers of relatively long *Helitron* sequences (<15 kb) were estimated as follows: (i) when each end almost perfectly matched the ends of a *Helitron* family's consensus sequence (identity >80%, pair-wise length >80 bp for each end), and when the sequence had a TC dinucleotide and a CTRR motif at the 5′- and 3′-ends, respectively, it was defined as a *Helitron* copy (Supplementary Fig. S1A–C). (ii) When each end almost perfectly matched (identity >80%, pair-wise length >80 bp) non-terminal regions of a *Helitron* family's consensus sequence and the sequence had no or one *Helitron* terminal sequence (either a 5′ TC or a 3′ CTRR), it was treated as two or more *Helitron* fragments (Supplementary Fig. S1D–F).

To better understand the composition and structure of *Helitron* sequences, the AT content of each *Helitron* family consensus sequence was estimated using BioEdit (http://www.mbio.ncsu.edu/bioedit/bioedit.html). UNAFOLD (http://mfold.rna.albany.edu/) was used to predict the Gibbs free energy (–dG) of each *Helitron*.^22^ Finally, all *Helitron* families were screened against the ISfinder (http://www-is.biotoul.fr/),^23^ RepBase (v. 17.08),^24^ and NCBI non-redundant (nr) databases to identify known families. Putative autonomous *Helitrons* were identified by using known autonomous *Helitrons* downloaded from RepBase as queries and performing TBLASTN searches against all the silkworm *Helitron* databases.

### 2.2. PCR validation of predicted *Helitrons*

Fifteen accessions (02–320, DaZao, Ri9, 872, Ou18, Yi16, YinDuSanMian, WuLin1Hao, BH863, YingWenXing, LuoSa, RiXian2Hao, ALiKeSi, SanMianBai, and Zhong4010) representing the four main geographic strains of silkworm (Chinese, Japanese, European, and tropical) were used for insertion validation. DNA was extracted from individual pupae and moths using a standard phenol–chloroform protocol.^25^ A *Helitron* (BmHel-8) was randomly selected for insertion validation, and primers (BmHel-8-S: 5′-ATTGTCAGTGGTATCGTTGCTCC-3′, BmHel-8-A: 5′-TAAGGGAATACAATAGAGCCGTG-3′) were designed based on the flanking insertion sites.

### 2.3. Estimates of insertion time and expansion events

To estimate *Helitron* age, all full-length sequences of each *Helitron* family were aligned using MUSCLE,^20^ and the amount of nucleotide substitution (*k*) between each *Helitron* and the family consensus sequence was calculated using Kimura 2-parameter (K2P) distance.^26^ The age of each *Helitron* was estimated using the formula *T* = *k*/2*r*, where *r* = 1.56 × 10^−8^, the neutral rate of substitutions per year in fruit fly, which has previously been used in silkworms.^27,28^ Neighbor-joining trees (pair-wise deletion of gaps and K2P substitution model) for *Helitron* families were reconstructed using MEGA4.^29^ Within each *Helitron* family, the frequency distribution of the number of pair-wise differences between sequences was calculated with Arlequin v. 3.11.^30^

### 2.4. Distribution of *Helitrons* on chromosomes

All identified members of each *Helitron* family were mapped onto chromosomes using SilkMap (http://silkworm.swu.edu.cn/silksoft/silkmap.html), and the copy number of each family on each chromosome was counted. We divided each chromosome into 100 uniformly-sized segments and estimated each *Helitron* family's distribution in these fragments using a Perl script. The positions of predicted genes in scaffolds and the lengths of scaffolds were downloaded from SilkDB (http://silkworm.swu.edu.cn/silkdb);^31^ a Perl script was used to identify the genes near to or containing *Helitrons*. To determine whether *Helitron* insertions into genes were due to chance, a computer simulation was performed.^32^

### 2.5. Gene fragment acquisition and contribution to transcripts

Gene fragments captured by *Helitrons* were identified by using all identified *Helitrons* in a BLASTX search against the NCBI nr protein database (as of 22 January 2013). Captured gene fragments were identified if a homologue had a maximum expected value of *e*^–10^ in the silkworm, or of *e*^–5^ in a species other than silkworm. TE-related proteins were discarded.

To evaluate the contributions of silkworm *Helitrons* to transcripts, we used all intact *Helitron* sequences as queries in BLASTN analyses against the expressed sequence tag (EST) database of the silkworm (http://silkworm.swu.edu.cn/silkdb). A match was defined when the fragments had at least 99% identity and *E* < *e*^−10^. All matching ESTs were classified as either parental transcripts (with a similarity between the EST and the parental genes of the captured fragment greater than that between the EST and the corresponding *Helitron*) or *Helitron* transcripts.

To estimate whether the silkworm *Helitrons* contributed to 5′ untranslated regions (UTRs), coding regions, or 3′-UTRs of the silkworm full-length cDNAs, we downloaded the silkworm full-length cDNA database from SilkBase (http://silkbase.ab.a.u-tokyo.ac.jp/cgi-bin/index.cgi). A Perl script was written to split each full-length cDNA into 5′-UTR, coding region, and 3′-UTR. Then, we performed BLASTN analyses against these three datasets with cut-off values of at least 99% identity and *E* < *e*^−10^. All matching transcripts were classified as either (i) parental transcripts (with the similarity between the transcript and parental gene greater than that between the transcript and corresponding *Helitron*); (ii) transcripts of *Helitron* transposase; or (iii) chimerical transcripts composed of other genes and *Helitron* fragments if a full-length *Helitron* copy matches a cDNA sequence and there is an overlapping region (matched region) between the position of the full-length cDNA sequence and the corresponding full-length *Helitron* copy in the silkworm genome.

## 3. Results

### 3.1. Identification, classification, and characterization of *Helitrons*

We used a structure-based approach to search for *Helitrons* in the silkworm genome (Fig. 1). First, we searched the silkworm genome for sequences with a characteristic *Helitron* end structure (Fig. 1A) and found 106 766 candidate sequences. We extended the sequence of each candidate upstream, clustered all candidates, and generated a consensus sequence for each cluster. In total, we obtained 1805 consensus sequences. Each consensus sequence was used as a query in BLASTN search against BmTEdb (http://202.202.1.217/BmTEdb/), and sequences that hit to other known non-*Helitron* TEs were discarded. The remaining 854 consensus sequences were used in a BLASTN search against the silkworm genome. We extracted no more than 20 most-similar sequences for each consensus sequence, aligned them, and manually defined the *Helitron* element boundaries (Supplementary Fig. S2). Finally, sequences were classified by similarity into families and subfamilies. This pipeline (Fig. 1B) identified 21 *Helitron* families (Table 1) that were designated BmHel-1 through BmHel-21.

**Table 1.** Summary information for the *Helitron* families in four lepidopteran species

Species	Family	Subfamily	Length (bp)	Copies	AT (%)	−dG	Annotation^ref^
*B. mori*	BmHel-1		198–781	514	56.6	33.8	Bm_283^BmTEdb^
	BmHel-2		822–9452	79	63.3	141.7	bm_691^BmTEdb^
	BmHel-3	BmHel-3a	196–8700	1386	63.9	30.5	Helisimi^33^
		BmHel-3b	206–10479	1995	69.4	144.5	Helisimi^33^
	BmHel-4		290–9930	661	64.0	69.0	Helianu^33^
	BmHel-5		3121–6696	80	68.7	535.3	Heliminu^33^
	BmHel-6		594–738	25	69.5	82.8	Novel
	BmHel-7		126–10099	6007	61.9	35.3	Novel
	BmHel-8		105–10644	14656	71.9	55.4	Novel
	BmHel-9		608–1256	11	68.9	52.6	Novel
	BmHel-10		455–935	21	63.6	72.0	Novel
	BmHel-11		96–9788	12206	65.4	21.9	Novel
	BmHel-12		152–10623	3138	59.3	62.6	Novel
	BmHel-13		285–8964	3428	66.9	43.3	Novel
	BmHel-14		258–2723	3431	69.9	69.7	Novel
	BmHel-15		142–10468	8297	67.1	71.2	Lep1^34^
	BmHel-16		136–10228	6537	65.9	25.9	Novel
	BmHel-17		288–683	645	66.9	57.5	Novel
	BmHel-18		113–9945	3768	64.7	26.8	Novel
	BmHel-19		300–2462	149	58.0	138.4	Novel
	BmHel-20		296–2355	144	65.9	43.8	HeligloriaAi^33^
	BmHel-21		149–6073	377	67.9	82.1	HeligloriaAii^33^
*H. mel*	HmHel-1		134–10671	6148	70.1	122.6	Lep1^34^
	HmHel-2		120–10381	6827	66.3	103.2	*Helitron*-5^17^
	HmHel-3		127–11371	3940	66.9	52.2	*Helitron*-4,7^17^
	HmHel-4		134–10068	5665	59.5	44.1	*Helitron*-15^17^
	HmHel-5		268–7417	4398	64.8	191.9	*Helitron*-6,11^17^
	HmHel-6		134–15055	15359	47.5	72.8	Novel
	HmHel-7		276–7883	7103	60.5	563.1	*Helitron*-13^17^
	HmHel-8		289–373	74	64.5	53.5	*Helitron*-16^17^
	HmHel-9		217–1404	621	67.8	37.4	*Helitron*-1^17^
	HmHel-10		192–5100	888	70.7	103.5	*Helitron*-9^17^
*D. ple*	DpHel-1		111–4232	1010	66.6	82.0	Novel
*M. ext*	MsHel-1		156–9446	6386	58.5	133.5	Lep1^34^
	MsHel-2		134–6559	2975	57.6	53.3	Novel
	MsHel-3	MsHel-3a	134–7735	3270	66.1	49.5	Novel
		MsHel-3b	119–9845	5632	60.4	33.0.	Novel
	MsHel-4		120–10147	697	60.3	91.1	Novel
	MsHel-5		120–3351	1957	62.1	49.2	Novel
	MsHel-6		122–9236	2574	65.5	31.4	Novel
	MsHel-7		107–3867	2691	62.3	119.2	Novel

–dG, average Gibbs energy (kcal/mol) for each *Helitron* family consensus sequence.

The silkworm *Helitron* families were annotated based on homology. Using a consensus sequence for each *Helitron* family as queries, we searched the BmTEdb, ISfinder, RepBase, and NCBI nr databases and found that eight families (BmHel-1, 2, 3 4, 5, 15, 20, and 21) had been previously identified. The other 13 families had no matches to any known *Helitron* (Table 1).

To estimate the abundance of these 21 families, we searched the silkworm genome. We identified 67 555 *Helitrons* in total, which constitute about 19.7 Mb (∼4.23%) of the silkworm genome. The insertion sites of these *Helitrons* into accession numbers (NCBI) are shown in Supplementary Table S1. Similar to previous reports on *Helitrons*,^7,8^ the silkworm *Helitron* size varied greatly both among and within a family; sizes ranged from 96 to 10 644 bp (Table 1). There were 202 very long (from 6000–10 644 bp) *Helitron* copies. The internal sequences of these *Helitrons* had at most 50% identity, but their ends (100 bp) had at least 80% identity. This pattern could be caused by different DNA sequences being captured either by the *Helitrons* or by the insertion of other TEs into the *Helitrons*. We identified 19 580 intact *Helitrons*, with a TC dinucleotide at the 5′-end and a CTRR motif at the 3′-end; they made up 10.7 Mb (∼2.30%) of the silkworm genome. Of these, 15 272 (∼78%) had at least 80% identity and 8615 (∼44%) had at least 90% identity.

The *Helitron* families were AT rich, with AT contents ranging from 56.61% to 71.9%. The average AT content of the silkworm genome is ∼62%. Four *Helitron* families (BmHel-1, 7, 12, and 19) had AT contents that did not exceed the genome average (Table 1). Almost all of the silkworm *Helitron* families had high predicted –dG values, indicating that most silkworm *Helitrons* can form stable secondary structures.

**Figure 2.**
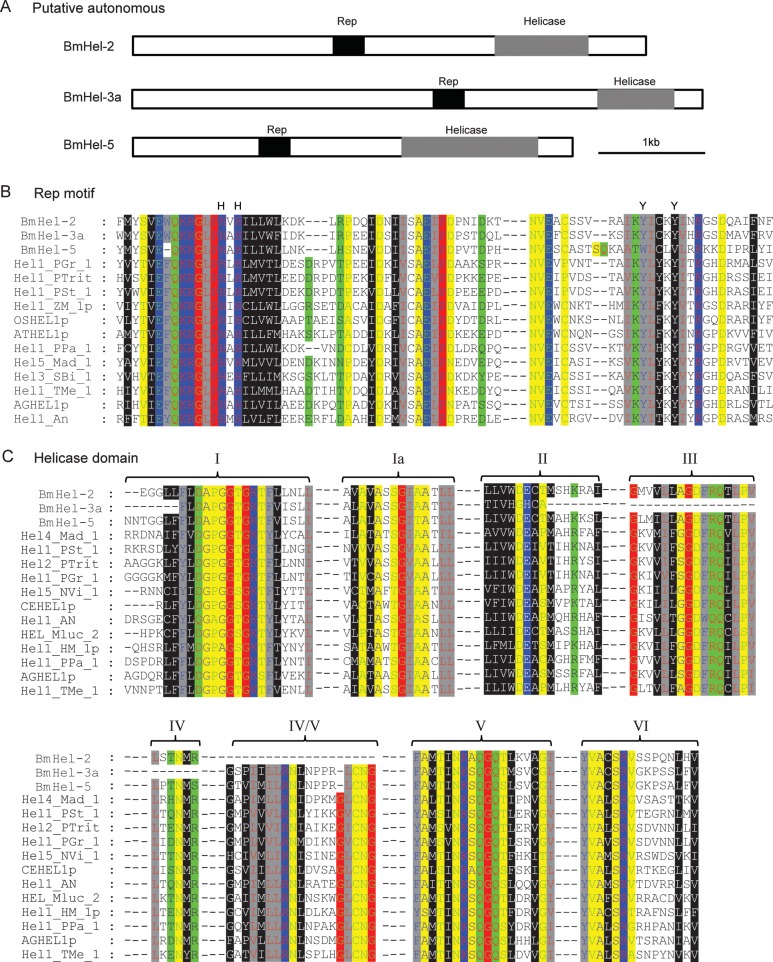
Three predicted putative autonomous elements in the silkworm based on protein domain. (A) A schematic representation of the putative autonomous *Helitrons*; Rep, rolling circle replication initiator motif; Helicase, region similar to SF1 superfamily of DNA helicases. (B) Alignment of REP motifs between silkworm and 12 other species. (C) Alignment of eight conserved motifs of the SF1 superfamily of DNA helicases.

Putative autonomous silkworm *Helitrons* were founded based on homology. An autonomous *Helitron* should encode a Rep/helicase protein, because both the Rep motif and DNA helicase domain are necessary for transposition. All 141 known autonomous *Helitrons* were downloaded from RepBase and screened against all intact silkworm *Helitrons*. Three silkworm *Helitron* families (BmHel-2, 3, and 5) were putatively autonomous (Fig. 2). These candidate autonomous elements encoded complete open reading frames (ORFs), in addition to a Rep motif and helicase domain. For example, the SilkDB accession numbers of transposase for BmHel-2, BmHel-3, and BmHel-5 were BGIBMGA003354-TA, BGIBMGA012372-TA, and BGIBMGA008616-TA, respectively. Furthermore, two of the three putative autonomous families had EST evidence; BGIBMGA003354-TA matched the EST BY927485 (identity, 0.98; length, 760 bp) and BGIBMGA012372-TA matched ESTs BB983132 (identity, 0.94; length, 681 bp), BY932007 (identity, 0.99; length, 702 bp), CK528421 (identity, 0.95; length, 632 bp), and BY916909 (identity, 0.96; length, 657 bp). Thus, we concluded that these elements could be active in the silkworm genome.

### 3.2. Validation of predicted *Helitrons*

A BmHel-8 insert site was selected for PCR verification in 15 silkworm accessions representing four main geographic strains. The results indicated that BmHel-8 was present in most of the strains, but absent in YinDuSanMian, YingWenXing, LuoSa, and RiXian2Hao (Supplementary Fig. S3). This polymorphism indicated that the *Helitron* was not fixed in the silkworm genome and verified the efficacy of our approach.

### 3.3. *Helitron* abundance in other lepidopteran genomes

**Figure 3.**
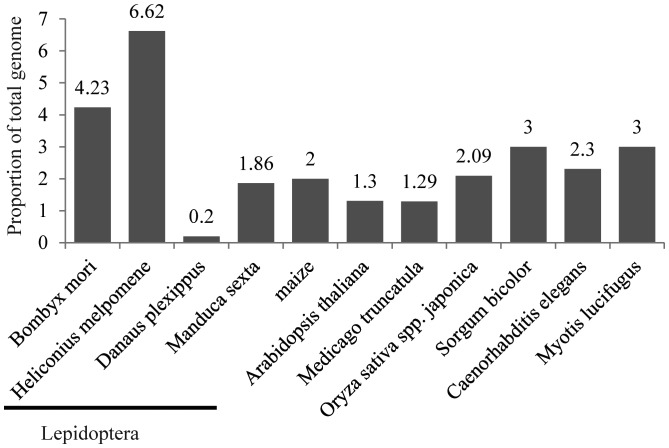
*Helitron* abundances in different organisms. Underlined *Helitron* contents were identified in this study, while others came from previous studies.^1,3–11^

To investigate whether *Helitrons* were pervasive in lepidopteran, three other recently released lepidopteran genomes were searched for *Helitrons. Helitron* abundance varied greatly among these genomes (Table 1 and Fig. 3). For instance, *H. melpomene* had 10 *Helitron* families that comprised ∼6.62% (17.1/260 Mb) of the genome. *Manduca sexta* harboured seven *Helitron* families that made up ∼1.86% (7.23/388 Mb) of the genome. However, *D. plexippus* had only one *Helitron* family that represented only ∼0.20% (0.48/237 Mb) of the genome. The locations of each *Helitron* in these three genomes are listed in Supplementary Table S2 (*H. melpomene*), Supplementary Table S3 (*D. plexippus*), and Supplementary Table S4 (*M. sexta*).

### 3.4. Distributions of *Helitrons* on chromosomes

The silkworm *Helitrons* were distributed on all 28 silkworm chromosomes and were uniformly distributed among chromosomes (*P*> 0.05; Supplementary Fig. S4). We also found that most silkworm *Helitrons* had no distinct insertion bias within chromosomes (Supplementary Fig. S5). When we examined whether the *Helitrons* preferentially inserted into or near genes, we discovered that their frequencies within introns and >1 kb from genes were significantly higher than expected (Supplementary Fig. S6), suggesting the silkworm *Helitrons* preferential insertion into these regions.

### 3.5. Insertion times and expansion patterns

**Figure 4.**
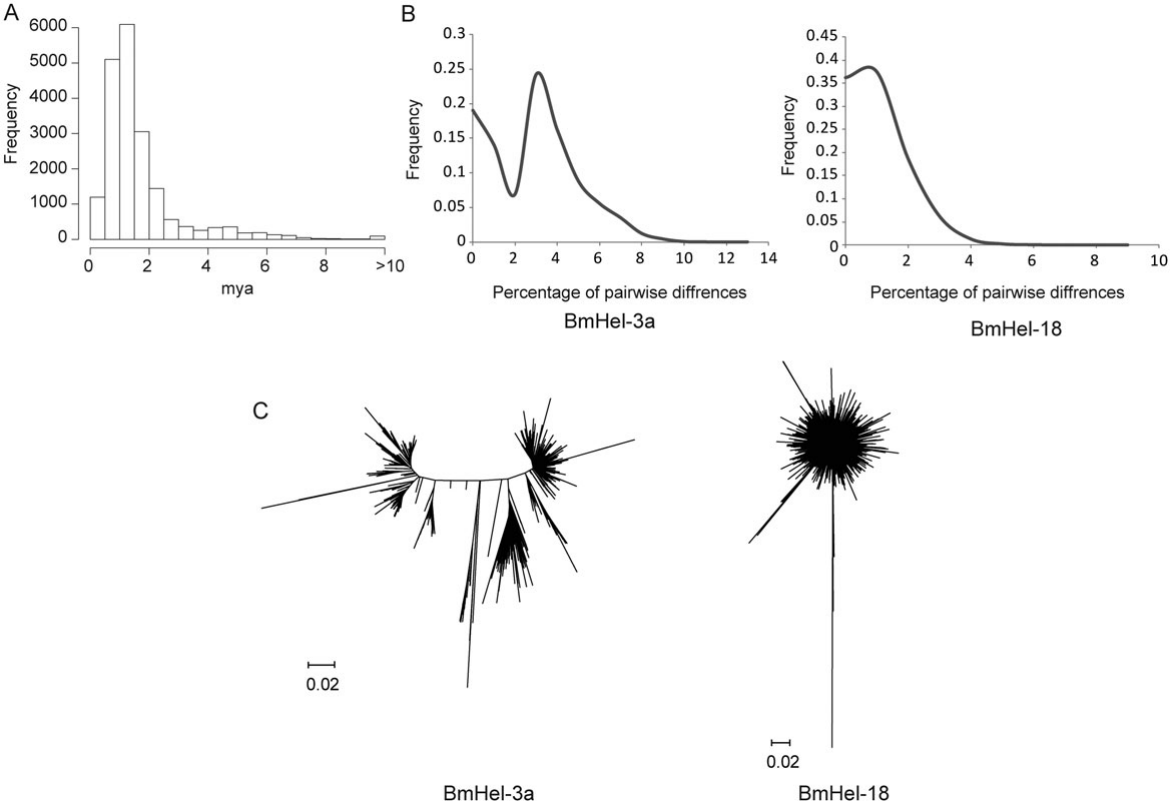
Evolutionary history of *Helitrons* in the silkworm. (A) *Helitron* amplification dates. (B) Distributions of pair-wise nucleotide diversity among full-length elements of BmHel-3a (with a bimodal distribution, suggesting more than one round of amplification) and BmHel-18 (with a unimodal distribution, suggesting one amplification burst). (C) Phylogenetic trees of BmHel-3a (bimodal pair-wise nucleotide diversity and more than one clade) and BmHel-18 (unimodal pair-wise nucleotide diversity and a single clade).

We estimated the age of each intact *Helitron* by first estimating *k* between each intact *Helitron* and its family consensus sequence based on K2P distances.^28^ The range was 0–0.69, but 15 443 copies (∼79% of the 19 580 copies) had *k*≤ 0.06. Insertion dates based on these *k-*values ranged from 0 to >10 mya (Fig. 4A), but most expansion events appeared to have happened within the most recent 2 my (corresponding to *k* = ∼0.06).

To investigate the history of *Helitron* expansion in silkworms, pair-wise nucleotide diversities of intact *Helitrons* were calculated and histograms were drawn for each *Helitron* family. Most histograms were wave-like (Fig. 4B and Supplementary Fig. S7). These histograms indicated that each family may have experienced a rapid population expansion (burst) during its evolutionary history.^35,36^ Thirteen families (BmHel-4, 7, 8, 10, 11, 12, 13, 14, 15, 16, 17, 20, and 21) of silkworm *Helitrons* had unimodal distributions, two (BmHel-2 and 3) had bimodal distributions, and the other six families displayed multimodal distributions (Supplementary Fig. S7), indicating that these *Helitron* families had experienced one, two, or multiple expansions, respectively.

To further investigate the histories of these *Helitron* families, phylogenetic trees were reconstructed (Fig. 4C and Supplementary Fig. S8). Families with unimodal histograms formed star-shaped clades, indicating a rapid amplification from a single master element. Those with bi- or multimodal distributions had more than one clade, providing evidence for amplification bursts at different times. Most silkworm *Helitron* families experienced a single evolutionary radiation.

### 3.6. Gene fragment acquisition and contribution to transcripts

**Figure 5.**
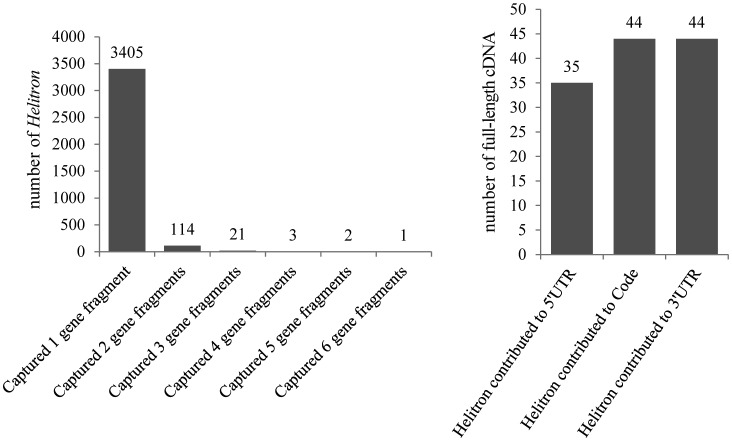
Silkworm *Helitrons* within genes. (A) Distribution of the number of gene fragments captured by silkworm *Helitrons*. (B) Silkworm *Helitrons* contributed to coding regions and to 5′- and 3′-UTRs of full-length cDNA.

To estimate the numbers of gene fragments captured by silkworm *Helitrons*, we performed a BLASTX search against the NCBI nr protein database. More than 18% (3546/19 580) of the intact elements captured one or more gene fragments (Fig. 5A). The number of captured gene fragments ranged from one to six. Most intact *Helitrons* (∼96%) captured no more than one gene fragment. A total of 3724 gene fragments from 268 genes were captured (Supplementary Table S5). Examples of genes captured by *Helitrons* are shown in Supplementary Fig. S9.

To investigate whether these *Helitrons* had transcriptional activity, we performed a BLASTN search against the silkworm EST database and discarded parental transcripts. We found that 1317 (∼6.7%) intact *Helitrons* had transcriptional activity, contributing to 1210 ESTs (Supplementary Table S6). Among these ESTs, five matched the transposases of BmHel-2 (BY927485) and BmHel-3a (BB983132, BY932007, CK528421, and BY916909), while 1205 matched 1317 intact *Helitrons* (Supplementary Table S6). We could not distinguish between *Helitron* transcripts and transcripts composed of *Helitron* fragments plus other genes (the 1205 ESTs), because most ESTs were too short.

To estimate whether the *Helitrons* contributed to 5′-UTRs, ORFs, and 3′-UTRs of full-length silkworm cDNAs, we performed BLASTN searches and discarded parental and *Helitron* transposase transcripts. The intact *Helitrons* contributed to the 5′­-UTRs of 35 full-length cDNAs, to the ORFs of 44, and to the 3′-UTRs of 44 (Fig. 5B; Supplementary Table S7–9). These donated fragments contributed to 123 full-length cDNAs, which represented ∼1.4% (123 of 8,654) of the silkworm full-length cDNAs. Examples are shown in Supplementary Fig. S10.

### 3.7. New *Helitron* creation through combinations of different *Helitrons*

**Figure 6.**
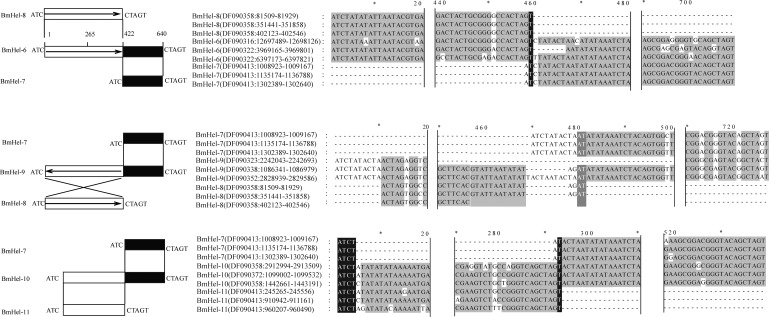
Possible mechanism of new *Helitron* generation through *Helitron* sequence acquisition and new end creation.

By clustering of all silkworm *Helitron* family consensus sequences with an all-versus-all BLAST search, we found that some distinct *Helitrons* had merged to form new *Helitrons*. Three examples are shown in Fig. 6: BmHel-6 was formed from forward-oriented segments of BmHel-7 and BmHel-8; BmHel-9 comprised forward BmHel-7 and reverse BmHel-8 segments; and BmHel-10 united forward segments of BmHel-7 and dBmHel-11.

## 4. Discussion

### 4.1. Identification and characterization of silkworm *Helitrons*

TEs in higher eukaryotic genomes are identified in two main ways: homology-based and structure-based methods. Homology-based methods are biased toward detecting previously identified families; its major limitation is that it cannot detect TEs that are very distinct in sequence from known TEs. In contrast, structure-based method use prior knowledge about the common structural features and can effectively identify unique TEs. However, a precondition for this method is that TEs have conserved sequence structures. Homology- and structure-based methods have been developed to identify *Helitrons*. The homology-based *Helitron*Finder has been used to identify *Helitrons* in the maize genome,^7^ but its applicability to other organisms is limited by the fact that *Helitrons* vary greatly among organisms. HelSearch is a structure-based programme.^11^

We developed a new structure-based pipeline to identify *Helitrons* in the silkworm genome. This method was fast and effective. We used Usearch to cluster sequences with *Helitron* end structures rather than BLASTALL, which greatly sped the clustering but also generated a consensus sequence for each cluster. In addition, we discarded false-positive matches to non-*Helitron* TEs when all candidate *Helitron* sequences were used to BLAST against the known silkworm TEs. A total of 951 such consensus sequences were discarded. Finally, the computational requirements of this method were very low; an ordinary PC machine could complete all of the work. Our pipeline was structure-based like HelSearch, so both methods should have similar efficacy.

Although the silkworm genome is rich in various types of TEs,^18^ few *Helitrons* have been identified. We performed a genome-wide structure-based scan for *Helitrons* and identified 21 *Helitron* families with a total of 67 555 copies. These *Helitron* families comprised ∼4.23% of the silkworm genome, a proportion higher than in other organisms. For instance, *Helitrons* occupied >2% of the genome sequence in *C. elegans*, >0.5% in frog, ∼3% in the *N. vectensis*, <0.1% in *A. nidulans*, ∼1.3% in *Arabidopsis thaliana*, ∼1.29% in *Medicago truncatula*, ∼3% in bat, and ∼2% in maize.^1,3–8,11^ Furthermore, our estimated proportion was much higher than the value of ∼0.1% previously reported for silkworm.^18^ This discrepancy may be because the previous study used an homology-based search. *Helitrons* vary greatly among organisms,^9,37^ so an homology-based search could greatly underestimate *Helitron* content.

We found that 13 of 21 *Helitron* families were new, while eight had been previously published in the BmTEdb or RepBase database or in papers.^33,34^ One silkworm *Helitron*-like family (Bm_1607) published in the BmTEdb was not identified in this study, because it does not have typical *Helitron* characteristics, such the 5′-TC dinucleotide and 3′-CTRR motif. These results, together with PCR verification, indicated that our approach was reliable and efficient at identifying *Helitrons*, but it could not identify structurally atypical *Helitron* families.

We found that the silkworm genome had 19 580 intact *Helitrons*, many more than the 1930 intact *Helitrons* in maize, the 281 in *Arabidopsis* and *C. elegans*, the 230 in *Medicago*, the 651 in rice, and the 608 in sorghum.^11^ Their abundance and high sequence identities (>90% in 8615 of 19 580 sequences, or ∼44%) in the silkworm genome implied a recent amplification history. Two *Helitron* families (BmHel-2 and -3) exhibited features of putative autonomous families. BmHel-5 (BmHel1p) was previously identified as putatively autonomous,^38^ suggesting that autonomous *Helitrons* may exist in the silkworm. However, BmHel-2 and -5 had very small copy numbers, just 79 and 80 copies, respectively. In contrast, some silkworm *Helitron* families with many copies did not have features of putative autonomous elements. A possible reason will be discussed below.

### 4.2. *Helitron* abundances in other lepidopterans

We analysed *Helitron* abundances in three recently released lepidopteran genomes using our approach. *Helitron* abundance varied strikingly among lepidopterans. For instance, there were 10 *Helitron* families that constituted ∼6.62% of the genome in *H. melpomene*. This proportion was much larger than the previously reported 5.37%,^16^ because we identified an additional big *Helitron* family (HmHel-6) that comprised ∼1.17% of the genome. However, we did not find the low-copy-number families of *Helitron*-like-2, 8, and 10, because they lacked the 5′-TC dinucleotide and 3′-CTRR motif. Unexpectedly, only one family, representing ∼0.20% of the *D. plexippus* genome, was identified. Thus, different lepidopteran genomes contained very different numbers of *Helitrons*, consistent with previous reports that *Helitron* content was highly variable even among closely-related species. For instance, *Helitrons* make up 1–5% of genomic DNA in different fruit fly species,^9^ 0–3% in mammals.^6,39^

Different *Helitron* abundances could be caused by three factors: (i) different rates of *Helitron* expansion or deletion in different lineages; (ii) vertical transfer with frequent diversification and extinction; and (iii) horizontal acquisition of *Helitrons*. For example, there were 21 *Helitron* families in the silkworm genome, but only one in the *D. plexippus* genome. These results could be caused by horizontal transfer. A previous study reported that five *Helitron* families (BmHel-3a, 4, 5, 20, and 21) experienced horizontal transfer in the silkworm.^33^ Furthermore, we found that an intact homologue of BmHel-16 was also present in the *Cotesia sesamiae* Kitale bracovirus genome. A phylogenetic analysis indicated that the silkworm BmHel-16 was more closely related to the *C. sesamiae* bracovirus copy than that to sequences from other Lepidoptera (Supplementary Fig. S8), suggesting horizontal transfer. However, BmHel-16 and the *C. sesamiae* bracovirus sequence had only 79% identity, implying that the horizontal transfer happened long ago.

### 4.3. Distribution of *Helitrons* on chromosomes

Previous studies indicated that *Helitrons* preferentially insert into gene-poor regions.^11^ For instance, *Helitrons* in *Arabidopsis* were rich in pericentromeric regions. Similarly, *Helitrons* in the *C. elegans* genome were most abundant in the terminal regions of each chromosome, which are often in the heterochromatin state. However, the silkworm *Helitrons* were randomly distributed on chromosomes. If *Helitrons* generally insert into heterochromatin regions, their random distribution in the silkworm genome is expected, because silkworm chromosomes are holocentromeres.

We also found that the numbers of *Helitrons* that inserted into introns and >1 kb away from genes were higher than expected. The reasons for this observation are not clear. A previous study proved that genes captured by *Helitrons* reshuffled the transcriptome of maize.^12^ Thus, preferential accumulation in intron regions could drive gene evolution through gene capture and exonization of *Helitrons*.

### 4.4 Massive expansions and diversity patterns

Our results indicated that major expansion events of silkworm *Helitrons* occurred in the past 2 my (Fig. 4A). Similarly, about 87% of *BaShos* insertions occurred within the most recent 5 my in *A. thaliana* and ∼71% of Hel1-105 elements and 69% of Hel-106 elements inserted within the past 1 my in maize.^7,40^

To investigate history of the silkworm *Helitron* amplifications, we estimated pair-wise nucleotide diversity and phylogenetic trees of the full-length *Helitrons* (Fig. 4B and C). Most *Helitron* families experienced single expansions. However, the mechanism was not clear. As discussed above, we did not find putative autonomous *Helitron* copies in some high-copy-number families (e.g. BmHel-8, 11, and 15). In contrast, some low-copy-number families (BmHel-2 and 5) appeared to be putative autonomous elements. These results were reminiscent of miniature inverted-repeat TEs (MITEs) that were highly transposable because of transposases encoded by distantly related and self-restrained autonomous elements in rice,^41^ a mechanism known as cross-mobilization. Thus, some non-autonomous *Helitrons* might move using transposases encoded by autonomous *Helitrons*. Whether this is true in silkworm *Helitrons* remains to be investigated.

Why some silkworm *Helitrons* experienced bursts of expansion is not clear. Most *Helitrons* probably remain inactive for most of their evolutionary histories, and they may be suddenly activated by ‘genome shock’, as observed in rice MITEs. For instance, *mPing* is known to be activated by irradiation, cell culture, and recent domestication.^42–44^ The silkworm was domesticated from wild Chinese silkworms about 5000 years ago.^45,46^ Whether some silkworm *Helitrons* were activated by domestication is an interesting question.

### 4.5. Gene fragments acquisition and contribution to transcripts

*Helitrons* vary greatly in sequence length, even within a family. One explanation is that these elements capture different gene fragments.^7,8,12,47^ Although some molecular mechanisms for gene capture have been proposed,^48–51^ clear experimental evidence for a particular mechanism is lacking. In this study, we found that 3546 intact *Helitrons* (>18% of all intact *Helitrons*) had captured one or more gene fragments, for a total of 3724 captured fragments. The average number of captured gene fragments per intact *Helitron* was 1.08, similar to the value (1.81) for maize *Helitrons*.^8^

Furthermore, we found that ∼6.7% of intact silkworm *Helitrons* (1317 of 19 580) had EST evidence. Based on a homology search against silkworm full-length cDNAs, we found that these intact *Helitrons* contributed to about 123 full-length cDNAs (∼1.4% of the published total) by donating one or more exons. A recent study suggested that ∼9% of maize *Helitrons* had EST evidence and could generate abundant transcripts through alternative splicing.^12^ Thus, *Helitrons* may play important roles in the evolution of silkworm transcripts.

### 4.6. Generation of new *Helitrons*

Previous studies indicated that *Helitrons* could acquire new sequences by recognizing either a new 3′ termination site or a new 5′ start site.^11,52^ A hairpin was proposed to serve as a stop signal during *Helitron* transposition. When this hairpin is destroyed by unknown mechanism, a new hairpin-like sequence could be acquired, perhaps from nearby *Helitrons*, to generate chimeric elements.^11^ Interestingly, we found that two *Helitrons* could combine to produce a new *Helitron* (Fig. 6). Similarly, in maize, *Helitron*_mc2 was composed of ZmHelA5 and *Helitron*_mc.^52^ Thus, new *Helitrons* can be generated in different ways, making them the most diverse class of transposons.

## 5. Conclusions

In present study, we developed a structure-based approach to identify *Helitrons* in a genome and analysed their presence in four Lepidoptera species. *Helitron* abundance and the number of families varied greatly among these insect genomes. One plausible explanation is that horizontal transfer caused these differences. A systematic analysis of silkworm *Helitrons* revealed that they accounted for ∼4.23% of the genome, much more than the previously reported ∼0.1%.^18^ A total of 21 *Helitron* families were identified in the silkworm, and 13 were new families. Most *Helitron* families expanded within the past 2 my in a single radiation. Furthermore, we found that *Helitrons* contributed to at least 1.4% of silkworm full-length cDNAs, indicating their important roles in the evolution of the silkworm genes. In addition, existent *Helitrons* could generate new families by combining. Our results provided insights into the generation and evolution of *Helitron* transposons as well as their contribution to transcripts.

## Authors' contribution

Z.Z. and M.J.H. designed the study. Y.H.S., M.S.X., H.Y.L., H.H.Z., and M.J.H. analysed the data. Z.Z. provided the platform for analysis. Z.Z. and M.J.H. drafted and revised the manuscript. All authors read and approved the final manuscript.

## Supplementary data

Supplementary data are available at www.dnaresearch.oxfordjournals.org.

## Funding

This work was supported by the Hi-Tech Research and Development (863) Program of China (2013AA102507), a grant from Natural Science Foundation Project of CQ CSTC (cstc2012jjB80007), and the Doctorial Innovation Fund of Southwest University (kb2010016).

## Supplementary Material

Supplementary Data
