# Supplementary material for: Identification and Evolution of the Silkworm Helitrons and their Contribution to Transcripts
Source: DNA Res. 2013 Jun 14;20(5):471–84. doi: 10.1093/dnares/dst024 (PMC3789558; doi:10.1093/dnares/dst024)
Supplement: Supplementary Data [file supp_20_5_471__index.html]

Identification and Evolution of the Silkworm Helitrons and their Contribution to Transcripts — Supplementary Data 

# Identification and Evolution of the Silkworm *Helitrons* and their Contribution to Transcripts

## 

Supplementary Data

**Files in this Data Supplement:**

- Supplementary Figures1-11 - pdf file
- Supplementary Table 1 - pdf file
- Supplementary Table 2 - pdf file
- Supplementary Table 3-9 - pdf file
