# Supplementary material for: Identification and Evolution of the Silkworm Helitrons and their Contribution to Transcripts
Source: DNA Res. 2013 Jun 14;20(5):471–84. doi: 10.1093/dnares/dst024 (PMC3789558; doi:10.1093/dnares/dst024)
Supplement: Supplementary Data [file supp_dst024_dst024supp_figs1-11.pdf]

Fig. S1. Estimated copy numbers of relatively long Helitron sequences. (A–C) Examples of single-copy sequences. (D-F) Examples of sequences with two fragments.

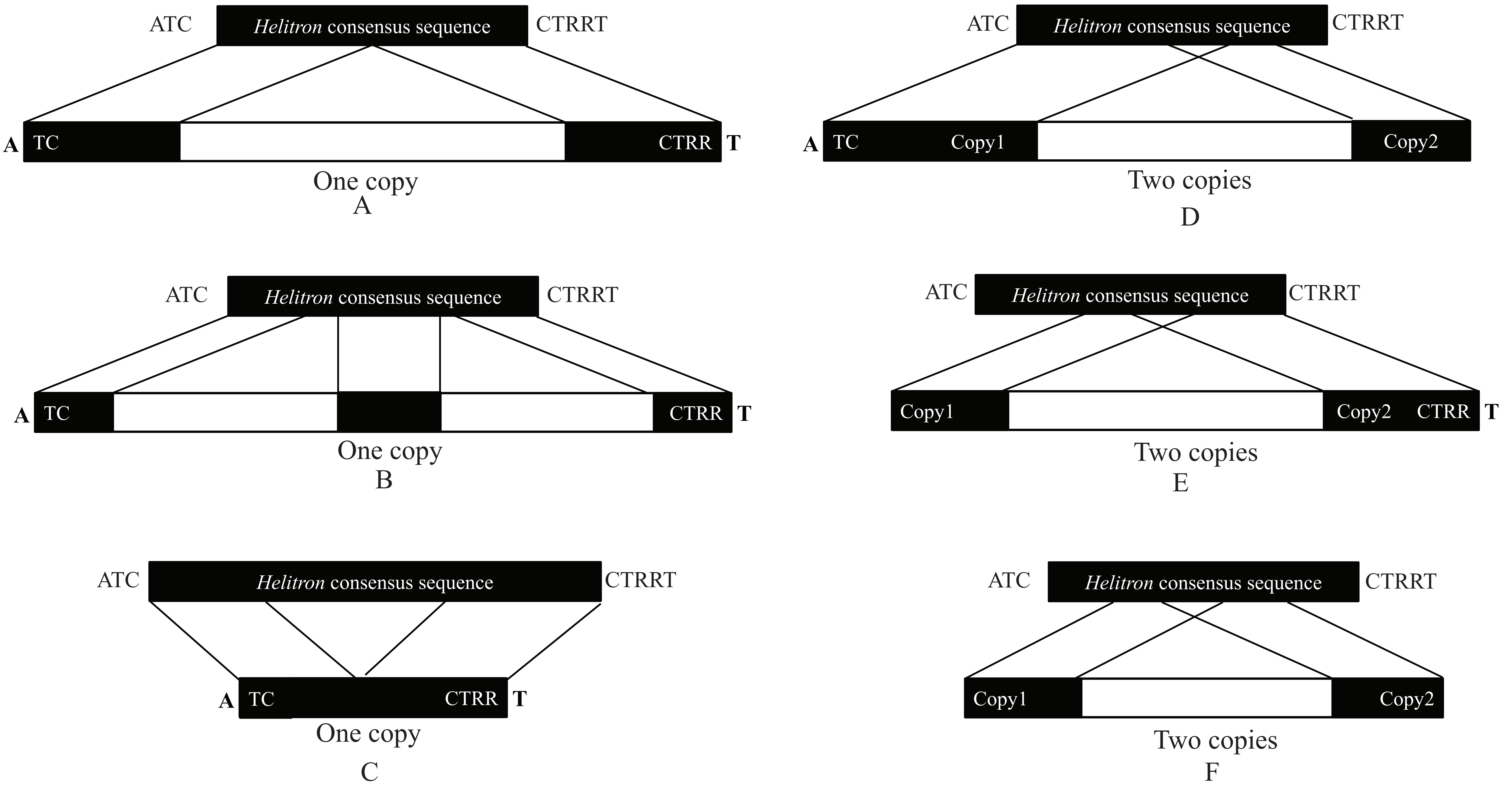

Fig. S2. Sequence alignments for each silkworm Helitron family. The 5' ATC, 3' CTAGT and flanking sequences are shown.

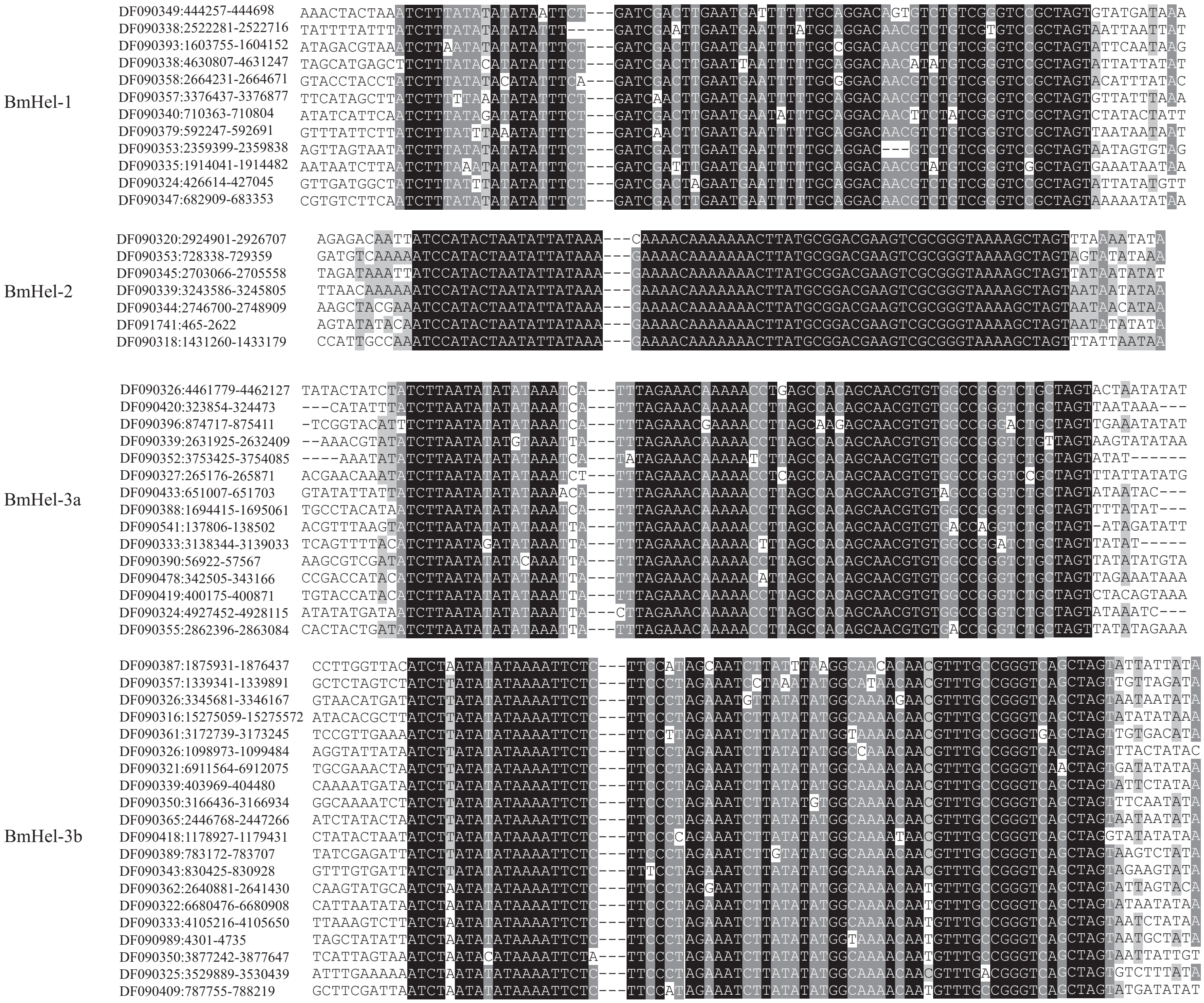

Fig. S2(continue). Sequence alignments for each silkworm Helitron family. The 5' ATC, 3' CTAGT and flanking sequences are shown.

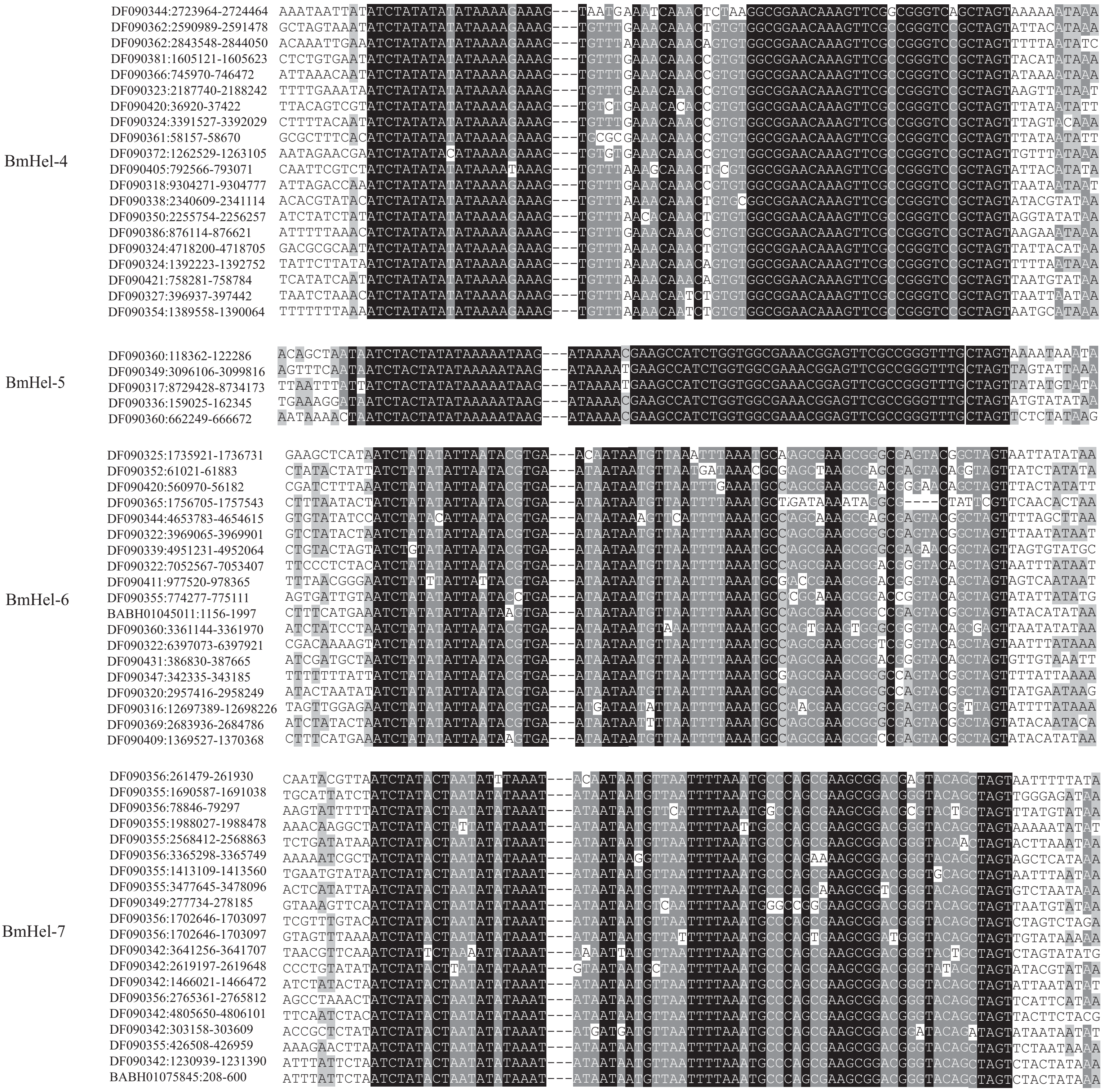

Fig. S2(continue). Sequence alignments for each silkworm Helitron family. The 5' ATC, 3' CTAGT and flanking sequences are shown.

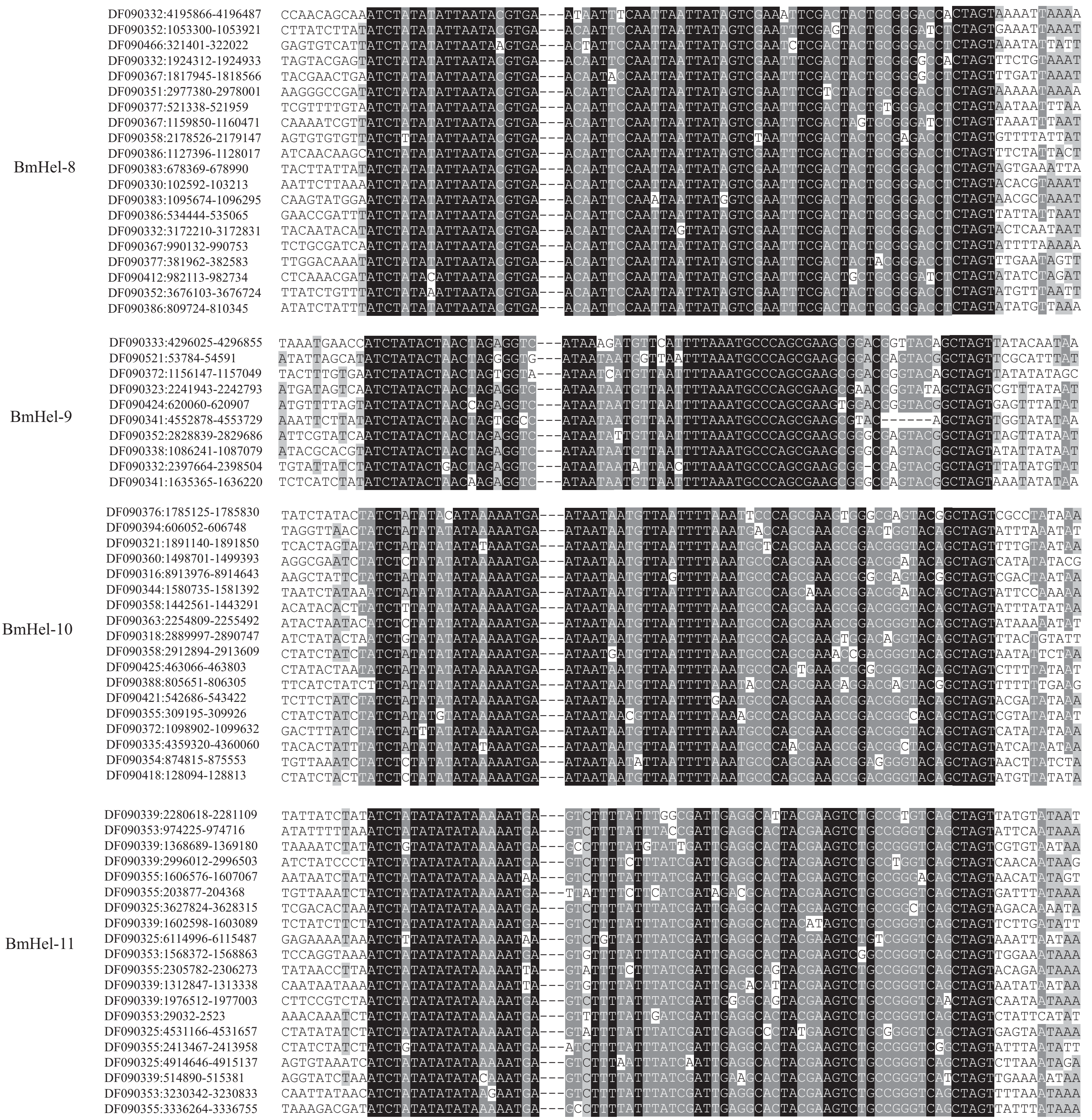

Fig. S2(continue). Sequence alignments for each silkworm Helitron family. The 5' ATC, 3' CTAGT and flanking sequences are shown.

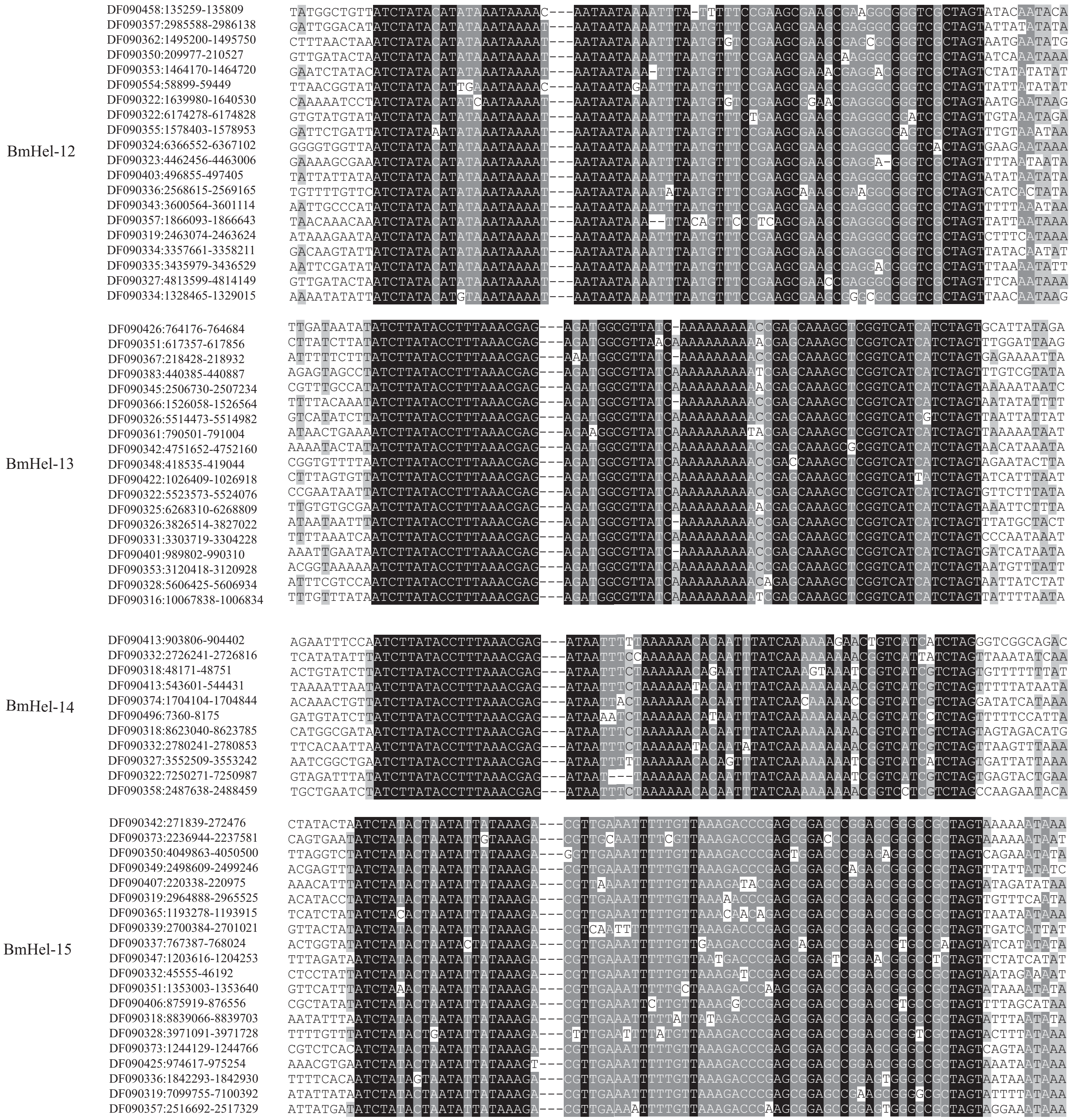

Fig. S2(continue). Sequence alignments for each silkworm Helitron family. The 5' ATC, 3' CTAGT and flanking sequences are shown.

BmHel-16

```
DF090347:1322429-1322842    AGTAGTCTATATCTATACTAATATTATAAA---TTCATGTGTGTTTTAATGTTTCCGAAGCGAAGCGAGGGCGGGTCGCTAGTATTATATTAA
DF090375:128578-128991      TAGGCTTAACATCTATACTAGTATTATAAA---TTCATGTG----TTAATGTTTCCGAAGCGAAGCGCGGGCTGGTCGCTAGTTTATTTATAA
DF090345:860982-861395      TGAATTTTAAATCTATACTAATATTATAAA---TTCATGTGTGTTTTAATGTTTCCGAAGCGAAGCGAGGGCGGGTCGCTAGTCATTCAATAA
DF090345:4286841-4287254    CTGAATTAAAATCTATACTAATATTATAAA---TTCATGTGTCTTTAATGTTTCCGAAGCGAAGCGAGGGCGGGTCGCTAGTTTCTACATAA
DF090383:525244-525657      GTTAGAAAACATCTATACTAATATTATAAA---TTCCTGTGTGTTTTAATGTTTCCGAAGCGAAACGAGGGCGGGTCGCTAGTTATTTTATAA
DF090357:1937551-1937964    TCAGACGGGAATCTATACTAATATTATAAA---TTCGTGTGTGTTTTAATGTTTCCGAAGCGAAGCGAGGGCGGATCGCTAGTATGGAATAAG
DF090392:1529631-1530044    AATTTAAATGATCTATACTAATATTATAAA---TTCATGTGTGTTTTAATGTTTCCGAAGCGAAGCGAGGGCAGGTCGCTAGTTCTCTTATAA
DF090372:2318877-2319290    CGTAGTCTATATCTATACTAATATTATAAA---TTCATGTGTGTTTTAATGTTTCCGAAGCGAAGCAAGGGCGGGTCGCTAGTAAAGCAATAA
DF090347:2678650-2679063    ATATGTCTTAATCTATACTAATATTATAAA---TTCATGTGTGTTTTAATGTTTCCGAAGCGAAGCGAGGGCGGGTCGCTAGTTATTTAGTAA
DF090351:1895627-1896040    CTAATATCTTATCTATACTAATATTATAAA---TTCATGTGTGTTTTAATGTTTCCGAAGCGAAGCGAGGGCGGGTCGCTAGTCGTTTGATAT
DF090335:2751401-2751811    ATACAGCTTTATCTATACTAATATTATAAA---TTCATGTGTGTTTTAATGTTTCCGAAGCGAAGCGAGGGCGGGTCGCTAGTCTATTATATC
DF090337:4533298-4533711    TAAATTATTTATCTATACTAATATTATAAA---TTCTTATGTGTTTTAATGTTTCCGAAGCGAAGCGAGGGTGGGTCGCTAGTTTTATATATT
DF090374:2066376-2066789    TATCTATACTATCTATACTAATATTATAAA---TTCATGTGTGTTTTAATGTTTCCGAAGCGAAGCGAGGGCGGGTCGCTAGTAAACAATACA
DF090365:25857-26270        TTGCGTACTAATCTATACTAATATTATAAA---TTCATGTGTGTTTTAATGTTTCCGAAGCGAAGCGAGGGCGGGTCGCTAGTAAATATATAA
DF090372:1008339-1008752    TTGAATCTATATCTATACTAATATTATAAA---TTCGTGTGTGTTTTAATGTTTCCGAAGCGAAGCGAGGGCGGGTCGCTAGTATTTAATAAT
DF090357:2427176-2427589    ATACTTTTGTATCTATACTAATATTATAAA---TTCATGTGTGTTTTAATGTTTCCGAAGCGAAGCGAGGGCGGGTCGCTAGTTTTAAAATAA
DF090369:442743-443156      TATTAATTTTATCTATACTAATATTATAAA---TTCATGTGTGTTTTAATGTTTCCGAAGCGAAGCGAGGGCGGGTCGCTAGTTCATTATAA
DF090390:1262833-1263246    CAACGTGTTTATCTATACTAATATTATAAA---TTCATGTGTATTTTAATGTTTCCGAAGCGAAGTAAGGGCGGGTCGCTAGTTACAAAAATA
DF090352:911488-911901      TTTTACAACTATCTATACTAATATTATAAA---TTCATGTGTGTTTTAATGTTTCCGAAGCGAAGCGAGGGCGGGTCGCTAGTGTATTCATAA
DF090381:1905137-1905550    CTTCTATACTATCTATACTAATATTATAAA---TTCATGTGTGTTTTAATGTTTCCGAAGCGAAGCGAGGGCGGGTCGCTAGTTTATTATAAA
```

BmHel-17

```
DF090395:1116556-1117438 :  TCCATCTAGACTCTAGAGCAGCGGTCGAGG---TTCATTGTACCCCATTTGGGGTAATTTACCCCGGTTCACAGACCGCTGATGAGCATTACG
DF090323:7976417-7977155 :  TACCTATACTATCTATATCAGCGGTCGGGG---TTAATTTTACCCCATTTGGGGTAATTTACCCCGGTTCCCCGACCGCTGATTATAGATAAC
DF090342:1302077-1302564 :  TTTCGTAACGATCTATATCAGCGGTCGGGG---TTCATTTTACCCCATTTGGGGTAATTTACCTCGGTTCCCCGCCCGCTGATTATAAATGTT
DF090363:2458310-2458799 :  TATTGAATTAATCTAGAGCAGCGGTCGGGG---TTCATTTTACCCTATTTGGGGTAACTTACCCCGATTCCCCGACCGCTGATGAGTATGGTA
DF090398:940364-940853 :    TTCTAAGTAGATCTAGAACAGCGGTCGGGG---TTAATTTTACTCCATTTGAGATAATTTACCCCGGTTCCCCGACCGCTGACGAAGCTATAG
DF090334:2753455-2754099 :  TTATCTAACGATCTAGATCAGCGGTCAGGG---TTCATTTTTCCCCATTTGGGGTAATTTACCCCGGTTCCCCGACCGCTGATGATGGACAGA
DF090362:518117-518786 :    TCGACGGTAGATCTAGATCAGCGGTCGGGG---TTCATTTTACCCCATTTGGGGTAATTTACCCCGGTTCCCCGACCGCTGATGATCTAGAAA
```

BmHel-18

```
DF090317:9152388-9152768 :  GACTTTAATCATCACTACATAGTATAAAAC---TTTAT-ATGTATAATAATATCCGAAGCGAAGCGAGGGCGGGTCGCTAGTAATCTAATAT
DF090414:954616-954967 :    TAAATAAAAAATCACTACGTAGTATAAAAC---TCAACAAGAAATTACAGTTTCCGAAGCGAAGCGAGGGCGGGTCGCTAGTATATTATAAT
DF090321:2829516-2829891 :  AGCGTACTTAATCACTACATAGTATAAAAC---ATAATAATAAATTACAGTTTCCGAAGCGAAGCGAGGGCTGGTCGCTAGTCCTAGATAAA
DF090390:1621180-1621530 :  GTTGATACTAATCACTACATAGTATAAACC---TCAATAATAAATTACAGTTTCCGAAGCGAAGCGAGGGCGGGTCGCTAGTACCAAATAAA
DF090326:2165246-2165558 :  TTAGGAATATATCACTACATAGTACAAAAC---TCAATAATAAATTACAGTTTCCGAAGCGAAGCGAGGGCGGGTCGCTAGTTGGTAAATAA
DF090441:138132-138503 :    TTTTATGTATATCACTACATAGTATAAAAC---TCAATAATAAGTTACAGTTTCCGAGGCGAAGCGAGGGCGGGTCACTAGTCAATAATAAA
DF090318:7430260-7430639 :  CATTTAAAACATCACTACATAGTATAAAAC---TCAATAATAAATTACAGTTTCCGAAGCGAAGCGAGGGCGGATCGCTAGTTTAAACTAAA
DF090342:3278214-3278595 :  ACTTCGTCTAGTCACTACATAGTATAAAAC---TCAATAATAAATTACATGTTCCGAAGTGAAGCGAGGGCGGATCGCTAGTCTTCGATAAA
DF090353:650676-651042 :    AATTGTGTTTATCACTACATAGTATAAAAC---TCAATAATAAATTACAGTTTCCGAAGCGAAGCGAGGGCGGGTCGCTAGTCTAAATATAT
DF090412:1161081-1161457 :  AAAGTTTAGTTTCACTACATAGTATAAAAC---TCAATAATAAATTACAGTTTCCGAAGCGAAGCGAGGGCGGGTCGCTAGTTAATTCTAAA
DF090352:898888-899261 :    TTTTTGCTTCATCACTACATAGTATAAAAC---TCAATAATAAATTACAGTATCCGAAGCGAAGCGAGGGCGGGTTGCTAGTATATTAATAT
DF090319:6833423-6833795 :  GTACCTCAATATCACTACATAGTATAAAAC---TCAATAATAAATTACAGTTTCCGAAGCGAAGCGAGGGCGGATCGCTAGTATAATACTAT
DF090413:525952-526321 :    GTGGGCATGCTTCACTACATAGTATAAAAC---TCAATAATAAATTACAGCTTCCGAAGCGAAGCGAGGGCGGGTCGCTAGTTATAAATAAA
DF090353:2661364-2661745 :  GAAACGCACCATCACTACATAGTATAAAAC---TCAATAATAAATTAGAGTTTCCGAAGCGAAGCGAGGGCGGGTCGCTAGTTTATTCTAAA
DF090328:5299153-5299516 :  AAAGTCATCCATCACTACATAGTATAAAAC---TCAATAATAAATTACAGTTTCCGAAGCGAAGCGAGGGCGGGTCGCTAGTATAAAATAAA
DF090421:621163-621537 :    TATCTTGATCATCACTGCATAGTATAAAAC---TCAATAATAAATTACAGTTTCTGAAGCGAAGCGAGGGCGGGTCGCTAGTTACACATAAA
DF090338:348456-348836 :    GTATGGCTTAATCACTATATAGTATAAAAC---TCAATAATAAATTACAGTTTCCGAAGCGAAGCCAGGGCGGGTCGCTAGTATAGTATAAA
DF090318:4479242-4479626 :  TTTAACTATTATCACTACATAGTATAAAAC---TCAATAATAAATTACAGTTTCCGAAACGAAGCGAGGGCGGGTCGCTAGTTTTTGTATAA
DF090322:403134-403514 :    AATATGATGTATCACTACATAGTATAAAAC---TCAATAATAAATTACAGTTTCCGAAGCGAAGCGAGGGCGGTTCGTTAGTTTACTATATA
DF090360:1765236-1765613 :  ACCAGTACACTTCACTACATAGTATAAAAC---TCAATAATAAATTACAGTTTCCGAAGCGAAGCGAGGGCGGGTCGCTAGTACCTAATAAT
```

BmHel-19

```
DF090410:436957-439205      GTTGGTTTATCTCATCAATACATATAATAA---AATCGTAACTGGTCGAGTCGCTATTCCACGCAGTCGCGGACACAGCTAGTGTTAAATA
DF090589:38378-40611        GTTGGTTTATCTCATCAATACATATAATAA---AATCGTAACTGGTCGAGTCGCTATTCCACGCAGTCGCGGACACAGCTAGTGTTAAATA
DF090434:350121-352268      TTAATTAATCATCATCAATACATATAATAA---AATCGTAACTGGTCGAGTCGCTATTCCACGCAGTCGCGGGCACAGCTAGTTAATATAA
DF090316:9611009-9613370    TGACAGTTTCTTCATCAATACATATAATAA---AATCGTAACTGGTCGAGTCGCTATTCCACGCCGTCGCGGGCACAGCTAGTTCGTAATA
DF090430:469695-471373      GTTTTAAATCATCATCAATACATATAATAA---AATCGTAACTGGTCGAGTCGCTATTCCACGCAGTCGCGGGCACAGCTAGTAAATAAAT
DF090323:666397-668787      CATCAATATCTTCATCAATACATATAATAA---AATCGTAACTGGTCGAGTCGCTATTCCACGCAGTCGCGGGCCCAGCTAGTTTCGGAAT
DF090574:50180-51261        TTCAATCAATATCATCAATACATATCATAA---AATCGTAACTGGTCGAGTCGCCATTCCACGCTGTCGCGGGCTCAGCTAGTTTCTAAAT
DF090322:4458598-4461259    CATCATCATCATCATCAATACATATAATAA---AATCGTAACTGGTCGAATCGCTATTCCACGCAGTCGCGGGCACAGCTAGTACTACCTA
DF090338:2312201-2312700    AGAATGCGACATCATCAATACATGTAATAA---AATCGTAACTGACCGAATCGCTATTCCACGCAGTCGCGGGCACAGCTAGTTTCTAATA
```

Fig. S2(continue). Sequence alignments for each silkworm Helitron family. The 5’ ATC, 3’ CTAGT and flanking sequences are shown.

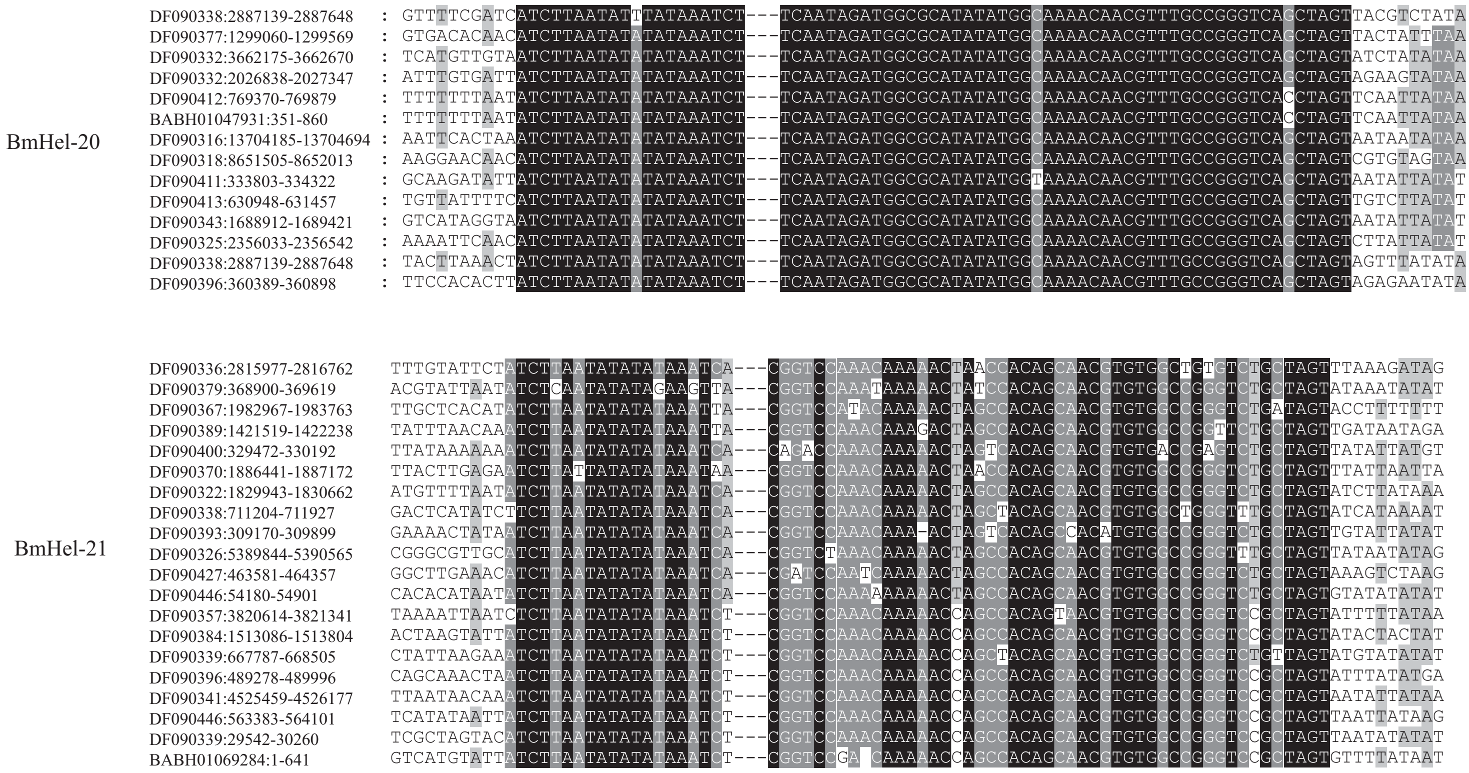

Fig. S3. PCR verification of BmHel-8 in 15 silkworm strains. Lanes M–15 are: DNA marker (DL2000), 02-320, DaZao, Ri9, 872, Ou18, Yi16, YinDuSanMian, WuLin1Hao, BH863, YingWenXing, LuoSa, RiXian2Hao, ALiKeSi, SanMianBai, and Zhong4010.

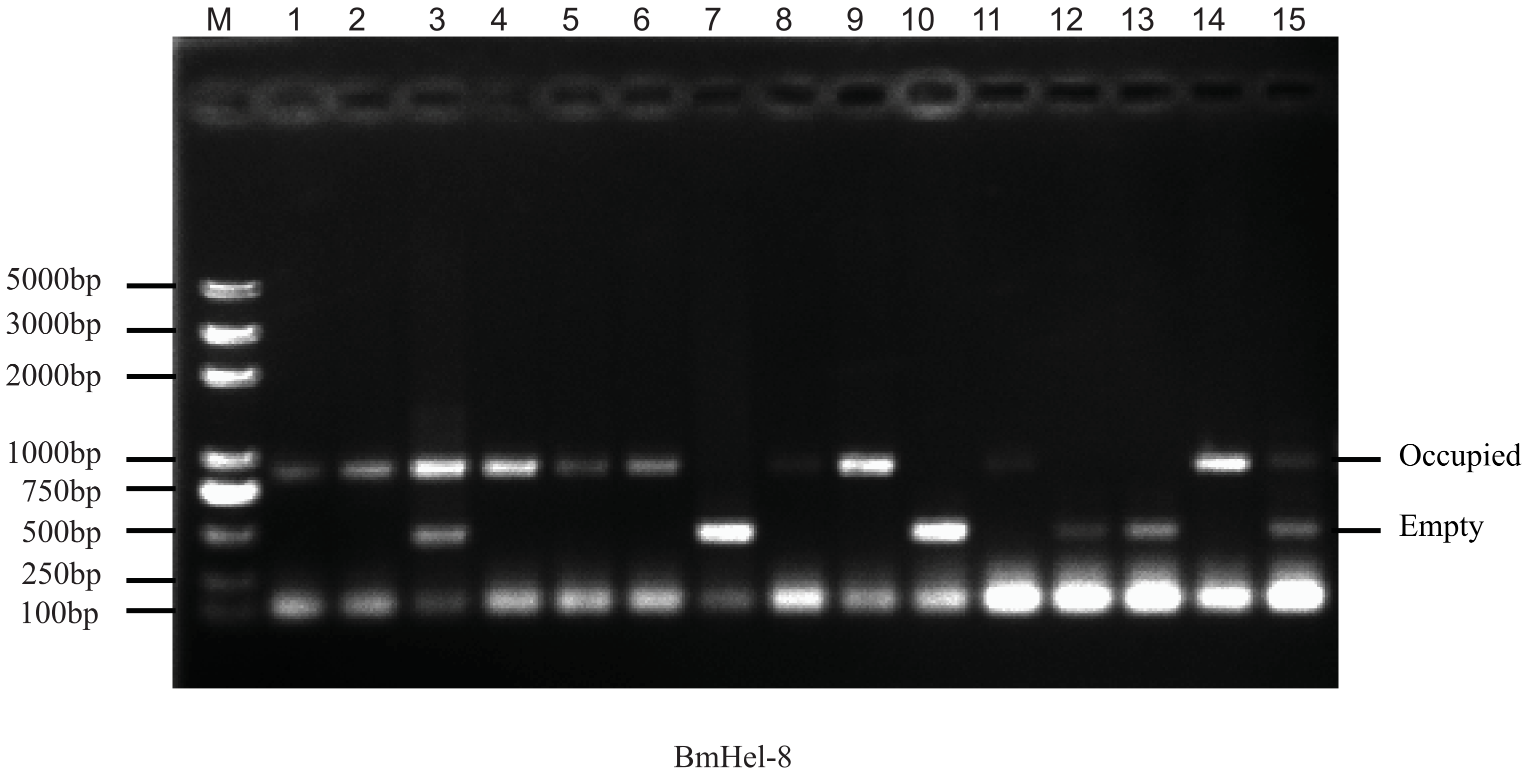

Fig. S4. Distribution of Helitrons on the 28 silkworm chromosomes. The observed distribution was no significantly different from the expected one based on the total length of 28 chromosomes (t = –6e-4, df = 27, P = 0.9996).

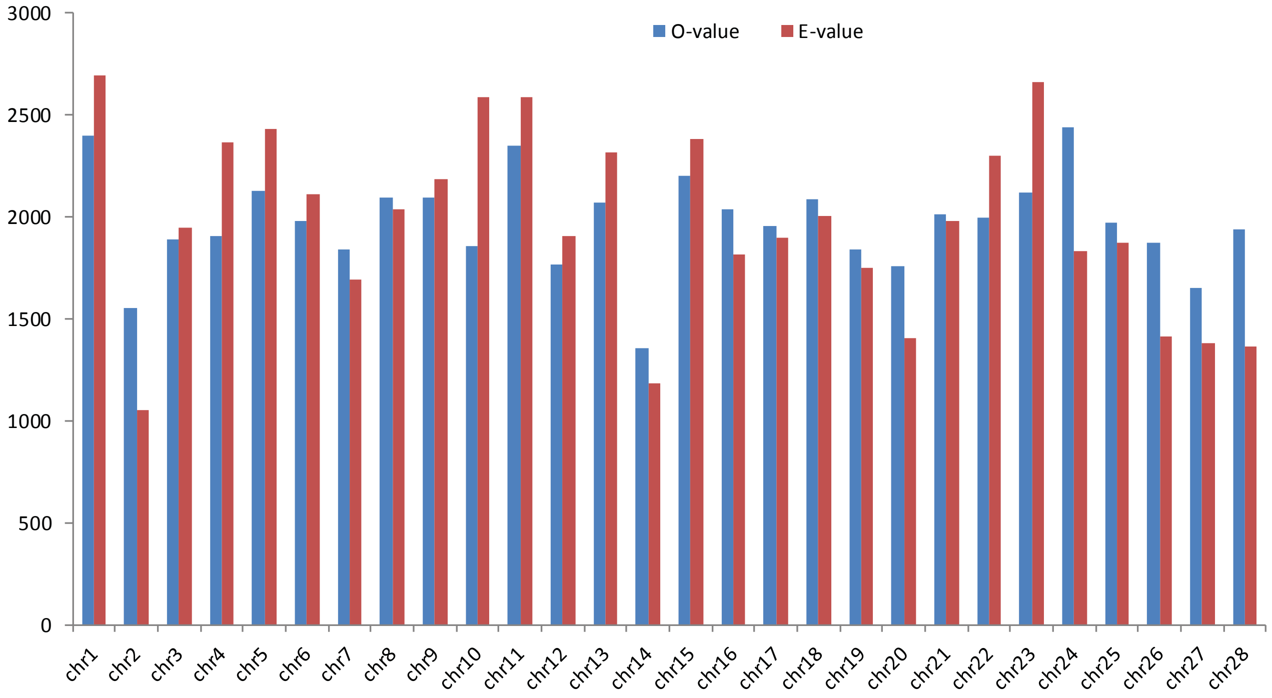

Fig. S5. Distributions of Helitrons within individual silkworm chromosomes. The x axis indicates chromosome locations while the y axis shows Helitron copy numbers (both intact and fragmented elements).

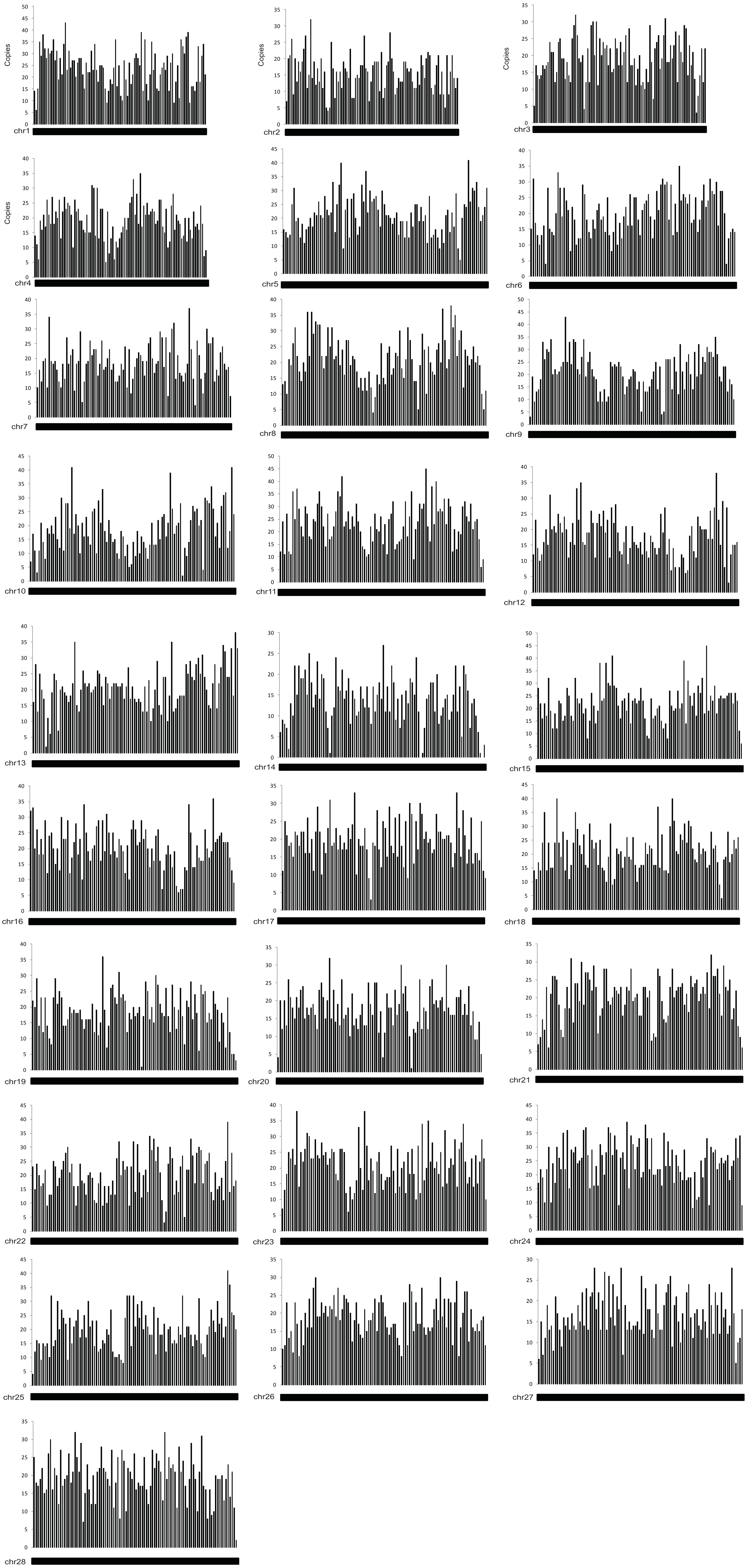

Fig. S6. Distribution of the silkworm Helitrons in gene regions.

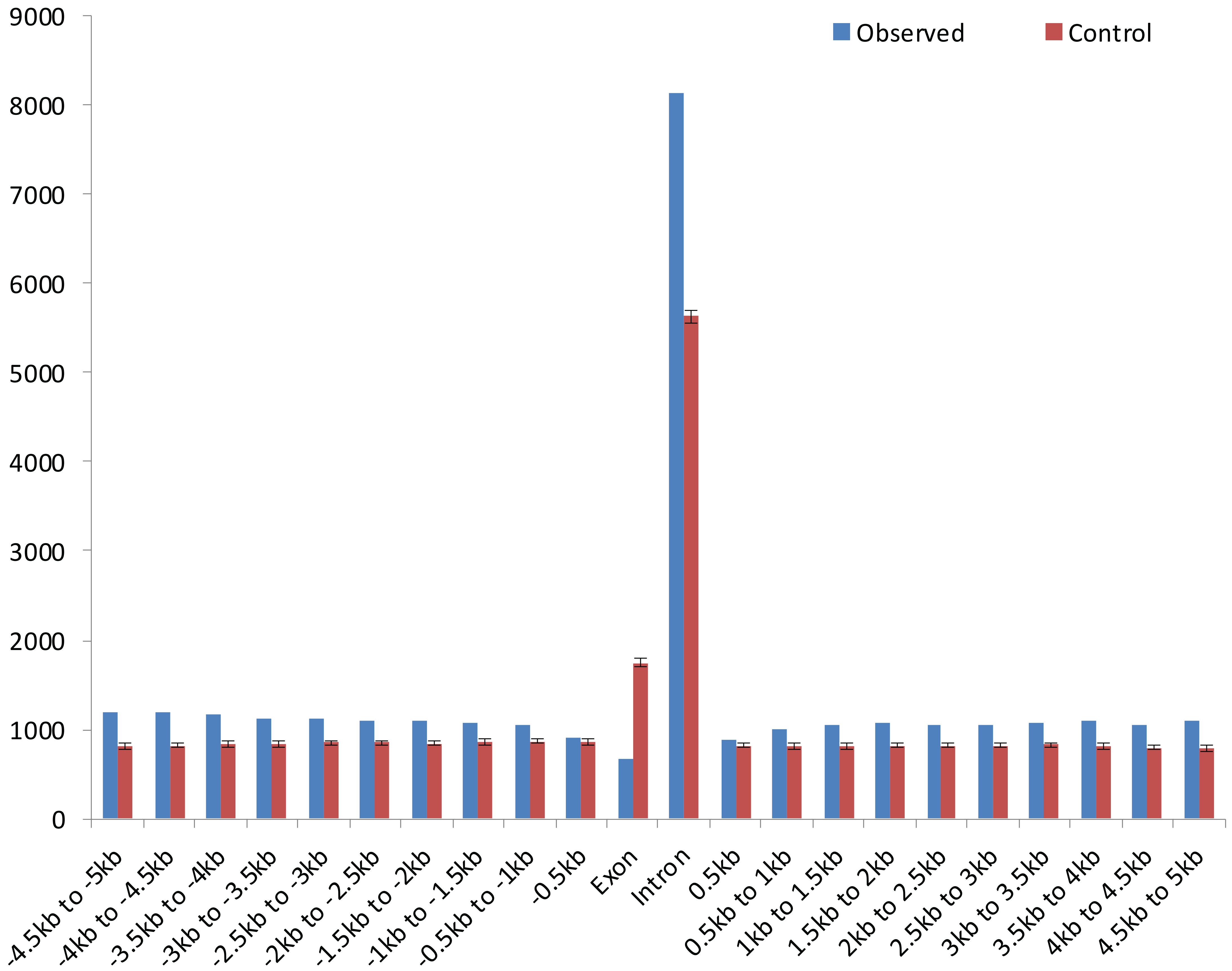

Fig. S7. Distribution of pairwise nucleotide diversity for 20 silkworm Helitron families.

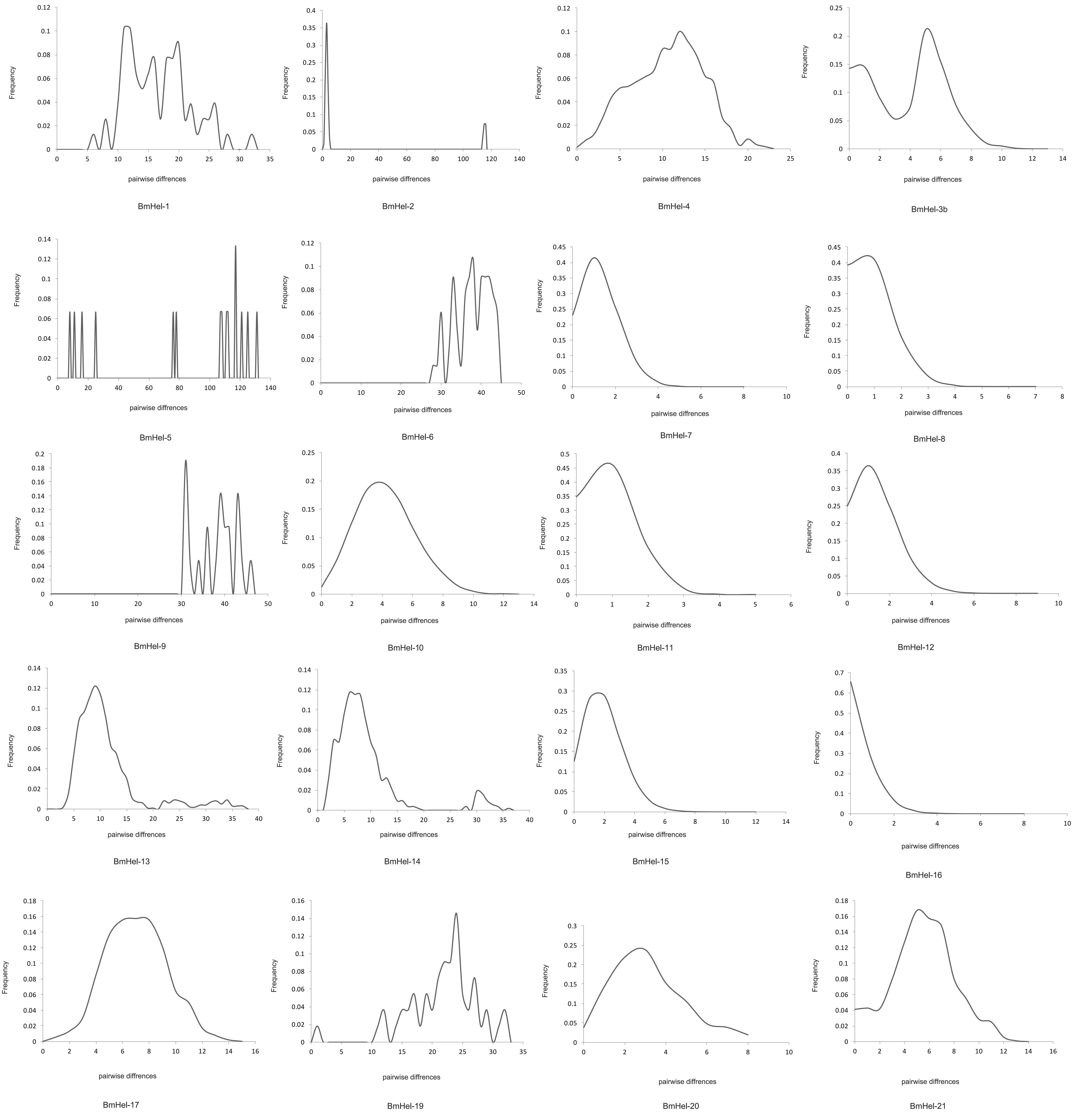

Fig. S8. Phylogenetic trees of 20 silkworm Helitron families.

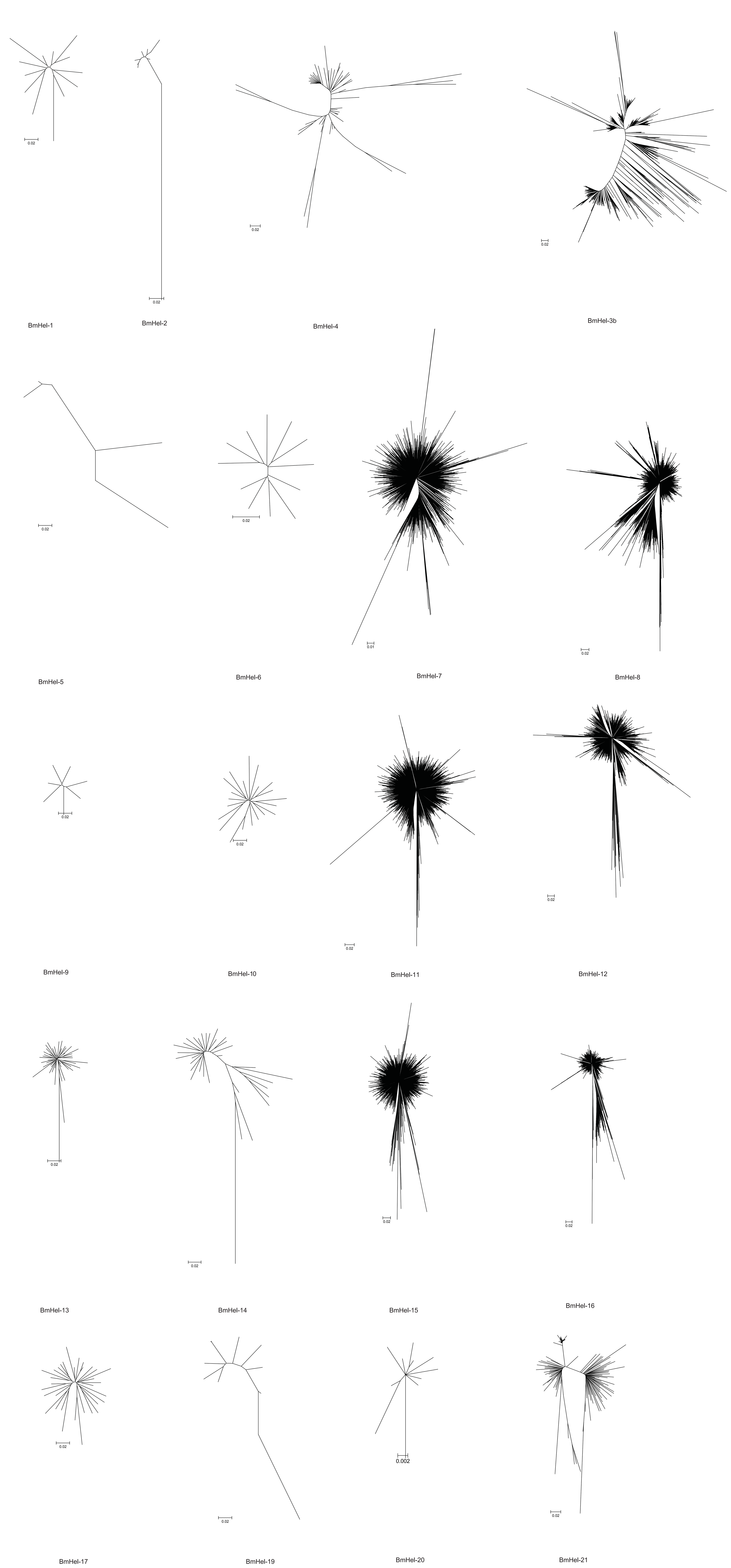

Fig. S9. Examples of Helitrons containing gene fragments.

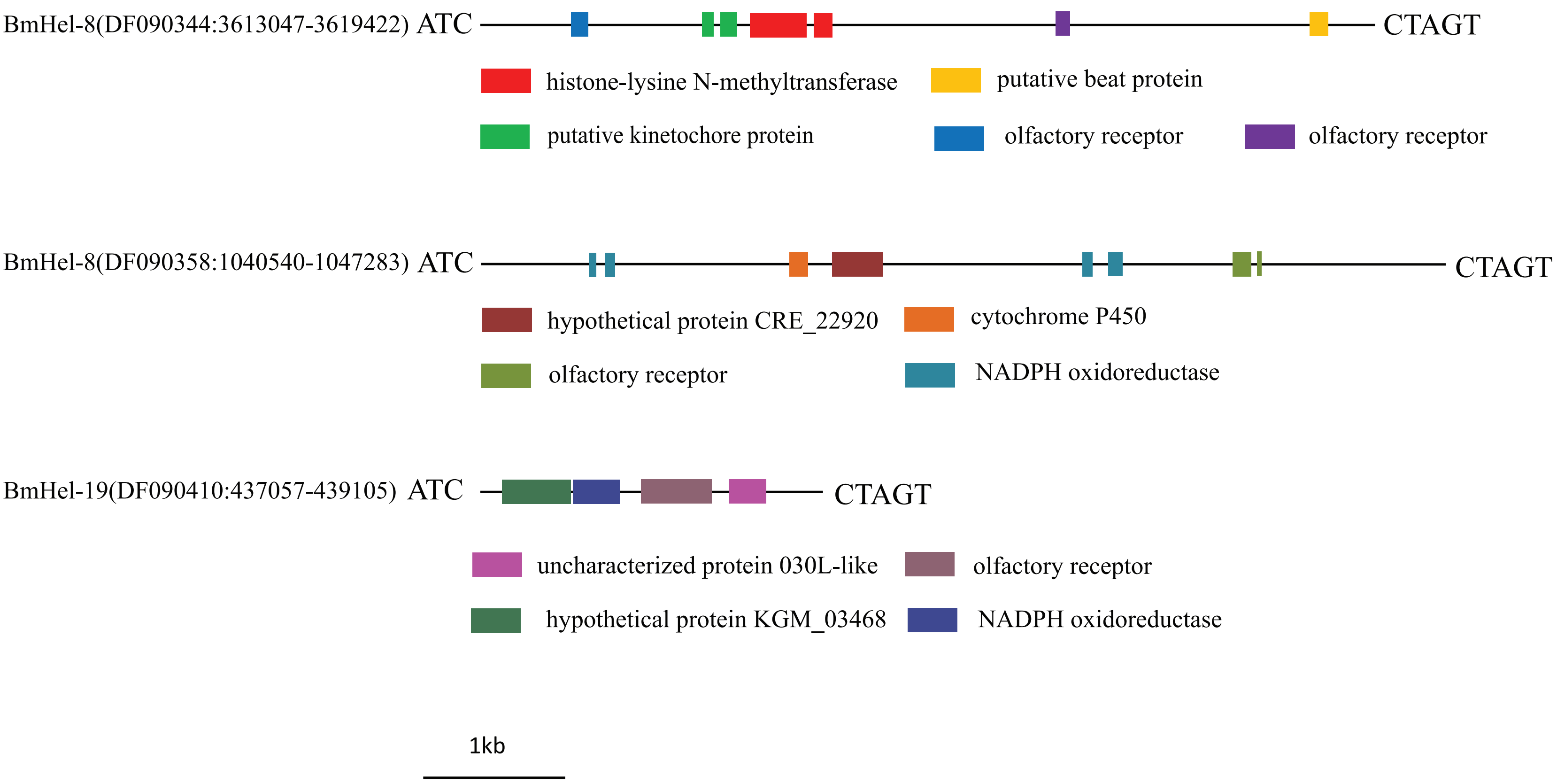

Fig. S10. Examples of Helitrons within full-length cDNAs. (A) Examples of Helitrons in 5' untranslated regions. (B) Examples of Helitrons in coding regions. (C) Examples of Helitrons in 3' untranslated regions.

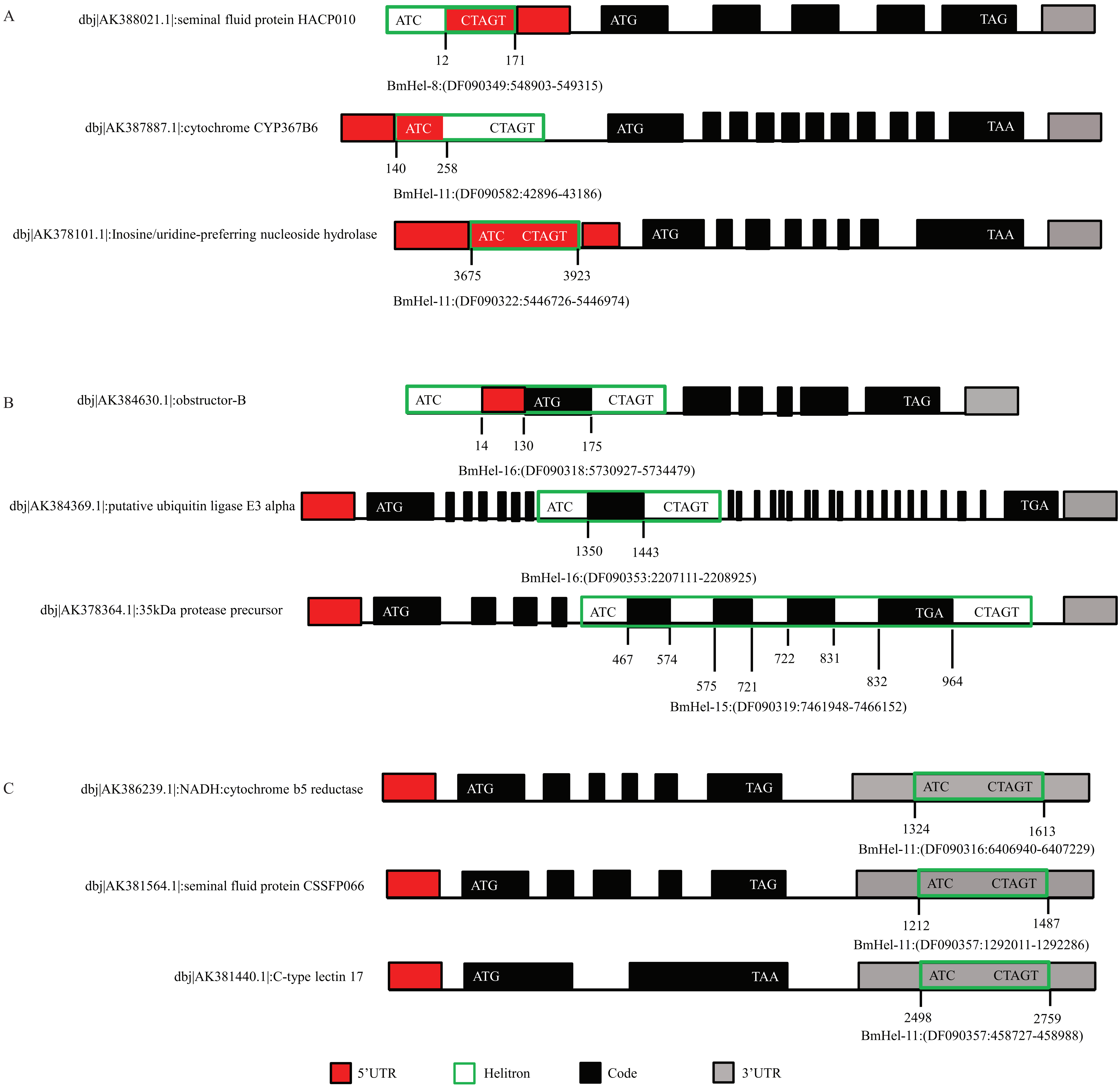

Fig. S11. Sequence alignment for an example of horizontal transfer (BmHel-16). S_fru, B_any, H_zea, P_dar, C_eur, H_arm, B_bet, C_plu_PDV, C_ses_KBV, and C_ses_MBV represent species of Spodoptera frugiperda, Bicyclus anynana, Helicoverpa zea, Papilio dardanus, Colias eurytheme, Helicoverpa armigera, Biston betularia, Cotesia plutellae polydnavirus, Cotesia sesamiae Kitale bracovirus, and Cotesia sesamiae Mombasa bracovirus, respectively.

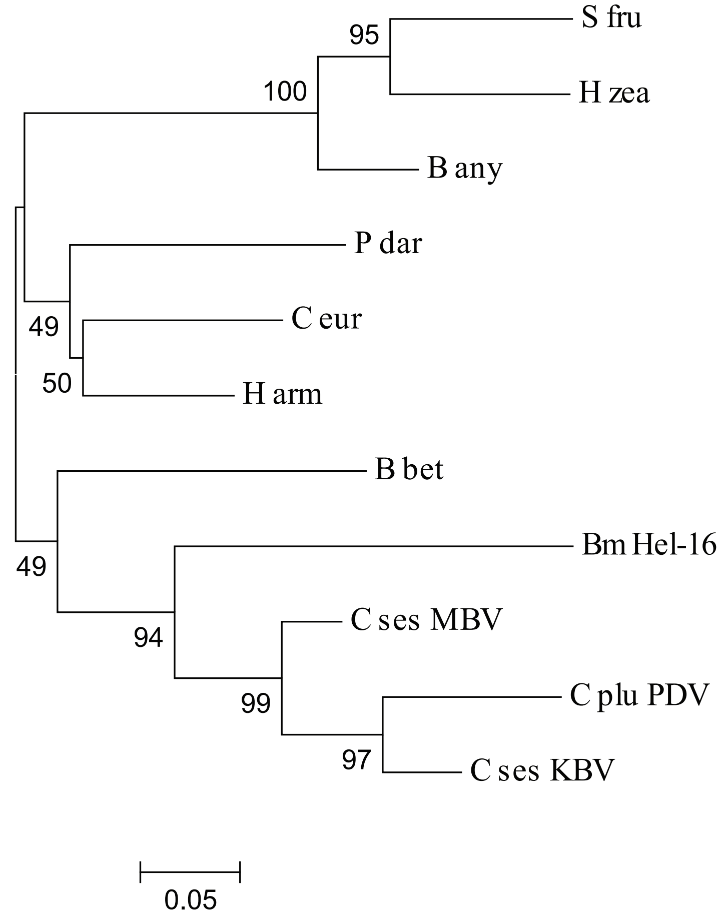


```
                             *       20         *       40         *       60         *       80         *      100         *
 BmHel-16 : ATCTATACTAATATTATAAAGAGGAAAGATTTGTTTGTTTGTTTGTTTCGAATAGGCTCCGAAACTACTGGACCGATTTGAAAAATTCTTTTTCCATTAGAAGCCGACATTGTCCCT
S_fru     : ATCTATACTAATATTATAAAGCTGAAGAGTTTGTTTGTTTGTTTGT-TTGAATAATCTCCGGAACTACTGGTCCGATTTGAATAATTATTTTTGTGTTGGATAGTGAATTTATCGAG
B_any     : ATCTATGATAATATTATAAAGCTGAAGAGTTTGTTTGTTTGTTTTTATTGAATAATCTCAGGAACTACTGGTCCGATTTGAAAAATTCTTTCAGTGTTAGATAGCCATATTATCGAG
H_zea     : ATCTATACTAATATTATAAAGCTGAAGAGTTTGTTTGTTTGTTTAT-TTGAATAATCTCAGGAACTACTGGTCCGATTTGAACAATTCTTTCGGTGTTAGATAGCCCATTTATCGAG
P_dar     : ATCTATCTTAATATTATAAAGAGAGAAACTTTGTTTGTATTTTTGTAATTAATTAACTCAAGAACTACTGGGCCGATTTCAAAAATTCTTTCACCATTAGAAAGCTACATTATCCCC
C_eur     : GTCTACACTAATATTATAAAGAGGAAAACTTTGTTTGTTTGATTGTAATGAATAGGCTCATAAACTACTGGACCGATTTTAAAAATTATTTCACCATTCGAAAGCTACATTATCCAC
H_arm     : ATCTATACTAATATTATAAAGAGGAAAACTTTGTTTGTTTGTTTGGAATGGATAAGCTCAAAAACTACTGGACCGATTTTAAAAATTCTTTCACCATTAGAAAGCTATATTATCCAC
B_bet     : ATCTA---TATTATTATAAAGAGGAAAGATTTGATTGTTTGTTTGCATTGAATAGGCTCTGAAACTACTGGACCGATTTCAAAAATTCTTTCTCTTTTGAAAAACTATACTATCCCC
C_plu_PDV : ATCTATACTAATATTATGAAAAGGGAATATTTATTTGTCTGTTTGCATTGAATAGGCTCCGAAACTACTGAACCGATTAAAAAAATTCATCCACTGATGGAAAGCTACAGTATCTCC
C_ses_KBV : ATCTATAATAATATTATAAAAAGGAAATATTTGTTTGTTTGTTTGCATTGAATAGGTTCCGAAACTACTGAATCGATTTAAAAAATTCTTTCACTGTTGAAAAGCTTCACTATCTCC
C_ses_MBV : ATATA---TAATATTATAAAGAGGAAAGATTTGTTTGTTTGTTTGCATTGAATAGGCTCCGAAACTACTGAACCGATTTGAAAAATTCTTTCACTGTTGAAAACCTACACTATCCCC
            aTcTA   TAaTATTATaAAg  gaA   TTTgtTTGTtTgtTTg    tgaATa  cTC   AACTACTG  cCGATTt AAaAATTctTtc     tT  A a c a a TaTC

            120         *       140         *       160         *       180         *       200         *
 BmHel-16 : GATGAACATAGGCTACTTTTTTTAATTTTTTTTTTTTTTTTTTTGGTTTCATGTGTGTTTTAATGTTTCCGAAGCGAAGCGAGGGCGGTCGCTAGT
S_fru     : GAAGGCTATAGGCTATAAAACATCACG-----------------CTATGACCAATAGGAGCCAAGCAGAGCG-GGTGAAACCCGCGGAAGTAGCTAGT
B_any     : GAAGGCTATAGGCTATATAT-------ATTTATCCCCGTATTCCTACGGGAACGGGAA------CCACGCG-GGTGAAACCCGCGGCGTCAGCTAGT
H_zea     : GAAGGCTATAGGCTACTTTTTATCCAGGTTCGTGCAGACGTTCCCACGTG-----------------ATGCG-GGTGAAACCGCTGGCAGAAGCTAGT
P_dar     : GAGTAACATAGGCTA----TTATTAT--TTAATTCC------------------------------ACGCG-GACGAAGTCGCGCACAACAGCTAGT
C_eur     : GAGTAATATAGGATAGGT-----------TTTATCCCGGAAATTCTACGGGAACGGGAA-CTATGCGACGCG-GGCGGAGCCCGGGCGGAAAGCTAGT
H_arm     : GAGTAACATAGGCTATATTTTAGTAT--TTTATCCCGG------TACGGGCAGTAGTTCCCACGGGACGCG-GGTGAAACCCGGGAAAACGGCTAGT
B_bet     : GGGTGAAATAGGCTT---------------TTTATCCCTGTATTTCCACGGGAACGGGAA------CTATGCT-GGTGAAACTCAAAAGGTTCGTTAGT
C_plu_PDV : AGGTAACATAAGCTATATTGTGTTATAATTAATTTTGAAATTTTTGTTTA---------------ACGCCCA-GGCAAAGCTAGGGCGGACTGCTAGT
C_ses_KBV : AAGTAACATAAGCTATATTATATTATAATTAATTTTGAAATTTTTGTTTA---------------ATACCCG-TGCAAAGCTAGTACGGACTGCTAGT
C_ses_MBV : GAGTGACATAGGCTATATTATAATATAATTAATTTTGAAATTTATGTTAA---------------GTACCCG-TGCGAAGCCGGACGGACTGCTAGT
            ga      ATAgGcTa               tt  t                                   Cg  g gaA c             GcTAGT
```
