# Supplementary material for: Identification and Evolution of the Silkworm Helitrons and their Contribution to Transcripts
Source: DNA Res. 2013 Jun 14;20(5):471–84. doi: 10.1093/dnares/dst024 (PMC3789558; doi:10.1093/dnares/dst024)
Supplement: Supplementary Data [file supp_dst024_dst024supp_table3-9.pdf]

TableS3 Positions of Helitron in Danaus plexippus genome

**Families**	**Accession numbers**
DpHel-1	DPSCF300143:708718-708828
DpHel-1	DPSCF300412:45108-45269
DpHel-1	DPSCF300354:210563-210739
DpHel-1	DPSCF300003:479300-479477
DpHel-1	DPSCF300121:165296-165473
DpHel-1	DPSCF300562:3329-3506
DpHel-1	DPSCF303474:216-395
DpHel-1	DPSCF300262:485484-485717
DpHel-1	DPSCF300222:569666-569956
DpHel-1	DPSCF300280:18668-18989
DpHel-1	DPSCF300088:1158123-1158455
DpHel-1	DPSCF300253:102455-102800
DpHel-1	DPSCF300302:89943-90290
DpHel-1	DPSCF300001:4140169-4140518
DpHel-1	DPSCF300012:1350317-1350702
DpHel-1	DPSCF300232:52890-53289
DpHel-1	DPSCF300071:904867-905272
DpHel-1	DPSCF300026:437680-438088
DpHel-1	DPSCF300069:506597-507009
DpHel-1	DPSCF300809:6358-6785
DpHel-1	DPSCF300058:494721-495157
DpHel-1	DPSCF300072:1278744-1279194
DpHel-1	DPSCF300001:3911802-3912256
DpHel-1	DPSCF300458:38071-38535
DpHel-1	DPSCF300128:157788-158256
DpHel-1	DPSCF300446:61450-61920
DpHel-1	DPSCF300497:40844-41315
DpHel-1	DPSCF300036:678020-678497
DpHel-1	DPSCF300136:302471-302949
DpHel-1	DPSCF300337:115012-115491
DpHel-1	DPSCF300170:450731-451215
DpHel-1	DPSCF300063:1059880-1060377
DpHel-1	DPSCF300065:130890-131394
DpHel-1	DPSCF300012:748576-749081
DpHel-1	DPSCF300098:52518-53023
DpHel-1	DPSCF300222:634386-634893
DpHel-1	DPSCF300088:1224339-1224856
DpHel-1	DPSCF300002:527682-528199
DpHel-1	DPSCF300241:169854-170372
DpHel-1	DPSCF300136:59665-60184
DpHel-1	DPSCF300035:1150993-1151529
DpHel-1	DPSCF300393:83139-83688
DpHel-1	DPSCF300182:294279-294831
DpHel-1	DPSCF300011:593876-594430
DpHel-1	DPSCF300123:206904-207458
DpHel-1	DPSCF300030:193084-193639

DpHel-1	DPSCF300600:5477-6032
DpHel-1	DPSCF300139:65653-66208
DpHel-1	DPSCF300043:512612-513170
DpHel-1	DPSCF301468:704-1262
DpHel-1	DPSCF300087:530987-531547
DpHel-1	DPSCF300286:153506-154070
DpHel-1	DPSCF300001:4464718-4465284
DpHel-1	DPSCF300031:59922-60489
DpHel-1	DPSCF300262:381735-382302
DpHel-1	DPSCF300298:57939-58508
DpHel-1	DPSCF300016:1010523-1011092
DpHel-1	DPSCF300130:314872-315442
DpHel-1	DPSCF300086:302382-302953
DpHel-1	DPSCF300341:72027-72598
DpHel-1	DPSCF300005:3074-3647
DpHel-1	DPSCF300063:692110-692684
DpHel-1	DPSCF300002:1722705-1723281
DpHel-1	DPSCF300967:3092-3669
DpHel-1	DPSCF300236:109145-109722
DpHel-1	DPSCF300117:1163638-1164216
DpHel-1	DPSCF300160:31649-32229
DpHel-1	DPSCF300222:457880-458460
DpHel-1	DPSCF300073:857721-858301
DpHel-1	DPSCF300108:354507-355087
DpHel-1	DPSCF300048:1266920-1267500
DpHel-1	DPSCF300023:32451-33032
DpHel-1	DPSCF300962:1294-1875
DpHel-1	DPSCF300172:305686-306268
DpHel-1	DPSCF300202:4162-4744
DpHel-1	DPSCF300300:143481-144063
DpHel-1	DPSCF300161:270518-271102
DpHel-1	DPSCF300318:157968-158555
DpHel-1	DPSCF300098:128664-129253
DpHel-1	DPSCF300212:308580-309170
DpHel-1	DPSCF300003:202753-203344
DpHel-1	DPSCF300083:305983-306575
DpHel-1	DPSCF300152:329343-329937
DpHel-1	DPSCF300086:764849-765443
DpHel-1	DPSCF300168:368314-368910
DpHel-1	DPSCF300006:420419-421015
DpHel-1	DPSCF300005:817545-818141
DpHel-1	DPSCF300131:421290-421887
DpHel-1	DPSCF300006:115989-116588
DpHel-1	DPSCF300273:265264-265863
DpHel-1	DPSCF300079:282107-282706
DpHel-1	DPSCF300060:247504-248104
DpHel-1	DPSCF300004:1370119-1370719
DpHel-1	DPSCF300001:2303807-2304408

DpHel-1	DPSCF300460:67010-67611
DpHel-1	DPSCF300027:543079-543681
DpHel-1	DPSCF300600:8153-8755
DpHel-1	DPSCF300024:426566-427169
DpHel-1	DPSCF300037:224329-224932
DpHel-1	DPSCF300061:717655-718258
DpHel-1	DPSCF300103:414117-414720
DpHel-1	DPSCF300237:33597-34201
DpHel-1	DPSCF300015:611177-611781
DpHel-1	DPSCF300042:1255535-1256139
DpHel-1	DPSCF300104:111263-111867
DpHel-1	DPSCF300472:9490-10094
DpHel-1	DPSCF300533:37994-38599
DpHel-1	DPSCF300090:760290-760895
DpHel-1	DPSCF300031:138914-139519
DpHel-1	DPSCF300329:56221-56827
DpHel-1	DPSCF300098:19521-20127
DpHel-1	DPSCF300022:298339-298945
DpHel-1	DPSCF300427:201811-202418
DpHel-1	DPSCF300108:404345-404952
DpHel-1	DPSCF300195:498454-499061
DpHel-1	DPSCF300218:292588-293195
DpHel-1	DPSCF300643:4912-5520
DpHel-1	DPSCF300042:593572-594180
DpHel-1	DPSCF300125:32362-32970
DpHel-1	DPSCF300069:414362-414970
DpHel-1	DPSCF300191:4071-4679
DpHel-1	DPSCF300028:1558910-1559519
DpHel-1	DPSCF300010:3236701-3237310
DpHel-1	DPSCF300487:43438-44047
DpHel-1	DPSCF300047:695729-696338
DpHel-1	DPSCF300100:72428-73037
DpHel-1	DPSCF300091:320706-321315
DpHel-1	DPSCF300201:2799-3409
DpHel-1	DPSCF300020:670677-671287
DpHel-1	DPSCF300028:339489-340099
DpHel-1	DPSCF300160:603476-604086
DpHel-1	DPSCF300143:544853-545463
DpHel-1	DPSCF300293:23748-24358
DpHel-1	DPSCF300282:26731-27342
DpHel-1	DPSCF300044:446597-447208
DpHel-1	DPSCF300191:1036585-1037196
DpHel-1	DPSCF300050:80986-81597
DpHel-1	DPSCF300262:473787-474399
DpHel-1	DPSCF300076:273005-273617
DpHel-1	DPSCF300290:119178-119790
DpHel-1	DPSCF300041:250653-251265
DpHel-1	DPSCF300143:180702-181314

DpHel-1	DPSCF300312:134302-134914
DpHel-1	DPSCF300049:302051-302663
DpHel-1	DPSCF300494:23509-24121
DpHel-1	DPSCF300191:700894-701506
DpHel-1	DPSCF300510:38322-38934
DpHel-1	DPSCF300200:75311-75923
DpHel-1	DPSCF300117:1023351-1023964
DpHel-1	DPSCF300196:810320-810933
DpHel-1	DPSCF300099:7420-8033
DpHel-1	DPSCF300085:237671-238284
DpHel-1	DPSCF300067:719012-719625
DpHel-1	DPSCF300134:42191-42804
DpHel-1	DPSCF300071:247317-247931
DpHel-1	DPSCF300063:864984-865598
DpHel-1	DPSCF300221:17504-18118
DpHel-1	DPSCF300071:725756-726370
DpHel-1	DPSCF300084:453600-454214
DpHel-1	DPSCF300168:78207-78821
DpHel-1	DPSCF300357:34820-35434
DpHel-1	DPSCF300006:712192-712807
DpHel-1	DPSCF300042:9983-10598
DpHel-1	DPSCF300010:3117849-3118464
DpHel-1	DPSCF300259:353826-354442
DpHel-1	DPSCF300827:4461-5077
DpHel-1	DPSCF300046:499274-499890
DpHel-1	DPSCF300408:75683-76299
DpHel-1	DPSCF300223:1148-1764
DpHel-1	DPSCF300108:828784-829400
DpHel-1	DPSCF300005:546945-547562
DpHel-1	DPSCF300051:503536-504153
DpHel-1	DPSCF300189:68393-69010
DpHel-1	DPSCF300005:124853-125470
DpHel-1	DPSCF300464:36307-36925
DpHel-1	DPSCF300258:82985-83603
DpHel-1	DPSCF300017:614661-615280
DpHel-1	DPSCF300001:3711893-3712513
DpHel-1	DPSCF300053:462073-462693
DpHel-1	DPSCF300297:200366-200986
DpHel-1	DPSCF300034:190769-191389
DpHel-1	DPSCF300386:9805-10425
DpHel-1	DPSCF300122:559014-559635
DpHel-1	DPSCF300001:4322684-4323305
DpHel-1	DPSCF300059:4880-5501
DpHel-1	DPSCF300429:77616-78237
DpHel-1	DPSCF300262:190066-190687
DpHel-1	DPSCF300842:3534-4155
DpHel-1	DPSCF300029:849375-849998
DpHel-1	DPSCF300027:772923-773547

DpHel-1	DPSCF300317:158355-158982
DpHel-1	DPSCF300281:388124-388751
DpHel-1	DPSCF300294:15476-16103
DpHel-1	DPSCF300042:1505628-1506255
DpHel-1	DPSCF300130:404148-404776
DpHel-1	DPSCF300063:489758-490386
DpHel-1	DPSCF300216:103374-104005
DpHel-1	DPSCF300122:189109-189742
DpHel-1	DPSCF300117:249840-250476
DpHel-1	DPSCF300010:1081914-1082553
DpHel-1	DPSCF300241:175516-176156
DpHel-1	DPSCF300007:986364-987008
DpHel-1	DPSCF300141:299836-300489
DpHel-1	DPSCF300090:612520-613173
DpHel-1	DPSCF300030:243587-244254
DpHel-1	DPSCF300104:383932-384627
DpHel-1	DPSCF300060:207485-208252
DpHel-1	DPSCF300181:38875-40104
DpHel-1	DPSCF300452:82171-85618
DpHel-1	DPSCF300166:31729-35960
DpHel-1	DPSCF300093:513604-514208
DpHel-1	DPSCF300268:156752-157373
DpHel-1	DPSCF300272:44967-45556
DpHel-1	DPSCF300028:20224-20841
DpHel-1	DPSCF300001:951913-952529
DpHel-1	DPSCF300107:40041-40652
DpHel-1	DPSCF300176:382546-383150
DpHel-1	DPSCF300108:502087-502704
DpHel-1	DPSCF300001:5046203-5046818
DpHel-1	DPSCF300430:84755-85370
DpHel-1	DPSCF300050:123308-123910
DpHel-1	DPSCF300028:1228754-1229366
DpHel-1	DPSCF300069:257465-258072
DpHel-1	DPSCF300001:638241-638853
DpHel-1	DPSCF300160:434969-435576
DpHel-1	DPSCF300135:71482-72086
DpHel-1	DPSCF300053:1258441-1259052
DpHel-1	DPSCF300020:667182-667791
DpHel-1	DPSCF300437:56157-56770
DpHel-1	DPSCF300417:39089-39692
DpHel-1	DPSCF300095:201617-202231
DpHel-1	DPSCF300027:296396-297006
DpHel-1	DPSCF300044:1078488-1079090
DpHel-1	DPSCF300093:357756-358371
DpHel-1	DPSCF300152:121766-122380
DpHel-1	DPSCF300063:444118-444717
DpHel-1	DPSCF300169:152805-153398
DpHel-1	DPSCF300033:531241-531849

DpHel-1	DPSCF300127:300358-300968
DpHel-1	DPSCF300002:816344-816941
DpHel-1	DPSCF300123:119475-120087
DpHel-1	DPSCF300048:1921664-1922283
DpHel-1	DPSCF300032:923934-924542
DpHel-1	DPSCF300030:645429-646040
DpHel-1	DPSCF301214:2364-2982
DpHel-1	DPSCF300253:73926-74539
DpHel-1	DPSCF300139:246028-246643
DpHel-1	DPSCF300255:205478-206086
DpHel-1	DPSCF300062:115951-116561
DpHel-1	DPSCF300290:418356-418962
DpHel-1	DPSCF300195:2130-2739
DpHel-1	DPSCF300012:878408-879008
DpHel-1	DPSCF300094:427505-428116
DpHel-1	DPSCF300401:50268-50877
DpHel-1	DPSCF300055:138550-139155
DpHel-1	DPSCF300212:54472-55078
DpHel-1	DPSCF300705:1896-2497
DpHel-1	DPSCF300484:48819-49363
DpHel-1	DPSCF300008:931059-931671
DpHel-1	DPSCF300090:595588-595705
DpHel-1	DPSCF300090:595072-595561
DpHel-1	DPSCF300210:211224-211832
DpHel-1	DPSCF300072:975249-975846
DpHel-1	DPSCF300260:118492-119094
DpHel-1	DPSCF300231:98825-98953
DpHel-1	DPSCF300231:98330-98802
DpHel-1	DPSCF300112:90205-90811
DpHel-1	DPSCF300182:51332-51947
DpHel-1	DPSCF300303:175180-175774
DpHel-1	DPSCF300001:6184496-6185102
DpHel-1	DPSCF300013:1172147-1172772
DpHel-1	DPSCF300076:849280-849879
DpHel-1	DPSCF300180:192067-192667
DpHel-1	DPSCF300009:543022-543627
DpHel-1	DPSCF300256:245670-246278
DpHel-1	DPSCF300143:723503-724101
DpHel-1	DPSCF300043:566326-566933
DpHel-1	DPSCF300011:348136-348745
DpHel-1	DPSCF300114:389050-389658
DpHel-1	DPSCF300069:553125-553738
DpHel-1	DPSCF300041:1947961-1948567
DpHel-1	DPSCF300369:14955-15567
DpHel-1	DPSCF300096:274384-274992
DpHel-1	DPSCF300173:59701-60307
DpHel-1	DPSCF300087:425258-425872
DpHel-1	DPSCF300515:13340-13939

DpHel-1	DPSCF300001:6130971-6131512
DpHel-1	DPSCF300098:354970-355582
DpHel-1	DPSCF300260:238056-238231
DpHel-1	DPSCF300260:238270-238700
DpHel-1	DPSCF300035:604031-604239
DpHel-1	DPSCF300035:604235-604622
DpHel-1	DPSCF300115:646746-647344
DpHel-1	DPSCF300124:309907-310399
DpHel-1	DPSCF300124:309793-309909
DpHel-1	DPSCF300300:158280-158430
DpHel-1	DPSCF300300:158452-158905
DpHel-1	DPSCF300041:991635-991755
DpHel-1	DPSCF300041:991766-992254
DpHel-1	DPSCF300176:317909-318513
DpHel-1	DPSCF300138:414860-415470
DpHel-1	DPSCF300491:56755-57091
DpHel-1	DPSCF300491:57127-57393
DpHel-1	DPSCF300365:55481-56083
DpHel-1	DPSCF300269:75776-76368
DpHel-1	DPSCF300269:33673-34269
DpHel-1	DPSCF300387:63898-64507
DpHel-1	DPSCF300014:1697226-1697764
DpHel-1	DPSCF300115:525102-525572
DpHel-1	DPSCF300115:524953-525083
DpHel-1	DPSCF300466:16682-17286
DpHel-1	DPSCF300021:538470-539058
DpHel-1	DPSCF300050:879242-879843
DpHel-1	DPSCF300110:147753-148347
DpHel-1	DPSCF300049:133973-134132
DpHel-1	DPSCF300049:133697-133940
DpHel-1	DPSCF300049:133493-133684
DpHel-1	DPSCF300322:332531-332972
DpHel-1	DPSCF300322:332379-332532
DpHel-1	DPSCF300061:32546-32803
DpHel-1	DPSCF300061:32919-33273
DpHel-1	DPSCF300217:30535-31134
DpHel-1	DPSCF300160:698149-698279
DpHel-1	DPSCF300160:697378-697842
DpHel-1	DPSCF300370:58053-58652
DpHel-1	DPSCF300472:28565-29165
DpHel-1	DPSCF300147:355859-356457
DpHel-1	DPSCF300017:1404187-1404773
DpHel-1	DPSCF300388:48852-49449
DpHel-1	DPSCF300061:1050710-1051310
DpHel-1	DPSCF300115:672723-673037
DpHel-1	DPSCF300115:672445-672723
DpHel-1	DPSCF300306:108740-109254
DpHel-1	DPSCF300306:108656-108740

DpHel-1	DPSCF300091:14280-14867
DpHel-1	DPSCF300079:57425-57569
DpHel-1	DPSCF300079:57586-58035
DpHel-1	DPSCF300136:374109-374703
DpHel-1	DPSCF300077:230399-230720
DpHel-1	DPSCF300077:230719-230983
DpHel-1	DPSCF300391:129651-130246
DpHel-1	DPSCF300046:187180-187770
DpHel-1	DPSCF300024:656039-656635
DpHel-1	DPSCF300043:383004-383592
DpHel-1	DPSCF300033:1101400-1101651
DpHel-1	DPSCF300033:1101687-1102032
DpHel-1	DPSCF300043:1090735-1090939
DpHel-1	DPSCF300043:1090936-1091246
DpHel-1	DPSCF300043:1092772-1092861
DpHel-1	DPSCF300067:99508-99842
DpHel-1	DPSCF300067:99255-99507
DpHel-1	DPSCF300089:188245-188605
DpHel-1	DPSCF300089:188016-188245
DpHel-1	DPSCF300099:370891-371475
DpHel-1	DPSCF300144:62627-63218
DpHel-1	DPSCF300222:83586-84179
DpHel-1	DPSCF300146:27285-27877
DpHel-1	DPSCF300097:180027-180305
DpHel-1	DPSCF300097:180493-180821
DpHel-1	DPSCF300053:783552-783729
DpHel-1	DPSCF300053:783730-784133
DpHel-1	DPSCF300063:297986-298332
DpHel-1	DPSCF300063:298340-298585
DpHel-1	DPSCF300527:38925-39353
DpHel-1	DPSCF300527:39355-39532
DpHel-1	DPSCF300030:1036423-1036906
DpHel-1	DPSCF300030:1036907-1037015
DpHel-1	DPSCF300095:347285-347875
DpHel-1	DPSCF300128:20082-20513
DpHel-1	DPSCF300128:19922-20080
DpHel-1	DPSCF300012:368168-368330
DpHel-1	DPSCF300012:368334-368763
DpHel-1	DPSCF300204:106721-107301
DpHel-1	DPSCF300632:4819-5274
DpHel-1	DPSCF300632:5573-5717
DpHel-1	DPSCF300235:403988-404120
DpHel-1	DPSCF300235:404113-404407
DpHel-1	DPSCF300235:404430-404592
DpHel-1	DPSCF300339:44243-44780
DpHel-1	DPSCF300050:733579-734172
DpHel-1	DPSCF300469:6803-7204
DpHel-1	DPSCF300469:6609-6803

DpHel-1	DPSCF300378:54214-54805
DpHel-1	DPSCF300042:273363-273956
DpHel-1	DPSCF300010:1147811-1148382
DpHel-1	DPSCF300371:23033-23621
DpHel-1	DPSCF300015:57526-58030
DpHel-1	DPSCF300015:57441-57523
DpHel-1	DPSCF300056:708515-709027
DpHel-1	DPSCF300372:90747-90947
DpHel-1	DPSCF300372:90954-91341
DpHel-1	DPSCF300302:68314-68566
DpHel-1	DPSCF300302:67926-68295
DpHel-1	DPSCF300212:104437-104787
DpHel-1	DPSCF300212:104184-104414
DpHel-1	DPSCF300146:421351-421935
DpHel-1	DPSCF300103:86930-87269
DpHel-1	DPSCF300103:88108-88228
DpHel-1	DPSCF300103:90755-90897
DpHel-1	DPSCF300419:69555-69942
DpHel-1	DPSCF300419:69360-69556
DpHel-1	DPSCF300218:302204-302709
DpHel-1	DPSCF300351:117474-117964
DpHel-1	DPSCF300351:117967-118058
DpHel-1	DPSCF300300:136714-137290
DpHel-1	DPSCF300002:1691537-1691978
DpHel-1	DPSCF300002:1691969-1692107
DpHel-1	DPSCF300009:907670-908255
DpHel-1	DPSCF300168:715104-715255
DpHel-1	DPSCF300168:715277-715460
DpHel-1	DPSCF300168:715618-715860
DpHel-1	DPSCF300358:25354-25839
DpHel-1	DPSCF300358:25861-25967
DpHel-1	DPSCF300164:319910-320207
DpHel-1	DPSCF300164:319625-319907
DpHel-1	DPSCF300221:447163-447745
DpHel-1	DPSCF300341:6802-7177
DpHel-1	DPSCF300341:7177-7386
DpHel-1	DPSCF300491:44776-45203
DpHel-1	DPSCF300491:45256-45399
DpHel-1	DPSCF300104:133251-133838
DpHel-1	DPSCF300222:503797-504304
DpHel-1	DPSCF300079:352899-353049
DpHel-1	DPSCF300079:353046-353475
DpHel-1	DPSCF300324:89328-89702
DpHel-1	DPSCF300324:89701-89913
DpHel-1	DPSCF300261:61549-61771
DpHel-1	DPSCF300261:61769-62129
DpHel-1	DPSCF300122:572208-572728
DpHel-1	DPSCF300013:711397-711552

DpHel-1	DPSCF300013:711562-711844
DpHel-1	DPSCF300013:711847-712019
DpHel-1	DPSCF300033:870361-870735
DpHel-1	DPSCF300033:870737-870938
DpHel-1	DPSCF300754:7440-7710
DpHel-1	DPSCF300754:7101-7414
DpHel-1	DPSCF300185:308057-308642
DpHel-1	DPSCF300072:1175133-1175710
DpHel-1	DPSCF300260:182632-183214
DpHel-1	DPSCF300507:5028-5369
DpHel-1	DPSCF300507:5371-5606
DpHel-1	DPSCF300160:683490-684070
DpHel-1	DPSCF300063:839810-840383
DpHel-1	DPSCF300001:5366314-5366515
DpHel-1	DPSCF300001:5366512-5366875
DpHel-1	DPSCF300147:338579-339157
DpHel-1	DPSCF300290:60952-61527
DpHel-1	DPSCF300179:87116-87693
DpHel-1	DPSCF300010:3097597-3097721
DpHel-1	DPSCF300010:3097742-3097881
DpHel-1	DPSCF300010:3097902-3098223
DpHel-1	DPSCF300010:523237-523811
DpHel-1	DPSCF300011:27924-28360
DpHel-1	DPSCF300011:28360-28494
DpHel-1	DPSCF300004:813839-814038
DpHel-1	DPSCF300004:813471-813840
DpHel-1	DPSCF300250:27136-27449
DpHel-1	DPSCF300250:27450-27705
DpHel-1	DPSCF300822:2579-3155
DpHel-1	DPSCF300043:243662-244163
DpHel-1	DPSCF300312:168688-169264
DpHel-1	DPSCF300019:78768-79078
DpHel-1	DPSCF300019:79085-79268
DpHel-1	DPSCF300019:79276-79356
DpHel-1	DPSCF300018:1333685-1334263
DpHel-1	DPSCF300018:1277227-1277801
DpHel-1	DPSCF300258:38864-39368
DpHel-1	DPSCF300082:441758-441862
DpHel-1	DPSCF300082:441615-441695
DpHel-1	DPSCF300082:441286-441619
DpHel-1	DPSCF300009:1704761-1704912
DpHel-1	DPSCF300009:1704327-1704747
DpHel-1	DPSCF300373:15492-16069
DpHel-1	DPSCF300019:218103-218677
DpHel-1	DPSCF301227:3302-3864
DpHel-1	DPSCF300108:735192-735768
DpHel-1	DPSCF300044:336099-336537
DpHel-1	DPSCF300078:810144-810718

DpHel-1	DPSCF300029:508968-509543
DpHel-1	DPSCF300055:73636-74213
DpHel-1	DPSCF300175:237412-237886
DpHel-1	DPSCF300175:237892-237991
DpHel-1	DPSCF300350:8066-8240
DpHel-1	DPSCF300350:7915-8019
DpHel-1	DPSCF300350:7600-7894
DpHel-1	DPSCF300014:960621-960904
DpHel-1	DPSCF300014:960904-961268
DpHel-1	DPSCF300050:918414-918951
DpHel-1	DPSCF300078:76902-77236
DpHel-1	DPSCF300078:76628-76887
DpHel-1	DPSCF300037:198970-199539
DpHel-1	DPSCF300270:29180-29599
DpHel-1	DPSCF300270:29033-29183
DpHel-1	DPSCF300426:108830-109271
DpHel-1	DPSCF300426:109270-109398
DpHel-1	DPSCF300072:1292061-1292218
DpHel-1	DPSCF300072:1292232-1292643
DpHel-1	DPSCF300255:6515-6672
DpHel-1	DPSCF300255:6686-7097
DpHel-1	DPSCF300406:8200-8746
DpHel-1	DPSCF300223:697176-697745
DpHel-1	DPSCF300117:587504-588079
DpHel-1	DPSCF300042:1071651-1072228
DpHel-1	DPSCF300507:30167-30747
DpHel-1	DPSCF300052:213672-214244
DpHel-1	DPSCF300176:436545-437055
DpHel-1	DPSCF300241:243529-244094
DpHel-1	DPSCF300168:238855-239235
DpHel-1	DPSCF300168:238639-238783
DpHel-1	DPSCF300127:333459-333541
DpHel-1	DPSCF300127:332939-333431
DpHel-1	DPSCF300427:44905-45202
DpHel-1	DPSCF300427:44651-44907
DpHel-1	DPSCF300082:955536-956034
DpHel-1	DPSCF300024:876599-877087
DpHel-1	DPSCF300024:877102-877186
DpHel-1	DPSCF300186:93241-93681
DpHel-1	DPSCF300186:93103-93222
DpHel-1	DPSCF300146:12408-12968
DpHel-1	DPSCF300039:438917-439481
DpHel-1	DPSCF300270:201012-201532
DpHel-1	DPSCF300074:638379-638496
DpHel-1	DPSCF300074:637904-638344
DpHel-1	DPSCF300157:286482-286689
DpHel-1	DPSCF300157:286120-286480
DpHel-1	DPSCF300226:32359-32928

DpHel-1	DPSCF300001:710700-711262
DpHel-1	DPSCF300367:98774-98881
DpHel-1	DPSCF300367:98309-98778
DpHel-1	DPSCF300147:455430-455734
DpHel-1	DPSCF300147:455730-455922
DpHel-1	DPSCF300157:88938-89057
DpHel-1	DPSCF300157:89068-89493
DpHel-1	DPSCF300083:568711-568950
DpHel-1	DPSCF300083:568379-568713
DpHel-1	DPSCF300110:172559-173125
DpHel-1	DPSCF300335:138186-138739
DpHel-1	DPSCF300063:659128-659569
DpHel-1	DPSCF300063:659573-659696
DpHel-1	DPSCF300031:999909-1000066
DpHel-1	DPSCF300031:1000080-1000358
DpHel-1	DPSCF300031:1000554-1000700
DpHel-1	DPSCF300032:632544-633050
DpHel-1	DPSCF300066:23504-24068
DpHel-1	DPSCF300073:491483-491591
DpHel-1	DPSCF300073:491603-491877
DpHel-1	DPSCF300073:491889-492064
DpHel-1	DPSCF300172:52237-52799
DpHel-1	DPSCF300275:84519-85081
DpHel-1	DPSCF300394:95569-96127
DpHel-1	DPSCF300228:200681-201233
DpHel-1	DPSCF300092:510338-510478
DpHel-1	DPSCF300092:510506-510922
DpHel-1	DPSCF300422:47370-47539
DpHel-1	DPSCF300422:46954-47347
DpHel-1	DPSCF300134:250256-250812
DpHel-1	DPSCF300354:128384-128596
DpHel-1	DPSCF300354:128608-128951
DpHel-1	DPSCF300683:12017-12133
DpHel-1	DPSCF300683:11558-12002
DpHel-1	DPSCF300120:442610-443161
DpHel-1	DPSCF300053:3636-3818
DpHel-1	DPSCF300053:3823-4054
DpHel-1	DPSCF300053:4053-4198
DpHel-1	DPSCF300014:359244-359538
DpHel-1	DPSCF300014:359561-359821
DpHel-1	DPSCF300084:271984-272371
DpHel-1	DPSCF300084:272371-272550
DpHel-1	DPSCF300121:188442-188903
DpHel-1	DPSCF300121:188334-188426
DpHel-1	DPSCF300007:905333-905727
DpHel-1	DPSCF300007:905242-905335
DpHel-1	DPSCF300797:1806-2136
DpHel-1	DPSCF300797:2135-2355

DpHel-1	DPSCF300191:898798-898892
DpHel-1	DPSCF300191:898915-899373
DpHel-1	DPSCF300053:663005-663555
DpHel-1	DPSCF300005:1250130-1250525
DpHel-1	DPSCF300005:1250616-1250769
DpHel-1	DPSCF300099:411519-411845
DpHel-1	DPSCF300099:411283-411511
DpHel-1	DPSCF300281:24005-24121
DpHel-1	DPSCF300281:23570-24005
DpHel-1	DPSCF300031:588475-588927
DpHel-1	DPSCF300031:588369-588469
DpHel-1	DPSCF300014:409652-410167
DpHel-1	DPSCF300010:198770-199124
DpHel-1	DPSCF300010:199128-199325
DpHel-1	DPSCF300312:92851-93408
DpHel-1	DPSCF300149:385677-385834
DpHel-1	DPSCF300149:385283-385676
DpHel-1	DPSCF300047:713389-713657
DpHel-1	DPSCF300047:713659-713936
DpHel-1	DPSCF300377:25651-25913
DpHel-1	DPSCF300377:25947-26235
DpHel-1	DPSCF300088:274030-274580
DpHel-1	DPSCF300424:116056-116607
DpHel-1	DPSCF300082:1247427-1247581
DpHel-1	DPSCF300082:1247238-1247425
DpHel-1	DPSCF300082:1247020-1247228
DpHel-1	DPSCF300018:995652-996196
DpHel-1	DPSCF300070:748646-748978
DpHel-1	DPSCF300070:748370-748597
DpHel-1	DPSCF300038:857326-857421
DpHel-1	DPSCF300038:857436-857625
DpHel-1	DPSCF300038:857629-857901
DpHel-1	DPSCF300163:172766-172997
DpHel-1	DPSCF300163:172998-173225
DpHel-1	DPSCF300163:173253-173342
DpHel-1	DPSCF300013:570638-570849
DpHel-1	DPSCF300013:570847-570943
DpHel-1	DPSCF300013:570992-571122
DpHel-1	DPSCF300018:645437-645983
DpHel-1	DPSCF300091:650425-650959
DpHel-1	DPSCF300130:479446-479693
DpHel-1	DPSCF300130:479692-479989
DpHel-1	DPSCF300033:756000-756544
DpHel-1	DPSCF300165:84452-84731
DpHel-1	DPSCF300165:84200-84453
DpHel-1	DPSCF300024:916264-916727
DpHel-1	DPSCF300024:916742-916826
DpHel-1	DPSCF300086:554673-554752

DpHel-1	DPSCF300086:554756-555159
DpHel-1	DPSCF300222:586575-587118
DpHel-1	DPSCF300182:328147-328622
DpHel-1	DPSCF300228:130948-131484
DpHel-1	DPSCF300149:36749-36921
DpHel-1	DPSCF300149:36334-36637
DpHel-1	DPSCF300214:314948-315375
DpHel-1	DPSCF300214:315389-315510
DpHel-1	DPSCF300041:2319188-2319435
DpHel-1	DPSCF300041:2318888-2319185
DpHel-1	DPSCF300024:188683-189223
DpHel-1	DPSCF300409:72916-73010
DpHel-1	DPSCF300409:72430-72880
DpHel-1	DPSCF300020:567806-568224
DpHel-1	DPSCF300020:567674-567789
DpHel-1	DPSCF300038:508471-508612
DpHel-1	DPSCF300038:508074-508472
DpHel-1	DPSCF300104:415497-416036
DpHel-1	DPSCF300047:844691-844843
DpHel-1	DPSCF300047:844485-844626
DpHel-1	DPSCF300047:844289-844486
DpHel-1	DPSCF300060:527200-527681
DpHel-1	DPSCF300198:59805-60109
DpHel-1	DPSCF300198:59567-59800
DpHel-1	DPSCF300093:4781-5314
DpHel-1	DPSCF300054:425703-426190
DpHel-1	DPSCF300230:402427-402568
DpHel-1	DPSCF300230:401953-402351
DpHel-1	DPSCF300253:247927-248342
DpHel-1	DPSCF300253:248371-248498
DpHel-1	DPSCF300117:416505-416967
DpHel-1	DPSCF300079:260576-261114
DpHel-1	DPSCF300075:518650-519181
DpHel-1	DPSCF300336:197074-197490
DpHel-1	DPSCF300336:196950-197066
DpHel-1	DPSCF300450:103412-103944
DpHel-1	DPSCF300015:1262300-1262481
DpHel-1	DPSCF300015:1262145-1262306
DpHel-1	DPSCF300015:1261957-1262135
DpHel-1	DPSCF300082:178310-178547
DpHel-1	DPSCF300082:180937-181239
DpHel-1	DPSCF300001:5866367-5866897
DpHel-1	DPSCF300001:1439405-1439545
DpHel-1	DPSCF300001:1439153-1439405
DpHel-1	DPSCF300001:1438918-1439062
DpHel-1	DPSCF300170:95731-95948
DpHel-1	DPSCF300170:95393-95716
DpHel-1	DPSCF300170:139892-140415

DpHel-1	DPSCF300001:4345316-4345844
DpHel-1	DPSCF300084:143772-144012
DpHel-1	DPSCF300084:144013-144303
DpHel-1	DPSCF300173:26297-26673
DpHel-1	DPSCF300173:26209-26293
DpHel-1	DPSCF300001:2825632-2826099
DpHel-1	DPSCF300039:67491-67841
DpHel-1	DPSCF300039:67841-68018
DpHel-1	DPSCF300008:1337326-1337515
DpHel-1	DPSCF300008:1337519-1337791
DpHel-1	DPSCF300410:15325-15812
DpHel-1	DPSCF300310:38110-38274
DpHel-1	DPSCF300310:38447-38601
DpHel-1	DPSCF300310:38605-38717
DpHel-1	DPSCF300094:332641-333166
DpHel-1	DPSCF300322:389267-389796
DpHel-1	DPSCF300001:5197453-5197857
DpHel-1	DPSCF300001:5197337-5197453
DpHel-1	DPSCF300003:214470-214888
DpHel-1	DPSCF300003:214349-214449
DpHel-1	DPSCF300150:535003-535527
DpHel-1	DPSCF300443:7765-8014
DpHel-1	DPSCF300443:8040-8316
DpHel-1	DPSCF300014:2124293-2124429
DpHel-1	DPSCF300014:2124435-2124665
DpHel-1	DPSCF300014:2124684-2124836
DpHel-1	DPSCF300090:242408-242736
DpHel-1	DPSCF300090:242864-243034
DpHel-1	DPSCF300444:68804-68990
DpHel-1	DPSCF300444:68458-68795
DpHel-1	DPSCF300007:1307507-1307839
DpHel-1	DPSCF300007:1307283-1307472
DpHel-1	DPSCF300176:261740-262106
DpHel-1	DPSCF300176:261549-261631
DpHel-1	DPSCF300108:680926-681448
DpHel-1	DPSCF300336:171259-171667
DpHel-1	DPSCF300336:170958-171069
DpHel-1	DPSCF300330:22582-22795
DpHel-1	DPSCF300330:22244-22581
DpHel-1	DPSCF300404:30872-31273
DpHel-1	DPSCF300404:31299-31414
DpHel-1	DPSCF300284:211345-211664
DpHel-1	DPSCF300284:211666-211818
DpHel-1	DPSCF300382:188756-189202
DpHel-1	DPSCF300386:55008-55472
DpHel-1	DPSCF300322:377935-378456
DpHel-1	DPSCF300146:31831-32158
DpHel-1	DPSCF300146:32164-32351

DpHel-1	DPSCF300277:270374-270559
DpHel-1	DPSCF300277:270041-270358
DpHel-1	DPSCF300498:64429-64546
DpHel-1	DPSCF300498:64007-64394
DpHel-1	DPSCF300082:1294874-1295028
DpHel-1	DPSCF300082:1294685-1294872
DpHel-1	DPSCF300082:1294506-1294675
DpHel-1	DPSCF300017:573209-573415
DpHel-1	DPSCF300017:572888-573193
DpHel-1	DPSCF300170:519483-519982
DpHel-1	DPSCF300190:242197-242304
DpHel-1	DPSCF300190:242310-242476
DpHel-1	DPSCF300190:242477-242711
DpHel-1	DPSCF300087:444394-444511
DpHel-1	DPSCF300087:443973-444364
DpHel-1	DPSCF300038:993624-993798
DpHel-1	DPSCF300038:993288-993622
DpHel-1	DPSCF300090:331228-331737
DpHel-1	DPSCF300355:58644-58849
DpHel-1	DPSCF300355:58869-59173
DpHel-1	DPSCF300164:200018-200533
DpHel-1	DPSCF300027:1423701-1424141
DpHel-1	DPSCF300337:92727-92872
DpHel-1	DPSCF300337:92891-93198
DpHel-1	DPSCF300137:1-94
DpHel-1	DPSCF300137:120-492
DpHel-1	DPSCF300204:171777-171978
DpHel-1	DPSCF300204:172054-172306
DpHel-1	DPSCF300128:439119-439623
DpHel-1	DPSCF300230:233418-233924
DpHel-1	DPSCF300528:7984-8294
DpHel-1	DPSCF300528:7526-7659
DpHel-1	DPSCF300070:436193-436576
DpHel-1	DPSCF300070:436585-436709
DpHel-1	DPSCF300124:325404-325652
DpHel-1	DPSCF300124:325216-325330
DpHel-1	DPSCF300001:5492621-5492772
DpHel-1	DPSCF300001:5492779-5492949
DpHel-1	DPSCF300001:5492948-5493126
DpHel-1	DPSCF300140:178901-179346
DpHel-1	DPSCF300033:1222462-1222959
DpHel-1	DPSCF300240:158350-158847
DpHel-1	DPSCF300135:191546-191890
DpHel-1	DPSCF300135:191370-191529
DpHel-1	DPSCF300593:12548-12892
DpHel-1	DPSCF300593:12372-12531
DpHel-1	DPSCF300268:15219-15326
DpHel-1	DPSCF300268:14755-15143

DpHel-1	DPSCF300256:296938-297431
DpHel-1	DPSCF300170:405186-405643
DpHel-1	DPSCF300188:397998-398123
DpHel-1	DPSCF300188:397839-397985
DpHel-1	DPSCF300188:397619-397836
DpHel-1	DPSCF300356:231287-231776
DpHel-1	DPSCF300083:189289-189740
DpHel-1	DPSCF300216:367096-367582
DpHel-1	DPSCF300100:411165-411657
DpHel-1	DPSCF300090:546857-546954
DpHel-1	DPSCF300090:546661-546832
DpHel-1	DPSCF300090:546430-546653
DpHel-1	DPSCF300035:252230-252718
DpHel-1	DPSCF300050:588223-588339
DpHel-1	DPSCF300050:588340-588709
DpHel-1	DPSCF300088:993440-993772
DpHel-1	DPSCF300088:993806-993894
DpHel-1	DPSCF300002:1468149-1468630
DpHel-1	DPSCF300345:238067-238343
DpHel-1	DPSCF300345:237855-238069
DpHel-1	DPSCF300113:241149-241458
DpHel-1	DPSCF300113:241462-241636
DpHel-1	DPSCF300262:97951-98281
DpHel-1	DPSCF300262:97835-97950
DpHel-1	DPSCF300065:283964-284061
DpHel-1	DPSCF300065:283768-283939
DpHel-1	DPSCF300065:283582-283760
DpHel-1	DPSCF300341:173997-174094
DpHel-1	DPSCF300341:174094-174482
DpHel-1	DPSCF300013:1775233-1775522
DpHel-1	DPSCF300013:1775525-1775713
DpHel-1	DPSCF300129:386331-386453
DpHel-1	DPSCF300129:385959-386322
DpHel-1	DPSCF300083:311512-311644
DpHel-1	DPSCF300083:311326-311506
DpHel-1	DPSCF300083:311151-311321
DpHel-1	DPSCF300104:95082-95552
DpHel-1	DPSCF300108:1046489-1046666
DpHel-1	DPSCF300108:1046665-1046969
DpHel-1	DPSCF300374:163117-163595
DpHel-1	DPSCF300203:116612-117089
DpHel-1	DPSCF300058:303419-303813
DpHel-1	DPSCF300191:362034-362192
DpHel-1	DPSCF300191:362206-362520
DpHel-1	DPSCF300102:542598-542838
DpHel-1	DPSCF300102:542362-542596
DpHel-1	DPSCF300661:4160-4452
DpHel-1	DPSCF300661:3979-4161

DpHel-1	DPSCF300340:116655-116951
DpHel-1	DPSCF300340:116472-116656
DpHel-1	DPSCF300147:248083-248188
DpHel-1	DPSCF300147:248195-248571
DpHel-1	DPSCF300012:794643-795071
DpHel-1	DPSCF300090:637858-638026
DpHel-1	DPSCF300090:638028-638332
DpHel-1	DPSCF300143:360364-360776
DpHel-1	DPSCF300027:96417-96526
DpHel-1	DPSCF300027:96314-96406
DpHel-1	DPSCF300027:96164-96310
DpHel-1	DPSCF300027:96044-96163
DpHel-1	DPSCF300336:158206-158415
DpHel-1	DPSCF300336:157914-158171
DpHel-1	DPSCF300218:260302-260460
DpHel-1	DPSCF300218:260492-260590
DpHel-1	DPSCF300218:260593-260845
DpHel-1	DPSCF300525:44802-45037
DpHel-1	DPSCF300525:45035-45317
DpHel-1	DPSCF300625:4298-4706
DpHel-1	DPSCF300111:356052-356325
DpHel-1	DPSCF300111:355564-355786
DpHel-1	DPSCF300232:24099-24564
DpHel-1	DPSCF300006:1275572-1275841
DpHel-1	DPSCF300006:1275841-1276038
DpHel-1	DPSCF300104:318736-319200
DpHel-1	DPSCF300004:156285-156484
DpHel-1	DPSCF300004:156490-156751
DpHel-1	DPSCF300001:4739059-4739456
DpHel-1	DPSCF300223:633697-634166
DpHel-1	DPSCF300379:157773-158192
DpHel-1	DPSCF300075:505015-505095
DpHel-1	DPSCF300075:504820-505013
DpHel-1	DPSCF300075:504708-504818
DpHel-1	DPSCF300012:1184089-1184244
DpHel-1	DPSCF300012:1184256-1184349
DpHel-1	DPSCF300012:1184350-1184557
DpHel-1	DPSCF300667:10142-10487
DpHel-1	DPSCF300667:10040-10125
DpHel-1	DPSCF300111:286168-286620
DpHel-1	DPSCF300273:325083-325294
DpHel-1	DPSCF300273:325392-325472
DpHel-1	DPSCF300273:325478-325589
DpHel-1	DPSCF300035:127023-127218
DpHel-1	DPSCF300035:127219-127375
DpHel-1	DPSCF300035:127369-127474
DpHel-1	DPSCF300020:997914-998279
DpHel-1	DPSCF300020:998386-998474

DpHel-1	DPSCF300042:1386968-1387165
DpHel-1	DPSCF300042:1386706-1386968
DpHel-1	DPSCF300460:32564-33018
DpHel-1	DPSCF300149:480999-481456
DpHel-1	DPSCF300001:5261256-5261712
DpHel-1	DPSCF300268:133135-133426
DpHel-1	DPSCF300268:132939-133024
DpHel-1	DPSCF300351:61453-61903
DpHel-1	DPSCF300048:1613808-1613962
DpHel-1	DPSCF300048:1613971-1614262
DpHel-1	DPSCF300179:183901-184137
DpHel-1	DPSCF300179:183683-183896
DpHel-1	DPSCF300193:309866-310124
DpHel-1	DPSCF300272:219303-219737
DpHel-1	DPSCF300044:637628-637902
DpHel-1	DPSCF300044:637911-638074
DpHel-1	DPSCF300087:591056-591340
DpHel-1	DPSCF300087:590896-591043
DpHel-1	DPSCF300286:5531-5815
DpHel-1	DPSCF300286:5371-5518
DpHel-1	DPSCF300087:98062-98240
DpHel-1	DPSCF300087:97831-98037
DpHel-1	DPSCF300143:10876-11016
DpHel-1	DPSCF300143:10581-10829
DpHel-1	DPSCF300419:51750-52093
DpHel-1	DPSCF300419:51237-51322
DpHel-1	DPSCF300423:81130-81552
DpHel-1	DPSCF300060:721447-721752
DpHel-1	DPSCF300060:721751-721895
DpHel-1	DPSCF301201:1117-1540
DpHel-1	DPSCF300065:175533-175959
DpHel-1	DPSCF300308:79428-79816
DpHel-1	DPSCF300059:121751-121925
DpHel-1	DPSCF300059:121578-121741
DpHel-1	DPSCF300059:121476-121556
DpHel-1	DPSCF300117:950827-951242
DpHel-1	DPSCF300106:396978-397252
DpHel-1	DPSCF300106:397272-397415
DpHel-1	DPSCF300001:3685811-3686044
DpHel-1	DPSCF300001:3686042-3686168
DpHel-1	DPSCF300115:508233-508648
DpHel-1	DPSCF300232:443883-444307
DpHel-1	DPSCF300232:456041-456465
DpHel-1	DPSCF300231:48252-48666
DpHel-1	DPSCF300028:1294695-1295032
DpHel-1	DPSCF300042:1776108-1776345
DpHel-1	DPSCF300042:1775929-1776106
DpHel-1	DPSCF300129:7429-7602

DpHel-1	DPSCF300129:7603-7705
DpHel-1	DPSCF300129:20378-20551
DpHel-1	DPSCF300129:20552-20654
DpHel-1	DPSCF300345:72573-72979
DpHel-1	DPSCF300078:1008401-1008494
DpHel-1	DPSCF300078:1008072-1008381
DpHel-1	DPSCF300505:4785-5107
DpHel-1	DPSCF300033:911090-911484
DpHel-1	DPSCF300052:223133-223218
DpHel-1	DPSCF300052:222769-223091
DpHel-1	DPSCF300231:370554-370948
DpHel-1	DPSCF300001:4016794-4017109
DpHel-1	DPSCF300001:4017111-4017199
DpHel-1	DPSCF300275:212002-212407
DpHel-1	DPSCF300005:537512-537591
DpHel-1	DPSCF300005:537254-537512
DpHel-1	DPSCF300041:1578333-1578530
DpHel-1	DPSCF300041:1578530-1578730
DpHel-1	DPSCF300156:209276-209410
DpHel-1	DPSCF300156:209461-209717
DpHel-1	DPSCF300371:103411-103729
DpHel-1	DPSCF300008:1544047-1544443
DpHel-1	DPSCF300059:480109-480237
DpHel-1	DPSCF300059:479964-480054
DpHel-1	DPSCF300059:479784-479967
DpHel-1	DPSCF300316:136376-136675
DpHel-1	DPSCF300316:136689-136785
DpHel-1	DPSCF300059:183170-183335
DpHel-1	DPSCF300059:182929-183150
DpHel-1	DPSCF300001:1414391-1414694
DpHel-1	DPSCF300001:1414308-1414391
DpHel-1	DPSCF300316:29660-30046
DpHel-1	DPSCF300138:743663-744047
DpHel-1	DPSCF300250:47740-48064
DpHel-1	DPSCF300508:18826-19104
DpHel-1	DPSCF300508:19129-19238
DpHel-1	DPSCF300018:642956-643342
DpHel-1	DPSCF300441:36809-37102
DpHel-1	DPSCF300441:36717-36806
DpHel-1	DPSCF300020:895339-895641
DpHel-1	DPSCF300020:895262-895344
DpHel-1	DPSCF300003:767085-767277
DpHel-1	DPSCF300003:766777-766917
DpHel-1	DPSCF300878:5458-5734
DpHel-1	DPSCF300878:5352-5460
DpHel-1	DPSCF300139:144511-144896
DpHel-1	DPSCF300013:1506318-1506646
DpHel-1	DPSCF300293:47227-47556

DpHel-1	DPSCF300021:1276168-1276361
DpHel-1	DPSCF300021:1276362-1276696
DpHel-1	DPSCF300013:10280-10656
DpHel-1	DPSCF300042:1741680-1741811
DpHel-1	DPSCF300042:1741810-1742052
DpHel-1	DPSCF300320:163400-163694
DpHel-1	DPSCF300320:163717-163799
DpHel-1	DPSCF300208:465929-466097
DpHel-1	DPSCF300208:470522-470981
DpHel-1	DPSCF300134:257664-257877
DpHel-1	DPSCF300134:257501-257663
DpHel-1	DPSCF300143:604359-604642
DpHel-1	DPSCF300143:604651-604744
DpHel-1	DPSCF300310:91925-92063
DpHel-1	DPSCF300310:91732-91844
DpHel-1	DPSCF300021:54142-54425
DpHel-1	DPSCF300021:54050-54141
DpHel-1	DPSCF300258:268472-268765
DpHel-1	DPSCF300258:268766-268847
DpHel-1	DPSCF300067:818498-818878
DpHel-1	DPSCF300062:699842-700223
DpHel-1	DPSCF300216:160965-161321
DpHel-1	DPSCF300159:370898-371008
DpHel-1	DPSCF300159:375830-376080
DpHel-1	DPSCF300655:11846-12027
DpHel-1	DPSCF300655:12065-12257
DpHel-1	DPSCF300058:80017-80382
DpHel-1	DPSCF300022:627210-627327
DpHel-1	DPSCF300022:627328-627570
DpHel-1	DPSCF300228:261197-261339
DpHel-1	DPSCF300228:261355-261576
DpHel-1	DPSCF300036:386082-386358
DpHel-1	DPSCF300036:385990-386079
DpHel-1	DPSCF300058:730844-730997
DpHel-1	DPSCF300058:730174-730334
DpHel-1	DPSCF300177:196327-196516
DpHel-1	DPSCF300177:196165-196325
DpHel-1	DPSCF300104:33890-34164
DpHel-1	DPSCF300104:33809-33890
DpHel-1	DPSCF300009:2040935-2041282
DpHel-1	DPSCF300010:2638429-2638517
DpHel-1	DPSCF300010:2638512-2638600
DpHel-1	DPSCF300010:2638618-2638794
DpHel-1	DPSCF300041:379405-379758
DpHel-1	DPSCF300138:674153-674507
DpHel-1	DPSCF300046:498687-498784
DpHel-1	DPSCF300046:498379-498631
DpHel-1	DPSCF300329:82834-83182

DpHel-1	DPSCF300266:209912-210088
DpHel-1	DPSCF300266:209745-209849
DpHel-1	DPSCF300001:1467224-1467417
DpHel-1	DPSCF300001:1467419-1467570
DpHel-1	DPSCF300031:493404-493507
DpHel-1	DPSCF300031:493556-493793
DpHel-1	DPSCF300001:4616409-4616675
DpHel-1	DPSCF300068:351709-351842
DpHel-1	DPSCF300068:351581-351699
DpHel-1	DPSCF300186:303593-303697
DpHel-1	DPSCF300186:303436-303580
DpHel-1	DPSCF300366:61233-61412
DpHel-1	DPSCF300366:61412-61570
DpHel-1	DPSCF300210:113772-113856
DpHel-1	DPSCF300210:113505-113768
DpHel-1	DPSCF300296:188482-188823
DpHel-1	DPSCF300028:543661-543824
DpHel-1	DPSCF300028:543474-543655
DpHel-1	DPSCF300053:42165-42417
DpHel-1	DPSCF300053:42017-42100
DpHel-1	DPSCF300391:209505-209628
DpHel-1	DPSCF300391:206827-207038
DpHel-1	DPSCF300068:132638-132855
DpHel-1	DPSCF300299:212989-213311
DpHel-1	DPSCF300002:363690-363798
DpHel-1	DPSCF300088:33562-33882
DpHel-1	DPSCF300173:60675-60831
DpHel-1	DPSCF300173:60986-61110
DpHel-1	DPSCF300109:121220-121424
DpHel-1	DPSCF300109:121457-121544
DpHel-1	DPSCF300043:839027-839285
DpHel-1	DPSCF300234:462281-462592
DpHel-1	DPSCF300234:613194-613505
DpHel-1	DPSCF300222:593342-593478
DpHel-1	DPSCF300222:593482-593670
DpHel-1	DPSCF300584:14168-14366
DpHel-1	DPSCF300584:14012-14139
DpHel-1	DPSCF300060:201247-201535
DpHel-1	DPSCF300177:69555-69875
DpHel-1	DPSCF300427:53422-53645
DpHel-1	DPSCF300427:53902-54000
DpHel-1	DPSCF300003:1673010-1673101
DpHel-1	DPSCF300003:1673099-1673310
DpHel-1	DPSCF300003:1673311-1673417
DpHel-1	DPSCF300296:8736-8888
DpHel-1	DPSCF300296:8927-9048
DpHel-1	DPSCF300382:42309-42625
DpHel-1	DPSCF300010:792564-792880

DpHel-1	DPSCF300196:474922-475075
DpHel-1	DPSCF300196:475087-475198
DpHel-1	DPSCF300446:70501-70807
DpHel-1	DPSCF300015:9097-9238
DpHel-1	DPSCF300015:9240-9369
DpHel-1	DPSCF300251:395838-396149
DpHel-1	DPSCF300064:1844804-1845062
DpHel-1	DPSCF300013:923899-924110
DpHel-1	DPSCF300013:923799-923897
DpHel-1	DPSCF300134:121062-121268
DpHel-1	DPSCF300344:84669-84748
DpHel-1	DPSCF300344:84749-84918
DpHel-1	DPSCF300075:32907-33140
DpHel-1	DPSCF300107:173343-173598
DpHel-1	DPSCF300002:1085485-1085706
DpHel-1	DPSCF300002:1085706-1085789
DpHel-1	DPSCF300291:192460-192757
DpHel-1	DPSCF300030:1154584-1154890
DpHel-1	DPSCF300003:1793503-1793742
DpHel-1	DPSCF300359:79163-79246
DpHel-1	DPSCF300359:79021-79167
DpHel-1	DPSCF300064:947709-947943
DpHel-1	DPSCF300082:811078-811378
DpHel-1	DPSCF300222:606830-607125
DpHel-1	DPSCF300072:1082627-1082771
DpHel-1	DPSCF300138:312980-313274
DpHel-1	DPSCF300116:143361-143487
DpHel-1	DPSCF300116:143066-143240
DpHel-1	DPSCF300073:251745-251889
DpHel-1	DPSCF300525:59794-60092
DpHel-1	DPSCF300310:155361-155505
DpHel-1	DPSCF300310:155107-155260
DpHel-1	DPSCF300156:6027-6321
DpHel-1	DPSCF300986:4690-4912
DpHel-1	DPSCF300089:542861-543151
DpHel-1	DPSCF300152:111466-111757
DpHel-1	DPSCF300038:943163-943362
DpHel-1	DPSCF300038:943948-944038
DpHel-1	DPSCF300130:41144-41431
DpHel-1	DPSCF300415:10739-10996
DpHel-1	DPSCF300121:46086-46168
DpHel-1	DPSCF300121:46184-46384
DpHel-1	DPSCF300187:40706-40930
DpHel-1	DPSCF300328:106511-106673
DpHel-1	DPSCF300328:106328-106444
DpHel-1	DPSCF300494:6871-7150
DpHel-1	DPSCF300162:261225-261389
DpHel-1	DPSCF300162:261123-261225

DpHel-1	DPSCF300010:1881756-1881838
DpHel-1	DPSCF300010:1881886-1882027
DpHel-1	DPSCF300221:350834-351056
DpHel-1	DPSCF300193:117975-118162
DpHel-1	DPSCF300193:118172-118257
DpHel-1	DPSCF300067:315362-315536
DpHel-1	DPSCF300067:315142-315328
DpHel-1	DPSCF300067:315059-315143
DpHel-1	DPSCF300230:27129-27323
DpHel-1	DPSCF300453:12176-12449
DpHel-1	DPSCF300028:1822108-1822220
DpHel-1	DPSCF300028:1821940-1822042
DpHel-1	DPSCF300048:1958952-1959213
DpHel-1	DPSCF300540:18260-18525
DpHel-1	DPSCF300046:6538-6800
DpHel-1	DPSCF300044:1042257-1042365
DpHel-1	DPSCF300044:1042384-1042538
DpHel-1	DPSCF300540:18730-18992
DpHel-1	DPSCF300048:735193-735462
DpHel-1	DPSCF300254:20132-20297
DpHel-1	DPSCF300254:20039-20127
DpHel-1	DPSCF300181:175098-175354
DpHel-1	DPSCF300072:46795-47058
DpHel-1	DPSCF300118:446008-446269
DpHel-1	DPSCF300287:353017-353098
DpHel-1	DPSCF300287:353118-353299
DpHel-1	DPSCF300338:128650-128788
DpHel-1	DPSCF300338:128502-128607
DpHel-1	DPSCF302450:354-618
DpHel-1	DPSCF300036:586227-586409
DpHel-1	DPSCF300039:567331-567510
DpHel-1	DPSCF300355:257194-257366
DpHel-1	DPSCF300232:316806-316919
DpHel-1	DPSCF300232:316673-316804
DpHel-1	DPSCF300035:547200-547379
DpHel-1	DPSCF300522:27036-27278
DpHel-1	DPSCF302920:272-517
DpHel-1	DPSCF300216:21322-21554
DpHel-1	DPSCF300205:115537-115632
DpHel-1	DPSCF300046:521843-522001
DpHel-1	DPSCF300148:179029-179261
DpHel-1	DPSCF300138:206706-206941
DpHel-1	DPSCF300064:601774-602003
DpHel-1	DPSCF300499:47982-48211
DpHel-1	DPSCF300096:101565-101799
DpHel-1	DPSCF300094:237193-237419
DpHel-1	DPSCF300498:7966-8186
DpHel-1	DPSCF300115:179444-179630

DpHel-1	DPSCF300222:283110-283331
DpHel-1	DPSCF300307:189307-189519
DpHel-1	DPSCF301161:4175-4333
DpHel-1	DPSCF300010:723213-723420
DpHel-1	DPSCF300201:34840-34994
DpHel-1	DPSCF300244:244894-245101
DpHel-1	DPSCF300805:1680-1891
DpHel-1	DPSCF300198:101170-101377
DpHel-1	DPSCF300322:181030-181239
DpHel-1	DPSCF300470:62374-62579
DpHel-1	DPSCF300301:76495-76701
DpHel-1	DPSCF300152:266994-267193
DpHel-1	DPSCF301775:754-946
DpHel-1	DPSCF301877:1-199
DpHel-1	DPSCF300191:193418-193614
DpHel-1	DPSCF300403:10508-10712
DpHel-1	DPSCF300029:353565-353715
DpHel-1	DPSCF300071:328422-328509
DpHel-1	DPSCF300071:328513-328618
DpHel-1	DPSCF300423:104809-104995
DpHel-1	DPSCF300001:4126876-4126989
DpHel-1	DPSCF300001:4126991-4127071
DpHel-1	DPSCF300003:808327-808427
DpHel-1	DPSCF300082:197225-197414
DpHel-1	DPSCF300290:34796-34988
DpHel-1	DPSCF300155:19794-19918
DpHel-1	DPSCF300155:20142-20235
DpHel-1	DPSCF300895:3085-3273
DpHel-1	DPSCF303697:232-418
DpHel-1	DPSCF300462:7281-7468
DpHel-1	DPSCF300254:8115-8210
DpHel-1	DPSCF300254:8230-8319
DpHel-1	DPSCF300048:1213267-1213443
DpHel-1	DPSCF300501:58623-58798
DpHel-1	DPSCF300334:13399-13574
DpHel-1	DPSCF300289:157001-157167
DpHel-1	DPSCF300273:218745-218913
DpHel-1	DPSCF300041:1470639-1470809
DpHel-1	DPSCF300028:1855174-1855342
DpHel-1	DPSCF300042:962705-962875
DpHel-1	DPSCF300068:125219-125383
DpHel-1	DPSCF300055:464074-464241
DpHel-1	DPSCF300013:889357-889523
DpHel-1	DPSCF300034:150403-150570
DpHel-1	DPSCF300078:120844-121011
DpHel-1	DPSCF300406:58703-58870
DpHel-1	DPSCF300008:1174164-1174335
DpHel-1	DPSCF300065:177163-177324

DpHel-1	DPSCF300086:745872-746039
DpHel-1	DPSCF300074:609169-609337
DpHel-1	DPSCF300181:248465-248633
DpHel-1	DPSCF300137:107524-107690
DpHel-1	DPSCF300188:177353-177516
DpHel-1	DPSCF300360:20468-20639
DpHel-1	DPSCF300445:29592-29759
DpHel-1	DPSCF301788:462-633
DpHel-1	DPSCF300181:212190-212357
DpHel-1	DPSCF303288:139-301
DpHel-1	DPSCF300211:230221-230385
DpHel-1	DPSCF300029:305166-305331
DpHel-1	DPSCF300176:259936-260103
DpHel-1	DPSCF300082:445060-445210
DpHel-1	DPSCF300231:292547-292663
DpHel-1	DPSCF300047:677323-677486
DpHel-1	DPSCF300218:362915-363074
DpHel-1	DPSCF300003:1388110-1388270
DpHel-1	DPSCF300208:290681-290844
DpHel-1	DPSCF300549:9592-9755
DpHel-1	DPSCF300384:88158-88320
DpHel-1	DPSCF300118:3880-4042
DpHel-1	DPSCF300014:1310183-1310345
DpHel-1	DPSCF300322:24893-25054
DpHel-1	DPSCF300099:93095-93256
DpHel-1	DPSCF300417:72902-73064
DpHel-1	DPSCF300114:417398-417557
DpHel-1	DPSCF300290:103418-103577
DpHel-1	DPSCF300438:67454-67614
DpHel-1	DPSCF300052:881465-881622
DpHel-1	DPSCF300036:361301-361460
DpHel-1	DPSCF300117:675299-675458
DpHel-1	DPSCF300128:430777-430930
DpHel-1	DPSCF300022:1301604-1301768
DpHel-1	DPSCF300137:70872-71021
DpHel-1	DPSCF301621:483-638
DpHel-1	DPSCF300500:12919-13073
DpHel-1	DPSCF300027:361023-361176
DpHel-1	DPSCF300233:7141-7294
DpHel-1	DPSCF300041:2037639-2037792
DpHel-1	DPSCF300174:132238-132328
DpHel-1	DPSCF300056:651066-651219
DpHel-1	DPSCF300048:772304-772457
DpHel-1	DPSCF300057:131432-131584
DpHel-1	DPSCF300483:40081-40182
DpHel-1	DPSCF300004:214585-214735
DpHel-1	DPSCF300356:310858-311014
DpHel-1	DPSCF303949:247-393

DpHel-1	DPSCF300241:158847-158992
DpHel-1	DPSCF300259:151488-151633
DpHel-1	DPSCF300127:275679-275825
DpHel-1	DPSCF300029:959430-959531
DpHel-1	DPSCF300163:88421-88567
DpHel-1	DPSCF300028:573665-573767
DpHel-1	DPSCF300109:548998-549132
DpHel-1	DPSCF300228:22536-22678
DpHel-1	DPSCF300035:726382-726526
DpHel-1	DPSCF300087:79253-79341
DpHel-1	DPSCF300266:141210-141344
DpHel-1	DPSCF300258:84767-84905
DpHel-1	DPSCF300006:30767-30903
DpHel-1	DPSCF300185:118677-118812
DpHel-1	DPSCF300193:99825-99964
DpHel-1	DPSCF300080:115501-115640
DpHel-1	DPSCF300439:30927-31058
DpHel-1	DPSCF300048:1255014-1255147
DpHel-1	DPSCF300086:164844-164981
DpHel-1	DPSCF300098:725816-725905
DpHel-1	DPSCF300315:514-644
DpHel-1	DPSCF300402:41655-41785
DpHel-1	DPSCF300345:214320-214451
DpHel-1	DPSCF300252:11327-11452
DpHel-1	DPSCF300166:109518-109651
DpHel-1	DPSCF300018:155655-155781
DpHel-1	DPSCF304501:227-350
DpHel-1	DPSCF302125:610-734
DpHel-1	DPSCF300052:134507-134631
DpHel-1	DPSCF300057:61214-61338
DpHel-1	DPSCF300130:223303-223426
DpHel-1	DPSCF300044:459993-460111
DpHel-1	DPSCF300022:91833-91956
DpHel-1	DPSCF300182:107787-107907
DpHel-1	DPSCF300001:630800-630919
DpHel-1	DPSCF300200:1-118
DpHel-1	DPSCF300215:127385-127503
DpHel-1	DPSCF300397:29802-29915
DpHel-1	DPSCF300009:2001472-2001586
DpHel-1	DPSCF305107:201-314
DpHel-1	DPSCF300426:200416-200524
DpHel-1	DPSCF300861:2945-3053
DpHel-1	DPSCF300219:5340-5448
DpHel-1	DPSCF300914:3369-3480
DpHel-1	DPSCF300955:5235-5344
DpHel-1	DPSCF300182:390303-390411
DpHel-1	DPSCF300009:1066674-1066781
DpHel-1	DPSCF300361:111303-111409

DpHel-1	DPSCF300361:71612-71718
DpHel-1	DPSCF300023:987503-987608
DpHel-1	DPSCF300019:1107128-1107227
DpHel-1	DPSCF304743:234-335
DpHel-1	DPSCF300050:138981-139064
DpHel-1	DPSCF300502:1162-1263
DpHel-1	DPSCF300138:215448-215549
DpHel-1	DPSCF300422:91623-91724
DpHel-1	DPSCF300541:443-538
DpHel-1	DPSCF300882:4851-4947
DpHel-1	DPSCF300074:194750-194849
DpHel-1	DPSCF300035:546929-547027
DpHel-1	DPSCF300175:153657-153754
DpHel-1	DPSCF300254:1-95
DpHel-1	DPSCF300055:29031-29125
DpHel-1	DPSCF300511:32144-32237
DpHel-1	DPSCF300025:1001281-1001373
DpHel-1	DPSCF301070:1-96
DpHel-1	DPSCF304843:238-329
DpHel-1	DPSCF300012:912528-912616
DpHel-1	DPSCF300027:289611-289700
DpHel-1	DPSCF302445:1-89
DpHel-1	DPSCF300012:1265767-1265853
DpHel-1	DPSCF302885:11-94
DpHel-1	DPSCF300244:244784-244868
DpHel-1	DPSCF304255:284-367
DpHel-1	DPSCF305187:227-309
DpHel-1	DPSCF300893:1134-1216
DpHel-1	DPSCF300086:153167-153248
DpHel-1	DPSCF300173:195823-195904
DpHel-1	DPSCF300139:202676-202757
DpHel-1	DPSCF304502:271-350
DpHel-1	DPSCF300078:195732-195813

TableS4 Positions of Helitron in Manduca sexta genome

**Families**	**Accession numbers**
MsHel-1	gb|AIXA01003152.1|:90745-90938
MsHel-1	gb|AIXA01003152.1|:136991-137819
MsHel-1	gb|AIXA01004482.1|:15426-19437
MsHel-1	gb|AIXA01008667.1|:23171-23540
MsHel-1	gb|AIXA01000823.1|:37215-37598
MsHel-1	gb|AIXA01000823.1|:55620-55739
MsHel-1	gb|AIXA01000388.1|:1986-2676
MsHel-1	gb|AIXA01000388.1|:27695-28517
MsHel-1	gb|AIXA01002309.1|:23807-24639
MsHel-1	gb|AIXA01000910.1|:1431-2263
MsHel-1	gb|AIXA01005763.1|:13514-14341
MsHel-1	gb|AIXA01002232.1|:35414-35910
MsHel-1	gb|AIXA01001593.1|:21982-22825
MsHel-1	gb|AIXA01014913.1|:13516-14348
MsHel-1	gb|AIXA01007659.1|:3563-4396
MsHel-1	gb|AIXA01004552.1|:15540-16245
MsHel-1	gb|AIXA01003117.1|:2783-3614
MsHel-1	gb|AIXA01001032.1|:58408-59242
MsHel-1	gb|AIXA01007584.1|:14310-15134
MsHel-1	gb|AIXA01003872.1|:16229-17052
MsHel-1	gb|AIXA01003872.1|:31602-32433
MsHel-1	gb|AIXA01001415.1|:7594-7690
MsHel-1	gb|AIXA01001415.1|:30653-32269
MsHel-1	gb|AIXA01001408.1|:17172-17291
MsHel-1	gb|AIXA01005415.1|:27352-27923
MsHel-1	gb|AIXA01006057.1|:25352-25844
MsHel-1	gb|AIXA01001366.1|:33068-33901
MsHel-1	gb|AIXA01000725.1|:44040-44873
MsHel-1	gb|AIXA01015888.1|:7461-8297
MsHel-1	gb|AIXA01014224.1|:9978-10799
MsHel-1	gb|AIXA01012253.1|:37875-38717
MsHel-1	gb|AIXA01002613.1|:11809-12337
MsHel-1	gb|AIXA01007552.1|:35575-36408
MsHel-1	gb|AIXA01006726.1|:5859-6567
MsHel-1	gb|AIXA01006726.1|:33595-34426
MsHel-1	gb|AIXA01001511.1|:24722-24893
MsHel-1	gb|AIXA01001511.1|:102016-102848
MsHel-1	gb|AIXA01017675.1|:7508-8001
MsHel-1	gb|AIXA01004110.1|:78-286
MsHel-1	gb|AIXA01017072.1|:5318-6151
MsHel-1	gb|AIXA01002650.1|:1-357
MsHel-1	gb|AIXA01012581.1|:18699-19154
MsHel-1	gb|AIXA01012581.1|:46776-47607
MsHel-1	gb|AIXA01004381.1|:4747-4960
MsHel-1	gb|AIXA01001957.1|:16716-17211
MsHel-1	gb|AIXA01004806.1|:61348-62175

MsHel-1	gb|AIXA01002041.1|:7868-8006
MsHel-1	gb|AIXA01002041.1|:24644-25479
MsHel-1	gb|AIXA01000242.1|:55394-56212
MsHel-1	gb|AIXA01004417.1|:11308-12141
MsHel-1	gb|AIXA01008468.1|:3327-4160
MsHel-1	gb|AIXA01002600.1|:51104-51687
MsHel-1	gb|AIXA01010468.1|:10160-10652
MsHel-1	gb|AIXA01002186.1|:39101-39928
MsHel-1	gb|AIXA01001539.1|:10975-11806
MsHel-1	gb|AIXA01005295.1|:1-531
MsHel-1	gb|AIXA01013067.1|:23227-24058
MsHel-1	gb|AIXA01010771.1|:35300-36132
MsHel-1	gb|AIXA01006713.1|:26776-27602
MsHel-1	gb|AIXA01005785.1|:28059-28889
MsHel-1	gb|AIXA01004187.1|:44128-44714
MsHel-1	gb|AIXA01003541.1|:8144-8977
MsHel-1	gb|AIXA01000825.1|:11669-12165
MsHel-1	gb|AIXA01010593.1|:13647-14711
MsHel-1	gb|AIXA01010593.1|:27136-27964
MsHel-1	gb|AIXA01002847.1|:31271-31492
MsHel-1	gb|AIXA01002813.1|:2984-3812
MsHel-1	gb|AIXA01002215.1|:18818-19645
MsHel-1	gb|AIXA01006693.1|:9286-10116
MsHel-1	gb|AIXA01010681.1|:15252-16086
MsHel-1	gb|AIXA01007596.1|:12352-13175
MsHel-1	gb|AIXA01000005.1|:2497-3317
MsHel-1	gb|AIXA01000005.1|:134649-135481
MsHel-1	gb|AIXA01000005.1|:153026-153422
MsHel-1	gb|AIXA01003176.1|:28598-29425
MsHel-1	gb|AIXA01003176.1|:66362-67170
MsHel-1	gb|AIXA01001814.1|:2315-3159
MsHel-1	gb|AIXA01010773.1|:24063-24536
MsHel-1	gb|AIXA01007854.1|:39434-40259
MsHel-1	gb|AIXA01007854.1|:60824-61319
MsHel-1	gb|AIXA01007078.1|:1145-2770
MsHel-1	gb|AIXA01008385.1|:18706-19285
MsHel-1	gb|AIXA01007595.1|:36579-37145
MsHel-1	gb|AIXA01004298.1|:16968-17202
MsHel-1	gb|AIXA01014222.1|:9947-10536
MsHel-1	gb|AIXA01003628.1|:15421-15790
MsHel-1	gb|AIXA01000951.1|:11453-12272
MsHel-1	gb|AIXA01007663.1|:33221-34048
MsHel-1	gb|AIXA01004313.1|:10259-11085
MsHel-1	gb|AIXA01012810.1|:58181-59016
MsHel-1	gb|AIXA01007681.1|:93733-94768
MsHel-1	gb|AIXA01003308.1|:5265-5761
MsHel-1	gb|AIXA01003308.1|:50976-51157
MsHel-1	gb|AIXA01000205.1|:18395-19231

MsHel-1	gb|AIXA01000205.1|:58075-58376
MsHel-1	gb|AIXA01008423.1|:58954-59635
MsHel-1	gb|AIXA01006276.1|:1-823
MsHel-1	gb|AIXA01001752.1|:10227-11061
MsHel-1	gb|AIXA01007205.1|:28941-29775
MsHel-1	gb|AIXA01003576.1|:22629-23462
MsHel-1	gb|AIXA01003576.1|:36653-37087
MsHel-1	gb|AIXA01014980.1|:4103-4599
MsHel-1	gb|AIXA01012143.1|:6951-7361
MsHel-1	gb|AIXA01005021.1|:802-1390
MsHel-1	gb|AIXA01003362.1|:17063-19169
MsHel-1	gb|AIXA01003317.1|:47115-47286
MsHel-1	gb|AIXA01003317.1|:60138-60675
MsHel-1	gb|AIXA01002283.1|:49607-49807
MsHel-1	gb|AIXA01001451.1|:2113-2947
MsHel-1	gb|AIXA01001700.1|:3871-4705
MsHel-1	gb|AIXA01001595.1|:38669-39502
MsHel-1	gb|AIXA01015427.1|:3409-12925
MsHel-1	gb|AIXA01007428.1|:1569-2396
MsHel-1	gb|AIXA01007850.1|:23177-24004
MsHel-1	gb|AIXA01006881.1|:46741-47194
MsHel-1	gb|AIXA01006769.1|:7295-7791
MsHel-1	gb|AIXA01006036.1|:33328-33824
MsHel-1	gb|AIXA01004430.1|:5783-6611
MsHel-1	gb|AIXA01012674.1|:7311-8146
MsHel-1	gb|AIXA01010659.1|:10653-11473
MsHel-1	gb|AIXA01006837.1|:40099-40198
MsHel-1	gb|AIXA01018927.1|:2716-3547
MsHel-1	gb|AIXA01013827.1|:1-242
MsHel-1	gb|AIXA01013827.1|:19508-19998
MsHel-1	gb|AIXA01007807.1|:1140-5465
MsHel-1	gb|AIXA01002631.1|:2970-3775
MsHel-1	gb|AIXA01007132.1|:19898-20394
MsHel-1	gb|AIXA01001847.1|:29444-30269
MsHel-1	gb|AIXA01015727.1|:3985-4950
MsHel-1	gb|AIXA01013130.1|:3632-4454
MsHel-1	gb|AIXA01010848.1|:63053-63843
MsHel-1	gb|AIXA01000149.1|:43296-43754
MsHel-1	gb|AIXA01009773.1|:19155-19982
MsHel-1	gb|AIXA01006519.1|:20800-20995
MsHel-1	gb|AIXA01006519.1|:40773-40891
MsHel-1	gb|AIXA01005321.1|:6860-6980
MsHel-1	gb|AIXA01005321.1|:78036-78867
MsHel-1	gb|AIXA01003875.1|:13186-14012
MsHel-1	gb|AIXA01010340.1|:1440-2274
MsHel-1	gb|AIXA01008429.1|:5411-5612
MsHel-1	gb|AIXA01007533.1|:2980-3812
MsHel-1	gb|AIXA01006885.1|:67936-68058

MsHel-1	gb|AIXA01006885.1|:91753-92412
MsHel-1	gb|AIXA01006885.1|:155349-155507
MsHel-1	gb|AIXA01002390.1|:3790-4615
MsHel-1	gb|AIXA01002390.1|:57633-58329
MsHel-1	gb|AIXA01007037.1|:7909-8405
MsHel-1	gb|AIXA01006705.1|:22217-23051
MsHel-1	gb|AIXA01001213.1|:12326-13159
MsHel-1	gb|AIXA01010166.1|:3248-3744
MsHel-1	gb|AIXA01009837.1|:71978-72807
MsHel-1	gb|AIXA01009229.1|:19296-20129
MsHel-1	gb|AIXA01008363.1|:176983-177821
MsHel-1	gb|AIXA01007848.1|:5945-6767
MsHel-1	gb|AIXA01007602.1|:6322-7162
MsHel-1	gb|AIXA01006316.1|:4735-11317
MsHel-1	gb|AIXA01006316.1|:24991-25821
MsHel-1	gb|AIXA01005406.1|:5405-5553
MsHel-1	gb|AIXA01004042.1|:180-7942
MsHel-1	gb|AIXA01008428.1|:5688-6183
MsHel-1	gb|AIXA01007228.1|:55412-55819
MsHel-1	gb|AIXA01007262.1|:26835-27307
MsHel-1	gb|AIXA01003363.1|:2376-3202
MsHel-1	gb|AIXA01003306.1|:15715-16540
MsHel-1	gb|AIXA01000650.1|:47565-48278
MsHel-1	gb|AIXA01000650.1|:133840-134332
MsHel-1	gb|AIXA01000650.1|:163425-164246
MsHel-1	gb|AIXA01014037.1|:18020-18198
MsHel-1	gb|AIXA01014037.1|:41023-41860
MsHel-1	gb|AIXA01000627.1|:8973-9798
MsHel-1	gb|AIXA01000627.1|:35071-36039
MsHel-1	gb|AIXA01006018.1|:4326-4583
MsHel-1	gb|AIXA01005494.1|:33096-33647
MsHel-1	gb|AIXA01005494.1|:48435-49243
MsHel-1	gb|AIXA01001995.1|:2631-3455
MsHel-1	gb|AIXA01000020.1|:16290-16873
MsHel-1	gb|AIXA01017770.1|:2145-3201
MsHel-1	gb|AIXA01009350.1|:13161-13352
MsHel-1	gb|AIXA01009350.1|:60589-61020
MsHel-1	gb|AIXA01002952.1|:9018-9841
MsHel-1	gb|AIXA01010189.1|:37890-38715
MsHel-1	gb|AIXA01005130.1|:16634-17130
MsHel-1	gb|AIXA01004683.1|:37832-38676
MsHel-1	gb|AIXA01011320.1|:17972-18791
MsHel-1	gb|AIXA01009039.1|:22878-22992
MsHel-1	gb|AIXA01007487.1|:14577-15411
MsHel-1	gb|AIXA01006380.1|:43026-43869
MsHel-1	gb|AIXA01004583.1|:35520-36014
MsHel-1	gb|AIXA01000327.1|:60421-61254
MsHel-1	gb|AIXA01012662.1|:1-468

MsHel-1	gb|AIXA01007783.1|:69486-70079
MsHel-1	gb|AIXA01002239.1|:20091-20585
MsHel-1	gb|AIXA01007451.1|:47569-47716
MsHel-1	gb|AIXA01004809.1|:84458-84954
MsHel-1	gb|AIXA01004809.1|:119049-119249
MsHel-1	gb|AIXA01007236.1|:9453-10210
MsHel-1	gb|AIXA01002728.1|:4977-5797
MsHel-1	gb|AIXA01000160.1|:3139-3635
MsHel-1	gb|AIXA01021052.1|:533-1368
MsHel-1	gb|AIXA01012644.1|:10342-10981
MsHel-1	gb|AIXA01012644.1|:28496-29077
MsHel-1	gb|AIXA01012644.1|:50918-51736
MsHel-1	gb|AIXA01011247.1|:14556-15142
MsHel-1	gb|AIXA01007626.1|:18184-21039
MsHel-1	gb|AIXA01007626.1|:98317-98480
MsHel-1	gb|AIXA01007626.1|:130749-131016
MsHel-1	gb|AIXA01000819.1|:2405-3116
MsHel-1	gb|AIXA01000819.1|:75795-76633
MsHel-1	gb|AIXA01006438.1|:353-501
MsHel-1	gb|AIXA01002365.1|:17491-18327
MsHel-1	gb|AIXA01000754.1|:46653-47475
MsHel-1	gb|AIXA01008628.1|:4161-4413
MsHel-1	gb|AIXA01007460.1|:6041-6565
MsHel-1	gb|AIXA01007460.1|:35044-35527
MsHel-1	gb|AIXA01007460.1|:64832-64925
MsHel-1	gb|AIXA01007460.1|:84137-84646
MsHel-1	gb|AIXA01004695.1|:15441-15939
MsHel-1	gb|AIXA01002937.1|:2230-2828
MsHel-1	gb|AIXA01012706.1|:15248-16075
MsHel-1	gb|AIXA01012240.1|:20891-21796
MsHel-1	gb|AIXA01006481.1|:8587-9416
MsHel-1	gb|AIXA01005538.1|:54516-61206
MsHel-1	gb|AIXA01002391.1|:12981-13686
MsHel-1	gb|AIXA01000892.1|:21513-21625
MsHel-1	gb|AIXA01009713.1|:35898-36304
MsHel-1	gb|AIXA01005862.1|:8008-8505
MsHel-1	gb|AIXA01005862.1|:22023-22480
MsHel-1	gb|AIXA01005841.1|:15997-16822
MsHel-1	gb|AIXA01002580.1|:54220-54707
MsHel-1	gb|AIXA01007966.1|:1075-2992
MsHel-1	gb|AIXA01007966.1|:51576-52290
MsHel-1	gb|AIXA01005067.1|:88955-89451
MsHel-1	gb|AIXA01008095.1|:46319-47152
MsHel-1	gb|AIXA01015075.1|:2350-3177
MsHel-1	gb|AIXA01004520.1|:79067-79850
MsHel-1	gb|AIXA01004550.1|:63375-64208
MsHel-1	gb|AIXA01004549.1|:2323-3156
MsHel-1	gb|AIXA01002936.1|:29712-29904

MsHel-1	gb|AIXA01000430.1|:1-496
MsHel-1	gb|AIXA01000430.1|:47351-48436
MsHel-1	gb|AIXA01017051.1|:15683-16179
MsHel-1	gb|AIXA01014695.1|:8077-8906
MsHel-1	gb|AIXA01008111.1|:58486-59295
MsHel-1	gb|AIXA01006950.1|:15608-16448
MsHel-1	gb|AIXA01001106.1|:11665-12493
MsHel-1	gb|AIXA01022246.1|:1-368
MsHel-1	gb|AIXA01013057.1|:5916-6742
MsHel-1	gb|AIXA01012528.1|:1376-1872
MsHel-1	gb|AIXA01004274.1|:602-1437
MsHel-1	gb|AIXA01002230.1|:83863-84698
MsHel-1	gb|AIXA01011722.1|:5863-6696
MsHel-1	gb|AIXA01007351.1|:7538-8243
MsHel-1	gb|AIXA01000399.1|:1551-2097
MsHel-1	gb|AIXA01000399.1|:72737-72937
MsHel-1	gb|AIXA01010590.1|:4-171
MsHel-1	gb|AIXA01010590.1|:40257-40692
MsHel-1	gb|AIXA01010590.1|:76753-76995
MsHel-1	gb|AIXA01007206.1|:4741-5576
MsHel-1	gb|AIXA01011466.1|:21941-23028
MsHel-1	gb|AIXA01002296.1|:92344-92516
MsHel-1	gb|AIXA01001469.1|:5181-6024
MsHel-1	gb|AIXA01016760.1|:56-882
MsHel-1	gb|AIXA01003374.1|:17905-18080
MsHel-1	gb|AIXA01003142.1|:24099-24804
MsHel-1	gb|AIXA01016282.1|:3957-4778
MsHel-1	gb|AIXA01010905.1|:17367-18171
MsHel-1	gb|AIXA01006991.1|:2-648
MsHel-1	gb|AIXA01005661.1|:15905-16722
MsHel-1	gb|AIXA01005661.1|:61309-61510
MsHel-1	gb|AIXA01002950.1|:19579-20073
MsHel-1	gb|AIXA01002640.1|:20084-20672
MsHel-1	gb|AIXA01002640.1|:54092-55681
MsHel-1	gb|AIXA01001682.1|:1-282
MsHel-1	gb|AIXA01001682.1|:14599-15429
MsHel-1	gb|AIXA01004803.1|:3005-4898
MsHel-1	gb|AIXA01004652.1|:4-432
MsHel-1	gb|AIXA01002130.1|:10063-10876
MsHel-1	gb|AIXA01000048.1|:18547-18677
MsHel-1	gb|AIXA01000048.1|:60076-60225
MsHel-1	gb|AIXA01000048.1|:72404-72538
MsHel-1	gb|AIXA01015097.1|:21720-22682
MsHel-1	gb|AIXA01005490.1|:7662-8080
MsHel-1	gb|AIXA01002701.1|:12564-13395
MsHel-1	gb|AIXA01016312.1|:1978-2474
MsHel-1	gb|AIXA01009287.1|:39750-41031
MsHel-1	gb|AIXA01006880.1|:8183-8641

MsHel-1	gb|AIXA01010543.1|:26535-26939
MsHel-1	gb|AIXA01006140.1|:30175-31003
MsHel-1	gb|AIXA01005324.1|:9241-9816
MsHel-1	gb|AIXA01004433.1|:44230-44356
MsHel-1	gb|AIXA01003989.1|:36242-36456
MsHel-1	gb|AIXA01003989.1|:52430-53017
MsHel-1	gb|AIXA01003967.1|:109600-110300
MsHel-1	gb|AIXA01010854.1|:61750-62454
MsHel-1	gb|AIXA01010854.1|:85119-85578
MsHel-1	gb|AIXA01004437.1|:13573-14408
MsHel-1	gb|AIXA01000905.1|:17505-18717
MsHel-1	gb|AIXA01002228.1|:1-643
MsHel-1	gb|AIXA01012260.1|:10898-12137
MsHel-1	gb|AIXA01008431.1|:13435-14143
MsHel-1	gb|AIXA01006956.1|:15656-16023
MsHel-1	gb|AIXA01004533.1|:6192-6899
MsHel-1	gb|AIXA01003018.1|:4417-5215
MsHel-1	gb|AIXA01014964.1|:31784-32618
MsHel-1	gb|AIXA01012785.1|:10351-11176
MsHel-1	gb|AIXA01007470.1|:21606-22102
MsHel-1	gb|AIXA01001490.1|:3890-4731
MsHel-1	gb|AIXA01000655.1|:2195-2928
MsHel-1	gb|AIXA01000655.1|:35177-35818
MsHel-1	gb|AIXA01000655.1|:196620-196718
MsHel-1	gb|AIXA01015193.1|:4379-5204
MsHel-1	gb|AIXA01007324.1|:6573-7154
MsHel-1	gb|AIXA01007324.1|:17038-17534
MsHel-1	gb|AIXA01006895.1|:11807-12631
MsHel-1	gb|AIXA01002398.1|:15122-15915
MsHel-1	gb|AIXA01013549.1|:9956-10452
MsHel-1	gb|AIXA01013328.1|:15189-15562
MsHel-1	gb|AIXA01013328.1|:44449-45289
MsHel-1	gb|AIXA01011489.1|:2593-3289
MsHel-1	gb|AIXA01011489.1|:33546-37024
MsHel-1	gb|AIXA01003928.1|:51258-52086
MsHel-1	gb|AIXA01003116.1|:7280-8221
MsHel-1	gb|AIXA01001132.1|:3882-4577
MsHel-1	gb|AIXA01001128.1|:30020-31111
MsHel-1	gb|AIXA01001128.1|:58374-59106
MsHel-1	gb|AIXA01017016.1|:8666-9491
MsHel-1	gb|AIXA01011734.1|:22893-23725
MsHel-1	gb|AIXA01009189.1|:19414-20119
MsHel-1	gb|AIXA01005082.1|:21764-22590
MsHel-1	gb|AIXA01000200.1|:1559-2403
MsHel-1	gb|AIXA01001115.1|:41096-42110
MsHel-1	gb|AIXA01009062.1|:2014-2967
MsHel-1	gb|AIXA01008566.1|:3-114
MsHel-1	gb|AIXA01008566.1|:65606-65743

MsHel-1	gb|AIXA01006007.1|:78743-79567
MsHel-1	gb|AIXA01001518.1|:22021-22856
MsHel-1	gb|AIXA01018024.1|:3624-4121
MsHel-1	gb|AIXA01007496.1|:31563-32148
MsHel-1	gb|AIXA01005435.1|:39141-39637
MsHel-1	gb|AIXA01013753.1|:13-522
MsHel-1	gb|AIXA01011562.1|:29005-29807
MsHel-1	gb|AIXA01009864.1|:21978-22806
MsHel-1	gb|AIXA01009864.1|:52953-53789
MsHel-1	gb|AIXA01009864.1|:73384-74164
MsHel-1	gb|AIXA01008417.1|:37525-38346
MsHel-1	gb|AIXA01006590.1|:32976-34036
MsHel-1	gb|AIXA01006590.1|:49739-50443
MsHel-1	gb|AIXA01004948.1|:24244-25070
MsHel-1	gb|AIXA01003739.1|:36625-36767
MsHel-1	gb|AIXA01014547.1|:875-1718
MsHel-1	gb|AIXA01004799.1|:8746-9453
MsHel-1	gb|AIXA01002579.1|:6789-7416
MsHel-1	gb|AIXA01001243.1|:235385-235477
MsHel-1	gb|AIXA01004693.1|:78083-78902
MsHel-1	gb|AIXA01003605.1|:3222-3742
MsHel-1	gb|AIXA01003605.1|:45108-45653
MsHel-1	gb|AIXA01002805.1|:48441-49275
MsHel-1	gb|AIXA01002671.1|:40552-41258
MsHel-1	gb|AIXA01002671.1|:109265-109563
MsHel-1	gb|AIXA01002140.1|:1-730
MsHel-1	gb|AIXA01011460.1|:26678-27503
MsHel-1	gb|AIXA01010881.1|:4183-5011
MsHel-1	gb|AIXA01009054.1|:9447-10264
MsHel-1	gb|AIXA01007679.1|:5843-6339
MsHel-1	gb|AIXA01007175.1|:58480-59175
MsHel-1	gb|AIXA01007718.1|:9570-10282
MsHel-1	gb|AIXA01003190.1|:1568-2384
MsHel-1	gb|AIXA01002386.1|:39480-40009
MsHel-1	gb|AIXA01007070.1|:35587-36432
MsHel-1	gb|AIXA01006132.1|:3167-3663
MsHel-1	gb|AIXA01005946.1|:44828-45231
MsHel-1	gb|AIXA01007001.1|:53182-53888
MsHel-1	gb|AIXA01005016.1|:40407-40904
MsHel-1	gb|AIXA01013521.1|:36814-38173
MsHel-1	gb|AIXA01013010.1|:54519-54728
MsHel-1	gb|AIXA01012483.1|:6219-6923
MsHel-1	gb|AIXA01011652.1|:5822-6635
MsHel-1	gb|AIXA01004355.1|:22387-23092
MsHel-1	gb|AIXA01002607.1|:4795-5499
MsHel-1	gb|AIXA01006602.1|:10197-10386
MsHel-1	gb|AIXA01005558.1|:21963-22182
MsHel-1	gb|AIXA01005558.1|:74909-75380

MsHel-1	gb|AIXA01002115.1|:3-729
MsHel-1	gb|AIXA01000755.1|:132666-133488
MsHel-1	gb|AIXA01016001.1|:5094-5917
MsHel-1	gb|AIXA01001858.1|:24131-24624
MsHel-1	gb|AIXA01001858.1|:51936-52459
MsHel-1	gb|AIXA01001693.1|:62664-63520
MsHel-1	gb|AIXA01012473.1|:8748-9453
MsHel-1	gb|AIXA01007375.1|:6246-6413
MsHel-1	gb|AIXA01003329.1|:11306-11515
MsHel-1	gb|AIXA01003329.1|:61428-62133
MsHel-1	gb|AIXA01015527.1|:9112-9956
MsHel-1	gb|AIXA01014408.1|:3529-4229
MsHel-1	gb|AIXA01010185.1|:51924-52632
MsHel-1	gb|AIXA01010185.1|:83591-88335
MsHel-1	gb|AIXA01004762.1|:18458-19464
MsHel-1	gb|AIXA01004762.1|:46276-46852
MsHel-1	gb|AIXA01001856.1|:2583-3409
MsHel-1	gb|AIXA01001204.1|:80688-81394
MsHel-1	gb|AIXA01000117.1|:5007-5305
MsHel-1	gb|AIXA01013807.1|:1-103
MsHel-1	gb|AIXA01013807.1|:56708-57541
MsHel-1	gb|AIXA01008622.1|:4797-5611
MsHel-1	gb|AIXA01007869.1|:3938-4752
MsHel-1	gb|AIXA01003276.1|:47794-48511
MsHel-1	gb|AIXA01001675.1|:9489-9948
MsHel-1	gb|AIXA01008254.1|:14960-16006
MsHel-1	gb|AIXA01001319.1|:74407-74902
MsHel-1	gb|AIXA01011810.1|:24225-24930
MsHel-1	gb|AIXA01011531.1|:4416-5120
MsHel-1	gb|AIXA01010667.1|:8824-9607
MsHel-1	gb|AIXA01005452.1|:4830-5515
MsHel-1	gb|AIXA01005452.1|:24779-25246
MsHel-1	gb|AIXA01005452.1|:65320-66131
MsHel-1	gb|AIXA01005347.1|:91277-91776
MsHel-1	gb|AIXA01001224.1|:11755-12515
MsHel-1	gb|AIXA01001224.1|:29979-30196
MsHel-1	gb|AIXA01000255.1|:27632-28474
MsHel-1	gb|AIXA01004104.1|:3021-3144
MsHel-1	gb|AIXA01004104.1|:13758-14448
MsHel-1	gb|AIXA01004104.1|:30683-32016
MsHel-1	gb|AIXA01012020.1|:14166-14981
MsHel-1	gb|AIXA01005609.1|:26244-27058
MsHel-1	gb|AIXA01000542.1|:20223-20827
MsHel-1	gb|AIXA01000542.1|:57159-57861
MsHel-1	gb|AIXA01000542.1|:154799-155502
MsHel-1	gb|AIXA01000266.1|:11113-11244
MsHel-1	gb|AIXA01001912.1|:53137-54102
MsHel-1	gb|AIXA01001048.1|:74319-75168

MsHel-1	gb|AIXA01006754.1|:34437-35375
MsHel-1	gb|AIXA01003287.1|:3932-4775
MsHel-1	gb|AIXA01002703.1|:60907-61733
MsHel-1	gb|AIXA01010151.1|:50574-50708
MsHel-1	gb|AIXA01010151.1|:117303-119823
MsHel-1	gb|AIXA01004751.1|:5135-5972
MsHel-1	gb|AIXA01005655.1|:9221-10041
MsHel-1	gb|AIXA01002218.1|:10175-11006
MsHel-1	gb|AIXA01001746.1|:25960-26807
MsHel-1	gb|AIXA01016280.1|:1967-2673
MsHel-1	gb|AIXA01009399.1|:33293-33998
MsHel-1	gb|AIXA01006346.1|:27992-28481
MsHel-1	gb|AIXA01004076.1|:35389-36095
MsHel-1	gb|AIXA01007597.1|:4784-5489
MsHel-1	gb|AIXA01002895.1|:321-1026
MsHel-1	gb|AIXA01002194.1|:1561-2034
MsHel-1	gb|AIXA01002194.1|:13521-13699
MsHel-1	gb|AIXA01001053.1|:54434-55806
MsHel-1	gb|AIXA01007560.1|:49797-50070
MsHel-1	gb|AIXA01001453.1|:1111-1205
MsHel-1	gb|AIXA01001453.1|:11892-12718
MsHel-1	gb|AIXA01014887.1|:2574-4189
MsHel-1	gb|AIXA01011510.1|:5358-6201
MsHel-1	gb|AIXA01009268.1|:11246-11420
MsHel-1	gb|AIXA01009268.1|:39656-40071
MsHel-1	gb|AIXA01004084.1|:1816-2657
MsHel-1	gb|AIXA01000701.1|:202-788
MsHel-1	gb|AIXA01000183.1|:26036-26879
MsHel-1	gb|AIXA01000183.1|:82804-82965
MsHel-1	gb|AIXA01005407.1|:21963-22669
MsHel-1	gb|AIXA01005407.1|:43837-44547
MsHel-1	gb|AIXA01005194.1|:2598-3521
MsHel-1	gb|AIXA01005194.1|:35063-36195
MsHel-1	gb|AIXA01002775.1|:46734-47440
MsHel-1	gb|AIXA01013382.1|:17240-18447
MsHel-1	gb|AIXA01008034.1|:89-837
MsHel-1	gb|AIXA01006752.1|:15409-15576
MsHel-1	gb|AIXA01000703.1|:1-142
MsHel-1	gb|AIXA01000703.1|:12786-13687
MsHel-1	gb|AIXA01000613.1|:35665-35894
MsHel-1	gb|AIXA01009407.1|:29546-30778
MsHel-1	gb|AIXA01005779.1|:1636-2475
MsHel-1	gb|AIXA01005779.1|:65927-66388
MsHel-1	gb|AIXA01005738.1|:10951-12372
MsHel-1	gb|AIXA01005738.1|:26895-27549
MsHel-1	gb|AIXA01005629.1|:42266-43037
MsHel-1	gb|AIXA01003124.1|:84559-85773
MsHel-1	gb|AIXA01006872.1|:61923-62626

MsHel-1	gb|AIXA01006731.1|:15078-15689
MsHel-1	gb|AIXA01006731.1|:102200-103021
MsHel-1	gb|AIXA01006029.1|:32668-33449
MsHel-1	gb|AIXA01010467.1|:5619-6325
MsHel-1	gb|AIXA01008578.1|:10484-11746
MsHel-1	gb|AIXA01008578.1|:26496-27201
MsHel-1	gb|AIXA01008578.1|:46436-46523
MsHel-1	gb|AIXA01008488.1|:4338-4932
MsHel-1	gb|AIXA01008767.1|:14842-15547
MsHel-1	gb|AIXA01008767.1|:67906-68112
MsHel-1	gb|AIXA01006757.1|:5563-6267
MsHel-1	gb|AIXA01006496.1|:29066-29771
MsHel-1	gb|AIXA01004758.1|:13065-13854
MsHel-1	gb|AIXA01004758.1|:38175-39242
MsHel-1	gb|AIXA01001286.1|:46344-47264
MsHel-1	gb|AIXA01001627.1|:48849-49552
MsHel-1	gb|AIXA01010698.1|:55275-56116
MsHel-1	gb|AIXA01006204.1|:8941-9648
MsHel-1	gb|AIXA01005008.1|:20852-21336
MsHel-1	gb|AIXA01013645.1|:2459-3165
MsHel-1	gb|AIXA01010802.1|:12078-13018
MsHel-1	gb|AIXA01010802.1|:29096-29912
MsHel-1	gb|AIXA01009720.1|:21033-21782
MsHel-1	gb|AIXA01009212.1|:31081-31783
MsHel-1	gb|AIXA01009212.1|:52064-52740
MsHel-1	gb|AIXA01009212.1|:93231-93930
MsHel-1	gb|AIXA01008192.1|:6533-7371
MsHel-1	gb|AIXA01000862.1|:12082-12669
MsHel-1	gb|AIXA01000862.1|:45101-45806
MsHel-1	gb|AIXA01000862.1|:76292-76897
MsHel-1	gb|AIXA01007865.1|:2106-2909
MsHel-1	gb|AIXA01005960.1|:17540-18501
MsHel-1	gb|AIXA01016043.1|:3726-4429
MsHel-1	gb|AIXA01007800.1|:55063-55790
MsHel-1	gb|AIXA01007800.1|:84649-85448
MsHel-1	gb|AIXA01007253.1|:33136-33378
MsHel-1	gb|AIXA01006385.1|:6888-7744
MsHel-1	gb|AIXA01006385.1|:23059-23621
MsHel-1	gb|AIXA01006385.1|:47652-48280
MsHel-1	gb|AIXA01002908.1|:23709-24413
MsHel-1	gb|AIXA01002440.1|:11826-12323
MsHel-1	gb|AIXA01000207.1|:48606-49917
MsHel-1	gb|AIXA01000129.1|:6780-7811
MsHel-1	gb|AIXA01009117.1|:16186-16888
MsHel-1	gb|AIXA01009016.1|:2311-3437
MsHel-1	gb|AIXA01001758.1|:11503-11643
MsHel-1	gb|AIXA01001758.1|:119074-119783
MsHel-1	gb|AIXA01015057.1|:25060-25192

MsHel-1	gb|AIXA01007721.1|:8392-9101
MsHel-1	gb|AIXA01003747.1|:82935-83056
MsHel-1	gb|AIXA01003747.1|:119396-120360
MsHel-1	gb|AIXA01002224.1|:93014-93852
MsHel-1	gb|AIXA01002224.1|:181051-181759
MsHel-1	gb|AIXA01001484.1|:42813-43646
MsHel-1	gb|AIXA01009177.1|:41968-42815
MsHel-1	gb|AIXA01009177.1|:106109-106912
MsHel-1	gb|AIXA01004827.1|:1752-2593
MsHel-1	gb|AIXA01002835.1|:5608-6449
MsHel-1	gb|AIXA01002384.1|:10969-11406
MsHel-1	gb|AIXA01002350.1|:141190-142155
MsHel-1	gb|AIXA01014372.1|:46171-47257
MsHel-1	gb|AIXA01014984.1|:19976-21206
MsHel-1	gb|AIXA01005138.1|:6490-6985
MsHel-1	gb|AIXA01003979.1|:1936-2406
MsHel-1	gb|AIXA01000828.1|:1-520
MsHel-1	gb|AIXA01000828.1|:16984-17699
MsHel-1	gb|AIXA01000507.1|:17051-17732
MsHel-1	gb|AIXA01014501.1|:3434-4268
MsHel-1	gb|AIXA01011131.1|:1726-2422
MsHel-1	gb|AIXA01010211.1|:23412-23848
MsHel-1	gb|AIXA01009305.1|:37709-38150
MsHel-1	gb|AIXA01000022.1|:62968-63333
MsHel-1	gb|AIXA01009512.1|:13393-14081
MsHel-1	gb|AIXA01003993.1|:8955-9781
MsHel-1	gb|AIXA01001060.1|:5581-6291
MsHel-1	gb|AIXA01000934.1|:29909-30563
MsHel-1	gb|AIXA01000934.1|:73033-73705
MsHel-1	gb|AIXA01019294.1|:1-430
MsHel-1	gb|AIXA01011821.1|:13-318
MsHel-1	gb|AIXA01009641.1|:22999-23919
MsHel-1	gb|AIXA01004738.1|:7831-8510
MsHel-1	gb|AIXA01016666.1|:8203-8892
MsHel-1	gb|AIXA01012479.1|:1319-2020
MsHel-1	gb|AIXA01012479.1|:20880-21092
MsHel-1	gb|AIXA01012352.1|:1-589
MsHel-1	gb|AIXA01009550.1|:31627-32905
MsHel-1	gb|AIXA01006390.1|:22978-23938
MsHel-1	gb|AIXA01004731.1|:34537-34635
MsHel-1	gb|AIXA01002308.1|:43744-44445
MsHel-1	gb|AIXA01000139.1|:12898-13395
MsHel-1	gb|AIXA01009548.1|:26683-26835
MsHel-1	gb|AIXA01006714.1|:41653-42358
MsHel-1	gb|AIXA01006700.1|:18014-18718
MsHel-1	gb|AIXA01006163.1|:17271-18109
MsHel-1	gb|AIXA01013773.1|:2631-3411
MsHel-1	gb|AIXA01009697.1|:17085-17926

MsHel-1	gb|AIXA01005009.1|:6806-7303
MsHel-1	gb|AIXA01005620.1|:9768-10582
MsHel-1	gb|AIXA01004810.1|:2535-3031
MsHel-1	gb|AIXA01003478.1|:20540-22113
MsHel-1	gb|AIXA01002488.1|:1756-2453
MsHel-1	gb|AIXA01013154.1|:1090-2127
MsHel-1	gb|AIXA01013154.1|:12772-13093
MsHel-1	gb|AIXA01011530.1|:1142-1848
MsHel-1	gb|AIXA01003105.1|:9109-9949
MsHel-1	gb|AIXA01030097.1|:1-746
MsHel-1	gb|AIXA01011285.1|:118429-119245
MsHel-1	gb|AIXA01010176.1|:2091-2785
MsHel-1	gb|AIXA01001968.1|:78520-78954
MsHel-1	gb|AIXA01005388.1|:44385-45086
MsHel-1	gb|AIXA01002551.1|:39003-39392
MsHel-1	gb|AIXA01002551.1|:89339-90043
MsHel-1	gb|AIXA01009218.1|:5846-6645
MsHel-1	gb|AIXA01003191.1|:3643-4730
MsHel-1	gb|AIXA01002827.1|:6653-7983
MsHel-1	gb|AIXA01001239.1|:50250-50893
MsHel-1	gb|AIXA01001039.1|:14902-15746
MsHel-1	gb|AIXA01009786.1|:41583-42551
MsHel-1	gb|AIXA01006930.1|:6417-7082
MsHel-1	gb|AIXA01006396.1|:88243-89332
MsHel-1	gb|AIXA01004747.1|:25893-26858
MsHel-1	gb|AIXA01001250.1|:286-835
MsHel-1	gb|AIXA01014992.1|:9406-10370
MsHel-1	gb|AIXA01013383.1|:14723-14850
MsHel-1	gb|AIXA01004251.1|:1409-1905
MsHel-1	gb|AIXA01003750.1|:232-1072
MsHel-1	gb|AIXA01011035.1|:187-1027
MsHel-1	gb|AIXA01006671.1|:21017-21716
MsHel-1	gb|AIXA01006666.1|:2848-4712
MsHel-1	gb|AIXA01000827.1|:144010-144099
MsHel-1	gb|AIXA01010302.1|:14-511
MsHel-1	gb|AIXA01003931.1|:6726-7432
MsHel-1	gb|AIXA01001289.1|:14249-14945
MsHel-1	gb|AIXA01013026.1|:8798-9887
MsHel-1	gb|AIXA01007499.1|:22169-22458
MsHel-1	gb|AIXA01007499.1|:33635-34340
MsHel-1	gb|AIXA01007216.1|:14792-15127
MsHel-1	gb|AIXA01007216.1|:26183-32182
MsHel-1	gb|AIXA01000801.1|:25452-34230
MsHel-1	gb|AIXA01014694.1|:10351-11052
MsHel-1	gb|AIXA01007111.1|:3977-4257
MsHel-1	gb|AIXA01007111.1|:32982-33691
MsHel-1	gb|AIXA01005601.1|:18355-19217
MsHel-1	gb|AIXA01002248.1|:72790-73486

MsHel-1	gb|AIXA01002248.1|:101135-101291
MsHel-1	gb|AIXA01001771.1|:140861-141592
MsHel-1	gb|AIXA01003431.1|:11011-11847
MsHel-1	gb|AIXA01010482.1|:897-1049
MsHel-1	gb|AIXA01008674.1|:8065-9130
MsHel-1	gb|AIXA01007524.1|:7325-7488
MsHel-1	gb|AIXA01004579.1|:39307-40004
MsHel-1	gb|AIXA01003288.1|:13144-13744
MsHel-1	gb|AIXA01003288.1|:114172-114706
MsHel-1	gb|AIXA01001690.1|:13327-14016
MsHel-1	gb|AIXA01029834.1|:1-681
MsHel-1	gb|AIXA01015575.1|:11851-12693
MsHel-1	gb|AIXA01008406.1|:53530-54219
MsHel-1	gb|AIXA01008406.1|:157885-161631
MsHel-1	gb|AIXA01004685.1|:23162-24881
MsHel-1	gb|AIXA01016852.1|:1978-2786
MsHel-1	gb|AIXA01008825.1|:555-1249
MsHel-1	gb|AIXA01008030.1|:2-182
MsHel-1	gb|AIXA01007527.1|:60490-61190
MsHel-1	gb|AIXA01001642.1|:15796-16636
MsHel-1	gb|AIXA01000707.1|:18986-19905
MsHel-1	gb|AIXA01016850.1|:2375-3066
MsHel-1	gb|AIXA01015261.1|:3643-4476
MsHel-1	gb|AIXA01006935.1|:6128-7092
MsHel-1	gb|AIXA01007991.1|:23420-24122
MsHel-1	gb|AIXA01007032.1|:33393-34087
MsHel-1	gb|AIXA01007032.1|:45143-46067
MsHel-1	gb|AIXA01006659.1|:3265-3966
MsHel-1	gb|AIXA01006659.1|:20140-21707
MsHel-1	gb|AIXA01000791.1|:8431-9308
MsHel-1	gb|AIXA01008253.1|:6451-7409
MsHel-1	gb|AIXA01007926.1|:384-589
MsHel-1	gb|AIXA01001384.1|:40706-41520
MsHel-1	gb|AIXA01000998.1|:25042-25745
MsHel-1	gb|AIXA01023644.1|:1-1015
MsHel-1	gb|AIXA01012668.1|:6390-6809
MsHel-1	gb|AIXA01011321.1|:1682-2324
MsHel-1	gb|AIXA01003173.1|:40957-41821
MsHel-1	gb|AIXA01002726.1|:26452-27148
MsHel-1	gb|AIXA01002726.1|:39974-40056
MsHel-1	gb|AIXA01012510.1|:9612-10429
MsHel-1	gb|AIXA01010110.1|:37539-38510
MsHel-1	gb|AIXA01009505.1|:1-683
MsHel-1	gb|AIXA01005920.1|:40620-41386
MsHel-1	gb|AIXA01005525.1|:3-451
MsHel-1	gb|AIXA01005525.1|:22695-22826
MsHel-1	gb|AIXA01005094.1|:2196-3765
MsHel-1	gb|AIXA01004635.1|:2373-3890

MsHel-1	gb|AIXA01000201.1|:73978-77315
MsHel-1	gb|AIXA01030589.1|:1-726
MsHel-1	gb|AIXA01002128.1|:24660-25015
MsHel-1	gb|AIXA01000212.1|:69151-69993
MsHel-1	gb|AIXA01006392.1|:2-493
MsHel-1	gb|AIXA01006392.1|:12502-13489
MsHel-1	gb|AIXA01006202.1|:7218-8297
MsHel-1	gb|AIXA01000955.1|:13944-14648
MsHel-1	gb|AIXA01013142.1|:11947-12660
MsHel-1	gb|AIXA01001869.1|:22137-22945
MsHel-1	gb|AIXA01006893.1|:34318-35015
MsHel-1	gb|AIXA01001866.1|:26590-27298
MsHel-1	gb|AIXA01001190.1|:2290-3039
MsHel-1	gb|AIXA01005053.1|:7041-7698
MsHel-1	gb|AIXA01004479.1|:8718-9399
MsHel-1	gb|AIXA01002372.1|:6356-7061
MsHel-1	gb|AIXA01009115.1|:13974-15064
MsHel-1	gb|AIXA01007711.1|:68372-68458
MsHel-1	gb|AIXA01005593.1|:11326-12102
MsHel-1	gb|AIXA01012784.1|:13869-14705
MsHel-1	gb|AIXA01001271.1|:78344-78923
MsHel-1	gb|AIXA01012494.1|:183-990
MsHel-1	gb|AIXA01009372.1|:4346-5050
MsHel-1	gb|AIXA01007247.1|:16455-17231
MsHel-1	gb|AIXA01007164.1|:56086-56791
MsHel-1	gb|AIXA01006876.1|:37745-38409
MsHel-1	gb|AIXA01004314.1|:922-1627
MsHel-1	gb|AIXA01004306.1|:1577-3261
MsHel-1	gb|AIXA01003798.1|:25830-26458
MsHel-1	gb|AIXA01002349.1|:20813-21652
MsHel-1	gb|AIXA01002057.1|:12012-12915
MsHel-1	gb|AIXA01002057.1|:32096-32682
MsHel-1	gb|AIXA01010076.1|:54145-54957
MsHel-1	gb|AIXA01004096.1|:4067-4827
MsHel-1	gb|AIXA01004045.1|:6437-6750
MsHel-1	gb|AIXA01001066.1|:168-586
MsHel-1	gb|AIXA01001066.1|:20856-21819
MsHel-1	gb|AIXA01030374.1|:4-645
MsHel-1	gb|AIXA01013331.1|:21225-21927
MsHel-1	gb|AIXA01006824.1|:21426-22641
MsHel-1	gb|AIXA01004260.1|:43536-43696
MsHel-1	gb|AIXA01004260.1|:63245-63373
MsHel-1	gb|AIXA01000718.1|:13315-13538
MsHel-1	gb|AIXA01000718.1|:29876-30587
MsHel-1	gb|AIXA01000226.1|:1-718
MsHel-1	gb|AIXA01014394.1|:5081-6022
MsHel-1	gb|AIXA01002665.1|:15775-16418
MsHel-1	gb|AIXA01016066.1|:3294-3995

MsHel-1	gb|AIXA01009811.1|:7700-8408
MsHel-1	gb|AIXA01007517.1|:1-700
MsHel-1	gb|AIXA01003970.1|:40795-41017
MsHel-1	gb|AIXA01013044.1|:1764-2705
MsHel-1	gb|AIXA01009949.1|:2307-2999
MsHel-1	gb|AIXA01008850.1|:1799-2810
MsHel-1	gb|AIXA01001225.1|:7544-8180
MsHel-1	gb|AIXA01009133.1|:30599-31184
MsHel-1	gb|AIXA01003041.1|:3414-4764
MsHel-1	gb|AIXA01002460.1|:1127-1831
MsHel-1	gb|AIXA01007493.1|:30226-37477
MsHel-1	gb|AIXA01003939.1|:3199-4001
MsHel-1	gb|AIXA01003939.1|:14099-14277
MsHel-1	gb|AIXA01007058.1|:82-786
MsHel-1	gb|AIXA01007058.1|:27001-27274
MsHel-1	gb|AIXA01006474.1|:1177-1302
MsHel-1	gb|AIXA01006474.1|:25117-25393
MsHel-1	gb|AIXA01006474.1|:46257-47320
MsHel-1	gb|AIXA01001721.1|:3638-4843
MsHel-1	gb|AIXA01001123.1|:16634-16832
MsHel-1	gb|AIXA01017979.1|:5999-6703
MsHel-1	gb|AIXA01011234.1|:8162-8866
MsHel-1	gb|AIXA01010772.1|:2277-2764
MsHel-1	gb|AIXA01008791.1|:2-180
MsHel-1	gb|AIXA01005782.1|:18046-18147
MsHel-1	gb|AIXA01003682.1|:58132-58312
MsHel-1	gb|AIXA01003334.1|:9372-10437
MsHel-1	gb|AIXA01002990.1|:26510-26834
MsHel-1	gb|AIXA01002620.1|:3600-4305
MsHel-1	gb|AIXA01017274.1|:1853-2566
MsHel-1	gb|AIXA01010976.1|:1521-1636
MsHel-1	gb|AIXA01007579.1|:24768-24895
MsHel-1	gb|AIXA01007579.1|:64212-64901
MsHel-1	gb|AIXA01007429.1|:1605-2926
MsHel-1	gb|AIXA01007179.1|:1-694
MsHel-1	gb|AIXA01007179.1|:27430-27665
MsHel-1	gb|AIXA01005020.1|:18758-19116
MsHel-1	gb|AIXA01012649.1|:3413-4632
MsHel-1	gb|AIXA01012649.1|:15312-15618
MsHel-1	gb|AIXA01011754.1|:23726-24600
MsHel-1	gb|AIXA01005356.1|:16581-17637
MsHel-1	gb|AIXA01000136.1|:1068-2010
MsHel-1	gb|AIXA01008531.1|:1-440
MsHel-1	gb|AIXA01003712.1|:12751-13463
MsHel-1	gb|AIXA01008416.1|:11917-12744
MsHel-1	gb|AIXA01006482.1|:31898-32593
MsHel-1	gb|AIXA01006042.1|:10057-14555
MsHel-1	gb|AIXA01010551.1|:7782-8824

MsHel-1	gb|AIXA01010480.1|:28530-29219
MsHel-1	gb|AIXA01010396.1|:14009-15005
MsHel-1	gb|AIXA01006509.1|:46926-47631
MsHel-1	gb|AIXA01003982.1|:9481-10185
MsHel-1	gb|AIXA01027440.1|:171-868
MsHel-1	gb|AIXA01018149.1|:10607-11195
MsHel-1	gb|AIXA01012024.1|:23348-25059
MsHel-1	gb|AIXA01002523.1|:10336-11025
MsHel-1	gb|AIXA01001296.1|:187452-188048
MsHel-1	gb|AIXA01001296.1|:214547-215127
MsHel-1	gb|AIXA01001258.1|:14145-14859
MsHel-1	gb|AIXA01006856.1|:4101-4597
MsHel-1	gb|AIXA01017031.1|:4732-6032
MsHel-1	gb|AIXA01011535.1|:9083-9259
MsHel-1	gb|AIXA01007320.1|:16705-17293
MsHel-1	gb|AIXA01006854.1|:71253-71964
MsHel-1	gb|AIXA01004411.1|:27214-27904
MsHel-1	gb|AIXA01004389.1|:2924-3542
MsHel-1	gb|AIXA01007053.1|:61521-62396
MsHel-1	gb|AIXA01002429.1|:1-462
MsHel-1	gb|AIXA01007270.1|:20452-21140
MsHel-1	gb|AIXA01004504.1|:7343-8061
MsHel-1	gb|AIXA01013664.1|:7298-7773
MsHel-1	gb|AIXA01013454.1|:3897-4610
MsHel-1	gb|AIXA01005664.1|:10221-10806
MsHel-1	gb|AIXA01005664.1|:30605-31071
MsHel-1	gb|AIXA01001397.1|:1-628
MsHel-1	gb|AIXA01001098.1|:26924-27999
MsHel-1	gb|AIXA01000874.1|:63093-63809
MsHel-1	gb|AIXA01014587.1|:28155-28743
MsHel-1	gb|AIXA01006621.1|:8240-8917
MsHel-1	gb|AIXA01006413.1|:18989-19568
MsHel-1	gb|AIXA01007686.1|:11056-11819
MsHel-1	gb|AIXA01008046.1|:36889-37602
MsHel-1	gb|AIXA01002942.1|:935-1499
MsHel-1	gb|AIXA01001294.1|:47229-47941
MsHel-1	gb|AIXA01000237.1|:33715-33982
MsHel-1	gb|AIXA01000237.1|:80678-81391
MsHel-1	gb|AIXA01000099.1|:27584-27738
MsHel-1	gb|AIXA01000157.1|:274-975
MsHel-1	gb|AIXA01015050.1|:1122-1708
MsHel-1	gb|AIXA01012485.1|:15502-16466
MsHel-1	gb|AIXA01011969.1|:39687-40440
MsHel-1	gb|AIXA01012119.1|:39508-40221
MsHel-1	gb|AIXA01007417.1|:1626-2367
MsHel-1	gb|AIXA01003673.1|:54005-61559
MsHel-1	gb|AIXA01001221.1|:35902-36614
MsHel-1	gb|AIXA01000868.1|:11145-11979

MsHel-1	gb|AIXA01000868.1|:26692-26908
MsHel-1	gb|AIXA01015777.1|:13221-13807
MsHel-1	gb|AIXA01012246.1|:1-659
MsHel-1	gb|AIXA01006174.1|:15171-15758
MsHel-1	gb|AIXA01000214.1|:6155-6870
MsHel-1	gb|AIXA01000214.1|:22343-23072
MsHel-1	gb|AIXA01018206.1|:1-620
MsHel-1	gb|AIXA01007453.1|:6587-6860
MsHel-1	gb|AIXA01005608.1|:9853-10737
MsHel-1	gb|AIXA01003614.1|:98397-98486
MsHel-1	gb|AIXA01002581.1|:8203-8912
MsHel-1	gb|AIXA01001798.1|:39962-40655
MsHel-1	gb|AIXA01001798.1|:64165-64478
MsHel-1	gb|AIXA01009515.1|:35454-36040
MsHel-1	gb|AIXA01005951.1|:1-644
MsHel-1	gb|AIXA01005951.1|:40197-40293
MsHel-1	gb|AIXA01004671.1|:2415-3001
MsHel-1	gb|AIXA01003881.1|:44472-45058
MsHel-1	gb|AIXA01001962.1|:58179-58644
MsHel-1	gb|AIXA01004112.1|:19518-19605
MsHel-1	gb|AIXA01015893.1|:1238-1853
MsHel-1	gb|AIXA01010431.1|:12-616
MsHel-1	gb|AIXA01001041.1|:9643-10229
MsHel-1	gb|AIXA01000809.1|:36885-37600
MsHel-1	gb|AIXA01000809.1|:121053-121938
MsHel-1	gb|AIXA01007598.1|:90387-90585
MsHel-1	gb|AIXA01004659.1|:19867-20765
MsHel-1	gb|AIXA01003692.1|:2637-3223
MsHel-1	gb|AIXA01010627.1|:3-666
MsHel-1	gb|AIXA01023218.1|:2-637
MsHel-1	gb|AIXA01013438.1|:2242-2830
MsHel-1	gb|AIXA01006967.1|:20282-20991
MsHel-1	gb|AIXA01015049.1|:16584-16983
MsHel-1	gb|AIXA01014928.1|:147-862
MsHel-1	gb|AIXA01005217.1|:24137-24752
MsHel-1	gb|AIXA01003182.1|:6808-7425
MsHel-1	gb|AIXA01002068.1|:33819-34875
MsHel-1	gb|AIXA01001483.1|:66777-67196
MsHel-1	gb|AIXA01001029.1|:7771-8604
MsHel-1	gb|AIXA01015281.1|:31139-31725
MsHel-1	gb|AIXA01010291.1|:8190-8830
MsHel-1	gb|AIXA01008376.1|:9934-10539
MsHel-1	gb|AIXA01003265.1|:4946-5836
MsHel-1	gb|AIXA01013340.1|:1-83
MsHel-1	gb|AIXA01013340.1|:22068-22584
MsHel-1	gb|AIXA01005227.1|:16235-16813
MsHel-1	gb|AIXA01004990.1|:73514-74781
MsHel-1	gb|AIXA01013433.1|:308-1139

MsHel-1	gb|AIXA01012684.1|:4096-4808
MsHel-1	gb|AIXA01007208.1|:8168-8752
MsHel-1	gb|AIXA01002552.1|:35697-35818
MsHel-1	gb|AIXA01002552.1|:78690-79434
MsHel-1	gb|AIXA01002189.1|:1-588
MsHel-1	gb|AIXA01017537.1|:3193-4011
MsHel-1	gb|AIXA01011244.1|:30517-31104
MsHel-1	gb|AIXA01004831.1|:77015-77598
MsHel-1	gb|AIXA01001017.1|:53051-54131
MsHel-1	gb|AIXA01008393.1|:25001-25588
MsHel-1	gb|AIXA01003294.1|:19024-19611
MsHel-1	gb|AIXA01008097.1|:9020-9604
MsHel-1	gb|AIXA01007009.1|:1-740
MsHel-1	gb|AIXA01006236.1|:8834-11332
MsHel-1	gb|AIXA01001261.1|:3282-3993
MsHel-1	gb|AIXA01021551.1|:548-1134
MsHel-1	gb|AIXA01009590.1|:55609-55797
MsHel-1	gb|AIXA01009489.1|:39611-40997
MsHel-1	gb|AIXA01009191.1|:1530-2116
MsHel-1	gb|AIXA01007197.1|:6705-7292
MsHel-1	gb|AIXA01006182.1|:13394-13515
MsHel-1	gb|AIXA01006143.1|:4872-5366
MsHel-1	gb|AIXA01005555.1|:1-395
MsHel-1	gb|AIXA01002846.1|:67600-68187
MsHel-1	gb|AIXA01002747.1|:74100-74705
MsHel-1	gb|AIXA01012635.1|:4637-4763
MsHel-1	gb|AIXA01008827.1|:3074-3538
MsHel-1	gb|AIXA01006932.1|:839-1548
MsHel-1	gb|AIXA01012434.1|:76632-77217
MsHel-1	gb|AIXA01009101.1|:10875-11219
MsHel-1	gb|AIXA01009101.1|:21269-22353
MsHel-1	gb|AIXA01009101.1|:44785-44958
MsHel-1	gb|AIXA01012884.1|:298-885
MsHel-1	gb|AIXA01009112.1|:16478-17065
MsHel-1	gb|AIXA01002658.1|:67249-67422
MsHel-1	gb|AIXA01018750.1|:1-652
MsHel-1	gb|AIXA01010658.1|:25290-25978
MsHel-1	gb|AIXA01005430.1|:3118-3689
MsHel-1	gb|AIXA01002924.1|:6380-7101
MsHel-1	gb|AIXA01000713.1|:56779-56910
MsHel-1	gb|AIXA01008752.1|:976-1588
MsHel-1	gb|AIXA01006161.1|:2178-2759
MsHel-1	gb|AIXA01005029.1|:30888-31597
MsHel-1	gb|AIXA01004291.1|:33798-34583
MsHel-1	gb|AIXA01001594.1|:86638-87223
MsHel-1	gb|AIXA01016642.1|:1-623
MsHel-1	gb|AIXA01011654.1|:3376-3566
MsHel-1	gb|AIXA01011313.1|:1-610

MsHel-1	gb|AIXA01010744.1|:11701-12413
MsHel-1	gb|AIXA01007454.1|:13128-13845
MsHel-1	gb|AIXA01007241.1|:106924-107590
MsHel-1	gb|AIXA01003569.1|:2257-2837
MsHel-1	gb|AIXA01002127.1|:20276-20892
MsHel-1	gb|AIXA01002127.1|:66494-66657
MsHel-1	gb|AIXA01001551.1|:28502-29210
MsHel-1	gb|AIXA01013430.1|:1896-2091
MsHel-1	gb|AIXA01009408.1|:1342-2564
MsHel-1	gb|AIXA01005578.1|:28935-29745
MsHel-1	gb|AIXA01003631.1|:28118-28833
MsHel-1	gb|AIXA01016253.1|:1696-2273
MsHel-1	gb|AIXA01012549.1|:29764-30584
MsHel-1	gb|AIXA01012549.1|:62529-62672
MsHel-1	gb|AIXA01009329.1|:1260-1847
MsHel-1	gb|AIXA01002560.1|:41240-41530
MsHel-1	gb|AIXA01013903.1|:1014-1692
MsHel-1	gb|AIXA01010336.1|:3938-4521
MsHel-1	gb|AIXA01007293.1|:370-1752
MsHel-1	gb|AIXA01004937.1|:33127-33712
MsHel-1	gb|AIXA01004595.1|:50282-50964
MsHel-1	gb|AIXA01000405.1|:17990-18574
MsHel-1	gb|AIXA01013441.1|:3672-4519
MsHel-1	gb|AIXA01006552.1|:2689-3274
MsHel-1	gb|AIXA01002705.1|:28338-28625
MsHel-1	gb|AIXA01000683.1|:5082-5673
MsHel-1	gb|AIXA01034064.1|:1-530
MsHel-1	gb|AIXA01009343.1|:33630-34331
MsHel-1	gb|AIXA01008573.1|:759-1474
MsHel-1	gb|AIXA01023476.1|:749-1380
MsHel-1	gb|AIXA01001085.1|:44224-44767
MsHel-1	gb|AIXA01017897.1|:7114-7700
MsHel-1	gb|AIXA01008796.1|:1-568
MsHel-1	gb|AIXA01010500.1|:7328-8106
MsHel-1	gb|AIXA01009508.1|:53956-54662
MsHel-1	gb|AIXA01009102.1|:7953-9020
MsHel-1	gb|AIXA01007691.1|:34288-34474
MsHel-1	gb|AIXA01003052.1|:80210-80975
MsHel-1	gb|AIXA01001953.1|:9373-9945
MsHel-1	gb|AIXA01001144.1|:115962-116685
MsHel-1	gb|AIXA01010003.1|:44974-45750
MsHel-1	gb|AIXA01001787.1|:38687-39383
MsHel-1	gb|AIXA01006710.1|:1-580
MsHel-1	gb|AIXA01007355.1|:16208-16798
MsHel-1	gb|AIXA01007044.1|:4-603
MsHel-1	gb|AIXA01003358.1|:4991-5578
MsHel-1	gb|AIXA01002405.1|:7743-8318
MsHel-1	gb|AIXA01001237.1|:17793-18296

MsHel-1	gb|AIXA01011840.1|:5718-6579
MsHel-1	gb|AIXA01007547.1|:2011-2597
MsHel-1	gb|AIXA01007023.1|:51041-51627
MsHel-1	gb|AIXA01005582.1|:5483-6069
MsHel-1	gb|AIXA01005479.1|:567-652
MsHel-1	gb|AIXA01005479.1|:24013-24696
MsHel-1	gb|AIXA01002105.1|:9669-10337
MsHel-1	gb|AIXA01034135.1|:53-629
MsHel-1	gb|AIXA01007920.1|:8257-8416
MsHel-1	gb|AIXA01005892.1|:44465-44560
MsHel-1	gb|AIXA01005892.1|:68122-68695
MsHel-1	gb|AIXA01012188.1|:5342-6616
MsHel-1	gb|AIXA01009230.1|:20244-21241
MsHel-1	gb|AIXA01018681.1|:5550-6214
MsHel-1	gb|AIXA01009235.1|:31149-39411
MsHel-1	gb|AIXA01009235.1|:49598-50083
MsHel-1	gb|AIXA01006620.1|:8173-9313
MsHel-1	gb|AIXA01016192.1|:2150-2736
MsHel-1	gb|AIXA01012554.1|:20-522
MsHel-1	gb|AIXA01012486.1|:3156-3866
MsHel-1	gb|AIXA01003107.1|:1-577
MsHel-1	gb|AIXA01005595.1|:70618-71302
MsHel-1	gb|AIXA01005521.1|:129168-129665
MsHel-1	gb|AIXA01004092.1|:36849-37428
MsHel-1	gb|AIXA01015447.1|:3799-4342
MsHel-1	gb|AIXA01001481.1|:18450-19040
MsHel-1	gb|AIXA01012711.1|:40933-41518
MsHel-1	gb|AIXA01009857.1|:19442-20018
MsHel-1	gb|AIXA01008997.1|:1-572
MsHel-1	gb|AIXA01008481.1|:15951-16413
MsHel-1	gb|AIXA01001978.1|:40863-41862
MsHel-1	gb|AIXA01000904.1|:52586-53261
MsHel-1	gb|AIXA01035056.1|:1-537
MsHel-1	gb|AIXA01012310.1|:4005-4498
MsHel-1	gb|AIXA01008366.1|:1006-1603
MsHel-1	gb|AIXA01002796.1|:28724-29774
MsHel-1	gb|AIXA01015776.1|:1-729
MsHel-1	gb|AIXA01004941.1|:56804-57300
MsHel-1	gb|AIXA01001740.1|:18340-18903
MsHel-1	gb|AIXA01001265.1|:14953-15531
MsHel-1	gb|AIXA01000046.1|:25913-26016
MsHel-1	gb|AIXA01008118.1|:5867-6419
MsHel-1	gb|AIXA01007033.1|:52935-53092
MsHel-1	gb|AIXA01001685.1|:64317-64530
MsHel-1	gb|AIXA01010099.1|:44064-44774
MsHel-1	gb|AIXA01009971.1|:2147-2852
MsHel-1	gb|AIXA01009596.1|:8864-9443
MsHel-1	gb|AIXA01007509.1|:3697-3841

MsHel-1	gb|AIXA01007509.1|:34696-34796
MsHel-1	gb|AIXA01003684.1|:1-534
MsHel-1	gb|AIXA01027472.1|:200-788
MsHel-1	gb|AIXA01015125.1|:14-533
MsHel-1	gb|AIXA01008409.1|:48492-49673
MsHel-1	gb|AIXA01006853.1|:43058-43633
MsHel-1	gb|AIXA01006815.1|:35717-36204
MsHel-1	gb|AIXA01009556.1|:1617-2200
MsHel-1	gb|AIXA01003800.1|:72708-73363
MsHel-1	gb|AIXA01020519.1|:1-505
MsHel-1	gb|AIXA01011711.1|:1-748
MsHel-1	gb|AIXA01006115.1|:30874-31437
MsHel-1	gb|AIXA01007435.1|:51012-51580
MsHel-1	gb|AIXA01004956.1|:2-1810
MsHel-1	gb|AIXA01001932.1|:1-96
MsHel-1	gb|AIXA01008266.1|:14313-15123
MsHel-1	gb|AIXA01003335.1|:3125-3622
MsHel-1	gb|AIXA01002213.1|:28200-28922
MsHel-1	gb|AIXA01002178.1|:13237-13496
MsHel-1	gb|AIXA01002178.1|:29243-29835
MsHel-1	gb|AIXA01010883.1|:5415-5922
MsHel-1	gb|AIXA01000654.1|:363-948
MsHel-1	gb|AIXA01000654.1|:45574-45701
MsHel-1	gb|AIXA01018067.1|:1316-1898
MsHel-1	gb|AIXA01002784.1|:29607-29751
MsHel-1	gb|AIXA01002022.1|:609-1210
MsHel-1	gb|AIXA01000224.1|:25266-25972
MsHel-1	gb|AIXA01018823.1|:1-565
MsHel-1	gb|AIXA01015778.1|:4-505
MsHel-1	gb|AIXA01008533.1|:20860-21040
MsHel-1	gb|AIXA01007801.1|:13679-14395
MsHel-1	gb|AIXA01007801.1|:65252-65818
MsHel-1	gb|AIXA01005864.1|:51114-51696
MsHel-1	gb|AIXA01001757.1|:10016-10598
MsHel-1	gb|AIXA01001757.1|:95172-95633
MsHel-1	gb|AIXA01029270.1|:1-466
MsHel-1	gb|AIXA01000844.1|:26635-27107
MsHel-1	gb|AIXA01000844.1|:38774-39361
MsHel-1	gb|AIXA01015321.1|:25316-25407
MsHel-1	gb|AIXA01000831.1|:49116-50023
MsHel-1	gb|AIXA01010552.1|:21308-21388
MsHel-1	gb|AIXA01008104.1|:23733-24423
MsHel-1	gb|AIXA01000285.1|:19886-20307
MsHel-1	gb|AIXA01004690.1|:1-554
MsHel-1	gb|AIXA01002928.1|:7812-8315
MsHel-1	gb|AIXA01000470.1|:1-541
MsHel-1	gb|AIXA01010902.1|:22025-22606
MsHel-1	gb|AIXA01009634.1|:3-716

MsHel-1	gb|AIXA01009214.1|:3935-4781
MsHel-1	gb|AIXA01009214.1|:35532-36275
MsHel-1	gb|AIXA01025168.1|:1059-1257
MsHel-1	gb|AIXA01010082.1|:1049-1623
MsHel-1	gb|AIXA01009936.1|:14528-14911
MsHel-1	gb|AIXA01004697.1|:62796-63382
MsHel-1	gb|AIXA01029209.1|:1-544
MsHel-1	gb|AIXA01009354.1|:3697-4377
MsHel-1	gb|AIXA01008028.1|:23228-23809
MsHel-1	gb|AIXA01000521.1|:93359-93935
MsHel-1	gb|AIXA01013393.1|:41-636
MsHel-1	gb|AIXA01009324.1|:4036-4662
MsHel-1	gb|AIXA01003320.1|:22030-22615
MsHel-1	gb|AIXA01031267.1|:1-561
MsHel-1	gb|AIXA01014272.1|:3081-5547
MsHel-1	gb|AIXA01018577.1|:1261-1812
MsHel-1	gb|AIXA01009699.1|:20516-21672
MsHel-1	gb|AIXA01005903.1|:40768-41289
MsHel-1	gb|AIXA01005903.1|:75378-75718
MsHel-1	gb|AIXA01011563.1|:2-504
MsHel-1	gb|AIXA01003330.1|:168132-168628
MsHel-1	gb|AIXA01001658.1|:1-646
MsHel-1	gb|AIXA01001658.1|:67499-67794
MsHel-1	gb|AIXA01016433.1|:10783-11376
MsHel-1	gb|AIXA01012702.1|:1-529
MsHel-1	gb|AIXA01010058.1|:5315-6552
MsHel-1	gb|AIXA01009765.1|:27353-28172
MsHel-1	gb|AIXA01007608.1|:11179-11770
MsHel-1	gb|AIXA01005253.1|:2-488
MsHel-1	gb|AIXA01011508.1|:5355-6168
MsHel-1	gb|AIXA01008824.1|:25782-26377
MsHel-1	gb|AIXA01000003.1|:31509-32004
MsHel-1	gb|AIXA01000003.1|:63467-64129
MsHel-1	gb|AIXA01014277.1|:2198-3145
MsHel-1	gb|AIXA01009564.1|:2-499
MsHel-1	gb|AIXA01007832.1|:6151-6647
MsHel-1	gb|AIXA01003301.1|:1904-3207
MsHel-1	gb|AIXA01008475.1|:31368-31886
MsHel-1	gb|AIXA01000533.1|:2528-3017
MsHel-1	gb|AIXA01003028.1|:13533-14460
MsHel-1	gb|AIXA01001496.1|:56746-57306
MsHel-1	gb|AIXA01026967.1|:1-516
MsHel-1	gb|AIXA01013947.1|:32490-33064
MsHel-1	gb|AIXA01008115.1|:4060-4556
MsHel-1	gb|AIXA01006123.1|:4922-5471
MsHel-1	gb|AIXA01004629.1|:20014-20957
MsHel-1	gb|AIXA01002683.1|:5771-5973
MsHel-1	gb|AIXA01008053.1|:17901-18364

MsHel-1	gb|AIXA01007163.1|:18908-19479
MsHel-1	gb|AIXA01007067.1|:13540-14208
MsHel-1	gb|AIXA01006638.1|:1993-2457
MsHel-1	gb|AIXA01006420.1|:41511-42080
MsHel-1	gb|AIXA01005766.1|:52706-52876
MsHel-1	gb|AIXA01004445.1|:5045-5541
MsHel-1	gb|AIXA01002989.1|:10800-11392
MsHel-1	gb|AIXA01001797.1|:389850-390274
MsHel-1	gb|AIXA01000531.1|:8003-8212
MsHel-1	gb|AIXA01000531.1|:50062-50182
MsHel-1	gb|AIXA01008520.1|:19450-20043
MsHel-1	gb|AIXA01023639.1|:1-472
MsHel-1	gb|AIXA01015729.1|:3-604
MsHel-1	gb|AIXA01012616.1|:2076-2833
MsHel-1	gb|AIXA01002196.1|:10412-10493
MsHel-1	gb|AIXA01006795.1|:1-603
MsHel-1	gb|AIXA01006795.1|:90537-90747
MsHel-1	gb|AIXA01000444.1|:10715-11500
MsHel-1	gb|AIXA01000444.1|:35772-36099
MsHel-1	gb|AIXA01000436.1|:16595-17074
MsHel-1	gb|AIXA01008398.1|:35699-36183
MsHel-1	gb|AIXA01007304.1|:28920-29594
MsHel-1	gb|AIXA01007217.1|:38169-39299
MsHel-1	gb|AIXA01005725.1|:1-611
MsHel-1	gb|AIXA01002870.1|:156398-156554
MsHel-1	gb|AIXA01026525.1|:468-1047
MsHel-1	gb|AIXA01010511.1|:3-531
MsHel-1	gb|AIXA01009603.1|:16729-17324
MsHel-1	gb|AIXA01008544.1|:15433-15979
MsHel-1	gb|AIXA01007283.1|:36976-37549
MsHel-1	gb|AIXA01004475.1|:8541-9134
MsHel-1	gb|AIXA01031493.1|:1-480
MsHel-1	gb|AIXA01002811.1|:8572-9166
MsHel-1	gb|AIXA01001077.1|:388-961
MsHel-1	gb|AIXA01000429.1|:1-483
MsHel-1	gb|AIXA01009984.1|:14834-14960
MsHel-1	gb|AIXA01009624.1|:16033-16233
MsHel-1	gb|AIXA01023426.1|:5-507
MsHel-1	gb|AIXA01006235.1|:4568-5062
MsHel-1	gb|AIXA01005448.1|:41358-42156
MsHel-1	gb|AIXA01004727.1|:53134-53986
MsHel-1	gb|AIXA01014447.1|:17256-18120
MsHel-1	gb|AIXA01013752.1|:132-726
MsHel-1	gb|AIXA01003166.1|:23776-24363
MsHel-1	gb|AIXA01002740.1|:22200-22725
MsHel-1	gb|AIXA01001877.1|:47016-47692
MsHel-1	gb|AIXA01010917.1|:2-667
MsHel-1	gb|AIXA01017720.1|:15694-16286

MsHel-1	gb|AIXA01014187.1|:1-504
MsHel-1	gb|AIXA01011484.1|:17735-18198
MsHel-1	gb|AIXA01024053.1|:275-870
MsHel-1	gb|AIXA01011512.1|:3-484
MsHel-1	gb|AIXA01004335.1|:88832-89035
MsHel-1	gb|AIXA01008894.1|:16667-17153
MsHel-1	gb|AIXA01008738.1|:12933-13960
MsHel-1	gb|AIXA01007790.1|:33823-34320
MsHel-1	gb|AIXA01006162.1|:24957-25552
MsHel-1	gb|AIXA01004774.1|:33640-34148
MsHel-1	gb|AIXA01003868.1|:25776-26265
MsHel-1	gb|AIXA01001924.1|:4510-4688
MsHel-1	gb|AIXA01001558.1|:22957-23071
MsHel-1	gb|AIXA01001558.1|:92999-93512
MsHel-1	gb|AIXA01026951.1|:102-699
MsHel-1	gb|AIXA01016590.1|:9719-10155
MsHel-1	gb|AIXA01016384.1|:3841-4410
MsHel-1	gb|AIXA01007486.1|:41793-42731
MsHel-1	gb|AIXA01004996.1|:51558-52233
MsHel-1	gb|AIXA01004761.1|:9839-10433
MsHel-1	gb|AIXA01004634.1|:10007-10124
MsHel-1	gb|AIXA01004634.1|:22815-23051
MsHel-1	gb|AIXA01004634.1|:64208-64779
MsHel-1	gb|AIXA01003713.1|:42798-42901
MsHel-1	gb|AIXA01003713.1|:54107-54812
MsHel-1	gb|AIXA01000777.1|:6011-6663
MsHel-1	gb|AIXA01025843.1|:165-628
MsHel-1	gb|AIXA01024514.1|:1-812
MsHel-1	gb|AIXA01009320.1|:1-547
MsHel-1	gb|AIXA01009216.1|:87206-87670
MsHel-1	gb|AIXA01001139.1|:1945-2514
MsHel-1	gb|AIXA01001054.1|:1513-2082
MsHel-1	gb|AIXA01000813.1|:53076-53542
MsHel-1	gb|AIXA01003201.1|:2-492
MsHel-1	gb|AIXA01011984.1|:15466-15917
MsHel-1	gb|AIXA01008169.1|:7736-7873
MsHel-1	gb|AIXA01005739.1|:1-488
MsHel-1	gb|AIXA01000294.1|:2750-3477
MsHel-1	gb|AIXA01000294.1|:80830-80914
MsHel-1	gb|AIXA01009491.1|:7339-7806
MsHel-1	gb|AIXA01004051.1|:6193-6660
MsHel-1	gb|AIXA01000306.1|:1707-2088
MsHel-1	gb|AIXA01018597.1|:509-1103
MsHel-1	gb|AIXA01017379.1|:7-616
MsHel-1	gb|AIXA01010117.1|:5099-5561
MsHel-1	gb|AIXA01008711.1|:8158-8683
MsHel-1	gb|AIXA01002598.1|:171-738
MsHel-1	gb|AIXA01002598.1|:63252-63739

MsHel-1	gb|AIXA01004647.1|:637-1099
MsHel-1	gb|AIXA01004647.1|:43030-43509
MsHel-1	gb|AIXA01009336.1|:46688-47184
MsHel-1	gb|AIXA01007510.1|:12919-13383
MsHel-1	gb|AIXA01004822.1|:315-779
MsHel-1	gb|AIXA01013048.1|:780-1243
MsHel-1	gb|AIXA01011370.1|:14567-15031
MsHel-1	gb|AIXA01002716.1|:23354-23805
MsHel-1	gb|AIXA01001498.1|:57939-58075
MsHel-1	gb|AIXA01008123.1|:12495-13113
MsHel-1	gb|AIXA01006971.1|:65758-65837
MsHel-1	gb|AIXA01026206.1|:1-433
MsHel-1	gb|AIXA01015554.1|:1266-1728
MsHel-1	gb|AIXA01009865.1|:68975-69111
MsHel-1	gb|AIXA01009865.1|:114413-115008
MsHel-1	gb|AIXA01009406.1|:76450-76943
MsHel-1	gb|AIXA01006293.1|:20929-21523
MsHel-1	gb|AIXA01005392.1|:12645-13142
MsHel-1	gb|AIXA01003690.1|:106087-107768
MsHel-1	gb|AIXA01002570.1|:20263-20857
MsHel-1	gb|AIXA01000456.1|:65835-66348
MsHel-1	gb|AIXA01008056.1|:18841-19306
MsHel-1	gb|AIXA01002583.1|:9085-9274
MsHel-1	gb|AIXA01028005.1|:1-693
MsHel-1	gb|AIXA01015682.1|:699-1393
MsHel-1	gb|AIXA01009183.1|:16664-17406
MsHel-1	gb|AIXA01006745.1|:2744-3208
MsHel-1	gb|AIXA01004480.1|:835-1412
MsHel-1	gb|AIXA01002754.1|:23277-23738
MsHel-1	gb|AIXA01001282.1|:89957-90551
MsHel-1	gb|AIXA01010956.1|:13406-13562
MsHel-1	gb|AIXA01002135.1|:14541-15007
MsHel-1	gb|AIXA01022576.1|:1-454
MsHel-1	gb|AIXA01013964.1|:1325-1788
MsHel-1	gb|AIXA01009535.1|:3133-3615
MsHel-1	gb|AIXA01011429.1|:1-494
MsHel-1	gb|AIXA01008182.1|:9427-10550
MsHel-1	gb|AIXA01003273.1|:929-1424
MsHel-1	gb|AIXA01002735.1|:6090-6640
MsHel-1	gb|AIXA01000043.1|:2329-2924
MsHel-1	gb|AIXA01026919.1|:30-494
MsHel-1	gb|AIXA01018741.1|:571-1034
MsHel-1	gb|AIXA01008670.1|:4-87
MsHel-1	gb|AIXA01008670.1|:19078-19850
MsHel-1	gb|AIXA01002718.1|:71165-71636
MsHel-1	gb|AIXA01012712.1|:35890-36996
MsHel-1	gb|AIXA01004861.1|:4755-5576
MsHel-1	gb|AIXA01016156.1|:3745-4196

MsHel-1	gb|AIXA01009238.1|:5010-5472
MsHel-1	gb|AIXA01007558.1|:6161-6747
MsHel-1	gb|AIXA01001708.1|:12549-13066
MsHel-1	gb|AIXA01008526.1|:6758-7348
MsHel-1	gb|AIXA01006756.1|:5425-5974
MsHel-1	gb|AIXA01005483.1|:1-433
MsHel-1	gb|AIXA01004763.1|:17728-18271
MsHel-1	gb|AIXA01001650.1|:24023-24652
MsHel-1	gb|AIXA01000077.1|:66956-67560
MsHel-1	gb|AIXA01006855.1|:45160-45500
MsHel-1	gb|AIXA01004777.1|:88385-88944
MsHel-1	gb|AIXA01003924.1|:72531-72730
MsHel-1	gb|AIXA01003924.1|:131149-131268
MsHel-1	gb|AIXA01001966.1|:4406-4998
MsHel-1	gb|AIXA01001574.1|:6622-7625
MsHel-1	gb|AIXA01011796.1|:1-518
MsHel-1	gb|AIXA01007419.1|:1-610
MsHel-1	gb|AIXA01007021.1|:1-507
MsHel-1	gb|AIXA01003021.1|:15255-15515
MsHel-1	gb|AIXA01003021.1|:103467-104227
MsHel-1	gb|AIXA01009219.1|:48037-48581
MsHel-1	gb|AIXA01007974.1|:5775-11996
MsHel-1	gb|AIXA01001189.1|:12579-13101
MsHel-1	gb|AIXA01027683.1|:197-795
MsHel-1	gb|AIXA01010589.1|:7057-7512
MsHel-1	gb|AIXA01007332.1|:176-665
MsHel-1	gb|AIXA01006723.1|:16539-17151
MsHel-1	gb|AIXA01000763.1|:28802-28976
MsHel-1	gb|AIXA01000763.1|:67429-67980
MsHel-1	gb|AIXA01000630.1|:12201-12753
MsHel-1	gb|AIXA01012319.1|:18824-19287
MsHel-1	gb|AIXA01005017.1|:4172-4713
MsHel-1	gb|AIXA01002988.1|:3345-3609
MsHel-1	gb|AIXA01002988.1|:61746-62558
MsHel-1	gb|AIXA01011192.1|:5227-5898
MsHel-1	gb|AIXA01009285.1|:23102-23680
MsHel-1	gb|AIXA01005882.1|:12285-12879
MsHel-1	gb|AIXA01001692.1|:7044-7225
MsHel-1	gb|AIXA01028433.1|:1-453
MsHel-1	gb|AIXA01013045.1|:6645-6860
MsHel-1	gb|AIXA01010805.1|:34-466
MsHel-1	gb|AIXA01007909.1|:47456-48240
MsHel-1	gb|AIXA01007218.1|:2317-2782
MsHel-1	gb|AIXA01017467.1|:1-587
MsHel-1	gb|AIXA01013020.1|:8924-9743
MsHel-1	gb|AIXA01012946.1|:26918-28354
MsHel-1	gb|AIXA01009125.1|:13610-14038
MsHel-1	gb|AIXA01008965.1|:92048-92511

MsHel-1	gb|AIXA01004461.1|:28141-28763
MsHel-1	gb|AIXA01000722.1|:4694-5288
MsHel-1	gb|AIXA01004838.1|:4577-5172
MsHel-1	gb|AIXA01001899.1|:27723-28458
MsHel-1	gb|AIXA01014191.1|:1225-1680
MsHel-1	gb|AIXA01008088.1|:66859-66939
MsHel-1	gb|AIXA01006945.1|:10638-11682
MsHel-1	gb|AIXA01000211.1|:52850-53404
MsHel-1	gb|AIXA01037045.1|:1-421
MsHel-1	gb|AIXA01024872.1|:130-598
MsHel-1	gb|AIXA01017919.1|:1-441
MsHel-1	gb|AIXA01003935.1|:36071-36303
MsHel-1	gb|AIXA01033218.1|:235-695
MsHel-1	gb|AIXA01010918.1|:1-447
MsHel-1	gb|AIXA01008054.1|:18771-19225
MsHel-1	gb|AIXA01000385.1|:50557-51073
MsHel-1	gb|AIXA01011979.1|:22615-23208
MsHel-1	gb|AIXA01009488.1|:65115-65793
MsHel-1	gb|AIXA01006520.1|:1-403
MsHel-1	gb|AIXA01005027.1|:5045-5922
MsHel-1	gb|AIXA01005027.1|:41047-42038
MsHel-1	gb|AIXA01003112.1|:63912-64399
MsHel-1	gb|AIXA01008636.1|:57914-58356
MsHel-1	gb|AIXA01005941.1|:2254-2675
MsHel-1	gb|AIXA01004699.1|:2807-3931
MsHel-1	gb|AIXA01004542.1|:1992-2576
MsHel-1	gb|AIXA01027731.1|:1-417
MsHel-1	gb|AIXA01000649.1|:32109-32234
MsHel-1	gb|AIXA01019930.1|:1903-2499
MsHel-1	gb|AIXA01019894.1|:1542-2212
MsHel-1	gb|AIXA01015400.1|:3933-4982
MsHel-1	gb|AIXA01012947.1|:2-426
MsHel-1	gb|AIXA01012384.1|:1-498
MsHel-1	gb|AIXA01024380.1|:6-516
MsHel-1	gb|AIXA01006488.1|:27389-27937
MsHel-1	gb|AIXA01001917.1|:36127-36662
MsHel-1	gb|AIXA01005880.1|:2714-3160
MsHel-1	gb|AIXA01004167.1|:9721-10618
MsHel-1	gb|AIXA01002157.1|:7201-7636
MsHel-1	gb|AIXA01012618.1|:36846-37471
MsHel-1	gb|AIXA01009781.1|:66720-67893
MsHel-1	gb|AIXA01000938.1|:53602-53815
MsHel-1	gb|AIXA01000181.1|:10023-10481
MsHel-1	gb|AIXA01034341.1|:2-423
MsHel-1	gb|AIXA01006622.1|:16177-16634
MsHel-1	gb|AIXA01004470.1|:93215-93377
MsHel-1	gb|AIXA01002984.1|:2073-2857
MsHel-1	gb|AIXA01013436.1|:50750-50888

MsHel-1	gb|AIXA01013219.1|:4274-4733
MsHel-1	gb|AIXA01010509.1|:8267-8785
MsHel-1	gb|AIXA01008369.1|:7228-7672
MsHel-1	gb|AIXA01007354.1|:124511-124755
MsHel-1	gb|AIXA01016211.1|:8-408
MsHel-1	gb|AIXA01015302.1|:8610-9063
MsHel-1	gb|AIXA01006478.1|:108853-109537
MsHel-1	gb|AIXA01033455.1|:184-674
MsHel-1	gb|AIXA01032829.1|:5-577
MsHel-1	gb|AIXA01007463.1|:15780-15898
MsHel-1	gb|AIXA01009517.1|:1-539
MsHel-1	gb|AIXA01004302.1|:47708-48142
MsHel-1	gb|AIXA01011104.1|:16169-16764
MsHel-1	gb|AIXA01009162.1|:1-373
MsHel-1	gb|AIXA01001625.1|:19831-20298
MsHel-1	gb|AIXA01007717.1|:2-401
MsHel-1	gb|AIXA01002394.1|:16953-17385
MsHel-1	gb|AIXA01002369.1|:5857-6357
MsHel-1	gb|AIXA01022757.1|:1-409
MsHel-1	gb|AIXA01014567.1|:5411-5799
MsHel-1	gb|AIXA01010819.1|:7818-8322
MsHel-1	gb|AIXA01006471.1|:6891-7309
MsHel-1	gb|AIXA01006423.1|:84706-85690
MsHel-1	gb|AIXA01006423.1|:148748-148904
MsHel-1	gb|AIXA01003380.1|:7413-7530
MsHel-1	gb|AIXA01003380.1|:25521-26037
MsHel-1	gb|AIXA01003054.1|:25799-25993
MsHel-1	gb|AIXA01001288.1|:2079-2293
MsHel-1	gb|AIXA01001288.1|:61924-62696
MsHel-1	gb|AIXA01013800.1|:3969-4581
MsHel-1	gb|AIXA01002859.1|:1198-1619
MsHel-1	gb|AIXA01033136.1|:1-402
MsHel-1	gb|AIXA01007859.1|:5316-6045
MsHel-1	gb|AIXA01003658.1|:16732-17296
MsHel-1	gb|AIXA01013002.1|:9606-10008
MsHel-1	gb|AIXA01014039.1|:3337-4063
MsHel-1	gb|AIXA01013123.1|:7812-8390
MsHel-1	gb|AIXA01007028.1|:29423-29839
MsHel-1	gb|AIXA01005110.1|:1-417
MsHel-1	gb|AIXA01003921.1|:33889-34504
MsHel-1	gb|AIXA01017176.1|:2-410
MsHel-1	gb|AIXA01002044.1|:73511-73775
MsHel-1	gb|AIXA01002044.1|:109038-109204
MsHel-1	gb|AIXA01007795.1|:7469-7657
MsHel-1	gb|AIXA01007795.1|:23731-24315
MsHel-1	gb|AIXA01000961.1|:15954-16104
MsHel-1	gb|AIXA01000961.1|:80449-80853
MsHel-1	gb|AIXA01017356.1|:11028-11485

MsHel-1	gb|AIXA01005278.1|:5962-6743
MsHel-1	gb|AIXA01001599.1|:37019-37491
MsHel-1	gb|AIXA01035233.1|:1-360
MsHel-1	gb|AIXA01025271.1|:8-638
MsHel-1	gb|AIXA01023917.1|:987-1411
MsHel-1	gb|AIXA01006894.1|:1-563
MsHel-1	gb|AIXA01004928.1|:8714-9002
MsHel-1	gb|AIXA01002941.1|:27690-28377
MsHel-1	gb|AIXA01007091.1|:23181-23816
MsHel-1	gb|AIXA01002242.1|:14367-15994
MsHel-1	gb|AIXA01001687.1|:21409-21812
MsHel-1	gb|AIXA01000547.1|:9811-10230
MsHel-1	gb|AIXA01034215.1|:5-445
MsHel-1	gb|AIXA01019283.1|:1-435
MsHel-1	gb|AIXA01014666.1|:1560-1930
MsHel-1	gb|AIXA01007305.1|:1-414
MsHel-1	gb|AIXA01003749.1|:25491-25886
MsHel-1	gb|AIXA01003282.1|:3-381
MsHel-1	gb|AIXA01002408.1|:24468-25078
MsHel-1	gb|AIXA01019679.1|:3718-4108
MsHel-1	gb|AIXA01014145.1|:4585-5057
MsHel-1	gb|AIXA01001482.1|:2470-3328
MsHel-1	gb|AIXA01006923.1|:23639-24078
MsHel-1	gb|AIXA01027562.1|:596-1016
MsHel-1	gb|AIXA01009692.1|:28683-29150
MsHel-1	gb|AIXA01036011.1|:1-355
MsHel-1	gb|AIXA01011650.1|:67940-68175
MsHel-1	gb|AIXA01005028.1|:4060-4451
MsHel-1	gb|AIXA01003603.1|:4194-4275
MsHel-1	gb|AIXA01003603.1|:17027-17411
MsHel-1	gb|AIXA01008313.1|:2-177
MsHel-1	gb|AIXA01000338.1|:25857-26329
MsHel-1	gb|AIXA01000093.1|:23236-23948
MsHel-1	gb|AIXA01037454.1|:1-346
MsHel-1	gb|AIXA01031349.1|:346-787
MsHel-1	gb|AIXA01005944.1|:2990-3595
MsHel-1	gb|AIXA01026895.1|:661-1071
MsHel-1	gb|AIXA01008673.1|:5518-5766
MsHel-1	gb|AIXA01004351.1|:46684-47153
MsHel-1	gb|AIXA01032172.1|:5-497
MsHel-1	gb|AIXA01027772.1|:1-370
MsHel-1	gb|AIXA01006285.1|:11298-11724
MsHel-1	gb|AIXA01010330.1|:49723-50189
MsHel-1	gb|AIXA01014903.1|:523-1260
MsHel-1	gb|AIXA01006648.1|:70226-70594
MsHel-1	gb|AIXA01002362.1|:1-144
MsHel-1	gb|AIXA01002362.1|:93306-93736
MsHel-1	gb|AIXA01005492.1|:16842-17652

MsHel-1	gb|AIXA01014301.1|:11708-12161
MsHel-1	gb|AIXA01004548.1|:35710-36177
MsHel-1	gb|AIXA01004394.1|:2566-3037
MsHel-1	gb|AIXA01003125.1|:33978-34746
MsHel-1	gb|AIXA01001806.1|:12777-13249
MsHel-1	gb|AIXA01001806.1|:23676-23768
MsHel-1	gb|AIXA01001465.1|:4964-5431
MsHel-1	gb|AIXA01005606.1|:1729-2794
MsHel-1	gb|AIXA01000679.1|:23859-24239
MsHel-1	gb|AIXA01038192.1|:13-381
MsHel-1	gb|AIXA01004733.1|:16462-16657
MsHel-1	gb|AIXA01001070.1|:25422-25950
MsHel-1	gb|AIXA01015905.1|:12797-14279
MsHel-1	gb|AIXA01010394.1|:30919-31087
MsHel-1	gb|AIXA01010394.1|:56214-56342
MsHel-1	gb|AIXA01000403.1|:149407-149828
MsHel-1	gb|AIXA01018292.1|:5979-6416
MsHel-1	gb|AIXA01018035.1|:5366-5838
MsHel-1	gb|AIXA01010906.1|:9162-9632
MsHel-1	gb|AIXA01009382.1|:44810-45282
MsHel-1	gb|AIXA01001321.1|:576-1051
MsHel-1	gb|AIXA01000884.1|:28979-29497
MsHel-1	gb|AIXA01032581.1|:381-725
MsHel-1	gb|AIXA01010019.1|:52065-52512
MsHel-1	gb|AIXA01005051.1|:34577-35044
MsHel-1	gb|AIXA01024903.1|:111-683
MsHel-1	gb|AIXA01009366.1|:70261-70855
MsHel-1	gb|AIXA01009195.1|:4270-4741
MsHel-1	gb|AIXA01007625.1|:53746-53941
MsHel-1	gb|AIXA01007531.1|:6703-7180
MsHel-1	gb|AIXA01004740.1|:2-497
MsHel-1	gb|AIXA01001222.1|:36864-37049
MsHel-1	gb|AIXA01001222.1|:70594-71059
MsHel-1	gb|AIXA01000596.1|:1737-2209
MsHel-1	gb|AIXA01012475.1|:1-358
MsHel-1	gb|AIXA01004121.1|:2217-2732
MsHel-1	gb|AIXA01002062.1|:7983-8485
MsHel-1	gb|AIXA01015972.1|:2052-2517
MsHel-1	gb|AIXA01008907.1|:20508-21124
MsHel-1	gb|AIXA01003581.1|:1-360
MsHel-1	gb|AIXA01016154.1|:1636-2107
MsHel-1	gb|AIXA01006772.1|:11929-12406
MsHel-1	gb|AIXA01003808.1|:15825-16509
MsHel-1	gb|AIXA01012048.1|:1125-1250
MsHel-1	gb|AIXA01012048.1|:38789-38945
MsHel-1	gb|AIXA01012048.1|:68921-69321
MsHel-1	gb|AIXA01005311.1|:12927-13307
MsHel-1	gb|AIXA01017221.1|:1-373

MsHel-1	gb|AIXA01011831.1|:4074-5084
MsHel-1	gb|AIXA01002331.1|:60602-61324
MsHel-1	gb|AIXA01000586.1|:1382-2552
MsHel-1	gb|AIXA01025108.1|:248-722
MsHel-1	gb|AIXA01005969.1|:3062-3477
MsHel-1	gb|AIXA01014843.1|:15016-15615
MsHel-1	gb|AIXA01010626.1|:3384-3732
MsHel-1	gb|AIXA01005576.1|:38490-48422
MsHel-1	gb|AIXA01004519.1|:1-292
MsHel-1	gb|AIXA01003873.1|:20240-20679
MsHel-1	gb|AIXA01003873.1|:52987-53121
MsHel-1	gb|AIXA01008224.1|:3441-3795
MsHel-1	gb|AIXA01004224.1|:17725-18192
MsHel-1	gb|AIXA01002563.1|:801-1270
MsHel-1	gb|AIXA01030916.1|:10-342
MsHel-1	gb|AIXA01015056.1|:3-434
MsHel-1	gb|AIXA01007246.1|:786-1159
MsHel-1	gb|AIXA01002851.1|:12153-12618
MsHel-1	gb|AIXA01001804.1|:3-361
MsHel-1	gb|AIXA01005100.1|:7376-7851
MsHel-1	gb|AIXA01016768.1|:12350-12763
MsHel-1	gb|AIXA01005888.1|:694-1166
MsHel-1	gb|AIXA01011449.1|:4780-4946
MsHel-1	gb|AIXA01008915.1|:22567-23040
MsHel-1	gb|AIXA01010781.1|:16839-17319
MsHel-1	gb|AIXA01007530.1|:43820-44168
MsHel-1	gb|AIXA01005574.1|:43219-43517
MsHel-1	gb|AIXA01004704.1|:7624-8094
MsHel-1	gb|AIXA01001933.1|:2760-3097
MsHel-1	gb|AIXA01000948.1|:23740-24168
MsHel-1	gb|AIXA01010187.1|:40185-40893
MsHel-1	gb|AIXA01010341.1|:2330-2772
MsHel-1	gb|AIXA01001920.1|:26750-27222
MsHel-1	gb|AIXA01008509.1|:41836-42280
MsHel-1	gb|AIXA01007210.1|:3-360
MsHel-1	gb|AIXA01007210.1|:14775-14879
MsHel-1	gb|AIXA01007210.1|:33522-33794
MsHel-1	gb|AIXA01019884.1|:3173-3646
MsHel-1	gb|AIXA01018293.1|:1-242
MsHel-1	gb|AIXA01000428.1|:273-621
MsHel-1	gb|AIXA01000187.1|:3529-4326
MsHel-1	gb|AIXA01010519.1|:12039-12682
MsHel-1	gb|AIXA01003829.1|:2353-3054
MsHel-1	gb|AIXA01015384.1|:1-295
MsHel-1	gb|AIXA01014339.1|:2627-3098
MsHel-1	gb|AIXA01008040.1|:6383-6675
MsHel-1	gb|AIXA01008040.1|:23407-23619
MsHel-1	gb|AIXA01006793.1|:7132-7216

MsHel-1	gb|AIXA01006793.1|:37203-37686
MsHel-1	gb|AIXA01005543.1|:1711-2302
MsHel-1	gb|AIXA01001292.1|:87551-87704
MsHel-1	gb|AIXA01033209.1|:345-694
MsHel-1	gb|AIXA01018438.1|:1255-1677
MsHel-1	gb|AIXA01011251.1|:2174-2511
MsHel-1	gb|AIXA01009597.1|:1-549
MsHel-1	gb|AIXA01000761.1|:11904-12380
MsHel-1	gb|AIXA01008505.1|:82956-83492
MsHel-1	gb|AIXA01007534.1|:36504-41476
MsHel-1	gb|AIXA01015593.1|:1-342
MsHel-1	gb|AIXA01014641.1|:13805-13933
MsHel-1	gb|AIXA01009792.1|:16159-16732
MsHel-1	gb|AIXA01005904.1|:103586-103869
MsHel-1	gb|AIXA01005904.1|:124779-124900
MsHel-1	gb|AIXA01001134.1|:7114-7687
MsHel-1	gb|AIXA01007443.1|:30561-31016
MsHel-1	gb|AIXA01009710.1|:25476-25947
MsHel-1	gb|AIXA01005881.1|:1272-1623
MsHel-1	gb|AIXA01000094.1|:1-306
MsHel-1	gb|AIXA01007008.1|:26684-26984
MsHel-1	gb|AIXA01004561.1|:473-697
MsHel-1	gb|AIXA01004561.1|:11490-11619
MsHel-1	gb|AIXA01001707.1|:3-192
MsHel-1	gb|AIXA01011528.1|:11511-11932
MsHel-1	gb|AIXA01009942.1|:38288-38753
MsHel-1	gb|AIXA01031784.1|:1-561
MsHel-1	gb|AIXA01005205.1|:1-347
MsHel-1	gb|AIXA01035156.1|:281-605
MsHel-1	gb|AIXA01010098.1|:9670-9974
MsHel-1	gb|AIXA01007121.1|:5837-6304
MsHel-1	gb|AIXA01005624.1|:4951-5363
MsHel-1	gb|AIXA01030664.1|:532-822
MsHel-1	gb|AIXA01035708.1|:265-589
MsHel-1	gb|AIXA01029393.1|:597-901
MsHel-1	gb|AIXA01024481.1|:1028-1339
MsHel-1	gb|AIXA01009982.1|:1-130
MsHel-1	gb|AIXA01009129.1|:11408-11820
MsHel-1	gb|AIXA01005433.1|:47931-48402
MsHel-1	gb|AIXA01031620.1|:1-294
MsHel-1	gb|AIXA01014896.1|:2934-3392
MsHel-1	gb|AIXA01006821.1|:12181-12697
MsHel-1	gb|AIXA01004481.1|:41859-42324
MsHel-1	gb|AIXA01018088.1|:2797-3141
MsHel-1	gb|AIXA01005228.1|:77793-78119
MsHel-1	gb|AIXA01005228.1|:124891-125400
MsHel-1	gb|AIXA01004680.1|:10154-10735
MsHel-1	gb|AIXA01036712.1|:1-278

MsHel-1	gb|AIXA01034919.1|:230-615
MsHel-1	gb|AIXA01007570.1|:1752-2220
MsHel-1	gb|AIXA01002376.1|:1-408
MsHel-1	gb|AIXA01026775.1|:820-1092
MsHel-1	gb|AIXA01021303.1|:564-1455
MsHel-1	gb|AIXA01011464.1|:14797-14899
MsHel-1	gb|AIXA01007178.1|:1242-1512
MsHel-1	gb|AIXA01002762.1|:6172-6695
MsHel-1	gb|AIXA01001127.1|:18604-19050
MsHel-1	gb|AIXA01001127.1|:59298-59731
MsHel-1	gb|AIXA01015046.1|:8099-8380
MsHel-1	gb|AIXA01003245.1|:3410-3793
MsHel-1	gb|AIXA01013805.1|:1271-1732
MsHel-1	gb|AIXA01003248.1|:6781-7093
MsHel-1	gb|AIXA01019563.1|:593-832
MsHel-1	gb|AIXA01011268.1|:34345-34608
MsHel-1	gb|AIXA01008173.1|:10004-10464
MsHel-1	gb|AIXA01001949.1|:2-292
MsHel-1	gb|AIXA01003597.1|:1-347
MsHel-1	gb|AIXA01003228.1|:1-592
MsHel-1	gb|AIXA01003113.1|:4-416
MsHel-1	gb|AIXA01002898.1|:2804-2975
MsHel-1	gb|AIXA01002898.1|:13013-13401
MsHel-1	gb|AIXA01002227.1|:641-909
MsHel-1	gb|AIXA01024986.1|:990-1275
MsHel-1	gb|AIXA01010568.1|:2250-2709
MsHel-1	gb|AIXA01005737.1|:35458-35696
MsHel-1	gb|AIXA01034291.1|:348-642
MsHel-1	gb|AIXA01018854.1|:7793-8051
MsHel-1	gb|AIXA01004786.1|:1-441
MsHel-1	gb|AIXA01002785.1|:1-263
MsHel-1	gb|AIXA01002785.1|:40078-40244
MsHel-1	gb|AIXA01011760.1|:3725-4147
MsHel-1	gb|AIXA01030760.1|:1-394
MsHel-1	gb|AIXA01003878.1|:19927-20005
MsHel-1	gb|AIXA01001885.1|:41802-42051
MsHel-1	gb|AIXA01001349.1|:39361-39914
MsHel-1	gb|AIXA01002629.1|:131655-132119
MsHel-1	gb|AIXA01014058.1|:1-251
MsHel-1	gb|AIXA01005566.1|:12755-13432
MsHel-1	gb|AIXA01005566.1|:93402-93485
MsHel-1	gb|AIXA01014254.1|:122-328
MsHel-1	gb|AIXA01010690.1|:9671-10122
MsHel-1	gb|AIXA01002174.1|:65173-65382
MsHel-1	gb|AIXA01002174.1|:176127-176433
MsHel-1	gb|AIXA01005083.1|:22031-22492
MsHel-1	gb|AIXA01002626.1|:15765-16179
MsHel-1	gb|AIXA01000508.1|:13265-13531

MsHel-1	gb|AIXA01010703.1|:33772-34015
MsHel-1	gb|AIXA01001799.1|:1-259
MsHel-1	gb|AIXA01026728.1|:2-341
MsHel-1	gb|AIXA01009141.1|:3-241
MsHel-1	gb|AIXA01002680.1|:2751-2863
MsHel-1	gb|AIXA01024192.1|:1045-1373
MsHel-1	gb|AIXA01012910.1|:1809-2070
MsHel-1	gb|AIXA01010118.1|:1-261
MsHel-1	gb|AIXA01035001.1|:14-270
MsHel-1	gb|AIXA01026985.1|:817-1076
MsHel-1	gb|AIXA01016075.1|:29372-30602
MsHel-1	gb|AIXA01002152.1|:1-263
MsHel-1	gb|AIXA01010745.1|:1-286
MsHel-1	gb|AIXA01029537.1|:611-891
MsHel-1	gb|AIXA01024737.1|:1018-1274
MsHel-1	gb|AIXA01009504.1|:41977-42249
MsHel-1	gb|AIXA01032030.1|:435-754
MsHel-1	gb|AIXA01001099.1|:2085-2332
MsHel-1	gb|AIXA01001117.1|:4758-5139
MsHel-1	gb|AIXA01006727.1|:46091-46369
MsHel-1	gb|AIXA01012049.1|:12136-12376
MsHel-1	gb|AIXA01000660.1|:17868-18120
MsHel-1	gb|AIXA01026666.1|:857-1104
MsHel-1	gb|AIXA01015140.1|:1-252
MsHel-1	gb|AIXA01013813.1|:1-116
MsHel-1	gb|AIXA01007743.1|:3-258
MsHel-1	gb|AIXA01003624.1|:10142-10638
MsHel-1	gb|AIXA01003070.1|:28562-28762
MsHel-1	gb|AIXA01035835.1|:327-586
MsHel-1	gb|AIXA01033227.1|:1-279
MsHel-1	gb|AIXA01020823.1|:2-252
MsHel-1	gb|AIXA01005734.1|:31409-31667
MsHel-1	gb|AIXA01016323.1|:3780-4250
MsHel-1	gb|AIXA01002599.1|:61915-62629
MsHel-1	gb|AIXA01002599.1|:98455-98639
MsHel-1	gb|AIXA01004867.1|:178-862
MsHel-1	gb|AIXA01006942.1|:1-294
MsHel-1	gb|AIXA01006527.1|:15218-15492
MsHel-1	gb|AIXA01010512.1|:844-1108
MsHel-1	gb|AIXA01004321.1|:1871-2276
MsHel-1	gb|AIXA01003230.1|:4258-4639
MsHel-1	gb|AIXA01002138.1|:24175-24734
MsHel-1	gb|AIXA01012659.1|:19383-19539
MsHel-1	gb|AIXA01001870.1|:9223-9694
MsHel-1	gb|AIXA01017534.1|:7333-7575
MsHel-1	gb|AIXA01008958.1|:1-476
MsHel-1	gb|AIXA01005636.1|:47653-47930
MsHel-1	gb|AIXA01005252.1|:28713-29182

MsHel-1	gb|AIXA01004135.1|:4647-4873
MsHel-1	gb|AIXA01002484.1|:22651-22902
MsHel-1	gb|AIXA01001997.1|:4706-5037
MsHel-1	gb|AIXA01023950.1|:1161-1412
MsHel-1	gb|AIXA01023762.1|:1-267
MsHel-1	gb|AIXA01023099.1|:1302-1561
MsHel-1	gb|AIXA01003633.1|:2646-3201
MsHel-1	gb|AIXA01037627.1|:1-368
MsHel-1	gb|AIXA01035384.1|:321-601
MsHel-1	gb|AIXA01009323.1|:18393-18931
MsHel-1	gb|AIXA01002417.1|:2796-3030
MsHel-1	gb|AIXA01016261.1|:11941-12249
MsHel-1	gb|AIXA01008177.1|:1-352
MsHel-1	gb|AIXA01006228.1|:37371-37615
MsHel-1	gb|AIXA01007649.1|:40542-40799
MsHel-1	gb|AIXA01007546.1|:13951-14288
MsHel-1	gb|AIXA01009335.1|:61-1922
MsHel-1	gb|AIXA01004618.1|:11766-12218
MsHel-1	gb|AIXA01000817.1|:60061-60274
MsHel-1	gb|AIXA01003723.1|:38-320
MsHel-1	gb|AIXA01021518.1|:5-353
MsHel-1	gb|AIXA01020196.1|:2775-2990
MsHel-1	gb|AIXA01017319.1|:4644-4876
MsHel-1	gb|AIXA01014128.1|:813-1073
MsHel-1	gb|AIXA01009924.1|:6-233
MsHel-1	gb|AIXA01002151.1|:3585-3812
MsHel-1	gb|AIXA01007523.1|:372-698
MsHel-1	gb|AIXA01001315.1|:10646-10876
MsHel-1	gb|AIXA01025743.1|:1-233
MsHel-1	gb|AIXA01002231.1|:42089-42542
MsHel-1	gb|AIXA01009326.1|:1-214
MsHel-1	gb|AIXA01004456.1|:17740-17920
MsHel-1	gb|AIXA01004456.1|:53089-53394
MsHel-1	gb|AIXA01020688.1|:2203-2440
MsHel-1	gb|AIXA01005277.1|:13-217
MsHel-1	gb|AIXA01003647.1|:14591-14807
MsHel-1	gb|AIXA01011269.1|:1-249
MsHel-1	gb|AIXA01005505.1|:11526-12108
MsHel-1	gb|AIXA01004824.1|:1-208
MsHel-1	gb|AIXA01036643.1|:1-524
MsHel-1	gb|AIXA01011718.1|:34430-34729
MsHel-1	gb|AIXA01009646.1|:74-275
MsHel-1	gb|AIXA01005857.1|:1-251
MsHel-1	gb|AIXA01006658.1|:18504-18895
MsHel-1	gb|AIXA01004592.1|:25827-26027
MsHel-1	gb|AIXA01025249.1|:8-305
MsHel-1	gb|AIXA01014665.1|:6362-6650
MsHel-1	gb|AIXA01011759.1|:1199-1656

MsHel-1	gb|AIXA01002528.1|:60918-61055
MsHel-1	gb|AIXA01035292.1|:405-603
MsHel-1	gb|AIXA01022358.1|:1-218
MsHel-1	gb|AIXA01015669.1|:2753-2971
MsHel-1	gb|AIXA01011663.1|:18716-18917
MsHel-1	gb|AIXA01005077.1|:25800-26001
MsHel-1	gb|AIXA01003005.1|:15205-15406
MsHel-1	gb|AIXA01031633.1|:564-773
MsHel-1	gb|AIXA01013552.1|:5816-6016
MsHel-1	gb|AIXA01035999.1|:316-579
MsHel-1	gb|AIXA01007834.1|:47-255
MsHel-1	gb|AIXA01004016.1|:90055-90438
MsHel-1	gb|AIXA01009128.1|:13625-13826
MsHel-1	gb|AIXA01002584.1|:2-257
MsHel-1	gb|AIXA01034675.1|:1-202
MsHel-1	gb|AIXA01034077.1|:451-655
MsHel-1	gb|AIXA01013334.1|:2-556
MsHel-1	gb|AIXA01001883.1|:40036-40506
MsHel-1	gb|AIXA01000267.1|:1-392
MsHel-1	gb|AIXA01035768.1|:379-587
MsHel-1	gb|AIXA01030204.1|:14-222
MsHel-1	gb|AIXA01024804.1|:1073-1296
MsHel-1	gb|AIXA01029190.1|:665-915
MsHel-1	gb|AIXA01011684.1|:10713-10920
MsHel-1	gb|AIXA01000109.1|:309-566
MsHel-1	gb|AIXA01027656.1|:800-1017
MsHel-1	gb|AIXA01008367.1|:8-220
MsHel-1	gb|AIXA01034338.1|:417-645
MsHel-1	gb|AIXA01027218.1|:805-1005
MsHel-1	gb|AIXA01018902.1|:3567-3804
MsHel-1	gb|AIXA01017891.1|:12079-12279
MsHel-1	gb|AIXA01016475.1|:30067-30403
MsHel-1	gb|AIXA01008379.1|:310-515
MsHel-1	gb|AIXA01006673.1|:3163-3875
MsHel-1	gb|AIXA01000762.1|:33648-33848
MsHel-1	gb|AIXA01038326.1|:294-502
MsHel-1	gb|AIXA01035976.1|:358-580
MsHel-1	gb|AIXA01033311.1|:2-213
MsHel-1	gb|AIXA01023496.1|:1223-1489
MsHel-1	gb|AIXA01022263.1|:1505-1740
MsHel-1	gb|AIXA01003238.1|:1-427
MsHel-1	gb|AIXA01015592.1|:5180-5395
MsHel-1	gb|AIXA01012355.1|:2041-2400
MsHel-1	gb|AIXA01005256.1|:25102-25329
MsHel-1	gb|AIXA01001546.1|:35149-35396
MsHel-1	gb|AIXA01001546.1|:58753-58960
MsHel-1	gb|AIXA01038229.1|:300-501
MsHel-1	gb|AIXA01016134.1|:1059-1310

MsHel-1	gb|AIXA01015223.1|:28-226
MsHel-1	gb|AIXA01003683.1|:125959-126165
MsHel-1	gb|AIXA01002399.1|:48499-48863
MsHel-1	gb|AIXA01026914.1|:885-1082
MsHel-1	gb|AIXA01009312.1|:3825-4269
MsHel-1	gb|AIXA01003293.1|:9-222
MsHel-1	gb|AIXA01023295.1|:1340-1523
MsHel-1	gb|AIXA01012248.1|:9305-9533
MsHel-1	gb|AIXA01009976.1|:49373-49600
MsHel-1	gb|AIXA01008087.1|:32805-33016
MsHel-1	gb|AIXA01000812.1|:1-191
MsHel-1	gb|AIXA01010143.1|:11892-12089
MsHel-1	gb|AIXA01011086.1|:32266-32468
MsHel-1	gb|AIXA01007708.1|:1-193
MsHel-1	gb|AIXA01007421.1|:20204-20570
MsHel-1	gb|AIXA01007212.1|:9008-9190
MsHel-1	gb|AIXA01009940.1|:9509-9694
MsHel-1	gb|AIXA01014840.1|:2-299
MsHel-1	gb|AIXA01036806.1|:19-237
MsHel-1	gb|AIXA01029639.1|:697-879
MsHel-1	gb|AIXA01011308.1|:4817-4928
MsHel-1	gb|AIXA01008226.1|:49471-49653
MsHel-1	gb|AIXA01020035.1|:2-293
MsHel-1	gb|AIXA01009532.1|:51931-52404
MsHel-1	gb|AIXA01026883.1|:3-179
MsHel-1	gb|AIXA01003580.1|:19225-19405
MsHel-1	gb|AIXA01000261.1|:22967-23882
MsHel-1	gb|AIXA01034527.1|:449-632
MsHel-1	gb|AIXA01025537.1|:1-184
MsHel-1	gb|AIXA01025359.1|:460-689
MsHel-1	gb|AIXA01011918.1|:1249-1423
MsHel-1	gb|AIXA01006882.1|:1843-2017
MsHel-1	gb|AIXA01002418.1|:1-544
MsHel-1	gb|AIXA01002368.1|:11771-11982
MsHel-1	gb|AIXA01023412.1|:1-291
MsHel-1	gb|AIXA01019557.1|:5-197
MsHel-1	gb|AIXA01017542.1|:7521-7899
MsHel-1	gb|AIXA01007213.1|:1-194
MsHel-1	gb|AIXA01007095.1|:895-1239
MsHel-1	gb|AIXA01007043.1|:6323-6504
MsHel-1	gb|AIXA01028276.1|:6-491
MsHel-1	gb|AIXA01007981.1|:5-177
MsHel-1	gb|AIXA01000075.1|:1669-2076
MsHel-1	gb|AIXA01019826.1|:1-196
MsHel-1	gb|AIXA01011823.1|:1-176
MsHel-1	gb|AIXA01006265.1|:3-174
MsHel-1	gb|AIXA01004004.1|:17719-17889
MsHel-1	gb|AIXA01036326.1|:2-172

MsHel-1	gb|AIXA01026240.1|:965-1146
MsHel-1	gb|AIXA01020849.1|:2147-2317
MsHel-1	gb|AIXA01006667.1|:10758-10947
MsHel-1	gb|AIXA01006667.1|:26134-26272
MsHel-1	gb|AIXA01007516.1|:136139-136312
MsHel-1	gb|AIXA01004154.1|:1-279
MsHel-1	gb|AIXA01021875.1|:665-955
MsHel-1	gb|AIXA01015139.1|:19666-19867
MsHel-1	gb|AIXA01008669.1|:25840-26012
MsHel-1	gb|AIXA01026739.1|:2-290
MsHel-1	gb|AIXA01001531.1|:24192-24322
MsHel-1	gb|AIXA01001531.1|:69520-69839
MsHel-1	gb|AIXA01000916.1|:8-183
MsHel-1	gb|AIXA01012652.1|:11-177
MsHel-1	gb|AIXA01004826.1|:8848-9022
MsHel-1	gb|AIXA01000367.1|:50662-50844
MsHel-1	gb|AIXA01021168.1|:1-170
MsHel-1	gb|AIXA01012200.1|:78015-78325
MsHel-1	gb|AIXA01004402.1|:9734-10121
MsHel-1	gb|AIXA01035858.1|:341-497
MsHel-1	gb|AIXA01006394.1|:46209-46385
MsHel-1	gb|AIXA01024248.1|:1-176
MsHel-1	gb|AIXA01016940.1|:5800-6051
MsHel-1	gb|AIXA01007055.1|:3-279
MsHel-1	gb|AIXA01022081.1|:1618-1782
MsHel-1	gb|AIXA01007382.1|:8196-8359
MsHel-1	gb|AIXA01005732.1|:20956-21290
MsHel-1	gb|AIXA01004580.1|:84691-85021
MsHel-1	gb|AIXA01000101.1|:1870-2080
MsHel-1	gb|AIXA01028233.1|:814-979
MsHel-1	gb|AIXA01025487.1|:1066-1223
MsHel-1	gb|AIXA01021789.1|:104-296
MsHel-1	gb|AIXA01006284.1|:2-170
MsHel-1	gb|AIXA01023301.1|:1-164
MsHel-1	gb|AIXA01021996.1|:1500-1742
MsHel-1	gb|AIXA01009845.1|:13514-13669
MsHel-1	gb|AIXA01005591.1|:20410-20573
MsHel-1	gb|AIXA01004929.1|:12475-12615
MsHel-1	gb|AIXA01002059.1|:3123-3670
MsHel-1	gb|AIXA01006623.1|:16629-16783
MsHel-1	gb|AIXA01007255.1|:7253-7466
MsHel-1	gb|AIXA01006484.1|:4-430
MsHel-1	gb|AIXA01000158.1|:9886-10031
MsHel-1	gb|AIXA01000040.1|:39636-39793
MsHel-1	gb|AIXA01035742.1|:1-287
MsHel-1	gb|AIXA01032692.1|:1-258
MsHel-1	gb|AIXA01004323.1|:36604-36763
MsHel-1	gb|AIXA01007576.1|:17192-17387

MsHel-1	gb|AIXA01007576.1|:62570-62736
MsHel-1	gb|AIXA01015234.1|:6081-6324
MsHel-1	gb|AIXA01010144.1|:2-155
MsHel-1	gb|AIXA01007980.1|:16263-16412
MsHel-1	gb|AIXA01000325.1|:32113-32270
MsHel-1	gb|AIXA01007577.1|:49950-50094
MsHel-1	gb|AIXA01006709.1|:5504-5652
MsHel-1	gb|AIXA01002837.1|:24-192
MsHel-1	gb|AIXA01000251.1|:1-189
MsHel-1	gb|AIXA01031937.1|:587-759
MsHel-1	gb|AIXA01003271.1|:7913-8129
MsHel-1	gb|AIXA01013655.1|:11488-11651
MsHel-1	gb|AIXA01009224.1|:26689-26778
MsHel-1	gb|AIXA01032749.1|:1-149
MsHel-1	gb|AIXA01026627.1|:960-1108
MsHel-1	gb|AIXA01024829.1|:1-157
MsHel-1	gb|AIXA01008575.1|:42341-42483
MsHel-1	gb|AIXA01000199.1|:39845-40089
MsHel-1	gb|AIXA01036731.1|:2-258
MsHel-1	gb|AIXA01025469.1|:1-160
MsHel-1	gb|AIXA01011826.1|:2510-2661
MsHel-1	gb|AIXA01011249.1|:11163-11287
MsHel-1	gb|AIXA01007505.1|:8653-8949
MsHel-1	gb|AIXA01002480.1|:13050-13460
MsHel-1	gb|AIXA01028857.1|:783-935
MsHel-1	gb|AIXA01004640.1|:76620-76943
MsHel-1	gb|AIXA01003351.1|:15159-15532
MsHel-1	gb|AIXA01001428.1|:3476-3612
MsHel-1	gb|AIXA01017867.1|:3511-3646
MsHel-1	gb|AIXA01011418.1|:1-253
MsHel-1	gb|AIXA01008033.1|:4480-4574
MsHel-1	gb|AIXA01008033.1|:70093-70247
MsHel-1	gb|AIXA01018169.1|:2035-2353
MsHel-1	gb|AIXA01009594.1|:9847-10088
MsHel-1	gb|AIXA01023688.1|:1293-1456
MsHel-1	gb|AIXA01019554.1|:4692-4833
MsHel-1	gb|AIXA01002263.1|:1-154
MsHel-1	gb|AIXA01032026.1|:607-754
MsHel-1	gb|AIXA01030271.1|:698-845
MsHel-1	gb|AIXA01020836.1|:2170-2322
MsHel-1	gb|AIXA01017894.1|:9463-9639
MsHel-1	gb|AIXA01015059.1|:11859-12007
MsHel-1	gb|AIXA01007495.1|:7568-7802
MsHel-1	gb|AIXA01015583.1|:20528-20748
MsHel-1	gb|AIXA01007303.1|:26877-35290
MsHel-1	gb|AIXA01029799.1|:1-224
MsHel-1	gb|AIXA01001835.1|:1-135
MsHel-1	gb|AIXA01018508.1|:9230-9386

MsHel-1	gb|AIXA01005355.1|:962-1355
MsHel-1	gb|AIXA01037159.1|:411-539
MsHel-1	gb|AIXA01013107.1|:4734-4911
MsHel-1	gb|AIXA01006331.1|:6317-6461
MsHel-1	gb|AIXA01024979.1|:637-844
MsHel-1	gb|AIXA01016382.1|:8831-8958
MsHel-1	gb|AIXA01012177.1|:1-139
MsHel-1	gb|AIXA01009987.1|:1078-1349
MsHel-1	gb|AIXA01009539.1|:67022-67155
MsHel-1	gb|AIXA01007000.1|:14351-14713
MsHel-1	gb|AIXA01006382.1|:83340-83466
MsHel-1	gb|AIXA01025054.1|:1129-1266
MsHel-1	gb|AIXA01015728.1|:462-617
MsHel-1	gb|AIXA01013271.1|:5982-6195
MsHel-1	gb|AIXA01008859.1|:30510-30717
MsHel-1	gb|AIXA01006937.1|:2-218
MsHel-1	gb|AIXA01003109.1|:18216-18432
MsHel-1	gb|AIXA01034465.1|:502-638
MsHel-1	gb|AIXA01030684.1|:683-822
MsHel-1	gb|AIXA01012412.1|:15868-16228
MsHel-1	gb|AIXA01010345.1|:7676-8543
MsHel-1	gb|AIXA01008378.1|:33598-34079
MsHel-1	gb|AIXA01029548.1|:1-221
MsHel-1	gb|AIXA01020152.1|:2932-3060
MsHel-1	gb|AIXA01014721.1|:1177-1656
MsHel-1	gb|AIXA01007383.1|:1-253
MsHel-1	gb|AIXA01007093.1|:15827-15962
MsHel-1	gb|AIXA01001834.1|:3176-3305
MsHel-1	gb|AIXA01015597.1|:14402-14532
MsHel-1	gb|AIXA01009846.1|:3-149
MsHel-1	gb|AIXA01037150.1|:5-185
MsHel-1	gb|AIXA01014275.1|:1658-1775
MsHel-1	gb|AIXA01013731.1|:2864-3016
MsHel-1	gb|AIXA01010088.1|:15548-15799
MsHel-1	gb|AIXA01010088.1|:33850-33967
MsHel-1	gb|AIXA01000818.1|:17940-18057
MsHel-1	gb|AIXA01000236.1|:4896-5033
MsHel-1	gb|AIXA01023226.1|:1401-1532
MsHel-1	gb|AIXA01034897.1|:1-254
MsHel-1	gb|AIXA01029123.1|:738-861
MsHel-1	gb|AIXA01016235.1|:5851-6136
MsHel-1	gb|AIXA01013384.1|:297-4437
MsHel-1	gb|AIXA01008554.1|:33816-39845
MsHel-1	gb|AIXA01017855.1|:1666-1984
MsHel-1	gb|AIXA01014772.1|:1-135
MsHel-1	gb|AIXA01000995.1|:27912-28034
MsHel-1	gb|AIXA01035550.1|:1-234
MsHel-1	gb|AIXA01028626.1|:832-949

MsHel-1	gb|AIXA01009024.1|:117-242
MsHel-1	gb|AIXA01003043.1|:41058-41183
MsHel-1	gb|AIXA01002441.1|:1-130
MsHel-1	gb|AIXA01012110.1|:1-137
MsHel-1	gb|AIXA01012110.1|:18791-20526
MsHel-1	gb|AIXA01010193.1|:1-201
MsHel-1	gb|AIXA01005948.1|:4137-4419
MsHel-1	gb|AIXA01003237.1|:7671-7811
MsHel-1	gb|AIXA01017340.1|:3891-4162
MsHel-1	gb|AIXA01007968.1|:1-116
MsHel-1	gb|AIXA01007590.1|:1-233
MsHel-1	gb|AIXA01004779.1|:38865-38992
MsHel-1	gb|AIXA01003203.1|:12470-12620
MsHel-1	gb|AIXA01000963.1|:109640-109980
MsHel-1	gb|AIXA01031151.1|:657-799
MsHel-1	gb|AIXA01019382.1|:2584-2705
MsHel-1	gb|AIXA01016320.1|:6256-6382
MsHel-1	gb|AIXA01009040.1|:60011-60380
MsHel-1	gb|AIXA01003227.1|:38264-38410
MsHel-1	gb|AIXA01035398.1|:493-601
MsHel-1	gb|AIXA01017338.1|:7012-7542
MsHel-1	gb|AIXA01017093.1|:3971-4343
MsHel-1	gb|AIXA01011450.1|:580-869
MsHel-1	gb|AIXA01007707.1|:54513-54625
MsHel-1	gb|AIXA01003973.1|:83621-83882
MsHel-1	gb|AIXA01023633.1|:1347-1466
MsHel-1	gb|AIXA01023422.1|:497-1300
MsHel-1	gb|AIXA01013053.1|:1-120
MsHel-1	gb|AIXA01012622.1|:1-116
MsHel-1	gb|AIXA01009519.1|:22461-22675
MsHel-1	gb|AIXA01007379.1|:1-211
MsHel-1	gb|AIXA01005631.1|:8537-8691
MsHel-1	gb|AIXA01005631.1|:20220-20339
MsHel-1	gb|AIXA01005592.1|:1-96
MsHel-1	gb|AIXA01005592.1|:31115-31319
MsHel-1	gb|AIXA01004358.1|:8683-8887
MsHel-1	gb|AIXA01000771.1|:33012-33384
MsHel-1	gb|AIXA01025972.1|:642-902
MsHel-1	gb|AIXA01016485.1|:2877-3835
MsHel-1	gb|AIXA01006624.1|:30276-30737
MsHel-1	gb|AIXA01002848.1|:163-372
MsHel-1	gb|AIXA01023025.1|:1466-1571
MsHel-1	gb|AIXA01013440.1|:4225-4411
MsHel-1	gb|AIXA01015055.1|:47562-47683
MsHel-1	gb|AIXA01011747.1|:21157-21345
MsHel-1	gb|AIXA01008381.1|:3788-4055
MsHel-1	gb|AIXA01008010.1|:16383-16667
MsHel-1	gb|AIXA01006253.1|:10365-10459

MsHel-1	gb|AIXA01032438.1|:1-104
MsHel-1	gb|AIXA01032239.1|:642-745
MsHel-1	gb|AIXA01025671.1|:524-664
MsHel-1	gb|AIXA01019400.1|:4041-4152
MsHel-1	gb|AIXA01010748.1|:116151-116260
MsHel-1	gb|AIXA01008802.1|:5753-6152
MsHel-1	gb|AIXA01002933.1|:10721-11199
MsHel-1	gb|AIXA01000287.1|:23746-24059
MsHel-1	gb|AIXA01017424.1|:1846-1959
MsHel-1	gb|AIXA01017238.1|:161-554
MsHel-1	gb|AIXA01010571.1|:602-905
MsHel-1	gb|AIXA01004081.1|:1319-1613
MsHel-1	gb|AIXA01033436.1|:557-685
MsHel-1	gb|AIXA01024494.1|:494-719
MsHel-1	gb|AIXA01006386.1|:27617-27851
MsHel-1	gb|AIXA01005878.1|:433-553
MsHel-1	gb|AIXA01004106.1|:1740-1873
MsHel-1	gb|AIXA01004106.1|:16139-16291
MsHel-1	gb|AIXA01014736.1|:2-132
MsHel-1	gb|AIXA01009070.1|:1029-1318
MsHel-1	gb|AIXA01037412.1|:421-531
MsHel-1	gb|AIXA01028892.1|:819-933
MsHel-1	gb|AIXA01017270.1|:3154-3260
MsHel-1	gb|AIXA01006319.1|:9376-9566
MsHel-1	gb|AIXA01005276.1|:438-540
MsHel-1	gb|AIXA01034785.1|:1-201
MsHel-1	gb|AIXA01007147.1|:35686-35855
MsHel-1	gb|AIXA01036719.1|:1-109
MsHel-1	gb|AIXA01031636.1|:5-117
MsHel-1	gb|AIXA01019558.1|:1780-1969
MsHel-1	gb|AIXA01014216.1|:6483-6864
MsHel-1	gb|AIXA01008925.1|:7249-7868
MsHel-1	gb|AIXA01000140.1|:19979-20180
MsHel-1	gb|AIXA01035899.1|:471-582
MsHel-1	gb|AIXA01026623.1|:1000-1107
MsHel-1	gb|AIXA01023464.1|:1400-1495
MsHel-1	gb|AIXA01014213.1|:10648-10781
MsHel-1	gb|AIXA01014213.1|:25854-26195
MsHel-1	gb|AIXA01002566.1|:11637-11840
MsHel-1	gb|AIXA01030454.1|:384-639
MsHel-1	gb|AIXA01022158.1|:1031-1154
MsHel-1	gb|AIXA01017554.1|:64-339
MsHel-1	gb|AIXA01008762.1|:50448-50553
MsHel-1	gb|AIXA01000944.1|:58064-58269
MsHel-1	gb|AIXA01036891.1|:1-203
MsHel-1	gb|AIXA01033750.1|:569-670
MsHel-1	gb|AIXA01016396.1|:1157-1534
MsHel-1	gb|AIXA01014800.1|:3423-3708

MsHel-1	gb|AIXA01013304.1|:4937-5125
MsHel-1	gb|AIXA01003151.1|:71905-72089
MsHel-1	gb|AIXA01001822.1|:14408-14534
MsHel-1	gb|AIXA01030865.1|:217-567
MsHel-1	gb|AIXA01021545.1|:878-1114
MsHel-1	gb|AIXA01015250.1|:10057-10149
MsHel-1	gb|AIXA01006225.1|:1794-2101
MsHel-1	gb|AIXA01005352.1|:78825-79112
MsHel-1	gb|AIXA01003194.1|:15007-15758
MsHel-1	gb|AIXA01003004.1|:4162-4362
MsHel-1	gb|AIXA01003004.1|:26629-27010
MsHel-1	gb|AIXA01002635.1|:43766-44067
MsHel-1	gb|AIXA01000459.1|:12682-12901
MsHel-1	gb|AIXA01000459.1|:45823-45978
MsHel-1	gb|AIXA01026556.1|:3-144
MsHel-1	gb|AIXA01013027.1|:22392-22602
MsHel-1	gb|AIXA01009301.1|:4110-6404
MsHel-1	gb|AIXA01026501.1|:2-193
MsHel-1	gb|AIXA01012231.1|:22366-22484
MsHel-1	gb|AIXA01012231.1|:33881-35265
MsHel-1	gb|AIXA01011788.1|:846-952
MsHel-1	gb|AIXA01007736.1|:715-901
MsHel-1	gb|AIXA01005777.1|:238-487
MsHel-1	gb|AIXA01015382.1|:6195-6383
MsHel-1	gb|AIXA01012642.1|:5718-6014
MsHel-1	gb|AIXA01010406.1|:1357-1641
MsHel-1	gb|AIXA01010406.1|:56480-56744
MsHel-1	gb|AIXA01008257.1|:22425-22522
MsHel-1	gb|AIXA01007751.1|:1539-1896
MsHel-1	gb|AIXA01005563.1|:36676-36849
MsHel-1	gb|AIXA01005563.1|:76427-76657
MsHel-1	gb|AIXA01005030.1|:96560-96751
MsHel-1	gb|AIXA01000767.1|:25845-26051
MsHel-1	gb|AIXA01036492.1|:470-562
MsHel-1	gb|AIXA01031570.1|:670-770
MsHel-1	gb|AIXA01021849.1|:1754-1850
MsHel-1	gb|AIXA01013854.1|:4380-4583
MsHel-1	gb|AIXA01013621.1|:20505-20724
MsHel-1	gb|AIXA01010885.1|:28121-28302
MsHel-1	gb|AIXA01010885.1|:43933-44120
MsHel-1	gb|AIXA01010885.1|:58141-58335
MsHel-1	gb|AIXA01010795.1|:11511-11599
MsHel-1	gb|AIXA01008380.1|:3335-3634
MsHel-1	gb|AIXA01004190.1|:47921-48017
MsHel-1	gb|AIXA01000522.1|:3874-4055
MsHel-1	gb|AIXA01034667.1|:1-92
MsHel-1	gb|AIXA01018351.1|:6499-6601
MsHel-1	gb|AIXA01009790.1|:7056-7231

MsHel-1	gb|AIXA01006318.1|:7667-7861
MsHel-1	gb|AIXA01002244.1|:22582-30581
MsHel-1	gb|AIXA01000246.1|:54962-55053
MsHel-1	gb|AIXA01023179.1|:201-452
MsHel-1	gb|AIXA01012537.1|:8780-9152
MsHel-1	gb|AIXA01008574.1|:60203-60438
MsHel-1	gb|AIXA01023392.1|:1-90
MsHel-1	gb|AIXA01020949.1|:867-1146
MsHel-1	gb|AIXA01020311.1|:3686-3779
MsHel-1	gb|AIXA01010278.1|:1961-2187
MsHel-1	gb|AIXA01000765.1|:8022-8178
MsHel-1	gb|AIXA01033891.1|:256-435
MsHel-1	gb|AIXA01019542.1|:2520-2684
MsHel-1	gb|AIXA01013796.1|:19338-19524
MsHel-1	gb|AIXA01005950.1|:3729-3825
MsHel-1	gb|AIXA01003150.1|:12621-12901
MsHel-1	gb|AIXA01001795.1|:32332-32629
MsHel-1	gb|AIXA01000517.1|:42778-43028
MsHel-1	gb|AIXA01019568.1|:2175-2298
MsHel-1	gb|AIXA01017935.1|:4856-4948
MsHel-1	gb|AIXA01013963.1|:1-1204
MsHel-1	gb|AIXA01013632.1|:17652-17960
MsHel-1	gb|AIXA01011672.1|:4902-5093
MsHel-1	gb|AIXA01009384.1|:9353-9440
MsHel-1	gb|AIXA01004997.1|:10539-10702
MsHel-1	gb|AIXA01004100.1|:25528-25723
MsHel-1	gb|AIXA01004100.1|:41482-41591
MsHel-1	gb|AIXA01004100.1|:72204-72374
MsHel-1	gb|AIXA01003057.1|:99006-99307
MsHel-1	gb|AIXA01001492.1|:83875-84086
MsHel-1	gb|AIXA01023622.1|:18-193
MsHel-1	gb|AIXA01022861.1|:1455-1606
MsHel-1	gb|AIXA01014700.1|:2023-2292
MsHel-1	gb|AIXA01011608.1|:1-103
MsHel-1	gb|AIXA01010137.1|:3747-4125
MsHel-1	gb|AIXA01003042.1|:160291-160381
MsHel-1	gb|AIXA01002816.1|:10-185
MsHel-1	gb|AIXA01001951.1|:28359-28519
MsHel-1	gb|AIXA01023502.1|:32-284
MsHel-1	gb|AIXA01017574.1|:3177-5048
MsHel-1	gb|AIXA01013754.1|:1086-1282
MsHel-1	gb|AIXA01013754.1|:31375-32358
MsHel-1	gb|AIXA01012380.1|:6281-6402
MsHel-1	gb|AIXA01011331.1|:5144-5308
MsHel-1	gb|AIXA01010402.1|:189-389
MsHel-1	gb|AIXA01010132.1|:21239-21439
MsHel-1	gb|AIXA01008171.1|:5-186
MsHel-1	gb|AIXA01008124.1|:44743-45872

MsHel-1	gb|AIXA01001509.1|:75468-75673
MsHel-1	gb|AIXA01000440.1|:65831-66055
MsHel-1	gb|AIXA01000061.1|:32475-32625
MsHel-1	gb|AIXA01029745.1|:2-143
MsHel-1	gb|AIXA01017316.1|:1189-1498
MsHel-1	gb|AIXA01016394.1|:1-81
MsHel-1	gb|AIXA01016138.1|:18394-25253
MsHel-1	gb|AIXA01015862.1|:19211-19411
MsHel-1	gb|AIXA01009946.1|:15754-15874
MsHel-1	gb|AIXA01009126.1|:18749-18920
MsHel-1	gb|AIXA01008044.1|:39205-39373
MsHel-1	gb|AIXA01006689.1|:53899-54047
MsHel-1	gb|AIXA01005654.1|:36520-36636
MsHel-1	gb|AIXA01004047.1|:26861-27077
MsHel-1	gb|AIXA01003160.1|:23286-23610
MsHel-1	gb|AIXA01015469.1|:2497-2612
MsHel-1	gb|AIXA01015132.1|:47946-48091
MsHel-1	gb|AIXA01014276.1|:1-88
MsHel-1	gb|AIXA01013343.1|:4842-4929
MsHel-1	gb|AIXA01007390.1|:1009-1268
MsHel-1	gb|AIXA01006619.1|:1478-1573
MsHel-1	gb|AIXA01029617.1|:319-621
MsHel-1	gb|AIXA01017358.1|:706-951
MsHel-1	gb|AIXA01017178.1|:3231-3408
MsHel-1	gb|AIXA01008568.1|:564-798
MsHel-1	gb|AIXA01004724.1|:51833-52138
MsHel-1	gb|AIXA01003198.1|:27266-27468
MsHel-1	gb|AIXA01000895.1|:47938-48398
MsHel-1	gb|AIXA01028933.1|:562-742
MsHel-1	gb|AIXA01020606.1|:1-86
MsHel-1	gb|AIXA01018211.1|:1431-1620
MsHel-1	gb|AIXA01011457.1|:19371-19531
MsHel-1	gb|AIXA01006699.1|:22213-22445
MsHel-1	gb|AIXA01003063.1|:31247-31494
MsHel-1	gb|AIXA01002155.1|:13476-13681
MsHel-1	gb|AIXA01024320.1|:1281-1361
MsHel-1	gb|AIXA01015713.1|:1240-1521
MsHel-1	gb|AIXA01015301.1|:1492-1861
MsHel-1	gb|AIXA01015083.1|:383-603
MsHel-1	gb|AIXA01013846.1|:2382-2644
MsHel-1	gb|AIXA01010620.1|:88-918
MsHel-1	gb|AIXA01009184.1|:13512-13895
MsHel-1	gb|AIXA01006130.1|:8679-8881
MsHel-1	gb|AIXA01005887.1|:6000-6184
MsHel-1	gb|AIXA01005735.1|:7-99
MsHel-1	gb|AIXA01005542.1|:33555-33695
MsHel-1	gb|AIXA01020945.1|:2095-2254
MsHel-1	gb|AIXA01015275.1|:2064-2426

MsHel-1	gb|AIXA01002690.1|:4881-5047
MsHel-1	gb|AIXA01001273.1|:62506-62589
MsHel-1	gb|AIXA01000363.1|:10445-10739
MsHel-1	gb|AIXA01028309.1|:890-972
MsHel-1	gb|AIXA01011379.1|:3-89
MsHel-1	gb|AIXA01010481.1|:15653-15846
MsHel-1	gb|AIXA01008773.1|:17141-17275
MsHel-1	gb|AIXA01006988.1|:10367-10584
MsHel-1	gb|AIXA01005822.1|:20738-21027
MsHel-1	gb|AIXA01005683.1|:5204-5338
MsHel-1	gb|AIXA01001681.1|:60978-61064
MsHel-1	gb|AIXA01000394.1|:9697-10073
MsHel-1	gb|AIXA01023980.1|:682-1053
MsHel-1	gb|AIXA01017089.1|:4055-4374
MsHel-1	gb|AIXA01010183.1|:2424-2755
MsHel-1	gb|AIXA01006587.1|:1716-1852
MsHel-1	gb|AIXA01005360.1|:1261-1487
MsHel-1	gb|AIXA01005312.1|:84558-84639
MsHel-1	gb|AIXA01005107.1|:2698-10094
MsHel-1	gb|AIXA01002510.1|:38258-38461
MsHel-1	gb|AIXA01002396.1|:2936-3211
MsHel-1	gb|AIXA01002240.1|:19428-19596
MsHel-1	gb|AIXA01001963.1|:6098-6358
MsHel-1	gb|AIXA01031783.1|:549-685
MsHel-1	gb|AIXA01027415.1|:14-210
MsHel-1	gb|AIXA01026611.1|:980-1109
MsHel-1	gb|AIXA01020181.1|:2503-2873
MsHel-1	gb|AIXA01015296.1|:8903-9097
MsHel-1	gb|AIXA01014849.1|:14267-14639
MsHel-1	gb|AIXA01010663.1|:43080-43171
MsHel-1	gb|AIXA01009215.1|:5391-5658
MsHel-1	gb|AIXA01004447.1|:3015-3106
MsHel-1	gb|AIXA01002183.1|:8767-8930
MsHel-1	gb|AIXA01001266.1|:44594-44809
MsHel-1	gb|AIXA01001266.1|:88699-88915
MsHel-1	gb|AIXA01000512.1|:5186-5277
MsHel-1	gb|AIXA01000512.1|:59409-59622
MsHel-1	gb|AIXA01018611.1|:142-705
MsHel-1	gb|AIXA01017553.1|:52-329
MsHel-1	gb|AIXA01016500.1|:2717-2844
MsHel-1	gb|AIXA01015244.1|:10-412
MsHel-1	gb|AIXA01015093.1|:7846-8153
MsHel-1	gb|AIXA01013762.1|:6690-7053
MsHel-1	gb|AIXA01013406.1|:337-504
MsHel-1	gb|AIXA01012399.1|:33298-33753
MsHel-1	gb|AIXA01005613.1|:11462-11738
MsHel-1	gb|AIXA01001939.1|:40945-41109
MsHel-1	gb|AIXA01001398.1|:10174-10356

MsHel-1	gb|AIXA01014040.1|:24713-24888
MsHel-1	gb|AIXA01008919.1|:2737-2916
MsHel-1	gb|AIXA01006222.1|:23851-23933
MsHel-1	gb|AIXA01005495.1|:1-83
MsHel-1	gb|AIXA01005132.1|:31074-31288
MsHel-1	gb|AIXA01004678.1|:16120-16420
MsHel-1	gb|AIXA01003178.1|:1-83
MsHel-1	gb|AIXA01003103.1|:46356-46507
MsHel-1	gb|AIXA01000641.1|:60764-61065
MsHel-1	gb|AIXA01000544.1|:27988-28205
MsHel-1	gb|AIXA01013210.1|:95-288
MsHel-1	gb|AIXA01008110.1|:55622-55742
MsHel-1	gb|AIXA01008110.1|:68539-68712
MsHel-1	gb|AIXA01007344.1|:35010-35468
MsHel-1	gb|AIXA01005128.1|:30216-30319
MsHel-1	gb|AIXA01004976.1|:73474-73683
MsHel-1	gb|AIXA01004492.1|:7424-7547
MsHel-1	gb|AIXA01003249.1|:1-178
MsHel-1	gb|AIXA01003058.1|:17000-17190
MsHel-1	gb|AIXA01002850.1|:110975-111149
MsHel-1	gb|AIXA01002361.1|:28803-29020
MsHel-1	gb|AIXA01037800.1|:437-517
MsHel-1	gb|AIXA01031116.1|:29-622
MsHel-1	gb|AIXA01003158.1|:10787-11035
MsHel-1	gb|AIXA01002485.1|:6689-7009
MsHel-1	gb|AIXA01002187.1|:1-125
MsHel-1	gb|AIXA01000903.1|:20460-20665
MsHel-1	gb|AIXA01015654.1|:9118-9216
MsHel-1	gb|AIXA01013244.1|:220-399
MsHel-1	gb|AIXA01012633.1|:12259-12547
MsHel-1	gb|AIXA01012204.1|:23758-23891
MsHel-1	gb|AIXA01011194.1|:10775-11034
MsHel-1	gb|AIXA01007557.1|:43228-43378
MsHel-1	gb|AIXA01005465.1|:48728-48934
MsHel-1	gb|AIXA01002788.1|:17279-17485
MsHel-1	gb|AIXA01001067.1|:11306-11466
MsHel-1	gb|AIXA01001057.1|:74928-75149
MsHel-1	gb|AIXA01012403.1|:74125-74289
MsHel-1	gb|AIXA01011682.1|:35343-35542
MsHel-1	gb|AIXA01011454.1|:26268-26403
MsHel-1	gb|AIXA01011387.1|:14622-14834
MsHel-1	gb|AIXA01005855.1|:84013-84098
MsHel-1	gb|AIXA01004691.1|:1-83
MsHel-1	gb|AIXA01003360.1|:27631-27814
MsHel-1	gb|AIXA01003122.1|:13412-13527
MsHel-1	gb|AIXA01003122.1|:24902-25147
MsHel-1	gb|AIXA01002917.1|:38054-38256
MsHel-1	gb|AIXA01002830.1|:16903-17103

MsHel-1	gb|AIXA01000810.1|:309424-309601
MsHel-1	gb|AIXA01026192.1|:560-705
MsHel-1	gb|AIXA01024623.1|:1219-1320
MsHel-1	gb|AIXA01020798.1|:156-639
MsHel-1	gb|AIXA01015834.1|:7069-7354
MsHel-1	gb|AIXA01011782.1|:121-354
MsHel-1	gb|AIXA01011197.1|:77303-77480
MsHel-1	gb|AIXA01010342.1|:16278-16489
MsHel-1	gb|AIXA01006834.1|:104604-104841
MsHel-1	gb|AIXA01005605.1|:39726-39861
MsHel-1	gb|AIXA01002170.1|:61410-61742
MsHel-1	gb|AIXA01000732.1|:73023-73140
MsHel-1	gb|AIXA01021748.1|:1631-1886
MsHel-1	gb|AIXA01016208.1|:24294-24512
MsHel-1	gb|AIXA01013681.1|:1003-1823
MsHel-1	gb|AIXA01013015.1|:45458-45599
MsHel-1	gb|AIXA01012588.1|:14623-14823
MsHel-1	gb|AIXA01011649.1|:11819-11979
MsHel-1	gb|AIXA01010344.1|:297-433
MsHel-1	gb|AIXA01009257.1|:18072-18351
MsHel-1	gb|AIXA01008167.1|:44248-44354
MsHel-1	gb|AIXA01008167.1|:74374-74475
MsHel-1	gb|AIXA01007632.1|:5711-5831
MsHel-1	gb|AIXA01007017.1|:59977-60111
MsHel-1	gb|AIXA01007011.1|:10886-11089
MsHel-1	gb|AIXA01005612.1|:55926-56060
MsHel-1	gb|AIXA01001985.1|:23812-24088
MsHel-1	gb|AIXA01000126.1|:46817-46975
MsHel-1	gb|AIXA01021145.1|:218-397
MsHel-1	gb|AIXA01019504.1|:668-1016
MsHel-1	gb|AIXA01017746.1|:10463-10663
MsHel-1	gb|AIXA01015600.1|:4659-4964
MsHel-1	gb|AIXA01014211.1|:2410-2594
MsHel-1	gb|AIXA01012548.1|:7558-7733
MsHel-1	gb|AIXA01009098.1|:2092-2288
MsHel-1	gb|AIXA01008480.1|:27613-27804
MsHel-1	gb|AIXA01008155.1|:2345-2472
MsHel-1	gb|AIXA01007732.1|:4800-4926
MsHel-1	gb|AIXA01007242.1|:6668-6747
MsHel-1	gb|AIXA01007204.1|:2186-2357
MsHel-1	gb|AIXA01006264.1|:41476-41805
MsHel-1	gb|AIXA01005835.1|:1219-1346
MsHel-1	gb|AIXA01005410.1|:15840-16035
MsHel-1	gb|AIXA01003876.1|:125816-125939
MsHel-1	gb|AIXA01003758.1|:8207-8445
MsHel-1	gb|AIXA01003560.1|:1610-1813
MsHel-1	gb|AIXA01003239.1|:9250-9522
MsHel-1	gb|AIXA01001679.1|:6673-6952

MsHel-1	gb|AIXA01033778.1|:328-478
MsHel-1	gb|AIXA01015325.1|:90-204
MsHel-1	gb|AIXA01014467.1|:7519-7606
MsHel-1	gb|AIXA01013314.1|:1368-1581
MsHel-1	gb|AIXA01012114.1|:9306-9601
MsHel-1	gb|AIXA01009856.1|:63490-63690
MsHel-1	gb|AIXA01008871.1|:22267-22714
MsHel-1	gb|AIXA01008739.1|:14821-15082
MsHel-1	gb|AIXA01004656.1|:135185-135319
MsHel-1	gb|AIXA01004465.1|:56615-56818
MsHel-1	gb|AIXA01002040.1|:2597-2771
MsHel-1	gb|AIXA01000847.1|:1450-1638
MsHel-1	gb|AIXA01000356.1|:20904-21062
MsHel-1	gb|AIXA01037243.1|:37-255
MsHel-1	gb|AIXA01015334.1|:450-547
MsHel-1	gb|AIXA01013089.1|:488-694
MsHel-1	gb|AIXA01012167.1|:5291-5564
MsHel-1	gb|AIXA01012005.1|:28522-28689
MsHel-1	gb|AIXA01011273.1|:40935-41202
MsHel-1	gb|AIXA01009037.1|:1114-1276
MsHel-1	gb|AIXA01005918.1|:62189-65449
MsHel-1	gb|AIXA01004115.1|:105089-105194
MsHel-1	gb|AIXA01002737.1|:43808-44010
MsHel-1	gb|AIXA01002412.1|:15888-16028
MsHel-1	gb|AIXA01002119.1|:10559-10708
MsHel-1	gb|AIXA01000408.1|:44646-44950
MsHel-1	gb|AIXA01022248.1|:248-484
MsHel-1	gb|AIXA01022176.1|:590-702
MsHel-1	gb|AIXA01020182.1|:721-932
MsHel-1	gb|AIXA01018244.1|:3541-3669
MsHel-1	gb|AIXA01017355.1|:1-128
MsHel-1	gb|AIXA01015381.1|:3291-3505
MsHel-1	gb|AIXA01014951.1|:1209-1482
MsHel-1	gb|AIXA01014804.1|:155-289
MsHel-1	gb|AIXA01013385.1|:6997-7077
MsHel-1	gb|AIXA01011995.1|:25697-25873
MsHel-1	gb|AIXA01009369.1|:10859-11082
MsHel-1	gb|AIXA01009245.1|:5171-5336
MsHel-1	gb|AIXA01009239.1|:317-429
MsHel-1	gb|AIXA01007488.1|:1-78
MsHel-1	gb|AIXA01004720.1|:13688-13829
MsHel-1	gb|AIXA01003617.1|:6565-6647
MsHel-1	gb|AIXA01001823.1|:51994-52128
MsHel-1	gb|AIXA01001095.1|:4391-4543
MsHel-1	gb|AIXA01001034.1|:9-144
MsHel-1	gb|AIXA01032163.1|:68-173
MsHel-1	gb|AIXA01016265.1|:9081-9231
MsHel-1	gb|AIXA01014492.1|:9217-9436

MsHel-1	gb|AIXA01014398.1|:2255-2533
MsHel-1	gb|AIXA01014008.1|:386-524
MsHel-1	gb|AIXA01012678.1|:12017-12228
MsHel-1	gb|AIXA01009983.1|:14223-14334
MsHel-1	gb|AIXA01007642.1|:109152-109283
MsHel-1	gb|AIXA01002633.1|:8192-8347
MsHel-1	gb|AIXA01001913.1|:5156-5338
MsHel-1	gb|AIXA01020460.1|:1846-1969
MsHel-1	gb|AIXA01019837.1|:24-104
MsHel-1	gb|AIXA01017219.1|:8625-8806
MsHel-1	gb|AIXA01015067.1|:765-875
MsHel-1	gb|AIXA01014124.1|:19773-19998
MsHel-1	gb|AIXA01013407.1|:8959-9119
MsHel-1	gb|AIXA01012148.1|:35218-35397
MsHel-1	gb|AIXA01012034.1|:17172-17455
MsHel-1	gb|AIXA01010962.1|:8455-8637
MsHel-1	gb|AIXA01008923.1|:68367-68511
MsHel-1	gb|AIXA01007967.1|:157-523
MsHel-1	gb|AIXA01007350.1|:7117-7220
MsHel-1	gb|AIXA01003346.1|:1-156
MsHel-1	gb|AIXA01003253.1|:21828-22040
MsHel-1	gb|AIXA01001890.1|:28716-28810
MsHel-1	gb|AIXA01001401.1|:13057-13276
MsHel-1	gb|AIXA01001058.1|:16091-16429
MsHel-1	gb|AIXA01000623.1|:15383-15549
MsHel-1	gb|AIXA01017926.1|:3800-3999
MsHel-1	gb|AIXA01010002.1|:13642-13815
MsHel-1	gb|AIXA01009763.1|:39985-40111
MsHel-1	gb|AIXA01008927.1|:11618-11759
MsHel-1	gb|AIXA01005113.1|:7688-7862
MsHel-1	gb|AIXA01004558.1|:13995-14144
MsHel-1	gb|AIXA01001770.1|:40659-40792
MsHel-1	gb|AIXA01000218.1|:12558-12766
MsHel-1	gb|AIXA01000091.1|:20306-20658
MsHel-1	gb|AIXA01000024.1|:22076-22297
MsHel-1	gb|AIXA01021307.1|:75-192
MsHel-1	gb|AIXA01020871.1|:1890-2087
MsHel-1	gb|AIXA01018112.1|:11704-11839
MsHel-1	gb|AIXA01017635.1|:10918-12637
MsHel-1	gb|AIXA01015820.1|:1262-1377
MsHel-1	gb|AIXA01015818.1|:3156-3277
MsHel-1	gb|AIXA01013320.1|:4500-4616
MsHel-1	gb|AIXA01012544.1|:4559-4892
MsHel-1	gb|AIXA01012164.1|:6613-6760
MsHel-1	gb|AIXA01010779.1|:12159-12315
MsHel-1	gb|AIXA01010429.1|:4896-5030
MsHel-1	gb|AIXA01009822.1|:10095-10227
MsHel-1	gb|AIXA01007090.1|:14867-15059

MsHel-1	gb|AIXA01006458.1|:2136-2361
MsHel-1	gb|AIXA01004137.1|:136612-136693
MsHel-1	gb|AIXA01004061.1|:50008-50183
MsHel-1	gb|AIXA01003927.1|:19654-19896
MsHel-1	gb|AIXA01003678.1|:66739-66873
MsHel-1	gb|AIXA01001983.1|:36997-37122
MsHel-1	gb|AIXA01001775.1|:66262-66445
MsHel-1	gb|AIXA01000395.1|:2565-2699
MsHel-1	gb|AIXA01000171.1|:16138-16257
MsHel-1	gb|AIXA01013794.1|:7461-7584
MsHel-1	gb|AIXA01010758.1|:20227-20362
MsHel-1	gb|AIXA01008854.1|:1475-1589
MsHel-1	gb|AIXA01008853.1|:3068-3182
MsHel-1	gb|AIXA01005391.1|:14555-14885
MsHel-1	gb|AIXA01005391.1|:54695-54806
MsHel-1	gb|AIXA01004286.1|:11145-11443
MsHel-1	gb|AIXA01003852.1|:7011-7282
MsHel-1	gb|AIXA01002852.1|:27329-27553
MsHel-1	gb|AIXA01002527.1|:155-237
MsHel-1	gb|AIXA01001218.1|:7671-7885
MsHel-1	gb|AIXA01001119.1|:34222-34321
MsHel-1	gb|AIXA01001005.1|:15219-15416
MsHel-1	gb|AIXA01031963.1|:13-107
MsHel-1	gb|AIXA01013360.1|:837-1200
MsHel-1	gb|AIXA01013359.1|:871-1151
MsHel-1	gb|AIXA01010742.1|:3095-3185
MsHel-1	gb|AIXA01010674.1|:2163-2332
MsHel-1	gb|AIXA01009948.1|:6286-6380
MsHel-1	gb|AIXA01009499.1|:5519-5752
MsHel-1	gb|AIXA01009019.1|:58416-59041
MsHel-1	gb|AIXA01008390.1|:10887-11028
MsHel-1	gb|AIXA01008237.1|:2122-2951
MsHel-1	gb|AIXA01007887.1|:9602-9724
MsHel-1	gb|AIXA01006970.1|:6187-6415
MsHel-1	gb|AIXA01004528.1|:23628-23722
MsHel-1	gb|AIXA01002582.1|:112066-112228
MsHel-1	gb|AIXA01002004.1|:18160-18306
MsHel-1	gb|AIXA01001919.1|:7039-7401
MsHel-1	gb|AIXA01001790.1|:25411-25617
MsHel-1	gb|AIXA01001476.1|:4352-4643
MsHel-1	gb|AIXA01000698.1|:38284-38489
MsHel-1	gb|AIXA01000574.1|:13412-13617
MsHel-1	gb|AIXA01014552.1|:5238-5435
MsHel-1	gb|AIXA01013651.1|:14874-14967
MsHel-1	gb|AIXA01012533.1|:15911-15993
MsHel-1	gb|AIXA01011679.1|:10336-10420
MsHel-1	gb|AIXA01011534.1|:7332-7555
MsHel-1	gb|AIXA01011030.1|:16307-16400

MsHel-1	gb|AIXA01010770.1|:550-711
MsHel-1	gb|AIXA01010769.1|:6565-9933
MsHel-1	gb|AIXA01009711.1|:3537-3739
MsHel-1	gb|AIXA01007603.1|:13985-14193
MsHel-1	gb|AIXA01005603.1|:15219-15338
MsHel-1	gb|AIXA01005603.1|:29040-29153
MsHel-1	gb|AIXA01005443.1|:8573-8950
MsHel-1	gb|AIXA01005068.1|:15591-15756
MsHel-1	gb|AIXA01004175.1|:4472-4580
MsHel-1	gb|AIXA01003204.1|:8-1678
MsHel-1	gb|AIXA01002673.1|:17555-17834
MsHel-1	gb|AIXA01002168.1|:18066-18147
MsHel-1	gb|AIXA01000132.1|:10080-10413
MsHel-1	gb|AIXA01000006.1|:41810-42010
MsHel-1	gb|AIXA01016197.1|:926-1378
MsHel-1	gb|AIXA01015819.1|:2863-2978
MsHel-1	gb|AIXA01013945.1|:31193-31423
MsHel-1	gb|AIXA01013560.1|:6944-7032
MsHel-1	gb|AIXA01012650.1|:8229-8357
MsHel-1	gb|AIXA01012531.1|:2617-2800
MsHel-1	gb|AIXA01010461.1|:1710-1902
MsHel-1	gb|AIXA01009319.1|:14837-15038
MsHel-1	gb|AIXA01006964.1|:1-127
MsHel-1	gb|AIXA01002311.1|:8500-8672
MsHel-1	gb|AIXA01002109.1|:7340-7602
MsHel-1	gb|AIXA01001850.1|:22021-22168
MsHel-1	gb|AIXA01001050.1|:18707-18863
MsHel-1	gb|AIXA01000964.1|:30593-30704
MsHel-1	gb|AIXA01000437.1|:155701-155915
MsHel-1	gb|AIXA01000155.1|:36916-37061
MsHel-1	gb|AIXA01000155.1|:49687-49813
MsHel-1	gb|AIXA01032237.1|:305-428
MsHel-1	gb|AIXA01031236.1|:86-185
MsHel-1	gb|AIXA01017813.1|:6581-6828
MsHel-1	gb|AIXA01016843.1|:200-319
MsHel-1	gb|AIXA01016207.1|:1662-1783
MsHel-1	gb|AIXA01013756.1|:1638-1753
MsHel-1	gb|AIXA01013661.1|:597-760
MsHel-1	gb|AIXA01013602.1|:121-319
MsHel-1	gb|AIXA01012285.1|:19064-19222
MsHel-1	gb|AIXA01012023.1|:1364-1941
MsHel-1	gb|AIXA01012003.1|:6327-6418
MsHel-1	gb|AIXA01011669.1|:5159-5260
MsHel-1	gb|AIXA01010853.1|:1356-1523
MsHel-1	gb|AIXA01007731.1|:66199-66297
MsHel-1	gb|AIXA01007567.1|:31489-31651
MsHel-1	gb|AIXA01003583.1|:7387-7466
MsHel-1	gb|AIXA01003510.1|:13249-13348

MsHel-1	gb|AIXA01000876.1|:24450-24578
MsHel-1	gb|AIXA01000719.1|:7386-7581
MsHel-1	gb|AIXA01020096.1|:821-1007
MsHel-1	gb|AIXA01012978.1|:3067-3181
MsHel-1	gb|AIXA01012685.1|:1601-1717
MsHel-1	gb|AIXA01012373.1|:5713-5856
MsHel-1	gb|AIXA01012264.1|:4854-5041
MsHel-1	gb|AIXA01009348.1|:25101-25303
MsHel-1	gb|AIXA01007118.1|:22367-22508
MsHel-1	gb|AIXA01007110.1|:15476-15677
MsHel-1	gb|AIXA01006827.1|:41360-41563
MsHel-1	gb|AIXA01004267.1|:4429-4555
MsHel-1	gb|AIXA01004179.1|:42-277
MsHel-1	gb|AIXA01004090.1|:17233-17373
MsHel-1	gb|AIXA01003575.1|:62-415
MsHel-1	gb|AIXA01002857.1|:32304-32408
MsHel-1	gb|AIXA01002732.1|:61200-61319
MsHel-1	gb|AIXA01002706.1|:16402-16532
MsHel-1	gb|AIXA01002513.1|:23501-23707
MsHel-1	gb|AIXA01000993.1|:19737-19871
MsHel-1	gb|AIXA01014199.1|:8557-8731
MsHel-1	gb|AIXA01014180.1|:965-1098
MsHel-1	gb|AIXA01013849.1|:1184-1338
MsHel-1	gb|AIXA01012798.1|:8423-8539
MsHel-1	gb|AIXA01012529.1|:38788-38909
MsHel-1	gb|AIXA01011494.1|:17475-17593
MsHel-1	gb|AIXA01010281.1|:7846-7984
MsHel-1	gb|AIXA01005997.1|:7341-7478
MsHel-1	gb|AIXA01005783.1|:26739-26928
MsHel-1	gb|AIXA01004122.1|:10851-10997
MsHel-1	gb|AIXA01003357.1|:18420-18600
MsHel-1	gb|AIXA01002006.1|:27054-27168
MsHel-1	gb|AIXA01001699.1|:73043-73175
MsHel-1	gb|AIXA01001046.1|:9640-9828
MsHel-1	gb|AIXA01000918.1|:615-727
MsHel-1	gb|AIXA01000468.1|:599-756
MsHel-1	gb|AIXA01027950.1|:158-394
MsHel-1	gb|AIXA01018652.1|:245-447
MsHel-1	gb|AIXA01017868.1|:1752-1859
MsHel-1	gb|AIXA01017779.1|:16673-16840
MsHel-1	gb|AIXA01014751.1|:5368-5468
MsHel-1	gb|AIXA01014307.1|:2492-2767
MsHel-1	gb|AIXA01010914.1|:2429-2563
MsHel-1	gb|AIXA01009542.1|:2806-2959
MsHel-1	gb|AIXA01009015.1|:41536-41619
MsHel-1	gb|AIXA01007386.1|:5631-5828
MsHel-1	gb|AIXA01006033.1|:5298-5454
MsHel-1	gb|AIXA01004884.1|:130-264

MsHel-1	gb|AIXA01004037.1|:16154-16332
MsHel-1	gb|AIXA01003214.1|:64165-64383
MsHel-1	gb|AIXA01001926.1|:7298-7418
MsHel-1	gb|AIXA01001849.1|:4859-4991
MsHel-1	gb|AIXA01001472.1|:32114-32286
MsHel-1	gb|AIXA01001082.1|:6191-6404
MsHel-1	gb|AIXA01001082.1|:23690-23817
MsHel-1	gb|AIXA01000614.1|:472-573
MsHel-1	gb|AIXA01017242.1|:658-777
MsHel-1	gb|AIXA01016466.1|:3414-3521
MsHel-1	gb|AIXA01016081.1|:10773-10872
MsHel-1	gb|AIXA01014208.1|:7137-7228
MsHel-1	gb|AIXA01011992.1|:48690-48786
MsHel-1	gb|AIXA01010868.1|:3429-3520
MsHel-1	gb|AIXA01009213.1|:22327-22670
MsHel-1	gb|AIXA01006963.1|:6980-7165
MsHel-1	gb|AIXA01005895.1|:2579-2666
MsHel-1	gb|AIXA01003661.1|:168771-168901
MsHel-1	gb|AIXA01002999.1|:9633-9779
MsHel-1	gb|AIXA01001943.1|:15392-15591
MsHel-1	gb|AIXA01000841.1|:33487-33678
MsHel-1	gb|AIXA01000662.1|:89782-89901
MsHel-1	gb|AIXA01024714.1|:480-1164
MsHel-1	gb|AIXA01016767.1|:21151-21297
MsHel-1	gb|AIXA01016736.1|:5355-5505
MsHel-1	gb|AIXA01016534.1|:1681-1807
MsHel-1	gb|AIXA01009208.1|:21139-21323
MsHel-1	gb|AIXA01009208.1|:50242-50338
MsHel-1	gb|AIXA01007975.1|:34378-34563
MsHel-1	gb|AIXA01007652.1|:10670-11941
MsHel-1	gb|AIXA01006388.1|:15747-15879
MsHel-1	gb|AIXA01004708.1|:104665-104789
MsHel-1	gb|AIXA01004708.1|:139921-140121
MsHel-1	gb|AIXA01004139.1|:2332-2426
MsHel-1	gb|AIXA01003643.1|:28015-28279
MsHel-1	gb|AIXA01002897.1|:1985-2209
MsHel-1	gb|AIXA01002712.1|:16774-16887
MsHel-1	gb|AIXA01001357.1|:6058-6164
MsHel-1	gb|AIXA01001151.1|:22986-23141
MsHel-1	gb|AIXA01000914.1|:117450-117579
MsHel-1	gb|AIXA01000909.1|:86144-86244
MsHel-1	gb|AIXA01000320.1|:1368-1522
MsHel-1	gb|AIXA01021388.1|:1699-1988
MsHel-1	gb|AIXA01019493.1|:3937-4126
MsHel-1	gb|AIXA01018767.1|:6960-7080
MsHel-1	gb|AIXA01015062.1|:5024-5188
MsHel-1	gb|AIXA01014764.1|:1910-2482
MsHel-1	gb|AIXA01014610.1|:9044-9165

MsHel-1	gb|AIXA01013631.1|:59-211
MsHel-1	gb|AIXA01012853.1|:10306-10477
MsHel-1	gb|AIXA01012587.1|:2371-2555
MsHel-1	gb|AIXA01007964.1|:3042-3158
MsHel-1	gb|AIXA01007964.1|:23201-23469
MsHel-1	gb|AIXA01007856.1|:66344-66436
MsHel-1	gb|AIXA01007418.1|:865-998
MsHel-1	gb|AIXA01006035.1|:64309-64489
MsHel-1	gb|AIXA01005509.1|:22417-22559
MsHel-1	gb|AIXA01005328.1|:51564-51653
MsHel-1	gb|AIXA01005002.1|:8331-8464
MsHel-1	gb|AIXA01004275.1|:47-175
MsHel-1	gb|AIXA01003636.1|:2012-2184
MsHel-1	gb|AIXA01002069.1|:20145-20285
MsHel-1	gb|AIXA01002069.1|:66634-66746
MsHel-1	gb|AIXA01001413.1|:295-752
MsHel-1	gb|AIXA01001035.1|:4173-4278
MsHel-1	gb|AIXA01032537.1|:108-212
MsHel-1	gb|AIXA01021276.1|:796-950
MsHel-1	gb|AIXA01015564.1|:2413-2578
MsHel-1	gb|AIXA01012350.1|:12857-13634
MsHel-1	gb|AIXA01011281.1|:8740-8884
MsHel-1	gb|AIXA01010894.1|:5088-5204
MsHel-1	gb|AIXA01010752.1|:2732-2906
MsHel-1	gb|AIXA01010063.1|:11907-12018
MsHel-1	gb|AIXA01007979.1|:3841-4050
MsHel-1	gb|AIXA01006865.1|:10528-10652
MsHel-1	gb|AIXA01002147.1|:6461-6608
MsHel-1	gb|AIXA01034714.1|:488-595
MsHel-1	gb|AIXA01028614.1|:525-696
MsHel-1	gb|AIXA01020745.1|:1165-1284
MsHel-1	gb|AIXA01014676.1|:8028-8338
MsHel-1	gb|AIXA01013522.1|:1-128
MsHel-1	gb|AIXA01012583.1|:10359-10493
MsHel-1	gb|AIXA01012466.1|:23194-23280
MsHel-1	gb|AIXA01012150.1|:15368-15494
MsHel-1	gb|AIXA01011405.1|:14972-15137
MsHel-1	gb|AIXA01011231.1|:39-209
MsHel-1	gb|AIXA01011116.1|:4423-4535
MsHel-1	gb|AIXA01003845.1|:50297-50436
MsHel-1	gb|AIXA01003001.1|:41476-41750
MsHel-1	gb|AIXA01026140.1|:108-278
MsHel-1	gb|AIXA01020230.1|:49-198
MsHel-1	gb|AIXA01019233.1|:465-600
MsHel-1	gb|AIXA01017669.1|:1969-2054
MsHel-1	gb|AIXA01017229.1|:5327-5453
MsHel-1	gb|AIXA01016454.1|:3153-3287
MsHel-1	gb|AIXA01016073.1|:10008-10141

MsHel-1	gb|AIXA01015528.1|:20145-20272
MsHel-1	gb|AIXA01013342.1|:42441-42527
MsHel-1	gb|AIXA01013115.1|:5978-6197
MsHel-1	gb|AIXA01012695.1|:2745-2859
MsHel-1	gb|AIXA01011126.1|:8019-18084
MsHel-1	gb|AIXA01009093.1|:2621-3124
MsHel-1	gb|AIXA01008803.1|:889-1054
MsHel-1	gb|AIXA01008436.1|:6959-7128
MsHel-1	gb|AIXA01007749.1|:4149-4302
MsHel-1	gb|AIXA01006449.1|:93494-93584
MsHel-1	gb|AIXA01006019.1|:20707-20999
MsHel-1	gb|AIXA01005241.1|:7762-8174
MsHel-1	gb|AIXA01001673.1|:30647-30730
MsHel-1	gb|AIXA01001501.1|:119-328
MsHel-1	gb|AIXA01000448.1|:109755-109850
MsHel-1	gb|AIXA01000448.1|:133944-134034
MsHel-1	gb|AIXA01019618.1|:4023-4193
MsHel-1	gb|AIXA01015420.1|:3549-3675
MsHel-1	gb|AIXA01007457.1|:7112-7238
MsHel-1	gb|AIXA01006543.1|:14910-15212
MsHel-1	gb|AIXA01005834.1|:12124-12256
MsHel-1	gb|AIXA01005344.1|:28223-28304
MsHel-1	gb|AIXA01004277.1|:23196-23487
MsHel-1	gb|AIXA01003332.1|:117944-118037
MsHel-1	gb|AIXA01002065.1|:39902-40078
MsHel-1	gb|AIXA01001828.1|:4576-4701
MsHel-1	gb|AIXA01001493.1|:43588-43804
MsHel-1	gb|AIXA01001399.1|:21698-21777
MsHel-1	gb|AIXA01001359.1|:23204-23313
MsHel-1	gb|AIXA01000489.1|:41272-41558
MsHel-1	gb|AIXA01014185.1|:2764-2965
MsHel-1	gb|AIXA01013565.1|:33715-33832
MsHel-1	gb|AIXA01012323.1|:3671-3786
MsHel-1	gb|AIXA01011246.1|:9872-10174
MsHel-1	gb|AIXA01011233.1|:46407-46489
MsHel-1	gb|AIXA01009671.1|:9385-9763
MsHel-1	gb|AIXA01007816.1|:300-476
MsHel-1	gb|AIXA01007606.1|:17186-17411
MsHel-1	gb|AIXA01007260.1|:19365-19465
MsHel-1	gb|AIXA01006592.1|:17926-18042
MsHel-1	gb|AIXA01005687.1|:238-438
MsHel-1	gb|AIXA01005686.1|:238-357
MsHel-1	gb|AIXA01004958.1|:46313-46408
MsHel-1	gb|AIXA01004322.1|:22143-22231
MsHel-1	gb|AIXA01002555.1|:4829-5095
MsHel-1	gb|AIXA01002525.1|:3319-3468
MsHel-1	gb|AIXA01002439.1|:30942-31121
MsHel-1	gb|AIXA01001730.1|:30775-30953

MsHel-1	gb|AIXA01000490.1|:4788-4923
MsHel-1	gb|AIXA01019813.1|:366-664
MsHel-1	gb|AIXA01018201.1|:2020-2118
MsHel-1	gb|AIXA01013029.1|:4850-4985
MsHel-1	gb|AIXA01012493.1|:6785-6893
MsHel-1	gb|AIXA01012353.1|:1744-2082
MsHel-1	gb|AIXA01010322.1|:18704-18787
MsHel-1	gb|AIXA01009804.1|:15537-15722
MsHel-1	gb|AIXA01008487.1|:11811-11918
MsHel-1	gb|AIXA01007582.1|:2783-3107
MsHel-1	gb|AIXA01007438.1|:430-509
MsHel-1	gb|AIXA01005044.1|:15404-15524
MsHel-1	gb|AIXA01004614.1|:2200-2362
MsHel-1	gb|AIXA01003889.1|:76637-76748
MsHel-1	gb|AIXA01003814.1|:226-365
MsHel-1	gb|AIXA01002595.1|:34415-34536
MsHel-1	gb|AIXA01002402.1|:30283-30370
MsHel-1	gb|AIXA01002146.1|:5773-5904
MsHel-1	gb|AIXA01002060.1|:5149-5382
MsHel-1	gb|AIXA01000900.1|:23437-23564
MsHel-1	gb|AIXA01000424.1|:76124-76238
MsHel-1	gb|AIXA01000196.1|:13850-13933
MsHel-1	gb|AIXA01020796.1|:288-406
MsHel-1	gb|AIXA01018968.1|:720-815
MsHel-1	gb|AIXA01016335.1|:2299-2379
MsHel-1	gb|AIXA01013028.1|:25748-25918
MsHel-1	gb|AIXA01012786.1|:10749-10847
MsHel-1	gb|AIXA01012205.1|:313-486
MsHel-1	gb|AIXA01010778.1|:11106-11242
MsHel-1	gb|AIXA01006630.1|:8716-8818
MsHel-1	gb|AIXA01006243.1|:3638-3843
MsHel-1	gb|AIXA01006210.1|:16170-16251
MsHel-1	gb|AIXA01005985.1|:496-599
MsHel-1	gb|AIXA01004230.1|:60162-60310
MsHel-1	gb|AIXA01000175.1|:28295-28397
MsHel-1	gb|AIXA01030777.1|:584-739
MsHel-1	gb|AIXA01030591.1|:1-82
MsHel-1	gb|AIXA01023020.1|:1258-1377
MsHel-1	gb|AIXA01017579.1|:3847-3948
MsHel-1	gb|AIXA01007891.1|:39449-47322
MsHel-1	gb|AIXA01005843.1|:11941-12070
MsHel-1	gb|AIXA01005730.1|:54204-54310
MsHel-1	gb|AIXA01005615.1|:14141-14253
MsHel-1	gb|AIXA01005293.1|:1232-1333
MsHel-1	gb|AIXA01005266.1|:36113-36210
MsHel-1	gb|AIXA01000372.1|:83302-83439
MsHel-1	gb|AIXA01018365.1|:2786-2886
MsHel-1	gb|AIXA01017698.1|:3843-3988

MsHel-1	gb|AIXA01016008.1|:969-1057
MsHel-1	gb|AIXA01014762.1|:3475-3815
MsHel-1	gb|AIXA01012977.1|:4126-4372
MsHel-1	gb|AIXA01012021.1|:61977-62073
MsHel-1	gb|AIXA01011487.1|:2785-2980
MsHel-1	gb|AIXA01004427.1|:765-881
MsHel-1	gb|AIXA01004382.1|:7269-7370
MsHel-1	gb|AIXA01003416.1|:321-586
MsHel-1	gb|AIXA01003415.1|:2624-2889
MsHel-1	gb|AIXA01001841.1|:54248-54384
MsHel-1	gb|AIXA01000486.1|:65793-65905
MsHel-1	gb|AIXA01000135.1|:13287-13417
MsHel-1	gb|AIXA01000033.1|:19916-20015
MsHel-1	gb|AIXA01018340.1|:2045-2210
MsHel-1	gb|AIXA01016929.1|:8134-8212
MsHel-1	gb|AIXA01016155.1|:2700-2787
MsHel-1	gb|AIXA01012018.1|:3386-3496
MsHel-1	gb|AIXA01011732.1|:17702-17817
MsHel-1	gb|AIXA01009209.1|:6269-6377
MsHel-1	gb|AIXA01008405.1|:7184-7540
MsHel-1	gb|AIXA01005111.1|:6908-7015
MsHel-1	gb|AIXA01004844.1|:15138-15245
MsHel-1	gb|AIXA01004712.1|:7727-7891
MsHel-1	gb|AIXA01004271.1|:3725-3816
MsHel-1	gb|AIXA01004069.1|:17397-17488
MsHel-1	gb|AIXA01003623.1|:23343-23479
MsHel-1	gb|AIXA01002612.1|:28579-28699
MsHel-1	gb|AIXA01001686.1|:6712-6795
MsHel-1	gb|AIXA01000276.1|:6469-6587
MsHel-1	gb|AIXA01000008.1|:38115-38210
MsHel-1	gb|AIXA01035207.1|:31-122
MsHel-1	gb|AIXA01012980.1|:2030-2124
MsHel-1	gb|AIXA01011498.1|:18804-18899
MsHel-1	gb|AIXA01011343.1|:3335-3460
MsHel-1	gb|AIXA01008521.1|:6452-6603
MsHel-1	gb|AIXA01006860.1|:46897-47031
MsHel-1	gb|AIXA01006655.1|:51687-51834
MsHel-1	gb|AIXA01005764.1|:28932-29054
MsHel-1	gb|AIXA01004527.1|:72605-72662
MsHel-1	gb|AIXA01002770.1|:12046-12132
MsHel-1	gb|AIXA01002675.1|:3414-3613
MsHel-1	gb|AIXA01002321.1|:35729-35907
MsHel-1	gb|AIXA01001568.1|:1599-1745
MsHel-1	gb|AIXA01001006.1|:831-1147
MsHel-1	gb|AIXA01027930.1|:575-690
MsHel-1	gb|AIXA01016123.1|:14930-15047
MsHel-1	gb|AIXA01015263.1|:1346-1435
MsHel-1	gb|AIXA01012398.1|:50949-51038

MsHel-1	gb|AIXA01010339.1|:3253-3365
MsHel-1	gb|AIXA01009744.1|:2805-2923
MsHel-1	gb|AIXA01008233.1|:4641-4738
MsHel-1	gb|AIXA01006518.1|:106560-106677
MsHel-1	gb|AIXA01006507.1|:14961-15062
MsHel-1	gb|AIXA01006476.1|:13057-13265
MsHel-1	gb|AIXA01005669.1|:22860-22949
MsHel-1	gb|AIXA01003189.1|:27664-27777
MsHel-1	gb|AIXA01002379.1|:58785-58870
MsHel-1	gb|AIXA01002254.1|:50027-50124
MsHel-1	gb|AIXA01000872.1|:1692-1901
MsHel-1	gb|AIXA01000066.1|:15559-15673
MsHel-1	gb|AIXA01000037.1|:35741-35826
MsHel-1	gb|AIXA01017410.1|:6543-6658
MsHel-1	gb|AIXA01014527.1|:6040-6146
MsHel-1	gb|AIXA01008355.1|:970-1066
MsHel-1	gb|AIXA01007694.1|:27190-27309
MsHel-1	gb|AIXA01007245.1|:1-119
MsHel-1	gb|AIXA01005943.1|:16787-16899
MsHel-1	gb|AIXA01005491.1|:37018-37110
MsHel-1	gb|AIXA01001202.1|:22494-22599
MsHel-1	gb|AIXA01023007.1|:108-257
MsHel-1	gb|AIXA01015693.1|:4279-4523
MsHel-1	gb|AIXA01013618.1|:16022-16368
MsHel-1	gb|AIXA01013155.1|:2194-2293
MsHel-1	gb|AIXA01012987.1|:810-953
MsHel-1	gb|AIXA01009819.1|:22001-22139
MsHel-1	gb|AIXA01005488.1|:2171-2345
MsHel-1	gb|AIXA01005292.1|:7695-7886
MsHel-1	gb|AIXA01004415.1|:61078-61161
MsHel-1	gb|AIXA01017362.1|:3068-3154
MsHel-1	gb|AIXA01007759.1|:1188-1302
MsHel-2	gb|AIXA01006922.1|:15002-15300
MsHel-2	gb|AIXA01014851.1|:743-1046
MsHel-2	gb|AIXA01004483.1|:7614-7740
MsHel-2	gb|AIXA01004483.1|:21701-22004
MsHel-2	gb|AIXA01015050.1|:1708-2010
MsHel-2	gb|AIXA01005808.1|:8901-9202
MsHel-2	gb|AIXA01014443.1|:41540-41840
MsHel-2	gb|AIXA01008392.1|:8179-8482
MsHel-2	gb|AIXA01008028.1|:36047-36176
MsHel-2	gb|AIXA01008028.1|:48783-49086
MsHel-2	gb|AIXA01002579.1|:67750-68053
MsHel-2	gb|AIXA01016762.1|:1326-1626
MsHel-2	gb|AIXA01007966.1|:73325-73417
MsHel-2	gb|AIXA01007966.1|:86650-86770
MsHel-2	gb|AIXA01005380.1|:11629-11933

MsHel-2	gb|AIXA01015736.1|:1317-1620
MsHel-2	gb|AIXA01005891.1|:1276-1579
MsHel-2	gb|AIXA01004404.1|:7191-7489
MsHel-2	gb|AIXA01002990.1|:18180-18285
MsHel-2	gb|AIXA01002340.1|:67025-67151
MsHel-2	gb|AIXA01002340.1|:94270-94376
MsHel-2	gb|AIXA01002340.1|:120901-121204
MsHel-2	gb|AIXA01008104.1|:4827-5129
MsHel-2	gb|AIXA01021770.1|:981-1281
MsHel-2	gb|AIXA01017993.1|:3404-3702
MsHel-2	gb|AIXA01004978.1|:54627-54927
MsHel-2	gb|AIXA01002717.1|:13212-13512
MsHel-2	gb|AIXA01017860.1|:4806-5101
MsHel-2	gb|AIXA01015649.1|:12332-12635
MsHel-2	gb|AIXA01009634.1|:5027-5148
MsHel-2	gb|AIXA01009634.1|:20125-20428
MsHel-2	gb|AIXA01007220.1|:7601-7904
MsHel-2	gb|AIXA01001546.1|:57856-58159
MsHel-2	gb|AIXA01000904.1|:20324-20622
MsHel-2	gb|AIXA01000161.1|:18670-18793
MsHel-2	gb|AIXA01014907.1|:18360-18671
MsHel-2	gb|AIXA01007229.1|:98198-98494
MsHel-2	gb|AIXA01024945.1|:631-934
MsHel-2	gb|AIXA01005510.1|:8130-8433
MsHel-2	gb|AIXA01003367.1|:7116-7419
MsHel-2	gb|AIXA01003320.1|:14904-15204
MsHel-2	gb|AIXA01003320.1|:102058-102188
MsHel-2	gb|AIXA01002377.1|:151273-151363
MsHel-2	gb|AIXA01001043.1|:63582-63870
MsHel-2	gb|AIXA01001043.1|:107898-108027
MsHel-2	gb|AIXA01000892.1|:58344-58473
MsHel-2	gb|AIXA01002999.1|:82779-88985
MsHel-2	gb|AIXA01013184.1|:7314-7617
MsHel-2	gb|AIXA01017099.1|:26-324
MsHel-2	gb|AIXA01016561.1|:19504-19798
MsHel-2	gb|AIXA01005097.1|:16978-17275
MsHel-2	gb|AIXA01011501.1|:34491-34607
MsHel-2	gb|AIXA01010563.1|:3521-3821
MsHel-2	gb|AIXA01009518.1|:22383-22684
MsHel-2	gb|AIXA01009518.1|:78326-78454
MsHel-2	gb|AIXA01003139.1|:5831-6120
MsHel-2	gb|AIXA01006954.1|:7684-7985
MsHel-2	gb|AIXA01004330.1|:18266-22449
MsHel-2	gb|AIXA01007107.1|:31006-31137
MsHel-2	gb|AIXA01007107.1|:54526-54825
MsHel-2	gb|AIXA01002288.1|:2287-2587
MsHel-2	gb|AIXA01020258.1|:2163-2465
MsHel-2	gb|AIXA01000041.1|:94561-94687

MsHel-2	gb|AIXA01011710.1|:587-881
MsHel-2	gb|AIXA01000946.1|:8991-9270
MsHel-2	gb|AIXA01016386.1|:1433-1734
MsHel-2	gb|AIXA01011969.1|:14300-14600
MsHel-2	gb|AIXA01017627.1|:2245-2545
MsHel-2	gb|AIXA01014145.1|:38067-38368
MsHel-2	gb|AIXA01012631.1|:1663-1961
MsHel-2	gb|AIXA01008923.1|:28979-29279
MsHel-2	gb|AIXA01007176.1|:36356-36652
MsHel-2	gb|AIXA01014556.1|:4447-4743
MsHel-2	gb|AIXA01000601.1|:4809-5112
MsHel-2	gb|AIXA01002067.1|:1573-1871
MsHel-2	gb|AIXA01001467.1|:18673-18968
MsHel-2	gb|AIXA01005323.1|:11664-11794
MsHel-2	gb|AIXA01012114.1|:38118-38425
MsHel-2	gb|AIXA01010748.1|:3098-3208
MsHel-2	gb|AIXA01010748.1|:32045-32348
MsHel-2	gb|AIXA01010748.1|:149217-149337
MsHel-2	gb|AIXA01014014.1|:2210-2484
MsHel-2	gb|AIXA01007172.1|:1017-1287
MsHel-2	gb|AIXA01002313.1|:11733-12040
MsHel-2	gb|AIXA01007968.1|:9217-9328
MsHel-2	gb|AIXA01001270.1|:2365-2489
MsHel-2	gb|AIXA01001270.1|:14539-14835
MsHel-2	gb|AIXA01020460.1|:1179-1485
MsHel-2	gb|AIXA01002185.1|:63765-64060
MsHel-2	gb|AIXA01000185.1|:10622-10915
MsHel-2	gb|AIXA01005132.1|:28410-28710
MsHel-2	gb|AIXA01004528.1|:9944-10241
MsHel-2	gb|AIXA01012319.1|:18123-18227
MsHel-2	gb|AIXA01011992.1|:6671-6975
MsHel-2	gb|AIXA01008536.1|:41885-41991
MsHel-2	gb|AIXA01015151.1|:23321-23419
MsHel-2	gb|AIXA01015151.1|:61720-62021
MsHel-2	gb|AIXA01013138.1|:4569-4871
MsHel-2	gb|AIXA01012741.1|:69655-69954
MsHel-2	gb|AIXA01007015.1|:42696-42998
MsHel-2	gb|AIXA01000064.1|:16599-16891
MsHel-2	gb|AIXA01004459.1|:87459-87756
MsHel-2	gb|AIXA01004128.1|:35344-35468
MsHel-2	gb|AIXA01004128.1|:65292-65573
MsHel-2	gb|AIXA01021835.1|:548-844
MsHel-2	gb|AIXA01006515.1|:27273-27371
MsHel-2	gb|AIXA01010185.1|:11752-12046
MsHel-2	gb|AIXA01007472.1|:46359-46665
MsHel-2	gb|AIXA01002311.1|:831-1118
MsHel-2	gb|AIXA01002311.1|:63462-63764
MsHel-2	gb|AIXA01002311.1|:152542-152671

MsHel-2	gb|AIXA01001050.1|:15897-16026
MsHel-2	gb|AIXA01001050.1|:29677-29802
MsHel-2	gb|AIXA01001050.1|:95283-95407
MsHel-2	gb|AIXA01000411.1|:13857-14153
MsHel-2	gb|AIXA01003151.1|:38977-39279
MsHel-2	gb|AIXA01003151.1|:70956-71084
MsHel-2	gb|AIXA01002658.1|:80722-80974
MsHel-2	gb|AIXA01002658.1|:131304-131429
MsHel-2	gb|AIXA01009233.1|:1-290
MsHel-2	gb|AIXA01006518.1|:79965-80266
MsHel-2	gb|AIXA01007499.1|:32180-32480
MsHel-2	gb|AIXA01020071.1|:1638-1938
MsHel-2	gb|AIXA01010854.1|:62834-63130
MsHel-2	gb|AIXA01002480.1|:7773-8059
MsHel-2	gb|AIXA01001543.1|:30472-30776
MsHel-2	gb|AIXA01007614.1|:15570-15873
MsHel-2	gb|AIXA01007614.1|:38229-38329
MsHel-2	gb|AIXA01010873.1|:21082-21354
MsHel-2	gb|AIXA01022330.1|:11-301
MsHel-2	gb|AIXA01005067.1|:83930-84225
MsHel-2	gb|AIXA01001045.1|:27121-27369
MsHel-2	gb|AIXA01012699.1|:1469-1772
MsHel-2	gb|AIXA01009829.1|:17914-18217
MsHel-2	gb|AIXA01008116.1|:2993-3296
MsHel-2	gb|AIXA01010394.1|:41731-41993
MsHel-2	gb|AIXA01004373.1|:1-282
MsHel-2	gb|AIXA01008287.1|:8853-9153
MsHel-2	gb|AIXA01011340.1|:34415-34711
MsHel-2	gb|AIXA01014023.1|:4786-4914
MsHel-2	gb|AIXA01014023.1|:18417-18876
MsHel-2	gb|AIXA01004066.1|:12748-13051
MsHel-2	gb|AIXA01009184.1|:3141-3434
MsHel-2	gb|AIXA01006423.1|:148602-149134
MsHel-2	gb|AIXA01002671.1|:153312-153441
MsHel-2	gb|AIXA01001558.1|:21839-21946
MsHel-2	gb|AIXA01001558.1|:34817-34939
MsHel-2	gb|AIXA01017841.1|:5288-5584
MsHel-2	gb|AIXA01003913.1|:1218-1347
MsHel-2	gb|AIXA01003913.1|:28208-28509
MsHel-2	gb|AIXA01004064.1|:9687-9768
MsHel-2	gb|AIXA01018371.1|:9102-9398
MsHel-2	gb|AIXA01007359.1|:25724-26014
MsHel-2	gb|AIXA01007359.1|:38833-38926
MsHel-2	gb|AIXA01002941.1|:2679-2980
MsHel-2	gb|AIXA01000251.1|:1717-1846
MsHel-2	gb|AIXA01000251.1|:109334-109460
MsHel-2	gb|AIXA01000251.1|:232396-232522
MsHel-2	gb|AIXA01012784.1|:27184-27487

MsHel-2	gb|AIXA01005068.1|:14946-15879
MsHel-2	gb|AIXA01002519.1|:22191-22494
MsHel-2	gb|AIXA01002519.1|:33117-33229
MsHel-2	gb|AIXA01002519.1|:59023-59152
MsHel-2	gb|AIXA01001840.1|:6882-7159
MsHel-2	gb|AIXA01033835.1|:364-666
MsHel-2	gb|AIXA01012325.1|:11733-12018
MsHel-2	gb|AIXA01016989.1|:1075-1368
MsHel-2	gb|AIXA01002732.1|:60901-61200
MsHel-2	gb|AIXA01029925.1|:1-240
MsHel-2	gb|AIXA01005483.1|:7890-8120
MsHel-2	gb|AIXA01003889.1|:136406-136535
MsHel-2	gb|AIXA01017844.1|:42-259
MsHel-2	gb|AIXA01004108.1|:69802-70097
MsHel-2	gb|AIXA01002293.1|:46602-46701
MsHel-2	gb|AIXA01000408.1|:42610-42907
MsHel-2	gb|AIXA01007607.1|:1-225
MsHel-2	gb|AIXA01004236.1|:11190-11491
MsHel-2	gb|AIXA01000809.1|:7587-7718
MsHel-2	gb|AIXA01000809.1|:26299-26425
MsHel-2	gb|AIXA01000809.1|:53933-54212
MsHel-2	gb|AIXA01000809.1|:72969-73087
MsHel-2	gb|AIXA01010208.1|:891-1193
MsHel-2	gb|AIXA01008398.1|:32241-32363
MsHel-2	gb|AIXA01022717.1|:69-370
MsHel-2	gb|AIXA01011650.1|:39934-40232
MsHel-2	gb|AIXA01009773.1|:6859-7156
MsHel-2	gb|AIXA01032826.1|:311-590
MsHel-2	gb|AIXA01013962.1|:1-221
MsHel-2	gb|AIXA01014296.1|:9842-10118
MsHel-2	gb|AIXA01012963.1|:2398-2700
MsHel-2	gb|AIXA01011724.1|:13006-13296
MsHel-2	gb|AIXA01007874.1|:23-299
MsHel-2	gb|AIXA01003924.1|:22729-23027
MsHel-2	gb|AIXA01003924.1|:111765-111894
MsHel-2	gb|AIXA01011560.1|:2495-2791
MsHel-2	gb|AIXA01011560.1|:43391-43514
MsHel-2	gb|AIXA01001048.1|:1721-1848
MsHel-2	gb|AIXA01001048.1|:12095-12214
MsHel-2	gb|AIXA01001048.1|:123773-124051
MsHel-2	gb|AIXA01000267.1|:51120-51420
MsHel-2	gb|AIXA01014249.1|:10330-10627
MsHel-2	gb|AIXA01007042.1|:26911-27214
MsHel-2	gb|AIXA01006199.1|:1-298
MsHel-2	gb|AIXA01019837.1|:447-743
MsHel-2	gb|AIXA01006834.1|:41939-42230
MsHel-2	gb|AIXA01005541.1|:1322-1612
MsHel-2	gb|AIXA01038195.1|:256-507

MsHel-2	gb|AIXA01011450.1|:28924-29185
MsHel-2	gb|AIXA01006055.1|:15811-16109
MsHel-2	gb|AIXA01005056.1|:11377-11674
MsHel-2	gb|AIXA01001469.1|:6318-6619
MsHel-2	gb|AIXA01003981.1|:28411-28691
MsHel-2	gb|AIXA01000569.1|:11226-11523
MsHel-2	gb|AIXA01006212.1|:2468-2917
MsHel-2	gb|AIXA01001196.1|:23192-23469
MsHel-2	gb|AIXA01012097.1|:8039-8279
MsHel-2	gb|AIXA01010343.1|:25782-25883
MsHel-2	gb|AIXA01002662.1|:34447-34706
MsHel-2	gb|AIXA01008025.1|:43034-43335
MsHel-2	gb|AIXA01008382.1|:34027-34290
MsHel-2	gb|AIXA01004489.1|:26820-26949
MsHel-2	gb|AIXA01004489.1|:52786-53782
MsHel-2	gb|AIXA01002500.1|:154401-154630
MsHel-2	gb|AIXA01002388.1|:32161-32434
MsHel-2	gb|AIXA01001213.1|:35274-35548
MsHel-2	gb|AIXA01014301.1|:7542-7782
MsHel-2	gb|AIXA01014301.1|:34360-34470
MsHel-2	gb|AIXA01004230.1|:33264-33390
MsHel-2	gb|AIXA01004230.1|:44307-44607
MsHel-2	gb|AIXA01004230.1|:61060-61191
MsHel-2	gb|AIXA01000459.1|:16927-17207
MsHel-2	gb|AIXA01000459.1|:39162-39290
MsHel-2	gb|AIXA01010551.1|:44259-44551
MsHel-2	gb|AIXA01009649.1|:13559-13664
MsHel-2	gb|AIXA01009649.1|:41878-42157
MsHel-2	gb|AIXA01009142.1|:13010-13125
MsHel-2	gb|AIXA01009142.1|:30799-31095
MsHel-2	gb|AIXA01009040.1|:39323-39525
MsHel-2	gb|AIXA01009040.1|:151490-151582
MsHel-2	gb|AIXA01004285.1|:318-525
MsHel-2	gb|AIXA01014242.1|:6207-6501
MsHel-2	gb|AIXA01006321.1|:6644-6773
MsHel-2	gb|AIXA01004155.1|:22561-22859
MsHel-2	gb|AIXA01012909.1|:1-265
MsHel-2	gb|AIXA01007314.1|:20254-20553
MsHel-2	gb|AIXA01018341.1|:4704-5018
MsHel-2	gb|AIXA01015622.1|:10513-10809
MsHel-2	gb|AIXA01031057.1|:11-254
MsHel-2	gb|AIXA01008505.1|:43750-44012
MsHel-2	gb|AIXA01002599.1|:57143-57326
MsHel-2	gb|AIXA01015348.1|:6234-6532
MsHel-2	gb|AIXA01002975.1|:100073-100372
MsHel-2	gb|AIXA01012324.1|:16848-17146
MsHel-2	gb|AIXA01007920.1|:12277-12565
MsHel-2	gb|AIXA01035030.1|:37-326

MsHel-2	gb|AIXA01012690.1|:7187-7478
MsHel-2	gb|AIXA01006939.1|:30125-30396
MsHel-2	gb|AIXA01000905.1|:28294-28537
MsHel-2	gb|AIXA01006915.1|:6194-6477
MsHel-2	gb|AIXA01003293.1|:17246-17362
MsHel-2	gb|AIXA01018997.1|:3747-4041
MsHel-2	gb|AIXA01008385.1|:26998-27241
MsHel-2	gb|AIXA01007595.1|:13690-13982
MsHel-2	gb|AIXA01007525.1|:12039-12378
MsHel-2	gb|AIXA01006871.1|:50869-51158
MsHel-2	gb|AIXA01003021.1|:41058-41184
MsHel-2	gb|AIXA01003108.1|:3302-3601
MsHel-2	gb|AIXA01004470.1|:21915-22038
MsHel-2	gb|AIXA01004470.1|:66624-66907
MsHel-2	gb|AIXA01004482.1|:9057-9149
MsHel-2	gb|AIXA01000066.1|:12885-13096
MsHel-2	gb|AIXA01032276.1|:588-739
MsHel-2	gb|AIXA01010340.1|:2274-2441
MsHel-2	gb|AIXA01014903.1|:4842-5115
MsHel-2	gb|AIXA01011628.1|:3-259
MsHel-2	gb|AIXA01009340.1|:52688-52963
MsHel-2	gb|AIXA01034335.1|:1-144
MsHel-2	gb|AIXA01002350.1|:24382-24658
MsHel-2	gb|AIXA01002350.1|:128214-128337
MsHel-2	gb|AIXA01012785.1|:11253-11411
MsHel-2	gb|AIXA01003698.1|:49602-49869
MsHel-2	gb|AIXA01009936.1|:14081-14391
MsHel-2	gb|AIXA01033447.1|:1-144
MsHel-2	gb|AIXA01004386.1|:83702-83824
MsHel-2	gb|AIXA01025746.1|:166-392
MsHel-2	gb|AIXA01009021.1|:10442-10716
MsHel-2	gb|AIXA01010870.1|:224-497
MsHel-2	gb|AIXA01002114.1|:64885-65137
MsHel-2	gb|AIXA01000931.1|:38077-38206
MsHel-2	gb|AIXA01005642.1|:1576-1693
MsHel-2	gb|AIXA01005642.1|:78610-78732
MsHel-2	gb|AIXA01005479.1|:326-452
MsHel-2	gb|AIXA01005314.1|:28717-28952
MsHel-2	gb|AIXA01002619.1|:30227-30507
MsHel-2	gb|AIXA01000591.1|:1-255
MsHel-2	gb|AIXA01000838.1|:56434-56563
MsHel-2	gb|AIXA01000838.1|:142187-142449
MsHel-2	gb|AIXA01013090.1|:10891-11039
MsHel-2	gb|AIXA01000494.1|:43225-43525
MsHel-2	gb|AIXA01009232.1|:2331-2614
MsHel-2	gb|AIXA01008335.1|:19174-19411
MsHel-2	gb|AIXA01001571.1|:115538-115652
MsHel-2	gb|AIXA01003979.1|:1760-1886

MsHel-2	gb|AIXA01000672.1|:3288-3508
MsHel-2	gb|AIXA01010855.1|:5741-6029
MsHel-2	gb|AIXA01008442.1|:1952-2260
MsHel-2	gb|AIXA01008442.1|:25753-25839
MsHel-2	gb|AIXA01008442.1|:71283-71411
MsHel-2	gb|AIXA01009307.1|:5631-5941
MsHel-2	gb|AIXA01006896.1|:8324-8606
MsHel-2	gb|AIXA01007104.1|:16757-16874
MsHel-2	gb|AIXA01007104.1|:40729-41011
MsHel-2	gb|AIXA01001830.1|:14532-14813
MsHel-2	gb|AIXA01007329.1|:29867-30162
MsHel-2	gb|AIXA01007063.1|:2471-2732
MsHel-2	gb|AIXA01002687.1|:686-813
MsHel-2	gb|AIXA01002687.1|:31020-31227
MsHel-2	gb|AIXA01017002.1|:18-169
MsHel-2	gb|AIXA01004264.1|:8902-10796
MsHel-2	gb|AIXA01001082.1|:6373-6461
MsHel-2	gb|AIXA01019673.1|:2311-2590
MsHel-2	gb|AIXA01002933.1|:10553-10678
MsHel-2	gb|AIXA01010678.1|:24935-25222
MsHel-2	gb|AIXA01004618.1|:11608-11732
MsHel-2	gb|AIXA01002453.1|:36975-37250
MsHel-2	gb|AIXA01009537.1|:73250-73449
MsHel-2	gb|AIXA01006848.1|:5730-5992
MsHel-2	gb|AIXA01016207.1|:1503-1814
MsHel-2	gb|AIXA01013847.1|:19119-19335
MsHel-2	gb|AIXA01000752.1|:16142-16263
MsHel-2	gb|AIXA01007970.1|:17709-17986
MsHel-2	gb|AIXA01014086.1|:18869-18996
MsHel-2	gb|AIXA01008635.1|:13974-14186
MsHel-2	gb|AIXA01004135.1|:33856-33977
MsHel-2	gb|AIXA01003311.1|:3016-3146
MsHel-2	gb|AIXA01008329.1|:35066-35332
MsHel-2	gb|AIXA01011494.1|:17315-17628
MsHel-2	gb|AIXA01010003.1|:13188-13450
MsHel-2	gb|AIXA01005350.1|:45266-46582
MsHel-2	gb|AIXA01005572.1|:37312-37596
MsHel-2	gb|AIXA01016138.1|:18236-18547
MsHel-2	gb|AIXA01002820.1|:44738-44842
MsHel-2	gb|AIXA01005895.1|:2349-2468
MsHel-2	gb|AIXA01014535.1|:11-295
MsHel-2	gb|AIXA01012048.1|:38623-38749
MsHel-2	gb|AIXA01010806.1|:7814-7940
MsHel-2	gb|AIXA01008296.1|:18348-18636
MsHel-2	gb|AIXA01008227.1|:46067-46352
MsHel-2	gb|AIXA01008155.1|:17881-18152
MsHel-2	gb|AIXA01001496.1|:57208-57291
MsHel-2	gb|AIXA01001534.1|:24790-24918

MsHel-2	gb|AIXA01009093.1|:2451-2574
MsHel-2	gb|AIXA01014388.1|:18692-18814
MsHel-2	gb|AIXA01006684.1|:4304-4431
MsHel-2	gb|AIXA01022176.1|:702-827
MsHel-2	gb|AIXA01013945.1|:31022-31142
MsHel-2	gb|AIXA01002989.1|:11377-11502
MsHel-2	gb|AIXA01000485.1|:36005-36294
MsHel-2	gb|AIXA01016208.1|:24121-24247
MsHel-2	gb|AIXA01007593.1|:21435-21562
MsHel-2	gb|AIXA01002525.1|:3150-3276
MsHel-2	gb|AIXA01035550.1|:234-359
MsHel-2	gb|AIXA01012787.1|:2629-2750
MsHel-2	gb|AIXA01011331.1|:2389-2490
MsHel-2	gb|AIXA01007902.1|:13404-13698
MsHel-2	gb|AIXA01002351.1|:2450-2575
MsHel-2	gb|AIXA01033753.1|:428-557
MsHel-2	gb|AIXA01002713.1|:5438-5567
MsHel-2	gb|AIXA01023082.1|:10-101
MsHel-2	gb|AIXA01010673.1|:11511-11816
MsHel-2	gb|AIXA01006681.1|:1777-1898
MsHel-2	gb|AIXA01003124.1|:13678-18212
MsHel-2	gb|AIXA01001829.1|:15836-15958
MsHel-2	gb|AIXA01016468.1|:3067-3180
MsHel-2	gb|AIXA01000201.1|:8820-8912
MsHel-2	gb|AIXA01024020.1|:814-1005
MsHel-2	gb|AIXA01020832.1|:1622-1716
MsHel-2	gb|AIXA01013684.1|:1197-1324
MsHel-2	gb|AIXA01010432.1|:11344-11467
MsHel-2	gb|AIXA01003002.1|:6450-6569
MsHel-2	gb|AIXA01000969.1|:37504-37623
MsHel-2	gb|AIXA01009855.1|:663-788
MsHel-2	gb|AIXA01006885.1|:67777-67902
MsHel-2	gb|AIXA01000269.1|:15983-16111
MsHel-2	gb|AIXA01011296.1|:4352-4480
MsHel-2	gb|AIXA01010558.1|:4563-4685
MsHel-2	gb|AIXA01008792.1|:6990-7115
MsHel-2	gb|AIXA01028245.1|:361-490
MsHel-2	gb|AIXA01014762.1|:11120-11246
MsHel-2	gb|AIXA01012200.1|:77861-77977
MsHel-2	gb|AIXA01010656.1|:27873-28405
MsHel-2	gb|AIXA01010069.1|:20657-20784
MsHel-2	gb|AIXA01009766.1|:12183-12308
MsHel-2	gb|AIXA01006545.1|:6729-6858
MsHel-2	gb|AIXA01001553.1|:14202-14331
MsHel-2	gb|AIXA01001264.1|:20056-20185
MsHel-2	gb|AIXA01016327.1|:6267-6554
MsHel-2	gb|AIXA01000132.1|:9915-10030
MsHel-2	gb|AIXA01000046.1|:48927-49042

MsHel-2	gb|AIXA01000046.1|:80815-80943
MsHel-2	gb|AIXA01013561.1|:4368-4653
MsHel-2	gb|AIXA01012352.1|:28114-28240
MsHel-2	gb|AIXA01003661.1|:85062-85188
MsHel-2	gb|AIXA01002069.1|:19976-20102
MsHel-2	gb|AIXA01001658.1|:64875-65001
MsHel-2	gb|AIXA01017229.1|:8150-8276
MsHel-2	gb|AIXA01013867.1|:7290-7408
MsHel-2	gb|AIXA01012195.1|:8587-8709
MsHel-2	gb|AIXA01010279.1|:2221-2796
MsHel-2	gb|AIXA01008294.1|:45673-45803
MsHel-2	gb|AIXA01007887.1|:9452-9570
MsHel-2	gb|AIXA01006420.1|:24412-24540
MsHel-2	gb|AIXA01006420.1|:71030-71160
MsHel-2	gb|AIXA01004670.1|:155723-155847
MsHel-2	gb|AIXA01002128.1|:24448-24570
MsHel-2	gb|AIXA01035647.1|:446-575
MsHel-2	gb|AIXA01032017.1|:359-488
MsHel-2	gb|AIXA01017253.1|:1161-1290
MsHel-2	gb|AIXA01016537.1|:15481-15610
MsHel-2	gb|AIXA01009981.1|:3528-3643
MsHel-2	gb|AIXA01009084.1|:16421-16539
MsHel-2	gb|AIXA01008957.1|:9651-9756
MsHel-2	gb|AIXA01007964.1|:10176-10304
MsHel-2	gb|AIXA01006299.1|:114764-114893
MsHel-2	gb|AIXA01002994.1|:16343-16462
MsHel-2	gb|AIXA01001692.1|:41316-41445
MsHel-2	gb|AIXA01001645.1|:37231-37356
MsHel-2	gb|AIXA01001645.1|:65267-65396
MsHel-2	gb|AIXA01000821.1|:109382-109510
MsHel-2	gb|AIXA01021890.1|:1602-1725
MsHel-2	gb|AIXA01007689.1|:40880-40988
MsHel-2	gb|AIXA01002466.1|:32420-32544
MsHel-2	gb|AIXA01013436.1|:10465-10576
MsHel-2	gb|AIXA01009972.1|:32092-32219
MsHel-2	gb|AIXA01009970.1|:10974-11101
MsHel-2	gb|AIXA01009427.1|:15467-15570
MsHel-2	gb|AIXA01009427.1|:26538-26665
MsHel-2	gb|AIXA01005792.1|:17122-17248
MsHel-2	gb|AIXA01004908.1|:7874-7997
MsHel-2	gb|AIXA01004893.1|:64229-64355
MsHel-2	gb|AIXA01004325.1|:28228-28355
MsHel-2	gb|AIXA01002972.1|:38917-43259
MsHel-2	gb|AIXA01002743.1|:12895-13021
MsHel-2	gb|AIXA01031539.1|:583-708
MsHel-2	gb|AIXA01020269.1|:1952-2076
MsHel-2	gb|AIXA01020025.1|:2180-2304
MsHel-2	gb|AIXA01019490.1|:7-105

MsHel-2	gb|AIXA01015144.1|:17190-17312
MsHel-2	gb|AIXA01012649.1|:16784-16900
MsHel-2	gb|AIXA01007888.1|:63348-63474
MsHel-2	gb|AIXA01007228.1|:100406-100528
MsHel-2	gb|AIXA01002712.1|:20083-20209
MsHel-2	gb|AIXA01001973.1|:12492-12616
MsHel-2	gb|AIXA01001520.1|:1194-1323
MsHel-2	gb|AIXA01001323.1|:2973-3073
MsHel-2	gb|AIXA01000627.1|:41781-42061
MsHel-2	gb|AIXA01023032.1|:578-706
MsHel-2	gb|AIXA01018508.1|:1931-2060
MsHel-2	gb|AIXA01017382.1|:6338-6459
MsHel-2	gb|AIXA01015485.1|:4768-4893
MsHel-2	gb|AIXA01013596.1|:14-138
MsHel-2	gb|AIXA01011842.1|:14196-14325
MsHel-2	gb|AIXA01011338.1|:6287-6408
MsHel-2	gb|AIXA01010211.1|:22237-22362
MsHel-2	gb|AIXA01009117.1|:23581-23681
MsHel-2	gb|AIXA01007712.1|:41826-41952
MsHel-2	gb|AIXA01006154.1|:13667-13788
MsHel-2	gb|AIXA01006141.1|:22026-22155
MsHel-2	gb|AIXA01006141.1|:105976-106095
MsHel-2	gb|AIXA01005566.1|:20541-20670
MsHel-2	gb|AIXA01004313.1|:50909-51031
MsHel-2	gb|AIXA01004160.1|:23054-23183
MsHel-2	gb|AIXA01003146.1|:18427-18550
MsHel-2	gb|AIXA01002469.1|:33790-33919
MsHel-2	gb|AIXA01001561.1|:10352-10481
MsHel-2	gb|AIXA01001022.1|:15504-15633
MsHel-2	gb|AIXA01000792.1|:14126-14255
MsHel-2	gb|AIXA01000356.1|:9895-10024
MsHel-2	gb|AIXA01032320.1|:1-105
MsHel-2	gb|AIXA01017576.1|:13943-14047
MsHel-2	gb|AIXA01013458.1|:15918-16037
MsHel-2	gb|AIXA01013458.1|:50141-50224
MsHel-2	gb|AIXA01012742.1|:3569-3685
MsHel-2	gb|AIXA01012697.1|:4423-4534
MsHel-2	gb|AIXA01010757.1|:13948-14072
MsHel-2	gb|AIXA01006287.1|:1850-1974
MsHel-2	gb|AIXA01005850.1|:28596-28711
MsHel-2	gb|AIXA01002281.1|:23322-23441
MsHel-2	gb|AIXA01017659.1|:1742-1865
MsHel-2	gb|AIXA01015155.1|:17688-17814
MsHel-2	gb|AIXA01014972.1|:183-308
MsHel-2	gb|AIXA01013947.1|:20724-20850
MsHel-2	gb|AIXA01012798.1|:25057-25151
MsHel-2	gb|AIXA01011449.1|:4616-4742
MsHel-2	gb|AIXA01008405.1|:7027-7145

MsHel-2	gb|AIXA01007346.1|:47102-47229
MsHel-2	gb|AIXA01006934.1|:2583-2709
MsHel-2	gb|AIXA01006729.1|:2729-2856
MsHel-2	gb|AIXA01006279.1|:7719-7846
MsHel-2	gb|AIXA01005228.1|:125155-125269
MsHel-2	gb|AIXA01005049.1|:20351-20477
MsHel-2	gb|AIXA01004958.1|:50176-50299
MsHel-2	gb|AIXA01002460.1|:92565-92693
MsHel-2	gb|AIXA01001804.1|:7214-7331
MsHel-2	gb|AIXA01001593.1|:5232-5353
MsHel-2	gb|AIXA01001362.1|:3875-3969
MsHel-2	gb|AIXA01001049.1|:17780-17907
MsHel-2	gb|AIXA01000891.1|:28089-28216
MsHel-2	gb|AIXA01032845.1|:210-340
MsHel-2	gb|AIXA01029000.1|:791-917
MsHel-2	gb|AIXA01023220.1|:1335-1448
MsHel-2	gb|AIXA01008999.1|:10508-10638
MsHel-2	gb|AIXA01006616.1|:8894-9021
MsHel-2	gb|AIXA01003249.1|:22618-22748
MsHel-2	gb|AIXA01003057.1|:60358-60452
MsHel-2	gb|AIXA01002339.1|:11260-11386
MsHel-2	gb|AIXA01002339.1|:39203-39328
MsHel-2	gb|AIXA01002330.1|:8039-8164
MsHel-2	gb|AIXA01001653.1|:19888-20015
MsHel-2	gb|AIXA01000482.1|:80303-80429
MsHel-2	gb|AIXA01000192.1|:16577-16697
MsHel-2	gb|AIXA01000011.1|:537-663
MsHel-2	gb|AIXA01036782.1|:155-284
MsHel-2	gb|AIXA01021508.1|:1608-1731
MsHel-2	gb|AIXA01015919.1|:73-194
MsHel-2	gb|AIXA01013644.1|:409-526
MsHel-2	gb|AIXA01008073.1|:24335-24464
MsHel-2	gb|AIXA01008022.1|:41937-42056
MsHel-2	gb|AIXA01007870.1|:14780-14897
MsHel-2	gb|AIXA01007719.1|:25447-25576
MsHel-2	gb|AIXA01006663.1|:2192-2297
MsHel-2	gb|AIXA01005516.1|:5593-5722
MsHel-2	gb|AIXA01005034.1|:33668-33770
MsHel-2	gb|AIXA01005034.1|:81530-81653
MsHel-2	gb|AIXA01004929.1|:49005-49130
MsHel-2	gb|AIXA01004487.1|:5085-5206
MsHel-2	gb|AIXA01004357.1|:2778-2903
MsHel-2	gb|AIXA01004331.1|:18608-18872
MsHel-2	gb|AIXA01004118.1|:4847-4976
MsHel-2	gb|AIXA01004113.1|:112892-113021
MsHel-2	gb|AIXA01002509.1|:6758-6860
MsHel-2	gb|AIXA01002509.1|:23268-23397
MsHel-2	gb|AIXA01001319.1|:11203-11332

MsHel-2	gb|AIXA01001304.1|:16812-16933
MsHel-2	gb|AIXA01001051.1|:59314-59434
MsHel-2	gb|AIXA01000939.1|:894-1011
MsHel-2	gb|AIXA01000939.1|:14932-15053
MsHel-2	gb|AIXA01000694.1|:80520-80649
MsHel-2	gb|AIXA01000437.1|:116638-116763
MsHel-2	gb|AIXA01000398.1|:23550-23679
MsHel-2	gb|AIXA01020715.1|:465-556
MsHel-2	gb|AIXA01017339.1|:162-274
MsHel-2	gb|AIXA01014037.1|:3568-3696
MsHel-2	gb|AIXA01013560.1|:6718-6832
MsHel-2	gb|AIXA01011303.1|:19287-19401
MsHel-2	gb|AIXA01011303.1|:62562-62686
MsHel-2	gb|AIXA01010109.1|:24799-24910
MsHel-2	gb|AIXA01010109.1|:38599-38730
MsHel-2	gb|AIXA01007223.1|:17282-17409
MsHel-2	gb|AIXA01007037.1|:13376-13503
MsHel-2	gb|AIXA01007037.1|:28659-28777
MsHel-2	gb|AIXA01005127.1|:28103-28231
MsHel-2	gb|AIXA01003329.1|:41753-41880
MsHel-2	gb|AIXA01003319.1|:24216-24337
MsHel-2	gb|AIXA01002995.1|:14277-14389
MsHel-2	gb|AIXA01002797.1|:8114-8214
MsHel-2	gb|AIXA01001675.1|:45914-46006
MsHel-2	gb|AIXA01030638.1|:216-339
MsHel-2	gb|AIXA01018595.1|:41-167
MsHel-2	gb|AIXA01017117.1|:7330-7458
MsHel-2	gb|AIXA01016291.1|:3176-3303
MsHel-2	gb|AIXA01015777.1|:33061-33188
MsHel-2	gb|AIXA01014467.1|:1537-1677
MsHel-2	gb|AIXA01014467.1|:48108-48234
MsHel-2	gb|AIXA01011986.1|:9726-9851
MsHel-2	gb|AIXA01011821.1|:10295-10417
MsHel-2	gb|AIXA01011593.1|:6867-6994
MsHel-2	gb|AIXA01010808.1|:1303-1429
MsHel-2	gb|AIXA01009162.1|:93006-93127
MsHel-2	gb|AIXA01006181.1|:5863-5978
MsHel-2	gb|AIXA01004567.1|:9925-10020
MsHel-2	gb|AIXA01003070.1|:18445-18571
MsHel-2	gb|AIXA01002774.1|:4620-4824
MsHel-2	gb|AIXA01002240.1|:28156-28282
MsHel-2	gb|AIXA01000813.1|:45541-45661
MsHel-2	gb|AIXA01000199.1|:4700-4830
MsHel-2	gb|AIXA01017982.1|:10809-10931
MsHel-2	gb|AIXA01012350.1|:17423-17533
MsHel-2	gb|AIXA01011361.1|:44226-44354
MsHel-2	gb|AIXA01008043.1|:92507-92619
MsHel-2	gb|AIXA01006983.1|:126019-126149

MsHel-2	gb|AIXA01006641.1|:16272-16402
MsHel-2	gb|AIXA01005247.1|:53043-53169
MsHel-2	gb|AIXA01005209.1|:2730-2856
MsHel-2	gb|AIXA01002722.1|:6462-6585
MsHel-2	gb|AIXA01002722.1|:18221-18348
MsHel-2	gb|AIXA01001518.1|:14066-14191
MsHel-2	gb|AIXA01001239.1|:143399-143500
MsHel-2	gb|AIXA01000843.1|:5909-6038
MsHel-2	gb|AIXA01000843.1|:85349-85476
MsHel-2	gb|AIXA01000359.1|:87181-87304
MsHel-2	gb|AIXA01000048.1|:39890-39993
MsHel-2	gb|AIXA01037367.1|:302-427
MsHel-2	gb|AIXA01023928.1|:949-1078
MsHel-2	gb|AIXA01020162.1|:1518-1647
MsHel-2	gb|AIXA01020040.1|:2148-2275
MsHel-2	gb|AIXA01018015.1|:896-1001
MsHel-2	gb|AIXA01017098.1|:10859-10988
MsHel-2	gb|AIXA01016314.1|:4925-5030
MsHel-2	gb|AIXA01014666.1|:3017-3110
MsHel-2	gb|AIXA01013067.1|:49465-49594
MsHel-2	gb|AIXA01012913.1|:14009-14133
MsHel-2	gb|AIXA01011712.1|:2623-2716
MsHel-2	gb|AIXA01011141.1|:3665-3794
MsHel-2	gb|AIXA01009135.1|:4999-5128
MsHel-2	gb|AIXA01009052.1|:3104-3209
MsHel-2	gb|AIXA01008417.1|:21832-21945
MsHel-2	gb|AIXA01008393.1|:32170-32287
MsHel-2	gb|AIXA01008393.1|:61377-61506
MsHel-2	gb|AIXA01007835.1|:94548-94677
MsHel-2	gb|AIXA01007808.1|:34094-34198
MsHel-2	gb|AIXA01006790.1|:7876-8005
MsHel-2	gb|AIXA01006690.1|:14769-14898
MsHel-2	gb|AIXA01006041.1|:6053-6174
MsHel-2	gb|AIXA01005339.1|:10756-10870
MsHel-2	gb|AIXA01005339.1|:34201-34330
MsHel-2	gb|AIXA01004862.1|:23392-23511
MsHel-2	gb|AIXA01004862.1|:58196-58324
MsHel-2	gb|AIXA01004354.1|:37147-37267
MsHel-2	gb|AIXA01003851.1|:3340-3464
MsHel-2	gb|AIXA01003673.1|:2585-2714
MsHel-2	gb|AIXA01003673.1|:56788-56912
MsHel-2	gb|AIXA01003330.1|:36522-36651
MsHel-2	gb|AIXA01002998.1|:43442-43543
MsHel-2	gb|AIXA01002996.1|:23535-23653
MsHel-2	gb|AIXA01002996.1|:60769-60888
MsHel-2	gb|AIXA01002653.1|:84-185
MsHel-2	gb|AIXA01002331.1|:718-843
MsHel-2	gb|AIXA01002187.1|:19894-20023

MsHel-2	gb|AIXA01001656.1|:39721-39845
MsHel-2	gb|AIXA01001656.1|:61854-61945
MsHel-2	gb|AIXA01001531.1|:56825-56951
MsHel-2	gb|AIXA01001265.1|:12493-12614
MsHel-2	gb|AIXA01000559.1|:8257-8377
MsHel-2	gb|AIXA01000508.1|:11990-12095
MsHel-2	gb|AIXA01000454.1|:18360-18489
MsHel-2	gb|AIXA01000188.1|:37009-37138
MsHel-2	gb|AIXA01023890.1|:1177-1305
MsHel-2	gb|AIXA01016545.1|:11607-11699
MsHel-2	gb|AIXA01015734.1|:1967-2095
MsHel-2	gb|AIXA01012398.1|:3025-3232
MsHel-2	gb|AIXA01011594.1|:907-1035
MsHel-2	gb|AIXA01007664.1|:5215-5339
MsHel-2	gb|AIXA01006514.1|:48567-48691
MsHel-2	gb|AIXA01005504.1|:47703-47830
MsHel-2	gb|AIXA01005406.1|:5207-5290
MsHel-2	gb|AIXA01005252.1|:35226-35348
MsHel-2	gb|AIXA01004905.1|:1549-1649
MsHel-2	gb|AIXA01004300.1|:23907-24040
MsHel-2	gb|AIXA01000561.1|:18951-19069
MsHel-2	gb|AIXA01000223.1|:8972-9091
MsHel-2	gb|AIXA01000127.1|:5123-5251
MsHel-2	gb|AIXA01000069.1|:122994-123083
MsHel-2	gb|AIXA01025282.1|:399-524
MsHel-2	gb|AIXA01016282.1|:2148-2274
MsHel-2	gb|AIXA01016021.1|:18566-18693
MsHel-2	gb|AIXA01015043.1|:906-1033
MsHel-2	gb|AIXA01012012.1|:8966-9092
MsHel-2	gb|AIXA01011085.1|:5848-5974
MsHel-2	gb|AIXA01010631.1|:2017-2140
MsHel-2	gb|AIXA01009719.1|:30120-30251
MsHel-2	gb|AIXA01008705.1|:11034-11157
MsHel-2	gb|AIXA01007842.1|:18462-18589
MsHel-2	gb|AIXA01005938.1|:17221-17342
MsHel-2	gb|AIXA01004956.1|:119830-119955
MsHel-2	gb|AIXA01004011.1|:13410-13537
MsHel-2	gb|AIXA01003858.1|:2835-2956
MsHel-2	gb|AIXA01001994.1|:25976-26098
MsHel-2	gb|AIXA01001189.1|:6684-6784
MsHel-2	gb|AIXA01000456.1|:15296-15412
MsHel-2	gb|AIXA01000397.1|:1839-1941
MsHel-2	gb|AIXA01000050.1|:61087-65083
MsHel-2	gb|AIXA01020949.1|:1111-1217
MsHel-2	gb|AIXA01019061.1|:1267-1399
MsHel-2	gb|AIXA01017540.1|:2266-2399
MsHel-2	gb|AIXA01017061.1|:16296-16422
MsHel-2	gb|AIXA01016376.1|:21779-21905

MsHel-2	gb|AIXA01014216.1|:6823-6941
MsHel-2	gb|AIXA01011371.1|:4778-5408
MsHel-2	gb|AIXA01009854.1|:170-294
MsHel-2	gb|AIXA01009315.1|:44600-44713
MsHel-2	gb|AIXA01009152.1|:7771-7897
MsHel-2	gb|AIXA01006529.1|:49101-49222
MsHel-2	gb|AIXA01004982.1|:98280-98410
MsHel-2	gb|AIXA01004841.1|:15560-15686
MsHel-2	gb|AIXA01004067.1|:26425-26543
MsHel-2	gb|AIXA01000984.1|:24750-24855
MsHel-2	gb|AIXA01037524.1|:257-370
MsHel-2	gb|AIXA01031120.1|:503-623
MsHel-2	gb|AIXA01022283.1|:1497-1614
MsHel-2	gb|AIXA01018818.1|:114-243
MsHel-2	gb|AIXA01018506.1|:2098-2194
MsHel-2	gb|AIXA01013942.1|:15001-15130
MsHel-2	gb|AIXA01012656.1|:3710-3819
MsHel-2	gb|AIXA01012544.1|:2335-2464
MsHel-2	gb|AIXA01011253.1|:2941-3065
MsHel-2	gb|AIXA01010913.1|:11552-11681
MsHel-2	gb|AIXA01009837.1|:11520-11625
MsHel-2	gb|AIXA01008463.1|:120-245
MsHel-2	gb|AIXA01008423.1|:59712-59841
MsHel-2	gb|AIXA01008106.1|:10963-11091
MsHel-2	gb|AIXA01007494.1|:5629-5729
MsHel-2	gb|AIXA01007494.1|:33898-34017
MsHel-2	gb|AIXA01007182.1|:8770-8899
MsHel-2	gb|AIXA01006854.1|:44578-48704
MsHel-2	gb|AIXA01006350.1|:45411-45537
MsHel-2	gb|AIXA01005488.1|:12570-12699
MsHel-2	gb|AIXA01005488.1|:26731-26851
MsHel-2	gb|AIXA01005035.1|:59750-59864
MsHel-2	gb|AIXA01004852.1|:97191-97316
MsHel-2	gb|AIXA01004829.1|:40678-40806
MsHel-2	gb|AIXA01004754.1|:27286-27414
MsHel-2	gb|AIXA01004402.1|:13690-13795
MsHel-2	gb|AIXA01004097.1|:22593-22722
MsHel-2	gb|AIXA01002472.1|:24976-25105
MsHel-2	gb|AIXA01001982.1|:34516-34645
MsHel-2	gb|AIXA01001775.1|:58533-58654
MsHel-2	gb|AIXA01001619.1|:18273-18402
MsHel-2	gb|AIXA01001612.1|:27457-27583
MsHel-2	gb|AIXA01001566.1|:27269-27397
MsHel-2	gb|AIXA01000562.1|:154174-154302
MsHel-2	gb|AIXA01000160.1|:39194-39323
MsHel-2	gb|AIXA01000099.1|:32896-33025
MsHel-2	gb|AIXA01035244.1|:223-315
MsHel-2	gb|AIXA01016884.1|:2468-2576

MsHel-2	gb|AIXA01015985.1|:23880-24008
MsHel-2	gb|AIXA01009532.1|:692-808
MsHel-2	gb|AIXA01009216.1|:86635-86727
MsHel-2	gb|AIXA01009078.1|:49351-49467
MsHel-2	gb|AIXA01006178.1|:8550-8675
MsHel-2	gb|AIXA01003653.1|:71138-71250
MsHel-2	gb|AIXA01003651.1|:49058-49166
MsHel-2	gb|AIXA01003152.1|:137918-138015
MsHel-2	gb|AIXA01002168.1|:13331-13449
MsHel-2	gb|AIXA01001786.1|:43033-43142
MsHel-2	gb|AIXA01001532.1|:65510-65598
MsHel-2	gb|AIXA01000554.1|:1709-1837
MsHel-2	gb|AIXA01000452.1|:15136-15255
MsHel-2	gb|AIXA01000307.1|:1956-2080
MsHel-2	gb|AIXA01000276.1|:10495-10623
MsHel-2	gb|AIXA01027375.1|:717-851
MsHel-2	gb|AIXA01024878.1|:314-402
MsHel-2	gb|AIXA01017682.1|:935-1062
MsHel-2	gb|AIXA01015132.1|:47797-48220
MsHel-2	gb|AIXA01015103.1|:8651-8772
MsHel-2	gb|AIXA01014867.1|:24093-24213
MsHel-2	gb|AIXA01013916.1|:13786-13909
MsHel-2	gb|AIXA01013223.1|:4950-5069
MsHel-2	gb|AIXA01013223.1|:29535-29680
MsHel-2	gb|AIXA01012196.1|:10117-10238
MsHel-2	gb|AIXA01009539.1|:74215-74317
MsHel-2	gb|AIXA01008922.1|:5372-5491
MsHel-2	gb|AIXA01008562.1|:30185-30296
MsHel-2	gb|AIXA01008220.1|:16001-16131
MsHel-2	gb|AIXA01007984.1|:4281-4380
MsHel-2	gb|AIXA01007515.1|:82644-82739
MsHel-2	gb|AIXA01006190.1|:19080-19195
MsHel-2	gb|AIXA01006091.1|:1455-1586
MsHel-2	gb|AIXA01004809.1|:83617-83737
MsHel-2	gb|AIXA01004068.1|:77436-77559
MsHel-2	gb|AIXA01003657.1|:2555-2685
MsHel-2	gb|AIXA01003190.1|:33288-33376
MsHel-2	gb|AIXA01003028.1|:15253-15371
MsHel-2	gb|AIXA01002672.1|:4765-4891
MsHel-2	gb|AIXA01001660.1|:32570-32696
MsHel-2	gb|AIXA01001605.1|:49934-50061
MsHel-2	gb|AIXA01001271.1|:57084-57180
MsHel-2	gb|AIXA01001271.1|:81597-81724
MsHel-2	gb|AIXA01001243.1|:175490-175612
MsHel-2	gb|AIXA01001243.1|:216282-216389
MsHel-2	gb|AIXA01000275.1|:25589-25714
MsHel-2	gb|AIXA01000275.1|:71348-71447
MsHel-2	gb|AIXA01026309.1|:800-922

MsHel-2	gb|AIXA01023697.1|:1089-1210
MsHel-2	gb|AIXA01021834.1|:1293-1419
MsHel-2	gb|AIXA01018540.1|:365-495
MsHel-2	gb|AIXA01017894.1|:5829-5952
MsHel-2	gb|AIXA01014513.1|:2049-2174
MsHel-2	gb|AIXA01011205.1|:614-736
MsHel-2	gb|AIXA01010960.1|:22933-23035
MsHel-2	gb|AIXA01010151.1|:117330-117424
MsHel-2	gb|AIXA01010151.1|:155996-156122
MsHel-2	gb|AIXA01009346.1|:34345-34471
MsHel-2	gb|AIXA01008187.1|:9772-9898
MsHel-2	gb|AIXA01008187.1|:55370-55482
MsHel-2	gb|AIXA01008123.1|:22240-22370
MsHel-2	gb|AIXA01006682.1|:21752-21879
MsHel-2	gb|AIXA01005088.1|:34434-34556
MsHel-2	gb|AIXA01004311.1|:8008-8138
MsHel-2	gb|AIXA01003949.1|:8309-8435
MsHel-2	gb|AIXA01003588.1|:14465-14594
MsHel-2	gb|AIXA01003008.1|:11203-11330
MsHel-2	gb|AIXA01002103.1|:21968-22066
MsHel-2	gb|AIXA01001995.1|:15810-15932
MsHel-2	gb|AIXA01001797.1|:58772-58875
MsHel-2	gb|AIXA01001667.1|:38603-38730
MsHel-2	gb|AIXA01001071.1|:94678-94795
MsHel-2	gb|AIXA01000909.1|:127439-127553
MsHel-2	gb|AIXA01031220.1|:22-150
MsHel-2	gb|AIXA01028129.1|:72-189
MsHel-2	gb|AIXA01022068.1|:983-1100
MsHel-2	gb|AIXA01019605.1|:1536-1661
MsHel-2	gb|AIXA01018933.1|:200-329
MsHel-2	gb|AIXA01017756.1|:2402-2518
MsHel-2	gb|AIXA01017702.1|:15362-15491
MsHel-2	gb|AIXA01017187.1|:3993-4122
MsHel-2	gb|AIXA01017047.1|:4733-4834
MsHel-2	gb|AIXA01015708.1|:3677-3806
MsHel-2	gb|AIXA01012652.1|:8511-16981
MsHel-2	gb|AIXA01012364.1|:12639-12738
MsHel-2	gb|AIXA01011126.1|:18049-18167
MsHel-2	gb|AIXA01011126.1|:38151-38280
MsHel-2	gb|AIXA01010871.1|:62-186
MsHel-2	gb|AIXA01009925.1|:60501-60625
MsHel-2	gb|AIXA01009405.1|:31011-31142
MsHel-2	gb|AIXA01009291.1|:32305-32426
MsHel-2	gb|AIXA01009039.1|:22947-23076
MsHel-2	gb|AIXA01008728.1|:5564-5693
MsHel-2	gb|AIXA01008226.1|:30292-30411
MsHel-2	gb|AIXA01008222.1|:18753-18874
MsHel-2	gb|AIXA01008076.1|:26485-26603

MsHel-2	gb|AIXA01007766.1|:4253-4382
MsHel-2	gb|AIXA01007576.1|:104208-104309
MsHel-2	gb|AIXA01007408.1|:2372-2491
MsHel-2	gb|AIXA01007398.1|:99-222
MsHel-2	gb|AIXA01007237.1|:174-275
MsHel-2	gb|AIXA01006123.1|:64161-64285
MsHel-2	gb|AIXA01005534.1|:16636-16758
MsHel-2	gb|AIXA01005508.1|:27024-27153
MsHel-2	gb|AIXA01005407.1|:183379-183479
MsHel-2	gb|AIXA01005203.1|:36348-36465
MsHel-2	gb|AIXA01004980.1|:35942-36073
MsHel-2	gb|AIXA01004950.1|:3268-3388
MsHel-2	gb|AIXA01004896.1|:40369-40496
MsHel-2	gb|AIXA01004896.1|:56584-56711
MsHel-2	gb|AIXA01004730.1|:22816-22944
MsHel-2	gb|AIXA01004360.1|:4571-4700
MsHel-2	gb|AIXA01004341.1|:2692-2812
MsHel-2	gb|AIXA01002621.1|:56163-56282
MsHel-2	gb|AIXA01002621.1|:92175-92304
MsHel-2	gb|AIXA01002620.1|:7977-8100
MsHel-2	gb|AIXA01002560.1|:32110-32223
MsHel-2	gb|AIXA01002477.1|:29182-33182
MsHel-2	gb|AIXA01002244.1|:12547-12652
MsHel-2	gb|AIXA01002174.1|:76540-76643
MsHel-2	gb|AIXA01001630.1|:26019-26117
MsHel-2	gb|AIXA01001525.1|:5508-5620
MsHel-2	gb|AIXA01001505.1|:53724-53852
MsHel-2	gb|AIXA01001366.1|:66295-66415
MsHel-2	gb|AIXA01001356.1|:32002-32131
MsHel-2	gb|AIXA01001210.1|:36953-37082
MsHel-2	gb|AIXA01000688.1|:87683-87785
MsHel-2	gb|AIXA01000688.1|:107375-107570
MsHel-2	gb|AIXA01000422.1|:73076-73203
MsHel-2	gb|AIXA01022008.1|:548-672
MsHel-2	gb|AIXA01021900.1|:1023-1115
MsHel-2	gb|AIXA01019132.1|:1433-1552
MsHel-2	gb|AIXA01018590.1|:1537-1660
MsHel-2	gb|AIXA01018181.1|:14601-14725
MsHel-2	gb|AIXA01015790.1|:4078-4166
MsHel-2	gb|AIXA01015424.1|:2069-2194
MsHel-2	gb|AIXA01013078.1|:4025-4150
MsHel-2	gb|AIXA01011968.1|:19369-19505
MsHel-2	gb|AIXA01010358.1|:16544-16672
MsHel-2	gb|AIXA01009752.1|:15650-15779
MsHel-2	gb|AIXA01009350.1|:3564-3688
MsHel-2	gb|AIXA01009131.1|:19405-19501
MsHel-2	gb|AIXA01007785.1|:6313-6439
MsHel-2	gb|AIXA01007785.1|:17480-17601

MsHel-2	gb|AIXA01007589.1|:24295-24423
MsHel-2	gb|AIXA01006874.1|:16724-16843
MsHel-2	gb|AIXA01005865.1|:75471-75597
MsHel-2	gb|AIXA01005730.1|:28391-28498
MsHel-2	gb|AIXA01005394.1|:42802-43117
MsHel-2	gb|AIXA01005229.1|:3332-3440
MsHel-2	gb|AIXA01005044.1|:13938-14036
MsHel-2	gb|AIXA01005033.1|:14700-14798
MsHel-2	gb|AIXA01004220.1|:19057-19181
MsHel-2	gb|AIXA01004220.1|:38153-38276
MsHel-2	gb|AIXA01003678.1|:64374-64502
MsHel-2	gb|AIXA01003621.1|:106694-106814
MsHel-2	gb|AIXA01003283.1|:6808-6911
MsHel-2	gb|AIXA01003112.1|:27704-27798
MsHel-2	gb|AIXA01002543.1|:4878-4966
MsHel-2	gb|AIXA01002308.1|:52031-52157
MsHel-2	gb|AIXA01000684.1|:25838-25921
MsHel-2	gb|AIXA01038179.1|:349-476
MsHel-2	gb|AIXA01020334.1|:2453-2556
MsHel-2	gb|AIXA01018917.1|:470-596
MsHel-2	gb|AIXA01018162.1|:4994-5112
MsHel-2	gb|AIXA01016250.1|:1468-1559
MsHel-2	gb|AIXA01015372.1|:59-166
MsHel-2	gb|AIXA01014780.1|:8501-8630
MsHel-2	gb|AIXA01014243.1|:39199-39325
MsHel-2	gb|AIXA01011322.1|:35189-35315
MsHel-2	gb|AIXA01010703.1|:26809-26935
MsHel-2	gb|AIXA01010319.1|:8112-8231
MsHel-2	gb|AIXA01007890.1|:1000-1113
MsHel-2	gb|AIXA01007296.1|:138773-138899
MsHel-2	gb|AIXA01006999.1|:45785-45910
MsHel-2	gb|AIXA01006705.1|:36744-36871
MsHel-2	gb|AIXA01006654.1|:8091-8214
MsHel-2	gb|AIXA01005822.1|:31094-31220
MsHel-2	gb|AIXA01005413.1|:17242-17364
MsHel-2	gb|AIXA01005096.1|:11421-11515
MsHel-2	gb|AIXA01005096.1|:50179-50276
MsHel-2	gb|AIXA01004946.1|:56952-57077
MsHel-2	gb|AIXA01004683.1|:56531-56629
MsHel-2	gb|AIXA01004683.1|:84195-84315
MsHel-2	gb|AIXA01004619.1|:31682-31801
MsHel-2	gb|AIXA01004184.1|:127655-127782
MsHel-2	gb|AIXA01004150.1|:4956-5056
MsHel-2	gb|AIXA01004150.1|:20607-20734
MsHel-2	gb|AIXA01004150.1|:51283-51411
MsHel-2	gb|AIXA01003876.1|:22388-22514
MsHel-2	gb|AIXA01003825.1|:27106-27210
MsHel-2	gb|AIXA01003650.1|:12626-12746

MsHel-2	gb|AIXA01003408.1|:3710-3833
MsHel-2	gb|AIXA01003129.1|:1008-1117
MsHel-2	gb|AIXA01003106.1|:15968-16066
MsHel-2	gb|AIXA01002718.1|:19486-19608
MsHel-2	gb|AIXA01002705.1|:28590-28677
MsHel-2	gb|AIXA01002612.1|:36085-37527
MsHel-2	gb|AIXA01001841.1|:10895-11021
MsHel-2	gb|AIXA01001841.1|:34651-34772
MsHel-2	gb|AIXA01001509.1|:28292-28413
MsHel-2	gb|AIXA01001257.1|:828-954
MsHel-2	gb|AIXA01000871.1|:6543-6650
MsHel-2	gb|AIXA01000871.1|:28194-28299
MsHel-2	gb|AIXA01032686.1|:262-384
MsHel-2	gb|AIXA01025567.1|:797-931
MsHel-2	gb|AIXA01015720.1|:8575-8680
MsHel-2	gb|AIXA01014707.1|:4568-4681
MsHel-2	gb|AIXA01014644.1|:24737-24839
MsHel-2	gb|AIXA01010995.1|:9696-9800
MsHel-2	gb|AIXA01010956.1|:59167-59253
MsHel-2	gb|AIXA01010691.1|:7595-7721
MsHel-2	gb|AIXA01010398.1|:30035-30157
MsHel-2	gb|AIXA01009919.1|:2072-2197
MsHel-2	gb|AIXA01009636.1|:2089-2272
MsHel-2	gb|AIXA01008711.1|:23628-23755
MsHel-2	gb|AIXA01008047.1|:5034-5147
MsHel-2	gb|AIXA01007944.1|:4217-4315
MsHel-2	gb|AIXA01007520.1|:2104-2194
MsHel-2	gb|AIXA01006696.1|:5176-5301
MsHel-2	gb|AIXA01006352.1|:3043-3133
MsHel-2	gb|AIXA01005391.1|:66203-66325
MsHel-2	gb|AIXA01005123.1|:26049-26175
MsHel-2	gb|AIXA01004580.1|:98548-98638
MsHel-2	gb|AIXA01004222.1|:69131-69245
MsHel-2	gb|AIXA01004094.1|:5265-5351
MsHel-2	gb|AIXA01003176.1|:43320-43450
MsHel-2	gb|AIXA01003176.1|:84290-84418
MsHel-2	gb|AIXA01002580.1|:109759-109852
MsHel-2	gb|AIXA01001032.1|:27163-27293
MsHel-2	gb|AIXA01000661.1|:18172-18299
MsHel-2	gb|AIXA01000661.1|:52851-52957
MsHel-2	gb|AIXA01000150.1|:7962-8073
MsHel-2	gb|AIXA01000129.1|:348-449
MsHel-2	gb|AIXA01024167.1|:422-551
MsHel-2	gb|AIXA01019129.1|:1731-1858
MsHel-2	gb|AIXA01018493.1|:5422-5515
MsHel-2	gb|AIXA01015447.1|:1295-1412
MsHel-2	gb|AIXA01013473.1|:10456-10577
MsHel-2	gb|AIXA01010756.1|:32801-32911

MsHel-2	gb|AIXA01009380.1|:23366-23495
MsHel-2	gb|AIXA01008793.1|:9607-9712
MsHel-2	gb|AIXA01008491.1|:16551-16680
MsHel-2	gb|AIXA01007577.1|:23329-23422
MsHel-2	gb|AIXA01007265.1|:26263-26390
MsHel-2	gb|AIXA01006980.1|:5070-5181
MsHel-2	gb|AIXA01006980.1|:27821-27949
MsHel-2	gb|AIXA01006736.1|:15081-15210
MsHel-2	gb|AIXA01006651.1|:62929-63054
MsHel-2	gb|AIXA01006080.1|:11164-11293
MsHel-2	gb|AIXA01005644.1|:588-707
MsHel-2	gb|AIXA01005329.1|:80-205
MsHel-2	gb|AIXA01005220.1|:1646-1771
MsHel-2	gb|AIXA01004546.1|:23767-23896
MsHel-2	gb|AIXA01004416.1|:30936-31065
MsHel-2	gb|AIXA01004337.1|:9199-9327
MsHel-2	gb|AIXA01003286.1|:2229-2354
MsHel-2	gb|AIXA01002992.1|:20413-20505
MsHel-2	gb|AIXA01002352.1|:27424-27537
MsHel-2	gb|AIXA01001847.1|:15810-15932
MsHel-2	gb|AIXA01001806.1|:21722-21848
MsHel-2	gb|AIXA01001695.1|:13862-13963
MsHel-2	gb|AIXA01001618.1|:46062-46171
MsHel-2	gb|AIXA01000641.1|:28796-28925
MsHel-2	gb|AIXA01000448.1|:135354-135482
MsHel-2	gb|AIXA01033225.1|:510-594
MsHel-2	gb|AIXA01025449.1|:215-334
MsHel-2	gb|AIXA01024093.1|:530-654
MsHel-2	gb|AIXA01021117.1|:1118-1226
MsHel-2	gb|AIXA01018591.1|:1537-1660
MsHel-2	gb|AIXA01017208.1|:2575-2703
MsHel-2	gb|AIXA01017198.1|:3038-3157
MsHel-2	gb|AIXA01013328.1|:15517-15638
MsHel-2	gb|AIXA01012527.1|:26581-26704
MsHel-2	gb|AIXA01010811.1|:6267-6390
MsHel-2	gb|AIXA01009584.1|:40959-41079
MsHel-2	gb|AIXA01009584.1|:73706-73835
MsHel-2	gb|AIXA01009047.1|:18361-18489
MsHel-2	gb|AIXA01008526.1|:7429-7557
MsHel-2	gb|AIXA01008520.1|:20124-20252
MsHel-2	gb|AIXA01007046.1|:26190-26302
MsHel-2	gb|AIXA01006998.1|:1306-1428
MsHel-2	gb|AIXA01006337.1|:39296-39397
MsHel-2	gb|AIXA01006184.1|:16391-16480
MsHel-2	gb|AIXA01004435.1|:9234-9338
MsHel-2	gb|AIXA01003901.1|:33534-33659
MsHel-2	gb|AIXA01003683.1|:122398-122514
MsHel-2	gb|AIXA01001863.1|:29882-29995

MsHel-2	gb|AIXA01001026.1|:29131-30682
MsHel-2	gb|AIXA01001001.1|:2906-3033
MsHel-2	gb|AIXA01000441.1|:15452-15576
MsHel-2	gb|AIXA01000440.1|:59108-66198
MsHel-2	gb|AIXA01000365.1|:13188-13284
MsHel-2	gb|AIXA01000273.1|:1557-1685
MsHel-2	gb|AIXA01029549.1|:181-276
MsHel-2	gb|AIXA01016590.1|:23711-23838
MsHel-2	gb|AIXA01016491.1|:314-1242
MsHel-2	gb|AIXA01013012.1|:3608-3737
MsHel-2	gb|AIXA01012659.1|:19504-19587
MsHel-2	gb|AIXA01012172.1|:9754-9875
MsHel-2	gb|AIXA01011987.1|:16876-16963
MsHel-2	gb|AIXA01011592.1|:6460-6576
MsHel-2	gb|AIXA01011318.1|:2983-3078
MsHel-2	gb|AIXA01010885.1|:23294-23409
MsHel-2	gb|AIXA01010475.1|:26863-26989
MsHel-2	gb|AIXA01008987.1|:1928-2022
MsHel-2	gb|AIXA01007555.1|:3015-3110
MsHel-2	gb|AIXA01005435.1|:6267-6390
MsHel-2	gb|AIXA01004694.1|:57182-57300
MsHel-2	gb|AIXA01004471.1|:44232-44359
MsHel-2	gb|AIXA01004412.1|:12750-12871
MsHel-2	gb|AIXA01003857.1|:16598-16724
MsHel-2	gb|AIXA01003615.1|:66741-66867
MsHel-2	gb|AIXA01002459.1|:6010-6137
MsHel-2	gb|AIXA01001993.1|:15422-15549
MsHel-2	gb|AIXA01001601.1|:21086-21213
MsHel-2	gb|AIXA01001282.1|:43262-43389
MsHel-2	gb|AIXA01000755.1|:106007-106131
MsHel-2	gb|AIXA01000685.1|:14595-14720
MsHel-2	gb|AIXA01000670.1|:39802-39925
MsHel-2	gb|AIXA01000078.1|:7926-8052
MsHel-2	gb|AIXA01027937.1|:80-207
MsHel-2	gb|AIXA01018937.1|:3142-3253
MsHel-2	gb|AIXA01017362.1|:7709-7815
MsHel-2	gb|AIXA01017333.1|:9102-9205
MsHel-2	gb|AIXA01016345.1|:1544-1642
MsHel-2	gb|AIXA01015716.1|:935-1053
MsHel-2	gb|AIXA01015028.1|:586-688
MsHel-2	gb|AIXA01014744.1|:2117-2221
MsHel-2	gb|AIXA01014041.1|:20802-20923
MsHel-2	gb|AIXA01013595.1|:6418-6542
MsHel-2	gb|AIXA01012654.1|:6023-6137
MsHel-2	gb|AIXA01012413.1|:4356-4454
MsHel-2	gb|AIXA01009942.1|:1614-1739
MsHel-2	gb|AIXA01009862.1|:948-1054
MsHel-2	gb|AIXA01008670.1|:27724-27830

MsHel-2	gb|AIXA01006590.1|:135670-135794
MsHel-2	gb|AIXA01006399.1|:4405-4528
MsHel-2	gb|AIXA01005474.1|:14065-14167
MsHel-2	gb|AIXA01004865.1|:23452-23580
MsHel-2	gb|AIXA01004634.1|:109817-109937
MsHel-2	gb|AIXA01004554.1|:13941-14066
MsHel-2	gb|AIXA01004464.1|:16993-17131
MsHel-2	gb|AIXA01003992.1|:19503-19619
MsHel-2	gb|AIXA01003114.1|:29678-29776
MsHel-2	gb|AIXA01003052.1|:28107-28228
MsHel-2	gb|AIXA01001826.1|:2471-2584
MsHel-2	gb|AIXA01001650.1|:26191-26292
MsHel-2	gb|AIXA01001610.1|:2070-2196
MsHel-2	gb|AIXA01000058.1|:3505-3615
MsHel-2	gb|AIXA01028696.1|:344-472
MsHel-2	gb|AIXA01025122.1|:727-836
MsHel-2	gb|AIXA01019850.1|:740-868
MsHel-2	gb|AIXA01018268.1|:1291-1420
MsHel-2	gb|AIXA01017992.1|:4935-5064
MsHel-2	gb|AIXA01017926.1|:3965-4094
MsHel-2	gb|AIXA01016786.1|:5012-5137
MsHel-2	gb|AIXA01016365.1|:157-262
MsHel-2	gb|AIXA01016204.1|:6267-6395
MsHel-2	gb|AIXA01015929.1|:26370-26484
MsHel-2	gb|AIXA01015446.1|:11316-11433
MsHel-2	gb|AIXA01012786.1|:13784-13880
MsHel-2	gb|AIXA01011739.1|:14479-14607
MsHel-2	gb|AIXA01010481.1|:15894-16023
MsHel-2	gb|AIXA01009945.1|:8778-8871
MsHel-2	gb|AIXA01009426.1|:2882-3003
MsHel-2	gb|AIXA01009127.1|:19140-19269
MsHel-2	gb|AIXA01009071.1|:41031-41159
MsHel-2	gb|AIXA01008364.1|:41775-41900
MsHel-2	gb|AIXA01007875.1|:50122-50251
MsHel-2	gb|AIXA01007613.1|:36052-36181
MsHel-2	gb|AIXA01005500.1|:75-204
MsHel-2	gb|AIXA01005319.1|:55690-55804
MsHel-2	gb|AIXA01005317.1|:4014-4143
MsHel-2	gb|AIXA01005187.1|:30755-30852
MsHel-2	gb|AIXA01004226.1|:5406-5520
MsHel-2	gb|AIXA01004164.1|:49108-49225
MsHel-2	gb|AIXA01004100.1|:16731-16860
MsHel-2	gb|AIXA01004042.1|:7897-8026
MsHel-2	gb|AIXA01003234.1|:59756-59856
MsHel-2	gb|AIXA01003027.1|:61918-62290
MsHel-2	gb|AIXA01002635.1|:44022-44149
MsHel-2	gb|AIXA01002635.1|:63722-63851
MsHel-2	gb|AIXA01001843.1|:26794-26909

MsHel-2	gb|AIXA01001803.1|:3867-3983
MsHel-2	gb|AIXA01001328.1|:11884-11969
MsHel-2	gb|AIXA01000741.1|:17672-17801
MsHel-2	gb|AIXA01000476.1|:30588-30715
MsHel-2	gb|AIXA01000403.1|:196477-196605
MsHel-2	gb|AIXA01000070.1|:30906-31027
MsHel-2	gb|AIXA01021701.1|:1130-1241
MsHel-2	gb|AIXA01021063.1|:1372-1476
MsHel-2	gb|AIXA01019771.1|:1902-2030
MsHel-2	gb|AIXA01016921.1|:5519-5642
MsHel-2	gb|AIXA01016735.1|:7701-7825
MsHel-2	gb|AIXA01016181.1|:10611-10740
MsHel-2	gb|AIXA01013982.1|:3550-3670
MsHel-2	gb|AIXA01010786.1|:51351-51458
MsHel-2	gb|AIXA01009798.1|:6209-6329
MsHel-2	gb|AIXA01009798.1|:24486-24613
MsHel-2	gb|AIXA01009144.1|:10188-10291
MsHel-2	gb|AIXA01009106.1|:7434-7535
MsHel-2	gb|AIXA01007881.1|:47573-47695
MsHel-2	gb|AIXA01007453.1|:6818-6942
MsHel-2	gb|AIXA01006855.1|:35770-35889
MsHel-2	gb|AIXA01006053.1|:45697-45805
MsHel-2	gb|AIXA01004861.1|:11537-11665
MsHel-2	gb|AIXA01004660.1|:57003-57127
MsHel-2	gb|AIXA01003994.1|:10421-10545
MsHel-2	gb|AIXA01003274.1|:997-1088
MsHel-2	gb|AIXA01003030.1|:26748-26863
MsHel-2	gb|AIXA01002651.1|:17085-17181
MsHel-2	gb|AIXA01000483.1|:33164-33260
MsHel-2	gb|AIXA01000296.1|:53640-53768
MsHel-2	gb|AIXA01033617.1|:471-598
MsHel-2	gb|AIXA01019762.1|:579-705
MsHel-2	gb|AIXA01018502.1|:345-469
MsHel-2	gb|AIXA01018319.1|:4525-4636
MsHel-2	gb|AIXA01015999.1|:14231-14333
MsHel-2	gb|AIXA01013682.1|:12144-12235
MsHel-2	gb|AIXA01009246.1|:10698-10823
MsHel-2	gb|AIXA01008195.1|:23223-23343
MsHel-2	gb|AIXA01007435.1|:7638-7781
MsHel-2	gb|AIXA01006906.1|:8870-8965
MsHel-2	gb|AIXA01005563.1|:28489-28616
MsHel-2	gb|AIXA01005558.1|:22137-22264
MsHel-2	gb|AIXA01005491.1|:30551-30642
MsHel-2	gb|AIXA01005396.1|:26725-26811
MsHel-2	gb|AIXA01005396.1|:53590-53713
MsHel-2	gb|AIXA01005349.1|:82078-82160
MsHel-2	gb|AIXA01004596.1|:47327-47422
MsHel-2	gb|AIXA01004430.1|:21860-21978

MsHel-2	gb|AIXA01003713.1|:39067-39174
MsHel-2	gb|AIXA01001991.1|:11976-12083
MsHel-2	gb|AIXA01001674.1|:65447-65534
MsHel-2	gb|AIXA01001273.1|:4086-4209
MsHel-2	gb|AIXA01021569.1|:429-553
MsHel-2	gb|AIXA01019077.1|:728-826
MsHel-2	gb|AIXA01017631.1|:2823-2904
MsHel-2	gb|AIXA01017241.1|:4930-5024
MsHel-2	gb|AIXA01016615.1|:5250-5363
MsHel-2	gb|AIXA01015423.1|:480-607
MsHel-2	gb|AIXA01015055.1|:19884-19977
MsHel-2	gb|AIXA01010737.1|:19486-19615
MsHel-2	gb|AIXA01010189.1|:543-658
MsHel-2	gb|AIXA01010189.1|:17364-17462
MsHel-2	gb|AIXA01009035.1|:17724-17854
MsHel-2	gb|AIXA01008554.1|:64394-64524
MsHel-2	gb|AIXA01008400.1|:34698-34830
MsHel-2	gb|AIXA01008049.1|:35198-35320
MsHel-2	gb|AIXA01008040.1|:6303-6815
MsHel-2	gb|AIXA01006860.1|:27159-27285
MsHel-2	gb|AIXA01006329.1|:8541-8665
MsHel-2	gb|AIXA01006135.1|:3982-4092
MsHel-2	gb|AIXA01002396.1|:2805-6447
MsHel-2	gb|AIXA01002395.1|:25612-25738
MsHel-2	gb|AIXA01001613.1|:12288-12400
MsHel-2	gb|AIXA01001613.1|:74586-74710
MsHel-2	gb|AIXA01001594.1|:7840-7967
MsHel-2	gb|AIXA01001313.1|:13096-13185
MsHel-2	gb|AIXA01001278.1|:30564-30690
MsHel-2	gb|AIXA01000636.1|:3851-3953
MsHel-2	gb|AIXA01000480.1|:58126-58248
MsHel-2	gb|AIXA01000261.1|:41736-41862
MsHel-2	gb|AIXA01037269.1|:158-287
MsHel-2	gb|AIXA01037243.1|:213-337
MsHel-2	gb|AIXA01029781.1|:641-757
MsHel-2	gb|AIXA01024515.1|:243-359
MsHel-2	gb|AIXA01021259.1|:1892-2020
MsHel-2	gb|AIXA01019728.1|:4564-4692
MsHel-2	gb|AIXA01017475.1|:3980-4096
MsHel-2	gb|AIXA01016755.1|:4198-4326
MsHel-2	gb|AIXA01015804.1|:6475-6594
MsHel-2	gb|AIXA01015770.1|:23831-23958
MsHel-2	gb|AIXA01013943.1|:45-167
MsHel-2	gb|AIXA01012903.1|:1824-1940
MsHel-2	gb|AIXA01012412.1|:4930-11849
MsHel-2	gb|AIXA01012235.1|:2726-2846
MsHel-2	gb|AIXA01011722.1|:24925-25047
MsHel-2	gb|AIXA01011722.1|:57557-57657

MsHel-2	gb|AIXA01009599.1|:37154-37282
MsHel-2	gb|AIXA01008972.1|:3782-3879
MsHel-2	gb|AIXA01008428.1|:9723-9834
MsHel-2	gb|AIXA01008313.1|:77257-77370
MsHel-2	gb|AIXA01008263.1|:10053-10181
MsHel-2	gb|AIXA01008013.1|:14395-14524
MsHel-2	gb|AIXA01007949.1|:10328-10445
MsHel-2	gb|AIXA01007949.1|:21437-21532
MsHel-2	gb|AIXA01007602.1|:44296-44414
MsHel-2	gb|AIXA01007588.1|:8162-8293
MsHel-2	gb|AIXA01007522.1|:12062-12186
MsHel-2	gb|AIXA01007027.1|:1316-1426
MsHel-2	gb|AIXA01005278.1|:19423-19552
MsHel-2	gb|AIXA01005278.1|:50301-50392
MsHel-2	gb|AIXA01004345.1|:20687-20788
MsHel-2	gb|AIXA01004057.1|:6790-6918
MsHel-2	gb|AIXA01003859.1|:23896-24001
MsHel-2	gb|AIXA01003846.1|:69356-69477
MsHel-2	gb|AIXA01003687.1|:543-644
MsHel-2	gb|AIXA01002643.1|:5804-5926
MsHel-2	gb|AIXA01002385.1|:42073-42193
MsHel-2	gb|AIXA01001952.1|:2436-2565
MsHel-2	gb|AIXA01001595.1|:36497-36617
MsHel-2	gb|AIXA01001021.1|:4290-4391
MsHel-2	gb|AIXA01020695.1|:2815-2934
MsHel-2	gb|AIXA01017807.1|:1010-1113
MsHel-2	gb|AIXA01015471.1|:11781-11873
MsHel-2	gb|AIXA01011354.1|:46396-46520
MsHel-2	gb|AIXA01009031.1|:26147-26276
MsHel-2	gb|AIXA01008871.1|:22086-22618
MsHel-2	gb|AIXA01008568.1|:768-885
MsHel-2	gb|AIXA01008258.1|:4605-4702
MsHel-2	gb|AIXA01007947.1|:53154-53275
MsHel-2	gb|AIXA01007568.1|:31570-31711
MsHel-2	gb|AIXA01005062.1|:36286-36415
MsHel-2	gb|AIXA01005009.1|:22406-22525
MsHel-2	gb|AIXA01004342.1|:26867-26958
MsHel-2	gb|AIXA01004139.1|:2381-2473
MsHel-2	gb|AIXA01003315.1|:35430-35554
MsHel-2	gb|AIXA01001331.1|:303-4743
MsHel-2	gb|AIXA01000771.1|:1391-1516
MsHel-2	gb|AIXA01000758.1|:1600-1712
MsHel-2	gb|AIXA01000740.1|:12184-12299
MsHel-2	gb|AIXA01021776.1|:1346-1441
MsHel-2	gb|AIXA01014934.1|:3824-3911
MsHel-2	gb|AIXA01012523.1|:5573-5671
MsHel-2	gb|AIXA01009940.1|:7065-7158
MsHel-2	gb|AIXA01007034.1|:39069-39190

MsHel-2	gb|AIXA01007034.1|:69292-69426
MsHel-2	gb|AIXA01006282.1|:6484-6587
MsHel-2	gb|AIXA01005618.1|:91738-91821
MsHel-2	gb|AIXA01005322.1|:34227-34350
MsHel-2	gb|AIXA01005086.1|:1337-1463
MsHel-2	gb|AIXA01003798.1|:996-1082
MsHel-2	gb|AIXA01003726.1|:3006-3128
MsHel-2	gb|AIXA01003185.1|:16658-16761
MsHel-2	gb|AIXA01001627.1|:35319-35445
MsHel-2	gb|AIXA01001501.1|:14463-16860
MsHel-2	gb|AIXA01001040.1|:20126-20236
MsHel-2	gb|AIXA01000972.1|:52036-56854
MsHel-2	gb|AIXA01032537.1|:309-414
MsHel-2	gb|AIXA01019619.1|:5473-5585
MsHel-2	gb|AIXA01018837.1|:6744-6864
MsHel-2	gb|AIXA01016156.1|:8124-8218
MsHel-2	gb|AIXA01015219.1|:2216-2316
MsHel-2	gb|AIXA01014936.1|:3246-3372
MsHel-2	gb|AIXA01014735.1|:5156-6626
MsHel-2	gb|AIXA01012404.1|:13018-13145
MsHel-2	gb|AIXA01012301.1|:14525-14654
MsHel-2	gb|AIXA01009804.1|:20340-20465
MsHel-2	gb|AIXA01006937.1|:1526-1620
MsHel-2	gb|AIXA01006932.1|:1856-1955
MsHel-2	gb|AIXA01006508.1|:25021-25109
MsHel-2	gb|AIXA01006139.1|:16873-16983
MsHel-2	gb|AIXA01005997.1|:4527-4651
MsHel-2	gb|AIXA01004334.1|:3753-3878
MsHel-2	gb|AIXA01003568.1|:5865-5978
MsHel-2	gb|AIXA01002785.1|:40206-40328
MsHel-2	gb|AIXA01002415.1|:36562-36652
MsHel-2	gb|AIXA01002361.1|:28975-29069
MsHel-2	gb|AIXA01002216.1|:3058-3168
MsHel-2	gb|AIXA01002003.1|:3628-3731
MsHel-2	gb|AIXA01001631.1|:49996-50103
MsHel-2	gb|AIXA01001556.1|:3771-3989
MsHel-2	gb|AIXA01000198.1|:78709-78800
MsHel-2	gb|AIXA01035517.1|:293-397
MsHel-2	gb|AIXA01024029.1|:651-730
MsHel-2	gb|AIXA01021730.1|:377-478
MsHel-2	gb|AIXA01019935.1|:1486-1579
MsHel-2	gb|AIXA01018802.1|:1650-1792
MsHel-2	gb|AIXA01018667.1|:1045-1170
MsHel-2	gb|AIXA01017885.1|:3288-3413
MsHel-2	gb|AIXA01017884.1|:564-689
MsHel-2	gb|AIXA01017872.1|:373-474
MsHel-2	gb|AIXA01016325.1|:9235-9346
MsHel-2	gb|AIXA01015956.1|:26973-27062

MsHel-2	gb|AIXA01015786.1|:5386-5507
MsHel-2	gb|AIXA01015731.1|:4771-4884
MsHel-2	gb|AIXA01013103.1|:28204-28296
MsHel-2	gb|AIXA01012537.1|:9107-9236
MsHel-2	gb|AIXA01011563.1|:1523-1643
MsHel-2	gb|AIXA01011563.1|:15023-15144
MsHel-2	gb|AIXA01010962.1|:22265-22393
MsHel-2	gb|AIXA01010117.1|:2295-2422
MsHel-2	gb|AIXA01009528.1|:16371-16476
MsHel-2	gb|AIXA01009376.1|:4973-5066
MsHel-2	gb|AIXA01007731.1|:41309-41397
MsHel-2	gb|AIXA01007538.1|:30399-30496
MsHel-2	gb|AIXA01007164.1|:70251-70370
MsHel-2	gb|AIXA01006833.1|:28278-28382
MsHel-2	gb|AIXA01006781.1|:29944-30068
MsHel-2	gb|AIXA01006316.1|:5044-11835
MsHel-2	gb|AIXA01005789.1|:15242-15359
MsHel-2	gb|AIXA01005353.1|:5381-5475
MsHel-2	gb|AIXA01004676.1|:96509-96638
MsHel-2	gb|AIXA01004085.1|:3411-3504
MsHel-2	gb|AIXA01002614.1|:20161-20289
MsHel-2	gb|AIXA01001163.1|:21875-22000
MsHel-2	gb|AIXA01000872.1|:1855-1977
MsHel-2	gb|AIXA01000787.1|:66866-66966
MsHel-2	gb|AIXA01000489.1|:41517-41646
MsHel-2	gb|AIXA01018516.1|:4902-4994
MsHel-2	gb|AIXA01018364.1|:3505-3601
MsHel-2	gb|AIXA01016981.1|:554-662
MsHel-2	gb|AIXA01012125.1|:7956-8060
MsHel-2	gb|AIXA01012103.1|:18199-18299
MsHel-2	gb|AIXA01011904.1|:2599-2706
MsHel-2	gb|AIXA01009984.1|:46829-46920
MsHel-2	gb|AIXA01009658.1|:6215-6315
MsHel-2	gb|AIXA01009596.1|:116-217
MsHel-2	gb|AIXA01009455.1|:5754-5878
MsHel-2	gb|AIXA01009154.1|:21986-22111
MsHel-2	gb|AIXA01008475.1|:13136-13262
MsHel-2	gb|AIXA01006710.1|:1644-1755
MsHel-2	gb|AIXA01005524.1|:6722-6813
MsHel-2	gb|AIXA01005045.1|:58381-58503
MsHel-2	gb|AIXA01004698.1|:8364-8460
MsHel-2	gb|AIXA01004277.1|:23442-32965
MsHel-2	gb|AIXA01003874.1|:8062-8162
MsHel-2	gb|AIXA01003351.1|:15487-15600
MsHel-2	gb|AIXA01003175.1|:28942-29026
MsHel-2	gb|AIXA01003001.1|:41715-41799
MsHel-2	gb|AIXA01002454.1|:53806-53935
MsHel-2	gb|AIXA01001222.1|:77593-77685

MsHel-2	gb|AIXA01000863.1|:7722-7810
MsHel-2	gb|AIXA01000770.1|:67515-67608
MsHel-2	gb|AIXA01000152.1|:9014-9126
MsHel-2	gb|AIXA01000135.1|:4054-4150
MsHel-2	gb|AIXA01000068.1|:77547-77659
MsHel-2	gb|AIXA01020914.1|:1609-1722
MsHel-2	gb|AIXA01018543.1|:9630-9721
MsHel-2	gb|AIXA01017620.1|:7565-7660
MsHel-2	gb|AIXA01017031.1|:5911-6032
MsHel-2	gb|AIXA01016781.1|:17628-17742
MsHel-2	gb|AIXA01010758.1|:34465-34556
MsHel-2	gb|AIXA01010291.1|:6044-6162
MsHel-2	gb|AIXA01008915.1|:33997-34088
MsHel-2	gb|AIXA01007477.1|:25887-26198
MsHel-2	gb|AIXA01006166.1|:7961-8052
MsHel-2	gb|AIXA01005126.1|:34363-34481
MsHel-2	gb|AIXA01005126.1|:54415-54510
MsHel-2	gb|AIXA01004780.1|:356-454
MsHel-2	gb|AIXA01004592.1|:27472-27590
MsHel-2	gb|AIXA01004583.1|:31741-31840
MsHel-2	gb|AIXA01004212.1|:22512-22640
MsHel-2	gb|AIXA01003997.1|:80390-80497
MsHel-2	gb|AIXA01000237.1|:58244-58363
MsHel-2	gb|AIXA01036564.1|:261-353
MsHel-2	gb|AIXA01029802.1|:626-754
MsHel-2	gb|AIXA01018955.1|:202-332
MsHel-2	gb|AIXA01018554.1|:4813-4905
MsHel-2	gb|AIXA01015719.1|:3564-3658
MsHel-2	gb|AIXA01015297.1|:2552-2672
MsHel-2	gb|AIXA01014383.1|:11412-11533
MsHel-2	gb|AIXA01013631.1|:177-267
MsHel-2	gb|AIXA01008289.1|:1516-1615
MsHel-2	gb|AIXA01007634.1|:24765-24864
MsHel-2	gb|AIXA01007114.1|:29693-29785
MsHel-2	gb|AIXA01006509.1|:2771-2860
MsHel-2	gb|AIXA01005636.1|:32362-32452
MsHel-2	gb|AIXA01005100.1|:4352-4442
MsHel-2	gb|AIXA01003773.1|:3816-3942
MsHel-2	gb|AIXA01003288.1|:122195-122281
MsHel-2	gb|AIXA01002906.1|:25581-25695
MsHel-2	gb|AIXA01002255.1|:16545-16639
MsHel-2	gb|AIXA01000961.1|:16144-16274
MsHel-2	gb|AIXA01000653.1|:21762-22117
MsHel-2	gb|AIXA01000294.1|:15146-15268
MsHel-2	gb|AIXA01034081.1|:549-654
MsHel-2	gb|AIXA01022552.1|:2-113
MsHel-2	gb|AIXA01018112.1|:11882-11975
MsHel-2	gb|AIXA01018021.1|:1526-1645

MsHel-2	gb|AIXA01016041.1|:19556-19698
MsHel-2	gb|AIXA01012987.1|:1044-1173
MsHel-2	gb|AIXA01012351.1|:5952-6055
MsHel-2	gb|AIXA01012294.1|:3798-3923
MsHel-2	gb|AIXA01010541.1|:26462-26543
MsHel-2	gb|AIXA01005037.1|:1111-1208
MsHel-2	gb|AIXA01002503.1|:38758-38850
MsHel-2	gb|AIXA01024979.1|:966-1054
MsHel-2	gb|AIXA01014764.1|:2448-2540
MsHel-2	gb|AIXA01013807.1|:28440-28573
MsHel-2	gb|AIXA01013454.1|:4474-4560
MsHel-2	gb|AIXA01012948.1|:8025-8158
MsHel-2	gb|AIXA01012479.1|:21057-21180
MsHel-2	gb|AIXA01010908.1|:51701-51828
MsHel-2	gb|AIXA01010830.1|:11761-11865
MsHel-2	gb|AIXA01010561.1|:10753-10853
MsHel-2	gb|AIXA01010304.1|:4492-4608
MsHel-2	gb|AIXA01008825.1|:5523-5597
MsHel-2	gb|AIXA01007975.1|:69443-69522
MsHel-2	gb|AIXA01005251.1|:1006-1110
MsHel-2	gb|AIXA01002814.1|:33699-33825
MsHel-2	gb|AIXA01002254.1|:10653-10778
MsHel-2	gb|AIXA01002057.1|:12779-12865
MsHel-2	gb|AIXA01001598.1|:49618-49749
MsHel-2	gb|AIXA01001017.1|:54737-54855
MsHel-2	gb|AIXA01000689.1|:4703-4804
MsHel-2	gb|AIXA01026028.1|:99-209
MsHel-2	gb|AIXA01015530.1|:6207-6298
MsHel-2	gb|AIXA01011405.1|:15092-15187
MsHel-2	gb|AIXA01009355.1|:5202-5292
MsHel-2	gb|AIXA01006933.1|:14923-15053
MsHel-2	gb|AIXA01006909.1|:12396-19447
MsHel-2	gb|AIXA01006683.1|:21022-21149
MsHel-2	gb|AIXA01004758.1|:13724-13809
MsHel-2	gb|AIXA01001117.1|:5228-5331
MsHel-2	gb|AIXA01027440.1|:749-868
MsHel-2	gb|AIXA01018617.1|:1951-2073
MsHel-2	gb|AIXA01018131.1|:3476-3567
MsHel-2	gb|AIXA01012198.1|:856-967
MsHel-2	gb|AIXA01012173.1|:8233-8327
MsHel-2	gb|AIXA01012164.1|:6816-6938
MsHel-2	gb|AIXA01010082.1|:1503-1623
MsHel-2	gb|AIXA01009243.1|:32847-32933
MsHel-2	gb|AIXA01008639.1|:25771-25891
MsHel-2	gb|AIXA01008521.1|:20189-20291
MsHel-2	gb|AIXA01007605.1|:3739-3825
MsHel-2	gb|AIXA01007301.1|:8829-8949
MsHel-2	gb|AIXA01005942.1|:29398-29519

MsHel-2	gb|AIXA01003680.1|:4661-4770
MsHel-2	gb|AIXA01003209.1|:12771-12895
MsHel-2	gb|AIXA01019973.1|:2241-2347
MsHel-2	gb|AIXA01017635.1|:12592-12682
MsHel-2	gb|AIXA01015922.1|:543-671
MsHel-2	gb|AIXA01013314.1|:1537-1646
MsHel-2	gb|AIXA01008752.1|:4744-4861
MsHel-2	gb|AIXA01006015.1|:25528-25642
MsHel-2	gb|AIXA01005497.1|:30519-30619
MsHel-2	gb|AIXA01005281.1|:13407-13492
MsHel-2	gb|AIXA01004209.1|:16970-17067
MsHel-2	gb|AIXA01003013.1|:14763-14856
MsHel-2	gb|AIXA01000982.1|:83569-83684
MsHel-2	gb|AIXA01023174.1|:1193-1289
MsHel-2	gb|AIXA01019722.1|:1920-2048
MsHel-2	gb|AIXA01014348.1|:4493-4592
MsHel-2	gb|AIXA01013965.1|:3333-3417
MsHel-2	gb|AIXA01010698.1|:55981-56055
MsHel-2	gb|AIXA01009550.1|:32769-32855
MsHel-2	gb|AIXA01006405.1|:18460-18559
MsHel-2	gb|AIXA01005631.1|:8803-8895
MsHel-2	gb|AIXA01004362.1|:7037-7137
MsHel-2	gb|AIXA01016295.1|:4221-4325
MsHel-2	gb|AIXA01015729.1|:469-554
MsHel-2	gb|AIXA01014984.1|:22468-22591
MsHel-2	gb|AIXA01009703.1|:14060-14177
MsHel-2	gb|AIXA01009298.1|:34766-34863
MsHel-2	gb|AIXA01008553.1|:30000-30110
MsHel-2	gb|AIXA01005805.1|:4992-5092
MsHel-2	gb|AIXA01005725.1|:476-561
MsHel-2	gb|AIXA01026525.1|:928-1047
MsHel-2	gb|AIXA01013015.1|:45480-45599
MsHel-2	gb|AIXA01011695.1|:4297-4391
MsHel-2	gb|AIXA01011484.1|:81610-81719
MsHel-2	gb|AIXA01002268.1|:137-245
MsHel-2	gb|AIXA01002232.1|:51121-51227
MsHel-2	gb|AIXA01001307.1|:30624-30717
MsHel-2	gb|AIXA01027465.1|:578-671
MsHel-2	gb|AIXA01017256.1|:1721-1810
MsHel-2	gb|AIXA01015693.1|:4478-4567
MsHel-3a	gb|AIXA01016572.1|:8907-9190
MsHel-3a	gb|AIXA01016295.1|:3067-3355
MsHel-3a	gb|AIXA01014695.1|:28124-28405
MsHel-3a	gb|AIXA01010396.1|:6862-7145
MsHel-3a	gb|AIXA01000809.1|:130505-130790
MsHel-3a	gb|AIXA01035912.1|:55-174
MsHel-3a	gb|AIXA01024267.1|:1152-1271

MsHel-3a	gb|AIXA01018012.1|:94-213
MsHel-3a	gb|AIXA01017542.1|:11295-11581
MsHel-3a	gb|AIXA01016075.1|:43455-43735
MsHel-3a	gb|AIXA01015280.1|:283-567
MsHel-3a	gb|AIXA01012367.1|:11604-11882
MsHel-3a	gb|AIXA01011985.1|:9994-10273
MsHel-3a	gb|AIXA01011493.1|:91401-91595
MsHel-3a	gb|AIXA01011448.1|:2827-3112
MsHel-3a	gb|AIXA01010909.1|:94229-94508
MsHel-3a	gb|AIXA01010568.1|:4712-4996
MsHel-3a	gb|AIXA01010432.1|:11058-11344
MsHel-3a	gb|AIXA01009865.1|:30134-30422
MsHel-3a	gb|AIXA01009468.1|:9601-9889
MsHel-3a	gb|AIXA01009401.1|:46279-46560
MsHel-3a	gb|AIXA01009401.1|:118429-118568
MsHel-3a	gb|AIXA01007966.1|:75884-76172
MsHel-3a	gb|AIXA01007966.1|:94189-94308
MsHel-3a	gb|AIXA01007966.1|:161100-161366
MsHel-3a	gb|AIXA01007579.1|:31792-31902
MsHel-3a	gb|AIXA01007579.1|:49094-49367
MsHel-3a	gb|AIXA01006861.1|:7494-7779
MsHel-3a	gb|AIXA01005918.1|:63582-63801
MsHel-3a	gb|AIXA01005104.1|:6255-6544
MsHel-3a	gb|AIXA01005104.1|:22172-22289
MsHel-3a	gb|AIXA01004915.1|:4652-4939
MsHel-3a	gb|AIXA01004593.1|:27955-28217
MsHel-3a	gb|AIXA01004126.1|:5379-5653
MsHel-3a	gb|AIXA01003912.1|:4950-5228
MsHel-3a	gb|AIXA01003853.1|:41872-42151
MsHel-3a	gb|AIXA01003853.1|:81384-81674
MsHel-3a	gb|AIXA01003850.1|:11058-11347
MsHel-3a	gb|AIXA01003822.1|:12449-12723
MsHel-3a	gb|AIXA01003243.1|:1977-2170
MsHel-3a	gb|AIXA01003144.1|:9515-9793
MsHel-3a	gb|AIXA01002568.1|:799-1071
MsHel-3a	gb|AIXA01002561.1|:1401-1520
MsHel-3a	gb|AIXA01002561.1|:12488-12773
MsHel-3a	gb|AIXA01002561.1|:76382-76607
MsHel-3a	gb|AIXA01001640.1|:4138-4423
MsHel-3a	gb|AIXA01014420.1|:12438-12701
MsHel-3a	gb|AIXA01004478.1|:14927-15194
MsHel-3a	gb|AIXA01004051.1|:556-831
MsHel-3a	gb|AIXA01004051.1|:16578-16851
MsHel-3a	gb|AIXA01011351.1|:1928-2213
MsHel-3a	gb|AIXA01001446.1|:15713-15994
MsHel-3a	gb|AIXA01027723.1|:1-117
MsHel-3a	gb|AIXA01011986.1|:5015-5291
MsHel-3a	gb|AIXA01020306.1|:1-280

MsHel-3a	gb|AIXA01019501.1|:3527-3840
MsHel-3a	gb|AIXA01019257.1|:3547-3811
MsHel-3a	gb|AIXA01016604.1|:1973-2248
MsHel-3a	gb|AIXA01016232.1|:11936-12220
MsHel-3a	gb|AIXA01015528.1|:15299-15586
MsHel-3a	gb|AIXA01015404.1|:4598-4864
MsHel-3a	gb|AIXA01015191.1|:5189-5412
MsHel-3a	gb|AIXA01015191.1|:30795-31059
MsHel-3a	gb|AIXA01014497.1|:40379-40667
MsHel-3a	gb|AIXA01014412.1|:337-627
MsHel-3a	gb|AIXA01014172.1|:23-312
MsHel-3a	gb|AIXA01014147.1|:2504-2790
MsHel-3a	gb|AIXA01014145.1|:3615-3901
MsHel-3a	gb|AIXA01014145.1|:21645-21922
MsHel-3a	gb|AIXA01014145.1|:36255-36528
MsHel-3a	gb|AIXA01013943.1|:593-880
MsHel-3a	gb|AIXA01013632.1|:10192-10440
MsHel-3a	gb|AIXA01013565.1|:29189-29475
MsHel-3a	gb|AIXA01013459.1|:2723-3001
MsHel-3a	gb|AIXA01012574.1|:1457-1576
MsHel-3a	gb|AIXA01012196.1|:1487-1772
MsHel-3a	gb|AIXA01011949.1|:7913-8203
MsHel-3a	gb|AIXA01011819.1|:896-1181
MsHel-3a	gb|AIXA01011758.1|:1574-1844
MsHel-3a	gb|AIXA01011457.1|:21207-21438
MsHel-3a	gb|AIXA01010773.1|:24539-24824
MsHel-3a	gb|AIXA01010536.1|:18987-19272
MsHel-3a	gb|AIXA01010406.1|:46726-46993
MsHel-3a	gb|AIXA01010406.1|:69948-70226
MsHel-3a	gb|AIXA01010330.1|:40865-41127
MsHel-3a	gb|AIXA01010330.1|:63227-63500
MsHel-3a	gb|AIXA01009936.1|:6105-6390
MsHel-3a	gb|AIXA01009807.1|:1740-2019
MsHel-3a	gb|AIXA01009407.1|:2250-2533
MsHel-3a	gb|AIXA01009162.1|:13582-13670
MsHel-3a	gb|AIXA01009162.1|:26210-26498
MsHel-3a	gb|AIXA01009162.1|:142336-142422
MsHel-3a	gb|AIXA01009040.1|:125650-125940
MsHel-3a	gb|AIXA01008393.1|:16290-16494
MsHel-3a	gb|AIXA01008393.1|:36048-36312
MsHel-3a	gb|AIXA01007978.1|:8542-8834
MsHel-3a	gb|AIXA01007023.1|:8943-9233
MsHel-3a	gb|AIXA01006687.1|:12841-13107
MsHel-3a	gb|AIXA01006672.1|:7721-8005
MsHel-3a	gb|AIXA01006401.1|:10074-10359
MsHel-3a	gb|AIXA01006155.1|:8564-8852
MsHel-3a	gb|AIXA01006141.1|:52999-53241
MsHel-3a	gb|AIXA01006141.1|:81213-81498

MsHel-3a	gb|AIXA01006141.1|:108531-108783
MsHel-3a	gb|AIXA01006046.1|:11201-11490
MsHel-3a	gb|AIXA01005505.1|:16574-16778
MsHel-3a	gb|AIXA01005505.1|:49836-50109
MsHel-3a	gb|AIXA01005492.1|:12937-13219
MsHel-3a	gb|AIXA01005437.1|:8686-8952
MsHel-3a	gb|AIXA01005067.1|:11715-11834
MsHel-3a	gb|AIXA01005062.1|:3627-3746
MsHel-3a	gb|AIXA01004893.1|:22669-22946
MsHel-3a	gb|AIXA01004893.1|:37840-38124
MsHel-3a	gb|AIXA01004865.1|:4881-5126
MsHel-3a	gb|AIXA01004865.1|:46058-46338
MsHel-3a	gb|AIXA01004742.1|:38189-38476
MsHel-3a	gb|AIXA01004094.1|:13189-13480
MsHel-3a	gb|AIXA01004069.1|:23442-23721
MsHel-3a	gb|AIXA01004061.1|:24166-24452
MsHel-3a	gb|AIXA01003928.1|:10759-10838
MsHel-3a	gb|AIXA01003928.1|:82070-82355
MsHel-3a	gb|AIXA01003928.1|:126558-126809
MsHel-3a	gb|AIXA01003660.1|:31700-31981
MsHel-3a	gb|AIXA01002811.1|:46554-46839
MsHel-3a	gb|AIXA01002629.1|:30736-31010
MsHel-3a	gb|AIXA01002540.1|:11830-12112
MsHel-3a	gb|AIXA01002533.1|:12859-13144
MsHel-3a	gb|AIXA01002303.1|:26443-26732
MsHel-3a	gb|AIXA01001844.1|:23202-23533
MsHel-3a	gb|AIXA01001844.1|:53205-53487
MsHel-3a	gb|AIXA01001662.1|:39218-39509
MsHel-3a	gb|AIXA01001636.1|:82828-83094
MsHel-3a	gb|AIXA01000892.1|:36486-36770
MsHel-3a	gb|AIXA01000877.1|:7489-7777
MsHel-3a	gb|AIXA01000732.1|:128574-128864
MsHel-3a	gb|AIXA01000732.1|:139198-139469
MsHel-3a	gb|AIXA01000692.1|:187624-187885
MsHel-3a	gb|AIXA01000224.1|:36063-36329
MsHel-3a	gb|AIXA01000036.1|:7684-7957
MsHel-3a	gb|AIXA01022687.1|:902-1184
MsHel-3a	gb|AIXA01018189.1|:7793-8079
MsHel-3a	gb|AIXA01014257.1|:3507-3771
MsHel-3a	gb|AIXA01014114.1|:1491-1793
MsHel-3a	gb|AIXA01013942.1|:4897-5185
MsHel-3a	gb|AIXA01013521.1|:18134-18414
MsHel-3a	gb|AIXA01013521.1|:32323-32600
MsHel-3a	gb|AIXA01012651.1|:5663-5947
MsHel-3a	gb|AIXA01007576.1|:14349-14639
MsHel-3a	gb|AIXA01007576.1|:41346-41632
MsHel-3a	gb|AIXA01007522.1|:47873-48152
MsHel-3a	gb|AIXA01006007.1|:37654-37932

MsHel-3a	gb|AIXA01005892.1|:77363-77651
MsHel-3a	gb|AIXA01005506.1|:2117-2395
MsHel-3a	gb|AIXA01003151.1|:687-2297
MsHel-3a	gb|AIXA01003151.1|:38323-38441
MsHel-3a	gb|AIXA01001948.1|:2659-2957
MsHel-3a	gb|AIXA01001948.1|:25434-25716
MsHel-3a	gb|AIXA01001948.1|:36176-36458
MsHel-3a	gb|AIXA01000938.1|:30473-30739
MsHel-3a	gb|AIXA01000938.1|:50394-50679
MsHel-3a	gb|AIXA01016082.1|:12625-12742
MsHel-3a	gb|AIXA01014629.1|:12392-12673
MsHel-3a	gb|AIXA01013127.1|:359-641
MsHel-3a	gb|AIXA01011993.1|:10910-11179
MsHel-3a	gb|AIXA01008112.1|:53272-53389
MsHel-3a	gb|AIXA01007688.1|:11349-11604
MsHel-3a	gb|AIXA01006015.1|:8701-8982
MsHel-3a	gb|AIXA01005126.1|:17756-18043
MsHel-3a	gb|AIXA01005126.1|:61358-61640
MsHel-3a	gb|AIXA01004486.1|:17225-17504
MsHel-3a	gb|AIXA01001304.1|:18321-18602
MsHel-3a	gb|AIXA01000844.1|:27600-27930
MsHel-3a	gb|AIXA01000824.1|:13231-13482
MsHel-3a	gb|AIXA01000423.1|:16152-16439
MsHel-3a	gb|AIXA01028132.1|:355-639
MsHel-3a	gb|AIXA01016648.1|:8806-9082
MsHel-3a	gb|AIXA01009030.1|:7-267
MsHel-3a	gb|AIXA01006841.1|:8989-9313
MsHel-3a	gb|AIXA01006841.1|:162782-162995
MsHel-3a	gb|AIXA01006815.1|:56727-57271
MsHel-3a	gb|AIXA01005347.1|:9601-9882
MsHel-3a	gb|AIXA01001522.1|:4062-4337
MsHel-3a	gb|AIXA01001273.1|:39574-39845
MsHel-3a	gb|AIXA01001273.1|:127459-127733
MsHel-3a	gb|AIXA01000560.1|:77953-78271
MsHel-3a	gb|AIXA01036055.1|:11-214
MsHel-3a	gb|AIXA01023930.1|:1048-1167
MsHel-3a	gb|AIXA01022570.1|:222-501
MsHel-3a	gb|AIXA01021158.1|:129-392
MsHel-3a	gb|AIXA01020510.1|:63-182
MsHel-3a	gb|AIXA01020444.1|:299-584
MsHel-3a	gb|AIXA01019758.1|:126-392
MsHel-3a	gb|AIXA01019212.1|:1180-1462
MsHel-3a	gb|AIXA01018722.1|:1617-1892
MsHel-3a	gb|AIXA01018540.1|:4658-5131
MsHel-3a	gb|AIXA01018493.1|:5831-6099
MsHel-3a	gb|AIXA01018042.1|:5987-6101
MsHel-3a	gb|AIXA01016545.1|:20450-20729
MsHel-3a	gb|AIXA01015284.1|:17769-18053

MsHel-3a	gb|AIXA01014968.1|:616-883
MsHel-3a	gb|AIXA01014644.1|:19058-19332
MsHel-3a	gb|AIXA01013938.1|:5655-5928
MsHel-3a	gb|AIXA01013449.1|:7067-7186
MsHel-3a	gb|AIXA01013185.1|:34767-35053
MsHel-3a	gb|AIXA01013129.1|:9951-10237
MsHel-3a	gb|AIXA01013129.1|:31097-31383
MsHel-3a	gb|AIXA01012544.1|:1707-1985
MsHel-3a	gb|AIXA01012381.1|:10459-10745
MsHel-3a	gb|AIXA01012350.1|:4619-4855
MsHel-3a	gb|AIXA01012350.1|:15643-15918
MsHel-3a	gb|AIXA01012200.1|:78336-78605
MsHel-3a	gb|AIXA01012198.1|:13204-13445
MsHel-3a	gb|AIXA01012198.1|:26495-26778
MsHel-3a	gb|AIXA01011650.1|:15605-15724
MsHel-3a	gb|AIXA01011322.1|:27634-27893
MsHel-3a	gb|AIXA01011293.1|:21200-21486
MsHel-3a	gb|AIXA01010885.1|:20915-21139
MsHel-3a	gb|AIXA01010885.1|:53554-53820
MsHel-3a	gb|AIXA01010850.1|:24007-24290
MsHel-3a	gb|AIXA01010809.1|:7773-8052
MsHel-3a	gb|AIXA01010794.1|:2525-2805
MsHel-3a	gb|AIXA01010531.1|:687-966
MsHel-3a	gb|AIXA01010152.1|:17072-17334
MsHel-3a	gb|AIXA01009972.1|:30991-31279
MsHel-3a	gb|AIXA01009703.1|:755-1020
MsHel-3a	gb|AIXA01009696.1|:27975-28263
MsHel-3a	gb|AIXA01009646.1|:2604-2871
MsHel-3a	gb|AIXA01009585.1|:7056-7481
MsHel-3a	gb|AIXA01009543.1|:2-211
MsHel-3a	gb|AIXA01009511.1|:1208-1479
MsHel-3a	gb|AIXA01009379.1|:113-232
MsHel-3a	gb|AIXA01009084.1|:25015-25300
MsHel-3a	gb|AIXA01009033.1|:7961-8250
MsHel-3a	gb|AIXA01008995.1|:7393-7677
MsHel-3a	gb|AIXA01008507.1|:1960-2245
MsHel-3a	gb|AIXA01008480.1|:31319-31588
MsHel-3a	gb|AIXA01008406.1|:41457-41744
MsHel-3a	gb|AIXA01008399.1|:32144-32430
MsHel-3a	gb|AIXA01007968.1|:1103-5157
MsHel-3a	gb|AIXA01007968.1|:21121-21405
MsHel-3a	gb|AIXA01007856.1|:58916-59200
MsHel-3a	gb|AIXA01007612.1|:44553-44832
MsHel-3a	gb|AIXA01007049.1|:13-297
MsHel-3a	gb|AIXA01006977.1|:29832-30041
MsHel-3a	gb|AIXA01006867.1|:12382-12669
MsHel-3a	gb|AIXA01006867.1|:40832-41121
MsHel-3a	gb|AIXA01006867.1|:63496-63783

MsHel-3a	gb|AIXA01006859.1|:3119-3405
MsHel-3a	gb|AIXA01006847.1|:14728-14923
MsHel-3a	gb|AIXA01006847.1|:43247-43493
MsHel-3a	gb|AIXA01006807.1|:2179-2457
MsHel-3a	gb|AIXA01005904.1|:154308-154595
MsHel-3a	gb|AIXA01005782.1|:1893-2177
MsHel-3a	gb|AIXA01005515.1|:6696-6986
MsHel-3a	gb|AIXA01005281.1|:34166-34440
MsHel-3a	gb|AIXA01005203.1|:13387-13631
MsHel-3a	gb|AIXA01005203.1|:28244-28509
MsHel-3a	gb|AIXA01004943.1|:3796-4083
MsHel-3a	gb|AIXA01004731.1|:43404-43685
MsHel-3a	gb|AIXA01004695.1|:3586-3878
MsHel-3a	gb|AIXA01004082.1|:68672-68954
MsHel-3a	gb|AIXA01003878.1|:33149-33410
MsHel-3a	gb|AIXA01003878.1|:90902-91113
MsHel-3a	gb|AIXA01003843.1|:2234-2517
MsHel-3a	gb|AIXA01003834.1|:7588-7865
MsHel-3a	gb|AIXA01003741.1|:57817-58098
MsHel-3a	gb|AIXA01003140.1|:92914-93192
MsHel-3a	gb|AIXA01003132.1|:14523-14784
MsHel-3a	gb|AIXA01003132.1|:41943-42208
MsHel-3a	gb|AIXA01003000.1|:37377-37660
MsHel-3a	gb|AIXA01002646.1|:4107-4397
MsHel-3a	gb|AIXA01002646.1|:24671-24957
MsHel-3a	gb|AIXA01002560.1|:14564-14853
MsHel-3a	gb|AIXA01002362.1|:20610-20888
MsHel-3a	gb|AIXA01002326.1|:25934-26218
MsHel-3a	gb|AIXA01002311.1|:98485-98727
MsHel-3a	gb|AIXA01001866.1|:3648-12277
MsHel-3a	gb|AIXA01001664.1|:20255-20538
MsHel-3a	gb|AIXA01001250.1|:2418-2702
MsHel-3a	gb|AIXA01001239.1|:117135-117418
MsHel-3a	gb|AIXA01001239.1|:134331-134539
MsHel-3a	gb|AIXA01001025.1|:22994-23281
MsHel-3a	gb|AIXA01000851.1|:67854-68135
MsHel-3a	gb|AIXA01000796.1|:45692-45967
MsHel-3a	gb|AIXA01000431.1|:13252-13539
MsHel-3a	gb|AIXA01000098.1|:12379-12650
MsHel-3a	gb|AIXA01000098.1|:34693-34980
MsHel-3a	gb|AIXA01031764.1|:239-489
MsHel-3a	gb|AIXA01021642.1|:866-1147
MsHel-3a	gb|AIXA01018794.1|:3893-4172
MsHel-3a	gb|AIXA01012652.1|:3407-3681
MsHel-3a	gb|AIXA01012652.1|:35137-35254
MsHel-3a	gb|AIXA01011823.1|:12333-12623
MsHel-3a	gb|AIXA01011350.1|:24420-24700
MsHel-3a	gb|AIXA01010574.1|:8603-8717

MsHel-3a	gb|AIXA01006721.1|:33715-34000
MsHel-3a	gb|AIXA01005408.1|:10456-10659
MsHel-3a	gb|AIXA01003609.1|:124289-124564
MsHel-3a	gb|AIXA01001810.1|:20736-21023
MsHel-3a	gb|AIXA01000661.1|:60153-60379
MsHel-3a	gb|AIXA01000661.1|:170175-170451
MsHel-3a	gb|AIXA01000235.1|:37165-37424
MsHel-3a	gb|AIXA01019279.1|:2585-2863
MsHel-3a	gb|AIXA01013621.1|:1703-1946
MsHel-3a	gb|AIXA01011808.1|:111-349
MsHel-3a	gb|AIXA01010877.1|:4633-4909
MsHel-3a	gb|AIXA01009285.1|:39086-39364
MsHel-3a	gb|AIXA01009126.1|:28266-28527
MsHel-3a	gb|AIXA01006855.1|:26594-26819
MsHel-3a	gb|AIXA01006649.1|:46824-47096
MsHel-3a	gb|AIXA01004327.1|:27067-27301
MsHel-3a	gb|AIXA01004327.1|:70796-71066
MsHel-3a	gb|AIXA01003329.1|:285-398
MsHel-3a	gb|AIXA01003316.1|:48433-48714
MsHel-3a	gb|AIXA01002001.1|:49476-49720
MsHel-3a	gb|AIXA01002001.1|:69810-70093
MsHel-3a	gb|AIXA01001761.1|:4695-4972
MsHel-3a	gb|AIXA01001558.1|:29-222
MsHel-3a	gb|AIXA01001558.1|:31121-31403
MsHel-3a	gb|AIXA01001558.1|:41933-42155
MsHel-3a	gb|AIXA01001378.1|:1376-1649
MsHel-3a	gb|AIXA01000105.1|:45022-45287
MsHel-3a	gb|AIXA01022903.1|:1224-1519
MsHel-3a	gb|AIXA01016487.1|:519-783
MsHel-3a	gb|AIXA01014447.1|:31292-31572
MsHel-3a	gb|AIXA01012685.1|:3280-3552
MsHel-3a	gb|AIXA01012685.1|:17620-17898
MsHel-3a	gb|AIXA01011364.1|:44818-44938
MsHel-3a	gb|AIXA01007228.1|:16169-16438
MsHel-3a	gb|AIXA01007168.1|:38242-38522
MsHel-3a	gb|AIXA01006985.1|:21742-22009
MsHel-3a	gb|AIXA01006836.1|:6659-6945
MsHel-3a	gb|AIXA01001504.1|:4981-5270
MsHel-3a	gb|AIXA01001504.1|:40007-40123
MsHel-3a	gb|AIXA01001307.1|:16320-16585
MsHel-3a	gb|AIXA01033951.1|:37-323
MsHel-3a	gb|AIXA01032501.1|:32-317
MsHel-3a	gb|AIXA01030778.1|:33-300
MsHel-3a	gb|AIXA01030680.1|:124-741
MsHel-3a	gb|AIXA01028697.1|:243-532
MsHel-3a	gb|AIXA01028016.1|:475-758
MsHel-3a	gb|AIXA01019364.1|:574-844
MsHel-3a	gb|AIXA01019298.1|:4391-4661

MsHel-3a	gb|AIXA01017917.1|:10723-11003
MsHel-3a	gb|AIXA01017893.1|:6845-7130
MsHel-3a	gb|AIXA01017331.1|:671-960
MsHel-3a	gb|AIXA01017061.1|:13466-13741
MsHel-3a	gb|AIXA01017018.1|:4018-4299
MsHel-3a	gb|AIXA01016811.1|:4928-5212
MsHel-3a	gb|AIXA01016699.1|:3340-3619
MsHel-3a	gb|AIXA01016669.1|:4158-4438
MsHel-3a	gb|AIXA01016468.1|:6464-6751
MsHel-3a	gb|AIXA01016307.1|:10454-10723
MsHel-3a	gb|AIXA01016261.1|:963-1259
MsHel-3a	gb|AIXA01015230.1|:10883-11172
MsHel-3a	gb|AIXA01015179.1|:8560-8839
MsHel-3a	gb|AIXA01014867.1|:35200-35488
MsHel-3a	gb|AIXA01014559.1|:1278-1553
MsHel-3a	gb|AIXA01014372.1|:14198-14461
MsHel-3a	gb|AIXA01013847.1|:14906-15195
MsHel-3a	gb|AIXA01013204.1|:1549-1831
MsHel-3a	gb|AIXA01013123.1|:9877-10167
MsHel-3a	gb|AIXA01012646.1|:404-690
MsHel-3a	gb|AIXA01012628.1|:27-300
MsHel-3a	gb|AIXA01012538.1|:15343-15619
MsHel-3a	gb|AIXA01012354.1|:5348-5591
MsHel-3a	gb|AIXA01011987.1|:39381-39658
MsHel-3a	gb|AIXA01011987.1|:53588-53831
MsHel-3a	gb|AIXA01011840.1|:9677-9963
MsHel-3a	gb|AIXA01011624.1|:7495-7769
MsHel-3a	gb|AIXA01011340.1|:16009-16296
MsHel-3a	gb|AIXA01011340.1|:32260-32482
MsHel-3a	gb|AIXA01010882.1|:1-281
MsHel-3a	gb|AIXA01010692.1|:25269-25550
MsHel-3a	gb|AIXA01010583.1|:21284-21558
MsHel-3a	gb|AIXA01010394.1|:54398-54681
MsHel-3a	gb|AIXA01009709.1|:898-1193
MsHel-3a	gb|AIXA01009248.1|:5085-5373
MsHel-3a	gb|AIXA01009153.1|:18336-18624
MsHel-3a	gb|AIXA01008901.1|:9433-9715
MsHel-3a	gb|AIXA01008226.1|:24767-25052
MsHel-3a	gb|AIXA01008226.1|:53835-54075
MsHel-3a	gb|AIXA01008043.1|:74953-75241
MsHel-3a	gb|AIXA01007572.1|:1264-1551
MsHel-3a	gb|AIXA01007299.1|:25377-25582
MsHel-3a	gb|AIXA01007100.1|:6486-6741
MsHel-3a	gb|AIXA01007078.1|:3-9881
MsHel-3a	gb|AIXA01007053.1|:45063-45301
MsHel-3a	gb|AIXA01006906.1|:9619-9738
MsHel-3a	gb|AIXA01006876.1|:41097-41383
MsHel-3a	gb|AIXA01006858.1|:3687-4022

MsHel-3a	gb|AIXA01006853.1|:14381-14500
MsHel-3a	gb|AIXA01006811.1|:16258-16544
MsHel-3a	gb|AIXA01006743.1|:22321-22425
MsHel-3a	gb|AIXA01006702.1|:27812-28099
MsHel-3a	gb|AIXA01006410.1|:460-731
MsHel-3a	gb|AIXA01006410.1|:11316-11601
MsHel-3a	gb|AIXA01006316.1|:1531-1805
MsHel-3a	gb|AIXA01006142.1|:3868-3987
MsHel-3a	gb|AIXA01005862.1|:6943-7224
MsHel-3a	gb|AIXA01005664.1|:21620-21901
MsHel-3a	gb|AIXA01005609.1|:1669-1951
MsHel-3a	gb|AIXA01005494.1|:3077-3346
MsHel-3a	gb|AIXA01005494.1|:37587-37871
MsHel-3a	gb|AIXA01005396.1|:20012-20267
MsHel-3a	gb|AIXA01005391.1|:65506-65746
MsHel-3a	gb|AIXA01005189.1|:16540-16822
MsHel-3a	gb|AIXA01005134.1|:3-5351
MsHel-3a	gb|AIXA01005051.1|:57586-57863
MsHel-3a	gb|AIXA01005034.1|:28956-29233
MsHel-3a	gb|AIXA01005034.1|:115503-115783
MsHel-3a	gb|AIXA01004980.1|:37597-37887
MsHel-3a	gb|AIXA01004925.1|:20521-20801
MsHel-3a	gb|AIXA01004925.1|:73681-73944
MsHel-3a	gb|AIXA01004846.1|:91-210
MsHel-3a	gb|AIXA01004474.1|:5634-5915
MsHel-3a	gb|AIXA01004474.1|:54205-54489
MsHel-3a	gb|AIXA01004381.1|:4034-4301
MsHel-3a	gb|AIXA01004347.1|:83-202
MsHel-3a	gb|AIXA01004257.1|:36795-37066
MsHel-3a	gb|AIXA01004184.1|:144225-144340
MsHel-3a	gb|AIXA01004169.1|:9782-10070
MsHel-3a	gb|AIXA01003973.1|:877-992
MsHel-3a	gb|AIXA01003716.1|:4607-4893
MsHel-3a	gb|AIXA01003591.1|:21425-21701
MsHel-3a	gb|AIXA01003591.1|:141425-141694
MsHel-3a	gb|AIXA01003332.1|:118118-118402
MsHel-3a	gb|AIXA01002998.1|:4693-4978
MsHel-3a	gb|AIXA01002993.1|:10846-11130
MsHel-3a	gb|AIXA01002806.1|:40177-40296
MsHel-3a	gb|AIXA01002570.1|:30410-30698
MsHel-3a	gb|AIXA01002453.1|:76312-76550
MsHel-3a	gb|AIXA01002361.1|:24442-24723
MsHel-3a	gb|AIXA01002296.1|:101455-101731
MsHel-3a	gb|AIXA01002069.1|:35411-35618
MsHel-3a	gb|AIXA01001850.1|:21480-21761
MsHel-3a	gb|AIXA01001829.1|:28802-29082
MsHel-3a	gb|AIXA01001677.1|:99343-99609
MsHel-3a	gb|AIXA01001544.1|:24607-24706

MsHel-3a	gb|AIXA01001532.1|:38947-39059
MsHel-3a	gb|AIXA01001532.1|:97207-97494
MsHel-3a	gb|AIXA01001520.1|:24422-24693
MsHel-3a	gb|AIXA01001520.1|:40963-41245
MsHel-3a	gb|AIXA01001465.1|:22175-22462
MsHel-3a	gb|AIXA01001193.1|:39-325
MsHel-3a	gb|AIXA01001111.1|:1295-1576
MsHel-3a	gb|AIXA01001061.1|:25450-25741
MsHel-3a	gb|AIXA01000864.1|:163-274
MsHel-3a	gb|AIXA01000561.1|:75487-75772
MsHel-3a	gb|AIXA01000272.1|:30622-30906
MsHel-3a	gb|AIXA01000178.1|:22105-22374
MsHel-3a	gb|AIXA01000178.1|:33042-33332
MsHel-3a	gb|AIXA01000070.1|:19434-19680
MsHel-3a	gb|AIXA01000070.1|:32242-32519
MsHel-3a	gb|AIXA01000069.1|:121614-121860
MsHel-3a	gb|AIXA01000064.1|:23469-23587
MsHel-3a	gb|AIXA01000064.1|:35178-35455
MsHel-3a	gb|AIXA01000064.1|:48660-48741
MsHel-3a	gb|AIXA01015805.1|:840-1130
MsHel-3a	gb|AIXA01015281.1|:47982-48261
MsHel-3a	gb|AIXA01013541.1|:4140-4426
MsHel-3a	gb|AIXA01009045.1|:6688-6953
MsHel-3a	gb|AIXA01004855.1|:3884-4131
MsHel-3a	gb|AIXA01004076.1|:23697-23965
MsHel-3a	gb|AIXA01002850.1|:88136-88422
MsHel-3a	gb|AIXA01001986.1|:1930-2217
MsHel-3a	gb|AIXA01017968.1|:4630-4909
MsHel-3a	gb|AIXA01014213.1|:25525-25794
MsHel-3a	gb|AIXA01014195.1|:37-320
MsHel-3a	gb|AIXA01011303.1|:27432-27714
MsHel-3a	gb|AIXA01011303.1|:50254-50531
MsHel-3a	gb|AIXA01011239.1|:31570-31855
MsHel-3a	gb|AIXA01011239.1|:47233-47396
MsHel-3a	gb|AIXA01010953.1|:37822-38108
MsHel-3a	gb|AIXA01010866.1|:2184-2471
MsHel-3a	gb|AIXA01010132.1|:33455-33726
MsHel-3a	gb|AIXA01007616.1|:8807-9046
MsHel-3a	gb|AIXA01007377.1|:8622-8877
MsHel-3a	gb|AIXA01007139.1|:54379-54492
MsHel-3a	gb|AIXA01005538.1|:62858-63103
MsHel-3a	gb|AIXA01005511.1|:13167-13451
MsHel-3a	gb|AIXA01005378.1|:90895-90998
MsHel-3a	gb|AIXA01005105.1|:2080-2357
MsHel-3a	gb|AIXA01005105.1|:22459-22572
MsHel-3a	gb|AIXA01003817.1|:13054-13323
MsHel-3a	gb|AIXA01001283.1|:2459-2715
MsHel-3a	gb|AIXA01001117.1|:14512-14795

MsHel-3a	gb|AIXA01021923.1|:754-1023
MsHel-3a	gb|AIXA01016779.1|:4869-5156
MsHel-3a	gb|AIXA01015531.1|:3810-4087
MsHel-3a	gb|AIXA01015167.1|:38-234
MsHel-3a	gb|AIXA01014467.1|:9314-9600
MsHel-3a	gb|AIXA01014467.1|:21756-22034
MsHel-3a	gb|AIXA01014298.1|:14693-14984
MsHel-3a	gb|AIXA01012554.1|:39775-40019
MsHel-3a	gb|AIXA01011807.1|:13202-13448
MsHel-3a	gb|AIXA01011629.1|:19142-19300
MsHel-3a	gb|AIXA01011629.1|:49671-49932
MsHel-3a	gb|AIXA01010404.1|:39334-39450
MsHel-3a	gb|AIXA01010404.1|:51761-51917
MsHel-3a	gb|AIXA01009654.1|:3290-3572
MsHel-3a	gb|AIXA01009642.1|:16333-16619
MsHel-3a	gb|AIXA01009489.1|:27980-28209
MsHel-3a	gb|AIXA01009488.1|:7089-7334
MsHel-3a	gb|AIXA01009488.1|:55675-55959
MsHel-3a	gb|AIXA01009152.1|:9447-9900
MsHel-3a	gb|AIXA01007449.1|:18365-18645
MsHel-3a	gb|AIXA01007438.1|:8462-8738
MsHel-3a	gb|AIXA01006878.1|:13592-13836
MsHel-3a	gb|AIXA01006627.1|:48731-49010
MsHel-3a	gb|AIXA01005113.1|:10040-10325
MsHel-3a	gb|AIXA01005110.1|:4440-4636
MsHel-3a	gb|AIXA01004969.1|:4250-4527
MsHel-3a	gb|AIXA01003803.1|:50658-50943
MsHel-3a	gb|AIXA01002497.1|:23621-23899
MsHel-3a	gb|AIXA01001609.1|:5-277
MsHel-3a	gb|AIXA01001546.1|:2113-2395
MsHel-3a	gb|AIXA01001317.1|:3100-3386
MsHel-3a	gb|AIXA01000926.1|:15322-15482
MsHel-3a	gb|AIXA01000926.1|:40955-41226
MsHel-3a	gb|AIXA01000754.1|:57622-57910
MsHel-3a	gb|AIXA01000621.1|:53-314
MsHel-3a	gb|AIXA01000196.1|:25497-25783
MsHel-3a	gb|AIXA01035537.1|:191-475
MsHel-3a	gb|AIXA01033916.1|:71-359
MsHel-3a	gb|AIXA01028955.1|:91-212
MsHel-3a	gb|AIXA01026577.1|:233-522
MsHel-3a	gb|AIXA01025304.1|:395-654
MsHel-3a	gb|AIXA01019115.1|:2775-3062
MsHel-3a	gb|AIXA01018495.1|:1-280
MsHel-3a	gb|AIXA01018426.1|:2583-2861
MsHel-3a	gb|AIXA01018324.1|:1-279
MsHel-3a	gb|AIXA01018007.1|:161-441
MsHel-3a	gb|AIXA01017620.1|:6003-6284
MsHel-3a	gb|AIXA01016084.1|:7202-7489

MsHel-3a	gb|AIXA01015659.1|:17048-17335
MsHel-3a	gb|AIXA01015446.1|:6169-6427
MsHel-3a	gb|AIXA01015407.1|:48892-49176
MsHel-3a	gb|AIXA01015055.1|:12334-12596
MsHel-3a	gb|AIXA01014900.1|:13577-13840
MsHel-3a	gb|AIXA01014496.1|:1075-1348
MsHel-3a	gb|AIXA01014010.1|:22830-23115
MsHel-3a	gb|AIXA01013583.1|:2710-2991
MsHel-3a	gb|AIXA01012911.1|:5323-5603
MsHel-3a	gb|AIXA01012798.1|:14896-15165
MsHel-3a	gb|AIXA01012747.1|:5008-5292
MsHel-3a	gb|AIXA01012377.1|:27-313
MsHel-3a	gb|AIXA01012328.1|:5577-5854
MsHel-3a	gb|AIXA01012307.1|:11936-12217
MsHel-3a	gb|AIXA01012097.1|:3793-4076
MsHel-3a	gb|AIXA01011756.1|:4601-4813
MsHel-3a	gb|AIXA01011452.1|:7264-7549
MsHel-3a	gb|AIXA01011347.1|:11637-11893
MsHel-3a	gb|AIXA01011314.1|:2323-2609
MsHel-3a	gb|AIXA01011253.1|:22795-23067
MsHel-3a	gb|AIXA01011202.1|:40196-40487
MsHel-3a	gb|AIXA01010911.1|:7155-7439
MsHel-3a	gb|AIXA01010577.1|:6274-6563
MsHel-3a	gb|AIXA01010300.1|:1081-1364
MsHel-3a	gb|AIXA01009973.1|:11950-12236
MsHel-3a	gb|AIXA01009127.1|:18267-18542
MsHel-3a	gb|AIXA01008793.1|:38965-39232
MsHel-3a	gb|AIXA01008396.1|:3666-3946
MsHel-3a	gb|AIXA01008227.1|:51553-52012
MsHel-3a	gb|AIXA01008022.1|:35591-35875
MsHel-3a	gb|AIXA01007871.1|:7600-7719
MsHel-3a	gb|AIXA01007620.1|:51233-51513
MsHel-3a	gb|AIXA01007367.1|:10203-10493
MsHel-3a	gb|AIXA01007058.1|:30054-30341
MsHel-3a	gb|AIXA01006863.1|:19767-19969
MsHel-3a	gb|AIXA01006648.1|:61828-62118
MsHel-3a	gb|AIXA01006123.1|:38092-38211
MsHel-3a	gb|AIXA01006123.1|:61376-61637
MsHel-3a	gb|AIXA01006053.1|:18858-19138
MsHel-3a	gb|AIXA01006053.1|:30354-30637
MsHel-3a	gb|AIXA01005635.1|:13175-13278
MsHel-3a	gb|AIXA01005548.1|:157-276
MsHel-3a	gb|AIXA01005452.1|:4483-5595
MsHel-3a	gb|AIXA01005452.1|:86156-86406
MsHel-3a	gb|AIXA01005279.1|:29941-30228
MsHel-3a	gb|AIXA01005088.1|:21359-21566
MsHel-3a	gb|AIXA01005058.1|:2088-2356
MsHel-3a	gb|AIXA01004408.1|:18980-19265

MsHel-3a	gb|AIXA01004226.1|:31166-31455
MsHel-3a	gb|AIXA01004093.1|:35400-35676
MsHel-3a	gb|AIXA01004089.1|:13904-14182
MsHel-3a	gb|AIXA01004089.1|:97529-97818
MsHel-3a	gb|AIXA01003315.1|:1526-1815
MsHel-3a	gb|AIXA01002805.1|:14700-14971
MsHel-3a	gb|AIXA01002552.1|:5622-5872
MsHel-3a	gb|AIXA01002552.1|:76101-76380
MsHel-3a	gb|AIXA01002552.1|:132125-132592
MsHel-3a	gb|AIXA01002528.1|:92466-92738
MsHel-3a	gb|AIXA01002217.1|:267-539
MsHel-3a	gb|AIXA01002208.1|:465-744
MsHel-3a	gb|AIXA01002092.1|:2935-3220
MsHel-3a	gb|AIXA01002041.1|:30697-30937
MsHel-3a	gb|AIXA01001965.1|:6-291
MsHel-3a	gb|AIXA01001965.1|:61425-61702
MsHel-3a	gb|AIXA01001840.1|:15867-16117
MsHel-3a	gb|AIXA01001675.1|:13650-13937
MsHel-3a	gb|AIXA01001434.1|:6124-6412
MsHel-3a	gb|AIXA01001415.1|:3804-4083
MsHel-3a	gb|AIXA01001395.1|:19144-19430
MsHel-3a	gb|AIXA01001289.1|:55326-55599
MsHel-3a	gb|AIXA01000842.1|:21737-22002
MsHel-3a	gb|AIXA01000842.1|:55474-55724
MsHel-3a	gb|AIXA01000747.1|:81260-81547
MsHel-3a	gb|AIXA01000687.1|:14533-14817
MsHel-3a	gb|AIXA01000542.1|:31950-32227
MsHel-3a	gb|AIXA01000430.1|:9994-10260
MsHel-3a	gb|AIXA01000430.1|:44472-44753
MsHel-3a	gb|AIXA01000398.1|:20532-20819
MsHel-3a	gb|AIXA01000375.1|:95528-95804
MsHel-3a	gb|AIXA01000310.1|:1435-1722
MsHel-3a	gb|AIXA01000242.1|:39049-39333
MsHel-3a	gb|AIXA01000107.1|:189448-189713
MsHel-3a	gb|AIXA01026638.1|:13-119
MsHel-3a	gb|AIXA01015067.1|:116-218
MsHel-3a	gb|AIXA01014544.1|:1-199
MsHel-3a	gb|AIXA01014541.1|:1824-2114
MsHel-3a	gb|AIXA01014540.1|:2534-2823
MsHel-3a	gb|AIXA01011863.1|:8479-8748
MsHel-3a	gb|AIXA01010250.1|:11292-11586
MsHel-3a	gb|AIXA01008565.1|:48239-48523
MsHel-3a	gb|AIXA01007801.1|:10691-10972
MsHel-3a	gb|AIXA01006674.1|:166-386
MsHel-3a	gb|AIXA01006297.1|:5513-5784
MsHel-3a	gb|AIXA01006154.1|:43169-43457
MsHel-3a	gb|AIXA01005631.1|:14476-14748
MsHel-3a	gb|AIXA01005596.1|:93640-93915

MsHel-3a	gb|AIXA01005388.1|:43913-44181
MsHel-3a	gb|AIXA01005056.1|:10640-10869
MsHel-3a	gb|AIXA01005056.1|:42741-42890
MsHel-3a	gb|AIXA01004338.1|:10105-10376
MsHel-3a	gb|AIXA01003814.1|:5026-5302
MsHel-3a	gb|AIXA01002321.1|:4875-5167
MsHel-3a	gb|AIXA01001045.1|:5074-5354
MsHel-3a	gb|AIXA01001017.1|:42518-42797
MsHel-3a	gb|AIXA01000838.1|:88927-89205
MsHel-3a	gb|AIXA01000838.1|:193493-193746
MsHel-3a	gb|AIXA01036790.1|:11-128
MsHel-3a	gb|AIXA01034605.1|:205-479
MsHel-3a	gb|AIXA01034004.1|:10-212
MsHel-3a	gb|AIXA01023468.1|:774-1052
MsHel-3a	gb|AIXA01016138.1|:17946-18216
MsHel-3a	gb|AIXA01014094.1|:5012-5298
MsHel-3a	gb|AIXA01011450.1|:24308-24571
MsHel-3a	gb|AIXA01010844.1|:6197-6466
MsHel-3a	gb|AIXA01010377.1|:1730-2009
MsHel-3a	gb|AIXA01010155.1|:24092-24380
MsHel-3a	gb|AIXA01007515.1|:20689-20967
MsHel-3a	gb|AIXA01007296.1|:124089-124373
MsHel-3a	gb|AIXA01007200.1|:10956-11235
MsHel-3a	gb|AIXA01006129.1|:4738-5016
MsHel-3a	gb|AIXA01006040.1|:1723-2000
MsHel-3a	gb|AIXA01005721.1|:35687-35866
MsHel-3a	gb|AIXA01005045.1|:39074-39352
MsHel-3a	gb|AIXA01003014.1|:7277-7562
MsHel-3a	gb|AIXA01002619.1|:47933-48220
MsHel-3a	gb|AIXA01002551.1|:74787-75059
MsHel-3a	gb|AIXA01001653.1|:2604-2847
MsHel-3a	gb|AIXA01001653.1|:45810-46079
MsHel-3a	gb|AIXA01001098.1|:20452-20734
MsHel-3a	gb|AIXA01021215.1|:922-1163
MsHel-3a	gb|AIXA01018362.1|:2565-2846
MsHel-3a	gb|AIXA01015166.1|:32-234
MsHel-3a	gb|AIXA01014446.1|:294-584
MsHel-3a	gb|AIXA01013826.1|:1992-2224
MsHel-3a	gb|AIXA01011709.1|:37247-37467
MsHel-3a	gb|AIXA01011709.1|:69080-69365
MsHel-3a	gb|AIXA01009653.1|:6483-6765
MsHel-3a	gb|AIXA01008157.1|:6436-6715
MsHel-3a	gb|AIXA01007621.1|:18383-18655
MsHel-3a	gb|AIXA01006912.1|:6715-6827
MsHel-3a	gb|AIXA01006014.1|:6039-6319
MsHel-3a	gb|AIXA01005047.1|:7159-7433
MsHel-3a	gb|AIXA01005047.1|:36137-36417
MsHel-3a	gb|AIXA01004134.1|:4480-4752

MsHel-3a	gb|AIXA01004134.1|:57002-57274
MsHel-3a	gb|AIXA01003789.1|:4448-4735
MsHel-3a	gb|AIXA01002975.1|:46098-46295
MsHel-3a	gb|AIXA01002975.1|:100772-101057
MsHel-3a	gb|AIXA01002283.1|:6588-6857
MsHel-3a	gb|AIXA01000245.1|:17443-17710
MsHel-3a	gb|AIXA01000234.1|:14556-14836
MsHel-3a	gb|AIXA01000234.1|:35233-35503
MsHel-3a	gb|AIXA01034934.1|:61-339
MsHel-3a	gb|AIXA01031763.1|:46-165
MsHel-3a	gb|AIXA01026666.1|:338-624
MsHel-3a	gb|AIXA01023980.1|:77-196
MsHel-3a	gb|AIXA01022276.1|:1128-1415
MsHel-3a	gb|AIXA01022098.1|:1429-1712
MsHel-3a	gb|AIXA01021929.1|:553-840
MsHel-3a	gb|AIXA01021828.1|:910-1199
MsHel-3a	gb|AIXA01021495.1|:453-734
MsHel-3a	gb|AIXA01021409.1|:941-1223
MsHel-3a	gb|AIXA01021402.1|:23-308
MsHel-3a	gb|AIXA01020973.1|:372-649
MsHel-3a	gb|AIXA01019123.1|:1754-2028
MsHel-3a	gb|AIXA01019002.1|:64-182
MsHel-3a	gb|AIXA01018924.1|:2120-2239
MsHel-3a	gb|AIXA01018789.1|:5451-5726
MsHel-3a	gb|AIXA01018554.1|:6513-6790
MsHel-3a	gb|AIXA01017113.1|:5275-5560
MsHel-3a	gb|AIXA01017042.1|:2043-2331
MsHel-3a	gb|AIXA01016431.1|:7577-7845
MsHel-3a	gb|AIXA01015874.1|:1853-2143
MsHel-3a	gb|AIXA01015401.1|:1-284
MsHel-3a	gb|AIXA01015379.1|:3733-4013
MsHel-3a	gb|AIXA01015311.1|:5105-5386
MsHel-3a	gb|AIXA01014537.1|:44294-44529
MsHel-3a	gb|AIXA01014528.1|:8768-9044
MsHel-3a	gb|AIXA01014246.1|:41947-42233
MsHel-3a	gb|AIXA01013994.1|:6046-6328
MsHel-3a	gb|AIXA01013524.1|:79175-79465
MsHel-3a	gb|AIXA01013132.1|:10804-11079
MsHel-3a	gb|AIXA01012698.1|:2956-3232
MsHel-3a	gb|AIXA01012240.1|:9822-10099
MsHel-3a	gb|AIXA01011270.1|:2658-2929
MsHel-3a	gb|AIXA01011270.1|:15707-15996
MsHel-3a	gb|AIXA01011243.1|:30904-31176
MsHel-3a	gb|AIXA01011233.1|:18781-19052
MsHel-3a	gb|AIXA01010966.1|:916-1196
MsHel-3a	gb|AIXA01010905.1|:11341-11602
MsHel-3a	gb|AIXA01010770.1|:10418-10691
MsHel-3a	gb|AIXA01010358.1|:6408-6690

MsHel-3a	gb|AIXA01010110.1|:3221-3478
MsHel-3a	gb|AIXA01009832.1|:24535-24765
MsHel-3a	gb|AIXA01009822.1|:3013-3132
MsHel-3a	gb|AIXA01009800.1|:650-923
MsHel-3a	gb|AIXA01009607.1|:26784-27038
MsHel-3a	gb|AIXA01009396.1|:8605-8874
MsHel-3a	gb|AIXA01009370.1|:13953-14243
MsHel-3a	gb|AIXA01009019.1|:28470-28585
MsHel-3a	gb|AIXA01008363.1|:48-322
MsHel-3a	gb|AIXA01008110.1|:63664-63907
MsHel-3a	gb|AIXA01008076.1|:56561-56847
MsHel-3a	gb|AIXA01007674.1|:2730-2992
MsHel-3a	gb|AIXA01007593.1|:101309-101406
MsHel-3a	gb|AIXA01007488.1|:4775-5057
MsHel-3a	gb|AIXA01007350.1|:85923-86204
MsHel-3a	gb|AIXA01007027.1|:80674-80957
MsHel-3a	gb|AIXA01006875.1|:66-357
MsHel-3a	gb|AIXA01006875.1|:27483-27769
MsHel-3a	gb|AIXA01006842.1|:742-1017
MsHel-3a	gb|AIXA01006780.1|:53522-53798
MsHel-3a	gb|AIXA01005901.1|:57592-57853
MsHel-3a	gb|AIXA01005085.1|:26131-26236
MsHel-3a	gb|AIXA01004137.1|:53073-53328
MsHel-3a	gb|AIXA01004137.1|:124035-124319
MsHel-3a	gb|AIXA01003708.1|:20-123
MsHel-3a	gb|AIXA01003682.1|:61824-62084
MsHel-3a	gb|AIXA01003295.1|:6518-6788
MsHel-3a	gb|AIXA01003283.1|:29828-30112
MsHel-3a	gb|AIXA01003248.1|:5733-6001
MsHel-3a	gb|AIXA01003015.1|:33076-33336
MsHel-3a	gb|AIXA01002889.1|:7817-7935
MsHel-3a	gb|AIXA01002517.1|:2160-2437
MsHel-3a	gb|AIXA01002304.1|:18245-18517
MsHel-3a	gb|AIXA01002168.1|:12675-12958
MsHel-3a	gb|AIXA01002168.1|:30343-30623
MsHel-3a	gb|AIXA01001797.1|:108816-108933
MsHel-3a	gb|AIXA01001613.1|:43142-43396
MsHel-3a	gb|AIXA01001594.1|:6769-7056
MsHel-3a	gb|AIXA01001518.1|:9529-9817
MsHel-3a	gb|AIXA01001358.1|:43199-43484
MsHel-3a	gb|AIXA01001048.1|:44726-45008
MsHel-3a	gb|AIXA01001048.1|:57162-57449
MsHel-3a	gb|AIXA01000532.1|:12223-12458
MsHel-3a	gb|AIXA01000337.1|:18801-19092
MsHel-3a	gb|AIXA01000337.1|:67297-67401
MsHel-3a	gb|AIXA01000278.1|:8086-8373
MsHel-3a	gb|AIXA01000278.1|:47254-47499
MsHel-3a	gb|AIXA01000207.1|:23717-23831

MsHel-3a	gb|AIXA01000207.1|:47273-47560
MsHel-3a	gb|AIXA01000055.1|:1267-1561
MsHel-3a	gb|AIXA01000055.1|:51111-51358
MsHel-3a	gb|AIXA01000030.1|:39635-39896
MsHel-3a	gb|AIXA01000005.1|:179360-179596
MsHel-3a	gb|AIXA01019288.1|:1557-1843
MsHel-3a	gb|AIXA01018017.1|:2249-2522
MsHel-3a	gb|AIXA01016758.1|:470-758
MsHel-3a	gb|AIXA01015193.1|:138-231
MsHel-3a	gb|AIXA01014539.1|:1856-2121
MsHel-3a	gb|AIXA01013945.1|:534-827
MsHel-3a	gb|AIXA01012741.1|:17384-17669
MsHel-3a	gb|AIXA01010473.1|:8598-8875
MsHel-3a	gb|AIXA01010185.1|:49836-50116
MsHel-3a	gb|AIXA01009423.1|:5215-5493
MsHel-3a	gb|AIXA01009423.1|:44218-44471
MsHel-3a	gb|AIXA01008229.1|:7886-8138
MsHel-3a	gb|AIXA01007331.1|:2581-2860
MsHel-3a	gb|AIXA01006900.1|:23997-24241
MsHel-3a	gb|AIXA01006509.1|:81206-81318
MsHel-3a	gb|AIXA01005615.1|:31509-31783
MsHel-3a	gb|AIXA01005406.1|:24687-24968
MsHel-3a	gb|AIXA01004933.1|:11527-11780
MsHel-3a	gb|AIXA01004857.1|:13298-13558
MsHel-3a	gb|AIXA01003357.1|:13663-13945
MsHel-3a	gb|AIXA01003168.1|:17434-17724
MsHel-3a	gb|AIXA01003168.1|:52960-53161
MsHel-3a	gb|AIXA01003168.1|:97303-97544
MsHel-3a	gb|AIXA01001823.1|:27609-27893
MsHel-3a	gb|AIXA01001441.1|:44451-44724
MsHel-3a	gb|AIXA01001147.1|:7846-8133
MsHel-3a	gb|AIXA01000469.1|:10140-10258
MsHel-3a	gb|AIXA01037805.1|:12-282
MsHel-3a	gb|AIXA01033740.1|:100-216
MsHel-3a	gb|AIXA01015457.1|:3259-3522
MsHel-3a	gb|AIXA01013677.1|:8252-8535
MsHel-3a	gb|AIXA01012535.1|:1776-2030
MsHel-3a	gb|AIXA01011984.1|:24852-25095
MsHel-3a	gb|AIXA01011370.1|:41268-41541
MsHel-3a	gb|AIXA01011328.1|:7575-7854
MsHel-3a	gb|AIXA01010785.1|:11986-12263
MsHel-3a	gb|AIXA01010785.1|:62421-62694
MsHel-3a	gb|AIXA01009430.1|:51391-51669
MsHel-3a	gb|AIXA01009430.1|:79067-79282
MsHel-3a	gb|AIXA01008234.1|:14967-15229
MsHel-3a	gb|AIXA01007955.1|:5701-5817
MsHel-3a	gb|AIXA01006731.1|:35629-35960
MsHel-3a	gb|AIXA01005585.1|:71047-71329

MsHel-3a	gb|AIXA01005345.1|:28136-28418
MsHel-3a	gb|AIXA01004242.1|:28276-28553
MsHel-3a	gb|AIXA01003247.1|:37828-38091
MsHel-3a	gb|AIXA01002350.1|:26153-26788
MsHel-3a	gb|AIXA01002350.1|:130480-130724
MsHel-3a	gb|AIXA01001811.1|:1200-1482
MsHel-3a	gb|AIXA01001811.1|:30832-30949
MsHel-3a	gb|AIXA01001002.1|:7661-7943
MsHel-3a	gb|AIXA01000148.1|:13758-14037
MsHel-3a	gb|AIXA01034860.1|:138-424
MsHel-3a	gb|AIXA01031000.1|:442-712
MsHel-3a	gb|AIXA01019742.1|:871-1157
MsHel-3a	gb|AIXA01018723.1|:1008-1293
MsHel-3a	gb|AIXA01018267.1|:4920-5185
MsHel-3a	gb|AIXA01012533.1|:12282-12570
MsHel-3a	gb|AIXA01010908.1|:21183-21450
MsHel-3a	gb|AIXA01010541.1|:29320-29609
MsHel-3a	gb|AIXA01009100.1|:18285-18573
MsHel-3a	gb|AIXA01009100.1|:52234-52513
MsHel-3a	gb|AIXA01008222.1|:3939-4216
MsHel-3a	gb|AIXA01007944.1|:30-130
MsHel-3a	gb|AIXA01007444.1|:6375-6651
MsHel-3a	gb|AIXA01006845.1|:490-601
MsHel-3a	gb|AIXA01006740.1|:114656-114914
MsHel-3a	gb|AIXA01006139.1|:16272-16550
MsHel-3a	gb|AIXA01005524.1|:516-780
MsHel-3a	gb|AIXA01005441.1|:81-193
MsHel-3a	gb|AIXA01004697.1|:7634-7914
MsHel-3a	gb|AIXA01004697.1|:56036-56319
MsHel-3a	gb|AIXA01004252.1|:19438-19939
MsHel-3a	gb|AIXA01004230.1|:71394-71816
MsHel-3a	gb|AIXA01003227.1|:21506-21786
MsHel-3a	gb|AIXA01001566.1|:4910-5142
MsHel-3a	gb|AIXA01001566.1|:20845-21116
MsHel-3a	gb|AIXA01001243.1|:41751-42037
MsHel-3a	gb|AIXA01001243.1|:176731-177062
MsHel-3a	gb|AIXA01001006.1|:24606-24881
MsHel-3a	gb|AIXA01000516.1|:12809-13090
MsHel-3a	gb|AIXA01000327.1|:95912-96137
MsHel-3a	gb|AIXA01000201.1|:27138-27380
MsHel-3a	gb|AIXA01000060.1|:86-201
MsHel-3a	gb|AIXA01036716.1|:102-384
MsHel-3a	gb|AIXA01017890.1|:1394-1680
MsHel-3a	gb|AIXA01017850.1|:7888-8179
MsHel-3a	gb|AIXA01017439.1|:9889-10116
MsHel-3a	gb|AIXA01016927.1|:1750-2036
MsHel-3a	gb|AIXA01016740.1|:13816-13938
MsHel-3a	gb|AIXA01015132.1|:731-929

MsHel-3a	gb|AIXA01014143.1|:783-1105
MsHel-3a	gb|AIXA01014018.1|:3250-3541
MsHel-3a	gb|AIXA01013133.1|:14608-14891
MsHel-3a	gb|AIXA01011742.1|:1815-2062
MsHel-3a	gb|AIXA01011568.1|:7595-7879
MsHel-3a	gb|AIXA01010853.1|:24841-25118
MsHel-3a	gb|AIXA01010706.1|:7909-8194
MsHel-3a	gb|AIXA01009273.1|:26-310
MsHel-3a	gb|AIXA01008795.1|:25540-25827
MsHel-3a	gb|AIXA01008488.1|:4045-4295
MsHel-3a	gb|AIXA01008409.1|:10262-10488
MsHel-3a	gb|AIXA01008409.1|:24617-24843
MsHel-3a	gb|AIXA01008044.1|:44975-45091
MsHel-3a	gb|AIXA01007961.1|:7423-7691
MsHel-3a	gb|AIXA01007885.1|:22120-22404
MsHel-3a	gb|AIXA01007030.1|:7904-8185
MsHel-3a	gb|AIXA01006940.1|:9163-9435
MsHel-3a	gb|AIXA01006860.1|:35-245
MsHel-3a	gb|AIXA01006860.1|:55021-55265
MsHel-3a	gb|AIXA01006478.1|:107508-107765
MsHel-3a	gb|AIXA01006423.1|:5049-5337
MsHel-3a	gb|AIXA01006423.1|:180076-180328
MsHel-3a	gb|AIXA01005410.1|:24351-24635
MsHel-3a	gb|AIXA01005285.1|:1221-1497
MsHel-3a	gb|AIXA01004990.1|:62660-62949
MsHel-3a	gb|AIXA01004528.1|:7085-7369
MsHel-3a	gb|AIXA01003939.1|:19836-20116
MsHel-3a	gb|AIXA01003002.1|:96552-96671
MsHel-3a	gb|AIXA01002793.1|:2-280
MsHel-3a	gb|AIXA01002532.1|:48665-48867
MsHel-3a	gb|AIXA01002248.1|:58912-59167
MsHel-3a	gb|AIXA01001722.1|:958-1161
MsHel-3a	gb|AIXA01001665.1|:47083-47371
MsHel-3a	gb|AIXA01001656.1|:100496-100782
MsHel-3a	gb|AIXA01001414.1|:25107-25357
MsHel-3a	gb|AIXA01001196.1|:17-293
MsHel-3a	gb|AIXA01001050.1|:57266-57557
MsHel-3a	gb|AIXA01000847.1|:8164-8447
MsHel-3a	gb|AIXA01000847.1|:27137-27392
MsHel-3a	gb|AIXA01000841.1|:10491-10802
MsHel-3a	gb|AIXA01000275.1|:33930-34207
MsHel-3a	gb|AIXA01000251.1|:169319-169432
MsHel-3a	gb|AIXA01000251.1|:198359-198643
MsHel-3a	gb|AIXA01028930.1|:229-516
MsHel-3a	gb|AIXA01020338.1|:2556-2828
MsHel-3a	gb|AIXA01017960.1|:5078-5347
MsHel-3a	gb|AIXA01017177.1|:9774-10057
MsHel-3a	gb|AIXA01016396.1|:590-862

MsHel-3a	gb|AIXA01013450.1|:6773-7056
MsHel-3a	gb|AIXA01010789.1|:1108-1353
MsHel-3a	gb|AIXA01010789.1|:16539-16824
MsHel-3a	gb|AIXA01009647.1|:2959-3238
MsHel-3a	gb|AIXA01009133.1|:33-298
MsHel-3a	gb|AIXA01009133.1|:27621-27882
MsHel-3a	gb|AIXA01009130.1|:6025-6305
MsHel-3a	gb|AIXA01008027.1|:15665-15895
MsHel-3a	gb|AIXA01007690.1|:43088-43371
MsHel-3a	gb|AIXA01005389.1|:35555-35842
MsHel-3a	gb|AIXA01005063.1|:15567-15718
MsHel-3a	gb|AIXA01004470.1|:7983-8243
MsHel-3a	gb|AIXA01004470.1|:33928-34027
MsHel-3a	gb|AIXA01003600.1|:20112-20389
MsHel-3a	gb|AIXA01002500.1|:153676-153951
MsHel-3a	gb|AIXA01001935.1|:10212-10490
MsHel-3a	gb|AIXA01000285.1|:17769-19688
MsHel-3a	gb|AIXA01000285.1|:45029-45307
MsHel-3a	gb|AIXA01000091.1|:24126-24411
MsHel-3a	gb|AIXA01000029.1|:118880-119157
MsHel-3a	gb|AIXA01027554.1|:2-115
MsHel-3a	gb|AIXA01027421.1|:108-224
MsHel-3a	gb|AIXA01021626.1|:20-300
MsHel-3a	gb|AIXA01018545.1|:4529-4760
MsHel-3a	gb|AIXA01017678.1|:1454-1570
MsHel-3a	gb|AIXA01016342.1|:3157-3443
MsHel-3a	gb|AIXA01013664.1|:1-203
MsHel-3a	gb|AIXA01012704.1|:2193-2634
MsHel-3a	gb|AIXA01012331.1|:2741-3030
MsHel-3a	gb|AIXA01011249.1|:41187-41468
MsHel-3a	gb|AIXA01010801.1|:72-189
MsHel-3a	gb|AIXA01010076.1|:75499-75670
MsHel-3a	gb|AIXA01008037.1|:11086-11370
MsHel-3a	gb|AIXA01008037.1|:36601-36690
MsHel-3a	gb|AIXA01006850.1|:7183-7469
MsHel-3a	gb|AIXA01005841.1|:6008-6295
MsHel-3a	gb|AIXA01005779.1|:209-472
MsHel-3a	gb|AIXA01005491.1|:16954-17187
MsHel-3a	gb|AIXA01005491.1|:48864-49136
MsHel-3a	gb|AIXA01004335.1|:65507-65795
MsHel-3a	gb|AIXA01002050.1|:18375-18485
MsHel-3a	gb|AIXA01002009.1|:5379-5665
MsHel-3a	gb|AIXA01001985.1|:9235-9494
MsHel-3a	gb|AIXA01001423.1|:1173-1270
MsHel-3a	gb|AIXA01001266.1|:60063-60336
MsHel-3a	gb|AIXA01000261.1|:40144-40418
MsHel-3a	gb|AIXA01024714.1|:172-448
MsHel-3a	gb|AIXA01020752.1|:32-120

MsHel-3a	gb|AIXA01019387.1|:2091-2378
MsHel-3a	gb|AIXA01019373.1|:617-899
MsHel-3a	gb|AIXA01018250.1|:21-301
MsHel-3a	gb|AIXA01013165.1|:2098-2371
MsHel-3a	gb|AIXA01012649.1|:13318-13602
MsHel-3a	gb|AIXA01012483.1|:20-306
MsHel-3a	gb|AIXA01010811.1|:6916-7201
MsHel-3a	gb|AIXA01010514.1|:26591-26847
MsHel-3a	gb|AIXA01010251.1|:8299-8563
MsHel-3a	gb|AIXA01010220.1|:4710-4988
MsHel-3a	gb|AIXA01009336.1|:14276-14536
MsHel-3a	gb|AIXA01008402.1|:1018-1308
MsHel-3a	gb|AIXA01007319.1|:285-559
MsHel-3a	gb|AIXA01006505.1|:55972-56259
MsHel-3a	gb|AIXA01005263.1|:29192-29466
MsHel-3a	gb|AIXA01004108.1|:109590-109853
MsHel-3a	gb|AIXA01003873.1|:5869-6113
MsHel-3a	gb|AIXA01003873.1|:26833-27090
MsHel-3a	gb|AIXA01003306.1|:12997-13288
MsHel-3a	gb|AIXA01003138.1|:41418-41677
MsHel-3a	gb|AIXA01002601.1|:6910-7192
MsHel-3a	gb|AIXA01002047.1|:30046-30307
MsHel-3a	gb|AIXA01032983.1|:438-689
MsHel-3a	gb|AIXA01025571.1|:63-350
MsHel-3a	gb|AIXA01025372.1|:19-222
MsHel-3a	gb|AIXA01020256.1|:74-186
MsHel-3a	gb|AIXA01017922.1|:1597-1877
MsHel-3a	gb|AIXA01017725.1|:7334-7622
MsHel-3a	gb|AIXA01017720.1|:8038-8324
MsHel-3a	gb|AIXA01015468.1|:10557-10877
MsHel-3a	gb|AIXA01015468.1|:28389-28674
MsHel-3a	gb|AIXA01014704.1|:8246-8341
MsHel-3a	gb|AIXA01013867.1|:3779-4062
MsHel-3a	gb|AIXA01013825.1|:20924-21201
MsHel-3a	gb|AIXA01013825.1|:46099-46390
MsHel-3a	gb|AIXA01012209.1|:24386-24641
MsHel-3a	gb|AIXA01011968.1|:5190-5476
MsHel-3a	gb|AIXA01010538.1|:3952-4071
MsHel-3a	gb|AIXA01010402.1|:12691-12976
MsHel-3a	gb|AIXA01009715.1|:13709-13996
MsHel-3a	gb|AIXA01009371.1|:752-1023
MsHel-3a	gb|AIXA01008871.1|:21769-22059
MsHel-3a	gb|AIXA01008490.1|:1824-2115
MsHel-3a	gb|AIXA01007903.1|:12893-13175
MsHel-3a	gb|AIXA01007721.1|:9720-10008
MsHel-3a	gb|AIXA01007721.1|:21485-21766
MsHel-3a	gb|AIXA01007721.1|:67431-67714
MsHel-3a	gb|AIXA01007626.1|:105985-106238

MsHel-3a	gb|AIXA01007298.1|:21393-21673
MsHel-3a	gb|AIXA01005586.1|:2117-2407
MsHel-3a	gb|AIXA01005395.1|:9104-9392
MsHel-3a	gb|AIXA01005312.1|:82994-83255
MsHel-3a	gb|AIXA01004929.1|:26196-26434
MsHel-3a	gb|AIXA01004831.1|:44-288
MsHel-3a	gb|AIXA01004809.1|:93474-93700
MsHel-3a	gb|AIXA01004059.1|:5498-5748
MsHel-3a	gb|AIXA01003839.1|:38163-38260
MsHel-3a	gb|AIXA01003695.1|:3991-4273
MsHel-3a	gb|AIXA01003585.1|:96103-96390
MsHel-3a	gb|AIXA01002344.1|:3433-3552
MsHel-3a	gb|AIXA01002264.1|:105047-105334
MsHel-3a	gb|AIXA01001973.1|:13085-13353
MsHel-3a	gb|AIXA01001486.1|:25805-25900
MsHel-3a	gb|AIXA01000206.1|:11078-11325
MsHel-3a	gb|AIXA01000206.1|:80067-80356
MsHel-3a	gb|AIXA01036721.1|:1-203
MsHel-3a	gb|AIXA01032709.1|:1-107
MsHel-3a	gb|AIXA01029123.1|:26-116
MsHel-3a	gb|AIXA01024128.1|:262-523
MsHel-3a	gb|AIXA01020346.1|:2411-2671
MsHel-3a	gb|AIXA01018040.1|:878-1156
MsHel-3a	gb|AIXA01017751.1|:1618-1876
MsHel-3a	gb|AIXA01016744.1|:604-850
MsHel-3a	gb|AIXA01016207.1|:512-797
MsHel-3a	gb|AIXA01010748.1|:79484-79735
MsHel-3a	gb|AIXA01009230.1|:20020-21343
MsHel-3a	gb|AIXA01007732.1|:8742-8932
MsHel-3a	gb|AIXA01006286.1|:17645-17897
MsHel-3a	gb|AIXA01006286.1|:34391-34605
MsHel-3a	gb|AIXA01005322.1|:29941-30310
MsHel-3a	gb|AIXA01005228.1|:80399-80873
MsHel-3a	gb|AIXA01005132.1|:7-288
MsHel-3a	gb|AIXA01005097.1|:6293-6591
MsHel-3a	gb|AIXA01005083.1|:62413-62698
MsHel-3a	gb|AIXA01004459.1|:81323-81529
MsHel-3a	gb|AIXA01004269.1|:59658-59855
MsHel-3a	gb|AIXA01003011.1|:12352-12622
MsHel-3a	gb|AIXA01002582.1|:84812-85061
MsHel-3a	gb|AIXA01002267.1|:114479-114728
MsHel-3a	gb|AIXA01001956.1|:53006-53278
MsHel-3a	gb|AIXA01001731.1|:34811-35056
MsHel-3a	gb|AIXA01001718.1|:27944-28221
MsHel-3a	gb|AIXA01029273.1|:601-874
MsHel-3a	gb|AIXA01016208.1|:89-202
MsHel-3a	gb|AIXA01014533.1|:3436-3676
MsHel-3a	gb|AIXA01013347.1|:5405-5691

MsHel-3a	gb|AIXA01012656.1|:6100-6381
MsHel-3a	gb|AIXA01012458.1|:19905-20170
MsHel-3a	gb|AIXA01012127.1|:3122-3386
MsHel-3a	gb|AIXA01010797.1|:2710-2975
MsHel-3a	gb|AIXA01010334.1|:1626-1742
MsHel-3a	gb|AIXA01010334.1|:15759-16045
MsHel-3a	gb|AIXA01009043.1|:20487-20750
MsHel-3a	gb|AIXA01006444.1|:6780-6886
MsHel-3a	gb|AIXA01004353.1|:25007-25300
MsHel-3a	gb|AIXA01004244.1|:476-589
MsHel-3a	gb|AIXA01002830.1|:10316-10585
MsHel-3a	gb|AIXA01002527.1|:991-1260
MsHel-3a	gb|AIXA01002527.1|:49020-49247
MsHel-3a	gb|AIXA01002298.1|:19531-19647
MsHel-3a	gb|AIXA01001525.1|:3073-3344
MsHel-3a	gb|AIXA01000787.1|:92882-93125
MsHel-3a	gb|AIXA01000165.1|:66-335
MsHel-3a	gb|AIXA01019045.1|:4215-4500
MsHel-3a	gb|AIXA01018174.1|:3381-3665
MsHel-3a	gb|AIXA01012355.1|:8035-8306
MsHel-3a	gb|AIXA01009864.1|:44363-44630
MsHel-3a	gb|AIXA01007478.1|:27887-28169
MsHel-3a	gb|AIXA01007218.1|:2022-2822
MsHel-3a	gb|AIXA01007054.1|:1088-1370
MsHel-3a	gb|AIXA01006299.1|:113114-113230
MsHel-3a	gb|AIXA01004863.1|:17920-18203
MsHel-3a	gb|AIXA01003932.1|:1148-1420
MsHel-3a	gb|AIXA01003694.1|:28231-28512
MsHel-3a	gb|AIXA01002507.1|:7080-7321
MsHel-3a	gb|AIXA01001825.1|:12920-13191
MsHel-3a	gb|AIXA01001663.1|:25159-25423
MsHel-3a	gb|AIXA01000801.1|:19166-19434
MsHel-3a	gb|AIXA01018862.1|:7620-7908
MsHel-3a	gb|AIXA01018494.1|:4623-4883
MsHel-3a	gb|AIXA01018488.1|:702-985
MsHel-3a	gb|AIXA01017935.1|:9843-10079
MsHel-3a	gb|AIXA01017233.1|:8068-8353
MsHel-3a	gb|AIXA01014969.1|:6565-6848
MsHel-3a	gb|AIXA01013458.1|:42509-42795
MsHel-3a	gb|AIXA01012398.1|:7690-7967
MsHel-3a	gb|AIXA01010756.1|:19679-19966
MsHel-3a	gb|AIXA01010756.1|:42916-43169
MsHel-3a	gb|AIXA01010118.1|:261-361
MsHel-3a	gb|AIXA01010117.1|:4601-5661
MsHel-3a	gb|AIXA01009235.1|:17371-17655
MsHel-3a	gb|AIXA01008328.1|:3603-3872
MsHel-3a	gb|AIXA01007470.1|:6281-6557
MsHel-3a	gb|AIXA01006654.1|:11125-11414

MsHel-3a	gb|AIXA01005551.1|:17187-17454
MsHel-3a	gb|AIXA01005131.1|:35836-36119
MsHel-3a	gb|AIXA01003881.1|:63513-63799
MsHel-3a	gb|AIXA01003711.1|:154090-154341
MsHel-3a	gb|AIXA01003582.1|:10938-11189
MsHel-3a	gb|AIXA01003124.1|:51866-52111
MsHel-3a	gb|AIXA01002544.1|:23919-24192
MsHel-3a	gb|AIXA01002339.1|:44151-44437
MsHel-3a	gb|AIXA01002045.1|:14942-15218
MsHel-3a	gb|AIXA01001001.1|:38011-38265
MsHel-3a	gb|AIXA01000772.1|:17-294
MsHel-3a	gb|AIXA01000051.1|:3252-3515
MsHel-3a	gb|AIXA01019243.1|:1485-1730
MsHel-3a	gb|AIXA01013741.1|:258-545
MsHel-3a	gb|AIXA01009763.1|:8216-8481
MsHel-3a	gb|AIXA01009617.1|:707-991
MsHel-3a	gb|AIXA01009433.1|:8314-8559
MsHel-3a	gb|AIXA01009129.1|:9728-9993
MsHel-3a	gb|AIXA01008148.1|:43117-43372
MsHel-3a	gb|AIXA01007197.1|:1357-1837
MsHel-3a	gb|AIXA01006838.1|:2382-7128
MsHel-3a	gb|AIXA01006838.1|:68398-68679
MsHel-3a	gb|AIXA01006350.1|:23913-24165
MsHel-3a	gb|AIXA01006350.1|:73782-74048
MsHel-3a	gb|AIXA01005531.1|:4869-5158
MsHel-3a	gb|AIXA01004954.1|:10332-10588
MsHel-3a	gb|AIXA01003678.1|:69324-69566
MsHel-3a	gb|AIXA01003209.1|:6862-7152
MsHel-3a	gb|AIXA01001862.1|:2060-2325
MsHel-3a	gb|AIXA01001401.1|:11746-12017
MsHel-3a	gb|AIXA01000195.1|:79274-79545
MsHel-3a	gb|AIXA01022684.1|:716-995
MsHel-3a	gb|AIXA01019063.1|:33-308
MsHel-3a	gb|AIXA01017937.1|:2945-3225
MsHel-3a	gb|AIXA01015519.1|:1653-1893
MsHel-3a	gb|AIXA01014708.1|:15111-15397
MsHel-3a	gb|AIXA01011821.1|:377-487
MsHel-3a	gb|AIXA01009138.1|:13263-13505
MsHel-3a	gb|AIXA01008933.1|:6817-7085
MsHel-3a	gb|AIXA01008403.1|:10966-11249
MsHel-3a	gb|AIXA01008398.1|:8302-8384
MsHel-3a	gb|AIXA01008398.1|:39621-39861
MsHel-3a	gb|AIXA01007472.1|:94834-95122
MsHel-3a	gb|AIXA01007046.1|:31197-31468
MsHel-3a	gb|AIXA01006695.1|:398-678
MsHel-3a	gb|AIXA01006548.1|:19549-19828
MsHel-3a	gb|AIXA01005640.1|:4291-4579
MsHel-3a	gb|AIXA01002157.1|:36206-36323

MsHel-3a	gb|AIXA01001481.1|:33760-34043
MsHel-3a	gb|AIXA01001237.1|:4653-4932
MsHel-3a	gb|AIXA01000508.1|:22570-22847
MsHel-3a	gb|AIXA01000061.1|:19426-19673
MsHel-3a	gb|AIXA01000044.1|:15702-15978
MsHel-3a	gb|AIXA01023486.1|:359-625
MsHel-3a	gb|AIXA01023383.1|:379-644
MsHel-3a	gb|AIXA01023284.1|:1160-1435
MsHel-3a	gb|AIXA01022149.1|:136-413
MsHel-3a	gb|AIXA01015185.1|:3052-3328
MsHel-3a	gb|AIXA01013089.1|:10854-11146
MsHel-3a	gb|AIXA01009657.1|:4539-4801
MsHel-3a	gb|AIXA01008561.1|:34687-34928
MsHel-3a	gb|AIXA01006862.1|:11645-11935
MsHel-3a	gb|AIXA01005595.1|:124665-124907
MsHel-3a	gb|AIXA01005349.1|:14138-14387
MsHel-3a	gb|AIXA01004220.1|:24862-25150
MsHel-3a	gb|AIXA01002812.1|:13941-14227
MsHel-3a	gb|AIXA01002812.1|:57134-57409
MsHel-3a	gb|AIXA01002785.1|:42876-43155
MsHel-3a	gb|AIXA01002448.1|:4409-4690
MsHel-3a	gb|AIXA01001988.1|:11935-12051
MsHel-3a	gb|AIXA01000808.1|:46-163
MsHel-3a	gb|AIXA01026302.1|:787-1046
MsHel-3a	gb|AIXA01018550.1|:2056-2345
MsHel-3a	gb|AIXA01017746.1|:1305-1388
MsHel-3a	gb|AIXA01011779.1|:19684-19923
MsHel-3a	gb|AIXA01008252.1|:20945-21214
MsHel-3a	gb|AIXA01008182.1|:28893-29009
MsHel-3a	gb|AIXA01007195.1|:38980-39236
MsHel-3a	gb|AIXA01007111.1|:10718-10985
MsHel-3a	gb|AIXA01006270.1|:9360-9641
MsHel-3a	gb|AIXA01003742.1|:7514-7783
MsHel-3a	gb|AIXA01003182.1|:36315-36588
MsHel-3a	gb|AIXA01003174.1|:12371-12627
MsHel-3a	gb|AIXA01003155.1|:12166-12454
MsHel-3a	gb|AIXA01002671.1|:155369-155643
MsHel-3a	gb|AIXA01002531.1|:48824-49095
MsHel-3a	gb|AIXA01002357.1|:858-1146
MsHel-3a	gb|AIXA01001962.1|:40039-40321
MsHel-3a	gb|AIXA01000183.1|:63923-64192
MsHel-3a	gb|AIXA01020312.1|:1-91
MsHel-3a	gb|AIXA01020177.1|:2646-2932
MsHel-3a	gb|AIXA01017318.1|:4830-5486
MsHel-3a	gb|AIXA01015786.1|:6442-6692
MsHel-3a	gb|AIXA01013899.1|:4432-4704
MsHel-3a	gb|AIXA01013047.1|:17269-17514
MsHel-3a	gb|AIXA01011722.1|:29023-29266

MsHel-3a	gb|AIXA01011722.1|:70648-70900
MsHel-3a	gb|AIXA01008280.1|:51770-51880
MsHel-3a	gb|AIXA01008108.1|:4252-4536
MsHel-3a	gb|AIXA01006424.1|:3047-3160
MsHel-3a	gb|AIXA01005977.1|:1-95
MsHel-3a	gb|AIXA01005691.1|:4221-4492
MsHel-3a	gb|AIXA01005576.1|:39314-39544
MsHel-3a	gb|AIXA01003353.1|:27574-27826
MsHel-3a	gb|AIXA01001682.1|:25098-25384
MsHel-3a	gb|AIXA01001370.1|:49192-49474
MsHel-3a	gb|AIXA01000385.1|:50313-51164
MsHel-3a	gb|AIXA01000145.1|:30712-30993
MsHel-3a	gb|AIXA01033161.1|:178-460
MsHel-3a	gb|AIXA01027371.1|:141-428
MsHel-3a	gb|AIXA01013523.1|:31393-31678
MsHel-3a	gb|AIXA01011446.1|:4283-4547
MsHel-3a	gb|AIXA01010691.1|:17509-17776
MsHel-3a	gb|AIXA01009697.1|:18114-18206
MsHel-3a	gb|AIXA01006785.1|:15081-15331
MsHel-3a	gb|AIXA01003910.1|:2456-2541
MsHel-3a	gb|AIXA01003722.1|:43389-43672
MsHel-3a	gb|AIXA01001926.1|:36543-37160
MsHel-3a	gb|AIXA01001605.1|:10504-10764
MsHel-3a	gb|AIXA01001605.1|:39932-40011
MsHel-3a	gb|AIXA01001271.1|:83857-84067
MsHel-3a	gb|AIXA01001271.1|:113918-114184
MsHel-3a	gb|AIXA01021974.1|:194-474
MsHel-3a	gb|AIXA01021535.1|:1643-1900
MsHel-3a	gb|AIXA01018557.1|:380-653
MsHel-3a	gb|AIXA01016202.1|:990-1257
MsHel-3a	gb|AIXA01016193.1|:480-674
MsHel-3a	gb|AIXA01015529.1|:7315-7589
MsHel-3a	gb|AIXA01013911.1|:3894-4011
MsHel-3a	gb|AIXA01012375.1|:10950-11222
MsHel-3a	gb|AIXA01012351.1|:3167-3295
MsHel-3a	gb|AIXA01010481.1|:22548-22810
MsHel-3a	gb|AIXA01003285.1|:42301-42570
MsHel-3a	gb|AIXA01001748.1|:13070-13346
MsHel-3a	gb|AIXA01001608.1|:13419-13698
MsHel-3a	gb|AIXA01001194.1|:5691-5980
MsHel-3a	gb|AIXA01000909.1|:33078-33169
MsHel-3a	gb|AIXA01000909.1|:82492-82776
MsHel-3a	gb|AIXA01032793.1|:29-224
MsHel-3a	gb|AIXA01024458.1|:492-610
MsHel-3a	gb|AIXA01022590.1|:114-387
MsHel-3a	gb|AIXA01020327.1|:1097-1329
MsHel-3a	gb|AIXA01018845.1|:3586-3822
MsHel-3a	gb|AIXA01017383.1|:34-280

MsHel-3a	gb|AIXA01015243.1|:4088-4175
MsHel-3a	gb|AIXA01014211.1|:4615-4899
MsHel-3a	gb|AIXA01011611.1|:17406-17697
MsHel-3a	gb|AIXA01010815.1|:3239-3503
MsHel-3a	gb|AIXA01009945.1|:13577-13860
MsHel-3a	gb|AIXA01009071.1|:26924-27197
MsHel-3a	gb|AIXA01008919.1|:9342-9635
MsHel-3a	gb|AIXA01007516.1|:48615-48887
MsHel-3a	gb|AIXA01006380.1|:45917-46032
MsHel-3a	gb|AIXA01003661.1|:183567-183849
MsHel-3a	gb|AIXA01001551.1|:14492-14777
MsHel-3a	gb|AIXA01000441.1|:29466-29739
MsHel-3a	gb|AIXA01000050.1|:42471-42735
MsHel-3a	gb|AIXA01030445.1|:530-769
MsHel-3a	gb|AIXA01018899.1|:2771-3023
MsHel-3a	gb|AIXA01018737.1|:1083-1364
MsHel-3a	gb|AIXA01016489.1|:4239-4518
MsHel-3a	gb|AIXA01014253.1|:10612-10782
MsHel-3a	gb|AIXA01013864.1|:12356-12639
MsHel-3a	gb|AIXA01011727.1|:390-677
MsHel-3a	gb|AIXA01008111.1|:40884-41127
MsHel-3a	gb|AIXA01008111.1|:56325-56426
MsHel-3a	gb|AIXA01007970.1|:53-312
MsHel-3a	gb|AIXA01007259.1|:3743-3969
MsHel-3a	gb|AIXA01005093.1|:3168-3440
MsHel-3a	gb|AIXA01004153.1|:2784-3060
MsHel-3a	gb|AIXA01001941.1|:94180-94468
MsHel-3a	gb|AIXA01001517.1|:7895-8138
MsHel-3a	gb|AIXA01001514.1|:11399-11645
MsHel-3a	gb|AIXA01001458.1|:8974-9254
MsHel-3a	gb|AIXA01029076.1|:24-226
MsHel-3a	gb|AIXA01026841.1|:382-660
MsHel-3a	gb|AIXA01026412.1|:253-493
MsHel-3a	gb|AIXA01019323.1|:527-793
MsHel-3a	gb|AIXA01018629.1|:5019-5300
MsHel-3a	gb|AIXA01018524.1|:1975-2177
MsHel-3a	gb|AIXA01016038.1|:460-706
MsHel-3a	gb|AIXA01014337.1|:8190-8444
MsHel-3a	gb|AIXA01014244.1|:18807-19078
MsHel-3a	gb|AIXA01013446.1|:2653-2914
MsHel-3a	gb|AIXA01012324.1|:10636-10925
MsHel-3a	gb|AIXA01012324.1|:26896-27165
MsHel-3a	gb|AIXA01012304.1|:1567-1840
MsHel-3a	gb|AIXA01012030.1|:5697-5984
MsHel-3a	gb|AIXA01011121.1|:25843-26126
MsHel-3a	gb|AIXA01010504.1|:168-415
MsHel-3a	gb|AIXA01010342.1|:12-296
MsHel-3a	gb|AIXA01009970.1|:10076-10161

MsHel-3a	gb|AIXA01009925.1|:118432-118708
MsHel-3a	gb|AIXA01009389.1|:683-971
MsHel-3a	gb|AIXA01009362.1|:24-117
MsHel-3a	gb|AIXA01008376.1|:25465-25857
MsHel-3a	gb|AIXA01007887.1|:2579-6721
MsHel-3a	gb|AIXA01006169.1|:1740-2028
MsHel-3a	gb|AIXA01006035.1|:51538-51630
MsHel-3a	gb|AIXA01004125.1|:1566-1853
MsHel-3a	gb|AIXA01002712.1|:18555-18842
MsHel-3a	gb|AIXA01033415.1|:1-253
MsHel-3a	gb|AIXA01027876.1|:679-924
MsHel-3a	gb|AIXA01023196.1|:468-736
MsHel-3a	gb|AIXA01022515.1|:1260-1492
MsHel-3a	gb|AIXA01022030.1|:90-203
MsHel-3a	gb|AIXA01021905.1|:384-636
MsHel-3a	gb|AIXA01021223.1|:1748-1852
MsHel-3a	gb|AIXA01013940.1|:3059-3310
MsHel-3a	gb|AIXA01010192.1|:25800-26058
MsHel-3a	gb|AIXA01008484.1|:10778-11043
MsHel-3a	gb|AIXA01007075.1|:33830-34283
MsHel-3a	gb|AIXA01006978.1|:11277-11821
MsHel-3a	gb|AIXA01006575.1|:39417-39708
MsHel-3a	gb|AIXA01006325.1|:1355-1570
MsHel-3a	gb|AIXA01006214.1|:3299-3538
MsHel-3a	gb|AIXA01005855.1|:66108-66379
MsHel-3a	gb|AIXA01005855.1|:84240-84479
MsHel-3a	gb|AIXA01005128.1|:7998-8255
MsHel-3a	gb|AIXA01002673.1|:15629-15922
MsHel-3a	gb|AIXA01002408.1|:25516-25771
MsHel-3a	gb|AIXA01001574.1|:11800-12018
MsHel-3a	gb|AIXA01000003.1|:46866-46982
MsHel-3a	gb|AIXA01032166.1|:411-690
MsHel-3a	gb|AIXA01026829.1|:891-1002
MsHel-3a	gb|AIXA01025444.1|:46-310
MsHel-3a	gb|AIXA01022852.1|:152-397
MsHel-3a	gb|AIXA01021543.1|:614-898
MsHel-3a	gb|AIXA01018360.1|:656-917
MsHel-3a	gb|AIXA01018165.1|:673-914
MsHel-3a	gb|AIXA01017339.1|:3613-3730
MsHel-3a	gb|AIXA01015928.1|:1574-1854
MsHel-3a	gb|AIXA01015920.1|:652-894
MsHel-3a	gb|AIXA01015177.1|:3805-4092
MsHel-3a	gb|AIXA01011716.1|:7687-7966
MsHel-3a	gb|AIXA01009649.1|:39217-39423
MsHel-3a	gb|AIXA01009643.1|:7374-7639
MsHel-3a	gb|AIXA01009099.1|:17290-17480
MsHel-3a	gb|AIXA01008917.1|:49518-49759
MsHel-3a	gb|AIXA01008510.1|:2845-3064

MsHel-3a	gb|AIXA01008370.1|:1205-1485
MsHel-3a	gb|AIXA01008258.1|:21073-21341
MsHel-3a	gb|AIXA01007845.1|:3571-3808
MsHel-3a	gb|AIXA01007636.1|:14462-14732
MsHel-3a	gb|AIXA01006914.1|:4194-4481
MsHel-3a	gb|AIXA01006909.1|:1142-1390
MsHel-3a	gb|AIXA01006808.1|:5052-5295
MsHel-3a	gb|AIXA01005991.1|:5104-5285
MsHel-3a	gb|AIXA01005265.1|:11853-12078
MsHel-3a	gb|AIXA01004758.1|:41200-41452
MsHel-3a	gb|AIXA01004585.1|:4330-4587
MsHel-3a	gb|AIXA01004583.1|:57526-57748
MsHel-3a	gb|AIXA01003749.1|:3104-3372
MsHel-3a	gb|AIXA01003157.1|:48046-48338
MsHel-3a	gb|AIXA01002959.1|:7189-7405
MsHel-3a	gb|AIXA01002756.1|:2792-3070
MsHel-3a	gb|AIXA01002242.1|:13549-13793
MsHel-3a	gb|AIXA01001960.1|:63410-63631
MsHel-3a	gb|AIXA01001593.1|:4769-5055
MsHel-3a	gb|AIXA01001268.1|:9814-10058
MsHel-3a	gb|AIXA01001032.1|:55168-55449
MsHel-3a	gb|AIXA01001018.1|:21113-21328
MsHel-3a	gb|AIXA01001012.1|:9375-9614
MsHel-3a	gb|AIXA01031133.1|:518-745
MsHel-3a	gb|AIXA01028067.1|:668-916
MsHel-3a	gb|AIXA01026337.1|:378-651
MsHel-3a	gb|AIXA01022048.1|:1290-1575
MsHel-3a	gb|AIXA01016978.1|:2021-2300
MsHel-3a	gb|AIXA01014692.1|:2922-3204
MsHel-3a	gb|AIXA01006884.1|:95028-95320
MsHel-3a	gb|AIXA01004064.1|:17557-17820
MsHel-3a	gb|AIXA01003949.1|:8474-8710
MsHel-3a	gb|AIXA01003816.1|:86429-86676
MsHel-3a	gb|AIXA01003336.1|:1215-1463
MsHel-3a	gb|AIXA01002363.1|:21948-22067
MsHel-3a	gb|AIXA01000359.1|:76531-76814
MsHel-3a	gb|AIXA01000168.1|:38169-38427
MsHel-3a	gb|AIXA01030388.1|:68-186
MsHel-3a	gb|AIXA01030163.1|:489-751
MsHel-3a	gb|AIXA01026859.1|:872-1038
MsHel-3a	gb|AIXA01018369.1|:1334-1594
MsHel-3a	gb|AIXA01018194.1|:913-1201
MsHel-3a	gb|AIXA01017425.1|:99-344
MsHel-3a	gb|AIXA01017105.1|:3398-3509
MsHel-3a	gb|AIXA01015343.1|:2625-2739
MsHel-3a	gb|AIXA01014641.1|:30811-31053
MsHel-3a	gb|AIXA01014417.1|:5876-6055
MsHel-3a	gb|AIXA01013624.1|:4377-4660

MsHel-3a	gb|AIXA01013472.1|:1834-2119
MsHel-3a	gb|AIXA01012924.1|:2172-2388
MsHel-3a	gb|AIXA01012363.1|:2312-2546
MsHel-3a	gb|AIXA01012117.1|:8858-9111
MsHel-3a	gb|AIXA01011536.1|:3907-4184
MsHel-3a	gb|AIXA01010829.1|:886-1166
MsHel-3a	gb|AIXA01010398.1|:28171-28447
MsHel-3a	gb|AIXA01009662.1|:651-919
MsHel-3a	gb|AIXA01009636.1|:13287-13486
MsHel-3a	gb|AIXA01009355.1|:48169-48454
MsHel-3a	gb|AIXA01009353.1|:5859-6131
MsHel-3a	gb|AIXA01009304.1|:21059-21295
MsHel-3a	gb|AIXA01008329.1|:34304-34807
MsHel-3a	gb|AIXA01006394.1|:13535-13732
MsHel-3a	gb|AIXA01005597.1|:10365-10608
MsHel-3a	gb|AIXA01005479.1|:32307-32512
MsHel-3a	gb|AIXA01004424.1|:71836-72056
MsHel-3a	gb|AIXA01003953.1|:5067-5356
MsHel-3a	gb|AIXA01003715.1|:3689-3982
MsHel-3a	gb|AIXA01003652.1|:12268-13098
MsHel-3a	gb|AIXA01003121.1|:2879-3158
MsHel-3a	gb|AIXA01001997.1|:1124-1404
MsHel-3a	gb|AIXA01001804.1|:46108-46379
MsHel-3a	gb|AIXA01001478.1|:15192-15429
MsHel-3a	gb|AIXA01001016.1|:62210-62455
MsHel-3a	gb|AIXA01000468.1|:344-456
MsHel-3a	gb|AIXA01000468.1|:62128-62367
MsHel-3a	gb|AIXA01000023.1|:1004-1284
MsHel-3a	gb|AIXA01038146.1|:322-434
MsHel-3a	gb|AIXA01032713.1|:548-660
MsHel-3a	gb|AIXA01018244.1|:3721-3981
MsHel-3a	gb|AIXA01016244.1|:5500-5736
MsHel-3a	gb|AIXA01015483.1|:4890-5169
MsHel-3a	gb|AIXA01012852.1|:1288-1502
MsHel-3a	gb|AIXA01012114.1|:33871-34303
MsHel-3a	gb|AIXA01011311.1|:13880-14146
MsHel-3a	gb|AIXA01009307.1|:670-939
MsHel-3a	gb|AIXA01008926.1|:13901-14135
MsHel-3a	gb|AIXA01006419.1|:18460-18719
MsHel-3a	gb|AIXA01005766.1|:38966-39508
MsHel-3a	gb|AIXA01005332.1|:685-924
MsHel-3a	gb|AIXA01004546.1|:59995-60248
MsHel-3a	gb|AIXA01003681.1|:40120-40367
MsHel-3a	gb|AIXA01003681.1|:51979-52239
MsHel-3a	gb|AIXA01003339.1|:50059-50281
MsHel-3a	gb|AIXA01003148.1|:5485-5751
MsHel-3a	gb|AIXA01002395.1|:8567-8801
MsHel-3a	gb|AIXA01001584.1|:3965-4259

MsHel-3a	gb|AIXA01001453.1|:10187-10472
MsHel-3a	gb|AIXA01001220.1|:16798-16915
MsHel-3a	gb|AIXA01000736.1|:2365-2622
MsHel-3a	gb|AIXA01029491.1|:53-333
MsHel-3a	gb|AIXA01028934.1|:653-818
MsHel-3a	gb|AIXA01027496.1|:266-468
MsHel-3a	gb|AIXA01021513.1|:1623-1709
MsHel-3a	gb|AIXA01019570.1|:1819-1935
MsHel-3a	gb|AIXA01018801.1|:5216-5332
MsHel-3a	gb|AIXA01018043.1|:3303-3580
MsHel-3a	gb|AIXA01016021.1|:7960-8250
MsHel-3a	gb|AIXA01015510.1|:567-786
MsHel-3a	gb|AIXA01014882.1|:1129-1372
MsHel-3a	gb|AIXA01014611.1|:11261-11477
MsHel-3a	gb|AIXA01014508.1|:4927-5160
MsHel-3a	gb|AIXA01014443.1|:8295-8530
MsHel-3a	gb|AIXA01013301.1|:4514-4791
MsHel-3a	gb|AIXA01011947.1|:149-394
MsHel-3a	gb|AIXA01011197.1|:27056-27173
MsHel-3a	gb|AIXA01011197.1|:82621-82862
MsHel-3a	gb|AIXA01010077.1|:12301-12541
MsHel-3a	gb|AIXA01009775.1|:22-113
MsHel-3a	gb|AIXA01009217.1|:65330-65562
MsHel-3a	gb|AIXA01009125.1|:8978-9260
MsHel-3a	gb|AIXA01009078.1|:48663-48932
MsHel-3a	gb|AIXA01008711.1|:13661-13902
MsHel-3a	gb|AIXA01007761.1|:3141-3640
MsHel-3a	gb|AIXA01007237.1|:12888-13006
MsHel-3a	gb|AIXA01006068.1|:18593-18847
MsHel-3a	gb|AIXA01005571.1|:17699-17943
MsHel-3a	gb|AIXA01005264.1|:518-756
MsHel-3a	gb|AIXA01005106.1|:4425-4714
MsHel-3a	gb|AIXA01004935.1|:45309-45591
MsHel-3a	gb|AIXA01004629.1|:13123-13368
MsHel-3a	gb|AIXA01004629.1|:35081-35363
MsHel-3a	gb|AIXA01004421.1|:36475-36764
MsHel-3a	gb|AIXA01004345.1|:394-616
MsHel-3a	gb|AIXA01004320.1|:2278-2549
MsHel-3a	gb|AIXA01003831.1|:1067-1305
MsHel-3a	gb|AIXA01003747.1|:100667-100917
MsHel-3a	gb|AIXA01002996.1|:100366-100602
MsHel-3a	gb|AIXA01002707.1|:744-986
MsHel-3a	gb|AIXA01001892.1|:2236-2481
MsHel-3a	gb|AIXA01001755.1|:78329-78611
MsHel-3a	gb|AIXA01001547.1|:22005-22123
MsHel-3a	gb|AIXA01001169.1|:436-719
MsHel-3a	gb|AIXA01000907.1|:1407-1486
MsHel-3a	gb|AIXA01000185.1|:44676-44792

MsHel-3a	gb|AIXA01037087.1|:354-469
MsHel-3a	gb|AIXA01036615.1|:171-455
MsHel-3a	gb|AIXA01035146.1|:443-562
MsHel-3a	gb|AIXA01034236.1|:482-601
MsHel-3a	gb|AIXA01017282.1|:8266-8477
MsHel-3a	gb|AIXA01017096.1|:2810-3047
MsHel-3a	gb|AIXA01016598.1|:57-172
MsHel-3a	gb|AIXA01012904.1|:19908-20164
MsHel-3a	gb|AIXA01011950.1|:6832-7114
MsHel-3a	gb|AIXA01011247.1|:19792-20070
MsHel-3a	gb|AIXA01010196.1|:50490-50724
MsHel-3a	gb|AIXA01009184.1|:14965-15183
MsHel-3a	gb|AIXA01008245.1|:5548-5819
MsHel-3a	gb|AIXA01007815.1|:4387-4679
MsHel-3a	gb|AIXA01007598.1|:131789-132071
MsHel-3a	gb|AIXA01006837.1|:16211-16451
MsHel-3a	gb|AIXA01005521.1|:73516-73797
MsHel-3a	gb|AIXA01005095.1|:13829-14106
MsHel-3a	gb|AIXA01005095.1|:46475-46710
MsHel-3a	gb|AIXA01005037.1|:6182-6416
MsHel-3a	gb|AIXA01004222.1|:41844-42124
MsHel-3a	gb|AIXA01003907.1|:50084-50355
MsHel-3a	gb|AIXA01003065.1|:1-244
MsHel-3a	gb|AIXA01002802.1|:4723-4961
MsHel-3a	gb|AIXA01002653.1|:10267-10490
MsHel-3a	gb|AIXA01002463.1|:59572-59690
MsHel-3a	gb|AIXA01002463.1|:70151-71408
MsHel-3a	gb|AIXA01001877.1|:156475-156716
MsHel-3a	gb|AIXA01001233.1|:8018-8260
MsHel-3a	gb|AIXA01000780.1|:3836-3996
MsHel-3a	gb|AIXA01000740.1|:12567-12855
MsHel-3a	gb|AIXA01000046.1|:11188-11440
MsHel-3a	gb|AIXA01035207.1|:121-359
MsHel-3a	gb|AIXA01034402.1|:35-305
MsHel-3a	gb|AIXA01034190.1|:237-491
MsHel-3a	gb|AIXA01033383.1|:115-395
MsHel-3a	gb|AIXA01026734.1|:298-541
MsHel-3a	gb|AIXA01024414.1|:840-1076
MsHel-3a	gb|AIXA01018618.1|:1358-1585
MsHel-3a	gb|AIXA01016805.1|:8436-8554
MsHel-3a	gb|AIXA01016493.1|:1428-1712
MsHel-3a	gb|AIXA01015935.1|:1570-1858
MsHel-3a	gb|AIXA01015902.1|:4162-4417
MsHel-3a	gb|AIXA01015810.1|:4920-5166
MsHel-3a	gb|AIXA01015420.1|:5110-5389
MsHel-3a	gb|AIXA01015390.1|:5160-5445
MsHel-3a	gb|AIXA01014738.1|:380-657
MsHel-3a	gb|AIXA01013805.1|:1065-2087

MsHel-3a	gb|AIXA01011502.1|:11566-11854
MsHel-3a	gb|AIXA01010964.1|:22343-22584
MsHel-3a	gb|AIXA01010470.1|:6129-6379
MsHel-3a	gb|AIXA01010261.1|:4050-4293
MsHel-3a	gb|AIXA01009492.1|:5271-5533
MsHel-3a	gb|AIXA01008897.1|:14212-14451
MsHel-3a	gb|AIXA01008554.1|:32803-33090
MsHel-3a	gb|AIXA01008131.1|:5848-6095
MsHel-3a	gb|AIXA01007969.1|:1079-1360
MsHel-3a	gb|AIXA01007783.1|:3573-3809
MsHel-3a	gb|AIXA01007220.1|:6195-6477
MsHel-3a	gb|AIXA01007021.1|:21567-21677
MsHel-3a	gb|AIXA01006817.1|:28702-28892
MsHel-3a	gb|AIXA01006682.1|:24482-24716
MsHel-3a	gb|AIXA01006508.1|:36645-36912
MsHel-3a	gb|AIXA01006499.1|:10527-10764
MsHel-3a	gb|AIXA01006337.1|:60048-60302
MsHel-3a	gb|AIXA01006066.1|:48709-48985
MsHel-3a	gb|AIXA01005318.1|:25611-25831
MsHel-3a	gb|AIXA01005256.1|:14598-14843
MsHel-3a	gb|AIXA01005140.1|:1265-1384
MsHel-3a	gb|AIXA01004930.1|:6664-6902
MsHel-3a	gb|AIXA01004225.1|:38-156
MsHel-3a	gb|AIXA01003858.1|:16326-16539
MsHel-3a	gb|AIXA01003858.1|:40203-40489
MsHel-3a	gb|AIXA01003845.1|:20339-20608
MsHel-3a	gb|AIXA01002817.1|:42973-43197
MsHel-3a	gb|AIXA01002768.1|:330-603
MsHel-3a	gb|AIXA01002521.1|:42209-42438
MsHel-3a	gb|AIXA01001899.1|:142628-142860
MsHel-3a	gb|AIXA01001805.1|:17037-17323
MsHel-3a	gb|AIXA01001657.1|:195549-195809
MsHel-3a	gb|AIXA01001632.1|:673-918
MsHel-3a	gb|AIXA01001611.1|:28664-28905
MsHel-3a	gb|AIXA01001515.1|:19883-20125
MsHel-3a	gb|AIXA01001190.1|:5072-5359
MsHel-3a	gb|AIXA01000928.1|:5853-6144
MsHel-3a	gb|AIXA01000562.1|:21232-21510
MsHel-3a	gb|AIXA01000437.1|:145380-145848
MsHel-3a	gb|AIXA01000270.1|:18568-18815
MsHel-3a	gb|AIXA01000035.1|:7027-7270
MsHel-3a	gb|AIXA01000024.1|:11177-11295
MsHel-3a	gb|AIXA01031648.1|:315-579
MsHel-3a	gb|AIXA01027081.1|:20-217
MsHel-3a	gb|AIXA01018914.1|:3627-3739
MsHel-3a	gb|AIXA01018358.1|:7118-7233
MsHel-3a	gb|AIXA01017054.1|:3124-3425
MsHel-3a	gb|AIXA01016438.1|:37146-37386

MsHel-3a	gb|AIXA01014760.1|:1744-2023
MsHel-3a	gb|AIXA01014667.1|:9120-9385
MsHel-3a	gb|AIXA01013305.1|:8079-8343
MsHel-3a	gb|AIXA01012618.1|:3754-3854
MsHel-3a	gb|AIXA01010808.1|:8047-8256
MsHel-3a	gb|AIXA01010343.1|:29806-30076
MsHel-3a	gb|AIXA01008931.1|:10917-11196
MsHel-3a	gb|AIXA01008323.1|:1560-1837
MsHel-3a	gb|AIXA01008025.1|:43335-43611
MsHel-3a	gb|AIXA01007157.1|:6307-6587
MsHel-3a	gb|AIXA01006852.1|:13613-13854
MsHel-3a	gb|AIXA01004155.1|:45604-45860
MsHel-3a	gb|AIXA01003040.1|:34705-34983
MsHel-3a	gb|AIXA01002951.1|:64467-64741
MsHel-3a	gb|AIXA01002782.1|:12501-12746
MsHel-3a	gb|AIXA01002747.1|:50763-50995
MsHel-3a	gb|AIXA01002726.1|:9440-9679
MsHel-3a	gb|AIXA01002458.1|:12705-12987
MsHel-3a	gb|AIXA01001852.1|:6167-6373
MsHel-3a	gb|AIXA01001817.1|:31825-32063
MsHel-3a	gb|AIXA01001621.1|:5127-5313
MsHel-3a	gb|AIXA01001513.1|:10851-10965
MsHel-3a	gb|AIXA01001418.1|:42846-43088
MsHel-3a	gb|AIXA01001397.1|:10772-11020
MsHel-3a	gb|AIXA01001200.1|:10685-10958
MsHel-3a	gb|AIXA01000828.1|:72386-72653
MsHel-3a	gb|AIXA01000604.1|:10809-11085
MsHel-3a	gb|AIXA01000536.1|:28523-28735
MsHel-3a	gb|AIXA01036650.1|:311-515
MsHel-3a	gb|AIXA01028379.1|:672-879
MsHel-3a	gb|AIXA01024739.1|:5-117
MsHel-3a	gb|AIXA01018729.1|:2082-2327
MsHel-3a	gb|AIXA01018679.1|:1505-1695
MsHel-3a	gb|AIXA01018644.1|:964-1199
MsHel-3a	gb|AIXA01017654.1|:12777-12867
MsHel-3a	gb|AIXA01017338.1|:19590-19878
MsHel-3a	gb|AIXA01016742.1|:2054-2223
MsHel-3a	gb|AIXA01016387.1|:943-1216
MsHel-3a	gb|AIXA01016161.1|:1720-1952
MsHel-3a	gb|AIXA01015972.1|:15417-15702
MsHel-3a	gb|AIXA01015500.1|:11599-11840
MsHel-3a	gb|AIXA01015424.1|:1321-1923
MsHel-3a	gb|AIXA01015086.1|:3141-3236
MsHel-3a	gb|AIXA01013992.1|:5886-6536
MsHel-3a	gb|AIXA01013947.1|:23519-23753
MsHel-3a	gb|AIXA01013679.1|:6653-6765
MsHel-3a	gb|AIXA01012556.1|:428-544
MsHel-3a	gb|AIXA01011820.1|:7480-7595

MsHel-3a	gb|AIXA01011620.1|:19763-20046
MsHel-3a	gb|AIXA01010795.1|:1882-2968
MsHel-3a	gb|AIXA01010663.1|:20363-20640
MsHel-3a	gb|AIXA01009650.1|:1911-2190
MsHel-3a	gb|AIXA01008883.1|:8037-8299
MsHel-3a	gb|AIXA01008426.1|:13894-14174
MsHel-3a	gb|AIXA01008023.1|:70782-71021
MsHel-3a	gb|AIXA01007694.1|:640-880
MsHel-3a	gb|AIXA01006954.1|:26279-26513
MsHel-3a	gb|AIXA01006898.1|:44294-44536
MsHel-3a	gb|AIXA01006713.1|:7104-7387
MsHel-3a	gb|AIXA01006693.1|:22522-22749
MsHel-3a	gb|AIXA01006311.1|:25322-25601
MsHel-3a	gb|AIXA01005621.1|:43468-43744
MsHel-3a	gb|AIXA01005484.1|:8802-9088
MsHel-3a	gb|AIXA01005092.1|:48295-48573
MsHel-3a	gb|AIXA01003813.1|:13898-14143
MsHel-3a	gb|AIXA01003605.1|:44877-45811
MsHel-3a	gb|AIXA01003173.1|:10063-10329
MsHel-3a	gb|AIXA01003105.1|:18972-19191
MsHel-3a	gb|AIXA01002995.1|:4694-4938
MsHel-3a	gb|AIXA01002804.1|:184-452
MsHel-3a	gb|AIXA01002804.1|:11461-11744
MsHel-3a	gb|AIXA01002522.1|:20583-20885
MsHel-3a	gb|AIXA01001949.1|:60312-60588
MsHel-3a	gb|AIXA01001284.1|:29447-29650
MsHel-3a	gb|AIXA01000929.1|:9654-9880
MsHel-3a	gb|AIXA01000785.1|:18408-19313
MsHel-3a	gb|AIXA01000163.1|:555-797
MsHel-3a	gb|AIXA01000020.1|:22121-22361
MsHel-3a	gb|AIXA01037832.1|:143-373
MsHel-3a	gb|AIXA01037232.1|:417-523
MsHel-3a	gb|AIXA01029764.1|:37-148
MsHel-3a	gb|AIXA01023639.1|:1086-1313
MsHel-3a	gb|AIXA01022514.1|:1561-1672
MsHel-3a	gb|AIXA01018684.1|:5884-6003
MsHel-3a	gb|AIXA01017979.1|:4913-5146
MsHel-3a	gb|AIXA01014892.1|:17553-17818
MsHel-3a	gb|AIXA01014837.1|:3193-3310
MsHel-3a	gb|AIXA01014836.1|:4343-4588
MsHel-3a	gb|AIXA01013385.1|:5284-5521
MsHel-3a	gb|AIXA01012159.1|:519-802
MsHel-3a	gb|AIXA01011846.1|:530-769
MsHel-3a	gb|AIXA01010727.1|:2616-2833
MsHel-3a	gb|AIXA01010581.1|:1397-1654
MsHel-3a	gb|AIXA01008686.1|:5549-5836
MsHel-3a	gb|AIXA01008147.1|:7994-8112
MsHel-3a	gb|AIXA01007712.1|:14130-16193

MsHel-3a	gb|AIXA01006518.1|:84462-84746
MsHel-3a	gb|AIXA01006420.1|:6493-6763
MsHel-3a	gb|AIXA01004245.1|:656-891
MsHel-3a	gb|AIXA01003125.1|:62848-63080
MsHel-3a	gb|AIXA01002594.1|:6814-6930
MsHel-3a	gb|AIXA01002044.1|:109516-109636
MsHel-3a	gb|AIXA01001598.1|:9883-10174
MsHel-3a	gb|AIXA01001598.1|:34430-34674
MsHel-3a	gb|AIXA01001526.1|:30479-30720
MsHel-3a	gb|AIXA01001292.1|:40627-40864
MsHel-3a	gb|AIXA01000883.1|:2668-2898
MsHel-3a	gb|AIXA01000531.1|:42190-42407
MsHel-3a	gb|AIXA01000399.1|:72943-73151
MsHel-3a	gb|AIXA01000200.1|:8591-8767
MsHel-3a	gb|AIXA01034066.1|:125-370
MsHel-3a	gb|AIXA01029787.1|:456-744
MsHel-3a	gb|AIXA01024611.1|:286-531
MsHel-3a	gb|AIXA01018841.1|:6339-6629
MsHel-3a	gb|AIXA01018674.1|:514-755
MsHel-3a	gb|AIXA01018083.1|:1701-1819
MsHel-3a	gb|AIXA01016666.1|:34226-34500
MsHel-3a	gb|AIXA01015382.1|:38819-39048
MsHel-3a	gb|AIXA01013839.1|:7584-7702
MsHel-3a	gb|AIXA01013638.1|:3362-3651
MsHel-3a	gb|AIXA01013470.1|:513-757
MsHel-3a	gb|AIXA01012541.1|:4186-4474
MsHel-3a	gb|AIXA01012419.1|:35713-35834
MsHel-3a	gb|AIXA01012156.1|:1531-1803
MsHel-3a	gb|AIXA01011943.1|:1665-1945
MsHel-3a	gb|AIXA01011498.1|:4803-5042
MsHel-3a	gb|AIXA01011285.1|:42827-43109
MsHel-3a	gb|AIXA01010212.1|:4873-5156
MsHel-3a	gb|AIXA01009929.1|:2720-3000
MsHel-3a	gb|AIXA01009857.1|:40510-40643
MsHel-3a	gb|AIXA01009765.1|:34914-35162
MsHel-3a	gb|AIXA01007974.1|:75609-75828
MsHel-3a	gb|AIXA01007606.1|:24721-24994
MsHel-3a	gb|AIXA01007310.1|:1718-1799
MsHel-3a	gb|AIXA01007242.1|:5392-5634
MsHel-3a	gb|AIXA01007233.1|:21374-21651
MsHel-3a	gb|AIXA01007160.1|:13563-13774
MsHel-3a	gb|AIXA01006924.1|:34329-34611
MsHel-3a	gb|AIXA01004800.1|:84507-84697
MsHel-3a	gb|AIXA01003998.1|:18399-18641
MsHel-3a	gb|AIXA01003957.1|:3993-4107
MsHel-3a	gb|AIXA01003509.1|:2051-2297
MsHel-3a	gb|AIXA01003380.1|:10678-10917
MsHel-3a	gb|AIXA01003190.1|:38385-38610

MsHel-3a	gb|AIXA01001876.1|:33022-33402
MsHel-3a	gb|AIXA01001489.1|:8390-8676
MsHel-3a	gb|AIXA01000230.1|:8143-8388
MsHel-3a	gb|AIXA01028863.1|:272-554
MsHel-3a	gb|AIXA01025055.1|:324-588
MsHel-3a	gb|AIXA01016797.1|:5806-6100
MsHel-3a	gb|AIXA01015151.1|:8579-8823
MsHel-3a	gb|AIXA01013388.1|:391-657
MsHel-3a	gb|AIXA01012584.1|:60034-60293
MsHel-3a	gb|AIXA01006987.1|:16464-16753
MsHel-3a	gb|AIXA01006013.1|:14937-15176
MsHel-3a	gb|AIXA01005804.1|:1094-1333
MsHel-3a	gb|AIXA01005136.1|:7707-7976
MsHel-3a	gb|AIXA01005111.1|:1059-1170
MsHel-3a	gb|AIXA01004319.1|:34346-34578
MsHel-3a	gb|AIXA01004009.1|:473-748
MsHel-3a	gb|AIXA01003820.1|:20420-20691
MsHel-3a	gb|AIXA01002672.1|:1863-2102
MsHel-3a	gb|AIXA01002308.1|:16567-16837
MsHel-3a	gb|AIXA01002308.1|:40705-40970
MsHel-3a	gb|AIXA01002247.1|:39-323
MsHel-3a	gb|AIXA01002231.1|:10050-10296
MsHel-3a	gb|AIXA01002230.1|:30209-30487
MsHel-3a	gb|AIXA01001650.1|:16506-16738
MsHel-3a	gb|AIXA01001529.1|:3372-3595
MsHel-3a	gb|AIXA01000991.1|:3006-3245
MsHel-3a	gb|AIXA01000170.1|:35421-35655
MsHel-3a	gb|AIXA01029739.1|:612-880
MsHel-3a	gb|AIXA01028076.1|:755-991
MsHel-3a	gb|AIXA01019370.1|:1190-1436
MsHel-3a	gb|AIXA01015117.1|:8222-8459
MsHel-3a	gb|AIXA01013782.1|:16912-17034
MsHel-3a	gb|AIXA01011267.1|:6426-6714
MsHel-3a	gb|AIXA01010660.1|:2863-3130
MsHel-3a	gb|AIXA01010197.1|:34758-35064
MsHel-3a	gb|AIXA01009506.1|:809-1071
MsHel-3a	gb|AIXA01009296.1|:26880-27133
MsHel-3a	gb|AIXA01009124.1|:8-103
MsHel-3a	gb|AIXA01008894.1|:17814-18103
MsHel-3a	gb|AIXA01008195.1|:8140-8415
MsHel-3a	gb|AIXA01008089.1|:1596-1849
MsHel-3a	gb|AIXA01006925.1|:13576-13865
MsHel-3a	gb|AIXA01006797.1|:51366-51606
MsHel-3a	gb|AIXA01006174.1|:23334-23631
MsHel-3a	gb|AIXA01006003.1|:2130-2366
MsHel-3a	gb|AIXA01005679.1|:7018-7260
MsHel-3a	gb|AIXA01005217.1|:30422-31399
MsHel-3a	gb|AIXA01005071.1|:40050-40330

MsHel-3a	gb|AIXA01004267.1|:3074-3349
MsHel-3a	gb|AIXA01003836.1|:2335-2587
MsHel-3a	gb|AIXA01003724.1|:356-593
MsHel-3a	gb|AIXA01003245.1|:4152-4417
MsHel-3a	gb|AIXA01000422.1|:20122-20407
MsHel-3a	gb|AIXA01000161.1|:25254-25490
MsHel-3a	gb|AIXA01022445.1|:1460-1697
MsHel-3a	gb|AIXA01018719.1|:4506-4740
MsHel-3a	gb|AIXA01017996.1|:2456-2699
MsHel-3a	gb|AIXA01012204.1|:1021-1309
MsHel-3a	gb|AIXA01005448.1|:42306-42534
MsHel-3a	gb|AIXA01005397.1|:2260-2485
MsHel-3a	gb|AIXA01004533.1|:15718-15998
MsHel-3a	gb|AIXA01004304.1|:31525-31794
MsHel-3a	gb|AIXA01003884.1|:22838-23074
MsHel-3a	gb|AIXA01003532.1|:7012-7185
MsHel-3a	gb|AIXA01001242.1|:9749-10019
MsHel-3a	gb|AIXA01001108.1|:40110-40393
MsHel-3a	gb|AIXA01000884.1|:16327-16552
MsHel-3a	gb|AIXA01000204.1|:23821-24059
MsHel-3a	gb|AIXA01000182.1|:3876-4134
MsHel-3a	gb|AIXA01000095.1|:86845-87126
MsHel-3a	gb|AIXA01031327.1|:516-787
MsHel-3a	gb|AIXA01024798.1|:486-753
MsHel-3a	gb|AIXA01021508.1|:637-755
MsHel-3a	gb|AIXA01016743.1|:1514-1736
MsHel-3a	gb|AIXA01012692.1|:5483-5772
MsHel-3a	gb|AIXA01012017.1|:49014-49281
MsHel-3a	gb|AIXA01011552.1|:6476-6733
MsHel-3a	gb|AIXA01010588.1|:6503-6776
MsHel-3a	gb|AIXA01010298.1|:7780-8029
MsHel-3a	gb|AIXA01010153.1|:4418-4600
MsHel-3a	gb|AIXA01009655.1|:10-252
MsHel-3a	gb|AIXA01008738.1|:4126-4351
MsHel-3a	gb|AIXA01008212.1|:93471-93755
MsHel-3a	gb|AIXA01007633.1|:4207-4447
MsHel-3a	gb|AIXA01007041.1|:13165-13409
MsHel-3a	gb|AIXA01006904.1|:11496-11768
MsHel-3a	gb|AIXA01006208.1|:2221-2494
MsHel-3a	gb|AIXA01006045.1|:2044-2281
MsHel-3a	gb|AIXA01005642.1|:51126-51369
MsHel-3a	gb|AIXA01005530.1|:25526-25736
MsHel-3a	gb|AIXA01005336.1|:11310-11537
MsHel-3a	gb|AIXA01004711.1|:6865-7108
MsHel-3a	gb|AIXA01004632.1|:6051-6267
MsHel-3a	gb|AIXA01004112.1|:22565-22851
MsHel-3a	gb|AIXA01004068.1|:49699-49944
MsHel-3a	gb|AIXA01003994.1|:4480-4766

MsHel-3a	gb|AIXA01002796.1|:12875-13164
MsHel-3a	gb|AIXA01002789.1|:4752-4989
MsHel-3a	gb|AIXA01002753.1|:67368-67481
MsHel-3a	gb|AIXA01002509.1|:22861-23105
MsHel-3a	gb|AIXA01002005.1|:44275-45328
MsHel-3a	gb|AIXA01001288.1|:38756-38876
MsHel-3a	gb|AIXA01000908.1|:8305-8592
MsHel-3a	gb|AIXA01000908.1|:38124-38240
MsHel-3a	gb|AIXA01000194.1|:7714-7951
MsHel-3a	gb|AIXA01000040.1|:946-1225
MsHel-3a	gb|AIXA01036390.1|:3-263
MsHel-3a	gb|AIXA01024241.1|:1224-1337
MsHel-3a	gb|AIXA01015393.1|:3957-4160
MsHel-3a	gb|AIXA01013778.1|:1504-1769
MsHel-3a	gb|AIXA01013663.1|:9342-9462
MsHel-3a	gb|AIXA01011964.1|:67642-67934
MsHel-3a	gb|AIXA01009139.1|:3493-3732
MsHel-3a	gb|AIXA01008862.1|:13375-13646
MsHel-3a	gb|AIXA01007713.1|:22779-23013
MsHel-3a	gb|AIXA01006193.1|:2785-3027
MsHel-3a	gb|AIXA01004160.1|:2066-2294
MsHel-3a	gb|AIXA01004160.1|:48067-48320
MsHel-3a	gb|AIXA01002390.1|:74365-74471
MsHel-3a	gb|AIXA01001766.1|:8258-8350
MsHel-3a	gb|AIXA01001359.1|:11865-12097
MsHel-3a	gb|AIXA01000078.1|:8128-8405
MsHel-3a	gb|AIXA01000048.1|:104772-105034
MsHel-3a	gb|AIXA01028190.1|:836-950
MsHel-3a	gb|AIXA01026039.1|:182-404
MsHel-3a	gb|AIXA01011501.1|:43755-44005
MsHel-3a	gb|AIXA01010656.1|:19837-20074
MsHel-3a	gb|AIXA01010656.1|:77362-77619
MsHel-3a	gb|AIXA01010480.1|:3531-3821
MsHel-3a	gb|AIXA01009094.1|:13026-13138
MsHel-3a	gb|AIXA01008465.1|:9608-9836
MsHel-3a	gb|AIXA01006514.1|:50881-51124
MsHel-3a	gb|AIXA01005867.1|:5411-5490
MsHel-3a	gb|AIXA01005331.1|:8486-8725
MsHel-3a	gb|AIXA01005292.1|:7118-7358
MsHel-3a	gb|AIXA01005036.1|:11565-11780
MsHel-3a	gb|AIXA01004849.1|:12811-13055
MsHel-3a	gb|AIXA01004715.1|:14924-15150
MsHel-3a	gb|AIXA01003859.1|:3698-3954
MsHel-3a	gb|AIXA01003823.1|:22117-22345
MsHel-3a	gb|AIXA01003548.1|:7695-7935
MsHel-3a	gb|AIXA01002537.1|:12249-12491
MsHel-3a	gb|AIXA01002029.1|:38335-38426
MsHel-3a	gb|AIXA01001843.1|:17227-17510

MsHel-3a	gb|AIXA01018232.1|:18-274
MsHel-3a	gb|AIXA01014879.1|:442-711
MsHel-3a	gb|AIXA01013295.1|:31229-31465
MsHel-3a	gb|AIXA01011652.1|:5414-5695
MsHel-3a	gb|AIXA01010786.1|:12670-12831
MsHel-3a	gb|AIXA01010775.1|:5849-6100
MsHel-3a	gb|AIXA01008934.1|:3096-3385
MsHel-3a	gb|AIXA01007585.1|:1965-2190
MsHel-3a	gb|AIXA01004920.1|:2711-2948
MsHel-3a	gb|AIXA01004391.1|:62097-62326
MsHel-3a	gb|AIXA01003139.1|:53147-53374
MsHel-3a	gb|AIXA01036964.1|:19-137
MsHel-3a	gb|AIXA01030404.1|:178-292
MsHel-3a	gb|AIXA01025313.1|:618-882
MsHel-3a	gb|AIXA01020239.1|:1911-2138
MsHel-3a	gb|AIXA01013611.1|:4480-4594
MsHel-3a	gb|AIXA01012446.1|:1122-1354
MsHel-3a	gb|AIXA01011654.1|:3596-3714
MsHel-3a	gb|AIXA01010124.1|:1287-1567
MsHel-3a	gb|AIXA01009546.1|:2262-2501
MsHel-3a	gb|AIXA01007595.1|:35459-35688
MsHel-3a	gb|AIXA01003616.1|:10844-11140
MsHel-3a	gb|AIXA01001666.1|:60865-60983
MsHel-3a	gb|AIXA01001279.1|:1160-1260
MsHel-3a	gb|AIXA01031419.1|:598-709
MsHel-3a	gb|AIXA01014970.1|:1489-1715
MsHel-3a	gb|AIXA01012190.1|:10192-10474
MsHel-3a	gb|AIXA01011516.1|:749-1000
MsHel-3a	gb|AIXA01011108.1|:5070-5347
MsHel-3a	gb|AIXA01005101.1|:620-825
MsHel-3a	gb|AIXA01001968.1|:58508-58607
MsHel-3a	gb|AIXA01030003.1|:594-862
MsHel-3a	gb|AIXA01023688.1|:225-342
MsHel-3a	gb|AIXA01022897.1|:145-260
MsHel-3a	gb|AIXA01018746.1|:1218-1454
MsHel-3a	gb|AIXA01014980.1|:6710-6943
MsHel-3a	gb|AIXA01014680.1|:7004-7206
MsHel-3a	gb|AIXA01010180.1|:24790-24997
MsHel-3a	gb|AIXA01009954.1|:2376-2597
MsHel-3a	gb|AIXA01006950.1|:345-573
MsHel-3a	gb|AIXA01006185.1|:7000-7263
MsHel-3a	gb|AIXA01004683.1|:28833-28949
MsHel-3a	gb|AIXA01004250.1|:13169-13279
MsHel-3a	gb|AIXA01004250.1|:36718-36954
MsHel-3a	gb|AIXA01003721.1|:11125-11391
MsHel-3a	gb|AIXA01002502.1|:2892-3377
MsHel-3a	gb|AIXA01034676.1|:354-627
MsHel-3a	gb|AIXA01011733.1|:52756-53010

MsHel-3a	gb|AIXA01003292.1|:20109-20310
MsHel-3a	gb|AIXA01001754.1|:24513-24749
MsHel-3a	gb|AIXA01018849.1|:2322-2534
MsHel-3a	gb|AIXA01011179.1|:3605-3850
MsHel-3a	gb|AIXA01007070.1|:9298-9519
MsHel-3a	gb|AIXA01002642.1|:9621-9833
MsHel-3a	gb|AIXA01021112.1|:258-519
MsHel-3a	gb|AIXA01018235.1|:1-223
MsHel-3a	gb|AIXA01010482.1|:10222-10470
MsHel-3a	gb|AIXA01008104.1|:29152-29247
MsHel-3a	gb|AIXA01002620.1|:194-283
MsHel-3a	gb|AIXA01000906.1|:5956-6073
MsHel-3a	gb|AIXA01034485.1|:72-168
MsHel-3a	gb|AIXA01017483.1|:6173-6382
MsHel-3a	gb|AIXA01007538.1|:40342-40531
MsHel-3a	gb|AIXA01007373.1|:8997-9235
MsHel-3a	gb|AIXA01004587.1|:4269-4513
MsHel-3a	gb|AIXA01032371.1|:312-541
MsHel-3a	gb|AIXA01028637.1|:5-114
MsHel-3a	gb|AIXA01018977.1|:1645-1860
MsHel-3a	gb|AIXA01014394.1|:16296-16552
MsHel-3a	gb|AIXA01013941.1|:8599-8709
MsHel-3a	gb|AIXA01011104.1|:2396-2558
MsHel-3a	gb|AIXA01009981.1|:4009-4281
MsHel-3a	gb|AIXA01009156.1|:5635-5850
MsHel-3a	gb|AIXA01008509.1|:59918-60027
MsHel-3a	gb|AIXA01005478.1|:14299-14534
MsHel-3a	gb|AIXA01004317.1|:1601-1701
MsHel-3a	gb|AIXA01032050.1|:396-656
MsHel-3a	gb|AIXA01026082.1|:3-85
MsHel-3a	gb|AIXA01016546.1|:704-949
MsHel-3a	gb|AIXA01012103.1|:13455-13660
MsHel-3a	gb|AIXA01010736.1|:15017-15145
MsHel-3a	gb|AIXA01009119.1|:17380-17505
MsHel-3a	gb|AIXA01008306.1|:26350-26621
MsHel-3a	gb|AIXA01004807.1|:7263-7346
MsHel-3a	gb|AIXA01003578.1|:34790-35073
MsHel-3a	gb|AIXA01001280.1|:8-90
MsHel-3a	gb|AIXA01018361.1|:855-970
MsHel-3a	gb|AIXA01009095.1|:1-660
MsHel-3a	gb|AIXA01008873.1|:18406-18632
MsHel-3a	gb|AIXA01008579.1|:193-440
MsHel-3a	gb|AIXA01006202.1|:25267-25466
MsHel-3a	gb|AIXA01004092.1|:8457-8555
MsHel-3a	gb|AIXA01003838.1|:24973-25204
MsHel-3a	gb|AIXA01003611.1|:3677-3908
MsHel-3a	gb|AIXA01002245.1|:16946-17178
MsHel-3a	gb|AIXA01012231.1|:82664-82924

MsHel-3a	gb|AIXA01011329.1|:1986-2080
MsHel-3a	gb|AIXA01003142.1|:25946-26154
MsHel-3a	gb|AIXA01000878.1|:8603-8821
MsHel-3a	gb|AIXA01031340.1|:1-212
MsHel-3a	gb|AIXA01017167.1|:4097-4341
MsHel-3a	gb|AIXA01003847.1|:6643-6889
MsHel-3a	gb|AIXA01015511.1|:2082-2313
MsHel-3a	gb|AIXA01007630.1|:36763-36867
MsHel-3a	gb|AIXA01004589.1|:3677-3947
MsHel-3a	gb|AIXA01001298.1|:1917-2143
MsHel-3a	gb|AIXA01033663.1|:467-598
MsHel-3a	gb|AIXA01008047.1|:6506-6714
MsHel-3a	gb|AIXA01006930.1|:6097-6202
MsHel-3a	gb|AIXA01000062.1|:34-270
MsHel-3a	gb|AIXA01011369.1|:34482-34657
MsHel-3a	gb|AIXA01010050.1|:5154-5356
MsHel-3a	gb|AIXA01008246.1|:2088-2305
MsHel-3a	gb|AIXA01002994.1|:45775-45993
MsHel-3a	gb|AIXA01036455.1|:82-305
MsHel-3a	gb|AIXA01003043.1|:8630-8865
MsHel-3a	gb|AIXA01026978.1|:903-992
MsHel-3a	gb|AIXA01001837.1|:13856-14057
MsHel-3a	gb|AIXA01014040.1|:12819-12909
MsHel-3a	gb|AIXA01000960.1|:169353-169589
MsHel-3a	gb|AIXA01026347.1|:298-566
MsHel-3a	gb|AIXA01000014.1|:9109-9343
MsHel-4	gb|AIXA01013916.1|:7937-8475
MsHel-4	gb|AIXA01002626.1|:21745-22289
MsHel-4	gb|AIXA01007957.1|:18681-19222
MsHel-4	gb|AIXA01001234.1|:13677-14219
MsHel-4	gb|AIXA01012652.1|:16438-16981
MsHel-4	gb|AIXA01006419.1|:1464-1999
MsHel-4	gb|AIXA01000337.1|:64764-65316
MsHel-4	gb|AIXA01012704.1|:1104-1637
MsHel-4	gb|AIXA01004105.1|:6857-7400
MsHel-4	gb|AIXA01001492.1|:15998-16543
MsHel-4	gb|AIXA01010342.1|:12844-13386
MsHel-4	gb|AIXA01009285.1|:4028-4564
MsHel-4	gb|AIXA01013754.1|:30557-31090
MsHel-4	gb|AIXA01003045.1|:24582-24920
MsHel-4	gb|AIXA01018186.1|:63-519
MsHel-4	gb|AIXA01000460.1|:10331-10856
MsHel-4	gb|AIXA01006382.1|:71296-71800
MsHel-4	gb|AIXA01016448.1|:662-1187
MsHel-4	gb|AIXA01012284.1|:47808-48177
MsHel-4	gb|AIXA01007860.1|:15600-16047
MsHel-4	gb|AIXA01000061.1|:13163-13696

MsHel-4	gb|AIXA01004836.1|:16235-16749
MsHel-4	gb|AIXA01012741.1|:46951-47704
MsHel-4	gb|AIXA01021166.1|:1422-1954
MsHel-4	gb|AIXA01009838.1|:572-1102
MsHel-4	gb|AIXA01014930.1|:1663-2079
MsHel-4	gb|AIXA01010253.1|:43135-43549
MsHel-4	gb|AIXA01001314.1|:1096-1507
MsHel-4	gb|AIXA01003316.1|:10159-11865
MsHel-4	gb|AIXA01011104.1|:26762-27184
MsHel-4	gb|AIXA01009593.1|:5902-6353
MsHel-4	gb|AIXA01015457.1|:14865-15265
MsHel-4	gb|AIXA01003661.1|:15274-15675
MsHel-4	gb|AIXA01003112.1|:4605-5034
MsHel-4	gb|AIXA01002641.1|:118-530
MsHel-4	gb|AIXA01008773.1|:32810-33210
MsHel-4	gb|AIXA01016603.1|:4023-4425
MsHel-4	gb|AIXA01012130.1|:19153-19563
MsHel-4	gb|AIXA01007259.1|:17744-18090
MsHel-4	gb|AIXA01005548.1|:2077-2532
MsHel-4	gb|AIXA01002531.1|:18822-19219
MsHel-4	gb|AIXA01003595.1|:46653-47027
MsHel-4	gb|AIXA01008406.1|:64984-65346
MsHel-4	gb|AIXA01008406.1|:192572-192982
MsHel-4	gb|AIXA01016438.1|:3464-3865
MsHel-4	gb|AIXA01012649.1|:9903-10311
MsHel-4	gb|AIXA01037975.1|:107-511
MsHel-4	gb|AIXA01012470.1|:3739-4153
MsHel-4	gb|AIXA01011635.1|:11310-11722
MsHel-4	gb|AIXA01004548.1|:29345-29755
MsHel-4	gb|AIXA01010402.1|:3819-4243
MsHel-4	gb|AIXA01000437.1|:93944-94324
MsHel-4	gb|AIXA01000198.1|:13777-14187
MsHel-4	gb|AIXA01008865.1|:2870-3286
MsHel-4	gb|AIXA01001605.1|:8217-8620
MsHel-4	gb|AIXA01010881.1|:82598-83123
MsHel-4	gb|AIXA01007205.1|:59755-60161
MsHel-4	gb|AIXA01012398.1|:3242-3656
MsHel-4	gb|AIXA01012398.1|:35586-35924
MsHel-4	gb|AIXA01015600.1|:6825-7234
MsHel-4	gb|AIXA01017387.1|:17-422
MsHel-4	gb|AIXA01002350.1|:43187-43596
MsHel-4	gb|AIXA01002350.1|:122267-122605
MsHel-4	gb|AIXA01011197.1|:89597-89935
MsHel-4	gb|AIXA01003273.1|:22066-22459
MsHel-4	gb|AIXA01009177.1|:124065-124580
MsHel-4	gb|AIXA01014929.1|:1393-1803
MsHel-4	gb|AIXA01007493.1|:68227-68637
MsHel-4	gb|AIXA01002427.1|:53562-53899

MsHel-4	gb|AIXA01001865.1|:8488-8883
MsHel-4	gb|AIXA01006809.1|:10858-11267
MsHel-4	gb|AIXA01035184.1|:343-609
MsHel-4	gb|AIXA01010468.1|:364-778
MsHel-4	gb|AIXA01010185.1|:19299-19776
MsHel-4	gb|AIXA01023050.1|:558-955
MsHel-4	gb|AIXA01012994.1|:6810-7212
MsHel-4	gb|AIXA01011449.1|:15504-15751
MsHel-4	gb|AIXA01004632.1|:1648-2042
MsHel-4	gb|AIXA01009826.1|:14352-14665
MsHel-4	gb|AIXA01000684.1|:7840-8206
MsHel-4	gb|AIXA01020407.1|:930-1324
MsHel-4	gb|AIXA01005505.1|:13233-13620
MsHel-4	gb|AIXA01003968.1|:3031-3427
MsHel-4	gb|AIXA01012901.1|:25410-25656
MsHel-4	gb|AIXA01033299.1|:435-690
MsHel-4	gb|AIXA01009217.1|:519-911
MsHel-4	gb|AIXA01032843.1|:1-204
MsHel-4	gb|AIXA01014724.1|:3474-3818
MsHel-4	gb|AIXA01011450.1|:19039-29185
MsHel-4	gb|AIXA01014236.1|:54-440
MsHel-4	gb|AIXA01034922.1|:10-216
MsHel-4	gb|AIXA01003222.1|:44014-44268
MsHel-4	gb|AIXA01011033.1|:2311-2499
MsHel-4	gb|AIXA01008307.1|:8684-9100
MsHel-4	gb|AIXA01001251.1|:15217-15406
MsHel-4	gb|AIXA01022114.1|:1572-1777
MsHel-4	gb|AIXA01023438.1|:1136-1418
MsHel-4	gb|AIXA01002579.1|:87146-87436
MsHel-4	gb|AIXA01016545.1|:2679-2948
MsHel-4	gb|AIXA01037539.1|:233-522
MsHel-4	gb|AIXA01020052.1|:1096-1372
MsHel-4	gb|AIXA01002679.1|:65150-65432
MsHel-4	gb|AIXA01017868.1|:11559-11820
MsHel-4	gb|AIXA01002807.1|:12667-12926
MsHel-4	gb|AIXA01001526.1|:16045-16240
MsHel-4	gb|AIXA01013094.1|:1-242
MsHel-4	gb|AIXA01012412.1|:11555-11849
MsHel-4	gb|AIXA01017706.1|:4091-4429
MsHel-4	gb|AIXA01015097.1|:3412-3750
MsHel-4	gb|AIXA01011432.1|:2520-2858
MsHel-4	gb|AIXA01010802.1|:29916-30254
MsHel-4	gb|AIXA01009976.1|:59012-59350
MsHel-4	gb|AIXA01009786.1|:21466-21804
MsHel-4	gb|AIXA01009104.1|:4493-4831
MsHel-4	gb|AIXA01008227.1|:2063-2235
MsHel-4	gb|AIXA01008220.1|:18791-19093
MsHel-4	gb|AIXA01006136.1|:39941-40279

MsHel-4	gb|AIXA01004852.1|:72459-72771
MsHel-4	gb|AIXA01004164.1|:25408-25746
MsHel-4	gb|AIXA01003653.1|:10437-10775
MsHel-4	gb|AIXA01002253.1|:1300-1638
MsHel-4	gb|AIXA01001990.1|:93144-93482
MsHel-4	gb|AIXA01001544.1|:17299-17637
MsHel-4	gb|AIXA01001533.1|:2052-2390
MsHel-4	gb|AIXA01001243.1|:164189-164527
MsHel-4	gb|AIXA01000740.1|:13686-14024
MsHel-4	gb|AIXA01000459.1|:27350-27688
MsHel-4	gb|AIXA01000242.1|:38748-39046
MsHel-4	gb|AIXA01000048.1|:68046-68384
MsHel-4	gb|AIXA01009184.1|:3439-3831
MsHel-4	gb|AIXA01029674.1|:4-152
MsHel-4	gb|AIXA01017976.1|:5-312
MsHel-4	gb|AIXA01016262.1|:10063-10346
MsHel-4	gb|AIXA01011318.1|:2563-2901
MsHel-4	gb|AIXA01010747.1|:8687-9025
MsHel-4	gb|AIXA01009108.1|:15711-16047
MsHel-4	gb|AIXA01008395.1|:1889-2227
MsHel-4	gb|AIXA01006702.1|:10779-11117
MsHel-4	gb|AIXA01005224.1|:13014-13352
MsHel-4	gb|AIXA01005047.1|:24714-25028
MsHel-4	gb|AIXA01004484.1|:64380-64718
MsHel-4	gb|AIXA01004324.1|:14646-14984
MsHel-4	gb|AIXA01004087.1|:10271-10609
MsHel-4	gb|AIXA01002657.1|:6144-6482
MsHel-4	gb|AIXA01001961.1|:1962-2300
MsHel-4	gb|AIXA01000721.1|:31-369
MsHel-4	gb|AIXA01000501.1|:32549-32887
MsHel-4	gb|AIXA01000246.1|:16123-16463
MsHel-4	gb|AIXA01009123.1|:3990-4258
MsHel-4	gb|AIXA01011244.1|:4215-4554
MsHel-4	gb|AIXA01001930.1|:16592-16931
MsHel-4	gb|AIXA01006708.1|:22579-22893
MsHel-4	gb|AIXA01006655.1|:39667-40004
MsHel-4	gb|AIXA01005083.1|:63880-64218
MsHel-4	gb|AIXA01003270.1|:75213-75551
MsHel-4	gb|AIXA01001536.1|:8487-8825
MsHel-4	gb|AIXA01001152.1|:50493-50831
MsHel-4	gb|AIXA01004933.1|:12363-12701
MsHel-4	gb|AIXA01002885.1|:3973-4310
MsHel-4	gb|AIXA01002339.1|:53291-53629
MsHel-4	gb|AIXA01008529.1|:13336-13497
MsHel-4	gb|AIXA01010591.1|:7830-8171
MsHel-4	gb|AIXA01007286.1|:9960-10238
MsHel-4	gb|AIXA01002164.1|:19229-19510
MsHel-4	gb|AIXA01008428.1|:1-254

MsHel-4	gb|AIXA01014243.1|:11464-11747
MsHel-4	gb|AIXA01010406.1|:42918-43199
MsHel-4	gb|AIXA01002172.1|:83766-84096
MsHel-4	gb|AIXA01033316.1|:1-119
MsHel-4	gb|AIXA01024776.1|:1-177
MsHel-4	gb|AIXA01014564.1|:1-224
MsHel-4	gb|AIXA01008970.1|:6-166
MsHel-4	gb|AIXA01005489.1|:26-148
MsHel-4	gb|AIXA01000473.1|:5932-6114
MsHel-4	gb|AIXA01015929.1|:1-304
MsHel-4	gb|AIXA01004999.1|:10648-10813
MsHel-4	gb|AIXA01004277.1|:32791-32965
MsHel-4	gb|AIXA01002344.1|:17-139
MsHel-4	gb|AIXA01011458.1|:1-123
MsHel-4	gb|AIXA01004400.1|:52198-52312
MsHel-4	gb|AIXA01004307.1|:18-208
MsHel-4	gb|AIXA01017869.1|:49-209
MsHel-4	gb|AIXA01012626.1|:1748-1842
MsHel-4	gb|AIXA01027694.1|:922-1018
MsHel-4	gb|AIXA01001506.1|:1-94
MsHel-4	gb|AIXA01007088.1|:1-81
MsHel-4	gb|AIXA01000594.1|:1-81
MsHel-4	gb|AIXA01002564.1|:1906-1997
MsHel-4	gb|AIXA01004378.1|:6286-6371
MsHel-4	gb|AIXA01002982.1|:16005-16166
MsHel-4	gb|AIXA01005882.1|:5547-5637
MsHel-4	gb|AIXA01001414.1|:14733-14824
MsHel-4	gb|AIXA01007813.1|:15087-15161
MsHel-4	gb|AIXA01011682.1|:11026-11112
MsHel-5	gb|AIXA01001643.1|:26060-26357
MsHel-5	gb|AIXA01020406.1|:732-1027
MsHel-5	gb|AIXA01004928.1|:31172-31469
MsHel-5	gb|AIXA01008947.1|:9836-10132
MsHel-5	gb|AIXA01005034.1|:114097-114393
MsHel-5	gb|AIXA01014862.1|:8938-9233
MsHel-5	gb|AIXA01000969.1|:36630-36924
MsHel-5	gb|AIXA01002558.1|:33692-33989
MsHel-5	gb|AIXA01002521.1|:19368-19664
MsHel-5	gb|AIXA01014900.1|:26427-26721
MsHel-5	gb|AIXA01012531.1|:878-1172
MsHel-5	gb|AIXA01009162.1|:200405-200710
MsHel-5	gb|AIXA01000698.1|:25574-25872
MsHel-5	gb|AIXA01014050.1|:1579-1872
MsHel-5	gb|AIXA01009235.1|:2643-2939
MsHel-5	gb|AIXA01007371.1|:10989-11286
MsHel-5	gb|AIXA01005082.1|:27698-27995
MsHel-5	gb|AIXA01001631.1|:4593-4785

MsHel-5	gb|AIXA01000024.1|:12802-12988
MsHel-5	gb|AIXA01002579.1|:73315-73611
MsHel-5	gb|AIXA01026189.1|:170-463
MsHel-5	gb|AIXA01017227.1|:1057-1354
MsHel-5	gb|AIXA01003846.1|:52899-53196
MsHel-5	gb|AIXA01003846.1|:70946-71133
MsHel-5	gb|AIXA01000443.1|:328-624
MsHel-5	gb|AIXA01009650.1|:8204-8500
MsHel-5	gb|AIXA01000809.1|:11359-11655
MsHel-5	gb|AIXA01000809.1|:55710-55895
MsHel-5	gb|AIXA01012350.1|:13215-13508
MsHel-5	gb|AIXA01011987.1|:16020-16413
MsHel-5	gb|AIXA01004289.1|:4752-5050
MsHel-5	gb|AIXA01014020.1|:16048-16333
MsHel-5	gb|AIXA01009602.1|:25522-25820
MsHel-5	gb|AIXA01019684.1|:431-723
MsHel-5	gb|AIXA01008415.1|:16234-16530
MsHel-5	gb|AIXA01006730.1|:3708-4004
MsHel-5	gb|AIXA01003943.1|:2715-3011
MsHel-5	gb|AIXA01004337.1|:30989-31284
MsHel-5	gb|AIXA01004337.1|:52798-52995
MsHel-5	gb|AIXA01003728.1|:8597-8893
MsHel-5	gb|AIXA01017540.1|:4684-4978
MsHel-5	gb|AIXA01012797.1|:8834-9128
MsHel-5	gb|AIXA01009028.1|:27487-27781
MsHel-5	gb|AIXA01006671.1|:3042-3334
MsHel-5	gb|AIXA01005441.1|:210-502
MsHel-5	gb|AIXA01005435.1|:891-1185
MsHel-5	gb|AIXA01005435.1|:16436-16729
MsHel-5	gb|AIXA01003528.1|:29795-30088
MsHel-5	gb|AIXA01000791.1|:7378-7672
MsHel-5	gb|AIXA01018288.1|:3095-3279
MsHel-5	gb|AIXA01013941.1|:3762-4051
MsHel-5	gb|AIXA01012198.1|:17348-17614
MsHel-5	gb|AIXA01012198.1|:27934-28171
MsHel-5	gb|AIXA01007966.1|:31897-32149
MsHel-5	gb|AIXA01007966.1|:60180-60474
MsHel-5	gb|AIXA01007966.1|:95776-95970
MsHel-5	gb|AIXA01002244.1|:70226-70524
MsHel-5	gb|AIXA01012932.1|:363-659
MsHel-5	gb|AIXA01010109.1|:33746-34041
MsHel-5	gb|AIXA01009601.1|:3614-3905
MsHel-5	gb|AIXA01008645.1|:1957-2252
MsHel-5	gb|AIXA01004968.1|:15781-16077
MsHel-5	gb|AIXA01002620.1|:5854-6143
MsHel-5	gb|AIXA01001548.1|:15211-15506
MsHel-5	gb|AIXA01017045.1|:4250-4544
MsHel-5	gb|AIXA01014211.1|:586-880

MsHel-5	gb|AIXA01010332.1|:5862-6156
MsHel-5	gb|AIXA01007968.1|:14768-15058
MsHel-5	gb|AIXA01007968.1|:31247-31541
MsHel-5	gb|AIXA01006684.1|:9260-9554
MsHel-5	gb|AIXA01001846.1|:21084-21378
MsHel-5	gb|AIXA01018211.1|:3601-3899
MsHel-5	gb|AIXA01014022.1|:9458-9655
MsHel-5	gb|AIXA01006908.1|:23888-24182
MsHel-5	gb|AIXA01001571.1|:5507-5811
MsHel-5	gb|AIXA01000995.1|:2774-3066
MsHel-5	gb|AIXA01000909.1|:67321-67610
MsHel-5	gb|AIXA01012361.1|:13923-14225
MsHel-5	gb|AIXA01027625.1|:248-551
MsHel-5	gb|AIXA01014743.1|:457-751
MsHel-5	gb|AIXA01012119.1|:40981-41272
MsHel-5	gb|AIXA01009125.1|:197-500
MsHel-5	gb|AIXA01008570.1|:13120-13402
MsHel-5	gb|AIXA01005103.1|:1452-1745
MsHel-5	gb|AIXA01026483.1|:469-762
MsHel-5	gb|AIXA01012351.1|:2479-2770
MsHel-5	gb|AIXA01001831.1|:24044-24342
MsHel-5	gb|AIXA01001750.1|:6860-7145
MsHel-5	gb|AIXA01001050.1|:60083-60381
MsHel-5	gb|AIXA01000622.1|:19003-19296
MsHel-5	gb|AIXA01012694.1|:6244-6541
MsHel-5	gb|AIXA01012096.1|:5626-5917
MsHel-5	gb|AIXA01011293.1|:10284-10575
MsHel-5	gb|AIXA01003949.1|:18463-18759
MsHel-5	gb|AIXA01002466.1|:34418-34717
MsHel-5	gb|AIXA01013942.1|:5242-5529
MsHel-5	gb|AIXA01003332.1|:24471-24664
MsHel-5	gb|AIXA01003332.1|:100484-100786
MsHel-5	gb|AIXA01000891.1|:29557-29861
MsHel-5	gb|AIXA01003011.1|:15445-15724
MsHel-5	gb|AIXA01001848.1|:660-954
MsHel-5	gb|AIXA01000618.1|:65384-65659
MsHel-5	gb|AIXA01000188.1|:65599-65874
MsHel-5	gb|AIXA01015509.1|:7403-7705
MsHel-5	gb|AIXA01011326.1|:5598-5896
MsHel-5	gb|AIXA01003353.1|:27379-27572
MsHel-5	gb|AIXA01004389.1|:14518-14812
MsHel-5	gb|AIXA01003002.1|:31252-31450
MsHel-5	gb|AIXA01003002.1|:98705-99006
MsHel-5	gb|AIXA01014764.1|:2079-2364
MsHel-5	gb|AIXA01010573.1|:4117-4417
MsHel-5	gb|AIXA01002632.1|:10-174
MsHel-5	gb|AIXA01000051.1|:442-741
MsHel-5	gb|AIXA01027457.1|:416-718

MsHel-5	gb|AIXA01014420.1|:5647-5949
MsHel-5	gb|AIXA01014397.1|:2091-2381
MsHel-5	gb|AIXA01009796.1|:28731-28923
MsHel-5	gb|AIXA01009591.1|:12173-12468
MsHel-5	gb|AIXA01009151.1|:4734-5036
MsHel-5	gb|AIXA01007267.1|:8157-8459
MsHel-5	gb|AIXA01014214.1|:3567-3856
MsHel-5	gb|AIXA01014884.1|:42787-43080
MsHel-5	gb|AIXA01012741.1|:78529-78832
MsHel-5	gb|AIXA01008211.1|:5248-5548
MsHel-5	gb|AIXA01022324.1|:777-1080
MsHel-5	gb|AIXA01014372.1|:8256-8453
MsHel-5	gb|AIXA01014372.1|:45995-47342
MsHel-5	gb|AIXA01012657.1|:20722-21025
MsHel-5	gb|AIXA01000486.1|:83518-83812
MsHel-5	gb|AIXA01015151.1|:62068-62257
MsHel-5	gb|AIXA01013945.1|:10557-10850
MsHel-5	gb|AIXA01013782.1|:10117-10408
MsHel-5	gb|AIXA01013782.1|:21772-22064
MsHel-5	gb|AIXA01011547.1|:16135-16437
MsHel-5	gb|AIXA01008023.1|:42998-43225
MsHel-5	gb|AIXA01006067.1|:10579-10872
MsHel-5	gb|AIXA01005504.1|:69999-70301
MsHel-5	gb|AIXA01005424.1|:57149-57450
MsHel-5	gb|AIXA01003007.1|:45865-46148
MsHel-5	gb|AIXA01002069.1|:25575-25875
MsHel-5	gb|AIXA01001758.1|:21092-21394
MsHel-5	gb|AIXA01001758.1|:145242-145544
MsHel-5	gb|AIXA01034905.1|:331-619
MsHel-5	gb|AIXA01015915.1|:3102-3403
MsHel-5	gb|AIXA01014611.1|:10354-10651
MsHel-5	gb|AIXA01012153.1|:11683-11984
MsHel-5	gb|AIXA01011493.1|:17595-17892
MsHel-5	gb|AIXA01006204.1|:4873-5174
MsHel-5	gb|AIXA01004941.1|:24901-25179
MsHel-5	gb|AIXA01004941.1|:60274-60572
MsHel-5	gb|AIXA01002459.1|:14157-14457
MsHel-5	gb|AIXA01000650.1|:94818-95101
MsHel-5	gb|AIXA01024963.1|:130-420
MsHel-5	gb|AIXA01011997.1|:7314-7613
MsHel-5	gb|AIXA01010574.1|:38177-38487
MsHel-5	gb|AIXA01009697.1|:4922-5134
MsHel-5	gb|AIXA01009697.1|:22484-22673
MsHel-5	gb|AIXA01007262.1|:25554-25843
MsHel-5	gb|AIXA01004579.1|:44842-45140
MsHel-5	gb|AIXA01004425.1|:1333-1637
MsHel-5	gb|AIXA01002514.1|:35659-35964
MsHel-5	gb|AIXA01001023.1|:21793-22092

MsHel-5	gb|AIXA01014610.1|:8850-9042
MsHel-5	gb|AIXA01006865.1|:51240-51539
MsHel-5	gb|AIXA01006689.1|:23910-24197
MsHel-5	gb|AIXA01006689.1|:81980-82274
MsHel-5	gb|AIXA01005707.1|:850-1149
MsHel-5	gb|AIXA01004482.1|:3494-3788
MsHel-5	gb|AIXA01002974.1|:35309-35603
MsHel-5	gb|AIXA01002522.1|:10132-10426
MsHel-5	gb|AIXA01001614.1|:1298-1597
MsHel-5	gb|AIXA01000844.1|:43345-43645
MsHel-5	gb|AIXA01000315.1|:37848-38154
MsHel-5	gb|AIXA01014665.1|:530-832
MsHel-5	gb|AIXA01011986.1|:2798-3084
MsHel-5	gb|AIXA01011122.1|:915-1217
MsHel-5	gb|AIXA01010909.1|:56-357
MsHel-5	gb|AIXA01010909.1|:103377-103679
MsHel-5	gb|AIXA01010401.1|:8762-9064
MsHel-5	gb|AIXA01007595.1|:32666-32968
MsHel-5	gb|AIXA01006983.1|:51932-52229
MsHel-5	gb|AIXA01005521.1|:70750-71048
MsHel-5	gb|AIXA01005493.1|:56229-56531
MsHel-5	gb|AIXA01005188.1|:5540-5836
MsHel-5	gb|AIXA01004108.1|:14761-15003
MsHel-5	gb|AIXA01004108.1|:164877-165179
MsHel-5	gb|AIXA01002686.1|:2813-3115
MsHel-5	gb|AIXA01023238.1|:778-1079
MsHel-5	gb|AIXA01022669.1|:279-468
MsHel-5	gb|AIXA01013560.1|:3874-4175
MsHel-5	gb|AIXA01012634.1|:1841-2139
MsHel-5	gb|AIXA01011328.1|:5356-5647
MsHel-5	gb|AIXA01006982.1|:20815-21113
MsHel-5	gb|AIXA01005096.1|:1965-2162
MsHel-5	gb|AIXA01005096.1|:76398-76699
MsHel-5	gb|AIXA01005064.1|:2485-2786
MsHel-5	gb|AIXA01003180.1|:8989-9286
MsHel-5	gb|AIXA01002999.1|:83114-83415
MsHel-5	gb|AIXA01001939.1|:55228-55527
MsHel-5	gb|AIXA01001590.1|:17104-17401
MsHel-5	gb|AIXA01003712.1|:44057-44357
MsHel-5	gb|AIXA01011842.1|:4669-4971
MsHel-5	gb|AIXA01010111.1|:3276-3472
MsHel-5	gb|AIXA01008648.1|:754-1048
MsHel-5	gb|AIXA01007229.1|:35451-35533
MsHel-5	gb|AIXA01005514.1|:9259-9562
MsHel-5	gb|AIXA01004563.1|:12167-12470
MsHel-5	gb|AIXA01002703.1|:25367-25671
MsHel-5	gb|AIXA01019645.1|:1886-2188
MsHel-5	gb|AIXA01009366.1|:9977-10279

MsHel-5	gb|AIXA01008565.1|:47181-47474
MsHel-5	gb|AIXA01007974.1|:1721-1918
MsHel-5	gb|AIXA01007974.1|:69655-69849
MsHel-5	gb|AIXA01006845.1|:3009-3311
MsHel-5	gb|AIXA01005051.1|:76585-76886
MsHel-5	gb|AIXA01004034.1|:63256-63558
MsHel-5	gb|AIXA01004034.1|:185574-185762
MsHel-5	gb|AIXA01003015.1|:25199-25500
MsHel-5	gb|AIXA01001880.1|:1826-2121
MsHel-5	gb|AIXA01001816.1|:23060-23361
MsHel-5	gb|AIXA01000741.1|:15271-15464
MsHel-5	gb|AIXA01000741.1|:43860-44162
MsHel-5	gb|AIXA01000164.1|:21506-21810
MsHel-5	gb|AIXA01027605.1|:632-934
MsHel-5	gb|AIXA01014772.1|:3889-4190
MsHel-5	gb|AIXA01007970.1|:17419-17709
MsHel-5	gb|AIXA01006215.1|:22495-22796
MsHel-5	gb|AIXA01006215.1|:56971-57272
MsHel-5	gb|AIXA01005995.1|:15927-16228
MsHel-5	gb|AIXA01004140.1|:26822-27121
MsHel-5	gb|AIXA01000892.1|:3695-3996
MsHel-5	gb|AIXA01037775.1|:101-394
MsHel-5	gb|AIXA01017964.1|:6794-7097
MsHel-5	gb|AIXA01015475.1|:2318-2610
MsHel-5	gb|AIXA01005629.1|:44098-44397
MsHel-5	gb|AIXA01005595.1|:172877-173180
MsHel-5	gb|AIXA01003609.1|:130058-130333
MsHel-5	gb|AIXA01002510.1|:57631-57933
MsHel-5	gb|AIXA01000012.1|:78267-78571
MsHel-5	gb|AIXA01026734.1|:542-841
MsHel-5	gb|AIXA01008927.1|:29863-30166
MsHel-5	gb|AIXA01002990.1|:60624-60923
MsHel-5	gb|AIXA01002975.1|:13204-13489
MsHel-5	gb|AIXA01001642.1|:5118-5412
MsHel-5	gb|AIXA01001642.1|:27494-27586
MsHel-5	gb|AIXA01000843.1|:106532-106828
MsHel-5	gb|AIXA01000597.1|:42278-42581
MsHel-5	gb|AIXA01036271.1|:51-353
MsHel-5	gb|AIXA01016551.1|:32928-33225
MsHel-5	gb|AIXA01012412.1|:19269-19571
MsHel-5	gb|AIXA01012311.1|:1357-1658
MsHel-5	gb|AIXA01011984.1|:25095-25378
MsHel-5	gb|AIXA01010536.1|:11495-11797
MsHel-5	gb|AIXA01008424.1|:6808-7109
MsHel-5	gb|AIXA01008234.1|:22559-22860
MsHel-5	gb|AIXA01007732.1|:6472-6774
MsHel-5	gb|AIXA01002740.1|:64978-65280
MsHel-5	gb|AIXA01001514.1|:2531-2818

MsHel-5	gb|AIXA01000692.1|:82014-82313
MsHel-5	gb|AIXA01000692.1|:106790-106989
MsHel-5	gb|AIXA01030114.1|:130-431
MsHel-5	gb|AIXA01012652.1|:6822-6959
MsHel-5	gb|AIXA01012652.1|:48904-49058
MsHel-5	gb|AIXA01012398.1|:31872-32169
MsHel-5	gb|AIXA01007469.1|:14768-15069
MsHel-5	gb|AIXA01007354.1|:68967-69112
MsHel-5	gb|AIXA01006129.1|:10776-11065
MsHel-5	gb|AIXA01005685.1|:236-537
MsHel-5	gb|AIXA01001926.1|:41512-41812
MsHel-5	gb|AIXA01001328.1|:15984-16290
MsHel-5	gb|AIXA01000792.1|:40338-40532
MsHel-5	gb|AIXA01000398.1|:6818-7119
MsHel-5	gb|AIXA01037046.1|:271-542
MsHel-5	gb|AIXA01023376.1|:723-1017
MsHel-5	gb|AIXA01013690.1|:3824-4124
MsHel-5	gb|AIXA01011325.1|:5907-6192
MsHel-5	gb|AIXA01009391.1|:10733-11034
MsHel-5	gb|AIXA01004345.1|:3078-3383
MsHel-5	gb|AIXA01017916.1|:15107-15406
MsHel-5	gb|AIXA01008282.1|:34016-34316
MsHel-5	gb|AIXA01006679.1|:8652-8954
MsHel-5	gb|AIXA01002214.1|:74377-74682
MsHel-5	gb|AIXA01001885.1|:4261-4372
MsHel-5	gb|AIXA01001885.1|:30560-30859
MsHel-5	gb|AIXA01000099.1|:32516-32815
MsHel-5	gb|AIXA01018471.1|:4596-4898
MsHel-5	gb|AIXA01011331.1|:6114-6409
MsHel-5	gb|AIXA01010402.1|:1419-1721
MsHel-5	gb|AIXA01009590.1|:21585-21845
MsHel-5	gb|AIXA01009044.1|:116556-116748
MsHel-5	gb|AIXA01005256.1|:6894-7192
MsHel-5	gb|AIXA01005061.1|:7278-7579
MsHel-5	gb|AIXA01002225.1|:6568-6879
MsHel-5	gb|AIXA01002031.1|:18-316
MsHel-5	gb|AIXA01033527.1|:393-681
MsHel-5	gb|AIXA01013224.1|:2652-2939
MsHel-5	gb|AIXA01007449.1|:20235-20536
MsHel-5	gb|AIXA01006066.1|:37011-37311
MsHel-5	gb|AIXA01005387.1|:6488-6781
MsHel-5	gb|AIXA01004094.1|:5511-5811
MsHel-5	gb|AIXA01000979.1|:7186-7484
MsHel-5	gb|AIXA01000783.1|:31830-32129
MsHel-5	gb|AIXA01000394.1|:6517-6717
MsHel-5	gb|AIXA01000394.1|:61492-61793
MsHel-5	gb|AIXA01014638.1|:18529-18784
MsHel-5	gb|AIXA01009511.1|:5174-5473

MsHel-5	gb|AIXA01006595.1|:23886-24184
MsHel-5	gb|AIXA01004753.1|:22309-22611
MsHel-5	gb|AIXA01004753.1|:55267-55562
MsHel-5	gb|AIXA01000764.1|:9186-9490
MsHel-5	gb|AIXA01010867.1|:18877-19181
MsHel-5	gb|AIXA01004487.1|:1951-2253
MsHel-5	gb|AIXA01003183.1|:115768-115986
MsHel-5	gb|AIXA01000725.1|:23215-23518
MsHel-5	gb|AIXA01000448.1|:37297-37600
MsHel-5	gb|AIXA01009292.1|:52447-52644
MsHel-5	gb|AIXA01009047.1|:13882-14187
MsHel-5	gb|AIXA01008187.1|:20620-20824
MsHel-5	gb|AIXA01004295.1|:30740-31042
MsHel-5	gb|AIXA01003743.1|:43690-43982
MsHel-5	gb|AIXA01001273.1|:6567-6765
MsHel-5	gb|AIXA01000296.1|:27739-28040
MsHel-5	gb|AIXA01000173.1|:565-870
MsHel-5	gb|AIXA01018066.1|:756-1057
MsHel-5	gb|AIXA01013814.1|:15425-15729
MsHel-5	gb|AIXA01008226.1|:17171-17471
MsHel-5	gb|AIXA01007023.1|:94396-94697
MsHel-5	gb|AIXA01003033.1|:898-1186
MsHel-5	gb|AIXA01001509.1|:2731-2991
MsHel-5	gb|AIXA01003021.1|:118423-118575
MsHel-5	gb|AIXA01016426.1|:1897-2196
MsHel-5	gb|AIXA01016262.1|:30725-30914
MsHel-5	gb|AIXA01013013.1|:4971-5274
MsHel-5	gb|AIXA01006748.1|:4076-4379
MsHel-5	gb|AIXA01002551.1|:66334-66637
MsHel-5	gb|AIXA01000688.1|:46615-46918
MsHel-5	gb|AIXA01009936.1|:24560-24862
MsHel-5	gb|AIXA01007586.1|:3337-3616
MsHel-5	gb|AIXA01006225.1|:21825-22124
MsHel-5	gb|AIXA01000143.1|:3766-4056
MsHel-5	gb|AIXA01016209.1|:14862-15160
MsHel-5	gb|AIXA01012294.1|:3403-3704
MsHel-5	gb|AIXA01009981.1|:2855-3140
MsHel-5	gb|AIXA01005684.1|:6511-6812
MsHel-5	gb|AIXA01005312.1|:75983-76577
MsHel-5	gb|AIXA01003023.1|:31359-31654
MsHel-5	gb|AIXA01003023.1|:51882-52036
MsHel-5	gb|AIXA01002507.1|:12198-12354
MsHel-5	gb|AIXA01002507.1|:63890-64191
MsHel-5	gb|AIXA01000569.1|:12334-12633
MsHel-5	gb|AIXA01012194.1|:5880-6177
MsHel-5	gb|AIXA01009342.1|:737-1033
MsHel-5	gb|AIXA01004790.1|:35511-35773
MsHel-5	gb|AIXA01009432.1|:8397-8694

MsHel-5	gb|AIXA01006701.1|:33595-33895
MsHel-5	gb|AIXA01006514.1|:16261-16564
MsHel-5	gb|AIXA01002702.1|:3997-4299
MsHel-5	gb|AIXA01002388.1|:139341-139640
MsHel-5	gb|AIXA01001001.1|:39902-40198
MsHel-5	gb|AIXA01000441.1|:27956-28258
MsHel-5	gb|AIXA01028045.1|:1-266
MsHel-5	gb|AIXA01004209.1|:20040-20338
MsHel-5	gb|AIXA01018184.1|:510-803
MsHel-5	gb|AIXA01014124.1|:15183-15490
MsHel-5	gb|AIXA01016208.1|:5976-6258
MsHel-5	gb|AIXA01020587.1|:1387-1696
MsHel-5	gb|AIXA01009580.1|:749-1032
MsHel-5	gb|AIXA01005319.1|:7311-7593
MsHel-5	gb|AIXA01003982.1|:19291-19580
MsHel-5	gb|AIXA01003645.1|:10909-11208
MsHel-5	gb|AIXA01003427.1|:13886-14189
MsHel-5	gb|AIXA01001968.1|:45747-46031
MsHel-5	gb|AIXA01001695.1|:39387-39686
MsHel-5	gb|AIXA01014030.1|:31810-32096
MsHel-5	gb|AIXA01011980.1|:5912-6208
MsHel-5	gb|AIXA01008487.1|:20310-20608
MsHel-5	gb|AIXA01002571.1|:706-1011
MsHel-5	gb|AIXA01001578.1|:1702-2002
MsHel-5	gb|AIXA01015508.1|:12672-12956
MsHel-5	gb|AIXA01001633.1|:89-335
MsHel-5	gb|AIXA01011357.1|:35-238
MsHel-5	gb|AIXA01005893.1|:1527-1826
MsHel-5	gb|AIXA01001598.1|:42515-42759
MsHel-5	gb|AIXA01020651.1|:817-1108
MsHel-5	gb|AIXA01010849.1|:31130-31423
MsHel-5	gb|AIXA01010849.1|:43624-43821
MsHel-5	gb|AIXA01005993.1|:3554-3855
MsHel-5	gb|AIXA01004349.1|:26815-27112
MsHel-5	gb|AIXA01003214.1|:52250-52552
MsHel-5	gb|AIXA01019090.1|:2434-2738
MsHel-5	gb|AIXA01018138.1|:11094-11398
MsHel-5	gb|AIXA01009024.1|:16727-16915
MsHel-5	gb|AIXA01009024.1|:59283-59584
MsHel-5	gb|AIXA01006491.1|:11912-12206
MsHel-5	gb|AIXA01002231.1|:78609-78915
MsHel-5	gb|AIXA01002215.1|:25287-25582
MsHel-5	gb|AIXA01016674.1|:3322-3618
MsHel-5	gb|AIXA01015956.1|:17636-17936
MsHel-5	gb|AIXA01012912.1|:8606-8862
MsHel-5	gb|AIXA01004374.1|:8594-8898
MsHel-5	gb|AIXA01001712.1|:14012-14352
MsHel-5	gb|AIXA01000847.1|:49483-49773

MsHel-5	gb|AIXA01000481.1|:43678-43974
MsHel-5	gb|AIXA01026611.1|:1-188
MsHel-5	gb|AIXA01019269.1|:3340-3667
MsHel-5	gb|AIXA01007520.1|:23-221
MsHel-5	gb|AIXA01000568.1|:1225-1503
MsHel-5	gb|AIXA01000568.1|:40487-40679
MsHel-5	gb|AIXA01010784.1|:13114-13400
MsHel-5	gb|AIXA01007249.1|:892-1193
MsHel-5	gb|AIXA01001736.1|:8823-9011
MsHel-5	gb|AIXA01010053.1|:9-213
MsHel-5	gb|AIXA01007576.1|:16294-16456
MsHel-5	gb|AIXA01007576.1|:93849-94318
MsHel-5	gb|AIXA01004342.1|:28822-29102
MsHel-5	gb|AIXA01013777.1|:1450-1745
MsHel-5	gb|AIXA01005603.1|:7680-7926
MsHel-5	gb|AIXA01004191.1|:19533-19767
MsHel-5	gb|AIXA01008443.1|:30361-30651
MsHel-5	gb|AIXA01005614.1|:21345-21615
MsHel-5	gb|AIXA01019154.1|:4431-4730
MsHel-5	gb|AIXA01002497.1|:59794-60004
MsHel-5	gb|AIXA01001757.1|:21530-21818
MsHel-5	gb|AIXA01007784.1|:22694-22887
MsHel-5	gb|AIXA01003003.1|:42169-42472
MsHel-5	gb|AIXA01000510.1|:14717-15017
MsHel-5	gb|AIXA01009976.1|:61949-62246
MsHel-5	gb|AIXA01006466.1|:9685-9979
MsHel-5	gb|AIXA01015923.1|:6209-6406
MsHel-5	gb|AIXA01005185.1|:2607-2853
MsHel-5	gb|AIXA01024133.1|:149-452
MsHel-5	gb|AIXA01005086.1|:1012-1256
MsHel-5	gb|AIXA01010658.1|:19027-19243
MsHel-5	gb|AIXA01010406.1|:13014-13205
MsHel-5	gb|AIXA01014695.1|:977-1185
MsHel-5	gb|AIXA01014695.1|:24311-24524
MsHel-5	gb|AIXA01010151.1|:130117-130313
MsHel-5	gb|AIXA01010151.1|:163716-164016
MsHel-5	gb|AIXA01030830.1|:26-297
MsHel-5	gb|AIXA01018328.1|:1113-1375
MsHel-5	gb|AIXA01006517.1|:4-194
MsHel-5	gb|AIXA01001725.1|:23115-23412
MsHel-5	gb|AIXA01001685.1|:20921-21220
MsHel-5	gb|AIXA01001032.1|:54681-54937
MsHel-5	gb|AIXA01013132.1|:8281-8572
MsHel-5	gb|AIXA01000148.1|:3127-3381
MsHel-5	gb|AIXA01001671.1|:3107-3408
MsHel-5	gb|AIXA01012124.1|:12859-13154
MsHel-5	gb|AIXA01011655.1|:32416-32711
MsHel-5	gb|AIXA01010577.1|:3259-4391

MsHel-5	gb|AIXA01002170.1|:61484-61648
MsHel-5	gb|AIXA01001224.1|:12519-12683
MsHel-5	gb|AIXA01000044.1|:1244-1438
MsHel-5	gb|AIXA01000044.1|:16516-16791
MsHel-5	gb|AIXA01002671.1|:133350-133544
MsHel-5	gb|AIXA01026125.1|:206-412
MsHel-5	gb|AIXA01012653.1|:3222-3420
MsHel-5	gb|AIXA01009809.1|:3597-3876
MsHel-5	gb|AIXA01014733.1|:986-1735
MsHel-5	gb|AIXA01003030.1|:35321-35590
MsHel-5	gb|AIXA01000424.1|:77551-77760
MsHel-5	gb|AIXA01031778.1|:1-200
MsHel-5	gb|AIXA01010471.1|:1134-1427
MsHel-5	gb|AIXA01009636.1|:37470-37804
MsHel-5	gb|AIXA01012305.1|:138-343
MsHel-5	gb|AIXA01010117.1|:3519-3785
MsHel-5	gb|AIXA01007597.1|:4603-5659
MsHel-5	gb|AIXA01017945.1|:2657-2851
MsHel-5	gb|AIXA01010076.1|:96364-96581
MsHel-5	gb|AIXA01013449.1|:12037-12322
MsHel-5	gb|AIXA01011449.1|:10560-10769
MsHel-5	gb|AIXA01008541.1|:4931-5224
MsHel-5	gb|AIXA01001502.1|:68579-68884
MsHel-5	gb|AIXA01007609.1|:1-158
MsHel-5	gb|AIXA01007152.1|:70525-70821
MsHel-5	gb|AIXA01000069.1|:142196-142388
MsHel-5	gb|AIXA01000069.1|:184881-185140
MsHel-5	gb|AIXA01007975.1|:27744-27907
MsHel-5	gb|AIXA01007975.1|:62682-62908
MsHel-5	gb|AIXA01004327.1|:124696-124897
MsHel-5	gb|AIXA01003634.1|:58761-59023
MsHel-5	gb|AIXA01000796.1|:2546-2819
MsHel-5	gb|AIXA01011656.1|:455-717
MsHel-5	gb|AIXA01008930.1|:95212-95442
MsHel-5	gb|AIXA01000271.1|:64085-64371
MsHel-5	gb|AIXA01008376.1|:13964-14267
MsHel-5	gb|AIXA01025824.1|:2-157
MsHel-5	gb|AIXA01015877.1|:9693-9908
MsHel-5	gb|AIXA01010854.1|:27153-27409
MsHel-5	gb|AIXA01003139.1|:7586-7801
MsHel-5	gb|AIXA01008665.1|:12132-12356
MsHel-5	gb|AIXA01014629.1|:30230-30386
MsHel-5	gb|AIXA01012301.1|:1880-2104
MsHel-5	gb|AIXA01003120.1|:130758-130951
MsHel-5	gb|AIXA01027791.1|:1-152
MsHel-5	gb|AIXA01003008.1|:40865-41081
MsHel-5	gb|AIXA01003008.1|:61792-62022
MsHel-5	gb|AIXA01001296.1|:118639-118829

MsHel-5	gb|AIXA01001296.1|:205678-205892
MsHel-5	gb|AIXA01011501.1|:11478-11698
MsHel-5	gb|AIXA01005391.1|:33809-34002
MsHel-5	gb|AIXA01004779.1|:37974-38174
MsHel-5	gb|AIXA01004307.1|:7447-7685
MsHel-5	gb|AIXA01004307.1|:70920-71114
MsHel-5	gb|AIXA01003272.1|:120880-121073
MsHel-5	gb|AIXA01000086.1|:22885-23087
MsHel-5	gb|AIXA01010599.1|:4717-4933
MsHel-5	gb|AIXA01006863.1|:1-135
MsHel-5	gb|AIXA01006863.1|:23106-23255
MsHel-5	gb|AIXA01031240.1|:473-681
MsHel-5	gb|AIXA01028477.1|:786-959
MsHel-5	gb|AIXA01020514.1|:291-484
MsHel-5	gb|AIXA01006699.1|:50365-50558
MsHel-5	gb|AIXA01003829.1|:2135-2289
MsHel-5	gb|AIXA01001672.1|:33206-33477
MsHel-5	gb|AIXA01000627.1|:7771-7964
MsHel-5	gb|AIXA01007712.1|:20827-21019
MsHel-5	gb|AIXA01005231.1|:17515-17707
MsHel-5	gb|AIXA01000854.1|:12674-12864
MsHel-5	gb|AIXA01011724.1|:26263-26456
MsHel-5	gb|AIXA01031686.1|:374-559
MsHel-5	gb|AIXA01015507.1|:5342-5460
MsHel-5	gb|AIXA01010831.1|:14242-14435
MsHel-5	gb|AIXA01005350.1|:58341-58558
MsHel-5	gb|AIXA01028084.1|:1-123
MsHel-5	gb|AIXA01013985.1|:2870-3063
MsHel-5	gb|AIXA01012536.1|:7154-7374
MsHel-5	gb|AIXA01005422.1|:44926-45119
MsHel-5	gb|AIXA01004724.1|:52715-52908
MsHel-5	gb|AIXA01002649.1|:1111-1304
MsHel-5	gb|AIXA01002515.1|:35902-36056
MsHel-5	gb|AIXA01002048.1|:14654-14869
MsHel-5	gb|AIXA01018076.1|:1754-1948
MsHel-5	gb|AIXA01017917.1|:5953-6145
MsHel-5	gb|AIXA01008228.1|:28736-28934
MsHel-5	gb|AIXA01007308.1|:319-511
MsHel-5	gb|AIXA01007118.1|:29516-29641
MsHel-5	gb|AIXA01005444.1|:5982-6172
MsHel-5	gb|AIXA01004186.1|:16013-16230
MsHel-5	gb|AIXA01003334.1|:2865-3030
MsHel-5	gb|AIXA01002673.1|:19143-19337
MsHel-5	gb|AIXA01002659.1|:15087-15275
MsHel-5	gb|AIXA01000982.1|:7900-8088
MsHel-5	gb|AIXA01000982.1|:60239-60433
MsHel-5	gb|AIXA01008088.1|:20361-20520
MsHel-5	gb|AIXA01014986.1|:9656-9869

MsHel-5	gb|AIXA01014176.1|:855-1052
MsHel-5	gb|AIXA01010395.1|:3226-3426
MsHel-5	gb|AIXA01004462.1|:12168-12389
MsHel-5	gb|AIXA01022471.1|:125-317
MsHel-5	gb|AIXA01022375.1|:550-741
MsHel-5	gb|AIXA01016574.1|:20885-21078
MsHel-5	gb|AIXA01010881.1|:76154-76346
MsHel-5	gb|AIXA01008139.1|:7858-8050
MsHel-5	gb|AIXA01007362.1|:14229-14443
MsHel-5	gb|AIXA01007303.1|:401-592
MsHel-5	gb|AIXA01006516.1|:12628-12814
MsHel-5	gb|AIXA01005105.1|:2366-2559
MsHel-5	gb|AIXA01004084.1|:69598-69791
MsHel-5	gb|AIXA01004056.1|:5753-5907
MsHel-5	gb|AIXA01001630.1|:12385-12580
MsHel-5	gb|AIXA01000155.1|:28053-28241
MsHel-5	gb|AIXA01030916.1|:645-812
MsHel-5	gb|AIXA01019423.1|:3702-3891
MsHel-5	gb|AIXA01011637.1|:21358-21549
MsHel-5	gb|AIXA01009787.1|:23781-23986
MsHel-5	gb|AIXA01004587.1|:38068-38260
MsHel-5	gb|AIXA01004335.1|:13506-13700
MsHel-5	gb|AIXA01003677.1|:50838-51029
MsHel-5	gb|AIXA01001899.1|:201593-201785
MsHel-5	gb|AIXA01000924.1|:1854-2048
MsHel-5	gb|AIXA01000769.1|:1778-1968
MsHel-5	gb|AIXA01000265.1|:959-1112
MsHel-5	gb|AIXA01028859.1|:1-121
MsHel-5	gb|AIXA01016740.1|:11246-11405
MsHel-5	gb|AIXA01015970.1|:17648-17845
MsHel-5	gb|AIXA01014981.1|:7-200
MsHel-5	gb|AIXA01013743.1|:460-651
MsHel-5	gb|AIXA01012690.1|:6994-9059
MsHel-5	gb|AIXA01012049.1|:42293-42509
MsHel-5	gb|AIXA01006686.1|:3587-3778
MsHel-5	gb|AIXA01006053.1|:42645-42838
MsHel-5	gb|AIXA01005338.1|:12425-12618
MsHel-5	gb|AIXA01002379.1|:5659-5852
MsHel-5	gb|AIXA01000787.1|:34823-35021
MsHel-5	gb|AIXA01004951.1|:10139-10333
MsHel-5	gb|AIXA01002996.1|:5907-6099
MsHel-5	gb|AIXA01001043.1|:91851-92041
MsHel-5	gb|AIXA01016204.1|:7758-7887
MsHel-5	gb|AIXA01014643.1|:39895-40088
MsHel-5	gb|AIXA01012695.1|:1493-1682
MsHel-5	gb|AIXA01005592.1|:19616-19803
MsHel-5	gb|AIXA01004772.1|:8857-9050
MsHel-5	gb|AIXA01003125.1|:29381-29573

MsHel-5	gb|AIXA01002582.1|:57049-57241
MsHel-5	gb|AIXA01002331.1|:41329-41522
MsHel-5	gb|AIXA01000943.1|:18482-18675
MsHel-5	gb|AIXA01033985.1|:2-123
MsHel-5	gb|AIXA01019115.1|:3439-3627
MsHel-5	gb|AIXA01009427.1|:403-592
MsHel-5	gb|AIXA01009427.1|:30324-30477
MsHel-5	gb|AIXA01008410.1|:12614-12808
MsHel-5	gb|AIXA01007552.1|:51182-51372
MsHel-5	gb|AIXA01005104.1|:11690-11884
MsHel-5	gb|AIXA01004893.1|:18145-18336
MsHel-5	gb|AIXA01004708.1|:143250-143442
MsHel-5	gb|AIXA01004228.1|:17149-17349
MsHel-5	gb|AIXA01003579.1|:1649-1846
MsHel-5	gb|AIXA01003359.1|:234-426
MsHel-5	gb|AIXA01003330.1|:131967-132161
MsHel-5	gb|AIXA01003042.1|:192968-193159
MsHel-5	gb|AIXA01002844.1|:5683-5880
MsHel-5	gb|AIXA01002004.1|:26167-26358
MsHel-5	gb|AIXA01001998.1|:1838-2032
MsHel-5	gb|AIXA01001615.1|:10872-11186
MsHel-5	gb|AIXA01018419.1|:7421-7639
MsHel-5	gb|AIXA01014768.1|:10257-10389
MsHel-5	gb|AIXA01012005.1|:6172-6330
MsHel-5	gb|AIXA01012005.1|:22522-22637
MsHel-5	gb|AIXA01010058.1|:10495-10688
MsHel-5	gb|AIXA01006507.1|:10294-10510
MsHel-5	gb|AIXA01005035.1|:89150-89343
MsHel-5	gb|AIXA01003859.1|:7781-7989
MsHel-5	gb|AIXA01002650.1|:41193-41419
MsHel-5	gb|AIXA01014034.1|:4782-4976
MsHel-5	gb|AIXA01011454.1|:20546-20731
MsHel-5	gb|AIXA01009156.1|:20481-20674
MsHel-5	gb|AIXA01005420.1|:17330-17605
MsHel-5	gb|AIXA01004489.1|:72628-72778
MsHel-5	gb|AIXA01004484.1|:63777-63970
MsHel-5	gb|AIXA01004279.1|:953-1146
MsHel-5	gb|AIXA01002612.1|:11791-11976
MsHel-5	gb|AIXA01002612.1|:28760-28951
MsHel-5	gb|AIXA01002325.1|:1-124
MsHel-5	gb|AIXA01023866.1|:1264-1418
MsHel-5	gb|AIXA01009382.1|:29252-29446
MsHel-5	gb|AIXA01008266.1|:27077-27270
MsHel-5	gb|AIXA01008108.1|:2460-2648
MsHel-5	gb|AIXA01004424.1|:85541-85788
MsHel-5	gb|AIXA01003644.1|:9476-9669
MsHel-5	gb|AIXA01001662.1|:39023-39218
MsHel-5	gb|AIXA01021122.1|:1671-1865

MsHel-5	gb|AIXA01020768.1|:586-775
MsHel-5	gb|AIXA01016504.1|:860-1052
MsHel-5	gb|AIXA01015531.1|:4914-5139
MsHel-5	gb|AIXA01015400.1|:5445-5637
MsHel-5	gb|AIXA01014763.1|:5266-5452
MsHel-5	gb|AIXA01014518.1|:10209-10403
MsHel-5	gb|AIXA01012120.1|:6518-6781
MsHel-5	gb|AIXA01012100.1|:14849-15009
MsHel-5	gb|AIXA01011295.1|:8714-8906
MsHel-5	gb|AIXA01009860.1|:37832-37992
MsHel-5	gb|AIXA01008271.1|:17477-17667
MsHel-5	gb|AIXA01008263.1|:38704-38896
MsHel-5	gb|AIXA01006184.1|:7893-8087
MsHel-5	gb|AIXA01001301.1|:17736-17872
MsHel-5	gb|AIXA01015191.1|:10197-10395
MsHel-5	gb|AIXA01013549.1|:2800-2981
MsHel-5	gb|AIXA01010342.1|:21782-22002
MsHel-5	gb|AIXA01005050.1|:9799-9987
MsHel-5	gb|AIXA01003494.1|:13796-14012
MsHel-5	gb|AIXA01003239.1|:7237-7438
MsHel-5	gb|AIXA01019611.1|:1979-2168
MsHel-5	gb|AIXA01018793.1|:3056-3245
MsHel-5	gb|AIXA01018622.1|:6773-6963
MsHel-5	gb|AIXA01017951.1|:5629-5779
MsHel-5	gb|AIXA01010387.1|:3059-3252
MsHel-5	gb|AIXA01008634.1|:4979-5173
MsHel-5	gb|AIXA01007642.1|:5723-5913
MsHel-5	gb|AIXA01005228.1|:80577-80771
MsHel-5	gb|AIXA01005155.1|:6988-7179
MsHel-5	gb|AIXA01003285.1|:28684-28840
MsHel-5	gb|AIXA01000936.1|:9566-9760
MsHel-5	gb|AIXA01000728.1|:8768-8962
MsHel-5	gb|AIXA01032096.1|:342-530
MsHel-5	gb|AIXA01010962.1|:12756-12948
MsHel-5	gb|AIXA01005813.1|:71708-71898
MsHel-5	gb|AIXA01004102.1|:9094-9287
MsHel-5	gb|AIXA01003551.1|:367-558
MsHel-5	gb|AIXA01003174.1|:19351-19544
MsHel-5	gb|AIXA01001728.1|:1082-1243
MsHel-5	gb|AIXA01025771.1|:828-1018
MsHel-5	gb|AIXA01019189.1|:2073-2269
MsHel-5	gb|AIXA01018043.1|:2990-3150
MsHel-5	gb|AIXA01015379.1|:7640-7804
MsHel-5	gb|AIXA01011785.1|:5714-5918
MsHel-5	gb|AIXA01007875.1|:639-830
MsHel-5	gb|AIXA01006190.1|:93218-93410
MsHel-5	gb|AIXA01004132.1|:31394-31588
MsHel-5	gb|AIXA01000427.1|:9414-9611

MsHel-5	gb|AIXA01016428.1|:5168-5366
MsHel-5	gb|AIXA01012136.1|:3440-3638
MsHel-5	gb|AIXA01011970.1|:13181-13332
MsHel-5	gb|AIXA01011535.1|:8576-8769
MsHel-5	gb|AIXA01011308.1|:6429-6608
MsHel-5	gb|AIXA01011308.1|:46391-46549
MsHel-5	gb|AIXA01010181.1|:315-505
MsHel-5	gb|AIXA01010072.1|:4281-4479
MsHel-5	gb|AIXA01007305.1|:1165-1323
MsHel-5	gb|AIXA01006442.1|:21349-21513
MsHel-5	gb|AIXA01005208.1|:52665-52856
MsHel-5	gb|AIXA01003613.1|:13566-13723
MsHel-5	gb|AIXA01003179.1|:58572-58765
MsHel-5	gb|AIXA01022362.1|:1542-1705
MsHel-5	gb|AIXA01015471.1|:6435-6624
MsHel-5	gb|AIXA01014297.1|:21776-21981
MsHel-5	gb|AIXA01007496.1|:28625-28817
MsHel-5	gb|AIXA01004939.1|:4334-4623
MsHel-5	gb|AIXA01002008.1|:28087-28281
MsHel-5	gb|AIXA01001282.1|:34554-34751
MsHel-5	gb|AIXA01000001.1|:2526-2725
MsHel-5	gb|AIXA01018543.1|:570-760
MsHel-5	gb|AIXA01014990.1|:9211-9405
MsHel-5	gb|AIXA01014587.1|:24285-26111
MsHel-5	gb|AIXA01005617.1|:7125-7319
MsHel-5	gb|AIXA01001665.1|:7240-7429
MsHel-5	gb|AIXA01001025.1|:26869-27066
MsHel-5	gb|AIXA01017184.1|:561-758
MsHel-5	gb|AIXA01013436.1|:36642-36838
MsHel-5	gb|AIXA01006159.1|:1-110
MsHel-5	gb|AIXA01005332.1|:2400-2593
MsHel-5	gb|AIXA01004527.1|:36204-36395
MsHel-5	gb|AIXA01002598.1|:2068-2263
MsHel-5	gb|AIXA01001645.1|:15525-15683
MsHel-5	gb|AIXA01028059.1|:66-256
MsHel-5	gb|AIXA01013936.1|:2313-2413
MsHel-5	gb|AIXA01013523.1|:43993-44183
MsHel-5	gb|AIXA01013144.1|:5123-5321
MsHel-5	gb|AIXA01012145.1|:76578-76671
MsHel-5	gb|AIXA01011985.1|:10819-11008
MsHel-5	gb|AIXA01009375.1|:36828-37018
MsHel-5	gb|AIXA01006978.1|:30073-30271
MsHel-5	gb|AIXA01006529.1|:63192-63382
MsHel-5	gb|AIXA01004987.1|:25407-25605
MsHel-5	gb|AIXA01003929.1|:16788-16952
MsHel-5	gb|AIXA01003929.1|:58767-59065
MsHel-5	gb|AIXA01003621.1|:32898-33094
MsHel-5	gb|AIXA01001740.1|:38932-39096

MsHel-5	gb|AIXA01000095.1|:86547-86744
MsHel-5	gb|AIXA01015530.1|:19759-19954
MsHel-5	gb|AIXA01014221.1|:9385-9582
MsHel-5	gb|AIXA01011371.1|:27546-27709
MsHel-5	gb|AIXA01010176.1|:27282-27468
MsHel-5	gb|AIXA01008300.1|:2305-2500
MsHel-5	gb|AIXA01005474.1|:22154-22351
MsHel-5	gb|AIXA01005406.1|:42683-42880
MsHel-5	gb|AIXA01001990.1|:28624-28787
MsHel-5	gb|AIXA01001356.1|:47156-47346
MsHel-5	gb|AIXA01026393.1|:941-1131
MsHel-5	gb|AIXA01023651.1|:1291-1453
MsHel-5	gb|AIXA01015790.1|:10790-10985
MsHel-5	gb|AIXA01003839.1|:60467-60624
MsHel-5	gb|AIXA01001498.1|:54006-54194
MsHel-5	gb|AIXA01001271.1|:8557-8730
MsHel-5	gb|AIXA01000655.1|:71384-71576
MsHel-5	gb|AIXA01010576.1|:9740-9964
MsHel-5	gb|AIXA01007527.1|:17570-17759
MsHel-5	gb|AIXA01006910.1|:3235-3430
MsHel-5	gb|AIXA01005413.1|:54806-54903
MsHel-5	gb|AIXA01003876.1|:112890-113079
MsHel-5	gb|AIXA01001653.1|:30324-30515
MsHel-5	gb|AIXA01029174.1|:719-911
MsHel-5	gb|AIXA01019179.1|:2551-2749
MsHel-5	gb|AIXA01017013.1|:14853-15051
MsHel-5	gb|AIXA01016327.1|:147-343
MsHel-5	gb|AIXA01014467.1|:24682-24878
MsHel-5	gb|AIXA01013524.1|:62985-63173
MsHel-5	gb|AIXA01011110.1|:1071-1288
MsHel-5	gb|AIXA01009035.1|:3893-4041
MsHel-5	gb|AIXA01005345.1|:25649-25845
MsHel-5	gb|AIXA01003889.1|:12727-12920
MsHel-5	gb|AIXA01003658.1|:12853-13069
MsHel-5	gb|AIXA01003363.1|:20554-20717
MsHel-5	gb|AIXA01020052.1|:1-175
MsHel-5	gb|AIXA01018818.1|:3511-3704
MsHel-5	gb|AIXA01018311.1|:8976-9173
MsHel-5	gb|AIXA01017842.1|:1294-1496
MsHel-5	gb|AIXA01013521.1|:26727-26921
MsHel-5	gb|AIXA01013420.1|:12777-12972
MsHel-5	gb|AIXA01011710.1|:390-587
MsHel-5	gb|AIXA01007577.1|:34281-34478
MsHel-5	gb|AIXA01004906.1|:1-100
MsHel-5	gb|AIXA01004157.1|:4983-5141
MsHel-5	gb|AIXA01003726.1|:14690-14825
MsHel-5	gb|AIXA01000046.1|:4675-4857
MsHel-5	gb|AIXA01012317.1|:4015-4205

MsHel-5	gb|AIXA01010785.1|:75991-76183
MsHel-5	gb|AIXA01010495.1|:3682-3878
MsHel-5	gb|AIXA01036690.1|:265-466
MsHel-5	gb|AIXA01025661.1|:1075-1200
MsHel-5	gb|AIXA01015620.1|:368-567
MsHel-5	gb|AIXA01014641.1|:7125-7322
MsHel-5	gb|AIXA01011446.1|:12280-12471
MsHel-5	gb|AIXA01010590.1|:26508-26751
MsHel-5	gb|AIXA01007721.1|:12201-12392
MsHel-5	gb|AIXA01006708.1|:5436-5637
MsHel-5	gb|AIXA01006680.1|:7400-7553
MsHel-5	gb|AIXA01006292.1|:9521-9722
MsHel-5	gb|AIXA01004287.1|:352-553
MsHel-5	gb|AIXA01003531.1|:2641-2840
MsHel-5	gb|AIXA01032066.1|:95-285
MsHel-5	gb|AIXA01015409.1|:1696-1894
MsHel-5	gb|AIXA01012700.1|:3164-3302
MsHel-5	gb|AIXA01005089.1|:28521-28709
MsHel-5	gb|AIXA01004480.1|:20158-20322
MsHel-5	gb|AIXA01003999.1|:43015-43205
MsHel-5	gb|AIXA01003343.1|:25532-25730
MsHel-5	gb|AIXA01002340.1|:114129-114326
MsHel-5	gb|AIXA01030898.1|:14-128
MsHel-5	gb|AIXA01027598.1|:1-92
MsHel-5	gb|AIXA01008106.1|:6529-6681
MsHel-5	gb|AIXA01000707.1|:1-100
MsHel-5	gb|AIXA01029886.1|:739-869
MsHel-5	gb|AIXA01017362.1|:8384-8585
MsHel-5	gb|AIXA01007886.1|:3888-4022
MsHel-5	gb|AIXA01004053.1|:8119-8293
MsHel-5	gb|AIXA01003061.1|:6465-6649
MsHel-5	gb|AIXA01002442.1|:8495-8597
MsHel-5	gb|AIXA01001658.1|:35104-35188
MsHel-5	gb|AIXA01001658.1|:70056-70254
MsHel-5	gb|AIXA01011300.1|:2002-2167
MsHel-5	gb|AIXA01007273.1|:4815-5009
MsHel-5	gb|AIXA01006720.1|:1810-2007
MsHel-5	gb|AIXA01004755.1|:13781-13984
MsHel-5	gb|AIXA01000440.1|:60097-60299
MsHel-5	gb|AIXA01000135.1|:8915-9099
MsHel-5	gb|AIXA01016427.1|:538-702
MsHel-5	gb|AIXA01014618.1|:868-1054
MsHel-5	gb|AIXA01010105.1|:4471-4635
MsHel-5	gb|AIXA01008474.1|:23850-24048
MsHel-5	gb|AIXA01008112.1|:37441-37630
MsHel-5	gb|AIXA01007762.1|:6232-6429
MsHel-5	gb|AIXA01001199.1|:7192-7355
MsHel-5	gb|AIXA01017804.1|:2523-2720

MsHel-5	gb|AIXA01013233.1|:9-125
MsHel-5	gb|AIXA01007346.1|:17105-17245
MsHel-5	gb|AIXA01004845.1|:2577-2771
MsHel-5	gb|AIXA01004001.1|:12864-13072
MsHel-5	gb|AIXA01002300.1|:27797-27994
MsHel-5	gb|AIXA01016218.1|:3008-3196
MsHel-5	gb|AIXA01008287.1|:28-216
MsHel-5	gb|AIXA01006055.1|:12326-12452
MsHel-5	gb|AIXA01002599.1|:32450-32724
MsHel-5	gb|AIXA01001243.1|:148135-148328
MsHel-5	gb|AIXA01026639.1|:1-106
MsHel-5	gb|AIXA01009539.1|:47834-48033
MsHel-5	gb|AIXA01003891.1|:21741-21907
MsHel-5	gb|AIXA01001985.1|:44645-44730
MsHel-5	gb|AIXA01001965.1|:53343-53602
MsHel-5	gb|AIXA01001656.1|:44484-44683
MsHel-5	gb|AIXA01016774.1|:2621-2970
MsHel-5	gb|AIXA01013976.1|:1-97
MsHel-5	gb|AIXA01012554.1|:59255-59449
MsHel-5	gb|AIXA01010725.1|:5239-5323
MsHel-5	gb|AIXA01005207.1|:34657-34847
MsHel-5	gb|AIXA01024998.1|:1161-1276
MsHel-5	gb|AIXA01010119.1|:24772-24966
MsHel-5	gb|AIXA01009026.1|:1-104
MsHel-5	gb|AIXA01004676.1|:35305-35503
MsHel-5	gb|AIXA01001520.1|:19357-19553
MsHel-5	gb|AIXA01012692.1|:1087-1271
MsHel-5	gb|AIXA01007049.1|:25527-25692
MsHel-5	gb|AIXA01005483.1|:433-518
MsHel-5	gb|AIXA01003986.1|:32908-33070
MsHel-5	gb|AIXA01002622.1|:37279-37473
MsHel-5	gb|AIXA01001862.1|:147-328
MsHel-5	gb|AIXA01000810.1|:160818-161007
MsHel-5	gb|AIXA01017914.1|:3308-3500
MsHel-5	gb|AIXA01016348.1|:2775-2859
MsHel-5	gb|AIXA01015008.1|:16558-16751
MsHel-5	gb|AIXA01007972.1|:3557-3754
MsHel-5	gb|AIXA01007967.1|:289-373
MsHel-5	gb|AIXA01007415.1|:40000-40197
MsHel-5	gb|AIXA01006165.1|:27680-27874
MsHel-5	gb|AIXA01004909.1|:66893-67084
MsHel-5	gb|AIXA01004647.1|:34102-34186
MsHel-5	gb|AIXA01003650.1|:16766-16961
MsHel-5	gb|AIXA01001559.1|:1802-2000
MsHel-5	gb|AIXA01000192.1|:8531-8717
MsHel-5	gb|AIXA01024743.1|:1175-1306
MsHel-5	gb|AIXA01016078.1|:6985-7179
MsHel-5	gb|AIXA01010069.1|:14017-14199

MsHel-5	gb|AIXA01009133.1|:45625-45819
MsHel-5	gb|AIXA01003990.1|:2834-3032
MsHel-5	gb|AIXA01015972.1|:41275-41449
MsHel-5	gb|AIXA01009856.1|:40483-40795
MsHel-5	gb|AIXA01009788.1|:9-95
MsHel-5	gb|AIXA01006952.1|:20843-21005
MsHel-5	gb|AIXA01004619.1|:40942-41155
MsHel-5	gb|AIXA01004237.1|:1338-1489
MsHel-5	gb|AIXA01003113.1|:14703-14903
MsHel-5	gb|AIXA01002014.1|:6603-6782
MsHel-5	gb|AIXA01010870.1|:10442-10632
MsHel-5	gb|AIXA01008393.1|:31541-31743
MsHel-5	gb|AIXA01006862.1|:46910-47048
MsHel-5	gb|AIXA01005784.1|:11271-11356
MsHel-5	gb|AIXA01019000.1|:1008-1208
MsHel-5	gb|AIXA01016347.1|:9816-9900
MsHel-5	gb|AIXA01009330.1|:15178-15334
MsHel-5	gb|AIXA01008293.1|:17366-17450
MsHel-5	gb|AIXA01028495.1|:5-84
MsHel-5	gb|AIXA01018671.1|:370-513
MsHel-5	gb|AIXA01017901.1|:13532-13736
MsHel-5	gb|AIXA01006853.1|:63402-63484
MsHel-5	gb|AIXA01000660.1|:12369-12552
MsHel-5	gb|AIXA01014967.1|:8391-8579
MsHel-5	gb|AIXA01013383.1|:24391-24523
MsHel-5	gb|AIXA01005594.1|:21416-21508
MsHel-5	gb|AIXA01005594.1|:32497-32689
MsHel-5	gb|AIXA01001189.1|:17888-17970
MsHel-5	gb|AIXA01032392.1|:194-384
MsHel-5	gb|AIXA01015613.1|:9864-9949
MsHel-5	gb|AIXA01003284.1|:42044-42223
MsHel-5	gb|AIXA01025448.1|:1-101
MsHel-5	gb|AIXA01009106.1|:12828-13017
MsHel-5	gb|AIXA01002560.1|:15811-16005
MsHel-5	gb|AIXA01013010.1|:32846-33042
MsHel-5	gb|AIXA01007608.1|:19929-20008
MsHel-5	gb|AIXA01011937.1|:2680-2869
MsHel-5	gb|AIXA01007230.1|:20253-20411
MsHel-5	gb|AIXA01005480.1|:42-189
MsHel-5	gb|AIXA01017242.1|:2059-2241
MsHel-5	gb|AIXA01015157.1|:2599-2800
MsHel-5	gb|AIXA01010370.1|:5419-5534
MsHel-5	gb|AIXA01010192.1|:29881-30022
MsHel-5	gb|AIXA01003156.1|:12476-12641
MsHel-5	gb|AIXA01029812.1|:654-850
MsHel-5	gb|AIXA01008417.1|:49973-50239
MsHel-5	gb|AIXA01003339.1|:46915-47083
MsHel-5	gb|AIXA01000905.1|:23011-23210

MsHel-5	gb|AIXA01000375.1|:1682-1885
MsHel-5	gb|AIXA01014443.1|:68814-68954
MsHel-5	gb|AIXA01007301.1|:10587-10744
MsHel-5	gb|AIXA01024261.1|:1231-1368
MsHel-5	gb|AIXA01010108.1|:5782-5973
MsHel-5	gb|AIXA01008103.1|:41-175
MsHel-5	gb|AIXA01004354.1|:40592-40793
MsHel-5	gb|AIXA01000625.1|:35757-36194
MsHel-5	gb|AIXA01004428.1|:5710-5875
MsHel-5	gb|AIXA01002678.1|:10777-10934
MsHel-5	gb|AIXA01010488.1|:784-944
MsHel-5	gb|AIXA01002808.1|:18357-18444
MsHel-5	gb|AIXA01002723.1|:38441-38614
MsHel-5	gb|AIXA01010019.1|:15392-15589
MsHel-5	gb|AIXA01003874.1|:16616-16746
MsHel-5	gb|AIXA01003190.1|:9-108
MsHel-5	gb|AIXA01000839.1|:21183-21371
MsHel-5	gb|AIXA01000508.1|:40812-41218
MsHel-5	gb|AIXA01011720.1|:19802-20000
MsHel-5	gb|AIXA01005520.1|:24306-24474
MsHel-5	gb|AIXA01015513.1|:2154-2318
MsHel-5	gb|AIXA01011811.1|:12864-13061
MsHel-5	gb|AIXA01002779.1|:52-257
MsHel-5	gb|AIXA01001300.1|:11582-11768
MsHel-5	gb|AIXA01010664.1|:12244-12338
MsHel-5	gb|AIXA01005036.1|:1-100
MsHel-5	gb|AIXA01003847.1|:14028-14163
MsHel-5	gb|AIXA01002003.1|:1-96
MsHel-5	gb|AIXA01027213.1|:429-517
MsHel-5	gb|AIXA01014451.1|:7615-7718
MsHel-5	gb|AIXA01014398.1|:2726-2868
MsHel-5	gb|AIXA01010518.1|:10620-10712
MsHel-5	gb|AIXA01009048.1|:606-773
MsHel-5	gb|AIXA01008084.1|:689-887
MsHel-5	gb|AIXA01004129.1|:90579-90720
MsHel-5	gb|AIXA01013629.1|:1730-1911
MsHel-5	gb|AIXA01005266.1|:36330-37355
MsHel-5	gb|AIXA01003651.1|:64500-64684
MsHel-5	gb|AIXA01029742.1|:786-878
MsHel-5	gb|AIXA01034957.1|:1-147
MsHel-5	gb|AIXA01024988.1|:1166-1249
MsHel-5	gb|AIXA01004137.1|:74378-74512
MsHel-5	gb|AIXA01029391.1|:18-108
MsHel-5	gb|AIXA01019404.1|:5882-5974
MsHel-5	gb|AIXA01018079.1|:2315-2433
MsHel-5	gb|AIXA01005326.1|:66210-66363
MsHel-5	gb|AIXA01003687.1|:104344-104484
MsHel-5	gb|AIXA01016109.1|:1819-1985

MsHel-5	gb|AIXA01017936.1|:8218-8314
MsHel-5	gb|AIXA01005482.1|:4970-5064
MsHel-5	gb|AIXA01000797.1|:1510-1616
MsHel-5	gb|AIXA01022197.1|:1-78
MsHel-5	gb|AIXA01026110.1|:6-88
MsHel-6	gb|AIXA01018583.1|:1249-1509
MsHel-6	gb|AIXA01003000.1|:35306-35572
MsHel-6	gb|AIXA01008329.1|:22681-22942
MsHel-6	gb|AIXA01008329.1|:35848-36058
MsHel-6	gb|AIXA01002552.1|:38510-38776
MsHel-6	gb|AIXA01000337.1|:60402-65316
MsHel-6	gb|AIXA01009648.1|:3464-3719
MsHel-6	gb|AIXA01007984.1|:64126-64397
MsHel-6	gb|AIXA01015486.1|:2343-2602
MsHel-6	gb|AIXA01005057.1|:2267-2523
MsHel-6	gb|AIXA01000091.1|:1092-1361
MsHel-6	gb|AIXA01000091.1|:131784-132039
MsHel-6	gb|AIXA01014121.1|:213-480
MsHel-6	gb|AIXA01008116.1|:50358-50622
MsHel-6	gb|AIXA01004158.1|:33944-34197
MsHel-6	gb|AIXA01004158.1|:44746-44950
MsHel-6	gb|AIXA01000838.1|:14126-14389
MsHel-6	gb|AIXA01000747.1|:24379-24629
MsHel-6	gb|AIXA01004135.1|:57415-57677
MsHel-6	gb|AIXA01002817.1|:20654-20918
MsHel-6	gb|AIXA01001246.1|:3875-4142
MsHel-6	gb|AIXA01006842.1|:11879-12145
MsHel-6	gb|AIXA01003599.1|:13357-13620
MsHel-6	gb|AIXA01003329.1|:6199-6449
MsHel-6	gb|AIXA01003329.1|:129578-129816
MsHel-6	gb|AIXA01013103.1|:1097-1262
MsHel-6	gb|AIXA01005184.1|:2002-2262
MsHel-6	gb|AIXA01005000.1|:21356-21626
MsHel-6	gb|AIXA01007961.1|:13549-13988
MsHel-6	gb|AIXA01006927.1|:29423-29684
MsHel-6	gb|AIXA01006595.1|:108253-108517
MsHel-6	gb|AIXA01000745.1|:5761-6024
MsHel-6	gb|AIXA01002512.1|:1777-2038
MsHel-6	gb|AIXA01006740.1|:88913-89167
MsHel-6	gb|AIXA01002551.1|:86009-86218
MsHel-6	gb|AIXA01035415.1|:62-323
MsHel-6	gb|AIXA01007694.1|:19112-19370
MsHel-6	gb|AIXA01002155.1|:25243-25505
MsHel-6	gb|AIXA01000005.1|:45711-45964
MsHel-6	gb|AIXA01000005.1|:59001-59262
MsHel-6	gb|AIXA01000948.1|:15313-15566
MsHel-6	gb|AIXA01000237.1|:13657-13925

MsHel-6	gb|AIXA01000237.1|:84588-84778
MsHel-6	gb|AIXA01008114.1|:9773-10035
MsHel-6	gb|AIXA01006824.1|:5035-5306
MsHel-6	gb|AIXA01000251.1|:160450-160716
MsHel-6	gb|AIXA01018554.1|:6183-6438
MsHel-6	gb|AIXA01010430.1|:4483-4749
MsHel-6	gb|AIXA01002232.1|:10638-10847
MsHel-6	gb|AIXA01000692.1|:64297-64492
MsHel-6	gb|AIXA01020269.1|:998-1260
MsHel-6	gb|AIXA01006474.1|:11863-12127
MsHel-6	gb|AIXA01002629.1|:11395-11847
MsHel-6	gb|AIXA01002629.1|:44856-45244
MsHel-6	gb|AIXA01001755.1|:73475-73733
MsHel-6	gb|AIXA01020883.1|:291-552
MsHel-6	gb|AIXA01012657.1|:16010-16269
MsHel-6	gb|AIXA01002983.1|:13266-13527
MsHel-6	gb|AIXA01032241.1|:191-447
MsHel-6	gb|AIXA01008562.1|:24952-25128
MsHel-6	gb|AIXA01008562.1|:53683-53933
MsHel-6	gb|AIXA01003850.1|:3916-4178
MsHel-6	gb|AIXA01003850.1|:133991-134159
MsHel-6	gb|AIXA01002972.1|:4869-5125
MsHel-6	gb|AIXA01002972.1|:43005-43259
MsHel-6	gb|AIXA01002493.1|:10211-10477
MsHel-6	gb|AIXA01001262.1|:12121-12334
MsHel-6	gb|AIXA01007816.1|:2659-2917
MsHel-6	gb|AIXA01004482.1|:47202-47455
MsHel-6	gb|AIXA01003260.1|:1323-1578
MsHel-6	gb|AIXA01032196.1|:288-537
MsHel-6	gb|AIXA01016958.1|:3940-4206
MsHel-6	gb|AIXA01015929.1|:35520-35737
MsHel-6	gb|AIXA01015749.1|:6876-7130
MsHel-6	gb|AIXA01004746.1|:56269-56523
MsHel-6	gb|AIXA01001237.1|:31179-31440
MsHel-6	gb|AIXA01000055.1|:31326-31584
MsHel-6	gb|AIXA01015713.1|:1618-1875
MsHel-6	gb|AIXA01013634.1|:914-1173
MsHel-6	gb|AIXA01010212.1|:11224-11487
MsHel-6	gb|AIXA01008493.1|:13923-14179
MsHel-6	gb|AIXA01004780.1|:70-278
MsHel-6	gb|AIXA01003970.1|:47276-47488
MsHel-6	gb|AIXA01000168.1|:6893-7097
MsHel-6	gb|AIXA01006316.1|:11587-11835
MsHel-6	gb|AIXA01006316.1|:26092-26340
MsHel-6	gb|AIXA01003661.1|:161474-161662
MsHel-6	gb|AIXA01002507.1|:22419-22617
MsHel-6	gb|AIXA01001599.1|:103235-103491
MsHel-6	gb|AIXA01013235.1|:602-810

MsHel-6	gb|AIXA01012538.1|:1416-1678
MsHel-6	gb|AIXA01012145.1|:3962-4202
MsHel-6	gb|AIXA01012145.1|:60043-60315
MsHel-6	gb|AIXA01010720.1|:12363-12568
MsHel-6	gb|AIXA01008148.1|:120292-120544
MsHel-6	gb|AIXA01001965.1|:19045-19294
MsHel-6	gb|AIXA01001965.1|:71072-71328
MsHel-6	gb|AIXA01000459.1|:11082-17207
MsHel-6	gb|AIXA01030352.1|:223-481
MsHel-6	gb|AIXA01018618.1|:1076-1334
MsHel-6	gb|AIXA01014990.1|:9575-9826
MsHel-6	gb|AIXA01004051.1|:5423-5644
MsHel-6	gb|AIXA01003290.1|:24542-24790
MsHel-6	gb|AIXA01001642.1|:3148-3356
MsHel-6	gb|AIXA01010542.1|:1522-1773
MsHel-6	gb|AIXA01010542.1|:38263-38535
MsHel-6	gb|AIXA01007337.1|:24448-24659
MsHel-6	gb|AIXA01006518.1|:4660-4919
MsHel-6	gb|AIXA01006518.1|:78949-79208
MsHel-6	gb|AIXA01014354.1|:3854-4118
MsHel-6	gb|AIXA01013564.1|:10548-10820
MsHel-6	gb|AIXA01009101.1|:34171-34393
MsHel-6	gb|AIXA01008475.1|:7862-8121
MsHel-6	gb|AIXA01017708.1|:20315-20520
MsHel-6	gb|AIXA01008325.1|:30023-30193
MsHel-6	gb|AIXA01003070.1|:27302-27557
MsHel-6	gb|AIXA01002599.1|:96227-96494
MsHel-6	gb|AIXA01002477.1|:32969-33182
MsHel-6	gb|AIXA01001962.1|:34050-34311
MsHel-6	gb|AIXA01027140.1|:573-776
MsHel-6	gb|AIXA01016666.1|:35407-35603
MsHel-6	gb|AIXA01016579.1|:6178-6436
MsHel-6	gb|AIXA01016574.1|:11949-12162
MsHel-6	gb|AIXA01016491.1|:988-1242
MsHel-6	gb|AIXA01011979.1|:3112-3376
MsHel-6	gb|AIXA01011979.1|:55714-56157
MsHel-6	gb|AIXA01010885.1|:45368-45483
MsHel-6	gb|AIXA01010885.1|:59332-59547
MsHel-6	gb|AIXA01010885.1|:72893-73146
MsHel-6	gb|AIXA01007688.1|:12987-13245
MsHel-6	gb|AIXA01005643.1|:6450-6708
MsHel-6	gb|AIXA01004778.1|:5947-6206
MsHel-6	gb|AIXA01004595.1|:33623-33836
MsHel-6	gb|AIXA01002794.1|:42755-43018
MsHel-6	gb|AIXA01002330.1|:24017-24215
MsHel-6	gb|AIXA01000636.1|:4270-4516
MsHel-6	gb|AIXA01033111.1|:55-311
MsHel-6	gb|AIXA01032711.1|:466-666

MsHel-6	gb|AIXA01016714.1|:17045-17305
MsHel-6	gb|AIXA01015507.1|:1-143
MsHel-6	gb|AIXA01015201.1|:1107-1371
MsHel-6	gb|AIXA01004334.1|:8282-8538
MsHel-6	gb|AIXA01003712.1|:36953-37208
MsHel-6	gb|AIXA01001653.1|:44064-44318
MsHel-6	gb|AIXA01000768.1|:1257-1479
MsHel-6	gb|AIXA01000031.1|:13764-14015
MsHel-6	gb|AIXA01033685.1|:55-310
MsHel-6	gb|AIXA01016301.1|:1311-1575
MsHel-6	gb|AIXA01015715.1|:488-748
MsHel-6	gb|AIXA01015618.1|:197-392
MsHel-6	gb|AIXA01011818.1|:4154-4312
MsHel-6	gb|AIXA01010281.1|:7406-7671
MsHel-6	gb|AIXA01008773.1|:27438-33210
MsHel-6	gb|AIXA01005047.1|:40821-40978
MsHel-6	gb|AIXA01017943.1|:5215-5444
MsHel-6	gb|AIXA01015635.1|:3263-3473
MsHel-6	gb|AIXA01012759.1|:3505-3756
MsHel-6	gb|AIXA01012759.1|:38530-38789
MsHel-6	gb|AIXA01011126.1|:28580-38280
MsHel-6	gb|AIXA01010782.1|:3118-3297
MsHel-6	gb|AIXA01010782.1|:13948-14154
MsHel-6	gb|AIXA01007561.1|:12563-12818
MsHel-6	gb|AIXA01007411.1|:2333-2595
MsHel-6	gb|AIXA01003023.1|:3588-3838
MsHel-6	gb|AIXA01003023.1|:68054-68248
MsHel-6	gb|AIXA01002167.1|:275-532
MsHel-6	gb|AIXA01001991.1|:1439-1682
MsHel-6	gb|AIXA01000443.1|:7103-7358
MsHel-6	gb|AIXA01020097.1|:1097-1351
MsHel-6	gb|AIXA01011922.1|:1680-1903
MsHel-6	gb|AIXA01008076.1|:77795-78130
MsHel-6	gb|AIXA01006136.1|:75391-75640
MsHel-6	gb|AIXA01005503.1|:42811-43266
MsHel-6	gb|AIXA01004931.1|:21284-21488
MsHel-6	gb|AIXA01004335.1|:20028-20289
MsHel-6	gb|AIXA01004335.1|:32056-32177
MsHel-6	gb|AIXA01004285.1|:21330-21595
MsHel-6	gb|AIXA01004093.1|:63382-63646
MsHel-6	gb|AIXA01001602.1|:40169-40366
MsHel-6	gb|AIXA01014029.1|:6113-6362
MsHel-6	gb|AIXA01012324.1|:2509-2655
MsHel-6	gb|AIXA01012324.1|:26099-26355
MsHel-6	gb|AIXA01008575.1|:77340-77851
MsHel-6	gb|AIXA01004132.1|:4149-4396
MsHel-6	gb|AIXA01000810.1|:46189-46408
MsHel-6	gb|AIXA01000457.1|:34382-34582

MsHel-6	gb|AIXA01000437.1|:31670-31833
MsHel-6	gb|AIXA01000437.1|:78933-79190
MsHel-6	gb|AIXA01023564.1|:567-820
MsHel-6	gb|AIXA01023250.1|:111-362
MsHel-6	gb|AIXA01019373.1|:1978-2371
MsHel-6	gb|AIXA01019367.1|:664-915
MsHel-6	gb|AIXA01017244.1|:3978-4242
MsHel-6	gb|AIXA01007698.1|:13414-13621
MsHel-6	gb|AIXA01006860.1|:23004-23204
MsHel-6	gb|AIXA01002612.1|:37094-37527
MsHel-6	gb|AIXA01001514.1|:19657-20610
MsHel-6	gb|AIXA01000444.1|:24500-24640
MsHel-6	gb|AIXA01015090.1|:5841-6096
MsHel-6	gb|AIXA01009080.1|:30403-30602
MsHel-6	gb|AIXA01005396.1|:20424-20676
MsHel-6	gb|AIXA01004134.1|:46772-46906
MsHel-6	gb|AIXA01004134.1|:83528-83776
MsHel-6	gb|AIXA01003858.1|:67531-67671
MsHel-6	gb|AIXA01003816.1|:69593-69846
MsHel-6	gb|AIXA01002812.1|:19015-19274
MsHel-6	gb|AIXA01002563.1|:2534-2743
MsHel-6	gb|AIXA01002398.1|:92400-92598
MsHel-6	gb|AIXA01017947.1|:7691-7937
MsHel-6	gb|AIXA01014552.1|:10060-10314
MsHel-6	gb|AIXA01013928.1|:499-696
MsHel-6	gb|AIXA01013529.1|:11150-11411
MsHel-6	gb|AIXA01013529.1|:64452-64681
MsHel-6	gb|AIXA01012628.1|:14205-14445
MsHel-6	gb|AIXA01006878.1|:51102-51310
MsHel-6	gb|AIXA01006861.1|:11295-11503
MsHel-6	gb|AIXA01005339.1|:13659-14125
MsHel-6	gb|AIXA01004935.1|:40977-41234
MsHel-6	gb|AIXA01003813.1|:6111-6373
MsHel-6	gb|AIXA01002160.1|:26016-26342
MsHel-6	gb|AIXA01000149.1|:16145-16405
MsHel-6	gb|AIXA01019878.1|:1634-1890
MsHel-6	gb|AIXA01017418.1|:7486-7753
MsHel-6	gb|AIXA01015191.1|:14834-15031
MsHel-6	gb|AIXA01014028.1|:3793-4011
MsHel-6	gb|AIXA01013010.1|:21161-21605
MsHel-6	gb|AIXA01012741.1|:4517-4780
MsHel-6	gb|AIXA01012741.1|:52464-52670
MsHel-6	gb|AIXA01012282.1|:6570-6825
MsHel-6	gb|AIXA01011970.1|:6234-6696
MsHel-6	gb|AIXA01011337.1|:605-801
MsHel-6	gb|AIXA01009107.1|:1851-2047
MsHel-6	gb|AIXA01008402.1|:7559-7733
MsHel-6	gb|AIXA01008402.1|:36492-36753

MsHel-6	gb|AIXA01006809.1|:27333-27529
MsHel-6	gb|AIXA01005379.1|:34389-34638
MsHel-6	gb|AIXA01005237.1|:41804-42068
MsHel-6	gb|AIXA01004568.1|:10081-10340
MsHel-6	gb|AIXA01004342.1|:10020-10270
MsHel-6	gb|AIXA01004342.1|:64595-64854
MsHel-6	gb|AIXA01027182.1|:578-777
MsHel-6	gb|AIXA01015406.1|:2733-2981
MsHel-6	gb|AIXA01015230.1|:17085-17338
MsHel-6	gb|AIXA01014840.1|:4808-5246
MsHel-6	gb|AIXA01012530.1|:4365-4562
MsHel-6	gb|AIXA01010792.1|:375-630
MsHel-6	gb|AIXA01010753.1|:38835-39038
MsHel-6	gb|AIXA01010152.1|:37280-37536
MsHel-6	gb|AIXA01008730.1|:7882-8088
MsHel-6	gb|AIXA01008670.1|:7259-7468
MsHel-6	gb|AIXA01001502.1|:64609-64770
MsHel-6	gb|AIXA01000627.1|:78515-78764
MsHel-6	gb|AIXA01024799.1|:838-1031
MsHel-6	gb|AIXA01020060.1|:201-456
MsHel-6	gb|AIXA01018549.1|:6979-7182
MsHel-6	gb|AIXA01015168.1|:5530-5768
MsHel-6	gb|AIXA01011538.1|:15230-15482
MsHel-6	gb|AIXA01011197.1|:48903-49329
MsHel-6	gb|AIXA01009923.1|:30647-30915
MsHel-6	gb|AIXA01009584.1|:89827-89979
MsHel-6	gb|AIXA01008725.1|:22849-23102
MsHel-6	gb|AIXA01008551.1|:22446-22695
MsHel-6	gb|AIXA01007808.1|:33805-34198
MsHel-6	gb|AIXA01006871.1|:13076-13328
MsHel-6	gb|AIXA01006731.1|:44804-45047
MsHel-6	gb|AIXA01006043.1|:4425-4680
MsHel-6	gb|AIXA01004633.1|:26647-26903
MsHel-6	gb|AIXA01001799.1|:8494-8758
MsHel-6	gb|AIXA01001799.1|:34376-34598
MsHel-6	gb|AIXA01001645.1|:52380-52621
MsHel-6	gb|AIXA01000960.1|:11976-12239
MsHel-6	gb|AIXA01000960.1|:51431-51680
MsHel-6	gb|AIXA01000960.1|:129690-129831
MsHel-6	gb|AIXA01026553.1|:632-877
MsHel-6	gb|AIXA01021314.1|:1789-2046
MsHel-6	gb|AIXA01016314.1|:4465-5030
MsHel-6	gb|AIXA01015119.1|:10848-11106
MsHel-6	gb|AIXA01015119.1|:45000-45242
MsHel-6	gb|AIXA01013222.1|:6376-6640
MsHel-6	gb|AIXA01011323.1|:3008-3142
MsHel-6	gb|AIXA01011323.1|:16597-16843
MsHel-6	gb|AIXA01011314.1|:7095-7348

MsHel-6	gb|AIXA01010323.1|:12201-12408
MsHel-6	gb|AIXA01010323.1|:82903-83158
MsHel-6	gb|AIXA01008741.1|:7680-7885
MsHel-6	gb|AIXA01007358.1|:1137-1369
MsHel-6	gb|AIXA01006857.1|:24963-25187
MsHel-6	gb|AIXA01005653.1|:3976-4188
MsHel-6	gb|AIXA01005220.1|:19640-19890
MsHel-6	gb|AIXA01003783.1|:3334-3539
MsHel-6	gb|AIXA01003297.1|:8707-8968
MsHel-6	gb|AIXA01001526.1|:27134-27389
MsHel-6	gb|AIXA01001254.1|:1529-1785
MsHel-6	gb|AIXA01001254.1|:23071-23287
MsHel-6	gb|AIXA01000909.1|:13226-13483
MsHel-6	gb|AIXA01026688.1|:852-1068
MsHel-6	gb|AIXA01021767.1|:488-738
MsHel-6	gb|AIXA01013912.1|:8758-9012
MsHel-6	gb|AIXA01013889.1|:6439-6699
MsHel-6	gb|AIXA01011814.1|:171-368
MsHel-6	gb|AIXA01011325.1|:820-1028
MsHel-6	gb|AIXA01010772.1|:41607-41814
MsHel-6	gb|AIXA01010076.1|:64127-64533
MsHel-6	gb|AIXA01009697.1|:28852-29052
MsHel-6	gb|AIXA01008824.1|:25-403
MsHel-6	gb|AIXA01008824.1|:21105-21313
MsHel-6	gb|AIXA01008108.1|:11770-12020
MsHel-6	gb|AIXA01007355.1|:32406-32657
MsHel-6	gb|AIXA01006478.1|:84008-84276
MsHel-6	gb|AIXA01006284.1|:8129-8345
MsHel-6	gb|AIXA01004676.1|:96247-96454
MsHel-6	gb|AIXA01004424.1|:6032-6279
MsHel-6	gb|AIXA01003651.1|:29785-30035
MsHel-6	gb|AIXA01001779.1|:4749-5007
MsHel-6	gb|AIXA01001650.1|:14978-15166
MsHel-6	gb|AIXA01001032.1|:63155-63306
MsHel-6	gb|AIXA01000809.1|:44944-45158
MsHel-6	gb|AIXA01020389.1|:1911-2350
MsHel-6	gb|AIXA01015371.1|:3731-3972
MsHel-6	gb|AIXA01013588.1|:1975-2225
MsHel-6	gb|AIXA01012031.1|:5941-6179
MsHel-6	gb|AIXA01010748.1|:5918-6013
MsHel-6	gb|AIXA01010748.1|:77275-80273
MsHel-6	gb|AIXA01010748.1|:158903-159151
MsHel-6	gb|AIXA01009566.1|:12789-13048
MsHel-6	gb|AIXA01007494.1|:23127-23342
MsHel-6	gb|AIXA01005095.1|:52376-52619
MsHel-6	gb|AIXA01004694.1|:13339-13600
MsHel-6	gb|AIXA01003609.1|:48502-48734
MsHel-6	gb|AIXA01001618.1|:39603-39861

MsHel-6	gb|AIXA01000897.1|:27368-27571
MsHel-6	gb|AIXA01000552.1|:645-902
MsHel-6	gb|AIXA01018376.1|:422-708
MsHel-6	gb|AIXA01016657.1|:18585-18788
MsHel-6	gb|AIXA01014401.1|:3899-4060
MsHel-6	gb|AIXA01013682.1|:6653-6902
MsHel-6	gb|AIXA01013502.1|:3127-3322
MsHel-6	gb|AIXA01011824.1|:2262-2516
MsHel-6	gb|AIXA01010886.1|:4980-5262
MsHel-6	gb|AIXA01010124.1|:2960-3211
MsHel-6	gb|AIXA01009642.1|:15686-15892
MsHel-6	gb|AIXA01009268.1|:17354-17868
MsHel-6	gb|AIXA01007966.1|:80297-80388
MsHel-6	gb|AIXA01007966.1|:105143-105277
MsHel-6	gb|AIXA01007966.1|:154646-154884
MsHel-6	gb|AIXA01005316.1|:5286-5462
MsHel-6	gb|AIXA01004167.1|:58493-58752
MsHel-6	gb|AIXA01001648.1|:30720-30885
MsHel-6	gb|AIXA01001511.1|:24396-24670
MsHel-6	gb|AIXA01001373.1|:1536-1726
MsHel-6	gb|AIXA01000944.1|:21285-21543
MsHel-6	gb|AIXA01000758.1|:2459-2718
MsHel-6	gb|AIXA01000717.1|:37513-37703
MsHel-6	gb|AIXA01000637.1|:5158-5420
MsHel-6	gb|AIXA01000613.1|:3021-3206
MsHel-6	gb|AIXA01000069.1|:39717-39921
MsHel-6	gb|AIXA01000069.1|:80463-80576
MsHel-6	gb|AIXA01000069.1|:95356-95583
MsHel-6	gb|AIXA01017755.1|:12201-12453
MsHel-6	gb|AIXA01013999.1|:4030-4282
MsHel-6	gb|AIXA01013565.1|:45489-45726
MsHel-6	gb|AIXA01013424.1|:7926-8176
MsHel-6	gb|AIXA01012523.1|:502-699
MsHel-6	gb|AIXA01012357.1|:4703-4919
MsHel-6	gb|AIXA01008934.1|:14536-14734
MsHel-6	gb|AIXA01007421.1|:52382-53050
MsHel-6	gb|AIXA01006808.1|:1496-1743
MsHel-6	gb|AIXA01006716.1|:5411-5611
MsHel-6	gb|AIXA01004212.1|:25479-25736
MsHel-6	gb|AIXA01003928.1|:41239-41495
MsHel-6	gb|AIXA01003124.1|:17957-18212
MsHel-6	gb|AIXA01002646.1|:3521-3722
MsHel-6	gb|AIXA01001651.1|:7939-8199
MsHel-6	gb|AIXA01001621.1|:5721-5971
MsHel-6	gb|AIXA01001196.1|:29792-29974
MsHel-6	gb|AIXA01000796.1|:36185-36409
MsHel-6	gb|AIXA01000796.1|:47206-47457
MsHel-6	gb|AIXA01000706.1|:74551-74738

MsHel-6	gb|AIXA01000570.1|:18567-18832
MsHel-6	gb|AIXA01034700.1|:272-468
MsHel-6	gb|AIXA01018822.1|:3740-3993
MsHel-6	gb|AIXA01015801.1|:7029-7288
MsHel-6	gb|AIXA01014739.1|:28696-28946
MsHel-6	gb|AIXA01012404.1|:4403-4660
MsHel-6	gb|AIXA01012306.1|:5703-6134
MsHel-6	gb|AIXA01011986.1|:3086-3518
MsHel-6	gb|AIXA01011234.1|:4645-4925
MsHel-6	gb|AIXA01009644.1|:1361-1563
MsHel-6	gb|AIXA01007757.1|:5589-5848
MsHel-6	gb|AIXA01006923.1|:4305-4674
MsHel-6	gb|AIXA01006676.1|:8498-8932
MsHel-6	gb|AIXA01005494.1|:1526-1970
MsHel-6	gb|AIXA01003836.1|:8533-8736
MsHel-6	gb|AIXA01003045.1|:50407-50661
MsHel-6	gb|AIXA01003042.1|:120102-120294
MsHel-6	gb|AIXA01002805.1|:59435-59664
MsHel-6	gb|AIXA01002805.1|:111730-111934
MsHel-6	gb|AIXA01000434.1|:2883-3147
MsHel-6	gb|AIXA01000422.1|:69145-69336
MsHel-6	gb|AIXA01000235.1|:1436-1615
MsHel-6	gb|AIXA01031004.1|:127-321
MsHel-6	gb|AIXA01029115.1|:435-634
MsHel-6	gb|AIXA01027184.1|:114-317
MsHel-6	gb|AIXA01017411.1|:2167-2556
MsHel-6	gb|AIXA01015983.1|:10174-10403
MsHel-6	gb|AIXA01015125.1|:7516-7759
MsHel-6	gb|AIXA01013523.1|:25135-25355
MsHel-6	gb|AIXA01011946.1|:992-1243
MsHel-6	gb|AIXA01010737.1|:11032-11291
MsHel-6	gb|AIXA01010378.1|:2290-2623
MsHel-6	gb|AIXA01005537.1|:3662-4088
MsHel-6	gb|AIXA01005310.1|:2317-2554
MsHel-6	gb|AIXA01005262.1|:579-794
MsHel-6	gb|AIXA01005062.1|:40642-40841
MsHel-6	gb|AIXA01000155.1|:43961-44217
MsHel-6	gb|AIXA01019066.1|:2225-2481
MsHel-6	gb|AIXA01017107.1|:3104-3301
MsHel-6	gb|AIXA01016946.1|:2633-2882
MsHel-6	gb|AIXA01016345.1|:926-1140
MsHel-6	gb|AIXA01013697.1|:496-682
MsHel-6	gb|AIXA01012346.1|:6617-6868
MsHel-6	gb|AIXA01010997.1|:3195-3450
MsHel-6	gb|AIXA01007478.1|:37995-38249
MsHel-6	gb|AIXA01002579.1|:100355-101016
MsHel-6	gb|AIXA01001679.1|:11542-11808
MsHel-6	gb|AIXA01001278.1|:926-1401

MsHel-6	gb|AIXA01001239.1|:130793-130954
MsHel-6	gb|AIXA01000494.1|:51465-51864
MsHel-6	gb|AIXA01018190.1|:3069-3308
MsHel-6	gb|AIXA01017433.1|:9690-10194
MsHel-6	gb|AIXA01015529.1|:5287-5524
MsHel-6	gb|AIXA01015447.1|:2088-2278
MsHel-6	gb|AIXA01014735.1|:6371-6626
MsHel-6	gb|AIXA01014021.1|:2986-3199
MsHel-6	gb|AIXA01013047.1|:19287-19481
MsHel-6	gb|AIXA01007654.1|:23555-23751
MsHel-6	gb|AIXA01006266.1|:29769-30019
MsHel-6	gb|AIXA01006021.1|:30347-30568
MsHel-6	gb|AIXA01006021.1|:69221-69695
MsHel-6	gb|AIXA01005497.1|:30077-30286
MsHel-6	gb|AIXA01005488.1|:8968-12699
MsHel-6	gb|AIXA01004775.1|:50109-50269
MsHel-6	gb|AIXA01004643.1|:46046-46301
MsHel-6	gb|AIXA01004483.1|:34896-35149
MsHel-6	gb|AIXA01002990.1|:17780-18285
MsHel-6	gb|AIXA01001665.1|:65853-66068
MsHel-6	gb|AIXA01030554.1|:28-284
MsHel-6	gb|AIXA01019906.1|:717-905
MsHel-6	gb|AIXA01014708.1|:4667-4925
MsHel-6	gb|AIXA01014695.1|:744-943
MsHel-6	gb|AIXA01014268.1|:28-284
MsHel-6	gb|AIXA01013521.1|:20001-20192
MsHel-6	gb|AIXA01011659.1|:5230-5488
MsHel-6	gb|AIXA01009427.1|:25901-26665
MsHel-6	gb|AIXA01005049.1|:2973-3157
MsHel-6	gb|AIXA01004336.1|:7087-7329
MsHel-6	gb|AIXA01003677.1|:7696-8137
MsHel-6	gb|AIXA01003677.1|:32328-32545
MsHel-6	gb|AIXA01002381.1|:83136-83398
MsHel-6	gb|AIXA01033474.1|:80-290
MsHel-6	gb|AIXA01017631.1|:8742-8904
MsHel-6	gb|AIXA01009610.1|:14522-14736
MsHel-6	gb|AIXA01008263.1|:40941-41214
MsHel-6	gb|AIXA01003698.1|:97685-97939
MsHel-6	gb|AIXA01003283.1|:44382-44639
MsHel-6	gb|AIXA01002951.1|:63740-63996
MsHel-6	gb|AIXA01002008.1|:102920-103160
MsHel-6	gb|AIXA01000228.1|:17550-17805
MsHel-6	gb|AIXA01019645.1|:621-831
MsHel-6	gb|AIXA01014467.1|:4506-4765
MsHel-6	gb|AIXA01013680.1|:21275-21440
MsHel-6	gb|AIXA01013557.1|:1802-2020
MsHel-6	gb|AIXA01013052.1|:4414-4578
MsHel-6	gb|AIXA01012158.1|:3469-3653

MsHel-6	gb|AIXA01011969.1|:38377-38634
MsHel-6	gb|AIXA01011273.1|:25882-26103
MsHel-6	gb|AIXA01009408.1|:22982-23290
MsHel-6	gb|AIXA01009126.1|:380-819
MsHel-6	gb|AIXA01008444.1|:56959-57141
MsHel-6	gb|AIXA01007105.1|:7877-8071
MsHel-6	gb|AIXA01007032.1|:42177-42431
MsHel-6	gb|AIXA01005104.1|:22439-22719
MsHel-6	gb|AIXA01004895.1|:4448-4637
MsHel-6	gb|AIXA01004895.1|:42663-42847
MsHel-6	gb|AIXA01004552.1|:48831-49098
MsHel-6	gb|AIXA01004487.1|:3198-3460
MsHel-6	gb|AIXA01003002.1|:73861-74088
MsHel-6	gb|AIXA01002655.1|:9566-9775
MsHel-6	gb|AIXA01001831.1|:5012-5198
MsHel-6	gb|AIXA01032105.1|:34-284
MsHel-6	gb|AIXA01017365.1|:4904-5126
MsHel-6	gb|AIXA01012784.1|:52970-53220
MsHel-6	gb|AIXA01011117.1|:6115-6316
MsHel-6	gb|AIXA01010733.1|:16059-16270
MsHel-6	gb|AIXA01010200.1|:925-1116
MsHel-6	gb|AIXA01009865.1|:105830-105990
MsHel-6	gb|AIXA01008793.1|:9327-9712
MsHel-6	gb|AIXA01008767.1|:18644-18905
MsHel-6	gb|AIXA01008521.1|:19743-19944
MsHel-6	gb|AIXA01008342.1|:13746-14008
MsHel-6	gb|AIXA01006821.1|:6076-6324
MsHel-6	gb|AIXA01005889.1|:8030-8182
MsHel-6	gb|AIXA01005406.1|:5735-5941
MsHel-6	gb|AIXA01005348.1|:22255-22492
MsHel-6	gb|AIXA01004160.1|:39534-39738
MsHel-6	gb|AIXA01001739.1|:17954-18214
MsHel-6	gb|AIXA01024267.1|:315-560
MsHel-6	gb|AIXA01018777.1|:3266-3523
MsHel-6	gb|AIXA01016082.1|:4395-4651
MsHel-6	gb|AIXA01014920.1|:4797-5053
MsHel-6	gb|AIXA01014386.1|:7466-7721
MsHel-6	gb|AIXA01010659.1|:4429-4674
MsHel-6	gb|AIXA01010538.1|:24734-24994
MsHel-6	gb|AIXA01009411.1|:11094-11285
MsHel-6	gb|AIXA01009370.1|:5440-5948
MsHel-6	gb|AIXA01008404.1|:21920-22117
MsHel-6	gb|AIXA01007350.1|:27928-28179
MsHel-6	gb|AIXA01007059.1|:8393-8642
MsHel-6	gb|AIXA01005289.1|:1519-1769
MsHel-6	gb|AIXA01004484.1|:10781-11036
MsHel-6	gb|AIXA01004230.1|:45650-45739
MsHel-6	gb|AIXA01004154.1|:13268-13520

MsHel-6	gb|AIXA01003825.1|:26705-27210
MsHel-6	gb|AIXA01002527.1|:1407-1654
MsHel-6	gb|AIXA01001532.1|:56191-56459
MsHel-6	gb|AIXA01000046.1|:44582-44803
MsHel-6	gb|AIXA01022939.1|:1303-1559
MsHel-6	gb|AIXA01021724.1|:1527-1710
MsHel-6	gb|AIXA01017285.1|:630-893
MsHel-6	gb|AIXA01016973.1|:1143-1402
MsHel-6	gb|AIXA01011707.1|:4271-4529
MsHel-6	gb|AIXA01010287.1|:942-1113
MsHel-6	gb|AIXA01008164.1|:13712-13893
MsHel-6	gb|AIXA01007513.1|:52267-52454
MsHel-6	gb|AIXA01007306.1|:6687-6941
MsHel-6	gb|AIXA01007099.1|:23304-23769
MsHel-6	gb|AIXA01006781.1|:63431-63690
MsHel-6	gb|AIXA01004867.1|:26480-26936
MsHel-6	gb|AIXA01004128.1|:8105-8536
MsHel-6	gb|AIXA01001520.1|:23885-24105
MsHel-6	gb|AIXA01001520.1|:39424-39869
MsHel-6	gb|AIXA01000586.1|:418-667
MsHel-6	gb|AIXA01000047.1|:49575-49833
MsHel-6	gb|AIXA01017991.1|:6838-7114
MsHel-6	gb|AIXA01016979.1|:2785-2975
MsHel-6	gb|AIXA01014245.1|:19033-19317
MsHel-6	gb|AIXA01011842.1|:17987-18228
MsHel-6	gb|AIXA01010075.1|:6914-7162
MsHel-6	gb|AIXA01008313.1|:65616-65830
MsHel-6	gb|AIXA01007104.1|:45902-46059
MsHel-6	gb|AIXA01006928.1|:1564-1781
MsHel-6	gb|AIXA01006874.1|:27692-27905
MsHel-6	gb|AIXA01005652.1|:11148-11349
MsHel-6	gb|AIXA01005596.1|:76254-76461
MsHel-6	gb|AIXA01003152.1|:99453-99704
MsHel-6	gb|AIXA01003152.1|:136329-138015
MsHel-6	gb|AIXA01002568.1|:23628-23841
MsHel-6	gb|AIXA01000922.1|:23343-23597
MsHel-6	gb|AIXA01027229.1|:54-248
MsHel-6	gb|AIXA01021158.1|:1658-1859
MsHel-6	gb|AIXA01018786.1|:4824-5031
MsHel-6	gb|AIXA01018099.1|:3779-3984
MsHel-6	gb|AIXA01016601.1|:688-883
MsHel-6	gb|AIXA01016018.1|:12963-13198
MsHel-6	gb|AIXA01015579.1|:42260-42693
MsHel-6	gb|AIXA01015158.1|:18718-18958
MsHel-6	gb|AIXA01014208.1|:12655-13124
MsHel-6	gb|AIXA01013041.1|:4806-5026
MsHel-6	gb|AIXA01013041.1|:44430-44624
MsHel-6	gb|AIXA01012004.1|:569-1046

MsHel-6	gb|AIXA01011104.1|:8180-8430
MsHel-6	gb|AIXA01011104.1|:48408-48637
MsHel-6	gb|AIXA01006854.1|:69198-70796
MsHel-6	gb|AIXA01006847.1|:5210-9400
MsHel-6	gb|AIXA01005538.1|:59561-66053
MsHel-6	gb|AIXA01005347.1|:83628-84057
MsHel-6	gb|AIXA01005110.1|:5238-5635
MsHel-6	gb|AIXA01004656.1|:135799-136000
MsHel-6	gb|AIXA01004077.1|:35580-35899
MsHel-6	gb|AIXA01002998.1|:43162-43543
MsHel-6	gb|AIXA01002340.1|:14423-14628
MsHel-6	gb|AIXA01002228.1|:15738-15939
MsHel-6	gb|AIXA01002003.1|:3222-3411
MsHel-6	gb|AIXA01001816.1|:11298-11487
MsHel-6	gb|AIXA01000561.1|:720-979
MsHel-6	gb|AIXA01000561.1|:120463-120684
MsHel-6	gb|AIXA01000019.1|:45371-45624
MsHel-6	gb|AIXA01000017.1|:4384-4647
MsHel-6	gb|AIXA01027772.1|:457-707
MsHel-6	gb|AIXA01024160.1|:821-1049
MsHel-6	gb|AIXA01021730.1|:1-144
MsHel-6	gb|AIXA01020174.1|:1415-1663
MsHel-6	gb|AIXA01018697.1|:449-701
MsHel-6	gb|AIXA01018696.1|:557-809
MsHel-6	gb|AIXA01017579.1|:2551-2800
MsHel-6	gb|AIXA01015812.1|:9417-10013
MsHel-6	gb|AIXA01013089.1|:5965-6216
MsHel-6	gb|AIXA01013089.1|:18369-18585
MsHel-6	gb|AIXA01012931.1|:7140-7398
MsHel-6	gb|AIXA01012451.1|:1214-1435
MsHel-6	gb|AIXA01011624.1|:5595-5857
MsHel-6	gb|AIXA01007963.1|:4156-4657
MsHel-6	gb|AIXA01007947.1|:42380-42487
MsHel-6	gb|AIXA01006908.1|:12819-13382
MsHel-6	gb|AIXA01006055.1|:69611-69859
MsHel-6	gb|AIXA01005125.1|:9551-9751
MsHel-6	gb|AIXA01004672.1|:16358-16546
MsHel-6	gb|AIXA01003849.1|:8819-9032
MsHel-6	gb|AIXA01002392.1|:5609-5858
MsHel-6	gb|AIXA01001985.1|:38809-39060
MsHel-6	gb|AIXA01000792.1|:26507-27009
MsHel-6	gb|AIXA01019791.1|:1960-2201
MsHel-6	gb|AIXA01017916.1|:9640-9806
MsHel-6	gb|AIXA01016376.1|:25720-25971
MsHel-6	gb|AIXA01015389.1|:5623-5873
MsHel-6	gb|AIXA01015147.1|:3124-3315
MsHel-6	gb|AIXA01013331.1|:9812-10067
MsHel-6	gb|AIXA01012495.1|:13197-13367

MsHel-6	gb|AIXA01011239.1|:58712-58886
MsHel-6	gb|AIXA01010379.1|:2295-2628
MsHel-6	gb|AIXA01010188.1|:1248-1519
MsHel-6	gb|AIXA01007854.1|:23897-24148
MsHel-6	gb|AIXA01006917.1|:108937-109121
MsHel-6	gb|AIXA01004697.1|:34123-34305
MsHel-6	gb|AIXA01003687.1|:151-309
MsHel-6	gb|AIXA01003536.1|:7479-7738
MsHel-6	gb|AIXA01003028.1|:34574-34813
MsHel-6	gb|AIXA01002021.1|:6436-6707
MsHel-6	gb|AIXA01001841.1|:53214-53357
MsHel-6	gb|AIXA01000654.1|:43879-44042
MsHel-6	gb|AIXA01000072.1|:17053-17497
MsHel-6	gb|AIXA01000072.1|:107952-108154
MsHel-6	gb|AIXA01014544.1|:8566-8811
MsHel-6	gb|AIXA01013970.1|:522-716
MsHel-6	gb|AIXA01013850.1|:39890-40142
MsHel-6	gb|AIXA01013135.1|:11742-11952
MsHel-6	gb|AIXA01010330.1|:25113-25286
MsHel-6	gb|AIXA01010330.1|:61637-61827
MsHel-6	gb|AIXA01010330.1|:71989-72433
MsHel-6	gb|AIXA01005084.1|:1590-1787
MsHel-6	gb|AIXA01004911.1|:20608-20858
MsHel-6	gb|AIXA01001886.1|:8774-8996
MsHel-6	gb|AIXA01001886.1|:40864-41029
MsHel-6	gb|AIXA01001695.1|:115350-115539
MsHel-6	gb|AIXA01001090.1|:26657-26915
MsHel-6	gb|AIXA01001010.1|:30867-31125
MsHel-6	gb|AIXA01000359.1|:88444-88702
MsHel-6	gb|AIXA01024285.1|:182-372
MsHel-6	gb|AIXA01019516.1|:723-947
MsHel-6	gb|AIXA01016208.1|:3920-4121
MsHel-6	gb|AIXA01013092.1|:31193-31645
MsHel-6	gb|AIXA01009837.1|:11096-11625
MsHel-6	gb|AIXA01009833.1|:2808-3009
MsHel-6	gb|AIXA01008291.1|:475-725
MsHel-6	gb|AIXA01007024.1|:122430-122671
MsHel-6	gb|AIXA01007024.1|:150534-150785
MsHel-6	gb|AIXA01006973.1|:6938-7143
MsHel-6	gb|AIXA01006833.1|:27867-28382
MsHel-6	gb|AIXA01006742.1|:18178-18450
MsHel-6	gb|AIXA01005073.1|:22465-22659
MsHel-6	gb|AIXA01004277.1|:41251-41448
MsHel-6	gb|AIXA01004084.1|:40719-40932
MsHel-6	gb|AIXA01003853.1|:39417-39668
MsHel-6	gb|AIXA01002526.1|:1169-1390
MsHel-6	gb|AIXA01002384.1|:76626-76902
MsHel-6	gb|AIXA01001865.1|:4558-4759

MsHel-6	gb|AIXA01000472.1|:3197-3404
MsHel-6	gb|AIXA01016020.1|:1199-1394
MsHel-6	gb|AIXA01012007.1|:34905-35080
MsHel-6	gb|AIXA01010714.1|:47747-47992
MsHel-6	gb|AIXA01010714.1|:99343-99812
MsHel-6	gb|AIXA01010667.1|:8227-8424
MsHel-6	gb|AIXA01009543.1|:4319-4574
MsHel-6	gb|AIXA01009230.1|:10665-10832
MsHel-6	gb|AIXA01008762.1|:7147-7364
MsHel-6	gb|AIXA01006909.1|:19185-19447
MsHel-6	gb|AIXA01005668.1|:4486-4740
MsHel-6	gb|AIXA01004220.1|:41782-41969
MsHel-6	gb|AIXA01004153.1|:114782-114978
MsHel-6	gb|AIXA01002656.1|:15767-15922
MsHel-6	gb|AIXA01002614.1|:59648-59905
MsHel-6	gb|AIXA01001234.1|:11219-11439
MsHel-6	gb|AIXA01000440.1|:210-457
MsHel-6	gb|AIXA01019479.1|:2456-2610
MsHel-6	gb|AIXA01018739.1|:7792-7946
MsHel-6	gb|AIXA01018052.1|:440-646
MsHel-6	gb|AIXA01017872.1|:2-144
MsHel-6	gb|AIXA01017114.1|:11248-11440
MsHel-6	gb|AIXA01010802.1|:17557-17769
MsHel-6	gb|AIXA01009663.1|:6034-6253
MsHel-6	gb|AIXA01005476.1|:540-698
MsHel-6	gb|AIXA01002680.1|:14465-14719
MsHel-6	gb|AIXA01015711.1|:11-160
MsHel-6	gb|AIXA01013945.1|:8813-8978
MsHel-6	gb|AIXA01013013.1|:21610-21825
MsHel-6	gb|AIXA01011242.1|:3372-3569
MsHel-6	gb|AIXA01010730.1|:10766-10964
MsHel-6	gb|AIXA01010442.1|:17795-18068
MsHel-6	gb|AIXA01009711.1|:22330-22548
MsHel-6	gb|AIXA01007964.1|:5612-10304
MsHel-6	gb|AIXA01006442.1|:38607-38873
MsHel-6	gb|AIXA01005610.1|:4217-4716
MsHel-6	gb|AIXA01004486.1|:44604-44818
MsHel-6	gb|AIXA01004324.1|:11139-14984
MsHel-6	gb|AIXA01004181.1|:29367-29833
MsHel-6	gb|AIXA01003722.1|:52025-52206
MsHel-6	gb|AIXA01003722.1|:63123-63203
MsHel-6	gb|AIXA01003637.1|:20225-20471
MsHel-6	gb|AIXA01003325.1|:9625-9820
MsHel-6	gb|AIXA01001935.1|:579-795
MsHel-6	gb|AIXA01001332.1|:16876-17121
MsHel-6	gb|AIXA01000972.1|:63953-64202
MsHel-6	gb|AIXA01037352.1|:152-399
MsHel-6	gb|AIXA01022217.1|:1325-1574

MsHel-6	gb|AIXA01019309.1|:3613-3865
MsHel-6	gb|AIXA01016438.1|:1480-3865
MsHel-6	gb|AIXA01015382.1|:23058-23325
MsHel-6	gb|AIXA01012348.1|:19374-19581
MsHel-6	gb|AIXA01009924.1|:6752-7000
MsHel-6	gb|AIXA01007303.1|:24327-24825
MsHel-6	gb|AIXA01005616.1|:9700-9972
MsHel-6	gb|AIXA01003129.1|:3700-3950
MsHel-6	gb|AIXA01002993.1|:5701-6229
MsHel-6	gb|AIXA01002519.1|:3767-3899
MsHel-6	gb|AIXA01002396.1|:6001-6206
MsHel-6	gb|AIXA01002240.1|:18905-28282
MsHel-6	gb|AIXA01002214.1|:1074-1294
MsHel-6	gb|AIXA01002078.1|:424-681
MsHel-6	gb|AIXA01001048.1|:88219-88479
MsHel-6	gb|AIXA01000639.1|:8889-9082
MsHel-6	gb|AIXA01020557.1|:451-607
MsHel-6	gb|AIXA01016883.1|:1806-2048
MsHel-6	gb|AIXA01016132.1|:1373-1652
MsHel-6	gb|AIXA01014172.1|:5974-6130
MsHel-6	gb|AIXA01012541.1|:11035-11174
MsHel-6	gb|AIXA01012398.1|:26689-35924
MsHel-6	gb|AIXA01011310.1|:1852-2027
MsHel-6	gb|AIXA01010758.1|:366-562
MsHel-6	gb|AIXA01006881.1|:39884-40085
MsHel-6	gb|AIXA01005322.1|:22049-22301
MsHel-6	gb|AIXA01004894.1|:38284-38480
MsHel-6	gb|AIXA01004865.1|:33278-33715
MsHel-6	gb|AIXA01004150.1|:52943-53199
MsHel-6	gb|AIXA01002753.1|:68360-68582
MsHel-6	gb|AIXA01002069.1|:54053-54216
MsHel-6	gb|AIXA01000439.1|:9648-9914
MsHel-6	gb|AIXA01032055.1|:153-392
MsHel-6	gb|AIXA01026199.1|:429-647
MsHel-6	gb|AIXA01024308.1|:755-1185
MsHel-6	gb|AIXA01022935.1|:157-346
MsHel-6	gb|AIXA01017942.1|:3794-4008
MsHel-6	gb|AIXA01013612.1|:13298-13758
MsHel-6	gb|AIXA01013004.1|:5155-5337
MsHel-6	gb|AIXA01012583.1|:4295-4652
MsHel-6	gb|AIXA01012366.1|:3655-3900
MsHel-6	gb|AIXA01011724.1|:22926-23174
MsHel-6	gb|AIXA01011360.1|:30128-30423
MsHel-6	gb|AIXA01009656.1|:790-1034
MsHel-6	gb|AIXA01009649.1|:33341-33591
MsHel-6	gb|AIXA01009477.1|:1506-1762
MsHel-6	gb|AIXA01008915.1|:58572-58738
MsHel-6	gb|AIXA01008688.1|:2574-2732

MsHel-6	gb|AIXA01008393.1|:65375-65587
MsHel-6	gb|AIXA01006926.1|:12308-12665
MsHel-6	gb|AIXA01006889.1|:22052-22250
MsHel-6	gb|AIXA01005437.1|:9036-9446
MsHel-6	gb|AIXA01003633.1|:36745-37001
MsHel-6	gb|AIXA01002607.1|:18841-19085
MsHel-6	gb|AIXA01002241.1|:25450-25894
MsHel-6	gb|AIXA01001690.1|:66118-66367
MsHel-6	gb|AIXA01000508.1|:11465-12095
MsHel-6	gb|AIXA01000278.1|:29384-29633
MsHel-6	gb|AIXA01018151.1|:6147-6547
MsHel-6	gb|AIXA01016583.1|:2355-2525
MsHel-6	gb|AIXA01014968.1|:11670-11851
MsHel-6	gb|AIXA01010744.1|:13508-13720
MsHel-6	gb|AIXA01010284.1|:488-710
MsHel-6	gb|AIXA01008099.1|:9678-9839
MsHel-6	gb|AIXA01007405.1|:756-1006
MsHel-6	gb|AIXA01007049.1|:71857-72103
MsHel-6	gb|AIXA01006682.1|:4992-5133
MsHel-6	gb|AIXA01005245.1|:25-236
MsHel-6	gb|AIXA01005071.1|:5523-5982
MsHel-6	gb|AIXA01004861.1|:3846-4096
MsHel-6	gb|AIXA01004520.1|:168-406
MsHel-6	gb|AIXA01004451.1|:10678-10895
MsHel-6	gb|AIXA01003216.1|:2130-2319
MsHel-6	gb|AIXA01002758.1|:3028-3221
MsHel-6	gb|AIXA01001366.1|:55322-55566
MsHel-6	gb|AIXA01000713.1|:40945-41123
MsHel-6	gb|AIXA01000225.1|:9241-9437
MsHel-6	gb|AIXA01029285.1|:118-363
MsHel-6	gb|AIXA01027452.1|:681-953
MsHel-6	gb|AIXA01020691.1|:833-1050
MsHel-6	gb|AIXA01018979.1|:488-646
MsHel-6	gb|AIXA01016411.1|:7140-7267
MsHel-6	gb|AIXA01012796.1|:30588-30777
MsHel-6	gb|AIXA01012534.1|:5339-5513
MsHel-6	gb|AIXA01011304.1|:27990-28178
MsHel-6	gb|AIXA01008001.1|:4063-4352
MsHel-6	gb|AIXA01006389.1|:132-290
MsHel-6	gb|AIXA01002077.1|:10799-10994
MsHel-6	gb|AIXA01001144.1|:109858-110089
MsHel-6	gb|AIXA01001135.1|:5767-6027
MsHel-6	gb|AIXA01000931.1|:8919-9183
MsHel-6	gb|AIXA01000481.1|:15296-15469
MsHel-6	gb|AIXA01000120.1|:110557-110755
MsHel-6	gb|AIXA01029247.1|:10-255
MsHel-6	gb|AIXA01020907.1|:17-189
MsHel-6	gb|AIXA01019774.1|:908-1109

MsHel-6	gb|AIXA01018566.1|:445-639
MsHel-6	gb|AIXA01018184.1|:804-992
MsHel-6	gb|AIXA01016991.1|:1736-1929
MsHel-6	gb|AIXA01016781.1|:6946-7135
MsHel-6	gb|AIXA01015350.1|:478-722
MsHel-6	gb|AIXA01012626.1|:6269-6466
MsHel-6	gb|AIXA01012183.1|:285-536
MsHel-6	gb|AIXA01011008.1|:10910-11121
MsHel-6	gb|AIXA01011008.1|:21728-21982
MsHel-6	gb|AIXA01010405.1|:2106-2321
MsHel-6	gb|AIXA01010405.1|:12455-12811
MsHel-6	gb|AIXA01009599.1|:13-144
MsHel-6	gb|AIXA01009388.1|:14184-14425
MsHel-6	gb|AIXA01008667.1|:28915-29093
MsHel-6	gb|AIXA01008222.1|:15400-15587
MsHel-6	gb|AIXA01007603.1|:17750-18007
MsHel-6	gb|AIXA01006265.1|:26045-26217
MsHel-6	gb|AIXA01006057.1|:28056-28224
MsHel-6	gb|AIXA01005972.1|:903-1370
MsHel-6	gb|AIXA01005498.1|:79642-79818
MsHel-6	gb|AIXA01003317.1|:78811-79011
MsHel-6	gb|AIXA01003015.1|:66565-66766
MsHel-6	gb|AIXA01002705.1|:10401-10941
MsHel-6	gb|AIXA01002521.1|:48115-48333
MsHel-6	gb|AIXA01029280.1|:338-579
MsHel-6	gb|AIXA01029071.1|:49-198
MsHel-6	gb|AIXA01018231.1|:12-170
MsHel-6	gb|AIXA01017972.1|:978-1140
MsHel-6	gb|AIXA01016561.1|:30980-31515
MsHel-6	gb|AIXA01013391.1|:823-1019
MsHel-6	gb|AIXA01013344.1|:7057-7198
MsHel-6	gb|AIXA01008395.1|:12192-12847
MsHel-6	gb|AIXA01020027.1|:960-1085
MsHel-6	gb|AIXA01015687.1|:3429-3639
MsHel-6	gb|AIXA01014991.1|:18747-18994
MsHel-6	gb|AIXA01014462.1|:5799-6074
MsHel-6	gb|AIXA01014135.1|:1668-2193
MsHel-6	gb|AIXA01011937.1|:15375-15854
MsHel-6	gb|AIXA01008533.1|:42447-42632
MsHel-6	gb|AIXA01007676.1|:13662-13855
MsHel-6	gb|AIXA01006190.1|:94330-94534
MsHel-6	gb|AIXA01005113.1|:10743-10922
MsHel-6	gb|AIXA01004546.1|:30666-30832
MsHel-6	gb|AIXA01004242.1|:15652-15813
MsHel-6	gb|AIXA01002114.1|:32495-32660
MsHel-6	gb|AIXA01002045.1|:16238-16416
MsHel-6	gb|AIXA01001132.1|:36144-36395
MsHel-6	gb|AIXA01018700.1|:2-188

MsHel-6	gb|AIXA01018409.1|:757-3331
MsHel-6	gb|AIXA01013328.1|:16605-16834
MsHel-6	gb|AIXA01011947.1|:1-128
MsHel-6	gb|AIXA01011947.1|:14455-14664
MsHel-6	gb|AIXA01011942.1|:18329-18543
MsHel-6	gb|AIXA01007189.1|:104-363
MsHel-6	gb|AIXA01004222.1|:51670-51891
MsHel-6	gb|AIXA01003113.1|:5105-5276
MsHel-6	gb|AIXA01002601.1|:34440-34620
MsHel-6	gb|AIXA01002266.1|:14186-14433
MsHel-6	gb|AIXA01002042.1|:48793-48994
MsHel-6	gb|AIXA01001102.1|:44560-44759
MsHel-6	gb|AIXA01001026.1|:27171-32030
MsHel-6	gb|AIXA01000556.1|:6543-6700
MsHel-6	gb|AIXA01000454.1|:27047-27224
MsHel-6	gb|AIXA01016865.1|:13944-14106
MsHel-6	gb|AIXA01014405.1|:2922-3186
MsHel-6	gb|AIXA01010933.1|:2776-3032
MsHel-6	gb|AIXA01010475.1|:1183-1359
MsHel-6	gb|AIXA01009305.1|:15666-15868
MsHel-6	gb|AIXA01009216.1|:84972-86727
MsHel-6	gb|AIXA01009216.1|:129174-129394
MsHel-6	gb|AIXA01009109.1|:28392-28643
MsHel-6	gb|AIXA01008535.1|:32839-33095
MsHel-6	gb|AIXA01007444.1|:5721-5984
MsHel-6	gb|AIXA01005787.1|:7315-7563
MsHel-6	gb|AIXA01003680.1|:1303-1557
MsHel-6	gb|AIXA01002198.1|:4643-4830
MsHel-6	gb|AIXA01001809.1|:21904-22144
MsHel-6	gb|AIXA01001747.1|:8421-8624
MsHel-6	gb|AIXA01001033.1|:1374-1628
MsHel-6	gb|AIXA01000795.1|:5067-5324
MsHel-6	gb|AIXA01000224.1|:36683-36858
MsHel-6	gb|AIXA01000201.1|:6406-7281
MsHel-6	gb|AIXA01019293.1|:4294-4507
MsHel-6	gb|AIXA01017852.1|:52-261
MsHel-6	gb|AIXA01014228.1|:23789-24030
MsHel-6	gb|AIXA01012051.1|:1409-1632
MsHel-6	gb|AIXA01009985.1|:27956-28210
MsHel-6	gb|AIXA01009575.1|:21986-22134
MsHel-6	gb|AIXA01007658.1|:20405-20555
MsHel-6	gb|AIXA01006121.1|:13944-14143
MsHel-6	gb|AIXA01005312.1|:52796-52994
MsHel-6	gb|AIXA01003047.1|:2056-2325
MsHel-6	gb|AIXA01027062.1|:566-771
MsHel-6	gb|AIXA01018648.1|:4269-4533
MsHel-6	gb|AIXA01009752.1|:14507-15779
MsHel-6	gb|AIXA01009345.1|:841-1043

MsHel-6	gb|AIXA01007092.1|:56116-56326
MsHel-6	gb|AIXA01005761.1|:31189-31354
MsHel-6	gb|AIXA01004990.1|:151460-151640
MsHel-6	gb|AIXA01003953.1|:28756-28954
MsHel-6	gb|AIXA01001083.1|:23209-23470
MsHel-6	gb|AIXA01000234.1|:76188-76383
MsHel-6	gb|AIXA01019179.1|:2826-2991
MsHel-6	gb|AIXA01017653.1|:11464-11620
MsHel-6	gb|AIXA01016151.1|:5506-5697
MsHel-6	gb|AIXA01014184.1|:40-196
MsHel-6	gb|AIXA01009130.1|:3868-4122
MsHel-6	gb|AIXA01008839.1|:24492-24683
MsHel-6	gb|AIXA01006420.1|:5811-6249
MsHel-6	gb|AIXA01006420.1|:36683-36867
MsHel-6	gb|AIXA01005902.1|:1860-2110
MsHel-6	gb|AIXA01002244.1|:12144-12652
MsHel-6	gb|AIXA01001630.1|:54279-54446
MsHel-6	gb|AIXA01000188.1|:54560-54763
MsHel-6	gb|AIXA01024617.1|:276-561
MsHel-6	gb|AIXA01016765.1|:3884-4146
MsHel-6	gb|AIXA01010927.1|:3532-3691
MsHel-6	gb|AIXA01009661.1|:3274-3413
MsHel-6	gb|AIXA01009346.1|:55239-55467
MsHel-6	gb|AIXA01009179.1|:59026-59201
MsHel-6	gb|AIXA01007262.1|:28242-28507
MsHel-6	gb|AIXA01006855.1|:24096-24268
MsHel-6	gb|AIXA01004980.1|:27542-27905
MsHel-6	gb|AIXA01004164.1|:22261-25746
MsHel-6	gb|AIXA01002750.1|:33349-37720
MsHel-6	gb|AIXA01002284.1|:180182-180393
MsHel-6	gb|AIXA01001349.1|:42313-42560
MsHel-6	gb|AIXA01020226.1|:1026-1179
MsHel-6	gb|AIXA01012491.1|:7116-7338
MsHel-6	gb|AIXA01011629.1|:43533-43714
MsHel-6	gb|AIXA01010663.1|:33814-34009
MsHel-6	gb|AIXA01009374.1|:11699-11966
MsHel-6	gb|AIXA01008110.1|:53238-53460
MsHel-6	gb|AIXA01007888.1|:45149-45299
MsHel-6	gb|AIXA01006837.1|:19278-19497
MsHel-6	gb|AIXA01006796.1|:68345-68516
MsHel-6	gb|AIXA01005521.1|:44044-44287
MsHel-6	gb|AIXA01005521.1|:115869-116070
MsHel-6	gb|AIXA01003054.1|:14013-14251
MsHel-6	gb|AIXA01003001.1|:14073-14318
MsHel-6	gb|AIXA01000652.1|:19715-19958
MsHel-6	gb|AIXA01000317.1|:29896-30172
MsHel-6	gb|AIXA01036219.1|:407-570
MsHel-6	gb|AIXA01016421.1|:3104-3249

MsHel-6	gb|AIXA01015894.1|:383-568
MsHel-6	gb|AIXA01012190.1|:31973-32165
MsHel-6	gb|AIXA01010779.1|:7833-8031
MsHel-6	gb|AIXA01008372.1|:17353-17847
MsHel-6	gb|AIXA01007266.1|:1653-1863
MsHel-6	gb|AIXA01005847.1|:3756-3996
MsHel-6	gb|AIXA01004924.1|:15821-16038
MsHel-6	gb|AIXA01001536.1|:1003-8825
MsHel-6	gb|AIXA01000331.1|:38919-39126
MsHel-6	gb|AIXA01000161.1|:14190-18793
MsHel-6	gb|AIXA01023200.1|:737-917
MsHel-6	gb|AIXA01015736.1|:4979-5228
MsHel-6	gb|AIXA01013617.1|:15112-15269
MsHel-6	gb|AIXA01010848.1|:73877-74071
MsHel-6	gb|AIXA01009798.1|:8516-8715
MsHel-6	gb|AIXA01009539.1|:73622-74317
MsHel-6	gb|AIXA01009298.1|:9015-9171
MsHel-6	gb|AIXA01006663.1|:2084-2297
MsHel-6	gb|AIXA01006663.1|:13767-14016
MsHel-6	gb|AIXA01005511.1|:36871-37318
MsHel-6	gb|AIXA01005087.1|:10896-11139
MsHel-6	gb|AIXA01005082.1|:8726-8869
MsHel-6	gb|AIXA01000969.1|:4869-5042
MsHel-6	gb|AIXA01036139.1|:226-425
MsHel-6	gb|AIXA01016454.1|:7067-7273
MsHel-6	gb|AIXA01012132.1|:1250-1390
MsHel-6	gb|AIXA01008962.1|:124384-124640
MsHel-6	gb|AIXA01004257.1|:46053-46251
MsHel-6	gb|AIXA01003127.1|:38031-38306
MsHel-6	gb|AIXA01001609.1|:46689-46895
MsHel-6	gb|AIXA01001370.1|:94173-94351
MsHel-6	gb|AIXA01000808.1|:2315-2546
MsHel-6	gb|AIXA01027976.1|:1-188
MsHel-6	gb|AIXA01025352.1|:435-632
MsHel-6	gb|AIXA01022885.1|:1471-1601
MsHel-6	gb|AIXA01020959.1|:1-236
MsHel-6	gb|AIXA01018669.1|:973-1228
MsHel-6	gb|AIXA01014721.1|:2645-2939
MsHel-6	gb|AIXA01014183.1|:3096-3316
MsHel-6	gb|AIXA01010413.1|:660-832
MsHel-6	gb|AIXA01009702.1|:34015-38428
MsHel-6	gb|AIXA01008318.1|:26645-26852
MsHel-6	gb|AIXA01005942.1|:14878-15122
MsHel-6	gb|AIXA01003585.1|:95652-95870
MsHel-6	gb|AIXA01001535.1|:2454-2668
MsHel-6	gb|AIXA01001414.1|:13949-14130
MsHel-6	gb|AIXA01014346.1|:50573-50731
MsHel-6	gb|AIXA01008028.1|:1625-2047

MsHel-6	gb|AIXA01007015.1|:53906-54161
MsHel-6	gb|AIXA01006181.1|:5649-5805
MsHel-6	gb|AIXA01001675.1|:42176-42431
MsHel-6	gb|AIXA01000709.1|:3366-3591
MsHel-6	gb|AIXA01015126.1|:7750-7903
MsHel-6	gb|AIXA01008938.1|:18426-18691
MsHel-6	gb|AIXA01007705.1|:20289-20539
MsHel-6	gb|AIXA01001521.1|:15887-16119
MsHel-6	gb|AIXA01001317.1|:5857-5993
MsHel-6	gb|AIXA01001188.1|:2076-2325
MsHel-6	gb|AIXA01000814.1|:21285-21638
MsHel-6	gb|AIXA01000631.1|:3234-3430
MsHel-6	gb|AIXA01026404.1|:623-1052
MsHel-6	gb|AIXA01022421.1|:701-841
MsHel-6	gb|AIXA01021440.1|:804-984
MsHel-6	gb|AIXA01013642.1|:1097-1262
MsHel-6	gb|AIXA01013639.1|:12673-12838
MsHel-6	gb|AIXA01008862.1|:7300-7473
MsHel-6	gb|AIXA01007147.1|:6170-6318
MsHel-6	gb|AIXA01006482.1|:40603-40895
MsHel-6	gb|AIXA01006074.1|:3980-4228
MsHel-6	gb|AIXA01005126.1|:41479-41727
MsHel-6	gb|AIXA01003889.1|:12983-13155
MsHel-6	gb|AIXA01003681.1|:35294-35510
MsHel-6	gb|AIXA01003288.1|:6685-6912
MsHel-6	gb|AIXA01001839.1|:5899-6114
MsHel-6	gb|AIXA01018174.1|:7835-8041
MsHel-6	gb|AIXA01013132.1|:4467-4939
MsHel-6	gb|AIXA01011264.1|:26718-26872
MsHel-6	gb|AIXA01008992.1|:27521-27852
MsHel-6	gb|AIXA01007612.1|:25741-25892
MsHel-6	gb|AIXA01004958.1|:51027-51237
MsHel-6	gb|AIXA01003176.1|:74-213
MsHel-6	gb|AIXA01003176.1|:81131-81333
MsHel-6	gb|AIXA01001515.1|:28984-29462
MsHel-6	gb|AIXA01000833.1|:189-340
MsHel-6	gb|AIXA01020259.1|:1016-1257
MsHel-6	gb|AIXA01018405.1|:441-630
MsHel-6	gb|AIXA01012798.1|:21527-21783
MsHel-6	gb|AIXA01012384.1|:26102-26394
MsHel-6	gb|AIXA01012148.1|:32435-32649
MsHel-6	gb|AIXA01010658.1|:3536-3782
MsHel-6	gb|AIXA01007944.1|:3698-4315
MsHel-6	gb|AIXA01002606.1|:4213-4466
MsHel-6	gb|AIXA01014911.1|:4010-4462
MsHel-6	gb|AIXA01006953.1|:27125-27346
MsHel-6	gb|AIXA01005530.1|:8332-8753
MsHel-6	gb|AIXA01038064.1|:349-492

MsHel-6	gb|AIXA01023863.1|:1211-1407
MsHel-6	gb|AIXA01016566.1|:6279-8354
MsHel-6	gb|AIXA01015400.1|:46708-47145
MsHel-6	gb|AIXA01013026.1|:9890-10061
MsHel-6	gb|AIXA01012651.1|:20008-20232
MsHel-6	gb|AIXA01008027.1|:10198-10448
MsHel-6	gb|AIXA01004625.1|:40887-41425
MsHel-6	gb|AIXA01002056.1|:1-112
MsHel-6	gb|AIXA01000914.1|:81886-82026
MsHel-6	gb|AIXA01000377.1|:2440-2670
MsHel-6	gb|AIXA01035830.1|:2-116
MsHel-6	gb|AIXA01017945.1|:1471-1685
MsHel-6	gb|AIXA01017009.1|:7726-7934
MsHel-6	gb|AIXA01016041.1|:18364-18588
MsHel-6	gb|AIXA01011626.1|:19948-20221
MsHel-6	gb|AIXA01009162.1|:67724-67914
MsHel-6	gb|AIXA01008957.1|:9273-9756
MsHel-6	gb|AIXA01008167.1|:29755-30003
MsHel-6	gb|AIXA01001501.1|:16442-16860
MsHel-6	gb|AIXA01028681.1|:3-217
MsHel-6	gb|AIXA01018790.1|:565-2433
MsHel-6	gb|AIXA01006207.1|:15608-15912
MsHel-6	gb|AIXA01002439.1|:11230-11416
MsHel-6	gb|AIXA01000037.1|:35882-36031
MsHel-6	gb|AIXA01021720.1|:1732-1874
MsHel-6	gb|AIXA01015431.1|:33380-33620
MsHel-6	gb|AIXA01010789.1|:3490-3682
MsHel-6	gb|AIXA01010541.1|:26087-26219
MsHel-6	gb|AIXA01007925.1|:22284-22445
MsHel-6	gb|AIXA01005505.1|:40123-40271
MsHel-6	gb|AIXA01004978.1|:43755-44003
MsHel-6	gb|AIXA01004408.1|:36307-36444
MsHel-6	gb|AIXA01004337.1|:65629-65811
MsHel-6	gb|AIXA01001572.1|:41775-42010
MsHel-6	gb|AIXA01034759.1|:178-404
MsHel-6	gb|AIXA01019672.1|:1460-1710
MsHel-6	gb|AIXA01019617.1|:922-1066
MsHel-6	gb|AIXA01010668.1|:7694-7873
MsHel-6	gb|AIXA01009466.1|:29955-30215
MsHel-6	gb|AIXA01008417.1|:2621-2999
MsHel-6	gb|AIXA01006132.1|:18662-18811
MsHel-6	gb|AIXA01005525.1|:18972-19250
MsHel-6	gb|AIXA01005419.1|:10707-10899
MsHel-6	gb|AIXA01004948.1|:58770-58960
MsHel-6	gb|AIXA01003326.1|:71989-72182
MsHel-6	gb|AIXA01003320.1|:102814-103060
MsHel-6	gb|AIXA01003234.1|:59203-59369
MsHel-6	gb|AIXA01020239.1|:96-304

MsHel-6	gb|AIXA01013425.1|:20402-20597
MsHel-6	gb|AIXA01013383.1|:27144-27637
MsHel-6	gb|AIXA01009670.1|:10940-11095
MsHel-6	gb|AIXA01008909.1|:5787-5965
MsHel-6	gb|AIXA01006329.1|:7350-7574
MsHel-6	gb|AIXA01002532.1|:33028-33126
MsHel-6	gb|AIXA01001498.1|:28622-28910
MsHel-6	gb|AIXA01021406.1|:599-732
MsHel-6	gb|AIXA01014195.1|:43426-43577
MsHel-6	gb|AIXA01005249.1|:250-457
MsHel-6	gb|AIXA01000204.1|:22391-22520
MsHel-6	gb|AIXA01008266.1|:38018-38118
MsHel-6	gb|AIXA01005009.1|:22008-22195
MsHel-6	gb|AIXA01017672.1|:8393-8552
MsHel-6	gb|AIXA01011817.1|:2821-2937
MsHel-6	gb|AIXA01010576.1|:8940-9429
MsHel-6	gb|AIXA01005480.1|:5223-5438
MsHel-6	gb|AIXA01002415.1|:36298-36652
MsHel-6	gb|AIXA01018007.1|:4625-4831
MsHel-6	gb|AIXA01006200.1|:23038-23252
MsHel-6	gb|AIXA01004796.1|:10476-10801
MsHel-6	gb|AIXA01003173.1|:31808-31995
MsHel-6	gb|AIXA01000765.1|:12184-12305
MsHel-6	gb|AIXA01011828.1|:295-478
MsHel-6	gb|AIXA01009884.1|:2845-3051
MsHel-6	gb|AIXA01007304.1|:33134-33544
MsHel-6	gb|AIXA01002776.1|:4-300
MsHel-6	gb|AIXA01001946.1|:5780-5895
MsHel-6	gb|AIXA01017135.1|:1210-1399
MsHel-6	gb|AIXA01009488.1|:62953-63061
MsHel-6	gb|AIXA01008037.1|:66776-67305
MsHel-6	gb|AIXA01002189.1|:50960-51146
MsHel-6	gb|AIXA01019424.1|:4806-4965
MsHel-6	gb|AIXA01008884.1|:4890-5069
MsHel-6	gb|AIXA01008873.1|:28769-28967
MsHel-6	gb|AIXA01007813.1|:4130-4365
MsHel-6	gb|AIXA01004389.1|:13055-13212
MsHel-6	gb|AIXA01001125.1|:10211-10394
MsHel-6	gb|AIXA01026337.1|:1036-1138
MsHel-6	gb|AIXA01006208.1|:22861-23040
MsHel-6	gb|AIXA01003175.1|:39461-39596
MsHel-6	gb|AIXA01002288.1|:43627-43851
MsHel-6	gb|AIXA01001955.1|:8771-9023
MsHel-6	gb|AIXA01008448.1|:1003-1125
MsHel-6	gb|AIXA01006911.1|:8055-8162
MsHel-6	gb|AIXA01001261.1|:5280-5479
MsHel-6	gb|AIXA01000371.1|:9937-10043
MsHel-6	gb|AIXA01024427.1|:735-880

MsHel-6	gb|AIXA01015049.1|:12104-12192
MsHel-6	gb|AIXA01012017.1|:18834-19114
MsHel-6	gb|AIXA01010920.1|:2116-2243
MsHel-6	gb|AIXA01010333.1|:7583-8266
MsHel-6	gb|AIXA01007102.1|:3427-3779
MsHel-6	gb|AIXA01002227.1|:137-576
MsHel-6	gb|AIXA01011547.1|:1775-1892
MsHel-6	gb|AIXA01004738.1|:16931-17081
MsHel-6	gb|AIXA01016118.1|:7561-7785
MsHel-6	gb|AIXA01015999.1|:13980-14085
MsHel-6	gb|AIXA01003852.1|:14675-14817
MsHel-6	gb|AIXA01003693.1|:8322-8428
MsHel-6	gb|AIXA01000347.1|:56027-56214
MsHel-6	gb|AIXA01000129.1|:5-449
MsHel-6	gb|AIXA01023263.1|:1350-1490
MsHel-6	gb|AIXA01015541.1|:5242-5433
MsHel-6	gb|AIXA01005576.1|:26228-26385
MsHel-6	gb|AIXA01004491.1|:11101-11381
MsHel-6	gb|AIXA01003356.1|:15309-15514
MsHel-6	gb|AIXA01022419.1|:527-664
MsHel-6	gb|AIXA01011125.1|:4106-4374
MsHel-6	gb|AIXA01007559.1|:41088-41553
MsHel-6	gb|AIXA01002162.1|:9212-9366
MsHel-6	gb|AIXA01001053.1|:8672-9243
MsHel-6	gb|AIXA01010922.1|:1-80
MsHel-6	gb|AIXA01005077.1|:9081-9232
MsHel-6	gb|AIXA01004166.1|:471-782
MsHel-6	gb|AIXA01000368.1|:20577-20812
MsHel-6	gb|AIXA01013061.1|:12327-12503
MsHel-6	gb|AIXA01012781.1|:2424-2665
MsHel-6	gb|AIXA01007146.1|:32471-32720
MsHel-6	gb|AIXA01014530.1|:4730-5146
MsHel-6	gb|AIXA01016835.1|:3427-3632
MsHel-6	gb|AIXA01003160.1|:13520-13702
MsHel-6	gb|AIXA01002454.1|:112218-112412
MsHel-6	gb|AIXA01011682.1|:10387-10586
MsHel-6	gb|AIXA01011269.1|:3100-3315
MsHel-6	gb|AIXA01009595.1|:4953-5048
MsHel-6	gb|AIXA01032064.1|:3-138
MsHel-6	gb|AIXA01003997.1|:18865-19317
MsHel-6	gb|AIXA01002851.1|:29164-29258
MsHel-6	gb|AIXA01001314.1|:13354-13596
MsHel-6	gb|AIXA01001057.1|:76221-76307
MsHel-6	gb|AIXA01000819.1|:85361-85664
MsHel-6	gb|AIXA01035933.1|:269-350
MsHel-6	gb|AIXA01020594.1|:557-679
MsHel-6	gb|AIXA01006931.1|:4253-4359
MsHel-6	gb|AIXA01006061.1|:28193-28274

MsHel-6	gb|AIXA01001571.1|:105668-115652
MsHel-6	gb|AIXA01013824.1|:1338-1575
MsHel-6	gb|AIXA01008378.1|:40020-40911
MsHel-6	gb|AIXA01005473.1|:24502-24593
MsHel-6	gb|AIXA01001505.1|:55394-55627
MsHel-6	gb|AIXA01000025.1|:24905-25096
MsHel-6	gb|AIXA01008565.1|:38334-38509
MsHel-6	gb|AIXA01006155.1|:1569-1791
MsHel-6	gb|AIXA01004882.1|:13629-13863
MsHel-6	gb|AIXA01003109.1|:11676-11906
MsHel-6	gb|AIXA01000412.1|:1493-1745
MsHel-6	gb|AIXA01037278.1|:1-124
MsHel-6	gb|AIXA01004417.1|:19470-19673
MsHel-6	gb|AIXA01004386.1|:23098-23534
MsHel-6	gb|AIXA01002105.1|:52021-52690
MsHel-6	gb|AIXA01000175.1|:31827-32077
MsHel-6	gb|AIXA01016971.1|:11277-11470
MsHel-6	gb|AIXA01009518.1|:306-479
MsHel-6	gb|AIXA01000044.1|:9320-9556
MsHel-6	gb|AIXA01011493.1|:78483-78651
MsHel-6	gb|AIXA01010817.1|:3760-4022
MsHel-6	gb|AIXA01009826.1|:7350-7517
MsHel-6	gb|AIXA01009489.1|:39238-39371
MsHel-6	gb|AIXA01006117.1|:2817-2929
MsHel-6	gb|AIXA01004355.1|:4021-4135
MsHel-6	gb|AIXA01003798.1|:58300-58396
MsHel-6	gb|AIXA01002561.1|:119278-119374
MsHel-6	gb|AIXA01010077.1|:60675-60932
MsHel-6	gb|AIXA01008308.1|:24228-24644
MsHel-6	gb|AIXA01006862.1|:2341-2725
MsHel-6	gb|AIXA01000345.1|:21557-21685
MsHel-6	gb|AIXA01038121.1|:242-446
MsHel-6	gb|AIXA01015096.1|:986-1092
MsHel-6	gb|AIXA01009713.1|:41339-41470
MsHel-6	gb|AIXA01008337.1|:10939-11030
MsHel-6	gb|AIXA01006785.1|:16101-16272
MsHel-6	gb|AIXA01005766.1|:46186-46331
MsHel-6	gb|AIXA01003114.1|:33451-33611
MsHel-6	gb|AIXA01020149.1|:2957-3041
MsHel-6	gb|AIXA01011308.1|:12536-12958
MsHel-6	gb|AIXA01004965.1|:99617-100340
MsHel-6	gb|AIXA01016796.1|:2122-2227
MsHel-6	gb|AIXA01005207.1|:33856-33999
MsHel-6	gb|AIXA01000593.1|:18045-18215
MsHel-6	gb|AIXA01008449.1|:337-587
MsHel-6	gb|AIXA01005250.1|:13148-13266
MsHel-6	gb|AIXA01004928.1|:15722-15853
MsHel-6	gb|AIXA01015246.1|:3664-3775

MsHel-6	gb|AIXA01012569.1|:4981-5135
MsHel-6	gb|AIXA01006602.1|:34332-34520
MsHel-6	gb|AIXA01005033.1|:14582-14798
MsHel-6	gb|AIXA01001941.1|:89899-90015
MsHel-6	gb|AIXA01019098.1|:4547-4628
MsHel-6	gb|AIXA01017934.1|:1-315
MsHel-6	gb|AIXA01014421.1|:1-307
MsHel-6	gb|AIXA01030737.1|:1-111
MsHel-6	gb|AIXA01022655.1|:1513-1648
MsHel-6	gb|AIXA01007113.1|:8224-8728
MsHel-6	gb|AIXA01021956.1|:819-1005
MsHel-6	gb|AIXA01004068.1|:85592-85711
MsHel-6	gb|AIXA01035517.1|:2-84
MsHel-6	gb|AIXA01013583.1|:6122-6213
MsHel-6	gb|AIXA01012570.1|:180-334
MsHel-6	gb|AIXA01013382.1|:9424-9583
MsHel-6	gb|AIXA01008127.1|:28525-28809
MsHel-6	gb|AIXA01006797.1|:89325-89773
MsHel-6	gb|AIXA01005360.1|:23154-23475
MsHel-6	gb|AIXA01002580.1|:108318-108912
MsHel-6	gb|AIXA01012820.1|:337-508
MsHel-6	gb|AIXA01012352.1|:22562-28240
MsHel-6	gb|AIXA01011331.1|:1843-2490
MsHel-6	gb|AIXA01009692.1|:103005-103192
MsHel-6	gb|AIXA01006319.1|:6151-6602
MsHel-6	gb|AIXA01005763.1|:20131-20312
MsHel-6	gb|AIXA01002333.1|:10770-19820
MsHel-6	gb|AIXA01037377.1|:350-455
MsHel-6	gb|AIXA01022190.1|:1644-1728
MsHel-6	gb|AIXA01004929.1|:61016-61238
MsHel-6	gb|AIXA01034664.1|:2-160
MsHel-6	gb|AIXA01001466.1|:6662-6847
MsHel-6	gb|AIXA01012151.1|:15799-15882
MsHel-6	gb|AIXA01006876.1|:34258-34558
MsHel-6	gb|AIXA01005244.1|:33531-33770
MsHel-6	gb|AIXA01019086.1|:2393-2491
MsHel-6	gb|AIXA01007168.1|:30744-30834
MsHel-6	gb|AIXA01011031.1|:8857-8953
MsHel-6	gb|AIXA01034178.1|:5-115
MsHel-6	gb|AIXA01023926.1|:361-749
MsHel-6	gb|AIXA01006039.1|:138-518
MsHel-6	gb|AIXA01005980.1|:1394-1498
MsHel-6	gb|AIXA01001139.1|:19892-20001
MsHel-6	gb|AIXA01012305.1|:30-138
MsHel-6	gb|AIXA01006423.1|:61336-61562
MsHel-6	gb|AIXA01000871.1|:27862-27935
MsHel-6	gb|AIXA01000128.1|:135154-135232
MsHel-6	gb|AIXA01021604.1|:1739-1839

MsHel-6	gb|AIXA01011267.1|:2704-3165
MsHel-6	gb|AIXA01006358.1|:3447-3546
MsHel-6	gb|AIXA01005394.1|:42709-43117
MsHel-6	gb|AIXA01002264.1|:50734-50839
MsHel-6	gb|AIXA01017891.1|:11552-11781
MsHel-6	gb|AIXA01002650.1|:3859-4363
MsHel-6	gb|AIXA01007636.1|:15448-15666
MsHel-6	gb|AIXA01007481.1|:49085-49310
MsHel-6	gb|AIXA01014644.1|:24597-24839
MsHel-6	gb|AIXA01003723.1|:321-407
MsHel-3b	gb|AIXA01007346.1|:47015-47229
MsHel-3b	gb|AIXA01001692.1|:41229-41445
MsHel-3b	gb|AIXA01001531.1|:24252-24368
MsHel-3b	gb|AIXA01001531.1|:56742-56951
MsHel-3b	gb|AIXA01013436.1|:10378-10576
MsHel-3b	gb|AIXA01013436.1|:79665-79822
MsHel-3b	gb|AIXA01011449.1|:4529-4742
MsHel-3b	gb|AIXA01008999.1|:10425-10638
MsHel-3b	gb|AIXA01012195.1|:8501-8709
MsHel-3b	gb|AIXA01010109.1|:24711-24910
MsHel-3b	gb|AIXA01010109.1|:38516-38730
MsHel-3b	gb|AIXA01009936.1|:14247-14391
MsHel-3b	gb|AIXA01006690.1|:14682-14898
MsHel-3b	gb|AIXA01001645.1|:9309-9481
MsHel-3b	gb|AIXA01001645.1|:37144-37356
MsHel-3b	gb|AIXA01001645.1|:65186-65396
MsHel-3b	gb|AIXA01001553.1|:14115-14331
MsHel-3b	gb|AIXA01014762.1|:11048-11246
MsHel-3b	gb|AIXA01015777.1|:32975-33188
MsHel-3b	gb|AIXA01004313.1|:26185-26323
MsHel-3b	gb|AIXA01004313.1|:50844-51031
MsHel-3b	gb|AIXA01002330.1|:7952-8164
MsHel-3b	gb|AIXA01035647.1|:359-575
MsHel-3b	gb|AIXA01017253.1|:1074-1290
MsHel-3b	gb|AIXA01010406.1|:56706-59444
MsHel-3b	gb|AIXA01010406.1|:79598-82316
MsHel-3b	gb|AIXA01009981.1|:3477-3643
MsHel-3b	gb|AIXA01007472.1|:41289-46665
MsHel-3b	gb|AIXA01007472.1|:73519-73686
MsHel-3b	gb|AIXA01007472.1|:98918-99133
MsHel-3b	gb|AIXA01006141.1|:21939-22155
MsHel-3b	gb|AIXA01006141.1|:91960-92043
MsHel-3b	gb|AIXA01006141.1|:105897-106095
MsHel-3b	gb|AIXA01004113.1|:112805-113021
MsHel-3b	gb|AIXA01000821.1|:109295-109510
MsHel-3b	gb|AIXA01000398.1|:23467-23679
MsHel-3b	gb|AIXA01008393.1|:22620-32287

MsHel-3b	gb|AIXA01008393.1|:61295-61506
MsHel-3b	gb|AIXA01009972.1|:32005-32219
MsHel-3b	gb|AIXA01009970.1|:10887-11101
MsHel-3b	gb|AIXA01008073.1|:24250-24464
MsHel-3b	gb|AIXA01002509.1|:6647-6860
MsHel-3b	gb|AIXA01002509.1|:23186-23397
MsHel-3b	gb|AIXA01000046.1|:48838-49042
MsHel-3b	gb|AIXA01000046.1|:80729-80943
MsHel-3b	gb|AIXA01010211.1|:22160-22362
MsHel-3b	gb|AIXA01003021.1|:15551-15743
MsHel-3b	gb|AIXA01003021.1|:36195-36408
MsHel-3b	gb|AIXA01003021.1|:136056-136136
MsHel-3b	gb|AIXA01001362.1|:3786-3969
MsHel-3b	gb|AIXA01017576.1|:13875-14047
MsHel-3b	gb|AIXA01017187.1|:3906-4122
MsHel-3b	gb|AIXA01015246.1|:5701-5783
MsHel-3b	gb|AIXA01014037.1|:3480-3696
MsHel-3b	gb|AIXA01013644.1|:326-526
MsHel-3b	gb|AIXA01009291.1|:32210-32426
MsHel-3b	gb|AIXA01008043.1|:92408-92619
MsHel-3b	gb|AIXA01002713.1|:5359-5567
MsHel-3b	gb|AIXA01002519.1|:22368-22494
MsHel-3b	gb|AIXA01002519.1|:33030-33229
MsHel-3b	gb|AIXA01002519.1|:58936-59152
MsHel-3b	gb|AIXA01002460.1|:92479-92693
MsHel-3b	gb|AIXA01001775.1|:58438-58654
MsHel-3b	gb|AIXA01001264.1|:19969-20185
MsHel-3b	gb|AIXA01008566.1|:37245-37451
MsHel-3b	gb|AIXA01008131.1|:4792-4935
MsHel-3b	gb|AIXA01007037.1|:13289-13503
MsHel-3b	gb|AIXA01007037.1|:28588-28777
MsHel-3b	gb|AIXA01017682.1|:852-1062
MsHel-3b	gb|AIXA01011141.1|:3580-3794
MsHel-3b	gb|AIXA01005049.1|:20264-20477
MsHel-3b	gb|AIXA01003653.1|:71044-71250
MsHel-3b	gb|AIXA01007104.1|:16670-16874
MsHel-3b	gb|AIXA01007104.1|:48254-48464
MsHel-3b	gb|AIXA01002350.1|:24534-24658
MsHel-3b	gb|AIXA01002350.1|:128128-128337
MsHel-3b	gb|AIXA01000251.1|:1676-1846
MsHel-3b	gb|AIXA01000251.1|:109250-109460
MsHel-3b	gb|AIXA01000251.1|:209308-209523
MsHel-3b	gb|AIXA01000251.1|:232309-232522
MsHel-3b	gb|AIXA01029000.1|:712-917
MsHel-3b	gb|AIXA01012544.1|:2252-2464
MsHel-3b	gb|AIXA01006514.1|:48483-48691
MsHel-3b	gb|AIXA01005339.1|:10756-10870
MsHel-3b	gb|AIXA01005339.1|:34114-34330

MsHel-3b	gb|AIXA01004841.1|:15482-15686
MsHel-3b	gb|AIXA01004489.1|:26737-26949
MsHel-3b	gb|AIXA01004489.1|:49177-53782
MsHel-3b	gb|AIXA01003673.1|:2498-2714
MsHel-3b	gb|AIXA01003673.1|:56782-56912
MsHel-3b	gb|AIXA01000234.1|:78794-78879
MsHel-3b	gb|AIXA01023032.1|:489-706
MsHel-3b	gb|AIXA01011842.1|:14115-14325
MsHel-3b	gb|AIXA01011498.1|:37811-37929
MsHel-3b	gb|AIXA01004128.1|:35257-35468
MsHel-3b	gb|AIXA01004128.1|:65448-65573
MsHel-3b	gb|AIXA01001612.1|:27379-27583
MsHel-3b	gb|AIXA01029549.1|:94-276
MsHel-3b	gb|AIXA01022008.1|:462-672
MsHel-3b	gb|AIXA01014972.1|:96-308
MsHel-3b	gb|AIXA01014467.1|:1453-1677
MsHel-3b	gb|AIXA01014467.1|:48021-48234
MsHel-3b	gb|AIXA01004067.1|:26338-26543
MsHel-3b	gb|AIXA01002459.1|:5923-6137
MsHel-3b	gb|AIXA01002459.1|:37472-37655
MsHel-3b	gb|AIXA01001675.1|:45911-46006
MsHel-3b	gb|AIXA01001546.1|:58033-58159
MsHel-3b	gb|AIXA01017982.1|:10722-10931
MsHel-3b	gb|AIXA01017659.1|:1655-1865
MsHel-3b	gb|AIXA01017229.1|:2728-2858
MsHel-3b	gb|AIXA01012352.1|:28028-28240
MsHel-3b	gb|AIXA01009366.1|:26192-26330
MsHel-3b	gb|AIXA01005494.1|:9085-9258
MsHel-3b	gb|AIXA01004068.1|:9146-9529
MsHel-3b	gb|AIXA01004068.1|:77350-77559
MsHel-3b	gb|AIXA01002996.1|:23447-23653
MsHel-3b	gb|AIXA01002996.1|:60688-60888
MsHel-3b	gb|AIXA01023928.1|:874-1078
MsHel-3b	gb|AIXA01016545.1|:11549-11699
MsHel-3b	gb|AIXA01015716.1|:845-1053
MsHel-3b	gb|AIXA01015155.1|:17607-17814
MsHel-3b	gb|AIXA01015003.1|:7990-8073
MsHel-3b	gb|AIXA01012697.1|:4343-4534
MsHel-3b	gb|AIXA01006681.1|:1694-1898
MsHel-3b	gb|AIXA01004487.1|:5014-5206
MsHel-3b	gb|AIXA01004337.1|:9112-9327
MsHel-3b	gb|AIXA01003924.1|:22373-23027
MsHel-3b	gb|AIXA01003924.1|:70532-70713
MsHel-3b	gb|AIXA01003924.1|:111698-111894
MsHel-3b	gb|AIXA01002990.1|:18102-18285
MsHel-3b	gb|AIXA01002351.1|:2377-2575
MsHel-3b	gb|AIXA01000201.1|:8743-8912
MsHel-3b	gb|AIXA01000201.1|:77245-77332

MsHel-3b	gb|AIXA01037524.1|:171-370
MsHel-3b	gb|AIXA01025449.1|:128-334
MsHel-3b	gb|AIXA01013916.1|:13698-13909
MsHel-3b	gb|AIXA01006807.1|:2459-2622
MsHel-3b	gb|AIXA01001048.1|:1701-1848
MsHel-3b	gb|AIXA01001048.1|:12003-12214
MsHel-3b	gb|AIXA01001048.1|:84409-88479
MsHel-3b	gb|AIXA01001048.1|:123953-124051
MsHel-3b	gb|AIXA01018181.1|:14515-14725
MsHel-3b	gb|AIXA01009127.1|:19055-19269
MsHel-3b	gb|AIXA01007888.1|:63261-63474
MsHel-3b	gb|AIXA01006705.1|:36657-36871
MsHel-3b	gb|AIXA01005435.1|:6188-6390
MsHel-3b	gb|AIXA01005435.1|:38106-38218
MsHel-3b	gb|AIXA01005247.1|:52967-53169
MsHel-3b	gb|AIXA01004862.1|:23303-23511
MsHel-3b	gb|AIXA01004862.1|:58111-58324
MsHel-3b	gb|AIXA01003329.1|:41706-41880
MsHel-3b	gb|AIXA01003329.1|:95417-95556
MsHel-3b	gb|AIXA01030638.1|:133-339
MsHel-3b	gb|AIXA01018540.1|:290-495
MsHel-3b	gb|AIXA01017061.1|:16209-16422
MsHel-3b	gb|AIXA01016603.1|:5714-5871
MsHel-3b	gb|AIXA01010069.1|:7811-7895
MsHel-3b	gb|AIXA01010069.1|:20570-20784
MsHel-3b	gb|AIXA01008670.1|:15942-16056
MsHel-3b	gb|AIXA01008670.1|:27613-27830
MsHel-3b	gb|AIXA01004222.1|:12491-12643
MsHel-3b	gb|AIXA01004222.1|:69032-69245
MsHel-3b	gb|AIXA01004222.1|:136407-136568
MsHel-3b	gb|AIXA01003124.1|:13678-18212
MsHel-3b	gb|AIXA01003070.1|:18363-18571
MsHel-3b	gb|AIXA01002658.1|:67465-67557
MsHel-3b	gb|AIXA01002658.1|:80849-80974
MsHel-3b	gb|AIXA01002658.1|:131213-131429
MsHel-3b	gb|AIXA01001593.1|:5152-5353
MsHel-3b	gb|AIXA01001593.1|:47022-47170
MsHel-3b	gb|AIXA01001518.1|:13974-14191
MsHel-3b	gb|AIXA01001271.1|:57084-57180
MsHel-3b	gb|AIXA01001271.1|:81510-81724
MsHel-3b	gb|AIXA01018818.1|:27-243
MsHel-3b	gb|AIXA01018508.1|:1844-2060
MsHel-3b	gb|AIXA01017992.1|:4856-5064
MsHel-3b	gb|AIXA01012364.1|:7386-7600
MsHel-3b	gb|AIXA01009719.1|:30035-30251
MsHel-3b	gb|AIXA01009649.1|:13523-13664
MsHel-3b	gb|AIXA01009649.1|:42030-42157
MsHel-3b	gb|AIXA01009152.1|:7681-7897

MsHel-3b	gb|AIXA01008406.1|:60494-65346
MsHel-3b	gb|AIXA01007593.1|:21374-21562
MsHel-3b	gb|AIXA01006651.1|:62842-63054
MsHel-3b	gb|AIXA01002103.1|:21878-22066
MsHel-3b	gb|AIXA01000909.1|:127345-127553
MsHel-3b	gb|AIXA01000694.1|:80433-80649
MsHel-3b	gb|AIXA01000192.1|:16511-16697
MsHel-3b	gb|AIXA01017208.1|:2492-2703
MsHel-3b	gb|AIXA01015485.1|:4681-4893
MsHel-3b	gb|AIXA01015043.1|:820-1033
MsHel-3b	gb|AIXA01009925.1|:60414-60625
MsHel-3b	gb|AIXA01005493.1|:58433-58606
MsHel-3b	gb|AIXA01004482.1|:8970-9149
MsHel-3b	gb|AIXA01000892.1|:58281-58473
MsHel-3b	gb|AIXA01016021.1|:18483-18693
MsHel-3b	gb|AIXA01014666.1|:1163-1341
MsHel-3b	gb|AIXA01011338.1|:6222-6408
MsHel-3b	gb|AIXA01009855.1|:583-788
MsHel-3b	gb|AIXA01006872.1|:57572-57732
MsHel-3b	gb|AIXA01006860.1|:27075-27285
MsHel-3b	gb|AIXA01006654.1|:8008-8214
MsHel-3b	gb|AIXA01005127.1|:28020-28231
MsHel-3b	gb|AIXA01004956.1|:119781-119955
MsHel-3b	gb|AIXA01004011.1|:13335-13537
MsHel-3b	gb|AIXA01002994.1|:16257-16462
MsHel-3b	gb|AIXA01002469.1|:33709-33919
MsHel-3b	gb|AIXA01001520.1|:1118-1323
MsHel-3b	gb|AIXA01013458.1|:15831-16037
MsHel-3b	gb|AIXA01013458.1|:50141-50224
MsHel-3b	gb|AIXA01011592.1|:6366-6576
MsHel-3b	gb|AIXA01008554.1|:64307-64524
MsHel-3b	gb|AIXA01008123.1|:22153-22370
MsHel-3b	gb|AIXA01007168.1|:129942-130172
MsHel-3b	gb|AIXA01006729.1|:2643-2856
MsHel-3b	gb|AIXA01006279.1|:7639-7846
MsHel-3b	gb|AIXA01005126.1|:34276-34481
MsHel-3b	gb|AIXA01005126.1|:54351-54510
MsHel-3b	gb|AIXA01003878.1|:20067-20199
MsHel-3b	gb|AIXA01001243.1|:108776-108859
MsHel-3b	gb|AIXA01001243.1|:175403-175612
MsHel-3b	gb|AIXA01001243.1|:216222-216389
MsHel-3b	gb|AIXA01000359.1|:87089-87304
MsHel-3b	gb|AIXA01033753.1|:175-557
MsHel-3b	gb|AIXA01032845.1|:132-340
MsHel-3b	gb|AIXA01017362.1|:7622-7815
MsHel-3b	gb|AIXA01013942.1|:14921-15130
MsHel-3b	gb|AIXA01013473.1|:10369-10577
MsHel-3b	gb|AIXA01009162.1|:92927-93127

MsHel-3b	gb|AIXA01009162.1|:178904-179120
MsHel-3b	gb|AIXA01007842.1|:18385-18589
MsHel-3b	gb|AIXA01007398.1|:12-222
MsHel-3b	gb|AIXA01006648.1|:23452-23647
MsHel-3b	gb|AIXA01003356.1|:14747-15514
MsHel-3b	gb|AIXA01003320.1|:15079-15204
MsHel-3b	gb|AIXA01003320.1|:101973-102188
MsHel-3b	gb|AIXA01002712.1|:20000-20209
MsHel-3b	gb|AIXA01002472.1|:24889-25105
MsHel-3b	gb|AIXA01002339.1|:11170-11386
MsHel-3b	gb|AIXA01002339.1|:39126-39328
MsHel-3b	gb|AIXA01001619.1|:18189-18402
MsHel-3b	gb|AIXA01001323.1|:2957-3073
MsHel-3b	gb|AIXA01001323.1|:46641-46855
MsHel-3b	gb|AIXA01000891.1|:28002-28216
MsHel-3b	gb|AIXA01024515.1|:153-359
MsHel-3b	gb|AIXA01018268.1|:1209-1420
MsHel-3b	gb|AIXA01008222.1|:18664-18874
MsHel-3b	gb|AIXA01007576.1|:17418-17525
MsHel-3b	gb|AIXA01007576.1|:28452-28634
MsHel-3b	gb|AIXA01007576.1|:53538-53663
MsHel-3b	gb|AIXA01007576.1|:104125-104309
MsHel-3b	gb|AIXA01005516.1|:5510-5722
MsHel-3b	gb|AIXA01002331.1|:670-843
MsHel-3b	gb|AIXA01002331.1|:28997-29183
MsHel-3b	gb|AIXA01002308.1|:51953-52157
MsHel-3b	gb|AIXA01002187.1|:19816-20023
MsHel-3b	gb|AIXA01001656.1|:29789-29991
MsHel-3b	gb|AIXA01001656.1|:61852-61945
MsHel-3b	gb|AIXA01001319.1|:11120-11332
MsHel-3b	gb|AIXA01000456.1|:15220-15412
MsHel-3b	gb|AIXA01000437.1|:21224-21430
MsHel-3b	gb|AIXA01000437.1|:116557-116763
MsHel-3b	gb|AIXA01000437.1|:149667-149789
MsHel-3b	gb|AIXA01000267.1|:51292-51420
MsHel-3b	gb|AIXA01000267.1|:76357-76555
MsHel-3b	gb|AIXA01000267.1|:109139-109222
MsHel-3b	gb|AIXA01019132.1|:1351-1552
MsHel-3b	gb|AIXA01012172.1|:9667-9875
MsHel-3b	gb|AIXA01011318.1|:2908-3078
MsHel-3b	gb|AIXA01009216.1|:86533-86727
MsHel-3b	gb|AIXA01006352.1|:2951-3133
MsHel-3b	gb|AIXA01004483.1|:7535-7740
MsHel-3b	gb|AIXA01004483.1|:21879-22004
MsHel-3b	gb|AIXA01003650.1|:12598-12746
MsHel-3b	gb|AIXA01003286.1|:2138-2354
MsHel-3b	gb|AIXA01003057.1|:99237-99338
MsHel-3b	gb|AIXA01002743.1|:12811-13021

MsHel-3b	gb|AIXA01001817.1|:13412-13557
MsHel-3b	gb|AIXA01001605.1|:49847-50061
MsHel-3b	gb|AIXA01001509.1|:28208-28413
MsHel-3b	gb|AIXA01000627.1|:41939-42061
MsHel-3b	gb|AIXA01016345.1|:1438-1642
MsHel-3b	gb|AIXA01014023.1|:4729-4914
MsHel-3b	gb|AIXA01014023.1|:18589-18876
MsHel-3b	gb|AIXA01010151.1|:117251-117424
MsHel-3b	gb|AIXA01010151.1|:155912-156122
MsHel-3b	gb|AIXA01009919.1|:1985-2197
MsHel-3b	gb|AIXA01006682.1|:21681-21879
MsHel-3b	gb|AIXA01004330.1|:18181-22449
MsHel-3b	gb|AIXA01004330.1|:79826-80037
MsHel-3b	gb|AIXA01004319.1|:41301-41504
MsHel-3b	gb|AIXA01001830.1|:6396-14813
MsHel-3b	gb|AIXA01001561.1|:6039-6249
MsHel-3b	gb|AIXA01015919.1|:5-194
MsHel-3b	gb|AIXA01007228.1|:100361-100528
MsHel-3b	gb|AIXA01006154.1|:13580-13788
MsHel-3b	gb|AIXA01005808.1|:9077-9202
MsHel-3b	gb|AIXA01002718.1|:19403-19608
MsHel-3b	gb|AIXA01001898.1|:136985-137065
MsHel-3b	gb|AIXA01001051.1|:59259-59434
MsHel-3b	gb|AIXA01000661.1|:18091-18299
MsHel-3b	gb|AIXA01000661.1|:52828-52957
MsHel-3b	gb|AIXA01000661.1|:126496-126661
MsHel-3b	gb|AIXA01000276.1|:10407-10623
MsHel-3b	gb|AIXA01011371.1|:4730-5408
MsHel-3b	gb|AIXA01011205.1|:524-736
MsHel-3b	gb|AIXA01008227.1|:46226-46352
MsHel-3b	gb|AIXA01008116.1|:3170-3296
MsHel-3b	gb|AIXA01007835.1|:94474-94677
MsHel-3b	gb|AIXA01002797.1|:8027-8214
MsHel-3b	gb|AIXA01002687.1|:598-813
MsHel-3b	gb|AIXA01002687.1|:31124-31227
MsHel-3b	gb|AIXA01001532.1|:65423-65598
MsHel-3b	gb|AIXA01018319.1|:4422-4636
MsHel-3b	gb|AIXA01008922.1|:5285-5491
MsHel-3b	gb|AIXA01008187.1|:9680-9898
MsHel-3b	gb|AIXA01008187.1|:55287-55482
MsHel-3b	gb|AIXA01007785.1|:6225-6439
MsHel-3b	gb|AIXA01007785.1|:17393-17601
MsHel-3b	gb|AIXA01006837.1|:37748-37869
MsHel-3b	gb|AIXA01006350.1|:45328-45537
MsHel-3b	gb|AIXA01006337.1|:23607-23815
MsHel-3b	gb|AIXA01006337.1|:39211-39397
MsHel-3b	gb|AIXA01005096.1|:11411-11515
MsHel-3b	gb|AIXA01005096.1|:21926-22072

MsHel-3b	gb|AIXA01005096.1|:50102-50276
MsHel-3b	gb|AIXA01004354.1|:37054-37267
MsHel-3b	gb|AIXA01004354.1|:57476-57602
MsHel-3b	gb|AIXA01004230.1|:33188-33390
MsHel-3b	gb|AIXA01004230.1|:44482-44607
MsHel-3b	gb|AIXA01004230.1|:60981-61191
MsHel-3b	gb|AIXA01001601.1|:20999-21213
MsHel-3b	gb|AIXA01001571.1|:115454-115652
MsHel-3b	gb|AIXA01001534.1|:24707-24918
MsHel-3b	gb|AIXA01001189.1|:6598-6784
MsHel-3b	gb|AIXA01016291.1|:3090-3303
MsHel-3b	gb|AIXA01015144.1|:17107-17312
MsHel-3b	gb|AIXA01014867.1|:24008-24213
MsHel-3b	gb|AIXA01010773.1|:24398-24486
MsHel-3b	gb|AIXA01009532.1|:635-808
MsHel-3b	gb|AIXA01009051.1|:595-804
MsHel-3b	gb|AIXA01007613.1|:35968-36181
MsHel-3b	gb|AIXA01007175.1|:57902-58097
MsHel-3b	gb|AIXA01007107.1|:30956-31137
MsHel-3b	gb|AIXA01007107.1|:54700-54825
MsHel-3b	gb|AIXA01006080.1|:7078-7280
MsHel-3b	gb|AIXA01005524.1|:6637-6813
MsHel-3b	gb|AIXA01005350.1|:46376-46582
MsHel-3b	gb|AIXA01005035.1|:27660-27841
MsHel-3b	gb|AIXA01005035.1|:59663-59864
MsHel-3b	gb|AIXA01003858.1|:2748-2956
MsHel-3b	gb|AIXA01003858.1|:40534-40670
MsHel-3b	gb|AIXA01003214.1|:64402-64488
MsHel-3b	gb|AIXA01002722.1|:6372-6585
MsHel-3b	gb|AIXA01002722.1|:18167-18348
MsHel-3b	gb|AIXA01001594.1|:7754-7967
MsHel-3b	gb|AIXA01031539.1|:499-708
MsHel-3b	gb|AIXA01022283.1|:1419-1614
MsHel-3b	gb|AIXA01017047.1|:4654-4834
MsHel-3b	gb|AIXA01015681.1|:2632-2846
MsHel-3b	gb|AIXA01015423.1|:397-607
MsHel-3b	gb|AIXA01013947.1|:20640-20850
MsHel-3b	gb|AIXA01010786.1|:24073-24278
MsHel-3b	gb|AIXA01010786.1|:51268-51458
MsHel-3b	gb|AIXA01010208.1|:1067-1193
MsHel-3b	gb|AIXA01010152.1|:16480-16572
MsHel-3b	gb|AIXA01009047.1|:18273-18489
MsHel-3b	gb|AIXA01009031.1|:26060-26276
MsHel-3b	gb|AIXA01008987.1|:1830-2022
MsHel-3b	gb|AIXA01008226.1|:30227-30411
MsHel-3b	gb|AIXA01008226.1|:40640-40786
MsHel-3b	gb|AIXA01008194.1|:7510-7666
MsHel-3b	gb|AIXA01007966.1|:73271-73417

MsHel-3b	gb|AIXA01007966.1|:86562-86770
MsHel-3b	gb|AIXA01007966.1|:156226-156593
MsHel-3b	gb|AIXA01007494.1|:5609-5729
MsHel-3b	gb|AIXA01007494.1|:33811-34017
MsHel-3b	gb|AIXA01005220.1|:1563-1771
MsHel-3b	gb|AIXA01005220.1|:18066-18262
MsHel-3b	gb|AIXA01004929.1|:12669-12811
MsHel-3b	gb|AIXA01004929.1|:48954-49130
MsHel-3b	gb|AIXA01004546.1|:23680-23896
MsHel-3b	gb|AIXA01004470.1|:21831-22038
MsHel-3b	gb|AIXA01004470.1|:69946-70162
MsHel-3b	gb|AIXA01003176.1|:43234-43450
MsHel-3b	gb|AIXA01003176.1|:59384-59530
MsHel-3b	gb|AIXA01003176.1|:84203-84418
MsHel-3b	gb|AIXA01001356.1|:31927-32131
MsHel-3b	gb|AIXA01000792.1|:14048-14255
MsHel-3b	gb|AIXA01028245.1|:307-490
MsHel-3b	gb|AIXA01028153.1|:547-767
MsHel-3b	gb|AIXA01012652.1|:2713-2860
MsHel-3b	gb|AIXA01012133.1|:13519-13614
MsHel-3b	gb|AIXA01012133.1|:41050-41182
MsHel-3b	gb|AIXA01012012.1|:8882-9092
MsHel-3b	gb|AIXA01010673.1|:10344-10553
MsHel-3b	gb|AIXA01010673.1|:25123-25206
MsHel-3b	gb|AIXA01009596.1|:10-217
MsHel-3b	gb|AIXA01009078.1|:30141-30323
MsHel-3b	gb|AIXA01009078.1|:49264-49467
MsHel-3b	gb|AIXA01008767.1|:15416-18905
MsHel-3b	gb|AIXA01008417.1|:21741-21945
MsHel-3b	gb|AIXA01008417.1|:35444-35536
MsHel-3b	gb|AIXA01007520.1|:2016-2194
MsHel-3b	gb|AIXA01007046.1|:26107-26302
MsHel-3b	gb|AIXA01007023.1|:27992-28155
MsHel-3b	gb|AIXA01006329.1|:8454-8665
MsHel-3b	gb|AIXA01006123.1|:64074-64285
MsHel-3b	gb|AIXA01004465.1|:44320-44524
MsHel-3b	gb|AIXA01003190.1|:33204-33376
MsHel-3b	gb|AIXA01002621.1|:56076-56282
MsHel-3b	gb|AIXA01002621.1|:92097-92304
MsHel-3b	gb|AIXA01000939.1|:832-1011
MsHel-3b	gb|AIXA01000939.1|:14878-15053
MsHel-3b	gb|AIXA01000685.1|:14507-14720
MsHel-3b	gb|AIXA01000482.1|:80246-80429
MsHel-3b	gb|AIXA01019061.1|:620-821
MsHel-3b	gb|AIXA01016282.1|:2097-2274
MsHel-3b	gb|AIXA01013067.1|:49412-49594
MsHel-3b	gb|AIXA01012523.1|:5486-5671
MsHel-3b	gb|AIXA01010757.1|:13898-14072

MsHel-3b	gb|AIXA01008106.1|:10879-11091
MsHel-3b	gb|AIXA01006816.1|:15608-15816
MsHel-3b	gb|AIXA01006529.1|:49013-49222
MsHel-3b	gb|AIXA01006529.1|:67229-67422
MsHel-3b	gb|AIXA01006287.1|:1772-1974
MsHel-3b	gb|AIXA01005792.1|:17047-17248
MsHel-3b	gb|AIXA01005278.1|:19357-19552
MsHel-3b	gb|AIXA01005278.1|:50301-50392
MsHel-3b	gb|AIXA01004670.1|:115283-115482
MsHel-3b	gb|AIXA01004670.1|:155650-155847
MsHel-3b	gb|AIXA01004567.1|:9840-10020
MsHel-3b	gb|AIXA01004567.1|:38457-38624
MsHel-3b	gb|AIXA01002293.1|:46522-46701
MsHel-3b	gb|AIXA01001804.1|:7127-7331
MsHel-3b	gb|AIXA01001511.1|:95307-95532
MsHel-3b	gb|AIXA01000048.1|:39796-39993
MsHel-3b	gb|AIXA01000048.1|:51936-52043
MsHel-3b	gb|AIXA01000048.1|:76694-76904
MsHel-3b	gb|AIXA01019077.1|:637-826
MsHel-3b	gb|AIXA01016884.1|:2363-2576
MsHel-3b	gb|AIXA01014018.1|:512-593
MsHel-3b	gb|AIXA01013867.1|:7203-7408
MsHel-3b	gb|AIXA01008711.1|:23542-23755
MsHel-3b	gb|AIXA01006641.1|:16193-16402
MsHel-3b	gb|AIXA01005037.1|:1023-1208
MsHel-3b	gb|AIXA01003658.1|:78523-78673
MsHel-3b	gb|AIXA01003146.1|:18343-18550
MsHel-3b	gb|AIXA01001952.1|:2348-2565
MsHel-3b	gb|AIXA01001554.1|:17034-17224
MsHel-3b	gb|AIXA01000767.1|:60186-60278
MsHel-3b	gb|AIXA01000188.1|:36925-37138
MsHel-3b	gb|AIXA01017117.1|:7252-7458
MsHel-3b	gb|AIXA01016590.1|:23632-23838
MsHel-3b	gb|AIXA01016468.1|:3013-3180
MsHel-3b	gb|AIXA01010748.1|:3010-3141
MsHel-3b	gb|AIXA01010748.1|:32223-32348
MsHel-3b	gb|AIXA01010748.1|:80170-80273
MsHel-3b	gb|AIXA01010748.1|:149129-149337
MsHel-3b	gb|AIXA01009766.1|:12104-12308
MsHel-3b	gb|AIXA01009346.1|:34267-34471
MsHel-3b	gb|AIXA01009135.1|:4956-5128
MsHel-3b	gb|AIXA01008234.1|:16837-16985
MsHel-3b	gb|AIXA01007870.1|:14688-14897
MsHel-3b	gb|AIXA01005488.1|:611-827
MsHel-3b	gb|AIXA01005488.1|:12491-12699
MsHel-3b	gb|AIXA01005488.1|:26731-26851
MsHel-3b	gb|AIXA01005351.1|:67712-67918
MsHel-3b	gb|AIXA01000969.1|:1721-1890

MsHel-3b	gb|AIXA01000969.1|:37438-37623
MsHel-3b	gb|AIXA01000838.1|:30609-30812
MsHel-3b	gb|AIXA01000838.1|:56352-56563
MsHel-3b	gb|AIXA01000838.1|:142339-142449
MsHel-3b	gb|AIXA01000562.1|:34088-34275
MsHel-3b	gb|AIXA01000562.1|:154098-154302
MsHel-3b	gb|AIXA01000562.1|:171122-171229
MsHel-3b	gb|AIXA01000559.1|:8182-8377
MsHel-3b	gb|AIXA01024029.1|:565-730
MsHel-3b	gb|AIXA01020715.1|:387-556
MsHel-3b	gb|AIXA01017702.1|:15296-15491
MsHel-3b	gb|AIXA01011722.1|:24876-25047
MsHel-3b	gb|AIXA01011722.1|:57474-57657
MsHel-3b	gb|AIXA01011361.1|:44139-44354
MsHel-3b	gb|AIXA01009634.1|:4953-5148
MsHel-3b	gb|AIXA01009634.1|:20302-20428
MsHel-3b	gb|AIXA01008705.1|:10946-11157
MsHel-3b	gb|AIXA01007968.1|:9166-9328
MsHel-3b	gb|AIXA01007164.1|:70164-70370
MsHel-3b	gb|AIXA01004160.1|:2952-3047
MsHel-3b	gb|AIXA01004160.1|:23004-23183
MsHel-3b	gb|AIXA01004160.1|:93763-93887
MsHel-3b	gb|AIXA01003993.1|:89382-89552
MsHel-3b	gb|AIXA01002313.1|:11911-12040
MsHel-3b	gb|AIXA01000068.1|:77460-77659
MsHel-3b	gb|AIXA01000011.1|:484-663
MsHel-3b	gb|AIXA01038179.1|:263-476
MsHel-3b	gb|AIXA01021900.1|:945-1115
MsHel-3b	gb|AIXA01016537.1|:15440-15610
MsHel-3b	gb|AIXA01016491.1|:236-1242
MsHel-3b	gb|AIXA01015731.1|:4681-4884
MsHel-3b	gb|AIXA01013684.1|:1151-1324
MsHel-3b	gb|AIXA01013223.1|:4865-5069
MsHel-3b	gb|AIXA01013223.1|:29589-29680
MsHel-3b	gb|AIXA01009079.1|:11703-11853
MsHel-3b	gb|AIXA01006874.1|:16631-16843
MsHel-3b	gb|AIXA01006874.1|:35705-35828
MsHel-3b	gb|AIXA01005841.1|:17538-17626
MsHel-3b	gb|AIXA01005841.1|:52539-52680
MsHel-3b	gb|AIXA01004905.1|:1495-1649
MsHel-3b	gb|AIXA01004893.1|:64162-64355
MsHel-3b	gb|AIXA01004865.1|:23364-23580
MsHel-3b	gb|AIXA01004311.1|:7936-8138
MsHel-3b	gb|AIXA01003889.1|:118370-118554
MsHel-3b	gb|AIXA01003889.1|:136357-136535
MsHel-3b	gb|AIXA01003713.1|:38980-39174
MsHel-3b	gb|AIXA01003661.1|:84999-85188
MsHel-3b	gb|AIXA01003234.1|:59656-59856

MsHel-3b	gb|AIXA01001847.1|:15742-15932
MsHel-3b	gb|AIXA01001366.1|:66295-66415
MsHel-3b	gb|AIXA01001021.1|:4177-4391
MsHel-3b	gb|AIXA01000185.1|:655-861
MsHel-3b	gb|AIXA01000127.1|:5045-5251
MsHel-3b	gb|AIXA01017894.1|:5746-5952
MsHel-3b	gb|AIXA01016376.1|:21689-21905
MsHel-3b	gb|AIXA01015400.1|:49844-50001
MsHel-3b	gb|AIXA01014771.1|:3577-3751
MsHel-3b	gb|AIXA01010475.1|:26796-26989
MsHel-3b	gb|AIXA01009804.1|:20253-20465
MsHel-3b	gb|AIXA01008364.1|:41692-41900
MsHel-3b	gb|AIXA01007890.1|:921-1113
MsHel-3b	gb|AIXA01006736.1|:14997-15210
MsHel-3b	gb|AIXA01006299.1|:69004-69115
MsHel-3b	gb|AIXA01006299.1|:114733-114893
MsHel-3b	gb|AIXA01006135.1|:3895-4092
MsHel-3b	gb|AIXA01005228.1|:125330-125448
MsHel-3b	gb|AIXA01004212.1|:22427-22640
MsHel-3b	gb|AIXA01004184.1|:127577-127782
MsHel-3b	gb|AIXA01003657.1|:2473-2685
MsHel-3b	gb|AIXA01003028.1|:15179-15371
MsHel-3b	gb|AIXA01002168.1|:13244-13449
MsHel-3b	gb|AIXA01001994.1|:25889-26098
MsHel-3b	gb|AIXA01001340.1|:24478-24601
MsHel-3b	gb|AIXA01001037.1|:9913-10037
MsHel-3b	gb|AIXA01001037.1|:27175-27380
MsHel-3b	gb|AIXA01001001.1|:2822-3033
MsHel-3b	gb|AIXA01000931.1|:3476-9183
MsHel-3b	gb|AIXA01000931.1|:38006-38206
MsHel-3b	gb|AIXA01000459.1|:17081-17207
MsHel-3b	gb|AIXA01000459.1|:39078-39290
MsHel-3b	gb|AIXA01000242.1|:43010-43221
MsHel-3b	gb|AIXA01027375.1|:634-851
MsHel-3b	gb|AIXA01025567.1|:710-931
MsHel-3b	gb|AIXA01021063.1|:1288-1476
MsHel-3b	gb|AIXA01019850.1|:657-868
MsHel-3b	gb|AIXA01017241.1|:4852-5024
MsHel-3b	gb|AIXA01017098.1|:10808-10988
MsHel-3b	gb|AIXA01015770.1|:23752-23958
MsHel-3b	gb|AIXA01015219.1|:2137-2316
MsHel-3b	gb|AIXA01012913.1|:13926-14133
MsHel-3b	gb|AIXA01012742.1|:3510-3685
MsHel-3b	gb|AIXA01012235.1|:2640-2846
MsHel-3b	gb|AIXA01012097.1|:8154-8279
MsHel-3b	gb|AIXA01010960.1|:22851-23035
MsHel-3b	gb|AIXA01010117.1|:2214-2422
MsHel-3b	gb|AIXA01009405.1|:30924-31142

MsHel-3b	gb|AIXA01008294.1|:45631-45803
MsHel-3b	gb|AIXA01007944.1|:4112-4315
MsHel-3b	gb|AIXA01007182.1|:8727-8899
MsHel-3b	gb|AIXA01006545.1|:6706-6858
MsHel-3b	gb|AIXA01005508.1|:26944-27153
MsHel-3b	gb|AIXA01004809.1|:83530-83737
MsHel-3b	gb|AIXA01004694.1|:57090-57300
MsHel-3b	gb|AIXA01004325.1|:28150-28355
MsHel-3b	gb|AIXA01003749.1|:15651-15751
MsHel-3b	gb|AIXA01001894.1|:6114-6260
MsHel-3b	gb|AIXA01001829.1|:15789-15958
MsHel-3b	gb|AIXA01001613.1|:12192-12400
MsHel-3b	gb|AIXA01001613.1|:74504-74710
MsHel-3b	gb|AIXA01000069.1|:122908-123083
MsHel-3b	gb|AIXA01000069.1|:152074-152158
MsHel-3b	gb|AIXA01036411.1|:233-377
MsHel-3b	gb|AIXA01021834.1|:1244-1419
MsHel-3b	gb|AIXA01021701.1|:1043-1241
MsHel-3b	gb|AIXA01015457.1|:11336-15265
MsHel-3b	gb|AIXA01009942.1|:1537-1739
MsHel-3b	gb|AIXA01009455.1|:5675-5878
MsHel-3b	gb|AIXA01009427.1|:15414-15570
MsHel-3b	gb|AIXA01009427.1|:26494-26665
MsHel-3b	gb|AIXA01009040.1|:17445-17564
MsHel-3b	gb|AIXA01009040.1|:147451-147545
MsHel-3b	gb|AIXA01008636.1|:49889-50042
MsHel-3b	gb|AIXA01004471.1|:16775-16989
MsHel-3b	gb|AIXA01004471.1|:44149-44359
MsHel-3b	gb|AIXA01003846.1|:69266-69477
MsHel-3b	gb|AIXA01003803.1|:28070-28195
MsHel-3b	gb|AIXA01003803.1|:58052-58265
MsHel-3b	gb|AIXA01003651.1|:48963-49166
MsHel-3b	gb|AIXA01003339.1|:13325-13526
MsHel-3b	gb|AIXA01003152.1|:90972-99704
MsHel-3b	gb|AIXA01003152.1|:137819-138015
MsHel-3b	gb|AIXA01001841.1|:10830-11021
MsHel-3b	gb|AIXA01001841.1|:34564-34772
MsHel-3b	gb|AIXA01001841.1|:54323-54404
MsHel-3b	gb|AIXA01001797.1|:58701-58875
MsHel-3b	gb|AIXA01001797.1|:138009-138099
MsHel-3b	gb|AIXA01001627.1|:35229-35445
MsHel-3b	gb|AIXA01000843.1|:5832-6038
MsHel-3b	gb|AIXA01000843.1|:85348-85476
MsHel-3b	gb|AIXA01000483.1|:33078-33260
MsHel-3b	gb|AIXA01000452.1|:15049-15255
MsHel-3b	gb|AIXA01000051.1|:38485-38586
MsHel-3b	gb|AIXA01036782.1|:102-284
MsHel-3b	gb|AIXA01019461.1|:4517-4605

MsHel-3b	gb|AIXA01011739.1|:14394-14607
MsHel-3b	gb|AIXA01011085.1|:5761-5974
MsHel-3b	gb|AIXA01010885.1|:10322-10514
MsHel-3b	gb|AIXA01010885.1|:23222-23409
MsHel-3b	gb|AIXA01010885.1|:94235-94406
MsHel-3b	gb|AIXA01010398.1|:4250-4428
MsHel-3b	gb|AIXA01010398.1|:29950-30157
MsHel-3b	gb|AIXA01008667.1|:29337-29546
MsHel-3b	gb|AIXA01008663.1|:34655-34852
MsHel-3b	gb|AIXA01008111.1|:24826-25003
MsHel-3b	gb|AIXA01006719.1|:11498-11630
MsHel-3b	gb|AIXA01006719.1|:37847-37968
MsHel-3b	gb|AIXA01005644.1|:505-707
MsHel-3b	gb|AIXA01005396.1|:26653-26811
MsHel-3b	gb|AIXA01005396.1|:53503-53713
MsHel-3b	gb|AIXA01004580.1|:49994-50207
MsHel-3b	gb|AIXA01004580.1|:98437-98638
MsHel-3b	gb|AIXA01004327.1|:91853-92050
MsHel-3b	gb|AIXA01003330.1|:36453-36651
MsHel-3b	gb|AIXA01003330.1|:71519-71723
MsHel-3b	gb|AIXA01003315.1|:35347-35554
MsHel-3b	gb|AIXA01002620.1|:7900-8100
MsHel-3b	gb|AIXA01002541.1|:14073-14177
MsHel-3b	gb|AIXA01002240.1|:28069-28282
MsHel-3b	gb|AIXA01000925.1|:24830-24969
MsHel-3b	gb|AIXA01000554.1|:1639-1837
MsHel-3b	gb|AIXA01000454.1|:18291-18489
MsHel-3b	gb|AIXA01000307.1|:1865-2080
MsHel-3b	gb|AIXA01027937.1|:1-207
MsHel-3b	gb|AIXA01018917.1|:387-596
MsHel-3b	gb|AIXA01017318.1|:15279-15408
MsHel-3b	gb|AIXA01016084.1|:6763-6917
MsHel-3b	gb|AIXA01014243.1|:39120-39325
MsHel-3b	gb|AIXA01011987.1|:16818-16963
MsHel-3b	gb|AIXA01010330.1|:45372-45510
MsHel-3b	gb|AIXA01008022.1|:41850-42056
MsHel-3b	gb|AIXA01006937.1|:1439-1620
MsHel-3b	gb|AIXA01005491.1|:30462-30642
MsHel-3b	gb|AIXA01004829.1|:40630-40806
MsHel-3b	gb|AIXA01002814.1|:33612-33825
MsHel-3b	gb|AIXA01002567.1|:14133-14301
MsHel-3b	gb|AIXA01002352.1|:27328-27537
MsHel-3b	gb|AIXA01001806.1|:21642-21848
MsHel-3b	gb|AIXA01001610.1|:1990-2196
MsHel-3b	gb|AIXA01001328.1|:11816-11969
MsHel-3b	gb|AIXA01000058.1|:3418-3615
MsHel-3b	gb|AIXA01032017.1|:279-488
MsHel-3b	gb|AIXA01023697.1|:1039-1210

MsHel-3b	gb|AIXA01016204.1|:7888-8089
MsHel-3b	gb|AIXA01015719.1|:3478-3658
MsHel-3b	gb|AIXA01015708.1|:3602-3806
MsHel-3b	gb|AIXA01014443.1|:35557-35739
MsHel-3b	gb|AIXA01011303.1|:19204-19401
MsHel-3b	gb|AIXA01011303.1|:62512-62686
MsHel-3b	gb|AIXA01010703.1|:26721-26935
MsHel-3b	gb|AIXA01010342.1|:26328-26480
MsHel-3b	gb|AIXA01009144.1|:10076-10291
MsHel-3b	gb|AIXA01008773.1|:27803-27986
MsHel-3b	gb|AIXA01008013.1|:14316-14524
MsHel-3b	gb|AIXA01006184.1|:16309-16480
MsHel-3b	gb|AIXA01005997.1|:4438-4651
MsHel-3b	gb|AIXA01005789.1|:15156-15359
MsHel-3b	gb|AIXA01004896.1|:40286-40496
MsHel-3b	gb|AIXA01004896.1|:56553-56711
MsHel-3b	gb|AIXA01004150.1|:4889-5056
MsHel-3b	gb|AIXA01004150.1|:20558-20734
MsHel-3b	gb|AIXA01004150.1|:51196-51411
MsHel-3b	gb|AIXA01004135.1|:33773-33977
MsHel-3b	gb|AIXA01004057.1|:6711-6918
MsHel-3b	gb|AIXA01003816.1|:68535-69846
MsHel-3b	gb|AIXA01003125.1|:29596-29794
MsHel-3b	gb|AIXA01002811.1|:43712-43919
MsHel-3b	gb|AIXA01001786.1|:42956-43142
MsHel-3b	gb|AIXA01001313.1|:13013-13185
MsHel-3b	gb|AIXA01001222.1|:77505-77685
MsHel-3b	gb|AIXA01000758.1|:1524-1712
MsHel-3b	gb|AIXA01031120.1|:420-623
MsHel-3b	gb|AIXA01016325.1|:9148-9346
MsHel-3b	gb|AIXA01014392.1|:2470-2558
MsHel-3b	gb|AIXA01011968.1|:19286-19505
MsHel-3b	gb|AIXA01008972.1|:3696-3879
MsHel-3b	gb|AIXA01008491.1|:3226-3317
MsHel-3b	gb|AIXA01008491.1|:16475-16680
MsHel-3b	gb|AIXA01007791.1|:51523-51690
MsHel-3b	gb|AIXA01007766.1|:4170-4382
MsHel-3b	gb|AIXA01007347.1|:20109-20294
MsHel-3b	gb|AIXA01007114.1|:10859-10996
MsHel-3b	gb|AIXA01007114.1|:29606-29785
MsHel-3b	gb|AIXA01006934.1|:2506-2709
MsHel-3b	gb|AIXA01006684.1|:4304-4431
MsHel-3b	gb|AIXA01005534.1|:16583-16758
MsHel-3b	gb|AIXA01005474.1|:13981-14167
MsHel-3b	gb|AIXA01005217.1|:31864-32086
MsHel-3b	gb|AIXA01005203.1|:36278-36465
MsHel-3b	gb|AIXA01004852.1|:97105-97316
MsHel-3b	gb|AIXA01004386.1|:23362-23534

MsHel-3b	gb|AIXA01004386.1|:83618-83824
MsHel-3b	gb|AIXA01003582.1|:12059-12267
MsHel-3b	gb|AIXA01003002.1|:6394-6569
MsHel-3b	gb|AIXA01002311.1|:1008-1118
MsHel-3b	gb|AIXA01002311.1|:63638-63764
MsHel-3b	gb|AIXA01002311.1|:152460-152671
MsHel-3b	gb|AIXA01001278.1|:27198-27388
MsHel-3b	gb|AIXA01001210.1|:36903-37082
MsHel-3b	gb|AIXA01000984.1|:10713-10891
MsHel-3b	gb|AIXA01000984.1|:24663-24855
MsHel-3b	gb|AIXA01000641.1|:28718-28925
MsHel-3b	gb|AIXA01000641.1|:61021-61153
MsHel-3b	gb|AIXA01000294.1|:15061-15268
MsHel-3b	gb|AIXA01000005.1|:29500-29704
MsHel-3b	gb|AIXA01026942.1|:919-1049
MsHel-3b	gb|AIXA01017382.1|:6264-6459
MsHel-3b	gb|AIXA01015530.1|:6120-6298
MsHel-3b	gb|AIXA01013682.1|:12061-12235
MsHel-3b	gb|AIXA01011501.1|:34409-34607
MsHel-3b	gb|AIXA01011322.1|:35142-35315
MsHel-3b	gb|AIXA01010696.1|:8954-9116
MsHel-3b	gb|AIXA01008934.1|:10587-10796
MsHel-3b	gb|AIXA01008398.1|:32160-32363
MsHel-3b	gb|AIXA01007881.1|:25761-25885
MsHel-3b	gb|AIXA01007881.1|:47498-47695
MsHel-3b	gb|AIXA01007875.1|:50040-50251
MsHel-3b	gb|AIXA01006683.1|:20943-21149
MsHel-3b	gb|AIXA01005034.1|:33625-33770
MsHel-3b	gb|AIXA01005034.1|:81477-81653
MsHel-3b	gb|AIXA01003030.1|:3664-3750
MsHel-3b	gb|AIXA01003030.1|:26669-26863
MsHel-3b	gb|AIXA01001991.1|:11865-12083
MsHel-3b	gb|AIXA01001832.1|:5768-5975
MsHel-3b	gb|AIXA01001296.1|:167148-167251
MsHel-3b	gb|AIXA01000441.1|:15373-15576
MsHel-3b	gb|AIXA01000272.1|:18509-18718
MsHel-3b	gb|AIXA01035244.1|:186-315
MsHel-3b	gb|AIXA01018955.1|:119-332
MsHel-3b	gb|AIXA01018933.1|:128-329
MsHel-3b	gb|AIXA01017136.1|:26046-26176
MsHel-3b	gb|AIXA01015804.1|:6400-6594
MsHel-3b	gb|AIXA01014707.1|:4482-4681
MsHel-3b	gb|AIXA01014041.1|:20715-20923
MsHel-3b	gb|AIXA01011300.1|:12471-12581
MsHel-3b	gb|AIXA01010631.1|:1959-2140
MsHel-3b	gb|AIXA01009945.1|:8686-8871
MsHel-3b	gb|AIXA01009580.1|:23948-24155
MsHel-3b	gb|AIXA01009580.1|:36700-36796

MsHel-3b	gb|AIXA01009102.1|:8857-8970
MsHel-3b	gb|AIXA01008220.1|:15950-16131
MsHel-3b	gb|AIXA01007265.1|:11200-11396
MsHel-3b	gb|AIXA01007265.1|:26176-26390
MsHel-3b	gb|AIXA01004100.1|:16650-16860
MsHel-3b	gb|AIXA01002815.1|:60048-60205
MsHel-3b	gb|AIXA01001816.1|:1994-2079
MsHel-3b	gb|AIXA01000813.1|:45463-45661
MsHel-3b	gb|AIXA01000810.1|:176059-176142
MsHel-3b	gb|AIXA01000415.1|:28416-28561
MsHel-3b	gb|AIXA01021259.1|:1805-2020
MsHel-3b	gb|AIXA01018493.1|:5371-5515
MsHel-3b	gb|AIXA01017198.1|:2954-3157
MsHel-3b	gb|AIXA01010962.1|:22182-22393
MsHel-3b	gb|AIXA01010372.1|:3673-3876
MsHel-3b	gb|AIXA01009528.1|:16288-16476
MsHel-3b	gb|AIXA01009071.1|:40944-41159
MsHel-3b	gb|AIXA01009052.1|:3050-3209
MsHel-3b	gb|AIXA01008428.1|:21099-21184
MsHel-3b	gb|AIXA01008313.1|:77154-77370
MsHel-3b	gb|AIXA01007589.1|:24224-24423
MsHel-3b	gb|AIXA01007510.1|:4042-4201
MsHel-3b	gb|AIXA01006190.1|:19019-19195
MsHel-3b	gb|AIXA01006190.1|:106838-107013
MsHel-3b	gb|AIXA01004754.1|:27243-27414
MsHel-3b	gb|AIXA01004357.1|:8517-8719
MsHel-3b	gb|AIXA01004226.1|:5327-5520
MsHel-3b	gb|AIXA01003678.1|:31322-31408
MsHel-3b	gb|AIXA01003678.1|:64298-64502
MsHel-3b	gb|AIXA01003615.1|:66659-66867
MsHel-3b	gb|AIXA01002340.1|:66787-66996
MsHel-3b	gb|AIXA01002340.1|:94174-94376
MsHel-3b	gb|AIXA01002340.1|:121079-121204
MsHel-3b	gb|AIXA01002296.1|:92585-92713
MsHel-3b	gb|AIXA01001973.1|:12414-12616
MsHel-3b	gb|AIXA01001674.1|:65362-65534
MsHel-3b	gb|AIXA01001598.1|:49539-49749
MsHel-3b	gb|AIXA01000273.1|:1474-1685
MsHel-3b	gb|AIXA01000099.1|:26302-27822
MsHel-3b	gb|AIXA01020269.1|:1865-2076
MsHel-3b	gb|AIXA01017935.1|:2632-5011
MsHel-3b	gb|AIXA01016735.1|:7614-7825
MsHel-3b	gb|AIXA01015786.1|:5303-5507
MsHel-3b	gb|AIXA01014934.1|:3717-3911
MsHel-3b	gb|AIXA01012656.1|:7304-7464
MsHel-3b	gb|AIXA01012196.1|:10042-10238
MsHel-3b	gb|AIXA01010102.1|:7464-7560
MsHel-3b	gb|AIXA01008526.1|:7349-7557

MsHel-3b	gb|AIXA01008520.1|:20044-20252
MsHel-3b	gb|AIXA01008110.1|:55868-56003
MsHel-3b	gb|AIXA01008076.1|:26393-26603
MsHel-3b	gb|AIXA01008028.1|:35964-36176
MsHel-3b	gb|AIXA01008028.1|:48961-49086
MsHel-3b	gb|AIXA01007947.1|:53072-53275
MsHel-3b	gb|AIXA01007577.1|:23252-23422
MsHel-3b	gb|AIXA01007515.1|:18814-18981
MsHel-3b	gb|AIXA01007515.1|:82601-82739
MsHel-3b	gb|AIXA01007111.1|:32153-32332
MsHel-3b	gb|AIXA01006482.1|:38214-38433
MsHel-3b	gb|AIXA01006420.1|:24349-24540
MsHel-3b	gb|AIXA01006420.1|:70996-71160
MsHel-3b	gb|AIXA01005088.1|:34381-34556
MsHel-3b	gb|AIXA01004412.1|:12671-12871
MsHel-3b	gb|AIXA01004214.1|:24427-24593
MsHel-3b	gb|AIXA01003209.1|:11504-12895
MsHel-3b	gb|AIXA01003151.1|:39152-39279
MsHel-3b	gb|AIXA01003151.1|:70869-71084
MsHel-3b	gb|AIXA01002782.1|:68094-68241
MsHel-3b	gb|AIXA01001661.1|:10195-10402
MsHel-3b	gb|AIXA01001071.1|:42763-42969
MsHel-3b	gb|AIXA01001071.1|:94635-94795
MsHel-3b	gb|AIXA01001049.1|:17749-17907
MsHel-3b	gb|AIXA01000356.1|:9865-10024
MsHel-3b	gb|AIXA01028141.1|:41-200
MsHel-3b	gb|AIXA01024878.1|:226-402
MsHel-3b	gb|AIXA01019935.1|:1425-1579
MsHel-3b	gb|AIXA01018590.1|:1459-1660
MsHel-3b	gb|AIXA01017620.1|:7478-7660
MsHel-3b	gb|AIXA01016786.1|:4959-5137
MsHel-3b	gb|AIXA01016389.1|:685-889
MsHel-3b	gb|AIXA01015446.1|:11242-11433
MsHel-3b	gb|AIXA01012798.1|:24969-25151
MsHel-3b	gb|AIXA01012404.1|:12931-13145
MsHel-3b	gb|AIXA01010291.1|:5957-6162
MsHel-3b	gb|AIXA01009854.1|:90-294
MsHel-3b	gb|AIXA01009752.1|:15572-15779
MsHel-3b	gb|AIXA01009584.1|:40923-41079
MsHel-3b	gb|AIXA01009584.1|:73623-73835
MsHel-3b	gb|AIXA01009426.1|:2829-3003
MsHel-3b	gb|AIXA01009426.1|:30531-30696
MsHel-3b	gb|AIXA01009376.1|:4912-5066
MsHel-3b	gb|AIXA01008423.1|:59636-59841
MsHel-3b	gb|AIXA01008261.1|:4545-4695
MsHel-3b	gb|AIXA01007964.1|:10110-10304
MsHel-3b	gb|AIXA01006998.1|:1219-1428
MsHel-3b	gb|AIXA01006321.1|:8440-8651

MsHel-3b	gb|AIXA01006091.1|:1379-1586
MsHel-3b	gb|AIXA01006041.1|:5996-6174
MsHel-3b	gb|AIXA01005439.1|:10827-10948
MsHel-3b	gb|AIXA01005045.1|:58291-58503
MsHel-3b	gb|AIXA01004980.1|:35859-36073
MsHel-3b	gb|AIXA01004342.1|:26776-26958
MsHel-3b	gb|AIXA01004220.1|:18982-19181
MsHel-3b	gb|AIXA01004220.1|:38103-38276
MsHel-3b	gb|AIXA01003825.1|:27032-27210
MsHel-3b	gb|AIXA01002454.1|:53722-53935
MsHel-3b	gb|AIXA01001982.1|:6911-7030
MsHel-3b	gb|AIXA01001982.1|:34437-34645
MsHel-3b	gb|AIXA01001525.1|:5435-5620
MsHel-3b	gb|AIXA01001505.1|:53635-53852
MsHel-3b	gb|AIXA01001163.1|:21789-22000
MsHel-3b	gb|AIXA01001032.1|:27103-27293
MsHel-3b	gb|AIXA01000770.1|:67441-67608
MsHel-3b	gb|AIXA01000684.1|:25742-25921
MsHel-3b	gb|AIXA01028696.1|:263-472
MsHel-3b	gb|AIXA01023497.1|:843-1055
MsHel-3b	gb|AIXA01020162.1|:1430-1647
MsHel-3b	gb|AIXA01019129.1|:1657-1858
MsHel-3b	gb|AIXA01018802.1|:1563-1792
MsHel-3b	gb|AIXA01017885.1|:3204-3413
MsHel-3b	gb|AIXA01017884.1|:480-689
MsHel-3b	gb|AIXA01015720.1|:8471-8680
MsHel-3b	gb|AIXA01014585.1|:8929-9111
MsHel-3b	gb|AIXA01012350.1|:17343-17533
MsHel-3b	gb|AIXA01011560.1|:2665-2791
MsHel-3b	gb|AIXA01011560.1|:43316-43514
MsHel-3b	gb|AIXA01010345.1|:6526-6672
MsHel-3b	gb|AIXA01010319.1|:8062-8231
MsHel-3b	gb|AIXA01008854.1|:1629-1717
MsHel-3b	gb|AIXA01006399.1|:4315-4528
MsHel-3b	gb|AIXA01005504.1|:47658-47830
MsHel-3b	gb|AIXA01004982.1|:98204-98410
MsHel-3b	gb|AIXA01004806.1|:24420-24501
MsHel-3b	gb|AIXA01004360.1|:4491-4700
MsHel-3b	gb|AIXA01002175.1|:10961-11158
MsHel-3b	gb|AIXA01001551.1|:29072-29160
MsHel-3b	gb|AIXA01001235.1|:26874-26959
MsHel-3b	gb|AIXA01001235.1|:38688-38824
MsHel-3b	gb|AIXA01001235.1|:78940-79066
MsHel-3b	gb|AIXA01000809.1|:7526-7718
MsHel-3b	gb|AIXA01000809.1|:26216-26425
MsHel-3b	gb|AIXA01000809.1|:54086-54212
MsHel-3b	gb|AIXA01000809.1|:72886-73087
MsHel-3b	gb|AIXA01000741.1|:17588-17801

MsHel-3b	gb|AIXA01000561.1|:18865-19069
MsHel-3b	gb|AIXA01000275.1|:25513-25714
MsHel-3b	gb|AIXA01000275.1|:49652-49860
MsHel-3b	gb|AIXA01000275.1|:71269-71447
MsHel-3b	gb|AIXA01024167.1|:342-551
MsHel-3b	gb|AIXA01023146.1|:717-913
MsHel-3b	gb|AIXA01017339.1|:86-274
MsHel-3b	gb|AIXA01016250.1|:1390-1559
MsHel-3b	gb|AIXA01010956.1|:20096-20186
MsHel-3b	gb|AIXA01010956.1|:59081-59253
MsHel-3b	gb|AIXA01010913.1|:11480-11681
MsHel-3b	gb|AIXA01010666.1|:12407-12594
MsHel-3b	gb|AIXA01008728.1|:5477-5693
MsHel-3b	gb|AIXA01006848.1|:5892-5992
MsHel-3b	gb|AIXA01006282.1|:6376-6587
MsHel-3b	gb|AIXA01004908.1|:7826-7997
MsHel-3b	gb|AIXA01004730.1|:22733-22944
MsHel-3b	gb|AIXA01002629.1|:87209-87291
MsHel-3b	gb|AIXA01002629.1|:97736-97950
MsHel-3b	gb|AIXA01002507.1|:11142-11278
MsHel-3b	gb|AIXA01002507.1|:46933-47148
MsHel-3b	gb|AIXA01002500.1|:154504-154630
MsHel-3b	gb|AIXA01002309.1|:11938-12085
MsHel-3b	gb|AIXA01002281.1|:23270-23441
MsHel-3b	gb|AIXA01001304.1|:16762-16933
MsHel-3b	gb|AIXA01001257.1|:750-954
MsHel-3b	gb|AIXA01000747.1|:72716-72872
MsHel-3b	gb|AIXA01013982.1|:3499-3670
MsHel-3b	gb|AIXA01012306.1|:10625-10736
MsHel-3b	gb|AIXA01008957.1|:9594-9756
MsHel-3b	gb|AIXA01008639.1|:25684-25891
MsHel-3b	gb|AIXA01008463.1|:41-245
MsHel-3b	gb|AIXA01008442.1|:2126-2260
MsHel-3b	gb|AIXA01008442.1|:25753-25839
MsHel-3b	gb|AIXA01008442.1|:71188-71411
MsHel-3b	gb|AIXA01007664.1|:5134-5339
MsHel-3b	gb|AIXA01006178.1|:8523-8675
MsHel-3b	gb|AIXA01006132.1|:18968-19099
MsHel-3b	gb|AIXA01005865.1|:75384-75597
MsHel-3b	gb|AIXA01005391.1|:25984-26132
MsHel-3b	gb|AIXA01005391.1|:66162-66325
MsHel-3b	gb|AIXA01005251.1|:920-1110
MsHel-3b	gb|AIXA01004219.1|:5887-6021
MsHel-3b	gb|AIXA01004209.1|:16887-17067
MsHel-3b	gb|AIXA01003944.1|:8709-8896
MsHel-3b	gb|AIXA01002651.1|:17014-17181
MsHel-3b	gb|AIXA01002473.1|:16145-16239
MsHel-3b	gb|AIXA01002356.1|:16252-16469

MsHel-3b	gb|AIXA01001501.1|:14392-16860
MsHel-3b	gb|AIXA01001270.1|:2314-2489
MsHel-3b	gb|AIXA01001270.1|:14717-14835
MsHel-3b	gb|AIXA01001196.1|:23344-23469
MsHel-3b	gb|AIXA01000320.1|:33022-33201
MsHel-3b	gb|AIXA01024093.1|:439-654
MsHel-3b	gb|AIXA01015447.1|:1221-1412
MsHel-3b	gb|AIXA01012741.1|:69829-69954
MsHel-3b	gb|AIXA01009131.1|:19288-19501
MsHel-3b	gb|AIXA01006863.1|:14458-14605
MsHel-3b	gb|AIXA01005095.1|:41728-41814
MsHel-3b	gb|AIXA01004861.1|:11491-11665
MsHel-3b	gb|AIXA01004596.1|:47243-47422
MsHel-3b	gb|AIXA01003699.1|:3068-3282
MsHel-3b	gb|AIXA01003621.1|:22081-22172
MsHel-3b	gb|AIXA01003621.1|:106633-106814
MsHel-3b	gb|AIXA01003588.1|:6906-14594
MsHel-3b	gb|AIXA01002975.1|:100245-100372
MsHel-3b	gb|AIXA01001957.1|:17542-18270
MsHel-3b	gb|AIXA01001796.1|:30611-30781
MsHel-3b	gb|AIXA01001599.1|:2465-2584
MsHel-3b	gb|AIXA01000154.1|:5276-5478
MsHel-3b	gb|AIXA01037269.1|:70-287
MsHel-3b	gb|AIXA01036627.1|:359-544
MsHel-3b	gb|AIXA01018595.1|:7-167
MsHel-3b	gb|AIXA01018162.1|:4944-5112
MsHel-3b	gb|AIXA01016615.1|:5166-5363
MsHel-3b	gb|AIXA01014513.1|:1998-2174
MsHel-3b	gb|AIXA01013065.1|:1103-1243
MsHel-3b	gb|AIXA01011695.1|:4219-4391
MsHel-3b	gb|AIXA01009508.1|:54524-59092
MsHel-3b	gb|AIXA01008295.1|:2268-2457
MsHel-3b	gb|AIXA01008049.1|:35135-35320
MsHel-3b	gb|AIXA01007974.1|:25800-25926
MsHel-3b	gb|AIXA01007974.1|:74132-74339
MsHel-3b	gb|AIXA01007139.1|:10540-10705
MsHel-3b	gb|AIXA01006158.1|:10215-10380
MsHel-3b	gb|AIXA01005566.1|:20517-20670
MsHel-3b	gb|AIXA01005407.1|:86618-86702
MsHel-3b	gb|AIXA01005407.1|:183301-183479
MsHel-3b	gb|AIXA01005209.1|:2651-2856
MsHel-3b	gb|AIXA01004066.1|:15403-15598
MsHel-3b	gb|AIXA01003857.1|:16512-16724
MsHel-3b	gb|AIXA01003857.1|:82949-83052
MsHel-3b	gb|AIXA01002672.1|:4680-4891
MsHel-3b	gb|AIXA01001995.1|:15733-15932
MsHel-3b	gb|AIXA01001660.1|:32485-32696
MsHel-3b	gb|AIXA01001602.1|:38893-39038

MsHel-3b	gb|AIXA01001022.1|:21143-21340
MsHel-3b	gb|AIXA01000508.1|:11935-12095
MsHel-3b	gb|AIXA01000448.1|:79069-79205
MsHel-3b	gb|AIXA01000448.1|:135280-135482
MsHel-3b	gb|AIXA01000422.1|:73018-73203
MsHel-3b	gb|AIXA01037367.1|:233-427
MsHel-3b	gb|AIXA01035818.1|:125-336
MsHel-3b	gb|AIXA01035517.1|:215-397
MsHel-3b	gb|AIXA01032164.1|:469-601
MsHel-3b	gb|AIXA01021912.1|:173-375
MsHel-3b	gb|AIXA01017343.1|:7066-7215
MsHel-3b	gb|AIXA01014780.1|:8425-8630
MsHel-3b	gb|AIXA01014388.1|:18598-18814
MsHel-3b	gb|AIXA01013595.1|:6362-6542
MsHel-3b	gb|AIXA01011354.1|:20391-20552
MsHel-3b	gb|AIXA01011354.1|:46312-46520
MsHel-3b	gb|AIXA01010691.1|:7513-7721
MsHel-3b	gb|AIXA01009576.1|:4999-5131
MsHel-3b	gb|AIXA01009380.1|:23302-27950
MsHel-3b	gb|AIXA01009084.1|:16393-16539
MsHel-3b	gb|AIXA01007902.1|:13575-13698
MsHel-3b	gb|AIXA01007015.1|:39986-40200
MsHel-3b	gb|AIXA01006999.1|:37794-38005
MsHel-3b	gb|AIXA01005323.1|:11582-11794
MsHel-3b	gb|AIXA01004349.1|:15646-15854
MsHel-3b	gb|AIXA01004300.1|:23817-24040
MsHel-3b	gb|AIXA01003249.1|:22576-22748
MsHel-3b	gb|AIXA01002477.1|:29094-33182
MsHel-3b	gb|AIXA01001508.1|:34379-34546
MsHel-3b	gb|AIXA01000688.1|:87605-87785
MsHel-3b	gb|AIXA01000688.1|:122516-122660
MsHel-3b	gb|AIXA01000650.1|:52563-52667
MsHel-3b	gb|AIXA01000494.1|:43400-43525
MsHel-3b	gb|AIXA01000476.1|:30537-30715
MsHel-3b	gb|AIXA01000397.1|:1785-1941
MsHel-3b	gb|AIXA01000161.1|:18584-18793
MsHel-3b	gb|AIXA01000092.1|:19054-19270
MsHel-3b	gb|AIXA01018591.1|:1458-1660
MsHel-3b	gb|AIXA01017926.1|:3937-4094
MsHel-3b	gb|AIXA01013807.1|:28362-28573
MsHel-3b	gb|AIXA01013596.1|:4-138
MsHel-3b	gb|AIXA01011712.1|:2573-2716
MsHel-3b	gb|AIXA01011385.1|:33456-33592
MsHel-3b	gb|AIXA01011253.1|:2852-3065
MsHel-3b	gb|AIXA01010222.1|:4839-4982
MsHel-3b	gb|AIXA01009940.1|:6985-7158
MsHel-3b	gb|AIXA01009798.1|:6134-6329
MsHel-3b	gb|AIXA01009142.1|:12958-13125

MsHel-3b	gb|AIXA01009142.1|:30974-31095
MsHel-3b	gb|AIXA01008743.1|:965-1108
MsHel-3b	gb|AIXA01007616.1|:8677-8760
MsHel-3b	gb|AIXA01007588.1|:8087-8293
MsHel-3b	gb|AIXA01007163.1|:14441-14524
MsHel-3b	gb|AIXA01006508.1|:24934-25109
MsHel-3b	gb|AIXA01004785.1|:30986-31208
MsHel-3b	gb|AIXA01004118.1|:4757-4976
MsHel-3b	gb|AIXA01003283.1|:6708-6911
MsHel-3b	gb|AIXA01003185.1|:16585-16761
MsHel-3b	gb|AIXA01002268.1|:54-245
MsHel-3b	gb|AIXA01002174.1|:65411-65543
MsHel-3b	gb|AIXA01002174.1|:76460-76643
MsHel-3b	gb|AIXA01001803.1|:3785-3983
MsHel-3b	gb|AIXA01001050.1|:15861-16026
MsHel-3b	gb|AIXA01001050.1|:29590-29802
MsHel-3b	gb|AIXA01001050.1|:95192-95407
MsHel-3b	gb|AIXA01028129.1|:1-189
MsHel-3b	gb|AIXA01018937.1|:3064-3253
MsHel-3b	gb|AIXA01016045.1|:1860-2029
MsHel-3b	gb|AIXA01015424.1|:1994-2194
MsHel-3b	gb|AIXA01015297.1|:2477-2672
MsHel-3b	gb|AIXA01014017.1|:4142-4294
MsHel-3b	gb|AIXA01011126.1|:17962-18167
MsHel-3b	gb|AIXA01011126.1|:38119-38280
MsHel-3b	gb|AIXA01010394.1|:32740-41993
MsHel-3b	gb|AIXA01008540.1|:9556-9711
MsHel-3b	gb|AIXA01007329.1|:35692-35897
MsHel-3b	gb|AIXA01006731.1|:143774-143985
MsHel-3b	gb|AIXA01005642.1|:1490-1693
MsHel-3b	gb|AIXA01005642.1|:33185-33334
MsHel-3b	gb|AIXA01005642.1|:70212-70416
MsHel-3b	gb|AIXA01005538.1|:65912-66053
MsHel-3b	gb|AIXA01005252.1|:35140-35348
MsHel-3b	gb|AIXA01004958.1|:63-154
MsHel-3b	gb|AIXA01004958.1|:47633-51237
MsHel-3b	gb|AIXA01004715.1|:68822-68906
MsHel-3b	gb|AIXA01004420.1|:3389-3555
MsHel-3b	gb|AIXA01003851.1|:3262-3464
MsHel-3b	gb|AIXA01002999.1|:9904-9993
MsHel-3b	gb|AIXA01002999.1|:59657-59747
MsHel-3b	gb|AIXA01002999.1|:82712-88985
MsHel-3b	gb|AIXA01001273.1|:4035-4209
MsHel-3b	gb|AIXA01000107.1|:34332-34489
MsHel-3b	gb|AIXA01000107.1|:144031-144121
MsHel-3b	gb|AIXA01000070.1|:30873-31027
MsHel-3b	gb|AIXA01021569.1|:354-553
MsHel-3b	gb|AIXA01018667.1|:959-1170

MsHel-3b	gb|AIXA01017988.1|:868-1005
MsHel-3b	gb|AIXA01015929.1|:26319-26484
MsHel-3b	gb|AIXA01012319.1|:18055-18227
MsHel-3b	gb|AIXA01012173.1|:8155-8327
MsHel-3b	gb|AIXA01012173.1|:22575-22691
MsHel-3b	gb|AIXA01011304.1|:18503-18678
MsHel-3b	gb|AIXA01010756.1|:32732-32911
MsHel-3b	gb|AIXA01010737.1|:19433-19615
MsHel-3b	gb|AIXA01010279.1|:2587-2796
MsHel-3b	gb|AIXA01006983.1|:125977-126149
MsHel-3b	gb|AIXA01005505.1|:8283-13620
MsHel-3b	gb|AIXA01005317.1|:3928-4143
MsHel-3b	gb|AIXA01005062.1|:36202-36415
MsHel-3b	gb|AIXA01004927.1|:13038-13199
MsHel-3b	gb|AIXA01004155.1|:22731-22859
MsHel-3b	gb|AIXA01003859.1|:23833-24001
MsHel-3b	gb|AIXA01003568.1|:5778-5978
MsHel-3b	gb|AIXA01003408.1|:3627-3833
MsHel-3b	gb|AIXA01002776.1|:75305-75437
MsHel-3b	gb|AIXA01002283.1|:42147-42348
MsHel-3b	gb|AIXA01001837.1|:24802-25006
MsHel-3b	gb|AIXA01001043.1|:63760-63870
MsHel-3b	gb|AIXA01001043.1|:103534-103730
MsHel-3b	gb|AIXA01000269.1|:15730-16111
MsHel-3b	gb|AIXA01021508.1|:1535-1731
MsHel-3b	gb|AIXA01019308.1|:119-239
MsHel-3b	gb|AIXA01017631.1|:2732-2904
MsHel-3b	gb|AIXA01014967.1|:11898-12046
MsHel-3b	gb|AIXA01014685.1|:1709-1845
MsHel-3b	gb|AIXA01013103.1|:28113-28296
MsHel-3b	gb|AIXA01012198.1|:776-967
MsHel-3b	gb|AIXA01012198.1|:33112-33280
MsHel-3b	gb|AIXA01011538.1|:25491-25642
MsHel-3b	gb|AIXA01010558.1|:4545-4685
MsHel-3b	gb|AIXA01008752.1|:4685-4861
MsHel-3b	gb|AIXA01008287.1|:9028-9153
MsHel-3b	gb|AIXA01008104.1|:5008-5129
MsHel-3b	gb|AIXA01007948.1|:44479-44678
MsHel-3b	gb|AIXA01007453.1|:6790-6942
MsHel-3b	gb|AIXA01006922.1|:15175-15300
MsHel-3b	gb|AIXA01006590.1|:135633-135794
MsHel-3b	gb|AIXA01006166.1|:7912-8052
MsHel-3b	gb|AIXA01005462.1|:13866-14073
MsHel-3b	gb|AIXA01003687.1|:461-644
MsHel-3b	gb|AIXA01003614.1|:73651-73739
MsHel-3b	gb|AIXA01003129.1|:925-1117
MsHel-3b	gb|AIXA01002703.1|:5547-5758
MsHel-3b	gb|AIXA01001650.1|:26105-26292

MsHel-3b	gb|AIXA01001026.1|:11841-11921
MsHel-3b	gb|AIXA01001026.1|:29064-30682
MsHel-3b	gb|AIXA01000949.1|:11665-11869
MsHel-3b	gb|AIXA01000152.1|:8926-9126
MsHel-3b	gb|AIXA01033288.1|:523-689
MsHel-3b	gb|AIXA01033225.1|:425-594
MsHel-3b	gb|AIXA01024963.1|:1090-1213
MsHel-3b	gb|AIXA01010730.1|:5340-5522
MsHel-3b	gb|AIXA01007689.1|:40539-40988
MsHel-3b	gb|AIXA01007522.1|:11979-12186
MsHel-3b	gb|AIXA01007408.1|:2322-2491
MsHel-3b	gb|AIXA01005393.1|:2951-3104
MsHel-3b	gb|AIXA01001653.1|:19816-20015
MsHel-3b	gb|AIXA01001566.1|:27183-27397
MsHel-3b	gb|AIXA01000261.1|:41655-41862
MsHel-3b	gb|AIXA01000199.1|:4648-4830
MsHel-3b	gb|AIXA01019771.1|:1844-2030
MsHel-3b	gb|AIXA01019387.1|:11-193
MsHel-3b	gb|AIXA01017593.1|:3588-3822
MsHel-3b	gb|AIXA01016006.1|:3575-3785
MsHel-3b	gb|AIXA01013346.1|:15938-16140
MsHel-3b	gb|AIXA01013328.1|:22949-23144
MsHel-3b	gb|AIXA01013012.1|:3527-3737
MsHel-3b	gb|AIXA01010908.1|:51623-51828
MsHel-3b	gb|AIXA01010871.1|:1-186
MsHel-3b	gb|AIXA01010358.1|:16467-16672
MsHel-3b	gb|AIXA01009605.1|:5585-5735
MsHel-3b	gb|AIXA01009360.1|:61900-62060
MsHel-3b	gb|AIXA01009360.1|:88356-88436
MsHel-3b	gb|AIXA01009163.1|:2693-2819
MsHel-3b	gb|AIXA01007857.1|:81844-81932
MsHel-3b	gb|AIXA01007712.1|:61-191
MsHel-3b	gb|AIXA01007712.1|:41742-41952
MsHel-3b	gb|AIXA01007602.1|:44208-44414
MsHel-3b	gb|AIXA01006924.1|:15197-15333
MsHel-3b	gb|AIXA01006855.1|:35716-35889
MsHel-3b	gb|AIXA01005563.1|:28440-28616
MsHel-3b	gb|AIXA01005563.1|:76598-76741
MsHel-3b	gb|AIXA01005495.1|:27341-27421
MsHel-3b	gb|AIXA01004402.1|:13590-13795
MsHel-3b	gb|AIXA01003874.1|:7975-8162
MsHel-3b	gb|AIXA01003726.1|:2959-3128
MsHel-3b	gb|AIXA01003683.1|:122296-122514
MsHel-3b	gb|AIXA01001009.1|:14112-14277
MsHel-3b	gb|AIXA01000981.1|:7116-7334
MsHel-3b	gb|AIXA01000771.1|:1312-1516
MsHel-3b	gb|AIXA01025942.1|:700-809
MsHel-3b	gb|AIXA01022717.1|:244-370

MsHel-3b	gb|AIXA01020914.1|:1534-1722
MsHel-3b	gb|AIXA01018015.1|:860-1001
MsHel-3b	gb|AIXA01016981.1|:469-662
MsHel-3b	gb|AIXA01014641.1|:13861-14013
MsHel-3b	gb|AIXA01014383.1|:11332-11533
MsHel-3b	gb|AIXA01012294.1|:3710-3923
MsHel-3b	gb|AIXA01011563.1|:1467-1643
MsHel-3b	gb|AIXA01011563.1|:14941-15144
MsHel-3b	gb|AIXA01009067.1|:30492-30691
MsHel-3b	gb|AIXA01008025.1|:45733-45896
MsHel-3b	gb|AIXA01005963.1|:4950-5161
MsHel-3b	gb|AIXA01005279.1|:10639-10786
MsHel-3b	gb|AIXA01004345.1|:20589-20788
MsHel-3b	gb|AIXA01029781.1|:553-757
MsHel-3b	gb|AIXA01026309.1|:750-922
MsHel-3b	gb|AIXA01020071.1|:1813-1938
MsHel-3b	gb|AIXA01019605.1|:1485-1661
MsHel-3b	gb|AIXA01018502.1|:260-469
MsHel-3b	gb|AIXA01012784.1|:27363-27487
MsHel-3b	gb|AIXA01011818.1|:4473-4561
MsHel-3b	gb|AIXA01010881.1|:25194-25312
MsHel-3b	gb|AIXA01009035.1|:17645-17854
MsHel-3b	gb|AIXA01008195.1|:23143-23343
MsHel-3b	gb|AIXA01007607.1|:1773-1975
MsHel-3b	gb|AIXA01007085.1|:10323-10515
MsHel-3b	gb|AIXA01006954.1|:7860-7985
MsHel-3b	gb|AIXA01006867.1|:2135-2349
MsHel-3b	gb|AIXA01006710.1|:1580-1755
MsHel-3b	gb|AIXA01005322.1|:34143-34350
MsHel-3b	gb|AIXA01005009.1|:22350-22525
MsHel-3b	gb|AIXA01004579.1|:29967-30150
MsHel-3b	gb|AIXA01003773.1|:3762-3942
MsHel-3b	gb|AIXA01002992.1|:20362-20505
MsHel-3b	gb|AIXA01002552.1|:35834-38776
MsHel-3b	gb|AIXA01002466.1|:32362-32544
MsHel-3b	gb|AIXA01001844.1|:12936-18605
MsHel-3b	gb|AIXA01001558.1|:21747-21946
MsHel-3b	gb|AIXA01000851.1|:78718-78930
MsHel-3b	gb|AIXA01000403.1|:196402-196605
MsHel-3b	gb|AIXA01000315.1|:62819-62900
MsHel-3b	gb|AIXA01000150.1|:7922-8073
MsHel-3b	gb|AIXA01000129.1|:275-449
MsHel-3b	gb|AIXA01028614.1|:825-952
MsHel-3b	gb|AIXA01025122.1|:642-836
MsHel-3b	gb|AIXA01020844.1|:960-1145
MsHel-3b	gb|AIXA01019728.1|:4510-4692
MsHel-3b	gb|AIXA01018617.1|:1865-2073
MsHel-3b	gb|AIXA01015970.1|:10323-10429

MsHel-3b	gb|AIXA01014686.1|:2345-2554
MsHel-3b	gb|AIXA01012413.1|:4271-4454
MsHel-3b	gb|AIXA01011612.1|:6398-6593
MsHel-3b	gb|AIXA01010830.1|:11679-11865
MsHel-3b	gb|AIXA01007649.1|:25003-25180
MsHel-3b	gb|AIXA01007620.1|:11455-11663
MsHel-3b	gb|AIXA01007286.1|:36129-36299
MsHel-3b	gb|AIXA01006980.1|:4982-5181
MsHel-3b	gb|AIXA01006980.1|:27782-27949
MsHel-3b	gb|AIXA01006698.1|:14282-14425
MsHel-3b	gb|AIXA01005822.1|:31011-31220
MsHel-3b	gb|AIXA01005123.1|:25963-26175
MsHel-3b	gb|AIXA01004950.1|:3182-3388
MsHel-3b	gb|AIXA01004683.1|:56455-56629
MsHel-3b	gb|AIXA01004683.1|:84106-84315
MsHel-3b	gb|AIXA01004592.1|:27388-27590
MsHel-3b	gb|AIXA01004554.1|:13866-14066
MsHel-3b	gb|AIXA01003783.1|:3690-3848
MsHel-3b	gb|AIXA01003311.1|:2939-3146
MsHel-3b	gb|AIXA01003170.1|:22427-22579
MsHel-3b	gb|AIXA01003113.1|:428-630
MsHel-3b	gb|AIXA01003027.1|:39983-40069
MsHel-3b	gb|AIXA01003027.1|:61885-62290
MsHel-3b	gb|AIXA01001771.1|:54170-54353
MsHel-3b	gb|AIXA01001631.1|:49943-50103
MsHel-3b	gb|AIXA01001556.1|:3681-3989
MsHel-3b	gb|AIXA01000941.1|:236-384
MsHel-3b	gb|AIXA01000941.1|:14804-14951
MsHel-3b	gb|AIXA01000616.1|:16111-16322
MsHel-3b	gb|AIXA01000480.1|:58023-58248
MsHel-3b	gb|AIXA01031220.1|:1-150
MsHel-3b	gb|AIXA01018554.1|:4741-4905
MsHel-3b	gb|AIXA01016262.1|:6674-6787
MsHel-3b	gb|AIXA01015152.1|:1260-1433
MsHel-3b	gb|AIXA01009539.1|:41647-41771
MsHel-3b	gb|AIXA01009539.1|:74150-74317
MsHel-3b	gb|AIXA01008289.1|:1439-1615
MsHel-3b	gb|AIXA01007477.1|:25800-26198
MsHel-3b	gb|AIXA01007054.1|:5159-5281
MsHel-3b	gb|AIXA01005422.1|:28910-29113
MsHel-3b	gb|AIXA01005413.1|:17165-17364
MsHel-3b	gb|AIXA01005189.1|:38056-38281
MsHel-3b	gb|AIXA01005115.1|:4073-4259
MsHel-3b	gb|AIXA01004997.1|:1941-2091
MsHel-3b	gb|AIXA01003360.1|:34062-34199
MsHel-3b	gb|AIXA01003319.1|:24166-24337
MsHel-3b	gb|AIXA01002635.1|:43994-44149
MsHel-3b	gb|AIXA01002635.1|:63644-63851

MsHel-3b	gb|AIXA01001040.1|:20071-20236
MsHel-3b	gb|AIXA01000686.1|:8279-8453
MsHel-3b	gb|AIXA01000440.1|:59021-66198
MsHel-3b	gb|AIXA01000237.1|:58169-58363
MsHel-3b	gb|AIXA01000237.1|:81253-81330
MsHel-3b	gb|AIXA01000078.1|:7839-8052
MsHel-3b	gb|AIXA01037745.1|:56-221
MsHel-3b	gb|AIXA01018341.1|:4892-5018
MsHel-3b	gb|AIXA01016041.1|:19469-19698
MsHel-3b	gb|AIXA01014932.1|:12236-12449
MsHel-3b	gb|AIXA01009636.1|:2032-2272
MsHel-3b	gb|AIXA01009636.1|:29249-29479
MsHel-3b	gb|AIXA01007641.1|:61992-62074
MsHel-3b	gb|AIXA01007538.1|:30303-30496
MsHel-3b	gb|AIXA01007435.1|:7551-7781
MsHel-3b	gb|AIXA01007435.1|:45852-46060
MsHel-3b	gb|AIXA01006192.1|:70879-71062
MsHel-3b	gb|AIXA01005047.1|:6783-6968
MsHel-3b	gb|AIXA01003997.1|:80289-80497
MsHel-3b	gb|AIXA01003913.1|:1167-1347
MsHel-3b	gb|AIXA01003913.1|:28413-28509
MsHel-3b	gb|AIXA01002689.1|:61290-61491
MsHel-3b	gb|AIXA01002515.1|:41221-41304
MsHel-3b	gb|AIXA01002385.1|:41992-42193
MsHel-3b	gb|AIXA01000643.1|:23954-24168
MsHel-3b	gb|AIXA01034335.1|:19-144
MsHel-3b	gb|AIXA01029802.1|:543-754
MsHel-3b	gb|AIXA01021770.1|:1156-1281
MsHel-3b	gb|AIXA01020448.1|:207-368
MsHel-3b	gb|AIXA01020040.1|:2134-2275
MsHel-3b	gb|AIXA01019619.1|:5388-5585
MsHel-3b	gb|AIXA01017844.1|:134-259
MsHel-3b	gb|AIXA01016314.1|:4847-5030
MsHel-3b	gb|AIXA01014958.1|:4763-4970
MsHel-3b	gb|AIXA01014851.1|:921-1046
MsHel-3b	gb|AIXA01013018.1|:2891-3102
MsHel-3b	gb|AIXA01008967.1|:6080-6230
MsHel-3b	gb|AIXA01008475.1|:13057-13262
MsHel-3b	gb|AIXA01008475.1|:27288-27402
MsHel-3b	gb|AIXA01008382.1|:34165-34290
MsHel-3b	gb|AIXA01007296.1|:123718-123864
MsHel-3b	gb|AIXA01007296.1|:138715-138899
MsHel-3b	gb|AIXA01005086.1|:1272-1463
MsHel-3b	gb|AIXA01004416.1|:30893-31065
MsHel-3b	gb|AIXA01004089.1|:6531-6688
MsHel-3b	gb|AIXA01003994.1|:10336-10545
MsHel-3b	gb|AIXA01003698.1|:49744-49869
MsHel-3b	gb|AIXA01003357.1|:8649-8815

MsHel-3b	gb|AIXA01003290.1|:31343-31518
MsHel-3b	gb|AIXA01002216.1|:3020-3168
MsHel-3b	gb|AIXA01001843.1|:5101-5294
MsHel-3b	gb|AIXA01001843.1|:26743-26909
MsHel-3b	gb|AIXA01000963.1|:109931-110026
MsHel-3b	gb|AIXA01000789.1|:1824-1912
MsHel-3b	gb|AIXA01000755.1|:34665-34844
MsHel-3b	gb|AIXA01000755.1|:91635-91778
MsHel-3b	gb|AIXA01000755.1|:105955-106131
MsHel-3b	gb|AIXA01000160.1|:37568-37762
MsHel-3b	gb|AIXA01018543.1|:9553-9721
MsHel-3b	gb|AIXA01017425.1|:1496-1638
MsHel-3b	gb|AIXA01017094.1|:10067-10229
MsHel-3b	gb|AIXA01016921.1|:5417-5642
MsHel-3b	gb|AIXA01016431.1|:4743-4866
MsHel-3b	gb|AIXA01016253.1|:895-1102
MsHel-3b	gb|AIXA01015261.1|:3282-3433
MsHel-3b	gb|AIXA01013551.1|:1218-1372
MsHel-3b	gb|AIXA01012412.1|:4843-11849
MsHel-3b	gb|AIXA01012209.1|:40291-40440
MsHel-3b	gb|AIXA01011308.1|:12786-12958
MsHel-3b	gb|AIXA01011308.1|:41278-41415
MsHel-3b	gb|AIXA01011243.1|:29588-29699
MsHel-3b	gb|AIXA01010561.1|:10679-10853
MsHel-3b	gb|AIXA01010402.1|:14255-14413
MsHel-3b	gb|AIXA01008691.1|:18503-18658
MsHel-3b	gb|AIXA01008553.1|:29920-30110
MsHel-3b	gb|AIXA01008233.1|:16904-17019
MsHel-3b	gb|AIXA01007719.1|:25410-25576
MsHel-3b	gb|AIXA01007642.1|:109243-109355
MsHel-3b	gb|AIXA01006781.1|:29858-30068
MsHel-3b	gb|AIXA01006181.1|:5824-5978
MsHel-3b	gb|AIXA01005500.1|:33-204
MsHel-3b	gb|AIXA01005380.1|:11807-11933
MsHel-3b	gb|AIXA01005097.1|:17150-17275
MsHel-3b	gb|AIXA01022629.1|:658-800
MsHel-3b	gb|AIXA01019824.1|:3334-3459
MsHel-3b	gb|AIXA01019490.1|:7-105
MsHel-3b	gb|AIXA01019102.1|:1493-1677
MsHel-3b	gb|AIXA01016928.1|:661-816
MsHel-3b	gb|AIXA01015622.1|:965-10809
MsHel-3b	gb|AIXA01015404.1|:12174-12380
MsHel-3b	gb|AIXA01014005.1|:7071-7217
MsHel-3b	gb|AIXA01009837.1|:11468-11625
MsHel-3b	gb|AIXA01009763.1|:16157-16275
MsHel-3b	gb|AIXA01009755.1|:31288-31422
MsHel-3b	gb|AIXA01008915.1|:33921-34088
MsHel-3b	gb|AIXA01008405.1|:21573-21753

MsHel-3b	gb|AIXA01008405.1|:36410-36551
MsHel-3b	gb|AIXA01007721.1|:25424-25591
MsHel-3b	gb|AIXA01006906.1|:8796-8965
MsHel-3b	gb|AIXA01006854.1|:8940-9050
MsHel-3b	gb|AIXA01006854.1|:44495-48704
MsHel-3b	gb|AIXA01006854.1|:70331-70796
MsHel-3b	gb|AIXA01006212.1|:2794-2917
MsHel-3b	gb|AIXA01005187.1|:30668-30852
MsHel-3b	gb|AIXA01003928.1|:134801-134940
MsHel-3b	gb|AIXA01002984.1|:12037-12175
MsHel-3b	gb|AIXA01002580.1|:109638-109852
MsHel-3b	gb|AIXA01002497.1|:52148-52297
MsHel-3b	gb|AIXA01002232.1|:51034-51227
MsHel-3b	gb|AIXA01001282.1|:43222-43389
MsHel-3b	gb|AIXA01000787.1|:66781-66966
MsHel-3b	gb|AIXA01000733.1|:2355-2565
MsHel-3b	gb|AIXA01000214.1|:23032-23117
MsHel-3b	gb|AIXA01000198.1|:3016-3202
MsHel-3b	gb|AIXA01000198.1|:53233-53429
MsHel-3b	gb|AIXA01000198.1|:65476-65588
MsHel-3b	gb|AIXA01000198.1|:78661-78800
MsHel-3b	gb|AIXA01000198.1|:107223-107308
MsHel-3b	gb|AIXA01000145.1|:263-408
MsHel-3b	gb|AIXA01025282.1|:328-524
MsHel-3b	gb|AIXA01024180.1|:678-803
MsHel-3b	gb|AIXA01023174.1|:1140-1289
MsHel-3b	gb|AIXA01016181.1|:10558-10740
MsHel-3b	gb|AIXA01015734.1|:1954-2095
MsHel-3b	gb|AIXA01015372.1|:5-166
MsHel-3b	gb|AIXA01010854.1|:63005-63130
MsHel-3b	gb|AIXA01010736.1|:29920-30069
MsHel-3b	gb|AIXA01010189.1|:456-658
MsHel-3b	gb|AIXA01010189.1|:17281-17462
MsHel-3b	gb|AIXA01010118.1|:11602-11758
MsHel-3b	gb|AIXA01009984.1|:46750-46920
MsHel-3b	gb|AIXA01009537.1|:63297-73449
MsHel-3b	gb|AIXA01009184.1|:3308-3434
MsHel-3b	gb|AIXA01007499.1|:32354-32480
MsHel-3b	gb|AIXA01007237.1|:104-275
MsHel-3b	gb|AIXA01006139.1|:16830-16983
MsHel-3b	gb|AIXA01004676.1|:96477-96638
MsHel-3b	gb|AIXA01004565.1|:14549-14725
MsHel-3b	gb|AIXA01004565.1|:40942-41141
MsHel-3b	gb|AIXA01004430.1|:21764-21978
MsHel-3b	gb|AIXA01003617.1|:2199-2340
MsHel-3b	gb|AIXA01002748.1|:3739-3904
MsHel-3b	gb|AIXA01002671.1|:39049-39130
MsHel-3b	gb|AIXA01002671.1|:109491-109586

MsHel-3b	gb|AIXA01002671.1|:153228-153441
MsHel-3b	gb|AIXA01002377.1|:151178-151363
MsHel-3b	gb|AIXA01000034.1|:60362-60502
MsHel-3b	gb|AIXA01035030.1|:201-326
MsHel-3b	gb|AIXA01033447.1|:19-144
MsHel-3b	gb|AIXA01032320.1|:1-105
MsHel-3b	gb|AIXA01027956.1|:350-467
MsHel-3b	gb|AIXA01019673.1|:777-2590
MsHel-3b	gb|AIXA01018997.1|:3916-4041
MsHel-3b	gb|AIXA01016781.1|:17567-17742
MsHel-3b	gb|AIXA01016755.1|:4112-4326
MsHel-3b	gb|AIXA01016561.1|:19673-19798
MsHel-3b	gb|AIXA01015579.1|:45590-45742
MsHel-3b	gb|AIXA01014556.1|:1370-1571
MsHel-3b	gb|AIXA01013383.1|:12375-12487
MsHel-3b	gb|AIXA01012017.1|:76719-76934
MsHel-3b	gb|AIXA01010870.1|:372-497
MsHel-3b	gb|AIXA01009510.1|:71998-72141
MsHel-3b	gb|AIXA01008385.1|:27116-27241
MsHel-3b	gb|AIXA01008263.1|:10006-10181
MsHel-3b	gb|AIXA01007949.1|:10279-10445
MsHel-3b	gb|AIXA01007949.1|:21351-21532
MsHel-3b	gb|AIXA01006708.1|:73822-73988
MsHel-3b	gb|AIXA01005483.1|:7995-8120
MsHel-3b	gb|AIXA01005395.1|:32425-32643
MsHel-3b	gb|AIXA01004755.1|:31696-31798
MsHel-3b	gb|AIXA01004689.1|:27489-27629
MsHel-3b	gb|AIXA01004285.1|:400-525
MsHel-3b	gb|AIXA01004108.1|:29497-29668
MsHel-3b	gb|AIXA01004108.1|:165188-165321
MsHel-3b	gb|AIXA01004108.1|:182018-182152
MsHel-3b	gb|AIXA01004108.1|:200083-200176
MsHel-3b	gb|AIXA01003977.1|:65-173
MsHel-3b	gb|AIXA01003747.1|:81484-81575
MsHel-3b	gb|AIXA01003174.1|:181-372
MsHel-3b	gb|AIXA01002995.1|:14277-14389
MsHel-3b	gb|AIXA01002977.1|:47263-47395
MsHel-3b	gb|AIXA01002579.1|:67928-68053
MsHel-3b	gb|AIXA01002579.1|:98051-101016
MsHel-3b	gb|AIXA01002560.1|:32017-32223
MsHel-3b	gb|AIXA01001764.1|:30920-31023
MsHel-3b	gb|AIXA01000946.1|:3351-9270
MsHel-3b	gb|AIXA01000670.1|:39732-39925
MsHel-3b	gb|AIXA01000041.1|:94465-94687
MsHel-3b	gb|AIXA01015985.1|:23869-24008
MsHel-3b	gb|AIXA01015471.1|:11730-11873
MsHel-3b	gb|AIXA01015382.1|:3230-3439
MsHel-3b	gb|AIXA01014296.1|:9993-10118

MsHel-3b	gb|AIXA01014014.1|:2360-2484
MsHel-3b	gb|AIXA01014002.1|:3623-3722
MsHel-3b	gb|AIXA01012527.1|:26523-26704
MsHel-3b	gb|AIXA01011593.1|:6774-6994
MsHel-3b	gb|AIXA01011340.1|:34592-34711
MsHel-3b	gb|AIXA01009290.1|:34601-34728
MsHel-3b	gb|AIXA01007223.1|:17267-17409
MsHel-3b	gb|AIXA01007223.1|:54007-54087
MsHel-3b	gb|AIXA01006838.1|:41326-41431
MsHel-3b	gb|AIXA01006838.1|:69505-69717
MsHel-3b	gb|AIXA01006255.1|:34604-34694
MsHel-3b	gb|AIXA01006066.1|:44606-44725
MsHel-3b	gb|AIXA01005137.1|:31810-32023
MsHel-3b	gb|AIXA01004634.1|:109760-109937
MsHel-3b	gb|AIXA01004085.1|:3324-3504
MsHel-3b	gb|AIXA01004064.1|:4418-4589
MsHel-3b	gb|AIXA01004012.1|:32309-32477
MsHel-3b	gb|AIXA01002612.1|:35995-37527
MsHel-3b	gb|AIXA01000820.1|:15037-15121
MsHel-3b	gb|AIXA01000820.1|:32970-33190
MsHel-3b	gb|AIXA01032686.1|:230-384
MsHel-3b	gb|AIXA01017807.1|:923-1113
MsHel-3b	gb|AIXA01013522.1|:80-177
MsHel-3b	gb|AIXA01011364.1|:46227-46432
MsHel-3b	gb|AIXA01009862.1|:893-1054
MsHel-3b	gb|AIXA01009350.1|:3485-3688
MsHel-3b	gb|AIXA01009350.1|:40312-40399
MsHel-3b	gb|AIXA01006932.1|:1776-1955
MsHel-3b	gb|AIXA01006909.1|:12311-12503
MsHel-3b	gb|AIXA01006833.1|:28207-28382
MsHel-3b	gb|AIXA01006616.1|:8852-9021
MsHel-3b	gb|AIXA01006498.1|:15454-15561
MsHel-3b	gb|AIXA01005850.1|:26031-26264
MsHel-3b	gb|AIXA01004205.1|:3014-3217
MsHel-3b	gb|AIXA01003853.1|:36895-39668
MsHel-3b	gb|AIXA01003853.1|:79208-79348
MsHel-3b	gb|AIXA01003292.1|:49139-49339
MsHel-3b	gb|AIXA01003139.1|:6009-6120
MsHel-3b	gb|AIXA01001544.1|:21153-21234
MsHel-3b	gb|AIXA01001017.1|:54652-54855
MsHel-3b	gb|AIXA01000905.1|:28416-28537
MsHel-3b	gb|AIXA01000732.1|:32376-32486
MsHel-3b	gb|AIXA01020025.1|:2162-2304
MsHel-3b	gb|AIXA01017256.1|:1669-1810
MsHel-3b	gb|AIXA01016151.1|:5996-6101
MsHel-3b	gb|AIXA01013345.1|:19105-19265
MsHel-3b	gb|AIXA01010995.1|:9652-9800
MsHel-3b	gb|AIXA01009340.1|:52837-52963

MsHel-3b	gb|AIXA01008505.1|:43886-44012
MsHel-3b	gb|AIXA01008402.1|:17922-18531
MsHel-3b	gb|AIXA01008400.1|:34675-34830
MsHel-3b	gb|AIXA01006790.1|:7819-8005
MsHel-3b	gb|AIXA01006727.1|:12318-12528
MsHel-3b	gb|AIXA01006663.1|:2192-2297
MsHel-3b	gb|AIXA01006515.1|:27198-27371
MsHel-3b	gb|AIXA01006515.1|:60999-61212
MsHel-3b	gb|AIXA01006101.1|:19606-19734
MsHel-3b	gb|AIXA01005239.1|:2667-2778
MsHel-3b	gb|AIXA01005208.1|:7793-7934
MsHel-3b	gb|AIXA01004464.1|:16892-17131
MsHel-3b	gb|AIXA01004362.1|:6954-7137
MsHel-3b	gb|AIXA01002906.1|:25478-25695
MsHel-3b	gb|AIXA01002762.1|:83767-83964
MsHel-3b	gb|AIXA01001865.1|:4905-5033
MsHel-3b	gb|AIXA01001752.1|:6281-6473
MsHel-3b	gb|AIXA01001677.1|:57930-58015
MsHel-3b	gb|AIXA01001677.1|:90423-90548
MsHel-3b	gb|AIXA01001666.1|:9688-17729
MsHel-3b	gb|AIXA01001484.1|:43508-43596
MsHel-3b	gb|AIXA01001265.1|:12414-12614
MsHel-3b	gb|AIXA01000972.1|:51958-52167
MsHel-3b	gb|AIXA01024945.1|:809-934
MsHel-3b	gb|AIXA01024321.1|:582-719
MsHel-3b	gb|AIXA01023890.1|:1105-1305
MsHel-3b	gb|AIXA01022330.1|:176-301
MsHel-3b	gb|AIXA01021146.1|:202-415
MsHel-3b	gb|AIXA01017860.1|:4980-5101
MsHel-3b	gb|AIXA01016762.1|:1501-1626
MsHel-3b	gb|AIXA01015736.1|:1495-1620
MsHel-3b	gb|AIXA01015649.1|:12518-12635
MsHel-3b	gb|AIXA01015055.1|:19834-19977
MsHel-3b	gb|AIXA01015050.1|:1885-2010
MsHel-3b	gb|AIXA01014865.1|:45-258
MsHel-3b	gb|AIXA01014145.1|:12141-12277
MsHel-3b	gb|AIXA01014145.1|:38245-38368
MsHel-3b	gb|AIXA01013184.1|:7492-7617
MsHel-3b	gb|AIXA01012631.1|:1837-1961
MsHel-3b	gb|AIXA01012192.1|:3664-3872
MsHel-3b	gb|AIXA01012135.1|:48788-48901
MsHel-3b	gb|AIXA01011922.1|:9023-9147
MsHel-3b	gb|AIXA01011710.1|:756-881
MsHel-3b	gb|AIXA01011594.1|:807-1035
MsHel-3b	gb|AIXA01009518.1|:22559-22684
MsHel-3b	gb|AIXA01009518.1|:78329-78454
MsHel-3b	gb|AIXA01008962.1|:132519-132708
MsHel-3b	gb|AIXA01008923.1|:29154-29279

MsHel-3b	gb|AIXA01008392.1|:8357-8482
MsHel-3b	gb|AIXA01008037.1|:67151-67305
MsHel-3b	gb|AIXA01007550.1|:6496-6706
MsHel-3b	gb|AIXA01007428.1|:13330-13412
MsHel-3b	gb|AIXA01007428.1|:110450-110599
MsHel-3b	gb|AIXA01006993.1|:16155-16360
MsHel-3b	gb|AIXA01006423.1|:61481-61562
MsHel-3b	gb|AIXA01006423.1|:149033-149134
MsHel-3b	gb|AIXA01005510.1|:8308-8433
MsHel-3b	gb|AIXA01004978.1|:54802-54927
MsHel-3b	gb|AIXA01004404.1|:7365-7489
MsHel-3b	gb|AIXA01003367.1|:7294-7419
MsHel-3b	gb|AIXA01003272.1|:46256-46470
MsHel-3b	gb|AIXA01003272.1|:70499-70635
MsHel-3b	gb|AIXA01002619.1|:30382-30507
MsHel-3b	gb|AIXA01002395.1|:25558-25738
MsHel-3b	gb|AIXA01002185.1|:63935-64060
MsHel-3b	gb|AIXA01001899.1|:201786-201992
MsHel-3b	gb|AIXA01001695.1|:13755-13963
MsHel-3b	gb|AIXA01001667.1|:38565-38730
MsHel-3b	gb|AIXA01000752.1|:16142-16263
MsHel-3b	gb|AIXA01000064.1|:16766-16891
MsHel-3b	gb|AIXA01021553.1|:833-967
MsHel-3b	gb|AIXA01018865.1|:8870-9042
MsHel-3b	gb|AIXA01018371.1|:9271-9398
MsHel-3b	gb|AIXA01018364.1|:3458-3601
MsHel-3b	gb|AIXA01015103.1|:8634-8772
MsHel-3b	gb|AIXA01012659.1|:19476-19587
MsHel-3b	gb|AIXA01012654.1|:2935-6137
MsHel-3b	gb|AIXA01012654.1|:28062-28165
MsHel-3b	gb|AIXA01012114.1|:38301-38425
MsHel-3b	gb|AIXA01011352.1|:4409-4614
MsHel-3b	gb|AIXA01010563.1|:3699-3821
MsHel-3b	gb|AIXA01010404.1|:28442-28651
MsHel-3b	gb|AIXA01009769.1|:1390-1509
MsHel-3b	gb|AIXA01008995.1|:5571-5753
MsHel-3b	gb|AIXA01008258.1|:4543-4702
MsHel-3b	gb|AIXA01007801.1|:28994-29117
MsHel-3b	gb|AIXA01007801.1|:65680-65768
MsHel-3b	gb|AIXA01006209.1|:14595-14766
MsHel-3b	gb|AIXA01006055.1|:15981-16109
MsHel-3b	gb|AIXA01002717.1|:13388-13512
MsHel-3b	gb|AIXA01001993.1|:15374-15549
MsHel-3b	gb|AIXA01001895.1|:968-1158
MsHel-3b	gb|AIXA01001221.1|:41844-41958
MsHel-3b	gb|AIXA01001221.1|:55848-57056
MsHel-3b	gb|AIXA01000740.1|:12098-12299
MsHel-3b	gb|AIXA01000050.1|:61021-61185

MsHel-3b	gb|AIXA01027925.1|:376-514
MsHel-3b	gb|AIXA01026028.1|:44-209
MsHel-3b	gb|AIXA01022630.1|:348-526
MsHel-3b	gb|AIXA01022140.1|:1627-1768
MsHel-3b	gb|AIXA01019722.1|:1862-2048
MsHel-3b	gb|AIXA01016923.1|:1916-2126
MsHel-3b	gb|AIXA01014216.1|:6799-6941
MsHel-3b	gb|AIXA01014065.1|:3806-3931
MsHel-3b	gb|AIXA01011370.1|:54946-55099
MsHel-3b	gb|AIXA01008782.1|:53924-54010
MsHel-3b	gb|AIXA01007795.1|:49871-50019
MsHel-3b	gb|AIXA01007303.1|:24672-24825
MsHel-3b	gb|AIXA01007239.1|:24442-24649
MsHel-3b	gb|AIXA01006891.1|:58315-58489
MsHel-3b	gb|AIXA01006284.1|:8509-8682
MsHel-3b	gb|AIXA01005277.1|:7250-7389
MsHel-3b	gb|AIXA01004522.1|:16575-16709
MsHel-3b	gb|AIXA01004164.1|:49019-49225
MsHel-3b	gb|AIXA01003901.1|:33447-33659
MsHel-3b	gb|AIXA01002998.1|:22115-22200
MsHel-3b	gb|AIXA01002998.1|:43418-43543
MsHel-3b	gb|AIXA01002774.1|:18659-18781
MsHel-3b	gb|AIXA01002527.1|:17600-17803
MsHel-3b	gb|AIXA01001826.1|:2407-2584
MsHel-3b	gb|AIXA01001117.1|:5228-5331
MsHel-3b	gb|AIXA01001117.1|:30257-30466
MsHel-3b	gb|AIXA01001117.1|:49249-49382
MsHel-3b	gb|AIXA01000934.1|:33548-33690
MsHel-3b	gb|AIXA01000795.1|:26305-26408
MsHel-3b	gb|AIXA01000031.1|:7118-14015
MsHel-3b	gb|AIXA01021134.1|:1-194
MsHel-3b	gb|AIXA01015049.1|:2317-2418
MsHel-3b	gb|AIXA01013078.1|:4016-4150
MsHel-3b	gb|AIXA01012699.1|:1646-1772
MsHel-3b	gb|AIXA01012434.1|:21436-21620
MsHel-3b	gb|AIXA01012434.1|:58386-58491
MsHel-3b	gb|AIXA01011650.1|:40106-40232
MsHel-3b	gb|AIXA01008443.1|:59735-59822
MsHel-3b	gb|AIXA01008229.1|:21752-21935
MsHel-3b	gb|AIXA01007859.1|:3343-3464
MsHel-3b	gb|AIXA01007808.1|:34094-34198
MsHel-3b	gb|AIXA01007568.1|:31502-31711
MsHel-3b	gb|AIXA01007301.1|:8750-8949
MsHel-3b	gb|AIXA01006424.1|:5427-5568
MsHel-3b	gb|AIXA01006199.1|:172-298
MsHel-3b	gb|AIXA01005497.1|:30448-30619
MsHel-3b	gb|AIXA01005465.1|:35594-35752
MsHel-3b	gb|AIXA01005465.1|:46756-46867

MsHel-3b	gb|AIXA01004097.1|:22450-22722
MsHel-3b	gb|AIXA01003992.1|:19413-19619
MsHel-3b	gb|AIXA01001840.1|:7037-7159
MsHel-3b	gb|AIXA01001636.1|:64668-65351
MsHel-3b	gb|AIXA01001492.1|:16624-16708
MsHel-3b	gb|AIXA01001492.1|:84145-84242
MsHel-3b	gb|AIXA01000982.1|:83482-83684
MsHel-3b	gb|AIXA01000091.1|:20604-20756
MsHel-3b	gb|AIXA01000091.1|:76798-76924
MsHel-3b	gb|AIXA01034915.1|:298-390
MsHel-3b	gb|AIXA01034081.1|:457-654
MsHel-3b	gb|AIXA01033835.1|:541-666
MsHel-3b	gb|AIXA01020460.1|:1360-1485
MsHel-3b	gb|AIXA01020258.1|:2341-2465
MsHel-3b	gb|AIXA01017993.1|:3578-3702
MsHel-3b	gb|AIXA01017901.1|:10287-10502
MsHel-3b	gb|AIXA01015151.1|:23266-23419
MsHel-3b	gb|AIXA01015151.1|:61896-62021
MsHel-3b	gb|AIXA01011992.1|:6850-6975
MsHel-3b	gb|AIXA01011985.1|:9648-9748
MsHel-3b	gb|AIXA01011450.1|:941-1065
MsHel-3b	gb|AIXA01011450.1|:29060-29185
MsHel-3b	gb|AIXA01010304.1|:1297-1457
MsHel-3b	gb|AIXA01010133.1|:34032-34148
MsHel-3b	gb|AIXA01009243.1|:32790-32933
MsHel-3b	gb|AIXA01007874.1|:174-299
MsHel-3b	gb|AIXA01007229.1|:98369-98494
MsHel-3b	gb|AIXA01007176.1|:36531-36652
MsHel-3b	gb|AIXA01007071.1|:13812-13984
MsHel-3b	gb|AIXA01007071.1|:32647-32781
MsHel-3b	gb|AIXA01006085.1|:6781-6908
MsHel-3b	gb|AIXA01005394.1|:13940-14053
MsHel-3b	gb|AIXA01005394.1|:42777-43117
MsHel-3b	gb|AIXA01005319.1|:55603-55804
MsHel-3b	gb|AIXA01004459.1|:11118-11236
MsHel-3b	gb|AIXA01004459.1|:87633-87756
MsHel-3b	gb|AIXA01003873.1|:53064-53161
MsHel-3b	gb|AIXA01003677.1|:40642-40855
MsHel-3b	gb|AIXA01003175.1|:28869-29026
MsHel-3b	gb|AIXA01003122.1|:919-1054
MsHel-3b	gb|AIXA01003122.1|:39439-39559
MsHel-3b	gb|AIXA01003008.1|:11185-11330
MsHel-3b	gb|AIXA01001863.1|:29849-29995
MsHel-3b	gb|AIXA01001467.1|:18843-18968
MsHel-3b	gb|AIXA01001303.1|:10035-10164
MsHel-3b	gb|AIXA01001292.1|:99240-99325
MsHel-3b	gb|AIXA01000653.1|:21678-22117
MsHel-3b	gb|AIXA01000408.1|:44893-45032

MsHel-3b	gb|AIXA01038318.1|:75-186
MsHel-3b	gb|AIXA01022129.1|:1093-1260
MsHel-3b	gb|AIXA01020695.1|:2736-2934
MsHel-3b	gb|AIXA01018837.1|:6690-6864
MsHel-3b	gb|AIXA01014907.1|:18567-18671
MsHel-3b	gb|AIXA01014015.1|:9189-9349
MsHel-3b	gb|AIXA01012377.1|:4666-4852
MsHel-3b	gb|AIXA01012103.1|:18120-18299
MsHel-3b	gb|AIXA01010873.1|:6641-6745
MsHel-3b	gb|AIXA01010873.1|:21254-21354
MsHel-3b	gb|AIXA01009698.1|:5370-5497
MsHel-3b	gb|AIXA01009154.1|:25892-26081
MsHel-3b	gb|AIXA01009015.1|:34025-34258
MsHel-3b	gb|AIXA01008325.1|:18907-19096
MsHel-3b	gb|AIXA01006991.1|:32152-32279
MsHel-3b	gb|AIXA01005244.1|:176-363
MsHel-3b	gb|AIXA01005125.1|:13826-14912
MsHel-3b	gb|AIXA01004660.1|:56956-57127
MsHel-3b	gb|AIXA01004105.1|:8364-8567
MsHel-3b	gb|AIXA01004094.1|:5263-5351
MsHel-3b	gb|AIXA01003377.1|:3411-3567
MsHel-3b	gb|AIXA01002543.1|:4855-4966
MsHel-3b	gb|AIXA01002172.1|:4454-4694
MsHel-3b	gb|AIXA01001370.1|:87498-87648
MsHel-3b	gb|AIXA01001173.1|:2926-3100
MsHel-3b	gb|AIXA01000819.1|:76502-85664
MsHel-3b	gb|AIXA01018021.1|:1447-1645
MsHel-3b	gb|AIXA01017823.1|:8345-8549
MsHel-3b	gb|AIXA01016418.1|:11210-11413
MsHel-3b	gb|AIXA01011643.1|:3087-3294
MsHel-3b	gb|AIXA01010185.1|:11923-12046
MsHel-3b	gb|AIXA01010185.1|:88429-88516
MsHel-3b	gb|AIXA01009039.1|:22946-23076
MsHel-3b	gb|AIXA01007626.1|:49351-49480
MsHel-3b	gb|AIXA01007626.1|:134058-134191
MsHel-3b	gb|AIXA01007220.1|:7781-7904
MsHel-3b	gb|AIXA01006696.1|:5130-5301
MsHel-3b	gb|AIXA01004528.1|:10118-10241
MsHel-3b	gb|AIXA01004334.1|:3702-3878
MsHel-3b	gb|AIXA01003949.1|:8244-8435
MsHel-3b	gb|AIXA01001630.1|:13759-14014
MsHel-3b	gb|AIXA01016914.1|:8885-9073
MsHel-3b	gb|AIXA01015999.1|:14149-14333
MsHel-3b	gb|AIXA01014936.1|:3211-3372
MsHel-3b	gb|AIXA01014587.1|:33401-33606
MsHel-3b	gb|AIXA01012268.1|:296-389
MsHel-3b	gb|AIXA01011638.1|:3840-4014
MsHel-3b	gb|AIXA01011056.1|:2099-2259

MsHel-3b	gb|AIXA01010551.1|:44425-44551
MsHel-3b	gb|AIXA01009658.1|:6113-6315
MsHel-3b	gb|AIXA01008537.1|:82252-82464
MsHel-3b	gb|AIXA01008130.1|:3615-3770
MsHel-3b	gb|AIXA01008054.1|:26376-26585
MsHel-3b	gb|AIXA01006319.1|:9600-9756
MsHel-3b	gb|AIXA01005805.1|:4911-5092
MsHel-3b	gb|AIXA01005795.1|:22085-22275
MsHel-3b	gb|AIXA01005541.1|:1487-1612
MsHel-3b	gb|AIXA01005329.1|:68-205
MsHel-3b	gb|AIXA01004583.1|:31658-31840
MsHel-3b	gb|AIXA01003326.1|:40371-40547
MsHel-3b	gb|AIXA01002653.1|:84-185
MsHel-3b	gb|AIXA01001307.1|:30545-30717
MsHel-3b	gb|AIXA01000904.1|:20502-20622
MsHel-3b	gb|AIXA01000223.1|:8908-9091
MsHel-3b	gb|AIXA01029925.1|:123-240
MsHel-3b	gb|AIXA01027569.1|:591-779
MsHel-3b	gb|AIXA01020949.1|:1097-1217
MsHel-3b	gb|AIXA01017756.1|:2323-2518
MsHel-3b	gb|AIXA01017099.1|:199-324
MsHel-3b	gb|AIXA01014301.1|:34338-34470
MsHel-3b	gb|AIXA01013962.1|:100-221
MsHel-3b	gb|AIXA01013180.1|:1271-1379
MsHel-3b	gb|AIXA01011950.1|:3469-3882
MsHel-3b	gb|AIXA01011852.1|:168-342
MsHel-3b	gb|AIXA01011408.1|:23673-23858
MsHel-3b	gb|AIXA01010855.1|:5904-6029
MsHel-3b	gb|AIXA01010656.1|:28204-28405
MsHel-3b	gb|AIXA01010656.1|:134386-134531
MsHel-3b	gb|AIXA01009232.1|:2489-2614
MsHel-3b	gb|AIXA01008521.1|:20109-20291
MsHel-3b	gb|AIXA01007928.1|:7240-7408
MsHel-3b	gb|AIXA01005891.1|:1454-1579
MsHel-3b	gb|AIXA01004844.1|:40391-40553
MsHel-3b	gb|AIXA01004625.1|:41294-41425
MsHel-3b	gb|AIXA01003656.1|:17354-17561
MsHel-3b	gb|AIXA01002573.1|:5637-5769
MsHel-3b	gb|AIXA01001990.1|:41516-41609
MsHel-3b	gb|AIXA01001572.1|:42445-42604
MsHel-3b	gb|AIXA01000601.1|:4987-5112
MsHel-3b	gb|AIXA01000489.1|:4387-4569
MsHel-3b	gb|AIXA01000489.1|:41514-41646
MsHel-3b	gb|AIXA01016103.1|:37354-37435
MsHel-3b	gb|AIXA01008792.1|:6987-7115
MsHel-3b	gb|AIXA01005349.1|:81995-82160
MsHel-3b	gb|AIXA01004946.1|:56936-57077
MsHel-3b	gb|AIXA01004114.1|:48957-49145

MsHel-3b	gb|AIXA01003826.1|:8702-8809
MsHel-3b	gb|AIXA01002928.1|:15773-15952
MsHel-3b	gb|AIXA01002009.1|:26751-26854
MsHel-3b	gb|AIXA01001814.1|:6744-6991
MsHel-3b	gb|AIXA01001658.1|:64874-65001
MsHel-3b	gb|AIXA01000206.1|:79605-79708
MsHel-3b	gb|AIXA01025991.1|:901-987
MsHel-3b	gb|AIXA01025451.1|:845-969
MsHel-3b	gb|AIXA01024513.1|:633-755
MsHel-3b	gb|AIXA01019762.1|:528-705
MsHel-3b	gb|AIXA01017650.1|:2771-2897
MsHel-3b	gb|AIXA01013946.1|:23390-23496
MsHel-3b	gb|AIXA01010758.1|:34418-34556
MsHel-3b	gb|AIXA01010541.1|:26387-26543
MsHel-3b	gb|AIXA01010343.1|:25735-25883
MsHel-3b	gb|AIXA01009355.1|:5179-5292
MsHel-3b	gb|AIXA01008489.1|:7930-8044
MsHel-3b	gb|AIXA01007349.1|:1-122
MsHel-3b	gb|AIXA01006927.1|:9571-9677
MsHel-3b	gb|AIXA01005730.1|:28389-28498
MsHel-3b	gb|AIXA01004933.1|:5326-12701
MsHel-3b	gb|AIXA01004435.1|:9170-9338
MsHel-3b	gb|AIXA01004260.1|:98685-98772
MsHel-3b	gb|AIXA01004260.1|:135650-135846
MsHel-3b	gb|AIXA01004167.1|:29682-29772
MsHel-3b	gb|AIXA01004167.1|:50890-58752
MsHel-3b	gb|AIXA01003293.1|:17163-17362
MsHel-3b	gb|AIXA01003210.1|:7455-7583
MsHel-3b	gb|AIXA01003210.1|:17979-18148
MsHel-3b	gb|AIXA01003210.1|:71235-71407
MsHel-3b	gb|AIXA01033617.1|:422-598
MsHel-3b	gb|AIXA01029652.1|:258-383
MsHel-3b	gb|AIXA01022068.1|:983-1100
MsHel-3b	gb|AIXA01019837.1|:617-743
MsHel-3b	gb|AIXA01019580.1|:311-486
MsHel-3b	gb|AIXA01017841.1|:5458-5584
MsHel-3b	gb|AIXA01012903.1|:1754-1940
MsHel-3b	gb|AIXA01010003.1|:13325-13450
MsHel-3b	gb|AIXA01009829.1|:7215-7407
MsHel-3b	gb|AIXA01009829.1|:18091-18217
MsHel-3b	gb|AIXA01009044.1|:32946-33135
MsHel-3b	gb|AIXA01008536.1|:41803-41991
MsHel-3b	gb|AIXA01007614.1|:15747-15873
MsHel-3b	gb|AIXA01007614.1|:38133-38329
MsHel-3b	gb|AIXA01007070.1|:21389-21471
MsHel-3b	gb|AIXA01007070.1|:61751-61831
MsHel-3b	gb|AIXA01007027.1|:1255-1426
MsHel-3b	gb|AIXA01007027.1|:64895-64978

MsHel-3b	gb|AIXA01006871.1|:51032-51158
MsHel-3b	gb|AIXA01005056.1|:11548-11674
MsHel-3b	gb|AIXA01005036.1|:5842-5974
MsHel-3b	gb|AIXA01004236.1|:11365-11491
MsHel-3b	gb|AIXA01003876.1|:22374-22514
MsHel-3b	gb|AIXA01003876.1|:88145-88233
MsHel-3b	gb|AIXA01003106.1|:15908-16066
MsHel-3b	gb|AIXA01002750.1|:37577-37720
MsHel-3b	gb|AIXA01002564.1|:1814-1997
MsHel-3b	gb|AIXA01002393.1|:4610-4835
MsHel-3b	gb|AIXA01002244.1|:232-414
MsHel-3b	gb|AIXA01002244.1|:12492-12652
MsHel-3b	gb|AIXA01002244.1|:22731-22872
MsHel-3b	gb|AIXA01001679.1|:6882-6967
MsHel-3b	gb|AIXA01001618.1|:46062-46171
MsHel-3b	gb|AIXA01001466.1|:6998-7158
MsHel-3b	gb|AIXA01022057.1|:29-226
MsHel-3b	gb|AIXA01017627.1|:2420-2545
MsHel-3b	gb|AIXA01017475.1|:3897-4096
MsHel-3b	gb|AIXA01016989.1|:1243-1368
MsHel-3b	gb|AIXA01016080.1|:178-374
MsHel-3b	gb|AIXA01015132.1|:48117-48220
MsHel-3b	gb|AIXA01014765.1|:3888-3980
MsHel-3b	gb|AIXA01013179.1|:851-995
MsHel-3b	gb|AIXA01012299.1|:14381-14549
MsHel-3b	gb|AIXA01011980.1|:15467-15686
MsHel-3b	gb|AIXA01010811.1|:6190-6390
MsHel-3b	gb|AIXA01009043.1|:11178-11286
MsHel-3b	gb|AIXA01009010.1|:936-1087
MsHel-3b	gb|AIXA01008236.1|:25106-25222
MsHel-3b	gb|AIXA01007034.1|:38986-39190
MsHel-3b	gb|AIXA01007034.1|:69248-69426
MsHel-3b	gb|AIXA01006896.1|:8481-8606
MsHel-3b	gb|AIXA01004373.1|:158-282
MsHel-3b	gb|AIXA01004296.1|:9838-10017
MsHel-3b	gb|AIXA01003052.1|:28034-28228
MsHel-3b	gb|AIXA01003052.1|:79580-79673
MsHel-3b	gb|AIXA01002662.1|:34588-34706
MsHel-3b	gb|AIXA01002288.1|:2462-2587
MsHel-3b	gb|AIXA01000066.1|:12966-13096
MsHel-3b	gb|AIXA01019360.1|:52-200
MsHel-3b	gb|AIXA01015185.1|:3335-3457
MsHel-3b	gb|AIXA01014535.1|:181-295
MsHel-3b	gb|AIXA01014515.1|:2044-2283
MsHel-3b	gb|AIXA01011724.1|:13172-13296
MsHel-3b	gb|AIXA01007460.1|:30410-30609
MsHel-3b	gb|AIXA01005636.1|:32325-32452
MsHel-3b	gb|AIXA01005229.1|:3234-3440

MsHel-3b	gb|AIXA01003981.1|:28563-28691
MsHel-3b	gb|AIXA01002989.1|:11374-11502
MsHel-3b	gb|AIXA01002801.1|:40743-40944
MsHel-3b	gb|AIXA01002614.1|:20162-20289
MsHel-3b	gb|AIXA01002614.1|:70732-70935
MsHel-3b	gb|AIXA01001763.1|:9851-10005
MsHel-3b	gb|AIXA01000871.1|:6543-6650
MsHel-3b	gb|AIXA01000871.1|:28156-28299
MsHel-3b	gb|AIXA01000722.1|:7110-7269
MsHel-3b	gb|AIXA01027614.1|:360-493
MsHel-3b	gb|AIXA01019998.1|:2974-3064
MsHel-3b	gb|AIXA01017872.1|:314-474
MsHel-3b	gb|AIXA01015507.1|:12718-12841
MsHel-3b	gb|AIXA01014348.1|:4451-4592
MsHel-3b	gb|AIXA01012324.1|:17025-17146
MsHel-3b	gb|AIXA01012324.1|:37227-37330
MsHel-3b	gb|AIXA01012297.1|:4013-4149
MsHel-3b	gb|AIXA01010079.1|:28829-28913
MsHel-3b	gb|AIXA01008793.1|:9607-9712
MsHel-3b	gb|AIXA01006884.1|:72980-73089
MsHel-3b	gb|AIXA01006884.1|:131740-131890
MsHel-3b	gb|AIXA01005347.1|:43098-43216
MsHel-3b	gb|AIXA01004889.1|:7932-8057
MsHel-3b	gb|AIXA01003579.1|:680-770
MsHel-3b	gb|AIXA01002003.1|:3573-3731
MsHel-3b	gb|AIXA01001129.1|:53969-54083
MsHel-3b	gb|AIXA01031057.1|:128-254
MsHel-3b	gb|AIXA01021835.1|:746-844
MsHel-3b	gb|AIXA01021117.1|:1056-1226
MsHel-3b	gb|AIXA01020443.1|:215-368
MsHel-3b	gb|AIXA01017430.1|:4962-5064
MsHel-3b	gb|AIXA01015028.1|:502-688
MsHel-3b	gb|AIXA01012351.1|:5876-6055
MsHel-3b	gb|AIXA01011369.1|:23945-24062
MsHel-3b	gb|AIXA01009974.1|:4372-4485
MsHel-3b	gb|AIXA01009246.1|:10615-10823
MsHel-3b	gb|AIXA01007555.1|:2759-3110
MsHel-3b	gb|AIXA01007540.1|:1868-1994
MsHel-3b	gb|AIXA01005068.1|:15791-15879
MsHel-3b	gb|AIXA01004777.1|:72563-72802
MsHel-3b	gb|AIXA01004264.1|:10673-10796
MsHel-3b	gb|AIXA01004166.1|:701-782
MsHel-3b	gb|AIXA01002972.1|:38879-43259
MsHel-3b	gb|AIXA01001331.1|:217-4743
MsHel-3b	gb|AIXA01000914.1|:29630-29739
MsHel-3b	gb|AIXA01000914.1|:62580-62669
MsHel-3b	gb|AIXA01000423.1|:30399-30546
MsHel-3b	gb|AIXA01028140.1|:207-358

MsHel-3b	gb|AIXA01023994.1|:523-725
MsHel-3b	gb|AIXA01021730.1|:320-478
MsHel-3b	gb|AIXA01018484.1|:5095-5266
MsHel-3b	gb|AIXA01017626.1|:11439-11654
MsHel-3b	gb|AIXA01016386.1|:1609-1734
MsHel-3b	gb|AIXA01016365.1|:143-262
MsHel-3b	gb|AIXA01015644.1|:14591-14731
MsHel-3b	gb|AIXA01014744.1|:2067-2221
MsHel-3b	gb|AIXA01013265.1|:5304-5487
MsHel-3b	gb|AIXA01012301.1|:14499-14654
MsHel-3b	gb|AIXA01011813.1|:3633-3741
MsHel-3b	gb|AIXA01006841.1|:56626-56839
MsHel-3b	gb|AIXA01006053.1|:45621-45805
MsHel-3b	gb|AIXA01005995.1|:13452-13647
MsHel-3b	gb|AIXA01005938.1|:17202-17342
MsHel-3b	gb|AIXA01004842.1|:32919-33056
MsHel-3b	gb|AIXA01004042.1|:7894-8026
MsHel-3b	gb|AIXA01003681.1|:4142-4230
MsHel-3b	gb|AIXA01001971.1|:12717-12825
MsHel-3b	gb|AIXA01001469.1|:6494-6619
MsHel-3b	gb|AIXA01000365.1|:13188-13284
MsHel-3b	gb|AIXA01000296.1|:53640-53768
MsHel-3b	gb|AIXA01028465.1|:160-279
MsHel-3b	gb|AIXA01025273.1|:546-666
MsHel-3b	gb|AIXA01019504.1|:967-1054
MsHel-3b	gb|AIXA01017672.1|:8807-8952
MsHel-3b	gb|AIXA01015922.1|:497-671
MsHel-3b	gb|AIXA01009599.1|:37129-37282
MsHel-3b	gb|AIXA01007984.1|:4245-4380
MsHel-3b	gb|AIXA01007970.1|:17874-17986
MsHel-3b	gb|AIXA01004082.1|:41447-41558
MsHel-3b	gb|AIXA01004082.1|:76258-76350
MsHel-3b	gb|AIXA01003274.1|:994-1088
MsHel-3b	gb|AIXA01002732.1|:43366-43495
MsHel-3b	gb|AIXA01002732.1|:61077-61200
MsHel-3b	gb|AIXA01002611.1|:42120-42261
MsHel-3b	gb|AIXA01001082.1|:6345-6461
MsHel-3b	gb|AIXA01000961.1|:16141-16274
MsHel-3b	gb|AIXA01000961.1|:64579-64782
MsHel-3b	gb|AIXA01036078.1|:398-484
MsHel-3b	gb|AIXA01025445.1|:123-287
MsHel-3b	gb|AIXA01024020.1|:894-1005
MsHel-3b	gb|AIXA01023082.1|:10-101
MsHel-3b	gb|AIXA01020605.1|:989-1071
MsHel-3b	gb|AIXA01017882.1|:2907-3099
MsHel-3b	gb|AIXA01016812.1|:24975-25133
MsHel-3b	gb|AIXA01016681.1|:3602-3757
MsHel-3b	gb|AIXA01015956.1|:26882-27062

MsHel-3b	gb|AIXA01014644.1|:16648-24839
MsHel-3b	gb|AIXA01012786.1|:13708-13880
MsHel-3b	gb|AIXA01012472.1|:47215-47405
MsHel-3b	gb|AIXA01012125.1|:7946-8060
MsHel-3b	gb|AIXA01012110.1|:13612-13739
MsHel-3b	gb|AIXA01011493.1|:85358-85448
MsHel-3b	gb|AIXA01011326.1|:1470-1594
MsHel-3b	gb|AIXA01010678.1|:25101-25222
MsHel-3b	gb|AIXA01008502.1|:1200-1347
MsHel-3b	gb|AIXA01007359.1|:25895-26014
MsHel-3b	gb|AIXA01007359.1|:38766-38926
MsHel-3b	gb|AIXA01006509.1|:2771-2860
MsHel-3b	gb|AIXA01006015.1|:25457-25642
MsHel-3b	gb|AIXA01005558.1|:22134-22264
MsHel-3b	gb|AIXA01005132.1|:28587-28710
MsHel-3b	gb|AIXA01005002.1|:1328-1500
MsHel-3b	gb|AIXA01003112.1|:27624-27798
MsHel-3b	gb|AIXA01002705.1|:10764-10941
MsHel-3b	gb|AIXA01002705.1|:28562-28677
MsHel-3b	gb|AIXA01002361.1|:28947-29069
MsHel-3b	gb|AIXA01002249.1|:141-221
MsHel-3b	gb|AIXA01002067.1|:1748-1871
MsHel-3b	gb|AIXA01001044.1|:11841-11983
MsHel-3b	gb|AIXA01014903.1|:5002-5115
MsHel-3b	gb|AIXA01013138.1|:4745-4871
MsHel-3b	gb|AIXA01011969.1|:14475-14600
MsHel-3b	gb|AIXA01009106.1|:7427-7535
MsHel-3b	gb|AIXA01003013.1|:14709-14856
MsHel-3b	gb|AIXA01002114.1|:65011-65137
MsHel-3b	gb|AIXA01001228.1|:12619-12740
MsHel-3b	gb|AIXA01018506.1|:1963-2194
MsHel-3b	gb|AIXA01011966.1|:11853-12050
MsHel-3b	gb|AIXA01011301.1|:8523-8659
MsHel-3b	gb|AIXA01010867.1|:19183-19413
MsHel-3b	gb|AIXA01010852.1|:3632-3807
MsHel-3b	gb|AIXA01010714.1|:16302-16406
MsHel-3b	gb|AIXA01010714.1|:71439-71559
MsHel-3b	gb|AIXA01009693.1|:859-1060
MsHel-3b	gb|AIXA01009233.1|:166-290
MsHel-3b	gb|AIXA01008635.1|:14073-14186
MsHel-3b	gb|AIXA01008575.1|:77667-77851
MsHel-3b	gb|AIXA01008341.1|:8703-8864
MsHel-3b	gb|AIXA01007595.1|:13858-13982
MsHel-3b	gb|AIXA01007410.1|:7753-7869
MsHel-3b	gb|AIXA01007129.1|:19762-19850
MsHel-3b	gb|AIXA01007063.1|:2611-2732
MsHel-3b	gb|AIXA01006672.1|:7552-7636
MsHel-3b	gb|AIXA01006375.1|:9739-9895

MsHel-3b	gb|AIXA01006317.1|:7161-8335
MsHel-3b	gb|AIXA01003990.1|:3763-4526
MsHel-3b	gb|AIXA01002760.1|:27810-27941
MsHel-3b	gb|AIXA01002302.1|:20224-20344
MsHel-3b	gb|AIXA01002286.1|:7134-7689
MsHel-3b	gb|AIXA01001657.1|:72908-73130
MsHel-3b	gb|AIXA01000444.1|:36026-36108
MsHel-3b	gb|AIXA01000444.1|:58599-58710
MsHel-3b	gb|AIXA01000411.1|:14032-14153
MsHel-3b	gb|AIXA01026341.1|:1026-1134
MsHel-3b	gb|AIXA01020334.1|:2453-2556
MsHel-3b	gb|AIXA01018899.1|:3049-3136
MsHel-3b	gb|AIXA01014735.1|:5079-6626
MsHel-3b	gb|AIXA01012537.1|:9093-9236
MsHel-3b	gb|AIXA01009400.1|:17343-17785
MsHel-3b	gb|AIXA01008047.1|:4951-5147
MsHel-3b	gb|AIXA01007920.1|:12445-12565
MsHel-3b	gb|AIXA01005785.1|:1118-1245
MsHel-3b	gb|AIXA01005067.1|:84101-84225
MsHel-3b	gb|AIXA01004731.1|:34731-34870
MsHel-3b	gb|AIXA01003445.1|:6199-6287
MsHel-3b	gb|AIXA01003001.1|:41712-41799
MsHel-3b	gb|AIXA01003001.1|:58120-58216
MsHel-3b	gb|AIXA01001284.1|:85861-86049
MsHel-3b	gb|AIXA01001238.1|:3482-3638
MsHel-3b	gb|AIXA01001180.1|:14179-14353
MsHel-3b	gb|AIXA01000630.1|:6342-6425
MsHel-3b	gb|AIXA01000512.1|:5372-5499
MsHel-3b	gb|AIXA01000512.1|:59657-59793
MsHel-3b	gb|AIXA01023575.1|:1330-1478
MsHel-3b	gb|AIXA01021776.1|:1343-1441
MsHel-3b	gb|AIXA01020663.1|:2589-2747
MsHel-3b	gb|AIXA01014249.1|:10503-10627
MsHel-3b	gb|AIXA01012904.1|:19126-19262
MsHel-3b	gb|AIXA01009021.1|:3367-3500
MsHel-3b	gb|AIXA01008562.1|:30104-30296
MsHel-3b	gb|AIXA01006722.1|:50569-50651
MsHel-3b	gb|AIXA01006518.1|:92029-92185
MsHel-3b	gb|AIXA01005618.1|:91735-91821
MsHel-3b	gb|AIXA01004341.1|:2347-2812
MsHel-3b	gb|AIXA01003114.1|:29678-29776
MsHel-3b	gb|AIXA01002147.1|:2512-2824
MsHel-3b	gb|AIXA01025389.1|:828-1013
MsHel-3b	gb|AIXA01024416.1|:446-644
MsHel-3b	gb|AIXA01021027.1|:2123-2223
MsHel-3b	gb|AIXA01014781.1|:278-422
MsHel-3b	gb|AIXA01013692.1|:6690-6832
MsHel-3b	gb|AIXA01011331.1|:2389-2490

MsHel-3b	gb|AIXA01009860.1|:48890-48970
MsHel-3b	gb|AIXA01009298.1|:34734-34863
MsHel-3b	gb|AIXA01005892.1|:44642-44739
MsHel-3b	gb|AIXA01005626.1|:12649-12814
MsHel-3b	gb|AIXA01004691.1|:7081-7182
MsHel-3b	gb|AIXA01002197.1|:32953-33044
MsHel-3b	gb|AIXA01002160.1|:26257-26342
MsHel-3b	gb|AIXA01001414.1|:14642-14761
MsHel-3b	gb|AIXA01000569.1|:11397-11523
MsHel-3b	gb|AIXA01015348.1|:6404-6532
MsHel-3b	gb|AIXA01014764.1|:2420-2540
MsHel-3b	gb|AIXA01014242.1|:6376-6501
MsHel-3b	gb|AIXA01013943.1|:42-167
MsHel-3b	gb|AIXA01013222.1|:10219-10355
MsHel-3b	gb|AIXA01011031.1|:32025-32165
MsHel-3b	gb|AIXA01007605.1|:3725-3825
MsHel-3b	gb|AIXA01005281.1|:13353-13492
MsHel-3b	gb|AIXA01004796.1|:10700-10801
MsHel-3b	gb|AIXA01004708.1|:11265-11409
MsHel-3b	gb|AIXA01001514.1|:11727-11877
MsHel-3b	gb|AIXA01001477.1|:5691-5817
MsHel-3b	gb|AIXA01001045.1|:27288-27369
MsHel-3b	gb|AIXA01000944.1|:38773-38962
MsHel-3b	gb|AIXA01000872.1|:1856-1977
MsHel-3b	gb|AIXA01000863.1|:7722-7810
MsHel-3b	gb|AIXA01000701.1|:28220-28393
MsHel-3b	gb|AIXA01000591.1|:148-255
MsHel-3b	gb|AIXA01000207.1|:15027-15271
MsHel-3b	gb|AIXA01020412.1|:54-148
MsHel-3b	gb|AIXA01019768.1|:546-698
MsHel-3b	gb|AIXA01012987.1|:1030-1173
MsHel-3b	gb|AIXA01010744.1|:2694-2879
MsHel-3b	gb|AIXA01009315.1|:44518-44713
MsHel-3b	gb|AIXA01009107.1|:2275-2369
MsHel-3b	gb|AIXA01007172.1|:1191-1287
MsHel-3b	gb|AIXA01004323.1|:26963-27066
MsHel-3b	gb|AIXA01003966.1|:48140-48270
MsHel-3b	gb|AIXA01002941.1|:2851-2980
MsHel-3b	gb|AIXA01002388.1|:32338-32434
MsHel-3b	gb|AIXA01001127.1|:55087-55245
MsHel-3b	gb|AIXA01000928.1|:2571-2756
MsHel-3b	gb|AIXA01032643.1|:332-422
MsHel-3b	gb|AIXA01019823.1|:1049-1138
MsHel-3b	gb|AIXA01017859.1|:8698-8808
MsHel-3b	gb|AIXA01014643.1|:33014-33165
MsHel-3b	gb|AIXA01014228.1|:8316-8423
MsHel-3b	gb|AIXA01013825.1|:4420-4547
MsHel-3b	gb|AIXA01013618.1|:17522-17604

MsHel-3b	gb|AIXA01012850.1|:806-952
MsHel-3b	gb|AIXA01011122.1|:10096-10251
MsHel-3b	gb|AIXA01010932.1|:10040-10182
MsHel-3b	gb|AIXA01009663.1|:1549-6253
MsHel-3b	gb|AIXA01007525.1|:12216-12378
MsHel-3b	gb|AIXA01006595.1|:36969-37236
MsHel-3b	gb|AIXA01004139.1|:2367-2473
MsHel-3b	gb|AIXA01002785.1|:132-9199
MsHel-3b	gb|AIXA01002785.1|:40206-40328
MsHel-3b	gb|AIXA01002480.1|:7945-8059
MsHel-3b	gb|AIXA01001595.1|:36497-36617
MsHel-3b	gb|AIXA01038210.1|:84-289
MsHel-3b	gb|AIXA01014382.1|:11654-11845
MsHel-3b	gb|AIXA01012963.1|:2575-2700
MsHel-3b	gb|AIXA01012368.1|:4870-4991
MsHel-3b	gb|AIXA01011723.1|:7932-8045
MsHel-3b	gb|AIXA01010481.1|:15894-16023
MsHel-3b	gb|AIXA01009773.1|:7034-7156
MsHel-3b	gb|AIXA01008182.1|:11230-11319
MsHel-3b	gb|AIXA01007260.1|:42073-42197
MsHel-3b	gb|AIXA01006061.1|:13268-21202
MsHel-3b	gb|AIXA01005855.1|:18092-18287
MsHel-3b	gb|AIXA01005353.1|:5292-5475
MsHel-3b	gb|AIXA01005254.1|:8720-8869
MsHel-3b	gb|AIXA01005044.1|:13938-14036
MsHel-3b	gb|AIXA01004619.1|:31626-31801
MsHel-3b	gb|AIXA01003335.1|:5881-5970
MsHel-3b	gb|AIXA01001237.1|:23191-23386
MsHel-3b	gb|AIXA01000231.1|:3961-4109
MsHel-3b	gb|AIXA01000135.1|:4037-4150
MsHel-3b	gb|AIXA01037243.1|:210-337
MsHel-3b	gb|AIXA01017635.1|:12589-12682
MsHel-3b	gb|AIXA01017333.1|:9010-9205
MsHel-3b	gb|AIXA01014255.1|:137-236
MsHel-3b	gb|AIXA01013047.1|:18021-18130
MsHel-3b	gb|AIXA01012334.1|:1216-1311
MsHel-3b	gb|AIXA01011904.1|:649-776
MsHel-3b	gb|AIXA01009307.1|:5805-5941
MsHel-3b	gb|AIXA01006915.1|:6391-6477
MsHel-3b	gb|AIXA01005835.1|:1608-1761
MsHel-3b	gb|AIXA01004698.1|:8271-8460
MsHel-3b	gb|AIXA01003680.1|:4603-4770
MsHel-3b	gb|AIXA01002255.1|:16543-16639
MsHel-3b	gb|AIXA01000128.1|:76032-76111
MsHel-3b	gb|AIXA01000128.1|:107740-107825
MsHel-3b	gb|AIXA01017735.1|:13865-14029
MsHel-3b	gb|AIXA01013850.1|:28423-28595
MsHel-3b	gb|AIXA01011405.1|:15072-15187

MsHel-3b	gb|AIXA01008568.1|:767-885
MsHel-3b	gb|AIXA01007042.1|:27084-27214
MsHel-3b	gb|AIXA01006933.1|:14923-15053
MsHel-3b	gb|AIXA01006073.1|:5161-5292
MsHel-3b	gb|AIXA01005107.1|:164-279
MsHel-3b	gb|AIXA01004780.1|:356-454
MsHel-3b	gb|AIXA01004331.1|:18762-18872
MsHel-3b	gb|AIXA01004162.1|:8018-8101
MsHel-3b	gb|AIXA01000589.1|:2964-3202
MsHel-3b	gb|AIXA01000531.1|:8262-8373
MsHel-3b	gb|AIXA01018516.1|:4900-4994
MsHel-3b	gb|AIXA01016156.1|:8096-8218
MsHel-3b	gb|AIXA01013854.1|:4645-4735
MsHel-3b	gb|AIXA01013757.1|:6606-6732
MsHel-3b	gb|AIXA01011837.1|:2325-2438
MsHel-3b	gb|AIXA01010192.1|:11150-11334
MsHel-3b	gb|AIXA01009407.1|:4685-4866
MsHel-3b	gb|AIXA01008472.1|:57012-57106
MsHel-3b	gb|AIXA01007816.1|:854-1000
MsHel-3b	gb|AIXA01006723.1|:12089-12199
MsHel-3b	gb|AIXA01003721.1|:5409-5527
MsHel-3b	gb|AIXA01003671.1|:20048-20130
MsHel-3b	gb|AIXA01003160.1|:14012-14145
MsHel-3b	gb|AIXA01023332.1|:542-626
MsHel-3b	gb|AIXA01015093.1|:8094-8199
MsHel-3b	gb|AIXA01012909.1|:148-265
MsHel-3b	gb|AIXA01011682.1|:10934-11050
MsHel-3b	gb|AIXA01010298.1|:6079-6201
MsHel-3b	gb|AIXA01005033.1|:14654-14798
MsHel-3b	gb|AIXA01004338.1|:70-167
MsHel-3b	gb|AIXA01002510.1|:38423-38548
MsHel-3b	gb|AIXA01001232.1|:19103-19208
MsHel-3b	gb|AIXA01036564.1|:221-353
MsHel-3b	gb|AIXA01024785.1|:287-377
MsHel-3b	gb|AIXA01018112.1|:11883-11975
MsHel-3b	gb|AIXA01017083.1|:7456-7613
MsHel-3b	gb|AIXA01013045.1|:6806-6913
MsHel-3b	gb|AIXA01009331.1|:16121-16240
MsHel-3b	gb|AIXA01008159.1|:22110-22304
MsHel-3b	gb|AIXA01006862.1|:12278-12619
MsHel-3b	gb|AIXA01004456.1|:53322-53478
MsHel-3b	gb|AIXA01002650.1|:1433-4363
MsHel-3b	gb|AIXA01000689.1|:4703-4804
MsHel-3b	gb|AIXA01000635.1|:12807-12938
MsHel-3b	gb|AIXA01000485.1|:36170-36294
MsHel-3b	gb|AIXA01034797.1|:541-624
MsHel-3b	gb|AIXA01028571.1|:264-385
MsHel-3b	gb|AIXA01024979.1|:967-1054

MsHel-3b	gb|AIXA01016563.1|:1666-1818
MsHel-3b	gb|AIXA01015790.1|:4007-4166
MsHel-3b	gb|AIXA01015693.1|:4464-4567
MsHel-3b	gb|AIXA01014984.1|:21827-22591
MsHel-3b	gb|AIXA01013827.1|:9788-9887
MsHel-3b	gb|AIXA01013586.1|:322-504
MsHel-3b	gb|AIXA01011771.1|:184-362
MsHel-3b	gb|AIXA01011478.1|:13296-13424
MsHel-3b	gb|AIXA01010802.1|:8676-17769
MsHel-3b	gb|AIXA01010802.1|:29781-30254
MsHel-3b	gb|AIXA01010379.1|:2549-2628
MsHel-3b	gb|AIXA01010378.1|:2544-2623
MsHel-3b	gb|AIXA01007086.1|:9930-10012
MsHel-3b	gb|AIXA01006699.1|:22581-22684
MsHel-3b	gb|AIXA01006699.1|:82074-82171
MsHel-3b	gb|AIXA01006466.1|:18506-18589
MsHel-3b	gb|AIXA01002813.1|:103025-103123
MsHel-3b	gb|AIXA01001220.1|:7189-7360
MsHel-3b	gb|AIXA01032826.1|:490-590
MsHel-3b	gb|AIXA01019654.1|:2413-2617
MsHel-3b	gb|AIXA01019618.1|:4255-4345
MsHel-3b	gb|AIXA01016843.1|:6135-6225
MsHel-3b	gb|AIXA01016073.1|:10093-10223
MsHel-3b	gb|AIXA01014964.1|:15606-15696
MsHel-3b	gb|AIXA01014964.1|:30516-32568
MsHel-3b	gb|AIXA01013631.1|:177-267
MsHel-3b	gb|AIXA01012325.1|:11912-12018
MsHel-3b	gb|AIXA01011266.1|:8172-8544
MsHel-3b	gb|AIXA01008994.1|:18541-18667
MsHel-3b	gb|AIXA01008815.1|:4928-5099
MsHel-3b	gb|AIXA01007216.1|:15065-15172
MsHel-3b	gb|AIXA01006847.1|:5362-9400
MsHel-3b	gb|AIXA01004433.1|:44284-44434
MsHel-3b	gb|AIXA01003150.1|:12842-12959
MsHel-3b	gb|AIXA01002415.1|:36562-36652
MsHel-3b	gb|AIXA01001524.1|:3146-3493
MsHel-3b	gb|AIXA01019137.1|:6825-6907
MsHel-3b	gb|AIXA01016075.1|:42707-42796
MsHel-3b	gb|AIXA01012948.1|:8012-8158
MsHel-3b	gb|AIXA01011628.1|:157-259
MsHel-3b	gb|AIXA01011424.1|:409-495
MsHel-3b	gb|AIXA01008378.1|:40430-40911
MsHel-3b	gb|AIXA01007891.1|:39655-39776
MsHel-3b	gb|AIXA01007110.1|:23706-23893
MsHel-3b	gb|AIXA01006685.1|:1529-1662
MsHel-3b	gb|AIXA01005016.1|:34224-34384
MsHel-3b	gb|AIXA01003689.1|:12345-12433
MsHel-3b	gb|AIXA01002643.1|:5731-5926

MsHel-3b	gb|AIXA01002503.1|:38502-38631
MsHel-3b	gb|AIXA01001951.1|:28618-28703
MsHel-3b	gb|AIXA01001896.1|:18657-18794
MsHel-3b	gb|AIXA01001673.1|:30661-30747
MsHel-3b	gb|AIXA01001673.1|:46045-46201
MsHel-3b	gb|AIXA01001496.1|:57203-57291
MsHel-3b	gb|AIXA01000876.1|:24543-24672
MsHel-3b	gb|AIXA01000168.1|:53486-53660
MsHel-3b	gb|AIXA01018439.1|:5189-5279
MsHel-3b	gb|AIXA01017542.1|:7826-7922
MsHel-3b	gb|AIXA01017387.1|:3190-3290
MsHel-3b	gb|AIXA01014800.1|:3649-3749
MsHel-3b	gb|AIXA01009653.1|:3612-3751
MsHel-3b	gb|AIXA01009153.1|:3764-3890
MsHel-3b	gb|AIXA01008799.1|:329-418
MsHel-3b	gb|AIXA01007963.1|:4573-4657
MsHel-3b	gb|AIXA01007314.1|:20431-20553
MsHel-3b	gb|AIXA01006926.1|:12577-12665
MsHel-3b	gb|AIXA01005825.1|:7812-7984
MsHel-3b	gb|AIXA01005100.1|:4303-4442
MsHel-3b	gb|AIXA01004277.1|:23439-32965
MsHel-3b	gb|AIXA01004178.1|:11818-11948
MsHel-3b	gb|AIXA01004178.1|:149754-149978
MsHel-3b	gb|AIXA01003881.1|:43730-43822
MsHel-3b	gb|AIXA01001276.1|:9877-10009
MsHel-3b	gb|AIXA01000895.1|:48266-48398
MsHel-3b	gb|AIXA01000713.1|:110474-110678
MsHel-3b	gb|AIXA01018131.1|:3473-3567
MsHel-3b	gb|AIXA01017863.1|:863-986
MsHel-3b	gb|AIXA01016421.1|:3434-3528
MsHel-3b	gb|AIXA01016310.1|:6448-6539
MsHel-3b	gb|AIXA01013965.1|:3330-3417
MsHel-3b	gb|AIXA01011984.1|:15995-16082
MsHel-3b	gb|AIXA01011620.1|:24004-24140
MsHel-3b	gb|AIXA01008641.1|:2088-2182
MsHel-3b	gb|AIXA01007877.1|:1668-1755
MsHel-3b	gb|AIXA01007691.1|:31492-31624
MsHel-3b	gb|AIXA01007632.1|:5953-6076
MsHel-3b	gb|AIXA01006908.1|:13181-13382
MsHel-3b	gb|AIXA01005364.1|:1686-1769
MsHel-3b	gb|AIXA01004928.1|:8965-9077
MsHel-3b	gb|AIXA01004132.1|:12373-12456
MsHel-3b	gb|AIXA01003416.1|:444-535
MsHel-3b	gb|AIXA01003415.1|:2747-2838
MsHel-3b	gb|AIXA01003351.1|:15484-15600
MsHel-3b	gb|AIXA01003316.1|:48898-48982
MsHel-3b	gb|AIXA01003264.1|:4240-4363
MsHel-3b	gb|AIXA01002254.1|:10652-10778

MsHel-3b	gb|AIXA01002254.1|:126986-127070
MsHel-3b	gb|AIXA01000947.1|:50123-50254
MsHel-3b	gb|AIXA01000847.1|:1700-1826
MsHel-3b	gb|AIXA01000847.1|:15537-15641
MsHel-3b	gb|AIXA01000460.1|:6318-10856
MsHel-3b	gb|AIXA01030109.1|:748-857
MsHel-3b	gb|AIXA01016295.1|:4117-4325
MsHel-3b	gb|AIXA01015454.1|:137-322
MsHel-3b	gb|AIXA01014977.1|:4153-4256
MsHel-3b	gb|AIXA01012538.1|:10498-10588
MsHel-3b	gb|AIXA01008395.1|:12649-12847
MsHel-3b	gb|AIXA01008069.1|:19389-19487
MsHel-3b	gb|AIXA01007883.1|:18985-19102
MsHel-3b	gb|AIXA01007751.1|:1837-1987
MsHel-3b	gb|AIXA01004157.1|:13184-13278
MsHel-3b	gb|AIXA01004069.1|:17551-17641
MsHel-3b	gb|AIXA01003585.1|:31057-31195
MsHel-3b	gb|AIXA01003585.1|:41554-41638
MsHel-3b	gb|AIXA01003585.1|:61876-62008
MsHel-3b	gb|AIXA01002655.1|:12957-13039
MsHel-3b	gb|AIXA01001919.1|:7419-7505
MsHel-3b	gb|AIXA01001812.1|:2433-2514
MsHel-3b	gb|AIXA01001448.1|:8046-8171
MsHel-3b	gb|AIXA01001190.1|:69604-69808
MsHel-3b	gb|AIXA01025499.1|:948-1070
MsHel-3b	gb|AIXA01023482.1|:289-392
MsHel-3b	gb|AIXA01017581.1|:17117-17245
MsHel-3b	gb|AIXA01017184.1|:1359-1440
MsHel-3b	gb|AIXA01016804.1|:4409-9583
MsHel-3b	gb|AIXA01015854.1|:3494-3574
MsHel-3b	gb|AIXA01014900.1|:21532-21610
MsHel-3b	gb|AIXA01013314.1|:1537-1646
MsHel-3b	gb|AIXA01010036.1|:10066-10147
MsHel-3b	gb|AIXA01009857.1|:15377-15466
MsHel-3b	gb|AIXA01009318.1|:17576-17689
MsHel-3b	gb|AIXA01008628.1|:4256-4341
MsHel-3b	gb|AIXA01008192.1|:7946-8098
MsHel-3b	gb|AIXA01007731.1|:41308-41397
MsHel-3b	gb|AIXA01007288.1|:43372-43466
MsHel-3b	gb|AIXA01004831.1|:81256-81376
MsHel-3b	gb|AIXA01004134.1|:65407-65511
MsHel-3b	gb|AIXA01002988.1|:4618-4754
MsHel-3b	gb|AIXA01002915.1|:4086-4183
MsHel-3b	gb|AIXA01001795.1|:32559-32656
MsHel-3b	gb|AIXA01000623.1|:15662-15747
MsHel-3b	gb|AIXA01022552.1|:2-113
MsHel-3b	gb|AIXA01014124.1|:19975-20107
MsHel-3b	gb|AIXA01012357.1|:2381-2538

MsHel-3b	gb|AIXA01011233.1|:46624-46756
MsHel-3b	gb|AIXA01008483.1|:9610-9742
MsHel-3b	gb|AIXA01008169.1|:7817-7909
MsHel-3b	gb|AIXA01007979.1|:4007-4138
MsHel-3b	gb|AIXA01006655.1|:31580-40004
MsHel-3b	gb|AIXA01006655.1|:51689-51785
MsHel-3b	gb|AIXA01005578.1|:45316-45531
MsHel-3b	gb|AIXA01005417.1|:4346-4453
MsHel-3b	gb|AIXA01004910.1|:1119-1220
MsHel-3b	gb|AIXA01004047.1|:27014-27114
MsHel-3b	gb|AIXA01002069.1|:2688-2857
MsHel-3b	gb|AIXA01001984.1|:1250-1362
MsHel-3b	gb|AIXA01001327.1|:30819-30903
MsHel-3b	gb|AIXA01000394.1|:10033-10157
MsHel-3b	gb|AIXA01000117.1|:24469-24564
MsHel-3b	gb|AIXA01000090.1|:53393-53501
MsHel-3b	gb|AIXA01023502.1|:225-308
MsHel-3b	gb|AIXA01016905.1|:7716-7799
MsHel-3b	gb|AIXA01016588.1|:8329-11227
MsHel-3b	gb|AIXA01013788.1|:6692-6868
MsHel-3b	gb|AIXA01011679.1|:10487-10617
MsHel-3b	gb|AIXA01009822.1|:10179-10274
MsHel-3b	gb|AIXA01009301.1|:6348-6489
MsHel-3b	gb|AIXA01007090.1|:15158-15237
MsHel-3b	gb|AIXA01005631.1|:8804-8895
MsHel-3b	gb|AIXA01004774.1|:60627-60762
MsHel-3b	gb|AIXA01000691.1|:20676-20806
MsHel-3b	gb|AIXA01000655.1|:196794-196891
MsHel-3b	gb|AIXA01000655.1|:255606-255704
MsHel-3b	gb|AIXA01000171.1|:16209-16315
MsHel-3b	gb|AIXA01000072.1|:76306-76444
MsHel-3b	gb|AIXA01019424.1|:5218-5365
MsHel-3b	gb|AIXA01017899.1|:1624-1735
MsHel-3b	gb|AIXA01017847.1|:86-171
MsHel-3b	gb|AIXA01015500.1|:8844-8926
MsHel-3b	gb|AIXA01014008.1|:572-702
MsHel-3b	gb|AIXA01013849.1|:1293-1414
MsHel-3b	gb|AIXA01012947.1|:295-2973
MsHel-3b	gb|AIXA01012398.1|:3141-3232
MsHel-3b	gb|AIXA01012021.1|:62201-62311
MsHel-3b	gb|AIXA01011484.1|:81610-81719
MsHel-3b	gb|AIXA01011281.1|:8970-9089
MsHel-3b	gb|AIXA01010495.1|:18670-18759
MsHel-3b	gb|AIXA01008391.1|:5835-5973
MsHel-3b	gb|AIXA01008155.1|:2470-2596
MsHel-3b	gb|AIXA01008155.1|:18057-18152
MsHel-3b	gb|AIXA01007030.1|:8425-8575
MsHel-3b	gb|AIXA01005901.1|:38819-38917

MsHel-3b	gb|AIXA01005615.1|:28355-28556
MsHel-3b	gb|AIXA01005610.1|:4563-4716
MsHel-3b	gb|AIXA01004712.1|:7978-8105
MsHel-3b	gb|AIXA01004463.1|:14354-14484
MsHel-3b	gb|AIXA01002673.1|:17761-17919
MsHel-3b	gb|AIXA01002603.1|:16993-17142
MsHel-3b	gb|AIXA01002530.1|:13029-13123
MsHel-3b	gb|AIXA01000098.1|:3604-3721
MsHel-3b	gb|AIXA01000098.1|:72468-72558
MsHel-3b	gb|AIXA01031993.1|:199-296
MsHel-3b	gb|AIXA01027772.1|:241-707
MsHel-3b	gb|AIXA01026139.1|:552-636
MsHel-3b	gb|AIXA01017868.1|:1980-2074
MsHel-3b	gb|AIXA01015798.1|:2208-2293
MsHel-3b	gb|AIXA01014398.1|:2460-2606
MsHel-3b	gb|AIXA01012678.1|:12165-12321
MsHel-3b	gb|AIXA01012549.1|:61819-61932
MsHel-3b	gb|AIXA01010937.1|:2608-2790
MsHel-3b	gb|AIXA01010806.1|:1189-1300
MsHel-3b	gb|AIXA01010594.1|:24559-24675
MsHel-3b	gb|AIXA01010116.1|:4786-4867
MsHel-3b	gb|AIXA01009594.1|:2902-3031
MsHel-3b	gb|AIXA01008634.1|:2448-2543
MsHel-3b	gb|AIXA01008167.1|:44447-44525
MsHel-3b	gb|AIXA01007926.1|:530-627
MsHel-3b	gb|AIXA01006316.1|:5030-11835
MsHel-3b	gb|AIXA01005175.1|:9581-9659
MsHel-3b	gb|AIXA01004724.1|:52081-52218
MsHel-3b	gb|AIXA01004678.1|:16345-16498
MsHel-3b	gb|AIXA01003691.1|:21579-21660
MsHel-3b	gb|AIXA01003643.1|:28238-28345
MsHel-3b	gb|AIXA01003288.1|:122196-122281
MsHel-3b	gb|AIXA01002379.1|:3987-4072
MsHel-3b	gb|AIXA01002379.1|:58951-59035
MsHel-3b	gb|AIXA01002170.1|:61754-61841
MsHel-3b	gb|AIXA01000636.1|:3873-3953
MsHel-3b	gb|AIXA01000503.1|:35378-35459
MsHel-3b	gb|AIXA01034947.1|:1-81
MsHel-3b	gb|AIXA01021311.1|:631-782
MsHel-3b	gb|AIXA01016714.1|:11837-17305
MsHel-3b	gb|AIXA01015738.1|:4-84
MsHel-3b	gb|AIXA01015409.1|:2895-3086
MsHel-3b	gb|AIXA01014849.1|:14594-14731
MsHel-3b	gb|AIXA01014208.1|:7334-7460
MsHel-3b	gb|AIXA01013107.1|:3825-3909
MsHel-3b	gb|AIXA01012264.1|:4984-5087
MsHel-3b	gb|AIXA01007622.1|:16204-16288
MsHel-3b	gb|AIXA01006939.1|:30299-30396

MsHel-3b	gb|AIXA01006447.1|:30640-30737
MsHel-3b	gb|AIXA01005942.1|:16343-16423
MsHel-3b	gb|AIXA01005942.1|:29398-29519
MsHel-3b	gb|AIXA01005589.1|:2979-3087
MsHel-3b	gb|AIXA01004472.1|:14736-14820
MsHel-3b	gb|AIXA01004045.1|:6691-6799
MsHel-3b	gb|AIXA01003839.1|:51787-51914
MsHel-3b	gb|AIXA01003682.1|:58377-58461
MsHel-3b	gb|AIXA01000613.1|:35857-35984
MsHel-3b	gb|AIXA01032537.1|:294-414
MsHel-3b	gb|AIXA01024133.1|:682-780
MsHel-3b	gb|AIXA01023222.1|:219-306
MsHel-3b	gb|AIXA01017701.1|:7000-7086
MsHel-3b	gb|AIXA01017093.1|:4303-4386
MsHel-3b	gb|AIXA01015449.1|:8074-8188
MsHel-3b	gb|AIXA01015303.1|:659-746
MsHel-3b	gb|AIXA01015275.1|:2389-2484
MsHel-3b	gb|AIXA01014372.1|:17869-17990
MsHel-3b	gb|AIXA01013799.1|:8794-8907
MsHel-3b	gb|AIXA01011883.1|:790-933
MsHel-3b	gb|AIXA01010132.1|:21480-21583
MsHel-3b	gb|AIXA01009765.1|:31679-31801
MsHel-3b	gb|AIXA01009681.1|:20011-20102
MsHel-3b	gb|AIXA01007634.1|:24688-24864
MsHel-3b	gb|AIXA01007298.1|:10139-10223
MsHel-3b	gb|AIXA01005241.1|:8225-8359
MsHel-3b	gb|AIXA01004775.1|:50347-50461
MsHel-3b	gb|AIXA01003140.1|:95821-95909
MsHel-3b	gb|AIXA01003004.1|:848-959
MsHel-3b	gb|AIXA01002926.1|:5513-5604
MsHel-3b	gb|AIXA01002443.1|:44342-44447
MsHel-3b	gb|AIXA01002398.1|:18027-18149
MsHel-3b	gb|AIXA01001415.1|:37949-38084
MsHel-3b	gb|AIXA01000607.1|:1290-1433
MsHel-3b	gb|AIXA01000218.1|:12721-12866
MsHel-3b	gb|AIXA01023105.1|:860-945
MsHel-3b	gb|AIXA01019973.1|:2238-2347
MsHel-3b	gb|AIXA01019558.1|:2101-2183
MsHel-3b	gb|AIXA01014148.1|:12-97
MsHel-3b	gb|AIXA01013796.1|:50267-50357
MsHel-3b	gb|AIXA01012645.1|:2143-2296
MsHel-3b	gb|AIXA01012642.1|:5475-5561
MsHel-3b	gb|AIXA01012148.1|:41755-41830
MsHel-3b	gb|AIXA01010906.1|:16039-16156
MsHel-3b	gb|AIXA01009268.1|:17714-17868
MsHel-3b	gb|AIXA01007386.1|:5903-6053
MsHel-3b	gb|AIXA01006507.1|:15236-15318
MsHel-3b	gb|AIXA01005972.1|:1251-1370

MsHel-3b	gb|AIXA01005768.1|:27761-27850
MsHel-3b	gb|AIXA01004566.1|:32025-32183
MsHel-3b	gb|AIXA01004154.1|:26788-26912
MsHel-3b	gb|AIXA01003970.1|:47632-47774
MsHel-3b	gb|AIXA01003771.1|:1314-1405
MsHel-3b	gb|AIXA01002737.1|:44069-44159
MsHel-3b	gb|AIXA01000918.1|:1956-2073
MsHel-3b	gb|AIXA01033828.1|:341-493
MsHel-3b	gb|AIXA01027465.1|:578-671
MsHel-3b	gb|AIXA01023980.1|:980-1136
MsHel-3b	gb|AIXA01021388.1|:1925-2010
MsHel-3b	gb|AIXA01016767.1|:21238-21354
MsHel-3b	gb|AIXA01015739.1|:3-84
MsHel-3b	gb|AIXA01013474.1|:3-92
MsHel-3b	gb|AIXA01013360.1|:1163-1269
MsHel-3b	gb|AIXA01011654.1|:46260-46357
MsHel-3b	gb|AIXA01010772.1|:24653-34458
MsHel-3b	gb|AIXA01009208.1|:50263-50369
MsHel-3b	gb|AIXA01009094.1|:1584-1704
MsHel-3b	gb|AIXA01006752.1|:15648-15733
MsHel-3b	gb|AIXA01006474.1|:1363-1464
MsHel-3b	gb|AIXA01006388.1|:12093-12219
MsHel-3b	gb|AIXA01006363.1|:5461-5563
MsHel-3b	gb|AIXA01006118.1|:25345-25462
MsHel-3b	gb|AIXA01005325.1|:2120-2291
MsHel-3b	gb|AIXA01004081.1|:1554-1662
MsHel-3b	gb|AIXA01003567.1|:9181-9262
MsHel-3b	gb|AIXA01003165.1|:9419-9573
MsHel-3b	gb|AIXA01001685.1|:64640-64726
MsHel-3b	gb|AIXA01001498.1|:10697-10853
MsHel-3b	gb|AIXA01001213.1|:35451-35548
MsHel-3b	gb|AIXA01001135.1|:21738-21821
MsHel-3b	gb|AIXA01000948.1|:8913-15566
MsHel-3b	gb|AIXA01022248.1|:411-562
MsHel-3b	gb|AIXA01018533.1|:9301-9385
MsHel-3b	gb|AIXA01015088.1|:1445-1533
MsHel-3b	gb|AIXA01013911.1|:4079-4159
MsHel-3b	gb|AIXA01013026.1|:7864-8001
MsHel-3b	gb|AIXA01011492.1|:3881-4000
MsHel-3b	gb|AIXA01009348.1|:25326-25478
MsHel-3b	gb|AIXA01009017.1|:2058-2166
MsHel-3b	gb|AIXA01007603.1|:14227-18007
MsHel-3b	gb|AIXA01007421.1|:39420-39509
MsHel-3b	gb|AIXA01007418.1|:1020-1132
MsHel-3b	gb|AIXA01005360.1|:1502-1614
MsHel-3b	gb|AIXA01004640.1|:76898-76990
MsHel-3b	gb|AIXA01004633.1|:10079-10204
MsHel-3b	gb|AIXA01002850.1|:111211-111308

MsHel-3b	gb|AIXA01002680.1|:2908-3004
MsHel-3b	gb|AIXA01002433.1|:105168-105260
MsHel-3b	gb|AIXA01001757.1|:33399-33480
MsHel-3b	gb|AIXA01001198.1|:90089-90185
MsHel-3b	gb|AIXA01029097.1|:36-123
MsHel-3b	gb|AIXA01017484.1|:2230-2349
MsHel-3b	gb|AIXA01016593.1|:1520-1653
MsHel-3b	gb|AIXA01012253.1|:35170-35304
MsHel-3b	gb|AIXA01012003.1|:6524-6634
MsHel-3b	gb|AIXA01011882.1|:1993-2136
MsHel-3b	gb|AIXA01011672.1|:5127-5209
MsHel-3b	gb|AIXA01011274.1|:749-836
MsHel-3b	gb|AIXA01011246.1|:10115-10258
MsHel-3b	gb|AIXA01009257.1|:18395-18489
MsHel-3b	gb|AIXA01008237.1|:3202-3292
MsHel-3b	gb|AIXA01007582.1|:3048-3187
MsHel-3b	gb|AIXA01007509.1|:34918-34997
MsHel-3b	gb|AIXA01006977.1|:22623-22756
MsHel-3b	gb|AIXA01004378.1|:6257-6371
MsHel-3b	gb|AIXA01003557.1|:4921-5012
MsHel-3b	gb|AIXA01002155.1|:13620-13703
MsHel-3b	gb|AIXA01001949.1|:8833-8947
MsHel-3b	gb|AIXA01001204.1|:81687-81834
MsHel-3b	gb|AIXA01000544.1|:28154-28284
MsHel-3b	gb|AIXA01025972.1|:854-940
MsHel-3b	gb|AIXA01018618.1|:2942-3024
MsHel-3b	gb|AIXA01016855.1|:12409-12488
MsHel-3b	gb|AIXA01016090.1|:4526-4710
MsHel-3b	gb|AIXA01015381.1|:3449-3550
MsHel-3b	gb|AIXA01012588.1|:14871-14964
MsHel-3b	gb|AIXA01012164.1|:6801-6938
MsHel-3b	gb|AIXA01011945.1|:2465-2547
MsHel-3b	gb|AIXA01011814.1|:2781-2866
MsHel-3b	gb|AIXA01010076.1|:24419-24509
MsHel-3b	gb|AIXA01007250.1|:6858-6948
MsHel-3b	gb|AIXA01006162.1|:45188-45274
MsHel-3b	gb|AIXA01005206.1|:6525-6609
MsHel-3b	gb|AIXA01003852.1|:7243-7383
MsHel-3b	gb|AIXA01003063.1|:40281-40375
MsHel-3b	gb|AIXA01002882.1|:3260-3339
MsHel-3b	gb|AIXA01001878.1|:22936-23052
MsHel-3b	gb|AIXA01001747.1|:28878-28964
MsHel-3b	gb|AIXA01000868.1|:6572-6679
MsHel-3b	gb|AIXA01020211.1|:15-114
MsHel-3b	gb|AIXA01016123.1|:15327-15452
MsHel-3b	gb|AIXA01013430.1|:2018-2098
MsHel-3b	gb|AIXA01013416.1|:1968-2122
MsHel-3b	gb|AIXA01013359.1|:1114-1220

MsHel-3b	gb|AIXA01012466.1|:46545-46674
MsHel-3b	gb|AIXA01012167.1|:5580-5665
MsHel-3b	gb|AIXA01009334.1|:634-719
MsHel-3b	gb|AIXA01009058.1|:11978-12092
MsHel-3b	gb|AIXA01008335.1|:19314-19411
MsHel-3b	gb|AIXA01007807.1|:1689-1790
MsHel-3b	gb|AIXA01007282.1|:61482-61592
MsHel-3b	gb|AIXA01007067.1|:42374-42458
MsHel-3b	gb|AIXA01007049.1|:16556-16665
MsHel-3b	gb|AIXA01004266.1|:18497-18605
MsHel-3b	gb|AIXA01003798.1|:1749-1858
MsHel-3b	gb|AIXA01001709.1|:21338-21418
MsHel-3b	gb|AIXA01000455.1|:1401-1509
MsHel-3b	gb|AIXA01000029.1|:6448-6573
MsHel-3b	gb|AIXA01027440.1|:746-868
MsHel-3b	gb|AIXA01019517.1|:2572-2656
MsHel-3b	gb|AIXA01016327.1|:6418-6554
MsHel-3b	gb|AIXA01015087.1|:6949-7037
MsHel-3b	gb|AIXA01014591.1|:4646-4766
MsHel-3b	gb|AIXA01013561.1|:4517-4653
MsHel-3b	gb|AIXA01012262.1|:6104-6295
MsHel-3b	gb|AIXA01008853.1|:3222-3310
MsHel-3b	gb|AIXA01006376.1|:2394-2474
MsHel-3b	gb|AIXA01006149.1|:37756-37852
MsHel-3b	gb|AIXA01003907.1|:23564-23653
MsHel-3b	gb|AIXA01003758.1|:8400-8517
MsHel-3b	gb|AIXA01003251.1|:35086-35194
MsHel-3b	gb|AIXA01000793.1|:15366-15446
MsHel-3b	gb|AIXA01000723.1|:19985-20069
MsHel-3b	gb|AIXA01000375.1|:80380-80468
MsHel-3b	gb|AIXA01000089.1|:39462-39554
MsHel-3b	gb|AIXA01037723.1|:399-510
MsHel-3b	gb|AIXA01019996.1|:12-104
MsHel-3b	gb|AIXA01019493.1|:4095-4192
MsHel-3b	gb|AIXA01019193.1|:5588-5687
MsHel-3b	gb|AIXA01018993.1|:952-1035
MsHel-3b	gb|AIXA01015597.1|:8142-8237
MsHel-3b	gb|AIXA01013846.1|:2693-2776
MsHel-3b	gb|AIXA01010420.1|:12125-12247
MsHel-3b	gb|AIXA01010278.1|:2160-2247
MsHel-3b	gb|AIXA01007975.1|:69434-69522
MsHel-3b	gb|AIXA01007287.1|:13804-13891
MsHel-3b	gb|AIXA01006916.1|:1761-1852
MsHel-3b	gb|AIXA01006191.1|:29709-29800
MsHel-3b	gb|AIXA01004396.1|:26557-26713
MsHel-3b	gb|AIXA01004216.1|:17823-17902
MsHel-3b	gb|AIXA01004021.1|:11656-11811
MsHel-3b	gb|AIXA01003979.1|:2262-8633

MsHel-3b	gb|AIXA01003575.1|:382-469
MsHel-3b	gb|AIXA01003245.1|:3736-3847
MsHel-3b	gb|AIXA01003116.1|:18235-18319
MsHel-3b	gb|AIXA01003116.1|:52070-52160
MsHel-3b	gb|AIXA01002787.1|:24963-25077
MsHel-3b	gb|AIXA01002402.1|:30426-30529
MsHel-3b	gb|AIXA01022668.1|:913-995
MsHel-3b	gb|AIXA01021858.1|:100-198
MsHel-3b	gb|AIXA01018277.1|:940-1056
MsHel-3b	gb|AIXA01017394.1|:17722-17812
MsHel-3b	gb|AIXA01016666.1|:13765-13905
MsHel-3b	gb|AIXA01016500.1|:2785-2883
MsHel-3b	gb|AIXA01013454.1|:4475-4560
MsHel-3b	gb|AIXA01012024.1|:13791-13881
MsHel-3b	gb|AIXA01011194.1|:10965-11063
MsHel-3b	gb|AIXA01010698.1|:55978-56055
MsHel-3b	gb|AIXA01008363.1|:23259-23379
MsHel-3b	gb|AIXA01007444.1|:5906-5984
MsHel-3b	gb|AIXA01007262.1|:27759-28507
MsHel-3b	gb|AIXA01006881.1|:10670-10763
MsHel-3b	gb|AIXA01005725.1|:448-561
MsHel-3b	gb|AIXA01004034.1|:82854-82935
MsHel-3b	gb|AIXA01002536.1|:21826-21907
MsHel-3b	gb|AIXA01002057.1|:12780-12865
MsHel-3b	gb|AIXA01001408.1|:17290-17372
MsHel-3b	gb|AIXA01000639.1|:39130-39280
MsHel-3b	gb|AIXA01000140.1|:13154-13237
MsHel-3b	gb|AIXA01000015.1|:52708-52809
MsHel-3b	gb|AIXA01025249.1|:167-255
MsHel-3b	gb|AIXA01019715.1|:1594-1679
MsHel-3b	gb|AIXA01016485.1|:3212-3293
MsHel-3b	gb|AIXA01015729.1|:466-554
MsHel-3b	gb|AIXA01014919.1|:38912-39020
MsHel-3b	gb|AIXA01014369.1|:3922-4003
MsHel-3b	gb|AIXA01013752.1|:588-676
MsHel-3b	gb|AIXA01012633.1|:12479-12578
MsHel-3b	gb|AIXA01009781.1|:76606-76687
MsHel-3b	gb|AIXA01008713.1|:312-388
MsHel-3b	gb|AIXA01007560.1|:50162-50246
MsHel-3b	gb|AIXA01007241.1|:107452-107540
MsHel-3b	gb|AIXA01006285.1|:12045-12125
MsHel-3b	gb|AIXA01005888.1|:7615-7769
MsHel-3b	gb|AIXA01005013.1|:10560-10674
MsHel-3b	gb|AIXA01004758.1|:13725-13809
MsHel-3b	gb|AIXA01002933.1|:11052-11149
MsHel-3b	gb|AIXA01002852.1|:27494-27608
MsHel-3b	gb|AIXA01002409.1|:40379-40468
MsHel-3b	gb|AIXA01002032.1|:1466-1583

MsHel-3b	gb|AIXA01002001.1|:59987-60087
MsHel-3b	gb|AIXA01001920.1|:27084-27172
MsHel-3b	gb|AIXA01001869.1|:22807-22895
MsHel-3b	gb|AIXA01030977.1|:214-298
MsHel-3b	gb|AIXA01026525.1|:925-1047
MsHel-3b	gb|AIXA01024068.1|:453-555
MsHel-3b	gb|AIXA01023231.1|:215-295
MsHel-3b	gb|AIXA01014389.1|:19293-19372
MsHel-3b	gb|AIXA01014300.1|:3299-3382
MsHel-3b	gb|AIXA01013373.1|:533-629
MsHel-3b	gb|AIXA01013015.1|:45477-45599
MsHel-3b	gb|AIXA01012007.1|:50369-50453
MsHel-3b	gb|AIXA01011231.1|:166-278
MsHel-3b	gb|AIXA01010616.1|:4986-5072
MsHel-3b	gb|AIXA01008669.1|:25407-25490
MsHel-3b	gb|AIXA01007004.1|:15450-15587
MsHel-3b	gb|AIXA01006167.1|:26910-27033
MsHel-3b	gb|AIXA01004930.1|:4147-4480
MsHel-3b	gb|AIXA01004181.1|:29921-30000
MsHel-3b	gb|AIXA01003017.1|:32027-32115
MsHel-3b	gb|AIXA01002675.1|:19697-19809
MsHel-3b	gb|AIXA01001699.1|:73103-73198
MsHel-3b	gb|AIXA01000170.1|:31621-31705
MsHel-3b	gb|AIXA01035198.1|:360-455
MsHel-3b	gb|AIXA01018128.1|:2097-2179
MsHel-3b	gb|AIXA01017720.1|:16123-16236
MsHel-3b	gb|AIXA01017349.1|:2988-3071
MsHel-3b	gb|AIXA01015523.1|:6935-7051
MsHel-3b	gb|AIXA01015397.1|:7143-7230
MsHel-3b	gb|AIXA01011534.1|:7490-7569
MsHel-3b	gb|AIXA01009527.1|:47073-47195
MsHel-3b	gb|AIXA01009473.1|:5777-5872
MsHel-3b	gb|AIXA01008574.1|:60389-60496
MsHel-3b	gb|AIXA01007732.1|:5405-5484
MsHel-3b	gb|AIXA01007732.1|:27971-28366
MsHel-3b	gb|AIXA01006630.1|:8900-9023
MsHel-3b	gb|AIXA01004720.1|:13839-13922
MsHel-3b	gb|AIXA01004298.1|:279-591
MsHel-3b	gb|AIXA01003972.1|:16516-16599
MsHel-3b	gb|AIXA01002868.1|:21134-21225
MsHel-3b	gb|AIXA01002387.1|:26421-26535
MsHel-3b	gb|AIXA01002186.1|:33963-34072
MsHel-3b	gb|AIXA01002183.1|:8987-9109
MsHel-3b	gb|AIXA01001849.1|:5032-5160
MsHel-3b	gb|AIXA01000917.1|:16517-16624
MsHel-3b	gb|AIXA01000322.1|:16616-16707
MsHel-3b	gb|AIXA01000037.1|:36333-36415
MsHel-3b	gb|AIXA01027930.1|:795-876

MsHel-3b	gb|AIXA01019884.1|:212-326
MsHel-3b	gb|AIXA01015459.1|:2056-2143
MsHel-3b	gb|AIXA01014537.1|:4134-4220
MsHel-3b	gb|AIXA01014277.1|:2655-3095
MsHel-3b	gb|AIXA01012668.1|:6678-8551
MsHel-3b	gb|AIXA01011684.1|:2861-2975
MsHel-3b	gb|AIXA01010912.1|:146-232
MsHel-3b	gb|AIXA01010461.1|:1943-2070
MsHel-3b	gb|AIXA01009513.1|:4581-4671
MsHel-3b	gb|AIXA01008796.1|:29509-29599
MsHel-3b	gb|AIXA01008653.1|:1268-1394
MsHel-3b	gb|AIXA01007236.1|:10079-11276
MsHel-3b	gb|AIXA01006772.1|:15668-15779
MsHel-3b	gb|AIXA01006756.1|:5836-5924
MsHel-3b	gb|AIXA01006223.1|:491-597
MsHel-3b	gb|AIXA01003143.1|:9730-9883
MsHel-3b	gb|AIXA01002980.1|:27866-27953
MsHel-3b	gb|AIXA01002729.1|:6760-6909
MsHel-3b	gb|AIXA01002439.1|:18736-18848
MsHel-3b	gb|AIXA01001697.1|:12882-13017
MsHel-3b	gb|AIXA01001258.1|:14721-17036
MsHel-3b	gb|AIXA01000662.1|:29903-30008
MsHel-3b	gb|AIXA01000570.1|:25940-26022
MsHel-3b	gb|AIXA01000205.1|:58333-58423
MsHel-3b	gb|AIXA01000108.1|:38964-39094
MsHel-3b	gb|AIXA01013239.1|:1511-1636
MsHel-3b	gb|AIXA01013135.1|:9913-9993
MsHel-3b	gb|AIXA01011700.1|:8287-8367
MsHel-3b	gb|AIXA01009949.1|:733-2905
MsHel-3b	gb|AIXA01005605.1|:39924-40005
MsHel-3b	gb|AIXA01001218.1|:7964-8053
MsHel-3b	gb|AIXA01000523.1|:15416-15497
MsHel-3b	gb|AIXA01009744.1|:3029-3120
MsHel-3b	gb|AIXA01009703.1|:14060-14177
MsHel-3b	gb|AIXA01004714.1|:18675-18754
MsHel-3b	gb|AIXA01004269.1|:8605-8685
MsHel-3b	gb|AIXA01004009.1|:4873-4961
MsHel-3b	gb|AIXA01003055.1|:10219-10303
MsHel-3b	gb|AIXA01002808.1|:50038-50129
MsHel-7	gb|AIXA01004100.1|:31897-32739
MsHel-7	gb|AIXA01007259.1|:16402-17248
MsHel-7	gb|AIXA01005538.1|:3854-4359
MsHel-7	gb|AIXA01005538.1|:51589-52443
MsHel-7	gb|AIXA01004996.1|:50458-51300
MsHel-7	gb|AIXA01000670.1|:13649-14487
MsHel-7	gb|AIXA01000670.1|:29914-30629
MsHel-7	gb|AIXA01007090.1|:12241-13100

MsHel-7	gb|AIXA01001129.1|:15999-16857
MsHel-7	gb|AIXA01008170.1|:8851-9190
MsHel-7	gb|AIXA01008170.1|:21930-22148
MsHel-7	gb|AIXA01011407.1|:35053-35294
MsHel-7	gb|AIXA01010338.1|:2779-3679
MsHel-7	gb|AIXA01002338.1|:1099-1953
MsHel-7	gb|AIXA01002130.1|:7001-7847
MsHel-7	gb|AIXA01006537.1|:3048-3893
MsHel-7	gb|AIXA01012877.1|:3626-4482
MsHel-7	gb|AIXA01012866.1|:24633-25434
MsHel-7	gb|AIXA01012103.1|:24411-25257
MsHel-7	gb|AIXA01010508.1|:1331-2179
MsHel-7	gb|AIXA01009100.1|:102951-103797
MsHel-7	gb|AIXA01008013.1|:11580-12281
MsHel-7	gb|AIXA01003057.1|:46402-47274
MsHel-7	gb|AIXA01001897.1|:13449-14299
MsHel-7	gb|AIXA01001883.1|:38053-38923
MsHel-7	gb|AIXA01001883.1|:50948-51784
MsHel-7	gb|AIXA01004815.1|:36342-37281
MsHel-7	gb|AIXA01012549.1|:73436-74102
MsHel-7	gb|AIXA01008500.1|:16746-17596
MsHel-7	gb|AIXA01007795.1|:16431-17242
MsHel-7	gb|AIXA01003223.1|:22555-23415
MsHel-7	gb|AIXA01001117.1|:91675-92511
MsHel-7	gb|AIXA01009116.1|:15296-16148
MsHel-7	gb|AIXA01007923.1|:1882-2723
MsHel-7	gb|AIXA01006061.1|:28336-29194
MsHel-7	gb|AIXA01005067.1|:38078-38931
MsHel-7	gb|AIXA01000627.1|:56256-57094
MsHel-7	gb|AIXA01012529.1|:27069-27908
MsHel-7	gb|AIXA01006860.1|:28508-29171
MsHel-7	gb|AIXA01006209.1|:17218-18059
MsHel-7	gb|AIXA01003183.1|:154689-155546
MsHel-7	gb|AIXA01011682.1|:17694-18546
MsHel-7	gb|AIXA01008124.1|:103078-103877
MsHel-7	gb|AIXA01000688.1|:99962-100813
MsHel-7	gb|AIXA01015119.1|:33690-34570
MsHel-7	gb|AIXA01011987.1|:56191-56788
MsHel-7	gb|AIXA01010524.1|:2052-2894
MsHel-7	gb|AIXA01008382.1|:48716-49570
MsHel-7	gb|AIXA01006880.1|:71340-72182
MsHel-7	gb|AIXA01006880.1|:128268-128767
MsHel-7	gb|AIXA01006602.1|:993-1835
MsHel-7	gb|AIXA01004165.1|:52318-52927
MsHel-7	gb|AIXA01002248.1|:97691-98478
MsHel-7	gb|AIXA01005862.1|:41392-42243
MsHel-7	gb|AIXA01004809.1|:115657-116394
MsHel-7	gb|AIXA01014919.1|:34120-34999

MsHel-7	gb|AIXA01001514.1|:19803-20505
MsHel-7	gb|AIXA01009318.1|:15191-16029
MsHel-7	gb|AIXA01008184.1|:7579-8424
MsHel-7	gb|AIXA01007681.1|:53147-53973
MsHel-7	gb|AIXA01003997.1|:12552-13369
MsHel-7	gb|AIXA01003997.1|:38521-39029
MsHel-7	gb|AIXA01002194.1|:6849-7686
MsHel-7	gb|AIXA01001554.1|:10584-11441
MsHel-7	gb|AIXA01006888.1|:28318-29184
MsHel-7	gb|AIXA01003229.1|:13128-13965
MsHel-7	gb|AIXA01000198.1|:50587-51295
MsHel-7	gb|AIXA01015304.1|:3370-4246
MsHel-7	gb|AIXA01011387.1|:3308-4148
MsHel-7	gb|AIXA01009217.1|:38031-38879
MsHel-7	gb|AIXA01003316.1|:47083-47939
MsHel-7	gb|AIXA01001677.1|:31199-32061
MsHel-7	gb|AIXA01001677.1|:102315-103181
MsHel-7	gb|AIXA01014659.1|:8631-9485
MsHel-7	gb|AIXA01007066.1|:51809-52559
MsHel-7	gb|AIXA01005029.1|:25931-26780
MsHel-7	gb|AIXA01003722.1|:58788-59623
MsHel-7	gb|AIXA01002985.1|:5901-6743
MsHel-7	gb|AIXA01002985.1|:27250-27478
MsHel-7	gb|AIXA01000988.1|:68988-69824
MsHel-7	gb|AIXA01004307.1|:119269-120123
MsHel-7	gb|AIXA01002500.1|:179904-180075
MsHel-7	gb|AIXA01000359.1|:2459-3314
MsHel-7	gb|AIXA01000318.1|:42904-43412
MsHel-7	gb|AIXA01001296.1|:43192-43578
MsHel-7	gb|AIXA01001296.1|:214023-214548
MsHel-7	gb|AIXA01012584.1|:19595-20441
MsHel-7	gb|AIXA01008248.1|:37041-37921
MsHel-7	gb|AIXA01006665.1|:1379-2228
MsHel-7	gb|AIXA01002464.1|:38613-39452
MsHel-7	gb|AIXA01000663.1|:4305-5178
MsHel-7	gb|AIXA01009591.1|:47402-48265
MsHel-7	gb|AIXA01005737.1|:29897-30737
MsHel-7	gb|AIXA01004188.1|:14149-14996
MsHel-7	gb|AIXA01000675.1|:17646-18498
MsHel-7	gb|AIXA01004184.1|:80932-81794
MsHel-7	gb|AIXA01000467.1|:5201-5972
MsHel-7	gb|AIXA01014346.1|:43896-44888
MsHel-7	gb|AIXA01004061.1|:37989-38832
MsHel-7	gb|AIXA01003683.1|:8969-9818
MsHel-7	gb|AIXA01017061.1|:4348-5184
MsHel-7	gb|AIXA01017061.1|:18014-18259
MsHel-7	gb|AIXA01008442.1|:31770-32614
MsHel-7	gb|AIXA01007129.1|:6035-6218

MsHel-7	gb|AIXA01007129.1|:18306-19147
MsHel-7	gb|AIXA01006048.1|:7877-8726
MsHel-7	gb|AIXA01004025.1|:21101-21941
MsHel-7	gb|AIXA01001102.1|:58886-59774
MsHel-7	gb|AIXA01001083.1|:838-1679
MsHel-7	gb|AIXA01001083.1|:18252-19071
MsHel-7	gb|AIXA01000929.1|:8343-9197
MsHel-7	gb|AIXA01013680.1|:3478-4444
MsHel-7	gb|AIXA01013680.1|:17679-18032
MsHel-7	gb|AIXA01007971.1|:26673-27521
MsHel-7	gb|AIXA01005610.1|:23112-23993
MsHel-7	gb|AIXA01004209.1|:15460-16218
MsHel-7	gb|AIXA01017239.1|:445-1280
MsHel-7	gb|AIXA01001810.1|:11059-11559
MsHel-7	gb|AIXA01011741.1|:209-1048
MsHel-7	gb|AIXA01008999.1|:298-1138
MsHel-7	gb|AIXA01007328.1|:6982-7823
MsHel-7	gb|AIXA01012962.1|:7884-8721
MsHel-7	gb|AIXA01010907.1|:15627-16332
MsHel-7	gb|AIXA01009537.1|:9895-10746
MsHel-7	gb|AIXA01009537.1|:55322-55512
MsHel-7	gb|AIXA01009537.1|:76109-76470
MsHel-7	gb|AIXA01006214.1|:2182-2980
MsHel-7	gb|AIXA01005641.1|:19038-19879
MsHel-7	gb|AIXA01002376.1|:4236-5113
MsHel-7	gb|AIXA01002296.1|:8033-8863
MsHel-7	gb|AIXA01002142.1|:1054-1896
MsHel-7	gb|AIXA01000074.1|:11121-11957
MsHel-7	gb|AIXA01002850.1|:145816-146507
MsHel-7	gb|AIXA01008393.1|:33183-33858
MsHel-7	gb|AIXA01005558.1|:8999-9841
MsHel-7	gb|AIXA01005558.1|:41590-42091
MsHel-7	gb|AIXA01005558.1|:56225-56577
MsHel-7	gb|AIXA01009179.1|:61610-62457
MsHel-7	gb|AIXA01009060.1|:5292-6106
MsHel-7	gb|AIXA01004227.1|:38320-39189
MsHel-7	gb|AIXA01011747.1|:26607-27444
MsHel-7	gb|AIXA01018128.1|:3871-4716
MsHel-7	gb|AIXA01005031.1|:6271-7096
MsHel-7	gb|AIXA01012679.1|:6478-7322
MsHel-7	gb|AIXA01001697.1|:51055-51265
MsHel-7	gb|AIXA01007199.1|:17284-18108
MsHel-7	gb|AIXA01006773.1|:3968-4816
MsHel-7	gb|AIXA01006053.1|:10887-11745
MsHel-7	gb|AIXA01006053.1|:25273-25757
MsHel-7	gb|AIXA01005615.1|:3687-4583
MsHel-7	gb|AIXA01011561.1|:38295-39000
MsHel-7	gb|AIXA01010536.1|:20203-21049

MsHel-7	gb|AIXA01004250.1|:38353-39196
MsHel-7	gb|AIXA01003198.1|:50865-51703
MsHel-7	gb|AIXA01003486.1|:15581-16276
MsHel-7	gb|AIXA01011611.1|:2597-2967
MsHel-7	gb|AIXA01011611.1|:20661-21500
MsHel-7	gb|AIXA01010667.1|:13124-13979
MsHel-7	gb|AIXA01005207.1|:4578-5407
MsHel-7	gb|AIXA01014467.1|:22052-22903
MsHel-7	gb|AIXA01013616.1|:35573-36417
MsHel-7	gb|AIXA01003048.1|:1002-1983
MsHel-7	gb|AIXA01000828.1|:58458-59127
MsHel-7	gb|AIXA01007460.1|:5512-6042
MsHel-7	gb|AIXA01007460.1|:55441-56281
MsHel-7	gb|AIXA01012049.1|:48515-49343
MsHel-7	gb|AIXA01002189.1|:19987-20838
MsHel-7	gb|AIXA01006474.1|:5120-5921
MsHel-7	gb|AIXA01004438.1|:18486-19328
MsHel-7	gb|AIXA01010691.1|:20017-20845
MsHel-7	gb|AIXA01007027.1|:35734-36581
MsHel-7	gb|AIXA01009544.1|:3-666
MsHel-7	gb|AIXA01008492.1|:63606-64566
MsHel-7	gb|AIXA01012191.1|:10478-11270
MsHel-7	gb|AIXA01001546.1|:36159-36990
MsHel-7	gb|AIXA01000792.1|:22094-23071
MsHel-7	gb|AIXA01000528.1|:72468-72968
MsHel-7	gb|AIXA01007462.1|:30057-31030
MsHel-7	gb|AIXA01012633.1|:3361-4194
MsHel-7	gb|AIXA01014124.1|:15-666
MsHel-7	gb|AIXA01014124.1|:20236-20543
MsHel-7	gb|AIXA01013049.1|:9770-10392
MsHel-7	gb|AIXA01009380.1|:6826-7594
MsHel-7	gb|AIXA01000375.1|:74731-75533
MsHel-7	gb|AIXA01001342.1|:1-633
MsHel-7	gb|AIXA01032247.1|:22-666
MsHel-7	gb|AIXA01001362.1|:10780-11524
MsHel-7	gb|AIXA01009703.1|:2500-3068
MsHel-7	gb|AIXA01009703.1|:15778-16691
MsHel-7	gb|AIXA01015409.1|:1-619
MsHel-7	gb|AIXA01001289.1|:1-545
MsHel-7	gb|AIXA01001289.1|:52234-52845
MsHel-7	gb|AIXA01006132.1|:1286-2088
MsHel-7	gb|AIXA01011728.1|:1-583
MsHel-7	gb|AIXA01001286.1|:20660-21503
MsHel-7	gb|AIXA01017289.1|:6562-7275
MsHel-7	gb|AIXA01005279.1|:17378-17947
MsHel-7	gb|AIXA01012949.1|:698-1765
MsHel-7	gb|AIXA01002411.1|:24405-24901
MsHel-7	gb|AIXA01013065.1|:1-587

MsHel-7	gb|AIXA01007139.1|:61302-62125
MsHel-7	gb|AIXA01001505.1|:40688-41409
MsHel-7	gb|AIXA01017291.1|:1-578
MsHel-7	gb|AIXA01005021.1|:12072-12891
MsHel-7	gb|AIXA01010019.1|:23891-24703
MsHel-7	gb|AIXA01018535.1|:4560-5136
MsHel-7	gb|AIXA01014086.1|:20550-21381
MsHel-7	gb|AIXA01007242.1|:5896-6606
MsHel-7	gb|AIXA01005125.1|:882-1712
MsHel-7	gb|AIXA01004161.1|:16964-17789
MsHel-7	gb|AIXA01001380.1|:1313-2158
MsHel-7	gb|AIXA01010155.1|:21880-22917
MsHel-7	gb|AIXA01014297.1|:1241-2270
MsHel-7	gb|AIXA01018233.1|:8596-9415
MsHel-7	gb|AIXA01010696.1|:6613-7255
MsHel-7	gb|AIXA01000102.1|:57771-58452
MsHel-7	gb|AIXA01000211.1|:91871-92677
MsHel-7	gb|AIXA01017967.1|:914-1732
MsHel-7	gb|AIXA01014041.1|:9250-10366
MsHel-7	gb|AIXA01012759.1|:8159-8992
MsHel-7	gb|AIXA01000827.1|:45976-46520
MsHel-7	gb|AIXA01018488.1|:5451-6684
MsHel-7	gb|AIXA01012573.1|:19359-20249
MsHel-7	gb|AIXA01007470.1|:32940-33575
MsHel-7	gb|AIXA01008931.1|:4494-5355
MsHel-7	gb|AIXA01008417.1|:20658-21480
MsHel-7	gb|AIXA01007931.1|:20967-21798
MsHel-7	gb|AIXA01005595.1|:40376-41207
MsHel-7	gb|AIXA01000950.1|:14731-15562
MsHel-7	gb|AIXA01007639.1|:5345-6152
MsHel-7	gb|AIXA01016691.1|:24273-25096
MsHel-7	gb|AIXA01012299.1|:13579-14371
MsHel-7	gb|AIXA01011106.1|:1-303
MsHel-7	gb|AIXA01008220.1|:4113-4952
MsHel-7	gb|AIXA01006648.1|:59653-60417
MsHel-7	gb|AIXA01023101.1|:59-876
MsHel-7	gb|AIXA01010406.1|:14665-15491
MsHel-7	gb|AIXA01007690.1|:28556-29390
MsHel-7	gb|AIXA01000361.1|:14971-15804
MsHel-7	gb|AIXA01013277.1|:27-709
MsHel-7	gb|AIXA01004775.1|:13089-13847
MsHel-7	gb|AIXA01004685.1|:96747-97569
MsHel-7	gb|AIXA01002953.1|:105458-106277
MsHel-7	gb|AIXA01002917.1|:55066-55761
MsHel-7	gb|AIXA01001396.1|:15073-15727
MsHel-7	gb|AIXA01024118.1|:708-1386
MsHel-7	gb|AIXA01013814.1|:7374-8218
MsHel-7	gb|AIXA01006386.1|:30223-31023

MsHel-7	gb|AIXA01004895.1|:17475-18455
MsHel-7	gb|AIXA01028523.1|:1-570
MsHel-7	gb|AIXA01019296.1|:5286-6116
MsHel-7	gb|AIXA01007022.1|:24910-25702
MsHel-7	gb|AIXA01011318.1|:23624-24470
MsHel-7	gb|AIXA01010785.1|:13-787
MsHel-7	gb|AIXA01010090.1|:10257-10990
MsHel-7	gb|AIXA01016438.1|:17806-18635
MsHel-7	gb|AIXA01012288.1|:5113-5939
MsHel-7	gb|AIXA01011540.1|:64677-65503
MsHel-7	gb|AIXA01007723.1|:13645-14467
MsHel-7	gb|AIXA01007287.1|:84339-85173
MsHel-7	gb|AIXA01004618.1|:4596-5421
MsHel-7	gb|AIXA01001973.1|:3325-4150
MsHel-7	gb|AIXA01000850.1|:90605-91428
MsHel-7	gb|AIXA01013650.1|:3797-4457
MsHel-7	gb|AIXA01013445.1|:35077-35952
MsHel-7	gb|AIXA01009340.1|:3133-4605
MsHel-7	gb|AIXA01002971.1|:117803-118334
MsHel-7	gb|AIXA01007543.1|:2827-4082
MsHel-7	gb|AIXA01001876.1|:5578-6225
MsHel-7	gb|AIXA01019486.1|:3052-3735
MsHel-7	gb|AIXA01014715.1|:1-860
MsHel-7	gb|AIXA01009269.1|:25388-26275
MsHel-7	gb|AIXA01009269.1|:36779-37522
MsHel-7	gb|AIXA01006230.1|:16198-17001
MsHel-7	gb|AIXA01004951.1|:8315-9102
MsHel-7	gb|AIXA01000329.1|:8290-9120
MsHel-7	gb|AIXA01000270.1|:15140-15673
MsHel-7	gb|AIXA01010077.1|:8680-9496
MsHel-7	gb|AIXA01008926.1|:8955-9778
MsHel-7	gb|AIXA01006231.1|:14845-15633
MsHel-7	gb|AIXA01001492.1|:47710-48532
MsHel-7	gb|AIXA01001282.1|:23934-24715
MsHel-7	gb|AIXA01001282.1|:55456-56582
MsHel-7	gb|AIXA01000297.1|:69826-70482
MsHel-7	gb|AIXA01023662.1|:43-700
MsHel-7	gb|AIXA01008930.1|:1-392
MsHel-7	gb|AIXA01000014.1|:13191-14025
MsHel-7	gb|AIXA01016081.1|:14625-15486
MsHel-7	gb|AIXA01007516.1|:11726-12546
MsHel-7	gb|AIXA01006983.1|:42755-43559
MsHel-7	gb|AIXA01002351.1|:15890-16716
MsHel-7	gb|AIXA01002267.1|:53743-54543
MsHel-7	gb|AIXA01002085.1|:21259-22051
MsHel-7	gb|AIXA01001243.1|:171574-172389
MsHel-7	gb|AIXA01000660.1|:18912-19606
MsHel-7	gb|AIXA01008249.1|:20-691

MsHel-7	gb|AIXA01004157.1|:2128-2785
MsHel-7	gb|AIXA01002950.1|:27189-28032
MsHel-7	gb|AIXA01002665.1|:29922-30428
MsHel-7	gb|AIXA01005311.1|:37321-40335
MsHel-7	gb|AIXA01003234.1|:22774-23604
MsHel-7	gb|AIXA01008068.1|:46565-47358
MsHel-7	gb|AIXA01001558.1|:103051-103776
MsHel-7	gb|AIXA01012536.1|:12203-13156
MsHel-7	gb|AIXA01005079.1|:40580-41320
MsHel-7	gb|AIXA01005076.1|:7750-8640
MsHel-7	gb|AIXA01000998.1|:25766-26647
MsHel-7	gb|AIXA01000649.1|:91742-92560
MsHel-7	gb|AIXA01020077.1|:775-1602
MsHel-7	gb|AIXA01005563.1|:62920-63625
MsHel-7	gb|AIXA01004252.1|:14779-15617
MsHel-7	gb|AIXA01002419.1|:39779-40602
MsHel-7	gb|AIXA01023799.1|:1-555
MsHel-7	gb|AIXA01008673.1|:2489-2601
MsHel-7	gb|AIXA01032959.1|:9-527
MsHel-7	gb|AIXA01007530.1|:39644-40464
MsHel-7	gb|AIXA01004670.1|:127601-128426
MsHel-7	gb|AIXA01003622.1|:18363-19184
MsHel-7	gb|AIXA01003214.1|:49686-50347
MsHel-7	gb|AIXA01002730.1|:41813-42601
MsHel-7	gb|AIXA01002730.1|:75512-76383
MsHel-7	gb|AIXA01002366.1|:24334-25414
MsHel-7	gb|AIXA01014489.1|:2203-3021
MsHel-7	gb|AIXA01008254.1|:17690-18513
MsHel-7	gb|AIXA01003996.1|:18067-18886
MsHel-7	gb|AIXA01003118.1|:27133-28473
MsHel-7	gb|AIXA01012904.1|:26750-27278
MsHel-7	gb|AIXA01007659.1|:11323-12184
MsHel-7	gb|AIXA01005180.1|:11615-12286
MsHel-7	gb|AIXA01025973.1|:505-1159
MsHel-7	gb|AIXA01014401.1|:1-526
MsHel-7	gb|AIXA01011734.1|:22173-23898
MsHel-7	gb|AIXA01010900.1|:30927-31736
MsHel-7	gb|AIXA01001026.1|:32035-32854
MsHel-7	gb|AIXA01000917.1|:7844-8673
MsHel-7	gb|AIXA01000517.1|:47860-48663
MsHel-7	gb|AIXA01000069.1|:309036-309841
MsHel-7	gb|AIXA01011367.1|:9810-10913
MsHel-7	gb|AIXA01006847.1|:8186-9023
MsHel-7	gb|AIXA01002776.1|:27976-28818
MsHel-7	gb|AIXA01002591.1|:12377-13180
MsHel-7	gb|AIXA01002591.1|:29586-30128
MsHel-7	gb|AIXA01005944.1|:15366-15929
MsHel-7	gb|AIXA01005865.1|:88209-89029

MsHel-7	gb|AIXA01004370.1|:44418-45215
MsHel-7	gb|AIXA01003693.1|:10666-11514
MsHel-7	gb|AIXA01021497.1|:669-1491
MsHel-7	gb|AIXA01004372.1|:4604-5423
MsHel-7	gb|AIXA01001685.1|:33314-34131
MsHel-7	gb|AIXA01004852.1|:50924-51390
MsHel-7	gb|AIXA01003748.1|:827-1457
MsHel-7	gb|AIXA01003105.1|:20593-21420
MsHel-7	gb|AIXA01012391.1|:6399-7031
MsHel-7	gb|AIXA01002527.1|:3260-4753
MsHel-7	gb|AIXA01001939.1|:20211-21008
MsHel-7	gb|AIXA01001939.1|:33674-34182
MsHel-7	gb|AIXA01007127.1|:86088-86750
MsHel-7	gb|AIXA01008396.1|:6637-7461
MsHel-7	gb|AIXA01001357.1|:1-635
MsHel-7	gb|AIXA01014443.1|:59406-60225
MsHel-7	gb|AIXA01009017.1|:4349-5419
MsHel-7	gb|AIXA01007247.1|:13881-14730
MsHel-7	gb|AIXA01000128.1|:54470-55207
MsHel-7	gb|AIXA01021156.1|:1250-2092
MsHel-7	gb|AIXA01011364.1|:1-496
MsHel-7	gb|AIXA01007359.1|:57564-58220
MsHel-7	gb|AIXA01000907.1|:45393-46210
MsHel-7	gb|AIXA01006698.1|:4997-5824
MsHel-7	gb|AIXA01018198.1|:11312-12097
MsHel-7	gb|AIXA01002294.1|:1-484
MsHel-7	gb|AIXA01000761.1|:11189-11857
MsHel-7	gb|AIXA01006421.1|:13034-13644
MsHel-7	gb|AIXA01004300.1|:20716-21394
MsHel-7	gb|AIXA01016301.1|:2118-2943
MsHel-7	gb|AIXA01000077.1|:66296-66909
MsHel-7	gb|AIXA01011197.1|:21152-21745
MsHel-7	gb|AIXA01004268.1|:28676-29423
MsHel-7	gb|AIXA01003449.1|:5359-6289
MsHel-7	gb|AIXA01010756.1|:25436-26269
MsHel-7	gb|AIXA01001707.1|:51143-51630
MsHel-7	gb|AIXA01010865.1|:32730-33492
MsHel-7	gb|AIXA01004636.1|:1-527
MsHel-7	gb|AIXA01011031.1|:25447-26276
MsHel-7	gb|AIXA01008360.1|:3090-3913
MsHel-7	gb|AIXA01008103.1|:25803-26559
MsHel-7	gb|AIXA01003469.1|:19148-19817
MsHel-7	gb|AIXA01001933.1|:2326-2760
MsHel-7	gb|AIXA01003977.1|:17441-18218
MsHel-7	gb|AIXA01002404.1|:477-1588
MsHel-7	gb|AIXA01008531.1|:28747-29410
MsHel-7	gb|AIXA01008738.1|:15162-15742
MsHel-7	gb|AIXA01008738.1|:33616-34102

MsHel-7	gb|AIXA01006891.1|:35121-35634
MsHel-7	gb|AIXA01006891.1|:53767-54598
MsHel-7	gb|AIXA01003679.1|:10924-11622
MsHel-7	gb|AIXA01002451.1|:10368-11188
MsHel-7	gb|AIXA01003490.1|:36649-37476
MsHel-7	gb|AIXA01009864.1|:31486-32072
MsHel-7	gb|AIXA01009864.1|:72868-73203
MsHel-7	gb|AIXA01000216.1|:12314-13128
MsHel-7	gb|AIXA01006121.1|:17630-18225
MsHel-7	gb|AIXA01001675.1|:49085-49917
MsHel-7	gb|AIXA01000327.1|:2510-3047
MsHel-7	gb|AIXA01009659.1|:7957-8555
MsHel-7	gb|AIXA01011370.1|:11256-12024
MsHel-7	gb|AIXA01007527.1|:28178-29012
MsHel-7	gb|AIXA01026321.1|:1-701
MsHel-7	gb|AIXA01008095.1|:25630-26372
MsHel-7	gb|AIXA01003880.1|:1-444
MsHel-7	gb|AIXA01000838.1|:2-610
MsHel-7	gb|AIXA01010777.1|:1-502
MsHel-7	gb|AIXA01000255.1|:35284-35780
MsHel-7	gb|AIXA01006682.1|:2798-4083
MsHel-7	gb|AIXA01004312.1|:30129-30780
MsHel-7	gb|AIXA01008337.1|:1-474
MsHel-7	gb|AIXA01007584.1|:12162-12906
MsHel-7	gb|AIXA01004754.1|:10926-11751
MsHel-7	gb|AIXA01004105.1|:2491-2948
MsHel-7	gb|AIXA01012841.1|:7342-7934
MsHel-7	gb|AIXA01023151.1|:961-1548
MsHel-7	gb|AIXA01009061.1|:57817-58321
MsHel-7	gb|AIXA01009061.1|:111089-111897
MsHel-7	gb|AIXA01002700.1|:18-614
MsHel-7	gb|AIXA01000590.1|:5717-6293
MsHel-7	gb|AIXA01009634.1|:14188-14737
MsHel-7	gb|AIXA01003673.1|:53393-53952
MsHel-7	gb|AIXA01024743.1|:3-437
MsHel-7	gb|AIXA01003358.1|:4452-4940
MsHel-7	gb|AIXA01003358.1|:16015-16710
MsHel-7	gb|AIXA01010656.1|:17331-18029
MsHel-7	gb|AIXA01000892.1|:107270-109220
MsHel-7	gb|AIXA01007608.1|:10554-11134
MsHel-7	gb|AIXA01004867.1|:1002-1307
MsHel-7	gb|AIXA01003634.1|:1353-1807
MsHel-7	gb|AIXA01007614.1|:9940-10505
MsHel-7	gb|AIXA01022530.1|:1112-1675
MsHel-7	gb|AIXA01009403.1|:1575-2485
MsHel-7	gb|AIXA01006241.1|:15566-16192
MsHel-7	gb|AIXA01009865.1|:28464-29011
MsHel-7	gb|AIXA01002384.1|:10448-10568

MsHel-7	gb|AIXA01000939.1|:34646-35610
MsHel-7	gb|AIXA01000511.1|:8060-8813
MsHel-7	gb|AIXA01015593.1|:1722-2157
MsHel-7	gb|AIXA01022555.1|:7-565
MsHel-7	gb|AIXA01002961.1|:33629-34417
MsHel-7	gb|AIXA01002977.1|:31459-32248
MsHel-7	gb|AIXA01029453.1|:363-897
MsHel-7	gb|AIXA01005849.1|:16057-16709
MsHel-7	gb|AIXA01006834.1|:91725-92422
MsHel-7	gb|AIXA01006893.1|:9275-9624
MsHel-7	gb|AIXA01006285.1|:10554-11108
MsHel-7	gb|AIXA01004988.1|:1362-2268
MsHel-7	gb|AIXA01008296.1|:9982-10470
MsHel-7	gb|AIXA01003023.1|:25561-26031
MsHel-7	gb|AIXA01007005.1|:1-413
MsHel-7	gb|AIXA01007926.1|:32374-32944
MsHel-7	gb|AIXA01010187.1|:39464-40011
MsHel-7	gb|AIXA01001322.1|:32884-33431
MsHel-7	gb|AIXA01011269.1|:3315-3852
MsHel-7	gb|AIXA01012644.1|:27606-28433
MsHel-7	gb|AIXA01001535.1|:18090-18745
MsHel-7	gb|AIXA01009343.1|:39860-41487
MsHel-7	gb|AIXA01007210.1|:17880-18385
MsHel-7	gb|AIXA01007210.1|:32794-33313
MsHel-7	gb|AIXA01004274.1|:46-432
MsHel-7	gb|AIXA01001949.1|:6898-7420
MsHel-7	gb|AIXA01014982.1|:28840-29620
MsHel-7	gb|AIXA01009870.1|:1-829
MsHel-7	gb|AIXA01007928.1|:6565-7230
MsHel-7	gb|AIXA01006736.1|:1-396
MsHel-7	gb|AIXA01012465.1|:1-399
MsHel-7	gb|AIXA01002362.1|:92280-92811
MsHel-7	gb|AIXA01012188.1|:4627-5160
MsHel-7	gb|AIXA01009308.1|:12393-12923
MsHel-7	gb|AIXA01008416.1|:11204-11727
MsHel-7	gb|AIXA01005256.1|:24528-25050
MsHel-7	gb|AIXA01003808.1|:19416-20226
MsHel-7	gb|AIXA01002186.1|:40012-40539
MsHel-7	gb|AIXA01001239.1|:49471-50062
MsHel-7	gb|AIXA01004929.1|:51231-51760
MsHel-7	gb|AIXA01017981.1|:3562-4088
MsHel-7	gb|AIXA01001133.1|:1-940
MsHel-7	gb|AIXA01005864.1|:50589-51115
MsHel-7	gb|AIXA01005971.1|:39720-40241
MsHel-7	gb|AIXA01003613.1|:19069-19568
MsHel-7	gb|AIXA01018634.1|:1-399
MsHel-7	gb|AIXA01006520.1|:5395-6050
MsHel-7	gb|AIXA01037511.1|:9-419

MsHel-7	gb|AIXA01024398.1|:1-406
MsHel-7	gb|AIXA01004121.1|:1515-3179
MsHel-7	gb|AIXA01005904.1|:124250-124780
MsHel-7	gb|AIXA01002762.1|:5651-6173
MsHel-7	gb|AIXA01002372.1|:5846-6357
MsHel-7	gb|AIXA01000956.1|:63748-64300
MsHel-7	gb|AIXA01000713.1|:19421-19973
MsHel-7	gb|AIXA01000181.1|:9498-10024
MsHel-7	gb|AIXA01004490.1|:3-390
MsHel-7	gb|AIXA01012741.1|:85041-85838
MsHel-7	gb|AIXA01012741.1|:112377-112829
MsHel-7	gb|AIXA01010099.1|:43339-43875
MsHel-7	gb|AIXA01003798.1|:25324-25831
MsHel-7	gb|AIXA01003798.1|:47210-47787
MsHel-7	gb|AIXA01003320.1|:21314-21844
MsHel-7	gb|AIXA01001866.1|:25880-26407
MsHel-7	gb|AIXA01000101.1|:1149-1683
MsHel-7	gb|AIXA01012196.1|:24177-24674
MsHel-7	gb|AIXA01005351.1|:23924-24821
MsHel-7	gb|AIXA01010683.1|:9716-10655
MsHel-7	gb|AIXA01007652.1|:20437-20970
MsHel-7	gb|AIXA01004800.1|:33703-34524
MsHel-7	gb|AIXA01022861.1|:740-1275
MsHel-7	gb|AIXA01006659.1|:19417-19948
MsHel-7	gb|AIXA01006217.1|:1456-1963
MsHel-7	gb|AIXA01001757.1|:9508-10010
MsHel-7	gb|AIXA01013219.1|:3748-4275
MsHel-7	gb|AIXA01008029.1|:19090-19640
MsHel-7	gb|AIXA01008028.1|:22723-23229
MsHel-7	gb|AIXA01002551.1|:88811-89340
MsHel-7	gb|AIXA01000862.1|:44576-45102
MsHel-7	gb|AIXA01005080.1|:26782-27578
MsHel-7	gb|AIXA01037724.1|:47-398
MsHel-7	gb|AIXA01000423.1|:31792-32243
MsHel-7	gb|AIXA01032684.1|:211-722
MsHel-7	gb|AIXA01008120.1|:12389-12880
MsHel-7	gb|AIXA01006509.1|:46401-46927
MsHel-7	gb|AIXA01001107.1|:28405-28961
MsHel-7	gb|AIXA01027080.1|:1-526
MsHel-7	gb|AIXA01006482.1|:31376-31890
MsHel-7	gb|AIXA01006194.1|:24701-25121
MsHel-7	gb|AIXA01010110.1|:3859-4515
MsHel-7	gb|AIXA01032999.1|:1-544
MsHel-7	gb|AIXA01005375.1|:48047-48538
MsHel-7	gb|AIXA01005003.1|:1-375
MsHel-7	gb|AIXA01008043.1|:31247-31793
MsHel-7	gb|AIXA01005339.1|:24154-25073
MsHel-7	gb|AIXA01021630.1|:1-512

MsHel-7	gb|AIXA01004374.1|:555-1218
MsHel-7	gb|AIXA01007692.1|:19495-20001
MsHel-7	gb|AIXA01012329.1|:14550-15053
MsHel-7	gb|AIXA01005376.1|:1-345
MsHel-7	gb|AIXA01004051.1|:1-341
MsHel-7	gb|AIXA01002460.1|:621-1128
MsHel-7	gb|AIXA01007454.1|:725-1408
MsHel-7	gb|AIXA01000388.1|:1485-1987
MsHel-7	gb|AIXA01001038.1|:1971-2962
MsHel-7	gb|AIXA01007582.1|:19226-19775
MsHel-7	gb|AIXA01006123.1|:4417-4923
MsHel-7	gb|AIXA01003478.1|:34650-35788
MsHel-7	gb|AIXA01010504.1|:462-1042
MsHel-7	gb|AIXA01008444.1|:55094-56030
MsHel-7	gb|AIXA01015923.1|:1653-2163
MsHel-7	gb|AIXA01011942.1|:9034-9519
MsHel-7	gb|AIXA01006595.1|:59732-60068
MsHel-7	gb|AIXA01004427.1|:1-341
MsHel-7	gb|AIXA01010916.1|:2144-3079
MsHel-7	gb|AIXA01009355.1|:34832-35370
MsHel-7	gb|AIXA01008896.1|:3942-4426
MsHel-7	gb|AIXA01002337.1|:10563-10900
MsHel-7	gb|AIXA01001283.1|:15424-15943
MsHel-7	gb|AIXA01026979.1|:2-361
MsHel-7	gb|AIXA01005089.1|:5762-6552
MsHel-7	gb|AIXA01018536.1|:9003-9488
MsHel-7	gb|AIXA01003868.1|:126203-126639
MsHel-7	gb|AIXA01000881.1|:18584-19197
MsHel-7	gb|AIXA01000085.1|:15-504
MsHel-7	gb|AIXA01001017.1|:52423-52867
MsHel-7	gb|AIXA01023496.1|:551-1054
MsHel-7	gb|AIXA01013591.1|:2-487
MsHel-7	gb|AIXA01009267.1|:10162-10935
MsHel-7	gb|AIXA01015554.1|:641-1089
MsHel-7	gb|AIXA01001905.1|:15503-15980
MsHel-7	gb|AIXA01001228.1|:11426-12023
MsHel-7	gb|AIXA01003749.1|:3-328
MsHel-7	gb|AIXA01007121.1|:28298-28923
MsHel-7	gb|AIXA01033451.1|:1-325
MsHel-7	gb|AIXA01015083.1|:13691-14362
MsHel-7	gb|AIXA01007175.1|:91007-91520
MsHel-7	gb|AIXA01005432.1|:4869-5575
MsHel-7	gb|AIXA01004965.1|:1-480
MsHel-7	gb|AIXA01004465.1|:151544-152126
MsHel-7	gb|AIXA01009894.1|:11436-12223
MsHel-7	gb|AIXA01007429.1|:645-1425
MsHel-7	gb|AIXA01002264.1|:43552-44005
MsHel-7	gb|AIXA01007428.1|:117319-117822

MsHel-7	gb|AIXA01001124.1|:11073-11749
MsHel-7	gb|AIXA01009679.1|:1-355
MsHel-7	gb|AIXA01007015.1|:48133-48787
MsHel-7	gb|AIXA01006381.1|:21483-21910
MsHel-7	gb|AIXA01007476.1|:1653-2468
MsHel-7	gb|AIXA01021042.1|:1-299
MsHel-7	gb|AIXA01000717.1|:23532-24610
MsHel-7	gb|AIXA01000989.1|:1017-1435
MsHel-7	gb|AIXA01014322.1|:1209-1619
MsHel-7	gb|AIXA01001532.1|:2-277
MsHel-7	gb|AIXA01003257.1|:18741-19161
MsHel-7	gb|AIXA01001970.1|:1-367
MsHel-7	gb|AIXA01001046.1|:40083-40609
MsHel-7	gb|AIXA01002311.1|:1121-1396
MsHel-7	gb|AIXA01036785.1|:1-298
MsHel-7	gb|AIXA01007966.1|:6833-7333
MsHel-7	gb|AIXA01037065.1|:1-278
MsHel-7	gb|AIXA01026119.1|:3-463
MsHel-7	gb|AIXA01000714.1|:10140-10553
MsHel-7	gb|AIXA01003594.1|:15365-15805
MsHel-7	gb|AIXA01001221.1|:67057-67524
MsHel-7	gb|AIXA01002567.1|:13671-14123
MsHel-7	gb|AIXA01000437.1|:63720-64100
MsHel-7	gb|AIXA01010758.1|:10099-10486
MsHel-7	gb|AIXA01004964.1|:1-286
MsHel-7	gb|AIXA01004964.1|:13182-13303
MsHel-7	gb|AIXA01001929.1|:1-417
MsHel-7	gb|AIXA01010747.1|:23879-25362
MsHel-7	gb|AIXA01006196.1|:203-487
MsHel-7	gb|AIXA01031287.1|:373-792
MsHel-7	gb|AIXA01007164.1|:55817-56087
MsHel-7	gb|AIXA01006270.1|:8096-8487
MsHel-7	gb|AIXA01008215.1|:23165-23628
MsHel-7	gb|AIXA01002599.1|:1-265
MsHel-7	gb|AIXA01002620.1|:3013-3601
MsHel-7	gb|AIXA01014101.1|:29647-30036
MsHel-7	gb|AIXA01013527.1|:22917-23386
MsHel-7	gb|AIXA01033437.1|:293-685
MsHel-7	gb|AIXA01029796.1|:470-873
MsHel-7	gb|AIXA01002956.1|:16077-16461
MsHel-7	gb|AIXA01014886.1|:1-255
MsHel-7	gb|AIXA01008169.1|:9313-10001
MsHel-7	gb|AIXA01006519.1|:23749-24142
MsHel-7	gb|AIXA01003825.1|:30462-31187
MsHel-7	gb|AIXA01008342.1|:1-423
MsHel-7	gb|AIXA01004239.1|:1757-2223
MsHel-7	gb|AIXA01008247.1|:7358-7964
MsHel-7	gb|AIXA01014326.1|:9435-10151

MsHel-7	gb|AIXA01003605.1|:2657-3084
MsHel-7	gb|AIXA01009361.1|:10379-10855
MsHel-7	gb|AIXA01005001.1|:43857-44470
MsHel-7	gb|AIXA01008335.1|:38397-38784
MsHel-7	gb|AIXA01012375.1|:37610-38116
MsHel-7	gb|AIXA01007750.1|:23678-24218
MsHel-7	gb|AIXA01016850.1|:1943-2376
MsHel-7	gb|AIXA01014734.1|:4297-5125
MsHel-7	gb|AIXA01008024.1|:4-380
MsHel-7	gb|AIXA01022910.1|:1207-1594
MsHel-7	gb|AIXA01012399.1|:15037-15404
MsHel-7	gb|AIXA01001278.1|:28363-28738
MsHel-7	gb|AIXA01008232.1|:22-264
MsHel-7	gb|AIXA01009014.1|:11583-11996
MsHel-7	gb|AIXA01001601.1|:9019-9510
MsHel-7	gb|AIXA01000641.1|:67010-67535
MsHel-7	gb|AIXA01007581.1|:8919-9414
MsHel-7	gb|AIXA01004963.1|:10002-10220
MsHel-7	gb|AIXA01003042.1|:159917-160289
MsHel-7	gb|AIXA01019512.1|:301-806
MsHel-7	gb|AIXA01026376.1|:1-357
MsHel-7	gb|AIXA01015485.1|:8941-9341
MsHel-7	gb|AIXA01037619.1|:106-522
MsHel-7	gb|AIXA01007982.1|:5852-6339
MsHel-7	gb|AIXA01004294.1|:915-1482
MsHel-7	gb|AIXA01002763.1|:17229-17720
MsHel-7	gb|AIXA01001482.1|:1601-2173
MsHel-7	gb|AIXA01003879.1|:16535-16882
MsHel-7	gb|AIXA01002287.1|:4807-5176
MsHel-7	gb|AIXA01008578.1|:27219-27700
MsHel-7	gb|AIXA01007060.1|:2756-3249
MsHel-7	gb|AIXA01002057.1|:35721-35958
MsHel-7	gb|AIXA01005263.1|:1840-2452
MsHel-7	gb|AIXA01007324.1|:6355-6574
MsHel-7	gb|AIXA01004400.1|:42946-43448
MsHel-7	gb|AIXA01022061.1|:1395-1739
MsHel-7	gb|AIXA01015887.1|:12955-13327
MsHel-7	gb|AIXA01006219.1|:1-502
MsHel-7	gb|AIXA01001504.1|:25778-26302
MsHel-7	gb|AIXA01011285.1|:17925-18419
MsHel-7	gb|AIXA01011285.1|:162156-162680
MsHel-7	gb|AIXA01000147.1|:19311-19815
MsHel-7	gb|AIXA01000144.1|:19500-20004
MsHel-7	gb|AIXA01022178.1|:17-379
MsHel-7	gb|AIXA01013794.1|:5662-6166
MsHel-7	gb|AIXA01009711.1|:5810-6319
MsHel-7	gb|AIXA01006922.1|:25478-26060
MsHel-7	gb|AIXA01004802.1|:27755-28241

MsHel-7	gb|AIXA01004230.1|:62218-62717
MsHel-7	gb|AIXA01000415.1|:25551-26056
MsHel-7	gb|AIXA01005399.1|:14410-14931
MsHel-7	gb|AIXA01004793.1|:5330-5685
MsHel-7	gb|AIXA01013105.1|:23-385
MsHel-7	gb|AIXA01011840.1|:6642-7103
MsHel-7	gb|AIXA01009518.1|:11207-11713
MsHel-7	gb|AIXA01003610.1|:15-362
MsHel-7	gb|AIXA01021757.1|:2-486
MsHel-7	gb|AIXA01012417.1|:1-460
MsHel-7	gb|AIXA01008996.1|:16324-16841
MsHel-7	gb|AIXA01004948.1|:31858-32363
MsHel-7	gb|AIXA01002331.1|:3748-4251
MsHel-7	gb|AIXA01026014.1|:829-1163
MsHel-7	gb|AIXA01016326.1|:5493-5839
MsHel-7	gb|AIXA01008636.1|:82788-83271
MsHel-7	gb|AIXA01029445.1|:1-212
MsHel-7	gb|AIXA01017156.1|:118-618
MsHel-7	gb|AIXA01012581.1|:3089-3596
MsHel-7	gb|AIXA01008966.1|:18629-19138
MsHel-7	gb|AIXA01007352.1|:13787-14288
MsHel-7	gb|AIXA01001486.1|:404-905
MsHel-7	gb|AIXA01001176.1|:16360-16857
MsHel-7	gb|AIXA01009951.1|:7149-7838
MsHel-7	gb|AIXA01005536.1|:1-492
MsHel-7	gb|AIXA01009508.1|:28780-29278
MsHel-7	gb|AIXA01028826.1|:1-352
MsHel-7	gb|AIXA01007333.1|:1475-1988
MsHel-7	gb|AIXA01005095.1|:2069-2503
MsHel-7	gb|AIXA01004990.1|:107614-108038
MsHel-7	gb|AIXA01014996.1|:32030-32394
MsHel-7	gb|AIXA01006890.1|:2-205
MsHel-7	gb|AIXA01003176.1|:32290-32638
MsHel-7	gb|AIXA01018938.1|:2467-2830
MsHel-7	gb|AIXA01009287.1|:28632-28872
MsHel-7	gb|AIXA01010957.1|:6607-6965
MsHel-7	gb|AIXA01027565.1|:113-623
MsHel-7	gb|AIXA01012279.1|:38114-38613
MsHel-7	gb|AIXA01010739.1|:3193-3531
MsHel-7	gb|AIXA01027944.1|:1-258
MsHel-7	gb|AIXA01026475.1|:1-204
MsHel-7	gb|AIXA01017070.1|:15597-15945
MsHel-7	gb|AIXA01012458.1|:34467-34986
MsHel-7	gb|AIXA01007801.1|:13203-13680
MsHel-7	gb|AIXA01007493.1|:36521-37644
MsHel-7	gb|AIXA01000772.1|:6423-6893
MsHel-7	gb|AIXA01000542.1|:168463-168973
MsHel-7	gb|AIXA01014681.1|:4-336

MsHel-7	gb|AIXA01011860.1|:27-221
MsHel-7	gb|AIXA01000600.1|:1-296
MsHel-7	gb|AIXA01018595.1|:327-755
MsHel-7	gb|AIXA01005592.1|:10357-10862
MsHel-7	gb|AIXA01013407.1|:21367-21863
MsHel-7	gb|AIXA01007790.1|:39491-39668
MsHel-7	gb|AIXA01024917.1|:987-1285
MsHel-7	gb|AIXA01005827.1|:48628-49147
MsHel-7	gb|AIXA01012499.1|:2-504
MsHel-7	gb|AIXA01008328.1|:2-177
MsHel-7	gb|AIXA01005471.1|:25723-26223
MsHel-7	gb|AIXA01000555.1|:4751-5466
MsHel-7	gb|AIXA01022372.1|:1383-1710
MsHel-7	gb|AIXA01015631.1|:2385-2555
MsHel-7	gb|AIXA01005719.1|:1981-2277
MsHel-7	gb|AIXA01004368.1|:2534-2862
MsHel-7	gb|AIXA01001483.1|:56017-56602
MsHel-7	gb|AIXA01013092.1|:32642-32988
MsHel-7	gb|AIXA01011897.1|:908-1421
MsHel-7	gb|AIXA01010111.1|:1871-2398
MsHel-7	gb|AIXA01007451.1|:5779-6280
MsHel-7	gb|AIXA01006805.1|:21186-21620
MsHel-7	gb|AIXA01005692.1|:10811-11311
MsHel-7	gb|AIXA01003848.1|:4535-5033
MsHel-7	gb|AIXA01003588.1|:35762-36101
MsHel-7	gb|AIXA01001182.1|:37474-37974
MsHel-7	gb|AIXA01009761.1|:8864-9363
MsHel-7	gb|AIXA01013420.1|:6513-6622
MsHel-7	gb|AIXA01008872.1|:14645-15132
MsHel-7	gb|AIXA01004314.1|:741-923
MsHel-7	gb|AIXA01003967.1|:23560-24012
MsHel-7	gb|AIXA01003651.1|:115608-115891
MsHel-7	gb|AIXA01000247.1|:6-179
MsHel-7	gb|AIXA01028501.1|:1-169
MsHel-7	gb|AIXA01015648.1|:236-396
MsHel-7	gb|AIXA01011964.1|:10528-11031
MsHel-7	gb|AIXA01010760.1|:11128-11629
MsHel-7	gb|AIXA01009752.1|:20639-21138
MsHel-7	gb|AIXA01008509.1|:51581-52045
MsHel-7	gb|AIXA01008505.1|:20323-20825
MsHel-7	gb|AIXA01008234.1|:8348-8848
MsHel-7	gb|AIXA01008054.1|:16129-16629
MsHel-7	gb|AIXA01007955.1|:6157-6679
MsHel-7	gb|AIXA01007606.1|:55043-55543
MsHel-7	gb|AIXA01006011.1|:23526-24026
MsHel-7	gb|AIXA01005548.1|:28838-29337
MsHel-7	gb|AIXA01005097.1|:8261-8765
MsHel-7	gb|AIXA01004936.1|:38855-39194

MsHel-7	gb|AIXA01004413.1|:45145-45645
MsHel-7	gb|AIXA01002592.1|:2-325
MsHel-7	gb|AIXA01001312.1|:6444-6948
MsHel-7	gb|AIXA01001251.1|:15902-16402
MsHel-7	gb|AIXA01013255.1|:24950-25452
MsHel-7	gb|AIXA01001132.1|:1-167
MsHel-7	gb|AIXA01013328.1|:43488-44004
MsHel-7	gb|AIXA01003055.1|:15506-15986
MsHel-7	gb|AIXA01001708.1|:6007-6512
MsHel-7	gb|AIXA01001555.1|:17781-18285
MsHel-7	gb|AIXA01000103.1|:554-715
MsHel-7	gb|AIXA01035051.1|:10-445
MsHel-7	gb|AIXA01014125.1|:252-412
MsHel-7	gb|AIXA01011852.1|:1-157
MsHel-7	gb|AIXA01009133.1|:11861-12361
MsHel-7	gb|AIXA01007631.1|:8447-8943
MsHel-7	gb|AIXA01007211.1|:11998-12498
MsHel-7	gb|AIXA01007168.1|:116407-116909
MsHel-7	gb|AIXA01006881.1|:46208-46708
MsHel-7	gb|AIXA01006493.1|:73484-73827
MsHel-7	gb|AIXA01005847.1|:21344-21843
MsHel-7	gb|AIXA01001667.1|:94255-94594
MsHel-7	gb|AIXA01001022.1|:120-620
MsHel-7	gb|AIXA01000091.1|:126298-126798
MsHel-7	gb|AIXA01035622.1|:7-170
MsHel-7	gb|AIXA01034573.1|:1-360
MsHel-7	gb|AIXA01005378.1|:125990-126201
MsHel-7	gb|AIXA01004738.1|:15903-16386
MsHel-7	gb|AIXA01000466.1|:2-168
MsHel-7	gb|AIXA01010322.1|:18823-19157
MsHel-7	gb|AIXA01020984.1|:823-975
MsHel-7	gb|AIXA01011707.1|:20293-20792
MsHel-7	gb|AIXA01010590.1|:31673-31996
MsHel-7	gb|AIXA01009832.1|:7502-8003
MsHel-7	gb|AIXA01008927.1|:48295-48798
MsHel-7	gb|AIXA01004891.1|:1-165
MsHel-7	gb|AIXA01003365.1|:35835-36268
MsHel-7	gb|AIXA01001731.1|:20934-21434
MsHel-7	gb|AIXA01001613.1|:80347-80810
MsHel-7	gb|AIXA01000117.1|:21881-22380
MsHel-7	gb|AIXA01011233.1|:15368-15872
MsHel-7	gb|AIXA01001919.1|:17833-18337
MsHel-7	gb|AIXA01021245.1|:1821-2125
MsHel-7	gb|AIXA01014005.1|:15506-15873
MsHel-7	gb|AIXA01011650.1|:67405-67905
MsHel-7	gb|AIXA01003121.1|:40153-40650
MsHel-7	gb|AIXA01003037.1|:47240-47739
MsHel-7	gb|AIXA01014490.1|:115-269

MsHel-7	gb|AIXA01011992.1|:40898-41228
MsHel-7	gb|AIXA01002658.1|:85407-85637
MsHel-7	gb|AIXA01000990.1|:910-1076
MsHel-7	gb|AIXA01009835.1|:29175-29336
MsHel-7	gb|AIXA01007216.1|:118326-118623
MsHel-7	gb|AIXA01004135.1|:27686-27980
MsHel-7	gb|AIXA01003953.1|:33840-34150
MsHel-7	gb|AIXA01029024.1|:7-186
MsHel-7	gb|AIXA01012527.1|:8106-8602
MsHel-7	gb|AIXA01008294.1|:11028-11525
MsHel-7	gb|AIXA01001061.1|:14695-15196
MsHel-7	gb|AIXA01006604.1|:19866-20370
MsHel-7	gb|AIXA01004868.1|:16292-16636
MsHel-7	gb|AIXA01002832.1|:218-523
MsHel-7	gb|AIXA01002531.1|:63008-63511
MsHel-7	gb|AIXA01038052.1|:34-227
MsHel-7	gb|AIXA01017159.1|:1-146
MsHel-7	gb|AIXA01004559.1|:42-370
MsHel-7	gb|AIXA01011226.1|:1853-2157
MsHel-7	gb|AIXA01032428.1|:141-446
MsHel-7	gb|AIXA01023357.1|:1092-1513
MsHel-7	gb|AIXA01018939.1|:1-147
MsHel-7	gb|AIXA01009492.1|:2-351
MsHel-7	gb|AIXA01005092.1|:11618-12229
MsHel-7	gb|AIXA01000982.1|:45558-46044
MsHel-7	gb|AIXA01002209.1|:722-1020
MsHel-7	gb|AIXA01003249.1|:317-623
MsHel-7	gb|AIXA01008171.1|:323-628
MsHel-7	gb|AIXA01009691.1|:2-302
MsHel-7	gb|AIXA01006302.1|:5703-6256
MsHel-7	gb|AIXA01006484.1|:570-874
MsHel-7	gb|AIXA01005437.1|:2-145
MsHel-7	gb|AIXA01001389.1|:1-172
MsHel-7	gb|AIXA01017906.1|:1-147
MsHel-7	gb|AIXA01003591.1|:11994-12422
MsHel-7	gb|AIXA01036040.1|:2-202
MsHel-7	gb|AIXA01035446.1|:1-168
MsHel-7	gb|AIXA01016761.1|:1118-1596
MsHel-7	gb|AIXA01034396.1|:7-310
MsHel-7	gb|AIXA01003253.1|:11199-11772
MsHel-7	gb|AIXA01034668.1|:344-630
MsHel-7	gb|AIXA01003989.1|:35960-36236
MsHel-7	gb|AIXA01036087.1|:316-573
MsHel-7	gb|AIXA01014123.1|:3327-3523
MsHel-7	gb|AIXA01013510.1|:13136-13321
MsHel-7	gb|AIXA01009324.1|:22221-22501
MsHel-7	gb|AIXA01007620.1|:20857-21058
MsHel-7	gb|AIXA01005278.1|:107002-107123

MsHel-7	gb|AIXA01001877.1|:194627-194893
MsHel-7	gb|AIXA01001831.1|:13091-13624
MsHel-7	gb|AIXA01000352.1|:1-142
MsHel-7	gb|AIXA01000281.1|:22773-22998
MsHel-7	gb|AIXA01027673.1|:805-1021
MsHel-7	gb|AIXA01009309.1|:1-306
MsHel-7	gb|AIXA01001531.1|:27299-27549
MsHel-7	gb|AIXA01001531.1|:72954-73141
MsHel-7	gb|AIXA01000655.1|:1918-2038
MsHel-7	gb|AIXA01000364.1|:5382-5502
MsHel-7	gb|AIXA01014885.1|:1817-2046
MsHel-7	gb|AIXA01004090.1|:19065-19572
MsHel-7	gb|AIXA01001571.1|:38812-39115
MsHel-7	gb|AIXA01038342.1|:304-425
MsHel-7	gb|AIXA01032938.1|:561-682
MsHel-7	gb|AIXA01030789.1|:662-783
MsHel-7	gb|AIXA01029600.1|:765-886
MsHel-7	gb|AIXA01016514.1|:2897-3018
MsHel-7	gb|AIXA01015812.1|:15614-15859
MsHel-7	gb|AIXA01011875.1|:1193-1314
MsHel-7	gb|AIXA01011014.1|:2654-2883
MsHel-7	gb|AIXA01007914.1|:36862-38678
MsHel-7	gb|AIXA01007836.1|:13075-13196
MsHel-7	gb|AIXA01005951.1|:3181-3502
MsHel-7	gb|AIXA01004097.1|:28485-28606
MsHel-7	gb|AIXA01002988.1|:82500-82776
MsHel-7	gb|AIXA01002293.1|:47434-47555
MsHel-7	gb|AIXA01002178.1|:11798-12001
MsHel-7	gb|AIXA01001840.1|:8026-8230
MsHel-7	gb|AIXA01000597.1|:48171-48292
MsHel-7	gb|AIXA01030823.1|:670-790
MsHel-7	gb|AIXA01030328.1|:561-843
MsHel-7	gb|AIXA01011720.1|:13217-13563
MsHel-7	gb|AIXA01035109.1|:214-496
MsHel-7	gb|AIXA01028407.1|:750-869
MsHel-7	gb|AIXA01004841.1|:90622-90741
MsHel-7	gb|AIXA01015421.1|:2970-3088
MsHel-7	gb|AIXA01000770.1|:20166-20444
MsHel-7	gb|AIXA01028349.1|:848-969
MsHel-7	gb|AIXA01025981.1|:951-1134
MsHel-7	gb|AIXA01021066.1|:1696-1899
MsHel-7	gb|AIXA01018071.1|:3528-3649
MsHel-7	gb|AIXA01016794.1|:6532-6710
MsHel-7	gb|AIXA01012853.1|:18688-18909
MsHel-7	gb|AIXA01009976.1|:49023-49373
MsHel-7	gb|AIXA01009665.1|:17533-17654
MsHel-7	gb|AIXA01006130.1|:18510-18685
MsHel-7	gb|AIXA01005270.1|:17799-17920

MsHel-7	gb|AIXA01003860.1|:39769-39890
MsHel-7	gb|AIXA01003713.1|:42553-42787
MsHel-7	gb|AIXA01002736.1|:15137-15477
MsHel-7	gb|AIXA01001747.1|:50418-50625
MsHel-7	gb|AIXA01001692.1|:46951-47072
MsHel-7	gb|AIXA01001673.1|:72677-72894
MsHel-7	gb|AIXA01000097.1|:16553-16674
MsHel-7	gb|AIXA01034742.1|:505-625
MsHel-7	gb|AIXA01033202.1|:1-232
MsHel-7	gb|AIXA01032694.1|:576-696
MsHel-7	gb|AIXA01031683.1|:622-742
MsHel-7	gb|AIXA01009235.1|:6517-6742
MsHel-7	gb|AIXA01008331.1|:3115-3378
MsHel-7	gb|AIXA01007239.1|:42064-42184
MsHel-7	gb|AIXA01006889.1|:32924-33044
MsHel-7	gb|AIXA01005002.1|:23257-23377
MsHel-7	gb|AIXA01002699.1|:33583-33695
MsHel-7	gb|AIXA01000948.1|:41305-41421
MsHel-7	gb|AIXA01000949.1|:3-122
MsHel-7	gb|AIXA01000599.1|:6696-6815
MsHel-7	gb|AIXA01037884.1|:310-511
MsHel-7	gb|AIXA01027649.1|:901-1019
MsHel-7	gb|AIXA01016222.1|:1453-1575
MsHel-7	gb|AIXA01011363.1|:19043-19297
MsHel-7	gb|AIXA01009028.1|:32432-32542
MsHel-7	gb|AIXA01002868.1|:23165-23470
MsHel-7	gb|AIXA01036019.1|:361-578
MsHel-7	gb|AIXA01034790.1|:375-624
MsHel-7	gb|AIXA01033819.1|:1-572
MsHel-7	gb|AIXA01012117.1|:66340-66461
MsHel-7	gb|AIXA01011105.1|:2-260
MsHel-7	gb|AIXA01011105.1|:21674-21795
MsHel-7	gb|AIXA01008529.1|:26042-26151
MsHel-7	gb|AIXA01008166.1|:7261-7802
MsHel-7	gb|AIXA01005856.1|:21179-21296
MsHel-7	gb|AIXA01003842.1|:6658-6779
MsHel-7	gb|AIXA01003292.1|:59078-59269
MsHel-7	gb|AIXA01032362.1|:1-270
MsHel-7	gb|AIXA01009674.1|:1533-1653
MsHel-7	gb|AIXA01004063.1|:5093-5366
MsHel-7	gb|AIXA01002918.1|:1-117
MsHel-7	gb|AIXA01002980.1|:9406-9708
MsHel-7	gb|AIXA01035422.1|:2-124
MsHel-7	gb|AIXA01023315.1|:731-850
MsHel-7	gb|AIXA01007028.1|:29164-29423
MsHel-7	gb|AIXA01006464.1|:1155-1273
MsHel-7	gb|AIXA01001045.1|:48972-49174
MsHel-7	gb|AIXA01000837.1|:11912-12034

MsHel-7	gb|AIXA01000530.1|:25469-25593
MsHel-7	gb|AIXA01000109.1|:1-123
MsHel-7	gb|AIXA01016849.1|:2814-2996
MsHel-7	gb|AIXA01013565.1|:49676-49897
MsHel-7	gb|AIXA01012976.1|:13812-14039
MsHel-7	gb|AIXA01011392.1|:10375-10496
MsHel-7	gb|AIXA01005860.1|:34974-35095
MsHel-7	gb|AIXA01000237.1|:38095-38320
MsHel-7	gb|AIXA01015983.1|:1342-1809
MsHel-7	gb|AIXA01013782.1|:16692-16809
MsHel-7	gb|AIXA01005109.1|:7269-7381
MsHel-7	gb|AIXA01001928.1|:7541-7658
MsHel-7	gb|AIXA01037107.1|:429-531
MsHel-7	gb|AIXA01013785.1|:4247-4349
MsHel-7	gb|AIXA01009596.1|:15313-15437
MsHel-7	gb|AIXA01001105.1|:23262-23388
MsHel-7	gb|AIXA01012464.1|:2460-2712
MsHel-7	gb|AIXA01017203.1|:13246-13458
MsHel-7	gb|AIXA01014144.1|:2252-2356
MsHel-7	gb|AIXA01005391.1|:44993-45093
MsHel-7	gb|AIXA01037878.1|:1-123
MsHel-7	gb|AIXA01001341.1|:109559-109656
MsHel-7	gb|AIXA01017110.1|:4924-5020
MsHel-7	gb|AIXA01001969.1|:14996-15100
MsHel-7	gb|AIXA01006589.1|:9759-9862
MsHel-7	gb|AIXA01004831.1|:147054-147149
MsHel-7	gb|AIXA01006722.1|:74938-75214
MsHel-7	gb|AIXA01000465.1|:8074-8167
MsHel-7	gb|AIXA01018924.1|:3018-3110
MsHel-7	gb|AIXA01013111.1|:1915-2158
MsHel-7	gb|AIXA01024647.1|:1-100
MsHel-7	gb|AIXA01004489.1|:94122-94229
MsHel-7	gb|AIXA01013535.1|:1-103
MsHel-7	gb|AIXA01004673.1|:10963-11061
MsHel-7	gb|AIXA01037753.1|:422-519
MsHel-7	gb|AIXA01005242.1|:8723-8812
MsHel-7	gb|AIXA01004221.1|:3250-3523
MsHel-7	gb|AIXA01001073.1|:1-101
MsHel-7	gb|AIXA01035943.1|:1-261
MsHel-7	gb|AIXA01019001.1|:140-403
MsHel-7	gb|AIXA01007209.1|:3647-3738
MsHel-7	gb|AIXA01001273.1|:62256-62504
MsHel-7	gb|AIXA01009666.1|:1-95
MsHel-7	gb|AIXA01006728.1|:3224-3313
MsHel-7	gb|AIXA01005535.1|:31647-31745
MsHel-7	gb|AIXA01034860.1|:534-619
MsHel-7	gb|AIXA01008128.1|:7661-7758
MsHel-7	gb|AIXA01003965.1|:28387-28502

MsHel-7	gb|AIXA01001106.1|:2-253
MsHel-7	gb|AIXA01014928.1|:867-1017
MsHel-7	gb|AIXA01009869.1|:4440-4622
MsHel-7	gb|AIXA01005640.1|:7736-7830
MsHel-7	gb|AIXA01016399.1|:8475-8561
MsHel-7	gb|AIXA01013701.1|:1-95
MsHel-7	gb|AIXA01027683.1|:932-1021
MsHel-7	gb|AIXA01005030.1|:65538-65861
MsHel-7	gb|AIXA01008176.1|:11445-11573
MsHel-7	gb|AIXA01019884.1|:4-154
MsHel-7	gb|AIXA01010370.1|:1-103
MsHel-7	gb|AIXA01004932.1|:5390-5472
MsHel-7	gb|AIXA01034895.1|:535-620
MsHel-7	gb|AIXA01010509.1|:7421-8946
MsHel-7	gb|AIXA01002757.1|:6-95
MsHel-7	gb|AIXA01011678.1|:7119-7276
MsHel-7	gb|AIXA01001635.1|:19371-19450
MsHel-7	gb|AIXA01008807.1|:1-90
MsHel-7	gb|AIXA01015107.1|:4264-4597
MsHel-7	gb|AIXA01002812.1|:73367-73486
MsHel-7	gb|AIXA01035983.1|:1-81
MsHel-7	gb|AIXA01011633.1|:1-129
MsHel-7	gb|AIXA01028517.1|:1-234
MsHel-7	gb|AIXA01005257.1|:170-429
MsHel-7	gb|AIXA01000598.1|:1-232
MsHel-7	gb|AIXA01002398.1|:101319-101451
MsHel-7	gb|AIXA01027933.1|:924-1001
MsHel-7	gb|AIXA01011993.1|:29069-29289
MsHel-7	gb|AIXA01004807.1|:3674-3924
MsHel-7	gb|AIXA01015262.1|:1-224
MsHel-7	gb|AIXA01037714.1|:1-225
MsHel-7	gb|AIXA01029683.1|:1-510
MsHel-7	gb|AIXA01034141.1|:548-652
MsHel-7	gb|AIXA01028703.1|:12-208
MsHel-7	gb|AIXA01008167.1|:1-193
MsHel-7	gb|AIXA01021502.1|:1-202
MsHel-7	gb|AIXA01013002.1|:1-197
MsHel-7	gb|AIXA01009972.1|:13067-13273
MsHel-7	gb|AIXA01006590.1|:6-202
MsHel-7	gb|AIXA01006122.1|:1-199
MsHel-7	gb|AIXA01004546.1|:73009-73230
MsHel-7	gb|AIXA01003843.1|:2-219
MsHel-1	gb|AIXA01008158.1|:37174-37670
MsHel-1	gb|AIXA01008158.1|:13414-15533
MsHel-1	gb|AIXA01000885.1|:15675-16077
MsHel-1	gb|AIXA01004482.1|:44620-45431
MsHel-1	gb|AIXA01008667.1|:8968-9232
MsHel-1	gb|AIXA01000823.1|:36104-36937

MsHel-1	gb|AIXA01000388.1|:31622-32118
MsHel-1	gb|AIXA01005309.1|:37125-37959
MsHel-1	gb|AIXA01005763.1|:22597-22833
MsHel-1	gb|AIXA01002232.1|:23012-24974
MsHel-1	gb|AIXA01001593.1|:69444-70137
MsHel-1	gb|AIXA01007071.1|:29322-34659
MsHel-1	gb|AIXA01007071.1|:3977-4804
MsHel-1	gb|AIXA01014629.1|:29307-30139
MsHel-1	gb|AIXA01008972.1|:4733-5558
MsHel-1	gb|AIXA01004552.1|:37915-38748
MsHel-1	gb|AIXA01003117.1|:15775-16477
MsHel-1	gb|AIXA01007584.1|:34501-35335
MsHel-1	gb|AIXA01007584.1|:17946-18811
MsHel-1	gb|AIXA01001415.1|:38610-38769
MsHel-1	gb|AIXA01001408.1|:40434-41269
MsHel-1	gb|AIXA01000150.1|:28503-29329
MsHel-1	gb|AIXA01005415.1|:98295-98717
MsHel-1	gb|AIXA01005415.1|:17890-18576
MsHel-1	gb|AIXA01000725.1|:45662-45943
MsHel-1	gb|AIXA01000725.1|:570-1295
MsHel-1	gb|AIXA01009221.1|:1948-2764
MsHel-1	gb|AIXA01001610.1|:18907-19734
MsHel-1	gb|AIXA01012253.1|:33748-34590
MsHel-1	gb|AIXA01002613.1|:1100-1933
MsHel-1	gb|AIXA01004070.1|:1819-2652
MsHel-1	gb|AIXA01002614.1|:8157-8945
MsHel-1	gb|AIXA01004644.1|:2511-3341
MsHel-1	gb|AIXA01002650.1|:6488-7321
MsHel-1	gb|AIXA01008185.1|:13315-14147
MsHel-1	gb|AIXA01005154.1|:4640-5473
MsHel-1	gb|AIXA01004381.1|:9167-10000
MsHel-1	gb|AIXA01004806.1|:4641-5607
MsHel-1	gb|AIXA01013112.1|:4559-5384
MsHel-1	gb|AIXA01000984.1|:39899-40394
MsHel-1	gb|AIXA01005827.1|:11778-12610
MsHel-1	gb|AIXA01018005.1|:4904-7440
MsHel-1	gb|AIXA01014643.1|:32498-32657
MsHel-1	gb|AIXA01009466.1|:15595-16420
MsHel-1	gb|AIXA01002182.1|:41585-42417
MsHel-1	gb|AIXA01002600.1|:24241-25071
MsHel-1	gb|AIXA01008562.1|:59267-59763
MsHel-1	gb|AIXA01007630.1|:99228-99724
MsHel-1	gb|AIXA01007630.1|:56221-56412
MsHel-1	gb|AIXA01013067.1|:7001-7201
MsHel-1	gb|AIXA01011785.1|:6822-8352
MsHel-1	gb|AIXA01009777.1|:19083-19915
MsHel-1	gb|AIXA01004187.1|:95369-95562
MsHel-1	gb|AIXA01004187.1|:76295-77133

MsHel-1	gb|AIXA01001076.1|:2991-3823
MsHel-1	gb|AIXA01018303.1|:4184-4674
MsHel-1	gb|AIXA01010593.1|:11745-12306
MsHel-1	gb|AIXA01006841.1|:40464-41291
MsHel-1	gb|AIXA01006215.1|:73350-74157
MsHel-1	gb|AIXA01002847.1|:20134-20971
MsHel-1	gb|AIXA01002215.1|:31082-31543
MsHel-1	gb|AIXA01001305.1|:939-1890
MsHel-1	gb|AIXA01006693.1|:32949-33700
MsHel-1	gb|AIXA01000503.1|:40248-41078
MsHel-1	gb|AIXA01001367.1|:2349-3183
MsHel-1	gb|AIXA01000005.1|:163201-163292
MsHel-1	gb|AIXA01000005.1|:1588-2166
MsHel-1	gb|AIXA01010773.1|:56106-56938
MsHel-1	gb|AIXA01007854.1|:107986-108070
MsHel-1	gb|AIXA01007854.1|:65851-66020
MsHel-1	gb|AIXA01006595.1|:20528-21355
MsHel-1	gb|AIXA01008385.1|:34611-35211
MsHel-1	gb|AIXA01008385.1|:17306-18135
MsHel-1	gb|AIXA01007595.1|:55215-56048
MsHel-1	gb|AIXA01014222.1|:17987-18820
MsHel-1	gb|AIXA01013992.1|:3757-4592
MsHel-1	gb|AIXA01006952.1|:40203-41030
MsHel-1	gb|AIXA01007663.1|:339-451
MsHel-1	gb|AIXA01007681.1|:106517-107128
MsHel-1	gb|AIXA01007681.1|:33583-34401
MsHel-1	gb|AIXA01003308.1|:46802-47324
MsHel-1	gb|AIXA01003308.1|:26062-26892
MsHel-1	gb|AIXA01000205.1|:13602-14436
MsHel-1	gb|AIXA01015557.1|:456-3078
MsHel-1	gb|AIXA01008423.1|:31223-32055
MsHel-1	gb|AIXA01007692.1|:951-1785
MsHel-1	gb|AIXA01005554.1|:38152-38982
MsHel-1	gb|AIXA01007205.1|:1101-1299
MsHel-1	gb|AIXA01003576.1|:12972-13054
MsHel-1	gb|AIXA01009593.1|:35248-36075
MsHel-1	gb|AIXA01009593.1|:8441-8522
MsHel-1	gb|AIXA01007903.1|:2722-3545
MsHel-1	gb|AIXA01005021.1|:10995-11822
MsHel-1	gb|AIXA01003317.1|:21837-22671
MsHel-1	gb|AIXA01018543.1|:4573-5413
MsHel-1	gb|AIXA01005571.1|:4298-5128
MsHel-1	gb|AIXA01002283.1|:52152-52977
MsHel-1	gb|AIXA01004378.1|:3843-4676
MsHel-1	gb|AIXA01015427.1|:14620-15443
MsHel-1	gb|AIXA01007668.1|:6102-6934
MsHel-1	gb|AIXA01007428.1|:121278-121652
MsHel-1	gb|AIXA01006241.1|:34643-34769

MsHel-1	gb|AIXA01005873.1|:8295-9128
MsHel-1	gb|AIXA01005831.1|:11932-12764
MsHel-1	gb|AIXA01004462.1|:13413-13565
MsHel-1	gb|AIXA01004462.1|:634-1467
MsHel-1	gb|AIXA01006769.1|:5626-5717
MsHel-1	gb|AIXA01006036.1|:59720-59837
MsHel-1	gb|AIXA01002913.1|:53311-53708
MsHel-1	gb|AIXA01002913.1|:36813-37638
MsHel-1	gb|AIXA01006837.1|:22297-23300
MsHel-1	gb|AIXA01006837.1|:11220-12061
MsHel-1	gb|AIXA01004945.1|:25007-25839
MsHel-1	gb|AIXA01002162.1|:9385-10214
MsHel-1	gb|AIXA01001045.1|:44178-45008
MsHel-1	gb|AIXA01016526.1|:7349-8170
MsHel-1	gb|AIXA01007813.1|:2180-3003
MsHel-1	gb|AIXA01007807.1|:9933-10201
MsHel-1	gb|AIXA01007092.1|:47123-47950
MsHel-1	gb|AIXA01003981.1|:22464-23301
MsHel-1	gb|AIXA01000690.1|:48129-48955
MsHel-1	gb|AIXA01015727.1|:8196-8689
MsHel-1	gb|AIXA01000149.1|:13567-14406
MsHel-1	gb|AIXA01018199.1|:8320-9148
MsHel-1	gb|AIXA01011303.1|:55553-55796
MsHel-1	gb|AIXA01011303.1|:29799-31763
MsHel-1	gb|AIXA01011303.1|:4491-5322
MsHel-1	gb|AIXA01006519.1|:29065-29895
MsHel-1	gb|AIXA01003745.1|:21301-22131
MsHel-1	gb|AIXA01009123.1|:1612-2447
MsHel-1	gb|AIXA01008429.1|:20619-20938
MsHel-1	gb|AIXA01007513.1|:19532-20352
MsHel-1	gb|AIXA01006838.1|:5491-5983
MsHel-1	gb|AIXA01006706.1|:3130-3948
MsHel-1	gb|AIXA01000896.1|:26937-27434
MsHel-1	gb|AIXA01000896.1|:11380-11621
MsHel-1	gb|AIXA01008157.1|:3748-4048
MsHel-1	gb|AIXA01007696.1|:603-1428
MsHel-1	gb|AIXA01007533.1|:14618-15444
MsHel-1	gb|AIXA01006885.1|:111928-112399
MsHel-1	gb|AIXA01006885.1|:41993-42818
MsHel-1	gb|AIXA01005273.1|:42052-42234
MsHel-1	gb|AIXA01005273.1|:2887-3723
MsHel-1	gb|AIXA01015762.1|:8854-9686
MsHel-1	gb|AIXA01007848.1|:5658-7401
MsHel-1	gb|AIXA01007641.1|:82858-83145
MsHel-1	gb|AIXA01007641.1|:47095-47928
MsHel-1	gb|AIXA01005406.1|:30092-30588
MsHel-1	gb|AIXA01004042.1|:6209-7038
MsHel-1	gb|AIXA01003832.1|:11087-11917

MsHel-1	gb|AIXA01001071.1|:91387-92220
MsHel-1	gb|AIXA01001071.1|:12966-13675
MsHel-1	gb|AIXA01014079.1|:31630-32122
MsHel-1	gb|AIXA01007228.1|:134061-134614
MsHel-1	gb|AIXA01007228.1|:71968-72174
MsHel-1	gb|AIXA01007228.1|:60934-61587
MsHel-1	gb|AIXA01006442.1|:53178-53671
MsHel-1	gb|AIXA01005469.1|:4689-6023
MsHel-1	gb|AIXA01004800.1|:8364-9245
MsHel-1	gb|AIXA01001105.1|:11017-11841
MsHel-1	gb|AIXA01007262.1|:26278-26754
MsHel-1	gb|AIXA01004567.1|:39929-40764
MsHel-1	gb|AIXA01003306.1|:23976-24819
MsHel-1	gb|AIXA01001557.1|:4441-4591
MsHel-1	gb|AIXA01014037.1|:23072-23746
MsHel-1	gb|AIXA01005544.1|:14400-15234
MsHel-1	gb|AIXA01005494.1|:42213-42707
MsHel-1	gb|AIXA01009350.1|:35417-35788
MsHel-1	gb|AIXA01003819.1|:39903-40730
MsHel-1	gb|AIXA01002911.1|:33926-42456
MsHel-1	gb|AIXA01004683.1|:59448-60607
MsHel-1	gb|AIXA01018087.1|:4060-7159
MsHel-1	gb|AIXA01003711.1|:229228-229463
MsHel-1	gb|AIXA01003711.1|:135105-135913
MsHel-1	gb|AIXA01002662.1|:103339-104174
MsHel-1	gb|AIXA01000327.1|:83332-83679
MsHel-1	gb|AIXA01000327.1|:69157-70007
MsHel-1	gb|AIXA01024780.1|:791-1282
MsHel-1	gb|AIXA01012662.1|:7956-8782
MsHel-1	gb|AIXA01007783.1|:40709-41536
MsHel-1	gb|AIXA01002375.1|:7189-7608
MsHel-1	gb|AIXA01015649.1|:10447-11280
MsHel-1	gb|AIXA01008632.1|:5209-6041
MsHel-1	gb|AIXA01005126.1|:7770-7861
MsHel-1	gb|AIXA01004809.1|:136520-136773
MsHel-1	gb|AIXA01016949.1|:3053-3822
MsHel-1	gb|AIXA01008442.1|:51795-51913
MsHel-1	gb|AIXA01008442.1|:27972-28805
MsHel-1	gb|AIXA01002449.1|:4580-5075
MsHel-1	gb|AIXA01012644.1|:47555-48207
MsHel-1	gb|AIXA01012644.1|:34292-37498
MsHel-1	gb|AIXA01012644.1|:19467-20062
MsHel-1	gb|AIXA01011247.1|:7729-8560
MsHel-1	gb|AIXA01008305.1|:1110-1941
MsHel-1	gb|AIXA01007626.1|:72663-72762
MsHel-1	gb|AIXA01008384.1|:11488-12315
MsHel-1	gb|AIXA01006438.1|:16106-16933
MsHel-1	gb|AIXA01000494.1|:36373-36767

MsHel-1	gb|AIXA01000494.1|:21627-22449
MsHel-1	gb|AIXA01007117.1|:5538-6368
MsHel-1	gb|AIXA01008628.1|:15861-16690
MsHel-1	gb|AIXA01004695.1|:27937-28132
MsHel-1	gb|AIXA01002719.1|:21124-21944
MsHel-1	gb|AIXA01010308.1|:3724-4557
MsHel-1	gb|AIXA01005040.1|:3056-4528
MsHel-1	gb|AIXA01002391.1|:18544-19040
MsHel-1	gb|AIXA01000892.1|:73123-73952
MsHel-1	gb|AIXA01010880.1|:32062-32890
MsHel-1	gb|AIXA01010880.1|:5420-5927
MsHel-1	gb|AIXA01010536.1|:24042-24538
MsHel-1	gb|AIXA01009713.1|:44793-46334
MsHel-1	gb|AIXA01008430.1|:3182-3678
MsHel-1	gb|AIXA01006847.1|:85389-86212
MsHel-1	gb|AIXA01006847.1|:47481-48184
MsHel-1	gb|AIXA01005862.1|:33367-34073
MsHel-1	gb|AIXA01002580.1|:39591-40699
MsHel-1	gb|AIXA01002580.1|:5322-5522
MsHel-1	gb|AIXA01005067.1|:82419-82678
MsHel-1	gb|AIXA01005067.1|:40788-41285
MsHel-1	gb|AIXA01010642.1|:4596-5423
MsHel-1	gb|AIXA01006180.1|:25321-25931
MsHel-1	gb|AIXA01006180.1|:8344-8838
MsHel-1	gb|AIXA01000864.1|:5027-5512
MsHel-1	gb|AIXA01008939.1|:6199-7034
MsHel-1	gb|AIXA01004550.1|:82011-82405
MsHel-1	gb|AIXA01009402.1|:28417-28913
MsHel-1	gb|AIXA01004692.1|:76909-77748
MsHel-1	gb|AIXA01004692.1|:24923-25748
MsHel-1	gb|AIXA01014695.1|:23771-24213
MsHel-1	gb|AIXA01010575.1|:285-1116
MsHel-1	gb|AIXA01020919.1|:1762-2252
MsHel-1	gb|AIXA01013057.1|:8599-8701
MsHel-1	gb|AIXA01002230.1|:18152-18855
MsHel-1	gb|AIXA01011722.1|:25603-25811
MsHel-1	gb|AIXA01000622.1|:27426-28254
MsHel-1	gb|AIXA01000399.1|:22095-23062
MsHel-1	gb|AIXA01010590.1|:37344-37835
MsHel-1	gb|AIXA01005614.1|:1010-2134
MsHel-1	gb|AIXA01001344.1|:12086-12927
MsHel-1	gb|AIXA01003408.1|:25341-26164
MsHel-1	gb|AIXA01011466.1|:10180-10767
MsHel-1	gb|AIXA01002296.1|:80887-81851
MsHel-1	gb|AIXA01007928.1|:43959-44791
MsHel-1	gb|AIXA01007928.1|:32152-32979
MsHel-1	gb|AIXA01003142.1|:3679-3884
MsHel-1	gb|AIXA01010905.1|:26668-27164

MsHel-1	gb|AIXA01006991.1|:47414-47897
MsHel-1	gb|AIXA01006991.1|:28114-28955
MsHel-1	gb|AIXA01005813.1|:23145-24372
MsHel-1	gb|AIXA01004068.1|:115089-115248
MsHel-1	gb|AIXA01004068.1|:4739-5554
MsHel-1	gb|AIXA01002500.1|:168516-169354
MsHel-1	gb|AIXA01002500.1|:110719-111386
MsHel-1	gb|AIXA01002500.1|:16203-16797
MsHel-1	gb|AIXA01001023.1|:16621-17426
MsHel-1	gb|AIXA01015092.1|:11508-11975
MsHel-1	gb|AIXA01004652.1|:65234-65733
MsHel-1	gb|AIXA01000048.1|:34885-35381
MsHel-1	gb|AIXA01015097.1|:33683-34150
MsHel-1	gb|AIXA01006683.1|:14363-15198
MsHel-1	gb|AIXA01002977.1|:62353-62941
MsHel-1	gb|AIXA01002977.1|:28218-29110
MsHel-1	gb|AIXA01008624.1|:14741-15445
MsHel-1	gb|AIXA01007791.1|:24392-25223
MsHel-1	gb|AIXA01006880.1|:77315-77969
MsHel-1	gb|AIXA01006880.1|:63544-64248
MsHel-1	gb|AIXA01006811.1|:18336-19041
MsHel-1	gb|AIXA01010543.1|:16157-16974
MsHel-1	gb|AIXA01009316.1|:2259-3615
MsHel-1	gb|AIXA01004433.1|:156468-157296
MsHel-1	gb|AIXA01003989.1|:7736-8555
MsHel-1	gb|AIXA01003967.1|:107581-108400
MsHel-1	gb|AIXA01011733.1|:49151-49343
MsHel-1	gb|AIXA01011733.1|:2307-3148
MsHel-1	gb|AIXA01010854.1|:100610-101260
MsHel-1	gb|AIXA01004597.1|:60942-62261
MsHel-1	gb|AIXA01000015.1|:15171-16137
MsHel-1	gb|AIXA01008271.1|:13715-14210
MsHel-1	gb|AIXA01002228.1|:48812-49240
MsHel-1	gb|AIXA01002228.1|:9171-10007
MsHel-1	gb|AIXA01009610.1|:13517-14459
MsHel-1	gb|AIXA01008431.1|:7705-8282
MsHel-1	gb|AIXA01006956.1|:6756-7595
MsHel-1	gb|AIXA01004533.1|:19444-19940
MsHel-1	gb|AIXA01000271.1|:32813-33517
MsHel-1	gb|AIXA01012089.1|:650-1491
MsHel-1	gb|AIXA01003414.1|:7831-8587
MsHel-1	gb|AIXA01000655.1|:83696-83864
MsHel-1	gb|AIXA01000655.1|:65343-66171
MsHel-1	gb|AIXA01000655.1|:27982-28581
MsHel-1	gb|AIXA01003292.1|:47418-48236
MsHel-1	gb|AIXA01003292.1|:1-312
MsHel-1	gb|AIXA01002943.1|:55870-56663
MsHel-1	gb|AIXA01004628.1|:42569-43386

MsHel-1	gb|AIXA01003928.1|:69754-70056
MsHel-1	gb|AIXA01003116.1|:21027-21676
MsHel-1	gb|AIXA01002420.1|:28522-29342
MsHel-1	gb|AIXA01001132.1|:84046-84872
MsHel-1	gb|AIXA01001132.1|:28202-28654
MsHel-1	gb|AIXA01016342.1|:4049-4882
MsHel-1	gb|AIXA01005206.1|:9496-10336
MsHel-1	gb|AIXA01008566.1|:12487-13258
MsHel-1	gb|AIXA01006007.1|:73637-74233
MsHel-1	gb|AIXA01018024.1|:6534-6667
MsHel-1	gb|AIXA01007496.1|:27525-28231
MsHel-1	gb|AIXA01007496.1|:12864-13117
MsHel-1	gb|AIXA01003823.1|:27078-27784
MsHel-1	gb|AIXA01003823.1|:2408-3311
MsHel-1	gb|AIXA01000610.1|:42168-42992
MsHel-1	gb|AIXA01014127.1|:7124-7829
MsHel-1	gb|AIXA01013753.1|:15319-16681
MsHel-1	gb|AIXA01011562.1|:13506-14108
MsHel-1	gb|AIXA01009864.1|:59657-60067
MsHel-1	gb|AIXA01008417.1|:86004-87091
MsHel-1	gb|AIXA01005493.1|:18815-19519
MsHel-1	gb|AIXA01012657.1|:11337-12241
MsHel-1	gb|AIXA01007082.1|:10188-10684
MsHel-1	gb|AIXA01003739.1|:51609-52428
MsHel-1	gb|AIXA01002946.1|:1161-1981
MsHel-1	gb|AIXA01000588.1|:45730-46547
MsHel-1	gb|AIXA01005350.1|:43968-44436
MsHel-1	gb|AIXA01005350.1|:19166-19980
MsHel-1	gb|AIXA01002579.1|:137829-137913
MsHel-1	gb|AIXA01002579.1|:48049-48895
MsHel-1	gb|AIXA01001243.1|:207221-207713
MsHel-1	gb|AIXA01001243.1|:132314-132424
MsHel-1	gb|AIXA01001243.1|:54530-54727
MsHel-1	gb|AIXA01001243.1|:39005-39098
MsHel-1	gb|AIXA01001243.1|:15225-15493
MsHel-1	gb|AIXA01008439.1|:10427-11269
MsHel-1	gb|AIXA01003605.1|:10847-11343
MsHel-1	gb|AIXA01002805.1|:93122-93710
MsHel-1	gb|AIXA01002671.1|:76663-77434
MsHel-1	gb|AIXA01002671.1|:61532-61732
MsHel-1	gb|AIXA01002671.1|:10796-11391
MsHel-1	gb|AIXA01011460.1|:84703-91088
MsHel-1	gb|AIXA01010881.1|:84181-84487
MsHel-1	gb|AIXA01010881.1|:66920-67016
MsHel-1	gb|AIXA01010881.1|:43246-44186
MsHel-1	gb|AIXA01007175.1|:63022-63856
MsHel-1	gb|AIXA01007718.1|:1570-2411
MsHel-1	gb|AIXA01004265.1|:3249-4089

MsHel-1	gb|AIXA01002386.1|:32406-32869
MsHel-1	gb|AIXA01002061.1|:22379-23220
MsHel-1	gb|AIXA01009513.1|:2229-3065
MsHel-1	gb|AIXA01009496.1|:10843-11425
MsHel-1	gb|AIXA01007070.1|:10055-10430
MsHel-1	gb|AIXA01006132.1|:41200-41295
MsHel-1	gb|AIXA01006132.1|:14748-14979
MsHel-1	gb|AIXA01016551.1|:19846-30338
MsHel-1	gb|AIXA01005016.1|:29230-29519
MsHel-1	gb|AIXA01004798.1|:16234-17174
MsHel-1	gb|AIXA01002885.1|:2282-2408
MsHel-1	gb|AIXA01013521.1|:8366-8512
MsHel-1	gb|AIXA01013010.1|:42350-43172
MsHel-1	gb|AIXA01011652.1|:2228-2347
MsHel-1	gb|AIXA01000631.1|:5059-6025
MsHel-1	gb|AIXA01006602.1|:69272-69765
MsHel-1	gb|AIXA01005558.1|:2291-2787
MsHel-1	gb|AIXA01000421.1|:10603-11420
MsHel-1	gb|AIXA01012705.1|:3623-4441
MsHel-1	gb|AIXA01000755.1|:125438-125905
MsHel-1	gb|AIXA01011255.1|:6809-7300
MsHel-1	gb|AIXA01004881.1|:23017-23513
MsHel-1	gb|AIXA01004328.1|:5893-6710
MsHel-1	gb|AIXA01004114.1|:66468-75933
MsHel-1	gb|AIXA01001693.1|:23438-23533
MsHel-1	gb|AIXA01001693.1|:9287-10044
MsHel-1	gb|AIXA01000922.1|:59326-59817
MsHel-1	gb|AIXA01008234.1|:34465-35169
MsHel-1	gb|AIXA01008234.1|:17504-18099
MsHel-1	gb|AIXA01007375.1|:9798-10502
MsHel-1	gb|AIXA01005851.1|:26962-27457
MsHel-1	gb|AIXA01001204.1|:66049-66789
MsHel-1	gb|AIXA01000117.1|:14893-15125
MsHel-1	gb|AIXA01006549.1|:7293-8022
MsHel-1	gb|AIXA01001675.1|:10588-11428
MsHel-1	gb|AIXA01003866.1|:20642-21477
MsHel-1	gb|AIXA01002748.1|:122841-123679
MsHel-1	gb|AIXA01002468.1|:6673-7635
MsHel-1	gb|AIXA01011810.1|:486-608
MsHel-1	gb|AIXA01010719.1|:6495-7200
MsHel-1	gb|AIXA01010667.1|:1-147
MsHel-1	gb|AIXA01005347.1|:65073-65162
MsHel-1	gb|AIXA01012020.1|:15090-15566
MsHel-1	gb|AIXA01009423.1|:38033-38876
MsHel-1	gb|AIXA01009423.1|:24393-24567
MsHel-1	gb|AIXA01008962.1|:29046-29859
MsHel-1	gb|AIXA01000266.1|:6703-7537
MsHel-1	gb|AIXA01013994.1|:4433-5261

MsHel-1	gb|AIXA01006440.1|:47221-47672
MsHel-1	gb|AIXA01010437.1|:3161-3865
MsHel-1	gb|AIXA01007827.1|:1340-2043
MsHel-1	gb|AIXA01005565.1|:1417-1914
MsHel-1	gb|AIXA01004013.1|:8345-9050
MsHel-1	gb|AIXA01003287.1|:5316-5435
MsHel-1	gb|AIXA01003224.1|:7543-8246
MsHel-1	gb|AIXA01001585.1|:1-741
MsHel-1	gb|AIXA01000487.1|:12032-12727
MsHel-1	gb|AIXA01000245.1|:86868-87708
MsHel-1	gb|AIXA01010151.1|:165957-166078
MsHel-1	gb|AIXA01010151.1|:140071-140767
MsHel-1	gb|AIXA01010151.1|:64887-65714
MsHel-1	gb|AIXA01005001.1|:72303-73120
MsHel-1	gb|AIXA01005001.1|:41016-41151
MsHel-1	gb|AIXA01004517.1|:6617-7430
MsHel-1	gb|AIXA01012487.1|:41417-42263
MsHel-1	gb|AIXA01013313.1|:3486-4644
MsHel-1	gb|AIXA01009399.1|:41722-41972
MsHel-1	gb|AIXA01000899.1|:17428-19817
MsHel-1	gb|AIXA01000460.1|:19591-20054
MsHel-1	gb|AIXA01006008.1|:869-1574
MsHel-1	gb|AIXA01002185.1|:210-914
MsHel-1	gb|AIXA01001053.1|:40969-41465
MsHel-1	gb|AIXA01001053.1|:6937-7134
MsHel-1	gb|AIXA01007560.1|:71629-72450
MsHel-1	gb|AIXA01005597.1|:614-1376
MsHel-1	gb|AIXA01001948.1|:29100-29921
MsHel-1	gb|AIXA01001453.1|:15046-15336
MsHel-1	gb|AIXA01011510.1|:4769-5240
MsHel-1	gb|AIXA01009510.1|:19230-19727
MsHel-1	gb|AIXA01000183.1|:49094-49719
MsHel-1	gb|AIXA01022845.1|:881-1607
MsHel-1	gb|AIXA01006907.1|:20358-21068
MsHel-1	gb|AIXA01006907.1|:6482-6734
MsHel-1	gb|AIXA01005194.1|:33597-34256
MsHel-1	gb|AIXA01022611.1|:605-1439
MsHel-1	gb|AIXA01007561.1|:26812-27504
MsHel-1	gb|AIXA01006752.1|:27358-28068
MsHel-1	gb|AIXA01006752.1|:8613-8803
MsHel-1	gb|AIXA01003632.1|:40444-41148
MsHel-1	gb|AIXA01002591.1|:27460-28247
MsHel-1	gb|AIXA01002591.1|:2082-2420
MsHel-1	gb|AIXA01000703.1|:49473-49615
MsHel-1	gb|AIXA01000703.1|:22711-23797
MsHel-1	gb|AIXA01000613.1|:7115-8080
MsHel-1	gb|AIXA01009407.1|:35153-35649
MsHel-1	gb|AIXA01007744.1|:1168-1997

MsHel-1	gb|AIXA01004665.1|:3051-3886
MsHel-1	gb|AIXA01003124.1|:65280-65455
MsHel-1	gb|AIXA01003124.1|:42931-43031
MsHel-1	gb|AIXA01006731.1|:57650-58360
MsHel-1	gb|AIXA01002388.1|:160287-160454
MsHel-1	gb|AIXA01002388.1|:106600-107412
MsHel-1	gb|AIXA01017533.1|:10019-11106
MsHel-1	gb|AIXA01013548.1|:3138-3844
MsHel-1	gb|AIXA01012375.1|:28928-30013
MsHel-1	gb|AIXA01011493.1|:65907-66612
MsHel-1	gb|AIXA01009785.1|:1153-1859
MsHel-1	gb|AIXA01008578.1|:33322-41474
MsHel-1	gb|AIXA01001272.1|:8224-9192
MsHel-1	gb|AIXA01016061.1|:26-731
MsHel-1	gb|AIXA01011622.1|:15254-15923
MsHel-1	gb|AIXA01008767.1|:60796-61002
MsHel-1	gb|AIXA01008767.1|:40417-41383
MsHel-1	gb|AIXA01002985.1|:18649-19466
MsHel-1	gb|AIXA01001286.1|:22055-22508
MsHel-1	gb|AIXA01001286.1|:6192-7010
MsHel-1	gb|AIXA01000259.1|:21022-21961
MsHel-1	gb|AIXA01009275.1|:5280-5989
MsHel-1	gb|AIXA01001627.1|:53145-53324
MsHel-1	gb|AIXA01004633.1|:5330-6028
MsHel-1	gb|AIXA01026574.1|:385-1114
MsHel-1	gb|AIXA01009212.1|:68868-69027
MsHel-1	gb|AIXA01009119.1|:4522-4787
MsHel-1	gb|AIXA01000862.1|:69148-69854
MsHel-1	gb|AIXA01015838.1|:7629-8466
MsHel-1	gb|AIXA01006712.1|:96279-96984
MsHel-1	gb|AIXA01006712.1|:67639-67848
MsHel-1	gb|AIXA01005960.1|:1690-2108
MsHel-1	gb|AIXA01002820.1|:27791-28499
MsHel-1	gb|AIXA01002400.1|:15274-15979
MsHel-1	gb|AIXA01007253.1|:2077-4385
MsHel-1	gb|AIXA01002908.1|:2-107
MsHel-1	gb|AIXA01002519.1|:139991-140488
MsHel-1	gb|AIXA01002440.1|:10563-11535
MsHel-1	gb|AIXA01017382.1|:3601-4298
MsHel-1	gb|AIXA01006833.1|:30085-30782
MsHel-1	gb|AIXA01005326.1|:80843-82053
MsHel-1	gb|AIXA01005326.1|:32709-33424
MsHel-1	gb|AIXA01005326.1|:2669-3375
MsHel-1	gb|AIXA01000207.1|:55718-56553
MsHel-1	gb|AIXA01000119.1|:22073-22257
MsHel-1	gb|AIXA01000119.1|:6283-6987
MsHel-1	gb|AIXA01011397.1|:41808-42514
MsHel-1	gb|AIXA01005635.1|:7923-8737

MsHel-1	gb|AIXA01004841.1|:6296-6998
MsHel-1	gb|AIXA01004483.1|:45537-46231
MsHel-1	gb|AIXA01003243.1|:5318-6006
MsHel-1	gb|AIXA01002543.1|:657-1358
MsHel-1	gb|AIXA01001758.1|:39995-40101
MsHel-1	gb|AIXA01000907.1|:13296-14385
MsHel-1	gb|AIXA01014500.1|:16177-17015
MsHel-1	gb|AIXA01013595.1|:5567-6272
MsHel-1	gb|AIXA01007168.1|:79023-79728
MsHel-1	gb|AIXA01004192.1|:23349-24054
MsHel-1	gb|AIXA01004192.1|:7323-7804
MsHel-1	gb|AIXA01003747.1|:149691-150779
MsHel-1	gb|AIXA01001484.1|:93587-94058
MsHel-1	gb|AIXA01001484.1|:8066-8334
MsHel-1	gb|AIXA01009177.1|:108730-109302
MsHel-1	gb|AIXA01007808.1|:16254-16949
MsHel-1	gb|AIXA01003365.1|:94113-94822
MsHel-1	gb|AIXA01002384.1|:5787-6493
MsHel-1	gb|AIXA01002350.1|:148695-153051
MsHel-1	gb|AIXA01007994.1|:11261-12075
MsHel-1	gb|AIXA01007853.1|:3077-4038
MsHel-1	gb|AIXA01004868.1|:1411-2113
MsHel-1	gb|AIXA01001226.1|:54046-55381
MsHel-1	gb|AIXA01014984.1|:28463-28579
MsHel-1	gb|AIXA01009793.1|:5420-6392
MsHel-1	gb|AIXA01007851.1|:4777-6436
MsHel-1	gb|AIXA01005265.1|:10928-11633
MsHel-1	gb|AIXA01004304.1|:33875-34580
MsHel-1	gb|AIXA01004304.1|:1-147
MsHel-1	gb|AIXA01000507.1|:18797-19501
MsHel-1	gb|AIXA01018605.1|:7981-8684
MsHel-1	gb|AIXA01018245.1|:2166-2869
MsHel-1	gb|AIXA01010211.1|:7211-7914
MsHel-1	gb|AIXA01009629.1|:1056-1902
MsHel-1	gb|AIXA01009305.1|:33547-34043
MsHel-1	gb|AIXA01006263.1|:12498-13203
MsHel-1	gb|AIXA01005274.1|:2908-3613
MsHel-1	gb|AIXA01004593.1|:10669-11509
MsHel-1	gb|AIXA01000684.1|:11345-12049
MsHel-1	gb|AIXA01000415.1|:2798-3501
MsHel-1	gb|AIXA01000022.1|:22996-23699
MsHel-1	gb|AIXA01003993.1|:83451-83612
MsHel-1	gb|AIXA01001060.1|:10953-11995
MsHel-1	gb|AIXA01004738.1|:10169-10537
MsHel-1	gb|AIXA01004330.1|:80995-81704
MsHel-1	gb|AIXA01016666.1|:3050-3228
MsHel-1	gb|AIXA01009550.1|:731-1697
MsHel-1	gb|AIXA01006390.1|:5374-5458

MsHel-1	gb|AIXA01004731.1|:35578-36980
MsHel-1	gb|AIXA01004731.1|:6480-6775
MsHel-1	gb|AIXA01000139.1|:18099-18516
MsHel-1	gb|AIXA01013315.1|:1968-2677
MsHel-1	gb|AIXA01009548.1|:37854-38266
MsHel-1	gb|AIXA01009548.1|:15695-15795
MsHel-1	gb|AIXA01008920.1|:26017-26722
MsHel-1	gb|AIXA01005385.1|:6529-12306
MsHel-1	gb|AIXA01011018.1|:6237-6941
MsHel-1	gb|AIXA01010684.1|:11222-11935
MsHel-1	gb|AIXA01009697.1|:10377-10639
MsHel-1	gb|AIXA01005009.1|:13139-13837
MsHel-1	gb|AIXA01004745.1|:647-1354
MsHel-1	gb|AIXA01003478.1|:39687-40394
MsHel-1	gb|AIXA01002708.1|:52312-53401
MsHel-1	gb|AIXA01000222.1|:7163-8001
MsHel-1	gb|AIXA01011530.1|:11251-11623
MsHel-1	gb|AIXA01011503.1|:20463-21169
MsHel-1	gb|AIXA01003105.1|:30954-31448
MsHel-1	gb|AIXA01015313.1|:3026-3850
MsHel-1	gb|AIXA01011285.1|:89674-90243
MsHel-1	gb|AIXA01007461.1|:5903-6567
MsHel-1	gb|AIXA01005050.1|:11918-12617
MsHel-1	gb|AIXA01007249.1|:1299-2163
MsHel-1	gb|AIXA01001239.1|:32258-33101
MsHel-1	gb|AIXA01015652.1|:3871-4807
MsHel-1	gb|AIXA01012951.1|:9755-10257
MsHel-1	gb|AIXA01006930.1|:33119-36658
MsHel-1	gb|AIXA01006396.1|:74121-74921
MsHel-1	gb|AIXA01003863.1|:3902-4860
MsHel-1	gb|AIXA01001250.1|:6917-7118
MsHel-1	gb|AIXA01013383.1|:18112-18516
MsHel-1	gb|AIXA01009217.1|:2558-3262
MsHel-1	gb|AIXA01004657.1|:2304-3106
MsHel-1	gb|AIXA01002983.1|:1570-2786
MsHel-1	gb|AIXA01010321.1|:3246-3944
MsHel-1	gb|AIXA01007445.1|:83093-83803
MsHel-1	gb|AIXA01006671.1|:22042-22440
MsHel-1	gb|AIXA01000827.1|:138702-139400
MsHel-1	gb|AIXA01015284.1|:13882-14962
MsHel-1	gb|AIXA01009361.1|:29606-30593
MsHel-1	gb|AIXA01007268.1|:83660-84110
MsHel-1	gb|AIXA01007268.1|:28996-29702
MsHel-1	gb|AIXA01002387.1|:24877-25577
MsHel-1	gb|AIXA01000801.1|:50251-50463
MsHel-1	gb|AIXA01011436.1|:10464-11167
MsHel-1	gb|AIXA01002248.1|:88344-88705
MsHel-1	gb|AIXA01002248.1|:49635-50339

MsHel-1	gb|AIXA01001562.1|:4934-5755
MsHel-1	gb|AIXA01000413.1|:3125-4041
MsHel-1	gb|AIXA01016009.1|:5117-7008
MsHel-1	gb|AIXA01010482.1|:3580-5541
MsHel-1	gb|AIXA01008287.1|:16863-17697
MsHel-1	gb|AIXA01007524.1|:9574-9706
MsHel-1	gb|AIXA01004579.1|:47557-47669
MsHel-1	gb|AIXA01004368.1|:48831-48951
MsHel-1	gb|AIXA01004368.1|:24324-25172
MsHel-1	gb|AIXA01003288.1|:52542-53128
MsHel-1	gb|AIXA01003288.1|:16555-16797
MsHel-1	gb|AIXA01002518.1|:36328-37035
MsHel-1	gb|AIXA01000973.1|:630-1467
MsHel-1	gb|AIXA01013445.1|:29771-30712
MsHel-1	gb|AIXA01008406.1|:33259-33958
MsHel-1	gb|AIXA01008825.1|:11-198
MsHel-1	gb|AIXA01008032.1|:38843-39640
MsHel-1	gb|AIXA01001642.1|:21392-21480
MsHel-1	gb|AIXA01001301.1|:302-1117
MsHel-1	gb|AIXA01000707.1|:59435-60136
MsHel-1	gb|AIXA01011034.1|:6471-7306
MsHel-1	gb|AIXA01008325.1|:39484-39982
MsHel-1	gb|AIXA01004308.1|:2565-3773
MsHel-1	gb|AIXA01000501.1|:10950-11779
MsHel-1	gb|AIXA01000111.1|:117275-117979
MsHel-1	gb|AIXA01003734.1|:6029-6723
MsHel-1	gb|AIXA01001927.1|:7247-8311
MsHel-1	gb|AIXA01009518.1|:97938-98647
MsHel-1	gb|AIXA01008253.1|:10414-10689
MsHel-1	gb|AIXA01007926.1|:26415-27119
MsHel-1	gb|AIXA01005095.1|:57982-58455
MsHel-1	gb|AIXA01005095.1|:36238-36943
MsHel-1	gb|AIXA01004478.1|:10203-11030
MsHel-1	gb|AIXA01002799.1|:5746-6448
MsHel-1	gb|AIXA01002689.1|:74763-75467
MsHel-1	gb|AIXA01001384.1|:46080-46186
MsHel-1	gb|AIXA01001384.1|:25382-25613
MsHel-1	gb|AIXA01001384.1|:10947-11760
MsHel-1	gb|AIXA01000998.1|:9377-9978
MsHel-1	gb|AIXA01015603.1|:2056-2813
MsHel-1	gb|AIXA01008665.1|:26771-27607
MsHel-1	gb|AIXA01008665.1|:5208-5924
MsHel-1	gb|AIXA01003817.1|:57480-58317
MsHel-1	gb|AIXA01003817.1|:7932-8108
MsHel-1	gb|AIXA01003173.1|:13976-15136
MsHel-1	gb|AIXA01001544.1|:64895-65594
MsHel-1	gb|AIXA01012191.1|:3531-4227
MsHel-1	gb|AIXA01005920.1|:12381-12485

MsHel-1	gb|AIXA01005920.1|:777-928
MsHel-1	gb|AIXA01002679.1|:46212-47134
MsHel-1	gb|AIXA01000615.1|:20211-20502
MsHel-1	gb|AIXA01000615.1|:2127-2726
MsHel-1	gb|AIXA01000582.1|:17781-18478
MsHel-1	gb|AIXA01000201.1|:101153-101850
MsHel-1	gb|AIXA01012197.1|:38-744
MsHel-1	gb|AIXA01011461.1|:848-1546
MsHel-1	gb|AIXA01002128.1|:66783-67421
MsHel-1	gb|AIXA01000212.1|:91797-92218
MsHel-1	gb|AIXA01000212.1|:1920-2054
MsHel-1	gb|AIXA01015133.1|:6604-7308
MsHel-1	gb|AIXA01006202.1|:28600-28789
MsHel-1	gb|AIXA01006202.1|:6916-8869
MsHel-1	gb|AIXA01002442.1|:7701-8197
MsHel-1	gb|AIXA01001396.1|:15874-17063
MsHel-1	gb|AIXA01013812.1|:38182-38379
MsHel-1	gb|AIXA01013812.1|:19263-20037
MsHel-1	gb|AIXA01011027.1|:58720-59471
MsHel-1	gb|AIXA01011027.1|:36225-36430
MsHel-1	gb|AIXA01011027.1|:2143-2366
MsHel-1	gb|AIXA01006870.1|:8100-8794
MsHel-1	gb|AIXA01004020.1|:26882-27849
MsHel-1	gb|AIXA01000669.1|:12654-13289
MsHel-1	gb|AIXA01011031.1|:7844-8684
MsHel-1	gb|AIXA01007287.1|:52443-53278
MsHel-1	gb|AIXA01005504.1|:82511-82609
MsHel-1	gb|AIXA01005504.1|:34431-35274
MsHel-1	gb|AIXA01005504.1|:12835-13048
MsHel-1	gb|AIXA01002982.1|:60014-61101
MsHel-1	gb|AIXA01002055.1|:25473-26439
MsHel-1	gb|AIXA01016084.1|:516-1564
MsHel-1	gb|AIXA01007620.1|:54264-55099
MsHel-1	gb|AIXA01006893.1|:53323-53680
MsHel-1	gb|AIXA01005413.1|:21219-22308
MsHel-1	gb|AIXA01011560.1|:40496-41200
MsHel-1	gb|AIXA01008270.1|:1986-2691
MsHel-1	gb|AIXA01007886.1|:3080-3779
MsHel-1	gb|AIXA01002512.1|:5348-6053
MsHel-1	gb|AIXA01007711.1|:19180-19281
MsHel-1	gb|AIXA01007711.1|:4866-5667
MsHel-1	gb|AIXA01012784.1|:20879-29752
MsHel-1	gb|AIXA01004395.1|:5595-6352
MsHel-1	gb|AIXA01003966.1|:102968-104054
MsHel-1	gb|AIXA01001717.1|:29485-30195
MsHel-1	gb|AIXA01001271.1|:24012-24975
MsHel-1	gb|AIXA01006876.1|:12608-12895
MsHel-1	gb|AIXA01004303.1|:7031-7988

MsHel-1	gb|AIXA01001395.1|:1507-2214
MsHel-1	gb|AIXA01003798.1|:69139-69715
MsHel-1	gb|AIXA01005833.1|:16591-17562
MsHel-1	gb|AIXA01004045.1|:40021-40814
MsHel-1	gb|AIXA01000600.1|:83143-83823
MsHel-1	gb|AIXA01017479.1|:1342-2432
MsHel-1	gb|AIXA01005860.1|:14834-14978
MsHel-1	gb|AIXA01005860.1|:2750-3813
MsHel-1	gb|AIXA01004260.1|:136761-137599
MsHel-1	gb|AIXA01004260.1|:60660-60743
MsHel-1	gb|AIXA01017038.1|:3181-4010
MsHel-1	gb|AIXA01007937.1|:3416-4116
MsHel-1	gb|AIXA01003970.1|:31990-32217
MsHel-1	gb|AIXA01003970.1|:506-1219
MsHel-1	gb|AIXA01008850.1|:21720-22491
MsHel-1	gb|AIXA01030396.1|:176-838
MsHel-1	gb|AIXA01016561.1|:5730-6531
MsHel-1	gb|AIXA01011544.1|:2129-2834
MsHel-1	gb|AIXA01011050.1|:4361-5062
MsHel-1	gb|AIXA01010477.1|:26407-29410
MsHel-1	gb|AIXA01009133.1|:41286-41604
MsHel-1	gb|AIXA01009133.1|:8487-9186
MsHel-1	gb|AIXA01004622.1|:36301-37264
MsHel-1	gb|AIXA01004369.1|:20124-20838
MsHel-1	gb|AIXA01002460.1|:114567-115061
MsHel-1	gb|AIXA01001851.1|:1365-3035
MsHel-1	gb|AIXA01009059.1|:11554-12257
MsHel-1	gb|AIXA01006474.1|:29194-29772
MsHel-1	gb|AIXA01001123.1|:7547-8260
MsHel-1	gb|AIXA01010772.1|:80469-80589
MsHel-1	gb|AIXA01008791.1|:12026-12772
MsHel-1	gb|AIXA01007723.1|:14775-15612
MsHel-1	gb|AIXA01005782.1|:12733-13332
MsHel-1	gb|AIXA01003682.1|:70441-71017
MsHel-1	gb|AIXA01003334.1|:12422-13016
MsHel-1	gb|AIXA01002990.1|:38451-39156
MsHel-1	gb|AIXA01002620.1|:6318-6396
MsHel-1	gb|AIXA01000549.1|:8467-9178
MsHel-1	gb|AIXA01028654.1|:272-948
MsHel-1	gb|AIXA01017274.1|:1-407
MsHel-1	gb|AIXA01010976.1|:4526-5310
MsHel-1	gb|AIXA01009543.1|:27497-28141
MsHel-1	gb|AIXA01007809.1|:21491-22204
MsHel-1	gb|AIXA01001674.1|:33350-34057
MsHel-1	gb|AIXA01007579.1|:61126-61355
MsHel-1	gb|AIXA01002340.1|:121297-121993
MsHel-1	gb|AIXA01005020.1|:6760-7702
MsHel-1	gb|AIXA01011754.1|:3319-4155

MsHel-1	gb|AIXA01006503.1|:63281-64063
MsHel-1	gb|AIXA01000136.1|:5576-6415
MsHel-1	gb|AIXA01015800.1|:21755-22560
MsHel-1	gb|AIXA01008531.1|:25954-26660
MsHel-1	gb|AIXA01011163.1|:9118-10037
MsHel-1	gb|AIXA01012247.1|:14178-14764
MsHel-1	gb|AIXA01012247.1|:1902-2082
MsHel-1	gb|AIXA01010480.1|:36070-36555
MsHel-1	gb|AIXA01007571.1|:8-649
MsHel-1	gb|AIXA01006509.1|:54931-55301
MsHel-1	gb|AIXA01003799.1|:9415-10012
MsHel-1	gb|AIXA01001669.1|:39889-40689
MsHel-1	gb|AIXA01006020.1|:16436-17144
MsHel-1	gb|AIXA01008477.1|:82585-83300
MsHel-1	gb|AIXA01008477.1|:62080-62352
MsHel-1	gb|AIXA01008477.1|:42572-43143
MsHel-1	gb|AIXA01001296.1|:197160-197982
MsHel-1	gb|AIXA01007508.1|:48827-49638
MsHel-1	gb|AIXA01006856.1|:1-85
MsHel-1	gb|AIXA01001489.1|:35688-36776
MsHel-1	gb|AIXA01017156.1|:2965-3677
MsHel-1	gb|AIXA01013516.1|:5583-6922
MsHel-1	gb|AIXA01011535.1|:6021-6610
MsHel-1	gb|AIXA01007320.1|:52685-53592
MsHel-1	gb|AIXA01006999.1|:47566-48415
MsHel-1	gb|AIXA01005642.1|:29631-31459
MsHel-1	gb|AIXA01004411.1|:17819-18714
MsHel-1	gb|AIXA01003764.1|:2362-3017
MsHel-1	gb|AIXA01009844.1|:3425-12799
MsHel-1	gb|AIXA01002429.1|:7964-8156
MsHel-1	gb|AIXA01023475.1|:882-1496
MsHel-1	gb|AIXA01013826.1|:4463-5050
MsHel-1	gb|AIXA01007270.1|:4074-4782
MsHel-1	gb|AIXA01006550.1|:16992-17821
MsHel-1	gb|AIXA01003312.1|:32558-33145
MsHel-1	gb|AIXA01002693.1|:2372-3033
MsHel-1	gb|AIXA01002367.1|:22145-22862
MsHel-1	gb|AIXA01012839.1|:40420-41037
MsHel-1	gb|AIXA01012839.1|:3393-3567
MsHel-1	gb|AIXA01011709.1|:99971-100088
MsHel-1	gb|AIXA01011709.1|:70691-71276
MsHel-1	gb|AIXA01008245.1|:24710-25465
MsHel-1	gb|AIXA01001397.1|:13379-13579
MsHel-1	gb|AIXA01000874.1|:72039-72325
MsHel-1	gb|AIXA01022945.1|:938-1588
MsHel-1	gb|AIXA01017668.1|:3460-4361
MsHel-1	gb|AIXA01014587.1|:14817-15514
MsHel-1	gb|AIXA01013151.1|:2342-3063

MsHel-1	gb|AIXA01012643.1|:25311-25899
MsHel-1	gb|AIXA01009179.1|:24926-25769
MsHel-1	gb|AIXA01001278.1|:48227-48743
MsHel-1	gb|AIXA01001278.1|:25221-26279
MsHel-1	gb|AIXA01009999.1|:18339-18542
MsHel-1	gb|AIXA01006413.1|:30071-30255
MsHel-1	gb|AIXA01003941.1|:3170-3860
MsHel-1	gb|AIXA01002324.1|:17996-18995
MsHel-1	gb|AIXA01011739.1|:11095-11803
MsHel-1	gb|AIXA01007932.1|:17438-17841
MsHel-1	gb|AIXA01000099.1|:36851-37549
MsHel-1	gb|AIXA01004299.1|:16507-17212
MsHel-1	gb|AIXA01000157.1|:8674-9174
MsHel-1	gb|AIXA01012485.1|:30352-30546
MsHel-1	gb|AIXA01002371.1|:56089-56778
MsHel-1	gb|AIXA01000164.1|:6282-6975
MsHel-1	gb|AIXA01000089.1|:29979-31195
MsHel-1	gb|AIXA01014846.1|:20593-21814
MsHel-1	gb|AIXA01011835.1|:4811-5523
MsHel-1	gb|AIXA01005762.1|:60364-61076
MsHel-1	gb|AIXA01002348.1|:2108-2823
MsHel-1	gb|AIXA01001701.1|:4134-4752
MsHel-1	gb|AIXA01000611.1|:54306-54426
MsHel-1	gb|AIXA01000611.1|:43615-43903
MsHel-1	gb|AIXA01000611.1|:8595-9512
MsHel-1	gb|AIXA01012246.1|:1658-2081
MsHel-1	gb|AIXA01006850.1|:11173-11922
MsHel-1	gb|AIXA01000775.1|:6631-7433
MsHel-1	gb|AIXA01000214.1|:23330-23933
MsHel-1	gb|AIXA01007971.1|:11764-12469
MsHel-1	gb|AIXA01007453.1|:23002-23675
MsHel-1	gb|AIXA01007453.1|:1-748
MsHel-1	gb|AIXA01007314.1|:10093-10806
MsHel-1	gb|AIXA01005473.1|:15437-15886
MsHel-1	gb|AIXA01004279.1|:6199-7680
MsHel-1	gb|AIXA01003614.1|:52143-52856
MsHel-1	gb|AIXA01011628.1|:18329-19042
MsHel-1	gb|AIXA01009515.1|:31362-31748
MsHel-1	gb|AIXA01005951.1|:58995-59225
MsHel-1	gb|AIXA01003881.1|:93933-94282
MsHel-1	gb|AIXA01003609.1|:14992-15579
MsHel-1	gb|AIXA01002067.1|:6361-7130
MsHel-1	gb|AIXA01001962.1|:56064-56756
MsHel-1	gb|AIXA01001731.1|:50016-50759
MsHel-1	gb|AIXA01001485.1|:1167-1850
MsHel-1	gb|AIXA01004112.1|:27681-28437
MsHel-1	gb|AIXA01001041.1|:3718-3906
MsHel-1	gb|AIXA01008009.1|:1637-2240

MsHel-1	gb|AIXA01000809.1|:82619-83237
MsHel-1	gb|AIXA01000524.1|:28199-28911
MsHel-1	gb|AIXA01025675.1|:493-1158
MsHel-1	gb|AIXA01022783.1|:1005-1621
MsHel-1	gb|AIXA01009534.1|:9872-10589
MsHel-1	gb|AIXA01009339.1|:3407-4025
MsHel-1	gb|AIXA01007598.1|:31616-32202
MsHel-1	gb|AIXA01001645.1|:83446-84032
MsHel-1	gb|AIXA01018937.1|:2093-2684
MsHel-1	gb|AIXA01009533.1|:10938-11527
MsHel-1	gb|AIXA01007022.1|:16051-16765
MsHel-1	gb|AIXA01017466.1|:1346-2058
MsHel-1	gb|AIXA01006967.1|:26017-26157
MsHel-1	gb|AIXA01022335.1|:1013-1600
MsHel-1	gb|AIXA01002068.1|:21564-22135
MsHel-1	gb|AIXA01002068.1|:6233-6904
MsHel-1	gb|AIXA01001483.1|:15563-16776
MsHel-1	gb|AIXA01010756.1|:18833-19420
MsHel-1	gb|AIXA01010625.1|:6289-6883
MsHel-1	gb|AIXA01010287.1|:12778-13364
MsHel-1	gb|AIXA01008376.1|:8963-9688
MsHel-1	gb|AIXA01002764.1|:47927-48514
MsHel-1	gb|AIXA01001504.1|:29769-30354
MsHel-1	gb|AIXA01021554.1|:1365-1982
MsHel-1	gb|AIXA01013340.1|:15018-16094
MsHel-1	gb|AIXA01005227.1|:30976-31078
MsHel-1	gb|AIXA01004990.1|:192086-192377
MsHel-1	gb|AIXA01016618.1|:1996-2644
MsHel-1	gb|AIXA01013433.1|:8-203
MsHel-1	gb|AIXA01012636.1|:1062-1748
MsHel-1	gb|AIXA01007215.1|:131-819
MsHel-1	gb|AIXA01007208.1|:9683-9895
MsHel-1	gb|AIXA01002552.1|:66600-67201
MsHel-1	gb|AIXA01016727.1|:2154-2741
MsHel-1	gb|AIXA01008239.1|:66034-66657
MsHel-1	gb|AIXA01008239.1|:19474-19566
MsHel-1	gb|AIXA01006504.1|:504-1463
MsHel-1	gb|AIXA01001017.1|:46016-46230
MsHel-1	gb|AIXA01027128.1|:485-1066
MsHel-1	gb|AIXA01016655.1|:927-1514
MsHel-1	gb|AIXA01004925.1|:13275-13916
MsHel-1	gb|AIXA01002971.1|:107298-107884
MsHel-1	gb|AIXA01002971.1|:95279-95533
MsHel-1	gb|AIXA01002971.1|:36311-36660
MsHel-1	gb|AIXA01002971.1|:23356-23453
MsHel-1	gb|AIXA01000584.1|:3830-4539
MsHel-1	gb|AIXA01023982.1|:118-1397
MsHel-1	gb|AIXA01015119.1|:32592-33276

MsHel-1	gb|AIXA01003298.1|:12723-13413
MsHel-1	gb|AIXA01009067.1|:11437-12012
MsHel-1	gb|AIXA01003999.1|:66768-67390
MsHel-1	gb|AIXA01029619.1|:133-888
MsHel-1	gb|AIXA01010343.1|:29057-29746
MsHel-1	gb|AIXA01010343.1|:10939-11536
MsHel-1	gb|AIXA01033673.1|:108-674
MsHel-1	gb|AIXA01009590.1|:35800-36184
MsHel-1	gb|AIXA01009590.1|:5923-6510
MsHel-1	gb|AIXA01007197.1|:10901-11621
MsHel-1	gb|AIXA01006669.1|:6503-7199
MsHel-1	gb|AIXA01006182.1|:30621-31207
MsHel-1	gb|AIXA01005555.1|:627-1192
MsHel-1	gb|AIXA01002846.1|:92587-92721
MsHel-1	gb|AIXA01002846.1|:33714-33800
MsHel-1	gb|AIXA01002846.1|:16476-16578
MsHel-1	gb|AIXA01033295.1|:76-692
MsHel-1	gb|AIXA01012635.1|:10199-10911
MsHel-1	gb|AIXA01008827.1|:2023-2717
MsHel-1	gb|AIXA01005733.1|:7558-8119
MsHel-1	gb|AIXA01003264.1|:31626-31912
MsHel-1	gb|AIXA01003264.1|:11716-12305
MsHel-1	gb|AIXA01003670.1|:20318-20906
MsHel-1	gb|AIXA01009112.1|:23125-23781
MsHel-1	gb|AIXA01008282.1|:30154-32925
MsHel-1	gb|AIXA01002658.1|:121699-121874
MsHel-1	gb|AIXA01002658.1|:13666-14254
MsHel-1	gb|AIXA01013423.1|:11289-11875
MsHel-1	gb|AIXA01007480.1|:5161-5728
MsHel-1	gb|AIXA01001536.1|:42401-42968
MsHel-1	gb|AIXA01000713.1|:38893-39479
MsHel-1	gb|AIXA01000713.1|:24220-24342
MsHel-1	gb|AIXA01005029.1|:27625-28112
MsHel-1	gb|AIXA01001594.1|:91323-91900
MsHel-1	gb|AIXA01014564.1|:12447-13027
MsHel-1	gb|AIXA01010744.1|:19910-20207
MsHel-1	gb|AIXA01007241.1|:41340-41615
MsHel-1	gb|AIXA01003567.1|:6733-7313
MsHel-1	gb|AIXA01013430.1|:1203-1791
MsHel-1	gb|AIXA01011728.1|:21542-22544
MsHel-1	gb|AIXA01009408.1|:44137-45022
MsHel-1	gb|AIXA01001525.1|:3720-4308
MsHel-1	gb|AIXA01016147.1|:13577-14508
MsHel-1	gb|AIXA01012193.1|:490-1077
MsHel-1	gb|AIXA01011091.1|:15596-16183
MsHel-1	gb|AIXA01008474.1|:28648-29217
MsHel-1	gb|AIXA01002560.1|:39102-39809
MsHel-1	gb|AIXA01006351.1|:7654-8353

MsHel-1	gb|AIXA01002190.1|:5297-5856
MsHel-1	gb|AIXA01014122.1|:4929-5643
MsHel-1	gb|AIXA01012520.1|:3811-4391
MsHel-1	gb|AIXA01004627.1|:2709-3505
MsHel-1	gb|AIXA01004595.1|:57138-57805
MsHel-1	gb|AIXA01003067.1|:9862-10510
MsHel-1	gb|AIXA01001260.1|:9720-10430
MsHel-1	gb|AIXA01005967.1|:13078-13660
MsHel-1	gb|AIXA01006552.1|:9572-10160
MsHel-1	gb|AIXA01006175.1|:74078-74760
MsHel-1	gb|AIXA01006175.1|:57457-57976
MsHel-1	gb|AIXA01005899.1|:25016-27547
MsHel-1	gb|AIXA01002705.1|:51762-52334
MsHel-1	gb|AIXA01000858.1|:53918-54029
MsHel-1	gb|AIXA01000858.1|:23545-24121
MsHel-1	gb|AIXA01010865.1|:38874-39461
MsHel-1	gb|AIXA01009343.1|:18768-18933
MsHel-1	gb|AIXA01008573.1|:7808-8547
MsHel-1	gb|AIXA01007864.1|:1409-1999
MsHel-1	gb|AIXA01021831.1|:1295-1847
MsHel-1	gb|AIXA01007426.1|:52036-52738
MsHel-1	gb|AIXA01003134.1|:1291-2231
MsHel-1	gb|AIXA01011999.1|:28718-29059
MsHel-1	gb|AIXA01009508.1|:79100-79656
MsHel-1	gb|AIXA01007691.1|:64864-65381
MsHel-1	gb|AIXA01005550.1|:34955-35477
MsHel-1	gb|AIXA01005550.1|:5213-5800
MsHel-1	gb|AIXA01003805.1|:6536-7112
MsHel-1	gb|AIXA01003052.1|:83004-83589
MsHel-1	gb|AIXA01001144.1|:114761-115231
MsHel-1	gb|AIXA01001144.1|:95648-95883
MsHel-1	gb|AIXA01004586.1|:6493-7259
MsHel-1	gb|AIXA01004660.1|:41615-42194
MsHel-1	gb|AIXA01009884.1|:3682-4402
MsHel-1	gb|AIXA01009116.1|:12384-12967
MsHel-1	gb|AIXA01006237.1|:16277-16959
MsHel-1	gb|AIXA01000678.1|:4061-4649
MsHel-1	gb|AIXA01011840.1|:14249-14849
MsHel-1	gb|AIXA01005479.1|:23063-23194
MsHel-1	gb|AIXA01004178.1|:154503-155107
MsHel-1	gb|AIXA01010656.1|:64298-64769
MsHel-1	gb|AIXA01010656.1|:28606-30047
MsHel-1	gb|AIXA01007920.1|:5248-6069
MsHel-1	gb|AIXA01007637.1|:12908-13488
MsHel-1	gb|AIXA01005892.1|:31774-31877
MsHel-1	gb|AIXA01003389.1|:4107-4903
MsHel-1	gb|AIXA01022747.1|:1002-1631
MsHel-1	gb|AIXA01009291.1|:56132-56719

MsHel-1	gb|AIXA01012554.1|:31459-31539
MsHel-1	gb|AIXA01012486.1|:1012-1476
MsHel-1	gb|AIXA01009315.1|:16157-16742
MsHel-1	gb|AIXA01006446.1|:20845-21435
MsHel-1	gb|AIXA01004546.1|:32946-33762
MsHel-1	gb|AIXA01004546.1|:16852-17076
MsHel-1	gb|AIXA01015508.1|:14236-14939
MsHel-1	gb|AIXA01008936.1|:18254-18819
MsHel-1	gb|AIXA01007581.1|:12482-13532
MsHel-1	gb|AIXA01005595.1|:140976-141188
MsHel-1	gb|AIXA01005595.1|:55339-55507
MsHel-1	gb|AIXA01000926.1|:52706-53413
MsHel-1	gb|AIXA01029641.1|:305-885
MsHel-1	gb|AIXA01018334.1|:460-1172
MsHel-1	gb|AIXA01008548.1|:1544-2078
MsHel-1	gb|AIXA01001475.1|:37428-38398
MsHel-1	gb|AIXA01001475.1|:2-351
MsHel-1	gb|AIXA01027526.1|:505-1032
MsHel-1	gb|AIXA01006660.1|:2661-3203
MsHel-1	gb|AIXA01003637.1|:18275-19151
MsHel-1	gb|AIXA01008481.1|:63664-64180
MsHel-1	gb|AIXA01007927.1|:1010-3632
MsHel-1	gb|AIXA01022502.1|:1097-1678
MsHel-1	gb|AIXA01012310.1|:7932-8395
MsHel-1	gb|AIXA01006679.1|:125-704
MsHel-1	gb|AIXA01002978.1|:41463-42044
MsHel-1	gb|AIXA01002796.1|:32088-32207
MsHel-1	gb|AIXA01002531.1|:32120-32713
MsHel-1	gb|AIXA01008476.1|:53549-54128
MsHel-1	gb|AIXA01004941.1|:19230-19568
MsHel-1	gb|AIXA01001740.1|:1-149
MsHel-1	gb|AIXA01000046.1|:81351-81473
MsHel-1	gb|AIXA01000046.1|:42662-43922
MsHel-1	gb|AIXA01001264.1|:21802-23011
MsHel-1	gb|AIXA01008118.1|:49615-50201
MsHel-1	gb|AIXA01007033.1|:76174-76823
MsHel-1	gb|AIXA01007033.1|:18339-18908
MsHel-1	gb|AIXA01007033.1|:5890-6017
MsHel-1	gb|AIXA01001685.1|:26371-26953
MsHel-1	gb|AIXA01008127.1|:21891-22711
MsHel-1	gb|AIXA01001980.1|:52548-53136
MsHel-1	gb|AIXA01001061.1|:9631-10148
MsHel-1	gb|AIXA01007509.1|:44721-45254
MsHel-1	gb|AIXA01007509.1|:3-185
MsHel-1	gb|AIXA01003403.1|:835-1538
MsHel-1	gb|AIXA01034408.1|:76-642
MsHel-1	gb|AIXA01006853.1|:40502-40968
MsHel-1	gb|AIXA01006853.1|:1-173

MsHel-1	gb|AIXA01029466.1|:352-893
MsHel-1	gb|AIXA01006815.1|:7491-8078
MsHel-1	gb|AIXA01002782.1|:24525-25433
MsHel-1	gb|AIXA01001974.1|:8883-9445
MsHel-1	gb|AIXA01026715.1|:440-1099
MsHel-1	gb|AIXA01010504.1|:6771-7044
MsHel-1	gb|AIXA01009337.1|:5882-6456
MsHel-1	gb|AIXA01005659.1|:33566-34077
MsHel-1	gb|AIXA01011997.1|:2421-2997
MsHel-1	gb|AIXA01007943.1|:23598-23735
MsHel-1	gb|AIXA01007943.1|:8445-9225
MsHel-1	gb|AIXA01002741.1|:3710-4366
MsHel-1	gb|AIXA01001932.1|:28754-29379
MsHel-1	gb|AIXA01024852.1|:715-1292
MsHel-1	gb|AIXA01011253.1|:16657-17477
MsHel-1	gb|AIXA01001677.1|:76773-77466
MsHel-1	gb|AIXA01001328.1|:33635-34187
MsHel-1	gb|AIXA01014080.1|:16408-16996
MsHel-1	gb|AIXA01004421.1|:34861-35455
MsHel-1	gb|AIXA01002784.1|:5157-5734
MsHel-1	gb|AIXA01008533.1|:36576-36919
MsHel-1	gb|AIXA01006864.1|:4817-5410
MsHel-1	gb|AIXA01001757.1|:121860-122106
MsHel-1	gb|AIXA01004243.1|:3977-4557
MsHel-1	gb|AIXA01032573.1|:88-677
MsHel-1	gb|AIXA01015321.1|:18195-23990
MsHel-1	gb|AIXA01015321.1|:3485-3724
MsHel-1	gb|AIXA01004222.1|:15744-16866
MsHel-1	gb|AIXA01003073.1|:8274-8770
MsHel-1	gb|AIXA01000831.1|:28770-28968
MsHel-1	gb|AIXA01027428.1|:570-1040
MsHel-1	gb|AIXA01010552.1|:7384-8015
MsHel-1	gb|AIXA01007465.1|:1-635
MsHel-1	gb|AIXA01002624.1|:23983-24533
MsHel-1	gb|AIXA01002548.1|:1-599
MsHel-1	gb|AIXA01000285.1|:23882-24679
MsHel-1	gb|AIXA01020403.1|:1019-1610
MsHel-1	gb|AIXA01003689.1|:36481-37020
MsHel-1	gb|AIXA01002858.1|:28889-29354
MsHel-1	gb|AIXA01028962.1|:446-928
MsHel-1	gb|AIXA01025168.1|:523-1056
MsHel-1	gb|AIXA01009936.1|:16992-17563
MsHel-1	gb|AIXA01010697.1|:10547-11137
MsHel-1	gb|AIXA01009354.1|:13246-13826
MsHel-1	gb|AIXA01008426.1|:15103-15665
MsHel-1	gb|AIXA01005457.1|:60491-61241
MsHel-1	gb|AIXA01005022.1|:8333-9106
MsHel-1	gb|AIXA01003320.1|:43871-44514

MsHel-1	gb|AIXA01014345.1|:15120-16551
MsHel-1	gb|AIXA01012203.1|:546-1147
MsHel-1	gb|AIXA01007201.1|:5209-5709
MsHel-1	gb|AIXA01007102.1|:1782-2377
MsHel-1	gb|AIXA01018101.1|:1915-2685
MsHel-1	gb|AIXA01011730.1|:2848-3549
MsHel-1	gb|AIXA01000746.1|:27998-28752
MsHel-1	gb|AIXA01007334.1|:29052-29647
MsHel-1	gb|AIXA01005071.1|:44627-45222
MsHel-1	gb|AIXA01002148.1|:68297-68923
MsHel-1	gb|AIXA01007608.1|:17426-17593
MsHel-1	gb|AIXA01007608.1|:4582-4688
MsHel-1	gb|AIXA01005253.1|:3438-5115
MsHel-1	gb|AIXA01020555.1|:2023-2546
MsHel-1	gb|AIXA01008258.1|:23320-24231
MsHel-1	gb|AIXA01006551.1|:2-586
MsHel-1	gb|AIXA01003130.1|:1144-1733
MsHel-1	gb|AIXA01007969.1|:14541-15339
MsHel-1	gb|AIXA01007207.1|:9821-10353
MsHel-1	gb|AIXA01004966.1|:4042-5119
MsHel-1	gb|AIXA01004642.1|:12044-12635
MsHel-1	gb|AIXA01008020.1|:4907-5371
MsHel-1	gb|AIXA01032659.1|:167-723
MsHel-1	gb|AIXA01008475.1|:24910-25411
MsHel-1	gb|AIXA01015051.1|:1680-2275
MsHel-1	gb|AIXA01010784.1|:20484-21711
MsHel-1	gb|AIXA01007628.1|:48861-49509
MsHel-1	gb|AIXA01007628.1|:34021-34623
MsHel-1	gb|AIXA01005724.1|:12762-13354
MsHel-1	gb|AIXA01036330.1|:83-568
MsHel-1	gb|AIXA01014907.1|:15497-16317
MsHel-1	gb|AIXA01011837.1|:13775-13974
MsHel-1	gb|AIXA01006123.1|:42885-43355
MsHel-1	gb|AIXA01005229.1|:19172-22001
MsHel-1	gb|AIXA01002683.1|:37931-38427
MsHel-1	gb|AIXA01002683.1|:27277-27489
MsHel-1	gb|AIXA01034894.1|:138-620
MsHel-1	gb|AIXA01014568.1|:7906-8413
MsHel-1	gb|AIXA01007163.1|:13494-14065
MsHel-1	gb|AIXA01007067.1|:4026-4203
MsHel-1	gb|AIXA01006420.1|:32995-33588
MsHel-1	gb|AIXA01005766.1|:46673-47174
MsHel-1	gb|AIXA01001797.1|:191998-192356
MsHel-1	gb|AIXA01001797.1|:76612-76757
MsHel-1	gb|AIXA01000531.1|:45915-46408
MsHel-1	gb|AIXA01015316.1|:7834-8429
MsHel-1	gb|AIXA01012392.1|:2959-3456
MsHel-1	gb|AIXA01008520.1|:29429-29599

MsHel-1	gb|AIXA01007601.1|:5283-5878
MsHel-1	gb|AIXA01035060.1|:98-614
MsHel-1	gb|AIXA01014024.1|:3463-4333
MsHel-1	gb|AIXA01012228.1|:13732-14252
MsHel-1	gb|AIXA01011576.1|:5044-5508
MsHel-1	gb|AIXA01005203.1|:32272-32832
MsHel-1	gb|AIXA01004767.1|:36384-37150
MsHel-1	gb|AIXA01002196.1|:11749-12348
MsHel-1	gb|AIXA01018462.1|:2679-3143
MsHel-1	gb|AIXA01010197.1|:9287-10054
MsHel-1	gb|AIXA01006640.1|:3250-3746
MsHel-1	gb|AIXA01000969.1|:16285-17242
MsHel-1	gb|AIXA01008361.1|:41968-42636
MsHel-1	gb|AIXA01008361.1|:26309-26904
MsHel-1	gb|AIXA01007304.1|:11603-12123
MsHel-1	gb|AIXA01002870.1|:35540-36233
MsHel-1	gb|AIXA01001624.1|:31558-31748
MsHel-1	gb|AIXA01001624.1|:9742-10235
MsHel-1	gb|AIXA01005877.1|:23760-24257
MsHel-1	gb|AIXA01005877.1|:5267-5371
MsHel-1	gb|AIXA01005216.1|:4190-4782
MsHel-1	gb|AIXA01004587.1|:26620-27226
MsHel-1	gb|AIXA01004218.1|:3194-3711
MsHel-1	gb|AIXA01007898.1|:105404-105908
MsHel-1	gb|AIXA01006781.1|:6746-7339
MsHel-1	gb|AIXA01005853.1|:9964-10563
MsHel-1	gb|AIXA01004319.1|:53611-54204
MsHel-1	gb|AIXA01012245.1|:1158-1750
MsHel-1	gb|AIXA01009984.1|:9361-9825
MsHel-1	gb|AIXA01009624.1|:3455-3970
MsHel-1	gb|AIXA01005952.1|:4945-5540
MsHel-1	gb|AIXA01006960.1|:16397-16708
MsHel-1	gb|AIXA01006960.1|:2750-3277
MsHel-1	gb|AIXA01006235.1|:6500-7002
MsHel-1	gb|AIXA01005448.1|:28918-29539
MsHel-1	gb|AIXA01004727.1|:69180-69400
MsHel-1	gb|AIXA01031388.1|:256-786
MsHel-1	gb|AIXA01029572.1|:419-890
MsHel-1	gb|AIXA01008262.1|:35716-36290
MsHel-1	gb|AIXA01003166.1|:52156-52240
MsHel-1	gb|AIXA01003166.1|:5162-5552
MsHel-1	gb|AIXA01005434.1|:24257-24753
MsHel-1	gb|AIXA01002981.1|:3535-4585
MsHel-1	gb|AIXA01033633.1|:165-668
MsHel-1	gb|AIXA01014780.1|:5848-6418
MsHel-1	gb|AIXA01011484.1|:26355-26711
MsHel-1	gb|AIXA01007857.1|:2707-3370
MsHel-1	gb|AIXA01011512.1|:3089-3754

MsHel-1	gb|AIXA01004619.1|:44458-45312
MsHel-1	gb|AIXA01004335.1|:84861-85431
MsHel-1	gb|AIXA01000567.1|:14721-15735
MsHel-1	gb|AIXA01014225.1|:7117-7599
MsHel-1	gb|AIXA01008738.1|:34693-34910
MsHel-1	gb|AIXA01008738.1|:11522-11635
MsHel-1	gb|AIXA01007790.1|:96482-96576
MsHel-1	gb|AIXA01004774.1|:15132-16554
MsHel-1	gb|AIXA01004670.1|:80728-81519
MsHel-1	gb|AIXA01004670.1|:49955-50550
MsHel-1	gb|AIXA01003868.1|:4224-4921
MsHel-1	gb|AIXA01001924.1|:3772-4331
MsHel-1	gb|AIXA01000102.1|:54531-54992
MsHel-1	gb|AIXA01026186.1|:116-591
MsHel-1	gb|AIXA01006211.1|:19295-20130
MsHel-1	gb|AIXA01004634.1|:13771-13890
MsHel-1	gb|AIXA01003713.1|:17612-18203
MsHel-1	gb|AIXA01010687.1|:8985-9492
MsHel-1	gb|AIXA01000813.1|:35028-35247
MsHel-1	gb|AIXA01000813.1|:22368-22831
MsHel-1	gb|AIXA01028396.1|:350-965
MsHel-1	gb|AIXA01009304.1|:14691-15687
MsHel-1	gb|AIXA01007661.1|:15261-15855
MsHel-1	gb|AIXA01003201.1|:2240-2751
MsHel-1	gb|AIXA01018619.1|:4616-5210
MsHel-1	gb|AIXA01011984.1|:25623-26216
MsHel-1	gb|AIXA01008169.1|:36495-36991
MsHel-1	gb|AIXA01006896.1|:9687-10280
MsHel-1	gb|AIXA01000747.1|:139594-140276
MsHel-1	gb|AIXA01000747.1|:14116-14204
MsHel-1	gb|AIXA01000294.1|:17477-18096
MsHel-1	gb|AIXA01013106.1|:4647-5143
MsHel-1	gb|AIXA01002884.1|:17952-18396
MsHel-1	gb|AIXA01000306.1|:11211-12076
MsHel-1	gb|AIXA01017435.1|:578-1042
MsHel-1	gb|AIXA01013898.1|:16088-16691
MsHel-1	gb|AIXA01013068.1|:1579-2167
MsHel-1	gb|AIXA01011964.1|:47765-47991
MsHel-1	gb|AIXA01011964.1|:303-766
MsHel-1	gb|AIXA01007152.1|:35720-36206
MsHel-1	gb|AIXA01003660.1|:8515-9108
MsHel-1	gb|AIXA01024508.1|:848-1337
MsHel-1	gb|AIXA01001947.1|:5614-6077
MsHel-1	gb|AIXA01025033.1|:728-1269
MsHel-1	gb|AIXA01008966.1|:5813-6308
MsHel-1	gb|AIXA01008101.1|:3936-6098
MsHel-1	gb|AIXA01001990.1|:4118-4717
MsHel-1	gb|AIXA01025000.1|:804-1274

MsHel-1	gb|AIXA01016771.1|:6026-6614
MsHel-1	gb|AIXA01001498.1|:12480-12945
MsHel-1	gb|AIXA01006971.1|:68792-69967
MsHel-1	gb|AIXA01036196.1|:52-568
MsHel-1	gb|AIXA01015407.1|:4529-5180
MsHel-1	gb|AIXA01009865.1|:121595-122376
MsHel-1	gb|AIXA01009865.1|:83493-83878
MsHel-1	gb|AIXA01009406.1|:4863-5050
MsHel-1	gb|AIXA01008994.1|:58422-59230
MsHel-1	gb|AIXA01003690.1|:116463-116663
MsHel-1	gb|AIXA01003690.1|:94626-94858
MsHel-1	gb|AIXA01003690.1|:71433-72193
MsHel-1	gb|AIXA01002570.1|:18793-19061
MsHel-1	gb|AIXA01001566.1|:12919-13514
MsHel-1	gb|AIXA01010692.1|:14044-14698
MsHel-1	gb|AIXA01004261.1|:2058-2841
MsHel-1	gb|AIXA01003234.1|:73344-73423
MsHel-1	gb|AIXA01003234.1|:14286-15128
MsHel-1	gb|AIXA01002583.1|:4950-5412
MsHel-1	gb|AIXA01001902.1|:34558-35022
MsHel-1	gb|AIXA01001807.1|:911-1873
MsHel-1	gb|AIXA01001104.1|:55131-55730
MsHel-1	gb|AIXA01016310.1|:3282-3746
MsHel-1	gb|AIXA01006012.1|:35661-36125
MsHel-1	gb|AIXA01004342.1|:18737-19238
MsHel-1	gb|AIXA01001660.1|:45213-45801
MsHel-1	gb|AIXA01001480.1|:16960-17559
MsHel-1	gb|AIXA01001282.1|:10030-10135
MsHel-1	gb|AIXA01000637.1|:9235-10101
MsHel-1	gb|AIXA01017904.1|:1305-1779
MsHel-1	gb|AIXA01010956.1|:3611-4208
MsHel-1	gb|AIXA01006866.1|:12-604
MsHel-1	gb|AIXA01006695.1|:6316-6811
MsHel-1	gb|AIXA01032464.1|:187-651
MsHel-1	gb|AIXA01018744.1|:44-540
MsHel-1	gb|AIXA01008182.1|:49911-50170
MsHel-1	gb|AIXA01007858.1|:25228-25693
MsHel-1	gb|AIXA01007420.1|:39424-39826
MsHel-1	gb|AIXA01007420.1|:26748-27299
MsHel-1	gb|AIXA01002903.1|:2863-3323
MsHel-1	gb|AIXA01002294.1|:15577-16038
MsHel-1	gb|AIXA01000404.1|:29855-30433
MsHel-1	gb|AIXA01011237.1|:2660-3120
MsHel-1	gb|AIXA01002718.1|:99147-99504
MsHel-1	gb|AIXA01002718.1|:10483-10588
MsHel-1	gb|AIXA01001586.1|:1356-1814
MsHel-1	gb|AIXA01001383.1|:30946-31531
MsHel-1	gb|AIXA01001383.1|:8293-8420

MsHel-1	gb|AIXA01017290.1|:228-1184
MsHel-1	gb|AIXA01007875.1|:11738-12565
MsHel-1	gb|AIXA01015641.1|:15874-16529
MsHel-1	gb|AIXA01015641.1|:2201-2363
MsHel-1	gb|AIXA01006087.1|:10985-11442
MsHel-1	gb|AIXA01003093.1|:1634-2437
MsHel-1	gb|AIXA01009681.1|:16053-16648
MsHel-1	gb|AIXA01006756.1|:19452-19950
MsHel-1	gb|AIXA01004763.1|:21936-22067
MsHel-1	gb|AIXA01031740.1|:135-632
MsHel-1	gb|AIXA01008970.1|:18774-19238
MsHel-1	gb|AIXA01006855.1|:48121-48572
MsHel-1	gb|AIXA01003924.1|:63375-63913
MsHel-1	gb|AIXA01033630.1|:198-677
MsHel-1	gb|AIXA01005024.1|:98751-99181
MsHel-1	gb|AIXA01000636.1|:12214-12793
MsHel-1	gb|AIXA01016246.1|:10757-11218
MsHel-1	gb|AIXA01009219.1|:55217-55344
MsHel-1	gb|AIXA01003072.1|:90072-90582
MsHel-1	gb|AIXA01001553.1|:6816-7996
MsHel-1	gb|AIXA01000286.1|:30798-31586
MsHel-1	gb|AIXA01021417.1|:13-520
MsHel-1	gb|AIXA01017994.1|:2291-2799
MsHel-1	gb|AIXA01007883.1|:4241-4700
MsHel-1	gb|AIXA01006723.1|:34197-34362
MsHel-1	gb|AIXA01004821.1|:23348-23937
MsHel-1	gb|AIXA01015560.1|:7148-7722
MsHel-1	gb|AIXA01010765.1|:1177-1772
MsHel-1	gb|AIXA01009285.1|:14080-14779
MsHel-1	gb|AIXA01005882.1|:2617-2790
MsHel-1	gb|AIXA01001692.1|:27185-27649
MsHel-1	gb|AIXA01032381.1|:234-699
MsHel-1	gb|AIXA01013045.1|:11133-11597
MsHel-1	gb|AIXA01004878.1|:11688-12174
MsHel-1	gb|AIXA01013129.1|:32999-33459
MsHel-1	gb|AIXA01012946.1|:14525-15218
MsHel-1	gb|AIXA01011987.1|:68135-68703
MsHel-1	gb|AIXA01011987.1|:39774-39967
MsHel-1	gb|AIXA01011987.1|:5545-6012
MsHel-1	gb|AIXA01009125.1|:17216-17855
MsHel-1	gb|AIXA01004461.1|:3541-3694
MsHel-1	gb|AIXA01001526.1|:4573-5031
MsHel-1	gb|AIXA01014299.1|:13263-13861
MsHel-1	gb|AIXA01013803.1|:523-1118
MsHel-1	gb|AIXA01001899.1|:179-535
MsHel-1	gb|AIXA01014799.1|:8987-9497
MsHel-1	gb|AIXA01008088.1|:26749-27382
MsHel-1	gb|AIXA01019012.1|:4678-5191

MsHel-1	gb|AIXA01016630.1|:4074-4317
MsHel-1	gb|AIXA01003935.1|:24194-24659
MsHel-1	gb|AIXA01002717.1|:37892-38351
MsHel-1	gb|AIXA01000134.1|:4899-5444
MsHel-1	gb|AIXA01013403.1|:4241-4773
MsHel-1	gb|AIXA01006266.1|:3225-3810
MsHel-1	gb|AIXA01004554.1|:19999-20462
MsHel-1	gb|AIXA01002882.1|:8227-8818
MsHel-1	gb|AIXA01024723.1|:911-1305
MsHel-1	gb|AIXA01021313.1|:1660-2092
MsHel-1	gb|AIXA01009488.1|:32205-32352
MsHel-1	gb|AIXA01009404.1|:24597-25082
MsHel-1	gb|AIXA01003112.1|:15217-15812
MsHel-1	gb|AIXA01004699.1|:32758-33151
MsHel-1	gb|AIXA01002103.1|:71040-72555
MsHel-1	gb|AIXA01030142.1|:446-853
MsHel-1	gb|AIXA01005014.1|:37294-37733
MsHel-1	gb|AIXA01003347.1|:20672-21144
MsHel-1	gb|AIXA01000390.1|:6956-7477
MsHel-1	gb|AIXA01009029.1|:19500-19924
MsHel-1	gb|AIXA01008921.1|:41121-41708
MsHel-1	gb|AIXA01000649.1|:93281-93783
MsHel-1	gb|AIXA01000649.1|:15917-16564
MsHel-1	gb|AIXA01000649.1|:2998-3643
MsHel-1	gb|AIXA01000027.1|:16259-16722
MsHel-1	gb|AIXA01036090.1|:188-577
MsHel-1	gb|AIXA01015400.1|:7208-7802
MsHel-1	gb|AIXA01008396.1|:2073-2620
MsHel-1	gb|AIXA01007849.1|:18394-19341
MsHel-1	gb|AIXA01003644.1|:12243-12635
MsHel-1	gb|AIXA01037229.1|:83-537
MsHel-1	gb|AIXA01006582.1|:3750-4314
MsHel-1	gb|AIXA01000821.1|:95865-96323
MsHel-1	gb|AIXA01000103.1|:21280-22066
MsHel-1	gb|AIXA01023795.1|:995-1439
MsHel-1	gb|AIXA01005141.1|:15009-15536
MsHel-1	gb|AIXA01028327.1|:574-969
MsHel-1	gb|AIXA01017628.1|:14185-14778
MsHel-1	gb|AIXA01016279.1|:40895-41440
MsHel-1	gb|AIXA01000938.1|:28235-28769
MsHel-1	gb|AIXA01012267.1|:2709-3165
MsHel-1	gb|AIXA01001074.1|:9546-9978
MsHel-1	gb|AIXA01020283.1|:2403-2843
MsHel-1	gb|AIXA01006622.1|:32041-32492
MsHel-1	gb|AIXA01004470.1|:64223-64727
MsHel-1	gb|AIXA01004470.1|:47737-47904
MsHel-1	gb|AIXA01003504.1|:26594-27617
MsHel-1	gb|AIXA01028756.1|:5-420

MsHel-1	gb|AIXA01018562.1|:1693-2368
MsHel-1	gb|AIXA01013436.1|:35843-36374
MsHel-1	gb|AIXA01013436.1|:18950-19486
MsHel-1	gb|AIXA01007354.1|:125429-125626
MsHel-1	gb|AIXA01007354.1|:51348-51979
MsHel-1	gb|AIXA01006311.1|:15285-15746
MsHel-1	gb|AIXA01000161.1|:38577-38762
MsHel-1	gb|AIXA01000161.1|:27436-28105
MsHel-1	gb|AIXA01010152.1|:46632-47025
MsHel-1	gb|AIXA01019378.1|:1106-1565
MsHel-1	gb|AIXA01009237.1|:2080-11533
MsHel-1	gb|AIXA01002451.1|:2830-3420
MsHel-1	gb|AIXA01001025.1|:13464-13926
MsHel-1	gb|AIXA01030965.1|:367-803
MsHel-1	gb|AIXA01014553.1|:427-887
MsHel-1	gb|AIXA01008438.1|:825-1271
MsHel-1	gb|AIXA01005773.1|:23668-24241
MsHel-1	gb|AIXA01000481.1|:77484-78048
MsHel-1	gb|AIXA01007463.1|:71-635
MsHel-1	gb|AIXA01005560.1|:2-440
MsHel-1	gb|AIXA01006898.1|:21878-22299
MsHel-1	gb|AIXA01006767.1|:2982-3359
MsHel-1	gb|AIXA01031008.1|:217-805
MsHel-1	gb|AIXA01000442.1|:1648-2563
MsHel-1	gb|AIXA01025116.1|:760-1260
MsHel-1	gb|AIXA01007717.1|:32827-33502
MsHel-1	gb|AIXA01007717.1|:17859-18217
MsHel-1	gb|AIXA01009667.1|:3-440
MsHel-1	gb|AIXA01007054.1|:20165-20649
MsHel-1	gb|AIXA01006759.1|:10082-10511
MsHel-1	gb|AIXA01000688.1|:108029-108498
MsHel-1	gb|AIXA01006423.1|:189681-189849
MsHel-1	gb|AIXA01005559.1|:71207-71623
MsHel-1	gb|AIXA01003054.1|:21207-21655
MsHel-1	gb|AIXA01002427.1|:68834-69226
MsHel-1	gb|AIXA01000539.1|:11149-11636
MsHel-1	gb|AIXA01000069.1|:22475-22883
MsHel-1	gb|AIXA01013800.1|:1909-2146
MsHel-1	gb|AIXA01009290.1|:14271-14725
MsHel-1	gb|AIXA01007542.1|:32871-33338
MsHel-1	gb|AIXA01001456.1|:29009-29256
MsHel-1	gb|AIXA01001456.1|:3882-4331
MsHel-1	gb|AIXA01019260.1|:371-789
MsHel-1	gb|AIXA01008243.1|:14813-15625
MsHel-1	gb|AIXA01006729.1|:43925-44014
MsHel-1	gb|AIXA01006729.1|:8301-8741
MsHel-1	gb|AIXA01004101.1|:14640-15394
MsHel-1	gb|AIXA01002953.1|:76158-76668

MsHel-1	gb|AIXA01002953.1|:26578-26712
MsHel-1	gb|AIXA01018162.1|:10581-11152
MsHel-1	gb|AIXA01006664.1|:6962-7390
MsHel-1	gb|AIXA01002044.1|:93175-93649
MsHel-1	gb|AIXA01002044.1|:28180-28607
MsHel-1	gb|AIXA01002044.1|:14351-17434
MsHel-1	gb|AIXA01002044.1|:1653-1813
MsHel-1	gb|AIXA01001907.1|:6995-7397
MsHel-1	gb|AIXA01028375.1|:538-933
MsHel-1	gb|AIXA01012630.1|:5619-6077
MsHel-1	gb|AIXA01011312.1|:14342-14719
MsHel-1	gb|AIXA01005728.1|:13432-13861
MsHel-1	gb|AIXA01028788.1|:533-940
MsHel-1	gb|AIXA01009114.1|:9958-10330
MsHel-1	gb|AIXA01034943.1|:4-444
MsHel-1	gb|AIXA01004928.1|:49982-50360
MsHel-1	gb|AIXA01005088.1|:10417-10832
MsHel-1	gb|AIXA01003687.1|:12008-12584
MsHel-1	gb|AIXA01000090.1|:29464-29854
MsHel-1	gb|AIXA01036633.1|:185-557
MsHel-1	gb|AIXA01031762.1|:215-759
MsHel-1	gb|AIXA01004643.1|:68371-68793
MsHel-1	gb|AIXA01003886.1|:1846-2283
MsHel-1	gb|AIXA01002804.1|:15014-15411
MsHel-1	gb|AIXA01002489.1|:5084-5543
MsHel-1	gb|AIXA01002175.1|:4425-4858
MsHel-1	gb|AIXA01016747.1|:1593-2001
MsHel-1	gb|AIXA01008851.1|:35294-35820
MsHel-1	gb|AIXA01033359.1|:194-686
MsHel-1	gb|AIXA01002408.1|:41076-41315
MsHel-1	gb|AIXA01001863.1|:20972-21432
MsHel-1	gb|AIXA01004632.1|:5351-5817
MsHel-1	gb|AIXA01012346.1|:48019-48583
MsHel-1	gb|AIXA01012346.1|:7491-13347
MsHel-1	gb|AIXA01002458.1|:1-373
MsHel-1	gb|AIXA01005964.1|:4893-5472
MsHel-1	gb|AIXA01004755.1|:39112-39714
MsHel-1	gb|AIXA01004755.1|:2880-2973
MsHel-1	gb|AIXA01004337.1|:7658-8078
MsHel-1	gb|AIXA01016714.1|:29255-29694
MsHel-1	gb|AIXA01013338.1|:466-949
MsHel-1	gb|AIXA01011650.1|:59321-59616
MsHel-1	gb|AIXA01011650.1|:5511-6054
MsHel-1	gb|AIXA01002864.1|:32153-32521
MsHel-1	gb|AIXA01008313.1|:49211-49723
MsHel-1	gb|AIXA01008313.1|:12001-14951
MsHel-1	gb|AIXA01006678.1|:490-925
MsHel-1	gb|AIXA01006395.1|:12019-12398

MsHel-1	gb|AIXA01004993.1|:5055-5527
MsHel-1	gb|AIXA01013814.1|:14151-14694
MsHel-1	gb|AIXA01006862.1|:38636-39123
MsHel-1	gb|AIXA01009980.1|:2119-2584
MsHel-1	gb|AIXA01005944.1|:3-299
MsHel-1	gb|AIXA01004089.1|:101428-101706
MsHel-1	gb|AIXA01004089.1|:19879-20412
MsHel-1	gb|AIXA01003422.1|:20594-20970
MsHel-1	gb|AIXA01002221.1|:4286-4734
MsHel-1	gb|AIXA01000805.1|:1187-2041
MsHel-1	gb|AIXA01012796.1|:4318-4921
MsHel-1	gb|AIXA01007645.1|:2081-2553
MsHel-1	gb|AIXA01005045.1|:40741-41117
MsHel-1	gb|AIXA01004285.1|:14103-14754
MsHel-1	gb|AIXA01026432.1|:745-1128
MsHel-1	gb|AIXA01015999.1|:5868-6781
MsHel-1	gb|AIXA01010330.1|:24306-24811
MsHel-1	gb|AIXA01010330.1|:415-502
MsHel-1	gb|AIXA01008522.1|:16662-17071
MsHel-1	gb|AIXA01007196.1|:1271-1834
MsHel-1	gb|AIXA01002838.1|:9607-10154
MsHel-1	gb|AIXA01001146.1|:7886-8550
MsHel-1	gb|AIXA01010717.1|:5876-6250
MsHel-1	gb|AIXA01010184.1|:11127-11573
MsHel-1	gb|AIXA01006133.1|:1-438
MsHel-1	gb|AIXA01004733.1|:47053-47724
MsHel-1	gb|AIXA01036314.1|:225-568
MsHel-1	gb|AIXA01010394.1|:55553-56021
MsHel-1	gb|AIXA01000403.1|:78573-78752
MsHel-1	gb|AIXA01009382.1|:31115-31249
MsHel-1	gb|AIXA01006931.1|:6-400
MsHel-1	gb|AIXA01003535.1|:12602-13051
MsHel-1	gb|AIXA01000814.1|:30100-30557
MsHel-1	gb|AIXA01015147.1|:7194-7752
MsHel-1	gb|AIXA01005051.1|:15188-15332
MsHel-1	gb|AIXA01009366.1|:48132-48820
MsHel-1	gb|AIXA01008218.1|:5704-6377
MsHel-1	gb|AIXA01007625.1|:41525-41989
MsHel-1	gb|AIXA01007531.1|:2639-2986
MsHel-1	gb|AIXA01003858.1|:15231-15703
MsHel-1	gb|AIXA01031335.1|:439-786
MsHel-1	gb|AIXA01021053.1|:1866-2213
MsHel-1	gb|AIXA01015972.1|:24067-24181
MsHel-1	gb|AIXA01005444.1|:33916-34600
MsHel-1	gb|AIXA01002907.1|:11688-12039
MsHel-1	gb|AIXA01008932.1|:5777-6244
MsHel-1	gb|AIXA01000562.1|:106020-106487
MsHel-1	gb|AIXA01011159.1|:10593-11273

MsHel-1	gb|AIXA01004675.1|:9169-9855
MsHel-1	gb|AIXA01011112.1|:9382-9841
MsHel-1	gb|AIXA01008433.1|:37929-38231
MsHel-1	gb|AIXA01008433.1|:25418-25991
MsHel-1	gb|AIXA01006780.1|:51489-51771
MsHel-1	gb|AIXA01006780.1|:9245-9974
MsHel-1	gb|AIXA01000923.1|:8119-8614
MsHel-1	gb|AIXA01013679.1|:3408-3844
MsHel-1	gb|AIXA01008403.1|:10259-10706
MsHel-1	gb|AIXA01006299.1|:41257-41911
MsHel-1	gb|AIXA01004327.1|:113423-113517
MsHel-1	gb|AIXA01004327.1|:83926-84286
MsHel-1	gb|AIXA01024954.1|:890-1262
MsHel-1	gb|AIXA01008883.1|:2746-3217
MsHel-1	gb|AIXA01022555.1|:1259-1662
MsHel-1	gb|AIXA01013138.1|:826-1612
MsHel-1	gb|AIXA01004857.1|:1423-3351
MsHel-1	gb|AIXA01003121.1|:33249-33697
MsHel-1	gb|AIXA01009537.1|:62649-63222
MsHel-1	gb|AIXA01009537.1|:23950-24104
MsHel-1	gb|AIXA01005576.1|:20316-20696
MsHel-1	gb|AIXA01004519.1|:26749-27078
MsHel-1	gb|AIXA01003873.1|:32757-32874
MsHel-1	gb|AIXA01012419.1|:25170-25633
MsHel-1	gb|AIXA01003942.1|:13913-14439
MsHel-1	gb|AIXA01002590.1|:16063-16577
MsHel-1	gb|AIXA01019137.1|:7467-7805
MsHel-1	gb|AIXA01009835.1|:15829-16235
MsHel-1	gb|AIXA01001970.1|:17683-17992
MsHel-1	gb|AIXA01001941.1|:58560-59127
MsHel-1	gb|AIXA01001508.1|:36327-36794
MsHel-1	gb|AIXA01014446.1|:3305-3880
MsHel-1	gb|AIXA01007613.1|:3600-4067
MsHel-1	gb|AIXA01013176.1|:3848-4191
MsHel-1	gb|AIXA01005100.1|:17451-17795
MsHel-1	gb|AIXA01000867.1|:2-346
MsHel-1	gb|AIXA01017696.1|:5772-6082
MsHel-1	gb|AIXA01011449.1|:11844-12288
MsHel-1	gb|AIXA01011111.1|:2172-2594
MsHel-1	gb|AIXA01010781.1|:2316-2694
MsHel-1	gb|AIXA01007199.1|:28331-28704
MsHel-1	gb|AIXA01005574.1|:27484-28054
MsHel-1	gb|AIXA01004624.1|:4007-4410
MsHel-1	gb|AIXA01010187.1|:11203-11370
MsHel-1	gb|AIXA01007612.1|:28088-28479
MsHel-1	gb|AIXA01006353.1|:10179-10501
MsHel-1	gb|AIXA01008509.1|:53984-54151
MsHel-1	gb|AIXA01007462.1|:13134-13607

MsHel-1	gb|AIXA01000334.1|:3291-3612
MsHel-1	gb|AIXA01018293.1|:3401-3743
MsHel-1	gb|AIXA01005770.1|:24138-24969
MsHel-1	gb|AIXA01000729.1|:1-349
MsHel-1	gb|AIXA01000498.1|:2918-3365
MsHel-1	gb|AIXA01010785.1|:87675-88146
MsHel-1	gb|AIXA01010785.1|:53421-53627
MsHel-1	gb|AIXA01006617.1|:1-340
MsHel-1	gb|AIXA01011318.1|:39540-40010
MsHel-1	gb|AIXA01008040.1|:10310-10846
MsHel-1	gb|AIXA01005549.1|:31297-31765
MsHel-1	gb|AIXA01001292.1|:36123-36518
MsHel-1	gb|AIXA01026428.1|:678-1129
MsHel-1	gb|AIXA01018794.1|:259-721
MsHel-1	gb|AIXA01014273.1|:10933-11249
MsHel-1	gb|AIXA01011251.1|:2509-2590
MsHel-1	gb|AIXA01005389.1|:8932-9449
MsHel-1	gb|AIXA01021431.1|:1710-2041
MsHel-1	gb|AIXA01018550.1|:5688-6579
MsHel-1	gb|AIXA01009271.1|:54017-55318
MsHel-1	gb|AIXA01003960.1|:2071-2532
MsHel-1	gb|AIXA01003853.1|:22396-22768
MsHel-1	gb|AIXA01003820.1|:68555-68952
MsHel-1	gb|AIXA01000376.1|:37673-38143
MsHel-1	gb|AIXA01015579.1|:33006-33479
MsHel-1	gb|AIXA01007534.1|:13406-13605
MsHel-1	gb|AIXA01005456.1|:2200-2912
MsHel-1	gb|AIXA01003118.1|:65150-65615
MsHel-1	gb|AIXA01001556.1|:8520-9228
MsHel-1	gb|AIXA01014641.1|:46477-47056
MsHel-1	gb|AIXA01005904.1|:212569-212912
MsHel-1	gb|AIXA01005904.1|:152598-153406
MsHel-1	gb|AIXA01019589.1|:1861-2263
MsHel-1	gb|AIXA01016772.1|:1-447
MsHel-1	gb|AIXA01011055.1|:1407-1932
MsHel-1	gb|AIXA01007442.1|:2605-3063
MsHel-1	gb|AIXA01005881.1|:29698-30062
MsHel-1	gb|AIXA01005881.1|:16358-16492
MsHel-1	gb|AIXA01011674.1|:27434-27955
MsHel-1	gb|AIXA01011674.1|:3768-4965
MsHel-1	gb|AIXA01007715.1|:36970-38440
MsHel-1	gb|AIXA01005119.1|:6560-7204
MsHel-1	gb|AIXA01004561.1|:35943-36547
MsHel-1	gb|AIXA01003681.1|:29901-30339
MsHel-1	gb|AIXA01003014.1|:15998-16532
MsHel-1	gb|AIXA01002007.1|:1878-2242
MsHel-1	gb|AIXA01001707.1|:62451-62754
MsHel-1	gb|AIXA01022889.1|:1217-1533

MsHel-1	gb|AIXA01011528.1|:23615-23821
MsHel-1	gb|AIXA01001362.1|:42486-42781
MsHel-1	gb|AIXA01021677.1|:4-314
MsHel-1	gb|AIXA01000291.1|:1-367
MsHel-1	gb|AIXA01016790.1|:9218-9867
MsHel-1	gb|AIXA01008023.1|:76482-76964
MsHel-1	gb|AIXA01028718.1|:623-943
MsHel-1	gb|AIXA01010098.1|:5501-5599
MsHel-1	gb|AIXA01007125.1|:10525-10983
MsHel-1	gb|AIXA01018102.1|:1-335
MsHel-1	gb|AIXA01008769.1|:10906-11346
MsHel-1	gb|AIXA01008342.1|:17248-17691
MsHel-1	gb|AIXA01007121.1|:15160-15326
MsHel-1	gb|AIXA01008511.1|:14525-14966
MsHel-1	gb|AIXA01004655.1|:2676-3167
MsHel-1	gb|AIXA01018618.1|:6297-6772
MsHel-1	gb|AIXA01004863.1|:1200-1587
MsHel-1	gb|AIXA01004439.1|:2360-3087
MsHel-1	gb|AIXA01030160.1|:557-853
MsHel-1	gb|AIXA01009982.1|:4751-5360
MsHel-1	gb|AIXA01009511.1|:15794-16270
MsHel-1	gb|AIXA01002526.1|:15324-16032
MsHel-1	gb|AIXA01000369.1|:13758-14086
MsHel-1	gb|AIXA01022673.1|:715-1137
MsHel-1	gb|AIXA01015944.1|:1970-2244
MsHel-1	gb|AIXA01003968.1|:19742-20199
MsHel-1	gb|AIXA01002266.1|:14797-15196
MsHel-1	gb|AIXA01026958.1|:750-1079
MsHel-1	gb|AIXA01003548.1|:3094-3532
MsHel-1	gb|AIXA01012915.1|:8-345
MsHel-1	gb|AIXA01019237.1|:2364-2631
MsHel-1	gb|AIXA01008388.1|:5860-6145
MsHel-1	gb|AIXA01001611.1|:14476-15217
MsHel-1	gb|AIXA01012145.1|:57064-57497
MsHel-1	gb|AIXA01007069.1|:55774-56405
MsHel-1	gb|AIXA01003476.1|:11477-11885
MsHel-1	gb|AIXA01028453.1|:1-272
MsHel-1	gb|AIXA01011464.1|:14968-15170
MsHel-1	gb|AIXA01011464.1|:14-297
MsHel-1	gb|AIXA01015194.1|:6068-6398
MsHel-1	gb|AIXA01010502.1|:1-291
MsHel-1	gb|AIXA01004894.1|:23437-24064
MsHel-1	gb|AIXA01002762.1|:71542-71660
MsHel-1	gb|AIXA01008856.1|:8034-8483
MsHel-1	gb|AIXA01003245.1|:1-282
MsHel-1	gb|AIXA01011561.1|:3-333
MsHel-1	gb|AIXA01006028.1|:1-309
MsHel-1	gb|AIXA01004132.1|:1-269

MsHel-1	gb|AIXA01002535.1|:1-285
MsHel-1	gb|AIXA01008247.1|:26400-26874
MsHel-1	gb|AIXA01017955.1|:429-1008
MsHel-1	gb|AIXA01003597.1|:63290-63560
MsHel-1	gb|AIXA01003597.1|:530-613
MsHel-1	gb|AIXA01003113.1|:2127-2532
MsHel-1	gb|AIXA01013775.1|:6765-7042
MsHel-1	gb|AIXA01005737.1|:27091-27376
MsHel-1	gb|AIXA01006227.1|:3-275
MsHel-1	gb|AIXA01033532.1|:239-680
MsHel-1	gb|AIXA01020765.1|:2093-2372
MsHel-1	gb|AIXA01014486.1|:6549-6835
MsHel-1	gb|AIXA01009787.1|:7089-7440
MsHel-1	gb|AIXA01007614.1|:28126-28494
MsHel-1	gb|AIXA01006556.1|:3992-4555
MsHel-1	gb|AIXA01005394.1|:50329-50745
MsHel-1	gb|AIXA01016537.1|:25160-25598
MsHel-1	gb|AIXA01031294.1|:1-310
MsHel-1	gb|AIXA01005845.1|:4028-4604
MsHel-1	gb|AIXA01022874.1|:1053-1508
MsHel-1	gb|AIXA01006539.1|:7470-7725
MsHel-1	gb|AIXA01003878.1|:20543-21205
MsHel-1	gb|AIXA01001885.1|:73-3875
MsHel-1	gb|AIXA01034631.1|:171-572
MsHel-1	gb|AIXA01013248.1|:1-320
MsHel-1	gb|AIXA01011019.1|:1-336
MsHel-1	gb|AIXA01002629.1|:136256-136474
MsHel-1	gb|AIXA01015996.1|:2-355
MsHel-1	gb|AIXA01011282.1|:86500-87118
MsHel-1	gb|AIXA01001314.1|:28662-28999
MsHel-1	gb|AIXA01033381.1|:21-282
MsHel-1	gb|AIXA01019088.1|:794-1109
MsHel-1	gb|AIXA01014254.1|:4240-4505
MsHel-1	gb|AIXA01009796.1|:10678-11219
MsHel-1	gb|AIXA01029924.1|:547-867
MsHel-1	gb|AIXA01008268.1|:1-286
MsHel-1	gb|AIXA01000508.1|:22012-22280
MsHel-1	gb|AIXA01009862.1|:14860-15324
MsHel-1	gb|AIXA01002680.1|:33005-33288
MsHel-1	gb|AIXA01037685.1|:256-522
MsHel-1	gb|AIXA01012109.1|:1-294
MsHel-1	gb|AIXA01008093.1|:13270-15208
MsHel-1	gb|AIXA01012686.1|:8229-8935
MsHel-1	gb|AIXA01012948.1|:22587-23165
MsHel-1	gb|AIXA01003501.1|:2368-2938
MsHel-1	gb|AIXA01003765.1|:30-294
MsHel-1	gb|AIXA01031134.1|:410-798
MsHel-1	gb|AIXA01013034.1|:1-261

MsHel-1	gb|AIXA01011586.1|:1-301
MsHel-1	gb|AIXA01009355.1|:35747-36015
MsHel-1	gb|AIXA01007261.1|:3-275
MsHel-1	gb|AIXA01020299.1|:3-274
MsHel-1	gb|AIXA01006232.1|:2475-3031
MsHel-1	gb|AIXA01029363.1|:594-902
MsHel-1	gb|AIXA01017572.1|:1507-1781
MsHel-1	gb|AIXA01010782.1|:36041-36488
MsHel-1	gb|AIXA01008770.1|:1-261
MsHel-1	gb|AIXA01002191.1|:1-276
MsHel-1	gb|AIXA01003893.1|:48545-48802
MsHel-1	gb|AIXA01000860.1|:9606-9871
MsHel-1	gb|AIXA01012049.1|:49983-50082
MsHel-1	gb|AIXA01012049.1|:22053-22516
MsHel-1	gb|AIXA01005089.1|:30350-30704
MsHel-1	gb|AIXA01036912.1|:1-260
MsHel-1	gb|AIXA01013813.1|:116-380
MsHel-1	gb|AIXA01003070.1|:44757-45149
MsHel-1	gb|AIXA01003070.1|:1-169
MsHel-1	gb|AIXA01003048.1|:4058-4405
MsHel-1	gb|AIXA01005734.1|:18058-19619
MsHel-1	gb|AIXA01010957.1|:1-250
MsHel-1	gb|AIXA01004867.1|:53259-53370
MsHel-1	gb|AIXA01006942.1|:23647-23761
MsHel-1	gb|AIXA01015578.1|:1-308
MsHel-1	gb|AIXA01006661.1|:1-250
MsHel-1	gb|AIXA01032755.1|:382-719
MsHel-1	gb|AIXA01022244.1|:1-1747
MsHel-1	gb|AIXA01012659.1|:19765-20033
MsHel-1	gb|AIXA01004744.1|:305-541
MsHel-1	gb|AIXA01004077.1|:1-265
MsHel-1	gb|AIXA01020323.1|:1-257
MsHel-1	gb|AIXA01035174.1|:1-299
MsHel-1	gb|AIXA01027320.1|:4-230
MsHel-1	gb|AIXA01011745.1|:1814-2270
MsHel-1	gb|AIXA01007254.1|:963-1974
MsHel-1	gb|AIXA01004135.1|:47098-47293
MsHel-1	gb|AIXA01006224.1|:290-738
MsHel-1	gb|AIXA01005428.1|:14484-15080
MsHel-1	gb|AIXA01003565.1|:2521-2865
MsHel-1	gb|AIXA01031741.1|:3-275
MsHel-1	gb|AIXA01025343.1|:1-264
MsHel-1	gb|AIXA01016795.1|:2-297
MsHel-1	gb|AIXA01003738.1|:1444-1727
MsHel-1	gb|AIXA01007629.1|:1-267
MsHel-1	gb|AIXA01006228.1|:8905-9254
MsHel-1	gb|AIXA01007649.1|:38177-38630
MsHel-1	gb|AIXA01001149.1|:2389-2837

MsHel-1	gb|AIXA01004994.1|:12610-12923
MsHel-1	gb|AIXA01001910.1|:725-1278
MsHel-1	gb|AIXA01008248.1|:7-277
MsHel-1	gb|AIXA01002150.1|:15720-15969
MsHel-1	gb|AIXA01001075.1|:1-268
MsHel-1	gb|AIXA01000537.1|:18188-18634
MsHel-1	gb|AIXA01013008.1|:2270-2483
MsHel-1	gb|AIXA01008482.1|:1-327
MsHel-1	gb|AIXA01004702.1|:38038-38282
MsHel-1	gb|AIXA01006027.1|:5964-6504
MsHel-1	gb|AIXA01004250.1|:78323-78886
MsHel-1	gb|AIXA01004250.1|:268-409
MsHel-1	gb|AIXA01028540.1|:346-956
MsHel-1	gb|AIXA01011820.1|:7609-7871
MsHel-1	gb|AIXA01011092.1|:14049-14335
MsHel-1	gb|AIXA01010569.1|:12655-13139
MsHel-1	gb|AIXA01010438.1|:24986-25201
MsHel-1	gb|AIXA01030628.1|:584-826
MsHel-1	gb|AIXA01025490.1|:978-1223
MsHel-1	gb|AIXA01006616.1|:11572-11814
MsHel-1	gb|AIXA01002428.1|:1-227
MsHel-1	gb|AIXA01007049.1|:79312-79646
MsHel-1	gb|AIXA01008019.1|:16894-17114
MsHel-1	gb|AIXA01023770.1|:1223-1442
MsHel-1	gb|AIXA01005090.1|:1-216
MsHel-1	gb|AIXA01002509.1|:37175-37476
MsHel-1	gb|AIXA01004456.1|:34518-35038
MsHel-1	gb|AIXA01035055.1|:375-615
MsHel-1	gb|AIXA01007466.1|:13164-13257
MsHel-1	gb|AIXA01007466.1|:1-217
MsHel-1	gb|AIXA01012025.1|:7248-7470
MsHel-1	gb|AIXA01004366.1|:3-241
MsHel-1	gb|AIXA01032486.1|:1-262
MsHel-1	gb|AIXA01024752.1|:1-243
MsHel-1	gb|AIXA01017214.1|:2748-2949
MsHel-1	gb|AIXA01000780.1|:4900-5101
MsHel-1	gb|AIXA01030724.1|:611-820
MsHel-1	gb|AIXA01023790.1|:1-261
MsHel-1	gb|AIXA01013477.1|:66680-66937
MsHel-1	gb|AIXA01035074.1|:382-614
MsHel-1	gb|AIXA01026784.1|:864-1091
MsHel-1	gb|AIXA01022900.1|:1-246
MsHel-1	gb|AIXA01020003.1|:1-213
MsHel-1	gb|AIXA01034298.1|:401-643
MsHel-1	gb|AIXA01013619.1|:22034-22470
MsHel-1	gb|AIXA01007257.1|:2093-2322
MsHel-1	gb|AIXA01004082.1|:6078-6387
MsHel-1	gb|AIXA01002528.1|:107366-107501

MsHel-1	gb|AIXA01002528.1|:24790-25059
MsHel-1	gb|AIXA01031688.1|:8-222
MsHel-1	gb|AIXA01010080.1|:4794-5068
MsHel-1	gb|AIXA01000804.1|:1-227
MsHel-1	gb|AIXA01034741.1|:11-268
MsHel-1	gb|AIXA01017796.1|:1-226
MsHel-1	gb|AIXA01013720.1|:10207-10407
MsHel-1	gb|AIXA01035999.1|:4-224
MsHel-1	gb|AIXA01017688.1|:935-1158
MsHel-1	gb|AIXA01010000.1|:1-222
MsHel-1	gb|AIXA01006038.1|:4045-4270
MsHel-1	gb|AIXA01005431.1|:2918-3254
MsHel-1	gb|AIXA01036810.1|:1-208
MsHel-1	gb|AIXA01032871.1|:1-208
MsHel-1	gb|AIXA01012694.1|:8610-8972
MsHel-1	gb|AIXA01023269.1|:1-211
MsHel-1	gb|AIXA01012782.1|:2263-2464
MsHel-1	gb|AIXA01011407.1|:25013-25213
MsHel-1	gb|AIXA01011399.1|:17794-18184
MsHel-1	gb|AIXA01000267.1|:84531-84761
MsHel-1	gb|AIXA01032988.1|:424-709
MsHel-1	gb|AIXA01003887.1|:3-207
MsHel-1	gb|AIXA01019526.1|:1-229
MsHel-1	gb|AIXA01000031.1|:2544-2931
MsHel-1	gb|AIXA01027746.1|:1-219
MsHel-1	gb|AIXA01017728.1|:7500-8078
MsHel-1	gb|AIXA01005900.1|:1-202
MsHel-1	gb|AIXA01001166.1|:528-821
MsHel-1	gb|AIXA01000762.1|:7232-7525
MsHel-1	gb|AIXA01032294.1|:1-224
MsHel-1	gb|AIXA01026502.1|:845-1123
MsHel-1	gb|AIXA01026187.1|:1-199
MsHel-1	gb|AIXA01011753.1|:22558-23013
MsHel-1	gb|AIXA01004612.1|:2385-2567
MsHel-1	gb|AIXA01004416.1|:36443-36644
MsHel-1	gb|AIXA01003683.1|:65078-65229
MsHel-1	gb|AIXA01003266.1|:15072-15270
MsHel-1	gb|AIXA01002399.1|:53753-54015
MsHel-1	gb|AIXA01024987.1|:637-935
MsHel-1	gb|AIXA01014813.1|:3652-3925
MsHel-1	gb|AIXA01006775.1|:2482-2790
MsHel-1	gb|AIXA01003291.1|:8289-8575
MsHel-1	gb|AIXA01013126.1|:3103-3340
MsHel-1	gb|AIXA01008937.1|:1-189
MsHel-1	gb|AIXA01002198.1|:3-191
MsHel-1	gb|AIXA01000482.1|:1-193
MsHel-1	gb|AIXA01028964.1|:1-224
MsHel-1	gb|AIXA01001712.1|:12-227

MsHel-1	gb|AIXA01021774.1|:1-227
MsHel-1	gb|AIXA01015060.1|:2523-2723
MsHel-1	gb|AIXA01010299.1|:19760-19995
MsHel-1	gb|AIXA01004021.1|:38351-38536
MsHel-1	gb|AIXA01007136.1|:4-200
MsHel-1	gb|AIXA01001347.1|:4-236
MsHel-1	gb|AIXA01007708.1|:8223-8464
MsHel-1	gb|AIXA01007421.1|:1-91
MsHel-1	gb|AIXA01002292.1|:1-215
MsHel-1	gb|AIXA01025077.1|:52-285
MsHel-1	gb|AIXA01009940.1|:8940-9074
MsHel-1	gb|AIXA01006768.1|:1-182
MsHel-1	gb|AIXA01000667.1|:17572-17790
MsHel-1	gb|AIXA01017292.1|:10294-11058
MsHel-1	gb|AIXA01012743.1|:1168-1477
MsHel-1	gb|AIXA01012496.1|:1-189
MsHel-1	gb|AIXA01011308.1|:14340-14544
MsHel-1	gb|AIXA01008579.1|:1-193
MsHel-1	gb|AIXA01005884.1|:329-800
MsHel-1	gb|AIXA01038110.1|:2-184
MsHel-1	gb|AIXA01024974.1|:1-188
MsHel-1	gb|AIXA01008267.1|:14360-14535
MsHel-1	gb|AIXA01001903.1|:1-201
MsHel-1	gb|AIXA01001859.1|:3-178
MsHel-1	gb|AIXA01000351.1|:3-230
MsHel-1	gb|AIXA01020063.1|:2944-3240
MsHel-1	gb|AIXA01018320.1|:12808-12977
MsHel-1	gb|AIXA01027025.1|:775-1072
MsHel-1	gb|AIXA01016602.1|:1-205
MsHel-1	gb|AIXA01008336.1|:20992-21211
MsHel-1	gb|AIXA01007034.1|:56484-56794
MsHel-1	gb|AIXA01007034.1|:1-221
MsHel-1	gb|AIXA01006796.1|:76595-76894
MsHel-1	gb|AIXA01003299.1|:1-173
MsHel-1	gb|AIXA01017104.1|:1308-1524
MsHel-1	gb|AIXA01006667.1|:20213-20443
MsHel-1	gb|AIXA01007516.1|:63576-63944
MsHel-1	gb|AIXA01004406.1|:11379-12226
MsHel-1	gb|AIXA01003319.1|:41783-42262
MsHel-1	gb|AIXA01001531.1|:55166-55342
MsHel-1	gb|AIXA01012652.1|:48468-48699
MsHel-1	gb|AIXA01005449.1|:28420-28774
MsHel-1	gb|AIXA01001739.1|:21520-21682
MsHel-1	gb|AIXA01033787.1|:384-666
MsHel-1	gb|AIXA01013523.1|:729-924
MsHel-1	gb|AIXA01011687.1|:45-246
MsHel-1	gb|AIXA01000263.1|:14051-14398
MsHel-1	gb|AIXA01000263.1|:2-166

MsHel-1	gb|AIXA01026649.1|:741-1105
MsHel-1	gb|AIXA01002096.1|:7-193
MsHel-1	gb|AIXA01006050.1|:1-216
MsHel-1	gb|AIXA01004580.1|:40984-41096
MsHel-1	gb|AIXA01015040.1|:9618-9887
MsHel-1	gb|AIXA01011811.1|:8909-9307
MsHel-1	gb|AIXA01009465.1|:4102-4582
MsHel-1	gb|AIXA01009111.1|:25707-26127
MsHel-1	gb|AIXA01006284.1|:11867-11982
MsHel-1	gb|AIXA01005192.1|:1-165
MsHel-1	gb|AIXA01004183.1|:25239-25665
MsHel-1	gb|AIXA01000800.1|:1942-2544
MsHel-1	gb|AIXA01028981.1|:5-193
MsHel-1	gb|AIXA01015072.1|:8741-8900
MsHel-1	gb|AIXA01007983.1|:9325-9581
MsHel-1	gb|AIXA01004929.1|:1-180
MsHel-1	gb|AIXA01026874.1|:1-199
MsHel-1	gb|AIXA01013708.1|:432-827
MsHel-1	gb|AIXA01008930.1|:115962-116139
MsHel-1	gb|AIXA01008930.1|:62548-63022
MsHel-1	gb|AIXA01036777.1|:299-552
MsHel-1	gb|AIXA01034617.1|:481-633
MsHel-1	gb|AIXA01019250.1|:1-157
MsHel-1	gb|AIXA01002865.1|:1-145
MsHel-1	gb|AIXA01011996.1|:6995-7162
MsHel-1	gb|AIXA01005307.1|:11604-11880
MsHel-1	gb|AIXA01007576.1|:42137-45887
MsHel-1	gb|AIXA01007576.1|:5503-5793
MsHel-1	gb|AIXA01008496.1|:1-166
MsHel-1	gb|AIXA01007980.1|:3841-4096
MsHel-1	gb|AIXA01007388.1|:1-158
MsHel-1	gb|AIXA01014571.1|:1-199
MsHel-1	gb|AIXA01010158.1|:9483-9690
MsHel-1	gb|AIXA01000383.1|:2326-2761
MsHel-1	gb|AIXA01032754.1|:1-147
MsHel-1	gb|AIXA01013655.1|:1-169
MsHel-1	gb|AIXA01009224.1|:41182-41366
MsHel-1	gb|AIXA01010111.1|:1116-1428
MsHel-1	gb|AIXA01008575.1|:88022-88174
MsHel-1	gb|AIXA01008575.1|:68201-68443
MsHel-1	gb|AIXA01020224.1|:306-504
MsHel-1	gb|AIXA01011389.1|:22767-22934
MsHel-1	gb|AIXA01011249.1|:59997-60266
MsHel-1	gb|AIXA01009122.1|:714-893
MsHel-1	gb|AIXA01007868.1|:1-144
MsHel-1	gb|AIXA01007505.1|:9494-9760
MsHel-1	gb|AIXA01038302.1|:151-313
MsHel-1	gb|AIXA01031867.1|:2-170

MsHel-1	gb|AIXA01011963.1|:1-143
MsHel-1	gb|AIXA01011500.1|:7939-8173
MsHel-1	gb|AIXA01010977.1|:8-200
MsHel-1	gb|AIXA01009121.1|:11211-11392
MsHel-1	gb|AIXA01006508.1|:29562-29707
MsHel-1	gb|AIXA01012233.1|:1217-1468
MsHel-1	gb|AIXA01004640.1|:88124-88308
MsHel-1	gb|AIXA01009156.1|:4735-5101
MsHel-1	gb|AIXA01002422.1|:5247-6193
MsHel-1	gb|AIXA01034482.1|:1-293
MsHel-1	gb|AIXA01015717.1|:1-157
MsHel-1	gb|AIXA01014423.1|:4111-4407
MsHel-1	gb|AIXA01008033.1|:16826-17085
MsHel-1	gb|AIXA01024907.1|:1151-1285
MsHel-1	gb|AIXA01007214.1|:3695-3829
MsHel-1	gb|AIXA01008690.1|:38032-38212
MsHel-1	gb|AIXA01024902.1|:981-1117
MsHel-1	gb|AIXA01017500.1|:1736-1976
MsHel-1	gb|AIXA01008691.1|:7-155
MsHel-1	gb|AIXA01007984.1|:18190-18342
MsHel-1	gb|AIXA01007984.1|:1-133
MsHel-1	gb|AIXA01027036.1|:1-155
MsHel-1	gb|AIXA01015583.1|:23888-24069
MsHel-1	gb|AIXA01011686.1|:86404-86596
MsHel-1	gb|AIXA01011376.1|:11794-12266
MsHel-1	gb|AIXA01007303.1|:4866-5207
MsHel-1	gb|AIXA01002688.1|:54-209
MsHel-1	gb|AIXA01012524.1|:17516-17649
MsHel-1	gb|AIXA01008688.1|:57138-57280
MsHel-1	gb|AIXA01024692.1|:1-142
MsHel-1	gb|AIXA01003044.1|:1222-1634
MsHel-1	gb|AIXA01033157.1|:474-699
MsHel-1	gb|AIXA01027542.1|:1-129
MsHel-1	gb|AIXA01012234.1|:932-1175
MsHel-1	gb|AIXA01002374.1|:1-129
MsHel-1	gb|AIXA01018159.1|:327-526
MsHel-1	gb|AIXA01017103.1|:4980-5131
MsHel-1	gb|AIXA01013609.1|:185-389
MsHel-1	gb|AIXA01011851.1|:2-162
MsHel-1	gb|AIXA01003153.1|:24534-24839
MsHel-1	gb|AIXA01002534.1|:5020-5147
MsHel-1	gb|AIXA01009539.1|:24075-24441
MsHel-1	gb|AIXA01006382.1|:65211-65302
MsHel-1	gb|AIXA01017268.1|:3479-3771
MsHel-1	gb|AIXA01008859.1|:8110-12056
MsHel-1	gb|AIXA01003578.1|:46033-46301
MsHel-1	gb|AIXA01030671.1|:691-823
MsHel-1	gb|AIXA01026714.1|:1-149

MsHel-1	gb|AIXA01010542.1|:43903-44194
MsHel-1	gb|AIXA01008378.1|:5460-5701
MsHel-1	gb|AIXA01007716.1|:10967-11204
MsHel-1	gb|AIXA01005968.1|:1-133
MsHel-1	gb|AIXA01033041.1|:1-169
MsHel-1	gb|AIXA01030862.1|:602-812
MsHel-1	gb|AIXA01023845.1|:1213-1348
MsHel-1	gb|AIXA01018613.1|:9733-10491
MsHel-1	gb|AIXA01025751.1|:1059-1193
MsHel-1	gb|AIXA01008427.1|:1-195
MsHel-1	gb|AIXA01000297.1|:78926-79347
MsHel-1	gb|AIXA01029665.1|:646-884
MsHel-1	gb|AIXA01006871.1|:2-119
MsHel-1	gb|AIXA01025825.1|:1057-1184
MsHel-1	gb|AIXA01010157.1|:3445-3573
MsHel-1	gb|AIXA01027700.1|:147-274
MsHel-1	gb|AIXA01025792.1|:1069-1188
MsHel-1	gb|AIXA01025335.1|:604-782
MsHel-1	gb|AIXA01008554.1|:31526-31621
MsHel-1	gb|AIXA01003106.1|:1-116
MsHel-1	gb|AIXA01019038.1|:5299-5600
MsHel-1	gb|AIXA01017295.1|:8232-8358
MsHel-1	gb|AIXA01006149.1|:40673-41051
MsHel-1	gb|AIXA01001817.1|:1147-1513
MsHel-1	gb|AIXA01035213.1|:380-600
MsHel-1	gb|AIXA01015280.1|:3632-3765
MsHel-1	gb|AIXA01009024.1|:69255-69466
MsHel-1	gb|AIXA01018738.1|:3901-4081
MsHel-1	gb|AIXA01017936.1|:2607-3022
MsHel-1	gb|AIXA01010036.1|:7204-7497
MsHel-1	gb|AIXA01008516.1|:8792-9005
MsHel-1	gb|AIXA01006548.1|:13158-13537
MsHel-1	gb|AIXA01003155.1|:1-129
MsHel-1	gb|AIXA01002957.1|:1672-2038
MsHel-1	gb|AIXA01037560.1|:1-140
MsHel-1	gb|AIXA01015716.1|:4135-4424
MsHel-1	gb|AIXA01000963.1|:47133-47330
MsHel-1	gb|AIXA01017091.1|:9649-9860
MsHel-1	gb|AIXA01016013.1|:4-122
MsHel-1	gb|AIXA01005474.1|:8930-9851
MsHel-1	gb|AIXA01023044.1|:7-115
MsHel-1	gb|AIXA01014197.1|:3-111
MsHel-1	gb|AIXA01001233.1|:23479-23606
MsHel-1	gb|AIXA01022051.1|:1647-1790
MsHel-1	gb|AIXA01007379.1|:6190-6454
MsHel-1	gb|AIXA01000771.1|:3003-3094
MsHel-1	gb|AIXA01030083.1|:1-107
MsHel-1	gb|AIXA01015563.1|:12471-12857

MsHel-1	gb|AIXA01012424.1|:17620-17761
MsHel-1	gb|AIXA01008705.1|:12332-12634
MsHel-1	gb|AIXA01006598.1|:7638-7917
MsHel-1	gb|AIXA01004451.1|:1-127
MsHel-1	gb|AIXA01002848.1|:2-116
MsHel-1	gb|AIXA01001603.1|:14112-14226
MsHel-1	gb|AIXA01028753.1|:3-120
MsHel-1	gb|AIXA01017310.1|:5122-5380
MsHel-1	gb|AIXA01015995.1|:336-445
MsHel-1	gb|AIXA01014739.1|:22665-22961
MsHel-1	gb|AIXA01002085.1|:24676-24882
MsHel-1	gb|AIXA01028761.1|:822-942
MsHel-1	gb|AIXA01021750.1|:4-112
MsHel-1	gb|AIXA01015055.1|:30012-30257
MsHel-1	gb|AIXA01014867.1|:18583-18947
MsHel-1	gb|AIXA01008010.1|:5-113
MsHel-1	gb|AIXA01006253.1|:6380-6585
MsHel-1	gb|AIXA01000527.1|:11974-12110
MsHel-1	gb|AIXA01018971.1|:3096-3560
MsHel-1	gb|AIXA01012741.1|:49265-49452
MsHel-1	gb|AIXA01010748.1|:77880-78104
MsHel-1	gb|AIXA01010748.1|:43863-43974
MsHel-1	gb|AIXA01002744.1|:7168-7472
MsHel-1	gb|AIXA01001875.1|:12948-13194
MsHel-1	gb|AIXA01037980.1|:1-115
MsHel-1	gb|AIXA01025455.1|:218-685
MsHel-1	gb|AIXA01004386.1|:85438-85845
MsHel-1	gb|AIXA01000298.1|:15843-16178
MsHel-1	gb|AIXA01012026.1|:1-114
MsHel-1	gb|AIXA01001346.1|:18192-18292
MsHel-1	gb|AIXA01005535.1|:13441-13663
MsHel-1	gb|AIXA01004802.1|:90599-90917
MsHel-1	gb|AIXA01030907.1|:689-810
MsHel-1	gb|AIXA01016421.1|:3811-3913
MsHel-1	gb|AIXA01009527.1|:1-107
MsHel-1	gb|AIXA01004539.1|:2-115
MsHel-1	gb|AIXA01008495.1|:23012-23209
MsHel-1	gb|AIXA01003285.1|:40644-40859
MsHel-1	gb|AIXA01001358.1|:79349-79629
MsHel-1	gb|AIXA01000140.1|:6871-7069
MsHel-1	gb|AIXA01029573.1|:496-779
MsHel-1	gb|AIXA01014129.1|:11118-11309
MsHel-1	gb|AIXA01013650.1|:547-745
MsHel-1	gb|AIXA01009003.1|:21402-21681
MsHel-1	gb|AIXA01006209.1|:14898-15148
MsHel-1	gb|AIXA01004263.1|:2182-2458
MsHel-1	gb|AIXA01012349.1|:9597-9941
MsHel-1	gb|AIXA01011202.1|:39202-39469

MsHel-1	gb|AIXA01010029.1|:40304-40595
MsHel-1	gb|AIXA01010029.1|:1-81
MsHel-1	gb|AIXA01002628.1|:2950-3147
MsHel-1	gb|AIXA01000944.1|:26916-27096
MsHel-1	gb|AIXA01019087.1|:1316-1500
MsHel-1	gb|AIXA01018984.1|:5138-5319
MsHel-1	gb|AIXA01014578.1|:4171-4294
MsHel-1	gb|AIXA01009149.1|:775-1068
MsHel-1	gb|AIXA01008629.1|:410-642
MsHel-1	gb|AIXA01005533.1|:62582-62701
MsHel-1	gb|AIXA01005533.1|:3-104
MsHel-1	gb|AIXA01005395.1|:25-154
MsHel-1	gb|AIXA01003186.1|:41335-41641
MsHel-1	gb|AIXA01029582.1|:706-889
MsHel-1	gb|AIXA01007594.1|:55565-55746
MsHel-1	gb|AIXA01005352.1|:54437-54572
MsHel-1	gb|AIXA01004022.1|:1-105
MsHel-1	gb|AIXA01003477.1|:1-124
MsHel-1	gb|AIXA01014683.1|:45230-45333
MsHel-1	gb|AIXA01005330.1|:2036-2199
MsHel-1	gb|AIXA01004450.1|:1-96
MsHel-1	gb|AIXA01004003.1|:10930-11177
MsHel-1	gb|AIXA01000776.1|:5850-6202
MsHel-1	gb|AIXA01024069.1|:133-523
MsHel-1	gb|AIXA01019289.1|:1-95
MsHel-1	gb|AIXA01017387.1|:1697-1899
MsHel-1	gb|AIXA01013144.1|:1244-1533
MsHel-1	gb|AIXA01007745.1|:8001-8099
MsHel-1	gb|AIXA01005996.1|:620-1005
MsHel-1	gb|AIXA01005548.1|:27665-27896
MsHel-1	gb|AIXA01003566.1|:1-95
MsHel-1	gb|AIXA01020657.1|:2004-2294
MsHel-1	gb|AIXA01011250.1|:17671-17860
MsHel-1	gb|AIXA01010406.1|:47726-48037
MsHel-1	gb|AIXA01007648.1|:25910-26377
MsHel-1	gb|AIXA01005563.1|:79179-79362
MsHel-1	gb|AIXA01003011.1|:20548-20912
MsHel-1	gb|AIXA01037190.1|:50-162
MsHel-1	gb|AIXA01027039.1|:962-1074
MsHel-1	gb|AIXA01010885.1|:21957-22316
MsHel-1	gb|AIXA01010736.1|:4902-5262
MsHel-1	gb|AIXA01006164.1|:4604-4704
MsHel-1	gb|AIXA01005242.1|:4519-4654
MsHel-1	gb|AIXA01001664.1|:16978-17265
MsHel-1	gb|AIXA01000522.1|:12721-12890
MsHel-1	gb|AIXA01030730.1|:707-794
MsHel-1	gb|AIXA01008788.1|:33758-33845
MsHel-1	gb|AIXA01008788.1|:7909-8137

MsHel-1	gb|AIXA01007750.1|:17175-17430
MsHel-1	gb|AIXA01005889.1|:10149-10599
MsHel-1	gb|AIXA01004338.1|:7375-8731
MsHel-1	gb|AIXA01003463.1|:5223-5497
MsHel-1	gb|AIXA01002244.1|:25601-25820
MsHel-1	gb|AIXA01000728.1|:51202-51371
MsHel-1	gb|AIXA01000313.1|:29692-29990
MsHel-1	gb|AIXA01000313.1|:1209-1387
MsHel-1	gb|AIXA01035537.1|:1-99
MsHel-1	gb|AIXA01018554.1|:1374-1569
MsHel-1	gb|AIXA01006177.1|:10392-10486
MsHel-1	gb|AIXA01001107.1|:74553-74651
MsHel-1	gb|AIXA01022297.1|:1190-1497
MsHel-1	gb|AIXA01018802.1|:128-229
MsHel-1	gb|AIXA01017956.1|:3756-3893
MsHel-1	gb|AIXA01016851.1|:581-860
MsHel-1	gb|AIXA01014015.1|:4811-4993
MsHel-1	gb|AIXA01008726.1|:6816-7190
MsHel-1	gb|AIXA01008382.1|:49628-49990
MsHel-1	gb|AIXA01004532.1|:21724-21981
MsHel-1	gb|AIXA01030617.1|:1-93
MsHel-1	gb|AIXA01027127.1|:433-738
MsHel-1	gb|AIXA01013796.1|:38626-38816
MsHel-1	gb|AIXA01012386.1|:1778-1926
MsHel-1	gb|AIXA01007130.1|:76012-76140
MsHel-1	gb|AIXA01007130.1|:13867-14070
MsHel-1	gb|AIXA01006529.1|:83945-84133
MsHel-1	gb|AIXA01006529.1|:60833-60925
MsHel-1	gb|AIXA01037358.1|:428-531
MsHel-1	gb|AIXA01025159.1|:43-134
MsHel-1	gb|AIXA01017935.1|:7634-8413
MsHel-1	gb|AIXA01014768.1|:10389-10518
MsHel-1	gb|AIXA01009627.1|:3450-3593
MsHel-1	gb|AIXA01009520.1|:14824-14918
MsHel-1	gb|AIXA01008194.1|:2388-2845
MsHel-1	gb|AIXA01007464.1|:18426-18558
MsHel-1	gb|AIXA01004034.1|:214237-214441
MsHel-1	gb|AIXA01009782.1|:1586-1767
MsHel-1	gb|AIXA01000620.1|:15503-15780
MsHel-1	gb|AIXA01032507.1|:443-580
MsHel-1	gb|AIXA01023106.1|:1471-1560
MsHel-1	gb|AIXA01016367.1|:12798-12963
MsHel-1	gb|AIXA01013754.1|:23832-24287
MsHel-1	gb|AIXA01012380.1|:8399-8784
MsHel-1	gb|AIXA01012103.1|:22489-22700
MsHel-1	gb|AIXA01011331.1|:22374-22608
MsHel-1	gb|AIXA01008124.1|:60285-60511
MsHel-1	gb|AIXA01001422.1|:1116-1205

MsHel-1	gb|AIXA01000061.1|:1-120
MsHel-1	gb|AIXA01017188.1|:4484-4576
MsHel-1	gb|AIXA01016746.1|:2987-3369
MsHel-1	gb|AIXA01011463.1|:998-1290
MsHel-1	gb|AIXA01008044.1|:28966-29157
MsHel-1	gb|AIXA01006689.1|:56236-56368
MsHel-1	gb|AIXA01005281.1|:26698-26990
MsHel-1	gb|AIXA01001055.1|:13970-14140
MsHel-1	gb|AIXA01000764.1|:1548-1669
MsHel-1	gb|AIXA01000455.1|:1-89
MsHel-1	gb|AIXA01022541.1|:1201-1431
MsHel-1	gb|AIXA01016048.1|:11964-12217
MsHel-1	gb|AIXA01015469.1|:1-92
MsHel-1	gb|AIXA01015132.1|:34779-35246
MsHel-1	gb|AIXA01007029.1|:30388-30679
MsHel-1	gb|AIXA01006517.1|:8277-8571
MsHel-1	gb|AIXA01004459.1|:60916-61475
MsHel-1	gb|AIXA01037308.1|:1-99
MsHel-1	gb|AIXA01027291.1|:15-170
MsHel-1	gb|AIXA01014171.1|:4107-4232
MsHel-1	gb|AIXA01011819.1|:7968-8702
MsHel-1	gb|AIXA01009109.1|:26854-27072
MsHel-1	gb|AIXA01006013.1|:37019-37194
MsHel-1	gb|AIXA01003198.1|:54680-54774
MsHel-1	gb|AIXA01003198.1|:331-643
MsHel-1	gb|AIXA01002378.1|:9718-9872
MsHel-1	gb|AIXA01032910.1|:437-680
MsHel-1	gb|AIXA01022959.1|:1397-1581
MsHel-1	gb|AIXA01018987.1|:640-765
MsHel-1	gb|AIXA01012651.1|:14304-15065
MsHel-1	gb|AIXA01012651.1|:765-853
MsHel-1	gb|AIXA01011457.1|:35310-35538
MsHel-1	gb|AIXA01008768.1|:88179-88353
MsHel-1	gb|AIXA01006447.1|:22-127
MsHel-1	gb|AIXA01005640.1|:6891-7152
MsHel-1	gb|AIXA01004565.1|:46875-47142
MsHel-1	gb|AIXA01003063.1|:45179-45314
MsHel-1	gb|AIXA01000347.1|:43451-43743
MsHel-1	gb|AIXA01030842.1|:659-814
MsHel-1	gb|AIXA01025893.1|:1090-1178
MsHel-1	gb|AIXA01020441.1|:356-729
MsHel-1	gb|AIXA01018551.1|:10816-10904
MsHel-1	gb|AIXA01018060.1|:5421-5620
MsHel-1	gb|AIXA01017432.1|:2199-2443
MsHel-1	gb|AIXA01012311.1|:7947-8148
MsHel-1	gb|AIXA01011452.1|:1-93
MsHel-1	gb|AIXA01008949.1|:4097-4559
MsHel-1	gb|AIXA01007522.1|:38644-38887

MsHel-1	gb|AIXA01006750.1|:25189-25512
MsHel-1	gb|AIXA01006194.1|:15651-15743
MsHel-1	gb|AIXA01005887.1|:4303-4420
MsHel-1	gb|AIXA01005634.1|:1486-1652
MsHel-1	gb|AIXA01003006.1|:962-1156
MsHel-1	gb|AIXA01000724.1|:7614-7821
MsHel-1	gb|AIXA01000535.1|:20766-21061
MsHel-1	gb|AIXA01024733.1|:1173-1308
MsHel-1	gb|AIXA01019001.1|:1-89
MsHel-1	gb|AIXA01013339.1|:1-84
MsHel-1	gb|AIXA01012857.1|:5558-5743
MsHel-1	gb|AIXA01005527.1|:16109-16389
MsHel-1	gb|AIXA01003336.1|:400-737
MsHel-1	gb|AIXA01002899.1|:31762-31968
MsHel-1	gb|AIXA01002197.1|:44444-44538
MsHel-1	gb|AIXA01002197.1|:10793-10896
MsHel-1	gb|AIXA01002030.1|:24599-24868
MsHel-1	gb|AIXA01023936.1|:789-979
MsHel-1	gb|AIXA01019922.1|:3284-3492
MsHel-1	gb|AIXA01011379.1|:2420-2716
MsHel-1	gb|AIXA01011158.1|:9837-9951
MsHel-1	gb|AIXA01008773.1|:39235-39446
MsHel-1	gb|AIXA01002313.1|:40630-41016
MsHel-1	gb|AIXA01000394.1|:33876-33960
MsHel-1	gb|AIXA01024744.1|:21-238
MsHel-1	gb|AIXA01019630.1|:361-605
MsHel-1	gb|AIXA01008227.1|:23831-24529
MsHel-1	gb|AIXA01007300.1|:19690-20068
MsHel-1	gb|AIXA01001963.1|:38243-38454
MsHel-1	gb|AIXA01022428.1|:876-1036
MsHel-1	gb|AIXA01015045.1|:653-879
MsHel-1	gb|AIXA01011937.1|:4440-4572
MsHel-1	gb|AIXA01009529.1|:22177-22349
MsHel-1	gb|AIXA01007515.1|:85594-85797
MsHel-1	gb|AIXA01005847.1|:28203-28394
MsHel-1	gb|AIXA01005078.1|:25372-25654
MsHel-1	gb|AIXA01015093.1|:2812-2921
MsHel-1	gb|AIXA01009274.1|:10261-10396
MsHel-1	gb|AIXA01006230.1|:15308-15589
MsHel-1	gb|AIXA01005585.1|:75410-75577
MsHel-1	gb|AIXA01003143.1|:3460-3645
MsHel-1	gb|AIXA01027996.1|:349-570
MsHel-1	gb|AIXA01025291.1|:198-392
MsHel-1	gb|AIXA01018614.1|:1514-1799
MsHel-1	gb|AIXA01017113.1|:6955-7198
MsHel-1	gb|AIXA01012504.1|:6385-6704
MsHel-1	gb|AIXA01010329.1|:2015-2356
MsHel-1	gb|AIXA01003178.1|:10991-11147

MsHel-1	gb|AIXA01022048.1|:26-177
MsHel-1	gb|AIXA01014737.1|:504-698
MsHel-1	gb|AIXA01014596.1|:4809-5180
MsHel-1	gb|AIXA01010126.1|:6664-7000
MsHel-1	gb|AIXA01007344.1|:46918-47191
MsHel-1	gb|AIXA01007282.1|:50668-50853
MsHel-1	gb|AIXA01007271.1|:3710-3895
MsHel-1	gb|AIXA01005896.1|:3096-3257
MsHel-1	gb|AIXA01005621.1|:54729-54854
MsHel-1	gb|AIXA01005621.1|:11968-12337
MsHel-1	gb|AIXA01005331.1|:45497-45856
MsHel-1	gb|AIXA01002850.1|:103182-103367
MsHel-1	gb|AIXA01002050.1|:69749-70043
MsHel-1	gb|AIXA01002001.1|:7986-8192
MsHel-1	gb|AIXA01000560.1|:83804-84070
MsHel-1	gb|AIXA01026471.1|:638-773
MsHel-1	gb|AIXA01013033.1|:10393-10536
MsHel-1	gb|AIXA01008634.1|:16663-16859
MsHel-1	gb|AIXA01004927.1|:15244-15324
MsHel-1	gb|AIXA01004409.1|:29725-30072
MsHel-1	gb|AIXA01003850.1|:13745-13906
MsHel-1	gb|AIXA01002547.1|:11251-11340
MsHel-1	gb|AIXA01002284.1|:195810-195894
MsHel-1	gb|AIXA01002284.1|:70094-70383
MsHel-1	gb|AIXA01000612.1|:22418-22595
MsHel-1	gb|AIXA01016829.1|:5887-6111
MsHel-1	gb|AIXA01016766.1|:2012-2287
MsHel-1	gb|AIXA01015243.1|:4270-4444
MsHel-1	gb|AIXA01014374.1|:1136-1274
MsHel-1	gb|AIXA01013065.1|:20875-21083
MsHel-1	gb|AIXA01012633.1|:6033-6196
MsHel-1	gb|AIXA01011402.1|:1-84
MsHel-1	gb|AIXA01009497.1|:1849-2036
MsHel-1	gb|AIXA01007267.1|:9451-9756
MsHel-1	gb|AIXA01005287.1|:3995-4347
MsHel-1	gb|AIXA01004594.1|:14884-15046
MsHel-1	gb|AIXA01003733.1|:9164-9441
MsHel-1	gb|AIXA01003536.1|:19021-19263
MsHel-1	gb|AIXA01003136.1|:956-1188
MsHel-1	gb|AIXA01002788.1|:5861-6043
MsHel-1	gb|AIXA01017265.1|:578-712
MsHel-1	gb|AIXA01013825.1|:59139-59407
MsHel-1	gb|AIXA01013652.1|:23259-23434
MsHel-1	gb|AIXA01009478.1|:2535-2696
MsHel-1	gb|AIXA01009341.1|:69121-69230
MsHel-1	gb|AIXA01009281.1|:33661-33828
MsHel-1	gb|AIXA01009281.1|:14288-14548
MsHel-1	gb|AIXA01008639.1|:24052-24253

MsHel-1	gb|AIXA01008561.1|:35547-35905
MsHel-1	gb|AIXA01007946.1|:68801-68941
MsHel-1	gb|AIXA01007946.1|:12271-12374
MsHel-1	gb|AIXA01004488.1|:12346-12713
MsHel-1	gb|AIXA01000988.1|:11703-11982
MsHel-1	gb|AIXA01023942.1|:323-551
MsHel-1	gb|AIXA01019962.1|:600-843
MsHel-1	gb|AIXA01018908.1|:2205-2394
MsHel-1	gb|AIXA01018021.1|:1992-2237
MsHel-1	gb|AIXA01015158.1|:9354-9527
MsHel-1	gb|AIXA01012302.1|:586-879
MsHel-1	gb|AIXA01011198.1|:751-901
MsHel-1	gb|AIXA01011197.1|:73887-74128
MsHel-1	gb|AIXA01010900.1|:24111-24310
MsHel-1	gb|AIXA01009282.1|:1458-1624
MsHel-1	gb|AIXA01008786.1|:26151-26324
MsHel-1	gb|AIXA01008113.1|:36450-36803
MsHel-1	gb|AIXA01007706.1|:23765-24054
MsHel-1	gb|AIXA01007040.1|:6329-6447
MsHel-1	gb|AIXA01006834.1|:83924-84112
MsHel-1	gb|AIXA01006409.1|:31207-31292
MsHel-1	gb|AIXA01006409.1|:3944-4217
MsHel-1	gb|AIXA01004621.1|:64922-65232
MsHel-1	gb|AIXA01002938.1|:22714-22881
MsHel-1	gb|AIXA01002170.1|:1283-1437
MsHel-1	gb|AIXA01001915.1|:18580-18788
MsHel-1	gb|AIXA01015869.1|:1063-1251
MsHel-1	gb|AIXA01015111.1|:6880-6996
MsHel-1	gb|AIXA01014154.1|:3299-3482
MsHel-1	gb|AIXA01013681.1|:12457-12747
MsHel-1	gb|AIXA01012237.1|:2005-2210
MsHel-1	gb|AIXA01011556.1|:9525-9693
MsHel-1	gb|AIXA01009847.1|:6077-6256
MsHel-1	gb|AIXA01009004.1|:16058-16288
MsHel-1	gb|AIXA01008167.1|:6394-6566
MsHel-1	gb|AIXA01007497.1|:885-1014
MsHel-1	gb|AIXA01007115.1|:12471-12587
MsHel-1	gb|AIXA01005612.1|:16093-16188
MsHel-1	gb|AIXA01005518.1|:8602-8821
MsHel-1	gb|AIXA01003962.1|:39035-39219
MsHel-1	gb|AIXA01003743.1|:54060-54219
MsHel-1	gb|AIXA01003263.1|:9392-9820
MsHel-1	gb|AIXA01001644.1|:11844-12177
MsHel-1	gb|AIXA01001387.1|:3455-3584
MsHel-1	gb|AIXA01000126.1|:51926-52056
MsHel-1	gb|AIXA01018830.1|:3250-3451
MsHel-1	gb|AIXA01015041.1|:15628-15835
MsHel-1	gb|AIXA01012095.1|:681-884

MsHel-1	gb|AIXA01011266.1|:4259-4417
MsHel-1	gb|AIXA01010678.1|:4751-5029
MsHel-1	gb|AIXA01009089.1|:2994-3227
MsHel-1	gb|AIXA01006628.1|:6222-6455
MsHel-1	gb|AIXA01004815.1|:9631-9793
MsHel-1	gb|AIXA01003876.1|:72893-73089
MsHel-1	gb|AIXA01001679.1|:50615-50830
MsHel-1	gb|AIXA01001192.1|:39574-39653
MsHel-1	gb|AIXA01000783.1|:32362-32549
MsHel-1	gb|AIXA01000030.1|:40059-40203
MsHel-1	gb|AIXA01015719.1|:8453-8653
MsHel-1	gb|AIXA01014442.1|:2995-3224
MsHel-1	gb|AIXA01013298.1|:10259-10471
MsHel-1	gb|AIXA01012114.1|:14678-14890
MsHel-1	gb|AIXA01012011.1|:6747-7846
MsHel-1	gb|AIXA01010074.1|:194-288
MsHel-1	gb|AIXA01009880.1|:9302-9918
MsHel-1	gb|AIXA01009875.1|:8-347
MsHel-1	gb|AIXA01008739.1|:70444-70669
MsHel-1	gb|AIXA01007593.1|:108528-108740
MsHel-1	gb|AIXA01007593.1|:66764-66886
MsHel-1	gb|AIXA01007298.1|:8237-8526
MsHel-1	gb|AIXA01006997.1|:23408-23608
MsHel-1	gb|AIXA01006995.1|:1472-1672
MsHel-1	gb|AIXA01006904.1|:10689-10802
MsHel-1	gb|AIXA01003036.1|:3339-3498
MsHel-1	gb|AIXA01001309.1|:69932-70258
MsHel-1	gb|AIXA01001309.1|:18212-18501
MsHel-1	gb|AIXA01018667.1|:2228-5567
MsHel-1	gb|AIXA01015327.1|:10618-10788
MsHel-1	gb|AIXA01014760.1|:529-808
MsHel-1	gb|AIXA01013721.1|:600-843
MsHel-1	gb|AIXA01013089.1|:8613-8738
MsHel-1	gb|AIXA01010541.1|:3510-3644
MsHel-1	gb|AIXA01009600.1|:54224-54397
MsHel-1	gb|AIXA01009309.1|:5573-5941
MsHel-1	gb|AIXA01008420.1|:29786-30011
MsHel-1	gb|AIXA01006583.1|:9041-9175
MsHel-1	gb|AIXA01005858.1|:3738-3895
MsHel-1	gb|AIXA01005381.1|:13129-13315
MsHel-1	gb|AIXA01004115.1|:72114-72396
MsHel-1	gb|AIXA01001409.1|:5405-5617
MsHel-1	gb|AIXA01001379.1|:7793-8332
MsHel-1	gb|AIXA01037209.1|:383-536
MsHel-1	gb|AIXA01035918.1|:467-580
MsHel-1	gb|AIXA01021440.1|:240-439
MsHel-1	gb|AIXA01018341.1|:1131-1285
MsHel-1	gb|AIXA01018276.1|:4601-4719

MsHel-1	gb|AIXA01012196.1|:25127-25419
MsHel-1	gb|AIXA01012040.1|:7045-7160
MsHel-1	gb|AIXA01011995.1|:90738-90949
MsHel-1	gb|AIXA01008860.1|:11382-11575
MsHel-1	gb|AIXA01006817.1|:24717-24851
MsHel-1	gb|AIXA01006809.1|:24344-24468
MsHel-1	gb|AIXA01005370.1|:13073-13187
MsHel-1	gb|AIXA01004108.1|:105277-105485
MsHel-1	gb|AIXA01003938.1|:18786-18992
MsHel-1	gb|AIXA01003426.1|:12937-13090
MsHel-1	gb|AIXA01003033.1|:5563-5698
MsHel-1	gb|AIXA01001823.1|:39100-39215
MsHel-1	gb|AIXA01001538.1|:9065-9294
MsHel-1	gb|AIXA01026612.1|:958-1106
MsHel-1	gb|AIXA01016598.1|:1528-1728
MsHel-1	gb|AIXA01015580.1|:6063-6243
MsHel-1	gb|AIXA01014388.1|:9780-9970
MsHel-1	gb|AIXA01014041.1|:3509-3664
MsHel-1	gb|AIXA01012312.1|:4311-4516
MsHel-1	gb|AIXA01009267.1|:11141-11275
MsHel-1	gb|AIXA01007665.1|:17793-18062
MsHel-1	gb|AIXA01007642.1|:116320-116487
MsHel-1	gb|AIXA01007642.1|:96165-96287
MsHel-1	gb|AIXA01007224.1|:46012-46168
MsHel-1	gb|AIXA01006410.1|:2273-2411
MsHel-1	gb|AIXA01001848.1|:19385-19759
MsHel-1	gb|AIXA01001404.1|:14409-14532
MsHel-1	gb|AIXA01000063.1|:11710-11861
MsHel-1	gb|AIXA01032437.1|:170-557
MsHel-1	gb|AIXA01022280.1|:1448-1642
MsHel-1	gb|AIXA01015444.1|:3525-3807
MsHel-1	gb|AIXA01012522.1|:16075-16295
MsHel-1	gb|AIXA01009197.1|:1711-1853
MsHel-1	gb|AIXA01008492.1|:40376-40542
MsHel-1	gb|AIXA01008252.1|:29352-29644
MsHel-1	gb|AIXA01005933.1|:11604-11778
MsHel-1	gb|AIXA01005447.1|:11913-12208
MsHel-1	gb|AIXA01004651.1|:3863-4096
MsHel-1	gb|AIXA01002003.1|:41772-41866
MsHel-1	gb|AIXA01002003.1|:829-1129
MsHel-1	gb|AIXA01001813.1|:42350-42508
MsHel-1	gb|AIXA01001813.1|:12850-12978
MsHel-1	gb|AIXA01000156.1|:2521-2650
MsHel-1	gb|AIXA01031934.1|:627-757
MsHel-1	gb|AIXA01028475.1|:475-687
MsHel-1	gb|AIXA01016140.1|:1088-1688
MsHel-1	gb|AIXA01015919.1|:1922-2039
MsHel-1	gb|AIXA01015584.1|:567-748

MsHel-1	gb|AIXA01008616.1|:887-1050
MsHel-1	gb|AIXA01007146.1|:867-984
MsHel-1	gb|AIXA01007019.1|:3393-3515
MsHel-1	gb|AIXA01002900.1|:8886-9174
MsHel-1	gb|AIXA01002750.1|:93898-94169
MsHel-1	gb|AIXA01000870.1|:39789-39973
MsHel-1	gb|AIXA01000870.1|:28492-28687
MsHel-1	gb|AIXA01000091.1|:89233-89603
MsHel-1	gb|AIXA01033095.1|:297-506
MsHel-1	gb|AIXA01022615.1|:1001-1221
MsHel-1	gb|AIXA01018540.1|:3191-3327
MsHel-1	gb|AIXA01013153.1|:6908-7042
MsHel-1	gb|AIXA01012190.1|:29015-29284
MsHel-1	gb|AIXA01010855.1|:21338-21482
MsHel-1	gb|AIXA01010028.1|:2948-3222
MsHel-1	gb|AIXA01009826.1|:12665-12933
MsHel-1	gb|AIXA01009456.1|:5257-5391
MsHel-1	gb|AIXA01008558.1|:13050-13193
MsHel-1	gb|AIXA01008283.1|:4576-4806
MsHel-1	gb|AIXA01004840.1|:3681-3815
MsHel-1	gb|AIXA01004591.1|:38218-38329
MsHel-1	gb|AIXA01004204.1|:5861-6130
MsHel-1	gb|AIXA01004186.1|:35888-36262
MsHel-1	gb|AIXA01004061.1|:39606-39778
MsHel-1	gb|AIXA01003927.1|:3053-3261
MsHel-1	gb|AIXA01003678.1|:27950-28073
MsHel-1	gb|AIXA01003559.1|:1217-1313
MsHel-1	gb|AIXA01000195.1|:113693-113935
MsHel-1	gb|AIXA01025968.1|:907-1159
MsHel-1	gb|AIXA01018547.1|:7200-7687
MsHel-1	gb|AIXA01016544.1|:4258-4358
MsHel-1	gb|AIXA01014745.1|:1265-1533
MsHel-1	gb|AIXA01012128.1|:13800-13914
MsHel-1	gb|AIXA01009592.1|:27226-27385
MsHel-1	gb|AIXA01002914.1|:1-81
MsHel-1	gb|AIXA01002527.1|:38469-38610
MsHel-1	gb|AIXA01001733.1|:15945-16103
MsHel-1	gb|AIXA01001119.1|:18262-18541
MsHel-1	gb|AIXA01000083.1|:7662-7974
MsHel-1	gb|AIXA01037246.1|:216-355
MsHel-1	gb|AIXA01019131.1|:1180-1412
MsHel-1	gb|AIXA01015116.1|:17816-17935
MsHel-1	gb|AIXA01014468.1|:168-347
MsHel-1	gb|AIXA01010742.1|:64575-64757
MsHel-1	gb|AIXA01010348.1|:17908-18054
MsHel-1	gb|AIXA01008717.1|:12808-13044
MsHel-1	gb|AIXA01006791.1|:21374-21591
MsHel-1	gb|AIXA01005364.1|:3929-4051

MsHel-1	gb|AIXA01004007.1|:12424-12554
MsHel-1	gb|AIXA01033761.1|:51-236
MsHel-1	gb|AIXA01018514.1|:3298-3445
MsHel-1	gb|AIXA01017768.1|:6611-6730
MsHel-1	gb|AIXA01014919.1|:33921-34037
MsHel-1	gb|AIXA01014152.1|:24184-24342
MsHel-1	gb|AIXA01011679.1|:15671-15885
MsHel-1	gb|AIXA01009820.1|:7915-8183
MsHel-1	gb|AIXA01008500.1|:8846-9103
MsHel-1	gb|AIXA01007296.1|:30487-30732
MsHel-1	gb|AIXA01006917.1|:115139-115340
MsHel-1	gb|AIXA01004965.1|:83135-83257
MsHel-1	gb|AIXA01004893.1|:39371-39501
MsHel-1	gb|AIXA01014281.1|:7989-8177
MsHel-1	gb|AIXA01013816.1|:7436-7592
MsHel-1	gb|AIXA01013317.1|:11555-11855
MsHel-1	gb|AIXA01010119.1|:9505-9625
MsHel-1	gb|AIXA01010060.1|:1420-1721
MsHel-1	gb|AIXA01006831.1|:10273-10393
MsHel-1	gb|AIXA01006279.1|:17657-17777
MsHel-1	gb|AIXA01006061.1|:49712-49852
MsHel-1	gb|AIXA01006061.1|:23074-23213
MsHel-1	gb|AIXA01005349.1|:7621-7754
MsHel-1	gb|AIXA01002537.1|:210-379
MsHel-1	gb|AIXA01002311.1|:32370-32503
MsHel-1	gb|AIXA01002042.1|:9720-9856
MsHel-1	gb|AIXA01001332.1|:16027-16171
MsHel-1	gb|AIXA01001182.1|:37061-37195
MsHel-1	gb|AIXA01001050.1|:35124-35247
MsHel-1	gb|AIXA01000738.1|:14804-14998
MsHel-1	gb|AIXA01000437.1|:42205-42506
MsHel-1	gb|AIXA01000155.1|:53985-54552
MsHel-1	gb|AIXA01032757.1|:573-672
MsHel-1	gb|AIXA01017590.1|:5715-5825
MsHel-1	gb|AIXA01013661.1|:3345-3455
MsHel-1	gb|AIXA01011935.1|:348-451
MsHel-1	gb|AIXA01007123.1|:21538-21747
MsHel-1	gb|AIXA01007123.1|:3195-3278
MsHel-1	gb|AIXA01004515.1|:30471-30736
MsHel-1	gb|AIXA01004495.1|:3856-3987
MsHel-1	gb|AIXA01019152.1|:3978-4184
MsHel-1	gb|AIXA01016420.1|:129-276
MsHel-1	gb|AIXA01015519.1|:16967-17134
MsHel-1	gb|AIXA01014138.1|:2501-2687
MsHel-1	gb|AIXA01012536.1|:3559-3645
MsHel-1	gb|AIXA01010666.1|:32800-32927
MsHel-1	gb|AIXA01010420.1|:31233-31350
MsHel-1	gb|AIXA01010102.1|:114509-114679

MsHel-1	gb|AIXA01008668.1|:14005-14091
MsHel-1	gb|AIXA01008532.1|:37313-37614
MsHel-1	gb|AIXA01007762.1|:6922-7130
MsHel-1	gb|AIXA01007444.1|:32039-32292
MsHel-1	gb|AIXA01007118.1|:24654-24796
MsHel-1	gb|AIXA01007012.1|:3043-3378
MsHel-1	gb|AIXA01006290.1|:10640-10878
MsHel-1	gb|AIXA01004431.1|:34597-34710
MsHel-1	gb|AIXA01004111.1|:6481-6630
MsHel-1	gb|AIXA01002857.1|:28075-28357
MsHel-1	gb|AIXA01002533.1|:2534-2644
MsHel-1	gb|AIXA01002339.1|:27300-27502
MsHel-1	gb|AIXA01022564.1|:486-602
MsHel-1	gb|AIXA01012798.1|:7618-7812
MsHel-1	gb|AIXA01012120.1|:6216-6313
MsHel-1	gb|AIXA01010680.1|:3846-4090
MsHel-1	gb|AIXA01009775.1|:12685-13410
MsHel-1	gb|AIXA01008960.1|:445-625
MsHel-1	gb|AIXA01008280.1|:25134-25359
MsHel-1	gb|AIXA01005496.1|:2151-2530
MsHel-1	gb|AIXA01001879.1|:34937-35022
MsHel-1	gb|AIXA01026674.1|:784-911
MsHel-1	gb|AIXA01024237.1|:877-969
MsHel-1	gb|AIXA01018583.1|:4169-4365
MsHel-1	gb|AIXA01018084.1|:3838-3970
MsHel-1	gb|AIXA01017948.1|:505-827
MsHel-1	gb|AIXA01015510.1|:1952-2036
MsHel-1	gb|AIXA01014986.1|:12218-12334
MsHel-1	gb|AIXA01013902.1|:17657-17791
MsHel-1	gb|AIXA01011306.1|:1087-1203
MsHel-1	gb|AIXA01009498.1|:524-652
MsHel-1	gb|AIXA01009015.1|:19708-19921
MsHel-1	gb|AIXA01008792.1|:5809-5929
MsHel-1	gb|AIXA01006546.1|:4368-4491
MsHel-1	gb|AIXA01006021.1|:68236-68449
MsHel-1	gb|AIXA01005901.1|:67216-67304
MsHel-1	gb|AIXA01005523.1|:9849-13145
MsHel-1	gb|AIXA01005239.1|:4897-4994
MsHel-1	gb|AIXA01004935.1|:72444-72659
MsHel-1	gb|AIXA01003860.1|:12235-12316
MsHel-1	gb|AIXA01003813.1|:31819-32011
MsHel-1	gb|AIXA01003214.1|:18816-18920
MsHel-1	gb|AIXA01002739.1|:7168-7413
MsHel-1	gb|AIXA01001973.1|:34734-34868
MsHel-1	gb|AIXA01018796.1|:775-881
MsHel-1	gb|AIXA01018502.1|:616-719
MsHel-1	gb|AIXA01017731.1|:12412-12539
MsHel-1	gb|AIXA01011292.1|:1400-1600

MsHel-1	gb|AIXA01009405.1|:22265-22383
MsHel-1	gb|AIXA01009405.1|:9011-9131
MsHel-1	gb|AIXA01009393.1|:4322-10511
MsHel-1	gb|AIXA01008281.1|:35446-35545
MsHel-1	gb|AIXA01007431.1|:20997-21131
MsHel-1	gb|AIXA01005484.1|:44865-45034
MsHel-1	gb|AIXA01005387.1|:7323-7521
MsHel-1	gb|AIXA01004817.1|:27344-27531
MsHel-1	gb|AIXA01004760.1|:2964-3103
MsHel-1	gb|AIXA01004038.1|:34977-35126
MsHel-1	gb|AIXA01002958.1|:31419-31958
MsHel-1	gb|AIXA01002542.1|:17461-17597
MsHel-1	gb|AIXA01034478.1|:339-460
MsHel-1	gb|AIXA01031551.1|:543-649
MsHel-1	gb|AIXA01022404.1|:301-557
MsHel-1	gb|AIXA01016998.1|:431-1202
MsHel-1	gb|AIXA01013418.1|:4513-4664
MsHel-1	gb|AIXA01009935.1|:13727-13881
MsHel-1	gb|AIXA01009872.1|:5856-5962
MsHel-1	gb|AIXA01009731.1|:2490-2600
MsHel-1	gb|AIXA01007975.1|:67401-67494
MsHel-1	gb|AIXA01006625.1|:15265-15469
MsHel-1	gb|AIXA01004708.1|:113923-114128
MsHel-1	gb|AIXA01004124.1|:73744-73861
MsHel-1	gb|AIXA01003315.1|:7032-7134
MsHel-1	gb|AIXA01002076.1|:5224-5345
MsHel-1	gb|AIXA01001502.1|:51133-51402
MsHel-1	gb|AIXA01001477.1|:3595-3807
MsHel-1	gb|AIXA01001441.1|:54184-54290
MsHel-1	gb|AIXA01001198.1|:13098-13898
MsHel-1	gb|AIXA01000909.1|:42206-42484
MsHel-1	gb|AIXA01000496.1|:13074-13224
MsHel-1	gb|AIXA01000386.1|:962-1084
MsHel-1	gb|AIXA01027492.1|:360-513
MsHel-1	gb|AIXA01011284.1|:806-932
MsHel-1	gb|AIXA01011102.1|:1004-1105
MsHel-1	gb|AIXA01011101.1|:9542-9643
MsHel-1	gb|AIXA01009841.1|:3722-3899
MsHel-1	gb|AIXA01007856.1|:29779-29946
MsHel-1	gb|AIXA01007856.1|:14071-14335
MsHel-1	gb|AIXA01007476.1|:22416-22598
MsHel-1	gb|AIXA01007232.1|:7410-7582
MsHel-1	gb|AIXA01007122.1|:11833-11953
MsHel-1	gb|AIXA01006843.1|:6223-6352
MsHel-1	gb|AIXA01005073.1|:20411-20569
MsHel-1	gb|AIXA01003140.1|:96918-97136
MsHel-1	gb|AIXA01003140.1|:6240-6474
MsHel-1	gb|AIXA01002069.1|:12317-12546

MsHel-1	gb|AIXA01001571.1|:74047-74275
MsHel-1	gb|AIXA01000972.1|:593-797
MsHel-1	gb|AIXA01000739.1|:5622-5811
MsHel-1	gb|AIXA01000638.1|:31750-31855
MsHel-1	gb|AIXA01036718.1|:308-451
MsHel-1	gb|AIXA01031476.1|:702-782
MsHel-1	gb|AIXA01025770.1|:486-595
MsHel-1	gb|AIXA01019464.1|:1764-1898
MsHel-1	gb|AIXA01019188.1|:1091-1583
MsHel-1	gb|AIXA01017872.1|:2273-2373
MsHel-1	gb|AIXA01015245.1|:8702-8806
MsHel-1	gb|AIXA01014932.1|:8681-9266
MsHel-1	gb|AIXA01014311.1|:390-510
MsHel-1	gb|AIXA01012730.1|:2699-2839
MsHel-1	gb|AIXA01011333.1|:5757-5855
MsHel-1	gb|AIXA01010506.1|:7876-8044
MsHel-1	gb|AIXA01009331.1|:39966-40098
MsHel-1	gb|AIXA01008443.1|:14034-14217
MsHel-1	gb|AIXA01007979.1|:18229-18313
MsHel-1	gb|AIXA01007347.1|:61930-62029
MsHel-1	gb|AIXA01005626.1|:11102-11253
MsHel-1	gb|AIXA01004297.1|:3680-3788
MsHel-1	gb|AIXA01003929.1|:1013-1128
MsHel-1	gb|AIXA01003275.1|:12457-12699
MsHel-1	gb|AIXA01001766.1|:23748-23876
MsHel-1	gb|AIXA01000692.1|:40264-40363
MsHel-1	gb|AIXA01019319.1|:560-667
MsHel-1	gb|AIXA01016525.1|:1927-2050
MsHel-1	gb|AIXA01015532.1|:1475-1609
MsHel-1	gb|AIXA01012583.1|:19885-20036
MsHel-1	gb|AIXA01009691.1|:33679-33820
MsHel-1	gb|AIXA01009349.1|:6390-6477
MsHel-1	gb|AIXA01008368.1|:6096-6231
MsHel-1	gb|AIXA01006665.1|:2954-3242
MsHel-1	gb|AIXA01006383.1|:1801-1996
MsHel-1	gb|AIXA01005516.1|:29180-29267
MsHel-1	gb|AIXA01005218.1|:6835-7044
MsHel-1	gb|AIXA01003314.1|:4359-4494
MsHel-1	gb|AIXA01003110.1|:1050-1148
MsHel-1	gb|AIXA01001710.1|:22450-22561
MsHel-1	gb|AIXA01000982.1|:62536-62675
MsHel-1	gb|AIXA01000167.1|:1272-1398
MsHel-1	gb|AIXA01020499.1|:1160-1333
MsHel-1	gb|AIXA01019628.1|:2302-2384
MsHel-1	gb|AIXA01014096.1|:23547-23745
MsHel-1	gb|AIXA01013342.1|:43405-43488
MsHel-1	gb|AIXA01011304.1|:5131-5244
MsHel-1	gb|AIXA01009249.1|:1322-1520

MsHel-1	gb|AIXA01009107.1|:3716-3855
MsHel-1	gb|AIXA01007844.1|:6326-6472
MsHel-1	gb|AIXA01007414.1|:14152-14265
MsHel-1	gb|AIXA01006983.1|:48920-49035
MsHel-1	gb|AIXA01006449.1|:177042-177149
MsHel-1	gb|AIXA01005422.1|:46114-46208
MsHel-1	gb|AIXA01004252.1|:75296-75425
MsHel-1	gb|AIXA01004252.1|:57607-57757
MsHel-1	gb|AIXA01000792.1|:38830-38932
MsHel-1	gb|AIXA01000213.1|:15167-15372
MsHel-1	gb|AIXA01022806.1|:250-331
MsHel-1	gb|AIXA01017933.1|:13845-13943
MsHel-1	gb|AIXA01017708.1|:81-203
MsHel-1	gb|AIXA01015715.1|:6165-6288
MsHel-1	gb|AIXA01014956.1|:11505-11590
MsHel-1	gb|AIXA01013546.1|:14219-14300
MsHel-1	gb|AIXA01010987.1|:3722-4024
MsHel-1	gb|AIXA01010823.1|:727-831
MsHel-1	gb|AIXA01010388.1|:342-855
MsHel-1	gb|AIXA01008437.1|:6286-6383
MsHel-1	gb|AIXA01008291.1|:36809-37060
MsHel-1	gb|AIXA01008242.1|:5903-6191
MsHel-1	gb|AIXA01006126.1|:20951-21077
MsHel-1	gb|AIXA01005995.1|:864-1022
MsHel-1	gb|AIXA01004522.1|:6016-6171
MsHel-1	gb|AIXA01004367.1|:6260-6420
MsHel-1	gb|AIXA01004354.1|:46927-47024
MsHel-1	gb|AIXA01003998.1|:6901-7037
MsHel-1	gb|AIXA01003671.1|:8096-8353
MsHel-1	gb|AIXA01002993.1|:14591-14784
MsHel-1	gb|AIXA01002452.1|:45976-46268
MsHel-1	gb|AIXA01001399.1|:13561-13674
MsHel-1	gb|AIXA01029702.1|:134-262
MsHel-1	gb|AIXA01016603.1|:1511-1638
MsHel-1	gb|AIXA01016506.1|:674-782
MsHel-1	gb|AIXA01011235.1|:5317-5449
MsHel-1	gb|AIXA01009346.1|:7336-7445
MsHel-1	gb|AIXA01009278.1|:7050-7130
MsHel-1	gb|AIXA01008517.1|:1-51
MsHel-1	gb|AIXA01005607.1|:3865-3965
MsHel-1	gb|AIXA01005432.1|:15060-15267
MsHel-1	gb|AIXA01004689.1|:9073-9353
MsHel-1	gb|AIXA01004688.1|:22254-22641
MsHel-1	gb|AIXA01004661.1|:10706-11413
MsHel-1	gb|AIXA01004307.1|:92636-92745
MsHel-1	gb|AIXA01002439.1|:1406-1517
MsHel-1	gb|AIXA01001402.1|:1856-2001
MsHel-1	gb|AIXA01001145.1|:16853-17034

MsHel-1	gb|AIXA01001090.1|:18394-18585
MsHel-1	gb|AIXA01000959.1|:53203-53363
MsHel-1	gb|AIXA01000597.1|:13343-13440
MsHel-1	gb|AIXA01000012.1|:78145-78264
MsHel-1	gb|AIXA01015637.1|:3820-3988
MsHel-1	gb|AIXA01013125.1|:806-918
MsHel-1	gb|AIXA01010882.1|:1267-1346
MsHel-1	gb|AIXA01010470.1|:10897-10999
MsHel-1	gb|AIXA01006687.1|:13239-13421
MsHel-1	gb|AIXA01005383.1|:27958-28232
MsHel-1	gb|AIXA01005131.1|:26595-26730
MsHel-1	gb|AIXA01004117.1|:8408-8535
MsHel-1	gb|AIXA01002281.1|:14985-15067
MsHel-1	gb|AIXA01002091.1|:1-92
MsHel-1	gb|AIXA01001494.1|:58360-58505
MsHel-1	gb|AIXA01023481.1|:244-358
MsHel-1	gb|AIXA01015396.1|:22322-22526
MsHel-1	gb|AIXA01013850.1|:18418-18532
MsHel-1	gb|AIXA01011818.1|:8084-8189
MsHel-1	gb|AIXA01011812.1|:770-1098
MsHel-1	gb|AIXA01009879.1|:12287-12402
MsHel-1	gb|AIXA01008917.1|:27012-27150
MsHel-1	gb|AIXA01008782.1|:56628-56872
MsHel-1	gb|AIXA01004985.1|:74862-74977
MsHel-1	gb|AIXA01004102.1|:16918-17128
MsHel-1	gb|AIXA01003986.1|:9446-9666
MsHel-1	gb|AIXA01002364.1|:89399-89513
MsHel-1	gb|AIXA01002217.1|:50385-50564
MsHel-1	gb|AIXA01001878.1|:17771-17857
MsHel-1	gb|AIXA01001052.1|:45117-45257
MsHel-1	gb|AIXA01000324.1|:6796-6913
MsHel-1	gb|AIXA01016311.1|:4109-4417
MsHel-1	gb|AIXA01015629.1|:12044-12159
MsHel-1	gb|AIXA01013920.1|:359-472
MsHel-1	gb|AIXA01013919.1|:11717-11830
MsHel-1	gb|AIXA01012406.1|:1094-1215
MsHel-1	gb|AIXA01010963.1|:4384-4477
MsHel-1	gb|AIXA01010109.1|:274-645
MsHel-1	gb|AIXA01009380.1|:3358-3439
MsHel-1	gb|AIXA01008961.1|:13264-13363
MsHel-1	gb|AIXA01008615.1|:2089-2250
MsHel-1	gb|AIXA01008241.1|:11084-11215
MsHel-1	gb|AIXA01007556.1|:25405-25493
MsHel-1	gb|AIXA01004974.1|:6130-6267
MsHel-1	gb|AIXA01004923.1|:552-633
MsHel-1	gb|AIXA01004888.1|:187-425
MsHel-1	gb|AIXA01002731.1|:14276-14533
MsHel-1	gb|AIXA01002264.1|:2390-2503

MsHel-1	gb|AIXA01001898.1|:231716-231834
MsHel-1	gb|AIXA01001898.1|:15267-15382
MsHel-1	gb|AIXA01001555.1|:3762-3867
MsHel-1	gb|AIXA01001532.1|:95135-95225
MsHel-1	gb|AIXA01000041.1|:69064-69162
MsHel-1	gb|AIXA01026328.1|:692-896
MsHel-1	gb|AIXA01024094.1|:735-827
MsHel-1	gb|AIXA01012589.1|:5780-5965
MsHel-1	gb|AIXA01009673.1|:1513-1669
MsHel-1	gb|AIXA01009481.1|:11862-12032
MsHel-1	gb|AIXA01008959.1|:16507-16606
MsHel-1	gb|AIXA01008418.1|:59326-59387
MsHel-1	gb|AIXA01005471.1|:4162-4262
MsHel-1	gb|AIXA01004876.1|:2835-2934
MsHel-1	gb|AIXA01003222.1|:19737-19853
MsHel-1	gb|AIXA01002090.1|:13279-13405
MsHel-1	gb|AIXA01001667.1|:38872-38984
MsHel-1	gb|AIXA01000060.1|:15766-15858
MsHel-1	gb|AIXA01021672.1|:1182-1305
MsHel-1	gb|AIXA01018869.1|:42-228
MsHel-1	gb|AIXA01009977.1|:5341-5576
MsHel-1	gb|AIXA01008778.1|:19467-19550
MsHel-1	gb|AIXA01008146.1|:513-642
MsHel-1	gb|AIXA01007050.1|:14042-14138
MsHel-1	gb|AIXA01006982.1|:2696-2824
MsHel-1	gb|AIXA01006586.1|:275-394
MsHel-1	gb|AIXA01006255.1|:94688-94915
MsHel-1	gb|AIXA01003053.1|:16765-16851
MsHel-1	gb|AIXA01002302.1|:16375-16509
MsHel-1	gb|AIXA01000358.1|:374-485
MsHel-1	gb|AIXA01022653.1|:1528-1648
MsHel-1	gb|AIXA01018033.1|:6637-6738
MsHel-1	gb|AIXA01017293.1|:2243-2345
MsHel-1	gb|AIXA01015029.1|:3673-3770
MsHel-1	gb|AIXA01014673.1|:1161-1335
MsHel-1	gb|AIXA01014642.1|:3575-3661
MsHel-1	gb|AIXA01014537.1|:8732-8850
MsHel-1	gb|AIXA01013913.1|:7800-7882
MsHel-1	gb|AIXA01010908.1|:22747-22865
MsHel-1	gb|AIXA01009234.1|:7628-7755
MsHel-1	gb|AIXA01005037.1|:30298-30433
MsHel-1	gb|AIXA01005035.1|:2081-2240
MsHel-1	gb|AIXA01004281.1|:24270-24384
MsHel-1	gb|AIXA01002906.1|:95178-95337
MsHel-1	gb|AIXA01001786.1|:149684-149842
MsHel-1	gb|AIXA01001768.1|:13830-14021
MsHel-1	gb|AIXA01001613.1|:92734-92816
MsHel-1	gb|AIXA01000796.1|:22184-22278

MsHel-1	gb|AIXA01000534.1|:25935-26605
MsHel-1	gb|AIXA01036232.1|:46-146
MsHel-1	gb|AIXA01032853.1|:366-459
MsHel-1	gb|AIXA01030681.1|:79-268
MsHel-1	gb|AIXA01025768.1|:6-119
MsHel-1	gb|AIXA01016044.1|:1926-2120
MsHel-1	gb|AIXA01015486.1|:5348-5453
MsHel-1	gb|AIXA01010913.1|:6053-6257
MsHel-1	gb|AIXA01008884.1|:26863-26981
MsHel-1	gb|AIXA01006800.1|:7581-7711
MsHel-1	gb|AIXA01006701.1|:23326-23464
MsHel-1	gb|AIXA01005883.1|:1575-1676
MsHel-1	gb|AIXA01004654.1|:49681-49782
MsHel-1	gb|AIXA01002972.1|:65231-65317
MsHel-1	gb|AIXA01002145.1|:11210-11321
MsHel-1	gb|AIXA01022526.1|:565-673
MsHel-1	gb|AIXA01019556.1|:1499-1635
MsHel-1	gb|AIXA01018298.1|:85-308
MsHel-1	gb|AIXA01014411.1|:710-828
MsHel-1	gb|AIXA01013539.1|:1-51
MsHel-1	gb|AIXA01012117.1|:2894-3047
MsHel-1	gb|AIXA01010487.1|:34419-34591
MsHel-1	gb|AIXA01004185.1|:3609-3717
MsHel-1	gb|AIXA01003895.1|:55782-55949
MsHel-1	gb|AIXA01003305.1|:1073-1199
MsHel-1	gb|AIXA01000790.1|:17508-17604
MsHel-1	gb|AIXA01032725.1|:90-180
MsHel-1	gb|AIXA01015240.1|:1862-1948
MsHel-1	gb|AIXA01013467.1|:31838-31921
MsHel-1	gb|AIXA01011121.1|:28511-28631
MsHel-1	gb|AIXA01009296.1|:22741-22847
MsHel-1	gb|AIXA01003840.1|:7474-7585
MsHel-1	gb|AIXA01002916.1|:8665-8788
MsHel-1	gb|AIXA01001805.1|:31162-31290
MsHel-1	gb|AIXA01001002.1|:11994-12084
MsHel-1	gb|AIXA01018449.1|:2391-2473
MsHel-1	gb|AIXA01008897.1|:5936-6013
MsHel-1	gb|AIXA01005765.1|:13449-13563
MsHel-1	gb|AIXA01005497.1|:8169-8263
MsHel-1	gb|AIXA01002625.1|:4034-4116
MsHel-1	gb|AIXA01002574.1|:16156-16329
MsHel-1	gb|AIXA01002514.1|:43445-43527
MsHel-1	gb|AIXA01001474.1|:41081-41330
MsHel-1	gb|AIXA01014603.1|:12101-12218
MsHel-1	gb|AIXA01013612.1|:15653-15785
MsHel-1	gb|AIXA01011096.1|:53357-53466
MsHel-1	gb|AIXA01009158.1|:8136-8264
MsHel-1	gb|AIXA01008383.1|:1173-1281

MsHel-1	gb|AIXA01008156.1|:2412-2504
MsHel-1	gb|AIXA01002814.1|:19968-20057
MsHel-2	gb|AIXA01005585.1|:63067-63370
MsHel-2	gb|AIXA01011818.1|:8259-8561
MsHel-2	gb|AIXA01000789.1|:2637-2937
MsHel-2	gb|AIXA01005321.1|:71883-72186
MsHel-2	gb|AIXA01005321.1|:9799-9890
MsHel-2	gb|AIXA01002566.1|:8679-8975
MsHel-2	gb|AIXA01005808.1|:34810-34939
MsHel-2	gb|AIXA01014443.1|:64596-64725
MsHel-2	gb|AIXA01014443.1|:21042-21137
MsHel-2	gb|AIXA01001147.1|:10428-10728
MsHel-2	gb|AIXA01009354.1|:10112-10415
MsHel-2	gb|AIXA01008962.1|:159639-159942
MsHel-2	gb|AIXA01002579.1|:120233-120325
MsHel-2	gb|AIXA01007966.1|:100572-100868
MsHel-2	gb|AIXA01007966.1|:39268-48247
MsHel-2	gb|AIXA01003814.1|:7273-7573
MsHel-2	gb|AIXA01001575.1|:190-494
MsHel-2	gb|AIXA01007580.1|:38584-38886
MsHel-2	gb|AIXA01002990.1|:57643-57946
MsHel-2	gb|AIXA01002340.1|:130135-130260
MsHel-2	gb|AIXA01002367.1|:1646-1949
MsHel-2	gb|AIXA01001546.1|:27058-27182
MsHel-2	gb|AIXA01001546.1|:14044-14171
MsHel-2	gb|AIXA01000161.1|:29004-29307
MsHel-2	gb|AIXA01018951.1|:3019-3317
MsHel-2	gb|AIXA01015732.1|:449-748
MsHel-2	gb|AIXA01011894.1|:1875-2177
MsHel-2	gb|AIXA01019243.1|:4061-4362
MsHel-2	gb|AIXA01010077.1|:47547-47847
MsHel-2	gb|AIXA01007229.1|:42553-42824
MsHel-2	gb|AIXA01005914.1|:9816-10112
MsHel-2	gb|AIXA01004923.1|:4989-5289
MsHel-2	gb|AIXA01001813.1|:44940-45042
MsHel-2	gb|AIXA01001813.1|:33334-33634
MsHel-2	gb|AIXA01003616.1|:8996-9299
MsHel-2	gb|AIXA01002377.1|:195767-196070
MsHel-2	gb|AIXA01001140.1|:4861-5148
MsHel-2	gb|AIXA01001043.1|:78656-78958
MsHel-2	gb|AIXA01000892.1|:77779-77896
MsHel-2	gb|AIXA01000892.1|:20375-20678
MsHel-2	gb|AIXA01008995.1|:46851-47154
MsHel-2	gb|AIXA01008320.1|:1407-1707
MsHel-2	gb|AIXA01005050.1|:1964-2264
MsHel-2	gb|AIXA01002999.1|:97577-97880
MsHel-2	gb|AIXA01000907.1|:32044-32154
MsHel-2	gb|AIXA01000907.1|:7513-7815

MsHel-2	gb|AIXA01012329.1|:15342-15646
MsHel-2	gb|AIXA01003747.1|:38133-38433
MsHel-2	gb|AIXA01000549.1|:15317-15622
MsHel-2	gb|AIXA01014533.1|:2783-3086
MsHel-2	gb|AIXA01001990.1|:19311-19614
MsHel-2	gb|AIXA01011970.1|:40393-40690
MsHel-2	gb|AIXA01005097.1|:7463-7585
MsHel-2	gb|AIXA01014420.1|:6286-6576
MsHel-2	gb|AIXA01011501.1|:20438-20735
MsHel-2	gb|AIXA01009661.1|:617-914
MsHel-2	gb|AIXA01030254.1|:379-678
MsHel-2	gb|AIXA01010690.1|:787-1088
MsHel-2	gb|AIXA01006863.1|:5161-5461
MsHel-2	gb|AIXA01001292.1|:37987-38275
MsHel-2	gb|AIXA01000077.1|:68553-68853
MsHel-2	gb|AIXA01013529.1|:65285-65387
MsHel-2	gb|AIXA01013529.1|:28402-28705
MsHel-2	gb|AIXA01012119.1|:1183-1486
MsHel-2	gb|AIXA01011810.1|:979-1280
MsHel-2	gb|AIXA01008862.1|:13945-14244
MsHel-2	gb|AIXA01004330.1|:80693-80995
MsHel-2	gb|AIXA01004330.1|:39793-39920
MsHel-2	gb|AIXA01004330.1|:17411-17540
MsHel-2	gb|AIXA01007107.1|:9400-9524
MsHel-2	gb|AIXA01002721.1|:6459-6760
MsHel-2	gb|AIXA01008232.1|:2920-3222
MsHel-2	gb|AIXA01003576.1|:61548-61672
MsHel-2	gb|AIXA01003576.1|:42-345
MsHel-2	gb|AIXA01001965.1|:60062-60365
MsHel-2	gb|AIXA01000846.1|:512-813
MsHel-2	gb|AIXA01000041.1|:37090-37370
MsHel-2	gb|AIXA01000041.1|:19884-20187
MsHel-2	gb|AIXA01012018.1|:14612-14919
MsHel-2	gb|AIXA01005639.1|:2371-7950
MsHel-2	gb|AIXA01029228.1|:300-603
MsHel-2	gb|AIXA01018669.1|:7247-7547
MsHel-2	gb|AIXA01011456.1|:497-797
MsHel-2	gb|AIXA01007072.1|:19508-19808
MsHel-2	gb|AIXA01002304.1|:36191-36494
MsHel-2	gb|AIXA01002304.1|:6928-7215
MsHel-2	gb|AIXA01012099.1|:2011-2307
MsHel-2	gb|AIXA01005770.1|:14375-14678
MsHel-2	gb|AIXA01003138.1|:33878-34178
MsHel-2	gb|AIXA01008443.1|:56143-56440
MsHel-2	gb|AIXA01007153.1|:50865-51160
MsHel-2	gb|AIXA01010804.1|:8487-8787
MsHel-2	gb|AIXA01035433.1|:163-458
MsHel-2	gb|AIXA01009742.1|:1864-2160

MsHel-2	gb|AIXA01005323.1|:31943-32244
MsHel-2	gb|AIXA01016168.1|:9444-9747
MsHel-2	gb|AIXA01010748.1|:131159-131268
MsHel-2	gb|AIXA01004153.1|:91109-91411
MsHel-2	gb|AIXA01004153.1|:80289-80413
MsHel-2	gb|AIXA01004141.1|:38146-38441
MsHel-2	gb|AIXA01003942.1|:37751-37872
MsHel-2	gb|AIXA01003942.1|:224-505
MsHel-2	gb|AIXA01002313.1|:24611-24702
MsHel-2	gb|AIXA01007968.1|:1533-1832
MsHel-2	gb|AIXA01001270.1|:58974-59144
MsHel-2	gb|AIXA01001270.1|:22414-22538
MsHel-2	gb|AIXA01014147.1|:8119-8411
MsHel-2	gb|AIXA01008665.1|:392-676
MsHel-2	gb|AIXA01001883.1|:4765-5062
MsHel-2	gb|AIXA01007688.1|:1094-1396
MsHel-2	gb|AIXA01003132.1|:44875-45174
MsHel-2	gb|AIXA01017004.1|:1487-1792
MsHel-2	gb|AIXA01013522.1|:4390-4688
MsHel-2	gb|AIXA01003873.1|:32446-32747
MsHel-2	gb|AIXA01002794.1|:54872-61430
MsHel-2	gb|AIXA01012319.1|:9347-9646
MsHel-2	gb|AIXA01008536.1|:28424-28723
MsHel-2	gb|AIXA01002552.1|:98926-99236
MsHel-2	gb|AIXA01028923.1|:310-613
MsHel-2	gb|AIXA01012741.1|:111142-111271
MsHel-2	gb|AIXA01012741.1|:59902-60031
MsHel-2	gb|AIXA01000064.1|:41043-41143
MsHel-2	gb|AIXA01015742.1|:3178-3476
MsHel-2	gb|AIXA01006515.1|:19630-19899
MsHel-2	gb|AIXA01004367.1|:19414-19715
MsHel-2	gb|AIXA01002545.1|:10990-11290
MsHel-2	gb|AIXA01007472.1|:54981-55110
MsHel-2	gb|AIXA01002311.1|:172759-172887
MsHel-2	gb|AIXA01002311.1|:4815-4940
MsHel-2	gb|AIXA01001050.1|:51719-52024
MsHel-2	gb|AIXA01001050.1|:34143-34235
MsHel-2	gb|AIXA01011249.1|:5948-6238
MsHel-2	gb|AIXA01005867.1|:9590-9893
MsHel-2	gb|AIXA01002679.1|:53067-53370
MsHel-2	gb|AIXA01002658.1|:27114-27406
MsHel-2	gb|AIXA01001764.1|:2778-3075
MsHel-2	gb|AIXA01001286.1|:18943-19245
MsHel-2	gb|AIXA01009016.1|:5766-6060
MsHel-2	gb|AIXA01010854.1|:70672-70797
MsHel-2	gb|AIXA01008632.1|:8308-8609
MsHel-2	gb|AIXA01001543.1|:26796-26960
MsHel-2	gb|AIXA01013483.1|:2900-3203

MsHel-2	gb|AIXA01036157.1|:53-350
MsHel-2	gb|AIXA01002404.1|:12883-13192
MsHel-2	gb|AIXA01010330.1|:45561-45682
MsHel-2	gb|AIXA01010330.1|:20463-20763
MsHel-2	gb|AIXA01010330.1|:339-460
MsHel-2	gb|AIXA01008194.1|:874-1169
MsHel-2	gb|AIXA01010483.1|:1286-1562
MsHel-2	gb|AIXA01008116.1|:1203-1294
MsHel-2	gb|AIXA01016326.1|:7083-7376
MsHel-2	gb|AIXA01002050.1|:39429-39684
MsHel-2	gb|AIXA01009826.1|:29206-29507
MsHel-2	gb|AIXA01005579.1|:33759-34055
MsHel-2	gb|AIXA01037135.1|:141-444
MsHel-2	gb|AIXA01006423.1|:8405-8697
MsHel-2	gb|AIXA01002671.1|:142837-143132
MsHel-2	gb|AIXA01002671.1|:60867-61020
MsHel-2	gb|AIXA01007971.1|:20748-21048
MsHel-2	gb|AIXA01006051.1|:10935-11238
MsHel-2	gb|AIXA01003365.1|:59174-59477
MsHel-2	gb|AIXA01007978.1|:17900-18174
MsHel-2	gb|AIXA01001636.1|:86999-87294
MsHel-2	gb|AIXA01001636.1|:24780-24900
MsHel-2	gb|AIXA01001558.1|:102023-102330
MsHel-2	gb|AIXA01006351.1|:1025-1334
MsHel-2	gb|AIXA01017841.1|:3188-3462
MsHel-2	gb|AIXA01007344.1|:45121-45383
MsHel-2	gb|AIXA01004064.1|:28453-28750
MsHel-2	gb|AIXA01000251.1|:183185-183489
MsHel-2	gb|AIXA01012784.1|:1475-1586
MsHel-2	gb|AIXA01009133.1|:36465-36684
MsHel-2	gb|AIXA01008563.1|:7223-7525
MsHel-2	gb|AIXA01005068.1|:20123-20418
MsHel-2	gb|AIXA01009825.1|:4610-4914
MsHel-2	gb|AIXA01037867.1|:1-280
MsHel-2	gb|AIXA01003231.1|:12021-12336
MsHel-2	gb|AIXA01002159.1|:14226-14525
MsHel-2	gb|AIXA01000005.1|:68476-68776
MsHel-2	gb|AIXA01000005.1|:50684-50780
MsHel-2	gb|AIXA01010473.1|:3672-3964
MsHel-2	gb|AIXA01001602.1|:52268-52566
MsHel-2	gb|AIXA01031781.1|:405-700
MsHel-2	gb|AIXA01003889.1|:107940-108238
MsHel-2	gb|AIXA01004108.1|:33244-33469
MsHel-2	gb|AIXA01002293.1|:26919-27232
MsHel-2	gb|AIXA01000408.1|:31841-32140
MsHel-2	gb|AIXA01006856.1|:5883-6179
MsHel-2	gb|AIXA01010208.1|:15187-15313
MsHel-2	gb|AIXA01008398.1|:6318-6618

MsHel-2	gb|AIXA01009109.1|:26674-27232
MsHel-2	gb|AIXA01016756.1|:1363-1672
MsHel-2	gb|AIXA01006671.1|:15859-15983
MsHel-2	gb|AIXA01006671.1|:417-702
MsHel-2	gb|AIXA01004943.1|:36540-36841
MsHel-2	gb|AIXA01004943.1|:7771-7900
MsHel-2	gb|AIXA01004115.1|:84783-85072
MsHel-2	gb|AIXA01014296.1|:6614-6697
MsHel-2	gb|AIXA01003924.1|:118981-119107
MsHel-2	gb|AIXA01010773.1|:49835-49929
MsHel-2	gb|AIXA01010773.1|:23243-23542
MsHel-2	gb|AIXA01010773.1|:4725-4843
MsHel-2	gb|AIXA01001048.1|:63990-64111
MsHel-2	gb|AIXA01000267.1|:98772-98885
MsHel-2	gb|AIXA01000267.1|:19284-19585
MsHel-2	gb|AIXA01000258.1|:4611-4911
MsHel-2	gb|AIXA01005724.1|:17174-17452
MsHel-2	gb|AIXA01003183.1|:142135-142264
MsHel-2	gb|AIXA01003183.1|:114991-115283
MsHel-2	gb|AIXA01015508.1|:10834-11124
MsHel-2	gb|AIXA01002674.1|:5928-6227
MsHel-2	gb|AIXA01005873.1|:5176-5473
MsHel-2	gb|AIXA01018306.1|:4404-4693
MsHel-2	gb|AIXA01012097.1|:5982-6101
MsHel-2	gb|AIXA01010343.1|:15744-16039
MsHel-2	gb|AIXA01002403.1|:2773-3035
MsHel-2	gb|AIXA01009420.1|:17465-17761
MsHel-2	gb|AIXA01015575.1|:9035-9331
MsHel-2	gb|AIXA01018899.1|:898-1162
MsHel-2	gb|AIXA01004091.1|:11098-11387
MsHel-2	gb|AIXA01011554.1|:1276-1567
MsHel-2	gb|AIXA01002500.1|:143085-149337
MsHel-2	gb|AIXA01002500.1|:46491-46591
MsHel-2	gb|AIXA01010689.1|:4613-6080
MsHel-2	gb|AIXA01009040.1|:135706-135820
MsHel-2	gb|AIXA01014242.1|:13093-13524
MsHel-2	gb|AIXA01016947.1|:9957-10278
MsHel-2	gb|AIXA01010694.1|:5298-5568
MsHel-2	gb|AIXA01018341.1|:1399-1522
MsHel-2	gb|AIXA01011031.1|:30018-30320
MsHel-2	gb|AIXA01016453.1|:1017-1303
MsHel-2	gb|AIXA01006861.1|:25105-25396
MsHel-2	gb|AIXA01002975.1|:124837-124964
MsHel-2	gb|AIXA01000214.1|:20083-20389
MsHel-2	gb|AIXA01002624.1|:31112-31329
MsHel-2	gb|AIXA01020880.1|:612-963
MsHel-2	gb|AIXA01007083.1|:68743-69060
MsHel-2	gb|AIXA01004130.1|:4598-4884

MsHel-2	gb|AIXA01017727.1|:15813-16131
MsHel-2	gb|AIXA01004218.1|:5725-5942
MsHel-2	gb|AIXA01009741.1|:6231-6523
MsHel-2	gb|AIXA01005498.1|:37967-38176
MsHel-2	gb|AIXA01001266.1|:79124-79248
MsHel-2	gb|AIXA01009411.1|:12088-12406
MsHel-2	gb|AIXA01006915.1|:3073-3181
MsHel-2	gb|AIXA01003293.1|:24898-25184
MsHel-2	gb|AIXA01001528.1|:3105-3400
MsHel-2	gb|AIXA01003021.1|:137139-137261
MsHel-2	gb|AIXA01003021.1|:85010-85177
MsHel-2	gb|AIXA01017455.1|:5430-5679
MsHel-2	gb|AIXA01008730.1|:5245-5467
MsHel-2	gb|AIXA01002155.1|:54919-55183
MsHel-2	gb|AIXA01004482.1|:17513-17641
MsHel-2	gb|AIXA01004482.1|:4580-4854
MsHel-2	gb|AIXA01001945.1|:5885-6163
MsHel-2	gb|AIXA01034839.1|:479-622
MsHel-2	gb|AIXA01003074.1|:9634-9901
MsHel-2	gb|AIXA01012247.1|:28365-28651
MsHel-2	gb|AIXA01001858.1|:47432-47727
MsHel-2	gb|AIXA01004753.1|:32755-33041
MsHel-2	gb|AIXA01002350.1|:148609-148730
MsHel-2	gb|AIXA01002350.1|:106000-106127
MsHel-2	gb|AIXA01002350.1|:69928-70056
MsHel-2	gb|AIXA01002350.1|:30142-30268
MsHel-2	gb|AIXA01009936.1|:8222-8346
MsHel-2	gb|AIXA01002813.1|:71663-71941
MsHel-2	gb|AIXA01001272.1|:13630-13933
MsHel-2	gb|AIXA01004386.1|:82639-82917
MsHel-2	gb|AIXA01009402.1|:54731-55005
MsHel-2	gb|AIXA01009402.1|:16998-17082
MsHel-2	gb|AIXA01001401.1|:21391-21541
MsHel-2	gb|AIXA01011295.1|:21025-21320
MsHel-2	gb|AIXA01005549.1|:18927-19210
MsHel-2	gb|AIXA01008725.1|:103158-103306
MsHel-2	gb|AIXA01008725.1|:10374-10459
MsHel-2	gb|AIXA01000931.1|:65694-65945
MsHel-2	gb|AIXA01016604.1|:9533-9793
MsHel-2	gb|AIXA01011635.1|:25350-25554
MsHel-2	gb|AIXA01008110.1|:67120-67412
MsHel-2	gb|AIXA01008110.1|:24496-24626
MsHel-2	gb|AIXA01005642.1|:71389-71670
MsHel-2	gb|AIXA01005479.1|:21797-22057
MsHel-2	gb|AIXA01013790.1|:2120-2391
MsHel-2	gb|AIXA01007521.1|:16778-16984
MsHel-2	gb|AIXA01003180.1|:6369-6630
MsHel-2	gb|AIXA01000838.1|:71196-71320

MsHel-2	gb|AIXA01000836.1|:199-488
MsHel-2	gb|AIXA01004087.1|:28817-29089
MsHel-2	gb|AIXA01000494.1|:50403-50528
MsHel-2	gb|AIXA01000494.1|:22818-22945
MsHel-2	gb|AIXA01011500.1|:26190-26481
MsHel-2	gb|AIXA01003703.1|:22872-23129
MsHel-2	gb|AIXA01018414.1|:8063-8307
MsHel-2	gb|AIXA01003977.1|:47524-47799
MsHel-2	gb|AIXA01001571.1|:92417-92705
MsHel-2	gb|AIXA01017886.1|:84-345
MsHel-2	gb|AIXA01010855.1|:20782-20906
MsHel-2	gb|AIXA01008442.1|:51959-52062
MsHel-2	gb|AIXA01003012.1|:12955-13212
MsHel-2	gb|AIXA01007104.1|:16334-16447
MsHel-2	gb|AIXA01007104.1|:2972-3258
MsHel-2	gb|AIXA01002386.1|:114679-114959
MsHel-2	gb|AIXA01001830.1|:44525-44646
MsHel-2	gb|AIXA01001830.1|:26269-26390
MsHel-2	gb|AIXA01010593.1|:8199-8325
MsHel-2	gb|AIXA01002586.1|:5432-5674
MsHel-2	gb|AIXA01036788.1|:2-234
MsHel-2	gb|AIXA01019346.1|:1396-1678
MsHel-2	gb|AIXA01013445.1|:18994-19262
MsHel-2	gb|AIXA01011327.1|:2015-2309
MsHel-2	gb|AIXA01007319.1|:4084-4405
MsHel-2	gb|AIXA01010770.1|:14796-15087
MsHel-2	gb|AIXA01001082.1|:6741-7029
MsHel-2	gb|AIXA01007626.1|:113288-113495
MsHel-2	gb|AIXA01007626.1|:97686-97786
MsHel-2	gb|AIXA01003426.1|:8761-9057
MsHel-2	gb|AIXA01018426.1|:3152-3403
MsHel-2	gb|AIXA01009360.1|:10043-10316
MsHel-2	gb|AIXA01008859.1|:8624-8751
MsHel-2	gb|AIXA01004702.1|:50112-50413
MsHel-2	gb|AIXA01009088.1|:4184-4386
MsHel-2	gb|AIXA01010079.1|:37931-38120
MsHel-2	gb|AIXA01005595.1|:141232-141337
MsHel-2	gb|AIXA01003207.1|:44690-46275
MsHel-2	gb|AIXA01004135.1|:50817-50945
MsHel-2	gb|AIXA01004135.1|:4398-4527
MsHel-2	gb|AIXA01003311.1|:5820-6142
MsHel-2	gb|AIXA01005604.1|:19467-19579
MsHel-2	gb|AIXA01005604.1|:3419-3709
MsHel-2	gb|AIXA01005350.1|:4391-4519
MsHel-2	gb|AIXA01013049.1|:12443-12732
MsHel-2	gb|AIXA01003380.1|:1699-1950
MsHel-2	gb|AIXA01002273.1|:27709-27921
MsHel-2	gb|AIXA01014537.1|:8945-9060

MsHel-2	gb|AIXA01006556.1|:454-4723
MsHel-2	gb|AIXA01003836.1|:19447-21018
MsHel-2	gb|AIXA01012660.1|:5495-5621
MsHel-2	gb|AIXA01008296.1|:20441-20564
MsHel-2	gb|AIXA01008227.1|:14444-14573
MsHel-2	gb|AIXA01004817.1|:50855-50956
MsHel-2	gb|AIXA01004817.1|:27238-27755
MsHel-2	gb|AIXA01003986.1|:9701-9827
MsHel-2	gb|AIXA01001496.1|:7110-7404
MsHel-2	gb|AIXA01001534.1|:2636-2885
MsHel-2	gb|AIXA01018380.1|:3331-3458
MsHel-2	gb|AIXA01006684.1|:13099-13199
MsHel-2	gb|AIXA01001641.1|:512-626
MsHel-2	gb|AIXA01001296.1|:171729-171852
MsHel-2	gb|AIXA01004183.1|:25711-25832
MsHel-2	gb|AIXA01012922.1|:4-209
MsHel-2	gb|AIXA01001092.1|:11434-11712
MsHel-2	gb|AIXA01015680.1|:1782-1907
MsHel-2	gb|AIXA01009592.1|:27422-27549
MsHel-2	gb|AIXA01003044.1|:1727-1839
MsHel-2	gb|AIXA01000713.1|:38732-38891
MsHel-2	gb|AIXA01019188.1|:1630-1752
MsHel-2	gb|AIXA01014641.1|:35733-35859
MsHel-2	gb|AIXA01014641.1|:5837-5958
MsHel-2	gb|AIXA01013523.1|:32504-32602
MsHel-2	gb|AIXA01013523.1|:17633-17760
MsHel-2	gb|AIXA01009393.1|:4518-4644
MsHel-2	gb|AIXA01007593.1|:66733-67046
MsHel-2	gb|AIXA01007593.1|:33720-33847
MsHel-2	gb|AIXA01005518.1|:8864-8989
MsHel-2	gb|AIXA01000767.1|:35323-35437
MsHel-2	gb|AIXA01000767.1|:22471-22597
MsHel-2	gb|AIXA01009706.1|:17522-17643
MsHel-2	gb|AIXA01007902.1|:12478-12578
MsHel-2	gb|AIXA01005355.1|:3193-3441
MsHel-2	gb|AIXA01002351.1|:34152-34442
MsHel-2	gb|AIXA01002351.1|:17347-17471
MsHel-2	gb|AIXA01002351.1|:1706-1832
MsHel-2	gb|AIXA01000974.1|:4473-4762
MsHel-2	gb|AIXA01005612.1|:22249-22378
MsHel-2	gb|AIXA01004629.1|:27368-27480
MsHel-2	gb|AIXA01006681.1|:31118-31240
MsHel-2	gb|AIXA01003124.1|:38078-38338
MsHel-2	gb|AIXA01018241.1|:2772-2890
MsHel-2	gb|AIXA01012377.1|:9354-9619
MsHel-2	gb|AIXA01001829.1|:39888-40017
MsHel-2	gb|AIXA01016140.1|:1731-1848
MsHel-2	gb|AIXA01016076.1|:25332-25458

MsHel-2	gb|AIXA01011243.1|:39986-40111
MsHel-2	gb|AIXA01038158.1|:159-288
MsHel-2	gb|AIXA01017402.1|:4064-4193
MsHel-2	gb|AIXA01016695.1|:1075-1204
MsHel-2	gb|AIXA01004369.1|:12422-12551
MsHel-2	gb|AIXA01002318.1|:20587-20715
MsHel-2	gb|AIXA01001402.1|:1739-1835
MsHel-2	gb|AIXA01001317.1|:8454-8583
MsHel-2	gb|AIXA01000028.1|:22351-22480
MsHel-2	gb|AIXA01003002.1|:12855-12978
MsHel-2	gb|AIXA01017708.1|:237-363
MsHel-2	gb|AIXA01016197.1|:462-581
MsHel-2	gb|AIXA01011131.1|:15659-15785
MsHel-2	gb|AIXA01009807.1|:9670-9788
MsHel-2	gb|AIXA01007783.1|:60234-60355
MsHel-2	gb|AIXA01007783.1|:26481-26610
MsHel-2	gb|AIXA01004548.1|:34855-34979
MsHel-2	gb|AIXA01004158.1|:5392-10010
MsHel-2	gb|AIXA01001548.1|:21671-21795
MsHel-2	gb|AIXA01018866.1|:3387-3512
MsHel-2	gb|AIXA01014762.1|:7306-7435
MsHel-2	gb|AIXA01011333.1|:5712-6014
MsHel-2	gb|AIXA01011157.1|:2195-2275
MsHel-2	gb|AIXA01010406.1|:67909-68036
MsHel-2	gb|AIXA01010406.1|:41930-42059
MsHel-2	gb|AIXA01009766.1|:3954-4082
MsHel-2	gb|AIXA01009366.1|:51896-52019
MsHel-2	gb|AIXA01009366.1|:15038-15167
MsHel-2	gb|AIXA01008088.1|:31787-31913
MsHel-2	gb|AIXA01006817.1|:14720-14849
MsHel-2	gb|AIXA01002473.1|:6144-6272
MsHel-2	gb|AIXA01002238.1|:23992-24121
MsHel-2	gb|AIXA01009865.1|:83913-84040
MsHel-2	gb|AIXA01007856.1|:84403-84531
MsHel-2	gb|AIXA01004857.1|:3394-3517
MsHel-2	gb|AIXA01000132.1|:2473-2600
MsHel-2	gb|AIXA01000046.1|:74007-74388
MsHel-2	gb|AIXA01014333.1|:95-217
MsHel-2	gb|AIXA01012197.1|:24370-24497
MsHel-2	gb|AIXA01010348.1|:12288-12414
MsHel-2	gb|AIXA01003993.1|:87137-87255
MsHel-2	gb|AIXA01003661.1|:46292-46415
MsHel-2	gb|AIXA01019794.1|:1763-2113
MsHel-2	gb|AIXA01018070.1|:1107-1232
MsHel-2	gb|AIXA01017761.1|:461-582
MsHel-2	gb|AIXA01006837.1|:46770-47325
MsHel-2	gb|AIXA01006689.1|:30424-30549
MsHel-2	gb|AIXA01006689.1|:8879-9008

MsHel-2	gb|AIXA01003214.1|:17170-17292
MsHel-2	gb|AIXA01000916.1|:1542-1667
MsHel-2	gb|AIXA01023942.1|:189-692
MsHel-2	gb|AIXA01011919.1|:11037-11150
MsHel-2	gb|AIXA01011846.1|:4097-4226
MsHel-2	gb|AIXA01009981.1|:27440-27569
MsHel-2	gb|AIXA01009442.1|:10325-10454
MsHel-2	gb|AIXA01009084.1|:4189-4302
MsHel-2	gb|AIXA01008532.1|:16275-16563
MsHel-2	gb|AIXA01006299.1|:28808-28934
MsHel-2	gb|AIXA01002994.1|:15412-15537
MsHel-2	gb|AIXA01002971.1|:95157-95705
MsHel-2	gb|AIXA01001645.1|:78356-78468
MsHel-2	gb|AIXA01000821.1|:42638-42766
MsHel-2	gb|AIXA01017270.1|:1130-1249
MsHel-2	gb|AIXA01016674.1|:5079-5195
MsHel-2	gb|AIXA01010598.1|:1306-1426
MsHel-2	gb|AIXA01009285.1|:42051-42175
MsHel-2	gb|AIXA01007077.1|:7033-7152
MsHel-2	gb|AIXA01004368.1|:48985-49108
MsHel-2	gb|AIXA01002248.1|:57163-57291
MsHel-2	gb|AIXA01001823.1|:42401-43093
MsHel-2	gb|AIXA01001823.1|:26696-26825
MsHel-2	gb|AIXA01001823.1|:4006-4134
MsHel-2	gb|AIXA01000725.1|:43796-43925
MsHel-2	gb|AIXA01000725.1|:16133-16261
MsHel-2	gb|AIXA01037276.1|:27-153
MsHel-2	gb|AIXA01018627.1|:1838-1963
MsHel-2	gb|AIXA01016698.1|:17066-17193
MsHel-2	gb|AIXA01009864.1|:60457-60566
MsHel-2	gb|AIXA01009864.1|:39487-39591
MsHel-2	gb|AIXA01008319.1|:6406-6533
MsHel-2	gb|AIXA01005464.1|:70863-70991
MsHel-2	gb|AIXA01005029.1|:28167-28277
MsHel-2	gb|AIXA01004908.1|:10706-10835
MsHel-2	gb|AIXA01004893.1|:2890-2990
MsHel-2	gb|AIXA01003739.1|:49441-49567
MsHel-2	gb|AIXA01002972.1|:42273-42395
MsHel-2	gb|AIXA01001810.1|:5790-13980
MsHel-2	gb|AIXA01001665.1|:17317-17444
MsHel-2	gb|AIXA01001511.1|:103448-103571
MsHel-2	gb|AIXA01020399.1|:141-266
MsHel-2	gb|AIXA01015144.1|:3647-3763
MsHel-2	gb|AIXA01014622.1|:4678-4808
MsHel-2	gb|AIXA01012649.1|:6895-7014
MsHel-2	gb|AIXA01010853.1|:7056-7186
MsHel-2	gb|AIXA01007888.1|:60998-61127
MsHel-2	gb|AIXA01007681.1|:61873-62003

MsHel-2	gb|AIXA01007228.1|:108961-109087
MsHel-2	gb|AIXA01007228.1|:72872-72998
MsHel-2	gb|AIXA01005571.1|:16649-16774
MsHel-2	gb|AIXA01004965.1|:83295-83414
MsHel-2	gb|AIXA01001323.1|:47049-47175
MsHel-2	gb|AIXA01000627.1|:62836-62962
MsHel-2	gb|AIXA01000450.1|:7682-7812
MsHel-2	gb|AIXA01030372.1|:169-298
MsHel-2	gb|AIXA01018593.1|:2012-2141
MsHel-2	gb|AIXA01016299.1|:5587-5716
MsHel-2	gb|AIXA01012133.1|:41883-42000
MsHel-2	gb|AIXA01012133.1|:1631-1752
MsHel-2	gb|AIXA01009575.1|:24337-24458
MsHel-2	gb|AIXA01009117.1|:27714-27839
MsHel-2	gb|AIXA01009001.1|:22283-22411
MsHel-2	gb|AIXA01006141.1|:51790-51916
MsHel-2	gb|AIXA01006141.1|:14743-14850
MsHel-2	gb|AIXA01005993.1|:25561-25690
MsHel-2	gb|AIXA01005493.1|:4466-4595
MsHel-2	gb|AIXA01004672.1|:42556-42685
MsHel-2	gb|AIXA01004313.1|:8798-8927
MsHel-2	gb|AIXA01004188.1|:6501-6630
MsHel-2	gb|AIXA01004160.1|:9258-9358
MsHel-2	gb|AIXA01004056.1|:31710-31839
MsHel-2	gb|AIXA01001753.1|:3786-3915
MsHel-2	gb|AIXA01026903.1|:368-488
MsHel-2	gb|AIXA01017576.1|:7205-7329
MsHel-2	gb|AIXA01017387.1|:1578-2045
MsHel-2	gb|AIXA01014387.1|:1841-1944
MsHel-2	gb|AIXA01011387.1|:4292-4414
MsHel-2	gb|AIXA01010757.1|:10355-10449
MsHel-2	gb|AIXA01009671.1|:5521-5645
MsHel-2	gb|AIXA01005576.1|:37293-37410
MsHel-2	gb|AIXA01005576.1|:11960-12087
MsHel-2	gb|AIXA01005567.1|:19009-19132
MsHel-2	gb|AIXA01002231.1|:69158-69286
MsHel-2	gb|AIXA01030522.1|:505-628
MsHel-2	gb|AIXA01014190.1|:3583-3709
MsHel-2	gb|AIXA01013947.1|:5566-5671
MsHel-2	gb|AIXA01012943.1|:6955-7082
MsHel-2	gb|AIXA01012798.1|:26640-26763
MsHel-2	gb|AIXA01010336.1|:2000-2126
MsHel-2	gb|AIXA01008785.1|:11618-11739
MsHel-2	gb|AIXA01007362.1|:17224-17343
MsHel-2	gb|AIXA01005228.1|:32793-32918
MsHel-2	gb|AIXA01005049.1|:8020-8149
MsHel-2	gb|AIXA01004958.1|:39573-39700
MsHel-2	gb|AIXA01004552.1|:30043-30171

MsHel-2	gb|AIXA01003820.1|:62115-62224
MsHel-2	gb|AIXA01002561.1|:8696-8811
MsHel-2	gb|AIXA01002460.1|:80704-80789
MsHel-2	gb|AIXA01001968.1|:86563-86689
MsHel-2	gb|AIXA01001804.1|:14396-14519
MsHel-2	gb|AIXA01001593.1|:40099-40225
MsHel-2	gb|AIXA01001362.1|:24468-24591
MsHel-2	gb|AIXA01000966.1|:64055-64181
MsHel-2	gb|AIXA01000966.1|:3420-3549
MsHel-2	gb|AIXA01018071.1|:1715-1840
MsHel-2	gb|AIXA01016047.1|:815-939
MsHel-2	gb|AIXA01011853.1|:11424-11554
MsHel-2	gb|AIXA01011776.1|:459-577
MsHel-2	gb|AIXA01010881.1|:115948-116067
MsHel-2	gb|AIXA01010881.1|:84097-84218
MsHel-2	gb|AIXA01010881.1|:36967-39918
MsHel-2	gb|AIXA01008275.1|:80286-80412
MsHel-2	gb|AIXA01007832.1|:20574-20704
MsHel-2	gb|AIXA01007115.1|:1500-1624
MsHel-2	gb|AIXA01006816.1|:27081-27203
MsHel-2	gb|AIXA01005710.1|:744-846
MsHel-2	gb|AIXA01005092.1|:48694-48824
MsHel-2	gb|AIXA01003057.1|:10178-10296
MsHel-2	gb|AIXA01002339.1|:19970-20070
MsHel-2	gb|AIXA01002339.1|:2538-2667
MsHel-2	gb|AIXA01001653.1|:9222-9319
MsHel-2	gb|AIXA01001621.1|:9253-9377
MsHel-2	gb|AIXA01000091.1|:11166-11286
MsHel-2	gb|AIXA01037851.1|:193-322
MsHel-2	gb|AIXA01021631.1|:1652-1781
MsHel-2	gb|AIXA01019161.1|:894-1023
MsHel-2	gb|AIXA01018912.1|:1883-2012
MsHel-2	gb|AIXA01018307.1|:2089-2218
MsHel-2	gb|AIXA01018030.1|:650-779
MsHel-2	gb|AIXA01013668.1|:2789-2894
MsHel-2	gb|AIXA01013255.1|:13666-13771
MsHel-2	gb|AIXA01011067.1|:4337-4462
MsHel-2	gb|AIXA01010782.1|:68317-68444
MsHel-2	gb|AIXA01010676.1|:5876-6006
MsHel-2	gb|AIXA01009650.1|:124-245
MsHel-2	gb|AIXA01006914.1|:7757-7866
MsHel-2	gb|AIXA01006875.1|:49592-49721
MsHel-2	gb|AIXA01005034.1|:10877-11006
MsHel-2	gb|AIXA01003749.1|:18250-18355
MsHel-2	gb|AIXA01003662.1|:1051-1180
MsHel-2	gb|AIXA01000997.1|:47739-47860
MsHel-2	gb|AIXA01000437.1|:35962-36076
MsHel-2	gb|AIXA01000398.1|:16992-17121

MsHel-2	gb|AIXA01000051.1|:25679-25805
MsHel-2	gb|AIXA01024502.1|:947-1070
MsHel-2	gb|AIXA01018705.1|:3520-3648
MsHel-2	gb|AIXA01018042.1|:6847-6970
MsHel-2	gb|AIXA01013560.1|:5483-5609
MsHel-2	gb|AIXA01011303.1|:14953-15068
MsHel-2	gb|AIXA01010109.1|:48468-48591
MsHel-2	gb|AIXA01009835.1|:31582-31704
MsHel-2	gb|AIXA01009792.1|:30242-30370
MsHel-2	gb|AIXA01007601.1|:6958-7058
MsHel-2	gb|AIXA01007037.1|:10124-10219
MsHel-2	gb|AIXA01005594.1|:36320-36448
MsHel-2	gb|AIXA01005415.1|:68722-68850
MsHel-2	gb|AIXA01004327.1|:108776-108886
MsHel-2	gb|AIXA01004327.1|:94400-94522
MsHel-2	gb|AIXA01003329.1|:132519-132639
MsHel-2	gb|AIXA01001817.1|:16623-16727
MsHel-2	gb|AIXA01001675.1|:33997-34125
MsHel-2	gb|AIXA01001675.1|:10649-10723
MsHel-2	gb|AIXA01024217.1|:517-644
MsHel-2	gb|AIXA01021444.1|:1823-1941
MsHel-2	gb|AIXA01019279.1|:7360-7482
MsHel-2	gb|AIXA01017979.1|:9687-9814
MsHel-2	gb|AIXA01016565.1|:1199-1317
MsHel-2	gb|AIXA01016291.1|:2202-2315
MsHel-2	gb|AIXA01015168.1|:3751-3853
MsHel-2	gb|AIXA01014467.1|:15639-15763
MsHel-2	gb|AIXA01011426.1|:17522-17649
MsHel-2	gb|AIXA01010808.1|:3973-4099
MsHel-2	gb|AIXA01009162.1|:1699-1822
MsHel-2	gb|AIXA01008112.1|:4430-4652
MsHel-2	gb|AIXA01005530.1|:18998-19124
MsHel-2	gb|AIXA01005001.1|:1241-1367
MsHel-2	gb|AIXA01004806.1|:42844-42966
MsHel-2	gb|AIXA01004693.1|:70142-70243
MsHel-2	gb|AIXA01004693.1|:19963-20089
MsHel-2	gb|AIXA01004567.1|:17559-17685
MsHel-2	gb|AIXA01004490.1|:17327-17453
MsHel-2	gb|AIXA01004281.1|:21837-21960
MsHel-2	gb|AIXA01004281.1|:3145-3271
MsHel-2	gb|AIXA01004213.1|:6891-7009
MsHel-2	gb|AIXA01002898.1|:9064-9185
MsHel-2	gb|AIXA01001349.1|:32094-32220
MsHel-2	gb|AIXA01001069.1|:849-944
MsHel-2	gb|AIXA01000813.1|:30591-30710
MsHel-2	gb|AIXA01029440.1|:394-519
MsHel-2	gb|AIXA01018504.1|:2761-2891
MsHel-2	gb|AIXA01017982.1|:4961-9158

MsHel-2	gb|AIXA01017113.1|:6804-7337
MsHel-2	gb|AIXA01016958.1|:7114-7238
MsHel-2	gb|AIXA01011361.1|:19441-19561
MsHel-2	gb|AIXA01009432.1|:13024-13145
MsHel-2	gb|AIXA01009108.1|:25471-25596
MsHel-2	gb|AIXA01009061.1|:113199-113325
MsHel-2	gb|AIXA01008043.1|:82719-82841
MsHel-2	gb|AIXA01007718.1|:11900-12018
MsHel-2	gb|AIXA01006983.1|:51623-51747
MsHel-2	gb|AIXA01005841.1|:2160-2284
MsHel-2	gb|AIXA01005247.1|:16541-16654
MsHel-2	gb|AIXA01002683.1|:272-396
MsHel-2	gb|AIXA01002463.1|:74767-74893
MsHel-2	gb|AIXA01002297.1|:17150-17276
MsHel-2	gb|AIXA01001620.1|:30790-30912
MsHel-2	gb|AIXA01001239.1|:86435-86555
MsHel-2	gb|AIXA01000560.1|:46688-46813
MsHel-2	gb|AIXA01000560.1|:21416-21545
MsHel-2	gb|AIXA01000359.1|:159627-159745
MsHel-2	gb|AIXA01000153.1|:1466-1593
MsHel-2	gb|AIXA01000048.1|:47165-47287
MsHel-2	gb|AIXA01034847.1|:231-336
MsHel-2	gb|AIXA01034056.1|:112-233
MsHel-2	gb|AIXA01022696.1|:1077-1186
MsHel-2	gb|AIXA01019289.1|:1529-1650
MsHel-2	gb|AIXA01017690.1|:3644-3772
MsHel-2	gb|AIXA01017416.1|:404-529
MsHel-2	gb|AIXA01016653.1|:175-287
MsHel-2	gb|AIXA01012628.1|:19558-19685
MsHel-2	gb|AIXA01012628.1|:8496-8617
MsHel-2	gb|AIXA01011121.1|:26529-26653
MsHel-2	gb|AIXA01009326.1|:25813-25937
MsHel-2	gb|AIXA01008403.1|:4213-4342
MsHel-2	gb|AIXA01008393.1|:56069-56202
MsHel-2	gb|AIXA01008111.1|:4981-5086
MsHel-2	gb|AIXA01008057.1|:36740-36869
MsHel-2	gb|AIXA01007598.1|:23501-23620
MsHel-2	gb|AIXA01007175.1|:61002-61130
MsHel-2	gb|AIXA01006807.1|:644-773
MsHel-2	gb|AIXA01006690.1|:24488-24617
MsHel-2	gb|AIXA01006642.1|:19559-19688
MsHel-2	gb|AIXA01005485.1|:35459-35562
MsHel-2	gb|AIXA01005485.1|:18544-18661
MsHel-2	gb|AIXA01005339.1|:30944-31062
MsHel-2	gb|AIXA01005246.1|:6604-6733
MsHel-2	gb|AIXA01002996.1|:59878-59978
MsHel-2	gb|AIXA01002996.1|:46661-46775
MsHel-2	gb|AIXA01002707.1|:44085-44214

MsHel-2	gb|AIXA01002471.1|:10466-10586
MsHel-2	gb|AIXA01002362.1|:68770-68899
MsHel-2	gb|AIXA01002362.1|:47003-47131
MsHel-2	gb|AIXA01002331.1|:64444-64530
MsHel-2	gb|AIXA01002331.1|:46466-46583
MsHel-2	gb|AIXA01002205.1|:1518-1623
MsHel-2	gb|AIXA01002187.1|:33358-33489
MsHel-2	gb|AIXA01001656.1|:98288-98380
MsHel-2	gb|AIXA01001656.1|:4804-4923
MsHel-2	gb|AIXA01000085.1|:673-796
MsHel-2	gb|AIXA01019187.1|:1741-1878
MsHel-2	gb|AIXA01016545.1|:5059-5163
MsHel-2	gb|AIXA01013702.1|:1016-1144
MsHel-2	gb|AIXA01012304.1|:13632-13755
MsHel-2	gb|AIXA01012212.1|:738-871
MsHel-2	gb|AIXA01010865.1|:28734-28839
MsHel-2	gb|AIXA01010568.1|:429-521
MsHel-2	gb|AIXA01008033.1|:47404-47528
MsHel-2	gb|AIXA01007692.1|:12359-12487
MsHel-2	gb|AIXA01007218.1|:65838-65964
MsHel-2	gb|AIXA01006514.1|:12395-12526
MsHel-2	gb|AIXA01005504.1|:113810-113912
MsHel-2	gb|AIXA01005504.1|:83083-83204
MsHel-2	gb|AIXA01004053.1|:10921-11041
MsHel-2	gb|AIXA01003850.1|:127786-127913
MsHel-2	gb|AIXA01003850.1|:85163-85256
MsHel-2	gb|AIXA01003275.1|:12254-12362
MsHel-2	gb|AIXA01003243.1|:10456-10584
MsHel-2	gb|AIXA01003170.1|:30132-30248
MsHel-2	gb|AIXA01003140.1|:6508-6627
MsHel-2	gb|AIXA01001811.1|:13822-18624
MsHel-2	gb|AIXA01000703.1|:23866-23966
MsHel-2	gb|AIXA01000561.1|:6000-6112
MsHel-2	gb|AIXA01000069.1|:284096-284220
MsHel-2	gb|AIXA01000069.1|:196359-196458
MsHel-2	gb|AIXA01000069.1|:128577-128658
MsHel-2	gb|AIXA01000069.1|:65545-65636
MsHel-2	gb|AIXA01036850.1|:268-392
MsHel-2	gb|AIXA01018652.1|:9521-9645
MsHel-2	gb|AIXA01015003.1|:12342-12445
MsHel-2	gb|AIXA01014648.1|:5353-5479
MsHel-2	gb|AIXA01012306.1|:8708-8837
MsHel-2	gb|AIXA01010627.1|:7951-8074
MsHel-2	gb|AIXA01009643.1|:12185-12303
MsHel-2	gb|AIXA01009603.1|:17957-18084
MsHel-2	gb|AIXA01009202.1|:4882-5009
MsHel-2	gb|AIXA01008754.1|:10354-10479
MsHel-2	gb|AIXA01008705.1|:12283-12375

MsHel-2	gb|AIXA01008566.1|:73286-73387
MsHel-2	gb|AIXA01008566.1|:1480-1617
MsHel-2	gb|AIXA01008406.1|:187726-187855
MsHel-2	gb|AIXA01008406.1|:62115-62219
MsHel-2	gb|AIXA01007946.1|:79022-79145
MsHel-2	gb|AIXA01007915.1|:1881-2008
MsHel-2	gb|AIXA01006318.1|:9113-9239
MsHel-2	gb|AIXA01006156.1|:21514-21621
MsHel-2	gb|AIXA01004956.1|:113040-113165
MsHel-2	gb|AIXA01004956.1|:94507-94595
MsHel-2	gb|AIXA01003600.1|:29546-29672
MsHel-2	gb|AIXA01002541.1|:4267-4393
MsHel-2	gb|AIXA01001983.1|:25611-25738
MsHel-2	gb|AIXA01001844.1|:11439-11557
MsHel-2	gb|AIXA01001189.1|:19493-19616
MsHel-2	gb|AIXA01001047.1|:6691-6810
MsHel-2	gb|AIXA01000397.1|:203-297
MsHel-2	gb|AIXA01000234.1|:85266-85388
MsHel-2	gb|AIXA01000050.1|:4260-4386
MsHel-2	gb|AIXA01015971.1|:4214-4341
MsHel-2	gb|AIXA01015119.1|:43430-43546
MsHel-2	gb|AIXA01011371.1|:17684-17814
MsHel-2	gb|AIXA01011323.1|:16308-16430
MsHel-2	gb|AIXA01009217.1|:34416-34542
MsHel-2	gb|AIXA01009217.1|:218-336
MsHel-2	gb|AIXA01008118.1|:35699-35824
MsHel-2	gb|AIXA01008023.1|:80583-80668
MsHel-2	gb|AIXA01008023.1|:31370-31488
MsHel-2	gb|AIXA01007690.1|:30055-30175
MsHel-2	gb|AIXA01007649.1|:4240-4366
MsHel-2	gb|AIXA01006566.1|:35477-35607
MsHel-2	gb|AIXA01005494.1|:36621-36751
MsHel-2	gb|AIXA01004692.1|:34625-34735
MsHel-2	gb|AIXA01004665.1|:1776-1903
MsHel-2	gb|AIXA01004067.1|:34609-34727
MsHel-2	gb|AIXA01004067.1|:1601-1731
MsHel-2	gb|AIXA01003860.1|:36494-36618
MsHel-2	gb|AIXA01001599.1|:75618-75743
MsHel-2	gb|AIXA01001037.1|:20423-20548
MsHel-2	gb|AIXA01025484.1|:498-599
MsHel-2	gb|AIXA01018991.1|:141-270
MsHel-2	gb|AIXA01018919.1|:411-536
MsHel-2	gb|AIXA01018623.1|:3414-3531
MsHel-2	gb|AIXA01018151.1|:3716-3837
MsHel-2	gb|AIXA01017638.1|:10192-10293
MsHel-2	gb|AIXA01017582.1|:19689-19818
MsHel-2	gb|AIXA01016352.1|:20783-20904
MsHel-2	gb|AIXA01015246.1|:21007-21136

MsHel-2	gb|AIXA01014982.1|:23486-23615
MsHel-2	gb|AIXA01014529.1|:13020-13121
MsHel-2	gb|AIXA01012629.1|:240-369
MsHel-2	gb|AIXA01011840.1|:34846-34975
MsHel-2	gb|AIXA01011638.1|:14173-14290
MsHel-2	gb|AIXA01011030.1|:15255-15384
MsHel-2	gb|AIXA01010953.1|:39653-39782
MsHel-2	gb|AIXA01010335.1|:4329-4450
MsHel-2	gb|AIXA01008148.1|:93892-94021
MsHel-2	gb|AIXA01008060.1|:7473-7602
MsHel-2	gb|AIXA01007854.1|:65592-65721
MsHel-2	gb|AIXA01007854.1|:48058-48177
MsHel-2	gb|AIXA01007854.1|:34814-34939
MsHel-2	gb|AIXA01007506.1|:82339-82444
MsHel-2	gb|AIXA01007494.1|:28008-28125
MsHel-2	gb|AIXA01007130.1|:90378-90507
MsHel-2	gb|AIXA01006350.1|:71439-71570
MsHel-2	gb|AIXA01006319.1|:16147-16267
MsHel-2	gb|AIXA01006253.1|:24940-25068
MsHel-2	gb|AIXA01005925.1|:5991-6120
MsHel-2	gb|AIXA01005668.1|:7206-7307
MsHel-2	gb|AIXA01005423.1|:855-984
MsHel-2	gb|AIXA01005224.1|:27338-27462
MsHel-2	gb|AIXA01005035.1|:88803-88923
MsHel-2	gb|AIXA01005035.1|:54093-54222
MsHel-2	gb|AIXA01005035.1|:23030-23159
MsHel-2	gb|AIXA01004550.1|:76573-76696
MsHel-2	gb|AIXA01004319.1|:7007-7100
MsHel-2	gb|AIXA01003494.1|:25751-25876
MsHel-2	gb|AIXA01003037.1|:12364-12493
MsHel-2	gb|AIXA01002366.1|:17847-17976
MsHel-2	gb|AIXA01002200.1|:7473-7601
MsHel-2	gb|AIXA01001640.1|:1516-1645
MsHel-2	gb|AIXA01001612.1|:32652-32777
MsHel-2	gb|AIXA01001566.1|:29634-29753
MsHel-2	gb|AIXA01001566.1|:19432-19546
MsHel-2	gb|AIXA01000468.1|:35945-36074
MsHel-2	gb|AIXA01018069.1|:6467-6570
MsHel-2	gb|AIXA01008131.1|:5332-5456
MsHel-2	gb|AIXA01006593.1|:25588-25699
MsHel-2	gb|AIXA01006593.1|:8820-8936
MsHel-2	gb|AIXA01005592.1|:30431-30535
MsHel-2	gb|AIXA01004225.1|:35889-35989
MsHel-2	gb|AIXA01003653.1|:63293-63414
MsHel-2	gb|AIXA01002622.1|:32805-32933
MsHel-2	gb|AIXA01002491.1|:608-728
MsHel-2	gb|AIXA01002168.1|:35134-35253
MsHel-2	gb|AIXA01002164.1|:5194-5314

MsHel-2	gb|AIXA01001898.1|:168115-168237
MsHel-2	gb|AIXA01001898.1|:61283-61411
MsHel-2	gb|AIXA01001354.1|:8137-8260
MsHel-2	gb|AIXA01000993.1|:64165-64273
MsHel-2	gb|AIXA01000057.1|:11868-11967
MsHel-2	gb|AIXA01027670.1|:714-839
MsHel-2	gb|AIXA01022563.1|:102-229
MsHel-2	gb|AIXA01021362.1|:1861-1982
MsHel-2	gb|AIXA01020094.1|:1780-1891
MsHel-2	gb|AIXA01015686.1|:1017-1138
MsHel-2	gb|AIXA01015394.1|:1543-1666
MsHel-2	gb|AIXA01015371.1|:349-475
MsHel-2	gb|AIXA01015132.1|:34875-35411
MsHel-2	gb|AIXA01014760.1|:3249-3331
MsHel-2	gb|AIXA01014346.1|:29348-29471
MsHel-2	gb|AIXA01014004.1|:247-360
MsHel-2	gb|AIXA01012760.1|:5630-5757
MsHel-2	gb|AIXA01012196.1|:26049-26164
MsHel-2	gb|AIXA01012162.1|:14696-14822
MsHel-2	gb|AIXA01010666.1|:32966-33067
MsHel-2	gb|AIXA01009797.1|:796-923
MsHel-2	gb|AIXA01009588.1|:3817-3943
MsHel-2	gb|AIXA01009539.1|:37060-37186
MsHel-2	gb|AIXA01009539.1|:1442-1554
MsHel-2	gb|AIXA01007860.1|:11392-11507
MsHel-2	gb|AIXA01006923.1|:41632-41757
MsHel-2	gb|AIXA01006719.1|:8093-8220
MsHel-2	gb|AIXA01006330.1|:11647-11766
MsHel-2	gb|AIXA01005509.1|:15376-15501
MsHel-2	gb|AIXA01005505.1|:18179-18306
MsHel-2	gb|AIXA01005221.1|:40956-41060
MsHel-2	gb|AIXA01004809.1|:16059-16182
MsHel-2	gb|AIXA01004224.1|:6265-6388
MsHel-2	gb|AIXA01003190.1|:8022-8144
MsHel-2	gb|AIXA01002730.1|:55568-55680
MsHel-2	gb|AIXA01002381.1|:12576-12679
MsHel-2	gb|AIXA01002296.1|:169522-169638
MsHel-2	gb|AIXA01002296.1|:6280-6364
MsHel-2	gb|AIXA01001925.1|:2566-2685
MsHel-2	gb|AIXA01001714.1|:8825-8948
MsHel-2	gb|AIXA01001554.1|:674-804
MsHel-2	gb|AIXA01001271.1|:113483-113601
MsHel-2	gb|AIXA01001243.1|:167338-167464
MsHel-2	gb|AIXA01001243.1|:147965-148060
MsHel-2	gb|AIXA01000275.1|:20772-20901
MsHel-2	gb|AIXA01000275.1|:5349-5451
MsHel-2	gb|AIXA01000044.1|:1480-1582
MsHel-2	gb|AIXA01016011.1|:2587-2707

MsHel-2	gb|AIXA01011995.1|:68536-68663
MsHel-2	gb|AIXA01010152.1|:6394-6500
MsHel-2	gb|AIXA01009504.1|:31352-31492
MsHel-2	gb|AIXA01009351.1|:9397-9503
MsHel-2	gb|AIXA01009267.1|:24134-24256
MsHel-2	gb|AIXA01007327.1|:271-397
MsHel-2	gb|AIXA01006893.1|:53230-53352
MsHel-2	gb|AIXA01006682.1|:9509-9631
MsHel-2	gb|AIXA01005440.1|:1083-1200
MsHel-2	gb|AIXA01005207.1|:46759-46865
MsHel-2	gb|AIXA01005087.1|:17847-17957
MsHel-2	gb|AIXA01004667.1|:145346-145464
MsHel-2	gb|AIXA01004465.1|:54100-54226
MsHel-2	gb|AIXA01004247.1|:17435-17533
MsHel-2	gb|AIXA01004247.1|:6671-6764
MsHel-2	gb|AIXA01004025.1|:397-527
MsHel-2	gb|AIXA01003818.1|:23346-23456
MsHel-2	gb|AIXA01003474.1|:11433-11543
MsHel-2	gb|AIXA01001797.1|:144822-144936
MsHel-2	gb|AIXA01001551.1|:2627-2740
MsHel-2	gb|AIXA01001542.1|:24572-24672
MsHel-2	gb|AIXA01001071.1|:87371-87501
MsHel-2	gb|AIXA01000925.1|:35703-35829
MsHel-2	gb|AIXA01000501.1|:7813-7943
MsHel-2	gb|AIXA01000167.1|:1485-1586
MsHel-2	gb|AIXA01000093.1|:17011-17133
MsHel-2	gb|AIXA01023302.1|:1272-1392
MsHel-2	gb|AIXA01020255.1|:1821-1950
MsHel-2	gb|AIXA01019456.1|:141-270
MsHel-2	gb|AIXA01019021.1|:1449-1578
MsHel-2	gb|AIXA01017704.1|:825-951
MsHel-2	gb|AIXA01016592.1|:3153-3282
MsHel-2	gb|AIXA01015473.1|:12155-12270
MsHel-2	gb|AIXA01013973.1|:15401-15529
MsHel-2	gb|AIXA01012652.1|:18021-18147
MsHel-2	gb|AIXA01012522.1|:15991-16120
MsHel-2	gb|AIXA01012364.1|:238-366
MsHel-2	gb|AIXA01012168.1|:4874-5003
MsHel-2	gb|AIXA01011562.1|:13424-13542
MsHel-2	gb|AIXA01011244.1|:7237-7366
MsHel-2	gb|AIXA01010776.1|:21338-21459
MsHel-2	gb|AIXA01009925.1|:110851-110967
MsHel-2	gb|AIXA01009925.1|:70102-70219
MsHel-2	gb|AIXA01009357.1|:11008-11137
MsHel-2	gb|AIXA01009177.1|:24471-24600
MsHel-2	gb|AIXA01008488.1|:3024-3129
MsHel-2	gb|AIXA01008226.1|:10748-10832
MsHel-2	gb|AIXA01007576.1|:120624-120737

MsHel-2	gb|AIXA01007576.1|:42056-42181
MsHel-2	gb|AIXA01007428.1|:64807-64921
MsHel-2	gb|AIXA01007168.1|:126828-126956
MsHel-2	gb|AIXA01007168.1|:52963-53072
MsHel-2	gb|AIXA01007033.1|:44594-44723
MsHel-2	gb|AIXA01006872.1|:4220-4345
MsHel-2	gb|AIXA01006706.1|:2216-2317
MsHel-2	gb|AIXA01006648.1|:65127-65256
MsHel-2	gb|AIXA01006123.1|:32572-32689
MsHel-2	gb|AIXA01005608.1|:41429-41549
MsHel-2	gb|AIXA01005536.1|:9165-9307
MsHel-2	gb|AIXA01005534.1|:1399-1528
MsHel-2	gb|AIXA01005495.1|:27022-27147
MsHel-2	gb|AIXA01005311.1|:5901-6026
MsHel-2	gb|AIXA01004980.1|:101875-101980
MsHel-2	gb|AIXA01004896.1|:35298-35403
MsHel-2	gb|AIXA01004515.1|:21060-21189
MsHel-2	gb|AIXA01004415.1|:45893-46023
MsHel-2	gb|AIXA01003711.1|:149495-149601
MsHel-2	gb|AIXA01003711.1|:84207-84337
MsHel-2	gb|AIXA01002620.1|:2811-2939
MsHel-2	gb|AIXA01002560.1|:46756-46880
MsHel-2	gb|AIXA01002468.1|:19579-19708
MsHel-2	gb|AIXA01001494.1|:35719-35820
MsHel-2	gb|AIXA01001366.1|:39147-39269
MsHel-2	gb|AIXA01001356.1|:27369-27469
MsHel-2	gb|AIXA01001244.1|:3288-3417
MsHel-2	gb|AIXA01000952.1|:19314-19432
MsHel-2	gb|AIXA01000810.1|:347686-347812
MsHel-2	gb|AIXA01000810.1|:110350-110470
MsHel-2	gb|AIXA01000422.1|:26728-26857
MsHel-2	gb|AIXA01000242.1|:78205-78325
MsHel-2	gb|AIXA01000242.1|:53501-53598
MsHel-2	gb|AIXA01025141.1|:781-905
MsHel-2	gb|AIXA01022558.1|:537-633
MsHel-2	gb|AIXA01018288.1|:6076-6195
MsHel-2	gb|AIXA01016791.1|:2600-2723
MsHel-2	gb|AIXA01012100.1|:15565-15681
MsHel-2	gb|AIXA01011538.1|:14973-15064
MsHel-2	gb|AIXA01011374.1|:8320-8445
MsHel-2	gb|AIXA01010909.1|:30260-30347
MsHel-2	gb|AIXA01010837.1|:1141-1263
MsHel-2	gb|AIXA01010829.1|:566-686
MsHel-2	gb|AIXA01009752.1|:25776-25879
MsHel-2	gb|AIXA01009350.1|:35344-35458
MsHel-2	gb|AIXA01008431.1|:5586-5706
MsHel-2	gb|AIXA01005793.1|:10433-10548
MsHel-2	gb|AIXA01005394.1|:48962-49079

MsHel-2	gb|AIXA01005351.1|:71273-71397
MsHel-2	gb|AIXA01004529.1|:34045-34173
MsHel-2	gb|AIXA01004220.1|:10095-10206
MsHel-2	gb|AIXA01004151.1|:4392-4500
MsHel-2	gb|AIXA01003817.1|:60189-60313
MsHel-2	gb|AIXA01003817.1|:16976-17095
MsHel-2	gb|AIXA01003621.1|:38815-38941
MsHel-2	gb|AIXA01003283.1|:80741-80862
MsHel-2	gb|AIXA01002817.1|:24942-25070
MsHel-2	gb|AIXA01002514.1|:8742-8834
MsHel-2	gb|AIXA01000206.1|:52732-52850
MsHel-2	gb|AIXA01000196.1|:56549-56650
MsHel-2	gb|AIXA01000196.1|:45144-45267
MsHel-2	gb|AIXA01028897.1|:530-657
MsHel-2	gb|AIXA01020334.1|:2689-2792
MsHel-2	gb|AIXA01018914.1|:1771-1862
MsHel-2	gb|AIXA01014392.1|:12193-12294
MsHel-2	gb|AIXA01014392.1|:1860-1981
MsHel-2	gb|AIXA01013120.1|:10999-11120
MsHel-2	gb|AIXA01011177.1|:4193-4320
MsHel-2	gb|AIXA01010019.1|:39813-39913
MsHel-2	gb|AIXA01009236.1|:1063-1190
MsHel-2	gb|AIXA01007616.1|:27067-27172
MsHel-2	gb|AIXA01007392.1|:23228-23317
MsHel-2	gb|AIXA01005439.1|:8232-8353
MsHel-2	gb|AIXA01005135.1|:27628-27755
MsHel-2	gb|AIXA01005135.1|:12875-12967
MsHel-2	gb|AIXA01005105.1|:33088-33206
MsHel-2	gb|AIXA01005096.1|:21646-21775
MsHel-2	gb|AIXA01003650.1|:30011-30139
MsHel-2	gb|AIXA01003490.1|:44587-44702
MsHel-2	gb|AIXA01002770.1|:9274-9397
MsHel-2	gb|AIXA01001841.1|:22129-22256
MsHel-2	gb|AIXA01001822.1|:19947-20073
MsHel-2	gb|AIXA01001509.1|:56653-56764
MsHel-2	gb|AIXA01000438.1|:84-218
MsHel-2	gb|AIXA01000430.1|:36035-36161
MsHel-2	gb|AIXA01000226.1|:40850-40941
MsHel-2	gb|AIXA01034743.1|:407-532
MsHel-2	gb|AIXA01028679.1|:790-887
MsHel-2	gb|AIXA01024313.1|:1188-1304
MsHel-2	gb|AIXA01015674.1|:2625-2751
MsHel-2	gb|AIXA01014544.1|:6868-6991
MsHel-2	gb|AIXA01013902.1|:3639-3764
MsHel-2	gb|AIXA01010956.1|:42885-42998
MsHel-2	gb|AIXA01009401.1|:65247-65373
MsHel-2	gb|AIXA01009043.1|:16081-16231
MsHel-2	gb|AIXA01008711.1|:2089-2218

MsHel-2	gb|AIXA01008668.1|:13494-13613
MsHel-2	gb|AIXA01007944.1|:4867-4970
MsHel-2	gb|AIXA01007552.1|:63262-63384
MsHel-2	gb|AIXA01006952.1|:49026-49123
MsHel-2	gb|AIXA01006878.1|:42272-42395
MsHel-2	gb|AIXA01005548.1|:27223-27627
MsHel-2	gb|AIXA01005449.1|:11434-11540
MsHel-2	gb|AIXA01004422.1|:16846-16976
MsHel-2	gb|AIXA01004222.1|:100963-101068
MsHel-2	gb|AIXA01003819.1|:24266-24392
MsHel-2	gb|AIXA01003355.1|:61894-62006
MsHel-2	gb|AIXA01003355.1|:31139-31237
MsHel-2	gb|AIXA01002640.1|:17024-17140
MsHel-2	gb|AIXA01002606.1|:8890-9020
MsHel-2	gb|AIXA01000747.1|:146938-147052
MsHel-2	gb|AIXA01000150.1|:14-136
MsHel-2	gb|AIXA01034544.1|:289-402
MsHel-2	gb|AIXA01025526.1|:467-576
MsHel-2	gb|AIXA01021096.1|:927-1044
MsHel-2	gb|AIXA01019406.1|:1681-1806
MsHel-2	gb|AIXA01018655.1|:3438-3567
MsHel-2	gb|AIXA01014932.1|:9598-9722
MsHel-2	gb|AIXA01014284.1|:7815-7924
MsHel-2	gb|AIXA01014003.1|:2190-2316
MsHel-2	gb|AIXA01013500.1|:1802-1931
MsHel-2	gb|AIXA01011819.1|:3573-3698
MsHel-2	gb|AIXA01011669.1|:13776-13892
MsHel-2	gb|AIXA01011493.1|:84282-84375
MsHel-2	gb|AIXA01010756.1|:37236-37344
MsHel-2	gb|AIXA01009381.1|:22702-22827
MsHel-2	gb|AIXA01008037.1|:63847-63967
MsHel-2	gb|AIXA01007665.1|:17663-18159
MsHel-2	gb|AIXA01007294.1|:9390-9519
MsHel-2	gb|AIXA01007193.1|:511-640
MsHel-2	gb|AIXA01006688.1|:6979-7096
MsHel-2	gb|AIXA01006498.1|:13429-13558
MsHel-2	gb|AIXA01005617.1|:7959-8088
MsHel-2	gb|AIXA01005336.1|:726-830
MsHel-2	gb|AIXA01005093.1|:11656-11741
MsHel-2	gb|AIXA01004204.1|:5726-6296
MsHel-2	gb|AIXA01003884.1|:24021-24126
MsHel-2	gb|AIXA01003356.1|:65669-65798
MsHel-2	gb|AIXA01003286.1|:10866-10991
MsHel-2	gb|AIXA01002911.1|:104947-105068
MsHel-2	gb|AIXA01002811.1|:61709-61828
MsHel-2	gb|AIXA01001847.1|:32879-33004
MsHel-2	gb|AIXA01001806.1|:22108-22216
MsHel-2	gb|AIXA01001695.1|:162-291

MsHel-2	gb|AIXA01001680.1|:39965-40090
MsHel-2	gb|AIXA01001337.1|:446-575
MsHel-2	gb|AIXA01001309.1|:49212-49333
MsHel-2	gb|AIXA01001267.1|:12613-12742
MsHel-2	gb|AIXA01000650.1|:115927-116047
MsHel-2	gb|AIXA01000650.1|:93930-94025
MsHel-2	gb|AIXA01000448.1|:46413-46530
MsHel-2	gb|AIXA01000204.1|:10997-11126
MsHel-2	gb|AIXA01033356.1|:212-312
MsHel-2	gb|AIXA01028354.1|:668-788
MsHel-2	gb|AIXA01025404.1|:939-1066
MsHel-2	gb|AIXA01021504.1|:1372-1476
MsHel-2	gb|AIXA01021288.1|:557-657
MsHel-2	gb|AIXA01014299.1|:12854-12945
MsHel-2	gb|AIXA01014084.1|:4312-4417
MsHel-2	gb|AIXA01013328.1|:26513-26637
MsHel-2	gb|AIXA01012960.1|:322-441
MsHel-2	gb|AIXA01011304.1|:19036-19150
MsHel-2	gb|AIXA01009696.1|:20682-20802
MsHel-2	gb|AIXA01009047.1|:104169-104263
MsHel-2	gb|AIXA01007046.1|:28234-28346
MsHel-2	gb|AIXA01006184.1|:12194-12316
MsHel-2	gb|AIXA01006086.1|:8348-8443
MsHel-2	gb|AIXA01005599.1|:10297-10401
MsHel-2	gb|AIXA01005236.1|:2740-2848
MsHel-2	gb|AIXA01004121.1|:28141-28269
MsHel-2	gb|AIXA01003943.1|:18309-18425
MsHel-2	gb|AIXA01003647.1|:821-944
MsHel-2	gb|AIXA01003000.1|:27343-27439
MsHel-2	gb|AIXA01001026.1|:62676-62755
MsHel-2	gb|AIXA01000938.1|:52348-52462
MsHel-2	gb|AIXA01037436.1|:153-279
MsHel-2	gb|AIXA01030845.1|:520-619
MsHel-2	gb|AIXA01027880.1|:583-686
MsHel-2	gb|AIXA01024291.1|:280-371
MsHel-2	gb|AIXA01019461.1|:3971-4101
MsHel-2	gb|AIXA01017774.1|:934-1062
MsHel-2	gb|AIXA01017307.1|:4414-4550
MsHel-2	gb|AIXA01016263.1|:806-933
MsHel-2	gb|AIXA01016082.1|:13331-13457
MsHel-2	gb|AIXA01015407.1|:8399-8501
MsHel-2	gb|AIXA01014990.1|:4178-4301
MsHel-2	gb|AIXA01013564.1|:13400-13503
MsHel-2	gb|AIXA01013395.1|:936-1058
MsHel-2	gb|AIXA01011784.1|:7418-7561
MsHel-2	gb|AIXA01011674.1|:7464-7564
MsHel-2	gb|AIXA01011498.1|:30867-30984
MsHel-2	gb|AIXA01011364.1|:21692-21816

MsHel-2	gb|AIXA01011300.1|:6520-6646
MsHel-2	gb|AIXA01010251.1|:9791-9917
MsHel-2	gb|AIXA01009977.1|:17982-18101
MsHel-2	gb|AIXA01008767.1|:71029-71154
MsHel-2	gb|AIXA01007800.1|:81723-81849
MsHel-2	gb|AIXA01007286.1|:60894-61010
MsHel-2	gb|AIXA01006987.1|:4903-5026
MsHel-2	gb|AIXA01006924.1|:21475-21601
MsHel-2	gb|AIXA01005377.1|:6239-6365
MsHel-2	gb|AIXA01005326.1|:87249-87377
MsHel-2	gb|AIXA01004569.1|:19334-19460
MsHel-2	gb|AIXA01003871.1|:6587-6712
MsHel-2	gb|AIXA01003857.1|:72215-72337
MsHel-2	gb|AIXA01003615.1|:1965-2084
MsHel-2	gb|AIXA01003276.1|:89546-89673
MsHel-2	gb|AIXA01002459.1|:14985-15119
MsHel-2	gb|AIXA01001234.1|:8878-8968
MsHel-2	gb|AIXA01000755.1|:23402-23543
MsHel-2	gb|AIXA01000054.1|:2943-3049
MsHel-2	gb|AIXA01017318.1|:18970-19079
MsHel-2	gb|AIXA01017318.1|:6267-6389
MsHel-2	gb|AIXA01017136.1|:7825-7939
MsHel-2	gb|AIXA01014819.1|:1925-2047
MsHel-2	gb|AIXA01014152.1|:27023-27149
MsHel-2	gb|AIXA01011324.1|:297-386
MsHel-2	gb|AIXA01011273.1|:44762-44883
MsHel-2	gb|AIXA01010334.1|:25931-26057
MsHel-2	gb|AIXA01009642.1|:5440-5531
MsHel-2	gb|AIXA01009641.1|:14372-14495
MsHel-2	gb|AIXA01009610.1|:841-955
MsHel-2	gb|AIXA01009476.1|:13023-13153
MsHel-2	gb|AIXA01009028.1|:21046-21168
MsHel-2	gb|AIXA01007642.1|:15262-15377
MsHel-2	gb|AIXA01007355.1|:4072-4171
MsHel-2	gb|AIXA01006590.1|:144047-144172
MsHel-2	gb|AIXA01006449.1|:176666-176796
MsHel-2	gb|AIXA01006132.1|:37791-37889
MsHel-2	gb|AIXA01005486.1|:46251-46343
MsHel-2	gb|AIXA01005486.1|:29246-29345
MsHel-2	gb|AIXA01005131.1|:26061-26191
MsHel-2	gb|AIXA01004865.1|:48464-48562
MsHel-2	gb|AIXA01004634.1|:1738-1845
MsHel-2	gb|AIXA01004099.1|:47643-47733
MsHel-2	gb|AIXA01003966.1|:33388-33507
MsHel-2	gb|AIXA01003658.1|:43557-43676
MsHel-2	gb|AIXA01002230.1|:94972-95096
MsHel-2	gb|AIXA01001939.1|:49115-49233
MsHel-2	gb|AIXA01001864.1|:2911-3022

MsHel-2	gb|AIXA01001826.1|:3773-3898
MsHel-2	gb|AIXA01001650.1|:16100-16231
MsHel-2	gb|AIXA01001254.1|:16264-16358
MsHel-2	gb|AIXA01000555.1|:250-373
MsHel-2	gb|AIXA01000155.1|:53905-54029
MsHel-2	gb|AIXA01000012.1|:79486-79611
MsHel-2	gb|AIXA01033760.1|:84-210
MsHel-2	gb|AIXA01027974.1|:17-134
MsHel-2	gb|AIXA01025455.1|:1019-1148
MsHel-2	gb|AIXA01017926.1|:6965-7092
MsHel-2	gb|AIXA01016222.1|:1108-1236
MsHel-2	gb|AIXA01015970.1|:7477-7596
MsHel-2	gb|AIXA01015929.1|:22969-23099
MsHel-2	gb|AIXA01015824.1|:23459-23564
MsHel-2	gb|AIXA01014893.1|:17935-18060
MsHel-2	gb|AIXA01014618.1|:5350-5442
MsHel-2	gb|AIXA01014263.1|:6476-6605
MsHel-2	gb|AIXA01010197.1|:6508-6593
MsHel-2	gb|AIXA01009179.1|:105809-105929
MsHel-2	gb|AIXA01009163.1|:18049-18177
MsHel-2	gb|AIXA01009126.1|:25650-30743
MsHel-2	gb|AIXA01009033.1|:5938-6067
MsHel-2	gb|AIXA01008501.1|:10739-10846
MsHel-2	gb|AIXA01008291.1|:27881-27998
MsHel-2	gb|AIXA01006853.1|:61049-62686
MsHel-2	gb|AIXA01006832.1|:17731-17832
MsHel-2	gb|AIXA01006780.1|:51413-51535
MsHel-2	gb|AIXA01006667.1|:6504-6633
MsHel-2	gb|AIXA01005108.1|:1388-1481
MsHel-2	gb|AIXA01004842.1|:17314-17437
MsHel-2	gb|AIXA01004591.1|:7620-7721
MsHel-2	gb|AIXA01004017.1|:10793-10882
MsHel-2	gb|AIXA01002457.1|:3378-3494
MsHel-2	gb|AIXA01001894.1|:36347-36476
MsHel-2	gb|AIXA01001803.1|:13842-13964
MsHel-2	gb|AIXA01001724.1|:3034-3166
MsHel-2	gb|AIXA01000819.1|:6117-6243
MsHel-2	gb|AIXA01000741.1|:9668-9791
MsHel-2	gb|AIXA01000285.1|:81956-82073
MsHel-2	gb|AIXA01000195.1|:49934-50054
MsHel-2	gb|AIXA01000070.1|:886-1014
MsHel-2	gb|AIXA01032876.1|:178-282
MsHel-2	gb|AIXA01017338.1|:18514-18637
MsHel-2	gb|AIXA01017213.1|:10296-10421
MsHel-2	gb|AIXA01012959.1|:28034-28153
MsHel-2	gb|AIXA01012657.1|:24270-24398
MsHel-2	gb|AIXA01012653.1|:6792-6900
MsHel-2	gb|AIXA01012625.1|:3773-3873

MsHel-2	gb|AIXA01012209.1|:15294-15394
MsHel-2	gb|AIXA01010815.1|:5688-5815
MsHel-2	gb|AIXA01010372.1|:2835-2939
MsHel-2	gb|AIXA01008663.1|:5179-5303
MsHel-2	gb|AIXA01007881.1|:6650-6753
MsHel-2	gb|AIXA01007617.1|:11612-11728
MsHel-2	gb|AIXA01007615.1|:6632-6748
MsHel-2	gb|AIXA01007396.1|:2999-3126
MsHel-2	gb|AIXA01007376.1|:18214-18338
MsHel-2	gb|AIXA01006930.1|:71359-71467
MsHel-2	gb|AIXA01006182.1|:28650-28773
MsHel-2	gb|AIXA01005533.1|:29140-29265
MsHel-2	gb|AIXA01005427.1|:10132-10224
MsHel-2	gb|AIXA01005270.1|:11124-11208
MsHel-2	gb|AIXA01005095.1|:34823-34923
MsHel-2	gb|AIXA01004000.1|:3016-3112
MsHel-2	gb|AIXA01002968.1|:5414-5513
MsHel-2	gb|AIXA01002642.1|:17184-17317
MsHel-2	gb|AIXA01002333.1|:9884-9988
MsHel-2	gb|AIXA01002249.1|:32778-32897
MsHel-2	gb|AIXA01001915.1|:25751-25886
MsHel-2	gb|AIXA01001277.1|:15248-15341
MsHel-2	gb|AIXA01000087.1|:9365-9465
MsHel-2	gb|AIXA01000021.1|:19933-20055
MsHel-2	gb|AIXA01021801.1|:424-519
MsHel-2	gb|AIXA01017938.1|:622-708
MsHel-2	gb|AIXA01016262.1|:677-803
MsHel-2	gb|AIXA01014938.1|:14203-14311
MsHel-2	gb|AIXA01013499.1|:9792-9899
MsHel-2	gb|AIXA01013118.1|:19025-19122
MsHel-2	gb|AIXA01013010.1|:4790-4911
MsHel-2	gb|AIXA01011445.1|:4593-4696
MsHel-2	gb|AIXA01011268.1|:26624-26723
MsHel-2	gb|AIXA01011197.1|:43820-43912
MsHel-2	gb|AIXA01011073.1|:3557-3634
MsHel-2	gb|AIXA01010102.1|:93660-93776
MsHel-2	gb|AIXA01010102.1|:82846-82938
MsHel-2	gb|AIXA01008533.1|:1607-1730
MsHel-2	gb|AIXA01007871.1|:11418-11545
MsHel-2	gb|AIXA01007694.1|:26928-27025
MsHel-2	gb|AIXA01007270.1|:25678-25796
MsHel-2	gb|AIXA01007270.1|:2746-2835
MsHel-2	gb|AIXA01006904.1|:7658-7761
MsHel-2	gb|AIXA01005987.1|:2936-3062
MsHel-2	gb|AIXA01005951.1|:22911-22998
MsHel-2	gb|AIXA01005521.1|:64760-64870
MsHel-2	gb|AIXA01005491.1|:9626-9721
MsHel-2	gb|AIXA01005349.1|:5087-5200

MsHel-2	gb|AIXA01004431.1|:16655-16774
MsHel-2	gb|AIXA01004219.1|:6722-6836
MsHel-2	gb|AIXA01003595.1|:123363-123490
MsHel-2	gb|AIXA01003595.1|:87664-87784
MsHel-2	gb|AIXA01003441.1|:15631-15759
MsHel-2	gb|AIXA01003103.1|:47760-47884
MsHel-2	gb|AIXA01002799.1|:5545-5659
MsHel-2	gb|AIXA01002659.1|:7167-7265
MsHel-2	gb|AIXA01002567.1|:18464-18584
MsHel-2	gb|AIXA01002175.1|:1615-1726
MsHel-2	gb|AIXA01001297.1|:11933-12061
MsHel-2	gb|AIXA01000103.1|:1670-1785
MsHel-2	gb|AIXA01035334.1|:320-420
MsHel-2	gb|AIXA01017268.1|:16609-16711
MsHel-2	gb|AIXA01016578.1|:1185-1283
MsHel-2	gb|AIXA01015427.1|:7315-7437
MsHel-2	gb|AIXA01013044.1|:18862-18992
MsHel-2	gb|AIXA01011648.1|:5549-5666
MsHel-2	gb|AIXA01011585.1|:636-761
MsHel-2	gb|AIXA01010839.1|:2726-2853
MsHel-2	gb|AIXA01010294.1|:18995-19125
MsHel-2	gb|AIXA01009508.1|:47486-47611
MsHel-2	gb|AIXA01009102.1|:4550-4647
MsHel-2	gb|AIXA01007932.1|:2661-7276
MsHel-2	gb|AIXA01007085.1|:52909-53028
MsHel-2	gb|AIXA01006815.1|:33968-34074
MsHel-2	gb|AIXA01006731.1|:63043-63130
MsHel-2	gb|AIXA01006632.1|:12812-12942
MsHel-2	gb|AIXA01006160.1|:3961-4087
MsHel-2	gb|AIXA01006135.1|:5645-6010
MsHel-2	gb|AIXA01006064.1|:18374-18497
MsHel-2	gb|AIXA01005287.1|:3936-4030
MsHel-2	gb|AIXA01004999.1|:37039-37165
MsHel-2	gb|AIXA01003970.1|:32261-32350
MsHel-2	gb|AIXA01003929.1|:24256-24370
MsHel-2	gb|AIXA01003816.1|:32451-32579
MsHel-2	gb|AIXA01001836.1|:2400-2519
MsHel-2	gb|AIXA01001816.1|:40751-40880
MsHel-2	gb|AIXA01001816.1|:8001-8127
MsHel-2	gb|AIXA01001305.1|:4256-4365
MsHel-2	gb|AIXA01001278.1|:36256-36353
MsHel-2	gb|AIXA01000317.1|:9429-9556
MsHel-2	gb|AIXA01031074.1|:598-727
MsHel-2	gb|AIXA01024474.1|:320-439
MsHel-2	gb|AIXA01018932.1|:3651-3771
MsHel-2	gb|AIXA01016742.1|:3267-3396
MsHel-2	gb|AIXA01016395.1|:3848-3943
MsHel-2	gb|AIXA01015637.1|:6697-6820

MsHel-2	gb|AIXA01014694.1|:7024-7125
MsHel-2	gb|AIXA01013211.1|:16069-16198
MsHel-2	gb|AIXA01012412.1|:10770-10899
MsHel-2	gb|AIXA01011722.1|:16167-16280
MsHel-2	gb|AIXA01010583.1|:15145-15259
MsHel-2	gb|AIXA01010201.1|:3616-3736
MsHel-2	gb|AIXA01009425.1|:14782-14911
MsHel-2	gb|AIXA01009082.1|:625-754
MsHel-2	gb|AIXA01008992.1|:31825-31950
MsHel-2	gb|AIXA01008636.1|:50728-50837
MsHel-2	gb|AIXA01007231.1|:5300-5429
MsHel-2	gb|AIXA01006800.1|:7717-7837
MsHel-2	gb|AIXA01006045.1|:5261-5369
MsHel-2	gb|AIXA01004520.1|:95400-95521
MsHel-2	gb|AIXA01003687.1|:190128-190219
MsHel-2	gb|AIXA01003633.1|:35999-36124
MsHel-2	gb|AIXA01003327.1|:18502-18630
MsHel-2	gb|AIXA01003142.1|:35593-35677
MsHel-2	gb|AIXA01002309.1|:16004-16104
MsHel-2	gb|AIXA01002283.1|:55103-55214
MsHel-2	gb|AIXA01002197.1|:13776-13881
MsHel-2	gb|AIXA01001848.1|:32392-32496
MsHel-2	gb|AIXA01000842.1|:37572-37700
MsHel-2	gb|AIXA01000842.1|:13911-13997
MsHel-2	gb|AIXA01000692.1|:102857-102946
MsHel-2	gb|AIXA01037019.1|:284-383
MsHel-2	gb|AIXA01025709.1|:506-610
MsHel-2	gb|AIXA01022615.1|:853-1330
MsHel-2	gb|AIXA01021888.1|:637-729
MsHel-2	gb|AIXA01020947.1|:1265-1393
MsHel-2	gb|AIXA01020741.1|:308-413
MsHel-2	gb|AIXA01018731.1|:3170-3266
MsHel-2	gb|AIXA01017963.1|:41-133
MsHel-2	gb|AIXA01017103.1|:2704-2805
MsHel-2	gb|AIXA01016970.1|:4682-4774
MsHel-2	gb|AIXA01016600.1|:1267-1359
MsHel-2	gb|AIXA01015343.1|:2376-2503
MsHel-2	gb|AIXA01014291.1|:2133-2256
MsHel-2	gb|AIXA01010848.1|:7941-8033
MsHel-2	gb|AIXA01010778.1|:5606-5698
MsHel-2	gb|AIXA01010722.1|:5664-5786
MsHel-2	gb|AIXA01010329.1|:1682-1773
MsHel-2	gb|AIXA01009820.1|:41447-41575
MsHel-2	gb|AIXA01009100.1|:107459-107565
MsHel-2	gb|AIXA01009031.1|:17150-17266
MsHel-2	gb|AIXA01009031.1|:1362-1462
MsHel-2	gb|AIXA01009024.1|:69204-69296
MsHel-2	gb|AIXA01007980.1|:13436-13558

MsHel-2	gb|AIXA01007414.1|:8907-9011
MsHel-2	gb|AIXA01007070.1|:9971-10099
MsHel-2	gb|AIXA01007044.1|:53919-54032
MsHel-2	gb|AIXA01005417.1|:13687-13774
MsHel-2	gb|AIXA01005083.1|:61476-61584
MsHel-2	gb|AIXA01005062.1|:9818-9929
MsHel-2	gb|AIXA01005010.1|:16959-17085
MsHel-2	gb|AIXA01004786.1|:8345-8466
MsHel-2	gb|AIXA01003315.1|:37418-37545
MsHel-2	gb|AIXA01001846.1|:15363-15480
MsHel-2	gb|AIXA01001662.1|:6253-6353
MsHel-2	gb|AIXA01001544.1|:57963-58087
MsHel-2	gb|AIXA01001492.1|:22087-22204
MsHel-2	gb|AIXA01001456.1|:28809-28937
MsHel-2	gb|AIXA01001137.1|:31359-31467
MsHel-2	gb|AIXA01000960.1|:77877-77969
MsHel-2	gb|AIXA01000791.1|:6775-7128
MsHel-2	gb|AIXA01000139.1|:14582-14666
MsHel-2	gb|AIXA01037025.1|:307-410
MsHel-2	gb|AIXA01029529.1|:637-756
MsHel-2	gb|AIXA01028241.1|:526-609
MsHel-2	gb|AIXA01024434.1|:439-537
MsHel-2	gb|AIXA01019982.1|:687-810
MsHel-2	gb|AIXA01018234.1|:2041-2132
MsHel-2	gb|AIXA01014934.1|:6751-6853
MsHel-2	gb|AIXA01014675.1|:2075-2198
MsHel-2	gb|AIXA01013628.1|:10000-10127
MsHel-2	gb|AIXA01012171.1|:14284-14410
MsHel-2	gb|AIXA01011485.1|:9632-9757
MsHel-2	gb|AIXA01010324.1|:10864-10967
MsHel-2	gb|AIXA01010188.1|:26578-26705
MsHel-2	gb|AIXA01009140.1|:4406-4501
MsHel-2	gb|AIXA01006470.1|:10108-10234
MsHel-2	gb|AIXA01006200.1|:29513-29641
MsHel-2	gb|AIXA01005618.1|:21779-21869
MsHel-2	gb|AIXA01005618.1|:8435-8569
MsHel-2	gb|AIXA01005356.1|:6987-7106
MsHel-2	gb|AIXA01005184.1|:25149-25264
MsHel-2	gb|AIXA01004990.1|:192005-192131
MsHel-2	gb|AIXA01004339.1|:14194-14317
MsHel-2	gb|AIXA01002051.1|:11953-12079
MsHel-2	gb|AIXA01001627.1|:60344-60459
MsHel-2	gb|AIXA01001627.1|:5008-5107
MsHel-2	gb|AIXA01032730.1|:99-217
MsHel-2	gb|AIXA01025428.1|:1088-1171
MsHel-2	gb|AIXA01021662.1|:1401-1523
MsHel-2	gb|AIXA01017130.1|:3628-3753
MsHel-2	gb|AIXA01017070.1|:9732-9826

MsHel-2	gb|AIXA01015218.1|:9369-9469
MsHel-2	gb|AIXA01014020.1|:17589-17690
MsHel-2	gb|AIXA01014015.1|:1445-1575
MsHel-2	gb|AIXA01013065.1|:23742-23868
MsHel-2	gb|AIXA01011267.1|:7672-7770
MsHel-2	gb|AIXA01008938.1|:346-460
MsHel-2	gb|AIXA01008873.1|:11983-12108
MsHel-2	gb|AIXA01008843.1|:5408-5522
MsHel-2	gb|AIXA01008402.1|:39496-42369
MsHel-2	gb|AIXA01007698.1|:11409-11517
MsHel-2	gb|AIXA01007384.1|:3094-3213
MsHel-2	gb|AIXA01007267.1|:9363-9489
MsHel-2	gb|AIXA01006708.1|:48753-48882
MsHel-2	gb|AIXA01006508.1|:15196-15317
MsHel-2	gb|AIXA01006029.1|:31367-31492
MsHel-2	gb|AIXA01005876.1|:239-347
MsHel-2	gb|AIXA01004715.1|:85544-85669
MsHel-2	gb|AIXA01004385.1|:170-292
MsHel-2	gb|AIXA01004176.1|:5888-6015
MsHel-2	gb|AIXA01004140.1|:6323-6446
MsHel-2	gb|AIXA01004102.1|:16784-17303
MsHel-2	gb|AIXA01003504.1|:12361-12466
MsHel-2	gb|AIXA01003198.1|:39833-39948
MsHel-2	gb|AIXA01003011.1|:33703-33802
MsHel-2	gb|AIXA01002891.1|:7703-7830
MsHel-2	gb|AIXA01002703.1|:61759-61870
MsHel-2	gb|AIXA01001538.1|:70816-72059
MsHel-2	gb|AIXA01001340.1|:11410-11513
MsHel-2	gb|AIXA01000765.1|:31423-31553
MsHel-2	gb|AIXA01000690.1|:3785-3915
MsHel-2	gb|AIXA01033254.1|:81-210
MsHel-2	gb|AIXA01029007.1|:595-704
MsHel-2	gb|AIXA01020134.1|:1446-1559
MsHel-2	gb|AIXA01018667.1|:5324-5454
MsHel-2	gb|AIXA01018226.1|:1672-1766
MsHel-2	gb|AIXA01017342.1|:1913-2035
MsHel-2	gb|AIXA01015406.1|:21059-21181
MsHel-2	gb|AIXA01013966.1|:10121-10207
MsHel-2	gb|AIXA01013156.1|:12895-12992
MsHel-2	gb|AIXA01013148.1|:55974-56098
MsHel-2	gb|AIXA01012755.1|:5456-5557
MsHel-2	gb|AIXA01012730.1|:2492-2608
MsHel-2	gb|AIXA01012700.1|:8561-8661
MsHel-2	gb|AIXA01011849.1|:11215-11307
MsHel-2	gb|AIXA01009753.1|:307-416
MsHel-2	gb|AIXA01009423.1|:24310-33394
MsHel-2	gb|AIXA01009297.1|:9558-9659
MsHel-2	gb|AIXA01008173.1|:3178-3307

MsHel-2	gb|AIXA01007543.1|:7161-7290
MsHel-2	gb|AIXA01007466.1|:13007-13129
MsHel-2	gb|AIXA01007192.1|:3664-3761
MsHel-2	gb|AIXA01007023.1|:19445-19530
MsHel-2	gb|AIXA01006986.1|:35521-35641
MsHel-2	gb|AIXA01006517.1|:8191-8320
MsHel-2	gb|AIXA01006477.1|:300-429
MsHel-2	gb|AIXA01006138.1|:37689-37818
MsHel-2	gb|AIXA01006106.1|:7318-7447
MsHel-2	gb|AIXA01004621.1|:64858-64967
MsHel-2	gb|AIXA01002697.1|:21905-22018
MsHel-2	gb|AIXA01001831.1|:23392-23517
MsHel-2	gb|AIXA01001414.1|:12230-12343
MsHel-2	gb|AIXA01000043.1|:27969-28085
MsHel-2	gb|AIXA01028552.1|:765-876
MsHel-2	gb|AIXA01019690.1|:1369-1457
MsHel-2	gb|AIXA01019057.1|:3932-4057
MsHel-2	gb|AIXA01018620.1|:2831-2931
MsHel-2	gb|AIXA01017287.1|:6024-6121
MsHel-2	gb|AIXA01017122.1|:561-661
MsHel-2	gb|AIXA01016383.1|:10121-10229
MsHel-2	gb|AIXA01015296.1|:9327-9449
MsHel-2	gb|AIXA01014598.1|:2158-2258
MsHel-2	gb|AIXA01011610.1|:35248-35367
MsHel-2	gb|AIXA01011389.1|:16371-16483
MsHel-2	gb|AIXA01011090.1|:14568-14685
MsHel-2	gb|AIXA01009590.1|:32523-35846
MsHel-2	gb|AIXA01009123.1|:8483-8614
MsHel-2	gb|AIXA01009014.1|:8848-8965
MsHel-2	gb|AIXA01007493.1|:112257-112353
MsHel-2	gb|AIXA01007163.1|:9723-9805
MsHel-2	gb|AIXA01006982.1|:12738-12838
MsHel-2	gb|AIXA01005892.1|:31874-31997
MsHel-2	gb|AIXA01005607.1|:3637-3762
MsHel-2	gb|AIXA01004340.1|:9438-9538
MsHel-2	gb|AIXA01002382.1|:1713-1801
MsHel-2	gb|AIXA01000963.1|:62067-62163
MsHel-2	gb|AIXA01000068.1|:55983-56074
MsHel-2	gb|AIXA01000068.1|:35636-35765
MsHel-2	gb|AIXA01031011.1|:197-331
MsHel-2	gb|AIXA01023722.1|:1127-1218
MsHel-2	gb|AIXA01018244.1|:268-394
MsHel-2	gb|AIXA01017766.1|:1079-1192
MsHel-2	gb|AIXA01016574.1|:18924-19027
MsHel-2	gb|AIXA01015458.1|:8553-8672
MsHel-2	gb|AIXA01013679.1|:13006-13133
MsHel-2	gb|AIXA01011753.1|:30270-30373
MsHel-2	gb|AIXA01009923.1|:16700-16831

MsHel-2	gb|AIXA01008868.1|:9757-9844
MsHel-2	gb|AIXA01006600.1|:365-492
MsHel-2	gb|AIXA01006595.1|:46853-46949
MsHel-2	gb|AIXA01004583.1|:45924-46037
MsHel-2	gb|AIXA01003529.1|:12817-12926
MsHel-2	gb|AIXA01000911.1|:16918-17037
MsHel-2	gb|AIXA01019501.1|:2775-2869
MsHel-2	gb|AIXA01013166.1|:2632-2730
MsHel-2	gb|AIXA01010111.1|:1116-1232
MsHel-2	gb|AIXA01009501.1|:499-633
MsHel-2	gb|AIXA01009226.1|:14446-14572
MsHel-2	gb|AIXA01008722.1|:27683-27781
MsHel-2	gb|AIXA01007853.1|:51769-51868
MsHel-2	gb|AIXA01007141.1|:4507-4617
MsHel-2	gb|AIXA01006509.1|:35720-43227
MsHel-2	gb|AIXA01005908.1|:2438-2547
MsHel-2	gb|AIXA01005309.1|:30078-30175
MsHel-2	gb|AIXA01003301.1|:31157-31255
MsHel-2	gb|AIXA01003186.1|:41253-41377
MsHel-2	gb|AIXA01001910.1|:725-845
MsHel-2	gb|AIXA01001562.1|:4934-5054
MsHel-2	gb|AIXA01000655.1|:8813-8934
MsHel-2	gb|AIXA01024524.1|:924-1052
MsHel-2	gb|AIXA01018895.1|:933-1032
MsHel-2	gb|AIXA01017939.1|:4133-4226
MsHel-2	gb|AIXA01015510.1|:1863-1986
MsHel-2	gb|AIXA01014585.1|:4049-4138
MsHel-2	gb|AIXA01013671.1|:3261-3386
MsHel-2	gb|AIXA01011499.1|:17174-17274
MsHel-2	gb|AIXA01010841.1|:1959-2044
MsHel-2	gb|AIXA01010541.1|:3462-3555
MsHel-2	gb|AIXA01009502.1|:384-511
MsHel-2	gb|AIXA01007791.1|:9683-9805
MsHel-2	gb|AIXA01007638.1|:8078-8206
MsHel-2	gb|AIXA01007631.1|:22574-22679
MsHel-2	gb|AIXA01005794.1|:10164-10283
MsHel-2	gb|AIXA01005359.1|:15285-15375
MsHel-2	gb|AIXA01004561.1|:3807-3917
MsHel-2	gb|AIXA01004038.1|:34881-35010
MsHel-2	gb|AIXA01003257.1|:13253-13388
MsHel-2	gb|AIXA01003174.1|:20092-20185
MsHel-2	gb|AIXA01002264.1|:20007-20136
MsHel-2	gb|AIXA01001477.1|:8086-8215
MsHel-2	gb|AIXA01000783.1|:32157-32285
MsHel-2	gb|AIXA01029887.1|:669-761
MsHel-2	gb|AIXA01024587.1|:783-921
MsHel-2	gb|AIXA01019307.1|:703-797
MsHel-2	gb|AIXA01015041.1|:15538-15665

MsHel-2	gb|AIXA01014560.1|:3809-3901
MsHel-2	gb|AIXA01013978.1|:1075-1203
MsHel-2	gb|AIXA01013342.1|:43322-43447
MsHel-2	gb|AIXA01013317.1|:11479-11600
MsHel-2	gb|AIXA01011285.1|:36997-37114
MsHel-2	gb|AIXA01009953.1|:2812-2916
MsHel-2	gb|AIXA01006652.1|:1337-1456
MsHel-2	gb|AIXA01006625.1|:15173-15296
MsHel-2	gb|AIXA01006013.1|:37069-37155
MsHel-2	gb|AIXA01005527.1|:42199-42291
MsHel-2	gb|AIXA01004675.1|:9219-9305
MsHel-2	gb|AIXA01004462.1|:13234-13326
MsHel-2	gb|AIXA01004303.1|:7092-7166
MsHel-2	gb|AIXA01004265.1|:3310-3384
MsHel-2	gb|AIXA01004110.1|:1089-1184
MsHel-2	gb|AIXA01004109.1|:9674-9775
MsHel-2	gb|AIXA01003130.1|:1188-1274
MsHel-2	gb|AIXA01001955.1|:3103-3195
MsHel-2	gb|AIXA01000162.1|:10327-10448
MsHel-2	gb|AIXA01000105.1|:53106-53210
MsHel-2	gb|AIXA01013873.1|:522-644
MsHel-2	gb|AIXA01007436.1|:1257-1381
MsHel-2	gb|AIXA01006909.1|:6985-7101
MsHel-2	gb|AIXA01003581.1|:9396-9496
MsHel-2	gb|AIXA01001015.1|:39462-39585
MsHel-2	gb|AIXA01000080.1|:7964-8068
MsHel-2	gb|AIXA01000014.1|:22182-24295
MsHel-2	gb|AIXA01033268.1|:190-303
MsHel-2	gb|AIXA01028505.1|:670-800
MsHel-2	gb|AIXA01023505.1|:538-657
MsHel-2	gb|AIXA01019898.1|:3279-3371
MsHel-2	gb|AIXA01016048.1|:21278-21363
MsHel-2	gb|AIXA01014218.1|:1961-2052
MsHel-2	gb|AIXA01013876.1|:58-175
MsHel-2	gb|AIXA01011075.1|:10963-11053
MsHel-2	gb|AIXA01010569.1|:4261-4362
MsHel-2	gb|AIXA01010477.1|:26407-26527
MsHel-2	gb|AIXA01009225.1|:49244-49362
MsHel-2	gb|AIXA01008342.1|:17248-17367
MsHel-2	gb|AIXA01003358.1|:2680-2782
MsHel-2	gb|AIXA01003209.1|:4724-4810
MsHel-2	gb|AIXA01003134.1|:1291-1411
MsHel-2	gb|AIXA01000956.1|:27340-27434
MsHel-2	gb|AIXA01000652.1|:30703-30820
MsHel-2	gb|AIXA01000031.1|:36266-36380
MsHel-2	gb|AIXA01037209.1|:444-519
MsHel-2	gb|AIXA01020746.1|:175-292
MsHel-2	gb|AIXA01018770.1|:3436-3550

MsHel-2	gb|AIXA01016679.1|:2004-2118
MsHel-2	gb|AIXA01014683.1|:9083-9204
MsHel-2	gb|AIXA01013382.1|:20299-20416
MsHel-2	gb|AIXA01010388.1|:265-384
MsHel-2	gb|AIXA01010233.1|:25165-25277
MsHel-2	gb|AIXA01009775.1|:12609-12730
MsHel-2	gb|AIXA01009327.1|:29838-29956
MsHel-2	gb|AIXA01008920.1|:74-184
MsHel-2	gb|AIXA01002967.1|:13393-13522
MsHel-2	gb|AIXA01001683.1|:953-1040
MsHel-2	gb|AIXA01028540.1|:396-482
MsHel-2	gb|AIXA01025881.1|:695-804
MsHel-2	gb|AIXA01022959.1|:1458-1532
MsHel-2	gb|AIXA01022546.1|:1476-1560
MsHel-2	gb|AIXA01020764.1|:1089-1217
MsHel-2	gb|AIXA01019019.1|:255-351
MsHel-2	gb|AIXA01017969.1|:1571-1662
MsHel-2	gb|AIXA01015838.1|:7690-7764
MsHel-2	gb|AIXA01010295.1|:1427-1546
MsHel-2	gb|AIXA01008929.1|:20586-20709
MsHel-2	gb|AIXA01007625.1|:41584-41658
MsHel-2	gb|AIXA01005991.1|:734-850
MsHel-2	gb|AIXA01005833.1|:16641-16727
MsHel-2	gb|AIXA01002085.1|:24726-24812
MsHel-2	gb|AIXA01001770.1|:33167-33299
MsHel-2	gb|AIXA01000800.1|:1984-2070
MsHel-2	gb|AIXA01000404.1|:24081-24185
MsHel-2	gb|AIXA01023124.1|:232-342
MsHel-2	gb|AIXA01021618.1|:549-668
MsHel-2	gb|AIXA01012974.1|:6751-6838
MsHel-2	gb|AIXA01008726.1|:6732-6856
MsHel-2	gb|AIXA01008629.1|:8094-8188
MsHel-2	gb|AIXA01003694.1|:8478-8605
MsHel-2	gb|AIXA01002708.1|:19292-19374
MsHel-2	gb|AIXA01002568.1|:6725-7095
MsHel-2	gb|AIXA01001616.1|:3443-3581
MsHel-2	gb|AIXA01000584.1|:3880-3965
MsHel-2	gb|AIXA01026715.1|:440-559
MsHel-2	gb|AIXA01012380.1|:5969-6076
MsHel-2	gb|AIXA01007717.1|:19113-19214
MsHel-2	gb|AIXA01006984.1|:10461-10587
MsHel-2	gb|AIXA01006599.1|:2890-2996
MsHel-2	gb|AIXA01004987.1|:10200-10302
MsHel-2	gb|AIXA01004887.1|:19360-19478
MsHel-2	gb|AIXA01003053.1|:16696-16810
MsHel-2	gb|AIXA01002570.1|:29833-29933
MsHel-2	gb|AIXA01000901.1|:4309-4400
MsHel-2	gb|AIXA01011719.1|:1569-1666

MsHel-2	gb|AIXA01002858.1|:23757-23846
MsHel-3a	gb|AIXA01018027.1|:2610-2813
MsHel-3a	gb|AIXA01014695.1|:2631-2907
MsHel-3a	gb|AIXA01012901.1|:20527-22209
MsHel-3a	gb|AIXA01012901.1|:5793-6083
MsHel-3a	gb|AIXA01012759.1|:38009-38271
MsHel-3a	gb|AIXA01012759.1|:5903-6163
MsHel-3a	gb|AIXA01024267.1|:889-1008
MsHel-3a	gb|AIXA01021640.1|:1331-1614
MsHel-3a	gb|AIXA01020785.1|:436-722
MsHel-3a	gb|AIXA01017542.1|:5188-5470
MsHel-3a	gb|AIXA01016645.1|:8901-9191
MsHel-3a	gb|AIXA01014021.1|:12959-13246
MsHel-3a	gb|AIXA01013324.1|:5090-5378
MsHel-3a	gb|AIXA01013124.1|:4042-4326
MsHel-3a	gb|AIXA01013115.1|:6682-6970
MsHel-3a	gb|AIXA01012697.1|:4691-4967
MsHel-3a	gb|AIXA01012694.1|:12739-12858
MsHel-3a	gb|AIXA01012694.1|:1942-2228
MsHel-3a	gb|AIXA01011493.1|:64886-65133
MsHel-3a	gb|AIXA01011493.1|:20403-20672
MsHel-3a	gb|AIXA01009648.1|:19872-20147
MsHel-3a	gb|AIXA01009411.1|:24252-24370
MsHel-3a	gb|AIXA01009411.1|:9226-9514
MsHel-3a	gb|AIXA01007966.1|:162280-162551
MsHel-3a	gb|AIXA01007966.1|:36647-36882
MsHel-3a	gb|AIXA01007579.1|:40787-41074
MsHel-3a	gb|AIXA01005918.1|:67067-67260
MsHel-3a	gb|AIXA01005493.1|:42455-42740
MsHel-3a	gb|AIXA01005104.1|:37892-38177
MsHel-3a	gb|AIXA01004593.1|:29801-30089
MsHel-3a	gb|AIXA01003912.1|:29929-30205
MsHel-3a	gb|AIXA01003853.1|:64534-64821
MsHel-3a	gb|AIXA01003850.1|:125759-125841
MsHel-3a	gb|AIXA01003850.1|:61354-61591
MsHel-3a	gb|AIXA01002568.1|:32707-32995
MsHel-3a	gb|AIXA01001361.1|:13908-14189
MsHel-3a	gb|AIXA01000988.1|:38254-38541
MsHel-3a	gb|AIXA01000643.1|:28496-28761
MsHel-3a	gb|AIXA01014420.1|:15000-15267
MsHel-3a	gb|AIXA01004051.1|:5859-6142
MsHel-3a	gb|AIXA01003679.1|:23824-24099
MsHel-3a	gb|AIXA01007627.1|:72955-73068
MsHel-3a	gb|AIXA01007627.1|:46166-46449
MsHel-3a	gb|AIXA01011986.1|:8681-8943
MsHel-3a	gb|AIXA01002978.1|:7645-7757
MsHel-3a	gb|AIXA01025472.1|:609-893
MsHel-3a	gb|AIXA01019411.1|:2-275

MsHel-3a	gb|AIXA01018544.1|:487-775
MsHel-3a	gb|AIXA01017134.1|:3482-3772
MsHel-3a	gb|AIXA01017081.1|:2-275
MsHel-3a	gb|AIXA01016103.1|:17315-17601
MsHel-3a	gb|AIXA01015406.1|:17800-18090
MsHel-3a	gb|AIXA01015406.1|:176-435
MsHel-3a	gb|AIXA01015404.1|:9725-9998
MsHel-3a	gb|AIXA01015191.1|:25464-25725
MsHel-3a	gb|AIXA01015182.1|:765-1045
MsHel-3a	gb|AIXA01014497.1|:27548-27837
MsHel-3a	gb|AIXA01014023.1|:15773-16059
MsHel-3a	gb|AIXA01013632.1|:16667-16949
MsHel-3a	gb|AIXA01013565.1|:7985-8226
MsHel-3a	gb|AIXA01012096.1|:7439-7726
MsHel-3a	gb|AIXA01011949.1|:9193-9433
MsHel-3a	gb|AIXA01011819.1|:5443-5719
MsHel-3a	gb|AIXA01011723.1|:29396-29682
MsHel-3a	gb|AIXA01011499.1|:35380-35652
MsHel-3a	gb|AIXA01011457.1|:33849-34100
MsHel-3a	gb|AIXA01011457.1|:19588-19868
MsHel-3a	gb|AIXA01011457.1|:557-844
MsHel-3a	gb|AIXA01010582.1|:286-571
MsHel-3a	gb|AIXA01010406.1|:50104-50386
MsHel-3a	gb|AIXA01010406.1|:12015-12283
MsHel-3a	gb|AIXA01010330.1|:54058-54337
MsHel-3a	gb|AIXA01010330.1|:19593-19879
MsHel-3a	gb|AIXA01009807.1|:9105-9386
MsHel-3a	gb|AIXA01009407.1|:58377-58656
MsHel-3a	gb|AIXA01009162.1|:60444-60730
MsHel-3a	gb|AIXA01008965.1|:68456-68741
MsHel-3a	gb|AIXA01008310.1|:40417-40705
MsHel-3a	gb|AIXA01007605.1|:50183-50470
MsHel-3a	gb|AIXA01007530.1|:71407-71670
MsHel-3a	gb|AIXA01007130.1|:86707-86826
MsHel-3a	gb|AIXA01006155.1|:30826-31110
MsHel-3a	gb|AIXA01006144.1|:3562-3849
MsHel-3a	gb|AIXA01006141.1|:79410-79699
MsHel-3a	gb|AIXA01006141.1|:60448-60729
MsHel-3a	gb|AIXA01006141.1|:2882-3157
MsHel-3a	gb|AIXA01005505.1|:22125-22409
MsHel-3a	gb|AIXA01005505.1|:5456-5575
MsHel-3a	gb|AIXA01005437.1|:3391-3675
MsHel-3a	gb|AIXA01005067.1|:34845-35099
MsHel-3a	gb|AIXA01004865.1|:7762-8046
MsHel-3a	gb|AIXA01004370.1|:92185-92466
MsHel-3a	gb|AIXA01004370.1|:26991-27264
MsHel-3a	gb|AIXA01004356.1|:371-651
MsHel-3a	gb|AIXA01004158.1|:10995-11284

MsHel-3a	gb|AIXA01004094.1|:38729-39016
MsHel-3a	gb|AIXA01004094.1|:6191-6502
MsHel-3a	gb|AIXA01004069.1|:36143-36420
MsHel-3a	gb|AIXA01004061.1|:52551-52832
MsHel-3a	gb|AIXA01003928.1|:19006-19120
MsHel-3a	gb|AIXA01003349.1|:48755-49053
MsHel-3a	gb|AIXA01003016.1|:11862-12151
MsHel-3a	gb|AIXA01002811.1|:16233-16509
MsHel-3a	gb|AIXA01002718.1|:39353-39557
MsHel-3a	gb|AIXA01002629.1|:70329-70614
MsHel-3a	gb|AIXA01002499.1|:4355-4602
MsHel-3a	gb|AIXA01002391.1|:22710-22998
MsHel-3a	gb|AIXA01002303.1|:28168-28410
MsHel-3a	gb|AIXA01001636.1|:32176-32460
MsHel-3a	gb|AIXA01001319.1|:58963-59251
MsHel-3a	gb|AIXA01000934.1|:95941-96229
MsHel-3a	gb|AIXA01000934.1|:75861-76142
MsHel-3a	gb|AIXA01000934.1|:34843-35130
MsHel-3a	gb|AIXA01000892.1|:113345-113449
MsHel-3a	gb|AIXA01000692.1|:84785-85074
MsHel-3a	gb|AIXA01000692.1|:70237-70517
MsHel-3a	gb|AIXA01000356.1|:12351-12628
MsHel-3a	gb|AIXA01000224.1|:21797-22082
MsHel-3a	gb|AIXA01000036.1|:1205-1426
MsHel-3a	gb|AIXA01022475.1|:486-806
MsHel-3a	gb|AIXA01013942.1|:15784-16039
MsHel-3a	gb|AIXA01013521.1|:36473-36751
MsHel-3a	gb|AIXA01013521.1|:25706-25990
MsHel-3a	gb|AIXA01013521.1|:5768-6020
MsHel-3a	gb|AIXA01013107.1|:4418-4696
MsHel-3a	gb|AIXA01012993.1|:3093-3378
MsHel-3a	gb|AIXA01009438.1|:10179-10405
MsHel-3a	gb|AIXA01007522.1|:35738-36017
MsHel-3a	gb|AIXA01005892.1|:54482-54753
MsHel-3a	gb|AIXA01003151.1|:30077-30359
MsHel-3a	gb|AIXA01000763.1|:18492-18773
MsHel-3a	gb|AIXA01028900.1|:203-452
MsHel-3a	gb|AIXA01023774.1|:198-482
MsHel-3a	gb|AIXA01016885.1|:5188-5466
MsHel-3a	gb|AIXA01008112.1|:19835-19990
MsHel-3a	gb|AIXA01007717.1|:31674-31958
MsHel-3a	gb|AIXA01005126.1|:69554-69838
MsHel-3a	gb|AIXA01005126.1|:58596-58870
MsHel-3a	gb|AIXA01005126.1|:39434-39710
MsHel-3a	gb|AIXA01004486.1|:10353-10611
MsHel-3a	gb|AIXA01003888.1|:10273-10549
MsHel-3a	gb|AIXA01002388.1|:91306-91585
MsHel-3a	gb|AIXA01016648.1|:11695-11963

MsHel-3a	gb|AIXA01015400.1|:40628-40896
MsHel-3a	gb|AIXA01015400.1|:17932-18016
MsHel-3a	gb|AIXA01014697.1|:4473-4750
MsHel-3a	gb|AIXA01010848.1|:7463-7749
MsHel-3a	gb|AIXA01008632.1|:49218-49510
MsHel-3a	gb|AIXA01008632.1|:4921-5203
MsHel-3a	gb|AIXA01006841.1|:144010-144295
MsHel-3a	gb|AIXA01005347.1|:21665-21772
MsHel-3a	gb|AIXA01001540.1|:3510-3789
MsHel-3a	gb|AIXA01001234.1|:23164-23425
MsHel-3a	gb|AIXA01000560.1|:42847-43095
MsHel-3a	gb|AIXA01030842.1|:65-318
MsHel-3a	gb|AIXA01026536.1|:375-647
MsHel-3a	gb|AIXA01020428.1|:65-342
MsHel-3a	gb|AIXA01018916.1|:358-635
MsHel-3a	gb|AIXA01018540.1|:1492-1680
MsHel-3a	gb|AIXA01018493.1|:3397-3591
MsHel-3a	gb|AIXA01018042.1|:4137-4415
MsHel-3a	gb|AIXA01017079.1|:4565-4764
MsHel-3a	gb|AIXA01016714.1|:26305-26580
MsHel-3a	gb|AIXA01016545.1|:9526-9709
MsHel-3a	gb|AIXA01014992.1|:10605-10886
MsHel-3a	gb|AIXA01014379.1|:8205-8408
MsHel-3a	gb|AIXA01013946.1|:7626-7913
MsHel-3a	gb|AIXA01013796.1|:7850-8133
MsHel-3a	gb|AIXA01013629.1|:4323-4442
MsHel-3a	gb|AIXA01013185.1|:22605-22852
MsHel-3a	gb|AIXA01012810.1|:49976-50249
MsHel-3a	gb|AIXA01012810.1|:37281-37555
MsHel-3a	gb|AIXA01012745.1|:1188-1467
MsHel-3a	gb|AIXA01012395.1|:22738-23015
MsHel-3a	gb|AIXA01012350.1|:19807-20074
MsHel-3a	gb|AIXA01012350.1|:5384-5662
MsHel-3a	gb|AIXA01012337.1|:11915-12200
MsHel-3a	gb|AIXA01012200.1|:43698-43973
MsHel-3a	gb|AIXA01012198.1|:12728-13009
MsHel-3a	gb|AIXA01012094.1|:15954-16221
MsHel-3a	gb|AIXA01011849.1|:10168-10451
MsHel-3a	gb|AIXA01011811.1|:12476-12762
MsHel-3a	gb|AIXA01011650.1|:68694-68970
MsHel-3a	gb|AIXA01011638.1|:31085-31378
MsHel-3a	gb|AIXA01011322.1|:54611-54854
MsHel-3a	gb|AIXA01011322.1|:28996-29253
MsHel-3a	gb|AIXA01011322.1|:6495-6774
MsHel-3a	gb|AIXA01011293.1|:11827-12111
MsHel-3a	gb|AIXA01011119.1|:5189-5474
MsHel-3a	gb|AIXA01011097.1|:24723-25013
MsHel-3a	gb|AIXA01010930.1|:1601-1891

MsHel-3a	gb|AIXA01010885.1|:56811-57054
MsHel-3a	gb|AIXA01010885.1|:3162-3444
MsHel-3a	gb|AIXA01010850.1|:8276-8521
MsHel-3a	gb|AIXA01010809.1|:5366-5639
MsHel-3a	gb|AIXA01010733.1|:7992-8282
MsHel-3a	gb|AIXA01009837.1|:26496-26771
MsHel-3a	gb|AIXA01009703.1|:30750-31032
MsHel-3a	gb|AIXA01009696.1|:19858-20139
MsHel-3a	gb|AIXA01009661.1|:3458-3725
MsHel-3a	gb|AIXA01009641.1|:1405-1687
MsHel-3a	gb|AIXA01009543.1|:12226-12494
MsHel-3a	gb|AIXA01009543.1|:1591-1839
MsHel-3a	gb|AIXA01009364.1|:15716-15985
MsHel-3a	gb|AIXA01008406.1|:66711-66998
MsHel-3a	gb|AIXA01008399.1|:35683-35927
MsHel-3a	gb|AIXA01007968.1|:23452-23729
MsHel-3a	gb|AIXA01007856.1|:70973-71255
MsHel-3a	gb|AIXA01007613.1|:26491-26780
MsHel-3a	gb|AIXA01006977.1|:38368-38621
MsHel-3a	gb|AIXA01006867.1|:44524-44758
MsHel-3a	gb|AIXA01006859.1|:69-336
MsHel-3a	gb|AIXA01006847.1|:10979-11262
MsHel-3a	gb|AIXA01006676.1|:10801-11075
MsHel-3a	gb|AIXA01005904.1|:102594-103107
MsHel-3a	gb|AIXA01005813.1|:43160-43416
MsHel-3a	gb|AIXA01005813.1|:31600-31889
MsHel-3a	gb|AIXA01005782.1|:26830-27090
MsHel-3a	gb|AIXA01005731.1|:4465-4749
MsHel-3a	gb|AIXA01005488.1|:27970-28253
MsHel-3a	gb|AIXA01005035.1|:65725-65991
MsHel-3a	gb|AIXA01004731.1|:60788-61024
MsHel-3a	gb|AIXA01004731.1|:40945-41227
MsHel-3a	gb|AIXA01004685.1|:39493-39612
MsHel-3a	gb|AIXA01004636.1|:14507-14787
MsHel-3a	gb|AIXA01004378.1|:45684-45960
MsHel-3a	gb|AIXA01004378.1|:6429-6686
MsHel-3a	gb|AIXA01004150.1|:14767-15053
MsHel-3a	gb|AIXA01004150.1|:2752-3031
MsHel-3a	gb|AIXA01004091.1|:9140-9417
MsHel-3a	gb|AIXA01004084.1|:77496-77779
MsHel-3a	gb|AIXA01004082.1|:66221-66340
MsHel-3a	gb|AIXA01003741.1|:21351-21641
MsHel-3a	gb|AIXA01003356.1|:79502-79782
MsHel-3a	gb|AIXA01003356.1|:33241-33533
MsHel-3a	gb|AIXA01003140.1|:74652-74934
MsHel-3a	gb|AIXA01003103.1|:34773-35057
MsHel-3a	gb|AIXA01003000.1|:74890-76458
MsHel-3a	gb|AIXA01003000.1|:56273-56532

MsHel-3a	gb|AIXA01003000.1|:21883-22169
MsHel-3a	gb|AIXA01002970.1|:101694-101966
MsHel-3a	gb|AIXA01002739.1|:69499-69777
MsHel-3a	gb|AIXA01002739.1|:52436-52724
MsHel-3a	gb|AIXA01002640.1|:3955-4247
MsHel-3a	gb|AIXA01002560.1|:16969-17222
MsHel-3a	gb|AIXA01002476.1|:6286-6569
MsHel-3a	gb|AIXA01002311.1|:60864-60975
MsHel-3a	gb|AIXA01001878.1|:12538-12833
MsHel-3a	gb|AIXA01001866.1|:12717-12977
MsHel-3a	gb|AIXA01001842.1|:6806-7125
MsHel-3a	gb|AIXA01001758.1|:157100-157390
MsHel-3a	gb|AIXA01001758.1|:76068-76353
MsHel-3a	gb|AIXA01001531.1|:37333-37605
MsHel-3a	gb|AIXA01001531.1|:18440-18727
MsHel-3a	gb|AIXA01001466.1|:702-992
MsHel-3a	gb|AIXA01001119.1|:29291-29579
MsHel-3a	gb|AIXA01000796.1|:48919-49025
MsHel-3a	gb|AIXA01000770.1|:59313-59591
MsHel-3a	gb|AIXA01000670.1|:17825-18125
MsHel-3a	gb|AIXA01000320.1|:15703-15956
MsHel-3a	gb|AIXA01000034.1|:41574-41816
MsHel-3a	gb|AIXA01000034.1|:11183-11469
MsHel-3a	gb|AIXA01034703.1|:468-586
MsHel-3a	gb|AIXA01023850.1|:398-675
MsHel-3a	gb|AIXA01019841.1|:2817-3086
MsHel-3a	gb|AIXA01018247.1|:1200-1446
MsHel-3a	gb|AIXA01015653.1|:2805-3075
MsHel-3a	gb|AIXA01014502.1|:2456-2743
MsHel-3a	gb|AIXA01012652.1|:44433-44707
MsHel-3a	gb|AIXA01012652.1|:26692-26975
MsHel-3a	gb|AIXA01011823.1|:4938-5205
MsHel-3a	gb|AIXA01003996.1|:21893-22086
MsHel-3a	gb|AIXA01003656.1|:50969-51245
MsHel-3a	gb|AIXA01003656.1|:4890-5171
MsHel-3a	gb|AIXA01002716.1|:20099-20365
MsHel-3a	gb|AIXA01001816.1|:10064-10351
MsHel-3a	gb|AIXA01001746.1|:2611-2889
MsHel-3a	gb|AIXA01001222.1|:84497-84774
MsHel-3a	gb|AIXA01036703.1|:169-409
MsHel-3a	gb|AIXA01033762.1|:326-439
MsHel-3a	gb|AIXA01020389.1|:1230-1482
MsHel-3a	gb|AIXA01018146.1|:3826-4115
MsHel-3a	gb|AIXA01014933.1|:458-741
MsHel-3a	gb|AIXA01014840.1|:4126-4379
MsHel-3a	gb|AIXA01012903.1|:27512-27790
MsHel-3a	gb|AIXA01008385.1|:17151-18469
MsHel-3a	gb|AIXA01006855.1|:28274-28559

MsHel-3a	gb|AIXA01006649.1|:15431-15678
MsHel-3a	gb|AIXA01006182.1|:33631-33748
MsHel-3a	gb|AIXA01004489.1|:59401-59679
MsHel-3a	gb|AIXA01004327.1|:85440-85726
MsHel-3a	gb|AIXA01003122.1|:30445-30716
MsHel-3a	gb|AIXA01001558.1|:81670-81787
MsHel-3a	gb|AIXA01001558.1|:66380-66668
MsHel-3a	gb|AIXA01001558.1|:24828-25115
MsHel-3a	gb|AIXA01001135.1|:19830-20080
MsHel-3a	gb|AIXA01000868.1|:15471-15757
MsHel-3a	gb|AIXA01000011.1|:8063-8339
MsHel-3a	gb|AIXA01022922.1|:1275-1564
MsHel-3a	gb|AIXA01012701.1|:939-1216
MsHel-3a	gb|AIXA01011979.1|:4036-4324
MsHel-3a	gb|AIXA01008927.1|:38512-38801
MsHel-3a	gb|AIXA01008578.1|:37336-37456
MsHel-3a	gb|AIXA01007410.1|:17915-18027
MsHel-3a	gb|AIXA01007168.1|:25102-25368
MsHel-3a	gb|AIXA01007088.1|:7567-7847
MsHel-3a	gb|AIXA01005564.1|:17313-17602
MsHel-3a	gb|AIXA01005387.1|:84721-84833
MsHel-3a	gb|AIXA01003274.1|:3951-4225
MsHel-3a	gb|AIXA01001307.1|:7139-7406
MsHel-3a	gb|AIXA01036953.1|:129-416
MsHel-3a	gb|AIXA01033195.1|:199-482
MsHel-3a	gb|AIXA01031934.1|:349-627
MsHel-3a	gb|AIXA01029104.1|:337-623
MsHel-3a	gb|AIXA01028359.1|:142-418
MsHel-3a	gb|AIXA01022751.1|:1330-1598
MsHel-3a	gb|AIXA01021388.1|:1193-1473
MsHel-3a	gb|AIXA01019780.1|:2381-2669
MsHel-3a	gb|AIXA01018966.1|:737-1028
MsHel-3a	gb|AIXA01018825.1|:827-946
MsHel-3a	gb|AIXA01018731.1|:3415-3704
MsHel-3a	gb|AIXA01018288.1|:3859-4131
MsHel-3a	gb|AIXA01017917.1|:11173-11394
MsHel-3a	gb|AIXA01016905.1|:9981-10100
MsHel-3a	gb|AIXA01016811.1|:14232-14517
MsHel-3a	gb|AIXA01016253.1|:1368-1645
MsHel-3a	gb|AIXA01015747.1|:5569-5856
MsHel-3a	gb|AIXA01015244.1|:507-761
MsHel-3a	gb|AIXA01015230.1|:2661-2902
MsHel-3a	gb|AIXA01015179.1|:3623-3899
MsHel-3a	gb|AIXA01014766.1|:3009-3298
MsHel-3a	gb|AIXA01014372.1|:48410-48699
MsHel-3a	gb|AIXA01014372.1|:1727-2010
MsHel-3a	gb|AIXA01014299.1|:2197-2396
MsHel-3a	gb|AIXA01014206.1|:2613-2899

MsHel-3a	gb|AIXA01013776.1|:776-1063
MsHel-3a	gb|AIXA01012628.1|:12354-12473
MsHel-3a	gb|AIXA01012306.1|:5-270
MsHel-3a	gb|AIXA01012145.1|:21067-21349
MsHel-3a	gb|AIXA01011987.1|:65130-65637
MsHel-3a	gb|AIXA01011987.1|:38004-38286
MsHel-3a	gb|AIXA01011340.1|:11230-11517
MsHel-3a	gb|AIXA01011255.1|:441-726
MsHel-3a	gb|AIXA01010855.1|:32066-32355
MsHel-3a	gb|AIXA01010804.1|:3303-3422
MsHel-3a	gb|AIXA01010788.1|:8113-8402
MsHel-3a	gb|AIXA01010714.1|:38637-38881
MsHel-3a	gb|AIXA01010583.1|:25214-25436
MsHel-3a	gb|AIXA01010441.1|:59219-59513
MsHel-3a	gb|AIXA01010394.1|:43796-44063
MsHel-3a	gb|AIXA01010260.1|:5058-5320
MsHel-3a	gb|AIXA01010109.1|:63381-63637
MsHel-3a	gb|AIXA01010109.1|:15784-16073
MsHel-3a	gb|AIXA01008566.1|:71116-71403
MsHel-3a	gb|AIXA01008043.1|:31812-32062
MsHel-3a	gb|AIXA01007888.1|:46865-47279
MsHel-3a	gb|AIXA01007800.1|:73139-73402
MsHel-3a	gb|AIXA01007078.1|:18985-19211
MsHel-3a	gb|AIXA01007053.1|:42383-42658
MsHel-3a	gb|AIXA01007051.1|:825-944
MsHel-3a	gb|AIXA01006876.1|:19041-19300
MsHel-3a	gb|AIXA01006865.1|:9918-10204
MsHel-3a	gb|AIXA01006853.1|:46144-46436
MsHel-3a	gb|AIXA01006853.1|:12374-12657
MsHel-3a	gb|AIXA01006812.1|:1499-1785
MsHel-3a	gb|AIXA01006771.1|:2899-3167
MsHel-3a	gb|AIXA01006743.1|:16379-16614
MsHel-3a	gb|AIXA01005507.1|:13719-14004
MsHel-3a	gb|AIXA01005494.1|:41748-42021
MsHel-3a	gb|AIXA01005494.1|:2255-2442
MsHel-3a	gb|AIXA01005475.1|:3801-4089
MsHel-3a	gb|AIXA01005396.1|:65907-66026
MsHel-3a	gb|AIXA01005391.1|:37700-37931
MsHel-3a	gb|AIXA01005391.1|:21028-21291
MsHel-3a	gb|AIXA01005034.1|:12428-12714
MsHel-3a	gb|AIXA01004474.1|:24723-25010
MsHel-3a	gb|AIXA01004265.1|:8505-8774
MsHel-3a	gb|AIXA01004169.1|:3588-3874
MsHel-3a	gb|AIXA01003555.1|:4130-4417
MsHel-3a	gb|AIXA01003431.1|:14420-14702
MsHel-3a	gb|AIXA01003272.1|:52078-52364
MsHel-3a	gb|AIXA01003114.1|:619-906
MsHel-3a	gb|AIXA01003112.1|:9278-9397

MsHel-3a	gb|AIXA01002998.1|:12946-13230
MsHel-3a	gb|AIXA01002870.1|:74456-74727
MsHel-3a	gb|AIXA01002808.1|:18902-19188
MsHel-3a	gb|AIXA01002757.1|:2757-3031
MsHel-3a	gb|AIXA01002675.1|:4338-4457
MsHel-3a	gb|AIXA01002453.1|:16779-17066
MsHel-3a	gb|AIXA01002296.1|:140283-140562
MsHel-3a	gb|AIXA01002174.1|:30135-30424
MsHel-3a	gb|AIXA01002030.1|:20988-21263
MsHel-3a	gb|AIXA01001863.1|:20073-20352
MsHel-3a	gb|AIXA01001850.1|:32807-33111
MsHel-3a	gb|AIXA01001846.1|:12694-12813
MsHel-3a	gb|AIXA01001846.1|:961-1240
MsHel-3a	gb|AIXA01001829.1|:8073-8274
MsHel-3a	gb|AIXA01001677.1|:45315-45589
MsHel-3a	gb|AIXA01001544.1|:2723-3001
MsHel-3a	gb|AIXA01001465.1|:25714-26003
MsHel-3a	gb|AIXA01001278.1|:2781-2986
MsHel-3a	gb|AIXA01001144.1|:132483-132766
MsHel-3a	gb|AIXA01001144.1|:9930-10210
MsHel-3a	gb|AIXA01001010.1|:10464-10665
MsHel-3a	gb|AIXA01000561.1|:35535-35813
MsHel-3a	gb|AIXA01000561.1|:20344-20627
MsHel-3a	gb|AIXA01000272.1|:49498-49689
MsHel-3a	gb|AIXA01000178.1|:4381-4491
MsHel-3a	gb|AIXA01000069.1|:262496-262804
MsHel-3a	gb|AIXA01000069.1|:210187-210399
MsHel-3a	gb|AIXA01000069.1|:112211-115432
MsHel-3a	gb|AIXA01000069.1|:21216-21492
MsHel-3a	gb|AIXA01037112.1|:96-373
MsHel-3a	gb|AIXA01020648.1|:782-1065
MsHel-3a	gb|AIXA01014772.1|:6432-6711
MsHel-3a	gb|AIXA01012526.1|:17010-17293
MsHel-3a	gb|AIXA01010316.1|:1136-1250
MsHel-3a	gb|AIXA01006690.1|:21979-22263
MsHel-3a	gb|AIXA01003153.1|:66799-67078
MsHel-3a	gb|AIXA01000166.1|:4865-5143
MsHel-3a	gb|AIXA01018948.1|:1-273
MsHel-3a	gb|AIXA01015155.1|:18432-18549
MsHel-3a	gb|AIXA01015155.1|:5676-5963
MsHel-3a	gb|AIXA01014213.1|:15566-15843
MsHel-3a	gb|AIXA01014195.1|:50067-50330
MsHel-3a	gb|AIXA01014190.1|:14636-14925
MsHel-3a	gb|AIXA01012170.1|:898-1171
MsHel-3a	gb|AIXA01011303.1|:49239-49517
MsHel-3a	gb|AIXA01011303.1|:11404-11680
MsHel-3a	gb|AIXA01011239.1|:13522-13799
MsHel-3a	gb|AIXA01010299.1|:1244-1522

MsHel-3a	gb|AIXA01008489.1|:8258-8375
MsHel-3a	gb|AIXA01005562.1|:7968-8247
MsHel-3a	gb|AIXA01005538.1|:53466-53752
MsHel-3a	gb|AIXA01005538.1|:40844-41133
MsHel-3a	gb|AIXA01005538.1|:25576-25809
MsHel-3a	gb|AIXA01005511.1|:36012-36125
MsHel-3a	gb|AIXA01005511.1|:3108-3381
MsHel-3a	gb|AIXA01005378.1|:12710-12994
MsHel-3a	gb|AIXA01005105.1|:41331-41449
MsHel-3a	gb|AIXA01005105.1|:30511-30754
MsHel-3a	gb|AIXA01005105.1|:11013-11301
MsHel-3a	gb|AIXA01004630.1|:33619-33898
MsHel-3a	gb|AIXA01003252.1|:30272-30556
MsHel-3a	gb|AIXA01002732.1|:5684-5967
MsHel-3a	gb|AIXA01001283.1|:29348-29581
MsHel-3a	gb|AIXA01000776.1|:14863-15131
MsHel-3a	gb|AIXA01024670.1|:745-1031
MsHel-3a	gb|AIXA01020079.1|:3094-3194
MsHel-3a	gb|AIXA01019018.1|:460-737
MsHel-3a	gb|AIXA01012554.1|:1185-1448
MsHel-3a	gb|AIXA01011807.1|:4302-4583
MsHel-3a	gb|AIXA01011629.1|:41765-42045
MsHel-3a	gb|AIXA01010772.1|:76703-76823
MsHel-3a	gb|AIXA01010772.1|:50695-50985
MsHel-3a	gb|AIXA01010404.1|:21262-21546
MsHel-3a	gb|AIXA01010256.1|:3401-3690
MsHel-3a	gb|AIXA01009489.1|:22342-22616
MsHel-3a	gb|AIXA01008103.1|:16174-16448
MsHel-3a	gb|AIXA01007301.1|:10304-10588
MsHel-3a	gb|AIXA01007072.1|:50309-50596
MsHel-3a	gb|AIXA01002497.1|:53353-53641
MsHel-3a	gb|AIXA01001806.1|:19541-19798
MsHel-3a	gb|AIXA01001609.1|:31718-32195
MsHel-3a	gb|AIXA01001609.1|:17632-17847
MsHel-3a	gb|AIXA01001550.1|:3643-3920
MsHel-3a	gb|AIXA01001546.1|:16244-16513
MsHel-3a	gb|AIXA01001385.1|:1832-2108
MsHel-3a	gb|AIXA01001317.1|:8972-9227
MsHel-3a	gb|AIXA01000811.1|:7228-7513
MsHel-3a	gb|AIXA01000396.1|:3084-3352
MsHel-3a	gb|AIXA01000196.1|:48985-49230
MsHel-3a	gb|AIXA01035718.1|:228-506
MsHel-3a	gb|AIXA01031397.1|:640-759
MsHel-3a	gb|AIXA01028955.1|:779-882
MsHel-3a	gb|AIXA01028434.1|:214-481
MsHel-3a	gb|AIXA01019516.1|:13-300
MsHel-3a	gb|AIXA01017620.1|:5657-5776
MsHel-3a	gb|AIXA01016676.1|:5759-6044

MsHel-3a	gb|AIXA01015749.1|:9155-9437
MsHel-3a	gb|AIXA01015446.1|:2547-2823
MsHel-3a	gb|AIXA01015389.1|:3827-4114
MsHel-3a	gb|AIXA01015348.1|:2677-2796
MsHel-3a	gb|AIXA01014853.1|:3670-3949
MsHel-3a	gb|AIXA01014214.1|:10021-10136
MsHel-3a	gb|AIXA01014076.1|:3803-4084
MsHel-3a	gb|AIXA01014010.1|:3535-3775
MsHel-3a	gb|AIXA01013712.1|:14782-15066
MsHel-3a	gb|AIXA01013010.1|:50934-51206
MsHel-3a	gb|AIXA01012798.1|:337-8359
MsHel-3a	gb|AIXA01012700.1|:8810-9092
MsHel-3a	gb|AIXA01012534.1|:2120-2401
MsHel-3a	gb|AIXA01012191.1|:26679-26964
MsHel-3a	gb|AIXA01012191.1|:10313-11394
MsHel-3a	gb|AIXA01012097.1|:31084-31328
MsHel-3a	gb|AIXA01011839.1|:7854-8135
MsHel-3a	gb|AIXA01011756.1|:3224-3426
MsHel-3a	gb|AIXA01011653.1|:17462-17743
MsHel-3a	gb|AIXA01011452.1|:4728-5003
MsHel-3a	gb|AIXA01011253.1|:34405-34689
MsHel-3a	gb|AIXA01010911.1|:1-208
MsHel-3a	gb|AIXA01010849.1|:21080-21363
MsHel-3a	gb|AIXA01010810.1|:4154-4431
MsHel-3a	gb|AIXA01010777.1|:22258-22546
MsHel-3a	gb|AIXA01010268.1|:3171-3449
MsHel-3a	gb|AIXA01010211.1|:10845-11112
MsHel-3a	gb|AIXA01010119.1|:1100-1373
MsHel-3a	gb|AIXA01010057.1|:4881-5166
MsHel-3a	gb|AIXA01009948.1|:1713-1985
MsHel-3a	gb|AIXA01009420.1|:17192-17455
MsHel-3a	gb|AIXA01008861.1|:10630-10895
MsHel-3a	gb|AIXA01008564.1|:6713-6998
MsHel-3a	gb|AIXA01008401.1|:5152-5422
MsHel-3a	gb|AIXA01008396.1|:5224-5514
MsHel-3a	gb|AIXA01008274.1|:14873-15142
MsHel-3a	gb|AIXA01008243.1|:7111-7401
MsHel-3a	gb|AIXA01008227.1|:2802-3090
MsHel-3a	gb|AIXA01008022.1|:21308-21588
MsHel-3a	gb|AIXA01007223.1|:47655-47897
MsHel-3a	gb|AIXA01007104.1|:12063-12308
MsHel-3a	gb|AIXA01007058.1|:13925-14156
MsHel-3a	gb|AIXA01006689.1|:32644-32928
MsHel-3a	gb|AIXA01006684.1|:47907-48193
MsHel-3a	gb|AIXA01006590.1|:55908-56189
MsHel-3a	gb|AIXA01006123.1|:36178-43903
MsHel-3a	gb|AIXA01006053.1|:27852-28134
MsHel-3a	gb|AIXA01005668.1|:17373-17656

MsHel-3a	gb|AIXA01005668.1|:5408-5634
MsHel-3a	gb|AIXA01005452.1|:18678-18957
MsHel-3a	gb|AIXA01005420.1|:27117-27403
MsHel-3a	gb|AIXA01005407.1|:202211-202529
MsHel-3a	gb|AIXA01005407.1|:167789-168071
MsHel-3a	gb|AIXA01005251.1|:60376-60663
MsHel-3a	gb|AIXA01005088.1|:19867-20126
MsHel-3a	gb|AIXA01004944.1|:11478-11765
MsHel-3a	gb|AIXA01004597.1|:39590-39870
MsHel-3a	gb|AIXA01004597.1|:4028-4312
MsHel-3a	gb|AIXA01004093.1|:74970-75089
MsHel-3a	gb|AIXA01004093.1|:12270-12555
MsHel-3a	gb|AIXA01004089.1|:24190-24467
MsHel-3a	gb|AIXA01004089.1|:6035-6318
MsHel-3a	gb|AIXA01004039.1|:8917-9036
MsHel-3a	gb|AIXA01003999.1|:23395-23684
MsHel-3a	gb|AIXA01003369.1|:767-970
MsHel-3a	gb|AIXA01003256.1|:25-299
MsHel-3a	gb|AIXA01003249.1|:3971-4271
MsHel-3a	gb|AIXA01002967.1|:2931-3221
MsHel-3a	gb|AIXA01002776.1|:31280-31559
MsHel-3a	gb|AIXA01002552.1|:40237-40508
MsHel-3a	gb|AIXA01002552.1|:5022-5308
MsHel-3a	gb|AIXA01002468.1|:3693-3980
MsHel-3a	gb|AIXA01002071.1|:4118-4397
MsHel-3a	gb|AIXA01002041.1|:45983-46247
MsHel-3a	gb|AIXA01001883.1|:2017-2307
MsHel-3a	gb|AIXA01001840.1|:9813-9932
MsHel-3a	gb|AIXA01001762.1|:46994-47269
MsHel-3a	gb|AIXA01001652.1|:629-924
MsHel-3a	gb|AIXA01001620.1|:28805-29075
MsHel-3a	gb|AIXA01001620.1|:1087-1374
MsHel-3a	gb|AIXA01001543.1|:46112-46394
MsHel-3a	gb|AIXA01001543.1|:10787-11057
MsHel-3a	gb|AIXA01001434.1|:2070-2189
MsHel-3a	gb|AIXA01001286.1|:33877-34161
MsHel-3a	gb|AIXA01000443.1|:60110-60313
MsHel-3a	gb|AIXA01000443.1|:23165-23450
MsHel-3a	gb|AIXA01000375.1|:113860-114139
MsHel-3a	gb|AIXA01000198.1|:114409-114698
MsHel-3a	gb|AIXA01000107.1|:174627-174910
MsHel-3a	gb|AIXA01018035.1|:10900-11161
MsHel-3a	gb|AIXA01015067.1|:1154-1269
MsHel-3a	gb|AIXA01014928.1|:9257-9543
MsHel-3a	gb|AIXA01013011.1|:2851-3112
MsHel-3a	gb|AIXA01008565.1|:70702-70958
MsHel-3a	gb|AIXA01007975.1|:17201-17478
MsHel-3a	gb|AIXA01006686.1|:13102-13393

MsHel-3a	gb|AIXA01005519.1|:9205-9480
MsHel-3a	gb|AIXA01005490.1|:43724-44002
MsHel-3a	gb|AIXA01005490.1|:30453-30713
MsHel-3a	gb|AIXA01005356.1|:7892-8162
MsHel-3a	gb|AIXA01005056.1|:37330-37608
MsHel-3a	gb|AIXA01004338.1|:15135-15411
MsHel-3a	gb|AIXA01001809.1|:23615-23890
MsHel-3a	gb|AIXA01001809.1|:3141-3257
MsHel-3a	gb|AIXA01000838.1|:127920-128199
MsHel-3a	gb|AIXA01000626.1|:5483-5741
MsHel-3a	gb|AIXA01033077.1|:602-703
MsHel-3a	gb|AIXA01031457.1|:610-729
MsHel-3a	gb|AIXA01023822.1|:1007-1280
MsHel-3a	gb|AIXA01018525.1|:4709-4996
MsHel-3a	gb|AIXA01017278.1|:1107-1400
MsHel-3a	gb|AIXA01016179.1|:476-753
MsHel-3a	gb|AIXA01015186.1|:3295-3551
MsHel-3a	gb|AIXA01015139.1|:12180-12459
MsHel-3a	gb|AIXA01014411.1|:2827-3103
MsHel-3a	gb|AIXA01014243.1|:34221-37185
MsHel-3a	gb|AIXA01014012.1|:3627-3883
MsHel-3a	gb|AIXA01013302.1|:20451-20714
MsHel-3a	gb|AIXA01012112.1|:8963-9212
MsHel-3a	gb|AIXA01011450.1|:17693-17974
MsHel-3a	gb|AIXA01010155.1|:20288-20571
MsHel-3a	gb|AIXA01005721.1|:19503-19620
MsHel-3a	gb|AIXA01005053.1|:19205-19482
MsHel-3a	gb|AIXA01005045.1|:40588-41239
MsHel-3a	gb|AIXA01005045.1|:8064-8352
MsHel-3a	gb|AIXA01002999.1|:61214-61455
MsHel-3a	gb|AIXA01002965.1|:2383-2670
MsHel-3a	gb|AIXA01002619.1|:31766-32022
MsHel-3a	gb|AIXA01002551.1|:61861-62137
MsHel-3a	gb|AIXA01002551.1|:44572-44802
MsHel-3a	gb|AIXA01001653.1|:46560-46839
MsHel-3a	gb|AIXA01001083.1|:10619-10887
MsHel-3a	gb|AIXA01001034.1|:16030-16299
MsHel-3a	gb|AIXA01000199.1|:38250-38534
MsHel-3a	gb|AIXA01000085.1|:33949-34220
MsHel-3a	gb|AIXA01035015.1|:1-218
MsHel-3a	gb|AIXA01020108.1|:902-1185
MsHel-3a	gb|AIXA01019010.1|:1553-1736
MsHel-3a	gb|AIXA01018225.1|:2173-2464
MsHel-3a	gb|AIXA01013826.1|:8701-8985
MsHel-3a	gb|AIXA01012650.1|:6605-6881
MsHel-3a	gb|AIXA01011709.1|:99204-99488
MsHel-3a	gb|AIXA01011709.1|:82778-83055
MsHel-3a	gb|AIXA01011131.1|:19453-19727

MsHel-3a	gb|AIXA01008593.1|:4418-4694
MsHel-3a	gb|AIXA01008135.1|:1202-1625
MsHel-3a	gb|AIXA01007971.1|:11491-11764
MsHel-3a	gb|AIXA01007678.1|:19973-20261
MsHel-3a	gb|AIXA01006897.1|:16938-17224
MsHel-3a	gb|AIXA01006014.1|:838-1080
MsHel-3a	gb|AIXA01005833.1|:2597-2884
MsHel-3a	gb|AIXA01005639.1|:12633-12879
MsHel-3a	gb|AIXA01002975.1|:39936-40172
MsHel-3a	gb|AIXA01002917.1|:34801-35089
MsHel-3a	gb|AIXA01002741.1|:10586-10870
MsHel-3a	gb|AIXA01000282.1|:10039-10303
MsHel-3a	gb|AIXA01000234.1|:94379-94655
MsHel-3a	gb|AIXA01000234.1|:53246-53523
MsHel-3a	gb|AIXA01036819.1|:219-501
MsHel-3a	gb|AIXA01031396.1|:593-708
MsHel-3a	gb|AIXA01031203.1|:153-408
MsHel-3a	gb|AIXA01029209.1|:554-836
MsHel-3a	gb|AIXA01022133.1|:833-1117
MsHel-3a	gb|AIXA01019750.1|:1584-1838
MsHel-3a	gb|AIXA01019123.1|:2403-2681
MsHel-3a	gb|AIXA01018454.1|:2962-3246
MsHel-3a	gb|AIXA01018110.1|:673-945
MsHel-3a	gb|AIXA01017427.1|:4153-4271
MsHel-3a	gb|AIXA01017241.1|:11298-11501
MsHel-3a	gb|AIXA01016327.1|:7380-7655
MsHel-3a	gb|AIXA01015311.1|:6778-7060
MsHel-3a	gb|AIXA01015051.1|:1406-1680
MsHel-3a	gb|AIXA01014537.1|:34058-34325
MsHel-3a	gb|AIXA01014484.1|:9961-10252
MsHel-3a	gb|AIXA01014228.1|:42603-42867
MsHel-3a	gb|AIXA01014228.1|:11895-14327
MsHel-3a	gb|AIXA01013884.1|:12501-12830
MsHel-3a	gb|AIXA01012850.1|:2835-3114
MsHel-3a	gb|AIXA01012122.1|:12354-12630
MsHel-3a	gb|AIXA01010699.1|:5014-5133
MsHel-3a	gb|AIXA01010110.1|:35867-36149
MsHel-3a	gb|AIXA01009832.1|:25501-25785
MsHel-3a	gb|AIXA01009832.1|:718-1003
MsHel-3a	gb|AIXA01009360.1|:75395-75674
MsHel-3a	gb|AIXA01009324.1|:15356-15475
MsHel-3a	gb|AIXA01009166.1|:24850-25053
MsHel-3a	gb|AIXA01009019.1|:40545-40832
MsHel-3a	gb|AIXA01008992.1|:34602-34891
MsHel-3a	gb|AIXA01008950.1|:5020-5304
MsHel-3a	gb|AIXA01008481.1|:38189-38470
MsHel-3a	gb|AIXA01008261.1|:16688-16966
MsHel-3a	gb|AIXA01008261.1|:3429-3664

MsHel-3a	gb|AIXA01008225.1|:2191-2480
MsHel-3a	gb|AIXA01008110.1|:56333-56620
MsHel-3a	gb|AIXA01008076.1|:39077-39322
MsHel-3a	gb|AIXA01007964.1|:18743-19046
MsHel-3a	gb|AIXA01007593.1|:24076-24345
MsHel-3a	gb|AIXA01007523.1|:7212-7498
MsHel-3a	gb|AIXA01006907.1|:5939-6225
MsHel-3a	gb|AIXA01006875.1|:18387-18618
MsHel-3a	gb|AIXA01006244.1|:23999-24286
MsHel-3a	gb|AIXA01006201.1|:2609-2728
MsHel-3a	gb|AIXA01005901.1|:20681-20768
MsHel-3a	gb|AIXA01005899.1|:35913-36188
MsHel-3a	gb|AIXA01005805.1|:2387-2663
MsHel-3a	gb|AIXA01005085.1|:13885-14154
MsHel-3a	gb|AIXA01004956.1|:82084-82344
MsHel-3a	gb|AIXA01004956.1|:21053-21158
MsHel-3a	gb|AIXA01004619.1|:44177-44460
MsHel-3a	gb|AIXA01004351.1|:37880-38167
MsHel-3a	gb|AIXA01004229.1|:9033-9313
MsHel-3a	gb|AIXA01003673.1|:41800-42089
MsHel-3a	gb|AIXA01003320.1|:76625-76911
MsHel-3a	gb|AIXA01003037.1|:3882-4164
MsHel-3a	gb|AIXA01003015.1|:41925-42190
MsHel-3a	gb|AIXA01002911.1|:23533-23761
MsHel-3a	gb|AIXA01002725.1|:22176-22452
MsHel-3a	gb|AIXA01002514.1|:11429-11711
MsHel-3a	gb|AIXA01002304.1|:36501-36766
MsHel-3a	gb|AIXA01001826.1|:298-588
MsHel-3a	gb|AIXA01001797.1|:182563-182879
MsHel-3a	gb|AIXA01001797.1|:31112-31399
MsHel-3a	gb|AIXA01001642.1|:5971-6259
MsHel-3a	gb|AIXA01001613.1|:92896-93142
MsHel-3a	gb|AIXA01001613.1|:33054-33333
MsHel-3a	gb|AIXA01001613.1|:10507-10708
MsHel-3a	gb|AIXA01001594.1|:35982-36273
MsHel-3a	gb|AIXA01001297.1|:7468-7757
MsHel-3a	gb|AIXA01001104.1|:44886-45168
MsHel-3a	gb|AIXA01001048.1|:92954-93072
MsHel-3a	gb|AIXA01001048.1|:15692-15967
MsHel-3a	gb|AIXA01001011.1|:692-964
MsHel-3a	gb|AIXA01001007.1|:460-741
MsHel-3a	gb|AIXA01000812.1|:5849-6134
MsHel-3a	gb|AIXA01000214.1|:20882-21001
MsHel-3a	gb|AIXA01000055.1|:39229-39495
MsHel-3a	gb|AIXA01000030.1|:30703-30988
MsHel-3a	gb|AIXA01037045.1|:441-543
MsHel-3a	gb|AIXA01018203.1|:4739-5004
MsHel-3a	gb|AIXA01017888.1|:693-975

MsHel-3a	gb|AIXA01016667.1|:1931-2219
MsHel-3a	gb|AIXA01015193.1|:3676-3954
MsHel-3a	gb|AIXA01014296.1|:11353-11628
MsHel-3a	gb|AIXA01013945.1|:22568-22815
MsHel-3a	gb|AIXA01013900.1|:14028-14277
MsHel-3a	gb|AIXA01012741.1|:12697-12933
MsHel-3a	gb|AIXA01010593.1|:14723-15011
MsHel-3a	gb|AIXA01010185.1|:89455-89729
MsHel-3a	gb|AIXA01009101.1|:27956-28649
MsHel-3a	gb|AIXA01007892.1|:71978-72236
MsHel-3a	gb|AIXA01007445.1|:79568-79856
MsHel-3a	gb|AIXA01007253.1|:20805-20999
MsHel-3a	gb|AIXA01006900.1|:20302-20585
MsHel-3a	gb|AIXA01006509.1|:78847-79105
MsHel-3a	gb|AIXA01006509.1|:38562-38844
MsHel-3a	gb|AIXA01006402.1|:163085-163318
MsHel-3a	gb|AIXA01005629.1|:22391-22661
MsHel-3a	gb|AIXA01005406.1|:35561-35846
MsHel-3a	gb|AIXA01004857.1|:561-854
MsHel-3a	gb|AIXA01004330.1|:31216-31449
MsHel-3a	gb|AIXA01004330.1|:8536-9147
MsHel-3a	gb|AIXA01003168.1|:4377-4655
MsHel-3a	gb|AIXA01002717.1|:6851-7125
MsHel-3a	gb|AIXA01002457.1|:35343-35578
MsHel-3a	gb|AIXA01002457.1|:18560-18795
MsHel-3a	gb|AIXA01001823.1|:20508-20774
MsHel-3a	gb|AIXA01001673.1|:66133-71763
MsHel-3a	gb|AIXA01028007.1|:423-701
MsHel-3a	gb|AIXA01019684.1|:1638-1911
MsHel-3a	gb|AIXA01015698.1|:4940-5199
MsHel-3a	gb|AIXA01015457.1|:10112-10390
MsHel-3a	gb|AIXA01015448.1|:3969-4248
MsHel-3a	gb|AIXA01013984.1|:13944-14231
MsHel-3a	gb|AIXA01013529.1|:5020-5303
MsHel-3a	gb|AIXA01012399.1|:17983-18261
MsHel-3a	gb|AIXA01011984.1|:2410-2686
MsHel-3a	gb|AIXA01011370.1|:48055-48343
MsHel-3a	gb|AIXA01010913.1|:12425-12706
MsHel-3a	gb|AIXA01010785.1|:41226-41468
MsHel-3a	gb|AIXA01009430.1|:35021-35132
MsHel-3a	gb|AIXA01008690.1|:36366-36675
MsHel-3a	gb|AIXA01008405.1|:30730-31001
MsHel-3a	gb|AIXA01007951.1|:441-726
MsHel-3a	gb|AIXA01007287.1|:72277-72560
MsHel-3a	gb|AIXA01006866.1|:7355-7641
MsHel-3a	gb|AIXA01006731.1|:130007-130264
MsHel-3a	gb|AIXA01006158.1|:6850-7115
MsHel-3a	gb|AIXA01005585.1|:28585-28867

MsHel-3a	gb|AIXA01003284.1|:48825-49117
MsHel-3a	gb|AIXA01003108.1|:7149-7432
MsHel-3a	gb|AIXA01002625.1|:72768-72881
MsHel-3a	gb|AIXA01002625.1|:54654-54959
MsHel-3a	gb|AIXA01002617.1|:10610-10891
MsHel-3a	gb|AIXA01002612.1|:9833-10093
MsHel-3a	gb|AIXA01002371.1|:95185-95468
MsHel-3a	gb|AIXA01002371.1|:12914-13146
MsHel-3a	gb|AIXA01002350.1|:19769-20053
MsHel-3a	gb|AIXA01002350.1|:1143-1408
MsHel-3a	gb|AIXA01002241.1|:3862-4109
MsHel-3a	gb|AIXA01001811.1|:18784-19019
MsHel-3a	gb|AIXA01001590.1|:35391-35633
MsHel-3a	gb|AIXA01000120.1|:2120-2394
MsHel-3a	gb|AIXA01032040.1|:432-679
MsHel-3a	gb|AIXA01030015.1|:578-686
MsHel-3a	gb|AIXA01020295.1|:1115-1401
MsHel-3a	gb|AIXA01017014.1|:12107-12393
MsHel-3a	gb|AIXA01016988.1|:4748-5031
MsHel-3a	gb|AIXA01016799.1|:4760-5014
MsHel-3a	gb|AIXA01016615.1|:6100-6384
MsHel-3a	gb|AIXA01014217.1|:4220-4442
MsHel-3a	gb|AIXA01012124.1|:43764-44049
MsHel-3a	gb|AIXA01011818.1|:5951-6222
MsHel-3a	gb|AIXA01011071.1|:4605-4881
MsHel-3a	gb|AIXA01010550.1|:16062-16347
MsHel-3a	gb|AIXA01010541.1|:22550-22837
MsHel-3a	gb|AIXA01009821.1|:3698-3987
MsHel-3a	gb|AIXA01009100.1|:94051-94313
MsHel-3a	gb|AIXA01008222.1|:18197-18451
MsHel-3a	gb|AIXA01006796.1|:1894-2072
MsHel-3a	gb|AIXA01006284.1|:18241-18528
MsHel-3a	gb|AIXA01004252.1|:33458-33744
MsHel-3a	gb|AIXA01003698.1|:104071-104336
MsHel-3a	gb|AIXA01002354.1|:19401-19658
MsHel-3a	gb|AIXA01002216.1|:3443-3724
MsHel-3a	gb|AIXA01001243.1|:152889-153102
MsHel-3a	gb|AIXA01001243.1|:80962-81241
MsHel-3a	gb|AIXA01001009.1|:23390-23665
MsHel-3a	gb|AIXA01001009.1|:5176-5293
MsHel-3a	gb|AIXA01030357.1|:484-772
MsHel-3a	gb|AIXA01029933.1|:343-612
MsHel-3a	gb|AIXA01025838.1|:795-914
MsHel-3a	gb|AIXA01019847.1|:1747-1842
MsHel-3a	gb|AIXA01018262.1|:5424-5543
MsHel-3a	gb|AIXA01017439.1|:8147-8433
MsHel-3a	gb|AIXA01015132.1|:2118-2232
MsHel-3a	gb|AIXA01015066.1|:10988-11271

MsHel-3a	gb|AIXA01013880.1|:1530-1814
MsHel-3a	gb|AIXA01013824.1|:4604-4723
MsHel-3a	gb|AIXA01013152.1|:12303-12422
MsHel-3a	gb|AIXA01012797.1|:2064-2353
MsHel-3a	gb|AIXA01011244.1|:11088-11207
MsHel-3a	gb|AIXA01010987.1|:10669-10949
MsHel-3a	gb|AIXA01010853.1|:26351-26637
MsHel-3a	gb|AIXA01010846.1|:5991-6279
MsHel-3a	gb|AIXA01010107.1|:2830-3107
MsHel-3a	gb|AIXA01009836.1|:5264-5551
MsHel-3a	gb|AIXA01009123.1|:441-724
MsHel-3a	gb|AIXA01008957.1|:8647-8934
MsHel-3a	gb|AIXA01008799.1|:14106-14385
MsHel-3a	gb|AIXA01008795.1|:35828-36070
MsHel-3a	gb|AIXA01008044.1|:15053-15338
MsHel-3a	gb|AIXA01007581.1|:16317-16590
MsHel-3a	gb|AIXA01007513.1|:34100-34341
MsHel-3a	gb|AIXA01007030.1|:7158-7398
MsHel-3a	gb|AIXA01006940.1|:30845-31086
MsHel-3a	gb|AIXA01006860.1|:25381-25615
MsHel-3a	gb|AIXA01006860.1|:15041-15298
MsHel-3a	gb|AIXA01006423.1|:182667-182935
MsHel-3a	gb|AIXA01006423.1|:121214-121486
MsHel-3a	gb|AIXA01006162.1|:44456-44686
MsHel-3a	gb|AIXA01006162.1|:22217-22509
MsHel-3a	gb|AIXA01006025.1|:63938-64217
MsHel-3a	gb|AIXA01005259.1|:5141-5432
MsHel-3a	gb|AIXA01005060.1|:13028-13305
MsHel-3a	gb|AIXA01004990.1|:52529-52711
MsHel-3a	gb|AIXA01004621.1|:39155-39431
MsHel-3a	gb|AIXA01004216.1|:15178-15455
MsHel-3a	gb|AIXA01004136.1|:36343-36631
MsHel-3a	gb|AIXA01003902.1|:13315-13601
MsHel-3a	gb|AIXA01003648.1|:3238-3513
MsHel-3a	gb|AIXA01003002.1|:4587-4864
MsHel-3a	gb|AIXA01002858.1|:29393-29727
MsHel-3a	gb|AIXA01002813.1|:35605-35892
MsHel-3a	gb|AIXA01002613.1|:9555-9835
MsHel-3a	gb|AIXA01002555.1|:23330-23622
MsHel-3a	gb|AIXA01002377.1|:151582-151860
MsHel-3a	gb|AIXA01002232.1|:3942-4202
MsHel-3a	gb|AIXA01001665.1|:22016-22299
MsHel-3a	gb|AIXA01001656.1|:86482-86759
MsHel-3a	gb|AIXA01001656.1|:47312-47588
MsHel-3a	gb|AIXA01000822.1|:17544-17834
MsHel-3a	gb|AIXA01000459.1|:18506-18793
MsHel-3a	gb|AIXA01000454.1|:41603-41891
MsHel-3a	gb|AIXA01000251.1|:181900-182015

MsHel-3a	gb|AIXA01021316.1|:1654-1928
MsHel-3a	gb|AIXA01020235.1|:2836-2930
MsHel-3a	gb|AIXA01018211.1|:4135-4405
MsHel-3a	gb|AIXA01017013.1|:2286-2558
MsHel-3a	gb|AIXA01014382.1|:17711-17976
MsHel-3a	gb|AIXA01014382.1|:2469-2756
MsHel-3a	gb|AIXA01013039.1|:7352-7603
MsHel-3a	gb|AIXA01012785.1|:13321-13594
MsHel-3a	gb|AIXA01010789.1|:14231-14436
MsHel-3a	gb|AIXA01010294.1|:11892-12002
MsHel-3a	gb|AIXA01009130.1|:18323-18576
MsHel-3a	gb|AIXA01008723.1|:28644-28922
MsHel-3a	gb|AIXA01008533.1|:82589-82878
MsHel-3a	gb|AIXA01008533.1|:9301-9537
MsHel-3a	gb|AIXA01006851.1|:3442-3548
MsHel-3a	gb|AIXA01005389.1|:3616-3892
MsHel-3a	gb|AIXA01005063.1|:4006-4293
MsHel-3a	gb|AIXA01004470.1|:76838-77101
MsHel-3a	gb|AIXA01004470.1|:24050-24335
MsHel-3a	gb|AIXA01004470.1|:9305-9589
MsHel-3a	gb|AIXA01004187.1|:111714-111996
MsHel-3a	gb|AIXA01002626.1|:16535-16820
MsHel-3a	gb|AIXA01001356.1|:61636-61911
MsHel-3a	gb|AIXA01000285.1|:23598-23882
MsHel-3a	gb|AIXA01000091.1|:81930-82208
MsHel-3a	gb|AIXA01000091.1|:21614-21892
MsHel-3a	gb|AIXA01000029.1|:129266-129538
MsHel-3a	gb|AIXA01027012.1|:688-952
MsHel-3a	gb|AIXA01026520.1|:744-923
MsHel-3a	gb|AIXA01023668.1|:440-719
MsHel-3a	gb|AIXA01018545.1|:5897-6185
MsHel-3a	gb|AIXA01014945.1|:3381-3680
MsHel-3a	gb|AIXA01010801.1|:15742-16035
MsHel-3a	gb|AIXA01010076.1|:96710-96994
MsHel-3a	gb|AIXA01010076.1|:62317-62616
MsHel-3a	gb|AIXA01009750.1|:2023-2308
MsHel-3a	gb|AIXA01008228.1|:7862-8143
MsHel-3a	gb|AIXA01008037.1|:9057-9334
MsHel-3a	gb|AIXA01005779.1|:55707-55982
MsHel-3a	gb|AIXA01005491.1|:31348-31634
MsHel-3a	gb|AIXA01004861.1|:2120-2401
MsHel-3a	gb|AIXA01004568.1|:31484-31769
MsHel-3a	gb|AIXA01004568.1|:21156-21440
MsHel-3a	gb|AIXA01004368.1|:29222-29505
MsHel-3a	gb|AIXA01004368.1|:16606-16828
MsHel-3a	gb|AIXA01002050.1|:106895-107178
MsHel-3a	gb|AIXA01002050.1|:31338-31624
MsHel-3a	gb|AIXA01002050.1|:7436-7517

MsHel-3a	gb|AIXA01002009.1|:28203-28495
MsHel-3a	gb|AIXA01001563.1|:3950-4197
MsHel-3a	gb|AIXA01001562.1|:5808-6085
MsHel-3a	gb|AIXA01000486.1|:17619-17887
MsHel-3a	gb|AIXA01000389.1|:14981-15181
MsHel-3a	gb|AIXA01000261.1|:54477-54593
MsHel-3a	gb|AIXA01018568.1|:4116-4403
MsHel-3a	gb|AIXA01017151.1|:2340-2632
MsHel-3a	gb|AIXA01016600.1|:1529-1794
MsHel-3a	gb|AIXA01015856.1|:3547-3654
MsHel-3a	gb|AIXA01012992.1|:2859-3125
MsHel-3a	gb|AIXA01012649.1|:8238-8451
MsHel-3a	gb|AIXA01012411.1|:4428-4688
MsHel-3a	gb|AIXA01009014.1|:2554-2845
MsHel-3a	gb|AIXA01005765.1|:17170-17433
MsHel-3a	gb|AIXA01004108.1|:36414-36647
MsHel-3a	gb|AIXA01003873.1|:19518-19799
MsHel-3a	gb|AIXA01003613.1|:11484-11766
MsHel-3a	gb|AIXA01003598.1|:26836-27128
MsHel-3a	gb|AIXA01003138.1|:18839-18959
MsHel-3a	gb|AIXA01001822.1|:12430-12695
MsHel-3a	gb|AIXA01029686.1|:792-883
MsHel-3a	gb|AIXA01026717.1|:892-1091
MsHel-3a	gb|AIXA01018724.1|:764-1039
MsHel-3a	gb|AIXA01016326.1|:6530-6816
MsHel-3a	gb|AIXA01015476.1|:9614-9873
MsHel-3a	gb|AIXA01014660.1|:5373-5653
MsHel-3a	gb|AIXA01014020.1|:27358-27644
MsHel-3a	gb|AIXA01012209.1|:41384-41625
MsHel-3a	gb|AIXA01011968.1|:1441-1727
MsHel-3a	gb|AIXA01011292.1|:574-866
MsHel-3a	gb|AIXA01011191.1|:15335-15630
MsHel-3a	gb|AIXA01010402.1|:25657-25921
MsHel-3a	gb|AIXA01010402.1|:10033-10300
MsHel-3a	gb|AIXA01010151.1|:139962-140071
MsHel-3a	gb|AIXA01010151.1|:124976-125257
MsHel-3a	gb|AIXA01010029.1|:537-816
MsHel-3a	gb|AIXA01009544.1|:21126-21346
MsHel-3a	gb|AIXA01009371.1|:33569-33853
MsHel-3a	gb|AIXA01009371.1|:14119-14364
MsHel-3a	gb|AIXA01009366.1|:57390-57671
MsHel-3a	gb|AIXA01009366.1|:19772-20063
MsHel-3a	gb|AIXA01008553.1|:43766-44052
MsHel-3a	gb|AIXA01008373.1|:238-524
MsHel-3a	gb|AIXA01008124.1|:120069-120261
MsHel-3a	gb|AIXA01007721.1|:17295-18100
MsHel-3a	gb|AIXA01007626.1|:123132-123383
MsHel-3a	gb|AIXA01007546.1|:52521-52796

MsHel-3a	gb|AIXA01007545.1|:21220-21493
MsHel-3a	gb|AIXA01007545.1|:9752-9997
MsHel-3a	gb|AIXA01007476.1|:25761-26047
MsHel-3a	gb|AIXA01007009.1|:43190-43470
MsHel-3a	gb|AIXA01006136.1|:43929-44217
MsHel-3a	gb|AIXA01005817.1|:4800-5091
MsHel-3a	gb|AIXA01004929.1|:36072-36348
MsHel-3a	gb|AIXA01004809.1|:114955-115246
MsHel-3a	gb|AIXA01004488.1|:4615-4905
MsHel-3a	gb|AIXA01004482.1|:27028-27296
MsHel-3a	gb|AIXA01004260.1|:50697-50961
MsHel-3a	gb|AIXA01004059.1|:4202-4491
MsHel-3a	gb|AIXA01003839.1|:47850-48090
MsHel-3a	gb|AIXA01003294.1|:51712-51995
MsHel-3a	gb|AIXA01003234.1|:3764-3963
MsHel-3a	gb|AIXA01002734.1|:17989-18275
MsHel-3a	gb|AIXA01002704.1|:7616-7906
MsHel-3a	gb|AIXA01002264.1|:119992-120109
MsHel-3a	gb|AIXA01001973.1|:17289-17540
MsHel-3a	gb|AIXA01001807.1|:6350-6636
MsHel-3a	gb|AIXA01000937.1|:41290-41409
MsHel-3a	gb|AIXA01000206.1|:98782-98901
MsHel-3a	gb|AIXA01021695.1|:1001-1100
MsHel-3a	gb|AIXA01021519.1|:1089-1374
MsHel-3a	gb|AIXA01018040.1|:1405-1691
MsHel-3a	gb|AIXA01014676.1|:5608-5888
MsHel-3a	gb|AIXA01011531.1|:2402-3012
MsHel-3a	gb|AIXA01010748.1|:53165-53427
MsHel-3a	gb|AIXA01010748.1|:24362-24646
MsHel-3a	gb|AIXA01007732.1|:58878-59126
MsHel-3a	gb|AIXA01006286.1|:9851-10046
MsHel-3a	gb|AIXA01005860.1|:2648-4049
MsHel-3a	gb|AIXA01005322.1|:36896-37148
MsHel-3a	gb|AIXA01004269.1|:2139-2389
MsHel-3a	gb|AIXA01003154.1|:6328-6602
MsHel-3a	gb|AIXA01003051.1|:26327-26605
MsHel-3a	gb|AIXA01003026.1|:23657-23943
MsHel-3a	gb|AIXA01002582.1|:90501-90951
MsHel-3a	gb|AIXA01002267.1|:15084-15327
MsHel-3a	gb|AIXA01001956.1|:69374-69472
MsHel-3a	gb|AIXA01001731.1|:34340-34503
MsHel-3a	gb|AIXA01000836.1|:3972-4261
MsHel-3a	gb|AIXA01027390.1|:469-748
MsHel-3a	gb|AIXA01019038.1|:5961-6242
MsHel-3a	gb|AIXA01017950.1|:1970-2088
MsHel-3a	gb|AIXA01016208.1|:16878-17090
MsHel-3a	gb|AIXA01015421.1|:2340-2614
MsHel-3a	gb|AIXA01013118.1|:3903-4167

MsHel-3a	gb|AIXA01010961.1|:9265-9444
MsHel-3a	gb|AIXA01010854.1|:35360-35452
MsHel-3a	gb|AIXA01010334.1|:3702-3977
MsHel-3a	gb|AIXA01008943.1|:10784-11050
MsHel-3a	gb|AIXA01007355.1|:4325-4611
MsHel-3a	gb|AIXA01006680.1|:5877-6135
MsHel-3a	gb|AIXA01006595.1|:43590-43877
MsHel-3a	gb|AIXA01005932.1|:903-1188
MsHel-3a	gb|AIXA01002527.1|:16544-16641
MsHel-3a	gb|AIXA01000787.1|:42937-43199
MsHel-3a	gb|AIXA01000114.1|:33465-33562
MsHel-3a	gb|AIXA01017928.1|:8903-9020
MsHel-3a	gb|AIXA01014709.1|:1545-1797
MsHel-3a	gb|AIXA01013676.1|:13939-14224
MsHel-3a	gb|AIXA01013448.1|:2784-2876
MsHel-3a	gb|AIXA01013424.1|:20301-20594
MsHel-3a	gb|AIXA01012529.1|:26541-26815
MsHel-3a	gb|AIXA01012529.1|:1471-1727
MsHel-3a	gb|AIXA01011533.1|:2258-2943
MsHel-3a	gb|AIXA01010678.1|:3700-3886
MsHel-3a	gb|AIXA01010620.1|:5377-5638
MsHel-3a	gb|AIXA01010395.1|:5226-5484
MsHel-3a	gb|AIXA01008872.1|:5808-6092
MsHel-3a	gb|AIXA01008816.1|:4512-4701
MsHel-3a	gb|AIXA01008701.1|:6087-6370
MsHel-3a	gb|AIXA01007478.1|:749-829
MsHel-3a	gb|AIXA01007218.1|:18507-18764
MsHel-3a	gb|AIXA01007166.1|:44023-44300
MsHel-3a	gb|AIXA01006520.1|:6061-6718
MsHel-3a	gb|AIXA01006299.1|:68240-68354
MsHel-3a	gb|AIXA01006299.1|:43945-44228
MsHel-3a	gb|AIXA01005527.1|:45574-45843
MsHel-3a	gb|AIXA01002507.1|:82564-82850
MsHel-3a	gb|AIXA01002507.1|:14091-14891
MsHel-3a	gb|AIXA01001993.1|:20249-20519
MsHel-3a	gb|AIXA01001825.1|:5728-6016
MsHel-3a	gb|AIXA01001366.1|:44688-44975
MsHel-3a	gb|AIXA01000394.1|:64059-64350
MsHel-3a	gb|AIXA01037843.1|:314-497
MsHel-3a	gb|AIXA01029937.1|:526-809
MsHel-3a	gb|AIXA01026307.1|:116-374
MsHel-3a	gb|AIXA01024502.1|:106-393
MsHel-3a	gb|AIXA01021022.1|:837-959
MsHel-3a	gb|AIXA01017935.1|:10863-11142
MsHel-3a	gb|AIXA01017679.1|:712-984
MsHel-3a	gb|AIXA01014424.1|:2174-2480
MsHel-3a	gb|AIXA01013665.1|:11448-11701
MsHel-3a	gb|AIXA01010756.1|:46161-46983

MsHel-3a	gb|AIXA01010559.1|:361-614
MsHel-3a	gb|AIXA01010118.1|:15438-15606
MsHel-3a	gb|AIXA01009539.1|:89424-89671
MsHel-3a	gb|AIXA01009539.1|:52785-53017
MsHel-3a	gb|AIXA01009539.1|:19771-20039
MsHel-3a	gb|AIXA01009235.1|:8930-9221
MsHel-3a	gb|AIXA01009140.1|:3646-3920
MsHel-3a	gb|AIXA01007813.1|:4507-4781
MsHel-3a	gb|AIXA01006196.1|:10508-10615
MsHel-3a	gb|AIXA01005324.1|:27946-28065
MsHel-3a	gb|AIXA01004313.1|:4191-4468
MsHel-3a	gb|AIXA01003582.1|:22308-22561
MsHel-3a	gb|AIXA01003494.1|:25954-26207
MsHel-3a	gb|AIXA01003124.1|:77635-77922
MsHel-3a	gb|AIXA01002969.1|:34523-34799
MsHel-3a	gb|AIXA01002057.1|:5725-6007
MsHel-3a	gb|AIXA01001658.1|:89478-89760
MsHel-3a	gb|AIXA01001275.1|:1613-1842
MsHel-3a	gb|AIXA01001001.1|:29822-30091
MsHel-3a	gb|AIXA01000971.1|:24522-24633
MsHel-3a	gb|AIXA01000967.1|:22602-22882
MsHel-3a	gb|AIXA01000051.1|:55192-55457
MsHel-3a	gb|AIXA01031560.1|:124-397
MsHel-3a	gb|AIXA01019124.1|:1900-2145
MsHel-3a	gb|AIXA01017914.1|:17607-17839
MsHel-3a	gb|AIXA01017914.1|:917-1148
MsHel-3a	gb|AIXA01015119.1|:46932-47202
MsHel-3a	gb|AIXA01015119.1|:19143-19411
MsHel-3a	gb|AIXA01012523.1|:8942-9231
MsHel-3a	gb|AIXA01011623.1|:4515-4778
MsHel-3a	gb|AIXA01010751.1|:2150-2675
MsHel-3a	gb|AIXA01009129.1|:1221-1496
MsHel-3a	gb|AIXA01009073.1|:2302-2652
MsHel-3a	gb|AIXA01006592.1|:6576-6859
MsHel-3a	gb|AIXA01006350.1|:48731-48968
MsHel-3a	gb|AIXA01005531.1|:323-1664
MsHel-3a	gb|AIXA01000664.1|:5351-5456
MsHel-3a	gb|AIXA01000208.1|:19410-19691
MsHel-3a	gb|AIXA01000195.1|:7856-8120
MsHel-3a	gb|AIXA01020022.1|:1309-1590
MsHel-3a	gb|AIXA01018791.1|:3261-3541
MsHel-3a	gb|AIXA01018471.1|:7333-7606
MsHel-3a	gb|AIXA01016872.1|:451-731
MsHel-3a	gb|AIXA01015508.1|:17749-18034
MsHel-3a	gb|AIXA01015508.1|:1925-2214
MsHel-3a	gb|AIXA01008403.1|:1715-1955
MsHel-3a	gb|AIXA01008398.1|:11415-11689
MsHel-3a	gb|AIXA01007707.1|:30805-31047

MsHel-3a	gb|AIXA01007472.1|:2993-3237
MsHel-3a	gb|AIXA01007229.1|:30288-30490
MsHel-3a	gb|AIXA01004894.1|:25749-26022
MsHel-3a	gb|AIXA01004105.1|:1569-1824
MsHel-3a	gb|AIXA01002157.1|:43497-43777
MsHel-3a	gb|AIXA01001830.1|:31084-31367
MsHel-3a	gb|AIXA01001830.1|:11366-11641
MsHel-3a	gb|AIXA01001481.1|:17318-17565
MsHel-3a	gb|AIXA01001237.1|:38716-38990
MsHel-3a	gb|AIXA01000507.1|:8119-8397
MsHel-3a	gb|AIXA01000421.1|:13541-13798
MsHel-3a	gb|AIXA01000061.1|:26204-26462
MsHel-3a	gb|AIXA01000059.1|:28112-28400
MsHel-3a	gb|AIXA01021425.1|:885-1171
MsHel-3a	gb|AIXA01020441.1|:1077-1910
MsHel-3a	gb|AIXA01018270.1|:1889-2101
MsHel-3a	gb|AIXA01017918.1|:1727-1846
MsHel-3a	gb|AIXA01015579.1|:12493-12601
MsHel-3a	gb|AIXA01015579.1|:1506-1771
MsHel-3a	gb|AIXA01015185.1|:447-729
MsHel-3a	gb|AIXA01013618.1|:9860-10137
MsHel-3a	gb|AIXA01009348.1|:10597-10879
MsHel-3a	gb|AIXA01008378.1|:12622-12722
MsHel-3a	gb|AIXA01008233.1|:14808-15091
MsHel-3a	gb|AIXA01001296.1|:170705-170933
MsHel-3a	gb|AIXA01000404.1|:37879-38170
MsHel-3a	gb|AIXA01000271.1|:38872-39106
MsHel-3a	gb|AIXA01000271.1|:10285-10561
MsHel-3a	gb|AIXA01000164.1|:28878-29121
MsHel-3a	gb|AIXA01000031.1|:51854-52098
MsHel-3a	gb|AIXA01000031.1|:25031-25279
MsHel-3a	gb|AIXA01000031.1|:7869-8133
MsHel-3a	gb|AIXA01018834.1|:1866-2155
MsHel-3a	gb|AIXA01014936.1|:623-900
MsHel-3a	gb|AIXA01012435.1|:9975-10233
MsHel-3a	gb|AIXA01012051.1|:11334-11612
MsHel-3a	gb|AIXA01011779.1|:11096-11311
MsHel-3a	gb|AIXA01007691.1|:8814-9106
MsHel-3a	gb|AIXA01005282.1|:1057-1343
MsHel-3a	gb|AIXA01003005.1|:8518-8985
MsHel-3a	gb|AIXA01002671.1|:107020-107307
MsHel-3a	gb|AIXA01001808.1|:1792-2068
MsHel-3a	gb|AIXA01020032.1|:1781-2058
MsHel-3a	gb|AIXA01017424.1|:1508-1761
MsHel-3a	gb|AIXA01013828.1|:82-358
MsHel-3a	gb|AIXA01013766.1|:2191-2293
MsHel-3a	gb|AIXA01005112.1|:13056-13165
MsHel-3a	gb|AIXA01003531.1|:17916-18185

MsHel-3a	gb|AIXA01003072.1|:40027-40313
MsHel-3a	gb|AIXA01002727.1|:28438-28823
MsHel-3a	gb|AIXA01014252.1|:15063-15325
MsHel-3a	gb|AIXA01013523.1|:29038-29156
MsHel-3a	gb|AIXA01012674.1|:12118-12403
MsHel-3a	gb|AIXA01009697.1|:87687-87962
MsHel-3a	gb|AIXA01009528.1|:11316-11549
MsHel-3a	gb|AIXA01002764.1|:61328-61579
MsHel-3a	gb|AIXA01001605.1|:14478-14721
MsHel-3a	gb|AIXA01001580.1|:13900-14016
MsHel-3a	gb|AIXA01001026.1|:12576-12741
MsHel-3a	gb|AIXA01029401.1|:603-868
MsHel-3a	gb|AIXA01023997.1|:1187-1275
MsHel-3a	gb|AIXA01018557.1|:6936-7119
MsHel-3a	gb|AIXA01012139.1|:2701-2797
MsHel-3a	gb|AIXA01011333.1|:6019-6305
MsHel-3a	gb|AIXA01011266.1|:3978-4255
MsHel-3a	gb|AIXA01010481.1|:27870-28124
MsHel-3a	gb|AIXA01009906.1|:6613-6884
MsHel-3a	gb|AIXA01008291.1|:65865-65955
MsHel-3a	gb|AIXA01008291.1|:50974-51246
MsHel-3a	gb|AIXA01007959.1|:1048-1340
MsHel-3a	gb|AIXA01007481.1|:80739-81008
MsHel-3a	gb|AIXA01007392.1|:21143-21415
MsHel-3a	gb|AIXA01006409.1|:25453-25569
MsHel-3a	gb|AIXA01002366.1|:23045-23335
MsHel-3a	gb|AIXA01000810.1|:265964-266225
MsHel-3a	gb|AIXA01030719.1|:159-406
MsHel-3a	gb|AIXA01022106.1|:693-937
MsHel-3a	gb|AIXA01021749.1|:90-256
MsHel-3a	gb|AIXA01021194.1|:180-467
MsHel-3a	gb|AIXA01018845.1|:2047-2326
MsHel-3a	gb|AIXA01016073.1|:8222-8501
MsHel-3a	gb|AIXA01015969.1|:4776-5034
MsHel-3a	gb|AIXA01015173.1|:2380-2651
MsHel-3a	gb|AIXA01011111.1|:10105-10224
MsHel-3a	gb|AIXA01009575.1|:13303-13565
MsHel-3a	gb|AIXA01008859.1|:42107-42338
MsHel-3a	gb|AIXA01008859.1|:5204-5466
MsHel-3a	gb|AIXA01008413.1|:11133-11245
MsHel-3a	gb|AIXA01008105.1|:6349-6633
MsHel-3a	gb|AIXA01007594.1|:14787-15004
MsHel-3a	gb|AIXA01007516.1|:61225-61489
MsHel-3a	gb|AIXA01007278.1|:852-971
MsHel-3a	gb|AIXA01006890.1|:14318-14585
MsHel-3a	gb|AIXA01005518.1|:4916-5152
MsHel-3a	gb|AIXA01003661.1|:89607-89837
MsHel-3a	gb|AIXA01003052.1|:46365-46657

MsHel-3a	gb|AIXA01002040.1|:4381-4639
MsHel-3a	gb|AIXA01001551.1|:24780-25055
MsHel-3a	gb|AIXA01001511.1|:50553-50828
MsHel-3a	gb|AIXA01035936.1|:37-156
MsHel-3a	gb|AIXA01029318.1|:453-724
MsHel-3a	gb|AIXA01018908.1|:1727-2011
MsHel-3a	gb|AIXA01017236.1|:4955-5221
MsHel-3a	gb|AIXA01017016.1|:1236-1330
MsHel-3a	gb|AIXA01011922.1|:9807-10091
MsHel-3a	gb|AIXA01011816.1|:1239-1515
MsHel-3a	gb|AIXA01011679.1|:14563-14818
MsHel-3a	gb|AIXA01009533.1|:47113-47396
MsHel-3a	gb|AIXA01008111.1|:50792-51033
MsHel-3a	gb|AIXA01007267.1|:18714-18951
MsHel-3a	gb|AIXA01007259.1|:32023-32743
MsHel-3a	gb|AIXA01004153.1|:67512-67745
MsHel-3a	gb|AIXA01004153.1|:43019-43247
MsHel-3a	gb|AIXA01002920.1|:2450-2709
MsHel-3a	gb|AIXA01002454.1|:140488-140762
MsHel-3a	gb|AIXA01002314.1|:9393-9645
MsHel-3a	gb|AIXA01001514.1|:21542-21806
MsHel-3a	gb|AIXA01001514.1|:5282-5565
MsHel-3a	gb|AIXA01001363.1|:10000-10259
MsHel-3a	gb|AIXA01029682.1|:135-404
MsHel-3a	gb|AIXA01022806.1|:1532-1617
MsHel-3a	gb|AIXA01021707.1|:1315-1599
MsHel-3a	gb|AIXA01018524.1|:6278-6523
MsHel-3a	gb|AIXA01017933.1|:10588-10867
MsHel-3a	gb|AIXA01016081.1|:14031-14319
MsHel-3a	gb|AIXA01015414.1|:7028-7273
MsHel-3a	gb|AIXA01015124.1|:8597-8859
MsHel-3a	gb|AIXA01015049.1|:3698-3976
MsHel-3a	gb|AIXA01014765.1|:1422-1704
MsHel-3a	gb|AIXA01013696.1|:3281-3520
MsHel-3a	gb|AIXA01013383.1|:1469-1744
MsHel-3a	gb|AIXA01012581.1|:65744-65982
MsHel-3a	gb|AIXA01012324.1|:25222-25464
MsHel-3a	gb|AIXA01011842.1|:2884-3150
MsHel-3a	gb|AIXA01011304.1|:34490-34773
MsHel-3a	gb|AIXA01010754.1|:9371-9648
MsHel-3a	gb|AIXA01010342.1|:22664-22875
MsHel-3a	gb|AIXA01009537.1|:77534-77779
MsHel-3a	gb|AIXA01006039.1|:895-1069
MsHel-3a	gb|AIXA01006035.1|:72625-72913
MsHel-3a	gb|AIXA01005769.1|:215-401
MsHel-3a	gb|AIXA01002455.1|:1-269
MsHel-3a	gb|AIXA01024946.1|:173-419
MsHel-3a	gb|AIXA01019461.1|:1703-1924

MsHel-3a	gb|AIXA01016746.1|:1672-1930
MsHel-3a	gb|AIXA01011655.1|:7536-7798
MsHel-3a	gb|AIXA01010322.1|:22930-23193
MsHel-3a	gb|AIXA01007224.1|:31057-31332
MsHel-3a	gb|AIXA01007103.1|:923-1011
MsHel-3a	gb|AIXA01006257.1|:6633-6880
MsHel-3a	gb|AIXA01005128.1|:31139-31408
MsHel-3a	gb|AIXA01004401.1|:27241-27470
MsHel-3a	gb|AIXA01003214.1|:57805-58059
MsHel-3a	gb|AIXA01002971.1|:94374-94646
MsHel-3a	gb|AIXA01001548.1|:25535-25790
MsHel-3a	gb|AIXA01001312.1|:5881-7134
MsHel-3a	gb|AIXA01000850.1|:15124-15391
MsHel-3a	gb|AIXA01000154.1|:46638-46903
MsHel-3a	gb|AIXA01000003.1|:57785-58068
MsHel-3a	gb|AIXA01019937.1|:2535-2777
MsHel-3a	gb|AIXA01019453.1|:6429-6665
MsHel-3a	gb|AIXA01019179.1|:3387-3620
MsHel-3a	gb|AIXA01018669.1|:4808-5049
MsHel-3a	gb|AIXA01016076.1|:250-495
MsHel-3a	gb|AIXA01014215.1|:3193-3467
MsHel-3a	gb|AIXA01014093.1|:28500-28782
MsHel-3a	gb|AIXA01011030.1|:12450-13196
MsHel-3a	gb|AIXA01010880.1|:29770-30063
MsHel-3a	gb|AIXA01010865.1|:29266-29540
MsHel-3a	gb|AIXA01010019.1|:43235-43478
MsHel-3a	gb|AIXA01009856.1|:62208-62463
MsHel-3a	gb|AIXA01009856.1|:39620-39907
MsHel-3a	gb|AIXA01009649.1|:3268-3553
MsHel-3a	gb|AIXA01008878.1|:20572-20845
MsHel-3a	gb|AIXA01008635.1|:4507-4754
MsHel-3a	gb|AIXA01008293.1|:23108-23354
MsHel-3a	gb|AIXA01007028.1|:15999-16285
MsHel-3a	gb|AIXA01006956.1|:13562-13851
MsHel-3a	gb|AIXA01006922.1|:191-402
MsHel-3a	gb|AIXA01006502.1|:9900-9983
MsHel-3a	gb|AIXA01006126.1|:644-911
MsHel-3a	gb|AIXA01004758.1|:11855-12117
MsHel-3a	gb|AIXA01004708.1|:140301-140561
MsHel-3a	gb|AIXA01004708.1|:54238-54525
MsHel-3a	gb|AIXA01004644.1|:2266-2511
MsHel-3a	gb|AIXA01003297.1|:57811-58057
MsHel-3a	gb|AIXA01002992.1|:41345-41576
MsHel-3a	gb|AIXA01002857.1|:7866-8150
MsHel-3a	gb|AIXA01002104.1|:14456-14831
MsHel-3a	gb|AIXA01001619.1|:12717-20123
MsHel-3a	gb|AIXA01001408.1|:10880-11125
MsHel-3a	gb|AIXA01001046.1|:56809-57097

MsHel-3a	gb|AIXA01001018.1|:13239-13471
MsHel-3a	gb|AIXA01000734.1|:12341-12620
MsHel-3a	gb|AIXA01000655.1|:211761-212048
MsHel-3a	gb|AIXA01000453.1|:3320-3617
MsHel-3a	gb|AIXA01021861.1|:1552-1838
MsHel-3a	gb|AIXA01017683.1|:3362-3642
MsHel-3a	gb|AIXA01013044.1|:43111-43400
MsHel-3a	gb|AIXA01013044.1|:11269-11537
MsHel-3a	gb|AIXA01012131.1|:14039-14337
MsHel-3a	gb|AIXA01009061.1|:119394-119650
MsHel-3a	gb|AIXA01008879.1|:10021-10232
MsHel-3a	gb|AIXA01008313.1|:17454-17696
MsHel-3a	gb|AIXA01006111.1|:18016-18285
MsHel-3a	gb|AIXA01005643.1|:26617-26902
MsHel-3a	gb|AIXA01004417.1|:31552-31797
MsHel-3a	gb|AIXA01004417.1|:18009-18283
MsHel-3a	gb|AIXA01004392.1|:1513-1753
MsHel-3a	gb|AIXA01003534.1|:22896-23185
MsHel-3a	gb|AIXA01003336.1|:17639-17874
MsHel-3a	gb|AIXA01002730.1|:77700-80939
MsHel-3a	gb|AIXA01000168.1|:2747-2858
MsHel-3a	gb|AIXA01037985.1|:281-513
MsHel-3a	gb|AIXA01037742.1|:4-228
MsHel-3a	gb|AIXA01026257.1|:36-154
MsHel-3a	gb|AIXA01019207.1|:905-1191
MsHel-3a	gb|AIXA01018917.1|:5214-5458
MsHel-3a	gb|AIXA01017667.1|:1117-1366
MsHel-3a	gb|AIXA01017323.1|:1-243
MsHel-3a	gb|AIXA01015879.1|:801-1043
MsHel-3a	gb|AIXA01015459.1|:847-1085
MsHel-3a	gb|AIXA01015214.1|:4924-5187
MsHel-3a	gb|AIXA01013176.1|:3353-3599
MsHel-3a	gb|AIXA01012788.1|:708-984
MsHel-3a	gb|AIXA01011782.1|:2344-2584
MsHel-3a	gb|AIXA01011058.1|:89-311
MsHel-3a	gb|AIXA01009365.1|:4651-4892
MsHel-3a	gb|AIXA01009304.1|:17461-17744
MsHel-3a	gb|AIXA01008329.1|:27789-28042
MsHel-3a	gb|AIXA01008053.1|:3090-3330
MsHel-3a	gb|AIXA01006183.1|:32276-32506
MsHel-3a	gb|AIXA01005597.1|:151-263
MsHel-3a	gb|AIXA01005479.1|:40390-40634
MsHel-3a	gb|AIXA01005479.1|:22775-23064
MsHel-3a	gb|AIXA01004164.1|:44623-45776
MsHel-3a	gb|AIXA01003929.1|:72016-72215
MsHel-3a	gb|AIXA01003567.1|:5481-5735
MsHel-3a	gb|AIXA01002189.1|:7870-8104
MsHel-3a	gb|AIXA01001994.1|:22510-22791

MsHel-3a	gb|AIXA01001861.1|:3762-4037
MsHel-3a	gb|AIXA01001717.1|:19505-19787
MsHel-3a	gb|AIXA01001617.1|:1679-1922
MsHel-3a	gb|AIXA01001571.1|:8423-8710
MsHel-3a	gb|AIXA01001354.1|:12664-13403
MsHel-3a	gb|AIXA01000944.1|:5242-5516
MsHel-3a	gb|AIXA01000930.1|:1-233
MsHel-3a	gb|AIXA01000457.1|:51911-54440
MsHel-3a	gb|AIXA01035487.1|:76-317
MsHel-3a	gb|AIXA01032646.1|:12-268
MsHel-3a	gb|AIXA01030130.1|:219-502
MsHel-3a	gb|AIXA01027402.1|:836-1020
MsHel-3a	gb|AIXA01024380.1|:1090-1348
MsHel-3a	gb|AIXA01022371.1|:596-868
MsHel-3a	gb|AIXA01019824.1|:2911-3142
MsHel-3a	gb|AIXA01019409.1|:4379-4660
MsHel-3a	gb|AIXA01017759.1|:1064-1345
MsHel-3a	gb|AIXA01016332.1|:3136-3410
MsHel-3a	gb|AIXA01015378.1|:7335-7581
MsHel-3a	gb|AIXA01015309.1|:3785-4051
MsHel-3a	gb|AIXA01013702.1|:2395-2672
MsHel-3a	gb|AIXA01012002.1|:10393-10656
MsHel-3a	gb|AIXA01010522.1|:13388-13603
MsHel-3a	gb|AIXA01010427.1|:15358-15644
MsHel-3a	gb|AIXA01010323.1|:96491-96769
MsHel-3a	gb|AIXA01009582.1|:15109-15377
MsHel-3a	gb|AIXA01009484.1|:17126-17366
MsHel-3a	gb|AIXA01007946.1|:37725-37987
MsHel-3a	gb|AIXA01007692.1|:21630-21900
MsHel-3a	gb|AIXA01006651.1|:21062-21324
MsHel-3a	gb|AIXA01005955.1|:1356-1613
MsHel-3a	gb|AIXA01005776.1|:4279-4537
MsHel-3a	gb|AIXA01005766.1|:36977-37215
MsHel-3a	gb|AIXA01005107.1|:9180-9422
MsHel-3a	gb|AIXA01004964.1|:2505-2747
MsHel-3a	gb|AIXA01004952.1|:42458-42738
MsHel-3a	gb|AIXA01004546.1|:46563-46777
MsHel-3a	gb|AIXA01004395.1|:4806-5067
MsHel-3a	gb|AIXA01003699.1|:11787-12043
MsHel-3a	gb|AIXA01003371.1|:11325-11611
MsHel-3a	gb|AIXA01002883.1|:1453-1532
MsHel-3a	gb|AIXA01002614.1|:74244-75180
MsHel-3a	gb|AIXA01002459.1|:29464-37198
MsHel-3a	gb|AIXA01002395.1|:1545-1779
MsHel-3a	gb|AIXA01002352.1|:23338-23586
MsHel-3a	gb|AIXA01001584.1|:3151-3388
MsHel-3a	gb|AIXA01001043.1|:45491-45728
MsHel-3a	gb|AIXA01019945.1|:2472-2771

MsHel-3a	gb|AIXA01018738.1|:1770-2034
MsHel-3a	gb|AIXA01018591.1|:40-193
MsHel-3a	gb|AIXA01018590.1|:40-193
MsHel-3a	gb|AIXA01018242.1|:132-412
MsHel-3a	gb|AIXA01018191.1|:48-298
MsHel-3a	gb|AIXA01017698.1|:3267-3543
MsHel-3a	gb|AIXA01017114.1|:7021-7302
MsHel-3a	gb|AIXA01015867.1|:1950-2232
MsHel-3a	gb|AIXA01015808.1|:7182-7426
MsHel-3a	gb|AIXA01015222.1|:403-636
MsHel-3a	gb|AIXA01013013.1|:18110-22908
MsHel-3a	gb|AIXA01012796.1|:29737-29951
MsHel-3a	gb|AIXA01012386.1|:20848-21441
MsHel-3a	gb|AIXA01011947.1|:5804-6085
MsHel-3a	gb|AIXA01011783.1|:2626-2866
MsHel-3a	gb|AIXA01011197.1|:83365-83603
MsHel-3a	gb|AIXA01011197.1|:50849-51137
MsHel-3a	gb|AIXA01010077.1|:50625-50868
MsHel-3a	gb|AIXA01009267.1|:29909-30199
MsHel-3a	gb|AIXA01007853.1|:64017-64261
MsHel-3a	gb|AIXA01007237.1|:25895-26164
MsHel-3a	gb|AIXA01006854.1|:54906-55166
MsHel-3a	gb|AIXA01006854.1|:21862-22104
MsHel-3a	gb|AIXA01005509.1|:25004-25238
MsHel-3a	gb|AIXA01004940.1|:24523-24769
MsHel-3a	gb|AIXA01004627.1|:12148-12431
MsHel-3a	gb|AIXA01004113.1|:82653-82933
MsHel-3a	gb|AIXA01003900.1|:30485-30775
MsHel-3a	gb|AIXA01003747.1|:77345-77517
MsHel-3a	gb|AIXA01003566.1|:8771-9009
MsHel-3a	gb|AIXA01003365.1|:66322-66414
MsHel-3a	gb|AIXA01002733.1|:2096-2380
MsHel-3a	gb|AIXA01002389.1|:3644-3920
MsHel-3a	gb|AIXA01002011.1|:5955-6195
MsHel-3a	gb|AIXA01001971.1|:20572-20815
MsHel-3a	gb|AIXA01001924.1|:3108-3386
MsHel-3a	gb|AIXA01001634.1|:16672-16905
MsHel-3a	gb|AIXA01001384.1|:18205-18468
MsHel-3a	gb|AIXA01000553.1|:10068-10309
MsHel-3a	gb|AIXA01000460.1|:15008-15298
MsHel-3a	gb|AIXA01000416.1|:19651-19920
MsHel-3a	gb|AIXA01000102.1|:40632-40906
MsHel-3a	gb|AIXA01000102.1|:5669-11796
MsHel-3a	gb|AIXA01028201.1|:101-390
MsHel-3a	gb|AIXA01027946.1|:626-862
MsHel-3a	gb|AIXA01018111.1|:1044-1288
MsHel-3a	gb|AIXA01017092.1|:7966-8209
MsHel-3a	gb|AIXA01016453.1|:23493-23722

MsHel-3a	gb|AIXA01016159.1|:832-1123
MsHel-3a	gb|AIXA01013645.1|:4605-4829
MsHel-3a	gb|AIXA01013643.1|:3188-3436
MsHel-3a	gb|AIXA01011981.1|:1963-2243
MsHel-3a	gb|AIXA01010069.1|:5727-6023
MsHel-3a	gb|AIXA01008543.1|:1484-1728
MsHel-3a	gb|AIXA01007882.1|:4062-4420
MsHel-3a	gb|AIXA01006837.1|:17460-17679
MsHel-3a	gb|AIXA01005485.1|:40381-40595
MsHel-3a	gb|AIXA01005095.1|:67477-67721
MsHel-3a	gb|AIXA01005095.1|:19479-19758
MsHel-3a	gb|AIXA01004706.1|:944-1235
MsHel-3a	gb|AIXA01003634.1|:75768-76014
MsHel-3a	gb|AIXA01002714.1|:12317-12531
MsHel-3a	gb|AIXA01002579.1|:104651-104914
MsHel-3a	gb|AIXA01002014.1|:5854-6139
MsHel-3a	gb|AIXA01001500.1|:1390-1492
MsHel-3a	gb|AIXA01000970.1|:2-222
MsHel-3a	gb|AIXA01000407.1|:8073-8337
MsHel-3a	gb|AIXA01037886.1|:1-237
MsHel-3a	gb|AIXA01027191.1|:112-230
MsHel-3a	gb|AIXA01021793.1|:1623-1868
MsHel-3a	gb|AIXA01020464.1|:2446-2564
MsHel-3a	gb|AIXA01019267.1|:2152-2240
MsHel-3a	gb|AIXA01017201.1|:1674-1792
MsHel-3a	gb|AIXA01015902.1|:3556-3801
MsHel-3a	gb|AIXA01015256.1|:3766-4010
MsHel-3a	gb|AIXA01014004.1|:2292-2537
MsHel-3a	gb|AIXA01013733.1|:1321-1520
MsHel-3a	gb|AIXA01012473.1|:4921-5165
MsHel-3a	gb|AIXA01011937.1|:3612-3859
MsHel-3a	gb|AIXA01010969.1|:15954-16195
MsHel-3a	gb|AIXA01010881.1|:31190-31466
MsHel-3a	gb|AIXA01010696.1|:5642-5916
MsHel-3a	gb|AIXA01009182.1|:17773-18048
MsHel-3a	gb|AIXA01007876.1|:3179-3444
MsHel-3a	gb|AIXA01007245.1|:4286-4571
MsHel-3a	gb|AIXA01006682.1|:8061-8338
MsHel-3a	gb|AIXA01006260.1|:3698-3933
MsHel-3a	gb|AIXA01005318.1|:23953-24216
MsHel-3a	gb|AIXA01005318.1|:9263-9535
MsHel-3a	gb|AIXA01005140.1|:23963-24206
MsHel-3a	gb|AIXA01004717.1|:1634-1875
MsHel-3a	gb|AIXA01004647.1|:16392-16635
MsHel-3a	gb|AIXA01004628.1|:847-1113
MsHel-3a	gb|AIXA01004527.1|:65484-65727
MsHel-3a	gb|AIXA01004058.1|:17023-17262
MsHel-3a	gb|AIXA01003991.1|:12395-12668

MsHel-3a	gb|AIXA01003845.1|:6724-7003
MsHel-3a	gb|AIXA01003628.1|:5694-5937
MsHel-3a	gb|AIXA01003204.1|:12756-12973
MsHel-3a	gb|AIXA01003170.1|:34282-34524
MsHel-3a	gb|AIXA01003022.1|:23056-23340
MsHel-3a	gb|AIXA01002288.1|:43080-43363
MsHel-3a	gb|AIXA01002056.1|:2624-2725
MsHel-3a	gb|AIXA01001841.1|:7270-7503
MsHel-3a	gb|AIXA01001730.1|:38024-38309
MsHel-3a	gb|AIXA01001676.1|:10877-11132
MsHel-3a	gb|AIXA01001657.1|:227437-227680
MsHel-3a	gb|AIXA01001515.1|:11287-11518
MsHel-3a	gb|AIXA01001330.1|:36564-36832
MsHel-3a	gb|AIXA01001235.1|:80170-80452
MsHel-3a	gb|AIXA01000928.1|:16080-16315
MsHel-3a	gb|AIXA01000662.1|:131491-131736
MsHel-3a	gb|AIXA01000437.1|:78078-78311
MsHel-3a	gb|AIXA01000071.1|:3674-3958
MsHel-3a	gb|AIXA01030785.1|:211-469
MsHel-3a	gb|AIXA01030255.1|:161-395
MsHel-3a	gb|AIXA01029325.1|:304-554
MsHel-3a	gb|AIXA01027572.1|:693-935
MsHel-3a	gb|AIXA01027478.1|:932-1031
MsHel-3a	gb|AIXA01024796.1|:83-363
MsHel-3a	gb|AIXA01024518.1|:1156-1255
MsHel-3a	gb|AIXA01018662.1|:7964-8206
MsHel-3a	gb|AIXA01015245.1|:18219-18454
MsHel-3a	gb|AIXA01012618.1|:27205-27472
MsHel-3a	gb|AIXA01011563.1|:7971-8223
MsHel-3a	gb|AIXA01011524.1|:2301-2561
MsHel-3a	gb|AIXA01010687.1|:25408-25648
MsHel-3a	gb|AIXA01007736.1|:2184-2440
MsHel-3a	gb|AIXA01007076.1|:37262-37524
MsHel-3a	gb|AIXA01006143.1|:18625-18897
MsHel-3a	gb|AIXA01005828.1|:7573-7830
MsHel-3a	gb|AIXA01003997.1|:51861-52098
MsHel-3a	gb|AIXA01002782.1|:22644-22886
MsHel-3a	gb|AIXA01002295.1|:6881-7449
MsHel-3a	gb|AIXA01001581.1|:105-197
MsHel-3a	gb|AIXA01000757.1|:6996-7283
MsHel-3a	gb|AIXA01036841.1|:285-525
MsHel-3a	gb|AIXA01036226.1|:1-222
MsHel-3a	gb|AIXA01025319.1|:29-147
MsHel-3a	gb|AIXA01022837.1|:69-186
MsHel-3a	gb|AIXA01022525.1|:798-1041
MsHel-3a	gb|AIXA01022498.1|:1295-1579
MsHel-3a	gb|AIXA01018931.1|:306-544
MsHel-3a	gb|AIXA01018729.1|:1439-1707

MsHel-3a	gb|AIXA01018498.1|:1322-1612
MsHel-3a	gb|AIXA01018301.1|:761-975
MsHel-3a	gb|AIXA01017731.1|:6272-6514
MsHel-3a	gb|AIXA01017654.1|:1312-1597
MsHel-3a	gb|AIXA01015591.1|:672-891
MsHel-3a	gb|AIXA01014690.1|:3177-3443
MsHel-3a	gb|AIXA01013818.1|:19215-19479
MsHel-3a	gb|AIXA01012197.1|:20264-20548
MsHel-3a	gb|AIXA01011744.1|:3453-3690
MsHel-3a	gb|AIXA01011658.1|:847-1132
MsHel-3a	gb|AIXA01011628.1|:3270-3514
MsHel-3a	gb|AIXA01011388.1|:1455-1572
MsHel-3a	gb|AIXA01009707.1|:14149-14415
MsHel-3a	gb|AIXA01009532.1|:37342-37622
MsHel-3a	gb|AIXA01009478.1|:2162-2249
MsHel-3a	gb|AIXA01009219.1|:23384-23643
MsHel-3a	gb|AIXA01007694.1|:10255-10369
MsHel-3a	gb|AIXA01007179.1|:11698-11939
MsHel-3a	gb|AIXA01006594.1|:502-760
MsHel-3a	gb|AIXA01006472.1|:12777-13013
MsHel-3a	gb|AIXA01006191.1|:34586-34926
MsHel-3a	gb|AIXA01006150.1|:12105-12383
MsHel-3a	gb|AIXA01005881.1|:12973-13174
MsHel-3a	gb|AIXA01005484.1|:22633-22938
MsHel-3a	gb|AIXA01005484.1|:4534-4810
MsHel-3a	gb|AIXA01005153.1|:2572-2690
MsHel-3a	gb|AIXA01005091.1|:732-967
MsHel-3a	gb|AIXA01004399.1|:29287-29547
MsHel-3a	gb|AIXA01003798.1|:23559-23788
MsHel-3a	gb|AIXA01003607.1|:15526-15782
MsHel-3a	gb|AIXA01002294.1|:8942-9184
MsHel-3a	gb|AIXA01002215.1|:73676-73788
MsHel-3a	gb|AIXA01002049.1|:3382-3661
MsHel-3a	gb|AIXA01002023.1|:12086-12375
MsHel-3a	gb|AIXA01001569.1|:27540-28125
MsHel-3a	gb|AIXA01001569.1|:1616-1838
MsHel-3a	gb|AIXA01001516.1|:5485-5716
MsHel-3a	gb|AIXA01000929.1|:7711-7817
MsHel-3a	gb|AIXA01000634.1|:2047-2286
MsHel-3a	gb|AIXA01000432.1|:7925-8153
MsHel-3a	gb|AIXA01000156.1|:16220-16459
MsHel-3a	gb|AIXA01029816.1|:618-852
MsHel-3a	gb|AIXA01028987.1|:42-311
MsHel-3a	gb|AIXA01020465.1|:14-291
MsHel-3a	gb|AIXA01018248.1|:523-814
MsHel-3a	gb|AIXA01016204.1|:4452-4691
MsHel-3a	gb|AIXA01015221.1|:9868-10145
MsHel-3a	gb|AIXA01014572.1|:3212-3401

MsHel-3a	gb|AIXA01014013.1|:875-1147
MsHel-3a	gb|AIXA01012500.1|:7357-7630
MsHel-3a	gb|AIXA01011724.1|:27306-27662
MsHel-3a	gb|AIXA01011366.1|:4617-4860
MsHel-3a	gb|AIXA01010870.1|:7726-7968
MsHel-3a	gb|AIXA01009103.1|:4535-4777
MsHel-3a	gb|AIXA01008967.1|:5604-5849
MsHel-3a	gb|AIXA01007719.1|:2673-2878
MsHel-3a	gb|AIXA01007175.1|:101834-102120
MsHel-3a	gb|AIXA01007175.1|:67014-67247
MsHel-3a	gb|AIXA01005578.1|:78717-78955
MsHel-3a	gb|AIXA01005337.1|:25943-26217
MsHel-3a	gb|AIXA01005321.1|:38774-39021
MsHel-3a	gb|AIXA01003355.1|:38640-38871
MsHel-3a	gb|AIXA01001292.1|:33756-34007
MsHel-3a	gb|AIXA01000956.1|:83479-83751
MsHel-3a	gb|AIXA01000559.1|:42918-43037
MsHel-3a	gb|AIXA01000559.1|:7115-7344
MsHel-3a	gb|AIXA01037103.1|:248-472
MsHel-3a	gb|AIXA01033303.1|:310-471
MsHel-3a	gb|AIXA01030186.1|:636-852
MsHel-3a	gb|AIXA01023853.1|:177-415
MsHel-3a	gb|AIXA01021542.1|:1356-1596
MsHel-3a	gb|AIXA01019312.1|:492-696
MsHel-3a	gb|AIXA01019039.1|:1917-2179
MsHel-3a	gb|AIXA01018884.1|:3454-3717
MsHel-3a	gb|AIXA01018409.1|:2462-2662
MsHel-3a	gb|AIXA01017237.1|:1670-1965
MsHel-3a	gb|AIXA01017093.1|:3166-3450
MsHel-3a	gb|AIXA01015947.1|:99-364
MsHel-3a	gb|AIXA01015114.1|:9949-10189
MsHel-3a	gb|AIXA01014305.1|:8185-8437
MsHel-3a	gb|AIXA01014037.1|:38831-39068
MsHel-3a	gb|AIXA01013767.1|:347-576
MsHel-3a	gb|AIXA01013103.1|:18044-18265
MsHel-3a	gb|AIXA01012305.1|:2944-3179
MsHel-3a	gb|AIXA01012194.1|:8410-8650
MsHel-3a	gb|AIXA01011712.1|:911-1149
MsHel-3a	gb|AIXA01011657.1|:15247-15530
MsHel-3a	gb|AIXA01011331.1|:3949-4170
MsHel-3a	gb|AIXA01011295.1|:30317-31329
MsHel-3a	gb|AIXA01011295.1|:3842-4083
MsHel-3a	gb|AIXA01011285.1|:92315-92550
MsHel-3a	gb|AIXA01011285.1|:55265-55497
MsHel-3a	gb|AIXA01011285.1|:37915-38153
MsHel-3a	gb|AIXA01009765.1|:36550-36786
MsHel-3a	gb|AIXA01009508.1|:44730-45013
MsHel-3a	gb|AIXA01008922.1|:8623-8856

MsHel-3a	gb|AIXA01008078.1|:9240-9542
MsHel-3a	gb|AIXA01007974.1|:88501-88767
MsHel-3a	gb|AIXA01007898.1|:18806-19048
MsHel-3a	gb|AIXA01007033.1|:2036-2264
MsHel-3a	gb|AIXA01004841.1|:49930-50215
MsHel-3a	gb|AIXA01004307.1|:42803-43044
MsHel-3a	gb|AIXA01003612.1|:4821-5050
MsHel-3a	gb|AIXA01003281.1|:4512-4756
MsHel-3a	gb|AIXA01003190.1|:7160-7441
MsHel-3a	gb|AIXA01002775.1|:43586-43873
MsHel-3a	gb|AIXA01002525.1|:24875-25138
MsHel-3a	gb|AIXA01002384.1|:40109-40398
MsHel-3a	gb|AIXA01002309.1|:24967-25211
MsHel-3a	gb|AIXA01002309.1|:1797-2034
MsHel-3a	gb|AIXA01002249.1|:17330-17568
MsHel-3a	gb|AIXA01002072.1|:1194-1467
MsHel-3a	gb|AIXA01002012.1|:1387-1662
MsHel-3a	gb|AIXA01001545.1|:31-331
MsHel-3a	gb|AIXA01001151.1|:42239-43600
MsHel-3a	gb|AIXA01000947.1|:29103-29347
MsHel-3a	gb|AIXA01000713.1|:104500-104736
MsHel-3a	gb|AIXA01000043.1|:4646-4931
MsHel-3a	gb|AIXA01018543.1|:7846-8139
MsHel-3a	gb|AIXA01016296.1|:130-366
MsHel-3a	gb|AIXA01015800.1|:19767-20004
MsHel-3a	gb|AIXA01014391.1|:3492-3757
MsHel-3a	gb|AIXA01013935.1|:4111-4380
MsHel-3a	gb|AIXA01013137.1|:17933-18165
MsHel-3a	gb|AIXA01012455.1|:3730-3847
MsHel-3a	gb|AIXA01012099.1|:8974-9188
MsHel-3a	gb|AIXA01011637.1|:29558-29828
MsHel-3a	gb|AIXA01011076.1|:4681-4963
MsHel-3a	gb|AIXA01009663.1|:5094-5332
MsHel-3a	gb|AIXA01009402.1|:21527-21778
MsHel-3a	gb|AIXA01008423.1|:53059-53298
MsHel-3a	gb|AIXA01006529.1|:54230-54469
MsHel-3a	gb|AIXA01006199.1|:6815-7040
MsHel-3a	gb|AIXA01004586.1|:41257-41371
MsHel-3a	gb|AIXA01004412.1|:5938-6250
MsHel-3a	gb|AIXA01004357.1|:13195-13427
MsHel-3a	gb|AIXA01003857.1|:13641-13879
MsHel-3a	gb|AIXA01003818.1|:5746-5973
MsHel-3a	gb|AIXA01002195.1|:35762-35979
MsHel-3a	gb|AIXA01001896.1|:33725-33952
MsHel-3a	gb|AIXA01001671.1|:43021-43291
MsHel-3a	gb|AIXA01001553.1|:3160-3441
MsHel-3a	gb|AIXA01001529.1|:2093-2354
MsHel-3a	gb|AIXA01001448.1|:989-1217

MsHel-3a	gb|AIXA01029522.1|:376-622
MsHel-3a	gb|AIXA01025088.1|:922-1207
MsHel-3a	gb|AIXA01020697.1|:78-313
MsHel-3a	gb|AIXA01018631.1|:1640-1880
MsHel-3a	gb|AIXA01018473.1|:718-830
MsHel-3a	gb|AIXA01015117.1|:2968-3051
MsHel-3a	gb|AIXA01014520.1|:1776-2016
MsHel-3a	gb|AIXA01014317.1|:2869-3080
MsHel-3a	gb|AIXA01014304.1|:2525-2813
MsHel-3a	gb|AIXA01014099.1|:1200-1318
MsHel-3a	gb|AIXA01013782.1|:1786-2030
MsHel-3a	gb|AIXA01013681.1|:17001-17279
MsHel-3a	gb|AIXA01012572.1|:47542-47784
MsHel-3a	gb|AIXA01009380.1|:9156-9427
MsHel-3a	gb|AIXA01009163.1|:46831-47113
MsHel-3a	gb|AIXA01009006.1|:12630-12864
MsHel-3a	gb|AIXA01008715.1|:7134-7377
MsHel-3a	gb|AIXA01006659.1|:7644-7918
MsHel-3a	gb|AIXA01005250.1|:20393-21321
MsHel-3a	gb|AIXA01004787.1|:16323-16556
MsHel-3a	gb|AIXA01003812.1|:14628-14874
MsHel-3a	gb|AIXA01002680.1|:32093-32337
MsHel-3a	gb|AIXA01000827.1|:103926-104172
MsHel-3a	gb|AIXA01023557.1|:21-137
MsHel-3a	gb|AIXA01021152.1|:135-369
MsHel-3a	gb|AIXA01019581.1|:6017-6280
MsHel-3a	gb|AIXA01018792.1|:3172-3442
MsHel-3a	gb|AIXA01017992.1|:5975-6208
MsHel-3a	gb|AIXA01017554.1|:959-1764
MsHel-3a	gb|AIXA01015919.1|:3318-3554
MsHel-3a	gb|AIXA01015704.1|:4076-4313
MsHel-3a	gb|AIXA01013014.1|:188-420
MsHel-3a	gb|AIXA01012288.1|:13849-14117
MsHel-3a	gb|AIXA01009895.1|:2571-2807
MsHel-3a	gb|AIXA01008492.1|:85394-85639
MsHel-3a	gb|AIXA01007543.1|:32699-32944
MsHel-3a	gb|AIXA01007346.1|:15126-15420
MsHel-3a	gb|AIXA01003874.1|:10076-10353
MsHel-3a	gb|AIXA01002452.1|:47732-47970
MsHel-3a	gb|AIXA01001349.1|:25065-25326
MsHel-3a	gb|AIXA01000549.1|:2836-3110
MsHel-3a	gb|AIXA01000269.1|:54254-54519
MsHel-3a	gb|AIXA01037931.1|:1-237
MsHel-3a	gb|AIXA01036780.1|:144-359
MsHel-3a	gb|AIXA01024822.1|:429-716
MsHel-3a	gb|AIXA01024370.1|:347-559
MsHel-3a	gb|AIXA01015887.1|:8483-8776
MsHel-3a	gb|AIXA01014219.1|:25529-25772

MsHel-3a	gb|AIXA01013622.1|:5871-6114
MsHel-3a	gb|AIXA01012959.1|:16021-16300
MsHel-3a	gb|AIXA01011494.1|:15235-15475
MsHel-3a	gb|AIXA01010590.1|:49027-49764
MsHel-3a	gb|AIXA01010215.1|:13702-13949
MsHel-3a	gb|AIXA01009131.1|:3133-3421
MsHel-3a	gb|AIXA01009082.1|:2789-3006
MsHel-3a	gb|AIXA01007706.1|:35577-35795
MsHel-3a	gb|AIXA01007706.1|:21089-21208
MsHel-3a	gb|AIXA01007050.1|:21519-21637
MsHel-3a	gb|AIXA01006904.1|:13249-13926
MsHel-3a	gb|AIXA01006256.1|:20800-21047
MsHel-3a	gb|AIXA01005642.1|:35649-35761
MsHel-3a	gb|AIXA01005100.1|:3349-3603
MsHel-3a	gb|AIXA01004591.1|:10616-16412
MsHel-3a	gb|AIXA01003624.1|:11285-11568
MsHel-3a	gb|AIXA01003344.1|:5032-5308
MsHel-3a	gb|AIXA01002759.1|:23663-23742
MsHel-3a	gb|AIXA01001856.1|:4729-4971
MsHel-3a	gb|AIXA01001660.1|:57077-57351
MsHel-3a	gb|AIXA01001530.1|:179-448
MsHel-3a	gb|AIXA01000846.1|:30260-30533
MsHel-3a	gb|AIXA01000728.1|:42881-43119
MsHel-3a	gb|AIXA01000346.1|:671-916
MsHel-3a	gb|AIXA01000072.1|:135296-135541
MsHel-3a	gb|AIXA01000040.1|:39368-39577
MsHel-3a	gb|AIXA01031959.1|:564-736
MsHel-3a	gb|AIXA01019719.1|:2715-2953
MsHel-3a	gb|AIXA01018060.1|:286-504
MsHel-3a	gb|AIXA01016551.1|:27628-27862
MsHel-3a	gb|AIXA01016068.1|:5902-6187
MsHel-3a	gb|AIXA01013680.1|:25622-25738
MsHel-3a	gb|AIXA01013680.1|:6108-6350
MsHel-3a	gb|AIXA01012809.1|:3270-3481
MsHel-3a	gb|AIXA01009572.1|:2247-2356
MsHel-3a	gb|AIXA01009504.1|:41686-41795
MsHel-3a	gb|AIXA01008595.1|:2141-2354
MsHel-3a	gb|AIXA01007713.1|:2114-2380
MsHel-3a	gb|AIXA01007260.1|:18969-19239
MsHel-3a	gb|AIXA01006864.1|:1225-1461
MsHel-3a	gb|AIXA01004835.1|:2560-2802
MsHel-3a	gb|AIXA01004775.1|:16500-16789
MsHel-3a	gb|AIXA01004360.1|:28799-29042
MsHel-3a	gb|AIXA01003559.1|:21064-21265
MsHel-3a	gb|AIXA01003042.1|:230806-231086
MsHel-3a	gb|AIXA01002972.1|:58585-58825
MsHel-3a	gb|AIXA01002412.1|:2782-3030
MsHel-3a	gb|AIXA01001444.1|:8625-8885

MsHel-3a	gb|AIXA01001252.1|:265-487
MsHel-3a	gb|AIXA01000641.1|:19284-19528
MsHel-3a	gb|AIXA01000048.1|:75676-75929
MsHel-3a	gb|AIXA01025591.1|:96-214
MsHel-3a	gb|AIXA01024964.1|:626-885
MsHel-3a	gb|AIXA01022226.1|:1-175
MsHel-3a	gb|AIXA01015866.1|:1-225
MsHel-3a	gb|AIXA01012095.1|:18-138
MsHel-3a	gb|AIXA01010297.1|:691-918
MsHel-3a	gb|AIXA01009658.1|:3543-3789
MsHel-3a	gb|AIXA01008194.1|:5286-5484
MsHel-3a	gb|AIXA01008083.1|:17396-17680
MsHel-3a	gb|AIXA01007108.1|:372-598
MsHel-3a	gb|AIXA01006514.1|:3150-3383
MsHel-3a	gb|AIXA01006135.1|:6768-7000
MsHel-3a	gb|AIXA01005041.1|:7629-7905
MsHel-3a	gb|AIXA01003893.1|:25094-25363
MsHel-3a	gb|AIXA01003463.1|:12805-13045
MsHel-3a	gb|AIXA01002194.1|:56201-56428
MsHel-3a	gb|AIXA01001833.1|:7230-7309
MsHel-3a	gb|AIXA01000594.1|:67305-67564
MsHel-3a	gb|AIXA01030141.1|:590-834
MsHel-3a	gb|AIXA01018166.1|:4915-5197
MsHel-3a	gb|AIXA01017311.1|:138-420
MsHel-3a	gb|AIXA01016606.1|:3772-4037
MsHel-3a	gb|AIXA01016540.1|:26-134
MsHel-3a	gb|AIXA01014210.1|:16843-17136
MsHel-3a	gb|AIXA01013058.1|:1128-1367
MsHel-3a	gb|AIXA01012853.1|:14978-15213
MsHel-3a	gb|AIXA01010786.1|:30896-31179
MsHel-3a	gb|AIXA01007663.1|:74074-74308
MsHel-3a	gb|AIXA01004391.1|:31531-32161
MsHel-3a	gb|AIXA01004391.1|:8548-8666
MsHel-3a	gb|AIXA01001685.1|:56458-56564
MsHel-3a	gb|AIXA01001255.1|:13749-13988
MsHel-3a	gb|AIXA01001221.1|:44258-44480
MsHel-3a	gb|AIXA01000707.1|:25526-25812
MsHel-3a	gb|AIXA01000037.1|:8063-8357
MsHel-3a	gb|AIXA01030055.1|:151-432
MsHel-3a	gb|AIXA01019030.1|:3250-3477
MsHel-3a	gb|AIXA01017770.1|:3446-3719
MsHel-3a	gb|AIXA01014092.1|:8279-8522
MsHel-3a	gb|AIXA01011732.1|:33356-33636
MsHel-3a	gb|AIXA01010068.1|:6044-6318
MsHel-3a	gb|AIXA01009710.1|:20802-21044
MsHel-3a	gb|AIXA01008719.1|:3211-3300
MsHel-3a	gb|AIXA01008185.1|:7736-8022
MsHel-3a	gb|AIXA01007920.1|:15032-15295

MsHel-3a	gb|AIXA01007614.1|:21607-21816
MsHel-3a	gb|AIXA01007553.1|:4907-5111
MsHel-3a	gb|AIXA01005096.1|:58475-58712
MsHel-3a	gb|AIXA01004909.1|:72510-72754
MsHel-3a	gb|AIXA01003616.1|:5064-5146
MsHel-3a	gb|AIXA01003189.1|:45629-45909
MsHel-3a	gb|AIXA01001290.1|:9256-9478
MsHel-3a	gb|AIXA01000766.1|:656-744
MsHel-3a	gb|AIXA01022951.1|:981-1268
MsHel-3a	gb|AIXA01015853.1|:992-1258
MsHel-3a	gb|AIXA01014448.1|:2622-2737
MsHel-3a	gb|AIXA01014126.1|:1210-1496
MsHel-3a	gb|AIXA01013623.1|:20126-20402
MsHel-3a	gb|AIXA01010800.1|:10458-10694
MsHel-3a	gb|AIXA01010367.1|:16139-16367
MsHel-3a	gb|AIXA01009134.1|:43204-43319
MsHel-3a	gb|AIXA01007460.1|:76607-76798
MsHel-3a	gb|AIXA01007024.1|:48445-48683
MsHel-3a	gb|AIXA01007024.1|:12382-12640
MsHel-3a	gb|AIXA01005768.1|:35572-35688
MsHel-3a	gb|AIXA01003677.1|:20150-20382
MsHel-3a	gb|AIXA01024895.1|:76-190
MsHel-3a	gb|AIXA01017841.1|:5956-6190
MsHel-3a	gb|AIXA01016641.1|:151-312
MsHel-3a	gb|AIXA01015736.1|:6476-6745
MsHel-3a	gb|AIXA01013213.1|:605-867
MsHel-3a	gb|AIXA01008054.1|:22631-22910
MsHel-3a	gb|AIXA01007068.1|:9924-10165
MsHel-3a	gb|AIXA01004817.1|:27125-27238
MsHel-3a	gb|AIXA01004693.1|:75922-76155
MsHel-3a	gb|AIXA01004100.1|:5009-5215
MsHel-3a	gb|AIXA01001549.1|:18102-18359
MsHel-3a	gb|AIXA01000289.1|:5885-6169
MsHel-3a	gb|AIXA01024841.1|:1043-1283
MsHel-3a	gb|AIXA01019420.1|:1645-1884
MsHel-3a	gb|AIXA01014780.1|:5567-5848
MsHel-3a	gb|AIXA01005280.1|:25053-25306
MsHel-3a	gb|AIXA01005089.1|:20699-20986
MsHel-3a	gb|AIXA01005077.1|:4505-4595
MsHel-3a	gb|AIXA01004481.1|:40172-40452
MsHel-3a	gb|AIXA01004350.1|:3657-3895
MsHel-3a	gb|AIXA01001813.1|:35020-35291
MsHel-3a	gb|AIXA01029476.1|:651-741
MsHel-3a	gb|AIXA01028596.1|:524-639
MsHel-3a	gb|AIXA01017852.1|:873-1243
MsHel-3a	gb|AIXA01010758.1|:31758-32032
MsHel-3a	gb|AIXA01007790.1|:49905-50154
MsHel-3a	gb|AIXA01004296.1|:13887-14124

MsHel-3a	gb|AIXA01002113.1|:26958-27244
MsHel-3a	gb|AIXA01025823.1|:932-1167
MsHel-3a	gb|AIXA01019162.1|:853-1060
MsHel-3a	gb|AIXA01006868.1|:14375-14621
MsHel-3a	gb|AIXA01005685.1|:8707-8966
MsHel-3a	gb|AIXA01003895.1|:59846-59935
MsHel-3a	gb|AIXA01001707.1|:49296-49523
MsHel-3a	gb|AIXA01000762.1|:16206-16434
MsHel-3a	gb|AIXA01018221.1|:8357-8514
MsHel-3a	gb|AIXA01011346.1|:1253-1519
MsHel-3a	gb|AIXA01009405.1|:39046-39244
MsHel-3a	gb|AIXA01003807.1|:5102-5185
MsHel-3a	gb|AIXA01028778.1|:744-927
MsHel-3a	gb|AIXA01015527.1|:13188-13388
MsHel-3a	gb|AIXA01007676.1|:18845-19067
MsHel-3a	gb|AIXA01006681.1|:29599-29839
MsHel-3a	gb|AIXA01006243.1|:3207-3289
MsHel-3a	gb|AIXA01005257.1|:16138-16349
MsHel-3a	gb|AIXA01002723.1|:51066-51306
MsHel-3a	gb|AIXA01002550.1|:7595-7822
MsHel-3a	gb|AIXA01000130.1|:1003-1226
MsHel-3a	gb|AIXA01017553.1|:935-1667
MsHel-3a	gb|AIXA01014596.1|:10286-10520
MsHel-3a	gb|AIXA01009118.1|:616-856
MsHel-3a	gb|AIXA01006734.1|:1368-1623
MsHel-3a	gb|AIXA01006615.1|:2295-2506
MsHel-3a	gb|AIXA01006471.1|:10204-10320
MsHel-3a	gb|AIXA01005138.1|:213-444
MsHel-3a	gb|AIXA01000021.1|:44813-45071
MsHel-3a	gb|AIXA01016420.1|:11402-11608
MsHel-3a	gb|AIXA01005563.1|:50710-50917
MsHel-3a	gb|AIXA01007071.1|:19179-19383
MsHel-3a	gb|AIXA01002777.1|:9149-9762
MsHel-3a	gb|AIXA01000205.1|:6608-6806
MsHel-3a	gb|AIXA01015491.1|:1589-1670
MsHel-3a	gb|AIXA01010551.1|:17625-17911
MsHel-3a	gb|AIXA01006124.1|:3097-3324
MsHel-3a	gb|AIXA01001957.1|:194-287
MsHel-3a	gb|AIXA01015192.1|:7218-7328
MsHel-3a	gb|AIXA01012659.1|:7088-7305
MsHel-3a	gb|AIXA01007932.1|:18660-18742
MsHel-3a	gb|AIXA01004589.1|:3218-3300
MsHel-3a	gb|AIXA01004266.1|:9765-9936
MsHel-3a	gb|AIXA01007309.1|:4576-4801
MsHel-3a	gb|AIXA01000099.1|:37596-37676
MsHel-3a	gb|AIXA01008936.1|:5233-5437
MsHel-3a	gb|AIXA01009705.1|:9682-9904
MsHel-3a	gb|AIXA01004896.1|:14193-14287

MsHel-3a	gb|AIXA01000786.1|:1106-1305
MsHel-3a	gb|AIXA01000227.1|:3383-3622
MsHel-3a	gb|AIXA01002690.1|:30757-30984
MsHel-3a	gb|AIXA01002310.1|:13257-13336
MsHel-3a	gb|AIXA01014535.1|:12983-13186
MsHel-3a	gb|AIXA01002046.1|:6-116
MsHel-3a	gb|AIXA01007230.1|:15565-15807
MsHel-3a	gb|AIXA01006207.1|:24648-24890
MsHel-3a	gb|AIXA01003595.1|:17310-17557
MsHel-3a	gb|AIXA01001750.1|:13851-13936
MsHel-3a	gb|AIXA01001847.1|:19581-19720
MsHel-3a	gb|AIXA01005774.1|:45041-45132
MsHel-3a	gb|AIXA01005215.1|:12231-12463
MsHel-3a	gb|AIXA01008638.1|:2005-2084
MsHel-3a	gb|AIXA01017084.1|:2566-2644
MsHel-3a	gb|AIXA01031458.1|:659-752
MsHel-4	gb|AIXA01000440.1|:40928-41464
MsHel-4	gb|AIXA01006025.1|:80578-81123
MsHel-4	gb|AIXA01014960.1|:10702-11236
MsHel-4	gb|AIXA01009590.1|:32523-33067
MsHel-4	gb|AIXA01014224.1|:13287-13832
MsHel-4	gb|AIXA01005095.1|:43690-44234
MsHel-4	gb|AIXA01008252.1|:28667-29205
MsHel-4	gb|AIXA01002612.1|:30966-31499
MsHel-4	gb|AIXA01012976.1|:359-882
MsHel-4	gb|AIXA01012364.1|:16978-17522
MsHel-4	gb|AIXA01004767.1|:24401-24952
MsHel-4	gb|AIXA01014693.1|:1215-1741
MsHel-4	gb|AIXA01001349.1|:32094-38627
MsHel-4	gb|AIXA01022377.1|:124-655
MsHel-4	gb|AIXA01001266.1|:80802-81350
MsHel-4	gb|AIXA01003198.1|:103603-104142
MsHel-4	gb|AIXA01015144.1|:21659-22204
MsHel-4	gb|AIXA01005766.1|:71784-72291
MsHel-4	gb|AIXA01003045.1|:21707-22224
MsHel-4	gb|AIXA01002124.1|:30586-31132
MsHel-4	gb|AIXA01016648.1|:14070-14565
MsHel-4	gb|AIXA01013529.1|:14571-15091
MsHel-4	gb|AIXA01003816.1|:337-750
MsHel-4	gb|AIXA01001176.1|:16961-17452
MsHel-4	gb|AIXA01004829.1|:48906-49340
MsHel-4	gb|AIXA01015151.1|:51493-51961
MsHel-4	gb|AIXA01028010.1|:570-991
MsHel-4	gb|AIXA01010905.1|:12837-13337
MsHel-4	gb|AIXA01002671.1|:47472-48003
MsHel-4	gb|AIXA01007860.1|:6429-6512
MsHel-4	gb|AIXA01002285.1|:3782-4505
MsHel-4	gb|AIXA01021151.1|:206-743

MsHel-4	gb|AIXA01005065.1|:86329-86728
MsHel-4	gb|AIXA01005065.1|:61574-62108
MsHel-4	gb|AIXA01001314.1|:2620-2959
MsHel-4	gb|AIXA01002137.1|:7462-7988
MsHel-4	gb|AIXA01001863.1|:21833-22145
MsHel-4	gb|AIXA01001682.1|:30007-30417
MsHel-4	gb|AIXA01018301.1|:9311-9718
MsHel-4	gb|AIXA01004229.1|:37379-37782
MsHel-4	gb|AIXA01011815.1|:2552-2863
MsHel-4	gb|AIXA01006719.1|:34462-34873
MsHel-4	gb|AIXA01015790.1|:14365-14775
MsHel-4	gb|AIXA01005110.1|:15221-15628
MsHel-4	gb|AIXA01019315.1|:2171-2554
MsHel-4	gb|AIXA01005470.1|:10798-11198
MsHel-4	gb|AIXA01002042.1|:20140-20548
MsHel-4	gb|AIXA01002857.1|:16595-16979
MsHel-4	gb|AIXA01000590.1|:12549-12950
MsHel-4	gb|AIXA01005536.1|:6517-6918
MsHel-4	gb|AIXA01002954.1|:5134-6043
MsHel-4	gb|AIXA01007801.1|:11221-11628
MsHel-4	gb|AIXA01001965.1|:1443-1857
MsHel-4	gb|AIXA01013193.1|:28777-29246
MsHel-4	gb|AIXA01005936.1|:4504-4912
MsHel-4	gb|AIXA01005060.1|:18021-18432
MsHel-4	gb|AIXA01016058.1|:2472-2883
MsHel-4	gb|AIXA01011533.1|:4871-5146
MsHel-4	gb|AIXA01008484.1|:45308-45710
MsHel-4	gb|AIXA01002938.1|:50096-50650
MsHel-4	gb|AIXA01011679.1|:5512-5922
MsHel-4	gb|AIXA01006381.1|:8635-9049
MsHel-4	gb|AIXA01011430.1|:10423-10831
MsHel-4	gb|AIXA01035607.1|:1-256
MsHel-4	gb|AIXA01002524.1|:21884-22276
MsHel-4	gb|AIXA01001239.1|:137221-137628
MsHel-4	gb|AIXA01013733.1|:6870-7267
MsHel-4	gb|AIXA01000034.1|:2772-3189
MsHel-4	gb|AIXA01029490.1|:381-785
MsHel-4	gb|AIXA01006421.1|:6218-6600
MsHel-4	gb|AIXA01009646.1|:727-1138
MsHel-4	gb|AIXA01002427.1|:12809-13313
MsHel-4	gb|AIXA01015691.1|:9772-10041
MsHel-4	gb|AIXA01012375.1|:11892-12207
MsHel-4	gb|AIXA01013383.1|:4766-5172
MsHel-4	gb|AIXA01002420.1|:27612-28083
MsHel-4	gb|AIXA01029849.1|:380-791
MsHel-4	gb|AIXA01020104.1|:2-405
MsHel-4	gb|AIXA01018608.1|:516-791
MsHel-4	gb|AIXA01000234.1|:29462-29876

MsHel-4	gb|AIXA01012654.1|:25147-25544
MsHel-4	gb|AIXA01000953.1|:890-1236
MsHel-4	gb|AIXA01018684.1|:8760-9016
MsHel-4	gb|AIXA01016647.1|:868-1266
MsHel-4	gb|AIXA01015095.1|:697-966
MsHel-4	gb|AIXA01004110.1|:71616-71845
MsHel-4	gb|AIXA01003621.1|:110332-111034
MsHel-4	gb|AIXA01010573.1|:849-1265
MsHel-4	gb|AIXA01008994.1|:1976-2351
MsHel-4	gb|AIXA01003982.1|:42757-43165
MsHel-4	gb|AIXA01006830.1|:11116-11372
MsHel-4	gb|AIXA01000931.1|:64256-64713
MsHel-4	gb|AIXA01029762.1|:223-611
MsHel-4	gb|AIXA01002792.1|:7429-7802
MsHel-4	gb|AIXA01004621.1|:79236-79458
MsHel-4	gb|AIXA01007211.1|:2000-2509
MsHel-4	gb|AIXA01007922.1|:4351-4711
MsHel-4	gb|AIXA01022355.1|:1-389
MsHel-4	gb|AIXA01010776.1|:10780-11117
MsHel-4	gb|AIXA01016540.1|:2392-2784
MsHel-4	gb|AIXA01010394.1|:10992-11345
MsHel-4	gb|AIXA01016179.1|:2517-2753
MsHel-4	gb|AIXA01014142.1|:7157-7378
MsHel-4	gb|AIXA01020079.1|:1083-1450
MsHel-4	gb|AIXA01028099.1|:591-989
MsHel-4	gb|AIXA01002753.1|:79086-79466
MsHel-4	gb|AIXA01008632.1|:30956-31706
MsHel-4	gb|AIXA01017577.1|:1688-1957
MsHel-4	gb|AIXA01011984.1|:64853-65129
MsHel-4	gb|AIXA01006658.1|:49358-49855
MsHel-4	gb|AIXA01004996.1|:39118-39323
MsHel-4	gb|AIXA01009162.1|:1699-2054
MsHel-4	gb|AIXA01008001.1|:7838-8152
MsHel-4	gb|AIXA01001018.1|:6276-6554
MsHel-4	gb|AIXA01016749.1|:3445-3783
MsHel-4	gb|AIXA01014584.1|:13724-14062
MsHel-4	gb|AIXA01011980.1|:16580-16918
MsHel-4	gb|AIXA01011540.1|:34808-35146
MsHel-4	gb|AIXA01010358.1|:6703-7041
MsHel-4	gb|AIXA01009842.1|:2065-2403
MsHel-4	gb|AIXA01009067.1|:53307-53645
MsHel-4	gb|AIXA01008477.1|:86052-86390
MsHel-4	gb|AIXA01008227.1|:34276-34614
MsHel-4	gb|AIXA01008156.1|:12193-12524
MsHel-4	gb|AIXA01008057.1|:552-890
MsHel-4	gb|AIXA01007872.1|:45893-46231
MsHel-4	gb|AIXA01006404.1|:44991-45329
MsHel-4	gb|AIXA01005529.1|:1584-1922

MsHel-4	gb|AIXA01004428.1|:27421-27759
MsHel-4	gb|AIXA01004067.1|:17792-18130
MsHel-4	gb|AIXA01003140.1|:6630-6968
MsHel-4	gb|AIXA01002979.1|:4741-5079
MsHel-4	gb|AIXA01002431.1|:27467-27805
MsHel-4	gb|AIXA01001877.1|:111338-111676
MsHel-4	gb|AIXA01001517.1|:9790-10128
MsHel-4	gb|AIXA01001243.1|:95611-95949
MsHel-4	gb|AIXA01002404.1|:5866-6351
MsHel-4	gb|AIXA01012199.1|:14178-14517
MsHel-4	gb|AIXA01015824.1|:7965-8243
MsHel-4	gb|AIXA01012810.1|:63630-63968
MsHel-4	gb|AIXA01012473.1|:2626-2964
MsHel-4	gb|AIXA01011609.1|:11410-11688
MsHel-4	gb|AIXA01008922.1|:7853-8191
MsHel-4	gb|AIXA01008568.1|:6930-7268
MsHel-4	gb|AIXA01006960.1|:11921-12259
MsHel-4	gb|AIXA01006085.1|:2757-3095
MsHel-4	gb|AIXA01005822.1|:23049-23387
MsHel-4	gb|AIXA01005642.1|:29839-30177
MsHel-4	gb|AIXA01005435.1|:26112-26450
MsHel-4	gb|AIXA01004978.1|:38155-38487
MsHel-4	gb|AIXA01004860.1|:15891-16229
MsHel-4	gb|AIXA01004242.1|:15210-15548
MsHel-4	gb|AIXA01002532.1|:22527-22865
MsHel-4	gb|AIXA01002443.1|:26126-26408
MsHel-4	gb|AIXA01001695.1|:40411-40749
MsHel-4	gb|AIXA01001570.1|:22832-23170
MsHel-4	gb|AIXA01001319.1|:53583-53921
MsHel-4	gb|AIXA01000680.1|:5394-5732
MsHel-4	gb|AIXA01015053.1|:4136-4399
MsHel-4	gb|AIXA01008399.1|:32760-32995
MsHel-4	gb|AIXA01014039.1|:18328-18604
MsHel-4	gb|AIXA01008258.1|:30777-31115
MsHel-4	gb|AIXA01007800.1|:63290-63627
MsHel-4	gb|AIXA01007508.1|:3684-4039
MsHel-4	gb|AIXA01004753.1|:33276-33614
MsHel-4	gb|AIXA01003737.1|:48270-48608
MsHel-4	gb|AIXA01001486.1|:21909-22247
MsHel-4	gb|AIXA01000950.1|:46201-46539
MsHel-4	gb|AIXA01016747.1|:3103-3260
MsHel-4	gb|AIXA01009360.1|:64223-64490
MsHel-4	gb|AIXA01009820.1|:28736-29074
MsHel-4	gb|AIXA01000251.1|:20378-20653
MsHel-4	gb|AIXA01009003.1|:31803-32083
MsHel-4	gb|AIXA01034247.1|:369-647
MsHel-4	gb|AIXA01034018.1|:457-659
MsHel-4	gb|AIXA01004161.1|:25494-25855

MsHel-4	gb|AIXA01016903.1|:3979-4256
MsHel-4	gb|AIXA01019798.1|:3737-3892
MsHel-4	gb|AIXA01009179.1|:105056-105292
MsHel-4	gb|AIXA01005238.1|:6000-6189
MsHel-4	gb|AIXA01004418.1|:946-1140
MsHel-4	gb|AIXA01004342.1|:14715-14965
MsHel-4	gb|AIXA01038334.1|:117-329
MsHel-4	gb|AIXA01003932.1|:42707-42885
MsHel-4	gb|AIXA01034909.1|:502-619
MsHel-4	gb|AIXA01008051.1|:10701-10821
MsHel-4	gb|AIXA01006027.1|:57421-57538
MsHel-4	gb|AIXA01000592.1|:17221-17385
MsHel-4	gb|AIXA01030861.1|:681-814
MsHel-4	gb|AIXA01004742.1|:70234-70337
MsHel-4	gb|AIXA01000078.1|:36693-36796
MsHel-4	gb|AIXA01002662.1|:12446-12704
MsHel-4	gb|AIXA01034962.1|:516-616
MsHel-4	gb|AIXA01015919.1|:11345-11431
MsHel-4	gb|AIXA01002913.1|:20532-20623
MsHel-4	gb|AIXA01009543.1|:14374-14474
MsHel-4	gb|AIXA01034730.1|:547-627
MsHel-4	gb|AIXA01006907.1|:17255-17346
MsHel-4	gb|AIXA01013255.1|:20804-20889
MsHel-4	gb|AIXA01006676.1|:2273-2373
MsHel-4	gb|AIXA01006548.1|:19189-19278
MsHel-4	gb|AIXA01004328.1|:45068-45187
MsHel-4	gb|AIXA01000778.1|:7494-7608
MsHel-4	gb|AIXA01004951.1|:14828-14952
MsHel-4	gb|AIXA01015695.1|:3619-3707
MsHel-5	gb|AIXA01001531.1|:49202-49499
MsHel-5	gb|AIXA01001978.1|:989-1286
MsHel-5	gb|AIXA01006891.1|:57329-57625
MsHel-5	gb|AIXA01006891.1|:44872-45160
MsHel-5	gb|AIXA01006891.1|:14537-14827
MsHel-5	gb|AIXA01004928.1|:17048-17246
MsHel-5	gb|AIXA01005034.1|:10564-10710
MsHel-5	gb|AIXA01000969.1|:8117-8310
MsHel-5	gb|AIXA01000897.1|:6659-6952
MsHel-5	gb|AIXA01007223.1|:16616-16913
MsHel-5	gb|AIXA01005440.1|:11062-11363
MsHel-5	gb|AIXA01012529.1|:43103-43397
MsHel-5	gb|AIXA01009162.1|:198473-198775
MsHel-5	gb|AIXA01009162.1|:167314-167596
MsHel-5	gb|AIXA01009162.1|:52620-52941
MsHel-5	gb|AIXA01009162.1|:33869-34172
MsHel-5	gb|AIXA01000061.1|:26823-27116
MsHel-5	gb|AIXA01010542.1|:26191-26488
MsHel-5	gb|AIXA01004461.1|:9272-9565

MsHel-5	gb|AIXA01001805.1|:240-537
MsHel-5	gb|AIXA01001631.1|:54781-55077
MsHel-5	gb|AIXA01000024.1|:27275-27572
MsHel-5	gb|AIXA01005808.1|:38367-38523
MsHel-5	gb|AIXA01005808.1|:27580-27876
MsHel-5	gb|AIXA01002782.1|:56001-56297
MsHel-5	gb|AIXA01002782.1|:35516-35817
MsHel-5	gb|AIXA01002782.1|:17211-17505
MsHel-5	gb|AIXA01016246.1|:26754-27048
MsHel-5	gb|AIXA01001797.1|:337345-337640
MsHel-5	gb|AIXA01001797.1|:49062-49364
MsHel-5	gb|AIXA01017406.1|:8329-8625
MsHel-5	gb|AIXA01014626.1|:8012-8304
MsHel-5	gb|AIXA01009487.1|:870-1166
MsHel-5	gb|AIXA01019285.1|:684-980
MsHel-5	gb|AIXA01017028.1|:2244-2539
MsHel-5	gb|AIXA01014500.1|:9785-10077
MsHel-5	gb|AIXA01014296.1|:17344-17639
MsHel-5	gb|AIXA01015342.1|:10359-10653
MsHel-5	gb|AIXA01011987.1|:61394-61687
MsHel-5	gb|AIXA01010330.1|:68308-68602
MsHel-5	gb|AIXA01020986.1|:748-1041
MsHel-5	gb|AIXA01004322.1|:22911-23208
MsHel-5	gb|AIXA01004322.1|:8957-9115
MsHel-5	gb|AIXA01008687.1|:29107-29406
MsHel-5	gb|AIXA01000159.1|:17011-17206
MsHel-5	gb|AIXA01010811.1|:7634-7928
MsHel-5	gb|AIXA01000144.1|:27040-27336
MsHel-5	gb|AIXA01018288.1|:490-781
MsHel-5	gb|AIXA01015246.1|:17477-17770
MsHel-5	gb|AIXA01011350.1|:19092-19390
MsHel-5	gb|AIXA01007966.1|:61603-61906
MsHel-5	gb|AIXA01005448.1|:39210-39504
MsHel-5	gb|AIXA01002244.1|:7629-7922
MsHel-5	gb|AIXA01001679.1|:2894-3188
MsHel-5	gb|AIXA01014552.1|:18771-19067
MsHel-5	gb|AIXA01010109.1|:66792-67091
MsHel-5	gb|AIXA01007298.1|:9187-9478
MsHel-5	gb|AIXA01006141.1|:7282-7578
MsHel-5	gb|AIXA01001866.1|:13583-13879
MsHel-5	gb|AIXA01001866.1|:1305-1488
MsHel-5	gb|AIXA01019543.1|:2915-3209
MsHel-5	gb|AIXA01017044.1|:1959-2253
MsHel-5	gb|AIXA01009119.1|:15506-15800
MsHel-5	gb|AIXA01007968.1|:33443-33743
MsHel-5	gb|AIXA01007968.1|:8911-9155
MsHel-5	gb|AIXA01006684.1|:29346-29465
MsHel-5	gb|AIXA01005247.1|:11509-11803

MsHel-5	gb|AIXA01014022.1|:40238-40417
MsHel-5	gb|AIXA01014022.1|:15142-15435
MsHel-5	gb|AIXA01011925.1|:3568-3856
MsHel-5	gb|AIXA01001571.1|:21225-21366
MsHel-5	gb|AIXA01000931.1|:5541-5834
MsHel-5	gb|AIXA01000909.1|:89247-89409
MsHel-5	gb|AIXA01012361.1|:23837-24029
MsHel-5	gb|AIXA01006073.1|:26545-26834
MsHel-5	gb|AIXA01005108.1|:830-1116
MsHel-5	gb|AIXA01004956.1|:15303-15595
MsHel-5	gb|AIXA01029450.1|:308-602
MsHel-5	gb|AIXA01019686.1|:2007-2299
MsHel-5	gb|AIXA01016448.1|:3785-4079
MsHel-5	gb|AIXA01015051.1|:961-1255
MsHel-5	gb|AIXA01009350.1|:32119-32410
MsHel-5	gb|AIXA01009125.1|:9754-10495
MsHel-5	gb|AIXA01002553.1|:40682-40945
MsHel-5	gb|AIXA01011304.1|:31189-31482
MsHel-5	gb|AIXA01003114.1|:20145-20337
MsHel-5	gb|AIXA01003114.1|:1-282
MsHel-5	gb|AIXA01001515.1|:27324-27615
MsHel-5	gb|AIXA01017011.1|:2614-2909
MsHel-5	gb|AIXA01012694.1|:9007-9207
MsHel-5	gb|AIXA01009315.1|:54075-54370
MsHel-5	gb|AIXA01005189.1|:57027-57326
MsHel-5	gb|AIXA01001618.1|:54212-54423
MsHel-5	gb|AIXA01001618.1|:14331-14525
MsHel-5	gb|AIXA01000903.1|:19672-19956
MsHel-5	gb|AIXA01008923.1|:52016-52321
MsHel-5	gb|AIXA01003332.1|:70249-70548
MsHel-5	gb|AIXA01015230.1|:24333-24623
MsHel-5	gb|AIXA01014739.1|:23341-23634
MsHel-5	gb|AIXA01006887.1|:9338-9631
MsHel-5	gb|AIXA01001848.1|:20437-20733
MsHel-5	gb|AIXA01027126.1|:707-1003
MsHel-5	gb|AIXA01014392.1|:2062-2358
MsHel-5	gb|AIXA01012313.1|:216-506
MsHel-5	gb|AIXA01007957.1|:181-465
MsHel-5	gb|AIXA01004486.1|:17820-18122
MsHel-5	gb|AIXA01003353.1|:17287-17579
MsHel-5	gb|AIXA01001330.1|:42222-42512
MsHel-5	gb|AIXA01029751.1|:113-405
MsHel-5	gb|AIXA01014219.1|:2753-3045
MsHel-5	gb|AIXA01013829.1|:6798-7087
MsHel-5	gb|AIXA01002632.1|:10638-10938
MsHel-5	gb|AIXA01012188.1|:22514-22816
MsHel-5	gb|AIXA01009796.1|:4175-4477
MsHel-5	gb|AIXA01008777.1|:491-788

MsHel-5	gb|AIXA01004470.1|:17201-17503
MsHel-5	gb|AIXA01001894.1|:25847-26149
MsHel-5	gb|AIXA01000662.1|:11134-11430
MsHel-5	gb|AIXA01000198.1|:77552-77854
MsHel-5	gb|AIXA01000198.1|:28335-28637
MsHel-5	gb|AIXA01016744.1|:1685-1973
MsHel-5	gb|AIXA01010074.1|:8922-9223
MsHel-5	gb|AIXA01011206.1|:1079-1383
MsHel-5	gb|AIXA01008074.1|:1150-1451
MsHel-5	gb|AIXA01006850.1|:17357-17657
MsHel-5	gb|AIXA01006850.1|:6878-7087
MsHel-5	gb|AIXA01002303.1|:35850-36154
MsHel-5	gb|AIXA01002303.1|:5-167
MsHel-5	gb|AIXA01016438.1|:9371-9660
MsHel-5	gb|AIXA01014372.1|:12890-13194
MsHel-5	gb|AIXA01012310.1|:9908-10201
MsHel-5	gb|AIXA01003275.1|:52710-53003
MsHel-5	gb|AIXA01000934.1|:54970-55264
MsHel-5	gb|AIXA01019178.1|:4279-4572
MsHel-5	gb|AIXA01015151.1|:55301-55602
MsHel-5	gb|AIXA01013106.1|:1148-1445
MsHel-5	gb|AIXA01012362.1|:8590-8892
MsHel-5	gb|AIXA01012199.1|:5174-5476
MsHel-5	gb|AIXA01008023.1|:66749-66939
MsHel-5	gb|AIXA01007447.1|:1539-1841
MsHel-5	gb|AIXA01004115.1|:83682-83984
MsHel-5	gb|AIXA01004115.1|:11196-11339
MsHel-5	gb|AIXA01003007.1|:16469-16770
MsHel-5	gb|AIXA01002640.1|:35218-35520
MsHel-5	gb|AIXA01000586.1|:10362-10664
MsHel-5	gb|AIXA01020434.1|:494-795
MsHel-5	gb|AIXA01011493.1|:56411-56603
MsHel-5	gb|AIXA01009397.1|:1659-1958
MsHel-5	gb|AIXA01005415.1|:86301-86499
MsHel-5	gb|AIXA01005415.1|:32162-32455
MsHel-5	gb|AIXA01004941.1|:57902-58203
MsHel-5	gb|AIXA01003928.1|:82864-83165
MsHel-5	gb|AIXA01000650.1|:16915-17216
MsHel-5	gb|AIXA01019409.1|:2373-2673
MsHel-5	gb|AIXA01014956.1|:7144-7444
MsHel-5	gb|AIXA01009697.1|:64694-64994
MsHel-5	gb|AIXA01009697.1|:11705-11866
MsHel-5	gb|AIXA01009346.1|:31138-31431
MsHel-5	gb|AIXA01007262.1|:41787-41984
MsHel-5	gb|AIXA01000457.1|:50614-50914
MsHel-5	gb|AIXA01000457.1|:9085-9381
MsHel-5	gb|AIXA01014610.1|:11618-11908
MsHel-5	gb|AIXA01009429.1|:20826-21115

MsHel-5	gb|AIXA01006902.1|:1708-2006
MsHel-5	gb|AIXA01002974.1|:47344-47636
MsHel-5	gb|AIXA01033894.1|:110-412
MsHel-5	gb|AIXA01014774.1|:20523-20820
MsHel-5	gb|AIXA01014075.1|:11292-11595
MsHel-5	gb|AIXA01012583.1|:3919-4221
MsHel-5	gb|AIXA01011256.1|:6324-6626
MsHel-5	gb|AIXA01009584.1|:342-644
MsHel-5	gb|AIXA01007612.1|:35319-35621
MsHel-5	gb|AIXA01007612.1|:6327-6521
MsHel-5	gb|AIXA01005493.1|:1294-1456
MsHel-5	gb|AIXA01005403.1|:6662-6964
MsHel-5	gb|AIXA01005033.1|:5545-5837
MsHel-5	gb|AIXA01002635.1|:12258-12560
MsHel-5	gb|AIXA01002365.1|:29618-29920
MsHel-5	gb|AIXA01002023.1|:14209-14507
MsHel-5	gb|AIXA01001644.1|:9877-10179
MsHel-5	gb|AIXA01001529.1|:9272-9574
MsHel-5	gb|AIXA01001225.1|:5503-5805
MsHel-5	gb|AIXA01000267.1|:70842-71144
MsHel-5	gb|AIXA01022669.1|:702-999
MsHel-5	gb|AIXA01017341.1|:2753-3054
MsHel-5	gb|AIXA01016123.1|:9815-10114
MsHel-5	gb|AIXA01010126.1|:4166-4463
MsHel-5	gb|AIXA01006718.1|:18255-18556
MsHel-5	gb|AIXA01002452.1|:44106-44408
MsHel-5	gb|AIXA01001144.1|:75246-75550
MsHel-5	gb|AIXA01001066.1|:14532-14834
MsHel-5	gb|AIXA01001036.1|:3973-4274
MsHel-5	gb|AIXA01017071.1|:1637-1925
MsHel-5	gb|AIXA01007479.1|:5104-5404
MsHel-5	gb|AIXA01007379.1|:7785-8085
MsHel-5	gb|AIXA01006175.1|:52516-52815
MsHel-5	gb|AIXA01003176.1|:135834-136022
MsHel-5	gb|AIXA01003176.1|:17821-18118
MsHel-5	gb|AIXA01035494.1|:23-326
MsHel-5	gb|AIXA01016609.1|:724-1022
MsHel-5	gb|AIXA01016598.1|:331-634
MsHel-5	gb|AIXA01010111.1|:5158-5461
MsHel-5	gb|AIXA01009800.1|:2577-2831
MsHel-5	gb|AIXA01009634.1|:14785-15092
MsHel-5	gb|AIXA01007229.1|:58927-59236
MsHel-5	gb|AIXA01004402.1|:35882-36181
MsHel-5	gb|AIXA01002703.1|:10029-10314
MsHel-5	gb|AIXA01014690.1|:1071-1369
MsHel-5	gb|AIXA01012148.1|:6508-6810
MsHel-5	gb|AIXA01011786.1|:10490-10792
MsHel-5	gb|AIXA01010687.1|:36292-36416

MsHel-5	gb|AIXA01010687.1|:16885-17184
MsHel-5	gb|AIXA01010468.1|:14129-14430
MsHel-5	gb|AIXA01009508.1|:41642-41944
MsHel-5	gb|AIXA01008227.1|:49159-49461
MsHel-5	gb|AIXA01008227.1|:7935-8126
MsHel-5	gb|AIXA01007974.1|:29722-29920
MsHel-5	gb|AIXA01005642.1|:48493-48795
MsHel-5	gb|AIXA01005538.1|:31757-31916
MsHel-5	gb|AIXA01005538.1|:19248-19550
MsHel-5	gb|AIXA01005244.1|:6793-7095
MsHel-5	gb|AIXA01004068.1|:89283-89584
MsHel-5	gb|AIXA01003168.1|:104338-104639
MsHel-5	gb|AIXA01002803.1|:20101-20402
MsHel-5	gb|AIXA01002776.1|:4127-4425
MsHel-5	gb|AIXA01001816.1|:43503-43785
MsHel-5	gb|AIXA01000741.1|:46433-46633
MsHel-5	gb|AIXA01034948.1|:300-601
MsHel-5	gb|AIXA01018652.1|:6681-6982
MsHel-5	gb|AIXA01014959.1|:6235-6532
MsHel-5	gb|AIXA01014772.1|:135-265
MsHel-5	gb|AIXA01012021.1|:62469-62770
MsHel-5	gb|AIXA01011502.1|:26993-27294
MsHel-5	gb|AIXA01008995.1|:7967-8268
MsHel-5	gb|AIXA01007460.1|:24021-24322
MsHel-5	gb|AIXA01004586.1|:53269-53462
MsHel-5	gb|AIXA01004586.1|:37126-37427
MsHel-5	gb|AIXA01004475.1|:15187-15447
MsHel-5	gb|AIXA01004140.1|:28973-29274
MsHel-5	gb|AIXA01003713.1|:33360-33659
MsHel-5	gb|AIXA01002611.1|:36901-37201
MsHel-5	gb|AIXA01002611.1|:25628-25925
MsHel-5	gb|AIXA01002148.1|:70658-70959
MsHel-5	gb|AIXA01000892.1|:120126-120430
MsHel-5	gb|AIXA01000892.1|:67627-67820
MsHel-5	gb|AIXA01014867.1|:27913-28213
MsHel-5	gb|AIXA01014781.1|:1849-2138
MsHel-5	gb|AIXA01014602.1|:8547-8848
MsHel-5	gb|AIXA01012783.1|:2675-2975
MsHel-5	gb|AIXA01005595.1|:131937-132097
MsHel-5	gb|AIXA01005509.1|:14134-14432
MsHel-5	gb|AIXA01002510.1|:4424-4688
MsHel-5	gb|AIXA01001708.1|:27136-27428
MsHel-5	gb|AIXA01025766.1|:279-582
MsHel-5	gb|AIXA01012306.1|:11758-12049
MsHel-5	gb|AIXA01010068.1|:7269-7573
MsHel-5	gb|AIXA01002327.1|:8938-9228
MsHel-5	gb|AIXA01001642.1|:661-961
MsHel-5	gb|AIXA01024747.1|:369-659

MsHel-5	gb|AIXA01012798.1|:13114-13404
MsHel-5	gb|AIXA01011984.1|:49017-49200
MsHel-5	gb|AIXA01011984.1|:13741-14042
MsHel-5	gb|AIXA01008309.1|:5406-5708
MsHel-5	gb|AIXA01006174.1|:26189-26490
MsHel-5	gb|AIXA01005785.1|:24315-24617
MsHel-5	gb|AIXA01003478.1|:15828-16130
MsHel-5	gb|AIXA01003288.1|:41748-42049
MsHel-5	gb|AIXA01002396.1|:5149-5450
MsHel-5	gb|AIXA01013892.1|:568-869
MsHel-5	gb|AIXA01012652.1|:45817-46117
MsHel-5	gb|AIXA01007354.1|:84635-84828
MsHel-5	gb|AIXA01006872.1|:66911-67211
MsHel-5	gb|AIXA01001627.1|:8027-8333
MsHel-5	gb|AIXA01001328.1|:20993-21896
MsHel-5	gb|AIXA01000786.1|:26370-26671
MsHel-5	gb|AIXA01000689.1|:5157-5450
MsHel-5	gb|AIXA01000485.1|:14608-14909
MsHel-5	gb|AIXA01013772.1|:860-1157
MsHel-5	gb|AIXA01013697.1|:6831-7131
MsHel-5	gb|AIXA01011325.1|:1296-1508
MsHel-5	gb|AIXA01009807.1|:13214-13512
MsHel-5	gb|AIXA01008940.1|:10679-10938
MsHel-5	gb|AIXA01008272.1|:1363-1656
MsHel-5	gb|AIXA01002753.1|:80009-80305
MsHel-5	gb|AIXA01002561.1|:66060-66324
MsHel-5	gb|AIXA01002278.1|:3299-3491
MsHel-5	gb|AIXA01013376.1|:404-707
MsHel-5	gb|AIXA01008949.1|:18650-18953
MsHel-5	gb|AIXA01008856.1|:8630-8930
MsHel-5	gb|AIXA01006043.1|:13231-13538
MsHel-5	gb|AIXA01005099.1|:1222-1532
MsHel-5	gb|AIXA01001326.1|:16542-16842
MsHel-5	gb|AIXA01000805.1|:19971-20274
MsHel-5	gb|AIXA01022532.1|:825-1126
MsHel-5	gb|AIXA01015373.1|:4147-4449
MsHel-5	gb|AIXA01014195.1|:38004-38297
MsHel-5	gb|AIXA01013946.1|:48587-48889
MsHel-5	gb|AIXA01013764.1|:5940-6241
MsHel-5	gb|AIXA01011331.1|:5634-5804
MsHel-5	gb|AIXA01011327.1|:6048-6350
MsHel-5	gb|AIXA01010017.1|:1672-1974
MsHel-5	gb|AIXA01009590.1|:52590-52891
MsHel-5	gb|AIXA01009044.1|:110934-111146
MsHel-5	gb|AIXA01007027.1|:9977-10279
MsHel-5	gb|AIXA01003799.1|:74208-74424
MsHel-5	gb|AIXA01003799.1|:2828-3129
MsHel-5	gb|AIXA01002513.1|:21713-22015

MsHel-5	gb|AIXA01001366.1|:60225-60526
MsHel-5	gb|AIXA01016647.1|:3625-3922
MsHel-5	gb|AIXA01008885.1|:4270-4571
MsHel-5	gb|AIXA01020317.1|:652-955
MsHel-5	gb|AIXA01017072.1|:17415-17710
MsHel-5	gb|AIXA01016273.1|:13817-14105
MsHel-5	gb|AIXA01013147.1|:20331-20524
MsHel-5	gb|AIXA01011003.1|:11278-11562
MsHel-5	gb|AIXA01007033.1|:16546-16847
MsHel-5	gb|AIXA01023009.1|:713-1007
MsHel-5	gb|AIXA01004929.1|:42658-42957
MsHel-5	gb|AIXA01004093.1|:42383-42682
MsHel-5	gb|AIXA01003183.1|:137513-137812
MsHel-5	gb|AIXA01003183.1|:117457-117648
MsHel-5	gb|AIXA01022952.1|:779-1081
MsHel-5	gb|AIXA01016650.1|:5839-6138
MsHel-5	gb|AIXA01010671.1|:9000-9301
MsHel-5	gb|AIXA01008187.1|:48851-49153
MsHel-5	gb|AIXA01007717.1|:37039-37337
MsHel-5	gb|AIXA01005491.1|:61993-62295
MsHel-5	gb|AIXA01004076.1|:62192-62501
MsHel-5	gb|AIXA01002310.1|:3305-3605
MsHel-5	gb|AIXA01001273.1|:118610-118911
MsHel-5	gb|AIXA01015872.1|:491-791
MsHel-5	gb|AIXA01015097.1|:49192-49491
MsHel-5	gb|AIXA01012250.1|:48281-48576
MsHel-5	gb|AIXA01009972.1|:20419-20700
MsHel-5	gb|AIXA01007602.1|:61567-61870
MsHel-5	gb|AIXA01007500.1|:9896-10196
MsHel-5	gb|AIXA01005272.1|:15362-15621
MsHel-5	gb|AIXA01004227.1|:2400-2705
MsHel-5	gb|AIXA01000661.1|:129387-129570
MsHel-5	gb|AIXA01000661.1|:45399-45700
MsHel-5	gb|AIXA01014659.1|:16944-17191
MsHel-5	gb|AIXA01006837.1|:41205-41506
MsHel-5	gb|AIXA01003021.1|:120339-120549
MsHel-5	gb|AIXA01003021.1|:46418-46712
MsHel-5	gb|AIXA01016262.1|:3717-4020
MsHel-5	gb|AIXA01013877.1|:5683-5982
MsHel-5	gb|AIXA01011587.1|:6677-6973
MsHel-5	gb|AIXA01006695.1|:4409-4709
MsHel-5	gb|AIXA01001046.1|:86942-87245
MsHel-5	gb|AIXA01001045.1|:34332-34633
MsHel-5	gb|AIXA01000688.1|:130096-130294
MsHel-5	gb|AIXA01000014.1|:16877-17182
MsHel-5	gb|AIXA01036073.1|:294-555
MsHel-5	gb|AIXA01007586.1|:43473-43664
MsHel-5	gb|AIXA01007586.1|:21209-21405

MsHel-5	gb|AIXA01007140.1|:27210-27515
MsHel-5	gb|AIXA01007020.1|:4790-5083
MsHel-5	gb|AIXA01005820.1|:43245-43546
MsHel-5	gb|AIXA01001785.1|:955-1259
MsHel-5	gb|AIXA01000385.1|:48027-48332
MsHel-5	gb|AIXA01017677.1|:8283-8581
MsHel-5	gb|AIXA01007698.1|:8775-9075
MsHel-5	gb|AIXA01004007.1|:29927-30227
MsHel-5	gb|AIXA01003556.1|:6323-6620
MsHel-5	gb|AIXA01002507.1|:87615-87767
MsHel-5	gb|AIXA01002507.1|:59510-59722
MsHel-5	gb|AIXA01013067.1|:8931-9030
MsHel-5	gb|AIXA01006177.1|:24728-24987
MsHel-5	gb|AIXA01004684.1|:5527-6093
MsHel-5	gb|AIXA01002293.1|:39313-39616
MsHel-5	gb|AIXA01002032.1|:11243-11546
MsHel-5	gb|AIXA01000947.1|:49462-49757
MsHel-5	gb|AIXA01000441.1|:34026-34313
MsHel-5	gb|AIXA01008025.1|:17938-18248
MsHel-5	gb|AIXA01018628.1|:156-449
MsHel-5	gb|AIXA01014147.1|:6440-6744
MsHel-5	gb|AIXA01014124.1|:19417-19612
MsHel-5	gb|AIXA01001316.1|:17834-18134
MsHel-5	gb|AIXA01000251.1|:86649-86953
MsHel-5	gb|AIXA01011638.1|:50375-50680
MsHel-5	gb|AIXA01004178.1|:92499-92794
MsHel-5	gb|AIXA01009789.1|:22239-22542
MsHel-5	gb|AIXA01009580.1|:19220-19413
MsHel-5	gb|AIXA01006063.1|:33288-33481
MsHel-5	gb|AIXA01006063.1|:6739-7039
MsHel-5	gb|AIXA01001947.1|:2164-2460
MsHel-5	gb|AIXA01001695.1|:134297-134475
MsHel-5	gb|AIXA01015371.1|:4951-5253
MsHel-5	gb|AIXA01007752.1|:8309-8606
MsHel-5	gb|AIXA01004323.1|:27553-27863
MsHel-5	gb|AIXA01001235.1|:11585-11879
MsHel-5	gb|AIXA01019804.1|:153-485
MsHel-5	gb|AIXA01000212.1|:26911-27206
MsHel-5	gb|AIXA01002972.1|:26952-27262
MsHel-5	gb|AIXA01028551.1|:660-952
MsHel-5	gb|AIXA01013779.1|:342-553
MsHel-5	gb|AIXA01006473.1|:38255-38559
MsHel-5	gb|AIXA01001598.1|:11791-11988
MsHel-5	gb|AIXA01002567.1|:25465-25764
MsHel-5	gb|AIXA01001292.1|:60992-61288
MsHel-5	gb|AIXA01021483.1|:1745-1985
MsHel-5	gb|AIXA01011552.1|:2925-3226
MsHel-5	gb|AIXA01004067.1|:2605-2901

MsHel-5	gb|AIXA01004805.1|:19347-19585
MsHel-5	gb|AIXA01009336.1|:34782-35085
MsHel-5	gb|AIXA01007100.1|:16529-16828
MsHel-5	gb|AIXA01001898.1|:215272-215564
MsHel-5	gb|AIXA01012685.1|:10429-10687
MsHel-5	gb|AIXA01012363.1|:9481-9713
MsHel-5	gb|AIXA01007392.1|:9159-9460
MsHel-5	gb|AIXA01006136.1|:37189-37482
MsHel-5	gb|AIXA01002044.1|:14917-15206
MsHel-5	gb|AIXA01001736.1|:14836-15136
MsHel-5	gb|AIXA01011457.1|:26113-26408
MsHel-5	gb|AIXA01011457.1|:10004-10165
MsHel-5	gb|AIXA01006162.1|:2879-3180
MsHel-5	gb|AIXA01017584.1|:1228-1516
MsHel-5	gb|AIXA01004331.1|:11481-11830
MsHel-5	gb|AIXA01008443.1|:15511-15714
MsHel-5	gb|AIXA01007904.1|:8007-8310
MsHel-5	gb|AIXA01005095.1|:45188-45379
MsHel-5	gb|AIXA01005095.1|:20901-21116
MsHel-5	gb|AIXA01002680.1|:6923-7198
MsHel-5	gb|AIXA01012018.1|:2344-2650
MsHel-5	gb|AIXA01018787.1|:575-867
MsHel-5	gb|AIXA01007784.1|:14311-14511
MsHel-5	gb|AIXA01004421.1|:26056-26352
MsHel-5	gb|AIXA01004160.1|:64441-64740
MsHel-5	gb|AIXA01003997.1|:18977-19236
MsHel-5	gb|AIXA01010397.1|:7787-8081
MsHel-5	gb|AIXA01007575.1|:15565-15745
MsHel-5	gb|AIXA01029593.1|:694-888
MsHel-5	gb|AIXA01005566.1|:33370-33630
MsHel-5	gb|AIXA01000454.1|:94536-94777
MsHel-5	gb|AIXA01000454.1|:11240-11435
MsHel-5	gb|AIXA01015923.1|:5913-6111
MsHel-5	gb|AIXA01015296.1|:32502-32799
MsHel-5	gb|AIXA01008561.1|:12111-12407
MsHel-5	gb|AIXA01012405.1|:3322-3624
MsHel-5	gb|AIXA01008922.1|:20216-20519
MsHel-5	gb|AIXA01008922.1|:1-212
MsHel-5	gb|AIXA01011709.1|:26318-26587
MsHel-5	gb|AIXA01003803.1|:81033-81321
MsHel-5	gb|AIXA01002813.1|:67409-67677
MsHel-5	gb|AIXA01000949.1|:29707-30010
MsHel-5	gb|AIXA01002155.1|:28101-28394
MsHel-5	gb|AIXA01010658.1|:23299-23596
MsHel-5	gb|AIXA01010406.1|:63577-63771
MsHel-5	gb|AIXA01007538.1|:44753-44994
MsHel-5	gb|AIXA01027824.1|:5-290
MsHel-5	gb|AIXA01007979.1|:10701-10999

MsHel-5	gb|AIXA01005217.1|:63505-63807
MsHel-5	gb|AIXA01018328.1|:5803-5995
MsHel-5	gb|AIXA01023561.1|:518-685
MsHel-5	gb|AIXA01021968.1|:1616-1814
MsHel-5	gb|AIXA01017009.1|:6429-6730
MsHel-5	gb|AIXA01003711.1|:201327-201586
MsHel-5	gb|AIXA01014009.1|:2237-2529
MsHel-5	gb|AIXA01007352.1|:13627-14392
MsHel-5	gb|AIXA01006211.1|:7986-8284
MsHel-5	gb|AIXA01001323.1|:3831-4090
MsHel-5	gb|AIXA01010714.1|:69862-70056
MsHel-5	gb|AIXA01007426.1|:87162-87456
MsHel-5	gb|AIXA01014943.1|:179-392
MsHel-5	gb|AIXA01014155.1|:10982-11284
MsHel-5	gb|AIXA01011810.1|:24012-25028
MsHel-5	gb|AIXA01002009.1|:1-190
MsHel-5	gb|AIXA01031440.1|:586-784
MsHel-5	gb|AIXA01002671.1|:94563-94759
MsHel-5	gb|AIXA01000319.1|:1441-1727
MsHel-5	gb|AIXA01035741.1|:1-227
MsHel-5	gb|AIXA01017721.1|:3006-3219
MsHel-5	gb|AIXA01012916.1|:3877-4175
MsHel-5	gb|AIXA01012653.1|:4291-4497
MsHel-5	gb|AIXA01031778.1|:628-765
MsHel-5	gb|AIXA01009636.1|:17790-17985
MsHel-5	gb|AIXA01004697.1|:42113-42394
MsHel-5	gb|AIXA01006731.1|:106573-106874
MsHel-5	gb|AIXA01003837.1|:7837-8068
MsHel-5	gb|AIXA01002005.1|:46011-46173
MsHel-5	gb|AIXA01000145.1|:13254-13504
MsHel-5	gb|AIXA01017945.1|:6041-6339
MsHel-5	gb|AIXA01036188.1|:343-507
MsHel-5	gb|AIXA01021915.1|:838-1120
MsHel-5	gb|AIXA01011688.1|:46663-46875
MsHel-5	gb|AIXA01023596.1|:288-506
MsHel-5	gb|AIXA01017292.1|:10488-10705
MsHel-5	gb|AIXA01011496.1|:16287-16492
MsHel-5	gb|AIXA01010187.1|:13906-14228
MsHel-5	gb|AIXA01007124.1|:19767-20058
MsHel-5	gb|AIXA01004312.1|:48876-49081
MsHel-5	gb|AIXA01000456.1|:57656-57836
MsHel-5	gb|AIXA01024057.1|:1213-1396
MsHel-5	gb|AIXA01014734.1|:24973-25188
MsHel-5	gb|AIXA01001769.1|:1749-1965
MsHel-5	gb|AIXA01001772.1|:3617-4626
MsHel-5	gb|AIXA01003821.1|:697-845
MsHel-5	gb|AIXA01010854.1|:19506-19675
MsHel-5	gb|AIXA01003139.1|:37507-37704

MsHel-5	gb|AIXA01003139.1|:20231-20427
MsHel-5	gb|AIXA01002172.1|:28091-28359
MsHel-5	gb|AIXA01023570.1|:3-200
MsHel-5	gb|AIXA01013314.1|:10106-10304
MsHel-5	gb|AIXA01011104.1|:7462-7777
MsHel-5	gb|AIXA01003120.1|:235913-236237
MsHel-5	gb|AIXA01003120.1|:33267-33461
MsHel-5	gb|AIXA01002386.1|:1-191
MsHel-5	gb|AIXA01023734.1|:1299-1445
MsHel-5	gb|AIXA01014996.1|:6824-7014
MsHel-5	gb|AIXA01011268.1|:15256-15466
MsHel-5	gb|AIXA01005102.1|:7530-7684
MsHel-5	gb|AIXA01004370.1|:28985-29275
MsHel-5	gb|AIXA01003272.1|:113896-114090
MsHel-5	gb|AIXA01002689.1|:43763-43917
MsHel-5	gb|AIXA01001566.1|:17566-17731
MsHel-5	gb|AIXA01011635.1|:15244-15436
MsHel-5	gb|AIXA01006702.1|:28190-28379
MsHel-5	gb|AIXA01001641.1|:5962-6108
MsHel-5	gb|AIXA01007589.1|:26649-26864
MsHel-5	gb|AIXA01015137.1|:2012-2282
MsHel-5	gb|AIXA01019461.1|:4181-4374
MsHel-5	gb|AIXA01012348.1|:18917-19110
MsHel-5	gb|AIXA01012324.1|:7730-7923
MsHel-5	gb|AIXA01009662.1|:4916-5109
MsHel-5	gb|AIXA01001620.1|:26025-26343
MsHel-5	gb|AIXA01000195.1|:100693-100886
MsHel-5	gb|AIXA01010805.1|:11557-11749
MsHel-5	gb|AIXA01004930.1|:8637-8829
MsHel-5	gb|AIXA01001483.1|:59773-59965
MsHel-5	gb|AIXA01008636.1|:74117-74329
MsHel-5	gb|AIXA01003360.1|:34964-35107
MsHel-5	gb|AIXA01015507.1|:411-626
MsHel-5	gb|AIXA01014287.1|:3051-3241
MsHel-5	gb|AIXA01010831.1|:3426-3581
MsHel-5	gb|AIXA01015125.1|:9630-9823
MsHel-5	gb|AIXA01014531.1|:1880-2073
MsHel-5	gb|AIXA01014369.1|:3276-3469
MsHel-5	gb|AIXA01013014.1|:6977-7265
MsHel-5	gb|AIXA01010885.1|:69562-69813
MsHel-5	gb|AIXA01008691.1|:14577-14770
MsHel-5	gb|AIXA01005375.1|:19939-20132
MsHel-5	gb|AIXA01005226.1|:38958-39151
MsHel-5	gb|AIXA01004243.1|:16301-16494
MsHel-5	gb|AIXA01003276.1|:47086-47279
MsHel-5	gb|AIXA01001319.1|:81680-81873
MsHel-5	gb|AIXA01001098.1|:24552-24745
MsHel-5	gb|AIXA01000907.1|:105030-105223

MsHel-5	gb|AIXA01000447.1|:42115-42249
MsHel-5	gb|AIXA01013328.1|:47436-47630
MsHel-5	gb|AIXA01013328.1|:31863-32063
MsHel-5	gb|AIXA01009100.1|:64308-64500
MsHel-5	gb|AIXA01008228.1|:35003-35195
MsHel-5	gb|AIXA01004943.1|:27387-27588
MsHel-5	gb|AIXA01003178.1|:25889-26049
MsHel-5	gb|AIXA01003049.1|:17557-17838
MsHel-5	gb|AIXA01001008.1|:3822-4014
MsHel-5	gb|AIXA01000982.1|:88577-88741
MsHel-5	gb|AIXA01000166.1|:1-197
MsHel-5	gb|AIXA01026335.1|:1013-1137
MsHel-5	gb|AIXA01013477.1|:34005-34137
MsHel-5	gb|AIXA01013092.1|:4881-5075
MsHel-5	gb|AIXA01008477.1|:37451-37644
MsHel-5	gb|AIXA01003934.1|:1-213
MsHel-5	gb|AIXA01007649.1|:8709-8871
MsHel-5	gb|AIXA01017762.1|:10376-10569
MsHel-5	gb|AIXA01009924.1|:8028-8221
MsHel-5	gb|AIXA01008076.1|:48484-48677
MsHel-5	gb|AIXA01005633.1|:9408-9601
MsHel-5	gb|AIXA01004896.1|:67340-67533
MsHel-5	gb|AIXA01004112.1|:9351-9544
MsHel-5	gb|AIXA01003548.1|:8545-8738
MsHel-5	gb|AIXA01001830.1|:12860-13053
MsHel-5	gb|AIXA01001630.1|:5123-5316
MsHel-5	gb|AIXA01000322.1|:10183-10376
MsHel-5	gb|AIXA01029289.1|:455-647
MsHel-5	gb|AIXA01023855.1|:1120-1312
MsHel-5	gb|AIXA01014907.1|:21599-21791
MsHel-5	gb|AIXA01012805.1|:2234-2394
MsHel-5	gb|AIXA01010886.1|:2442-2595
MsHel-5	gb|AIXA01008915.1|:39118-39309
MsHel-5	gb|AIXA01008674.1|:7502-7662
MsHel-5	gb|AIXA01005184.1|:51391-51578
MsHel-5	gb|AIXA01003320.1|:34311-34503
MsHel-5	gb|AIXA01003170.1|:24118-24316
MsHel-5	gb|AIXA01003170.1|:8720-8912
MsHel-5	gb|AIXA01002566.1|:8155-8347
MsHel-5	gb|AIXA01000841.1|:13342-13495
MsHel-5	gb|AIXA01022564.1|:1259-1470
MsHel-5	gb|AIXA01018212.1|:7549-7677
MsHel-5	gb|AIXA01015574.1|:2822-3012
MsHel-5	gb|AIXA01010594.1|:23898-24114
MsHel-5	gb|AIXA01010579.1|:5481-5625
MsHel-5	gb|AIXA01004311.1|:916-1110
MsHel-5	gb|AIXA01001284.1|:13196-13355
MsHel-5	gb|AIXA01000787.1|:59297-59587

MsHel-5	gb|AIXA01017553.1|:1379-1568
MsHel-5	gb|AIXA01005535.1|:14690-14884
MsHel-5	gb|AIXA01005535.1|:208-368
MsHel-5	gb|AIXA01003140.1|:92436-92622
MsHel-5	gb|AIXA01003140.1|:80973-81159
MsHel-5	gb|AIXA01001043.1|:109297-109487
MsHel-5	gb|AIXA01029437.1|:1-186
MsHel-5	gb|AIXA01014419.1|:10115-10308
MsHel-5	gb|AIXA01008574.1|:7118-7407
MsHel-5	gb|AIXA01007711.1|:28564-28757
MsHel-5	gb|AIXA01006254.1|:22982-23181
MsHel-5	gb|AIXA01001718.1|:17730-17923
MsHel-5	gb|AIXA01001613.1|:8241-8434
MsHel-5	gb|AIXA01000943.1|:6065-6258
MsHel-5	gb|AIXA01033290.1|:501-693
MsHel-5	gb|AIXA01017950.1|:1729-1918
MsHel-5	gb|AIXA01016936.1|:44-305
MsHel-5	gb|AIXA01014415.1|:636-830
MsHel-5	gb|AIXA01009951.1|:127-317
MsHel-5	gb|AIXA01008405.1|:29747-29939
MsHel-5	gb|AIXA01006290.1|:6169-6361
MsHel-5	gb|AIXA01005494.1|:45600-45794
MsHel-5	gb|AIXA01004727.1|:13211-13403
MsHel-5	gb|AIXA01003330.1|:112521-112707
MsHel-5	gb|AIXA01001340.1|:2125-2313
MsHel-5	gb|AIXA01034462.1|:511-639
MsHel-5	gb|AIXA01018581.1|:703-894
MsHel-5	gb|AIXA01015758.1|:6897-7222
MsHel-5	gb|AIXA01014768.1|:7823-8016
MsHel-5	gb|AIXA01010058.1|:7532-7665
MsHel-5	gb|AIXA01009131.1|:16115-16949
MsHel-5	gb|AIXA01008564.1|:9136-9329
MsHel-5	gb|AIXA01008283.1|:4404-4563
MsHel-5	gb|AIXA01007834.1|:10533-10692
MsHel-5	gb|AIXA01007513.1|:50699-50888
MsHel-5	gb|AIXA01005563.1|:20182-20396
MsHel-5	gb|AIXA01005035.1|:73941-74074
MsHel-5	gb|AIXA01005035.1|:20499-20696
MsHel-5	gb|AIXA01004490.1|:36602-36793
MsHel-5	gb|AIXA01004474.1|:31463-31622
MsHel-5	gb|AIXA01004474.1|:1-205
MsHel-5	gb|AIXA01002301.1|:13667-13779
MsHel-5	gb|AIXA01024598.1|:1206-1325
MsHel-5	gb|AIXA01011454.1|:20948-21140
MsHel-5	gb|AIXA01010820.1|:3606-3800
MsHel-5	gb|AIXA01010070.1|:1193-1408
MsHel-5	gb|AIXA01007350.1|:57020-57214
MsHel-5	gb|AIXA01005533.1|:22473-22676

MsHel-5	gb|AIXA01004489.1|:67327-67525
MsHel-5	gb|AIXA01001192.1|:1-196
MsHel-5	gb|AIXA01018093.1|:206-398
MsHel-5	gb|AIXA01014762.1|:4719-4914
MsHel-5	gb|AIXA01013135.1|:8360-8513
MsHel-5	gb|AIXA01004583.1|:21208-21361
MsHel-5	gb|AIXA01004242.1|:24894-25048
MsHel-5	gb|AIXA01003660.1|:33821-34014
MsHel-5	gb|AIXA01002825.1|:5141-5328
MsHel-5	gb|AIXA01016504.1|:17-106
MsHel-5	gb|AIXA01014948.1|:1519-1709
MsHel-5	gb|AIXA01013285.1|:2992-3180
MsHel-5	gb|AIXA01012100.1|:7778-7971
MsHel-5	gb|AIXA01009360.1|:57598-57789
MsHel-5	gb|AIXA01008271.1|:4991-5179
MsHel-5	gb|AIXA01003533.1|:2065-2256
MsHel-5	gb|AIXA01003124.1|:81530-81723
MsHel-5	gb|AIXA01023535.1|:88-271
MsHel-5	gb|AIXA01017554.1|:1400-1585
MsHel-5	gb|AIXA01016082.1|:3212-3371
MsHel-5	gb|AIXA01015191.1|:31342-31544
MsHel-5	gb|AIXA01011450.1|:3744-3942
MsHel-5	gb|AIXA01010342.1|:29090-29246
MsHel-5	gb|AIXA01010342.1|:18375-18578
MsHel-5	gb|AIXA01007011.1|:21330-21528
MsHel-5	gb|AIXA01006683.1|:22271-22464
MsHel-5	gb|AIXA01006654.1|:5964-6154
MsHel-5	gb|AIXA01005044.1|:20823-21084
MsHel-5	gb|AIXA01002311.1|:97616-97814
MsHel-5	gb|AIXA01001558.1|:62797-62949
MsHel-5	gb|AIXA01000838.1|:51375-51558
MsHel-5	gb|AIXA01024109.1|:1-152
MsHel-5	gb|AIXA01019887.1|:1876-2068
MsHel-5	gb|AIXA01004483.1|:4435-4753
MsHel-5	gb|AIXA01001352.1|:4441-4635
MsHel-5	gb|AIXA01000150.1|:14744-14942
MsHel-5	gb|AIXA01007369.1|:2391-2580
MsHel-5	gb|AIXA01006922.1|:43742-43937
MsHel-5	gb|AIXA01002174.1|:208557-208746
MsHel-5	gb|AIXA01000893.1|:2-215
MsHel-5	gb|AIXA01031987.1|:215-402
MsHel-5	gb|AIXA01024035.1|:332-524
MsHel-5	gb|AIXA01015379.1|:17515-17709
MsHel-5	gb|AIXA01011265.1|:5713-5907
MsHel-5	gb|AIXA01006888.1|:33836-34033
MsHel-5	gb|AIXA01006888.1|:22191-22389
MsHel-5	gb|AIXA01006888.1|:498-690
MsHel-5	gb|AIXA01006499.1|:20096-20290

MsHel-5	gb|AIXA01006061.1|:44556-44716
MsHel-5	gb|AIXA01002505.1|:8187-8380
MsHel-5	gb|AIXA01028644.1|:592-790
MsHel-5	gb|AIXA01027671.1|:564-715
MsHel-5	gb|AIXA01018501.1|:6554-6750
MsHel-5	gb|AIXA01017856.1|:918-1108
MsHel-5	gb|AIXA01013015.1|:50500-50648
MsHel-5	gb|AIXA01011308.1|:12112-12298
MsHel-5	gb|AIXA01010663.1|:13088-13285
MsHel-5	gb|AIXA01010558.1|:3612-3809
MsHel-5	gb|AIXA01006154.1|:71922-72118
MsHel-5	gb|AIXA01005208.1|:7495-7688
MsHel-5	gb|AIXA01025770.1|:1037-1190
MsHel-5	gb|AIXA01018539.1|:1174-1315
MsHel-5	gb|AIXA01011900.1|:18254-18445
MsHel-5	gb|AIXA01010748.1|:117294-117483
MsHel-5	gb|AIXA01010748.1|:64645-64811
MsHel-5	gb|AIXA01005609.1|:6910-7066
MsHel-5	gb|AIXA01004338.1|:9205-9409
MsHel-5	gb|AIXA01003280.1|:16401-16592
MsHel-5	gb|AIXA01002343.1|:12252-12367
MsHel-5	gb|AIXA01018203.1|:5951-6139
MsHel-5	gb|AIXA01004861.1|:10682-10966
MsHel-5	gb|AIXA01003004.1|:23384-23579
MsHel-5	gb|AIXA01002658.1|:8046-8252
MsHel-5	gb|AIXA01026564.1|:445-633
MsHel-5	gb|AIXA01008724.1|:13322-13633
MsHel-5	gb|AIXA01006048.1|:18255-18447
MsHel-5	gb|AIXA01001857.1|:11030-11221
MsHel-5	gb|AIXA01001645.1|:174701-174886
MsHel-5	gb|AIXA01000450.1|:15993-16102
MsHel-5	gb|AIXA01017702.1|:12271-12467
MsHel-5	gb|AIXA01015922.1|:2302-2438
MsHel-5	gb|AIXA01015579.1|:10399-10597
MsHel-5	gb|AIXA01013447.1|:712-960
MsHel-5	gb|AIXA01012145.1|:20614-20812
MsHel-5	gb|AIXA01011985.1|:6643-6833
MsHel-5	gb|AIXA01008154.1|:340-585
MsHel-5	gb|AIXA01007760.1|:713-910
MsHel-5	gb|AIXA01007551.1|:4481-4679
MsHel-5	gb|AIXA01007535.1|:1-194
MsHel-5	gb|AIXA01006978.1|:4364-4560
MsHel-5	gb|AIXA01006821.1|:431-595
MsHel-5	gb|AIXA01006519.1|:30481-30679
MsHel-5	gb|AIXA01004113.1|:29605-29790
MsHel-5	gb|AIXA01003621.1|:39525-39704
MsHel-5	gb|AIXA01003012.1|:48952-49150
MsHel-5	gb|AIXA01000942.1|:8145-8337

MsHel-5	gb|AIXA01000653.1|:26484-26648
MsHel-5	gb|AIXA01000050.1|:76188-76339
MsHel-5	gb|AIXA01012000.1|:5252-5407
MsHel-5	gb|AIXA01010396.1|:30493-30684
MsHel-5	gb|AIXA01009089.1|:2158-2355
MsHel-5	gb|AIXA01008110.1|:64356-64545
MsHel-5	gb|AIXA01008110.1|:39798-40044
MsHel-5	gb|AIXA01007075.1|:6022-6213
MsHel-5	gb|AIXA01005379.1|:8165-8362
MsHel-5	gb|AIXA01001990.1|:75837-75947
MsHel-5	gb|AIXA01001356.1|:88409-88600
MsHel-5	gb|AIXA01021864.1|:779-975
MsHel-5	gb|AIXA01019286.1|:670-852
MsHel-5	gb|AIXA01016294.1|:2490-2588
MsHel-5	gb|AIXA01013830.1|:1-135
MsHel-5	gb|AIXA01007060.1|:1234-1389
MsHel-5	gb|AIXA01003747.1|:165879-166087
MsHel-5	gb|AIXA01003747.1|:116244-116444
MsHel-5	gb|AIXA01003530.1|:1516-1707
MsHel-5	gb|AIXA01001037.1|:17406-17593
MsHel-5	gb|AIXA01015533.1|:6453-6654
MsHel-5	gb|AIXA01009528.1|:7486-7687
MsHel-5	gb|AIXA01007527.1|:47832-48031
MsHel-5	gb|AIXA01007366.1|:20022-20216
MsHel-5	gb|AIXA01006289.1|:13110-13596
MsHel-5	gb|AIXA01005413.1|:60201-60397
MsHel-5	gb|AIXA01003852.1|:12890-13087
MsHel-5	gb|AIXA01030722.1|:219-415
MsHel-5	gb|AIXA01018914.1|:7089-7277
MsHel-5	gb|AIXA01017885.1|:6061-6225
MsHel-5	gb|AIXA01013761.1|:7131-7295
MsHel-5	gb|AIXA01011819.1|:8079-8251
MsHel-5	gb|AIXA01006046.1|:15277-15441
MsHel-5	gb|AIXA01005643.1|:70023-70221
MsHel-5	gb|AIXA01003658.1|:69287-69487
MsHel-5	gb|AIXA01002628.1|:15765-15916
MsHel-5	gb|AIXA01001311.1|:448-646
MsHel-5	gb|AIXA01000278.1|:50670-50861
MsHel-5	gb|AIXA01020510.1|:815-953
MsHel-5	gb|AIXA01018855.1|:3232-3440
MsHel-5	gb|AIXA01010538.1|:12772-12938
MsHel-5	gb|AIXA01008177.1|:2382-2577
MsHel-5	gb|AIXA01007168.1|:91967-92158
MsHel-5	gb|AIXA01004685.1|:55594-55785
MsHel-5	gb|AIXA01003895.1|:70801-71295
MsHel-5	gb|AIXA01016593.1|:6865-7063
MsHel-5	gb|AIXA01010541.1|:24366-24582
MsHel-5	gb|AIXA01001717.1|:63984-64151

MsHel-5	gb|AIXA01007949.1|:42681-42869
MsHel-5	gb|AIXA01007949.1|:8071-8231
MsHel-5	gb|AIXA01005449.1|:30691-30853
MsHel-5	gb|AIXA01000559.1|:7346-7545
MsHel-5	gb|AIXA01018461.1|:7014-7212
MsHel-5	gb|AIXA01015428.1|:6929-7125
MsHel-5	gb|AIXA01014412.1|:8793-8991
MsHel-5	gb|AIXA01012542.1|:2110-2303
MsHel-5	gb|AIXA01009837.1|:5761-5924
MsHel-5	gb|AIXA01009407.1|:19826-20024
MsHel-5	gb|AIXA01006118.1|:4864-5062
MsHel-5	gb|AIXA01005585.1|:17049-17247
MsHel-5	gb|AIXA01005242.1|:1-170
MsHel-5	gb|AIXA01000308.1|:1-129
MsHel-5	gb|AIXA01033671.1|:550-649
MsHel-5	gb|AIXA01032829.1|:579-693
MsHel-5	gb|AIXA01016376.1|:16585-16782
MsHel-5	gb|AIXA01011725.1|:11103-11292
MsHel-5	gb|AIXA01011214.1|:34101-34298
MsHel-5	gb|AIXA01008318.1|:8783-8979
MsHel-5	gb|AIXA01006809.1|:54460-54623
MsHel-5	gb|AIXA01004134.1|:84972-85135
MsHel-5	gb|AIXA01002213.1|:55674-55909
MsHel-5	gb|AIXA01011826.1|:1-191
MsHel-5	gb|AIXA01000512.1|:61113-61300
MsHel-5	gb|AIXA01023303.1|:1142-1331
MsHel-5	gb|AIXA01023208.1|:546-743
MsHel-5	gb|AIXA01005336.1|:17528-17727
MsHel-5	gb|AIXA01003857.1|:14185-14373
MsHel-5	gb|AIXA01002344.1|:139-272
MsHel-5	gb|AIXA01020360.1|:454-652
MsHel-5	gb|AIXA01020232.1|:1139-1658
MsHel-5	gb|AIXA01013103.1|:21995-22190
MsHel-5	gb|AIXA01009544.1|:24812-25012
MsHel-5	gb|AIXA01007856.1|:78486-78684
MsHel-5	gb|AIXA01004895.1|:17361-18597
MsHel-5	gb|AIXA01002664.1|:19747-19945
MsHel-5	gb|AIXA01018438.1|:3389-3586
MsHel-5	gb|AIXA01018149.1|:15417-15567
MsHel-5	gb|AIXA01012580.1|:7206-7403
MsHel-5	gb|AIXA01011818.1|:8561-8733
MsHel-5	gb|AIXA01011690.1|:36185-36382
MsHel-5	gb|AIXA01007192.1|:3879-4026
MsHel-5	gb|AIXA01006255.1|:2556-2750
MsHel-5	gb|AIXA01002693.1|:48288-48486
MsHel-5	gb|AIXA01013066.1|:3083-3236
MsHel-5	gb|AIXA01021639.1|:1-182
MsHel-5	gb|AIXA01018252.1|:7-92

MsHel-5	gb|AIXA01014850.1|:1889-2077
MsHel-5	gb|AIXA01011215.1|:912-1074
MsHel-5	gb|AIXA01010343.1|:16041-16229
MsHel-5	gb|AIXA01004045.1|:88556-88755
MsHel-5	gb|AIXA01000928.1|:14899-15091
MsHel-5	gb|AIXA01000785.1|:10813-11008
MsHel-5	gb|AIXA01010802.1|:39603-39797
MsHel-5	gb|AIXA01010544.1|:11363-11561
MsHel-5	gb|AIXA01007736.1|:1395-1563
MsHel-5	gb|AIXA01006759.1|:9630-9793
MsHel-5	gb|AIXA01005085.1|:11228-11426
MsHel-5	gb|AIXA01004719.1|:249-1222
MsHel-5	gb|AIXA01004150.1|:39830-40032
MsHel-5	gb|AIXA01008256.1|:1970-2170
MsHel-5	gb|AIXA01003400.1|:5616-5742
MsHel-5	gb|AIXA01002006.1|:33-164
MsHel-5	gb|AIXA01017104.1|:3552-3708
MsHel-5	gb|AIXA01001798.1|:37097-37288
MsHel-5	gb|AIXA01033770.1|:25-110
MsHel-5	gb|AIXA01029469.1|:1-114
MsHel-5	gb|AIXA01014846.1|:4711-4884
MsHel-5	gb|AIXA01012368.1|:13-98
MsHel-5	gb|AIXA01008233.1|:15979-16177
MsHel-5	gb|AIXA01007464.1|:11063-11255
MsHel-5	gb|AIXA01007216.1|:117308-117503
MsHel-5	gb|AIXA01006807.1|:7158-7349
MsHel-5	gb|AIXA01004958.1|:7176-10526
MsHel-5	gb|AIXA01004313.1|:3454-3653
MsHel-5	gb|AIXA01003893.1|:18887-19076
MsHel-5	gb|AIXA01000795.1|:1-174
MsHel-5	gb|AIXA01000383.1|:35919-36004
MsHel-5	gb|AIXA01036861.1|:205-414
MsHel-5	gb|AIXA01012354.1|:5079-5243
MsHel-5	gb|AIXA01002010.1|:6156-6355
MsHel-5	gb|AIXA01000755.1|:113369-113567
MsHel-5	gb|AIXA01027559.1|:7-151
MsHel-5	gb|AIXA01009385.1|:327-527
MsHel-5	gb|AIXA01036331.1|:1-159
MsHel-5	gb|AIXA01024012.1|:1-153
MsHel-5	gb|AIXA01017714.1|:4713-4907
MsHel-5	gb|AIXA01003113.1|:14962-15055
MsHel-5	gb|AIXA01000811.1|:6-135
MsHel-5	gb|AIXA01015809.1|:3693-3868
MsHel-5	gb|AIXA01008778.1|:9479-9640
MsHel-5	gb|AIXA01007800.1|:41546-41686
MsHel-5	gb|AIXA01006700.1|:1-82
MsHel-5	gb|AIXA01006688.1|:12893-13049
MsHel-5	gb|AIXA01005838.1|:1606-1795

MsHel-5	gb|AIXA01003800.1|:57589-57744
MsHel-5	gb|AIXA01003000.1|:27511-27649
MsHel-5	gb|AIXA01001841.1|:38042-38232
MsHel-5	gb|AIXA01027086.1|:700-893
MsHel-5	gb|AIXA01023481.1|:469-663
MsHel-5	gb|AIXA01014421.1|:1270-1408
MsHel-5	gb|AIXA01012319.1|:13692-13879
MsHel-5	gb|AIXA01011527.1|:5399-5589
MsHel-5	gb|AIXA01010754.1|:20-104
MsHel-5	gb|AIXA01001666.1|:61832-62026
MsHel-5	gb|AIXA01030004.1|:1-132
MsHel-5	gb|AIXA01017061.1|:9780-9979
MsHel-5	gb|AIXA01003822.1|:31-114
MsHel-5	gb|AIXA01003361.1|:413-508
MsHel-5	gb|AIXA01016978.1|:332-498
MsHel-5	gb|AIXA01016665.1|:9687-9826
MsHel-5	gb|AIXA01009042.1|:2708-2897
MsHel-5	gb|AIXA01004479.1|:3301-3407
MsHel-5	gb|AIXA01017854.1|:16-101
MsHel-5	gb|AIXA01014529.1|:4538-4712
MsHel-5	gb|AIXA01004433.1|:3406-3618
MsHel-5	gb|AIXA01008679.1|:3897-4079
MsHel-5	gb|AIXA01006980.1|:51507-51591
MsHel-5	gb|AIXA01003251.1|:29158-29352
MsHel-5	gb|AIXA01000208.1|:41913-42105
MsHel-5	gb|AIXA01005510.1|:2-81
MsHel-5	gb|AIXA01016561.1|:10377-10561
MsHel-5	gb|AIXA01026649.1|:65-203
MsHel-5	gb|AIXA01006847.1|:18048-18129
MsHel-5	gb|AIXA01003329.1|:53455-53593
MsHel-5	gb|AIXA01017070.1|:12991-13181
MsHel-5	gb|AIXA01008666.1|:34575-34780
MsHel-5	gb|AIXA01006106.1|:10725-10913
MsHel-5	gb|AIXA01004965.1|:27032-27231
MsHel-5	gb|AIXA01004654.1|:12-101
MsHel-5	gb|AIXA01012029.1|:6056-6236
MsHel-5	gb|AIXA01010116.1|:993-1114
MsHel-5	gb|AIXA01003643.1|:10936-11130
MsHel-5	gb|AIXA01002296.1|:41893-42091
MsHel-5	gb|AIXA01018165.1|:6-148
MsHel-5	gb|AIXA01015427.1|:17760-17957
MsHel-5	gb|AIXA01013382.1|:1-173
MsHel-5	gb|AIXA01010253.1|:48148-48234
MsHel-5	gb|AIXA01007795.1|:39988-40148
MsHel-5	gb|AIXA01005070.1|:1-195
MsHel-5	gb|AIXA01002648.1|:6346-6484
MsHel-5	gb|AIXA01002255.1|:10208-10295
MsHel-5	gb|AIXA01016649.1|:9-97

MsHel-5	gb|AIXA01024216.1|:641-838
MsHel-5	gb|AIXA01016203.1|:652-738
MsHel-5	gb|AIXA01009907.1|:8754-8916
MsHel-5	gb|AIXA01008033.1|:56286-56372
MsHel-5	gb|AIXA01003663.1|:40210-40373
MsHel-5	gb|AIXA01013123.1|:9508-9646
MsHel-5	gb|AIXA01005479.1|:13814-20440
MsHel-5	gb|AIXA01002662.1|:22-115
MsHel-5	gb|AIXA01002527.1|:50241-50445
MsHel-5	gb|AIXA01028455.1|:861-962
MsHel-5	gb|AIXA01019657.1|:1-143
MsHel-5	gb|AIXA01015245.1|:18458-18636
MsHel-5	gb|AIXA01012697.1|:1-138
MsHel-5	gb|AIXA01004230.1|:4405-4601
MsHel-5	gb|AIXA01033339.1|:395-555
MsHel-5	gb|AIXA01030076.1|:747-845
MsHel-5	gb|AIXA01013073.1|:441-712
MsHel-5	gb|AIXA01011190.1|:15242-15440
MsHel-5	gb|AIXA01009490.1|:35159-35252
MsHel-5	gb|AIXA01005627.1|:24019-24148
MsHel-5	gb|AIXA01009355.1|:28197-28293
MsHel-5	gb|AIXA01006860.1|:31040-31229
MsHel-5	gb|AIXA01005470.1|:41385-41485
MsHel-5	gb|AIXA01002866.1|:14447-14535
MsHel-5	gb|AIXA01014108.1|:1-158
MsHel-5	gb|AIXA01003118.1|:49006-49140
MsHel-5	gb|AIXA01002783.1|:6019-6214
MsHel-5	gb|AIXA01021921.1|:805-904
MsHel-5	gb|AIXA01001663.1|:17090-17262
MsHel-5	gb|AIXA01036129.1|:1-88
MsHel-5	gb|AIXA01008109.1|:50095-50267
MsHel-5	gb|AIXA01034010.1|:522-621
MsHel-5	gb|AIXA01007476.1|:19918-20015
MsHel-5	gb|AIXA01000451.1|:14-107
MsHel-5	gb|AIXA01007295.1|:22553-22653
MsHel-5	gb|AIXA01037743.1|:415-506
MsHel-5	gb|AIXA01033906.1|:572-663
MsHel-5	gb|AIXA01018280.1|:1-88
MsHel-5	gb|AIXA01012099.1|:6-95
MsHel-5	gb|AIXA01011451.1|:2802-2895
MsHel-5	gb|AIXA01009956.1|:2189-2266
MsHel-5	gb|AIXA01007789.1|:31-123
MsHel-5	gb|AIXA01001117.1|:74223-74397
MsHel-5	gb|AIXA01017103.1|:1017-1119
MsHel-5	gb|AIXA01014983.1|:20662-20741
MsHel-5	gb|AIXA01036951.1|:461-545
MsHel-5	gb|AIXA01009445.1|:4088-4177
MsHel-5	gb|AIXA01007946.1|:95591-95678

MsHel-5	gb|AIXA01006123.1|:4340-5556
MsHel-5	gb|AIXA01018418.1|:2248-2336
MsHel-5	gb|AIXA01026387.1|:1045-1132
MsHel-5	gb|AIXA01004336.1|:22693-22781
MsHel-6	gb|AIXA01013450.1|:15906-16172
MsHel-6	gb|AIXA01005230.1|:15171-15439
MsHel-6	gb|AIXA01008226.1|:41269-41531
MsHel-6	gb|AIXA01008226.1|:29962-30211
MsHel-6	gb|AIXA01000337.1|:51233-51498
MsHel-6	gb|AIXA01009648.1|:22165-22435
MsHel-6	gb|AIXA01007984.1|:15741-15988
MsHel-6	gb|AIXA01000783.1|:35747-36008
MsHel-6	gb|AIXA01006509.1|:35720-35986
MsHel-6	gb|AIXA01012810.1|:19721-19986
MsHel-6	gb|AIXA01000091.1|:83312-83518
MsHel-6	gb|AIXA01000091.1|:49411-49627
MsHel-6	gb|AIXA01004158.1|:62426-62675
MsHel-6	gb|AIXA01001291.1|:5771-6032
MsHel-6	gb|AIXA01000838.1|:141253-141500
MsHel-6	gb|AIXA01000838.1|:87280-88426
MsHel-6	gb|AIXA01003599.1|:35830-36073
MsHel-6	gb|AIXA01003329.1|:120736-120991
MsHel-6	gb|AIXA01000959.1|:45362-45617
MsHel-6	gb|AIXA01013103.1|:16920-17184
MsHel-6	gb|AIXA01009040.1|:134059-134213
MsHel-6	gb|AIXA01009040.1|:101843-102104
MsHel-6	gb|AIXA01002693.1|:11402-11659
MsHel-6	gb|AIXA01000105.1|:53638-53908
MsHel-6	gb|AIXA01015390.1|:8822-9074
MsHel-6	gb|AIXA01005333.1|:6739-7002
MsHel-6	gb|AIXA01002717.1|:24317-24525
MsHel-6	gb|AIXA01001558.1|:74955-75150
MsHel-6	gb|AIXA01001558.1|:33454-33708
MsHel-6	gb|AIXA01005486.1|:29419-29689
MsHel-6	gb|AIXA01001797.1|:252741-253006
MsHel-6	gb|AIXA01001836.1|:8103-8360
MsHel-6	gb|AIXA01000948.1|:31507-31692
MsHel-6	gb|AIXA01000436.1|:18669-18932
MsHel-6	gb|AIXA01017681.1|:190-448
MsHel-6	gb|AIXA01018554.1|:2156-2403
MsHel-6	gb|AIXA01015706.1|:971-1229
MsHel-6	gb|AIXA01013643.1|:2073-2339
MsHel-6	gb|AIXA01012079.1|:4247-7737
MsHel-6	gb|AIXA01005351.1|:60239-60490
MsHel-6	gb|AIXA01001823.1|:42401-42656
MsHel-6	gb|AIXA01001823.1|:28147-28392
MsHel-6	gb|AIXA01005353.1|:15649-15914
MsHel-6	gb|AIXA01004329.1|:6229-6442

MsHel-6	gb|AIXA01002629.1|:9161-9404
MsHel-6	gb|AIXA01001755.1|:106686-106853
MsHel-6	gb|AIXA01010185.1|:56525-56793
MsHel-6	gb|AIXA01007856.1|:77847-78203
MsHel-6	gb|AIXA01001759.1|:1738-2001
MsHel-6	gb|AIXA01008562.1|:27861-28109
MsHel-6	gb|AIXA01007114.1|:11513-11766
MsHel-6	gb|AIXA01024735.1|:154-407
MsHel-6	gb|AIXA01009129.1|:551-773
MsHel-6	gb|AIXA01003921.1|:27341-27599
MsHel-6	gb|AIXA01003884.1|:24021-24567
MsHel-6	gb|AIXA01015929.1|:40636-40890
MsHel-6	gb|AIXA01011303.1|:39042-39297
MsHel-6	gb|AIXA01004140.1|:18503-18770
MsHel-6	gb|AIXA01006111.1|:996-1216
MsHel-6	gb|AIXA01001830.1|:32422-32680
MsHel-6	gb|AIXA01001412.1|:12255-12461
MsHel-6	gb|AIXA01031811.1|:470-669
MsHel-6	gb|AIXA01005896.1|:11152-11418
MsHel-6	gb|AIXA01003661.1|:176658-176917
MsHel-6	gb|AIXA01003661.1|:100403-100665
MsHel-6	gb|AIXA01003661.1|:62594-62852
MsHel-6	gb|AIXA01002507.1|:93785-93996
MsHel-6	gb|AIXA01001599.1|:51689-51880
MsHel-6	gb|AIXA01001599.1|:30573-31127
MsHel-6	gb|AIXA01012538.1|:18036-18212
MsHel-6	gb|AIXA01007545.1|:22649-22920
MsHel-6	gb|AIXA01014990.1|:5210-5472
MsHel-6	gb|AIXA01011554.1|:14658-14916
MsHel-6	gb|AIXA01008488.1|:3024-3545
MsHel-6	gb|AIXA01004051.1|:12245-12450
MsHel-6	gb|AIXA01000761.1|:9905-10155
MsHel-6	gb|AIXA01000688.1|:110713-110971
MsHel-6	gb|AIXA01000688.1|:97313-97515
MsHel-6	gb|AIXA01000688.1|:6759-7021
MsHel-6	gb|AIXA01010542.1|:10275-10407
MsHel-6	gb|AIXA01006518.1|:84779-85023
MsHel-6	gb|AIXA01005391.1|:34326-34576
MsHel-6	gb|AIXA01005217.1|:56723-56977
MsHel-6	gb|AIXA01005217.1|:28949-29127
MsHel-6	gb|AIXA01005004.1|:38190-38463
MsHel-6	gb|AIXA01001323.1|:40493-40755
MsHel-6	gb|AIXA01000089.1|:54155-54352
MsHel-6	gb|AIXA01007033.1|:43048-43300
MsHel-6	gb|AIXA01000934.1|:45729-45985
MsHel-6	gb|AIXA01000231.1|:10010-10273
MsHel-6	gb|AIXA01012907.1|:5626-5881
MsHel-6	gb|AIXA01011568.1|:7352-7591

MsHel-6	gb|AIXA01008325.1|:34899-35158
MsHel-6	gb|AIXA01007477.1|:4929-5191
MsHel-6	gb|AIXA01003070.1|:38253-38519
MsHel-6	gb|AIXA01002651.1|:33207-33475
MsHel-6	gb|AIXA01002477.1|:15011-15163
MsHel-6	gb|AIXA01018251.1|:4899-5154
MsHel-6	gb|AIXA01016400.1|:11970-12167
MsHel-6	gb|AIXA01011979.1|:11762-12206
MsHel-6	gb|AIXA01010885.1|:66076-66231
MsHel-6	gb|AIXA01010816.1|:6945-7197
MsHel-6	gb|AIXA01009428.1|:10306-10527
MsHel-6	gb|AIXA01005643.1|:38524-38976
MsHel-6	gb|AIXA01004595.1|:45075-45332
MsHel-6	gb|AIXA01003725.1|:6910-7168
MsHel-6	gb|AIXA01002794.1|:54872-55138
MsHel-6	gb|AIXA01002330.1|:7541-7792
MsHel-6	gb|AIXA01001190.1|:17656-17860
MsHel-6	gb|AIXA01026831.1|:78-509
MsHel-6	gb|AIXA01015507.1|:15221-15440
MsHel-6	gb|AIXA01012617.1|:11023-11274
MsHel-6	gb|AIXA01012371.1|:9492-9702
MsHel-6	gb|AIXA01006726.1|:29146-29398
MsHel-6	gb|AIXA01004334.1|:8831-9023
MsHel-6	gb|AIXA01003712.1|:572-786
MsHel-6	gb|AIXA01003154.1|:26892-27275
MsHel-6	gb|AIXA01002469.1|:14338-14599
MsHel-6	gb|AIXA01001653.1|:24840-25070
MsHel-6	gb|AIXA01001653.1|:11588-11722
MsHel-6	gb|AIXA01001566.1|:33119-33371
MsHel-6	gb|AIXA01001566.1|:10167-10374
MsHel-6	gb|AIXA01000768.1|:569-830
MsHel-6	gb|AIXA01016959.1|:6863-7061
MsHel-6	gb|AIXA01014660.1|:1625-1878
MsHel-6	gb|AIXA01009002.1|:4923-5187
MsHel-6	gb|AIXA01008773.1|:20465-20660
MsHel-6	gb|AIXA01005047.1|:53390-53649
MsHel-6	gb|AIXA01004968.1|:9821-10077
MsHel-6	gb|AIXA01003052.1|:18018-18274
MsHel-6	gb|AIXA01002707.1|:49399-49636
MsHel-6	gb|AIXA01035937.1|:187-451
MsHel-6	gb|AIXA01016638.1|:254-458
MsHel-6	gb|AIXA01013913.1|:1113-1323
MsHel-6	gb|AIXA01011326.1|:3550-3797
MsHel-6	gb|AIXA01011126.1|:614-866
MsHel-6	gb|AIXA01008271.1|:11823-12086
MsHel-6	gb|AIXA01007579.1|:53066-53268
MsHel-6	gb|AIXA01007579.1|:29495-33678
MsHel-6	gb|AIXA01007551.1|:8986-9252

MsHel-6	gb|AIXA01004802.1|:88231-88493
MsHel-6	gb|AIXA01003023.1|:1372-1478
MsHel-6	gb|AIXA01002782.1|:21006-21265
MsHel-6	gb|AIXA01000455.1|:1841-2043
MsHel-6	gb|AIXA01013037.1|:5385-5639
MsHel-6	gb|AIXA01012974.1|:7564-7781
MsHel-6	gb|AIXA01012835.1|:2308-2513
MsHel-6	gb|AIXA01012135.1|:41671-41923
MsHel-6	gb|AIXA01011623.1|:1443-1884
MsHel-6	gb|AIXA01009772.1|:2137-2395
MsHel-6	gb|AIXA01008965.1|:14916-15159
MsHel-6	gb|AIXA01006136.1|:68279-68526
MsHel-6	gb|AIXA01006136.1|:33860-34059
MsHel-6	gb|AIXA01006136.1|:22168-22630
MsHel-6	gb|AIXA01004931.1|:20119-20308
MsHel-6	gb|AIXA01003292.1|:49842-50099
MsHel-6	gb|AIXA01003292.1|:32609-32815
MsHel-6	gb|AIXA01003285.1|:34686-34883
MsHel-6	gb|AIXA01000997.1|:23379-23638
MsHel-6	gb|AIXA01015339.1|:6661-6912
MsHel-6	gb|AIXA01015222.1|:3022-3223
MsHel-6	gb|AIXA01010108.1|:14750-14991
MsHel-6	gb|AIXA01009231.1|:5433-5708
MsHel-6	gb|AIXA01007369.1|:3706-3955
MsHel-6	gb|AIXA01004132.1|:16053-16273
MsHel-6	gb|AIXA01003331.1|:25477-25722
MsHel-6	gb|AIXA01002252.1|:3226-3487
MsHel-6	gb|AIXA01000810.1|:136131-136335
MsHel-6	gb|AIXA01000810.1|:99151-99375
MsHel-6	gb|AIXA01000437.1|:107629-107771
MsHel-6	gb|AIXA01000437.1|:62180-62418
MsHel-6	gb|AIXA01000090.1|:53779-54035
MsHel-6	gb|AIXA01011450.1|:104-350
MsHel-6	gb|AIXA01011369.1|:24245-24465
MsHel-6	gb|AIXA01010276.1|:1065-1318
MsHel-6	gb|AIXA01006860.1|:30301-30739
MsHel-6	gb|AIXA01004943.1|:19339-19554
MsHel-6	gb|AIXA01004478.1|:7107-7334
MsHel-6	gb|AIXA01003741.1|:32607-32867
MsHel-6	gb|AIXA01002248.1|:75631-75885
MsHel-6	gb|AIXA01001514.1|:6705-6850
MsHel-6	gb|AIXA01000770.1|:34631-34887
MsHel-6	gb|AIXA01000444.1|:66290-66553
MsHel-6	gb|AIXA01000444.1|:27116-27372
MsHel-6	gb|AIXA01018780.1|:3965-4220
MsHel-6	gb|AIXA01016544.1|:5793-6045
MsHel-6	gb|AIXA01010858.1|:1665-1871
MsHel-6	gb|AIXA01009080.1|:48261-48517

MsHel-6	gb|AIXA01004060.1|:1998-2241
MsHel-6	gb|AIXA01003858.1|:63275-63789
MsHel-6	gb|AIXA01003816.1|:41378-41629
MsHel-6	gb|AIXA01002398.1|:67418-67645
MsHel-6	gb|AIXA01001327.1|:25553-25811
MsHel-6	gb|AIXA01016762.1|:16583-16800
MsHel-6	gb|AIXA01016564.1|:5048-5252
MsHel-6	gb|AIXA01014213.1|:23437-23690
MsHel-6	gb|AIXA01013529.1|:42020-42216
MsHel-6	gb|AIXA01013458.1|:41402-41651
MsHel-6	gb|AIXA01011268.1|:28560-28797
MsHel-6	gb|AIXA01010294.1|:8322-8577
MsHel-6	gb|AIXA01008632.1|:53518-53708
MsHel-6	gb|AIXA01008632.1|:7404-7656
MsHel-6	gb|AIXA01006888.1|:9896-10153
MsHel-6	gb|AIXA01006878.1|:34619-34819
MsHel-6	gb|AIXA01006878.1|:21990-22254
MsHel-6	gb|AIXA01006861.1|:25105-25890
MsHel-6	gb|AIXA01004708.1|:102361-102585
MsHel-6	gb|AIXA01004708.1|:8595-8756
MsHel-6	gb|AIXA01004693.1|:17337-17513
MsHel-6	gb|AIXA01004433.1|:169620-169874
MsHel-6	gb|AIXA01002160.1|:22160-22368
MsHel-6	gb|AIXA01002157.1|:1971-2216
MsHel-6	gb|AIXA01001235.1|:46596-46813
MsHel-6	gb|AIXA01000016.1|:47906-48161
MsHel-6	gb|AIXA01018194.1|:1430-1678
MsHel-6	gb|AIXA01016869.1|:10096-10292
MsHel-6	gb|AIXA01016262.1|:2722-2970
MsHel-6	gb|AIXA01014764.1|:200-394
MsHel-6	gb|AIXA01014417.1|:3513-3762
MsHel-6	gb|AIXA01008706.1|:1277-1518
MsHel-6	gb|AIXA01008402.1|:16402-16644
MsHel-6	gb|AIXA01006809.1|:54189-54419
MsHel-6	gb|AIXA01005433.1|:50755-51026
MsHel-6	gb|AIXA01005433.1|:21704-21974
MsHel-6	gb|AIXA01005237.1|:18026-18279
MsHel-6	gb|AIXA01004568.1|:19630-19777
MsHel-6	gb|AIXA01004527.1|:57373-57613
MsHel-6	gb|AIXA01004527.1|:14342-14596
MsHel-6	gb|AIXA01004342.1|:7184-7442
MsHel-6	gb|AIXA01003895.1|:56025-56249
MsHel-6	gb|AIXA01002065.1|:34780-35492
MsHel-6	gb|AIXA01000171.1|:35436-35713
MsHel-6	gb|AIXA01000104.1|:6851-7110
MsHel-6	gb|AIXA01020113.1|:1070-1313
MsHel-6	gb|AIXA01017640.1|:5035-5292
MsHel-6	gb|AIXA01014079.1|:30868-31121

MsHel-6	gb|AIXA01013710.1|:2593-2800
MsHel-6	gb|AIXA01007107.1|:22959-23215
MsHel-6	gb|AIXA01006872.1|:2780-2995
MsHel-6	gb|AIXA01005439.1|:2589-2804
MsHel-6	gb|AIXA01003992.1|:20473-20738
MsHel-6	gb|AIXA01002582.1|:164813-164985
MsHel-6	gb|AIXA01002582.1|:132971-133231
MsHel-6	gb|AIXA01001502.1|:22775-23049
MsHel-6	gb|AIXA01001502.1|:8925-9127
MsHel-6	gb|AIXA01001313.1|:2522-2692
MsHel-6	gb|AIXA01032629.1|:171-427
MsHel-6	gb|AIXA01015168.1|:6830-7016
MsHel-6	gb|AIXA01011197.1|:67234-67488
MsHel-6	gb|AIXA01010918.1|:14035-14298
MsHel-6	gb|AIXA01009584.1|:51604-51848
MsHel-6	gb|AIXA01009083.1|:6519-6783
MsHel-6	gb|AIXA01006731.1|:28759-28915
MsHel-6	gb|AIXA01006731.1|:14192-14393
MsHel-6	gb|AIXA01006043.1|:6585-6738
MsHel-6	gb|AIXA01005599.1|:63209-63305
MsHel-6	gb|AIXA01005599.1|:24937-25099
MsHel-6	gb|AIXA01005599.1|:10472-10666
MsHel-6	gb|AIXA01004022.1|:4072-4326
MsHel-6	gb|AIXA01002213.1|:54913-55246
MsHel-6	gb|AIXA01001300.1|:10569-10776
MsHel-6	gb|AIXA01000727.1|:6837-7050
MsHel-6	gb|AIXA01011323.1|:21823-22043
MsHel-6	gb|AIXA01010323.1|:104537-104758
MsHel-6	gb|AIXA01006848.1|:6255-6496
MsHel-6	gb|AIXA01005504.1|:134218-134672
MsHel-6	gb|AIXA01005504.1|:113810-114138
MsHel-6	gb|AIXA01005221.1|:40956-41353
MsHel-6	gb|AIXA01004124.1|:64476-64731
MsHel-6	gb|AIXA01004057.1|:9076-9340
MsHel-6	gb|AIXA01003632.1|:53073-53331
MsHel-6	gb|AIXA01001526.1|:23738-23939
MsHel-6	gb|AIXA01001254.1|:33997-34186
MsHel-6	gb|AIXA01000909.1|:39413-39560
MsHel-6	gb|AIXA01000701.1|:62262-62661
MsHel-6	gb|AIXA01000661.1|:33213-33461
MsHel-6	gb|AIXA01000500.1|:4007-4210
MsHel-6	gb|AIXA01000319.1|:2495-2779
MsHel-6	gb|AIXA01000277.1|:16448-16713
MsHel-6	gb|AIXA01000021.1|:56815-56967
MsHel-6	gb|AIXA01000021.1|:17114-17377
MsHel-6	gb|AIXA01015252.1|:8143-8399
MsHel-6	gb|AIXA01014772.1|:1296-1456
MsHel-6	gb|AIXA01012351.1|:14135-14581

MsHel-6	gb|AIXA01012325.1|:10176-10627
MsHel-6	gb|AIXA01011726.1|:2774-3318
MsHel-6	gb|AIXA01010568.1|:4054-4251
MsHel-6	gb|AIXA01010118.1|:31465-31709
MsHel-6	gb|AIXA01006907.1|:17255-17992
MsHel-6	gb|AIXA01006793.1|:24283-24758
MsHel-6	gb|AIXA01005533.1|:4170-4363
MsHel-6	gb|AIXA01004424.1|:65529-65724
MsHel-6	gb|AIXA01001032.1|:65464-65716
MsHel-6	gb|AIXA01019575.1|:4378-4575
MsHel-6	gb|AIXA01019221.1|:4029-4276
MsHel-6	gb|AIXA01019132.1|:2796-2995
MsHel-6	gb|AIXA01016164.1|:944-1112
MsHel-6	gb|AIXA01013947.1|:5916-6118
MsHel-6	gb|AIXA01013807.1|:38425-38626
MsHel-6	gb|AIXA01012196.1|:45365-45568
MsHel-6	gb|AIXA01012196.1|:26387-26544
MsHel-6	gb|AIXA01010748.1|:62647-62891
MsHel-6	gb|AIXA01010748.1|:27246-27675
MsHel-6	gb|AIXA01009047.1|:49144-49368
MsHel-6	gb|AIXA01007506.1|:82339-82901
MsHel-6	gb|AIXA01005711.1|:13197-13468
MsHel-6	gb|AIXA01005095.1|:61461-61713
MsHel-6	gb|AIXA01004073.1|:1042-1326
MsHel-6	gb|AIXA01003749.1|:18250-18799
MsHel-6	gb|AIXA01003068.1|:9387-9652
MsHel-6	gb|AIXA01001968.1|:30506-30709
MsHel-6	gb|AIXA01001618.1|:14105-14288
MsHel-6	gb|AIXA01001348.1|:7099-7340
MsHel-6	gb|AIXA01000892.1|:70016-70235
MsHel-6	gb|AIXA01000892.1|:52847-53101
MsHel-6	gb|AIXA01000552.1|:3958-4208
MsHel-6	gb|AIXA01015052.1|:3788-4009
MsHel-6	gb|AIXA01013812.1|:26887-27048
MsHel-6	gb|AIXA01012889.1|:6761-6966
MsHel-6	gb|AIXA01012643.1|:27798-27980
MsHel-6	gb|AIXA01011995.1|:16737-17207
MsHel-6	gb|AIXA01010880.1|:8560-8768
MsHel-6	gb|AIXA01009796.1|:2769-3015
MsHel-6	gb|AIXA01009490.1|:10741-10935
MsHel-6	gb|AIXA01009430.1|:22687-22954
MsHel-6	gb|AIXA01009425.1|:6222-6436
MsHel-6	gb|AIXA01007966.1|:130265-130368
MsHel-6	gb|AIXA01007966.1|:16296-16441
MsHel-6	gb|AIXA01007218.1|:11926-12140
MsHel-6	gb|AIXA01005003.1|:24337-24595
MsHel-6	gb|AIXA01004562.1|:2231-3148
MsHel-6	gb|AIXA01003334.1|:2113-2358

MsHel-6	gb|AIXA01002913.1|:20532-21278
MsHel-6	gb|AIXA01002503.1|:5550-5804
MsHel-6	gb|AIXA01002313.1|:5876-6125
MsHel-6	gb|AIXA01001648.1|:57519-57664
MsHel-6	gb|AIXA01001648.1|:39986-40157
MsHel-6	gb|AIXA01000655.1|:91405-91664
MsHel-6	gb|AIXA01000655.1|:5485-6096
MsHel-6	gb|AIXA01000611.1|:57161-57432
MsHel-6	gb|AIXA01000577.1|:16298-16549
MsHel-6	gb|AIXA01000069.1|:267375-267587
MsHel-6	gb|AIXA01000069.1|:3078-3331
MsHel-6	gb|AIXA01028066.1|:773-986
MsHel-6	gb|AIXA01013695.1|:3652-3856
MsHel-6	gb|AIXA01011987.1|:35969-36196
MsHel-6	gb|AIXA01011610.1|:41456-41739
MsHel-6	gb|AIXA01011407.1|:24576-24777
MsHel-6	gb|AIXA01009696.1|:21799-22057
MsHel-6	gb|AIXA01008593.1|:9034-9296
MsHel-6	gb|AIXA01008294.1|:39566-39829
MsHel-6	gb|AIXA01006808.1|:142-339
MsHel-6	gb|AIXA01005028.1|:3401-3648
MsHel-6	gb|AIXA01004952.1|:27013-27236
MsHel-6	gb|AIXA01003928.1|:84838-85099
MsHel-6	gb|AIXA01003692.1|:20711-20962
MsHel-6	gb|AIXA01002674.1|:5928-14877
MsHel-6	gb|AIXA01001196.1|:27970-28228
MsHel-6	gb|AIXA01034672.1|:135-400
MsHel-6	gb|AIXA01023902.1|:645-894
MsHel-6	gb|AIXA01015157.1|:1270-1467
MsHel-6	gb|AIXA01014372.1|:8576-8983
MsHel-6	gb|AIXA01012306.1|:1214-1409
MsHel-6	gb|AIXA01011986.1|:3649-3840
MsHel-6	gb|AIXA01007354.1|:24932-25152
MsHel-6	gb|AIXA01006141.1|:56005-56259
MsHel-6	gb|AIXA01006141.1|:12075-12260
MsHel-6	gb|AIXA01005494.1|:29699-29905
MsHel-6	gb|AIXA01005309.1|:12328-12583
MsHel-6	gb|AIXA01004670.1|:160178-160429
MsHel-6	gb|AIXA01004028.1|:8864-9117
MsHel-6	gb|AIXA01003836.1|:10477-10673
MsHel-6	gb|AIXA01003611.1|:15533-15729
MsHel-6	gb|AIXA01003611.1|:2340-2537
MsHel-6	gb|AIXA01003376.1|:2503-3512
MsHel-6	gb|AIXA01003045.1|:43514-43715
MsHel-6	gb|AIXA01001446.1|:26663-27108
MsHel-6	gb|AIXA01000235.1|:49280-49543
MsHel-6	gb|AIXA01019823.1|:760-976
MsHel-6	gb|AIXA01017184.1|:5624-5873

MsHel-6	gb|AIXA01016329.1|:3828-4116
MsHel-6	gb|AIXA01013134.1|:4971-5166
MsHel-6	gb|AIXA01010797.1|:4002-4272
MsHel-6	gb|AIXA01010110.1|:16186-16409
MsHel-6	gb|AIXA01003328.1|:8745-8945
MsHel-6	gb|AIXA01001222.1|:89498-89656
MsHel-6	gb|AIXA01001222.1|:42153-42401
MsHel-6	gb|AIXA01000155.1|:27814-28034
MsHel-6	gb|AIXA01000111.1|:34875-35060
MsHel-6	gb|AIXA01019095.1|:1519-1737
MsHel-6	gb|AIXA01017038.1|:13307-13572
MsHel-6	gb|AIXA01016081.1|:2063-2258
MsHel-6	gb|AIXA01012477.1|:28113-28388
MsHel-6	gb|AIXA01007713.1|:13841-14092
MsHel-6	gb|AIXA01007344.1|:48884-49321
MsHel-6	gb|AIXA01002870.1|:2391-2556
MsHel-6	gb|AIXA01002579.1|:123919-124173
MsHel-6	gb|AIXA01001239.1|:123978-124195
MsHel-6	gb|AIXA01001239.1|:79971-80361
MsHel-6	gb|AIXA01000650.1|:93930-94298
MsHel-6	gb|AIXA01000006.1|:20371-20589
MsHel-6	gb|AIXA01028635.1|:97-363
MsHel-6	gb|AIXA01027543.1|:361-621
MsHel-6	gb|AIXA01017935.1|:10155-10401
MsHel-6	gb|AIXA01016066.1|:1768-1986
MsHel-6	gb|AIXA01015221.1|:10229-10418
MsHel-6	gb|AIXA01013942.1|:17030-17218
MsHel-6	gb|AIXA01013626.1|:708-909
MsHel-6	gb|AIXA01011812.1|:10365-11697
MsHel-6	gb|AIXA01009860.1|:14831-15259
MsHel-6	gb|AIXA01007265.1|:18470-18724
MsHel-6	gb|AIXA01005639.1|:2371-2646
MsHel-6	gb|AIXA01004483.1|:8836-9096
MsHel-6	gb|AIXA01004421.1|:9574-9937
MsHel-6	gb|AIXA01001811.1|:13822-14076
MsHel-6	gb|AIXA01001665.1|:12623-13085
MsHel-6	gb|AIXA01000947.1|:23558-23816
MsHel-6	gb|AIXA01029447.1|:641-886
MsHel-6	gb|AIXA01020518.1|:522-786
MsHel-6	gb|AIXA01017440.1|:1783-1999
MsHel-6	gb|AIXA01016603.1|:753-1004
MsHel-6	gb|AIXA01014695.1|:3708-3902
MsHel-6	gb|AIXA01013521.1|:24555-24745
MsHel-6	gb|AIXA01012311.1|:4023-4207
MsHel-6	gb|AIXA01009699.1|:27220-27440
MsHel-6	gb|AIXA01009580.1|:30033-30296
MsHel-6	gb|AIXA01009427.1|:23083-23334
MsHel-6	gb|AIXA01009405.1|:14332-14582

MsHel-6	gb|AIXA01009132.1|:1954-2151
MsHel-6	gb|AIXA01007761.1|:1694-1949
MsHel-6	gb|AIXA01006279.1|:1985-2144
MsHel-6	gb|AIXA01005881.1|:3871-4071
MsHel-6	gb|AIXA01004915.1|:540-728
MsHel-6	gb|AIXA01004113.1|:108923-109160
MsHel-6	gb|AIXA01004113.1|:72512-72727
MsHel-6	gb|AIXA01003682.1|:29060-29302
MsHel-6	gb|AIXA01003677.1|:53086-53302
MsHel-6	gb|AIXA01002500.1|:160217-160432
MsHel-6	gb|AIXA01002500.1|:143085-143537
MsHel-6	gb|AIXA01002500.1|:83711-83970
MsHel-6	gb|AIXA01002489.1|:12961-13221
MsHel-6	gb|AIXA01002381.1|:12576-12855
MsHel-6	gb|AIXA01001292.1|:16919-17177
MsHel-6	gb|AIXA01018303.1|:6557-6815
MsHel-6	gb|AIXA01017620.1|:1891-2080
MsHel-6	gb|AIXA01015448.1|:11683-11936
MsHel-6	gb|AIXA01013938.1|:8061-8223
MsHel-6	gb|AIXA01011241.1|:11287-11499
MsHel-6	gb|AIXA01010358.1|:8907-9117
MsHel-6	gb|AIXA01010275.1|:1021-1274
MsHel-6	gb|AIXA01009922.1|:13144-13367
MsHel-6	gb|AIXA01008263.1|:56107-56317
MsHel-6	gb|AIXA01008021.1|:11328-11529
MsHel-6	gb|AIXA01005531.1|:24444-24640
MsHel-6	gb|AIXA01005349.1|:61233-61440
MsHel-6	gb|AIXA01004909.1|:1348-1601
MsHel-6	gb|AIXA01004805.1|:25437-25694
MsHel-6	gb|AIXA01004512.1|:76-322
MsHel-6	gb|AIXA01004351.1|:41492-41745
MsHel-6	gb|AIXA01003283.1|:31374-31619
MsHel-6	gb|AIXA01003033.1|:4814-5063
MsHel-6	gb|AIXA01001408.1|:584-791
MsHel-6	gb|AIXA01001299.1|:433-677
MsHel-6	gb|AIXA01001297.1|:5915-6110
MsHel-6	gb|AIXA01029289.1|:161-384
MsHel-6	gb|AIXA01022451.1|:1337-1587
MsHel-6	gb|AIXA01017568.1|:9572-9823
MsHel-6	gb|AIXA01012645.1|:6347-6571
MsHel-6	gb|AIXA01012158.1|:14303-14493
MsHel-6	gb|AIXA01011273.1|:44762-45473
MsHel-6	gb|AIXA01010855.1|:21493-21640
MsHel-6	gb|AIXA01010855.1|:8065-8230
MsHel-6	gb|AIXA01009126.1|:25650-25865
MsHel-6	gb|AIXA01009126.1|:8451-9434
MsHel-6	gb|AIXA01007601.1|:6958-7509
MsHel-6	gb|AIXA01005407.1|:30099-30347

MsHel-6	gb|AIXA01003876.1|:127731-128172
MsHel-6	gb|AIXA01003876.1|:7405-7654
MsHel-6	gb|AIXA01003002.1|:71859-72086
MsHel-6	gb|AIXA01002593.1|:11312-11563
MsHel-6	gb|AIXA01001926.1|:71069-71188
MsHel-6	gb|AIXA01001926.1|:15868-16063
MsHel-6	gb|AIXA01001825.1|:19521-19722
MsHel-6	gb|AIXA01001825.1|:4507-4667
MsHel-6	gb|AIXA01001762.1|:34296-34464
MsHel-6	gb|AIXA01001762.1|:15950-16195
MsHel-6	gb|AIXA01000211.1|:25693-25956
MsHel-6	gb|AIXA01000071.1|:3112-3376
MsHel-6	gb|AIXA01027904.1|:525-717
MsHel-6	gb|AIXA01025944.1|:577-778
MsHel-6	gb|AIXA01017028.1|:12594-12781
MsHel-6	gb|AIXA01011589.1|:2956-3215
MsHel-6	gb|AIXA01010733.1|:6675-6887
MsHel-6	gb|AIXA01009865.1|:3463-3687
MsHel-6	gb|AIXA01008767.1|:73938-74200
MsHel-6	gb|AIXA01007783.1|:58335-58583
MsHel-6	gb|AIXA01007281.1|:4165-4412
MsHel-6	gb|AIXA01006143.1|:14566-14808
MsHel-6	gb|AIXA01005701.1|:1822-2344
MsHel-6	gb|AIXA01005404.1|:98-289
MsHel-6	gb|AIXA01005105.1|:12821-13042
MsHel-6	gb|AIXA01004987.1|:79201-79386
MsHel-6	gb|AIXA01004976.1|:326-529
MsHel-6	gb|AIXA01004160.1|:51634-51887
MsHel-6	gb|AIXA01004160.1|:28794-29030
MsHel-6	gb|AIXA01003112.1|:37331-37585
MsHel-6	gb|AIXA01002652.1|:20450-20725
MsHel-6	gb|AIXA01000905.1|:43614-43827
MsHel-6	gb|AIXA01019298.1|:1672-1888
MsHel-6	gb|AIXA01018307.1|:2734-2946
MsHel-6	gb|AIXA01017109.1|:7803-8059
MsHel-6	gb|AIXA01015925.1|:5387-5563
MsHel-6	gb|AIXA01012915.1|:1473-1727
MsHel-6	gb|AIXA01008195.1|:18317-18529
MsHel-6	gb|AIXA01007790.1|:103543-103722
MsHel-6	gb|AIXA01007350.1|:92394-92591
MsHel-6	gb|AIXA01005793.1|:10433-10972
MsHel-6	gb|AIXA01005659.1|:53266-53434
MsHel-6	gb|AIXA01004565.1|:31690-31942
MsHel-6	gb|AIXA01004230.1|:43441-43637
MsHel-6	gb|AIXA01004154.1|:45159-45322
MsHel-6	gb|AIXA01002399.1|:8511-8745
MsHel-6	gb|AIXA01002386.1|:20037-20280
MsHel-6	gb|AIXA01002215.1|:37858-38062

MsHel-6	gb|AIXA01002006.1|:13217-13643
MsHel-6	gb|AIXA01001860.1|:9853-10061
MsHel-6	gb|AIXA01001729.1|:1781-1988
MsHel-6	gb|AIXA01001694.1|:89127-89387
MsHel-6	gb|AIXA01001014.1|:8984-9228
MsHel-6	gb|AIXA01000790.1|:1867-2333
MsHel-6	gb|AIXA01034030.1|:59-222
MsHel-6	gb|AIXA01030656.1|:19-277
MsHel-6	gb|AIXA01022972.1|:33-289
MsHel-6	gb|AIXA01022375.1|:254-469
MsHel-6	gb|AIXA01020220.1|:2644-2893
MsHel-6	gb|AIXA01016160.1|:481-698
MsHel-6	gb|AIXA01010109.1|:9286-9497
MsHel-6	gb|AIXA01009835.1|:29564-29774
MsHel-6	gb|AIXA01008635.1|:3760-4010
MsHel-6	gb|AIXA01006506.1|:5690-5933
MsHel-6	gb|AIXA01006175.1|:91737-91953
MsHel-6	gb|AIXA01006175.1|:20629-20871
MsHel-6	gb|AIXA01005592.1|:30431-30784
MsHel-6	gb|AIXA01003935.1|:35557-35923
MsHel-6	gb|AIXA01003896.1|:2333-2546
MsHel-6	gb|AIXA01003051.1|:28966-29222
MsHel-6	gb|AIXA01003012.1|:49228-49488
MsHel-6	gb|AIXA01002999.1|:39570-39826
MsHel-6	gb|AIXA01002235.1|:31823-32070
MsHel-6	gb|AIXA01002044.1|:112537-112792
MsHel-6	gb|AIXA01001520.1|:32581-32798
MsHel-6	gb|AIXA01001351.1|:30755-31007
MsHel-6	gb|AIXA01001296.1|:171729-174245
MsHel-6	gb|AIXA01000968.1|:7485-7732
MsHel-6	gb|AIXA01033968.1|:103-264
MsHel-6	gb|AIXA01015193.1|:5230-10126
MsHel-6	gb|AIXA01014673.1|:5039-5289
MsHel-6	gb|AIXA01011718.1|:31855-32073
MsHel-6	gb|AIXA01011498.1|:33496-33658
MsHel-6	gb|AIXA01007104.1|:22041-22310
MsHel-6	gb|AIXA01006656.1|:3642-3840
MsHel-6	gb|AIXA01005573.1|:12477-12737
MsHel-6	gb|AIXA01005273.1|:4165-4602
MsHel-6	gb|AIXA01005096.1|:29614-29993
MsHel-6	gb|AIXA01004330.1|:51628-51881
MsHel-6	gb|AIXA01004247.1|:13210-13371
MsHel-6	gb|AIXA01004089.1|:40840-41060
MsHel-6	gb|AIXA01003516.1|:10506-10764
MsHel-6	gb|AIXA01001129.1|:46296-46493
MsHel-6	gb|AIXA01000596.1|:7488-7749
MsHel-6	gb|AIXA01000102.1|:40261-40483
MsHel-6	gb|AIXA01025057.1|:403-656

MsHel-6	gb|AIXA01024335.1|:152-348
MsHel-6	gb|AIXA01021015.1|:1942-2131
MsHel-6	gb|AIXA01020036.1|:403-594
MsHel-6	gb|AIXA01017831.1|:11958-12150
MsHel-6	gb|AIXA01014617.1|:3560-3938
MsHel-6	gb|AIXA01013041.1|:40664-40867
MsHel-6	gb|AIXA01012544.1|:4058-4217
MsHel-6	gb|AIXA01011104.1|:10156-10321
MsHel-6	gb|AIXA01008112.1|:47448-47686
MsHel-6	gb|AIXA01008111.1|:4981-5519
MsHel-6	gb|AIXA01006854.1|:31528-31959
MsHel-6	gb|AIXA01006854.1|:19978-20174
MsHel-6	gb|AIXA01006847.1|:17697-21412
MsHel-6	gb|AIXA01006403.1|:79520-79723
MsHel-6	gb|AIXA01004597.1|:65068-65249
MsHel-6	gb|AIXA01002312.1|:1387-1603
MsHel-6	gb|AIXA01001912.1|:37299-37547
MsHel-6	gb|AIXA01001699.1|:2484-2685
MsHel-6	gb|AIXA01001613.1|:7979-8139
MsHel-6	gb|AIXA01001518.1|:19983-20423
MsHel-6	gb|AIXA01001355.1|:41155-41348
MsHel-6	gb|AIXA01000766.1|:3244-3690
MsHel-6	gb|AIXA01000315.1|:27996-28256
MsHel-6	gb|AIXA01021288.1|:557-1266
MsHel-6	gb|AIXA01017122.1|:2546-2735
MsHel-6	gb|AIXA01016904.1|:4077-4291
MsHel-6	gb|AIXA01016323.1|:9600-9806
MsHel-6	gb|AIXA01015684.1|:6098-6295
MsHel-6	gb|AIXA01014761.1|:2694-2881
MsHel-6	gb|AIXA01014084.1|:4312-5144
MsHel-6	gb|AIXA01011624.1|:19457-19675
MsHel-6	gb|AIXA01009223.1|:87-500
MsHel-6	gb|AIXA01008897.1|:14893-15155
MsHel-6	gb|AIXA01008492.1|:16916-17172
MsHel-6	gb|AIXA01007947.1|:32080-32274
MsHel-6	gb|AIXA01006967.1|:1125-1344
MsHel-6	gb|AIXA01005535.1|:10717-10914
MsHel-6	gb|AIXA01003839.1|:10901-11159
MsHel-6	gb|AIXA01002671.1|:7985-8443
MsHel-6	gb|AIXA01002625.1|:63511-63764
MsHel-6	gb|AIXA01001990.1|:27611-27862
MsHel-6	gb|AIXA01000908.1|:23871-24068
MsHel-6	gb|AIXA01000064.1|:29365-29625
MsHel-6	gb|AIXA01015854.1|:4294-4545
MsHel-6	gb|AIXA01013331.1|:49471-49716
MsHel-6	gb|AIXA01011822.1|:75-254
MsHel-6	gb|AIXA01011243.1|:38476-38726
MsHel-6	gb|AIXA01011239.1|:47991-48429

MsHel-6	gb|AIXA01009102.1|:11681-11937
MsHel-6	gb|AIXA01004554.1|:34135-34391
MsHel-6	gb|AIXA01002242.1|:37042-37286
MsHel-6	gb|AIXA01001841.1|:14250-14446
MsHel-6	gb|AIXA01001610.1|:3477-3736
MsHel-6	gb|AIXA01000990.1|:23592-23845
MsHel-6	gb|AIXA01036848.1|:31-218
MsHel-6	gb|AIXA01018771.1|:1216-1438
MsHel-6	gb|AIXA01014825.1|:4049-4251
MsHel-6	gb|AIXA01011734.1|:27753-27951
MsHel-6	gb|AIXA01010308.1|:819-1001
MsHel-6	gb|AIXA01008988.1|:13994-14197
MsHel-6	gb|AIXA01008815.1|:3960-4142
MsHel-6	gb|AIXA01008690.1|:19519-19693
MsHel-6	gb|AIXA01007052.1|:5195-5360
MsHel-6	gb|AIXA01005692.1|:19439-19653
MsHel-6	gb|AIXA01005506.1|:2753-3007
MsHel-6	gb|AIXA01004470.1|:3368-3563
MsHel-6	gb|AIXA01003881.1|:55810-56015
MsHel-6	gb|AIXA01003318.1|:12722-12920
MsHel-6	gb|AIXA01003024.1|:661-913
MsHel-6	gb|AIXA01001090.1|:15523-15758
MsHel-6	gb|AIXA01024474.1|:320-827
MsHel-6	gb|AIXA01022465.1|:1306-1454
MsHel-6	gb|AIXA01018924.1|:115-814
MsHel-6	gb|AIXA01016633.1|:1885-2082
MsHel-6	gb|AIXA01015621.1|:1160-1422
MsHel-6	gb|AIXA01015274.1|:5075-5327
MsHel-6	gb|AIXA01015192.1|:7565-8174
MsHel-6	gb|AIXA01012656.1|:5163-5409
MsHel-6	gb|AIXA01007730.1|:7968-8191
MsHel-6	gb|AIXA01007101.1|:494-750
MsHel-6	gb|AIXA01006864.1|:8074-8332
MsHel-6	gb|AIXA01004541.1|:3207-3462
MsHel-6	gb|AIXA01004260.1|:148699-148987
MsHel-6	gb|AIXA01004084.1|:62464-62652
MsHel-6	gb|AIXA01003996.1|:11144-11308
MsHel-6	gb|AIXA01001627.1|:59328-59524
MsHel-6	gb|AIXA01001544.1|:29375-29627
MsHel-6	gb|AIXA01001045.1|:12919-13128
MsHel-6	gb|AIXA01000801.1|:28552-28744
MsHel-6	gb|AIXA01016925.1|:974-1173
MsHel-6	gb|AIXA01016418.1|:15364-15557
MsHel-6	gb|AIXA01015608.1|:1357-1605
MsHel-6	gb|AIXA01012715.1|:5921-6445
MsHel-6	gb|AIXA01012209.1|:15294-15816
MsHel-6	gb|AIXA01012197.1|:24370-27697
MsHel-6	gb|AIXA01009396.1|:3132-3377

MsHel-6	gb|AIXA01009357.1|:33230-33441
MsHel-6	gb|AIXA01008406.1|:80577-80825
MsHel-6	gb|AIXA01005668.1|:7206-7736
MsHel-6	gb|AIXA01005490.1|:22970-23184
MsHel-6	gb|AIXA01002698.1|:15412-15665
MsHel-6	gb|AIXA01001963.1|:48001-48165
MsHel-6	gb|AIXA01001963.1|:7446-7699
MsHel-6	gb|AIXA01000662.1|:127983-128250
MsHel-6	gb|AIXA01000662.1|:97454-97615
MsHel-6	gb|AIXA01000440.1|:41651-41915
MsHel-6	gb|AIXA01014980.1|:18857-19107
MsHel-6	gb|AIXA01012554.1|:40451-40699
MsHel-6	gb|AIXA01011621.1|:4862-5086
MsHel-6	gb|AIXA01009719.1|:25706-26148
MsHel-6	gb|AIXA01007634.1|:24352-24609
MsHel-6	gb|AIXA01007100.1|:28578-28789
MsHel-6	gb|AIXA01006498.1|:17455-17629
MsHel-6	gb|AIXA01004586.1|:22753-22895
MsHel-6	gb|AIXA01003857.1|:47025-47216
MsHel-6	gb|AIXA01003857.1|:7625-7808
MsHel-6	gb|AIXA01003079.1|:3902-4105
MsHel-6	gb|AIXA01002922.1|:56584-56840
MsHel-6	gb|AIXA01001786.1|:32064-32282
MsHel-6	gb|AIXA01000424.1|:46983-47196
MsHel-6	gb|AIXA01020354.1|:1619-1787
MsHel-6	gb|AIXA01019764.1|:2835-3070
MsHel-6	gb|AIXA01018907.1|:1339-1569
MsHel-6	gb|AIXA01018880.1|:46-243
MsHel-6	gb|AIXA01011366.1|:2120-2385
MsHel-6	gb|AIXA01010442.1|:14252-14507
MsHel-6	gb|AIXA01009407.1|:18423-18686
MsHel-6	gb|AIXA01009328.1|:15120-15378
MsHel-6	gb|AIXA01008566.1|:73286-73904
MsHel-6	gb|AIXA01008328.1|:5585-5852
MsHel-6	gb|AIXA01008083.1|:21316-21567
MsHel-6	gb|AIXA01007157.1|:4797-4983
MsHel-6	gb|AIXA01006548.1|:19189-20186
MsHel-6	gb|AIXA01004486.1|:20499-20656
MsHel-6	gb|AIXA01004395.1|:12224-12446
MsHel-6	gb|AIXA01002596.1|:16427-16584
MsHel-6	gb|AIXA01001935.1|:14976-15171
MsHel-6	gb|AIXA01001820.1|:4873-5064
MsHel-6	gb|AIXA01034070.1|:31-220
MsHel-6	gb|AIXA01031199.1|:565-784
MsHel-6	gb|AIXA01024651.1|:977-1234
MsHel-6	gb|AIXA01019900.1|:2738-2911
MsHel-6	gb|AIXA01019323.1|:1165-1330
MsHel-6	gb|AIXA01018189.1|:5244-5497

MsHel-6	gb|AIXA01015620.1|:4444-4706
MsHel-6	gb|AIXA01013301.1|:17594-17858
MsHel-6	gb|AIXA01012653.1|:1667-1853
MsHel-6	gb|AIXA01012348.1|:23-239
MsHel-6	gb|AIXA01011985.1|:13345-13501
MsHel-6	gb|AIXA01010406.1|:82727-82986
MsHel-6	gb|AIXA01008398.1|:41079-41322
MsHel-6	gb|AIXA01006853.1|:61049-61483
MsHel-6	gb|AIXA01005319.1|:44356-44549
MsHel-6	gb|AIXA01004755.1|:50253-50469
MsHel-6	gb|AIXA01004457.1|:30071-30219
MsHel-6	gb|AIXA01004374.1|:9354-9564
MsHel-6	gb|AIXA01004082.1|:33341-33543
MsHel-6	gb|AIXA01004082.1|:11325-11571
MsHel-6	gb|AIXA01004082.1|:515-631
MsHel-6	gb|AIXA01002994.1|:78839-79101
MsHel-6	gb|AIXA01002350.1|:128637-128794
MsHel-6	gb|AIXA01001048.1|:115687-115924
MsHel-6	gb|AIXA01000048.1|:73106-73238
MsHel-6	gb|AIXA01026643.1|:674-1107
MsHel-6	gb|AIXA01017183.1|:12-275
MsHel-6	gb|AIXA01014781.1|:3169-3301
MsHel-6	gb|AIXA01013524.1|:20735-20933
MsHel-6	gb|AIXA01009841.1|:4195-4354
MsHel-6	gb|AIXA01009371.1|:16519-16759
MsHel-6	gb|AIXA01004894.1|:37768-38023
MsHel-6	gb|AIXA01001289.1|:1916-2129
MsHel-6	gb|AIXA01000787.1|:26783-27008
MsHel-6	gb|AIXA01000787.1|:11265-11513
MsHel-6	gb|AIXA01020316.1|:168-541
MsHel-6	gb|AIXA01017885.1|:6308-6548
MsHel-6	gb|AIXA01016106.1|:4566-4761
MsHel-6	gb|AIXA01009812.1|:18659-18913
MsHel-6	gb|AIXA01008393.1|:19562-19752
MsHel-6	gb|AIXA01005892.1|:28214-28380
MsHel-6	gb|AIXA01005563.1|:17471-17723
MsHel-6	gb|AIXA01004275.1|:5734-5980
MsHel-6	gb|AIXA01003249.1|:28607-28770
MsHel-6	gb|AIXA01002658.1|:53976-54227
MsHel-6	gb|AIXA01002351.1|:547-741
MsHel-6	gb|AIXA01001989.1|:1766-2016
MsHel-6	gb|AIXA01000278.1|:13831-14054
MsHel-6	gb|AIXA01022250.1|:672-866
MsHel-6	gb|AIXA01018699.1|:2468-2659
MsHel-6	gb|AIXA01016265.1|:25510-25711
MsHel-6	gb|AIXA01010929.1|:2522-2930
MsHel-6	gb|AIXA01010690.1|:787-2335
MsHel-6	gb|AIXA01008634.1|:15148-15342

MsHel-6	gb|AIXA01006798.1|:5306-5559
MsHel-6	gb|AIXA01006682.1|:37830-37995
MsHel-6	gb|AIXA01006086.1|:8652-8881
MsHel-6	gb|AIXA01005993.1|:23099-23363
MsHel-6	gb|AIXA01005629.1|:53312-53579
MsHel-6	gb|AIXA01005071.1|:3468-4008
MsHel-6	gb|AIXA01004939.1|:482-662
MsHel-6	gb|AIXA01003927.1|:34271-34516
MsHel-6	gb|AIXA01000107.1|:135494-135732
MsHel-6	gb|AIXA01018789.1|:540-726
MsHel-6	gb|AIXA01016411.1|:1891-2126
MsHel-6	gb|AIXA01016392.1|:1910-2151
MsHel-6	gb|AIXA01016257.1|:1990-2235
MsHel-6	gb|AIXA01015219.1|:367-617
MsHel-6	gb|AIXA01014846.1|:23716-23958
MsHel-6	gb|AIXA01014126.1|:9917-10298
MsHel-6	gb|AIXA01013886.1|:2140-2335
MsHel-6	gb|AIXA01013811.1|:13784-13978
MsHel-6	gb|AIXA01012536.1|:11343-11502
MsHel-6	gb|AIXA01012534.1|:10739-10952
MsHel-6	gb|AIXA01011499.1|:13388-13578
MsHel-6	gb|AIXA01010900.1|:66178-66480
MsHel-6	gb|AIXA01010900.1|:13552-13806
MsHel-6	gb|AIXA01010870.1|:7475-7726
MsHel-6	gb|AIXA01009538.1|:22437-22589
MsHel-6	gb|AIXA01009116.1|:1641-1879
MsHel-6	gb|AIXA01008384.1|:6965-7123
MsHel-6	gb|AIXA01008376.1|:23002-23202
MsHel-6	gb|AIXA01007630.1|:78653-78913
MsHel-6	gb|AIXA01006430.1|:825-1047
MsHel-6	gb|AIXA01004465.1|:59370-59586
MsHel-6	gb|AIXA01004440.1|:2181-2432
MsHel-6	gb|AIXA01003277.1|:23077-23332
MsHel-6	gb|AIXA01002971.1|:29919-30163
MsHel-6	gb|AIXA01002909.1|:15584-16203
MsHel-6	gb|AIXA01002530.1|:6451-10541
MsHel-6	gb|AIXA01001465.1|:20737-20949
MsHel-6	gb|AIXA01000481.1|:537-736
MsHel-6	gb|AIXA01000320.1|:52250-52503
MsHel-6	gb|AIXA01018647.1|:2903-3123
MsHel-6	gb|AIXA01017625.1|:5753-5917
MsHel-6	gb|AIXA01016880.1|:242-441
MsHel-6	gb|AIXA01015151.1|:17529-17962
MsHel-6	gb|AIXA01008931.1|:17202-17463
MsHel-6	gb|AIXA01008327.1|:8641-8892
MsHel-6	gb|AIXA01008019.1|:15261-16466
MsHel-6	gb|AIXA01007926.1|:46961-47180
MsHel-6	gb|AIXA01004168.1|:2344-2599

MsHel-6	gb|AIXA01003301.1|:31157-31699
MsHel-6	gb|AIXA01001757.1|:29345-29565
MsHel-6	gb|AIXA01001657.1|:229885-230019
MsHel-6	gb|AIXA01001657.1|:155293-155476
MsHel-6	gb|AIXA01001290.1|:19862-20134
MsHel-6	gb|AIXA01001001.1|:21681-21932
MsHel-6	gb|AIXA01028798.1|:21-387
MsHel-6	gb|AIXA01025949.1|:4-189
MsHel-6	gb|AIXA01022014.1|:136-350
MsHel-6	gb|AIXA01020741.1|:308-795
MsHel-6	gb|AIXA01019386.1|:1859-2095
MsHel-6	gb|AIXA01017477.1|:1364-1543
MsHel-6	gb|AIXA01015806.1|:6214-6472
MsHel-6	gb|AIXA01012649.1|:14634-14833
MsHel-6	gb|AIXA01010321.1|:7370-7546
MsHel-6	gb|AIXA01008789.1|:27976-28232
MsHel-6	gb|AIXA01007233.1|:27588-27828
MsHel-6	gb|AIXA01005627.1|:4806-5085
MsHel-6	gb|AIXA01004099.1|:20678-20856
MsHel-6	gb|AIXA01002730.1|:73530-73748
MsHel-6	gb|AIXA01001643.1|:1212-1369
MsHel-6	gb|AIXA01001266.1|:28049-28318
MsHel-6	gb|AIXA01000555.1|:4166-5623
MsHel-6	gb|AIXA01021020.1|:1968-2130
MsHel-6	gb|AIXA01019677.1|:2187-2423
MsHel-6	gb|AIXA01017611.1|:8023-8282
MsHel-6	gb|AIXA01015402.1|:732-927
MsHel-6	gb|AIXA01014991.1|:3951-4126
MsHel-6	gb|AIXA01013625.1|:361-608
MsHel-6	gb|AIXA01011307.1|:758-923
MsHel-6	gb|AIXA01009350.1|:4586-5025
MsHel-6	gb|AIXA01009024.1|:55683-55930
MsHel-6	gb|AIXA01008533.1|:70288-70507
MsHel-6	gb|AIXA01006401.1|:14019-14277
MsHel-6	gb|AIXA01005872.1|:9758-9940
MsHel-6	gb|AIXA01002706.1|:54743-54993
MsHel-6	gb|AIXA01001688.1|:36686-36935
MsHel-6	gb|AIXA01020817.1|:598-806
MsHel-6	gb|AIXA01018977.1|:2679-2905
MsHel-6	gb|AIXA01016487.1|:10173-10421
MsHel-6	gb|AIXA01015664.1|:12714-12955
MsHel-6	gb|AIXA01015583.1|:14021-14224
MsHel-6	gb|AIXA01015395.1|:5147-5552
MsHel-6	gb|AIXA01014700.1|:2592-2847
MsHel-6	gb|AIXA01014443.1|:21359-21523
MsHel-6	gb|AIXA01005855.1|:55966-56136
MsHel-6	gb|AIXA01005617.1|:24918-25079
MsHel-6	gb|AIXA01004862.1|:10432-10585

MsHel-6	gb|AIXA01004678.1|:23082-23217
MsHel-6	gb|AIXA01004678.1|:4982-5186
MsHel-6	gb|AIXA01004472.1|:19837-20060
MsHel-6	gb|AIXA01003601.1|:17032-17190
MsHel-6	gb|AIXA01003140.1|:46955-47139
MsHel-6	gb|AIXA01002601.1|:57732-57917
MsHel-6	gb|AIXA01002358.1|:21686-21899
MsHel-6	gb|AIXA01002281.1|:15960-16180
MsHel-6	gb|AIXA01000186.1|:6876-7084
MsHel-6	gb|AIXA01037087.1|:75-300
MsHel-6	gb|AIXA01025752.1|:170-323
MsHel-6	gb|AIXA01018252.1|:1072-1478
MsHel-6	gb|AIXA01017134.1|:2178-2377
MsHel-6	gb|AIXA01016584.1|:2201-2391
MsHel-6	gb|AIXA01009216.1|:132843-132965
MsHel-6	gb|AIXA01008976.1|:371-560
MsHel-6	gb|AIXA01007731.1|:42397-42828
MsHel-6	gb|AIXA01007731.1|:5128-5396
MsHel-6	gb|AIXA01006890.1|:17394-17845
MsHel-6	gb|AIXA01006653.1|:594-789
MsHel-6	gb|AIXA01005787.1|:41615-41808
MsHel-6	gb|AIXA01005483.1|:726-974
MsHel-6	gb|AIXA01003770.1|:482-757
MsHel-6	gb|AIXA01003701.1|:55068-55317
MsHel-6	gb|AIXA01002198.1|:5350-5591
MsHel-6	gb|AIXA01002072.1|:2270-2467
MsHel-6	gb|AIXA01000562.1|:32644-33101
MsHel-6	gb|AIXA01000498.1|:5470-5860
MsHel-6	gb|AIXA01000395.1|:6461-6612
MsHel-6	gb|AIXA01000224.1|:23087-23248
MsHel-6	gb|AIXA01000201.1|:62898-63025
MsHel-6	gb|AIXA01016858.1|:12403-12561
MsHel-6	gb|AIXA01015242.1|:13995-14189
MsHel-6	gb|AIXA01014017.1|:5015-5174
MsHel-6	gb|AIXA01012360.1|:17111-17353
MsHel-6	gb|AIXA01012051.1|:14704-14938
MsHel-6	gb|AIXA01007170.1|:41647-41891
MsHel-6	gb|AIXA01007170.1|:3710-3802
MsHel-6	gb|AIXA01001611.1|:10031-10260
MsHel-6	gb|AIXA01001176.1|:10898-11089
MsHel-6	gb|AIXA01001104.1|:66617-66793
MsHel-6	gb|AIXA01000559.1|:12925-13173
MsHel-6	gb|AIXA01000196.1|:56667-56831
MsHel-6	gb|AIXA01000154.1|:9964-10163
MsHel-6	gb|AIXA01023706.1|:3-140
MsHel-6	gb|AIXA01021696.1|:735-896
MsHel-6	gb|AIXA01013709.1|:878-1083
MsHel-6	gb|AIXA01009864.1|:39487-39956

MsHel-6	gb|AIXA01009820.1|:32596-32841
MsHel-6	gb|AIXA01009804.1|:22030-22192
MsHel-6	gb|AIXA01008637.1|:4262-4451
MsHel-6	gb|AIXA01008232.1|:14086-14481
MsHel-6	gb|AIXA01006925.1|:4254-4704
MsHel-6	gb|AIXA01006253.1|:24940-30673
MsHel-6	gb|AIXA01002475.1|:3355-3547
MsHel-6	gb|AIXA01000970.1|:21797-22003
MsHel-6	gb|AIXA01000884.1|:7807-8229
MsHel-6	gb|AIXA01000206.1|:72947-73112
MsHel-6	gb|AIXA01000206.1|:31208-31996
MsHel-6	gb|AIXA01027317.1|:522-686
MsHel-6	gb|AIXA01017980.1|:5548-5973
MsHel-6	gb|AIXA01015422.1|:3657-3846
MsHel-6	gb|AIXA01013606.1|:38425-38950
MsHel-6	gb|AIXA01012509.1|:4175-4376
MsHel-6	gb|AIXA01011367.1|:807-1065
MsHel-6	gb|AIXA01009532.1|:44219-44422
MsHel-6	gb|AIXA01009127.1|:12384-12620
MsHel-6	gb|AIXA01007844.1|:28512-28855
MsHel-6	gb|AIXA01007177.1|:8560-8753
MsHel-6	gb|AIXA01006684.1|:46217-46651
MsHel-6	gb|AIXA01005108.1|:3482-3676
MsHel-6	gb|AIXA01005023.1|:6741-6953
MsHel-6	gb|AIXA01003027.1|:38528-38745
MsHel-6	gb|AIXA01002600.1|:43447-43673
MsHel-6	gb|AIXA01002471.1|:10668-15990
MsHel-6	gb|AIXA01001630.1|:31837-32046
MsHel-6	gb|AIXA01001630.1|:3072-3245
MsHel-6	gb|AIXA01000703.1|:23866-24395
MsHel-6	gb|AIXA01000216.1|:27375-27614
MsHel-6	gb|AIXA01000052.1|:2957-3110
MsHel-6	gb|AIXA01022407.1|:262-511
MsHel-6	gb|AIXA01021630.1|:525-730
MsHel-6	gb|AIXA01017011.1|:11816-12076
MsHel-6	gb|AIXA01015784.1|:3530-3795
MsHel-6	gb|AIXA01012206.1|:31467-31691
MsHel-6	gb|AIXA01009661.1|:617-4550
MsHel-6	gb|AIXA01007309.1|:6101-6354
MsHel-6	gb|AIXA01006255.1|:76069-76323
MsHel-6	gb|AIXA01006053.1|:47839-48036
MsHel-6	gb|AIXA01004164.1|:20122-20253
MsHel-6	gb|AIXA01002071.1|:4904-5373
MsHel-6	gb|AIXA01001864.1|:539-701
MsHel-6	gb|AIXA01020445.1|:369-591
MsHel-6	gb|AIXA01018781.1|:1306-1465
MsHel-6	gb|AIXA01016881.1|:797-998
MsHel-6	gb|AIXA01012581.1|:22662-22815

MsHel-6	gb|AIXA01011635.1|:3773-3966
MsHel-6	gb|AIXA01010578.1|:103-345
MsHel-6	gb|AIXA01007691.1|:44218-44428
MsHel-6	gb|AIXA01007250.1|:2148-2321
MsHel-6	gb|AIXA01006837.1|:30904-31010
MsHel-6	gb|AIXA01003346.1|:27701-28207
MsHel-6	gb|AIXA01001677.1|:11652-11908
MsHel-6	gb|AIXA01028347.1|:299-507
MsHel-6	gb|AIXA01014189.1|:3609-4079
MsHel-6	gb|AIXA01008864.1|:4046-4229
MsHel-6	gb|AIXA01007451.1|:47213-47406
MsHel-6	gb|AIXA01006184.1|:12397-12618
MsHel-6	gb|AIXA01005847.1|:24570-24954
MsHel-6	gb|AIXA01004365.1|:20155-20357
MsHel-6	gb|AIXA01003769.1|:5534-5744
MsHel-6	gb|AIXA01002245.1|:4044-4199
MsHel-6	gb|AIXA01001271.1|:69761-70012
MsHel-6	gb|AIXA01014952.1|:7268-7483
MsHel-6	gb|AIXA01014214.1|:7769-7962
MsHel-6	gb|AIXA01013743.1|:21806-21947
MsHel-6	gb|AIXA01013255.1|:13666-21544
MsHel-6	gb|AIXA01012864.1|:1240-1729
MsHel-6	gb|AIXA01009539.1|:36474-36619
MsHel-6	gb|AIXA01007359.1|:69823-70005
MsHel-6	gb|AIXA01006075.1|:22759-22974
MsHel-6	gb|AIXA01002621.1|:38905-39094
MsHel-6	gb|AIXA01002621.1|:18293-18459
MsHel-6	gb|AIXA01001859.1|:8115-8392
MsHel-6	gb|AIXA01001716.1|:49839-50114
MsHel-6	gb|AIXA01008289.1|:6568-6745
MsHel-6	gb|AIXA01006507.1|:4831-5079
MsHel-6	gb|AIXA01003375.1|:1089-1346
MsHel-6	gb|AIXA01003276.1|:44257-44506
MsHel-6	gb|AIXA01002771.1|:14447-14603
MsHel-6	gb|AIXA01001527.1|:9797-10057
MsHel-6	gb|AIXA01019551.1|:3029-3274
MsHel-6	gb|AIXA01019144.1|:1977-2251
MsHel-6	gb|AIXA01017964.1|:7811-7961
MsHel-6	gb|AIXA01012895.1|:1859-2106
MsHel-6	gb|AIXA01011709.1|:101394-101574
MsHel-6	gb|AIXA01011270.1|:16050-16220
MsHel-6	gb|AIXA01008633.1|:8755-9254
MsHel-6	gb|AIXA01007542.1|:9030-9233
MsHel-6	gb|AIXA01006475.1|:4678-4971
MsHel-6	gb|AIXA01002068.1|:28890-29064
MsHel-6	gb|AIXA01001414.1|:13197-13613
MsHel-6	gb|AIXA01027324.1|:667-903
MsHel-6	gb|AIXA01021899.1|:107-350

MsHel-6	gb|AIXA01019876.1|:1873-2022
MsHel-6	gb|AIXA01013782.1|:8852-9125
MsHel-6	gb|AIXA01013449.1|:26839-26996
MsHel-6	gb|AIXA01009004.1|:11877-12103
MsHel-6	gb|AIXA01008399.1|:3890-13514
MsHel-6	gb|AIXA01008028.1|:48029-48289
MsHel-6	gb|AIXA01008028.1|:19891-20127
MsHel-6	gb|AIXA01006118.1|:18836-19274
MsHel-6	gb|AIXA01005002.1|:3063-3310
MsHel-6	gb|AIXA01004357.1|:15165-15505
MsHel-6	gb|AIXA01002560.1|:46756-50852
MsHel-6	gb|AIXA01002285.1|:878-1138
MsHel-6	gb|AIXA01000851.1|:72033-72230
MsHel-6	gb|AIXA01017197.1|:794-1044
MsHel-6	gb|AIXA01016327.1|:9920-10072
MsHel-6	gb|AIXA01013840.1|:2275-2496
MsHel-6	gb|AIXA01007681.1|:63813-64060
MsHel-6	gb|AIXA01007681.1|:34620-34849
MsHel-6	gb|AIXA01005868.1|:14106-14300
MsHel-6	gb|AIXA01005187.1|:78441-78702
MsHel-6	gb|AIXA01002004.1|:20571-20920
MsHel-6	gb|AIXA01001790.1|:50744-50957
MsHel-6	gb|AIXA01017403.1|:13833-14105
MsHel-6	gb|AIXA01017014.1|:11766-11911
MsHel-6	gb|AIXA01011753.1|:30621-30772
MsHel-6	gb|AIXA01010102.1|:41760-41949
MsHel-6	gb|AIXA01006665.1|:6754-6973
MsHel-6	gb|AIXA01005895.1|:5590-5736
MsHel-6	gb|AIXA01003889.1|:22079-22285
MsHel-6	gb|AIXA01003681.1|:48743-48933
MsHel-6	gb|AIXA01001205.1|:3850-4033
MsHel-6	gb|AIXA01000361.1|:28158-28352
MsHel-6	gb|AIXA01015570.1|:8469-8655
MsHel-6	gb|AIXA01013219.1|:15376-15592
MsHel-6	gb|AIXA01012756.1|:937-1100
MsHel-6	gb|AIXA01011001.1|:4156-4396
MsHel-6	gb|AIXA01010184.1|:430-668
MsHel-6	gb|AIXA01008992.1|:2-196
MsHel-6	gb|AIXA01004634.1|:2123-2271
MsHel-6	gb|AIXA01001660.1|:68581-68776
MsHel-6	gb|AIXA01034182.1|:164-414
MsHel-6	gb|AIXA01012755.1|:6447-6609
MsHel-6	gb|AIXA01010854.1|:26985-27102
MsHel-6	gb|AIXA01010658.1|:13009-13106
MsHel-6	gb|AIXA01007175.1|:76104-76313
MsHel-6	gb|AIXA01006529.1|:1054-1219
MsHel-6	gb|AIXA01005083.1|:19272-19455
MsHel-6	gb|AIXA01004779.1|:30823-30928

MsHel-6	gb|AIXA01002197.1|:40602-40840
MsHel-6	gb|AIXA01002197.1|:13953-14179
MsHel-6	gb|AIXA01000904.1|:1233-1450
MsHel-6	gb|AIXA01014040.1|:34257-34465
MsHel-6	gb|AIXA01013303.1|:4453-4676
MsHel-6	gb|AIXA01012459.1|:215-477
MsHel-6	gb|AIXA01010236.1|:784-990
MsHel-6	gb|AIXA01006953.1|:20798-20905
MsHel-6	gb|AIXA01005388.1|:13687-13904
MsHel-6	gb|AIXA01004427.1|:11147-11320
MsHel-6	gb|AIXA01003907.1|:31928-32168
MsHel-6	gb|AIXA01002119.1|:148-349
MsHel-6	gb|AIXA01002043.1|:9217-9651
MsHel-6	gb|AIXA01033697.1|:434-621
MsHel-6	gb|AIXA01016075.1|:42981-43226
MsHel-6	gb|AIXA01010785.1|:10746-10931
MsHel-6	gb|AIXA01009691.1|:6674-6876
MsHel-6	gb|AIXA01006706.1|:2216-2796
MsHel-6	gb|AIXA01002239.1|:26723-27091
MsHel-6	gb|AIXA01001191.1|:46183-46365
MsHel-6	gb|AIXA01024849.1|:692-913
MsHel-6	gb|AIXA01009360.1|:98938-99097
MsHel-6	gb|AIXA01007027.1|:62407-62658
MsHel-6	gb|AIXA01006350.1|:60534-61142
MsHel-6	gb|AIXA01004066.1|:2885-3104
MsHel-6	gb|AIXA01000070.1|:34928-35094
MsHel-6	gb|AIXA01025558.1|:829-1065
MsHel-6	gb|AIXA01017263.1|:1179-1632
MsHel-6	gb|AIXA01009210.1|:10888-11121
MsHel-6	gb|AIXA01006795.1|:5055-5410
MsHel-6	gb|AIXA01005266.1|:26445-26634
MsHel-6	gb|AIXA01001638.1|:34163-34414
MsHel-6	gb|AIXA01000643.1|:69778-70025
MsHel-6	gb|AIXA01000469.1|:9041-9230
MsHel-6	gb|AIXA01000004.1|:291-421
MsHel-6	gb|AIXA01018861.1|:2432-2802
MsHel-6	gb|AIXA01018499.1|:1840-3783
MsHel-6	gb|AIXA01011977.1|:2726-2974
MsHel-6	gb|AIXA01007925.1|:42787-43291
MsHel-6	gb|AIXA01005505.1|:21071-21636
MsHel-6	gb|AIXA01004777.1|:2456-2724
MsHel-6	gb|AIXA01004408.1|:45321-45477
MsHel-6	gb|AIXA01035089.1|:421-599
MsHel-6	gb|AIXA01022284.1|:264-509
MsHel-6	gb|AIXA01021106.1|:1875-2122
MsHel-6	gb|AIXA01018203.1|:6432-6657
MsHel-6	gb|AIXA01009544.1|:32001-32161
MsHel-6	gb|AIXA01006867.1|:40097-40340

MsHel-6	gb|AIXA01003190.1|:8022-10951
MsHel-6	gb|AIXA01002968.1|:5414-6074
MsHel-6	gb|AIXA01028637.1|:704-901
MsHel-6	gb|AIXA01014020.1|:17153-17311
MsHel-6	gb|AIXA01010416.1|:1519-1646
MsHel-6	gb|AIXA01009775.1|:12868-13005
MsHel-6	gb|AIXA01005321.1|:20477-20680
MsHel-6	gb|AIXA01000553.1|:1728-1870
MsHel-6	gb|AIXA01026863.1|:904-1086
MsHel-6	gb|AIXA01026173.1|:426-614
MsHel-6	gb|AIXA01011650.1|:818-996
MsHel-6	gb|AIXA01003624.1|:54419-54558
MsHel-6	gb|AIXA01032876.1|:516-644
MsHel-6	gb|AIXA01018641.1|:130-296
MsHel-6	gb|AIXA01015499.1|:1648-1801
MsHel-6	gb|AIXA01007224.1|:47578-48029
MsHel-6	gb|AIXA01002678.1|:4351-4550
MsHel-6	gb|AIXA01001315.1|:18475-18741
MsHel-6	gb|AIXA01016585.1|:1192-1341
MsHel-6	gb|AIXA01015716.1|:1-173
MsHel-6	gb|AIXA01014424.1|:1489-1699
MsHel-6	gb|AIXA01013302.1|:22327-22814
MsHel-6	gb|AIXA01012774.1|:6387-6571
MsHel-6	gb|AIXA01004956.1|:10689-11108
MsHel-6	gb|AIXA01000207.1|:48377-48540
MsHel-6	gb|AIXA01018540.1|:4095-4202
MsHel-6	gb|AIXA01006689.1|:84220-84409
MsHel-6	gb|AIXA01003147.1|:4915-5065
MsHel-6	gb|AIXA01002535.1|:13021-13202
MsHel-6	gb|AIXA01002502.1|:3547-4014
MsHel-6	gb|AIXA01001538.1|:70816-71006
MsHel-6	gb|AIXA01001538.1|:38375-38473
MsHel-6	gb|AIXA01018908.1|:5349-5594
MsHel-6	gb|AIXA01017589.1|:698-840
MsHel-6	gb|AIXA01010687.1|:39078-39175
MsHel-6	gb|AIXA01008529.1|:25828-26013
MsHel-6	gb|AIXA01000014.1|:22182-22397
MsHel-6	gb|AIXA01013652.1|:14882-15095
MsHel-6	gb|AIXA01002747.1|:92916-93059
MsHel-6	gb|AIXA01018412.1|:1-125
MsHel-6	gb|AIXA01015824.1|:23459-23993
MsHel-6	gb|AIXA01013226.1|:24302-24429
MsHel-6	gb|AIXA01007334.1|:40571-40755
MsHel-6	gb|AIXA01005720.1|:15212-15451
MsHel-6	gb|AIXA01005500.1|:65599-65991
MsHel-6	gb|AIXA01005420.1|:27719-27831
MsHel-6	gb|AIXA01000767.1|:83897-84079
MsHel-6	gb|AIXA01036399.1|:377-475

MsHel-6	gb|AIXA01025302.1|:927-1197
MsHel-6	gb|AIXA01016065.1|:1467-1633
MsHel-6	gb|AIXA01003528.1|:4791-5028
MsHel-6	gb|AIXA01003507.1|:9223-9399
MsHel-6	gb|AIXA01003238.1|:14390-14624
MsHel-6	gb|AIXA01023741.1|:450-649
MsHel-6	gb|AIXA01015870.1|:5566-6079
MsHel-6	gb|AIXA01011984.1|:30683-31099
MsHel-6	gb|AIXA01011943.1|:5292-5405
MsHel-6	gb|AIXA01010742.1|:67926-68075
MsHel-6	gb|AIXA01010334.1|:2122-2659
MsHel-6	gb|AIXA01010189.1|:27641-27762
MsHel-6	gb|AIXA01006192.1|:18453-18906
MsHel-6	gb|AIXA01000358.1|:3280-3738
MsHel-6	gb|AIXA01000003.1|:37993-38254
MsHel-6	gb|AIXA01012903.1|:28986-29214
MsHel-6	gb|AIXA01012017.1|:10379-10579
MsHel-6	gb|AIXA01004583.1|:38883-39153
MsHel-6	gb|AIXA01002250.1|:39646-39861
MsHel-6	gb|AIXA01022689.1|:760-923
MsHel-6	gb|AIXA01021495.1|:1748-1883
MsHel-6	gb|AIXA01020756.1|:1553-1810
MsHel-6	gb|AIXA01016549.1|:29276-29476
MsHel-6	gb|AIXA01012028.1|:17622-17713
MsHel-6	gb|AIXA01006382.1|:44599-44800
MsHel-6	gb|AIXA01004413.1|:7709-7935
MsHel-6	gb|AIXA01004354.1|:10946-11195
MsHel-6	gb|AIXA01002766.1|:25163-25417
MsHel-6	gb|AIXA01009166.1|:23998-24212
MsHel-6	gb|AIXA01007271.1|:166-404
MsHel-6	gb|AIXA01005338.1|:4624-4873
MsHel-6	gb|AIXA01004199.1|:9880-10051
MsHel-6	gb|AIXA01004092.1|:2234-2409
MsHel-6	gb|AIXA01002018.1|:8388-8519
MsHel-6	gb|AIXA01011611.1|:19012-19228
MsHel-6	gb|AIXA01006156.1|:9490-9749
MsHel-6	gb|AIXA01005248.1|:1417-1664
MsHel-6	gb|AIXA01004300.1|:51418-51567
MsHel-6	gb|AIXA01001858.1|:60173-60350
MsHel-6	gb|AIXA01014281.1|:1-89
MsHel-6	gb|AIXA01006681.1|:20699-21030
MsHel-6	gb|AIXA01005781.1|:23123-23263
MsHel-6	gb|AIXA01003275.1|:23588-23763
MsHel-6	gb|AIXA01015045.1|:320-484
MsHel-6	gb|AIXA01008489.1|:28697-28899
MsHel-6	gb|AIXA01005015.1|:18966-19136
MsHel-6	gb|AIXA01001284.1|:77595-77871
MsHel-6	gb|AIXA01001070.1|:20177-20365

MsHel-6	gb|AIXA01007028.1|:11807-11998
MsHel-6	gb|AIXA01002480.1|:8335-8562
MsHel-6	gb|AIXA01001517.1|:13809-14042
MsHel-6	gb|AIXA01000576.1|:14257-14501
MsHel-6	gb|AIXA01000576.1|:1310-1440
MsHel-6	gb|AIXA01016275.1|:1-199
MsHel-6	gb|AIXA01011006.1|:180-439
MsHel-6	gb|AIXA01009400.1|:14868-15001
MsHel-6	gb|AIXA01008999.1|:19684-19798
MsHel-6	gb|AIXA01004790.1|:84938-85204
MsHel-6	gb|AIXA01004102.1|:16784-19721
MsHel-6	gb|AIXA01003595.1|:57494-57685
MsHel-6	gb|AIXA01002793.1|:11573-11787
MsHel-6	gb|AIXA01000813.1|:48198-48371
MsHel-6	gb|AIXA01023100.1|:695-794
MsHel-6	gb|AIXA01017058.1|:7529-7625
MsHel-6	gb|AIXA01014945.1|:2154-2313
MsHel-6	gb|AIXA01015215.1|:735-818
MsHel-6	gb|AIXA01013419.1|:7958-8554
MsHel-6	gb|AIXA01011247.1|:21489-21604
MsHel-6	gb|AIXA01004654.1|:17358-17491
MsHel-6	gb|AIXA01011674.1|:7464-8177
MsHel-6	gb|AIXA01010676.1|:2796-2885
MsHel-6	gb|AIXA01008892.1|:2214-2403
MsHel-6	gb|AIXA01000026.1|:5791-6195
MsHel-6	gb|AIXA01011440.1|:5032-5180
MsHel-6	gb|AIXA01009019.1|:13759-13848
MsHel-6	gb|AIXA01007664.1|:40085-40235
MsHel-6	gb|AIXA01007115.1|:1500-3008
MsHel-6	gb|AIXA01006505.1|:25101-25324
MsHel-6	gb|AIXA01010656.1|:73914-74076
MsHel-6	gb|AIXA01017574.1|:1830-2555
MsHel-6	gb|AIXA01008270.1|:6322-6511
MsHel-6	gb|AIXA01006977.1|:30744-37185
MsHel-6	gb|AIXA01001915.1|:25751-26107
MsHel-6	gb|AIXA01012625.1|:3773-4223
MsHel-6	gb|AIXA01011349.1|:21671-21854
MsHel-6	gb|AIXA01008051.1|:5971-6182
MsHel-6	gb|AIXA01004152.1|:40803-41026
MsHel-6	gb|AIXA01002298.1|:7397-7762
MsHel-6	gb|AIXA01010788.1|:10125-10327
MsHel-6	gb|AIXA01015292.1|:4993-5228
MsHel-6	gb|AIXA01005710.1|:744-1891
MsHel-6	gb|AIXA01004155.1|:37851-41844
MsHel-6	gb|AIXA01003199.1|:12496-12743
MsHel-6	gb|AIXA01002764.1|:25972-26230
MsHel-6	gb|AIXA01000755.1|:41363-41756
MsHel-6	gb|AIXA01009208.1|:50392-50562

MsHel-6	gb|AIXA01007853.1|:51769-52311
MsHel-6	gb|AIXA01025470.1|:267-577
MsHel-6	gb|AIXA01021970.1|:421-600
MsHel-6	gb|AIXA01018439.1|:6106-6514
MsHel-6	gb|AIXA01016141.1|:1540-1635
MsHel-6	gb|AIXA01009712.1|:4643-5155
MsHel-6	gb|AIXA01009401.1|:103935-104049
MsHel-6	gb|AIXA01000430.1|:51621-51805
MsHel-6	gb|AIXA01012329.1|:6912-7082
MsHel-6	gb|AIXA01008247.1|:5687-5914
MsHel-6	gb|AIXA01005424.1|:3024-3117
MsHel-6	gb|AIXA01004550.1|:76573-81969
MsHel-6	gb|AIXA01004550.1|:66034-66190
MsHel-6	gb|AIXA01004511.1|:76-385
MsHel-6	gb|AIXA01003578.1|:70411-70549
MsHel-6	gb|AIXA01033994.1|:330-661
MsHel-6	gb|AIXA01024301.1|:236-319
MsHel-6	gb|AIXA01010756.1|:8331-8564
MsHel-6	gb|AIXA01009834.1|:5723-5899
MsHel-6	gb|AIXA01007488.1|:48964-49170
MsHel-6	gb|AIXA01001474.1|:68866-69061
MsHel-6	gb|AIXA01001009.1|:15641-15724
MsHel-6	gb|AIXA01021045.1|:1916-2111
MsHel-6	gb|AIXA01010798.1|:670-1309
MsHel-6	gb|AIXA01001941.1|:45782-45912
MsHel-6	gb|AIXA01001496.1|:15901-16031
MsHel-6	gb|AIXA01016228.1|:1279-1372
MsHel-6	gb|AIXA01007932.1|:13384-13634
MsHel-6	gb|AIXA01005876.1|:918-1046
MsHel-6	gb|AIXA01009504.1|:34663-34760
MsHel-6	gb|AIXA01013430.1|:6776-7083
MsHel-6	gb|AIXA01004666.1|:7028-7514
MsHel-6	gb|AIXA01002510.1|:1-152
MsHel-6	gb|AIXA01002205.1|:1518-1794
MsHel-6	gb|AIXA01007339.1|:7068-7185
MsHel-6	gb|AIXA01000099.1|:17491-17617
MsHel-6	gb|AIXA01007179.1|:7924-8049
MsHel-6	gb|AIXA01002362.1|:23814-24033
MsHel-6	gb|AIXA01012352.1|:11781-11981
MsHel-6	gb|AIXA01005593.1|:17048-17340
MsHel-6	gb|AIXA01018970.1|:1-112
MsHel-6	gb|AIXA01012052.1|:1-151
MsHel-6	gb|AIXA01000907.1|:7513-7925
MsHel-6	gb|AIXA01009067.1|:25683-25914
MsHel-6	gb|AIXA01007626.1|:97966-98067
MsHel-6	gb|AIXA01001494.1|:35719-36246
MsHel-6	gb|AIXA01014086.1|:10621-10727
MsHel-6	gb|AIXA01011034.1|:11086-11361

MsHel-6	gb|AIXA01001128.1|:55182-55731
MsHel-6	gb|AIXA01000882.1|:9109-9815
MsHel-6	gb|AIXA01000728.1|:34194-34420
MsHel-6	gb|AIXA01017323.1|:1942-2065
MsHel-6	gb|AIXA01017267.1|:2-112
MsHel-6	gb|AIXA01007500.1|:42081-42193
MsHel-6	gb|AIXA01026729.1|:1-106
MsHel-6	gb|AIXA01004115.1|:128318-128459
MsHel-6	gb|AIXA01010866.1|:7801-8784
MsHel-6	gb|AIXA01031166.1|:690-797
MsHel-6	gb|AIXA01003738.1|:5118-5318
MsHel-6	gb|AIXA01026201.1|:883-1095
MsHel-6	gb|AIXA01022612.1|:684-770
MsHel-6	gb|AIXA01007295.1|:29336-29473
MsHel-6	gb|AIXA01013120.1|:4874-4995
MsHel-6	gb|AIXA01010773.1|:14896-15089
MsHel-6	gb|AIXA01009100.1|:11929-12158
MsHel-6	gb|AIXA01001674.1|:19277-19384
MsHel-6	gb|AIXA01000075.1|:26009-26130
MsHel-6	gb|AIXA01032213.1|:1-104
MsHel-6	gb|AIXA01013395.1|:936-1699
MsHel-6	gb|AIXA01005914.1|:9816-14869
MsHel-6	gb|AIXA01024853.1|:166-301
MsHel-6	gb|AIXA01005153.1|:3475-3564
MsHel-6	gb|AIXA01006971.1|:67586-67703
MsHel-6	gb|AIXA01001445.1|:3410-3559
MsHel-3b	gb|AIXA01002318.1|:20587-20802
MsHel-3b	gb|AIXA01001531.1|:54965-55122
MsHel-3b	gb|AIXA01012943.1|:6955-7166
MsHel-3b	gb|AIXA01013436.1|:74839-74923
MsHel-3b	gb|AIXA01011449.1|:299-425
MsHel-3b	gb|AIXA01002463.1|:74767-74980
MsHel-3b	gb|AIXA01038158.1|:159-363
MsHel-3b	gb|AIXA01010109.1|:48468-48630
MsHel-3b	gb|AIXA01010109.1|:254-333
MsHel-3b	gb|AIXA01016076.1|:25332-25545
MsHel-3b	gb|AIXA01009936.1|:55178-55277
MsHel-3b	gb|AIXA01009936.1|:8222-8433
MsHel-3b	gb|AIXA01006690.1|:24488-24704
MsHel-3b	gb|AIXA01001645.1|:78356-78542
MsHel-3b	gb|AIXA01001317.1|:8454-8670
MsHel-3b	gb|AIXA01015680.1|:1782-1988
MsHel-3b	gb|AIXA01014762.1|:7306-7510
MsHel-3b	gb|AIXA01004313.1|:53454-53618
MsHel-3b	gb|AIXA01004313.1|:8798-9012
MsHel-3b	gb|AIXA01001542.1|:24572-24755
MsHel-3b	gb|AIXA01010406.1|:67909-68114
MsHel-3b	gb|AIXA01010406.1|:41930-42142

MsHel-3b	gb|AIXA01009981.1|:27440-27656
MsHel-3b	gb|AIXA01007856.1|:84403-84619
MsHel-3b	gb|AIXA01007856.1|:29725-29848
MsHel-3b	gb|AIXA01007472.1|:128360-128544
MsHel-3b	gb|AIXA01007472.1|:84281-84417
MsHel-3b	gb|AIXA01007472.1|:54981-55189
MsHel-3b	gb|AIXA01006141.1|:51790-51997
MsHel-3b	gb|AIXA01006141.1|:14743-14950
MsHel-3b	gb|AIXA01004188.1|:6501-6717
MsHel-3b	gb|AIXA01000821.1|:42638-42810
MsHel-3b	gb|AIXA01000398.1|:16992-17208
MsHel-3b	gb|AIXA01008393.1|:56069-56287
MsHel-3b	gb|AIXA01000046.1|:74007-74470
MsHel-3b	gb|AIXA01030522.1|:505-718
MsHel-3b	gb|AIXA01013523.1|:32504-32689
MsHel-3b	gb|AIXA01013523.1|:17633-17839
MsHel-3b	gb|AIXA01003021.1|:137139-137348
MsHel-3b	gb|AIXA01003021.1|:85010-85135
MsHel-3b	gb|AIXA01003021.1|:55906-56068
MsHel-3b	gb|AIXA01001362.1|:24468-24673
MsHel-3b	gb|AIXA01020255.1|:1821-2037
MsHel-3b	gb|AIXA01017576.1|:7205-7405
MsHel-3b	gb|AIXA01015246.1|:21007-21223
MsHel-3b	gb|AIXA01011846.1|:4097-4309
MsHel-3b	gb|AIXA01008043.1|:82719-82935
MsHel-3b	gb|AIXA01008043.1|:3844-4002
MsHel-3b	gb|AIXA01006817.1|:14720-14928
MsHel-3b	gb|AIXA01002460.1|:128819-128988
MsHel-3b	gb|AIXA01002460.1|:80704-80868
MsHel-3b	gb|AIXA01002460.1|:10669-10866
MsHel-3b	gb|AIXA01012629.1|:240-455
MsHel-3b	gb|AIXA01008566.1|:73286-73387
MsHel-3b	gb|AIXA01008566.1|:1480-1704
MsHel-3b	gb|AIXA01008131.1|:5332-5543
MsHel-3b	gb|AIXA01007037.1|:10124-10306
MsHel-3b	gb|AIXA01004281.1|:21837-22044
MsHel-3b	gb|AIXA01004281.1|:3145-3353
MsHel-3b	gb|AIXA01008088.1|:31787-32000
MsHel-3b	gb|AIXA01005049.1|:8020-8196
MsHel-3b	gb|AIXA01004056.1|:31710-31924
MsHel-3b	gb|AIXA01004056.1|:3878-4341
MsHel-3b	gb|AIXA01003653.1|:63293-63501
MsHel-3b	gb|AIXA01003653.1|:2115-2194
MsHel-3b	gb|AIXA01001823.1|:42401-43092
MsHel-3b	gb|AIXA01001823.1|:26696-26901
MsHel-3b	gb|AIXA01001823.1|:4006-4219
MsHel-3b	gb|AIXA01018705.1|:3520-3734
MsHel-3b	gb|AIXA01017416.1|:404-606

MsHel-3b	gb|AIXA01011995.1|:98646-98742
MsHel-3b	gb|AIXA01011995.1|:68536-68750
MsHel-3b	gb|AIXA01007104.1|:16334-16527
MsHel-3b	gb|AIXA01005423.1|:855-1060
MsHel-3b	gb|AIXA01002350.1|:148609-148817
MsHel-3b	gb|AIXA01002350.1|:106000-106200
MsHel-3b	gb|AIXA01002350.1|:69928-70135
MsHel-3b	gb|AIXA01002350.1|:30142-30311
MsHel-3b	gb|AIXA01002238.1|:23992-24177
MsHel-3b	gb|AIXA01000251.1|:183185-183311
MsHel-3b	gb|AIXA01000251.1|:20378-25072
MsHel-3b	gb|AIXA01012162.1|:14696-14907
MsHel-3b	gb|AIXA01012162.1|:394-518
MsHel-3b	gb|AIXA01006514.1|:12395-12613
MsHel-3b	gb|AIXA01005339.1|:30944-31161
MsHel-3b	gb|AIXA01003576.1|:61548-61764
MsHel-3b	gb|AIXA01003576.1|:11622-11814
MsHel-3b	gb|AIXA01003576.1|:42-167
MsHel-3b	gb|AIXA01000234.1|:85266-85481
MsHel-3b	gb|AIXA01011498.1|:30867-31070
MsHel-3b	gb|AIXA01010953.1|:39653-39860
MsHel-3b	gb|AIXA01008060.1|:7473-7693
MsHel-3b	gb|AIXA01001612.1|:32652-32863
MsHel-3b	gb|AIXA01018380.1|:3331-3540
MsHel-3b	gb|AIXA01014467.1|:15639-15842
MsHel-3b	gb|AIXA01011177.1|:4193-4407
MsHel-3b	gb|AIXA01004067.1|:34609-34748
MsHel-3b	gb|AIXA01004067.1|:1601-1815
MsHel-3b	gb|AIXA01002459.1|:14985-15207
MsHel-3b	gb|AIXA01001675.1|:33997-34211
MsHel-3b	gb|AIXA01001675.1|:10649-10726
MsHel-3b	gb|AIXA01001546.1|:50665-50835
MsHel-3b	gb|AIXA01001546.1|:27058-27243
MsHel-3b	gb|AIXA01001546.1|:14044-14209
MsHel-3b	gb|AIXA01018919.1|:411-613
MsHel-3b	gb|AIXA01017982.1|:4961-9245
MsHel-3b	gb|AIXA01012352.1|:16768-16888
MsHel-3b	gb|AIXA01009366.1|:51896-52080
MsHel-3b	gb|AIXA01009366.1|:15038-15250
MsHel-3b	gb|AIXA01005494.1|:36621-36834
MsHel-3b	gb|AIXA01005415.1|:68722-68936
MsHel-3b	gb|AIXA01004068.1|:8242-8321
MsHel-3b	gb|AIXA01002996.1|:59878-60061
MsHel-3b	gb|AIXA01002996.1|:46661-46826
MsHel-3b	gb|AIXA01002231.1|:69158-69351
MsHel-3b	gb|AIXA01001811.1|:13822-18701
MsHel-3b	gb|AIXA01016545.1|:5059-5231
MsHel-3b	gb|AIXA01015155.1|:6248-6401

MsHel-3b	gb|AIXA01015003.1|:12342-12526
MsHel-3b	gb|AIXA01006681.1|:31118-31313
MsHel-3b	gb|AIXA01005207.1|:46759-46943
MsHel-3b	gb|AIXA01003924.1|:118981-119196
MsHel-3b	gb|AIXA01002990.1|:57643-57768
MsHel-3b	gb|AIXA01002683.1|:272-483
MsHel-3b	gb|AIXA01002351.1|:34152-34275
MsHel-3b	gb|AIXA01002351.1|:17347-17559
MsHel-3b	gb|AIXA01002351.1|:1706-1911
MsHel-3b	gb|AIXA01015486.1|:525-736
MsHel-3b	gb|AIXA01012197.1|:24370-24541
MsHel-3b	gb|AIXA01011121.1|:26529-26738
MsHel-3b	gb|AIXA01011067.1|:4337-4545
MsHel-3b	gb|AIXA01008319.1|:6406-6605
MsHel-3b	gb|AIXA01008033.1|:47404-47599
MsHel-3b	gb|AIXA01007376.1|:18214-18425
MsHel-3b	gb|AIXA01006807.1|:644-859
MsHel-3b	gb|AIXA01001048.1|:63990-64196
MsHel-3b	gb|AIXA01000725.1|:43796-43964
MsHel-3b	gb|AIXA01000725.1|:16133-16328
MsHel-3b	gb|AIXA01020094.1|:1780-1977
MsHel-3b	gb|AIXA01018288.1|:6076-6289
MsHel-3b	gb|AIXA01011131.1|:15659-15868
MsHel-3b	gb|AIXA01010598.1|:1306-1508
MsHel-3b	gb|AIXA01009671.1|:5521-5728
MsHel-3b	gb|AIXA01007888.1|:81066-81217
MsHel-3b	gb|AIXA01007888.1|:60998-61163
MsHel-3b	gb|AIXA01006705.1|:21988-22092
MsHel-3b	gb|AIXA01005247.1|:16541-16730
MsHel-3b	gb|AIXA01004862.1|:43048-43247
MsHel-3b	gb|AIXA01003329.1|:132519-132713
MsHel-3b	gb|AIXA01002770.1|:9274-9480
MsHel-3b	gb|AIXA01016695.1|:1075-1292
MsHel-3b	gb|AIXA01010197.1|:6508-6678
MsHel-3b	gb|AIXA01009807.1|:9670-9839
MsHel-3b	gb|AIXA01009001.1|:22283-22475
MsHel-3b	gb|AIXA01006688.1|:6979-7180
MsHel-3b	gb|AIXA01004222.1|:148094-148310
MsHel-3b	gb|AIXA01004222.1|:100963-101068
MsHel-3b	gb|AIXA01003183.1|:135948-136165
MsHel-3b	gb|AIXA01003183.1|:114991-115110
MsHel-3b	gb|AIXA01003124.1|:65176-65305
MsHel-3b	gb|AIXA01003124.1|:54827-54911
MsHel-3b	gb|AIXA01003124.1|:42821-42945
MsHel-3b	gb|AIXA01002658.1|:27114-27239
MsHel-3b	gb|AIXA01001593.1|:40099-40311
MsHel-3b	gb|AIXA01001518.1|:5431-13341
MsHel-3b	gb|AIXA01001271.1|:113483-113719

MsHel-3b	gb|AIXA01034056.1|:112-327
MsHel-3b	gb|AIXA01017992.1|:6587-6741
MsHel-3b	gb|AIXA01017761.1|:461-672
MsHel-3b	gb|AIXA01012628.1|:19558-19767
MsHel-3b	gb|AIXA01012364.1|:238-453
MsHel-3b	gb|AIXA01011919.1|:11037-11234
MsHel-3b	gb|AIXA01009649.1|:43207-43412
MsHel-3b	gb|AIXA01008406.1|:187726-187942
MsHel-3b	gb|AIXA01008406.1|:153887-154014
MsHel-3b	gb|AIXA01008406.1|:62115-62305
MsHel-3b	gb|AIXA01007681.1|:61873-62085
MsHel-3b	gb|AIXA01007593.1|:90071-90272
MsHel-3b	gb|AIXA01007593.1|:39774-39959
MsHel-3b	gb|AIXA01006689.1|:40335-40544
MsHel-3b	gb|AIXA01006689.1|:8879-9091
MsHel-3b	gb|AIXA01004369.1|:12422-12630
MsHel-3b	gb|AIXA01002468.1|:19579-19787
MsHel-3b	gb|AIXA01001548.1|:21671-21874
MsHel-3b	gb|AIXA01000997.1|:47739-47955
MsHel-3b	gb|AIXA01000909.1|:42161-42253
MsHel-3b	gb|AIXA01018307.1|:2089-2301
MsHel-3b	gb|AIXA01015168.1|:3751-3937
MsHel-3b	gb|AIXA01013560.1|:5483-5695
MsHel-3b	gb|AIXA01009925.1|:110851-111045
MsHel-3b	gb|AIXA01009925.1|:70102-70306
MsHel-3b	gb|AIXA01005493.1|:4466-4670
MsHel-3b	gb|AIXA01005440.1|:1083-1281
MsHel-3b	gb|AIXA01005105.1|:40703-40925
MsHel-3b	gb|AIXA01004482.1|:17513-17715
MsHel-3b	gb|AIXA01004482.1|:4580-4695
MsHel-3b	gb|AIXA01004153.1|:91109-91234
MsHel-3b	gb|AIXA01004153.1|:80289-80488
MsHel-3b	gb|AIXA01002471.1|:10466-10668
MsHel-3b	gb|AIXA01000892.1|:116849-116940
MsHel-3b	gb|AIXA01000892.1|:77779-77991
MsHel-3b	gb|AIXA01000892.1|:20375-20500
MsHel-3b	gb|AIXA01027880.1|:583-772
MsHel-3b	gb|AIXA01024217.1|:517-727
MsHel-3b	gb|AIXA01017402.1|:4064-4254
MsHel-3b	gb|AIXA01009588.1|:3817-4030
MsHel-3b	gb|AIXA01006872.1|:4220-4432
MsHel-3b	gb|AIXA01004956.1|:113040-113250
MsHel-3b	gb|AIXA01004956.1|:94507-94686
MsHel-3b	gb|AIXA01003818.1|:23346-23553
MsHel-3b	gb|AIXA01002994.1|:68186-68355
MsHel-3b	gb|AIXA01002994.1|:37717-37835
MsHel-3b	gb|AIXA01002994.1|:19245-19445
MsHel-3b	gb|AIXA01001813.1|:44940-45126

MsHel-3b	gb|AIXA01001813.1|:33334-33459
MsHel-3b	gb|AIXA01012304.1|:13632-13841
MsHel-3b	gb|AIXA01011853.1|:11424-11629
MsHel-3b	gb|AIXA01011840.1|:34846-35059
MsHel-3b	gb|AIXA01007168.1|:126828-127044
MsHel-3b	gb|AIXA01007168.1|:52963-53127
MsHel-3b	gb|AIXA01006279.1|:17573-17670
MsHel-3b	gb|AIXA01005571.1|:16649-16861
MsHel-3b	gb|AIXA01003878.1|:92114-92315
MsHel-3b	gb|AIXA01003662.1|:1051-1268
MsHel-3b	gb|AIXA01003647.1|:821-1038
MsHel-3b	gb|AIXA01001243.1|:201503-201638
MsHel-3b	gb|AIXA01001243.1|:167338-167528
MsHel-3b	gb|AIXA01001243.1|:147965-148060
MsHel-3b	gb|AIXA01001243.1|:38958-39075
MsHel-3b	gb|AIXA01001243.1|:4605-4754
MsHel-3b	gb|AIXA01000359.1|:159627-159825
MsHel-3b	gb|AIXA01017638.1|:10192-10396
MsHel-3b	gb|AIXA01013255.1|:20804-20981
MsHel-3b	gb|AIXA01011030.1|:15255-15471
MsHel-3b	gb|AIXA01009236.1|:1063-1275
MsHel-3b	gb|AIXA01009162.1|:1699-1830
MsHel-3b	gb|AIXA01007946.1|:79022-79230
MsHel-3b	gb|AIXA01006648.1|:65127-65343
MsHel-3b	gb|AIXA01006648.1|:36417-36532
MsHel-3b	gb|AIXA01004692.1|:34625-34806
MsHel-3b	gb|AIXA01003356.1|:65669-65885
MsHel-3b	gb|AIXA01002339.1|:19970-20156
MsHel-3b	gb|AIXA01002339.1|:2538-2742
MsHel-3b	gb|AIXA01001640.1|:1516-1724
MsHel-3b	gb|AIXA01001323.1|:47049-47262
MsHel-3b	gb|AIXA01000952.1|:19314-19531
MsHel-3b	gb|AIXA01037851.1|:193-400
MsHel-3b	gb|AIXA01030372.1|:169-381
MsHel-3b	gb|AIXA01016299.1|:5587-5793
MsHel-3b	gb|AIXA01007718.1|:11900-12096
MsHel-3b	gb|AIXA01007617.1|:11612-11811
MsHel-3b	gb|AIXA01007615.1|:6632-6831
MsHel-3b	gb|AIXA01007576.1|:120624-120823
MsHel-3b	gb|AIXA01007576.1|:42056-42181
MsHel-3b	gb|AIXA01005576.1|:37293-37505
MsHel-3b	gb|AIXA01005576.1|:11960-12162
MsHel-3b	gb|AIXA01005516.1|:29130-29253
MsHel-3b	gb|AIXA01002331.1|:64444-64581
MsHel-3b	gb|AIXA01002331.1|:46466-46645
MsHel-3b	gb|AIXA01002187.1|:33358-33540
MsHel-3b	gb|AIXA01001656.1|:98288-98462
MsHel-3b	gb|AIXA01001656.1|:4804-5006

MsHel-3b	gb|AIXA01000437.1|:35962-36150
MsHel-3b	gb|AIXA01000267.1|:98772-98971
MsHel-3b	gb|AIXA01000267.1|:18347-18548
MsHel-3b	gb|AIXA01037276.1|:27-237
MsHel-3b	gb|AIXA01019279.1|:7360-7569
MsHel-3b	gb|AIXA01017704.1|:825-1034
MsHel-3b	gb|AIXA01010676.1|:5876-6092
MsHel-3b	gb|AIXA01009642.1|:5440-5617
MsHel-3b	gb|AIXA01004124.1|:73478-73577
MsHel-3b	gb|AIXA01003711.1|:206450-206664
MsHel-3b	gb|AIXA01003711.1|:149495-149700
MsHel-3b	gb|AIXA01003711.1|:84207-84423
MsHel-3b	gb|AIXA01003711.1|:40823-41004
MsHel-3b	gb|AIXA01003650.1|:30011-30221
MsHel-3b	gb|AIXA01003286.1|:10866-11076
MsHel-3b	gb|AIXA01003057.1|:71946-73778
MsHel-3b	gb|AIXA01003057.1|:10178-10383
MsHel-3b	gb|AIXA01001817.1|:16623-16804
MsHel-3b	gb|AIXA01001509.1|:56653-56859
MsHel-3b	gb|AIXA01001297.1|:11933-12151
MsHel-3b	gb|AIXA01000627.1|:62836-63041
MsHel-3b	gb|AIXA01000468.1|:35945-36159
MsHel-3b	gb|AIXA01000044.1|:1480-1649
MsHel-3b	gb|AIXA01019161.1|:894-1104
MsHel-3b	gb|AIXA01010294.1|:12679-12890
MsHel-3b	gb|AIXA01010151.1|:58000-58080
MsHel-3b	gb|AIXA01009357.1|:11008-11222
MsHel-3b	gb|AIXA01008785.1|:11618-11826
MsHel-3b	gb|AIXA01006682.1|:9509-9708
MsHel-3b	gb|AIXA01005131.1|:26061-26274
MsHel-3b	gb|AIXA01004330.1|:80693-80818
MsHel-3b	gb|AIXA01004330.1|:39793-39965
MsHel-3b	gb|AIXA01004330.1|:17411-17596
MsHel-3b	gb|AIXA01004319.1|:46348-46563
MsHel-3b	gb|AIXA01004319.1|:7007-7185
MsHel-3b	gb|AIXA01004247.1|:17435-17620
MsHel-3b	gb|AIXA01004247.1|:6671-6877
MsHel-3b	gb|AIXA01004213.1|:6891-7095
MsHel-3b	gb|AIXA01003817.1|:60189-60397
MsHel-3b	gb|AIXA01003817.1|:16976-17179
MsHel-3b	gb|AIXA01001830.1|:44525-44731
MsHel-3b	gb|AIXA01001830.1|:26269-26477
MsHel-3b	gb|AIXA01001830.1|:12296-12387
MsHel-3b	gb|AIXA01000450.1|:7682-7879
MsHel-3b	gb|AIXA01015919.1|:10309-10436
MsHel-3b	gb|AIXA01009977.1|:17982-18189
MsHel-3b	gb|AIXA01009217.1|:34416-34628
MsHel-3b	gb|AIXA01009217.1|:218-402

MsHel-3b	gb|AIXA01008403.1|:5118-5310
MsHel-3b	gb|AIXA01007228.1|:133919-135982
MsHel-3b	gb|AIXA01007228.1|:108961-109175
MsHel-3b	gb|AIXA01007228.1|:72872-73086
MsHel-3b	gb|AIXA01006154.1|:68901-68991
MsHel-3b	gb|AIXA01006106.1|:7318-7534
MsHel-3b	gb|AIXA01005808.1|:34810-35026
MsHel-3b	gb|AIXA01004999.1|:37039-37247
MsHel-3b	gb|AIXA01004548.1|:34855-35062
MsHel-3b	gb|AIXA01004415.1|:45893-46110
MsHel-3b	gb|AIXA01001898.1|:231630-231775
MsHel-3b	gb|AIXA01001898.1|:168136-168311
MsHel-3b	gb|AIXA01001898.1|:110635-110853
MsHel-3b	gb|AIXA01001898.1|:61283-61495
MsHel-3b	gb|AIXA01001354.1|:8137-8341
MsHel-3b	gb|AIXA01001337.1|:446-662
MsHel-3b	gb|AIXA01000661.1|:65637-65853
MsHel-3b	gb|AIXA01000661.1|:13595-13681
MsHel-3b	gb|AIXA01029440.1|:394-605
MsHel-3b	gb|AIXA01022563.1|:102-305
MsHel-3b	gb|AIXA01013395.1|:936-1142
MsHel-3b	gb|AIXA01011371.1|:17684-17891
MsHel-3b	gb|AIXA01010829.1|:566-773
MsHel-3b	gb|AIXA01010335.1|:4329-4533
MsHel-3b	gb|AIXA01009285.1|:42051-42257
MsHel-3b	gb|AIXA01008227.1|:14444-14648
MsHel-3b	gb|AIXA01008116.1|:1203-1380
MsHel-3b	gb|AIXA01007362.1|:17224-17428
MsHel-3b	gb|AIXA01006923.1|:41632-41841
MsHel-3b	gb|AIXA01005993.1|:25561-25740
MsHel-3b	gb|AIXA01005710.1|:744-955
MsHel-3b	gb|AIXA01004693.1|:70142-70330
MsHel-3b	gb|AIXA01004693.1|:19963-20174
MsHel-3b	gb|AIXA01001983.1|:43046-43137
MsHel-3b	gb|AIXA01001983.1|:25611-25825
MsHel-3b	gb|AIXA01037436.1|:153-358
MsHel-3b	gb|AIXA01017270.1|:1130-1332
MsHel-3b	gb|AIXA01012171.1|:14284-14497
MsHel-3b	gb|AIXA01011456.1|:6386-6577
MsHel-3b	gb|AIXA01010251.1|:10314-10527
MsHel-3b	gb|AIXA01009797.1|:796-974
MsHel-3b	gb|AIXA01009442.1|:10325-10526
MsHel-3b	gb|AIXA01009061.1|:113199-113377
MsHel-3b	gb|AIXA01007396.1|:2999-3212
MsHel-3b	gb|AIXA01007266.1|:14764-14929
MsHel-3b	gb|AIXA01006837.1|:46770-46967
MsHel-3b	gb|AIXA01006350.1|:64407-64609
MsHel-3b	gb|AIXA01006337.1|:36472-36688

MsHel-3b	gb|AIXA01005108.1|:1388-1566
MsHel-3b	gb|AIXA01005096.1|:21646-21789
MsHel-3b	gb|AIXA01004354.1|:60812-60924
MsHel-3b	gb|AIXA01004354.1|:46650-46772
MsHel-3b	gb|AIXA01003871.1|:6587-6799
MsHel-3b	gb|AIXA01003739.1|:49441-49654
MsHel-3b	gb|AIXA01001571.1|:92417-92542
MsHel-3b	gb|AIXA01001189.1|:19493-19702
MsHel-3b	gb|AIXA01000907.1|:56787-56883
MsHel-3b	gb|AIXA01000907.1|:32044-32225
MsHel-3b	gb|AIXA01000907.1|:7513-7639
MsHel-3b	gb|AIXA01018912.1|:1883-2097
MsHel-3b	gb|AIXA01018866.1|:3387-3572
MsHel-3b	gb|AIXA01016291.1|:2202-2402
MsHel-3b	gb|AIXA01015144.1|:3647-3845
MsHel-3b	gb|AIXA01014867.1|:30661-30799
MsHel-3b	gb|AIXA01014867.1|:18525-18645
MsHel-3b	gb|AIXA01014544.1|:6868-7078
MsHel-3b	gb|AIXA01012168.1|:4874-5083
MsHel-3b	gb|AIXA01010773.1|:49835-49932
MsHel-3b	gb|AIXA01010773.1|:23243-23365
MsHel-3b	gb|AIXA01008965.1|:73345-73543
MsHel-3b	gb|AIXA01007613.1|:15605-15818
MsHel-3b	gb|AIXA01007175.1|:61002-61210
MsHel-3b	gb|AIXA01007107.1|:9400-9605
MsHel-3b	gb|AIXA01005350.1|:4391-4595
MsHel-3b	gb|AIXA01005092.1|:48694-48898
MsHel-3b	gb|AIXA01005092.1|:33337-33523
MsHel-3b	gb|AIXA01005092.1|:9947-10076
MsHel-3b	gb|AIXA01005035.1|:88803-89002
MsHel-3b	gb|AIXA01005035.1|:54093-54292
MsHel-3b	gb|AIXA01005035.1|:23030-23245
MsHel-3b	gb|AIXA01003858.1|:63275-63427
MsHel-3b	gb|AIXA01003243.1|:10456-10667
MsHel-3b	gb|AIXA01003214.1|:17170-17383
MsHel-3b	gb|AIXA01002561.1|:8696-8909
MsHel-3b	gb|AIXA01002065.1|:40225-40430
MsHel-3b	gb|AIXA01001665.1|:17317-17494
MsHel-3b	gb|AIXA01000501.1|:7813-8030
MsHel-3b	gb|AIXA01035334.1|:320-507
MsHel-3b	gb|AIXA01019289.1|:1529-1718
MsHel-3b	gb|AIXA01016674.1|:5079-5254
MsHel-3b	gb|AIXA01013947.1|:5566-5749
MsHel-3b	gb|AIXA01011273.1|:44762-44973
MsHel-3b	gb|AIXA01010208.1|:15187-15404
MsHel-3b	gb|AIXA01010152.1|:45668-45788
MsHel-3b	gb|AIXA01010152.1|:6394-6574
MsHel-3b	gb|AIXA01009047.1|:104169-104353

MsHel-3b	gb|AIXA01009031.1|:17150-17357
MsHel-3b	gb|AIXA01009031.1|:1362-1535
MsHel-3b	gb|AIXA01008275.1|:80286-80498
MsHel-3b	gb|AIXA01008226.1|:10748-10919
MsHel-3b	gb|AIXA01008194.1|:874-992
MsHel-3b	gb|AIXA01007966.1|:100572-100697
MsHel-3b	gb|AIXA01007966.1|:39268-48301
MsHel-3b	gb|AIXA01007692.1|:12359-12559
MsHel-3b	gb|AIXA01007494.1|:28008-28208
MsHel-3b	gb|AIXA01006914.1|:7757-7948
MsHel-3b	gb|AIXA01005612.1|:44968-45138
MsHel-3b	gb|AIXA01005612.1|:22249-22465
MsHel-3b	gb|AIXA01004470.1|:40067-40152
MsHel-3b	gb|AIXA01004051.1|:9334-9502
MsHel-3b	gb|AIXA01003176.1|:9333-9526
MsHel-3b	gb|AIXA01001356.1|:27369-27556
MsHel-3b	gb|AIXA01000317.1|:9429-9642
MsHel-3b	gb|AIXA01019982.1|:687-894
MsHel-3b	gb|AIXA01019406.1|:1681-1889
MsHel-3b	gb|AIXA01014004.1|:247-443
MsHel-3b	gb|AIXA01012652.1|:18021-18231
MsHel-3b	gb|AIXA01012133.1|:41883-42078
MsHel-3b	gb|AIXA01012133.1|:1631-1802
MsHel-3b	gb|AIXA01011387.1|:4292-4497
MsHel-3b	gb|AIXA01011197.1|:43820-44007
MsHel-3b	gb|AIXA01009326.1|:37505-37585
MsHel-3b	gb|AIXA01009326.1|:25813-26020
MsHel-3b	gb|AIXA01008767.1|:71029-71242
MsHel-3b	gb|AIXA01008767.1|:40467-40555
MsHel-3b	gb|AIXA01007915.1|:1881-2096
MsHel-3b	gb|AIXA01007046.1|:28234-28374
MsHel-3b	gb|AIXA01007046.1|:12358-12441
MsHel-3b	gb|AIXA01007023.1|:19445-19588
MsHel-3b	gb|AIXA01006123.1|:32572-32739
MsHel-3b	gb|AIXA01005050.1|:7638-7853
MsHel-3b	gb|AIXA01004665.1|:1776-1976
MsHel-3b	gb|AIXA01004569.1|:19334-19540
MsHel-3b	gb|AIXA01004465.1|:54100-54302
MsHel-3b	gb|AIXA01003929.1|:49802-50000
MsHel-3b	gb|AIXA01003929.1|:24256-24458
MsHel-3b	gb|AIXA01003929.1|:1605-1706
MsHel-3b	gb|AIXA01003820.1|:62115-62315
MsHel-3b	gb|AIXA01003190.1|:8022-8232
MsHel-3b	gb|AIXA01002621.1|:38721-38824
MsHel-3b	gb|AIXA01001968.1|:86563-86745
MsHel-3b	gb|AIXA01001621.1|:9253-9463
MsHel-3b	gb|AIXA01001320.1|:539-751
MsHel-3b	gb|AIXA01026903.1|:368-546

MsHel-3b	gb|AIXA01019309.1|:1915-2085
MsHel-3b	gb|AIXA01013067.1|:6813-6995
MsHel-3b	gb|AIXA01011776.1|:459-629
MsHel-3b	gb|AIXA01010757.1|:10355-10532
MsHel-3b	gb|AIXA01006875.1|:49592-49798
MsHel-3b	gb|AIXA01006816.1|:27081-27278
MsHel-3b	gb|AIXA01006529.1|:720-878
MsHel-3b	gb|AIXA01006287.1|:73047-73185
MsHel-3b	gb|AIXA01005509.1|:15376-15588
MsHel-3b	gb|AIXA01005278.1|:74289-74480
MsHel-3b	gb|AIXA01005278.1|:41504-41656
MsHel-3b	gb|AIXA01005087.1|:17847-18029
MsHel-3b	gb|AIXA01004567.1|:34964-35056
MsHel-3b	gb|AIXA01004567.1|:17559-17771
MsHel-3b	gb|AIXA01002293.1|:26919-30254
MsHel-3b	gb|AIXA01001804.1|:14396-14520
MsHel-3b	gb|AIXA01001511.1|:103448-103622
MsHel-3b	gb|AIXA01000048.1|:47165-47374
MsHel-3b	gb|AIXA01017342.1|:1913-2105
MsHel-3b	gb|AIXA01016698.1|:17066-17251
MsHel-3b	gb|AIXA01015971.1|:4214-4427
MsHel-3b	gb|AIXA01014333.1|:95-300
MsHel-3b	gb|AIXA01014018.1|:4737-4881
MsHel-3b	gb|AIXA01011669.1|:13776-13980
MsHel-3b	gb|AIXA01008711.1|:2089-2293
MsHel-3b	gb|AIXA01006593.1|:25588-25699
MsHel-3b	gb|AIXA01006593.1|:8820-9026
MsHel-3b	gb|AIXA01005135.1|:27628-27840
MsHel-3b	gb|AIXA01005135.1|:12875-13021
MsHel-3b	gb|AIXA01005037.1|:30210-30353
MsHel-3b	gb|AIXA01003658.1|:43557-43771
MsHel-3b	gb|AIXA01002817.1|:24942-25155
MsHel-3b	gb|AIXA01002362.1|:68770-68971
MsHel-3b	gb|AIXA01002362.1|:47003-47217
MsHel-3b	gb|AIXA01002362.1|:23814-23966
MsHel-3b	gb|AIXA01001753.1|:3786-3996
MsHel-3b	gb|AIXA01001554.1|:674-886
MsHel-3b	gb|AIXA01000767.1|:35323-35510
MsHel-3b	gb|AIXA01000767.1|:22471-22651
MsHel-3b	gb|AIXA01000767.1|:11702-11845
MsHel-3b	gb|AIXA01018151.1|:3716-3912
MsHel-3b	gb|AIXA01017130.1|:3628-3836
MsHel-3b	gb|AIXA01011648.1|:5549-5749
MsHel-3b	gb|AIXA01010839.1|:2726-2927
MsHel-3b	gb|AIXA01010748.1|:131159-131325
MsHel-3b	gb|AIXA01010748.1|:77866-83143
MsHel-3b	gb|AIXA01010748.1|:27246-27338
MsHel-3b	gb|AIXA01009766.1|:3954-4096

MsHel-3b	gb|AIXA01009346.1|:95388-95468
MsHel-3b	gb|AIXA01009135.1|:42839-43056
MsHel-3b	gb|AIXA01007231.1|:5300-5516
MsHel-3b	gb|AIXA01005793.1|:10433-10644
MsHel-3b	gb|AIXA01005536.1|:9165-9350
MsHel-3b	gb|AIXA01005351.1|:71273-71477
MsHel-3b	gb|AIXA01005351.1|:29581-29746
MsHel-3b	gb|AIXA01005093.1|:11656-11827
MsHel-3b	gb|AIXA01004529.1|:34045-34261
MsHel-3b	gb|AIXA01000838.1|:93716-93892
MsHel-3b	gb|AIXA01000838.1|:71196-71412
MsHel-3b	gb|AIXA01000560.1|:46688-46899
MsHel-3b	gb|AIXA01000560.1|:21416-21629
MsHel-3b	gb|AIXA01026245.1|:207-414
MsHel-3b	gb|AIXA01018627.1|:1838-2040
MsHel-3b	gb|AIXA01018069.1|:6467-6645
MsHel-3b	gb|AIXA01016742.1|:3267-3479
MsHel-3b	gb|AIXA01011722.1|:69809-70019
MsHel-3b	gb|AIXA01011722.1|:16167-16366
MsHel-3b	gb|AIXA01011361.1|:19441-19617
MsHel-3b	gb|AIXA01009575.1|:24337-24508
MsHel-3b	gb|AIXA01009381.1|:22702-22897
MsHel-3b	gb|AIXA01008705.1|:12283-12380
MsHel-3b	gb|AIXA01007968.1|:1533-1658
MsHel-3b	gb|AIXA01007543.1|:7161-7375
MsHel-3b	gb|AIXA01004160.1|:9258-9443
MsHel-3b	gb|AIXA01003993.1|:87137-87332
MsHel-3b	gb|AIXA01002491.1|:608-815
MsHel-3b	gb|AIXA01002313.1|:24611-24789
MsHel-3b	gb|AIXA01000093.1|:17011-17222
MsHel-3b	gb|AIXA01000068.1|:55983-56163
MsHel-3b	gb|AIXA01000068.1|:35636-35848
MsHel-3b	gb|AIXA01018234.1|:2041-2218
MsHel-3b	gb|AIXA01014611.1|:7470-7612
MsHel-3b	gb|AIXA01013702.1|:1016-1227
MsHel-3b	gb|AIXA01013211.1|:7199-7409
MsHel-3b	gb|AIXA01008725.1|:10374-10540
MsHel-3b	gb|AIXA01008134.1|:13545-13750
MsHel-3b	gb|AIXA01006402.1|:218851-219064
MsHel-3b	gb|AIXA01005841.1|:2160-2362
MsHel-3b	gb|AIXA01004893.1|:2890-3077
MsHel-3b	gb|AIXA01004865.1|:48464-48646
MsHel-3b	gb|AIXA01004865.1|:12164-12311
MsHel-3b	gb|AIXA01004740.1|:135215-135422
MsHel-3b	gb|AIXA01003889.1|:107940-108466
MsHel-3b	gb|AIXA01003889.1|:55184-55270
MsHel-3b	gb|AIXA01003661.1|:46292-46505
MsHel-3b	gb|AIXA01002707.1|:50748-50938

MsHel-3b	gb|AIXA01002707.1|:21169-21324
MsHel-3b	gb|AIXA01002604.1|:582-790
MsHel-3b	gb|AIXA01001847.1|:32879-33086
MsHel-3b	gb|AIXA01001366.1|:39147-39354
MsHel-3b	gb|AIXA01001015.1|:39462-39672
MsHel-3b	gb|AIXA01016222.1|:1108-1321
MsHel-3b	gb|AIXA01014622.1|:4678-4887
MsHel-3b	gb|AIXA01013934.1|:7391-7597
MsHel-3b	gb|AIXA01008364.1|:9334-9542
MsHel-3b	gb|AIXA01006632.1|:12812-13013
MsHel-3b	gb|AIXA01006299.1|:28808-29012
MsHel-3b	gb|AIXA01006135.1|:5645-6104
MsHel-3b	gb|AIXA01005228.1|:32793-33001
MsHel-3b	gb|AIXA01004672.1|:42556-42725
MsHel-3b	gb|AIXA01004053.1|:10921-11119
MsHel-3b	gb|AIXA01003028.1|:36344-36485
MsHel-3b	gb|AIXA01002168.1|:35134-35278
MsHel-3b	gb|AIXA01001994.1|:41058-41152
MsHel-3b	gb|AIXA01001994.1|:21529-21631
MsHel-3b	gb|AIXA01001340.1|:11410-11597
MsHel-3b	gb|AIXA01001037.1|:20423-20631
MsHel-3b	gb|AIXA01000966.1|:64055-64267
MsHel-3b	gb|AIXA01000966.1|:3420-3624
MsHel-3b	gb|AIXA01000931.1|:64256-64430
MsHel-3b	gb|AIXA01000555.1|:250-462
MsHel-3b	gb|AIXA01000242.1|:78205-78376
MsHel-3b	gb|AIXA01000242.1|:53501-53652
MsHel-3b	gb|AIXA01000224.1|:25017-25229
MsHel-3b	gb|AIXA01000013.1|:5375-5544
MsHel-3b	gb|AIXA01032730.1|:99-311
MsHel-3b	gb|AIXA01025709.1|:506-700
MsHel-3b	gb|AIXA01021063.1|:445-646
MsHel-3b	gb|AIXA01018652.1|:9521-9705
MsHel-3b	gb|AIXA01018583.1|:343-551
MsHel-3b	gb|AIXA01018504.1|:2761-2945
MsHel-3b	gb|AIXA01017979.1|:9687-9899
MsHel-3b	gb|AIXA01017774.1|:934-1145
MsHel-3b	gb|AIXA01015427.1|:14711-19571
MsHel-3b	gb|AIXA01015218.1|:9369-9548
MsHel-3b	gb|AIXA01012097.1|:5982-6198
MsHel-3b	gb|AIXA01008992.1|:31825-32029
MsHel-3b	gb|AIXA01008294.1|:10065-10244
MsHel-3b	gb|AIXA01007944.1|:4867-5075
MsHel-3b	gb|AIXA01005309.1|:30078-30249
MsHel-3b	gb|AIXA01004943.1|:36540-36666
MsHel-3b	gb|AIXA01004943.1|:7771-7978
MsHel-3b	gb|AIXA01004809.1|:16059-16262
MsHel-3b	gb|AIXA01003749.1|:18250-18432

MsHel-3b	gb|AIXA01002891.1|:7703-7913
MsHel-3b	gb|AIXA01001894.1|:36347-36563
MsHel-3b	gb|AIXA01001829.1|:39888-40084
MsHel-3b	gb|AIXA01000069.1|:284096-284310
MsHel-3b	gb|AIXA01000069.1|:196359-196539
MsHel-3b	gb|AIXA01000069.1|:128577-128738
MsHel-3b	gb|AIXA01000069.1|:65545-65683
MsHel-3b	gb|AIXA01015457.1|:6132-6216
MsHel-3b	gb|AIXA01009942.1|:27665-27847
MsHel-3b	gb|AIXA01009427.1|:22672-22821
MsHel-3b	gb|AIXA01009040.1|:135706-135913
MsHel-3b	gb|AIXA01009040.1|:5785-5868
MsHel-3b	gb|AIXA01008636.1|:50728-50928
MsHel-3b	gb|AIXA01007598.1|:23501-23687
MsHel-3b	gb|AIXA01007270.1|:25678-25888
MsHel-3b	gb|AIXA01007270.1|:2746-2863
MsHel-3b	gb|AIXA01004151.1|:4392-4578
MsHel-3b	gb|AIXA01002230.1|:94972-95182
MsHel-3b	gb|AIXA01001841.1|:45545-45693
MsHel-3b	gb|AIXA01001841.1|:22129-22346
MsHel-3b	gb|AIXA01001810.1|:21177-21393
MsHel-3b	gb|AIXA01001810.1|:5790-5972
MsHel-3b	gb|AIXA01001797.1|:190430-190518
MsHel-3b	gb|AIXA01001797.1|:144822-145021
MsHel-3b	gb|AIXA01001797.1|:101765-101958
MsHel-3b	gb|AIXA01001627.1|:60344-60543
MsHel-3b	gb|AIXA01001627.1|:40965-41099
MsHel-3b	gb|AIXA01001627.1|:5008-5165
MsHel-3b	gb|AIXA01000960.1|:77877-78056
MsHel-3b	gb|AIXA01000960.1|:43336-43431
MsHel-3b	gb|AIXA01000051.1|:25679-25890
MsHel-3b	gb|AIXA01019461.1|:3971-4179
MsHel-3b	gb|AIXA01018030.1|:650-816
MsHel-3b	gb|AIXA01016970.1|:4682-4863
MsHel-3b	gb|AIXA01016565.1|:1199-1394
MsHel-3b	gb|AIXA01016263.1|:806-984
MsHel-3b	gb|AIXA01014893.1|:17935-18147
MsHel-3b	gb|AIXA01014152.1|:27023-27233
MsHel-3b	gb|AIXA01011739.1|:13139-13219
MsHel-3b	gb|AIXA01010885.1|:85706-85787
MsHel-3b	gb|AIXA01010188.1|:26578-26792
MsHel-3b	gb|AIXA01009603.1|:17957-18162
MsHel-3b	gb|AIXA01009108.1|:25471-25687
MsHel-3b	gb|AIXA01008663.1|:5179-5382
MsHel-3b	gb|AIXA01008431.1|:5586-5796
MsHel-3b	gb|AIXA01008111.1|:4981-5158
MsHel-3b	gb|AIXA01007601.1|:6958-7139
MsHel-3b	gb|AIXA01006719.1|:8093-8275

MsHel-3b	gb|AIXA01006566.1|:35477-35690
MsHel-3b	gb|AIXA01006318.1|:9113-9323
MsHel-3b	gb|AIXA01005925.1|:5991-6205
MsHel-3b	gb|AIXA01005617.1|:7959-8165
MsHel-3b	gb|AIXA01005356.1|:17934-18023
MsHel-3b	gb|AIXA01005356.1|:6987-7180
MsHel-3b	gb|AIXA01004381.1|:16852-17061
MsHel-3b	gb|AIXA01004327.1|:108776-108911
MsHel-3b	gb|AIXA01004327.1|:94400-94606
MsHel-3b	gb|AIXA01004225.1|:35889-36101
MsHel-3b	gb|AIXA01003315.1|:37418-37631
MsHel-3b	gb|AIXA01002620.1|:2811-3006
MsHel-3b	gb|AIXA01002541.1|:4267-4479
MsHel-3b	gb|AIXA01002240.1|:44186-44360
MsHel-3b	gb|AIXA01000925.1|:35703-35906
MsHel-3b	gb|AIXA01000248.1|:1479-1633
MsHel-3b	gb|AIXA01025693.1|:656-813
MsHel-3b	gb|AIXA01017318.1|:18970-19160
MsHel-3b	gb|AIXA01017318.1|:6267-6474
MsHel-3b	gb|AIXA01016082.1|:13331-13507
MsHel-3b	gb|AIXA01014346.1|:29348-29567
MsHel-3b	gb|AIXA01014243.1|:7469-7600
MsHel-3b	gb|AIXA01010330.1|:45561-45685
MsHel-3b	gb|AIXA01010330.1|:20463-20585
MsHel-3b	gb|AIXA01010330.1|:1866-2019
MsHel-3b	gb|AIXA01009028.1|:21046-21255
MsHel-3b	gb|AIXA01008291.1|:27881-28083
MsHel-3b	gb|AIXA01007854.1|:69244-69456
MsHel-3b	gb|AIXA01007854.1|:48058-48266
MsHel-3b	gb|AIXA01007854.1|:34814-34995
MsHel-3b	gb|AIXA01005491.1|:9626-9807
MsHel-3b	gb|AIXA01002567.1|:18464-18662
MsHel-3b	gb|AIXA01001806.1|:22108-22293
MsHel-3b	gb|AIXA01001806.1|:1441-1523
MsHel-3b	gb|AIXA01000153.1|:1466-1636
MsHel-3b	gb|AIXA01019178.1|:3-127
MsHel-3b	gb|AIXA01019021.1|:1449-1653
MsHel-3b	gb|AIXA01017582.1|:19689-19893
MsHel-3b	gb|AIXA01015214.1|:23110-23306
MsHel-3b	gb|AIXA01014443.1|:64596-64759
MsHel-3b	gb|AIXA01014443.1|:45524-45705
MsHel-3b	gb|AIXA01014443.1|:21042-21191
MsHel-3b	gb|AIXA01014263.1|:6476-6687
MsHel-3b	gb|AIXA01013564.1|:13400-13610
MsHel-3b	gb|AIXA01011374.1|:8320-8511
MsHel-3b	gb|AIXA01011303.1|:14953-15138
MsHel-3b	gb|AIXA01011244.1|:7237-7453
MsHel-3b	gb|AIXA01010841.1|:1959-2131

MsHel-3b	gb|AIXA01010568.1|:429-594
MsHel-3b	gb|AIXA01008773.1|:39052-39138
MsHel-3b	gb|AIXA01007980.1|:13436-13643
MsHel-3b	gb|AIXA01006832.1|:17731-17938
MsHel-3b	gb|AIXA01006184.1|:12194-12396
MsHel-3b	gb|AIXA01005997.1|:27605-27748
MsHel-3b	gb|AIXA01004896.1|:35298-35486
MsHel-3b	gb|AIXA01004135.1|:4398-4605
MsHel-3b	gb|AIXA01003884.1|:24021-24204
MsHel-3b	gb|AIXA01003816.1|:32451-32666
MsHel-3b	gb|AIXA01003327.1|:18502-18717
MsHel-3b	gb|AIXA01002811.1|:61709-61871
MsHel-3b	gb|AIXA01001955.1|:3103-3283
MsHel-3b	gb|AIXA01001786.1|:149621-149725
MsHel-3b	gb|AIXA01001309.1|:49212-49420
MsHel-3b	gb|AIXA01000916.1|:1542-1755
MsHel-3b	gb|AIXA01027670.1|:714-924
MsHel-3b	gb|AIXA01020060.1|:1667-1850
MsHel-3b	gb|AIXA01017690.1|:3644-3853
MsHel-3b	gb|AIXA01015371.1|:11574-11660
MsHel-3b	gb|AIXA01015371.1|:349-555
MsHel-3b	gb|AIXA01014392.1|:12193-12362
MsHel-3b	gb|AIXA01014392.1|:1860-2060
MsHel-3b	gb|AIXA01014291.1|:2133-2335
MsHel-3b	gb|AIXA01007791.1|:9683-9859
MsHel-3b	gb|AIXA01007347.1|:73121-73287
MsHel-3b	gb|AIXA01007193.1|:511-723
MsHel-3b	gb|AIXA01007166.1|:55202-55423
MsHel-3b	gb|AIXA01006684.1|:13099-13279
MsHel-3b	gb|AIXA01006029.1|:31367-31584
MsHel-3b	gb|AIXA01005534.1|:1399-1570
MsHel-3b	gb|AIXA01005474.1|:8985-9083
MsHel-3b	gb|AIXA01004386.1|:82639-82763
MsHel-3b	gb|AIXA01003002.1|:12855-12990
MsHel-3b	gb|AIXA01002366.1|:17847-18051
MsHel-3b	gb|AIXA01002311.1|:172759-172965
MsHel-3b	gb|AIXA01002311.1|:17100-17179
MsHel-3b	gb|AIXA01002311.1|:4815-5030
MsHel-3b	gb|AIXA01001717.1|:57276-57478
MsHel-3b	gb|AIXA01001278.1|:48277-48365
MsHel-3b	gb|AIXA01001278.1|:36256-36427
MsHel-3b	gb|AIXA01000641.1|:15423-15644
MsHel-3b	gb|AIXA01000294.1|:23750-23861
MsHel-3b	gb|AIXA01000028.1|:22351-22494
MsHel-3b	gb|AIXA01000028.1|:9456-9622
MsHel-3b	gb|AIXA01000005.1|:124860-124965
MsHel-3b	gb|AIXA01000005.1|:68476-68602
MsHel-3b	gb|AIXA01000005.1|:50684-50867

MsHel-3b	gb|AIXA01017766.1|:1079-1271
MsHel-3b	gb|AIXA01016740.1|:2227-2353
MsHel-3b	gb|AIXA01015125.1|:9492-9629
MsHel-3b	gb|AIXA01014190.1|:3583-3748
MsHel-3b	gb|AIXA01011501.1|:20438-20563
MsHel-3b	gb|AIXA01010336.1|:2000-2177
MsHel-3b	gb|AIXA01010334.1|:25931-26143
MsHel-3b	gb|AIXA01009696.1|:20682-20889
MsHel-3b	gb|AIXA01008398.1|:18596-18678
MsHel-3b	gb|AIXA01008398.1|:6318-6439
MsHel-3b	gb|AIXA01008057.1|:36740-36950
MsHel-3b	gb|AIXA01007881.1|:6650-6782
MsHel-3b	gb|AIXA01007875.1|:107976-108133
MsHel-3b	gb|AIXA01007875.1|:45012-45136
MsHel-3b	gb|AIXA01007875.1|:13106-13228
MsHel-3b	gb|AIXA01007694.1|:26928-27112
MsHel-3b	gb|AIXA01006987.1|:4903-5113
MsHel-3b	gb|AIXA01005530.1|:18998-19206
MsHel-3b	gb|AIXA01005034.1|:46860-46999
MsHel-3b	gb|AIXA01005034.1|:17239-17405
MsHel-3b	gb|AIXA01004490.1|:17327-17480
MsHel-3b	gb|AIXA01004431.1|:16655-16856
MsHel-3b	gb|AIXA01002799.1|:5545-5746
MsHel-3b	gb|AIXA01001296.1|:171729-171897
MsHel-3b	gb|AIXA01001227.1|:36471-36679
MsHel-3b	gb|AIXA01001069.1|:849-1024
MsHel-3b	gb|AIXA01000272.1|:73696-73916
MsHel-3b	gb|AIXA01000195.1|:49934-50143
MsHel-3b	gb|AIXA01000095.1|:84573-84657
MsHel-3b	gb|AIXA01000095.1|:58883-59049
MsHel-3b	gb|AIXA01022696.1|:1077-1234
MsHel-3b	gb|AIXA01018620.1|:2831-3004
MsHel-3b	gb|AIXA01017136.1|:7825-8025
MsHel-3b	gb|AIXA01014675.1|:2075-2284
MsHel-3b	gb|AIXA01014299.1|:12854-13031
MsHel-3b	gb|AIXA01014041.1|:3332-3458
MsHel-3b	gb|AIXA01012649.1|:6895-7065
MsHel-3b	gb|AIXA01012212.1|:738-957
MsHel-3b	gb|AIXA01011300.1|:6520-6729
MsHel-3b	gb|AIXA01010776.1|:25165-25361
MsHel-3b	gb|AIXA01010627.1|:7951-8132
MsHel-3b	gb|AIXA01009432.1|:13024-13220
MsHel-3b	gb|AIXA01009123.1|:8483-8689
MsHel-3b	gb|AIXA01009102.1|:4550-4728
MsHel-3b	gb|AIXA01009082.1|:625-833
MsHel-3b	gb|AIXA01008220.1|:21462-21546
MsHel-3b	gb|AIXA01005464.1|:87352-87455
MsHel-3b	gb|AIXA01005464.1|:70863-71025

MsHel-3b	gb|AIXA01004817.1|:50855-51047
MsHel-3b	gb|AIXA01004817.1|:27238-27322
MsHel-3b	gb|AIXA01004017.1|:10793-10970
MsHel-3b	gb|AIXA01003490.1|:44587-44788
MsHel-3b	gb|AIXA01002815.1|:42399-42579
MsHel-3b	gb|AIXA01001816.1|:40751-40944
MsHel-3b	gb|AIXA01001816.1|:22036-22255
MsHel-3b	gb|AIXA01001816.1|:8001-8177
MsHel-3b	gb|AIXA01001047.1|:6691-6905
MsHel-3b	gb|AIXA01000813.1|:30591-30787
MsHel-3b	gb|AIXA01000810.1|:347686-347898
MsHel-3b	gb|AIXA01000810.1|:318655-318804
MsHel-3b	gb|AIXA01000810.1|:240073-240174
MsHel-3b	gb|AIXA01000810.1|:110350-110517
MsHel-3b	gb|AIXA01000204.1|:10997-11210
MsHel-3b	gb|AIXA01021631.1|:1652-1840
MsHel-3b	gb|AIXA01021505.1|:475-603
MsHel-3b	gb|AIXA01011426.1|:17522-17733
MsHel-3b	gb|AIXA01010372.1|:2835-3027
MsHel-3b	gb|AIXA01008313.1|:14543-14653
MsHel-3b	gb|AIXA01008173.1|:3178-3386
MsHel-3b	gb|AIXA01007783.1|:60234-60355
MsHel-3b	gb|AIXA01007783.1|:26481-26697
MsHel-3b	gb|AIXA01007130.1|:90378-90554
MsHel-3b	gb|AIXA01005594.1|:36320-36530
MsHel-3b	gb|AIXA01005246.1|:6604-6784
MsHel-3b	gb|AIXA01003942.1|:37751-37959
MsHel-3b	gb|AIXA01003942.1|:224-327
MsHel-3b	gb|AIXA01003615.1|:40147-40287
MsHel-3b	gb|AIXA01003615.1|:1965-2175
MsHel-3b	gb|AIXA01003358.1|:2680-2860
MsHel-3b	gb|AIXA01002622.1|:32805-33216
MsHel-3b	gb|AIXA01002340.1|:130135-130347
MsHel-3b	gb|AIXA01002340.1|:97534-97717
MsHel-3b	gb|AIXA01002296.1|:169522-169675
MsHel-3b	gb|AIXA01002296.1|:154296-154512
MsHel-3b	gb|AIXA01001680.1|:39965-40141
MsHel-3b	gb|AIXA01001267.1|:12613-12828
MsHel-3b	gb|AIXA01001234.1|:8878-9062
MsHel-3b	gb|AIXA01000430.1|:36035-36250
MsHel-3b	gb|AIXA01000162.1|:10327-10520
MsHel-3b	gb|AIXA01033760.1|:84-296
MsHel-3b	gb|AIXA01028354.1|:668-864
MsHel-3b	gb|AIXA01023722.1|:1127-1299
MsHel-3b	gb|AIXA01021888.1|:637-780
MsHel-3b	gb|AIXA01018623.1|:3414-3622
MsHel-3b	gb|AIXA01017070.1|:9732-9911
MsHel-3b	gb|AIXA01016592.1|:3153-3360

MsHel-3b	gb|AIXA01015674.1|:2625-2800
MsHel-3b	gb|AIXA01014934.1|:6751-6940
MsHel-3b	gb|AIXA01013500.1|:1802-1981
MsHel-3b	gb|AIXA01012760.1|:5630-5840
MsHel-3b	gb|AIXA01012653.1|:6792-6947
MsHel-3b	gb|AIXA01012196.1|:26049-26228
MsHel-3b	gb|AIXA01010853.1|:7056-7231
MsHel-3b	gb|AIXA01010583.1|:15145-15338
MsHel-3b	gb|AIXA01010583.1|:1666-1806
MsHel-3b	gb|AIXA01010102.1|:93660-93826
MsHel-3b	gb|AIXA01010102.1|:82846-83033
MsHel-3b	gb|AIXA01010102.1|:33908-34033
MsHel-3b	gb|AIXA01009953.1|:2812-2996
MsHel-3b	gb|AIXA01009033.1|:5938-6125
MsHel-3b	gb|AIXA01008722.1|:27683-27867
MsHel-3b	gb|AIXA01008533.1|:1607-1794
MsHel-3b	gb|AIXA01008110.1|:24496-24696
MsHel-3b	gb|AIXA01008076.1|:13751-13835
MsHel-3b	gb|AIXA01007577.1|:29205-29317
MsHel-3b	gb|AIXA01006878.1|:42272-42478
MsHel-3b	gb|AIXA01006156.1|:21514-21701
MsHel-3b	gb|AIXA01005521.1|:64760-64962
MsHel-3b	gb|AIXA01005521.1|:46445-46779
MsHel-3b	gb|AIXA01005088.1|:14896-15344
MsHel-3b	gb|AIXA01003935.1|:35557-35643
MsHel-3b	gb|AIXA01003474.1|:11433-11612
MsHel-3b	gb|AIXA01003209.1|:4724-4887
MsHel-3b	gb|AIXA01003000.1|:27343-27507
MsHel-3b	gb|AIXA01002782.1|:21006-22102
MsHel-3b	gb|AIXA01001071.1|:87371-87500
MsHel-3b	gb|AIXA01000085.1|:673-878
MsHel-3b	gb|AIXA01029210.1|:158-327
MsHel-3b	gb|AIXA01022558.1|:537-718
MsHel-3b	gb|AIXA01016791.1|:2600-2796
MsHel-3b	gb|AIXA01014982.1|:23486-23701
MsHel-3b	gb|AIXA01014175.1|:2932-3127
MsHel-3b	gb|AIXA01013973.1|:15401-15607
MsHel-3b	gb|AIXA01013876.1|:58-256
MsHel-3b	gb|AIXA01013628.1|:10000-10202
MsHel-3b	gb|AIXA01012798.1|:26640-26847
MsHel-3b	gb|AIXA01009752.1|:25776-25939
MsHel-3b	gb|AIXA01009267.1|:24134-24343
MsHel-3b	gb|AIXA01007552.1|:63262-63443
MsHel-3b	gb|AIXA01006321.1|:269-475
MsHel-3b	gb|AIXA01005439.1|:8232-8436
MsHel-3b	gb|AIXA01005336.1|:726-907
MsHel-3b	gb|AIXA01004980.1|:101875-102069
MsHel-3b	gb|AIXA01004342.1|:14715-18868

MsHel-3b	gb|AIXA01004220.1|:10095-10251
MsHel-3b	gb|AIXA01003825.1|:19195-19340
MsHel-3b	gb|AIXA01003819.1|:24266-24460
MsHel-3b	gb|AIXA01003355.1|:61894-62055
MsHel-3b	gb|AIXA01003355.1|:31139-31339
MsHel-3b	gb|AIXA01002454.1|:69210-69344
MsHel-3b	gb|AIXA01002381.1|:38212-38342
MsHel-3b	gb|AIXA01002381.1|:12576-12757
MsHel-3b	gb|AIXA01002248.1|:87521-87702
MsHel-3b	gb|AIXA01002248.1|:57163-57325
MsHel-3b	gb|AIXA01001822.1|:19947-20124
MsHel-3b	gb|AIXA01001822.1|:3297-3415
MsHel-3b	gb|AIXA01000770.1|:11128-11274
MsHel-3b	gb|AIXA01000054.1|:2943-3127
MsHel-3b	gb|AIXA01024524.1|:924-1137
MsHel-3b	gb|AIXA01023710.1|:66-244
MsHel-3b	gb|AIXA01021504.1|:1372-1558
MsHel-3b	gb|AIXA01021444.1|:1823-1998
MsHel-3b	gb|AIXA01014585.1|:4049-4226
MsHel-3b	gb|AIXA01010782.1|:68317-68532
MsHel-3b	gb|AIXA01009610.1|:841-1006
MsHel-3b	gb|AIXA01009501.1|:499-721
MsHel-3b	gb|AIXA01008854.1|:30149-30310
MsHel-3b	gb|AIXA01006982.1|:12738-12943
MsHel-3b	gb|AIXA01006815.1|:33968-34142
MsHel-3b	gb|AIXA01006330.1|:11647-11848
MsHel-3b	gb|AIXA01005951.1|:22911-23084
MsHel-3b	gb|AIXA01005504.1|:113810-113912
MsHel-3b	gb|AIXA01005504.1|:83083-83324
MsHel-3b	gb|AIXA01004919.1|:11729-11943
MsHel-3b	gb|AIXA01004806.1|:55199-55294
MsHel-3b	gb|AIXA01004806.1|:42844-43059
MsHel-3b	gb|AIXA01003860.1|:36494-36702
MsHel-3b	gb|AIXA01002175.1|:1615-1726
MsHel-3b	gb|AIXA01001620.1|:30790-30951
MsHel-3b	gb|AIXA01001551.1|:2627-2826
MsHel-3b	gb|AIXA01001347.1|:14366-14527
MsHel-3b	gb|AIXA01001235.1|:73144-73281
MsHel-3b	gb|AIXA01000741.1|:9668-9866
MsHel-3b	gb|AIXA01000561.1|:47579-47663
MsHel-3b	gb|AIXA01000561.1|:6000-6185
MsHel-3b	gb|AIXA01000438.1|:84-305
MsHel-3b	gb|AIXA01000275.1|:20772-20978
MsHel-3b	gb|AIXA01000275.1|:5349-5525
MsHel-3b	gb|AIXA01034847.1|:231-390
MsHel-3b	gb|AIXA01018146.1|:2747-2879
MsHel-3b	gb|AIXA01010956.1|:42885-43100
MsHel-3b	gb|AIXA01009502.1|:384-598

MsHel-3b	gb|AIXA01007638.1|:8078-8293
MsHel-3b	gb|AIXA01007384.1|:3094-3300
MsHel-3b	gb|AIXA01007192.1|:3664-3840
MsHel-3b	gb|AIXA01006917.1|:111965-112048
MsHel-3b	gb|AIXA01006917.1|:36379-36586
MsHel-3b	gb|AIXA01006917.1|:265-447
MsHel-3b	gb|AIXA01006848.1|:6255-7250
MsHel-3b	gb|AIXA01006642.1|:19559-19750
MsHel-3b	gb|AIXA01006064.1|:18374-18587
MsHel-3b	gb|AIXA01005311.1|:5901-6094
MsHel-3b	gb|AIXA01004985.1|:33265-33409
MsHel-3b	gb|AIXA01004985.1|:15363-15544
MsHel-3b	gb|AIXA01004908.1|:10706-10902
MsHel-3b	gb|AIXA01003441.1|:15631-15836
MsHel-3b	gb|AIXA01002507.1|:82273-82425
MsHel-3b	gb|AIXA01002507.1|:69577-69737
MsHel-3b	gb|AIXA01002500.1|:143085-149426
MsHel-3b	gb|AIXA01002500.1|:48775-48963
MsHel-3b	gb|AIXA01002500.1|:8500-8602
MsHel-3b	gb|AIXA01002309.1|:16004-16191
MsHel-3b	gb|AIXA01002281.1|:15782-15863
MsHel-3b	gb|AIXA01001304.1|:15181-15267
MsHel-3b	gb|AIXA01000747.1|:146938-147136
MsHel-3b	gb|AIXA01000747.1|:68621-68773
MsHel-3b	gb|AIXA01000734.1|:10673-10813
MsHel-3b	gb|AIXA01029891.1|:484-686
MsHel-3b	gb|AIXA01020764.1|:1089-1296
MsHel-3b	gb|AIXA01014990.1|:4178-4388
MsHel-3b	gb|AIXA01014529.1|:13020-13167
MsHel-3b	gb|AIXA01013966.1|:1064-10291
MsHel-3b	gb|AIXA01012306.1|:8708-8886
MsHel-3b	gb|AIXA01009177.1|:24471-24659
MsHel-3b	gb|AIXA01008754.1|:10354-10534
MsHel-3b	gb|AIXA01007530.1|:48220-48422
MsHel-3b	gb|AIXA01007414.1|:8907-9098
MsHel-3b	gb|AIXA01006132.1|:37791-37955
MsHel-3b	gb|AIXA01006132.1|:14647-14772
MsHel-3b	gb|AIXA01005321.1|:71883-77467
MsHel-3b	gb|AIXA01005321.1|:9799-9973
MsHel-3b	gb|AIXA01004550.1|:76573-76755
MsHel-3b	gb|AIXA01004550.1|:66034-66124
MsHel-3b	gb|AIXA01004219.1|:6722-6913
MsHel-3b	gb|AIXA01003633.1|:51666-51776
MsHel-3b	gb|AIXA01003633.1|:35999-36198
MsHel-3b	gb|AIXA01002968.1|:5414-5620
MsHel-3b	gb|AIXA01002651.1|:10313-10455
MsHel-3b	gb|AIXA01002473.1|:6144-6282
MsHel-3b	gb|AIXA01002297.1|:17150-17305

MsHel-3b	gb|AIXA01002264.1|:20007-20221
MsHel-3b	gb|AIXA01001846.1|:15363-15531
MsHel-3b	gb|AIXA01001270.1|:45918-46072
MsHel-3b	gb|AIXA01001270.1|:22414-22625
MsHel-3b	gb|AIXA01001196.1|:55196-55403
MsHel-3b	gb|AIXA01000799.1|:841-980
MsHel-3b	gb|AIXA01037019.1|:284-474
MsHel-3b	gb|AIXA01018914.1|:1771-1917
MsHel-3b	gb|AIXA01018226.1|:1672-1844
MsHel-3b	gb|AIXA01015686.1|:1017-1228
MsHel-3b	gb|AIXA01015406.1|:21059-21265
MsHel-3b	gb|AIXA01015406.1|:7977-8123
MsHel-3b	gb|AIXA01014598.1|:2158-2345
MsHel-3b	gb|AIXA01013902.1|:3639-3849
MsHel-3b	gb|AIXA01013697.1|:5284-5410
MsHel-3b	gb|AIXA01013302.1|:22327-22504
MsHel-3b	gb|AIXA01012741.1|:111142-111329
MsHel-3b	gb|AIXA01012741.1|:59902-60112
MsHel-3b	gb|AIXA01012741.1|:27085-27229
MsHel-3b	gb|AIXA01012100.1|:15565-15735
MsHel-3b	gb|AIXA01009835.1|:31582-31782
MsHel-3b	gb|AIXA01008418.1|:95972-96075
MsHel-3b	gb|AIXA01008418.1|:63798-63900
MsHel-3b	gb|AIXA01008418.1|:28177-28308
MsHel-3b	gb|AIXA01007382.1|:7152-7278
MsHel-3b	gb|AIXA01006863.1|:2726-2935
MsHel-3b	gb|AIXA01005095.1|:66878-67085
MsHel-3b	gb|AIXA01005095.1|:34823-35002
MsHel-3b	gb|AIXA01004515.1|:21060-21274
MsHel-3b	gb|AIXA01004224.1|:6265-6457
MsHel-3b	gb|AIXA01003621.1|:61174-61340
MsHel-3b	gb|AIXA01003621.1|:38815-39021
MsHel-3b	gb|AIXA01003494.1|:25751-25944
MsHel-3b	gb|AIXA01002975.1|:124837-125048
MsHel-3b	gb|AIXA01002975.1|:74221-74425
MsHel-3b	gb|AIXA01002975.1|:25925-26060
MsHel-3b	gb|AIXA01002730.1|:55568-55776
MsHel-3b	gb|AIXA01001599.1|:75618-75820
MsHel-3b	gb|AIXA01001349.1|:32094-32271
MsHel-3b	gb|AIXA01001272.1|:9277-9460
MsHel-3b	gb|AIXA01000703.1|:23866-24047
MsHel-3b	gb|AIXA01000105.1|:53106-53304
MsHel-3b	gb|AIXA01000105.1|:31807-32015
MsHel-3b	gb|AIXA01028241.1|:526-704
MsHel-3b	gb|AIXA01025455.1|:1019-1200
MsHel-3b	gb|AIXA01021662.1|:1401-1578
MsHel-3b	gb|AIXA01018770.1|:3436-3629
MsHel-3b	gb|AIXA01018731.1|:3170-3333

MsHel-3b	gb|AIXA01013065.1|:23742-23952
MsHel-3b	gb|AIXA01012349.1|:10014-10207
MsHel-3b	gb|AIXA01009650.1|:124-282
MsHel-3b	gb|AIXA01009643.1|:12185-12380
MsHel-3b	gb|AIXA01009508.1|:47486-47695
MsHel-3b	gb|AIXA01008938.1|:346-542
MsHel-3b	gb|AIXA01008337.1|:14689-14886
MsHel-3b	gb|AIXA01006253.1|:24940-25159
MsHel-3b	gb|AIXA01005604.1|:19467-19659
MsHel-3b	gb|AIXA01005604.1|:3419-3545
MsHel-3b	gb|AIXA01005566.1|:41294-41504
MsHel-3b	gb|AIXA01005377.1|:6239-6448
MsHel-3b	gb|AIXA01005270.1|:11124-11288
MsHel-3b	gb|AIXA01004176.1|:5888-6102
MsHel-3b	gb|AIXA01004025.1|:397-574
MsHel-3b	gb|AIXA01003857.1|:72215-72427
MsHel-3b	gb|AIXA01003857.1|:24242-24336
MsHel-3b	gb|AIXA01003857.1|:6182-6314
MsHel-3b	gb|AIXA01001660.1|:71880-72088
MsHel-3b	gb|AIXA01001602.1|:52268-52393
MsHel-3b	gb|AIXA01001305.1|:4256-4445
MsHel-3b	gb|AIXA01001231.1|:14398-14584
MsHel-3b	gb|AIXA01001022.1|:10964-11094
MsHel-3b	gb|AIXA01000448.1|:46413-46588
MsHel-3b	gb|AIXA01000422.1|:26728-26918
MsHel-3b	gb|AIXA01025404.1|:939-1151
MsHel-3b	gb|AIXA01024474.1|:320-498
MsHel-3b	gb|AIXA01019098.1|:606-746
MsHel-3b	gb|AIXA01016383.1|:10121-10325
MsHel-3b	gb|AIXA01015394.1|:1543-1751
MsHel-3b	gb|AIXA01011964.1|:25661-25829
MsHel-3b	gb|AIXA01011451.1|:343-475
MsHel-3b	gb|AIXA01009576.1|:5853-6056
MsHel-3b	gb|AIXA01009084.1|:4189-4342
MsHel-3b	gb|AIXA01007902.1|:12478-12657
MsHel-3b	gb|AIXA01007218.1|:65838-66047
MsHel-3b	gb|AIXA01007015.1|:54269-54357
MsHel-3b	gb|AIXA01006652.1|:1337-1551
MsHel-3b	gb|AIXA01006160.1|:3961-4173
MsHel-3b	gb|AIXA01005323.1|:31943-32068
MsHel-3b	gb|AIXA01005323.1|:18098-18275
MsHel-3b	gb|AIXA01002477.1|:33703-33787
MsHel-3b	gb|AIXA01001239.1|:86435-86602
MsHel-3b	gb|AIXA01000911.1|:16918-17123
MsHel-3b	gb|AIXA01000650.1|:115927-116098
MsHel-3b	gb|AIXA01000650.1|:93930-94025
MsHel-3b	gb|AIXA01000611.1|:90239-90450
MsHel-3b	gb|AIXA01000494.1|:50403-50575

MsHel-3b	gb|AIXA01000494.1|:22818-23008
MsHel-3b	gb|AIXA01000397.1|:203-331
MsHel-3b	gb|AIXA01000161.1|:29004-29129
MsHel-3b	gb|AIXA01000102.1|:14434-14637
MsHel-3b	gb|AIXA01018932.1|:3651-3825
MsHel-3b	gb|AIXA01017985.1|:6321-6499
MsHel-3b	gb|AIXA01017969.1|:1571-1749
MsHel-3b	gb|AIXA01017926.1|:6965-7178
MsHel-3b	gb|AIXA01015220.1|:2193-2332
MsHel-3b	gb|AIXA01013807.1|:46341-46539
MsHel-3b	gb|AIXA01011445.1|:4593-4792
MsHel-3b	gb|AIXA01011385.1|:28745-28964
MsHel-3b	gb|AIXA01009624.1|:20100-20311
MsHel-3b	gb|AIXA01009142.1|:41973-42111
MsHel-3b	gb|AIXA01009117.1|:27714-27914
MsHel-3b	gb|AIXA01007616.1|:27067-27221
MsHel-3b	gb|AIXA01007451.1|:33076-33282
MsHel-3b	gb|AIXA01007355.1|:4072-4252
MsHel-3b	gb|AIXA01007163.1|:9723-9890
MsHel-3b	gb|AIXA01006508.1|:15196-15320
MsHel-3b	gb|AIXA01006182.1|:28650-28840
MsHel-3b	gb|AIXA01004422.1|:16846-17058
MsHel-3b	gb|AIXA01004121.1|:28141-28324
MsHel-3b	gb|AIXA01004099.1|:47643-47805
MsHel-3b	gb|AIXA01004099.1|:13498-13579
MsHel-3b	gb|AIXA01004000.1|:3016-3163
MsHel-3b	gb|AIXA01003850.1|:127786-127968
MsHel-3b	gb|AIXA01003850.1|:85163-85307
MsHel-3b	gb|AIXA01003850.1|:59363-59537
MsHel-3b	gb|AIXA01003283.1|:80741-80941
MsHel-3b	gb|AIXA01001939.1|:49115-49319
MsHel-3b	gb|AIXA01001803.1|:13842-14046
MsHel-3b	gb|AIXA01001050.1|:78534-78642
MsHel-3b	gb|AIXA01001050.1|:51719-51842
MsHel-3b	gb|AIXA01001050.1|:34143-34317
MsHel-3b	gb|AIXA01020399.1|:141-347
MsHel-3b	gb|AIXA01020177.1|:1848-2049
MsHel-3b	gb|AIXA01018937.1|:5824-5932
MsHel-3b	gb|AIXA01018655.1|:3438-3616
MsHel-3b	gb|AIXA01015424.1|:2592-2766
MsHel-3b	gb|AIXA01014648.1|:5353-5526
MsHel-3b	gb|AIXA01013978.1|:1075-1290
MsHel-3b	gb|AIXA01012194.1|:6236-6411
MsHel-3b	gb|AIXA01011587.1|:14001-14144
MsHel-3b	gb|AIXA01011126.1|:24856-25062
MsHel-3b	gb|AIXA01010394.1|:58060-58274
MsHel-3b	gb|AIXA01009792.1|:56635-56821
MsHel-3b	gb|AIXA01009792.1|:21181-21383

MsHel-3b	gb|AIXA01008665.1|:13058-13192
MsHel-3b	gb|AIXA01008665.1|:392-498
MsHel-3b	gb|AIXA01007436.1|:1257-1460
MsHel-3b	gb|AIXA01006731.1|:138382-138534
MsHel-3b	gb|AIXA01006731.1|:63043-63130
MsHel-3b	gb|AIXA01005642.1|:68384-68468
MsHel-3b	gb|AIXA01005538.1|:8145-8348
MsHel-3b	gb|AIXA01004958.1|:39573-39773
MsHel-3b	gb|AIXA01004715.1|:85544-85753
MsHel-3b	gb|AIXA01004715.1|:65270-65465
MsHel-3b	gb|AIXA01004667.1|:145346-145547
MsHel-3b	gb|AIXA01004667.1|:54983-55067
MsHel-3b	gb|AIXA01004385.1|:170-361
MsHel-3b	gb|AIXA01003600.1|:29546-29746
MsHel-3b	gb|AIXA01003504.1|:12361-12574
MsHel-3b	gb|AIXA01002999.1|:97577-97702
MsHel-3b	gb|AIXA01002386.1|:117082-117227
MsHel-3b	gb|AIXA01001277.1|:15248-15417
MsHel-3b	gb|AIXA01000070.1|:886-1068
MsHel-3b	gb|AIXA01020021.1|:115-249
MsHel-3b	gb|AIXA01018667.1|:5324-5482
MsHel-3b	gb|AIXA01017988.1|:2290-2425
MsHel-3b	gb|AIXA01017939.1|:4133-4303
MsHel-3b	gb|AIXA01016600.1|:1267-1424
MsHel-3b	gb|AIXA01015929.1|:22969-23166
MsHel-3b	gb|AIXA01013160.1|:1217-1350
MsHel-3b	gb|AIXA01012319.1|:9347-9472
MsHel-3b	gb|AIXA01011304.1|:19036-19231
MsHel-3b	gb|AIXA01010808.1|:3973-4182
MsHel-3b	gb|AIXA01010756.1|:37236-37396
MsHel-3b	gb|AIXA01010722.1|:5664-5869
MsHel-3b	gb|AIXA01010253.1|:18734-18912
MsHel-3b	gb|AIXA01008023.1|:80583-80668
MsHel-3b	gb|AIXA01008023.1|:31370-31523
MsHel-3b	gb|AIXA01007800.1|:81723-81924
MsHel-3b	gb|AIXA01007800.1|:64778-64869
MsHel-3b	gb|AIXA01006983.1|:51623-51820
MsHel-3b	gb|AIXA01006983.1|:21276-21493
MsHel-3b	gb|AIXA01006888.1|:24372-24501
MsHel-3b	gb|AIXA01005505.1|:18179-18366
MsHel-3b	gb|AIXA01005062.1|:9818-10012
MsHel-3b	gb|AIXA01004561.1|:3807-4005
MsHel-3b	gb|AIXA01004155.1|:37851-38055
MsHel-3b	gb|AIXA01002283.1|:55103-55315
MsHel-3b	gb|AIXA01001043.1|:78656-78781
MsHel-3b	gb|AIXA01020592.1|:507-635
MsHel-3b	gb|AIXA01016578.1|:1185-1360
MsHel-3b	gb|AIXA01011538.1|:14973-15116

MsHel-3b	gb|AIXA01011324.1|:297-469
MsHel-3b	gb|AIXA01008920.1|:74-274
MsHel-3b	gb|AIXA01008296.1|:20441-20623
MsHel-3b	gb|AIXA01008287.1|:86474-86650
MsHel-3b	gb|AIXA01007493.1|:112257-112441
MsHel-3b	gb|AIXA01007453.1|:39547-39762
MsHel-3b	gb|AIXA01007115.1|:1500-1705
MsHel-3b	gb|AIXA01006590.1|:144047-144249
MsHel-3b	gb|AIXA01006200.1|:29513-29728
MsHel-3b	gb|AIXA01006200.1|:9646-9730
MsHel-3b	gb|AIXA01005462.1|:18311-18421
MsHel-3b	gb|AIXA01003690.1|:94587-94699
MsHel-3b	gb|AIXA01003690.1|:7131-7338
MsHel-3b	gb|AIXA01003687.1|:224248-224351
MsHel-3b	gb|AIXA01003687.1|:190128-190247
MsHel-3b	gb|AIXA01003687.1|:119459-124326
MsHel-3b	gb|AIXA01003614.1|:60185-60386
MsHel-3b	gb|AIXA01003614.1|:45594-45716
MsHel-3b	gb|AIXA01002703.1|:61759-61904
MsHel-3b	gb|AIXA01001650.1|:16100-16316
MsHel-3b	gb|AIXA01001026.1|:51955-52101
MsHel-3b	gb|AIXA01000012.1|:79486-79653
MsHel-3b	gb|AIXA01023505.1|:538-744
MsHel-3b	gb|AIXA01014960.1|:21394-21579
MsHel-3b	gb|AIXA01014284.1|:7815-7999
MsHel-3b	gb|AIXA01012657.1|:24270-24766
MsHel-3b	gb|AIXA01007141.1|:4507-4688
MsHel-3b	gb|AIXA01004140.1|:6323-6517
MsHel-3b	gb|AIXA01003187.1|:69-274
MsHel-3b	gb|AIXA01003042.1|:49591-49731
MsHel-3b	gb|AIXA01003037.1|:12364-12527
MsHel-3b	gb|AIXA01002514.1|:27423-27599
MsHel-3b	gb|AIXA01002514.1|:8742-8914
MsHel-3b	gb|AIXA01002051.1|:11953-12165
MsHel-3b	gb|AIXA01001724.1|:3034-3249
MsHel-3b	gb|AIXA01001653.1|:9222-9405
MsHel-3b	gb|AIXA01001566.1|:19432-19589
MsHel-3b	gb|AIXA01036892.1|:225-358
MsHel-3b	gb|AIXA01030551.1|:228-410
MsHel-3b	gb|AIXA01025839.1|:1031-1185
MsHel-3b	gb|AIXA01023208.1|:378-536
MsHel-3b	gb|AIXA01018999.1|:3770-3983
MsHel-3b	gb|AIXA01013328.1|:26513-26683
MsHel-3b	gb|AIXA01013166.1|:2632-2808
MsHel-3b	gb|AIXA01011784.1|:7418-7648
MsHel-3b	gb|AIXA01010019.1|:39813-39996
MsHel-3b	gb|AIXA01010019.1|:16401-16488
MsHel-3b	gb|AIXA01009425.1|:14782-14985

MsHel-3b	gb|AIXA01009360.1|:44872-45012
MsHel-3b	gb|AIXA01009360.1|:25062-25261
MsHel-3b	gb|AIXA01009163.1|:18049-18262
MsHel-3b	gb|AIXA01008501.1|:11627-11828
MsHel-3b	gb|AIXA01007857.1|:50036-50185
MsHel-3b	gb|AIXA01007712.1|:28237-28423
MsHel-3b	gb|AIXA01007690.1|:30055-30251
MsHel-3b	gb|AIXA01006984.1|:10461-10670
MsHel-3b	gb|AIXA01006924.1|:21475-21687
MsHel-3b	gb|AIXA01006855.1|:13144-13307
MsHel-3b	gb|AIXA01006814.1|:20614-20823
MsHel-3b	gb|AIXA01005495.1|:27022-27180
MsHel-3b	gb|AIXA01004552.1|:30043-30212
MsHel-3b	gb|AIXA01004475.1|:24087-24220
MsHel-3b	gb|AIXA01003011.1|:33703-33890
MsHel-3b	gb|AIXA01002978.1|:26512-26699
MsHel-3b	gb|AIXA01032970.1|:445-586
MsHel-3b	gb|AIXA01028679.1|:790-939
MsHel-3b	gb|AIXA01025526.1|:467-662
MsHel-3b	gb|AIXA01020134.1|:1446-1647
MsHel-3b	gb|AIXA01014641.1|:35733-35869
MsHel-3b	gb|AIXA01014641.1|:5837-6034
MsHel-3b	gb|AIXA01006045.1|:5261-5453
MsHel-3b	gb|AIXA01005112.1|:6495-6642
MsHel-3b	gb|AIXA01003142.1|:35593-35762
MsHel-3b	gb|AIXA01002457.1|:3378-3542
MsHel-3b	gb|AIXA01036850.1|:268-465
MsHel-3b	gb|AIXA01034839.1|:479-604
MsHel-3b	gb|AIXA01034544.1|:289-489
MsHel-3b	gb|AIXA01018593.1|:2012-2144
MsHel-3b	gb|AIXA01016958.1|:7114-7331
MsHel-3b	gb|AIXA01013499.1|:9792-9967
MsHel-3b	gb|AIXA01013087.1|:7047-7243
MsHel-3b	gb|AIXA01012784.1|:1475-1664
MsHel-3b	gb|AIXA01012343.1|:1773-1988
MsHel-3b	gb|AIXA01012257.1|:1156-1353
MsHel-3b	gb|AIXA01011818.1|:8259-8384
MsHel-3b	gb|AIXA01010881.1|:115948-116159
MsHel-3b	gb|AIXA01010881.1|:84097-84252
MsHel-3b	gb|AIXA01010881.1|:36967-37151
MsHel-3b	gb|AIXA01010848.1|:7941-8119
MsHel-3b	gb|AIXA01009133.1|:36465-36590
MsHel-3b	gb|AIXA01007860.1|:11392-11563
MsHel-3b	gb|AIXA01007580.1|:38584-38709
MsHel-3b	gb|AIXA01007506.1|:82339-82524
MsHel-3b	gb|AIXA01007085.1|:52909-53109
MsHel-3b	gb|AIXA01006867.1|:40097-49424
MsHel-3b	gb|AIXA01006667.1|:6504-6692

MsHel-3b	gb|AIXA01005585.1|:63067-63192
MsHel-3b	gb|AIXA01003595.1|:123363-123493
MsHel-3b	gb|AIXA01003595.1|:87664-87825
MsHel-3b	gb|AIXA01002659.1|:7167-7348
MsHel-3b	gb|AIXA01002552.1|:98926-99051
MsHel-3b	gb|AIXA01002552.1|:41364-41481
MsHel-3b	gb|AIXA01001844.1|:11439-11607
MsHel-3b	gb|AIXA01001558.1|:102023-102140
MsHel-3b	gb|AIXA01001538.1|:70816-72146
MsHel-3b	gb|AIXA01001147.1|:10428-10553
MsHel-3b	gb|AIXA01000315.1|:76488-76697
MsHel-3b	gb|AIXA01000315.1|:47073-47157
MsHel-3b	gb|AIXA01000196.1|:56549-56650
MsHel-3b	gb|AIXA01000196.1|:45144-45331
MsHel-3b	gb|AIXA01000150.1|:14-220
MsHel-3b	gb|AIXA01024347.1|:929-1124
MsHel-3b	gb|AIXA01023005.1|:430-561
MsHel-3b	gb|AIXA01020947.1|:1265-1750
MsHel-3b	gb|AIXA01015970.1|:7477-7686
MsHel-3b	gb|AIXA01015473.1|:12155-12321
MsHel-3b	gb|AIXA01014003.1|:2190-2390
MsHel-3b	gb|AIXA01013668.1|:2789-2951
MsHel-3b	gb|AIXA01013185.1|:13322-13493
MsHel-3b	gb|AIXA01011457.1|:235-378
MsHel-3b	gb|AIXA01009826.1|:29206-29326
MsHel-3b	gb|AIXA01009641.1|:14372-14578
MsHel-3b	gb|AIXA01009018.1|:1752-1955
MsHel-3b	gb|AIXA01007649.1|:38124-38236
MsHel-3b	gb|AIXA01007649.1|:4240-4407
MsHel-3b	gb|AIXA01007286.1|:60894-61093
MsHel-3b	gb|AIXA01007077.1|:7033-7247
MsHel-3b	gb|AIXA01006796.1|:76894-77078
MsHel-3b	gb|AIXA01005406.1|:62528-62699
MsHel-3b	gb|AIXA01004683.1|:67366-67574
MsHel-3b	gb|AIXA01004554.1|:22419-22625
MsHel-3b	gb|AIXA01003311.1|:5820-5917
MsHel-3b	gb|AIXA01003276.1|:89546-89761
MsHel-3b	gb|AIXA01003170.1|:30132-30311
MsHel-3b	gb|AIXA01003027.1|:61785-61864
MsHel-3b	gb|AIXA01002642.1|:17184-17367
MsHel-3b	gb|AIXA01002568.1|:6725-7181
MsHel-3b	gb|AIXA01001771.1|:88472-88650
MsHel-3b	gb|AIXA01001714.1|:8825-9025
MsHel-3b	gb|AIXA01001556.1|:12686-12854
MsHel-3b	gb|AIXA01000043.1|:27969-28172
MsHel-3b	gb|AIXA01024291.1|:280-410
MsHel-3b	gb|AIXA01023302.1|:1272-1472
MsHel-3b	gb|AIXA01018244.1|:268-469

MsHel-3b	gb|AIXA01017338.1|:18514-18708
MsHel-3b	gb|AIXA01016262.1|:26916-36107
MsHel-3b	gb|AIXA01016262.1|:677-854
MsHel-3b	gb|AIXA01013044.1|:18862-19076
MsHel-3b	gb|AIXA01011268.1|:26624-26773
MsHel-3b	gb|AIXA01010785.1|:68443-68609
MsHel-3b	gb|AIXA01010785.1|:12742-12918
MsHel-3b	gb|AIXA01009539.1|:37060-37235
MsHel-3b	gb|AIXA01009539.1|:1442-1639
MsHel-3b	gb|AIXA01005486.1|:46251-46415
MsHel-3b	gb|AIXA01005486.1|:29246-29371
MsHel-3b	gb|AIXA01005413.1|:45189-45281
MsHel-3b	gb|AIXA01002635.1|:30017-30173
MsHel-3b	gb|AIXA01000237.1|:59837-59961
MsHel-3b	gb|AIXA01023124.1|:232-429
MsHel-3b	gb|AIXA01019629.1|:1-205
MsHel-3b	gb|AIXA01014932.1|:9598-9772
MsHel-3b	gb|AIXA01013620.1|:8963-9145
MsHel-3b	gb|AIXA01013156.1|:12895-13078
MsHel-3b	gb|AIXA01012015.1|:18854-19097
MsHel-3b	gb|AIXA01011485.1|:9632-9826
MsHel-3b	gb|AIXA01010219.1|:6898-7090
MsHel-3b	gb|AIXA01009697.1|:11522-11674
MsHel-3b	gb|AIXA01009226.1|:14446-14651
MsHel-3b	gb|AIXA01009126.1|:25650-30810
MsHel-3b	gb|AIXA01007641.1|:69426-69630
MsHel-3b	gb|AIXA01006051.1|:10935-11061
MsHel-3b	gb|AIXA01005449.1|:11434-11607
MsHel-3b	gb|AIXA01004965.1|:38273-38502
MsHel-3b	gb|AIXA01003015.1|:41277-41455
MsHel-3b	gb|AIXA01002515.1|:3981-4122
MsHel-3b	gb|AIXA01000175.1|:27113-27304
MsHel-3b	gb|AIXA01000103.1|:1670-1865
MsHel-3b	gb|AIXA01000021.1|:19933-20138
MsHel-3b	gb|AIXA01016604.1|:9533-9658
MsHel-3b	gb|AIXA01015742.1|:3178-3303
MsHel-3b	gb|AIXA01014760.1|:3249-3376
MsHel-3b	gb|AIXA01012959.1|:28034-28242
MsHel-3b	gb|AIXA01012329.1|:15342-15467
MsHel-3b	gb|AIXA01009923.1|:16700-16879
MsHel-3b	gb|AIXA01009228.1|:22850-23024
MsHel-3b	gb|AIXA01008873.1|:11983-12190
MsHel-3b	gb|AIXA01008475.1|:29248-29362
MsHel-3b	gb|AIXA01008320.1|:1407-1532
MsHel-3b	gb|AIXA01007832.1|:20574-20781
MsHel-3b	gb|AIXA01006649.1|:35595-35809
MsHel-3b	gb|AIXA01006594.1|:30874-31071
MsHel-3b	gb|AIXA01004845.1|:7050-7190

MsHel-3b	gb|AIXA01004674.1|:24189-24342
MsHel-3b	gb|AIXA01004416.1|:31656-31833
MsHel-3b	gb|AIXA01004089.1|:19402-19614
MsHel-3b	gb|AIXA01003999.1|:22317-22461
MsHel-3b	gb|AIXA01002312.1|:19568-19727
MsHel-3b	gb|AIXA01002304.1|:36191-36316
MsHel-3b	gb|AIXA01002304.1|:6928-7053
MsHel-3b	gb|AIXA01001945.1|:5885-6010
MsHel-3b	gb|AIXA01001254.1|:16264-16412
MsHel-3b	gb|AIXA01001244.1|:3288-3488
MsHel-3b	gb|AIXA01001140.1|:4861-4986
MsHel-3b	gb|AIXA01000963.1|:62067-62254
MsHel-3b	gb|AIXA01000789.1|:2637-2762
MsHel-3b	gb|AIXA01000755.1|:107044-107148
MsHel-3b	gb|AIXA01000755.1|:23402-23618
MsHel-3b	gb|AIXA01000690.1|:3785-3965
MsHel-3b	gb|AIXA01000465.1|:6686-6813
MsHel-3b	gb|AIXA01027974.1|:17-221
MsHel-3b	gb|AIXA01020746.1|:175-374
MsHel-3b	gb|AIXA01020741.1|:308-483
MsHel-3b	gb|AIXA01018991.1|:141-319
MsHel-3b	gb|AIXA01018241.1|:2772-3101
MsHel-3b	gb|AIXA01017231.1|:2800-2974
MsHel-3b	gb|AIXA01016393.1|:156-278
MsHel-3b	gb|AIXA01015772.1|:7000-7203
MsHel-3b	gb|AIXA01014683.1|:9083-9285
MsHel-3b	gb|AIXA01013382.1|:20299-20495
MsHel-3b	gb|AIXA01012412.1|:10770-10941
MsHel-3b	gb|AIXA01012209.1|:15294-15444
MsHel-3b	gb|AIXA01011819.1|:3573-3744
MsHel-3b	gb|AIXA01011719.1|:1569-1752
MsHel-3b	gb|AIXA01011243.1|:39986-40130
MsHel-3b	gb|AIXA01010837.1|:1141-1342
MsHel-3b	gb|AIXA01008691.1|:20136-20346
MsHel-3b	gb|AIXA01008469.1|:7457-7631
MsHel-3b	gb|AIXA01007892.1|:89384-89559
MsHel-3b	gb|AIXA01007892.1|:68273-68468
MsHel-3b	gb|AIXA01007892.1|:11772-12022
MsHel-3b	gb|AIXA01007642.1|:15262-15456
MsHel-3b	gb|AIXA01005533.1|:52714-52798
MsHel-3b	gb|AIXA01005533.1|:21714-21899
MsHel-3b	gb|AIXA01005097.1|:7463-7654
MsHel-3b	gb|AIXA01002898.1|:9064-9230
MsHel-3b	gb|AIXA01002766.1|:24953-25073
MsHel-3b	gb|AIXA01001770.1|:33167-33387
MsHel-3b	gb|AIXA01001269.1|:4493-4667
MsHel-3b	gb|AIXA01001266.1|:79124-79331
MsHel-3b	gb|AIXA01000842.1|:59205-59331

MsHel-3b	gb|AIXA01000842.1|:37572-37781
MsHel-3b	gb|AIXA01000424.1|:72393-72525
MsHel-3b	gb|AIXA01000404.1|:24081-24268
MsHel-3b	gb|AIXA01030254.1|:379-502
MsHel-3b	gb|AIXA01028923.1|:310-433
MsHel-3b	gb|AIXA01025691.1|:330-482
MsHel-3b	gb|AIXA01019690.1|:1369-1491
MsHel-3b	gb|AIXA01017574.1|:1830-2031
MsHel-3b	gb|AIXA01014492.1|:4075-4229
MsHel-3b	gb|AIXA01009796.1|:24909-34100
MsHel-3b	gb|AIXA01009796.1|:2769-10809
MsHel-3b	gb|AIXA01009755.1|:18679-18844
MsHel-3b	gb|AIXA01009297.1|:9558-9733
MsHel-3b	gb|AIXA01008915.1|:42394-42550
MsHel-3b	gb|AIXA01008488.1|:3024-3201
MsHel-3b	gb|AIXA01005914.1|:9816-9939
MsHel-3b	gb|AIXA01005326.1|:87249-87462
MsHel-3b	gb|AIXA01005187.1|:32526-32612
MsHel-3b	gb|AIXA01004339.1|:14194-14385
MsHel-3b	gb|AIXA01003928.1|:84838-88951
MsHel-3b	gb|AIXA01003928.1|:69735-69817
MsHel-3b	gb|AIXA01001836.1|:2400-2604
MsHel-3b	gb|AIXA01001282.1|:119277-119360
MsHel-3b	gb|AIXA01000787.1|:58058-58438
MsHel-3b	gb|AIXA01000214.1|:20083-20206
MsHel-3b	gb|AIXA01016047.1|:815-990
MsHel-3b	gb|AIXA01013704.1|:5300-5437
MsHel-3b	gb|AIXA01012028.1|:35949-36129
MsHel-3b	gb|AIXA01011585.1|:636-833
MsHel-3b	gb|AIXA01011323.1|:16308-16481
MsHel-3b	gb|AIXA01010854.1|:63738-63945
MsHel-3b	gb|AIXA01010693.1|:2391-2581
MsHel-3b	gb|AIXA01010189.1|:23610-23754
MsHel-3b	gb|AIXA01009202.1|:4882-5071
MsHel-3b	gb|AIXA01008425.1|:1306-1505
MsHel-3b	gb|AIXA01008099.1|:7053-7310
MsHel-3b	gb|AIXA01007499.1|:11698-11876
MsHel-3b	gb|AIXA01007237.1|:14429-14525
MsHel-3b	gb|AIXA01007052.1|:8132-8301
MsHel-3b	gb|AIXA01007044.1|:53919-54112
MsHel-3b	gb|AIXA01007044.1|:9967-10104
MsHel-3b	gb|AIXA01006986.1|:35521-35721
MsHel-3b	gb|AIXA01006904.1|:7658-7848
MsHel-3b	gb|AIXA01005867.1|:9590-9716
MsHel-3b	gb|AIXA01004565.1|:46699-46821
MsHel-3b	gb|AIXA01003132.1|:44875-45001
MsHel-3b	gb|AIXA01002671.1|:142837-142960
MsHel-3b	gb|AIXA01002671.1|:60867-60991

MsHel-3b	gb|AIXA01002671.1|:26687-26856
MsHel-3b	gb|AIXA01002671.1|:7985-10959
MsHel-3b	gb|AIXA01002640.1|:17024-17226
MsHel-3b	gb|AIXA01002377.1|:195767-195892
MsHel-3b	gb|AIXA01002278.1|:29664-29871
MsHel-3b	gb|AIXA01001543.1|:26796-26922
MsHel-3b	gb|AIXA01000765.1|:31423-31604
MsHel-3b	gb|AIXA01000584.1|:42452-42557
MsHel-3b	gb|AIXA01000584.1|:3880-3993
MsHel-3b	gb|AIXA01025428.1|:1088-1226
MsHel-3b	gb|AIXA01019243.1|:4061-4186
MsHel-3b	gb|AIXA01018951.1|:3019-3144
MsHel-3b	gb|AIXA01016925.1|:678-813
MsHel-3b	gb|AIXA01016395.1|:3848-3991
MsHel-3b	gb|AIXA01013383.1|:15908-21358
MsHel-3b	gb|AIXA01012864.1|:1240-1415
MsHel-3b	gb|AIXA01012119.1|:1183-1308
MsHel-3b	gb|AIXA01012017.1|:9922-10093
MsHel-3b	gb|AIXA01010690.1|:787-912
MsHel-3b	gb|AIXA01010077.1|:47547-47672
MsHel-3b	gb|AIXA01010077.1|:13395-13581
MsHel-3b	gb|AIXA01009002.1|:31787-31962
MsHel-3b	gb|AIXA01007949.1|:35500-35679
MsHel-3b	gb|AIXA01007294.1|:9390-9576
MsHel-3b	gb|AIXA01007253.1|:5330-5494
MsHel-3b	gb|AIXA01006708.1|:48753-48941
MsHel-3b	gb|AIXA01006254.1|:52313-52425
MsHel-3b	gb|AIXA01006138.1|:37689-37898
MsHel-3b	gb|AIXA01004923.1|:4989-5114
MsHel-3b	gb|AIXA01004755.1|:50253-54619
MsHel-3b	gb|AIXA01004755.1|:2715-2799
MsHel-3b	gb|AIXA01004753.1|:32755-32879
MsHel-3b	gb|AIXA01004689.1|:8910-9015
MsHel-3b	gb|AIXA01004218.1|:4680-4805
MsHel-3b	gb|AIXA01004108.1|:33244-40768
MsHel-3b	gb|AIXA01003977.1|:47524-47649
MsHel-3b	gb|AIXA01003747.1|:38133-38258
MsHel-3b	gb|AIXA01003257.1|:13253-13474
MsHel-3b	gb|AIXA01003174.1|:20092-20255
MsHel-3b	gb|AIXA01003138.1|:33878-33999
MsHel-3b	gb|AIXA01002721.1|:6459-6584
MsHel-3b	gb|AIXA01002704.1|:11418-11584
MsHel-3b	gb|AIXA01002579.1|:120233-120398
MsHel-3b	gb|AIXA01002566.1|:8679-8804
MsHel-3b	gb|AIXA01002560.1|:46756-46964
MsHel-3b	gb|AIXA01002367.1|:1646-1771
MsHel-3b	gb|AIXA01001965.1|:60062-60187
MsHel-3b	gb|AIXA01001864.1|:2911-3200

MsHel-3b	gb|AIXA01001764.1|:2778-2903
MsHel-3b	gb|AIXA01001401.1|:21391-21516
MsHel-3b	gb|AIXA01000041.1|:19884-20009
MsHel-3b	gb|AIXA01029529.1|:637-808
MsHel-3b	gb|AIXA01021288.1|:557-715
MsHel-3b	gb|AIXA01019456.1|:141-319
MsHel-3b	gb|AIXA01019002.1|:561-692
MsHel-3b	gb|AIXA01014704.1|:4986-5133
MsHel-3b	gb|AIXA01014296.1|:6614-6748
MsHel-3b	gb|AIXA01012635.1|:17827-18034
MsHel-3b	gb|AIXA01011340.1|:30061-30184
MsHel-3b	gb|AIXA01010094.1|:36073-36203
MsHel-3b	gb|AIXA01009292.1|:30881-31004
MsHel-3b	gb|AIXA01008896.1|:1026-1168
MsHel-3b	gb|AIXA01007579.1|:29495-29693
MsHel-3b	gb|AIXA01006255.1|:94483-94601
MsHel-3b	gb|AIXA01006255.1|:45414-45654
MsHel-3b	gb|AIXA01005575.1|:3969-4173
MsHel-3b	gb|AIXA01005184.1|:25149-25345
MsHel-3b	gb|AIXA01005030.1|:12641-12783
MsHel-3b	gb|AIXA01004634.1|:1738-1913
MsHel-3b	gb|AIXA01004158.1|:5392-5587
MsHel-3b	gb|AIXA01004142.1|:5098-5233
MsHel-3b	gb|AIXA01004064.1|:28453-28579
MsHel-3b	gb|AIXA01004012.1|:28984-29110
MsHel-3b	gb|AIXA01003301.1|:31157-31327
MsHel-3b	gb|AIXA01002224.1|:21592-21728
MsHel-3b	gb|AIXA01001576.1|:870-1057
MsHel-3b	gb|AIXA01001575.1|:190-319
MsHel-3b	gb|AIXA01000820.1|:42472-42567
MsHel-3b	gb|AIXA01000820.1|:4612-4816
MsHel-3b	gb|AIXA01028727.1|:254-400
MsHel-3b	gb|AIXA01022415.1|:896-1038
MsHel-3b	gb|AIXA01019307.1|:703-888
MsHel-3b	gb|AIXA01016048.1|:21278-21445
MsHel-3b	gb|AIXA01015925.1|:435-572
MsHel-3b	gb|AIXA01015732.1|:449-571
MsHel-3b	gb|AIXA01015119.1|:43430-43608
MsHel-3b	gb|AIXA01013522.1|:4390-4513
MsHel-3b	gb|AIXA01012700.1|:8561-8728
MsHel-3b	gb|AIXA01012585.1|:16980-17159
MsHel-3b	gb|AIXA01011364.1|:21692-21861
MsHel-3b	gb|AIXA01009350.1|:35344-35458
MsHel-3b	gb|AIXA01009179.1|:105809-105978
MsHel-3b	gb|AIXA01008906.1|:25874-26019
MsHel-3b	gb|AIXA01008410.1|:35334-35520
MsHel-3b	gb|AIXA01008410.1|:7734-7849
MsHel-3b	gb|AIXA01008148.1|:93892-94073

MsHel-3b	gb|AIXA01007066.1|:48684-48806
MsHel-3b	gb|AIXA01007033.1|:44594-44798
MsHel-3b	gb|AIXA01006909.1|:6985-7101
MsHel-3b	gb|AIXA01006498.1|:13429-13607
MsHel-3b	gb|AIXA01005001.1|:40863-40956
MsHel-3b	gb|AIXA01005001.1|:1241-1441
MsHel-3b	gb|AIXA01004520.1|:95400-95572
MsHel-3b	gb|AIXA01004087.1|:28817-28940
MsHel-3b	gb|AIXA01003387.1|:480-687
MsHel-3b	gb|AIXA01002397.1|:11150-11325
MsHel-3b	gb|AIXA01001861.1|:18795-18973
MsHel-3b	gb|AIXA01001831.1|:23392-23599
MsHel-3b	gb|AIXA01001544.1|:57963-58169
MsHel-3b	gb|AIXA01000732.1|:54024-54182
MsHel-3b	gb|AIXA01018006.1|:1528-1710
MsHel-3b	gb|AIXA01017963.1|:41-189
MsHel-3b	gb|AIXA01016679.1|:2004-2197
MsHel-3b	gb|AIXA01016326.1|:7083-7209
MsHel-3b	gb|AIXA01013764.1|:13296-13471
MsHel-3b	gb|AIXA01013483.1|:2900-3026
MsHel-3b	gb|AIXA01012474.1|:3102-3285
MsHel-3b	gb|AIXA01011285.1|:132703-132836
MsHel-3b	gb|AIXA01011285.1|:74882-75014
MsHel-3b	gb|AIXA01011285.1|:36997-37200
MsHel-3b	gb|AIXA01009811.1|:1-98
MsHel-3b	gb|AIXA01009100.1|:107459-107624
MsHel-3b	gb|AIXA01009100.1|:78178-78269
MsHel-3b	gb|AIXA01009100.1|:11929-12120
MsHel-3b	gb|AIXA01008402.1|:39496-42451
MsHel-3b	gb|AIXA01007978.1|:17900-18023
MsHel-3b	gb|AIXA01007327.1|:271-435
MsHel-3b	gb|AIXA01006515.1|:19630-19752
MsHel-3b	gb|AIXA01006515.1|:6457-6576
MsHel-3b	gb|AIXA01006086.1|:8348-8552
MsHel-3b	gb|AIXA01005770.1|:14375-14497
MsHel-3b	gb|AIXA01005485.1|:35459-35587
MsHel-3b	gb|AIXA01005485.1|:18544-18733
MsHel-3b	gb|AIXA01005239.1|:3646-3783
MsHel-3b	gb|AIXA01005208.1|:17290-17440
MsHel-3b	gb|AIXA01003722.1|:38818-38939
MsHel-3b	gb|AIXA01001865.1|:879-1012
MsHel-3b	gb|AIXA01001677.1|:96836-96941
MsHel-3b	gb|AIXA01001666.1|:24936-25145
MsHel-3b	gb|AIXA01001484.1|:87557-87733
MsHel-3b	gb|AIXA01001484.1|:2007-2134
MsHel-3b	gb|AIXA01019346.1|:1396-1521
MsHel-3b	gb|AIXA01016197.1|:462-661
MsHel-3b	gb|AIXA01015055.1|:29921-30069

MsHel-3b	gb|AIXA01014694.1|:7024-7168
MsHel-3b	gb|AIXA01012099.1|:2011-2136
MsHel-3b	gb|AIXA01011925.1|:2354-2567
MsHel-3b	gb|AIXA01011810.1|:28998-29124
MsHel-3b	gb|AIXA01011810.1|:979-1104
MsHel-3b	gb|AIXA01011635.1|:25350-25475
MsHel-3b	gb|AIXA01011635.1|:3467-3613
MsHel-3b	gb|AIXA01011249.1|:27471-27552
MsHel-3b	gb|AIXA01011249.1|:5948-6069
MsHel-3b	gb|AIXA01009354.1|:10112-10237
MsHel-3b	gb|AIXA01009351.1|:9397-9552
MsHel-3b	gb|AIXA01009324.1|:13906-14035
MsHel-3b	gb|AIXA01008962.1|:159639-159764
MsHel-3b	gb|AIXA01008862.1|:13945-14070
MsHel-3b	gb|AIXA01008037.1|:63847-64054
MsHel-3b	gb|AIXA01007688.1|:1094-1219
MsHel-3b	gb|AIXA01007428.1|:64807-64999
MsHel-3b	gb|AIXA01006470.1|:10108-10271
MsHel-3b	gb|AIXA01006423.1|:8405-8530
MsHel-3b	gb|AIXA01004978.1|:13723-13805
MsHel-3b	gb|AIXA01004141.1|:38146-38271
MsHel-3b	gb|AIXA01003275.1|:12254-12394
MsHel-3b	gb|AIXA01003272.1|:99173-99386
MsHel-3b	gb|AIXA01002404.1|:12883-13008
MsHel-3b	gb|AIXA01001695.1|:51144-51342
MsHel-3b	gb|AIXA01001695.1|:162-297
MsHel-3b	gb|AIXA01000846.1|:512-636
MsHel-3b	gb|AIXA01000668.1|:15628-15768
MsHel-3b	gb|AIXA01000549.1|:15317-15441
MsHel-3b	gb|AIXA01000064.1|:41043-41203
MsHel-3b	gb|AIXA01000057.1|:11868-12055
MsHel-3b	gb|AIXA01021362.1|:1861-1999
MsHel-3b	gb|AIXA01018895.1|:933-1091
MsHel-3b	gb|AIXA01014088.1|:3557-3704
MsHel-3b	gb|AIXA01012554.1|:22570-22709
MsHel-3b	gb|AIXA01010865.1|:28734-28894
MsHel-3b	gb|AIXA01008995.1|:46851-46979
MsHel-3b	gb|AIXA01008982.1|:13599-13806
MsHel-3b	gb|AIXA01008929.1|:20586-20779
MsHel-3b	gb|AIXA01006716.1|:14715-14855
MsHel-3b	gb|AIXA01005359.1|:15285-15437
MsHel-3b	gb|AIXA01003120.1|:174615-174701
MsHel-3b	gb|AIXA01003120.1|:136519-136725
MsHel-3b	gb|AIXA01001895.1|:28012-28183
MsHel-3b	gb|AIXA01001221.1|:45468-45673
MsHel-3b	gb|AIXA01000692.1|:102857-102996
MsHel-3b	gb|AIXA01000050.1|:4260-4462
MsHel-3b	gb|AIXA01029228.1|:300-403

MsHel-3b	gb|AIXA01028439.1|:462-600
MsHel-3b	gb|AIXA01018070.1|:1107-1287
MsHel-3b	gb|AIXA01016907.1|:10360-10554
MsHel-3b	gb|AIXA01015637.1|:6697-6873
MsHel-3b	gb|AIXA01015625.1|:7349-7535
MsHel-3b	gb|AIXA01014420.1|:6286-6405
MsHel-3b	gb|AIXA01013842.1|:4436-4619
MsHel-3b	gb|AIXA01013790.1|:2120-2243
MsHel-3b	gb|AIXA01013118.1|:19025-19173
MsHel-3b	gb|AIXA01011370.1|:50955-51065
MsHel-3b	gb|AIXA01009401.1|:65247-65393
MsHel-3b	gb|AIXA01008989.1|:681-827
MsHel-3b	gb|AIXA01008883.1|:6563-6689
MsHel-3b	gb|AIXA01008782.1|:60135-60249
MsHel-3b	gb|AIXA01008146.1|:51476-51611
MsHel-3b	gb|AIXA01008146.1|:419-500
MsHel-3b	gb|AIXA01008118.1|:35699-35875
MsHel-3b	gb|AIXA01007853.1|:51769-51947
MsHel-3b	gb|AIXA01007853.1|:10557-10692
MsHel-3b	gb|AIXA01006931.1|:45214-45332
MsHel-3b	gb|AIXA01006931.1|:9446-9624
MsHel-3b	gb|AIXA01006351.1|:1025-1154
MsHel-3b	gb|AIXA01006284.1|:11789-11921
MsHel-3b	gb|AIXA01005987.1|:2936-3113
MsHel-3b	gb|AIXA01005960.1|:17277-17474
MsHel-3b	gb|AIXA01005479.1|:28387-28563
MsHel-3b	gb|AIXA01004781.1|:2187-2387
MsHel-3b	gb|AIXA01004367.1|:19414-19537
MsHel-3b	gb|AIXA01004367.1|:9034-9269
MsHel-3b	gb|AIXA01004164.1|:25780-25974
MsHel-3b	gb|AIXA01004063.1|:2933-3114
MsHel-3b	gb|AIXA01001925.1|:2566-2728
MsHel-3b	gb|AIXA01001826.1|:3773-3980
MsHel-3b	gb|AIXA01001494.1|:35719-35906
MsHel-3b	gb|AIXA01001385.1|:6969-7145
MsHel-3b	gb|AIXA01001002.1|:25694-25816
MsHel-3b	gb|AIXA01001002.1|:11808-11904
MsHel-3b	gb|AIXA01000938.1|:52348-52528
MsHel-3b	gb|AIXA01000795.1|:11768-11942
MsHel-3b	gb|AIXA01000031.1|:36266-36416
MsHel-3b	gb|AIXA01033278.1|:216-412
MsHel-3b	gb|AIXA01022662.1|:88-215
MsHel-3b	gb|AIXA01019019.1|:255-404
MsHel-3b	gb|AIXA01017260.1|:2626-2754
MsHel-3b	gb|AIXA01016627.1|:1992-2104
MsHel-3b	gb|AIXA01016352.1|:20783-20904
MsHel-3b	gb|AIXA01013049.1|:12443-12569
MsHel-3b	gb|AIXA01012960.1|:322-486

MsHel-3b	gb|AIXA01011650.1|:1026-1110
MsHel-3b	gb|AIXA01010329.1|:1727-1860
MsHel-3b	gb|AIXA01008868.1|:9757-9931
MsHel-3b	gb|AIXA01008443.1|:56143-56269
MsHel-3b	gb|AIXA01006853.1|:62592-62768
MsHel-3b	gb|AIXA01004786.1|:8345-8543
MsHel-3b	gb|AIXA01003906.1|:21917-22035
MsHel-3b	gb|AIXA01003905.1|:4291-4438
MsHel-3b	gb|AIXA01003616.1|:8996-9118
MsHel-3b	gb|AIXA01003365.1|:59174-59300
MsHel-3b	gb|AIXA01002697.1|:21905-22062
MsHel-3b	gb|AIXA01002200.1|:7473-7668
MsHel-3b	gb|AIXA01002159.1|:14226-14351
MsHel-3b	gb|AIXA01001636.1|:86999-87125
MsHel-3b	gb|AIXA01001636.1|:24780-24952
MsHel-3b	gb|AIXA01001492.1|:22087-22244
MsHel-3b	gb|AIXA01000091.1|:11166-11293
MsHel-3b	gb|AIXA01000016.1|:32754-32885
MsHel-3b	gb|AIXA01028552.1|:765-955
MsHel-3b	gb|AIXA01026201.1|:883-1002
MsHel-3b	gb|AIXA01020704.1|:1343-1515
MsHel-3b	gb|AIXA01017968.1|:3623-3799
MsHel-3b	gb|AIXA01016168.1|:9444-9569
MsHel-3b	gb|AIXA01015151.1|:51493-55879
MsHel-3b	gb|AIXA01014147.1|:8119-14469
MsHel-3b	gb|AIXA01013529.1|:65285-65523
MsHel-3b	gb|AIXA01013529.1|:28402-28527
MsHel-3b	gb|AIXA01013148.1|:55974-56180
MsHel-3b	gb|AIXA01012380.1|:5969-6150
MsHel-3b	gb|AIXA01012247.1|:28365-28490
MsHel-3b	gb|AIXA01012247.1|:1851-1971
MsHel-3b	gb|AIXA01011500.1|:26190-26315
MsHel-3b	gb|AIXA01011500.1|:9802-9958
MsHel-3b	gb|AIXA01011295.1|:21025-21150
MsHel-3b	gb|AIXA01011202.1|:38056-38168
MsHel-3b	gb|AIXA01010133.1|:5017-5107
MsHel-3b	gb|AIXA01009310.1|:7672-7832
MsHel-3b	gb|AIXA01007229.1|:42553-42650
MsHel-3b	gb|AIXA01005549.1|:18927-19052
MsHel-3b	gb|AIXA01005531.1|:30165-30379
MsHel-3b	gb|AIXA01005394.1|:48962-49114
MsHel-3b	gb|AIXA01005394.1|:17753-17953
MsHel-3b	gb|AIXA01005355.1|:3193-3310
MsHel-3b	gb|AIXA01004428.1|:11832-12000
MsHel-3b	gb|AIXA01003873.1|:32446-32571
MsHel-3b	gb|AIXA01003873.1|:2647-2743
MsHel-3b	gb|AIXA01003008.1|:48689-48813
MsHel-3b	gb|AIXA01002354.1|:8278-8463

MsHel-3b	gb|AIXA01002241.1|:15390-15530
MsHel-3b	gb|AIXA01002164.1|:5194-5397
MsHel-3b	gb|AIXA01001292.1|:37987-38112
MsHel-3b	gb|AIXA01000791.1|:6775-7177
MsHel-3b	gb|AIXA01000408.1|:31841-31967
MsHel-3b	gb|AIXA01028506.1|:252-371
MsHel-3b	gb|AIXA01022067.1|:181-291
MsHel-3b	gb|AIXA01019343.1|:2819-3025
MsHel-3b	gb|AIXA01018837.1|:3531-3665
MsHel-3b	gb|AIXA01018198.1|:3816-3947
MsHel-3b	gb|AIXA01018062.1|:341-463
MsHel-3b	gb|AIXA01017307.1|:4414-4637
MsHel-3b	gb|AIXA01017103.1|:2704-2920
MsHel-3b	gb|AIXA01014015.1|:1445-1602
MsHel-3b	gb|AIXA01012625.1|:3773-3927
MsHel-3b	gb|AIXA01012377.1|:9354-9469
MsHel-3b	gb|AIXA01010873.1|:35760-35946
MsHel-3b	gb|AIXA01009706.1|:17522-17648
MsHel-3b	gb|AIXA01009423.1|:24310-33476
MsHel-3b	gb|AIXA01009154.1|:8660-8790
MsHel-3b	gb|AIXA01008404.1|:15764-15903
MsHel-3b	gb|AIXA01008329.1|:29820-30030
MsHel-3b	gb|AIXA01008325.1|:14699-15086
MsHel-3b	gb|AIXA01006991.1|:38884-39110
MsHel-3b	gb|AIXA01006193.1|:5136-5296
MsHel-3b	gb|AIXA01006169.1|:6424-6598
MsHel-3b	gb|AIXA01005794.1|:10164-10335
MsHel-3b	gb|AIXA01005608.1|:41429-41621
MsHel-3b	gb|AIXA01005608.1|:12809-12990
MsHel-3b	gb|AIXA01004094.1|:58262-58453
MsHel-3b	gb|AIXA01002574.1|:5032-5241
MsHel-3b	gb|AIXA01000819.1|:6117-6322
MsHel-3b	gb|AIXA01034743.1|:407-626
MsHel-3b	gb|AIXA01031723.1|:177-291
MsHel-3b	gb|AIXA01025075.1|:1-103
MsHel-3b	gb|AIXA01019898.1|:3279-3453
MsHel-3b	gb|AIXA01019622.1|:14-164
MsHel-3b	gb|AIXA01017938.1|:622-759
MsHel-3b	gb|AIXA01011753.1|:30270-30452
MsHel-3b	gb|AIXA01010185.1|:14139-14316
MsHel-3b	gb|AIXA01009661.1|:617-736
MsHel-3b	gb|AIXA01009402.1|:54731-54853
MsHel-3b	gb|AIXA01009402.1|:16998-17124
MsHel-3b	gb|AIXA01009140.1|:4406-4550
MsHel-3b	gb|AIXA01007626.1|:113288-113411
MsHel-3b	gb|AIXA01007626.1|:97686-97869
MsHel-3b	gb|AIXA01007626.1|:77676-77791
MsHel-3b	gb|AIXA01007480.1|:18414-18532

MsHel-3b	gb|AIXA01007480.1|:6839-7022
MsHel-3b	gb|AIXA01006671.1|:15859-16011
MsHel-3b	gb|AIXA01006671.1|:417-541
MsHel-3b	gb|AIXA01004110.1|:1089-1266
MsHel-3b	gb|AIXA01004109.1|:9674-9857
MsHel-3b	gb|AIXA01003949.1|:52119-52201
MsHel-3b	gb|AIXA01002610.1|:25579-25725
MsHel-3b	gb|AIXA01002606.1|:8890-9028
MsHel-3b	gb|AIXA01033254.1|:81-250
MsHel-3b	gb|AIXA01028897.1|:530-732
MsHel-3b	gb|AIXA01028294.1|:515-628
MsHel-3b	gb|AIXA01027069.1|:945-1046
MsHel-3b	gb|AIXA01022649.1|:88-215
MsHel-3b	gb|AIXA01021618.1|:549-754
MsHel-3b	gb|AIXA01020834.1|:1372-1481
MsHel-3b	gb|AIXA01011638.1|:14173-14290
MsHel-3b	gb|AIXA01009109.1|:26380-26498
MsHel-3b	gb|AIXA01008632.1|:8308-8431
MsHel-3b	gb|AIXA01008537.1|:92183-92280
MsHel-3b	gb|AIXA01007717.1|:19113-19304
MsHel-3b	gb|AIXA01007024.1|:68551-68742
MsHel-3b	gb|AIXA01006325.1|:572-661
MsHel-3b	gb|AIXA01006319.1|:16147-16326
MsHel-3b	gb|AIXA01005991.1|:734-936
MsHel-3b	gb|AIXA01005668.1|:7206-7307
MsHel-3b	gb|AIXA01005647.1|:1605-1734
MsHel-3b	gb|AIXA01005371.1|:4390-4600
MsHel-3b	gb|AIXA01004583.1|:45924-46064
MsHel-3b	gb|AIXA01004129.1|:36801-36954
MsHel-3b	gb|AIXA01004004.1|:18227-18422
MsHel-3b	gb|AIXA01003836.1|:19447-19573
MsHel-3b	gb|AIXA01003814.1|:7273-7395
MsHel-3b	gb|AIXA01003697.1|:17714-17839
MsHel-3b	gb|AIXA01003326.1|:78215-78416
MsHel-3b	gb|AIXA01002205.1|:1518-1623
MsHel-3b	gb|AIXA01000030.1|:39937-42968
MsHel-3b	gb|AIXA01035433.1|:163-288
MsHel-3b	gb|AIXA01035031.1|:6-129
MsHel-3b	gb|AIXA01027639.1|:637-843
MsHel-3b	gb|AIXA01018669.1|:7247-7371
MsHel-3b	gb|AIXA01017004.1|:1487-1612
MsHel-3b	gb|AIXA01016189.1|:4415-4610
MsHel-3b	gb|AIXA01015407.1|:26470-26613
MsHel-3b	gb|AIXA01015407.1|:8399-8650
MsHel-3b	gb|AIXA01014533.1|:2783-2908
MsHel-3b	gb|AIXA01014387.1|:1841-2341
MsHel-3b	gb|AIXA01012522.1|:15991-16123
MsHel-3b	gb|AIXA01010855.1|:20782-20930

MsHel-3b	gb|AIXA01010656.1|:73722-73850
MsHel-3b	gb|AIXA01010201.1|:3616-4024
MsHel-3b	gb|AIXA01009864.1|:59707-59829
MsHel-3b	gb|AIXA01009864.1|:39487-39591
MsHel-3b	gb|AIXA01009742.1|:1864-1989
MsHel-3b	gb|AIXA01009070.1|:13928-14146
MsHel-3b	gb|AIXA01008864.1|:3742-3885
MsHel-3b	gb|AIXA01007072.1|:19508-19633
MsHel-3b	gb|AIXA01006893.1|:53230-53386
MsHel-3b	gb|AIXA01005689.1|:11309-11495
MsHel-3b	gb|AIXA01003703.1|:22872-25416
MsHel-3b	gb|AIXA01002679.1|:53067-53192
MsHel-3b	gb|AIXA01002615.1|:11455-11634
MsHel-3b	gb|AIXA01002545.1|:10990-11115
MsHel-3b	gb|AIXA01002061.1|:3255-3433
MsHel-3b	gb|AIXA01001990.1|:19311-19436
MsHel-3b	gb|AIXA01000077.1|:68553-68678
MsHel-3b	gb|AIXA01000047.1|:3930-4045
MsHel-3b	gb|AIXA01017287.1|:6024-6171
MsHel-3b	gb|AIXA01016103.1|:2755-2878
MsHel-3b	gb|AIXA01014560.1|:3809-3968
MsHel-3b	gb|AIXA01011894.1|:1875-2004
MsHel-3b	gb|AIXA01011327.1|:2015-2137
MsHel-3b	gb|AIXA01011075.1|:10963-11106
MsHel-3b	gb|AIXA01010617.1|:9307-9499
MsHel-3b	gb|AIXA01010295.1|:1427-1597
MsHel-3b	gb|AIXA01008412.1|:200-400
MsHel-3b	gb|AIXA01007339.1|:6886-7038
MsHel-3b	gb|AIXA01006997.1|:16419-16570
MsHel-3b	gb|AIXA01006071.1|:2611-2734
MsHel-3b	gb|AIXA01005349.1|:51937-52034
MsHel-3b	gb|AIXA01005349.1|:5087-5285
MsHel-3b	gb|AIXA01005224.1|:27338-27523
MsHel-3b	gb|AIXA01004114.1|:7947-8048
MsHel-3b	gb|AIXA01002928.1|:26370-26454
MsHel-3b	gb|AIXA01002062.1|:46820-46917
MsHel-3b	gb|AIXA01002062.1|:36271-36449
MsHel-3b	gb|AIXA01001658.1|:25277-25420
MsHel-3b	gb|AIXA01000993.1|:64165-64367
MsHel-3b	gb|AIXA01000726.1|:550-661
MsHel-3b	gb|AIXA01000206.1|:92871-93028
MsHel-3b	gb|AIXA01000206.1|:52732-56678
MsHel-3b	gb|AIXA01025676.1|:3-110
MsHel-3b	gb|AIXA01024313.1|:1188-1348
MsHel-3b	gb|AIXA01022546.1|:1476-1621
MsHel-3b	gb|AIXA01018306.1|:4404-4519
MsHel-3b	gb|AIXA01018071.1|:1715-1840
MsHel-3b	gb|AIXA01017268.1|:16609-16750

MsHel-3b	gb|AIXA01015458.1|:8553-8691
MsHel-3b	gb|AIXA01011970.1|:40393-40515
MsHel-3b	gb|AIXA01010909.1|:30260-30421
MsHel-3b	gb|AIXA01010843.1|:21121-21326
MsHel-3b	gb|AIXA01010778.1|:5606-5775
MsHel-3b	gb|AIXA01010541.1|:3462-3583
MsHel-3b	gb|AIXA01010343.1|:15744-15865
MsHel-3b	gb|AIXA01009355.1|:3865-3995
MsHel-3b	gb|AIXA01007824.1|:12174-12372
MsHel-3b	gb|AIXA01004435.1|:12088-12255
MsHel-3b	gb|AIXA01004322.1|:21007-21205
MsHel-3b	gb|AIXA01004260.1|:145254-145425
MsHel-3b	gb|AIXA01004167.1|:7596-7682
MsHel-3b	gb|AIXA01003293.1|:24898-25016
MsHel-3b	gb|AIXA01002452.1|:45897-46035
MsHel-3b	gb|AIXA01000167.1|:1023-1150
MsHel-3b	gb|AIXA01037135.1|:141-267
MsHel-3b	gb|AIXA01036157.1|:53-179
MsHel-3b	gb|AIXA01031781.1|:405-531
MsHel-3b	gb|AIXA01025484.1|:498-599
MsHel-3b	gb|AIXA01023300.1|:508-605
MsHel-3b	gb|AIXA01017841.1|:3188-3314
MsHel-3b	gb|AIXA01013681.1|:13079-13219
MsHel-3b	gb|AIXA01010963.1|:546-667
MsHel-3b	gb|AIXA01010233.1|:25165-25364
MsHel-3b	gb|AIXA01008536.1|:28424-28550
MsHel-3b	gb|AIXA01007614.1|:29485-29583
MsHel-3b	gb|AIXA01007614.1|:2737-2821
MsHel-3b	gb|AIXA01007612.1|:40999-41161
MsHel-3b	gb|AIXA01007070.1|:9971-10120
MsHel-3b	gb|AIXA01007027.1|:985-1102
MsHel-3b	gb|AIXA01006929.1|:20704-20909
MsHel-3b	gb|AIXA01006706.1|:2216-3259
MsHel-3b	gb|AIXA01006472.1|:3063-3405
MsHel-3b	gb|AIXA01006174.1|:50736-50868
MsHel-3b	gb|AIXA01005579.1|:33759-33880
MsHel-3b	gb|AIXA01005036.1|:4244-4394
MsHel-3b	gb|AIXA01003876.1|:72854-72935
MsHel-3b	gb|AIXA01003180.1|:6369-6491
MsHel-3b	gb|AIXA01002917.1|:51105-51308
MsHel-3b	gb|AIXA01002917.1|:21115-21224
MsHel-3b	gb|AIXA01002911.1|:104947-105068
MsHel-3b	gb|AIXA01002624.1|:31112-31238
MsHel-3b	gb|AIXA01002586.1|:5432-5538
MsHel-3b	gb|AIXA01001679.1|:50512-50644
MsHel-3b	gb|AIXA01001679.1|:14221-14383
MsHel-3b	gb|AIXA01000974.1|:4473-4599
MsHel-3b	gb|AIXA01031011.1|:197-411

MsHel-3b	gb|AIXA01019745.1|:1579-1700
MsHel-3b	gb|AIXA01018996.1|:473-564
MsHel-3b	gb|AIXA01017627.1|:1584-1698
MsHel-3b	gb|AIXA01017530.1|:10816-10904
MsHel-3b	gb|AIXA01015673.1|:8471-8680
MsHel-3b	gb|AIXA01015378.1|:9930-10077
MsHel-3b	gb|AIXA01015132.1|:47248-47403
MsHel-3b	gb|AIXA01015132.1|:34840-34923
MsHel-3b	gb|AIXA01013873.1|:522-725
MsHel-3b	gb|AIXA01010770.1|:14796-14921
MsHel-3b	gb|AIXA01009411.1|:12088-12213
MsHel-3b	gb|AIXA01009043.1|:16081-16284
MsHel-3b	gb|AIXA01008232.1|:14086-15402
MsHel-3b	gb|AIXA01008232.1|:2920-3044
MsHel-3b	gb|AIXA01007737.1|:17815-18015
MsHel-3b	gb|AIXA01007034.1|:56546-56641
MsHel-3b	gb|AIXA01006048.1|:28175-28287
MsHel-3b	gb|AIXA01005592.1|:30431-30535
MsHel-3b	gb|AIXA01005383.1|:28884-29052
MsHel-3b	gb|AIXA01005236.1|:2740-2848
MsHel-3b	gb|AIXA01003915.1|:3212-3417
MsHel-3b	gb|AIXA01003052.1|:76283-76424
MsHel-3b	gb|AIXA01003052.1|:18018-25131
MsHel-3b	gb|AIXA01001502.1|:51092-52280
MsHel-3b	gb|AIXA01036765.1|:450-533
MsHel-3b	gb|AIXA01030845.1|:520-619
MsHel-3b	gb|AIXA01028011.1|:668-836
MsHel-3b	gb|AIXA01021893.1|:267-444
MsHel-3b	gb|AIXA01017122.1|:561-719
MsHel-3b	gb|AIXA01016806.1|:5983-6192
MsHel-3b	gb|AIXA01015257.1|:2441-2647
MsHel-3b	gb|AIXA01014739.1|:23930-24050
MsHel-3b	gb|AIXA01014535.1|:4718-4905
MsHel-3b	gb|AIXA01011502.1|:25441-25625
MsHel-3b	gb|AIXA01009825.1|:4610-4734
MsHel-3b	gb|AIXA01009016.1|:5766-5886
MsHel-3b	gb|AIXA01008224.1|:687-850
MsHel-3b	gb|AIXA01005724.1|:17174-17282
MsHel-3b	gb|AIXA01005427.1|:10132-10227
MsHel-3b	gb|AIXA01004987.1|:10200-10399
MsHel-3b	gb|AIXA01004948.1|:39627-39750
MsHel-3b	gb|AIXA01003598.1|:27172-27250
MsHel-3b	gb|AIXA01003598.1|:107-311
MsHel-3b	gb|AIXA01003581.1|:9396-9572
MsHel-3b	gb|AIXA01003206.1|:2388-2483
MsHel-3b	gb|AIXA01002801.1|:54921-55045
MsHel-3b	gb|AIXA01002614.1|:5650-5907
MsHel-3b	gb|AIXA01001137.1|:31359-31499

MsHel-3b	gb|AIXA01000722.1|:10259-10411
MsHel-3b	gb|AIXA01026410.1|:949-1059
MsHel-3b	gb|AIXA01023977.1|:1070-1180
MsHel-3b	gb|AIXA01020880.1|:612-707
MsHel-3b	gb|AIXA01018711.1|:3738-3883
MsHel-3b	gb|AIXA01015508.1|:10834-10948
MsHel-3b	gb|AIXA01013706.1|:2050-2263
MsHel-3b	gb|AIXA01011499.1|:42146-42259
MsHel-3b	gb|AIXA01011499.1|:17174-17325
MsHel-3b	gb|AIXA01010804.1|:8487-9212
MsHel-3b	gb|AIXA01009839.1|:12040-12177
MsHel-3b	gb|AIXA01009396.1|:2924-3130
MsHel-3b	gb|AIXA01009235.1|:33072-33186
MsHel-3b	gb|AIXA01009225.1|:49244-49409
MsHel-3b	gb|AIXA01008793.1|:24556-24690
MsHel-3b	gb|AIXA01008004.1|:28110-28325
MsHel-3b	gb|AIXA01007392.1|:23228-23354
MsHel-3b	gb|AIXA01007392.1|:2745-2828
MsHel-3b	gb|AIXA01007153.1|:50865-50989
MsHel-3b	gb|AIXA01006952.1|:49026-49297
MsHel-3b	gb|AIXA01005548.1|:27223-27313
MsHel-3b	gb|AIXA01005347.1|:83303-83402
MsHel-3b	gb|AIXA01005347.1|:23792-23872
MsHel-3b	gb|AIXA01005347.1|:4687-4768
MsHel-3b	gb|AIXA01004165.1|:6448-6563
MsHel-3b	gb|AIXA01002518.1|:40152-40344
MsHel-3b	gb|AIXA01002003.1|:745-870
MsHel-3b	gb|AIXA01001648.1|:22446-22572
MsHel-3b	gb|AIXA01001052.1|:44912-45113
MsHel-3b	gb|AIXA01000581.1|:4118-4360
MsHel-3b	gb|AIXA01000226.1|:49243-49451
MsHel-3b	gb|AIXA01035938.1|:19-223
MsHel-3b	gb|AIXA01021096.1|:927-1044
MsHel-3b	gb|AIXA01018684.1|:2136-2360
MsHel-3b	gb|AIXA01015824.1|:23459-23599
MsHel-3b	gb|AIXA01014769.1|:1682-1862
MsHel-3b	gb|AIXA01012351.1|:4582-4733
MsHel-3b	gb|AIXA01011849.1|:11215-11359
MsHel-3b	gb|AIXA01006861.1|:25105-25207
MsHel-3b	gb|AIXA01006800.1|:7717-7841
MsHel-3b	gb|AIXA01005287.1|:3936-4033
MsHel-3b	gb|AIXA01005068.1|:20123-20249
MsHel-3b	gb|AIXA01004424.1|:87768-87854
MsHel-3b	gb|AIXA01004424.1|:30607-30805
MsHel-3b	gb|AIXA01003943.1|:18309-18530
MsHel-3b	gb|AIXA01003380.1|:1699-1816
MsHel-3b	gb|AIXA01002913.1|:20532-20715
MsHel-3b	gb|AIXA01002674.1|:5928-6054

MsHel-3b	gb|AIXA01000964.1|:1272-1440
MsHel-3b	gb|AIXA01000914.1|:34973-35054
MsHel-3b	gb|AIXA01000423.1|:40255-40345
MsHel-3b	gb|AIXA01000285.1|:81956-82157
MsHel-3b	gb|AIXA01033356.1|:212-437
MsHel-3b	gb|AIXA01032876.1|:178-333
MsHel-3b	gb|AIXA01032227.1|:1-168
MsHel-3b	gb|AIXA01032094.1|:289-428
MsHel-3b	gb|AIXA01025141.1|:781-909
MsHel-3b	gb|AIXA01017237.1|:5032-5436
MsHel-3b	gb|AIXA01017213.1|:10296-10421
MsHel-3b	gb|AIXA01014084.1|:4312-4524
MsHel-3b	gb|AIXA01013844.1|:3821-3956
MsHel-3b	gb|AIXA01010815.1|:5688-5815
MsHel-3b	gb|AIXA01008738.1|:32410-32526
MsHel-3b	gb|AIXA01007987.1|:47609-47782
MsHel-3b	gb|AIXA01006780.1|:51413-51538
MsHel-3b	gb|AIXA01005908.1|:2438-2561
MsHel-3b	gb|AIXA01005599.1|:10297-10401
MsHel-3b	gb|AIXA01005024.1|:28894-29091
MsHel-3b	gb|AIXA01004842.1|:17314-17491
MsHel-3b	gb|AIXA01001828.1|:330-511
MsHel-3b	gb|AIXA01001016.1|:3127-3296
MsHel-3b	gb|AIXA01032784.1|:29-112
MsHel-3b	gb|AIXA01018478.1|:701-820
MsHel-3b	gb|AIXA01016764.1|:2931-3120
MsHel-3b	gb|AIXA01016261.1|:16879-16992
MsHel-3b	gb|AIXA01016015.1|:4112-4245
MsHel-3b	gb|AIXA01015773.1|:3209-3304
MsHel-3b	gb|AIXA01014981.1|:15005-15156
MsHel-3b	gb|AIXA01014022.1|:5158-5354
MsHel-3b	gb|AIXA01013736.1|:363-543
MsHel-3b	gb|AIXA01013134.1|:4704-4807
MsHel-3b	gb|AIXA01012644.1|:50553-50689
MsHel-3b	gb|AIXA01012644.1|:34426-34561
MsHel-3b	gb|AIXA01012644.1|:18915-19050
MsHel-3b	gb|AIXA01012018.1|:14612-14737
MsHel-3b	gb|AIXA01011112.1|:38842-38968
MsHel-3b	gb|AIXA01011112.1|:8906-9007
MsHel-3b	gb|AIXA01010110.1|:27709-27829
MsHel-3b	gb|AIXA01010110.1|:6946-10550
MsHel-3b	gb|AIXA01009599.1|:7184-7395
MsHel-3b	gb|AIXA01009024.1|:69204-69299
MsHel-3b	gb|AIXA01009014.1|:8848-9017
MsHel-3b	gb|AIXA01007665.1|:64351-64483
MsHel-3b	gb|AIXA01007665.1|:17663-17766
MsHel-3b	gb|AIXA01007333.1|:18083-18162
MsHel-3b	gb|AIXA01006477.1|:300-479

MsHel-3b	gb|AIXA01005643.1|:79649-79756
MsHel-3b	gb|AIXA01004562.1|:2231-2420
MsHel-3b	gb|AIXA01004082.1|:53706-53787
MsHel-3b	gb|AIXA01003103.1|:47760-47884
MsHel-3b	gb|AIXA01002453.1|:25574-25661
MsHel-3b	gb|AIXA01001858.1|:47432-47560
MsHel-3b	gb|AIXA01001082.1|:6741-6862
MsHel-3b	gb|AIXA01000666.1|:62906-63046
MsHel-3b	gb|AIXA01000080.1|:7964-8159
MsHel-3b	gb|AIXA01036973.1|:266-464
MsHel-3b	gb|AIXA01024882.1|:713-818
MsHel-3b	gb|AIXA01024287.1|:279-434
MsHel-3b	gb|AIXA01021801.1|:424-522
MsHel-3b	gb|AIXA01021755.1|:1023-1231
MsHel-3b	gb|AIXA01014819.1|:1925-2047
MsHel-3b	gb|AIXA01011493.1|:84282-84471
MsHel-3b	gb|AIXA01006509.1|:35720-43313
MsHel-3b	gb|AIXA01003112.1|:54493-54611
MsHel-3b	gb|AIXA01002249.1|:32778-32903
MsHel-3b	gb|AIXA01027323.1|:356-445
MsHel-3b	gb|AIXA01024358.1|:1148-1249
MsHel-3b	gb|AIXA01014695.1|:23817-23903
MsHel-3b	gb|AIXA01014695.1|:11585-11726
MsHel-3b	gb|AIXA01011981.1|:3340-3437
MsHel-3b	gb|AIXA01011554.1|:1276-2925
MsHel-3b	gb|AIXA01010473.1|:3672-3774
MsHel-3b	gb|AIXA01010324.1|:10864-11010
MsHel-3b	gb|AIXA01009476.1|:13023-13152
MsHel-3b	gb|AIXA01009042.1|:84312-84497
MsHel-3b	gb|AIXA01008563.1|:7223-7349
MsHel-3b	gb|AIXA01006449.1|:176666-176795
MsHel-3b	gb|AIXA01006398.1|:47305-47703
MsHel-3b	gb|AIXA01005873.1|:5176-5297
MsHel-3b	gb|AIXA01004969.1|:4060-4248
MsHel-3b	gb|AIXA01002114.1|:127651-127742
MsHel-3b	gb|AIXA01001842.1|:4417-4518
MsHel-3b	gb|AIXA01001504.1|:21866-22049
MsHel-3b	gb|AIXA01037964.1|:388-504
MsHel-3b	gb|AIXA01033927.1|:270-431
MsHel-3b	gb|AIXA01023241.1|:290-382
MsHel-3b	gb|AIXA01017914.1|:2208-2337
MsHel-3b	gb|AIXA01016691.1|:20243-20366
MsHel-3b	gb|AIXA01015575.1|:9035-9159
MsHel-3b	gb|AIXA01015343.1|:2376-2503
MsHel-3b	gb|AIXA01015110.1|:6788-6930
MsHel-3b	gb|AIXA01013835.1|:4174-4277
MsHel-3b	gb|AIXA01013661.1|:365-465
MsHel-3b	gb|AIXA01013227.1|:81162-81314

MsHel-3b	gb|AIXA01011562.1|:13424-13618
MsHel-3b	gb|AIXA01010714.1|:75958-76093
MsHel-3b	gb|AIXA01009753.1|:307-423
MsHel-3b	gb|AIXA01008668.1|:13494-13613
MsHel-3b	gb|AIXA01008575.1|:18427-18520
MsHel-3b	gb|AIXA01007871.1|:11418-11546
MsHel-3b	gb|AIXA01007756.1|:5504-5656
MsHel-3b	gb|AIXA01007595.1|:32170-32274
MsHel-3b	gb|AIXA01006856.1|:5883-6008
MsHel-3b	gb|AIXA01006665.1|:2904-3941
MsHel-3b	gb|AIXA01005567.1|:19009-19132
MsHel-3b	gb|AIXA01003996.1|:15780-15883
MsHel-3b	gb|AIXA01003426.1|:8761-8862
MsHel-3b	gb|AIXA01002403.1|:2773-2862
MsHel-3b	gb|AIXA01002286.1|:8157-8324
MsHel-3b	gb|AIXA01001915.1|:25751-25918
MsHel-3b	gb|AIXA01001144.1|:158637-158837
MsHel-3b	gb|AIXA01001144.1|:138816-138953
MsHel-3b	gb|AIXA01001144.1|:95470-95565
MsHel-3b	gb|AIXA01000444.1|:138733-138907
MsHel-3b	gb|AIXA01000139.1|:14116-14225
MsHel-3b	gb|AIXA01000014.1|:22182-24343
MsHel-3b	gb|AIXA01032311.1|:141-283
MsHel-3b	gb|AIXA01020334.1|:2689-2792
MsHel-3b	gb|AIXA01016453.1|:1017-1125
MsHel-3b	gb|AIXA01015735.1|:745-938
MsHel-3b	gb|AIXA01013671.1|:3261-3443
MsHel-3b	gb|AIXA01011073.1|:3557-3775
MsHel-3b	gb|AIXA01009400.1|:4308-4416
MsHel-3b	gb|AIXA01008047.1|:26445-26594
MsHel-3b	gb|AIXA01007319.1|:4084-4214
MsHel-3b	gb|AIXA01006517.1|:8191-8334
MsHel-3b	gb|AIXA01005067.1|:82342-82467
MsHel-3b	gb|AIXA01005067.1|:68624-68711
MsHel-3b	gb|AIXA01004621.1|:64858-64981
MsHel-3b	gb|AIXA01004250.1|:22528-22669
MsHel-3b	gb|AIXA01004115.1|:109396-109571
MsHel-3b	gb|AIXA01004115.1|:84783-84907
MsHel-3b	gb|AIXA01004115.1|:72029-72152
MsHel-3b	gb|AIXA01003741.1|:56502-56620
MsHel-3b	gb|AIXA01003741.1|:32607-36102
MsHel-3b	gb|AIXA01003445.1|:2489-2713
MsHel-3b	gb|AIXA01002576.1|:5423-5614
MsHel-3b	gb|AIXA01002005.1|:40132-40311
MsHel-3b	gb|AIXA01001348.1|:7099-7767
MsHel-3b	gb|AIXA01019057.1|:3932-4071
MsHel-3b	gb|AIXA01019023.1|:445-583
MsHel-3b	gb|AIXA01016232.1|:17530-17665

MsHel-3b	gb|AIXA01016191.1|:8281-8476
MsHel-3b	gb|AIXA01015237.1|:1073-1253
MsHel-3b	gb|AIXA01014126.1|:9917-10007
MsHel-3b	gb|AIXA01013317.1|:11479-11614
MsHel-3b	gb|AIXA01013120.1|:10999-11156
MsHel-3b	gb|AIXA01012904.1|:24819-24937
MsHel-3b	gb|AIXA01008843.1|:5408-5607
MsHel-3b	gb|AIXA01007227.1|:4077-4271
MsHel-3b	gb|AIXA01006930.1|:71359-71535
MsHel-3b	gb|AIXA01006177.1|:24041-24459
MsHel-3b	gb|AIXA01005618.1|:21779-21897
MsHel-3b	gb|AIXA01005618.1|:8435-8620
MsHel-3b	gb|AIXA01001762.1|:11145-11501
MsHel-3b	gb|AIXA01001662.1|:6253-6355
MsHel-3b	gb|AIXA01001616.1|:3443-3635
MsHel-3b	gb|AIXA01016011.1|:2587-3123
MsHel-3b	gb|AIXA01010006.1|:5896-6033
MsHel-3b	gb|AIXA01009860.1|:26992-27081
MsHel-3b	gb|AIXA01006990.1|:6917-7110
MsHel-3b	gb|AIXA01005892.1|:67473-67599
MsHel-3b	gb|AIXA01005892.1|:31874-32010
MsHel-3b	gb|AIXA01005639.1|:2371-7775
MsHel-3b	gb|AIXA01005607.1|:3637-3763
MsHel-3b	gb|AIXA01005221.1|:40956-41060
MsHel-3b	gb|AIXA01004894.1|:84900-85037
MsHel-3b	gb|AIXA01004591.1|:7620-7762
MsHel-3b	gb|AIXA01004421.1|:9574-9659
MsHel-3b	gb|AIXA01002333.1|:9884-10083
MsHel-3b	gb|AIXA01002197.1|:13776-13881
MsHel-3b	gb|AIXA01001414.1|:12230-12343
MsHel-3b	gb|AIXA01000828.1|:33661-33789
MsHel-3b	gb|AIXA01029887.1|:669-789
MsHel-3b	gb|AIXA01019501.1|:2775-2883
MsHel-3b	gb|AIXA01016987.1|:4772-4867
MsHel-3b	gb|AIXA01015296.1|:9327-9464
MsHel-3b	gb|AIXA01014242.1|:13093-13357
MsHel-3b	gb|AIXA01011188.1|:1495-1662
MsHel-3b	gb|AIXA01011031.1|:30018-30142
MsHel-3b	gb|AIXA01010689.1|:4613-5905
MsHel-3b	gb|AIXA01010483.1|:1286-1384
MsHel-3b	gb|AIXA01008399.1|:3890-4027
MsHel-3b	gb|AIXA01007300.1|:28253-28380
MsHel-3b	gb|AIXA01004708.1|:113840-113994
MsHel-3b	gb|AIXA01001477.1|:8086-8218
MsHel-3b	gb|AIXA01001066.1|:3657-3777
MsHel-3b	gb|AIXA01000944.1|:26731-26863
MsHel-3b	gb|AIXA01031074.1|:598-741
MsHel-3b	gb|AIXA01020742.1|:2097-2261

MsHel-3b	gb|AIXA01018809.1|:2094-2208
MsHel-3b	gb|AIXA01014864.1|:9705-9891
MsHel-3b	gb|AIXA01013147.1|:19484-19616
MsHel-3b	gb|AIXA01013010.1|:4790-5320
MsHel-3b	gb|AIXA01012310.1|:9814-9896
MsHel-3b	gb|AIXA01012288.1|:13527-13715
MsHel-3b	gb|AIXA01010744.1|:41703-41914
MsHel-3b	gb|AIXA01010479.1|:1502-1585
MsHel-3b	gb|AIXA01008086.1|:16444-16611
MsHel-3b	gb|AIXA01007083.1|:68743-68883
MsHel-3b	gb|AIXA01006962.1|:22044-22248
MsHel-3b	gb|AIXA01005895.1|:24290-24462
MsHel-3b	gb|AIXA01004442.1|:2066-2161
MsHel-3b	gb|AIXA01004093.1|:73759-73889
MsHel-3b	gb|AIXA01004093.1|:34491-34616
MsHel-3b	gb|AIXA01003966.1|:33388-33507
MsHel-3b	gb|AIXA01002388.1|:16812-16971
MsHel-3b	gb|AIXA01024502.1|:947-1102
MsHel-3b	gb|AIXA01017591.1|:5122-5506
MsHel-3b	gb|AIXA01017310.1|:13661-13743
MsHel-3b	gb|AIXA01016475.1|:29125-29396
MsHel-3b	gb|AIXA01013825.1|:59065-59188
MsHel-3b	gb|AIXA01013623.1|:36366-36551
MsHel-3b	gb|AIXA01012581.1|:52666-52949
MsHel-3b	gb|AIXA01007267.1|:9363-13335
MsHel-3b	gb|AIXA01006595.1|:46853-47055
MsHel-3b	gb|AIXA01003012.1|:12955-13079
MsHel-3b	gb|AIXA01002273.1|:27709-27796
MsHel-3b	gb|AIXA01001595.1|:7541-11766
MsHel-3b	gb|AIXA01000823.1|:48018-48184
MsHel-3b	gb|AIXA01037344.1|:249-469
MsHel-3b	gb|AIXA01012755.1|:5456-5557
MsHel-3b	gb|AIXA01012536.1|:3547-3632
MsHel-3b	gb|AIXA01011674.1|:7464-7949
MsHel-3b	gb|AIXA01007752.1|:5812-5961
MsHel-3b	gb|AIXA01007631.1|:22574-22679
MsHel-3b	gb|AIXA01007224.1|:45874-47738
MsHel-3b	gb|AIXA01007103.1|:5419-5619
MsHel-3b	gb|AIXA01006869.1|:22758-22847
MsHel-3b	gb|AIXA01006625.1|:15173-15313
MsHel-3b	gb|AIXA01006556.1|:454-603
MsHel-3b	gb|AIXA01004624.1|:1633-1784
MsHel-3b	gb|AIXA01004340.1|:9438-9588
MsHel-3b	gb|AIXA01004204.1|:5726-12938
MsHel-3b	gb|AIXA01004091.1|:11098-11215
MsHel-3b	gb|AIXA01003712.1|:45211-45343
MsHel-3b	gb|AIXA01003154.1|:26892-26994
MsHel-3b	gb|AIXA01001883.1|:4765-4884

MsHel-3b	gb|AIXA01000763.1|:88657-96614
MsHel-3b	gb|AIXA01000258.1|:4611-4737
MsHel-3b	gb|AIXA01000231.1|:8789-8915
MsHel-3b	gb|AIXA01000155.1|:53905-54029
MsHel-3b	gb|AIXA01030932.1|:84-257
MsHel-3b	gb|AIXA01016756.1|:1363-1495
MsHel-3b	gb|AIXA01016065.1|:1204-1339
MsHel-3b	gb|AIXA01013573.1|:6895-6987
MsHel-3b	gb|AIXA01012730.1|:2492-2611
MsHel-3b	gb|AIXA01007466.1|:13007-13132
MsHel-3b	gb|AIXA01007217.1|:40900-41082
MsHel-3b	gb|AIXA01006915.1|:3073-3195
MsHel-3b	gb|AIXA01006600.1|:365-497
MsHel-3b	gb|AIXA01004626.1|:26207-26287
MsHel-3b	gb|AIXA01004130.1|:4598-4714
MsHel-3b	gb|AIXA01003053.1|:16696-16824
MsHel-3b	gb|AIXA01001286.1|:18943-19067
MsHel-3b	gb|AIXA01000568.1|:7308-7419
MsHel-3b	gb|AIXA01000128.1|:83485-83573
MsHel-3b	gb|AIXA01037025.1|:307-410
MsHel-3b	gb|AIXA01021005.1|:1791-1890
MsHel-3b	gb|AIXA01019094.1|:2319-2523
MsHel-3b	gb|AIXA01017056.1|:13743-13878
MsHel-3b	gb|AIXA01016574.1|:18924-19114
MsHel-3b	gb|AIXA01015877.1|:7153-7353
MsHel-3b	gb|AIXA01015510.1|:1863-2000
MsHel-3b	gb|AIXA01013850.1|:18215-18307
MsHel-3b	gb|AIXA01013342.1|:43322-43461
MsHel-3b	gb|AIXA01009327.1|:29838-29966
MsHel-3b	gb|AIXA01008573.1|:8180-8271
MsHel-3b	gb|AIXA01008477.1|:61904-61995
MsHel-3b	gb|AIXA01006953.1|:29059-29178
MsHel-3b	gb|AIXA01006396.1|:62739-62859
MsHel-3b	gb|AIXA01005880.1|:1472-1690
MsHel-3b	gb|AIXA01005342.1|:2946-3029
MsHel-3b	gb|AIXA01005249.1|:14627-14738
MsHel-3b	gb|AIXA01002723.1|:7050-7235
MsHel-3b	gb|AIXA01001289.1|:37675-37872
MsHel-3b	gb|AIXA01000783.1|:32157-32304
MsHel-3b	gb|AIXA01000486.1|:61326-61456
MsHel-3b	gb|AIXA01035756.1|:151-309
MsHel-3b	gb|AIXA01031277.1|:620-765
MsHel-3b	gb|AIXA01018376.1|:260-349
MsHel-3b	gb|AIXA01017915.1|:1-186
MsHel-3b	gb|AIXA01015041.1|:15538-15663
MsHel-3b	gb|AIXA01014618.1|:5350-5524
MsHel-3b	gb|AIXA01011837.1|:13623-13718
MsHel-3b	gb|AIXA01011267.1|:7672-7837

MsHel-3b	gb|AIXA01009741.1|:6231-6334
MsHel-3b	gb|AIXA01007844.1|:28512-28603
MsHel-3b	gb|AIXA01007844.1|:6104-6232
MsHel-3b	gb|AIXA01007344.1|:45121-45213
MsHel-3b	gb|AIXA01005876.1|:239-359
MsHel-3b	gb|AIXA01005392.1|:45253-45436
MsHel-3b	gb|AIXA01005010.1|:16959-17162
MsHel-3b	gb|AIXA01004990.1|:192005-192142
MsHel-3b	gb|AIXA01003671.1|:7982-8114
MsHel-3b	gb|AIXA01003548.1|:10551-10633
MsHel-3b	gb|AIXA01002831.1|:9872-9959
MsHel-3b	gb|AIXA01000087.1|:9365-14383
MsHel-3b	gb|AIXA01029007.1|:595-799
MsHel-3b	gb|AIXA01023807.1|:1139-1236
MsHel-3b	gb|AIXA01023131.1|:354-536
MsHel-3b	gb|AIXA01021232.1|:984-1149
MsHel-3b	gb|AIXA01016044.1|:1812-1969
MsHel-3b	gb|AIXA01010480.1|:24911-25033
MsHel-3b	gb|AIXA01010388.1|:265-384
MsHel-3b	gb|AIXA01009775.1|:12609-12730
MsHel-3b	gb|AIXA01009712.1|:4643-4803
MsHel-3b	gb|AIXA01009420.1|:17465-17594
MsHel-3b	gb|AIXA01006907.1|:17255-17437
MsHel-3b	gb|AIXA01006898.1|:44998-45182
MsHel-3b	gb|AIXA01006555.1|:7019-7148
MsHel-3b	gb|AIXA01006548.1|:19189-19367
MsHel-3b	gb|AIXA01004628.1|:32840-32925
MsHel-3b	gb|AIXA01004628.1|:7099-7283
MsHel-3b	gb|AIXA01004338.1|:7375-7504
MsHel-3b	gb|AIXA01004038.1|:34881-35010
MsHel-3b	gb|AIXA01001603.1|:2027-2164
MsHel-3b	gb|AIXA01001232.1|:12511-12593
MsHel-3b	gb|AIXA01000622.1|:73812-74150
MsHel-3b	gb|AIXA01000622.1|:14235-14316
MsHel-3b	gb|AIXA01000347.1|:43354-43483
MsHel-3b	gb|AIXA01028505.1|:670-814
MsHel-3b	gb|AIXA01024619.1|:499-591
MsHel-3b	gb|AIXA01019845.1|:1309-1399
MsHel-3b	gb|AIXA01018924.1|:115-251
MsHel-3b	gb|AIXA01017083.1|:10207-10361
MsHel-3b	gb|AIXA01016947.1|:9957-10104
MsHel-3b	gb|AIXA01016540.1|:2392-3733
MsHel-3b	gb|AIXA01014219.1|:21348-21432
MsHel-3b	gb|AIXA01013872.1|:9531-9678
MsHel-3b	gb|AIXA01013612.1|:15452-15576
MsHel-3b	gb|AIXA01013057.1|:8342-8430
MsHel-3b	gb|AIXA01011389.1|:16371-16483
MsHel-3b	gb|AIXA01008629.1|:8094-8201

MsHel-3b	gb|AIXA01008518.1|:5499-5583
MsHel-3b	gb|AIXA01006701.1|:23228-23360
MsHel-3b	gb|AIXA01005265.1|:38136-38244
MsHel-3b	gb|AIXA01004456.1|:25019-25139
MsHel-3b	gb|AIXA01004186.1|:35844-35943
MsHel-3b	gb|AIXA01002858.1|:23757-23873
MsHel-3b	gb|AIXA01002570.1|:29833-29992
MsHel-3b	gb|AIXA01002050.1|:39429-39510
MsHel-3b	gb|AIXA01032077.1|:557-736
MsHel-3b	gb|AIXA01024434.1|:439-601
MsHel-3b	gb|AIXA01023846.1|:205-284
MsHel-3b	gb|AIXA01014984.1|:28279-28362
MsHel-3b	gb|AIXA01013144.1|:1669-1796
MsHel-3b	gb|AIXA01011106.1|:10253-10385
MsHel-3b	gb|AIXA01011104.1|:24999-25094
MsHel-3b	gb|AIXA01009820.1|:41447-41577
MsHel-3b	gb|AIXA01009820.1|:7803-7928
MsHel-3b	gb|AIXA01008789.1|:27976-29458
MsHel-3b	gb|AIXA01002971.1|:95157-95245
MsHel-3b	gb|AIXA01002971.1|:29919-36359
MsHel-3b	gb|AIXA01002971.1|:14951-15054
MsHel-3b	gb|AIXA01002967.1|:13393-13536
MsHel-3b	gb|AIXA01002813.1|:71663-71764
MsHel-3b	gb|AIXA01001194.1|:32205-32301
MsHel-3b	gb|AIXA01000908.1|:35324-35459
MsHel-3b	gb|AIXA01000882.1|:9109-9289
MsHel-3b	gb|AIXA01025651.1|:957-1082
MsHel-3b	gb|AIXA01021312.1|:382-472
MsHel-3b	gb|AIXA01018870.1|:5810-5909
MsHel-3b	gb|AIXA01017727.1|:15813-15952
MsHel-3b	gb|AIXA01016525.1|:1818-1915
MsHel-3b	gb|AIXA01016287.1|:4927-5111
MsHel-3b	gb|AIXA01015715.1|:5674-5768
MsHel-3b	gb|AIXA01014938.1|:14203-14311
MsHel-3b	gb|AIXA01009934.1|:115-222
MsHel-3b	gb|AIXA01007138.1|:10271-10384
MsHel-3b	gb|AIXA01005598.1|:22297-22407
MsHel-3b	gb|AIXA01004887.1|:19360-19478
MsHel-3b	gb|AIXA01004486.1|:8969-9072
MsHel-3b	gb|AIXA01004433.1|:152191-156599
MsHel-3b	gb|AIXA01003967.1|:107401-107696
MsHel-3b	gb|AIXA01003528.1|:4497-4626
MsHel-3b	gb|AIXA01000115.1|:91935-92162
MsHel-3b	gb|AIXA01025881.1|:695-813
MsHel-3b	gb|AIXA01016323.1|:12280-12482
MsHel-3b	gb|AIXA01014020.1|:17589-17950
MsHel-3b	gb|AIXA01012485.1|:30229-30342
MsHel-3b	gb|AIXA01011610.1|:35248-35377

MsHel-3b	gb|AIXA01011576.1|:4954-5044
MsHel-3b	gb|AIXA01010108.1|:14750-16652
MsHel-3b	gb|AIXA01009504.1|:31352-31536
MsHel-3b	gb|AIXA01008788.1|:7800-7889
MsHel-3b	gb|AIXA01008378.1|:5521-5598
MsHel-3b	gb|AIXA01008203.1|:67893-68040
MsHel-3b	gb|AIXA01007648.1|:31943-32089
MsHel-3b	gb|AIXA01006021.1|:68063-68220
MsHel-3b	gb|AIXA01005527.1|:42199-42292
MsHel-3b	gb|AIXA01004661.1|:11061-11149
MsHel-3b	gb|AIXA01003376.1|:3004-3186
MsHel-3b	gb|AIXA01002693.1|:66508-66597
MsHel-3b	gb|AIXA01002399.1|:30439-30540
MsHel-3b	gb|AIXA01001624.1|:31375-31480
MsHel-3b	gb|AIXA01001496.1|:6860-6946
MsHel-3b	gb|AIXA01031190.1|:503-689
MsHel-3b	gb|AIXA01019576.1|:2151-2232
MsHel-3b	gb|AIXA01017387.1|:1578-1664
MsHel-3b	gb|AIXA01016693.1|:14200-14340
MsHel-3b	gb|AIXA01012371.1|:9155-9247
MsHel-3b	gb|AIXA01011097.1|:17567-17651
MsHel-3b	gb|AIXA01010205.1|:1273-1373
MsHel-3b	gb|AIXA01009433.1|:206-563
MsHel-3b	gb|AIXA01008845.1|:7065-7157
MsHel-3b	gb|AIXA01008242.1|:5820-5945
MsHel-3b	gb|AIXA01007635.1|:3656-3860
MsHel-3b	gb|AIXA01007516.1|:82051-82205
MsHel-3b	gb|AIXA01007516.1|:63632-63743
MsHel-3b	gb|AIXA01007516.1|:4906-5007
MsHel-3b	gb|AIXA01005667.1|:20182-20297
MsHel-3b	gb|AIXA01003881.1|:94408-94512
MsHel-3b	gb|AIXA01002726.1|:41643-41815
MsHel-3b	gb|AIXA01001597.1|:6152-6232
MsHel-3b	gb|AIXA01000895.1|:46686-46823
MsHel-3b	gb|AIXA01000713.1|:24022-24113
MsHel-3b	gb|AIXA01000268.1|:718-806
MsHel-3b	gb|AIXA01034302.1|:532-623
MsHel-3b	gb|AIXA01032853.1|:27-135
MsHel-3b	gb|AIXA01022046.1|:937-1125
MsHel-3b	gb|AIXA01016851.1|:507-636
MsHel-3b	gb|AIXA01016839.1|:2177-2349
MsHel-3b	gb|AIXA01016411.1|:22132-22311
MsHel-3b	gb|AIXA01012974.1|:6751-7272
MsHel-3b	gb|AIXA01012796.1|:3764-3851
MsHel-3b	gb|AIXA01010900.1|:24014-24102
MsHel-3b	gb|AIXA01010060.1|:1375-1479
MsHel-3b	gb|AIXA01009593.1|:393-532
MsHel-3b	gb|AIXA01009281.1|:20225-20349

MsHel-3b	gb|AIXA01007691.1|:33105-33188
MsHel-3b	gb|AIXA01006809.1|:25740-25831
MsHel-3b	gb|AIXA01006599.1|:2890-3001
MsHel-3b	gb|AIXA01006202.1|:28376-28495
MsHel-3b	gb|AIXA01005484.1|:9166-9305
MsHel-3b	gb|AIXA01004928.1|:12894-12997
MsHel-3b	gb|AIXA01004462.1|:13234-13329
MsHel-3b	gb|AIXA01004293.1|:6133-6263
MsHel-3b	gb|AIXA01003536.1|:18840-18951
MsHel-3b	gb|AIXA01003443.1|:34452-34653
MsHel-3b	gb|AIXA01003316.1|:55869-55951
MsHel-3b	gb|AIXA01003264.1|:19759-20363
MsHel-3b	gb|AIXA01002044.1|:42861-43033
MsHel-3b	gb|AIXA01001683.1|:953-1057
MsHel-3b	gb|AIXA01001293.1|:5157-5297
MsHel-3b	gb|AIXA01000878.1|:54374-54479
MsHel-3b	gb|AIXA01031956.1|:583-716
MsHel-3b	gb|AIXA01021679.1|:610-692
MsHel-3b	gb|AIXA01016903.1|:3979-8579
MsHel-3b	gb|AIXA01015111.1|:273-460
MsHel-3b	gb|AIXA01012976.1|:1040-1162
MsHel-3b	gb|AIXA01009841.1|:3687-3777
MsHel-3b	gb|AIXA01007340.1|:61642-61745
MsHel-3b	gb|AIXA01006834.1|:83880-83962
MsHel-3b	gb|AIXA01006691.1|:7253-7368
MsHel-3b	gb|AIXA01005083.1|:61476-61583
MsHel-3b	gb|AIXA01004798.1|:3609-3773
MsHel-3b	gb|AIXA01003743.1|:53884-54014
MsHel-3b	gb|AIXA01003198.1|:39833-39948
MsHel-3b	gb|AIXA01002938.1|:22674-22757
MsHel-3b	gb|AIXA01002794.1|:54872-61252
MsHel-3b	gb|AIXA01002213.1|:54913-54999
MsHel-3b	gb|AIXA01001963.1|:47373-47525
MsHel-3b	gb|AIXA01001456.1|:28809-28935
MsHel-3b	gb|AIXA01000959.1|:45362-53064
MsHel-3b	gb|AIXA01000827.1|:47007-47093
MsHel-3b	gb|AIXA01028798.1|:21-102
MsHel-3b	gb|AIXA01020966.1|:641-824
MsHel-3b	gb|AIXA01017945.1|:5393-5474
MsHel-3b	gb|AIXA01014971.1|:7888-8052
MsHel-3b	gb|AIXA01013679.1|:13006-13135
MsHel-3b	gb|AIXA01012698.1|:1758-1950
MsHel-3b	gb|AIXA01012551.1|:64109-64202
MsHel-3b	gb|AIXA01012170.1|:304-404
MsHel-3b	gb|AIXA01011726.1|:2774-2943
MsHel-3b	gb|AIXA01011247.1|:21138-21253
MsHel-3b	gb|AIXA01010036.1|:7173-7266
MsHel-3b	gb|AIXA01009481.1|:11693-11822

MsHel-3b	gb|AIXA01008688.1|:5760-5890
MsHel-3b	gb|AIXA01008192.1|:2356-2438
MsHel-3b	gb|AIXA01004831.1|:66817-66934
MsHel-3b	gb|AIXA01003231.1|:12021-12155
MsHel-3b	gb|AIXA01002988.1|:59154-59248
MsHel-3b	gb|AIXA01000893.1|:7544-7797
MsHel-3b	gb|AIXA01025795.1|:507-707
MsHel-3b	gb|AIXA01018414.1|:8063-8165
MsHel-3b	gb|AIXA01018138.1|:6682-6802
MsHel-3b	gb|AIXA01018132.1|:2932-3039
MsHel-3b	gb|AIXA01012344.1|:9529-9657
MsHel-3b	gb|AIXA01011893.1|:594-674
MsHel-3b	gb|AIXA01011637.1|:23227-23569
MsHel-3b	gb|AIXA01010702.1|:1929-2036
MsHel-3b	gb|AIXA01008884.1|:26776-26857
MsHel-3b	gb|AIXA01008558.1|:12887-12979
MsHel-3b	gb|AIXA01007776.1|:9142-9330
MsHel-3b	gb|AIXA01005417.1|:13687-13790
MsHel-3b	gb|AIXA01004192.1|:7376-7493
MsHel-3b	gb|AIXA01003463.1|:5107-5239
MsHel-3b	gb|AIXA01002382.1|:1713-1801
MsHel-3b	gb|AIXA01001379.1|:8046-8130
MsHel-3b	gb|AIXA01000796.1|:7506-7717
MsHel-3b	gb|AIXA01000597.1|:13150-13252
MsHel-3b	gb|AIXA01000117.1|:14779-14874
MsHel-3b	gb|AIXA01000060.1|:15294-15391
MsHel-3b	gb|AIXA01028475.1|:383-513
MsHel-3b	gb|AIXA01016588.1|:1592-1710
MsHel-3b	gb|AIXA01016321.1|:996-1095
MsHel-3b	gb|AIXA01014569.1|:1414-1513
MsHel-3b	gb|AIXA01014218.1|:1961-2055
MsHel-3b	gb|AIXA01010692.1|:16440-16567
MsHel-3b	gb|AIXA01010569.1|:4261-4750
MsHel-3b	gb|AIXA01010348.1|:17862-17961
MsHel-3b	gb|AIXA01007113.1|:36376-36510
MsHel-3b	gb|AIXA01005974.1|:103641-103744
MsHel-3b	gb|AIXA01005974.1|:3420-3748
MsHel-3b	gb|AIXA01005352.1|:65828-65910
MsHel-3b	gb|AIXA01004177.1|:19748-19831
MsHel-3b	gb|AIXA01003694.1|:8478-8605
MsHel-3b	gb|AIXA01002909.1|:15584-15958
MsHel-3b	gb|AIXA01002047.1|:14326-14421
MsHel-3b	gb|AIXA01001853.1|:32081-32218
MsHel-3b	gb|AIXA01000655.1|:83375-83479
MsHel-3b	gb|AIXA01000655.1|:59603-59802
MsHel-3b	gb|AIXA01000655.1|:8813-9167
MsHel-3b	gb|AIXA01000528.1|:46787-46877
MsHel-3b	gb|AIXA01000358.1|:102-189

MsHel-3b	gb|AIXA01000241.1|:13148-13270
MsHel-3b	gb|AIXA01017085.1|:12311-12392
MsHel-3b	gb|AIXA01015711.1|:4035-4445
MsHel-3b	gb|AIXA01013738.1|:21986-22182
MsHel-3b	gb|AIXA01009943.1|:96-177
MsHel-3b	gb|AIXA01008964.1|:12244-12346
MsHel-3b	gb|AIXA01006843.1|:6018-6104
MsHel-3b	gb|AIXA01006144.1|:1655-1761
MsHel-3b	gb|AIXA01005153.1|:3206-3304
MsHel-3b	gb|AIXA01004370.1|:31656-31784
MsHel-3b	gb|AIXA01004060.1|:571-7802
MsHel-3b	gb|AIXA01003186.1|:41253-41380
MsHel-3b	gb|AIXA01003153.1|:24481-24587
MsHel-3b	gb|AIXA01002744.1|:7121-7213
MsHel-3b	gb|AIXA01002008.1|:25293-25387
MsHel-3b	gb|AIXA01001738.1|:234-328
MsHel-3b	gb|AIXA01001092.1|:11434-11553
MsHel-3b	gb|AIXA01028981.1|:762-851
MsHel-3b	gb|AIXA01018834.1|:2344-2553
MsHel-3b	gb|AIXA01016971.1|:3978-4074
MsHel-3b	gb|AIXA01015096.1|:138-224
MsHel-3b	gb|AIXA01012706.1|:14469-14648
MsHel-3b	gb|AIXA01012533.1|:11064-11175
MsHel-3b	gb|AIXA01006755.1|:10513-10705
MsHel-3b	gb|AIXA01006230.1|:15226-15367
MsHel-3b	gb|AIXA01006130.1|:27494-27615
MsHel-3b	gb|AIXA01004527.1|:34087-34188
MsHel-3b	gb|AIXA01004391.1|:47788-47873
MsHel-3b	gb|AIXA01003989.1|:43416-43632
MsHel-3b	gb|AIXA01003624.1|:7329-7442
MsHel-3b	gb|AIXA01003288.1|:16510-16600
MsHel-3b	gb|AIXA01003056.1|:28465-28593
MsHel-3b	gb|AIXA01000884.1|:33228-33361
MsHel-3b	gb|AIXA01000884.1|:7807-7935
MsHel-3b	gb|AIXA01000739.1|:7875-7967
MsHel-3b	gb|AIXA01030562.1|:116-208
MsHel-3b	gb|AIXA01025216.1|:522-654
MsHel-3b	gb|AIXA01024587.1|:783-944
MsHel-3b	gb|AIXA01021626.1|:958-1046
MsHel-3b	gb|AIXA01017137.1|:4585-4665
MsHel-3b	gb|AIXA01015819.1|:3234-3326
MsHel-3b	gb|AIXA01015695.1|:3679-3781
MsHel-3b	gb|AIXA01014213.1|:6176-6253
MsHel-3b	gb|AIXA01013089.1|:12294-12383
MsHel-3b	gb|AIXA01012061.1|:8293-8372
MsHel-3b	gb|AIXA01011407.1|:24320-24411
MsHel-3b	gb|AIXA01008726.1|:6732-6868
MsHel-3b	gb|AIXA01007299.1|:24933-25020

MsHel-3b	gb|AIXA01006757.1|:7496-7616
MsHel-3b	gb|AIXA01006503.1|:12741-12825
MsHel-3b	gb|AIXA01006418.1|:2792-2876
MsHel-3b	gb|AIXA01003806.1|:18821-18901
MsHel-3b	gb|AIXA01003682.1|:11729-11831
MsHel-3b	gb|AIXA01003540.1|:1840-1932
MsHel-3b	gb|AIXA01001642.1|:10278-10382
MsHel-3b	gb|AIXA01000901.1|:4309-8785
MsHel-3b	gb|AIXA01000453.1|:1618-1714
MsHel-3b	gb|AIXA01026224.1|:12-95
MsHel-3b	gb|AIXA01023942.1|:142-225
MsHel-3b	gb|AIXA01017113.1|:6757-6840
MsHel-3b	gb|AIXA01016266.1|:1-123
MsHel-3b	gb|AIXA01015584.1|:387-498
MsHel-3b	gb|AIXA01015193.1|:5230-5989
MsHel-3b	gb|AIXA01013141.1|:1294-1373
MsHel-3b	gb|AIXA01012504.1|:6294-6416
MsHel-3b	gb|AIXA01011559.1|:6435-6517
MsHel-3b	gb|AIXA01009875.1|:8-151
MsHel-3b	gb|AIXA01009530.1|:13042-13124
MsHel-3b	gb|AIXA01009406.1|:4645-4723
MsHel-3b	gb|AIXA01007634.1|:23937-24025
MsHel-3b	gb|AIXA01007298.1|:14534-14699
MsHel-3b	gb|AIXA01004937.1|:62376-62471
MsHel-3b	gb|AIXA01004484.1|:60112-60442
MsHel-3b	gb|AIXA01004191.1|:27536-27619
MsHel-3b	gb|AIXA01003529.1|:12817-12926
MsHel-3b	gb|AIXA01003270.1|:68979-69080
MsHel-3b	gb|AIXA01003148.1|:6642-6721
MsHel-3b	gb|AIXA01002250.1|:39095-39202
MsHel-3b	gb|AIXA01001848.1|:32392-32496
MsHel-3b	gb|AIXA01000764.1|:3838-3928
MsHel-3b	gb|AIXA01036134.1|:286-384
MsHel-3b	gb|AIXA01029702.1|:390-480
MsHel-3b	gb|AIXA01022974.1|:1072-1209
MsHel-3b	gb|AIXA01021672.1|:1152-1234
MsHel-3b	gb|AIXA01017455.1|:5430-5526
MsHel-3b	gb|AIXA01016959.1|:9204-9383
MsHel-3b	gb|AIXA01015583.1|:23713-23823
MsHel-3b	gb|AIXA01014883.1|:20552-20638
MsHel-3b	gb|AIXA01013733.1|:6243-6325
MsHel-3b	gb|AIXA01012502.1|:3310-3424
MsHel-3b	gb|AIXA01011312.1|:6540-6697
MsHel-3b	gb|AIXA01011293.1|:22961-23041
MsHel-3b	gb|AIXA01009879.1|:12194-12351
MsHel-3b	gb|AIXA01008078.1|:8279-8352
MsHel-3b	gb|AIXA01007488.1|:43882-43980
MsHel-3b	gb|AIXA01007379.1|:6131-6216

MsHel-3b	gb|AIXA01006740.1|:120668-120750
MsHel-3b	gb|AIXA01003597.1|:63227-63336
MsHel-3b	gb|AIXA01003597.1|:20334-20459
MsHel-3b	gb|AIXA01002411.1|:11023-11149
MsHel-3b	gb|AIXA01002371.1|:47898-47988
MsHel-3b	gb|AIXA01001839.1|:7156-15980
MsHel-3b	gb|AIXA01000289.1|:11663-11749
MsHel-3b	gb|AIXA01000228.1|:4472-4643
MsHel-3b	gb|AIXA01023936.1|:601-732
MsHel-3b	gb|AIXA01020441.1|:308-429
MsHel-3b	gb|AIXA01020276.1|:73-158
MsHel-3b	gb|AIXA01015126.1|:1393-1510
MsHel-3b	gb|AIXA01013650.1|:349-477
MsHel-3b	gb|AIXA01013445.1|:18994-19091
MsHel-3b	gb|AIXA01013123.1|:7587-7686
MsHel-3b	gb|AIXA01012400.1|:15719-15886
MsHel-3b	gb|AIXA01011265.1|:2561-2660
MsHel-3b	gb|AIXA01008271.1|:11823-13272
MsHel-3b	gb|AIXA01008257.1|:2910-2995
MsHel-3b	gb|AIXA01007556.1|:25214-25331
MsHel-3b	gb|AIXA01007179.1|:7837-7918
MsHel-3b	gb|AIXA01006752.1|:8604-8690
MsHel-3b	gb|AIXA01006712.1|:67466-67579
MsHel-3b	gb|AIXA01006474.1|:19426-29325
MsHel-3b	gb|AIXA01006288.1|:16586-16678
MsHel-3b	gb|AIXA01006288.1|:4495-4576
MsHel-3b	gb|AIXA01003962.1|:28622-28719
MsHel-3b	gb|AIXA01002747.1|:63343-63424
MsHel-3b	gb|AIXA01001563.1|:12109-12194
MsHel-3b	gb|AIXA01034040.1|:493-607
MsHel-3b	gb|AIXA01031089.1|:524-671
MsHel-3b	gb|AIXA01019730.1|:1769-1885
MsHel-3b	gb|AIXA01015478.1|:128-212
MsHel-3b	gb|AIXA01013427.1|:4170-4262
MsHel-3b	gb|AIXA01012278.1|:6704-6783
MsHel-3b	gb|AIXA01012144.1|:4481-4571
MsHel-3b	gb|AIXA01011482.1|:834-914
MsHel-3b	gb|AIXA01011349.1|:25578-25709
MsHel-3b	gb|AIXA01011027.1|:25786-25882
MsHel-3b	gb|AIXA01010781.1|:28253-28348
MsHel-3b	gb|AIXA01010694.1|:5298-5395
MsHel-3b	gb|AIXA01010158.1|:13711-13848
MsHel-3b	gb|AIXA01009750.1|:19816-19963
MsHel-3b	gb|AIXA01009710.1|:31490-31586
MsHel-3b	gb|AIXA01008578.1|:3238-3322
MsHel-3b	gb|AIXA01006126.1|:20914-21014
MsHel-3b	gb|AIXA01004935.1|:72277-72409
MsHel-3b	gb|AIXA01004695.1|:27753-27876

MsHel-3b	gb|AIXA01004647.1|:33955-34035
MsHel-3b	gb|AIXA01004549.1|:4685-4768
MsHel-3b	gb|AIXA01002018.1|:4184-4268
MsHel-3b	gb|AIXA01000926.1|:61610-61696
MsHel-3b	gb|AIXA01000926.1|:939-1051
MsHel-3b	gb|AIXA01000233.1|:5200-5284
MsHel-3b	gb|AIXA01024253.1|:669-752
MsHel-3b	gb|AIXA01019234.1|:852-947
MsHel-3b	gb|AIXA01010111.1|:1116-5719
MsHel-3b	gb|AIXA01009590.1|:32523-35845
MsHel-3b	gb|AIXA01009564.1|:3494-3604
MsHel-3b	gb|AIXA01009341.1|:68975-69069
MsHel-3b	gb|AIXA01008237.1|:17316-17403
MsHel-3b	gb|AIXA01007806.1|:6553-6636
MsHel-3b	gb|AIXA01007059.1|:9670-9784
MsHel-3b	gb|AIXA01005365.1|:17464-17575
MsHel-3b	gb|AIXA01005328.1|:51288-51375
MsHel-3b	gb|AIXA01005003.1|:14696-14807
MsHel-3b	gb|AIXA01004597.1|:51757-51836
MsHel-3b	gb|AIXA01004532.1|:21667-21778
MsHel-3b	gb|AIXA01003723.1|:20028-20122
MsHel-3b	gb|AIXA01003144.1|:28113-28228
MsHel-3b	gb|AIXA01003016.1|:17872-17983
MsHel-3b	gb|AIXA01002582.1|:109324-109429
MsHel-3b	gb|AIXA01002155.1|:54919-55009
MsHel-3b	gb|AIXA01001325.1|:7584-7715
MsHel-3b	gb|AIXA01000652.1|:30703-30820
MsHel-3b	gb|AIXA01000386.1|:791-910
MsHel-3b	gb|AIXA01026803.1|:472-558
MsHel-3b	gb|AIXA01018551.1|:7366-7506
MsHel-3b	gb|AIXA01017933.1|:5275-12059
MsHel-3b	gb|AIXA01017566.1|:10427-10529
MsHel-3b	gb|AIXA01015820.1|:1633-1723
MsHel-3b	gb|AIXA01014040.1|:25583-25673
MsHel-3b	gb|AIXA01012386.1|:1610-1696
MsHel-3b	gb|AIXA01012219.1|:8605-8703
MsHel-3b	gb|AIXA01010593.1|:7772-7894
MsHel-3b	gb|AIXA01008561.1|:35497-35591
MsHel-3b	gb|AIXA01008280.1|:25034-25157
MsHel-3b	gb|AIXA01007750.1|:17029-17111
MsHel-3b	gb|AIXA01004685.1|:106556-106638
MsHel-3b	gb|AIXA01004651.1|:3802-3898
MsHel-3b	gb|AIXA01004303.1|:7092-7169
MsHel-3b	gb|AIXA01004265.1|:3310-3387
MsHel-3b	gb|AIXA01003207.1|:44690-46128
MsHel-3b	gb|AIXA01001878.1|:17730-17819
MsHel-3b	gb|AIXA01001219.1|:35703-35874
MsHel-3b	gb|AIXA01000868.1|:7575-7657

MsHel-3b	gb|AIXA01000707.1|:90925-91014
MsHel-3b	gb|AIXA01000707.1|:67963-68048
MsHel-3b	gb|AIXA01015083.1|:15058-15139
MsHel-3b	gb|AIXA01013430.1|:34319-34433
MsHel-3b	gb|AIXA01012346.1|:7431-7528
MsHel-3b	gb|AIXA01011283.1|:8132-8250
MsHel-3b	gb|AIXA01010026.1|:19057-19184
MsHel-3b	gb|AIXA01009274.1|:9779-9893
MsHel-3b	gb|AIXA01009156.1|:4701-4786
MsHel-3b	gb|AIXA01009086.1|:1291-1376
MsHel-3b	gb|AIXA01007121.1|:14984-15065
MsHel-3b	gb|AIXA01005344.1|:56682-56763
MsHel-3b	gb|AIXA01003322.1|:4960-5052
MsHel-3b	gb|AIXA01002847.1|:17392-17489
MsHel-3b	gb|AIXA01002542.1|:14513-14599
MsHel-3b	gb|AIXA01001453.1|:15013-15107
MsHel-3b	gb|AIXA01001358.1|:79320-79416
MsHel-3b	gb|AIXA01000776.1|:5822-5923
MsHel-3b	gb|AIXA01030415.1|:101-657
MsHel-3b	gb|AIXA01026107.1|:457-589
MsHel-3b	gb|AIXA01019954.1|:3372-3460
MsHel-3b	gb|AIXA01017697.1|:3293-3395
MsHel-3b	gb|AIXA01016397.1|:4001-4112
MsHel-3b	gb|AIXA01016307.1|:9135-9243
MsHel-3b	gb|AIXA01016154.1|:12984-13072
MsHel-3b	gb|AIXA01016140.1|:1064-1156
MsHel-3b	gb|AIXA01015321.1|:18159-18254
MsHel-3b	gb|AIXA01015321.1|:3434-3554
MsHel-3b	gb|AIXA01014986.1|:12177-12277
MsHel-3b	gb|AIXA01014090.1|:12120-12238
MsHel-3b	gb|AIXA01013219.1|:19237-19369
MsHel-3b	gb|AIXA01012120.1|:5801-5925
MsHel-3b	gb|AIXA01011733.1|:48965-49079
MsHel-3b	gb|AIXA01011733.1|:3217-3299
MsHel-3b	gb|AIXA01010847.1|:23881-24046
MsHel-3b	gb|AIXA01010081.1|:3559-3668
MsHel-3b	gb|AIXA01009852.1|:2725-2813
MsHel-3b	gb|AIXA01009393.1|:10099-10207
MsHel-3b	gb|AIXA01007478.1|:14618-14711
MsHel-3b	gb|AIXA01007375.1|:9891-13586
MsHel-3b	gb|AIXA01006596.1|:18021-18140
MsHel-3b	gb|AIXA01006143.1|:24960-25044
MsHel-3b	gb|AIXA01004815.1|:9451-9543
MsHel-3b	gb|AIXA01004263.1|:2104-2211
MsHel-3b	gb|AIXA01004028.1|:8864-14231
MsHel-3b	gb|AIXA01003503.1|:4054-4148
MsHel-3b	gb|AIXA01003338.1|:17925-18009
MsHel-3b	gb|AIXA01003278.1|:93088-93179

MsHel-3b	gb|AIXA01003251.1|:12501-12592
MsHel-3b	gb|AIXA01003074.1|:9634-9731
MsHel-3b	gb|AIXA01002846.1|:64345-64432
MsHel-3b	gb|AIXA01002838.1|:9607-9730
MsHel-3b	gb|AIXA01002303.1|:7998-8086
MsHel-3b	gb|AIXA01001487.1|:2885-2969
MsHel-3b	gb|AIXA01001090.1|:15523-18394
MsHel-3b	gb|AIXA01001061.1|:23564-23648
MsHel-3b	gb|AIXA01000894.1|:3790-3925
MsHel-3b	gb|AIXA01021705.1|:1160-1239
MsHel-3b	gb|AIXA01017936.1|:9765-9864
MsHel-3b	gb|AIXA01015838.1|:7690-7792
MsHel-3b	gb|AIXA01014244.1|:20863-20958
MsHel-3b	gb|AIXA01012776.1|:5634-5713
MsHel-3b	gb|AIXA01010882.1|:738-866
MsHel-3b	gb|AIXA01010713.1|:10980-11106
MsHel-3b	gb|AIXA01010705.1|:808-906
MsHel-3b	gb|AIXA01009149.1|:338-421
MsHel-3b	gb|AIXA01008334.1|:10061-10144
MsHel-3b	gb|AIXA01007197.1|:17804-17934
MsHel-3b	gb|AIXA01006791.1|:21285-21415
MsHel-3b	gb|AIXA01005781.1|:13917-14021
MsHel-3b	gb|AIXA01005128.1|:45951-46078
MsHel-3b	gb|AIXA01004888.1|:75-224
MsHel-3b	gb|AIXA01004654.1|:49624-49743
MsHel-3b	gb|AIXA01004598.1|:14407-14506
MsHel-3b	gb|AIXA01003983.1|:3961-4040
MsHel-3b	gb|AIXA01003887.1|:8530-8661
MsHel-3b	gb|AIXA01003833.1|:8587-8710
MsHel-3b	gb|AIXA01003805.1|:5434-6667
MsHel-3b	gb|AIXA01002708.1|:19292-19389
MsHel-3b	gb|AIXA01002505.1|:21186-21263
MsHel-3b	gb|AIXA01001671.1|:12447-12550
MsHel-3b	gb|AIXA01001562.1|:4934-5057
MsHel-3b	gb|AIXA01001112.1|:5840-5927
MsHel-3b	gb|AIXA01000083.1|:7641-7720
MsHel-3b	gb|AIXA01025555.1|:834-920
MsHel-3b	gb|AIXA01022959.1|:1458-1535
MsHel-3b	gb|AIXA01022284.1|:264-1230
MsHel-3b	gb|AIXA01015274.1|:10091-10173
MsHel-3b	gb|AIXA01015252.1|:8143-8967
MsHel-3b	gb|AIXA01013217.1|:741-868
MsHel-3b	gb|AIXA01009662.1|:8655-8737
MsHel-3b	gb|AIXA01009049.1|:14740-14825
MsHel-3b	gb|AIXA01007630.1|:56064-56146
MsHel-3b	gb|AIXA01007334.1|:39058-39199
MsHel-3b	gb|AIXA01007262.1|:29054-29143
MsHel-3b	gb|AIXA01006944.1|:15208-15290

MsHel-3b	gb|AIXA01006413.1|:30029-30116
MsHel-3b	gb|AIXA01006238.1|:2102-2219
MsHel-3b	gb|AIXA01006013.1|:37069-37154
MsHel-3b	gb|AIXA01005734.1|:10851-11301
MsHel-3b	gb|AIXA01005535.1|:13393-13495
MsHel-3b	gb|AIXA01004034.1|:214137-214233
MsHel-3b	gb|AIXA01003130.1|:1188-1273
MsHel-3b	gb|AIXA01002057.1|:51976-52074
MsHel-3b	gb|AIXA01000045.1|:125-215
MsHel-3b	gb|AIXA01033747.1|:83-165
MsHel-3b	gb|AIXA01032437.1|:130-211
MsHel-3b	gb|AIXA01023269.1|:1252-1353
MsHel-3b	gb|AIXA01019653.1|:822-927
MsHel-3b	gb|AIXA01017148.1|:1126-1247
MsHel-3b	gb|AIXA01015347.1|:7177-7314
MsHel-3b	gb|AIXA01014154.1|:3033-3127
MsHel-3b	gb|AIXA01012190.1|:24364-24446
MsHel-3b	gb|AIXA01011835.1|:4861-4949
MsHel-3b	gb|AIXA01011313.1|:35462-35550
MsHel-3b	gb|AIXA01009296.1|:22684-22773
MsHel-3b	gb|AIXA01006241.1|:13219-13328
MsHel-3b	gb|AIXA01006237.1|:11307-11395
MsHel-3b	gb|AIXA01005640.1|:6805-6913
MsHel-3b	gb|AIXA01004688.1|:22227-22311
MsHel-3b	gb|AIXA01003314.1|:4271-4418
MsHel-3b	gb|AIXA01001970.1|:5041-5174
MsHel-3b	gb|AIXA01000507.1|:18730-18853
MsHel-3b	gb|AIXA01000255.1|:869-1052
MsHel-3b	gb|AIXA01026715.1|:440-562
MsHel-3b	gb|AIXA01013709.1|:15477-15567
MsHel-3b	gb|AIXA01013433.1|:1-81
MsHel-3b	gb|AIXA01013389.1|:1786-1866
MsHel-3b	gb|AIXA01013388.1|:2057-2137
MsHel-3b	gb|AIXA01009982.1|:3745-6055
MsHel-3b	gb|AIXA01009529.1|:15511-15595
MsHel-3b	gb|AIXA01009166.1|:11596-11676
MsHel-3b	gb|AIXA01008859.1|:8176-8256
MsHel-3b	gb|AIXA01008565.1|:58047-58138
MsHel-3b	gb|AIXA01008238.1|:16796-16876
MsHel-3b	gb|AIXA01007637.1|:4623-4707
MsHel-3b	gb|AIXA01007010.1|:1275-1354
MsHel-3b	gb|AIXA01006430.1|:69761-69843
MsHel-3b	gb|AIXA01005073.1|:20324-20444
MsHel-3b	gb|AIXA01037209.1|:444-518
MsHel-3b	gb|AIXA01030887.1|:514-597
MsHel-3b	gb|AIXA01026018.1|:365-448
MsHel-3b	gb|AIXA01024171.1|:787-874
MsHel-3b	gb|AIXA01017292.1|:10346-10432

MsHel-3b	gb|AIXA01016184.1|:6855-6942
MsHel-3b	gb|AIXA01016013.1|:8942-9025
MsHel-3b	gb|AIXA01014096.1|:23426-23509
MsHel-3b	gb|AIXA01012008.1|:8405-8517
MsHel-3b	gb|AIXA01011090.1|:14568-14685
MsHel-3b	gb|AIXA01010987.1|:3679-3770
MsHel-3b	gb|AIXA01009249.1|:1193-1276
MsHel-3b	gb|AIXA01007234.1|:9410-9521
MsHel-3b	gb|AIXA01006175.1|:70462-70541
MsHel-3b	gb|AIXA01006008.1|:24601-24695
MsHel-3b	gb|AIXA01005884.1|:329-450
MsHel-3b	gb|AIXA01005701.1|:1822-1979
MsHel-3b	gb|AIXA01002735.1|:13447-13534
MsHel-3b	gb|AIXA01001287.1|:18976-19061
MsHel-3b	gb|AIXA01000956.1|:27340-27443
MsHel-3b	gb|AIXA01024678.1|:71-165
MsHel-3b	gb|AIXA01017466.1|:1396-1484
MsHel-3b	gb|AIXA01017156.1|:3026-3103
MsHel-3b	gb|AIXA01016748.1|:3766-3856
MsHel-3b	gb|AIXA01016180.1|:6763-6891
MsHel-3b	gb|AIXA01015316.1|:7895-7972
MsHel-3b	gb|AIXA01012049.1|:22130-22212
MsHel-3b	gb|AIXA01011584.1|:3970-4071
MsHel-3b	gb|AIXA01009465.1|:4150-4238
MsHel-3b	gb|AIXA01009158.1|:8049-8166
MsHel-3b	gb|AIXA01007513.1|:82713-82807
MsHel-3b	gb|AIXA01007323.1|:21222-21317
MsHel-3b	gb|AIXA01006152.1|:42839-42993
MsHel-3b	gb|AIXA01005952.1|:5006-5083
MsHel-3b	gb|AIXA01005833.1|:16641-16726
MsHel-3b	gb|AIXA01005827.1|:37741-37862
MsHel-3b	gb|AIXA01004198.1|:40-127
MsHel-3b	gb|AIXA01003347.1|:48744-48847
MsHel-3b	gb|AIXA01003271.1|:6977-7059
MsHel-3b	gb|AIXA01002336.1|:9913-10007
MsHel-3b	gb|AIXA01002085.1|:24726-24811
MsHel-3b	gb|AIXA01000800.1|:1984-2069
MsHel-3b	gb|AIXA01015662.1|:17067-17147
MsHel-3b	gb|AIXA01009831.1|:1354-1450
MsHel-3b	gb|AIXA01008248.1|:29165-29246
MsHel-3b	gb|AIXA01007706.1|:23730-23828
MsHel-3b	gb|AIXA01007123.1|:3020-3098
MsHel-3b	gb|AIXA01006549.1|:2370-2475
MsHel-3b	gb|AIXA01003832.1|:19497-19594
MsHel-3b	gb|AIXA01003072.1|:10524-10644
MsHel-3b	gb|AIXA01000602.1|:17945-18025
MsHel-3b	gb|AIXA01023481.1|:47-138
MsHel-3b	gb|AIXA01018451.1|:1939-2046

MsHel-3b	gb|AIXA01017568.1|:9572-12971
MsHel-3b	gb|AIXA01013748.1|:15312-15392
MsHel-3b	gb|AIXA01007991.1|:20817-20921
MsHel-3b	gb|AIXA01004317.1|:7932-8016
MsHel-3b	gb|AIXA01002716.1|:32092-32171
MsHel-3b	gb|AIXA01000794.1|:355-439
MsHel-3b	gb|AIXA01000311.1|:6655-6771
MsHel-3b	gb|AIXA01000063.1|:11555-11639
MsHel-7	gb|AIXA01000810.1|:241466-242328
MsHel-7	gb|AIXA01005946.1|:14748-15625
MsHel-7	gb|AIXA01005538.1|:2325-2829
MsHel-7	gb|AIXA01005228.1|:135980-136826
MsHel-7	gb|AIXA01003205.1|:9839-10685
MsHel-7	gb|AIXA01003182.1|:25737-26582
MsHel-7	gb|AIXA01000824.1|:12029-12859
MsHel-7	gb|AIXA01000611.1|:63976-64821
MsHel-7	gb|AIXA01008170.1|:19365-20217
MsHel-7	gb|AIXA01011407.1|:4647-5486
MsHel-7	gb|AIXA01010632.1|:8575-9424
MsHel-7	gb|AIXA01012866.1|:7665-8515
MsHel-7	gb|AIXA01009401.1|:92015-92868
MsHel-7	gb|AIXA01007357.1|:6526-7394
MsHel-7	gb|AIXA01003376.1|:11615-12461
MsHel-7	gb|AIXA01004815.1|:35422-36121
MsHel-7	gb|AIXA01002431.1|:12410-13262
MsHel-7	gb|AIXA01023891.1|:19-869
MsHel-7	gb|AIXA01012549.1|:33230-34071
MsHel-7	gb|AIXA01011030.1|:17078-17779
MsHel-7	gb|AIXA01008782.1|:58266-59108
MsHel-7	gb|AIXA01008263.1|:36481-36966
MsHel-7	gb|AIXA01008263.1|:21150-22004
MsHel-7	gb|AIXA01007949.1|:15417-16271
MsHel-7	gb|AIXA01005099.1|:4644-5501
MsHel-7	gb|AIXA01000443.1|:56657-57503
MsHel-7	gb|AIXA01009906.1|:3492-4363
MsHel-7	gb|AIXA01012302.1|:5672-6518
MsHel-7	gb|AIXA01009780.1|:2551-3401
MsHel-7	gb|AIXA01007197.1|:50584-51417
MsHel-7	gb|AIXA01006061.1|:68503-69324
MsHel-7	gb|AIXA01005067.1|:85804-86628
MsHel-7	gb|AIXA01004818.1|:78041-78901
MsHel-7	gb|AIXA01004818.1|:67147-67268
MsHel-7	gb|AIXA01003690.1|:55229-56063
MsHel-7	gb|AIXA01003614.1|:2247-2948
MsHel-7	gb|AIXA01002967.1|:7634-8469
MsHel-7	gb|AIXA01002390.1|:5827-6665
MsHel-7	gb|AIXA01000706.1|:18422-19266
MsHel-7	gb|AIXA01000627.1|:63275-64122

MsHel-7	gb|AIXA01006860.1|:23788-24480
MsHel-7	gb|AIXA01005346.1|:23199-24063
MsHel-7	gb|AIXA01004595.1|:9293-10141
MsHel-7	gb|AIXA01003847.1|:16149-16992
MsHel-7	gb|AIXA01006271.1|:34416-35264
MsHel-7	gb|AIXA01005266.1|:36454-37286
MsHel-7	gb|AIXA01000688.1|:111582-112398
MsHel-7	gb|AIXA01012231.1|:35404-36105
MsHel-7	gb|AIXA01011987.1|:32218-33083
MsHel-7	gb|AIXA01011987.1|:798-1311
MsHel-7	gb|AIXA01010197.1|:13262-14140
MsHel-7	gb|AIXA01008262.1|:36289-36552
MsHel-7	gb|AIXA01008262.1|:870-1723
MsHel-7	gb|AIXA01005226.1|:16831-17682
MsHel-7	gb|AIXA01004165.1|:17770-18615
MsHel-7	gb|AIXA01002605.1|:5664-6508
MsHel-7	gb|AIXA01002248.1|:50338-50848
MsHel-7	gb|AIXA01007019.1|:13337-14177
MsHel-7	gb|AIXA01004809.1|:53213-54080
MsHel-7	gb|AIXA01014919.1|:16052-16908
MsHel-7	gb|AIXA01011693.1|:2930-3789
MsHel-7	gb|AIXA01005738.1|:1071-1834
MsHel-7	gb|AIXA01001514.1|:5871-6701
MsHel-7	gb|AIXA01011995.1|:7353-8207
MsHel-7	gb|AIXA01008403.1|:1955-2797
MsHel-7	gb|AIXA01007681.1|:95120-95616
MsHel-7	gb|AIXA01007642.1|:56124-56985
MsHel-7	gb|AIXA01006856.1|:6849-7691
MsHel-7	gb|AIXA01006413.1|:20492-21333
MsHel-7	gb|AIXA01006054.1|:679-1520
MsHel-7	gb|AIXA01004642.1|:5296-6140
MsHel-7	gb|AIXA01003997.1|:33133-33936
MsHel-7	gb|AIXA01003997.1|:1056-1890
MsHel-7	gb|AIXA01003117.1|:7586-8467
MsHel-7	gb|AIXA01002788.1|:371-1237
MsHel-7	gb|AIXA01002047.1|:11894-12739
MsHel-7	gb|AIXA01000557.1|:10809-11651
MsHel-7	gb|AIXA01012497.1|:2527-3373
MsHel-7	gb|AIXA01021363.1|:591-1426
MsHel-7	gb|AIXA01000198.1|:48141-48999
MsHel-7	gb|AIXA01002969.1|:55938-56780
MsHel-7	gb|AIXA01024319.1|:441-1341
MsHel-7	gb|AIXA01014296.1|:915-1777
MsHel-7	gb|AIXA01012032.1|:2158-3008
MsHel-7	gb|AIXA01003628.1|:4216-5059
MsHel-7	gb|AIXA01003349.1|:40054-40895
MsHel-7	gb|AIXA01003187.1|:9741-10440
MsHel-7	gb|AIXA01001991.1|:13587-14432

MsHel-7	gb|AIXA01000775.1|:43711-44560
MsHel-7	gb|AIXA01002500.1|:135702-136542
MsHel-7	gb|AIXA01001123.1|:23144-23639
MsHel-7	gb|AIXA01001123.1|:9120-9962
MsHel-7	gb|AIXA01000318.1|:44843-45679
MsHel-7	gb|AIXA01009360.1|:86055-86895
MsHel-7	gb|AIXA01001136.1|:13122-13979
MsHel-7	gb|AIXA01018499.1|:2791-3630
MsHel-7	gb|AIXA01003689.1|:32250-33089
MsHel-7	gb|AIXA01001296.1|:107485-108331
MsHel-7	gb|AIXA01023873.1|:407-1242
MsHel-7	gb|AIXA01009298.1|:266-1071
MsHel-7	gb|AIXA01008248.1|:31338-32288
MsHel-7	gb|AIXA01007205.1|:62389-63235
MsHel-7	gb|AIXA01004082.1|:58949-59784
MsHel-7	gb|AIXA01000196.1|:48143-48977
MsHel-7	gb|AIXA01015122.1|:4163-5011
MsHel-7	gb|AIXA01008075.1|:4341-5133
MsHel-7	gb|AIXA01001370.1|:81926-82765
MsHel-7	gb|AIXA01018767.1|:1734-2579
MsHel-7	gb|AIXA01004564.1|:10218-11049
MsHel-7	gb|AIXA01014346.1|:31018-31518
MsHel-7	gb|AIXA01012547.1|:9352-10191
MsHel-7	gb|AIXA01017511.1|:6190-7017
MsHel-7	gb|AIXA01008442.1|:28848-29180
MsHel-7	gb|AIXA01004680.1|:2435-3267
MsHel-7	gb|AIXA01002748.1|:141164-142016
MsHel-7	gb|AIXA01000738.1|:12101-12941
MsHel-7	gb|AIXA01000763.1|:17609-18459
MsHel-7	gb|AIXA01017076.1|:5663-6515
MsHel-7	gb|AIXA01001810.1|:18624-19441
MsHel-7	gb|AIXA01007328.1|:5049-5164
MsHel-7	gb|AIXA01004011.1|:9538-10387
MsHel-7	gb|AIXA01003219.1|:8524-9360
MsHel-7	gb|AIXA01018479.1|:2371-3205
MsHel-7	gb|AIXA01002968.1|:38551-39250
MsHel-7	gb|AIXA01001494.1|:44988-45827
MsHel-7	gb|AIXA01009060.1|:6113-6653
MsHel-7	gb|AIXA01006697.1|:1293-2177
MsHel-7	gb|AIXA01011747.1|:2274-3133
MsHel-7	gb|AIXA01008378.1|:32566-33455
MsHel-7	gb|AIXA01005194.1|:18530-19386
MsHel-7	gb|AIXA01003781.1|:1293-2128
MsHel-7	gb|AIXA01002819.1|:11479-12340
MsHel-7	gb|AIXA01011496.1|:2667-3526
MsHel-7	gb|AIXA01004879.1|:196-1038
MsHel-7	gb|AIXA01002701.1|:33267-34134
MsHel-7	gb|AIXA01000972.1|:38759-39596

MsHel-7	gb|AIXA01001697.1|:32619-33455
MsHel-7	gb|AIXA01004980.1|:86282-87118
MsHel-7	gb|AIXA01000877.1|:24169-25023
MsHel-7	gb|AIXA01012436.1|:7279-8117
MsHel-7	gb|AIXA01009775.1|:35216-36041
MsHel-7	gb|AIXA01006979.1|:2624-3601
MsHel-7	gb|AIXA01006405.1|:1013-1855
MsHel-7	gb|AIXA01000251.1|:135014-135845
MsHel-7	gb|AIXA01006855.1|:4244-5065
MsHel-7	gb|AIXA01010076.1|:43977-44835
MsHel-7	gb|AIXA01008251.1|:3671-4355
MsHel-7	gb|AIXA01002469.1|:30175-31005
MsHel-7	gb|AIXA01010101.1|:10784-11447
MsHel-7	gb|AIXA01021964.1|:205-1058
MsHel-7	gb|AIXA01005485.1|:4092-4937
MsHel-7	gb|AIXA01000828.1|:74673-75509
MsHel-7	gb|AIXA01009168.1|:25981-26841
MsHel-7	gb|AIXA01006940.1|:31122-31780
MsHel-7	gb|AIXA01012049.1|:53658-54179
MsHel-7	gb|AIXA01004158.1|:798-1651
MsHel-7	gb|AIXA01004099.1|:11601-12400
MsHel-7	gb|AIXA01025492.1|:2-845
MsHel-7	gb|AIXA01023517.1|:442-1427
MsHel-7	gb|AIXA01009338.1|:5365-6213
MsHel-7	gb|AIXA01007371.1|:2845-3492
MsHel-7	gb|AIXA01012191.1|:24424-25504
MsHel-7	gb|AIXA01012191.1|:4410-4944
MsHel-7	gb|AIXA01000528.1|:5190-6045
MsHel-7	gb|AIXA01007461.1|:4812-5657
MsHel-7	gb|AIXA01012633.1|:8328-9147
MsHel-7	gb|AIXA01006021.1|:46958-47786
MsHel-7	gb|AIXA01023241.1|:892-1532
MsHel-7	gb|AIXA01002232.1|:27897-28919
MsHel-7	gb|AIXA01001362.1|:13581-14080
MsHel-7	gb|AIXA01003624.1|:50738-51600
MsHel-7	gb|AIXA01009703.1|:49329-50431
MsHel-7	gb|AIXA01009703.1|:2218-3497
MsHel-7	gb|AIXA01003873.1|:4638-5458
MsHel-7	gb|AIXA01007513.1|:22103-23041
MsHel-7	gb|AIXA01030938.1|:118-770
MsHel-7	gb|AIXA01008363.1|:174753-175445
MsHel-7	gb|AIXA01008363.1|:129626-130122
MsHel-7	gb|AIXA01008363.1|:29738-30287
MsHel-7	gb|AIXA01004456.1|:56359-56959
MsHel-7	gb|AIXA01011728.1|:23933-26050
MsHel-7	gb|AIXA01018979.1|:1898-2644
MsHel-7	gb|AIXA01004758.1|:8621-9259
MsHel-7	gb|AIXA01000819.1|:56213-57041

MsHel-7	gb|AIXA01013585.1|:720-1327
MsHel-7	gb|AIXA01004482.1|:39913-40682
MsHel-7	gb|AIXA01002411.1|:12047-12803
MsHel-7	gb|AIXA01007139.1|:33388-33888
MsHel-7	gb|AIXA01007139.1|:9048-9653
MsHel-7	gb|AIXA01004271.1|:13879-14696
MsHel-7	gb|AIXA01011760.1|:1638-2460
MsHel-7	gb|AIXA01013430.1|:24262-25078
MsHel-7	gb|AIXA01010019.1|:7431-8178
MsHel-7	gb|AIXA01010152.1|:29627-30589
MsHel-7	gb|AIXA01007034.1|:26113-27321
MsHel-7	gb|AIXA01002626.1|:10310-11155
MsHel-7	gb|AIXA01008313.1|:32786-33375
MsHel-7	gb|AIXA01002651.1|:24077-24927
MsHel-7	gb|AIXA01000215.1|:19271-20149
MsHel-7	gb|AIXA01006563.1|:1766-2324
MsHel-7	gb|AIXA01006898.1|:30339-31051
MsHel-7	gb|AIXA01005595.1|:3432-4273
MsHel-7	gb|AIXA01004108.1|:158650-159491
MsHel-7	gb|AIXA01004108.1|:108241-108698
MsHel-7	gb|AIXA01004108.1|:8210-8796
MsHel-7	gb|AIXA01002313.1|:41195-42013
MsHel-7	gb|AIXA01000030.1|:20275-21089
MsHel-7	gb|AIXA01007070.1|:17242-18068
MsHel-7	gb|AIXA01005779.1|:12130-12944
MsHel-7	gb|AIXA01009522.1|:1-703
MsHel-7	gb|AIXA01007639.1|:6194-7404
MsHel-7	gb|AIXA01014245.1|:33012-33714
MsHel-7	gb|AIXA01011106.1|:18222-18775
MsHel-7	gb|AIXA01006581.1|:25174-26000
MsHel-7	gb|AIXA01002718.1|:93435-94277
MsHel-7	gb|AIXA01002718.1|:68034-68534
MsHel-7	gb|AIXA01002615.1|:13832-14669
MsHel-7	gb|AIXA01023733.1|:854-1444
MsHel-7	gb|AIXA01005594.1|:1871-2687
MsHel-7	gb|AIXA01000963.1|:65344-66183
MsHel-7	gb|AIXA01026016.1|:629-1165
MsHel-7	gb|AIXA01008395.1|:20613-21164
MsHel-7	gb|AIXA01001898.1|:16185-17057
MsHel-7	gb|AIXA01010904.1|:2043-2842
MsHel-7	gb|AIXA01009322.1|:17816-18480
MsHel-7	gb|AIXA01008665.1|:9482-10344
MsHel-7	gb|AIXA01007286.1|:38195-39077
MsHel-7	gb|AIXA01005741.1|:6039-6861
MsHel-7	gb|AIXA01004894.1|:90013-90287
MsHel-7	gb|AIXA01004894.1|:29441-30269
MsHel-7	gb|AIXA01002737.1|:44993-45843
MsHel-7	gb|AIXA01001007.1|:4741-5560

MsHel-7	gb|AIXA01000480.1|:32343-33134
MsHel-7	gb|AIXA01030501.1|:275-832
MsHel-7	gb|AIXA01008915.1|:24296-25124
MsHel-7	gb|AIXA01001118.1|:4142-4968
MsHel-7	gb|AIXA01013676.1|:788-1588
MsHel-7	gb|AIXA01007723.1|:12433-16299
MsHel-7	gb|AIXA01006863.1|:8518-9527
MsHel-7	gb|AIXA01004989.1|:26421-27311
MsHel-7	gb|AIXA01004989.1|:1-107
MsHel-7	gb|AIXA01013650.1|:25533-26607
MsHel-7	gb|AIXA01002971.1|:19219-20044
MsHel-7	gb|AIXA01002971.1|:6074-6700
MsHel-7	gb|AIXA01002377.1|:111089-111909
MsHel-7	gb|AIXA01002377.1|:77653-78473
MsHel-7	gb|AIXA01000603.1|:9682-11045
MsHel-7	gb|AIXA01032227.1|:178-745
MsHel-7	gb|AIXA01016056.1|:12497-13339
MsHel-7	gb|AIXA01019486.1|:1-90
MsHel-7	gb|AIXA01000747.1|:7841-8700
MsHel-7	gb|AIXA01002379.1|:28732-29510
MsHel-7	gb|AIXA01007798.1|:23555-24295
MsHel-7	gb|AIXA01002401.1|:35479-36304
MsHel-7	gb|AIXA01000297.1|:72644-73459
MsHel-7	gb|AIXA01008930.1|:46429-47343
MsHel-7	gb|AIXA01007706.1|:24311-25035
MsHel-7	gb|AIXA01007516.1|:53715-54535
MsHel-7	gb|AIXA01006841.1|:25774-26658
MsHel-7	gb|AIXA01001243.1|:136391-136782
MsHel-7	gb|AIXA01028809.1|:126-791
MsHel-7	gb|AIXA01022224.1|:1048-1730
MsHel-7	gb|AIXA01017576.1|:1379-2197
MsHel-7	gb|AIXA01008965.1|:23273-23812
MsHel-7	gb|AIXA01007318.1|:4623-5452
MsHel-7	gb|AIXA01005831.1|:1851-2686
MsHel-7	gb|AIXA01002665.1|:32162-32992
MsHel-7	gb|AIXA01001538.1|:51759-52583
MsHel-7	gb|AIXA01001538.1|:12442-13097
MsHel-7	gb|AIXA01017148.1|:6734-7555
MsHel-7	gb|AIXA01012514.1|:4528-5358
MsHel-7	gb|AIXA01004851.1|:1866-2423
MsHel-7	gb|AIXA01001558.1|:68795-69094
MsHel-7	gb|AIXA01001558.1|:8693-9187
MsHel-7	gb|AIXA01000399.1|:7416-8303
MsHel-7	gb|AIXA01005834.1|:4176-5003
MsHel-7	gb|AIXA01003603.1|:16001-17027
MsHel-7	gb|AIXA01003294.1|:68278-69133
MsHel-7	gb|AIXA01017112.1|:1001-2195
MsHel-7	gb|AIXA01009233.1|:7551-8374

MsHel-7	gb|AIXA01007615.1|:8120-8785
MsHel-7	gb|AIXA01009019.1|:15049-15894
MsHel-7	gb|AIXA01004252.1|:92863-92995
MsHel-7	gb|AIXA01009291.1|:30483-31307
MsHel-7	gb|AIXA01008673.1|:3005-3765
MsHel-7	gb|AIXA01008575.1|:68481-69307
MsHel-7	gb|AIXA01003622.1|:27421-28112
MsHel-7	gb|AIXA01002972.1|:57227-57842
MsHel-7	gb|AIXA01002730.1|:90429-91400
MsHel-7	gb|AIXA01001143.1|:26543-27370
MsHel-7	gb|AIXA01018149.1|:8062-8934
MsHel-7	gb|AIXA01009533.1|:22382-23169
MsHel-7	gb|AIXA01002386.1|:58391-59222
MsHel-7	gb|AIXA01002127.1|:36679-37194
MsHel-7	gb|AIXA01012923.1|:1295-2110
MsHel-7	gb|AIXA01007544.1|:6003-7013
MsHel-7	gb|AIXA01006308.1|:5960-6469
MsHel-7	gb|AIXA01005544.1|:35089-35935
MsHel-7	gb|AIXA01005544.1|:5969-6927
MsHel-7	gb|AIXA01000069.1|:262804-263304
MsHel-7	gb|AIXA01002591.1|:14400-14736
MsHel-7	gb|AIXA01002423.1|:6804-7621
MsHel-7	gb|AIXA01004893.1|:21829-22190
MsHel-7	gb|AIXA01004893.1|:5038-5990
MsHel-7	gb|AIXA01001032.1|:33402-38179
MsHel-7	gb|AIXA01016258.1|:6865-7690
MsHel-7	gb|AIXA01007933.1|:6438-7270
MsHel-7	gb|AIXA01007593.1|:34425-35252
MsHel-7	gb|AIXA01005197.1|:20152-22383
MsHel-7	gb|AIXA01000909.1|:133018-133827
MsHel-7	gb|AIXA01010323.1|:103113-103771
MsHel-7	gb|AIXA01006667.1|:20447-21095
MsHel-7	gb|AIXA01001685.1|:26952-27454
MsHel-7	gb|AIXA01000703.1|:67316-68152
MsHel-7	gb|AIXA01000075.1|:4547-5543
MsHel-7	gb|AIXA01016481.1|:5252-6095
MsHel-7	gb|AIXA01004852.1|:73375-74195
MsHel-7	gb|AIXA01004852.1|:77-197
MsHel-7	gb|AIXA01030952.1|:287-811
MsHel-7	gb|AIXA01004619.1|:51714-52446
MsHel-7	gb|AIXA01000293.1|:658-1559
MsHel-7	gb|AIXA01004183.1|:265-1138
MsHel-7	gb|AIXA01003993.1|:86282-87105
MsHel-7	gb|AIXA01011979.1|:31569-32363
MsHel-7	gb|AIXA01009169.1|:20305-21129
MsHel-7	gb|AIXA01002375.1|:20495-21313
MsHel-7	gb|AIXA01011065.1|:10922-11740
MsHel-7	gb|AIXA01004406.1|:10225-11222

MsHel-7	gb|AIXA01007362.1|:20714-21492
MsHel-7	gb|AIXA01003595.1|:85349-86149
MsHel-7	gb|AIXA01000907.1|:111823-112386
MsHel-7	gb|AIXA01008037.1|:2292-3443
MsHel-7	gb|AIXA01004596.1|:48783-49614
MsHel-7	gb|AIXA01028434.1|:481-964
MsHel-7	gb|AIXA01010849.1|:83665-84496
MsHel-7	gb|AIXA01004300.1|:52426-53216
MsHel-7	gb|AIXA01000668.1|:23192-23915
MsHel-7	gb|AIXA01011197.1|:20575-21067
MsHel-7	gb|AIXA01004179.1|:2212-2703
MsHel-7	gb|AIXA01011501.1|:19062-19657
MsHel-7	gb|AIXA01009297.1|:841-1634
MsHel-7	gb|AIXA01002503.1|:21139-21989
MsHel-7	gb|AIXA01011389.1|:3907-4539
MsHel-7	gb|AIXA01010865.1|:28933-29271
MsHel-7	gb|AIXA01004089.1|:3569-4206
MsHel-7	gb|AIXA01011253.1|:29542-30152
MsHel-7	gb|AIXA01003469.1|:8998-9931
MsHel-7	gb|AIXA01001933.1|:30588-31467
MsHel-7	gb|AIXA01002858.1|:38441-39335
MsHel-7	gb|AIXA01000975.1|:13670-14469
MsHel-7	gb|AIXA01008256.1|:1-620
MsHel-7	gb|AIXA01001173.1|:505-1911
MsHel-7	gb|AIXA01010578.1|:2847-3686
MsHel-7	gb|AIXA01006207.1|:20192-21318
MsHel-7	gb|AIXA01002451.1|:3608-3934
MsHel-7	gb|AIXA01036039.1|:102-576
MsHel-7	gb|AIXA01022051.1|:1179-1647
MsHel-7	gb|AIXA01009701.1|:5830-6565
MsHel-7	gb|AIXA01006837.1|:4775-5428
MsHel-7	gb|AIXA01006548.1|:23909-24893
MsHel-7	gb|AIXA01011362.1|:2-509
MsHel-7	gb|AIXA01001675.1|:63700-63935
MsHel-7	gb|AIXA01000327.1|:22443-23063
MsHel-7	gb|AIXA01002697.1|:7618-8232
MsHel-7	gb|AIXA01008663.1|:20590-21413
MsHel-7	gb|AIXA01004153.1|:46834-47737
MsHel-7	gb|AIXA01006720.1|:23323-23941
MsHel-7	gb|AIXA01002580.1|:94152-94750
MsHel-7	gb|AIXA01005896.1|:16668-17131
MsHel-7	gb|AIXA01007630.1|:34461-35451
MsHel-7	gb|AIXA01013054.1|:1880-2400
MsHel-7	gb|AIXA01001543.1|:10081-10663
MsHel-7	gb|AIXA01000255.1|:14234-14954
MsHel-7	gb|AIXA01000255.1|:1-114
MsHel-7	gb|AIXA01000212.1|:14627-15385
MsHel-7	gb|AIXA01006754.1|:22896-23613

MsHel-7	gb|AIXA01012097.1|:21143-21623
MsHel-7	gb|AIXA01009162.1|:40108-40849
MsHel-7	gb|AIXA01025970.1|:1-586
MsHel-7	gb|AIXA01023644.1|:1018-1464
MsHel-7	gb|AIXA01021424.1|:607-1104
MsHel-7	gb|AIXA01001825.1|:16052-16679
MsHel-7	gb|AIXA01000049.1|:2496-3609
MsHel-7	gb|AIXA01031060.1|:368-801
MsHel-7	gb|AIXA01037551.1|:54-523
MsHel-7	gb|AIXA01004628.1|:24486-25059
MsHel-7	gb|AIXA01004628.1|:11247-11789
MsHel-7	gb|AIXA01011655.1|:13154-13884
MsHel-7	gb|AIXA01000098.1|:77666-78610
MsHel-7	gb|AIXA01000900.1|:15861-16429
MsHel-7	gb|AIXA01010656.1|:30241-30770
MsHel-7	gb|AIXA01009344.1|:967-1564
MsHel-7	gb|AIXA01004293.1|:21828-22324
MsHel-7	gb|AIXA01004464.1|:1-585
MsHel-7	gb|AIXA01015762.1|:9737-10307
MsHel-7	gb|AIXA01004867.1|:63049-63615
MsHel-7	gb|AIXA01003634.1|:16816-17381
MsHel-7	gb|AIXA01007225.1|:28572-29168
MsHel-7	gb|AIXA01009403.1|:2137-2366
MsHel-7	gb|AIXA01003815.1|:10700-11793
MsHel-7	gb|AIXA01027213.1|:653-1057
MsHel-7	gb|AIXA01002847.1|:24367-25071
MsHel-7	gb|AIXA01026176.1|:687-1149
MsHel-7	gb|AIXA01002384.1|:38635-39470
MsHel-7	gb|AIXA01008491.1|:33857-34546
MsHel-7	gb|AIXA01004656.1|:110955-111495
MsHel-7	gb|AIXA01007645.1|:2756-3295
MsHel-7	gb|AIXA01006796.1|:60900-61739
MsHel-7	gb|AIXA01002977.1|:62988-63546
MsHel-7	gb|AIXA01013619.1|:22663-23224
MsHel-7	gb|AIXA01007927.1|:3835-4398
MsHel-7	gb|AIXA01006893.1|:36483-37256
MsHel-7	gb|AIXA01004988.1|:1925-7309
MsHel-7	gb|AIXA01002532.1|:1-573
MsHel-7	gb|AIXA01003070.1|:44324-44617
MsHel-7	gb|AIXA01003070.1|:4503-5293
MsHel-7	gb|AIXA01034390.1|:1-468
MsHel-7	gb|AIXA01006930.1|:34012-34539
MsHel-7	gb|AIXA01006529.1|:60948-61490
MsHel-7	gb|AIXA01002286.1|:8334-8785
MsHel-7	gb|AIXA01036500.1|:137-562
MsHel-7	gb|AIXA01002170.1|:48489-49179
MsHel-7	gb|AIXA01000111.1|:118177-118728
MsHel-7	gb|AIXA01003578.1|:8710-9816

MsHel-7	gb|AIXA01006772.1|:23460-24025
MsHel-7	gb|AIXA01018036.1|:138-660
MsHel-7	gb|AIXA01010261.1|:4443-5136
MsHel-7	gb|AIXA01009511.1|:16452-16838
MsHel-7	gb|AIXA01007210.1|:13618-13958
MsHel-7	gb|AIXA01007117.1|:6565-7129
MsHel-7	gb|AIXA01007092.1|:53420-54161
MsHel-7	gb|AIXA01006215.1|:44210-44670
MsHel-7	gb|AIXA01001071.1|:12194-14407
MsHel-7	gb|AIXA01007055.1|:2170-2750
MsHel-7	gb|AIXA01014024.1|:4456-5508
MsHel-7	gb|AIXA01009721.1|:3001-3528
MsHel-7	gb|AIXA01007987.1|:47792-48230
MsHel-7	gb|AIXA01011754.1|:2873-4864
MsHel-7	gb|AIXA01011544.1|:2833-3343
MsHel-7	gb|AIXA01003413.1|:2057-2763
MsHel-7	gb|AIXA01000337.1|:55183-55862
MsHel-7	gb|AIXA01015468.1|:19101-19624
MsHel-7	gb|AIXA01007509.1|:370-891
MsHel-7	gb|AIXA01005504.1|:33996-36006
MsHel-7	gb|AIXA01000199.1|:30870-31692
MsHel-7	gb|AIXA01011674.1|:46859-47071
MsHel-7	gb|AIXA01011674.1|:3323-5684
MsHel-7	gb|AIXA01008372.1|:23050-23603
MsHel-7	gb|AIXA01001053.1|:1640-2412
MsHel-7	gb|AIXA01001133.1|:4284-4792
MsHel-7	gb|AIXA01012928.1|:8175-8772
MsHel-7	gb|AIXA01008546.1|:8943-9621
MsHel-7	gb|AIXA01002512.1|:6052-6562
MsHel-7	gb|AIXA01008406.1|:33963-34493
MsHel-7	gb|AIXA01003613.1|:34440-35137
MsHel-7	gb|AIXA01003071.1|:16-540
MsHel-7	gb|AIXA01001660.1|:62940-63326
MsHel-7	gb|AIXA01001346.1|:7659-8302
MsHel-7	gb|AIXA01021271.1|:1296-2116
MsHel-7	gb|AIXA01003319.1|:42439-42965
MsHel-7	gb|AIXA01012839.1|:3566-4100
MsHel-7	gb|AIXA01008218.1|:6376-6901
MsHel-7	gb|AIXA01007052.1|:1-519
MsHel-7	gb|AIXA01002978.1|:42043-42557
MsHel-7	gb|AIXA01002371.1|:56777-57275
MsHel-7	gb|AIXA01002371.1|:36011-36359
MsHel-7	gb|AIXA01010465.1|:11756-12329
MsHel-7	gb|AIXA01008404.1|:35121-35638
MsHel-7	gb|AIXA01006655.1|:1-501
MsHel-7	gb|AIXA01007241.1|:11976-12493
MsHel-7	gb|AIXA01004517.1|:32039-32716
MsHel-7	gb|AIXA01004517.1|:7608-8139

MsHel-7	gb|AIXA01015133.1|:7307-7828
MsHel-7	gb|AIXA01012196.1|:4856-5204
MsHel-7	gb|AIXA01011282.1|:87117-87627
MsHel-7	gb|AIXA01002756.1|:26515-27066
MsHel-7	gb|AIXA01001674.1|:34056-34554
MsHel-7	gb|AIXA01027429.1|:1-502
MsHel-7	gb|AIXA01010683.1|:4990-5642
MsHel-7	gb|AIXA01007652.1|:20045-20422
MsHel-7	gb|AIXA01007502.1|:1-501
MsHel-7	gb|AIXA01006887.1|:13039-13856
MsHel-7	gb|AIXA01004800.1|:63195-63406
MsHel-7	gb|AIXA01007270.1|:4001-5494
MsHel-7	gb|AIXA01006087.1|:11649-12178
MsHel-7	gb|AIXA01005416.1|:1-542
MsHel-7	gb|AIXA01000593.1|:41188-41673
MsHel-7	gb|AIXA01012784.1|:21581-22098
MsHel-7	gb|AIXA01009059.1|:12256-12778
MsHel-7	gb|AIXA01008028.1|:14884-15384
MsHel-7	gb|AIXA01005383.1|:29062-29540
MsHel-7	gb|AIXA01004925.1|:13919-14421
MsHel-7	gb|AIXA01004522.1|:36-542
MsHel-7	gb|AIXA01004114.1|:66805-67311
MsHel-7	gb|AIXA01000476.1|:17835-18226
MsHel-7	gb|AIXA01010477.1|:55874-56937
MsHel-7	gb|AIXA01010477.1|:25962-27915
MsHel-7	gb|AIXA01003878.1|:21381-21901
MsHel-7	gb|AIXA01002659.1|:1-493
MsHel-7	gb|AIXA01021654.1|:833-1314
MsHel-7	gb|AIXA01001885.1|:3874-4235
MsHel-7	gb|AIXA01010730.1|:20873-21065
MsHel-7	gb|AIXA01010730.1|:9448-10276
MsHel-7	gb|AIXA01001529.1|:19278-19649
MsHel-7	gb|AIXA01009778.1|:22253-22743
MsHel-7	gb|AIXA01034441.1|:265-638
MsHel-7	gb|AIXA01013151.1|:3062-3568
MsHel-7	gb|AIXA01006194.1|:15751-16269
MsHel-7	gb|AIXA01002668.1|:31397-32176
MsHel-7	gb|AIXA01012939.1|:4834-5196
MsHel-7	gb|AIXA01004461.1|:151-683
MsHel-7	gb|AIXA01017727.1|:16131-16973
MsHel-7	gb|AIXA01005956.1|:244-1174
MsHel-7	gb|AIXA01005326.1|:3374-3719
MsHel-7	gb|AIXA01001499.1|:40432-40978
MsHel-7	gb|AIXA01000502.1|:5726-6078
MsHel-7	gb|AIXA01033725.1|:1-475
MsHel-7	gb|AIXA01002990.1|:63571-63748
MsHel-7	gb|AIXA01002990.1|:39155-39680
MsHel-7	gb|AIXA01002505.1|:1274-2120

MsHel-7	gb|AIXA01012402.1|:3589-4418
MsHel-7	gb|AIXA01005053.1|:42314-42671
MsHel-7	gb|AIXA01010208.1|:15644-15988
MsHel-7	gb|AIXA01021091.1|:1853-2192
MsHel-7	gb|AIXA01036495.1|:1-562
MsHel-7	gb|AIXA01000396.1|:1-492
MsHel-7	gb|AIXA01035797.1|:1-492
MsHel-7	gb|AIXA01002462.1|:4-475
MsHel-7	gb|AIXA01000938.1|:28930-29402
MsHel-7	gb|AIXA01018669.1|:8313-8646
MsHel-7	gb|AIXA01018089.1|:3377-3724
MsHel-7	gb|AIXA01010189.1|:20650-21123
MsHel-7	gb|AIXA01001457.1|:7199-7813
MsHel-7	gb|AIXA01000871.1|:30132-30609
MsHel-7	gb|AIXA01022764.1|:1285-1624
MsHel-7	gb|AIXA01036128.1|:2-472
MsHel-7	gb|AIXA01017771.1|:3762-4145
MsHel-7	gb|AIXA01007994.1|:12268-12784
MsHel-7	gb|AIXA01020281.1|:1-488
MsHel-7	gb|AIXA01022101.1|:1-491
MsHel-7	gb|AIXA01002692.1|:18518-18828
MsHel-7	gb|AIXA01008632.1|:23093-23555
MsHel-7	gb|AIXA01008270.1|:2842-3308
MsHel-7	gb|AIXA01037762.1|:161-515
MsHel-7	gb|AIXA01001889.1|:3942-4285
MsHel-7	gb|AIXA01002671.1|:149267-149787
MsHel-7	gb|AIXA01004516.1|:2-479
MsHel-7	gb|AIXA01005473.1|:11204-11683
MsHel-7	gb|AIXA01009828.1|:13870-14200
MsHel-7	gb|AIXA01008310.1|:42284-42610
MsHel-7	gb|AIXA01030123.1|:1-455
MsHel-7	gb|AIXA01007850.1|:12710-13371
MsHel-7	gb|AIXA01007439.1|:2203-2941
MsHel-7	gb|AIXA01007121.1|:7146-7701
MsHel-7	gb|AIXA01003585.1|:85641-86091
MsHel-7	gb|AIXA01003134.1|:2361-2919
MsHel-7	gb|AIXA01009773.1|:29919-30650
MsHel-7	gb|AIXA01012946.1|:28353-28685
MsHel-7	gb|AIXA01005432.1|:1-477
MsHel-7	gb|AIXA01004465.1|:2-291
MsHel-7	gb|AIXA01034877.1|:168-619
MsHel-7	gb|AIXA01033764.1|:326-667
MsHel-7	gb|AIXA01008289.1|:31991-32438
MsHel-7	gb|AIXA01036586.1|:255-558
MsHel-7	gb|AIXA01010151.1|:2-441
MsHel-7	gb|AIXA01002261.1|:1444-1826
MsHel-7	gb|AIXA01011427.1|:244-708
MsHel-7	gb|AIXA01011426.1|:15762-16226

MsHel-7	gb|AIXA01008770.1|:2948-3255
MsHel-7	gb|AIXA01007429.1|:1-472
MsHel-7	gb|AIXA01007428.1|:121699-122016
MsHel-7	gb|AIXA01009755.1|:6646-7190
MsHel-7	gb|AIXA01006673.1|:1-946
MsHel-7	gb|AIXA01006381.1|:4-263
MsHel-7	gb|AIXA01012660.1|:2-411
MsHel-7	gb|AIXA01028505.1|:1-429
MsHel-7	gb|AIXA01010205.1|:1-407
MsHel-7	gb|AIXA01004731.1|:23458-23985
MsHel-7	gb|AIXA01002496.1|:1-454
MsHel-7	gb|AIXA01000762.1|:10108-10637
MsHel-7	gb|AIXA01012409.1|:4254-4832
MsHel-7	gb|AIXA01010866.1|:8964-10679
MsHel-7	gb|AIXA01019621.1|:1662-2134
MsHel-7	gb|AIXA01005347.1|:54550-54956
MsHel-7	gb|AIXA01000351.1|:229-820
MsHel-7	gb|AIXA01004643.1|:1-394
MsHel-7	gb|AIXA01023484.1|:824-1236
MsHel-7	gb|AIXA01009932.1|:11069-11532
MsHel-7	gb|AIXA01005880.1|:2188-2591
MsHel-7	gb|AIXA01001066.1|:1152-1605
MsHel-7	gb|AIXA01013623.1|:20807-21386
MsHel-7	gb|AIXA01003924.1|:56755-57591
MsHel-7	gb|AIXA01018868.1|:7384-7665
MsHel-7	gb|AIXA01007784.1|:3034-3512
MsHel-7	gb|AIXA01001474.1|:69199-69500
MsHel-7	gb|AIXA01002712.1|:33695-33982
MsHel-7	gb|AIXA01023713.1|:1007-1443
MsHel-7	gb|AIXA01000767.1|:52709-53102
MsHel-7	gb|AIXA01002911.1|:26050-26301
MsHel-7	gb|AIXA01010343.1|:28322-28603
MsHel-7	gb|AIXA01001714.1|:1-410
MsHel-7	gb|AIXA01015748.1|:1-414
MsHel-7	gb|AIXA01008522.1|:16378-16662
MsHel-7	gb|AIXA01038051.1|:100-511
MsHel-7	gb|AIXA01019356.1|:1771-2181
MsHel-7	gb|AIXA01013132.1|:11840-12245
MsHel-7	gb|AIXA01001699.1|:16429-16884
MsHel-7	gb|AIXA01004515.1|:31292-31708
MsHel-7	gb|AIXA01024420.1|:537-1348
MsHel-7	gb|AIXA01027721.1|:741-1016
MsHel-7	gb|AIXA01001983.1|:16243-16626
MsHel-7	gb|AIXA01006046.1|:6010-6517
MsHel-7	gb|AIXA01017604.1|:2-414
MsHel-7	gb|AIXA01013617.1|:4-477
MsHel-7	gb|AIXA01006113.1|:34284-34608
MsHel-7	gb|AIXA01013265.1|:6732-7155

MsHel-7	gb|AIXA01013340.1|:16270-16559
MsHel-7	gb|AIXA01011229.1|:2165-2600
MsHel-7	gb|AIXA01013677.1|:8775-9028
MsHel-7	gb|AIXA01000928.1|:1-414
MsHel-7	gb|AIXA01001743.1|:15529-15998
MsHel-7	gb|AIXA01002473.1|:10184-10454
MsHel-7	gb|AIXA01010430.1|:8306-8786
MsHel-7	gb|AIXA01008774.1|:4382-5499
MsHel-7	gb|AIXA01031261.1|:1-426
MsHel-7	gb|AIXA01008432.1|:1323-1815
MsHel-7	gb|AIXA01008739.1|:75379-75777
MsHel-7	gb|AIXA01009262.1|:13061-13453
MsHel-7	gb|AIXA01011686.1|:27330-27824
MsHel-7	gb|AIXA01005578.1|:59012-59646
MsHel-7	gb|AIXA01000803.1|:5044-5279
MsHel-7	gb|AIXA01030911.1|:1-421
MsHel-7	gb|AIXA01021263.1|:1536-2023
MsHel-7	gb|AIXA01017523.1|:5579-5813
MsHel-7	gb|AIXA01004180.1|:1-372
MsHel-7	gb|AIXA01010326.1|:2418-2662
MsHel-7	gb|AIXA01032898.1|:1-419
MsHel-7	gb|AIXA01009299.1|:40-571
MsHel-7	gb|AIXA01002987.1|:9321-9730
MsHel-7	gb|AIXA01004963.1|:1-366
MsHel-7	gb|AIXA01018702.1|:2593-2835
MsHel-7	gb|AIXA01013184.1|:14476-14989
MsHel-7	gb|AIXA01007478.1|:29401-29875
MsHel-7	gb|AIXA01000041.1|:72656-73157
MsHel-7	gb|AIXA01004985.1|:55639-56052
MsHel-7	gb|AIXA01035636.1|:3-347
MsHel-7	gb|AIXA01007817.1|:1-374
MsHel-7	gb|AIXA01002995.1|:21932-22343
MsHel-7	gb|AIXA01001548.1|:25793-26061
MsHel-7	gb|AIXA01010114.1|:9751-10232
MsHel-7	gb|AIXA01001012.1|:9918-10409
MsHel-7	gb|AIXA01004102.1|:22590-23052
MsHel-7	gb|AIXA01002287.1|:1-108
MsHel-7	gb|AIXA01036816.1|:320-550
MsHel-7	gb|AIXA01012509.1|:1-372
MsHel-7	gb|AIXA01010773.1|:1-376
MsHel-7	gb|AIXA01007100.1|:29714-30196
MsHel-7	gb|AIXA01006208.1|:14224-14565
MsHel-7	gb|AIXA01003045.1|:76446-76944
MsHel-7	gb|AIXA01001395.1|:30765-31264
MsHel-7	gb|AIXA01009345.1|:1-385
MsHel-7	gb|AIXA01022679.1|:2-380
MsHel-7	gb|AIXA01019461.1|:2404-2906
MsHel-7	gb|AIXA01011283.1|:10819-11050

MsHel-7	gb|AIXA01002422.1|:5450-5944
MsHel-7	gb|AIXA01007686.1|:43909-44395
MsHel-7	gb|AIXA01000662.1|:108752-109252
MsHel-7	gb|AIXA01000662.1|:88258-88793
MsHel-7	gb|AIXA01028163.1|:480-981
MsHel-7	gb|AIXA01005661.1|:14365-14867
MsHel-7	gb|AIXA01004785.1|:41458-41661
MsHel-7	gb|AIXA01000861.1|:2-365
MsHel-7	gb|AIXA01000230.1|:23419-23918
MsHel-7	gb|AIXA01007585.1|:2318-2799
MsHel-7	gb|AIXA01008443.1|:22313-22813
MsHel-7	gb|AIXA01008018.1|:6414-6931
MsHel-7	gb|AIXA01016442.1|:3576-4093
MsHel-7	gb|AIXA01013621.1|:17234-17577
MsHel-7	gb|AIXA01012415.1|:13321-14984
MsHel-7	gb|AIXA01012415.1|:891-1387
MsHel-7	gb|AIXA01011980.1|:16217-16562
MsHel-7	gb|AIXA01007725.1|:1846-2350
MsHel-7	gb|AIXA01006929.1|:1053-1553
MsHel-7	gb|AIXA01004683.1|:15557-16051
MsHel-7	gb|AIXA01002807.1|:5946-6445
MsHel-7	gb|AIXA01000910.1|:24994-25499
MsHel-7	gb|AIXA01009833.1|:9118-9345
MsHel-7	gb|AIXA01006513.1|:12767-13165
MsHel-7	gb|AIXA01000265.1|:6663-7066
MsHel-7	gb|AIXA01031295.1|:445-788
MsHel-7	gb|AIXA01003986.1|:28036-28546
MsHel-7	gb|AIXA01000959.1|:46663-47175
MsHel-7	gb|AIXA01011732.1|:42139-42638
MsHel-7	gb|AIXA01011732.1|:11685-12185
MsHel-7	gb|AIXA01009283.1|:14805-15305
MsHel-7	gb|AIXA01007077.1|:10996-11496
MsHel-7	gb|AIXA01006143.1|:8508-9005
MsHel-7	gb|AIXA01005899.1|:1-406
MsHel-7	gb|AIXA01004796.1|:12142-12655
MsHel-7	gb|AIXA01001872.1|:4855-5066
MsHel-7	gb|AIXA01001832.1|:12184-12689
MsHel-7	gb|AIXA01001486.1|:10594-10947
MsHel-7	gb|AIXA01009951.1|:16-125
MsHel-7	gb|AIXA01001908.1|:45562-45981
MsHel-7	gb|AIXA01001908.1|:1-92
MsHel-7	gb|AIXA01012952.1|:8779-8979
MsHel-7	gb|AIXA01012124.1|:16480-16978
MsHel-7	gb|AIXA01011409.1|:3420-3922
MsHel-7	gb|AIXA01001219.1|:32690-33131
MsHel-7	gb|AIXA01013484.1|:339-840
MsHel-7	gb|AIXA01011250.1|:1036-1541
MsHel-7	gb|AIXA01002504.1|:4797-5302

MsHel-7	gb|AIXA01006919.1|:5713-6221
MsHel-7	gb|AIXA01006377.1|:22217-22693
MsHel-7	gb|AIXA01001358.1|:15403-16078
MsHel-7	gb|AIXA01000544.1|:3637-4111
MsHel-7	gb|AIXA01007648.1|:5168-5674
MsHel-7	gb|AIXA01002864.1|:25984-26484
MsHel-7	gb|AIXA01019592.1|:2458-2960
MsHel-7	gb|AIXA01010102.1|:99445-99942
MsHel-7	gb|AIXA01032930.1|:514-710
MsHel-7	gb|AIXA01027374.1|:841-1044
MsHel-7	gb|AIXA01009550.1|:19674-20188
MsHel-7	gb|AIXA01004110.1|:40663-41167
MsHel-7	gb|AIXA01002915.1|:30270-30770
MsHel-7	gb|AIXA01001226.1|:71839-72269
MsHel-7	gb|AIXA01007217.1|:34816-35315
MsHel-7	gb|AIXA01006826.1|:20700-21209
MsHel-7	gb|AIXA01006753.1|:17904-18412
MsHel-7	gb|AIXA01036947.1|:152-338
MsHel-7	gb|AIXA01016172.1|:4248-4425
MsHel-7	gb|AIXA01005878.1|:1-310
MsHel-7	gb|AIXA01003318.1|:10337-10764
MsHel-7	gb|AIXA01001695.1|:22793-23287
MsHel-7	gb|AIXA01004621.1|:43239-43658
MsHel-7	gb|AIXA01003052.1|:64714-65236
MsHel-7	gb|AIXA01000708.1|:15846-16213
MsHel-7	gb|AIXA01023015.1|:1372-1558
MsHel-7	gb|AIXA01004993.1|:3684-4199
MsHel-7	gb|AIXA01016665.1|:20477-20666
MsHel-7	gb|AIXA01009218.1|:11315-11814
MsHel-7	gb|AIXA01008048.1|:73248-73748
MsHel-7	gb|AIXA01007840.1|:28793-29297
MsHel-7	gb|AIXA01007689.1|:20877-21377
MsHel-7	gb|AIXA01007072.1|:9930-10430
MsHel-7	gb|AIXA01006867.1|:50467-50967
MsHel-7	gb|AIXA01006136.1|:73169-73673
MsHel-7	gb|AIXA01005925.1|:2564-3946
MsHel-7	gb|AIXA01005189.1|:20492-20952
MsHel-7	gb|AIXA01004829.1|:53820-54320
MsHel-7	gb|AIXA01004667.1|:57299-57638
MsHel-7	gb|AIXA01004650.1|:9961-10461
MsHel-7	gb|AIXA01004480.1|:4269-4769
MsHel-7	gb|AIXA01004260.1|:56885-57385
MsHel-7	gb|AIXA01003747.1|:24547-25042
MsHel-7	gb|AIXA01002046.1|:13502-14002
MsHel-7	gb|AIXA01001823.1|:46898-47402
MsHel-7	gb|AIXA01001515.1|:14797-15292
MsHel-7	gb|AIXA01001137.1|:34136-34636
MsHel-7	gb|AIXA01000631.1|:7986-8486

MsHel-7	gb|AIXA01000453.1|:9183-9656
MsHel-7	gb|AIXA01000003.1|:22254-22743
MsHel-7	gb|AIXA01018333.1|:1-337
MsHel-7	gb|AIXA01001752.1|:9960-11249
MsHel-7	gb|AIXA01013420.1|:7071-7454
MsHel-7	gb|AIXA01019055.1|:1054-1242
MsHel-7	gb|AIXA01008478.1|:10319-10818
MsHel-7	gb|AIXA01022763.1|:1207-1574
MsHel-7	gb|AIXA01013880.1|:6960-7298
MsHel-7	gb|AIXA01010473.1|:3964-4463
MsHel-7	gb|AIXA01009002.1|:25275-25775
MsHel-7	gb|AIXA01008505.1|:68-189
MsHel-7	gb|AIXA01008225.1|:4332-4836
MsHel-7	gb|AIXA01007492.1|:42963-43462
MsHel-7	gb|AIXA01006854.1|:61709-62891
MsHel-7	gb|AIXA01006430.1|:68229-68696
MsHel-7	gb|AIXA01005734.1|:18266-18765
MsHel-7	gb|AIXA01005272.1|:35096-35607
MsHel-7	gb|AIXA01005117.1|:15857-16357
MsHel-7	gb|AIXA01004944.1|:30678-31178
MsHel-7	gb|AIXA01002461.1|:3382-3550
MsHel-7	gb|AIXA01002442.1|:44309-44810
MsHel-7	gb|AIXA01001566.1|:18432-18928
MsHel-7	gb|AIXA01007881.1|:29503-29930
MsHel-7	gb|AIXA01021347.1|:1902-2079
MsHel-7	gb|AIXA01015155.1|:19232-19401
MsHel-7	gb|AIXA01014392.1|:533-995
MsHel-7	gb|AIXA01008091.1|:2597-3101
MsHel-7	gb|AIXA01007166.1|:34305-34806
MsHel-7	gb|AIXA01000681.1|:28895-29056
MsHel-7	gb|AIXA01013803.1|:4694-5061
MsHel-7	gb|AIXA01013655.1|:4886-5225
MsHel-7	gb|AIXA01011008.1|:45434-45935
MsHel-7	gb|AIXA01007346.1|:579-1079
MsHel-7	gb|AIXA01006623.1|:16422-16586
MsHel-7	gb|AIXA01004598.1|:19501-19823
MsHel-7	gb|AIXA01001920.1|:23272-23772
MsHel-7	gb|AIXA01001284.1|:87120-87623
MsHel-7	gb|AIXA01031169.1|:2-302
MsHel-7	gb|AIXA01016540.1|:1996-2198
MsHel-7	gb|AIXA01009120.1|:3335-3531
MsHel-7	gb|AIXA01003972.1|:23812-24176
MsHel-7	gb|AIXA01033511.1|:480-682
MsHel-7	gb|AIXA01008361.1|:49386-49540
MsHel-7	gb|AIXA01008361.1|:9191-9491
MsHel-7	gb|AIXA01023786.1|:1-292
MsHel-7	gb|AIXA01005918.1|:30728-31229
MsHel-7	gb|AIXA01000562.1|:79005-79503

MsHel-7	gb|AIXA01011665.1|:14065-14565
MsHel-7	gb|AIXA01005071.1|:27004-27371
MsHel-7	gb|AIXA01006935.1|:34562-34725
MsHel-7	gb|AIXA01006935.1|:37-157
MsHel-7	gb|AIXA01007893.1|:59271-59429
MsHel-7	gb|AIXA01004075.1|:12564-12933
MsHel-7	gb|AIXA01002407.1|:304-469
MsHel-7	gb|AIXA01011528.1|:9387-9888
MsHel-7	gb|AIXA01003853.1|:47595-48099
MsHel-7	gb|AIXA01002374.1|:3299-3815
MsHel-7	gb|AIXA01001147.1|:21865-22017
MsHel-7	gb|AIXA01003054.1|:20643-20948
MsHel-7	gb|AIXA01032414.1|:577-735
MsHel-7	gb|AIXA01014172.1|:17434-17600
MsHel-7	gb|AIXA01002658.1|:134029-134207
MsHel-7	gb|AIXA01013993.1|:4773-5013
MsHel-7	gb|AIXA01006220.1|:4762-5107
MsHel-7	gb|AIXA01005433.1|:16917-17423
MsHel-7	gb|AIXA01003976.1|:1-283
MsHel-7	gb|AIXA01002117.1|:44158-44583
MsHel-7	gb|AIXA01012527.1|:50072-50453
MsHel-7	gb|AIXA01008022.1|:49566-50066
MsHel-7	gb|AIXA01006934.1|:9748-10038
MsHel-7	gb|AIXA01005604.1|:32009-32508
MsHel-7	gb|AIXA01036075.1|:225-576
MsHel-7	gb|AIXA01011730.1|:2403-2708
MsHel-7	gb|AIXA01011695.1|:1-248
MsHel-7	gb|AIXA01007336.1|:619-1160
MsHel-7	gb|AIXA01008540.1|:1-301
MsHel-7	gb|AIXA01007675.1|:23733-24233
MsHel-7	gb|AIXA01006791.1|:38281-38781
MsHel-7	gb|AIXA01002631.1|:15151-15651
MsHel-7	gb|AIXA01026612.1|:513-818
MsHel-7	gb|AIXA01009629.1|:611-916
MsHel-7	gb|AIXA01007499.1|:35468-35974
MsHel-7	gb|AIXA01005850.1|:4-288
MsHel-7	gb|AIXA01004327.1|:18192-18471
MsHel-7	gb|AIXA01003670.1|:44985-45401
MsHel-7	gb|AIXA01025956.1|:971-1171
MsHel-7	gb|AIXA01011097.1|:29967-30392
MsHel-7	gb|AIXA01003745.1|:19783-20129
MsHel-7	gb|AIXA01027936.1|:830-997
MsHel-7	gb|AIXA01011369.1|:14132-14411
MsHel-7	gb|AIXA01003607.1|:20213-20572
MsHel-7	gb|AIXA01032579.1|:530-727
MsHel-7	gb|AIXA01015261.1|:1-319
MsHel-7	gb|AIXA01009695.1|:27416-27825
MsHel-7	gb|AIXA01016186.1|:3558-3846

MsHel-7	gb|AIXA01029811.1|:1-407
MsHel-7	gb|AIXA01007576.1|:92937-93220
MsHel-7	gb|AIXA01036087.1|:2-259
MsHel-7	gb|AIXA01035580.1|:1-274
MsHel-7	gb|AIXA01021829.1|:1-334
MsHel-7	gb|AIXA01019656.1|:34-155
MsHel-7	gb|AIXA01015997.1|:2-218
MsHel-7	gb|AIXA01014938.1|:34-155
MsHel-7	gb|AIXA01013980.1|:27-148
MsHel-7	gb|AIXA01009316.1|:2-123
MsHel-7	gb|AIXA01006549.1|:15-136
MsHel-7	gb|AIXA01004660.1|:25-146
MsHel-7	gb|AIXA01002478.1|:50-171
MsHel-7	gb|AIXA01001568.1|:3413-3879
MsHel-7	gb|AIXA01001498.1|:13197-13318
MsHel-7	gb|AIXA01001348.1|:7777-7906
MsHel-7	gb|AIXA01000926.1|:26945-27352
MsHel-7	gb|AIXA01010031.1|:16297-16632
MsHel-7	gb|AIXA01007889.1|:842-982
MsHel-7	gb|AIXA01005556.1|:65-185
MsHel-7	gb|AIXA01002028.1|:18429-18564
MsHel-7	gb|AIXA01034298.1|:9-130
MsHel-7	gb|AIXA01031457.1|:1-264
MsHel-7	gb|AIXA01016262.1|:7847-8048
MsHel-7	gb|AIXA01014130.1|:1-198
MsHel-7	gb|AIXA01013994.1|:1-235
MsHel-7	gb|AIXA01010693.1|:37-158
MsHel-7	gb|AIXA01010116.1|:1-210
MsHel-7	gb|AIXA01007909.1|:29621-29891
MsHel-7	gb|AIXA01006765.1|:39-160
MsHel-7	gb|AIXA01005828.1|:32-153
MsHel-7	gb|AIXA01005054.1|:28-149
MsHel-7	gb|AIXA01003671.1|:25-146
MsHel-7	gb|AIXA01002991.1|:20-141
MsHel-7	gb|AIXA01002988.1|:25-143
MsHel-7	gb|AIXA01002435.1|:6-127
MsHel-7	gb|AIXA01001298.1|:1-270
MsHel-7	gb|AIXA01000275.1|:31-152
MsHel-7	gb|AIXA01010698.1|:53-173
MsHel-7	gb|AIXA01009825.1|:1-273
MsHel-7	gb|AIXA01007917.1|:1-327
MsHel-7	gb|AIXA01004839.1|:1-344
MsHel-7	gb|AIXA01002406.1|:1-272
MsHel-7	gb|AIXA01034514.1|:1-272
MsHel-7	gb|AIXA01031096.1|:2-123
MsHel-7	gb|AIXA01030333.1|:13-134
MsHel-7	gb|AIXA01026983.1|:33-154
MsHel-7	gb|AIXA01024757.1|:1144-1273

MsHel-7	gb|AIXA01024719.1|:6-127
MsHel-7	gb|AIXA01014683.1|:42612-42922
MsHel-7	gb|AIXA01009534.1|:10588-10943
MsHel-7	gb|AIXA01008398.1|:1-256
MsHel-7	gb|AIXA01007818.1|:23-140
MsHel-7	gb|AIXA01005897.1|:46-167
MsHel-7	gb|AIXA01005425.1|:4-216
MsHel-7	gb|AIXA01001481.1|:8-129
MsHel-7	gb|AIXA01001108.1|:1-218
MsHel-7	gb|AIXA01034097.1|:20-140
MsHel-7	gb|AIXA01031789.1|:2-114
MsHel-7	gb|AIXA01028962.1|:35-155
MsHel-7	gb|AIXA01025797.1|:32-290
MsHel-7	gb|AIXA01022847.1|:8-128
MsHel-7	gb|AIXA01022184.1|:21-141
MsHel-7	gb|AIXA01006853.1|:51937-52170
MsHel-7	gb|AIXA01005384.1|:1-117
MsHel-7	gb|AIXA01002292.1|:423-543
MsHel-7	gb|AIXA01001530.1|:59-179
MsHel-7	gb|AIXA01001458.1|:71-326
MsHel-7	gb|AIXA01000059.1|:1-293
MsHel-7	gb|AIXA01034683.1|:1-254
MsHel-7	gb|AIXA01009240.1|:4-114
MsHel-7	gb|AIXA01009123.1|:72-190
MsHel-7	gb|AIXA01036637.1|:1-235
MsHel-7	gb|AIXA01035700.1|:34-155
MsHel-7	gb|AIXA01029807.1|:1-187
MsHel-7	gb|AIXA01027932.1|:14-135
MsHel-7	gb|AIXA01017501.1|:41-162
MsHel-7	gb|AIXA01005128.1|:12-133
MsHel-7	gb|AIXA01004694.1|:26-147
MsHel-7	gb|AIXA01002069.1|:21818-21939
MsHel-7	gb|AIXA01000896.1|:41318-41461
MsHel-7	gb|AIXA01000896.1|:1-219
MsHel-7	gb|AIXA01016801.1|:7-127
MsHel-7	gb|AIXA01010966.1|:28-144
MsHel-7	gb|AIXA01005898.1|:3089-3316
MsHel-7	gb|AIXA01002408.1|:389-497
MsHel-7	gb|AIXA01005855.1|:49151-49433
MsHel-7	gb|AIXA01006936.1|:2-120
MsHel-7	gb|AIXA01001349.1|:1-107
MsHel-7	gb|AIXA01037313.1|:413-534
MsHel-7	gb|AIXA01031402.1|:3-212
MsHel-7	gb|AIXA01029709.1|:1-114
MsHel-7	gb|AIXA01028375.1|:25-148
MsHel-7	gb|AIXA01023483.1|:1-227
MsHel-7	gb|AIXA01003623.1|:13-134
MsHel-7	gb|AIXA01001070.1|:1-122

MsHel-7	gb|AIXA01022501.1|:4-198
MsHel-7	gb|AIXA01016532.1|:4788-5043
MsHel-7	gb|AIXA01003966.1|:50106-50380
MsHel-7	gb|AIXA01002516.1|:26333-26450
MsHel-7	gb|AIXA01008792.1|:5-223
MsHel-7	gb|AIXA01001299.1|:15268-15550
MsHel-7	gb|AIXA01013748.1|:27587-27849
MsHel-7	gb|AIXA01009580.1|:1-171
MsHel-7	gb|AIXA01007948.1|:16963-17141
MsHel-7	gb|AIXA01002391.1|:17-140
MsHel-7	gb|AIXA01000115.1|:15764-16004
MsHel-7	gb|AIXA01035793.1|:293-587
MsHel-7	gb|AIXA01026776.1|:829-1094
MsHel-7	gb|AIXA01007506.1|:49797-50115
MsHel-7	gb|AIXA01007472.1|:140058-140175
MsHel-7	gb|AIXA01005388.1|:20620-20942
MsHel-7	gb|AIXA01002994.1|:23949-24142
MsHel-7	gb|AIXA01002474.1|:34498-34761
MsHel-7	gb|AIXA01001270.1|:90899-91202
MsHel-7	gb|AIXA01038164.1|:2-123
MsHel-7	gb|AIXA01021838.1|:5-209
MsHel-7	gb|AIXA01010757.1|:18600-19053
MsHel-7	gb|AIXA01022691.1|:20-139
MsHel-7	gb|AIXA01010204.1|:5333-5447
MsHel-7	gb|AIXA01030622.1|:235-347
MsHel-7	gb|AIXA01009504.1|:33806-34017
MsHel-7	gb|AIXA01033751.1|:565-669
MsHel-7	gb|AIXA01013833.1|:3-130
MsHel-7	gb|AIXA01001069.1|:20705-20813
MsHel-7	gb|AIXA01000798.1|:6100-6208
MsHel-7	gb|AIXA01009354.1|:1-104
MsHel-7	gb|AIXA01003774.1|:2603-2706
MsHel-7	gb|AIXA01009200.1|:1-99
MsHel-7	gb|AIXA01037259.1|:1-236
MsHel-7	gb|AIXA01028413.1|:865-965
MsHel-7	gb|AIXA01014399.1|:3825-3933
MsHel-7	gb|AIXA01011675.1|:1-105
MsHel-7	gb|AIXA01004299.1|:17264-17364
MsHel-7	gb|AIXA01002676.1|:1-97
MsHel-7	gb|AIXA01024647.1|:1078-1314
MsHel-7	gb|AIXA01003479.1|:1-92
MsHel-7	gb|AIXA01011107.1|:7-97
MsHel-7	gb|AIXA01007244.1|:6115-6497
MsHel-7	gb|AIXA01001255.1|:10597-10844
MsHel-7	gb|AIXA01001907.1|:49203-49453
MsHel-7	gb|AIXA01031422.1|:683-783
MsHel-7	gb|AIXA01010328.1|:1-89
MsHel-7	gb|AIXA01011073.1|:9078-9366

MsHel-7	gb|AIXA01038284.1|:281-503
MsHel-7	gb|AIXA01033888.1|:1-86
MsHel-7	gb|AIXA01026628.1|:1-97
MsHel-7	gb|AIXA01016368.1|:1-90
MsHel-7	gb|AIXA01007921.1|:3-96
MsHel-7	gb|AIXA01017787.1|:13791-13887
MsHel-7	gb|AIXA01002633.1|:5-101
MsHel-7	gb|AIXA01006010.1|:33-170
MsHel-7	gb|AIXA01001526.1|:5211-5346
MsHel-7	gb|AIXA01006683.1|:19720-19844
MsHel-7	gb|AIXA01003326.1|:33921-34198
MsHel-7	gb|AIXA01019211.1|:937-1022
MsHel-7	gb|AIXA01010466.1|:2-87
MsHel-7	gb|AIXA01000705.1|:16832-17076
MsHel-7	gb|AIXA01010841.1|:1-89
MsHel-7	gb|AIXA01004481.1|:42629-42729
MsHel-7	gb|AIXA01007920.1|:18060-18598
MsHel-7	gb|AIXA01006287.1|:83271-83517
MsHel-7	gb|AIXA01003885.1|:6620-6967
MsHel-7	gb|AIXA01009341.1|:22469-22553
MsHel-7	gb|AIXA01017737.1|:1-81
MsHel-7	gb|AIXA01007951.1|:2-101
MsHel-7	gb|AIXA01008903.1|:1-84
MsHel-7	gb|AIXA01034485.1|:239-508
MsHel-7	gb|AIXA01005127.1|:31090-31330
MsHel-7	gb|AIXA01005058.1|:10730-10976
MsHel-7	gb|AIXA01024591.1|:1235-1324
MsHel-7	gb|AIXA01005431.1|:2603-2839
MsHel-7	gb|AIXA01003621.1|:62737-62821
MsHel-7	gb|AIXA01013901.1|:2848-3116
MsHel-7	gb|AIXA01003819.1|:46055-46135
MsHel-7	gb|AIXA01023533.1|:1250-1485
MsHel-7	gb|AIXA01023544.1|:1210-1483
MsHel-7	gb|AIXA01007370.1|:1-112
MsHel-7	gb|AIXA01011654.1|:24579-24664
MsHel-7	gb|AIXA01035387.1|:361-596
MsHel-7	gb|AIXA01002359.1|:13086-13316
MsHel-7	gb|AIXA01000895.1|:55778-55961
MsHel-7	gb|AIXA01007430.1|:19066-19256
MsHel-7	gb|AIXA01003733.1|:5825-5901
MsHel-7	gb|AIXA01013087.1|:14237-14471
MsHel-7	gb|AIXA01009977.1|:4543-4649
MsHel-7	gb|AIXA01008034.1|:16018-16249
MsHel-7	gb|AIXA01001590.1|:6126-6337
MsHel-7	gb|AIXA01031686.1|:582-766
MsHel-7	gb|AIXA01006536.1|:2769-2955
MsHel-7	gb|AIXA01015535.1|:8420-8645
MsHel-7	gb|AIXA01018185.1|:1750-1953

MsHel-7	gb|AIXA01011359.1|:15462-15579
MsHel-7	gb|AIXA01029408.1|:704-899

TableS5 Gene fragment acquisitions by silkworm intact Helitron

Helitron family(accession number):Q_start-Q_end	subject_ID:S_start-S_end	identity
BmHel-1(DF090340:710463-710704):8-157	gb|EHJ73820.1|:685-733	50
BmHel-1(DF090353:2359499-2359738):24-158	gb|EHJ73820.1|:685-729	62
BmHel-10(DF090318:2890097-2890647):124-315	gb|AAO33922.1|:14-77	82
BmHel-10(DF090321:1891240-1891750):126-284	gb|AAO33922.1|:17-69	75
BmHel-10(DF090323:7975572-7976500):441-637	dbj|BAH66344.1|:45-107	54
BmHel-10(DF090335:4359420-4359960):145-309	gb|AAO33922.1|:17-71	69
BmHel-10(DF090354:874915-875453):124-307	gb|AAO33922.1|:17-77	64
BmHel-10(DF090355:309295-309826):121-219	gb|AAO33922.1|:45-77	81
BmHel-10(DF090358:1442661-1443191):146-282	gb|AAO33922.1|:22-65	62
BmHel-10(DF090358:2912994-2913509):124-283	gb|AAO33922.1|:17-77	64.5
BmHel-10(DF090372:1099002-1099532):138-299	gb|AAO33922.1|:17-70	77
BmHel-10(DF090376:1785225-1785730):124-288	gb|AAO33922.1|:23-77	81
BmHel-10(DF090393:1554683-1555617):124-225	gb|AAO33922.1|:44-77	94
BmHel-10(DF090421:542786-543322):153-305	gb|AAO33922.1|:17-67	68
BmHel-10(DF090425:463166-463703):124-306	gb|AAO33922.1|:17-77	72
BmHel-11(BABH01042926:11001-11301):123-300	gb|AAO33922.1|:22-77	66.5
BmHel-11(BABH01043610:14232-14523):124-291	gb|AAO33922.1|:22-77	87
BmHel-11(BABH01043618:11865-12119):124-254	gb|AAO33922.1|:22-77	71
BmHel-11(BABH01044028:8811-9092):123-281	gb|AAO33922.1|:22-77	85
BmHel-11(BABH01044232:10189-10479):124-290	gb|AAO33922.1|:22-77	61.5
BmHel-11(BABH01045038:3715-4006):124-291	gb|AAO33922.1|:22-77	76
BmHel-11(BABH01045079:1512-1803):124-291	gb|AAO33922.1|:22-77	85
BmHel-11(BABH01045299:5159-5426):106-267	gb|AAO33922.1|:22-75	87
BmHel-11(BABH01045341:1107-1395):160-288	gb|AAO33922.1|:22-65	84
BmHel-11(BABH01045757:3287-3573):124-286	gb|AAO33922.1|:22-77	64.5
BmHel-11(BABH01045809:756-1049):153-293	gb|AAO33922.1|:22-71	74
BmHel-11(BABH01046158:55-347):161-292	gb|AAO33922.1|:22-65	95
BmHel-11(BABH01046480:2155-2445):123-290	gb|AAO33922.1|:22-77	89
BmHel-11(BABH01046693:2030-2320):123-290	gb|AAO33922.1|:22-77	92
BmHel-11(BABH01046700:1091-1383):158-292	gb|AAO33922.1|:22-66	93
BmHel-11(BABH01047081:1652-1948):150-296	gb|AAO33922.1|:22-77	69
BmHel-11(BABH01047188:358-620):124-262	gb|AAO33922.1|:22-77	52.5
BmHel-11(BABH01047257:336-630):128-286	gb|AAO33922.1|:25-77	81
BmHel-11(BABH01047346:351-650):192-299	gb|AAO33922.1|:22-57	83
BmHel-11(BABH01047413:963-1474):166-278	ref|NP_001157648.1|:213-250	59
BmHel-11(BABH01047565:725-992):127-267	gb|AAO33922.1|:22-77	67
BmHel-11(BABH01048038:853-1148):128-295	gb|AAO33922.1|:22-77	70
BmHel-11(BABH01048053:340-619):124-279	gb|AAO33922.1|:22-77	62
BmHel-11(BABH01048151:461-748):120-287	gb|AAO33922.1|:22-77	75
BmHel-11(BABH01048282:139-382):124-243	gb|AAO33922.1|:22-77	69
BmHel-11(BABH01048372:821-1086):140-265	gb|AAO33922.1|:22-63	85
BmHel-11(BABH01048401:715-1006):124-291	gb|AAO33922.1|:22-77	89
BmHel-11(BABH01048604:859-1150):124-291	gb|AAO33922.1|:22-77	85
BmHel-11(BABH01048734:919-1161):124-242	gb|AAO33922.1|:22-77	68.5
BmHel-11(BABH01048763:14-289):124-276	gb|AAO33922.1|:22-77	80
BmHel-11(BABH01048951:412-703):124-291	gb|AAO33922.1|:22-77	80

BmHel-11(BABH01049118:437-728):139-291	gb|AAO33922.1|:22-72	82
BmHel-11(BABH01049208:694-937):124-243	gb|AAO33922.1|:22-77	66
BmHel-11(BABH01050185:238-530):125-292	gb|AAO33922.1|:22-77	62
BmHel-11(BABH01050312:271-530):164-259	gb|AAO33922.1|:22-53	90
BmHel-11(BABH01050537:146-439):150-293	gb|AAO33922.1|:22-69	70
BmHel-11(BABH01050538:621-850):104-229	gb|AAO33922.1|:22-77	64
BmHel-11(BABH01050603:165-459):124-294	gb|AAO33922.1|:22-77	87
BmHel-11(BABH01051291:469-698):104-229	gb|AAO33922.1|:22-77	64
BmHel-11(BABH01051293:83-382):123-299	gb|AAO33922.1|:22-77	61
BmHel-11(BABH01051688:562-852):123-290	gb|AAO33922.1|:22-77	85
BmHel-11(BABH01051867:125-416):160-291	gb|AAO33922.1|:22-65	88
BmHel-11(BABH01052004:496-790):169-294	gb|AAO33922.1|:22-63	95
BmHel-11(BABH01055020:332-622):123-290	gb|AAO33922.1|:22-77	96
BmHel-11(BABH01055439:242-527):142-285	gb|AAO33922.1|:22-71	78
BmHel-11(BABH01056570:290-581):160-291	gb|AAO33922.1|:22-65	93
BmHel-11(BABH01059575:117-408):124-291	gb|AAO33922.1|:22-77	89
BmHel-11(BABH01059727:160-450):123-290	gb|AAO33922.1|:22-77	91
BmHel-11(BABH01060459:389-676):147-287	gb|AAO33922.1|:22-68	85
BmHel-11(BABH01072144:183-426):124-243	gb|AAO33922.1|:22-77	67
BmHel-11(BABH01074640:315-598):122-283	gb|AAO33922.1|:22-75	85
BmHel-11(DF090316:10060189-10060480):160-291	gb|AAO33922.1|:22-65	88
BmHel-11(DF090316:10242609-10242900):157-291	gb|AAO33922.1|:22-66	86
BmHel-11(DF090316:10376447-10380567):574-2528	ref|XP_003725633.1|:281-1123	35
BmHel-11(DF090316:10507932-10508227):155-295	gb|AAO33922.1|:22-67	72
BmHel-11(DF090316:10551283-10551575):155-292	gb|AAO33922.1|:22-67	69
BmHel-11(DF090316:10581627-10581922):158-295	gb|AAO33922.1|:22-67	80
BmHel-11(DF090316:10753036-10753323):124-222	gb|AAO33922.1|:45-77	84
BmHel-11(DF090316:10864826-10865117):124-291	gb|AAO33922.1|:22-77	87
BmHel-11(DF090316:10925322-10925613):160-291	gb|AAO33922.1|:22-65	77
BmHel-11(DF090316:11175441-11175736):131-295	gb|AAO33922.1|:22-76	87
BmHel-11(DF090316:11425752-11426019):145-267	gb|AAO33922.1|:22-71	70
BmHel-11(DF090316:11853034-11853303):126-269	gb|AAO33922.1|:22-69	83
BmHel-11(DF090316:11990841-11991132):124-291	gb|AAO33922.1|:22-77	87
BmHel-11(DF090316:12100612-12100901):143-289	gb|AAO33922.1|:22-71	64
BmHel-11(DF090316:12173548-12173839):154-291	gb|AAO33922.1|:22-67	86
BmHel-11(DF090316:12186326-12186617):124-291	gb|AAO33922.1|:22-77	83
BmHel-11(DF090316:12304736-12305027):127-291	gb|AAO33922.1|:22-76	85
BmHel-11(DF090316:12574269-12574535):180-266	gb|AAO33922.1|:22-50	89
BmHel-11(DF090316:12613688-12613978):126-290	gb|AAO33922.1|:22-76	89
BmHel-11(DF090316:12665366-12665659):144-293	gb|AAO33922.1|:22-71	94
BmHel-11(DF090316:12785265-12785551):122-286	gb|AAO33922.1|:22-69	61
BmHel-11(DF090316:13085312-13085603):152-292	gb|AAO33922.1|:22-68	78
BmHel-11(DF090316:13117417-13117707):153-290	gb|AAO33922.1|:22-67	65
BmHel-11(DF090316:13198659-13198940):115-281	gb|AAO33922.1|:22-77	67
BmHel-11(DF090316:13250030-13259336):811-960	gb|AAO33922.1|:22-71	80
BmHel-11(DF090316:13346008-13346300):158-292	gb|AAO33922.1|:22-66	73
BmHel-11(DF090316:13403701-13404000):156-299	gb|AAO33922.1|:22-67	77
BmHel-11(DF090316:13671267-13671559):155-292	gb|AAO33922.1|:22-67	86

BmHel-11(DF090316:13767896-13768191):152-295	gb|AAO33922.1|:22-69	89
BmHel-11(DF090316:13923512-13923807):124-295	gb|AAO33922.1|:22-77	63.5
BmHel-11(DF090316:1402566-1402859):126-293	gb|AAO33922.1|:22-77	60
BmHel-11(DF090316:14075111-14075398):156-287	gb|AAO33922.1|:22-65	88
BmHel-11(DF090316:14532981-14533272):124-291	gb|AAO33922.1|:22-77	82
BmHel-11(DF090316:14597246-14597536):123-290	gb|AAO33922.1|:22-77	85
BmHel-11(DF090316:14709892-14710183):124-291	gb|AAO33922.1|:22-77	87
BmHel-11(DF090316:15015084-15015656):155-253	gb|AAO33922.1|:35-67	84
BmHel-11(DF090316:15087325-15087590):122-265	gb|AAO33922.1|:22-69	81
BmHel-11(DF090316:1513572-1513861):152-289	gb|AAO33922.1|:22-69	62
BmHel-11(DF090316:15498724-15498969):124-245	gb|AAO33922.1|:22-77	68.5
BmHel-11(DF090316:15561306-15561597):124-291	gb|AAO33922.1|:22-77	87
BmHel-11(DF090316:15640994-15641242):141-248	gb|AAO33922.1|:22-57	83
BmHel-11(DF090316:1796005-1796298):160-293	gb|AAO33922.1|:22-65	61
BmHel-11(DF090316:1832357-1832648):124-291	gb|AAO33922.1|:22-77	89
BmHel-11(DF090316:1879831-1880074):124-243	gb|AAO33922.1|:22-77	64
BmHel-11(DF090316:2096898-2097180):118-282	gb|AAO33922.1|:22-76	83
BmHel-11(DF090316:212156-212457):158-301	gb|AAO33922.1|:22-69	83
BmHel-11(DF090316:2126757-2127050):124-216	gb|AAO33922.1|:47-77	77
BmHel-11(DF090316:2178625-2178919):124-294	gb|AAO33922.1|:22-77	89
BmHel-11(DF090316:2198791-2199082):124-291	gb|AAO33922.1|:22-77	85
BmHel-11(DF090316:2237401-2237659):139-258	gb|AAO33922.1|:22-61	80
BmHel-11(DF090316:2482465-2482761):165-296	gb|AAO33922.1|:22-65	88
BmHel-11(DF090316:2674946-2675237):124-291	gb|AAO33922.1|:22-77	66
BmHel-11(DF090316:2892160-2892451):124-291	gb|AAO33922.1|:22-77	87
BmHel-11(DF090316:3314171-3314457):125-286	gb|AAO33922.1|:22-77	62
BmHel-11(DF090316:3541117-3541407):124-291	gb|AAO33922.1|:22-77	66
BmHel-11(DF090316:3682970-3683227):127-257	gb|AAO33922.1|:22-76	65
BmHel-11(DF090316:3715387-3715678):142-291	gb|AAO33922.1|:22-71	92
BmHel-11(DF090316:3863177-3863448):154-271	gb|AAO33922.1|:22-64	57.5
BmHel-11(DF090316:4013797-4014078):120-281	gb|AAO33922.1|:22-75	87
BmHel-11(DF090316:4156045-4156335):153-290	gb|AAO33922.1|:22-67	84
BmHel-11(DF090316:4228300-4228585):160-285	gb|AAO33922.1|:22-65	93
BmHel-11(DF090316:4365960-4366251):124-291	gb|AAO33922.1|:22-77	87
BmHel-11(DF090316:4366777-4367061):144-284	gb|AAO33922.1|:22-77	71
BmHel-11(DF090316:4430179-4430470):160-291	gb|AAO33922.1|:22-65	88
BmHel-11(DF090316:4465322-4465613):124-291	gb|AAO33922.1|:22-77	85
BmHel-11(DF090316:4488097-4488387):159-290	gb|AAO33922.1|:22-65	86
BmHel-11(DF090316:4548988-4549277):125-289	gb|AAO33922.1|:22-77	82
BmHel-11(DF090316:4779573-4779811):97-238	gb|AAO33922.1|:22-69	67.5
BmHel-11(DF090316:4846833-4847129):156-296	gb|AAO33922.1|:22-66	70
BmHel-11(DF090316:4997479-4997770):124-291	gb|AAO33922.1|:22-77	87
BmHel-11(DF090316:5046927-5047226):126-299	gb|AAO33922.1|:22-76	70
BmHel-11(DF090316:585767-587089):866-1000	gb|AAO33922.1|:22-66	75
BmHel-11(DF090316:585767-587089):296-823	gb|ADC53707.1|:221-396	28
BmHel-11(DF090316:6406940-6407229):123-289	gb|AAO33922.1|:22-77	63
BmHel-11(DF090316:6418883-6419288):124-305	gb|AAO33922.1|:22-77	60.5
BmHel-11(DF090316:674387-680918):508-1265	sp|P21329|RTJK_DROFU:676-907	33.5

BmHel-11(DF090316:6763257-6763549):125-292	gb|AAO33922.1|:22-77	87
BmHel-11(DF090316:7113095-7113389):127-294	gb|AAO33922.1|:22-77	87
BmHel-11(DF090316:725655-725946):160-291	gb|AAO33922.1|:22-65	84
BmHel-11(DF090316:7432627-7432880):110-253	gb|AAO33922.1|:22-69	85
BmHel-11(DF090316:751704-751995):127-291	gb|AAO33922.1|:22-76	81
BmHel-11(DF090316:7976948-7977239):160-291	gb|AAO33922.1|:22-65	93
BmHel-11(DF090316:8105515-8105809):160-294	gb|AAO33922.1|:22-65	77
BmHel-11(DF090316:816996-817281):118-285	gb|AAO33922.1|:22-77	89
BmHel-11(DF090316:8191479-8191743):124-264	gb|AAO33922.1|:22-77	64
BmHel-11(DF090316:8515991-8516291):124-300	gb|AAO33922.1|:22-77	77
BmHel-11(DF090316:8718314-8718573):113-259	gb|AAO33922.1|:22-70	89
BmHel-11(DF090316:8917091-8917383):152-292	gb|AAO33922.1|:22-68	89
BmHel-11(DF090316:8933897-8934190):168-293	gb|AAO33922.1|:22-63	80
BmHel-11(DF090316:9064310-9064601):160-291	gb|AAO33922.1|:22-65	88
BmHel-11(DF090316:9119162-9119449):138-287	gb|AAO33922.1|:22-71	78
BmHel-11(DF090316:9302364-9302592):136-228	gb|AAO33922.1|:22-52	93
BmHel-11(DF090316:9340559-9340843):123-284	gb|AAO33922.1|:22-77	78
BmHel-11(DF090316:9382038-9383712):124-231	gb|AAO33922.1|:42-77	72
BmHel-11(DF090316:9382038-9383712):875-1303	dbj|BAM20202.1|:70-198	38
BmHel-11(DF090316:9473244-9473537):126-293	gb|AAO33922.1|:22-77	85
BmHel-11(DF090316:9492610-9492921):180-311	gb|AAO33922.1|:22-65	86
BmHel-11(DF090316:9670985-9671282):149-280	gb|AAO33922.1|:27-70	65
BmHel-11(DF090316:9709030-9709306):139-276	gb|AAO33922.1|:22-67	89
BmHel-11(DF090316:9790731-9791023):155-292	gb|AAO33922.1|:22-67	82
BmHel-11(DF090316:9916090-9916381):124-291	gb|AAO33922.1|:22-77	91
BmHel-11(DF090317:10362240-10362512):124-272	gb|AAO33922.1|:22-76	66
BmHel-11(DF090317:10523469-10523761):161-292	gb|AAO33922.1|:22-65	88
BmHel-11(DF090317:10802563-10802816):127-253	gb|AAO33922.1|:22-76	63
BmHel-11(DF090317:10825998-10826293):152-295	gb|AAO33922.1|:22-69	64
BmHel-11(DF090317:10839405-10841189):628-1107	gb|EFA11941.1|:632-1130	33.5
BmHel-11(DF090317:10839405-10841189):1119-1229	gb|AAO33922.1|:22-58	81
BmHel-11(DF090317:10853829-10854117):139-288	gb|AAO33922.1|:22-72	78
BmHel-11(DF090317:10868053-10868348):125-295	gb|AAO33922.1|:22-77	75
BmHel-11(DF090317:11131496-11131787):124-291	gb|AAO33922.1|:22-77	87
BmHel-11(DF090317:1185164-1185455):124-291	gb|AAO33922.1|:22-77	83
BmHel-11(DF090317:1294834-1295128):157-294	gb|AAO33922.1|:22-67	89
BmHel-11(DF090317:150939-151210):164-271	gb|AAO33922.1|:22-57	86
BmHel-11(DF090317:1607570-1607813):124-243	gb|AAO33922.1|:22-77	66
BmHel-11(DF090317:1615473-1615763):156-290	gb|AAO33922.1|:22-66	84
BmHel-11(DF090317:1646137-1646427):159-290	gb|AAO33922.1|:22-65	86
BmHel-11(DF090317:1668099-1668390):124-291	gb|AAO33922.1|:22-77	78
BmHel-11(DF090317:1744161-1744433):183-272	gb|AAO33922.1|:22-51	96
BmHel-11(DF090317:1764616-1764906):150-290	gb|AAO33922.1|:22-68	82
BmHel-11(DF090317:190371-190658):120-287	gb|AAO33922.1|:22-77	87
BmHel-11(DF090317:2016918-2017209):154-291	gb|AAO33922.1|:22-67	69
BmHel-11(DF090317:2117057-2117309):126-252	gb|AAO33922.1|:22-76	53
BmHel-11(DF090317:2159893-2160152):126-259	gb|AAO33922.1|:22-77	59
BmHel-11(DF090317:2173976-2174254):142-278	gb|AAO33922.1|:22-71	64.5

BmHel-11(DF090317:2200432-2200699):124-267	gb|AAO33922.1|:22-77	69
BmHel-11(DF090317:2311542-2311771):137-229	gb|AAO33922.1|:22-52	96
BmHel-11(DF090317:2336419-2336710):123-291	gb|AAO33922.1|:22-77	66
BmHel-11(DF090317:2637952-2638243):124-291	gb|AAO33922.1|:22-77	80
BmHel-11(DF090317:2872269-2872558):123-215	gb|AAO33922.1|:47-77	87
BmHel-11(DF090317:3007982-3008253):160-271	gb|AAO33922.1|:22-65	64.5
BmHel-11(DF090317:3009779-3010069):123-290	gb|AAO33922.1|:22-77	82
BmHel-11(DF090317:3663054-3663305):124-251	gb|AAO33922.1|:22-77	57
BmHel-11(DF090317:4013800-4014057):127-257	gb|AAO33922.1|:22-76	60
BmHel-11(DF090317:4067671-4067962):124-291	gb|AAO33922.1|:22-77	87
BmHel-11(DF090317:4068541-4068832):124-291	gb|AAO33922.1|:22-77	78
BmHel-11(DF090317:4735926-4736217):124-291	gb|AAO33922.1|:22-77	83
BmHel-11(DF090317:4870931-4871199):125-268	gb|AAO33922.1|:22-77	69
BmHel-11(DF090317:4883739-4884023):124-284	gb|AAO33922.1|:22-77	62
BmHel-11(DF090317:53084-53374):156-290	gb|AAO33922.1|:22-66	93
BmHel-11(DF090317:5372289-5372575):123-278	gb|AAO33922.1|:25-77	68
BmHel-11(DF090317:5425218-5425506):157-288	gb|AAO33922.1|:22-65	63
BmHel-11(DF090317:5547051-5547337):122-286	gb|AAO33922.1|:22-75	65
BmHel-11(DF090317:5565159-5565450):142-291	gb|AAO33922.1|:22-71	88
BmHel-11(DF090317:588532-588939):240-407	gb|AAO33922.1|:22-77	91
BmHel-11(DF090317:5910102-5910381):124-279	gb|AAO33922.1|:22-77	76
BmHel-11(DF090317:6061459-6061752):129-293	gb|AAO33922.1|:22-76	85
BmHel-11(DF090317:6134589-6134891):150-302	gb|AAO33922.1|:22-71	82
BmHel-11(DF090317:6571661-6572363):601-702	gb|AAO33922.1|:22-55	91
BmHel-11(DF090317:6683831-6684090):122-259	gb|AAO33922.1|:22-69	81
BmHel-11(DF090317:6722134-6722424):123-290	gb|AAO33922.1|:22-77	82
BmHel-11(DF090317:6811790-6812089):133-240	gb|AAO33922.1|:42-77	86
BmHel-11(DF090317:6840071-6840357):152-286	gb|AAO33922.1|:22-67	69
BmHel-11(DF090317:6863314-6863620):169-306	gb|AAO33922.1|:22-67	71
BmHel-11(DF090317:6949095-6949390):128-295	gb|AAO33922.1|:22-77	83
BmHel-11(DF090317:7029131-7029423):155-292	gb|AAO33922.1|:22-67	80
BmHel-11(DF090317:7036057-7036347):126-290	gb|AAO33922.1|:22-77	60
BmHel-11(DF090317:7060664-7062192):760-1356	ref|XP_003103275.1|:473-675	26
BmHel-11(DF090317:7102725-7103018):124-271	gb|AAO33922.1|:29-77	55
BmHel-11(DF090317:7141988-7142285):169-297	gb|AAO33922.1|:22-64	88
BmHel-11(DF090317:7219728-7220019):160-291	gb|AAO33922.1|:22-65	84
BmHel-11(DF090317:7240201-7240492):160-291	gb|AAO33922.1|:22-65	88
BmHel-11(DF090317:7258486-7258764):111-278	gb|AAO33922.1|:22-77	80
BmHel-11(DF090317:7271319-7271609):123-290	gb|AAO33922.1|:22-77	85
BmHel-11(DF090317:734281-734559):111-278	gb|AAO33922.1|:22-77	83
BmHel-11(DF090317:74580-74879):192-299	gb|AAO33922.1|:22-57	86
BmHel-11(DF090317:7464420-7464711):124-291	gb|AAO33922.1|:22-77	64
BmHel-11(DF090317:7466600-7466891):124-291	gb|AAO33922.1|:22-77	80
BmHel-11(DF090317:7533913-7534209):159-296	gb|AAO33922.1|:22-67	89
BmHel-11(DF090317:7815507-7815798):124-291	gb|AAO33922.1|:22-77	89
BmHel-11(DF090317:8386787-8390534):1824-1993	gb|AAO33922.1|:22-77	62.5
BmHel-11(DF090317:8386787-8390534):597-1460	ref|XP_003491781.1|:146-450	22
BmHel-11(DF090317:844772-845038):125-266	gb|AAO33922.1|:22-77	71

BmHel-11(DF090317:8545040-8545322):124-282	gb|AAO33922.1|:22-77	64
BmHel-11(DF090317:8580203-8580480):164-277	gb|AAO33922.1|:22-59	81
BmHel-11(DF090317:8747668-8747952):124-276	gb|AAO33922.1|:25-77	79
BmHel-11(DF090317:8761337-8761634):163-297	gb|AAO33922.1|:22-68	78
BmHel-11(DF090317:8902871-8903162):124-291	gb|AAO33922.1|:22-77	82
BmHel-11(DF090317:8943791-8944077):123-286	gb|AAO33922.1|:22-77	60
BmHel-11(DF090317:9128745-9129036):160-291	gb|AAO33922.1|:22-65	84
BmHel-11(DF090317:9313576-9313869):132-293	gb|AAO33922.1|:22-75	88
BmHel-11(DF090317:9331337-9331628):124-291	gb|AAO33922.1|:22-77	82
BmHel-11(DF090317:9410561-9410852):124-291	gb|AAO33922.1|:22-77	78
BmHel-11(DF090317:9465081-9465373):155-292	gb|AAO33922.1|:22-67	82
BmHel-11(DF090317:952421-952712):124-291	gb|AAO33922.1|:22-77	78
BmHel-11(DF090317:9745406-9745694):123-251	gb|AAO33922.1|:35-77	86
BmHel-11(DF090318:1026795-1027087):124-292	gb|AAO33922.1|:22-77	64
BmHel-11(DF090318:1089709-1089999):123-290	gb|AAO33922.1|:22-77	87
BmHel-11(DF090318:1377092-1377390):123-298	gb|AAO33922.1|:22-77	67.5
BmHel-11(DF090318:138210-138501):124-291	gb|AAO33922.1|:22-77	83
BmHel-11(DF090318:1881024-1881313):124-246	gb|AAO33922.1|:37-77	82
BmHel-11(DF090318:1905392-1905684):143-292	gb|AAO33922.1|:22-72	76
BmHel-11(DF090318:2087586-2087877):124-291	gb|AAO33922.1|:22-77	89
BmHel-11(DF090318:2411005-2411296):124-291	gb|AAO33922.1|:22-77	87
BmHel-11(DF090318:2442412-2442698):122-286	gb|AAO33922.1|:22-77	64
BmHel-11(DF090318:2453518-2453808):174-290	gb|AAO33922.1|:22-60	89
BmHel-11(DF090318:2499834-2500123):123-290	gb|AAO33922.1|:22-77	87
BmHel-11(DF090318:2852108-2852351):127-243	gb|AAO33922.1|:22-76	63
BmHel-11(DF090318:315253-315544):160-291	gb|AAO33922.1|:22-65	86
BmHel-11(DF090318:3314644-3314935):124-291	gb|AAO33922.1|:22-77	80
BmHel-11(DF090318:3366004-3366298):127-294	gb|AAO33922.1|:22-77	80
BmHel-11(DF090318:3396613-3396904):124-291	gb|AAO33922.1|:22-77	89
BmHel-11(DF090318:3452669-3452960):160-291	gb|AAO33922.1|:22-65	84
BmHel-11(DF090318:3501475-3501765):165-290	gb|AAO33922.1|:22-62	71
BmHel-11(DF090318:3586592-3586883):124-291	gb|AAO33922.1|:22-77	85
BmHel-11(DF090318:3636863-3637148):124-285	gb|AAO33922.1|:22-77	82
BmHel-11(DF090318:3656422-3656715):156-293	gb|AAO33922.1|:22-67	80
BmHel-11(DF090318:3816926-3817220):160-294	gb|AAO33922.1|:22-65	71
BmHel-11(DF090318:4040471-4040762):124-291	gb|AAO33922.1|:22-77	82
BmHel-11(DF090318:4067656-4067923):97-267	gb|AAO33922.1|:22-76	64
BmHel-11(DF090318:4444289-4444580):124-291	gb|AAO33922.1|:22-77	89
BmHel-11(DF090318:4631196-4631464):115-268	gb|AAO33922.1|:22-76	65.5
BmHel-11(DF090318:4812545-4812835):126-290	gb|AAO33922.1|:22-77	57
BmHel-11(DF090318:5035564-5035855):124-291	gb|AAO33922.1|:22-77	92
BmHel-11(DF090318:5048016-5048308):155-292	gb|AAO33922.1|:22-67	67
BmHel-11(DF090318:5051654-5051948):130-294	gb|AAO33922.1|:22-76	81
BmHel-11(DF090318:520748-521036):124-288	gb|AAO33922.1|:22-77	67
BmHel-11(DF090318:5918868-5919159):126-291	gb|AAO33922.1|:22-77	62
BmHel-11(DF090318:5952888-5953182):124-294	gb|AAO33922.1|:22-77	87
BmHel-11(DF090318:5971520-5971810):153-290	gb|AAO33922.1|:22-76	65
BmHel-11(DF090318:6254464-6254756):155-292	gb|AAO33922.1|:22-67	84

BmHel-11(DF090318:6350690-6350985):188-295	gb|AAO33922.1|:22-57	80
BmHel-11(DF090318:6430904-6431171):124-267	gb|AAO33922.1|:22-77	67
BmHel-11(DF090318:6490266-6490556):123-290	gb|AAO33922.1|:22-77	92
BmHel-11(DF090318:6670793-6671040):124-248	gb|AAO33922.1|:22-77	61.5
BmHel-11(DF090318:674-975):124-301	gb|AAO33922.1|:22-77	66
BmHel-11(DF090318:6811831-6812123):143-292	gb|AAO33922.1|:22-71	84
BmHel-11(DF090318:6923347-6923632):119-253	gb|AAO33922.1|:33-77	82
BmHel-11(DF090318:732956-733247):124-291	gb|AAO33922.1|:22-77	89
BmHel-11(DF090318:7609258-7609541):149-283	gb|AAO33922.1|:22-66	75
BmHel-11(DF090318:7743285-7743577):155-292	gb|AAO33922.1|:22-67	89
BmHel-11(DF090318:7958469-7958761):131-292	gb|AAO33922.1|:22-75	83
BmHel-11(DF090318:7969660-7969985):161-325	gb|AAO33922.1|:22-77	85
BmHel-11(DF090318:808399-808688):123-281	gb|AAO33922.1|:25-77	73
BmHel-11(DF090318:8123022-8123310):123-281	gb|AAO33922.1|:25-77	84
BmHel-11(DF090318:8188947-8189238):160-291	gb|AAO33922.1|:22-65	84
BmHel-11(DF090318:8256370-8256646):124-276	gb|AAO33922.1|:22-77	80
BmHel-11(DF090318:8463984-8464269):163-285	gb|AAO33922.1|:22-62	85
BmHel-11(DF090318:8846686-8846977):124-291	gb|AAO33922.1|:22-77	85
BmHel-11(DF090318:9021634-9021924):123-290	gb|AAO33922.1|:22-77	80
BmHel-11(DF090318:9462769-9463060):124-291	gb|AAO33922.1|:22-77	76
BmHel-11(DF090318:9828125-9828396):128-271	gb|AAO33922.1|:22-76	65
BmHel-11(DF090319:1034803-1035094):124-291	gb|AAO33922.1|:22-77	83
BmHel-11(DF090319:117442-117696):125-254	gb|AAO33922.1|:22-77	66
BmHel-11(DF090319:1320429-1320720):124-291	gb|AAO33922.1|:22-77	91
BmHel-11(DF090319:1409113-1409404):124-291	gb|AAO33922.1|:22-77	80
BmHel-11(DF090319:1892039-1892305):124-266	gb|AAO33922.1|:22-77	60.5
BmHel-11(DF090319:2023340-2023630):123-290	gb|AAO33922.1|:22-77	82
BmHel-11(DF090319:2124913-2125205):125-292	gb|AAO33922.1|:22-77	83
BmHel-11(DF090319:2205858-2206149):124-291	gb|AAO33922.1|:22-77	92
BmHel-11(DF090319:2275887-2276169):115-282	gb|AAO33922.1|:22-77	83
BmHel-11(DF090319:2300505-2300800):124-295	gb|AAO33922.1|:22-77	64
BmHel-11(DF090319:2352764-2353051):133-287	gb|AAO33922.1|:22-74	54
BmHel-11(DF090319:2488627-2488916):135-289	gb|AAO33922.1|:22-74	60
BmHel-11(DF090319:2656985-2657257):150-272	gb|AAO33922.1|:22-62	80
BmHel-11(DF090319:2700111-2700406):128-295	gb|AAO33922.1|:22-77	57
BmHel-11(DF090319:271137-271350):67-213	gb|AAO33922.1|:22-70	91
BmHel-11(DF090319:272034-272324):153-290	gb|AAO33922.1|:22-67	78
BmHel-11(DF090319:2742294-2742588):136-294	gb|AAO33922.1|:22-74	60
BmHel-11(DF090319:2977278-2977542):144-264	gb|AAO33922.1|:22-64	59.5
BmHel-11(DF090319:300190-300477):124-287	gb|AAO33922.1|:22-77	60
BmHel-11(DF090319:3032316-3032607):124-291	gb|AAO33922.1|:22-77	82
BmHel-11(DF090319:3050513-3050741):139-228	gb|AAO33922.1|:22-51	93
BmHel-11(DF090319:3059677-3059934):124-257	gb|AAO33922.1|:22-77	67.5
BmHel-11(DF090319:3610087-3610356):150-269	gb|AAO33922.1|:22-61	82
BmHel-11(DF090319:3627630-3627919):114-289	gb|AAO33922.1|:22-77	53
BmHel-11(DF090319:3653561-3653852):163-291	gb|AAO33922.1|:22-64	88
BmHel-11(DF090319:3666161-3666454):126-293	gb|AAO33922.1|:22-77	87
BmHel-11(DF090319:3706527-3706817):141-290	gb|AAO33922.1|:22-70	66

BmHel-11(DF090319:3795636-3795927):124-291	gb|AAO33922.1|:22-77	76
BmHel-11(DF090319:3830206-3830496):165-290	gb|AAO33922.1|:22-63	88
BmHel-11(DF090319:3979404-3979703):162-299	gb|AAO33922.1|:22-67	78
BmHel-11(DF090319:418950-419131):68-181	gb|AAO33922.1|:22-59	89
BmHel-11(DF090319:4385818-4386111):126-293	gb|AAO33922.1|:22-77	85
BmHel-11(DF090319:4420252-4420542):138-290	gb|AAO33922.1|:22-72	70
BmHel-11(DF090319:4861078-4861335):122-257	gb|AAO33922.1|:22-66	58.5
BmHel-11(DF090319:4880620-4880909):124-289	gb|AAO33922.1|:22-77	67
BmHel-11(DF090319:5099446-5099673):72-227	gb|AAO33922.1|:22-73	80
BmHel-11(DF090319:5126255-5126515):114-260	gb|AAO33922.1|:22-69	65
BmHel-11(DF090319:5142249-5142535):124-286	gb|AAO33922.1|:22-77	65
BmHel-11(DF090319:521076-521364):124-288	gb|AAO33922.1|:22-77	66
BmHel-11(DF090319:5279117-5279363):124-246	gb|AAO33922.1|:22-77	64
BmHel-11(DF090319:5596545-5596836):124-291	gb|AAO33922.1|:22-77	80
BmHel-11(DF090319:5687689-5687976):120-287	gb|AAO33922.1|:22-77	92
BmHel-11(DF090319:573728-573987):150-259	gb|AAO33922.1|:22-70	57
BmHel-11(DF090319:5764459-5764716):144-257	gb|AAO33922.1|:22-71	68
BmHel-11(DF090319:5915806-5916093):120-287	gb|AAO33922.1|:22-77	91
BmHel-11(DF090319:6126105-6126385):151-280	gb|AAO33922.1|:22-68	60
BmHel-11(DF090319:6147562-6147819):129-258	gb|AAO33922.1|:22-64	62
BmHel-11(DF090319:6161461-6161729):119-268	gb|AAO33922.1|:22-69	80
BmHel-11(DF090319:6184010-6184302):155-292	gb|AAO33922.1|:22-67	73
BmHel-11(DF090319:6548316-6548589):124-273	gb|AAO33922.1|:22-77	75
BmHel-11(DF090319:6964505-6964784):123-279	gb|AAO33922.1|:22-77	62.5
BmHel-11(DF090319:7004357-7004621):97-264	gb|AAO33922.1|:22-77	83
BmHel-11(DF090319:7145589-7145886):143-297	gb|AAO33922.1|:22-72	61.5
BmHel-11(DF090319:7246857-7247148):160-291	gb|AAO33922.1|:22-65	86
BmHel-11(DF090319:7643934-7644225):124-291	gb|AAO33922.1|:22-77	92
BmHel-11(DF090319:7662533-7662825):125-292	gb|AAO33922.1|:22-77	82
BmHel-11(DF090319:7891421-7891706):121-285	gb|AAO33922.1|:22-76	85
BmHel-11(DF090319:8203904-8205855):1518-1661	gb|AAO33922.1|:22-69	83
BmHel-11(DF090319:8203904-8205855):40-149	ref|NP_001157648.1|:214-250	68.5
BmHel-11(DF090319:8203904-8205855):214-345	dbj|BAH66363.1|:5-48	86
BmHel-11(DF090319:8413409-8413699):126-290	gb|AAO33922.1|:22-77	76
BmHel-11(DF090319:8500113-8500397):124-284	gb|AAO33922.1|:22-77	59.5
BmHel-11(DF090319:882598-882890):161-292	gb|AAO33922.1|:22-65	86
BmHel-11(DF090320:1248355-1248646):124-291	gb|AAO33922.1|:22-77	83
BmHel-11(DF090320:1372617-1372902):121-285	gb|AAO33922.1|:22-76	85
BmHel-11(DF090320:1430258-1430548):150-290	gb|AAO33922.1|:22-68	85
BmHel-11(DF090320:1567079-1567332):124-253	gb|AAO33922.1|:22-77	71
BmHel-11(DF090320:1630580-1630866):119-286	gb|AAO33922.1|:22-77	76
BmHel-11(DF090320:174010-174297):124-258	gb|AAO33922.1|:33-77	75
BmHel-11(DF090320:1796161-1796452):124-291	gb|AAO33922.1|:22-77	92
BmHel-11(DF090320:1856408-1856709):124-301	gb|AAO33922.1|:22-77	68.5
BmHel-11(DF090320:1927941-1928234):126-293	gb|AAO33922.1|:22-77	87
BmHel-11(DF090320:2089734-2090014):113-280	gb|AAO33922.1|:22-77	62
BmHel-11(DF090320:2177972-2178242):133-270	gb|AAO33922.1|:22-67	84
BmHel-11(DF090320:2224166-2224410):137-244	gb|AAO33922.1|:22-57	86

BmHel-11(DF090320:2269155-2269446):123-239	gb|AAO33922.1|:39-77	84
BmHel-11(DF090320:2352387-2352655):128-268	gb|AAO33922.1|:22-70	67
BmHel-11(DF090320:2482349-2482610):124-261	gb|AAO33922.1|:22-77	71
BmHel-11(DF090320:2533560-2533823):123-263	gb|AAO33922.1|:22-68	87
BmHel-11(DF090320:2590683-2590974):142-291	gb|AAO33922.1|:22-71	88
BmHel-11(DF090320:2708622-2708907):124-285	gb|AAO33922.1|:22-77	87
BmHel-11(DF090320:2722647-2722936):158-289	gb|AAO33922.1|:22-65	84
BmHel-11(DF090320:2927711-2927985):124-274	gb|AAO33922.1|:22-77	62.5
BmHel-11(DF090320:2998025-2998316):124-291	gb|AAO33922.1|:22-77	80
BmHel-11(DF090320:3023095-3023387):125-292	gb|AAO33922.1|:22-76	78
BmHel-11(DF090320:3073934-3074162):136-228	gb|AAO33922.1|:22-52	93
BmHel-11(DF090320:3596684-3596976):158-292	gb|AAO33922.1|:22-66	84
BmHel-11(DF090320:3679630-3679923):126-293	gb|AAO33922.1|:22-77	82
BmHel-11(DF090320:3695692-3695982):126-290	gb|AAO33922.1|:22-77	60
BmHel-11(DF090320:3730210-3730473):124-263	gb|AAO33922.1|:22-77	68
BmHel-11(DF090320:4037645-4037877):116-232	gb|AAO33922.1|:22-76	65
BmHel-11(DF090320:4131703-4131993):159-290	gb|AAO33922.1|:22-65	86
BmHel-11(DF090320:4191526-4191820):160-294	gb|AAO33922.1|:22-65	86
BmHel-11(DF090320:4295915-4296209):127-294	gb|AAO33922.1|:22-77	85
BmHel-11(DF090320:4412364-4412654):123-290	gb|AAO33922.1|:22-77	80
BmHel-11(DF090320:4561820-4562099):127-279	gb|AAO33922.1|:22-77	76
BmHel-11(DF090320:4673807-4676123):1465-1629	gb|AAO33922.1|:22-76	87
BmHel-11(DF090320:495929-496219):153-290	gb|AAO33922.1|:22-67	78
BmHel-11(DF090320:5002740-5003033):126-293	gb|AAO33922.1|:22-77	85
BmHel-11(DF090320:5014564-5014868):133-304	gb|AAO33922.1|:22-76	55
BmHel-11(DF090320:5341014-5341305):124-291	gb|AAO33922.1|:22-77	91
BmHel-11(DF090320:5505733-5505975):124-242	gb|AAO33922.1|:22-77	54
BmHel-11(DF090320:5668591-5668873):166-282	gb|AAO33922.1|:22-60	69
BmHel-11(DF090320:5809773-5810144):127-306	gb|AAO33922.1|:14-76	52
BmHel-11(DF090320:5889555-5889847):124-292	gb|AAO33922.1|:22-77	62
BmHel-11(DF090320:6149244-6149535):124-291	gb|AAO33922.1|:22-77	89
BmHel-11(DF090320:6168118-6168404):119-286	gb|AAO33922.1|:22-77	89
BmHel-11(DF090320:6202241-6202533):155-292	gb|AAO33922.1|:22-67	84
BmHel-11(DF090320:6251771-6252062):124-291	gb|AAO33922.1|:22-77	83
BmHel-11(DF090320:6542977-6543282):124-305	gb|AAO33922.1|:22-77	56
BmHel-11(DF090320:668563-668842):118-279	gb|AAO33922.1|:22-75	81
BmHel-11(DF090320:6757489-6757780):124-291	gb|AAO33922.1|:22-77	87
BmHel-11(DF090320:6799149-6799440):160-291	gb|AAO33922.1|:22-65	86
BmHel-11(DF090320:6888634-6888933):171-299	gb|AAO33922.1|:22-64	93
BmHel-11(DF090320:7046543-7046870):126-327	gb|AAO33922.1|:22-77	66.5
BmHel-11(DF090320:7087257-7087547):153-290	gb|AAO33922.1|:22-67	82
BmHel-11(DF090320:737520-737803):123-283	gb|AAO33922.1|:22-77	58
BmHel-11(DF090320:7504447-7504733):124-286	gb|AAO33922.1|:22-77	66
BmHel-11(DF090320:7616896-7617187):160-291	gb|AAO33922.1|:22-65	88
BmHel-11(DF090320:7619689-7619972):131-283	gb|AAO33922.1|:22-74	81
BmHel-11(DF090320:7630464-7630755):124-291	gb|AAO33922.1|:22-77	82
BmHel-11(DF090320:7748185-7748455):124-270	gb|AAO33922.1|:22-77	66
BmHel-11(DF090320:8094504-8094661):65-157	gb|AAO33922.1|:22-52	93

BmHel-11(DF090320:8099028-8099315):126-287	gb|AAO33922.1|:22-77	58
BmHel-11(DF090320:818145-818429):160-285	gb|AAO33922.1|:22-65	70
BmHel-11(DF090321:1215096-1215378):115-282	gb|AAO33922.1|:22-77	87
BmHel-11(DF090321:1228624-1228883):124-259	gb|AAO33922.1|:22-77	51
BmHel-11(DF090321:1425478-1425760):161-282	gb|AAO33922.1|:22-65	59
BmHel-11(DF090321:1768856-1769149):124-293	gb|AAO33922.1|:22-77	59.5
BmHel-11(DF090321:1784902-1785186):127-284	gb|AAO33922.1|:22-76	66.5
BmHel-11(DF090321:1934039-1934323):117-284	gb|AAO33922.1|:22-77	83
BmHel-11(DF090321:249000-249274):119-274	gb|AAO33922.1|:22-69	67
BmHel-11(DF090321:257847-258137):123-290	gb|AAO33922.1|:22-77	76
BmHel-11(DF090321:2720275-2720566):124-291	gb|AAO33922.1|:22-77	85
BmHel-11(DF090321:2728461-2728748):138-287	gb|AAO33922.1|:22-71	90
BmHel-11(DF090321:2793268-2793566):131-298	gb|AAO33922.1|:22-77	68
BmHel-11(DF090321:2931841-2932132):124-291	gb|AAO33922.1|:22-77	94
BmHel-11(DF090321:3292204-3293212):783-980	ref|XP_002075098.1|:13-284	42.5
BmHel-11(DF090321:3396331-3396621):124-290	gb|AAO33922.1|:22-77	63
BmHel-11(DF090321:3573311-3573602):124-291	gb|AAO33922.1|:22-77	83
BmHel-11(DF090321:3574775-3575061):126-286	gb|AAO33922.1|:22-77	65
BmHel-11(DF090321:3624572-3624862):159-290	gb|AAO33922.1|:22-65	90
BmHel-11(DF090321:3801873-3802164):157-291	gb|AAO33922.1|:22-66	82
BmHel-11(DF090321:3857994-3858250):149-256	gb|AAO33922.1|:22-57	88
BmHel-11(DF090321:3925668-3925947):124-279	gb|AAO33922.1|:22-77	78
BmHel-11(DF090321:3952993-3953284):160-291	gb|AAO33922.1|:22-65	88
BmHel-11(DF090321:4128008-4128297):152-289	gb|AAO33922.1|:22-67	71
BmHel-11(DF090321:4143775-4144068):126-293	gb|AAO33922.1|:22-77	64
BmHel-11(DF090321:4237638-4237917):124-279	gb|AAO33922.1|:22-77	75
BmHel-11(DF090321:4246759-4247050):124-291	gb|AAO33922.1|:22-77	83
BmHel-11(DF090321:4281247-4281540):126-293	gb|AAO33922.1|:22-77	55
BmHel-11(DF090321:4308055-4310065):163-687	gb|EFA08522.1|:59-446	35
BmHel-11(DF090321:4308055-4310065):1068-1217	gb|AAO33922.1|:22-71	92
BmHel-11(DF090321:4308055-4310065):915-1025	dbj|BAH66325.1|:121-156	83
BmHel-11(DF090321:4833173-4833467):194-295	gb|AAO33922.1|:22-55	82
BmHel-11(DF090321:4905262-4905505):124-243	gb|AAO33922.1|:22-77	64
BmHel-11(DF090321:4957723-4958008):121-285	gb|AAO33922.1|:22-76	85
BmHel-11(DF090321:5073369-5073662):153-293	gb|AAO33922.1|:22-68	87
BmHel-11(DF090321:5126012-5126303):124-291	gb|AAO33922.1|:22-77	87
BmHel-11(DF090321:5486518-5486761):124-243	gb|AAO33922.1|:22-77	66
BmHel-11(DF090321:5533232-5533526):127-294	gb|AAO33922.1|:22-77	51
BmHel-11(DF090321:5866790-5867026):105-236	gb|AAO33922.1|:22-65	90
BmHel-11(DF090321:5993728-5994005):167-277	gb|AAO33922.1|:22-58	86
BmHel-11(DF090321:6256735-6257020):121-285	gb|AAO33922.1|:22-76	76
BmHel-11(DF090321:6533079-6534334):1112-1255	gb|AAO33922.1|:22-70	79
BmHel-11(DF090321:6584780-6585056):148-276	gb|AAO33922.1|:22-64	83
BmHel-11(DF090321:6588644-6588935):142-291	gb|AAO33922.1|:22-71	92
BmHel-11(DF090321:6701638-6701929):124-291	gb|AAO33922.1|:22-77	82
BmHel-11(DF090321:7026839-7027129):186-290	gb|AAO33922.1|:22-56	88
BmHel-11(DF090321:7206662-7206944):157-282	gb|AAO33922.1|:22-63	90
BmHel-11(DF090321:7393186-7393472):124-270	gb|AAO33922.1|:29-77	87

BmHel-11(DF090321:7655432-7655723):133-291	gb|AAO33922.1|:22-74	83
BmHel-11(DF090321:7833232-7833499):107-267	gb|AAO33922.1|:22-75	52
BmHel-11(DF090321:7849072-7849349):155-277	gb|AAO33922.1|:22-67	76
BmHel-11(DF090321:796886-797171):124-285	gb|AAO33922.1|:22-77	75
BmHel-11(DF090321:838700-838991):124-291	gb|AAO33922.1|:22-77	78
BmHel-11(DF090321:873567-873856):122-289	gb|AAO33922.1|:22-77	87
BmHel-11(DF090322:1117868-1118134):116-266	gb|AAO33922.1|:22-71	67.5
BmHel-11(DF090322:1144449-1144699):164-250	gb|AAO33922.1|:22-50	93
BmHel-11(DF090322:118170-118445):124-275	gb|AAO33922.1|:22-77	61
BmHel-11(DF090322:1275869-1276131):101-262	gb|AAO33922.1|:22-76	78
BmHel-11(DF090322:1329587-1329876):124-234	gb|AAO33922.1|:41-77	78
BmHel-11(DF090322:138182-138472):123-290	gb|AAO33922.1|:22-77	87
BmHel-11(DF090322:1454096-1454361):122-265	gb|AAO33922.1|:22-69	81
BmHel-11(DF090322:1493869-1494117):124-248	gb|AAO33922.1|:22-77	65
BmHel-11(DF090322:1550436-1550727):124-291	gb|AAO33922.1|:22-77	92
BmHel-11(DF090322:1598982-1599273):160-291	gb|AAO33922.1|:22-65	88
BmHel-11(DF090322:1644649-1644939):147-290	gb|AAO33922.1|:22-69	83
BmHel-11(DF090322:1755566-1755867):124-301	gb|AAO33922.1|:22-77	68
BmHel-11(DF090322:2073286-2073554):125-268	gb|AAO33922.1|:22-69	91
BmHel-11(DF090322:210725-211020):179-295	gb|AAO33922.1|:22-60	89
BmHel-11(DF090322:2237769-2238050):117-281	gb|AAO33922.1|:22-76	80
BmHel-11(DF090322:2479929-2480176):134-247	gb|AAO33922.1|:22-74	56
BmHel-11(DF090322:2655563-2656162):459-599	gb|AAO33922.1|:22-68	74
BmHel-11(DF090322:3082654-3082945):130-291	gb|AAO33922.1|:22-75	87
BmHel-11(DF090322:3485900-3486190):123-290	gb|AAO33922.1|:22-77	85
BmHel-11(DF090322:3591117-3591408):124-291	gb|AAO33922.1|:22-77	85
BmHel-11(DF090322:3637335-3637636):200-301	gb|AAO33922.1|:22-55	88
BmHel-11(DF090322:3721816-3722079):124-255	gb|AAO33922.1|:25-77	69
BmHel-11(DF090322:3757624-3757915):160-291	gb|AAO33922.1|:22-65	81
BmHel-11(DF090322:3769718-3770009):124-291	gb|AAO33922.1|:22-77	85
BmHel-11(DF090322:4243450-4243728):126-278	gb|AAO33922.1|:22-77	64
BmHel-11(DF090322:4495279-4495571):125-292	gb|AAO33922.1|:22-77	58
BmHel-11(DF090322:4558153-4558674):343-444	dbj|BAH66325.1|:119-152	88
BmHel-11(DF090322:4614274-4621067):6441-6608	gb|AAO33922.1|:22-77	87
BmHel-11(DF090322:4614274-4621067):4995-5863	ref|XP_003705684.1|:7-339	36
BmHel-11(DF090322:4718228-4718501):124-273	gb|AAO33922.1|:22-77	57
BmHel-11(DF090322:4816788-4817080):125-292	gb|AAO33922.1|:22-77	80
BmHel-11(DF090322:5036006-5037539):1387-1533	gb|AAO33922.1|:22-70	85
BmHel-11(DF090322:5036006-5037539):516-637	ref|NP_001157648.1|:210-250	65
BmHel-11(DF090322:5036006-5037539):711-1136	ref|XP_318459.5|:1598-1742	40
BmHel-11(DF090322:5304625-5304916):124-291	gb|AAO33922.1|:22-77	89
BmHel-11(DF090322:5332259-5332550):129-291	gb|AAO33922.1|:22-77	63.5
BmHel-11(DF090322:5419671-5419965):124-294	gb|AAO33922.1|:22-77	87
BmHel-11(DF090322:547419-547688):162-269	gb|AAO33922.1|:22-57	80
BmHel-11(DF090322:5479680-5479998):124-318	gb|AAO33922.1|:22-77	76
BmHel-11(DF090322:5528295-5528586):160-291	gb|AAO33922.1|:22-65	86
BmHel-11(DF090322:5614752-5615043):124-291	gb|AAO33922.1|:22-77	83
BmHel-11(DF090322:5784269-5784533):132-264	gb|AAO33922.1|:22-75	63.5

BmHel-11(DF090322:5830897-5831157):105-260	gb|AAO33922.1|:22-73	61
BmHel-11(DF090322:5858543-5858838):124-295	gb|AAO33922.1|:22-77	63
BmHel-11(DF090322:6162606-6162882):112-276	gb|AAO33922.1|:22-77	75
BmHel-11(DF090322:6283118-6283416):125-298	gb|AAO33922.1|:22-77	56
BmHel-11(DF090322:6529851-6530139):124-288	gb|AAO33922.1|:22-77	85
BmHel-11(DF090322:6550106-6550399):126-293	gb|AAO33922.1|:22-77	83
BmHel-11(DF090322:6561845-6562136):160-291	gb|AAO33922.1|:22-65	77
BmHel-11(DF090322:6856476-6856771):128-295	gb|AAO33922.1|:22-77	71
BmHel-11(DF090322:6957359-6957651):155-292	gb|AAO33922.1|:22-67	89
BmHel-11(DF090322:7043734-7044013):116-280	gb|AAO33922.1|:22-76	87
BmHel-11(DF090322:7712183-7712465):126-282	gb|AAO33922.1|:22-75	56
BmHel-11(DF090322:7908991-7909279):121-288	gb|AAO33922.1|:22-77	51
BmHel-11(DF090322:7984909-7985191):116-282	gb|AAO33922.1|:22-76	54
BmHel-11(DF090322:8101334-8101625):124-291	gb|AAO33922.1|:22-77	85
BmHel-11(DF090322:8150097-8150389):155-292	gb|AAO33922.1|:22-67	89
BmHel-11(DF090322:8194885-8195178):180-293	gb|AAO33922.1|:22-59	94
BmHel-11(DF090322:8223519-8223793):124-274	gb|AAO33922.1|:22-77	61
BmHel-11(DF090322:852769-853014):132-245	gb|AAO33922.1|:22-59	84
BmHel-11(DF090322:940956-941248):131-292	gb|AAO33922.1|:22-75	85
BmHel-11(DF090323:1043345-1043635):126-290	gb|AAO33922.1|:22-77	64
BmHel-11(DF090323:1322016-1322305):116-289	gb|AAO33922.1|:22-77	82
BmHel-11(DF090323:1342217-1342508):124-291	gb|AAO33922.1|:22-77	57
BmHel-11(DF090323:1378367-1378657):126-290	gb|AAO33922.1|:22-77	53
BmHel-11(DF090323:1433093-1433379):161-286	gb|AAO33922.1|:22-63	88
BmHel-11(DF090323:1734979-1735263):150-284	gb|AAO33922.1|:22-69	83
BmHel-11(DF090323:1740202-1740493):124-291	gb|AAO33922.1|:22-77	82
BmHel-11(DF090323:1749630-1749919):124-289	gb|AAO33922.1|:22-77	67.5
BmHel-11(DF090323:1805325-1805616):124-291	gb|AAO33922.1|:22-77	89
BmHel-11(DF090323:1958500-1958791):160-291	gb|AAO33922.1|:22-65	88
BmHel-11(DF090323:2091185-2091475):165-290	gb|AAO33922.1|:22-63	83
BmHel-11(DF090323:2206585-2206890):174-305	gb|AAO33922.1|:22-65	79
BmHel-11(DF090323:2273093-2273381):121-288	gb|AAO33922.1|:22-77	87
BmHel-11(DF090323:2751437-2751738):124-301	gb|AAO33922.1|:22-77	71
BmHel-11(DF090323:2862045-2870662):5569-5685	dbj|BAH66325.1|:116-154	79
BmHel-11(DF090323:2862045-2870662):7337-7639	emb|CAX36785.1|:107-212	64
BmHel-11(DF090323:2961669-2962148):125-259	gb|AAO33922.1|:23-69	78
BmHel-11(DF090323:2987617-2987908):124-291	gb|AAO33922.1|:22-77	87
BmHel-11(DF090323:3024063-3024322):122-259	gb|AAO33922.1|:22-69	83
BmHel-11(DF090323:3416349-3416635):146-286	gb|AAO33922.1|:22-68	74
BmHel-11(DF090323:3597290-3597580):154-282	gb|AAO33922.1|:25-67	76
BmHel-11(DF090323:3727456-3727745):164-289	gb|AAO33922.1|:22-63	73
BmHel-11(DF090323:392235-392526):124-291	gb|AAO33922.1|:22-77	85
BmHel-11(DF090323:4198964-4199192):136-228	gb|AAO33922.1|:22-52	90
BmHel-11(DF090323:4275363-4275652):123-289	gb|AAO33922.1|:22-77	67.5
BmHel-11(DF090323:437263-437554):160-291	gb|AAO33922.1|:22-65	90
BmHel-11(DF090323:4763553-4763837):126-284	gb|AAO33922.1|:22-77	75
BmHel-11(DF090323:5034520-5034817):127-297	gb|AAO33922.1|:22-76	78
BmHel-11(DF090323:5135167-5135458):124-291	gb|AAO33922.1|:22-77	89

BmHel-11(DF090323:5576560-5576854):130-294	gb|AAO33922.1|:22-77	80
BmHel-11(DF090323:5699375-5699664):122-289	gb|AAO33922.1|:22-77	67
BmHel-11(DF090323:577383-578151):247-407	dbj|BAH66344.1|:45-95	52
BmHel-11(DF090323:577383-578151):601-768	gb|AAO33922.1|:22-77	91
BmHel-11(DF090323:6531392-6531685):126-293	gb|AAO33922.1|:22-77	85
BmHel-11(DF090323:6568205-6568504):123-299	gb|AAO33922.1|:22-77	61
BmHel-11(DF090323:6984078-6984364):124-286	gb|AAO33922.1|:22-77	64
BmHel-11(DF090323:7066588-7066879):124-291	gb|AAO33922.1|:22-77	66
BmHel-11(DF090323:7547874-7548154):125-280	gb|AAO33922.1|:22-77	80
BmHel-11(DF090323:7561833-7562104):104-271	gb|AAO33922.1|:22-77	83
BmHel-11(DF090323:7599257-7599543):122-286	gb|AAO33922.1|:22-76	80
BmHel-11(DF090323:7640924-7641215):124-291	gb|AAO33922.1|:22-77	83
BmHel-11(DF090323:8000185-8000476):124-291	gb|AAO33922.1|:22-77	83
BmHel-11(DF090323:84764-85053):150-281	gb|AAO33922.1|:25-68	79
BmHel-11(DF090324:1028774-1029065):157-291	gb|AAO33922.1|:22-66	86
BmHel-11(DF090324:1726799-1727074):123-275	gb|AAO33922.1|:22-77	73
BmHel-11(DF090324:1739510-1739793):116-283	gb|AAO33922.1|:22-77	85
BmHel-11(DF090324:1833010-1833301):124-291	gb|AAO33922.1|:22-77	82
BmHel-11(DF090324:2036880-2037168):123-257	gb|AAO33922.1|:33-77	86
BmHel-11(DF090324:239344-239617):124-273	gb|AAO33922.1|:22-77	71
BmHel-11(DF090324:2448238-2448529):124-291	gb|AAO33922.1|:22-77	87
BmHel-11(DF090324:2461484-2461775):160-291	gb|AAO33922.1|:22-65	77
BmHel-11(DF090324:2686962-2687255):126-293	gb|AAO33922.1|:22-77	89
BmHel-11(DF090324:2721680-2721953):106-273	gb|AAO33922.1|:22-77	78
BmHel-11(DF090324:2781654-2781948):123-269	gb|AAO33922.1|:29-77	85
BmHel-11(DF090324:3042129-3042419):126-290	gb|AAO33922.1|:22-77	62
BmHel-11(DF090324:3256593-3256885):124-292	gb|AAO33922.1|:22-77	65
BmHel-11(DF090324:3285621-3285914):151-293	gb|AAO33922.1|:22-77	57.5
BmHel-11(DF090324:3419083-3419373):142-243	gb|AAO33922.1|:38-71	82
BmHel-11(DF090324:3463902-3464209):143-307	gb|AAO33922.1|:22-77	78
BmHel-11(DF090324:3493086-3493395):187-309	gb|AAO33922.1|:22-62	82
BmHel-11(DF090324:3612512-3612803):160-291	gb|AAO33922.1|:22-65	84
BmHel-11(DF090324:3842044-3842331):165-287	gb|AAO33922.1|:22-62	75
BmHel-11(DF090324:4067512-4067804):134-292	gb|AAO33922.1|:22-74	69
BmHel-11(DF090324:4259429-4259717):124-288	gb|AAO33922.1|:22-77	83
BmHel-11(DF090324:4339274-4339560):137-286	gb|AAO33922.1|:22-71	66
BmHel-11(DF090324:4523981-4524223):123-242	gb|AAO33922.1|:22-77	64
BmHel-11(DF090324:462182-462476):130-294	gb|AAO33922.1|:22-76	83
BmHel-11(DF090324:4632522-4632812):123-290	gb|AAO33922.1|:22-77	72
BmHel-11(DF090324:4983139-4983434):124-295	gb|AAO33922.1|:22-77	66.5
BmHel-11(DF090324:5185497-5185784):124-287	gb|AAO33922.1|:22-77	63.5
BmHel-11(DF090324:5484087-5484377):153-290	gb|AAO33922.1|:22-67	82
BmHel-11(DF090324:551619-551910):124-291	gb|AAO33922.1|:22-77	85
BmHel-11(DF090324:5655908-5656336):76-204	dbj|BAH66325.1|:111-153	67
BmHel-11(DF090324:5655908-5656336):267-428	gb|AAO33922.1|:22-75	83
BmHel-11(DF090324:5722773-5723064):124-291	gb|AAO33922.1|:22-77	94
BmHel-11(DF090324:6192357-6192646):122-289	gb|AAO33922.1|:22-77	89
BmHel-11(DF090324:6307051-6307301):124-250	gb|AAO33922.1|:22-77	58.5

BmHel-11(DF090324:6954201-6954494):156-293	gb|AAO33922.1|:22-67	86
BmHel-11(DF090324:703586-703876):124-290	gb|AAO33922.1|:22-77	62.5
BmHel-11(DF090324:7284792-7285083):124-291	gb|AAO33922.1|:22-77	76
BmHel-11(DF090324:7708237-7708530):144-293	gb|AAO33922.1|:22-71	90
BmHel-11(DF090324:779724-779988):125-264	gb|AAO33922.1|:22-77	65
BmHel-11(DF090324:7810170-7810462):134-292	gb|AAO33922.1|:22-74	83
BmHel-11(DF090324:7978393-7978641):123-248	gb|AAO33922.1|:22-77	64
BmHel-11(DF090324:973242-973531):124-289	gb|AAO33922.1|:22-77	58.5
BmHel-11(DF090325:1071639-1071966):202-327	gb|AAO33922.1|:22-63	92
BmHel-11(DF090325:1091782-1092064):142-282	gb|AAO33922.1|:22-71	86
BmHel-11(DF090325:1132856-1133143):165-287	gb|AAO33922.1|:22-62	90
BmHel-11(DF090325:1611653-1611910):124-257	gb|AAO33922.1|:22-77	71
BmHel-11(DF090325:1659778-1660071):150-293	gb|AAO33922.1|:22-69	72
BmHel-11(DF090325:1737523-1737774):124-243	gb|AAO33922.1|:38-77	67
BmHel-11(DF090325:2268226-2268476):124-250	gb|AAO33922.1|:22-77	65.5
BmHel-11(DF090325:230453-230745):157-292	gb|AAO33922.1|:22-66	60
BmHel-11(DF090325:2438342-2438633):124-291	gb|AAO33922.1|:22-77	87
BmHel-11(DF090325:2712646-2712935):121-289	gb|AAO33922.1|:22-75	65
BmHel-11(DF090325:3098299-3098580):114-281	gb|AAO33922.1|:22-77	80
BmHel-11(DF090325:3167571-3167863):131-292	gb|AAO33922.1|:22-77	57
BmHel-11(DF090325:3225706-3226192):361-486	gb|AAO33922.1|:22-70	70
BmHel-11(DF090325:3398946-3399239):124-204	gb|AAO33922.1|:51-77	92
BmHel-11(DF090325:3523326-3523595):105-269	gb|AAO33922.1|:22-76	81
BmHel-11(DF090325:3616367-3616632):146-265	gb|AAO33922.1|:22-61	75
BmHel-11(DF090325:3627924-3628215):124-291	gb|AAO33922.1|:22-77	92
BmHel-11(DF090325:3722880-3723172):143-292	gb|AAO33922.1|:22-71	90
BmHel-11(DF090325:4111888-4112178):153-290	gb|AAO33922.1|:22-67	73
BmHel-11(DF090325:4139001-4139292):124-291	gb|AAO33922.1|:22-77	82
BmHel-11(DF090325:4356536-4356829):126-293	gb|AAO33922.1|:22-77	60
BmHel-11(DF090325:4467458-4467749):124-291	gb|AAO33922.1|:22-77	85
BmHel-11(DF090325:4531266-4531557):124-291	gb|AAO33922.1|:22-77	83
BmHel-11(DF090325:4540131-4540423):155-292	gb|AAO33922.1|:22-67	80
BmHel-11(DF090325:4568118-4568373):124-255	gb|AAO33922.1|:22-77	69
BmHel-11(DF090325:4732550-4732821):117-271	gb|AAO33922.1|:22-77	60
BmHel-11(DF090325:4864230-4864527):133-297	gb|AAO33922.1|:22-76	80
BmHel-11(DF090325:4914746-4915037):124-291	gb|AAO33922.1|:22-77	89
BmHel-11(DF090325:4930607-4930898):124-291	gb|AAO33922.1|:22-77	82
BmHel-11(DF090325:4985143-4985439):126-296	gb|AAO33922.1|:22-77	84
BmHel-11(DF090325:5089266-5089555):127-289	gb|AAO33922.1|:22-76	61.5
BmHel-11(DF090325:5150511-5150802):124-291	gb|AAO33922.1|:22-77	82
BmHel-11(DF090325:5181131-5181421):162-290	gb|AAO33922.1|:22-64	83
BmHel-11(DF090325:5365618-5366301):516-683	gb|AAO33922.1|:22-77	75
BmHel-11(DF090325:5472565-5472860):164-295	gb|AAO33922.1|:22-64	86
BmHel-11(DF090325:5528833-5529125):155-292	gb|AAO33922.1|:22-67	82
BmHel-11(DF090325:6054491-6054785):124-294	gb|AAO33922.1|:22-77	80
BmHel-11(DF090325:6115096-6115387):142-291	gb|AAO33922.1|:22-71	88
BmHel-11(DF090325:6126991-6127278):171-287	gb|AAO33922.1|:22-60	89
BmHel-11(DF090325:6323387-6323677):123-290	gb|AAO33922.1|:22-77	94

BmHel-11(DF090325:6540846-6541130):124-284	gb|AAO33922.1|:22-77	62
BmHel-11(DF090325:722752-723039):122-287	gb|AAO33922.1|:22-77	58.5
BmHel-11(DF090325:7256897-7257186):186-290	gb|AAO33922.1|:22-56	85
BmHel-11(DF090325:7287102-7287386):117-284	gb|AAO33922.1|:22-77	87
BmHel-11(DF090325:744777-745056):124-279	gb|AAO33922.1|:22-77	76
BmHel-11(DF090325:829342-829570):106-228	gb|AAO33922.1|:22-62	82
BmHel-11(DF090325:92241-92536):155-295	gb|AAO33922.1|:22-67	78
BmHel-11(DF090326:110303-110594):166-291	gb|AAO33922.1|:22-63	90
BmHel-11(DF090326:1444455-1444744):122-289	gb|AAO33922.1|:22-77	78
BmHel-11(DF090326:1512399-1512641):120-242	gb|AAO33922.1|:22-62	90
BmHel-11(DF090326:1682813-1683104):124-291	gb|AAO33922.1|:22-77	91
BmHel-11(DF090326:1737246-1737535):122-289	gb|AAO33922.1|:22-77	85
BmHel-11(DF090326:2057245-2057537):169-292	gb|AAO33922.1|:22-65	67.5
BmHel-11(DF090326:2242176-2242485):151-309	gb|AAO33922.1|:22-74	84
BmHel-11(DF090326:2285756-2286045):124-289	gb|AAO33922.1|:22-77	64
BmHel-11(DF090326:2316912-2317203):124-291	gb|AAO33922.1|:22-77	85
BmHel-11(DF090326:2520060-2520312):110-220	gb|AAO33922.1|:32-68	75
BmHel-11(DF090326:2868835-2869125):160-291	gb|AAO33922.1|:22-65	93
BmHel-11(DF090326:2961163-2961452):123-290	gb|AAO33922.1|:22-77	91
BmHel-11(DF090326:3085182-3085474):125-292	gb|AAO33922.1|:22-77	82
BmHel-11(DF090326:3186364-3186637):107-274	gb|AAO33922.1|:22-77	91
BmHel-11(DF090326:3346735-3347019):133-284	gb|AAO33922.1|:22-75	64
BmHel-11(DF090326:3825909-3826200):124-291	gb|AAO33922.1|:22-77	87
BmHel-11(DF090326:4227765-4228045):143-280	gb|AAO33922.1|:22-67	78
BmHel-11(DF090326:436680-436971):148-291	gb|AAO33922.1|:22-69	68
BmHel-11(DF090326:4530785-4531078):144-293	gb|AAO33922.1|:22-71	82
BmHel-11(DF090326:4577044-4577335):124-291	gb|AAO33922.1|:22-77	82
BmHel-11(DF090326:4865188-4865468):124-280	gb|AAO33922.1|:22-77	64.5
BmHel-11(DF090326:4922420-4922711):124-291	gb|AAO33922.1|:22-77	89
BmHel-11(DF090326:4927758-4928039):123-281	gb|AAO33922.1|:22-77	83
BmHel-11(DF090326:4951964-4952254):156-290	gb|AAO33922.1|:22-67	71
BmHel-11(DF090326:5074265-5074521):119-256	gb|AAO33922.1|:22-77	67
BmHel-11(DF090326:5107735-5108000):122-265	gb|AAO33922.1|:22-69	62
BmHel-11(DF090326:5224464-5224732):124-243	gb|AAO33922.1|:29-77	71
BmHel-11(DF090326:5301857-5302123):123-266	gb|AAO33922.1|:22-69	60
BmHel-11(DF090326:5405242-5405533):160-291	gb|AAO33922.1|:22-65	84
BmHel-11(DF090326:5470144-5470580):50-166	dbj|BAH66325.1|:114-152	66
BmHel-11(DF090326:5470144-5470580):269-436	gb|AAO33922.1|:22-75	62
BmHel-11(DF090326:5551190-5551497):197-307	gb|AAO33922.1|:22-58	86
BmHel-11(DF090326:5561544-5561835):154-291	gb|AAO33922.1|:22-67	86
BmHel-11(DF090326:5639249-5639536):150-287	gb|AAO33922.1|:22-67	86
BmHel-11(DF090326:5657337-5657591):124-254	gb|AAO33922.1|:22-77	66
BmHel-11(DF090326:5682789-5683078):167-281	gb|AAO33922.1|:25-66	59
BmHel-11(DF090326:5712559-5712850):124-291	gb|AAO33922.1|:22-77	85
BmHel-11(DF090326:5858669-5858948):124-279	gb|AAO33922.1|:22-77	83
BmHel-11(DF090326:5921138-5921384):145-246	gb|AAO33922.1|:22-71	64
BmHel-11(DF090326:6226998-6227287):125-289	gb|AAO33922.1|:22-77	62
BmHel-11(DF090326:624949-625191):124-242	gb|AAO33922.1|:22-77	68.5

BmHel-11(DF090326:6311575-6311865):165-290	gb|AAO33922.1|:22-63	85
BmHel-11(DF090326:6442393-6442693):118-300	gb|AAO33922.1|:22-77	81
BmHel-11(DF090326:6534144-6534435):124-291	gb|AAO33922.1|:22-77	87
BmHel-11(DF090326:6592051-6592342):160-291	gb|AAO33922.1|:22-65	88
BmHel-11(DF090326:6836760-6837049):134-289	gb|AAO33922.1|:22-73	73
BmHel-11(DF090326:6891205-6891495):165-290	gb|AAO33922.1|:22-63	85
BmHel-11(DF090327:109379-109655):139-276	gb|AAO33922.1|:22-67	80
BmHel-11(DF090327:1226014-1226317):123-303	gb|AAO33922.1|:22-77	62
BmHel-11(DF090327:123625-123916):124-291	gb|AAO33922.1|:22-77	83
BmHel-11(DF090327:1341371-1341666):124-295	gb|AAO33922.1|:22-77	50
BmHel-11(DF090327:1721076-1721358):115-282	gb|AAO33922.1|:22-77	94
BmHel-11(DF090327:1970143-1970431):166-288	gb|AAO33922.1|:22-62	80
BmHel-11(DF090327:1987608-1987896):124-288	gb|AAO33922.1|:22-77	87
BmHel-11(DF090327:2274994-2275284):124-290	gb|AAO33922.1|:22-77	65.5
BmHel-11(DF090327:2444536-2444793):135-257	gb|AAO33922.1|:22-62	85
BmHel-11(DF090327:2907432-2907723):121-291	gb|AAO33922.1|:22-77	64
BmHel-11(DF090327:2924203-2924494):124-291	gb|AAO33922.1|:22-77	87
BmHel-11(DF090327:3184490-3184746):126-256	gb|AAO33922.1|:22-76	59.5
BmHel-11(DF090327:3604759-3605033):143-274	gb|AAO33922.1|:22-65	88
BmHel-11(DF090327:3895984-3896227):124-243	gb|AAO33922.1|:22-77	69
BmHel-11(DF090327:4053917-4054415):124-231	gb|AAO33922.1|:42-77	80
BmHel-11(DF090327:4312422-4312667):84-245	gb|AAO33922.1|:22-77	89
BmHel-11(DF090327:4409169-4409462):127-293	gb|AAO33922.1|:22-76	65
BmHel-11(DF090327:441306-441596):123-290	gb|AAO33922.1|:22-77	87
BmHel-11(DF090327:461118-461383):122-265	gb|AAO33922.1|:22-69	89
BmHel-11(DF090327:4848801-4849092):160-291	gb|AAO33922.1|:22-65	88
BmHel-11(DF090327:5074019-5074310):130-291	gb|AAO33922.1|:22-77	57
BmHel-11(DF090327:5518618-5519352):368-525	dbj|BAH66344.1|:45-94	68
BmHel-11(DF090327:5599812-5600106):157-294	gb|AAO33922.1|:22-67	91
BmHel-11(DF090327:5649892-5650163):107-271	gb|AAO33922.1|:22-76	83
BmHel-11(DF090327:5682082-5682374):124-240	gb|AAO33922.1|:39-77	84
BmHel-11(DF090327:5788968-5789258):123-290	gb|AAO33922.1|:22-77	83
BmHel-11(DF090327:6188135-6188424):124-234	gb|AAO33922.1|:41-77	86
BmHel-11(DF090327:876652-876942):159-290	gb|AAO33922.1|:22-65	86
BmHel-11(DF090328:1054798-1055064):126-266	gb|AAO33922.1|:22-69	87
BmHel-11(DF090328:1206573-1206823):119-250	gb|AAO33922.1|:22-71	68
BmHel-11(DF090328:121529-121796):118-267	gb|AAO33922.1|:22-71	72
BmHel-11(DF090328:1377813-1378109):138-296	gb|AAO33922.1|:22-74	73
BmHel-11(DF090328:1646681-1646973):155-292	gb|AAO33922.1|:22-67	73
BmHel-11(DF090328:1756906-1757197):124-291	gb|AAO33922.1|:22-77	87
BmHel-11(DF090328:1778246-1778520):149-274	gb|AAO33922.1|:22-69	77
BmHel-11(DF090328:1925205-1925490):124-285	gb|AAO33922.1|:22-77	78
BmHel-11(DF090328:1957331-1957666):204-335	gb|AAO33922.1|:22-65	79
BmHel-11(DF090328:2002137-2002427):123-290	gb|AAO33922.1|:22-77	85
BmHel-11(DF090328:214900-215207):128-307	gb|AAO33922.1|:22-77	76
BmHel-11(DF090328:2406829-2407110):117-281	gb|AAO33922.1|:22-77	62
BmHel-11(DF090328:2443623-2443911):121-288	gb|AAO33922.1|:22-77	91
BmHel-11(DF090328:2621389-2621672):116-283	gb|AAO33922.1|:22-77	85

BmHel-11(DF090328:2730850-2731123):123-273	gb|AAO33922.1|:22-77	59
BmHel-11(DF090328:2740886-2741161):125-275	gb|AAO33922.1|:22-77	57.5
BmHel-11(DF090328:2918123-2919130):742-966	ref|XP_002075098.1|:4-284	43
BmHel-11(DF090328:2974798-2975089):124-291	gb|AAO33922.1|:22-77	82
BmHel-11(DF090328:3086418-3086708):153-290	gb|AAO33922.1|:22-67	63
BmHel-11(DF090328:3219952-3220241):158-289	gb|AAO33922.1|:22-65	88
BmHel-11(DF090328:3270340-3270623):124-283	gb|AAO33922.1|:22-77	51.5
BmHel-11(DF090328:3279011-3279324):138-313	gb|AAO33922.1|:22-77	66.5
BmHel-11(DF090328:3310925-3311173):130-248	gb|AAO33922.1|:22-77	66
BmHel-11(DF090328:3410982-3411264):151-282	gb|AAO33922.1|:22-65	84
BmHel-11(DF090328:3565487-3565777):123-290	gb|AAO33922.1|:22-77	82
BmHel-11(DF090328:3594889-3595180):142-291	gb|AAO33922.1|:22-71	86
BmHel-11(DF090328:3622724-3623039):144-315	gb|AAO33922.1|:22-71	63
BmHel-11(DF090328:3900944-3901235):124-291	gb|AAO33922.1|:22-77	89
BmHel-11(DF090328:4050170-4050461):124-291	gb|AAO33922.1|:22-77	83
BmHel-11(DF090328:4133111-4133402):124-291	gb|AAO33922.1|:22-77	78
BmHel-11(DF090328:419142-419434):146-292	gb|AAO33922.1|:22-70	89
BmHel-11(DF090328:4241576-4241865):127-289	gb|AAO33922.1|:22-76	68
BmHel-11(DF090328:4524968-4525259):160-291	gb|AAO33922.1|:22-65	84
BmHel-11(DF090328:4739186-4739477):124-291	gb|AAO33922.1|:22-77	91
BmHel-11(DF090328:4878329-4878617):124-288	gb|AAO33922.1|:22-77	66
BmHel-11(DF090328:4967649-4967935):137-286	gb|AAO33922.1|:22-71	88
BmHel-11(DF090328:5169276-5169519):124-243	gb|AAO33922.1|:22-77	67
BmHel-11(DF090328:5382789-5383081):124-292	gb|AAO33922.1|:22-77	61.5
BmHel-11(DF090328:5554150-5554442):125-292	gb|AAO33922.1|:22-77	85
BmHel-11(DF090328:5796042-5796285):124-243	gb|AAO33922.1|:22-77	66
BmHel-11(DF090328:5882611-5882853):150-242	gb|AAO33922.1|:22-52	96
BmHel-11(DF090328:6121709-6121994):152-286	gb|AAO33922.1|:22-66	84
BmHel-11(DF090328:6353441-6353735):163-294	gb|AAO33922.1|:22-65	79
BmHel-11(DF090328:6688750-6689006):124-257	gb|AAO33922.1|:22-77	64
BmHel-11(DF090328:708555-708847):134-292	gb|AAO33922.1|:22-74	86
BmHel-11(DF090328:88297-88588):124-291	gb|AAO33922.1|:22-77	83
BmHel-11(DF090328:889699-889986):124-287	gb|AAO33922.1|:22-77	58.5
BmHel-11(DF090329:1184552-1184841):150-233	gb|AAO33922.1|:41-68	96
BmHel-11(DF090329:1402255-1402549):128-295	gb|AAO33922.1|:22-77	87
BmHel-11(DF090329:1770322-1770611):134-289	gb|AAO33922.1|:22-73	80
BmHel-11(DF090329:1912544-1912860):152-316	gb|AAO33922.1|:22-76	76
BmHel-11(DF090329:1943022-1943313):124-291	gb|AAO33922.1|:22-77	89
BmHel-11(DF090329:2369475-2369763):148-288	gb|AAO33922.1|:22-68	89
BmHel-11(DF090329:2382303-2382593):124-290	gb|AAO33922.1|:22-77	71
BmHel-11(DF090329:2404895-2405186):124-291	gb|AAO33922.1|:22-77	89
BmHel-11(DF090329:2411407-2411700):126-293	gb|AAO33922.1|:22-77	60
BmHel-11(DF090329:2486397-2486687):153-290	gb|AAO33922.1|:22-67	78
BmHel-11(DF090329:2674427-2674723):147-296	gb|AAO33922.1|:22-71	88
BmHel-11(DF090329:2843516-2843807):160-291	gb|AAO33922.1|:22-65	86
BmHel-11(DF090329:2993348-2993591):124-243	gb|AAO33922.1|:22-77	66
BmHel-11(DF090329:3133557-3133848):124-291	gb|AAO33922.1|:22-77	91
BmHel-11(DF090329:3463946-3464236):126-290	gb|AAO33922.1|:22-77	62

BmHel-11(DF090329:3555244-3555493):124-249	gb|AAO33922.1|:22-77	64
BmHel-11(DF090329:4075228-4075522):127-294	gb|AAO33922.1|:22-76	80
BmHel-11(DF090329:4091607-4091895):169-288	gb|AAO33922.1|:22-61	90
BmHel-11(DF090329:4206580-4206871):157-291	gb|AAO33922.1|:22-66	82
BmHel-11(DF090329:421961-422251):128-229	gb|AAO33922.1|:44-77	82
BmHel-11(DF090329:4248376-4248617):134-241	gb|AAO33922.1|:22-57	88
BmHel-11(DF090329:4400755-4401046):142-291	gb|AAO33922.1|:22-71	82
BmHel-11(DF090329:4571591-4571882):124-291	gb|AAO33922.1|:22-77	75
BmHel-11(DF090329:4675917-4676206):127-260	gb|AAO33922.1|:33-77	63.5
BmHel-11(DF090329:4826737-4827028):124-291	gb|AAO33922.1|:22-77	91
BmHel-11(DF090329:4918556-4918840):161-284	gb|AAO33922.1|:22-65	63.5
BmHel-11(DF090329:4986753-4987011):92-258	gb|AAO33922.1|:22-76	67
BmHel-11(DF090329:5232561-5232818):135-257	gb|AAO33922.1|:22-74	64
BmHel-11(DF090329:5278922-5279214):149-292	gb|AAO33922.1|:22-69	79
BmHel-11(DF090329:5313867-5314144):125-277	gb|AAO33922.1|:22-71	76
BmHel-11(DF090329:5337487-5337778):124-291	gb|AAO33922.1|:22-77	85
BmHel-11(DF090329:5609419-5609705):125-286	gb|AAO33922.1|:22-77	76
BmHel-11(DF090329:5630067-5630358):160-291	gb|AAO33922.1|:22-65	90
BmHel-11(DF090329:5967183-5967476):126-293	gb|AAO33922.1|:22-77	75
BmHel-11(DF090329:6076966-6077259):150-293	gb|AAO33922.1|:22-69	85
BmHel-11(DF090329:6092826-6093117):124-291	gb|AAO33922.1|:22-77	76
BmHel-11(DF090329:6126353-6126650):133-297	gb|AAO33922.1|:22-76	81
BmHel-11(DF090329:6213917-6214206):160-289	gb|AAO33922.1|:22-65	71
BmHel-11(DF090329:6265323-6265614):127-291	gb|AAO33922.1|:22-76	87
BmHel-11(DF090329:6547141-6547416):114-275	gb|AAO33922.1|:22-77	73
BmHel-11(DF090329:685559-685851):155-292	gb|AAO33922.1|:22-67	76
BmHel-11(DF090329:796361-796640):124-279	gb|AAO33922.1|:22-77	62
BmHel-11(DF090329:821999-822289):123-290	gb|AAO33922.1|:22-77	89
BmHel-11(DF090329:84173-84464):124-291	gb|AAO33922.1|:22-77	87
BmHel-11(DF090330:1151456-1151754):131-298	gb|AAO33922.1|:22-77	87
BmHel-11(DF090330:1223111-1223401):122-238	gb|AAO33922.1|:39-77	92
BmHel-11(DF090330:1349131-1349422):124-291	gb|AAO33922.1|:22-77	92
BmHel-11(DF090330:1445265-1445562):133-297	gb|AAO33922.1|:22-77	58
BmHel-11(DF090330:147999-148268):126-269	gb|AAO33922.1|:22-69	81
BmHel-11(DF090330:1711108-1711399):142-291	gb|AAO33922.1|:22-71	86
BmHel-11(DF090330:1760026-1760325):134-299	gb|AAO33922.1|:22-74	65
BmHel-11(DF090330:1815776-1816065):122-289	gb|AAO33922.1|:22-77	57
BmHel-11(DF090330:1893372-1893661):122-289	gb|AAO33922.1|:22-77	85
BmHel-11(DF090330:1930932-1931221):164-289	gb|AAO33922.1|:22-63	69
BmHel-11(DF090330:2017685-2017976):124-291	gb|AAO33922.1|:22-77	87
BmHel-11(DF090330:2097646-2097943):130-297	gb|AAO33922.1|:22-77	91
BmHel-11(DF090330:2145941-2146126):75-185	gb|AAO33922.1|:22-58	86
BmHel-11(DF090330:2268424-2268719):164-295	gb|AAO33922.1|:22-65	88
BmHel-11(DF090330:2382597-2382863):124-266	gb|AAO33922.1|:22-77	64
BmHel-11(DF090330:2386252-2386545):150-293	gb|AAO33922.1|:22-69	81
BmHel-11(DF090330:2573031-2573287):137-256	gb|AAO33922.1|:22-61	85
BmHel-11(DF090330:3005408-3005652):125-244	gb|AAO33922.1|:22-77	67
BmHel-11(DF090330:3017744-3018038):128-286	gb|AAO33922.1|:25-77	81

BmHel-11(DF090330:3032175-3032461):124-286	gb|AAO33922.1|:22-77	56
BmHel-11(DF090330:3068004-3068232):136-228	gb|AAO33922.1|:22-52	96
BmHel-11(DF090330:3108907-3109224):177-317	gb|AAO33922.1|:22-68	87
BmHel-11(DF090330:3160228-3160511):128-283	gb|AAO33922.1|:22-74	60
BmHel-11(DF090330:3180136-3180436):124-300	gb|AAO33922.1|:22-77	77
BmHel-11(DF090330:3215589-3215875):131-286	gb|AAO33922.1|:22-73	92
BmHel-11(DF090330:3257632-3257922):141-290	gb|AAO33922.1|:22-71	88
BmHel-11(DF090330:3442314-3442606):155-292	gb|AAO33922.1|:22-67	80
BmHel-11(DF090330:3466153-3466444):124-291	gb|AAO33922.1|:22-77	94
BmHel-11(DF090330:3588615-3588858):142-243	gb|AAO33922.1|:22-71	64
BmHel-11(DF090330:3655260-3655541):124-281	gb|AAO33922.1|:22-77	66
BmHel-11(DF090330:3671903-3672170):172-267	gb|AAO33922.1|:22-53	90
BmHel-11(DF090330:3697729-3697989):124-260	gb|AAO33922.1|:22-77	64
BmHel-11(DF090330:3744466-3744756):174-290	gb|AAO33922.1|:22-60	82
BmHel-11(DF090330:376167-376468):127-301	gb|AAO33922.1|:22-76	63.5
BmHel-11(DF090330:3828195-3828486):154-291	gb|AAO33922.1|:22-67	89
BmHel-11(DF090330:3917664-3917955):124-291	gb|AAO33922.1|:22-77	87
BmHel-11(DF090330:3996950-3997241):124-291	gb|AAO33922.1|:22-77	87
BmHel-11(DF090330:402012-402311):132-299	gb|AAO33922.1|:22-77	91
BmHel-11(DF090330:4030308-4030607):124-299	gb|AAO33922.1|:22-77	67
BmHel-11(DF090330:4077117-4077360):91-243	gb|AAO33922.1|:22-68	78
BmHel-11(DF090330:4152480-4152782):123-302	gb|AAO33922.1|:22-77	85
BmHel-11(DF090330:4336481-4336770):163-289	gb|AAO33922.1|:22-64	64
BmHel-11(DF090330:4434717-4435014):124-297	gb|AAO33922.1|:22-77	63
BmHel-11(DF090330:4556700-4556979):123-279	gb|AAO33922.1|:22-77	61.5
BmHel-11(DF090330:466473-466764):124-291	gb|AAO33922.1|:22-77	92
BmHel-11(DF090330:4786049-4786338):124-289	gb|AAO33922.1|:22-77	62
BmHel-11(DF090330:4823366-4823653):132-287	gb|AAO33922.1|:22-73	90
BmHel-11(DF090330:5401462-5401756):126-251	gb|AAO33922.1|:36-77	80
BmHel-11(DF090330:5460495-5460770):144-275	gb|AAO33922.1|:22-67	82
BmHel-11(DF090330:5559532-5559823):160-291	gb|AAO33922.1|:22-65	93
BmHel-11(DF090330:5604463-5604762):134-250	gb|AAO33922.1|:36-74	87
BmHel-11(DF090330:5872803-5873094):124-291	gb|AAO33922.1|:22-77	87
BmHel-11(DF090330:604529-604820):160-291	gb|AAO33922.1|:22-65	86
BmHel-11(DF090330:6262411-6262702):160-291	gb|AAO33922.1|:22-65	90
BmHel-11(DF090330:6287454-6287741):124-287	gb|AAO33922.1|:22-77	68.5
BmHel-11(DF090330:6304908-6305157):123-249	gb|AAO33922.1|:22-77	64
BmHel-11(DF090330:6332359-6332640):157-281	gb|AAO33922.1|:22-66	69
BmHel-11(DF090330:867491-867770):123-279	gb|AAO33922.1|:22-77	66.5
BmHel-11(DF090331:1010137-1010422):124-285	gb|AAO33922.1|:22-77	83
BmHel-11(DF090331:1117600-1117843):124-243	gb|AAO33922.1|:22-77	64
BmHel-11(DF090331:1132585-1132875):123-290	gb|AAO33922.1|:22-77	82
BmHel-11(DF090331:1627218-1627507):128-289	gb|AAO33922.1|:22-75	79
BmHel-11(DF090331:2096031-2096322):124-291	gb|AAO33922.1|:22-77	87
BmHel-11(DF090331:2120767-2121416):168-331	dbj|BAH66344.1|:45-96	65.5
BmHel-11(DF090331:2166398-2166656):148-258	gb|AAO33922.1|:22-70	69
BmHel-11(DF090331:2207013-2207299):143-286	gb|AAO33922.1|:22-71	86
BmHel-11(DF090331:2409301-2409591):123-290	gb|AAO33922.1|:22-77	91

BmHel-11(DF090331:2719936-2720218):124-282	gb|AAO33922.1|:22-77	57
BmHel-11(DF090331:2729863-2730121):115-258	gb|AAO33922.1|:22-69	83
BmHel-11(DF090331:2824605-2824866):124-261	gb|AAO33922.1|:22-77	71
BmHel-11(DF090331:3126096-3126390):127-294	gb|AAO33922.1|:22-77	91
BmHel-11(DF090331:3357912-3358201):125-289	gb|AAO33922.1|:22-76	80
BmHel-11(DF090331:3700932-3701223):124-291	gb|AAO33922.1|:22-77	89
BmHel-11(DF090331:3889801-3890096):128-295	gb|AAO33922.1|:22-77	87
BmHel-11(DF090331:4139637-4139927):153-290	gb|AAO33922.1|:22-67	67
BmHel-11(DF090331:4157253-4157544):124-291	gb|AAO33922.1|:22-77	87
BmHel-11(DF090331:4685439-4685731):155-292	gb|AAO33922.1|:22-67	73
BmHel-11(DF090331:4709637-4709934):124-297	gb|AAO33922.1|:22-77	60
BmHel-11(DF090331:4732102-4732389):165-287	gb|AAO33922.1|:22-62	82
BmHel-11(DF090331:4776044-4776335):160-291	gb|AAO33922.1|:22-65	90
BmHel-11(DF090331:5031204-5031498):123-294	gb|AAO33922.1|:22-77	61.5
BmHel-11(DF090331:5084637-5084930):157-252	gb|AAO33922.1|:36-67	87
BmHel-11(DF090331:524013-524257):137-244	gb|AAO33922.1|:22-57	88
BmHel-11(DF090331:5277327-5277612):129-285	gb|AAO33922.1|:22-77	65
BmHel-11(DF090331:5424728-5425019):124-291	gb|AAO33922.1|:22-77	85
BmHel-11(DF090331:5712870-5713160):153-290	gb|AAO33922.1|:22-67	71
BmHel-11(DF090331:5817163-5817443):125-280	gb|AAO33922.1|:22-77	69
BmHel-11(DF090331:5920145-5920438):126-293	gb|AAO33922.1|:22-77	76
BmHel-11(DF090331:626105-626352):111-248	gb|AAO33922.1|:22-77	67
BmHel-11(DF090331:901003-901430):274-401	gb|AAO33922.1|:32-77	68.5
BmHel-11(DF090332:1063133-1063418):157-285	gb|AAO33922.1|:22-64	88
BmHel-11(DF090332:1191073-1191365):125-292	gb|AAO33922.1|:22-77	82
BmHel-11(DF090332:1259535-1259840):123-305	gb|AAO33922.1|:22-77	65
BmHel-11(DF090332:1321318-1321569):165-251	gb|AAO33922.1|:22-50	96
BmHel-11(DF090332:1488481-1488744):126-263	gb|AAO33922.1|:22-77	58
BmHel-11(DF090332:1537714-1538006):155-292	gb|AAO33922.1|:22-67	82
BmHel-11(DF090332:1564382-1564675):132-293	gb|AAO33922.1|:22-75	81
BmHel-11(DF090332:17039-17333):163-294	gb|AAO33922.1|:22-65	93
BmHel-11(DF090332:1709869-1710151):160-282	gb|AAO33922.1|:22-62	87
BmHel-11(DF090332:1820234-1820544):179-310	gb|AAO33922.1|:22-65	72
BmHel-11(DF090332:1888903-1889195):125-292	gb|AAO33922.1|:22-77	82
BmHel-11(DF090332:19947-20216):119-208	gb|AAO33922.1|:42-71	86
BmHel-11(DF090332:2071827-2072106):116-280	gb|AAO33922.1|:22-76	85
BmHel-11(DF090332:2435283-2435579):124-296	gb|AAO33922.1|:22-77	61.5
BmHel-11(DF090332:2701861-2702152):124-291	gb|AAO33922.1|:22-77	89
BmHel-11(DF090332:2814782-2815073):142-291	gb|AAO33922.1|:22-71	88
BmHel-11(DF090332:2855086-2855371):112-285	gb|AAO33922.1|:22-77	55
BmHel-11(DF090332:2885610-2885897):174-287	gb|AAO33922.1|:22-59	92
BmHel-11(DF090332:2917354-2917645):124-291	gb|AAO33922.1|:22-77	82
BmHel-11(DF090332:311488-311778):124-290	gb|AAO33922.1|:22-77	71
BmHel-11(DF090332:3141386-3141629):124-243	gb|AAO33922.1|:22-77	67
BmHel-11(DF090332:3274873-3275157):142-284	gb|AAO33922.1|:22-71	61.5
BmHel-11(DF090332:3339464-3339767):172-303	gb|AAO33922.1|:22-65	86
BmHel-11(DF090332:3572519-3572811):161-292	gb|AAO33922.1|:22-65	88
BmHel-11(DF090332:4154335-4154625):127-290	gb|AAO33922.1|:22-76	44

BmHel-11(DF090332:4580380-4580670):123-290	gb|AAO33922.1|:22-77	91
BmHel-11(DF090332:4707317-4707611):124-294	gb|AAO33922.1|:22-77	75
BmHel-11(DF090332:4930687-4930975):160-288	gb|AAO33922.1|:22-64	93
BmHel-11(DF090332:4941068-4941360):124-292	gb|AAO33922.1|:22-77	67
BmHel-11(DF090332:4971071-4971362):124-291	gb|AAO33922.1|:22-77	91
BmHel-11(DF090332:5000917-5001207):124-290	gb|AAO33922.1|:22-77	61
BmHel-11(DF090332:5031256-5031528):159-272	gb|AAO33922.1|:22-65	77
BmHel-11(DF090332:5171929-5172219):159-290	gb|AAO33922.1|:22-65	93
BmHel-11(DF090332:5634305-5634594):123-289	gb|AAO33922.1|:22-77	59.5
BmHel-11(DF090332:568901-569160):124-259	gb|AAO33922.1|:22-77	48.5
BmHel-11(DF090332:5839300-5839585):118-285	gb|AAO33922.1|:22-77	80
BmHel-11(DF090332:5903409-5903671):125-262	gb|AAO33922.1|:22-67	89
BmHel-11(DF090332:5967793-5968082):122-289	gb|AAO33922.1|:22-77	89
BmHel-11(DF090332:6006954-6007243):124-289	gb|AAO33922.1|:22-77	64
BmHel-11(DF090332:6216400-6216691):124-291	gb|AAO33922.1|:22-77	87
BmHel-11(DF090332:6275592-6275868):124-276	gb|AAO33922.1|:22-77	76
BmHel-11(DF090332:743772-744081):152-309	gb|AAO33922.1|:22-67	62
BmHel-11(DF090332:872369-872659):123-290	gb|AAO33922.1|:22-77	87
BmHel-11(DF090332:907713-907991):136-278	gb|AAO33922.1|:22-71	61
BmHel-11(DF090332:92708-92981):124-273	gb|AAO33922.1|:22-77	60
BmHel-11(DF090333:1320961-1321252):160-291	gb|AAO33922.1|:22-65	84
BmHel-11(DF090333:1513254-1513545):124-291	gb|AAO33922.1|:22-77	87
BmHel-11(DF090333:1532568-1532859):160-291	gb|AAO33922.1|:22-65	90
BmHel-11(DF090333:1769153-1769454):134-301	gb|AAO33922.1|:22-77	71
BmHel-11(DF090333:1852283-1852532):151-249	gb|AAO33922.1|:22-68	63
BmHel-11(DF090333:1998780-1999071):124-291	gb|AAO33922.1|:22-77	80
BmHel-11(DF090333:2432543-2432822):123-280	gb|AAO33922.1|:22-77	66
BmHel-11(DF090333:2513973-2514152):84-179	gb|AAO33922.1|:22-53	93
BmHel-11(DF090333:2808366-2808656):123-290	gb|AAO33922.1|:22-77	83
BmHel-11(DF090333:3128825-3129092):124-267	gb|AAO33922.1|:22-77	76
BmHel-11(DF090333:3144103-3144400):124-297	gb|AAO33922.1|:22-77	86
BmHel-11(DF090333:3182126-3182528):153-302	gb|AAO33922.1|:22-71	84
BmHel-11(DF090333:3376439-3376730):125-292	gb|AAO33922.1|:22-77	85
BmHel-11(DF090333:3467125-3467412):123-287	gb|AAO33922.1|:22-77	80
BmHel-11(DF090333:3501619-3501911):158-292	gb|AAO33922.1|:22-66	86
BmHel-11(DF090333:3552725-3553115):253-390	gb|AAO33922.1|:22-67	65
BmHel-11(DF090333:3682478-3682768):123-290	gb|AAO33922.1|:22-77	89
BmHel-11(DF090333:3697268-3697559):124-291	gb|AAO33922.1|:22-77	85
BmHel-11(DF090333:3724539-3724867):222-329	gb|AAO33922.1|:22-57	86
BmHel-11(DF090333:3791331-3791621):159-290	gb|AAO33922.1|:22-65	88
BmHel-11(DF090333:3878316-3878608):155-292	gb|AAO33922.1|:22-67	73
BmHel-11(DF090333:38795-39086):124-291	gb|AAO33922.1|:22-77	85
BmHel-11(DF090333:3998569-3998839):127-270	gb|AAO33922.1|:22-77	73
BmHel-11(DF090333:400906-401192):119-286	gb|AAO33922.1|:22-77	87
BmHel-11(DF090333:4116801-4117094):123-293	gb|AAO33922.1|:22-77	77
BmHel-11(DF090333:4271368-4271660):155-292	gb|AAO33922.1|:22-67	89
BmHel-11(DF090333:4279471-4279759):126-288	gb|AAO33922.1|:22-77	64
BmHel-11(DF090333:4291491-4291775):126-284	gb|AAO33922.1|:22-76	83

BmHel-11(DF090333:4760533-4760824):169-291	gb|AAO33922.1|:22-62	82
BmHel-11(DF090333:4890047-4890338):124-291	gb|AAO33922.1|:22-77	80
BmHel-11(DF090333:5058649-5058940):124-291	gb|AAO33922.1|:22-77	85
BmHel-11(DF090333:510577-510865):139-288	gb|AAO33922.1|:22-71	90
BmHel-11(DF090333:5107412-5107704):124-292	gb|AAO33922.1|:22-77	71
BmHel-11(DF090333:5370236-5370523):121-216	gb|AAO33922.1|:46-77	93
BmHel-11(DF090333:5374973-5375264):124-291	gb|AAO33922.1|:22-77	83
BmHel-11(DF090333:5414265-5414558):162-293	gb|AAO33922.1|:22-65	84
BmHel-11(DF090333:5460322-5460613):133-291	gb|AAO33922.1|:22-74	71
BmHel-11(DF090333:559591-559830):102-239	gb|AAO33922.1|:22-67	82
BmHel-11(DF090333:5674719-5675009):136-290	gb|AAO33922.1|:22-74	66
BmHel-11(DF090333:570766-571036):112-270	gb|AAO33922.1|:22-77	80
BmHel-11(DF090333:5718756-5719047):160-291	gb|AAO33922.1|:22-65	88
BmHel-11(DF090333:855657-855949):125-292	gb|AAO33922.1|:22-77	85
BmHel-11(DF090334:1148664-1148955):160-291	gb|AAO33922.1|:22-65	86
BmHel-11(DF090334:1209958-1210248):168-290	gb|AAO33922.1|:22-62	87
BmHel-11(DF090334:126176-126451):108-275	gb|AAO33922.1|:22-77	76
BmHel-11(DF090334:1398525-1398853):167-328	gb|AAO33922.1|:22-75	81
BmHel-11(DF090334:1625020-1625278):124-258	gb|AAO33922.1|:22-77	66
BmHel-11(DF090334:2045546-2045816):124-270	gb|AAO33922.1|:22-77	66
BmHel-11(DF090334:2181859-2182127):146-268	gb|AAO33922.1|:22-67	80
BmHel-11(DF090334:2201747-2202037):159-290	gb|AAO33922.1|:22-65	77
BmHel-11(DF090334:2312592-2312885):174-293	gb|AAO33922.1|:22-61	90
BmHel-11(DF090334:2456487-2456778):124-291	gb|AAO33922.1|:22-77	91
BmHel-11(DF090334:2774546-2774824):124-278	gb|AAO33922.1|:22-77	64
BmHel-11(DF090334:2889127-2889421):160-294	gb|AAO33922.1|:22-65	86
BmHel-11(DF090334:289510-289786):139-276	gb|AAO33922.1|:22-67	73
BmHel-11(DF090334:2920895-2921181):128-286	gb|AAO33922.1|:22-74	69
BmHel-11(DF090334:3128502-3128745):124-243	gb|AAO33922.1|:22-77	66
BmHel-11(DF090334:3204779-3205078):118-299	gb|AAO33922.1|:22-77	60
BmHel-11(DF090334:3335043-3335334):124-291	gb|AAO33922.1|:22-77	83
BmHel-11(DF090334:355465-355754):125-289	gb|AAO33922.1|:22-77	78
BmHel-11(DF090334:367280-367574):157-294	gb|AAO33922.1|:22-66	84
BmHel-11(DF090334:3692933-3693217):120-284	gb|AAO33922.1|:22-77	71
BmHel-11(DF090334:3819605-3819873):131-268	gb|AAO33922.1|:22-67	91
BmHel-11(DF090334:3843643-3843933):153-290	gb|AAO33922.1|:22-67	80
BmHel-11(DF090334:4037057-4037355):128-298	gb|AAO33922.1|:22-76	80
BmHel-11(DF090334:4087972-4088267):155-295	gb|AAO33922.1|:22-67	89
BmHel-11(DF090334:4168751-4169042):163-291	gb|AAO33922.1|:22-64	79
BmHel-11(DF090334:4543973-4544266):125-250	gb|AAO33922.1|:36-77	83
BmHel-11(DF090334:4894682-4894973):124-291	gb|AAO33922.1|:22-77	89
BmHel-11(DF090334:4909447-4909701):124-254	gb|AAO33922.1|:22-77	54
BmHel-11(DF090334:5078152-5078418):177-266	gb|AAO33922.1|:22-51	93
BmHel-11(DF090334:5103087-5103375):125-288	gb|AAO33922.1|:22-77	50.5
BmHel-11(DF090334:5218594-5218890):155-296	gb|AAO33922.1|:22-67	61
BmHel-11(DF090334:5295101-5295383):115-282	gb|AAO33922.1|:22-77	92
BmHel-11(DF090334:5322777-5323068):124-291	gb|AAO33922.1|:22-77	89
BmHel-11(DF090334:5365990-5366282):125-292	gb|AAO33922.1|:22-77	83

BmHel-11(DF090334:5369651-5370164):373-513	gb|AAO33922.1|:22-68	76
BmHel-11(DF090334:5369651-5370164):138-292	ref|NP_001157648.1|:198-250	59
BmHel-11(DF090334:5477730-5478020):126-290	gb|AAO33922.1|:22-77	57
BmHel-11(DF090334:5618961-5619223):94-262	gb|AAO33922.1|:22-77	65
BmHel-11(DF090334:5903752-5904038):125-286	gb|AAO33922.1|:22-77	87
BmHel-11(DF090334:803986-804299):125-313	gb|AAO33922.1|:22-77	60
BmHel-11(DF090334:906125-906416):124-291	gb|AAO33922.1|:22-77	67
BmHel-11(DF090335:1417897-1418196):147-299	gb|AAO33922.1|:22-76	78
BmHel-11(DF090335:1469752-1470365):509-613	gb|AAO33922.1|:22-56	82
BmHel-11(DF090335:1469752-1470365):348-499	dbj|BAH66344.1|:45-92	53.5
BmHel-11(DF090335:1922811-1923102):160-291	gb|AAO33922.1|:22-65	86
BmHel-11(DF090335:1960475-1960745):147-270	gb|AAO33922.1|:22-65	61.5
BmHel-11(DF090335:2047546-2047818):124-264	gb|AAO33922.1|:25-77	77
BmHel-11(DF090335:2186878-2187145):160-267	gb|AAO33922.1|:22-65	70
BmHel-11(DF090335:2235888-2236178):123-290	gb|AAO33922.1|:22-77	83
BmHel-11(DF090335:2439477-2439768):160-291	gb|AAO33922.1|:22-65	86
BmHel-11(DF090335:2466783-2467073):186-290	gb|AAO33922.1|:22-56	85
BmHel-11(DF090335:2548301-2548576):144-275	gb|AAO33922.1|:22-65	86
BmHel-11(DF090335:2721061-2721319):128-258	gb|AAO33922.1|:22-76	58.5
BmHel-11(DF090335:2787697-2787981):135-284	gb|AAO33922.1|:22-71	92
BmHel-11(DF090335:2913440-2913717):128-277	gb|AAO33922.1|:22-77	69
BmHel-11(DF090335:309469-309750):123-281	gb|AAO33922.1|:22-74	90
BmHel-11(DF090335:3104574-3104864):124-261	gb|AAO33922.1|:33-77	82
BmHel-11(DF090335:3171495-3171789):143-294	gb|AAO33922.1|:22-70	68
BmHel-11(DF090335:3201109-3201399):126-290	gb|AAO33922.1|:22-77	62
BmHel-11(DF090335:3297603-3297894):124-291	gb|AAO33922.1|:22-77	89
BmHel-11(DF090335:3596672-3596963):124-291	gb|AAO33922.1|:22-77	85
BmHel-11(DF090335:4152161-4152373):72-212	gb|AAO33922.1|:22-68	80
BmHel-11(DF090335:4208168-4208456):142-270	gb|AAO33922.1|:29-71	88
BmHel-11(DF090335:445532-445822):124-290	gb|AAO33922.1|:22-77	71
BmHel-11(DF090335:4471645-4471937):155-292	gb|AAO33922.1|:22-67	63
BmHel-11(DF090335:4553003-4553294):160-291	gb|AAO33922.1|:22-65	88
BmHel-11(DF090335:4603597-4603887):123-290	gb|AAO33922.1|:22-77	85
BmHel-11(DF090335:488250-488499):145-249	gb|AAO33922.1|:22-70	61
BmHel-11(DF090335:5264371-5264663):125-292	gb|AAO33922.1|:22-77	85
BmHel-11(DF090335:5326726-5327016):123-290	gb|AAO33922.1|:22-77	91
BmHel-11(DF090335:5452816-5453106):153-290	gb|AAO33922.1|:22-67	67
BmHel-11(DF090335:5794304-5794576):116-272	gb|AAO33922.1|:22-77	59
BmHel-11(DF090335:5827969-5828250):117-281	gb|AAO33922.1|:22-77	82
BmHel-11(DF090335:927690-927971):121-281	gb|AAO33922.1|:22-76	61
BmHel-11(DF090335:936705-936996):124-291	gb|AAO33922.1|:22-77	89
BmHel-11(DF090336:1177001-1177291):159-290	gb|AAO33922.1|:22-65	88
BmHel-11(DF090336:1219295-1219577):123-282	gb|AAO33922.1|:22-77	65
BmHel-11(DF090336:1258540-1258831):124-291	gb|AAO33922.1|:22-77	89
BmHel-11(DF090336:1601294-1601578):165-284	gb|AAO33922.1|:22-61	85
BmHel-11(DF090336:1758929-1759220):160-291	gb|AAO33922.1|:22-65	93
BmHel-11(DF090336:1859319-1859599):127-280	gb|AAO33922.1|:22-76	62.5
BmHel-11(DF090336:1890126-1890416):153-290	gb|AAO33922.1|:22-67	93

BmHel-11(DF090336:1891220-1891510):123-290	gb|AAO33922.1|:22-77	89
BmHel-11(DF090336:200216-200506):126-290	gb|AAO33922.1|:22-77	66
BmHel-11(DF090336:2155516-2155811):188-295	gb|AAO33922.1|:22-57	91
BmHel-11(DF090336:2221340-2221630):123-290	gb|AAO33922.1|:22-77	91
BmHel-11(DF090336:2301029-2301283):159-254	gb|AAO33922.1|:22-53	93
BmHel-11(DF090336:2516452-2516726):107-274	gb|AAO33922.1|:22-77	89
BmHel-11(DF090336:2735060-2735353):123-293	gb|AAO33922.1|:22-77	80
BmHel-11(DF090336:2806046-2806335):152-289	gb|AAO33922.1|:22-67	67
BmHel-11(DF090336:2821099-2821393):124-294	gb|AAO33922.1|:22-77	84
BmHel-11(DF090336:2836966-2837257):124-291	gb|AAO33922.1|:22-77	82
BmHel-11(DF090336:2841850-2842141):124-291	gb|AAO33922.1|:22-77	71
BmHel-11(DF090336:3312784-3313069):121-285	gb|AAO33922.1|:22-77	78
BmHel-11(DF090336:336222-336491):102-269	gb|AAO33922.1|:22-77	85
BmHel-11(DF090336:3482609-3482867):124-258	gb|AAO33922.1|:22-77	75
BmHel-11(DF090336:3831862-3832155):126-293	gb|AAO33922.1|:22-77	73
BmHel-11(DF090336:4205960-4206248):124-288	gb|AAO33922.1|:22-76	78
BmHel-11(DF090336:4251792-4252086):175-294	gb|AAO33922.1|:22-61	85
BmHel-11(DF090336:42560-42788):136-228	gb|AAO33922.1|:22-52	90
BmHel-11(DF090336:4322634-4322888):122-254	gb|AAO33922.1|:22-75	59
BmHel-11(DF090336:4370083-4370366):161-283	gb|AAO33922.1|:22-64	65
BmHel-11(DF090336:4383168-4383462):127-294	gb|AAO33922.1|:22-77	55
BmHel-11(DF090336:4416615-4416903):124-288	gb|AAO33922.1|:22-77	83
BmHel-11(DF090336:4517018-4517292):146-274	gb|AAO33922.1|:22-70	75
BmHel-11(DF090336:4643802-4644094):131-292	gb|AAO33922.1|:22-75	92
BmHel-11(DF090336:4838865-4839156):124-291	gb|AAO33922.1|:22-77	89
BmHel-11(DF090336:4987895-4988186):124-291	gb|AAO33922.1|:22-77	91
BmHel-11(DF090336:5003819-5004081):137-262	gb|AAO33922.1|:22-63	85
BmHel-11(DF090336:5084363-5084667):197-304	gb|AAO33922.1|:22-57	88
BmHel-11(DF090336:5464902-5465195):144-293	gb|AAO33922.1|:22-71	84
BmHel-11(DF090336:5509587-5509979):225-392	gb|AAO33922.1|:22-77	85
BmHel-11(DF090336:5550212-5550459):124-247	gb|AAO33922.1|:22-77	62.5
BmHel-11(DF090336:627853-628144):124-291	gb|AAO33922.1|:22-77	94
BmHel-11(DF090336:729807-730100):126-293	gb|AAO33922.1|:22-77	60
BmHel-11(DF090336:744302-747701):2555-2695	gb|AAO33922.1|:22-68	72
BmHel-11(DF090336:914777-915074):140-268	gb|AAO33922.1|:33-76	75
BmHel-11(DF090336:945263-945548):154-285	gb|AAO33922.1|:22-65	86
BmHel-11(DF090337:1038485-1038775):123-290	gb|AAO33922.1|:22-77	87
BmHel-11(DF090337:1115527-1115816):115-289	gb|AAO33922.1|:22-77	66.5
BmHel-11(DF090337:1332305-1332596):124-291	gb|AAO33922.1|:22-77	75
BmHel-11(DF090337:1442611-1442900):122-289	gb|AAO33922.1|:22-77	87
BmHel-11(DF090337:1559581-1559871):123-290	gb|AAO33922.1|:22-77	83
BmHel-11(DF090337:1730732-1730995):122-263	gb|AAO33922.1|:22-68	64
BmHel-11(DF090337:2472073-2472363):160-290	gb|AAO33922.1|:22-65	71
BmHel-11(DF090337:2758571-2759199):493-628	gb|AAO33922.1|:22-77	62.5
BmHel-11(DF090337:2876641-2876915):107-274	gb|AAO33922.1|:22-77	89
BmHel-11(DF090337:3158878-3159171):147-293	gb|AAO33922.1|:22-69	89
BmHel-11(DF090337:3491114-3491362):118-248	gb|AAO33922.1|:22-77	66.5
BmHel-11(DF090337:3542504-3542761):135-257	gb|AAO33922.1|:22-70	69

BmHel-11(DF090337:3692115-3692406):124-291	gb|AAO33922.1|:22-77	82
BmHel-11(DF090337:3920077-3920367):160-290	gb|AAO33922.1|:22-65	65.5
BmHel-11(DF090337:4342865-4343155):124-252	gb|AAO33922.1|:35-77	81
BmHel-11(DF090337:4563681-4563973):155-292	gb|AAO33922.1|:22-67	80
BmHel-11(DF090337:4731491-4731782):124-291	gb|AAO33922.1|:22-77	89
BmHel-11(DF090337:4831767-4832060):126-293	gb|AAO33922.1|:22-77	85
BmHel-11(DF090337:4959687-4959980):150-293	gb|AAO33922.1|:22-69	64
BmHel-11(DF090337:5049824-5050119):164-295	gb|AAO33922.1|:22-65	90
BmHel-11(DF090338:1049982-1050273):142-291	gb|AAO33922.1|:22-71	90
BmHel-11(DF090338:1323882-1324175):132-293	gb|AAO33922.1|:22-75	88
BmHel-11(DF090338:1567874-1568164):189-290	gb|AAO33922.1|:22-55	88
BmHel-11(DF090338:1599367-1599658):160-291	gb|AAO33922.1|:22-65	84
BmHel-11(DF090338:2010024-2010315):124-291	gb|AAO33922.1|:22-77	89
BmHel-11(DF090338:2101346-2101635):122-289	gb|AAO33922.1|:22-77	64
BmHel-11(DF090338:2171369-2171660):124-291	gb|AAO33922.1|:22-77	87
BmHel-11(DF090338:2264168-2264459):124-291	gb|AAO33922.1|:22-77	87
BmHel-11(DF090338:2290802-2291089):144-287	gb|AAO33922.1|:22-69	85
BmHel-11(DF090338:2939346-2939639):123-293	gb|AAO33922.1|:22-77	80
BmHel-11(DF090338:3068170-3068460):157-291	gb|AAO33922.1|:22-66	84
BmHel-11(DF090338:3629432-3629704):135-272	gb|AAO33922.1|:22-67	71
BmHel-11(DF090338:3828978-3829259):192-281	gb|AAO33922.1|:22-51	93
BmHel-11(DF090338:3942029-3942320):160-291	gb|AAO33922.1|:22-65	95
BmHel-11(DF090338:3971737-3972020):125-283	gb|AAO33922.1|:22-77	69
BmHel-11(DF090338:4095244-4095533):158-289	gb|AAO33922.1|:22-65	88
BmHel-11(DF090338:4125841-4126108):121-267	gb|AAO33922.1|:22-74	81
BmHel-11(DF090338:4132010-4132291):123-281	gb|AAO33922.1|:22-69	69
BmHel-11(DF090338:4161765-4162068):136-303	gb|AAO33922.1|:22-77	61
BmHel-11(DF090338:4297514-4297805):154-291	gb|AAO33922.1|:22-67	82
BmHel-11(DF090338:4545602-4545867):124-265	gb|AAO33922.1|:22-77	67
BmHel-11(DF090338:4650448-4650744):159-296	gb|AAO33922.1|:22-67	69
BmHel-11(DF090338:4919778-4920084):84-206	gb|AAO33922.1|:22-62	92
BmHel-11(DF090338:5080587-5080882):131-295	gb|AAO33922.1|:22-75	83
BmHel-11(DF090338:659868-660159):124-291	gb|AAO33922.1|:22-77	83
BmHel-11(DF090338:6775-7066):124-291	gb|AAO33922.1|:22-77	82
BmHel-11(DF090338:735005-735308):136-303	gb|AAO33922.1|:22-76	64
BmHel-11(DF090338:753564-753855):124-291	gb|AAO33922.1|:22-77	85
BmHel-11(DF090338:845525-845820):124-295	gb|AAO33922.1|:22-77	65
BmHel-11(DF090339:1095203-1095497):124-294	gb|AAO33922.1|:22-77	89
BmHel-11(DF090339:119810-120091):114-281	gb|AAO33922.1|:22-77	89
BmHel-11(DF090339:1283273-1283564):124-291	gb|AAO33922.1|:22-77	85
BmHel-11(DF090339:1283961-1284236):111-275	gb|AAO33922.1|:22-76	87
BmHel-11(DF090339:1312947-1313238):124-291	gb|AAO33922.1|:22-77	89
BmHel-11(DF090339:1368789-1369080):124-291	gb|AAO33922.1|:22-77	85
BmHel-11(DF090339:1602698-1602989):124-291	gb|AAO33922.1|:22-77	85
BmHel-11(DF090339:167451-167717):124-266	gb|AAO33922.1|:22-77	66
BmHel-11(DF090339:1772430-1772676):124-246	gb|AAO33922.1|:22-77	60
BmHel-11(DF090339:1818825-1819115):123-290	gb|AAO33922.1|:22-77	83
BmHel-11(DF090339:1824563-1824855):125-292	gb|AAO33922.1|:22-77	87

BmHel-11(DF090339:1976612-1976903):124-291	gb|AAO33922.1|:22-77	85
BmHel-11(DF090339:1994538-1994824):124-286	gb|AAO33922.1|:22-77	58
BmHel-11(DF090339:2280718-2281009):124-291	gb|AAO33922.1|:22-77	82
BmHel-11(DF090339:2386864-2387154):124-282	gb|AAO33922.1|:25-77	60
BmHel-11(DF090339:2390416-2390699):149-283	gb|AAO33922.1|:22-66	77
BmHel-11(DF090339:2511132-2511419):126-287	gb|AAO33922.1|:22-77	71
BmHel-11(DF090339:2529789-2530362):415-573	gb|AAO33922.1|:22-77	75
BmHel-11(DF090339:2555568-2555859):163-291	gb|AAO33922.1|:22-64	90
BmHel-11(DF090339:2571008-2571290):151-282	gb|AAO33922.1|:22-65	88
BmHel-11(DF090339:2644624-2644916):155-292	gb|AAO33922.1|:22-67	80
BmHel-11(DF090339:2692910-2693458):117-229	ref|NP_001157648.1|:213-250	50
BmHel-11(DF090339:2692910-2693458):382-549	gb|AAO33922.1|:22-77	82
BmHel-11(DF090339:2996112-2996403):124-291	gb|AAO33922.1|:22-77	87
BmHel-11(DF090339:3123865-3124156):124-291	gb|AAO33922.1|:22-77	91
BmHel-11(DF090339:3159677-3159968):124-291	gb|AAO33922.1|:22-77	89
BmHel-11(DF090339:3319818-3320112):163-294	gb|AAO33922.1|:22-65	88
BmHel-11(DF090339:3341316-3341601):160-285	gb|AAO33922.1|:22-63	83
BmHel-11(DF090339:4255790-4256075):124-285	gb|AAO33922.1|:22-77	83
BmHel-11(DF090339:4259946-4260200):124-254	gb|AAO33922.1|:22-77	61
BmHel-11(DF090339:4441149-4441440):124-291	gb|AAO33922.1|:22-77	91
BmHel-11(DF090339:4562544-4562837):127-225	gb|AAO33922.1|:45-77	90
BmHel-11(DF090339:4867243-4867485):123-242	gb|AAO33922.1|:22-77	66
BmHel-11(DF090339:4895982-4896275):150-293	gb|AAO33922.1|:22-69	66
BmHel-11(DF090339:4950767-4951062):130-282	gb|AAO33922.1|:26-77	73
BmHel-11(DF090339:4971169-4971457):121-288	gb|AAO33922.1|:22-77	75
BmHel-11(DF090339:4989270-4989539):144-269	gb|AAO33922.1|:22-63	66
BmHel-11(DF090339:514990-515281):160-291	gb|AAO33922.1|:22-65	90
BmHel-11(DF090339:603137-603427):123-290	gb|AAO33922.1|:22-77	85
BmHel-11(DF090339:722972-723262):123-290	gb|AAO33922.1|:22-77	87
BmHel-11(DF090339:989594-989886):161-292	gb|AAO33922.1|:22-65	88
BmHel-11(DF090340:1003155-1003447):179-292	gb|AAO33922.1|:22-59	86
BmHel-11(DF090340:1082352-1082649):160-297	gb|AAO33922.1|:22-65	71
BmHel-11(DF090340:1218060-1218353):162-293	gb|AAO33922.1|:22-65	84
BmHel-11(DF090340:1415348-1415639):124-291	gb|AAO33922.1|:22-77	85
BmHel-11(DF090340:1727224-1727515):123-263	gb|AAO33922.1|:31-77	87
BmHel-11(DF090340:1742746-1743037):163-291	gb|AAO33922.1|:22-64	95
BmHel-11(DF090340:1757-2048):124-291	gb|AAO33922.1|:22-77	85
BmHel-11(DF090340:1955757-1956045):123-288	gb|AAO33922.1|:22-77	57
BmHel-11(DF090340:2179059-2179353):163-294	gb|AAO33922.1|:22-65	90
BmHel-11(DF090340:2453968-2454259):124-291	gb|AAO33922.1|:22-77	87
BmHel-11(DF090340:2563389-2563675):131-286	gb|AAO33922.1|:22-77	67
BmHel-11(DF090340:2892492-2892783):124-291	gb|AAO33922.1|:22-77	87
BmHel-11(DF090340:304344-304602):115-258	gb|AAO33922.1|:22-70	64
BmHel-11(DF090340:3089388-3089679):124-291	gb|AAO33922.1|:22-77	87
BmHel-11(DF090340:3375933-3376223):123-290	gb|AAO33922.1|:22-77	89
BmHel-11(DF090340:3581508-3581805):127-297	gb|AAO33922.1|:22-76	82
BmHel-11(DF090340:3698767-3699060):135-293	gb|AAO33922.1|:22-74	83
BmHel-11(DF090340:3806540-3806831):124-291	gb|AAO33922.1|:22-77	85

BmHel-11(DF090340:3847186-3847476):123-290	gb|AAO33922.1|:22-77	87
BmHel-11(DF090340:3848251-3848542):124-291	gb|AAO33922.1|:22-77	87
BmHel-11(DF090340:4008038-4008330):161-292	gb|AAO33922.1|:22-65	93
BmHel-11(DF090340:4067879-4068189):141-310	gb|AAO33922.1|:22-73	62.5
BmHel-11(DF090340:4258197-4258464):124-267	gb|AAO33922.1|:22-77	76
BmHel-11(DF090340:4262704-4262994):162-290	gb|AAO33922.1|:22-64	90
BmHel-11(DF090340:4321157-4321436):157-279	gb|AAO33922.1|:22-67	73
BmHel-11(DF090340:4342971-4343263):146-292	gb|AAO33922.1|:22-70	77
BmHel-11(DF090340:4496649-4496891):124-242	gb|AAO33922.1|:22-77	64
BmHel-11(DF090340:4569970-4570257):162-287	gb|AAO33922.1|:22-63	85
BmHel-11(DF090340:4617367-4617659):125-292	gb|AAO33922.1|:22-77	89
BmHel-11(DF090340:4637000-4637292):125-292	gb|AAO33922.1|:22-77	89
BmHel-11(DF090340:4718303-4718599):150-296	gb|AAO33922.1|:22-77	60
BmHel-11(DF090340:483204-483496):128-292	gb|AAO33922.1|:22-76	85
BmHel-11(DF090340:4896613-4896894):134-281	gb|AAO33922.1|:22-74	64.5
BmHel-11(DF090340:494849-495340):124-231	gb|AAO33922.1|:42-77	88
BmHel-11(DF090340:55206-55502):195-296	gb|AAO33922.1|:22-55	88
BmHel-11(DF090340:764671-765240):146-250	gb|AAO33922.1|:35-69	91
BmHel-11(DF090340:801325-801618):127-285	gb|AAO33922.1|:25-76	79
BmHel-11(DF090340:813861-814148):123-287	gb|AAO33922.1|:22-76	89
BmHel-11(DF090340:905634-905916):124-282	gb|AAO33922.1|:22-77	82
BmHel-11(DF090341:1210436-1210727):124-291	gb|AAO33922.1|:22-77	87
BmHel-11(DF090341:1439776-1440071):124-295	gb|AAO33922.1|:22-77	67
BmHel-11(DF090341:1472902-1473194):124-292	gb|AAO33922.1|:22-77	63.5
BmHel-11(DF090341:1705218-1705509):169-291	gb|AAO33922.1|:22-62	78
BmHel-11(DF090341:1997586-1997983):101-220	gb|AAO33922.1|:22-61	87
BmHel-11(DF090341:2615188-2615479):124-291	gb|AAO33922.1|:22-77	85
BmHel-11(DF090341:2704361-2704626):122-265	gb|AAO33922.1|:22-69	89
BmHel-11(DF090341:2716117-2716506):124-241	gb|AAO33922.1|:22-77	71
BmHel-11(DF090341:2765376-2765751):250-375	gb|AAO33922.1|:22-62	69
BmHel-11(DF090341:2945293-2945576):124-283	gb|AAO33922.1|:22-77	66
BmHel-11(DF090341:3252168-3252459):163-291	gb|AAO33922.1|:22-64	83
BmHel-11(DF090341:345000-345291):124-291	gb|AAO33922.1|:22-77	82
BmHel-11(DF090341:3822852-3823137):118-285	gb|AAO33922.1|:22-77	75
BmHel-11(DF090341:3851581-3851871):123-290	gb|AAO33922.1|:22-77	87
BmHel-11(DF090341:3981967-3982259):125-292	gb|AAO33922.1|:22-77	85
BmHel-11(DF090341:4160009-4160299):127-290	gb|AAO33922.1|:22-76	59.5
BmHel-11(DF090341:4320595-4320885):153-290	gb|AAO33922.1|:22-67	67
BmHel-11(DF090341:4727545-4727841):129-296	gb|AAO33922.1|:22-77	89
BmHel-11(DF090342:1120367-1120658):124-291	gb|AAO33922.1|:22-77	83
BmHel-11(DF090342:1226859-1227154):128-295	gb|AAO33922.1|:22-77	94
BmHel-11(DF090342:1259350-1259639):123-289	gb|AAO33922.1|:22-77	64
BmHel-11(DF090342:1402311-1402585):128-274	gb|AAO33922.1|:22-77	67
BmHel-11(DF090342:1449743-1450021):111-278	gb|AAO33922.1|:22-77	91
BmHel-11(DF090342:1475029-1475322):126-293	gb|AAO33922.1|:22-77	64
BmHel-11(DF090342:1512214-1512505):124-291	gb|AAO33922.1|:22-77	92
BmHel-11(DF090342:1559209-1559500):124-291	gb|AAO33922.1|:22-77	87
BmHel-11(DF090342:1562645-1562938):160-261	gb|AAO33922.1|:32-65	85

BmHel-11(DF090342:1749427-1749655):136-228	gb|AAO33922.1|:22-52	96
BmHel-11(DF090342:1753877-1754168):160-291	gb|AAO33922.1|:22-65	88
BmHel-11(DF090342:1836358-1836601):99-243	gb|AAO33922.1|:22-77	53
BmHel-11(DF090342:1971479-1971771):134-292	gb|AAO33922.1|:22-74	83
BmHel-11(DF090342:1993733-1994024):124-291	gb|AAO33922.1|:22-77	87
BmHel-11(DF090342:2161836-2162126):153-290	gb|AAO33922.1|:22-67	84
BmHel-11(DF090342:2183122-2183415):126-293	gb|AAO33922.1|:22-77	55
BmHel-11(DF090342:2196859-2197149):123-290	gb|AAO33922.1|:22-77	58
BmHel-11(DF090342:2260712-2261002):124-225	gb|AAO33922.1|:44-77	88
BmHel-11(DF090342:2637824-2638107):123-283	gb|AAO33922.1|:22-77	62
BmHel-11(DF090342:2910053-2910327):125-274	gb|AAO33922.1|:22-71	86
BmHel-11(DF090342:3116324-3116614):165-290	gb|AAO33922.1|:22-63	92
BmHel-11(DF090342:3253444-3253735):160-291	gb|AAO33922.1|:22-65	93
BmHel-11(DF090342:3379959-3380256):133-297	gb|AAO33922.1|:22-76	83
BmHel-11(DF090342:3409641-3409932):124-291	gb|AAO33922.1|:22-77	91
BmHel-11(DF090342:3572484-3572791):212-307	gb|AAO33922.1|:22-53	84
BmHel-11(DF090342:3610411-3610702):124-291	gb|AAO33922.1|:22-77	91
BmHel-11(DF090342:3649933-3650216):122-283	gb|AAO33922.1|:22-69	59
BmHel-11(DF090342:3678888-3688675):1401-1923	dbj|BAB21761.1|:36-211	70
BmHel-11(DF090342:4009283-4009575):125-292	gb|AAO33922.1|:22-77	62
BmHel-11(DF090342:408948-409239):160-291	gb|AAO33922.1|:22-65	88
BmHel-11(DF090342:4110298-4110588):153-290	gb|AAO33922.1|:22-67	71
BmHel-11(DF090342:422884-423176):155-292	gb|AAO33922.1|:22-67	67
BmHel-11(DF090342:4254302-4254596):124-294	gb|AAO33922.1|:22-77	87
BmHel-11(DF090342:433251-433539):125-288	gb|AAO33922.1|:22-77	66
BmHel-11(DF090342:4339329-4339624):124-270	gb|AAO33922.1|:29-77	87
BmHel-11(DF090342:4626313-4626605):155-292	gb|AAO33922.1|:22-67	78
BmHel-11(DF090342:4764367-4764661):190-294	gb|AAO33922.1|:22-56	85
BmHel-11(DF090342:4777978-4778262):115-284	gb|AAO33922.1|:22-77	62.5
BmHel-11(DF090342:658056-658348):125-292	gb|AAO33922.1|:22-77	83
BmHel-11(DF090342:689919-690205):126-286	gb|AAO33922.1|:22-77	59
BmHel-11(DF090342:808960-809221):94-261	gb|AAO33922.1|:22-77	87
BmHel-11(DF090342:978152-978439):120-287	gb|AAO33922.1|:22-77	85
BmHel-11(DF090342:987179-987470):124-291	gb|AAO33922.1|:22-77	85
BmHel-11(DF090343:1025516-1025806):124-290	gb|AAO33922.1|:22-77	48
BmHel-11(DF090343:115316-115599):124-283	gb|AAO33922.1|:22-77	64
BmHel-11(DF090343:1268992-1269269):111-277	gb|AAO33922.1|:22-77	63
BmHel-11(DF090343:1509912-1510204):149-292	gb|AAO33922.1|:22-69	81
BmHel-11(DF090343:1529448-1529743):149-295	gb|AAO33922.1|:22-69	83
BmHel-11(DF090343:160735-161121):247-386	gb|AAO33922.1|:22-71	58.5
BmHel-11(DF090343:1619505-1619934):262-429	gb|AAO33922.1|:22-77	83
BmHel-11(DF090343:1769325-1769615):123-290	gb|AAO33922.1|:22-77	80
BmHel-11(DF090343:1899674-1899917):124-243	gb|AAO33922.1|:22-77	62
BmHel-11(DF090343:1919046-1919337):124-291	gb|AAO33922.1|:22-77	85
BmHel-11(DF090343:2194805-2195099):136-294	gb|AAO33922.1|:22-74	84
BmHel-11(DF090343:2650064-2650355):142-291	gb|AAO33922.1|:22-71	88
BmHel-11(DF090343:3075293-3075597):124-304	gb|AAO33922.1|:22-77	59
BmHel-11(DF090343:3204154-3204446):161-292	gb|AAO33922.1|:22-65	90

BmHel-11(DF090343:3217000-3217293):126-293	gb|AAO33922.1|:22-77	75
BmHel-11(DF090343:3249359-3249649):126-290	gb|AAO33922.1|:22-77	60
BmHel-11(DF090343:3280980-3281276):147-296	gb|AAO33922.1|:22-71	84
BmHel-11(DF090343:3327984-3328272):117-288	gb|AAO33922.1|:22-76	67
BmHel-11(DF090343:3345109-3345419):155-310	gb|AAO33922.1|:22-73	84
BmHel-11(DF090343:3370761-3371053):149-292	gb|AAO33922.1|:22-69	87
BmHel-11(DF090343:3768019-3768298):124-279	gb|AAO33922.1|:22-77	83
BmHel-11(DF090343:3907880-3908175):158-295	gb|AAO33922.1|:22-67	73
BmHel-11(DF090343:4012239-4012530):160-291	gb|AAO33922.1|:22-65	79
BmHel-11(DF090343:4054057-4054347):124-291	gb|AAO33922.1|:22-77	91
BmHel-11(DF090343:4197903-4198190):126-287	gb|AAO33922.1|:22-77	61
BmHel-11(DF090343:4365684-4365971):153-287	gb|AAO33922.1|:22-66	84
BmHel-11(DF090343:4392861-4393183):124-222	gb|AAO33922.1|:22-77	55
BmHel-11(DF090343:4439972-4440263):161-291	gb|AAO33922.1|:22-65	64
BmHel-11(DF090343:4634236-4634505):109-269	gb|AAO33922.1|:22-77	68
BmHel-11(DF090343:4651724-4652015):160-291	gb|AAO33922.1|:22-65	86
BmHel-11(DF090343:4670653-4670943):159-290	gb|AAO33922.1|:22-65	90
BmHel-11(DF090343:477652-477961):142-309	gb|AAO33922.1|:22-77	76
BmHel-11(DF090343:636440-636728):175-288	gb|AAO33922.1|:22-59	81
BmHel-11(DF090343:763999-764290):160-291	gb|AAO33922.1|:22-65	84
BmHel-11(DF090343:862947-863236):142-289	gb|AAO33922.1|:22-71	71
BmHel-11(DF090343:874489-874641):9-152	gb|AAO33922.1|:22-69	60
BmHel-11(DF090344:1048140-1048433):126-293	gb|AAO33922.1|:22-77	85
BmHel-11(DF090344:1366474-1366758):126-284	gb|AAO33922.1|:22-77	60
BmHel-11(DF090344:1456403-1456688):160-285	gb|AAO33922.1|:22-63	88
BmHel-11(DF090344:1494475-1494766):124-291	gb|AAO33922.1|:22-77	89
BmHel-11(DF090344:1710734-1711019):121-285	gb|AAO33922.1|:22-76	87
BmHel-11(DF090344:1838794-1839078):124-284	gb|AAO33922.1|:22-77	59
BmHel-11(DF090344:2013998-2014279):134-281	gb|AAO33922.1|:22-74	64.5
BmHel-11(DF090344:2321930-2322221):124-291	gb|AAO33922.1|:22-77	83
BmHel-11(DF090344:2368956-2369247):160-291	gb|AAO33922.1|:22-65	88
BmHel-11(DF090344:2514144-2514435):124-291	gb|AAO33922.1|:22-77	91
BmHel-11(DF090344:252851-253206):138-355	gb|AAO33922.1|:22-71	67
BmHel-11(DF090344:2594752-2595040):157-288	gb|AAO33922.1|:22-65	84
BmHel-11(DF090344:2695467-2695759):167-292	gb|AAO33922.1|:22-63	85
BmHel-11(DF090344:2768786-2769077):124-291	gb|AAO33922.1|:22-77	85
BmHel-11(DF090344:3209995-3210286):124-291	gb|AAO33922.1|:22-77	91
BmHel-11(DF090344:3293196-3293486):123-290	gb|AAO33922.1|:22-77	83
BmHel-11(DF090344:3734199-3734491):155-292	gb|AAO33922.1|:22-67	93
BmHel-11(DF090344:3963364-3963654):159-290	gb|AAO33922.1|:22-65	86
BmHel-11(DF090344:3970737-3971325):448-588	gb|AAO33922.1|:22-67	89
BmHel-11(DF090344:4020059-4020357):191-298	gb|AAO33922.1|:22-57	91
BmHel-11(DF090344:4148312-4148577):122-265	gb|AAO33922.1|:22-77	55
BmHel-11(DF090344:4195282-4195570):124-288	gb|AAO33922.1|:22-77	85
BmHel-11(DF090344:4214606-4214860):159-254	gb|AAO33922.1|:22-53	87
BmHel-11(DF090344:4277683-4277976):155-283	gb|AAO33922.1|:25-67	81
BmHel-11(DF090344:4489448-4489735):141-287	gb|AAO33922.1|:22-71	66
BmHel-11(DF090344:4646189-4646471):115-282	gb|AAO33922.1|:22-77	89

BmHel-11(DF090344:4666356-4666646):153-290	gb|AAO33922.1|:22-67	67
BmHel-11(DF090344:473314-473602):124-288	gb|AAO33922.1|:22-76	83
BmHel-11(DF090344:500548-500840):125-292	gb|AAO33922.1|:22-77	87
BmHel-11(DF090344:522445-522732):156-287	gb|AAO33922.1|:22-65	81
BmHel-11(DF090344:557362-557652):123-290	gb|AAO33922.1|:22-77	92
BmHel-11(DF090344:568169-568486):150-317	gb|AAO33922.1|:22-77	85
BmHel-11(DF090344:702928-703218):174-290	gb|AAO33922.1|:22-60	79
BmHel-11(DF090344:703507-703779):116-217	gb|AAO33922.1|:41-75	85
BmHel-11(DF090344:863369-863661):161-292	gb|AAO33922.1|:22-65	86
BmHel-11(DF090344:910941-911229):127-288	gb|AAO33922.1|:22-76	78
BmHel-11(DF090345:1275813-1276104):124-291	gb|AAO33922.1|:22-77	87
BmHel-11(DF090345:1351251-1351541):141-290	gb|AAO33922.1|:22-71	90
BmHel-11(DF090345:1602446-1602736):123-290	gb|AAO33922.1|:22-77	83
BmHel-11(DF090345:2051289-2051580):124-291	gb|AAO33922.1|:22-77	87
BmHel-11(DF090345:2081904-2082234):163-330	gb|AAO33922.1|:22-77	58
BmHel-11(DF090345:2395162-2395449):156-287	gb|AAO33922.1|:22-67	89
BmHel-11(DF090345:2454740-2454989):124-249	gb|AAO33922.1|:22-77	60
BmHel-11(DF090345:2519362-2519659):130-297	gb|AAO33922.1|:22-77	82
BmHel-11(DF090345:2609856-2610142):160-286	gb|AAO33922.1|:22-65	71
BmHel-11(DF090345:269232-269513):144-281	gb|AAO33922.1|:22-67	84
BmHel-11(DF090345:3243739-3244030):124-291	gb|AAO33922.1|:22-77	83
BmHel-11(DF090345:3708819-3709101):124-282	gb|AAO33922.1|:22-77	85
BmHel-11(DF090345:3915360-3915655):128-295	gb|AAO33922.1|:22-77	89
BmHel-11(DF090345:4059333-4059621):122-288	gb|AAO33922.1|:22-77	60
BmHel-11(DF090345:4215712-4216002):124-261	gb|AAO33922.1|:32-77	80
BmHel-11(DF090345:4305240-4305637):230-397	gb|AAO33922.1|:22-77	85
BmHel-11(DF090345:584934-585225):124-291	gb|AAO33922.1|:22-77	83
BmHel-11(DF090345:799762-800052):124-291	gb|AAO33922.1|:22-77	91
BmHel-11(DF090345:954850-955148):161-298	gb|AAO33922.1|:22-67	84
BmHel-11(DF090346:1023480-1023770):123-290	gb|AAO33922.1|:22-77	85
BmHel-11(DF090346:1082126-1082414):124-288	gb|AAO33922.1|:22-76	81
BmHel-11(DF090346:108480-108766):124-225	gb|AAO33922.1|:44-77	85
BmHel-11(DF090346:1217339-1217630):160-291	gb|AAO33922.1|:22-65	88
BmHel-11(DF090346:1279061-1279352):166-291	gb|AAO33922.1|:22-63	85
BmHel-11(DF090346:1855192-1855484):128-292	gb|AAO33922.1|:22-76	85
BmHel-11(DF090346:2002488-2002776):130-288	gb|AAO33922.1|:22-76	65
BmHel-11(DF090346:2070840-2071097):123-257	gb|AAO33922.1|:22-77	67
BmHel-11(DF090346:2082367-2082656):131-289	gb|AAO33922.1|:22-74	81
BmHel-11(DF090346:2372348-2373506):1027-1158	gb|AAO33922.1|:22-65	86
BmHel-11(DF090346:240942-241233):124-291	gb|AAO33922.1|:22-77	89
BmHel-11(DF090346:2419322-2419607):160-285	gb|AAO33922.1|:22-65	88
BmHel-11(DF090346:2531298-2531587):126-260	gb|AAO33922.1|:33-77	80
BmHel-11(DF090346:268573-268861):151-288	gb|AAO33922.1|:22-67	73
BmHel-11(DF090346:2716298-2716549):124-251	gb|AAO33922.1|:22-77	62
BmHel-11(DF090346:2736168-2736463):123-230	gb|AAO33922.1|:42-77	88
BmHel-11(DF090346:2850540-2850803):125-263	gb|AAO33922.1|:22-77	64
BmHel-11(DF090346:3169952-3170238):122-286	gb|AAO33922.1|:22-76	81
BmHel-11(DF090346:3210122-3210405):124-283	gb|AAO33922.1|:22-77	69

BmHel-11(DF090346:3283202-3283491):123-289	gb|AAO33922.1|:22-77	52
BmHel-11(DF090346:3460589-3460882):141-293	gb|AAO33922.1|:22-72	74
BmHel-11(DF090346:3616508-3616799):124-291	gb|AAO33922.1|:22-77	85
BmHel-11(DF090346:3648529-3648820):163-291	gb|AAO33922.1|:22-64	83
BmHel-11(DF090346:3774079-3774364):121-285	gb|AAO33922.1|:22-77	87
BmHel-11(DF090346:3899301-3899595):160-294	gb|AAO33922.1|:22-66	84
BmHel-11(DF090346:3910327-3910618):124-291	gb|AAO33922.1|:22-77	85
BmHel-11(DF090346:3934685-3934977):125-292	gb|AAO33922.1|:22-77	89
BmHel-11(DF090346:4003-4295):134-292	gb|AAO33922.1|:22-74	83
BmHel-11(DF090346:4003671-4003966):125-295	gb|AAO33922.1|:22-77	68
BmHel-11(DF090346:401740-402018):105-278	gb|AAO33922.1|:22-77	63
BmHel-11(DF090346:4039659-4039950):124-291	gb|AAO33922.1|:22-77	85
BmHel-11(DF090347:102449-102741):155-292	gb|AAO33922.1|:22-67	65
BmHel-11(DF090347:1120974-1121265):124-291	gb|AAO33922.1|:22-77	92
BmHel-11(DF090347:1161800-1162084):117-284	gb|AAO33922.1|:22-77	67
BmHel-11(DF090347:1219013-1219298):118-285	gb|AAO33922.1|:22-77	91
BmHel-11(DF090347:1250765-1251008):124-243	gb|AAO33922.1|:22-77	67
BmHel-11(DF090347:1334078-1334369):124-291	gb|AAO33922.1|:22-77	91
BmHel-11(DF090347:1539198-1539493):173-295	gb|AAO33922.1|:22-62	87
BmHel-11(DF090347:1743258-1743549):124-291	gb|AAO33922.1|:22-77	80
BmHel-11(DF090347:1746638-1746910):124-272	gb|AAO33922.1|:22-77	71
BmHel-11(DF090347:179617-179883):147-266	gb|AAO33922.1|:22-67	78
BmHel-11(DF090347:1830372-1830662):124-291	gb|AAO33922.1|:22-77	83
BmHel-11(DF090347:1841387-1841677):123-290	gb|AAO33922.1|:22-77	85
BmHel-11(DF090347:2013979-2014268):125-289	gb|AAO33922.1|:22-76	90
BmHel-11(DF090347:2116074-2116354):116-280	gb|AAO33922.1|:22-76	83
BmHel-11(DF090347:2189642-2189875):123-233	gb|AAO33922.1|:22-58	75
BmHel-11(DF090347:2252178-2252469):124-291	gb|AAO33922.1|:22-77	83
BmHel-11(DF090347:228811-229100):125-281	gb|AAO33922.1|:25-77	63.5
BmHel-11(DF090347:2636934-2637224):123-290	gb|AAO33922.1|:22-77	85
BmHel-11(DF090347:2757254-2757577):138-323	gb|AAO33922.1|:22-77	80
BmHel-11(DF090347:2904039-2904337):124-298	gb|AAO33922.1|:22-77	64
BmHel-11(DF090347:3296450-3296746):150-296	gb|AAO33922.1|:22-70	81
BmHel-11(DF090347:3333738-3334060):124-240	gb|AAO33922.1|:39-77	76
BmHel-11(DF090347:3340126-3340405):124-279	gb|AAO33922.1|:22-77	75
BmHel-11(DF090347:3397122-3397414):125-292	gb|AAO33922.1|:22-77	85
BmHel-11(DF090347:343369-343659):156-290	gb|AAO33922.1|:22-66	84
BmHel-11(DF090347:3624400-3624744):213-344	gb|AAO33922.1|:22-65	93
BmHel-11(DF090347:3686631-3686874):124-243	gb|AAO33922.1|:22-77	67
BmHel-11(DF090347:3790814-3791551):467-601	gb|AAO33922.1|:22-68	78
BmHel-11(DF090347:3832817-3833111):124-294	gb|AAO33922.1|:22-77	84
BmHel-11(DF090347:3894117-3894419):135-302	gb|AAO33922.1|:22-77	85
BmHel-11(DF090347:3966791-3967085):124-294	gb|AAO33922.1|:22-77	75
BmHel-11(DF090347:4026137-4026427):123-290	gb|AAO33922.1|:22-77	89
BmHel-11(DF090347:4053802-4054079):144-277	gb|AAO33922.1|:22-71	62.5
BmHel-11(DF090347:4086883-4087175):142-292	gb|AAO33922.1|:22-71	61
BmHel-11(DF090347:4361949-4362228):112-279	gb|AAO33922.1|:22-77	91
BmHel-11(DF090347:524885-525175):124-291	gb|AAO33922.1|:22-77	91

BmHel-11(DF090347:891088-891379):124-291	gb|AAO33922.1|:22-77	89
BmHel-11(DF090347:988353-988642):122-289	gb|AAO33922.1|:22-77	62
BmHel-11(DF090348:10698-10991):126-293	gb|AAO33922.1|:22-77	57
BmHel-11(DF090348:1251799-1252089):165-290	gb|AAO33922.1|:22-63	76
BmHel-11(DF090348:130402-130694):161-292	gb|AAO33922.1|:22-65	88
BmHel-11(DF090348:1322127-1322412):161-285	gb|AAO33922.1|:22-65	66.5
BmHel-11(DF090348:148099-148388):122-289	gb|AAO33922.1|:22-77	91
BmHel-11(DF090348:1557156-1557447):136-291	gb|AAO33922.1|:22-73	84
BmHel-11(DF090348:1842547-1842836):124-231	gb|AAO33922.1|:42-77	83
BmHel-11(DF090348:1899427-1899718):124-291	gb|AAO33922.1|:22-77	89
BmHel-11(DF090348:1931146-1931436):150-290	gb|AAO33922.1|:22-68	85
BmHel-11(DF090348:2059225-2059513):123-288	gb|AAO33922.1|:22-77	58.5
BmHel-11(DF090348:215233-215520):121-231	gb|AAO33922.1|:41-77	81
BmHel-11(DF090348:2164705-2164992):120-287	gb|AAO33922.1|:22-77	89
BmHel-11(DF090348:2372710-2373000):166-276	gb|AAO33922.1|:27-63	89
BmHel-11(DF090348:237695-237985):123-290	gb|AAO33922.1|:22-77	85
BmHel-11(DF090348:2481678-2482083):77-220	dbj|BAH66325.1|:105-152	68
BmHel-11(DF090348:2481678-2482083):274-405	gb|AAO33922.1|:22-65	95
BmHel-11(DF090348:2519064-2519356):125-292	gb|AAO33922.1|:22-77	83
BmHel-11(DF090348:2612799-2613091):134-292	gb|AAO33922.1|:22-74	83
BmHel-11(DF090348:2909086-2909377):124-291	gb|AAO33922.1|:22-77	87
BmHel-11(DF090348:3063684-3063974):123-290	gb|AAO33922.1|:22-77	83
BmHel-11(DF090348:3385532-3385822):162-290	gb|AAO33922.1|:22-64	86
BmHel-11(DF090348:3573144-3573431):121-287	gb|AAO33922.1|:22-77	48.5
BmHel-11(DF090348:3696201-3696487):149-286	gb|AAO33922.1|:22-67	80
BmHel-11(DF090348:3793472-3793754):151-282	gb|AAO33922.1|:22-65	84
BmHel-11(DF090348:3819849-3820146):151-297	gb|AAO33922.1|:22-77	64
BmHel-11(DF090348:3985573-3985847):146-274	gb|AAO33922.1|:22-70	75
BmHel-11(DF090348:4104141-4104430):135-290	gb|AAO33922.1|:22-73	82
BmHel-11(DF090348:4315761-4316051):123-290	gb|AAO33922.1|:22-77	92
BmHel-11(DF090348:4355825-4356116):124-291	gb|AAO33922.1|:22-77	89
BmHel-11(DF090348:470896-471164):124-268	gb|AAO33922.1|:22-77	62
BmHel-11(DF090348:535176-535463):120-287	gb|AAO33922.1|:22-77	87
BmHel-11(DF090348:618138-618432):124-294	gb|AAO33922.1|:22-77	84
BmHel-11(DF090348:899207-899497):123-290	gb|AAO33922.1|:22-77	85
BmHel-11(DF090348:949960-950254):127-294	gb|AAO33922.1|:22-77	87
BmHel-11(DF090348:991143-991435):155-292	gb|AAO33922.1|:22-67	78
BmHel-11(DF090349:1075981-1076276):124-295	gb|AAO33922.1|:22-77	68
BmHel-11(DF090349:1079653-1079944):124-291	gb|AAO33922.1|:22-77	85
BmHel-11(DF090349:1190702-1190994):124-292	gb|AAO33922.1|:22-77	68
BmHel-11(DF090349:1239766-1240021):127-255	gb|AAO33922.1|:22-76	67
BmHel-11(DF090349:1428801-1429095):157-294	gb|AAO33922.1|:22-67	76
BmHel-11(DF090349:1495656-1495925):119-269	gb|AAO33922.1|:22-77	61
BmHel-11(DF090349:1549704-1550004):133-300	gb|AAO33922.1|:22-77	87
BmHel-11(DF090349:1673624-1673915):124-291	gb|AAO33922.1|:22-77	85
BmHel-11(DF090349:1746730-1747020):123-290	gb|AAO33922.1|:22-77	85
BmHel-11(DF090349:1792297-1792588):127-291	gb|AAO33922.1|:22-76	92
BmHel-11(DF090349:1809350-1809640):126-290	gb|AAO33922.1|:22-77	80

BmHel-11(DF090349:1838782-1839074):143-292	gb|AAO33922.1|:22-71	84
BmHel-11(DF090349:204081-204372):154-291	gb|AAO33922.1|:22-67	82
BmHel-11(DF090349:2041366-2041631):170-265	gb|AAO33922.1|:22-53	96
BmHel-11(DF090349:222918-223207):127-289	gb|AAO33922.1|:22-77	60
BmHel-11(DF090349:2269013-2269258):127-245	gb|AAO33922.1|:22-76	71
BmHel-11(DF090349:2297704-2297995):151-291	gb|AAO33922.1|:22-68	87
BmHel-11(DF090349:2400540-2400832):125-292	gb|AAO33922.1|:22-77	85
BmHel-11(DF090349:2520746-2521037):124-291	gb|AAO33922.1|:22-77	89
BmHel-11(DF090349:2534378-2534688):143-310	gb|AAO33922.1|:22-77	89
BmHel-11(DF090349:2633674-2633965):151-291	gb|AAO33922.1|:22-77	75
BmHel-11(DF090349:2906752-2907042):123-290	gb|AAO33922.1|:22-77	92
BmHel-11(DF090349:3260500-3260760):114-260	gb|AAO33922.1|:22-70	79
BmHel-11(DF090349:3267782-3268072):123-290	gb|AAO33922.1|:22-77	91
BmHel-11(DF090349:3283537-3283831):157-294	gb|AAO33922.1|:22-67	89
BmHel-11(DF090349:3397549-3397829):146-280	gb|AAO33922.1|:22-68	70
BmHel-11(DF090349:3606568-3606860):130-292	gb|AAO33922.1|:22-77	62
BmHel-11(DF090349:3626023-3626390):136-267	gb|AAO33922.1|:22-77	73
BmHel-11(DF090349:3849075-3849368):126-293	gb|AAO33922.1|:22-77	55
BmHel-11(DF090349:3915711-3916003):124-292	gb|AAO33922.1|:22-77	64
BmHel-11(DF090349:3955965-3956257):128-292	gb|AAO33922.1|:22-76	85
BmHel-11(DF090349:3988935-3989227):130-292	gb|AAO33922.1|:22-75	62
BmHel-11(DF090349:400724-401016):160-279	gb|AAO33922.1|:26-65	82
BmHel-11(DF090349:4012360-4012638):127-278	gb|AAO33922.1|:22-76	64.5
BmHel-11(DF090349:4014636-4014927):124-291	gb|AAO33922.1|:22-77	87
BmHel-11(DF090349:4100607-4100896):144-289	gb|AAO33922.1|:22-71	71
BmHel-11(DF090349:4175801-4176094):124-293	gb|AAO33922.1|:22-77	50.5
BmHel-11(DF090349:4223000-4223296):150-296	gb|AAO33922.1|:22-77	62
BmHel-11(DF090349:4262333-4262621):160-288	gb|AAO33922.1|:22-65	84
BmHel-11(DF090349:890255-890545):141-290	gb|AAO33922.1|:22-71	84
BmHel-11(DF090349:9968-10259):124-291	gb|AAO33922.1|:22-77	89
BmHel-11(DF090350:1259663-1259954):127-291	gb|AAO33922.1|:22-76	92
BmHel-11(DF090350:1291094-1291385):172-291	gb|AAO33922.1|:22-61	90
BmHel-11(DF090350:1305619-1305909):159-290	gb|AAO33922.1|:22-65	88
BmHel-11(DF090350:1374084-1374370):140-286	gb|AAO33922.1|:22-71	70
BmHel-11(DF090350:1523189-1523457):134-268	gb|AAO33922.1|:22-74	66
BmHel-11(DF090350:1630154-1630431):167-277	gb|AAO33922.1|:22-58	86
BmHel-11(DF090350:1861314-1861606):124-249	gb|AAO33922.1|:36-77	71
BmHel-11(DF090350:2150823-2151118):128-295	gb|AAO33922.1|:22-77	91
BmHel-11(DF090350:2456808-2457057):124-249	gb|AAO33922.1|:22-77	67
BmHel-11(DF090350:2548781-2549073):124-292	gb|AAO33922.1|:22-77	63.5
BmHel-11(DF090350:280079-280370):124-291	gb|AAO33922.1|:22-77	85
BmHel-11(DF090350:2805932-2806229):160-297	gb|AAO33922.1|:22-65	86
BmHel-11(DF090350:30297-30567):124-270	gb|AAO33922.1|:22-76	81
BmHel-11(DF090350:3117703-3118213):126-269	gb|AAO33922.1|:30-77	81
BmHel-11(DF090350:325823-326114):160-291	gb|AAO33922.1|:22-65	81
BmHel-11(DF090350:4138868-4139164):135-296	gb|AAO33922.1|:22-77	53
BmHel-11(DF090350:556801-557059):121-258	gb|AAO33922.1|:22-67	82
BmHel-11(DF090351:1090784-1091084):131-286	gb|AAO33922.1|:26-77	82

BmHel-11(DF090351:1288616-1288917):137-301	gb|AAO33922.1|:22-77	80
BmHel-11(DF090351:1357587-1357876):123-203	gb|AAO33922.1|:51-77	92
BmHel-11(DF090351:1413777-1414068):160-291	gb|AAO33922.1|:22-65	95
BmHel-11(DF090351:1470364-1470655):124-291	gb|AAO33922.1|:22-77	83
BmHel-11(DF090351:1485485-1485776):124-291	gb|AAO33922.1|:22-77	87
BmHel-11(DF090351:1599387-1599642):115-255	gb|AAO33922.1|:22-68	85
BmHel-11(DF090351:163046-163336):123-290	gb|AAO33922.1|:22-77	87
BmHel-11(DF090351:1634917-1635206):134-289	gb|AAO33922.1|:22-73	84
BmHel-11(DF090351:1715602-1715892):126-290	gb|AAO33922.1|:22-77	56
BmHel-11(DF090351:1780998-1781291):123-293	gb|AAO33922.1|:22-77	56
BmHel-11(DF090351:1874548-1874839):124-291	gb|AAO33922.1|:22-77	89
BmHel-11(DF090351:1990211-1990504):144-293	gb|AAO33922.1|:22-71	68
BmHel-11(DF090351:2154025-2154321):150-296	gb|AAO33922.1|:22-68	87
BmHel-11(DF090351:2234404-2234674):127-270	gb|AAO33922.1|:22-69	75
BmHel-11(DF090351:2282408-2282697):152-289	gb|AAO33922.1|:22-67	76
BmHel-11(DF090351:2342028-2342319):124-291	gb|AAO33922.1|:22-77	85
BmHel-11(DF090351:2342986-2343273):120-287	gb|AAO33922.1|:22-77	83
BmHel-11(DF090351:2373990-2374279):125-289	gb|AAO33922.1|:22-77	53
BmHel-11(DF090351:2636144-2636433):152-289	gb|AAO33922.1|:22-67	89
BmHel-11(DF090351:2777209-2777500):160-291	gb|AAO33922.1|:22-65	84
BmHel-11(DF090351:2961099-2961395):124-296	gb|AAO33922.1|:22-77	55.5
BmHel-11(DF090351:303206-303451):124-245	gb|AAO33922.1|:22-77	59.5
BmHel-11(DF090351:3218368-3218654):124-264	gb|AAO33922.1|:31-77	91
BmHel-11(DF090351:3313737-3314004):130-267	gb|AAO33922.1|:22-77	71
BmHel-11(DF090351:3442827-3443119):149-292	gb|AAO33922.1|:22-69	83
BmHel-11(DF090351:3511675-3511965):160-291	gb|AAO33922.1|:22-65	90
BmHel-11(DF090351:3526718-3527005):163-279	gb|AAO33922.1|:25-63	76
BmHel-11(DF090351:3580385-3580660):138-275	gb|AAO33922.1|:22-67	86
BmHel-11(DF090351:3635521-3636358):606-737	gb|AAO33922.1|:22-65	93
BmHel-11(DF090351:3635521-3636358):240-459	dbj|BAH66344.1|:23-96	38
BmHel-11(DF090351:3978863-3979137):116-274	gb|AAO33922.1|:22-74	77
BmHel-11(DF090351:4034115-4034407):161-292	gb|AAO33922.1|:22-65	90
BmHel-11(DF090351:4039371-4039662):160-291	gb|AAO33922.1|:22-65	90
BmHel-11(DF090351:4198568-4198878):160-310	gb|AAO33922.1|:22-65	62.5
BmHel-11(DF090351:429598-429874):144-276	gb|AAO33922.1|:22-71	62
BmHel-11(DF090351:640802-641095):132-293	gb|AAO33922.1|:22-75	90
BmHel-11(DF090351:683533-683802):108-269	gb|AAO33922.1|:22-77	71
BmHel-11(DF090352:1108450-1108729):163-279	gb|AAO33922.1|:22-64	83
BmHel-11(DF090352:138645-138922):129-277	gb|AAO33922.1|:22-76	71
BmHel-11(DF090352:1510301-1510593):155-292	gb|AAO33922.1|:22-67	86
BmHel-11(DF090352:164755-165041):126-286	gb|AAO33922.1|:22-77	66.5
BmHel-11(DF090352:1657295-1657586):124-291	gb|AAO33922.1|:22-77	85
BmHel-11(DF090352:1860264-1860552):148-288	gb|AAO33922.1|:22-68	85
BmHel-11(DF090352:2465576-2465864):122-289	gb|AAO33922.1|:22-77	71
BmHel-11(DF090352:2482900-2484688):656-1213	gb|AAT47767.1|:20-196	23
BmHel-11(DF090352:2648605-2648842):124-237	gb|AAO33922.1|:22-77	62
BmHel-11(DF090352:2661074-2661364):156-290	gb|AAO33922.1|:22-66	84
BmHel-11(DF090352:2664504-2664747):124-243	gb|AAO33922.1|:22-77	62

BmHel-11(DF090352:2703217-2703507):123-290	gb|AAO33922.1|:22-77	87
BmHel-11(DF090352:2855030-2855352):158-322	gb|AAO33922.1|:22-75	63
BmHel-11(DF090352:2929980-2930272):134-292	gb|AAO33922.1|:22-74	83
BmHel-11(DF090352:3065118-3065386):128-268	gb|AAO33922.1|:22-67	76
BmHel-11(DF090352:3151152-3151438):143-286	gb|AAO33922.1|:22-71	88
BmHel-11(DF090352:3168649-3168941):125-292	gb|AAO33922.1|:22-77	87
BmHel-11(DF090352:343311-343603):125-292	gb|AAO33922.1|:22-77	85
BmHel-11(DF090352:3768768-3769058):159-290	gb|AAO33922.1|:22-65	79
BmHel-11(DF090352:3792341-3792635):139-294	gb|AAO33922.1|:22-73	82
BmHel-11(DF090352:3930864-3931155):160-291	gb|AAO33922.1|:22-65	86
BmHel-11(DF090352:4120736-4120992):117-256	gb|AAO33922.1|:22-68	61.5
BmHel-11(DF090352:436853-437141):124-288	gb|AAO33922.1|:22-76	76
BmHel-11(DF090352:565969-566261):158-292	gb|AAO33922.1|:22-66	93
BmHel-11(DF090352:616417-616709):155-292	gb|AAO33922.1|:22-67	80
BmHel-11(DF090352:641190-641481):157-291	gb|AAO33922.1|:22-66	88
BmHel-11(DF090352:73217-73491):179-274	gb|AAO33922.1|:22-53	84
BmHel-11(DF090352:871757-872038):123-281	gb|AAO33922.1|:22-77	80
BmHel-11(DF090353:1019017-1019276):124-259	gb|AAO33922.1|:22-77	56.5
BmHel-11(DF090353:1062369-1062661):155-292	gb|AAO33922.1|:22-67	82
BmHel-11(DF090353:1165483-1165772):131-289	gb|AAO33922.1|:22-75	83
BmHel-11(DF090353:118344-118636):124-292	gb|AAO33922.1|:22-77	55
BmHel-11(DF090353:1200848-1201139):142-291	gb|AAO33922.1|:22-71	84
BmHel-11(DF090353:1286919-1287205):156-286	gb|AAO33922.1|:22-67	65
BmHel-11(DF090353:1392086-1392373):147-287	gb|AAO33922.1|:22-70	79
BmHel-11(DF090353:1464045-1464270):98-225	gb|AAO33922.1|:22-77	62
BmHel-11(DF090353:1529745-1530036):124-291	gb|AAO33922.1|:22-77	91
BmHel-11(DF090353:1568472-1568763):127-291	gb|AAO33922.1|:22-76	89
BmHel-11(DF090353:1572898-1573181):143-283	gb|AAO33922.1|:22-68	70
BmHel-11(DF090353:1579253-1579545):125-292	gb|AAO33922.1|:22-77	87
BmHel-11(DF090353:1588975-1589256):180-281	gb|AAO33922.1|:22-55	82
BmHel-11(DF090353:1643541-1643809):157-268	gb|AAO33922.1|:22-66	60.5
BmHel-11(DF090353:2152800-2153019):78-219	gb|AAO33922.1|:22-76	63.5
BmHel-11(DF090353:2667881-2668172):154-291	gb|AAO33922.1|:22-67	91
BmHel-11(DF090353:2776333-2776612):115-279	gb|AAO33922.1|:22-76	83
BmHel-11(DF090353:29132-29423):126-248	gb|AAO33922.1|:36-77	71
BmHel-11(DF090353:2970863-2971199):124-336	gb|AAO33922.1|:22-77	54
BmHel-11(DF090353:3052613-3052901):157-288	gb|AAO33922.1|:22-65	88
BmHel-11(DF090353:3165382-3165688):175-306	gb|AAO33922.1|:22-65	77
BmHel-11(DF090353:3230442-3230733):124-291	gb|AAO33922.1|:22-77	83
BmHel-11(DF090353:3245448-3245744):129-296	gb|AAO33922.1|:22-76	82
BmHel-11(DF090353:3407525-3407799):124-274	gb|AAO33922.1|:22-77	59
BmHel-11(DF090353:3500609-3500896):120-287	gb|AAO33922.1|:22-77	89
BmHel-11(DF090353:3516407-3516682):177-275	gb|AAO33922.1|:22-54	93
BmHel-11(DF090353:3544694-3544985):124-291	gb|AAO33922.1|:22-77	87
BmHel-11(DF090353:3634623-3634914):124-291	gb|AAO33922.1|:22-77	83
BmHel-11(DF090353:3821975-3822265):123-290	gb|AAO33922.1|:22-77	87
BmHel-11(DF090353:3921317-3921591):129-274	gb|AAO33922.1|:22-73	67
BmHel-11(DF090353:3941847-3942137):153-290	gb|AAO33922.1|:22-67	78

BmHel-11(DF090353:4001172-4001461):122-289	gb|AAO33922.1|:22-77	62
BmHel-11(DF090353:501350-501641):124-291	gb|AAO33922.1|:22-77	85
BmHel-11(DF090353:580445-580735):153-290	gb|AAO33922.1|:22-67	82
BmHel-11(DF090353:648195-648458):174-263	gb|AAO33922.1|:22-51	90
BmHel-11(DF090353:974325-974616):154-291	gb|AAO33922.1|:22-67	89
BmHel-11(DF090354:1067229-1067523):130-294	gb|AAO33922.1|:22-76	89
BmHel-11(DF090354:1091622-1091916):124-294	gb|AAO33922.1|:22-77	71
BmHel-11(DF090354:1122106-1122398):149-292	gb|AAO33922.1|:22-67	77
BmHel-11(DF090354:1514212-1514505):126-293	gb|AAO33922.1|:22-77	71
BmHel-11(DF090354:1704337-1704576):126-239	gb|AAO33922.1|:22-74	62
BmHel-11(DF090354:1706847-1707141):124-294	gb|AAO33922.1|:22-77	82
BmHel-11(DF090354:1746410-1746705):131-295	gb|AAO33922.1|:22-76	83
BmHel-11(DF090354:1818339-1818593):117-254	gb|AAO33922.1|:22-67	63
BmHel-11(DF090354:2355453-2355744):163-291	gb|AAO33922.1|:22-64	90
BmHel-11(DF090354:2393837-2394092):133-255	gb|AAO33922.1|:22-62	90
BmHel-11(DF090354:2637130-2637422):125-292	gb|AAO33922.1|:22-77	89
BmHel-11(DF090354:2748767-2749058):124-291	gb|AAO33922.1|:22-77	80
BmHel-11(DF090354:278129-278404):144-275	gb|AAO33922.1|:22-69	83
BmHel-11(DF090354:2847463-2847737):104-274	gb|AAO33922.1|:22-77	71
BmHel-11(DF090354:2907057-2907274):125-217	gb|AAO33922.1|:22-52	93
BmHel-11(DF090354:2923267-2923560):156-293	gb|AAO33922.1|:22-67	76
BmHel-11(DF090354:2947744-2948033):124-289	gb|AAO33922.1|:22-77	59.5
BmHel-11(DF090354:2961069-2961361):125-292	gb|AAO33922.1|:22-77	91
BmHel-11(DF090354:3202651-3202932):171-281	gb|AAO33922.1|:22-64	74
BmHel-11(DF090354:3235043-3235328):135-285	gb|AAO33922.1|:22-76	63.5
BmHel-11(DF090354:3339304-3339592):166-288	gb|AAO33922.1|:22-62	82
BmHel-11(DF090354:3497170-3497469):124-299	gb|AAO33922.1|:22-77	63.5
BmHel-11(DF090354:475347-475642):131-295	gb|AAO33922.1|:22-76	81
BmHel-11(DF090354:491764-492019):124-255	gb|AAO33922.1|:22-77	71
BmHel-11(DF090354:534581-534872):124-291	gb|AAO33922.1|:22-77	85
BmHel-11(DF090354:778374-778641):148-267	gb|AAO33922.1|:22-61	72
BmHel-11(DF090354:978366-978655):124-289	gb|AAO33922.1|:22-77	52
BmHel-11(DF090355:110742-111034):125-292	gb|AAO33922.1|:22-77	87
BmHel-11(DF090355:1453147-1453434):156-287	gb|AAO33922.1|:22-65	90
BmHel-11(DF090355:1464891-1465182):125-292	gb|AAO33922.1|:22-77	82
BmHel-11(DF090355:1606676-1606967):124-291	gb|AAO33922.1|:22-77	89
BmHel-11(DF090355:203155-203442):123-287	gb|AAO33922.1|:22-77	83
BmHel-11(DF090355:203977-204268):124-291	gb|AAO33922.1|:22-77	73
BmHel-11(DF090355:2157464-2157754):168-290	gb|AAO33922.1|:22-62	70
BmHel-11(DF090355:2172895-2173123):136-228	gb|AAO33922.1|:22-52	93
BmHel-11(DF090355:2305882-2306173):124-291	gb|AAO33922.1|:22-77	55
BmHel-11(DF090355:2413567-2413858):124-291	gb|AAO33922.1|:22-77	87
BmHel-11(DF090355:2501864-2502157):126-293	gb|AAO33922.1|:22-77	82
BmHel-11(DF090355:2704131-2704410):127-279	gb|AAO33922.1|:22-77	57
BmHel-11(DF090355:2956287-2956577):153-290	gb|AAO33922.1|:22-67	89
BmHel-11(DF090355:2980629-2980920):124-291	gb|AAO33922.1|:22-77	85
BmHel-11(DF090355:3012935-3013222):189-287	gb|AAO33922.1|:22-54	87
BmHel-11(DF090355:3129272-3129570):137-298	gb|AAO33922.1|:22-75	83

BmHel-11(DF090355:3194518-3194806):121-288	gb|AAO33922.1|:22-77	85
BmHel-11(DF090355:3274167-3274460):123-293	gb|AAO33922.1|:22-77	85
BmHel-11(DF090355:3336364-3336655):160-291	gb|AAO33922.1|:22-65	90
BmHel-11(DF090355:3393118-3393408):123-290	gb|AAO33922.1|:22-77	91
BmHel-11(DF090355:3435518-3435761):127-243	gb|AAO33922.1|:22-76	63
BmHel-11(DF090355:3508072-3508351):124-279	gb|AAO33922.1|:22-77	78
BmHel-11(DF090355:3692766-3693062):124-296	gb|AAO33922.1|:22-77	61
BmHel-11(DF090355:3749104-3749399):126-295	gb|AAO33922.1|:22-77	65
BmHel-11(DF090355:3753857-3754092):122-235	gb|AAO33922.1|:22-69	72
BmHel-11(DF090355:734527-734838):124-243	gb|AAO33922.1|:38-77	77
BmHel-11(DF090356:1039761-1040019):124-258	gb|AAO33922.1|:22-77	60
BmHel-11(DF090356:1067246-1067489):124-243	gb|AAO33922.1|:22-77	62
BmHel-11(DF090356:1132999-1133292):162-293	gb|AAO33922.1|:22-65	93
BmHel-11(DF090356:1485717-1485940):62-223	gb|AAO33922.1|:22-75	81
BmHel-11(DF090356:1508549-1508839):123-290	gb|AAO33922.1|:22-77	92
BmHel-11(DF090356:1668439-1668729):126-290	gb|AAO33922.1|:22-77	62
BmHel-11(DF090356:167142-167432):159-290	gb|AAO33922.1|:22-65	90
BmHel-11(DF090356:1732354-1732647):126-293	gb|AAO33922.1|:22-77	80
BmHel-11(DF090356:2062557-2062849):125-292	gb|AAO33922.1|:22-77	82
BmHel-11(DF090356:2212418-2212708):153-290	gb|AAO33922.1|:22-67	80
BmHel-11(DF090356:2349850-2350140):124-290	gb|AAO33922.1|:22-77	63
BmHel-11(DF090356:2621729-2621982):116-253	gb|AAO33922.1|:22-67	80
BmHel-11(DF090356:2895048-2895346):124-298	gb|AAO33922.1|:22-77	62
BmHel-11(DF090356:2907779-2908378):483-599	gb|AAO33922.1|:22-62	80
BmHel-11(DF090356:3194582-3194872):125-290	gb|AAO33922.1|:22-77	61.5
BmHel-11(DF090356:3226213-3226500):120-287	gb|AAO33922.1|:22-77	55
BmHel-11(DF090356:3343716-3344010):130-294	gb|AAO33922.1|:22-76	89
BmHel-11(DF090356:3408119-3408411):155-292	gb|AAO33922.1|:22-67	84
BmHel-11(DF090356:3426693-3426984):148-291	gb|AAO33922.1|:22-69	81
BmHel-11(DF090356:3566275-3566538):125-263	gb|AAO33922.1|:22-77	61.5
BmHel-11(DF090356:386967-387195):136-228	gb|AAO33922.1|:22-52	90
BmHel-11(DF090356:483903-484194):124-291	gb|AAO33922.1|:22-77	87
BmHel-11(DF090356:930966-931267):134-301	gb|AAO33922.1|:22-77	73
BmHel-11(DF090357:1009324-1009616):155-292	gb|AAO33922.1|:22-67	76
BmHel-11(DF090357:1178094-1178375):117-281	gb|AAO33922.1|:22-76	60
BmHel-11(DF090357:1292011-1292286):124-275	gb|AAO33922.1|:22-77	51.5
BmHel-11(DF090357:1445287-1445541):124-254	gb|AAO33922.1|:22-77	71
BmHel-11(DF090357:1500223-1500512):124-289	gb|AAO33922.1|:22-77	60
BmHel-11(DF090357:1585255-1585537):115-282	gb|AAO33922.1|:22-77	73
BmHel-11(DF090357:1784005-1784295):124-264	gb|AAO33922.1|:31-77	89
BmHel-11(DF090357:178832-179124):161-292	gb|AAO33922.1|:22-65	86
BmHel-11(DF090357:203472-203770):145-298	gb|AAO33922.1|:22-71	61
BmHel-11(DF090357:2075854-2076145):124-291	gb|AAO33922.1|:22-77	83
BmHel-11(DF090357:2094756-2095046):135-290	gb|AAO33922.1|:22-73	84
BmHel-11(DF090357:2118817-2119102):118-285	gb|AAO33922.1|:22-77	75
BmHel-11(DF090357:2283117-2283397):123-280	gb|AAO33922.1|:22-77	55.5
BmHel-11(DF090357:2482600-2482829):76-210	gb|AAO33922.1|:29-76	66
BmHel-11(DF090357:2706072-2706369):161-297	gb|AAO33922.1|:22-65	67

BmHel-11(DF090357:2750393-2750672):111-248	gb|AAO33922.1|:32-77	84
BmHel-11(DF090357:2882855-2883148):135-293	gb|AAO33922.1|:22-74	77
BmHel-11(DF090357:34062-34319):117-257	gb|AAO33922.1|:22-68	82
BmHel-11(DF090357:3451161-3451452):124-291	gb|AAO33922.1|:22-77	85
BmHel-11(DF090357:345465-345753):124-288	gb|AAO33922.1|:22-77	82
BmHel-11(DF090357:3621612-3625399):1144-1440	gb|ELU16645.1|:8-281	36.5
BmHel-11(DF090357:3621612-3625399):2656-2793	gb|AAO33922.1|:22-67	91
BmHel-11(DF090357:3657916-3658206):123-290	gb|AAO33922.1|:22-77	85
BmHel-11(DF090357:458727-458988):124-261	gb|AAO33922.1|:22-77	65
BmHel-11(DF090357:493457-493754):134-232	gb|AAO33922.1|:42-74	81
BmHel-11(DF090357:526832-527097):164-265	gb|AAO33922.1|:22-55	88
BmHel-11(DF090357:608291-608583):155-292	gb|AAO33922.1|:22-67	86
BmHel-11(DF090357:629446-629738):125-292	gb|AAO33922.1|:22-77	83
BmHel-11(DF090357:811687-811971):124-284	gb|AAO33922.1|:22-77	71
BmHel-11(DF090357:857047-857291):125-244	gb|AAO33922.1|:22-77	66
BmHel-11(DF090357:860047-860340):127-225	gb|AAO33922.1|:45-77	78
BmHel-11(DF090357:925652-925936):147-284	gb|AAO33922.1|:22-67	63
BmHel-11(DF090358:116375-116667):161-292	gb|AAO33922.1|:22-65	84
BmHel-11(DF090358:1203176-1203471):134-295	gb|AAO33922.1|:22-75	85
BmHel-11(DF090358:1512537-1512794):124-257	gb|AAO33922.1|:22-77	57.5
BmHel-11(DF090358:1544722-1545012):126-290	gb|AAO33922.1|:22-77	58
BmHel-11(DF090358:195572-195861):122-289	gb|AAO33922.1|:22-77	83
BmHel-11(DF090358:1967150-1967393):134-243	gb|AAO33922.1|:22-74	65.5
BmHel-11(DF090358:2018940-2019233):153-293	gb|AAO33922.1|:22-68	78
BmHel-11(DF090358:2032575-2032867):125-292	gb|AAO33922.1|:22-77	83
BmHel-11(DF090358:2376153-2376439):119-286	gb|AAO33922.1|:22-76	75
BmHel-11(DF090358:2399115-2399391):112-276	gb|AAO33922.1|:22-76	90
BmHel-11(DF090358:2439956-2440248):127-292	gb|AAO33922.1|:22-76	71
BmHel-11(DF090358:2632380-2632671):124-291	gb|AAO33922.1|:22-77	87
BmHel-11(DF090358:2739622-2739858):141-236	gb|AAO33922.1|:22-53	96
BmHel-11(DF090358:3234790-3235081):124-291	gb|AAO33922.1|:22-77	87
BmHel-11(DF090358:3320181-3320472):151-291	gb|AAO33922.1|:22-68	93
BmHel-11(DF090358:385421-385712):124-291	gb|AAO33922.1|:22-77	87
BmHel-11(DF090358:480081-480301):128-220	gb|AAO33922.1|:22-52	87
BmHel-11(DF090358:511967-512257):123-290	gb|AAO33922.1|:22-77	87
BmHel-11(DF090358:51564-51849):163-285	gb|AAO33922.1|:22-62	78
BmHel-11(DF090358:568734-568977):124-243	gb|AAO33922.1|:22-77	66
BmHel-11(DF090358:734485-734768):116-283	gb|AAO33922.1|:22-77	87
BmHel-11(DF090358:997095-997386):124-291	gb|AAO33922.1|:22-77	83
BmHel-11(DF090359:1013506-1013748):124-242	gb|AAO33922.1|:22-77	71
BmHel-11(DF090359:1037878-1038176):144-298	gb|AAO33922.1|:22-70	71
BmHel-11(DF090359:1140029-1140320):124-291	gb|AAO33922.1|:22-77	87
BmHel-11(DF090359:1318157-1318448):163-291	gb|AAO33922.1|:22-64	93
BmHel-11(DF090359:1369694-1369984):168-290	gb|AAO33922.1|:22-62	78
BmHel-11(DF090359:1383897-1384171):124-274	gb|AAO33922.1|:22-77	62.5
BmHel-11(DF090359:1527639-1527924):124-285	gb|AAO33922.1|:22-77	76
BmHel-11(DF090359:1696375-1696666):127-291	gb|AAO33922.1|:22-76	90
BmHel-11(DF090359:1706551-1706842):154-291	gb|AAO33922.1|:22-67	91

BmHel-11(DF090359:1799465-1799769):123-304	gb|AAO33922.1|:22-77	67.5
BmHel-11(DF090359:1830534-1830826):155-292	gb|AAO33922.1|:22-67	71
BmHel-11(DF090359:1902700-1902991):124-291	gb|AAO33922.1|:22-77	75
BmHel-11(DF090359:2002696-2002982):155-286	gb|AAO33922.1|:22-67	82
BmHel-11(DF090359:2085844-2086121):152-277	gb|AAO33922.1|:22-67	78
BmHel-11(DF090359:2476234-2476510):172-276	gb|AAO33922.1|:22-56	82
BmHel-11(DF090359:2600974-2601235):124-261	gb|AAO33922.1|:22-77	71
BmHel-11(DF090359:2635298-2635589):125-220	gb|AAO33922.1|:46-77	81
BmHel-11(DF090359:266385-266676):160-291	gb|AAO33922.1|:22-65	77
BmHel-11(DF090359:2682264-2682551):141-287	gb|AAO33922.1|:22-70	95
BmHel-11(DF090359:2811038-2811328):141-290	gb|AAO33922.1|:22-71	92
BmHel-11(DF090359:3050688-3050979):124-291	gb|AAO33922.1|:22-77	91
BmHel-11(DF090359:580537-580796):153-259	gb|AAO33922.1|:22-68	65
BmHel-11(DF090359:649328-649590):125-262	gb|AAO33922.1|:22-77	67
BmHel-11(DF090360:1153253-1153531):144-278	gb|AAO33922.1|:22-68	68
BmHel-11(DF090360:1235776-1236064):123-224	gb|AAO33922.1|:43-76	85
BmHel-11(DF090360:1488565-1488855):123-290	gb|AAO33922.1|:22-77	85
BmHel-11(DF090360:1610003-1610294):154-291	gb|AAO33922.1|:22-67	84
BmHel-11(DF090360:2032464-2032753):122-289	gb|AAO33922.1|:22-77	83
BmHel-11(DF090360:204752-205043):124-291	gb|AAO33922.1|:22-77	82
BmHel-11(DF090360:2151810-2152055):126-245	gb|AAO33922.1|:22-77	60
BmHel-11(DF090360:229997-230276):122-279	gb|AAO33922.1|:22-75	63.5
BmHel-11(DF090360:2707565-2707832):124-267	gb|AAO33922.1|:22-77	73
BmHel-11(DF090360:2914357-2914973):452-616	gb|AAO33922.1|:22-77	73
BmHel-11(DF090360:2933261-2933545):160-284	gb|AAO33922.1|:22-65	65
BmHel-11(DF090360:2961342-2961633):124-291	gb|AAO33922.1|:22-77	85
BmHel-11(DF090360:3203200-3203492):155-292	gb|AAO33922.1|:22-67	78
BmHel-11(DF090360:40297-40593):159-296	gb|AAO33922.1|:22-65	84
BmHel-11(DF090360:469536-469827):124-291	gb|AAO33922.1|:22-77	87
BmHel-11(DF090360:706222-706513):124-291	gb|AAO33922.1|:22-77	89
BmHel-11(DF090360:834520-834754):67-234	gb|AAO33922.1|:22-77	83
BmHel-11(DF090360:913179-913503):127-324	gb|AAO33922.1|:22-77	72
BmHel-11(DF090361:1006315-1006614):124-299	gb|AAO33922.1|:22-77	63
BmHel-11(DF090361:1119095-1119347):157-252	gb|AAO33922.1|:22-53	90
BmHel-11(DF090361:1162811-1163092):114-281	gb|AAO33922.1|:22-77	89
BmHel-11(DF090361:1212735-1213019):156-284	gb|AAO33922.1|:22-64	88
BmHel-11(DF090361:1348410-1348697):120-287	gb|AAO33922.1|:22-77	85
BmHel-11(DF090361:137101-137387):124-286	gb|AAO33922.1|:22-77	67.5
BmHel-11(DF090361:1530570-1530816):100-246	gb|AAO33922.1|:22-70	85
BmHel-11(DF090361:1593211-1593613):175-302	gb|AAO33922.1|:22-65	59
BmHel-11(DF090361:1624234-1624531):160-297	gb|AAO33922.1|:22-65	84
BmHel-11(DF090361:1837431-1837722):142-291	gb|AAO33922.1|:22-71	88
BmHel-11(DF090361:1938999-1939294):128-295	gb|AAO33922.1|:22-77	83
BmHel-11(DF090361:2005860-2006088):136-228	gb|AAO33922.1|:22-52	90
BmHel-11(DF090361:207201-207492):154-291	gb|AAO33922.1|:22-67	78
BmHel-11(DF090361:2079168-2079461):156-293	gb|AAO33922.1|:22-67	67
BmHel-11(DF090361:2109840-2110123):158-283	gb|AAO33922.1|:22-69	66
BmHel-11(DF090361:2145339-2145632):126-293	gb|AAO33922.1|:22-77	82

BmHel-11(DF090361:2188997-2189288):160-291	gb|AAO33922.1|:22-65	81
BmHel-11(DF090361:2432674-2432965):124-291	gb|AAO33922.1|:22-77	87
BmHel-11(DF090361:2574132-2574426):127-294	gb|AAO33922.1|:22-77	85
BmHel-11(DF090361:2639654-2639946):155-292	gb|AAO33922.1|:22-67	76
BmHel-11(DF090361:3067928-3068219):124-291	gb|AAO33922.1|:22-77	80
BmHel-11(DF090361:3173557-3173860):129-230	gb|AAO33922.1|:44-77	85
BmHel-11(DF090361:368609-368899):122-290	gb|AAO33922.1|:22-77	65
BmHel-11(DF090361:433888-434178):123-290	gb|AAO33922.1|:22-77	87
BmHel-11(DF090361:531037-531330):144-293	gb|AAO33922.1|:22-71	92
BmHel-11(DF090361:566199-566489):123-290	gb|AAO33922.1|:22-77	80
BmHel-11(DF090361:772191-772482):124-291	gb|AAO33922.1|:22-77	87
BmHel-11(DF090361:786670-788053):912-1079	gb|AAO33922.1|:22-77	83
BmHel-11(DF090362:1197919-1198209):141-290	gb|AAO33922.1|:22-71	94
BmHel-11(DF090362:14458-14744):122-286	gb|AAO33922.1|:22-77	60
BmHel-11(DF090362:157720-158011):124-291	gb|AAO33922.1|:22-77	91
BmHel-11(DF090362:1588700-1588990):123-290	gb|AAO33922.1|:22-77	85
BmHel-11(DF090362:1645432-1645719):126-287	gb|AAO33922.1|:22-77	58
BmHel-11(DF090362:1812873-1813163):128-290	gb|AAO33922.1|:22-76	52.5
BmHel-11(DF090362:1953510-1953801):124-291	gb|AAO33922.1|:22-77	87
BmHel-11(DF090362:1997523-1997818):126-295	gb|AAO33922.1|:22-77	69
BmHel-11(DF090362:2133014-2133305):124-291	gb|AAO33922.1|:22-77	89
BmHel-11(DF090362:2261781-2262072):124-291	gb|AAO33922.1|:22-77	89
BmHel-11(DF090362:2303092-2303384):124-234	gb|AAO33922.1|:41-77	89
BmHel-11(DF090362:2397630-2405140):124-3859	gb|AAO33922.1|:22-77	53
BmHel-11(DF090362:2493825-2494103):174-278	gb|AAO33922.1|:22-56	85
BmHel-11(DF090362:2507909-2508136):135-227	gb|AAO33922.1|:22-52	93
BmHel-11(DF090362:265833-266118):121-285	gb|AAO33922.1|:22-76	85
BmHel-11(DF090362:2789646-2789928):145-282	gb|AAO33922.1|:22-67	89
BmHel-11(DF090362:302705-302995):123-290	gb|AAO33922.1|:22-77	83
BmHel-11(DF090362:3147029-3147319):153-290	gb|AAO33922.1|:22-67	80
BmHel-11(DF090362:325408-325698):123-290	gb|AAO33922.1|:22-77	80
BmHel-11(DF090362:492705-492996):124-291	gb|AAO33922.1|:22-77	83
BmHel-11(DF090362:494058-494349):160-291	gb|AAO33922.1|:22-65	84
BmHel-11(DF090362:644128-644419):154-291	gb|AAO33922.1|:22-67	73
BmHel-11(DF090362:666888-667405):127-261	gb|AAO33922.1|:33-77	86
BmHel-11(DF090362:707579-707868):128-289	gb|AAO33922.1|:22-75	75
BmHel-11(DF090362:782546-782837):157-291	gb|AAO33922.1|:22-66	88
BmHel-11(DF090363:1039435-1039726):124-291	gb|AAO33922.1|:22-77	85
BmHel-11(DF090363:106992-107275):125-283	gb|AAO33922.1|:22-77	75
BmHel-11(DF090363:107743-111771):124-234	gb|AAO33922.1|:41-77	75
BmHel-11(DF090363:107743-111771):628-1233	emb|CAA32198.1|:308-509	39
BmHel-11(DF090363:107743-111771):1278-1508	ref|XP_003490608.1|:35-138	34
BmHel-11(DF090363:1137555-1137846):124-291	gb|AAO33922.1|:22-77	85
BmHel-11(DF090363:1155806-1156083):146-277	gb|AAO33922.1|:22-65	77
BmHel-11(DF090363:1441867-1442158):124-291	gb|AAO33922.1|:22-77	83
BmHel-11(DF090363:1485401-1485692):123-233	gb|AAO33922.1|:41-77	81
BmHel-11(DF090363:1580872-1581168):150-296	gb|AAO33922.1|:22-77	64
BmHel-11(DF090363:204421-204712):124-291	gb|AAO33922.1|:22-77	85

BmHel-11(DF090363:212418-212698):137-280	gb|AAO33922.1|:22-69	93
BmHel-11(DF090363:2129608-2129900):161-292	gb|AAO33922.1|:22-65	86
BmHel-11(DF090363:2249407-2249672):124-265	gb|AAO33922.1|:22-77	67
BmHel-11(DF090363:2284942-2285240):131-298	gb|AAO33922.1|:22-77	89
BmHel-11(DF090363:2466769-2467060):124-291	gb|AAO33922.1|:22-77	82
BmHel-11(DF090363:2536464-2536755):124-291	gb|AAO33922.1|:22-77	85
BmHel-11(DF090363:2603548-2603840):125-292	gb|AAO33922.1|:22-77	71
BmHel-11(DF090363:2614777-2615034):124-213	gb|AAO33922.1|:48-77	80
BmHel-11(DF090363:2745890-2746174):124-284	gb|AAO33922.1|:22-77	67.5
BmHel-11(DF090363:2902906-2903197):160-291	gb|AAO33922.1|:22-65	86
BmHel-11(DF090363:495268-495521):127-253	gb|AAO33922.1|:22-76	67
BmHel-11(DF090363:591588-591879):157-291	gb|AAO33922.1|:22-66	86
BmHel-11(DF090363:653499-653784):124-285	gb|AAO33922.1|:22-77	76
BmHel-11(DF090363:973851-974095):127-244	gb|AAO33922.1|:22-76	71
BmHel-11(DF090364:1080113-1080385):148-272	gb|AAO33922.1|:22-66	62
BmHel-11(DF090364:1220872-1221152):124-280	gb|AAO33922.1|:22-77	66.5
BmHel-11(DF090364:136813-137095):115-282	gb|AAO33922.1|:22-77	78
BmHel-11(DF090364:1459174-1459464):147-290	gb|AAO33922.1|:22-69	66
BmHel-11(DF090364:1506022-1506282):123-260	gb|AAO33922.1|:22-77	73
BmHel-11(DF090364:1591313-1591554):84-241	gb|AAO33922.1|:22-77	59
BmHel-11(DF090364:1715708-1716109):245-401	gb|AAO33922.1|:22-71	65
BmHel-11(DF090364:183159-183458):192-299	gb|AAO33922.1|:22-57	88
BmHel-11(DF090364:1851715-1852006):157-291	gb|AAO33922.1|:22-66	82
BmHel-11(DF090364:1884275-1884565):123-290	gb|AAO33922.1|:22-77	80
BmHel-11(DF090364:1904429-1904705):124-276	gb|AAO33922.1|:22-77	76
BmHel-11(DF090364:2059466-2059730):163-264	gb|AAO33922.1|:22-65	75
BmHel-11(DF090364:2441087-2441373):146-286	gb|AAO33922.1|:22-68	89
BmHel-11(DF090364:2499432-2499723):124-291	gb|AAO33922.1|:22-77	83
BmHel-11(DF090364:2648583-2648875):122-292	gb|AAO33922.1|:22-77	57
BmHel-11(DF090364:2905442-2905733):124-291	gb|AAO33922.1|:22-77	66
BmHel-11(DF090364:2918217-2918508):124-291	gb|AAO33922.1|:22-77	87
BmHel-11(DF090364:305523-305812):124-289	gb|AAO33922.1|:22-77	62
BmHel-11(DF090364:339950-340240):123-290	gb|AAO33922.1|:22-77	85
BmHel-11(DF090364:372113-372399):125-286	gb|AAO33922.1|:22-75	85
BmHel-11(DF090364:64375-64655):124-280	gb|AAO33922.1|:22-77	53.5
BmHel-11(DF090364:651551-651839):130-288	gb|AAO33922.1|:22-74	79
BmHel-11(DF090364:718361-718604):124-243	gb|AAO33922.1|:22-77	60
BmHel-11(DF090364:736966-737258):125-292	gb|AAO33922.1|:22-77	91
BmHel-11(DF090364:836010-836301):124-291	gb|AAO33922.1|:22-77	85
BmHel-11(DF090365:1072475-1072763):124-288	gb|AAO33922.1|:22-77	80
BmHel-11(DF090365:1149682-1149968):119-286	gb|AAO33922.1|:22-77	91
BmHel-11(DF090365:1223166-1223433):124-267	gb|AAO33922.1|:22-77	76
BmHel-11(DF090365:1225152-1225574):279-422	gb|AAO33922.1|:22-77	69
BmHel-11(DF090365:1304140-1304431):124-291	gb|AAO33922.1|:22-77	89
BmHel-11(DF090365:1367468-1367741):148-273	gb|AAO33922.1|:22-63	80
BmHel-11(DF090365:1420475-1420722):107-247	gb|AAO33922.1|:22-68	89
BmHel-11(DF090365:154273-154568):152-295	gb|AAO33922.1|:22-69	83
BmHel-11(DF090365:1759114-1759410):150-296	gb|AAO33922.1|:22-70	85

BmHel-11(DF090365:1825768-1826039):124-271	gb|AAO33922.1|:22-77	48
BmHel-11(DF090365:1858711-1859000):152-289	gb|AAO33922.1|:22-67	78
BmHel-11(DF090365:2050910-2051205):155-295	gb|AAO33922.1|:22-67	68
BmHel-11(DF090365:2089367-2089636):124-222	gb|AAO33922.1|:45-77	87
BmHel-11(DF090365:2136201-2136452):84-251	gb|AAO33922.1|:22-77	89
BmHel-11(DF090365:2146206-2146497):142-291	gb|AAO33922.1|:22-71	90
BmHel-11(DF090365:2247098-2247389):124-291	gb|AAO33922.1|:22-77	92
BmHel-11(DF090365:2283077-2283379):157-302	gb|AAO33922.1|:22-66	64
BmHel-11(DF090365:2398377-2398668):157-291	gb|AAO33922.1|:22-66	93
BmHel-11(DF090365:2405693-2405936):136-243	gb|AAO33922.1|:22-57	91
BmHel-11(DF090365:2414005-2414276):110-271	gb|AAO33922.1|:22-77	83
BmHel-11(DF090365:2492390-2492661):103-195	gb|AAO33922.1|:47-77	90
BmHel-11(DF090365:280549-280835):124-286	gb|AAO33922.1|:22-77	64
BmHel-11(DF090365:2824699-2824992):150-293	gb|AAO33922.1|:22-69	68
BmHel-11(DF090365:2866087-2866374):120-287	gb|AAO33922.1|:22-77	83
BmHel-11(DF090365:2932468-2932758):174-290	gb|AAO33922.1|:22-60	89
BmHel-11(DF090365:2943129-2943411):157-282	gb|AAO33922.1|:22-66	82
BmHel-11(DF090365:460848-461140):155-292	gb|AAO33922.1|:22-67	89
BmHel-11(DF090365:649586-649877):124-291	gb|AAO33922.1|:22-77	80
BmHel-11(DF090365:677720-678011):124-291	gb|AAO33922.1|:22-77	91
BmHel-11(DF090365:787847-788140):126-293	gb|AAO33922.1|:22-77	91
BmHel-11(DF090366:104414-104706):124-292	gb|AAO33922.1|:22-77	66
BmHel-11(DF090366:1178504-1178794):165-290	gb|AAO33922.1|:22-63	69
BmHel-11(DF090366:132214-132476):123-262	gb|AAO33922.1|:22-74	55.5
BmHel-11(DF090366:1458942-1459233):124-291	gb|AAO33922.1|:22-77	83
BmHel-11(DF090366:1548914-1549168):123-254	gb|AAO33922.1|:22-77	71
BmHel-11(DF090366:1663916-1664207):124-291	gb|AAO33922.1|:22-77	67
BmHel-11(DF090366:1746168-1746459):124-291	gb|AAO33922.1|:22-77	91
BmHel-11(DF090366:1836608-1836898):159-290	gb|AAO33922.1|:22-65	86
BmHel-11(DF090366:1896811-1897102):160-291	gb|AAO33922.1|:22-65	81
BmHel-11(DF090366:1973465-1973735):121-270	gb|AAO33922.1|:22-71	90
BmHel-11(DF090366:2338134-2338423):185-289	gb|AAO33922.1|:22-56	91
BmHel-11(DF090366:241022-241313):124-291	gb|AAO33922.1|:22-77	91
BmHel-11(DF090366:268577-268868):124-291	gb|AAO33922.1|:22-77	91
BmHel-11(DF090366:287965-288252):117-287	gb|AAO33922.1|:22-77	56
BmHel-11(DF090366:290826-291116):123-290	gb|AAO33922.1|:22-77	85
BmHel-11(DF090366:303991-304282):124-291	gb|AAO33922.1|:22-77	75
BmHel-11(DF090366:578391-578666):124-276	gb|AAO33922.1|:22-77	80
BmHel-11(DF090366:631429-631720):124-291	gb|AAO33922.1|:22-77	89
BmHel-11(DF090366:875175-875466):124-291	gb|AAO33922.1|:22-77	83
BmHel-11(DF090366:884840-885131):124-291	gb|AAO33922.1|:22-77	78
BmHel-11(DF090366:987235-987525):135-290	gb|AAO33922.1|:22-73	92
BmHel-11(DF090367:1023890-1024185):128-295	gb|AAO33922.1|:22-77	85
BmHel-11(DF090367:1146755-1147023):124-268	gb|AAO33922.1|:22-77	68
BmHel-11(DF090367:1289464-1289755):124-291	gb|AAO33922.1|:22-77	87
BmHel-11(DF090367:1369215-1369485):115-270	gb|AAO33922.1|:22-77	75
BmHel-11(DF090367:1379558-1379851):153-293	gb|AAO33922.1|:22-68	82
BmHel-11(DF090367:1415470-1416209):261-421	dbj|BAH66344.1|:45-95	55

BmHel-11(DF090367:151612-151905):144-293	gb|AAO33922.1|:22-71	88
BmHel-11(DF090367:1597330-1597616):122-286	gb|AAO33922.1|:22-76	87
BmHel-11(DF090367:1979640-1979889):154-249	gb|AAO33922.1|:22-69	66
BmHel-11(DF090367:2300983-2301234):90-251	gb|AAO33922.1|:22-73	60
BmHel-11(DF090367:2369942-2370232):123-290	gb|AAO33922.1|:22-77	85
BmHel-11(DF090367:2645854-2646153):174-299	gb|AAO33922.1|:22-63	83
BmHel-11(DF090367:2703318-2703613):124-291	gb|AAO33922.1|:22-77	80
BmHel-11(DF090367:2775689-2775979):162-290	gb|AAO33922.1|:22-64	79
BmHel-11(DF090367:364793-365081):172-288	gb|AAO33922.1|:22-60	89
BmHel-11(DF090367:366498-366793):136-282	gb|AAO33922.1|:26-73	83
BmHel-11(DF090367:376408-376699):124-291	gb|AAO33922.1|:22-77	85
BmHel-11(DF090367:636297-636575):114-278	gb|AAO33922.1|:22-76	89
BmHel-11(DF090367:670160-670451):124-291	gb|AAO33922.1|:22-77	91
BmHel-11(DF090367:784530-784949):152-319	gb|AAO33922.1|:22-77	87
BmHel-11(DF090367:787652-787937):163-285	gb|AAO33922.1|:22-62	85
BmHel-11(DF090367:825854-826611):647-757	gb|AAO33922.1|:22-59	81
BmHel-11(DF090367:923498-923789):124-291	gb|AAO33922.1|:22-77	89
BmHel-11(DF090367:976797-977064):124-267	gb|AAO33922.1|:22-77	73
BmHel-11(DF090368:1054636-1054926):174-290	gb|AAO33922.1|:22-60	82
BmHel-11(DF090368:1185522-1185813):160-291	gb|AAO33922.1|:22-65	86
BmHel-11(DF090368:1291246-1291541):161-295	gb|AAO33922.1|:22-66	86
BmHel-11(DF090368:1388720-1389004):118-225	gb|AAO33922.1|:42-77	83
BmHel-11(DF090368:1599470-1599751):117-281	gb|AAO33922.1|:22-76	85
BmHel-11(DF090368:1719120-1719415):155-295	gb|AAO33922.1|:22-67	91
BmHel-11(DF090368:1732276-1732549):163-273	gb|AAO33922.1|:22-64	74
BmHel-11(DF090368:176195-176486):160-291	gb|AAO33922.1|:22-65	90
BmHel-11(DF090368:1905546-1905837):160-291	gb|AAO33922.1|:22-65	90
BmHel-11(DF090368:2025820-2026111):160-291	gb|AAO33922.1|:22-65	88
BmHel-11(DF090368:2203867-2204157):123-290	gb|AAO33922.1|:22-77	82
BmHel-11(DF090368:2225776-2226062):119-286	gb|AAO33922.1|:22-77	85
BmHel-11(DF090368:2350575-2350866):124-291	gb|AAO33922.1|:22-77	89
BmHel-11(DF090368:2428128-2428419):133-291	gb|AAO33922.1|:22-74	83
BmHel-11(DF090368:2447206-2447496):123-290	gb|AAO33922.1|:22-77	87
BmHel-11(DF090368:475516-475759):124-243	gb|AAO33922.1|:22-77	62
BmHel-11(DF090368:848465-848729):157-264	gb|AAO33922.1|:22-57	80
BmHel-11(DF090369:1070728-1070978):104-250	gb|AAO33922.1|:22-70	89
BmHel-11(DF090369:1172438-1172740):123-302	gb|AAO33922.1|:22-76	65
BmHel-11(DF090369:1365158-1365455):124-297	gb|AAO33922.1|:22-77	84
BmHel-11(DF090369:1489948-1490239):124-291	gb|AAO33922.1|:22-77	82
BmHel-11(DF090369:1618538-1618794):134-256	gb|AAO33922.1|:22-62	90
BmHel-11(DF090369:1699831-1700122):155-283	gb|AAO33922.1|:25-67	74
BmHel-11(DF090369:1728405-1728687):151-282	gb|AAO33922.1|:22-65	90
BmHel-11(DF090369:1770043-1770334):127-291	gb|AAO33922.1|:22-76	85
BmHel-11(DF090369:1810512-1810803):133-291	gb|AAO33922.1|:22-74	69
BmHel-11(DF090369:2102088-2102376):130-288	gb|AAO33922.1|:22-75	83
BmHel-11(DF090369:2317575-2317866):124-291	gb|AAO33922.1|:22-77	83
BmHel-11(DF090369:2453731-2454227):335-496	gb|AAO33922.1|:22-68	66
BmHel-11(DF090369:2494130-2494421):124-291	gb|AAO33922.1|:22-77	85

BmHel-11(DF090369:556503-556794):125-220	gb|AAO33922.1|:46-77	81
BmHel-11(DF090369:606374-606696):183-323	gb|AAO33922.1|:22-76	67
BmHel-11(DF090369:693811-694104):126-293	gb|AAO33922.1|:22-77	75
BmHel-11(DF090369:853665-853956):154-291	gb|AAO33922.1|:22-67	67
BmHel-11(DF090370:1323758-1324049):124-291	gb|AAO33922.1|:22-77	78
BmHel-11(DF090370:1361799-1362036):118-237	gb|AAO33922.1|:22-77	60
BmHel-11(DF090370:1562845-1563136):163-291	gb|AAO33922.1|:22-64	83
BmHel-11(DF090370:1666415-1666699):180-284	gb|AAO33922.1|:22-56	88
BmHel-11(DF090370:1769601-1769862):101-261	gb|AAO33922.1|:22-77	57
BmHel-11(DF090370:1771510-1771801):133-291	gb|AAO33922.1|:22-74	84
BmHel-11(DF090370:1908771-1909064):123-293	gb|AAO33922.1|:22-77	63
BmHel-11(DF090370:197626-197919):150-293	gb|AAO33922.1|:22-69	79
BmHel-11(DF090370:2113709-2113990):123-281	gb|AAO33922.1|:22-77	85
BmHel-11(DF090370:2251216-2251507):129-291	gb|AAO33922.1|:22-76	59
BmHel-11(DF090370:2408579-2408870):160-291	gb|AAO33922.1|:22-65	93
BmHel-11(DF090370:2556442-2556734):155-292	gb|AAO33922.1|:22-67	76
BmHel-11(DF090370:2583772-2584063):124-291	gb|AAO33922.1|:22-77	85
BmHel-11(DF090370:269435-269726):139-291	gb|AAO33922.1|:22-72	86
BmHel-11(DF090370:443431-443727):123-296	gb|AAO33922.1|:22-77	81
BmHel-11(DF090370:528857-529148):124-291	gb|AAO33922.1|:22-77	87
BmHel-11(DF090370:627611-627903):157-292	gb|AAO33922.1|:22-67	71
BmHel-11(DF090370:697267-697571):165-304	gb|AAO33922.1|:22-65	66
BmHel-11(DF090370:721822-722101):124-279	gb|AAO33922.1|:22-77	67
BmHel-11(DF090370:783815-784102):120-287	gb|AAO33922.1|:22-77	83
BmHel-11(DF090371:1121584-1121836):133-252	gb|AAO33922.1|:22-65	73
BmHel-11(DF090371:1243222-1243513):124-291	gb|AAO33922.1|:22-77	83
BmHel-11(DF090371:140227-140501):124-274	gb|AAO33922.1|:22-77	68
BmHel-11(DF090371:1541186-1541476):124-234	gb|AAO33922.1|:41-77	83
BmHel-11(DF090371:175125-175416):160-291	gb|AAO33922.1|:22-65	84
BmHel-11(DF090371:2031396-2031687):124-291	gb|AAO33922.1|:22-77	60
BmHel-11(DF090371:2088523-2088818):134-295	gb|AAO33922.1|:22-75	87
BmHel-11(DF090371:2135072-2135329):124-257	gb|AAO33922.1|:22-77	59
BmHel-11(DF090371:2240714-2241006):161-292	gb|AAO33922.1|:22-65	93
BmHel-11(DF090371:2260039-2260308):128-269	gb|AAO33922.1|:22-75	66
BmHel-11(DF090371:2265222-2265519):130-297	gb|AAO33922.1|:22-77	83
BmHel-11(DF090371:2411977-2412230):142-253	gb|AAO33922.1|:22-71	67
BmHel-11(DF090371:2566932-2567222):126-290	gb|AAO33922.1|:22-77	76
BmHel-11(DF090371:308110-308401):124-291	gb|AAO33922.1|:22-77	91
BmHel-11(DF090371:473667-473957):126-290	gb|AAO33922.1|:22-77	76
BmHel-11(DF090371:682291-682582):157-291	gb|AAO33922.1|:22-66	88
BmHel-11(DF090371:863370-863661):169-291	gb|AAO33922.1|:22-62	70
BmHel-11(DF090371:886632-886924):124-285	gb|AAO33922.1|:24-77	83
BmHel-11(DF090372:1071682-1071972):123-290	gb|AAO33922.1|:22-77	75
BmHel-11(DF090372:1196695-1196982):124-243	gb|AAO33922.1|:35-77	81
BmHel-11(DF090372:1684100-1684378):156-278	gb|AAO33922.1|:22-62	80
BmHel-11(DF090372:1835190-1835481):124-291	gb|AAO33922.1|:22-77	83
BmHel-11(DF090372:1892839-1893127):126-288	gb|AAO33922.1|:22-77	59.5
BmHel-11(DF090372:1936270-1936561):124-291	gb|AAO33922.1|:22-77	83

BmHel-11(DF090372:1965902-1966157):124-255	gb|AAO33922.1|:22-77	71
BmHel-11(DF090372:2078263-2078554):124-291	gb|AAO33922.1|:22-77	80
BmHel-11(DF090372:2322026-2322317):160-291	gb|AAO33922.1|:22-65	86
BmHel-11(DF090372:2467203-2467478):111-275	gb|AAO33922.1|:22-77	85
BmHel-11(DF090372:285988-286286):124-298	gb|AAO33922.1|:22-77	56.5
BmHel-11(DF090372:383149-383431):132-282	gb|AAO33922.1|:22-75	60.5
BmHel-11(DF090372:524645-524914):124-269	gb|AAO33922.1|:22-77	63.5
BmHel-11(DF090372:586952-587243):124-291	gb|AAO33922.1|:22-77	87
BmHel-11(DF090372:617818-618108):124-282	gb|AAO33922.1|:25-77	77
BmHel-11(DF090372:73225-73516):124-291	gb|AAO33922.1|:22-77	91
BmHel-11(DF090372:880688-880972):156-284	gb|AAO33922.1|:22-64	83
BmHel-11(DF090372:947785-948076):160-291	gb|AAO33922.1|:22-65	77
BmHel-11(DF090373:132637-132928):154-291	gb|AAO33922.1|:22-67	76
BmHel-11(DF090373:1402468-1402759):124-291	gb|AAO33922.1|:22-77	85
BmHel-11(DF090373:1643957-1644243):160-286	gb|AAO33922.1|:22-65	59.5
BmHel-11(DF090373:1719218-1719509):163-291	gb|AAO33922.1|:22-64	83
BmHel-11(DF090373:180580-180828):124-248	gb|AAO33922.1|:22-77	66
BmHel-11(DF090373:1991569-1991860):124-291	gb|AAO33922.1|:22-77	83
BmHel-11(DF090373:2049225-2049516):160-291	gb|AAO33922.1|:22-65	81
BmHel-11(DF090373:247019-247319):163-300	gb|AAO33922.1|:22-67	78
BmHel-11(DF090373:362752-363044):125-292	gb|AAO33922.1|:22-77	89
BmHel-11(DF090373:415230-415521):124-291	gb|AAO33922.1|:22-77	85
BmHel-11(DF090373:429813-430102):124-289	gb|AAO33922.1|:22-77	59.5
BmHel-11(DF090373:659711-660002):124-291	gb|AAO33922.1|:22-77	91
BmHel-11(DF090373:726007-726299):125-292	gb|AAO33922.1|:22-77	83
BmHel-11(DF090373:973519-973851):73-183	dbj|BAH66325.1|:116-152	83
BmHel-11(DF090374:1025465-1025756):160-291	gb|AAO33922.1|:22-65	84
BmHel-11(DF090374:1137955-1138250):164-295	gb|AAO33922.1|:22-65	90
BmHel-11(DF090374:1155868-1156159):124-291	gb|AAO33922.1|:22-77	85
BmHel-11(DF090374:1195438-1195728):123-290	gb|AAO33922.1|:22-77	80
BmHel-11(DF090374:1292947-1293149):95-202	gb|AAO33922.1|:22-57	91
BmHel-11(DF090374:1380898-1381143):124-245	gb|AAO33922.1|:22-77	71
BmHel-11(DF090374:1468060-1468337):110-277	gb|AAO33922.1|:22-77	85
BmHel-11(DF090374:1539180-1539450):117-270	gb|AAO33922.1|:22-74	55
BmHel-11(DF090374:1564179-1564470):124-291	gb|AAO33922.1|:22-77	85
BmHel-11(DF090374:212688-212979):163-291	gb|AAO33922.1|:22-64	88
BmHel-11(DF090374:300902-301192):162-290	gb|AAO33922.1|:22-64	83
BmHel-11(DF090374:335975-336265):123-290	gb|AAO33922.1|:22-77	85
BmHel-11(DF090374:336658-336948):153-290	gb|AAO33922.1|:22-67	76
BmHel-11(DF090374:574419-574709):123-290	gb|AAO33922.1|:22-77	91
BmHel-11(DF090374:666724-667013):119-289	gb|AAO33922.1|:22-77	84
BmHel-11(DF090374:686349-686645):153-296	gb|AAO33922.1|:22-69	93
BmHel-11(DF090374:910837-911127):126-290	gb|AAO33922.1|:22-76	85
BmHel-11(DF090374:975660-975952):155-292	gb|AAO33922.1|:22-67	65
BmHel-11(DF090375:105564-105856):155-292	gb|AAO33922.1|:22-67	71
BmHel-11(DF090375:1124565-1124856):161-292	gb|AAO33922.1|:22-65	86
BmHel-11(DF090375:1250323-1250611):160-288	gb|AAO33922.1|:22-65	86
BmHel-11(DF090375:132240-132530):123-290	gb|AAO33922.1|:22-77	80

BmHel-11(DF090375:1387240-1387520):117-281	gb|AAO33922.1|:22-76	89
BmHel-11(DF090375:1606523-1606801):126-278	gb|AAO33922.1|:22-77	64
BmHel-11(DF090375:1736824-1737116):155-292	gb|AAO33922.1|:22-67	82
BmHel-11(DF090375:1998021-1998302):117-281	gb|AAO33922.1|:22-76	89
BmHel-11(DF090375:291656-291948):149-292	gb|AAO33922.1|:22-69	81
BmHel-11(DF090375:523921-524201):169-280	gb|AAO33922.1|:22-62	68.5
BmHel-11(DF090375:567963-568242):124-279	gb|AAO33922.1|:22-77	73
BmHel-11(DF090375:695557-695834):158-277	gb|AAO33922.1|:22-61	82
BmHel-11(DF090376:1231307-1231574):172-267	gb|AAO33922.1|:22-53	93
BmHel-11(DF090376:1282052-1283020):124-273	gb|AAO33922.1|:28-77	80
BmHel-11(DF090376:1404760-1405051):124-291	gb|AAO33922.1|:22-77	80
BmHel-11(DF090376:146854-147123):124-269	gb|AAO33922.1|:22-77	58.5
BmHel-11(DF090376:1486458-1486735):129-277	gb|AAO33922.1|:22-75	64.5
BmHel-11(DF090376:1522557-1522840):133-283	gb|AAO33922.1|:22-71	68.5
BmHel-11(DF090376:1971504-1971778):110-274	gb|AAO33922.1|:22-77	57
BmHel-11(DF090376:2219536-2219827):154-291	gb|AAO33922.1|:22-67	80
BmHel-11(DF090376:2243057-2243346):131-289	gb|AAO33922.1|:22-74	64
BmHel-11(DF090376:409229-409524):128-295	gb|AAO33922.1|:22-77	82
BmHel-11(DF090377:1009521-1009812):124-291	gb|AAO33922.1|:22-77	91
BmHel-11(DF090377:1110150-1110441):124-291	gb|AAO33922.1|:22-77	87
BmHel-11(DF090377:1168356-1168599):124-243	gb|AAO33922.1|:22-77	64
BmHel-11(DF090377:1226035-1226322):124-288	gb|AAO33922.1|:22-77	66
BmHel-11(DF090377:1284204-1284495):124-291	gb|AAO33922.1|:22-77	82
BmHel-11(DF090377:1331685-1331977):125-292	gb|AAO33922.1|:22-77	78
BmHel-11(DF090377:1340805-1341092):117-287	gb|AAO33922.1|:22-77	80
BmHel-11(DF090377:1352426-1352713):123-287	gb|AAO33922.1|:22-77	87
BmHel-11(DF090377:1422851-1423142):160-291	gb|AAO33922.1|:22-65	88
BmHel-11(DF090377:1565976-1566251):159-275	gb|AAO33922.1|:22-67	78
BmHel-11(DF090377:1826200-1826490):153-290	gb|AAO33922.1|:22-67	91
BmHel-11(DF090377:1917572-1917861):122-289	gb|AAO33922.1|:22-77	73
BmHel-11(DF090377:319231-319521):159-290	gb|AAO33922.1|:22-65	90
BmHel-11(DF090377:362976-363255):112-279	gb|AAO33922.1|:22-77	92
BmHel-11(DF090377:448760-449044):124-284	gb|AAO33922.1|:22-77	53
BmHel-11(DF090377:462928-463219):160-291	gb|AAO33922.1|:22-65	90
BmHel-11(DF090377:523432-523712):124-280	gb|AAO33922.1|:22-77	64.5
BmHel-11(DF090377:642949-643239):159-290	gb|AAO33922.1|:22-65	79
BmHel-11(DF090377:692032-692323):167-291	gb|AAO33922.1|:22-63	51.5
BmHel-11(DF090377:801893-802157):121-264	gb|AAO33922.1|:22-69	87
BmHel-11(DF090377:895694-895984):124-243	gb|AAO33922.1|:38-77	85
BmHel-11(DF090378:1122676-1122967):124-291	gb|AAO33922.1|:22-77	82
BmHel-11(DF090378:1237186-1237480):130-294	gb|AAO33922.1|:22-76	87
BmHel-11(DF090378:1290711-1291005):163-294	gb|AAO33922.1|:22-64	79
BmHel-11(DF090378:1384546-1384843):151-297	gb|AAO33922.1|:22-77	60
BmHel-11(DF090378:279548-279835):123-287	gb|AAO33922.1|:22-77	57
BmHel-11(DF090378:283708-283999):124-291	gb|AAO33922.1|:22-77	83
BmHel-11(DF090378:308898-309186):124-288	gb|AAO33922.1|:22-77	82
BmHel-11(DF090378:352130-352421):124-291	gb|AAO33922.1|:22-77	89
BmHel-11(DF090378:534109-534401):123-292	gb|AAO33922.1|:22-77	62.5

BmHel-11(DF090378:659971-660261):126-290	gb|AAO33922.1|:22-77	62
BmHel-11(DF090378:76105-76393):124-288	gb|AAO33922.1|:22-77	82
BmHel-11(DF090378:87309-87631):185-322	gb|AAO33922.1|:22-67	82
BmHel-11(DF090378:941061-941326):119-265	gb|AAO33922.1|:22-69	67
BmHel-11(DF090379:1100248-1100536):121-288	gb|AAO33922.1|:22-77	89
BmHel-11(DF090379:1318399-1318690):124-291	gb|AAO33922.1|:22-77	87
BmHel-11(DF090379:1404343-1404634):124-291	gb|AAO33922.1|:22-77	85
BmHel-11(DF090379:1486270-1486554):159-284	gb|AAO33922.1|:22-63	83
BmHel-11(DF090379:1777071-1777361):186-290	gb|AAO33922.1|:22-56	85
BmHel-11(DF090379:1853627-1853885):136-258	gb|AAO33922.1|:22-65	79
BmHel-11(DF090379:237538-237829):124-291	gb|AAO33922.1|:22-77	83
BmHel-11(DF090379:334274-334566):124-292	gb|AAO33922.1|:22-77	71
BmHel-11(DF090379:362686-362950):88-264	gb|AAO33922.1|:22-76	55
BmHel-11(DF090379:386160-386451):124-291	gb|AAO33922.1|:22-77	78
BmHel-11(DF090379:415435-416309):712-874	gb|AAO33922.1|:22-77	61
BmHel-11(DF090379:632891-633182):124-291	gb|AAO33922.1|:22-77	78
BmHel-11(DF090379:714152-714425):127-273	gb|AAO33922.1|:22-77	73
BmHel-11(DF090379:842530-842820):159-290	gb|AAO33922.1|:22-70	69
BmHel-11(DF090379:884766-885044):138-278	gb|AAO33922.1|:22-68	87
BmHel-11(DF090380:1003600-1003894):127-294	gb|AAO33922.1|:22-77	87
BmHel-11(DF090380:110411-110643):113-232	gb|AAO33922.1|:22-77	64
BmHel-11(DF090380:1498034-1498324):159-290	gb|AAO33922.1|:22-65	86
BmHel-11(DF090380:1719888-1720163):132-275	gb|AAO33922.1|:22-69	81
BmHel-11(DF090380:1726121-1726412):124-291	gb|AAO33922.1|:22-77	87
BmHel-11(DF090380:1894651-1894942):124-291	gb|AAO33922.1|:22-77	85
BmHel-11(DF090380:2012410-2012701):142-291	gb|AAO33922.1|:22-71	92
BmHel-11(DF090380:2128318-2128602):124-284	gb|AAO33922.1|:22-77	61
BmHel-11(DF090380:272864-273121):127-257	gb|AAO33922.1|:22-76	58.5
BmHel-11(DF090380:419822-420113):124-291	gb|AAO33922.1|:22-77	85
BmHel-11(DF090380:867846-868139):126-293	gb|AAO33922.1|:22-77	78
BmHel-11(DF090381:1053510-1053801):124-291	gb|AAO33922.1|:22-77	91
BmHel-11(DF090381:1516125-1516404):112-279	gb|AAO33922.1|:22-77	92
BmHel-11(DF090381:1583398-1583689):124-291	gb|AAO33922.1|:22-77	87
BmHel-11(DF090381:163783-164074):124-291	gb|AAO33922.1|:22-77	92
BmHel-11(DF090381:1813524-1813815):124-291	gb|AAO33922.1|:22-77	89
BmHel-11(DF090381:1815759-1816050):160-291	gb|AAO33922.1|:22-65	84
BmHel-11(DF090381:1996727-1997020):162-293	gb|AAO33922.1|:22-65	84
BmHel-11(DF090381:327093-327380):124-258	gb|AAO33922.1|:33-77	66
BmHel-11(DF090381:686165-686456):160-291	gb|AAO33922.1|:22-65	88
BmHel-11(DF090381:726379-726670):160-291	gb|AAO33922.1|:22-65	86
BmHel-11(DF090381:813305-813596):124-291	gb|AAO33922.1|:22-77	82
BmHel-11(DF090381:87804-88090):119-286	gb|AAO33922.1|:22-77	85
BmHel-11(DF090381:936016-936307):124-291	gb|AAO33922.1|:22-77	85
BmHel-11(DF090382:1126116-1126406):153-290	gb|AAO33922.1|:22-67	73
BmHel-11(DF090382:1260818-1261105):146-262	gb|AAO33922.1|:32-70	87
BmHel-11(DF090382:1587104-1587370):123-266	gb|AAO33922.1|:22-77	73
BmHel-11(DF090382:1597116-1597404):154-288	gb|AAO33922.1|:22-66	73
BmHel-11(DF090382:1659659-1659951):149-292	gb|AAO33922.1|:22-69	83

BmHel-11(DF090382:1701614-1701906):124-216	gb|AAO33922.1|:47-77	90
BmHel-11(DF090382:1867646-1867918):177-272	gb|AAO33922.1|:22-53	90
BmHel-11(DF090382:1909358-1909650):161-292	gb|AAO33922.1|:22-65	86
BmHel-11(DF090382:2003093-2003383):126-290	gb|AAO33922.1|:22-77	66
BmHel-11(DF090382:347313-347568):124-255	gb|AAO33922.1|:22-77	71
BmHel-11(DF090382:533429-533720):159-291	gb|AAO33922.1|:22-65	66
BmHel-11(DF090382:589313-589610):124-297	gb|AAO33922.1|:22-77	60
BmHel-11(DF090382:847489-847779):123-290	gb|AAO33922.1|:22-77	83
BmHel-11(DF090383:1089651-1089942):124-291	gb|AAO33922.1|:22-77	91
BmHel-11(DF090383:1113048-1113339):154-291	gb|AAO33922.1|:22-67	82
BmHel-11(DF090383:1136990-1137281):124-291	gb|AAO33922.1|:22-77	83
BmHel-11(DF090383:1326896-1327162):102-266	gb|AAO33922.1|:22-75	65
BmHel-11(DF090383:1441019-1441305):128-286	gb|AAO33922.1|:22-74	88
BmHel-11(DF090383:152948-153241):141-293	gb|AAO33922.1|:22-72	80
BmHel-11(DF090383:1683119-1683400):126-281	gb|AAO33922.1|:22-77	63
BmHel-11(DF090383:1779523-1779813):123-290	gb|AAO33922.1|:22-77	89
BmHel-11(DF090383:1855117-1855410):156-293	gb|AAO33922.1|:22-67	80
BmHel-11(DF090383:2008803-2009093):123-290	gb|AAO33922.1|:22-77	83
BmHel-11(DF090383:31682-31971):179-289	gb|AAO33922.1|:22-58	91
BmHel-11(DF090383:452405-452698):162-293	gb|AAO33922.1|:22-65	88
BmHel-11(DF090383:498108-498399):124-291	gb|AAO33922.1|:22-77	89
BmHel-11(DF090383:695538-695832):127-294	gb|AAO33922.1|:22-77	91
BmHel-11(DF090383:980215-980503):145-288	gb|AAO33922.1|:22-69	83
BmHel-11(DF090384:1036568-1036855):120-287	gb|AAO33922.1|:22-77	89
BmHel-11(DF090384:1072166-1072456):124-291	gb|AAO33922.1|:22-77	87
BmHel-11(DF090384:1091334-1091632):162-298	gb|AAO33922.1|:22-65	66
BmHel-11(DF090384:1244086-1244389):127-303	gb|AAO33922.1|:22-77	76
BmHel-11(DF090384:1275687-1275930):124-243	gb|AAO33922.1|:22-77	62
BmHel-11(DF090384:1277917-1278209):160-292	gb|AAO33922.1|:22-65	62
BmHel-11(DF090384:146635-146922):120-287	gb|AAO33922.1|:22-77	69
BmHel-11(DF090384:1504902-1505190):121-288	gb|AAO33922.1|:22-77	76
BmHel-11(DF090384:1578461-1578713):133-252	gb|AAO33922.1|:22-74	58
BmHel-11(DF090384:1686209-1686496):132-287	gb|AAO33922.1|:22-73	88
BmHel-11(DF090384:1727927-1728218):160-291	gb|AAO33922.1|:22-65	88
BmHel-11(DF090384:1783517-1783806):146-289	gb|AAO33922.1|:22-69	87
BmHel-11(DF090384:188520-188808):148-288	gb|AAO33922.1|:22-68	85
BmHel-11(DF090384:1987846-1988138):155-292	gb|AAO33922.1|:22-67	71
BmHel-11(DF090384:2014239-2014517):135-278	gb|AAO33922.1|:22-70	73
BmHel-11(DF090384:216507-216809):124-302	gb|AAO33922.1|:22-77	68.5
BmHel-11(DF090384:293004-293286):123-282	gb|AAO33922.1|:22-77	67
BmHel-11(DF090384:416922-417215):156-293	gb|AAO33922.1|:22-67	84
BmHel-11(DF090384:478329-478621):155-292	gb|AAO33922.1|:22-67	84
BmHel-11(DF090384:582140-582431):154-291	gb|AAO33922.1|:22-67	86
BmHel-11(DF090384:845900-846191):124-291	gb|AAO33922.1|:22-77	83
BmHel-11(DF090385:1268622-1268911):149-289	gb|AAO33922.1|:22-68	65
BmHel-11(DF090385:1361955-1362208):127-253	gb|AAO33922.1|:22-76	65
BmHel-11(DF090385:1806689-1806987):131-298	gb|AAO33922.1|:22-77	85
BmHel-11(DF090385:189538-189829):124-291	gb|AAO33922.1|:22-77	89

BmHel-11(DF090385:316680-316973):150-293	gb|AAO33922.1|:22-69	87
BmHel-11(DF090385:339643-339934):163-291	gb|AAO33922.1|:22-64	93
BmHel-11(DF090385:497427-497721):127-294	gb|AAO33922.1|:22-77	83
BmHel-11(DF090385:966237-966523):119-286	gb|AAO33922.1|:22-77	83
BmHel-11(DF090385:99443-99734):124-291	gb|AAO33922.1|:22-77	91
BmHel-11(DF090386:1068543-1068833):124-225	gb|AAO33922.1|:44-77	76
BmHel-11(DF090386:1198160-1198449):122-289	gb|AAO33922.1|:22-77	91
BmHel-11(DF090386:1382809-1383099):123-290	gb|AAO33922.1|:22-77	87
BmHel-11(DF090386:140065-140356):160-291	gb|AAO33922.1|:22-65	88
BmHel-11(DF090386:1616349-1616652):125-303	gb|AAO33922.1|:22-77	66
BmHel-11(DF090386:1671703-1671993):159-290	gb|AAO33922.1|:22-65	88
BmHel-11(DF090386:1687105-1687396):124-291	gb|AAO33922.1|:22-77	91
BmHel-11(DF090386:1716790-1717086):150-296	gb|AAO33922.1|:22-77	62
BmHel-11(DF090386:1754743-1755034):124-291	gb|AAO33922.1|:22-77	91
BmHel-11(DF090386:1908842-1909135):126-293	gb|AAO33922.1|:22-77	82
BmHel-11(DF090386:2025408-2025657):124-249	gb|AAO33922.1|:22-77	64
BmHel-11(DF090386:313219-313511):134-292	gb|AAO33922.1|:22-74	83
BmHel-11(DF090386:459012-459306):163-294	gb|AAO33922.1|:22-65	90
BmHel-11(DF090386:494359-494648):124-289	gb|AAO33922.1|:22-77	60
BmHel-11(DF090386:547460-547726):126-266	gb|AAO33922.1|:22-68	89
BmHel-11(DF090386:56850-57142):155-292	gb|AAO33922.1|:22-67	84
BmHel-11(DF090386:698262-698557):128-295	gb|AAO33922.1|:22-77	76
BmHel-11(DF090386:733591-733880):152-289	gb|AAO33922.1|:22-67	71
BmHel-11(DF090386:765425-765712):141-287	gb|AAO33922.1|:22-71	80
BmHel-11(DF090386:831334-831625):124-291	gb|AAO33922.1|:22-77	87
BmHel-11(DF090386:873107-873394):165-287	gb|AAO33922.1|:22-62	82
BmHel-11(DF090386:937380-937673):126-293	gb|AAO33922.1|:22-77	85
BmHel-11(DF090387:1091099-1091388):124-289	gb|AAO33922.1|:22-77	60
BmHel-11(DF090387:1304590-1304882):125-292	gb|AAO33922.1|:22-77	64
BmHel-11(DF090387:1570038-1570332):154-294	gb|AAO33922.1|:22-67	72
BmHel-11(DF090387:1690867-1691158):160-291	gb|AAO33922.1|:22-65	84
BmHel-11(DF090387:439302-439592):150-290	gb|AAO33922.1|:22-68	80
BmHel-11(DF090387:616083-616345):173-262	gb|AAO33922.1|:22-51	90
BmHel-11(DF090387:802595-802883):163-288	gb|AAO33922.1|:22-63	78
BmHel-11(DF090387:868733-869021):158-262	gb|AAO33922.1|:31-65	82
BmHel-11(DF090387:998553-998844):127-291	gb|AAO33922.1|:22-76	83
BmHel-11(DF090388:1059621-1059878):124-257	gb|AAO33922.1|:22-77	67
BmHel-11(DF090388:1132571-1132862):124-291	gb|AAO33922.1|:22-77	91
BmHel-11(DF090388:1292549-1292839):157-290	gb|AAO33922.1|:22-66	60
BmHel-11(DF090388:1440267-1440559):155-292	gb|AAO33922.1|:22-67	86
BmHel-11(DF090388:1484140-1484424):126-284	gb|AAO33922.1|:22-77	62
BmHel-11(DF090388:1648465-1648749):141-284	gb|AAO33922.1|:22-69	89
BmHel-11(DF090388:1653161-1653452):124-291	gb|AAO33922.1|:22-77	85
BmHel-11(DF090388:1784017-1784303):155-286	gb|AAO33922.1|:22-67	69
BmHel-11(DF090388:262693-263420):272-435	dbj|BAH66344.1|:45-96	65.5
BmHel-11(DF090388:335024-335752):630-728	gb|AAO33922.1|:22-54	93
BmHel-11(DF090388:335024-335752):277-440	dbj|BAH66344.1|:45-96	68.5
BmHel-11(DF090388:492817-493108):124-291	gb|AAO33922.1|:22-77	76

BmHel-11(DF090388:517179-517470):142-291	gb|AAO33922.1|:22-71	90
BmHel-11(DF090388:542114-542405):124-291	gb|AAO33922.1|:22-77	78
BmHel-11(DF090388:563232-563521):122-289	gb|AAO33922.1|:22-77	69
BmHel-11(DF090388:615650-615942):125-292	gb|AAO33922.1|:22-77	78
BmHel-11(DF090388:951992-952277):121-285	gb|AAO33922.1|:22-76	83
BmHel-11(DF090389:1022207-1022497):159-290	gb|AAO33922.1|:22-65	90
BmHel-11(DF090389:1036274-1036565):124-291	gb|AAO33922.1|:22-77	89
BmHel-11(DF090389:1513218-1513503):148-285	gb|AAO33922.1|:22-67	84
BmHel-11(DF090389:23696-23988):158-292	gb|AAO33922.1|:22-66	93
BmHel-11(DF090389:37546-37833):189-287	gb|AAO33922.1|:22-54	84
BmHel-11(DF090389:431821-432111):153-290	gb|AAO33922.1|:22-67	73
BmHel-11(DF090389:586538-586782):122-244	gb|AAO33922.1|:22-62	90
BmHel-11(DF090389:771501-771788):156-287	gb|AAO33922.1|:22-65	90
BmHel-11(DF090389:982411-982701):123-290	gb|AAO33922.1|:22-77	83
BmHel-11(DF090389:996346-996637):124-291	gb|AAO33922.1|:22-77	89
BmHel-11(DF090390:1065487-1065778):124-291	gb|AAO33922.1|:22-77	89
BmHel-11(DF090390:1111366-1111656):124-234	gb|AAO33922.1|:41-77	81
BmHel-11(DF090390:1335949-1336240):163-291	gb|AAO33922.1|:22-64	90
BmHel-11(DF090390:1368203-1368495):161-292	gb|AAO33922.1|:22-65	93
BmHel-11(DF090390:407987-408283):124-296	gb|AAO33922.1|:22-77	61.5
BmHel-11(DF090390:465501-465788):150-287	gb|AAO33922.1|:22-68	87
BmHel-11(DF090390:609540-609828):123-288	gb|AAO33922.1|:22-77	66.5
BmHel-11(DF090390:636184-636474):124-219	gb|AAO33922.1|:46-77	87
BmHel-11(DF090390:792102-792393):124-291	gb|AAO33922.1|:22-77	87
BmHel-11(DF090390:860667-860963):123-296	gb|AAO33922.1|:22-77	86
BmHel-11(DF090391:171600-171891):124-291	gb|AAO33922.1|:22-77	82
BmHel-11(DF090391:250276-250567):124-291	gb|AAO33922.1|:22-77	87
BmHel-11(DF090391:278875-279167):152-292	gb|AAO33922.1|:22-68	80
BmHel-11(DF090391:403440-403750):124-252	gb|AAO33922.1|:35-77	83
BmHel-11(DF090391:529632-529905):160-273	gb|AAO33922.1|:22-65	75
BmHel-11(DF090391:725609-725871):123-262	gb|AAO33922.1|:22-77	60
BmHel-11(DF090391:761041-761332):124-291	gb|AAO33922.1|:22-77	87
BmHel-11(DF090391:934941-935226):124-285	gb|AAO33922.1|:22-77	85
BmHel-11(DF090392:134231-134521):160-290	gb|AAO33922.1|:22-65	57.5
BmHel-11(DF090392:1539846-1540136):153-290	gb|AAO33922.1|:22-67	67
BmHel-11(DF090392:1571404-1571694):123-290	gb|AAO33922.1|:22-77	91
BmHel-11(DF090392:1696381-1696672):124-291	gb|AAO33922.1|:22-77	91
BmHel-11(DF090392:1757044-1757286):124-242	gb|AAO33922.1|:22-77	71
BmHel-11(DF090392:338931-339255):181-324	gb|AAO33922.1|:22-69	85
BmHel-11(DF090392:537681-537975):123-294	gb|AAO33922.1|:22-77	63
BmHel-11(DF090392:600075-600359):153-284	gb|AAO33922.1|:22-65	90
BmHel-11(DF090392:787909-788192):122-283	gb|AAO33922.1|:22-77	82
BmHel-11(DF090392:993685-993981):150-296	gb|AAO33922.1|:22-70	87
BmHel-11(DF090393:1021702-1021945):124-243	gb|AAO33922.1|:22-77	62
BmHel-11(DF090393:114941-115219):115-278	gb|AAO33922.1|:22-77	54
BmHel-11(DF090393:1275134-1275427):156-293	gb|AAO33922.1|:22-67	67
BmHel-11(DF090393:1320017-1320314):157-297	gb|AAO33922.1|:22-67	76
BmHel-11(DF090393:1560627-1560909):145-282	gb|AAO33922.1|:22-67	91

BmHel-11(DF090394:1136906-1137203):128-297	gb|AAO33922.1|:22-77	71
BmHel-11(DF090394:1175231-1175522):124-291	gb|AAO33922.1|:22-77	82
BmHel-11(DF090394:1304073-1304357):126-284	gb|AAO33922.1|:22-77	78
BmHel-11(DF090394:1370075-1370365):123-290	gb|AAO33922.1|:22-77	87
BmHel-11(DF090394:1392049-1392329):127-280	gb|AAO33922.1|:22-76	68.5
BmHel-11(DF090394:1399357-1399648):133-291	gb|AAO33922.1|:22-74	75
BmHel-11(DF090394:142700-142953):106-253	gb|AAO33922.1|:22-77	59
BmHel-11(DF090394:1472671-1472961):123-290	gb|AAO33922.1|:22-77	89
BmHel-11(DF090394:1562027-1562316):124-289	gb|AAO33922.1|:22-77	65
BmHel-11(DF090394:542740-543031):157-291	gb|AAO33922.1|:22-66	91
BmHel-11(DF090394:956155-956446):151-291	gb|AAO33922.1|:22-68	95
BmHel-11(DF090395:1002382-1002673):124-291	gb|AAO33922.1|:22-77	83
BmHel-11(DF090395:1032676-1032966):162-290	gb|AAO33922.1|:22-65	86
BmHel-11(DF090395:1138581-1138869):121-288	gb|AAO33922.1|:22-77	66
BmHel-11(DF090395:1192201-1192494):129-293	gb|AAO33922.1|:22-76	76
BmHel-11(DF090395:1212397-1212688):154-291	gb|AAO33922.1|:22-67	78
BmHel-11(DF090395:1281357-1281654):148-297	gb|AAO33922.1|:22-71	88
BmHel-11(DF090395:141829-142049):137-220	gb|AAO33922.1|:22-49	96
BmHel-11(DF090395:1519432-1519723):124-291	gb|AAO33922.1|:22-77	85
BmHel-11(DF090395:230918-231427):123-278	gb|AAO33922.1|:28-77	73
BmHel-11(DF090395:244311-244584):124-273	gb|AAO33922.1|:22-77	75
BmHel-11(DF090395:388659-388999):191-340	gb|AAO33922.1|:22-71	88
BmHel-11(DF090395:468506-468797):145-291	gb|AAO33922.1|:22-70	85
BmHel-11(DF090395:657795-658085):123-290	gb|AAO33922.1|:22-77	89
BmHel-11(DF090395:974889-975179):160-291	gb|AAO33922.1|:22-65	88
BmHel-11(DF090396:1135920-1136211):124-291	gb|AAO33922.1|:22-77	67
BmHel-11(DF090396:1189266-1189553):126-287	gb|AAO33922.1|:22-76	80
BmHel-11(DF090396:1464603-1464895):164-292	gb|AAO33922.1|:22-64	83
BmHel-11(DF090396:1475232-1475524):158-292	gb|AAO33922.1|:22-66	86
BmHel-11(DF090396:1654-1925):124-271	gb|AAO33922.1|:22-77	63.5
BmHel-11(DF090396:232062-232348):122-286	gb|AAO33922.1|:22-76	56
BmHel-11(DF090396:514282-514564):124-282	gb|AAO33922.1|:22-77	62
BmHel-11(DF090396:571169-571460):124-291	gb|AAO33922.1|:22-77	85
BmHel-11(DF090396:667305-667598):156-293	gb|AAO33922.1|:22-67	89
BmHel-11(DF090396:784855-785139):144-284	gb|AAO33922.1|:22-67	65
BmHel-11(DF090396:87915-88207):134-292	gb|AAO33922.1|:22-74	81
BmHel-11(DF090397:1148987-1149276):122-289	gb|AAO33922.1|:22-77	89
BmHel-11(DF090397:1254517-1254813):123-296	gb|AAO33922.1|:22-77	58
BmHel-11(DF090397:1368390-1368682):134-292	gb|AAO33922.1|:22-74	71
BmHel-11(DF090397:345052-345343):124-291	gb|AAO33922.1|:22-77	87
BmHel-11(DF090397:492788-493082):127-294	gb|AAO33922.1|:22-77	85
BmHel-11(DF090397:640296-640587):160-291	gb|AAO33922.1|:22-65	88
BmHel-11(DF090397:652609-652898):152-289	gb|AAO33922.1|:22-67	80
BmHel-11(DF090397:721384-721628):124-244	gb|AAO33922.1|:22-77	67
BmHel-11(DF090397:997519-997810):124-291	gb|AAO33922.1|:22-77	87
BmHel-11(DF090398:110360-110650):123-290	gb|AAO33922.1|:22-77	85
BmHel-11(DF090398:1133591-1133882):124-291	gb|AAO33922.1|:22-77	91
BmHel-11(DF090398:1294601-1294901):124-300	gb|AAO33922.1|:22-77	84

BmHel-11(DF090398:187826-188115):124-289	gb|AAO33922.1|:22-77	71
BmHel-11(DF090398:270989-271280):124-291	gb|AAO33922.1|:22-77	85
BmHel-11(DF090398:633173-633431):118-258	gb|AAO33922.1|:22-77	64
BmHel-11(DF090398:727060-727348):154-288	gb|AAO33922.1|:22-67	71
BmHel-11(DF090399:1056620-1056909):158-289	gb|AAO33922.1|:22-65	86
BmHel-11(DF090399:1282281-1282563):155-282	gb|AAO33922.1|:22-67	59.5
BmHel-11(DF090399:1509531-1509822):127-291	gb|AAO33922.1|:22-76	81
BmHel-11(DF090399:192008-192298):123-290	gb|AAO33922.1|:22-77	87
BmHel-11(DF090399:23583-23872):124-290	gb|AAO33922.1|:22-77	60
BmHel-11(DF090399:284095-284379):141-284	gb|AAO33922.1|:22-69	87
BmHel-11(DF090399:551422-551717):123-227	gb|AAO33922.1|:43-77	80
BmHel-11(DF090399:652539-652833):124-294	gb|AAO33922.1|:22-77	85
BmHel-11(DF090399:943540-943845):165-305	gb|AAO33922.1|:22-68	87
BmHel-11(DF090400:1077839-1078114):117-275	gb|AAO33922.1|:22-77	55
BmHel-11(DF090400:1207552-1207843):124-291	gb|AAO33922.1|:22-77	83
BmHel-11(DF090400:1384121-1384412):124-291	gb|AAO33922.1|:22-77	83
BmHel-11(DF090400:1490853-1491147):124-294	gb|AAO33922.1|:22-77	87
BmHel-11(DF090400:1559676-1559911):68-235	gb|AAO33922.1|:22-77	91
BmHel-11(DF090400:288581-288872):124-291	gb|AAO33922.1|:22-77	89
BmHel-11(DF090400:688413-688698):154-285	gb|AAO33922.1|:22-65	79
BmHel-11(DF090400:780872-781163):127-291	gb|AAO33922.1|:22-76	81
BmHel-11(DF090400:810889-811180):124-291	gb|AAO33922.1|:22-77	91
BmHel-11(DF090400:884269-884560):124-291	gb|AAO33922.1|:22-77	91
BmHel-11(DF090401:1111526-1111802):109-276	gb|AAO33922.1|:22-77	85
BmHel-11(DF090401:1131684-1131974):123-290	gb|AAO33922.1|:22-77	87
BmHel-11(DF090401:1160171-1160453):133-282	gb|AAO33922.1|:22-71	82
BmHel-11(DF090401:1370898-1371188):164-290	gb|AAO33922.1|:22-65	61.5
BmHel-11(DF090401:1419656-1419969):124-313	gb|AAO33922.1|:22-77	61
BmHel-11(DF090401:424924-425214):159-290	gb|AAO33922.1|:22-65	88
BmHel-11(DF090401:480608-480899):124-291	gb|AAO33922.1|:22-77	92
BmHel-11(DF090401:500449-500743):163-294	gb|AAO33922.1|:22-65	90
BmHel-11(DF090401:820268-820541):124-273	gb|AAO33922.1|:22-77	73
BmHel-11(DF090401:842734-843011):124-277	gb|AAO33922.1|:22-77	61.5
BmHel-11(DF090402:1174917-1175208):160-291	gb|AAO33922.1|:22-65	88
BmHel-11(DF090402:1272680-1272968):124-288	gb|AAO33922.1|:22-77	64
BmHel-11(DF090402:1303591-1303882):124-291	gb|AAO33922.1|:22-77	82
BmHel-11(DF090402:1327913-1328209):150-296	gb|AAO33922.1|:22-77	60
BmHel-11(DF090402:310545-310836):124-291	gb|AAO33922.1|:22-77	85
BmHel-11(DF090402:370981-371272):166-291	gb|AAO33922.1|:22-63	90
BmHel-11(DF090402:474501-474788):126-287	gb|AAO33922.1|:22-77	53
BmHel-11(DF090402:493925-494221):126-296	gb|AAO33922.1|:22-77	56
BmHel-11(DF090402:517960-518251):160-291	gb|AAO33922.1|:22-65	93
BmHel-11(DF090402:594784-595058):124-274	gb|AAO33922.1|:22-77	68
BmHel-11(DF090402:636401-636658):124-257	gb|AAO33922.1|:22-77	68
BmHel-11(DF090402:886876-890788):221-781	dbj|BAA76303.1|:7-197	31
BmHel-11(DF090402:886876-890788):1188-1304	gb|AAO33922.1|:22-60	84
BmHel-11(DF090402:89103-89393):126-290	gb|AAO33922.1|:22-76	87
BmHel-11(DF090402:952420-952711):160-291	gb|AAO33922.1|:22-65	90

BmHel-11(DF090403:1066038-1066328):156-290	gb|AAO33922.1|:22-66	84
BmHel-11(DF090403:1141603-1141893):151-282	gb|AAO33922.1|:25-68	86
BmHel-11(DF090403:1161596-1161887):160-291	gb|AAO33922.1|:22-65	90
BmHel-11(DF090403:1279438-1279719):150-281	gb|AAO33922.1|:22-69	62
BmHel-11(DF090403:1565986-1566284):161-298	gb|AAO33922.1|:22-67	71
BmHel-11(DF090403:820487-820778):124-291	gb|AAO33922.1|:22-77	94
BmHel-11(DF090403:937837-938126):123-221	gb|AAO33922.1|:45-77	87
BmHel-11(DF090404:1044228-1044504):142-276	gb|AAO33922.1|:22-67	76
BmHel-11(DF090404:1125063-1125356):126-293	gb|AAO33922.1|:22-77	59
BmHel-11(DF090404:1144619-1144910):124-291	gb|AAO33922.1|:22-77	87
BmHel-11(DF090404:1358693-1359095):271-402	gb|AAO33922.1|:22-65	90
BmHel-11(DF090404:1477553-1477827):125-274	gb|AAO33922.1|:22-71	94
BmHel-11(DF090404:167414-167659):138-245	gb|AAO33922.1|:22-57	86
BmHel-11(DF090404:513895-514158):129-263	gb|AAO33922.1|:22-66	80
BmHel-11(DF090404:546029-546320):142-291	gb|AAO33922.1|:22-71	86
BmHel-11(DF090404:963655-963945):126-290	gb|AAO33922.1|:22-77	57
BmHel-11(DF090405:1134943-1135190):122-247	gb|AAO33922.1|:22-71	68
BmHel-11(DF090405:1153225-1153527):144-302	gb|AAO33922.1|:22-74	79
BmHel-11(DF090405:1370876-1371167):124-291	gb|AAO33922.1|:22-77	76
BmHel-11(DF090405:1409362-1409653):130-291	gb|AAO33922.1|:22-75	88
BmHel-11(DF090405:301609-301902):126-293	gb|AAO33922.1|:22-77	80
BmHel-11(DF090405:622595-622887):150-292	gb|AAO33922.1|:22-70	60
BmHel-11(DF090405:699867-700158):124-291	gb|AAO33922.1|:22-77	87
BmHel-11(DF090405:818638-818887):124-249	gb|AAO33922.1|:22-77	71
BmHel-11(DF090405:876448-876708):120-260	gb|AAO33922.1|:22-68	70
BmHel-11(DF090405:966077-966363):164-286	gb|AAO33922.1|:22-62	78
BmHel-11(DF090406:1101934-1102250):149-316	gb|AAO33922.1|:22-77	87
BmHel-11(DF090406:641684-642298):447-614	gb|AAO33922.1|:22-77	83
BmHel-11(DF090406:705997-706287):123-290	gb|AAO33922.1|:22-77	87
BmHel-11(DF090406:791101-791391):162-290	gb|AAO33922.1|:22-64	90
BmHel-11(DF090406:99746-100031):164-277	gb|AAO33922.1|:25-62	76
BmHel-11(DF090407:14004-14294):124-290	gb|AAO33922.1|:22-76	61.5
BmHel-11(DF090407:175593-175876):123-283	gb|AAO33922.1|:22-77	59
BmHel-11(DF090407:26751-27043):126-292	gb|AAO33922.1|:22-77	62
BmHel-11(DF090407:272059-272350):124-291	gb|AAO33922.1|:22-77	85
BmHel-11(DF090407:514590-514880):123-290	gb|AAO33922.1|:22-77	83
BmHel-11(DF090407:668717-669000):123-283	gb|AAO33922.1|:22-77	61
BmHel-11(DF090407:715789-716082):126-293	gb|AAO33922.1|:22-77	53
BmHel-11(DF090407:820808-821095):123-287	gb|AAO33922.1|:22-77	87
BmHel-11(DF090407:92212-92503):124-291	gb|AAO33922.1|:22-77	87
BmHel-11(DF090407:964814-965105):124-291	gb|AAO33922.1|:22-77	85
BmHel-11(DF090408:1009793-1010080):150-287	gb|AAO33922.1|:22-67	84
BmHel-11(DF090408:1074631-1074922):124-291	gb|AAO33922.1|:22-77	87
BmHel-11(DF090408:1351962-1352257):124-291	gb|AAO33922.1|:22-77	73
BmHel-11(DF090408:1410776-1411017):74-241	gb|AAO33922.1|:22-77	83
BmHel-11(DF090408:593086-593344):122-250	gb|AAO33922.1|:25-67	83
BmHel-11(DF090408:640651-640901):119-250	gb|AAO33922.1|:22-77	69
BmHel-11(DF090408:74328-74622):127-294	gb|AAO33922.1|:22-77	91

BmHel-11(DF090408:985084-985374):129-290	gb|AAO33922.1|:22-76	58
BmHel-11(DF090409:115780-116059):149-280	gb|AAO33922.1|:22-65	90
BmHel-11(DF090409:1383798-1384083):159-285	gb|AAO33922.1|:22-63	67.5
BmHel-11(DF090409:1410928-1411218):126-290	gb|AAO33922.1|:22-77	62
BmHel-11(DF090409:1484612-1484903):124-291	gb|AAO33922.1|:22-77	89
BmHel-11(DF090409:353509-353772):124-263	gb|AAO33922.1|:22-77	61
BmHel-11(DF090409:381275-381564):153-290	gb|AAO33922.1|:22-67	76
BmHel-11(DF090409:393703-393993):153-290	gb|AAO33922.1|:22-67	80
BmHel-11(DF090410:408070-408361):124-291	gb|AAO33922.1|:22-77	87
BmHel-11(DF090410:754066-754360):129-251	gb|AAO33922.1|:36-76	87
BmHel-11(DF090410:892740-893030):150-290	gb|AAO33922.1|:22-68	85
BmHel-11(DF090411:1061776-1062067):124-291	gb|AAO33922.1|:22-77	69
BmHel-11(DF090411:1136317-1136609):155-292	gb|AAO33922.1|:22-67	86
BmHel-11(DF090411:1340223-1340460):100-237	gb|AAO33922.1|:22-67	89
BmHel-11(DF090411:1403956-1404246):123-290	gb|AAO33922.1|:22-77	92
BmHel-11(DF090411:1443679-1443971):125-292	gb|AAO33922.1|:22-77	87
BmHel-11(DF090411:15699-15989):123-290	gb|AAO33922.1|:22-77	89
BmHel-11(DF090411:233223-233480):117-257	gb|AAO33922.1|:22-68	87
BmHel-11(DF090411:257558-257853):191-295	gb|AAO33922.1|:22-56	85
BmHel-11(DF090411:812438-812732):154-294	gb|AAO33922.1|:22-68	89
BmHel-11(DF090411:824339-824854):376-515	gb|AAO33922.1|:22-65	63.5
BmHel-11(DF090411:840479-840771):125-292	gb|AAO33922.1|:22-77	85
BmHel-11(DF090411:930170-930461):160-291	gb|AAO33922.1|:22-65	79
BmHel-11(DF090412:102721-102976):124-255	gb|AAO33922.1|:22-77	67
BmHel-11(DF090412:132157-132442):154-285	gb|AAO33922.1|:22-65	86
BmHel-11(DF090412:1361019-1361310):124-291	gb|AAO33922.1|:22-77	80
BmHel-11(DF090412:174317-174608):124-291	gb|AAO33922.1|:22-77	85
BmHel-11(DF090412:264993-265256):156-263	gb|AAO33922.1|:22-57	86
BmHel-11(DF090412:298324-298594):124-270	gb|AAO33922.1|:22-77	75
BmHel-11(DF090412:317134-317425):124-291	gb|AAO33922.1|:22-77	80
BmHel-11(DF090412:351300-351555):122-255	gb|AAO33922.1|:22-68	64
BmHel-11(DF090412:789374-789664):124-290	gb|AAO33922.1|:22-77	60
BmHel-11(DF090412:885107-885396):124-289	gb|AAO33922.1|:22-77	61.5
BmHel-11(DF090413:1023789-1024078):185-289	gb|AAO33922.1|:22-56	91
BmHel-11(DF090413:1029274-1029564):126-290	gb|AAO33922.1|:22-77	76
BmHel-11(DF090413:1108691-1108981):123-290	gb|AAO33922.1|:22-77	89
BmHel-11(DF090413:1144682-1144970):158-288	gb|AAO33922.1|:22-65	58.5
BmHel-11(DF090413:1165207-1165497):126-290	gb|AAO33922.1|:22-77	57
BmHel-11(DF090413:1196206-1196505):126-299	gb|AAO33922.1|:22-77	55
BmHel-11(DF090413:1302107-1302389):125-282	gb|AAO33922.1|:22-77	63.5
BmHel-11(DF090413:245265-245556):160-291	gb|AAO33922.1|:22-65	81
BmHel-11(DF090413:259934-260181):116-247	gb|AAO33922.1|:22-70	69
BmHel-11(DF090413:484414-484677):165-263	gb|AAO33922.1|:22-54	84
BmHel-11(DF090413:620806-621071):144-265	gb|AAO33922.1|:22-71	60.5
BmHel-11(DF090413:875718-875998):143-280	gb|AAO33922.1|:22-67	73
BmHel-11(DF090413:910942-911161):61-219	gb|AAO33922.1|:22-74	90
BmHel-11(DF090414:1167584-1167874):124-290	gb|AAO33922.1|:22-77	60
BmHel-11(DF090414:1307334-1307621):114-287	gb|AAO33922.1|:22-77	72

BmHel-11(DF090414:133298-134823):1130-1312	gb|AAO33922.1|:22-77	77
BmHel-11(DF090414:133298-134823):291-752	gb|EIE78900.1|:161-491	36.5
BmHel-11(DF090414:283573-283855):124-282	gb|AAO33922.1|:22-77	76
BmHel-11(DF090414:338830-339121):124-291	gb|AAO33922.1|:22-77	83
BmHel-11(DF090414:461068-461412):114-215	dbj|BAH66325.1|:119-152	91
BmHel-11(DF090414:596243-596533):141-290	gb|AAO33922.1|:22-71	88
BmHel-11(DF090414:63515-63795):116-280	gb|AAO33922.1|:22-76	85
BmHel-11(DF090414:711722-712015):150-293	gb|AAO33922.1|:22-69	75
BmHel-11(DF090415:1004640-1004930):153-290	gb|AAO33922.1|:22-67	67
BmHel-11(DF090415:1142624-1142920):168-296	gb|AAO33922.1|:22-64	74
BmHel-11(DF090415:1163338-1163640):162-302	gb|AAO33922.1|:22-68	87
BmHel-11(DF090415:206963-207255):128-292	gb|AAO33922.1|:22-77	55
BmHel-11(DF090415:312062-312354):128-292	gb|AAO33922.1|:22-76	83
BmHel-11(DF090415:432387-432675):121-288	gb|AAO33922.1|:22-69	71
BmHel-11(DF090415:480399-480684):121-285	gb|AAO33922.1|:22-76	85
BmHel-11(DF090415:504546-504800):124-254	gb|AAO33922.1|:22-77	68
BmHel-11(DF090415:944484-944738):115-237	gb|AAO33922.1|:22-62	90
BmHel-11(DF090416:115383-115672):124-289	gb|AAO33922.1|:22-77	66
BmHel-11(DF090416:1154813-1155092):154-279	gb|AAO33922.1|:22-67	84
BmHel-11(DF090416:326806-327100):130-294	gb|AAO33922.1|:22-76	78
BmHel-11(DF090416:413415-413695):143-280	gb|AAO33922.1|:22-67	80
BmHel-11(DF090416:511248-511542):125-250	gb|AAO33922.1|:36-77	76
BmHel-11(DF090416:522210-522453):124-243	gb|AAO33922.1|:22-77	62
BmHel-11(DF090416:537862-538150):121-288	gb|AAO33922.1|:22-77	89
BmHel-11(DF090416:572896-573191):188-295	gb|AAO33922.1|:22-57	80
BmHel-11(DF090416:628047-628337):153-290	gb|AAO33922.1|:22-67	71
BmHel-11(DF090416:649155-649446):124-291	gb|AAO33922.1|:22-77	85
BmHel-11(DF090416:672074-672366):125-292	gb|AAO33922.1|:22-77	94
BmHel-11(DF090416:829591-829865):170-274	gb|AAO33922.1|:22-56	82
BmHel-11(DF090416:854519-854810):124-291	gb|AAO33922.1|:22-77	57
BmHel-11(DF090416:906764-907061):124-297	gb|AAO33922.1|:22-77	84
BmHel-11(DF090417:163161-163430):159-269	gb|AAO33922.1|:22-58	83
BmHel-11(DF090417:164796-164978):33-182	gb|AAO33922.1|:22-73	63
BmHel-11(DF090417:218216-218498):160-282	gb|AAO33922.1|:22-65	77
BmHel-11(DF090417:795598-795875):124-277	gb|AAO33922.1|:22-77	66.5
BmHel-11(DF090417:810844-811117):127-273	gb|AAO33922.1|:22-76	61
BmHel-11(DF090417:857409-857699):160-291	gb|AAO33922.1|:22-65	88
BmHel-11(DF090417:868825-869116):163-291	gb|AAO33922.1|:22-64	86
BmHel-11(DF090417:879416-879710):157-294	gb|AAO33922.1|:22-67	76
BmHel-11(DF090417:919657-919944):145-288	gb|AAO33922.1|:22-69	85
BmHel-11(DF090418:1047406-1047699):126-293	gb|AAO33922.1|:22-77	83
BmHel-11(DF090418:1150547-1150812):124-265	gb|AAO33922.1|:22-77	67.5
BmHel-11(DF090418:1162931-1163224):126-293	gb|AAO33922.1|:22-77	85
BmHel-11(DF090418:124385-124679):124-294	gb|AAO33922.1|:22-77	77
BmHel-11(DF090418:259929-260212):125-283	gb|AAO33922.1|:22-76	69
BmHel-11(DF090418:93910-94204):127-294	gb|AAO33922.1|:22-77	66
BmHel-11(DF090418:978832-979123):151-291	gb|AAO33922.1|:22-68	93
BmHel-11(DF090419:645038-645282):125-244	gb|AAO33922.1|:22-77	64

BmHel-11(DF090419:686757-687048):160-291	gb|AAO33922.1|:22-65	84
BmHel-11(DF090419:772308-772553):126-245	gb|AAO33922.1|:22-75	64
BmHel-11(DF090419:833040-833341):164-301	gb|AAO33922.1|:22-67	84
BmHel-11(DF090419:838408-838699):124-291	gb|AAO33922.1|:22-77	83
BmHel-11(DF090419:919541-919803):122-262	gb|AAO33922.1|:22-68	85
BmHel-11(DF090419:987771-988057):124-286	gb|AAO33922.1|:22-77	61
BmHel-11(DF090420:322771-323063):125-292	gb|AAO33922.1|:22-77	87
BmHel-11(DF090420:424724-425015):124-291	gb|AAO33922.1|:22-77	83
BmHel-11(DF090420:625646-625935):152-289	gb|AAO33922.1|:22-67	86
BmHel-11(DF090420:749619-749901):124-282	gb|AAO33922.1|:22-77	75
BmHel-11(DF090420:883547-883838):124-291	gb|AAO33922.1|:22-77	89
BmHel-11(DF090421:1058835-1059126):124-291	gb|AAO33922.1|:22-77	91
BmHel-11(DF090421:321657-321950):126-293	gb|AAO33922.1|:22-77	80
BmHel-11(DF090421:43430-43721):160-291	gb|AAO33922.1|:22-65	90
BmHel-11(DF090421:591787-592078):124-291	gb|AAO33922.1|:22-77	87
BmHel-11(DF090421:746630-746923):150-293	gb|AAO33922.1|:22-69	87
BmHel-11(DF090421:788877-789144):142-267	gb|AAO33922.1|:22-63	90
BmHel-11(DF090421:872751-873042):124-291	gb|AAO33922.1|:22-77	85
BmHel-11(DF090421:919158-919448):153-290	gb|AAO33922.1|:22-67	71
BmHel-11(DF090422:135959-136250):160-291	gb|AAO33922.1|:22-65	88
BmHel-11(DF090422:336322-336593):104-271	gb|AAO33922.1|:22-77	87
BmHel-11(DF090422:339169-339459):165-290	gb|AAO33922.1|:22-63	80
BmHel-11(DF090422:392534-392826):124-240	gb|AAO33922.1|:39-77	76
BmHel-11(DF090422:548933-549183):124-250	gb|AAO33922.1|:22-77	65.5
BmHel-11(DF090422:802837-803133):123-296	gb|AAO33922.1|:22-77	84
BmHel-11(DF090422:865267-865559):163-292	gb|AAO33922.1|:22-64	71
BmHel-11(DF090422:95911-96202):124-291	gb|AAO33922.1|:22-77	80
BmHel-11(DF090423:233774-234055):162-281	gb|AAO33922.1|:22-65	84
BmHel-11(DF090423:50411-50703):161-292	gb|AAO33922.1|:22-65	90
BmHel-11(DF090423:650089-650379):124-219	gb|AAO33922.1|:46-77	93
BmHel-11(DF090423:709652-709928):115-276	gb|AAO33922.1|:22-75	87
BmHel-11(DF090423:723019-723319):124-300	gb|AAO33922.1|:22-77	52
BmHel-11(DF090423:867484-867779):131-295	gb|AAO33922.1|:22-77	80
BmHel-11(DF090423:917827-918106):124-279	gb|AAO33922.1|:22-77	80
BmHel-11(DF090423:982260-982488):136-228	gb|AAO33922.1|:22-52	90
BmHel-11(DF090424:113066-113632):124-267	gb|AAO33922.1|:30-77	89
BmHel-11(DF090424:197374-197664):177-290	gb|AAO33922.1|:22-59	86
BmHel-11(DF090424:336587-336878):160-291	gb|AAO33922.1|:22-65	90
BmHel-11(DF090424:383189-383455):127-266	gb|AAO33922.1|:22-76	60
BmHel-11(DF090424:657197-657488):160-291	gb|AAO33922.1|:22-65	88
BmHel-11(DF090424:964191-964481):159-290	gb|AAO33922.1|:22-65	86
BmHel-11(DF090425:168905-169159):95-254	gb|AAO33922.1|:22-77	48.5
BmHel-11(DF090425:193680-193970):123-290	gb|AAO33922.1|:22-77	91
BmHel-11(DF090425:323038-323331):126-293	gb|AAO33922.1|:22-77	58
BmHel-11(DF090425:363666-363928):173-262	gb|AAO33922.1|:22-51	90
BmHel-11(DF090425:436119-436409):123-290	gb|AAO33922.1|:22-77	85
BmHel-11(DF090425:490201-490492):160-291	gb|AAO33922.1|:22-65	86
BmHel-11(DF090425:763182-763474):126-292	gb|AAO33922.1|:22-77	59

BmHel-11(DF090425:854932-855224):125-292	gb|AAO33922.1|:22-77	92
BmHel-11(DF090425:926098-926378):143-280	gb|AAO33922.1|:22-67	78
BmHel-11(DF090425:970048-970330):124-282	gb|AAO33922.1|:22-77	78
BmHel-11(DF090426:109732-109997):122-265	gb|AAO33922.1|:22-69	81
BmHel-11(DF090426:279212-279503):124-291	gb|AAO33922.1|:22-77	85
BmHel-11(DF090426:501-789):126-288	gb|AAO33922.1|:22-76	64
BmHel-11(DF090426:558176-558467):154-291	gb|AAO33922.1|:22-67	89
BmHel-11(DF090426:573775-574072):124-297	gb|AAO33922.1|:22-77	84
BmHel-11(DF090426:654062-654324):122-262	gb|AAO33922.1|:22-69	64
BmHel-11(DF090427:11192-11423):100-231	gb|AAO33922.1|:22-74	69
BmHel-11(DF090427:248677-248965):121-288	gb|AAO33922.1|:22-77	82
BmHel-11(DF090427:294711-295002):124-291	gb|AAO33922.1|:22-77	87
BmHel-11(DF090427:444496-444787):151-291	gb|AAO33922.1|:22-68	85
BmHel-11(DF090427:476051-476345):145-294	gb|AAO33922.1|:22-71	94
BmHel-11(DF090427:565726-566017):124-291	gb|AAO33922.1|:22-77	67
BmHel-11(DF090427:654542-654827):123-233	gb|AAO33922.1|:41-77	91
BmHel-11(DF090427:672928-673902):124-234	gb|AAO33922.1|:41-77	86
BmHel-11(DF090427:756163-756435):138-272	gb|AAO33922.1|:22-71	86
BmHel-11(DF090427:896070-896350):131-280	gb|AAO33922.1|:22-71	78
BmHel-11(DF090427:899886-900177):124-291	gb|AAO33922.1|:22-77	85
BmHel-11(DF090427:930676-930962):124-249	gb|AAO33922.1|:36-77	92
BmHel-11(DF090428:366997-367288):124-291	gb|AAO33922.1|:22-77	78
BmHel-11(DF090428:587191-587482):124-291	gb|AAO33922.1|:22-77	91
BmHel-11(DF090428:766833-767124):124-291	gb|AAO33922.1|:22-77	85
BmHel-11(DF090429:324533-324787):168-254	gb|AAO33922.1|:22-50	93
BmHel-11(DF090429:425257-425539):123-282	gb|AAO33922.1|:22-77	62
BmHel-11(DF090429:463778-464063):118-285	gb|AAO33922.1|:22-77	82
BmHel-11(DF090429:565157-565448):124-291	gb|AAO33922.1|:22-77	85
BmHel-11(DF090429:679908-680203):124-295	gb|AAO33922.1|:22-77	63.5
BmHel-11(DF090429:864699-865023):126-324	gb|AAO33922.1|:22-77	54
BmHel-11(DF090429:886219-886513):124-294	gb|AAO33922.1|:22-77	87
BmHel-11(DF090429:92186-92471):163-285	gb|AAO33922.1|:22-62	80
BmHel-11(DF090430:166804-167067):132-263	gb|AAO33922.1|:22-75	72
BmHel-11(DF090430:679438-679724):165-286	gb|AAO33922.1|:22-61	63
BmHel-11(DF090431:165861-166117):90-227	gb|AAO33922.1|:33-77	84
BmHel-11(DF090431:288256-288750):161-265	gb|AAO33922.1|:33-67	85
BmHel-11(DF090431:325850-326141):124-291	gb|AAO33922.1|:22-77	78
BmHel-11(DF090431:359302-359595):126-293	gb|AAO33922.1|:22-77	91
BmHel-11(DF090431:511883-512173):147-290	gb|AAO33922.1|:22-69	83
BmHel-11(DF090431:565191-565439):129-248	gb|AAO33922.1|:22-66	80
BmHel-11(DF090431:775708-775972):136-264	gb|AAO33922.1|:22-64	86
BmHel-11(DF090432:288727-289004):110-277	gb|AAO33922.1|:22-77	63
BmHel-11(DF090432:343877-344167):165-290	gb|AAO33922.1|:22-62	73
BmHel-11(DF090432:415658-415909):123-251	gb|AAO33922.1|:22-77	67
BmHel-11(DF090432:445319-445612):133-293	gb|AAO33922.1|:22-73	59
BmHel-11(DF090432:576081-576372):124-291	gb|AAO33922.1|:22-77	85
BmHel-11(DF090432:609806-610097):160-291	gb|AAO33922.1|:22-65	90
BmHel-11(DF090433:297749-298045):150-296	gb|AAO33922.1|:22-77	64

BmHel-11(DF090433:409841-410129):132-257	gb|AAO33922.1|:33-74	80
BmHel-11(DF090433:431201-431492):124-291	gb|AAO33922.1|:22-77	87
BmHel-11(DF090433:569574-569855):135-281	gb|AAO33922.1|:22-70	93
BmHel-11(DF090433:649218-649497):124-279	gb|AAO33922.1|:22-77	80
BmHel-11(DF090433:678198-678531):165-263	gb|AAO33922.1|:44-76	90
BmHel-11(DF090434:104474-104768):158-295	gb|AAO33922.1|:22-67	86
BmHel-11(DF090434:133188-133480):125-292	gb|AAO33922.1|:22-77	91
BmHel-11(DF090434:374858-375148):123-290	gb|AAO33922.1|:22-77	89
BmHel-11(DF090434:427866-428157):124-291	gb|AAO33922.1|:22-77	83
BmHel-11(DF090435:362384-362675):154-291	gb|AAO33922.1|:22-67	93
BmHel-11(DF090435:406326-406620):124-294	gb|AAO33922.1|:22-77	82
BmHel-11(DF090435:409160-409435):125-275	gb|AAO33922.1|:22-77	65
BmHel-11(DF090435:46112-46402):126-290	gb|AAO33922.1|:22-77	56
BmHel-11(DF090435:526120-526363):124-243	gb|AAO33922.1|:22-77	67
BmHel-11(DF090435:565025-565315):123-290	gb|AAO33922.1|:22-77	85
BmHel-11(DF090435:92512-92804):149-292	gb|AAO33922.1|:22-69	91
BmHel-11(DF090436:3874-4156):124-282	gb|AAO33922.1|:22-77	67
BmHel-11(DF090436:448560-448838):129-278	gb|AAO33922.1|:22-77	78
BmHel-11(DF090437:284680-284967):143-287	gb|AAO33922.1|:22-71	57
BmHel-11(DF090437:31355-31649):124-294	gb|AAO33922.1|:22-77	87
BmHel-11(DF090437:493975-494267):143-292	gb|AAO33922.1|:22-71	94
BmHel-11(DF090438:106468-106762):157-294	gb|AAO33922.1|:22-67	63
BmHel-11(DF090438:1316-1608):155-292	gb|AAO33922.1|:22-67	91
BmHel-11(DF090438:496496-496787):160-291	gb|AAO33922.1|:22-65	88
BmHel-11(DF090438:507602-507848):124-246	gb|AAO33922.1|:22-77	58
BmHel-11(DF090438:541495-541784):152-289	gb|AAO33922.1|:22-67	67
BmHel-11(DF090438:660422-660698):139-276	gb|AAO33922.1|:22-67	78
BmHel-11(DF090438:674981-675272):124-291	gb|AAO33922.1|:22-77	87
BmHel-11(DF090439:108929-109222):126-293	gb|AAO33922.1|:22-77	80
BmHel-11(DF090439:242046-242324):145-278	gb|AAO33922.1|:22-71	71
BmHel-11(DF090439:337276-337564):121-288	gb|AAO33922.1|:22-77	94
BmHel-11(DF090439:47779-48070):127-291	gb|AAO33922.1|:22-76	89
BmHel-11(DF090439:500575-500867):155-292	gb|AAO33922.1|:22-67	84
BmHel-11(DF090440:457462-459118):357-728	ref|XP_002167237.1|:17-146	26
BmHel-11(DF090440:505454-505741):154-287	gb|AAO33922.1|:22-67	65
BmHel-11(DF090441:183183-183468):121-285	gb|AAO33922.1|:22-76	58
BmHel-11(DF090441:262171-262430):127-259	gb|AAO33922.1|:22-76	65
BmHel-11(DF090441:404492-404775):126-283	gb|AAO33922.1|:22-77	71
BmHel-11(DF090441:436265-436547):115-282	gb|AAO33922.1|:22-77	92
BmHel-11(DF090441:48738-49029):127-291	gb|AAO33922.1|:22-76	90
BmHel-11(DF090441:637739-638006):135-267	gb|AAO33922.1|:22-77	51
BmHel-11(DF090441:683544-683836):155-292	gb|AAO33922.1|:22-67	82
BmHel-11(DF090442:257871-258162):154-291	gb|AAO33922.1|:22-67	78
BmHel-11(DF090442:268353-268642):157-289	gb|AAO33922.1|:22-65	55
BmHel-11(DF090443:291766-292262):392-496	gb|AAO33922.1|:22-56	82
BmHel-11(DF090443:423548-423837):122-289	gb|AAO33922.1|:22-77	85
BmHel-11(DF090443:448546-448831):124-285	gb|AAO33922.1|:22-77	91
BmHel-11(DF090443:539194-539485):160-291	gb|AAO33922.1|:22-65	90

BmHel-11(DF090444:389862-390160):134-298	gb|AAO33922.1|:22-76	89
BmHel-11(DF090444:590705-590987):123-282	gb|AAO33922.1|:22-77	65
BmHel-11(DF090444:595529-595786):117-257	gb|AAO33922.1|:22-68	85
BmHel-11(DF090445:226440-226618):74-178	gb|AAO33922.1|:22-56	85
BmHel-11(DF090445:34379-34669):123-290	gb|AAO33922.1|:22-77	85
BmHel-11(DF090446:24262-24530):124-268	gb|AAO33922.1|:22-77	63.5
BmHel-11(DF090446:368885-369176):124-291	gb|AAO33922.1|:22-77	85
BmHel-11(DF090446:578523-578803):113-280	gb|AAO33922.1|:22-77	89
BmHel-11(DF090447:213948-214239):152-291	gb|AAO33922.1|:22-68	67.5
BmHel-11(DF090447:325702-325993):160-291	gb|AAO33922.1|:22-65	86
BmHel-11(DF090447:469175-469437):125-262	gb|AAO33922.1|:22-67	78
BmHel-11(DF090447:490304-490596):197-292	gb|AAO33922.1|:22-53	93
BmHel-11(DF090447:93601-93882):127-281	gb|AAO33922.1|:22-76	62
BmHel-11(DF090448:30385-30673):124-288	gb|AAO33922.1|:22-77	83
BmHel-11(DF090448:367131-367422):124-291	gb|AAO33922.1|:22-77	92
BmHel-11(DF090448:684963-685254):127-291	gb|AAO33922.1|:22-76	80
BmHel-11(DF090449:403102-403393):160-291	gb|AAO33922.1|:22-65	86
BmHel-11(DF090449:426209-426500):142-291	gb|AAO33922.1|:22-71	84
BmHel-11(DF090449:432001-432292):124-291	gb|AAO33922.1|:22-77	87
BmHel-11(DF090449:453124-453416):125-292	gb|AAO33922.1|:22-77	71
BmHel-11(DF090449:460779-461070):160-291	gb|AAO33922.1|:22-65	86
BmHel-11(DF090449:642386-642677):124-291	gb|AAO33922.1|:22-77	85
BmHel-11(DF090450:247397-247688):124-291	gb|AAO33922.1|:22-77	58
BmHel-11(DF090450:30263-30545):107-282	gb|AAO33922.1|:22-77	56
BmHel-11(DF090450:323239-323529):159-290	gb|AAO33922.1|:22-65	88
BmHel-11(DF090450:474247-474536):126-260	gb|AAO33922.1|:33-77	64
BmHel-11(DF090450:512411-512702):157-291	gb|AAO33922.1|:22-66	88
BmHel-11(DF090451:313094-313386):124-243	gb|AAO33922.1|:38-77	90
BmHel-11(DF090452:129928-130207):154-279	gb|AAO33922.1|:22-63	80
BmHel-11(DF090452:29882-30179):148-297	gb|AAO33922.1|:22-67	78
BmHel-11(DF090452:372979-373273):124-294	gb|AAO33922.1|:22-77	87
BmHel-11(DF090454:451139-451431):155-292	gb|AAO33922.1|:22-67	86
BmHel-11(DF090455:197972-198263):160-291	gb|AAO33922.1|:22-65	81
BmHel-11(DF090457:143910-144201):124-291	gb|AAO33922.1|:22-77	83
BmHel-11(DF090457:218165-218455):124-290	gb|AAO33922.1|:22-77	60
BmHel-11(DF090457:246490-246754):124-264	gb|AAO33922.1|:22-68	85
BmHel-11(DF090458:12086-12373):156-287	gb|AAO33922.1|:22-65	84
BmHel-11(DF090458:18246-18537):124-291	gb|AAO33922.1|:22-77	83
BmHel-11(DF090459:289467-289757):123-290	gb|AAO33922.1|:22-77	89
BmHel-11(DF090459:331839-332130):163-291	gb|AAO33922.1|:22-64	83
BmHel-11(DF090460:147092-147383):124-291	gb|AAO33922.1|:22-77	83
BmHel-11(DF090460:45524-45812):121-288	gb|AAO33922.1|:22-77	73
BmHel-11(DF090461:140151-140428):116-277	gb|AAO33922.1|:22-77	82
BmHel-11(DF090461:268341-268632):124-291	gb|AAO33922.1|:22-77	80
BmHel-11(DF090461:324142-324433):160-291	gb|AAO33922.1|:22-65	81
BmHel-11(DF090462:1718-2014):129-296	gb|AAO33922.1|:22-77	89
BmHel-11(DF090462:253256-253526):103-270	gb|AAO33922.1|:22-77	80
BmHel-11(DF090462:406020-406311):160-291	gb|AAO33922.1|:22-65	90

BmHel-11(DF090462:94010-94277):139-267	gb|AAO33922.1|:22-70	65
BmHel-11(DF090463:240029-240321):125-292	gb|AAO33922.1|:22-77	92
BmHel-11(DF090464:104598-104873):124-275	gb|AAO33922.1|:22-77	51
BmHel-11(DF090466:113880-114170):141-290	gb|AAO33922.1|:22-71	84
BmHel-11(DF090466:156762-157052):165-290	gb|AAO33922.1|:22-63	76
BmHel-11(DF090466:222875-223166):124-291	gb|AAO33922.1|:22-77	80
BmHel-11(DF090467:248739-249031):125-292	gb|AAO33922.1|:22-77	76
BmHel-11(DF090467:282754-283047):138-293	gb|AAO33922.1|:22-73	90
BmHel-11(DF090468:124595-124875):124-280	gb|AAO33922.1|:22-77	64
BmHel-11(DF090468:205572-205836):124-264	gb|AAO33922.1|:22-77	57
BmHel-11(DF090468:213508-213865):67-162	dbj|BAH66325.1|:121-152	87
BmHel-11(DF090468:265359-265650):124-291	gb|AAO33922.1|:22-77	83
BmHel-11(DF090468:84908-85199):124-291	gb|AAO33922.1|:22-77	83
BmHel-11(DF090469:262671-262920):142-249	gb|AAO33922.1|:22-71	62
BmHel-11(DF090469:353539-353831):155-292	gb|AAO33922.1|:22-67	78
BmHel-11(DF090472:175042-175336):160-294	gb|AAO33922.1|:22-65	91
BmHel-11(DF090472:269401-269699):123-298	gb|AAO33922.1|:22-77	57.5
BmHel-11(DF090472:42203-42498):125-295	gb|AAO33922.1|:22-77	91
BmHel-11(DF090472:58945-59236):124-291	gb|AAO33922.1|:22-77	85
BmHel-11(DF090472:88387-88679):155-292	gb|AAO33922.1|:22-67	78
BmHel-11(DF090473:156832-157124):155-292	gb|AAO33922.1|:22-67	76
BmHel-11(DF090473:237643-237935):131-292	gb|AAO33922.1|:22-75	90
BmHel-11(DF090473:352583-352875):124-292	gb|AAO33922.1|:22-77	59.5
BmHel-11(DF090475:239002-241101):532-1222	sp|Q9NBX4|RTXE_DROME:454-898	41.5
BmHel-11(DF090475:369-655):143-286	gb|AAO33922.1|:22-71	74
BmHel-11(DF090476:125765-126050):127-285	gb|AAO33922.1|:22-74	81
BmHel-11(DF090476:197461-197746):154-285	gb|AAO33922.1|:22-65	86
BmHel-11(DF090476:210731-210958):117-227	gb|AAO33922.1|:22-67	67
BmHel-11(DF090476:31383-31677):124-294	gb|AAO33922.1|:22-77	80
BmHel-11(DF090477:154491-154782):124-291	gb|AAO33922.1|:22-77	85
BmHel-11(DF090477:2693-2936):124-243	gb|AAO33922.1|:22-77	67
BmHel-11(DF090477:43525-43822):154-297	gb|AAO33922.1|:22-69	66
BmHel-11(DF090478:142269-142574):124-305	gb|AAO33922.1|:22-77	68
BmHel-11(DF090479:246406-246697):124-291	gb|AAO33922.1|:22-77	87
BmHel-11(DF090479:290666-290956):160-282	gb|AAO33922.1|:25-65	85
BmHel-11(DF090481:180053-180336):116-283	gb|AAO33922.1|:22-77	60
BmHel-11(DF090481:227745-228049):124-304	gb|AAO33922.1|:22-77	66
BmHel-11(DF090482:43469-43728):132-259	gb|AAO33922.1|:22-71	58.5
BmHel-11(DF090483:42160-42467):124-307	gb|AAO33922.1|:22-77	66
BmHel-11(DF090483:49590-49894):124-304	gb|AAO33922.1|:22-77	63.5
BmHel-11(DF090483:64332-64623):124-291	gb|AAO33922.1|:22-77	92
BmHel-11(DF090483:94607-94900):126-293	gb|AAO33922.1|:22-77	83
BmHel-11(DF090484:128054-128345):124-291	gb|AAO33922.1|:22-77	83
BmHel-11(DF090484:152442-152736):133-294	gb|AAO33922.1|:22-75	83
BmHel-11(DF090486:150632-150923):124-291	gb|AAO33922.1|:22-77	89
BmHel-11(DF090486:160162-160453):160-291	gb|AAO33922.1|:22-65	81
BmHel-11(DF090486:39584-39870):123-286	gb|AAO33922.1|:22-77	58.5
BmHel-11(DF090486:96354-96618):157-264	gb|AAO33922.1|:22-57	83

BmHel-11(DF090487:146870-147161):124-291	gb|AAO33922.1|:22-77	83
BmHel-11(DF090487:75003-75294):124-291	gb|AAO33922.1|:22-77	78
BmHel-11(DF090488:52214-52526):102-269	gb|AAO33922.1|:22-77	89
BmHel-11(DF090492:205587-205862):114-275	gb|AAO33922.1|:22-75	83
BmHel-11(DF090492:216043-216292):118-249	gb|AAO33922.1|:22-71	62
BmHel-11(DF090492:223300-223582):166-282	gb|AAO33922.1|:22-74	64
BmHel-11(DF090492:83222-83511):152-289	gb|AAO33922.1|:22-67	67
BmHel-11(DF090494:101630-101921):160-291	gb|AAO33922.1|:22-65	90
BmHel-11(DF090495:10431-10722):124-291	gb|AAO33922.1|:22-77	89
BmHel-11(DF090496:36711-37002):124-291	gb|AAO33922.1|:22-77	87
BmHel-11(DF090496:59190-59480):123-290	gb|AAO33922.1|:22-77	87
BmHel-11(DF090498:153234-153525):124-291	gb|AAO33922.1|:22-77	87
BmHel-11(DF090499:103596-103884):167-288	gb|AAO33922.1|:22-63	58
BmHel-11(DF090500:207040-207318):124-258	gb|AAO33922.1|:32-77	67
BmHel-11(DF090501:91256-91545):124-289	gb|AAO33922.1|:22-77	71
BmHel-11(DF090501:94208-98990):124-213	gb|AAO33922.1|:48-77	90
BmHel-11(DF090501:94208-98990):449-817	ref|XP_003093149.1|:1187-1310	30
BmHel-11(DF090502:111154-111445):124-291	gb|AAO33922.1|:22-77	87
BmHel-11(DF090503:114248-114489):104-241	gb|AAO33922.1|:22-67	86
BmHel-11(DF090503:49463-49755):134-292	gb|AAO33922.1|:22-74	88
BmHel-11(DF090510:29872-30162):124-264	gb|AAO33922.1|:31-77	87
BmHel-11(DF090511:14114-14684):124-252	gb|AAO33922.1|:35-77	88
BmHel-11(DF090517:44907-45199):155-292	gb|AAO33922.1|:22-67	80
BmHel-11(DF090518:11026-11317):160-291	gb|AAO33922.1|:22-65	88
BmHel-11(DF090518:82916-83207):124-291	gb|AAO33922.1|:22-77	83
BmHel-11(DF090520:151004-151292):146-288	gb|AAO33922.1|:22-71	63
BmHel-11(DF090520:46518-47069):409-551	gb|AAO33922.1|:22-68	59.5
BmHel-11(DF090520:895-1163):119-268	gb|AAO33922.1|:22-71	82
BmHel-11(DF090521:69607-69894):120-287	gb|AAO33922.1|:22-77	91
BmHel-11(DF090521:88513-88807):162-294	gb|AAO33922.1|:22-65	62
BmHel-11(DF090525:35402-35629):135-227	gb|AAO33922.1|:22-52	90
BmHel-11(DF090525:73909-74200):124-291	gb|AAO33922.1|:22-77	91
BmHel-11(DF090526:11480-11774):125-294	gb|AAO33922.1|:22-77	62
BmHel-11(DF090526:62701-62980):147-279	gb|AAO33922.1|:22-69	64.5
BmHel-11(DF090528:17545-17836):160-291	gb|AAO33922.1|:22-65	86
BmHel-11(DF090528:844-1123):157-279	gb|AAO33922.1|:22-62	87
BmHel-11(DF090536:25113-25404):124-291	gb|AAO33922.1|:22-77	83
BmHel-11(DF090537:6895-7171):160-276	gb|AAO33922.1|:22-68	70
BmHel-11(DF090539:18333-18625):124-228	gb|AAO33922.1|:43-77	88
BmHel-11(DF090539:69984-70280):126-296	gb|AAO33922.1|:22-77	66
BmHel-11(DF090540:57077-57321):122-244	gb|AAO33922.1|:22-62	90
BmHel-11(DF090543:44270-44561):124-291	gb|AAO33922.1|:22-77	83
BmHel-11(DF090544:47339-47638):195-299	gb|AAO33922.1|:22-56	94
BmHel-11(DF090549:61246-61503):124-257	gb|AAO33922.1|:22-77	59.5
BmHel-11(DF090557:54187-54482):149-295	gb|AAO33922.1|:22-70	83
BmHel-11(DF090558:46906-47172):135-266	gb|AAO33922.1|:22-66	84
BmHel-11(DF090561:28099-28397):150-230	gb|AAO33922.1|:43-69	96
BmHel-11(DF090562:57636-57932):150-296	gb|AAO33922.1|:22-77	66

BmHel-11(DF090570:218-509):160-291	gb|AAO33922.1|:22-65	81
BmHel-11(DF090570:51980-52237):120-257	gb|AAO33922.1|:22-67	71
BmHel-11(DF090571:37584-37875):157-291	gb|AAO33922.1|:22-66	88
BmHel-11(DF090571:5040-5328):128-288	gb|AAO33922.1|:22-77	58.5
BmHel-11(DF090573:10384-10673):122-289	gb|AAO33922.1|:22-77	82
BmHel-11(DF090573:21627-21918):124-291	gb|AAO33922.1|:22-77	85
BmHel-11(DF090577:34966-35258):125-292	gb|AAO33922.1|:22-77	92
BmHel-11(DF090579:48401-48694):131-293	gb|AAO33922.1|:22-77	53.5
BmHel-11(DF090579:53028-53320):125-292	gb|AAO33922.1|:22-77	87
BmHel-11(DF090580:43789-44082):162-293	gb|AAO33922.1|:22-65	86
BmHel-11(DF090582:42896-43186):123-290	gb|AAO33922.1|:22-77	73
BmHel-11(DF090587:23158-23410):124-252	gb|AAO33922.1|:22-77	69
BmHel-11(DF090590:34908-35199):160-291	gb|AAO33922.1|:22-65	86
BmHel-11(DF090591:47021-47287):123-266	gb|AAO33922.1|:22-77	76
BmHel-11(DF090592:31933-32224):124-291	gb|AAO33922.1|:22-77	89
BmHel-11(DF090592:4088-4373):119-285	gb|AAO33922.1|:22-75	61.5
BmHel-11(DF090596:11486-11749):126-263	gb|AAO33922.1|:22-77	64
BmHel-11(DF090598:37316-37590):145-274	gb|AAO33922.1|:22-65	71
BmHel-11(DF090608:18999-19356):268-357	gb|AAO33922.1|:22-51	93
BmHel-11(DF090622:4962-5210):141-248	gb|AAO33922.1|:22-71	66
BmHel-11(DF090622:815-1106):124-291	gb|AAO33922.1|:22-77	91
BmHel-11(DF090626:3033-3332):150-299	gb|AAO33922.1|:22-70	62
BmHel-11(DF090639:15509-15799):159-290	gb|AAO33922.1|:22-65	86
BmHel-11(DF090644:90892-91184):149-292	gb|AAO33922.1|:22-69	79
BmHel-11(DF090663:9719-9981):98-262	gb|AAO33922.1|:22-76	85
BmHel-11(DF090667:27887-28375):345-488	gb|AAO33922.1|:22-69	85
BmHel-11(DF090671:1907-2198):124-291	gb|AAO33922.1|:22-77	87
BmHel-11(DF090683:13520-13811):154-291	gb|AAO33922.1|:22-67	73
BmHel-11(DF090692:20309-20604):160-295	gb|AAO33922.1|:22-65	65.5
BmHel-11(DF090693:3734-4025):124-291	gb|AAO33922.1|:22-77	91
BmHel-11(DF090699:10532-10814):124-282	gb|AAO33922.1|:22-77	76
BmHel-11(DF090699:21088-21381):162-293	gb|AAO33922.1|:22-65	88
BmHel-11(DF090702:44859-45154):134-295	gb|AAO33922.1|:22-75	70
BmHel-11(DF090703:25357-25647):159-290	gb|AAO33922.1|:22-65	86
BmHel-11(DF090716:8510-8802):128-292	gb|AAO33922.1|:22-76	83
BmHel-11(DF090724:11585-11873):124-288	gb|AAO33922.1|:22-77	87
BmHel-11(DF090728:19907-20199):155-292	gb|AAO33922.1|:22-67	84
BmHel-11(DF090738:23882-29466):13-320	ref|NP_001108468.1|:78-177	49
BmHel-11(DF090738:23882-29466):993-1174	gb|AAO33922.1|:22-77	67.5
BmHel-11(DF090746:19850-20144):127-294	gb|AAO33922.1|:22-76	83
BmHel-11(DF090754:7176-7452):124-276	gb|AAO33922.1|:22-77	71
BmHel-11(DF090758:3667-3958):124-291	gb|AAO33922.1|:22-77	85
BmHel-11(DF090800:5020-5310):124-290	gb|AAO33922.1|:22-77	61.5
BmHel-11(DF090806:5147-5438):124-291	gb|AAO33922.1|:22-77	91
BmHel-11(DF090808:8200-8491):136-291	gb|AAO33922.1|:22-73	80
BmHel-11(DF090822:13231-13522):160-291	gb|AAO33922.1|:22-65	86
BmHel-11(DF090839:41194-41465):126-271	gb|AAO33922.1|:22-75	61.5
BmHel-11(DF090868:13836-14112):124-276	gb|AAO33922.1|:22-77	76

BmHel-11(DF090873:14236-14504):123-268	gb|AAO33922.1|:22-77	65.5
BmHel-11(DF090880:10575-10860):142-285	gb|AAO33922.1|:22-69	72
BmHel-11(DF090892:12242-12900):68-246	dbj|BAH66344.1|:45-101	58.5
BmHel-11(DF090892:12242-12900):509-658	gb|AAO33922.1|:22-71	80
BmHel-11(DF090895:24407-24698):124-291	gb|AAO33922.1|:22-77	87
BmHel-11(DF090898:1218-1510):134-292	gb|AAO33922.1|:22-74	71
BmHel-11(DF090905:6099-6389):162-290	gb|AAO33922.1|:22-64	88
BmHel-11(DF090944:366-655):120-212	gb|AAO33922.1|:47-77	93
BmHel-11(DF091006:6644-6929):123-285	gb|AAO33922.1|:22-77	54.5
BmHel-11(DF091017:995-1289):124-294	gb|AAO33922.1|:22-77	87
BmHel-11(DF091063:960-1251):126-248	gb|AAO33922.1|:36-76	80
BmHel-11(DF091083:3072-3369):157-297	gb|AAO33922.1|:22-65	74
BmHel-11(DF091090:3812-4112):169-300	gb|AAO33922.1|:22-65	90
BmHel-11(DF091096:3863-4092):104-229	gb|AAO33922.1|:22-77	67
BmHel-11(DF091135:6158-6449):157-291	gb|AAO33922.1|:22-66	86
BmHel-11(DF091137:33385-33675):123-290	gb|AAO33922.1|:22-77	94
BmHel-11(DF091147:1173-1443):145-270	gb|AAO33922.1|:22-63	78
BmHel-11(DF091194:4758-5787):430-555	gb|AAO33922.1|:22-63	90
BmHel-11(DF091216:3619-3917):155-298	gb|AAO33922.1|:22-67	81
BmHel-11(DF091225:12075-12366):124-291	gb|AAO33922.1|:22-77	82
BmHel-11(DF091245:4574-4855):153-281	gb|AAO33922.1|:22-64	86
BmHel-11(DF091269:3090-3383):156-293	gb|AAO33922.1|:22-67	76
BmHel-11(DF091273:561-850):167-289	gb|AAO33922.1|:22-62	80
BmHel-11(DF091359:3790-4059):121-269	gb|AAO33922.1|:22-77	71
BmHel-11(DF091431:3591-3825):124-234	gb|AAO33922.1|:22-77	60
BmHel-11(DF091533:496-765):126-269	gb|AAO33922.1|:22-69	83
BmHel-11(DF091903:103-394):160-291	gb|AAO33922.1|:22-65	81
BmHel-12(DF090316:1700249-1700641):67-196	ref|ZP_03293582.1|:391-441	47.5
BmHel-12(DF090316:7492878-7498843):3323-3604	dbj|BAM73877.1|:11-103	44
BmHel-12(DF090317:7069422-7070306):5-481	ref|XP_003737968.1|:186-352	30
BmHel-12(DF090318:9088405-9099027):209-535	ref|XP_003094987.1|:204-312	40
BmHel-12(DF090318:9088405-9099027):8044-8631	gb|EHJ72752.1|:24-216	56
BmHel-12(DF090319:5737697-5743437):816-1267	ref|NP_001157648.1|:98-232	51.5
BmHel-12(DF090319:6371615-6372042):114-257	gb|EFN77020.1|:11-59	56
BmHel-12(DF090320:3605896-3607307):830-1056	ref|XP_003244591.1|:1-76	44
BmHel-12(DF090320:5518336-5518735):82-195	ref|ZP_03293582.1|:393-441	51
BmHel-12(DF090320:7088343-7091049):582-2028	gb|AAC60281.1|:427-932	38
BmHel-12(DF090322:5411157-5412538):720-883	dbj|BAH66344.1|:45-96	68
BmHel-12(DF090322:7816005-7821602):1714-5019	dbj|BAH66363.1|:1-56	59
BmHel-12(DF090322:7859445-7859782):167-271	dbj|BAH66325.1|:115-149	74
BmHel-12(DF090324:4462017-4463930):1407-1582	ref|XP_003096072.1|:17-107	52
BmHel-12(DF090324:4463243-4463715):120-299	ref|XP_001746355.1|:221-303	52
BmHel-12(DF090324:4635503-4641607):240-1165	ref|XP_003095773.1|:7-338	40
BmHel-12(DF090324:4635503-4641607):4256-4423	dbj|BAH66363.1|:1-56	75
BmHel-12(DF090325:230403-232174):1486-1660	gb|AAO33922.1|:9-66	67.5
BmHel-12(DF090325:5648676-5650651):646-945	ref|XP_001620362.1|:3-111	34
BmHel-12(DF090327:2594432-2603550):7856-8005	gb|AAO33922.1|:17-66	84
BmHel-12(DF090327:3070954-3071357):96-203	ref|ZP_04658305.1|:357-416	48

BmHel-12(DF090327:5496383-5505946):2867-3142	dbj|BAM73877.1|:8-100	84
BmHel-12(DF090327:5496383-5505946):7795-8060	dbj|BAH66344.1|:45-130	42.5
BmHel-12(DF090329:2739628-2747675):2318-6683	dbj|BAH66363.1|:1-60	61
BmHel-12(DF090329:328060-333508):3423-3651	gb|AAO33922.1|:3-77	57
BmHel-12(DF090329:328060-333508):878-1009	dbj|BAH66363.1|:4-47	84
BmHel-12(DF090330:3795068-3799557):905-1092	gb|AAT38756.2|:39-104	47
BmHel-12(DF090330:3795068-3799557):2651-2794	dbj|BAH66363.1|:7-56	74
BmHel-12(DF090333:4967817-4968372):80-247	ref|XP_002034589.1|:122-197	53
BmHel-12(DF090338:68607-69598):280-642	ref|XP_003093476.1|:18-135	33
BmHel-12(DF090339:560653-561052):116-193	ref|XP_003096072.1|:63-96	57.5
BmHel-12(DF090342:189372-190860):208-354	ref|XP_003099082.1|:294-342	42
BmHel-12(DF090345:2158068-2158871):633-788	dbj|BAH66325.1|:107-152	65
BmHel-12(DF090347:101200-106077):1103-1355	ref|XP_003725312.1|:776-865	36.5
BmHel-12(DF090347:1155258-1155887):433-624	gb|AAO33922.1|:7-70	67
BmHel-12(DF090349:1990668-2000232):8326-8654	emb|CAX36785.1|:101-212	46
BmHel-12(DF090349:1990668-2000232):3755-3886	dbj|BAH66363.1|:13-56	79
BmHel-12(DF090349:3304411-3310116):4319-4512	ref|NP_001108468.1|:95-156	56
BmHel-12(DF090349:3304411-3310116):4882-5353	emb|CAX36785.1|:53-212	47.5
BmHel-12(DF090350:2499342-2506416):2381-2548	gb|AAO33922.1|:17-71	80
BmHel-12(DF090350:2499342-2506416):4987-5097	dbj|BAH66363.1|:2-38	83
BmHel-12(DF090351:2493194-2495588):1400-1906	ref|XP_646766.1|:38-212	33
BmHel-12(DF090351:3529933-3530732):328-496	dbj|BAH66363.1|:1-56	55
BmHel-12(DF090357:2186875-2190227):711-1727	ref|XP_003092184.1|:7-342	37.5
BmHel-12(DF090357:3131153-3132607):208-762	gb|AAN06610.1|AF461149_1:1-346	69.5
BmHel-12(DF090358:1697561-1705658):3124-3234	dbj|BAH66325.1|:116-152	91
BmHel-12(DF090358:1697561-1705658):1395-1553	gb|ACY06930.1|:21-73	100
BmHel-12(DF090358:1697561-1705658):3251-3364	dbj|BAH66363.1|:1-38	84
BmHel-12(DF090358:2064316-2065903):220-679	gb|AAN06610.1|AF461149_1:1-346	68.5
BmHel-12(DF090359:1782439-1783255):122-547	emb|CAX36785.1|:67-210	53
BmHel-12(DF090362:505054-506585):469-960	ref|XP_003241819.1|:4-166	30
BmHel-12(DF090363:1552928-1559978):5331-5515	dbj|BAH66344.1|:31-95	47
BmHel-12(DF090363:1552928-1559978):646-3498	gb|EAW72053.1|:116-443	39
BmHel-12(DF090363:164145-165882):198-620	ref|XP_003092956.1|:205-341	35
BmHel-12(DF090367:2380439-2382043):216-837	ref|XP_002159924.1|:14-347	40
BmHel-12(DF090369:1702413-1703162):245-357	ref|NP_001157648.1|:213-250	56
BmHel-12(DF090369:967530-969097):297-1119	ref|XP_002641086.1|:23-319	34
BmHel-12(DF090370:2327018-2329397):62-229	dbj|BAH66363.1|:1-56	66
BmHel-12(DF090370:335948-336424):191-301	dbj|BAH66325.1|:116-152	86
BmHel-12(DF090382:1408114-1412266):1443-1614	dbj|BAH66363.1|:1-55	57
BmHel-12(DF090382:1408114-1412266):548-701	gb|AAO33922.1|:18-67	54.5
BmHel-12(DF090382:1929646-1938360):4687-5292	gb|AAC60281.1|:770-969	25
BmHel-12(DF090386:121713-122452):186-341	dbj|BAH66325.1|:106-156	65
BmHel-12(DF090388:302841-304721):278-385	dbj|BAH66325.1|:119-154	83
BmHel-12(DF090391:139484-141491):208-1091	ref|XP_003094987.1|:96-312	40.5
BmHel-12(DF090393:807619-810378):200-380	dbj|BAH66363.1|:1-60	51
BmHel-12(DF090395:620827-629745):3479-3642	dbj|BAH66344.1|:45-96	68
BmHel-12(DF090396:1306047-1306668):186-287	ref|NP_001157648.1|:217-250	76
BmHel-12(DF090400:1469057-1470099):173-280	dbj|BAH66325.1|:115-150	80

BmHel-12(DF090400:1549349-1549935):419-575	gb|AAO33922.1|:9-77	55.5
BmHel-12(DF090400:1563324-1563861):239-370	dbj|BAH66325.1|:117-159	70
BmHel-12(DF090412:249340-251082):186-1255	gb|AAN06610.1|AF461149_1:2-346	56.5
BmHel-12(DF090418:382323-386077):473-734	gb|ACY06938.1|:1-84	63
BmHel-12(DF090428:693073-695307):536-760	gb|EFA13284.1|:191-265	29
BmHel-12(DF090431:775658-776282):446-613	gb|AAO33922.1|:9-64	69
BmHel-12(DF090433:387110-395107):4643-4795	gb|EHJ78355.1|:2-52	80
BmHel-12(DF090433:387110-395107):918-2411	dbj|BAH66325.1|:113-156	56.5
BmHel-12(DF090433:387110-395107):5485-5667	gb|AAO33922.1|:16-76	80
BmHel-12(DF090434:28369-28845):170-274	dbj|BAH66325.1|:118-152	85
BmHel-12(DF090442:424638-432862):1617-2057	ref|XP_002056696.1|:66-221	29
BmHel-12(DF090442:424638-432862):2236-7511	dbj|BAH66363.1|:1-56	59
BmHel-12(DF090443:545445-549466):232-512	ref|NP_001157648.1|:92-177	50.5
BmHel-12(DF090481:203319-208346):3690-3914	gb|AAT38756.2|:30-103	42
BmHel-12(DF090543:71292-72880):347-1023	ref|XP_003089587.1|:7-263	34
BmHel-12(DF090573:13113-14721):206-513	gb|EEZ97620.1|:197-306	38.5
BmHel-12(DF090599:42144-42962):225-394	dbj|BAH66344.1|:43-96	65
BmHel-12(DF090683:25851-26642):247-390	dbj|BAH66363.1|:1-48	91
BmHel-12(DF090683:25851-26642):71-183	ref|NP_001157648.1|:213-250	71
BmHel-13(DF090342:2146867-2147437):26-277	gb|AAT40504.2|:624-708	37
BmHel-13(DF090375:772351-781314):405-497	ref|XP_003241752.1|:522-552	64
BmHel-13(DF090375:772351-781314):260-403	gb|EFN65340.1|:1-48	56
BmHel-15(DF090316:4640683-4647901):1777-4958	gb|EHJ71591.1|:60-225	54.5
BmHel-15(DF090317:4783746-4793700):810-992	ref|XP_003423966.1|:173-237	58
BmHel-15(DF090317:4783746-4793700):8341-8496	gb|AAO33922.1|:16-67	71
BmHel-15(DF090319:7461948-7466152):404-2624	ref|NP_001037037.1|:150-313	70
BmHel-15(DF090321:5785047-5785597):201-313	ref|NP_001157648.1|:213-250	59
BmHel-15(DF090330:3067827-3068477):247-339	gb|AAO33922.1|:22-52	96
BmHel-15(DF090340:995106-1005573):4176-6191	gb|EHJ73385.1|:181-352	53.5
BmHel-15(DF090341:2811759-2822156):1898-2065	ref|ZP_11076087.1|:52-108	43
BmHel-15(DF090341:2811759-2822156):4207-5139	ref|XP_003093154.1|:97-453	27
BmHel-15(DF090343:186284-190502):2219-2323	dbj|BAH66325.1|:118-152	85
BmHel-15(DF090343:3632-12130):4676-5053	gb|EFA11647.1|:653-2111	35.5
BmHel-15(DF090343:3632-12130):3189-3752	gb|EFN60885.1|:14-222	21
BmHel-15(DF090343:3632-12130):3162-4118	ref|NP_006506.3|:354-681	36
BmHel-15(DF090343:3632-12130):2590-2996	ref|XP_003731244.1|:5-137	31.5
BmHel-15(DF090343:3632-12130):5678-5962	dbj|BAH66344.1|:31-125	48
BmHel-15(DF090344:3415301-3421863):3292-3464	gb|AAO33922.1|:18-75	64.5
BmHel-15(DF090344:610750-617696):688-777	gb|EHJ67551.1|:58-87	93
BmHel-15(DF090345:1106413-1107095):246-372	gb|AAO33922.1|:22-76	63
BmHel-15(DF090345:2615495-2619338):808-979	dbj|BAH66363.1|:1-56	58.5
BmHel-15(DF090348:1025468-1027291):465-977	ref|XP_003243973.1|:205-742	37.5
BmHel-15(DF090354:3162954-3163973):360-894	ref|XP_003243437.1|:1007-1181	41
BmHel-15(DF090354:3393346-3394045):529-627	dbj|BAH66325.1|:121-153	75
BmHel-15(DF090355:1270676-1271302):165-325	dbj|BAH66344.1|:45-95	52
BmHel-15(DF090357:3761569-3767221):695-891	dbj|BAH66344.1|:45-107	53.5
BmHel-15(DF090357:3761569-3767221):1061-1195	dbj|BAM73877.1|:59-103	64
BmHel-15(DF090359:2418465-2423715):618-4722	dbj|BAA74713.1|:10-1310	32

BmHel-15(DF090359:2539517-2544157):539-1130	gb|EFA12871.1|:79-290	35
BmHel-15(DF090359:2539517-2544157):1825-2830	gb|EEZ99102.1|:194-457	39.5
BmHel-15(DF090359:2539517-2544157):1825-2988	sp|P10394|POL4_DROME:302-687	20
BmHel-15(DF090359:2539517-2544157):1733-3186	gb|EFA13465.1|:550-1051	34
BmHel-15(DF090363:1958478-1960441):395-817	ref|XP_003243973.1|:73-684	34.5
BmHel-15(DF090369:2439547-2442093):1609-1909	ref|XP_001842594.1|:182-247	49
BmHel-15(DF090378:1510420-1512065):241-1265	gb|AAN06610.1|AF461149_1:1-346	63.5
BmHel-15(DF090384:1521910-1523370):80-788	ref|XP_004003398.1|:19-353	36
BmHel-15(DF090388:146140-147043):509-705	dbj|BAH66344.1|:45-107	49
BmHel-15(DF090388:804008-810677):3090-3202	ref|NP_001157648.1|:213-250	68.5
BmHel-15(DF090388:804008-810677):3239-5367	gb|EHJ67082.1|:225-349	61.5
BmHel-15(DF090411:518389-518953):369-491	dbj|BAH66325.1|:121-161	70
BmHel-15(DF090411:765560-772603):2763-2965	dbj|BAH66344.1|:45-109	52
BmHel-15(DF090413:1179353-1189423):992-1107	ref|NP_001157648.1|:212-250	68.5
BmHel-15(DF090424:60633-66846):5141-5254	dbj|BAH66325.1|:115-152	86
BmHel-15(DF090424:60633-66846):5356-5709	ref|XP_001523679.1|:337-463	43
BmHel-15(DF090424:60633-66846):4405-4521	ref|NP_001157648.1|:213-250	79
BmHel-15(DF090441:390251-398790):3784-3909	dbj|BAH66325.1|:116-156	85
BmHel-15(DF090441:390251-398790):5907-7607	dbj|BAH66363.1|:1-56	64
BmHel-15(DF090442:425262-431226):4197-4360	dbj|BAH66344.1|:45-96	53.5
BmHel-15(DF090447:101549-102991):524-909	gb|AAN06610.1|AF461149_1:218-346	68.5
BmHel-15(DF090455:194077-202037):4009-4152	gb|AAO33922.1|:18-65	79
BmHel-15(DF090455:194077-202037):6490-6600	dbj|BAH66325.1|:116-152	86
BmHel-15(DF090455:194077-202037):591-731	dbj|BAH66363.1|:2-48	76
BmHel-15(DF090493:165795-168140):473-1027	gb|EFA11798.1|:401-573	25
BmHel-15(DF090494:114117-117360):498-686	gb|EFN69706.1|:178-239	52
BmHel-15(DF090797:3256-11833):6763-7083	ref|XP_002592083.1|:68-169	33
BmHel-16(DF090316:12056015-12063386):2169-2399	ref|XP_003730492.1|:17-98	30
BmHel-16(DF090316:12875237-12877053):1030-1194	dbj|BAH66363.1|:1-55	70
BmHel-16(DF090316:4260356-4264835):4027-4139	ref|NP_001157648.1|:213-250	56
BmHel-16(DF090316:4260356-4264835):2266-2394	dbj|BAH66325.1|:115-156	69
BmHel-16(DF090316:7282047-7289793):1038-1145	dbj|BAH66325.1|:117-152	88
BmHel-16(DF090317:1698061-1703403):2120-4470	dbj|BAH66363.1|:1-56	53
BmHel-16(DF090317:1698061-1703403):4659-4772	dbj|BAH66325.1|:115-152	73
BmHel-16(DF090317:223451-228408):2167-2327	dbj|BAH66344.1|:45-95	55
BmHel-16(DF090317:223451-228408):1425-1520	dbj|BAH66325.1|:119-150	87
BmHel-16(DF090317:2644788-2649757):3864-4144	gb|EHJ77411.1|:6-99	38.5
BmHel-16(DF090317:3329257-3332526):434-574	gb|EHJ75389.1|:38-84	72
BmHel-16(DF090317:3329257-3332526):1732-2195	gb|AAN06610.1|AF461149_1:1-346	68.5
BmHel-16(DF090317:3329257-3332526):1063-1188	dbj|BAH66325.1|:116-156	80
BmHel-16(DF090318:5982700-5988156):643-753	dbj|BAH66325.1|:116-152	86
BmHel-16(DF090318:7758037-7765913):1054-3502	gb|EHJ69914.1|:99-730	50.5
BmHel-16(DF090318:7758037-7765913):5481-5780	gb|EFA12534.1|:659-961	37
BmHel-16(DF090319:4621027-4624608):1945-2255	ref|NP_001157648.1|:85-180	50
BmHel-16(DF090319:7099855-7107716):895-1091	dbj|BAH66344.1|:45-107	52.5
BmHel-16(DF090320:7919399-7924507):2973-4497	ref|XP_003242305.1|:115-241	44.5
BmHel-16(DF090320:7919399-7924507):2004-2114	dbj|BAH66325.1|:116-152	83
BmHel-16(DF090322:2828416-2834615):3622-4014	gb|EHJ71270.1|:1-130	79

BmHel-16(DF090322:4029433-4029784):208-303	dbj|BAH66325.1|:121-152	87
BmHel-16(DF090324:4215452-4225156):1050-1514	gb|EFA12068.1|:135-282	26
BmHel-16(DF090324:4215452-4225156):813-1370	gb|EFA08522.1|:112-463	33.5
BmHel-16(DF090325:2694607-2704726):3875-3985	dbj|BAH66325.1|:117-153	83
BmHel-16(DF090325:3562839-3570779):1629-1739	dbj|BAH66325.1|:116-152	86
BmHel-16(DF090329:2463740-2471942):2129-2284	dbj|BAH66363.1|:1-56	71
BmHel-16(DF090329:2463740-2471942):3787-3900	dbj|BAH66325.1|:116-153	81
BmHel-16(DF090329:4897437-4904544):413-1515	gb|EHJ66315.1|:321-721	39
BmHel-16(DF090330:1026079-1035431):439-619	dbj|BAH66363.1|:1-60	51
BmHel-16(DF090330:2748684-2757755):371-1159	ref|NP_001093308.1|:474-546	58
BmHel-16(DF090330:2748684-2757755):4218-8532	gb|EHJ74367.1|:78-402	58.5
BmHel-16(DF090330:2748684-2757755):5347-7614	ref|NP_001087873.1|:137-347	49.5
BmHel-16(DF090330:2836439-2840689):633-2441	ref|XP_002071952.1|:44-168	51.5
BmHel-16(DF090330:2836439-2840689):468-593	ref|XP_002735800.1|:65-106	50
BmHel-16(DF090332:4853041-4853701):65-163	dbj|BAH66325.1|:118-150	90
BmHel-16(DF090332:774512-784102):5796-6814	ref|XP_003093154.1|:97-453	35
BmHel-16(DF090333:4165650-4168469):1204-1623	ref|XP_003248246.1|:1508-1640	32
BmHel-16(DF090334:3090387-3092859):835-1063	gb|EHJ77256.1|:81-153	40.5
BmHel-16(DF090336:1654555-1660155):1437-1958	ref|XP_002159069.1|:633-810	28
BmHel-16(DF090336:3389069-3396865):4918-7618	ref|XP_003744767.1|:37-271	58
BmHel-16(DF090337:1372134-1377631):1723-2684	ref|XP_003264969.1|:341-681	37
BmHel-16(DF090338:1193720-1198106):584-733	gb|EHJ78355.1|:7-56	74
BmHel-16(DF090338:1675963-1685931):1515-1691	gb|EGI63872.1|:51-108	64
BmHel-16(DF090338:1675963-1685931):9234-9401	dbj|BAH66363.1|:1-56	67
BmHel-16(DF090338:2354724-2358595):2716-2844	dbj|BAH66325.1|:111-153	67
BmHel-16(DF090338:4349109-4356571):4817-6244	gb|EKX42347.1|:183-424	39
BmHel-16(DF090339:1591484-1593266):589-707	ref|NP_001157648.1|:211-250	56
BmHel-16(DF090339:1604985-1615038):9346-9531	gb|EFX62931.1|:45-111	41
BmHel-16(DF090339:1604985-1615038):5237-5781	ref|XP_003093476.1|:18-197	38.5
BmHel-16(DF090340:1730484-1740165):2334-2459	dbj|BAH66325.1|:116-156	78
BmHel-16(DF090342:4529207-4531517):1267-1434	dbj|BAH66363.1|:1-56	75
BmHel-16(DF090342:4529207-4531517):804-923	gb|EHJ64304.1|:172-211	72
BmHel-16(DF090343:3210124-3215152):2299-2451	gb|AAO33922.1|:22-72	86
BmHel-16(DF090345:1007855-1011019):1163-1399	ref|XP_003727690.1|:632-712	40
BmHel-16(DF090345:1007855-1011019):1868-2026	gb|EHJ76639.1|:117-169	50
BmHel-16(DF090345:1007855-1011019):1645-2017	ref|XP_002604366.1|:159-282	35
BmHel-16(DF090345:2122886-2129495):468-650	dbj|BAH66325.1|:114-164	59
BmHel-16(DF090345:2122886-2129495):4449-4568	ref|NP_001037023.1|:10-49	90
BmHel-16(DF090345:4347736-4357963):618-9468	gb|EHJ70608.1|:31-171	45
BmHel-16(DF090345:4506514-4515815):7885-7989	dbj|BAH66325.1|:118-152	82
BmHel-16(DF090346:857520-864626):3102-3221	dbj|BAH66325.1|:116-155	75
BmHel-16(DF090346:857520-864626):1259-2325	ref|XP_003092476.1|:493-921	37.5
BmHel-16(DF090346:857520-864626):4220-4603	gb|EIE78900.1|:189-491	36
BmHel-16(DF090347:2193477-2198774):1055-3699	dbj|BAH66363.1|:1-56	63.5
BmHel-16(DF090347:2533093-2541029):1671-4841	ref|XP_001893226.1|:18-304	41
BmHel-16(DF090347:2533093-2541029):5975-6138	dbj|BAH66344.1|:45-96	68
BmHel-16(DF090347:2945307-2951369):470-1683	ref|NP_001040259.1|:160-272	69.5
BmHel-16(DF090348:1547392-1554237):4862-5019	dbj|BAH66344.1|:45-94	62

BmHel-16(DF090348:1547392-1554237):1493-1624	gb|EHJ66271.1|:11-57	76
BmHel-16(DF090349:4178239-4181090):504-2588	gb|EHJ67005.1|:1-112	58.5
BmHel-16(DF090349:781406-783056):1181-1330	gb|EHJ78508.1|:5-54	96
BmHel-16(DF090352:1309604-1311520):1357-1791	ref|XP_003731244.1|:20-169	25
BmHel-16(DF090352:3755044-3761596):615-1979	gb|EHJ74301.1|:975-1129	64
BmHel-16(DF090352:3755044-3761596):105-338	emb|CAX36785.1|:135-212	43
BmHel-16(DF090352:3755044-3761596):4568-4994	ref|XP_003247334.1|:189-332	36
BmHel-16(DF090353:2207111-2208925):848-973	dbj|BAH66325.1|:115-156	71
BmHel-16(DF090355:1003047-1008502):3098-3346	ref|NP_001089923.1|:707-786	43
BmHel-16(DF090357:1369233-1378655):5960-6094	dbj|BAH66363.1|:16-60	73
BmHel-16(DF090357:1369233-1378655):2743-2869	gb|AAO33922.1|:23-66	66
BmHel-16(DF090358:1591183-1596466):3042-4699	dbj|BAH66325.1|:99-153	51
BmHel-16(DF090358:2632903-2641878):2697-2858	dbj|BAA33570.1|:156-209	83
BmHel-16(DF090358:2632903-2641878):6108-6701	ref|XP_002741128.1|:1-132	36.5
BmHel-16(DF090358:3412773-3420615):530-4256	ref|XP_001648445.1|:640-892	44.5
BmHel-16(DF090358:861190-864705):878-1065	dbj|BAH66344.1|:31-96	48
BmHel-16(DF090360:1834075-1839981):1399-1562	dbj|BAH66344.1|:45-96	53.5
BmHel-16(DF090360:2370022-2378521):636-799	dbj|BAH66344.1|:45-96	68
BmHel-16(DF090360:577450-582854):568-3822	dbj|BAM19485.1|:24-222	56
BmHel-16(DF090362:1001554-1004561):1061-1870	gb|AAT40504.2|:560-731	35.5
BmHel-16(DF090363:1016066-1018178):1229-1391	dbj|BAH66363.1|:2-56	64
BmHel-16(DF090363:1529226-1537964):1127-1370	ref|XP_002075098.1|:1-254	44.5
BmHel-16(DF090365:984146-993990):681-848	ref|XP_002735800.1|:51-106	51
BmHel-16(DF090365:984146-993990):2587-4602	dbj|BAH66363.1|:1-56	58.5
BmHel-16(DF090366:432884-441413):6493-6615	dbj|BAH66325.1|:117-156	82
BmHel-16(DF090366:432884-441413):4092-5534	ref|XP_003247261.1|:97-236	44
BmHel-16(DF090366:432884-441413):2499-2659	dbj|BAH66344.1|:45-95	53.5
BmHel-16(DF090369:2691667-2692334):216-379	dbj|BAH66344.1|:45-96	55
BmHel-16(DF090374:1644857-1645824):314-468	gb|EGI67902.1|:109-158	54
BmHel-16(DF090377:1843233-1846736):2279-2632	gb|EHJ70971.1|:2-128	44
BmHel-16(DF090378:1385335-1394506):1662-1823	gb|EHJ75926.1|:2-55	66
BmHel-16(DF090378:1385335-1394506):7362-7505	gb|AAO33922.1|:16-63	83
BmHel-16(DF090382:2097958-2100547):329-406	gb|EHJ63491.1|:2月27日	96
BmHel-16(DF090384:1925814-1930442):3015-3146	dbj|BAH66363.1|:13-56	75
BmHel-16(DF090387:1396973-1399458):741-1001	gb|EHJ69309.1|:1025-1110	55
BmHel-16(DF090387:1396973-1399458):1319-1461	gb|AAO33922.1|:18-67	63
BmHel-16(DF090388:1134997-1139700):506-643	dbj|BAH66363.1|:11-56	76
BmHel-16(DF090389:1795692-1798251):1710-1865	gb|EHJ67286.1|:260-311	51
BmHel-16(DF090389:1795692-1798251):781-990	emb|CAL25134.1|:7-78	37
BmHel-16(DF090391:1129879-1138507):5824-5944	dbj|BAH66325.1|:114-152	63.5
BmHel-16(DF090393:899894-908549):2859-8043	ref|XP_003704446.1|:172-389	47.5
BmHel-16(DF090396:336839-345895):3397-3560	gb|EFN69608.1|:1-56	62.5
BmHel-16(DF090401:515874-523944):6765-6928	dbj|BAH66344.1|:45-96	55
BmHel-16(DF090401:515874-523944):6291-6410	dbj|BAH66325.1|:118-156	85
BmHel-16(DF090401:515874-523944):6105-6359	dbj|BAM73877.1|:19-103	100
BmHel-16(DF090401:52805-54620):1134-1435	tpe|CAJ00236.1|:880-980	42
BmHel-16(DF090402:993291-1003260):2507-2665	dbj|BAH66363.1|:1-53	83
BmHel-16(DF090407:613093-623006):1370-1501	ref|NP_001157648.1|:209-250	77

BmHel-16(DF090407:613093-623006):9456-9575	dbj|BAH66325.1|:114-153	72
BmHel-16(DF090411:584200-591807):500-810	ref|NP_001157648.1|:85-180	49
BmHel-16(DF090412:1280803-1285096):3333-3482	dbj|BAH66325.1|:115-162	68
BmHel-16(DF090412:1280803-1285096):445-627	dbj|BAH66363.1|:13-73	57
BmHel-16(DF090412:1280803-1285096):1304-2419	ref|XP_003093476.1|:19-385	36
BmHel-16(DF090413:738345-742725):918-1091	gb|AAO33922.1|:20-77	84
BmHel-16(DF090414:349426-358753):2999-5844	emb|CCK70872.1|:276-457	46
BmHel-16(DF090414:349426-358753):1128-1291	dbj|BAH66344.1|:45-96	65.5
BmHel-16(DF090422:316613-323086):3111-3221	dbj|BAH66325.1|:116-152	83
BmHel-16(DF090422:316613-323086):1486-2775	dbj|BAH66363.1|:1-60	59
BmHel-16(DF090424:853801-862766):1574-5427	emb|CAB37562.1|:47-206	45
BmHel-16(DF090428:709025-712080):194-430	dbj|BAM73877.1|:15-95	46
BmHel-16(DF090441:390251-397207):5907-6067	dbj|BAH66363.1|:1-56	64
BmHel-16(DF090441:390251-397207):3784-3909	dbj|BAH66325.1|:116-156	85
BmHel-16(DF090443:470180-472585):899-1074	ref|NP_001157648.1|:196-250	52
BmHel-16(DF090472:111991-120989):2052-6250	gb|EFX76394.1|:834-1150	38
BmHel-16(DF090483:6494-10042):2564-2803	gb|EFA13284.1|:186-265	30
BmHel-16(DF090580:11135-13474):561-737	gb|AAO33922.1|:18-76	86
BmHel-16(DF090611:30956-31629):355-518	dbj|BAH66344.1|:45-96	68
BmHel-16(DF090717:15132-19908):2135-2500	emb|CAX36785.1|:91-212	54
BmHel-16(DF090756:2708-9846):1993-2249	dbj|BAH66344.1|:45-125	55
BmHel-16(DF090807:5879-13982):2427-2588	dbj|BAH66363.1|:1-60	61
BmHel-16(DF090807:5879-13982):3308-4149	gb|AAN06610.1|AF461149_1:1-339	54.5
BmHel-16(DF090807:5879-13982):4752-4880	dbj|BAM73877.1|:61-103	76
BmHel-18(BABH01042926:271-632):87-188	dbj|BAH66325.1|:119-152	88
BmHel-18(DF090316:11800178-11804121):1407-1570	dbj|BAH66344.1|:45-96	55
BmHel-18(DF090318:7843811-7845184):215-367	emb|CAX36785.1|:162-212	45
BmHel-18(DF090319:4269852-4275965):2884-3030	ref|XP_002735800.1|:58-106	55
BmHel-18(DF090319:4622493-4624608):479-789	ref|NP_001157648.1|:85-180	48
BmHel-18(DF090320:8093086-8096691):977-1164	dbj|BAH66344.1|:31-96	60
BmHel-18(DF090320:8093086-8096691):520-2130	ref|XP_003965775.1|:795-939	58.5
BmHel-18(DF090325:5380232-5380805):382-525	gb|AAO33922.1|:22-77	76
BmHel-18(DF090325:5695823-5705767):2565-2707	dbj|BAH66363.1|:1-48	58.5
BmHel-18(DF090328:1065691-1067838):372-542	gb|AAO33922.1|:9-65	66
BmHel-18(DF090333:1472591-1479995):611-6851	ref|XP_004093175.1|:511-1072	35.5
BmHel-18(DF090336:5227284-5227823):19-314	ref|NP_001157648.1|:90-180	49
BmHel-18(DF090338:1489115-1493483):1707-1835	ref|NP_001157648.1|:208-250	72
BmHel-18(DF090338:1599157-1599708):370-540	gb|AAO33922.1|:9-65	68
BmHel-18(DF090339:560653-565898):964-1119	gb|AAO33922.1|:22-73	84
BmHel-18(DF090340:1946379-1954459):4858-6910	dbj|BAH66325.1|:108-153	56.5
BmHel-18(DF090341:4055247-4055716):146-258	ref|NP_001157648.1|:213-250	50
BmHel-18(DF090342:139507-140012):290-457	gb|AAO33922.1|:22-77	76
BmHel-18(DF090342:4232782-4233261):198-310	ref|NP_001157648.1|:213-250	68.5
BmHel-18(DF090344:3565389-3565745):155-262	dbj|BAH66325.1|:117-152	83
BmHel-18(DF090345:1992536-1996976):1973-2137	gb|AAO33922.1|:13-68	67
BmHel-18(DF090345:3915195-3915701):293-460	gb|AAO33922.1|:22-77	89
BmHel-18(DF090347:907168-907669):70-390	ref|XP_002159661.1|:94-194	27
BmHel-18(DF090348:2164651-2165162):290-457	gb|AAO33922.1|:22-77	89

BmHel-18(DF090348:521774-528462):776-1222	gb|EFA13225.1|:6-147	31
BmHel-18(DF090349:339273-339827):66-233	dbj|BAH66363.1|:1-56	76
BmHel-18(DF090355:2582484-2584995):410-634	dbj|BAB21761.1|:1-75	84
BmHel-18(DF090356:525987-526502):82-351	ref|NP_001183823.1|:117-207	36
BmHel-18(DF090357:2264646-2265471):392-814	ref|XP_003115364.1|:886-1030	27
BmHel-18(DF090359:3080639-3081108):211-323	ref|NP_001157648.1|:213-250	59
BmHel-18(DF090362:1997359-1998427):290-465	gb|AAO33922.1|:20-77	67.5
BmHel-18(DF090362:2087054-2088958):540-1052	gb|EFA11941.1|:364-2481	36
BmHel-18(DF090366:861841-862597):53-751	ref|XP_003763597.1|:640-1227	35.5
BmHel-18(DF090367:1379510-1380319):331-761	gb|AAO33922.1|:22-68	63.5
BmHel-18(DF090368:1411428-1411981):151-263	ref|NP_001157648.1|:213-250	71
BmHel-18(DF090368:1411428-1411981):271-351	dbj|BAH66325.1|:122-148	88
BmHel-18(DF090371:308062-308567):290-457	gb|AAO33922.1|:22-77	91
BmHel-18(DF090373:2053430-2055377):1040-1317	ref|NP_001157648.1|:96-180	53.5
BmHel-18(DF090377:1870582-1871076):142-306	gb|AAO33922.1|:22-76	87
BmHel-18(DF090390:228674-229090):143-346	ref|YP_001650753.1|:32-99	52
BmHel-18(DF090390:295968-298784):551-1105	ref|XP_002167994.1|:14-205	27
BmHel-18(DF090401:1270094-1271713):197-964	ref|XP_001201605.2|:463-905	36.5
BmHel-18(DF090401:1270094-1271713):515-1012	gb|EFA11941.1|:376-2488	32
BmHel-18(DF090409:807351-808088):142-291	gb|AAO33922.1|:22-71	70
BmHel-18(DF090427:921476-922152):268-431	dbj|BAH66344.1|:45-96	53.5
BmHel-18(DF090428:3344-5509):991-1616	gb|EHJ67097.1|:45-257	38
BmHel-18(DF090428:3344-5509):225-530	ref|ZP_00373035.1|:1-108	37
BmHel-18(DF090436:471313-473113):133-489	ref|XP_003243973.1|:124-708	36.5
BmHel-18(DF090442:272865-274205):414-518	ref|XP_003249026.1|:175-209	57
BmHel-18(DF090442:431778-432862):50-162	ref|NP_001157648.1|:213-250	71
BmHel-18(DF090442:431778-432862):214-377	dbj|BAH66344.1|:45-96	65.5
BmHel-18(DF090446:282644-282989):131-244	dbj|BAH66325.1|:118-156	82
BmHel-18(DF090451:65117-67324):1040-1209	gb|AAO33922.1|:13-77	63.5
BmHel-18(DF090456:226060-231932):1488-1613	gb|EHJ64726.1|:1-42	54
BmHel-18(DF090476:32954-38251):788-951	dbj|BAH66344.1|:45-96	55
BmHel-18(DF090476:32954-38251):604-737	ref|NP_001157648.1|:207-250	56
BmHel-18(DF090547:65848-66854):800-913	emb|CAX36785.1|:14-51	47
BmHel-18(DF090579:54087-54414):47-151	dbj|BAH66325.1|:116-150	94
BmHel-18(DF090626:23848-26718):1272-1760	ref|NP_001107265.2|:165-318	29
BmHel-18(DF091074:4008-5555):163-315	ref|XP_003098078.1|:542-592	45
BmHel-19(DF090316:9611109-9613270):1196-1808	gb|EHJ73189.1|:1-109	41
BmHel-19(DF090316:9611109-9613270):1440-1863	ref|NP_001108468.1|:1-91	42
BmHel-19(DF090322:4446607-4448064):1127-1344	ref|XP_003738409.1|:38-106	42
BmHel-19(DF090322:4446607-4448064):850-1063	ref|NP_001108468.1|:1-77	51
BmHel-19(DF090322:4458698-4461159):1937-2130	ref|XP_002437537.1|:27-102	38
BmHel-19(DF090322:4458698-4461159):680-1770	ref|XP_002162585.1|:70-279	37.5
BmHel-19(DF090323:666497-668687):1329-1902	ref|NP_001108468.1|:1-91	64.5
BmHel-19(DF090410:437057-439105):976-1088	ref|NP_001157648.1|:213-250	68.5
BmHel-19(DF090410:437057-439105):1713-1918	ref|XP_003243636.1|:3-67	46
BmHel-19(DF090410:437057-439105):1279-1475	dbj|BAH66344.1|:45-107	54
BmHel-19(DF090410:437057-439105):594-967	gb|EHJ73189.1|:1-121	39.5
BmHel-19(DF090430:469795-471273):1154-1267	ref|XP_003248684.1|:24-61	50

BmHel-19(DF090430:469795-471273):625-963	gb|EHJ73189.1|:1-109	38
BmHel-19(DF090434:350221-352168):982-1296	gb|EHJ73189.1|:1-101	41
BmHel-19(DF090434:350221-352168):1455-1672	ref|XP_003326454.1|:202-271	38.5
BmHel-19(DF090574:50280-51161):547-767	ref|XP_003248684.1|:24-93	48
BmHel-19(DF090589:38478-40511):1698-1888	ref|XP_003747365.1|:38-97	46
BmHel-19(DF090589:38478-40511):1264-1460	dbj|BAH66344.1|:45-107	53
BmHel-19(DF090589:38478-40511):961-1073	ref|NP_001157648.1|:213-250	53
BmHel-19(DF090589:38478-40511):582-952	gb|EHJ73189.1|:2-121	39
BmHel-2(DF090344:2746800-2748809):597-713	ref|XP_002455091.1|:77-123	57
BmHel-2(DF090345:2703166-2705458):360-563	emb|CAB63155.1|:230-366	46.5
BmHel-2(DF090352:968456-970820):447-668	emb|CAB63155.1|:224-366	43.5
BmHel-2(DF091741:565-2522):456-692	gb|EJK56760.1|:76-160	34
BmHel-21(DF090342:3798722-3800915):426-1386	dbj|BAA92690.1|:247-587	41
BmHel-21(DF090478:54336-55641):304-465	dbj|BAH66363.1|:1-54	74
BmHel-3a(DF090316:11439612-11441399):712-915	ref|XP_003325915.1|:225-292	32
BmHel-3a(DF090316:11439612-11441399):425-846	ref|XP_003090903.1|:1777-2377	36.5
BmHel-3a(DF090316:13948236-13950204):469-720	gb|EFN69710.1|:78-155	38
BmHel-3a(DF090316:13948236-13950204):460-1025	gb|EGT52382.1|:1668-2607	48.5
BmHel-3a(DF090316:4119984-4124912):1909-2493	gb|EFA09320.1|:95-279	27
BmHel-3a(DF090316:8635134-8637058):330-668	ref|XP_003742376.1|:320-418	30
BmHel-3a(DF090317:2907116-2907828):316-428	ref|NP_001157648.1|:213-250	56
BmHel-3a(DF090317:5399272-5401256):908-1567	ref|XP_003402035.1|:1396-1820	42.5
BmHel-3a(DF090319:2585970-2587461):438-659	emb|CAB63155.1|:210-354	44
BmHel-3a(DF090319:3669253-3671026):446-891	ref|XP_003335986.1|:399-891	37.5
BmHel-3a(DF090321:2586031-2587792):555-866	emb|CAN60263.1|:1141-1837	39.5
BmHel-3a(DF090321:2586031-2587792):908-1111	gb|EHJ69627.1|:59-127	60
BmHel-3a(DF090322:8096964-8099097):488-1058	ref|XP_003335986.1|:400-944	36.5
BmHel-3a(DF090325:6014746-6016410):689-1182	ref|XP_003331018.2|:953-1135	32.5
BmHel-3a(DF090326:5509926-5511190):504-728	gb|EGT52382.1|:2520-2607	52
BmHel-3a(DF090326:6038773-6040978):482-643	gb|ABA93594.1|:432-482	38
BmHel-3a(DF090327:2875507-2877363):491-1190	ref|XP_003092647.1|:466-1158	34.5
BmHel-3a(DF090327:5003663-5005764):1012-1174	dbj|BAH66344.1|:45-96	64
BmHel-3a(DF090329:1155820-1157616):494-774	gb|EIW71217.1|:377-463	44
BmHel-3a(DF090329:3602746-3604070):804-947	dbj|BAH66325.1|:107-152	77
BmHel-3a(DF090331:5411091-5418355):4687-5772	ref|XP_003242682.1|:84-344	36
BmHel-3a(DF090331:5411091-5418355):2834-3364	gb|EHJ70308.1|:1-117	32
BmHel-3a(DF090336:2078193-2079755):787-950	dbj|BAH66344.1|:45-96	68
BmHel-3a(DF090341:125681-127722):707-870	dbj|BAH66344.1|:45-96	63
BmHel-3a(DF090341:2158988-2160585):350-626	ref|XP_002457854.1|:1-94	38.5
BmHel-3a(DF090341:582653-584628):485-807	gb|EFN84419.1|:8-108	38.5
BmHel-3a(DF090342:4346927-4348762):898-1076	dbj|BAH66363.1|:1-60	51
BmHel-3a(DF090343:1789958-1791253):668-1027	gb|EIE78900.1|:195-491	36
BmHel-3a(DF090347:3485477-3487150):1153-1365	emb|CAB63155.1|:214-354	44
BmHel-3a(DF090347:809442-813121):485-1718	ref|XP_003096407.1|:1744-2664	37
BmHel-3a(DF090350:2496479-2497581):492-863	gb|AEM44817.1|:278-400	34
BmHel-3a(DF090353:1834500-1836759):352-615	ref|XP_003615332.1|:152-248	36
BmHel-3a(DF090354:3202461-3203119):144-299	gb|AAO33922.1|:7-64	56
BmHel-3a(DF090355:1749986-1755656):475-1397	ref|XP_003335986.1|:399-1059	33.5

BmHel-3a(DF090358:1688071-1689637):1200-1363	dbj|BAH66344.1|:45-96	55
BmHel-3a(DF090358:2476172-2478934):455-622	dbj|BAH66363.1|:1-56	75
BmHel-3a(DF090360:682176-683728):240-602	gb|EFA12786.1|:271-390	31
BmHel-3a(DF090362:2751754-2752925):347-595	ref|XP_002437574.1|:31-122	40
BmHel-3a(DF090363:77242-77900):225-341	ref|NP_001157648.1|:213-249	71
BmHel-3a(DF090366:1721503-1722031):307-417	dbj|BAH66325.1|:118-154	78
BmHel-3a(DF090366:2186553-2188547):846-1319	gb|JAA54880.1|:293-449	24
BmHel-3a(DF090372:1765213-1771066):2208-2572	ref|XP_003402035.1|:1476-1599	40.5
BmHel-3a(DF090372:1765213-1771066):979-1503	ref|XP_002159375.1|:129-307	26
BmHel-3a(DF090378:696977-698023):252-458	ref|ZP_00373035.1|:2-76	32
BmHel-3a(DF090379:418921-420960):1324-1461	dbj|BAH66363.1|:11-56	73
BmHel-3a(DF090379:945633-948047):473-1662	ref|XP_003110294.1|:1743-2663	35.5
BmHel-3a(DF090384:1077500-1079576):426-555	ref|XP_003248684.1|:49-92	52
BmHel-3a(DF090385:1517738-1519989):503-1021	ref|XP_003326454.1|:15-253	25
BmHel-3a(DF090394:572364-574044):520-656	ref|XP_003249039.1|:61-106	51.5
BmHel-3a(DF090397:1254813-1260820):2322-5130	ref|XP_003096407.1|:1472-2664	34.5
BmHel-3a(DF090397:1254813-1260820):1420-4624	gb|EGT50892.1|:881-2426	34.5
BmHel-3a(DF090412:480439-486379):939-1141	dbj|BAH66344.1|:45-108	53.5
BmHel-3a(DF090412:480439-486379):1842-2450	ref|XP_003739482.1|:725-942	26
BmHel-3a(DF090413:1030418-1032939):973-1494	ref|NP_001108468.1|:97-234	40
BmHel-3a(DF090433:48530-50650):479-781	ref|XP_003329956.1|:734-841	34
BmHel-3a(DF090502:46944-49302):507-1455	ref|XP_001201605.2|:367-950	38.5
BmHel-3b(DF090316:11439612-11441399):425-846	ref|XP_003335986.1|:407-891	37
BmHel-3b(DF090316:13948236-13950204):460-1025	gb|EGT52382.1|:1668-2607	36
BmHel-3b(DF090316:4119984-4124912):1909-2493	gb|EFA09320.1|:95-279	27
BmHel-3b(DF090316:8635134-8637058):330-668	ref|XP_003742376.1|:320-418	30
BmHel-3b(DF090317:2907116-2907828):316-428	ref|NP_001157648.1|:213-250	56
BmHel-3b(DF090317:5399272-5401256):908-1567	ref|XP_003402035.1|:1396-1820	42.5
BmHel-3b(DF090319:2585970-2587461):438-659	emb|CAB63155.1|:210-354	44
BmHel-3b(DF090319:3669253-3671026):443-906	ref|XP_003336762.1|:10-396	39
BmHel-3b(DF090321:2586031-2587792):555-866	emb|CAN60263.1|:1141-1837	41
BmHel-3b(DF090321:2586031-2587792):908-1111	gb|EHJ69627.1|:59-127	60
BmHel-3b(DF090322:8096964-8099097):488-754	ref|XP_003097907.1|:1759-1848	39
BmHel-3b(DF090325:6014746-6016410):653-1176	ref|XP_003330494.1|:166-354	33.5
BmHel-3b(DF090326:5509926-5511190):504-728	gb|EGT52382.1|:2520-2607	52
BmHel-3b(DF090326:6038773-6040978):482-643	gb|ABA93594.1|:432-482	38
BmHel-3b(DF090327:2875507-2877363):497-1187	ref|XP_003090903.1|:1772-2461	35
BmHel-3b(DF090327:5003663-5005764):1012-1174	dbj|BAH66344.1|:45-96	52
BmHel-3b(DF090329:1155820-1157616):494-774	gb|EIW71217.1|:377-463	44
BmHel-3b(DF090329:3602746-3604070):804-947	dbj|BAH66325.1|:107-152	77
BmHel-3b(DF090331:5411091-5418355):2834-3364	gb|EHJ70308.1|:1-117	32
BmHel-3b(DF090331:5411091-5418355):4687-5772	ref|XP_003242682.1|:84-344	36
BmHel-3b(DF090336:2078193-2079755):787-950	dbj|BAH66344.1|:45-96	55
BmHel-3b(DF090341:125681-127722):707-870	dbj|BAH66344.1|:45-96	53.5
BmHel-3b(DF090341:582653-584628):479-1141	ref|XP_003323090.1|:519-1048	36.5
BmHel-3b(DF090342:4346927-4348762):898-1076	dbj|BAH66363.1|:1-60	51
BmHel-3b(DF090347:3485477-3487150):1153-1365	emb|CAB63155.1|:214-354	43
BmHel-3b(DF090347:809442-813121):485-1718	ref|XP_003110294.1|:1743-2663	37

BmHel-3b(DF090350:2496479-2497581):492-863	gb|AEM44817.1|:278-400	34
BmHel-3b(DF090353:1834500-1836759):352-615	ref|XP_003615332.1|:152-248	36
BmHel-3b(DF090354:3202461-3203119):144-299	gb|AAO33922.1|:7-64	56
BmHel-3b(DF090355:1749986-1755656):463-783	ref|XP_003742376.1|:320-418	39
BmHel-3b(DF090358:1688071-1689637):1200-1363	dbj|BAH66344.1|:45-96	65.5
BmHel-3b(DF090358:2476172-2478934):455-622	dbj|BAH66363.1|:1-56	75
BmHel-3b(DF090362:2751754-2752925):347-595	ref|XP_002437574.1|:31-122	40
BmHel-3b(DF090363:77242-77900):225-341	ref|NP_001157648.1|:213-249	71
BmHel-3b(DF090366:2186553-2188547):846-1319	gb|JAA54880.1|:293-449	24
BmHel-3b(DF090372:1765213-1771066):979-1503	ref|XP_002159375.1|:129-307	26
BmHel-3b(DF090372:1765213-1771066):2148-2566	gb|EHJ66095.1|:211-351	42.5
BmHel-3b(DF090378:696977-698023):252-458	ref|ZP_00373035.1|:2-76	32
BmHel-3b(DF090379:418921-420960):1324-1461	dbj|BAH66363.1|:11-56	73
BmHel-3b(DF090379:945633-948047):452-1662	gb|EGT50893.1|:1712-2651	36.5
BmHel-3b(DF090384:1077500-1079576):426-555	ref|XP_003249039.1|:63-106	54.5
BmHel-3b(DF090385:1517738-1519989):521-1030	emb|CAB63155.1|:93-354	35.5
BmHel-3b(DF090394:572364-574044):520-656	ref|XP_003248684.1|:47-92	53.5
BmHel-3b(DF090397:1254813-1260820):1420-5130	gb|EGT50893.1|:882-2651	34
BmHel-3b(DF090412:480439-486379):939-1141	dbj|BAH66344.1|:45-108	53.5
BmHel-3b(DF090412:480439-486379):1842-2450	ref|XP_003739482.1|:725-942	26
BmHel-3b(DF090413:1030418-1032939):973-1494	ref|NP_001108468.1|:97-234	40
BmHel-4(DF090322:3920779-3922460):322-858	ref|XP_001967311.1|:50-212	29
BmHel-4(DF090322:3920779-3922460):118-299	ref|NP_001108468.1|:95-152	56
BmHel-4(DF090347:1917378-1917867):125-319	ref|XP_002437537.1|:23-102	41
BmHel-4(DF090347:2859232-2859757):349-461	ref|NP_001157648.1|:213-250	56
BmHel-4(DF090356:693660-694173):118-357	gb|EGT43880.1|:404-488	36
BmHel-4(DF090363:1654339-1654814):77-305	ref|XP_003747365.1|:27-106	48
BmHel-4(DF090364:265484-275413):1056-1627	gb|EGT43880.1|:254-446	38.5
BmHel-5(DF090317:8729528-8734073):1014-3849	ref|XP_003097026.1|:306-1379	35
BmHel-5(DF090329:209059-215754):520-651	ref|XP_003738409.1|:63-106	75
BmHel-5(DF090336:159125-162245):2335-2656	ref|XP_003747365.1|:1-107	56
BmHel-5(DF090336:159125-162245):1595-2255	emb|CAN71277.1|:141-345	39
BmHel-5(DF090336:159125-162245):500-1584	ref|XP_003097907.1|:1677-2125	36
BmHel-5(DF090349:3096206-3099716):1230-2822	gb|EGT52382.1|:1404-2022	26
BmHel-5(DF090360:118462-122186):1461-1817	ref|XP_003110294.1|:2540-2676	35
BmHel-5(DF090360:662349-666572):586-3093	ref|XP_003091759.1|:394-1139	33
BmHel-7(DF090316:1427109-1427498):100-225	dbj|BAH66325.1|:111-152	69
BmHel-7(DF090316:5215611-5216422):176-610	gb|EFA13462.1|:534-678	36
BmHel-7(DF090317:7103724-7107872):462-602	dbj|BAA74713.1|:1-47	97
BmHel-7(DF090318:2256449-2256825):66-200	dbj|BAH66325.1|:116-160	75
BmHel-7(DF090319:3417314-3417684):64-190	dbj|BAH66325.1|:116-156	66
BmHel-7(DF090320:2232385-2232760):94-201	dbj|BAH66325.1|:116-152	81
BmHel-7(DF090322:1337263-1338528):314-670	gb|EHJ67097.1|:144-270	29
BmHel-7(DF090322:7519991-7526188):221-1811	ref|XP_003706662.1|:166-391	45
BmHel-7(DF090323:6777287-6778950):181-937	dbj|BAB21761.1|:588-923	57
BmHel-7(DF090324:1382593-1383043):53-165	ref|NP_001157648.1|:213-250	71
BmHel-7(DF090324:23553-24280):394-557	dbj|BAH66344.1|:45-96	68
BmHel-7(DF090326:6654619-6655883):171-903	sp|P10394|POL4_DROME:57-298	35

BmHel-7(DF090328:1074868-1075240):34-144	dbj|BAH66325.1|:116-152	89
BmHel-7(DF090330:1744790-1746339):951-1133	ref|XP_001999756.1|:252-350	46
BmHel-7(DF090331:3082334-3091408):7386-8114	gb|EFA11778.1|:690-905	36
BmHel-7(DF090334:4937636-4938319):332-495	dbj|BAH66344.1|:45-96	68
BmHel-7(DF090334:4937636-4938319):541-668	ref|NP_001157648.1|:207-249	62
BmHel-7(DF090340:1866704-1867204):49-168	dbj|BAH66363.1|:1-40	87
BmHel-7(DF090340:3354187-3354780):76-198	dbj|BAH66325.1|:117-156	73
BmHel-7(DF090340:3354187-3354780):130-468	emb|CAX36785.1|:105-212	45
BmHel-7(DF090342:3524452-3526064):264-719	gb|EFA11941.1|:512-2602	34
BmHel-7(DF090343:1614731-1615313):187-299	ref|NP_001157648.1|:213-250	53
BmHel-7(DF090343:1614731-1615313):352-512	dbj|BAH66344.1|:45-95	65
BmHel-7(DF090344:1344072-1344415):94-198	dbj|BAH66325.1|:118-152	91
BmHel-7(DF090347:1152553-1158976):3138-3329	gb|AAO33922.1|:7-70	67
BmHel-7(DF090347:1152553-1158976):1306-1422	dbj|BAH66325.1|:115-153	87
BmHel-7(DF090347:367104-370766):1124-1285	gb|ACY06938.1|:18-71	94
BmHel-7(DF090347:367104-370766):657-1087	emb|CAA32198.1|:202-326	43.5
BmHel-7(DF090356:1724260-1724954):96-221	dbj|BAH66325.1|:116-156	73
BmHel-7(DF090357:312470-315711):565-2496	ref|XP_003240988.1|:145-729	31
BmHel-7(DF090358:969716-972537):261-899	ref|XP_001983026.1|:57-254	25
BmHel-7(DF090359:2987912-2988356):36-155	ref|NP_001157648.1|:213-250	72
BmHel-7(DF090367:1939042-1949140):8142-8309	dbj|BAH66363.1|:1-56	66
BmHel-7(DF090367:969577-970267):330-493	dbj|BAH66344.1|:45-96	53.5
BmHel-7(DF090371:320585-321082):103-315	emb|CAX36785.1|:147-212	47
BmHel-7(DF090371:730616-731038):153-248	dbj|BAH66325.1|:122-153	78
BmHel-7(DF090382:1711467-1711872):167-295	dbj|BAH66325.1|:110-152	72
BmHel-7(DF090382:1796469-1797182):207-370	dbj|BAH66344.1|:45-96	68
BmHel-7(DF090384:1127495-1127875):44-154	dbj|BAH66325.1|:116-152	91
BmHel-7(DF090395:136053-137438):182-1171	ref|XP_003725312.1|:505-863	26
BmHel-7(DF090404:508289-508940):205-330	dbj|BAH66363.1|:15-56	69
BmHel-7(DF090406:1558565-1558931):208-324	dbj|BAH66325.1|:115-153	76
BmHel-7(DF090414:286308-290133):551-1098	gb|AAN06610.1|AF461149_1:1-346	70
BmHel-7(DF090414:286308-290133):1480-2188	gb|EHJ76019.1|:66-157	49.5
BmHel-7(DF090422:328164-330540):442-1035	ref|XP_001842594.1|:495-694	25
BmHel-7(DF090422:328164-330540):103-413	ref|NP_001157648.1|:85-180	49
BmHel-7(DF090423:120660-121047):53-154	dbj|BAH66325.1|:119-152	91
BmHel-7(DF090441:549112-549490):61-171	dbj|BAH66325.1|:118-154	70
BmHel-7(DF090446:392228-392563):97-234	dbj|BAH66325.1|:116-161	65
BmHel-7(DF091311:1966-2619):317-480	dbj|BAH66344.1|:45-96	65.5
BmHel-8(DF090316:2503678-2505748):1056-1280	ref|XP_002135939.1|:132-675	39.5
BmHel-8(DF090316:9843089-9845415):242-361	dbj|BAH66325.1|:118-156	82
BmHel-8(DF090316:9843089-9845415):353-1369	ref|XP_003089877.1|:503-1134	35.5
BmHel-8(DF090317:2446680-2454265):3855-4070	ref|XP_003089877.1|:1064-1134	32
BmHel-8(DF090317:2446680-2454265):2921-3863	dbj|BAH66325.1|:116-166	54.5
BmHel-8(DF090318:5212361-5220287):5458-5831	gb|AAF61856.1|AF189221_1:69-181	37
BmHel-8(DF090318:5357865-5360913):456-560	dbj|BAH66325.1|:118-152	82
BmHel-8(DF090318:7469530-7475453):1466-2622	gb|EHJ78696.1|:50-186	37.5
BmHel-8(DF090318:7469530-7475453):2770-3050	gb|EHJ73186.1|:1-94	46
BmHel-8(DF090319:3898659-3900745):631-1038	gb|EFA11941.1|:520-2591	38.5

BmHel-8(DF090319:5078658-5081688):440-691	gb|EFA12014.1|:315-395	26
BmHel-8(DF090319:5078658-5081688):629-1847	emb|CCD14323.1|:48-391	34.5
BmHel-8(DF090320:1559838-1561573):532-720	ref|XP_001225468.1|:268-337	45
BmHel-8(DF090320:1604276-1610841):837-1000	dbj|BAH66344.1|:45-96	53.5
BmHel-8(DF090320:1604276-1610841):107-211	dbj|BAH66325.1|:118-152	88
BmHel-8(DF090321:4325562-4335145):1518-8573	gb|AAO33922.1|:17-77	63.5
BmHel-8(DF090321:572229-575131):1910-2125	gb|EHJ68990.1|:97-168	51
BmHel-8(DF090321:572229-575131):2663-2811	dbj|BAH66344.1|:45-91	55
BmHel-8(DF090321:572229-575131):419-586	gb|AAO33922.1|:22-76	92
BmHel-8(DF090322:1692177-1702273):6332-6451	gb|AAO33922.1|:13-52	75
BmHel-8(DF090322:2131916-2133903):46-879	gb|AAN87272.1|:595-852	29
BmHel-8(DF090322:4503333-4508316):4547-4731	dbj|BAH66344.1|:45-103	55.5
BmHel-8(DF090322:5360085-5363455):1192-1496	gb|AAN06610.1|AF461149_1:1-346	53.5
BmHel-8(DF090322:7596542-7597253):487-636	gb|AAO33922.1|:18-67	60
BmHel-8(DF090323:4437511-4438853):135-302	gb|AAO33922.1|:22-77	58
BmHel-8(DF090323:465961-467152):166-483	ref|ZP_00373035.1|:1-108	36
BmHel-8(DF090323:5706353-5707204):262-425	dbj|BAH66344.1|:45-96	55
BmHel-8(DF090324:1431358-1434715):1157-1824	dbj|BAH66363.1|:1-60	66.5
BmHel-8(DF090324:4903966-4908332):703-876	ref|NP_001108468.1|:118-177	56
BmHel-8(DF090324:5515902-5517612):1209-1558	ref|XP_003738758.1|:1-116	34.5
BmHel-8(DF090325:3437133-3439323):234-428	gb|AAO33922.1|:7-65	69
BmHel-8(DF090325:3515637-3524712):751-6008	gb|EIE87815.1|:47-340	39.5
BmHel-8(DF090325:3515637-3524712):8634-8764	ref|NP_001157648.1|:209-250	62
BmHel-8(DF090325:3515637-3524712):7694-7885	gb|AAO33922.1|:13-76	73
BmHel-8(DF090325:4342532-4344332):476-1317	ref|XP_002162585.1|:44-336	37.5
BmHel-8(DF090325:4461195-4463037):637-1218	gb|EFA11778.1|:660-881	34
BmHel-8(DF090325:5182051-5182844):199-491	ref|NP_001157648.1|:91-180	49
BmHel-8(DF090326:6027414-6029720):273-773	gb|EFA09320.1|:538-707	30
BmHel-8(DF090326:6305701-6306472):230-501	ref|NP_001157648.1|:98-180	48
BmHel-8(DF090327:416184-425260):6762-6941	dbj|BAH66363.1|:1-60	63
BmHel-8(DF090327:416184-425260):4744-4911	gb|EHJ64726.1|:1-56	44
BmHel-8(DF090327:4316874-4318459):499-1286	ref|XP_002154601.1|:14-348	39
BmHel-8(DF090327:4316874-4318459):505-972	gb|EIE78900.1|:161-491	36.5
BmHel-8(DF090327:5810915-5813896):323-949	emb|CAX36785.1|:2-212	42
BmHel-8(DF090328:1990816-1992849):389-1602	gb|EFA12192.1|:26-558	36.5
BmHel-8(DF090332:5388312-5392678):1095-1328	ref|XP_002074394.1|:268-346	34
BmHel-8(DF090332:5388312-5392678):2966-3104	dbj|BAH66363.1|:1-47	66
BmHel-8(DF090332:6043175-6044056):184-365	dbj|BAH66344.1|:39-96	65.5
BmHel-8(DF090333:5488038-5497555):1847-2071	dbj|BAH66363.1|:12-81	50
BmHel-8(DF090333:5488038-5497555):5113-5402	dbj|BAM73877.1|:7-103	69
BmHel-8(DF090333:5488038-5497555):7557-7709	gb|AAO33922.1|:27-77	84
BmHel-8(DF090333:5488038-5497555):8209-8393	ref|NP_001157648.1|:188-250	56
BmHel-8(DF090333:5488038-5497555):7961-8158	dbj|BAH66344.1|:45-107	60
BmHel-8(DF090333:5488038-5497555):3454-4158	ref|XP_003092476.1|:613-922	25
BmHel-8(DF090333:5770902-5771479):355-465	dbj|BAH66363.1|:20-56	78
BmHel-8(DF090335:1312930-1313941):312-422	dbj|BAH66325.1|:116-152	89
BmHel-8(DF090335:2046541-2050345):2651-2812	gb|AAO33922.1|:18-77	71
BmHel-8(DF090336:3836817-3843416):759-1509	gb|EFN60481.1|:56-303	34

BmHel-8(DF090337:2091829-2093693):517-1090	ref|XP_002067712.1|:1390-1570	35
BmHel-8(DF090338:1349811-1350695):491-654	dbj|BAH66344.1|:45-96	53.5
BmHel-8(DF090338:4300200-4300956):120-392	ref|XP_002076001.1|:19-110	39
BmHel-8(DF090338:654307-663826):5685-7268	gb|AAO33922.1|:21-77	60
BmHel-8(DF090338:654307-663826):8832-8945	dbj|BAM73877.1|:66-103	92
BmHel-8(DF090338:770863-775287):1219-1467	gb|EHJ68245.1|:61-144	57
BmHel-8(DF090339:2529111-2531501):674-838	gb|AAO33922.1|:20-77	72
BmHel-8(DF090340:1145783-1155488):6555-6659	dbj|BAH66325.1|:115-149	77
BmHel-8(DF090340:1679984-1687196):2604-2794	gb|AAO33922.1|:15-77	65
BmHel-8(DF090340:2671526-2681284):3330-3616	gb|EHJ77256.1|:59-153	48.5
BmHel-8(DF090340:2840463-2840993):240-365	dbj|BAH66325.1|:112-153	66
BmHel-8(DF090341:1844329-1848421):991-1155	gb|AAO33922.1|:23-77	89
BmHel-8(DF090342:2000779-2008590):4003-4185	gb|AAO33922.1|:17-77	77
BmHel-8(DF090342:2836259-2846298):2327-4610	gb|EFA11937.1|:458-877	35.5
BmHel-8(DF090342:2983760-2984630):328-491	dbj|BAH66344.1|:45-96	50.5
BmHel-8(DF090342:3233534-3234386):389-796	ref|XP_003725312.1|:735-884	30
BmHel-8(DF090342:4216664-4221265):3063-3241	dbj|BAH66344.1|:40-96	68
BmHel-8(DF090343:4301065-4304694):1549-2405	gb|EGG04513.1|:139-301	39
BmHel-8(DF090344:3613047-3619422):5917-6051	gb|EHJ76716.1|:46-90	95
BmHel-8(DF090344:3613047-3619422):4025-4126	dbj|BAH66325.1|:119-152	82
BmHel-8(DF090344:3613047-3619422):649-768	dbj|BAH66363.1|:1-40	85
BmHel-8(DF090344:3613047-3619422):1597-1798	gb|EHJ69153.1|:119-186	62
BmHel-8(DF090344:3613047-3619422):2216-2711	ref|XP_003264969.1|:346-523	43
BmHel-8(DF090344:430493-433870):2148-2471	ref|XP_003726750.1|:7-126	34
BmHel-8(DF090346:2356439-2357297):439-837	ref|XP_003243437.1|:1028-1178	29
BmHel-8(DF090346:3558727-3559640):542-829	gb|EFA13284.1|:170-265	26
BmHel-8(DF090346:3934044-3934563):315-425	dbj|BAH66325.1|:116-152	81
BmHel-8(DF090347:1170331-1171771):183-605	ref|XP_003241752.1|:606-1324	39.5
BmHel-8(DF090347:480418-481639):157-708	ref|XP_002075181.1|:2-188	23
BmHel-8(DF090348:1177679-1178682):261-674	gb|EGI67037.1|:64-209	32
BmHel-8(DF090349:3618430-3628671):7729-7860	gb|AAO33922.1|:22-77	73
BmHel-8(DF090350:1600212-1603621):2324-2508	dbj|BAH66344.1|:31-95	68
BmHel-8(DF090350:2139785-2140691):255-418	dbj|BAH66344.1|:45-96	53.5
BmHel-8(DF090350:2139785-2140691):475-587	ref|NP_001157648.1|:213-250	59
BmHel-8(DF090350:3886975-3887988):367-771	ref|XP_003740256.1|:340-473	36
BmHel-8(DF090353:1525397-1526269):531-643	ref|NP_001157648.1|:213-250	50
BmHel-8(DF090353:1525397-1526269):316-479	dbj|BAH66344.1|:45-96	53.5
BmHel-8(DF090353:3590200-3595049):964-1124	dbj|BAH66344.1|:46-96	53
BmHel-8(DF090354:1273838-1279466):1411-1605	dbj|BAH66325.1|:88-154	52
BmHel-8(DF090354:1433174-1434807):380-1148	gb|EFA02770.1|:1-297	40
BmHel-8(DF090354:1632302-1632868):150-262	ref|NP_001157648.1|:213-250	71
BmHel-8(DF090354:285563-287442):1204-1467	gb|EFA11941.1|:577-2600	42.5
BmHel-8(DF090355:1425066-1429217):284-526	ref|XP_002028596.1|:381-477	27
BmHel-8(DF090358:1040540-1047283):5347-5512	dbj|BAH66344.1|:45-96	66
BmHel-8(DF090358:1040540-1047283):2199-2330	dbj|BAM73877.1|:60-103	72
BmHel-8(DF090358:1040540-1047283):5183-5295	ref|NP_001157648.1|:213-250	59
BmHel-8(DF090358:2178260-2179220):696-857	dbj|BAH66325.1|:116-169	55
BmHel-8(DF090358:2414899-2418395):1212-1355	dbj|BAH66363.1|:13-60	66

BmHel-8(DF090358:2414899-2418395):2263-2484	ref|XP_003725469.1|:223-296	35
BmHel-8(DF090359:3233765-3234566):246-556	ref|NP_001157648.1|:85-180	50
BmHel-8(DF090362:1107944-1108581):322-546	dbj|BAH66325.1|:76-152	46
BmHel-8(DF090362:1503803-1507072):1116-1494	ref|XP_003740256.1|:348-473	38
BmHel-8(DF090362:1526446-1528154):350-1134	ref|XP_002639513.1|:23-309	41.5
BmHel-8(DF090362:1698645-1699426):340-563	dbj|BAH66344.1|:45-116	47.5
BmHel-8(DF090362:47422-48293):480-643	dbj|BAH66344.1|:45-96	68
BmHel-8(DF090362:507126-511051):368-852	ref|XP_002769157.1|:133-294	40.5
BmHel-8(DF090362:886383-891014):657-1142	gb|JAA54880.1|:186-351	28
BmHel-8(DF090363:2074718-2075804):84-263	ref|XP_002075098.1|:4-269	41.5
BmHel-8(DF090363:2186554-2192550):2535-2744	dbj|BAM73877.1|:32-101	84
BmHel-8(DF090363:2186554-2192550):5184-5738	ref|NP_001108468.1|:95-230	54.5
BmHel-8(DF090364:1381304-1389664):1935-4694	ref|XP_003725633.1|:639-1337	36
BmHel-8(DF090365:2858832-2862999):3114-3257	dbj|BAH66363.1|:9-56	72
BmHel-8(DF090366:1329473-1329993):326-409	dbj|BAH66325.1|:117-144	89
BmHel-8(DF090366:177066-182305):3259-3371	ref|NP_001157648.1|:213-250	53.5
BmHel-8(DF090366:177066-182305):709-909	gb|EFR27951.1|:108-667	45.5
BmHel-8(DF090367:2517330-2518001):339-451	ref|NP_001157648.1|:213-250	59
BmHel-8(DF090369:1423832-1424374):156-305	dbj|BAH66325.1|:121-169	62
BmHel-8(DF090371:103949-109860):689-991	ref|NP_001157648.1|:100-194	49
BmHel-8(DF090371:2262337-2262910):224-336	ref|NP_001157648.1|:213-250	71
BmHel-8(DF090372:1456021-1456642):390-518	dbj|BAH66325.1|:116-154	72
BmHel-8(DF090372:1799369-1800151):413-703	dbj|BAM73877.1|:7-103	97
BmHel-8(DF090372:2055877-2062232):1729-1892	ref|XP_003705684.1|:1-48	49
BmHel-8(DF090372:2055877-2062232):4705-4857	dbj|BAH66363.1|:1-56	67
BmHel-8(DF090374:1447987-1457446):934-4979	ref|XP_003819840.1|:240-523	43.5
BmHel-8(DF090376:1874945-1878933):558-1192	dbj|BAA76303.1|:254-464	62
BmHel-8(DF090379:859829-862032):48-530	gb|AAT47767.1|:108-274	25
BmHel-8(DF090380:1761839-1770172):3960-4207	ref|XP_003402035.1|:1761-1848	40.5
BmHel-8(DF090382:2067930-2070438):397-977	dbj|BAB21761.1|:300-494	55.5
BmHel-8(DF090383:1334172-1335587):40-315	gb|EHJ64772.1|:36-127	51
BmHel-8(DF090386:1470182-1478296):1345-1836	ref|XP_003727690.1|:479-645	28
BmHel-8(DF090386:1470182-1478296):4056-4229	ref|XP_002040584.1|:160-217	65
BmHel-8(DF090387:1861869-1864788):452-705	ref|XP_002029246.1|:159-243	45
BmHel-8(DF090388:226928-227956):264-809	ref|XP_003241752.1|:1309-1571	45
BmHel-8(DF090391:703980-712232):5045-7562	dbj|BAM73877.1|:3-85	60.5
BmHel-8(DF090396:1092167-1097869):3149-3333	dbj|BAH66344.1|:45-103	49.5
BmHel-8(DF090396:1092167-1097869):2808-3008	gb|EHJ68448.1|:3-66	50
BmHel-8(DF090397:260047-261912):694-1154	gb|EGI60189.1|:30-224	36
BmHel-8(DF090398:286574-287493):543-647	dbj|BAH66325.1|:116-150	94
BmHel-8(DF090398:303249-304942):1111-1298	dbj|BAH66344.1|:31-96	49
BmHel-8(DF090399:1224385-1226894):369-862	gb|EGI67667.1|:4-220	38
BmHel-8(DF090399:1261543-1262088):107-217	dbj|BAH66325.1|:118-154	86
BmHel-8(DF090402:436635-440030):463-957	ref|XP_002135939.1|:42-675	40.5
BmHel-8(DF090402:585881-586484):282-395	gb|EHJ64726.1|:1-38	60
BmHel-8(DF090404:305133-305838):283-498	emb|CAL25134.1|:7-75	43
BmHel-8(DF090411:882729-889062):1860-2001	gb|EHJ64726.1|:1-48	42.5
BmHel-8(DF090413:482630-483965):292-620	gb|EHJ67097.1|:129-237	44

BmHel-8(DF090413:647341-649855):175-855	gb|EFA11647.1|:524-2095	32.5
BmHel-8(DF090413:932063-939710):3324-3686	gb|JAA54880.1|:410-518	27
BmHel-8(DF090413:932063-939710):5299-6447	dbj|BAH66325.1|:116-153	63
BmHel-8(DF090414:1031352-1032991):558-1474	ref|XP_003098078.1|:571-906	37.5
BmHel-8(DF090414:530758-532436):361-1165	gb|AAN06610.1|AF461149_1:1-346	69.5
BmHel-8(DF090415:979563-988440):7060-7164	dbj|BAH66325.1|:116-150	88
BmHel-8(DF090415:979563-988440):3619-3774	dbj|BAH66363.1|:1-56	64
BmHel-8(DF090416:564779-572212):5985-6092	gb|AAO33922.1|:22-57	86
BmHel-8(DF090416:564779-572212):2509-2619	dbj|BAH66325.1|:116-152	86
BmHel-8(DF090422:293198-296674):969-3022	gb|EHJ72386.1|:127-196	64
BmHel-8(DF090422:567178-577821):5804-5970	dbj|BAH66363.1|:1-56	55.5
BmHel-8(DF090423:55067-58018):692-1481	ref|XP_003095773.1|:79-338	37
BmHel-8(DF090425:81909-86099):330-965	dbj|BAA33575.1|:6-217	35
BmHel-8(DF090433:131834-133575):333-566	gb|EFA11950.1|:40-123	33
BmHel-8(DF090436:106109-106742):374-502	gb|EHJ64726.1|:1-43	53
BmHel-8(DF090436:439866-447065):2267-2821	emb|CAB53564.1|:21-205	27
BmHel-8(DF090462:171387-172099):351-569	ref|XP_003729256.1|:33-116	32
BmHel-8(DF090462:371175-379425):1744-1907	dbj|BAH66344.1|:45-96	55
BmHel-8(DF090475:181042-191264):7604-7723	dbj|BAH66325.1|:112-153	73
BmHel-8(DF090475:181042-191264):7305-7433	gb|AAO33922.1|:26-68	86
BmHel-8(DF090481:211800-212415):311-523	gb|AAT40504.2|:644-714	35
BmHel-8(DF090499:4937-5796):436-599	dbj|BAH66344.1|:45-96	52
BmHel-8(DF090547:70519-71763):870-1192	ref|NP_001157648.1|:85-184	47.5
BmHel-8(DF090573:29094-31275):1314-1772	ref|XP_002074394.1|:212-360	28
BmHel-8(DF090580:7441-8577):499-759	ref|XP_002075098.1|:4-296	41
BmHel-8(DF090807:9336-11320):535-1352	ref|XP_002157933.1|:14-338	33.5
BmHel-8(DF090972:5461-9435):2980-3150	ref|XP_974607.1|:197-254	39
BmHel-8(DF091102:1059-2770):14-192	ref|NP_001157648.1|:192-250	47
BmHel-8(DF091278:2422-3286):378-546	dbj|BAH66363.1|:1-56	61
BmHel-8(DF091430:489-2068):372-970	gb|AAN06610.1|AF461149_1:1-346	59.5

TableS6 Silkworm intact Helitrons contributed to EST

Family(accession number):Q_start-Q_end	Subject_ID:S_start-S_end	identity
BmHel-1(DF090340:3262915-3263695):619-703	gb|AU002038.1|:10-94	1
BmHel-1(DF090340:3262915-3263695):483-703	gb|CK555254.1|:78-238	0.9908
BmHel-10(DF090344:3507546-3508271):461-682	gb|AU003269.1|:426-647	0.9955
BmHel-11(DF090357:3290239-3290497):1-259	gb|BP118853.1|:275-558	0.9921
BmHel-11(DF090323:4385167-4385373):8-122	gb|AV405087.1|:319-433	0.9913
BmHel-11(DF090417:795598-795875):206-278	gb|BP117848.1|:269-341	1
BmHel-11(DF090325:5760072-5760748):203-626	gb|BY919661.1|:20-443	0.9976
BmHel-11(DF090322:2534818-2535461):230-356	gb|CK514259.1|:292-418	0.9921
BmHel-11(DF090324:398243-398490):199-248	gb|CK524795.1|:428-477	1
BmHel-11(DF090326:6539909-6540088):6-180	gb|AV400268.1|:167-452	0.9935
BmHel-11(DF090333:1769153-1769454):1-107	gb|BW998612.1|:225-331	0.9907
BmHel-11(DF090334:3204779-3205078):249-300	gb|CK521335.1|:563-614	1
BmHel-11(DF090367:801457-801887):2-307	gb|BP126076.1|:587-689	1
BmHel-11(DF090381:978813-979106):224-294	gb|BY915048.1|:511-581	1
BmHel-11(DF090357:1500223-1500512):1-290	gb|CK538613.1|:347-636	0.9931
BmHel-11(DF090436:154125-154341):60-122	gb|CN375063.1|:568-630	1
BmHel-11(DF090352:2482900-2484688):1166-1601	gb|BP178144.1|:1-436	1
BmHel-11(DF090321:4905262-4905505):6-90	gb|BW998267.1|:8-92	1
BmHel-11(DF090358:2376153-2376439):1-104	gb|AU005777.1|:1-104	1
BmHel-11(DF090321:5993728-5994005):201-278	gb|BY917287.1|:518-595	1
BmHel-11(DF090359:1725345-1725534):6-119	gb|BP126076.1|:591-704	1
BmHel-11(DF090374:1292947-1293149):1-53	gb|BB983980.1|:10-62	1
BmHel-11(DF090329:3167924-3168510):420-501	gb|BY933984.1|:421-502	1
BmHel-11(DF090347:1250765-1251008):194-244	gb|BY933063.1|:411-461	1
BmHel-11(DF090370:1666415-1666699):53-285	gb|BP179252.1|:304-542	0.9915
BmHel-11(DF090321:4308055-4310065):1599-1830	gb|BY944965.1|:43-274	1
BmHel-11(DF090475:239002-241101):190-436	gb|BP116150.1|:6-252	0.9919
BmHel-11(DF090367:825854-826611):435-642	gb|CK554551.1|:45-251	0.9944
BmHel-11(DF090388:1484140-1484424):16-285	gb|CK518816.1|:8-275	0.9926
BmHel-11(DF090420:749619-749901):197-283	gb|BY917381.1|:493-579	1
BmHel-11(DF090373:558027-558305):67-132	gb|CK556965.1|:439-504	1
BmHel-11(DF090357:1292011-1292286):1-276	gb|BB983884.1|:210-485	1
BmHel-11(DF090408:1351962-1352257):1-52	gb|BP120938.1|:421-472	1
BmHel-11(DF090447:325702-325993):53-292	gb|CK556965.1|:289-528	1
BmHel-11(DF090417:164796-164978):8-183	gb|BB991278.1|:405-621	0.992
BmHel-11(DF090332:1709869-1710151):11-52	gb|CK519893.1|:578-619	1
BmHel-11(DF090324:1784902-1785142):6-241	gb|BP178306.1|:168-369	0.9912
BmHel-11(DF090360:2914357-2914973):271-378	gb|CK539986.1|:419-526	1
BmHel-11(DF090327:6188135-6188424):1-290	gb|BB984136.1|:24-303	0.9958
BmHel-11(BABH01047457:1418-1620):122-203	gb|CK552190.1|:399-480	1
BmHel-11(DF090360:2914357-2914973):267-378	gb|CK522635.1|:244-355	0.9911
BmHel-11(DF090332:5125934-5126208):75-155	gb|CK526643.1|:276-356	1
BmHel-11(DF090562:8795-9040):198-246	gb|CK529823.1|:163-211	1
BmHel-11(DF090441:564481-565146):181-577	gb|BP118963.1|:1-361	0.9971
BmHel-11(DF090325:5760072-5760748):316-431	gb|BW998907.1|:516-631	1
BmHel-11(DF090414:502834-503187):139-188	gb|BJ988853.1|:27-76	1

BmHel-11(DF090331:4685439-4685731):17-287	gb|BY921474.1|:287-556	0.99
BmHel-11(DF090343:160735-161121):9-109	gb|AV401554.1|:324-424	0.9901
BmHel-11(DF090388:731955-732580):223-397	gb|CK518917.1|:462-636	0.9943
BmHel-11(DF090418:393405-393591):1-144	gb|AV399241.2|:17-141	1
BmHel-11(DF090343:160735-161121):13-387	gb|CK539268.1|:44-322	0.9926
BmHel-11(DF090317:8386787-8390534):1281-1814	gb|BP177830.1|:1-534	0.9906
BmHel-11(DF090424:383189-383455):20-98	gb|BP178574.1|:475-553	1
BmHel-11(DF090411:1443679-1443971):52-293	gb|CK521302.1|:338-579	0.9948
BmHel-11(DF090321:3574775-3575061):53-287	gb|CK526113.1|:265-447	1
BmHel-11(DF090349:2633674-2633965):56-292	gb|BP179252.1|:304-538	0.9934
BmHel-11(DF090411:930170-930461):53-292	gb|CK527430.1|:355-594	1
BmHel-11(DF090517:56979-57241):47-263	gb|BP121116.1|:276-473	0.9932
BmHel-11(DF090347:3790814-3791551):239-604	gb|BB992052.1|:1-281	1
BmHel-11(DF090325:5760072-5760748):203-609	gb|BP120006.1|:119-525	0.9902
BmHel-11(DF090322:2237769-2238050):6-124	gb|BP126076.1|:591-709	0.9916
BmHel-11(DF090317:6346306-6346544):53-106	gb|CK522002.1|:594-647	1
BmHel-11(DF090359:1013506-1013748):1-243	gb|CK551318.1|:359-601	1
BmHel-11(DF090319:6851664-6852004):236-313	gb|CK535207.1|:150-227	1
BmHel-11(DF090374:1539180-1539450):53-107	gb|CK524688.1|:222-276	1
BmHel-11(DF090324:193794-194027):185-234	gb|AU002748.1|:12-61	1
BmHel-11(DF090330:3108907-3109224):1-196	gb|AV399241.2|:8-141	1
BmHel-11(DF090414:461068-461412):112-218	gb|CK498216.1|:364-470	0.9907
BmHel-11(DF090340:2135635-2137082):401-875	gb|CK524713.1|:7-615	0.9945
BmHel-11(DF090347:2252178-2252469):1-292	gb|BP182864.1|:240-531	1
BmHel-11(BABH01048053:340-619):47-97	gb|CK522461.1|:523-573	1
BmHel-11(DF090402:682549-682828):2-122	gb|BP126076.1|:587-707	0.9917
BmHel-11(DF090342:4473551-4474109):235-451	gb|BY915951.1|:13-229	0.9954
BmHel-11(DF090339:1824563-1824855):76-157	gb|CK526643.1|:275-356	1
BmHel-11(DF090318:5072030-5072304):10-275	gb|BP178306.1|:168-365	0.9922
BmHel-11(DF090395:856970-857173):155-204	gb|CK555899.1|:401-450	1
BmHel-11(DF090468:124595-124875):4-90	gb|BW998267.1|:8-94	1
BmHel-11(DF090320:8094504-8094661):122-158	gb|BY925070.1|:547-583	1
BmHel-11(DF090379:415435-416309):44-181	gb|BP121762.1|:465-602	0.9928
BmHel-11(DF090399:652539-652833):53-295	gb|BP179252.1|:304-542	0.9941
BmHel-11(DF090357:166049-166295):47-247	gb|CK523558.1|:376-540	0.9906
BmHel-11(DF090340:3153042-3153288):79-247	gb|CN374825.1|:290-506	1
BmHel-11(DF090347:4361949-4362228):109-168	gb|AV402360.1|:1-60	1
BmHel-11(DF090511:17-228):154-212	gb|BP115352.1|:509-567	1
BmHel-11(DF090317:6863314-6863620):10-71	gb|CK555035.1|:50-111	1
BmHel-11(DF090379:415435-416309):184-408	gb|BY926396.1|:364-588	0.9956
BmHel-11(DF090339:2529789-2530362):197-312	gb|CK540853.1|:76-191	0.9914
BmHel-11(DF090322:7581321-7581566):197-246	gb|CK555665.1|:399-448	1
BmHel-11(DF090333:3724539-3724867):133-329	gb|CK496513.1|:336-533	0.9909
BmHel-11(DF090369:2453731-2454227):392-497	gb|CK524795.1|:428-533	0.9906
BmHel-11(DF090316:11425752-11426019):214-268	gb|CK550779.1|:374-428	1
BmHel-11(DF090328:2324786-2325034):200-249	gb|CK529823.1|:162-211	1
BmHel-11(DF090317:9825953-9826197):55-245	gb|CN375063.1|:402-636	1
BmHel-11(DF090352:2482900-2484688):489-655	gb|CK536302.1|:411-577	0.994

BmHel-11(DF090324:193794-194027):79-234	gb|CN374825.1|:290-506	1
BmHel-11(DF090422:443837-444101):220-264	gb|CK527109.1|:42-86	1
BmHel-11(DF090376:1522557-1522840):1-76	gb|AV404339.1|:1-76	1
BmHel-11(DF090330:2145941-2146126):37-75	gb|BY942425.1|:602-640	1
BmHel-11(DF090316:4196205-4196408):10-92	gb|CK555035.1|:29-111	1
BmHel-11(DF090328:4878329-4878617):1-289	gb|CK515357.1|:54-341	1
BmHel-11(DF090333:559591-559830):206-240	gb|CK519250.1|:558-592	1
BmHel-11(DF090325:1091782-1092064):194-251	gb|CK526635.1|:151-208	1
BmHel-11(DF090316:12162291-12162512):173-222	gb|CK555745.1|:123-172	1
BmHel-11(DF090324:4067512-4067804):16-293	gb|BY926407.1|:282-559	1
BmHel-11(DF090319:5450055-5450345):1-291	gb|AV399072.1|:47-337	0.9931
BmHel-11(DF090328:2918123-2919130):774-974	gb|BP120104.1|:594-794	0.99
BmHel-11(DF090358:35975-36220):196-246	gb|CK542087.1|:75-125	1
BmHel-11(DF090361:1530570-1530816):1-52	gb|CK519893.1|:568-619	1
BmHel-11(DF090352:2482900-2484688):126-655	gb|BY919104.1|:17-546	0.9925
BmHel-11(DF090369:381083-381456):62-183	gb|CK529862.1|:232-353	0.9918
BmHel-11(DF090361:1837431-1837722):165-288	gb|AU005741.1|:48-171	1
BmHel-11(DF090371:2135072-2135329):144-258	gb|CK522129.1|:5-119	0.9913
BmHel-11(DF090371:2260039-2260308):1-270	gb|BP118756.1|:206-475	1
BmHel-11(DF090400:780872-781163):53-292	gb|CK528357.1|:183-422	1
BmHel-11(DF090359:2869763-2870006):82-146	gb|CK528551.1|:40-104	1
BmHel-11(DF090380:110411-110643):173-230	gb|CK563474.1|:21-78	1
BmHel-11(DF090322:4558153-4558674):340-453	gb|BJ987298.1|:17-130	1
BmHel-11(DF090346:1082126-1082414):50-101	gb|CK527242.1|:146-197	1
BmHel-11(DF090369:1028309-1028519):1-211	gb|BY933961.1|:160-370	1
BmHel-11(BABH01087819:8-251):195-244	gb|CK521341.1|:545-594	1
BmHel-11(DF090319:6498947-6499173):150-227	gb|BP122019.1|:372-449	1
BmHel-11(DF090352:2482900-2484688):1166-1605	gb|BP120845.1|:50-490	0.9955
BmHel-11(DF090333:2258216-2258487):99-159	gb|CK519973.1|:105-165	1
BmHel-11(DF090398:817248-817539):220-292	gb|CK514307.1|:514-586	1
BmHel-11(DF090403:1513824-1514061):62-238	gb|CK523293.1|:364-507	1
BmHel-11(DF090394:1399357-1399648):60-292	gb|BY930078.1|:38-269	0.9921
BmHel-11(DF090338:1121454-1121664):153-211	gb|BY936674.1|:143-201	1
BmHel-11(DF090892:12242-12900):73-403	gb|BY923731.1|:73-403	0.9909
BmHel-11(DF090414:502834-503187):135-188	gb|BJ986930.1|:19-72	1
BmHel-11(BABH01044460:4547-4837):1-291	gb|BY919524.1|:498-788	1
BmHel-11(DF090325:5760072-5760748):204-617	gb|CK553102.1|:184-597	0.9976
BmHel-11(DF090335:5794304-5794576):108-273	gb|BY933063.1|:411-558	0.9924
BmHel-11(DF090371:1121584-1121836):10-190	gb|BY933275.1|:391-615	0.9901
BmHel-11(DF090328:2918123-2919130):429-564	gb|BP118064.1|:210-345	0.9926
BmHel-11(DF090366:132214-132476):151-263	gb|AU005678.1|:30-161	0.9907
BmHel-11(DF090317:10802563-10802816):6-114	gb|AV403035.1|:591-699	0.9908
BmHel-11(DF090361:2639654-2639946):1-293	gb|BP116148.1|:268-560	0.9932
BmHel-11(DF090358:2399115-2399391):43-189	gb|BP122569.1|:416-573	0.9921
BmHel-11(DF090336:744302-747701):1305-1422	gb|CK519772.1|:292-409	1
BmHel-11(DF090357:166049-166295):53-247	gb|CK551704.1|:394-589	0.9923
BmHel-11(DF090316:10376447-10380567):2698-309[illegible]	gb|CK524453.1|:82-483	0.995
BmHel-11(DF090447:453468-453714):166-247	gb|CK522809.1|:80-207	1

BmHel-11(DF090341:1210436-1210727):72-138	gb|CK523640.1|:508-574	1
BmHel-11(DF090365:787847-788140):1-214	gb|BP118407.1|:51-262	0.9932
BmHel-11(DF090414:133298-134823):706-1086	gb|CK553831.1|:8-627	0.9939
BmHel-11(DF090350:30297-30567):124-271	gb|AU002750.1|:12-158	0.9932
BmHel-11(DF090379:1853627-1853885):43-138	gb|BP120337.1|:305-419	1
BmHel-11(DF090364:2059466-2059730):229-265	gb|CK524710.1|:440-476	1
BmHel-11(DF090322:6856476-6856771):87-137	gb|CK551791.1|:577-627	1
BmHel-11(DF090324:181756-182035):48-280	gb|CK519331.1|:5-238	0.9903
BmHel-11(DF090365:154273-154568):71-114	gb|BY931538.1|:24-67	1
BmHel-11(DF090361:1102508-1102796):4-46	gb|CK554847.1|:419-461	1
BmHel-11(DF090419:919541-919803):2-37	gb|CK521502.1|:522-557	1
BmHel-11(DF090319:5684184-5684434):53-226	gb|BB982750.1|:1-149	0.9912
BmHel-11(DF090345:2665283-2665471):141-189	gb|CK524125.1|:451-499	1
BmHel-11(DF090343:160735-161121):112-174	gb|BB985534.1|:403-465	1
BmHel-11(DF090357:2094756-2095046):134-291	gb|CK554708.1|:6-163	0.9937
BmHel-11(DF090345:3022907-3023142):32-150	gb|BY942425.1|:515-646	0.9913
BmHel-11(DF090339:1095203-1095497):53-295	gb|CK524688.1|:66-276	0.9901
BmHel-11(DF090390:1463609-1464172):245-523	gb|BY921552.1|:34-312	0.9964
BmHel-11(DF090335:5483717-5483964):199-248	gb|CK515021.1|:532-581	1
BmHel-11(DF090361:3067928-3068219):53-292	gb|CK518794.1|:366-605	0.9958
BmHel-11(DF090321:3292204-3293212):288-731	gb|CK514965.1|:8-451	0.991
BmHel-11(DF090419:279258-279413):98-144	gb|CK521688.1|:368-414	1
BmHel-11(DF090378:856176-856421):179-246	gb|CK520582.1|:337-404	1
BmHel-11(DF090322:5479680-5479998):189-217	gb|CK524429.1|:253-281	1
BmHel-11(DF090392:787909-788192):1-284	gb|BP118853.1|:275-558	1
BmHel-11(DF090540:57077-57321):73-242	gb|BY931538.1|:26-238	1
BmHel-11(DF090319:271137-271350):67-100	gb|BB982765.1|:109-142	1
BmHel-11(DF090329:249684-249931):199-247	gb|CK527109.1|:38-86	1
BmHel-11(DF090339:4360539-4364384):119-751	gb|CK521411.1|:7-639	0.9953
BmHel-11(DF090354:1067229-1067523):1-132	gb|BY916933.1|:459-579	0.9915
BmHel-11(DF090431:288256-288750):265-462	gb|BB992838.1|:75-290	0.994
BmHel-11(DF090379:1853627-1853885):98-259	gb|CK529820.1|:41-212	1
BmHel-11(DF090430:166804-167067):216-264	gb|BY923127.1|:192-240	1
BmHel-11(DF090327:5979723-5979963):195-241	gb|CK552636.1|:399-445	1
BmHel-11(DF090388:731955-732580):223-304	gb|BY933391.1|:439-520	1
BmHel-11(DF090364:136813-137095):101-283	gb|CK523967.1|:426-608	1
BmHel-11(DF090316:6406940-6407229):55-290	gb|CK555613.1|:400-635	0.9958
BmHel-11(DF090324:3454607-3454712):64-106	gb|CK540245.1|:64-106	1
BmHel-11(DF090340:2135635-2137082):234-555	gb|CK526209.1|:31-352	0.9907
BmHel-11(DF090520:46518-47069):101-164	gb|CK512647.1|:516-579	1
BmHel-11(DF090352:206812-207074):53-263	gb|CK521958.1|:330-540	1
BmHel-11(DF090355:2157464-2157754):57-291	gb|CK520917.1|:118-288	0.9915
BmHel-11(DF090424:260068-260327):10-243	gb|BP115008.1|:5-266	0.9929
BmHel-11(DF090347:1250765-1251008):8-244	gb|BY934001.1|:387-603	1
BmHel-11(DF090317:7466600-7466891):56-292	gb|CK523132.1|:355-576	0.9921
BmHel-11(DF090322:4614274-4621067):3991-4049	gb|CK518073.1|:124-182	1
BmHel-11(DF090340:494849-495340):64-492	gb|CK495823.1|:1-429	0.9972
BmHel-11(DF090415:944484-944738):132-167	gb|CK524262.1|:366-401	1

BmHel-11(DF090384:3697-3943):53-247	gb|CK551704.1|:394-589	0.9931
BmHel-11(DF090354:2748767-2749058):1-292	gb|AU001431.1|:233-524	1
BmHel-11(DF090319:8203904-8205855):1076-1381	gb|BP177986.1|:1-305	0.9902
BmHel-11(DF090325:1014752-1015125):255-302	gb|CK520811.1|:84-131	1
BmHel-11(DF090328:2918123-2919130):862-974	gb|CK517646.1|:489-601	0.9912
BmHel-11(DF090768:285-622):11-68	gb|CK528814.1|:266-323	1
BmHel-11(DF090326:6539909-6540088):13-180	gb|BY919113.1|:459-695	0.9935
BmHel-11(DF090377:692032-692323):61-292	gb|CK527913.1|:223-453	0.9913
BmHel-11(DF090325:2712646-2712935):53-290	gb|CK527913.1|:223-460	0.9916
BmHel-11(BABH01047413:963-1474):171-407	gb|BB988132.1|:177-413	0.9916
BmHel-11(DF090332:2972239-2972506):212-268	gb|BP182545.1|:330-386	1
BmHel-11(DF090414:502834-503187):135-186	gb|BJ989055.1|:19-70	1
BmHel-11(DF090334:3204779-3205078):248-300	gb|BP117554.1|:244-296	1
BmHel-11(DF090355:3753857-3754092):4-46	gb|CK555351.1|:417-459	1
BmHel-11(DF090321:4308055-4310065):1480-1659	gb|CK523879.1|:460-640	0.9945
BmHel-11(DF090475:239002-241101):548-767	gb|CK552070.1|:325-544	0.9909
BmHel-11(DF090356:2907779-2908378):206-487	gb|CK528514.1|:301-582	0.9929
BmHel-11(DF090316:10376447-10380567):2746-309[illegible]	gb|CK529802.1|:244-597	0.9915
BmHel-11(DF090386:1792852-1793101):92-250	gb|CK555745.1|:123-326	1
BmHel-11(DF090439:1704-2017):66-314	gb|BY919367.1|:1-249	1
BmHel-11(DF090394:142700-142953):194-254	gb|CK524125.1|:451-511	1
BmHel-11(DF090316:6406940-6407229):1-232	gb|CK538838.1|:1-232	1
BmHel-11(DF090322:2993345-2993594):6-122	gb|AV403035.1|:591-707	0.9915
BmHel-11(DF090344:2695467-2695759):6-94	gb|BW998267.1|:4-92	1
BmHel-11(DF090413:245265-245556):66-292	gb|BY930445.1|:1-227	1
BmHel-11(DF090356:2907779-2908378):371-487	gb|BY919134.1|:637-753	1
BmHel-11(DF090608:18999-19356):8-358	gb|BY934001.1|:387-603	0.9914
BmHel-11(DF090353:3165382-3165688):1-113	gb|BY927056.1|:265-377	0.9912
BmHel-11(DF090371:1541186-1541476):53-291	gb|CK528760.1|:341-579	0.9916
BmHel-11(DF091194:4758-5787):772-897	gb|BY933162.1|:429-554	1
BmHel-11(DF090401:219802-220037):187-236	gb|BY923127.1|:192-241	1
BmHel-11(DF090317:7060664-7062192):490-1028	gb|BP119268.1|:1-539	0.9944
BmHel-11(DF090366:1882288-1882531):193-244	gb|CK521103.1|:488-539	1
BmHel-11(DF090317:4608348-4608640):2-103	gb|AV401239.2|:12-113	0.9902
BmHel-11(DF090361:1406812-1407750):258-394	gb|CK556666.1|:200-336	0.9927
BmHel-11(DF090347:3832817-3833111):54-295	gb|CK506839.1|:421-662	0.9959
BmHel-11(DF090356:1485717-1485940):4-51	gb|CK548724.1|:505-552	1
BmHel-11(DF090355:1987314-1987594):1-281	gb|BP183188.1|:97-377	0.9964
BmHel-11(DF090325:5760072-5760748):203-431	gb|BP120998.1|:360-588	1
BmHel-11(DF090335:5794304-5794576):7-273	gb|BY934001.1|:387-603	0.9939
BmHel-11(DF090326:3186364-3186637):53-274	gb|CK519891.1|:245-466	0.9942
BmHel-11(DF090332:19947-20216):48-174	gb|CK551587.1|:406-555	1
BmHel-11(DF090413:165999-166244):165-246	gb|CK524539.1|:272-353	1
BmHel-11(DF090379:714152-714425):159-274	gb|BY917381.1|:493-625	0.9914
BmHel-11(DF090475:239002-241101):1626-1978	gb|BY914805.1|:276-628	0.9915
BmHel-11(DF090379:415435-416309):45-181	gb|CK525499.1|:22-157	0.9927
BmHel-11(DF090370:829219-829732):201-467	gb|CK549730.1|:75-341	0.9963
BmHel-11(DF090322:2479929-2480176):48-248	gb|CK551587.1|:311-555	0.9909

BmHel-11(DF090317:3873746-3874049):236-304	gb|CK523214.1|:437-505	1
BmHel-11(DF090384:275439-282168):2103-2259	gb|CK564005.1|:196-352	0.9936
BmHel-11(DF090316:12162291-12162512):63-222	gb|CK524776.1|:444-672	1
BmHel-11(DF090371:473667-473957):12-114	gb|CK513752.1|:313-415	0.9903
BmHel-11(BABH01044729:537-783):198-247	gb|CK526335.1|:247-296	1
BmHel-11(DF090386:1375571-1375803):1-51	gb|CK535105.1|:21-71	1
BmHel-11(DF090492:223300-223582):225-283	gb|CK499481.1|:477-535	1
BmHel-11(DF090332:1469222-1469522):7-106	gb|BP120819.1|:33-132	0.99
BmHel-11(DF090324:2721680-2721953):238-274	gb|CK553885.1|:301-337	1
BmHel-11(DF090323:3727456-3727745):47-290	gb|CK527957.1|:372-615	0.9948
BmHel-11(DF090325:4985143-4985439):1-136	gb|AU234546.1|:160-295	0.9926
BmHel-11(DF090316:12269284-12269530):198-247	gb|CK553814.1|:384-433	1
BmHel-11(DF090337:2758571-2759199):135-352	gb|BY931517.1|:428-645	0.9908
BmHel-11(DF090331:5031204-5031498):216-295	gb|CK519250.1|:558-637	1
BmHel-11(DF090402:886876-890788):204-356	gb|CK524910.1|:29-181	0.9935
BmHel-11(DF090320:7717311-7717533):174-223	gb|CK522349.1|:373-422	1
BmHel-11(DF090447:213948-214239):76-282	gb|BY931538.1|:29-231	0.99
BmHel-11(DF090363:1520255-1520549):10-92	gb|CK555994.1|:12-94	1
BmHel-11(DF090414:461068-461412):113-218	gb|BJ986088.1|:165-270	0.9906
BmHel-11(DF090340:3375933-3376223):7-113	gb|AV401554.1|:322-428	0.9907
BmHel-11(DF090317:1527807-1528024):54-218	gb|CK556026.1|:402-638	1
BmHel-11(DF090369:381083-381456):316-372	gb|CK524669.1|:558-614	1
BmHel-11(DF090356:2907779-2908378):207-487	gb|BJ984371.1|:457-737	0.9929
BmHel-11(DF090338:4919778-4920084):183-219	gb|CK505975.1|:2-38	1
BmHel-11(DF090321:4308055-4310065):162-686	gb|BY928031.1|:19-543	0.9943
BmHel-11(DF090608:18999-19356):1-358	gb|CK498303.1|:30-319	0.9916
BmHel-11(DF090321:3292204-3293212):288-906	gb|BJ987723.1|:18-636	0.9919
BmHel-11(DF090332:5903409-5903671):1-122	gb|CK505838.1|:356-477	0.9918
BmHel-11(DF090342:3678888-3688675):1404-1650	gb|BY928668.1|:541-787	0.996
BmHel-11(DF090386:1792852-1793101):195-250	gb|CK552636.1|:399-454	1
BmHel-11(DF090354:3754058-3754315):53-258	gb|CK524429.1|:177-382	1
BmHel-11(DF090332:3532044-3532239):7-64	gb|BP180708.2|:369-426	1
BmHel-11(DF090342:4552065-4552360):10-92	gb|CK551299.1|:8-90	1
BmHel-11(DF090336:744302-747701):1594-1869	gb|BP118394.1|:1-276	1
BmHel-11(DF090327:3895984-3896227):99-244	gb|BY915462.1|:305-495	0.993
BmHel-11(DF090317:7060664-7062192):490-1094	gb|CK554809.1|:15-619	0.9934
BmHel-11(DF090381:1813524-1813815):1-292	gb|CK555157.1|:300-591	0.9966
BmHel-11(DF090418:393405-393591):149-187	gb|CK520405.1|:183-221	1
BmHel-11(DF090442:187094-187323):154-230	gb|CK556125.1|:469-545	1
BmHel-11(DF090389:1162438-1162731):1-292	gb|BY917048.1|:1-292	1
BmHel-11(DF090339:1095203-1095497):47-295	gb|CK523558.1|:376-540	0.9918
BmHel-11(DF090334:1931365-1931614):190-250	gb|BY936674.1|:141-201	1
BmHel-11(DF090356:1485717-1485940):1-50	gb|BY914063.1|:457-506	1
BmHel-11(DF090406:117333-117581):54-249	gb|CK552824.1|:394-588	0.9932
BmHel-11(DF090317:335766-336010):143-245	gb|CK540245.1|:5-106	0.9903
BmHel-11(DF090317:8386787-8390534):126-515	gb|BY919453.1|:19-408	0.9949
BmHel-11(DF090359:266385-266676):1-102	gb|CK562085.1|:534-635	1
BmHel-11(DF090346:1082126-1082414):162-289	gb|CK520622.1|:298-425	1

BmHel-11(DF090316:6406940-6407229):1-290	gb|CK498303.1|:30-319	1
BmHel-11(DF090378:941061-941326):87-136	gb|BY943534.1|:47-96	1
BmHel-11(DF090325:1071639-1071966):53-328	gb|CK526113.1|:265-447	0.9905
BmHel-12(DF090340:3671774-3672090):27-71	gb|CN211713.1|:79-123	1
BmHel-12(DF090415:235541-235893):315-353	gb|BB984263.1|:376-414	1
BmHel-12(DF090320:6845635-6846003):331-369	gb|CK526890.1|:246-284	1
BmHel-12(DF090327:2165318-2165671):317-354	gb|BY930683.1|:47-84	1
BmHel-12(DF090428:693073-695307):1605-1920	gb|CK513600.1|:24-340	0.9915
BmHel-12(DF090936:7161-7459):252-299	gb|CK556712.1|:155-202	1
BmHel-12(DF090495:3891-4267):350-377	gb|CK553844.1|:234-261	1
BmHel-12(DF090616:1563-1915):315-353	gb|CK517913.1|:87-125	1
BmHel-12(DF090358:2064316-2065903):81-143	gb|CK556107.1|:15-77	1
BmHel-12(DF090376:1423026-1423383):305-349	gb|BP116118.1|:37-81	1
BmHel-12(DF090391:139484-141491):75-1740	gb|BY915132.1|:283-457	0.9944
BmHel-12(DF090330:241499-241687):139-183	gb|BP118679.1|:200-244	1
BmHel-12(DF090351:1434265-1434563):269-299	gb|CK496268.1|:306-336	1
BmHel-12(DF090340:1612875-1613240):4-35	gb|CK517913.1|:47-78	1
BmHel-12(DF090363:164145-165882):1218-1460	gb|CK560227.1|:1-242	0.9918
BmHel-12(DF090316:3504439-3504791):180-230	gb|BW999083.1|:1-51	1
BmHel-12(DF090442:424638-432862):1464-2008	gb|CK525929.1|:52-597	0.9908
BmHel-12(DF090327:1310067-1310435):341-369	gb|BP119632.1|:413-441	1
BmHel-12(BABH01051435:611-864):223-254	gb|CK541157.1|:272-303	1
BmHel-12(DF090415:209008-209332):272-323	gb|BP182754.1|:178-229	1
BmHel-12(DF090338:1379143-1379415):120-176	gb|CK510437.1|:271-327	1
BmHel-12(DF090564:44852-45401):512-536	gb|CK500706.1|:8-32	1
BmHel-12(DF090316:7698585-7699428):291-407	gb|BP184046.1|:525-641	0.9915
BmHel-12(DF090322:247166-247518):4-44	gb|CK517913.1|:47-87	1
BmHel-12(DF090336:414556-414908):314-353	gb|AU002032.1|:437-476	1
BmHel-12(DF090543:71292-72880):74-141	gb|CK521604.1|:22-89	1
BmHel-12(DF090405:626075-626444):332-370	gb|CK523036.1|:465-503	1
BmHel-12(DF090374:885864-886150):249-287	gb|CN375976.1|:5-43	1
BmHel-12(DF090357:3131153-3132607):501-930	gb|CK551272.1|:15-649	0.9935
BmHel-12(DF090370:1460920-1461236):279-317	gb|CK554691.1|:194-232	1
BmHel-12(DF090321:508915-509295):328-373	gb|BP116118.1|:37-82	1
BmHel-12(DF090333:4967817-4968372):117-255	gb|CK530441.1|:413-551	1
BmHel-12(DF090378:738236-738581):308-346	gb|CK555489.1|:206-244	1
BmHel-12(DF090452:144695-145048):316-354	gb|CK516055.1|:164-202	1
BmHel-12(DF090407:724992-725356):327-365	gb|AV403785.1|:51-89	1
BmHel-12(DF090320:4829049-4829455):376-407	gb|CK496268.1|:305-336	1
BmHel-12(DF090400:1563324-1563861):406-441	gb|BP117429.1|:199-234	1
BmHel-12(DF090327:4583023-4583333):124-311	gb|BY932098.1|:541-765	0.9925
BmHel-12(DF090322:8166831-8167188):331-358	gb|CK553227.1|:151-178	1
BmHel-12(DF090335:2856234-2856597):153-204	gb|BP127744.1|:370-421	1
BmHel-12(DF090363:164145-165882):68-214	gb|AU006195.1|:1-147	0.9932
BmHel-12(DF090356:2461759-2462105):317-347	gb|CN375915.1|:328-358	1
BmHel-12(DF090353:3690356-3690718):325-363	gb|CK555529.1|:64-102	1
BmHel-12(DF090353:3441178-3441882):238-562	gb|CK515035.1|:10-383	0.9909
BmHel-12(DF090353:1694932-1697502):1935-2262	gb|CK519763.1|:254-581	1

BmHel-12(DF090451:383169-383505):289-337	gb|BP177840.1|:647-695	1
BmHel-12(DF090380:561698-562075):338-378	gb|CK529482.1|:135-175	1
BmHel-12(DF090377:1327455-1327811):307-357	gb|CK523421.1|:379-429	1
BmHel-12(DF090345:2158068-2158871):636-737	gb|BY930386.1|:26-127	0.9902
BmHel-12(DF090316:14624703-14625066):136-167	gb|CK510437.1|:372-403	1
BmHel-12(DF090357:1866193-1866543):163-351	gb|CK556712.1|:155-368	0.9902
BmHel-12(DF090357:3131153-3132607):78-224	gb|BY917423.1|:1-147	0.9932
BmHel-12(DF090367:1600466-1600765):106-158	gb|CK525831.1|:194-246	1
BmHel-12(DF090462:145042-145725):344-433	gb|CK529104.1|:508-597	1
BmHel-12(DF090363:164145-165882):1206-1460	gb|CK558872.1|:21-274	0.9922
BmHel-12(DF090384:814670-815023):316-354	gb|BY938105.1|:313-351	1
BmHel-12(DF090437:491600-491918):135-191	gb|CK538671.1|:481-537	1
BmHel-12(DF090425:959229-959572):317-344	gb|CK561624.1|:154-181	1
BmHel-12(DF090322:6620653-6621006):316-354	gb|DY231086.1|:295-333	1
BmHel-12(DF090339:86635-86996):324-362	gb|CN375915.1|:328-366	1
BmHel-12(DF090354:370113-371579):1431-1467	gb|CK518559.1|:192-228	1
BmHel-12(DF090316:4960407-4961019):457-508	gb|BW999083.1|:1-52	1
BmHel-12(DF090316:10127448-10128900):793-1002	gb|CK523881.1|:248-457	0.9905
BmHel-12(DF090335:4632381-4636595):836-1073	gb|BP119542.1|:170-407	0.9958
BmHel-12(DF090391:139484-141491):1919-2008	gb|BP116118.1|:1-90	1
BmHel-12(DF090332:3700503-3700850):311-348	gb|CK521428.1|:422-459	1
BmHel-12(DF090319:2463174-2463524):1-351	gb|CK524096.1|:138-488	0.9972
BmHel-12(DF090326:6519318-6519894):132-306	gb|BY931063.1|:120-294	0.9943
BmHel-12(DF090442:424638-432862):1360-1934	gb|CK548817.1|:1-577	0.9931
BmHel-12(DF090317:8993659-8994064):368-406	gb|CK525452.1|:173-211	1
BmHel-12(DF090369:1850865-1851237):335-373	gb|CK494583.1|:57-95	1
BmHel-12(DF090338:68607-69598):276-391	gb|BP178163.1|:480-596	0.9915
BmHel-12(DF090318:9088405-9099027):3691-4322	gb|BB993533.1|:22-653	0.9905
BmHel-12(DF090321:3836495-3836842):1-212	gb|BP118274.1|:303-514	1
BmHel-12(DF090401:1413899-1414833):290-490	gb|CK552366.1|:395-595	0.99
BmHel-12(DF090364:497191-497576):256-386	gb|BP177840.1|:647-761	1
BmHel-12(DF090324:6366652-6367002):125-207	gb|CK519432.1|:30-112	1
BmHel-12(DF090342:189372-190860):78-133	gb|CK517523.1|:5-60	1
BmHel-12(DF090362:1998079-1998427):317-349	gb|BP118278.1|:205-237	1
BmHel-12(DF090431:39069-39425):309-357	gb|BP184314.1|:92-140	1
BmHel-12(DF090428:693073-695307):276-390	gb|BP119576.1|:8-122	0.9913
BmHel-12(DF090363:912390-912685):256-296	gb|BP117616.1|:67-107	1
BmHel-12(DF090443:545445-549466):3526-3662	gb|CK516986.1|:8-144	0.9927
BmHel-12(DF090476:117698-119202):1106-1221	gb|BY914083.1|:539-654	0.9914
BmHel-12(DF090393:807619-810378):1108-1472	gb|BY915226.1|:2-366	1
BmHel-12(DF090483:14400-14743):112-215	gb|CK556080.1|:93-196	0.9904
BmHel-12(DF090420:980313-981167):122-617	gb|BP118988.1|:166-582	0.998
BmHel-12(DF090363:912390-912685):258-294	gb|CK529128.1|:349-385	1
BmHel-12(DF090316:12994620-12996148):1085-125[illegible]	gb|BJ984503.1|:1-167	0.994
BmHel-12(DF090342:189372-190860):251-351	gb|BY914251.1|:426-526	0.9901
BmHel-12(DF090317:2819422-2819766):295-345	gb|CK554043.1|:74-124	1
BmHel-12(DF090458:135359-135709):162-219	gb|CK518650.1|:34-91	1
BmHel-12(DF090322:1895960-1896258):261-299	gb|CK553227.1|:140-178	1

BmHel-12(DF090361:2300214-2300571):320-354	gb|CK559032.1|:287-321	1
BmHel-12(DF090443:545445-549466):1135-1498	gb|BJ982685.1|:103-571	0.9918
BmHel-12(DF090358:2064316-2065903):847-985	gb|CK509583.1|:486-624	0.9928
BmHel-12(DF090443:545445-549466):1044-1103	gb|BJ988087.1|:434-493	1
BmHel-12(DF090316:10127448-10128900):185-245	gb|CK519396.1|:14-74	1
BmHel-12(DF090325:5648676-5650651):230-496	gb|BP118349.1|:172-438	0.9925
BmHel-12(DF090442:424638-432862):1360-2000	gb|BY932979.1|:33-673	0.9907
BmHel-12(DF090347:2125678-2125997):282-320	gb|CK545543.1|:251-289	1
BmHel-12(DF090320:3605896-3607307):1258-1315	gb|CK519860.1|:10-67	1
BmHel-12(DF090412:231113-231484):1-314	gb|CK551200.1|:368-681	0.9936
BmHel-12(DF090391:139484-141491):183-1192	gb|CK525449.1|:29-536	0.9901
BmHel-12(DF090405:802864-803513):207-361	gb|CK554642.1|:8-110	1
BmHel-12(DF090318:952730-953085):13-63	gb|CN211713.1|:85-135	1
BmHel-12(DF090348:1214439-1214797):321-359	gb|CK525309.1|:22-60	1
BmHel-12(DF090332:774512-774861):1-109	gb|CK535896.1|:441-566	1
BmHel-12(DF090375:1016771-1017401):146-354	gb|BP181216.1|:1-209	0.9952
BmHel-12(DF090476:117698-119202):464-972	gb|CK555491.1|:19-662	0.9951
BmHel-12(DF090318:6332484-6332839):331-356	gb|CK522398.1|:171-196	1
BmHel-12(DF090320:8093086-8093432):309-347	gb|CN375914.1|:332-370	1
BmHel-12(DF091821:975-1960):50-219	gb|BP119304.1|:429-598	0.9941
BmHel-12(DF090364:357382-358027):127-309	gb|CK556374.1|:452-634	0.9945
BmHel-12(DF090359:1782439-1783255):212-322	gb|BY930692.1|:451-560	0.991
BmHel-12(DF090355:3146811-3147172):324-362	gb|CK527912.1|:1-39	1
BmHel-12(DF090389:905476-905827):314-352	gb|CK521428.1|:421-459	1
BmHel-12(DF090451:383169-383505):216-337	gb|BP184314.1|:19-140	1
BmHel-12(DF090329:5121916-5122536):75-109	gb|AU001174.1|:68-102	1
BmHel-12(DF090403:1106987-1107341):255-355	gb|BP117616.1|:7-107	0.9901
BmHel-12(DF090396:487630-488003):1-116	gb|CK535896.1|:441-568	0.9905
BmHel-12(DF090316:5680822-5681174):315-353	gb|CK518559.1|:190-228	1
BmHel-12(DF090378:343048-343406):321-359	gb|CK553844.1|:223-261	1
BmHel-12(DF090318:1326537-1326881):285-343	gb|BY916722.1|:514-572	1
BmHel-12(DF090344:1383649-1384021):86-118	gb|BP121757.1|:560-592	1
BmHel-12(DF090365:495974-496308):297-335	gb|BB982208.1|:44-82	1
BmHel-12(DF090318:9088405-9099027):3691-4437	gb|BY919652.1|:20-766	0.9906
BmHel-12(DF090326:1543237-1543593):318-357	gb|CK525452.1|:173-212	1
BmHel-12(DF090349:1107323-1107655):285-333	gb|BP181767.1|:427-475	1
BmHel-12(DF090357:3131153-3132607):74-141	gb|CK521502.1|:26-93	1
BmHel-12(DF090358:1697561-1705658):6211-6367	gb|CK539621.1|:468-624	0.9936
BmHel-12(DF090357:3131153-3132607):491-847	gb|CK521629.1|:8-568	1
BmHel-12(DF090334:6067165-6067517):315-353	gb|CK498549.1|:479-517	1
BmHel-12(DF090335:4348950-4349308):321-359	gb|CK554213.1|:460-498	1
BmHel-12(DF090334:3194900-3195224):237-265	gb|BP120143.1|:571-599	1
BmHel-12(DF090476:117698-119202):398-974	gb|BY928700.1|:2-712	0.9926
BmHel-12(DF090332:989964-990330):156-207	gb|BP118691.1|:327-378	1
BmHel-12(DF090424:672403-672731):291-329	gb|CK529651.1|:351-389	1
BmHel-12(DF090441:610659-611016):326-358	gb|CK542125.1|:49-81	1
BmHel-12(DF090325:5648676-5650651):1460-1599	gb|AU005690.1|:97-236	0.9929
BmHel-12(DF090336:919966-920231):66-182	gb|AU006149.1|:25-141	0.9915

BmHel-12(DF090442:424638-432862):2143-2541	gb|BY923317.1|:2-402	0.99
BmHel-12(DF090328:1358247-1358864):225-431	gb|CK533582.1|:491-697	0.9903
BmHel-12(DF091019:3904-4317):381-414	gb|BP119652.1|:454-487	1
BmHel-12(DF090380:1813092-1813469):162-243	gb|BP118691.1|:303-384	1
BmHel-12(DF090365:881616-881805):134-186	gb|BP118679.1|:193-245	1
BmHel-12(DF090543:71292-72880):75-141	gb|CK522730.1|:22-88	1
BmHel-12(DF090330:4300638-4300986):124-168	gb|CK542028.1|:18-62	1
BmHel-12(DF090382:1929646-1938360):2479-2641	gb|BP118257.1|:184-346	1
BmHel-12(DF090573:13113-14721):73-1359	gb|BP120211.1|:282-456	1
BmHel-12(DF090345:1000914-1001280):336-362	gb|BY914588.1|:89-115	1
BmHel-12(DF090338:68607-69598):663-716	gb|BY925942.1|:33-86	1
BmHel-12(DF090320:7088343-7091049):1005-1119	gb|CK524120.1|:7-121	1
BmHel-12(DF090319:6730026-6730375):318-350	gb|BP119632.1|:413-445	1
BmHel-12(DF090543:71292-72880):805-902	gb|CK525624.1|:5-355	0.9903
BmHel-12(DF090358:1592238-1592593):249-356	gb|BP177840.1|:647-754	1
BmHel-12(DF090393:807619-810378):508-907	gb|AU004348.1|:10-409	0.9917
BmHel-12(DF090316:10127448-10128900):793-1006	gb|CK552272.1|:393-606	0.9907
BmHel-12(DF090416:1199946-1200307):311-362	gb|BP181767.1|:427-478	1
BmHel-12(DF090320:5499666-5500021):169-356	gb|CK526566.1|:23-210	1
BmHel-12(DF090353:1694932-1697502):1935-2441	gb|BY927807.1|:33-539	0.9941
BmHel-12(DF090353:1694932-1697502):1935-2252	gb|CK504014.1|:268-585	0.9969
BmHel-12(DF090483:14400-14743):112-160	gb|CK542028.1|:18-66	1
BmHel-12(DF090321:6756069-6756422):316-354	gb|BW999470.1|:116-154	1
BmHel-12(DF090543:71292-72880):1240-1303	gb|CK556107.1|:14-77	1
BmHel-12(DF090321:1768298-1768669):334-372	gb|BY922441.1|:59-97	1
BmHel-12(DF090359:1782439-1783255):377-499	gb|BY921921.1|:2-124	0.9919
BmHel-12(DF090364:357382-358027):76-309	gb|BY914372.1|:16-249	0.9915
BmHel-12(DF090406:775002-775353):314-352	gb|BY930683.1|:46-84	1
BmHel-12(DF090420:980313-981167):122-601	gb|BP120000.1|:1-363	0.9943
BmHel-12(DF090411:1304487-1304835):1-349	gb|CK500446.1|:67-415	0.997
BmHel-12(DF090358:223749-225593):1347-1625	gb|AU000392.1|:378-657	0.9929
BmHel-13(DF090384:1564576-1564873):1-58	gb|CK509854.1|:522-591	1
BmHel-13(DF090321:5246387-5246726):1-167	gb|CK501681.1|:416-581	0.9931
BmHel-13(DF090326:3826614-3826922):1-273	gb|CK553729.1|:1-130	0.9914
BmHel-13(DF090342:2146867-2147437):1-366	gb|CK510168.1|:412-516	0.9911
BmHel-13(DF090328:5606525-5606834):1-157	gb|CK513191.1|:395-551	0.9936
BmHel-13(DF090358:3238923-3239217):1-74	gb|CK509261.1|:471-558	1
BmHel-13(DF090487:109528-109841):2-99	gb|AU006381.1|:1-82	1
BmHel-13(DF090429:486399-486697):1-141	gb|CK515390.1|:429-578	0.9907
BmHel-13(DF090375:772351-781314):8875-8927	gb|BP120466.1|:21-73	1
BmHel-13(DF090328:5606525-5606834):1-166	gb|CK501681.1|:416-581	0.994
BmHel-13(DF090384:1564576-1564873):1-261	gb|CK553729.1|:1-130	0.9917
BmHel-13(DF090401:989902-990210):1-69	gb|CK502173.1|:462-530	1
BmHel-13(DF090342:2146867-2147437):410-510	gb|CK520220.1|:5-105	0.9901
BmHel-14(DF090333:1235505-1235878):146-180	gb|CK520220.1|:6-40	1
BmHel-14(DF090341:2289862-2290589):541-650	gb|BP117669.1|:290-399	0.9909
BmHel-14(DF090341:2289862-2290589):553-650	gb|CK503418.1|:178-275	1
BmHel-14(DF090318:8623140-8623685):1-177	gb|CK502952.1|:351-532	0.9935

BmHel-14(DF090466:308443-309440):247-319	gb|CK539142.1|:14-86	1
BmHel-14(DF090332:2780341-2780753):280-325	gb|CK520039.1|:213-258	1
BmHel-14(DF090318:48271-48651):224-274	gb|CK529589.1|:4-54	1
BmHel-14(DF090392:1479449-1479986):148-191	gb|AU005994.1|:93-136	1
BmHel-14(DF090318:48271-48651):1-29	gb|CK502173.1|:462-490	1
BmHel-14(DF090318:48271-48651):99-149	gb|CK500657.1|:430-480	1
BmHel-14(DF090466:308443-309440):267-318	gb|CK552243.1|:4-55	1
BmHel-14(DF090341:2289862-2290589):1-34	gb|CK502173.1|:462-495	1
BmHel-14(DF090466:308443-309440):256-315	gb|CK512057.1|:4-63	1
BmHel-14(DF090367:1749776-1750033):1-111	gb|AU001815.1|:392-518	0.9915
BmHel-14(DF090391:577702-578048):1-128	gb|AV405121.1|:1-121	0.9907
BmHel-14(DF090318:8623140-8623685):1-152	gb|CK515390.1|:429-583	0.9922
BmHel-14(DF090367:1749776-1750033):1-110	gb|BP178323.1|:437-562	0.9912
BmHel-15(DF090388:804008-810677):4934-5235	gb|CK524979.1|:4-264	0.9925
BmHel-15(DF090330:25428-27419):68-564	gb|BB986195.1|:24-522	0.996
BmHel-15(DF090442:425262-431226):3175-3225	gb|BY927423.1|:674-724	1
BmHel-15(DF090340:995106-1005573):9698-10141	gb|CK549230.1|:1-444	0.991
BmHel-15(DF090341:2800594-2801032):312-341	gb|CK562463.1|:538-567	1
BmHel-15(DF090343:2055839-2057676):1152-1585	gb|AU002761.1|:1-434	0.9919
BmHel-15(DF090324:165770-166206):1-128	gb|CK528633.1|:100-239	0.9922
BmHel-15(DF090338:3855621-3856192):388-507	gb|BB982563.1|:27-146	0.9917
BmHel-15(DF090375:2363321-2363695):318-361	gb|AV398234.1|:59-102	1
BmHel-15(DF090386:429453-430587):149-515	gb|CN374586.1|:5-361	0.9913
BmHel-15(DF090378:1098251-1098906):304-370	gb|BB983650.1|:18-84	1
BmHel-15(DF090327:1080343-1080767):1-45	gb|CK554071.1|:6-50	1
BmHel-15(DF090318:2813472-2813904):366-420	gb|BP126035.1|:435-489	1
BmHel-15(DF090338:1786044-1787122):306-436	gb|BP117543.1|:1-131	1
BmHel-15(DF090369:893401-894211):255-361	gb|BP118064.1|:239-345	0.9907
BmHel-15(DF090363:1958478-1960441):1235-1638	gb|BP178283.1|:314-717	0.9926
BmHel-15(DF090342:2187416-2187861):2-99	gb|CK526303.1|:141-226	1
BmHel-15(DF090316:4640683-4647901):1329-1511	gb|BJ984865.1|:1-182	0.9945
BmHel-15(DF090386:429453-430587):149-252	gb|CK519892.1|:301-404	1
BmHel-15(DF090344:969983-971163):451-856	gb|BY927307.1|:98-503	0.9951
BmHel-15(DF090369:893401-894211):109-181	gb|CK554252.1|:10-82	1
BmHel-15(DF090411:518389-518953):396-500	gb|BJ987298.1|:23-127	1
BmHel-15(DF090363:1958478-1960441):78-388	gb|CK550182.1|:1-311	0.9968
BmHel-15(DF090378:1510420-1512065):1185-1411	gb|CK524016.1|:5-231	0.9912
BmHel-15(DF090325:5783422-5784075):246-466	gb|BY931613.1|:157-363	0.9947
BmHel-15(DF090343:2055839-2057676):751-987	gb|BY921184.1|:180-416	0.9916
BmHel-15(DF090344:3415301-3421863):5613-5667	gb|CK524339.1|:488-542	1
BmHel-15(DF090372:693591-694024):2-101	gb|BP120390.1|:653-752	0.99
BmHel-15(DF090343:2055839-2057676):750-987	gb|CK528955.1|:283-520	0.9958
BmHel-15(DF090335:4843793-4845300):1230-1328	gb|CK559903.1|:90-188	1
BmHel-15(DF090328:2870286-2870701):1-52	gb|CK519960.1|:22-73	1
BmHel-15(DF090321:5785047-5785597):185-292	gb|CK518996.1|:196-303	0.9907
BmHel-15(DF090316:4640683-4647901):717-774	gb|DN236889.1|:153-210	1
BmHel-15(DF090353:1437517-1437953):370-425	gb|CK551127.1|:33-88	1
BmHel-15(DF090340:23209-23647):379-439	gb|CK527257.1|:288-348	1

BmHel-15(DF090316:4640683-4647901):1327-1797	gb|BY914110.1|:2-472	0.9925
BmHel-15(DF090379:1402683-1403056):232-374	gb|BP120137.1|:633-768	0.9904
BmHel-15(DF090486:37294-37709):146-170	gb|CK557184.1|:157-181	1
BmHel-15(DF090377:73303-73737):321-421	gb|AV398234.1|:2-102	0.9901
BmHel-15(DF090316:408031-408478):1-448	gb|AV401263.1|:44-491	1
BmHel-15(DF090482:45073-45494):1-63	gb|CK519960.1|:11-73	1
BmHel-15(DF090344:969983-971163):73-205	gb|CK515511.1|:5-137	0.9925
BmHel-15(DF090322:7030559-7040318):532-971	gb|CK515719.1|:84-522	0.9932
BmHel-15(DF090344:425904-426633):330-558	gb|BP177740.1|:468-697	0.9913
BmHel-15(DF090327:5532702-5533638):712-816	gb|CK528546.1|:211-315	1
BmHel-15(DF090341:2800594-2801032):313-341	gb|CK542400.1|:189-217	1
BmHel-15(DF090413:1179353-1189423):8465-8688	gb|BY916823.1|:1-224	0.9911
BmHel-15(DF090348:1025468-1027291):1052-1106	gb|CN382063.1|:8-62	1
BmHel-15(DF090404:1094035-1094413):318-372	gb|CK542400.1|:239-293	1
BmHel-15(DF090329:3767217-3767646):1-78	gb|CK551294.1|:329-406	1
BmHel-15(DF090411:518389-518953):395-500	gb|CK551297.1|:152-257	1
BmHel-15(DF090414:1100309-1101446):232-332	gb|CK543370.1|:160-260	1
BmHel-15(DF090755:19749-20199):1-48	gb|CK553870.1|:314-361	1
BmHel-15(DF090348:416161-416861):368-454	gb|BY932101.1|:774-860	1
BmHel-15(DF090351:1295366-1297621):108-479	gb|AU003262.1|:7-378	0.9919
BmHel-15(DF090336:1654555-1654994):1-125	gb|CK519629.1|:7-131	0.992
BmHel-15(DF090407:624979-627754):2364-2510	gb|CK541510.1|:68-214	1
BmHel-15(DF090342:4371948-4372385):1-94	gb|CK526303.1|:141-226	1
BmHel-15(DF090318:3597060-3597501):1-112	gb|BP179727.1|:30-141	0.9911
BmHel-15(DF090364:294001-296705):122-253	gb|AU003412.1|:335-466	0.9924
BmHel-15(DF090388:804008-810677):4934-5300	gb|BB988633.1|:343-709	0.9918
BmHel-15(DF090317:9958243-9958679):370-437	gb|AV404607.1|:12-79	1
BmHel-15(DF090360:1645496-1645925):1-101	gb|BP120390.1|:653-752	0.9901
BmHel-15(DF090393:1643410-1643838):362-416	gb|AV402993.1|:435-489	1
BmHel-15(DF090318:5516469-5516877):107-323	gb|CK562463.1|:271-567	0.9928
BmHel-15(DF090466:39287-40000):1-47	gb|CK525452.1|:339-385	1
BmHel-15(DF090363:2870083-2870486):337-383	gb|CK521961.1|:121-167	1
BmHel-15(DF090386:429453-430587):297-832	gb|DY230982.1|:261-503	0.9918
BmHel-15(DF090354:1967759-1968001):1-230	gb|CK552476.1|:124-538	1
BmHel-15(DF090435:66311-66750):1-47	gb|CK555987.1|:288-334	1
BmHel-15(DF090351:1295366-1297621):310-479	gb|BW997805.1|:1-170	0.9941
BmHel-15(DF090422:501676-502112):299-431	gb|BP119847.1|:641-767	0.9908
BmHel-15(DF090327:6013485-6013917):366-415	gb|CK524153.1|:119-168	1
BmHel-15(DF090344:3643789-3645748):1338-1676	gb|AV401253.1|:57-393	0.9912
BmHel-15(DF090425:734316-734896):1-143	gb|CK551220.1|:13-155	1
BmHel-15(DF090379:1778562-1778994):374-416	gb|CK549831.1|:572-614	1
BmHel-15(DF090378:52204-52596):267-393	gb|BP182579.1|:34-159	0.9921
BmHel-15(DF090493:165795-168140):1548-1631	gb|CK540194.1|:475-558	1
BmHel-15(DF090361:3198073-3198482):1-47	gb|CK553844.1|:43-89	1
BmHel-15(DF090327:2716068-2716464):341-374	gb|BP121007.1|:565-598	1
BmHel-15(DF090331:1234523-1234953):286-431	gb|BP120137.1|:633-778	1
BmHel-15(DF090348:1025468-1027291):900-1385	gb|CK541817.1|:46-531	0.9979
BmHel-15(DF090348:1025468-1027291):307-389	gb|DN237397.1|:60-142	1

BmHel-15(DF090319:8405019-8405342):1-32	gb|CK523036.1|:291-322	1
BmHel-15(DF090447:101549-102991):1260-1342	gb|BB983596.1|:190-272	1
BmHel-15(DF090390:1234892-1235671):713-767	gb|CK549831.1|:568-622	1
BmHel-15(DF090507:90362-90777):349-416	gb|CK521961.1|:100-167	1
BmHel-15(DF090348:416161-416861):367-401	gb|AU001285.1|:68-102	1
BmHel-15(DF090322:7030559-7040318):7801-7936	gb|CK556136.1|:263-398	1
BmHel-15(DF090576:1509-11518):6325-6574	gb|CK555176.1|:8-257	0.992
BmHel-15(DF090330:25428-27419):68-385	gb|AV403028.1|:14-331	0.9906
BmHel-15(DF090319:8796061-8796478):1-72	gb|BP125692.1|:314-385	1
BmHel-15(DF090420:879821-880272):1-41	gb|CK551978.1|:21-61	1
BmHel-15(DF090400:1036619-1037360):502-619	gb|BY930692.1|:443-560	0.9915
BmHel-15(DF090338:1786044-1787122):732-956	gb|CK552259.1|:186-410	0.9911
BmHel-15(DF090316:4640683-4647901):716-777	gb|CK553245.1|:261-322	1
BmHel-15(DF090320:7919399-7920426):182-274	gb|BP183662.1|:50-142	1
BmHel-15(DF090327:6013485-6013917):1-27	gb|CK494583.1|:235-261	1
BmHel-15(DF090338:3855621-3856192):390-507	gb|BP117864.1|:208-325	0.9915
BmHel-15(DF090323:707278-707746):15-63	gb|CK522481.1|:100-148	1
BmHel-15(DF090319:4402909-4403296):1-41	gb|CK545543.1|:414-454	1
BmHel-15(DF090433:374258-374561):1-138	gb|CK509125.1|:9-274	1
BmHel-15(DF090348:1025468-1027291):900-1492	gb|BY928568.1|:27-619	0.9933
BmHel-15(DF090411:765560-772603):6004-6215	gb|CK504192.1|:231-442	1
BmHel-15(DF090320:7919399-7920426):184-274	gb|BY925348.1|:461-551	1
BmHel-15(DF090322:1849655-1850095):1-49	gb|CK556303.1|:2-50	1
BmHel-15(DF090420:297368-297799):359-414	gb|CK524631.1|:118-173	1
BmHel-15(DF090364:294001-296705):195-253	gb|BP178158.1|:1-59	1
BmHel-15(DF090360:3111397-3111833):370-426	gb|CK524153.1|:112-168	1
BmHel-15(DF090372:73973-74522):401-460	gb|CK528348.1|:5-64	1
BmHel-15(DF090369:1667725-1668400):115-181	gb|CK526425.1|:237-303	1
BmHel-15(DF090414:1100309-1101446):136-332	gb|AU005741.1|:470-662	0.9916
BmHel-15(DF090411:518389-518953):397-500	gb|CK528713.1|:288-391	1
BmHel-15(DF090507:90362-90777):10-132	gb|CK554528.1|:378-500	0.9919
BmHel-15(DF090343:4327785-4328214):1-96	gb|AV400824.1|:677-772	1
BmHel-15(DF090338:2357897-2358595):1-113	gb|BP179727.1|:30-142	0.9912
BmHel-16(DF090329:5513416-5518497):4560-4793	gb|CK563015.1|:5-238	1
BmHel-16(DF090335:3627062-3631211):3779-3943	gb|BJ988505.1|:22-186	0.9939
BmHel-16(DF090378:1502417-1502622):2-206	gb|BP120838.1|:102-317	0.9947
BmHel-16(DF090340:2471714-2471931):1-133	gb|CK556834.1|:43-175	0.9925
BmHel-16(DF090357:2902649-2902866):1-218	gb|CK519413.1|:150-366	0.9948
BmHel-16(DF090344:2366151-2366367):1-217	gb|BP121132.1|:430-647	0.9946
BmHel-16(DF090438:500685-500897):57-213	gb|CK555019.1|:428-588	0.9928
BmHel-16(DF090403:839645-839849):1-96	gb|CK524488.1|:13-108	1
BmHel-16(DF090336:3389069-3396865):2356-2482	gb|BY914730.1|:295-421	0.9921
BmHel-16(DF090319:4773563-4773774):1-212	gb|CK502381.1|:422-635	0.993
BmHel-16(DF090317:4245241-4245458):1-113	gb|BP120498.1|:3-115	0.9912
BmHel-16(DF090327:2411853-2412056):9-204	gb|CK525052.1|:264-456	0.9939
BmHel-16(DF091420:1635-2033):1-399	gb|CN375974.1|:389-600	0.9948
BmHel-16(DF090368:1943632-1943849):1-90	gb|BY915257.1|:5-94	1
BmHel-16(DF090339:3934020-3934223):1-190	gb|BP118236.1|:449-640	0.9941

BmHel-16(DF090326:4721723-4721916):1-194	gb|CK556184.1|:92-299	0.9942
BmHel-16(DF090319:7071373-7071570):20-198	gb|CK554409.1|:19-200	0.9939
BmHel-16(DF090345:2122886-2129495):2818-4551	gb|CK495732.1|:217-367	0.9935
BmHel-16(DF090443:421917-422134):1-115	gb|BP120498.1|:1-115	1
BmHel-16(DF090364:1896124-1896327):1-204	gb|BY930027.1|:61-265	0.9946
BmHel-16(DF090319:4621027-4624608):3088-3309	gb|BY914162.1|:170-391	0.9955
BmHel-16(DF090324:5185017-5185216):1-200	gb|CK520308.1|:226-441	0.9946
BmHel-16(DF090409:1574884-1575098):2-215	gb|CK515676.1|:79-282	0.9943
BmHel-16(DF090377:1722644-1722860):1-217	gb|BY930027.1|:61-265	0.9946
BmHel-16(DF090343:513921-514128):9-200	gb|BY922385.1|:2-201	0.9938
BmHel-16(DF090335:5670061-5670266):1-206	gb|CK528627.1|:138-350	0.9941
BmHel-16(DF090357:422507-422674):2-108	gb|CK555831.1|:111-217	0.9907
BmHel-16(DF090414:1111089-1111301):1-133	gb|CK513446.1|:26-158	0.9925
BmHel-16(DF090340:386355-392164):2549-2603	gb|BP120921.1|:43-97	1
BmHel-16(DF090325:2694607-2704726):1681-2121	gb|CK520159.1|:5-445	0.9932
BmHel-16(DF090332:326426-334306):4411-4640	gb|BJ981285.1|:322-551	0.9913
BmHel-16(DF090321:4114927-4115144):1-218	gb|CK529695.1|:43-260	0.9944
BmHel-16(DF090319:4754545-4754759):1-201	gb|BP118236.1|:448-640	0.9943
BmHel-16(DF090366:432884-441413):6024-6859	gb|BY920209.1|:20-825	0.9983
BmHel-16(DF090494:72272-72490):21-219	gb|CK554409.1|:19-200	0.9943
BmHel-16(DF090362:1001554-1004561):1490-1621	gb|BJ986594.1|:1-132	0.9924
BmHel-16(DF090322:2518631-2518843):1-111	gb|BP124248.1|:12-122	0.991
BmHel-16(DF090335:3627062-3631211):45-245	gb|BJ982415.1|:4-204	1
BmHel-16(DF090460:81796-82009):8-214	gb|CK538564.1|:42-251	0.9941
BmHel-16(DF090317:4442123-4442341):20-219	gb|CK556964.1|:5-186	0.9943
BmHel-16(DF090403:866487-866703):4-217	gb|CK519584.1|:7-222	1
BmHel-16(DF090396:1005084-1009316):3736-3980	gb|BY938309.1|:172-416	0.9918
BmHel-16(DF090347:2945307-2951369):476-2211	gb|CK510930.1|:262-616	0.9944
BmHel-16(DF090363:1798281-1798490):12-86	gb|CK516321.1|:9-83	1
BmHel-16(DF090345:3258139-3259525):146-700	gb|BY933795.1|:32-338	0.9938
BmHel-16(DF090332:4853041-4853701):142-268	gb|BP122564.1|:1-127	0.9921
BmHel-16(DF090333:5864069-5864275):2-122	gb|BP124248.1|:1-122	0.9918
BmHel-16(DF090330:5028981-5029192):160-212	gb|CK528918.1|:24-76	1
BmHel-16(DF090321:5562852-5563064):1-213	gb|BP121132.1|:430-646	0.9947
BmHel-16(DF090347:4018425-4018641):1-202	gb|CK552276.1|:20-222	0.9941
BmHel-16(DF090324:5515293-5515509):1-217	gb|CK528999.1|:163-379	0.9946
BmHel-16(DF090324:1455323-1455538):2-216	gb|CK495823.1|:168-374	0.9938
BmHel-16(DF090376:2244895-2245110):1-216	gb|BP121345.1|:255-472	0.9901
BmHel-16(DF090362:511479-511973):33-150	gb|CK518533.1|:6-123	0.9915
BmHel-16(DF090482:157260-157481):77-222	gb|BP117278.1|:1-145	0.9917
BmHel-16(DF090375:1457406-1457572):2-167	gb|CK528387.1|:140-341	1
BmHel-16(DF090320:4202148-4205717):1-3556	gb|BP118236.1|:449-640	0.9945
BmHel-16(DF090317:4100241-4100471):1-231	gb|BY931613.1|:159-363	0.9946
BmHel-16(DF090471:108366-108587):170-222	gb|CK526890.1|:246-298	1
BmHel-16(DF090346:857520-864626):2060-2493	gb|AV402400.1|:1-433	0.9908
BmHel-16(DF090412:1361725-1361934):1-210	gb|CK556296.1|:88-298	0.9949
BmHel-16(DF090318:7758037-7765913):6488-6887	gb|BJ988910.1|:17-417	0.995
BmHel-16(DF090321:1109962-1110174):1-140	gb|CK516741.1|:202-337	0.9915

BmHel-16(DF090318:5730927-5734479):2335-2473	gb|BP115118.1|:1-140	0.9929
BmHel-16(DF090323:7181360-7181524):1-165	gb|CK517913.1|:87-304	0.99
BmHel-16(DF090337:1372134-1377631):3675-4034	gb|CK532964.1|:186-521	0.9912
BmHel-16(DF090354:383915-384150):1-236	gb|CK552657.1|:348-561	0.9938
BmHel-16(DF090316:9350597-9350809):31-213	gb|CK529708.1|:220-391	0.9927
BmHel-16(DF090340:386355-392164):2535-2603	gb|BB993456.1|:20-88	1
BmHel-16(DF090336:2466995-2468232):868-1061	gb|BP119679.1|:523-716	0.9948
BmHel-16(DF090326:1454174-1457639):2480-2510	gb|CK527662.1|:19-49	1
BmHel-16(DF090337:760461-760676):1-216	gb|CK552877.1|:75-289	0.9945
BmHel-16(DF090330:2748684-2757755):1351-1679	gb|AV403051.1|:2-330	1
BmHel-16(DF090339:4137764-4137971):1-208	gb|CK552657.1|:348-561	0.9948
BmHel-16(DF090354:383915-384150):1-236	gb|CK554169.1|:108-316	0.9937
BmHel-16(DF090423:445873-446057):83-185	gb|CK551884.1|:9-148	1
BmHel-16(DF090460:81796-82009):8-214	gb|CK554691.1|:22-232	0.9935
BmHel-16(DF090319:1833698-1833917):1-92	gb|CK522890.1|:14-105	1
BmHel-16(DF090414:488732-488949):1-218	gb|CK520308.1|:226-441	0.9946
BmHel-16(DF090363:1552928-1553134):6-207	gb|CN375975.1|:201-409	0.9944
BmHel-16(DF090332:326426-334306):3439-3696	gb|BP122435.1|:256-513	0.9922
BmHel-16(DF090365:984146-993990):8349-8913	gb|CN212264.1|:8-572	0.998
BmHel-16(DF090663:4443-4644):155-202	gb|CK556332.1|:64-111	1
BmHel-16(DF090374:683165-683379):1-215	gb|CK495823.1|:168-374	0.9946
BmHel-16(DF090411:1115645-1115864):168-220	gb|CK556332.1|:64-116	1
BmHel-16(DF090343:4284658-4284875):1-218	gb|CK528609.1|:275-492	0.9947
BmHel-16(DF090336:1654555-1660155):2301-2511	gb|CK551576.1|:49-259	0.9905
BmHel-16(DF090343:1316612-1316834):174-223	gb|CK528707.1|:575-624	1
BmHel-16(DF090386:1571185-1571402):4-218	gb|CK519584.1|:7-222	0.9947
BmHel-16(DF090423:445873-446057):133-183	gb|CK553020.1|:387-437	1
BmHel-16(DF090611:30956-31629):197-604	gb|CN374654.1|:18-425	0.9926
BmHel-16(DF090404:356300-356530):1-229	gb|BP184218.1|:29-245	0.9946
BmHel-16(DF090441:744708-745300):151-520	gb|CK515035.1|:15-383	0.9919
BmHel-16(DF090590:35456-35663):1-208	gb|CK526171.1|:79-286	1
BmHel-16(DF090329:5513416-5518497):4559-4837	gb|BB993351.1|:55-333	0.9928
BmHel-16(DF090337:1372134-1377631):3696-4034	gb|BP119766.1|:30-367	0.9941
BmHel-16(DF090421:739593-739810):1-129	gb|CK522460.1|:5-133	0.9922
BmHel-16(DF090335:5167895-5168107):1-138	gb|CK513446.1|:21-158	0.9928
BmHel-16(DF090362:2194387-2201525):5599-5808	gb|CK551881.1|:300-509	0.9905
BmHel-16(DF090363:1640559-1640781):87-138	gb|BW999470.1|:38-89	1
BmHel-16(DF090319:4621027-4624608):2705-2923	gb|CK517281.1|:398-616	0.9954
BmHel-16(DF090484:219923-222083):151-341	gb|CK497895.1|:5-194	0.9948
BmHel-16(DF090331:5354645-5354865):1-221	gb|CK520308.1|:226-441	0.9947
BmHel-16(DF090337:2085697-2085917):2-133	gb|AU002659.1|:231-362	0.9924
BmHel-16(DF090391:565762-565980):12-87	gb|CK516321.1|:8-83	1
BmHel-16(DF090336:3389069-3396865):5082-6536	gb|BY927137.1|:3-690	0.9913
BmHel-16(DF090330:2748684-2757755):1813-2435	gb|CK516420.1|:6-628	0.9952
BmHel-16(DF091420:1635-2033):8-399	gb|CK554551.1|:45-251	0.9946
BmHel-16(DF090324:4215452-4225156):4868-4941	gb|BP117709.1|:183-256	1
BmHel-16(DF090371:1541476-1541677):1-202	gb|CK528760.1|:140-341	0.9945
BmHel-16(DF090330:2748684-2757755):3151-3750	gb|BY931808.1|:15-615	0.9982

BmHel-16(DF090326:4102636-4102850):1-215	gb|BP120838.1|:102-317	0.9948
BmHel-16(DF090362:768539-768760):1-136	gb|CK529823.1|:15-150	1
BmHel-16(DF090866:5814-12072):5613-6143	gb|BJ988366.1|:17-547	0.9944
BmHel-16(DF090340:1438792-1438993):1-202	gb|CK527662.1|:294-495	1
BmHel-16(DF090444:600452-600697):1-244	gb|BP184218.1|:29-245	0.9901
BmHel-16(DF090334:4056878-4057096):20-219	gb|CK553394.1|:5-185	0.994
BmHel-16(DF090359:2403365-2403595):9-114	gb|CK557184.1|:5-110	0.9906
BmHel-16(DF090329:5914381-5914596):3-132	gb|CK554720.1|:82-211	0.9923
BmHel-16(DF090411:584200-591807):1-137	gb|AU002659.1|:231-367	0.9927
BmHel-16(DF090392:1082594-1082803):1-109	gb|BP178123.1|:8-116	0.9908
BmHel-16(DF090318:6660308-6660527):168-220	gb|CK523724.1|:392-444	1
BmHel-16(DF090376:2244895-2245110):4-216	gb|CK519584.1|:7-222	0.995
BmHel-16(DF090321:3904731-3909839):1003-1035	gb|CK542982.1|:278-310	1
BmHel-16(DF090317:223451-228408):455-1011	gb|BP120561.1|:74-435	0.9971
BmHel-16(DF090434:459729-459944):1-93	gb|BY915257.1|:1-93	1
BmHel-16(DF090347:3789126-3789332):2-207	gb|BP120838.1|:102-317	0.9949
BmHel-16(DF090325:4688631-4688848):1-110	gb|BP178426.1|:3-112	0.9909
BmHel-16(DF090349:3447846-3448771):544-617	gb|BY915036.1|:603-676	1
BmHel-16(DF090361:1511433-1518281):623-764	gb|CK552125.1|:162-303	0.993
BmHel-16(DF090320:4202148-4205717):3041-3361	gb|BJ988838.1|:18-338	0.9907
BmHel-16(DF090338:1193720-1198106):3307-3878	gb|CK552489.1|:1-572	0.9948
BmHel-16(DF090342:1790991-1791177):1-187	gb|CN375975.1|:201-413	0.9946
BmHel-16(DF090397:1085296-1085514):1-111	gb|BP178426.1|:3-113	1
BmHel-16(DF090318:7758037-7765913):5499-5734	gb|BJ981172.1|:100-336	0.9958
BmHel-16(DF090320:3679990-3680198):1-209	gb|BB992838.1|:75-290	0.9935
BmHel-16(DF090346:857520-864626):3884-4200	gb|CK526029.1|:29-345	0.9905
BmHel-16(DF090332:326426-334306):4411-4641	gb|BJ982306.1|:3-233	0.9957
BmHel-16(DF090366:432884-441413):6469-6859	gb|CK533826.1|:4-394	1
BmHel-16(DF090366:432884-441413):5652-6443	gb|BP122211.1|:1-766	0.9945
BmHel-16(DF090330:2748684-2757755):1803-2258	gb|BP180786.1|:66-521	1
BmHel-16(DF090318:627532-629157):365-403	gb|BB992201.1|:690-728	1
BmHel-16(DF090370:2708651-2708865):1-94	gb|CK522890.1|:11-104	1
BmHel-16(DF090339:1591484-1593266):1-26	gb|CK555562.1|:120-145	1
BmHel-16(DF090317:223451-228408):439-963	gb|BB990197.1|:1-525	0.9943
BmHel-16(DF090638:33488-33669):1-49	gb|AU235166.1|:46-94	1
BmHel-16(DF090342:2663794-2663995):2-192	gb|CK526303.1|:23-226	0.9942
BmHel-16(DF090324:5193373-5193590):1-218	gb|CK519413.1|:150-366	0.9948
BmHel-16(DF090351:3443971-3444182):83-212	gb|CK551884.1|:9-148	1
BmHel-16(DF090344:3850198-3854511):1842-2054	gb|CK551881.1|:296-508	1
BmHel-16(DF090321:1278949-1279149):1-201	gb|CK545409.1|:298-435	1
BmHel-16(DF090326:5820434-5821620):786-1166	gb|CK527578.1|:5-386	0.9971
BmHel-16(DF090339:1920429-1920643):1-215	gb|BP118383.1|:430-648	0.9947
BmHel-16(DF090342:4856620-4857211):150-354	gb|BB982706.1|:409-613	1
BmHel-16(DF090317:1078052-1078269):1-218	gb|CK515948.1|:335-552	0.9948
BmHel-16(DF090459:277563-277779):12-130	gb|CK521480.1|:104-223	0.9917
BmHel-16(DF090332:4961195-4961561):63-193	gb|BB983590.1|:18-148	0.9924
BmHel-16(DF090318:9098813-9099027):1-137	gb|AV398676.1|:454-590	0.9927
BmHel-16(DF090330:2748684-2757755):3164-3674	gb|BB983413.1|:50-560	1

BmHel-16(DF090365:2314109-2314325):1-217	gb|CK495823.1|:168-374	0.9949
BmHel-16(DF090346:857520-864626):4419-4684	gb|CK509583.1|:359-624	0.9925
BmHel-16(DF090345:2122886-2129495):1303-1360	gb|BY923545.1|:122-179	1
BmHel-16(DF090323:241069-241274):1-206	gb|CK529695.1|:43-260	0.9944
BmHel-16(DF090330:2836439-2840689):1673-1897	gb|BY926057.1|:25-249	0.9911
BmHel-16(DF090325:3887361-3892587):2398-2432	gb|CK520358.1|:316-350	1
BmHel-16(DF090347:2600792-2601640):485-785	gb|BP120666.1|:38-340	0.9934
BmHel-16(DF090326:1454174-1457639):3415-3466	gb|BP121720.1|:616-667	1
BmHel-16(DF090342:434117-435323):547-611	gb|CK522278.1|:2-66	1
BmHel-16(DF090320:4202148-4205717):46-267	gb|CK494417.1|:47-268	1
BmHel-16(DF090349:1020821-1021022):2-98	gb|CK553666.1|:8-104	1
BmHel-16(DF090362:346313-346523):1-211	gb|CK556184.1|:91-299	0.9943
BmHel-16(DF090360:1986188-1986323):1-136	gb|CK557269.1|:96-315	0.9907
BmHel-16(DF090342:3971097-3971318):1-222	gb|CK502381.1|:422-635	0.994
BmHel-16(DF090446:456854-457070):2-217	gb|BP120838.1|:102-317	0.99
BmHel-16(DF090352:1309604-1311520):10-42	gb|CK521361.1|:15-47	1
BmHel-16(DF090336:172117-172325):1-209	gb|BP121567.1|:10-219	0.9945
BmHel-16(DF090326:3712457-3712662):35-206	gb|CK529708.1|:220-391	1
BmHel-16(DF090317:9488899-9496750):2909-3146	gb|BY919005.1|:20-257	0.9958
BmHel-16(DF090352:2223799-2224002):1-204	gb|BB992838.1|:75-290	0.9943
BmHel-16(DF090330:2748684-2757755):2906-2955	gb|CK502129.1|:18-67	1
BmHel-16(DF090342:4856620-4857211):150-472	gb|BY922539.1|:51-408	0.9967
BmHel-16(DF090413:738345-742725):1326-1572	gb|BP122326.1|:64-310	0.9919
BmHel-16(DF090371:830988-831201):1-214	gb|CK502381.1|:422-635	0.9946
BmHel-16(DF091420:1635-2033):1-399	gb|CK529695.1|:43-259	0.9947
BmHel-16(DF090339:1289451-1289668):1-94	gb|BY915257.1|:1-94	1
BmHel-16(DF090319:7193264-7193468):1-205	gb|CK539902.1|:191-395	1
BmHel-16(BABH01049282:51-264):18-214	gb|CN374644.1|:425-623	0.9935
BmHel-16(DF090320:7919399-7924507):182-274	gb|CK524751.1|:17-109	1
BmHel-16(DF090369:2691667-2692334):56-439	gb|BB985308.1|:30-411	0.9922
BmHel-16(DF090374:2263723-2263974):1-134	gb|CK524488.1|:17-108	1
BmHel-16(DF090409:301024-301239):1-216	gb|CK520308.1|:226-441	0.9948
BmHel-16(DF090417:688805-689012):1-138	gb|AV398676.1|:453-590	0.9928
BmHel-16(DF090484:219923-222083):151-2047	gb|BY922539.1|:51-408	0.9907
BmHel-16(DF090345:3258139-3259525):146-767	gb|BP118988.1|:168-577	0.9943
BmHel-16(DF090443:151026-151234):1-209	gb|CK556184.1|:91-299	1
BmHel-16(DF090428:709025-712080):205-352	gb|CK523719.1|:482-629	1
BmHel-16(DF090639:16533-16751):1-219	gb|CK517913.1|:87-305	1
BmHel-16(DF090320:4202148-4205717):46-469	gb|CK518621.1|:4-427	1
BmHel-16(DF090444:439544-439761):9-218	gb|CK525052.1|:264-456	0.9944
BmHel-16(DF090344:3850198-3854511):1840-2054	gb|BP182110.1|:418-632	0.9953
BmHel-16(DF090353:3232440-3241704):2144-2689	gb|CK522779.1|:7-552	1
BmHel-16(DF090362:768539-768760):1-108	gb|CK529262.1|:1-108	1
BmHel-16(DF090420:771648-771864):1-111	gb|BP119559.1|:1-111	0.991
BmHel-16(DF090613:71067-71289):82-138	gb|CK524254.1|:8-64	1
BmHel-16(DF090473:233557-233760):1-204	gb|CK515676.1|:79-282	1
BmHel-16(DF090318:6196784-6196998):1-215	gb|BP121291.1|:256-472	0.9944
BmHel-16(DF090361:1576881-1577098):1-218	gb|BY930027.1|:61-265	0.995

BmHel-16(DF090322:4029433-4029784):197-305	gb|BY915145.1|:411-519	0.9908
BmHel-16(DF090316:7731130-7731346):1-217	gb|CK516787.1|:118-334	1
BmHel-16(DF090410:1042932-1043457):59-347	gb|BJ988631.1|:25-313	0.9965
BmHel-16(DF090347:2287887-2288106):1-220	gb|CK557269.1|:96-315	1
BmHel-16(DF090324:30003-30140):1-138	gb|CK545409.1|:298-435	1
BmHel-16(DF090345:3258139-3259525):192-1315	gb|BB990197.1|:1-519	0.9967
BmHel-16(DF090349:3447846-3448771):747-882	gb|CK523553.1|:8-125	1
BmHel-16(DF090344:359348-359564):20-217	gb|CK553394.1|:5-185	0.994
BmHel-16(DF090374:2263723-2263974):1-157	gb|BP120498.1|:1-115	0.9919
BmHel-16(DF090365:2314109-2314325):1-217	gb|CK516787.1|:118-333	0.99
BmHel-16(DF090410:1042932-1043457):196-347	gb|BJ986403.1|:19-170	0.9934
BmHel-16(DF090362:2219194-2219414):1-221	gb|AV403785.1|:51-263	0.9938
BmHel-16(DF090321:5983403-5983608):1-95	gb|CK522890.1|:11-105	1
BmHel-16(DF090356:1468624-1468845):4-222	gb|CK554720.1|:82-295	1
BmHel-16(DF090379:1060041-1060261):176-221	gb|BB987350.1|:36-81	1
BmHel-16(DF090328:5684240-5684449):1-133	gb|CK551895.1|:49-180	0.9925
BmHel-16(DF090323:7631505-7639779):3378-3495	gb|CK504979.1|:380-497	0.9915
BmHel-16(DF090389:1795692-1798251):839-1021	gb|BP182496.1|:166-348	0.9945
BmHel-16(DF090324:30003-30140):86-138	gb|CK525309.1|:8-60	1
BmHel-16(DF090336:784364-784584):170-221	gb|BB982208.1|:31-82	1
BmHel-16(DF090345:2122886-2129495):5863-6342	gb|BY914806.1|:2-366	1
BmHel-16(BABH01046468:1955-2171):20-217	gb|CK553394.1|:5-185	0.9942
BmHel-16(DF090319:3084685-3084906):1-219	gb|CK526702.1|:23-236	0.9939
BmHel-16(DF090578:3658-3875):20-218	gb|CK553394.1|:5-185	0.9943
BmHel-16(DF090358:2895671-2896397):1-727	gb|BP120838.1|:102-317	0.9952
BmHel-16(DF090348:4029659-4029876):10-218	gb|CK555489.1|:34-244	0.9939
BmHel-16(DF090316:13402910-13403126):1-217	gb|CK528999.1|:163-379	0.9947
BmHel-16(DF090358:3412773-3420615):1209-1238	gb|BY920701.1|:317-346	1
BmHel-16(DF090338:2354724-2358595):2754-2781	gb|BP182169.1|:671-698	1
BmHel-16(DF090373:148971-149186):1-112	gb|BP178426.1|:1-112	0.9911
BmHel-16(DF090376:2244895-2245110):9-216	gb|CK525052.1|:264-456	0.9947
BmHel-16(DF090316:14283226-14283448):171-223	gb|CK529482.1|:135-187	1
BmHel-16(DF090345:3258139-3259525):146-745	gb|BP115107.1|:1-389	0.992
BmHel-16(DF090316:14283226-14283448):173-223	gb|DY231086.1|:295-345	1
BmHel-16(DF090347:2600792-2601640):294-409	gb|BP123539.1|:107-222	0.9914
BmHel-16(DF090389:1795692-1798251):2091-2174	gb|BY933446.1|:436-519	1
BmHel-16(DF091366:417-630):9-214	gb|CK554691.1|:22-232	0.9941
BmHel-16(DF090325:7029723-7029935):3-213	gb|CK554720.1|:82-295	0.9939
BmHel-16(DF090385:120602-129994):6690-7054	gb|BP120442.1|:1-364	0.9973
BmHel-16(DF090331:207470-207687):1-130	gb|CK540003.1|:432-561	0.9923
BmHel-16(DF090358:3412773-3420615):1209-1249	gb|CK493504.1|:189-229	1
BmHel-16(DF090317:3329257-3332526):1091-1216	gb|BP125634.1|:451-576	0.9921
BmHel-16(DF090364:2436309-2436512):152-204	gb|CK554213.1|:460-512	1
BmHel-16(DF090386:812478-813678):1-1201	gb|CK520308.1|:226-441	0.9958
BmHel-16(DF090449:820162-820375):1-214	gb|BB992838.1|:75-290	0.9944
BmHel-16(DF090337:1372134-1377631):3675-3754	gb|BY915036.1|:604-683	1
BmHel-16(DF090329:4786666-4787386):233-419	gb|DN237396.1|:1-187	0.9947
BmHel-16(DF090336:3389069-3396865):2355-2469	gb|CK526821.1|:66-180	0.9913

BmHel-16(DF090332:326426-334306):3246-3696	gb|BJ987499.1|:25-476	0.9978
BmHel-17(DF090395:1116656-1117338):446-507	gb|BP121175.1|:15-76	1
BmHel-17(DF090395:1116656-1117338):238-507	gb|BY922686.1|:419-700	0.9927
BmHel-17(DF090323:7976517-7977055):133-299	gb|BJ988993.1|:18-184	0.994
BmHel-17(DF090362:518217-518686):9-124	gb|AU000055.1|:111-226	1
BmHel-17(DF090323:7976517-7977055):135-299	gb|BY914911.1|:332-496	0.9939
BmHel-18(DF090339:473537-474125):2-589	gb|CK516418.1|:155-353	0.9912
BmHel-18(DF090333:4396867-4397880):886-941	gb|BY942799.1|:504-559	1
BmHel-18(DF090358:2945871-2946097):153-227	gb|CK553475.1|:317-391	1
BmHel-18(DF090443:331164-331379):179-216	gb|BP121567.1|:10-47	1
BmHel-18(DF090318:2562096-2562309):165-199	gb|CK497353.1|:11-45	1
BmHel-18(DF090320:499378-499601):192-224	gb|CK552877.1|:257-289	1
BmHel-18(DF090317:1775098-1775299):146-200	gb|BP117847.1|:297-351	1
BmHel-18(DF090318:5020983-5021212):181-216	gb|CK497353.1|:11-46	1
BmHel-18(DF090433:466705-468079):146-1253	gb|CK517895.1|:369-634	0.9903
BmHel-18(DF090360:656089-656311):111-136	gb|CK519983.1|:315-340	1
BmHel-18(DF090357:2264646-2265471):591-645	gb|CK527368.1|:464-518	1
BmHel-18(DF090343:4245544-4245764):146-221	gb|CK553475.1|:317-392	1
BmHel-18(DF090320:6449222-6449445):192-224	gb|CK556332.1|:64-96	1
BmHel-18(DF090442:431778-432862):55-292	gb|CK520069.1|:70-307	0.9916
BmHel-18(DF090360:883431-883862):1-432	gb|CN376202.1|:220-432	0.9936
BmHel-18(DF090321:7414925-7415139):15-45	gb|CK529531.1|:56-86	1
BmHel-18(DF090646:3097-3678):451-508	gb|BJ987257.1|:17-74	1
BmHel-18(DF090329:2425187-2425423):1-83	gb|CK563410.1|:146-240	1
BmHel-18(DF090395:1679165-1679709):105-530	gb|BP121032.1|:610-709	1
BmHel-18(DF090345:2324049-2324264):167-216	gb|BY931082.1|:569-618	1
BmHel-18(DF090328:2074505-2075079):95-235	gb|CK556501.1|:30-182	0.993
BmHel-18(DF090358:2154087-2154310):1-224	gb|CK542628.1|:373-584	0.9938
BmHel-18(DF090386:1971495-1971707):131-200	gb|CK519065.1|:285-354	1
BmHel-18(DF090318:3407574-3408144):390-447	gb|CK529890.1|:551-608	1
BmHel-18(DF090442:272865-274205):181-221	gb|CK497991.1|:22-62	1
BmHel-18(DF090394:555767-556245):340-402	gb|CK551420.1|:292-354	1
BmHel-18(DF090433:466705-468079):137-1253	gb|BJ984371.1|:463-737	0.9954
BmHel-18(DF090379:1152114-1152743):579-630	gb|BY931082.1|:569-620	1
BmHel-18(DF090321:3450967-3451190):198-224	gb|CK523610.1|:374-400	1
BmHel-18(DF090363:2023182-2023402):193-221	gb|CN374644.1|:425-453	1
BmHel-18(DF090324:4852595-4852802):125-190	gb|CK560495.1|:266-331	1
BmHel-18(DF090451:511615-512055):244-358	gb|CK508694.1|:17-131	0.9913
BmHel-18(DF090346:384582-384791):1-42	gb|CK494673.1|:12-53	1
BmHel-18(DF090377:950993-951207):1-215	gb|CK520708.1|:44-238	1
BmHel-18(DF090390:295968-298784):2785-2817	gb|CK555713.1|:411-443	1
BmHel-18(DF090351:1351458-1352045):475-588	gb|CK524779.1|:86-199	0.9912
BmHel-18(DF090394:555767-556245):263-386	gb|CK525086.1|:497-620	0.9919
BmHel-18(DF090356:525987-526502):132-364	gb|CK555928.1|:7-239	0.9957
BmHel-18(DF090343:1822675-1822871):2-83	gb|BP181828.1|:422-503	1
BmHel-18(DF090365:2954498-2954711):182-214	gb|CK528387.1|:140-172	1
BmHel-18(DF090334:5243945-5244139):1-195	gb|BP120078.1|:346-560	0.9904
BmHel-18(DF090322:6958813-6959035):104-131	gb|CK519983.1|:319-346	1

BmHel-18(DF090362:2131518-2131741):106-134	gb|CK519983.1|:317-345	1
BmHel-18(DF090344:4201767-4201989):140-206	gb|CK560495.1|:266-332	1
BmHel-18(DF090347:2945307-2945840):454-534	gb|CK553475.1|:317-397	1
BmHel-18(DF090400:1131213-1131428):1-216	gb|CK520708.1|:44-238	1
BmHel-18(DF090365:2692450-2692666):1-43	gb|CK494673.1|:12-54	1
BmHel-18(DF090318:7843811-7845184):164-272	gb|CK515517.1|:458-566	0.9908
BmHel-18(DF090344:2899219-2899434):184-216	gb|BW999470.1|:122-154	1
BmHel-18(DF090344:807089-808077):106-617	gb|BB990663.1|:4-482	0.9936
BmHel-18(DF090486:11693-12036):312-344	gb|CK556296.1|:88-120	1
BmHel-18(DF090365:2560341-2560791):134-173	gb|CK556855.1|:16-55	1
BmHel-18(BABH01044948:2925-3140):45-205	gb|CK522485.1|:306-465	0.9916
BmHel-18(DF090360:2509225-2509444):188-220	gb|BY922441.1|:65-97	1
BmHel-18(DF090376:1604055-1604281):112-227	gb|CK501049.1|:506-621	1
BmHel-18(DF090326:1454174-1454439):215-266	gb|BP119311.1|:617-668	1
BmHel-18(DF090433:260389-260601):38-72	gb|CK539075.1|:640-674	1
BmHel-18(DF090325:6606183-6607411):196-719	gb|BP117911.1|:1-619	0.9918
BmHel-18(DF090451:321033-321257):170-225	gb|CK497353.1|:5-60	1
BmHel-18(DF090322:3788778-3793503):4463-4612	gb|BP118204.1|:1-150	0.9933
BmHel-18(DF090375:1377604-1378784):440-581	gb|CK520634.1|:457-598	0.993
BmHel-18(DF090456:226060-231932):549-5873	gb|CK555019.1|:428-679	0.9904
BmHel-18(DF090418:502848-503058):180-211	gb|CK554213.1|:460-491	1
BmHel-18(DF090323:5027773-5028225):415-453	gb|CK556332.1|:64-102	1
BmHel-18(DF090334:2145148-2145693):155-546	gb|CK532324.1|:71-462	0.9969
BmHel-18(DF090404:576839-578431):1341-1483	gb|CK493098.1|:5-147	0.993
BmHel-18(DF090356:2547386-2547602):185-217	gb|BB982208.1|:50-82	1
BmHel-18(DF090357:31805-32024):191-220	gb|CK523610.1|:374-403	1
BmHel-18(DF090347:2945307-2945840):131-163	gb|CK526036.1|:212-244	1
BmHel-18(DF090328:3636877-3637083):41-73	gb|BY940166.1|:461-493	1
BmHel-18(DF090364:2351860-2352417):142-174	gb|BP118791.1|:454-486	1
BmHel-18(DF090330:471-2056):626-689	gb|CK552294.1|:381-444	1
BmHel-18(DF090451:336063-336416):142-211	gb|AV404692.1|:410-479	1
BmHel-18(DF090405:36204-36406):3-197	gb|CN374918.1|:98-296	0.9942
BmHel-18(DF090401:1270094-1271713):349-514	gb|BY918239.1|:1-166	0.994
BmHel-18(DF090449:686173-686431):208-259	gb|BP119558.1|:616-667	1
BmHel-18(DF090483:106993-107571):340-456	gb|BP120139.1|:602-718	0.9915
BmHel-18(DF090332:1145821-1146022):5-202	gb|CK496268.1|:137-336	0.9915
BmHel-18(BABH01042926:271-632):84-188	gb|BY920320.1|:23-127	0.9905
BmHel-18(DF090378:48401-48605):1-205	gb|CK541157.1|:272-479	0.9906
BmHel-18(DF090357:2264646-2265471):85-146	gb|CK520626.1|:290-351	1
BmHel-18(DF090317:293609-294186):199-440	gb|BP178973.1|:1-268	0.9908
BmHel-18(DF090322:7764298-7764564):1-70	gb|CK542400.1|:370-439	1
BmHel-18(DF090483:106993-107571):15-66	gb|CK529531.1|:35-86	1
BmHel-18(DF090327:2633349-2633572):192-224	gb|BP180457.1|:31-63	1
BmHel-18(DF090342:171164-174209):1105-2529	gb|BP117081.1|:174-710	0.9908
BmHel-18(DF090344:807089-808077):619-826	gb|BJ980712.1|:354-561	0.9952
BmHel-18(DF090341:2797888-2799179):846-1095	gb|BB984132.1|:222-471	1
BmHel-18(DF090388:713894-714103):1-210	gb|CK541157.1|:272-479	0.994
BmHel-18(DF090329:5825331-5825794):1-37	gb|BY914784.1|:550-586	1

BmHel-18(DF090345:1992536-1996976):2766-2923	gb|BP181130.1|:289-446	1
BmHel-18(DF090388:426146-426740):113-448	gb|BP119766.1|:30-364	0.9911
BmHel-18(DF090386:1449602-1449823):27-76	gb|CK553416.1|:306-355	1
BmHel-18(DF090424:191342-193740):932-1277	gb|BY924300.1|:62-407	0.9913
BmHel-18(DF090343:1394061-1394275):1-73	gb|CK542400.1|:367-439	1
BmHel-18(DF090359:2371724-2371937):1-214	gb|CK526036.1|:23-244	0.9907
BmHel-18(DF090626:23848-26718):1-2871	gb|CK520708.1|:44-238	0.9906
BmHel-18(DF090319:4622493-4624608):1622-1875	gb|BP120089.1|:495-749	0.9961
BmHel-18(DF090401:52300-52506):1-89	gb|CK563410.1|:143-239	1
BmHel-18(DF090317:293609-294186):172-256	gb|AU005412.1|:2-86	1
BmHel-18(DF090344:807089-808077):238-763	gb|CK525562.1|:1-525	0.9905
BmHel-18(BABH01058086:224-440):1-217	gb|CK490677.1|:184-398	0.9902
BmHel-18(DF090340:1214566-1215136):99-150	gb|BW997868.1|:1-52	1
BmHel-18(DF090353:3611962-3612147):136-172	gb|BY933016.1|:450-486	1
BmHel-18(DF090322:3788778-3793503):4203-4301	gb|CK552786.1|:589-687	1
BmHel-18(DF090387:1371472-1371695):4-93	gb|BP120021.1|:510-599	1
BmHel-18(DF090352:1926896-1927111):1-216	gb|CK520708.1|:44-238	0.9925
BmHel-18(DF090363:1435385-1435768):157-288	gb|BP120641.1|:546-678	0.9925
BmHel-18(DF090451:65117-67324):1833-1908	gb|CK489089.1|:5-80	1
BmHel-18(DF090341:2797888-2799179):360-922	gb|CK553756.1|:1-621	0.9914
BmHel-18(DF090358:1352049-1352256):176-208	gb|CN375914.1|:332-364	1
BmHel-18(DF090334:2145148-2145693):1-68	gb|AU000085.1|:698-765	1
BmHel-18(DF090322:3788778-3793503):134-340	gb|CK528478.1|:16-173	0.9938
BmHel-18(DF090318:6351885-6352093):177-209	gb|CN374644.1|:425-457	1
BmHel-18(DF090428:3344-5509):1953-2006	gb|CK519574.1|:9-62	1
BmHel-18(DF090322:6038763-6039366):148-250	gb|CK539677.1|:127-229	0.9903
BmHel-18(DF090338:1702161-1706375):395-736	gb|BP117796.1|:19-360	0.9912
BmHel-18(DF090363:2441086-2441309):4-157	gb|CK494928.1|:252-397	1
BmHel-18(DF090318:3069576-3069792):185-217	gb|CK554720.1|:263-295	1
BmHel-18(DF090327:2153647-2153868):197-222	gb|CK555029.1|:354-379	1
BmHel-18(DF090339:1831338-1831557):1-220	gb|CK529394.1|:207-426	1
BmHel-18(DF090361:2424692-2424911):1-220	gb|CK522994.1|:116-335	0.9909
BmHel-18(DF090424:191342-193740):1725-2175	gb|BP177967.1|:264-714	0.9911
BmHel-18(DF090349:1449778-1450278):144-301	gb|CN376685.1|:24-181	0.9937
BmHel-18(DF090439:433167-433384):99-128	gb|CK519983.1|:317-346	1
BmHel-18(DF090345:3699641-3699847):1-49	gb|CK494673.1|:5-53	1
BmHel-18(DF090362:2087054-2088958):41-171	gb|AV404532.1|:272-403	0.9924
BmHel-18(DF090328:4903461-4903685):58-91	gb|BY940166.1|:461-494	1
BmHel-18(DF090451:65117-67324):1833-1911	gb|CK562257.1|:92-170	1
BmHel-18(BABH01044948:2925-3140):136-202	gb|CK554516.1|:255-321	1
BmHel-18(DF090327:2276075-2276287):131-200	gb|CK556046.1|:252-321	1
BmHel-18(DF090357:2264646-2265471):94-146	gb|CK516738.1|:328-380	1
BmHel-18(DF090340:1214566-1215136):178-449	gb|AU005412.1|:6-240	1
BmHel-18(DF090329:1708339-1709568):1-1194	gb|CN376201.1|:458-637	0.9915
BmHel-18(DF090351:1351458-1352045):106-467	gb|AV400531.1|:95-456	0.9917
BmHel-18(DF090913:10518-10724):175-207	gb|CK555029.1|:354-386	1
BmHel-18(DF090416:1255860-1256092):157-218	gb|BY933016.1|:451-512	1
BmHel-18(DF090326:4177116-4177348):73-101	gb|CK521540.1|:41-69	1

BmHel-18(DF090330:2074907-2075126):1-125	gb|CK521540.1|:8-132	0.992
BmHel-18(DF090390:648526-648966):142-194	gb|CK525001.1|:41-93	1
BmHel-18(DF090320:8093086-8096691):916-944	gb|CK551220.1|:293-321	1
BmHel-18(DF090424:191342-193740):1725-1977	gb|CK552786.1|:435-687	1
BmHel-18(DF090319:2540717-2540937):4-107	gb|BP120021.1|:510-613	0.9904
BmHel-18(DF090361:2364721-2364923):121-184	gb|BY933016.1|:460-523	1
BmHel-18(DF090351:3109222-3109429):1-208	gb|CK520708.1|:44-238	0.9929
BmHel-18(DF090331:2598756-2598966):1-211	gb|CK520708.1|:44-238	0.9929
BmHel-18(DF090375:1377604-1378784):49-203	gb|BJ980595.1|:415-569	0.9935
BmHel-18(DF090373:2053430-2055377):1673-1826	gb|CK521661.1|:5-158	1
BmHel-18(DF090336:5227284-5227823):47-288	gb|CK534114.1|:345-586	0.9917
BmHel-18(DF090318:9407141-9407397):225-257	gb|BP121720.1|:635-667	1
BmHel-18(DF090428:3344-5509):1152-1749	gb|CK526380.1|:1-423	0.9932
BmHel-18(DF090317:7127881-7128086):119-195	gb|BY933016.1|:447-523	1
BmHel-18(BABH01045310:1895-2115):1-221	gb|CK524611.1|:81-293	0.9942
BmHel-18(DF090316:1458428-1458822):233-312	gb|CK555210.1|:54-133	1
BmHel-18(DF090405:36204-36406):1-203	gb|CK541157.1|:272-478	0.9944
BmHel-18(DF090323:279272-279471):1-76	gb|BY914784.1|:550-625	1
BmHel-18(DF090328:2074505-2075079):420-445	gb|BP118278.1|:1-26	1
BmHel-18(DF090322:563036-563251):26-216	gb|CK554831.1|:8-198	1
BmHel-18(DF090316:7159944-7160523):217-275	gb|CK555161.1|:573-631	1
BmHel-18(DF090344:807089-808077):93-192	gb|CK487641.1|:1-100	0.99
BmHel-18(DF090355:2582484-2584995):1471-1912	gb|CK518365.1|:78-519	1
BmHel-18(DF090384:999285-999480):51-196	gb|CK524187.1|:180-357	0.9922
BmHel-18(DF090379:997584-997814):199-231	gb|BP181767.1|:427-459	1
BmHel-18(DF090349:2526814-2527030):46-211	gb|CN374919.1|:126-295	0.9924
BmHel-18(DF090375:1377604-1378784):49-281	gb|BP180630.1|:1-233	0.9914
BmHel-18(DF090476:32954-38251):4757-5166	gb|BY928230.1|:2-412	0.9976
BmHel-18(DF090451:158701-158917):106-217	gb|BY933494.1|:575-686	0.9911
BmHel-18(DF090433:466705-468079):136-1037	gb|BP121614.1|:136-639	0.9958
BmHel-18(DF090355:2582484-2584995):1471-1684	gb|BY925535.1|:2-215	0.9953
BmHel-18(DF090333:2534209-2539483):328-594	gb|BY917899.1|:1-268	0.9925
BmHel-18(DF090333:1053129-1053344):134-202	gb|CK554516.1|:252-320	1
BmHel-18(DF090342:171164-174209):282-397	gb|CK513775.1|:475-590	0.9914
BmHel-18(DF090352:67814-68035):148-220	gb|BP117847.1|:297-369	1
BmHel-18(DF090325:4864627-4864849):1-84	gb|CK539075.1|:591-674	1
BmHel-18(DF090352:4165517-4165797):228-281	gb|BB992201.1|:675-728	1
BmHel-18(DF090367:364073-364285):1-50	gb|CK494673.1|:5-54	1
BmHel-18(DF090396:762793-763000):125-194	gb|CK554644.1|:212-281	1
BmHel-18(DF090379:1284159-1284946):312-399	gb|CK524301.1|:246-333	1
BmHel-18(DF090373:2053430-2055377):1306-1623	gb|BY933173.1|:27-345	0.9906
BmHel-18(DF090401:1270094-1271713):1333-1419	gb|BY918635.1|:1-87	1
BmHel-18(DF090351:1293312-1293570):168-259	gb|CK555029.1|:354-445	1
BmHel-18(DF090381:1542022-1542269):197-248	gb|BP119405.1|:616-667	1
BmHel-18(DF090394:555767-556245):361-400	gb|CK556128.1|:19-58	1
BmHel-18(DF091074:4008-5555):1436-1463	gb|BY919113.1|:21-48	1
BmHel-18(DF090416:1255860-1256092):110-218	gb|BP121032.1|:610-709	1
BmHel-18(DF090463:360311-361749):379-665	gb|CK555817.1|:398-684	0.993

BmHel-18(DF090375:1377604-1378784):440-803	gb|BJ982315.1|:406-645	0.9923
BmHel-18(DF090339:166843-167058):1-216	gb|CK522174.1|:213-428	1
BmHel-18(DF090356:2872033-2880661):920-1282	gb|AV400531.1|:90-452	0.9917
BmHel-18(DF090575:15227-15717):95-121	gb|AV405562.1|:468-494	1
BmHel-18(DF090334:2200537-2200793):206-257	gb|BP120143.1|:616-667	1
BmHel-18(DF090416:81904-83471):127-1488	gb|BP121704.1|:287-457	1
BmHel-18(DF090327:5291267-5291564):183-298	gb|CK554043.1|:9-124	1
BmHel-18(DF090436:471313-473113):300-477	gb|BP120577.1|:15-191	0.9944
BmHel-18(DF090355:2582484-2584995):1471-1841	gb|BP119807.1|:1-371	1
BmHel-18(DF090363:1435385-1435768):230-306	gb|CK527124.1|:211-287	1
BmHel-18(DF090348:849089-849619):69-173	gb|CK527528.1|:531-635	0.9905
BmHel-18(DF090349:3258938-3259512):150-201	gb|CK517678.1|:145-196	1
BmHel-18(DF090436:471313-473113):1222-1685	gb|BY931580.1|:49-511	0.9914
BmHel-18(DF090428:3344-5509):666-899	gb|CK556424.1|:42-291	0.9915
BmHel-18(DF090362:1112268-1112489):1-106	gb|BP181986.1|:171-276	0.9906
BmHel-18(DF090476:32954-38251):4762-5174	gb|CK542411.1|:5-417	1
BmHel-18(DF090353:1402410-1402631):4-108	gb|BP120021.1|:510-614	0.9905
BmHel-18(DF090440:520221-520431):13-68	gb|CK529531.1|:33-88	1
BmHel-18(DF090322:417506-417721):1-94	gb|CK523253.1|:18-111	1
BmHel-18(DF090329:4051944-4052676):45-490	gb|BJ987252.1|:18-463	0.991
BmHel-18(DF090436:471313-473113):218-479	gb|BJ982558.1|:4-264	0.9962
BmHel-18(DF090355:2582484-2584995):1474-1898	gb|BP117019.1|:1-425	0.9976
BmHel-18(DF090401:1270094-1271713):995-1158	gb|CK528567.1|:257-420	1
BmHel-18(DF090433:466705-468079):1100-1253	gb|BB989811.1|:10-163	0.9935
BmHel-18(DF090404:576839-578431):990-1371	gb|CK537503.1|:5-385	0.9921
BmHel-18(DF090378:514889-515096):38-74	gb|BY940166.1|:458-494	1
BmHel-18(DF090701:9350-9568):2-219	gb|BY919955.1|:112-311	0.9913
BmHel-18(DF090355:1308160-1308795):140-262	gb|CK525170.1|:406-528	0.9919
BmHel-18(BABH01044460:4296-4510):1-81	gb|BY940166.1|:414-494	1
BmHel-18(DF090356:3757563-3757775):131-200	gb|CK560495.1|:266-335	1
BmHel-18(DF090319:6555624-6555901):1-61	gb|AU002813.1|:58-118	1
BmHel-18(DF090362:2087054-2088958):44-213	gb|CK524681.1|:365-535	0.9942
BmHel-18(DF090348:4090951-4091169):2-207	gb|BB982343.1|:336-549	0.993
BmHel-18(DF090377:1913436-1913656):189-221	gb|CK521480.1|:28-60	1
BmHel-18(DF090451:511615-512055):245-358	gb|AU003225.1|:33-146	0.9912
BmHel-18(DF090353:1640080-1640346):215-267	gb|BP181767.1|:427-479	1
BmHel-18(DF090460:69226-69757):66-200	gb|CN376142.1|:5-139	0.9926
BmHel-18(DF090318:7843811-7845184):164-267	gb|BY915116.1|:545-648	0.9904
BmHel-18(DF090322:403234-403414):1-28	gb|CK497191.1|:426-453	1
BmHel-18(DF090352:3044724-3045228):2-505	gb|BP120041.1|:454-668	0.9913
BmHel-18(DF090379:1152114-1152743):192-246	gb|AU002781.1|:1-55	1
BmHel-18(DF090325:3092746-3093211):240-315	gb|CK518739.1|:498-573	1
BmHel-18(DF090341:3295804-3296026):140-209	gb|CK563265.1|:269-338	1
BmHel-18(DF090395:1208356-1208786):87-188	gb|BP120215.1|:613-714	0.9902
BmHel-18(DF090463:360311-361749):1076-1189	gb|BY914251.1|:413-526	0.9912
BmHel-18(DF090351:2375303-2375850):137-187	gb|CK556855.1|:5-55	1
BmHel-18(DF090483:106993-107571):94-241	gb|CK556501.1|:30-176	0.9932
BmHel-18(DF090547:65848-66854):499-614	gb|CK553001.1|:134-249	1

BmHel-18(DF090502:107625-107830):1-143	gb|CK525667.1|:403-556	0.9902
BmHel-19(DF090322:4458698-4461159):399-529	gb|BP121103.1|:6-136	0.9924
BmHel-19(DF090434:350221-352168):284-645	gb|CK532964.1|:184-521	0.9912
BmHel-19(DF090316:9611109-9613270):986-1017	gb|BP180808.1|:385-416	1
BmHel-2(DF090345:2703166-2705458):1-290	gb|CK564259.1|:7-292	0.9962
BmHel-2(DF090320:2925001-2926607):789-907	gb|BY930772.1|:407-525	0.9916
BmHel-21(DF090400:74518-75895):128-910	gb|BP118756.1|:1-202	0.9962
BmHel-21(DF090342:1500619-1501130):273-300	gb|AU002781.1|:28-55	1
BmHel-21(DF090339:4726498-4727461):348-672	gb|BB987992.1|:215-539	1
BmHel-21(DF090400:74518-75895):267-389	gb|CK524779.1|:208-330	0.9919
BmHel-21(DF090415:650193-651249):615-645	gb|CK515387.1|:450-480	1
BmHel-21(DF090339:4726498-4727461):348-662	gb|BP118929.1|:1-364	0.9937
BmHel-21(DF090415:650193-651249):618-643	gb|CK527664.1|:254-279	1
BmHel-21(DF090390:1141008-1141800):377-469	gb|CK528608.1|:542-634	1
BmHel-21(DF090342:3798722-3800915):188-264	gb|CK509261.1|:469-557	1
BmHel-3a(DF090329:3602746-3604070):73-325	gb|BP178125.1|:44-296	0.9921
BmHel-3a(DF090353:3415767-3418392):539-602	gb|BY945067.1|:520-583	1
BmHel-3a(DF090333:517360-517750):102-141	gb|CK505211.1|:439-478	1
BmHel-3a(DF090318:13272-14908):1-143	gb|BP118401.1|:356-498	0.993
BmHel-3a(DF090424:658921-660047):1065-1127	gb|CK526254.1|:440-502	1
BmHel-3a(DF090355:1749986-1755656):4092-4300	gb|CK524798.1|:346-554	0.9952
BmHel-3a(DF090353:3415767-3418392):539-658	gb|CK552247.1|:5-123	0.9917
BmHel-3a(DF090329:2624279-2626163):349-389	gb|CK552892.1|:431-471	1
BmHel-3a(DF090360:682176-683728):720-1088	gb|BP118688.1|:70-439	0.9946
BmHel-3a(DF090342:2690079-2691771):474-524	gb|CK551654.1|:175-225	1
BmHel-3a(DF090322:5945680-5946891):1-269	gb|AU001264.1|:49-318	0.9955
BmHel-3a(DF090419:181520-182799):2-161	gb|CK534428.1|:72-231	0.9938
BmHel-3a(DF090322:1631017-1632460):1-1444	gb|AU000491.1|:229-520	1
BmHel-3a(DF090317:5814141-5815021):156-680	gb|CK525562.1|:1-525	0.9905
BmHel-3a(DF090396:17993-19933):738-804	gb|AV401586.1|:423-489	1
BmHel-3a(DF090317:7326923-7329338):1359-1417	gb|CK526242.1|:29-87	1
BmHel-3a(DF090391:239889-240674):167-576	gb|CN376862.1|:124-498	0.9923
BmHel-3a(DF090330:1749094-1750328):1182-1235	gb|CK552957.1|:348-401	1
BmHel-3a(DF090321:1090152-1092360):1749-1890	gb|CK533823.1|:7-148	0.993
BmHel-3a(DF090397:1028882-1029265):1-162	gb|CK519845.1|:233-394	0.9938
BmHel-3a(DF090330:1183112-1184764):784-1059	gb|BY924745.1|:113-398	0.9918
BmHel-3a(DF090354:3578363-3578757):1-164	gb|CK519845.1|:234-397	1
BmHel-3a(DF090331:4885891-4887568):816-1296	gb|CK529836.1|:18-499	0.9924
BmHel-3a(DF090350:1233689-1234812):352-627	gb|BY914842.1|:24-418	1
BmHel-3a(DF090356:2401858-2403409):1158-1220	gb|CK528279.1|:5-67	1
BmHel-3a(DF090392:1567615-1568818):1-269	gb|BP118401.1|:356-624	0.991
BmHel-3a(DF090328:1027231-1029289):399-656	gb|CK527777.1|:392-649	0.9922
BmHel-3a(DF090356:615674-617715):1717-1774	gb|BY930503.1|:595-652	1
BmHel-3a(DF090329:3602746-3604070):577-731	gb|CK509302.1|:490-644	1
BmHel-3a(DF090327:872572-873514):501-549	gb|BY922321.1|:583-631	1
BmHel-3a(DF090402:795607-796924):351-811	gb|BP122315.1|:1-417	0.9918
BmHel-3a(DF090332:3946327-3947514):167-989	gb|CN376862.1|:118-498	1
BmHel-3a(DF090343:2407780-2408748):2-162	gb|CK534428.1|:71-231	0.9938

BmHel-3a(DF090395:69726-72875):463-519	gb|BP182940.1|:429-485	1
BmHel-3a(DF090322:6792512-6794249):462-884	gb|BB990197.1|:97-519	0.9953
BmHel-3a(DF090318:6415458-6417339):1-267	gb|CK519471.1|:37-303	0.991
BmHel-3a(DF090356:2946083-2946749):439-634	gb|CK501349.1|:1-196	0.9949
BmHel-3a(DF090332:401041-408094):460-523	gb|CK556735.1|:308-371	1
BmHel-3a(DF090396:17993-19933):1019-1152	gb|CK520572.1|:18-151	0.9925
BmHel-3a(DF090334:2314086-2315636):1-162	gb|CK502332.1|:341-502	0.9938
BmHel-3a(DF090355:1749986-1755656):4917-5019	gb|CK528524.1|:23-125	0.9903
BmHel-3a(DF090317:5399272-5401256):1448-1802	gb|CK520945.1|:5-359	1
BmHel-3a(DF090355:1749986-1755656):1345-1456	gb|BP118690.1|:15-126	0.9911
BmHel-3a(DF090333:1201847-1202609):187-551	gb|BP121469.1|:1-366	0.9945
BmHel-3a(DF090329:5093763-5095373):1295-1351	gb|CK522880.1|:519-575	1
BmHel-3a(DF090377:2186424-2187803):97-516	gb|BW999294.1|:112-531	0.9905
BmHel-3a(DF090332:3946327-3947514):168-989	gb|DY230981.1|:254-629	0.9904
BmHel-3a(DF090354:3806806-3807873):2-163	gb|CK534428.1|:70-231	0.9938
BmHel-3a(DF090551:22940-23330):101-141	gb|CK505211.1|:438-478	1
BmHel-3a(DF090353:1234867-1236446):1013-1062	gb|AV405149.1|:1-50	1
BmHel-3a(DF090326:1714021-1714765):117-450	gb|BJ987548.1|:20-353	0.991
BmHel-3a(DF090343:4529412-4530485):353-554	gb|CK555798.1|:50-217	0.9942
BmHel-3a(DF090316:4119984-4124912):4047-4465	gb|BJ988214.1|:14-432	0.9905
BmHel-3a(DF090330:1183112-1184764):783-1059	gb|BY924414.1|:158-444	0.9918
BmHel-3a(DF090353:3415767-3418392):900-956	gb|BY942412.1|:591-647	1
BmHel-3a(DF090317:5399272-5401256):1645-1927	gb|CK519400.1|:24-307	0.9965
BmHel-3a(DF090330:6382854-6384051):179-437	gb|BJ987097.1|:18-276	0.9961
BmHel-3a(DF090323:2857099-2859062):1858-1930	gb|BP126238.1|:1-73	1
BmHel-3a(DF090347:3485477-3487150):1084-1201	gb|BY922175.1|:1-118	0.9915
BmHel-3a(DF090391:239889-240674):170-576	gb|BY918117.1|:215-586	0.9923
BmHel-3a(DF090355:1749986-1755656):4093-4190	gb|DN237397.1|:45-142	1
BmHel-3a(DF090367:1400081-1400469):1-167	gb|CK519845.1|:233-399	0.994
BmHel-3a(DF090326:3354131-3354495):39-134	gb|BP121420.1|:6-101	1
BmHel-3a(DF090332:3946327-3947514):167-985	gb|CK515035.1|:10-385	0.9952
BmHel-3a(DF090328:1027231-1029289):179-656	gb|BJ988277.1|:19-496	0.9958
BmHel-3a(DF090323:2107204-2109792):1898-1962	gb|CK551837.1|:401-465	1
BmHel-3a(DF090389:1687854-1688923):541-645	gb|BP114824.1|:260-364	0.9905
BmHel-3a(DF090316:4119984-4124912):1377-1783	gb|BJ987061.1|:21-427	0.9902
BmHel-3a(DF090330:1183112-1184764):780-1059	gb|BY924467.1|:151-440	0.9919
BmHel-3a(DF090359:2960091-2961116):927-1026	gb|BY932939.1|:35-135	1
BmHel-3a(DF090403:1099268-1100502):1173-1235	gb|CK545303.1|:263-325	1
BmHel-3a(DF090322:6792512-6794249):604-884	gb|AV404339.1|:251-531	0.9964
BmHel-3a(BABH01081216:108-432):213-237	gb|CK552957.1|:300-324	1
BmHel-3a(DF090333:1201847-1202609):478-561	gb|AV401111.1|:1-84	1
BmHel-3a(DF090386:463138-463653):185-261	gb|BJ987162.1|:18-94	1
BmHel-3a(DF090316:5925611-5927035):1-1198	gb|CK519845.1|:233-397	0.9943
BmHel-3a(DF090324:6585897-6592382):5768-6202	gb|BB982932.1|:53-486	0.9971
BmHel-3a(DF090386:463138-463653):185-267	gb|BJ986978.1|:20-102	1
BmHel-3a(DF090323:2107204-2109792):1896-1962	gb|CK554221.1|:401-467	1
BmHel-3a(DF090323:2857099-2859062):1720-1933	gb|CK551881.1|:296-509	0.9907
BmHel-3a(DF090341:3284646-3285959):848-974	gb|BY919002.1|:682-808	0.9921

BmHel-3a(DF090379:1145521-1146768):1193-1248	gb|AU000988.1|:577-632	1
BmHel-3a(DF090372:580411-581183):377-734	gb|AU003263.1|:381-738	0.9916
BmHel-3a(DF090432:533076-533756):464-523	gb|CK505211.1|:419-478	1
BmHel-3a(DF090328:1027231-1029289):179-654	gb|BJ987621.1|:19-494	0.9958
BmHel-3b(DF090377:922113-922501):39-127	gb|BP121420.1|:13-101	1
BmHel-3b(DF090374:1239293-1239669):1-127	gb|CK519845.1|:233-359	0.9921
BmHel-3b(DF090316:4119984-4124912):1377-1783	gb|BJ987252.1|:18-424	0.9902
BmHel-3b(DF090340:237636-239937):2237-2302	gb|BY915133.1|:328-393	1
BmHel-3b(DF090333:1201847-1202609):1-167	gb|BP121757.1|:19-185	1
BmHel-3b(DF090340:2720286-2721878):211-266	gb|BP122945.1|:72-127	1
BmHel-3b(DF090318:2419698-2421688):567-1028	gb|BB992149.1|:10-471	0.9913
BmHel-3b(DF090317:5399272-5401256):670-1082	gb|CK521456.1|:5-419	0.9928
BmHel-3b(DF090316:5925611-5927035):1069-1130	gb|CK522421.1|:3-64	1
BmHel-3b(DF090329:4381273-4381646):90-118	gb|BP114824.1|:25-53	1
BmHel-3b(DF090322:5269357-5269737):88-115	gb|AU003225.1|:646-673	1
BmHel-3b(DF090330:6382854-6384051):179-287	gb|CK551698.1|:22-130	0.9908
BmHel-3b(DF090353:3415767-3418392):904-956	gb|BY938341.1|:666-718	1
BmHel-3b(DF090317:9743378-9745386):365-491	gb|CK520613.1|:24-149	0.9921
BmHel-3b(DF090316:4119984-4124912):4047-4465	gb|BJ986954.1|:16-434	0.9905
BmHel-3b(DF090319:2349555-2350718):352-737	gb|CK552892.1|:114-471	0.9906
BmHel-3b(DF090322:6792512-6794249):395-722	gb|BB992633.1|:422-749	0.997
BmHel-3b(DF090317:5399272-5401256):410-953	gb|CK518473.1|:4-547	0.9908
BmHel-3b(DF090339:1249527-1251084):1203-1236	gb|CK526719.1|:57-90	1
BmHel-3b(DF090380:1443753-1444118):1-133	gb|CK522892.1|:381-513	0.9925
BmHel-3b(DF090376:100016-101265):1197-1245	gb|CK519642.1|:571-619	1
BmHel-3b(DF090325:3481841-3482860):1-267	gb|BP117991.1|:193-459	0.9908
BmHel-3b(DF090328:1027231-1029289):684-787	gb|CK555456.1|:533-636	0.9904
BmHel-3b(DF090333:3693347-3693737):255-391	gb|AU002602.1|:1-136	0.992
BmHel-3b(DF090342:2524283-2525402):1-162	gb|CK519471.1|:142-303	0.9938
BmHel-3b(DF090355:1749986-1755656):3633-3985	gb|CK541817.1|:268-620	0.9972
BmHel-3b(DF090329:3602746-3604070):979-1091	gb|CK502142.1|:234-346	1
BmHel-3b(DF090386:463138-463653):192-267	gb|BJ987113.1|:21-96	1
BmHel-3b(DF090317:7735210-7736331):1069-1122	gb|CK554847.1|:366-419	1
BmHel-3b(DF090329:3602746-3604070):981-1091	gb|BY933494.1|:429-539	0.991
BmHel-3b(DF090324:6585897-6592382):6147-6202	gb|BP181922.1|:36-91	1
BmHel-3b(DF090328:4122952-4124206):478-859	gb|CK551654.1|:8-347	1
BmHel-3b(DF090317:2077951-2078349):6-321	gb|CK524931.1|:298-608	0.9924
BmHel-3b(DF090350:3877342-3877547):86-206	gb|CK519130.1|:30-330	0.9918
BmHel-3b(DF090413:45472-46499):85-247	gb|CK556388.1|:1-163	0.9906
BmHel-3b(DF090342:3075840-3076984):1-268	gb|BP117991.1|:194-461	0.9909
BmHel-3b(DF090317:7326923-7329338):1359-1417	gb|CK521956.1|:24-82	1
BmHel-3b(DF090395:69726-72875):463-562	gb|CK522002.1|:24-123	1
BmHel-3b(DF090335:2246147-2247400):1198-1254	gb|CK522207.1|:43-99	1
BmHel-3b(DF090379:279551-282265):1-163	gb|CK505759.1|:408-570	0.9939
BmHel-3b(DF090317:7326923-7329338):1754-1798	gb|CK554221.1|:423-467	1
BmHel-3b(DF090317:5399272-5401256):358-911	gb|BJ986407.1|:18-571	0.9982
BmHel-3b(DF090390:1324632-1326230):647-714	gb|CK547122.1|:504-571	1
BmHel-3b(DF090353:3415767-3418392):539-658	gb|CK553722.1|:5-124	1

BmHel-3b(DF090351:3015338-3016864):642-758	gb|BP178123.1|:1-117	0.9915
BmHel-3b(DF090317:5814141-5815021):149-453	gb|BJ988111.1|:163-467	0.9902
BmHel-3b(DF090324:6585897-6592382):5768-6202	gb|AV405729.1|:134-568	0.9914
BmHel-3b(DF090322:3833871-3835104):1-396	gb|BP117991.1|:193-588	0.9914
BmHel-3b(DF090351:3244913-3246456):1160-1212	gb|CK522395.1|:23-75	1
BmHel-3b(DF090322:5945680-5946891):114-269	gb|CK506001.1|:44-199	1
BmHel-3b(DF090317:512042-512573):324-442	gb|BJ987097.1|:26-144	0.9916
BmHel-3b(DF090365:1497183-1498753):1518-1571	gb|CK545303.1|:263-316	1
BmHel-3b(DF090359:2960091-2961116):349-542	gb|CK555798.1|:288-468	0.9914
BmHel-3b(DF090377:744314-753013):926-1002	gb|AU003225.1|:536-612	1
BmHel-3b(DF090367:2633106-2634671):208-265	gb|BP122945.1|:70-127	1
BmHel-3b(DF090355:2149960-2151704):332-959	gb|CK555019.1|:428-629	0.99
BmHel-3b(DF090322:6792512-6794249):440-884	gb|BP119350.1|:356-685	0.9968
BmHel-3b(DF090367:2633106-2634671):900-932	gb|CK493458.1|:29-61	1
BmHel-3b(DF090318:6415458-6417339):1821-1880	gb|CK519642.1|:568-627	1
BmHel-3b(DF090321:1090152-1092360):1461-1892	gb|BP122508.1|:175-607	0.9931
BmHel-3b(DF090316:12339936-12340322):87-138	gb|CK505211.1|:423-474	1
BmHel-3b(DF090386:1458905-1460776):206-265	gb|BP122945.1|:68-127	1
BmHel-3b(DF090322:1631017-1632460):487-1444	gb|CK542981.1|:221-454	1
BmHel-3b(DF090351:3244913-3246456):822-878	gb|BJ983856.1|:1-57	1
BmHel-3b(DF090353:1234867-1236446):955-1257	gb|BB983737.1|:213-515	0.9934
BmHel-3b(DF090386:463138-463653):185-267	gb|BJ988433.1|:19-101	1
BmHel-3b(DF090378:696977-698023):695-840	gb|BY919199.1|:644-789	0.9932
BmHel-3b(DF090425:418630-421195):2142-2312	gb|CK522880.1|:411-581	0.9903
BmHel-3b(DF090465:30890-32740):1540-1851	gb|CK555351.1|:106-417	1
BmHel-3b(DF090318:2419698-2421688):41-188	gb|BP118920.1|:302-449	0.9932
BmHel-3b(DF090329:3602746-3604070):979-1099	gb|CK502023.1|:211-331	0.9917
BmHel-3b(DF090413:45472-46499):327-583	gb|BY914842.1|:2-311	0.9927
BmHel-3b(DF090347:3485477-3487150):1085-1187	gb|CK518868.1|:151-253	1
BmHel-3b(DF090395:69726-72875):465-560	gb|BY916969.1|:456-551	1
BmHel-3b(DF090332:3946327-3947514):167-989	gb|CN376861.1|:107-487	0.9952
BmHel-3b(DF090328:1027231-1029289):179-605	gb|BJ988483.1|:18-444	0.9953
BmHel-3b(DF090327:872572-873514):501-549	gb|CK539187.1|:13-61	1
BmHel-3b(DF090350:2496479-2497581):237-345	gb|CK526770.1|:134-242	1
BmHel-3b(DF090339:630666-631897):1165-1232	gb|BY915133.1|:328-395	1
BmHel-3b(DF090412:480439-486379):2603-3202	gb|CK514159.1|:31-630	0.9917
BmHel-3b(DF090372:1782630-1783007):1-127	gb|CK557323.1|:144-270	0.9921
BmHel-3b(DF091188:12475-12868):257-394	gb|AU002602.1|:1-136	0.9921
BmHel-3b(DF090347:3485477-3487150):1084-1227	gb|AU003263.1|:595-738	0.9931
BmHel-3b(DF090332:3946327-3947514):579-973	gb|BP119195.1|:1-359	0.9913
BmHel-3b(DF090322:5945680-5946891):1-269	gb|CK502332.1|:341-609	0.9955
BmHel-4(DF090364:265484-275413):1193-1264	gb|CK520039.1|:7-78	1
BmHel-4(DF090341:3778708-3779293):236-267	gb|CK542633.1|:395-426	1
BmHel-4(DF090837:13171-13764):7-84	gb|BP120689.1|:540-617	1
BmHel-4(DF090355:2158290-2159048):620-670	gb|BJ984635.1|:5-55	1
BmHel-4(DF090364:265484-275413):1191-1295	gb|CK528911.1|:4-108	0.9905
BmHel-4(DF090341:3778708-3779293):182-206	gb|CK541395.1|:193-217	1
BmHel-4(DF090439:425746-426477):310-419	gb|CK554702.1|:8-117	0.9909

BmHel-4(DF090386:1517098-1517790):271-376	gb|BY932218.1|:29-134	0.9906
BmHel-4(DF090414:1318475-1319729):396-775	gb|CK552622.1|:228-614	0.9904
BmHel-4(DF090411:907851-909135):327-378	gb|CK546156.1|:372-423	1
BmHel-4(DF090318:9304371-9304677):235-297	gb|CK542633.1|:397-459	1
BmHel-6(DF090358:1155721-1156386):640-665	gb|CK504921.1|:176-201	1
BmHel-7(DF090331:32405-32656):15-92	gb|CK519470.1|:472-549	1
BmHel-7(DF090462:419757-420005):1-249	gb|AU005989.1|:29-277	0.992
BmHel-7(DF090680:4160-4760):314-420	gb|CK529675.1|:177-283	0.9907
BmHel-7(DF090317:327704-329167):1144-1324	gb|CK560320.1|:64-245	0.9945
BmHel-7(DF090367:1477208-1477434):68-125	gb|BP178625.1|:170-227	1
BmHel-7(DF090344:663358-663577):48-152	gb|BP120004.1|:1-105	0.9905
BmHel-7(DF090334:2516016-2516721):71-667	gb|BP178625.1|:172-314	0.9909
BmHel-7(DF090342:1521659-1524754):631-967	gb|CK524739.1|:43-379	0.9911
BmHel-7(BABH01050671:378-629):57-176	gb|CK559419.1|:32-151	0.9917
BmHel-7(DF090323:6777287-6778950):289-717	gb|BY923070.1|:112-540	0.993
BmHel-7(DF090439:561651-561870):192-220	gb|CK518622.1|:37-65	1
BmHel-7(DF090326:4129139-4129390):196-252	gb|CK518622.1|:9-65	1
BmHel-7(DF090369:2632947-2640362):247-521	gb|BJ984371.1|:463-737	0.9964
BmHel-7(DF090340:1760550-1760750):1-98	gb|CK522155.1|:455-603	0.99
BmHel-7(DF090374:1578336-1578842):178-406	gb|BP119800.1|:119-347	0.9956
BmHel-7(DF090317:327704-329167):1171-1324	gb|CK550405.1|:44-198	0.9935
BmHel-7(DF090342:3524452-3526064):1001-1465	gb|BJ980841.1|:35-499	0.9935
BmHel-7(DF090427:268270-268513):14-229	gb|AV404349.1|:403-592	0.9935
BmHel-7(DF090337:760198-760446):1-249	gb|CK552877.1|:304-552	1
BmHel-7(DF090381:1352093-1352341):36-123	gb|CK507865.1|:539-626	1
BmHel-7(DF090422:328164-330540):65-202	gb|CK528287.1|:8-145	0.9928
BmHel-7(DF090422:328164-330540):281-445	gb|CK498325.1|:502-666	0.9939
BmHel-7(DF090322:7519991-7526188):5572-5763	gb|CK537503.1|:423-614	0.9948
BmHel-7(DF090373:28281-28647):87-161	gb|BY939347.1|:1-75	1
BmHel-7(DF090388:1231520-1231771):1-252	gb|CK524519.1|:294-545	1
BmHel-7(DF090417:1040300-1040553):98-149	gb|CK556336.1|:11-62	1
BmHel-7(DF090381:1469596-1470013):394-418	gb|CK511417.1|:481-505	1
BmHel-7(DF090423:120660-121047):48-157	gb|BY927639.1|:331-440	1
BmHel-7(DF090423:120660-121047):50-157	gb|CK556959.1|:72-179	0.9907
BmHel-7(DF090386:533831-534027):1-126	gb|BP117488.1|:254-401	0.9915
BmHel-7(DF090330:1078177-1078424):34-133	gb|CK507865.1|:529-628	0.99
BmHel-7(DF090340:3333726-3334348):28-262	gb|CN376864.1|:5-239	1
BmHel-7(DF090384:1127495-1127875):45-148	gb|BY922175.1|:15-118	0.9904
BmHel-7(DF090336:4035129-4035380):99-224	gb|CK513121.1|:459-584	0.9921
BmHel-7(DF090330:2758377-2758598):23-92	gb|AV404351.1|:416-485	1
BmHel-7(DF090334:3824737-3824984):29-129	gb|CK507865.1|:534-634	0.9901
BmHel-7(DF090422:328164-330540):241-445	gb|BJ982315.1|:441-645	0.9902
BmHel-7(DF090414:286308-290133):1295-1790	gb|BP181075.1|:26-521	0.994
BmHel-7(DF090318:2505973-2506607):1-374	gb|AV404351.1|:400-773	1
BmHel-7(DF090342:3524452-3526064):1001-1427	gb|BJ982098.1|:1-427	0.9906
BmHel-7(DF090423:120660-121047):49-157	gb|CK508649.1|:183-291	0.9908
BmHel-7(DF090369:2632947-2640362):256-521	gb|CK517895.1|:369-634	0.9925
BmHel-7(DF090318:6508849-6510779):1287-1312	gb|CK518739.1|:165-190	1

BmHel-7(DF090378:818377-818858):233-357	gb|CK525086.1|:497-621	0.992
BmHel-7(DF090322:7519991-7526188):5539-5763	gb|CK538809.1|:379-603	1
BmHel-7(DF090325:3579108-3579337):70-191	gb|BP178625.1|:171-314	0.9919
BmHel-7(DF090322:8070956-8076999):5687-5832	gb|DN236976.1|:1-146	0.9932
BmHel-7(DF090340:3333726-3334348):29-393	gb|BP118963.1|:1-364	0.9973
BmHel-7(DF090370:2457056-2457260):98-185	gb|CK556336.1|:11-113	1
BmHel-7(DF090386:533831-534027):14-126	gb|CK526288.1|:188-322	0.9906
BmHel-7(DF090344:1344072-1344415):89-198	gb|BB988379.1|:667-776	0.9909
BmHel-7(DF090333:1166869-1167090):37-136	gb|CK511417.1|:579-678	0.99
BmHel-7(DF090322:7519991-7526188):517-648	gb|CK546851.1|:45-176	1
BmHel-7(DF090322:8070956-8076999):5671-5832	gb|AU000375.1|:621-782	1
BmHel-7(DF090388:1466980-1467544):206-450	gb|BP121253.1|:526-710	0.996
BmHel-7(DF090414:286308-290133):1258-1412	gb|CK555938.1|:4-158	0.9935
BmHel-7(DF090486:94349-94568):1-220	gb|CK557039.1|:38-257	1
BmHel-7(DF090367:1568655-1568905):190-251	gb|CK518622.1|:4-65	1
BmHel-7(DF090363:2822506-2823350):164-270	gb|BP122061.1|:475-581	0.9907
BmHel-7(DF090586:12792-16003):3045-3097	gb|BY925942.1|:33-85	1
BmHel-7(DF090441:549112-549490):61-163	gb|BY921730.1|:18-120	0.9903
BmHel-7(DF090395:136053-137438):1201-1249	gb|CK527822.1|:305-353	1
BmHel-7(DF090317:8501675-8501926):23-90	gb|CK542602.1|:442-509	1
BmHel-7(DF090323:6777287-6778950):1154-1570	gb|BJ988087.1|:17-433	0.9904
BmHel-7(DF090317:1535313-1535523):17-42	gb|CK524022.1|:188-213	1
BmHel-7(DF090317:7457897-7458147):8-91	gb|CK556296.1|:5-88	1
BmHel-7(DF090323:6777287-6778950):1115-1365	gb|CK520544.1|:194-444	0.992
BmHel-7(DF090340:3333726-3334348):354-403	gb|BB982069.1|:29-78	1
BmHel-7(DF090447:358668-358915):29-124	gb|CK559557.1|:27-122	1
BmHel-7(DF090378:818377-818858):332-367	gb|CK555854.1|:18-53	1
BmHel-7(BABH01070669:29-622):90-221	gb|CK515369.1|:450-581	1
BmHel-8(DF090378:1926625-1927057):1-107	gb|CK527387.1|:150-256	1
BmHel-8(DF090444:561610-562028):1-27	gb|CK519130.1|:3-29	1
BmHel-8(DF090317:10448499-10448897):290-316	gb|CK539986.1|:190-216	1
BmHel-8(DF090335:845566-845986):389-421	gb|CK547449.1|:99-131	1
BmHel-8(DF090332:6288861-6289285):375-425	gb|CK520451.1|:31-81	1
BmHel-8(DF090322:2131916-2133903):302-821	gb|BJ980408.1|:1-520	0.9981
BmHel-8(DF090572:1492-1911):341-420	gb|AV405210.1|:53-132	1
BmHel-8(DF090362:507126-511051):3445-3543	gb|CN212224.1|:5-103	1
BmHel-8(DF090346:3900039-3900463):318-425	gb|CK518664.1|:52-159	0.9907
BmHel-8(DF090317:8638454-8640659):420-460	gb|BB988626.1|:255-295	1
BmHel-8(DF090327:6354475-6355151):291-329	gb|CK556128.1|:19-57	1
BmHel-8(DF090365:1227508-1228427):265-421	gb|BY917067.1|:185-341	0.9936
BmHel-8(DF090375:1394642-1395083):1-122	gb|CK525170.1|:407-528	0.9918
BmHel-8(DF090365:993804-994306):108-156	gb|CK555562.1|:432-480	1
BmHel-8(DF090322:3367383-3368144):127-435	gb|BJ987126.1|:20-328	0.9935
BmHel-8(DF090340:2294119-2294492):337-374	gb|CK547449.1|:99-136	1
BmHel-8(DF090358:3088153-3088630):89-117	gb|AV398234.1|:117-145	1
BmHel-8(DF090327:5810915-5813896):272-411	gb|CK556962.1|:436-575	0.9929
BmHel-8(DF090366:1015642-1017448):135-572	gb|BJ986647.1|:17-454	0.9909
BmHel-8(DF090319:1280008-1281477):1025-1267	gb|BJ985772.1|:1-243	0.9918

BmHel-8(DF090321:1905841-1906253):261-291	gb|CK539986.1|:155-185	1
BmHel-8(DF090547:70519-71763):1086-1202	gb|CK527576.1|:8-124	0.9915
BmHel-8(DF090363:1777217-1777590):326-374	gb|CK547449.1|:99-147	1
BmHel-8(DF090379:347576-347944):101-130	gb|BP117991.1|:151-180	1
BmHel-8(DF090354:3554701-3555133):266-290	gb|CK545303.1|:546-570	1
BmHel-8(DF090322:1692177-1702273):3835-4015	gb|BP178415.1|:443-623	0.9945
BmHel-8(DF090322:5360085-5363455):1160-1220	gb|CK522468.1|:21-81	1
BmHel-8(DF090397:260047-261912):754-1470	gb|BY928700.1|:2-712	0.9913
BmHel-8(DF090336:730820-731238):32-91	gb|BP117991.1|:11-70	1
BmHel-8(DF090367:665689-666106):329-418	gb|CK514689.1|:35-124	1
BmHel-8(DF090342:3689075-3689496):1-123	gb|BY922165.1|:511-633	0.9919
BmHel-8(DF090324:2841263-2841987):343-575	gb|BB992575.1|:19-251	0.9914
BmHel-8(DF090391:834444-834856):1-184	gb|AV405232.1|:300-485	0.9937
BmHel-8(DF090321:1280630-1282211):847-877	gb|BY944965.1|:142-172	1
BmHel-8(DF090328:1865164-1865676):1-128	gb|CK552943.1|:314-441	1
BmHel-8(DF090331:719650-720348):393-501	gb|CK551720.1|:530-638	0.9908
BmHel-8(DF090372:1799369-1800151):467-590	gb|BY927708.1|:603-727	0.992
BmHel-8(DF090347:480418-481639):355-999	gb|CK555462.1|:7-651	0.9938
BmHel-8(DF090318:7198681-7199139):317-459	gb|CK518666.1|:16-120	0.9931
BmHel-8(DF090422:973353-974091):1-127	gb|BY922165.1|:511-637	0.9921
BmHel-8(DF090326:5044018-5044439):318-422	gb|BY928646.1|:1-105	0.9905
BmHel-8(DF090316:2346549-2348992):2327-2354	gb|CK484912.1|:40-67	1
BmHel-8(DF090354:1273838-1279466):296-706	gb|BP123298.1|:163-525	0.9913
BmHel-8(DF090414:1361598-1362351):124-391	gb|CK546317.1|:374-641	0.9963
BmHel-8(DF090335:5376589-5377246):293-512	gb|CK552349.1|:366-584	0.9947
BmHel-8(DF090324:4397293-4397989):449-660	gb|BY915951.1|:13-224	0.9906
BmHel-8(DF090397:1021922-1022341):318-420	gb|CK514689.1|:22-124	0.9903
BmHel-8(DF090327:4316874-4318459):373-427	gb|CK498448.1|:25-79	1
BmHel-8(DF090320:6927649-6932185):4052-4292	gb|CK553828.1|:396-636	0.9917
BmHel-8(DF090410:657095-661899):1493-1933	gb|CK525787.1|:68-509	0.9919
BmHel-8(DF090362:2163882-2168331):3985-4210	gb|AV405836.1|:74-299	1
BmHel-8(DF090429:515526-515946):348-421	gb|CK552276.1|:222-295	1
BmHel-8(DF090399:1224385-1226894):2218-2274	gb|CK522784.1|:547-603	1
BmHel-8(DF090379:949339-949726):247-274	gb|BP183662.1|:205-232	1
BmHel-8(DF090424:738042-738470):330-361	gb|CK528608.1|:539-570	1
BmHel-8(DF090436:420749-421176):318-356	gb|CK539986.1|:189-227	1
BmHel-8(DF090446:458351-458815):360-465	gb|CK554071.1|:50-129	1
BmHel-8(DF090341:1577533-1584040):637-834	gb|CK541758.1|:432-629	0.9949
BmHel-8(DF090319:1280008-1281477):1167-1265	gb|CK563741.1|:46-144	1
BmHel-8(DF090327:5810915-5813896):272-371	gb|BY918805.1|:23-122	0.99
BmHel-8(DF090325:4342532-4344332):1547-1597	gb|BY925942.1|:34-84	1
BmHel-8(DF090362:47422-48293):456-686	gb|AV399759.1|:4-234	0.9913
BmHel-8(DF090352:2480854-2481233):73-139	gb|BY915184.1|:530-596	1
BmHel-8(DF090356:2662684-2663750):591-769	gb|BY923414.1|:630-808	0.9944
BmHel-8(DF090449:686485-690520):3565-3683	gb|CK549528.1|:8-126	0.9916
BmHel-8(DF090329:6124102-6124777):160-299	gb|CK556071.1|:350-489	1
BmHel-8(DF090403:1107532-1111107):771-872	gb|CK519983.1|:161-262	0.9902
BmHel-8(DF090348:3703519-3703943):373-425	gb|CK520451.1|:29-81	1

BmHel-8(DF090318:7198681-7199139):335-459	gb|AV405210.1|:45-132	0.9921
BmHel-8(DF090377:1375487-1375909):1-28	gb|CK519130.1|:2-29	1
BmHel-8(DF090370:1137878-1138296):74-192	gb|CK519552.1|:25-144	0.9908
BmHel-8(DF090422:365974-366411):351-378	gb|CK519574.1|:108-135	1
BmHel-8(DF090411:719577-724350):227-545	gb|BJ987793.1|:19-337	1
BmHel-8(DF090362:886383-891014):228-336	gb|CN377652.1|:91-198	0.9908
BmHel-8(DF090370:2703954-2704384):36-60	gb|CK528955.1|:5-29	1
BmHel-8(DF090321:4157649-4158280):445-566	gb|BP124248.1|:1-123	0.9919
BmHel-8(DF090326:6285139-6285692):68-187	gb|BP119091.1|:545-664	0.9917
BmHel-8(DF090337:3858983-3859802):218-595	gb|CK551305.1|:179-556	0.9921
BmHel-8(DF090316:5175171-5175854):339-414	gb|CK555746.1|:204-279	1
BmHel-8(DF090394:1263130-1263771):95-323	gb|CK552349.1|:367-592	0.9949
BmHel-8(DF090326:5129812-5134509):243-352	gb|BP115715.1|:19-128	1
BmHel-8(DF090320:6927649-6932185):4135-4321	gb|DN237409.1|:261-447	0.9947
BmHel-8(DF090378:1999162-1999599):391-438	gb|CK551978.1|:94-141	1
BmHel-8(DF090337:2091829-2093693):1006-1410	gb|CK509421.1|:7-410	0.9901
BmHel-8(DF090411:1104574-1106740):2018-2082	gb|CK526254.1|:438-502	1
BmHel-8(DF090388:1039203-1039999):562-669	gb|CN373881.1|:5-112	1
BmHel-8(DF090982:10340-10812):429-465	gb|BP121427.1|:40-76	1
BmHel-8(DF090324:4903966-4908332):633-1096	gb|CK515387.1|:1-416	1
BmHel-8(DF090321:4325562-4335145):6448-7008	gb|AV404339.1|:251-519	0.9963
BmHel-8(DF090362:507126-511051):159-2502	gb|BY919408.1|:20-347	0.993
BmHel-8(DF090321:7379015-7379676):167-194	gb|BY931063.1|:248-275	1
BmHel-8(DF090384:1962199-1962681):331-359	gb|BP183662.1|:204-232	1
BmHel-8(DF090423:55067-58018):2301-2340	gb|BB982750.1|:680-719	1
BmHel-8(DF090391:842142-843905):217-616	gb|BW998551.1|:16-415	0.9975
BmHel-8(DF090339:1227385-1228431):596-756	gb|BY915826.1|:100-260	0.9938
BmHel-8(DF090377:487393-490043):2134-2163	gb|AU002659.1|:664-693	1
BmHel-8(DF090365:1956065-1956481):225-257	gb|CK517523.1|:190-222	1
BmHel-8(DF090316:2346549-2348992):238-266	gb|CK528999.1|:454-482	1
BmHel-8(DF090364:2073021-2073438):2-113	gb|CK554647.1|:513-624	0.9911
BmHel-8(DF090380:1761839-1770172):981-1300	gb|CK554617.1|:45-364	1
BmHel-8(DF090318:5060374-5060823):1-107	gb|BP121656.1|:256-350	1
BmHel-8(DF090373:2004658-2005315):499-524	gb|CN376142.1|:561-586	1
BmHel-8(DF090386:429106-430660):651-1332	gb|DY230981.1|:260-607	0.9901
BmHel-8(DF090323:5793164-5793609):316-347	gb|BP118257.1|:535-566	1
BmHel-8(DF091102:1059-2770):593-1442	gb|BP123298.1|:167-754	0.9925
BmHel-8(DF090338:2023564-2024943):1068-1313	gb|CK520268.1|:21-272	0.9942
BmHel-8(DF090349:3039623-3040386):297-456	gb|BB982617.1|:1-160	0.9938
BmHel-8(DF090343:126803-128721):229-693	gb|BB982604.1|:19-483	0.9957
BmHel-8(DF090319:5078658-5081688):1713-2425	gb|BJ980843.1|:1-598	0.9944
BmHel-8(DF090321:3652705-3653996):1022-1126	gb|CN374381.1|:155-259	0.9905
BmHel-8(DF090363:2074718-2075804):78-714	gb|BY920246.1|:23-748	0.9944
BmHel-8(DF090332:5388312-5392678):1145-1446	gb|CK518011.1|:8-309	0.9901
BmHel-8(DF090440:379977-380644):109-345	gb|BP119865.1|:175-411	0.9916
BmHel-8(DF090836:14839-16785):1-1722	gb|CK554842.1|:270-466	0.9921
BmHel-8(DF090341:4616941-4619657):2399-2645	gb|BJ988962.1|:16-262	1
BmHel-8(DF090316:8380530-8381531):671-888	gb|BY930027.1|:59-265	0.9948

BmHel-8(DF090348:1319600-1320026):335-427	gb|CK551978.1|:61-141	1
BmHel-8(DF090461:36547-36978):385-432	gb|CK556303.1|:83-130	1
BmHel-8(DF090350:1365393-1366403):160-387	gb|BP118066.1|:213-440	0.9912
BmHel-8(DF090323:465961-467152):630-842	gb|BY915826.1|:100-312	1
BmHel-8(DF090320:6927649-6932185):4052-4325	gb|BY934287.1|:26-299	0.9964
BmHel-8(DF090972:5461-9435):2361-2466	gb|BB990369.1|:686-791	0.9906
BmHel-8(DF090330:5624469-5624889):201-421	gb|BY914587.1|:355-575	1
BmHel-8(DF090396:463680-471572):7687-7763	gb|BY939650.1|:198-274	1
BmHel-8(DF090322:5360085-5363455):1159-1381	gb|BP116865.1|:375-597	0.991
BmHel-8(DF090413:482630-483965):810-1028	gb|BJ981528.1|:42-260	0.9909
BmHel-8(DF090348:1177679-1178682):636-750	gb|BY914083.1|:540-654	0.9913
BmHel-8(DF090331:4072649-4073635):141-626	gb|BJ988218.1|:18-503	0.9938
BmHel-8(BABH01046834:1492-2003):412-456	gb|CK526895.1|:385-429	1
BmHel-8(DF090357:3408612-3409031):319-344	gb|CK502023.1|:7-32	1
BmHel-8(DF090374:634593-639689):3478-3611	gb|AV398676.1|:452-585	0.9925
BmHel-8(DF090380:1761839-1770172):1077-1300	gb|BP179008.1|:1-224	1
BmHel-8(DF090342:526439-528001):322-647	gb|CK537265.1|:185-510	0.9908
BmHel-8(DF090316:3944830-3945231):207-315	gb|CK484912.1|:71-179	0.9908
BmHel-8(DF090344:430493-433870):2369-2533	gb|CK563861.1|:8-116	0.9906
BmHel-8(DF090358:1040540-1047283):4224-4544	gb|CN376141.1|:326-646	0.9907
BmHel-8(DF090344:818990-819411):353-422	gb|CK521138.1|:327-396	1
BmHel-8(DF090338:628097-628517):294-318	gb|AU002750.1|:630-654	1
BmHel-8(DF090321:4884516-4892629):5200-5507	gb|CK554455.1|:269-576	0.9903
BmHel-8(DF090329:6124102-6124777):1-73	gb|CK554971.1|:43-115	1
BmHel-8(DF090339:1227385-1228431):877-921	gb|CK497991.1|:18-62	1
BmHel-8(DF090449:686485-690520):3570-3682	gb|AU002770.1|:286-398	0.9912
BmHel-8(DF090322:4503333-4508316):2035-2095	gb|CK522455.1|:38-98	1
BmHel-8(DF090412:572646-573063):345-418	gb|CK555831.1|:217-290	1
BmHel-8(DF090317:8638454-8640659):1776-1861	gb|AV398321.1|:90-175	1
BmHel-8(DF090366:1015642-1017448):135-621	gb|BJ988512.1|:16-502	0.9918
BmHel-8(DF090613:70988-71624):530-637	gb|CK554071.1|:23-129	0.9907
BmHel-8(DF090322:2506288-2507628):723-839	gb|CK559990.1|:5-121	1
BmHel-8(DF090337:4172025-4172478):396-420	gb|AU002750.1|:557-581	1
BmHel-8(DF090325:4461195-4463037):267-503	gb|BY922962.1|:19-255	0.9916
BmHel-8(DF090417:403540-404224):374-473	gb|CK555947.1|:405-504	0.99
BmHel-8(DF090322:3367383-3368144):127-436	gb|BJ987316.1|:19-328	0.9968
BmHel-8(DF090332:6043175-6044056):76-504	gb|BB984324.1|:33-461	0.9907
BmHel-8(DF090342:3233534-3234386):615-799	gb|AU002454.1|:257-441	0.9946
BmHel-8(DF090411:115459-120386):271-631	gb|BY927807.1|:33-393	0.9972
BmHel-8(DF090556:5652-6643):891-919	gb|CK523255.1|:123-151	1
BmHel-8(DF090325:3276611-3279918):2843-2967	gb|DY230766.1|:845-969	0.992
BmHel-8(DF090436:106109-106742):368-536	gb|CK563861.1|:5-117	0.9909
BmHel-8(DF090374:634593-639689):3433-5097	gb|CK552276.1|:20-295	0.9917
BmHel-8(DF090325:3276611-3279918):2858-2967	gb|BP120748.1|:1-110	1
BmHel-8(DF090371:1499769-1500470):333-554	gb|BY942914.1|:452-673	0.9955
BmHel-8(DF090364:626292-626707):2-113	gb|CK551309.1|:510-621	0.9911
BmHel-8(DF090372:1983752-1985581):497-1704	gb|CK554635.1|:1-238	0.9947
BmHel-8(DF090327:5354635-5355589):693-723	gb|BY922165.1|:413-443	1

BmHel-8(DF090350:1365393-1366403):167-333	gb|CK516027.1|:99-265	0.994
BmHel-8(DF090440:379977-380644):110-345	gb|BY929129.1|:134-369	0.9958
BmHel-8(DF090317:6748659-6753512):653-762	gb|BP121625.1|:1-110	1
BmHel-8(DF090352:3024613-3025191):1-129	gb|BY922165.1|:511-639	0.9922
BmHel-8(DF090335:4826004-4826503):211-238	gb|CK555017.1|:179-206	1
BmHel-8(DF090317:8638454-8640659):421-460	gb|BP121821.1|:97-136	1
BmHel-8(DF090317:10589758-10590538):214-248	gb|CK524519.1|:550-584	1
BmHel-8(DF090342:969506-969988):49-110	gb|BP178619.1|:61-122	1
BmHel-8(DF090365:2649920-2653996):2630-2656	gb|BB983678.1|:41-67	1
BmHel-8(DF090328:4710284-4711325):708-733	gb|BP178889.1|:508-533	1
BmHel-8(DF090316:9843089-9845415):1539-1846	gb|BP120115.1|:1-308	0.9935
BmHel-8(DF090327:872260-873601):813-861	gb|CK554591.1|:17-65	1
BmHel-8(DF090335:2482387-2484313):276-307	gb|CK490677.1|:454-485	1
BmHel-8(DF090378:347334-348021):458-546	gb|BY931529.1|:117-205	1
BmHel-8(DF090352:3024613-3025191):441-492	gb|CK553227.1|:127-178	1
BmHel-8(DF090363:2186554-2192550):5396-5512	gb|CK547848.1|:437-553	1
BmHel-8(DF090433:352662-353013):262-352	gb|CK520896.1|:95-185	1
BmHel-8(DF090362:886383-891014):228-609	gb|BY926111.1|:70-452	0.9965
BmHel-8(DF090414:975389-975809):264-421	gb|CK555194.1|:7-164	1
BmHel-8(DF090316:6349579-6349975):161-185	gb|AV398234.1|:206-230	1
BmHel-8(DF090322:5360085-5363455):1162-1381	gb|CK493194.1|:200-419	0.9909
BmHel-8(DF090348:421944-425542):2217-2271	gb|CK552957.1|:348-402	1
BmHel-8(DF090316:4088184-4088727):232-325	gb|BJ984376.1|:1-94	1
BmHel-8(DF090325:708530-712359):92-460	gb|BP121221.1|:160-528	0.9919
BmHel-8(DF090348:1177679-1178682):196-324	gb|BY916156.1|:265-393	0.9922
BmHel-8(DF090344:3288309-3288812):168-195	gb|CK524681.1|:384-411	1
BmHel-8(DF090334:2329855-2330261):295-319	gb|CK502142.1|:12-36	1
BmHel-8(DF090433:131834-133575):191-216	gb|CK527966.1|:355-380	1
BmHel-8(DF090420:142716-143462):636-683	gb|CK498448.1|:301-348	1
BmHel-8(DF090362:886383-891014):462-609	gb|CK495766.1|:311-459	0.9933
BmHel-8(DF090341:2175006-2179683):781-1183	gb|BJ987253.1|:20-422	0.9926
BmHel-8(DF090337:3039234-3039626):341-393	gb|CK547449.1|:99-151	1
BmHel-8(DF090318:7198681-7199139):313-459	gb|CK518664.1|:50-159	0.9933
BmHel-8(DF090326:5129812-5134509):243-349	gb|BY928814.1|:12-118	0.9907
BmHel-8(DF090352:1151593-1152758):344-533	gb|BP121625.1|:133-322	0.9947
BmHel-8(DF090362:875311-880996):5110-5423	gb|BY919077.1|:1-314	0.9904
BmHel-8(DF090573:29094-31275):634-661	gb|BJ988522.1|:366-393	1
BmHel-8(DF090323:465961-467152):590-842	gb|CK556329.1|:22-273	0.996
BmHel-8(DF090323:4426301-4435530):2350-2531	gb|BY918838.1|:233-414	1
BmHel-8(DF090343:126803-128721):229-703	gb|BJ988993.1|:18-492	0.9937
BmHel-8(DF090413:482630-483965):810-844	gb|BY920334.1|:621-655	1
BmHel-8(DF090546:65747-66158):269-294	gb|AU002750.1|:667-692	1
BmHel-8(DF090321:242700-244323):183-208	gb|CK523002.1|:317-342	1
BmHel-8(DF090343:285109-285823):301-565	gb|BP119268.1|:275-539	0.9925
BmHel-8(DF090357:793971-794415):317-445	gb|BY928646.1|:1-105	0.9921
BmHel-8(DF090473:10603-11741):162-226	gb|CK557076.1|:41-105	1
BmHel-8(DF090386:429106-430660):1014-1334	gb|CK505253.1|:313-633	0.9969
BmHel-8(DF090381:1793122-1794058):806-937	gb|AV405210.1|:1-132	1

BmHel-8(DF090333:2476556-2478613):1483-1747	gb|CK521284.1|:367-631	0.9909
BmHel-8(DF090342:169484-170957):1123-1276	gb|CK564005.1|:199-352	0.9935
BmHel-8(DF090347:480418-481639):480-999	gb|BJ987754.1|:26-545	0.9942
BmHel-8(DF090350:1365393-1366403):628-903	gb|CK528514.1|:307-582	0.9964
BmHel-8(DF090375:255586-261282):279-363	gb|CK527822.1|:5-89	1
BmHel-8(DF090351:4131637-4135291):1303-1646	gb|BJ989189.1|:19-362	0.9971
BmHel-8(DF090377:1280637-1281038):299-402	gb|CK520896.1|:82-185	0.9904
BmHel-8(DF090352:1151593-1152758):383-1014	gb|BB994777.1|:1-537	0.996
BmHel-8(DF090404:906331-909800):2553-2625	gb|CK512647.1|:507-579	1
BmHel-8(DF090391:842142-843905):1348-1485	gb|CK499134.1|:498-635	1
BmHel-8(DF090320:5911638-5912077):1-99	gb|BP121656.1|:256-354	1
BmHel-8(DF090327:4316874-4318459):461-966	gb|AV399980.2|:4-507	0.9901
BmHel-8(DF090449:686485-690520):3565-3682	gb|BJ987089.1|:21-138	0.9915
BmHel-8(DF090343:126803-128721):1384-1689	gb|BJ982683.1|:159-464	1
BmHel-8(DF090425:905304-905709):342-366	gb|BP182579.1|:159-183	1
BmHel-8(DF090336:2730665-2731193):318-347	gb|CK505211.1|:335-364	1
BmHel-8(DF090441:559311-559716):1-185	gb|AV405232.1|:300-485	0.9943
BmHel-8(DF090321:3652705-3653996):302-804	gb|BY926731.1|:45-391	0.9964
BmHel-8(DF090335:1312930-1313941):318-422	gb|BP118994.1|:412-516	1
BmHel-8(DF090475:237083-237796):602-642	gb|CK521361.1|:15-55	1
BmHel-8(DF090324:5193257-5193926):329-553	gb|BP120838.1|:94-317	0.9951
BmHel-8(DF090322:2506288-2507628):723-1013	gb|AU004348.1|:120-410	0.9956
BmHel-8(DF090362:875311-880996):5354-5463	gb|BP122000.1|:1-110	1
BmHel-8(DF090433:131834-133575):322-502	gb|BP120783.1|:59-240	0.9945
BmHel-8(DF090319:5078658-5081688):1910-2512	gb|BJ980805.1|:2-489	0.9901
BmHel-8(DF090405:1097995-1098449):237-261	gb|BP183662.1|:154-178	1
BmHel-8(DF090327:5810915-5813896):272-586	gb|BB984206.1|:24-338	0.9937
BmHel-8(DF090425:953495-954490):508-645	gb|CK493458.1|:494-631	1
BmHel-8(DF090324:5193257-5193926):336-554	gb|BP121132.1|:429-647	0.995
BmHel-8(DF090344:2927731-2929485):638-1472	gb|BY919104.1|:17-747	0.9928
BmHel-8(DF090318:9477609-9481565):1084-1199	gb|CK519058.1|:166-281	0.9914
BmHel-8(DF090334:5781190-5781609):4-56	gb|CK528955.1|:5-57	1
BmHel-8(DF090379:347576-347944):14-46	gb|BP118232.1|:153-185	1
BmHel-8(DF090369:2128891-2129030):23-140	gb|BP122569.1|:1-396	0.9914
BmHel-8(DF090334:3767864-3768278):131-382	gb|CK517523.1|:59-301	0.9916
BmHel-8(DF090322:2506288-2507628):771-1012	gb|BY914728.1|:2-243	0.9944
BmHel-8(DF090337:4087281-4089203):322-425	gb|BY920670.1|:533-636	0.9904
BmHel-8(DF090395:1332576-1333384):173-292	gb|BP120337.1|:104-223	0.9917
BmHel-8(DF090326:4478055-4478469):2-116	gb|CK551309.1|:510-624	0.9913
BmHel-8(DF090410:657095-661899):1465-1570	gb|CK528911.1|:4-109	0.9906
BmHel-8(DF090350:1365393-1366403):166-199	gb|CK525145.1|:423-456	1
BmHel-8(DF090390:783965-785679):894-1319	gb|BY929938.1|:22-447	0.993
BmHel-8(DF090331:4072649-4073635):141-644	gb|BJ987065.1|:19-522	0.9901
BmHel-8(DF090328:4710284-4711325):341-403	gb|CK543773.1|:628-690	1
BmHel-8(DF090383:1260223-1261406):778-804	gb|BP121003.1|:650-676	1
BmHel-8(DF090340:2226452-2228369):1-75	gb|CK554971.1|:41-115	1
BmHel-8(DF090318:2964319-2966510):1493-1775	gb|CK553936.1|:346-628	0.9929
BmHel-8(BABH01056432:159-231):38-73	gb|BP120089.1|:109-144	1

BmHel-8(DF090399:1154973-1156712):1209-1536	gb|BY919981.1|:19-346	0.9926
BmHel-8(DF090399:1154973-1156712):366-485	gb|CN212224.1|:5-124	1
BmHel-8(DF090414:530758-532436):233-1465	gb|BY915132.1|:283-454	1
BmHel-8(DF090398:286574-287493):540-641	gb|BP118306.1|:7-108	0.9902
BmHel-8(DF090324:5193257-5193926):337-554	gb|BP121291.1|:255-472	0.99
BmHel-8(DF090399:1224385-1226894):2038-2270	gb|CK528253.1|:21-253	0.9914
BmHel-8(DF090349:3039623-3040386):299-382	gb|BY933198.1|:437-520	1
BmHel-8(DF090343:4199560-4199982):1-118	gb|CK551309.1|:510-627	1
BmHel-8(DF090414:530758-532436):654-1208	gb|CK551272.1|:15-649	0.9901
BmHel-8(DF090324:4600685-4605646):4462-4884	gb|BJ987091.1|:24-446	0.9905
BmHel-8(DF090344:2927731-2929485):496-1341	gb|BY927423.1|:2-743	0.9912
BmHel-8(DF090336:3381600-3383558):707-821	gb|CK521661.1|:249-376	0.9904
BmHel-8(DF090325:3515637-3524712):5291-5734	gb|BP182145.1|:1-469	0.991
BmHel-8(DF090346:3934044-3934563):312-412	gb|CK519576.1|:224-324	0.9901
BmHel-8(DF090351:824312-824724):1-151	gb|CK554842.1|:270-454	1
BmHel-8(DF090335:1312930-1313941):318-425	gb|CK551297.1|:150-257	1
BmHel-8(DF090354:3180234-3181017):547-602	gb|CK522395.1|:23-78	1
BmHel-8(DF090468:181706-182081):318-355	gb|BY914784.1|:377-414	1
BmHel-8(DF090417:403540-404224):374-474	gb|CK521050.1|:128-228	0.9901
BmHel-8(DF090342:169484-170957):272-672	gb|CK537265.1|:110-510	0.99
BmHel-8(DF090329:4999357-4999838):1-146	gb|CK527387.1|:150-258	1
BmHel-8(DF090333:4339602-4339927):41-73	gb|BP117991.1|:146-178	1
BmHel-8(DF090381:1752311-1752612):1-103	gb|CK554971.1|:13-115	0.9903
BmHel-8(DF090354:3180234-3181017):540-603	gb|CK528279.1|:5-68	1
BmHel-8(DF090331:3857842-3859218):337-960	gb|BP121614.1|:16-639	1
BmHel-8(DF090411:115459-120386):4339-4762	gb|BB982337.1|:62-486	0.9971
BmHel-8(DF090354:1113395-1113815):1-184	gb|CK552943.1|:314-502	0.9942
BmHel-8(DF090348:421944-425542):1437-1593	gb|BJ982247.1|:9-165	0.9936
BmHel-8(DF090341:4616941-4619657):2399-2644	gb|BJ987539.1|:22-267	1
BmHel-8(DF090322:5360085-5363455):1159-2668	gb|BY915132.1|:283-457	0.9945
BmHel-8(DF090319:7583768-7584505):245-454	gb|BY914789.1|:252-461	0.9905
BmHel-8(DF090325:3276611-3279918):253-375	gb|BP121123.1|:464-586	1
BmHel-8(DF090369:2276888-2277625):154-261	gb|CK529675.1|:179-286	0.9907
BmHel-8(DF090341:2175006-2179683):4017-4441	gb|BB982337.1|:64-488	0.9941
BmHel-8(DF090362:2163882-2168331):194-505	gb|CK492814.1|:63-374	0.9904
BmHel-8(BABH01044927:473-1142):462-581	gb|BP184046.1|:525-644	0.9917
BmHel-8(DF090316:9843089-9845415):1914-2115	gb|BY927406.1|:570-771	0.995
BmHel-8(DF090344:2927731-2929485):230-434	gb|CK524670.1|:89-293	0.9902
BmHel-8(DF090327:4316874-4318459):372-438	gb|BY914506.1|:603-669	1
BmHel-8(DF090414:1071623-1072079):219-248	gb|CK542981.1|:498-527	1
BmHel-8(DF090316:9843089-9845415):242-542	gb|BP118994.1|:216-516	0.99
BmHel-8(DF090411:719577-724350):223-545	gb|BJ988459.1|:23-345	0.9938
BmHel-8(DF090322:2131916-2133903):308-828	gb|BP180630.1|:1-521	0.9962
BmHel-9(DF090321:1315613-1316868):316-1116	gb|CK527387.1|:150-258	0.991
BmHel-9(DF090341:1635465-1636120):525-578	gb|CK513121.1|:531-584	1

TableS7 Silkworm Intact Helitrons contributed to 5UTR of full-length cDNA

Helitron family(accession number):Q_start-Q_end	Subject_ID:S_start-S_end	Identity
BmHel-18(DF090364:2327036-2332537):5294-5502	dbj|AK377568.1|:5-213	1
BmHel-8(DF090342:155496-155991):393-496	dbj|AK377599.1|:1-104	1
BmHel-11(DF090322:5446726-5446974):1-249	dbj|AK378101.1|:3675-3923	1
BmHel-16(DF090336:3389069-3396865):3801-3893	dbj|AK378172.1|:12-104	1
BmHel-16(DF090366:432884-441413):854-4196	dbj|AK385475.1|:1-395	0.9975
BmHel-8(DF090353:2717141-2720235):3047-3095	dbj|AK378951.1|:84-132	1
BmHel-8(DF090357:1638999-1639422):284-336	dbj|AK379294.1|:15-67	1
BmHel-7(DF090355:1988127-1988378):1-252	dbj|AK377669.1|:1009-1260	1
BmHel-8(DF090388:1284082-1284538):190-304	dbj|AK379359.1|:10-124	1
BmHel-8(DF090352:1011095-1011514):190-420	dbj|AK379585.1|:254-360	1
BmHel-11(BABH01044460:4547-4837):126-179	dbj|AK380214.1|:22-75	1
BmHel-8(DF090317:11078259-11078674):216-416	dbj|AK380222.1|:96-172	1
BmHel-8(DF090339:1956184-1956617):304-434	dbj|AK380233.1|:52-182	1
BmHel-15(DF090346:575803-576239):1-112	dbj|AK380528.1|:14-125	1
BmHel-8(DF090353:2717141-2720235):3047-3095	dbj|AK384808.1|:97-145	1
BmHel-11(DF090355:1987314-1987594):1-280	dbj|AK377669.1|:197-476	0.9964
BmHel-11(DF090354:2748767-2749058):229-292	dbj|AK380771.1|:18-81	1
BmHel-8(DF090349:3365134-3365556):1-123	dbj|AK380945.1|:458-580	1
BmHel-7(DF090414:286308-290133):2037-2750	dbj|AK380980.1|:364-638	1
BmHel-11(DF090350:3583885-3584127):1-118	dbj|AK381184.1|:68-185	1
BmHel-8(DF090348:127332-127747):329-422	dbj|AK381554.1|:10-103	1
BmHel-12(DF090451:383169-383505):5945-5992	dbj|AK381658.1|:2547-2592	1
BmHel-8(DF090356:1668989-1669828):612-756	dbj|AK381835.1|:87-231	1
BmHel-8(DF090353:2717141-2720235):3047-3095	dbj|AK382695.1|:86-134	1
BmHel-11(DF090362:2397630-2405140):7014-7090	dbj|AK383056.1|:2-78	1
BmHel-11(DF090400:933182-933475):16-81	dbj|AK385073.1|:4-69	1
BmHel-16(DF090366:432884-441413):852-894	dbj|AK385268.1|:3-45	1
BmHel-11(DF090317:5547051-5547337):1-287	dbj|AK387030.1|:1624-1910	1
BmHel-8(DF090375:768787-769175):280-389	dbj|AK387317.1|:11-120	1
BmHel-8(DF090399:1595829-1596240):331-412	dbj|AK387694.1|:10-91	1
BmHel-7(DF090338:4486893-4487112):1-220	dbj|AK387774.1|:234-453	1
BmHel-11(DF090582:42896-43186):1-119	dbj|AK387887.1|:140-258	1
BmHel-8(DF090349:548903-549315):254-413	dbj|AK388021.1|:12-171	1
BmHel-8(DF090318:4265220-4265638):218-419	dbj|AK388032.1|:47-124	1
BmHel-8(DF090344:779108-779505):190-398	dbj|AK388059.1|:113-321	1

TableS8 Silkworm Intact Helitrons contributed to code sequences of full-length cDNA

Helitron family(accession number):Q_start-Q_end	Subject:S_start-S_end	Identity
BmHel-3a(DF090398:697995-698384):212-390	dbj|AK384761.1|:760-938	1
BmHel-8(DF090333:5488038-5497555):7303-7366	dbj|AK382498.1|:185-248	1
BmHel-8(DF090357:238448-244737):628-1578	dbj|AK387382.1|:12-660	1
BmHel-11(DF090387:802595-802883):35-120	dbj|AK384525.1|:87-172	1
BmHel-16(DF090353:2207111-2208925):623-716	dbj|AK384369.1|:1350-1443	1
BmHel-16(DF090324:4215452-4225156):4780-4941	dbj|AK383012.1|:2-163	1
BmHel-8(DF090316:2503678-2505748):446-1023	dbj|AK380961.1|:12-589	1
BmHel-16(DF090316:11118562-11127452):6934-7072	dbj|AK380376.1|:12-150	1
BmHel-11(DF090320:3449103-3449393):126-291	dbj|AK380214.1|:22-187	1
BmHel-18(DF090338:1702161-1706375):1627-2176	dbj|AK379462.1|:12-561	1
BmHel-11(DF090378:551738-551964):14-227	dbj|AK377848.1|:584-797	1
BmHel-16(DF090330:2748684-2757755):369-1672	dbj|AK377256.1|:1714-2361	1
BmHel-16(DF090366:432884-441413):852-4753	dbj|AK385268.1|:3-2723	1
BmHel-16(DF090345:4347736-4357963):617-1663	dbj|AK378165.1|:359-695	0.9973
BmHel-16(DF090336:3389069-3396865):3801-7603	dbj|AK378172.1|:12-842	1
BmHel-15(DF090316:4640683-4647901):2275-5434	dbj|AK378214.1|:384-850	1
BmHel-8(DF090350:1600212-1603621):450-2766	dbj|AK378279.1|:152-515	1
BmHel-15(DF090319:7461948-7466152):402-2628	dbj|AK378364.1|:467-964	1
BmHel-16(DF090320:7919399-7924507):1512-4505	dbj|AK378891.1|:227-800	1
BmHel-16(DF090366:432884-441413):854-4753	dbj|AK385475.1|:1-2719	0.9981
BmHel-16(DF090347:2945307-2951369):476-3553	dbj|AK382421.1|:784-1452	0.9949
BmHel-16(DF090317:3329257-3332526):439-1567	dbj|AK387609.1|:444-670	1
BmHel-16(DF090347:2945307-2951369):476-3570	dbj|AK381010.1|:796-1481	1
BmHel-16(DF090345:2122886-2129495):2818-4551	dbj|AK385872.1|:119-269	1
BmHel-8(DF090343:4301065-4304694):1551-2378	dbj|AK386879.1|:670-1084	1
BmHel-12(DF090327:5496383-5505946):282-9119	dbj|AK381145.1|:2314-3916	0.9968
BmHel-11(DF090400:933182-933475):16-81	dbj|AK385073.1|:4-69	1
BmHel-12(DF090318:9088405-9099027):8055-8745	dbj|AK381695.1|:12-765	1
BmHel-11(DF090432:415658-415909):1-252	dbj|AK377997.1|:1418-1669	1
BmHel-8(DF090318:4265220-4265638):189-419	dbj|AK388032.1|:47-153	1
BmHel-11(DF090392:787909-788192):1-284	dbj|AK386827.1|:275-558	1
BmHel-8(DF090396:463680-471572):6342-7763	dbj|AK385563.1|:193-1881	1
BmHel-16(DF090318:5730927-5734479):2335-2496	dbj|AK384630.1|:14-175	0.9938
BmHel-12(DF090366:145031-151552):4604-4715	dbj|AK383499.1|:4-115	0.9911
BmHel-15(DF090317:4783746-4793700):808-993	dbj|AK380597.1|:889-1074	0.9946
BmHel-16(DF090347:2945307-2951369):4606-5490	dbj|AK377406.1|:1462-1840	1
BmHel-16(DF090377:1843233-1846736):2222-3185	dbj|AK377720.1|:2-965	0.9907
BmHel-16(DF090393:899894-908549):990-8359	dbj|AK380642.1|:703-1555	0.9989
BmHel-16(DF090317:3329257-3332526):439-1567	dbj|AK379549.1|:359-585	1
BmHel-16(DF090349:4178239-4181090):504-2557	dbj|AK380735.1|:207-505	1
BmHel-16(DF090330:2748684-2757755):3778-8534	dbj|AK378662.1|:525-1938	0.9951
BmHel-12(DF090363:1552928-1559978):647-6120	dbj|AK379000.1|:939-1536	1
BmHel-11(DF090362:2397630-2405140):6950-7090	dbj|AK383056.1|:2-142	1
BmHel-15(DF090317:4783746-4793700):811-993	dbj|AK388244.1|:1124-1306	0.9945

TableS9 Silkworm Intact Helitrons contributed to 3UTR of full-length cDNA

**Helitron family(accession number):Q_start-Q_end**	**Subject:S_start-S_end**	**Identity**
BmHel-16(DF090330:2748684-2757755):369-1672	dbj|AK377256.1|:1714-2361	1
BmHel-3a(DF090402:1385222-1386975):1-274	dbj|AK377855.1|:2192-2465	1
BmHel-7(DF090319:3570920-3571168):1-249	dbj|AK378000.1|:7651-7899	1
BmHel-11(DF090333:3724539-3724867):16-329	dbj|AK378067.1|:3436-3749	1
BmHel-16(DF090349:1766404-1766610):1-207	dbj|AK378240.1|:1213-1422	1
BmHel-11(DF090354:2748767-2749058):1-292	dbj|AK378241.1|:838-1129	1
BmHel-11(DF090319:5450055-5450345):1-291	dbj|AK378427.1|:1730-2020	1
BmHel-11(DF090317:5547051-5547337):1-287	dbj|AK378626.1|:3569-3855	1
BmHel-18(DF090338:1702161-1706375):1627-2176	dbj|AK379462.1|:12-561	1
BmHel-18(DF090344:954842-955056):1-215	dbj|AK387306.1|:2425-2639	1
BmHel-11(DF090332:4541116-4541332):1-217	dbj|AK380083.1|:1520-1736	1
BmHel-11(DF090356:1067246-1067489):1-80	dbj|AK380701.1|:766-845	1
BmHel-16(DF090347:2945307-2951369):476-3570	dbj|AK381010.1|:796-1481	1
BmHel-16(DF090380:390172-390377):1-206	dbj|AK381356.1|:1588-1793	1
BmHel-11(DF090357:458727-458988):1-262	dbj|AK381440.1|:2498-2759	1
BmHel-11(DF090357:1292011-1292286):1-276	dbj|AK381564.1|:1212-1487	1
BmHel-16(DF090347:2945307-2951369):476-3553	dbj|AK382421.1|:784-1452	0.9949
BmHel-11(DF090354:2748767-2749058):1-292	dbj|AK384353.1|:868-1159	1
BmHel-11(DF090319:6498947-6499173):1-227	dbj|AK384746.1|:2244-2470	1
BmHel-16(DF090357:3800935-3801136):20-202	dbj|AK385368.1|:1866-2048	1
BmHel-11(DF090324:4067512-4067804):16-161	dbj|AK385852.1|:2656-2801	1
BmHel-16(DF090378:1385335-1394506):1643-5166	dbj|AK385894.1|:352-706	1
BmHel-7(DF090391:1263093-1264541):1-1449	dbj|AK386563.1|:4406-5955	1
BmHel-11(DF090416:829591-829865):1-275	dbj|AK387052.1|:930-1204	0.9951
BmHel-7(DF090867:11339-11589):1-251	dbj|AK387140.1|:1498-1748	1
BmHel-11(DF090363:973851-974095):1-114	dbj|AK387174.1|:1863-1976	1
BmHel-8(DF090357:238448-244737):628-1578	dbj|AK387382.1|:12-660	1
BmHel-11(DF090316:13250030-13259336):164-434	dbj|AK388014.1|:551-821	1
BmHel-16(DF090347:2945307-2951369):4606-5490	dbj|AK377406.1|:1462-1840	1
BmHel-8(DF090319:704742-705102):162-361	dbj|AK378418.1|:2350-2549	1
BmHel-7(DF090390:53166-53415):1-250	dbj|AK378796.1|:1647-1896	1
BmHel-15(DF090475:252267-252702):383-436	dbj|AK379773.1|:597-650	1
BmHel-7(DF090330:3918349-3918588):1-240	dbj|AK381306.1|:351-590	1
BmHel-16(DF090319:7193264-7193468):21-205	dbj|AK382237.1|:1304-1488	1
BmHel-16(DF090332:6237321-6237537):1-217	dbj|AK382936.1|:1946-2162	1
BmHel-11(DF090369:1028309-1028519):1-211	dbj|AK385294.1|:681-891	1
BmHel-3b(DF090374:1060401-1060792):1-392	dbj|AK386044.1|:2065-2428	0.9912
BmHel-11(DF090316:6406940-6407229):1-290	dbj|AK386239.1|:1324-1613	1
BmHel-16(DF090319:7193264-7193468):21-205	dbj|AK386905.1|:1326-1509	0.994
BmHel-11(DF090361:2639654-2639946):1-293	dbj|AK386939.1|:2409-2701	0.9966
BmHel-18(DF090344:954842-955056):1-215	dbj|AK387405.1|:4922-5136	1
BmHel-8(DF090353:1493002-1493425):274-424	dbj|AK387599.1|:920-1070	1
BmHel-11(DF090361:2639654-2639946):1-293	dbj|AK388197.1|:2207-2499	0.9966
BmHel-18(DF090385:1182077-1182627):150-551	dbj|AK388330.1|:1015-1426	1
