# Supplementary material for: Identification and Evolution of the Silkworm Helitrons and their Contribution to Transcripts
Source: DNA Res. 2013 Jun 14;20(5):471–84. doi: 10.1093/dnares/dst024 (PMC3789558; doi:10.1093/dnares/dst024)
Supplement: Supplementary Data [file supp_dst024_dst024supp_table1.pdf]

TableS1 Positions of Helitrons in the silkworm genome

Families	Accession numbers:start-end
BmHel-1	DF090393:1603855-1604052
BmHel-1	DF090324:426714-426945
BmHel-1	DF090338:2522381-2522616
BmHel-1	DF090353:2359499-2359738
BmHel-1	DF090357:3376537-3376777
BmHel-1	DF090358:2664331-2664571
BmHel-1	DF090338:4630907-4631147
BmHel-1	DF090335:1914141-1914382
BmHel-1	DF090340:710463-710704
BmHel-1	DF090349:444357-444598
BmHel-1	DF090347:683009-683253
BmHel-1	DF090379:592347-592591
BmHel-1	DF090340:3262915-3263695
BmHel-1	DF090469:172567-172804
BmHel-1	DF090329:569097-569339
BmHel-1	DF090367:1982328-1982566
BmHel-1	DF090448:431846-432080
BmHel-1	DF090448:538086-538314
BmHel-1	DF090336:2789504-2789737
BmHel-1	DF090343:2867387-2867626
BmHel-1	DF090342:3354563-3354800
BmHel-1	DF090525:28859-29095
BmHel-1	DF090377:1347351-1347587
BmHel-1	DF090362:276870-277104
BmHel-1	DF090364:981176-981408
BmHel-1	DF090497:116185-116417
BmHel-1	DF090316:15868644-15868877
BmHel-1	DF090408:263864-264092
BmHel-1	DF090359:2347690-2347896
BmHel-1	DF090384:1896576-1896805
BmHel-1	DF090334:4551261-4551495
BmHel-1	DF090370:2320994-2321229
BmHel-1	DF090347:3119930-3120041
BmHel-1	DF090347:3119211-3119331
BmHel-1	DF090397:1369565-1369801
BmHel-1	DF090335:5555016-5555259
BmHel-1	DF090422:911284-911515
BmHel-1	DF090366:738480-738711
BmHel-1	DF090354:1922627-1922859
BmHel-1	DF090325:5365381-5365613
BmHel-1	DF090335:4604262-4604493
BmHel-1	DF090361:1940312-1940544
BmHel-1	DF090354:2306900-2307127
BmHel-1	DF090378:711510-711737
BmHel-1	DF090385:1616644-1616875
BmHel-1	DF090369:660846-661076

BmHel-1	DF090366:1649055-1649281
BmHel-1	DF090390:1573780-1573935
BmHel-1	DF090367:1470195-1470431
BmHel-1	DF090316:12373924-12374104
BmHel-1	DF090403:522647-522877
BmHel-1	DF090375:2354143-2354361
BmHel-1	DF090344:4661465-4661694
BmHel-1	DF090508:119292-119526
BmHel-1	DF090323:620230-620461
BmHel-1	DF090317:8848638-8848865
BmHel-1	DF090333:4325068-4325219
BmHel-1	DF090324:5172151-5172379
BmHel-1	DF090341:4590127-4590356
BmHel-1	DF090323:5311640-5311877
BmHel-1	DF090324:505869-506097
BmHel-1	DF090376:347131-347358
BmHel-1	DF090339:2873390-2873545
BmHel-1	DF090329:4571351-4571508
BmHel-1	DF090332:2044744-2044968
BmHel-1	DF090443:345224-345451
BmHel-1	DF090340:4343253-4343442
BmHel-1	DF090390:52937-53162
BmHel-1	DF090365:1459862-1460083
BmHel-1	DF090393:435351-435570
BmHel-1	DF090316:13997817-13997973
BmHel-1	DF090361:1593668-1593892
BmHel-1	BABH01044459:8656-8879
BmHel-1	DF090386:728451-728674
BmHel-1	DF090358:1104439-1104654
BmHel-1	DF090370:2337648-2337780
BmHel-1	DF090370:2337781-2337868
BmHel-1	DF090368:1344231-1344415
BmHel-1	DF090329:2003566-2003728
BmHel-1	DF090323:7792918-7793140
BmHel-1	DF090388:668771-668986
BmHel-1	DF090361:888144-888296
BmHel-1	DF090422:453781-453890
BmHel-1	DF090334:4945883-4946104
BmHel-1	DF090349:2370185-2370332
BmHel-1	DF090384:998651-998804
BmHel-1	DF090362:2853530-2853659
BmHel-1	DF090327:4027193-4027408
BmHel-1	DF090367:1888764-1888921
BmHel-1	DF090329:3865246-3865466
BmHel-1	DF090410:648551-648764
BmHel-1	DF090358:3207307-3207521
BmHel-1	DF090373:1533636-1533847
BmHel-1	DF090319:6081971-6082175

BmHel-1	DF090350:723373-723587
BmHel-1	DF090387:453294-453513
BmHel-1	DF090330:6149651-6149800
BmHel-1	DF090366:2068053-2068265
BmHel-1	DF090381:256120-256331
BmHel-1	DF090328:1765522-1765731
BmHel-1	DF090357:2755445-2755649
BmHel-1	DF090366:578178-578389
BmHel-1	DF090338:4751434-4751641
BmHel-1	DF090335:3087409-3087620
BmHel-1	DF090321:2105113-2105322
BmHel-1	DF090369:333747-333955
BmHel-1	DF090382:1694005-1694214
BmHel-1	BABH01059807:498-706
BmHel-1	DF090744:19954-20155
BmHel-1	DF090455:383773-383984
BmHel-1	DF090413:858990-859195
BmHel-1	DF090365:2777273-2777480
BmHel-1	DF090368:1084225-1084432
BmHel-1	DF090548:16058-16212
BmHel-1	DF090373:1093403-1093610
BmHel-1	DF090456:392281-392492
BmHel-1	DF090364:2094027-2094232
BmHel-1	DF090358:2871097-2871303
BmHel-1	DF090434:374644-374846
BmHel-1	DF090320:1978758-1978893
BmHel-1	DF090335:3213059-3213145
BmHel-1	DF090325:2532474-2532680
BmHel-1	DF090439:215071-215272
BmHel-1	DF090369:1323909-1324037
BmHel-1	DF090788:9018-9146
BmHel-1	DF090413:459265-459396
BmHel-1	DF090363:653786-653990
BmHel-1	DF090401:204943-205143
BmHel-1	DF090345:1247084-1247288
BmHel-1	DF090337:1005726-1005930
BmHel-1	BABH01046471:2330-2526
BmHel-1	BABH01046471:66-262
BmHel-1	DF090339:438762-438919
BmHel-1	DF090331:4484645-4484849
BmHel-1	DF090410:669810-669970
BmHel-1	DF090397:1275335-1275538
BmHel-1	DF090391:582149-582281
BmHel-1	DF090688:53203-53399
BmHel-1	DF090316:2980574-2980731
BmHel-1	DF090469:100817-101016
BmHel-1	DF090387:1184268-1184469
BmHel-1	DF090401:466561-466692

BmHel-1	DF090379:1575424-1575618
BmHel-1	DF090409:342770-342963
BmHel-1	DF090391:78913-79107
BmHel-1	DF090361:1232272-1232400
BmHel-1	DF090327:4427080-4427204
BmHel-1	DF090374:2310152-2310347
BmHel-1	DF090353:3312013-3312178
BmHel-1	DF090348:1607556-1607749
BmHel-1	DF090390:1019427-1019621
BmHel-1	DF090388:1358343-1358500
BmHel-1	DF090326:636103-636295
BmHel-1	DF090322:6595899-6596091
BmHel-1	BABH01055108:459-651
BmHel-1	DF090327:4694722-4694867
BmHel-1	DF090466:28064-28204
BmHel-1	DF090318:6949399-6949556
BmHel-1	DF090384:1566779-1566964
BmHel-1	DF090340:1967587-1967768
BmHel-1	DF090338:1349477-1349596
BmHel-1	DF090320:7341145-7341319
BmHel-1	DF090409:170495-170679
BmHel-1	DF090445:345308-345492
BmHel-1	DF090356:2396158-2396342
BmHel-1	DF090355:501223-501364
BmHel-1	DF090350:424096-424201
BmHel-1	DF090372:1881130-1881313
BmHel-1	DF090416:1083157-1083330
BmHel-1	DF090347:2913121-2913293
BmHel-1	DF090325:2385086-2385268
BmHel-1	DF090323:6043419-6043599
BmHel-1	DF090338:2950248-2950429
BmHel-1	DF090421:1065464-1065645
BmHel-1	DF090374:2024667-2024843
BmHel-1	DF090320:1203276-1203416
BmHel-1	DF090380:15718-15844
BmHel-1	DF090356:3644789-3644964
BmHel-1	DF090373:2105267-2105445
BmHel-1	DF090343:773035-773214
BmHel-1	DF090330:5305392-5305533
BmHel-1	DF090444:497180-497309
BmHel-1	DF090421:41068-41242
BmHel-1	DF090343:1220940-1221116
BmHel-1	DF090319:5049012-5049185
BmHel-1	DF090377:107223-107395
BmHel-1	DF090316:13202877-13203047
BmHel-1	DF090344:4139397-4139567
BmHel-1	DF090340:1182000-1182170
BmHel-1	DF090318:491447-491617

BmHel-1	DF090368:536884-537054
BmHel-1	DF090336:660161-660326
BmHel-1	DF090333:1992461-1992631
BmHel-1	DF090392:95694-95865
BmHel-1	DF090330:3018529-3018696
BmHel-1	DF090328:2847689-2847857
BmHel-1	DF090319:130333-130502
BmHel-1	DF090438:507937-508107
BmHel-1	DF090352:2213874-2214045
BmHel-1	DF090410:975998-976165
BmHel-1	DF090331:5133256-5133426
BmHel-1	DF090401:1027174-1027338
BmHel-1	DF090503:18389-18559
BmHel-1	DF090444:374606-374774
BmHel-1	DF090337:2425105-2425276
BmHel-1	DF090337:5135756-5135929
BmHel-1	BABH01047257:1121-1288
BmHel-1	DF090492:206023-206190
BmHel-1	DF091150:2386-2555
BmHel-1	DF090340:4432787-4432898
BmHel-1	DF090325:1881339-1881434
BmHel-1	DF090376:2132293-2132463
BmHel-1	DF090317:10040495-10040662
BmHel-1	DF090375:1390114-1390283
BmHel-1	DF090337:3661035-3661203
BmHel-1	DF090432:693243-693411
BmHel-1	DF090340:3053473-3053578
BmHel-1	DF090317:4164145-4164313
BmHel-1	DF090330:535184-535348
BmHel-1	DF090355:1562798-1562967
BmHel-1	DF090398:307127-307221
BmHel-1	DF090443:512696-512858
BmHel-1	DF090321:7691935-7692102
BmHel-1	DF090432:422992-423158
BmHel-1	DF090357:1405389-1405515
BmHel-1	DF090411:415204-415371
BmHel-1	DF090334:5956297-5956461
BmHel-1	DF090321:588552-588718
BmHel-1	DF090316:3974678-3974844
BmHel-1	DF090357:2398593-2398759
BmHel-1	DF090340:4387890-4388057
BmHel-1	DF090353:3788112-3788277
BmHel-1	DF090366:937967-938133
BmHel-1	DF090329:4381100-4381263
BmHel-1	DF090321:1604491-1604656
BmHel-1	DF090384:351494-351665
BmHel-1	DF090349:1999197-1999363
BmHel-1	DF090319:4041069-4041234

BmHel-1	DF090326:1444280-1444445
BmHel-1	DF090363:1039227-1039383
BmHel-1	DF090409:639503-639669
BmHel-1	DF090337:1674973-1675139
BmHel-1	DF090350:2568552-2568718
BmHel-1	DF090407:745473-745639
BmHel-1	DF090524:19856-20021
BmHel-1	DF090347:3752428-3752594
BmHel-1	DF090330:4124587-4124753
BmHel-1	DF090331:5104639-5104805
BmHel-1	DF090365:1906269-1906435
BmHel-1	BABH01044374:3245-3410
BmHel-1	DF090341:827573-827738
BmHel-1	DF090393:504912-505077
BmHel-1	DF090333:229753-229878
BmHel-1	DF090423:846592-846756
BmHel-1	DF090338:1673633-1673794
BmHel-1	DF090328:2580020-2580128
BmHel-1	DF090342:825135-825304
BmHel-1	DF090316:10435484-10435646
BmHel-1	DF090344:979114-979277
BmHel-1	DF090325:2595815-2595979
BmHel-1	DF090321:1686582-1686742
BmHel-1	DF090321:5237018-5237178
BmHel-1	DF090383:116234-116398
BmHel-1	DF090438:74899-75062
BmHel-1	DF090320:2568613-2568776
BmHel-1	DF090380:702511-702674
BmHel-1	DF090331:3187750-3187912
BmHel-1	DF090369:807154-807313
BmHel-1	DF090343:2701951-2702111
BmHel-1	DF090340:1801096-1801259
BmHel-1	DF090321:6548982-6549145
BmHel-1	DF090317:7043706-7043869
BmHel-1	DF090373:224050-224213
BmHel-1	DF090385:710437-710599
BmHel-1	DF090636:29276-29440
BmHel-1	DF090395:1626668-1626829
BmHel-1	DF090353:3422645-3422805
BmHel-1	DF090318:3949998-3950159
BmHel-1	DF090336:897804-897962
BmHel-1	DF090418:912070-912228
BmHel-1	DF090449:298198-298360
BmHel-1	DF090353:3897419-3897581
BmHel-1	DF090320:6537671-6537832
BmHel-1	DF090394:719074-719233
BmHel-1	DF090454:237807-237965
BmHel-1	DF090467:247839-247925

BmHel-1	DF090324:3279235-3279395
BmHel-1	DF090326:1324816-1324903
BmHel-1	DF090341:714639-714800
BmHel-1	DF090343:4086699-4086791
BmHel-1	DF090380:1731246-1731408
BmHel-1	DF090412:558752-558913
BmHel-1	DF090323:1089877-1090037
BmHel-1	DF090354:1673778-1673936
BmHel-1	DF090321:1749335-1749490
BmHel-1	DF090328:24387-24547
BmHel-1	DF090375:1070516-1070676
BmHel-1	DF090456:106653-106814
BmHel-1	DF090437:395280-395441
BmHel-1	DF090320:2618359-2618445
BmHel-1	DF090353:89791-89948
BmHel-1	DF090325:3683220-3683379
BmHel-1	DF090317:6839901-6840062
BmHel-1	DF090367:2750290-2750374
BmHel-1	DF090390:183998-184153
BmHel-1	DF090366:1118329-1118488
BmHel-1	DF090395:1304010-1304168
BmHel-1	DF090324:7953037-7953196
BmHel-1	DF090326:5854736-5854891
BmHel-1	DF090324:462482-462641
BmHel-1	DF090340:1533878-1534037
BmHel-1	DF090393:1342176-1342335
BmHel-1	DF090383:456178-456338
BmHel-1	DF090332:6028770-6028925
BmHel-1	DF090317:6312970-6313129
BmHel-1	DF090871:14845-14928
BmHel-1	DF090342:670510-670667
BmHel-1	DF090332:2352876-2353036
BmHel-1	DF090355:1251972-1252129
BmHel-1	BABH01086543:78-233
BmHel-1	DF090590:53419-53577
BmHel-1	DF090343:4230248-4230406
BmHel-1	DF090372:1835503-1835660
BmHel-1	DF090333:3920825-3920907
BmHel-1	DF090344:3976868-3977025
BmHel-1	DF090331:546253-546339
BmHel-1	DF090415:1206260-1206417
BmHel-1	DF090393:91714-91806
BmHel-1	DF090352:4144274-4144430
BmHel-1	DF090316:14529776-14529933
BmHel-1	DF090362:2739335-2739488
BmHel-1	DF090423:514614-514771
BmHel-1	DF090365:2403406-2403558
BmHel-1	DF090333:3570931-3571087

BmHel-1	DF090485:158402-158557
BmHel-1	DF090386:316643-316730
BmHel-1	DF090357:3390153-3390283
BmHel-1	DF090327:4013944-4014031
BmHel-1	DF090406:875037-875197
BmHel-1	DF090455:204094-204246
BmHel-1	DF090340:3455083-3455237
BmHel-1	DF090345:2321636-2321793
BmHel-1	DF090345:3620247-3620403
BmHel-1	DF090351:3920137-3920287
BmHel-1	DF090366:1018544-1018700
BmHel-1	DF090319:5866806-5866962
BmHel-1	DF090321:1731875-1732030
BmHel-1	DF090453:11915-12001
BmHel-1	DF090325:5819482-5819569
BmHel-1	DF090356:1671663-1671819
BmHel-1	DF090394:1505317-1505472
BmHel-1	DF090348:304411-304566
BmHel-1	DF090321:7261273-7261427
BmHel-1	DF090316:6480520-6480675
BmHel-1	DF090357:3753399-3753553
BmHel-1	DF090319:3536167-3536254
BmHel-1	DF090358:1986092-1986246
BmHel-1	DF090319:5714362-5714516
BmHel-1	DF090377:32466-32620
BmHel-1	DF090520:49934-50088
BmHel-1	DF090323:6629978-6630061
BmHel-1	DF090330:4909549-4909636
BmHel-1	DF090325:7268834-7268985
BmHel-1	DF090327:884041-884194
BmHel-1	DF090394:46088-46240
BmHel-1	DF090391:1522710-1522860
BmHel-1	DF090321:966467-966617
BmHel-1	BABH01043176:13311-13398
BmHel-1	DF090316:2053390-2053540
BmHel-1	DF090403:666657-666808
BmHel-1	DF090316:4119818-4119901
BmHel-1	DF090316:4820750-4820898
BmHel-1	DF090452:420781-420931
BmHel-1	DF090344:1116037-1116185
BmHel-1	DF090375:1323579-1323699
BmHel-1	DF090326:4756528-4756640
BmHel-1	DF090419:1193514-1193663
BmHel-1	DF090395:1414856-1415005
BmHel-1	DF090395:1237008-1237154
BmHel-1	DF090484:140796-140945
BmHel-1	DF090386:1994235-1994359
BmHel-1	DF090342:2592831-2592979

BmHel-1	DF090329:3036807-3036954
BmHel-1	DF090317:366149-366247
BmHel-1	DF090374:1756716-1756800
BmHel-1	DF090319:6804180-6804327
BmHel-1	DF090320:2137051-2137196
BmHel-1	DF090344:1938424-1938567
BmHel-1	DF090417:1099678-1099765
BmHel-1	DF090316:13040320-13040409
BmHel-1	DF090362:2501638-2501781
BmHel-1	DF090331:5891126-5891270
BmHel-1	DF090354:1806098-1806192
BmHel-1	DF090355:1093877-1094016
BmHel-1	DF090355:2650701-2650839
BmHel-1	DF090318:6368739-6368825
BmHel-1	DF090357:3507649-3507792
BmHel-1	DF090456:41389-41531
BmHel-1	DF090316:13452587-13452700
BmHel-1	DF090339:880082-880222
BmHel-1	DF090402:237742-237881
BmHel-1	DF090440:687524-687663
BmHel-1	DF090365:2029223-2029363
BmHel-1	DF090397:967309-967389
BmHel-1	DF090338:3752143-3752281
BmHel-1	DF090360:3162510-3162649
BmHel-1	DF090322:974737-974871
BmHel-1	DF090389:1000501-1000580
BmHel-1	DF090328:733338-733472
BmHel-1	DF090366:1199729-1199867
BmHel-1	DF090318:5423173-5423309
BmHel-1	DF090327:4045383-4045519
BmHel-1	DF090403:415776-415910
BmHel-1	BABH01047340:1635-1769
BmHel-1	DF090367:2382707-2382791
BmHel-1	DF090329:1294370-1294503
BmHel-1	DF090405:700182-700315
BmHel-1	DF090412:757847-757931
BmHel-1	DF090350:1867006-1867138
BmHel-1	DF090384:1323890-1323969
BmHel-1	DF090373:629522-629653
BmHel-1	DF090332:395467-395597
BmHel-1	DF090316:8883394-8883524
BmHel-1	BABH01057873:303-382
BmHel-1	DF090391:1349335-1349415
BmHel-1	DF090350:706679-706809
BmHel-1	DF090330:2332554-2332682
BmHel-1	DF090341:540335-540464
BmHel-1	DF090347:1715865-1715990
BmHel-1	DF090438:581216-581345

BmHel-1	DF090337:4940873-4941006
BmHel-1	DF090429:390019-390148
BmHel-1	DF090921:279-408
BmHel-1	DF090389:77520-77644
BmHel-1	DF090326:4195542-4195670
BmHel-1	DF090410:1281689-1281811
BmHel-1	DF090459:42968-43096
BmHel-1	DF090327:5377144-5377267
BmHel-1	DF090330:4147133-4147261
BmHel-1	DF090327:2273018-2273141
BmHel-1	DF090329:2375167-2375295
BmHel-1	DF090454:261652-261779
BmHel-1	DF090333:728414-728537
BmHel-1	DF090343:3981393-3981479
BmHel-1	DF090317:8684341-8684428
BmHel-1	DF090327:6040957-6041081
BmHel-1	BABH01055617:285-406
BmHel-1	DF090353:783063-783184
BmHel-1	DF090355:2488502-2488626
BmHel-1	DF090342:4060372-4060495
BmHel-1	DF090351:2016682-2016802
BmHel-1	DF090318:2311619-2311741
BmHel-1	DF090372:996433-996552
BmHel-1	DF090395:532494-532616
BmHel-1	DF090427:558458-558579
BmHel-1	DF090359:2180825-2180943
BmHel-1	DF090352:1431261-1431381
BmHel-1	DF090320:4129134-4129253
BmHel-1	BABH01048450:186-305
BmHel-1	DF090327:4777472-4777592
BmHel-1	DF090410:108780-108894
BmHel-1	DF090339:2807219-2807337
BmHel-1	DF090658:25485-25603
BmHel-1	DF090351:4062154-4062273
BmHel-1	DF090422:105301-105417
BmHel-1	DF090390:205191-205307
BmHel-1	DF090393:485225-485338
BmHel-1	DF090722:6163-6276
BmHel-1	DF090321:4108892-4109005
BmHel-1	DF090317:9629057-9629168
BmHel-1	DF090386:1430475-1430583
BmHel-1	DF090319:8227562-8227667
BmHel-1	DF090382:1533804-1533912
BmHel-1	DF090352:27327-27433
BmHel-1	DF090384:57145-57252
BmHel-1	DF090317:2545199-2545304
BmHel-1	DF090469:88995-89101
BmHel-1	DF090317:7718623-7718727

BmHel-1	DF090318:1537952-1538055
BmHel-1	DF090365:1010598-1010701
BmHel-1	DF090323:184860-184963
BmHel-1	DF090399:329658-329761
BmHel-1	DF090329:3751559-3751661
BmHel-1	DF090420:120053-120155
BmHel-1	BABH01062760:585-683
BmHel-1	DF090377:2110773-2110870
BmHel-1	DF090337:1211102-1211194
BmHel-1	DF090321:58067-58160
BmHel-1	DF090357:1376973-1377065
BmHel-1	DF090463:294869-294958
BmHel-1	DF090331:6236515-6236604
BmHel-1	DF090325:4631683-4631771
BmHel-1	DF090347:1561686-1561773
BmHel-1	DF090355:2273997-2274084
BmHel-1	DF090327:6355466-6355553
BmHel-1	DF090336:1715081-1715164
BmHel-1	DF090333:4815738-4815825
BmHel-1	DF090345:3730175-3730261
BmHel-1	DF090360:2665699-2665786
BmHel-1	DF090380:372494-372581
BmHel-1	BABH01059824:300-387
BmHel-1	DF091104:6924-7011
BmHel-1	DF091283:4950-5037
BmHel-1	DF090365:353537-353622
BmHel-1	DF090429:411776-411861
BmHel-1	DF090326:2389467-2389551
BmHel-1	DF090323:4572256-4572343
BmHel-1	DF090373:1360331-1360414
BmHel-1	DF090358:2418412-2418495
BmHel-1	DF090361:3140148-3140230
BmHel-1	DF090489:11130-11211
BmHel-1	DF090372:1151688-1151768
BmHel-1	DF090578:44386-44466
BmHel-1	DF090357:2716902-2716981
BmHel-2	DF090353:728438-729259
BmHel-2	DF090320:2925001-2926607
BmHel-2	DF090318:1431360-1433079
BmHel-2	DF091741:565-2522
BmHel-2	DF090344:2746800-2748809
BmHel-2	DF090339:3243686-3245705
BmHel-2	DF090345:2703166-2705458
BmHel-2	DF090352:968456-970820
BmHel-2	DF090357:767418-772673
BmHel-2	DF090457:308846-318297
BmHel-2	DF090385:1028351-1028789

BmHel-2	DF090385:1023486-1024007
BmHel-2	DF090350:3536925-3537371
BmHel-2	DF090350:3538611-3538956
BmHel-2	BABH01073719:46-568
BmHel-2	DF090367:2744521-2745043
BmHel-2	DF090378:1377059-1377580
BmHel-2	BABH01067127:84-605
BmHel-2	BABH01056842:222-738
BmHel-2	BABH01073604:99-615
BmHel-2	BABH01078248:87-583
BmHel-2	BABH01075396:80-522
BmHel-2	DF090355:701284-701726
BmHel-2	DF090319:4264760-4265201
BmHel-2	DF090319:2336785-2337227
BmHel-2	BABH01058083:147-589
BmHel-2	BABH01047312:1354-1796
BmHel-2	DF090333:4724106-4724548
BmHel-2	BABH01083963:52-498
BmHel-2	BABH01069047:32-478
BmHel-2	BABH01071515:2-448
BmHel-2	BABH01066923:131-577
BmHel-2	DF090436:211324-211770
BmHel-2	BABH01077873:36-474
BmHel-2	BABH01074932:9-447
BmHel-2	BABH01065224:54-492
BmHel-2	DF090441:359907-360345
BmHel-2	BABH01076577:60-510
BmHel-2	BABH01067368:165-615
BmHel-2	BABH01056279:286-736
BmHel-2	BABH01080468:61-511
BmHel-2	DF090421:68297-68747
BmHel-2	DF090430:861813-862261
BmHel-2	BABH01074756:168-604
BmHel-2	BABH01052646:407-837
BmHel-2	BABH01080515:1-433
BmHel-2	DF090355:550579-550989
BmHel-2	BABH01076661:1-421
BmHel-2	DF090316:6784979-6785383
BmHel-2	BABH01068895:1-395
BmHel-2	DF090418:204299-204674
BmHel-2	BABH01068669:274-645
BmHel-2	DF090318:572591-572961
BmHel-2	BABH01065401:1-371
BmHel-2	DF090324:7139628-7139928
BmHel-2	BABH01052681:485-835
BmHel-2	BABH01064089:323-673
BmHel-2	DF090332:586772-587116
BmHel-2	BABH01073140:279-618

BmHel-2	BABH01085613:1-335
BmHel-2	DF090423:163878-164212
BmHel-2	DF090455:446788-447114
BmHel-2	DF090474:26170-26437
BmHel-2	DF090332:611851-612153
BmHel-2	DF090428:175951-176249
BmHel-2	BABH01066529:375-658
BmHel-2	DF090665:1-257
BmHel-2	BABH01054169:38-291
BmHel-2	BABH01062879:432-682
BmHel-2	BABH01055550:509-758
BmHel-2	BABH01046149:2906-3132
BmHel-2	BABH01086588:13-181
BmHel-2	BABH01074639:441-604
BmHel-2	BABH01056993:613-732
BmHel-2	DF090364:184098-184198
BmHel-2	DF090335:3575886-3575982
BmHel-2	BABH01071139:531-630
BmHel-2	BABH01067326:567-653
BmHel-2	DF090403:295504-295586

BmHel-3a	DF090327:2595363-2595558
BmHel-3a	DF090350:3877342-3877547
BmHel-3a	DF090326:2370906-2371125
BmHel-3a	DF090369:882595-882817
BmHel-3a	DF090328:629615-629844
BmHel-3a	DF090322:6680576-6680808
BmHel-3a	DF090333:4105316-4105550
BmHel-3a	DF090989:4401-4635
BmHel-3a	DF090318:7699686-7699944
BmHel-3a	DF090409:787855-788119
BmHel-3a	DF090326:3345781-3346067
BmHel-3a	DF090350:3166536-3166834
BmHel-3a	DF090365:2446868-2447166
BmHel-3a	DF090343:830525-830828
BmHel-3a	DF090418:1179027-1179331
BmHel-3a	DF090387:1876031-1876337
BmHel-3a	DF090361:3172839-3173145
BmHel-3a	DF090339:404069-404380
BmHel-3a	DF090326:1099073-1099384
BmHel-3a	DF090321:6911664-6911975
BmHel-3a	DF090316:15275159-15275472
BmHel-3a	DF090355:761441-761755
BmHel-3a	BABH01081216:108-432
BmHel-3a	DF090389:1037029-1037361
BmHel-3a	DF090389:783272-783607
BmHel-3a	DF090317:2696138-2696482
BmHel-3a	DF090321:4773305-4773654

BmHel-3a	DF090362:2640981-2641330
BmHel-3a	DF090325:3529989-3530339
BmHel-3a	DF090357:1339441-1339791
BmHel-3a	DF090322:6733237-6733589
BmHel-3a	DF090395:923198-923552
BmHel-3a	DF090340:677369-677723
BmHel-3a	DF090329:2762030-2762386
BmHel-3a	DF090383:1588966-1589323
BmHel-3a	DF090375:816944-817301
BmHel-3a	DF090405:749066-749424
BmHel-3a	DF090464:78017-78376
BmHel-3a	DF090372:775197-775556
BmHel-3a	DF090411:535999-536358
BmHel-3a	DF090388:117061-117424
BmHel-3a	DF090354:2980552-2980915
BmHel-3a	DF090326:3354131-3354495
BmHel-3a	DF090343:3730128-3730493
BmHel-3a	DF090386:1983702-1984067
BmHel-3a	DF090372:728316-728681
BmHel-3a	DF090380:1799755-1800120
BmHel-3a	DF090380:1443753-1444118
BmHel-3a	DF090458:257870-258236
BmHel-3a	DF090342:2186624-2186990
BmHel-3a	DF090350:4120559-4120926
BmHel-3a	DF090397:892246-892613
BmHel-3a	DF090331:521998-522365
BmHel-3a	DF090380:1091091-1091458
BmHel-3a	DF090322:6704735-6705103
BmHel-3a	DF090318:6718085-6718454
BmHel-3a	DF090332:5496498-5496867
BmHel-3a	DF090318:6153532-6153901
BmHel-3a	DF090332:5911792-5912161
BmHel-3a	DF090354:2471796-2472166
BmHel-3a	DF090395:15310-15680
BmHel-3a	BABH01045077:4954-5324
BmHel-3a	DF090361:838199-838570
BmHel-3a	DF090317:869362-869733
BmHel-3a	DF090337:1495341-1495712
BmHel-3a	DF090388:1170478-1170849
BmHel-3a	DF090343:3890490-3890861
BmHel-3a	DF090405:847723-848095
BmHel-3a	DF090332:161055-161427
BmHel-3a	DF090449:746952-747324
BmHel-3a	DF090412:503743-504115
BmHel-3a	DF090329:4381273-4381646
BmHel-3a	DF090340:4190709-4191083
BmHel-3a	DF090342:3011071-3011445
BmHel-3a	DF090801:2941-3315

BmHel-3a	DF090386:147439-147813
BmHel-3a	DF090322:6661770-6662144
BmHel-3a	DF090517:38108-38482
BmHel-3a	DF090340:4602682-4603057
BmHel-3a	DF090537:18865-19240
BmHel-3a	DF090729:5449-5824
BmHel-3a	DF090471:278457-278832
BmHel-3a	DF090403:823762-824138
BmHel-3a	DF090374:1239293-1239669
BmHel-3a	DF090339:2633332-2633708
BmHel-3a	DF090324:4693808-4694184
BmHel-3a	DF090333:5328686-5329062
BmHel-3a	DF090336:1883419-1883796
BmHel-3a	DF090413:267334-267711
BmHel-3a	BABH01042899:5058-5435
BmHel-3a	DF090403:743487-743864
BmHel-3a	DF090367:754511-754888
BmHel-3a	DF090372:1782630-1783007
BmHel-3a	DF090346:2879750-2880127
BmHel-3a	DF090737:3475-3852
BmHel-3a	DF090350:3425488-3425866
BmHel-3a	DF090390:1452290-1452668
BmHel-3a	DF090355:1451321-1451699
BmHel-3a	DF090318:6199024-6199402
BmHel-3a	DF090423:409625-410003
BmHel-3a	DF090322:1446362-1446741
BmHel-3a	DF090391:877922-878301
BmHel-3a	DF090379:851878-852257
BmHel-3a	DF090322:5269357-5269737
BmHel-3a	DF090397:1549475-1549855
BmHel-3a	DF090340:4817532-4817912
BmHel-3a	DF090325:712360-712740
BmHel-3a	DF090388:888836-889216
BmHel-3a	BABH01044948:2071-2451
BmHel-3a	DF090445:123133-123513
BmHel-3a	DF090402:991840-992220
BmHel-3a	DF090333:3556304-3556684
BmHel-3a	DF090317:5918941-5919322
BmHel-3a	DF090356:3576755-3577136
BmHel-3a	DF090354:2511900-2512281
BmHel-3a	DF090388:901897-902278
BmHel-3a	DF090449:454737-455118
BmHel-3a	DF090409:573968-574349
BmHel-3a	DF090418:1061122-1061503
BmHel-3a	DF090367:2168671-2169052
BmHel-3a	DF090354:3226037-3226418
BmHel-3a	DF090754:3110-3491
BmHel-3a	DF090316:10605775-10606157

BmHel-3a	DF090338:4591180-4591562
BmHel-3a	DF090397:1138979-1139361
BmHel-3a	DF090318:234635-235017
BmHel-3a	DF090340:4554582-4554964
BmHel-3a	DF090462:225518-225900
BmHel-3a	DF090343:4550604-4550987
BmHel-3a	DF090397:1028882-1029265
BmHel-3a	DF090373:802359-802742
BmHel-3a	DF090344:936844-937227
BmHel-3a	DF090319:3081021-3081404
BmHel-3a	DF090317:6654554-6654938
BmHel-3a	DF090328:6351862-6352246
BmHel-3a	DF090361:722845-723229
BmHel-3a	DF090362:1272020-1272404
BmHel-3a	DF090347:4269257-4269641
BmHel-3a	DF090317:5558376-5558761
BmHel-3a	DF090349:100303-100688
BmHel-3a	DF090349:2989294-2989679
BmHel-3a	DF090421:528930-529315
BmHel-3a	DF090378:539580-539965
BmHel-3a	DF090392:1544714-1545099
BmHel-3a	DF090338:3752360-3752745
BmHel-3a	DF090316:2521830-2522215
BmHel-3a	DF090425:828438-828823
BmHel-3a	DF090356:2632983-2633368
BmHel-3a	DF090348:3607724-3608109
BmHel-3a	DF090332:1050024-1050409
BmHel-3a	DF090346:2095466-2095851
BmHel-3a	DF090333:374208-374593
BmHel-3a	DF090350:4058938-4059324
BmHel-3a	DF090360:1011104-1011490
BmHel-3a	DF090336:3152480-3152866
BmHel-3a	DF090341:4687423-4687809
BmHel-3a	DF090320:3377699-3378085
BmHel-3a	DF090318:478799-479185
BmHel-3a	DF090364:2573585-2573971
BmHel-3a	DF090322:5720588-5720974
BmHel-3a	DF090326:3907564-3907950
BmHel-3a	DF090316:12339936-12340322
BmHel-3a	DF090356:3507244-3507630
BmHel-3a	DF090401:617082-617468
BmHel-3a	DF090430:406341-406727
BmHel-3a	DF090705:1133-1519
BmHel-3a	DF090358:2404650-2405036
BmHel-3a	DF090341:787111-787497
BmHel-3a	DF090322:4680420-4680807
BmHel-3a	DF090350:755005-755392
BmHel-3a	DF090377:1844983-1845370

BmHel-3a	DF090352:211473-211860
BmHel-3a	DF090374:2077539-2077926
BmHel-3a	DF090355:3162177-3162564
BmHel-3a	DF090319:6529899-6530286
BmHel-3a	DF090334:3963453-3963840
BmHel-3a	DF090342:3456897-3457284
BmHel-3a	DF090565:110568-110955
BmHel-3a	DF090344:4011223-4011610
BmHel-3a	DF090317:7348195-7348582
BmHel-3a	DF090326:1671875-1672262
BmHel-3a	DF090394:1452480-1452867
BmHel-3a	DF090322:6830992-6831379
BmHel-3a	DF090322:2586647-2587034
BmHel-3a	DF090338:4278646-4279033
BmHel-3a	DF090338:2078922-2079309
BmHel-3a	DF090317:2828969-2829356
BmHel-3a	DF090337:5094170-5094557
BmHel-3a	DF090349:3826963-3827350
BmHel-3a	DF090336:2128864-2129251
BmHel-3a	DF090425:796319-796706
BmHel-3a	DF090425:122342-122729
BmHel-3a	DF090318:2720313-2720700
BmHel-3a	DF090318:1596073-1596460
BmHel-3a	DF090340:1961462-1961849
BmHel-3a	DF090391:546244-546631
BmHel-3a	DF090364:885509-885896
BmHel-3a	DF090347:1125606-1125993
BmHel-3a	DF090334:2931666-2932053
BmHel-3a	DF090382:365208-365595
BmHel-3a	BABH01059261:164-551
BmHel-3a	DF090459:158611-158998
BmHel-3a	DF090325:3478339-3478726
BmHel-3a	DF090351:3035029-3035416
BmHel-3a	DF090322:848832-849220
BmHel-3a	DF090361:1181954-1182342
BmHel-3a	DF090338:4758746-4759134
BmHel-3a	DF090317:10388433-10388821
BmHel-3a	DF090377:922113-922501
BmHel-3a	DF090377:1477379-1477767
BmHel-3a	DF090352:567644-568032
BmHel-3a	DF090352:2167970-2168358
BmHel-3a	DF090337:2283020-2283408
BmHel-3a	DF090326:1219384-1219772
BmHel-3a	DF090316:5357887-5358275
BmHel-3a	DF090316:14489326-14489714
BmHel-3a	DF090316:15880569-15880957
BmHel-3a	DF090349:1451820-1452208
BmHel-3a	DF090349:2454102-2454490

BmHel-3a	DF090348:1529097-1529485
BmHel-3a	DF090331:1060412-1060800
BmHel-3a	DF090323:7651293-7651681
BmHel-3a	DF090323:7708157-7708545
BmHel-3a	DF090321:2083081-2083469
BmHel-3a	DF090321:4593106-4593494
BmHel-3a	DF090466:129267-129655
BmHel-3a	DF090408:436340-436728
BmHel-3a	BABH01073241:45-433
BmHel-3a	DF090460:366247-366635
BmHel-3a	DF090385:576107-576495
BmHel-3a	DF090372:217792-218180
BmHel-3a	DF090365:1864062-1864450
BmHel-3a	DF090334:4925913-4926301
BmHel-3a	DF090346:373336-373724
BmHel-3a	DF090346:3469126-3469514
BmHel-3a	DF090342:3917545-3917933
BmHel-3a	DF090333:267920-268308
BmHel-3a	BABH01046054:818-1206
BmHel-3a	DF090480:30788-31176
BmHel-3a	DF090392:1238079-1238467
BmHel-3a	DF090434:103993-104381
BmHel-3a	DF090417:756106-756494
BmHel-3a	DF090373:315349-315737
BmHel-3a	DF090370:747348-747736
BmHel-3a	DF090370:1040087-1040475
BmHel-3a	DF090350:3681738-3682126
BmHel-3a	DF090350:3593515-3593903
BmHel-3a	DF090377:1212899-1213287
BmHel-3a	DF090367:2570632-2571020
BmHel-3a	DF090367:1400081-1400469
BmHel-3a	DF090358:385765-386153
BmHel-3a	DF090352:1816527-1816915
BmHel-3a	DF090352:1744604-1744992
BmHel-3a	DF090337:3119885-3120273
BmHel-3a	DF090316:5425281-5425669
BmHel-3a	DF090316:1196283-1196671
BmHel-3a	DF090336:4391098-4391486
BmHel-3a	DF090422:1076023-1076411
BmHel-3a	DF090405:1237965-1238353
BmHel-3a	DF090355:3527716-3528104
BmHel-3a	DF090354:1334265-1334653
BmHel-3a	DF090354:780877-781265
BmHel-3a	DF090339:1063476-1063864
BmHel-3a	DF090321:2763468-2763856
BmHel-3a	DF090319:3483561-3483949
BmHel-3a	DF090318:1210802-1211190
BmHel-3a	DF090340:4064652-4065040

BmHel-3a	DF090340:2233133-2233521
BmHel-3a	DF090391:1064359-1064747
BmHel-3a	DF090385:1860051-1860439
BmHel-3a	DF090362:2026114-2026502
BmHel-3a	DF090334:1441526-1441914
BmHel-3a	DF090332:3201273-3201661
BmHel-3a	DF090450:274501-274889
BmHel-3a	DF090440:285595-285983
BmHel-3a	DF090351:4016747-4017135
BmHel-3a	DF090472:144298-144686
BmHel-3a	BABH01047872:175-563
BmHel-3a	DF090328:3749061-3749449
BmHel-3a	DF090445:135944-136332
BmHel-3a	DF090360:2461969-2462357
BmHel-3a	DF090322:7811723-7812112
BmHel-3a	DF090317:10155852-10156241
BmHel-3a	DF090360:2030602-2030991
BmHel-3a	DF090337:2608401-2608790
BmHel-3a	DF090316:9252480-9252869
BmHel-3a	DF090316:12255554-12255943
BmHel-3a	DF090349:234747-235136
BmHel-3a	DF090349:3376973-3377362
BmHel-3a	DF090425:739519-739908
BmHel-3a	DF090422:910590-910979
BmHel-3a	DF090374:1133984-1134373
BmHel-3a	DF090356:2172664-2173053
BmHel-3a	DF090355:2288381-2288770
BmHel-3a	DF090339:692521-692910
BmHel-3a	DF090339:953043-953432
BmHel-3a	DF090339:1081556-1081945
BmHel-3a	DF090339:2329661-2330050
BmHel-3a	DF090339:3443752-3444141
BmHel-3a	DF090339:4070366-4070755
BmHel-3a	DF090339:4629264-4629653
BmHel-3a	DF090331:5627509-5627898
BmHel-3a	DF090329:4440022-4440411
BmHel-3a	DF090321:4826848-4827237
BmHel-3a	DF090319:1398920-1399309
BmHel-3a	DF090319:2114052-2114441
BmHel-3a	DF090319:2479370-2479759
BmHel-3a	DF090319:9062044-9062433
BmHel-3a	DF090460:349698-350087
BmHel-3a	DF090426:297982-298371
BmHel-3a	DF090395:717752-718141
BmHel-3a	DF090379:536473-536862
BmHel-3a	DF090362:1162365-1162754
BmHel-3a	DF090325:5850698-5851087
BmHel-3a	DF090324:7315701-7316090

BmHel-3a	DF090357:3657527-3657916
BmHel-3a	DF090342:2726556-2726945
BmHel-3a	DF090342:4650244-4650633
BmHel-3a	DF090333:685644-686033
BmHel-3a	DF090388:142862-143251
BmHel-3a	DF090378:2130869-2131258
BmHel-3a	DF090549:13460-13849
BmHel-3a	DF090450:379157-379546
BmHel-3a	DF090392:1386181-1386570
BmHel-3a	DF090490:107324-107713
BmHel-3a	DF090396:211287-211676
BmHel-3a	DF090481:223022-223411
BmHel-3a	DF091181:645-1034
BmHel-3a	DF090358:1510030-1510419
BmHel-3a	DF090422:788237-788626
BmHel-3a	DF090346:2074022-2074411
BmHel-3a	DF090456:356770-357159
BmHel-3a	DF090488:9816-10205
BmHel-3a	DF090317:3044550-3044939
BmHel-3a	DF090377:437651-438040
BmHel-3a	DF090374:29232-29621
BmHel-3a	DF090353:3877952-3878341
BmHel-3a	DF090348:970218-970607
BmHel-3a	DF090345:3704397-3704786
BmHel-3a	DF090339:2410417-2410806
BmHel-3a	DF090331:4070891-4071280
BmHel-3a	DF090321:3185550-3185939
BmHel-3a	DF090321:3155136-3155525
BmHel-3a	DF090319:7296056-7296445
BmHel-3a	DF090319:5153826-5154215
BmHel-3a	DF090319:1622596-1622985
BmHel-3a	DF090318:155633-156022
BmHel-3a	DF090340:4531469-4531858
BmHel-3a	DF090408:1150392-1150781
BmHel-3a	DF090363:1000925-1001314
BmHel-3a	DF090398:697995-698384
BmHel-3a	BABH01069069:98-487
BmHel-3a	DF090619:1330-1719
BmHel-3a	DF090596:42322-42711
BmHel-3a	DF090365:1998564-1998953
BmHel-3a	DF090332:2273683-2274072
BmHel-3a	DF090332:2031515-2031904
BmHel-3a	DF090324:524106-524495
BmHel-3a	DF090384:1615520-1615909
BmHel-3a	DF090376:1446506-1446895
BmHel-3a	BABH01082713:142-531
BmHel-3a	DF090459:278045-278434
BmHel-3a	DF090469:323005-323394

BmHel-3a	DF090322:634185-634575
BmHel-3a	DF090344:3108838-3109228
BmHel-3a	DF090338:1919140-1919530
BmHel-3a	DF090317:3018368-3018758
BmHel-3a	DF090358:2625013-2625403
BmHel-3a	DF090352:1257688-1258078
BmHel-3a	DF090337:4604742-4605132
BmHel-3a	DF090326:5325921-5326311
BmHel-3a	DF090316:14612279-14612669
BmHel-3a	BABH01053278:163-553
BmHel-3a	DF090425:975334-975724
BmHel-3a	DF090356:1444264-1444654
BmHel-3a	DF090355:2446642-2447032
BmHel-3a	DF090323:5817814-5818204
BmHel-3a	DF090321:2768596-2768986
BmHel-3a	DF090321:4344571-4344961
BmHel-3a	DF090321:4732335-4732725
BmHel-3a	DF090319:4967614-4968004
BmHel-3a	DF090318:5534018-5534408
BmHel-3a	DF090398:827663-828053
BmHel-3a	DF090431:673942-674332
BmHel-3a	DF090389:781107-781497
BmHel-3a	DF090372:1067923-1068313
BmHel-3a	DF090362:1780703-1781093
BmHel-3a	DF090334:1921708-1922098
BmHel-3a	DF090334:2521007-2521397
BmHel-3a	DF090334:4206569-4206959
BmHel-3a	DF090342:2836559-2836949
BmHel-3a	DF090333:517360-517750
BmHel-3a	BABH01045753:136-526
BmHel-3a	DF090680:25557-25947
BmHel-3a	DF090551:22940-23330
BmHel-3a	DF090411:814448-814838
BmHel-3a	DF090467:278961-279351
BmHel-3a	DF090374:1448320-1448710
BmHel-3a	DF090316:4212174-4212564
BmHel-3a	DF090498:164679-165069
BmHel-3a	DF090344:1388293-1388683
BmHel-3a	DF090338:2246619-2247009
BmHel-3a	DF090317:2607162-2607552
BmHel-3a	DF090360:1946084-1946474
BmHel-3a	DF090386:826376-826766
BmHel-3a	DF090352:4003224-4003614
BmHel-3a	DF090337:5299955-5300345
BmHel-3a	DF090330:5986833-5987223
BmHel-3a	DF090326:1398722-1399112
BmHel-3a	DF090327:6578859-6579249
BmHel-3a	DF090425:312424-312814

BmHel-3a	DF090341:529788-530178
BmHel-3a	DF090331:5346508-5346898
BmHel-3a	DF090321:2464546-2464936
BmHel-3a	BABH01047743:1002-1392
BmHel-3a	DF090436:652438-652828
BmHel-3a	DF090364:13696-14086
BmHel-3a	DF090362:2668654-2669044
BmHel-3a	DF090334:4886134-4886524
BmHel-3a	DF090382:1681647-1682037
BmHel-3a	DF090373:894149-894539
BmHel-3a	DF090333:3693347-3693737
BmHel-3a	DF090333:685003-685393
BmHel-3a	DF090368:1188171-1188561
BmHel-3a	DF090476:195693-196083
BmHel-3a	DF090949:467-857
BmHel-3a	DF090448:35566-35956
BmHel-3a	DF090366:103353-103743
BmHel-3a	DF090317:9757904-9758295
BmHel-3a	DF090377:658874-659265
BmHel-3a	DF090367:1946661-1947052
BmHel-3a	DF090316:681701-682092
BmHel-3a	DF090327:5050225-5050616
BmHel-3a	DF090421:901994-902385
BmHel-3a	DF090374:1060401-1060792
BmHel-3a	DF090321:2801133-2801524
BmHel-3a	DF090347:557740-558131
BmHel-3a	DF090332:4625454-4625845
BmHel-3a	DF090384:2009805-2010196
BmHel-3a	DF090333:5518708-5519099
BmHel-3a	DF090472:150916-151307
BmHel-3a	DF090328:1145678-1146069
BmHel-3a	DF090347:3484050-3484441
BmHel-3a	DF090344:4106827-4107218
BmHel-3a	DF090317:1699115-1699506
BmHel-3a	DF090386:1553187-1553578
BmHel-3a	DF090316:12474100-12474491
BmHel-3a	DF090374:1706525-1706916
BmHel-3a	DF090369:1865527-1865918
BmHel-3a	DF090355:2726166-2726557
BmHel-3a	DF090339:860428-860819
BmHel-3a	DF090339:565055-565446
BmHel-3a	DF090331:5603744-5604135
BmHel-3a	DF090340:3943506-3943897
BmHel-3a	DF090335:2392491-2392882
BmHel-3a	DF090365:634181-634572
BmHel-3a	DF090401:1052070-1052461
BmHel-3a	DF090384:281505-281896
BmHel-3a	DF090375:204225-204616

BmHel-3a	DF090461:198954-199345
BmHel-3a	DF090339:3266412-3266803
BmHel-3a	DF090331:1979139-1979530
BmHel-3a	DF090330:152088-152480
BmHel-3a	DF090330:3951837-3952229
BmHel-3a	DF090330:5347646-5348038
BmHel-3a	DF090316:13058673-13059065
BmHel-3a	DF090331:5002004-5002396
BmHel-3a	DF090321:4839372-4839764
BmHel-3a	DF090340:1970674-1971066
BmHel-3a	DF090521:28357-28749
BmHel-3a	DF090372:602374-602766
BmHel-3a	DF090342:1923336-1923728
BmHel-3a	DF090376:1899735-1900127
BmHel-3a	DF090317:7610048-7610440
BmHel-3a	DF090338:4030095-4030487
BmHel-3a	DF090367:1319281-1319673
BmHel-3a	DF090352:301580-301972
BmHel-3a	DF090316:2049453-2049845
BmHel-3a	DF090327:2257759-2258151
BmHel-3a	DF090431:668206-668598
BmHel-3a	DF090372:1857189-1857581
BmHel-3a	DF090451:429296-429688
BmHel-3a	DF090401:1165671-1166063
BmHel-3a	DF090400:1557995-1558387
BmHel-3a	DF090329:5306436-5306828
BmHel-3a	DF090348:2139829-2140222
BmHel-3a	DF090345:341105-341498
BmHel-3a	DF090381:2030070-2030463
BmHel-3a	DF090350:3760568-3760961
BmHel-3a	DF090317:9967195-9967588
BmHel-3a	DF090317:4101863-4102256
BmHel-3a	DF090316:14682616-14683009
BmHel-3a	DF090327:5492983-5493376
BmHel-3a	DF090354:2603324-2603717
BmHel-3a	DF090329:2133520-2133913
BmHel-3a	DF090372:1500022-1500415
BmHel-3a	DF090365:2058927-2059320
BmHel-3a	DF091188:12475-12868
BmHel-3a	BABH01042956:20353-20746
BmHel-3a	DF090484:145054-145448
BmHel-3a	DF090369:2232552-2232946
BmHel-3a	DF090355:832033-832427
BmHel-3a	DF090323:870951-871345
BmHel-3a	DF090318:9280329-9280723
BmHel-3a	DF090381:244318-244712
BmHel-3a	DF090377:104508-104902
BmHel-3a	DF090327:5471868-5472262

BmHel-3a	DF090341:4616054-4616448
BmHel-3a	DF090354:3578363-3578757
BmHel-3a	DF090353:1541782-1542176
BmHel-3a	DF090465:160617-161012
BmHel-3a	DF090358:1888441-1888836
BmHel-3a	DF090316:667373-667768
BmHel-3a	DF090335:3345234-3345629
BmHel-3a	DF090413:1304346-1304741
BmHel-3a	DF090347:53483-53878
BmHel-3a	DF090336:3062643-3063039
BmHel-3a	DF090345:2567045-2567441
BmHel-3a	DF090339:4633021-4633417
BmHel-3a	DF090333:3311642-3312038
BmHel-3a	DF090354:1662727-1663124
BmHel-3a	DF090318:4602150-4602547
BmHel-3a	DF090335:726987-727384
BmHel-3a	DF090357:2928885-2929282
BmHel-3a	DF090317:2077951-2078349
BmHel-3a	DF090401:992664-993062
BmHel-3a	DF091264:2154-2553
BmHel-3a	DF090328:3418957-3419357
BmHel-3a	DF090410:1376687-1377087
BmHel-3a	DF090317:2097910-2098311
BmHel-3a	DF090488:162778-163180
BmHel-3a	DF090337:4054794-4055196
BmHel-3a	DF090337:3245021-3245425
BmHel-3a	DF090342:3231329-3231735
BmHel-3a	DF090321:3034202-3034609
BmHel-3a	DF090337:1842617-1843024
BmHel-3a	DF090342:1534653-1535061
BmHel-3a	DF090319:6871523-6871932
BmHel-3a	DF090349:3548499-3548911
BmHel-3a	DF090343:1515424-1515837
BmHel-3a	DF090407:226249-226663
BmHel-3a	DF090326:4205248-4205673
BmHel-3a	DF090319:2279349-2279795
BmHel-3a	DF090348:3338454-3338903
BmHel-3a	DF090319:4507698-4508161
BmHel-3a	DF090358:754297-754789
BmHel-3a	DF090320:6250350-6250842
BmHel-3a	DF090475:140797-141289
BmHel-3a	DF090317:10033767-10034262
BmHel-3a	DF090361:1216232-1216746
BmHel-3a	DF090386:463138-463653
BmHel-3a	DF090366:1721503-1722031
BmHel-3a	DF090317:512042-512573
BmHel-3a	DF090437:100745-101351
BmHel-3a	DF090354:3202461-3203119

BmHel-3a	DF090363:77242-77900
BmHel-3a	DF090356:2946083-2946749
BmHel-3a	DF090422:664405-665073
BmHel-3a	DF090432:533076-533756
BmHel-3a	DF090317:2907116-2907828
BmHel-3a	DF090369:1013390-1014121
BmHel-3a	DF090460:65759-66493
BmHel-3a	DF090342:1197232-1197974
BmHel-3a	DF090326:1714021-1714765
BmHel-3a	DF090333:1201847-1202609
BmHel-3a	DF090372:580411-581183
BmHel-3a	DF090323:3416903-3417679
BmHel-3a	DF090391:239889-240674
BmHel-3a	DF090319:6001473-6002259
BmHel-3a	DF090342:3076984-3077796
BmHel-3a	DF090413:252881-253703
BmHel-3a	DF090346:1505962-1506818
BmHel-3a	DF090384:944151-945018
BmHel-3a	DF090400:1515066-1515933
BmHel-3a	DF090317:5814141-5815021
BmHel-3a	DF090412:25617-26502
BmHel-3a	DF091700:427-1323
BmHel-3a	DF091557:1279-2198
BmHel-3a	DF090403:495169-496097
BmHel-3a	DF090340:2637593-2638522
BmHel-3a	DF090359:2347898-2348832
BmHel-3a	DF090327:872572-873514
BmHel-3a	DF090319:3402384-3403331
BmHel-3a	DF090317:5069017-5069968
BmHel-3a	DF090344:3988651-3989610
BmHel-3a	DF090333:4746826-4747788
BmHel-3a	DF090343:2407780-2408748
BmHel-3a	DF090358:274371-275341
BmHel-3a	DF090338:3088101-3089074
BmHel-3a	DF090328:2600794-2601768
BmHel-3a	DF090522:82016-82990
BmHel-3a	DF090322:7293934-7294908
BmHel-3a	DF090338:2023875-2024856
BmHel-3a	DF090483:127452-128438
BmHel-3a	DF090358:345645-346634
BmHel-3a	DF090318:2008718-2009708
BmHel-3a	DF090350:2013504-2014501
BmHel-3a	DF090348:2677025-2678025
BmHel-3a	DF090598:17634-18635
BmHel-3a	DF090356:3299802-3300807
BmHel-3a	DF090420:135748-136764
BmHel-3a	DF090376:337243-338261
BmHel-3a	DF090325:3481841-3482860

BmHel-3a	DF090359:2960091-2961116
BmHel-3a	DF090351:3537292-3538334
BmHel-3a	DF090319:3677246-3678292
BmHel-3a	DF090378:696977-698023
BmHel-3a	DF090341:2110558-2111611
BmHel-3a	DF090342:2586152-2587209
BmHel-3a	DF090348:422280-423342
BmHel-3a	DF090319:8944012-8945074
BmHel-3a	DF090409:20683-21749
BmHel-3a	DF090354:3806806-3807873
BmHel-3a	DF090389:1687854-1688923
BmHel-3a	DF090426:927542-928612
BmHel-3a	DF090343:4529412-4530485
BmHel-3a	DF090330:577510-578587
BmHel-3a	DF090655:23569-24654
BmHel-3a	DF090340:1421754-1422841
BmHel-3a	DF090334:3873759-3874848
BmHel-3a	DF090330:4344055-4345146
BmHel-3a	DF090350:2496479-2497581
BmHel-3a	DF090346:3502672-3503778
BmHel-3a	DF090380:2103093-2104200
BmHel-3a	DF090336:3322129-3323237
BmHel-3a	DF090367:2008651-2009759
BmHel-3a	DF090316:15007049-15008161
BmHel-3a	DF090327:3713822-3714934
BmHel-3a	DF090317:7813636-7814750
BmHel-3a	DF090347:3940846-3941961
BmHel-3a	DF090331:5514667-5515784
BmHel-3a	DF090329:5466268-5467385
BmHel-3a	DF090385:157893-159010
BmHel-3a	DF090316:15871206-15872323
BmHel-3a	DF090350:3725058-3726176
BmHel-3a	DF090342:2524283-2525402
BmHel-3a	DF090374:2246394-2247514
BmHel-3a	DF090317:7735210-7736331
BmHel-3a	DF090353:613133-614255
BmHel-3a	DF090350:1233689-1234812
BmHel-3a	DF090342:2023565-2024689
BmHel-3a	DF090321:4573124-4574248
BmHel-3a	DF090349:1403709-1404834
BmHel-3a	DF090422:149526-150652
BmHel-3a	DF090424:658921-660047
BmHel-3a	DF090401:225923-227051
BmHel-3a	DF090320:2195738-2196868
BmHel-3a	DF090358:44370-45500
BmHel-3a	DF090323:4253762-4254893
BmHel-3a	DF090322:4623028-4624160
BmHel-3a	DF090348:2948692-2949829

BmHel-3a	DF090380:1942115-1943253
BmHel-3a	DF090343:3178841-3179982
BmHel-3a	DF090353:3321563-3322707
BmHel-3a	DF090365:2480453-2481598
BmHel-3a	DF090316:14395491-14396638
BmHel-3a	DF090343:2501681-2502833
BmHel-3a	DF090321:7271900-7273063
BmHel-3a	DF090319:2349555-2350718
BmHel-3a	DF090322:6293344-6294515
BmHel-3a	DF090362:2751754-2752925
BmHel-3a	DF090333:797328-798499
BmHel-3a	DF090378:1388006-1389178
BmHel-3a	DF090429:508428-509610
BmHel-3a	DF090332:3946327-3947514
BmHel-3a	DF090318:3903088-3904275
BmHel-3a	DF090408:1264352-1265542
BmHel-3a	DF090355:3150616-3151811
BmHel-3a	DF090330:6382854-6384051
BmHel-3a	DF090339:3399724-3400925
BmHel-3a	DF090392:1567615-1568818
BmHel-3a	DF090389:946037-947244
BmHel-3a	DF090322:1468462-1469671
BmHel-3a	DF090350:1483141-1484350
BmHel-3a	DF090322:5945680-5946891
BmHel-3a	DF090321:2157872-2159087
BmHel-3a	DF090328:5074427-5075643
BmHel-3a	DF090560:29541-30760
BmHel-3a	DF090335:2483000-2484220
BmHel-3a	DF090319:9113145-9114366
BmHel-3a	DF090333:4942799-4944022
BmHel-3a	DF090352:1992401-1993626
BmHel-3a	DF090328:6334036-6335261
BmHel-3a	DF090339:3237437-3238666
BmHel-3a	DF090678:94582-95811
BmHel-3a	DF090346:1326043-1327273
BmHel-3a	DF090322:3833871-3835104
BmHel-3a	DF090403:1099268-1100502
BmHel-3a	DF090330:1749094-1750328
BmHel-3a	DF090320:1605192-1606428
BmHel-3a	DF090454:406334-407570
BmHel-3a	DF090322:7240686-7241924
BmHel-3a	DF090326:2790033-2791271
BmHel-3a	DF090365:681062-682302
BmHel-3a	DF090405:485870-487110
BmHel-3a	DF090359:1775925-1777165
BmHel-3a	DF090386:602982-604223
BmHel-3a	DF090372:1958342-1959584
BmHel-3a	DF090347:634986-636228

BmHel-3a	DF090357:3722632-3723876
BmHel-3a	DF090320:2584424-2585671
BmHel-3a	DF090379:1145521-1146768
BmHel-3a	DF090335:3149037-3150285
BmHel-3a	DF090355:1617489-1618738
BmHel-3a	DF090388:927174-928423
BmHel-3a	DF090376:100016-101265
BmHel-3a	DF090317:7742887-7744139
BmHel-3a	DF090335:2246147-2247400
BmHel-3a	DF090382:1680382-1681635
BmHel-3a	DF090369:1584306-1585560
BmHel-3a	DF090355:3728497-3729751
BmHel-3a	DF090329:216590-217844
BmHel-3a	DF090328:4122952-4124206
BmHel-3a	DF090324:4036692-4037948
BmHel-3a	DF090383:1830332-1831588
BmHel-3a	DF090330:1120689-1121948
BmHel-3a	DF090347:1557773-1559033
BmHel-3a	DF090350:657015-658276
BmHel-3a	DF090386:1749457-1750719
BmHel-3a	DF090460:217558-218820
BmHel-3a	DF090326:5509926-5511190
BmHel-3a	DF090318:2579675-2580940
BmHel-3a	DF090373:743039-744306
BmHel-3a	DF090348:3003313-3004588
BmHel-3a	DF090346:475766-477042
BmHel-3a	DF090330:664345-665621
BmHel-3a	DF090352:1832558-1833835
BmHel-3a	DF090419:181520-182799
BmHel-3a	DF090317:9615089-9616371
BmHel-3a	DF090384:251812-253101
BmHel-3a	DF090343:1789958-1791253
BmHel-3a	DF090356:2416531-2417833
BmHel-3a	DF090370:420348-421651
BmHel-3a	DF090368:415684-416990
BmHel-3a	DF090341:3284646-3285959
BmHel-3a	DF090402:795607-796924
BmHel-3a	DF091458:1220-2540
BmHel-3a	DF090349:306993-308316
BmHel-3a	DF090329:3602746-3604070
BmHel-3a	DF090319:2070816-2072145
BmHel-3a	DF090344:201845-203184
BmHel-3a	DF090378:965141-966484
BmHel-3a	DF090666:26934-28290
BmHel-3a	DF090350:1369294-1370651
BmHel-3a	DF090366:369472-370835
BmHel-3a	DF090348:883138-884502
BmHel-3a	DF090316:10643304-10644673

BmHel-3a	DF090413:43052-44421
BmHel-3a	DF090384:419543-420913
BmHel-3a	DF090350:4144941-4146314
BmHel-3a	DF090334:2108904-2110279
BmHel-3a	DF090336:3599054-3600432
BmHel-3a	DF090377:2186424-2187803
BmHel-3a	DF090416:455274-456670
BmHel-3a	DF090324:4003850-4005249
BmHel-3a	DF090341:276234-277640
BmHel-3a	DF090356:2099932-2101340
BmHel-3a	DF090334:220726-222138
BmHel-3a	DF090362:1404812-1406227
BmHel-3a	DF090412:1375249-1376664
BmHel-3a	DF090316:5925611-5927035
BmHel-3a	DF090318:2249875-2251304
BmHel-3a	DF090322:1631017-1632460
BmHel-3a	DF090384:1745963-1747421
BmHel-3a	DF090338:1402374-1403854
BmHel-3a	DF090334:1839671-1841154
BmHel-3a	DF090422:407139-408623
BmHel-3a	DF090364:1218565-1220051
BmHel-3a	DF090414:1200608-1202098
BmHel-3a	DF090319:2585970-2587461
BmHel-3a	DF090361:2016255-2017773
BmHel-3a	DF090396:1207348-1208869
BmHel-3a	DF090351:3015338-3016864
BmHel-3a	DF090337:5009247-5010777
BmHel-3a	DF090408:890979-892511
BmHel-3a	DF090394:90700-92242
BmHel-3a	DF090351:3244913-3246456
BmHel-3a	DF090451:27578-29125
BmHel-3a	DF090334:2314086-2315636
BmHel-3a	DF090356:2401858-2403409
BmHel-3a	DF090344:1793569-1795121
BmHel-3a	DF090360:682176-683728
BmHel-3a	DF090339:1249527-1251084
BmHel-3a	DF090339:1138186-1139747
BmHel-3a	DF090336:2078193-2079755
BmHel-3a	DF090358:1688071-1689637
BmHel-3a	DF090365:1497183-1498753
BmHel-3a	DF090353:1234867-1236446
BmHel-3a	DF090328:4206984-4208575
BmHel-3a	DF090341:2158988-2160585
BmHel-3a	DF090347:809644-811242
BmHel-3a	DF090390:1324632-1326230
BmHel-3a	DF090329:5093763-5095373
BmHel-3a	DF090332:4909395-4911012
BmHel-3a	DF090327:4092938-4094566

BmHel-3a	DF090318:13272-14908
BmHel-3a	DF090427:458734-460373
BmHel-3a	DF090435:412598-414247
BmHel-3a	DF090333:2476868-2478519
BmHel-3a	DF090330:1183112-1184764
BmHel-3a	DF090325:6014746-6016410
BmHel-3a	DF090409:1324200-1325866
BmHel-3a	DF090347:3485477-3487150
BmHel-3a	DF090328:3390316-3391990
BmHel-3a	DF090406:489568-491245
BmHel-3a	DF090331:4885891-4887568
BmHel-3a	DF090394:572364-574044
BmHel-3a	DF090332:4858563-4860244
BmHel-3a	DF090385:1614953-1616639
BmHel-3a	DF090316:12115307-12116997
BmHel-3a	DF090342:2690079-2691771
BmHel-3a	DF090419:1038985-1040696
BmHel-3a	DF090341:4816319-4818031
BmHel-3a	DF090595:5825-7542
BmHel-3a	DF090322:6792512-6794249
BmHel-3a	DF090373:1798719-1800461
BmHel-3a	DF090355:2149960-2151704
BmHel-3a	DF090402:1385222-1386975
BmHel-3a	DF090411:1104661-1106415
BmHel-3a	DF090353:735230-736987
BmHel-3a	DF090324:5694890-5696648
BmHel-3a	DF090321:2586031-2587792
BmHel-3a	DF090522:92941-94704
BmHel-3a	DF090348:3758617-3760386
BmHel-3a	DF090319:3669253-3671026
BmHel-3a	DF090413:149462-151245
BmHel-3a	DF090316:11439612-11441399
BmHel-3a	DF090329:1155820-1157616
BmHel-3a	DF090380:248115-249921
BmHel-3a	DF090342:4346927-4348762
BmHel-3a	DF090321:1209841-1211685
BmHel-3a	DF090465:30890-32740
BmHel-3a	DF090362:1311817-1313667
BmHel-3a	DF090349:987151-989006
BmHel-3a	DF090327:2875507-2877363
BmHel-3a	DF090321:7271304-7273162
BmHel-3a	DF090348:170231-172091
BmHel-3a	DF090557:4323-6190
BmHel-3a	DF090386:1458905-1460776
BmHel-3a	DF090318:6415458-6417339
BmHel-3a	DF090329:2624279-2626163
BmHel-3a	DF090331:4881601-4883486
BmHel-3a	DF090364:1975687-1977580

BmHel-3a	DF090316:8635134-8637058
BmHel-3a	DF090321:6840549-6842479
BmHel-3a	DF090396:17993-19933
BmHel-3a	DF090323:2857099-2859062
BmHel-3a	DF090316:13948236-13950204
BmHel-3a	DF090355:1049286-1051258
BmHel-3a	DF090332:1432145-1434119
BmHel-3a	DF090402:1106219-1108193
BmHel-3a	DF090341:582653-584628
BmHel-3a	DF090341:4383443-4385422
BmHel-3a	DF090317:5399272-5401256
BmHel-3a	DF090366:2186553-2188547
BmHel-3a	DF090359:3218735-3220734
BmHel-3a	DF090331:2897727-2899729
BmHel-3a	DF090455:277136-279140
BmHel-3a	DF090317:9743378-9745386
BmHel-3a	DF090348:701775-703784
BmHel-3a	DF090379:418921-420960
BmHel-3a	DF090356:615674-617715
BmHel-3a	DF090341:125681-127722
BmHel-3a	DF090334:3673343-3675384
BmHel-3a	DF090328:1027231-1029289
BmHel-3a	DF090384:1077500-1079576
BmHel-3a	DF090327:5003663-5005764
BmHel-3a	DF090433:48530-50650
BmHel-3a	DF090322:8096964-8099097
BmHel-3a	DF090326:6038773-6040978
BmHel-3a	DF090321:1090152-1092360
BmHel-3a	DF090360:2405147-2407376
BmHel-3a	DF090385:1517738-1519989
BmHel-3a	DF090353:1834500-1836759
BmHel-3a	DF091548:23-2283
BmHel-3a	DF090372:1123022-1125290
BmHel-3a	DF090340:237636-239937
BmHel-3a	DF090395:104988-107300
BmHel-3a	DF090350:1480772-1483089
BmHel-3a	DF090502:46944-49302
BmHel-3a	DF090375:800493-802872
BmHel-3a	DF090379:945633-948047
BmHel-3a	DF090317:7326923-7329338
BmHel-3a	DF090328:2833907-2836354
BmHel-3a	DF090413:1030418-1032939
BmHel-3a	DF090425:418630-421195
BmHel-3a	DF090323:2107204-2109792
BmHel-3a	DF090353:3415767-3418392
BmHel-3a	DF090317:4676258-4678914
BmHel-3a	DF090379:279551-282265
BmHel-3a	DF090452:43790-46525

BmHel-3a	DF090358:2476172-2478934
BmHel-3a	DF090395:69726-72875
BmHel-3a	DF090347:809442-813121
BmHel-3a	DF090445:229082-233341
BmHel-3a	DF090316:4119984-4124912
BmHel-3a	DF090486:143504-148480
BmHel-3a	DF090355:1749986-1755656
BmHel-3a	DF090372:1765213-1771066
BmHel-3a	DF090412:480439-486379
BmHel-3a	DF090397:1254813-1260820
BmHel-3a	DF090324:6585897-6592382
BmHel-3a	DF090332:401041-408094
BmHel-3a	DF090331:5411091-5418355
BmHel-3a	DF090377:744314-753013
BmHel-3a	DF090387:774200-774362
BmHel-3a	DF090444:542416-542578
BmHel-3a	DF090339:632096-632258
BmHel-3a	DF090324:221758-221918
BmHel-3a	DF090375:1777493-1777655
BmHel-3a	BABH01072198:315-476
BmHel-3a	DF090360:648875-649035
BmHel-3a	DF090354:3342146-3342306
BmHel-3a	DF090398:1581617-1581777
BmHel-3a	DF090322:8044312-8044473
BmHel-3a	DF090360:2883244-2883407
BmHel-3a	DF090319:8480268-8480429
BmHel-3a	BABH01065067:282-444
BmHel-3a	DF090316:9397033-9397192
BmHel-3a	DF090421:510162-510321
BmHel-3a	DF090398:968447-968606
BmHel-3a	DF090395:1578833-1579002
BmHel-3a	DF090391:490624-490783
BmHel-3a	DF090372:1900187-1900346
BmHel-3a	DF090345:4413303-4413466
BmHel-3a	DF090342:4785577-4785738
BmHel-3a	DF090329:3557430-3557592
BmHel-3a	DF090419:268260-268422
BmHel-3a	DF090330:2778143-2778302
BmHel-3a	DF090369:1937992-1938153
BmHel-3a	DF090320:3985495-3985657
BmHel-3a	DF090317:1146028-1146185
BmHel-3a	DF090386:1837072-1837229
BmHel-3a	DF090316:15777529-15777690
BmHel-3a	DF090349:2340325-2340482
BmHel-3a	DF090425:216531-216688
BmHel-3a	DF090341:3785270-3785427
BmHel-3a	DF090329:4375666-4375823
BmHel-3a	DF090319:2913611-2913768

BmHel-3a	DF090408:606185-606342
BmHel-3a	DF090365:571565-571722
BmHel-3a	DF090364:2492785-2492943
BmHel-3a	DF090492:34385-34542
BmHel-3a	DF090357:303246-303403
BmHel-3a	DF090346:2000565-2000722
BmHel-3a	DF090412:12516-12672
BmHel-3a	DF090351:1677789-1677946
BmHel-3a	DF090415:978667-978824
BmHel-3a	DF090719:18907-19064
BmHel-3a	DF090316:15677841-15677999
BmHel-3a	DF090334:1436820-1436978
BmHel-3a	DF090324:833032-833192
BmHel-3a	DF090737:8505-8665
BmHel-3a	DF090344:1001597-1001754
BmHel-3a	DF090316:15314720-15314877
BmHel-3a	DF090318:1070617-1070774
BmHel-3a	DF090340:992548-992705
BmHel-3a	DF090338:3850580-3850736
BmHel-3a	DF090363:1957037-1957197
BmHel-3a	DF090376:458789-458945
BmHel-3a	DF090412:350234-350389
BmHel-3a	BABH01050309:589-751
BmHel-3a	DF090426:276826-276981
BmHel-3a	DF090344:2694725-2694884
BmHel-3a	DF090317:6425149-6425307
BmHel-3a	DF090317:8643187-8643341
BmHel-3a	DF090330:3426026-3426178
BmHel-3a	DF090319:6906118-6906273
BmHel-3a	DF090393:1555169-1555324
BmHel-3a	DF090428:306941-307092
BmHel-3a	DF091630:628-783
BmHel-3a	DF090340:1839377-1839534
BmHel-3a	DF090344:2365984-2366139
BmHel-3a	DF090404:841713-841868
BmHel-3a	DF090387:1752799-1752954
BmHel-3a	DF090317:10530513-10530669
BmHel-3a	DF090360:1718997-1719152
BmHel-3a	DF090363:1251173-1251328
BmHel-3a	DF090335:3459637-3459792
BmHel-3a	DF090344:3948143-3948304
BmHel-3a	DF090322:1223017-1223170
BmHel-3a	DF090320:5211226-5211383
BmHel-3a	DF090629:42745-42899
BmHel-3a	DF091109:5564-5718
BmHel-3a	DF090344:436435-436589
BmHel-3a	DF090339:3607513-3607667
BmHel-3a	DF090395:1211299-1211453

BmHel-3a	DF090434:680853-681007
BmHel-3a	DF090396:705974-706128
BmHel-3a	DF090319:3200328-3200486
BmHel-3a	DF090344:331673-331828
BmHel-3a	DF090438:195364-195517
BmHel-3a	DF090330:6210475-6210627
BmHel-3a	DF090317:3262504-3262657
BmHel-3a	DF090410:1294738-1294884
BmHel-3a	DF090352:3321578-3321731
BmHel-3a	DF090336:2522158-2522315
BmHel-3a	DF090324:480480-480634
BmHel-3a	DF090333:989774-989920
BmHel-3a	DF090337:780987-781149
BmHel-3a	DF090320:3562264-3562419
BmHel-3a	DF090349:3562185-3562346
BmHel-3a	DF090336:2498183-2498339
BmHel-3a	DF090321:3697872-3698003
BmHel-3a	BABH01074514:230-385
BmHel-3a	DF090326:4286491-4286648
BmHel-3a	DF090446:297455-297609
BmHel-3a	DF090331:639366-639527
BmHel-3a	DF091264:945-1096
BmHel-3a	DF090394:24350-24507
BmHel-3a	DF090321:1556295-1556442
BmHel-3a	DF090375:1457581-1457732
BmHel-3a	DF090384:1781627-1781778
BmHel-3a	DF091074:2283-2434
BmHel-3a	DF090322:3542698-3542849
BmHel-3a	DF090342:2810601-2810751
BmHel-3a	DF090383:516843-516994
BmHel-3a	DF090663:6146-6306
BmHel-3a	DF090349:3326237-3326390
BmHel-3a	DF090346:1605873-1606026
BmHel-3a	DF090424:686596-686746
BmHel-3a	DF090327:4981961-4982111
BmHel-3a	DF090402:781511-781664
BmHel-3a	DF090317:7502876-7503029
BmHel-3a	DF090341:4648287-4648437
BmHel-3a	DF090377:850541-850691
BmHel-3a	DF090370:1158546-1158699
BmHel-3a	DF090318:7935551-7935700
BmHel-3a	BABH01087010:1-149
BmHel-3a	DF090415:818995-819155
BmHel-3a	DF090406:42162-42327
BmHel-3a	DF090320:8553822-8553970
BmHel-3a	DF090356:2309680-2309834
BmHel-3a	DF090695:14977-15125
BmHel-3a	DF090339:491248-491405

BmHel-3a	DF090322:6333234-6333396
BmHel-3a	DF090338:2052562-2052724
BmHel-3a	DF090333:1556272-1556434
BmHel-3a	DF090335:1226807-1226954
BmHel-3a	DF090338:3426416-3426563
BmHel-3a	DF090349:2533245-2533392
BmHel-3a	DF090356:1674436-1674587
BmHel-3a	DF090342:656367-656527
BmHel-3a	DF090485:110725-110883
BmHel-3a	DF090394:239089-239238
BmHel-3a	DF090316:16045144-16045259
BmHel-3a	DF090394:228775-228926
BmHel-3a	DF090334:1635701-1635863
BmHel-3a	DF090344:337121-337277
BmHel-3a	DF090482:231988-232144
BmHel-3a	DF090441:241747-241898
BmHel-3a	DF090374:933773-933922
BmHel-3a	DF090344:744250-744396
BmHel-3a	DF090405:977348-977492
BmHel-3a	DF090362:2284009-2284169
BmHel-3a	DF090420:893112-893254
BmHel-3a	BABH01048626:7-155
BmHel-3a	DF090438:433542-433685
BmHel-3a	BABH01048445:565-722
BmHel-3a	DF090322:2214934-2215091
BmHel-3a	DF090393:571975-572076
BmHel-3a	DF090329:276926-277068
BmHel-3a	DF090405:1259264-1259406
BmHel-3a	DF090318:5492882-5493029
BmHel-3a	DF091188:503-618
BmHel-3a	DF090498:21017-21176
BmHel-3a	DF090342:1559050-1559205
BmHel-3a	DF090353:3914427-3914565
BmHel-3a	DF090358:3315904-3316064
BmHel-3a	DF090341:4632122-4632268
BmHel-3a	DF090364:175112-175248
BmHel-3a	DF090485:35420-35560
BmHel-3a	DF090456:94949-95091
BmHel-3a	DF090320:3622418-3622553
BmHel-3a	DF090331:3918433-3918590
BmHel-3a	DF090372:1067065-1067227
BmHel-3a	DF090349:3550652-3550785
BmHel-3a	DF090384:1107515-1107648
BmHel-3a	DF090370:1079483-1079616
BmHel-3a	DF090348:3674241-3674371
BmHel-3a	DF090476:174503-174641
BmHel-3a	DF090421:924089-924235
BmHel-3a	DF090325:2208958-2209120

BmHel-3a	DF090382:652072-652225
BmHel-3a	DF090354:46900-47044
BmHel-3a	DF090372:1598504-1598638
BmHel-3a	DF090320:4358662-4358793
BmHel-3a	DF090330:147857-147988
BmHel-3a	DF090418:196282-196414
BmHel-3a	DF090316:6903597-6903763
BmHel-3a	DF090317:253407-253566
BmHel-3a	DF090339:1001180-1001342
BmHel-3a	DF090338:2147009-2147160
BmHel-3a	DF090331:3955785-3955931
BmHel-3a	BABH01050139:629-791
BmHel-3a	DF090403:902343-902472
BmHel-3a	DF090672:3258-3386
BmHel-3a	DF090374:1499636-1499733
BmHel-3a	DF090414:1163149-1163273
BmHel-3a	DF090338:3115616-3115697
BmHel-3a	DF090358:3023085-3023210
BmHel-3a	DF090350:2498687-2498809
BmHel-3a	DF090401:1397596-1397712
BmHel-3a	DF090323:590236-590357
BmHel-3a	DF090316:5503043-5503164
BmHel-3a	DF090396:493650-493766
BmHel-3a	DF090327:2971476-2971609
BmHel-3a	DF090429:525112-525230
BmHel-3a	DF090432:285234-285352
BmHel-3a	DF090337:3968823-3968941
BmHel-3a	DF090318:2420529-2420646
BmHel-3a	DF090399:1219049-1219166
BmHel-3a	DF090470:103840-103955
BmHel-3a	DF090340:1429941-1430059
BmHel-3a	DF090326:6301039-6301157
BmHel-3a	DF090354:2404344-2404458
BmHel-3a	DF090343:1583745-1583875
BmHel-3a	DF090330:2790553-2790652
BmHel-3a	DF090349:2069089-2069190
BmHel-3a	DF090368:2239682-2239792
BmHel-3a	DF090345:2071731-2071867
BmHel-3a	DF090374:1099374-1099481
BmHel-3a	DF090365:46568-46671
BmHel-3a	DF090360:807491-807591
BmHel-3a	BABH01053528:675-775
BmHel-3a	BABH01068486:1-101
BmHel-3a	DF090317:135743-135905
BmHel-3a	DF090343:1102766-1102925
BmHel-3a	DF090330:1202985-1203147
BmHel-3a	DF090326:1951248-1951414
BmHel-3a	DF090326:115445-115608

BmHel-3a	DF090349:1715866-1716027
BmHel-3a	BABH01062105:432-594
BmHel-3a	BABH01048717:182-344
BmHel-3a	DF090355:470489-470651
BmHel-3a	DF090348:289527-289689
BmHel-3a	DF090341:4413392-4413554
BmHel-3a	DF090331:489658-489823
BmHel-3a	DF090320:5060654-5060816
BmHel-3a	DF090363:1432489-1432651
BmHel-3a	DF090398:220069-220232
BmHel-3a	DF090362:1205976-1206138
BmHel-3a	DF090347:1384017-1384179
BmHel-3a	DF090334:2610534-2610702
BmHel-3a	BABH01075756:303-465
BmHel-3a	DF090414:1177055-1177217
BmHel-3a	DF090394:807257-807419
BmHel-3a	DF090328:6479251-6479407
BmHel-3a	DF090364:68595-68693
BmHel-3a	DF090370:1424886-1425046
BmHel-3a	DF090361:1782367-1782527
BmHel-3a	DF090350:1234927-1235087
BmHel-3a	DF090338:4481840-4482000
BmHel-3a	DF090337:2129221-2129381
BmHel-3a	DF090337:3779807-3779967
BmHel-3a	DF090330:5210664-5210824
BmHel-3a	DF090326:6038984-6039144
BmHel-3a	DF090326:4652363-4652523
BmHel-3a	DF090349:1169597-1169757
BmHel-3a	DF090327:4908224-4908384
BmHel-3a	DF090355:2220508-2220668
BmHel-3a	DF090345:1570120-1570280
BmHel-3a	DF090345:2159916-2160076
BmHel-3a	DF090341:4472594-4472755
BmHel-3a	DF090339:1698853-1699013
BmHel-3a	DF090339:5001950-5002103
BmHel-3a	DF090329:744673-744833
BmHel-3a	DF090323:4438444-4438605
BmHel-3a	DF090320:6458234-6458394
BmHel-3a	DF090340:3859851-3860011
BmHel-3a	DF090340:2721718-2721878
BmHel-3a	DF090408:321801-321961
BmHel-3a	DF090363:1038322-1038482
BmHel-3a	DF090398:857983-858143
BmHel-3a	DF090372:2106901-2107061
BmHel-3a	DF090334:2214526-2214686
BmHel-3a	DF090325:1155372-1155532
BmHel-3a	DF090346:994934-995094
BmHel-3a	DF090384:1351505-1351665

BmHel-3a	DF090376:721503-721663
BmHel-3a	DF090333:1111632-1111792
BmHel-3a	DF090351:3896423-3896583
BmHel-3a	DF090437:499745-499904
BmHel-3a	DF090407:656002-656162
BmHel-3a	BABH01078780:24-184
BmHel-3a	BABH01075053:381-541
BmHel-3a	BABH01074317:177-337
BmHel-3a	BABH01087162:35-195
BmHel-3a	BABH01087053:59-219
BmHel-3a	BABH01086783:129-289
BmHel-3a	BABH01085566:51-211
BmHel-3a	BABH01084998:211-371
BmHel-3a	BABH01084761:15-175
BmHel-3a	BABH01082637:41-201
BmHel-3a	BABH01082579:310-470
BmHel-3a	BABH01082354:233-393
BmHel-3a	BABH01082353:39-199
BmHel-3a	BABH01081961:118-278
BmHel-3a	BABH01079995:18-178
BmHel-3a	BABH01078965:237-397
BmHel-3a	BABH01078011:336-496
BmHel-3a	BABH01077386:50-210
BmHel-3a	BABH01077062:91-251
BmHel-3a	BABH01077002:305-465
BmHel-3a	BABH01076336:42-202
BmHel-3a	BABH01074770:259-419
BmHel-3a	BABH01072825:361-521
BmHel-3a	BABH01072240:345-505
BmHel-3a	BABH01071934:412-572
BmHel-3a	BABH01071835:331-491
BmHel-3a	BABH01068886:432-592
BmHel-3a	BABH01068149:322-482
BmHel-3a	BABH01066971:359-519
BmHel-3a	BABH01065657:478-638
BmHel-3a	BABH01065270:440-600
BmHel-3a	BABH01063158:142-302
BmHel-3a	BABH01063046:470-630
BmHel-3a	BABH01062517:77-237
BmHel-3a	BABH01061986:87-247
BmHel-3a	BABH01058593:425-585
BmHel-3a	BABH01055683:447-607
BmHel-3a	BABH01054121:3-163
BmHel-3a	BABH01053361:117-277
BmHel-3a	BABH01053274:331-491
BmHel-3a	BABH01052963:421-581
BmHel-3a	BABH01050941:166-326
BmHel-3a	DF090824:107-266

BmHel-3a	BABH01087869:60-220
BmHel-3a	BABH01085549:187-347
BmHel-3a	BABH01084787:70-230
BmHel-3a	BABH01078512:335-495
BmHel-3a	BABH01077171:29-189
BmHel-3a	BABH01076803:409-569
BmHel-3a	BABH01075414:307-467
BmHel-3a	BABH01073187:384-544
BmHel-3a	BABH01072985:392-552
BmHel-3a	BABH01071686:440-600
BmHel-3a	BABH01067104:379-539
BmHel-3a	BABH01063868:417-577
BmHel-3a	BABH01063535:439-599
BmHel-3a	BABH01062973:121-281
BmHel-3a	BABH01062125:71-231
BmHel-3a	BABH01061407:380-540
BmHel-3a	BABH01058607:345-505
BmHel-3a	BABH01058218:53-213
BmHel-3a	BABH01054250:534-693
BmHel-3a	BABH01052582:11-171
BmHel-3a	BABH01050533:470-630
BmHel-3a	BABH01047499:176-336
BmHel-3a	BABH01068239:440-600
BmHel-3a	BABH01049165:805-966
BmHel-3a	BABH01046082:2734-2895
BmHel-3a	BABH01080620:351-511
BmHel-3a	BABH01080003:11-170
BmHel-3a	BABH01079946:36-196
BmHel-3a	BABH01075987:16-176
BmHel-3a	BABH01074721:399-559
BmHel-3a	BABH01073734:361-521
BmHel-3a	BABH01071543:373-533
BmHel-3a	BABH01062721:508-667
BmHel-3a	BABH01055813:241-401
BmHel-3a	BABH01050569:99-258
BmHel-3a	DF090704:18645-18805
BmHel-3a	BABH01071845:432-592
BmHel-3a	BABH01051452:447-607
BmHel-3a	BABH01055097:100-259
BmHel-3a	BABH01073891:454-613
BmHel-3a	DF090318:9139451-9139568
BmHel-3a	DF090335:5353033-5353131
BmHel-3a	BABH01084770:309-468
BmHel-3a	BABH01077757:289-448
BmHel-3a	BABH01070625:149-308
BmHel-3a	BABH01053038:226-385
BmHel-3a	BABH01083295:12-171
BmHel-3a	BABH01054719:603-762

BmHel-3a	BABH01061017:10-169
BmHel-3a	DF090426:579446-579605
BmHel-3a	DF090435:564125-564284
BmHel-3a	BABH01079342:298-457
BmHel-3a	DF090812:15548-15707
BmHel-3a	DF090441:110824-110982
BmHel-3a	BABH01049427:359-517
BmHel-3a	DF090337:1402654-1402812
BmHel-3a	BABH01068678:278-435
BmHel-3a	DF090316:12116784-12116941
BmHel-3a	DF090680:364-521
BmHel-3a	BABH01076029:5-162
BmHel-3a	BABH01059638:67-224
BmHel-3a	BABH01079057:317-474
BmHel-3a	BABH01057545:237-394
BmHel-3a	DF090318:254102-254258
BmHel-3a	DF090412:298598-298755
BmHel-3a	DF090322:1817610-1817705
BmHel-3a	DF090389:528440-528596
BmHel-3a	BABH01054191:195-351
BmHel-3a	BABH01076429:407-563
BmHel-3a	BABH01048650:381-537
BmHel-3a	BABH01057965:453-609
BmHel-3a	DF090470:156556-156649
BmHel-3a	DF090360:2750611-2750767
BmHel-3a	DF090401:560507-560663
BmHel-3a	DF090369:334911-335066
BmHel-3a	DF090319:2862132-2862287
BmHel-3a	DF090386:9981-10136
BmHel-3a	DF090337:835428-835583
BmHel-3a	BABH01084938:341-496
BmHel-3a	BABH01064078:66-220
BmHel-3a	BABH01067848:107-261
BmHel-3a	BABH01051955:1-155
BmHel-3a	DF090360:3289076-3289229
BmHel-3a	DF090430:204161-204318
BmHel-3a	DF090359:2856024-2856177
BmHel-3a	DF090318:3955055-3955144
BmHel-3a	BABH01075662:448-601
BmHel-3a	DF090328:343970-344122
BmHel-3a	DF090378:1748781-1748871
BmHel-3a	DF090351:3979942-3980032
BmHel-3a	BABH01067259:1-153
BmHel-3a	DF090388:296009-296127
BmHel-3a	BABH01059958:612-705
BmHel-3a	DF090336:3116861-3116958
BmHel-3a	DF090330:2832343-2832494
BmHel-3a	DF091011:19114-19265

BmHel-3a	BABH01068178:426-576
BmHel-3a	DF090345:3004472-3004626
BmHel-3a	DF090364:323543-323689
BmHel-3a	DF090340:3985628-3985714
BmHel-3a	BABH01074659:1-148
BmHel-3a	DF090348:2817574-2817721
BmHel-3a	DF090319:572924-573071
BmHel-3a	BABH01067586:16-162
BmHel-3a	BABH01059437:7-153
BmHel-3a	BABH01059234:1-145
BmHel-3a	BABH01064858:1-146
BmHel-3a	DF090468:248452-248534
BmHel-3a	BABH01057349:11-154
BmHel-3a	BABH01057599:589-728
BmHel-3a	BABH01058874:572-714
BmHel-3a	BABH01085705:1-80
BmHel-3a	BABH01054769:1-142
BmHel-3a	DF090364:2104362-2104497
BmHel-3a	DF090367:2634535-2634671
BmHel-3a	DF090352:3554257-3554393
BmHel-3a	DF090318:1152600-1152736
BmHel-3a	BABH01073272:412-548
BmHel-3a	DF090419:897494-897629
BmHel-3a	DF090321:3388006-3388145
BmHel-3a	DF090364:390685-390818
BmHel-3a	DF090401:184182-184311
BmHel-3a	BABH01063164:558-680
BmHel-3a	DF090326:4597905-4598000
BmHel-3a	DF090320:1919779-1919907
BmHel-3a	DF090368:323487-323615
BmHel-3a	DF090330:5254599-5254725
BmHel-3a	BABH01069330:511-633
BmHel-3a	BABH01071438:11-133
BmHel-3a	BABH01059748:6-128
BmHel-3a	BABH01056126:11-133
BmHel-3a	BABH01072160:504-624
BmHel-3a	BABH01080935:1-119
BmHel-3a	BABH01064175:1-118
BmHel-3a	DF090321:1418546-1418663
BmHel-3a	DF090339:940895-941009
BmHel-3a	BABH01085099:378-493
BmHel-3a	DF090316:7737159-7737272
BmHel-3a	DF090316:718281-718393
BmHel-3a	BABH01050935:311-423
BmHel-3a	BABH01065445:2-112
BmHel-3a	DF090317:58671-58780
BmHel-3a	BABH01056114:640-749
BmHel-3a	BABH01068483:539-646

BmHel-3a	BABH01067387:1-108
BmHel-3a	DF090317:2567489-2567594
BmHel-3a	DF090394:1423918-1424019
BmHel-3a	BABH01078177:1-97
BmHel-3a	BABH01075456:476-571
BmHel-3a	DF090379:945839-945931
BmHel-3a	DF090411:1354787-1354878
BmHel-3a	DF090329:1359187-1359277
BmHel-3a	DF090383:845670-845759
BmHel-3a	BABH01079598:1-86
BmHel-3a	DF090382:2026157-2026237
BmHel-3a	BABH01060330:592-672
BmHel-3a	BABH01050171:613-693
BmHel-3a	DF091776:945-1025
BmHel-3a	BABH01082903:403-482
BmHel-4	DF090362:2591089-2591378
BmHel-4	DF090344:2724064-2724364
BmHel-4	DF090362:2843648-2843950
BmHel-4	DF090420:37020-37322
BmHel-4	DF090324:3391627-3391929
BmHel-4	DF090381:1605221-1605523
BmHel-4	DF090323:2187840-2188142
BmHel-4	DF090366:746070-746372
BmHel-4	DF090421:758381-758684
BmHel-4	DF090350:2255854-2256157
BmHel-4	DF090405:792666-792971
BmHel-4	DF090338:2340709-2341014
BmHel-4	DF090327:397037-397342
BmHel-4	DF090324:4718300-4718605
BmHel-4	DF090318:9304371-9304677
BmHel-4	DF090354:1389658-1389964
BmHel-4	DF090386:876214-876521
BmHel-4	DF090361:58257-58570
BmHel-4	DF090324:1392323-1392652
BmHel-4	DF090372:1262629-1263005
BmHel-4	DF090413:490764-491140
BmHel-4	DF090332:1993106-1993486
BmHel-4	DF090343:3947385-3947821
BmHel-4	DF090363:1654339-1654814
BmHel-4	DF090347:1917378-1917867
BmHel-4	DF090356:693660-694173
BmHel-4	DF090329:4566430-4566952
BmHel-4	DF090347:2859232-2859757
BmHel-4	DF090511:4073-4600
BmHel-4	DF090466:163779-164320
BmHel-4	DF090322:4716305-4716857
BmHel-4	DF091000:4347-4910

BmHel-4	DF090344:3031317-3031883
BmHel-4	DF090322:3928728-3929296
BmHel-4	DF090322:8151770-8152338
BmHel-4	DF090382:526406-526975
BmHel-4	DF090321:6158250-6158823
BmHel-4	DF090321:1298870-1299447
BmHel-4	DF090356:3729613-3730190
BmHel-4	DF090328:3345675-3346253
BmHel-4	DF090354:2434344-2434922
BmHel-4	DF090322:2337545-2338123
BmHel-4	DF090467:15255-15836
BmHel-4	DF090337:4683102-4683685
BmHel-4	DF090341:3778708-3779293
BmHel-4	DF090382:1133152-1133737
BmHel-4	DF090319:2328404-2328992
BmHel-4	DF090349:3384786-3385377
BmHel-4	DF090837:13171-13764
BmHel-4	DF090326:811151-811746
BmHel-4	DF090427:672982-673667
BmHel-4	DF090386:1517098-1517790
BmHel-4	DF090439:425746-426477
BmHel-4	DF090355:2158290-2159048
BmHel-4	DF090339:284976-286210
BmHel-4	DF090414:1318475-1319729
BmHel-4	DF090411:907851-909135
BmHel-4	DF090322:3920779-3922460
BmHel-4	DF090364:265484-275413
BmHel-4	DF090391:857666-858252
BmHel-4	DF090358:2131445-2132022
BmHel-4	DF090341:3680903-3681479
BmHel-4	DF090386:298567-299139
BmHel-4	DF090378:544865-545440
BmHel-4	DF090326:151697-152266
BmHel-4	DF090330:4455077-4455662
BmHel-4	DF090352:2515282-2515419
BmHel-4	DF090352:2514576-2515013
BmHel-4	DF090411:235851-236423
BmHel-4	DF090358:3435773-3436352
BmHel-4	BABH01055464:111-690
BmHel-4	DF090344:3234927-3235506
BmHel-4	DF090370:10025-10608
BmHel-4	DF090748:20320-20894
BmHel-4	DF090345:984995-985559
BmHel-4	DF090322:2259388-2259966
BmHel-4	DF090412:96214-96800
BmHel-4	DF090355:165766-166344
BmHel-4	DF090371:1581118-1581696
BmHel-4	DF090689:7680-8264

BmHel-4	DF090384:914871-915451
BmHel-4	DF090363:321599-322179
BmHel-4	DF090409:287347-287933
BmHel-4	DF090317:9817656-9818226
BmHel-4	DF090342:1446985-1447558
BmHel-4	DF090383:379540-380135
BmHel-4	DF090394:5837-6071
BmHel-4	DF090394:5217-5559
BmHel-4	DF090330:3819769-3819859
BmHel-4	DF090330:3819875-3820352
BmHel-4	DF090344:1135475-1136050
BmHel-4	DF090326:724035-724606
BmHel-4	DF090389:467368-467935
BmHel-4	DF090449:262836-263400
BmHel-4	DF090325:3479838-3479957
BmHel-4	DF090325:3480234-3480715
BmHel-4	DF090327:1832630-1833204
BmHel-4	DF090330:1030059-1030634
BmHel-4	DF090340:3756633-3757208
BmHel-4	DF090316:7106573-7106802
BmHel-4	DF090316:7107286-7107629
BmHel-4	DF090333:3682779-3683356
BmHel-4	DF090381:648098-648325
BmHel-4	DF090381:649694-649779
BmHel-4	DF090381:649791-650054
BmHel-4	DF090346:1868695-1869270
BmHel-4	DF090389:1323731-1324297
BmHel-4	DF090365:1272329-1272895
BmHel-4	DF090355:631909-632087
BmHel-4	DF090355:631525-631926
BmHel-4	DF090365:550546-551111
BmHel-4	DF090687:23619-24184
BmHel-4	DF090396:1072797-1073394
BmHel-4	DF090357:3109218-3109783
BmHel-4	DF090343:2813307-2813477
BmHel-4	DF090343:2813460-2813872
BmHel-4	DF090411:567761-567935
BmHel-4	DF090411:567550-567778
BmHel-4	DF090411:567377-567530
BmHel-4	DF090441:346174-346352
BmHel-4	DF090441:345785-346191
BmHel-4	DF090320:296518-297089
BmHel-4	DF090316:14876672-14876757
BmHel-4	DF090316:14876761-14877235
BmHel-4	DF090318:8293983-8294115
BmHel-4	DF090318:8293551-8293986
BmHel-4	DF090329:4545850-4546255
BmHel-4	DF090329:4545690-4545850

BmHel-4	DF090364:1513770-1513879
BmHel-4	DF090364:1513912-1514365
BmHel-4	DF090329:5838516-5838948
BmHel-4	DF090329:5838951-5839104
BmHel-4	DF090356:877978-878408
BmHel-4	DF090356:877515-877665
BmHel-4	DF090371:1595974-1596133
BmHel-4	DF090371:1596136-1596546
BmHel-4	DF090329:6525965-6526130
BmHel-4	DF090329:6525587-6525982
BmHel-4	DF090319:6728629-6729130
BmHel-4	DF090790:18832-19331
BmHel-4	DF090335:4499216-4499638
BmHel-4	DF090335:4498746-4498882
BmHel-4	DF090356:3181947-3182154
BmHel-4	DF090356:3182295-3182521
BmHel-4	DF090356:3183468-3183610
BmHel-4	DF090362:2133855-2134031
BmHel-4	DF090362:2133467-2133868
BmHel-4	DF090336:1304437-1304542
BmHel-4	DF090336:1304597-1305064
BmHel-4	DF090319:5437390-5437567
BmHel-4	DF090319:5436951-5437186
BmHel-4	DF090319:5436771-5436930
BmHel-4	DF090318:6917406-6917807
BmHel-4	DF090318:6917244-6917406
BmHel-4	DF090527:83867-84283
BmHel-4	DF090527:84530-84679
BmHel-4	DF090330:6263367-6263797
BmHel-4	DF090330:6263929-6264060
BmHel-4	DF090320:7075018-7075326
BmHel-4	DF090320:7075393-7075601
BmHel-4	DF090339:4768352-4768859
BmHel-4	DF090409:1372804-1372919
BmHel-4	DF090409:1372454-1372782
BmHel-4	DF090409:1372346-1372461
BmHel-4	DF090318:7935438-7935542
BmHel-4	DF090318:7935109-7935395
BmHel-4	DF090318:7933851-7933960
BmHel-4	BABH01045011:4433-4548
BmHel-4	BABH01045011:4083-4411
BmHel-4	BABH01045011:3975-4090
BmHel-4	DF090373:244968-245363
BmHel-4	DF090373:245377-245538
BmHel-4	DF090318:7086290-7086523
BmHel-4	DF090318:7085906-7086272
BmHel-4	DF090387:524390-524482
BmHel-4	DF090387:523931-524388

BmHel-4	DF090348:653548-654112
BmHel-4	DF090410:239165-239597
BmHel-4	DF090410:238972-239090
BmHel-4	DF090586:38062-38366
BmHel-4	DF090586:37803-38055
BmHel-4	DF090316:7625255-7625367
BmHel-4	DF090316:7625017-7625228
BmHel-4	DF090316:7624931-7625017
BmHel-4	DF090316:7624769-7624928
BmHel-4	DF090384:2011572-2012049
BmHel-4	DF090384:2011491-2011571
BmHel-4	DF090551:75670-76175
BmHel-4	DF090376:1892697-1893086
BmHel-4	DF090376:1892552-1892643
BmHel-4	DF090369:149662-149771
BmHel-4	DF090369:148961-149331
BmHel-4	DF090329:6062475-6063021
BmHel-4	DF090364:2305787-2305912
BmHel-4	DF090364:2305918-2306364
BmHel-4	BABH01075480:59-603
BmHel-4	DF090352:650888-651126
BmHel-4	DF090352:652240-652561
BmHel-4	DF090405:1322006-1322194
BmHel-4	DF090405:1322204-1322548
BmHel-4	DF090337:4288098-4288301
BmHel-4	DF090337:4287522-4287816
BmHel-4	DF090419:840371-840618
BmHel-4	DF090419:841037-841199
BmHel-4	DF090419:841205-841292
BmHel-4	DF090317:1508784-1509322
BmHel-4	DF090341:4321752-4321836
BmHel-4	DF090341:4321836-4322134
BmHel-4	DF090341:4322151-4322313
BmHel-4	BABH01045295:1377-1786
BmHel-4	BABH01045295:1229-1354
BmHel-4	DF090323:7051513-7051637
BmHel-4	DF090323:7051637-7052051
BmHel-4	DF090375:2280476-2280887
BmHel-4	DF090375:2280328-2280445
BmHel-4	BABH01083568:1-532
BmHel-4	DF090389:945459-945997
BmHel-4	DF090329:4590210-4590385
BmHel-4	DF090329:4590396-4590745
BmHel-4	DF090544:16784-16886
BmHel-4	DF090544:16370-16562
BmHel-4	DF090544:16191-16350
BmHel-4	DF090336:5284707-5284816
BmHel-4	DF090336:5284286-5284699

BmHel-4	DF090372:2414519-2414898
BmHel-4	DF090372:2414348-2414495
BmHel-4	DF090442:582795-583035
BmHel-4	DF090442:583034-583324
BmHel-4	BABH01075272:3-522
BmHel-4	DF090322:7878586-7878759
BmHel-4	DF090322:7878742-7878966
BmHel-4	DF090322:7879034-7879150
BmHel-4	DF090352:1369154-1369352
BmHel-4	DF090352:1370237-1370332
BmHel-4	DF090352:1370364-1370515
BmHel-4	BABH01058555:331-717
BmHel-4	BABH01058555:183-308
BmHel-4	DF090329:3624267-3624543
BmHel-4	DF090329:3624173-3624260
BmHel-4	DF090329:3623927-3624072
BmHel-4	DF090337:2400556-2400850
BmHel-4	DF090337:2401210-2401301
BmHel-4	DF090445:467678-467855
BmHel-4	DF090445:467333-467668
BmHel-4	DF090319:3983955-3984457
BmHel-4	BABH01078591:1-495
BmHel-4	DF090394:854440-854896
BmHel-4	BABH01084211:28-524
BmHel-4	DF090555:58589-58788
BmHel-4	DF090555:58867-59115
BmHel-4	DF090555:59138-59263
BmHel-4	DF090400:1368839-1368957
BmHel-4	DF090400:1368986-1369248
BmHel-4	DF090400:1369250-1369357
BmHel-4	DF090363:838926-839262
BmHel-4	DF090363:837321-837608
BmHel-4	DF090459:22735-22916
BmHel-4	DF090459:22425-22752
BmHel-4	DF090349:3507646-3507797
BmHel-4	DF090349:3507371-3507573
BmHel-4	DF090349:3506874-3507027
BmHel-4	DF090368:1489674-1489775
BmHel-4	DF090368:1489567-1489693
BmHel-4	DF090368:1489140-1489426
BmHel-4	DF090350:1239006-1239332
BmHel-4	DF090350:1239904-1240056
BmHel-4	DF090321:2656444-2656546
BmHel-4	DF090321:2656549-2656920
BmHel-4	DF090333:977766-978019
BmHel-4	DF090333:978051-978277
BmHel-4	DF090345:3236376-3236856
BmHel-4	DF090419:307467-307922

BmHel-4	DF090336:436329-436788
BmHel-4	BABH01072582:1-459
BmHel-4	DF090337:1551388-1551613
BmHel-4	DF090337:1550852-1550935
BmHel-4	DF090335:290599-290888
BmHel-4	DF090335:290422-290599
BmHel-4	DF090368:725329-725504
BmHel-4	DF090368:725487-725598
BmHel-4	DF090368:725611-725771
BmHel-4	BABH01047844:1066-1500
BmHel-4	DF090349:410223-410524
BmHel-4	DF090349:410532-410652
BmHel-4	DF090360:1845167-1845591
BmHel-4	DF090371:5891-6117
BmHel-4	DF090371:5309-5416
BmHel-4	DF090318:9645776-9646105
BmHel-4	DF090322:399719-400045
BmHel-4	DF090319:4817692-4818018
BmHel-4	DF090352:1816197-1816527
BmHel-4	DF090396:1575956-1576285
BmHel-4	DF090322:2132083-2132487
BmHel-4	DF090420:994479-994809
BmHel-4	DF090422:1021304-1021457
BmHel-4	DF090422:1021150-1021306
BmHel-4	DF090319:1997885-1998114
BmHel-4	DF090319:1998203-1998363
BmHel-4	BABH01081601:172-555
BmHel-4	DF090391:1183577-1183738
BmHel-4	DF090391:1183763-1183913
BmHel-4	DF090342:2502268-2502627
BmHel-4	DF090362:211471-211819
BmHel-4	DF090468:202277-202474
BmHel-4	DF090468:201837-201986
BmHel-4	DF090341:753415-753626
BmHel-4	DF090341:753629-753731
BmHel-4	DF090318:3995199-3995541
BmHel-4	DF090317:10457263-10457445
BmHel-4	DF090353:3704996-3705124
BmHel-4	DF090353:3705494-3705654
BmHel-4	BABH01051769:142-371
BmHel-4	BABH01046084:1383-1612
BmHel-4	DF090319:714800-715026
BmHel-4	DF090319:714714-714794
BmHel-4	DF091157:2982-3206
BmHel-4	DF090320:8626658-8626887
BmHel-4	DF090316:6664394-6664625
BmHel-4	DF090325:6349114-6349294
BmHel-4	DF090325:6349277-6349371

BmHel-4	DF090344:4541451-4541706
BmHel-4	DF090318:1823560-1823730
BmHel-4	DF090318:1823480-1823577
BmHel-4	DF090330:5565686-5565861
BmHel-4	DF090330:5565609-5565703
BmHel-4	DF090340:2661328-2661503
BmHel-4	DF090340:2661251-2661345
BmHel-4	DF090405:824926-825144
BmHel-4	DF090344:3359513-3359757
BmHel-4	DF090339:1556123-1556375
BmHel-4	DF090338:3671347-3671602
BmHel-4	DF090417:1147582-1147830
BmHel-4	DF090422:973509-973796
BmHel-4	DF090320:3843975-3844225
BmHel-4	DF090349:192162-192293
BmHel-4	DF090349:192601-192691
BmHel-4	DF090352:996021-996264
BmHel-4	DF090347:2199188-2199437
BmHel-4	DF090373:1646523-1646770
BmHel-4	DF090340:4644839-4645088
BmHel-4	DF090362:2256859-2257073
BmHel-4	DF090358:909416-909663
BmHel-4	DF090366:298292-298544
BmHel-4	DF090344:2211511-2211709
BmHel-4	DF090349:1787995-1788093
BmHel-4	DF090349:1787849-1787974
BmHel-4	DF090342:1177983-1178181
BmHel-4	DF090321:4776207-4776376
BmHel-4	DF090358:1251105-1251375
BmHel-4	DF090344:763952-764229
BmHel-4	DF090344:2397659-2397922
BmHel-4	DF090326:1199854-1200124
BmHel-4	DF090386:1029091-1029354
BmHel-4	DF090365:1733527-1733790
BmHel-4	DF090341:3166888-3167158
BmHel-4	DF090327:4438297-4438570
BmHel-4	DF090330:1149482-1149752
BmHel-4	DF090330:5421061-5421331
BmHel-4	DF090319:3852428-3852698
BmHel-4	DF090319:4762574-4762844
BmHel-4	DF090319:4383676-4383943
BmHel-4	DF090412:491790-492062
BmHel-4	DF090320:4257448-4257721
BmHel-4	DF090320:6397396-6397666
BmHel-4	DF090340:1767374-1767644
BmHel-4	DF090340:604930-605200
BmHel-4	DF090329:3818782-3819047
BmHel-4	DF090357:824699-824972

BmHel-4	DF090357:2184911-2185181
BmHel-4	DF090357:817615-817884
BmHel-4	DF090349:165346-165616
BmHel-4	DF090414:510718-510988
BmHel-4	DF090414:209698-209975
BmHel-4	DF090337:2328713-2328983
BmHel-4	DF090336:3802902-3803172
BmHel-4	DF090336:3192803-3193081
BmHel-4	DF090336:2801095-2801365
BmHel-4	DF090336:2342890-2343161
BmHel-4	DF090396:1489420-1489685
BmHel-4	DF090323:6395589-6395859
BmHel-4	DF090323:7767162-7767432
BmHel-4	DF090318:5491593-5491866
BmHel-4	DF090335:2183692-2183962
BmHel-4	DF090451:415614-415884
BmHel-4	BABH01055562:323-593
BmHel-4	DF090432:304696-304966
BmHel-4	DF090407:164197-164471
BmHel-4	DF090402:897435-897705
BmHel-4	DF090368:951272-951539
BmHel-4	DF090368:1169726-1169996
BmHel-4	DF090403:1455554-1455824
BmHel-4	DF090331:814870-815138
BmHel-4	DF090331:2448140-2448410
BmHel-4	DF090331:3959559-3959828
BmHel-4	DF090324:856167-856437
BmHel-4	DF090408:631418-631688
BmHel-4	DF090338:830740-831008
BmHel-4	DF090468:256097-256367
BmHel-4	DF090381:69170-69440
BmHel-4	DF090421:783815-784089
BmHel-4	DF090415:496853-497126
BmHel-4	DF090813:4748-5025
BmHel-4	DF090332:899274-899543
BmHel-4	BABH01044278:4365-4634
BmHel-4	DF090370:911453-911722
BmHel-4	DF090353:775771-776039
BmHel-4	DF090328:5132745-5133011
BmHel-4	DF090369:1751935-1752195
BmHel-4	DF090343:3968084-3968344
BmHel-4	DF090353:1558351-1558623
BmHel-4	DF090430:750313-750573
BmHel-4	DF090324:3476326-3476444
BmHel-4	DF090324:3476158-3476306
BmHel-4	DF090344:3575214-3575481
BmHel-4	DF090328:4773424-4773700
BmHel-4	DF090326:2653417-2653684

BmHel-4	DF090365:1529925-1530192
BmHel-4	DF090342:3316602-3316877
BmHel-4	DF090322:1439876-1440143
BmHel-4	DF090355:2152863-2153130
BmHel-4	DF090352:2029059-2029326
BmHel-4	DF090362:848923-849184
BmHel-4	DF090407:1055994-1056261
BmHel-4	DF090338:1694046-1694314
BmHel-4	DF090366:1352351-1352619
BmHel-4	DF090787:13498-13763
BmHel-4	DF090374:1181252-1181524
BmHel-4	DF090386:841505-841770
BmHel-4	DF090340:3349619-3349886
BmHel-4	DF090337:2690918-2691184
BmHel-4	DF090353:3353545-3353662
BmHel-4	DF090353:3353880-3354027
BmHel-4	DF090431:212356-212621
BmHel-4	DF090391:1559593-1559859
BmHel-4	DF090340:752826-753092
BmHel-4	DF090316:6783132-6783393
BmHel-4	DF090318:5506132-5506397
BmHel-4	DF090343:3804259-3804523
BmHel-4	DF090343:3775901-3776165
BmHel-4	BABH01052910:311-576
BmHel-4	DF090378:476200-476382
BmHel-4	DF090378:476365-476459
BmHel-4	DF090342:137930-138112
BmHel-4	DF090342:138095-138192
BmHel-4	DF090322:6951774-6951956
BmHel-4	DF090322:6951701-6951791
BmHel-4	DF090316:11691929-11692111
BmHel-4	DF090316:11692094-11692188
BmHel-4	DF090349:1203887-1204076
BmHel-4	DF090349:1204059-1204153
BmHel-4	DF090343:4489734-4489912
BmHel-4	DF090343:4489895-4489978
BmHel-4	DF090376:1953876-1954057
BmHel-4	DF090376:1953799-1953893
BmHel-4	DF090558:42412-42594
BmHel-4	DF090558:42577-42674
BmHel-4	DF090352:2242532-2242805
BmHel-4	DF090339:2610455-2610719
BmHel-4	DF090362:1220439-1220696
BmHel-4	DF090327:3668597-3668867
BmHel-4	DF090357:1268676-1268938
BmHel-4	DF090335:1530626-1530889
BmHel-4	DF090369:1670108-1670365
BmHel-4	DF090346:2920467-2920645

BmHel-4	DF090346:2920629-2920713
BmHel-4	DF090402:406943-407121
BmHel-4	DF090402:406875-406959
BmHel-4	DF090353:3975863-3976041
BmHel-4	DF090353:3975795-3975879
BmHel-4	DF090692:13508-13771
BmHel-4	DF090324:1699559-1699819
BmHel-4	DF090329:2823917-2824099
BmHel-4	DF090362:244611-244870
BmHel-4	DF090428:403845-404114
BmHel-4	DF090357:2475108-2475369
BmHel-4	DF090394:942673-942848
BmHel-4	DF090394:942831-942925
BmHel-4	DF090322:7038212-7038323
BmHel-4	DF090322:7038049-7038202
BmHel-4	DF090316:9319089-9319346
BmHel-4	DF090438:663335-663596
BmHel-4	DF090353:2595794-2595972
BmHel-4	DF090353:2595959-2596053
BmHel-4	DF090394:68886-69067
BmHel-4	DF090327:4577622-4577801
BmHel-4	DF090327:4577784-4577868
BmHel-4	DF090321:4599904-4599993
BmHel-4	DF090321:4600445-4600625
BmHel-4	DF090316:1848733-1848991
BmHel-4	DF090316:12696563-12696742
BmHel-4	DF090316:12696726-12696810
BmHel-4	DF090367:1747107-1747281
BmHel-4	DF090367:1747265-1747349
BmHel-4	DF090329:1509432-1509601
BmHel-4	DF090329:1509584-1509669
BmHel-4	DF090398:952298-952560
BmHel-4	DF090382:2104062-2104170
BmHel-4	DF090382:2102800-2102969
BmHel-4	DF090368:1311494-1311752
BmHel-4	DF090352:4197305-4197557
BmHel-4	DF090386:589443-589615
BmHel-4	DF090386:589375-589460
BmHel-4	DF090363:399254-399512
BmHel-4	DF090417:982891-983039
BmHel-4	DF090417:982803-982908
BmHel-4	DF090333:3306850-3307032
BmHel-4	DF090333:3307015-3307101
BmHel-4	DF090344:4044713-4044849
BmHel-4	DF090422:555808-556063
BmHel-4	DF090358:204314-204569
BmHel-4	DF090409:762659-762916
BmHel-4	DF090356:2875667-2875925

BmHel-4	DF090377:41344-41600
BmHel-4	DF090452:97195-97450
BmHel-4	DF090359:651388-651643
BmHel-4	DF090334:3667053-3667307
BmHel-4	DF090348:3674959-3675072
BmHel-4	DF090348:3675076-3675217
BmHel-4	DF090316:2330687-2330861
BmHel-4	DF091337:1927-2050
BmHel-4	DF091337:2277-2407
BmHel-4	DF090317:2886373-2886624
BmHel-4	DF090346:471973-472227
BmHel-4	DF090330:4791632-4791754
BmHel-4	DF090330:4791753-4791884
BmHel-4	DF090317:2193933-2194059
BmHel-4	DF090317:2193773-2193933
BmHel-4	DF090360:3337435-3337688
BmHel-4	DF090441:126825-126996
BmHel-4	DF090441:126748-126842
BmHel-4	DF090391:823555-823807
BmHel-4	DF090338:4307765-4308017
BmHel-4	DF090535:75873-75965
BmHel-4	DF090535:76353-76510
BmHel-4	DF090417:627449-627665
BmHel-4	DF090350:2536145-2536290
BmHel-4	DF090350:2536035-2536140
BmHel-4	DF090336:195521-195773
BmHel-4	BABH01049769:1-246
BmHel-4	DF090316:13228308-13228557
BmHel-4	DF090439:440070-440321
BmHel-4	DF090363:1278086-1278263
BmHel-4	DF090318:4811962-4812059
BmHel-4	DF090318:4811791-4811940
BmHel-4	DF090348:1062120-1062267
BmHel-4	DF090348:1062556-1062654
BmHel-4	DF090340:3456410-3456517
BmHel-4	DF090340:3456517-3456655
BmHel-4	DF090353:3480755-3480836
BmHel-4	DF090353:3480853-3480941
BmHel-4	DF090443:617094-617191
BmHel-4	DF090443:616722-616871
BmHel-4	DF090321:771043-771225
BmHel-4	BABH01064898:404-651
BmHel-4	DF090340:3478478-3478691
BmHel-4	DF090316:9883133-9883345
BmHel-4	DF090394:1533405-1533616
BmHel-4	DF090323:3083093-3083306
BmHel-4	DF090331:786733-786945
BmHel-4	DF090366:1179806-1180019

BmHel-4	DF090334:4883608-4883821
BmHel-4	BABH01051746:240-484
BmHel-4	DF090326:2143169-2143380
BmHel-4	DF090379:1295548-1295758
BmHel-4	DF090324:313016-313228
BmHel-4	DF090366:2018959-2019168
BmHel-4	DF090376:1398266-1398477
BmHel-4	BABH01073094:378-618
BmHel-4	DF090394:1036753-1036911
BmHel-4	DF090363:1016287-1016378
BmHel-4	DF090363:1016433-1016527
BmHel-4	DF090824:9537-9778
BmHel-4	DF090331:1005091-1005244
BmHel-4	DF090331:1005227-1005321
BmHel-4	DF090330:5517584-5517818
BmHel-4	DF090350:1607002-1607235
BmHel-4	DF090354:3166192-3166425
BmHel-4	DF090334:3919050-3919213
BmHel-4	DF090374:2160992-2161164
BmHel-4	DF090344:1745679-1745921
BmHel-4	DF090399:454458-454591
BmHel-4	DF090399:454846-454942
BmHel-4	DF090342:1990722-1990883
BmHel-4	DF090336:1611587-1611789
BmHel-4	DF090336:1611495-1611604
BmHel-4	DF090330:4486656-4486857
BmHel-4	DF090330:4486841-4486950
BmHel-4	BABH01083592:401-532
BmHel-4	BABH01083592:16-112
BmHel-4	DF090316:13996726-13996845
BmHel-4	DF091450:3581-3809
BmHel-4	DF090363:2465085-2465239
BmHel-4	DF090403:743287-743429
BmHel-4	DF090403:743217-743302
BmHel-4	DF090353:988917-989113
BmHel-4	BABH01051778:652-876
BmHel-4	DF090318:6603572-6603755
BmHel-4	DF090351:1263443-1263625
BmHel-4	DF090338:968245-968466
BmHel-4	BABH01045450:4044-4265
BmHel-4	DF090492:176689-176840
BmHel-4	DF090840:14799-14977
BmHel-4	DF090324:6604589-6604733
BmHel-4	DF091006:4041-4271
BmHel-4	DF090451:10905-11064
BmHel-4	DF090370:599089-599303
BmHel-4	DF090335:3520111-3520321
BmHel-4	DF090413:210407-210620

BmHel-4	DF090472:89535-89742
BmHel-4	DF090355:2075841-2076040
BmHel-4	DF090355:2075762-2075858
BmHel-4	DF090362:2122049-2122245
BmHel-4	DF090362:2122228-2122328
BmHel-4	DF090418:753314-753470
BmHel-4	DF090332:1218249-1218427
BmHel-4	DF090347:1004845-1004953
BmHel-4	DF090347:1005198-1005297
BmHel-4	DF090332:5467876-5468072
BmHel-4	DF090332:5467775-5467869
BmHel-4	DF090587:25763-25924
BmHel-4	DF090372:615592-615768
BmHel-4	BABH01063200:1-190
BmHel-4	DF090457:447429-447562
BmHel-4	DF090361:1182348-1182526
BmHel-4	DF090398:1037674-1037778
BmHel-4	DF090329:6232947-6233116
BmHel-4	DF090487:44534-44702
BmHel-4	DF090323:5702035-5702193
BmHel-4	BABH01078797:1-158
BmHel-4	DF090360:2728310-2728406
BmHel-4	DF090339:4266943-4267095
BmHel-4	DF090367:932812-932935
BmHel-4	DF090322:1405280-1405424
BmHel-4	DF090341:4017197-4017338
BmHel-4	DF090358:2014713-2014820
BmHel-4	DF090476:243608-243756
BmHel-4	DF090340:4805606-4805695
BmHel-4	DF090316:2238359-2238455
BmHel-4	DF090336:3753261-3753403
BmHel-4	BABH01074547:1-139
BmHel-4	BABH01081831:1-138
BmHel-4	DF091104:6567-6702
BmHel-4	DF090405:195308-195441
BmHel-4	BABH01062107:53-186
BmHel-4	BABH01048406:867-1000
BmHel-4	BABH01048015:1085-1213
BmHel-4	BABH01088133:51-179
BmHel-4	DF090330:6291184-6291309
BmHel-4	DF090677:7559-7682
BmHel-4	BABH01064812:4-117
BmHel-4	DF090421:888007-888123
BmHel-4	DF090378:1549111-1549223
BmHel-4	BABH01078115:14-125
BmHel-4	BABH01061372:156-266
BmHel-4	DF090464:49501-49609
BmHel-4	DF090386:1082755-1082852

BmHel-4	BABH01076398:300-396
BmHel-4	BABH01067923:561-650
BmHel-4	BABH01081501:470-556
BmHel-4	DF090327:3619896-3619979
BmHel-4	DF090317:271368-271447

BmHel-3b	DF090350:3877342-3877547
BmHel-3b	DF090322:6680576-6680808
BmHel-3b	DF090333:4105316-4105550
BmHel-3b	DF090989:4401-4635
BmHel-3b	DF090409:787855-788119
BmHel-3b	DF090326:3345781-3346067
BmHel-3b	DF090350:3166536-3166834
BmHel-3b	DF090365:2446868-2447166
BmHel-3b	DF090343:830525-830828
BmHel-3b	DF090418:1179027-1179331
BmHel-3b	DF090361:3172839-3173145
BmHel-3b	DF090387:1876031-1876337
BmHel-3b	DF090339:404069-404380
BmHel-3b	DF090326:1099073-1099384
BmHel-3b	DF090321:6911664-6911975
BmHel-3b	DF090316:15275159-15275472
BmHel-3b	DF090389:783272-783607
BmHel-3b	DF090362:2640981-2641330
BmHel-3b	DF090325:3529989-3530339
BmHel-3b	DF090357:1339441-1339791
BmHel-3b	DF090395:923198-923552
BmHel-3b	DF090340:677369-677723
BmHel-3b	DF090329:2762030-2762386
BmHel-3b	DF090375:816944-817301
BmHel-3b	DF090405:749066-749424
BmHel-3b	DF090464:78017-78376
BmHel-3b	DF090372:775197-775556
BmHel-3b	DF090411:535999-536358
BmHel-3b	DF090388:117061-117424
BmHel-3b	DF090354:2980552-2980915
BmHel-3b	DF090343:3730128-3730493
BmHel-3b	DF090386:1983702-1984067
BmHel-3b	DF090372:728316-728681
BmHel-3b	DF090380:1443753-1444118
BmHel-3b	DF090342:2186624-2186990
BmHel-3b	DF090458:257870-258236
BmHel-3b	DF090331:521998-522365
BmHel-3b	DF090350:4120559-4120926
BmHel-3b	DF090397:892246-892613
BmHel-3b	DF090380:1091091-1091458
BmHel-3b	DF090322:6704735-6705103
BmHel-3b	DF090332:5496498-5496867

BmHel-3b	DF090318:6718085-6718454
BmHel-3b	DF090332:5911792-5912161
BmHel-3b	DF090318:6153532-6153901
BmHel-3b	DF090354:2471796-2472166
BmHel-3b	DF090395:15310-15680
BmHel-3b	BABH01045077:4954-5324
BmHel-3b	DF090317:869362-869733
BmHel-3b	DF090361:838199-838570
BmHel-3b	DF090337:1495341-1495712
BmHel-3b	DF090343:3890490-3890861
BmHel-3b	DF090332:161055-161427
BmHel-3b	DF090412:503743-504115
BmHel-3b	DF090449:746952-747324
BmHel-3b	DF090329:4381273-4381646
BmHel-3b	DF090342:3011071-3011445
BmHel-3b	DF090340:4190709-4191083
BmHel-3b	DF090801:2941-3315
BmHel-3b	DF090386:147439-147813
BmHel-3b	DF090322:6661770-6662144
BmHel-3b	DF090517:38108-38482
BmHel-3b	DF090340:4602682-4603057
BmHel-3b	DF090537:18865-19240
BmHel-3b	DF090471:278457-278832
BmHel-3b	DF090729:5449-5824
BmHel-3b	DF090403:823762-824138
BmHel-3b	DF090374:1239293-1239669
BmHel-3b	DF090324:4693808-4694184
BmHel-3b	DF090339:2633332-2633708
BmHel-3b	DF090333:5328686-5329062
BmHel-3b	DF090336:1883419-1883796
BmHel-3b	BABH01042899:5058-5435
BmHel-3b	DF090403:743487-743864
BmHel-3b	DF090372:1782630-1783007
BmHel-3b	DF090346:2879750-2880127
BmHel-3b	DF090367:754511-754888
BmHel-3b	DF090737:3475-3852
BmHel-3b	DF090350:3425488-3425866
BmHel-3b	DF090355:1451321-1451699
BmHel-3b	DF090318:6199024-6199402
BmHel-3b	DF090322:1446362-1446741
BmHel-3b	DF090391:877922-878301
BmHel-3b	DF090388:888836-889216
BmHel-3b	DF090322:5269357-5269737
BmHel-3b	DF090340:4817532-4817912
BmHel-3b	DF090325:712360-712740
BmHel-3b	DF090397:1549475-1549855
BmHel-3b	BABH01044948:2071-2451
BmHel-3b	DF090402:991840-992220

BmHel-3b	DF090445:123133-123513
BmHel-3b	DF090333:3556304-3556684
BmHel-3b	DF090354:2511900-2512281
BmHel-3b	DF090317:5918941-5919322
BmHel-3b	DF090409:573968-574349
BmHel-3b	DF090449:454737-455118
BmHel-3b	DF090418:1061122-1061503
BmHel-3b	DF090354:3226037-3226418
BmHel-3b	DF090754:3110-3491
BmHel-3b	DF090316:10605775-10606157
BmHel-3b	DF090338:4591180-4591562
BmHel-3b	DF090318:234635-235017
BmHel-3b	DF090397:1138979-1139361
BmHel-3b	DF090462:225518-225900
BmHel-3b	DF090343:4550604-4550987
BmHel-3b	DF090373:802359-802742
BmHel-3b	DF090328:6351862-6352246
BmHel-3b	DF090317:6654554-6654938
BmHel-3b	DF090347:4269257-4269641
BmHel-3b	DF090361:722845-723229
BmHel-3b	DF090362:1272020-1272404
BmHel-3b	DF090317:5558376-5558761
BmHel-3b	DF090349:100303-100688
BmHel-3b	DF090349:2989294-2989679
BmHel-3b	DF090392:1544714-1545099
BmHel-3b	DF090378:539580-539965
BmHel-3b	DF090421:528930-529315
BmHel-3b	DF090332:1050024-1050409
BmHel-3b	DF090356:2632983-2633368
BmHel-3b	DF090348:3607724-3608109
BmHel-3b	DF090346:2095466-2095851
BmHel-3b	DF090338:3752360-3752745
BmHel-3b	DF090333:374208-374593
BmHel-3b	DF090425:828438-828823
BmHel-3b	DF090320:3377699-3378085
BmHel-3b	DF090350:4058938-4059324
BmHel-3b	DF090364:2573585-2573971
BmHel-3b	DF090360:1011104-1011490
BmHel-3b	DF090336:3152480-3152866
BmHel-3b	DF090326:3907564-3907950
BmHel-3b	DF090316:12339936-12340322
BmHel-3b	DF090322:5720588-5720974
BmHel-3b	DF090401:617082-617468
BmHel-3b	DF090358:2404650-2405036
BmHel-3b	DF090341:787111-787497
BmHel-3b	DF090355:3162177-3162564
BmHel-3b	DF090342:3456897-3457284
BmHel-3b	DF090350:755005-755392

BmHel-3b	DF090322:4680420-4680807
BmHel-3b	DF090377:1844983-1845370
BmHel-3b	DF090352:211473-211860
BmHel-3b	DF090374:2077539-2077926
BmHel-3b	DF090565:110568-110955
BmHel-3b	DF090317:7348195-7348582
BmHel-3b	DF090344:4011223-4011610
BmHel-3b	DF090326:1671875-1672262
BmHel-3b	DF090394:1452480-1452867
BmHel-3b	DF090317:2828969-2829356
BmHel-3b	DF090382:365208-365595
BmHel-3b	DF090337:5094170-5094557
BmHel-3b	DF090334:2931666-2932053
BmHel-3b	DF090364:885509-885896
BmHel-3b	DF090349:3826963-3827350
BmHel-3b	DF090322:6830992-6831379
BmHel-3b	DF090322:2586647-2587034
BmHel-3b	DF090338:4278646-4279033
BmHel-3b	DF090338:2078922-2079309
BmHel-3b	DF090340:1961462-1961849
BmHel-3b	DF090425:796319-796706
BmHel-3b	DF090425:122342-122729
BmHel-3b	DF090391:546244-546631
BmHel-3b	BABH01059261:164-551
BmHel-3b	DF090351:3035029-3035416
BmHel-3b	DF090325:3478339-3478726
BmHel-3b	DF090323:7651293-7651681
BmHel-3b	DF090323:7708157-7708545
BmHel-3b	DF090372:217792-218180
BmHel-3b	DF090331:1060412-1060800
BmHel-3b	DF090342:3917545-3917933
BmHel-3b	DF090317:10388433-10388821
BmHel-3b	DF090348:1529097-1529485
BmHel-3b	DF090337:2283020-2283408
BmHel-3b	DF090334:4925913-4926301
BmHel-3b	DF090326:1219384-1219772
BmHel-3b	DF090460:366247-366635
BmHel-3b	DF090321:2083081-2083469
BmHel-3b	DF090321:4593106-4593494
BmHel-3b	DF090349:1451820-1452208
BmHel-3b	DF090349:2454102-2454490
BmHel-3b	DF090346:373336-373724
BmHel-3b	DF090346:3469126-3469514
BmHel-3b	DF090392:1238079-1238467
BmHel-3b	DF090316:5357887-5358275
BmHel-3b	DF090316:14489326-14489714
BmHel-3b	DF090322:848832-849220
BmHel-3b	DF090338:4758746-4759134

BmHel-3b	DF090361:1181954-1182342
BmHel-3b	DF090377:922113-922501
BmHel-3b	DF090377:1477379-1477767
BmHel-3b	DF090333:267920-268308
BmHel-3b	DF090365:1864062-1864450
BmHel-3b	DF090385:576107-576495
BmHel-3b	DF090352:567644-568032
BmHel-3b	DF090352:2167970-2168358
BmHel-3b	DF090408:436340-436728
BmHel-3b	DF090466:129267-129655
BmHel-3b	BABH01073241:45-433
BmHel-3b	BABH01046054:818-1206
BmHel-3b	DF090480:30788-31176
BmHel-3b	DF090434:103993-104381
BmHel-3b	DF090373:315349-315737
BmHel-3b	DF090417:756106-756494
BmHel-3b	DF090370:747348-747736
BmHel-3b	DF090354:1334265-1334653
BmHel-3b	DF090354:780877-781265
BmHel-3b	DF090328:3749061-3749449
BmHel-3b	DF090332:3201273-3201661
BmHel-3b	DF090355:3527716-3528104
BmHel-3b	DF090358:385765-386153
BmHel-3b	DF090422:1076023-1076411
BmHel-3b	DF090350:3681738-3682126
BmHel-3b	DF090339:1063476-1063864
BmHel-3b	DF090319:3483561-3483949
BmHel-3b	DF090337:3119885-3120273
BmHel-3b	DF090334:1441526-1441914
BmHel-3b	DF090321:2763468-2763856
BmHel-3b	DF090316:5425281-5425669
BmHel-3b	DF090316:1196283-1196671
BmHel-3b	DF090351:4016747-4017135
BmHel-3b	DF090377:1212899-1213287
BmHel-3b	DF090318:1210802-1211190
BmHel-3b	DF090336:4391098-4391486
BmHel-3b	DF090340:4064652-4065040
BmHel-3b	DF090340:2233133-2233521
BmHel-3b	DF090367:2570632-2571020
BmHel-3b	DF090367:1400081-1400469
BmHel-3b	DF090385:1860051-1860439
BmHel-3b	DF090352:1816527-1816915
BmHel-3b	DF090352:1744604-1744992
BmHel-3b	DF090405:1237965-1238353
BmHel-3b	DF090370:1040087-1040475
BmHel-3b	DF090362:2026114-2026502
BmHel-3b	DF090391:1064359-1064747
BmHel-3b	DF090472:144298-144686

BmHel-3b	DF090450:274501-274889
BmHel-3b	DF090440:285595-285983
BmHel-3b	BABH01047872:175-563
BmHel-3b	DF090360:2461969-2462357
BmHel-3b	DF090445:135944-136332
BmHel-3b	DF090329:4440022-4440411
BmHel-3b	DF090356:2172664-2173053
BmHel-3b	DF090331:5627509-5627898
BmHel-3b	DF090324:7315701-7316090
BmHel-3b	DF090395:717752-718141
BmHel-3b	DF090422:910590-910979
BmHel-3b	DF090342:2726556-2726945
BmHel-3b	DF090342:4650244-4650633
BmHel-3b	DF090339:692521-692910
BmHel-3b	DF090339:953043-953432
BmHel-3b	DF090339:1081556-1081945
BmHel-3b	DF090339:2329661-2330050
BmHel-3b	DF090339:3443752-3444141
BmHel-3b	DF090339:4070366-4070755
BmHel-3b	DF090339:4629264-4629653
BmHel-3b	DF090319:1398920-1399309
BmHel-3b	DF090319:2114052-2114441
BmHel-3b	DF090319:2479370-2479759
BmHel-3b	DF090319:9062044-9062433
BmHel-3b	DF090317:10155852-10156241
BmHel-3b	DF090337:2608401-2608790
BmHel-3b	DF090388:142862-143251
BmHel-3b	DF090460:349698-350087
BmHel-3b	DF090321:4826848-4827237
BmHel-3b	DF090349:234747-235136
BmHel-3b	DF090349:3376973-3377362
BmHel-3b	DF090392:1386181-1386570
BmHel-3b	DF090357:3657527-3657916
BmHel-3b	DF090316:9252480-9252869
BmHel-3b	DF090316:12255554-12255943
BmHel-3b	DF090322:7811723-7812112
BmHel-3b	DF090360:2030602-2030991
BmHel-3b	DF090333:685644-686033
BmHel-3b	DF090396:211287-211676
BmHel-3b	DF090325:5850698-5851087
BmHel-3b	DF090378:2130869-2131258
BmHel-3b	DF090379:536473-536862
BmHel-3b	DF090426:297982-298371
BmHel-3b	DF090362:1162365-1162754
BmHel-3b	DF090425:739519-739908
BmHel-3b	DF090374:1133984-1134373
BmHel-3b	DF090481:223022-223411
BmHel-3b	DF090450:379157-379546

BmHel-3b	DF090490:107324-107713
BmHel-3b	DF091181:645-1034
BmHel-3b	DF090358:1510030-1510419
BmHel-3b	DF090422:788237-788626
BmHel-3b	DF090346:2074022-2074411
BmHel-3b	DF090456:356770-357159
BmHel-3b	DF090345:3704397-3704786
BmHel-3b	DF090332:2273683-2274072
BmHel-3b	DF090332:2031515-2031904
BmHel-3b	DF090384:1615520-1615909
BmHel-3b	DF090331:4070891-4071280
BmHel-3b	DF090324:524106-524495
BmHel-3b	DF090376:1446506-1446895
BmHel-3b	DF090339:2410417-2410806
BmHel-3b	DF090319:7296056-7296445
BmHel-3b	DF090319:5153826-5154215
BmHel-3b	DF090319:1622596-1622985
BmHel-3b	DF090317:3044550-3044939
BmHel-3b	DF090321:3185550-3185939
BmHel-3b	DF090321:3155136-3155525
BmHel-3b	DF090353:3877952-3878341
BmHel-3b	DF090398:697995-698384
BmHel-3b	DF090377:437651-438040
BmHel-3b	DF090318:155633-156022
BmHel-3b	DF090340:4531469-4531858
BmHel-3b	DF090365:1998564-1998953
BmHel-3b	DF090363:1000925-1001314
BmHel-3b	DF090408:1150392-1150781
BmHel-3b	DF090374:29232-29621
BmHel-3b	DF090488:9816-10205
BmHel-3b	DF090619:1330-1719
BmHel-3b	BABH01069069:98-487
BmHel-3b	DF090459:278045-278434
BmHel-3b	BABH01082713:142-531
BmHel-3b	DF090469:323005-323394
BmHel-3b	DF090596:42322-42711
BmHel-3b	DF090323:5817814-5818204
BmHel-3b	DF090355:2446642-2447032
BmHel-3b	DF090372:1067923-1068313
BmHel-3b	DF090356:1444264-1444654
BmHel-3b	DF090358:2625013-2625403
BmHel-3b	DF090342:2836559-2836949
BmHel-3b	DF090319:4967614-4968004
BmHel-3b	DF090317:3018368-3018758
BmHel-3b	DF090337:4604742-4605132
BmHel-3b	DF090334:1921708-1922098
BmHel-3b	DF090334:2521007-2521397
BmHel-3b	DF090334:4206569-4206959

BmHel-3b	DF090326:5325921-5326311
BmHel-3b	DF090321:2768596-2768986
BmHel-3b	DF090321:4344571-4344961
BmHel-3b	DF090321:4732335-4732725
BmHel-3b	DF090316:14612279-14612669
BmHel-3b	DF090322:634185-634575
BmHel-3b	DF090338:1919140-1919530
BmHel-3b	DF090398:827663-828053
BmHel-3b	DF090318:5534018-5534408
BmHel-3b	DF090333:517360-517750
BmHel-3b	DF090352:1257688-1258078
BmHel-3b	DF090389:781107-781497
BmHel-3b	DF090362:1780703-1781093
BmHel-3b	DF090411:814448-814838
BmHel-3b	DF090680:25557-25947
BmHel-3b	BABH01053278:163-553
BmHel-3b	DF090551:22940-23330
BmHel-3b	DF090431:673942-674332
BmHel-3b	BABH01045753:136-526
BmHel-3b	DF090467:278961-279351
BmHel-3b	DF090374:1448320-1448710
BmHel-3b	DF090331:5346508-5346898
BmHel-3b	DF090330:5986833-5987223
BmHel-3b	DF090317:2607162-2607552
BmHel-3b	DF090382:1681647-1682037
BmHel-3b	DF090344:1388293-1388683
BmHel-3b	DF090386:826376-826766
BmHel-3b	DF090337:5299955-5300345
BmHel-3b	DF090334:4886134-4886524
BmHel-3b	DF090326:1398722-1399112
BmHel-3b	DF090321:2464546-2464936
BmHel-3b	DF090327:6578859-6579249
BmHel-3b	DF090364:13696-14086
BmHel-3b	DF090338:2246619-2247009
BmHel-3b	DF090360:1946084-1946474
BmHel-3b	DF090333:3693347-3693737
BmHel-3b	DF090333:685003-685393
BmHel-3b	DF090341:529788-530178
BmHel-3b	DF090352:4003224-4003614
BmHel-3b	DF090373:894149-894539
BmHel-3b	DF090366:103353-103743
BmHel-3b	DF090368:1188171-1188561
BmHel-3b	DF090425:312424-312814
BmHel-3b	DF090436:652438-652828
BmHel-3b	BABH01047743:1002-1392
BmHel-3b	DF090476:195693-196083
BmHel-3b	DF090949:467-857
BmHel-3b	DF090448:35566-35956

BmHel-3b	DF090328:1145678-1146069
BmHel-3b	DF090384:2009805-2010196
BmHel-3b	DF090347:557740-558131
BmHel-3b	DF090317:9757904-9758295
BmHel-3b	DF090321:2801133-2801524
BmHel-3b	DF090327:5050225-5050616
BmHel-3b	DF090316:681701-682092
BmHel-3b	DF090377:658874-659265
BmHel-3b	DF090333:5518708-5519099
BmHel-3b	DF090367:1946661-1947052
BmHel-3b	DF090374:1060401-1060792
BmHel-3b	DF090421:901994-902385
BmHel-3b	DF090472:150916-151307
BmHel-3b	DF090384:281505-281896
BmHel-3b	DF090355:2726166-2726557
BmHel-3b	DF090331:5603744-5604135
BmHel-3b	DF090335:2392491-2392882
BmHel-3b	DF090339:860428-860819
BmHel-3b	DF090339:565055-565446
BmHel-3b	DF090317:1699115-1699506
BmHel-3b	DF090344:4106827-4107218
BmHel-3b	DF090386:1553187-1553578
BmHel-3b	DF090375:204225-204616
BmHel-3b	DF090369:1865527-1865918
BmHel-3b	DF090316:12474100-12474491
BmHel-3b	DF090340:3943506-3943897
BmHel-3b	DF090365:634181-634572
BmHel-3b	DF090401:1052070-1052461
BmHel-3b	DF090374:1706525-1706916
BmHel-3b	DF090461:198954-199345
BmHel-3b	DF090331:1979139-1979530
BmHel-3b	DF090339:3266412-3266803
BmHel-3b	DF090372:602374-602766
BmHel-3b	DF090331:5002004-5002396
BmHel-3b	DF090376:1899735-1900127
BmHel-3b	DF090330:152088-152480
BmHel-3b	DF090330:5347646-5348038
BmHel-3b	DF090342:1923336-1923728
BmHel-3b	DF090321:4839372-4839764
BmHel-3b	DF090316:13058673-13059065
BmHel-3b	DF090340:1970674-1971066
BmHel-3b	DF090317:7610048-7610440
BmHel-3b	DF090372:1857189-1857581
BmHel-3b	DF090327:2257759-2258151
BmHel-3b	DF090316:2049453-2049845
BmHel-3b	DF090338:4030095-4030487
BmHel-3b	DF090367:1319281-1319673
BmHel-3b	DF090451:429296-429688

BmHel-3b	DF090352:301580-301972
BmHel-3b	DF090401:1165671-1166063
BmHel-3b	DF090431:668206-668598
BmHel-3b	DF090329:5306436-5306828
BmHel-3b	DF090348:2139829-2140222
BmHel-3b	DF090354:2603324-2603717
BmHel-3b	DF090329:2133520-2133913
BmHel-3b	DF090372:1500022-1500415
BmHel-3b	DF090350:3760568-3760961
BmHel-3b	DF090317:9967195-9967588
BmHel-3b	DF090317:4101863-4102256
BmHel-3b	DF090316:14682616-14683009
BmHel-3b	DF090365:2058927-2059320
BmHel-3b	DF091188:12475-12868
BmHel-3b	BABH01042956:20353-20746
BmHel-3b	DF090323:870951-871345
BmHel-3b	DF090355:832033-832427
BmHel-3b	DF090369:2232552-2232946
BmHel-3b	DF090318:9280329-9280723
BmHel-3b	DF090484:145054-145448
BmHel-3b	DF090381:244318-244712
BmHel-3b	DF090377:104508-104902
BmHel-3b	DF090341:4616054-4616448
BmHel-3b	DF090354:3578363-3578757
BmHel-3b	DF090353:1541782-1542176
BmHel-3b	DF090358:1888441-1888836
BmHel-3b	DF090347:53483-53878
BmHel-3b	DF090335:3345234-3345629
BmHel-3b	DF090316:667373-667768
BmHel-3b	DF090345:2567045-2567441
BmHel-3b	DF090339:4633021-4633417
BmHel-3b	DF090336:3062643-3063039
BmHel-3b	DF090333:3311642-3312038
BmHel-3b	DF090354:1662727-1663124
BmHel-3b	DF090335:726987-727384
BmHel-3b	DF090318:4602150-4602547
BmHel-3b	DF090357:2928885-2929282
BmHel-3b	DF090317:2077951-2078349
BmHel-3b	DF090401:992664-993062
BmHel-3b	DF091264:2154-2553
BmHel-3b	DF090410:1376687-1377087
BmHel-3b	DF090317:2097910-2098311
BmHel-3b	DF090337:4054794-4055196
BmHel-3b	DF090342:3231329-3231735
BmHel-3b	DF090321:3034202-3034609
BmHel-3b	DF090337:1842617-1843024
BmHel-3b	DF090349:3548499-3548911
BmHel-3b	DF090343:1515424-1515837

BmHel-3b	DF090407:226249-226663
BmHel-3b	DF090326:4205248-4205673
BmHel-3b	DF090319:2279349-2279795
BmHel-3b	DF090348:3338454-3338903
BmHel-3b	DF090319:4507698-4508161
BmHel-3b	DF090320:6250350-6250842
BmHel-3b	DF090358:754297-754789
BmHel-3b	DF090475:140797-141289
BmHel-3b	DF090317:10033767-10034262
BmHel-3b	DF090361:1216232-1216746
BmHel-3b	DF090386:463138-463653
BmHel-3b	DF090317:512042-512573
BmHel-3b	DF090437:100745-101351
BmHel-3b	DF090354:3202461-3203119
BmHel-3b	DF090363:77242-77900
BmHel-3b	DF090317:2907116-2907828
BmHel-3b	DF090369:1013390-1014121
BmHel-3b	DF090460:65759-66493
BmHel-3b	DF090342:1197232-1197974
BmHel-3b	DF090326:1714021-1714765
BmHel-3b	DF090333:1201847-1202609
BmHel-3b	DF090323:3416903-3417679
BmHel-3b	DF090319:6001473-6002259
BmHel-3b	DF090413:252881-253703
BmHel-3b	DF090346:1505962-1506818
BmHel-3b	DF090384:944151-945018
BmHel-3b	DF090400:1515066-1515933
BmHel-3b	DF090317:5814141-5815021
BmHel-3b	DF090412:25617-26502
BmHel-3b	DF091700:427-1323
BmHel-3b	DF091557:1279-2198
BmHel-3b	DF090403:495169-496097
BmHel-3b	DF090340:2637593-2638522
BmHel-3b	DF090359:2347898-2348832
BmHel-3b	DF090327:872572-873514
BmHel-3b	DF090319:3402384-3403331
BmHel-3b	DF090317:5069017-5069968
BmHel-3b	DF090344:3988651-3989610
BmHel-3b	DF090333:4746826-4747788
BmHel-3b	DF090343:2407780-2408748
BmHel-3b	DF090358:274371-275341
BmHel-3b	DF090338:3088101-3089074
BmHel-3b	DF090328:2600794-2601768
BmHel-3b	DF090522:82016-82990
BmHel-3b	DF090322:7293934-7294908
BmHel-3b	DF090338:2023875-2024856
BmHel-3b	DF090483:127452-128438
BmHel-3b	DF090358:345645-346634

BmHel-3b	DF090318:2008718-2009708
BmHel-3b	DF090350:2013504-2014501
BmHel-3b	DF090348:2677025-2678025
BmHel-3b	DF090598:17634-18635
BmHel-3b	DF090356:3299802-3300807
BmHel-3b	DF090420:135748-136764
BmHel-3b	DF090376:337243-338261
BmHel-3b	DF090325:3481841-3482860
BmHel-3b	DF090359:2960091-2961116
BmHel-3b	DF090413:45472-46499
BmHel-3b	DF090351:3537292-3538334
BmHel-3b	DF090319:3677246-3678292
BmHel-3b	DF090378:696977-698023
BmHel-3b	DF090341:2110558-2111611
BmHel-3b	DF090342:2586152-2587209
BmHel-3b	DF090348:422280-423342
BmHel-3b	DF090409:20683-21749
BmHel-3b	DF090354:3806806-3807873
BmHel-3b	DF090389:1687854-1688923
BmHel-3b	DF090426:927542-928612
BmHel-3b	DF090343:4529412-4530485
BmHel-3b	DF090330:577510-578587
BmHel-3b	DF090655:23569-24654
BmHel-3b	DF090340:1421754-1422841
BmHel-3b	DF090334:3873759-3874848
BmHel-3b	DF090330:4344055-4345146
BmHel-3b	DF090350:2496479-2497581
BmHel-3b	DF090346:3502672-3503778
BmHel-3b	DF090380:2103093-2104200
BmHel-3b	DF090336:3322129-3323237
BmHel-3b	DF090367:2008651-2009759
BmHel-3b	DF090316:15007049-15008161
BmHel-3b	DF090327:3713822-3714934
BmHel-3b	DF090317:7813636-7814750
BmHel-3b	DF090347:3940846-3941961
BmHel-3b	DF090329:5466268-5467385
BmHel-3b	DF090331:5514667-5515784
BmHel-3b	DF090385:157893-159010
BmHel-3b	DF090316:15871206-15872323
BmHel-3b	DF090350:3725058-3726176
BmHel-3b	DF090342:2524283-2525402
BmHel-3b	DF090374:2246394-2247514
BmHel-3b	DF090317:7735210-7736331
BmHel-3b	DF090353:613133-614255
BmHel-3b	DF090350:1233689-1234812
BmHel-3b	DF090342:2023565-2024689
BmHel-3b	DF090321:4573124-4574248
BmHel-3b	DF090349:1403709-1404834

BmHel-3b	DF090422:149526-150652
BmHel-3b	DF090424:658921-660047
BmHel-3b	DF090401:225923-227051
BmHel-3b	DF090320:2195738-2196868
BmHel-3b	DF090358:44370-45500
BmHel-3b	DF090323:4253762-4254893
BmHel-3b	DF090322:4623028-4624160
BmHel-3b	DF090380:1942115-1943253
BmHel-3b	DF090343:3178841-3179982
BmHel-3b	DF090353:3321563-3322707
BmHel-3b	DF090342:3075840-3076984
BmHel-3b	DF090365:2480453-2481598
BmHel-3b	DF090316:14395491-14396638
BmHel-3b	DF090343:2501681-2502833
BmHel-3b	DF090321:1210435-1211587
BmHel-3b	DF090319:2349555-2350718
BmHel-3b	DF090321:7271900-7273063
BmHel-3b	DF090322:6293344-6294515
BmHel-3b	DF090362:2751754-2752925
BmHel-3b	DF090333:797328-798499
BmHel-3b	DF090378:1388006-1389178
BmHel-3b	DF090429:508428-509610
BmHel-3b	DF090332:3946327-3947514
BmHel-3b	DF090318:3903088-3904275
BmHel-3b	DF090408:1264352-1265542
BmHel-3b	DF090355:3150616-3151811
BmHel-3b	DF090330:6382854-6384051
BmHel-3b	DF090339:3399724-3400925
BmHel-3b	DF090392:1567615-1568818
BmHel-3b	DF090389:946037-947244
BmHel-3b	DF090322:1468462-1469671
BmHel-3b	DF090322:5945680-5946891
BmHel-3b	DF090321:2157872-2159087
BmHel-3b	DF090328:5074427-5075643
BmHel-3b	DF090560:29541-30760
BmHel-3b	DF090335:2483000-2484220
BmHel-3b	DF090319:9113145-9114366
BmHel-3b	DF090333:4942799-4944022
BmHel-3b	DF090328:6334036-6335261
BmHel-3b	DF090352:1992401-1993626
BmHel-3b	DF090339:3237437-3238666
BmHel-3b	DF090678:94582-95811
BmHel-3b	DF090346:1326043-1327273
BmHel-3b	DF090339:630666-631897
BmHel-3b	DF090322:3833871-3835104
BmHel-3b	DF090403:1099268-1100502
BmHel-3b	DF090330:1749094-1750328
BmHel-3b	DF090320:1605192-1606428

BmHel-3b	DF090454:406334-407570
BmHel-3b	DF090326:2790033-2791271
BmHel-3b	DF090322:7240686-7241924
BmHel-3b	DF090365:681062-682302
BmHel-3b	DF090359:1775925-1777165
BmHel-3b	DF090405:485870-487110
BmHel-3b	DF090386:602982-604223
BmHel-3b	DF090372:1958342-1959584
BmHel-3b	DF090347:634986-636228
BmHel-3b	DF090357:3722632-3723876
BmHel-3b	DF090320:2584424-2585671
BmHel-3b	DF090379:1145521-1146768
BmHel-3b	DF090335:3149037-3150285
BmHel-3b	DF090355:1617489-1618738
BmHel-3b	DF090388:927174-928423
BmHel-3b	DF090376:100016-101265
BmHel-3b	DF090317:7742887-7744139
BmHel-3b	DF090335:2246147-2247400
BmHel-3b	DF090382:1680382-1681635
BmHel-3b	DF090355:3728497-3729751
BmHel-3b	DF090369:1584306-1585560
BmHel-3b	DF090328:4122952-4124206
BmHel-3b	DF090329:216590-217844
BmHel-3b	DF090324:4036692-4037948
BmHel-3b	DF090383:1830332-1831588
BmHel-3b	DF090330:1120689-1121948
BmHel-3b	DF090347:1557773-1559033
BmHel-3b	DF090350:657015-658276
BmHel-3b	DF090386:1749457-1750719
BmHel-3b	DF090460:217558-218820
BmHel-3b	DF090384:1351505-1352768
BmHel-3b	DF090326:5509926-5511190
BmHel-3b	DF090318:2579675-2580940
BmHel-3b	DF090373:743039-744306
BmHel-3b	DF090348:3003313-3004588
BmHel-3b	DF090346:475766-477042
BmHel-3b	DF090330:664345-665621
BmHel-3b	DF090352:1832558-1833835
BmHel-3b	DF090419:181520-182799
BmHel-3b	DF090317:9615089-9616371
BmHel-3b	DF090384:251812-253101
BmHel-3b	DF090356:2416531-2417833
BmHel-3b	DF090370:420348-421651
BmHel-3b	DF090368:415684-416990
BmHel-3b	DF090348:2943161-2944474
BmHel-3b	DF090341:3284646-3285959
BmHel-3b	DF090402:795607-796924
BmHel-3b	DF091458:1220-2540

BmHel-3b	DF090349:306993-308316
BmHel-3b	DF090329:3602746-3604070
BmHel-3b	DF090344:201845-203184
BmHel-3b	DF090378:965141-966484
BmHel-3b	DF090666:26934-28290
BmHel-3b	DF090350:1369294-1370651
BmHel-3b	DF090366:369472-370835
BmHel-3b	DF090348:883138-884502
BmHel-3b	DF090316:10643304-10644673
BmHel-3b	DF090384:419543-420913
BmHel-3b	DF090350:4144941-4146314
BmHel-3b	DF090334:2108904-2110279
BmHel-3b	DF090320:6458234-6459610
BmHel-3b	DF090336:3599054-3600432
BmHel-3b	DF090377:2186424-2187803
BmHel-3b	DF090324:4003850-4005249
BmHel-3b	DF090341:276234-277640
BmHel-3b	DF090356:2099932-2101340
BmHel-3b	DF090334:220726-222138
BmHel-3b	DF090412:1375249-1376664
BmHel-3b	DF090362:1404812-1406227
BmHel-3b	DF090316:5925611-5927035
BmHel-3b	DF090318:2249875-2251304
BmHel-3b	DF090322:1631017-1632460
BmHel-3b	DF090384:1745963-1747421
BmHel-3b	DF090338:1402374-1403854
BmHel-3b	DF090334:1839671-1841154
BmHel-3b	DF090422:407139-408623
BmHel-3b	DF090364:1218565-1220051
BmHel-3b	DF090414:1200608-1202098
BmHel-3b	DF090319:2585970-2587461
BmHel-3b	DF090361:2016255-2017773
BmHel-3b	DF090396:1207348-1208869
BmHel-3b	DF090351:3015338-3016864
BmHel-3b	DF090337:5009247-5010777
BmHel-3b	DF090408:890979-892511
BmHel-3b	DF090394:90700-92242
BmHel-3b	DF090351:3244913-3246456
BmHel-3b	DF090451:27578-29125
BmHel-3b	DF090334:2314086-2315636
BmHel-3b	DF090356:2401858-2403409
BmHel-3b	DF090344:1793569-1795121
BmHel-3b	DF090339:1249527-1251084
BmHel-3b	DF090339:1138186-1139747
BmHel-3b	DF090336:2078193-2079755
BmHel-3b	DF090367:2633106-2634671
BmHel-3b	DF090358:1688071-1689637
BmHel-3b	DF090365:1497183-1498753

BmHel-3b	DF090353:1234867-1236446
BmHel-3b	DF090328:4206984-4208575
BmHel-3b	DF090340:2720286-2721878
BmHel-3b	DF090347:809644-811242
BmHel-3b	DF090390:1324632-1326230
BmHel-3b	DF090329:5093763-5095373
BmHel-3b	DF090332:4909395-4911012
BmHel-3b	DF090327:4092938-4094566
BmHel-3b	DF090318:13272-14908
BmHel-3b	DF090427:458734-460373
BmHel-3b	DF090435:412598-414247
BmHel-3b	DF090333:2476868-2478519
BmHel-3b	DF090330:1183112-1184764
BmHel-3b	DF090325:6014746-6016410
BmHel-3b	DF090409:1324200-1325866
BmHel-3b	DF090347:3485477-3487150
BmHel-3b	DF090328:3390316-3391990
BmHel-3b	DF090406:489568-491245
BmHel-3b	DF090331:4885891-4887568
BmHel-3b	DF090394:572364-574044
BmHel-3b	DF090332:4858563-4860244
BmHel-3b	DF090385:1614953-1616639
BmHel-3b	DF090316:12115307-12116997
BmHel-3b	DF090376:721503-723195
BmHel-3b	DF090342:2690079-2691771
BmHel-3b	DF090419:1038985-1040696
BmHel-3b	DF090341:4816319-4818031
BmHel-3b	DF090595:5825-7542
BmHel-3b	DF090322:6792512-6794249
BmHel-3b	DF090373:1798719-1800461
BmHel-3b	DF090355:2149960-2151704
BmHel-3b	DF090402:1385222-1386975
BmHel-3b	DF090411:1104661-1106415
BmHel-3b	DF090353:735230-736987
BmHel-3b	DF090324:5694890-5696648
BmHel-3b	DF090321:2586031-2587792
BmHel-3b	DF090522:92941-94704
BmHel-3b	DF090348:3758617-3760386
BmHel-3b	DF090319:3669253-3671026
BmHel-3b	DF090413:149462-151245
BmHel-3b	DF090316:11439612-11441399
BmHel-3b	DF090329:1155820-1157616
BmHel-3b	DF090380:248115-249921
BmHel-3b	DF090342:4346927-4348762
BmHel-3b	DF090321:1209841-1211685
BmHel-3b	DF090465:30890-32740
BmHel-3b	DF090362:1311817-1313667
BmHel-3b	DF090349:987151-989006

BmHel-3b	DF090327:2875507-2877363
BmHel-3b	DF090321:7271304-7273162
BmHel-3b	DF090348:170231-172091
BmHel-3b	DF090557:4323-6190
BmHel-3b	DF090386:1458905-1460776
BmHel-3b	DF090318:6415458-6417339
BmHel-3b	DF090329:2624279-2626163
BmHel-3b	DF090331:4881601-4883486
BmHel-3b	DF090364:1975687-1977580
BmHel-3b	DF090316:8635134-8637058
BmHel-3b	DF090321:6840549-6842479
BmHel-3b	DF090396:17993-19933
BmHel-3b	DF090355:761757-763710
BmHel-3b	DF090316:13948236-13950204
BmHel-3b	DF090355:1049286-1051258
BmHel-3b	DF090332:1432145-1434119
BmHel-3b	DF090402:1106219-1108193
BmHel-3b	DF090341:582653-584628
BmHel-3b	DF090317:5399272-5401256
BmHel-3b	DF090318:2419698-2421688
BmHel-3b	DF090366:2186553-2188547
BmHel-3b	DF090359:3218735-3220734
BmHel-3b	DF090331:2897727-2899729
BmHel-3b	DF090317:9743378-9745386
BmHel-3b	DF090348:701775-703784
BmHel-3b	DF090379:418921-420960
BmHel-3b	DF090356:615674-617715
BmHel-3b	DF090334:3673343-3675384
BmHel-3b	DF090341:125681-127722
BmHel-3b	DF090328:1027231-1029289
BmHel-3b	DF090384:1077500-1079576
BmHel-3b	DF090327:5003663-5005764
BmHel-3b	DF090322:8096964-8099097
BmHel-3b	DF090326:6038773-6040978
BmHel-3b	DF090321:1090152-1092360
BmHel-3b	DF090360:2405147-2407376
BmHel-3b	DF090385:1517738-1519989
BmHel-3b	DF090353:1834500-1836759
BmHel-3b	DF091548:23-2283
BmHel-3b	DF090372:1123022-1125290
BmHel-3b	DF090340:237636-239937
BmHel-3b	DF090395:104988-107300
BmHel-3b	DF090502:46944-49302
BmHel-3b	DF090375:800493-802872
BmHel-3b	DF090379:945633-948047
BmHel-3b	DF090317:7326923-7329338
BmHel-3b	DF090328:2833907-2836354
BmHel-3b	DF090413:1030418-1032939

BmHel-3b	DF090425:418630-421195
BmHel-3b	DF090323:2107204-2109792
BmHel-3b	DF090353:3415767-3418392
BmHel-3b	DF090317:4676258-4678914
BmHel-3b	DF090379:279551-282265
BmHel-3b	DF090452:43790-46525
BmHel-3b	DF090358:2476172-2478934
BmHel-3b	DF090395:69726-72875
BmHel-3b	DF090347:809442-813121
BmHel-3b	DF090445:229082-233341
BmHel-3b	DF090316:4119984-4124912
BmHel-3b	DF090486:143504-148480
BmHel-3b	DF090355:1749986-1755656
BmHel-3b	DF090372:1765213-1771066
BmHel-3b	DF090412:480439-486379
BmHel-3b	DF090397:1254813-1260820
BmHel-3b	DF090324:6585897-6592382
BmHel-3b	DF090332:401041-408094
BmHel-3b	DF090331:5411091-5418355
BmHel-3b	DF090377:744314-753013
BmHel-3b	DF090326:4286491-4296969
BmHel-3b	DF090663:6147-6306
BmHel-3b	DF090663:5210-5711
BmHel-3b	DF090663:4746-4846
BmHel-3b	DF090337:835429-835583
BmHel-3b	DF090337:834343-834854
BmHel-3b	DF090320:8553822-8553970
BmHel-3b	DF090320:8552501-8553009
BmHel-3b	DF090320:8552359-8552463
BmHel-3b	DF090354:46900-47044
BmHel-3b	DF090354:45930-46437
BmHel-3b	DF090354:45788-45892
BmHel-3b	DF090394:24350-24506
BmHel-3b	DF090394:24953-25228
BmHel-3b	DF090394:25229-25431
BmHel-3b	DF090394:25472-25568
BmHel-3b	BABH01048626:7-154
BmHel-3b	BABH01048626:601-876
BmHel-3b	BABH01048626:877-1079
BmHel-3b	BABH01048626:1120-1216
BmHel-3b	DF090319:6906119-6906273
BmHel-3b	DF090319:6905044-6905546
BmHel-3b	DF090319:6904902-6905004
BmHel-3b	DF090334:4901054-4901375
BmHel-3b	DF090334:4901669-4901854
BmHel-3b	DF090334:4901893-4901996
BmHel-3b	DF090395:24598-24731
BmHel-3b	DF090395:20883-21259

BmHel-3b	DF090395:20744-20848
BmHel-3b	DF090398:251279-251708
BmHel-3b	DF090398:251746-251851
BmHel-3b	DF091630:628-782
BmHel-3b	DF091630:1271-1454
BmHel-3b	DF091630:1632-1772
BmHel-3b	DF091630:1810-1914
BmHel-3b	DF090737:8505-8660
BmHel-3b	DF090737:8870-9016
BmHel-3b	DF090737:9128-9306
BmHel-3b	DF090737:9313-9435
BmHel-3b	DF090737:9474-9574
BmHel-3b	DF090361:2943428-2943864
BmHel-3b	DF090361:2943286-2943388
BmHel-3b	DF090327:4061392-4061756
BmHel-3b	DF090327:4061756-4061885
BmHel-3b	DF090485:110725-110882
BmHel-3b	DF090485:111195-111370
BmHel-3b	DF090485:111463-111673
BmHel-3b	DF090375:1283992-1284428
BmHel-3b	DF090345:1570120-1570279
BmHel-3b	DF090345:1570595-1571101
BmHel-3b	DF090406:1322432-1322771
BmHel-3b	DF090406:1322264-1322367
BmHel-3b	DF090406:1322119-1322225
BmHel-3b	DF090482:231988-232143
BmHel-3b	DF090482:232488-232816
BmHel-3b	DF090482:232818-232944
BmHel-3b	DF090360:1089787-1090280
BmHel-3b	DF090369:769395-769906
BmHel-3b	DF090369:769936-770034
BmHel-3b	DF090355:2830659-2830904
BmHel-3b	DF090355:2830004-2830182
BmHel-3b	DF090346:2168870-2169195
BmHel-3b	DF090346:2168769-2168868
BmHel-3b	DF090346:2168604-2168703
BmHel-3b	DF090357:602223-602323
BmHel-3b	DF090357:602080-602204
BmHel-3b	DF090357:601974-602078
BmHel-3b	BABH01052582:11-170
BmHel-3b	BABH01052582:362-538
BmHel-3b	BABH01052582:631-839
BmHel-3b	DF090371:2189084-2189258
BmHel-3b	DF090371:2188934-2189067
BmHel-3b	DF090371:2188817-2188932
BmHel-3b	DF090337:2129221-2129380
BmHel-3b	DF090337:2129574-2129899
BmHel-3b	DF090337:2129901-2130025

BmHel-3b	DF090321:1418546-1418662
BmHel-3b	DF090321:1419106-1419367
BmHel-3b	DF090321:1419389-1419492
BmHel-3b	DF090553:14529-14637
BmHel-3b	DF090553:14650-14787
BmHel-3b	DF090553:14789-14915
BmHel-3b	DF090349:3891968-3892199
BmHel-3b	DF090349:3890579-3890740
BmHel-3b	DF090336:3454960-3455294
BmHel-3b	DF090336:3454818-3454900
BmHel-3b	DF090336:3454676-3454775
BmHel-3b	DF090319:6381544-6381863
BmHel-3b	DF090319:6381417-6381515
BmHel-3b	DF090385:1001953-1002293
BmHel-3b	DF090385:1002310-1002435
BmHel-3b	DF090318:1710373-1710630
BmHel-3b	DF090318:1710704-1710802
BmHel-3b	DF090372:145222-145549
BmHel-3b	DF090372:145093-145192
BmHel-3b	DF090320:8166126-8166381
BmHel-3b	DF090320:8166456-8166552
BmHel-3b	DF090349:3159378-3159503
BmHel-3b	DF090349:3158719-3159024
BmHel-3b	DF090354:1812384-1812614
BmHel-3b	DF090354:1812256-1812382
BmHel-3b	DF090354:1812121-1812225
BmHel-3b	BABH01048784:161-579
BmHel-3b	BABH01048784:18-122
BmHel-3b	DF090330:927596-927775
BmHel-3b	DF090330:927786-927924
BmHel-3b	DF090330:927997-928095
BmHel-3b	BABH01051494:255-757
BmHel-3b	DF090331:785167-785494
BmHel-3b	DF090331:785034-785133
BmHel-3b	DF090331:784892-784994
BmHel-3b	DF090319:2862132-2862287
BmHel-3b	DF090319:2862710-2862873
BmHel-3b	DF090319:2862890-2863102
BmHel-3b	DF090341:2874753-2875079
BmHel-3b	DF090341:2874574-2874670
BmHel-3b	DF090341:2874429-2874532
BmHel-3b	DF090542:46841-46951
BmHel-3b	DF090542:46690-46815
BmHel-3b	DF090542:46554-46680
BmHel-3b	DF090542:46417-46515
BmHel-3b	DF090344:1562869-1563042
BmHel-3b	DF090344:1562717-1562856
BmHel-3b	DF090344:1562589-1562715

BmHel-3b	BABH01050941:166-325
BmHel-3b	BABH01050941:517-776
BmHel-3b	BABH01050941:781-923
BmHel-3b	DF090327:4908225-4908384
BmHel-3b	DF090327:4907285-4907722
BmHel-3b	DF090317:8643188-8643341
BmHel-3b	DF090317:8642761-8642914
BmHel-3b	DF090317:8642545-8642756
BmHel-3b	DF090317:8642402-8642506
BmHel-3b	DF090777:7885-8210
BmHel-3b	DF090398:857984-858143
BmHel-3b	DF090398:857378-857485
BmHel-3b	DF090398:857227-857365
BmHel-3b	DF090398:857099-857196
BmHel-3b	DF090355:504483-504637
BmHel-3b	DF090355:504391-504486
BmHel-3b	DF090355:504219-504316
BmHel-3b	DF090355:504076-504177
BmHel-3b	DF090485:34974-35299
BmHel-3b	DF090319:8942907-8943301
BmHel-3b	DF090319:8944970-8945074
BmHel-3b	DF090344:436435-436588
BmHel-3b	DF090344:437496-437793
BmHel-3b	DF090344:437831-437934
BmHel-3b	DF090351:2566114-2566441
BmHel-3b	DF090351:2565814-2565906
BmHel-3b	BABH01060162:174-680
BmHel-3b	BABH01060162:31-135
BmHel-3b	DF090332:1708321-1708828
BmHel-3b	DF090332:1708867-1708972
BmHel-3b	BABH01061131:43-549
BmHel-3b	BABH01061131:587-689
BmHel-3b	BABH01055016:150-652
BmHel-3b	BABH01055016:8-111
BmHel-3b	BABH01066881:9-508
BmHel-3b	BABH01066881:546-649
BmHel-3b	DF090704:18645-18804
BmHel-3b	DF090704:19127-19301
BmHel-3b	DF090704:19318-19460
BmHel-3b	DF090322:287182-287361
BmHel-3b	DF090322:287062-287174
BmHel-3b	DF090322:286934-287060
BmHel-3b	DF090392:234387-234644
BmHel-3b	DF090367:10997-11220
BmHel-3b	DF090367:10886-10995
BmHel-3b	DF090367:10734-10832
BmHel-3b	BABH01052579:495-673
BmHel-3b	BABH01052579:156-483

BmHel-3b	BABH01052579:13-118
BmHel-3b	DF090333:3243669-3243847
BmHel-3b	DF090333:3243508-3243658
BmHel-3b	DF090333:3243345-3243443
BmHel-3b	DF090323:7594898-7595410
BmHel-3b	DF090323:7594731-7594821
BmHel-3b	DF090346:2730752-2731259
BmHel-3b	DF090346:2731310-2731400
BmHel-3b	DF090345:1696709-1697002
BmHel-3b	DF090345:1696580-1696709
BmHel-3b	BABH01056458:13-499
BmHel-3b	BABH01056458:538-642
BmHel-3b	DF090342:656368-656527
BmHel-3b	DF090342:655788-655979
BmHel-3b	DF090342:655666-655786
BmHel-3b	DF090329:1140977-1141413
BmHel-3b	DF090329:1551407-1551901
BmHel-3b	DF090329:1551939-1552040
BmHel-3b	BABH01069355:5-481
BmHel-3b	BABH01069355:521-623
BmHel-3b	DF090328:3124953-3125281
BmHel-3b	DF090328:3125357-3125454
BmHel-3b	DF090331:1198510-1198839
BmHel-3b	DF090331:1198956-1199053
BmHel-3b	DF090331:1199093-1199195
BmHel-3b	DF090337:1925354-1925598
BmHel-3b	DF090372:1598150-1598235
BmHel-3b	DF090372:1598250-1598381
BmHel-3b	DF090351:1369742-1370166
BmHel-3b	DF090351:1370205-1370308
BmHel-3b	BABH01057349:11-153
BmHel-3b	BABH01057349:347-731
BmHel-3b	BABH01065044:1-489
BmHel-3b	BABH01065044:527-631
BmHel-3b	DF090319:7470894-7471071
BmHel-3b	DF090319:7471079-7471265
BmHel-3b	DF090319:7471264-7471384
BmHel-3b	DF090319:7471425-7471529
BmHel-3b	BABH01071120:138-630
BmHel-3b	BABH01071120:1-101
BmHel-3b	BABH01071629:5-507
BmHel-3b	BABH01071629:547-627
BmHel-3b	DF090394:729592-729838
BmHel-3b	DF090394:729840-729966
BmHel-3b	DF090319:3081568-3081894
BmHel-3b	DF090342:886460-886638
BmHel-3b	DF090342:886649-886787
BmHel-3b	DF090342:886817-886919

BmHel-3b	DF090342:886939-887040
BmHel-3b	BABH01068508:67-486
BmHel-3b	BABH01068508:525-629
BmHel-3b	DF090451:41971-42225
BmHel-3b	DF090451:42301-42398
BmHel-3b	BABH01061218:75-578
BmHel-3b	BABH01069107:8-271
BmHel-3b	BABH01069107:276-487
BmHel-3b	BABH01069107:525-628
BmHel-3b	DF090347:1018459-1018625
BmHel-3b	DF090347:1017814-1017941
BmHel-3b	DF090347:1017670-1017771
BmHel-3b	DF090336:2408771-2409102
BmHel-3b	DF090336:2408642-2408740
BmHel-3b	DF090336:2408501-2408601
BmHel-3b	DF090374:1672271-1672444
BmHel-3b	DF090374:1672117-1672259
BmHel-3b	DF090374:1671944-1672042
BmHel-3b	DF090374:1671804-1671905
BmHel-3b	DF090323:4023481-4023574
BmHel-3b	DF090323:4023597-4023736
BmHel-3b	DF090323:4023811-4023909
BmHel-3b	DF090323:4023949-4024052
BmHel-3b	BABH01062827:176-598
BmHel-3b	BABH01062827:34-138
BmHel-3b	BABH01074201:1-475
BmHel-3b	BABH01074201:512-611
BmHel-3b	DF090333:4588610-4588874
BmHel-3b	DF090333:4588446-4588535
BmHel-3b	BABH01055507:62-225
BmHel-3b	BABH01055507:242-560
BmHel-3b	BABH01055507:603-696
BmHel-3b	BABH01051955:1-150
BmHel-3b	BABH01051955:459-815
BmHel-3b	DF090333:1111633-1111792
BmHel-3b	DF090333:1110916-1111183
BmHel-3b	DF090798:7219-7552
BmHel-3b	DF090798:7554-7680
BmHel-3b	BABH01057688:175-669
BmHel-3b	DF090317:7503176-7503429
BmHel-3b	BABH01064503:141-648
BmHel-3b	DF090405:862507-862663
BmHel-3b	DF090405:862357-862496
BmHel-3b	DF090405:862181-862283
BmHel-3b	DF090338:87004-87129
BmHel-3b	BABH01050833:408-580
BmHel-3b	BABH01050833:106-315
BmHel-3b	DF090498:164255-164515

BmHel-3b	DF090350:3492206-3492375
BmHel-3b	DF090350:3492019-3492138
BmHel-3b	DF090358:2741031-2741234
BmHel-3b	DF090358:2741309-2741405
BmHel-3b	DF090358:2741446-2741546
BmHel-3b	DF090379:1985004-1985112
BmHel-3b	DF090379:1985389-1985529
BmHel-3b	DF090379:1985531-1985657
BmHel-3b	DF090327:4981961-4982111
BmHel-3b	DF090327:4983019-4983257
BmHel-3b	DF090327:4983296-4983400
BmHel-3b	BABH01063056:190-681
BmHel-3b	BABH01077370:101-589
BmHel-3b	BABH01066345:427-659
BmHel-3b	BABH01066345:211-422
BmHel-3b	BABH01066345:68-172
BmHel-3b	DF090343:3032339-3032494
BmHel-3b	DF090343:3032205-3032338
BmHel-3b	DF090343:3032037-3032133
BmHel-3b	BABH01067484:168-543
BmHel-3b	BABH01067484:542-653
BmHel-3b	DF090316:5284078-5284395
BmHel-3b	DF090316:5284476-5284574
BmHel-3b	DF090316:5284599-5284703
BmHel-3b	BABH01060966:121-628
BmHel-3b	DF090392:769761-770060
BmHel-3b	DF090392:770071-770211
BmHel-3b	DF090392:770249-770352
BmHel-3b	DF090316:15376563-15377003
BmHel-3b	DF090316:8534238-8534340
BmHel-3b	DF090316:8534084-8534224
BmHel-3b	DF090316:8533956-8534082
BmHel-3b	BABH01062407:156-575
BmHel-3b	DF090340:993167-993323
BmHel-3b	DF090340:993890-994207
BmHel-3b	DF090340:994246-994349
BmHel-3b	BABH01072082:11-448
BmHel-3b	BABH01072082:486-590
BmHel-3b	BABH01052394:2-439
BmHel-3b	BABH01052394:464-566
BmHel-3b	BABH01057922:39-474
BmHel-3b	BABH01057922:513-616
BmHel-3b	BABH01072843:37-469
BmHel-3b	BABH01072843:508-609
BmHel-3b	BABH01064175:1-118
BmHel-3b	BABH01064175:311-574
BmHel-3b	BABH01064175:579-673
BmHel-3b	BABH01052728:11-388

BmHel-3b	BABH01071509:40-479
BmHel-3b	BABH01071509:517-620
BmHel-3b	BABH01067599:184-652
BmHel-3b	BABH01061973:81-586
BmHel-3b	BABH01068280:173-648
BmHel-3b	BABH01063010:109-616
BmHel-3b	BABH01062555:77-583
BmHel-3b	BABH01069840:202-638
BmHel-3b	BABH01069840:62-165
BmHel-3b	DF090399:1444817-1444896
BmHel-3b	DF090399:1445340-1445458
BmHel-3b	DF090399:1451347-1451510
BmHel-3b	DF090350:1923281-1923456
BmHel-3b	DF090350:1923131-1923270
BmHel-3b	DF090350:1922756-1922851
BmHel-3b	DF090316:12118322-12118409
BmHel-3b	DF090316:12117995-12118311
BmHel-3b	BABH01058414:299-719
BmHel-3b	BABH01069386:10-437
BmHel-3b	BABH01069386:474-576
BmHel-3b	BABH01072948:133-619
BmHel-3b	BABH01054579:10-429
BmHel-3b	DF090405:661737-661956
BmHel-3b	DF090405:661142-661302
BmHel-3b	DF090323:4438445-4438605
BmHel-3b	DF090323:4437884-4438125
BmHel-3b	DF090341:4472594-4472754
BmHel-3b	DF090341:4473074-4473315
BmHel-3b	BABH01049165:806-966
BmHel-3b	BABH01049165:245-486
BmHel-3b	BABH01069758:26-201
BmHel-3b	BABH01069758:294-505
BmHel-3b	DF090337:3393157-3393414
BmHel-3b	DF090337:3392983-3393081
BmHel-3b	DF090338:3115901-3116228
BmHel-3b	BABH01073063:125-554
BmHel-3b	BABH01065785:2-421
BmHel-3b	BABH01065785:459-563
BmHel-3b	BABH01068106:225-647
BmHel-3b	BABH01068106:85-188
BmHel-3b	BABH01076785:170-593
BmHel-3b	BABH01076785:29-132
BmHel-3b	BABH01068206:242-648
BmHel-3b	DF090326:5112906-5113321
BmHel-3b	DF090326:5113360-5113464
BmHel-3b	BABH01067387:1-107
BmHel-3b	BABH01067387:299-653
BmHel-3b	DF090357:977224-977322

BmHel-3b	DF090357:977071-977210
BmHel-3b	BABH01084549:10-492
BmHel-3b	BABH01057686:234-652
BmHel-3b	BABH01071918:1-397
BmHel-3b	BABH01075787:271-567
BmHel-3b	BABH01075787:142-264
BmHel-3b	BABH01075787:3-103
BmHel-3b	DF090351:2585320-2585578
BmHel-3b	DF090351:2585147-2585245
BmHel-3b	DF090351:2585009-2585106
BmHel-3b	BABH01079722:84-572
BmHel-3b	DF090316:502-829
BmHel-3b	DF090316:831-957
BmHel-3b	BABH01077635:176-587
BmHel-3b	BABH01077635:34-138
BmHel-3b	BABH01068723:540-629
BmHel-3b	BABH01068723:209-526
BmHel-3b	BABH01068723:71-173
BmHel-3b	DF090387:774673-774781
BmHel-3b	DF090387:774517-774662
BmHel-3b	BABH01065741:22-215
BmHel-3b	BABH01065741:220-431
BmHel-3b	BABH01065741:465-569
BmHel-3b	BABH01059638:67-224
BmHel-3b	BABH01059638:420-707
BmHel-3b	DF090348:3673427-3673605
BmHel-3b	DF090348:3673620-3673747
BmHel-3b	BABH01062972:112-375
BmHel-3b	BABH01062972:380-591
BmHel-3b	BABH01080233:377-567
BmHel-3b	BABH01080233:161-372
BmHel-3b	BABH01080233:20-123
BmHel-3b	BABH01065657:479-638
BmHel-3b	BABH01065657:1-285
BmHel-3b	BABH01073465:84-591
BmHel-3b	BABH01076380:31-538
BmHel-3b	DF090334:2214527-2214686
BmHel-3b	DF090334:2213986-2214206
BmHel-3b	BABH01063166:101-454
BmHel-3b	BABH01063166:2-94
BmHel-3b	BABH01057722:317-726
BmHel-3b	BABH01079993:43-550
BmHel-3b	BABH01067827:249-647
BmHel-3b	BABH01067827:124-227
BmHel-3b	BABH01070715:90-587
BmHel-3b	DF090365:2818373-2818464
BmHel-3b	DF090365:2818480-2818799
BmHel-3b	DF090365:2818843-2818936

BmHel-3b	DF090332:3375764-3375870
BmHel-3b	DF090332:3375646-3375744
BmHel-3b	DF090332:3375486-3375582
BmHel-3b	BABH01069414:67-503
BmHel-3b	BABH01079194:140-399
BmHel-3b	BABH01079194:404-576
BmHel-3b	BABH01066397:221-659
BmHel-3b	BABH01072319:232-623
BmHel-3b	BABH01072319:90-193
BmHel-3b	DF090332:746820-747147
BmHel-3b	BABH01056114:641-749
BmHel-3b	BABH01056114:4-326
BmHel-3b	BABH01067304:143-464
BmHel-3b	BABH01067304:466-590
BmHel-3b	BABH01071399:1-399
BmHel-3b	BABH01071399:441-532
BmHel-3b	DF091803:113-572
BmHel-3b	DF090361:290193-290525
BmHel-3b	DF090361:290720-290819
BmHel-3b	DF090432:285231-285352
BmHel-3b	DF090432:285826-285961
BmHel-3b	DF090432:285997-286095
BmHel-3b	BABH01069273:39-473
BmHel-3b	DF090363:648513-648839
BmHel-3b	DF090363:648344-648442
BmHel-3b	DF090366:2645435-2645650
BmHel-3b	DF090414:1325555-1325974
BmHel-3b	BABH01059646:1-382
BmHel-3b	BABH01059646:420-522
BmHel-3b	BABH01080370:1-362
BmHel-3b	BABH01078622:7-487
BmHel-3b	BABH01072678:45-523
BmHel-3b	BABH01057770:596-726
BmHel-3b	BABH01057770:1-282
BmHel-3b	DF092097:6923-7293
BmHel-3b	DF092097:7315-7418
BmHel-3b	BABH01066622:1-374
BmHel-3b	BABH01066622:412-515
BmHel-3b	BABH01054432:90-401
BmHel-3b	BABH01054432:440-544
BmHel-3b	BABH01079112:299-558
BmHel-3b	BABH01079112:83-294
BmHel-3b	BABH01061712:6-108
BmHel-3b	BABH01061712:434-690
BmHel-3b	DF090419:1252752-1252997
BmHel-3b	DF090419:1252618-1252714
BmHel-3b	BABH01080578:11-272
BmHel-3b	BABH01080578:355-500

BmHel-3b	BABH01054191:195-351
BmHel-3b	BABH01054191:543-787
BmHel-3b	BABH01054418:1-365
BmHel-3b	BABH01081427:1-235
BmHel-3b	BABH01081427:235-364
BmHel-3b	BABH01081427:406-505
BmHel-3b	DF090359:431462-431823
BmHel-3b	DF090359:431863-431965
BmHel-3b	BABH01084030:1-358
BmHel-3b	BABH01084030:395-498
BmHel-3b	BABH01072564:5-360
BmHel-3b	BABH01072564:400-502
BmHel-3b	BABH01057488:1-391
BmHel-3b	BABH01061622:311-546
BmHel-3b	BABH01061622:536-691
BmHel-3b	DF090332:5139817-5140052
BmHel-3b	DF090332:5140054-5140178
BmHel-3b	BABH01081642:201-551
BmHel-3b	BABH01081642:76-179
BmHel-3b	BABH01063613:40-434
BmHel-3b	BABH01055429:31-358
BmHel-3b	DF090335:425542-425657
BmHel-3b	DF090364:1060401-1060751
BmHel-3b	DF090364:1060260-1060364
BmHel-3b	BABH01067297:217-541
BmHel-3b	BABH01067297:89-215
BmHel-3b	BABH01076789:1-384
BmHel-3b	BABH01054769:1-141
BmHel-3b	BABH01054769:586-766
BmHel-3b	BABH01060384:260-585
BmHel-3b	DF090341:3412662-3412748
BmHel-3b	DF090341:3412508-3412610
BmHel-3b	BABH01059437:7-152
BmHel-3b	BABH01059437:466-642
BmHel-3b	BABH01071934:413-572
BmHel-3b	BABH01071934:1-219
BmHel-3b	DF090360:30869-31047
BmHel-3b	DF090360:30764-30855
BmHel-3b	BABH01058019:9-408
BmHel-3b	BABH01064509:293-509
BmHel-3b	BABH01064509:509-668
BmHel-3b	BABH01058590:106-360
BmHel-3b	BABH01058590:362-488
BmHel-3b	BABH01052438:377-807
BmHel-3b	BABH01058874:573-714
BmHel-3b	BABH01058874:81-188
BmHel-3b	BABH01082893:10-320
BmHel-3b	BABH01082893:322-448

BmHel-3b	DF090377:125779-125972
BmHel-3b	BABH01073432:120-373
BmHel-3b	BABH01073432:375-501
BmHel-3b	BABH01085962:55-487
BmHel-3b	BABH01059748:1-127
BmHel-3b	BABH01059748:444-622
BmHel-3b	BABH01057643:368-695
BmHel-3b	BABH01072862:357-619
BmHel-3b	BABH01075911:210-386
BmHel-3b	BABH01075911:1-194
BmHel-3b	BABH01082466:1-368
BmHel-3b	DF090432:533212-533294
BmHel-3b	DF090432:533076-533174
BmHel-3b	BABH01080935:1-118
BmHel-3b	BABH01080935:312-561
BmHel-3b	BABH01056058:44-405
BmHel-3b	BABH01057655:301-727
BmHel-3b	DF090320:113396-113715
BmHel-3b	DF090320:113254-113358
BmHel-3b	BABH01080668:3-315
BmHel-3b	BABH01080668:354-458
BmHel-3b	DF090330:5254937-5255109
BmHel-3b	DF090330:5254793-5254921
BmHel-3b	BABH01080497:3-321
BmHel-3b	BABH01080497:361-464
BmHel-3b	BABH01078901:17-338
BmHel-3b	BABH01078901:376-474
BmHel-3b	BABH01060283:1-311
BmHel-3b	BABH01083974:55-473
BmHel-3b	BABH01076772:1-319
BmHel-3b	BABH01076772:357-460
BmHel-3b	BABH01080300:194-550
BmHel-3b	DF090408:321801-321960
BmHel-3b	DF090408:322774-322865
BmHel-3b	DF090408:322940-323038
BmHel-3b	BABH01072373:176-352
BmHel-3b	BABH01072373:362-500
BmHel-3b	BABH01072373:502-600
BmHel-3b	DF090318:904232-904364
BmHel-3b	DF090318:904101-904203
BmHel-3b	BABH01056693:303-480
BmHel-3b	BABH01056693:494-585
BmHel-3b	BABH01069399:1-346
BmHel-3b	BABH01086321:1-303
BmHel-3b	BABH01086321:341-445
BmHel-3b	BABH01050426:5-306
BmHel-3b	BABH01050426:343-446
BmHel-3b	BABH01054372:568-783

BmHel-3b	DF090323:7379640-7379745
BmHel-3b	DF090323:7379763-7379896
BmHel-3b	DF090334:3029941-3030243
BmHel-3b	DF090334:3030281-3030385
BmHel-3b	BABH01063158:142-301
BmHel-3b	BABH01063158:493-672
BmHel-3b	BABH01075053:382-541
BmHel-3b	BABH01075053:10-190
BmHel-3b	BABH01081209:264-558
BmHel-3b	BABH01081209:122-226
BmHel-3b	BABH01058617:484-717
BmHel-3b	BABH01064970:1-296
BmHel-3b	BABH01059234:1-145
BmHel-3b	BABH01059234:588-711
BmHel-3b	BABH01077061:302-592
BmHel-3b	BABH01077061:177-280
BmHel-3b	BABH01067259:1-152
BmHel-3b	BABH01067259:467-646
BmHel-3b	BABH01084191:236-524
BmHel-3b	BABH01084191:94-198
BmHel-3b	BABH01072885:13-341
BmHel-3b	BABH01060972:367-695
BmHel-3b	BABH01055894:209-537
BmHel-3b	BABH01075073:312-605
BmHel-3b	BABH01075073:172-275
BmHel-3b	BABH01068113:204-532
BmHel-3b	BABH01054684:297-628
BmHel-3b	DF090435:7230-7515
BmHel-3b	DF090435:7088-7191
BmHel-3b	BABH01080480:276-565
BmHel-3b	BABH01080480:136-239
BmHel-3b	BABH01055236:157-484
BmHel-3b	DF090322:2438913-2439238
BmHel-3b	BABH01065276:373-548
BmHel-3b	BABH01065276:563-654
BmHel-3b	BABH01069504:22-346
BmHel-3b	BABH01062135:235-322
BmHel-3b	BABH01062135:339-478
BmHel-3b	BABH01062135:553-649
BmHel-3b	DF090340:3859851-3860010
BmHel-3b	DF090340:3860200-3860365
BmHel-3b	DF090346:2186585-2186824
BmHel-3b	BABH01060149:450-703
BmHel-3b	BABH01077609:1-276
BmHel-3b	BABH01077609:314-418
BmHel-3b	DF090410:289550-289724
BmHel-3b	DF090410:289799-289897
BmHel-3b	DF090536:39670-39867

BmHel-3b	DF090536:39544-39668
BmHel-3b	BABH01079276:393-571
BmHel-3b	BABH01069804:283-459
BmHel-3b	BABH01069804:469-607
BmHel-3b	BABH01068483:540-646
BmHel-3b	BABH01068483:46-223
BmHel-3b	BABH01066765:361-538
BmHel-3b	BABH01066765:551-642
BmHel-3b	BABH01068822:394-644
BmHel-3b	BABH01058607:346-505
BmHel-3b	BABH01058607:1-153
BmHel-3b	BABH01071438:1-132
BmHel-3b	BABH01071438:446-623
BmHel-3b	BABH01086308:1-246
BmHel-3b	DF090365:1277804-1278070
BmHel-3b	DF090365:1277662-1277766
BmHel-3b	BABH01055937:1-370
BmHel-3b	DF090359:2231201-2231413
BmHel-3b	BABH01068157:129-306
BmHel-3b	BABH01068157:320-411
BmHel-3b	BABH01068157:486-584
BmHel-3b	BABH01060041:10-187
BmHel-3b	BABH01060041:201-292
BmHel-3b	BABH01060041:367-465
BmHel-3b	BABH01056549:513-690
BmHel-3b	BABH01056549:408-499
BmHel-3b	BABH01056549:235-333
BmHel-3b	BABH01064360:60-237
BmHel-3b	BABH01064360:251-342
BmHel-3b	BABH01064360:417-515
BmHel-3b	BABH01066071:154-331
BmHel-3b	BABH01066071:345-436
BmHel-3b	BABH01066071:511-609
BmHel-3b	BABH01075208:127-295
BmHel-3b	BABH01075208:309-400
BmHel-3b	BABH01075208:475-573
BmHel-3b	BABH01085099:378-507
BmHel-3b	BABH01085099:1-179
BmHel-3b	BABH01050577:814-949
BmHel-3b	BABH01050577:685-814
BmHel-3b	BABH01050577:545-644
BmHel-3b	BABH01084835:264-509
BmHel-3b	BABH01069301:129-456
BmHel-3b	BABH01066626:128-455
BmHel-3b	BABH01065375:1-243
BmHel-3b	BABH01072507:1-244
BmHel-3b	BABH01085854:49-284
BmHel-3b	DF090323:1807631-1807869

BmHel-3b	BABH01070625:149-307
BmHel-3b	BABH01070625:499-633
BmHel-3b	BABH01057965:454-609
BmHel-3b	BABH01057965:2-139
BmHel-3b	BABH01071079:321-498
BmHel-3b	BABH01080667:104-315
BmHel-3b	DF090370:1424887-1425046
BmHel-3b	DF090370:1424566-1424696
BmHel-3b	DF090457:26800-26980
BmHel-3b	BABH01075044:347-605
BmHel-3b	BABH01073440:388-616
BmHel-3b	BABH01069196:396-642
BmHel-3b	BABH01069196:254-358
BmHel-3b	BABH01061986:87-246
BmHel-3b	BABH01061986:559-687
BmHel-3b	BABH01074659:1-147
BmHel-3b	BABH01074659:464-608
BmHel-3b	BABH01064078:66-220
BmHel-3b	BABH01064078:542-674
BmHel-3b	BABH01068149:323-482
BmHel-3b	BABH01068149:1-128
BmHel-3b	BABH01076912:1-287
BmHel-3b	BABH01075459:360-603
BmHel-3b	BABH01075459:216-321
BmHel-3b	BABH01073891:455-613
BmHel-3b	BABH01063535:440-599
BmHel-3b	BABH01063535:1-125
BmHel-3b	DF090388:1249905-1250083
BmHel-3b	DF090388:1249814-1249894
BmHel-3b	BABH01058218:53-212
BmHel-3b	BABH01064764:431-594
BmHel-3b	BABH01079584:292-573
BmHel-3b	BABH01072429:349-583
BmHel-3b	BABH01053038:226-384
BmHel-3b	BABH01053038:699-821
BmHel-3b	BABH01050533:470-629
BmHel-3b	BABH01050533:829-951
BmHel-3b	BABH01062721:509-667
BmHel-3b	DF090420:463747-463991
BmHel-3b	DF090397:507683-507920
BmHel-3b	DF090397:507542-507646
BmHel-3b	BABH01052601:164-497
BmHel-3b	BABH01057599:589-728
BmHel-3b	BABH01072416:304-481
BmHel-3b	BABH01072416:495-586
BmHel-3b	BABH01051914:455-749
BmHel-3b	BABH01075139:381-605
BmHel-3b	BABH01075139:240-344

BmHel-3b	BABH01076638:429-595
BmHel-3b	BABH01085461:1-265
BmHel-3b	BABH01053800:333-661
BmHel-3b	BABH01084323:197-522
BmHel-3b	BABH01080506:50-374
BmHel-3b	BABH01069729:1-110
BmHel-3b	BABH01069729:554-639
BmHel-3b	DF090709:15419-15565
BmHel-3b	BABH01068632:1-218
BmHel-3b	BABH01068632:256-360
BmHel-3b	BABH01079269:168-490
BmHel-3b	DF090352:324139-324366
BmHel-3b	DF090471:372816-372993
BmHel-3b	DF090471:373011-373146
BmHel-3b	DF090319:8894295-8894609
BmHel-3b	BABH01052723:490-744
BmHel-3b	BABH01069330:512-641
BmHel-3b	BABH01071485:434-611
BmHel-3b	BABH01071827:1-153
BmHel-3b	DF090349:1169598-1169757
BmHel-3b	DF090349:1168837-1168920
BmHel-3b	BABH01078858:460-578
BmHel-3b	BABH01082976:1-241
BmHel-3b	DF090411:1398200-1398394
BmHel-3b	DF090411:1398431-1398529
BmHel-3b	BABH01079683:22-197
BmHel-3b	BABH01083556:73-250
BmHel-3b	DF090360:2883247-2883407
BmHel-3b	DF090375:1777496-1777655
BmHel-3b	DF090444:542416-542575
BmHel-3b	BABH01072198:318-476
BmHel-3b	DF090320:3985498-3985657
BmHel-3b	DF090398:1581617-1581774
BmHel-3b	DF090360:648875-649032
BmHel-3b	DF090329:4375666-4375820
BmHel-3b	DF090386:1837072-1837226
BmHel-3b	DF090341:3785273-3785427
BmHel-3b	DF090415:978667-978821
BmHel-3b	DF090360:1719000-1719152
BmHel-3b	DF090372:1900190-1900346
BmHel-3b	DF090316:9397036-9397192
BmHel-3b	DF090398:968450-968606
BmHel-3b	DF090421:510162-510318
BmHel-3b	BABH01064933:339-524
BmHel-3b	BABH01064933:562-665
BmHel-3b	DF090543:14625-14810
BmHel-3b	DF090543:14482-14586
BmHel-3b	BABH01065806:275-459

BmHel-3b	BABH01065806:498-601
BmHel-3b	DF090419:268260-268419
BmHel-3b	DF090330:2778143-2778299
BmHel-3b	DF090404:841713-841865
BmHel-3b	BABH01084369:289-521
BmHel-3b	DF090369:1937992-1938150
BmHel-3b	DF090317:1146028-1146182
BmHel-3b	DF090351:1677789-1677943
BmHel-3b	BABH01046082:2734-2894
BmHel-3b	DF090338:3850583-3850736
BmHel-3b	DF090363:1251176-1251328
BmHel-3b	DF090319:3200331-3200486
BmHel-3b	DF090372:2106902-2107061
BmHel-3b	BABH01072825:362-521
BmHel-3b	BABH01065270:441-600
BmHel-3b	BABH01062517:77-236
BmHel-3b	BABH01054121:3-162
BmHel-3b	BABH01076803:410-569
BmHel-3b	BABH01071686:441-600
BmHel-3b	BABH01063868:418-577
BmHel-3b	BABH01063046:471-630
BmHel-3b	BABH01080003:11-169
BmHel-3b	DF090377:850541-850688
BmHel-3b	BABH01053723:613-792
BmHel-3b	BABH01062171:244-425
BmHel-3b	BABH01062171:465-568
BmHel-3b	BABH01074691:143-324
BmHel-3b	BABH01074691:5-106
BmHel-3b	BABH01074361:162-342
BmHel-3b	BABH01074361:19-123
BmHel-3b	BABH01059909:241-422
BmHel-3b	BABH01059909:99-202
BmHel-3b	BABH01077171:29-188
BmHel-3b	DF090329:744673-744832
BmHel-3b	BABH01076029:5-162
BmHel-3b	BABH01062125:71-230
BmHel-3b	BABH01061407:381-540
BmHel-3b	BABH01079995:18-177
BmHel-3b	BABH01079946:36-195
BmHel-3b	DF090383:516846-516994
BmHel-3b	BABH01064767:262-447
BmHel-3b	BABH01064767:108-210
BmHel-3b	BABH01076429:407-563
BmHel-3b	DF090389:528440-528596
BmHel-3b	DF090354:3342146-3342303
BmHel-3b	DF090351:3896424-3896583
BmHel-3b	BABH01066971:360-519
BmHel-3b	BABH01070205:282-467

BmHel-3b	BABH01070205:128-227
BmHel-3b	BABH01069065:294-479
BmHel-3b	BABH01069065:534-633
BmHel-3b	BABH01066050:143-328
BmHel-3b	BABH01066050:383-482
BmHel-3b	BABH01057348:111-296
BmHel-3b	BABH01057348:351-450
BmHel-3b	BABH01049427:359-517
BmHel-3b	DF090338:4481841-4482000
BmHel-3b	BABH01055813:241-400
BmHel-3b	DF090366:2671444-2671544
BmHel-3b	DF090366:2671318-2671442
BmHel-3b	BABH01048650:381-537
BmHel-3b	BABH01075662:449-601
BmHel-3b	BABH01081635:175-354
BmHel-3b	BABH01081635:409-512
BmHel-3b	BABH01075203:210-389
BmHel-3b	BABH01075203:444-547
BmHel-3b	BABH01047434:58-237
BmHel-3b	BABH01047434:293-397
BmHel-3b	BABH01078913:359-537
BmHel-3b	BABH01078913:199-303
BmHel-3b	BABH01077185:143-321
BmHel-3b	BABH01077185:377-481
BmHel-3b	BABH01078780:24-183
BmHel-3b	DF090346:2000565-2000719
BmHel-3b	BABH01067848:107-260
BmHel-3b	DF090344:2365987-2366139
BmHel-3b	BABH01067104:380-539
BmHel-3b	BABH01076336:42-201
BmHel-3b	DF090384:1781627-1781778
BmHel-3b	DF090344:744253-744396
BmHel-3b	BABH01077386:50-209
BmHel-3b	BABH01076876:483-593
BmHel-3b	BABH01048373:1124-1295
BmHel-3b	BABH01048373:982-1085
BmHel-3b	BABH01072240:346-505
BmHel-3b	DF090341:4632122-4632268
BmHel-3b	BABH01074514:230-382
BmHel-3b	BABH01082926:370-540
BmHel-3b	DF090351:3174006-3174177
BmHel-3b	DF090351:3173864-3173967
BmHel-3b	BABH01082637:41-200
BmHel-3b	DF090322:2214937-2215091
BmHel-3b	BABH01048445:565-719
BmHel-3b	BABH01052963:421-580
BmHel-3b	BABH01084761:15-174
BmHel-3b	BABH01075352:1-147

BmHel-3b	BABH01065067:282-418
BmHel-3b	DF090391:490624-490757
BmHel-3b	DF090329:3557456-3557592
BmHel-3b	BABH01070609:1-272
BmHel-3b	DF090408:606211-606342
BmHel-3b	BABH01082459:3-141
BmHel-3b	BABH01082459:143-269
BmHel-3b	DF090342:4785603-4785738
BmHel-3b	DF090316:15314720-15314852
BmHel-3b	DF090320:3622422-3622550
BmHel-3b	BABH01060891:1-129
BmHel-3b	BABH01060891:532-697
BmHel-3b	DF090344:1001621-1001754
BmHel-3b	DF090375:1457581-1457712
BmHel-3b	BABH01084938:342-496
BmHel-3b	BABH01085471:3-136
BmHel-3b	BABH01085471:138-264
BmHel-3b	BABH01078512:336-495
BmHel-3b	DF090412:12549-12672
BmHel-3b	BABH01060675:562-698
BmHel-3b	BABH01060675:1-83
BmHel-3b	DF090719:18933-19064
BmHel-3b	BABH01061553:1-202
BmHel-3b	BABH01061553:606-691
BmHel-3b	DF090436:213290-213372
BmHel-3b	DF090436:213374-213498
BmHel-3b	DF090405:977348-977479
BmHel-3b	BABH01082353:39-198
BmHel-3b	DF090418:196285-196406
BmHel-3b	BABH01048680:22-172
BmHel-3b	BABH01048680:211-314
BmHel-3b	DF090340:1429941-1430071
BmHel-3b	DF090344:3948143-3948271
BmHel-3b	DF090353:3914427-3914560
BmHel-3b	BABH01047441:1-152
BmHel-3b	BABH01047441:196-289
BmHel-3b	DF090379:435436-435580
BmHel-3b	DF090379:435622-435724
BmHel-3b	DF090323:301994-302138
BmHel-3b	DF090323:302178-302281
BmHel-3b	DF090321:3623020-3623162
BmHel-3b	DF090321:3623201-3623305
BmHel-3b	DF090345:2159916-2160075
BmHel-3b	DF090345:2160641-2160726
BmHel-3b	BABH01086144:236-481
BmHel-3b	DF090476:174505-174641
BmHel-3b	BABH01084137:348-525
BmHel-3b	BABH01085995:1-113

BmHel-3b	BABH01085468:323-499
BmHel-3b	DF090410:401835-402015
BmHel-3b	DF090343:1102766-1102922
BmHel-3b	DF090316:15880569-15880728
BmHel-3b	DF090341:4413392-4413551
BmHel-3b	BABH01056784:1-107
BmHel-3b	DF090345:341105-341264
BmHel-3b	DF090356:2946083-2946242
BmHel-3b	DF090356:3576755-3576914
BmHel-3b	DF090347:1384017-1384176
BmHel-3b	DF090422:664405-664564
BmHel-3b	DF090330:3951837-3951996
BmHel-3b	DF090339:1001183-1001342
BmHel-3b	DF090319:6529899-6530058
BmHel-3b	DF090317:135743-135902
BmHel-3b	DF090348:970448-970607
BmHel-3b	DF090337:780987-781146
BmHel-3b	DF090327:5472641-5472800
BmHel-3b	DF090349:1715869-1716027
BmHel-3b	DF090413:1304582-1304741
BmHel-3b	DF090316:4212174-4212333
BmHel-3b	DF090322:6333237-6333396
BmHel-3b	DF090338:2052562-2052721
BmHel-3b	DF090318:2720541-2720700
BmHel-3b	DF090318:1596301-1596460
BmHel-3b	DF090325:2208958-2209117
BmHel-3b	DF090362:1205976-1206135
BmHel-3b	BABH01062105:432-591
BmHel-3b	BABH01050139:629-788
BmHel-3b	BABH01048717:185-344
BmHel-3b	DF090391:239889-240048
BmHel-3b	DF090459:158839-158998
BmHel-3b	BABH01075756:303-462
BmHel-3b	DF090465:160617-160775
BmHel-3b	DF090412:298598-298752
BmHel-3b	BABH01085806:317-486
BmHel-3b	DF090331:3918433-3918587
BmHel-3b	DF090360:2750611-2750764
BmHel-3b	DF090335:3459640-3459792
BmHel-3b	DF090395:1211299-1211450
BmHel-3b	DF090363:708538-708619
BmHel-3b	DF090363:708661-708762
BmHel-3b	DF090318:204968-205134
BmHel-3b	DF090401:560507-560660
BmHel-3b	DF091700:1322-1422
BmHel-3b	DF090387:1752802-1752954
BmHel-3b	DF090350:3593744-3593903
BmHel-3b	DF090355:2220508-2220667

BmHel-3b	DF090339:1698853-1699012
BmHel-3b	DF090337:3779807-3779966
BmHel-3b	DF090326:6038984-6039143
BmHel-3b	DF090361:1782367-1782526
BmHel-3b	DF090325:1155372-1155531
BmHel-3b	DF090341:2158988-2159147
BmHel-3b	BABH01087162:35-194
BmHel-3b	BABH01087053:59-218
BmHel-3b	BABH01086783:130-289
BmHel-3b	BABH01082354:233-392
BmHel-3b	BABH01081961:119-278
BmHel-3b	BABH01078965:238-397
BmHel-3b	BABH01078011:337-496
BmHel-3b	BABH01077062:91-250
BmHel-3b	BABH01077002:306-465
BmHel-3b	BABH01074770:260-419
BmHel-3b	BABH01071835:332-491
BmHel-3b	BABH01058593:426-585
BmHel-3b	BABH01053274:331-490
BmHel-3b	BABH01087869:60-219
BmHel-3b	BABH01085566:51-210
BmHel-3b	BABH01085549:188-347
BmHel-3b	BABH01084998:212-371
BmHel-3b	BABH01084787:70-229
BmHel-3b	BABH01079342:298-457
BmHel-3b	BABH01075414:308-467
BmHel-3b	BABH01074317:177-336
BmHel-3b	BABH01072985:393-552
BmHel-3b	BABH01062973:122-281
BmHel-3b	BABH01047499:177-336
BmHel-3b	DF090812:15548-15707
BmHel-3b	BABH01082579:311-470
BmHel-3b	BABH01080620:352-511
BmHel-3b	BABH01055683:447-606
BmHel-3b	DF090455:278981-279140
BmHel-3b	BABH01074721:400-559
BmHel-3b	BABH01073272:389-548
BmHel-3b	BABH01055097:100-258
BmHel-3b	BABH01053361:118-277
BmHel-3b	BABH01051452:447-606
BmHel-3b	DF090425:975334-975493
BmHel-3b	BABH01084770:310-468
BmHel-3b	BABH01077757:289-447
BmHel-3b	BABH01083295:12-170
BmHel-3b	DF090680:364-521
BmHel-3b	BABH01057545:237-394
BmHel-3b	BABH01050171:613-769
BmHel-3b	BABH01079057:317-474

BmHel-3b	DF090334:1436820-1436978
BmHel-3b	BABH01071845:437-592
BmHel-3b	DF090824:108-266
BmHel-3b	DF090426:276826-276981
BmHel-3b	DF090342:781525-781635
BmHel-3b	BABH01061145:485-663
BmHel-3b	DF090319:8480268-8480349
BmHel-3b	DF090349:2340325-2340476
BmHel-3b	DF091074:2283-2434
BmHel-3b	DF090318:1070624-1070774
BmHel-3b	DF090341:4648288-4648437
BmHel-3b	DF090318:7935551-7935699
BmHel-3b	BABH01087010:1-148
BmHel-3b	BABH01050569:100-258
BmHel-3b	DF090335:1226807-1226954
BmHel-3b	DF090349:2533245-2533392
BmHel-3b	DF090320:5060680-5060816
BmHel-3b	DF090414:1177081-1177217
BmHel-3b	DF090333:1556272-1556408
BmHel-3b	DF090362:2668908-2669044
BmHel-3b	DF090344:337121-337277
BmHel-3b	DF090549:13460-13594
BmHel-3b	BABH01071543:373-532
BmHel-3b	DF091264:945-1024
BmHel-3b	DF090355:2288381-2288517
BmHel-3b	DF090381:2030070-2030206
BmHel-3b	BABH01054250:534-692
BmHel-3b	DF090331:489658-489787
BmHel-3b	DF090336:2129115-2129251
BmHel-3b	DF090446:297473-297609
BmHel-3b	DF090372:581047-581183
BmHel-3b	DF090415:818995-819131
BmHel-3b	BABH01073734:361-520
BmHel-3b	DF090405:1259264-1259403
BmHel-3b	DF090360:3289076-3289212
BmHel-3b	DF090352:3321604-3321731
BmHel-3b	DF090360:683594-683728
BmHel-3b	DF090350:1484211-1484350
BmHel-3b	DF090341:4687423-4687559
BmHel-3b	BABH01079239:299-466
BmHel-3b	DF090319:572924-573057
BmHel-3b	DF090340:1839404-1839534
BmHel-3b	DF090356:1674439-1674587
BmHel-3b	DF090320:4358662-4358793
BmHel-3b	BABH01056126:1-132
BmHel-3b	DF090491:59465-59563
BmHel-3b	DF090491:59606-59702
BmHel-3b	DF090407:656002-656161

BmHel-3b	DF090488:163044-163180
BmHel-3b	DF090331:639366-639494
BmHel-3b	DF090430:406599-406727
BmHel-3b	DF090384:1107519-1107647
BmHel-3b	DF090349:3550656-3550784
BmHel-3b	DF090347:1125868-1125993
BmHel-3b	BABH01064145:34-125
BmHel-3b	BABH01064145:200-298
BmHel-3b	BABH01053006:743-822
BmHel-3b	BABH01053006:602-705
BmHel-3b	BABH01072160:505-624
BmHel-3b	DF090350:2498688-2498815
BmHel-3b	BABH01072521:7-179
BmHel-3b	DF090345:3004472-3004553
BmHel-3b	DF090366:1721503-1721639
BmHel-3b	BABH01065445:1-111
BmHel-3b	DF090358:3023090-3023210
BmHel-3b	DF090392:1297415-1297536
BmHel-3b	BABH01058120:48-224
BmHel-3b	DF090330:4345145-4345245
BmHel-3b	BABH01065545:500-627
BmHel-3b	DF090398:220152-220232
BmHel-3b	DF090398:219950-220032
BmHel-3b	DF090323:2857099-2857258
BmHel-3b	DF090358:3315905-3316064
BmHel-3b	DF090330:5210664-5210823
BmHel-3b	DF090350:1234928-1235087
BmHel-3b	DF090317:253407-253565
BmHel-3b	DF090326:4652364-4652523
BmHel-3b	DF090346:994935-995094
BmHel-3b	DF090322:8044312-8044471
BmHel-3b	DF090394:807260-807419
BmHel-3b	DF090435:564125-564284
BmHel-3b	DF090437:499745-499903
BmHel-3b	DF090426:579446-579605
BmHel-3b	DF090363:1038323-1038482
BmHel-3b	DF090397:1028882-1029041
BmHel-3b	BABH01068886:432-591
BmHel-3b	BABH01073187:384-543
BmHel-3b	DF090498:21017-21176
BmHel-3b	DF090390:1452290-1452449
BmHel-3b	BABH01075987:17-176
BmHel-3b	BABH01081216:350-432
BmHel-3b	DF090347:3484050-3484208
BmHel-3b	BABH01054719:603-761
BmHel-3b	DF090316:15677841-15677999
BmHel-3b	DF090405:847723-847881
BmHel-3b	DF090441:110824-110982

BmHel-3b	DF090334:1013933-1014031
BmHel-3b	BABH01068678:278-435
BmHel-3b	DF090356:3507550-3507630
BmHel-3b	DF090330:1202985-1203065
BmHel-3b	DF090316:2522135-2522215
BmHel-3b	DF090316:2522049-2522128
BmHel-3b	DF090359:2856024-2856104
BmHel-3b	DF090326:2370906-2371062
BmHel-3b	DF090327:2595402-2595558
BmHel-3b	DF090372:1067145-1067227
BmHel-3b	DF090705:1437-1519
BmHel-3b	DF090344:331673-331828
BmHel-3b	BABH01050309:589-670
BmHel-3b	DF090386:9981-10135
BmHel-3b	DF090336:2498183-2498337
BmHel-3b	DF090343:1789958-1790038
BmHel-3b	DF090326:3354131-3354213
BmHel-3b	DF090374:2133263-2133359
BmHel-3b	DF090344:3108838-3108990
BmHel-3b	DF090324:833112-833192
BmHel-3b	DF090330:2832343-2832494
BmHel-3b	DF090340:2085449-2085550
BmHel-3b	DF090334:1635701-1635781
BmHel-3b	DF090367:2168970-2169052
BmHel-3b	DF090386:631571-631720
BmHel-3b	DF090357:303253-303403
BmHel-3b	DF090424:686596-686746
BmHel-3b	DF090334:3963453-3963533
BmHel-3b	DF090334:2610534-2610614
BmHel-3b	BABH01068178:426-575
BmHel-3b	DF090363:1432570-1432651
BmHel-3b	DF090406:42162-42244
BmHel-3b	DF090427:7179-7327
BmHel-3b	DF090379:852178-852257
BmHel-3b	DF090388:1170769-1170849
BmHel-3b	DF090348:2817574-2817721
BmHel-3b	DF090332:4625454-4625535
BmHel-3b	DF090326:4597919-4598000
BmHel-3b	DF090327:5493296-5493376
BmHel-3b	BABH01064858:1-145
BmHel-3b	BABH01055921:169-314
BmHel-3b	BABH01061999:544-688
BmHel-3b	BABH01085832:314-412
BmHel-3b	BABH01065012:497-595
BmHel-3b	BABH01082903:403-541
BmHel-3b	DF090364:2155647-2155785
BmHel-3b	DF090345:2071730-2071867
BmHel-3b	DF090327:2971475-2971609

BmHel-3b	DF090344:937147-937227
BmHel-3b	DF090319:6871852-6871932
BmHel-3b	DF090368:323487-323619
BmHel-3b	BABH01073333:468-600
BmHel-3b	DF090419:897549-897629
BmHel-3b	DF090317:58671-58798
BmHel-3b	DF090382:2026110-2026237
BmHel-3b	DF090320:1919779-1919859
BmHel-3b	DF090322:6733237-6733317
BmHel-3b	BABH01060330:592-701
BmHel-3b	DF091776:945-1050
BmHel-3b	DF090317:2567489-2567594
BmHel-3b	DF090419:267799-267903
BmHel-3b	DF090318:6966134-6966238
BmHel-3b	BABH01061804:587-690
BmHel-3b	DF090320:7185107-7185208
BmHel-3b	BABH01077681:35-134
BmHel-3b	DF090567:28309-28409
BmHel-3b	BABH01066991:14-112
BmHel-3b	DF090357:3711464-3711557
BmHel-3b	BABH01085093:1-93
BmHel-3b	DF090411:1354787-1354871
BmHel-3b	DF090336:4024251-4024335
BmHel-3b	DF090380:1800038-1800120
BmHel-3b	DF090413:267334-267416
BmHel-3b	DF090379:945839-945920
BmHel-3b	DF090421:924089-924171
BmHel-3b	BABH01067586:80-162
BmHel-3b	DF090383:1588966-1589047
BmHel-3b	DF090369:882595-882675
BmHel-3b	DF090394:1423939-1424019
BmHel-3b	BABH01075456:491-571
BmHel-3b	DF090326:1170917-1170996
BmHel-5	DF090336:159125-162245
BmHel-5	DF090349:3096206-3099716
BmHel-5	DF090360:118462-122186
BmHel-5	DF090360:662349-666572
BmHel-5	DF090317:8729528-8734073
BmHel-5	DF090329:209059-215754
BmHel-5	DF090355:2315727-2315823
BmHel-5	DF090355:2315349-2315713
BmHel-5	DF090355:2314912-2315084
BmHel-5	DF090355:2314599-2314772
BmHel-5	DF090355:2311438-2311629
BmHel-5	DF090355:2311112-2311410
BmHel-5	DF090355:2310323-2310412
BmHel-5	DF090355:2310116-2310309

BmHel-5	DF090355:2310020-2310102
BmHel-5	DF090320:6025805-6025937
BmHel-5	DF090320:6025445-6025530
BmHel-5	DF090320:6024969-6025367
BmHel-5	DF090320:6024678-6024948
BmHel-5	DF090320:6019696-6020001
BmHel-5	DF090398:528462-528594
BmHel-5	DF090398:528102-528187
BmHel-5	DF090398:527634-528024
BmHel-5	DF090398:527350-527613
BmHel-5	DF090405:213059-213310
BmHel-5	DF090405:214415-214577
BmHel-5	DF090405:214595-214784
BmHel-5	BABH01065148:7-405
BmHel-5	BABH01065148:426-664
BmHel-5	BABH01054495:94-357
BmHel-5	BABH01054495:370-666
BmHel-5	BABH01075197:123-521
BmHel-5	BABH01075197:1-102
BmHel-5	BABH01079259:23-550
BmHel-5	BABH01075691:390-552
BmHel-5	BABH01075691:22-376
BmHel-5	BABH01061374:212-610
BmHel-5	BABH01072945:96-494
BmHel-5	BABH01072945:515-615
BmHel-5	BABH01072417:71-156
BmHel-5	BABH01072417:234-622
BmHel-5	BABH01069201:105-267
BmHel-5	BABH01069201:281-568
BmHel-5	BABH01056443:15-157
BmHel-5	BABH01056443:169-465
BmHel-5	DF090660:17417-17549
BmHel-5	DF090660:18870-19172
BmHel-5	BABH01078933:1-141
BmHel-5	BABH01078933:155-442
BmHel-5	DF090360:140091-140251
BmHel-5	DF090360:140281-140429
BmHel-5	DF090360:140444-140526
BmHel-5	BABH01066118:357-661
BmHel-5	BABH01077998:280-585
BmHel-5	BABH01068224:202-411
BmHel-5	BABH01068224:8-181
BmHel-5	BABH01053359:535-744
BmHel-5	BABH01053359:341-514
BmHel-5	DF090320:6025370-6025623
BmHel-5	DF090360:120543-120796
BmHel-5	DF090360:661190-661450
BmHel-5	BABH01080856:238-491

BmHel-5	DF090398:528028-528280
BmHel-5	BABH01057107:485-734
BmHel-5	BABH01072417:7-231
BmHel-5	BABH01080856:331-416
BmHel-5	DF090336:159439-159624
BmHel-5	BABH01052271:12-174
BmHel-5	BABH01051749:2-175
BmHel-5	BABH01069204:474-634
BmHel-5	BABH01070242:1-113
BmHel-5	DF090660:18764-18867
BmHel-5	BABH01072945:1-93
BmHel-5	DF090349:3096556-3096638
BmHel-5	DF090329:215322-215404
BmHel-5	BABH01079259:372-454
BmHel-5	DF090317:8735904-8735986
BmHel-5	BABH01075691:118-200
BmHel-5	DF090355:2315455-2315537
BmHel-5	BABH01053287:653-735

BmHel-6	DF090446:517553-518146
BmHel-6	DF090325:1736021-1736631
BmHel-6	DF090360:3361244-3361870
BmHel-6	DF090344:4653883-4654515
BmHel-6	DF090320:2957516-2958149
BmHel-6	DF090339:4951331-4951964
BmHel-6	DF090355:774377-775011
BmHel-6	DF090431:386930-387565
BmHel-6	DF090322:3969165-3969801
BmHel-6	DF090316:12697489-12698126
BmHel-6	DF090365:1756805-1757443
BmHel-6	DF090322:7052667-7053307
BmHel-6	DF090409:1369627-1370268
BmHel-6	BABH01045011:1256-1897
BmHel-6	DF090411:977620-978265
BmHel-6	DF090331:5648552-5649199
BmHel-6	DF090322:6397173-6397821
BmHel-6	DF090347:342435-343085
BmHel-6	DF090369:2684036-2684686
BmHel-6	DF090420:561070-561729
BmHel-6	DF090352:61121-61783
BmHel-6	DF090358:1155721-1156386
BmHel-6	DF090384:24974-25643
BmHel-6	DF090317:79994-80699
BmHel-6	DF090322:8229782-8230519

BmHel-9	DF090521:53884-54491
BmHel-9	DF090333:4296125-4296755

BmHel-9	DF090338:1086341-1086979
BmHel-9	DF090332:2397764-2398404
BmHel-9	DF090424:620160-620807
BmHel-9	DF090352:2828939-2829586
BmHel-9	DF090323:2242043-2242693
BmHel-9	DF090341:4552978-4553629
BmHel-9	DF090341:1635465-1636120
BmHel-9	DF090372:1156247-1156949
BmHel-9	DF090321:1315613-1316868

BmHel-10	DF090388:805751-806205
BmHel-10	DF090344:1580835-1581292
BmHel-10	DF090316:8914076-8914543
BmHel-10	DF090363:2254909-2255392
BmHel-10	DF090360:1498801-1499293
BmHel-10	DF090394:606152-606648
BmHel-10	DF090376:1785225-1785730
BmHel-10	DF090321:1891240-1891750
BmHel-10	DF090358:2912994-2913509
BmHel-10	DF090418:128194-128713
BmHel-10	DF090372:1099002-1099532
BmHel-10	DF090358:1442661-1443191
BmHel-10	DF090355:309295-309826
BmHel-10	DF090421:542786-543322
BmHel-10	DF090425:463166-463703
BmHel-10	DF090354:874915-875453
BmHel-10	DF090335:4359420-4359960
BmHel-10	DF090318:2890097-2890647
BmHel-10	DF090344:3507546-3508271
BmHel-10	DF090323:7975572-7976500
BmHel-10	DF090393:1554683-1555617

BmHel-7	DF090366:179893-180018
BmHel-7	DF090509:30132-30266
BmHel-7	BABH01082432:318-452
BmHel-7	DF091274:223-357
BmHel-7	DF090358:1071952-1072092
BmHel-7	DF090401:719689-719841
BmHel-7	DF090364:2165407-2165565
BmHel-7	DF090420:989334-989493
BmHel-7	DF090402:1437646-1437806
BmHel-7	DF090378:1897690-1897853
BmHel-7	DF090326:6536384-6536547
BmHel-7	DF090322:6166724-6166887
BmHel-7	DF090347:1862201-1862371
BmHel-7	DF090664:11971-12148
BmHel-7	DF090343:1847022-1847199

BmHel-7	DF090317:10821212-10821392
BmHel-7	DF090339:3289379-3289561
BmHel-7	DF090363:1615048-1615238
BmHel-7	DF090320:1113109-1113299
BmHel-7	DF090347:313170-313362
BmHel-7	DF090331:3681389-3681582
BmHel-7	BABH01055606:290-483
BmHel-7	DF090343:1301928-1302122
BmHel-7	DF090351:3012859-3013053
BmHel-7	DF090320:3179050-3179245
BmHel-7	DF090386:533831-534027
BmHel-7	DF090322:5793951-5794148
BmHel-7	DF090322:5198960-5199158
BmHel-7	DF090321:7328416-7328614
BmHel-7	DF090316:11836175-11836374
BmHel-7	DF090391:160914-161114
BmHel-7	DF090340:1760550-1760750
BmHel-7	DF090325:6674363-6674563
BmHel-7	DF090336:4693752-4693952
BmHel-7	DF090323:7315791-7315992
BmHel-7	DF090389:1320265-1320466
BmHel-7	DF090366:1579245-1579446
BmHel-7	DF090316:6624774-6624975
BmHel-7	DF090393:440843-441045
BmHel-7	DF090371:2502799-2503002
BmHel-7	DF090352:3674148-3674351
BmHel-7	DF090320:6128285-6128488
BmHel-7	DF090375:374389-374592
BmHel-7	DF090598:41285-41488
BmHel-7	DF090319:7984089-7984292
BmHel-7	DF090377:288396-288599
BmHel-7	DF090334:2157602-2157805
BmHel-7	DF090338:756813-757016
BmHel-7	DF090367:1484138-1484342
BmHel-7	DF090370:2457056-2457260
BmHel-7	DF090401:407063-407267
BmHel-7	DF090331:1339992-1340196
BmHel-7	DF090322:1280921-1281126
BmHel-7	DF090345:3040747-3040952
BmHel-7	DF090410:204908-205113
BmHel-7	DF090344:388247-388453
BmHel-7	DF090416:181556-181762
BmHel-7	DF090324:8008657-8008863
BmHel-7	DF090401:234202-234408
BmHel-7	DF090316:15575358-15575564
BmHel-7	DF090333:1270797-1271003
BmHel-7	DF090404:1269616-1269822
BmHel-7	DF090343:1428233-1428440

BmHel-7	DF090349:3419894-3420101
BmHel-7	DF090349:1524877-1525085
BmHel-7	DF090326:5042665-5042873
BmHel-7	DF090402:499039-499247
BmHel-7	DF090360:263167-263376
BmHel-7	DF090336:3456494-3456703
BmHel-7	DF090366:1306900-1307109
BmHel-7	DF090329:3315923-3316133
BmHel-7	DF090317:1535313-1535523
BmHel-7	DF090416:758094-758305
BmHel-7	DF090401:1267306-1267517
BmHel-7	DF090517:93754-93965
BmHel-7	DF090363:352927-353138
BmHel-7	DF090328:571898-572110
BmHel-7	DF090321:5598720-5598932
BmHel-7	DF090369:1506666-1506878
BmHel-7	DF090316:4658799-4659011
BmHel-7	DF090334:1344448-1344660
BmHel-7	DF090337:4595634-4595846
BmHel-7	DF090355:1994467-1994679
BmHel-7	DF090469:314562-314774
BmHel-7	DF090469:132611-132823
BmHel-7	DF090328:1492327-1492540
BmHel-7	DF090326:3805211-3805425
BmHel-7	DF090343:2702322-2702536
BmHel-7	DF090321:6253766-6253980
BmHel-7	DF090369:2431184-2431398
BmHel-7	DF090319:5564704-5564918
BmHel-7	DF090383:548797-549011
BmHel-7	DF090367:2407582-2407796
BmHel-7	DF090318:2933870-2934084
BmHel-7	DF090358:1510523-1510737
BmHel-7	DF090321:8084698-8084912
BmHel-7	DF090405:1009111-1009326
BmHel-7	DF090354:2248221-2248436
BmHel-7	DF090316:15206844-15207059
BmHel-7	DF090328:100324-100539
BmHel-7	DF090328:2226861-2227076
BmHel-7	DF090350:755770-755985
BmHel-7	DF090317:2491454-2491669
BmHel-7	DF090616:18898-19113
BmHel-7	DF090364:461655-461870
BmHel-7	DF090330:5008301-5008516
BmHel-7	DF090402:829982-830197
BmHel-7	DF090388:759144-759359
BmHel-7	DF090359:2459692-2459907
BmHel-7	DF090370:198761-198976
BmHel-7	DF090370:163080-163295

BmHel-7	DF090500:48866-49081
BmHel-7	DF090325:3072640-3072856
BmHel-7	DF090352:900014-900230
BmHel-7	DF090378:1576021-1576237
BmHel-7	DF090317:1754435-1754651
BmHel-7	DF090404:1111777-1111993
BmHel-7	DF090370:1745663-1745879
BmHel-7	DF090351:1124669-1124885
BmHel-7	DF090320:6828667-6828883
BmHel-7	DF090320:6026983-6027199
BmHel-7	DF090320:1238673-1238889
BmHel-7	DF090350:1939637-1939853
BmHel-7	DF090317:7611230-7611446
BmHel-7	DF090383:568493-568709
BmHel-7	DF090369:2466433-2466649
BmHel-7	DF090349:2028898-2029115
BmHel-7	DF090349:3480615-3480832
BmHel-7	DF090344:1703062-1703279
BmHel-7	DF090316:5486651-5486868
BmHel-7	DF090352:930364-930581
BmHel-7	DF090352:1510615-1510832
BmHel-7	DF090318:2697241-2697458
BmHel-7	DF090419:738127-738344
BmHel-7	DF090350:351248-351465
BmHel-7	DF090338:2786720-2786937
BmHel-7	DF090349:2500466-2500683
BmHel-7	DF090319:7216922-7217139
BmHel-7	DF090377:400147-400364
BmHel-7	DF090334:2363161-2363378
BmHel-7	DF090351:3652828-3653045
BmHel-7	DF090332:1462399-1462616
BmHel-7	DF090412:846147-846364
BmHel-7	DF090406:152870-153087
BmHel-7	DF090340:4410225-4410442
BmHel-7	DF090355:811126-811344
BmHel-7	DF090341:1944562-1944780
BmHel-7	DF090346:20714-20932
BmHel-7	DF090346:403980-404198
BmHel-7	DF090343:1477085-1477303
BmHel-7	DF090343:1987581-1987799
BmHel-7	DF090372:2449527-2449745
BmHel-7	DF090352:3435465-3435683
BmHel-7	DF090320:6107231-6107449
BmHel-7	DF090333:4341094-4341312
BmHel-7	DF090339:1720699-1720917
BmHel-7	DF090353:595980-596198
BmHel-7	DF090398:518028-518246
BmHel-7	DF090361:2171309-2171527

BmHel-7	DF090380:682497-682715
BmHel-7	DF090403:467052-467270
BmHel-7	DF090406:1022903-1023121
BmHel-7	DF090677:9389-9607
BmHel-7	DF090794:9002-9220
BmHel-7	DF090355:3276059-3276277
BmHel-7	DF090316:10016004-10016222
BmHel-7	DF090316:14899241-14899459
BmHel-7	DF090350:289868-290086
BmHel-7	DF090318:7395036-7395254
BmHel-7	DF090339:3795940-3796158
BmHel-7	DF090373:1898394-1898612
BmHel-7	DF090345:2005376-2005594
BmHel-7	DF090578:20735-20953
BmHel-7	DF090382:708261-708479
BmHel-7	DF090356:986935-987153
BmHel-7	DF090322:4847300-4847518
BmHel-7	DF090329:4372685-4372903
BmHel-7	DF090329:3114226-3114444
BmHel-7	DF090353:697332-697550
BmHel-7	DF090361:2840591-2840809
BmHel-7	DF090441:267993-268211
BmHel-7	DF090416:795623-795841
BmHel-7	DF090381:1998822-1999040
BmHel-7	DF090362:2891954-2892172
BmHel-7	DF090459:326049-326267
BmHel-7	DF090436:99950-100168
BmHel-7	DF090447:579912-580130
BmHel-7	DF090342:3411350-3411568
BmHel-7	DF090322:5221280-5221498
BmHel-7	DF090352:775633-775851
BmHel-7	DF090327:4556958-4557176
BmHel-7	DF090373:404073-404291
BmHel-7	DF090361:826352-826570
BmHel-7	DF090358:726404-726622
BmHel-7	DF090362:3100870-3101088
BmHel-7	DF090362:308851-309069
BmHel-7	DF090362:288157-288375
BmHel-7	DF090331:1878560-1878778
BmHel-7	DF090417:555533-555751
BmHel-7	DF090409:1369637-1369855
BmHel-7	DF090426:527966-528184
BmHel-7	DF090778:5728-5946
BmHel-7	BABH01045011:1266-1484
BmHel-7	DF090322:79481-79699
BmHel-7	DF090348:2130588-2130806
BmHel-7	DF090381:102939-103157
BmHel-7	BABH01046134:1392-1610

BmHel-7	DF090356:1934053-1934272
BmHel-7	DF090355:2884424-2884643
BmHel-7	DF090349:246574-246793
BmHel-7	DF090349:1919297-1919516
BmHel-7	DF090341:709965-710184
BmHel-7	DF090341:4571300-4571519
BmHel-7	DF090344:663358-663577
BmHel-7	DF090367:372785-373004
BmHel-7	DF090354:965517-965736
BmHel-7	DF090354:1913654-1913873
BmHel-7	DF090319:296253-296472
BmHel-7	DF090319:3345723-3345942
BmHel-7	DF090319:4921187-4921406
BmHel-7	DF090319:6404671-6404890
BmHel-7	DF090326:2127602-2127821
BmHel-7	DF090316:3417170-3417389
BmHel-7	DF090316:3622898-3623117
BmHel-7	DF090316:5121817-5122036
BmHel-7	DF090316:13465393-13465612
BmHel-7	DF090402:354959-355178
BmHel-7	DF090402:508896-509115
BmHel-7	DF090402:570693-570912
BmHel-7	DF090388:888435-888654
BmHel-7	DF090364:580600-580819
BmHel-7	DF090346:57733-57952
BmHel-7	DF090343:196526-196745
BmHel-7	DF090343:1816171-1816390
BmHel-7	DF090343:1875104-1875323
BmHel-7	DF090343:3109086-3109305
BmHel-7	DF090340:1070919-1071138
BmHel-7	DF090334:3988128-3988347
BmHel-7	DF090328:2161527-2161746
BmHel-7	DF090325:5750723-5750942
BmHel-7	DF090323:1317722-1317941
BmHel-7	DF090323:7450306-7450525
BmHel-7	DF090322:5596247-5596466
BmHel-7	DF090347:2565016-2565235
BmHel-7	DF090352:608354-608573
BmHel-7	DF090352:2504163-2504382
BmHel-7	DF090320:570694-570913
BmHel-7	DF090384:1229272-1229491
BmHel-7	DF090350:1373865-1374084
BmHel-7	DF090337:912342-912561
BmHel-7	DF090333:4508139-4508358
BmHel-7	DF090318:5391335-5391554
BmHel-7	DF090318:6223952-6224171
BmHel-7	DF090318:9197996-9198215
BmHel-7	DF090407:89719-89938

BmHel-7	DF090360:1343486-1343705
BmHel-7	DF090360:3265265-3265484
BmHel-7	DF090353:3613822-3614041
BmHel-7	DF090398:658697-658916
BmHel-7	DF090363:153632-153851
BmHel-7	DF090361:2983551-2983770
BmHel-7	DF090357:2993193-2993412
BmHel-7	DF090422:411060-411279
BmHel-7	DF090399:306161-306380
BmHel-7	DF090366:1819973-1820192
BmHel-7	DF090358:710898-711117
BmHel-7	DF090358:1115530-1115749
BmHel-7	DF090317:6896397-6896616
BmHel-7	DF090421:730913-731132
BmHel-7	DF090335:3068479-3068698
BmHel-7	DF090335:3160738-3160957
BmHel-7	DF090335:3615246-3615465
BmHel-7	DF090321:4640856-4641075
BmHel-7	DF090321:4981703-4981922
BmHel-7	DF090383:209793-210012
BmHel-7	DF090383:1120049-1120268
BmHel-7	DF090381:919319-919538
BmHel-7	DF090381:1239320-1239539
BmHel-7	DF090338:702804-703023
BmHel-7	DF090338:1293078-1293297
BmHel-7	DF090338:4486893-4487112
BmHel-7	DF090338:4510940-4511159
BmHel-7	DF090386:888248-888467
BmHel-7	DF090374:1464415-1464634
BmHel-7	DF090362:63-282
BmHel-7	DF090362:1723254-1723473
BmHel-7	DF090331:536741-536960
BmHel-7	DF090369:1938761-1938980
BmHel-7	DF090403:1554426-1554645
BmHel-7	DF090437:288730-288949
BmHel-7	DF090431:207815-208034
BmHel-7	DF090423:467958-468177
BmHel-7	DF090382:196011-196230
BmHel-7	DF090382:1743608-1743827
BmHel-7	DF090359:3048015-3048234
BmHel-7	DF090439:561651-561870
BmHel-7	DF090428:487799-488018
BmHel-7	DF090476:102015-102234
BmHel-7	DF090508:110249-110468
BmHel-7	BABH01046083:705-924
BmHel-7	DF090583:24539-24758
BmHel-7	DF090668:6388-6607
BmHel-7	DF091322:929-1148

BmHel-7	DF090570:47672-47891
BmHel-7	DF090355:2275205-2275424
BmHel-7	DF090344:913063-913282
BmHel-7	DF090375:2162470-2162689
BmHel-7	DF090342:472338-472557
BmHel-7	DF090356:2057913-2058132
BmHel-7	DF090356:1521783-1522002
BmHel-7	DF090349:3790520-3790739
BmHel-7	DF090349:2918183-2918402
BmHel-7	DF090341:4632456-4632675
BmHel-7	DF090341:3695955-3696174
BmHel-7	DF090405:672488-672707
BmHel-7	DF090414:115121-115340
BmHel-7	DF090354:260415-260634
BmHel-7	DF090336:1887101-1887320
BmHel-7	DF090319:7117530-7117749
BmHel-7	DF090319:5090463-5090682
BmHel-7	DF090319:2737546-2737765
BmHel-7	DF090319:107151-107370
BmHel-7	DF090326:1714783-1715002
BmHel-7	DF090316:12878772-12878991
BmHel-7	DF090316:10971359-10971578
BmHel-7	DF090388:1733440-1733659
BmHel-7	DF090343:4420200-4420419
BmHel-7	DF090343:2772821-2773040
BmHel-7	DF090343:1972468-1972687
BmHel-7	DF090343:1359335-1359554
BmHel-7	DF090343:1103971-1104190
BmHel-7	DF090340:3656741-3656960
BmHel-7	DF090334:3199990-3200209
BmHel-7	DF090328:3652314-3652533
BmHel-7	DF090325:6061735-6061954
BmHel-7	DF090325:2862293-2862512
BmHel-7	DF090322:7283300-7283519
BmHel-7	DF090322:2870512-2870731
BmHel-7	DF090372:1901856-1902075
BmHel-7	DF090329:4581455-4581674
BmHel-7	DF090329:2415099-2415318
BmHel-7	DF090351:3554946-3555165
BmHel-7	DF090351:1672912-1673131
BmHel-7	DF090347:3505128-3505347
BmHel-7	DF090320:1431244-1431463
BmHel-7	DF090394:640517-640736
BmHel-7	DF090384:1356615-1356834
BmHel-7	DF090378:2157626-2157845
BmHel-7	DF090337:3849459-3849678
BmHel-7	DF090337:3727409-3727628
BmHel-7	DF090337:650925-651144

BmHel-7	DF090333:4190045-4190264
BmHel-7	DF090333:4098225-4098444
BmHel-7	DF090327:3603807-3604026
BmHel-7	DF090373:1845402-1845621
BmHel-7	DF090361:1253978-1254197
BmHel-7	DF090332:4070756-4070975
BmHel-7	DF090366:1093738-1093957
BmHel-7	DF090348:511902-512121
BmHel-7	DF090345:2874156-2874375
BmHel-7	DF090345:2633063-2633282
BmHel-7	DF090330:6330273-6330492
BmHel-7	DF090330:617861-618080
BmHel-7	DF090330:87475-87694
BmHel-7	DF090317:5569360-5569579
BmHel-7	DF090317:4924872-4925091
BmHel-7	DF090317:3106460-3106679
BmHel-7	DF090317:1352350-1352569
BmHel-7	DF090365:2031345-2031564
BmHel-7	DF090335:4490724-4490943
BmHel-7	DF090321:486621-486840
BmHel-7	DF090383:1416453-1416672
BmHel-7	DF090381:1829338-1829557
BmHel-7	DF090381:873782-874001
BmHel-7	DF090375:2251264-2251483
BmHel-7	DF090368:1890166-1890385
BmHel-7	DF090362:3160279-3160498
BmHel-7	DF090427:486540-486759
BmHel-7	DF090393:340307-340526
BmHel-7	DF090417:31442-31661
BmHel-7	DF090409:182591-182810
BmHel-7	DF090391:1481964-1482183
BmHel-7	DF090369:2620844-2621063
BmHel-7	DF090437:476480-476699
BmHel-7	DF090382:1211910-1212129
BmHel-7	DF090370:1480807-1481026
BmHel-7	DF090504:92977-93196
BmHel-7	DF090510:85505-85724
BmHel-7	DF091018:3765-3984
BmHel-7	DF090483:47321-47540
BmHel-7	DF091361:417-636
BmHel-7	DF090486:94349-94568
BmHel-7	DF090848:3467-3686
BmHel-7	BABH01069669:365-584
BmHel-7	DF090822:8814-9033
BmHel-7	BABH01042926:19634-19853
BmHel-7	DF090685:11745-11964
BmHel-7	BABH01042971:16297-16516
BmHel-7	DF090356:3005652-3005872

BmHel-7	DF090316:2918695-2918915
BmHel-7	DF090402:671623-671843
BmHel-7	DF090322:2296349-2296569
BmHel-7	DF090372:680511-680731
BmHel-7	DF090372:2076433-2076653
BmHel-7	DF090347:54795-55015
BmHel-7	DF090320:2456354-2456574
BmHel-7	DF090380:1807683-1807903
BmHel-7	DF090345:2622351-2622571
BmHel-7	DF090421:596421-596641
BmHel-7	DF090324:3097945-3098165
BmHel-7	DF090354:2911142-2911362
BmHel-7	DF090319:7661189-7661409
BmHel-7	DF090316:10726634-10726854
BmHel-7	DF090390:1309035-1309255
BmHel-7	DF090328:4918096-4918316
BmHel-7	DF090366:179320-179540
BmHel-7	DF090424:961538-961758
BmHel-7	DF090408:304532-304752
BmHel-7	DF090331:2077735-2077955
BmHel-7	DF090324:7695738-7695958
BmHel-7	DF090452:74229-74449
BmHel-7	DF090346:1289995-1290215
BmHel-7	DF090349:3143800-3144021
BmHel-7	DF090343:2651903-2652124
BmHel-7	DF090340:3175673-3175894
BmHel-7	DF090330:2758377-2758598
BmHel-7	DF090317:2535728-2535949
BmHel-7	DF090362:127190-127411
BmHel-7	DF090386:865222-865443
BmHel-7	DF090322:6872538-6872759
BmHel-7	DF090333:1166869-1167090
BmHel-7	DF090383:513717-513938
BmHel-7	DF090338:1537129-1537350
BmHel-7	DF090359:985098-985319
BmHel-7	DF090397:1473988-1474209
BmHel-7	DF090530:24204-24425
BmHel-7	DF090319:6551540-6551761
BmHel-7	DF090332:1417760-1417981
BmHel-7	DF090396:588559-588780
BmHel-7	DF090341:2173652-2173874
BmHel-7	DF090344:2982299-2982521
BmHel-7	DF090367:2568139-2568361
BmHel-7	DF090402:597013-597235
BmHel-7	DF090347:2481-2703
BmHel-7	DF090327:5928911-5929133
BmHel-7	DF090348:3669714-3669936
BmHel-7	DF090338:4467541-4467763

BmHel-7	DF090374:878098-878320
BmHel-7	DF090382:602107-602329
BmHel-7	DF090391:258089-258311
BmHel-7	DF090349:1722261-1722483
BmHel-7	DF090354:1935959-1936181
BmHel-7	DF090316:14889796-14890018
BmHel-7	DF090316:10517729-10517951
BmHel-7	DF090343:2796015-2796237
BmHel-7	BABH01047810:429-651
BmHel-7	DF090363:2687217-2687439
BmHel-7	DF090361:891888-892110
BmHel-7	DF090369:774318-774540
BmHel-7	DF090405:1310307-1310530
BmHel-7	DF090414:1006960-1007183
BmHel-7	DF090346:578852-579075
BmHel-7	DF090325:5265972-5266195
BmHel-7	DF090347:4246870-4247093
BmHel-7	DF090394:563299-563522
BmHel-7	DF090332:4284292-4284515
BmHel-7	DF090321:4020070-4020293
BmHel-7	DF090368:380233-380456
BmHel-7	DF090319:2391414-2391637
BmHel-7	DF090318:5217034-5217257
BmHel-7	DF090332:2384545-2384768
BmHel-7	DF090338:848339-848562
BmHel-7	DF090583:19997-20220
BmHel-7	DF090349:4203325-4203549
BmHel-7	DF090336:3914218-3914442
BmHel-7	DF090332:2815190-2815414
BmHel-7	DF090410:812425-812649
BmHel-7	DF090347:1684533-1684757
BmHel-7	DF090327:2330815-2331039
BmHel-7	DF090416:491068-491292
BmHel-7	DF090330:985843-986067
BmHel-7	DF090377:2116244-2116469
BmHel-7	DF090418:699165-699390
BmHel-7	DF090330:3860324-3860549
BmHel-7	DF090421:290624-290849
BmHel-7	DF090588:33310-33535
BmHel-7	DF090327:3294811-3295036
BmHel-7	DF090318:2855855-2856080
BmHel-7	DF090361:743223-743448
BmHel-7	DF090335:1229784-1230009
BmHel-7	DF090338:4918061-4918286
BmHel-7	DF090354:1934898-1935124
BmHel-7	DF090318:8351450-8351676
BmHel-7	DF090319:2954458-2954684
BmHel-7	DF090507:3894-4120

BmHel-7	DF090367:1477208-1477434
BmHel-7	DF090372:364342-364568
BmHel-7	DF090316:7277998-7278225
BmHel-7	DF090340:2644629-2644856
BmHel-7	DF090361:1862942-1863169
BmHel-7	DF090351:2918261-2918489
BmHel-7	DF090318:260490-260718
BmHel-7	DF090330:1979657-1979885
BmHel-7	DF090422:1103607-1103835
BmHel-7	DF090325:3579108-3579337
BmHel-7	DF090329:3206878-3207107
BmHel-7	DF090338:1543253-1543482
BmHel-7	DF090426:103389-103618
BmHel-7	DF090553:34232-34461
BmHel-7	DF090482:140801-141030
BmHel-7	DF090360:334192-334421
BmHel-7	DF090369:2213899-2214128
BmHel-7	DF090428:554997-555226
BmHel-7	DF090389:1207073-1207303
BmHel-7	DF090394:6508-6738
BmHel-7	DF090318:1067594-1067825
BmHel-7	DF090344:3234519-3234750
BmHel-7	DF090316:13054143-13054374
BmHel-7	DF090334:5590607-5590838
BmHel-7	DF090317:9291679-9291910
BmHel-7	DF090320:7859842-7860074
BmHel-7	DF090398:1413113-1413345
BmHel-7	DF090328:2263193-2263425
BmHel-7	DF090329:5152332-5152564
BmHel-7	DF090378:1620206-1620438
BmHel-7	DF090316:15997519-15997752
BmHel-7	DF090347:2944239-2944472
BmHel-7	DF090386:838232-838465
BmHel-7	DF090328:2290209-2290442
BmHel-7	DF090348:3109991-3110224
BmHel-7	DF090375:526144-526377
BmHel-7	DF090316:12920389-12920623
BmHel-7	DF090370:1330613-1330847
BmHel-7	DF090412:376924-377158
BmHel-7	DF090619:27040-27274
BmHel-7	DF090342:3632613-3632847
BmHel-7	DF090391:684511-684745
BmHel-7	DF090434:419249-419484
BmHel-7	DF090401:1505765-1506000
BmHel-7	DF090455:86853-87088
BmHel-7	DF091016:1441-1676
BmHel-7	DF090329:4974585-4974820
BmHel-7	DF090342:3259503-3259738

BmHel-7	DF090338:809618-809854
BmHel-7	DF090323:4778463-4778699
BmHel-7	DF090349:750600-750836
BmHel-7	DF090323:3633266-3633502
BmHel-7	DF090407:312922-313158
BmHel-7	DF090402:984080-984317
BmHel-7	DF090364:980349-980586
BmHel-7	DF090322:468712-468949
BmHel-7	DF090339:3592281-3592518
BmHel-7	DF090365:2593402-2593639
BmHel-7	DF090336:3516790-3517027
BmHel-7	DF090340:2932574-2932811
BmHel-7	DF090472:17837-18074
BmHel-7	DF090339:2070894-2071132
BmHel-7	DF090331:3312460-3312698
BmHel-7	BABH01052492:555-793
BmHel-7	DF090552:28802-29040
BmHel-7	DF090345:3208146-3208384
BmHel-7	DF090405:293100-293339
BmHel-7	DF090325:6711326-6711565
BmHel-7	DF090360:612920-613159
BmHel-7	DF090366:1070001-1070240
BmHel-7	DF090330:3918349-3918588
BmHel-7	DF090319:2306013-2306252
BmHel-7	DF090316:1277898-1278137
BmHel-7	DF090316:13116030-13116269
BmHel-7	DF090316:4872774-4873013
BmHel-7	DF090344:4308185-4308425
BmHel-7	DF090319:7275649-7275889
BmHel-7	DF090329:4550471-4550711
BmHel-7	DF090368:1679831-1680071
BmHel-7	DF090396:478241-478481
BmHel-7	DF090340:2203641-2203881
BmHel-7	DF090401:1023801-1024041
BmHel-7	DF090316:633354-633595
BmHel-7	DF090316:1987484-1987725
BmHel-7	DF090377:1567203-1567444
BmHel-7	DF090445:399644-399885
BmHel-7	DF090319:1334274-1334515
BmHel-7	DF090355:1093642-1093883
BmHel-7	DF090316:3907756-3907997
BmHel-7	DF090333:5525975-5526216
BmHel-7	DF090339:1992287-1992528
BmHel-7	DF090330:4411363-4411604
BmHel-7	DF090386:1097873-1098114
BmHel-7	DF090333:5147942-5148183
BmHel-7	DF090323:7973205-7973447
BmHel-7	DF090337:3672827-3673069

BmHel-7	DF090366:718978-719220
BmHel-7	DF090345:898046-898288
BmHel-7	DF090383:1142991-1143233
BmHel-7	DF090362:1047781-1048023
BmHel-7	DF090331:1263388-1263630
BmHel-7	DF090380:1677102-1677344
BmHel-7	DF090349:692990-693232
BmHel-7	DF090354:2323613-2323855
BmHel-7	DF090336:445605-445847
BmHel-7	DF090332:5658851-5659093
BmHel-7	DF090381:702509-702751
BmHel-7	DF090391:1243743-1243985
BmHel-7	DF090410:1031581-1031823
BmHel-7	DF090469:224887-225129
BmHel-7	DF090784:17140-17382
BmHel-7	DF090376:547729-547971
BmHel-7	DF090370:1575831-1576073
BmHel-7	DF090325:6879161-6879404
BmHel-7	DF090322:443119-443362
BmHel-7	DF090320:1245781-1246024
BmHel-7	DF090337:1424011-1424254
BmHel-7	DF090374:1250986-1251229
BmHel-7	DF090363:1165467-1165710
BmHel-7	DF090344:128499-128742
BmHel-7	DF090319:1181640-1181883
BmHel-7	DF090328:4072147-4072390
BmHel-7	DF090325:5169710-5169953
BmHel-7	DF090318:3259785-3260028
BmHel-7	DF090345:1617063-1617306
BmHel-7	DF090401:1316719-1316962
BmHel-7	DF090426:204821-205064
BmHel-7	DF090353:2383810-2384053
BmHel-7	DF090348:3855730-3855973
BmHel-7	DF090427:268270-268513
BmHel-7	DF090341:2593381-2593625
BmHel-7	DF090319:7491636-7491880
BmHel-7	DF090340:2619220-2619464
BmHel-7	DF090317:1553587-1553831
BmHel-7	DF090413:1008923-1009167
BmHel-7	DF090391:620436-620680
BmHel-7	DF090462:394624-394868
BmHel-7	DF090323:2797696-2797940
BmHel-7	DF090352:451989-452233
BmHel-7	DF090320:8251851-8252095
BmHel-7	DF090365:1229949-1230193
BmHel-7	DF090335:175673-175917
BmHel-7	DF090458:12779-13023
BmHel-7	DF090327:2948351-2948595

BmHel-7	DF090317:7145676-7145920
BmHel-7	DF090349:4177435-4177680
BmHel-7	DF090349:4435527-4435772
BmHel-7	DF090354:1366596-1366841
BmHel-7	DF090328:113071-113316
BmHel-7	DF090322:1388801-1389046
BmHel-7	DF090322:7834018-7834263
BmHel-7	DF090347:2590784-2591029
BmHel-7	DF090318:3537797-3538042
BmHel-7	DF090353:2537527-2537772
BmHel-7	DF090332:651071-651316
BmHel-7	DF090321:633067-633312
BmHel-7	DF090409:625956-626201
BmHel-7	DF090912:1916-2161
BmHel-7	DF090323:4139725-4139970
BmHel-7	DF090352:2487720-2487965
BmHel-7	DF090388:1320112-1320357
BmHel-7	DF090371:1226782-1227027
BmHel-7	DF090343:2330541-2330786
BmHel-7	DF090340:3837088-3837333
BmHel-7	DF090340:867674-867919
BmHel-7	DF090323:7382154-7382399
BmHel-7	DF090320:5852318-5852563
BmHel-7	DF090416:221487-221732
BmHel-7	DF090368:1998606-1998851
BmHel-7	DF090379:601973-602218
BmHel-7	DF090367:538838-539083
BmHel-7	DF090319:2502995-2503240
BmHel-7	DF090316:8323672-8323917
BmHel-7	DF090470:268811-269056
BmHel-7	DF090356:1803237-1803483
BmHel-7	DF090349:27503-27749
BmHel-7	DF090344:1427720-1427966
BmHel-7	DF090367:1410279-1410525
BmHel-7	DF090367:2491104-2491350
BmHel-7	DF090320:7615890-7616136
BmHel-7	DF090318:1517857-1518103
BmHel-7	DF090373:2173817-2174063
BmHel-7	DF090348:3224574-3224820
BmHel-7	DF090400:897623-897869
BmHel-7	DF090321:3696348-3696594
BmHel-7	DF090404:714116-714362
BmHel-7	DF090396:848384-848630
BmHel-7	DF090431:153810-154056
BmHel-7	DF090352:1522954-1523200
BmHel-7	DF090520:58338-58584
BmHel-7	DF090316:9908429-9908675
BmHel-7	DF090323:4514343-4514589

BmHel-7	DF090320:7222444-7222690
BmHel-7	DF090337:5165064-5165310
BmHel-7	DF090327:4118422-4118668
BmHel-7	DF090321:1650963-1651209
BmHel-7	DF090393:1056039-1056285
BmHel-7	DF090461:48370-48616
BmHel-7	DF090388:499363-499610
BmHel-7	DF090334:3824737-3824984
BmHel-7	DF090334:5527337-5527584
BmHel-7	DF090328:1276648-1276895
BmHel-7	DF090323:6741992-6742239
BmHel-7	DF090322:8139233-8139480
BmHel-7	DF090389:534462-534709
BmHel-7	DF090353:3128267-3128514
BmHel-7	DF090363:1696718-1696965
BmHel-7	DF090365:2861808-2862055
BmHel-7	DF090335:464586-464833
BmHel-7	DF090379:1124701-1124948
BmHel-7	DF090326:4309374-4309621
BmHel-7	DF090325:5753805-5754052
BmHel-7	DF090384:1191001-1191248
BmHel-7	DF090337:1430985-1431232
BmHel-7	DF090337:1404953-1405200
BmHel-7	DF090332:259351-259598
BmHel-7	DF090345:2197146-2197393
BmHel-7	DF090345:1704695-1704942
BmHel-7	DF090330:1078177-1078424
BmHel-7	DF090317:7414976-7415223
BmHel-7	DF090317:5492212-5492459
BmHel-7	DF090338:3162231-3162478
BmHel-7	DF090331:307514-307761
BmHel-7	DF091976:1810-2057
BmHel-7	DF090932:208-455
BmHel-7	DF090455:290814-291061
BmHel-7	DF090447:358668-358915
BmHel-7	DF090318:8439838-8440085
BmHel-7	DF090331:605886-606133
BmHel-7	DF090349:2677375-2677623
BmHel-7	DF090319:3570920-3571168
BmHel-7	DF090316:9372815-9373063
BmHel-7	DF090343:3889190-3889438
BmHel-7	DF090328:5207306-5207554
BmHel-7	DF090320:3013698-3013946
BmHel-7	DF090378:1049849-1050097
BmHel-7	DF090337:760198-760446
BmHel-7	DF090373:916723-916971
BmHel-7	DF090358:1144908-1145156
BmHel-7	DF090330:1295853-1296101

BmHel-7	DF090330:3990415-3990663
BmHel-7	DF090330:5285559-5285807
BmHel-7	DF090381:1352093-1352341
BmHel-7	DF090434:43180-43428
BmHel-7	DF090404:892987-893235
BmHel-7	DF090415:455909-456157
BmHel-7	DF090445:341657-341905
BmHel-7	DF090463:115684-115932
BmHel-7	BABH01065481:415-663
BmHel-7	BABH01073967:5-253
BmHel-7	DF090354:2307130-2307378
BmHel-7	DF090394:609658-609906
BmHel-7	DF090359:1287090-1287338
BmHel-7	DF090453:12085-12333
BmHel-7	DF090347:1587579-1587827
BmHel-7	DF090355:1928415-1928663
BmHel-7	DF090336:1722691-1722939
BmHel-7	DF090316:11068588-11068836
BmHel-7	DF090323:157991-158239
BmHel-7	DF090372:995588-995836
BmHel-7	DF090320:1364494-1364742
BmHel-7	DF090327:2016055-2016303
BmHel-7	DF090318:2464656-2464904
BmHel-7	DF090407:1552578-1552826
BmHel-7	DF090361:396627-396875
BmHel-7	DF090332:6123056-6123304
BmHel-7	DF090317:8329634-8329882
BmHel-7	DF090317:5220287-5220535
BmHel-7	DF090381:1782103-1782351
BmHel-7	DF090386:1775702-1775950
BmHel-7	DF090324:5106515-5106763
BmHel-7	DF090462:419757-420005
BmHel-7	DF090712:28520-28768
BmHel-7	DF090426:131012-131260
BmHel-7	BABH01047011:595-843
BmHel-7	DF090356:720875-721123
BmHel-7	DF090328:4812709-4812957
BmHel-7	DF090320:1537945-1538193
BmHel-7	DF090317:8237242-8237490
BmHel-7	DF090368:1813034-1813282
BmHel-7	DF090391:1629320-1629568
BmHel-7	DF090437:206833-207081
BmHel-7	DF090725:11139-11387
BmHel-7	DF090355:2193554-2193803
BmHel-7	DF090341:2065449-2065698
BmHel-7	DF090319:2972059-2972308
BmHel-7	DF090390:53166-53415
BmHel-7	DF090388:245076-245325

BmHel-7	DF090340:1472710-1472959
BmHel-7	DF090329:2774937-2775186
BmHel-7	DF090351:3730510-3730759
BmHel-7	DF090350:1866422-1866671
BmHel-7	DF090350:3235254-3235503
BmHel-7	DF090856:6234-6483
BmHel-7	DF090332:3093504-3093753
BmHel-7	DF090345:707803-708052
BmHel-7	DF090345:2151995-2152244
BmHel-7	DF090317:8810299-8810548
BmHel-7	DF090317:9013802-9014051
BmHel-7	DF090335:4948631-4948880
BmHel-7	DF090335:5197025-5197274
BmHel-7	DF090321:7937076-7937325
BmHel-7	DF090381:146338-146587
BmHel-7	DF090362:2117892-2118141
BmHel-7	DF090324:2866647-2866896
BmHel-7	DF090472:197087-197336
BmHel-7	DF090382:2026267-2026516
BmHel-7	DF090443:172613-172862
BmHel-7	DF090370:2636282-2636531
BmHel-7	DF090599:1337-1586
BmHel-7	DF090455:368172-368421
BmHel-7	BABH01051821:575-824
BmHel-7	DF090679:25834-26083
BmHel-7	DF090342:2908117-2908366
BmHel-7	DF090349:3881143-3881392
BmHel-7	DF090346:2458782-2459031
BmHel-7	DF090333:229960-230209
BmHel-7	DF090411:575514-575763
BmHel-7	DF090419:231263-231512
BmHel-7	DF090365:1302065-1302314
BmHel-7	DF090467:31488-31737
BmHel-7	DF090610:8478-8727
BmHel-7	DF090318:3358073-3358322
BmHel-7	DF090332:1867558-1867807
BmHel-7	DF090467:304618-304867
BmHel-7	DF090341:3477240-3477489
BmHel-7	DF090405:1111426-1111675
BmHel-7	DF090344:2790436-2790685
BmHel-7	DF090388:450912-451161
BmHel-7	DF090334:2933540-2933789
BmHel-7	DF090334:2721558-2721807
BmHel-7	DF090394:725172-725421
BmHel-7	DF090318:8598914-8599163
BmHel-7	DF090318:7545892-7546141
BmHel-7	DF090318:4682544-4682793
BmHel-7	DF090353:2697087-2697336

BmHel-7	DF090353:311545-311794
BmHel-7	DF090363:2848679-2848928
BmHel-7	DF090332:640358-640607
BmHel-7	DF090317:6903744-6903993
BmHel-7	DF090317:6473048-6473297
BmHel-7	DF090317:1057150-1057399
BmHel-7	DF090421:317174-317423
BmHel-7	DF090338:4093696-4093945
BmHel-7	DF090368:1669985-1670234
BmHel-7	DF090362:1843152-1843401
BmHel-7	DF090413:467699-467948
BmHel-7	DF090410:597706-597955
BmHel-7	DF090450:78890-79139
BmHel-7	DF090431:377163-377412
BmHel-7	DF090548:58926-59175
BmHel-7	DF090319:7189249-7189498
BmHel-7	DF090364:2866643-2866892
BmHel-7	DF090364:2691234-2691483
BmHel-7	DF090334:4784984-4785233
BmHel-7	DF090323:536424-536673
BmHel-7	DF090329:2503970-2504219
BmHel-7	DF090320:8204332-8204581
BmHel-7	DF090332:3374357-3374606
BmHel-7	DF090317:79973-80222
BmHel-7	DF090369:437441-437690
BmHel-7	DF090349:492000-492250
BmHel-7	DF090349:985621-985871
BmHel-7	DF090341:8258-8508
BmHel-7	DF090341:4732897-4733147
BmHel-7	DF090341:4820882-4821132
BmHel-7	DF090367:1496417-1496667
BmHel-7	DF090326:4883917-4884167
BmHel-7	DF090388:586610-586860
BmHel-7	DF090377:52273-52523
BmHel-7	DF090346:2532865-2533115
BmHel-7	DF090334:5076752-5077002
BmHel-7	DF090328:4085668-4085918
BmHel-7	DF090325:5687861-5688111
BmHel-7	DF090323:872013-872263
BmHel-7	DF090323:5901430-5901680
BmHel-7	DF090322:2444110-2444360
BmHel-7	DF090372:1397242-1397492
BmHel-7	DF090329:4581197-4581447
BmHel-7	DF090351:1608517-1608767
BmHel-7	DF090351:2723848-2724098
BmHel-7	DF090347:1082881-1083131
BmHel-7	DF090347:2964917-2965167
BmHel-7	DF090378:1520731-1520981

BmHel-7	DF090350:1761773-1762023
BmHel-7	DF090333:4369138-4369388
BmHel-7	DF090327:1342121-1342371
BmHel-7	DF090327:2455925-2456175
BmHel-7	DF090318:8950286-8950536
BmHel-7	DF090389:195502-195752
BmHel-7	DF090360:1452326-1452576
BmHel-7	DF090339:1741059-1741309
BmHel-7	DF090398:732685-732935
BmHel-7	DF090357:1113015-1113265
BmHel-7	DF090441:658561-658811
BmHel-7	DF090419:1128719-1128969
BmHel-7	DF090358:359593-359843
BmHel-7	DF090330:956807-957057
BmHel-7	DF090317:10262367-10262617
BmHel-7	DF090381:57253-57503
BmHel-7	DF090438:668285-668535
BmHel-7	DF090429:236044-236294
BmHel-7	DF090408:675183-675433
BmHel-7	DF090408:1107064-1107314
BmHel-7	DF090408:1120701-1120951
BmHel-7	DF090392:1708844-1709094
BmHel-7	DF090368:1910346-1910596
BmHel-7	DF090368:2067212-2067462
BmHel-7	DF090331:3460461-3460711
BmHel-7	DF090324:2135636-2135886
BmHel-7	DF090324:4040785-4041035
BmHel-7	BABH01046657:1351-1601
BmHel-7	DF090417:720094-720344
BmHel-7	DF090415:334518-334768
BmHel-7	DF090415:898771-899021
BmHel-7	DF090410:752425-752675
BmHel-7	DF090469:286874-287124
BmHel-7	DF090370:1512340-1512590
BmHel-7	DF090782:7288-7538
BmHel-7	DF090436:87763-88013
BmHel-7	DF090426:170332-170582
BmHel-7	BABH01049313:609-859
BmHel-7	DF090867:11339-11589
BmHel-7	DF090475:168594-168844
BmHel-7	DF090655:21894-22144
BmHel-7	DF090342:4415496-4415746
BmHel-7	DF090341:271596-271846
BmHel-7	DF090405:635324-635574
BmHel-7	DF090344:2069508-2069758
BmHel-7	DF090367:1043993-1044243
BmHel-7	DF090336:4406647-4406897
BmHel-7	DF090390:444755-445005

BmHel-7	DF090390:1601694-1601944
BmHel-7	DF090388:1391643-1391893
BmHel-7	DF090346:4084940-4085190
BmHel-7	DF090343:427089-427339
BmHel-7	DF090340:1264391-1264641
BmHel-7	DF090340:1856597-1856847
BmHel-7	DF090347:642668-642918
BmHel-7	DF090320:2575625-2575875
BmHel-7	DF090320:3487879-3488129
BmHel-7	DF090394:434993-435243
BmHel-7	DF090327:5315267-5315517
BmHel-7	DF090318:2271765-2272015
BmHel-7	DF090318:5601584-5601834
BmHel-7	DF090389:1224780-1225030
BmHel-7	DF090373:272366-272616
BmHel-7	DF090419:196457-196707
BmHel-7	DF090345:2768976-2769226
BmHel-7	DF090421:490546-490796
BmHel-7	DF090321:1560324-1560574
BmHel-7	DF090321:2842383-2842633
BmHel-7	DF090321:7398358-7398608
BmHel-7	DF090381:1262306-1262556
BmHel-7	DF090338:660646-660896
BmHel-7	DF090403:403316-403566
BmHel-7	DF090379:1154881-1155131
BmHel-7	DF090431:180942-181192
BmHel-7	BABH01044444:6649-6899
BmHel-7	BABH01072094:51-301
BmHel-7	DF091813:334-584
BmHel-7	BABH01045837:2220-2470
BmHel-7	BABH01047632:1334-1584
BmHel-7	DF090449:425895-426145
BmHel-7	DF090356:1126085-1126335
BmHel-7	DF090376:484198-484448
BmHel-7	DF090366:1452581-1452831
BmHel-7	DF090393:812922-813172
BmHel-7	DF090382:1193243-1193493
BmHel-7	DF090371:2240179-2240429
BmHel-7	DF090349:965460-965710
BmHel-7	DF090344:718733-718983
BmHel-7	DF090336:1155769-1156019
BmHel-7	DF090319:7045841-7046091
BmHel-7	DF090326:5415286-5415536
BmHel-7	DF090316:13987134-13987384
BmHel-7	DF090371:72532-72782
BmHel-7	DF090364:2409735-2409985
BmHel-7	DF090364:1195792-1196042
BmHel-7	DF090340:1201380-1201630

BmHel-7	DF090334:2066549-2066799
BmHel-7	DF090328:5748083-5748333
BmHel-7	DF090328:3210054-3210304
BmHel-7	DF090328:1591711-1591961
BmHel-7	DF090325:6176895-6177145
BmHel-7	DF090325:4580025-4580275
BmHel-7	DF090325:2228683-2228933
BmHel-7	DF090323:421729-421979
BmHel-7	DF090322:8307464-8307714
BmHel-7	DF090322:3713233-3713483
BmHel-7	DF090372:2167901-2168151
BmHel-7	DF090372:1434482-1434732
BmHel-7	DF090329:4132171-4132421
BmHel-7	DF090352:3944201-3944451
BmHel-7	DF090320:8184548-8184798
BmHel-7	DF090327:840069-840319
BmHel-7	DF090318:7383746-7383996
BmHel-7	DF090318:6364452-6364702
BmHel-7	DF090339:1136885-1137135
BmHel-7	DF090353:3632413-3632663
BmHel-7	DF090363:1221930-1222180
BmHel-7	DF090361:557481-557731
BmHel-7	DF090332:6298507-6298757
BmHel-7	DF090332:6181077-6181327
BmHel-7	DF090332:5596730-5596980
BmHel-7	BABH01051398:369-619
BmHel-7	DF090441:245839-246089
BmHel-7	DF090418:592737-592987
BmHel-7	DF090395:405092-405342
BmHel-7	DF090380:559092-559342
BmHel-7	DF090366:2287778-2288028
BmHel-7	DF090366:1649284-1649534
BmHel-7	DF090358:1842567-1842817
BmHel-7	DF090358:272603-272853
BmHel-7	DF090330:3856325-3856575
BmHel-7	DF090317:5974291-5974541
BmHel-7	DF090365:1852895-1853145
BmHel-7	DF090335:2620693-2620943
BmHel-7	DF090335:1544749-1544999
BmHel-7	DF090321:3388749-3388999
BmHel-7	DF090321:1127811-1128061
BmHel-7	DF090435:155562-155812
BmHel-7	DF090408:856241-856491
BmHel-7	DF090385:1335224-1335474
BmHel-7	DF090374:725994-726244
BmHel-7	DF090362:1294269-1294519
BmHel-7	DF090362:576615-576865
BmHel-7	DF090324:4933540-4933790

BmHel-7	DF090872:6865-7115
BmHel-7	DF090409:642470-642720
BmHel-7	DF090409:128967-129217
BmHel-7	DF090396:827978-828228
BmHel-7	DF090423:140325-140575
BmHel-7	BABH01079475:152-402
BmHel-7	DF090479:272448-272698
BmHel-7	DF091187:4440-4690
BmHel-7	DF090430:263819-264069
BmHel-7	BABH01047913:787-1037
BmHel-7	DF090603:18584-18834
BmHel-7	DF090349:4411998-4412248
BmHel-7	DF090341:2435710-2435960
BmHel-7	DF090344:1748414-1748664
BmHel-7	DF090414:486837-487087
BmHel-7	DF090367:2631497-2631747
BmHel-7	DF090367:1568655-1568905
BmHel-7	DF090316:6858038-6858288
BmHel-7	DF090390:649572-649822
BmHel-7	DF090377:2049941-2050191
BmHel-7	DF090343:769846-770096
BmHel-7	DF090340:2343599-2343849
BmHel-7	DF090328:6492865-6493115
BmHel-7	DF090328:3872956-3873206
BmHel-7	DF090322:6397151-6397401
BmHel-7	DF090372:1358892-1359142
BmHel-7	DF090320:3823748-3823998
BmHel-7	DF090378:1883494-1883744
BmHel-7	DF090327:1971521-1971771
BmHel-7	DF090339:2114109-2114359
BmHel-7	DF090363:1863172-1863422
BmHel-7	DF090361:67851-68101
BmHel-7	DF090332:4915015-4915265
BmHel-7	DF090358:2677328-2677578
BmHel-7	DF090330:4713234-4713484
BmHel-7	DF090317:7457897-7458147
BmHel-7	DF090317:4047546-4047796
BmHel-7	DF090317:2299636-2299886
BmHel-7	BABH01044830:6639-6889
BmHel-7	DF090335:2095254-2095504
BmHel-7	DF090386:411809-412059
BmHel-7	DF090368:1787541-1787791
BmHel-7	DF090331:3393725-3393975
BmHel-7	DF090324:3081434-3081684
BmHel-7	BABH01045936:210-460
BmHel-7	DF090521:10236-10486
BmHel-7	DF090347:815670-815920
BmHel-7	DF090416:854878-855128

BmHel-7	DF090324:375060-375310
BmHel-7	DF090415:339538-339788
BmHel-7	DF090459:189157-189407
BmHel-7	DF090414:981094-981344
BmHel-7	BABH01075845:308-559
BmHel-7	DF090342:303258-303509
BmHel-7	DF090342:1231039-1231290
BmHel-7	DF090342:1466121-1466372
BmHel-7	DF090342:2619297-2619548
BmHel-7	DF090342:3641356-3641607
BmHel-7	DF090342:4805750-4806001
BmHel-7	DF090356:78946-79197
BmHel-7	DF090356:261579-261830
BmHel-7	DF090356:1702746-1702997
BmHel-7	DF090356:2765461-2765712
BmHel-7	DF090356:3365398-3365649
BmHel-7	DF090355:426608-426859
BmHel-7	DF090355:1413209-1413460
BmHel-7	DF090355:1690687-1690938
BmHel-7	DF090355:1988127-1988378
BmHel-7	DF090355:2568512-2568763
BmHel-7	DF090355:3477745-3477996
BmHel-7	DF090349:277834-278085
BmHel-7	DF090349:1081276-1081527
BmHel-7	DF090349:2697335-2697586
BmHel-7	DF090349:3447595-3447846
BmHel-7	DF090349:3626922-3627173
BmHel-7	DF090341:419234-419485
BmHel-7	DF090341:3046090-3046341
BmHel-7	DF090405:265614-265865
BmHel-7	DF090344:861473-861724
BmHel-7	DF090344:1170787-1171038
BmHel-7	DF090414:245779-246030
BmHel-7	DF090414:1228259-1228510
BmHel-7	BABH01061386:288-539
BmHel-7	DF090367:1271967-1272218
BmHel-7	DF090367:2669442-2669693
BmHel-7	DF090354:1441703-1441954
BmHel-7	DF090354:1872480-1872731
BmHel-7	DF090354:2138258-2138509
BmHel-7	DF090336:2283702-2283953
BmHel-7	DF090336:3542876-3543127
BmHel-7	DF090336:3823109-3823360
BmHel-7	DF090336:3838223-3838474
BmHel-7	DF090336:4421919-4422170
BmHel-7	DF090336:5171867-5172118
BmHel-7	DF090336:5391855-5392106
BmHel-7	DF090319:1446570-1446821

BmHel-7	DF090319:2158350-2158601
BmHel-7	DF090319:2929837-2930088
BmHel-7	DF090319:4404675-4404926
BmHel-7	DF090319:5623102-5623353
BmHel-7	DF090319:6576898-6577149
BmHel-7	DF090319:6969485-6969736
BmHel-7	DF090319:8322543-8322794
BmHel-7	DF090326:1191305-1191556
BmHel-7	DF090326:6076643-6076894
BmHel-7	DF090316:743466-743717
BmHel-7	DF090316:7532417-7532668
BmHel-7	DF090316:7808242-7808493
BmHel-7	DF090316:11136141-11136392
BmHel-7	DF090316:11766992-11767243
BmHel-7	DF090390:593654-593905
BmHel-7	DF090390:884751-885002
BmHel-7	DF090390:1751302-1751553
BmHel-7	BABH01050671:378-629
BmHel-7	DF090470:304101-304352
BmHel-7	DF090402:822836-823087
BmHel-7	DF090388:950147-950398
BmHel-7	DF090388:1337165-1337416
BmHel-7	DF090388:1747687-1747938
BmHel-7	DF090377:598126-598377
BmHel-7	DF090371:198940-199191
BmHel-7	DF090371:593170-593421
BmHel-7	DF090364:17214-17465
BmHel-7	DF090364:918184-918435
BmHel-7	DF090364:1938942-1939193
BmHel-7	DF090346:883488-883739
BmHel-7	DF090346:1979017-1979268
BmHel-7	DF090346:2614073-2614324
BmHel-7	DF090346:3537620-3537871
BmHel-7	DF090346:3941937-3942188
BmHel-7	DF090343:1165891-1166142
BmHel-7	DF090343:1343742-1343993
BmHel-7	DF090343:1898716-1898967
BmHel-7	DF090343:4234634-4234885
BmHel-7	DF090343:4285805-4286056
BmHel-7	DF090340:1500370-1500621
BmHel-7	DF090340:3271691-3271942
BmHel-7	DF090340:4585713-4585964
BmHel-7	DF090334:5430278-5430529
BmHel-7	DF090328:1633539-1633790
BmHel-7	DF090328:2448056-2448307
BmHel-7	DF090325:5475241-5475492
BmHel-7	DF090325:7103235-7103486
BmHel-7	DF090323:175866-176117

BmHel-7	DF090323:1380924-1381175
BmHel-7	DF090323:1579598-1579849
BmHel-7	DF090323:2679042-2679293
BmHel-7	DF090322:1716018-1716269
BmHel-7	DF090322:2177213-2177464
BmHel-7	DF090322:2519910-2520161
BmHel-7	DF090322:4771162-4771413
BmHel-7	DF090322:5202317-5202568
BmHel-7	DF090322:6375964-6376215
BmHel-7	DF090322:8058700-8058951
BmHel-7	DF090372:1380440-1380691
BmHel-7	DF090372:2127602-2127853
BmHel-7	DF090372:2371299-2371550
BmHel-7	DF090329:5535481-5535732
BmHel-7	DF090351:1093721-1093972
BmHel-7	DF090351:1349770-1350021
BmHel-7	DF090351:1393219-1393470
BmHel-7	DF090351:1501338-1501589
BmHel-7	DF090347:253411-253662
BmHel-7	DF090347:766078-766329
BmHel-7	DF090347:2188125-2188376
BmHel-7	DF090347:3234390-3234641
BmHel-7	DF090352:104049-104300
BmHel-7	DF090352:341712-341963
BmHel-7	DF090352:1205606-1205857
BmHel-7	DF090352:2454148-2454399
BmHel-7	DF090320:699611-699862
BmHel-7	DF090320:712844-713095
BmHel-7	DF090320:1983226-1983477
BmHel-7	DF090320:4609816-4610067
BmHel-7	DF090320:5460417-5460668
BmHel-7	DF090320:6512764-6513015
BmHel-7	BABH01049771:330-581
BmHel-7	DF090384:1020254-1020505
BmHel-7	DF090378:1488537-1488788
BmHel-7	DF090378:1543740-1543991
BmHel-7	DF090378:2002441-2002692
BmHel-7	DF090350:2365841-2366092
BmHel-7	DF090350:3423440-3423691
BmHel-7	DF090337:3465901-3466152
BmHel-7	DF090337:4442950-4443201
BmHel-7	DF090327:1747926-1748177
BmHel-7	DF090327:2248268-2248519
BmHel-7	DF090327:3264102-3264353
BmHel-7	DF090327:3886960-3887211
BmHel-7	DF090318:1223452-1223703
BmHel-7	DF090318:2334470-2334721
BmHel-7	DF090318:6054470-6054721

BmHel-7	DF090318:6448468-6448719
BmHel-7	DF090318:6604400-6604651
BmHel-7	DF090407:191128-191379
BmHel-7	DF090339:2167420-2167671
BmHel-7	DF090339:2325887-2326138
BmHel-7	DF090353:28096-28347
BmHel-7	DF090353:2327126-2327377
BmHel-7	DF090353:2947578-2947829
BmHel-7	DF090411:35592-35843
BmHel-7	DF090398:902904-903155
BmHel-7	DF090398:1296795-1297046
BmHel-7	DF090398:1583001-1583252
BmHel-7	DF090373:1822464-1822715
BmHel-7	DF090363:1211293-1211544
BmHel-7	DF090363:1476944-1477195
BmHel-7	DF090363:1888232-1888483
BmHel-7	DF090361:138533-138784
BmHel-7	DF090361:895378-895629
BmHel-7	DF090361:1630748-1630999
BmHel-7	DF090361:2059704-2059955
BmHel-7	DF090361:2779704-2779955
BmHel-7	DF090332:271803-272054
BmHel-7	DF090332:1300541-1300792
BmHel-7	DF090564:39494-39745
BmHel-7	DF090441:142004-142255
BmHel-7	DF090422:360779-361030
BmHel-7	DF090418:567590-567841
BmHel-7	DF090416:129063-129314
BmHel-7	DF090395:1055927-1056178
BmHel-7	DF090395:1635905-1636156
BmHel-7	DF090380:494247-494498
BmHel-7	DF090380:2107947-2108198
BmHel-7	DF090366:1703210-1703461
BmHel-7	DF090358:186219-186470
BmHel-7	DF090358:1091734-1091985
BmHel-7	DF090348:1545414-1545665
BmHel-7	DF090348:1643810-1644061
BmHel-7	DF090348:2934169-2934420
BmHel-7	DF090348:3590845-3591096
BmHel-7	DF090348:3987642-3987893
BmHel-7	DF090348:4314840-4315091
BmHel-7	DF090345:1608128-1608379
BmHel-7	DF090345:3304022-3304273
BmHel-7	DF090330:1407895-1408146
BmHel-7	DF090317:3877469-3877720
BmHel-7	DF090317:4023639-4023890
BmHel-7	DF090317:4109589-4109840
BmHel-7	DF090317:6664382-6664633

BmHel-7	DF090317:6709329-6709580
BmHel-7	DF090317:7959958-7960209
BmHel-7	DF090317:8501675-8501926
BmHel-7	DF090317:8714237-8714488
BmHel-7	DF090317:10151650-10151901
BmHel-7	DF090317:10585368-10585619
BmHel-7	DF090400:655263-655514
BmHel-7	DF090400:1276125-1276376
BmHel-7	DF090365:178878-179129
BmHel-7	DF090365:1369979-1370230
BmHel-7	DF090365:2847302-2847553
BmHel-7	DF090335:29889-30140
BmHel-7	DF090335:3509046-3509297
BmHel-7	DF090321:2224655-2224906
BmHel-7	DF090321:3988035-3988286
BmHel-7	DF090321:6312061-6312312
BmHel-7	DF090383:1215274-1215525
BmHel-7	DF090338:452839-453090
BmHel-7	DF090338:1036387-1036638
BmHel-7	DF090338:3136187-3136438
BmHel-7	DF090338:3509753-3510004
BmHel-7	DF090661:13184-13435
BmHel-7	DF090617:33517-33768
BmHel-7	DF090438:194778-195029
BmHel-7	DF090434:407179-407430
BmHel-7	DF090429:9196-9447
BmHel-7	DF090404:1020899-1021150
BmHel-7	DF090392:759461-759712
BmHel-7	DF090386:107353-107604
BmHel-7	DF090386:1703693-1703944
BmHel-7	DF090385:1900876-1901127
BmHel-7	DF090375:1392796-1393047
BmHel-7	DF090375:1435549-1435800
BmHel-7	DF090374:1543957-1544208
BmHel-7	DF090362:1230344-1230595
BmHel-7	DF090362:1428692-1428943
BmHel-7	DF090362:1801434-1801685
BmHel-7	DF090362:3199621-3199872
BmHel-7	DF090331:1288293-1288544
BmHel-7	DF090331:4756138-4756389
BmHel-7	DF090331:5725715-5725966
BmHel-7	DF090331:5853505-5853756
BmHel-7	DF090331:6029189-6029440
BmHel-7	DF090324:55181-55432
BmHel-7	DF090324:2089410-2089661
BmHel-7	DF090324:5933768-5934019
BmHel-7	DF090324:6514770-6515021
BmHel-7	DF090427:156886-157137

BmHel-7	DF090427:656386-656637
BmHel-7	DF090427:938810-939061
BmHel-7	DF090393:826451-826702
BmHel-7	DF090805:17763-18014
BmHel-7	DF090795:10384-10635
BmHel-7	DF090472:144991-145242
BmHel-7	DF090417:435519-435770
BmHel-7	DF090417:598629-598880
BmHel-7	DF090409:847051-847302
BmHel-7	DF090401:695925-696176
BmHel-7	DF090396:68057-68308
BmHel-7	DF090396:522064-522315
BmHel-7	DF090396:888932-889183
BmHel-7	DF090391:744208-744459
BmHel-7	DF090403:543416-543667
BmHel-7	DF090403:1426732-1426983
BmHel-7	DF090415:312823-313074
BmHel-7	DF090415:731409-731660
BmHel-7	DF090379:902732-902983
BmHel-7	DF090410:67420-67671
BmHel-7	BABH01049892:535-786
BmHel-7	DF090469:111856-112107
BmHel-7	DF090459:86491-86742
BmHel-7	DF090450:34593-34844
BmHel-7	DF090423:395179-395430
BmHel-7	DF090443:153786-154037
BmHel-7	DF090488:145815-146066
BmHel-7	DF090370:422432-422683
BmHel-7	DF090370:1368374-1368625
BmHel-7	BABH01080090:280-531
BmHel-7	DF090461:348004-348255
BmHel-7	DF090458:41034-41285
BmHel-7	DF090504:118154-118405
BmHel-7	DF091002:9603-9854
BmHel-7	DF090738:955-1206
BmHel-7	DF090454:441105-441356
BmHel-7	DF090451:299641-299892
BmHel-7	DF090465:203604-203855
BmHel-7	DF090464:139379-139630
BmHel-7	DF090979:9317-9568
BmHel-7	DF091361:4176-4427
BmHel-7	BABH01058071:107-358
BmHel-7	DF090356:1476120-1476371
BmHel-7	DF090327:5272409-5272660
BmHel-7	DF090341:1667324-1667575
BmHel-7	BABH01085474:150-401
BmHel-7	BABH01076283:319-570
BmHel-7	DF090342:4468837-4469088

BmHel-7	DF090342:3726015-3726266
BmHel-7	DF090342:3575557-3575808
BmHel-7	DF090342:3271439-3271690
BmHel-7	DF090342:1264947-1265198
BmHel-7	DF090342:294818-295069
BmHel-7	DF090356:3828237-3828488
BmHel-7	DF090356:3707882-3708133
BmHel-7	DF090356:1308178-1308429
BmHel-7	DF090355:2231705-2231956
BmHel-7	DF090349:4162289-4162540
BmHel-7	DF090349:3819665-3819916
BmHel-7	DF090349:2440007-2440258
BmHel-7	DF090349:2272541-2272792
BmHel-7	DF090349:1686590-1686841
BmHel-7	DF090349:1153563-1153814
BmHel-7	DF090349:725153-725404
BmHel-7	DF090349:479031-479282
BmHel-7	DF090349:396913-397164
BmHel-7	DF090349:355384-355635
BmHel-7	DF090341:4657138-4657389
BmHel-7	DF090341:4118293-4118544
BmHel-7	DF090341:3548928-3549179
BmHel-7	DF090341:2474889-2475140
BmHel-7	DF090341:1575445-1575696
BmHel-7	DF090341:1113500-1113751
BmHel-7	DF090405:1074601-1074852
BmHel-7	DF090344:2754977-2755228
BmHel-7	DF090344:1865163-1865414
BmHel-7	DF090414:802190-802441
BmHel-7	DF090414:178132-178383
BmHel-7	DF090414:95684-95935
BmHel-7	DF090367:2798688-2798939
BmHel-7	DF090367:2106868-2107119
BmHel-7	DF090367:1767199-1767450
BmHel-7	DF090367:793310-793561
BmHel-7	DF090367:725078-725329
BmHel-7	DF090367:703477-703728
BmHel-7	DF090354:2496309-2496560
BmHel-7	DF090354:793504-793755
BmHel-7	DF090336:4035129-4035380
BmHel-7	DF090336:324627-324878
BmHel-7	DF090319:8300198-8300449
BmHel-7	DF090319:8124219-8124470
BmHel-7	DF090319:5714112-5714363
BmHel-7	DF090319:4532631-4532882
BmHel-7	DF090319:2172430-2172681
BmHel-7	DF090326:4487235-4487486
BmHel-7	DF090326:4129139-4129390

BmHel-7	DF090326:1618268-1618519
BmHel-7	DF090326:1544673-1544924
BmHel-7	DF090326:1478743-1478994
BmHel-7	DF090326:291936-292187
BmHel-7	DF090326:52033-52284
BmHel-7	DF090326:5873-6124
BmHel-7	DF090316:14157928-14158179
BmHel-7	DF090316:14105324-14105575
BmHel-7	DF090316:12741154-12741405
BmHel-7	DF090316:11099743-11099994
BmHel-7	DF090316:10785701-10785952
BmHel-7	DF090316:5421286-5421537
BmHel-7	DF090433:434629-434880
BmHel-7	DF090402:1223917-1224168
BmHel-7	DF090402:996255-996506
BmHel-7	DF090402:763084-763335
BmHel-7	DF090388:1231520-1231771
BmHel-7	DF090388:8-259
BmHel-7	DF090377:1719055-1719306
BmHel-7	DF090377:637531-637782
BmHel-7	DF090377:371338-371589
BmHel-7	DF090371:205812-206063
BmHel-7	DF090364:1917042-1917293
BmHel-7	DF090364:1565101-1565352
BmHel-7	DF090364:1368563-1368814
BmHel-7	DF090343:4521972-4522223
BmHel-7	DF090343:3321006-3321257
BmHel-7	DF090343:2030958-2031209
BmHel-7	DF090343:329915-330166
BmHel-7	DF090340:4339264-4339515
BmHel-7	DF090340:3105055-3105306
BmHel-7	DF090340:2631218-2631469
BmHel-7	DF090340:430142-430393
BmHel-7	DF090340:359924-360175
BmHel-7	DF090334:5837311-5837562
BmHel-7	DF090334:4902900-4903151
BmHel-7	DF090328:5304926-5305177
BmHel-7	DF090328:3922878-3923129
BmHel-7	DF090328:2693776-2694027
BmHel-7	DF090328:1413917-1414168
BmHel-7	DF090325:6856680-6856931
BmHel-7	DF090325:3598047-3598298
BmHel-7	DF090323:4685984-4686235
BmHel-7	DF090323:4385677-4385928
BmHel-7	DF090323:3525717-3525968
BmHel-7	DF090323:3330720-3330971
BmHel-7	DF090323:2353324-2353575
BmHel-7	DF090323:2010303-2010554

BmHel-7	DF090322:6075573-6075824
BmHel-7	DF090329:5175615-5175866
BmHel-7	DF090329:5112503-5112754
BmHel-7	DF090329:4735928-4736179
BmHel-7	DF090329:4078986-4079237
BmHel-7	DF090329:2394481-2394732
BmHel-7	DF090329:2107144-2107395
BmHel-7	DF090351:4175801-4176052
BmHel-7	DF090347:2715754-2716005
BmHel-7	DF090347:1518832-1519083
BmHel-7	DF090347:424935-425186
BmHel-7	DF090352:3531624-3531875
BmHel-7	DF090352:3327290-3327541
BmHel-7	DF090352:2387071-2387322
BmHel-7	DF090320:5786701-5786952
BmHel-7	DF090320:4705606-4705857
BmHel-7	DF090320:1701015-1701266
BmHel-7	DF090394:970843-971094
BmHel-7	DF090394:538508-538759
BmHel-7	DF090394:113373-113624
BmHel-7	DF090350:3068386-3068637
BmHel-7	DF090350:2720196-2720447
BmHel-7	DF090333:4603583-4603834
BmHel-7	DF090333:727154-727405
BmHel-7	DF090327:6158995-6159246
BmHel-7	DF090327:4026936-4027187
BmHel-7	DF090327:3911505-3911756
BmHel-7	DF090318:8322255-8322506
BmHel-7	DF090318:6101712-6101963
BmHel-7	DF090318:3450705-3450956
BmHel-7	DF090318:2945204-2945455
BmHel-7	DF090318:1286492-1286743
BmHel-7	DF090318:963943-964194
BmHel-7	DF090389:293886-294137
BmHel-7	DF090360:3166931-3167182
BmHel-7	DF090339:4845381-4845632
BmHel-7	DF090339:4610163-4610414
BmHel-7	DF090339:3821698-3821949
BmHel-7	DF090339:2550213-2550464
BmHel-7	DF090339:2537265-2537516
BmHel-7	DF090339:2192296-2192547
BmHel-7	DF090339:1047937-1048188
BmHel-7	DF090353:3930679-3930930
BmHel-7	DF090353:3395035-3395286
BmHel-7	DF090353:3321059-3321310
BmHel-7	DF090353:2201917-2202168
BmHel-7	DF090398:72516-72767
BmHel-7	DF090363:1235196-1235447

BmHel-7	DF090363:563762-564013
BmHel-7	DF090361:2037567-2037818
BmHel-7	DF090361:1758070-1758321
BmHel-7	DF090361:242352-242603
BmHel-7	DF090357:3135292-3135543
BmHel-7	DF090357:2483827-2484078
BmHel-7	DF090332:2070236-2070487
BmHel-7	DF090332:358904-359155
BmHel-7	DF090332:115714-115965
BmHel-7	BABH01043669:12574-12825
BmHel-7	DF090555:39437-39688
BmHel-7	DF090519:74620-74871
BmHel-7	DF090422:1140425-1140676
BmHel-7	DF090422:352567-352818
BmHel-7	DF090419:1015858-1016109
BmHel-7	DF090419:140201-140452
BmHel-7	DF090399:1567497-1567748
BmHel-7	DF090395:590078-590329
BmHel-7	DF090376:1556835-1557086
BmHel-7	DF090376:1542938-1543189
BmHel-7	DF090376:1238126-1238377
BmHel-7	DF090376:878510-878761
BmHel-7	DF090366:2461433-2461684
BmHel-7	DF090366:995878-996129
BmHel-7	DF090366:509056-509307
BmHel-7	DF090366:337171-337422
BmHel-7	DF090358:547226-547477
BmHel-7	DF090348:3905733-3905984
BmHel-7	DF090348:3832238-3832489
BmHel-7	DF090348:1474307-1474558
BmHel-7	DF090345:4278063-4278314
BmHel-7	DF090345:2377074-2377325
BmHel-7	DF090345:1799573-1799824
BmHel-7	DF090330:6171594-6171845
BmHel-7	DF090330:5146708-5146959
BmHel-7	DF090330:635743-635994
BmHel-7	DF090330:447655-447906
BmHel-7	DF090317:11167804-11168055
BmHel-7	DF090317:10433555-10433806
BmHel-7	DF090317:9540368-9540619
BmHel-7	DF090317:8109091-8109342
BmHel-7	DF090317:2004132-2004383
BmHel-7	DF090317:1746090-1746341
BmHel-7	DF090317:1680334-1680585
BmHel-7	DF090421:112737-112988
BmHel-7	DF090365:2092853-2093104
BmHel-7	DF090335:5574361-5574612
BmHel-7	DF090335:5368477-5368728

BmHel-7	DF090335:4083840-4084091
BmHel-7	DF090335:144559-144810
BmHel-7	DF090321:6584065-6584316
BmHel-7	DF090321:6085188-6085439
BmHel-7	DF090321:1692610-1692861
BmHel-7	DF090321:895708-895959
BmHel-7	DF090321:629859-630110
BmHel-7	DF090383:1855697-1855948
BmHel-7	DF090383:608031-608282
BmHel-7	DF090338:3268870-3269121
BmHel-7	DF090338:2018574-2018825
BmHel-7	DF090338:1601287-1601538
BmHel-7	DF091315:4436-4687
BmHel-7	DF090980:1096-1347
BmHel-7	DF090664:5160-5411
BmHel-7	DF090578:16922-17173
BmHel-7	DF090438:348157-348408
BmHel-7	DF090435:268843-269094
BmHel-7	DF090434:388335-388586
BmHel-7	DF090424:865806-866057
BmHel-7	DF090404:856601-856852
BmHel-7	DF090386:2008105-2008356
BmHel-7	DF090386:1861316-1861567
BmHel-7	DF090386:1683804-1684055
BmHel-7	DF090385:318107-318358
BmHel-7	DF090374:960215-960466
BmHel-7	DF090368:2240225-2240476
BmHel-7	DF090368:1845993-1846244
BmHel-7	DF090368:1533787-1534038
BmHel-7	DF090368:899758-900009
BmHel-7	DF090368:322177-322428
BmHel-7	DF090362:898808-899059
BmHel-7	DF090362:532870-533121
BmHel-7	DF090331:5756497-5756748
BmHel-7	DF090331:3186935-3187186
BmHel-7	DF090331:2016567-2016818
BmHel-7	DF090331:32405-32656
BmHel-7	DF090324:4344338-4344589
BmHel-7	DF090324:2861104-2861355
BmHel-7	DF090324:2737254-2737505
BmHel-7	DF090324:668022-668273
BmHel-7	DF090393:883983-884234
BmHel-7	DF090807:12357-12608
BmHel-7	DF090605:42256-42507
BmHel-7	DF090545:77813-78064
BmHel-7	DF090503:24840-25091
BmHel-7	DF090492:202710-202961
BmHel-7	DF090468:31364-31615

BmHel-7	DF090413:1302389-1302640
BmHel-7	DF090413:1176184-1176435
BmHel-7	DF090413:659596-659847
BmHel-7	DF090413:2404-2655
BmHel-7	DF090401:1376957-1377208
BmHel-7	DF090401:307157-307408
BmHel-7	DF090396:894253-894504
BmHel-7	DF090396:22673-22924
BmHel-7	DF090369:2337138-2337389
BmHel-7	BABH01083183:79-330
BmHel-7	DF090577:49067-49318
BmHel-7	DF090577:8573-8824
BmHel-7	DF090469:352532-352783
BmHel-7	DF090450:429901-430152
BmHel-7	DF090437:680636-680887
BmHel-7	DF090431:446120-446371
BmHel-7	DF090423:747465-747716
BmHel-7	DF090423:512520-512771
BmHel-7	DF090387:1405063-1405314
BmHel-7	DF090387:887166-887417
BmHel-7	DF090382:2060632-2060883
BmHel-7	DF090382:8289-8540
BmHel-7	DF090359:2360628-2360879
BmHel-7	DF090461:239801-240052
BmHel-7	DF090397:1311253-1311504
BmHel-7	BABH01058644:330-581
BmHel-7	DF090517:31940-32191
BmHel-7	DF090430:112185-112436
BmHel-7	DF090426:663362-663613
BmHel-7	DF090426:366395-366646
BmHel-7	DF090465:64979-65230
BmHel-7	BABH01044948:2465-2716
BmHel-7	DF090644:59586-59837
BmHel-7	DF090406:848417-848668
BmHel-7	DF090406:623388-623639
BmHel-7	BABH01055441:494-745
BmHel-7	BABH01049433:118-369
BmHel-7	DF090349:3177053-3177304
BmHel-7	DF090341:1876816-1877067
BmHel-7	DF090344:1114705-1114956
BmHel-7	DF090319:7799203-7799454
BmHel-7	DF090326:4148962-4149213
BmHel-7	DF090318:8026427-8026678
BmHel-7	DF090324:4967380-4967631
BmHel-7	DF090372:732278-732529
BmHel-7	DF090368:2185801-2186052
BmHel-7	DF090349:1844157-1844409
BmHel-7	DF090341:2523187-2523439

BmHel-7	DF090344:4631505-4631757
BmHel-7	DF090414:421258-421510
BmHel-7	DF090414:716627-716879
BmHel-7	DF090414:1298734-1298986
BmHel-7	DF090367:2810618-2810870
BmHel-7	DF090336:497079-497331
BmHel-7	DF090326:5095156-5095408
BmHel-7	DF090316:5240005-5240257
BmHel-7	DF090316:8067211-8067463
BmHel-7	DF090346:43931-44183
BmHel-7	DF090325:5175803-5176055
BmHel-7	DF090325:6835540-6835792
BmHel-7	DF090323:2816622-2816874
BmHel-7	DF090329:2199039-2199291
BmHel-7	DF090351:1279697-1279949
BmHel-7	DF090347:2539473-2539725
BmHel-7	DF090352:991355-991607
BmHel-7	DF090320:7628958-7629210
BmHel-7	DF090394:1236086-1236338
BmHel-7	DF090327:4057754-4058006
BmHel-7	DF090318:9077881-9078133
BmHel-7	DF090389:521851-522103
BmHel-7	DF090373:1114887-1115139
BmHel-7	DF090361:1865159-1865411
BmHel-7	DF090441:230274-230526
BmHel-7	DF090380:1028594-1028846
BmHel-7	DF090348:1346778-1347030
BmHel-7	DF090348:2604013-2604265
BmHel-7	DF090348:3689058-3689310
BmHel-7	DF090317:7088567-7088819
BmHel-7	DF090321:5114871-5115123
BmHel-7	DF090381:367258-367510
BmHel-7	DF090381:2019909-2020161
BmHel-7	DF090324:4625851-4626103
BmHel-7	DF090324:6736822-6737074
BmHel-7	DF090324:7432510-7432762
BmHel-7	DF090554:53969-54221
BmHel-7	DF090425:622285-622537
BmHel-7	DF090410:1339383-1339635
BmHel-7	DF090632:6866-7118
BmHel-7	DF090445:1708-1960
BmHel-7	DF090599:19995-20247
BmHel-7	BABH01045165:4924-5176
BmHel-7	DF090343:4405353-4405605
BmHel-7	DF090360:2498435-2498687
BmHel-7	DF090366:870525-870777
BmHel-7	DF090317:7592575-7592827
BmHel-7	DF090335:5241416-5241668

BmHel-7	BABH01079556:81-333
BmHel-7	DF090341:205362-205614
BmHel-7	DF090386:514436-514688
BmHel-7	DF090342:3778781-3779033
BmHel-7	DF090356:3693217-3693469
BmHel-7	DF090356:823117-823369
BmHel-7	DF090355:992314-992566
BmHel-7	DF090341:4293920-4294172
BmHel-7	DF090341:1340258-1340510
BmHel-7	DF090341:856065-856317
BmHel-7	DF090414:865274-865526
BmHel-7	DF090319:4904704-4904956
BmHel-7	DF090326:2322623-2322875
BmHel-7	DF090316:8237828-8238080
BmHel-7	DF090316:3674443-3674695
BmHel-7	DF090377:1916498-1916750
BmHel-7	DF090371:46321-46573
BmHel-7	DF090328:5516422-5516674
BmHel-7	DF090328:1171129-1171381
BmHel-7	DF090329:2566558-2566810
BmHel-7	DF090352:1461114-1461366
BmHel-7	DF090320:5881244-5881496
BmHel-7	DF090360:297280-297532
BmHel-7	DF090353:3983617-3983869
BmHel-7	DF090353:1326258-1326510
BmHel-7	DF090361:2541047-2541299
BmHel-7	DF090332:835138-835390
BmHel-7	DF090416:80344-80596
BmHel-7	DF090358:3200241-3200493
BmHel-7	DF090317:6731193-6731445
BmHel-7	DF090421:534051-534303
BmHel-7	DF090335:220406-220658
BmHel-7	DF090338:4857057-4857309
BmHel-7	DF090338:1416402-1416654
BmHel-7	DF090338:1097476-1097728
BmHel-7	DF090438:636651-636903
BmHel-7	DF090374:939611-939863
BmHel-7	DF090362:1463165-1463417
BmHel-7	DF090331:387654-387906
BmHel-7	DF090324:954040-954292
BmHel-7	DF090324:574687-574939
BmHel-7	DF090393:131323-131575
BmHel-7	DF090409:1592417-1592669
BmHel-7	DF090425:217085-217337
BmHel-7	DF090359:2308661-2308913
BmHel-7	DF091275:2227-2479
BmHel-7	DF090364:2175839-2176091
BmHel-7	DF090351:842556-842808

BmHel-7	DF090330:3932025-3932277
BmHel-7	DF090344:564649-564902
BmHel-7	DF090336:4108861-4109114
BmHel-7	DF090326:4720405-4720658
BmHel-7	DF090316:4313790-4314043
BmHel-7	DF090346:2491391-2491644
BmHel-7	DF090340:2123384-2123637
BmHel-7	DF090323:6015437-6015690
BmHel-7	DF090320:5651821-5652074
BmHel-7	DF090333:723523-723776
BmHel-7	DF090348:1659678-1659931
BmHel-7	DF090330:4576816-4577069
BmHel-7	DF090335:1989464-1989717
BmHel-7	DF090335:5534797-5535050
BmHel-7	DF090383:2044187-2044440
BmHel-7	DF090368:797520-797773
BmHel-7	DF090369:2533843-2534096
BmHel-7	DF090410:688745-688998
BmHel-7	DF090457:181276-181529
BmHel-7	DF090607:29812-30065
BmHel-7	DF090717:3087-3340
BmHel-7	DF090358:2315675-2315928
BmHel-7	DF090319:533516-533769
BmHel-7	DF090355:1164200-1164453
BmHel-7	DF090336:1046034-1046287
BmHel-7	DF090326:4812711-4812964
BmHel-7	DF090316:4153830-4154083
BmHel-7	DF090343:3541798-3542051
BmHel-7	DF090334:2783562-2783815
BmHel-7	DF090322:5978341-5978594
BmHel-7	DF090398:820759-821012
BmHel-7	DF090416:232155-232408
BmHel-7	DF090358:217424-217677
BmHel-7	DF090317:2995165-2995418
BmHel-7	DF090365:1605024-1605277
BmHel-7	DF090362:79989-80242
BmHel-7	DF090417:1040300-1040553
BmHel-7	DF090406:237105-237358
BmHel-7	DF090409:941915-942168
BmHel-7	DF090353:3221687-3221940
BmHel-7	DF090356:3562450-3562704
BmHel-7	DF090355:2136170-2136424
BmHel-7	DF090341:460193-460447
BmHel-7	DF090326:4752849-4753103
BmHel-7	DF090334:832702-832956
BmHel-7	DF090347:1110584-1110838
BmHel-7	DF090320:6624150-6624404
BmHel-7	DF090333:5551485-5551739

BmHel-7	DF090327:1785277-1785531
BmHel-7	DF090357:2848139-2848393
BmHel-7	DF090358:1366086-1366340
BmHel-7	DF090369:953427-953681
BmHel-7	DF090359:1637012-1637266
BmHel-7	DF090985:642-896
BmHel-7	DF090341:3361890-3362144
BmHel-7	DF090350:740402-740656
BmHel-7	DF090337:4974393-4974647
BmHel-7	DF090396:209954-210208
BmHel-7	DF090317:179140-179394
BmHel-7	DF090342:2453291-2453545
BmHel-7	DF090367:1997705-1997959
BmHel-7	DF090340:991431-991685
BmHel-7	DF090378:1781223-1781477
BmHel-7	DF090338:3884723-3884977
BmHel-7	DF090374:1337951-1338205
BmHel-7	DF090324:3266245-3266499
BmHel-7	DF090413:258731-258985
BmHel-7	DF090752:2316-2570
BmHel-7	BABH01044488:6884-7138
BmHel-7	DF090426:2735-2989
BmHel-7	DF090316:16046043-16046297
BmHel-7	DF090353:3554114-3554368
BmHel-7	DF090319:2689934-2690189
BmHel-7	DF090326:5519186-5519441
BmHel-7	DF090316:2831003-2831258
BmHel-7	DF090347:1297035-1297290
BmHel-7	DF090347:2152138-2152393
BmHel-7	DF090394:1530514-1530769
BmHel-7	DF090327:1026261-1026516
BmHel-7	DF090357:3275081-3275336
BmHel-7	DF090332:126549-126804
BmHel-7	DF090348:1260443-1260698
BmHel-7	DF090317:2404255-2404510
BmHel-7	DF090365:1871914-1872169
BmHel-7	DF090321:7745758-7746013
BmHel-7	DF090362:489460-489715
BmHel-7	DF090324:7461137-7461392
BmHel-7	DF090472:304056-304311
BmHel-7	BABH01086489:87-342
BmHel-7	DF090451:12386-12641
BmHel-7	DF090341:382420-382675
BmHel-7	DF090356:3283137-3283392
BmHel-7	DF090414:902010-902265
BmHel-7	DF090326:5126102-5126357
BmHel-7	DF090343:2868164-2868419
BmHel-7	DF090340:2992744-2992999

BmHel-7	DF090318:4954247-4954502
BmHel-7	DF090338:2333021-2333276
BmHel-7	DF090324:3945688-3945943
BmHel-7	DF090391:572472-572727
BmHel-7	DF090425:876986-877241
BmHel-7	DF090524:89896-90151
BmHel-7	DF090400:984302-984557
BmHel-7	DF090368:2556095-2556350
BmHel-7	DF090388:970626-970882
BmHel-7	DF090323:6848007-6848263
BmHel-7	DF090351:3570990-3571246
BmHel-7	DF090398:671543-671799
BmHel-7	DF090363:185850-186106
BmHel-7	DF090317:8760668-8760924
BmHel-7	DF090403:164836-165092
BmHel-7	DF090367:1217814-1218070
BmHel-7	DF090367:655744-656000
BmHel-7	DF090336:1524015-1524271
BmHel-7	DF090329:4100050-4100306
BmHel-7	DF090357:3111796-3112052
BmHel-7	DF090345:939638-939894
BmHel-7	DF090330:5215738-5215994
BmHel-7	DF090317:9702880-9703136
BmHel-7	DF090335:4787294-4787550
BmHel-7	DF090438:542013-542269
BmHel-7	DF090382:2085027-2085283
BmHel-7	DF090466:212722-212978
BmHel-7	DF090336:2313043-2313300
BmHel-7	DF090316:7695197-7695454
BmHel-7	DF090361:212207-212464
BmHel-7	DF090321:1440216-1440473
BmHel-7	DF090446:426440-426697
BmHel-7	DF090490:78550-78807
BmHel-7	DF090344:3630856-3631113
BmHel-7	DF090342:2851879-2852136
BmHel-7	DF090336:3195276-3195533
BmHel-7	DF090336:492786-493043
BmHel-7	DF090325:3319730-3319987
BmHel-7	DF090322:4506656-4506913
BmHel-7	DF090347:3027354-3027611
BmHel-7	DF090358:2307429-2307686
BmHel-7	DF090413:131359-131616
BmHel-7	DF090446:416138-416395
BmHel-7	BABH01053727:392-649
BmHel-7	DF091099:640-897
BmHel-7	DF090360:2685591-2685848
BmHel-7	DF090393:87080-87338
BmHel-7	DF090332:952621-952879

BmHel-7	DF090317:10927593-10927851
BmHel-7	DF090400:1105736-1105994
BmHel-7	DF090336:4797882-4798141
BmHel-7	DF090316:11079892-11080151
BmHel-7	DF090325:304635-304894
BmHel-7	DF090322:1240641-1240900
BmHel-7	DF090357:853463-853722
BmHel-7	DF090395:863106-863365
BmHel-7	DF090366:117291-117550
BmHel-7	DF090911:7067-7326
BmHel-7	DF090319:6012117-6012376
BmHel-7	DF090326:5181337-5181596
BmHel-7	DF090433:602035-602294
BmHel-7	DF090438:437660-437919
BmHel-7	DF090735:17971-18230
BmHel-7	DF090475:234547-234806
BmHel-7	DF090319:4976178-4976438
BmHel-7	DF090335:286183-286443
BmHel-7	DF090391:1404394-1404654
BmHel-7	DF090320:2197009-2197269
BmHel-7	DF090360:2629722-2629982
BmHel-7	DF090332:2321735-2321995
BmHel-7	DF090395:171388-171648
BmHel-7	DF090317:9870136-9870396
BmHel-7	DF090488:168059-168319
BmHel-7	DF090318:847688-847949
BmHel-7	DF090435:198447-198708
BmHel-7	DF090328:4525273-4525534
BmHel-7	DF090317:8740189-8740450
BmHel-7	DF090318:9046480-9046741
BmHel-7	DF090326:6893585-6893847
BmHel-7	DF090388:422804-423066
BmHel-7	DF090389:5476-5738
BmHel-7	DF090358:252008-252270
BmHel-7	DF090368:694035-694297
BmHel-7	DF090332:5691923-5692185
BmHel-7	DF090397:1570906-1571168
BmHel-7	DF090755:4476-4738
BmHel-7	DF090941:1076-1339
BmHel-7	DF090330:1450425-1450688
BmHel-7	DF090316:284870-285133
BmHel-7	DF090319:7673959-7674223
BmHel-7	DF090328:5347192-5347456
BmHel-7	DF090318:6752007-6752271
BmHel-7	DF090330:4609247-4609512
BmHel-7	DF090323:1846291-1846557
BmHel-7	DF090472:96146-96412
BmHel-7	DF090328:1371348-1371616

BmHel-7	DF090317:2337259-2337528
BmHel-7	DF090468:321802-322071
BmHel-7	DF090319:3419358-3419627
BmHel-7	DF090323:4854928-4855199
BmHel-7	DF090347:3542669-3542940
BmHel-7	DF090323:1610049-1610321
BmHel-7	DF090332:6259939-6260212
BmHel-7	DF090389:435939-436212
BmHel-7	DF090355:1313900-1314174
BmHel-7	DF090319:4248454-4248728
BmHel-7	DF090374:2150234-2150508
BmHel-7	DF090320:3422646-3422920
BmHel-7	DF090378:2093925-2094199
BmHel-7	DF090328:2606875-2607149
BmHel-7	DF090349:168868-169143
BmHel-7	DF090410:887397-887673
BmHel-7	DF091203:5133-5413
BmHel-7	DF090522:7176-7456
BmHel-7	DF090429:334339-334621
BmHel-7	DF090385:1171669-1171952
BmHel-7	DF090768:22873-23156
BmHel-7	DF090373:1284274-1284558
BmHel-7	DF090344:3460741-3461025
BmHel-7	DF090362:582218-582506
BmHel-7	DF090361:2312584-2312872
BmHel-7	DF090317:2710804-2711094
BmHel-7	DF090400:960363-960654
BmHel-7	DF090328:5137221-5137514
BmHel-7	DF090362:844698-845005
BmHel-7	DF090332:5623195-5623505
BmHel-7	BABH01062724:133-456
BmHel-7	DF090316:4643744-4644068
BmHel-7	BABH01076569:77-401
BmHel-7	DF090330:3473708-3474035
BmHel-7	DF090317:10660548-10660875
BmHel-7	DF090388:1093496-1093824
BmHel-7	DF090446:392228-392563
BmHel-7	DF090323:1469701-1470038
BmHel-7	DF090991:9001-9341
BmHel-7	DF090344:1344072-1344415
BmHel-7	DF090321:849304-849661
BmHel-7	DF090352:2423491-2423854
BmHel-7	DF090373:28281-28647
BmHel-7	DF090406:1558565-1558931
BmHel-7	DF090319:3417314-3417684
BmHel-7	DF090328:1074868-1075240
BmHel-7	DF090320:2232385-2232760
BmHel-7	DF090318:2256449-2256825

BmHel-7	DF090355:1658013-1658389
BmHel-7	DF090357:1804185-1804563
BmHel-7	DF090441:549112-549490
BmHel-7	DF090384:1127495-1127875
BmHel-7	DF090390:1352863-1353248
BmHel-7	DF090423:120660-121047
BmHel-7	DF090316:1427109-1427498
BmHel-7	DF090324:4204265-4204662
BmHel-7	DF090388:1137651-1138051
BmHel-7	DF090378:1533228-1533629
BmHel-7	DF090345:1273489-1273893
BmHel-7	DF090382:1711467-1711872
BmHel-7	DF090381:1469596-1470013
BmHel-7	DF090371:730616-731038
BmHel-7	DF090376:508329-508752
BmHel-7	DF090339:2871780-2872219
BmHel-7	DF090359:2987912-2988356
BmHel-7	DF090324:1382593-1383043
BmHel-7	DF090368:604415-604875
BmHel-7	BABH01044974:3801-4263
BmHel-7	DF090334:4873370-4873834
BmHel-7	DF090483:195941-196413
BmHel-7	DF090378:818377-818858
BmHel-7	DF090404:1284509-1284993
BmHel-7	DF090318:7109730-7110225
BmHel-7	DF090352:4145381-4145878
BmHel-7	DF090371:320585-321082
BmHel-7	DF090323:6623884-6624383
BmHel-7	DF090340:1866704-1867204
BmHel-7	DF090340:1976933-1977437
BmHel-7	DF090374:1578336-1578842
BmHel-7	DF090386:1634842-1635349
BmHel-7	DF090316:9203259-9203769
BmHel-7	DF090323:1015203-1015713
BmHel-7	DF090328:3460518-3461030
BmHel-7	DF090411:490662-491174
BmHel-7	DF090320:7918069-7918605
BmHel-7	DF090343:2904091-2904641
BmHel-7	DF090401:513218-513779
BmHel-7	DF090388:1466980-1467544
BmHel-7	DF090354:2089873-2090442
BmHel-7	DF090349:1989200-1989772
BmHel-7	DF090343:1614731-1615313
BmHel-7	DF090436:653026-653617
BmHel-7	DF090340:3354187-3354780
BmHel-7	BABH01070669:29-622
BmHel-7	DF090381:886551-887144
BmHel-7	DF090338:4960061-4960654

BmHel-7	DF090680:4160-4760
BmHel-7	DF090358:1155350-1155953
BmHel-7	DF090367:2603570-2604175
BmHel-7	DF090316:16083955-16084576
BmHel-7	DF090340:3333726-3334348
BmHel-7	DF090318:2505973-2506607
BmHel-7	DF090404:508289-508940
BmHel-7	DF090356:1754339-1754992
BmHel-7	DF091311:1966-2619
BmHel-7	DF090375:495541-496210
BmHel-7	DF090327:6585163-6585841
BmHel-7	DF090334:4937636-4938319
BmHel-7	DF090367:969577-970267
BmHel-7	DF090356:1724260-1724954
BmHel-7	DF090334:2516016-2516721
BmHel-7	DF090382:1796469-1797182
BmHel-7	DF090324:23553-24280
BmHel-7	DF090391:936842-937580
BmHel-7	DF090371:1640143-1640921
BmHel-7	DF090316:5215611-5216422
BmHel-7	DF090346:3730825-3731656
BmHel-7	DF090363:2822506-2823350
BmHel-7	DF090340:1901266-1902441
BmHel-7	DF090328:484593-485852
BmHel-7	DF090326:6654619-6655883
BmHel-7	DF090322:1337263-1338528
BmHel-7	DF090395:136053-137438
BmHel-7	DF090391:1263093-1264541
BmHel-7	DF090317:327704-329167
BmHel-7	DF090330:1744790-1746339
BmHel-7	DF090316:12519336-12520912
BmHel-7	DF090342:3524452-3526064
BmHel-7	DF090413:1135174-1136788
BmHel-7	DF090323:6777287-6778950
BmHel-7	DF090347:456826-458714
BmHel-7	DF090320:1388153-1390055
BmHel-7	DF090318:6508849-6510779
BmHel-7	DF090422:328164-330540
BmHel-7	DF090358:969716-972537
BmHel-7	DF090342:1521659-1524754
BmHel-7	DF090586:12792-16003
BmHel-7	DF090357:312470-315711
BmHel-7	DF090347:367104-370766
BmHel-7	DF090331:969210-972970
BmHel-7	DF090414:286308-290133
BmHel-7	DF090317:7103724-7107872
BmHel-7	DF090323:5682989-5687590
BmHel-7	DF090364:2086141-2090883

BmHel-7	DF090337:4253750-4259026
BmHel-7	DF090411:643462-649469
BmHel-7	DF090322:8070956-8076999
BmHel-7	DF090322:7519991-7526188
BmHel-7	DF090347:1152553-1158976
BmHel-7	DF090369:2632947-2640362
BmHel-7	DF090331:3082334-3091408
BmHel-7	DF090367:1939042-1949140
BmHel-7	DF090342:1160910-1161159
BmHel-7	DF090342:2924824-2925073
BmHel-7	DF090355:1262853-1263106
BmHel-7	DF090349:2362442-2362691
BmHel-7	DF090341:1528392-1528641
BmHel-7	DF090344:1154264-1154512
BmHel-7	DF091335:3070-3319
BmHel-7	DF090367:1359182-1359433
BmHel-7	DF090319:2404525-2404768
BmHel-7	DF090326:5016957-5017204
BmHel-7	DF090326:4503606-4503855
BmHel-7	DF090316:8563316-8563565
BmHel-7	DF090316:11747306-11747555
BmHel-7	DF090316:10737236-10737485
BmHel-7	DF090316:2998466-2998715
BmHel-7	DF090390:744512-744761
BmHel-7	DF090377:452591-452839
BmHel-7	DF090343:2538257-2538507
BmHel-7	DF090343:2595947-2596193
BmHel-7	DF090340:4916637-4916884
BmHel-7	DF090323:568951-569199
BmHel-7	DF090322:2454478-2454727
BmHel-7	DF090322:2627080-2627329
BmHel-7	DF090322:8199266-8199515
BmHel-7	DF090322:3036587-3036838
BmHel-7	DF090322:2235128-2235377
BmHel-7	DF090372:346118-346367
BmHel-7	DF090372:1555411-1555658
BmHel-7	DF090329:1017016-1017265
BmHel-7	DF090329:5675144-5675394
BmHel-7	DF090351:1306485-1306734
BmHel-7	DF090352:1374806-1375055
BmHel-7	DF090320:4158651-4158899
BmHel-7	DF090320:5878008-5878258
BmHel-7	DF090320:6118223-6118470
BmHel-7	DF090394:1291121-1291367
BmHel-7	DF090333:4755295-4755543
BmHel-7	DF090318:73335-73588
BmHel-7	DF090318:9740162-9740411
BmHel-7	DF090360:2049282-2049531

BmHel-7	DF090353:1965624-1965873
BmHel-7	DF090353:3371086-3371334
BmHel-7	DF090353:3289985-3290238
BmHel-7	DF090357:1294261-1294510
BmHel-7	DF090332:3176165-3176413
BmHel-7	DF090419:536250-536499
BmHel-7	DF090418:806551-806800
BmHel-7	DF090376:1635415-1635653
BmHel-7	DF090366:834936-835179
BmHel-7	DF090358:862218-862467
BmHel-7	DF090345:1311918-1312164
BmHel-7	DF090317:1593241-1593489
BmHel-7	DF090420:771180-771431
BmHel-7	DF090365:1043186-1043435
BmHel-7	DF090365:190123-190371
BmHel-7	DF090634:28674-28923
BmHel-7	DF090386:1491952-1492200
BmHel-7	DF090385:726765-727012
BmHel-7	DF090362:3190681-3190930
BmHel-7	DF090362:3059525-3059774
BmHel-7	DF090324:5723857-5724105
BmHel-7	DF090427:437210-437459
BmHel-7	DF090467:96743-96994
BmHel-7	DF090413:715241-715493
BmHel-7	DF090432:420779-421028
BmHel-7	DF090379:1628860-1629109
BmHel-7	DF090410:777949-778198
BmHel-7	DF090387:1197299-1197548
BmHel-7	DF090770:21769-22018
BmHel-7	DF090461:201224-201470
BmHel-7	DF090504:28100-28349
BmHel-7	DF090439:342516-342765
BmHel-7	DF090518:11840-12089
BmHel-7	DF090447:113917-114166
BmHel-7	DF090602:21779-22028
BmHel-7	DF090682:8920-9172
BmHel-7	DF090355:1376350-1376605
BmHel-7	DF090355:2374237-2374489
BmHel-7	DF090355:1958238-1958487
BmHel-7	DF090354:1470960-1471206
BmHel-7	DF090354:2202564-2202808
BmHel-7	DF090319:6842592-6842841
BmHel-7	DF090371:1602143-1602392
BmHel-7	DF090343:1873664-1873913
BmHel-7	DF090328:518474-518721
BmHel-7	DF090351:4148382-4148631
BmHel-7	DF090347:3682546-3682795
BmHel-7	DF090320:73532-73780

BmHel-7	DF090320:780171-780420
BmHel-7	DF090350:2722666-2722919
BmHel-7	DF090318:8422371-8422619
BmHel-7	DF090339:1829388-1829634
BmHel-7	DF090363:2850459-2850708
BmHel-7	DF090361:2347719-2347967
BmHel-7	DF090361:793382-793636
BmHel-7	DF090357:971390-971638
BmHel-7	DF090357:3416412-3416661
BmHel-7	DF090330:1577255-1577504
BmHel-7	DF090330:5208382-5208631
BmHel-7	DF090421:868613-868864
BmHel-7	DF090335:5626496-5626740
BmHel-7	DF090375:921891-922143
BmHel-7	DF090374:864034-864283
BmHel-7	DF090410:1120442-1120690
BmHel-7	DF090459:401014-401263
BmHel-7	DF090490:129207-129449
BmHel-7	DF090412:1203677-1203935
BmHel-7	DF090473:84626-84876
BmHel-7	DF090543:57296-57551
BmHel-7	DF090364:86151-86288
BmHel-7	DF090364:86022-86128
BmHel-7	DF090323:3762825-3763006
BmHel-7	DF090349:4030633-4030877
BmHel-7	DF090347:1715560-1715807
BmHel-7	DF090456:58851-59099
BmHel-7	DF090345:3874997-3875245
BmHel-7	DF091136:736-984
BmHel-7	DF090342:138482-138730
BmHel-7	DF090342:1583515-1583763
BmHel-7	DF090341:577608-577858
BmHel-7	DF090344:2097302-2097551
BmHel-7	DF090367:1615586-1615832
BmHel-7	DF090390:1520302-1520550
BmHel-7	DF090364:1958658-1958906
BmHel-7	DF090340:2980251-2980499
BmHel-7	DF090328:425217-425469
BmHel-7	DF090352:4076854-4077102
BmHel-7	DF090394:823075-823329
BmHel-7	DF090350:3170331-3170579
BmHel-7	DF090327:5608210-5608458
BmHel-7	DF090327:5394271-5394511
BmHel-7	DF090318:1153097-1153345
BmHel-7	DF090373:1552871-1553115
BmHel-7	DF090373:1975884-1976132
BmHel-7	DF090395:1004405-1004658
BmHel-7	DF090366:521844-522092

BmHel-7	DF090345:3804506-3804754
BmHel-7	DF090330:1971950-1972195
BmHel-7	DF090317:10470566-10470814
BmHel-7	DF090420:477230-477478
BmHel-7	DF090365:441517-441769
BmHel-7	DF090335:1105187-1105435
BmHel-7	DF090321:4422699-4422948
BmHel-7	DF090321:3584126-3584378
BmHel-7	DF090321:654472-654720
BmHel-7	DF090381:1273196-1273444
BmHel-7	DF090331:5155393-5155638
BmHel-7	DF090324:479968-480214
BmHel-7	DF090427:611900-612146
BmHel-7	DF090511:10446-10694
BmHel-7	DF090467:153962-154210
BmHel-7	DF090417:182344-182591
BmHel-7	DF090387:1518821-1519067
BmHel-7	DF090382:1519731-1519977
BmHel-7	DF090359:146279-146527
BmHel-7	DF090443:271545-271793
BmHel-7	DF090443:251432-251681
BmHel-7	DF090780:6979-7226
BmHel-7	DF090488:54548-54796
BmHel-7	DF090370:821961-822209
BmHel-7	DF090397:1224683-1224931
BmHel-7	DF090430:26011-26270
BmHel-7	DF090452:488010-488252
BmHel-7	DF090597:41508-41755
BmHel-7	DF090440:201023-201273
BmHel-7	DF090342:445667-445915
BmHel-7	DF090356:1864850-1865098
BmHel-7	DF090341:2276168-2276421
BmHel-7	DF090341:3287425-3287672
BmHel-7	DF090344:2492422-2492667
BmHel-7	DF090344:2823605-2823852
BmHel-7	DF090344:1955786-1956025
BmHel-7	DF090344:436190-436438
BmHel-7	DF090367:1097723-1097970
BmHel-7	DF090336:312445-312682
BmHel-7	DF090336:1754354-1754594
BmHel-7	DF090319:4423316-4423563
BmHel-7	DF090319:4721719-4721967
BmHel-7	DF090319:5334563-5334814
BmHel-7	DF090319:373503-373748
BmHel-7	DF090319:186657-186893
BmHel-7	DF090326:1325878-1326113
BmHel-7	DF090316:358138-358386
BmHel-7	DF090316:457530-457770

BmHel-7	DF090316:15368680-15368928
BmHel-7	DF090316:4792903-4793151
BmHel-7	DF090470:203934-204180
BmHel-7	DF090388:271624-271868
BmHel-7	DF090388:444920-445168
BmHel-7	DF090371:659928-660176
BmHel-7	DF090346:834458-834706
BmHel-7	DF090343:4700236-4700484
BmHel-7	DF090340:3668498-3668746
BmHel-7	DF090340:4255412-4255667
BmHel-7	DF090334:2409601-2409849
BmHel-7	DF090334:3165150-3165398
BmHel-7	DF090325:2358290-2358539
BmHel-7	DF090323:5649406-5649654
BmHel-7	DF090322:649933-650182
BmHel-7	DF090329:2313974-2314221
BmHel-7	DF090329:5123875-5124122
BmHel-7	DF090329:559596-559846
BmHel-7	DF090347:2828547-2828794
BmHel-7	DF090320:1267387-1267635
BmHel-7	DF090350:3257394-3257642
BmHel-7	DF090350:1569003-1569251
BmHel-7	DF090337:1184563-1184811
BmHel-7	DF090327:1646202-1646450
BmHel-7	DF090327:2285116-2285364
BmHel-7	DF090327:731082-731330
BmHel-7	DF090318:8156138-8156373
BmHel-7	DF090318:9103434-9103682
BmHel-7	DF090318:4769030-4769276
BmHel-7	DF090407:924306-924555
BmHel-7	DF090407:868372-868617
BmHel-7	DF090360:1331810-1332058
BmHel-7	DF090339:3513041-3513286
BmHel-7	DF090353:3733095-3733343
BmHel-7	DF090411:776585-776833
BmHel-7	DF090411:1130162-1130409
BmHel-7	DF090411:594480-594723
BmHel-7	DF090373:705751-705998
BmHel-7	DF090363:1291738-1291989
BmHel-7	DF090361:2446903-2447151
BmHel-7	DF090357:1608356-1608604
BmHel-7	DF090332:732205-732453
BmHel-7	DF090332:938954-939203
BmHel-7	DF090332:1118610-1118856
BmHel-7	DF090422:528807-529053
BmHel-7	DF090419:762529-762779
BmHel-7	DF090399:1165999-1166241
BmHel-7	DF090376:2055083-2055327

BmHel-7	DF090358:3027636-3027884
BmHel-7	DF090348:2446685-2446933
BmHel-7	DF090345:3667679-3667926
BmHel-7	DF090330:3324544-3324792
BmHel-7	DF090330:4367078-4367326
BmHel-7	DF090330:6423404-6423652
BmHel-7	DF090317:7189044-7189292
BmHel-7	DF090317:10861209-10861462
BmHel-7	DF090317:9179828-9180076
BmHel-7	DF090335:2733637-2733885
BmHel-7	DF090321:860283-860531
BmHel-7	DF090321:708274-708522
BmHel-7	DF090861:12205-12453
BmHel-7	DF090338:2420217-2420465
BmHel-7	DF090338:3487890-3488142
BmHel-7	DF090438:471639-471888
BmHel-7	DF090429:720229-720477
BmHel-7	DF090392:453100-453347
BmHel-7	DF090374:997384-997631
BmHel-7	DF090368:1151051-1151290
BmHel-7	DF090324:2664726-2664973
BmHel-7	DF090324:463685-463927
BmHel-7	DF090393:1281773-1282021
BmHel-7	DF090401:1462921-1463169
BmHel-7	DF090391:1445796-1446045
BmHel-7	DF090415:994633-994879
BmHel-7	DF090735:25287-25535
BmHel-7	DF090382:2088468-2088715
BmHel-7	BABH01061172:96-344
BmHel-7	DF090370:1502319-1502567
BmHel-7	DF091008:5018-5266
BmHel-7	DF090397:529772-530020
BmHel-7	DF090833:5065-5313
BmHel-7	BABH01056555:167-414
BmHel-7	DF090452:14766-15008
BmHel-7	DF091526:2350-2597
BmHel-7	BABH01088036:24-271
BmHel-7	DF091434:866-1112
BmHel-7	DF090535:5419-5665
BmHel-7	DF090647:9354-9594
BmHel-7	DF090337:882650-882850
BmHel-7	DF090316:6884808-6885055
BmHel-7	DF090384:1146356-1146602
BmHel-7	DF090332:5336827-5337067
BmHel-7	DF090399:276718-276953
BmHel-7	DF090585:57931-58180
BmHel-7	DF090354:1396447-1396699
BmHel-7	DF090336:4343544-4343791

BmHel-7	DF090319:4152842-4153090
BmHel-7	DF090326:6338046-6338292
BmHel-7	DF090326:3079371-3079622
BmHel-7	DF090316:11921773-11922012
BmHel-7	DF090343:4559585-4559844
BmHel-7	DF090322:1838045-1838292
BmHel-7	DF090347:1124889-1125143
BmHel-7	DF090320:3743186-3743433
BmHel-7	DF090318:2779103-2779350
BmHel-7	DF090357:3759352-3759599
BmHel-7	DF090332:2474036-2474283
BmHel-7	DF090332:5808691-5808936
BmHel-7	DF090335:2578728-2578975
BmHel-7	DF090434:11870-12116
BmHel-7	DF090434:501708-501955
BmHel-7	DF090362:2509847-2510094
BmHel-7	DF090401:170541-170788
BmHel-7	DF090387:1148299-1148546
BmHel-7	DF090446:79131-79376
BmHel-7	DF090412:955048-955299
BmHel-7	BABH01047377:804-1058
BmHel-7	DF090404:1340011-1340258
BmHel-7	DF090340:2434272-2434517
BmHel-7	DF090325:5426258-5426505
BmHel-7	DF090325:1234190-1234378
BmHel-7	DF090394:1428642-1428890
BmHel-7	DF090339:2272847-2273028
BmHel-7	DF090400:796973-797174
BmHel-7	DF090396:496257-496504
BmHel-7	DF090359:1314191-1314438
BmHel-7	DF090342:1919186-1919434
BmHel-7	DF090344:184665-184913
BmHel-7	DF090344:818328-818575
BmHel-7	DF090367:1666059-1666305
BmHel-7	DF090336:2448195-2448442
BmHel-7	DF090319:6054647-6054893
BmHel-7	DF090319:3276840-3277079
BmHel-7	DF090316:6033885-6034132
BmHel-7	DF090320:1120473-1120719
BmHel-7	DF090394:1039373-1039620
BmHel-7	DF090350:2113177-2113424
BmHel-7	DF090350:2947399-2947639
BmHel-7	DF090350:2995442-2995689
BmHel-7	DF090333:2596196-2596443
BmHel-7	DF090327:6058457-6058702
BmHel-7	DF090318:2562538-2562785
BmHel-7	DF090389:1630370-1630613
BmHel-7	DF090411:281982-282229

BmHel-7	DF090363:18259-18506
BmHel-7	DF090357:3579528-3579768
BmHel-7	DF090422:287740-287987
BmHel-7	DF090317:8916957-8917206
BmHel-7	DF090335:5162210-5162457
BmHel-7	DF090438:184731-184978
BmHel-7	DF090386:1876491-1876727
BmHel-7	DF090374:1732932-1733186
BmHel-7	DF090331:1864062-1864309
BmHel-7	DF090331:5818592-5818837
BmHel-7	DF090401:1333029-1333276
BmHel-7	DF090379:371733-371980
BmHel-7	DF090459:156663-156910
BmHel-7	DF090657:22698-22946
BmHel-7	DF090439:525338-525585
BmHel-7	BABH01045485:1384-1632
BmHel-7	DF090477:242232-242478
BmHel-7	DF090451:436596-436842
BmHel-7	DF090472:60697-60898
BmHel-7	DF090339:856903-857153
BmHel-7	DF090330:6154276-6154522
BmHel-7	DF090344:2930008-2930254
BmHel-7	DF090316:11461701-11461945
BmHel-7	DF090325:2532689-2532934
BmHel-7	DF090320:1817195-1817441
BmHel-7	DF090320:1616781-1617026
BmHel-7	DF090363:54619-54869
BmHel-7	DF090357:3367119-3367364
BmHel-7	DF090441:537611-537856
BmHel-7	DF090376:766109-766362
BmHel-7	DF090317:406593-406839
BmHel-7	DF090335:3245868-3245970
BmHel-7	DF090335:3245713-3245854
BmHel-7	DF090362:1482384-1482626
BmHel-7	DF090425:531807-532053
BmHel-7	DF090486:55567-55813
BmHel-7	DF090342:1755826-1755930
BmHel-7	DF090342:1756191-1756336
BmHel-7	DF090367:921099-921345
BmHel-7	DF090329:862431-862677
BmHel-7	DF090365:2594675-2594921
BmHel-7	DF090365:1204103-1204210
BmHel-7	DF090365:1203616-1203751
BmHel-7	DF090321:3932612-3932857
BmHel-7	DF090383:641544-641791
BmHel-7	DF090439:104321-104523
BmHel-7	BABH01055686:378-624
BmHel-7	BABH01050810:442-688

BmHel-7	DF090495:121510-121756
BmHel-7	DF090351:3776282-3776528
BmHel-7	DF090342:2987148-2987394
BmHel-7	DF090342:3457284-3457530
BmHel-7	DF090342:3697282-3697528
BmHel-7	DF090349:2383767-2384013
BmHel-7	DF090349:3385665-3385914
BmHel-7	DF090341:1389186-1389432
BmHel-7	DF090341:4816073-4816319
BmHel-7	DF090405:975006-975252
BmHel-7	DF090354:2579207-2579453
BmHel-7	DF090354:579467-579713
BmHel-7	DF090319:2215411-2215657
BmHel-7	DF090326:2052756-2053002
BmHel-7	DF090316:12350943-12351188
BmHel-7	DF090402:1032329-1032575
BmHel-7	DF090377:434960-435211
BmHel-7	DF090343:4379444-4379685
BmHel-7	DF090328:5882287-5882533
BmHel-7	DF090325:3452203-3452447
BmHel-7	DF090323:4687651-4687898
BmHel-7	DF090322:5955435-5955677
BmHel-7	DF090351:861236-861474
BmHel-7	DF090320:4668247-4668492
BmHel-7	DF090327:3650745-3650982
BmHel-7	DF090318:9506614-9506858
BmHel-7	DF090407:341275-341521
BmHel-7	DF090353:401749-401995
BmHel-7	DF090373:1961743-1961992
BmHel-7	DF090418:120172-120418
BmHel-7	DF090418:106360-106609
BmHel-7	DF090376:194336-194580
BmHel-7	DF090366:1260810-1261058
BmHel-7	DF090358:1888194-1888441
BmHel-7	DF090348:3396262-3396507
BmHel-7	DF090330:635383-635628
BmHel-7	DF090330:1184764-1185010
BmHel-7	DF090330:5267263-5267509
BmHel-7	DF090317:10017497-10017743
BmHel-7	DF090317:4091781-4092027
BmHel-7	DF090420:161723-161969
BmHel-7	DF090365:682302-682541
BmHel-7	DF090321:7778259-7778504
BmHel-7	DF090321:7309107-7309354
BmHel-7	DF090338:1203681-1203922
BmHel-7	DF090424:928841-929087
BmHel-7	DF090386:2028947-2029196
BmHel-7	DF090362:2313777-2314023

BmHel-7	DF090501:84575-84821
BmHel-7	DF090379:1379080-1379331
BmHel-7	DF090425:894414-894659
BmHel-7	DF090450:109569-109815
BmHel-7	DF090382:1281071-1281314
BmHel-7	DF090359:2383506-2383752
BmHel-7	DF090764:3083-3329
BmHel-7	BABH01043665:2297-2543
BmHel-7	DF090510:94809-95055
BmHel-7	BABH01045313:2568-2668
BmHel-7	BABH01045313:2722-2867
BmHel-7	DF090327:4100369-4100531
BmHel-7	DF090327:4100542-4100624
BmHel-7	DF090440:517621-517812
BmHel-7	DF090580:46258-46415
BmHel-7	DF090580:45823-45917
BmHel-7	DF090344:2166115-2166312
BmHel-7	DF090354:674023-674270
BmHel-7	DF090336:3356057-3356303
BmHel-7	DF090351:3418658-3418905
BmHel-7	DF090358:1413318-1413561
BmHel-7	DF090321:2765455-2765695
BmHel-7	DF090412:1251825-1252067
BmHel-7	DF090344:1656760-1656998
BmHel-7	DF090326:344873-345118
BmHel-7	DF090328:4287961-4288200
BmHel-7	DF090325:3646001-3646250
BmHel-7	DF090322:4630435-4630683
BmHel-7	DF090351:3454301-3454540
BmHel-7	DF090347:295126-295366
BmHel-7	DF090352:2256314-2256559
BmHel-7	DF090384:884036-884281
BmHel-7	DF090384:826015-826260
BmHel-7	DF090398:1365984-1366213
BmHel-7	DF090363:748283-748529
BmHel-7	DF090357:164949-165194
BmHel-7	DF090358:781330-781574
BmHel-7	DF090417:946269-946515
BmHel-7	DF090501:14236-14483
BmHel-7	DF090379:2032983-2033229
BmHel-7	DF091129:3356-3601
BmHel-7	DF090465:76297-76539
BmHel-7	DF090318:4284938-4285184
BmHel-7	DF090353:1839134-1839379
BmHel-7	DF090375:1279485-1279732
BmHel-7	DF090427:527668-527913
BmHel-7	DF090415:591763-592006
BmHel-7	DF090337:3623751-3623996

BmHel-7	DF090456:320374-320619
BmHel-7	DF090358:1254320-1254564
BmHel-7	DF090368:2289631-2289875
BmHel-7	DF090417:308936-309135
BmHel-7	DF090425:479349-479600
BmHel-7	DF090382:1860044-1860295
BmHel-7	DF090356:1904031-1904276
BmHel-7	DF090344:2200701-2200946
BmHel-7	DF090344:4044988-4045228
BmHel-7	DF090336:4060256-4060501
BmHel-7	DF090319:4881222-4881471
BmHel-7	DF090319:5912836-5913079
BmHel-7	DF090326:1296903-1297145
BmHel-7	DF090316:6732248-6732496
BmHel-7	DF090343:180555-180795
BmHel-7	DF090328:5414688-5414926
BmHel-7	DF090323:5977889-5978134
BmHel-7	DF090320:112994-113238
BmHel-7	DF090320:7041758-7042002
BmHel-7	DF090327:4144342-4144587
BmHel-7	DF090318:6804734-6804979
BmHel-7	DF090318:6608391-6608636
BmHel-7	DF090318:4153758-4154003
BmHel-7	DF090358:1867225-1867470
BmHel-7	DF090335:4968050-4968295
BmHel-7	DF090321:5485761-5486011
BmHel-7	DF090321:6883519-6883764
BmHel-7	DF090452:348105-348350
BmHel-7	DF090334:812563-812684
BmHel-7	DF090334:812428-812550
BmHel-7	DF090323:4982857-4983105
BmHel-7	DF090337:1800330-1800581
BmHel-7	DF090327:612963-613207
BmHel-7	DF090373:1190342-1190586
BmHel-7	DF090317:8854566-8854810
BmHel-7	DF090367:1601184-1601424
BmHel-7	DF090354:1695155-1695398
BmHel-7	DF090336:3590020-3590264
BmHel-7	DF090364:2198547-2198791
BmHel-7	DF090346:1275332-1275576
BmHel-7	DF090343:3041136-3041380
BmHel-7	DF090340:2694223-2694468
BmHel-7	DF090340:3626448-3626691
BmHel-7	DF090372:878436-878680
BmHel-7	DF090320:6195752-6195996
BmHel-7	DF090320:984250-984495
BmHel-7	DF090333:3934626-3934870
BmHel-7	DF090360:1885118-1885357

BmHel-7	DF090339:4156428-4156674
BmHel-7	DF090411:748096-748340
BmHel-7	DF090361:2877487-2877731
BmHel-7	DF090317:339239-339483
BmHel-7	DF090335:4032635-4032879
BmHel-7	DF090335:3154684-3154920
BmHel-7	DF090368:1589809-1590053
BmHel-7	DF090368:1700479-1700723
BmHel-7	DF090331:2909035-2909279
BmHel-7	DF090379:1393254-1393498
BmHel-7	DF090379:1014346-1014591
BmHel-7	DF090431:112021-112264
BmHel-7	DF091148:4270-4518
BmHel-7	DF090609:14586-14829
BmHel-7	DF090494:79132-79376
BmHel-7	DF091426:3026-3270
BmHel-7	DF090323:542147-542235
BmHel-7	DF090323:543453-543616
BmHel-7	DF090363:2600495-2600739
BmHel-7	DF090645:32050-32291
BmHel-7	DF090319:256638-256838
BmHel-7	DF090323:1761435-1761528
BmHel-7	DF090323:1761540-1761697
BmHel-7	DF090355:3773341-3773593
BmHel-7	DF090350:2295026-2295269
BmHel-7	DF090335:1205562-1205811
BmHel-7	DF090460:356906-357150
BmHel-7	DF090349:2409484-2409738
BmHel-7	DF090319:8534303-8534543
BmHel-7	DF090366:1216863-1217105
BmHel-7	DF090367:1264552-1264795
BmHel-7	DF090334:1975781-1976025
BmHel-7	DF090328:3620773-3621016
BmHel-7	DF090389:871842-872086
BmHel-7	DF090332:4716627-4716871
BmHel-7	BABH01043095:13836-14075
BmHel-7	DF090322:7151220-7151345
BmHel-7	DF090322:7151363-7151489
BmHel-7	DF090444:597068-597226
BmHel-7	DF090444:597428-597522
BmHel-7	DF090336:846390-846633
BmHel-7	DF090353:2979412-2979655
BmHel-7	DF090387:716978-717221
BmHel-7	DF090326:2604039-2604286
BmHel-7	DF090316:3216221-3216464
BmHel-7	DF090402:1145670-1145915
BmHel-7	DF090377:735632-735874
BmHel-7	DF090371:895408-895650

BmHel-7	DF090325:2980800-2981042
BmHel-7	DF090372:2303958-2304200
BmHel-7	DF090318:1187927-1188168
BmHel-7	DF090318:4472012-4472247
BmHel-7	DF090339:3117020-3117263
BmHel-7	DF090357:1281834-1282073
BmHel-7	DF090418:298081-298321
BmHel-7	DF090376:922711-922954
BmHel-7	DF090345:744651-744888
BmHel-7	DF090345:1285901-1286137
BmHel-7	DF090330:4765589-4765832
BmHel-7	DF090338:1161585-1161833
BmHel-7	DF090331:6231676-6231919
BmHel-7	DF090331:1627923-1628166
BmHel-7	DF090468:243405-243648
BmHel-7	DF090382:591585-591828
BmHel-7	DF090714:3159-3402
BmHel-7	DF090697:24874-25113
BmHel-7	DF090556:25668-25914
BmHel-7	DF090348:2470428-2470671
BmHel-7	DF090363:1120904-1121146
BmHel-7	DF090428:412425-412672
BmHel-7	DF090476:209570-209813
BmHel-7	BABH01087051:203-446
BmHel-7	DF090340:3583227-3583345
BmHel-7	DF090340:3583362-3583490
BmHel-7	DF090333:5027912-5028154
BmHel-7	DF090365:2135323-2135531
BmHel-7	DF090321:4381890-4382055
BmHel-7	BABH01043917:8223-8466
BmHel-7	DF090342:4276398-4276641
BmHel-7	DF090344:1812934-1813143
BmHel-7	DF090323:128576-128824
BmHel-7	DF090389:1354642-1354888
BmHel-7	DF090342:42606-42842
BmHel-7	DF090342:2572064-2572307
BmHel-7	DF090342:1904295-1904534
BmHel-7	DF090356:1406468-1406708
BmHel-7	DF090349:4193724-4193967
BmHel-7	DF090341:1278646-1278889
BmHel-7	DF090344:2184632-2184875
BmHel-7	DF090354:2830053-2830296
BmHel-7	DF090336:2597142-2597385
BmHel-7	DF090336:1425202-1425444
BmHel-7	DF090319:3150859-3151102
BmHel-7	DF090319:5612252-5612497
BmHel-7	DF090319:2887627-2887870
BmHel-7	DF090326:1846735-1846978

BmHel-7	DF090326:4296969-4297215
BmHel-7	DF090316:12265939-12266183
BmHel-7	DF090316:14929124-14929366
BmHel-7	DF090390:1163163-1163406
BmHel-7	DF090402:1425650-1425890
BmHel-7	DF090364:76501-76744
BmHel-7	DF090343:475901-476145
BmHel-7	DF090343:4244099-4244337
BmHel-7	DF090343:2383649-2383892
BmHel-7	DF090334:4375347-4375584
BmHel-7	DF090328:3025629-3025872
BmHel-7	DF090323:501913-502151
BmHel-7	DF090323:3832474-3832717
BmHel-7	DF090322:1540556-1540798
BmHel-7	DF090352:2396519-2396760
BmHel-7	DF090320:5893095-5893190
BmHel-7	DF090320:5893331-5893478
BmHel-7	DF090320:1575460-1575703
BmHel-7	DF090327:232697-232940
BmHel-7	DF090327:2820451-2820694
BmHel-7	DF090327:4604844-4605087
BmHel-7	DF090327:1439005-1439248
BmHel-7	DF090318:5963782-5964021
BmHel-7	DF090318:9835391-9835634
BmHel-7	DF090318:3927405-3927649
BmHel-7	DF090389:717366-717609
BmHel-7	DF090339:2923176-2923419
BmHel-7	DF090339:513612-513854
BmHel-7	DF090363:1901219-1901462
BmHel-7	DF090363:1088131-1088373
BmHel-7	DF090361:2646855-2647098
BmHel-7	DF090361:285698-285938
BmHel-7	DF090332:206844-207082
BmHel-7	DF090332:995017-995262
BmHel-7	DF090416:950725-950968
BmHel-7	DF090366:28537-28779
BmHel-7	DF090358:477706-477950
BmHel-7	DF090358:2423030-2423271
BmHel-7	DF090358:2499016-2499260
BmHel-7	DF090330:4596803-4597046
BmHel-7	DF090420:310137-310380
BmHel-7	DF090365:26170-26413
BmHel-7	DF090365:1010265-1010507
BmHel-7	DF090335:5450910-5451158
BmHel-7	DF090338:4320963-4321196
BmHel-7	DF090438:257505-257750
BmHel-7	DF090362:2567780-2568020
BmHel-7	DF090362:777128-777371

BmHel-7	DF090413:577238-577481
BmHel-7	DF090391:177636-177874
BmHel-7	DF090369:1176884-1177127
BmHel-7	DF090754:1560-1803
BmHel-7	DF090437:390643-390882
BmHel-7	DF090431:168021-168263
BmHel-7	DF090370:1282415-1282657
BmHel-7	DF090412:575957-576194
BmHel-7	DF090455:260151-260398
BmHel-7	BABH01071865:1-244
BmHel-7	DF090451:419347-419590
BmHel-7	BABH01078539:14-257
BmHel-7	BABH01048984:444-686
BmHel-7	DF091141:424-667
BmHel-7	DF090349:1888220-1888352
BmHel-7	DF090349:1887852-1887973
BmHel-7	DF090371:1237947-1238190
BmHel-7	DF090328:6634840-6635079
BmHel-7	DF090323:7628742-7628987
BmHel-7	DF090353:2117991-2118235
BmHel-7	DF090342:1623935-1624174
BmHel-7	DF090316:6851592-6851832
BmHel-7	DF090328:2702984-2703214
BmHel-7	DF090323:3343969-3344211
BmHel-7	DF090329:3662343-3662585
BmHel-7	DF090337:3683899-3684134
BmHel-7	DF090318:6197691-6197933
BmHel-7	DF090358:1915622-1915864
BmHel-7	DF090358:2261988-2262227
BmHel-7	DF090365:999447-999689
BmHel-7	DF090368:2746286-2746527
BmHel-7	DF090426:575533-575757
BmHel-7	DF090319:3934025-3934227
BmHel-7	DF090374:1442578-1442755
BmHel-7	DF090395:357813-357998
BmHel-7	DF090449:61740-61931
BmHel-7	DF090333:3588771-3589009
BmHel-7	DF090373:890341-890588
BmHel-7	DF090338:3697807-3697927
BmHel-7	DF090338:3697199-3697322
BmHel-7	DF090342:1988443-1988685
BmHel-7	DF090344:1860725-1860967
BmHel-7	DF090344:3179510-3179752
BmHel-7	DF090319:3625734-3625976
BmHel-7	DF090326:645615-645857
BmHel-7	DF090316:1602349-1602591
BmHel-7	DF090346:271418-271665
BmHel-7	DF090323:813484-813726

BmHel-7	DF090329:3619242-3619481
BmHel-7	DF090329:5878206-5878444
BmHel-7	DF090360:725500-725741
BmHel-7	DF090353:105803-106045
BmHel-7	DF090317:1952272-1952514
BmHel-7	DF090400:1400039-1400281
BmHel-7	DF090321:3727410-3727652
BmHel-7	DF090375:507335-507577
BmHel-7	DF090374:1471592-1471834
BmHel-7	DF090393:1227714-1227959
BmHel-7	DF090453:285698-285938
BmHel-7	BABH01044028:1609-1852
BmHel-7	DF090356:998044-998173
BmHel-7	DF090356:997917-998036
BmHel-7	DF090353:2086006-2086137
BmHel-7	DF090353:2086545-2086661
BmHel-7	DF090326:4859243-4859476
BmHel-7	DF090343:4674777-4675020
BmHel-7	DF090378:1118130-1118374
BmHel-7	DF090411:463389-463631
BmHel-7	DF090332:1765003-1765242
BmHel-7	DF090388:194878-195010
BmHel-7	DF090388:195446-195566
BmHel-7	DF090317:2522006-2522240
BmHel-7	DF090417:1031335-1031417
BmHel-7	DF090417:1032043-1032188
BmHel-7	DF090342:204793-205034
BmHel-7	DF090361:1541424-1541638
BmHel-7	DF090355:2405383-2405624
BmHel-7	DF090340:2844317-2844555
BmHel-7	DF090345:700106-700351
BmHel-7	DF090365:2903794-2904034
BmHel-7	DF090338:1402132-1402373
BmHel-7	DF090397:1129656-1129857
BmHel-7	DF090656:21750-21989
BmHel-7	DF090355:2937460-2937701
BmHel-7	DF090349:1404833-1405075
BmHel-7	DF090405:1189362-1189603
BmHel-7	DF090336:3722521-3722761
BmHel-7	DF090316:6995117-6995358
BmHel-7	DF090388:877019-877262
BmHel-7	DF090322:765959-766206
BmHel-7	DF090329:3463701-3463942
BmHel-7	DF090351:1347982-1348223
BmHel-7	DF090320:1470067-1470307
BmHel-7	DF090320:6220180-6220421
BmHel-7	DF090394:625010-625256
BmHel-7	DF090407:695058-695301

BmHel-7	DF090353:3388466-3388715
BmHel-7	DF090361:1953533-1953765
BmHel-7	DF090332:4443126-4443357
BmHel-7	DF090332:6250076-6250317
BmHel-7	DF090422:57270-57506
BmHel-7	DF090419:242219-242460
BmHel-7	DF090345:560693-560934
BmHel-7	DF090335:4925497-4925738
BmHel-7	DF090381:449156-449397
BmHel-7	DF090338:1886367-1886610
BmHel-7	DF090386:15412-15653
BmHel-7	DF090374:912887-913123
BmHel-7	DF090324:5661380-5661621
BmHel-7	DF090613:838-1079
BmHel-7	DF090538:46513-46754
BmHel-7	DF090393:1097752-1097999
BmHel-7	DF090391:489801-490040
BmHel-7	DF090410:1076588-1076831
BmHel-7	DF090443:373924-374165
BmHel-7	DF090455:174985-175226
BmHel-7	DF090428:440222-440464
BmHel-7	DF090810:17152-17393
BmHel-7	DF090444:101110-101350
BmHel-7	DF090471:223829-224065
BmHel-7	DF090448:49514-49748
BmHel-7	DF090760:3470-3662
BmHel-7	DF090417:860437-860677
BmHel-7	DF090367:1442699-1442944
BmHel-7	DF090361:2145092-2145332
BmHel-7	DF090330:2305061-2305302
BmHel-7	DF090321:2146866-2147106
BmHel-7	DF090383:867917-868158
BmHel-7	DF090359:749666-749907
BmHel-7	DF090451:408968-409208
BmHel-7	DF090395:159373-159614
BmHel-7	DF090407:165892-166132
BmHel-7	DF090385:1887654-1887887
BmHel-7	DF090425:752980-753073
BmHel-7	DF090425:753068-753214
BmHel-7	DF090330:1682578-1682676
BmHel-7	DF090330:1682222-1682374
BmHel-7	DF090797:1468-1708
BmHel-7	DF090349:4287132-4287374
BmHel-7	DF090316:438478-438688
BmHel-7	DF090330:5347138-5347378
BmHel-7	DF090434:421916-422157
BmHel-7	DF090426:454181-454421
BmHel-7	DF090350:2958518-2958757

BmHel-7	DF090368:200386-200622
BmHel-7	DF090349:1382519-1382760
BmHel-7	DF090405:445521-445761
BmHel-7	DF090344:4152251-4152491
BmHel-7	DF090414:574642-574880
BmHel-7	DF090354:1535046-1535280
BmHel-7	DF090319:1131022-1131262
BmHel-7	DF090316:4682858-4683098
BmHel-7	DF090340:2508688-2508928
BmHel-7	DF090340:3672912-3673152
BmHel-7	DF090325:2912089-2912329
BmHel-7	DF090323:5740044-5740283
BmHel-7	DF090323:4795833-4796070
BmHel-7	DF090323:511602-511842
BmHel-7	DF090329:4010712-4010952
BmHel-7	DF090351:2451687-2451927
BmHel-7	DF090352:2548270-2548510
BmHel-7	DF090352:3318885-3319125
BmHel-7	DF090394:84414-84654
BmHel-7	DF090350:1906877-1907117
BmHel-7	DF090350:976191-976431
BmHel-7	DF090407:696245-696483
BmHel-7	DF090339:788915-789154
BmHel-7	DF090353:703583-703823
BmHel-7	DF090353:2830961-2831201
BmHel-7	DF090398:1544159-1544399
BmHel-7	DF090332:4007760-4008000
BmHel-7	DF090317:3190059-3190305
BmHel-7	DF090335:3961161-3961400
BmHel-7	DF090338:3128138-3128378
BmHel-7	DF090435:177430-177670
BmHel-7	DF090324:2537254-2537494
BmHel-7	DF090401:808863-809103
BmHel-7	DF090415:354261-354501
BmHel-7	DF090382:1063700-1063940
BmHel-7	DF090397:1080410-1080647
BmHel-7	BABH01046158:1130-1364
BmHel-7	BABH01047169:289-383
BmHel-7	BABH01047169:401-548
BmHel-7	DF090391:239274-239509
BmHel-7	DF090384:1279225-1279464
BmHel-7	DF090324:4628509-4628747
BmHel-7	DF090317:8882978-8883162
BmHel-7	DF090342:1004751-1004983
BmHel-7	DF090335:5267510-5267748
BmHel-7	DF090316:1010355-1010591
BmHel-7	DF090405:939820-940059
BmHel-7	DF090414:1200369-1200608

BmHel-7	DF090343:2443383-2443471
BmHel-7	DF090343:2443590-2443740
BmHel-7	DF090335:1046775-1047014
BmHel-7	DF090344:2788425-2788659
BmHel-7	DF090316:12018217-12018456
BmHel-7	DF090316:12093330-12093569
BmHel-7	DF090346:3618824-3619022
BmHel-7	DF090332:4928509-4928748
BmHel-7	BABH01048505:680-918
BmHel-7	DF090316:9983957-9984194
BmHel-7	DF090319:5680416-5680656
BmHel-7	DF090343:3571554-3571796
BmHel-7	DF090340:1215800-1216039
BmHel-7	DF090340:968415-968654
BmHel-7	DF090334:6067523-6067761
BmHel-7	DF090325:5487797-5488036
BmHel-7	DF090372:1515298-1515536
BmHel-7	DF090329:2626164-2626406
BmHel-7	DF090351:1277060-1277299
BmHel-7	DF090394:964204-964447
BmHel-7	DF090378:572094-572333
BmHel-7	DF090389:555349-555588
BmHel-7	DF090357:2850381-2850613
BmHel-7	DF090422:398872-399113
BmHel-7	DF090338:1798277-1798517
BmHel-7	DF090432:532831-533072
BmHel-7	DF090473:325230-325477
BmHel-7	DF090483:166067-166302
BmHel-7	DF091171:4066-4304
BmHel-7	DF091056:1512-1751
BmHel-7	DF090356:1332424-1332664
BmHel-7	DF090323:1816086-1816323
BmHel-7	DF090329:1874947-1875185
BmHel-7	DF090378:1709008-1709242
BmHel-7	DF090327:2401413-2401642
BmHel-7	DF090411:695726-695974
BmHel-7	DF090391:1066043-1066280
BmHel-7	DF090333:5290239-5290420
BmHel-7	DF090360:1759218-1759456
BmHel-7	DF090359:159627-159865
BmHel-7	DF090327:5580901-5581139
BmHel-7	DF090351:804215-804450
BmHel-7	DF090394:730098-730331
BmHel-7	DF090367:822147-822385
BmHel-7	DF090420:209480-209573
BmHel-7	DF090420:213200-213352
BmHel-7	DF090331:2370897-2371053
BmHel-7	DF090331:2371061-2371148

BmHel-7	BABH01048147:1120-1351
BmHel-7	DF090378:1844876-1845110
BmHel-7	DF090367:2706347-2706577
BmHel-7	DF090374:773425-773663
BmHel-7	DF090342:3900672-3900910
BmHel-7	DF090316:14203201-14203439
BmHel-7	DF090368:1160951-1161192
BmHel-7	DF090586:46241-46479
BmHel-7	DF090358:1061084-1061322
BmHel-7	DF090342:4606662-4606900
BmHel-7	DF090355:2499902-2500138
BmHel-7	DF090355:1347636-1347878
BmHel-7	DF090349:3269309-3269547
BmHel-7	DF090344:1368480-1368718
BmHel-7	DF090319:2800834-2801075
BmHel-7	DF090319:6102369-6102607
BmHel-7	DF090319:5546309-5546547
BmHel-7	DF090316:10704939-10705177
BmHel-7	DF090364:2811648-2811885
BmHel-7	DF090334:3920436-3920671
BmHel-7	DF090334:3344229-3344467
BmHel-7	DF090323:3977466-3977704
BmHel-7	DF090372:775562-775800
BmHel-7	DF090320:7717904-7718141
BmHel-7	DF090333:3319623-3319861
BmHel-7	DF090398:1450789-1451026
BmHel-7	DF090363:258473-258710
BmHel-7	DF090357:444193-444432
BmHel-7	DF090332:1042848-1043082
BmHel-7	DF090366:2719472-2719709
BmHel-7	DF090366:930680-930918
BmHel-7	DF090358:2636766-2637004
BmHel-7	DF090345:2306415-2306659
BmHel-7	DF090335:2960007-2960238
BmHel-7	DF090338:748236-748474
BmHel-7	DF090338:1963520-1963759
BmHel-7	DF090424:774264-774504
BmHel-7	DF090408:849212-849443
BmHel-7	DF090375:2294484-2294723
BmHel-7	DF090368:2146793-2147031
BmHel-7	DF090362:1225638-1225876
BmHel-7	DF090415:548723-548961
BmHel-7	DF090431:591690-591925
BmHel-7	DF090534:25703-25941
BmHel-7	DF090731:6586-6827
BmHel-7	DF090609:29849-30088
BmHel-7	DF090317:3763122-3763238
BmHel-7	DF090317:3763256-3763374

BmHel-7	DF090316:15494250-15494487
BmHel-7	DF090327:343509-343746
BmHel-7	DF090348:3799994-3800225
BmHel-7	DF090330:3215036-3215272
BmHel-7	DF090356:3796672-3796853
BmHel-7	DF090319:8568979-8569097
BmHel-7	DF090319:8569095-8569213
BmHel-7	DF090316:390127-390358
BmHel-7	DF090335:4686289-4686409
BmHel-7	DF090435:553256-553435
BmHel-7	BABH01050691:700-936
BmHel-7	DF090343:554331-554568
BmHel-7	DF090418:1092701-1092800
BmHel-7	DF090418:1092566-1092703
BmHel-7	DF090327:6475636-6475873
BmHel-7	DF090443:231829-232065
BmHel-7	DF090354:807127-807364
BmHel-7	DF090316:474333-474421
BmHel-7	DF090316:474521-474681
BmHel-7	DF090320:1652108-1652221
BmHel-7	DF090320:1652237-1652357
BmHel-7	DF090317:5566154-5566361
BmHel-7	DF090457:18714-18951
BmHel-7	DF090391:47378-47498
BmHel-7	DF090391:47524-47650
BmHel-7	DF090356:3497132-3497369
BmHel-7	DF090333:5710388-5710628
BmHel-7	DF090454:274983-275180
BmHel-7	DF090449:341678-341846
BmHel-7	DF090390:548146-548339
BmHel-7	DF090322:4649413-4649650
BmHel-7	DF090330:4083386-4083623
BmHel-7	DF090393:1216581-1216672
BmHel-7	DF090393:1216688-1216830
BmHel-7	DF090356:1948249-1948486
BmHel-7	DF090390:1557336-1557573
BmHel-7	DF090329:3560308-3560545
BmHel-7	DF090347:1321284-1321521
BmHel-7	DF090345:164863-165100
BmHel-7	DF090324:978349-978585
BmHel-7	DF090403:425017-425252
BmHel-7	DF090379:945249-945486
BmHel-7	DF090358:419305-419408
BmHel-7	DF090358:419830-419982
BmHel-7	DF090319:4430482-4430717
BmHel-7	DF090343:1075551-1075783
BmHel-7	DF090347:1492522-1492758
BmHel-7	DF090327:253007-253243

BmHel-7	DF090389:1411298-1411531
BmHel-7	DF090416:425698-425934
BmHel-7	DF090335:383135-383372
BmHel-7	DF090321:8124167-8124402
BmHel-7	DF090408:1381451-1381685
BmHel-7	DF090331:5957209-5957429
BmHel-7	DF090422:112567-112803
BmHel-7	DF090412:817389-817592
BmHel-7	BABH01051307:404-585
BmHel-7	DF090354:1393344-1393577
BmHel-7	DF090332:1078800-1079003
BmHel-7	DF090429:502480-502673
BmHel-7	DF090335:2513179-2513415
BmHel-7	DF090358:2573487-2573723
BmHel-7	DF090375:1670-1906
BmHel-7	DF090323:4275682-4275918
BmHel-7	DF090544:68027-68261
BmHel-7	DF090438:524396-524538
BmHel-7	DF090438:524560-524653
BmHel-7	DF090330:2894102-2894337
BmHel-7	DF090533:41512-41640
BmHel-7	DF090533:41369-41482
BmHel-7	DF090429:564902-565142
BmHel-7	DF090331:4451516-4451752
BmHel-7	DF090408:78088-78324
BmHel-7	DF090342:1002611-1002847
BmHel-7	DF090414:395848-396086
BmHel-7	DF090319:6731621-6731865
BmHel-7	DF090340:3973368-3973604
BmHel-7	DF090350:1537672-1537908
BmHel-7	DF090317:1868734-1868970
BmHel-7	DF090321:7698872-7699108
BmHel-7	DF090408:85549-85785
BmHel-7	DF090324:6377469-6377702
BmHel-7	DF090329:3359118-3359353
BmHel-7	DF090343:1258930-1259165
BmHel-7	DF090329:3819306-3819541
BmHel-7	DF090351:1920358-1920593
BmHel-7	DF090327:503845-504080
BmHel-7	BABH01063359:19-254
BmHel-7	DF090338:1066415-1066625
BmHel-7	DF090343:2417574-2417666
BmHel-7	DF090343:2417990-2418141
BmHel-7	DF090322:7797699-7797934
BmHel-7	DF090391:1320822-1320927
BmHel-7	DF090391:1321054-1321199
BmHel-7	DF090335:2461867-2462102
BmHel-7	BABH01073671:288-523

BmHel-7	DF090319:2537447-2537680
BmHel-7	DF090441:377904-378087
BmHel-7	DF090326:4388922-4389154
BmHel-7	DF090415:136405-136583
BmHel-7	DF090344:2341308-2341543
BmHel-7	DF090334:5478690-5478805
BmHel-7	DF090334:5478825-5478947
BmHel-7	DF090321:957929-958164
BmHel-7	DF090322:2779425-2779664
BmHel-7	DF090539:24406-24641
BmHel-7	DF090444:130103-130338
BmHel-7	DF090394:1376255-1376493
BmHel-7	DF090340:3480615-3480850
BmHel-7	DF090329:3772522-3772759
BmHel-7	DF090333:568396-568632
BmHel-7	DF090327:4064861-4065096
BmHel-7	DF090361:663506-663741
BmHel-7	DF090358:2775185-2775420
BmHel-7	DF090321:116766-117001
BmHel-7	DF090321:268891-269126
BmHel-7	DF090434:132427-132676
BmHel-7	DF090375:726912-727147
BmHel-7	DF090415:371015-371249
BmHel-7	DF090709:17560-17793
BmHel-7	DF090377:110800-110977
BmHel-7	DF090324:536955-537079
BmHel-7	DF090324:536845-536956
BmHel-7	DF090333:3835895-3836062
BmHel-7	DF090332:2409108-2409342
BmHel-7	DF090317:1256864-1257098
BmHel-7	DF090356:2819269-2819511
BmHel-7	DF090319:2033383-2033620
BmHel-7	DF090319:314240-314473
BmHel-7	DF090340:2261484-2261715
BmHel-7	DF090325:5105135-5105369
BmHel-7	DF090323:3019102-3019334
BmHel-7	DF090329:4604949-4605181
BmHel-7	DF090417:802715-802950
BmHel-7	DF090338:1357138-1357372
BmHel-7	DF090325:2286898-2287038
BmHel-7	DF090325:2286790-2286885
BmHel-7	DF090327:6021695-6021882
BmHel-7	DF090328:2071018-2071252
BmHel-7	DF090362:3117379-3117548
BmHel-7	DF090316:9728116-9728350
BmHel-7	DF090325:1929649-1929879
BmHel-7	DF090337:2758035-2758259
BmHel-7	DF090399:762221-762457

BmHel-7	DF090321:534611-534845
BmHel-7	DF090321:6019399-6019633
BmHel-7	DF090368:1514753-1514987
BmHel-7	DF090427:202772-203006
BmHel-7	DF090413:224243-224483
BmHel-7	DF090428:923150-923259
BmHel-7	DF090428:923019-923138
BmHel-7	DF090447:403924-404159
BmHel-7	DF090394:719667-719842
BmHel-7	DF090433:181325-181456
BmHel-7	DF090433:181458-181559
BmHel-7	DF090318:7920529-7920717
BmHel-7	DF090336:3675614-3675846
BmHel-7	DF090316:5637213-5637446
BmHel-7	DF090346:1709667-1709903
BmHel-7	DF090334:3130459-3130690
BmHel-7	DF090351:739681-739914
BmHel-7	DF090317:4129935-4130168
BmHel-7	DF090365:791527-791762
BmHel-7	DF090324:7496077-7496307
BmHel-7	BABH01045169:5001-5233
BmHel-7	DF090342:1462269-1462502
BmHel-7	DF090374:1391098-1391310
BmHel-7	DF090345:3889733-3889967
BmHel-7	DF090523:42910-43140
BmHel-7	DF090380:982420-982654
BmHel-7	DF090363:1563110-1563343
BmHel-7	DF090342:4031264-4031498
BmHel-7	DF090349:3579213-3579449
BmHel-7	DF090319:2474929-2475160
BmHel-7	DF090362:214677-214910
BmHel-7	DF090324:2955846-2956079
BmHel-7	DF090369:1811344-1811578
BmHel-7	DF090358:3424863-3424965
BmHel-7	DF090358:3424735-3424858
BmHel-7	DF090680:27926-28157
BmHel-7	DF090350:413062-413294
BmHel-7	DF090330:3492231-3492466
BmHel-7	DF090468:96178-96410
BmHel-7	DF090321:1370283-1370509
BmHel-7	DF090692:21285-21517
BmHel-7	DF090325:7191720-7191825
BmHel-7	DF090325:7191822-7191948
BmHel-7	DF090441:425731-425908
BmHel-7	DF090354:1287909-1288141
BmHel-7	DF090326:1704993-1705225
BmHel-7	DF090320:4568901-4569133
BmHel-7	BABH01047633:639-871

BmHel-7	DF090899:10082-10314
BmHel-7	DF090349:2349031-2349266
BmHel-7	DF090347:2603099-2603321
BmHel-7	DF090317:10542009-10542238
BmHel-7	DF090427:346537-346768
BmHel-7	DF090393:388767-388999
BmHel-7	DF090327:4664422-4664654
BmHel-7	DF090317:8634978-8635115
BmHel-7	DF090317:8635113-8635208
BmHel-7	DF090338:2302887-2302981
BmHel-7	DF090338:2302732-2302868
BmHel-7	DF090353:915434-915665
BmHel-7	DF090368:1640388-1640626
BmHel-7	DF090516:53039-53153
BmHel-7	DF090516:53605-53747
BmHel-7	DF090319:346079-346261
BmHel-7	DF090364:1515077-1515315
BmHel-7	DF090354:3596600-3596831
BmHel-7	DF090325:5405194-5405425
BmHel-7	DF090332:915296-915527
BmHel-7	DF090338:3600339-3600567
BmHel-7	DF090338:3930288-3930376
BmHel-7	DF090338:3930616-3930760
BmHel-7	DF090342:690895-691122
BmHel-7	DF090367:427695-427926
BmHel-7	DF090325:7069785-7070016
BmHel-7	DF090323:2391181-2391411
BmHel-7	DF090320:2635099-2635327
BmHel-7	DF090353:1806354-1806585
BmHel-7	DF090361:2949771-2949997
BmHel-7	DF090332:3700863-3701094
BmHel-7	DF090330:537136-537367
BmHel-7	DF090339:4714695-4714811
BmHel-7	DF090339:4714808-4714921
BmHel-7	DF090354:3116720-3116817
BmHel-7	DF090354:3116812-3116937
BmHel-7	DF090356:307110-307339
BmHel-7	DF090344:2434992-2435217
BmHel-7	DF090377:1903834-1904064
BmHel-7	DF090347:3138396-3138625
BmHel-7	DF090327:2765800-2766033
BmHel-7	DF090389:630680-630910
BmHel-7	DF090331:4485632-4485862
BmHel-7	DF090367:2139787-2139983
BmHel-7	DF090325:6110824-6111054
BmHel-7	DF090325:7247260-7247490
BmHel-7	DF090353:2607781-2608012
BmHel-7	DF090358:2168412-2168642

BmHel-7	DF090343:4440400-4440630
BmHel-7	DF090328:3022476-3022707
BmHel-7	DF090356:1180000-1180230
BmHel-7	DF090349:4074321-4074548
BmHel-7	DF090323:2708551-2708781
BmHel-7	DF090333:758937-759167
BmHel-7	DF090327:1659988-1660218
BmHel-7	DF090318:1726042-1726272
BmHel-7	DF090318:2486427-2486658
BmHel-7	DF090363:990886-991116
BmHel-7	DF090441:19015-19245
BmHel-7	DF090395:410243-410473
BmHel-7	DF090424:66867-67096
BmHel-7	DF090375:242177-242410
BmHel-7	DF090409:1552145-1552375
BmHel-7	DF090431:406729-406959
BmHel-7	DF090464:71352-71582
BmHel-7	BABH01049417:834-1064
BmHel-7	DF090324:4909627-4909797
BmHel-7	DF090324:3385217-3385446
BmHel-7	DF090394:1523533-1523683
BmHel-7	DF090337:5290021-5290250
BmHel-7	DF090383:1918936-1919165
BmHel-7	DF090316:2900404-2900511
BmHel-7	DF090316:2899996-2900139
BmHel-7	DF090418:312223-312404
BmHel-7	DF090319:4650499-4650728
BmHel-7	DF090382:1058453-1058681
BmHel-7	DF090340:710217-710450
BmHel-7	DF090419:156741-156970
BmHel-7	DF090379:1088772-1089001
BmHel-7	DF091245:5155-5384
BmHel-7	DF090411:126965-127113
BmHel-7	DF090411:126883-126962
BmHel-7	DF090381:264711-264879
BmHel-7	DF090316:11409557-11409791
BmHel-7	DF090345:1494785-1495013
BmHel-7	DF090579:50972-51200
BmHel-7	DF090512:21598-21819
BmHel-7	DF090414:501497-501617
BmHel-7	DF090414:501391-501498
BmHel-7	DF090328:4980808-4980916
BmHel-7	DF090328:4980915-4981034
BmHel-7	DF090388:1432450-1432678
BmHel-7	DF090353:1064637-1064865
BmHel-7	DF090399:280080-280308
BmHel-7	DF090362:2752938-2753164
BmHel-7	DF090324:7803051-7803279

BmHel-7	DF090356:3757019-3757247
BmHel-7	DF090377:524582-524809
BmHel-7	DF090338:1865891-1866116
BmHel-7	DF090403:586178-586407
BmHel-7	DF090394:1128721-1128803
BmHel-7	DF090394:1128542-1128720
BmHel-7	DF090325:4006699-4006817
BmHel-7	DF090325:4006822-4006930
BmHel-7	DF090317:2814518-2814694
BmHel-7	DF090407:830799-830891
BmHel-7	DF090407:830661-830794
BmHel-7	DF090327:697187-697418
BmHel-7	DF090446:236181-236371
BmHel-7	DF090466:316723-316956
BmHel-7	DF090357:2339335-2339554
BmHel-7	DF090362:1780442-1780669
BmHel-7	DF090344:2023970-2024196
BmHel-7	DF090319:4184645-4184878
BmHel-7	DF090323:3692184-3692411
BmHel-7	DF090333:3651913-3652140
BmHel-7	DF090363:2283085-2283312
BmHel-7	DF090324:5894579-5894806
BmHel-7	DF091326:2908-3135
BmHel-7	BABH01051685:653-880
BmHel-7	DF090348:2201517-2201686
BmHel-7	DF090410:594195-594424
BmHel-7	DF090367:2302100-2302329
BmHel-7	DF090338:2596498-2596726
BmHel-7	DF090329:386375-386505
BmHel-7	DF090329:386520-386616
BmHel-7	DF090373:1138423-1138595
BmHel-7	DF090335:4005933-4006084
BmHel-7	DF090326:3139350-3139453
BmHel-7	DF090326:3139229-3139351
BmHel-7	DF090324:7190318-7190544
BmHel-7	DF090331:6016349-6016467
BmHel-7	DF090331:6016226-6016337
BmHel-7	DF090350:4082866-4083092
BmHel-7	DF090334:674206-674432
BmHel-7	DF090323:2456774-2457000
BmHel-7	DF090329:4166424-4166651
BmHel-7	BABH01047479:291-518
BmHel-7	BABH01080392:7-234
BmHel-7	DF091059:6684-6911
BmHel-7	DF091484:3759-3986
BmHel-7	DF090444:269519-269670
BmHel-7	DF090436:364031-364170
BmHel-7	DF090340:1170146-1170228

BmHel-7	DF090340:1170244-1170390
BmHel-7	DF090356:770557-770781
BmHel-7	DF090367:1889249-1889473
BmHel-7	DF090322:8236022-8236247
BmHel-7	DF090363:878994-879218
BmHel-7	DF090429:281862-282087
BmHel-7	DF090502:123417-123641
BmHel-7	DF090442:637366-637589
BmHel-7	DF090376:1957998-1958178
BmHel-7	DF090389:909185-909410
BmHel-7	DF090418:1178803-1179024
BmHel-7	DF090566:66808-66995
BmHel-7	DF090504:80916-81141
BmHel-7	DF090343:374049-374274
BmHel-7	DF090368:2327049-2327274
BmHel-7	DF090341:681330-681555
BmHel-7	DF090354:2017920-2018145
BmHel-7	DF090343:2672727-2672952
BmHel-7	DF090334:381967-382192
BmHel-7	DF090351:497220-497449
BmHel-7	DF090339:3712149-3712374
BmHel-7	DF090358:1545677-1545902
BmHel-7	DF090393:323284-323510
BmHel-7	BABH01075194:378-603
BmHel-7	DF090348:555675-555769
BmHel-7	DF090348:555545-555674
BmHel-7	DF090336:2438692-2438797
BmHel-7	DF090336:2438296-2438414
BmHel-7	DF090404:799389-799556
BmHel-7	DF090342:1835007-1835231
BmHel-7	DF090355:747110-747332
BmHel-7	DF090319:4019501-4019723
BmHel-7	DF090340:3166261-3166483
BmHel-7	DF090368:471829-471952
BmHel-7	DF090368:471950-472050
BmHel-7	DF090377:825504-825594
BmHel-7	DF090377:825221-825352
BmHel-7	DF090319:1876463-1876632
BmHel-7	DF090364:2380096-2380265
BmHel-7	DF090386:803969-804064
BmHel-7	DF090386:808341-808473
BmHel-7	DF090335:5269955-5270171
BmHel-7	DF090389:882936-883067
BmHel-7	DF090389:882591-882676
BmHel-7	DF090374:456792-456964
BmHel-7	DF090319:1736827-1737054
BmHel-7	DF090320:902664-902880
BmHel-7	DF090362:2534819-2535043

BmHel-7	DF090354:3626690-3626914
BmHel-7	DF090337:66576-66800
BmHel-7	DF090360:2458492-2458711
BmHel-7	DF090365:353307-353525
BmHel-7	DF090338:1904170-1904394
BmHel-7	BABH01045192:51-151
BmHel-7	DF090347:531777-531921
BmHel-7	DF090327:6132841-6133058
BmHel-7	DF090509:49187-49410
BmHel-7	DF090319:280540-280766
BmHel-7	DF090316:8137171-8137394
BmHel-7	DF090340:3542971-3543194
BmHel-7	DF090325:382925-383148
BmHel-7	DF090325:2160490-2160713
BmHel-7	DF090322:6557122-6557339
BmHel-7	DF090410:1042374-1042597
BmHel-7	DF090355:965604-965778
BmHel-7	DF090402:881606-881700
BmHel-7	DF090402:881706-881837
BmHel-7	DF090351:171307-171485
BmHel-7	DF090316:6841231-6841455
BmHel-7	DF090317:10482875-10482986
BmHel-7	DF090317:10482986-10483089
BmHel-7	DF091018:5805-6028
BmHel-7	DF090410:1081382-1081609
BmHel-7	DF090349:1414410-1414636
BmHel-7	DF090328:3368795-3369024
BmHel-7	DF090332:910907-911130
BmHel-7	DF090358:2474312-2474534
BmHel-7	DF090348:1931442-1931665
BmHel-7	DF090468:294967-295191
BmHel-7	DF090391:430571-430794
BmHel-7	DF090458:256127-256349
BmHel-7	BABH01059744:200-423
BmHel-7	DF090337:4043951-4044104
BmHel-7	DF090346:2412468-2412694
BmHel-7	DF090387:1422368-1422585
BmHel-7	DF090340:1312191-1312418
BmHel-7	DF090327:3174634-3174856
BmHel-7	DF090444:186508-186730
BmHel-7	DF090321:3835238-3835460
BmHel-7	DF090384:1657075-1657297
BmHel-7	DF090430:206176-206398
BmHel-7	DF090319:8510671-8510892
BmHel-7	DF090425:547975-548197
BmHel-7	DF090319:8225453-8225601
BmHel-7	DF090342:1711847-1712008
BmHel-7	DF090329:6359722-6359892

BmHel-7	DF090362:1856940-1857078
BmHel-7	DF090362:1857085-1857171
BmHel-7	DF090360:703650-703871
BmHel-7	DF090381:969053-969273
BmHel-7	DF090363:1332406-1332611
BmHel-7	DF090471:170886-171107
BmHel-7	DF090356:548561-548781
BmHel-7	BABH01085777:20-244
BmHel-7	DF090383:858060-858279
BmHel-7	DF090318:634622-634842
BmHel-7	DF090424:505656-505876
BmHel-7	DF090401:1290813-1291032
BmHel-7	DF090319:6663784-6664004
BmHel-7	DF090318:6398614-6398834
BmHel-7	DF090348:1910133-1910240
BmHel-7	DF090348:1910255-1910369
BmHel-7	DF090412:1287843-1288028
BmHel-7	DF090390:1247118-1247233
BmHel-7	DF090390:1247532-1247636
BmHel-7	DF090405:1435776-1436004
BmHel-7	BABH01087334:198-418
BmHel-7	DF090355:3631144-3631364
BmHel-7	DF090328:664441-664649
BmHel-7	DF090342:2569314-2569533
BmHel-7	DF090326:102146-102365
BmHel-7	DF090393:1119859-1120078
BmHel-7	DF090433:200462-200601
BmHel-7	DF090433:200622-200701
BmHel-7	DF090357:367388-367607
BmHel-7	DF090347:2531871-2532089
BmHel-7	DF090334:5951107-5951326
BmHel-7	BABH01052321:4-223
BmHel-7	DF090377:1174282-1174499
BmHel-7	DF090343:4168330-4168549
BmHel-7	DF090332:4557349-4557573
BmHel-7	DF090418:214129-214342
BmHel-7	DF090317:1286432-1286651
BmHel-7	DF090368:2413009-2413231
BmHel-7	DF090344:4603853-4603982
BmHel-7	DF090344:4603756-4603843
BmHel-7	DF091584:379-468
BmHel-7	DF090343:2485368-2485586
BmHel-7	DF090318:8495563-8495781
BmHel-7	DF090364:2049574-2049797
BmHel-7	DF090353:2020991-2021209
BmHel-7	DF090778:3393-3614
BmHel-7	DF090370:2511030-2511126
BmHel-7	DF090370:2510910-2511029

BmHel-7	DF090434:165597-165728
BmHel-7	DF090434:165509-165594
BmHel-7	DF090397:839296-839487
BmHel-7	DF090416:464340-464556
BmHel-7	DF090355:2532793-2533010
BmHel-7	DF090363:60059-60281
BmHel-7	BABH01045089:1279-1496
BmHel-7	BABH01045828:1173-1350
BmHel-7	DF090441:591211-591428
BmHel-7	DF090331:214529-214695
BmHel-7	DF090316:7264091-7264308
BmHel-7	DF090356:2706418-2706497
BmHel-7	DF090356:2706279-2706417
BmHel-7	DF090410:455241-455329
BmHel-7	DF090410:455115-455246
BmHel-7	DF090342:1402594-1402782
BmHel-7	DF090324:5187025-5187243
BmHel-7	DF090338:1843632-1843755
BmHel-7	DF090338:1843755-1843847
BmHel-7	DF090325:1526126-1526342
BmHel-7	DF090317:1369384-1369599
BmHel-7	DF090342:3370503-3370717
BmHel-7	DF090357:1765744-1765907
BmHel-7	DF090320:4338178-4338391
BmHel-7	DF090413:93493-93709
BmHel-7	DF090316:12831247-12831459
BmHel-7	DF090316:12654118-12654268
BmHel-7	DF090350:1295220-1295359
BmHel-7	DF090343:3922739-3922952
BmHel-7	DF090343:2576874-2577089
BmHel-7	DF090369:816545-816651
BmHel-7	DF090369:816440-816548
BmHel-7	DF090336:4483328-4483542
BmHel-7	DF090370:1478623-1478838
BmHel-7	DF090318:1239433-1239648
BmHel-7	DF090334:1657734-1657949
BmHel-7	DF090425:498248-498463
BmHel-7	DF090345:2283236-2283442
BmHel-7	DF090328:5335275-5335364
BmHel-7	DF090328:5335129-5335257
BmHel-7	DF090321:8039211-8039425
BmHel-7	DF090338:4997536-4997750
BmHel-7	DF090319:6191587-6191809
BmHel-7	DF090404:473293-473452
BmHel-7	DF090337:2554470-2554684
BmHel-7	DF090375:828476-828694
BmHel-7	DF090445:375967-376098
BmHel-7	DF090316:5742321-5742480

BmHel-7	DF090330:4886647-4886784
BmHel-7	DF090385:1539721-1539934
BmHel-7	DF090319:4102474-4102598
BmHel-7	DF090319:4102607-4102695
BmHel-7	DF090361:2415296-2415509
BmHel-7	DF090383:823845-824047
BmHel-7	DF090407:545599-545812
BmHel-7	DF090374:106920-107133
BmHel-7	DF090362:1147093-1147309
BmHel-7	DF090328:2531707-2531836
BmHel-7	DF090328:2531626-2531707
BmHel-7	DF090517:38650-38862
BmHel-7	DF090319:3405303-3405466
BmHel-7	DF090319:5018481-5018693
BmHel-7	DF090344:2926332-2926545
BmHel-7	DF090338:2220590-2220728
BmHel-7	DF090321:2310163-2310269
BmHel-7	DF090321:2310062-2310166
BmHel-7	DF090467:36266-36397
BmHel-7	DF090467:36189-36268
BmHel-7	BABH01045974:331-543
BmHel-7	DF090356:2876578-2876788
BmHel-7	DF090422:241017-241155
BmHel-7	DF090326:557342-557467
BmHel-7	DF090326:557505-557616
BmHel-7	DF090355:946505-946711
BmHel-7	DF090390:153923-154136
BmHel-7	DF090337:4181482-4181692
BmHel-7	DF090383:639861-640042
BmHel-7	DF090421:963428-963582
BmHel-7	DF090342:3254333-3254543
BmHel-7	DF090341:3829120-3829322
BmHel-7	DF090619:31065-31275
BmHel-7	DF090316:3835617-3835826
BmHel-7	DF090467:52363-52567
BmHel-7	BABH01047576:382-590
BmHel-7	DF090326:1100629-1100839
BmHel-7	DF090337:4063439-4063647
BmHel-7	DF090317:10103188-10103274
BmHel-7	DF090317:10103039-10103153
BmHel-7	DF090424:896874-897086
BmHel-7	DF090336:4154891-4155100
BmHel-7	DF090317:1444074-1444289
BmHel-7	DF090350:222452-222665
BmHel-7	DF090342:1435910-1436047
BmHel-7	DF090342:346432-346580
BmHel-7	DF090336:277835-278044
BmHel-7	DF090336:2046116-2046324

BmHel-7	DF090679:12507-12609
BmHel-7	DF090679:12613-12718
BmHel-7	DF090433:653822-653918
BmHel-7	DF090433:653916-654020
BmHel-7	DF090333:5443662-5443870
BmHel-7	DF090347:3350788-3350904
BmHel-7	DF090347:3350658-3350749
BmHel-7	DF090361:786421-786658
BmHel-7	DF090343:3736758-3736898
BmHel-7	DF090394:68079-68250
BmHel-7	BABH01052185:649-855
BmHel-7	DF090326:1911276-1911435
BmHel-7	DF090420:1148076-1148283
BmHel-7	DF090428:906033-906240
BmHel-7	DF090356:1176953-1177054
BmHel-7	DF090356:1177081-1177185
BmHel-7	DF090389:240627-240833
BmHel-7	DF090338:3522128-3522334
BmHel-7	DF090401:330333-330539
BmHel-7	DF090332:85774-85863
BmHel-7	DF090332:85654-85766
BmHel-7	BABH01057039:1-207
BmHel-7	DF090360:3052779-3052984
BmHel-7	DF090325:4709240-4709446
BmHel-7	DF090327:3836061-3836265
BmHel-7	DF090411:406311-406425
BmHel-7	DF090411:406207-406298
BmHel-7	DF090366:597346-597552
BmHel-7	DF090385:358620-358826
BmHel-7	DF090339:668556-668761
BmHel-7	DF090342:1068730-1068933
BmHel-7	DF090369:2366674-2366803
BmHel-7	DF090331:2612750-2612954
BmHel-7	DF090316:4446021-4446175
BmHel-7	DF090393:352219-352424
BmHel-7	DF090847:11816-12012
BmHel-7	DF090320:2008652-2008857
BmHel-7	DF090317:7859838-7860042
BmHel-7	DF090356:2125184-2125347
BmHel-7	DF090317:9556637-9556811
BmHel-7	DF090325:4357264-4357389
BmHel-7	DF090316:9186064-9186263
BmHel-7	DF090339:1604046-1604171
BmHel-7	DF090683:11622-11733
BmHel-7	DF090683:12920-13030
BmHel-7	DF090415:653762-653842
BmHel-7	DF090415:653857-653980
BmHel-7	DF090332:744495-744698

BmHel-7	DF090328:6182265-6182418
BmHel-7	DF090323:3649871-3650024
BmHel-7	DF090358:1914045-1914198
BmHel-7	DF090317:5938677-5938827
BmHel-7	DF090335:2658114-2658267
BmHel-7	DF090386:192352-192505
BmHel-7	DF090393:777210-777363
BmHel-7	DF090401:273343-273495
BmHel-7	DF090428:699473-699626
BmHel-7	DF090463:194835-194988
BmHel-7	DF090508:169410-169563
BmHel-7	DF090367:1418479-1418590
BmHel-7	DF090367:1418386-1418480
BmHel-7	BABH01048235:1147-1342
BmHel-7	DF090316:7352319-7352470
BmHel-7	DF090322:3676806-3676956
BmHel-7	DF090339:226899-227051
BmHel-7	DF090403:526570-526721
BmHel-7	DF090415:1062102-1062253
BmHel-7	DF090444:76970-77121
BmHel-7	DF090566:7987-8138
BmHel-7	DF090379:1672663-1672870
BmHel-7	DF090340:1388403-1388606
BmHel-7	DF090404:456771-456974
BmHel-7	DF090333:177964-178116
BmHel-7	DF090327:434045-434136
BmHel-7	DF090317:3040217-3040321
BmHel-7	DF090331:5231032-5231185
BmHel-7	DF090335:1659457-1659608
BmHel-7	DF090405:527555-527754
BmHel-7	DF090366:841484-841635
BmHel-7	DF090356:1780037-1780187
BmHel-7	DF090356:1636843-1636993
BmHel-7	DF090341:1490894-1491044
BmHel-7	DF090341:2417932-2418082
BmHel-7	DF090344:3896888-3897038
BmHel-7	DF090336:5248767-5248915
BmHel-7	DF090316:5578813-5578967
BmHel-7	DF090346:3860788-3860938
BmHel-7	DF090334:2020961-2021111
BmHel-7	DF090328:1894772-1894922
BmHel-7	DF090328:2464219-2464369
BmHel-7	DF090323:84279-84429
BmHel-7	DF090351:2889523-2889677
BmHel-7	DF090333:351874-352024
BmHel-7	DF090327:4490681-4490831
BmHel-7	DF090332:3545226-3545368
BmHel-7	DF090418:504018-504168

BmHel-7	DF090330:1988326-1988476
BmHel-7	DF090317:111674-111823
BmHel-7	DF090338:2659789-2659939
BmHel-7	DF090338:2764656-2764808
BmHel-7	DF090386:1207458-1207611
BmHel-7	DF090386:1995548-1995698
BmHel-7	DF090374:1407511-1407656
BmHel-7	DF090393:236359-236505
BmHel-7	DF090391:359818-359971
BmHel-7	DF090403:506315-506465
BmHel-7	DF090410:930200-930358
BmHel-7	DF090445:314345-314492
BmHel-7	DF090461:22403-22553
BmHel-7	BABH01044627:2264-2414
BmHel-7	DF090559:22228-22378
BmHel-7	BABH01050333:398-553
BmHel-7	DF090347:1325880-1326082
BmHel-7	DF090317:5993779-5993927
BmHel-7	DF090351:3116670-3116872
BmHel-7	DF090355:3791682-3791831
BmHel-7	DF090322:3518518-3518668
BmHel-7	BABH01054449:628-779
BmHel-7	DF090344:1774041-1774188
BmHel-7	DF090367:177449-177599
BmHel-7	DF090316:16152162-16152304
BmHel-7	DF090361:1649879-1650029
BmHel-7	DF090323:12758-12908
BmHel-7	DF090338:1375514-1375664
BmHel-7	DF090362:827098-827247
BmHel-7	DF090472:281496-281649
BmHel-7	DF090413:12597-12746
BmHel-7	DF090378:1562173-1562365
BmHel-7	DF090460:220650-220851
BmHel-7	DF090364:1881248-1881449
BmHel-7	DF090330:1355791-1355989
BmHel-7	DF090461:129035-129234
BmHel-7	DF090323:1502658-1502810
BmHel-7	DF090332:3766044-3766168
BmHel-7	DF090344:3620240-3620399
BmHel-7	DF090344:4498611-4498761
BmHel-7	DF090340:3865698-3865848
BmHel-7	DF090340:4179058-4179198
BmHel-7	DF090317:10464767-10464924
BmHel-7	DF090362:275980-276081
BmHel-7	DF090362:271877-271973
BmHel-7	DF090393:450534-450684
BmHel-7	DF090343:690005-690153
BmHel-7	DF090340:2037717-2037872

BmHel-7	DF090408:401430-401579
BmHel-7	DF090355:1580840-1580988
BmHel-7	DF090336:3294630-3294778
BmHel-7	DF090394:1158770-1158914
BmHel-7	DF090318:6033454-6033602
BmHel-7	DF090353:219155-219306
BmHel-7	DF090332:5331313-5331461
BmHel-7	DF090330:227109-227257
BmHel-7	DF090317:9175698-9175846
BmHel-7	DF090321:1592383-1592531
BmHel-7	DF090331:1978991-1979139
BmHel-7	DF090324:1409445-1409593
BmHel-7	DF090460:370833-370981
BmHel-7	BABH01050524:760-906
BmHel-7	DF091196:2306-2451
BmHel-7	DF090421:355043-355192
BmHel-7	DF090363:461368-461492
BmHel-7	DF090316:14483429-14483582
BmHel-7	DF090429:963178-963263
BmHel-7	DF090429:963328-963441
BmHel-7	DF090323:5471922-5472121
BmHel-7	DF090334:3119453-3119652
BmHel-7	BABH01047308:432-634
BmHel-7	DF090342:1424203-1424352
BmHel-7	DF090342:791485-791638
BmHel-7	DF090336:3481342-3481492
BmHel-7	DF090325:5198351-5198501
BmHel-7	DF090435:610007-610160
BmHel-7	DF090332:1357165-1357318
BmHel-7	DF090342:4943301-4943403
BmHel-7	DF090342:4943430-4943526
BmHel-7	DF090336:173791-173917
BmHel-7	DF090364:2631344-2631543
BmHel-7	DF090397:847233-847379
BmHel-7	DF090342:1939193-1939342
BmHel-7	DF090336:4217447-4217597
BmHel-7	DF090322:3323993-3324143
BmHel-7	DF090399:1057900-1058047
BmHel-7	DF090342:506973-507128
BmHel-7	DF090402:416291-416436
BmHel-7	DF090378:971326-971473
BmHel-7	DF090358:2553574-2553714
BmHel-7	DF090330:3003811-3003958
BmHel-7	DF090434:579579-579726
BmHel-7	DF090382:1775249-1775396
BmHel-7	DF090328:4879128-4879327
BmHel-7	DF090324:10452-10655
BmHel-7	DF090468:224650-224848

BmHel-7	DF090330:3812586-3812734
BmHel-7	DF090411:527660-527808
BmHel-7	DF090468:141129-141277
BmHel-7	DF090318:9290167-9290320
BmHel-7	DF090371:371278-371432
BmHel-7	DF090367:989068-989221
BmHel-7	DF090340:815623-815776
BmHel-7	DF090328:3301697-3301850
BmHel-7	DF090350:131208-131361
BmHel-7	DF090327:2295821-2295974
BmHel-7	DF090360:2322332-2322485
BmHel-7	DF090366:1662729-1662882
BmHel-7	DF090345:2929345-2929499
BmHel-7	DF090338:3796967-3797120
BmHel-7	DF090369:2687764-2687917
BmHel-7	DF090505:80091-80244
BmHel-7	DF090342:343633-343812
BmHel-7	DF090329:3372205-3372403
BmHel-7	DF090477:116878-117076
BmHel-7	DF090325:1514275-1514421
BmHel-7	DF090334:1501199-1501341
BmHel-7	DF090327:1323288-1323425
BmHel-7	DF090427:524171-524319
BmHel-7	DF090359:2433377-2433516
BmHel-7	DF090763:3827-3973
BmHel-7	DF090338:3829563-3829690
BmHel-7	DF090364:766209-766361
BmHel-7	DF090319:5820727-5820878
BmHel-7	DF090413:35638-35754
BmHel-7	DF090413:35554-35636
BmHel-7	DF090341:1794023-1794172
BmHel-7	DF090406:930020-930167
BmHel-7	DF090316:5776777-5776923
BmHel-7	DF090349:568739-568884
BmHel-7	DF090345:3744058-3744205
BmHel-7	DF090319:3281380-3281530
BmHel-7	DF090390:113727-113872
BmHel-7	DF090371:1179329-1179474
BmHel-7	DF090340:2832306-2832451
BmHel-7	DF090340:2660189-2660334
BmHel-7	DF090328:1204688-1204832
BmHel-7	DF090323:5796312-5796457
BmHel-7	DF090329:1524221-1524366
BmHel-7	DF090351:1525896-1526041
BmHel-7	DF090347:3255302-3255446
BmHel-7	DF090352:311717-311862
BmHel-7	DF090352:2947168-2947313
BmHel-7	DF090333:5353577-5353717

BmHel-7	DF090339:4027347-4027491
BmHel-7	DF090363:2407138-2407285
BmHel-7	DF090332:5896807-5896951
BmHel-7	DF090395:1656041-1656186
BmHel-7	DF090366:2317296-2317442
BmHel-7	DF090358:578030-578176
BmHel-7	DF090358:1271326-1271470
BmHel-7	DF090317:250485-250630
BmHel-7	DF090317:2050938-2051083
BmHel-7	DF090338:4673775-4673920
BmHel-7	DF090375:664967-665112
BmHel-7	DF090331:807475-807620
BmHel-7	DF090324:4112390-4112535
BmHel-7	DF090472:2876-3019
BmHel-7	DF090410:275381-275526
BmHel-7	DF090431:736230-736376
BmHel-7	DF090406:1195192-1195337
BmHel-7	DF090440:586419-586565
BmHel-7	BABH01047308:1004-1149
BmHel-7	DF090391:341143-341340
BmHel-7	DF090329:5656325-5656523
BmHel-7	DF090382:1618659-1618855
BmHel-7	DF090398:1464613-1464763
BmHel-7	DF090484:207318-207469
BmHel-7	DF090326:4462027-4462170
BmHel-7	DF090318:5093478-5093622
BmHel-7	DF090431:78703-78849
BmHel-7	DF090367:2026782-2026928
BmHel-7	DF090374:1567951-1568101
BmHel-7	DF090319:4042944-4043088
BmHel-7	DF090388:1773528-1773677
BmHel-7	DF090322:247522-247665
BmHel-7	DF090347:4347071-4347219
BmHel-7	DF090338:3725821-3725957
BmHel-7	DF090391:929582-929726
BmHel-7	DF090401:612596-612709
BmHel-7	DF090401:612726-612808
BmHel-7	DF090318:7059499-7059629
BmHel-7	DF090396:275550-275750
BmHel-7	DF090324:4249488-4249641
BmHel-7	DF090342:2857533-2857738
BmHel-7	DF090355:2052775-2052970
BmHel-7	DF090322:1229799-1229994
BmHel-7	DF090396:175130-175326
BmHel-7	DF090415:749517-749712
BmHel-7	DF090339:844391-844529
BmHel-7	DF090330:4856878-4857028
BmHel-7	DF090439:242830-242980

BmHel-7	DF090361:54163-54306
BmHel-7	DF090419:384438-384586
BmHel-7	DF090336:3801337-3801480
BmHel-7	DF090329:3874418-3874564
BmHel-7	DF090351:3632435-3632578
BmHel-7	DF090318:6883354-6883497
BmHel-7	DF090407:527685-527829
BmHel-7	DF090348:2328383-2328526
BmHel-7	DF090409:290045-290188
BmHel-7	BABH01043649:5570-5713
BmHel-7	BABH01048513:154-297
BmHel-7	DF090417:963106-963300
BmHel-7	DF090430:535886-536023
BmHel-7	DF090335:3205472-3205670
BmHel-7	BABH01079129:375-576
BmHel-7	DF090330:42756-42866
BmHel-7	DF090326:5896603-5896792
BmHel-7	DF090349:810498-810692
BmHel-7	DF090343:3711363-3711550
BmHel-7	DF090380:372258-372452
BmHel-7	DF090316:12669426-12669620
BmHel-7	DF090384:1352768-1352910
BmHel-7	DF090316:13766033-13766177
BmHel-7	DF090322:6947771-6947919
BmHel-7	DF090327:69531-69673
BmHel-7	DF090421:371399-371541
BmHel-7	DF090321:8088748-8088883
BmHel-7	DF090324:2310254-2310396
BmHel-7	DF090319:8555914-8556038
BmHel-7	DF090337:1596037-1596231
BmHel-7	DF090398:588724-588876
BmHel-7	DF090347:3836193-3836346
BmHel-7	DF090416:140029-140181
BmHel-7	DF090358:1179318-1179474
BmHel-7	DF090358:105492-105646
BmHel-7	DF090370:2198153-2198303
BmHel-7	DF090476:245094-245247
BmHel-7	DF090355:155403-155549
BmHel-7	DF090560:49358-49502
BmHel-7	DF090365:2446724-2446865
BmHel-7	DF090338:2446263-2446407
BmHel-7	DF090396:459369-459513
BmHel-7	DF090349:3432456-3432600
BmHel-7	DF090363:2082172-2082314
BmHel-7	DF090349:4092554-4092696
BmHel-7	DF090336:3321991-3322130
BmHel-7	DF090316:14790284-14790420
BmHel-7	DF090402:164563-164704

BmHel-7	DF090343:3077289-3077430
BmHel-7	DF090329:1246975-1247113
BmHel-7	DF090351:2098946-2099086
BmHel-7	DF090352:2013898-2014039
BmHel-7	DF090350:477925-478066
BmHel-7	DF090318:216136-216277
BmHel-7	DF090386:1348285-1348426
BmHel-7	DF090366:42762-42915
BmHel-7	DF090412:306910-307063
BmHel-7	DF090365:47590-47782
BmHel-7	DF091152:3635-3827
BmHel-7	DF090316:13166508-13166700
BmHel-7	DF090357:3753675-3753866
BmHel-7	DF090370:788733-788881
BmHel-7	DF090433:633372-633511
BmHel-7	DF090349:4313621-4313761
BmHel-7	DF090318:3389133-3389273
BmHel-7	DF090317:4321280-4321423
BmHel-7	DF090358:2165028-2165169
BmHel-7	DF090316:13441774-13441915
BmHel-7	DF090340:1347295-1347436
BmHel-7	DF090332:1803351-1803450
BmHel-7	DF090332:1803453-1803545
BmHel-7	DF090343:134862-135002
BmHel-7	DF090351:4212803-4212943
BmHel-7	DF090422:896085-896226
BmHel-7	DF090468:68757-68897
BmHel-7	DF090446:557931-558075
BmHel-7	DF090355:392639-392829
BmHel-7	DF090341:1837501-1837693
BmHel-7	DF090356:1081892-1082084
BmHel-7	DF090368:2003784-2003925
BmHel-7	DF090321:1509144-1509295
BmHel-7	DF090343:1504095-1504286
BmHel-7	DF090328:2850092-2850282
BmHel-7	DF090393:456943-457134
BmHel-7	DF090317:8615047-8615140
BmHel-7	DF090316:12532494-12532635
BmHel-7	DF090372:1990411-1990552
BmHel-7	DF090382:1499187-1499328
BmHel-7	DF090342:4644448-4644588
BmHel-7	DF090355:3364833-3364973
BmHel-7	DF090388:1670544-1670682
BmHel-7	DF090350:3866524-3866663
BmHel-7	DF090335:2269001-2269140
BmHel-7	DF090338:1475004-1475143
BmHel-7	DF090370:884156-884295
BmHel-7	DF090317:9678335-9678528

BmHel-7	DF090333:5804634-5804829
BmHel-7	DF090342:1966593-1966746
BmHel-7	DF090356:2808308-2808453
BmHel-7	DF090356:781224-781377
BmHel-7	DF090355:1269780-1269932
BmHel-7	DF090355:606811-606964
BmHel-7	DF090349:655432-655585
BmHel-7	DF090349:1997993-1998146
BmHel-7	DF090349:441493-441646
BmHel-7	DF090341:1842251-1842404
BmHel-7	DF090341:4054926-4055078
BmHel-7	DF090341:4154063-4154216
BmHel-7	DF090405:686629-686782
BmHel-7	DF090344:3349725-3349881
BmHel-7	DF090344:4461563-4461714
BmHel-7	DF090344:2420819-2420969
BmHel-7	DF090367:297868-298021
BmHel-7	DF090336:5183450-5183603
BmHel-7	DF090319:1459297-1459450
BmHel-7	DF090319:2766898-2767051
BmHel-7	DF090319:7288924-7289077
BmHel-7	DF090316:8272905-8273058
BmHel-7	DF090316:9065629-9065782
BmHel-7	DF090316:12786232-12786385
BmHel-7	DF090316:15409094-15409247
BmHel-7	DF090316:11125743-11125896
BmHel-7	DF090316:5078927-5079070
BmHel-7	DF090316:2926340-2926493
BmHel-7	DF090390:1635546-1635699
BmHel-7	DF090377:1853657-1853810
BmHel-7	DF090377:1513611-1513762
BmHel-7	DF090364:566700-566845
BmHel-7	DF090346:726325-726478
BmHel-7	DF090346:2217614-2217770
BmHel-7	DF090340:3086899-3087052
BmHel-7	DF090340:4733757-4733910
BmHel-7	DF090340:232741-232894
BmHel-7	DF090334:3027661-3027814
BmHel-7	DF090334:4346481-4346634
BmHel-7	DF090328:2383299-2383452
BmHel-7	DF090325:848707-848860
BmHel-7	DF090323:333751-333904
BmHel-7	DF090323:1827649-1827805
BmHel-7	DF090323:4622838-4622994
BmHel-7	DF090323:2950966-2951120
BmHel-7	DF090322:1553768-1553921
BmHel-7	DF090322:8119005-8119158
BmHel-7	DF090322:6661402-6661555

BmHel-7	DF090322:6272716-6272869
BmHel-7	DF090322:5037626-5037782
BmHel-7	DF090322:1966418-1966571
BmHel-7	DF090372:1793267-1793420
BmHel-7	DF090329:1222138-1222291
BmHel-7	DF090329:5746199-5746352
BmHel-7	DF090329:2962235-2962388
BmHel-7	DF090329:2457051-2457204
BmHel-7	DF090347:1313271-1313424
BmHel-7	DF090352:3028822-3028975
BmHel-7	DF090352:810257-810410
BmHel-7	DF090320:7207604-7207758
BmHel-7	DF090320:7589432-7589584
BmHel-7	DF090320:7011672-7011828
BmHel-7	DF090394:1097767-1097920
BmHel-7	DF090394:1004465-1004614
BmHel-7	DF090394:97344-97497
BmHel-7	DF090384:1782319-1782473
BmHel-7	DF090378:712362-712523
BmHel-7	DF090350:3716015-3716163
BmHel-7	DF090337:3650352-3650506
BmHel-7	DF090333:4971417-4971570
BmHel-7	DF090333:5045001-5045154
BmHel-7	DF090333:378541-378690
BmHel-7	DF090327:2944359-2944523
BmHel-7	DF090327:4418057-4418213
BmHel-7	DF090327:1726476-1726629
BmHel-7	DF090318:5469389-5469533
BmHel-7	DF090318:2308185-2308341
BmHel-7	DF090407:1571557-1571711
BmHel-7	DF090339:2416661-2416819
BmHel-7	DF090339:3032276-3032430
BmHel-7	DF090411:1025636-1025789
BmHel-7	DF090398:535543-535655
BmHel-7	DF090398:789185-789338
BmHel-7	DF090398:28805-28958
BmHel-7	DF090373:602752-602905
BmHel-7	DF090363:2952576-2952730
BmHel-7	DF090357:3259449-3259602
BmHel-7	DF090332:1791761-1791914
BmHel-7	DF090332:5884070-5884223
BmHel-7	DF090332:2844273-2844415
BmHel-7	DF090332:2796025-2796177
BmHel-7	DF090332:690100-690260
BmHel-7	DF090418:338128-338281
BmHel-7	DF090418:1200047-1200200
BmHel-7	DF090395:1308229-1308382
BmHel-7	DF090380:560992-561145

BmHel-7	DF090380:2111042-2111195
BmHel-7	DF090380:1535543-1535696
BmHel-7	DF090358:323216-323369
BmHel-7	DF090358:2246989-2247142
BmHel-7	DF090348:313478-313631
BmHel-7	DF090348:2308726-2308879
BmHel-7	DF090345:2449359-2449512
BmHel-7	DF090345:4306626-4306779
BmHel-7	DF090330:3313735-3313888
BmHel-7	DF090330:2852249-2852402
BmHel-7	DF090317:492248-492400
BmHel-7	DF090317:943570-943722
BmHel-7	DF090317:4494086-4494239
BmHel-7	DF090317:8029670-8029826
BmHel-7	DF090317:10673186-10673336
BmHel-7	DF090317:9584317-9584471
BmHel-7	DF090317:8655502-8655651
BmHel-7	DF090421:1008237-1008390
BmHel-7	DF090420:958070-958223
BmHel-7	DF090420:339965-340118
BmHel-7	DF090400:1305030-1305181
BmHel-7	DF090365:2679492-2679645
BmHel-7	DF090365:2688928-2689081
BmHel-7	DF090365:1926166-1926319
BmHel-7	DF090365:1912652-1912805
BmHel-7	DF090335:4406456-4406609
BmHel-7	DF090335:3430352-3430503
BmHel-7	DF090321:4049889-4050042
BmHel-7	DF090338:1375954-1376107
BmHel-7	DF090338:3366850-3367003
BmHel-7	DF090338:4661720-4661875
BmHel-7	DF090338:4877721-4877874
BmHel-7	DF090338:2354413-2354566
BmHel-7	DF090429:405537-405691
BmHel-7	DF090408:61982-62135
BmHel-7	DF090404:630347-630504
BmHel-7	DF090386:340964-341117
BmHel-7	DF090386:1790072-1790225
BmHel-7	DF090368:580729-580883
BmHel-7	DF090368:757686-757838
BmHel-7	DF090362:1525519-1525671
BmHel-7	DF090362:6327-6484
BmHel-7	DF090331:2032096-2032249
BmHel-7	DF090331:2453739-2453892
BmHel-7	DF090331:5611229-5611382
BmHel-7	DF090324:1427948-1428101
BmHel-7	DF090324:5772288-5772437
BmHel-7	DF090324:1127911-1128064

BmHel-7	DF090324:796523-796676
BmHel-7	DF090468:260955-261103
BmHel-7	DF090417:760994-761146
BmHel-7	DF090413:1096271-1096429
BmHel-7	DF090409:822991-823144
BmHel-7	DF090409:1542826-1542979
BmHel-7	DF090401:727562-727715
BmHel-7	DF090396:388626-388782
BmHel-7	DF090396:1573552-1573705
BmHel-7	DF090369:552972-553125
BmHel-7	DF090403:633417-633570
BmHel-7	DF090432:662688-662841
BmHel-7	DF090379:1130881-1131034
BmHel-7	DF090410:681141-681293
BmHel-7	DF090382:1906551-1906704
BmHel-7	DF090382:1125430-1125583
BmHel-7	DF090359:1780509-1780663
BmHel-7	DF090359:1350028-1350180
BmHel-7	DF090443:164879-165032
BmHel-7	DF090445:296132-296284
BmHel-7	DF090370:2476316-2476466
BmHel-7	DF090436:585541-585697
BmHel-7	DF090510:107900-108053
BmHel-7	DF090451:512192-512345
BmHel-7	DF090451:396576-396729
BmHel-7	DF090494:35938-36092
BmHel-7	DF090406:271204-271357
BmHel-7	DF090406:316303-316456
BmHel-7	DF090646:4310-4463
BmHel-7	BABH01045037:1703-1856
BmHel-7	DF090621:18257-18410
BmHel-7	DF090598:4889-5038
BmHel-7	DF090485:84514-84667
BmHel-7	DF090908:1965-2117
BmHel-7	DF090636:28057-28210
BmHel-7	DF090565:90376-90529
BmHel-7	DF090981:6857-7016
BmHel-7	BABH01047318:129-281
BmHel-7	BABH01046009:2731-2884
BmHel-7	DF090928:11835-11988
BmHel-7	DF090642:13116-13275
BmHel-7	DF090322:5691968-5692159
BmHel-7	DF090449:304716-304906
BmHel-7	DF090355:1342719-1342869
BmHel-7	DF090332:3446910-3447060
BmHel-7	DF090319:7448927-7449117
BmHel-7	DF090323:4819510-4819614
BmHel-7	DF090333:4007952-4008090

BmHel-7	DF090358:2974435-2974578
BmHel-7	BABH01071415:54-194
BmHel-7	DF090355:227194-227332
BmHel-7	DF090341:2888929-2889067
BmHel-7	DF090381:1962371-1962509
BmHel-7	DF090362:2496832-2496970
BmHel-7	DF090319:4990829-4990982
BmHel-7	DF090326:3013249-3013402
BmHel-7	DF090390:332973-333126
BmHel-7	DF090364:868615-868735
BmHel-7	DF090334:677202-677354
BmHel-7	DF090329:4538525-4538678
BmHel-7	DF090352:2731261-2731414
BmHel-7	DF090352:1786551-1786704
BmHel-7	DF090416:1006977-1007129
BmHel-7	DF090380:1723930-1724082
BmHel-7	DF090331:144490-144649
BmHel-7	DF090396:90143-90295
BmHel-7	DF090447:297031-297183
BmHel-7	DF090406:218451-218603
BmHel-7	BABH01058356:409-562
BmHel-7	BABH01049824:809-962
BmHel-7	DF090834:14039-14192
BmHel-7	DF090336:833717-833869
BmHel-7	DF090326:5648616-5648769
BmHel-7	DF090316:12323620-12323772
BmHel-7	DF090377:428252-428404
BmHel-7	DF090346:2269918-2270071
BmHel-7	DF090325:1717823-1717975
BmHel-7	DF090394:293347-293499
BmHel-7	DF090350:3110472-3110630
BmHel-7	DF090321:4315391-4315543
BmHel-7	DF090435:366721-366874
BmHel-7	DF090368:2171216-2171368
BmHel-7	DF090331:277455-277605
BmHel-7	DF090355:1474329-1474482
BmHel-7	DF090344:2861780-2861936
BmHel-7	DF090319:7765727-7765880
BmHel-7	DF090326:6598516-6598669
BmHel-7	DF090371:1380327-1380480
BmHel-7	DF090343:1411732-1411885
BmHel-7	DF090340:281561-281714
BmHel-7	DF090340:1330381-1330534
BmHel-7	DF090323:1786978-1787131
BmHel-7	DF090322:7856716-7856869
BmHel-7	DF090337:4078309-4078462
BmHel-7	DF090411:424696-424849
BmHel-7	DF090338:3960682-3960835

BmHel-7	DF090401:877309-877462
BmHel-7	DF090442:136210-136363
BmHel-7	DF090370:1631441-1631629
BmHel-7	DF090464:85111-85299
BmHel-7	DF090347:1170193-1170330
BmHel-7	DF090316:1544917-1545058
BmHel-7	DF090349:560218-560355
BmHel-7	DF090319:7816592-7816729
BmHel-7	DF090345:3461956-3462097
BmHel-7	DF090338:3753932-3754070
BmHel-7	DF090436:394025-394162
BmHel-7	DF090349:908641-908781
BmHel-7	DF090344:2407578-2407715
BmHel-7	DF090422:549766-549903
BmHel-7	DF090324:5577936-5578073
BmHel-7	DF090485:153548-153685
BmHel-7	DF090431:483919-484045
BmHel-7	DF090340:4231617-4231806
BmHel-7	DF090376:148098-148236
BmHel-7	DF090320:6160706-6160816
BmHel-7	BABH01048094:788-898
BmHel-7	DF091220:163-314
BmHel-7	DF090361:1404892-1405041
BmHel-7	DF090320:2404696-2404848
BmHel-7	DF090327:5557231-5557385
BmHel-7	DF090330:477114-477265
BmHel-7	DF090321:7228441-7228583
BmHel-7	DF090392:1531241-1531394
BmHel-7	DF090431:60920-61073
BmHel-7	DF090436:125772-125923
BmHel-7	DF090367:2046843-2046995
BmHel-7	DF090319:7870381-7870537
BmHel-7	DF090316:13349425-13349574
BmHel-7	DF090387:1339300-1339449
BmHel-7	DF090316:13383316-13383468
BmHel-7	DF090347:3551523-3551681
BmHel-7	DF090317:7870609-7870763
BmHel-7	DF090321:2494939-2495092
BmHel-7	DF090319:5322119-5322275
BmHel-7	DF090341:3310991-3311145
BmHel-7	DF090326:509532-509684
BmHel-7	DF090316:5707255-5707408
BmHel-7	DF090340:3496200-3496353
BmHel-7	DF090328:3607111-3607264
BmHel-7	DF090325:3912796-3912950
BmHel-7	DF090352:1626385-1626542
BmHel-7	DF090398:173171-173324
BmHel-7	DF090365:2261157-2261310

BmHel-7	DF090383:620694-620846
BmHel-7	DF090409:306961-307114
BmHel-7	DF090406:1060480-1060633
BmHel-7	DF090390:39115-39303
BmHel-7	DF090367:1323980-1324131
BmHel-7	DF090332:4320989-4321139
BmHel-7	DF090410:670859-671008
BmHel-7	DF090397:541043-541225
BmHel-7	DF090361:2669829-2669965
BmHel-7	DF090376:987132-987268
BmHel-7	DF090403:1131942-1132080
BmHel-7	DF090357:2525680-2525819
BmHel-7	DF090351:3516358-3516494
BmHel-7	DF090352:661316-661451
BmHel-7	DF090318:6852763-6852892
BmHel-7	DF090684:14052-14188
BmHel-7	DF090409:472071-472232
BmHel-7	DF090383:246000-246185
BmHel-7	DF090414:745666-745854
BmHel-7	DF090360:1256998-1257187
BmHel-7	DF090520:154839-155027
BmHel-7	BABH01051214:587-775
BmHel-7	DF090354:2872149-2872299
BmHel-7	DF090319:1397926-1398079
BmHel-7	DF090326:5311584-5311737
BmHel-7	DF090346:2128422-2128572
BmHel-7	DF090340:3160099-3160253
BmHel-7	DF090325:4669537-4669695
BmHel-7	DF090323:6566741-6566895
BmHel-7	DF090322:3575417-3575567
BmHel-7	DF090333:2642177-2642321
BmHel-7	DF090327:5866196-5866345
BmHel-7	DF090422:567928-568078
BmHel-7	DF090330:3637467-3637618
BmHel-7	DF090365:2570303-2570453
BmHel-7	DF090434:358821-358971
BmHel-7	DF090396:1409044-1409194
BmHel-7	DF090387:1390485-1390636
BmHel-7	DF090370:1714568-1714718
BmHel-7	DF090451:328144-328245
BmHel-7	DF090711:23443-23593
BmHel-7	DF090541:119254-119404
BmHel-7	DF090399:1374415-1374518
BmHel-7	BABH01072256:371-524
BmHel-7	DF090349:1781658-1781816
BmHel-7	DF090412:227386-227539
BmHel-7	DF090356:410008-410162
BmHel-7	DF090367:2540725-2540882

BmHel-7	DF090371:102104-102257
BmHel-7	DF090340:250595-250749
BmHel-7	DF090328:1749322-1749475
BmHel-7	DF090352:858204-858348
BmHel-7	DF090337:4663801-4663954
BmHel-7	DF090327:2573746-2573899
BmHel-7	DF090353:147410-147564
BmHel-7	DF090363:575525-575678
BmHel-7	DF090358:1629133-1629286
BmHel-7	DF090330:3778967-3779120
BmHel-7	DF090317:1804964-1805117
BmHel-7	DF090392:28375-28528
BmHel-7	DF090409:1105878-1106031
BmHel-7	DF090455:220985-221138
BmHel-7	DF090368:2102899-2103024
BmHel-7	DF090365:443495-443682
BmHel-7	DF090369:719929-720080
BmHel-7	DF090432:302884-303035
BmHel-7	DF090340:2359181-2359331
BmHel-7	DF090322:4543575-4543725
BmHel-7	DF090352:3394577-3394727
BmHel-7	DF090378:1024694-1024844
BmHel-7	DF090350:3773283-3773431
BmHel-7	DF090327:3560530-3560680
BmHel-7	DF090416:564184-564334
BmHel-7	DF090330:2365648-2365799
BmHel-7	DF090335:2875802-2875952
BmHel-7	DF090413:1224271-1224421
BmHel-7	DF090415:225317-225466
BmHel-7	DF090544:8931-9081
BmHel-7	DF090332:5094785-5094935
BmHel-7	DF090317:8702321-8702454
BmHel-7	DF090319:5753286-5753428
BmHel-7	DF090366:2274913-2275050
BmHel-7	DF090338:1654559-1654699
BmHel-7	DF090330:4785911-4786046
BmHel-7	DF090337:1480502-1480641
BmHel-7	DF090329:1938743-1938878
BmHel-7	DF090372:1605383-1605513
BmHel-7	DF090357:2743415-2743550
BmHel-7	DF090353:367498-367685
BmHel-7	DF090382:1453806-1453944
BmHel-7	BABH01076896:1-188
BmHel-7	DF090362:1552164-1552313
BmHel-7	DF090354:487819-487972
BmHel-7	DF090334:3715158-3715307
BmHel-7	DF090325:2029641-2029794
BmHel-7	DF090323:6500034-6500183

BmHel-7	DF090372:1867127-1867287
BmHel-7	DF090329:2836467-2836622
BmHel-7	DF090378:1034022-1034171
BmHel-7	DF090389:856463-856616
BmHel-7	DF090398:782734-782878
BmHel-7	DF090357:2021483-2021638
BmHel-7	DF090345:3371960-3372109
BmHel-7	DF090338:3562682-3562835
BmHel-7	DF090331:3328011-3328168
BmHel-7	DF090396:1437900-1438052
BmHel-7	DF090337:1445181-1445330
BmHel-7	DF090411:1197289-1197439
BmHel-7	DF090332:5287295-5287444
BmHel-7	DF090348:178095-178244
BmHel-7	BABH01051946:710-859
BmHel-7	DF090344:4663362-4663515
BmHel-7	DF090323:2513727-2513877
BmHel-7	DF090339:603955-604106
BmHel-7	DF090366:756267-756416
BmHel-7	DF090319:5653049-5653202
BmHel-7	DF090334:3401589-3401740
BmHel-7	DF090358:2392445-2392601
BmHel-7	DF090323:7259669-7259822
BmHel-7	DF090324:7607729-7607882
BmHel-7	DF090444:454547-454696
BmHel-7	DF090355:3699099-3699252
BmHel-7	DF090355:2073425-2073578
BmHel-7	DF090326:167761-167913
BmHel-7	DF090388:219282-219438
BmHel-7	DF090377:1581851-1582004
BmHel-7	DF090353:3259851-3260004
BmHel-7	DF090381:1189223-1189377
BmHel-7	DF090392:1190734-1190891
BmHel-7	DF090396:527954-528107
BmHel-7	DF090425:838222-838374
BmHel-7	DF090443:301648-301801
BmHel-7	DF090888:6210-6361
BmHel-7	DF090629:25327-25483
BmHel-7	DF090327:3105439-3105595
BmHel-7	DF090486:81033-81184
BmHel-7	BABH01073559:1-187
BmHel-7	DF090319:8778279-8778428
BmHel-7	DF090388:536781-536931
BmHel-7	DF090388:1396679-1396829
BmHel-7	DF090372:1290894-1291044
BmHel-7	DF090317:9719202-9719352
BmHel-7	DF090362:687277-687372
BmHel-7	DF090326:5701857-5702006

BmHel-7	DF090363:2746635-2746786
BmHel-7	DF090321:6500061-6500210
BmHel-7	DF090321:6158070-6158204
BmHel-7	DF090383:950570-950719
BmHel-7	DF090324:392384-392534
BmHel-7	DF090363:75605-75753
BmHel-7	DF090372:1707357-1707491
BmHel-7	DF090329:1294078-1294272
BmHel-7	DF090332:1367845-1367982
BmHel-7	DF090335:239715-239807
BmHel-7	DF090342:2373643-2373791
BmHel-7	DF090378:1401290-1401438
BmHel-7	DF090411:215251-215400
BmHel-7	DF090357:2664527-2664675
BmHel-7	DF090357:667279-667427
BmHel-7	DF090330:3761373-3761524
BmHel-7	DF090327:1313748-1313897
BmHel-7	DF090373:866915-867063
BmHel-7	DF090366:858838-858991
BmHel-7	DF090336:5006221-5006371
BmHel-7	DF090349:1427989-1428079
BmHel-7	DF090325:6347016-6347162
BmHel-7	DF090405:878187-878336
BmHel-7	DF090318:9071095-9071251
BmHel-7	DF090322:8201226-8201379
BmHel-7	DF090317:8313792-8313975
BmHel-7	DF090383:1325061-1325205
BmHel-7	DF090340:416969-417119
BmHel-7	DF090454:475696-475846
BmHel-7	DF090444:599690-599823
BmHel-7	DF090341:4334991-4335139
BmHel-7	DF090340:669297-669443
BmHel-7	DF090325:3609320-3609468
BmHel-7	DF090325:2101949-2102097
BmHel-7	DF090323:392087-392235
BmHel-7	DF090351:1308175-1308323
BmHel-7	DF090363:416514-416662
BmHel-7	DF090363:945083-945231
BmHel-7	DF090321:7029264-7029412
BmHel-7	DF090324:811577-811728
BmHel-7	DF090413:1165497-1165645
BmHel-7	DF090391:818143-818292
BmHel-7	DF090445:228934-229082
BmHel-7	BABH01049846:328-476
BmHel-7	DF090598:18635-18781
BmHel-7	BABH01050251:231-372
BmHel-7	DF090367:1839743-1839884
BmHel-7	DF090424:175138-175278

BmHel-7	DF090437:461468-461607
BmHel-7	DF090401:1185663-1185798
BmHel-7	DF090340:4406934-4407067
BmHel-7	DF090346:1061258-1061394
BmHel-7	DF090386:486114-486299
BmHel-7	DF090355:3749480-3749627
BmHel-7	DF090405:797336-797429
BmHel-7	DF090339:4771119-4771211
BmHel-7	DF090358:333508-333658
BmHel-7	DF090466:136737-136868
BmHel-7	DF090332:5421553-5421663
BmHel-7	DF090323:7049465-7049606
BmHel-7	BABH01068109:67-222
BmHel-7	DF090390:1380173-1380327
BmHel-7	DF090345:931396-931545
BmHel-7	DF090431:328905-329054
BmHel-7	BABH01049469:504-657
BmHel-7	DF090319:3535785-3535968
BmHel-7	DF090321:2584586-2584770
BmHel-7	DF090358:3359080-3359231
BmHel-7	DF090360:1187893-1188025
BmHel-7	DF090338:1322748-1322898
BmHel-7	DF090336:3053848-3053999
BmHel-7	DF090371:776891-777038
BmHel-7	DF090394:1505553-1505700
BmHel-7	DF090333:409058-409205
BmHel-7	DF090358:511813-511964
BmHel-7	DF090462:279057-279204
BmHel-7	DF090378:658545-658691
BmHel-7	DF090352:1693559-1693692
BmHel-7	DF090332:5748424-5748556
BmHel-7	DF090327:5726892-5727024
BmHel-7	DF090405:1309195-1309332
BmHel-7	DF090483:103509-103643
BmHel-7	DF090319:7327649-7327782
BmHel-7	DF090323:6647112-6647244
BmHel-7	DF090317:3473655-3473790
BmHel-7	DF090437:62484-62614
BmHel-7	BABH01062132:239-370
BmHel-7	DF090319:8006503-8006636
BmHel-7	DF090411:1150817-1150953
BmHel-7	DF090402:1102703-1102841
BmHel-7	DF090354:3444523-3444669
BmHel-7	DF090340:3309329-3309475
BmHel-7	DF090329:4694938-4695081
BmHel-7	DF090357:835838-835991
BmHel-7	DF090400:827495-827641
BmHel-7	DF090375:1520729-1520875

BmHel-7	DF090331:3495075-3495181
BmHel-7	DF091318:2603-2694
BmHel-7	DF090329:6099956-6100112
BmHel-7	DF090316:13072019-13072105
BmHel-7	DF090375:1134082-1134173
BmHel-7	DF090320:1441450-1441603
BmHel-7	DF090445:585095-585242
BmHel-7	DF091944:1192-1339
BmHel-7	DF090396:1279735-1279835
BmHel-7	DF090418:283088-283242
BmHel-7	BABH01045320:345-498
BmHel-7	DF090348:3288562-3288715
BmHel-7	DF090391:1224624-1224807
BmHel-7	DF090341:3841641-3841791
BmHel-7	DF090318:3305538-3305685
BmHel-7	DF090360:1169137-1169285
BmHel-7	DF090357:3439543-3439693
BmHel-7	DF090317:5645172-5645315
BmHel-7	DF090389:22391-22522
BmHel-7	DF090358:319639-319787
BmHel-7	DF090343:1545786-1545932
BmHel-7	DF090318:4003218-4003362
BmHel-7	DF090317:9485559-9485691
BmHel-7	DF090455:388028-388121
BmHel-7	DF090341:4707716-4707858
BmHel-7	DF090323:5873047-5873178
BmHel-7	DF090337:2273333-2273470
BmHel-7	DF090325:6611727-6611858
BmHel-7	DF090396:1501947-1502078
BmHel-7	DF090405:1153750-1153866
BmHel-7	DF090337:4275423-4275577
BmHel-7	DF090376:828286-828431
BmHel-7	DF090331:3160396-3160543
BmHel-7	DF090317:6308002-6308147
BmHel-7	DF090337:1017096-1017182
BmHel-7	DF090337:4287815-4287960
BmHel-7	DF090329:3107468-3107583
BmHel-7	DF090336:341546-341699
BmHel-7	DF090433:32580-32728
BmHel-7	DF090342:2055717-2055875
BmHel-7	DF090316:3567022-3567175
BmHel-7	DF090415:987016-987169
BmHel-7	DF090333:2918613-2918797
BmHel-7	DF090319:423427-423578
BmHel-7	DF090390:1372920-1373030
BmHel-7	DF090338:1336235-1336385
BmHel-7	DF090382:794178-794327
BmHel-7	DF090388:1162122-1162273

BmHel-7	DF090318:4970897-4971046
BmHel-7	DF090374:1098985-1099085
BmHel-7	DF090355:1032211-1032356
BmHel-7	DF090354:937362-937507
BmHel-7	DF090316:12142167-12142312
BmHel-7	DF090334:465431-465576
BmHel-7	DF090334:3582044-3582190
BmHel-7	DF090334:5514048-5514193
BmHel-7	DF090334:293963-294108
BmHel-7	DF090323:6286021-6286172
BmHel-7	DF090347:1823307-1823453
BmHel-7	DF090318:587333-587478
BmHel-7	DF090456:329674-329819
BmHel-7	DF090348:4355349-4355494
BmHel-7	DF090338:1575680-1575810
BmHel-7	DF090394:1329273-1329455
BmHel-7	DF090323:4703130-4703260
BmHel-7	DF090359:564325-564455
BmHel-7	DF090333:4944048-4944179
BmHel-7	DF090359:2951732-2951831
BmHel-7	DF090359:2951639-2951721
BmHel-7	DF090345:3396018-3396170
BmHel-7	DF090316:9330097-9330210
BmHel-7	DF090433:214842-214986
BmHel-7	DF090345:2643219-2643318
BmHel-7	DF090428:11435-11580
BmHel-7	DF090322:457191-457289
BmHel-7	DF090381:1873119-1873302
BmHel-7	DF090377:1666042-1666191
BmHel-7	DF090369:2477025-2477168
BmHel-7	DF090354:2658215-2658358
BmHel-7	BABH01077699:4-190
BmHel-7	DF090436:154930-155075
BmHel-7	DF090406:487776-487920
BmHel-7	DF090355:1252136-1252283
BmHel-7	DF090558:44579-44723
BmHel-7	DF090342:3222223-3222366
BmHel-7	DF090365:1841338-1841474
BmHel-7	DF090355:2785577-2785707
BmHel-7	DF090316:4091574-4091693
BmHel-7	DF090318:3392859-3392992
BmHel-7	DF090365:2232809-2232938
BmHel-7	BABH01050696:174-293
BmHel-7	DF090408:1196986-1197112
BmHel-7	DF090317:2717021-2717207
BmHel-7	DF090387:1047536-1047717
BmHel-7	DF090316:3959238-3959368
BmHel-7	DF090436:438207-438334

BmHel-7	DF090316:2066775-2066918
BmHel-7	DF090340:2164357-2164500
BmHel-7	DF090372:826420-826523
BmHel-7	DF090329:1713548-1713698
BmHel-7	DF090333:5130350-5130493
BmHel-7	DF090407:901772-901904
BmHel-7	DF090316:16029809-16029953
BmHel-7	DF090953:8048-8192
BmHel-7	DF090417:846111-846291
BmHel-7	DF090362:2298231-2298382
BmHel-7	DF090367:58765-58904
BmHel-7	DF090652:52398-52526
BmHel-7	DF090356:3055712-3055857
BmHel-7	DF090316:15163444-15163587
BmHel-7	DF090346:2519405-2519543
BmHel-7	DF090441:730493-730636
BmHel-7	DF090331:2007695-2007838
BmHel-7	DF090427:725983-726126
BmHel-7	DF090446:369947-370083
BmHel-7	DF090532:46392-46535
BmHel-7	DF090396:47972-48105
BmHel-7	DF090457:176104-176232
BmHel-7	BABH01044634:291-419
BmHel-7	DF090325:6792961-6793141
BmHel-7	DF090323:1320926-1321107
BmHel-7	DF090421:113992-114131
BmHel-7	DF090336:974923-975065
BmHel-7	DF090358:1580830-1580975
BmHel-7	DF090435:47416-47558
BmHel-7	DF090357:2870803-2870910
BmHel-7	DF090319:7488799-7488895
BmHel-7	DF090334:4850150-4850296
BmHel-7	DF090447:177329-177479
BmHel-7	DF090344:162697-162839
BmHel-7	DF090319:170869-170996
BmHel-7	DF090357:758170-758280
BmHel-7	DF090340:2794479-2794578
BmHel-7	DF090316:4923786-4923929
BmHel-7	DF090341:3926511-3926653
BmHel-7	DF090346:590373-590518
BmHel-7	DF090328:1568379-1568479
BmHel-7	DF090325:5719793-5719932
BmHel-7	DF090322:2496858-2497002
BmHel-7	DF090378:1565973-1566115
BmHel-7	DF090318:6436689-6436831
BmHel-7	DF090358:2195287-2195426
BmHel-7	DF090412:427288-427466
BmHel-7	DF090389:41151-41230

BmHel-7	DF090412:124755-124900
BmHel-7	DF090363:2204026-2204167
BmHel-7	DF090325:1594812-1594957
BmHel-7	DF090326:6840280-6840462
BmHel-7	DF090332:5473132-5473309
BmHel-7	DF090340:2481577-2481722
BmHel-7	DF090382:1833371-1833513
BmHel-7	DF090342:2056470-2056615
BmHel-7	DF090394:245948-246093
BmHel-7	DF090383:1814819-1814912
BmHel-7	DF090395:923557-923702
BmHel-7	DF090352:3802100-3802241
BmHel-7	DF090519:272-414
BmHel-7	DF090317:509285-509427
BmHel-7	DF090410:528547-528629
BmHel-7	DF090410:528681-528776
BmHel-7	DF090354:1859901-1860042
BmHel-7	DF090316:15687011-15687144
BmHel-7	DF090347:813120-813261
BmHel-7	DF090345:3319675-3319808
BmHel-7	DF090442:202455-202596
BmHel-7	DF090349:758137-758277
BmHel-7	DF090373:1263399-1263580
BmHel-7	DF090317:1022406-1022536
BmHel-7	DF090320:3875331-3875457
BmHel-7	DF090409:1372027-1372153
BmHel-7	BABH01045011:3656-3782
BmHel-7	DF090376:744344-744525
BmHel-7	DF090341:3429810-3429988
BmHel-7	DF090382:1292054-1292186
BmHel-7	DF090322:4174544-4174644
BmHel-7	DF090369:1289456-1289596
BmHel-7	DF090335:3894520-3894614
BmHel-7	DF090316:4381966-4382116
BmHel-7	DF090361:3010629-3010708
BmHel-7	DF090361:3010962-3011045
BmHel-7	DF090351:4138563-4138703
BmHel-7	DF090375:1456562-1456670
BmHel-7	DF090330:5307542-5307626
BmHel-7	DF090342:250343-250488
BmHel-7	DF090329:1928915-1929060
BmHel-7	DF090375:568252-568378
BmHel-7	DF090354:1078719-1078863
BmHel-7	DF090321:777737-777880
BmHel-7	DF090387:1589516-1589658
BmHel-7	DF090388:621951-622093
BmHel-7	DF090405:1385933-1386073
BmHel-7	DF090367:2645290-2645430

BmHel-7	DF090343:3196316-3196458
BmHel-7	DF090329:3806767-3806907
BmHel-7	DF090331:2666731-2666873
BmHel-7	DF090324:1260640-1260779
BmHel-7	DF090357:1771579-1771704
BmHel-7	DF090361:72426-72601
BmHel-7	DF091189:542-719
BmHel-7	DF090336:2871756-2871844
BmHel-7	DF090351:242201-242350
BmHel-7	DF090339:208341-208441
BmHel-7	DF090358:2820660-2820757
BmHel-7	DF090331:1502788-1502874
BmHel-7	DF090359:2693628-2693767
BmHel-7	DF090328:2520794-2520883
BmHel-7	DF090362:3069785-3069924
BmHel-7	DF090330:4793235-4793411
BmHel-7	DF090328:6443943-6444119
BmHel-7	DF090354:2205028-2205167
BmHel-7	DF090333:1299046-1299178
BmHel-7	DF090323:3003334-3003477
BmHel-7	DF090322:148446-148589
BmHel-7	DF090332:6166298-6166440
BmHel-7	DF090316:2501553-2501689
BmHel-7	DF090439:16287-16367
BmHel-7	DF090325:6780995-6781121
BmHel-7	DF090394:874930-875104
BmHel-7	DF090610:24071-24155
BmHel-7	DF090322:4232776-4232914
BmHel-7	DF090324:2344755-2344897
BmHel-7	DF090351:3525348-3525480
BmHel-7	DF090475:26410-26522
BmHel-7	DF090476:76559-76704
BmHel-7	DF090346:696863-697002
BmHel-7	DF090318:9004975-9005113
BmHel-7	DF090383:1321970-1322107
BmHel-7	DF090321:142821-142947
BmHel-7	DF090336:4190625-4190748
BmHel-7	DF090544:19942-20114
BmHel-7	DF090342:1743371-1743508
BmHel-7	DF090388:1027157-1027290
BmHel-7	DF090327:1495944-1496084
BmHel-7	DF090363:284243-284372
BmHel-7	DF090354:3743506-3743647
BmHel-7	DF090388:852756-852904
BmHel-7	DF090434:437995-438130
BmHel-7	DF090393:753843-753988
BmHel-7	BABH01045321:4153-4298
BmHel-7	DF090394:1558607-1558703

BmHel-7	DF090427:97192-97334
BmHel-7	DF090370:1801470-1801605
BmHel-7	DF090329:3753278-3753418
BmHel-7	DF090396:667608-667739
BmHel-7	DF090319:6256977-6257111
BmHel-7	DF090316:2649285-2649407
BmHel-7	DF090318:6114575-6114697
BmHel-7	DF090853:1980-2154
BmHel-7	DF090366:113446-113620
BmHel-7	DF090326:6671435-6671573
BmHel-7	DF090388:106017-106158
BmHel-7	DF090364:599324-599452
BmHel-7	DF090321:4349572-4349654
BmHel-7	DF090365:2614663-2614836
BmHel-7	DF090348:1607816-1607957
BmHel-7	DF090369:1229864-1229949
BmHel-7	DF090413:285469-285606
BmHel-7	DF090351:2494016-2494190
BmHel-7	DF090363:798073-798216
BmHel-7	DF090353:536642-536773
BmHel-7	DF090323:1246732-1246872
BmHel-7	DF090374:2236908-2237046
BmHel-7	DF090537:76262-76398
BmHel-7	DF090398:1277474-1277605
BmHel-7	DF090387:1108186-1108307
BmHel-7	DF090444:213414-213535
BmHel-7	DF090501:35011-35146
BmHel-7	DF090339:4810533-4810624
BmHel-7	DF090317:609679-609851
BmHel-7	DF090318:6903942-6904051
BmHel-7	DF090318:6597462-6597601
BmHel-7	DF090317:8443334-8443472
BmHel-7	DF090391:484165-484307
BmHel-7	DF090388:1250307-1250442
BmHel-7	DF090396:854294-854380
BmHel-7	DF090327:4456496-4456635
BmHel-7	DF090347:3743165-3743300
BmHel-7	DF090367:1570521-1570659
BmHel-7	DF090383:1198880-1199053
BmHel-7	DF090413:1257973-1258108
BmHel-7	BABH01049220:333-468
BmHel-7	DF090368:2053489-2053609
BmHel-7	DF090417:824076-824196
BmHel-7	BABH01072600:437-609
BmHel-7	BABH01051845:511-683
BmHel-7	DF090410:170091-170214
BmHel-7	DF090321:7583518-7583650
BmHel-7	DF090338:1723613-1723747

BmHel-7	DF090319:5525563-5525654
BmHel-7	DF090375:221901-222071
BmHel-7	DF090396:193166-193300
BmHel-7	DF090344:3772060-3772153
BmHel-7	DF090381:1941494-1941636
BmHel-7	DF090325:2599806-2599940
BmHel-7	DF091408:2688-2823
BmHel-7	DF090386:1090070-1090207
BmHel-7	DF090409:1609329-1609465
BmHel-7	DF090365:1810702-1810837
BmHel-7	DF090330:4118319-4118453
BmHel-7	DF090324:8006127-8006261
BmHel-7	DF090357:2821233-2821399
BmHel-7	DF090614:22435-22608
BmHel-7	DF090318:3409332-3409466
BmHel-7	DF090444:235248-235338
BmHel-7	DF090316:5194024-5194160
BmHel-7	DF090368:599434-599566
BmHel-7	BABH01066636:19-193
BmHel-7	DF090716:2411-2582
BmHel-7	DF090514:119157-119275
BmHel-7	DF090371:1103470-1103600
BmHel-7	DF090392:1671336-1671469
BmHel-7	DF090459:306824-306961
BmHel-7	DF090340:2503180-2503299
BmHel-7	BABH01079902:451-570
BmHel-7	DF090405:643190-643360
BmHel-7	DF090327:983385-983555
BmHel-7	DF090328:2591775-2591907
BmHel-7	DF090339:1225414-1225547
BmHel-7	DF090368:463457-463589
BmHel-7	DF091392:764-868
BmHel-7	DF091663:1137-1240
BmHel-7	DF090328:5440545-5440679
BmHel-7	DF090345:1486501-1486635
BmHel-7	DF090499:29963-30096
BmHel-7	DF090375:803197-803316
BmHel-7	DF090372:159355-159472
BmHel-7	DF090323:7257877-7258010
BmHel-7	DF090352:1943802-1943934
BmHel-7	DF090353:2687718-2687850
BmHel-7	DF090418:917829-917961
BmHel-7	DF090846:12046-12178
BmHel-7	DF090394:1171570-1171690
BmHel-7	DF090397:1410414-1410584
BmHel-7	DF090340:1718169-1718249
BmHel-7	DF090318:6475324-6475461
BmHel-7	DF090332:308142-308273

BmHel-7	DF090646:38515-38631
BmHel-7	DF090450:353314-353409
BmHel-7	DF090373:654653-654787
BmHel-7	DF090433:474773-474901
BmHel-7	DF090399:743511-743650
BmHel-7	DF090341:1889050-1889144
BmHel-7	DF090348:2649595-2649722
BmHel-7	DF090320:1096681-1096847
BmHel-7	DF090316:11329231-11329402
BmHel-7	DF090430:4477-4644
BmHel-7	DF090332:1280522-1280652
BmHel-7	DF090354:3099263-3099390
BmHel-7	DF090341:2616125-2616283
BmHel-7	DF090343:2810498-2810613
BmHel-7	DF090413:673571-673651
BmHel-7	DF090318:8070197-8070363
BmHel-7	DF090322:5800058-5800172
BmHel-7	DF090409:376788-376925
BmHel-7	DF090342:4776613-4776745
BmHel-7	DF090339:3392285-3392412
BmHel-7	DF091597:2711-2840
BmHel-7	DF090776:15508-15637
BmHel-7	BABH01051407:173-336
BmHel-7	DF090408:439623-439788
BmHel-7	DF090336:2522022-2522148
BmHel-7	DF090318:2205431-2205563
BmHel-7	DF090521:22206-22334
BmHel-7	DF090323:6888888-6889027
BmHel-7	DF090336:5116122-5116237
BmHel-7	DF090341:3499866-3500030
BmHel-7	DF090320:3121784-3121949
BmHel-7	DF091726:629-759
BmHel-7	DF090322:1493574-1493653
BmHel-7	DF090484:40068-40195
BmHel-7	DF090316:9300186-9300298
BmHel-7	BABH01080374:1-164
BmHel-7	DF090322:6711442-6711576
BmHel-7	DF090464:117672-117796
BmHel-7	DF090321:5058988-5059099
BmHel-7	DF090316:2643578-2643690
BmHel-7	DF090366:1094016-1094180
BmHel-7	BABH01079222:5-158
BmHel-7	BABH01075206:77-163
BmHel-7	DF090323:6719717-6719846
BmHel-7	DF090375:949320-949482
BmHel-7	DF090319:925640-925730
BmHel-7	DF090364:2442265-2442391
BmHel-7	DF090460:76969-77080

BmHel-7	DF090475:193513-193621
BmHel-7	DF090352:2820249-2820360
BmHel-7	DF090319:8259326-8259415
BmHel-7	DF090319:8259418-8259498
BmHel-7	DF090392:979220-979382
BmHel-7	DF090380:100701-100826
BmHel-7	DF090326:5976905-5977035
BmHel-7	DF090441:686120-686214
BmHel-7	DF090441:686530-686750
BmHel-7	DF090317:9047530-9047659
BmHel-7	DF090345:2173349-2173474
BmHel-7	DF090352:2547040-2547163
BmHel-7	DF090316:16175192-16175308
BmHel-7	DF090334:2247203-2247364
BmHel-7	DF090446:516983-517141
BmHel-7	DF090328:4496100-4496224
BmHel-7	DF090367:442167-442254
BmHel-7	DF090350:2575732-2575901
BmHel-7	DF090329:3610072-3610238
BmHel-7	DF090358:1397428-1397588
BmHel-7	DF090328:2658162-2658323
BmHel-7	DF090386:206992-207151
BmHel-7	DF090321:4608957-4609079
BmHel-7	DF090593:18459-18543
BmHel-7	DF091128:162-285
BmHel-7	DF090321:5825150-5825258
BmHel-7	BABH01087107:10-169
BmHel-7	DF090380:240436-240595
BmHel-7	BABH01054873:1-159
BmHel-7	BABH01085275:346-504
BmHel-7	DF090420:335784-335943
BmHel-7	DF090370:2683749-2683856
BmHel-7	DF090326:5919597-5919721
BmHel-7	DF090338:613160-613282
BmHel-7	DF090374:1914661-1914778
BmHel-7	DF090340:2638558-2638720
BmHel-7	BABH01076129:283-446
BmHel-7	DF091276:4414-4572
BmHel-7	DF090357:133791-133912
BmHel-7	BABH01046564:1170-1279
BmHel-7	DF090937:9110-9222
BmHel-7	DF090316:8757597-8757703
BmHel-7	DF090320:1951036-1951142
BmHel-7	DF090349:20771-20929
BmHel-7	DF090334:5099568-5099688
BmHel-7	DF090330:1832100-1832223
BmHel-7	DF090328:2277173-2277327
BmHel-7	DF090404:1089295-1089393

BmHel-7	DF090340:4555731-4555851
BmHel-7	DF090329:4752275-4752395
BmHel-7	DF090376:251489-251610
BmHel-7	DF090367:1118673-1118763
BmHel-7	DF090372:1421225-1421330
BmHel-7	BABH01066722:8-171
BmHel-7	DF090377:127322-127425
BmHel-7	DF090373:212646-212751
BmHel-7	BABH01070988:500-631
BmHel-7	DF091558:1492-1647
BmHel-7	DF090329:5095405-5095486
BmHel-7	DF090806:11655-11810
BmHel-7	DF090407:593938-594057
BmHel-7	DF090362:1961381-1961502
BmHel-7	DF090334:2656627-2656749
BmHel-7	DF090354:3370797-3370951
BmHel-7	DF090396:1336141-1336297
BmHel-7	DF090352:3884088-3884207
BmHel-7	DF090353:1730904-1731059
BmHel-7	DF090367:1517972-1518054
BmHel-7	DF091053:971-1090
BmHel-7	DF090336:2829783-2829865
BmHel-7	BABH01062228:1-154
BmHel-7	BABH01046290:1152-1273
BmHel-7	DF090441:245329-245449
BmHel-7	DF090422:841747-841862
BmHel-7	DF090337:886458-886612
BmHel-7	DF090343:540969-541086
BmHel-7	DF090342:76596-76750
BmHel-7	DF090342:288922-289076
BmHel-7	DF090342:1340978-1341131
BmHel-7	DF090342:3745052-3745203
BmHel-7	DF090342:4275286-4275439
BmHel-7	DF090342:4917987-4918142
BmHel-7	DF090356:607532-607685
BmHel-7	DF090356:1105980-1106133
BmHel-7	DF090356:1250448-1250600
BmHel-7	DF090355:744826-744980
BmHel-7	DF090355:3070159-3070312
BmHel-7	DF090349:75299-75452
BmHel-7	DF090349:2125205-2125358
BmHel-7	DF090349:4251397-4251554
BmHel-7	DF090349:3883008-3883164
BmHel-7	DF090341:3341126-3341280
BmHel-7	DF090341:4476625-4476778
BmHel-7	DF090341:2049048-2049200
BmHel-7	DF090344:1039378-1039529
BmHel-7	DF090344:4040210-4040365

BmHel-7	DF090344:2989174-2989325
BmHel-7	DF090367:1155371-1155527
BmHel-7	DF090354:2813317-2813469
BmHel-7	DF090336:1485424-1485577
BmHel-7	DF090336:4773745-4773898
BmHel-7	DF090336:5085568-5085721
BmHel-7	DF090336:4790454-4790605
BmHel-7	DF090336:923495-923647
BmHel-7	DF090319:5507422-5507575
BmHel-7	DF090319:6531973-6532119
BmHel-7	DF090319:7547311-7547464
BmHel-7	DF090319:8495309-8495462
BmHel-7	DF090319:6140400-6140553
BmHel-7	DF090319:5866014-5866158
BmHel-7	DF090316:614372-614524
BmHel-7	DF090316:15883497-15883650
BmHel-7	DF090316:13908708-13908861
BmHel-7	DF090316:13733653-13733806
BmHel-7	DF090316:12393961-12394118
BmHel-7	DF090316:10173125-10173278
BmHel-7	DF090316:6386402-6386559
BmHel-7	DF090316:5799683-5799836
BmHel-7	DF090316:4444714-4444862
BmHel-7	DF090316:811218-811376
BmHel-7	DF090390:214402-214555
BmHel-7	DF090388:1784720-1784872
BmHel-7	DF090388:1355462-1355615
BmHel-7	DF090377:1151289-1151442
BmHel-7	DF090371:236026-236179
BmHel-7	DF090371:1563407-1563556
BmHel-7	DF090364:2231154-2231307
BmHel-7	DF090364:2287047-2287200
BmHel-7	DF090346:1803013-1803166
BmHel-7	DF090346:456171-456324
BmHel-7	DF090343:1220759-1220912
BmHel-7	DF090343:2244618-2244771
BmHel-7	DF090343:4004819-4004972
BmHel-7	DF090340:397369-397527
BmHel-7	DF090340:4121960-4122113
BmHel-7	DF090340:4291030-4291183
BmHel-7	DF090340:1814107-1814260
BmHel-7	DF090328:283861-284014
BmHel-7	DF090328:5550880-5551033
BmHel-7	DF090328:629036-629189
BmHel-7	DF090325:764275-764428
BmHel-7	DF090325:6807501-6807654
BmHel-7	DF090325:2618316-2618469
BmHel-7	DF090325:2015117-2015269

BmHel-7	DF090325:538616-538773
BmHel-7	DF090323:1360802-1360955
BmHel-7	DF090323:3153113-3153266
BmHel-7	DF090323:6211070-6211223
BmHel-7	DF090322:882950-883103
BmHel-7	DF090322:7406768-7406921
BmHel-7	DF090322:4706687-4706840
BmHel-7	DF090322:1750696-1750848
BmHel-7	DF090329:3725556-3725709
BmHel-7	DF090329:2824675-2824828
BmHel-7	DF090351:3309429-3309582
BmHel-7	DF090347:101856-102003
BmHel-7	DF090352:628242-628395
BmHel-7	DF090352:3697746-3697899
BmHel-7	DF090352:4051652-4051804
BmHel-7	DF090352:2490805-2490958
BmHel-7	DF090320:3516378-3516531
BmHel-7	DF090320:5013885-5014044
BmHel-7	DF090320:6471447-6471600
BmHel-7	DF090320:3799551-3799704
BmHel-7	DF090384:1568011-1568161
BmHel-7	DF090378:1205976-1206136
BmHel-7	DF090350:1851774-1851927
BmHel-7	DF090333:383623-383776
BmHel-7	DF090333:2039242-2039395
BmHel-7	DF090327:5833835-5833988
BmHel-7	DF090327:4045218-4045371
BmHel-7	DF090327:2166344-2166496
BmHel-7	DF090318:544118-544271
BmHel-7	DF090318:4511776-4511929
BmHel-7	DF090318:7661624-7661777
BmHel-7	DF090318:8887829-8887982
BmHel-7	DF090318:7968799-7968952
BmHel-7	DF090318:7519323-7519476
BmHel-7	DF090389:45388-45541
BmHel-7	DF090339:44349-44502
BmHel-7	DF090339:4881819-4881976
BmHel-7	DF090339:1526597-1526750
BmHel-7	DF090353:1761445-1761598
BmHel-7	DF090353:3362367-3362525
BmHel-7	DF090353:1570316-1570467
BmHel-7	DF090456:49122-49273
BmHel-7	DF090398:1360028-1360181
BmHel-7	DF090373:1459867-1460017
BmHel-7	DF090373:1060737-1060890
BmHel-7	DF090363:1434476-1434628
BmHel-7	DF090361:1462049-1462202
BmHel-7	DF090357:1096473-1096626

BmHel-7	DF090332:515119-515272
BmHel-7	DF090332:1532492-1532644
BmHel-7	DF090332:2004431-2004584
BmHel-7	DF090332:755004-755157
BmHel-7	DF090441:720193-720346
BmHel-7	DF090422:811582-811737
BmHel-7	DF090419:190888-191041
BmHel-7	DF090418:13512-13665
BmHel-7	DF090418:561856-562009
BmHel-7	DF090380:711899-712051
BmHel-7	DF090380:1980006-1980159
BmHel-7	DF090380:1917007-1917160
BmHel-7	DF090366:1717050-1717202
BmHel-7	DF090366:295770-295923
BmHel-7	DF090358:1162998-1163155
BmHel-7	DF090358:1233593-1233741
BmHel-7	DF090358:1495038-1495189
BmHel-7	DF090358:1890599-1890752
BmHel-7	DF090358:1619200-1619356
BmHel-7	DF090348:2625244-2625395
BmHel-7	DF090348:3185391-3185542
BmHel-7	DF090348:3485164-3485317
BmHel-7	DF090348:2543724-2543877
BmHel-7	DF090345:813497-813646
BmHel-7	DF090345:1465571-1465723
BmHel-7	DF090345:4325163-4325316
BmHel-7	DF090345:3769946-3770099
BmHel-7	DF090330:141392-141545
BmHel-7	DF090330:6127305-6127463
BmHel-7	DF090317:4802920-4803073
BmHel-7	DF090317:9586802-9586955
BmHel-7	DF090317:10358074-10358227
BmHel-7	DF090317:9437025-9437178
BmHel-7	DF090317:9367941-9368091
BmHel-7	DF090317:4033942-4034095
BmHel-7	DF090317:2172089-2172242
BmHel-7	DF090317:678538-678691
BmHel-7	DF090421:991317-991470
BmHel-7	DF090400:1449950-1450103
BmHel-7	DF090400:137905-138057
BmHel-7	DF090365:748619-748772
BmHel-7	DF090335:3088115-3088266
BmHel-7	DF090335:5183683-5183836
BmHel-7	DF090335:5309292-5309445
BmHel-7	DF090321:4561205-4561358
BmHel-7	DF090383:311675-311828
BmHel-7	DF090383:895485-895638
BmHel-7	DF090383:714562-714714

BmHel-7	DF090381:583482-583633
BmHel-7	DF090338:557161-557314
BmHel-7	DF090338:901278-901426
BmHel-7	DF090338:2708893-2709047
BmHel-7	DF090338:3245618-3245771
BmHel-7	DF090338:4058590-4058742
BmHel-7	DF090338:4288346-4288499
BmHel-7	DF090338:4450394-4450547
BmHel-7	DF090438:519501-519646
BmHel-7	DF090434:127868-128021
BmHel-7	DF090434:689385-689533
BmHel-7	DF090424:796078-796231
BmHel-7	DF090408:659121-659274
BmHel-7	DF090404:274508-274661
BmHel-7	DF090404:1395607-1395760
BmHel-7	DF090404:955053-955212
BmHel-7	DF090392:1183068-1183221
BmHel-7	DF090385:1490426-1490579
BmHel-7	DF090385:458461-458614
BmHel-7	DF090368:300060-300213
BmHel-7	DF090362:2240395-2240548
BmHel-7	DF090362:172425-172578
BmHel-7	DF090331:4547119-4547273
BmHel-7	DF090331:5885529-5885685
BmHel-7	DF090324:2088333-2088486
BmHel-7	DF090427:629327-629483
BmHel-7	DF090467:214170-214326
BmHel-7	DF090409:676986-677139
BmHel-7	DF090401:1523107-1523260
BmHel-7	DF090401:1400538-1400691
BmHel-7	DF090401:1348508-1348660
BmHel-7	DF090391:399877-400030
BmHel-7	DF090391:1523362-1523514
BmHel-7	DF090369:878136-878283
BmHel-7	DF090403:1478553-1478706
BmHel-7	DF090415:968260-968409
BmHel-7	DF090410:236214-236367
BmHel-7	DF090459:359947-360106
BmHel-7	DF090437:12521-12674
BmHel-7	DF090382:1933939-1934092
BmHel-7	DF090382:1626850-1627005
BmHel-7	DF090359:2814044-2814196
BmHel-7	DF090359:3012113-3012266
BmHel-7	DF090359:1815897-1816050
BmHel-7	DF090443:615517-615670
BmHel-7	DF090428:495782-495935
BmHel-7	DF090539:5147-5300
BmHel-7	DF090430:425465-425618

BmHel-7	DF090451:339489-339642
BmHel-7	DF090465:15643-15797
BmHel-7	DF090466:36604-36757
BmHel-7	DF090483:133721-133874
BmHel-7	DF090509:121765-121928
BmHel-7	DF090496:135260-135413
BmHel-7	BABH01081294:242-395
BmHel-7	DF090648:18804-18957
BmHel-7	BABH01064763:396-549
BmHel-7	DF090906:1631-1788
BmHel-7	BABH01077814:433-586
BmHel-7	BABH01064748:319-472
BmHel-7	DF090528:24132-24285
BmHel-7	DF090528:27238-27396
BmHel-7	DF090482:39519-39672
BmHel-7	DF090982:7193-7349
BmHel-7	DF090480:93011-93167
BmHel-7	BABH01064670:509-661
BmHel-7	DF090640:31959-32112
BmHel-7	DF090726:17124-17274
BmHel-7	DF090925:3465-3618
BmHel-7	DF090500:74121-74281
BmHel-7	DF091635:222-374
BmHel-7	DF090888:13161-13314
BmHel-7	BABH01059831:389-540
BmHel-7	BABH01050554:343-502
BmHel-7	DF091130:2460-2616
BmHel-7	DF090774:21296-21449
BmHel-7	DF090751:1205-1359
BmHel-7	DF090590:12349-12505
BmHel-7	BABH01088596:17-165
BmHel-7	DF090331:2764293-2764446
BmHel-7	DF090379:834642-834795
BmHel-7	DF090403:786128-786257
BmHel-7	DF090347:1598538-1598641
BmHel-7	DF090327:4939633-4939742
BmHel-7	DF090600:33574-33685
BmHel-7	DF090349:2770197-2770349
BmHel-7	DF090341:3999260-3999413
BmHel-7	DF090319:5050367-5050523
BmHel-7	DF090319:5431378-5431530
BmHel-7	DF090319:7897946-7898098
BmHel-7	DF090319:5796218-5796369
BmHel-7	DF090326:6098863-6099011
BmHel-7	DF090316:746128-746280
BmHel-7	DF090390:1499174-1499326
BmHel-7	DF090343:942672-942824
BmHel-7	DF090329:2691609-2691761

BmHel-7	DF090378:313346-313498
BmHel-7	DF090350:3851662-3851812
BmHel-7	DF090318:2727107-2727259
BmHel-7	DF090318:5968551-5968703
BmHel-7	DF090360:1251779-1251931
BmHel-7	DF090339:1856884-1857038
BmHel-7	DF090339:4772836-4772988
BmHel-7	DF090373:169965-170117
BmHel-7	DF090363:1857369-1857521
BmHel-7	DF090361:1378173-1378326
BmHel-7	DF090345:1095674-1095827
BmHel-7	DF090330:852871-853023
BmHel-7	DF090400:191812-191964
BmHel-7	DF090365:1231290-1231444
BmHel-7	DF090335:2072724-2072876
BmHel-7	DF090338:4134765-4134917
BmHel-7	DF090324:1678181-1678333
BmHel-7	DF090391:347903-348055
BmHel-7	DF090469:140495-140647
BmHel-7	DF090387:1694221-1694373
BmHel-7	DF090359:2272411-2272563
BmHel-7	DF090370:1780668-1780822
BmHel-7	DF090341:750593-750750
BmHel-7	DF090344:2205297-2205449
BmHel-7	DF090316:14204258-14204410
BmHel-7	DF090322:5804950-5805102
BmHel-7	DF090347:847392-847548
BmHel-7	DF090350:3369384-3369536
BmHel-7	DF090335:5730085-5730230
BmHel-7	DF090409:900665-900815
BmHel-7	DF090415:1093639-1093790
BmHel-7	DF090452:447423-447575
BmHel-7	BABH01049659:885-1035
BmHel-7	DF090481:209451-209603
BmHel-7	DF090329:2161324-2161476
BmHel-7	DF090324:4598555-4598670
BmHel-7	DF090338:1295752-1295880
BmHel-7	DF090318:3764807-3764959
BmHel-7	DF090386:1899513-1899665
BmHel-7	DF090343:3383354-3383444
BmHel-7	DF090380:1162761-1162871
BmHel-7	DF090330:2782067-2782172
BmHel-7	DF090318:8411988-8412139
BmHel-7	DF090319:1840420-1840571
BmHel-7	DF090340:3608675-3608832
BmHel-7	DF090323:754960-755111
BmHel-7	DF090320:1848200-1848351
BmHel-7	DF090327:1852998-1853152

BmHel-7	DF090339:393344-393492
BmHel-7	DF090363:2156421-2156572
BmHel-7	DF090332:879893-880039
BmHel-7	DF090380:298137-298288
BmHel-7	DF090345:2952872-2953024
BmHel-7	DF090435:343660-343811
BmHel-7	DF090435:597983-598134
BmHel-7	DF090391:1333315-1333453
BmHel-7	DF090382:1694867-1695019
BmHel-7	DF090367:2530043-2530194
BmHel-7	DF090329:5281801-5281955
BmHel-7	DF090351:1237802-1237948
BmHel-7	DF090389:213872-214023
BmHel-7	DF090363:2786713-2786864
BmHel-7	DF090399:359883-360034
BmHel-7	DF090335:5508301-5508452
BmHel-7	DF090444:64891-65042
BmHel-7	DF090449:809164-809315
BmHel-7	BABH01061720:539-690
BmHel-7	BABH01085419:53-175
BmHel-7	DF090334:3958133-3958247
BmHel-7	DF090355:3244389-3244489
BmHel-7	DF090470:346478-346577
BmHel-7	DF090350:25364-25466
BmHel-7	DF090403:259320-259420
BmHel-7	DF090359:1060153-1060268
BmHel-7	DF090333:3794299-3794398
BmHel-7	DF090395:609754-609904
BmHel-7	DF090397:576651-576805
BmHel-7	DF090341:500014-500164
BmHel-7	DF090319:2495090-2495240
BmHel-7	DF090343:497796-497946
BmHel-7	DF090347:873131-873281
BmHel-7	DF090350:465369-465519
BmHel-7	DF090363:1983111-1983261
BmHel-7	DF090358:465535-465688
BmHel-7	DF090348:3766902-3767052
BmHel-7	DF090330:3380458-3380608
BmHel-7	DF090383:1543673-1543823
BmHel-7	DF090429:252724-252874
BmHel-7	DF090368:942562-942712
BmHel-7	DF090369:1168134-1168284
BmHel-7	DF090382:153900-154050
BmHel-7	DF090412:695065-695210
BmHel-7	DF090471:187941-188091
BmHel-7	DF090349:380452-380602
BmHel-7	DF090341:442122-442272
BmHel-7	DF090414:1184158-1184308

BmHel-7	DF090319:219439-219589
BmHel-7	DF090402:1321510-1321655
BmHel-7	DF090388:650074-650224
BmHel-7	DF090364:348741-348891
BmHel-7	DF090325:281260-281410
BmHel-7	DF090329:5653690-5653840
BmHel-7	DF090351:2626578-2626723
BmHel-7	DF090352:2951380-2951529
BmHel-7	DF090320:1254678-1254828
BmHel-7	DF090378:1158435-1158585
BmHel-7	DF090350:3845818-3845977
BmHel-7	DF090337:4904405-4904555
BmHel-7	DF090333:4036677-4036827
BmHel-7	DF090318:5774799-5774949
BmHel-7	DF090339:317077-317228
BmHel-7	DF090363:1191791-1191941
BmHel-7	DF090361:942158-942308
BmHel-7	DF090332:2194078-2194225
BmHel-7	DF090381:527145-527294
BmHel-7	DF090338:4839840-4839989
BmHel-7	DF090362:2215087-2215237
BmHel-7	DF090413:457363-457512
BmHel-7	DF090413:863958-864108
BmHel-7	DF090369:2354783-2354933
BmHel-7	DF090431:287832-287979
BmHel-7	DF091113:1412-1562
BmHel-7	BABH01045119:4399-4544
BmHel-7	DF090476:61738-61852
BmHel-7	DF090327:4973870-4973989
BmHel-7	DF090319:6555402-6555552
BmHel-7	DF090411:1330874-1331024
BmHel-7	DF090349:3646614-3646723
BmHel-7	DF090319:312988-313102
BmHel-7	DF090390:400077-400172
BmHel-7	DF090377:1448599-1448696
BmHel-7	DF090378:1451728-1451819
BmHel-7	DF090317:10772837-10772939
BmHel-7	DF090319:3730656-3730805
BmHel-7	DF090345:3819621-3819770
BmHel-7	DF090379:145376-145526
BmHel-7	DF090342:361834-361983
BmHel-7	DF090326:69925-70076
BmHel-7	DF090326:2522764-2522909
BmHel-7	DF090326:6193369-6193518
BmHel-7	DF090402:30856-31005
BmHel-7	DF090334:4342594-4342743
BmHel-7	DF090323:627287-627436
BmHel-7	DF090323:5008095-5008246

BmHel-7	DF090323:6316804-6316953
BmHel-7	DF090351:1762569-1762718
BmHel-7	DF090333:5406153-5406302
BmHel-7	DF090380:286329-286476
BmHel-7	DF090330:1927050-1927191
BmHel-7	DF090317:6419899-6420052
BmHel-7	DF090421:11982-12131
BmHel-7	DF090375:266471-266620
BmHel-7	DF090331:5691572-5691721
BmHel-7	DF090436:289280-289429
BmHel-7	DF090486:39882-40027
BmHel-7	DF090433:318634-318737
BmHel-7	DF090342:2123317-2123466
BmHel-7	DF090414:38852-39001
BmHel-7	DF090338:2434563-2434712
BmHel-7	DF090445:419712-419855
BmHel-7	DF091609:2395-2546
BmHel-7	DF090343:3178692-3178841
BmHel-7	DF090317:7403704-7403807
BmHel-7	DF090337:1294655-1294767
BmHel-7	BABH01043931:381-492
BmHel-7	DF090413:80458-80557
BmHel-7	DF090353:615743-615854
BmHel-7	DF090356:3578379-3578492
BmHel-7	BABH01050404:290-404
BmHel-7	DF090377:509231-509329
BmHel-7	DF090317:600050-600145
BmHel-7	DF090367:1729182-1729330
BmHel-7	DF090364:753925-754073
BmHel-7	DF090337:1672819-1672967
BmHel-7	DF090338:4615186-4615291
BmHel-7	DF090379:212591-212740
BmHel-7	DF090797:11358-11506
BmHel-7	DF090356:1121848-1121997
BmHel-7	DF090342:401543-401690
BmHel-7	DF090405:774742-774881
BmHel-7	DF090367:2271483-2271631
BmHel-7	DF090354:2462031-2462178
BmHel-7	DF090323:5015290-5015438
BmHel-7	DF090322:5570869-5571024
BmHel-7	DF090350:2154107-2154260
BmHel-7	DF090353:1924098-1924246
BmHel-7	DF090332:1924920-1925068
BmHel-7	DF090335:1844004-1844152
BmHel-7	DF090335:4657672-4657820
BmHel-7	DF090335:2116039-2116187
BmHel-7	DF090362:3089175-3089323
BmHel-7	BABH01045180:2791-2939

BmHel-7	DF090425:774871-774967
BmHel-7	DF090378:2147780-2147928
BmHel-7	BABH01088265:182-299
BmHel-7	DF090319:6804356-6804505
BmHel-7	DF090530:46749-46895
BmHel-7	DF090322:3760406-3760503
BmHel-7	DF090357:558377-558487
BmHel-7	DF090327:3123456-3123569
BmHel-7	DF090956:4897-4991
BmHel-7	DF090336:1130223-1130370
BmHel-7	DF090318:5466469-5466608
BmHel-7	DF090364:1577821-1577968
BmHel-7	DF090398:1031166-1031317
BmHel-7	BABH01045845:3204-3351
BmHel-7	DF090372:298691-298831
BmHel-7	DF090361:1581892-1582039
BmHel-7	DF090318:4175094-4175241
BmHel-7	DF090364:686848-686991
BmHel-7	DF090343:2218473-2218620
BmHel-7	DF090396:1359220-1359367
BmHel-7	DF090423:138822-138969
BmHel-7	DF090562:26087-26183
BmHel-7	DF090334:4770642-4770729
BmHel-7	DF090360:590310-590423
BmHel-7	DF090331:917960-918105
BmHel-7	DF090341:2630893-2631039
BmHel-7	DF090326:1757657-1757804
BmHel-7	DF090323:7453822-7453965
BmHel-7	DF090329:3477300-3477450
BmHel-7	DF090350:2522529-2522674
BmHel-7	DF090350:1699294-1699440
BmHel-7	DF090339:1968842-1968988
BmHel-7	DF090380:533174-533311
BmHel-7	DF090366:1628688-1628834
BmHel-7	DF090345:474199-474348
BmHel-7	DF090317:8953289-8953435
BmHel-7	DF090338:3232602-3232748
BmHel-7	DF090429:415099-415245
BmHel-7	DF090437:58724-58869
BmHel-7	DF090457:235816-235959
BmHel-7	DF090910:1042-1194
BmHel-7	DF091643:1762-1904
BmHel-7	DF090340:2880204-2880359
BmHel-7	DF090440:293124-293270
BmHel-7	DF090341:3892481-3892632
BmHel-7	DF090356:478207-478356
BmHel-7	DF090323:5930347-5930500
BmHel-7	DF090351:3584380-3584489

BmHel-7	DF090317:2628724-2628870
BmHel-7	DF090439:5202-5348
BmHel-7	DF090368:881335-881429
BmHel-7	DF090349:1242131-1242247
BmHel-7	DF090370:724922-725035
BmHel-7	DF090333:4257075-4257183
BmHel-7	DF090323:7324437-7324549
BmHel-7	DF090326:4239340-4239435
BmHel-7	DF090316:12813274-12813391
BmHel-7	DF090429:529595-529677
BmHel-7	DF090320:5223780-5223925
BmHel-7	DF090421:557674-557819
BmHel-7	DF090355:1451173-1451318
BmHel-7	DF090354:2248923-2249061
BmHel-7	DF090316:13221494-13221638
BmHel-7	DF090325:5873296-5873445
BmHel-7	DF090323:6217697-6217851
BmHel-7	DF090329:3545264-3545409
BmHel-7	DF090352:1162072-1162217
BmHel-7	DF090327:2971220-2971365
BmHel-7	DF090407:941592-941737
BmHel-7	DF090456:208579-208724
BmHel-7	DF090330:3242477-3242622
BmHel-7	DF090317:1269356-1269500
BmHel-7	DF090317:3260448-3260593
BmHel-7	DF090365:1037104-1037249
BmHel-7	DF090338:4948917-4949062
BmHel-7	DF090359:1844110-1844255
BmHel-7	DF090440:392145-392238
BmHel-7	DF090507:107922-108067
BmHel-7	DF090329:1875425-1875518
BmHel-7	DF090387:1773889-1773996
BmHel-7	DF090358:1791044-1791152
BmHel-7	DF090341:4322713-4322809
BmHel-7	BABH01077008:75-211
BmHel-7	DF090519:112246-112359
BmHel-7	DF090362:1931415-1931525
BmHel-7	DF090381:411752-411896
BmHel-7	DF090620:21037-21177
BmHel-7	DF090363:1135677-1135824
BmHel-7	DF090322:1464114-1464246
BmHel-7	DF090366:382611-382755
BmHel-7	BABH01070362:1-144
BmHel-7	BABH01049688:677-781
BmHel-7	DF090342:524226-524337
BmHel-7	DF090348:2598317-2598460
BmHel-7	DF090411:461880-462024
BmHel-7	DF090418:979581-979725

BmHel-7	BABH01053693:639-783
BmHel-7	DF090360:21865-22008
BmHel-7	BABH01067864:507-650
BmHel-7	DF090437:223064-223207
BmHel-7	DF090384:485701-485793
BmHel-7	DF090338:1938170-1938306
BmHel-7	DF090344:893351-893494
BmHel-7	DF090342:4857242-4857388
BmHel-7	DF090354:225128-225267
BmHel-7	DF090340:690693-690836
BmHel-7	DF090320:6065385-6065528
BmHel-7	DF090373:1486440-1486580
BmHel-7	DF090317:6426407-6426552
BmHel-7	DF090317:6955790-6955928
BmHel-7	DF090421:574746-574889
BmHel-7	DF090338:2105403-2105545
BmHel-7	DF090937:2284-2426
BmHel-7	DF090322:187413-187522
BmHel-7	DF090338:3306060-3306166
BmHel-7	DF090340:1653181-1653272
BmHel-7	BABH01057384:208-292
BmHel-7	BABH01054438:198-282
BmHel-7	DF090369:2673371-2673512
BmHel-7	DF090340:3134161-3134274
BmHel-7	DF090316:6941992-6942132
BmHel-7	DF090325:5857266-5857408
BmHel-7	DF090320:6256996-6257083
BmHel-7	DF090413:1030276-1030418
BmHel-7	DF090357:1792793-1792935
BmHel-7	DF091298:3947-4089
BmHel-7	DF090367:2224968-2225110
BmHel-7	DF090334:5281188-5281329
BmHel-7	DF090334:2840852-2840994
BmHel-7	DF090323:1960809-1960951
BmHel-7	DF090322:5646809-5646951
BmHel-7	DF090318:2789716-2789858
BmHel-7	DF090411:1284268-1284410
BmHel-7	DF090317:10619998-10620140
BmHel-7	DF090383:1055344-1055486
BmHel-7	DF090450:288390-288533
BmHel-7	DF090318:9698408-9698515
BmHel-7	DF090349:1646284-1646374
BmHel-7	DF090318:2100607-2100741
BmHel-7	DF090325:4888712-4888858
BmHel-7	BABH01076464:455-596
BmHel-7	DF090615:44721-44870
BmHel-7	DF090320:423519-423659
BmHel-7	DF090378:1680975-1681116

BmHel-7	DF090340:3900896-3901034
BmHel-7	DF090323:6232642-6232783
BmHel-7	DF090318:5358867-5359008
BmHel-7	DF090362:1013420-1013561
BmHel-7	DF090379:782034-782175
BmHel-7	DF090331:2804211-2804300
BmHel-7	DF090336:947426-947517
BmHel-7	DF090337:2302626-2302769
BmHel-7	DF090414:572835-572945
BmHel-7	DF090351:2565673-2565813
BmHel-7	DF090347:1781606-1781746
BmHel-7	DF090327:4268059-4268199
BmHel-7	DF090358:2818246-2818386
BmHel-7	DF090323:1106126-1106266
BmHel-7	DF090335:5771956-5772037
BmHel-7	DF090344:3928968-3929108
BmHel-7	DF090318:4926443-4926583
BmHel-7	DF090319:7069421-7069561
BmHel-7	DF090334:5343666-5343806
BmHel-7	DF090328:6239422-6239561
BmHel-7	DF090323:2205234-2205374
BmHel-7	DF090327:5535990-5536130
BmHel-7	DF090318:5307586-5307726
BmHel-7	DF090363:2540730-2540870
BmHel-7	DF090348:2720939-2721080
BmHel-7	DF090404:1411313-1411454
BmHel-7	BABH01081745:403-543
BmHel-7	DF090353:243323-243434
BmHel-7	DF090460:53441-53544
BmHel-7	DF090338:655462-655566
BmHel-7	DF090318:1586729-1586817
BmHel-7	DF090344:3687676-3687816
BmHel-7	DF090351:3170250-3170382
BmHel-7	DF090333:4981616-4981755
BmHel-7	DF090363:381568-381705
BmHel-7	DF090368:270078-270217
BmHel-7	DF090518:26549-26655
BmHel-7	DF090323:4309680-4309819
BmHel-7	DF090363:170437-170576
BmHel-7	DF090387:1589003-1589142
BmHel-7	DF090535:75626-75765
BmHel-7	DF090361:1525746-1525884
BmHel-7	DF090366:416982-417082
BmHel-7	DF090381:400125-400263
BmHel-7	DF090397:706258-706396
BmHel-7	DF090318:5943094-5943232
BmHel-7	DF090429:567392-567530
BmHel-7	DF090409:1156111-1156197

BmHel-7	DF090367:2008549-2008651
BmHel-7	DF090358:281943-282081
BmHel-7	DF090330:65913-66050
BmHel-7	DF090332:1434714-1434850
BmHel-7	BABH01048224:580-710
BmHel-7	DF090343:2390573-2390674
BmHel-7	DF090343:2948731-2948812
BmHel-7	DF090411:582224-582360
BmHel-7	BABH01059046:575-713
BmHel-7	DF090392:1216329-1216464
BmHel-7	DF090415:377829-377966
BmHel-7	DF090615:9901-10038
BmHel-7	BABH01052674:80-161
BmHel-7	DF090410:850311-850416
BmHel-7	DF090388:615444-615573
BmHel-7	DF090323:711197-711300
BmHel-7	DF090327:1877297-1877434
BmHel-7	DF090381:1711867-1712004
BmHel-7	DF090319:8145031-8145171
BmHel-7	DF090323:4572396-4572537
BmHel-7	DF090321:2201595-2201732
BmHel-7	BABH01066281:25-162
BmHel-7	DF090339:1692821-1692917
BmHel-7	DF090325:146923-147060
BmHel-7	DF090418:814325-814426
BmHel-7	DF090414:1116874-1117010
BmHel-7	DF090319:3810182-3810318
BmHel-7	DF090367:2171769-2171905
BmHel-7	DF090316:13831908-13832044
BmHel-7	DF090322:1210119-1210255
BmHel-7	DF090394:1581141-1581277
BmHel-7	DF090366:708207-708343
BmHel-7	BABH01051630:743-880
BmHel-7	DF090364:380651-380787
BmHel-7	DF090329:2062547-2062646
BmHel-7	DF090341:3228362-3228490
BmHel-7	DF090360:512495-512597
BmHel-7	DF090327:3764588-3764672
BmHel-7	DF090374:1686525-1686661
BmHel-7	DF090344:3818130-3818230
BmHel-7	DF090332:3526640-3526745
BmHel-7	DF090354:3496631-3496766
BmHel-7	DF090364:2247898-2248025
BmHel-7	DF090395:1381794-1381929
BmHel-7	DF090338:4702070-4702206
BmHel-7	DF090327:1476582-1476717
BmHel-7	DF090895:18864-18998
BmHel-7	BABH01060365:1-139

BmHel-7	DF090491:133278-133361
BmHel-7	DF090319:3355422-3355508
BmHel-7	DF090332:3386326-3386423
BmHel-7	DF090331:3961693-3961827
BmHel-7	BABH01058680:504-636
BmHel-7	DF090429:158658-158792
BmHel-7	DF090340:1920696-1920807
BmHel-7	DF090331:3827467-3827601
BmHel-7	DF090347:1469740-1469878
BmHel-7	DF090361:491401-491535
BmHel-7	DF090405:43406-43540
BmHel-7	DF090341:2511799-2511892
BmHel-7	DF090354:2097102-2097235
BmHel-7	DF090327:1265318-1265451
BmHel-7	DF090419:575043-575176
BmHel-7	DF090518:80321-80454
BmHel-7	DF090367:1760000-1760091
BmHel-7	DF090334:1805672-1805809
BmHel-7	DF090321:1709937-1710070
BmHel-7	DF090369:1561784-1561921
BmHel-7	BABH01059309:56-152
BmHel-7	BABH01048549:710-806
BmHel-7	DF090373:934301-934384
BmHel-7	DF090388:1287638-1287768
BmHel-7	DF090357:405441-405581
BmHel-7	DF090367:771748-771845
BmHel-7	DF090367:2383061-2383194
BmHel-7	DF090435:25498-25630
BmHel-7	DF090408:730935-731067
BmHel-7	DF090386:274818-274950
BmHel-7	DF090324:343532-343664
BmHel-7	DF090341:4100448-4100551
BmHel-7	DF090323:1185334-1185466
BmHel-7	DF090320:3366184-3366316
BmHel-7	DF090327:2130669-2130801
BmHel-7	DF090407:1118427-1118559
BmHel-7	DF090348:3365950-3366082
BmHel-7	DF090396:492811-492945
BmHel-7	BABH01044374:2757-2890
BmHel-7	DF090321:4448882-4448973
BmHel-7	DF090369:1369036-1369117
BmHel-7	DF090329:5502957-5503088
BmHel-7	DF090365:1174892-1175023
BmHel-7	BABH01081112:1-132
BmHel-7	BABH01079954:439-570
BmHel-7	DF090324:7090140-7090234
BmHel-7	DF090325:3779547-3779678
BmHel-7	DF090327:2888966-2889097

BmHel-7	DF090340:3741382-3741511
BmHel-7	DF090323:7979014-7979096
BmHel-7	BABH01081035:430-560
BmHel-7	DF090323:1234790-1234919
BmHel-7	DF090348:2695703-2695832
BmHel-7	DF090348:2797259-2797388
BmHel-7	DF090386:588265-588394
BmHel-7	DF090492:24085-24214
BmHel-7	DF091125:8825-8952
BmHel-7	DF090360:1236064-1236160
BmHel-7	DF090386:494648-494730
BmHel-7	DF090489:2026-2121
BmHel-7	DF090422:1048716-1048845
BmHel-7	DF090482:186960-187040
BmHel-7	DF090386:966436-966565
BmHel-7	DF090348:1570677-1570769
BmHel-7	DF090398:374500-374618
BmHel-7	DF090333:4815876-4816004
BmHel-7	DF090349:3256185-3256313
BmHel-7	DF090335:3316637-3316765
BmHel-7	DF090335:3550506-3550634
BmHel-7	DF090386:254683-254811
BmHel-7	DF090321:2152677-2152805
BmHel-7	DF091407:3359-3445
BmHel-7	BABH01088073:1-129
BmHel-7	DF090316:15834101-15834228
BmHel-7	DF090322:2675675-2675802
BmHel-7	BABH01070215:241-368
BmHel-7	BABH01064700:93-220
BmHel-7	DF091354:4812-4939
BmHel-7	BABH01070205:1-128
BmHel-7	DF090414:766957-767084
BmHel-7	BABH01079140:1-127
BmHel-7	DF090417:638228-638356
BmHel-7	DF090325:2004227-2004317
BmHel-7	DF090411:725395-725480
BmHel-7	DF090327:1941720-1941845
BmHel-7	DF090327:914227-914353
BmHel-7	DF090318:8112356-8112482
BmHel-7	DF090353:1474397-1474523
BmHel-7	DF090338:4163642-4163755
BmHel-7	DF090362:3011586-3011712
BmHel-7	DF090372:2097765-2097891
BmHel-7	DF090442:234150-234276
BmHel-7	BABH01044635:5318-5444
BmHel-7	DF090318:8641444-8641570
BmHel-7	DF090346:3745239-3745332
BmHel-7	BABH01068927:1-90

BmHel-7	DF090320:1336180-1336303
BmHel-7	BABH01045503:2-128
BmHel-7	BABH01059907:1-127
BmHel-7	DF090321:4471744-4471831
BmHel-7	DF090316:14672580-14672705
BmHel-7	DF090418:678510-678634
BmHel-7	DF090466:323042-323167
BmHel-7	DF090396:1152709-1152834
BmHel-7	DF090321:294150-294244
BmHel-7	DF090361:2265701-2265825
BmHel-7	DF090344:1736807-1736932
BmHel-7	DF090327:1214199-1214317
BmHel-7	DF090345:1734587-1734679
BmHel-7	DF090316:14299939-14300064
BmHel-7	DF090317:6784248-6784372
BmHel-7	DF090425:680468-680592
BmHel-7	DF090443:43420-43544
BmHel-7	BABH01048191:408-532
BmHel-7	DF090350:1603055-1603179
BmHel-7	DF091281:5803-5927
BmHel-7	DF090340:2918642-2918761
BmHel-7	DF090418:334019-334142
BmHel-7	DF090586:22691-22817
BmHel-7	DF090403:303471-303598
BmHel-7	DF090361:2994882-2995005
BmHel-7	DF090327:5277720-5277842
BmHel-7	DF090407:1187183-1187305
BmHel-7	DF090358:1543526-1543648
BmHel-7	DF090327:2912646-2912768
BmHel-7	DF090336:3392113-3392235
BmHel-7	DF090318:4283386-4283508
BmHel-7	DF090333:490964-491086
BmHel-7	DF090317:8459112-8459234
BmHel-7	DF090356:2085446-2085568
BmHel-7	DF090318:7013055-7013177
BmHel-7	DF090317:3416659-3416781
BmHel-7	DF090410:195866-195990
BmHel-7	DF090326:607962-608083
BmHel-7	DF090390:1230048-1230169
BmHel-7	DF090352:3339769-3339890
BmHel-7	DF090373:813276-813396
BmHel-7	DF090329:4523563-4523684
BmHel-7	BABH01081209:1-122
BmHel-7	DF090344:1904605-1904726
BmHel-7	DF090378:97477-97602
BmHel-7	BABH01053488:521-602
BmHel-7	DF090321:7699147-7699268
BmHel-7	DF090321:971249-971370

BmHel-7	DF090320:6577593-6577713
BmHel-7	BABH01052189:3-112
BmHel-7	DF090422:980935-981055
BmHel-7	DF090328:1674672-1674792
BmHel-7	DF090367:2236795-2236915
BmHel-7	DF090378:776031-776149
BmHel-7	DF090469:386982-387100
BmHel-7	DF090444:43210-43328
BmHel-7	BABH01073879:484-603
BmHel-7	DF090328:3577063-3577145
BmHel-7	DF090325:3132216-3132335
BmHel-7	BABH01046693:497-609
BmHel-7	DF090398:947469-947550
BmHel-7	DF090337:2824786-2824903
BmHel-7	DF090335:3485703-3485821
BmHel-7	DF090362:3203949-3204067
BmHel-7	DF090320:2293027-2293144
BmHel-7	DF090353:3170126-3170236
BmHel-7	DF090428:889092-889175
BmHel-7	DF090339:2758672-2758779
BmHel-7	DF090391:1246611-1246690
BmHel-7	DF090335:825900-825979
BmHel-7	DF090380:658691-658772
BmHel-7	BABH01082281:433-548
BmHel-7	DF090434:285837-285952
BmHel-7	DF090372:865729-865842
BmHel-7	DF090366:2311292-2311405
BmHel-7	DF090338:3814037-3814150
BmHel-7	DF090328:27506-27619
BmHel-7	DF090347:2679345-2679458
BmHel-7	DF090371:1407190-1407303
BmHel-7	BABH01068634:1-114
BmHel-7	DF090387:312983-313088
BmHel-7	BABH01053101:590-701
BmHel-7	DF090316:7431941-7432053
BmHel-7	BABH01060155:173-283
BmHel-7	DF090396:51422-51534
BmHel-7	DF090400:177871-177982
BmHel-7	DF090401:455914-456025
BmHel-7	DF090316:4232127-4232237
BmHel-7	DF090391:1423422-1423532
BmHel-7	DF090356:3075849-3075959
BmHel-7	DF090344:3722978-3723088
BmHel-7	DF090422:362824-362934
BmHel-7	DF090398:970850-970960
BmHel-7	DF090345:673283-673393
BmHel-7	DF090390:308912-309022
BmHel-7	DF090317:1648172-1648282

BmHel-7	BABH01046211:2701-2809
BmHel-7	DF090343:1141238-1141348
BmHel-7	DF090340:763455-763564
BmHel-7	BABH01047025:1-110
BmHel-7	DF090394:493446-493556
BmHel-7	DF090422:201073-201182
BmHel-7	BABH01052503:7-117
BmHel-7	DF090329:2737890-2737999
BmHel-7	DF090360:1956123-1956233
BmHel-7	DF090398:487153-487261
BmHel-7	BABH01045179:10-114
BmHel-7	DF090354:1295669-1295777
BmHel-7	DF090369:1359744-1359852
BmHel-7	BABH01045879:3077-3187
BmHel-7	BABH01071113:10-121
BmHel-7	DF091144:3871-3978
BmHel-7	DF090332:2617172-2617279
BmHel-7	DF090335:3142603-3142708
BmHel-7	DF090574:52928-53034
BmHel-7	DF090405:363634-363740
BmHel-7	DF090375:1018295-1018402
BmHel-7	BABH01082844:422-528
BmHel-7	DF090338:2291752-2291858
BmHel-7	DF090342:1066648-1066754
BmHel-7	DF090323:4945807-4945913
BmHel-7	DF090332:3310648-3310754
BmHel-7	DF090338:1060903-1061009
BmHel-7	DF090335:5588565-5588673
BmHel-7	DF090461:145829-145935
BmHel-7	DF090476:61615-61721
BmHel-7	DF090319:7511177-7511282
BmHel-7	DF090323:589174-589282
BmHel-7	DF090323:1678385-1678489
BmHel-7	DF090347:740983-741088
BmHel-7	DF090412:939656-939761
BmHel-7	DF090321:8136647-8136751
BmHel-7	DF090339:4086200-4086304
BmHel-7	DF090345:2415684-2415789
BmHel-7	DF090359:836158-836262
BmHel-7	DF090427:766258-766366
BmHel-7	BABH01057269:1-105
BmHel-7	DF090342:3690296-3690400
BmHel-7	DF090357:531852-531955
BmHel-7	BABH01086550:341-445
BmHel-7	BABH01069764:543-638
BmHel-7	DF090376:1525858-1525962
BmHel-7	DF090376:1143238-1143341
BmHel-7	DF090367:442071-442174

BmHel-7	DF090436:375125-375228
BmHel-7	DF090389:606493-606595
BmHel-7	DF090389:608322-608424
BmHel-7	DF090363:2663558-2663662
BmHel-7	DF090778:18653-18756
BmHel-7	BABH01050655:290-392
BmHel-7	DF090317:5082833-5082935
BmHel-7	DF090335:632479-632581
BmHel-7	DF090344:3014292-3014393
BmHel-7	DF090319:2863423-2863524
BmHel-7	DF090319:3841979-3842079
BmHel-7	BABH01078901:478-578
BmHel-7	DF090365:1307290-1307389
BmHel-7	DF090388:1716636-1716736
BmHel-7	DF090338:1763720-1763822
BmHel-7	BABH01074054:166-265
BmHel-7	DF091492:1949-2048
BmHel-7	DF090348:1371578-1371676
BmHel-7	DF090477:124902-124982
BmHel-7	BABH01058783:1-99
BmHel-7	DF090316:886521-886619
BmHel-7	DF090334:1606556-1606654
BmHel-7	DF090325:127828-127925
BmHel-7	DF090339:3274279-3274378
BmHel-7	DF090379:289729-289826
BmHel-7	DF090323:3748246-3748340
BmHel-7	DF090317:2270957-2271053
BmHel-7	DF090803:6629-6725
BmHel-7	BABH01065047:62-158
BmHel-7	BABH01069712:1-97
BmHel-7	DF090317:10641669-10641765
BmHel-7	DF090366:658465-658560
BmHel-7	DF090376:1483645-1483741
BmHel-7	BABH01076095:5-101
BmHel-7	DF090317:6697990-6698086
BmHel-7	DF090334:4229166-4229262
BmHel-7	DF090403:716400-716495
BmHel-7	BABH01061459:110-203
BmHel-7	BABH01083761:435-530
BmHel-7	DF090440:470650-470745
BmHel-7	DF090333:3163485-3163579
BmHel-7	BABH01080431:471-565
BmHel-7	DF090332:2940255-2940348
BmHel-7	DF090330:2171520-2171611
BmHel-7	BABH01084603:394-488
BmHel-7	BABH01085711:400-494
BmHel-7	BABH01066707:1-95
BmHel-7	DF090321:5157131-5157224

BmHel-7	BABH01048839:1068-1161
BmHel-7	DF090334:5559804-5559897
BmHel-7	BABH01050505:413-500
BmHel-7	DF090387:1976627-1976715
BmHel-7	DF090327:4573424-4573516
BmHel-7	DF090335:5657164-5657256
BmHel-7	DF090420:149240-149332
BmHel-7	DF090393:1004652-1004743
BmHel-7	DF090317:738834-738925
BmHel-7	DF090367:735933-736023
BmHel-7	DF090330:256389-256479
BmHel-7	BABH01052007:1-90
BmHel-7	BABH01061106:609-695
BmHel-7	DF090346:443301-443389
BmHel-7	DF090317:10322790-10322877
BmHel-7	BABH01071213:542-629
BmHel-7	DF090371:1792257-1792343
BmHel-7	DF090436:524371-524457
BmHel-7	DF090457:143292-143378
BmHel-7	DF090317:8810181-8810267
BmHel-7	DF090352:2511091-2511176
BmHel-7	DF090411:868258-868343
BmHel-7	BABH01080607:1-86
BmHel-7	DF090416:711809-711894
BmHel-7	DF090325:5841476-5841558
BmHel-7	BABH01055902:668-751
BmHel-7	DF090362:1197833-1197915
BmHel-7	DF090365:2766194-2766276
BmHel-7	DF090430:6602-6684
BmHel-7	DF090451:373058-373139
BmHel-7	DF090906:2217-2298
BmHel-7	DF090373:880449-880530
BmHel-7	DF090373:951431-951512
BmHel-7	BABH01083413:232-313
BmHel-7	BABH01082171:3-84
BmHel-7	DF090396:964670-964750
BmHel-7	BABH01049511:971-1051
BmHel-7	DF090363:3297-3376
BmHel-7	DF090446:250308-250387
BmHel-7	DF090406:1165187-1165266
BmHel-8	DF090357:3024695-3024799
BmHel-8	DF090317:7867510-7867633
BmHel-8	DF090385:1844098-1844222
BmHel-8	DF090329:3505245-3505369
BmHel-8	DF090369:2128891-2129030
BmHel-8	DF090339:302174-302323
BmHel-8	DF090373:2158613-2158793

BmHel-8	DF090322:7375171-7375357
BmHel-8	DF090469:357866-358053
BmHel-8	DF090337:4529401-4529610
BmHel-8	DF090384:1103267-1103500
BmHel-8	DF090339:325474-325707
BmHel-8	DF090336:4315226-4315461
BmHel-8	DF090320:314131-314366
BmHel-8	DF090333:4584652-4584888
BmHel-8	DF090471:135499-135745
BmHel-8	DF090329:3658721-3658967
BmHel-8	DF090383:653710-653962
BmHel-8	DF090384:7985-8241
BmHel-8	DF090439:430630-430887
BmHel-8	DF090392:547832-548090
BmHel-8	DF090365:2640359-2640621
BmHel-8	DF090780:17997-18259
BmHel-8	DF090398:208918-209181
BmHel-8	DF090327:4009345-4009611
BmHel-8	DF090326:5659222-5659489
BmHel-8	DF090380:283629-283899
BmHel-8	DF090328:5188098-5188372
BmHel-8	DF090335:1052262-1052541
BmHel-8	DF090353:1001155-1001435
BmHel-8	DF090328:6510585-6510869
BmHel-8	DF090318:3782333-3782617
BmHel-8	DF090443:71949-72240
BmHel-8	DF090366:21366-21660
BmHel-8	DF090499:117376-117671
BmHel-8	DF090323:2712917-2713212
BmHel-8	DF090322:5894564-5894861
BmHel-8	DF090331:3370285-3370583
BmHel-8	DF090339:2309709-2310008
BmHel-8	DF090361:582537-582837
BmHel-8	DF090317:6918345-6918646
BmHel-8	DF090381:1752311-1752612
BmHel-8	DF090324:7327567-7327876
BmHel-8	DF090423:421554-421863
BmHel-8	DF090346:1472659-1472969
BmHel-8	DF090366:2034127-2034439
BmHel-8	DF090429:181171-181485
BmHel-8	BABH01044348:8716-9030
BmHel-8	DF090351:4191009-4191327
BmHel-8	DF090325:2118976-2119295
BmHel-8	DF090373:355933-356255
BmHel-8	DF090344:3713074-3713398
BmHel-8	DF090351:4162285-4162610
BmHel-8	DF090333:4339602-4339927
BmHel-8	DF090325:3172621-3172946

BmHel-8	DF090349:672457-672784
BmHel-8	DF090382:1614602-1614932
BmHel-8	DF090322:6504532-6504863
BmHel-8	DF090340:4627026-4627357
BmHel-8	DF090327:465676-466007
BmHel-8	DF090348:1610346-1610677
BmHel-8	DF090412:1314122-1314455
BmHel-8	DF090324:4643063-4643397
BmHel-8	DF090346:1361339-1361673
BmHel-8	DF090316:3170461-3170796
BmHel-8	DF090345:232088-232423
BmHel-8	DF090393:1474141-1474476
BmHel-8	DF090361:385939-386274
BmHel-8	DF090379:500502-500837
BmHel-8	DF091066:6199-6535
BmHel-8	DF090319:1434412-1434749
BmHel-8	DF091423:3111-3448
BmHel-8	DF090329:5110731-5111068
BmHel-8	DF090317:9005407-9005746
BmHel-8	DF090366:380150-380489
BmHel-8	DF090323:335040-335381
BmHel-8	DF090337:2364875-2365217
BmHel-8	DF090365:229570-229912
BmHel-8	DF090347:619383-619726
BmHel-8	DF090342:3473417-3473760
BmHel-8	DF090446:293253-293596
BmHel-8	DF090344:2106748-2107094
BmHel-8	DF090712:19282-19628
BmHel-8	DF090336:654132-654478
BmHel-8	DF090405:48792-49139
BmHel-8	DF090365:1444997-1445345
BmHel-8	DF090351:3212221-3212569
BmHel-8	DF090380:662743-663093
BmHel-8	DF090419:125118-125468
BmHel-8	DF090433:352662-353013
BmHel-8	DF090354:3222328-3222679
BmHel-8	DF090321:4718811-4719163
BmHel-8	DF090362:3204067-3204420
BmHel-8	DF090316:7965098-7965451
BmHel-8	DF090341:2369781-2370134
BmHel-8	DF090323:1564416-1564770
BmHel-8	DF090322:3811854-3812208
BmHel-8	DF090353:3074973-3075327
BmHel-8	DF090347:623860-624214
BmHel-8	DF090393:1131601-1131956
BmHel-8	DF090420:97396-97752
BmHel-8	DF090701:3642-3998
BmHel-8	DF090344:727049-727406

BmHel-8	DF090415:36727-37084
BmHel-8	DF090375:1283495-1283852
BmHel-8	DF090416:372873-373231
BmHel-8	DF090364:1041804-1042163
BmHel-8	DF090339:473684-474043
BmHel-8	DF090330:5221887-5222247
BmHel-8	DF090332:3107261-3107621
BmHel-8	DF090331:5751091-5751451
BmHel-8	DF090319:704742-705102
BmHel-8	DF090352:684348-684709
BmHel-8	DF090316:11532538-11532900
BmHel-8	DF090352:1798172-1798534
BmHel-8	DF090484:194352-194714
BmHel-8	DF090317:6609326-6609688
BmHel-8	DF090332:5067862-5068225
BmHel-8	DF090325:1829028-1829391
BmHel-8	DF090330:2881442-2881806
BmHel-8	DF090436:312531-312895
BmHel-8	DF090316:9190671-9191035
BmHel-8	DF090343:2490083-2490447
BmHel-8	DF090326:3112522-3112887
BmHel-8	DF090496:131983-132348
BmHel-8	DF090340:4821618-4821984
BmHel-8	DF090332:3448388-3448755
BmHel-8	DF090332:6361223-6361590
BmHel-8	DF090402:850065-850432
BmHel-8	DF090401:1324376-1324743
BmHel-8	DF090348:1079066-1079433
BmHel-8	DF090378:1650996-1651363
BmHel-8	DF090330:3214244-3214612
BmHel-8	DF090336:2436489-2436857
BmHel-8	DF090339:662952-663320
BmHel-8	DF090801:9336-9704
BmHel-8	DF090357:1916939-1917307
BmHel-8	DF090329:4563897-4564265
BmHel-8	DF090379:347576-347944
BmHel-8	DF090317:1706597-1706966
BmHel-8	DF090366:106897-107266
BmHel-8	DF090418:1106204-1106573
BmHel-8	DF090386:1409779-1410148
BmHel-8	DF090319:4395288-4395658
BmHel-8	DF090383:1652529-1652899
BmHel-8	DF090371:338935-339305
BmHel-8	DF090332:2759596-2759967
BmHel-8	DF090347:3015717-3016088
BmHel-8	DF090333:5849080-5849452
BmHel-8	DF090363:2494346-2494718
BmHel-8	DF090336:2457805-2458177

BmHel-8	DF090388:1730522-1730894
BmHel-8	DF090337:3054172-3054544
BmHel-8	BABH01083007:154-526
BmHel-8	DF090340:2294119-2294492
BmHel-8	DF090316:15460500-15460873
BmHel-8	DF090363:1777217-1777590
BmHel-8	DF090420:143901-144274
BmHel-8	DF090318:7491114-7491487
BmHel-8	DF090374:2293602-2293976
BmHel-8	DF090346:3807156-3807530
BmHel-8	DF090357:1574186-1574560
BmHel-8	DF090408:278872-279246
BmHel-8	DF090479:231105-231480
BmHel-8	DF090468:181706-182081
BmHel-8	DF090393:1604132-1604507
BmHel-8	DF090347:1566354-1566730
BmHel-8	DF090333:1331775-1332151
BmHel-8	DF090368:456998-457374
BmHel-8	DF090384:318363-318739
BmHel-8	DF090344:590549-590926
BmHel-8	DF090408:29329-29706
BmHel-8	DF090333:3978074-3978451
BmHel-8	DF090342:4040353-4040730
BmHel-8	DF090414:1237285-1237662
BmHel-8	DF090393:1693214-1693591
BmHel-8	DF090409:929507-929885
BmHel-8	DF090318:3288388-3288766
BmHel-8	DF090322:1842081-1842459
BmHel-8	DF090322:1048733-1049111
BmHel-8	DF090317:7502028-7502406
BmHel-8	DF090331:1718238-1718616
BmHel-8	DF090404:1473113-1473491
BmHel-8	DF090352:2480854-2481233
BmHel-8	DF090336:4791986-4792365
BmHel-8	DF090338:2262181-2262560
BmHel-8	DF090349:1818027-1818406
BmHel-8	DF090392:1514326-1514705
BmHel-8	DF090370:1744256-1744635
BmHel-8	DF090318:4714124-4714503
BmHel-8	DF090338:2022411-2022790
BmHel-8	DF090475:236477-236856
BmHel-8	DF090389:1124981-1125360
BmHel-8	DF090317:9495416-9495796
BmHel-8	DF090345:1621431-1621811
BmHel-8	DF090526:51070-51450
BmHel-8	DF090352:3664465-3664845
BmHel-8	DF090363:991800-992180
BmHel-8	DF090362:1413996-1414376

BmHel-8	DF090316:3177323-3177703
BmHel-8	DF090326:2455469-2455849
BmHel-8	DF090418:507796-508176
BmHel-8	DF090354:3437395-3437775
BmHel-8	DF090484:148388-148768
BmHel-8	DF090352:1337335-1337716
BmHel-8	DF090344:2301722-2302103
BmHel-8	DF090508:149636-150017
BmHel-8	DF090340:642391-642772
BmHel-8	DF090436:650064-650445
BmHel-8	DF090444:222524-222905
BmHel-8	DF090412:893675-894057
BmHel-8	DF090323:3905012-3905394
BmHel-8	DF090319:3337257-3337639
BmHel-8	DF090316:14553187-14553569
BmHel-8	DF090335:1228217-1228599
BmHel-8	DF090324:1663929-1664311
BmHel-8	DF090333:1883443-1883825
BmHel-8	DF090316:14309792-14310174
BmHel-8	DF090351:3924956-3925338
BmHel-8	DF090465:126311-126693
BmHel-8	DF090352:803393-803776
BmHel-8	DF090330:954434-954817
BmHel-8	DF090340:3274582-3274965
BmHel-8	DF090338:1471690-1472073
BmHel-8	DF090319:6739832-6740215
BmHel-8	DF090316:12037116-12037499
BmHel-8	DF090337:832458-832841
BmHel-8	DF090350:1177350-1177734
BmHel-8	DF090329:3235571-3235955
BmHel-8	DF090349:3382655-3383039
BmHel-8	DF090349:3787168-3787552
BmHel-8	DF090376:1527790-1528174
BmHel-8	DF090364:1804024-1804408
BmHel-8	BABH01047125:981-1365
BmHel-8	DF090350:1308352-1308736
BmHel-8	DF090338:2676094-2676478
BmHel-8	DF090338:2062745-2063130
BmHel-8	DF090381:821408-821793
BmHel-8	DF090346:3187944-3188329
BmHel-8	DF090421:681051-681436
BmHel-8	DF090387:1832297-1832682
BmHel-8	DF090480:231727-232112
BmHel-8	DF090463:51300-51685
BmHel-8	DF090318:3659557-3659943
BmHel-8	DF090322:4481483-4481869
BmHel-8	DF090371:326357-326743
BmHel-8	DF090331:554943-555329

BmHel-8	DF090348:227711-228097
BmHel-8	DF090351:3302768-3303155
BmHel-8	DF090321:5926247-5926634
BmHel-8	DF090388:1142482-1142869
BmHel-8	DF090326:4846191-4846578
BmHel-8	DF090348:649047-649434
BmHel-8	DF090339:2951330-2951717
BmHel-8	DF090379:949339-949726
BmHel-8	DF090368:393460-393847
BmHel-8	DF090342:978573-978960
BmHel-8	DF090320:3069639-3070026
BmHel-8	DF090319:1626565-1626952
BmHel-8	DF090316:15329332-15329719
BmHel-8	DF090316:14860332-14860719
BmHel-8	DF090316:6045959-6046346
BmHel-8	DF090349:2877586-2877973
BmHel-8	DF090328:4061832-4062219
BmHel-8	DF090324:1886575-1886962
BmHel-8	DF090333:4753220-4753607
BmHel-8	DF090324:1335023-1335410
BmHel-8	DF090358:2649761-2650149
BmHel-8	DF090350:2948009-2948397
BmHel-8	DF090321:2449950-2450338
BmHel-8	DF090357:180809-181197
BmHel-8	DF090381:70588-70976
BmHel-8	DF090375:768787-769175
BmHel-8	DF090399:50154-50542
BmHel-8	DF090367:1472483-1472871
BmHel-8	DF090326:2966512-2966900
BmHel-8	DF090415:41200-41588
BmHel-8	DF090517:37540-37928
BmHel-8	DF090350:4269865-4270254
BmHel-8	DF090318:3920786-3921175
BmHel-8	DF090318:4732509-4732898
BmHel-8	DF090438:645625-646014
BmHel-8	DF090357:2737474-2737863
BmHel-8	DF090341:4818740-4819129
BmHel-8	DF090339:1108258-1108647
BmHel-8	DF090423:67448-67837
BmHel-8	DF090358:2964623-2965013
BmHel-8	DF090331:1205244-1205634
BmHel-8	DF090353:1271626-1272016
BmHel-8	DF090327:5697764-5698154
BmHel-8	DF090316:14583374-14583764
BmHel-8	DF090331:2403232-2403622
BmHel-8	DF090415:129682-130072
BmHel-8	DF090408:612173-612563
BmHel-8	BABH01046005:1711-2101

BmHel-8	DF090322:6360568-6360959
BmHel-8	DF090347:2299169-2299560
BmHel-8	DF090358:2532005-2532396
BmHel-8	DF090466:207366-207757
BmHel-8	DF090377:1413045-1413436
BmHel-8	DF090355:260086-260477
BmHel-8	DF090382:676027-676418
BmHel-8	DF090339:4997081-4997472
BmHel-8	DF090424:211489-211880
BmHel-8	DF090350:3676213-3676605
BmHel-8	DF090319:877666-878058
BmHel-8	DF090345:3372639-3373031
BmHel-8	DF090337:3039234-3039626
BmHel-8	DF090337:5203492-5203884
BmHel-8	DF090319:2193562-2193954
BmHel-8	DF090332:6255326-6255718
BmHel-8	DF090323:4521901-4522293
BmHel-8	DF090319:184727-185119
BmHel-8	DF090333:5064047-5064439
BmHel-8	DF090346:2744544-2744937
BmHel-8	DF090342:4681397-4681790
BmHel-8	DF090362:2981463-2981856
BmHel-8	DF090344:3629986-3630379
BmHel-8	DF090329:3604174-3604567
BmHel-8	DF090317:1548190-1548583
BmHel-8	DF090389:1351743-1352136
BmHel-8	DF090330:3211276-3211669
BmHel-8	DF090351:322151-322544
BmHel-8	DF090356:1254536-1254929
BmHel-8	DF090329:5138119-5138512
BmHel-8	DF090392:762936-763329
BmHel-8	DF090324:666365-666758
BmHel-8	DF090375:1231261-1231654
BmHel-8	DF090364:1509020-1509413
BmHel-8	DF090413:766797-767190
BmHel-8	DF090412:968650-969043
BmHel-8	DF090412:587032-587426
BmHel-8	DF090342:4298785-4299179
BmHel-8	DF090356:3232854-3233248
BmHel-8	DF090344:2679401-2679795
BmHel-8	DF090327:332833-333227
BmHel-8	DF090323:1199637-1200031
BmHel-8	DF090317:3957295-3957689
BmHel-8	DF090317:9352004-9352398
BmHel-8	DF090365:698358-698752
BmHel-8	DF090390:895595-895989
BmHel-8	DF090339:91007-91401
BmHel-8	DF091078:12771-13165

BmHel-8	DF090371:531622-532016
BmHel-8	DF090342:3554706-3555100
BmHel-8	DF090362:1357724-1358118
BmHel-8	DF090323:2020182-2020576
BmHel-8	DF090319:5768911-5769305
BmHel-8	DF090401:459087-459481
BmHel-8	DF090397:1328128-1328522
BmHel-8	DF090369:672199-672593
BmHel-8	DF090341:3619776-3620170
BmHel-8	DF090324:3415355-3415749
BmHel-8	DF090373:303599-303993
BmHel-8	DF090339:1848157-1848551
BmHel-8	DF090339:1451741-1452135
BmHel-8	DF090370:2170781-2171176
BmHel-8	DF090367:786410-786805
BmHel-8	DF090403:935593-935988
BmHel-8	DF090359:1524246-1524641
BmHel-8	DF090335:5430693-5431088
BmHel-8	DF090328:5186401-5186796
BmHel-8	DF090339:4634944-4635339
BmHel-8	DF090389:32099-32494
BmHel-8	DF090440:428237-428632
BmHel-8	DF090358:576972-577367
BmHel-8	DF090402:166610-167005
BmHel-8	DF090322:5464094-5464489
BmHel-8	DF090317:864396-864791
BmHel-8	DF090349:2857725-2858120
BmHel-8	DF090339:967330-967725
BmHel-8	DF090364:2703209-2703604
BmHel-8	BABH01062814:76-471
BmHel-8	BABH01060004:289-684
BmHel-8	DF091472:2274-2669
BmHel-8	DF090380:673981-674376
BmHel-8	DF090370:2299805-2300201
BmHel-8	DF090352:2465126-2465522
BmHel-8	DF090353:1462653-1463049
BmHel-8	DF090336:4416076-4416472
BmHel-8	DF090317:803952-804348
BmHel-8	DF090334:6063007-6063403
BmHel-8	DF090331:5038624-5039020
BmHel-8	DF090390:637821-638217
BmHel-8	DF090417:137612-138008
BmHel-8	DF090354:1903079-1903475
BmHel-8	DF090457:265448-265844
BmHel-8	DF090352:4083087-4083483
BmHel-8	DF090346:513674-514070
BmHel-8	DF090402:370395-370791
BmHel-8	DF090336:4040280-4040676

BmHel-8	DF090344:3574826-3575222
BmHel-8	DF090340:1800701-1801097
BmHel-8	DF090323:2277691-2278087
BmHel-8	DF090322:5573710-5574106
BmHel-8	DF090316:6349579-6349975
BmHel-8	DF090395:1039016-1039412
BmHel-8	DF090348:2483550-2483946
BmHel-8	DF090434:691507-691903
BmHel-8	DF090424:780796-781192
BmHel-8	DF090408:928499-928895
BmHel-8	DF090653:16804-17200
BmHel-8	BABH01047865:471-867
BmHel-8	DF090370:1169856-1170253
BmHel-8	DF090321:722061-722458
BmHel-8	DF090344:779108-779505
BmHel-8	DF090344:1545095-1545492
BmHel-8	DF090340:4730628-4731025
BmHel-8	DF090326:5509118-5509515
BmHel-8	DF090369:1352287-1352684
BmHel-8	DF090347:2558217-2558614
BmHel-8	DF090345:2275649-2276046
BmHel-8	DF090437:703997-704394
BmHel-8	DF090332:5763534-5763931
BmHel-8	DF090361:1799262-1799659
BmHel-8	DF090333:1315105-1315502
BmHel-8	DF090429:263036-263433
BmHel-8	DF090368:1684327-1684724
BmHel-8	DF090342:1407398-1407796
BmHel-8	DF090321:4844725-4845123
BmHel-8	DF090356:2511656-2512054
BmHel-8	DF090338:2343618-2344016
BmHel-8	DF090338:3853605-3854003
BmHel-8	DF090323:729851-730249
BmHel-8	DF090319:3636301-3636699
BmHel-8	DF090317:10448499-10448897
BmHel-8	DF090317:10709082-10709480
BmHel-8	DF090395:364120-364518
BmHel-8	DF090392:1346272-1346670
BmHel-8	DF090392:1654909-1655307
BmHel-8	DF090372:1247195-1247593
BmHel-8	DF090387:884493-884891
BmHel-8	DF090437:695113-695511
BmHel-8	DF090321:6331956-6332354
BmHel-8	DF090321:885001-885399
BmHel-8	DF090388:1269706-1270104
BmHel-8	DF090327:4660568-4660966
BmHel-8	DF090323:2185918-2186316
BmHel-8	DF090366:618697-619095

BmHel-8	DF090349:1966856-1967254
BmHel-8	DF090379:872984-873382
BmHel-8	DF090495:61850-62248
BmHel-8	DF090332:668495-668894
BmHel-8	DF090363:2326199-2326598
BmHel-8	DF090321:5879183-5879582
BmHel-8	DF090319:510953-511352
BmHel-8	DF090380:1816210-1816609
BmHel-8	DF090361:3025813-3026212
BmHel-8	DF090341:3626452-3626851
BmHel-8	DF090331:2751556-2751955
BmHel-8	DF090482:162171-162570
BmHel-8	DF090332:5490605-5491004
BmHel-8	DF090350:930320-930719
BmHel-8	DF090318:9505307-9505706
BmHel-8	DF090357:3429749-3430148
BmHel-8	DF090338:3322121-3322520
BmHel-8	DF090322:2487079-2487478
BmHel-8	DF090316:15488790-15489189
BmHel-8	DF090349:125033-125432
BmHel-8	DF090360:1762950-1763349
BmHel-8	DF090335:3326381-3326780
BmHel-8	DF090331:4433961-4434360
BmHel-8	DF090324:5220666-5221065
BmHel-8	DF090379:1726016-1726415
BmHel-8	DF090325:1265761-1266160
BmHel-8	DF090389:252628-253027
BmHel-8	DF090364:116531-116930
BmHel-8	DF090461:39855-40254
BmHel-8	DF090346:2955252-2955652
BmHel-8	DF090332:514057-514457
BmHel-8	DF090342:559051-559451
BmHel-8	DF090362:2034660-2035060
BmHel-8	DF090357:3264746-3265146
BmHel-8	DF090317:9972729-9973129
BmHel-8	DF090316:15330985-15331385
BmHel-8	DF090349:166442-166842
BmHel-8	DF090360:2069828-2070228
BmHel-8	DF090341:3869092-3869492
BmHel-8	DF090328:3292852-3293252
BmHel-8	DF090324:8048461-8048861
BmHel-8	DF090376:1494033-1494433
BmHel-8	DF090333:5643051-5643451
BmHel-8	DF090330:428945-429345
BmHel-8	DF090332:6182149-6182549
BmHel-8	DF090409:922062-922462
BmHel-8	DF090342:4076376-4076776
BmHel-8	DF090342:1048146-1048546

BmHel-8	DF090336:1762575-1762975
BmHel-8	DF090344:4486230-4486630
BmHel-8	DF090331:4095580-4095980
BmHel-8	DF090328:1453755-1454155
BmHel-8	DF090328:1215485-1215885
BmHel-8	DF090379:1204460-1204860
BmHel-8	DF090375:361241-361641
BmHel-8	BABH01047898:452-852
BmHel-8	DF090899:65-465
BmHel-8	DF090380:1767295-1767695
BmHel-8	DF090351:2213345-2213746
BmHel-8	DF090350:3040386-3040787
BmHel-8	DF090342:340225-340626
BmHel-8	DF090342:4694101-4694502
BmHel-8	DF090336:2558897-2559298
BmHel-8	DF090318:7685413-7685814
BmHel-8	DF090362:1548766-1549167
BmHel-8	DF090359:100168-100569
BmHel-8	DF090344:2691471-2691872
BmHel-8	DF090329:712086-712487
BmHel-8	DF090329:3548164-3548565
BmHel-8	DF090316:1747190-1747591
BmHel-8	DF090316:2991763-2992164
BmHel-8	DF090316:14008794-14009195
BmHel-8	DF090326:3290978-3291379
BmHel-8	DF090433:216644-217045
BmHel-8	DF090394:1108441-1108842
BmHel-8	DF090360:423862-424263
BmHel-8	DF090337:3234477-3234878
BmHel-8	DF090339:1943330-1943731
BmHel-8	DF090434:694596-694997
BmHel-8	DF090368:802752-803153
BmHel-8	DF090950:3561-3962
BmHel-8	DF090679:26232-26633
BmHel-8	DF090378:598708-599109
BmHel-8	DF090330:5813447-5813848
BmHel-8	DF090377:1280637-1281038
BmHel-8	DF090353:1206233-1206634
BmHel-8	DF090321:4377127-4377528
BmHel-8	DF090388:648497-648898
BmHel-8	DF090356:3235796-3236197
BmHel-8	DF090344:3978073-3978474
BmHel-8	DF090329:4814844-4815245
BmHel-8	DF090327:4627133-4627534
BmHel-8	DF090323:3086178-3086579
BmHel-8	DF090322:6384766-6385167
BmHel-8	DF090316:3944830-3945231
BmHel-8	DF090349:3688004-3688405

BmHel-8	DF090392:632459-632860
BmHel-8	DF090328:1026038-1026439
BmHel-8	DF090324:3880766-3881167
BmHel-8	DF090373:2335511-2335912
BmHel-8	DF090384:2013135-2013536
BmHel-8	DF090368:434350-434751
BmHel-8	DF090330:5522324-5522725
BmHel-8	DF090383:291921-292323
BmHel-8	DF090352:450144-450546
BmHel-8	DF090352:1505033-1505435
BmHel-8	DF090402:611429-611831
BmHel-8	DF090355:188585-188987
BmHel-8	DF090350:3172256-3172658
BmHel-8	DF090320:5675338-5675740
BmHel-8	DF090344:3569691-3570093
BmHel-8	DF090319:7437383-7437785
BmHel-8	DF090348:995304-995706
BmHel-8	DF090411:774772-775174
BmHel-8	DF090341:1901753-1902155
BmHel-8	DF090333:324855-325257
BmHel-8	DF090325:944697-945099
BmHel-8	DF090414:1299622-1300024
BmHel-8	DF090404:279203-279605
BmHel-8	DF090408:494997-495399
BmHel-8	DF090399:264774-265176
BmHel-8	DF090756:3337-3739
BmHel-8	DF090619:20674-21076
BmHel-8	DF090316:12849517-12849919
BmHel-8	DF090330:1171750-1172152
BmHel-8	DF090342:3018001-3018403
BmHel-8	DF090321:4829931-4830333
BmHel-8	DF090388:1669434-1669836
BmHel-8	DF090344:3714836-3715238
BmHel-8	DF090329:5798842-5799244
BmHel-8	DF090316:9544057-9544459
BmHel-8	DF090395:1639940-1640342
BmHel-8	DF090348:983792-984194
BmHel-8	DF090360:3112474-3112876
BmHel-8	DF090324:485615-486017
BmHel-8	DF090339:4929075-4929477
BmHel-8	DF090325:4761742-4762144
BmHel-8	DF090325:3760812-3761214
BmHel-8	DF090414:1015434-1015836
BmHel-8	DF090408:437339-437741
BmHel-8	DF090406:428639-429041
BmHel-8	DF090625:9573-9975
BmHel-8	BABH01048062:92-494
BmHel-8	BABH01048159:777-1179

BmHel-8	DF090325:229549-229951
BmHel-8	DF090358:1599973-1600376
BmHel-8	DF090383:1880966-1881369
BmHel-8	DF090351:1058324-1058727
BmHel-8	DF090332:4151270-4151673
BmHel-8	DF090321:975841-976244
BmHel-8	DF090403:867425-867828
BmHel-8	DF090340:3135203-3135606
BmHel-8	DF090338:2976628-2977031
BmHel-8	DF090319:5250412-5250815
BmHel-8	DF090316:1587222-1587625
BmHel-8	DF090365:962165-962568
BmHel-8	DF090371:666708-667111
BmHel-8	DF090335:2807020-2807423
BmHel-8	DF090324:2328910-2329313
BmHel-8	DF090425:121525-121928
BmHel-8	DF090543:79591-79994
BmHel-8	DF090332:2545347-2545750
BmHel-8	DF090321:1232007-1232410
BmHel-8	DF090321:505004-505407
BmHel-8	DF090320:7676874-7677277
BmHel-8	DF090362:2346819-2347222
BmHel-8	DF090317:6557997-6558400
BmHel-8	DF090316:6912742-6913145
BmHel-8	DF090382:1894217-1894620
BmHel-8	DF090347:2517593-2517996
BmHel-8	DF090341:3409427-3409830
BmHel-8	DF090339:2569235-2569638
BmHel-8	DF090333:5265704-5266107
BmHel-8	DF090417:947470-947873
BmHel-8	DF090415:24439-24842
BmHel-8	DF090501:46971-47374
BmHel-8	BABH01050755:128-531
BmHel-8	DF090321:1611883-1612286
BmHel-8	DF090338:3872474-3872877
BmHel-8	DF090318:7668191-7668595
BmHel-8	DF090329:5432701-5433105
BmHel-8	DF090319:2433232-2433636
BmHel-8	DF090316:15619118-15619522
BmHel-8	DF090349:451389-451793
BmHel-8	DF090372:1876178-1876582
BmHel-8	DF090360:2505774-2506178
BmHel-8	DF090339:4419429-4419833
BmHel-8	DF090364:2136330-2136734
BmHel-8	DF090507:79474-79878
BmHel-8	DF090435:313335-313739
BmHel-8	DF090448:221834-222238
BmHel-8	DF090448:379376-379780

BmHel-8	DF090561:3653-4057
BmHel-8	DF090629:9990-10394
BmHel-8	DF090330:4202901-4203305
BmHel-8	DF090351:3494402-3494806
BmHel-8	DF090332:3997755-3998159
BmHel-8	DF090421:702617-703021
BmHel-8	DF090329:2314680-2315084
BmHel-8	DF090319:5750223-5750627
BmHel-8	DF090394:1575691-1576095
BmHel-8	DF090361:1608632-1609036
BmHel-8	DF090347:3843632-3844036
BmHel-8	DF090331:5294602-5295006
BmHel-8	DF090373:646445-646849
BmHel-8	DF090325:3730287-3730691
BmHel-8	DF090431:685510-685914
BmHel-8	DF090472:312566-312970
BmHel-8	BABH01072057:129-533
BmHel-8	DF090330:6297659-6298064
BmHel-8	DF090355:538253-538658
BmHel-8	DF090321:4424419-4424824
BmHel-8	DF090320:6603276-6603681
BmHel-8	DF090441:623347-623752
BmHel-8	DF090359:2389768-2390173
BmHel-8	DF090338:2958511-2958916
BmHel-8	DF090391:1538443-1538848
BmHel-8	DF090347:715870-716275
BmHel-8	DF090335:1240909-1241314
BmHel-8	DF090334:2939454-2939859
BmHel-8	DF090494:49054-49459
BmHel-8	DF090425:905304-905709
BmHel-8	DF090379:1622844-1623249
BmHel-8	DF090429:248526-248931
BmHel-8	DF090518:57215-57620
BmHel-8	BABH01050018:359-764
BmHel-8	DF090332:3835448-3835853
BmHel-8	DF090342:799094-799499
BmHel-8	DF090333:4745608-4746013
BmHel-8	DF090352:3105506-3105911
BmHel-8	DF090346:2091331-2091736
BmHel-8	DF090402:494941-495346
BmHel-8	DF090350:1254562-1254967
BmHel-8	DF090441:559311-559716
BmHel-8	DF090388:355358-355763
BmHel-8	DF090322:4187075-4187480
BmHel-8	DF090319:6828161-6828566
BmHel-8	DF090366:544356-544761
BmHel-8	DF090349:2316282-2316687
BmHel-8	DF090335:2202973-2203378

BmHel-8	DF090334:2976202-2976607
BmHel-8	DF090462:135417-135822
BmHel-8	DF090405:688940-689345
BmHel-8	DF090456:26354-26759
BmHel-8	BABH01054049:256-661
BmHel-8	DF090330:1773445-1773851
BmHel-8	DF090352:1530755-1531161
BmHel-8	DF090346:3777862-3778268
BmHel-8	DF090421:649405-649811
BmHel-8	DF090342:4464368-4464774
BmHel-8	DF090342:4741738-4742144
BmHel-8	DF090344:1097393-1097799
BmHel-8	DF090323:1735815-1736221
BmHel-8	DF090319:5975622-5976028
BmHel-8	DF090317:4177311-4177717
BmHel-8	DF090316:15738780-15739186
BmHel-8	DF090324:4377487-4377893
BmHel-8	DF090339:3061874-3062280
BmHel-8	DF090364:553685-554091
BmHel-8	DF090350:1556377-1556783
BmHel-8	DF090364:2303570-2303976
BmHel-8	DF090437:386094-386500
BmHel-8	DF090332:6029681-6030087
BmHel-8	DF090409:1000764-1001170
BmHel-8	DF090329:2032742-2033148
BmHel-8	DF090323:2592631-2593037
BmHel-8	DF090322:1868231-1868637
BmHel-8	DF090322:939867-940273
BmHel-8	DF090319:6526481-6526887
BmHel-8	DF090317:7148444-7148850
BmHel-8	DF090439:434876-435282
BmHel-8	DF090401:534468-534874
BmHel-8	DF090334:2329855-2330261
BmHel-8	DF090337:317465-317871
BmHel-8	DF090343:3208340-3208746
BmHel-8	DF090376:1497501-1497907
BmHel-8	DF090431:56455-56861
BmHel-8	DF090404:1291425-1291831
BmHel-8	DF090406:798669-799075
BmHel-8	DF090447:110125-110531
BmHel-8	DF090322:3373944-3374350
BmHel-8	DF090450:90979-91385
BmHel-8	DF090358:3425858-3426265
BmHel-8	DF090351:4229758-4230165
BmHel-8	DF090318:6149505-6149912
BmHel-8	DF090320:2131771-2132178
BmHel-8	DF090323:5627082-5627489
BmHel-8	DF090322:2177829-2178236

BmHel-8	DF090395:488967-489374
BmHel-8	DF090326:6556274-6556681
BmHel-8	DF090391:1094862-1095269
BmHel-8	DF090372:2145617-2146024
BmHel-8	DF090341:2575661-2576068
BmHel-8	DF090381:810790-811197
BmHel-8	DF090444:598780-599187
BmHel-8	DF090431:82142-82549
BmHel-8	DF090451:125540-125947
BmHel-8	DF091041:3552-3959
BmHel-8	DF090768:17441-17848
BmHel-8	DF090421:614050-614457
BmHel-8	DF090421:546022-546429
BmHel-8	DF090329:2861027-2861434
BmHel-8	DF090323:7107192-7107599
BmHel-8	DF090322:6255662-6256069
BmHel-8	DF090319:6799897-6800304
BmHel-8	DF090316:8391586-8391993
BmHel-8	DF090316:3559691-3560098
BmHel-8	DF090365:1801545-1801952
BmHel-8	DF090348:1769267-1769674
BmHel-8	DF090360:764930-765337
BmHel-8	DF090334:3136512-3136919
BmHel-8	DF090337:4516735-4517142
BmHel-8	DF090333:1946270-1946677
BmHel-8	DF090378:412370-412777
BmHel-8	DF090408:313611-314018
BmHel-8	BABH01049939:74-481
BmHel-8	DF090352:1555909-1556317
BmHel-8	DF090351:2299498-2299906
BmHel-8	DF090332:6312605-6313013
BmHel-8	DF090377:1347975-1348383
BmHel-8	DF090367:1630061-1630469
BmHel-8	DF090363:3567-3975
BmHel-8	DF090350:743333-743741
BmHel-8	DF090321:3497443-3497851
BmHel-8	DF090321:4666245-4666653
BmHel-8	DF090320:4989630-4990038
BmHel-8	DF090441:196209-196617
BmHel-8	DF090420:282398-282806
BmHel-8	DF090400:1348343-1348751
BmHel-8	DF090357:1186781-1187189
BmHel-8	DF090340:4317417-4317825
BmHel-8	DF090338:2786312-2786720
BmHel-8	DF090323:1936841-1937249
BmHel-8	DF090317:3633074-3633482
BmHel-8	DF090366:403159-403567
BmHel-8	DF090422:1085952-1086360

BmHel-8	DF090401:1272908-1273316
BmHel-8	DF090396:1508436-1508844
BmHel-8	DF090382:1918784-1919192
BmHel-8	DF090337:4721263-4721671
BmHel-8	DF090337:5297614-5298022
BmHel-8	DF090375:907331-907739
BmHel-8	DF090375:1649233-1649641
BmHel-8	DF090375:2326266-2326674
BmHel-8	DF090438:170457-170865
BmHel-8	DF090408:304933-305341
BmHel-8	DF090598:41866-42274
BmHel-8	BABH01055711:113-521
BmHel-8	BABH01044911:3173-3581
BmHel-8	BABH01056383:211-619
BmHel-8	DF090330:1325915-1326323
BmHel-8	DF090344:3971730-3972138
BmHel-8	DF090330:4862757-4863165
BmHel-8	DF090332:4837993-4838401
BmHel-8	DF090353:1748549-1748957
BmHel-8	DF090321:160462-160870
BmHel-8	DF090318:6488081-6488489
BmHel-8	DF090344:145156-145564
BmHel-8	DF090329:4166754-4167162
BmHel-8	DF090327:4543008-4543416
BmHel-8	DF090322:5682194-5682602
BmHel-8	DF090316:12485448-12485856
BmHel-8	DF090316:11788850-11789258
BmHel-8	DF090316:3335409-3335817
BmHel-8	DF090365:1212013-1212421
BmHel-8	DF090411:1070954-1071362
BmHel-8	DF090361:410653-411061
BmHel-8	DF090335:1366139-1366547
BmHel-8	DF090328:378185-378593
BmHel-8	DF090339:3667555-3667963
BmHel-8	DF090339:1680154-1680562
BmHel-8	DF090339:42382-42790
BmHel-8	DF090416:512611-513019
BmHel-8	DF090354:2886102-2886510
BmHel-8	DF090459:165490-165898
BmHel-8	DF090465:355988-356396
BmHel-8	BABH01075299:40-448
BmHel-8	DF090317:4638847-4639255
BmHel-8	DF090328:4774453-4774861
BmHel-8	DF090332:2664660-2665069
BmHel-8	DF090367:1842660-1843069
BmHel-8	DF090363:1752049-1752458
BmHel-8	DF090353:2807824-2808233
BmHel-8	DF090342:3310667-3311076

BmHel-8	DF090321:6850775-6851184
BmHel-8	DF090362:2740105-2740514
BmHel-8	DF090319:5766243-5766652
BmHel-8	DF090316:15651236-15651645
BmHel-8	DF090410:125064-125473
BmHel-8	DF090349:312239-312648
BmHel-8	DF090349:1411253-1411662
BmHel-8	DF090326:5003588-5003997
BmHel-8	DF090348:3520581-3520990
BmHel-8	DF090439:30849-31258
BmHel-8	DF090391:358820-359229
BmHel-8	DF090360:1095314-1095723
BmHel-8	DF090341:4029821-4030230
BmHel-8	DF090328:5130127-5130536
BmHel-8	DF090425:1011391-1011800
BmHel-8	DF090375:1688904-1689313
BmHel-8	DF090364:1075579-1075988
BmHel-8	DF090429:335121-335530
BmHel-8	DF090415:466227-466636
BmHel-8	DF090471:172217-172626
BmHel-8	DF090450:186178-186587
BmHel-8	DF090358:2782155-2782564
BmHel-8	DF090420:421975-422384
BmHel-8	DF090338:4997750-4998159
BmHel-8	DF090327:6310197-6310606
BmHel-8	DF090323:1787995-1788404
BmHel-8	DF090319:7599005-7599414
BmHel-8	DF090319:7429880-7430289
BmHel-8	DF090316:14756511-14756920
BmHel-8	DF090382:664283-664692
BmHel-8	DF090369:556029-556438
BmHel-8	DF090361:698737-699146
BmHel-8	DF090345:2499304-2499713
BmHel-8	DF090345:1341048-1341457
BmHel-8	DF090334:1867486-1867895
BmHel-8	DF090325:5716666-5717075
BmHel-8	DF090354:2975050-2975459
BmHel-8	DF090457:279368-279777
BmHel-8	DF090685:12158-12567
BmHel-8	DF090316:6543566-6543975
BmHel-8	DF090462:211432-211841
BmHel-8	DF090330:2041250-2041660
BmHel-8	DF090346:1277336-1277746
BmHel-8	DF090346:1852369-1852779
BmHel-8	DF090367:2755832-2756242
BmHel-8	DF090363:334711-335121
BmHel-8	DF090342:222145-222555
BmHel-8	DF090321:4136691-4137101

BmHel-8	DF090344:873221-873631
BmHel-8	DF090329:2431660-2432070
BmHel-8	DF090322:6172370-6172780
BmHel-8	DF090317:3074772-3075182
BmHel-8	DF090365:2216468-2216878
BmHel-8	DF090365:2704605-2705015
BmHel-8	DF090348:2518491-2518901
BmHel-8	DF090360:685226-685636
BmHel-8	DF090335:2018940-2019350
BmHel-8	DF090334:5572024-5572434
BmHel-8	DF090385:684999-685409
BmHel-8	DF090333:588605-589015
BmHel-8	DF090333:4266013-4266423
BmHel-8	DF090333:4389777-4390187
BmHel-8	DF090368:2334384-2334794
BmHel-8	DF090370:682687-683097
BmHel-8	DF090352:3604381-3604791
BmHel-8	DF090377:1104245-1104655
BmHel-8	DF090342:3691841-3692251
BmHel-8	DF090488:39732-40142
BmHel-8	DF090388:336579-336989
BmHel-8	DF090359:1340315-1340725
BmHel-8	DF090356:3054519-3054929
BmHel-8	DF090344:1294763-1295173
BmHel-8	DF090338:957078-957488
BmHel-8	DF090329:5215493-5215903
BmHel-8	DF090323:6736388-6736798
BmHel-8	DF090322:7406262-7406672
BmHel-8	DF090322:5975804-5976214
BmHel-8	DF090319:2116959-2117369
BmHel-8	DF090316:6142962-6143372
BmHel-8	DF090391:587436-587846
BmHel-8	DF090360:3158652-3159062
BmHel-8	DF090328:6501204-6501614
BmHel-8	DF090364:393015-393425
BmHel-8	BABH01045036:3060-3470
BmHel-8	DF090330:3943275-3943686
BmHel-8	DF090370:1332542-1332953
BmHel-8	DF090383:1965427-1965838
BmHel-8	DF090352:929-1340
BmHel-8	DF090351:3076956-3077367
BmHel-8	DF090353:2585782-2586193
BmHel-8	DF090336:4586664-4587075
BmHel-8	DF090321:7247122-7247533
BmHel-8	DF090318:3859821-3860232
BmHel-8	DF090318:8925547-8925958
BmHel-8	DF090320:5982428-5982839
BmHel-8	DF090320:6526467-6526878

BmHel-8	DF090338:4224768-4225179
BmHel-8	DF090319:6467535-6467946
BmHel-8	DF090317:5424473-5424884
BmHel-8	DF090317:6342981-6343392
BmHel-8	DF090326:40621-41032
BmHel-8	DF090458:1444-1855
BmHel-8	DF090369:1619841-1620252
BmHel-8	DF090361:1994205-1994616
BmHel-8	DF090347:4187276-4187687
BmHel-8	DF090341:4056065-4056476
BmHel-8	DF090328:227545-227956
BmHel-8	DF090328:592855-593266
BmHel-8	DF090328:1892174-1892585
BmHel-8	DF090337:1690394-1690805
BmHel-8	DF090381:34629-35040
BmHel-8	DF090381:2000029-2000440
BmHel-8	DF090343:4409214-4409625
BmHel-8	DF090339:1671862-1672273
BmHel-8	DF090376:1839742-1840153
BmHel-8	DF090558:51575-51986
BmHel-8	DF090325:3605964-3606375
BmHel-8	DF090387:1097420-1097831
BmHel-8	DF090413:185932-186343
BmHel-8	DF090517:19602-20013
BmHel-8	DF090461:76313-76724
BmHel-8	DF090675:1698-2109
BmHel-8	DF090546:65747-66158
BmHel-8	DF090330:4428496-4428907
BmHel-8	DF090358:1842922-1843333
BmHel-8	DF090386:1284759-1285170
BmHel-8	DF090383:1798338-1798749
BmHel-8	DF090352:2322126-2322537
BmHel-8	DF090352:321896-322307
BmHel-8	DF090332:2978978-2979389
BmHel-8	DF090321:264924-265335
BmHel-8	DF090400:1351505-1351916
BmHel-8	DF090388:1164784-1165195
BmHel-8	DF090356:3268670-3269081
BmHel-8	DF090340:1372891-1373302
BmHel-8	DF090338:3912058-3912469
BmHel-8	DF090338:3253233-3253644
BmHel-8	DF090322:2525863-2526274
BmHel-8	DF090319:3197835-3198246
BmHel-8	DF090317:8541947-8542358
BmHel-8	DF090317:5231093-5231504
BmHel-8	DF090316:12762086-12762497
BmHel-8	DF090366:1674742-1675153
BmHel-8	DF090349:251841-252252

BmHel-8	DF090326:5145941-5146352
BmHel-8	DF090411:506110-506521
BmHel-8	DF090394:1713-2124
BmHel-8	DF090392:1431094-1431505
BmHel-8	DF090334:1767686-1768097
BmHel-8	DF090331:801470-801881
BmHel-8	DF090333:4049909-4050320
BmHel-8	DF090333:3233378-3233789
BmHel-8	DF090325:5855295-5855706
BmHel-8	DF090811:14096-14507
BmHel-8	DF090354:2835961-2836372
BmHel-8	DF090354:1993441-1993852
BmHel-8	DF090354:681296-681707
BmHel-8	BABH01056655:49-460
BmHel-8	DF090399:1595829-1596240
BmHel-8	DF090492:221921-222332
BmHel-8	BABH01044730:6165-6576
BmHel-8	BABH01048914:57-468
BmHel-8	DF090795:9067-9478
BmHel-8	DF090527:45867-46278
BmHel-8	DF090320:6667224-6667635
BmHel-8	DF090340:2029494-2029905
BmHel-8	DF090392:1188690-1189101
BmHel-8	DF090494:14567-14978
BmHel-8	DF090353:3520524-3520935
BmHel-8	DF090322:7612487-7612898
BmHel-8	DF090330:3650060-3650472
BmHel-8	DF090386:1365581-1365993
BmHel-8	DF090383:1005616-1006028
BmHel-8	DF090351:824312-824724
BmHel-8	DF090351:4095190-4095602
BmHel-8	DF090363:1249633-1250045
BmHel-8	DF090363:2220421-2220833
BmHel-8	DF090363:2631842-2632254
BmHel-8	DF090342:596724-597136
BmHel-8	DF090342:1898359-1898771
BmHel-8	DF090336:4259445-4259857
BmHel-8	DF090320:3181479-3181891
BmHel-8	DF090488:97798-98210
BmHel-8	DF090441:430989-431401
BmHel-8	DF090344:232865-233277
BmHel-8	DF090338:3669105-3669517
BmHel-8	DF090319:3417687-3418099
BmHel-8	DF090319:6928385-6928797
BmHel-8	DF090317:2082447-2082859
BmHel-8	DF090317:6907906-6908318
BmHel-8	DF090317:7991136-7991548
BmHel-8	DF090317:10649522-10649934

BmHel-8	DF090316:3350386-3350798
BmHel-8	DF090316:7590855-7591267
BmHel-8	DF090316:13337429-13337841
BmHel-8	DF090349:2231761-2232173
BmHel-8	DF090348:3560885-3561297
BmHel-8	DF090348:3757922-3758334
BmHel-8	DF090411:979351-979763
BmHel-8	DF090396:31356-31768
BmHel-8	DF090391:834444-834856
BmHel-8	DF090347:1103329-1103741
BmHel-8	DF090347:2601919-2602331
BmHel-8	DF090341:1249047-1249459
BmHel-8	DF090341:2953630-2954042
BmHel-8	DF090335:1758892-1759304
BmHel-8	DF090335:2657610-2658022
BmHel-8	DF090335:5728799-5729211
BmHel-8	DF090334:3573738-3574150
BmHel-8	DF090343:3999660-4000072
BmHel-8	DF090339:2900272-2900684
BmHel-8	DF090339:3701673-3702085
BmHel-8	DF090325:1083555-1083967
BmHel-8	DF090364:16509-16921
BmHel-8	DF090354:427276-427688
BmHel-8	DF090464:349064-349476
BmHel-8	DF090517:79286-79698
BmHel-8	DF090896:2960-3372
BmHel-8	BABH01056566:215-627
BmHel-8	DF090752:14212-14624
BmHel-8	DF090559:20169-20581
BmHel-8	DF090352:3996458-3996870
BmHel-8	DF090321:1905841-1906253
BmHel-8	DF090499:20904-21316
BmHel-8	DF090330:4182867-4183279
BmHel-8	DF090352:728356-728768
BmHel-8	DF090332:2371283-2371695
BmHel-8	DF090409:1333495-1333907
BmHel-8	DF090402:100621-101033
BmHel-8	DF090353:3253044-3253456
BmHel-8	DF090336:1042015-1042427
BmHel-8	DF090318:642043-642455
BmHel-8	DF090403:1156296-1156708
BmHel-8	DF090388:1317221-1317633
BmHel-8	DF090388:1116249-1116661
BmHel-8	DF090388:1056850-1057262
BmHel-8	DF090362:703497-703909
BmHel-8	DF090356:2672582-2672994
BmHel-8	DF090323:7050894-7051306
BmHel-8	DF090323:3209475-3209887

BmHel-8	DF090322:1003876-1004288
BmHel-8	DF090316:15123637-15124049
BmHel-8	DF090349:548903-549315
BmHel-8	DF090365:1480891-1481303
BmHel-8	DF090348:2956692-2957104
BmHel-8	DF090348:2851213-2851625
BmHel-8	DF090394:95390-95802
BmHel-8	DF090371:1000198-1000610
BmHel-8	DF090345:4215293-4215705
BmHel-8	DF090345:963672-964084
BmHel-8	DF090334:2516991-2517403
BmHel-8	DF090331:4703696-4704108
BmHel-8	DF090328:5123388-5123800
BmHel-8	DF090328:822712-823124
BmHel-8	DF090337:487669-488081
BmHel-8	DF090343:3307405-3307817
BmHel-8	DF090425:76169-76581
BmHel-8	DF090376:861504-861916
BmHel-8	DF090374:1097128-1097540
BmHel-8	DF090378:387468-387880
BmHel-8	DF090438:471927-472339
BmHel-8	DF090415:11958-12370
BmHel-8	DF090407:662550-662962
BmHel-8	BABH01044627:4061-4473
BmHel-8	BABH01086513:8-420
BmHel-8	BABH01046138:1934-2346
BmHel-8	DF090355:2341083-2341495
BmHel-8	DF090341:542957-543369
BmHel-8	DF090370:835122-835535
BmHel-8	DF090412:1201926-1202339
BmHel-8	DF090383:2073922-2074335
BmHel-8	DF090351:1383710-1384123
BmHel-8	DF090351:3064673-3065086
BmHel-8	DF090421:690962-691375
BmHel-8	DF090355:1308300-1308713
BmHel-8	DF090353:1158918-1159331
BmHel-8	DF090336:4206921-4207334
BmHel-8	DF090321:5734801-5735214
BmHel-8	DF090318:3560176-3560589
BmHel-8	DF090488:119080-119493
BmHel-8	DF090388:573793-574206
BmHel-8	DF090388:824499-824912
BmHel-8	DF090359:2107595-2108008
BmHel-8	DF090357:302590-303003
BmHel-8	DF090344:3872277-3872690
BmHel-8	DF090327:209839-210252
BmHel-8	DF090327:4950234-4950647
BmHel-8	DF090319:2801297-2801710

BmHel-8	DF090317:563522-563935
BmHel-8	DF090317:9680089-9680502
BmHel-8	DF090316:6546190-6546603
BmHel-8	DF090316:14959852-14960265
BmHel-8	DF090366:1784262-1784675
BmHel-8	DF090326:124749-125162
BmHel-8	DF090365:1607880-1608293
BmHel-8	DF090365:1924739-1925152
BmHel-8	DF090365:2928665-2929078
BmHel-8	DF090422:894419-894832
BmHel-8	DF090391:1221381-1221794
BmHel-8	DF090347:87418-87831
BmHel-8	DF090345:2602457-2602870
BmHel-8	DF090341:2185825-2186238
BmHel-8	DF090390:733349-733762
BmHel-8	DF090343:644010-644423
BmHel-8	DF090364:2522722-2523135
BmHel-8	BABH01072669:208-621
BmHel-8	DF090404:1413090-1413503
BmHel-8	DF090354:204957-205370
BmHel-8	DF090354:3488134-3488547
BmHel-8	DF090526:35742-36155
BmHel-8	DF090890:2399-2812
BmHel-8	DF090751:13266-13679
BmHel-8	BABH01046112:2414-2827
BmHel-8	DF090356:1003454-1003867
BmHel-8	DF090330:5375191-5375604
BmHel-8	DF090330:116455-116868
BmHel-8	DF090370:2383741-2384154
BmHel-8	DF090412:523199-523612
BmHel-8	DF090351:1317501-1317914
BmHel-8	DF090346:2220562-2220975
BmHel-8	DF090332:3066457-3066870
BmHel-8	DF090377:1151708-1152121
BmHel-8	DF090363:1215013-1215426
BmHel-8	DF090363:949052-949465
BmHel-8	DF090355:3364977-3365390
BmHel-8	DF090353:522186-522599
BmHel-8	DF090362:1189646-1190059
BmHel-8	DF090357:2155588-2156001
BmHel-8	DF090357:171283-171696
BmHel-8	DF090356:1701618-1702031
BmHel-8	DF090344:3302615-3303028
BmHel-8	DF090338:3824099-3824512
BmHel-8	DF090319:7842565-7842978
BmHel-8	DF090348:3890202-3890615
BmHel-8	DF090382:2102052-2102465
BmHel-8	DF090369:1479365-1479778

BmHel-8	DF090369:1318516-1318929
BmHel-8	DF090361:464714-465127
BmHel-8	DF090360:2904043-2904456
BmHel-8	DF090347:1248233-1248646
BmHel-8	DF090341:4592024-4592437
BmHel-8	DF090328:675139-675552
BmHel-8	DF090324:833992-834405
BmHel-8	DF090337:5298571-5298984
BmHel-8	DF090373:1212064-1212477
BmHel-8	DF090339:892709-893122
BmHel-8	DF090423:693489-693902
BmHel-8	DF090364:2480245-2480658
BmHel-8	DF090364:1466614-1467027
BmHel-8	DF090407:214709-215122
BmHel-8	BABH01081224:7-420
BmHel-8	DF090430:220939-221352
BmHel-8	DF090490:7041-7454
BmHel-8	DF090447:347876-348289
BmHel-8	DF090731:5951-6364
BmHel-8	DF090563:42664-43077
BmHel-8	DF090530:2934-3347
BmHel-8	DF090788:3625-4038
BmHel-8	DF090375:499501-499914
BmHel-8	DF090330:3048431-3048845
BmHel-8	DF090370:251407-251821
BmHel-8	DF090355:3758473-3758887
BmHel-8	DF090353:203976-204390
BmHel-8	DF090342:691161-691575
BmHel-8	DF090336:809017-809431
BmHel-8	DF090318:2450780-2451194
BmHel-8	DF090320:3047351-3047765
BmHel-8	DF090320:3915311-3915725
BmHel-8	DF090320:4555060-4555474
BmHel-8	DF090344:3389575-3389989
BmHel-8	DF090344:3648826-3649240
BmHel-8	DF090344:3760776-3761190
BmHel-8	DF090327:5622965-5623379
BmHel-8	DF090323:7983016-7983430
BmHel-8	DF090317:5795655-5796069
BmHel-8	DF090316:3625019-3625433
BmHel-8	DF090326:4478055-4478469
BmHel-8	DF090348:1608278-1608692
BmHel-8	DF090348:2437566-2437980
BmHel-8	DF090422:837955-838369
BmHel-8	DF090397:1373987-1374401
BmHel-8	DF090372:1893909-1894323
BmHel-8	DF090361:339961-340375
BmHel-8	DF090360:860588-861002

BmHel-8	DF090345:475132-475546
BmHel-8	DF090341:1474396-1474810
BmHel-8	DF090335:1263632-1264046
BmHel-8	DF090335:1932332-1932746
BmHel-8	DF090335:3821877-3822291
BmHel-8	DF090331:2263895-2264309
BmHel-8	DF090331:2875557-2875971
BmHel-8	DF090331:4822091-4822505
BmHel-8	DF090328:2814513-2814927
BmHel-8	DF090328:5315639-5316053
BmHel-8	DF090324:6305694-6306108
BmHel-8	DF090324:7960452-7960866
BmHel-8	DF090373:1885214-1885628
BmHel-8	DF090339:924149-924563
BmHel-8	DF090339:4981393-4981807
BmHel-8	DF090432:411693-412107
BmHel-8	DF090379:311054-311468
BmHel-8	DF090374:1620106-1620520
BmHel-8	DF090325:3372366-3372780
BmHel-8	DF090325:4120279-4120693
BmHel-8	DF090378:1207339-1207753
BmHel-8	DF090414:136485-136899
BmHel-8	DF090389:505280-505694
BmHel-8	DF090387:454543-454957
BmHel-8	BABH01054794:82-496
BmHel-8	DF090451:79058-79472
BmHel-8	DF090404:418016-418430
BmHel-8	DF090354:874080-874494
BmHel-8	DF090398:1309494-1309908
BmHel-8	BABH01077066:110-524
BmHel-8	DF091184:11465-11879
BmHel-8	DF090330:5988643-5989057
BmHel-8	DF090344:1991166-1991580
BmHel-8	DF090327:5342942-5343356
BmHel-8	DF090360:2092579-2092993
BmHel-8	DF090333:5259249-5259663
BmHel-8	DF090370:2235707-2236121
BmHel-8	DF090386:551577-551991
BmHel-8	DF090352:1643375-1643789
BmHel-8	DF090351:4237240-4237654
BmHel-8	DF090332:5446122-5446536
BmHel-8	DF090421:862040-862454
BmHel-8	DF090421:473395-473809
BmHel-8	DF090355:2223068-2223482
BmHel-8	DF090353:1344022-1344436
BmHel-8	DF090353:589211-589625
BmHel-8	DF090342:46003-46417
BmHel-8	DF090336:69499-69913

BmHel-8	DF090318:2045878-2046292
BmHel-8	DF090403:75075-75489
BmHel-8	DF090388:785672-786086
BmHel-8	DF090362:3038033-3038447
BmHel-8	DF090362:1074345-1074759
BmHel-8	DF090357:3610040-3610454
BmHel-8	DF090357:2745821-2746235
BmHel-8	DF090344:3903653-3904067
BmHel-8	DF090340:2893496-2893910
BmHel-8	DF090338:4149795-4150209
BmHel-8	DF090329:4917307-4917721
BmHel-8	DF090329:1246079-1246493
BmHel-8	DF090319:4952238-4952652
BmHel-8	DF090319:2511791-2512205
BmHel-8	DF090319:2341552-2341966
BmHel-8	DF090319:1893071-1893485
BmHel-8	DF090319:1336706-1337120
BmHel-8	DF090319:929601-930015
BmHel-8	DF090319:251602-252016
BmHel-8	DF090317:9770728-9771142
BmHel-8	DF090316:10923852-10924266
BmHel-8	DF090349:203053-203467
BmHel-8	DF090348:820345-820759
BmHel-8	DF090382:1489436-1489850
BmHel-8	DF090345:3412526-3412940
BmHel-8	DF090345:2412149-2412563
BmHel-8	DF090345:2046968-2047382
BmHel-8	DF090345:540743-541157
BmHel-8	DF090341:4686850-4687264
BmHel-8	DF090335:4703929-4704343
BmHel-8	DF090335:4544617-4545031
BmHel-8	DF090335:2953084-2953498
BmHel-8	DF090334:3767864-3768278
BmHel-8	DF090334:3167077-3167491
BmHel-8	DF090334:2722347-2722761
BmHel-8	DF090331:778128-778542
BmHel-8	DF090328:5965564-5965978
BmHel-8	DF090324:7673345-7673759
BmHel-8	DF090425:681771-682185
BmHel-8	DF090376:1367961-1368375
BmHel-8	DF090375:1541561-1541975
BmHel-8	DF090333:5411676-5412090
BmHel-8	DF090333:229019-229433
BmHel-8	DF090325:4426856-4427270
BmHel-8	DF090325:949323-949737
BmHel-8	BABH01067454:108-522
BmHel-8	DF090414:871806-872220
BmHel-8	DF090414:495287-495701

BmHel-8	DF090387:690025-690439
BmHel-8	DF090384:1989013-1989427
BmHel-8	DF090364:2650496-2650910
BmHel-8	DF090429:821536-821950
BmHel-8	DF090429:462640-463054
BmHel-8	DF090469:281725-282139
BmHel-8	BABH01046770:428-842
BmHel-8	BABH01075618:149-563
BmHel-8	BABH01047710:933-1347
BmHel-8	DF090506:193441-193855
BmHel-8	DF090358:772285-772699
BmHel-8	DF090351:3342679-3343093
BmHel-8	DF090317:6720210-6720624
BmHel-8	DF090366:47587-48001
BmHel-8	DF090326:6626356-6626770
BmHel-8	DF090343:4231358-4231772
BmHel-8	DF090467:311647-312061
BmHel-8	BABH01085896:1-415
BmHel-8	DF090330:5115636-5116051
BmHel-8	DF090330:5596624-5597039
BmHel-8	DF090330:6265582-6265997
BmHel-8	DF090358:1870123-1870538
BmHel-8	DF090358:2793373-2793788
BmHel-8	DF090386:121161-121576
BmHel-8	DF090386:401214-401629
BmHel-8	DF090386:1988496-1988911
BmHel-8	DF090352:2578468-2578883
BmHel-8	DF090352:2780616-2781031
BmHel-8	DF090352:3238343-3238758
BmHel-8	DF090351:1268375-1268790
BmHel-8	DF090332:786983-787398
BmHel-8	DF090466:86462-86877
BmHel-8	DF090402:531658-532073
BmHel-8	DF090402:1038982-1039397
BmHel-8	DF090377:34408-34823
BmHel-8	DF090355:2644969-2645384
BmHel-8	DF090350:2569583-2569998
BmHel-8	DF090342:2565704-2566119
BmHel-8	DF090342:4044083-4044498
BmHel-8	DF090336:1612986-1613401
BmHel-8	DF090336:3641452-3641867
BmHel-8	DF090321:4099504-4099919
BmHel-8	DF090318:8577646-8578061
BmHel-8	DF090441:443397-443812
BmHel-8	DF090362:1764615-1765030
BmHel-8	DF090359:1268595-1269010
BmHel-8	DF090357:2950132-2950547
BmHel-8	DF090356:2123187-2123602

BmHel-8	DF090356:3085884-3086299
BmHel-8	DF090340:3327561-3327976
BmHel-8	DF090338:1674171-1674586
BmHel-8	DF090329:1831869-1832284
BmHel-8	DF090329:1850950-1851365
BmHel-8	DF090327:5074980-5075395
BmHel-8	DF090327:5714070-5714485
BmHel-8	DF090322:2890274-2890689
BmHel-8	DF090322:3915772-3916187
BmHel-8	DF090322:4437064-4437479
BmHel-8	DF090319:4440347-4440762
BmHel-8	DF090319:5538033-5538448
BmHel-8	DF090319:6766337-6766752
BmHel-8	DF090317:6801631-6802046
BmHel-8	DF090317:6840706-6841121
BmHel-8	DF090317:10040080-10040495
BmHel-8	DF090317:11078259-11078674
BmHel-8	DF090316:7576297-7576712
BmHel-8	DF090316:9429838-9430253
BmHel-8	DF090316:15893428-15893843
BmHel-8	DF090326:6098004-6098419
BmHel-8	DF090365:2199451-2199866
BmHel-8	DF090348:459526-459941
BmHel-8	DF090411:1149193-1149608
BmHel-8	DF090401:47202-47617
BmHel-8	DF090396:860864-861279
BmHel-8	DF090396:1555095-1555510
BmHel-8	DF090372:676931-677346
BmHel-8	DF090360:1163647-1164062
BmHel-8	DF090360:2888165-2888580
BmHel-8	DF090360:3313181-3313596
BmHel-8	DF090347:789055-789470
BmHel-8	DF090341:2331369-2331784
BmHel-8	DF090334:4824852-4825267
BmHel-8	DF090334:5622180-5622595
BmHel-8	DF090331:249341-249756
BmHel-8	DF090331:687629-688044
BmHel-8	DF090331:4927378-4927793
BmHel-8	DF090331:5135779-5136194
BmHel-8	DF090328:123778-124193
BmHel-8	DF090324:646981-647396
BmHel-8	DF090343:694560-694975
BmHel-8	DF090343:829425-829840
BmHel-8	DF090339:778143-778558
BmHel-8	DF090339:4367325-4367740
BmHel-8	DF090432:446996-447411
BmHel-8	DF090379:136632-137047
BmHel-8	DF090379:669031-669446

BmHel-8	DF090333:3718704-3719119
BmHel-8	DF090325:6033241-6033656
BmHel-8	DF090623:24728-25143
BmHel-8	BABH01042583:7846-8261
BmHel-8	DF090389:391958-392373
BmHel-8	DF090389:987143-987558
BmHel-8	DF090384:1521051-1521466
BmHel-8	DF090364:626292-626707
BmHel-8	DF090364:975440-975855
BmHel-8	DF090364:2612829-2613244
BmHel-8	BABH01083921:64-479
BmHel-8	DF090415:640285-640700
BmHel-8	DF090472:239593-240008
BmHel-8	DF090398:518524-518939
BmHel-8	DF090407:106247-106662
BmHel-8	BABH01076931:31-446
BmHel-8	DF090459:369455-369870
BmHel-8	DF090419:1037764-1038179
BmHel-8	DF090520:153434-153849
BmHel-8	DF090455:136740-137155
BmHel-8	DF090792:15249-15664
BmHel-8	DF090574:35470-35885
BmHel-8	BABH01073892:6-421
BmHel-8	DF090463:43522-43937
BmHel-8	DF090463:109447-109862
BmHel-8	DF090430:571789-572204
BmHel-8	BABH01043997:2876-3291
BmHel-8	BABH01086557:49-464
BmHel-8	BABH01045064:5259-5674
BmHel-8	DF090742:18864-19279
BmHel-8	DF090318:3635052-3635467
BmHel-8	DF090318:7067804-7068219
BmHel-8	DF090422:1071525-1071940
BmHel-8	DF090325:2731305-2731720
BmHel-8	DF090412:1215271-1215686
BmHel-8	DF090412:392576-392991
BmHel-8	DF090351:1225813-1226228
BmHel-8	DF090346:3715304-3715719
BmHel-8	DF090346:1040956-1041371
BmHel-8	DF090346:373913-374328
BmHel-8	DF090402:1341457-1341872
BmHel-8	DF090377:1779475-1779890
BmHel-8	DF090377:1514626-1515041
BmHel-8	DF090377:449309-449724
BmHel-8	DF090377:290088-290503
BmHel-8	DF090353:3181870-3182285
BmHel-8	DF090350:4339293-4339708
BmHel-8	DF090350:3751023-3751438

BmHel-8	DF090350:1572376-1572791
BmHel-8	DF090342:3576883-3577298
BmHel-8	DF090342:3308925-3309340
BmHel-8	DF090342:2712811-2713226
BmHel-8	DF090321:8208768-8209183
BmHel-8	DF090321:5212432-5212847
BmHel-8	DF090321:1953846-1954261
BmHel-8	DF090321:1538695-1539110
BmHel-8	DF090321:1510770-1511185
BmHel-8	DF090318:6343221-6343636
BmHel-8	DF090320:6027742-6028157
BmHel-8	DF090320:4279348-4279763
BmHel-8	DF090320:2465755-2466170
BmHel-8	DF090443:335977-336392
BmHel-8	DF090420:113008-113423
BmHel-8	DF090388:726744-727159
BmHel-8	DF090388:300000-300415
BmHel-8	DF090362:2910074-2910489
BmHel-8	DF090362:2719994-2720409
BmHel-8	DF090362:2079810-2080225
BmHel-8	DF090362:1296861-1297276
BmHel-8	DF090357:3591811-3592226
BmHel-8	DF090357:3230441-3230856
BmHel-8	DF090357:2288445-2288860
BmHel-8	DF090357:1267990-1268405
BmHel-8	DF090344:3158765-3159180
BmHel-8	DF090344:2970711-2971126
BmHel-8	DF090344:2152134-2152549
BmHel-8	DF090344:1856447-1856862
BmHel-8	DF090329:5348040-5348455
BmHel-8	DF090329:1597885-1598300
BmHel-8	DF090323:7212200-7212615
BmHel-8	DF090323:3228508-3228923
BmHel-8	DF090323:547663-548078
BmHel-8	DF090323:241341-241756
BmHel-8	DF090322:4369811-4370226
BmHel-8	DF090319:412356-412771
BmHel-8	DF090319:123521-123936
BmHel-8	DF090317:10913616-10914031
BmHel-8	DF090317:7130726-7131141
BmHel-8	DF090317:2115944-2116359
BmHel-8	DF090317:1750330-1750745
BmHel-8	DF090317:648300-648715
BmHel-8	DF090316:15044270-15044685
BmHel-8	DF090316:4794031-4794446
BmHel-8	DF090366:919369-919784
BmHel-8	DF090349:3717031-3717446
BmHel-8	DF090349:2659152-2659567

BmHel-8	DF090326:1364304-1364719
BmHel-8	DF090348:127332-127747
BmHel-8	DF090422:1065801-1066216
BmHel-8	DF090411:395052-395467
BmHel-8	DF090401:1509469-1509884
BmHel-8	DF090396:1255175-1255590
BmHel-8	DF090391:816223-816638
BmHel-8	DF090372:915541-915956
BmHel-8	DF090371:167093-167508
BmHel-8	DF090360:1170968-1171383
BmHel-8	DF090360:864430-864845
BmHel-8	DF090360:291818-292233
BmHel-8	DF090347:4324330-4324745
BmHel-8	DF090347:671387-671802
BmHel-8	DF090341:1644074-1644489
BmHel-8	DF090335:1135049-1135464
BmHel-8	DF090334:3041865-3042280
BmHel-8	DF090334:1599401-1599816
BmHel-8	DF090331:3755603-3756018
BmHel-8	DF090328:755217-755632
BmHel-8	DF090324:5526358-5526773
BmHel-8	DF090337:1630039-1630454
BmHel-8	DF090337:967052-967467
BmHel-8	DF090373:780345-780760
BmHel-8	DF090343:1477954-1478369
BmHel-8	DF090405:51310-51725
BmHel-8	DF090375:1793336-1793751
BmHel-8	DF090325:2101158-2101573
BmHel-8	DF090378:1587896-1588311
BmHel-8	DF090580:9678-10093
BmHel-8	DF090414:544791-545206
BmHel-8	DF090387:1576033-1576448
BmHel-8	DF090384:224039-224454
BmHel-8	DF090429:949515-949930
BmHel-8	DF090404:145895-146310
BmHel-8	DF090354:2470875-2471290
BmHel-8	DF090354:1014184-1014599
BmHel-8	BABH01057605:309-724
BmHel-8	DF090446:386022-386437
BmHel-8	BABH01083623:75-490
BmHel-8	DF090628:7846-8261
BmHel-8	DF090517:2711-3126
BmHel-8	DF090419:738417-738832
BmHel-8	BABH01074411:140-555
BmHel-8	DF090520:2747-3162
BmHel-8	DF090393:1498133-1498548
BmHel-8	DF090471:114399-114814
BmHel-8	DF090461:345406-345821

BmHel-8	BABH01068238:7-422
BmHel-8	BABH01047705:220-635
BmHel-8	DF090457:63462-63877
BmHel-8	DF091038:6532-6947
BmHel-8	DF090913:8190-8605
BmHel-8	DF091016:2493-2908
BmHel-8	DF090960:4174-4589
BmHel-8	DF090458:388947-389362
BmHel-8	DF090378:613519-613934
BmHel-8	DF090318:4262387-4262802
BmHel-8	DF090330:1160177-1160593
BmHel-8	DF090351:1298051-1298467
BmHel-8	DF090346:2642482-2642898
BmHel-8	DF090346:2805190-2805606
BmHel-8	DF090332:1146345-1146761
BmHel-8	DF090332:5799655-5800071
BmHel-8	DF090421:554856-555272
BmHel-8	DF090367:2315573-2315989
BmHel-8	DF090363:1797532-1797948
BmHel-8	DF090355:2584497-2584913
BmHel-8	DF090355:3581809-3582225
BmHel-8	DF090353:3161692-3162108
BmHel-8	DF090350:2411761-2412177
BmHel-8	DF090350:2723430-2723846
BmHel-8	DF090342:4518240-4518656
BmHel-8	DF090336:4454618-4455034
BmHel-8	DF090336:5239224-5239640
BmHel-8	DF090321:1948524-1948940
BmHel-8	DF090321:3116602-3117018
BmHel-8	DF090321:3156179-3156595
BmHel-8	DF090318:1400442-1400858
BmHel-8	DF090318:2886785-2887201
BmHel-8	DF090318:6162615-6163031
BmHel-8	DF090318:9498468-9498884
BmHel-8	DF090362:1040413-1040829
BmHel-8	DF090359:1704061-1704477
BmHel-8	DF090357:1740558-1740974
BmHel-8	DF090344:1907673-1908089
BmHel-8	DF090329:535113-535529
BmHel-8	DF090329:4514774-4515190
BmHel-8	DF090327:1722665-1723081
BmHel-8	DF090327:4806302-4806718
BmHel-8	DF090323:2195814-2196230
BmHel-8	DF090323:5074356-5074772
BmHel-8	DF090322:1565612-1566028
BmHel-8	DF090322:1639289-1639705
BmHel-8	DF090322:7914939-7915355
BmHel-8	DF090319:2110745-2111161

BmHel-8	DF090319:3031255-3031671
BmHel-8	DF090319:3924337-3924753
BmHel-8	DF090317:545449-545865
BmHel-8	DF090317:1984208-1984624
BmHel-8	DF090317:2976298-2976714
BmHel-8	DF090317:9591175-9591591
BmHel-8	DF090316:1203359-1203775
BmHel-8	DF090316:3680044-3680460
BmHel-8	DF090316:12418056-12418472
BmHel-8	DF090316:13558028-13558444
BmHel-8	DF090316:14868228-14868644
BmHel-8	DF090316:15009451-15009867
BmHel-8	DF090366:577343-577759
BmHel-8	DF090349:2712717-2713133
BmHel-8	DF090349:4142632-4143048
BmHel-8	DF090365:1956065-1956481
BmHel-8	DF090348:3665078-3665494
BmHel-8	DF090411:1058848-1059264
BmHel-8	DF090396:1433312-1433728
BmHel-8	DF090347:530804-531220
BmHel-8	DF090347:1806282-1806698
BmHel-8	DF090345:4069444-4069860
BmHel-8	DF090341:396248-396664
BmHel-8	DF090341:4732423-4732839
BmHel-8	DF090335:1473489-1473905
BmHel-8	DF090335:2782495-2782911
BmHel-8	DF090334:164370-164786
BmHel-8	DF090334:3807076-3807492
BmHel-8	DF090331:438093-438509
BmHel-8	DF090331:4713249-4713665
BmHel-8	DF090328:22893-23309
BmHel-8	DF090324:739855-740271
BmHel-8	DF090324:1204139-1204555
BmHel-8	DF090337:2553502-2553918
BmHel-8	DF090390:343043-343459
BmHel-8	DF090449:747419-747835
BmHel-8	DF090373:2021923-2022339
BmHel-8	DF090343:3224665-3225081
BmHel-8	DF090425:487948-488364
BmHel-8	DF090379:497610-498026
BmHel-8	DF090376:919066-919482
BmHel-8	DF090376:2137401-2137817
BmHel-8	DF090475:84054-84470
BmHel-8	DF090445:482868-483284
BmHel-8	DF090434:435324-435740
BmHel-8	DF090431:430535-430951
BmHel-8	DF090417:903458-903874
BmHel-8	DF090416:770080-770496

BmHel-8	BABH01053531:92-508
BmHel-8	DF090398:949515-949931
BmHel-8	DF091346:3786-4202
BmHel-8	BABH01084871:83-499
BmHel-8	BABH01047203:669-1085
BmHel-8	BABH01043665:2567-2983
BmHel-8	BABH01083606:20-436
BmHel-8	DF090326:3475173-3475589
BmHel-8	DF090354:286730-287146
BmHel-8	DF090330:5641594-5642010
BmHel-8	DF090330:4733497-4733913
BmHel-8	DF090370:2427577-2427993
BmHel-8	DF090370:1668205-1668621
BmHel-8	DF090358:790192-790608
BmHel-8	DF090386:1902783-1903199
BmHel-8	DF090352:3828154-3828570
BmHel-8	DF090352:1556681-1557097
BmHel-8	DF090352:634249-634665
BmHel-8	DF090351:4189319-4189735
BmHel-8	DF090351:94746-95162
BmHel-8	DF090346:639398-639814
BmHel-8	DF090332:2395148-2395564
BmHel-8	DF090332:86104-86520
BmHel-8	DF090367:1991783-1992199
BmHel-8	DF090363:1847774-1848190
BmHel-8	DF090355:2818306-2818722
BmHel-8	DF090350:3944802-3945218
BmHel-8	DF090342:1996346-1996762
BmHel-8	DF090321:4580767-4581183
BmHel-8	DF090321:3123831-3124247
BmHel-8	DF090318:7726256-7726672
BmHel-8	DF090318:7557226-7557642
BmHel-8	DF090320:7660662-7661078
BmHel-8	DF090320:6541837-6542253
BmHel-8	DF090320:3313018-3313434
BmHel-8	DF090420:930842-931258
BmHel-8	DF090400:690713-691129
BmHel-8	DF090362:2754933-2755349
BmHel-8	DF090362:2547191-2547607
BmHel-8	DF090362:2360185-2360601
BmHel-8	DF090362:1369432-1369848
BmHel-8	DF090344:4338096-4338512
BmHel-8	DF090344:1100393-1100809
BmHel-8	DF090340:1348498-1348914
BmHel-8	DF090329:6111677-6112093
BmHel-8	DF090327:4583431-4583847
BmHel-8	DF090322:3560425-3560841
BmHel-8	DF090322:602439-602855

BmHel-8	DF090319:7654950-7655366
BmHel-8	DF090319:7063371-7063787
BmHel-8	DF090319:4818416-4818832
BmHel-8	DF090319:4485999-4486415
BmHel-8	DF090319:36752-37168
BmHel-8	DF090317:10412316-10412732
BmHel-8	DF090317:1664953-1665369
BmHel-8	DF090317:581994-582410
BmHel-8	DF090316:14277454-14277870
BmHel-8	DF090316:13332407-13332823
BmHel-8	DF090316:10733976-10734392
BmHel-8	DF090316:9029152-9029568
BmHel-8	DF090410:864497-864913
BmHel-8	DF090366:839719-840135
BmHel-8	DF090349:4208274-4208690
BmHel-8	DF090349:3596402-3596818
BmHel-8	DF090326:6391249-6391665
BmHel-8	DF090365:2937379-2937795
BmHel-8	DF090365:2332032-2332448
BmHel-8	DF090365:1524872-1525288
BmHel-8	DF090411:674800-675216
BmHel-8	DF090394:141927-142343
BmHel-8	DF090391:215609-216025
BmHel-8	DF090371:746554-746970
BmHel-8	DF090347:3314267-3314683
BmHel-8	DF090341:981329-981745
BmHel-8	DF090341:708264-708680
BmHel-8	DF090334:4929860-4930276
BmHel-8	DF090331:5800903-5801319
BmHel-8	DF090331:4254852-4255268
BmHel-8	DF090328:3673435-3673851
BmHel-8	DF090324:3537547-3537963
BmHel-8	DF090324:1276151-1276567
BmHel-8	DF090381:260041-260457
BmHel-8	DF090375:1877212-1877628
BmHel-8	DF090333:5084599-5085015
BmHel-8	DF090333:3931715-3932131
BmHel-8	DF090558:19633-20049
BmHel-8	DF090378:1752401-1752817
BmHel-8	DF090486:126810-127226
BmHel-8	DF090431:78053-78469
BmHel-8	DF090414:855305-855721
BmHel-8	DF090387:343006-343422
BmHel-8	DF090364:2850158-2850574
BmHel-8	DF090364:2185628-2186044
BmHel-8	DF090451:424666-425082
BmHel-8	DF090354:2566409-2566825
BmHel-8	DF090413:596370-596786

BmHel-8	DF090481:238106-238522
BmHel-8	BABH01069754:153-569
BmHel-8	DF090940:3123-3539
BmHel-8	DF090510:19567-19983
BmHel-8	BABH01055742:312-728
BmHel-8	DF091045:9804-10220
BmHel-8	DF090765:12637-13053
BmHel-8	DF090352:3578471-3578887
BmHel-8	DF090322:3664140-3664556
BmHel-8	DF090423:139080-139496
BmHel-8	DF090358:351441-351858
BmHel-8	DF090358:1475083-1475500
BmHel-8	DF090412:572646-573063
BmHel-8	DF090383:1290168-1290585
BmHel-8	DF090346:4402496-4402913
BmHel-8	DF090332:1250744-1251161
BmHel-8	DF090332:1981363-1981780
BmHel-8	DF090342:1037326-1037743
BmHel-8	DF090342:4339777-4340194
BmHel-8	DF090336:3947992-3948409
BmHel-8	DF090336:5082746-5083163
BmHel-8	DF090318:213705-214122
BmHel-8	DF090320:3329362-3329779
BmHel-8	DF090320:8583687-8584104
BmHel-8	DF090362:2552214-2552631
BmHel-8	DF090357:2537406-2537823
BmHel-8	DF090356:3386851-3387268
BmHel-8	DF090338:3645357-3645774
BmHel-8	DF090329:2349424-2349841
BmHel-8	DF090329:2790315-2790732
BmHel-8	DF090327:140910-141327
BmHel-8	DF090327:1953975-1954392
BmHel-8	DF090319:2415507-2415924
BmHel-8	DF090319:3717788-3718205
BmHel-8	DF090316:10427884-10428301
BmHel-8	DF090349:3224184-3224601
BmHel-8	DF090433:191368-191785
BmHel-8	DF090365:869484-869901
BmHel-8	DF090365:1534702-1535119
BmHel-8	DF090365:2384892-2385309
BmHel-8	DF090394:1560299-1560716
BmHel-8	DF090382:1935848-1936265
BmHel-8	DF090371:869253-869670
BmHel-8	DF090360:1869793-1870210
BmHel-8	DF090347:1507058-1507475
BmHel-8	DF090345:1216934-1217351
BmHel-8	DF090335:4073299-4073716
BmHel-8	DF090335:5361058-5361475

BmHel-8	DF090334:5141505-5141922
BmHel-8	DF090328:6611915-6612332
BmHel-8	DF090324:7754751-7755168
BmHel-8	DF090324:8021949-8022366
BmHel-8	DF090324:8065823-8066240
BmHel-8	DF090343:4498359-4498776
BmHel-8	DF090405:684879-685296
BmHel-8	DF090379:1052376-1052793
BmHel-8	DF090376:1387215-1387632
BmHel-8	DF090375:1523341-1523758
BmHel-8	DF090375:2259464-2259881
BmHel-8	DF090325:7164044-7164461
BmHel-8	DF090417:662671-663088
BmHel-8	DF090364:1203972-1204389
BmHel-8	DF090364:2073021-2073438
BmHel-8	BABH01071171:27-444
BmHel-8	DF090354:842341-842758
BmHel-8	DF090541:129936-130353
BmHel-8	DF090419:543719-544136
BmHel-8	DF090489:37794-38211
BmHel-8	BABH01045377:4133-4550
BmHel-8	DF090767:10302-10719
BmHel-8	DF090448:237916-238333
BmHel-8	DF090448:499372-499789
BmHel-8	DF090563:23122-23539
BmHel-8	DF090361:568894-569311
BmHel-8	DF090417:949323-949740
BmHel-8	BABH01043566:3820-4237
BmHel-8	BABH01050835:514-931
BmHel-8	DF090330:4386058-4386475
BmHel-8	DF090383:1277960-1278377
BmHel-8	DF090346:139257-139674
BmHel-8	DF090332:5047149-5047566
BmHel-8	DF090332:5015545-5015962
BmHel-8	DF090367:1872209-1872626
BmHel-8	DF090367:1537642-1538059
BmHel-8	DF090367:665689-666106
BmHel-8	DF090355:3013623-3014040
BmHel-8	DF090355:2415812-2416229
BmHel-8	DF090342:4485202-4485619
BmHel-8	DF090342:2336782-2337199
BmHel-8	DF090342:1890266-1890683
BmHel-8	DF090336:1743411-1743828
BmHel-8	DF090318:3962950-3963367
BmHel-8	DF090318:2871614-2872031
BmHel-8	DF090318:2769191-2769608
BmHel-8	DF090362:2788640-2789057
BmHel-8	DF090359:2184989-2185406

BmHel-8	DF090344:3403846-3404263
BmHel-8	DF090344:2432439-2432856
BmHel-8	DF090338:3333190-3333607
BmHel-8	DF090329:4804132-4804549
BmHel-8	DF090329:2082588-2083005
BmHel-8	DF090327:5163146-5163563
BmHel-8	DF090327:4638510-4638927
BmHel-8	DF090323:6491337-6491754
BmHel-8	DF090317:10867003-10867420
BmHel-8	DF090317:509854-510271
BmHel-8	DF090316:11389252-11389669
BmHel-8	DF090316:5060086-5060503
BmHel-8	DF090349:1954585-1955002
BmHel-8	DF090365:964977-965394
BmHel-8	DF090348:3607306-3607723
BmHel-8	DF090411:237487-237904
BmHel-8	DF090397:1192584-1193001
BmHel-8	DF090392:542049-542466
BmHel-8	DF090369:418305-418722
BmHel-8	DF090361:1631163-1631580
BmHel-8	DF090347:556909-557326
BmHel-8	DF090328:6625351-6625768
BmHel-8	DF090328:3685919-3686336
BmHel-8	DF090328:1580967-1581384
BmHel-8	DF090337:1064934-1065351
BmHel-8	DF090390:1547320-1547737
BmHel-8	DF090390:702260-702677
BmHel-8	DF090343:383621-384038
BmHel-8	DF090339:4786473-4786890
BmHel-8	DF090339:1296565-1296982
BmHel-8	DF090379:517802-518219
BmHel-8	DF090325:3668146-3668563
BmHel-8	DF090427:493480-493897
BmHel-8	DF090387:1864870-1865287
BmHel-8	DF090364:675236-675653
BmHel-8	BABH01055891:277-694
BmHel-8	DF090588:19761-20178
BmHel-8	DF090463:16640-17057
BmHel-8	DF090503:70820-71237
BmHel-8	DF090447:322313-322730
BmHel-8	DF090521:61675-62092
BmHel-8	DF090563:54506-54923
BmHel-8	DF090714:5309-5726
BmHel-8	DF090321:2403229-2403646
BmHel-8	DF090322:7321879-7322296
BmHel-8	DF090319:2058307-2058724
BmHel-8	DF090348:495435-495852
BmHel-8	DF090339:1933526-1933943

BmHel-8	DF090338:4279584-4280001
BmHel-8	DF090330:419216-419634
BmHel-8	DF090437:669167-669585
BmHel-8	DF090386:1040023-1040441
BmHel-8	DF090346:588208-588626
BmHel-8	DF090332:1168099-1168517
BmHel-8	DF090332:1679476-1679894
BmHel-8	DF090332:2881287-2881705
BmHel-8	DF090332:2962514-2962932
BmHel-8	DF090332:5654657-5655075
BmHel-8	DF090409:1124085-1124503
BmHel-8	DF090367:2738110-2738528
BmHel-8	DF090363:2146203-2146621
BmHel-8	DF090342:2742585-2743003
BmHel-8	DF090336:3106355-3106773
BmHel-8	DF090336:4290102-4290520
BmHel-8	DF090336:4330118-4330536
BmHel-8	DF090336:4821913-4822331
BmHel-8	DF090336:5619943-5620361
BmHel-8	DF090321:1755639-1756057
BmHel-8	DF090321:2474034-2474452
BmHel-8	DF090318:4265220-4265638
BmHel-8	DF090318:7173830-7174248
BmHel-8	DF090318:8657183-8657601
BmHel-8	DF090320:6083890-6084308
BmHel-8	DF090488:25026-25444
BmHel-8	DF090362:176076-176494
BmHel-8	DF090362:1364959-1365377
BmHel-8	DF090362:1475077-1475495
BmHel-8	DF090357:1927505-1927923
BmHel-8	DF090344:2368466-2368884
BmHel-8	DF090344:2580265-2580683
BmHel-8	DF090340:2897525-2897943
BmHel-8	DF090338:2144125-2144543
BmHel-8	DF090338:3840637-3841055
BmHel-8	DF090338:4549121-4549539
BmHel-8	DF090329:300137-300555
BmHel-8	DF090323:1142186-1142604
BmHel-8	DF090322:5547120-5547538
BmHel-8	DF090319:416235-416653
BmHel-8	DF090319:8675009-8675427
BmHel-8	DF090317:3377722-3378140
BmHel-8	DF090316:11353498-11353916
BmHel-8	DF090316:15359620-15360038
BmHel-8	DF090366:30601-31019
BmHel-8	DF090366:1538329-1538747
BmHel-8	DF090349:3817327-3817745
BmHel-8	DF090349:4318316-4318734

BmHel-8	DF090326:2705930-2706348
BmHel-8	DF090348:1595515-1595933
BmHel-8	DF090397:471125-471543
BmHel-8	DF090372:1923896-1924314
BmHel-8	DF090369:988907-989325
BmHel-8	DF090361:2148532-2148950
BmHel-8	DF090347:938570-938988
BmHel-8	DF090345:2043641-2044059
BmHel-8	DF090345:4394240-4394658
BmHel-8	DF090341:1225654-1226072
BmHel-8	DF090341:1732816-1733234
BmHel-8	DF090341:3900163-3900581
BmHel-8	DF090335:3561514-3561932
BmHel-8	DF090328:6677695-6678113
BmHel-8	DF090343:3897839-3898257
BmHel-8	DF090423:253353-253771
BmHel-8	DF090375:442068-442486
BmHel-8	DF090325:5917339-5917757
BmHel-8	DF090486:13563-13981
BmHel-8	DF090482:149820-150238
BmHel-8	DF090384:623873-624291
BmHel-8	DF090413:24259-24677
BmHel-8	DF090406:407629-408047
BmHel-8	DF090406:662452-662870
BmHel-8	DF090368:748224-748642
BmHel-8	DF090368:2431555-2431973
BmHel-8	DF090616:2550-2968
BmHel-8	DF090419:561396-561814
BmHel-8	DF090542:26636-27054
BmHel-8	DF090648:24649-25067
BmHel-8	DF091021:1881-2299
BmHel-8	DF090421:529626-530044
BmHel-8	DF090317:637894-638312
BmHel-8	DF090316:7758471-7758889
BmHel-8	DF090372:2289386-2289804
BmHel-8	DF090352:404814-405232
BmHel-8	DF090382:1749955-1750373
BmHel-8	DF090330:5212825-5213243
BmHel-8	DF090330:1947342-1947760
BmHel-8	DF090370:2017905-2018323
BmHel-8	DF090370:1137878-1138296
BmHel-8	DF090370:247719-248137
BmHel-8	DF090358:407273-407691
BmHel-8	DF090437:641264-641682
BmHel-8	DF090386:353026-353444
BmHel-8	DF090383:322667-323085
BmHel-8	DF090352:2510342-2510760
BmHel-8	DF090352:1437920-1438338

BmHel-8	DF090351:3315846-3316264
BmHel-8	DF090351:3050406-3050824
BmHel-8	DF090346:454733-455151
BmHel-8	DF090332:3836354-3836772
BmHel-8	DF090466:254530-254948
BmHel-8	DF090402:1277958-1278376
BmHel-8	DF090402:1181740-1182158
BmHel-8	DF090363:2646944-2647362
BmHel-8	DF090350:829963-830381
BmHel-8	DF090342:2736427-2736845
BmHel-8	DF090342:286734-287152
BmHel-8	DF090336:730820-731238
BmHel-8	DF090321:6878788-6879206
BmHel-8	DF090321:5579437-5579855
BmHel-8	DF090321:5233886-5234304
BmHel-8	DF090318:8183908-8184326
BmHel-8	DF090318:6442257-6442675
BmHel-8	DF090318:5815884-5816302
BmHel-8	DF090318:5133310-5133728
BmHel-8	DF090318:1295491-1295909
BmHel-8	DF090320:8520389-8520807
BmHel-8	DF090320:5970294-5970712
BmHel-8	DF090320:617309-617727
BmHel-8	DF090400:1113010-1113428
BmHel-8	DF090400:588095-588513
BmHel-8	DF090357:2872266-2872684
BmHel-8	DF090344:4457177-4457595
BmHel-8	DF090344:4218898-4219316
BmHel-8	DF090344:3528419-3528837
BmHel-8	DF090338:968483-968901
BmHel-8	DF090329:5526824-5527242
BmHel-8	DF090327:3277130-3277548
BmHel-8	DF090327:1072755-1073173
BmHel-8	DF090323:6054306-6054724
BmHel-8	DF090323:2772688-2773106
BmHel-8	DF090322:7983194-7983612
BmHel-8	DF090319:6869710-6870128
BmHel-8	DF090319:6554310-6554728
BmHel-8	DF090319:2857485-2857903
BmHel-8	DF090317:11040699-11041117
BmHel-8	DF090317:4725863-4726281
BmHel-8	DF090395:184443-184861
BmHel-8	DF090349:947738-948156
BmHel-8	DF090326:1702556-1702974
BmHel-8	DF090326:848640-849058
BmHel-8	DF090433:365688-366106
BmHel-8	DF090433:20662-21080
BmHel-8	DF090348:3715555-3715973

BmHel-8	DF090422:341156-341574
BmHel-8	DF090394:1527996-1528414
BmHel-8	DF090360:2210969-2211387
BmHel-8	DF090347:805893-806311
BmHel-8	DF090334:5619879-5620297
BmHel-8	DF090334:1560570-1560988
BmHel-8	DF090334:1549763-1550181
BmHel-8	DF090328:1766090-1766508
BmHel-8	DF090324:7055634-7056052
BmHel-8	DF090337:2469009-2469427
BmHel-8	DF090381:165589-166007
BmHel-8	DF090381:69777-70195
BmHel-8	DF090343:3164698-3165116
BmHel-8	DF090339:4952963-4953381
BmHel-8	DF090333:5246627-5247045
BmHel-8	DF090418:1143432-1143850
BmHel-8	DF090325:5981070-5981488
BmHel-8	DF090325:3591915-3592333
BmHel-8	DF090325:2705027-2705445
BmHel-8	DF090444:561610-562028
BmHel-8	DF090389:1711953-1712371
BmHel-8	DF090384:1000403-1000821
BmHel-8	DF090639:33985-34403
BmHel-8	DF090438:197074-197492
BmHel-8	DF090438:85632-86050
BmHel-8	DF090413:1276454-1276872
BmHel-8	DF090493:111968-112386
BmHel-8	DF090407:1170365-1170783
BmHel-8	DF090407:167605-168023
BmHel-8	DF090435:367081-367499
BmHel-8	DF090428:783876-784294
BmHel-8	DF090480:184099-184517
BmHel-8	DF090399:1030977-1031395
BmHel-8	BABH01048439:781-1199
BmHel-8	DF090342:1977777-1978195
BmHel-8	DF090336:5291565-5291983
BmHel-8	DF090318:8772232-8772650
BmHel-8	DF090323:5984386-5984804
BmHel-8	DF090323:3014029-3014447
BmHel-8	DF090391:1370276-1370694
BmHel-8	DF090334:1821781-1822199
BmHel-8	DF090459:370251-370669
BmHel-8	DF090330:792448-792867
BmHel-8	DF090358:851885-852304
BmHel-8	DF090386:512906-513325
BmHel-8	DF090386:1899062-1899481
BmHel-8	DF090383:1896329-1896748
BmHel-8	DF090352:14742-15161

BmHel-8	DF090352:174331-174750
BmHel-8	DF090352:3203809-3204228
BmHel-8	DF090351:375084-375503
BmHel-8	DF090351:902349-902768
BmHel-8	DF090351:2096703-2097122
BmHel-8	DF090351:2752775-2753194
BmHel-8	DF090351:2998388-2998807
BmHel-8	DF090351:4261449-4261868
BmHel-8	DF090346:3285466-3285885
BmHel-8	DF090409:1359718-1360137
BmHel-8	DF090377:546092-546511
BmHel-8	DF090377:2061149-2061568
BmHel-8	DF090363:1508819-1509238
BmHel-8	DF090355:2114108-2114527
BmHel-8	DF090353:488600-489019
BmHel-8	DF090353:1181058-1181477
BmHel-8	DF090350:682757-683176
BmHel-8	DF090350:1324636-1325055
BmHel-8	DF090342:1660035-1660454
BmHel-8	DF090342:2462749-2463168
BmHel-8	DF090342:3827423-3827842
BmHel-8	DF090342:3981313-3981732
BmHel-8	DF090342:4851705-4852124
BmHel-8	DF090336:5043150-5043569
BmHel-8	DF090321:2573910-2574329
BmHel-8	DF090321:3590808-3591227
BmHel-8	DF090321:7743926-7744345
BmHel-8	DF090318:6442957-6443376
BmHel-8	DF090318:7927289-7927708
BmHel-8	DF090320:2732077-2732496
BmHel-8	DF090320:5497564-5497983
BmHel-8	DF090320:7799932-7800351
BmHel-8	DF090420:495621-496040
BmHel-8	DF090403:1511201-1511620
BmHel-8	DF090400:394999-395418
BmHel-8	DF090400:1044265-1044684
BmHel-8	DF090362:2502302-2502721
BmHel-8	DF090356:3458925-3459344
BmHel-8	DF090344:2948062-2948481
BmHel-8	DF090344:3049486-3049905
BmHel-8	DF090344:3261748-3262167
BmHel-8	DF090344:3933875-3934294
BmHel-8	DF090340:305849-306268
BmHel-8	DF090340:1610253-1610672
BmHel-8	DF090340:2791285-2791704
BmHel-8	DF090340:4093939-4094358
BmHel-8	DF090338:978455-978874
BmHel-8	DF090329:5910743-5911162

BmHel-8	DF090327:928685-929104
BmHel-8	DF090327:5283084-5283503
BmHel-8	DF090322:1590075-1590494
BmHel-8	DF090322:2616487-2616906
BmHel-8	DF090322:2674919-2675338
BmHel-8	DF090322:4528958-4529377
BmHel-8	DF090322:5118189-5118608
BmHel-8	DF090322:7667327-7667746
BmHel-8	DF090319:2085909-2086328
BmHel-8	DF090319:2214698-2215117
BmHel-8	DF090319:4496156-4496575
BmHel-8	DF090319:4610873-4611292
BmHel-8	DF090319:5277543-5277962
BmHel-8	DF090317:237094-237513
BmHel-8	DF090317:5335856-5336275
BmHel-8	DF090317:5594919-5595338
BmHel-8	DF090317:6014510-6014929
BmHel-8	DF090317:7839071-7839490
BmHel-8	DF090317:9244694-9245113
BmHel-8	DF090317:9465825-9466244
BmHel-8	DF090317:10756896-10757315
BmHel-8	DF090316:4854503-4854922
BmHel-8	DF090316:7963551-7963970
BmHel-8	DF090316:8459875-8460294
BmHel-8	DF090316:14531896-14532315
BmHel-8	DF090316:14933145-14933564
BmHel-8	DF090316:16142960-16143379
BmHel-8	DF090395:626658-627077
BmHel-8	DF090366:358967-359386
BmHel-8	DF090349:1157330-1157749
BmHel-8	DF090349:2889941-2890360
BmHel-8	DF090349:3065483-3065902
BmHel-8	DF090349:4090384-4090803
BmHel-8	DF090326:2248929-2249348
BmHel-8	DF090326:4966583-4967002
BmHel-8	DF090365:1661531-1661950
BmHel-8	DF090348:492799-493218
BmHel-8	DF090348:1362986-1363405
BmHel-8	BABH01078507:15-434
BmHel-8	DF090422:155410-155829
BmHel-8	DF090422:542231-542650
BmHel-8	DF090422:1115140-1115559
BmHel-8	DF090411:491905-492324
BmHel-8	DF090397:1021922-1022341
BmHel-8	DF090394:1332639-1333058
BmHel-8	DF090392:1168917-1169336
BmHel-8	DF090382:1595596-1596015
BmHel-8	DF090380:795796-796215

BmHel-8	DF090380:1935754-1936173
BmHel-8	DF090380:2015871-2016290
BmHel-8	DF090369:1832636-1833055
BmHel-8	DF090369:2537493-2537912
BmHel-8	DF090361:1143011-1143430
BmHel-8	DF090361:1892764-1893183
BmHel-8	DF090360:933843-934262
BmHel-8	DF090341:888665-889084
BmHel-8	DF090335:799183-799602
BmHel-8	DF090335:5122239-5122658
BmHel-8	DF090334:5308434-5308853
BmHel-8	DF090331:472146-472565
BmHel-8	DF090331:647769-648188
BmHel-8	DF090331:3975354-3975773
BmHel-8	DF090331:4671583-4672002
BmHel-8	DF090331:5078234-5078653
BmHel-8	DF090328:1029441-1029860
BmHel-8	DF090328:2127087-2127506
BmHel-8	DF090328:6177087-6177506
BmHel-8	DF090324:444524-444943
BmHel-8	DF090449:810424-810843
BmHel-8	DF090436:417661-418080
BmHel-8	DF090343:682967-683386
BmHel-8	DF090343:3175990-3176409
BmHel-8	DF090343:3584562-3584981
BmHel-8	DF090339:4575359-4575778
BmHel-8	BABH01072237:17-436
BmHel-8	DF090460:377382-377801
BmHel-8	DF090376:1186615-1187034
BmHel-8	DF090376:1897399-1897818
BmHel-8	DF090375:941399-941818
BmHel-8	DF090375:997047-997466
BmHel-8	DF090333:3148005-3148424
BmHel-8	DF090333:4468763-4469182
BmHel-8	DF090333:5037665-5038084
BmHel-8	DF090418:579256-579675
BmHel-8	DF090418:1214734-1215153
BmHel-8	DF090325:906885-907304
BmHel-8	DF090325:5009981-5010400
BmHel-8	DF090427:880711-881130
BmHel-8	DF090378:1347819-1348238
BmHel-8	DF090444:41007-41426
BmHel-8	DF090434:50884-51303
BmHel-8	DF090431:53679-54098
BmHel-8	DF090414:208846-209265
BmHel-8	DF090387:873028-873447
BmHel-8	DF090451:515157-515576
BmHel-8	DF090398:971145-971564

BmHel-8	BABH01048513:296-715
BmHel-8	DF090481:69744-70163
BmHel-8	DF090627:2803-3222
BmHel-8	BABH01048470:158-577
BmHel-8	DF090560:9472-9891
BmHel-8	DF090710:17233-17652
BmHel-8	DF090695:12843-13262
BmHel-8	BABH01044987:4392-4811
BmHel-8	BABH01044775:5257-5676
BmHel-8	BABH01049048:188-607
BmHel-8	DF090496:66543-66962
BmHel-8	BABH01065663:7-426
BmHel-8	BABH01054178:35-454
BmHel-8	DF090441:635844-636263
BmHel-8	DF090326:2190296-2190715
BmHel-8	DF090331:3083335-3083754
BmHel-8	DF090352:3330647-3331066
BmHel-8	DF090388:763794-764213
BmHel-8	DF090388:1161284-1161703
BmHel-8	BABH01054378:364-783
BmHel-8	DF090374:185248-185667
BmHel-8	DF090330:3654202-3654621
BmHel-8	DF090330:254970-255389
BmHel-8	DF090358:3033336-3033755
BmHel-8	DF090358:2036975-2037394
BmHel-8	DF090358:2022699-2023118
BmHel-8	DF090358:61525-61944
BmHel-8	DF090386:1763423-1763842
BmHel-8	DF090386:1665933-1666352
BmHel-8	DF090386:83512-83931
BmHel-8	DF090383:1025050-1025469
BmHel-8	DF090352:3861970-3862389
BmHel-8	DF090352:3782862-3783281
BmHel-8	DF090352:2468455-2468874
BmHel-8	DF090352:1011095-1011514
BmHel-8	DF090351:2233449-2233868
BmHel-8	DF090346:3625025-3625444
BmHel-8	DF090332:5797461-5797880
BmHel-8	DF090332:5085551-5085970
BmHel-8	DF090332:2037638-2038057
BmHel-8	DF090332:117264-117683
BmHel-8	DF090377:1764887-1765306
BmHel-8	DF090377:1581240-1581659
BmHel-8	DF090367:2532177-2532596
BmHel-8	DF090363:1228184-1228603
BmHel-8	DF090363:244493-244912
BmHel-8	DF090350:2348929-2349348
BmHel-8	DF090350:2279772-2280191

BmHel-8	DF090342:2564373-2564792
BmHel-8	DF090342:806824-807243
BmHel-8	DF090342:564017-564436
BmHel-8	DF090342:195651-196070
BmHel-8	DF090342:109292-109711
BmHel-8	DF090336:5378704-5379123
BmHel-8	DF090336:3275163-3275582
BmHel-8	DF090336:168010-168429
BmHel-8	DF090321:7742124-7742543
BmHel-8	DF090321:6315739-6316158
BmHel-8	DF090321:5170115-5170534
BmHel-8	DF090321:3039062-3039481
BmHel-8	DF090321:902090-902509
BmHel-8	DF090318:5502759-5503178
BmHel-8	DF090318:4773859-4774278
BmHel-8	DF090318:3911179-3911598
BmHel-8	DF090318:653502-653921
BmHel-8	DF090320:75506-75925
BmHel-8	BABH01062128:256-675
BmHel-8	DF090441:694532-694951
BmHel-8	DF090441:484553-484972
BmHel-8	DF090388:152427-152846
BmHel-8	DF090362:1274173-1274592
BmHel-8	DF090357:3408612-3409031
BmHel-8	DF090357:543915-544334
BmHel-8	DF090356:1635423-1635842
BmHel-8	DF090344:4116930-4117349
BmHel-8	DF090344:3502256-3502675
BmHel-8	DF090344:3008365-3008784
BmHel-8	DF090340:4745996-4746415
BmHel-8	DF090340:4284194-4284613
BmHel-8	DF090340:4018454-4018873
BmHel-8	DF090340:3866286-3866705
BmHel-8	DF090340:3471473-3471892
BmHel-8	DF090329:1366409-1366828
BmHel-8	DF090327:916944-917363
BmHel-8	DF090327:32381-32800
BmHel-8	DF090323:6262118-6262537
BmHel-8	DF090322:7538915-7539334
BmHel-8	DF090322:7448265-7448684
BmHel-8	DF090322:6131307-6131726
BmHel-8	DF090322:3587736-3588155
BmHel-8	DF090322:1134636-1135055
BmHel-8	DF090319:4396524-4396943
BmHel-8	DF090319:3827141-3827560
BmHel-8	DF090319:2353820-2354239
BmHel-8	DF090319:499979-500398
BmHel-8	DF090317:9585019-9585438

BmHel-8	DF090317:8707233-8707652
BmHel-8	DF090317:2483037-2483456
BmHel-8	DF090317:349821-350240
BmHel-8	DF090316:3215053-3215472
BmHel-8	DF090316:1563070-1563489
BmHel-8	DF090366:1786690-1787109
BmHel-8	DF090366:1543756-1544175
BmHel-8	DF090349:2556745-2557164
BmHel-8	DF090349:2126535-2126954
BmHel-8	DF090349:1528800-1529219
BmHel-8	DF090349:961683-962102
BmHel-8	DF090348:2791606-2792025
BmHel-8	BABH01080995:67-486
BmHel-8	DF090458:321796-322215
BmHel-8	DF090422:1108389-1108808
BmHel-8	DF090401:692051-692470
BmHel-8	DF090396:1548563-1548982
BmHel-8	DF090396:1125986-1126405
BmHel-8	DF090394:66473-66892
BmHel-8	DF090380:2104631-2105050
BmHel-8	DF090380:2033155-2033574
BmHel-8	DF090380:230745-231164
BmHel-8	DF090372:2317415-2317834
BmHel-8	DF090372:1926517-1926936
BmHel-8	DF090371:2437744-2438163
BmHel-8	DF090369:1467328-1467747
BmHel-8	DF090361:1058276-1058695
BmHel-8	DF090360:3141571-3141990
BmHel-8	DF090360:2573260-2573679
BmHel-8	DF090360:435898-436317
BmHel-8	DF090360:318762-319181
BmHel-8	DF090345:3546202-3546621
BmHel-8	DF090345:673609-674028
BmHel-8	DF090341:4539170-4539589
BmHel-8	DF090341:4188988-4189407
BmHel-8	DF090341:4026346-4026765
BmHel-8	DF090341:2157695-2158114
BmHel-8	DF090341:772732-773151
BmHel-8	DF090335:5498065-5498484
BmHel-8	DF090335:2844140-2844559
BmHel-8	DF090335:1436926-1437345
BmHel-8	DF090334:5781190-5781609
BmHel-8	DF090334:4213928-4214347
BmHel-8	DF090334:1838319-1838738
BmHel-8	DF090331:5878514-5878933
BmHel-8	DF090331:2680561-2680980
BmHel-8	DF090331:988846-989265
BmHel-8	DF090328:3388539-3388958

BmHel-8	DF090328:1355179-1355598
BmHel-8	DF090328:1317239-1317658
BmHel-8	DF090328:264388-264807
BmHel-8	DF090324:6281137-6281556
BmHel-8	DF090324:6205168-6205587
BmHel-8	DF090324:4398383-4398802
BmHel-8	DF090343:3439780-3440199
BmHel-8	DF090343:1215228-1215647
BmHel-8	DF090339:2510537-2510956
BmHel-8	DF090462:43422-43841
BmHel-8	DF090385:1509536-1509955
BmHel-8	DF090379:2046188-2046607
BmHel-8	DF090379:957531-957950
BmHel-8	DF090379:214666-215085
BmHel-8	DF090379:126839-127258
BmHel-8	DF090376:2114636-2115055
BmHel-8	DF090376:1554384-1554803
BmHel-8	DF090375:619302-619721
BmHel-8	DF090375:104752-105171
BmHel-8	DF090333:2511689-2512108
BmHel-8	DF090333:2042876-2043295
BmHel-8	DF090325:5577408-5577827
BmHel-8	DF090325:1689831-1690250
BmHel-8	DF090378:2128793-2129212
BmHel-8	DF090378:1345144-1345563
BmHel-8	DF090378:336869-337288
BmHel-8	DF090387:1630907-1631326
BmHel-8	DF090438:673926-674345
BmHel-8	DF090438:356473-356892
BmHel-8	DF090415:955909-956328
BmHel-8	DF090413:507528-507947
BmHel-8	DF090408:9551-9970
BmHel-8	DF090398:417205-417624
BmHel-8	DF090407:113950-114369
BmHel-8	DF090435:509026-509445
BmHel-8	DF090419:1019644-1020063
BmHel-8	DF090572:1492-1911
BmHel-8	DF090560:34327-34746
BmHel-8	DF090463:138788-139207
BmHel-8	DF091320:3157-3576
BmHel-8	DF090370:2641470-2641889
BmHel-8	DF090336:1177739-1178158
BmHel-8	DF090323:5047885-5048304
BmHel-8	DF090391:889659-890078
BmHel-8	DF090324:7596749-7597168
BmHel-8	DF090324:4384175-4384594
BmHel-8	DF090390:608888-609307
BmHel-8	DF090343:535359-535778

BmHel-8	DF090375:981198-981617
BmHel-8	DF090354:2354384-2354803
BmHel-8	DF090372:2398057-2398476
BmHel-8	DF090330:311863-312283
BmHel-8	DF090330:1071678-1072098
BmHel-8	DF090330:1425835-1426255
BmHel-8	DF090330:5624469-5624889
BmHel-8	DF090330:5844006-5844426
BmHel-8	BABH01072255:41-461
BmHel-8	DF090370:1497521-1497941
BmHel-8	DF090370:2578664-2579084
BmHel-8	DF090370:2652968-2653388
BmHel-8	DF090358:81509-81929
BmHel-8	DF090358:818699-819119
BmHel-8	DF090358:2066762-2067182
BmHel-8	DF090437:588246-588666
BmHel-8	DF090437:599670-600090
BmHel-8	DF090412:759088-759508
BmHel-8	DF090412:915613-916033
BmHel-8	DF090386:145964-146384
BmHel-8	DF090386:1213973-1214393
BmHel-8	DF090386:1801693-1802113
BmHel-8	DF090383:1815327-1815747
BmHel-8	DF090383:1947551-1947971
BmHel-8	DF090352:1767014-1767434
BmHel-8	DF090352:1994337-1994757
BmHel-8	DF090352:3499617-3500037
BmHel-8	DF090352:4087428-4087848
BmHel-8	DF090351:641417-641837
BmHel-8	DF090351:866616-867036
BmHel-8	DF090351:2629304-2629724
BmHel-8	DF090351:2851500-2851920
BmHel-8	DF090351:3032305-3032725
BmHel-8	DF090346:816600-817020
BmHel-8	DF090346:1384631-1385051
BmHel-8	DF090346:2981058-2981478
BmHel-8	DF090346:3539124-3539544
BmHel-8	DF090332:362344-362764
BmHel-8	DF090332:467797-468217
BmHel-8	DF090332:491380-491800
BmHel-8	DF090332:3085102-3085522
BmHel-8	DF090332:6081170-6081590
BmHel-8	BABH01082007:97-517
BmHel-8	DF090470:67218-67638
BmHel-8	DF090421:568674-569094
BmHel-8	DF090402:386256-386676
BmHel-8	DF090377:1191682-1192102
BmHel-8	DF090377:1774609-1775029

BmHel-8	DF090367:1615981-1616401
BmHel-8	DF090367:1683839-1684259
BmHel-8	DF090367:1705478-1705898
BmHel-8	DF090367:1962775-1963195
BmHel-8	DF090363:2367131-2367551
BmHel-8	DF090363:2423732-2424152
BmHel-8	DF090363:2776231-2776651
BmHel-8	DF090355:704769-705189
BmHel-8	DF090355:2974183-2974603
BmHel-8	DF090355:3035183-3035603
BmHel-8	DF090355:3098714-3099134
BmHel-8	DF090355:3695757-3696177
BmHel-8	DF090353:3471691-3472111
BmHel-8	DF090342:14746-15166
BmHel-8	DF090342:1311107-1311527
BmHel-8	DF090342:2817309-2817729
BmHel-8	DF090342:3535530-3535950
BmHel-8	DF090342:4001960-4002380
BmHel-8	DF090321:1461594-1462014
BmHel-8	DF090321:1961086-1961506
BmHel-8	DF090321:2901362-2901782
BmHel-8	DF090321:4193664-4194084
BmHel-8	DF090321:4277580-4278000
BmHel-8	DF090321:7851925-7852345
BmHel-8	DF090318:2055067-2055487
BmHel-8	DF090318:5386200-5386620
BmHel-8	DF090318:6019826-6020246
BmHel-8	DF090318:6899402-6899822
BmHel-8	DF090318:6918865-6919285
BmHel-8	DF090318:7156532-7156952
BmHel-8	DF090318:8526277-8526697
BmHel-8	DF090318:8561327-8561747
BmHel-8	DF090318:8888007-8888427
BmHel-8	DF090320:1288399-1288819
BmHel-8	DF090320:4225280-4225700
BmHel-8	DF090320:5435549-5435969
BmHel-8	BABH01083074:2-422
BmHel-8	BABH01067268:109-529
BmHel-8	DF090729:18593-19013
BmHel-8	DF090488:150734-151154
BmHel-8	DF090420:651136-651556
BmHel-8	DF090403:132951-133371
BmHel-8	DF090403:179155-179575
BmHel-8	DF090403:1530253-1530673
BmHel-8	DF090362:2583316-2583736
BmHel-8	DF090362:2698227-2698647
BmHel-8	DF090359:1597860-1598280
BmHel-8	DF090357:912380-912800

BmHel-8	DF090357:1151258-1151678
BmHel-8	DF090357:1299398-1299818
BmHel-8	DF090357:3511091-3511511
BmHel-8	DF090356:813159-813579
BmHel-8	DF090356:1588366-1588786
BmHel-8	DF090356:2771767-2772187
BmHel-8	DF090344:1362823-1363243
BmHel-8	DF090344:1952598-1953018
BmHel-8	DF090344:3584730-3585150
BmHel-8	DF090344:4273158-4273578
BmHel-8	DF090344:4478269-4478689
BmHel-8	DF090340:261637-262057
BmHel-8	DF090340:400744-401164
BmHel-8	DF090340:541326-541746
BmHel-8	DF090340:1389478-1389898
BmHel-8	DF090340:1498981-1499401
BmHel-8	DF090340:4014602-4015022
BmHel-8	DF090340:4826140-4826560
BmHel-8	DF090338:1484921-1485341
BmHel-8	DF090338:1949472-1949892
BmHel-8	DF090338:3771251-3771671
BmHel-8	DF090329:3094919-3095339
BmHel-8	DF090329:4833417-4833837
BmHel-8	DF090329:6451282-6451702
BmHel-8	DF090327:5475093-5475513
BmHel-8	DF090323:2999666-3000086
BmHel-8	DF090323:5358698-5359118
BmHel-8	DF090323:7798006-7798426
BmHel-8	DF090322:757919-758339
BmHel-8	DF090322:3739271-3739691
BmHel-8	DF090322:4211789-4212209
BmHel-8	DF090322:5622592-5623012
BmHel-8	DF090322:6033402-6033822
BmHel-8	DF090322:6877864-6878284
BmHel-8	DF090322:7819099-7819519
BmHel-8	DF090319:914341-914761
BmHel-8	DF090319:2181150-2181570
BmHel-8	DF090319:2522572-2522992
BmHel-8	DF090317:1490643-1491063
BmHel-8	DF090317:1588887-1589307
BmHel-8	DF090317:2936271-2936691
BmHel-8	DF090317:3391624-3392044
BmHel-8	DF090317:3601168-3601588
BmHel-8	DF090317:8579071-8579491
BmHel-8	DF090317:8760025-8760445
BmHel-8	DF090317:8828949-8829369
BmHel-8	DF090317:9447977-9448397
BmHel-8	DF090316:2798629-2799049

BmHel-8	DF090316:4733856-4734276
BmHel-8	DF090316:7327596-7328016
BmHel-8	DF090316:9522016-9522436
BmHel-8	DF090316:12273304-12273724
BmHel-8	DF090316:13164144-13164564
BmHel-8	DF090316:13184288-13184708
BmHel-8	DF090316:15284539-15284959
BmHel-8	DF090316:15922266-15922686
BmHel-8	DF090410:292757-293177
BmHel-8	DF090395:1346636-1347056
BmHel-8	DF090349:906558-906978
BmHel-8	DF090349:2079377-2079797
BmHel-8	DF090349:2853553-2853973
BmHel-8	DF090349:3107254-3107674
BmHel-8	DF090349:3880440-3880860
BmHel-8	DF090326:1481633-1482053
BmHel-8	DF090326:5468930-5469350
BmHel-8	DF090365:38410-38830
BmHel-8	DF090365:1135527-1135947
BmHel-8	DF090348:1100806-1101226
BmHel-8	DF090348:2845064-2845484
BmHel-8	DF090348:2906666-2907086
BmHel-8	BABH01061020:2-422
BmHel-8	DF090609:21699-22119
BmHel-8	DF090422:240500-240920
BmHel-8	DF090422:518736-519156
BmHel-8	DF090422:1060202-1060622
BmHel-8	DF090401:783850-784270
BmHel-8	DF090401:841581-842001
BmHel-8	DF090397:853284-853704
BmHel-8	DF090396:152266-152686
BmHel-8	DF090396:417895-418315
BmHel-8	DF090396:1305341-1305761
BmHel-8	DF090391:1195579-1195999
BmHel-8	DF090382:223-643
BmHel-8	DF090382:1685898-1686318
BmHel-8	DF090380:182844-183264
BmHel-8	DF090380:465988-466408
BmHel-8	DF090372:1698999-1699419
BmHel-8	DF090372:2307576-2307996
BmHel-8	DF090371:7329-7749
BmHel-8	DF090371:420678-421098
BmHel-8	DF090371:489114-489534
BmHel-8	DF090371:571151-571571
BmHel-8	DF090371:1706306-1706726
BmHel-8	DF090369:1691205-1691625
BmHel-8	DF090361:242700-243120
BmHel-8	DF090361:551753-552173

BmHel-8	DF090361:1125509-1125929
BmHel-8	DF090361:1648136-1648556
BmHel-8	DF090361:2226906-2227326
BmHel-8	DF090360:600130-600550
BmHel-8	DF090360:967063-967483
BmHel-8	DF090360:1577512-1577932
BmHel-8	DF090360:2255123-2255543
BmHel-8	DF090347:755238-755658
BmHel-8	DF090347:1445675-1446095
BmHel-8	DF090347:2203676-2204096
BmHel-8	DF090347:2455887-2456307
BmHel-8	DF090347:3297674-3298094
BmHel-8	DF090345:1463747-1464167
BmHel-8	DF090345:3576849-3577269
BmHel-8	DF090341:1515791-1516211
BmHel-8	DF090341:1839472-1839892
BmHel-8	DF090335:4666247-4666667
BmHel-8	DF090334:4089307-4089727
BmHel-8	DF090331:658553-658973
BmHel-8	DF090331:2040958-2041378
BmHel-8	DF090331:4809909-4810329
BmHel-8	DF090328:499214-499634
BmHel-8	DF090328:2656407-2656827
BmHel-8	DF090328:3466013-3466433
BmHel-8	DF090324:316362-316782
BmHel-8	DF090324:2459416-2459836
BmHel-8	DF090324:4059907-4060327
BmHel-8	DF090324:7654992-7655412
BmHel-8	DF090390:1478477-1478897
BmHel-8	DF090449:352574-352994
BmHel-8	DF090449:503331-503751
BmHel-8	DF090436:512127-512547
BmHel-8	DF090381:529228-529648
BmHel-8	DF090381:1805845-1806265
BmHel-8	DF090381:2026067-2026487
BmHel-8	DF090373:190710-191130
BmHel-8	DF090373:1779315-1779735
BmHel-8	DF090343:83984-84404
BmHel-8	DF090343:310244-310664
BmHel-8	DF090343:625826-626246
BmHel-8	DF090343:2311950-2312370
BmHel-8	DF090343:4187832-4188252
BmHel-8	DF090339:489883-490303
BmHel-8	DF090339:1034774-1035194
BmHel-8	DF090339:1834320-1834740
BmHel-8	DF090339:1917474-1917894
BmHel-8	DF090339:2597610-2598030
BmHel-8	DF090339:3091187-3091607

BmHel-8	DF090339:4760048-4760468
BmHel-8	BABH01084194:45-465
BmHel-8	BABH01065920:155-575
BmHel-8	DF090462:201057-201477
BmHel-8	DF090432:312292-312712
BmHel-8	DF090432:630576-630996
BmHel-8	DF090425:316338-316758
BmHel-8	DF090425:829401-829821
BmHel-8	DF090405:62864-63284
BmHel-8	DF090379:219067-219487
BmHel-8	DF090379:1011113-1011533
BmHel-8	DF090379:1492981-1493401
BmHel-8	DF090375:113879-114299
BmHel-8	DF090375:211977-212397
BmHel-8	DF090375:1727616-1728036
BmHel-8	DF090375:2180310-2180730
BmHel-8	DF090374:864661-865081
BmHel-8	DF090374:1723215-1723635
BmHel-8	DF090418:133736-134156
BmHel-8	DF090325:1308443-1308863
BmHel-8	DF090325:2012254-2012674
BmHel-8	DF090325:2062091-2062511
BmHel-8	DF090325:3992647-3993067
BmHel-8	BABH01055914:192-612
BmHel-8	BABH01051791:6-426
BmHel-8	BABH01044374:1539-1959
BmHel-8	DF090445:334481-334901
BmHel-8	DF090444:434523-434943
BmHel-8	DF090444:578745-579165
BmHel-8	DF090431:6081-6501
BmHel-8	DF090417:353971-354391
BmHel-8	DF090417:631554-631974
BmHel-8	DF090414:975389-975809
BmHel-8	DF090387:1153077-1153497
BmHel-8	DF090384:664141-664561
BmHel-8	DF090384:1314378-1314798
BmHel-8	DF090364:1127183-1127603
BmHel-8	DF090364:2039958-2040378
BmHel-8	DF090429:515526-515946
BmHel-8	DF090429:966576-966996
BmHel-8	DF090354:1113395-1113815
BmHel-8	DF090354:1842557-1842977
BmHel-8	DF090464:180356-180776
BmHel-8	DF090456:186123-186543
BmHel-8	DF090456:352503-352923
BmHel-8	DF090368:922976-923396
BmHel-8	DF090643:23558-23978
BmHel-8	DF090407:12468-12888

BmHel-8	DF090407:478142-478562
BmHel-8	BABH01050221:51-471
BmHel-8	BABH01044780:2956-3376
BmHel-8	DF090468:326241-326661
BmHel-8	DF090428:846026-846446
BmHel-8	DF090393:1541472-1541892
BmHel-8	BABH01076392:87-507
BmHel-8	DF090634:7944-8364
BmHel-8	DF090948:3774-4194
BmHel-8	DF090465:17521-17941
BmHel-8	DF091150:7990-8410
BmHel-8	DF090421:671837-672257
BmHel-8	DF090356:599610-600030
BmHel-8	DF090317:1413566-1413986
BmHel-8	DF090411:305634-306054
BmHel-8	DF090339:3945136-3945556
BmHel-8	DF090320:7824292-7824712
BmHel-8	DF090398:479574-479994
BmHel-8	DF090330:6035272-6035692
BmHel-8	DF090330:4909051-4909471
BmHel-8	DF090330:4767864-4768284
BmHel-8	DF090330:838564-838984
BmHel-8	DF090330:140186-140606
BmHel-8	BABH01071479:107-527
BmHel-8	BABH01069829:104-524
BmHel-8	DF090370:880169-880589
BmHel-8	DF090358:3467006-3467426
BmHel-8	DF090358:2856936-2857356
BmHel-8	DF090358:1917789-1918209
BmHel-8	DF090358:1514244-1514664
BmHel-8	DF090358:931131-931551
BmHel-8	BABH01085481:37-457
BmHel-8	BABH01084869:13-433
BmHel-8	BABH01047502:5-425
BmHel-8	DF090437:462794-463214
BmHel-8	DF090437:183985-184405
BmHel-8	DF090386:2000234-2000654
BmHel-8	DF090386:1709382-1709802
BmHel-8	DF090386:213777-214197
BmHel-8	DF090383:431211-431631
BmHel-8	DF090352:3877518-3877938
BmHel-8	DF090352:3640767-3641187
BmHel-8	DF090352:1768036-1768456
BmHel-8	DF090352:1697733-1698153
BmHel-8	DF090352:1120592-1121012
BmHel-8	DF090352:368903-369323
BmHel-8	DF090352:293413-293833
BmHel-8	DF090351:3561305-3561725

BmHel-8	DF090351:2537485-2537905
BmHel-8	DF090351:2125147-2125567
BmHel-8	DF090351:1968401-1968821
BmHel-8	DF090346:3914388-3914808
BmHel-8	DF090332:3218119-3218539
BmHel-8	DF090332:1748772-1749192
BmHel-8	DF090332:248229-248649
BmHel-8	BABH01078395:9-429
BmHel-8	BABH01075778:85-505
BmHel-8	BABH01069667:71-491
BmHel-8	BABH01062601:68-488
BmHel-8	BABH01051738:142-562
BmHel-8	DF090409:1379466-1379886
BmHel-8	DF090409:1351792-1352212
BmHel-8	DF090377:957773-958193
BmHel-8	DF090377:378840-379260
BmHel-8	DF090377:60343-60763
BmHel-8	DF090367:2584690-2585110
BmHel-8	DF090367:2167093-2167513
BmHel-8	DF090367:1856326-1856746
BmHel-8	DF090367:832967-833387
BmHel-8	DF090367:244866-245286
BmHel-8	DF090363:2069425-2069845
BmHel-8	DF090363:1420140-1420560
BmHel-8	DF090363:1087216-1087636
BmHel-8	DF090355:3431009-3431429
BmHel-8	DF090355:2071318-2071738
BmHel-8	DF090355:296122-296542
BmHel-8	DF090355:281390-281810
BmHel-8	DF090353:2831638-2832058
BmHel-8	DF090353:2686407-2686827
BmHel-8	DF090353:1714545-1714965
BmHel-8	DF090350:137563-137983
BmHel-8	DF090342:3966807-3967227
BmHel-8	DF090342:1397918-1398338
BmHel-8	DF090342:904748-905168
BmHel-8	DF090336:341106-341526
BmHel-8	DF090321:4893107-4893527
BmHel-8	DF090321:2845946-2846366
BmHel-8	DF090321:2599293-2599713
BmHel-8	DF090321:1740491-1740911
BmHel-8	DF090321:1429067-1429487
BmHel-8	DF090318:7177102-7177522
BmHel-8	DF090320:5815492-5815912
BmHel-8	DF090320:3189806-3190226
BmHel-8	DF090320:2171908-2172328
BmHel-8	BABH01086983:10-430
BmHel-8	BABH01074687:141-561

BmHel-8	DF090499:88420-88840
BmHel-8	DF090443:596311-596731
BmHel-8	DF090441:594527-594947
BmHel-8	DF090441:390868-391288
BmHel-8	DF090403:885201-885621
BmHel-8	DF090400:1569068-1569488
BmHel-8	DF090388:950774-951194
BmHel-8	DF090388:528605-529025
BmHel-8	DF090362:2589882-2590302
BmHel-8	DF090362:1240780-1241200
BmHel-8	DF090362:816799-817219
BmHel-8	DF090362:540101-540521
BmHel-8	DF090359:2222386-2222806
BmHel-8	DF090357:3555842-3556262
BmHel-8	DF090357:2942447-2942867
BmHel-8	DF090357:2304091-2304511
BmHel-8	DF090357:1319044-1319464
BmHel-8	DF090357:790230-790650
BmHel-8	DF090357:64084-64504
BmHel-8	DF090356:1142373-1142793
BmHel-8	DF090344:4008289-4008709
BmHel-8	DF090344:3761602-3762022
BmHel-8	DF090344:3554995-3555415
BmHel-8	DF090344:3425545-3425965
BmHel-8	DF090344:2465473-2465893
BmHel-8	DF090344:1671673-1672093
BmHel-8	DF090344:1658346-1658766
BmHel-8	DF090344:866170-866590
BmHel-8	DF090340:2783919-2784339
BmHel-8	DF090340:2746358-2746778
BmHel-8	DF090340:2666333-2666753
BmHel-8	DF090340:1307706-1308126
BmHel-8	DF090340:1266167-1266587
BmHel-8	DF090340:392762-393182
BmHel-8	DF090338:4729009-4729429
BmHel-8	DF090338:3769692-3770112
BmHel-8	DF090338:3525186-3525606
BmHel-8	DF090338:2789435-2789855
BmHel-8	DF090338:1927885-1928305
BmHel-8	DF090338:904702-905122
BmHel-8	DF090338:221576-221996
BmHel-8	DF090329:4315219-4315639
BmHel-8	DF090329:2986328-2986748
BmHel-8	DF090329:2415533-2415953
BmHel-8	DF090327:5712603-5713023
BmHel-8	DF090327:4403008-4403428
BmHel-8	DF090327:3657925-3658345
BmHel-8	DF090327:2600353-2600773

BmHel-8	DF090327:1824187-1824607
BmHel-8	DF090323:7574087-7574507
BmHel-8	DF090323:7399118-7399538
BmHel-8	DF090323:7244803-7245223
BmHel-8	DF090323:4039945-4040365
BmHel-8	DF090322:6989688-6990108
BmHel-8	DF090322:5822847-5823267
BmHel-8	DF090322:3641151-3641571
BmHel-8	DF090319:8608966-8609386
BmHel-8	DF090319:8112074-8112494
BmHel-8	DF090319:7815417-7815837
BmHel-8	DF090319:7039148-7039568
BmHel-8	DF090319:6761671-6762091
BmHel-8	DF090319:6222003-6222423
BmHel-8	DF090319:5670232-5670652
BmHel-8	DF090319:3810923-3811343
BmHel-8	DF090319:3788508-3788928
BmHel-8	DF090319:2162209-2162629
BmHel-8	DF090319:1750824-1751244
BmHel-8	DF090319:1372590-1373010
BmHel-8	DF090319:734415-734835
BmHel-8	DF090319:233275-233695
BmHel-8	DF090317:10821982-10822402
BmHel-8	DF090317:10001893-10002313
BmHel-8	DF090317:9421637-9422057
BmHel-8	DF090317:8836024-8836444
BmHel-8	DF090317:8160976-8161396
BmHel-8	DF090317:5433604-5434024
BmHel-8	DF090317:4435542-4435962
BmHel-8	DF090317:3270111-3270531
BmHel-8	DF090317:1186295-1186715
BmHel-8	DF090316:14923630-14924050
BmHel-8	DF090316:14661642-14662062
BmHel-8	DF090316:11455626-11456046
BmHel-8	DF090316:11019915-11020335
BmHel-8	DF090316:7012830-7013250
BmHel-8	DF090316:4219357-4219777
BmHel-8	DF090316:2986195-2986615
BmHel-8	DF090316:1357715-1358135
BmHel-8	DF090410:1090717-1091137
BmHel-8	DF090395:261762-262182
BmHel-8	DF090366:2050449-2050869
BmHel-8	DF090366:746791-747211
BmHel-8	DF090349:3273240-3273660
BmHel-8	DF090349:2780381-2780801
BmHel-8	DF090349:2749183-2749603
BmHel-8	DF090349:1381247-1381667
BmHel-8	DF090349:1200892-1201312

BmHel-8	DF090349:239438-239858
BmHel-8	DF090349:52001-52421
BmHel-8	DF090326:2500666-2501086
BmHel-8	DF090326:2358064-2358484
BmHel-8	DF090326:833021-833441
BmHel-8	DF090326:118526-118946
BmHel-8	DF090433:387929-388349
BmHel-8	DF090365:2133350-2133770
BmHel-8	DF090365:1929680-1930100
BmHel-8	DF090365:1909346-1909766
BmHel-8	DF090365:1829760-1830180
BmHel-8	DF090365:64424-64844
BmHel-8	DF090348:2205072-2205492
BmHel-8	DF090348:1018519-1018939
BmHel-8	DF090348:173230-173650
BmHel-8	BABH01082118:6-426
BmHel-8	BABH01074787:179-599
BmHel-8	BABH01070333:183-603
BmHel-8	BABH01067792:61-481
BmHel-8	DF090439:586734-587154
BmHel-8	DF090439:202737-203157
BmHel-8	DF090411:736844-737264
BmHel-8	DF090396:1465170-1465590
BmHel-8	DF090396:782622-783042
BmHel-8	DF090394:1592268-1592688
BmHel-8	DF090394:614376-614796
BmHel-8	DF090392:608323-608743
BmHel-8	DF090391:758597-759017
BmHel-8	DF090391:161735-162155
BmHel-8	DF090382:1879170-1879590
BmHel-8	DF090382:747655-748075
BmHel-8	DF090372:2442724-2443144
BmHel-8	DF090372:2158244-2158664
BmHel-8	DF090372:1778188-1778608
BmHel-8	DF090372:1757734-1758154
BmHel-8	DF090372:1486983-1487403
BmHel-8	DF090372:465499-465919
BmHel-8	DF090371:479987-480407
BmHel-8	DF090369:634959-635379
BmHel-8	DF090369:601845-602265
BmHel-8	DF090369:527110-527530
BmHel-8	DF090361:2412811-2413231
BmHel-8	DF090361:1751736-1752156
BmHel-8	DF090361:746966-747386
BmHel-8	DF090360:3194454-3194874
BmHel-8	DF090347:250932-251352
BmHel-8	DF090345:4464642-4465062
BmHel-8	DF090345:3634541-3634961

BmHel-8	DF090345:3289669-3290089
BmHel-8	DF090345:1608552-1608972
BmHel-8	DF090345:1027235-1027655
BmHel-8	DF090341:4163391-4163811
BmHel-8	DF090335:3371466-3371886
BmHel-8	DF090335:1344286-1344706
BmHel-8	DF090335:899263-899683
BmHel-8	DF090335:845566-845986
BmHel-8	DF090334:5645298-5645718
BmHel-8	DF090334:2542204-2542624
BmHel-8	DF090334:1521151-1521571
BmHel-8	DF090331:5501518-5501938
BmHel-8	DF090331:4976362-4976782
BmHel-8	DF090331:4016486-4016906
BmHel-8	DF090331:2921479-2921899
BmHel-8	DF090331:2799747-2800167
BmHel-8	DF090331:2510865-2511285
BmHel-8	DF090328:1368939-1369359
BmHel-8	DF090328:865385-865805
BmHel-8	DF090328:506432-506852
BmHel-8	DF090324:4885760-4886180
BmHel-8	DF090324:4648173-4648593
BmHel-8	DF090324:1144896-1145316
BmHel-8	DF090324:1104825-1105245
BmHel-8	DF090324:755020-755440
BmHel-8	DF090337:4697211-4697631
BmHel-8	DF090337:4617035-4617455
BmHel-8	DF090337:2181223-2181643
BmHel-8	DF090494:87049-87469
BmHel-8	DF090537:10419-10839
BmHel-8	DF090381:109748-110168
BmHel-8	DF090373:144843-145263
BmHel-8	DF090373:99316-99736
BmHel-8	DF090343:3892183-3892603
BmHel-8	DF090343:3247359-3247779
BmHel-8	DF090343:3153337-3153757
BmHel-8	DF090343:2203141-2203561
BmHel-8	DF090343:700697-701117
BmHel-8	DF090343:36839-37259
BmHel-8	DF090339:3097380-3097800
BmHel-8	DF090339:2019566-2019986
BmHel-8	BABH01047370:1328-1748
BmHel-8	BABH01046022:1839-2259
BmHel-8	DF090575:33472-33892
BmHel-8	DF090405:35654-36074
BmHel-8	DF090385:152890-153310
BmHel-8	DF090379:1825051-1825471
BmHel-8	DF090379:1135281-1135701

BmHel-8	DF090379:450894-451314
BmHel-8	DF090379:142595-143015
BmHel-8	DF090376:1821121-1821541
BmHel-8	DF090376:480539-480959
BmHel-8	DF090375:667055-667475
BmHel-8	DF090374:576320-576740
BmHel-8	DF090374:519566-519986
BmHel-8	DF090333:5652547-5652967
BmHel-8	DF090333:4106630-4107050
BmHel-8	DF090333:2143543-2143963
BmHel-8	DF090325:6826699-6827119
BmHel-8	DF090325:5083521-5083941
BmHel-8	BABH01044520:6686-7106
BmHel-8	DF090378:2097029-2097449
BmHel-8	DF090378:1252357-1252777
BmHel-8	DF090378:1057874-1058294
BmHel-8	DF091262:5173-5593
BmHel-8	BABH01043098:10685-11105
BmHel-8	DF090417:706659-707079
BmHel-8	DF090416:737268-737688
BmHel-8	DF090416:12340-12760
BmHel-8	DF090414:1333761-1334181
BmHel-8	DF090414:704896-705316
BmHel-8	DF090387:1790518-1790938
BmHel-8	DF090387:1510721-1511141
BmHel-8	DF090384:1138578-1138998
BmHel-8	DF090364:2624081-2624501
BmHel-8	DF090487:158575-158995
BmHel-8	DF090424:971571-971991
BmHel-8	DF090404:1450871-1451291
BmHel-8	DF090354:3195756-3196176
BmHel-8	DF090354:2154817-2155237
BmHel-8	DF090354:958009-958429
BmHel-8	BABH01051726:411-831
BmHel-8	DF091987:2397-2817
BmHel-8	BABH01045837:1796-2216
BmHel-8	DF090621:34207-34627
BmHel-8	DF090541:122070-122490
BmHel-8	DF090415:89588-90008
BmHel-8	DF090408:104476-104896
BmHel-8	DF090483:8971-9391
BmHel-8	DF090684:13519-13939
BmHel-8	DF090446:452427-452847
BmHel-8	BABH01053639:159-579
BmHel-8	DF090658:8536-8956
BmHel-8	DF090468:155006-155426
BmHel-8	DF090539:35195-35615
BmHel-8	DF090520:63188-63608

BmHel-8	BABH01050272:31-451
BmHel-8	DF090450:288753-289173
BmHel-8	DF090801:7235-7655
BmHel-8	DF090531:13434-13854
BmHel-8	DF090440:676338-676758
BmHel-8	BABH01083200:15-435
BmHel-8	BABH01044769:221-641
BmHel-8	DF090363:1007154-1007574
BmHel-8	DF090336:5609403-5609823
BmHel-8	DF090336:2729850-2730270
BmHel-8	DF090326:4643635-4644055
BmHel-8	DF090328:6676394-6676814
BmHel-8	DF090375:74881-75301
BmHel-8	DF090342:2667247-2667667
BmHel-8	DF090338:628097-628517
BmHel-8	DF090330:102692-103113
BmHel-8	DF090358:2178626-2179047
BmHel-8	DF090412:982213-982634
BmHel-8	DF090386:534544-534965
BmHel-8	DF090386:809824-810245
BmHel-8	DF090386:1127496-1127917
BmHel-8	DF090383:678469-678890
BmHel-8	DF090383:1095774-1096195
BmHel-8	DF090352:1053400-1053821
BmHel-8	DF090352:3676203-3676624
BmHel-8	DF090351:2977480-2977901
BmHel-8	DF090332:1924412-1924833
BmHel-8	DF090332:3172310-3172731
BmHel-8	DF090332:4195966-4196387
BmHel-8	DF090466:321501-321922
BmHel-8	DF090377:382062-382483
BmHel-8	DF090377:521438-521859
BmHel-8	DF090367:990232-990653
BmHel-8	DF090367:1159950-1160371
BmHel-8	DF090367:1818045-1818466
BmHel-8	DF090367:2148478-2148899
BmHel-8	DF090363:1822570-1822991
BmHel-8	DF090355:2937700-2938121
BmHel-8	DF090355:3140138-3140559
BmHel-8	DF090353:3291205-3291626
BmHel-8	DF090353:3442322-3442743
BmHel-8	DF090353:3633832-3634253
BmHel-8	DF090350:2448133-2448554
BmHel-8	DF090342:253824-254245
BmHel-8	DF090342:1289473-1289894
BmHel-8	DF090342:2947200-2947621
BmHel-8	DF090342:3689075-3689496
BmHel-8	DF090342:4799447-4799868

BmHel-8	DF090336:1681820-1682241
BmHel-8	DF090336:2863077-2863498
BmHel-8	DF090336:3608111-3608532
BmHel-8	DF090336:5370245-5370666
BmHel-8	DF090321:1242880-1243301
BmHel-8	DF090321:6597677-6598098
BmHel-8	DF090318:806768-807189
BmHel-8	DF090320:6257949-6258370
BmHel-8	BABH01066396:24-445
BmHel-8	DF090443:275230-275651
BmHel-8	DF090403:1543047-1543468
BmHel-8	DF090388:318296-318717
BmHel-8	DF090388:1256721-1257142
BmHel-8	DF090362:798001-798422
BmHel-8	DF090362:821471-821892
BmHel-8	DF090359:1149395-1149816
BmHel-8	DF090359:2306044-2306465
BmHel-8	DF090356:448339-448760
BmHel-8	DF090356:1271639-1272060
BmHel-8	DF090356:2198475-2198896
BmHel-8	DF090356:2890777-2891198
BmHel-8	DF090356:3366017-3366438
BmHel-8	DF090344:1213778-1214199
BmHel-8	DF090344:2659091-2659512
BmHel-8	DF090344:3482909-3483330
BmHel-8	DF090344:4293397-4293818
BmHel-8	DF090344:4462390-4462811
BmHel-8	DF090340:1833345-1833766
BmHel-8	DF090340:3503956-3504377
BmHel-8	DF090340:4538641-4539062
BmHel-8	DF090329:1309863-1310284
BmHel-8	DF090329:5116420-5116841
BmHel-8	DF090329:5180634-5181055
BmHel-8	DF090329:5818029-5818450
BmHel-8	DF090327:462054-462475
BmHel-8	DF090327:5734405-5734826
BmHel-8	DF090323:1391478-1391899
BmHel-8	DF090323:2210470-2210891
BmHel-8	DF090322:1701286-1701707
BmHel-8	DF090322:2120144-2120565
BmHel-8	DF090322:3259672-3260093
BmHel-8	DF090322:3687015-3687436
BmHel-8	DF090322:4000919-4001340
BmHel-8	DF090322:5455399-5455820
BmHel-8	DF090322:7357093-7357514
BmHel-8	DF090319:765057-765478
BmHel-8	DF090319:1560603-1561024
BmHel-8	DF090319:4134360-4134781

BmHel-8	DF090319:7657005-7657426
BmHel-8	DF090319:8303398-8303819
BmHel-8	DF090319:8383699-8384120
BmHel-8	DF090317:4057102-4057523
BmHel-8	DF090317:6145662-6146083
BmHel-8	DF090317:8526491-8526912
BmHel-8	DF090316:1640734-1641155
BmHel-8	DF090316:7128628-7129049
BmHel-8	DF090316:10554745-10555166
BmHel-8	DF090316:12058436-12058857
BmHel-8	DF090366:214506-214927
BmHel-8	DF090349:927076-927497
BmHel-8	DF090349:2437769-2438190
BmHel-8	DF090326:2045218-2045639
BmHel-8	DF090326:2589485-2589906
BmHel-8	BABH01071656:188-609
BmHel-8	DF090365:445710-446131
BmHel-8	DF090348:735393-735814
BmHel-8	DF090401:202675-203096
BmHel-8	DF090401:256657-257078
BmHel-8	DF090397:1039127-1039548
BmHel-8	DF090394:687808-688229
BmHel-8	DF090391:1131371-1131792
BmHel-8	DF090391:1506970-1507391
BmHel-8	DF090382:1634478-1634899
BmHel-8	DF090372:165662-166083
BmHel-8	DF090372:1163310-1163731
BmHel-8	DF090369:1117252-1117673
BmHel-8	DF090361:1592477-1592898
BmHel-8	DF090361:2404115-2404536
BmHel-8	DF090360:331081-331502
BmHel-8	DF090347:3117512-3117933
BmHel-8	DF090345:3398135-3398556
BmHel-8	DF090341:597897-598318
BmHel-8	DF090341:4401054-4401475
BmHel-8	DF090335:2957949-2958370
BmHel-8	DF090335:5813359-5813780
BmHel-8	DF090334:1753954-1754375
BmHel-8	DF090334:2323418-2323839
BmHel-8	DF090334:3820541-3820962
BmHel-8	DF090334:5792367-5792788
BmHel-8	DF090331:4210307-4210728
BmHel-8	DF090331:5213148-5213569
BmHel-8	DF090331:5882526-5882947
BmHel-8	DF090328:4992407-4992828
BmHel-8	DF090337:909083-909504
BmHel-8	DF090337:3936806-3937227
BmHel-8	DF090343:1039076-1039497

BmHel-8	DF090343:3236117-3236538
BmHel-8	DF090343:3336974-3337395
BmHel-8	DF090343:3607497-3607918
BmHel-8	DF090343:4289527-4289948
BmHel-8	DF090339:310090-310511
BmHel-8	DF090339:766318-766739
BmHel-8	DF090339:2630239-2630660
BmHel-8	DF090339:3104824-3105245
BmHel-8	DF090462:215553-215974
BmHel-8	DF090385:78871-79292
BmHel-8	DF090374:498203-498624
BmHel-8	DF090374:631277-631698
BmHel-8	DF090374:2016831-2017252
BmHel-8	DF090333:4969424-4969845
BmHel-8	DF090333:5131242-5131663
BmHel-8	DF090325:1536960-1537381
BmHel-8	DF090325:6074358-6074779
BmHel-8	DF090378:539143-539564
BmHel-8	DF090417:730370-730791
BmHel-8	DF090414:1088524-1088945
BmHel-8	DF090389:956503-956924
BmHel-8	DF090389:1111038-1111459
BmHel-8	DF090387:1573659-1574080
BmHel-8	DF090384:1244693-1245114
BmHel-8	DF090384:1872538-1872959
BmHel-8	DF090364:2674794-2675215
BmHel-8	DF090364:2810640-2811061
BmHel-8	BABH01054499:1-422
BmHel-8	DF090528:43555-43976
BmHel-8	DF090404:625570-625991
BmHel-8	DF090354:1365063-1365484
BmHel-8	DF090438:683122-683543
BmHel-8	DF090438:695443-695864
BmHel-8	DF090413:426371-426792
BmHel-8	DF090406:863682-864103
BmHel-8	DF090398:1401679-1402100
BmHel-8	DF090398:1571157-1571578
BmHel-8	DF090507:14414-14835
BmHel-8	BABH01046266:2445-2866
BmHel-8	DF090368:961671-962092
BmHel-8	DF090368:1043623-1044044
BmHel-8	DF090446:427007-427428
BmHel-8	BABH01081274:79-500
BmHel-8	DF091227:2965-3386
BmHel-8	BABH01045196:3406-3827
BmHel-8	DF090461:21779-22200
BmHel-8	DF090399:509809-510230
BmHel-8	BABH01045784:1631-2052

BmHel-8	DF090590:3522-3943
BmHel-8	DF090426:713942-714363
BmHel-8	DF090411:1113570-1113991
BmHel-8	DF090382:1121696-1122117
BmHel-8	DF090330:2919340-2919761
BmHel-8	DF090358:2048201-2048622
BmHel-8	DF090358:1231854-1232275
BmHel-8	DF090358:215612-216033
BmHel-8	DF090383:194558-194979
BmHel-8	DF090352:2927952-2928373
BmHel-8	DF090351:4267656-4268077
BmHel-8	DF090351:2214728-2215149
BmHel-8	DF090346:2263782-2264203
BmHel-8	DF090332:4674127-4674548
BmHel-8	DF090332:2325078-2325499
BmHel-8	DF090332:859532-859953
BmHel-8	DF090332:649460-649881
BmHel-8	DF090421:764538-764959
BmHel-8	DF090402:694562-694983
BmHel-8	DF090402:296332-296753
BmHel-8	DF090367:2286304-2286725
BmHel-8	DF090367:2223464-2223885
BmHel-8	DF090367:1144110-1144531
BmHel-8	DF090363:1062242-1062663
BmHel-8	DF090355:2786399-2786820
BmHel-8	DF090355:384709-385130
BmHel-8	DF090353:4007672-4008093
BmHel-8	DF090353:1364728-1365149
BmHel-8	DF090342:4300169-4300590
BmHel-8	DF090342:1877238-1877659
BmHel-8	DF090342:1660631-1661052
BmHel-8	DF090336:3361218-3361639
BmHel-8	DF090321:8051546-8051967
BmHel-8	DF090321:3166845-3167266
BmHel-8	DF090321:2487736-2488157
BmHel-8	DF090318:4691356-4691777
BmHel-8	DF090318:1406124-1406545
BmHel-8	DF090318:1225828-1226249
BmHel-8	DF090318:356938-357359
BmHel-8	DF090320:5485030-5485451
BmHel-8	DF090443:41320-41741
BmHel-8	DF090441:546947-547368
BmHel-8	DF090388:840966-841387
BmHel-8	DF090362:2697167-2697588
BmHel-8	DF090362:2483509-2483930
BmHel-8	DF090362:2277333-2277754
BmHel-8	DF090362:128876-129297
BmHel-8	DF090359:2564831-2565252

BmHel-8	DF090359:2548766-2549187
BmHel-8	DF090356:3675002-3675423
BmHel-8	DF090344:4554928-4555349
BmHel-8	DF090344:818990-819411
BmHel-8	DF090340:3363092-3363513
BmHel-8	DF090340:2169667-2170088
BmHel-8	DF090338:3406142-3406563
BmHel-8	DF090329:3964609-3965030
BmHel-8	DF090329:2137132-2137553
BmHel-8	DF090322:7949385-7949806
BmHel-8	DF090322:6157722-6158143
BmHel-8	DF090322:5488878-5489299
BmHel-8	DF090322:4744320-4744741
BmHel-8	DF090322:3999752-4000173
BmHel-8	DF090322:3351265-3351686
BmHel-8	DF090319:8394532-8394953
BmHel-8	DF090319:7762797-7763218
BmHel-8	DF090319:7306454-7306875
BmHel-8	DF090319:3652409-3652830
BmHel-8	DF090319:1867083-1867504
BmHel-8	DF090317:9847130-9847551
BmHel-8	DF090316:15348042-15348463
BmHel-8	DF090316:11954252-11954673
BmHel-8	DF090316:7819663-7820084
BmHel-8	DF090316:6639825-6640246
BmHel-8	DF090316:5873946-5874367
BmHel-8	DF090410:844500-844921
BmHel-8	DF090349:1048110-1048531
BmHel-8	DF090326:6917794-6918215
BmHel-8	DF090326:5044018-5044439
BmHel-8	DF090365:877748-878169
BmHel-8	DF090348:3569057-3569478
BmHel-8	DF090348:2607307-2607728
BmHel-8	DF090348:2167995-2168416
BmHel-8	DF090439:631591-632012
BmHel-8	DF090422:839370-839791
BmHel-8	DF090396:1314182-1314603
BmHel-8	DF090394:430720-431141
BmHel-8	DF090392:1723203-1723624
BmHel-8	DF090391:543716-544137
BmHel-8	DF090382:1962455-1962876
BmHel-8	DF090372:2038839-2039260
BmHel-8	DF090372:1333603-1334024
BmHel-8	DF090371:1124060-1124481
BmHel-8	DF090361:2746270-2746691
BmHel-8	DF090360:1799445-1799866
BmHel-8	DF090347:3529833-3530254
BmHel-8	DF090347:536587-537008

BmHel-8	DF090347:269830-270251
BmHel-8	DF090345:3741493-3741914
BmHel-8	DF090345:2286588-2287009
BmHel-8	DF090345:1970197-1970618
BmHel-8	DF090345:1284362-1284783
BmHel-8	DF090341:3389352-3389773
BmHel-8	DF090341:2807754-2808175
BmHel-8	DF090335:5719102-5719523
BmHel-8	DF090334:6054780-6055201
BmHel-8	DF090334:2768355-2768776
BmHel-8	DF090331:5440200-5440621
BmHel-8	DF090331:2141611-2142032
BmHel-8	DF090328:892915-893336
BmHel-8	DF090324:4444009-4444430
BmHel-8	DF090324:2711779-2712200
BmHel-8	DF090324:768248-768669
BmHel-8	DF090324:409288-409709
BmHel-8	DF090390:969639-970060
BmHel-8	DF090390:442803-443224
BmHel-8	BABH01081828:123-544
BmHel-8	BABH01054272:159-580
BmHel-8	DF090449:41436-41857
BmHel-8	DF090436:449019-449440
BmHel-8	DF090436:71022-71443
BmHel-8	DF090381:656219-656640
BmHel-8	DF090381:329370-329791
BmHel-8	DF090381:48081-48502
BmHel-8	DF090373:73866-74287
BmHel-8	DF090343:3190489-3190910
BmHel-8	DF090343:1169873-1170294
BmHel-8	DF090343:814319-814740
BmHel-8	DF090343:451058-451479
BmHel-8	DF090460:277332-277753
BmHel-8	DF090405:1315377-1315798
BmHel-8	DF090405:1124636-1125057
BmHel-8	DF090333:5010907-5011328
BmHel-8	DF090325:4907033-4907454
BmHel-8	DF090325:1000104-1000525
BmHel-8	DF090378:678352-678773
BmHel-8	DF090416:886254-886675
BmHel-8	DF090414:617676-618097
BmHel-8	DF090414:570669-571090
BmHel-8	DF090387:1061129-1061550
BmHel-8	DF090384:1262089-1262510
BmHel-8	DF090384:842031-842452
BmHel-8	DF090364:2880446-2880867
BmHel-8	DF090364:2596153-2596574
BmHel-8	DF090364:629472-629893

BmHel-8	DF090608:14433-14854
BmHel-8	DF090528:39193-39614
BmHel-8	DF090404:1125367-1125788
BmHel-8	DF090438:702497-702918
BmHel-8	DF090438:385938-386359
BmHel-8	DF090415:805618-806039
BmHel-8	DF090413:576005-576426
BmHel-8	DF090408:373267-373688
BmHel-8	DF090368:489733-490154
BmHel-8	DF090368:315721-316142
BmHel-8	DF090627:9595-10016
BmHel-8	BABH01055556:66-487
BmHel-8	BABH01051576:273-694
BmHel-8	BABH01045382:4684-5105
BmHel-8	DF090692:27568-27989
BmHel-8	DF090459:262766-263187
BmHel-8	BABH01062018:10-431
BmHel-8	DF090756:6134-6555
BmHel-8	BABH01045639:1473-1894
BmHel-8	DF090342:4176376-4176797
BmHel-8	DF090401:1057874-1058295
BmHel-8	DF090372:1891563-1891984
BmHel-8	DF090347:187696-188117
BmHel-8	DF090366:1975295-1975716
BmHel-8	DF090330:142470-142892
BmHel-8	DF090330:5754284-5754706
BmHel-8	DF090330:6041051-6041473
BmHel-8	DF090370:1622132-1622554
BmHel-8	DF090358:1054287-1054709
BmHel-8	DF090358:1122844-1123266
BmHel-8	DF090358:2907259-2907681
BmHel-8	DF090383:1785116-1785538
BmHel-8	DF090352:480400-480822
BmHel-8	DF090402:741620-742042
BmHel-8	DF090377:943260-943682
BmHel-8	DF090342:2525405-2525827
BmHel-8	DF090342:2933303-2933725
BmHel-8	DF090342:3945635-3946057
BmHel-8	DF090336:3793746-3794168
BmHel-8	DF090321:6838452-6838874
BmHel-8	DF090318:3458125-3458547
BmHel-8	DF090318:4888650-4889072
BmHel-8	DF090320:4652487-4652909
BmHel-8	DF090320:6116366-6116788
BmHel-8	DF090388:307545-307967
BmHel-8	DF090362:2324782-2325204
BmHel-8	DF090362:2660963-2661385
BmHel-8	DF090362:3160511-3160933

BmHel-8	DF090359:1282688-1283110
BmHel-8	DF090356:2586980-2587402
BmHel-8	DF090338:3471788-3472210
BmHel-8	DF090329:5846880-5847302
BmHel-8	DF090323:7115682-7116104
BmHel-8	DF090322:2531344-2531766
BmHel-8	DF090322:4817403-4817825
BmHel-8	DF090319:4665833-4666255
BmHel-8	DF090319:5483320-5483742
BmHel-8	DF090319:7146153-7146575
BmHel-8	DF090317:6966775-6967197
BmHel-8	DF090317:7510717-7511139
BmHel-8	DF090317:9385623-9386045
BmHel-8	DF090316:9779217-9779639
BmHel-8	DF090316:11988223-11988645
BmHel-8	DF090316:13038920-13039342
BmHel-8	DF090316:14782072-14782494
BmHel-8	BABH01074895:9-431
BmHel-8	DF090366:1761120-1761542
BmHel-8	DF090326:5332985-5333407
BmHel-8	DF090365:2099536-2099958
BmHel-8	DF090348:1223122-1223544
BmHel-8	DF090396:1380194-1380616
BmHel-8	DF090382:1843863-1844285
BmHel-8	DF090372:1752752-1753174
BmHel-8	DF090369:1580286-1580708
BmHel-8	DF090361:2392389-2392811
BmHel-8	DF090347:3993421-3993843
BmHel-8	DF090345:1183711-1184133
BmHel-8	DF090345:2068932-2069354
BmHel-8	DF090341:1716073-1716495
BmHel-8	DF090335:5269022-5269444
BmHel-8	DF090334:582461-582883
BmHel-8	DF090334:1582205-1582627
BmHel-8	DF090331:4159832-4160254
BmHel-8	DF090324:4031806-4032228
BmHel-8	DF090337:2842602-2843024
BmHel-8	DF090375:83912-84334
BmHel-8	DF090375:674107-674529
BmHel-8	DF090333:2200709-2201131
BmHel-8	DF090333:4544285-4544707
BmHel-8	BABH01048078:974-1396
BmHel-8	DF090427:793337-793759
BmHel-8	DF090414:633681-634103
BmHel-8	DF090406:13274-13696
BmHel-8	BABH01048586:305-727
BmHel-8	DF090368:519844-520266
BmHel-8	DF090368:1440068-1440490

BmHel-8	BABH01048617:159-581
BmHel-8	DF090452:41153-41575
BmHel-8	DF090452:433654-434076
BmHel-8	DF090393:1714838-1715260
BmHel-8	BABH01073265:48-470
BmHel-8	DF090510:69317-69739
BmHel-8	DF090762:11797-12219
BmHel-8	DF090707:22592-23014
BmHel-8	DF090605:31008-31430
BmHel-8	DF090355:3360447-3360869
BmHel-8	DF090350:2590854-2591276
BmHel-8	DF090358:3439991-3440413
BmHel-8	DF090358:1394046-1394468
BmHel-8	DF090437:68953-69375
BmHel-8	DF090386:515570-515992
BmHel-8	DF090352:3900980-3901402
BmHel-8	DF090352:2958137-2958559
BmHel-8	DF090352:511333-511755
BmHel-8	DF090346:3701949-3702371
BmHel-8	DF090421:908124-908546
BmHel-8	DF090402:610219-610641
BmHel-8	DF090377:1375487-1375909
BmHel-8	DF090367:2432916-2433338
BmHel-8	DF090367:2118476-2118898
BmHel-8	DF090363:2565368-2565790
BmHel-8	DF090350:281189-281611
BmHel-8	DF090342:4760975-4761397
BmHel-8	DF090342:3401053-3401475
BmHel-8	DF090342:3209998-3210420
BmHel-8	DF090342:2592402-2592824
BmHel-8	DF090342:1005274-1005696
BmHel-8	DF090342:782587-783009
BmHel-8	DF090336:3813764-3814186
BmHel-8	DF090321:6282865-6283287
BmHel-8	DF090318:5528994-5529416
BmHel-8	DF090318:1016899-1017321
BmHel-8	DF090320:5679433-5679855
BmHel-8	DF090320:4882087-4882509
BmHel-8	DF090320:168481-168903
BmHel-8	DF090400:539772-540194
BmHel-8	DF090362:2257687-2258109
BmHel-8	DF090357:1443021-1443443
BmHel-8	DF090356:3185638-3186060
BmHel-8	DF090356:2581028-2581450
BmHel-8	DF090344:2574912-2575334
BmHel-8	DF090344:1174400-1174822
BmHel-8	DF090344:775634-776056
BmHel-8	DF090344:632176-632598

BmHel-8	DF090340:2699933-2700355
BmHel-8	DF090340:1936086-1936508
BmHel-8	DF090338:3973594-3974016
BmHel-8	DF090323:5338826-5339248
BmHel-8	DF090322:2204232-2204654
BmHel-8	DF090317:10394407-10394829
BmHel-8	DF090317:5348873-5349295
BmHel-8	DF090317:4998152-4998574
BmHel-8	DF090316:16109752-16110174
BmHel-8	DF090316:16081726-16082148
BmHel-8	DF090316:12833509-12833931
BmHel-8	DF090316:1966016-1966438
BmHel-8	DF090366:1532970-1533392
BmHel-8	DF090349:3365134-3365556
BmHel-8	DF090326:6336484-6336906
BmHel-8	DF090433:317388-317810
BmHel-8	DF090365:1162849-1163271
BmHel-8	DF090348:3234862-3235284
BmHel-8	DF090411:1002739-1003161
BmHel-8	DF090401:498873-499295
BmHel-8	DF090396:962137-962559
BmHel-8	DF090369:1703571-1703993
BmHel-8	DF090360:1661684-1662106
BmHel-8	DF090341:4269062-4269484
BmHel-8	DF090341:4039514-4039936
BmHel-8	DF090334:4133483-4133905
BmHel-8	DF090331:1140035-1140457
BmHel-8	DF090331:743109-743531
BmHel-8	DF090331:642703-643125
BmHel-8	DF090328:1953312-1953734
BmHel-8	DF090328:403611-404033
BmHel-8	DF090324:7434434-7434856
BmHel-8	DF090324:5964966-5965388
BmHel-8	BABH01048804:192-614
BmHel-8	DF090436:669730-670152
BmHel-8	DF090436:156921-157343
BmHel-8	DF090343:254485-254907
BmHel-8	DF090339:3485550-3485972
BmHel-8	DF090339:2599383-2599805
BmHel-8	DF090339:2258486-2258908
BmHel-8	DF090374:209873-210295
BmHel-8	DF090558:33602-34024
BmHel-8	DF090325:3501100-3501522
BmHel-8	DF090387:1652959-1653381
BmHel-8	DF090472:298829-299251
BmHel-8	DF090446:485294-485716
BmHel-8	DF090435:578062-578484
BmHel-8	DF090450:313105-313527

BmHel-8	DF090669:3355-3777
BmHel-8	DF090665:11111-11533
BmHel-8	BABH01046033:1669-2091
BmHel-8	BABH01074693:170-592
BmHel-8	DF090664:31253-31675
BmHel-8	DF090330:3023720-3024142
BmHel-8	DF090337:3506883-3507305
BmHel-8	DF090343:4199560-4199982
BmHel-8	DF090358:402123-402546
BmHel-8	DF090412:252754-253177
BmHel-8	DF090386:906031-906454
BmHel-8	DF090386:1828520-1828943
BmHel-8	DF090352:1980428-1980851
BmHel-8	DF090351:3497898-3498321
BmHel-8	DF090421:871203-871626
BmHel-8	DF090367:1412857-1413280
BmHel-8	DF090355:1501327-1501750
BmHel-8	DF090350:2182142-2182565
BmHel-8	DF090350:2646151-2646574
BmHel-8	DF090342:4199641-4200064
BmHel-8	DF090336:5162479-5162902
BmHel-8	DF090336:5404447-5404870
BmHel-8	DF090321:1298257-1298680
BmHel-8	DF090318:8390655-8391078
BmHel-8	DF090320:6722281-6722704
BmHel-8	DF090362:3071140-3071563
BmHel-8	DF090340:688952-689375
BmHel-8	DF090340:793296-793719
BmHel-8	DF090340:1402467-1402890
BmHel-8	DF090329:2448294-2448717
BmHel-8	DF090327:5867957-5868380
BmHel-8	DF090327:6653705-6654128
BmHel-8	DF090323:3247783-3248206
BmHel-8	DF090323:7873950-7874373
BmHel-8	DF090322:7969560-7969983
BmHel-8	DF090319:2158810-2159233
BmHel-8	DF090319:7303543-7303966
BmHel-8	DF090316:598671-599094
BmHel-8	DF090316:3420104-3420527
BmHel-8	DF090316:6289779-6290202
BmHel-8	DF090316:6944396-6944819
BmHel-8	DF090316:7025983-7026406
BmHel-8	DF090316:12468087-12468510
BmHel-8	DF090349:4424202-4424625
BmHel-8	DF090411:6392-6815
BmHel-8	DF090391:1065003-1065426
BmHel-8	DF090382:1137279-1137702
BmHel-8	DF090372:192840-193263

BmHel-8	DF090361:2489324-2489747
BmHel-8	DF090361:2700858-2701281
BmHel-8	DF090347:3462957-3463380
BmHel-8	DF090334:4230918-4231341
BmHel-8	DF090373:763336-763759
BmHel-8	DF090343:1783661-1784084
BmHel-8	DF090343:3284923-3285346
BmHel-8	DF090339:4615641-4616064
BmHel-8	DF090462:230258-230681
BmHel-8	DF090333:3701666-3702089
BmHel-8	BABH01054254:255-678
BmHel-8	DF090431:411150-411573
BmHel-8	DF090417:252686-253109
BmHel-8	DF090416:236704-237127
BmHel-8	DF090364:2469983-2470406
BmHel-8	DF090621:4142-4565
BmHel-8	DF090413:567125-567548
BmHel-8	BABH01045667:2906-3329
BmHel-8	DF090503:20098-20521
BmHel-8	BABH01049207:577-1000
BmHel-8	DF090516:81062-81485
BmHel-8	DF090600:4343-4766
BmHel-8	BABH01042926:4605-5028
BmHel-8	DF090357:1392075-1392498
BmHel-8	DF090319:6890512-6890935
BmHel-8	DF090333:2961847-2962270
BmHel-8	DF090414:468838-469261
BmHel-8	DF090330:488696-489119
BmHel-8	DF090358:2254873-2255296
BmHel-8	DF090358:1171180-1171603
BmHel-8	DF090386:1059890-1060313
BmHel-8	DF090352:3399626-3400049
BmHel-8	DF090352:1322683-1323106
BmHel-8	DF090346:1448180-1448603
BmHel-8	DF090332:5370184-5370607
BmHel-8	DF090332:2952833-2953256
BmHel-8	DF090332:372785-373208
BmHel-8	DF090421:593151-593574
BmHel-8	DF090377:1362529-1362952
BmHel-8	DF090377:579377-579800
BmHel-8	DF090355:3379673-3380096
BmHel-8	DF090350:970668-971091
BmHel-8	DF090336:2557973-2558396
BmHel-8	DF090321:2998826-2999249
BmHel-8	DF090320:6440085-6440508
BmHel-8	DF090320:2602551-2602974
BmHel-8	DF090320:2495063-2495486
BmHel-8	DF090388:1574513-1574936

BmHel-8	DF090362:1608140-1608563
BmHel-8	DF090362:1571818-1572241
BmHel-8	DF090362:1208932-1209355
BmHel-8	DF090359:2116322-2116745
BmHel-8	DF090357:2108450-2108873
BmHel-8	DF090357:1638999-1639422
BmHel-8	DF090356:1156582-1157005
BmHel-8	DF090327:4673262-4673685
BmHel-8	DF090323:3120076-3120499
BmHel-8	DF090322:6534787-6535210
BmHel-8	DF090319:5723495-5723918
BmHel-8	DF090319:4300819-4301242
BmHel-8	DF090317:8572267-8572690
BmHel-8	DF090317:5010759-5011182
BmHel-8	DF090317:2887704-2888127
BmHel-8	DF090316:3405458-3405881
BmHel-8	DF090349:2208974-2209397
BmHel-8	DF090326:3449431-3449854
BmHel-8	DF090396:1361678-1362101
BmHel-8	DF090392:324277-324700
BmHel-8	DF090382:1660658-1661081
BmHel-8	DF090360:1519535-1519958
BmHel-8	DF090347:373592-374015
BmHel-8	DF090345:2468413-2468836
BmHel-8	DF090328:4115511-4115934
BmHel-8	DF090324:7237578-7238001
BmHel-8	DF090324:6887054-6887477
BmHel-8	DF090324:5274078-5274501
BmHel-8	DF090333:4696919-4697342
BmHel-8	DF090333:4074420-4074843
BmHel-8	DF090333:2885933-2886356
BmHel-8	DF090325:4562607-4563030
BmHel-8	DF090434:43592-44015
BmHel-8	DF090389:1294495-1294918
BmHel-8	DF090364:2254096-2254519
BmHel-8	DF090429:682985-683408
BmHel-8	DF090415:272176-272599
BmHel-8	DF090413:51321-51744
BmHel-8	DF090406:491256-491679
BmHel-8	DF090368:1605918-1606341
BmHel-8	DF090407:313608-314031
BmHel-8	BABH01047373:1093-1516
BmHel-8	DF090534:117305-117728
BmHel-8	BABH01061118:272-695
BmHel-8	DF090484:222770-223193
BmHel-8	BABH01072836:82-505
BmHel-8	DF091063:4162-4585
BmHel-8	DF090426:337737-338160

BmHel-8	BABH01044768:2682-3105
BmHel-8	DF090716:56125-56548
BmHel-8	BABH01078105:149-572
BmHel-8	DF090353:1493002-1493425
BmHel-8	BABH01069565:74-497
BmHel-8	DF090386:1719912-1720336
BmHel-8	DF090352:1729971-1730395
BmHel-8	DF090352:2924761-2925185
BmHel-8	DF090351:1399623-1400047
BmHel-8	DF090351:3441011-3441435
BmHel-8	DF090332:408095-408519
BmHel-8	DF090332:3202070-3202494
BmHel-8	DF090332:5138842-5139266
BmHel-8	DF090332:6121826-6122250
BmHel-8	DF090363:2808960-2809384
BmHel-8	DF090355:604291-604715
BmHel-8	DF090353:2054309-2054733
BmHel-8	DF090342:4175420-4175844
BmHel-8	DF090336:1693554-1693978
BmHel-8	DF090321:7718191-7718615
BmHel-8	DF090318:2694116-2694540
BmHel-8	DF090320:4182025-4182449
BmHel-8	DF090441:556700-557124
BmHel-8	DF090362:2343725-2344149
BmHel-8	DF090359:351618-352042
BmHel-8	DF090359:2020771-2021195
BmHel-8	DF090359:2081068-2081492
BmHel-8	DF090359:2140013-2140437
BmHel-8	DF090357:2360885-2361309
BmHel-8	DF090344:2183003-2183427
BmHel-8	DF090344:4202124-4202548
BmHel-8	DF090340:1416906-1417330
BmHel-8	DF090340:4360972-4361396
BmHel-8	DF090327:1395183-1395607
BmHel-8	DF090327:4980213-4980637
BmHel-8	DF090323:3107935-3108359
BmHel-8	DF090322:3473391-3473815
BmHel-8	DF090322:3718545-3718969
BmHel-8	DF090322:5086297-5086721
BmHel-8	DF090322:5587687-5588111
BmHel-8	DF090322:7895812-7896236
BmHel-8	DF090319:1084420-1084844
BmHel-8	DF090319:4267400-4267824
BmHel-8	DF090316:10067131-10067555
BmHel-8	DF090395:873892-874316
BmHel-8	DF090366:920477-920901
BmHel-8	DF090366:1463414-1463838
BmHel-8	DF090326:783728-784152

BmHel-8	DF090326:6118763-6119187
BmHel-8	DF090348:272421-272845
BmHel-8	DF090348:1114345-1114769
BmHel-8	DF090348:3261497-3261921
BmHel-8	DF090396:50984-51408
BmHel-8	DF090391:199534-199958
BmHel-8	DF090391:872368-872792
BmHel-8	DF090371:1158106-1158530
BmHel-8	DF090341:2927528-2927952
BmHel-8	DF090331:5709663-5710087
BmHel-8	DF090337:356885-357309
BmHel-8	DF090390:1092688-1093112
BmHel-8	DF090373:293767-294191
BmHel-8	DF090343:3779346-3779770
BmHel-8	DF090339:613596-614020
BmHel-8	DF090376:2092548-2092972
BmHel-8	DF090333:2111177-2111601
BmHel-8	DF090434:335432-335856
BmHel-8	DF090429:676889-677313
BmHel-8	DF090408:1399126-1399550
BmHel-8	DF090406:701828-702252
BmHel-8	DF090517:49643-50067
BmHel-8	DF090442:432286-432710
BmHel-8	DF090741:5505-5929
BmHel-8	DF090663:27376-27800
BmHel-8	DF090409:965383-965807
BmHel-8	DF090353:3539033-3539457
BmHel-8	DF090330:6219225-6219649
BmHel-8	DF090330:6067201-6067625
BmHel-8	DF090330:92135-92559
BmHel-8	DF090358:357432-357856
BmHel-8	DF090352:1230753-1231177
BmHel-8	DF090351:3606533-3606957
BmHel-8	DF090346:3900039-3900463
BmHel-8	DF090332:6288861-6289285
BmHel-8	DF090466:171204-171628
BmHel-8	DF090367:1416949-1417373
BmHel-8	DF090363:2243251-2243675
BmHel-8	DF090353:69117-69541
BmHel-8	DF090350:2568136-2568560
BmHel-8	DF090350:1595046-1595470
BmHel-8	DF090342:2604659-2605083
BmHel-8	DF090318:7327463-7327887
BmHel-8	DF090320:991345-991769
BmHel-8	DF090359:325058-325482
BmHel-8	DF090357:2178472-2178896
BmHel-8	DF090344:3364857-3365281
BmHel-8	DF090340:4333250-4333674

BmHel-8	DF090340:1981707-1982131
BmHel-8	DF090340:333006-333430
BmHel-8	DF090323:2800326-2800750
BmHel-8	DF090322:6023227-6023651
BmHel-8	DF090322:5204158-5204582
BmHel-8	DF090322:4771905-4772329
BmHel-8	DF090322:1060048-1060472
BmHel-8	DF090319:3756567-3756991
BmHel-8	DF090319:906507-906931
BmHel-8	DF090317:7532427-7532851
BmHel-8	DF090316:15924913-15925337
BmHel-8	DF090316:14252858-14253282
BmHel-8	DF090316:11931252-11931676
BmHel-8	DF090395:1583719-1584143
BmHel-8	DF090349:1699891-1700315
BmHel-8	DF090326:3361175-3361599
BmHel-8	DF090326:2320015-2320439
BmHel-8	DF090326:1684888-1685312
BmHel-8	DF090348:3703519-3703943
BmHel-8	DF090411:281444-281868
BmHel-8	DF090396:238415-238839
BmHel-8	DF090382:354204-354628
BmHel-8	DF090372:1233812-1234236
BmHel-8	DF090372:739982-740406
BmHel-8	DF090361:554701-555125
BmHel-8	DF090347:1111983-1112407
BmHel-8	DF090345:927235-927659
BmHel-8	DF090341:4611438-4611862
BmHel-8	DF090341:3458582-3459006
BmHel-8	DF090335:5148462-5148886
BmHel-8	DF090335:4126436-4126860
BmHel-8	DF090331:2763404-2763828
BmHel-8	DF090324:2283449-2283873
BmHel-8	DF090343:1535482-1535906
BmHel-8	DF090343:332038-332462
BmHel-8	DF090379:720554-720978
BmHel-8	DF090375:2255140-2255564
BmHel-8	DF090325:1533627-1534051
BmHel-8	DF090431:272700-273124
BmHel-8	DF090639:4647-5071
BmHel-8	DF090354:1783729-1784153
BmHel-8	DF090413:336663-337087
BmHel-8	DF090408:916110-916534
BmHel-8	DF090368:2387933-2388357
BmHel-8	DF090648:19212-19636
BmHel-8	DF090521:17483-17907
BmHel-8	DF090320:8500970-8501394
BmHel-8	DF090317:8023435-8023859

BmHel-8	DF090330:6053873-6054298
BmHel-8	DF090351:1650432-1650857
BmHel-8	DF090409:1074420-1074845
BmHel-8	DF090363:2587889-2588314
BmHel-8	DF090342:302409-302834
BmHel-8	DF090342:1003907-1004332
BmHel-8	DF090342:1454294-1454719
BmHel-8	DF090336:5058377-5058802
BmHel-8	DF090321:1709510-1709935
BmHel-8	DF090318:170492-170917
BmHel-8	DF090318:4293503-4293928
BmHel-8	DF090318:6064218-6064643
BmHel-8	DF090318:6617854-6618279
BmHel-8	DF090320:517844-518269
BmHel-8	DF090327:1997920-1998345
BmHel-8	DF090327:2336115-2336540
BmHel-8	DF090323:2231860-2232285
BmHel-8	DF090322:2871975-2872400
BmHel-8	DF090317:274079-274504
BmHel-8	DF090317:1428291-1428716
BmHel-8	DF090317:8095094-8095519
BmHel-8	DF090317:8851902-8852327
BmHel-8	DF090316:3972938-3973363
BmHel-8	DF090316:12873413-12873838
BmHel-8	DF090349:3319085-3319510
BmHel-8	DF090382:923592-924017
BmHel-8	DF090380:505327-505752
BmHel-8	DF090369:1515016-1515441
BmHel-8	DF090335:4766069-4766494
BmHel-8	DF090334:1985995-1986420
BmHel-8	DF090390:1770858-1771283
BmHel-8	DF090343:1126090-1126515
BmHel-8	DF090375:1762483-1762908
BmHel-8	DF090333:2826759-2827184
BmHel-8	DF090325:6508052-6508477
BmHel-8	DF090414:606565-606990
BmHel-8	DF090389:795492-795917
BmHel-8	DF090364:746377-746802
BmHel-8	DF090429:930105-930530
BmHel-8	DF090406:681398-681823
BmHel-8	BABH01053618:297-722
BmHel-8	DF090419:272617-273042
BmHel-8	DF090450:116503-116928
BmHel-8	DF090590:40613-41038
BmHel-8	DF091238:84-509
BmHel-8	DF090947:6154-6579
BmHel-8	DF090504:71930-72355
BmHel-8	DF091007:2762-3187

BmHel-8	DF090318:6773554-6773979
BmHel-8	DF090327:6585376-6585801
BmHel-8	DF090391:1638310-1638735
BmHel-8	DF090373:1198-1623
BmHel-8	DF090330:232669-233094
BmHel-8	DF090370:1732284-1732709
BmHel-8	DF090383:1165423-1165848
BmHel-8	DF090352:3250280-3250705
BmHel-8	DF090351:1916515-1916940
BmHel-8	DF090346:3467857-3468282
BmHel-8	DF090332:218811-219236
BmHel-8	DF090350:3833317-3833742
BmHel-8	DF090350:2381559-2381984
BmHel-8	DF090318:7637305-7637730
BmHel-8	DF090318:5095271-5095696
BmHel-8	DF090320:6205845-6206270
BmHel-8	DF090388:1536796-1537221
BmHel-8	DF090338:4006347-4006772
BmHel-8	DF090329:3295979-3296404
BmHel-8	DF090323:6797081-6797506
BmHel-8	DF090323:4057678-4058103
BmHel-8	DF090319:6845962-6846387
BmHel-8	DF090316:2780867-2781292
BmHel-8	DF090326:2244301-2244726
BmHel-8	DF090326:1414742-1415167
BmHel-8	DF090365:644881-645306
BmHel-8	DF090396:880175-880600
BmHel-8	DF090361:1206769-1207194
BmHel-8	DF090361:1135452-1135877
BmHel-8	DF090347:3163461-3163886
BmHel-8	DF090328:1660650-1661075
BmHel-8	DF090379:168896-169321
BmHel-8	DF090333:3975877-3976302
BmHel-8	DF090325:1660483-1660908
BmHel-8	DF090417:245612-246037
BmHel-8	DF090384:501475-501900
BmHel-8	BABH01043665:14574-14999
BmHel-8	DF090906:6268-6693
BmHel-8	DF090755:20570-20995
BmHel-8	DF090325:5344191-5344616
BmHel-8	DF090358:2041932-2042358
BmHel-8	DF090352:2751772-2752198
BmHel-8	DF090352:4192305-4192731
BmHel-8	DF090332:68797-69223
BmHel-8	DF090332:2830007-2830433
BmHel-8	DF090332:5285523-5285949
BmHel-8	DF090363:1445265-1445691
BmHel-8	DF090355:1402411-1402837

BmHel-8	DF090355:2047386-2047812
BmHel-8	DF090355:2611674-2612100
BmHel-8	DF090353:3518068-3518494
BmHel-8	DF090336:672030-672456
BmHel-8	DF090388:1206220-1206646
BmHel-8	DF090356:3832425-3832851
BmHel-8	DF090344:7898-8324
BmHel-8	DF090344:72810-73236
BmHel-8	DF090322:3573840-3574266
BmHel-8	DF090317:7201122-7201548
BmHel-8	DF090317:8401457-8401883
BmHel-8	DF090316:5858192-5858618
BmHel-8	DF090326:6020000-6020426
BmHel-8	DF090365:2299867-2300293
BmHel-8	DF090365:3003127-3003553
BmHel-8	DF090382:1551982-1552408
BmHel-8	DF090369:540365-540791
BmHel-8	DF090361:225835-226261
BmHel-8	DF090345:1354850-1355276
BmHel-8	DF090341:802941-803367
BmHel-8	DF090335:4323187-4323613
BmHel-8	DF090334:3793409-3793835
BmHel-8	DF090373:2312033-2312459
BmHel-8	DF090339:3926364-3926790
BmHel-8	DF090387:322780-323206
BmHel-8	DF090407:492559-492985
BmHel-8	DF090517:3256-3682
BmHel-8	DF090938:3893-4319
BmHel-8	DF090452:103516-103942
BmHel-8	DF090671:26287-26713
BmHel-8	DF090393:1124327-1124753
BmHel-8	DF090447:403097-403523
BmHel-8	BABH01045039:6001-6427
BmHel-8	DF090562:38046-38472
BmHel-8	BABH01044747:270-696
BmHel-8	DF090330:3732667-3733093
BmHel-8	DF090352:1741175-1741601
BmHel-8	DF090355:2511079-2511505
BmHel-8	DF090350:3847866-3848292
BmHel-8	DF090350:3665150-3665576
BmHel-8	DF090350:3090844-3091270
BmHel-8	DF090350:2650013-2650439
BmHel-8	DF090320:5424014-5424440
BmHel-8	DF090340:4908872-4909298
BmHel-8	DF090316:15382747-15383173
BmHel-8	DF090316:14135219-14135645
BmHel-8	DF090316:12407745-12408171
BmHel-8	DF090326:3106200-3106626

BmHel-8	DF090326:3056038-3056464
BmHel-8	DF090326:1124559-1124985
BmHel-8	DF090365:21692-22118
BmHel-8	DF090348:1319600-1320026
BmHel-8	DF090382:1927302-1927728
BmHel-8	DF090382:650780-651206
BmHel-8	DF090371:1919408-1919834
BmHel-8	DF090360:3093774-3094200
BmHel-8	DF090331:3641197-3641623
BmHel-8	DF090328:2342925-2343351
BmHel-8	DF090343:4448938-4449364
BmHel-8	DF090339:2822588-2823014
BmHel-8	DF090339:2087068-2087494
BmHel-8	DF090460:43945-44371
BmHel-8	DF090425:225606-226032
BmHel-8	DF090405:534176-534602
BmHel-8	DF090580:23605-24031
BmHel-8	DF090417:687969-688395
BmHel-8	DF090438:259028-259454
BmHel-8	DF090330:6394291-6394718
BmHel-8	DF090351:79600-80027
BmHel-8	DF090402:290259-290686
BmHel-8	DF090336:5588601-5589028
BmHel-8	DF090321:3447575-3448002
BmHel-8	DF090356:1706431-1706858
BmHel-8	DF090338:4951517-4951944
BmHel-8	DF090323:1908280-1908707
BmHel-8	DF090323:2165318-2165745
BmHel-8	DF090322:618768-619195
BmHel-8	DF090319:7069826-7070253
BmHel-8	DF090319:7085897-7086324
BmHel-8	DF090317:9929709-9930136
BmHel-8	DF090365:269447-269874
BmHel-8	DF090371:741969-742396
BmHel-8	DF090324:3465766-3466193
BmHel-8	DF090373:168630-169057
BmHel-8	DF090339:4345695-4346122
BmHel-8	DF090374:1149750-1150177
BmHel-8	DF090333:5481341-5481768
BmHel-8	DF090325:4358531-4358958
BmHel-8	DF090414:826271-826698
BmHel-8	DF090398:233409-233836
BmHel-8	DF090467:250915-251342
BmHel-8	DF090435:113935-114362
BmHel-8	DF090393:883276-883703
BmHel-8	DF090418:820973-821400
BmHel-8	DF090330:4513613-4514040
BmHel-8	DF090358:3316090-3316517

BmHel-8	DF090383:2020163-2020590
BmHel-8	DF090332:4578997-4579424
BmHel-8	DF090363:45193-45620
BmHel-8	DF090355:751899-752326
BmHel-8	DF090353:455313-455740
BmHel-8	DF090342:2505146-2505573
BmHel-8	DF090321:2706815-2707242
BmHel-8	DF090318:4182306-4182733
BmHel-8	DF090400:451409-451836
BmHel-8	DF090388:75059-75486
BmHel-8	DF090359:3165100-3165527
BmHel-8	DF090344:40965-41392
BmHel-8	DF090340:4151885-4152312
BmHel-8	DF090317:3016260-3016687
BmHel-8	DF090316:6213491-6213918
BmHel-8	DF090316:5711138-5711565
BmHel-8	DF090326:4754087-4754514
BmHel-8	DF090365:404366-404793
BmHel-8	DF090396:403706-404133
BmHel-8	DF090391:830985-831412
BmHel-8	DF090369:2117222-2117649
BmHel-8	DF090361:1728127-1728554
BmHel-8	DF090345:4499072-4499499
BmHel-8	DF090341:2766764-2767191
BmHel-8	DF090436:420749-421176
BmHel-8	DF090373:558678-559105
BmHel-8	DF090339:1748103-1748530
BmHel-8	DF090425:17781-18208
BmHel-8	DF090379:185087-185514
BmHel-8	DF090374:276886-277313
BmHel-8	DF090416:947396-947823
BmHel-8	DF090416:339834-340261
BmHel-8	DF090409:644055-644482
BmHel-8	DF090332:1345459-1345887
BmHel-8	DF090332:4636659-4637087
BmHel-8	DF090367:1450378-1450806
BmHel-8	DF090320:7916083-7916511
BmHel-8	DF090362:9654-10082
BmHel-8	DF090362:1722454-1722882
BmHel-8	DF090357:2163352-2163780
BmHel-8	DF090356:2731996-2732424
BmHel-8	DF090323:6991877-6992305
BmHel-8	DF090322:2590633-2591061
BmHel-8	DF090326:6203328-6203756
BmHel-8	DF090345:2706913-2707341
BmHel-8	DF090331:760473-760901
BmHel-8	DF090405:1149168-1149596
BmHel-8	DF090384:1539388-1539816

BmHel-8	DF090424:738042-738470
BmHel-8	DF090404:1119259-1119687
BmHel-8	DF090923:4847-5275
BmHel-8	DF090667:13011-13439
BmHel-8	DF090424:611704-612132
BmHel-8	DF090386:1368398-1368826
BmHel-8	DF090352:2549361-2549789
BmHel-8	DF090421:430123-430551
BmHel-8	DF090377:437221-437649
BmHel-8	DF090350:2717370-2717798
BmHel-8	DF090321:6492586-6493014
BmHel-8	DF090443:234371-234799
BmHel-8	DF090441:613383-613811
BmHel-8	DF090388:692874-693302
BmHel-8	DF090329:5510627-5511055
BmHel-8	DF090327:2900442-2900870
BmHel-8	DF090322:1242538-1242966
BmHel-8	DF090317:9139243-9139671
BmHel-8	DF090317:8897848-8898276
BmHel-8	DF090349:3768826-3769254
BmHel-8	DF090326:6497960-6498388
BmHel-8	DF090394:901910-902338
BmHel-8	DF090394:493741-494169
BmHel-8	DF090361:316423-316851
BmHel-8	DF090341:4237997-4238425
BmHel-8	DF090328:3629302-3629730
BmHel-8	DF090328:3317669-3318097
BmHel-8	DF090333:4405407-4405835
BmHel-8	DF090407:951310-951738
BmHel-8	DF090524:39087-39515
BmHel-8	BABH01063254:42-470
BmHel-8	DF090330:3847278-3847707
BmHel-8	DF090352:2721764-2722193
BmHel-8	DF090352:3428309-3428738
BmHel-8	DF090351:4325626-4326055
BmHel-8	DF090377:739137-739566
BmHel-8	DF090353:2662721-2663150
BmHel-8	DF090336:742321-742750
BmHel-8	DF090388:893061-893490
BmHel-8	DF090362:2114770-2115199
BmHel-8	DF090359:696330-696759
BmHel-8	DF090359:1327205-1327634
BmHel-8	DF090344:1493175-1493604
BmHel-8	DF090338:4045389-4045818
BmHel-8	DF090329:1185715-1186144
BmHel-8	DF090329:5022931-5023360
BmHel-8	DF090323:7373312-7373741
BmHel-8	DF090322:8140440-8140869

BmHel-8	DF090319:3807739-3808168
BmHel-8	DF090317:4508518-4508947
BmHel-8	DF090317:6532846-6533275
BmHel-8	DF090316:9459268-9459697
BmHel-8	DF090349:368408-368837
BmHel-8	DF090348:3087353-3087782
BmHel-8	DF090369:1785541-1785970
BmHel-8	DF090331:601263-601692
BmHel-8	DF090331:5759528-5759957
BmHel-8	DF090417:618183-618612
BmHel-8	DF090364:193216-193645
BmHel-8	DF090364:2260787-2261216
BmHel-8	DF090455:32587-33016
BmHel-8	DF090636:1116-1545
BmHel-8	DF090383:214575-215004
BmHel-8	DF090352:4156677-4157106
BmHel-8	DF090346:1315649-1316078
BmHel-8	DF090421:654580-655009
BmHel-8	DF090318:5995996-5996425
BmHel-8	DF090359:2052535-2052964
BmHel-8	DF090356:1233729-1234158
BmHel-8	DF090327:5480141-5480570
BmHel-8	DF090317:1290115-1290544
BmHel-8	DF090316:1381892-1382321
BmHel-8	DF090366:567671-568100
BmHel-8	DF090397:1240925-1241354
BmHel-8	DF090369:2416771-2417200
BmHel-8	DF090361:2013175-2013604
BmHel-8	DF090345:2256723-2257152
BmHel-8	DF090341:555261-555690
BmHel-8	DF090328:1071091-1071520
BmHel-8	DF090373:658992-659421
BmHel-8	DF090325:6510641-6511070
BmHel-8	DF090354:406562-406991
BmHel-8	BABH01082561:8-437
BmHel-8	DF090624:2829-3258
BmHel-8	DF090336:1351140-1351569
BmHel-8	DF090358:1203912-1204342
BmHel-8	DF090352:4036694-4037124
BmHel-8	DF090351:2612968-2613398
BmHel-8	DF090350:470569-470999
BmHel-8	DF090350:960183-960613
BmHel-8	DF090350:1689203-1689633
BmHel-8	DF090342:1260776-1261206
BmHel-8	DF090356:1124679-1125109
BmHel-8	DF090329:5752891-5753321
BmHel-8	DF090319:3604349-3604779
BmHel-8	DF090319:7787386-7787816

BmHel-8	DF090348:1344663-1345093
BmHel-8	DF090380:1092319-1092749
BmHel-8	DF090380:1712868-1713298
BmHel-8	DF090331:1477398-1477828
BmHel-8	DF090328:6143186-6143616
BmHel-8	DF090337:2814877-2815307
BmHel-8	DF090373:1123858-1124288
BmHel-8	DF090325:1690702-1691132
BmHel-8	DF090543:47221-47651
BmHel-8	DF090382:1186863-1187293
BmHel-8	DF090373:75992-76422
BmHel-8	DF090370:2703954-2704384
BmHel-8	DF090346:1364237-1364667
BmHel-8	DF090336:96087-96517
BmHel-8	DF090329:2115533-2115963
BmHel-8	DF090323:4486068-4486498
BmHel-8	DF090323:1900158-1900588
BmHel-8	DF090319:2577548-2577978
BmHel-8	DF090317:4842577-4843007
BmHel-8	DF090365:1419769-1420199
BmHel-8	DF090439:531747-532177
BmHel-8	DF090397:1291109-1291539
BmHel-8	DF090394:990651-991081
BmHel-8	DF090382:1174922-1175352
BmHel-8	DF090341:4362829-4363259
BmHel-8	DF090341:1495480-1495910
BmHel-8	DF090331:5116078-5116508
BmHel-8	DF090328:528334-528764
BmHel-8	DF090373:1995215-1995645
BmHel-8	DF090339:1550796-1551226
BmHel-8	DF090405:1447132-1447562
BmHel-8	DF090333:947491-947921
BmHel-8	DF090354:3575010-3575440
BmHel-8	DF090324:5866392-5866822
BmHel-8	DF090332:3344682-3345113
BmHel-8	DF090420:400969-401400
BmHel-8	DF090349:134346-134777
BmHel-8	DF090361:3070723-3071154
BmHel-8	DF090341:4509751-4510182
BmHel-8	DF090431:773001-773432
BmHel-8	DF090387:1026561-1026992
BmHel-8	DF090461:36547-36978
BmHel-8	DF090690:13666-14097
BmHel-8	DF090495:12984-13415
BmHel-8	DF090992:1274-1705
BmHel-8	DF090638:3681-4112
BmHel-8	DF090352:89036-89467
BmHel-8	DF090346:1516297-1516728

BmHel-8	DF090332:2124290-2124721
BmHel-8	DF090353:3588907-3589338
BmHel-8	DF090353:3538210-3538641
BmHel-8	DF090443:142904-143335
BmHel-8	DF090327:2340320-2340751
BmHel-8	DF090319:6037628-6038059
BmHel-8	DF090395:1468154-1468585
BmHel-8	DF090326:4818486-4818917
BmHel-8	DF090354:459533-459964
BmHel-8	DF090447:129200-129631
BmHel-8	DF090359:504230-504661
BmHel-8	DF090324:7971661-7972092
BmHel-8	DF090358:2333076-2333508
BmHel-8	DF090346:3918320-3918752
BmHel-8	DF090332:1461931-1462363
BmHel-8	DF090350:3814488-3814920
BmHel-8	DF090321:3029766-3030198
BmHel-8	DF090321:6888605-6889037
BmHel-8	DF090340:3259026-3259458
BmHel-8	DF090319:4554666-4555098
BmHel-8	DF090317:362674-363106
BmHel-8	DF090349:647653-648085
BmHel-8	DF090365:2073590-2074022
BmHel-8	DF090341:1027615-1028047
BmHel-8	DF090378:1926625-1927057
BmHel-8	DF090434:34967-35399
BmHel-8	DF090384:1713959-1714391
BmHel-8	DF090404:1097709-1098141
BmHel-8	DF090332:3163025-3163457
BmHel-8	DF090377:1034144-1034576
BmHel-8	DF090363:2761105-2761537
BmHel-8	DF090329:449365-449797
BmHel-8	DF090327:536320-536752
BmHel-8	DF090319:6266296-6266728
BmHel-8	DF090317:3258430-3258862
BmHel-8	DF090348:4059925-4060357
BmHel-8	DF090371:670885-671317
BmHel-8	DF090341:2825332-2825764
BmHel-8	DF090331:29937-30369
BmHel-8	DF090343:1040850-1041282
BmHel-8	DF090375:1902079-1902511
BmHel-8	DF090378:1610300-1610732
BmHel-8	DF090445:625968-626400
BmHel-8	DF090354:3554701-3555133
BmHel-8	DF090455:146638-147070
BmHel-8	DF090321:2145254-2145687
BmHel-8	DF090321:2666609-2667042
BmHel-8	DF090327:4455371-4455804

BmHel-8	DF090319:8492264-8492697
BmHel-8	DF090349:589913-590346
BmHel-8	DF090380:1037580-1038013
BmHel-8	DF090369:915496-915929
BmHel-8	DF090328:2887844-2888277
BmHel-8	DF090328:3632349-3632782
BmHel-8	DF090450:330705-331138
BmHel-8	DF090338:705978-706411
BmHel-8	DF090330:5489273-5489706
BmHel-8	DF090386:2033462-2033895
BmHel-8	DF090346:2987984-2988417
BmHel-8	DF090346:2008687-2009120
BmHel-8	DF090321:7176104-7176537
BmHel-8	DF090320:5596794-5597227
BmHel-8	DF090320:4264398-4264831
BmHel-8	DF090388:682221-682654
BmHel-8	DF090338:3385151-3385584
BmHel-8	DF090319:6983007-6983440
BmHel-8	DF090366:1563292-1563725
BmHel-8	DF090326:3958319-3958752
BmHel-8	DF090348:3434245-3434678
BmHel-8	DF090396:440679-441112
BmHel-8	DF090382:1089621-1090054
BmHel-8	DF090347:2089709-2090142
BmHel-8	DF090339:1956184-1956617
BmHel-8	DF090364:352141-352574
BmHel-8	DF090452:69864-70297
BmHel-8	DF090772:12032-12465
BmHel-8	DF090330:3889408-3889842
BmHel-8	DF090351:2279599-2280033
BmHel-8	DF090362:934441-934875
BmHel-8	DF090356:1905948-1906382
BmHel-8	DF090340:1787316-1787750
BmHel-8	DF090323:1966157-1966591
BmHel-8	DF090319:4332173-4332607
BmHel-8	DF090316:9971543-9971977
BmHel-8	DF090365:2622235-2622669
BmHel-8	DF090411:1185083-1185517
BmHel-8	DF090337:4919952-4920386
BmHel-8	DF090333:5274424-5274858
BmHel-8	DF090325:3965219-3965653
BmHel-8	DF090427:201253-201687
BmHel-8	DF090490:130640-131074
BmHel-8	DF090351:3675326-3675760
BmHel-8	DF090330:1253323-1253757
BmHel-8	DF090383:108612-109046
BmHel-8	DF090346:679026-679460
BmHel-8	DF090336:5053798-5054232

BmHel-8	DF090362:2924912-2925346
BmHel-8	DF090322:3679413-3679847
BmHel-8	DF090339:592157-592591
BmHel-8	DF090374:2312102-2312536
BmHel-8	DF090346:4438557-4438992
BmHel-8	DF090342:4162873-4163308
BmHel-8	DF090336:4140724-4141159
BmHel-8	DF090338:949276-949711
BmHel-8	DF090329:6727758-6728193
BmHel-8	DF090319:2393792-2394227
BmHel-8	DF090319:8229265-8229700
BmHel-8	DF090316:2424757-2425192
BmHel-8	DF090316:16026080-16026515
BmHel-8	DF090347:3979245-3979680
BmHel-8	DF090341:15727-16162
BmHel-8	DF090341:2146957-2147392
BmHel-8	DF090429:78447-78882
BmHel-8	DF090393:1172171-1172606
BmHel-8	DF090339:4787445-4787880
BmHel-8	DF090383:1575877-1576312
BmHel-8	DF090359:1058933-1059368
BmHel-8	DF090340:3761283-3761718
BmHel-8	DF090340:3187725-3188160
BmHel-8	DF090395:816685-817120
BmHel-8	DF090326:3652751-3653186
BmHel-8	DF090361:2534395-2534830
BmHel-8	DF090345:2796189-2796624
BmHel-8	DF090324:4524609-4525044
BmHel-8	DF090419:13742-14177
BmHel-8	DF090463:365863-366298
BmHel-8	DF090409:335380-335815
BmHel-8	DF090402:512378-512814
BmHel-8	DF090336:2349596-2350032
BmHel-8	DF090319:1747806-1748242
BmHel-8	DF090317:8118869-8119305
BmHel-8	DF090410:238201-238637
BmHel-8	DF090395:673512-673948
BmHel-8	DF090382:1963052-1963488
BmHel-8	DF090347:3326754-3327190
BmHel-8	DF090347:3498242-3498678
BmHel-8	DF090398:780752-781188
BmHel-8	DF090435:692867-693303
BmHel-8	DF090463:83362-83798
BmHel-8	DF090490:106646-107082
BmHel-8	DF090386:456194-456630
BmHel-8	DF090409:285348-285784
BmHel-8	DF090320:1360955-1361391
BmHel-8	DF090356:1186148-1186584

BmHel-8	DF090327:1111548-1111984
BmHel-8	DF090319:2967766-2968202
BmHel-8	DF090319:1256002-1256438
BmHel-8	DF090317:5725428-5725864
BmHel-8	DF090349:2181727-2182163
BmHel-8	DF090371:1714852-1715288
BmHel-8	DF090343:1290570-1291006
BmHel-8	DF090374:2296984-2297420
BmHel-8	DF090389:1545715-1546151
BmHel-8	DF090344:310230-310666
BmHel-8	DF090370:1475660-1476097
BmHel-8	DF090346:3401703-3402140
BmHel-8	DF090332:2237854-2238291
BmHel-8	DF090367:1387627-1388064
BmHel-8	DF090321:1937885-1938322
BmHel-8	DF090320:1997364-1997801
BmHel-8	DF090316:4272249-4272686
BmHel-8	DF090396:216094-216531
BmHel-8	DF090341:383127-383564
BmHel-8	DF090335:1662772-1663209
BmHel-8	DF090406:1141363-1141800
BmHel-8	DF090844:1035-1472
BmHel-8	DF090346:4170272-4170709
BmHel-8	DF090355:807906-808343
BmHel-8	DF090362:1163394-1163831
BmHel-8	DF090359:2086513-2086950
BmHel-8	DF090422:365974-366411
BmHel-8	DF090347:2307511-2307948
BmHel-8	DF090341:4253617-4254054
BmHel-8	DF090337:4791139-4791576
BmHel-8	DF090390:183565-184002
BmHel-8	DF090374:2058437-2058874
BmHel-8	DF090378:1999162-1999599
BmHel-8	DF090406:1512589-1513026
BmHel-8	DF090368:2192361-2192798
BmHel-8	DF090330:6132251-6132689
BmHel-8	DF090352:3851816-3852254
BmHel-8	DF090363:226157-226595
BmHel-8	DF090355:518007-518445
BmHel-8	DF090380:1985888-1986326
BmHel-8	DF090360:2957998-2958436
BmHel-8	DF090345:3764555-3764993
BmHel-8	DF090376:1308372-1308810
BmHel-8	DF090364:2747415-2747853
BmHel-8	DF090879:16673-17111
BmHel-8	DF090819:7012-7450
BmHel-8	DF090377:136607-137045
BmHel-8	DF090355:2191772-2192210

BmHel-8	DF090388:116621-117059
BmHel-8	DF090395:1529493-1529931
BmHel-8	DF090382:815064-815502
BmHel-8	DF090424:184280-184718
BmHel-8	DF090368:919568-920006
BmHel-8	DF090487:5884-6322
BmHel-8	DF090367:2536826-2537265
BmHel-8	DF090320:5911638-5912077
BmHel-8	DF090329:202428-202867
BmHel-8	DF090328:1220490-1220929
BmHel-8	DF090328:1834458-1834897
BmHel-8	DF090316:10845385-10845824
BmHel-8	DF090398:989107-989546
BmHel-8	DF090342:1434074-1434513
BmHel-8	DF090327:125991-126430
BmHel-8	DF090323:7966494-7966933
BmHel-8	DF090317:3585447-3585886
BmHel-8	DF090365:2610303-2610742
BmHel-8	DF090345:1103771-1104210
BmHel-8	DF090415:874660-875099
BmHel-8	DF090492:25267-25706
BmHel-8	BABH01046264:2038-2477
BmHel-8	DF090967:50-489
BmHel-8	DF090317:2817668-2818107
BmHel-8	DF090617:18104-18543
BmHel-8	DF090332:3779579-3780019
BmHel-8	DF090355:722616-723056
BmHel-8	DF090320:7521017-7521457
BmHel-8	DF090420:168390-168830
BmHel-8	DF090362:1819172-1819612
BmHel-8	DF090329:5880903-5881343
BmHel-8	DF090323:146580-147020
BmHel-8	DF090394:1274841-1275281
BmHel-8	DF090360:2579752-2580192
BmHel-8	DF090328:1956487-1956927
BmHel-8	DF090362:1740981-1741421
BmHel-8	DF090317:2091742-2092182
BmHel-8	DF090316:12064997-12065437
BmHel-8	DF090337:1524638-1525078
BmHel-8	DF090374:820405-820845
BmHel-8	DF090387:864397-864837
BmHel-8	DF090393:1326178-1326618
BmHel-8	DF090351:3522135-3522576
BmHel-8	DF090421:445475-445916
BmHel-8	DF090340:4868445-4868886
BmHel-8	DF090322:2568416-2568857
BmHel-8	DF090316:6627896-6628337
BmHel-8	DF090375:1394642-1395083

BmHel-8	DF090340:4812903-4813344
BmHel-8	DF090330:3383093-3383534
BmHel-8	DF090402:805588-806029
BmHel-8	DF090318:5490414-5490855
BmHel-8	DF090318:2897834-2898275
BmHel-8	DF090322:1957789-1958230
BmHel-8	DF090335:1912964-1913405
BmHel-8	DF090333:3554516-3554957
BmHel-8	DF090325:4682461-4682902
BmHel-8	DF090364:1327134-1327575
BmHel-8	DF090510:428-869
BmHel-8	DF090490:93191-93632
BmHel-8	DF091440:533-974
BmHel-8	DF090331:1750855-1751296
BmHel-8	DF090336:632648-633090
BmHel-8	DF090336:2610109-2610551
BmHel-8	DF090318:3332481-3332923
BmHel-8	DF090441:148261-148703
BmHel-8	DF090357:2841348-2841790
BmHel-8	DF090329:2996489-2996931
BmHel-8	DF090317:1740021-1740463
BmHel-8	DF090316:12959996-12960438
BmHel-8	DF090328:548982-549424
BmHel-8	DF090325:6057759-6058201
BmHel-8	DF090463:6137-6579
BmHel-8	BABH01045899:615-1057
BmHel-8	DF090379:351997-352439
BmHel-8	DF090981:8606-9048
BmHel-8	DF090377:24603-25045
BmHel-8	DF090350:3962369-3962811
BmHel-8	DF090318:8606717-8607159
BmHel-8	DF090359:2166072-2166514
BmHel-8	DF090322:6640605-6641047
BmHel-8	DF090322:1774148-1774590
BmHel-8	DF090349:701775-702217
BmHel-8	DF090381:85151-85593
BmHel-8	DF090389:514903-515345
BmHel-8	DF090451:13997-14439
BmHel-8	BABH01048325:529-971
BmHel-8	DF090330:5338473-5338916
BmHel-8	DF090351:3974773-3975216
BmHel-8	DF090353:3115742-3116185
BmHel-8	DF090342:2110183-2110626
BmHel-8	DF090321:3374233-3374676
BmHel-8	DF090359:1554102-1554545
BmHel-8	DF090316:4527994-4528437
BmHel-8	DF090371:59787-60230
BmHel-8	DF090360:2271694-2272137

BmHel-8	DF090347:1997040-1997483
BmHel-8	DF090403:1271905-1272348
BmHel-8	DF090346:330656-331099
BmHel-8	DF090362:1468919-1469362
BmHel-8	DF090329:777913-778356
BmHel-8	DF090341:1167723-1168166
BmHel-8	DF090354:3539522-3539965
BmHel-8	DF090357:793971-794415
BmHel-8	DF090326:3275153-3275597
BmHel-8	DF090345:2989852-2990296
BmHel-8	DF090328:1406511-1406955
BmHel-8	DF090374:256123-256567
BmHel-8	DF090951:4227-4671
BmHel-8	DF090321:2513936-2514380
BmHel-8	DF090360:2045586-2046030
BmHel-8	DF090343:1228529-1228973
BmHel-8	DF090358:1584272-1584717
BmHel-8	DF090323:5793164-5793609
BmHel-8	DF090365:1512476-1512921
BmHel-8	DF090335:2142801-2143246
BmHel-8	DF090463:342808-343253
BmHel-8	DF091771:1284-1729
BmHel-8	DF090365:1430610-1431055
BmHel-8	DF090364:94021-94466
BmHel-8	DF090406:369785-370230
BmHel-8	DF090551:20874-21319
BmHel-8	DF090358:1323585-1324031
BmHel-8	DF090367:245534-245980
BmHel-8	DF090344:2275153-2275599
BmHel-8	DF090340:1258913-1259359
BmHel-8	DF090322:6315715-6316161
BmHel-8	DF090322:8102483-8102929
BmHel-8	DF090331:5420888-5421334
BmHel-8	DF090320:3472529-3472975
BmHel-8	DF090321:332400-332846
BmHel-8	DF090338:1090350-1090796
BmHel-8	DF090317:8397475-8397921
BmHel-8	DF090316:14165833-14166279
BmHel-8	DF090384:1843330-1843776
BmHel-8	DF090368:380521-380967
BmHel-8	DF090352:943806-944253
BmHel-8	DF090316:2926815-2927262
BmHel-8	DF090548:37414-37861
BmHel-8	DF090498:15688-16135
BmHel-8	DF090356:1949686-1950133
BmHel-8	DF090329:2772481-2772928
BmHel-8	DF090316:12625427-12625874
BmHel-8	DF090316:4580913-4581360

BmHel-8	DF090347:2572337-2572784
BmHel-8	DF090350:1160578-1161026
BmHel-8	DF090342:1795006-1795454
BmHel-8	DF090326:1893874-1894322
BmHel-8	DF090439:261019-261467
BmHel-8	DF090347:1215179-1215627
BmHel-8	DF090414:88320-88768
BmHel-8	DF090351:3408646-3409094
BmHel-8	DF090363:2009780-2010228
BmHel-8	DF090345:2729646-2730094
BmHel-8	DF090337:4842932-4843380
BmHel-8	DF090339:4231376-4231824
BmHel-8	DF090385:3533-3981
BmHel-8	DF090427:285357-285805
BmHel-8	DF090364:1224303-1224751
BmHel-8	DF090398:1368575-1369023
BmHel-8	DF090525:15585-16033
BmHel-8	DF090330:825530-825979
BmHel-8	DF090346:3972509-3972958
BmHel-8	DF090367:1148091-1148540
BmHel-8	DF090318:5060374-5060823
BmHel-8	DF090382:675343-675792
BmHel-8	DF090374:1017125-1017574
BmHel-8	DF090368:2503046-2503495
BmHel-8	DF090552:56816-57265
BmHel-8	DF090320:1854835-1855284
BmHel-8	DF090397:1206416-1206865
BmHel-8	DF090358:1808320-1808769
BmHel-8	DF090320:5945236-5945685
BmHel-8	DF090356:2947113-2947562
BmHel-8	DF090416:592113-592562
BmHel-8	DF090326:212090-212540
BmHel-8	DF090401:306609-307059
BmHel-8	DF090335:1791516-1791966
BmHel-8	DF090358:238142-238592
BmHel-8	DF090340:2879179-2879629
BmHel-8	DF090319:8132917-8133367
BmHel-8	DF090326:3707723-3708173
BmHel-8	DF090385:197115-197565
BmHel-8	DF090472:62517-62967
BmHel-8	DF091372:290-740
BmHel-8	DF090363:649290-649741
BmHel-8	DF090355:3641985-3642436
BmHel-8	DF090359:941695-942146
BmHel-8	DF090323:6199090-6199541
BmHel-8	DF090348:4044157-4044608
BmHel-8	DF090449:65693-66144
BmHel-8	DF090358:965767-966219

BmHel-8	DF090317:2632103-2632555
BmHel-8	DF090316:15856190-15856642
BmHel-8	DF090349:769613-770065
BmHel-8	DF090349:2991179-2991631
BmHel-8	DF090343:972517-972969
BmHel-8	DF090353:1518716-1519168
BmHel-8	DF090326:2652031-2652483
BmHel-8	DF090365:2955644-2956096
BmHel-8	DF090331:136347-136799
BmHel-8	DF090357:3213584-3214037
BmHel-8	DF090356:334546-334999
BmHel-8	DF090337:4172025-4172478
BmHel-8	DF090729:16569-17022
BmHel-8	DF090329:3173339-3173792
BmHel-8	DF090316:6776285-6776738
BmHel-8	DF090433:603023-603476
BmHel-8	BABH01044052:5826-6279
BmHel-8	DF090445:316886-317339
BmHel-8	DF090664:3235-3688
BmHel-8	DF090348:1292780-1293234
BmHel-8	DF090347:1408811-1409265
BmHel-8	DF090422:58646-59100
BmHel-8	DF090357:1954061-1954515
BmHel-8	DF090323:5237513-5237967
BmHel-8	DF090326:3675006-3675460
BmHel-8	DF090360:1296809-1297263
BmHel-8	DF090328:5740449-5740903
BmHel-8	DF090405:1097995-1098449
BmHel-8	BABH01044597:3456-3910
BmHel-8	DF090367:2462740-2463195
BmHel-8	DF090342:1066065-1066520
BmHel-8	DF090318:9067427-9067882
BmHel-8	DF090403:889648-890103
BmHel-8	DF090337:1957727-1958182
BmHel-8	DF090316:3148066-3148521
BmHel-8	DF090336:2236185-2236640
BmHel-8	DF090335:1849693-1850148
BmHel-8	DF090378:798849-799304
BmHel-8	DF090413:870854-871310
BmHel-8	DF090383:609987-610443
BmHel-8	DF090388:1284082-1284538
BmHel-8	DF090316:6717460-6717916
BmHel-8	DF090414:1071623-1072079
BmHel-8	DF090324:4754424-4754881
BmHel-8	DF090370:297260-297717
BmHel-8	DF090358:2663691-2664148
BmHel-8	DF090377:1564350-1564807
BmHel-8	DF090328:4680022-4680479

BmHel-8	DF090374:836395-836852
BmHel-8	DF090318:7198681-7199139
BmHel-8	DF090328:158746-159204
BmHel-8	DF090335:4996584-4997042
BmHel-8	DF090339:662353-662811
BmHel-8	DF090352:2562118-2562577
BmHel-8	DF090319:2498794-2499253
BmHel-8	DF090345:2949204-2949663
BmHel-8	DF090341:1569144-1569603
BmHel-8	DF090324:3218809-3219268
BmHel-8	DF090386:319740-320200
BmHel-8	DF090361:1953764-1954224
BmHel-8	DF090415:1052234-1052694
BmHel-8	DF090367:591739-592199
BmHel-8	DF090318:1028821-1029281
BmHel-8	DF090339:2105563-2106024
BmHel-8	DF090376:1031719-1032180
BmHel-8	DF090644:47550-48011
BmHel-8	DF090339:3890096-3890558
BmHel-8	DF090358:2624538-2625001
BmHel-8	DF090359:3122409-3122872
BmHel-8	DF090324:4936243-4936706
BmHel-8	DF090438:98912-99375
BmHel-8	DF090350:3927161-3927624
BmHel-8	DF090412:719261-719725
BmHel-8	DF090321:770170-770634
BmHel-8	DF090446:458351-458815
BmHel-8	DF090318:2704069-2704533
BmHel-8	DF090366:1942506-1942970
BmHel-8	DF090377:1002355-1002820
BmHel-8	DF090516:46880-47346
BmHel-8	DF090357:2863683-2864150
BmHel-8	DF090362:2234824-2235291
BmHel-8	DF090379:1327739-1328206
BmHel-8	DF090330:3562355-3562823
BmHel-8	DF090384:1462532-1463000
BmHel-8	DF090339:2286589-2287058
BmHel-8	DF090330:4700603-4701072
BmHel-8	DF090486:65542-66011
BmHel-8	DF090357:132146-132616
BmHel-8	DF090317:2010136-2010606
BmHel-8	DF090347:1791916-1792386
BmHel-8	DF090406:1355471-1355941
BmHel-8	DF090324:5812350-5812820
BmHel-8	DF090391:990861-991332
BmHel-8	DF090317:1279025-1279496
BmHel-8	DF090927:4724-5195
BmHel-8	DF090349:3642586-3643058

BmHel-8	DF090982:10340-10812
BmHel-8	DF090458:163628-164100
BmHel-8	DF090328:2695827-2696300
BmHel-8	DF090358:2403744-2404217
BmHel-8	DF090349:3662542-3663015
BmHel-8	DF090380:112619-113093
BmHel-8	DF090320:2096216-2096691
BmHel-8	DF090346:868322-868798
BmHel-8	DF090358:3088153-3088630
BmHel-8	DF090340:4593998-4594475
BmHel-8	DF090389:727850-728329
BmHel-8	DF090319:4776881-4777360
BmHel-8	DF090316:12146763-12147244
BmHel-8	DF090401:1398129-1398610
BmHel-8	DF090337:3621269-3621750
BmHel-8	DF090329:4999357-4999838
BmHel-8	DF090342:969506-969988
BmHel-8	DF090365:2333219-2333701
BmHel-8	DF090384:1962199-1962681
BmHel-8	DF090319:8744942-8745424
BmHel-8	DF090377:1016621-1017105
BmHel-8	DF090360:2664194-2664678
BmHel-8	DF090331:3708160-3708646
BmHel-8	DF090342:338984-339470
BmHel-8	DF090425:489293-489779
BmHel-8	DF090392:1536437-1536924
BmHel-8	DF090384:612187-612674
BmHel-8	DF090378:374491-374979
BmHel-8	BABH01061534:17-505
BmHel-8	DF090323:4103291-4103780
BmHel-8	DF090357:3361971-3362460
BmHel-8	DF090316:8873171-8873661
BmHel-8	DF090413:680296-680787
BmHel-8	DF090335:1186748-1187240
BmHel-8	DF090316:2809803-2810296
BmHel-8	DF090355:326785-327279
BmHel-8	DF090342:155496-155991
BmHel-8	DF090429:517818-518313
BmHel-8	DF090413:3367-3864
BmHel-8	DF090335:4826004-4826503
BmHel-8	DF090365:993804-994306
BmHel-8	DF090344:3288309-3288812
BmHel-8	DF090337:175935-176438
BmHel-8	DF090372:598943-599447
BmHel-8	DF091600:1619-2124
BmHel-8	DF090384:61196-61703
BmHel-8	DF090339:3922341-3922848
BmHel-8	DF090458:362926-363435

BmHel-8	DF090374:1139226-1139736
BmHel-8	BABH01046834:1492-2003
BmHel-8	DF090824:896-1407
BmHel-8	DF090438:41805-42316
BmHel-8	DF090322:381641-382153
BmHel-8	DF090328:1865164-1865676
BmHel-8	DF090319:6991529-6992042
BmHel-8	DF090370:1159223-1159737
BmHel-8	DF090387:1984075-1984589
BmHel-8	DF090344:2385034-2385548
BmHel-8	DF090344:109691-110206
BmHel-8	DF090346:3934044-3934563
BmHel-8	DF090366:1329473-1329993
BmHel-8	DF090338:3485635-3486156
BmHel-8	DF090368:2049560-2050082
BmHel-8	DF090344:251943-252465
BmHel-8	DF090322:3575649-3576175
BmHel-8	DF090336:2730665-2731193
BmHel-8	DF090320:3162839-3163367
BmHel-8	DF090391:576257-576786
BmHel-8	DF090359:2656386-2656916
BmHel-8	DF090340:2840463-2840993
BmHel-8	DF090432:815514-816045
BmHel-8	DF090334:926371-926905
BmHel-8	DF090467:246392-246926
BmHel-8	DF091030:4519-5053
BmHel-8	DF090441:670658-671193
BmHel-8	DF090422:152412-152948
BmHel-8	DF090393:908587-909124
BmHel-8	DF090357:317176-317713
BmHel-8	DF090322:7561457-7561995
BmHel-8	DF090379:547138-547676
BmHel-8	DF090344:1477375-1477914
BmHel-8	DF090316:15051377-15051917
BmHel-8	DF090336:2620713-2621253
BmHel-8	DF090325:6648175-6648716
BmHel-8	DF090430:3651-4193
BmHel-8	DF090369:1423832-1424374
BmHel-8	DF090364:37805-38347
BmHel-8	DF090356:3765513-3766056
BmHel-8	DF090317:9885015-9885558
BmHel-8	DF090316:4088184-4088727
BmHel-8	DF090399:1261543-1262088
BmHel-8	DF090318:8028245-8028791
BmHel-8	DF090388:228138-228686
BmHel-8	DF090388:238891-239440
BmHel-8	DF090367:99144-99696
BmHel-8	DF090326:6285139-6285692

BmHel-8	DF090528:69056-69614
BmHel-8	DF090319:3834870-3835432
BmHel-8	DF090388:1335395-1335959
BmHel-8	DF090425:601071-601637
BmHel-8	DF090354:1632302-1632868
BmHel-8	DF090316:6076701-6077269
BmHel-8	DF090339:1467440-1468010
BmHel-8	DF090321:2751539-2752110
BmHel-8	DF090346:3901700-3902273
BmHel-8	DF090371:2262337-2262910
BmHel-8	DF090333:5770902-5771479
BmHel-8	DF090352:3024613-3025191
BmHel-8	DF090368:359315-359897
BmHel-8	DF090342:4607192-4607775
BmHel-8	DF090791:16504-17091
BmHel-8	DF090377:526743-527333
BmHel-8	DF090330:3149988-3150584
BmHel-8	DF090402:585881-586484
BmHel-8	DF090393:1563930-1564536
BmHel-8	DF090548:2712-3318
BmHel-8	DF090323:1937835-1938441
BmHel-8	DF090331:5299867-5300482
BmHel-8	DF090481:211800-212415
BmHel-8	DF090316:12997004-12997619
BmHel-8	DF090326:3425223-3425841
BmHel-8	DF090344:2391404-2392023
BmHel-8	DF090325:7029621-7030240
BmHel-8	DF090366:2673700-2674321
BmHel-8	DF090372:1456021-1456642
BmHel-8	DF090358:3198181-3198803
BmHel-8	DF090414:1141930-1142552
BmHel-8	DF090374:1162132-1162754
BmHel-8	DF090316:2259278-2259901
BmHel-8	DF090342:3571372-3571995
BmHel-8	DF090344:1427967-1428592
BmHel-8	DF091173:4766-5391
BmHel-8	DF090348:3148885-3149511
BmHel-8	DF090318:2089922-2090549
BmHel-8	DF090340:4786466-4787093
BmHel-8	DF090323:6966764-6967391
BmHel-8	DF090366:1373176-1373803
BmHel-8	DF090339:1566237-1566865
BmHel-8	DF090333:177794-178422
BmHel-8	DF090388:290632-291261
BmHel-8	DF090424:882322-882951
BmHel-8	DF090387:1848132-1848762
BmHel-8	DF090418:30233-30863
BmHel-8	DF090321:4157649-4158280

BmHel-8	DF090386:178489-179120
BmHel-8	DF090352:442212-442843
BmHel-8	DF090332:4014727-4015359
BmHel-8	DF090411:772058-772690
BmHel-8	DF090324:4972022-4972654
BmHel-8	DF090388:659586-660219
BmHel-8	DF090436:106109-106742
BmHel-8	DF090357:337507-338140
BmHel-8	DF090324:6294488-6295122
BmHel-8	DF090331:3504027-3504662
BmHel-8	DF090327:3533149-3533784
BmHel-8	DF090397:1096332-1096967
BmHel-8	DF090335:5652335-5652971
BmHel-8	DF090613:70988-71624
BmHel-8	DF090332:6372357-6372994
BmHel-8	DF090357:3082279-3082916
BmHel-8	DF090329:2299120-2299757
BmHel-8	DF090316:7155065-7155702
BmHel-8	DF090337:4595356-4595993
BmHel-8	DF090418:692252-692889
BmHel-8	DF090362:1107944-1108581
BmHel-8	DF090324:5086994-5087631
BmHel-8	DF090359:1849423-1850061
BmHel-8	DF090405:8757-9395
BmHel-8	DF090344:4511676-4512315
BmHel-8	DF090317:2868014-2868653
BmHel-8	BABH01048455:210-849
BmHel-8	DF090364:2905823-2906463
BmHel-8	DF090394:1263130-1263771
BmHel-8	DF090349:24854-25496
BmHel-8	DF090376:1418943-1419586
BmHel-8	DF090343:2977872-2978516
BmHel-8	DF090319:8772570-8773218
BmHel-8	DF090466:24635-25284
BmHel-8	DF090347:3481123-3481772
BmHel-8	DF090319:181609-182261
BmHel-8	DF090336:1942439-1943093
BmHel-8	DF090627:35010-35665
BmHel-8	DF090335:5376589-5377246
BmHel-8	DF090373:2004658-2005315
BmHel-8	DF090368:304162-304819
BmHel-8	DF090377:1336835-1337495
BmHel-8	DF090321:7379015-7379676
BmHel-8	DF090390:299766-300430
BmHel-8	DF090401:1531342-1532007
BmHel-8	DF090381:1643757-1644422
BmHel-8	DF090342:4218674-4219340
BmHel-8	DF090344:2107309-2107976

BmHel-8	DF090440:379977-380644
BmHel-8	DF090330:1438687-1439355
BmHel-8	BABH01044927:473-1142
BmHel-8	DF090324:5193257-5193926
BmHel-8	DF090334:4069717-4070387
BmHel-8	DF090367:2517330-2518001
BmHel-8	DF090373:1315548-1316219
BmHel-8	DF090371:1141936-1142608
BmHel-8	DF090350:303398-304071
BmHel-8	DF090350:1286187-1286861
BmHel-8	DF090329:6124102-6124777
BmHel-8	DF090327:6354475-6355151
BmHel-8	DF090317:2402881-2403557
BmHel-8	DF090541:31511-32187
BmHel-8	DF090349:2267636-2268315
BmHel-8	DF090343:3802810-3803491
BmHel-8	DF090316:5175171-5175854
BmHel-8	DF090320:2728705-2729388
BmHel-8	DF090417:403540-404224
BmHel-8	DF090382:596914-597598
BmHel-8	DF091740:37445-38130
BmHel-8	DF090337:1624385-1625072
BmHel-8	DF090378:347334-348021
BmHel-8	DF090326:4388780-4389467
BmHel-8	DF090544:58125-58813
BmHel-8	DF090342:4652863-4653552
BmHel-8	DF090351:2685044-2685737
BmHel-8	DF090324:4397293-4397989
BmHel-8	DF090319:4937937-4938633
BmHel-8	DF090331:719650-720348
BmHel-8	DF090354:1389397-1390095
BmHel-8	DF090351:3413680-3414379
BmHel-8	DF090317:4111074-4111773
BmHel-8	DF090411:545647-546346
BmHel-8	DF090322:3230137-3230837
BmHel-8	DF090396:1582023-1582724
BmHel-8	DF090371:1499769-1500470
BmHel-8	DF090404:305133-305838
BmHel-8	DF090354:904647-905353
BmHel-8	DF090344:2364471-2365178
BmHel-8	DF090344:1331490-1332199
BmHel-8	DF090317:2822632-2823342
BmHel-8	DF090548:5024-5734
BmHel-8	DF090322:7596542-7597253
BmHel-8	DF090429:717885-718596
BmHel-8	DF090462:171387-172099
BmHel-8	DF090475:237083-237796
BmHel-8	DF090344:888770-889483

BmHel-8	DF090343:285109-285823
BmHel-8	DF090323:3082760-3083476
BmHel-8	DF090409:659780-660502
BmHel-8	DF090324:2841263-2841987
BmHel-8	DF090330:5027385-5028110
BmHel-8	DF090332:1195624-1196350
BmHel-8	DF090336:4739235-4739962
BmHel-8	DF090389:1037964-1038691
BmHel-8	DF090320:7126754-7127485
BmHel-8	DF090403:242390-243121
BmHel-8	DF090318:8803468-8804199
BmHel-8	DF090340:1419786-1420519
BmHel-8	DF090849:2906-3639
BmHel-8	DF090318:6603239-6603974
BmHel-8	DF090349:594582-595317
BmHel-8	DF090325:1045880-1046616
BmHel-8	DF090319:7583768-7584505
BmHel-8	DF090369:2276888-2277625
BmHel-8	DF090328:2243009-2243747
BmHel-8	DF090422:973353-974091
BmHel-8	DF090321:4204784-4205525
BmHel-8	DF090391:906368-907113
BmHel-8	DF090420:142716-143462
BmHel-8	DF090322:6548130-6548879
BmHel-8	DF090414:1361598-1362351
BmHel-8	DF090345:2553821-2554574
BmHel-8	DF090341:259907-260661
BmHel-8	DF090345:3515837-3516592
BmHel-8	DF090338:4300200-4300956
BmHel-8	DF090322:3367383-3368144
BmHel-8	DF090316:5151719-5152481
BmHel-8	DF090349:3039623-3040386
BmHel-8	DF090326:6305701-6306472
BmHel-8	DF090396:360402-361182
BmHel-8	DF090317:10589758-10590538
BmHel-8	DF090362:1698645-1699426
BmHel-8	DF090362:2632444-2633226
BmHel-8	DF090372:1799369-1800151
BmHel-8	DF090354:3180234-3181017
BmHel-8	DF090325:5182051-5182844
BmHel-8	DF090445:43976-44771
BmHel-8	DF090388:1039203-1039999
BmHel-8	DF090355:3527622-3528419
BmHel-8	DF090333:335741-336539
BmHel-8	DF090358:465758-466559
BmHel-8	DF090359:3233765-3234566
BmHel-8	DF090639:17649-18453
BmHel-8	DF090395:1332576-1333384

BmHel-8	DF090360:625715-626527
BmHel-8	DF090368:1954415-1955229
BmHel-8	DF090355:955278-956093
BmHel-8	DF090337:3858983-3859802
BmHel-8	DF090352:711619-712440
BmHel-8	DF090330:1913272-1914094
BmHel-8	DF090362:1765898-1766720
BmHel-8	DF090323:3970027-3970851
BmHel-8	DF090349:1839194-1840022
BmHel-8	DF090392:45463-46295
BmHel-8	DF090471:14334-15166
BmHel-8	DF090322:2969168-2970007
BmHel-8	DF090356:1668989-1669828
BmHel-8	DF090929:9880-10722
BmHel-8	DF090320:689350-690195
BmHel-8	DF090341:3510240-3511088
BmHel-8	DF090323:5706353-5707204
BmHel-8	DF090382:449802-450654
BmHel-8	DF090342:3233534-3234386
BmHel-8	DF090404:1147051-1147908
BmHel-8	DF090346:2356439-2357297
BmHel-8	DF090352:3635185-3636044
BmHel-8	DF090499:4937-5796
BmHel-8	DF090400:724132-724992
BmHel-8	DF090364:2926263-2927123
BmHel-8	DF090370:1364404-1365265
BmHel-8	DF090348:1215803-1216664
BmHel-8	DF090361:534967-535830
BmHel-8	DF091278:2422-3286
BmHel-8	DF090388:619810-620676
BmHel-8	DF090317:10653963-10654829
BmHel-8	DF090342:2983760-2984630
BmHel-8	DF090346:2372468-2373338
BmHel-8	DF090334:2801244-2802114
BmHel-8	DF090345:2101464-2102335
BmHel-8	DF090330:39789-40660
BmHel-8	DF090362:47422-48293
BmHel-8	DF090358:1105233-1106105
BmHel-8	DF090353:1525397-1526269
BmHel-8	DF090355:903268-904141
BmHel-8	DF090317:10229584-10230462
BmHel-8	DF090332:6043175-6044056
BmHel-8	DF090338:1349811-1350695
BmHel-8	DF090343:3988048-3988938
BmHel-8	DF090342:2143909-2144799
BmHel-8	DF090328:2098258-2099155
BmHel-8	DF090331:4674092-4674997
BmHel-8	DF090350:2139785-2140691

BmHel-8	DF090475:140711-141621
BmHel-8	DF090346:3558727-3559640
BmHel-8	DF090354:769926-770841
BmHel-8	DF090365:1227508-1228427
BmHel-8	DF090398:286574-287493
BmHel-8	DF090320:1434706-1435627
BmHel-8	DF090364:1909484-1910411
BmHel-8	DF090354:1662291-1663218
BmHel-8	DF090321:476735-477668
BmHel-8	DF090327:4145937-4146870
BmHel-8	DF090381:1793122-1794058
BmHel-8	DF090342:2549858-2550795
BmHel-8	DF090353:1216572-1217515
BmHel-8	DF090340:769802-770747
BmHel-8	DF090374:1498484-1499429
BmHel-8	DF090334:4841711-4842657
BmHel-8	DF090327:5354635-5355589
BmHel-8	DF090369:1965300-1966256
BmHel-8	DF090358:2178260-2179220
BmHel-8	DF090439:555467-556428
BmHel-8	DF090420:700887-701853
BmHel-8	DF090339:345908-346885
BmHel-8	DF090542:10328-11306
BmHel-8	DF090331:4072649-4073635
BmHel-8	DF090556:5652-6643
BmHel-8	DF090425:953495-954490
BmHel-8	DF090316:15209906-15210905
BmHel-8	DF090316:8380530-8381531
BmHel-8	DF090348:1177679-1178682
BmHel-8	DF090350:1365393-1366403
BmHel-8	DF090335:1312930-1313941
BmHel-8	DF090350:3886975-3887988
BmHel-8	DF090399:701758-702782
BmHel-8	DF090413:221905-222931
BmHel-8	DF090388:226928-227956
BmHel-8	DF090328:4710284-4711325
BmHel-8	DF090339:1227385-1228431
BmHel-8	DF090524:15416-16467
BmHel-8	DF090356:2662684-2663750
BmHel-8	DF090391:1029686-1030758
BmHel-8	DF090363:2074718-2075804
BmHel-8	DF090565:109921-111047
BmHel-8	DF090331:3146704-3147834
BmHel-8	DF090580:7441-8577
BmHel-8	DF090473:10603-11741
BmHel-8	DF090397:12006-13159
BmHel-8	DF090352:1151593-1152758
BmHel-8	DF090329:6673656-6674824

BmHel-8	DF090383:1260223-1261406
BmHel-8	DF090324:7884908-7886091
BmHel-8	DF090365:163468-164654
BmHel-8	DF091000:6336-7525
BmHel-8	DF090323:465961-467152
BmHel-8	DF090370:2147615-2148816
BmHel-8	DF090547:26335-27553
BmHel-8	DF090347:480418-481639
BmHel-8	DF090547:70519-71763
BmHel-8	DF090351:471500-472752
BmHel-8	DF090321:3652705-3653996
BmHel-8	DF090374:1634577-1635870
BmHel-8	DF090316:4059402-4060719
BmHel-8	DF090327:2391344-2392662
BmHel-8	DF091557:952-2275
BmHel-8	DF090358:3078096-3079429
BmHel-8	DF090413:482630-483965
BmHel-8	DF090322:2506288-2507628
BmHel-8	DF090327:872260-873601
BmHel-8	DF090323:4437511-4438853
BmHel-8	DF090340:114819-116165
BmHel-8	DF090316:14364927-14366281
BmHel-8	DF090331:3857842-3859218
BmHel-8	DF090338:2023564-2024943
BmHel-8	DF090336:2945327-2946729
BmHel-8	DF090358:274273-275677
BmHel-8	DF090351:2748128-2749533
BmHel-8	DF090383:1334172-1335587
BmHel-8	DF090363:1206396-1207829
BmHel-8	DF090347:1170331-1171771
BmHel-8	DF090461:137282-138743
BmHel-8	DF090352:3031046-3032510
BmHel-8	DF090319:1280008-1281477
BmHel-8	DF090325:3481709-3483179
BmHel-8	DF090342:169484-170957
BmHel-8	DF090425:992717-994226
BmHel-8	DF090353:2617613-2619134
BmHel-8	DF090357:2120410-2121945
BmHel-8	DF090330:2054147-2055696
BmHel-8	DF090344:3644130-3645681
BmHel-8	DF090386:429106-430660
BmHel-8	DF090377:1943661-1945223
BmHel-8	DF090342:526439-528001
BmHel-8	DF091430:489-2068
BmHel-8	DF090321:1280630-1282211
BmHel-8	DF090334:2305643-2307227
BmHel-8	DF090327:4316874-4318459
BmHel-8	DF090333:797241-798830

BmHel-8	DF090346:2705292-2706901
BmHel-8	DF090321:1906414-1908030
BmHel-8	DF090321:242700-244323
BmHel-8	DF090367:2294028-2295652
BmHel-8	DF090367:1547944-1549574
BmHel-8	DF090354:1433174-1434807
BmHel-8	DF090414:1031352-1032991
BmHel-8	DF090372:1958013-1959671
BmHel-8	DF090414:530758-532436
BmHel-8	DF090337:3755462-3757151
BmHel-8	DF090398:303249-304942
BmHel-8	DF090346:475433-477129
BmHel-8	DF090362:1526446-1528154
BmHel-8	DF090324:5515902-5517612
BmHel-8	DF091102:1059-2770
BmHel-8	DF090390:783965-785679
BmHel-8	DF090402:795290-797011
BmHel-8	DF090320:1559838-1561573
BmHel-8	DF090399:1154973-1156712
BmHel-8	DF090433:131834-133575
BmHel-8	DF090318:2246648-2248402
BmHel-8	DF090344:2927731-2929485
BmHel-8	DF090391:842142-843905
BmHel-8	DF090384:178921-180690
BmHel-8	DF090373:957779-959549
BmHel-8	DF090351:4027186-4028965
BmHel-8	DF090334:3028676-3030472
BmHel-8	DF090325:4342532-4344332
BmHel-8	DF090366:1015642-1017448
BmHel-8	DF090412:847309-849134
BmHel-8	DF090372:1983752-1985581
BmHel-8	DF090325:4461195-4463037
BmHel-8	DF090337:2091829-2093693
BmHel-8	DF090397:260047-261912
BmHel-8	DF090354:285563-287442
BmHel-8	DF090422:406824-408710
BmHel-8	DF090428:430251-432163
BmHel-8	DF090364:1060164-1062081
BmHel-8	DF090340:2226452-2228369
BmHel-8	DF090343:126803-128721
BmHel-8	DF090337:4087281-4089203
BmHel-8	DF090335:2482387-2484313
BmHel-8	DF090418:822902-824829
BmHel-8	DF090321:4563076-4565012
BmHel-8	DF090404:281915-283853
BmHel-8	DF090663:4646-6588
BmHel-8	DF090836:14839-16785
BmHel-8	DF090336:3381600-3383558

BmHel-8	DF090340:287857-289824
BmHel-8	DF090342:754393-756365
BmHel-8	DF090339:1137860-1139837
BmHel-8	DF090807:9336-11320
BmHel-8	DF090322:2131916-2133903
BmHel-8	DF090340:2720199-2722211
BmHel-8	DF090328:1990816-1992849
BmHel-8	DF090333:2476556-2478613
BmHel-8	DF090316:2503678-2505748
BmHel-8	DF090319:3898659-3900745
BmHel-8	DF090347:3415654-3417756
BmHel-8	DF090353:734916-737074
BmHel-8	DF090411:1104574-1106740
BmHel-8	DF090341:3248429-3250601
BmHel-8	DF090573:29094-31275
BmHel-8	DF090325:3437133-3439323
BmHel-8	DF090318:2964319-2966510
BmHel-8	DF090379:859829-862032
BmHel-8	DF090317:8638454-8640659
BmHel-8	DF090321:6751087-6753302
BmHel-8	DF090316:5389119-5391420
BmHel-8	DF090326:6027414-6029720
BmHel-8	DF090396:1564548-1566866
BmHel-8	DF090316:9843089-9845415
BmHel-8	DF090403:4271-6631
BmHel-8	DF090383:1042182-1044545
BmHel-8	DF090361:3060207-3062594
BmHel-8	DF090339:2529111-2531501
BmHel-8	DF090388:434650-437077
BmHel-8	DF090318:12796-15235
BmHel-8	DF090316:2346549-2348992
BmHel-8	DF090335:877807-880270
BmHel-8	DF090350:3382699-3385169
BmHel-8	DF090382:2067930-2070438
BmHel-8	DF090399:1224385-1226894
BmHel-8	DF090413:647341-649855
BmHel-8	DF090377:487393-490043
BmHel-8	DF090341:4616941-4619657
BmHel-8	DF090322:6227230-6229980
BmHel-8	DF090473:191183-193970
BmHel-8	DF090331:2097297-2100165
BmHel-8	DF090321:572229-575131
BmHel-8	DF090387:1861869-1864788
BmHel-8	DF090423:55067-58018
BmHel-8	DF090425:418303-421282
BmHel-8	DF090327:5810915-5813896
BmHel-8	DF090319:5078658-5081688
BmHel-8	DF090318:5357865-5360913

BmHel-8	DF090353:2717141-2720235
BmHel-8	DF090319:5204426-5207562
BmHel-8	DF090452:43703-46845
BmHel-8	DF090316:13934015-13937175
BmHel-8	DF090362:1503803-1507072
BmHel-8	DF090325:3276611-3279918
BmHel-8	DF090319:2048988-2052303
BmHel-8	DF090324:1431358-1434715
BmHel-8	DF090322:5360085-5363455
BmHel-8	DF090344:430493-433870
BmHel-8	DF090402:436635-440030
BmHel-8	DF090350:1600212-1603621
BmHel-8	DF090404:906331-909800
BmHel-8	DF090422:293198-296674
BmHel-8	DF090358:2414899-2418395
BmHel-8	DF090403:1107532-1111107
BmHel-8	DF090348:421944-425542
BmHel-8	DF090317:3893276-3896881
BmHel-8	DF090343:4301065-4304694
BmHel-8	DF090351:4131637-4135291
BmHel-8	DF090319:77325-81012
BmHel-8	DF090335:2046541-2050345
BmHel-8	DF090325:708530-712359
BmHel-8	DF090354:536249-540134
BmHel-8	DF090362:507126-511051
BmHel-8	DF090318:9477609-9481565
BmHel-8	DF090972:5461-9435
BmHel-8	DF090376:1874945-1878933
BmHel-8	DF090449:686485-690520
BmHel-8	DF090365:2649920-2653996
BmHel-8	DF090341:1844329-1848421
BmHel-8	DF090355:1425066-1429217
BmHel-8	DF090365:2858832-2862999
BmHel-8	DF090425:81909-86099
BmHel-8	DF090716:13139-17504
BmHel-8	DF090324:4903966-4908332
BmHel-8	DF090332:5388312-5392678
BmHel-8	DF090322:3328601-3332981
BmHel-8	DF090407:35323-39737
BmHel-8	DF090338:770863-775287
BmHel-8	DF090362:2163882-2168331
BmHel-8	DF090320:6927649-6932185
BmHel-8	DF090382:2010401-2014992
BmHel-8	DF090342:4216664-4221265
BmHel-8	DF090464:333924-338528
BmHel-8	DF090362:886383-891014
BmHel-8	DF090364:529783-534454
BmHel-8	DF090341:2175006-2179683

BmHel-8	DF090326:5129812-5134509
BmHel-8	DF090411:719577-724350
BmHel-8	DF090410:657095-661899
BmHel-8	DF090353:3590200-3595049
BmHel-8	DF090317:6748659-6753512
BmHel-8	DF090331:272537-277452
BmHel-8	DF090411:115459-120386
BmHel-8	DF090316:5606578-5611529
BmHel-8	DF090324:4600685-4605646
BmHel-8	DF090322:4503333-4508316
BmHel-8	DF090349:2602874-2607969
BmHel-8	DF090374:634593-639689
BmHel-8	DF090320:1810796-1815926
BmHel-8	DF090366:177066-182305
BmHel-8	DF090364:2327118-2332401
BmHel-8	DF090360:971566-976851
BmHel-8	DF090354:1273838-1279466
BmHel-8	DF090362:875311-880996
BmHel-8	DF090375:255586-261282
BmHel-8	DF090396:1092167-1097869
BmHel-8	DF090324:6877718-6883578
BmHel-8	DF090371:103949-109860
BmHel-8	DF090318:7469530-7475453
BmHel-8	DF090363:2186554-2192550
BmHel-8	DF090679:4099-10242
BmHel-8	DF090316:4294340-4300602
BmHel-8	DF090357:238448-244737
BmHel-8	DF090411:882729-889062
BmHel-8	DF090372:2055877-2062232
BmHel-8	DF090344:3613047-3619422
BmHel-8	DF090341:1577533-1584040
BmHel-8	DF090320:1604276-1610841
BmHel-8	DF090336:3836817-3843416
BmHel-8	DF090358:1040540-1047283
BmHel-8	DF090341:3812455-3819275
BmHel-8	DF090414:1103910-1110902
BmHel-8	DF090316:13657952-13665014
BmHel-8	DF090436:439866-447065
BmHel-8	DF090340:1679984-1687196
BmHel-8	DF090333:2417657-2424891
BmHel-8	DF090416:564779-572212
BmHel-8	DF090317:2446680-2454265
BmHel-8	DF090413:932063-939710
BmHel-8	DF090342:2000779-2008590
BmHel-8	DF090390:817088-824908
BmHel-8	DF090396:463680-471572
BmHel-8	DF090318:5212361-5220287
BmHel-8	DF090367:628120-636129

BmHel-8	DF090424:535459-543523
BmHel-8	DF090321:4884516-4892629
BmHel-8	DF090386:1470182-1478296
BmHel-8	DF090380:210425-218645
BmHel-8	DF090462:371175-379425
BmHel-8	DF090391:703980-712232
BmHel-8	DF090416:690763-699023
BmHel-8	DF090380:1761839-1770172
BmHel-8	DF090364:1381304-1389664
BmHel-8	DF090317:40252-48780
BmHel-8	DF090415:979563-988440
BmHel-8	DF090325:3515637-3524712
BmHel-8	DF090327:416184-425260
BmHel-8	DF090333:2710729-2719871
BmHel-8	DF090323:4426301-4435530
BmHel-8	DF090374:1447987-1457446
BmHel-8	DF090333:5488038-5497555
BmHel-8	DF090338:654307-663826
BmHel-8	DF090321:4325562-4335145
BmHel-8	DF090375:1445230-1454859
BmHel-8	DF090340:1145783-1155488
BmHel-8	DF090340:2671526-2681284
BmHel-8	DF090342:2836259-2846298
BmHel-8	DF090322:1692177-1702273
BmHel-8	DF090475:181042-191264
BmHel-8	DF090349:3618430-3628671
BmHel-8	DF090316:1985724-1996047
BmHel-8	DF090422:567178-577821
BmHel-8	DF090407:966993-967333
BmHel-8	DF090407:967343-967429
BmHel-8	DF090335:2820987-2821317
BmHel-8	DF090335:2820692-2820783
BmHel-8	DF090338:296319-296660
BmHel-8	BABH01067709:296-637
BmHel-8	DF090351:276817-277153
BmHel-8	DF090351:1092151-1092491
BmHel-8	DF090351:3327286-3327621
BmHel-8	DF090351:3575680-3576020
BmHel-8	DF090346:1318079-1318421
BmHel-8	DF090332:6198291-6198633
BmHel-8	DF090355:2084112-2084454
BmHel-8	DF090355:1052793-1053134
BmHel-8	DF090355:143804-144141
BmHel-8	DF090342:4921271-4921614
BmHel-8	DF090342:1954497-1954839
BmHel-8	DF090336:67939-68279
BmHel-8	DF090336:960079-960420
BmHel-8	BABH01080312:222-562

BmHel-8	BABH01063721:185-525
BmHel-8	DF090443:123978-124319
BmHel-8	DF090400:1525194-1525535
BmHel-8	DF090388:903798-904129
BmHel-8	DF090356:1623796-1624131
BmHel-8	DF090344:1582542-1582885
BmHel-8	DF090340:4445121-4445462
BmHel-8	DF090340:1743391-1743736
BmHel-8	DF090329:5416368-5416703
BmHel-8	DF090327:6341448-6341790
BmHel-8	DF090323:1575211-1575552
BmHel-8	DF090323:3214985-3215326
BmHel-8	DF090322:3947818-3948159
BmHel-8	DF090319:282444-282786
BmHel-8	DF090317:8757930-8758265
BmHel-8	DF090317:3204818-3205154
BmHel-8	DF090317:891199-891543
BmHel-8	DF090316:4325675-4326015
BmHel-8	DF090349:4230710-4231048
BmHel-8	DF090433:637472-637814
BmHel-8	DF090365:492749-493090
BmHel-8	DF090348:1448607-1448957
BmHel-8	DF090391:1291528-1291868
BmHel-8	DF090371:1015624-1015958
BmHel-8	DF090369:2373413-2373757
BmHel-8	DF090361:867346-867685
BmHel-8	DF090345:2600905-2601245
BmHel-8	DF090341:4088338-4088680
BmHel-8	DF090334:1658731-1659068
BmHel-8	DF090331:3801393-3801735
BmHel-8	DF090331:3801737-3801817
BmHel-8	DF090331:3882221-3882555
BmHel-8	DF090328:3343905-3344252
BmHel-8	DF090343:4635739-4636081
BmHel-8	DF090343:4635656-4635737
BmHel-8	DF090343:1904475-1904816
BmHel-8	BABH01086305:78-420
BmHel-8	DF090354:1624962-1625302
BmHel-8	DF090413:805492-805832
BmHel-8	DF090469:221390-221735
BmHel-8	DF090419:557000-557341
BmHel-8	BABH01043566:9175-9516
BmHel-8	DF090548:58310-58646
BmHel-8	DF090358:900368-900707
BmHel-8	DF090412:841918-842258
BmHel-8	DF090352:2505909-2506249
BmHel-8	DF090352:2506251-2506333
BmHel-8	DF090346:1688777-1689116

BmHel-8	DF090332:1596945-1597284
BmHel-8	DF090363:103908-104252
BmHel-8	DF090336:2941036-2941368
BmHel-8	DF090320:6844386-6844725
BmHel-8	DF090327:3947833-3948172
BmHel-8	DF090322:8300318-8300662
BmHel-8	DF090322:1793493-1793832
BmHel-8	DF090319:7352879-7353219
BmHel-8	DF090319:7453004-7453333
BmHel-8	DF090366:253184-253522
BmHel-8	DF090365:382446-382786
BmHel-8	DF090394:1220478-1220818
BmHel-8	DF090371:2463136-2463471
BmHel-8	DF090360:189953-190287
BmHel-8	DF090335:4339726-4340066
BmHel-8	DF090331:2507445-2507783
BmHel-8	DF090331:5395943-5396287
BmHel-8	DF090328:1894392-1894732
BmHel-8	DF090328:1514296-1514635
BmHel-8	DF090324:4716459-4716798
BmHel-8	DF090339:7115-7458
BmHel-8	DF090325:3538641-3538978
BmHel-8	BABH01054871:5-343
BmHel-8	DF090378:669227-669566
BmHel-8	DF090378:1051467-1051803
BmHel-8	DF090445:163396-163736
BmHel-8	DF090417:210937-211279
BmHel-8	DF090384:1512839-1513179
BmHel-8	DF090384:1512755-1512837
BmHel-8	DF090464:162388-162711
BmHel-8	DF090464:162304-162386
BmHel-8	BABH01073884:151-495
BmHel-8	DF090590:51262-51605
BmHel-8	BABH01046692:1327-1661
BmHel-8	BABH01080954:153-496
BmHel-8	DF090344:4615595-4615920
BmHel-8	DF090344:4616133-4616222
BmHel-8	DF090358:2531620-2531962
BmHel-8	DF090325:1638461-1638803
BmHel-8	DF090332:5534524-5534865
BmHel-8	BABH01064589:1-341
BmHel-8	DF090338:4668749-4669080
BmHel-8	DF090330:5787585-5787930
BmHel-8	DF090330:5036079-5036415
BmHel-8	DF090330:2303103-2303450
BmHel-8	DF090358:3182800-3183142
BmHel-8	DF090358:3255445-3255780
BmHel-8	DF090332:3511812-3512153

BmHel-8	DF090355:479836-480178
BmHel-8	DF090318:6947935-6948269
BmHel-8	DF090318:3610751-3611088
BmHel-8	DF090320:6290772-6291117
BmHel-8	DF090388:483102-483441
BmHel-8	DF090357:2475781-2476116
BmHel-8	DF090340:3440062-3440403
BmHel-8	DF090340:4117538-4117880
BmHel-8	DF090340:1254974-1255309
BmHel-8	DF090322:1724770-1725111
BmHel-8	DF090319:1175432-1175773
BmHel-8	DF090317:8366708-8367044
BmHel-8	DF090317:3936849-3937191
BmHel-8	DF090316:2561310-2561656
BmHel-8	DF090366:1022416-1022757
BmHel-8	DF090348:2542511-2542854
BmHel-8	DF090348:264838-265179
BmHel-8	DF090401:1148855-1149192
BmHel-8	DF090396:1221251-1221601
BmHel-8	DF090372:1879836-1880178
BmHel-8	DF090345:2324695-2325037
BmHel-8	DF090345:2324614-2324693
BmHel-8	DF090324:7002188-7002532
BmHel-8	DF090324:7274644-7274972
BmHel-8	BABH01072497:103-445
BmHel-8	BABH01072497:447-526
BmHel-8	DF090343:2277668-2278014
BmHel-8	DF090343:2278016-2278095
BmHel-8	DF090343:1126995-1127330
BmHel-8	DF090339:2312463-2312804
BmHel-8	DF090460:324172-324513
BmHel-8	DF090325:1395881-1396215
BmHel-8	BABH01064093:295-637
BmHel-8	BABH01064093:214-293
BmHel-8	DF090364:866466-866805
BmHel-8	DF090368:1877304-1877637
BmHel-8	DF090616:10628-10965
BmHel-8	DF090455:208932-209268
BmHel-8	DF090377:1546374-1546713
BmHel-8	DF090420:288047-288388
BmHel-8	DF090317:10786647-10786992
BmHel-8	DF090358:2136776-2137114
BmHel-8	DF090386:288347-288686
BmHel-8	DF090386:288688-288768
BmHel-8	DF090386:1726370-1726712
BmHel-8	DF090386:1081795-1082134
BmHel-8	DF090386:850534-850873
BmHel-8	DF090383:1071856-1072199

BmHel-8	DF090351:2075414-2075747
BmHel-8	DF090351:4341827-4342164
BmHel-8	DF090351:1542502-1542835
BmHel-8	DF090332:5744787-5745126
BmHel-8	DF090409:223643-223977
BmHel-8	DF090402:252828-253164
BmHel-8	DF090367:2206373-2206705
BmHel-8	DF090355:1016028-1016365
BmHel-8	DF090353:3420993-3421331
BmHel-8	DF090350:3003033-3003370
BmHel-8	DF090350:3817028-3817368
BmHel-8	DF090342:1055576-1055915
BmHel-8	DF090342:1422401-1422741
BmHel-8	DF090342:3890808-3891140
BmHel-8	DF090342:3891142-3891223
BmHel-8	DF090342:2426937-2427274
BmHel-8	DF090342:1677855-1678194
BmHel-8	DF090342:1139096-1139429
BmHel-8	DF090336:1354261-1354600
BmHel-8	DF090336:5623979-5624319
BmHel-8	DF090336:4980632-4980970
BmHel-8	DF090336:1644926-1645265
BmHel-8	DF090318:6523925-6524259
BmHel-8	DF090320:1046444-1046777
BmHel-8	DF090320:5130702-5131037
BmHel-8	DF090320:1912048-1912386
BmHel-8	BABH01081215:125-463
BmHel-8	DF090443:102450-102789
BmHel-8	DF090441:379940-380278
BmHel-8	DF090400:1368508-1368845
BmHel-8	DF090388:712993-713327
BmHel-8	DF090362:668951-669279
BmHel-8	DF090362:156593-156931
BmHel-8	DF090356:2500706-2501039
BmHel-8	DF090356:2065116-2065449
BmHel-8	DF090356:2065032-2065114
BmHel-8	DF090356:1870036-1870374
BmHel-8	DF090356:1869955-1870034
BmHel-8	DF090344:1289855-1290193
BmHel-8	DF090344:4590336-4590670
BmHel-8	DF090338:2093137-2093467
BmHel-8	DF090338:4667933-4668274
BmHel-8	DF090329:4158790-4159125
BmHel-8	DF090329:4195278-4195616
BmHel-8	DF090329:4195618-4195700
BmHel-8	DF090327:955019-955352
BmHel-8	DF090327:5586418-5586756
BmHel-8	DF090322:5557115-5557452

BmHel-8	DF090322:2860688-2861027
BmHel-8	DF090319:40405-40743
BmHel-8	DF090319:1899431-1899773
BmHel-8	DF090319:7935302-7935640
BmHel-8	DF090319:4833715-4834047
BmHel-8	DF090319:1053945-1054287
BmHel-8	DF090317:1288506-1288844
BmHel-8	DF090317:5234485-5234823
BmHel-8	DF090317:9265565-9265908
BmHel-8	DF090317:9265910-9265992
BmHel-8	DF090317:9827719-9828057
BmHel-8	DF090316:15081117-15081455
BmHel-8	DF090316:9147542-9147880
BmHel-8	DF090316:7281710-7282053
BmHel-8	DF090395:238355-238683
BmHel-8	DF090366:1906477-1906802
BmHel-8	DF090366:2527262-2527599
BmHel-8	DF090366:647343-647577
BmHel-8	DF090366:647186-647290
BmHel-8	DF090366:647102-647184
BmHel-8	DF090349:279624-279961
BmHel-8	DF090349:1908842-1909175
BmHel-8	BABH01064825:59-397
BmHel-8	DF090433:509581-509914
BmHel-8	DF090365:1645751-1646090
BmHel-8	DF090348:2444720-2445058
BmHel-8	DF090411:789430-789768
BmHel-8	DF090401:174491-174828
BmHel-8	DF090401:174410-174489
BmHel-8	DF090372:1940741-1941079
BmHel-8	DF090361:2715946-2716281
BmHel-8	DF090347:3901810-3902152
BmHel-8	DF090347:3265110-3265462
BmHel-8	DF090345:2629787-2630119
BmHel-8	DF090345:4514383-4514721
BmHel-8	DF090335:1578037-1578375
BmHel-8	DF090335:4576679-4577012
BmHel-8	DF090335:1320188-1320532
BmHel-8	DF090334:1715249-1715583
BmHel-8	DF090334:6024706-6025041
BmHel-8	DF090328:4689699-4690035
BmHel-8	DF090328:4690037-4690119
BmHel-8	DF090324:7974443-7974780
BmHel-8	DF090324:1600230-1600563
BmHel-8	DF090697:734-1072
BmHel-8	DF090436:240073-240411
BmHel-8	DF090373:1197912-1198249
BmHel-8	DF090373:1663973-1664312

BmHel-8	DF090343:4615999-4616341
BmHel-8	DF090343:1014437-1014777
BmHel-8	DF090343:211113-211451
BmHel-8	DF090339:1275781-1276119
BmHel-8	DF090339:1979821-1980159
BmHel-8	DF090339:4285221-4285559
BmHel-8	DF090339:4285139-4285219
BmHel-8	DF090339:3059764-3060096
BmHel-8	DF090425:774997-775336
BmHel-8	DF090423:528097-528438
BmHel-8	DF090405:21652-21985
BmHel-8	DF090379:106902-107247
BmHel-8	DF090376:1565302-1565640
BmHel-8	DF090374:496587-496925
BmHel-8	DF090333:1144202-1144541
BmHel-8	DF090418:447232-447565
BmHel-8	DF090325:348566-348908
BmHel-8	DF090378:297048-297386
BmHel-8	DF090445:31724-32060
BmHel-8	DF090389:1462873-1463210
BmHel-8	DF090387:794247-794585
BmHel-8	DF090364:324345-324676
BmHel-8	DF090364:1896338-1896675
BmHel-8	DF090487:121166-121505
BmHel-8	BABH01057596:370-707
BmHel-8	DF090429:889517-889850
BmHel-8	DF090404:437060-437399
BmHel-8	DF090354:1545459-1545807
BmHel-8	DF090354:2041704-2042039
BmHel-8	DF090354:2676624-2676963
BmHel-8	DF090354:2676965-2677047
BmHel-8	DF090438:425264-425598
BmHel-8	DF090415:1094790-1095127
BmHel-8	DF090415:440166-440502
BmHel-8	DF090413:194229-194565
BmHel-8	DF090368:1254191-1254525
BmHel-8	DF090368:1679074-1679413
BmHel-8	DF090481:68372-68706
BmHel-8	DF090435:622669-623000
BmHel-8	DF090739:2170-2509
BmHel-8	DF090671:3268-3601
BmHel-8	DF090393:28868-29203
BmHel-8	DF090792:12869-13202
BmHel-8	DF090440:632983-633326
BmHel-8	DF091149:6787-7123
BmHel-8	DF090521:33429-33764
BmHel-8	DF090960:5148-5490
BmHel-8	DF090762:1856-2194

BmHel-8	DF090512:70419-70747
BmHel-8	DF090340:2864352-2864693
BmHel-8	DF090321:1255205-1255549
BmHel-8	DF090329:6204982-6205325
BmHel-8	DF090407:798901-799234
BmHel-8	DF090316:1353352-1353690
BmHel-8	DF090402:410368-410700
BmHel-8	DF090367:1330311-1330655
BmHel-8	DF090318:7731822-7732158
BmHel-8	DF090359:2575085-2575427
BmHel-8	DF090338:3818513-3818856
BmHel-8	DF090329:3064549-3064888
BmHel-8	DF090327:4300527-4300867
BmHel-8	DF090323:6588437-6588773
BmHel-8	DF090317:2926650-2926992
BmHel-8	DF090365:2115195-2115540
BmHel-8	DF090331:3766888-3767229
BmHel-8	DF090376:1513489-1513830
BmHel-8	DF090472:89862-90197
BmHel-8	DF090533:36245-36586
BmHel-8	DF090383:1054928-1055262
BmHel-8	DF090353:1186698-1187039
BmHel-8	BABH01080918:223-561
BmHel-8	DF090352:1376735-1377076
BmHel-8	DF090336:4231065-4231403
BmHel-8	DF090499:99189-99527
BmHel-8	DF090388:1463035-1463219
BmHel-8	DF090388:1463362-1463507
BmHel-8	DF090344:2248364-2248687
BmHel-8	DF090329:1441992-1442332
BmHel-8	DF090322:2398463-2398800
BmHel-8	DF090411:379995-380329
BmHel-8	DF090391:330571-330909
BmHel-8	DF090333:5339315-5339396
BmHel-8	DF090333:5339517-5339771
BmHel-8	DF090330:6385056-6385389
BmHel-8	DF090370:705610-705941
BmHel-8	DF090358:1705970-1706307
BmHel-8	DF090383:1775799-1776137
BmHel-8	DF090383:1775715-1775797
BmHel-8	DF090352:3577222-3577555
BmHel-8	DF090351:2567066-2567397
BmHel-8	DF090351:2113927-2114263
BmHel-8	DF090332:422566-422904
BmHel-8	DF090409:1183461-1183794
BmHel-8	DF090377:1111522-1111857
BmHel-8	DF090355:1046832-1047165
BmHel-8	DF090355:315316-315657

BmHel-8	DF090353:3205425-3205766
BmHel-8	DF090353:1827452-1827780
BmHel-8	DF090353:658749-659089
BmHel-8	DF090353:658665-658747
BmHel-8	DF090342:507134-507473
BmHel-8	DF090342:1535066-1535404
BmHel-8	DF090342:4823589-4823925
BmHel-8	DF090342:3469198-3469529
BmHel-8	DF090342:2089350-2089688
BmHel-8	DF090336:3354803-3355135
BmHel-8	DF090321:6414045-6414384
BmHel-8	DF090321:6414386-6414467
BmHel-8	DF090318:8546899-8547241
BmHel-8	DF090318:2638801-2639137
BmHel-8	DF090388:848153-848491
BmHel-8	DF090362:413636-413966
BmHel-8	DF090357:3446178-3446514
BmHel-8	DF090356:3665767-3666110
BmHel-8	DF090356:3581545-3581868
BmHel-8	DF090340:1216122-1216459
BmHel-8	DF090329:2953305-2953644
BmHel-8	DF090329:4634541-4634878
BmHel-8	DF090327:2687888-2688226
BmHel-8	DF090323:5094167-5094500
BmHel-8	DF090323:4451310-4451647
BmHel-8	DF090323:4451226-4451308
BmHel-8	DF090323:3033914-3034247
BmHel-8	DF090323:301479-301817
BmHel-8	DF090322:2461937-2462272
BmHel-8	DF090322:5030014-5030344
BmHel-8	DF090322:3528152-3528493
BmHel-8	DF090319:2501791-2502115
BmHel-8	DF090317:2061377-2061715
BmHel-8	DF090317:4018209-4018536
BmHel-8	DF090316:4312424-4312765
BmHel-8	DF090316:4025134-4025470
BmHel-8	DF090349:3952837-3953174
BmHel-8	DF090433:168435-168772
BmHel-8	DF090433:355052-355384
BmHel-8	DF090433:693650-693982
BmHel-8	DF090365:2357624-2357959
BmHel-8	DF090365:2776516-2776857
BmHel-8	DF090348:783809-784136
BmHel-8	DF090394:1003264-1003601
BmHel-8	DF090369:2351708-2352048
BmHel-8	DF090361:1088510-1088847
BmHel-8	DF090345:559835-560173
BmHel-8	DF090334:6083345-6083683

BmHel-8	DF090331:4331108-4331442
BmHel-8	DF090331:4377380-4377713
BmHel-8	DF090328:1089040-1089377
BmHel-8	DF090328:1088956-1089038
BmHel-8	DF090324:3247064-3247396
BmHel-8	DF090390:999316-999658
BmHel-8	DF090373:1575165-1575502
BmHel-8	DF090373:1575504-1575584
BmHel-8	DF090339:391410-391747
BmHel-8	DF090425:626341-626677
BmHel-8	DF090325:6162573-6162915
BmHel-8	DF090364:169348-169686
BmHel-8	BABH01061508:1-339
BmHel-8	DF090354:3572951-3573289
BmHel-8	DF090438:491519-491857
BmHel-8	DF090438:491859-491939
BmHel-8	DF090415:14664-15001
BmHel-8	DF090413:913067-913405
BmHel-8	DF090413:912985-913065
BmHel-8	DF090406:956585-956934
BmHel-8	DF090534:121130-121467
BmHel-8	DF090469:238911-239247
BmHel-8	DF090393:1036743-1037079
BmHel-8	DF090471:60378-60702
BmHel-8	DF090447:227849-228181
BmHel-8	BABH01049439:631-957
BmHel-8	DF090631:22193-22523
BmHel-8	DF090351:2821230-2821566
BmHel-8	DF090332:5543257-5543598
BmHel-8	DF090329:2402629-2402974
BmHel-8	DF090319:5267009-5267351
BmHel-8	DF090416:1102505-1102756
BmHel-8	DF090416:1102796-1102883
BmHel-8	DF090342:253213-253551
BmHel-8	DF090334:5676944-5677281
BmHel-8	DF090365:462332-462662
BmHel-8	DF090335:2550962-2551304
BmHel-8	DF090334:3925834-3926174
BmHel-8	BABH01043095:12561-12896
BmHel-8	DF090320:946487-946821
BmHel-8	DF090329:1422357-1422699
BmHel-8	DF090322:7220257-7220597
BmHel-8	DF090365:1405856-1406189
BmHel-8	DF090345:2174977-2175317
BmHel-8	DF090390:1129949-1130287
BmHel-8	DF090373:1359819-1360159
BmHel-8	DF090373:1732604-1732945
BmHel-8	BABH01079464:75-416

BmHel-8	DF090376:938297-938637
BmHel-8	DF090389:432710-433047
BmHel-8	BABH01087436:3-342
BmHel-8	DF090375:1774149-1774486
BmHel-8	DF090375:1774061-1774144
BmHel-8	DF090414:186714-186899
BmHel-8	DF090414:189526-189683
BmHel-8	DF090414:189685-189767
BmHel-8	DF090808:5323-5661
BmHel-8	DF090319:2339231-2339449
BmHel-8	DF090319:2339481-2339600
BmHel-8	DF090317:8623523-8623859
BmHel-8	DF090397:1439412-1439750
BmHel-8	DF090418:1161868-1162207
BmHel-8	DF090378:1517741-1518077
BmHel-8	DF090479:264946-265284
BmHel-8	DF090372:1843285-1843375
BmHel-8	DF090372:1843580-1843828
BmHel-8	DF090330:806912-807245
BmHel-8	DF090330:3618020-3618356
BmHel-8	DF090370:2135985-2136311
BmHel-8	DF090351:1476721-1477056
BmHel-8	DF090351:3011195-3011532
BmHel-8	DF090332:2109345-2109687
BmHel-8	DF090367:1590169-1590506
BmHel-8	DF090367:2393021-2393366
BmHel-8	DF090363:2463691-2464027
BmHel-8	DF090355:1183965-1184304
BmHel-8	DF090355:1184306-1184388
BmHel-8	DF090342:1434562-1434897
BmHel-8	DF090342:3165042-3165367
BmHel-8	DF090342:3165369-3165451
BmHel-8	DF090342:4782432-4782768
BmHel-8	DF090342:3366363-3366696
BmHel-8	DF090342:3353026-3353361
BmHel-8	DF090336:2319847-2320178
BmHel-8	DF090318:1993254-1993588
BmHel-8	DF090318:7259090-7259426
BmHel-8	DF090318:3091938-3092255
BmHel-8	DF090320:5449274-5449602
BmHel-8	DF090320:4962281-4962613
BmHel-8	DF090441:706905-707245
BmHel-8	DF090403:63344-63675
BmHel-8	DF090362:2814645-2814984
BmHel-8	DF090356:1368831-1369165
BmHel-8	DF090356:1368749-1368829
BmHel-8	DF090340:2989830-2990161
BmHel-8	DF090340:3836362-3836701

BmHel-8	DF090329:4141665-4142001
BmHel-8	DF090323:7346165-7346497
BmHel-8	DF090322:6594506-6594840
BmHel-8	DF090322:3279903-3280240
BmHel-8	DF090319:1843051-1843383
BmHel-8	DF090319:4879089-4879424
BmHel-8	DF090319:1447966-1448302
BmHel-8	DF090317:2801800-2802130
BmHel-8	DF090316:812596-812923
BmHel-8	DF090395:636608-636940
BmHel-8	DF090395:636527-636606
BmHel-8	DF090349:231719-232055
BmHel-8	DF090365:1302791-1303127
BmHel-8	DF090401:1837-2174
BmHel-8	DF090401:2176-2256
BmHel-8	DF090401:1318726-1319065
BmHel-8	DF090397:988516-988852
BmHel-8	DF090396:2858-3191
BmHel-8	DF090382:494973-495301
BmHel-8	DF090369:1863188-1863515
BmHel-8	DF090369:1863104-1863186
BmHel-8	DF090347:1768375-1768711
BmHel-8	DF090345:1006045-1006383
BmHel-8	DF090341:1286886-1287223
BmHel-8	DF090341:1287225-1287305
BmHel-8	DF090335:2612947-2613282
BmHel-8	DF090343:3806551-3806888
BmHel-8	DF090375:173779-174114
BmHel-8	DF090333:2302363-2302701
BmHel-8	BABH01061147:229-564
BmHel-8	DF090325:6358939-6359276
BmHel-8	BABH01077273:203-534
BmHel-8	BABH01071893:138-469
BmHel-8	DF090354:1301605-1301940
BmHel-8	DF090415:920991-921325
BmHel-8	DF090368:723063-723394
BmHel-8	BABH01079635:1-337
BmHel-8	BABH01073386:130-467
BmHel-8	DF090644:57788-58127
BmHel-8	DF090450:461371-461703
BmHel-8	DF090759:4920-5250
BmHel-8	DF090332:6250316-6250657
BmHel-8	DF090388:1110015-1110351
BmHel-8	DF090338:4048859-4049199
BmHel-8	DF090344:2343386-2343725
BmHel-8	DF090466:64808-65145
BmHel-8	DF090402:1079858-1080193
BmHel-8	DF090320:4836920-4837264

BmHel-8	DF090340:4776200-4776532
BmHel-8	DF090317:4880033-4880369
BmHel-8	DF090317:2322350-2322681
BmHel-8	DF090316:6077824-6078166
BmHel-8	DF090316:12721738-12722085
BmHel-8	DF090316:7930099-7930432
BmHel-8	DF090337:4313206-4313545
BmHel-8	DF090339:1915451-1915795
BmHel-8	DF090558:20630-20964
BmHel-8	DF090413:173316-173653
BmHel-8	DF090517:80988-81329
BmHel-8	DF090455:118203-118539
BmHel-8	DF090353:3993521-3993855
BmHel-8	DF090353:3993436-3993515
BmHel-8	DF090325:3477900-3478234
BmHel-8	DF090316:12108858-12109196
BmHel-8	DF090325:3133207-3133545
BmHel-8	DF090325:5809408-5809738
BmHel-8	DF090424:226813-227150
BmHel-8	DF090352:3819530-3819875
BmHel-8	DF090318:8507387-8507719
BmHel-8	DF090359:1567933-1568279
BmHel-8	DF090439:355074-355409
BmHel-8	DF090439:355411-355495
BmHel-8	DF090372:767921-768253
BmHel-8	DF090324:1808885-1809218
BmHel-8	DF090364:1921832-1922157
BmHel-8	DF090429:496950-497289
BmHel-8	DF090398:1546487-1546821
BmHel-8	DF090393:1479949-1480280
BmHel-8	DF090399:1172440-1172784
BmHel-8	DF090332:5568087-5568422
BmHel-8	DF090350:2503060-2503264
BmHel-8	DF090350:2502922-2503048
BmHel-8	DF090319:4079288-4079628
BmHel-8	DF090319:5169306-5169642
BmHel-8	DF090319:5169645-5169726
BmHel-8	DF090382:1903976-1904316
BmHel-8	DF090374:2138330-2138671
BmHel-8	DF090330:4160330-4160666
BmHel-8	DF090330:279895-280231
BmHel-8	DF090358:282127-282462
BmHel-8	DF090358:2872607-2872942
BmHel-8	DF090358:2872944-2873024
BmHel-8	DF090358:2820126-2820462
BmHel-8	DF090437:659415-659751
BmHel-8	DF090352:1868812-1869147
BmHel-8	DF090352:2066882-2067218

BmHel-8	DF090377:542150-542486
BmHel-8	DF090363:2111813-2112145
BmHel-8	DF090363:2845288-2845624
BmHel-8	DF090363:551413-551752
BmHel-8	DF090355:923674-924010
BmHel-8	DF090353:1099028-1099358
BmHel-8	DF090353:3409283-3409618
BmHel-8	DF090353:3028684-3029017
BmHel-8	DF090350:3186163-3186492
BmHel-8	DF090342:542822-543156
BmHel-8	DF090342:1165290-1165626
BmHel-8	DF090342:1985587-1985924
BmHel-8	DF090342:1292676-1293013
BmHel-8	DF090336:2595106-2595446
BmHel-8	DF090321:3953362-3953692
BmHel-8	DF090318:8786478-8786813
BmHel-8	DF090318:9246891-9247220
BmHel-8	DF090320:5971000-5971329
BmHel-8	DF090388:139744-140079
BmHel-8	DF090357:1660114-1660454
BmHel-8	DF090357:1370095-1370426
BmHel-8	DF090344:4189450-4189793
BmHel-8	DF090340:3359743-3360085
BmHel-8	DF090329:2265811-2266141
BmHel-8	DF090329:2545179-2545512
BmHel-8	DF090329:1906660-1906996
BmHel-8	DF090323:2054505-2054837
BmHel-8	DF090323:7533441-7533776
BmHel-8	DF090323:7462045-7462379
BmHel-8	DF090323:353733-354069
BmHel-8	DF090322:864283-864618
BmHel-8	DF090317:1972234-1972568
BmHel-8	DF090317:9866049-9866384
BmHel-8	DF090317:9866386-9866467
BmHel-8	DF090317:2685358-2685688
BmHel-8	DF090316:3304887-3305222
BmHel-8	DF090316:16119375-16119709
BmHel-8	DF090316:15013005-15013340
BmHel-8	DF090316:15012922-15013003
BmHel-8	DF090316:2237994-2238338
BmHel-8	DF090395:1573447-1573777
BmHel-8	DF090366:1756645-1756979
BmHel-8	DF090349:228650-228979
BmHel-8	DF090394:1531553-1531888
BmHel-8	DF090392:830345-830671
BmHel-8	DF090372:839503-839838
BmHel-8	DF090372:1372192-1372526
BmHel-8	DF090361:727369-727705

BmHel-8	DF090360:202903-203238
BmHel-8	DF090360:3086993-3087328
BmHel-8	DF090345:3703052-3703388
BmHel-8	DF090328:1831047-1831381
BmHel-8	DF090328:1830964-1831045
BmHel-8	DF090328:1121193-1121523
BmHel-8	DF090324:1181216-1181552
BmHel-8	DF090494:46688-47023
BmHel-8	DF090449:488993-489325
BmHel-8	DF090373:1986444-1986775
BmHel-8	DF090343:305288-305625
BmHel-8	DF090339:1778997-1779338
BmHel-8	DF090339:3729631-3729961
BmHel-8	DF090376:1280349-1280683
BmHel-8	DF090325:2666669-2667005
BmHel-8	DF090325:3231030-3231369
BmHel-8	DF090431:697512-697847
BmHel-8	DF090387:1658372-1658708
BmHel-8	DF090384:1650428-1650757
BmHel-8	DF090384:379985-380309
BmHel-8	DF090364:1734948-1735283
BmHel-8	DF090429:615715-616044
BmHel-8	DF090354:1931642-1931977
BmHel-8	DF090438:126625-126961
BmHel-8	BABH01047756:1204-1539
BmHel-8	DF090468:313562-313891
BmHel-8	DF090671:20084-20422
BmHel-8	BABH01048343:463-798
BmHel-8	DF090480:137863-138194
BmHel-8	BABH01050212:262-597
BmHel-8	BABH01082154:1-335
BmHel-8	DF091207:5895-6225
BmHel-8	BABH01045619:2395-2731
BmHel-8	DF090383:1347016-1347351
BmHel-8	DF090329:5071248-5071576
BmHel-8	DF090323:944485-944818
BmHel-8	DF090358:1074499-1074833
BmHel-8	DF090356:2751224-2751569
BmHel-8	DF090323:7703525-7703865
BmHel-8	DF090520:35332-35669
BmHel-8	DF090348:1393910-1394007
BmHel-8	DF090348:1394151-1394396
BmHel-8	DF090362:2379149-2379479
BmHel-8	DF090337:2336827-2337167
BmHel-8	DF090355:1584524-1584869
BmHel-8	DF090362:1021983-1022333
BmHel-8	DF090331:5316015-5316350
BmHel-8	DF090378:1825045-1825269

BmHel-8	DF090378:1824784-1824900
BmHel-8	DF090407:613546-613887
BmHel-8	DF090349:2824906-2825242
BmHel-8	DF090371:1333847-1334183
BmHel-8	DF090346:3580407-3580748
BmHel-8	DF090332:4405507-4405841
BmHel-8	DF091051:13475-13816
BmHel-8	DF090409:1599188-1599528
BmHel-8	DF090402:1324295-1324636
BmHel-8	DF090363:2355979-2356320
BmHel-8	DF090318:7572757-7573100
BmHel-8	DF090320:6662997-6663333
BmHel-8	DF090320:1496837-1497182
BmHel-8	DF090400:1018076-1018421
BmHel-8	DF090344:760492-760823
BmHel-8	DF090340:1949907-1950248
BmHel-8	DF090327:4890775-4891114
BmHel-8	DF090323:6575698-6576040
BmHel-8	DF090322:2164670-2165017
BmHel-8	DF090317:11272035-11272371
BmHel-8	DF090348:3998047-3998372
BmHel-8	DF090397:958039-958365
BmHel-8	DF090394:710248-710590
BmHel-8	DF090380:1860452-1860788
BmHel-8	DF090360:1729012-1729349
BmHel-8	DF090341:4210169-4210510
BmHel-8	DF090334:5089087-5089426
BmHel-8	DF090331:6106280-6106619
BmHel-8	DF090390:1098557-1098901
BmHel-8	BABH01043649:14318-14659
BmHel-8	DF090820:14653-14994
BmHel-8	BABH01076411:174-511
BmHel-8	DF090416:1149337-1149668
BmHel-8	DF090387:1963016-1963354
BmHel-8	DF090387:1697325-1697667
BmHel-8	DF090354:3662259-3662604
BmHel-8	DF090435:76698-77032
BmHel-8	DF091065:5211-5548
BmHel-8	DF090341:863431-863764
BmHel-8	DF090341:863341-863422
BmHel-8	DF090383:138546-138884
BmHel-8	DF090332:1231153-1231487
BmHel-8	DF090342:2321992-2322326
BmHel-8	DF090336:2340252-2340586
BmHel-8	DF090336:2340164-2340246
BmHel-8	DF090338:979613-979833
BmHel-8	DF090338:979284-979406
BmHel-8	DF090319:3593414-3593752

BmHel-8	DF090317:3311387-3311719
BmHel-8	DF090317:5701044-5701380
BmHel-8	DF090348:2350890-2351228
BmHel-8	DF090372:1678897-1679231
BmHel-8	DF090361:223239-223574
BmHel-8	DF090347:291286-291621
BmHel-8	DF090347:2029222-2029439
BmHel-8	DF090347:2029083-2029202
BmHel-8	DF090335:1094210-1094548
BmHel-8	DF090449:252146-252420
BmHel-8	DF090386:1560195-1560534
BmHel-8	DF090320:1936409-1936631
BmHel-8	DF090320:1936252-1936356
BmHel-8	DF090319:5155625-5155962
BmHel-8	DF090316:2027660-2027999
BmHel-8	DF090382:932160-932488
BmHel-8	DF090341:4551179-4551518
BmHel-8	DF090334:4045591-4045927
BmHel-8	DF090374:1733739-1734077
BmHel-8	DF090374:1733655-1733734
BmHel-8	DF090367:1619615-1619950
BmHel-8	DF090342:1610548-1610807
BmHel-8	DF090388:754762-755092
BmHel-8	DF090319:6096461-6096802
BmHel-8	DF090365:1338839-1339174
BmHel-8	DF090365:1998474-1998564
BmHel-8	DF090365:1998952-1999191
BmHel-8	DF090411:551218-551552
BmHel-8	DF090360:2225532-2225871
BmHel-8	DF090360:2225449-2225530
BmHel-8	DF090330:623225-623559
BmHel-8	DF090370:1131998-1132332
BmHel-8	DF090412:822555-822885
BmHel-8	DF090352:2825981-2826305
BmHel-8	DF090352:2826307-2826389
BmHel-8	DF090346:1616218-1616552
BmHel-8	DF090346:1616554-1616634
BmHel-8	DF090466:193717-194051
BmHel-8	DF090402:941566-941899
BmHel-8	DF090363:2097620-2097956
BmHel-8	DF090355:1161197-1161531
BmHel-8	DF090353:3809612-3809943
BmHel-8	DF090342:3713994-3714323
BmHel-8	DF090342:1756942-1757277
BmHel-8	DF090320:2918790-2919120
BmHel-8	DF090359:2241038-2241372
BmHel-8	DF090357:2019543-2019879
BmHel-8	DF090344:2065178-2065509

BmHel-8	DF090344:3500892-3501226
BmHel-8	DF090344:3554156-3554484
BmHel-8	DF090344:3842615-3842949
BmHel-8	DF090340:2452913-2453249
BmHel-8	DF090340:798738-799072
BmHel-8	DF090338:2712859-2713193
BmHel-8	DF090327:5982269-5982602
BmHel-8	DF090319:736489-736819
BmHel-8	DF090319:632916-633255
BmHel-8	DF090317:10733720-10734049
BmHel-8	DF090316:13207512-13207841
BmHel-8	DF090366:1242370-1242700
BmHel-8	DF090326:238223-238557
BmHel-8	DF090326:3777713-3778042
BmHel-8	DF090348:3354210-3354542
BmHel-8	DF090380:1798985-1799320
BmHel-8	DF090372:1068555-1068888
BmHel-8	DF090361:1679302-1679635
BmHel-8	DF090361:1679219-1679300
BmHel-8	DF090347:354858-355188
BmHel-8	DF090328:1871270-1871595
BmHel-8	DF090328:1871597-1871678
BmHel-8	DF090328:1845081-1845415
BmHel-8	DF090324:45726-46061
BmHel-8	DF090324:539606-539935
BmHel-8	DF090537:71180-71514
BmHel-8	DF090449:845194-845519
BmHel-8	DF090343:2795280-2795605
BmHel-8	DF090343:2795607-2795689
BmHel-8	DF090462:28026-28359
BmHel-8	DF090460:146313-146647
BmHel-8	DF090375:1845603-1845932
BmHel-8	DF090375:1303420-1303747
BmHel-8	DF090325:1564930-1565264
BmHel-8	DF090623:29521-29855
BmHel-8	DF090479:277252-277585
BmHel-8	DF090424:921869-922203
BmHel-8	DF090354:1561619-1561950
BmHel-8	DF090415:1188952-1189281
BmHel-8	DF090690:18317-18652
BmHel-8	DF090338:3603752-3604071
BmHel-8	DF090316:11833999-11834331
BmHel-8	DF090334:5363289-5363622
BmHel-8	DF090359:3222961-3223300
BmHel-8	DF090329:2405360-2405703
BmHel-8	DF090319:1030743-1031086
BmHel-8	DF090319:6954912-6955006
BmHel-8	DF090319:6955215-6955454

BmHel-8	DF090345:2649234-2649567
BmHel-8	DF090352:718680-719024
BmHel-8	DF090352:1069729-1070067
BmHel-8	DF090377:1496995-1497338
BmHel-8	DF090363:1265061-1265406
BmHel-8	DF090342:2833756-2834097
BmHel-8	DF090342:1779140-1779481
BmHel-8	DF090338:3427875-3428213
BmHel-8	DF090338:4351677-4352018
BmHel-8	DF090329:5765332-5765676
BmHel-8	DF090323:803545-803886
BmHel-8	DF090316:3003155-3003496
BmHel-8	DF090348:4180854-4181188
BmHel-8	DF090348:2999778-3000115
BmHel-8	DF090397:1559914-1560256
BmHel-8	DF090361:1172422-1172758
BmHel-8	DF090345:3477566-3477897
BmHel-8	DF090335:1756690-1757031
BmHel-8	DF090331:3958493-3958834
BmHel-8	DF090339:1561210-1561549
BmHel-8	DF090978:8212-8553
BmHel-8	DF090375:1614353-1614693
BmHel-8	DF090364:2870680-2871015
BmHel-8	DF090408:659290-659630
BmHel-8	DF090428:812737-813078
BmHel-8	DF090892:9726-10067
BmHel-8	DF090362:3101982-3102324
BmHel-8	DF090562:10075-10320
BmHel-8	DF090562:9964-10065
BmHel-8	DF090342:4671680-4672009
BmHel-8	DF090366:100136-100464
BmHel-8	DF090343:3718591-3718894
BmHel-8	DF090622:36442-36593
BmHel-8	DF090622:37039-37231
BmHel-8	DF090344:1270109-1270446
BmHel-8	DF090316:14191715-14192052
BmHel-8	DF090411:488648-488980
BmHel-8	DF090345:1560309-1560499
BmHel-8	DF090345:1558844-1558995
BmHel-8	DF091218:1767-2104
BmHel-8	BABH01048539:817-1145
BmHel-8	DF090362:483225-483560
BmHel-8	DF090316:6272674-6272998
BmHel-8	DF090374:1545887-1546151
BmHel-8	DF090325:2538606-2538940
BmHel-8	DF090325:3861744-3862075
BmHel-8	DF090384:1619185-1619513
BmHel-8	BABH01074355:203-534

BmHel-8	DF090481:159621-159956
BmHel-8	DF090340:4911656-4911989
BmHel-8	DF090329:3452442-3452764
BmHel-8	DF090319:5366535-5366872
BmHel-8	DF090422:367879-368214
BmHel-8	DF090331:5428500-5428830
BmHel-8	DF090375:2063378-2063713
BmHel-8	DF090507:95623-95958
BmHel-8	DF090350:3599431-3599767
BmHel-8	DF090321:3732870-3733200
BmHel-8	DF090317:661572-661905
BmHel-8	DF090396:1111501-1111582
BmHel-8	DF090396:1112017-1112261
BmHel-8	DF090335:3197046-3197386
BmHel-8	DF090330:4367592-4367924
BmHel-8	DF090330:5440135-5440464
BmHel-8	DF090330:5025490-5025818
BmHel-8	DF090370:2702082-2702415
BmHel-8	DF090351:4075866-4076199
BmHel-8	DF090346:102453-102783
BmHel-8	DF090332:2704239-2704572
BmHel-8	DF090377:325690-326025
BmHel-8	DF090363:1293394-1293727
BmHel-8	DF090353:781360-781692
BmHel-8	DF090342:727377-727711
BmHel-8	DF090321:2803790-2804125
BmHel-8	DF090321:2804127-2804207
BmHel-8	DF090318:4744543-4744878
BmHel-8	DF090318:184136-184466
BmHel-8	DF090320:3085384-3085720
BmHel-8	DF090320:6873169-6873501
BmHel-8	DF090320:1514931-1515264
BmHel-8	DF090388:266741-267074
BmHel-8	DF090338:687038-687371
BmHel-8	DF090338:1161034-1161368
BmHel-8	DF090329:1001656-1001977
BmHel-8	DF090327:1932413-1932741
BmHel-8	DF090327:5842382-5842713
BmHel-8	DF090327:3085229-3085560
BmHel-8	DF090322:1037086-1037420
BmHel-8	DF090322:8110685-8111021
BmHel-8	DF090319:2976498-2976831
BmHel-8	DF090319:7224176-7224510
BmHel-8	DF090316:13410995-13411328
BmHel-8	DF090316:3591238-3591570
BmHel-8	DF090316:1893979-1894312
BmHel-8	DF090349:3441490-3441822
BmHel-8	DF090349:3441824-3441903

BmHel-8	DF090349:1977539-1977872
BmHel-8	DF090326:55738-56072
BmHel-8	BABH01075987:175-509
BmHel-8	DF090365:1076248-1076582
BmHel-8	DF090365:2366832-2367164
BmHel-8	DF090397:1069675-1070008
BmHel-8	DF090394:795475-795811
BmHel-8	DF090394:795813-795895
BmHel-8	DF090369:451800-452137
BmHel-8	DF090369:62522-62855
BmHel-8	DF090369:62438-62520
BmHel-8	DF090360:645187-645512
BmHel-8	DF090347:2209155-2209489
BmHel-8	DF090347:2117575-2117906
BmHel-8	DF090345:3751758-3752085
BmHel-8	DF090334:1419941-1420267
BmHel-8	DF090337:4762841-4763178
BmHel-8	DF090343:4037454-4037776
BmHel-8	DF090462:410502-410835
BmHel-8	DF090425:1000529-1000862
BmHel-8	DF090379:1308672-1309006
BmHel-8	DF090374:1595584-1595917
BmHel-8	DF090374:2073326-2073660
BmHel-8	DF090325:7007436-7007764
BmHel-8	DF090482:167019-167352
BmHel-8	DF090451:29124-29458
BmHel-8	DF090354:889856-890190
BmHel-8	DF090408:1124529-1124863
BmHel-8	DF090368:1558392-1558727
BmHel-8	DF090452:15423-15756
BmHel-8	BABH01053840:166-500
BmHel-8	BABH01050470:622-956
BmHel-8	DF090484:42636-42970
BmHel-8	DF090393:1352172-1352505
BmHel-8	DF090393:1262066-1262400
BmHel-8	DF090644:89999-90332
BmHel-8	BABH01086365:124-457
BmHel-8	DF090531:69125-69454
BmHel-8	DF090465:160278-160614
BmHel-8	DF090463:315440-315786
BmHel-8	DF090463:311578-311911
BmHel-8	DF090492:213657-213984
BmHel-8	DF090440:289568-289900
BmHel-8	BABH01043610:13359-13695
BmHel-8	DF090956:4220-4551
BmHel-8	DF090332:5698383-5698718
BmHel-8	DF090343:4603415-4603755
BmHel-8	DF090404:1319267-1319605

BmHel-8	BABH01045443:2152-2492
BmHel-8	DF090348:1259366-1259713
BmHel-8	DF090319:1603864-1604157
BmHel-8	DF090418:1169169-1169514
BmHel-8	DF090328:707826-708161
BmHel-8	DF090324:3960537-3960625
BmHel-8	DF090324:3960069-3960327
BmHel-8	DF090337:1544219-1544550
BmHel-8	DF090317:4640616-4640950
BmHel-8	DF090332:4759762-4760103
BmHel-8	DF090355:2344910-2345252
BmHel-8	DF090350:3234487-3234826
BmHel-8	DF090320:2388808-2389153
BmHel-8	DF090317:1670357-1670689
BmHel-8	DF090317:2390932-2391274
BmHel-8	DF090361:2085744-2086086
BmHel-8	DF090376:1682053-1682398
BmHel-8	DF090448:684283-684622
BmHel-8	DF090358:2028594-2028932
BmHel-8	DF090342:2693749-2694088
BmHel-8	DF090365:2412102-2412405
BmHel-8	DF090394:1322142-1322485
BmHel-8	DF090360:3291573-3291910
BmHel-8	DF090343:116506-116844
BmHel-8	DF090322:6410039-6410157
BmHel-8	DF090322:6410170-6410380
BmHel-8	DF090322:6410402-6410482
BmHel-8	DF090379:1672957-1673283
BmHel-8	DF090378:480136-480475
BmHel-8	DF090353:3834511-3834847
BmHel-8	DF090327:5952735-5953073
BmHel-8	DF090366:1709958-1710290
BmHel-8	DF090381:1190219-1190556
BmHel-8	DF090379:312667-313005
BmHel-8	DF090318:8756020-8756193
BmHel-8	DF090318:8757816-8757974
BmHel-8	DF090349:2160915-2161248
BmHel-8	DF090391:1389024-1389361
BmHel-8	DF090349:4191076-4191407
BmHel-8	DF090394:827912-828241
BmHel-8	DF090345:1048432-1048763
BmHel-8	DF090375:480841-481136
BmHel-8	DF090368:367635-367976
BmHel-8	DF090905:12501-12837
BmHel-8	DF090351:2642990-2643322
BmHel-8	DF090362:564834-565170
BmHel-8	DF090344:4161169-4161507
BmHel-8	DF090344:4161509-4161591

BmHel-8	DF090369:1769699-1770031
BmHel-8	DF090345:3504801-3505132
BmHel-8	DF090384:246272-246603
BmHel-8	DF090384:246186-246270
BmHel-8	DF090464:134283-134614
BmHel-8	DF090464:134616-134697
BmHel-8	BABH01082450:1-334
BmHel-8	DF090340:3583106-3583228
BmHel-8	DF090340:3582887-3583093
BmHel-8	DF090317:714619-714941
BmHel-8	DF090345:4301487-4301700
BmHel-8	DF090345:4301333-4301468
BmHel-8	DF090368:625470-625688
BmHel-8	DF090368:625712-625843
BmHel-8	DF090349:1263211-1263512
BmHel-8	DF090356:1930303-1930644
BmHel-8	DF090386:812774-813115
BmHel-8	DF090449:799135-799478
BmHel-8	DF090320:1666649-1666990
BmHel-8	DF090344:1391524-1391862
BmHel-8	DF090327:2427403-2427730
BmHel-8	DF090317:10162635-10162976
BmHel-8	DF090372:718904-719249
BmHel-8	DF090429:286186-286533
BmHel-8	DF090344:2962837-2963100
BmHel-8	DF090344:2963201-2963289
BmHel-8	DF090381:90535-90815
BmHel-8	DF090355:398692-399030
BmHel-8	DF090342:2033157-2033483
BmHel-8	DF090316:7992746-7993086
BmHel-8	DF090316:6793913-6794255
BmHel-8	DF090349:4055534-4055875
BmHel-8	DF090392:1299497-1299829
BmHel-8	DF090391:792843-793185
BmHel-8	DF090407:243384-243720
BmHel-8	DF090355:3263212-3263549
BmHel-8	DF090320:2022272-2022490
BmHel-8	DF090320:2021965-2022092
BmHel-8	DF090322:7711253-7711591
BmHel-8	DF090331:2496473-2496814
BmHel-8	DF090333:3640149-3640479
BmHel-8	DF090352:1864712-1865043
BmHel-8	DF090350:3479824-3480154
BmHel-8	DF090320:4604603-4604937
BmHel-8	DF090345:4219503-4219836
BmHel-8	DF090335:2728469-2728800
BmHel-8	DF090405:488356-488694
BmHel-8	DF090434:547867-548183

BmHel-8	DF090567:73049-73390
BmHel-8	DF090357:910088-910418
BmHel-8	DF090344:1120584-1120919
BmHel-8	DF090365:664925-665260
BmHel-8	DF090339:4799044-4799375
BmHel-8	DF090416:975733-976067
BmHel-8	DF090354:1722814-1723145
BmHel-8	DF090370:2193163-2193495
BmHel-8	DF090321:7912451-7912783
BmHel-8	DF090326:6920994-6921330
BmHel-8	DF090330:606072-606396
BmHel-8	DF090330:744386-744718
BmHel-8	DF090330:2155965-2156291
BmHel-8	DF090330:5233908-5234240
BmHel-8	DF090351:1633771-1634098
BmHel-8	DF090351:2662500-2662832
BmHel-8	DF090402:1380106-1380437
BmHel-8	DF090377:914437-914768
BmHel-8	DF090350:353184-353507
BmHel-8	DF090342:2200302-2200628
BmHel-8	DF090342:2371476-2371807
BmHel-8	DF090318:2021275-2021607
BmHel-8	DF090318:2021609-2021691
BmHel-8	DF090320:3168013-3168345
BmHel-8	DF090388:1599883-1600215
BmHel-8	DF090388:1600217-1600298
BmHel-8	DF090344:3333166-3333503
BmHel-8	DF090344:3333505-3333591
BmHel-8	DF090344:3451569-3451901
BmHel-8	DF090344:3985362-3985693
BmHel-8	DF090340:138739-139066
BmHel-8	DF090340:3816660-3816987
BmHel-8	DF090329:3851799-3852130
BmHel-8	DF090327:2273623-2273954
BmHel-8	DF090327:4053226-4053555
BmHel-8	DF090327:195222-195553
BmHel-8	DF090319:8592448-8592775
BmHel-8	DF090316:470922-471253
BmHel-8	DF090316:13759060-13759391
BmHel-8	DF090316:13403255-13403575
BmHel-8	DF090316:2311237-2311562
BmHel-8	DF090349:2975937-2976272
BmHel-8	DF090326:2549297-2549627
BmHel-8	DF090348:3854913-3855245
BmHel-8	DF090396:63414-63745
BmHel-8	DF090391:553329-553656
BmHel-8	DF090372:2455594-2455925
BmHel-8	DF090347:1949908-1950233

BmHel-8	DF090345:4286509-4286838
BmHel-8	DF090335:3586665-3586996
BmHel-8	DF090334:5031316-5031648
BmHel-8	DF090324:4364108-4364439
BmHel-8	DF090337:2940519-2940850
BmHel-8	DF090381:583922-584248
BmHel-8	DF090373:2254221-2254553
BmHel-8	DF090405:791483-791811
BmHel-8	DF090375:495646-495976
BmHel-8	DF090333:3485149-3485481
BmHel-8	DF090325:3461352-3461684
BmHel-8	DF090325:2811246-2811571
BmHel-8	DF090417:1160724-1161056
BmHel-8	DF090384:1916549-1916881
BmHel-8	BABH01083430:1-332
BmHel-8	DF090398:1101484-1101813
BmHel-8	BABH01083792:202-529
BmHel-8	DF090676:10094-10427
BmHel-8	DF090321:2646476-2646809
BmHel-8	DF090394:649258-649484
BmHel-8	DF090394:649498-649620
BmHel-8	DF090334:2224841-2224960
BmHel-8	DF090334:2224973-2225183
BmHel-8	DF090408:1080266-1080603
BmHel-8	DF090431:211790-212130
BmHel-8	DF090354:1039054-1039267
BmHel-8	DF090354:1045929-1046055
BmHel-8	BABH01073889:69-410
BmHel-8	BABH01056963:69-410
BmHel-8	DF090344:4608322-4608663
BmHel-8	DF090340:4803661-4804004
BmHel-8	DF090316:1925332-1925670
BmHel-8	DF090514:51484-51816
BmHel-8	DF090363:900491-900801
BmHel-8	DF090344:3513960-3514298
BmHel-8	DF090442:353903-354178
BmHel-8	DF090352:2078386-2078723
BmHel-8	DF090418:624784-624950
BmHel-8	DF090418:624170-624343
BmHel-8	DF090342:993248-993584
BmHel-8	DF090318:7860861-7861195
BmHel-8	DF090321:8975-9306
BmHel-8	DF090321:9312-9394
BmHel-8	DF090395:501335-501422
BmHel-8	DF090395:501657-501900
BmHel-8	DF090333:426732-427066
BmHel-8	DF090354:2608210-2608543
BmHel-8	DF090407:632893-633226

BmHel-8	DF090318:5518769-5518852
BmHel-8	DF090318:5519162-5519410
BmHel-8	DF090319:4227100-4227274
BmHel-8	DF090319:4226921-4227075
BmHel-8	DF090317:4507663-4507993
BmHel-8	DF090328:6270-6444
BmHel-8	DF090328:6083-6240
BmHel-8	DF090325:2946828-2947162
BmHel-8	DF090325:2947164-2947243
BmHel-8	DF090325:2270559-2270889
BmHel-8	DF090414:730991-731315
BmHel-8	DF090335:666310-666642
BmHel-8	DF090335:666229-666308
BmHel-8	DF090421:859852-860110
BmHel-8	DF090409:1578452-1578779
BmHel-8	DF090340:4866859-4867190
BmHel-8	DF090329:4284557-4284888
BmHel-8	DF090423:858210-858540
BmHel-8	DF090352:192293-192625
BmHel-8	DF090352:1938492-1938822
BmHel-8	DF090363:2442221-2442543
BmHel-8	DF090321:1941306-1941628
BmHel-8	DF090318:9336471-9336801
BmHel-8	DF090403:1590531-1590858
BmHel-8	DF090338:1612319-1612648
BmHel-8	DF090319:7262384-7262714
BmHel-8	DF090319:4799711-4800041
BmHel-8	DF090365:374546-374881
BmHel-8	DF090381:1974907-1975237
BmHel-8	DF090381:1141392-1141722
BmHel-8	DF090381:1141306-1141390
BmHel-8	DF090339:3084313-3084637
BmHel-8	DF090462:409789-410120
BmHel-8	DF090376:1375667-1375993
BmHel-8	DF090378:275628-275962
BmHel-8	DF090378:275964-276044
BmHel-8	BABH01046849:1-331
BmHel-8	DF090404:368488-368827
BmHel-8	DF090371:291993-292324
BmHel-8	DF090381:1816647-1816993
BmHel-8	DF090426:107097-107340
BmHel-8	DF090426:107812-107910
BmHel-8	DF090336:2058980-2059325
BmHel-8	DF090355:2169072-2169269
BmHel-8	DF090355:2168788-2168936
BmHel-8	DF090339:3954355-3954629
BmHel-8	DF090391:1209007-1209349
BmHel-8	DF090342:1356876-1357216

BmHel-8	DF090342:3765165-3765510
BmHel-8	DF090318:6366001-6366339
BmHel-8	DF090359:3150236-3150577
BmHel-8	DF090359:173797-174138
BmHel-8	DF090357:79937-80281
BmHel-8	DF090423:1003192-1003537
BmHel-8	DF090431:622350-622690
BmHel-8	DF090389:462521-462858
BmHel-8	DF090389:457188-457528
BmHel-8	DF090387:855423-855761
BmHel-8	DF090472:202901-203244
BmHel-8	DF090419:828830-829172
BmHel-8	DF090715:21982-22326
BmHel-8	DF090358:2997473-2997803
BmHel-8	DF090358:2997814-2997895
BmHel-8	DF090316:5069168-5069286
BmHel-8	DF090316:5069299-5069509
BmHel-8	DF090366:902936-903281
BmHel-8	DF090345:1191642-1191974
BmHel-8	DF090358:1616902-1616998
BmHel-8	DF090358:1617014-1617261
BmHel-8	DF090351:3779584-3779729
BmHel-8	DF090351:3779380-3779580
BmHel-8	DF090334:2932052-2932380
BmHel-8	DF090390:264045-264376
BmHel-8	DF090343:587790-588124
BmHel-8	DF090402:374175-374507
BmHel-8	DF090320:2638467-2638811
BmHel-8	DF090434:346550-346886
BmHel-8	DF090481:57441-57772
BmHel-8	DF090342:4408776-4409112
BmHel-8	DF090322:34231-34559
BmHel-8	DF090316:14514800-14515134
BmHel-8	DF090434:321293-321637
BmHel-8	DF090415:437878-438024
BmHel-8	DF090415:438781-438967
BmHel-8	DF090459:116938-117281
BmHel-8	DF090358:1105707-1106034
BmHel-8	DF090332:58985-59314
BmHel-8	DF090347:4018869-4018975
BmHel-8	DF090347:4018647-4018857
BmHel-8	DF090347:4018352-4018441
BmHel-8	DF090346:2031952-2032281
BmHel-8	DF090318:7267398-7267727
BmHel-8	DF090403:550158-550482
BmHel-8	DF090357:1607053-1607381
BmHel-8	DF090357:1606967-1607047
BmHel-8	DF090349:3042320-3042649

BmHel-8	DF090432:202242-202564
BmHel-8	BABH01079088:157-491
BmHel-8	BABH01060093:75-409
BmHel-8	DF090480:109236-109561
BmHel-8	DF090894:1095-1420
BmHel-8	DF090403:1134415-1134744
BmHel-8	DF090319:8595928-8596259
BmHel-8	DF090328:2914018-2914347
BmHel-8	DF090346:2681630-2681960
BmHel-8	DF090409:760206-760531
BmHel-8	DF090367:1973458-1973787
BmHel-8	DF090353:1045458-1045789
BmHel-8	DF090353:1045374-1045456
BmHel-8	DF090342:236172-236503
BmHel-8	DF090318:6314836-6315163
BmHel-8	DF090318:6314755-6314834
BmHel-8	DF090320:4909165-4909493
BmHel-8	DF090320:4909495-4909576
BmHel-8	DF090400:707673-708004
BmHel-8	DF090362:2016751-2017075
BmHel-8	DF090356:1108695-1109026
BmHel-8	DF090344:4628234-4628565
BmHel-8	DF090344:4628567-4628646
BmHel-8	DF090338:1492602-1492937
BmHel-8	DF090322:4532167-4532492
BmHel-8	DF090317:9372060-9372393
BmHel-8	DF090316:15424164-15424488
BmHel-8	DF090316:6165279-6165613
BmHel-8	DF090349:1866414-1866741
BmHel-8	DF090365:2587933-2588264
BmHel-8	DF090439:441054-441374
BmHel-8	DF090345:1260761-1261090
BmHel-8	DF090341:303447-303776
BmHel-8	DF090341:1212226-1212555
BmHel-8	DF090335:2983380-2983709
BmHel-8	DF090331:2305595-2305924
BmHel-8	DF090328:5537126-5537450
BmHel-8	DF090324:3923432-3923757
BmHel-8	DF090405:562440-562769
BmHel-8	DF090325:1186332-1186659
BmHel-8	DF090354:566738-567067
BmHel-8	DF090354:567069-567151
BmHel-8	DF090354:2184407-2184735
BmHel-8	DF090354:2136341-2136672
BmHel-8	BABH01079472:245-574
BmHel-8	BABH01077189:1-330
BmHel-8	DF090415:892832-893156
BmHel-8	DF090368:2313440-2313768

BmHel-8	BABH01049601:28-361
BmHel-8	DF091187:6558-6882
BmHel-8	DF090834:11963-12294
BmHel-8	DF090338:2521777-2522118
BmHel-8	DF090395:1654714-1654808
BmHel-8	DF090395:1654261-1654502
BmHel-8	BABH01077236:176-522
BmHel-8	DF090383:445399-445735
BmHel-8	DF090316:1340414-1340745
BmHel-8	DF090369:961570-961906
BmHel-8	DF090345:2827902-2828238
BmHel-8	DF090325:6678307-6678646
BmHel-8	DF090328:6630097-6630426
BmHel-8	DF090383:1397817-1398153
BmHel-8	DF090367:1945210-1945542
BmHel-8	DF090321:3563090-3563269
BmHel-8	DF090321:3563334-3563502
BmHel-8	DF090319:3179522-3179860
BmHel-8	DF090317:8153067-8153395
BmHel-8	DF090317:5256331-5256617
BmHel-8	DF090433:205825-206158
BmHel-8	DF090371:1580794-1581119
BmHel-8	DF090360:1581689-1582026
BmHel-8	DF090462:314924-315046
BmHel-8	DF090462:315058-315268
BmHel-8	DF090414:770575-770907
BmHel-8	DF090399:99767-100094
BmHel-8	DF090351:3438421-3438750
BmHel-8	DF090329:4737519-4737637
BmHel-8	DF090329:4737289-4737499
BmHel-8	DF090370:386302-386636
BmHel-8	DF090332:2026606-2026939
BmHel-8	DF090377:154616-154948
BmHel-8	DF090385:285998-286334
BmHel-8	DF090330:2329036-2329371
BmHel-8	DF090322:6071781-6072110
BmHel-8	DF090422:213374-213702
BmHel-8	DF090324:1168408-1168736
BmHel-8	DF090343:1518903-1519226
BmHel-8	DF090419:1256683-1257009
BmHel-8	DF091151:4301-4564
BmHel-8	DF090332:1473462-1473789
BmHel-8	DF090458:214003-214317
BmHel-8	DF090437:564144-564477
BmHel-8	DF090319:3928785-3929118
BmHel-8	DF090416:357757-358014
BmHel-8	BABH01086812:83-409
BmHel-8	BABH01084036:1-333

BmHel-8	DF090342:4785994-4786317
BmHel-8	DF090316:9734294-9734615
BmHel-8	DF090335:4705376-4705699
BmHel-8	DF090335:4721135-4721464
BmHel-8	DF090324:3124371-3124694
BmHel-8	DF090324:3126703-3126785
BmHel-8	DF090385:1677048-1677371
BmHel-8	DF090354:1590842-1591170
BmHel-8	DF090533:5244-5570
BmHel-8	DF090463:198504-198836
BmHel-8	DF090349:2158044-2158373
BmHel-8	DF090325:6270527-6270748
BmHel-8	DF090325:6270746-6270851
BmHel-8	DF090341:1622848-1623179
BmHel-8	DF090342:3184109-3184231
BmHel-8	DF090342:3183883-3184095
BmHel-8	DF090327:5780783-5780905
BmHel-8	DF090327:5780552-5780766
BmHel-8	DF090347:634653-634987
BmHel-8	DF090414:393900-394243
BmHel-8	DF090350:991620-991809
BmHel-8	DF090350:993563-993722
BmHel-8	DF090350:4359473-4359813
BmHel-8	DF090350:3787953-3788284
BmHel-8	DF090493:69-407
BmHel-8	BABH01085315:60-399
BmHel-8	DF090330:3174370-3174709
BmHel-8	DF090330:850112-850448
BmHel-8	DF090367:1179667-1180007
BmHel-8	DF090323:468399-468745
BmHel-8	DF090348:1864237-1864581
BmHel-8	BABH01067989:66-407
BmHel-8	DF090378:625232-625584
BmHel-8	DF090435:144744-145089
BmHel-8	DF091453:2721-3061
BmHel-8	DF090412:317899-318234
BmHel-8	DF090363:75162-75489
BmHel-8	DF090363:195811-196157
BmHel-8	DF090348:532529-532648
BmHel-8	DF090348:532306-532515
BmHel-8	DF090494:145716-145962
BmHel-8	DF090447:555994-556209
BmHel-8	DF090447:555859-555991
BmHel-8	DF090447:555774-555856
BmHel-8	DF090336:4376909-4377095
BmHel-8	DF090336:4373264-4373424
BmHel-8	DF090372:1266810-1267141
BmHel-8	DF090320:594842-594959

BmHel-8	DF090320:594971-595181
BmHel-8	DF090749:15172-15413
BmHel-8	DF090749:15052-15155
BmHel-8	DF090377:753012-753339
BmHel-8	DF090324:7706041-7706376
BmHel-8	DF090320:5767605-5767932
BmHel-8	DF090328:3728628-3728961
BmHel-8	DF090408:1066150-1066498
BmHel-8	DF090317:5076241-5076570
BmHel-8	DF090330:5339089-5339416
BmHel-8	DF090388:511653-511977
BmHel-8	DF090335:2192647-2192979
BmHel-8	DF090343:2137305-2137628
BmHel-8	BABH01044520:85-415
BmHel-8	DF090601:19075-19402
BmHel-8	DF090601:18990-19072
BmHel-8	BABH01043567:15042-15281
BmHel-8	BABH01043567:13565-13656
BmHel-8	DF090401:268018-268350
BmHel-8	DF090381:305627-305955
BmHel-8	DF090415:171065-171393
BmHel-8	DF090316:7133731-7134055
BmHel-8	DF090361:1736354-1736680
BmHel-8	DF090413:183228-183555
BmHel-8	BABH01047868:149-477
BmHel-8	BABH01047868:479-566
BmHel-8	DF090355:239394-239724
BmHel-8	DF090402:833646-833862
BmHel-8	DF090402:833540-833648
BmHel-8	DF090355:3174867-3174952
BmHel-8	DF090355:3174954-3175194
BmHel-8	DF090391:920169-920501
BmHel-8	DF090388:95213-95501
BmHel-8	DF090347:3940010-3940132
BmHel-8	DF090347:3939788-3939997
BmHel-8	DF090444:39879-40219
BmHel-8	DF090398:436375-436716
BmHel-8	DF090330:4770557-4770893
BmHel-8	BABH01066251:255-596
BmHel-8	DF090352:540254-540594
BmHel-8	DF090351:686824-687163
BmHel-8	DF090351:1759369-1759713
BmHel-8	DF090332:2678384-2678723
BmHel-8	DF090402:825723-826065
BmHel-8	DF090355:1687911-1688256
BmHel-8	DF090321:234985-235325
BmHel-8	DF090318:6335578-6335918
BmHel-8	DF090403:670917-671258

BmHel-8	DF090359:1541763-1542103
BmHel-8	DF090356:3694333-3694675
BmHel-8	DF090340:3959128-3959472
BmHel-8	DF090338:1051107-1051454
BmHel-8	DF090322:2605821-2606167
BmHel-8	DF090319:861460-861804
BmHel-8	DF090326:3252798-3253147
BmHel-8	DF090348:149972-150319
BmHel-8	DF090394:1345013-1345355
BmHel-8	DF090369:1067331-1067668
BmHel-8	DF090335:2584215-2584560
BmHel-8	DF090337:4029004-4029343
BmHel-8	DF090436:300663-301008
BmHel-8	DF090339:5093072-5093423
BmHel-8	DF090432:352867-353204
BmHel-8	DF090425:361613-361946
BmHel-8	DF090325:5828837-5829178
BmHel-8	BABH01046054:68-409
BmHel-8	DF090387:1694833-1695177
BmHel-8	DF090404:1147436-1147778
BmHel-8	DF090354:3319883-3320223
BmHel-8	DF090438:369576-369915
BmHel-8	DF090413:1150753-1151096
BmHel-8	DF090457:173279-173618
BmHel-8	BABH01047326:1382-1719
BmHel-8	BABH01046074:2475-2808
BmHel-8	DF090830:460-793
BmHel-8	BABH01048447:520-866
BmHel-8	DF090367:2703607-2703951
BmHel-8	DF090353:1119775-1119980
BmHel-8	DF090353:1119981-1120099
BmHel-8	DF090327:1982580-1982907
BmHel-8	DF090343:187135-187468
BmHel-8	DF090352:3490945-3491286
BmHel-8	DF090367:2308355-2308684
BmHel-8	DF090328:1468895-1469235
BmHel-8	DF090334:2409151-2409478
BmHel-8	DF090367:311516-311863
BmHel-8	DF090356:2750375-2750491
BmHel-8	DF090356:2750167-2750363
BmHel-8	DF090591:20188-20304
BmHel-8	DF090591:20301-20511
BmHel-8	DF090353:3489341-3489674
BmHel-8	DF090323:6739773-6740096
BmHel-8	DF090363:2590960-2591246
BmHel-8	DF090375:2382959-2383297
BmHel-8	DF091656:2370-2704
BmHel-8	DF090472:282823-283149

BmHel-8	BABH01059582:378-708
BmHel-8	DF090343:4400529-4400864
BmHel-8	DF090782:14547-14769
BmHel-8	DF090782:14780-14885
BmHel-8	DF090437:32835-32915
BmHel-8	DF090437:33110-33348
BmHel-8	DF090321:7689293-7689611
BmHel-8	BABH01086330:1-327
BmHel-8	DF090342:4142326-4142448
BmHel-8	DF090342:4142466-4142666
BmHel-8	DF090332:101664-102000
BmHel-8	DF090361:2190127-2190476
BmHel-8	DF090345:4349970-4350299
BmHel-8	DF090346:1444675-1444915
BmHel-8	DF090346:1444912-1445003
BmHel-8	DF090330:4057780-4058117
BmHel-8	DF090332:316234-316568
BmHel-8	DF090367:373858-374209
BmHel-8	DF090321:2782430-2782771
BmHel-8	DF090443:149988-150327
BmHel-8	DF090362:1574801-1575139
BmHel-8	DF090356:2825630-2825972
BmHel-8	DF090344:2027579-2027926
BmHel-8	DF090340:1469479-1469814
BmHel-8	DF090319:113817-113905
BmHel-8	DF090319:114121-114374
BmHel-8	DF090317:5620609-5620957
BmHel-8	DF090349:3914521-3914871
BmHel-8	DF090348:831776-832126
BmHel-8	DF090337:2387521-2387870
BmHel-8	DF090337:4936087-4936434
BmHel-8	DF090342:434299-434637
BmHel-8	DF090403:1440182-1440520
BmHel-8	DF090362:721091-721433
BmHel-8	DF090356:3664088-3664430
BmHel-8	DF090348:457807-458142
BmHel-8	DF090380:1561169-1561510
BmHel-8	DF090369:2430659-2431000
BmHel-8	DF090335:2521050-2521386
BmHel-8	DF090985:4123-4464
BmHel-8	DF090416:791504-791843
BmHel-8	BABH01055839:353-690
BmHel-8	DF090324:5184507-5184719
BmHel-8	DF090324:5184886-5185011
BmHel-8	DF090367:7340-7680
BmHel-8	DF090367:797473-797818
BmHel-8	DF090363:2527084-2527202
BmHel-8	DF090363:2527216-2527417

BmHel-8	DF090333:3776959-3777073
BmHel-8	DF090333:3777088-3777298
BmHel-8	DF090475:82756-82870
BmHel-8	DF090475:82539-82744
BmHel-8	DF090316:13440875-13440993
BmHel-8	DF090316:13441031-13441237
BmHel-8	DF090316:10469096-10469308
BmHel-8	DF090316:10468989-10469098
BmHel-8	DF090316:7894887-7895219
BmHel-8	DF090345:2086452-2086786
BmHel-8	DF090601:40187-40419
BmHel-8	DF090601:45384-45494
BmHel-8	DF090318:1466632-1466749
BmHel-8	DF090318:1466763-1466970
BmHel-8	DF090318:868726-869060
BmHel-8	DF090326:4958861-4959029
BmHel-8	DF090326:4958652-4958774
BmHel-8	DF090354:2425729-2425845
BmHel-8	DF090354:2425856-2426064
BmHel-8	DF090326:6600249-6600391
BmHel-8	DF090326:6599778-6599979
BmHel-8	DF090411:101811-102147
BmHel-8	BABH01046462:70-406
BmHel-8	DF090374:795321-795494
BmHel-8	DF090374:796340-796507
BmHel-8	DF090419:586129-586244
BmHel-8	DF090419:585456-585669
BmHel-8	DF090321:6280829-6280942
BmHel-8	DF090321:6281267-6281477
BmHel-8	DF090322:7670322-7670649
BmHel-8	DF090317:5202336-5202660
BmHel-8	DF090316:8562195-8562530
BmHel-8	DF090414:1319728-1320054
BmHel-8	DF090348:1840757-1841089
BmHel-8	DF090703:27396-27687
BmHel-8	DF090402:596566-596891
BmHel-8	DF090342:3336176-3336501
BmHel-8	DF090396:428338-428663
BmHel-8	DF090336:5504225-5504553
BmHel-8	DF090336:5504558-5504637
BmHel-8	DF090323:1545831-1546160
BmHel-8	DF090370:2088287-2088612
BmHel-8	DF090320:2595786-2596111
BmHel-8	DF090316:992938-993258
BmHel-8	DF090372:1125955-1126278
BmHel-8	DF090328:793840-794165
BmHel-8	BABH01066597:333-658
BmHel-8	DF090367:2404736-2404851

BmHel-8	DF090367:2404533-2404729
BmHel-8	DF090367:2404452-2404531
BmHel-8	DF090322:7191014-7191263
BmHel-8	DF090325:929700-929819
BmHel-8	DF090325:929472-929684
BmHel-8	DF090333:2293585-2293918
BmHel-8	DF090338:698290-698625
BmHel-8	DF090323:7551879-7552207
BmHel-8	DF090393:1679994-1680115
BmHel-8	DF090393:1679772-1679982
BmHel-8	DF090382:550903-551201
BmHel-8	DF090677:22073-22238
BmHel-8	DF090677:22492-22669
BmHel-8	DF090374:1563534-1563877
BmHel-8	DF090477:231854-232195
BmHel-8	DF090357:852493-852837
BmHel-8	DF090458:363562-363901
BmHel-8	DF090325:505882-506221
BmHel-8	DF090340:1662424-1662517
BmHel-8	DF090340:1661741-1661991
BmHel-8	DF090333:1407294-1407637
BmHel-8	DF090351:3095571-3095910
BmHel-8	DF090318:951809-952149
BmHel-8	DF090316:14936702-14937043
BmHel-8	DF090382:1504113-1504449
BmHel-8	BABH01045594:3-354
BmHel-8	DF090363:1583745-1583858
BmHel-8	DF090363:1583509-1583718
BmHel-8	DF090353:3973466-3973792
BmHel-8	DF090356:3042260-3042596
BmHel-8	DF090366:1726712-1726830
BmHel-8	DF090366:1726485-1726696
BmHel-8	DF090436:363006-363340
BmHel-8	DF090506:189509-189758
BmHel-8	DF090317:1923447-1923563
BmHel-8	DF090317:1923225-1923427
BmHel-8	DF090341:1876005-1876122
BmHel-8	DF090341:1876137-1876345
BmHel-8	DF090999:1799-1912
BmHel-8	DF090999:1578-1784
BmHel-8	DF090403:434978-435302
BmHel-8	DF090317:9401535-9401867
BmHel-8	DF090325:4651858-4652069
BmHel-8	DF090325:4652069-4652181
BmHel-8	DF090325:4652183-4652265
BmHel-8	DF090384:1814439-1814673
BmHel-8	DF090384:1814670-1814761
BmHel-8	DF090362:954853-955182

BmHel-8	DF090373:1842726-1842839
BmHel-8	DF090373:1842849-1843068
BmHel-8	DF090405:1395008-1395099
BmHel-8	DF090405:1395112-1395354
BmHel-8	DF090456:411409-411524
BmHel-8	DF090456:411848-412061
BmHel-8	DF090456:412063-412143
BmHel-8	DF090352:1993625-1993953
BmHel-8	DF090342:2874905-2875237
BmHel-8	DF090772:216-466
BmHel-8	DF090772:483-571
BmHel-8	DF090417:271554-271724
BmHel-8	DF090417:271749-271905
BmHel-8	DF090336:798459-798792
BmHel-8	DF090336:798377-798457
BmHel-8	DF090362:1066541-1066866
BmHel-8	DF090319:5985022-5985343
BmHel-8	DF090349:3565112-3565436
BmHel-8	DF090345:4009699-4010018
BmHel-8	DF090328:813655-813973
BmHel-8	DF090343:2799184-2799508
BmHel-8	DF090405:800201-800511
BmHel-8	BABH01064878:1-325
BmHel-8	DF090387:715204-715529
BmHel-8	BABH01049794:701-1019
BmHel-8	DF091217:2024-2347
BmHel-8	DF091217:2349-2431
BmHel-8	DF090341:4600170-4600289
BmHel-8	DF090341:4600304-4600509
BmHel-8	DF090330:5135004-5135124
BmHel-8	DF090330:5134790-5134991
BmHel-8	DF090339:4413917-4414259
BmHel-8	DF090330:894667-895008
BmHel-8	DF090321:3325188-3325532
BmHel-8	DF090316:14510479-14510827
BmHel-8	DF090411:1303113-1303454
BmHel-8	DF090343:1566690-1567033
BmHel-8	DF090461:244991-245332
BmHel-8	DF090358:2896694-2897034
BmHel-8	DF090383:653956-654283
BmHel-8	DF090388:735270-735382
BmHel-8	DF090388:735397-735607
BmHel-8	DF090362:780128-780470
BmHel-8	BABH01063106:280-618
BmHel-8	DF090345:2003194-2003541
BmHel-8	DF090405:328276-328615
BmHel-8	DF090373:1160269-1160592
BmHel-8	DF090339:4931749-4932072

BmHel-8	DF090338:2778725-2778906
BmHel-8	DF090338:2777622-2777789
BmHel-8	DF090332:4114714-4115051
BmHel-8	DF090365:478620-478937
BmHel-8	DF090391:1561553-1561887
BmHel-8	DF090382:696758-697092
BmHel-8	DF090338:1058146-1058475
BmHel-8	DF090467:47767-48024
BmHel-8	DF090461:296890-297002
BmHel-8	DF090461:296672-296877
BmHel-8	DF090355:3601851-3602118
BmHel-8	DF090316:6040622-6040957
BmHel-8	DF090349:1712245-1712576
BmHel-8	DF090339:3452154-3452482
BmHel-8	DF090324:3795062-3795392
BmHel-8	DF090368:1339202-1339527
BmHel-8	DF090398:631545-631864
BmHel-8	DF090322:4637462-4637787
BmHel-8	DF090325:4086492-4086816
BmHel-8	DF090330:6356556-6356878
BmHel-8	DF090367:2387788-2388111
BmHel-8	DF090324:4425789-4426106
BmHel-8	BABH01087555:1-319
BmHel-8	DF090363:2604877-2605220
BmHel-8	DF090327:1034546-1034889
BmHel-8	DF090317:2145252-2145439
BmHel-8	DF090317:2143800-2143962
BmHel-8	DF090316:14224943-14225058
BmHel-8	DF090316:14225073-14225284
BmHel-8	DF090335:5116299-5116634
BmHel-8	DF090362:2254065-2254399
BmHel-8	DF090377:415281-415501
BmHel-8	DF090377:415110-415230
BmHel-8	DF090370:2236565-2236904
BmHel-8	DF090355:1948701-1949034
BmHel-8	DF090397:601387-601715
BmHel-8	DF090369:2065171-2065332
BmHel-8	DF090369:2064621-2064805
BmHel-8	BABH01080765:163-499
BmHel-8	DF090338:4845194-4845531
BmHel-8	DF090639:20187-20527
BmHel-8	BABH01083558:137-472
BmHel-8	DF090330:4831481-4831805
BmHel-8	DF090317:4958036-4958375
BmHel-8	DF090354:3115935-3116273
BmHel-8	DF090319:931671-932003
BmHel-8	DF090318:6393727-6394062
BmHel-8	DF090322:4853930-4854190

BmHel-8	BABH01048326:1199-1310
BmHel-8	BABH01048326:974-1184
BmHel-8	DF090340:3991395-3991657
BmHel-8	DF090401:1524012-1524329
BmHel-8	DF090418:1125884-1126225
BmHel-8	DF090377:1263261-1263485
BmHel-8	DF090377:1263502-1263620
BmHel-8	DF090318:2114373-2114551
BmHel-8	DF090318:2121443-2121598
BmHel-8	DF090355:2855021-2855344
BmHel-8	DF090368:1170998-1171319
BmHel-8	DF090645:19290-19618
BmHel-8	DF090364:1738413-1738734
BmHel-8	DF090317:4806292-4806616
BmHel-8	DF090451:351749-352069
BmHel-8	DF090335:5695192-5695522
BmHel-8	DF090334:1772094-1772414
BmHel-8	DF090560:689-1010
BmHel-8	DF091083:4285-4607
BmHel-8	BABH01050246:82-405
BmHel-8	DF090388:428582-428676
BmHel-8	DF090388:428896-429139
BmHel-8	DF090704:13342-13459
BmHel-8	DF090704:13471-13677
BmHel-8	DF090373:1003201-1003355
BmHel-8	DF090373:1002713-1002904
BmHel-8	DF090320:1299037-1299158
BmHel-8	DF090320:1298944-1299025
BmHel-8	DF090320:1298633-1298764
BmHel-8	DF090419:294025-294353
BmHel-8	DF090367:2234651-2234984
BmHel-8	DF090346:835493-835834
BmHel-8	DF090409:1034311-1034648
BmHel-8	DF090335:2399158-2399344
BmHel-8	DF090335:2398964-2399086
BmHel-8	DF090390:945653-945993
BmHel-8	DF090418:148828-149166
BmHel-8	DF090325:3077465-3077551
BmHel-8	DF090325:3077615-3077873
BmHel-8	DF090330:75257-75599
BmHel-8	DF090386:590839-591178
BmHel-8	DF090334:4086263-4086602
BmHel-8	BABH01084495:67-408
BmHel-8	DF090338:2291408-2291750
BmHel-8	DF090329:1540776-1540895
BmHel-8	DF090329:1541037-1541248
BmHel-8	DF090433:279147-279471
BmHel-8	DF090341:4707998-4708263

BmHel-8	DF090373:1560177-1560512
BmHel-8	DF090335:3969943-3970053
BmHel-8	DF090335:3969715-3969925
BmHel-8	DF090320:2509476-2509811
BmHel-8	DF090366:1181078-1181405
BmHel-8	DF090359:1252503-1252822
BmHel-8	DF090362:1305596-1305923
BmHel-8	DF090349:2726654-2726991
BmHel-8	DF090331:5149517-5149837
BmHel-8	DF090452:258393-258716
BmHel-8	DF090484:219842-219923
BmHel-8	DF090484:222082-222326
BmHel-8	DF090374:1812395-1812721
BmHel-8	DF090326:2767550-2767873
BmHel-8	DF090319:7254083-7254408
BmHel-8	DF090472:268367-268690
BmHel-8	DF090472:268283-268365
BmHel-8	BABH01046569:470-792
BmHel-8	DF090332:3091267-3091556
BmHel-8	DF090320:6970508-6970759
BmHel-8	DF090320:6974770-6974871
BmHel-8	DF090551:76237-76500
BmHel-8	DF090343:344761-345109
BmHel-8	DF090323:3268271-3268612
BmHel-8	DF090322:5741050-5741391
BmHel-8	DF090395:1032001-1032343
BmHel-8	DF090392:437334-437672
BmHel-8	DF090337:1716751-1717089
BmHel-8	DF090424:368959-369266
BmHel-8	DF090348:3791347-3791705
BmHel-8	DF090329:3979236-3979574
BmHel-8	DF090377:368308-368650
BmHel-8	DF090350:1180107-1180455
BmHel-8	DF090317:3582357-3582698
BmHel-8	DF090396:1502962-1503305
BmHel-8	DF090413:1011322-1011657
BmHel-8	DF090317:549941-550272
BmHel-8	DF090343:1935715-1935824
BmHel-8	DF090343:1935836-1936041
BmHel-8	DF090399:1506732-1507063
BmHel-8	DF090358:1542479-1542816
BmHel-8	DF090376:2244491-2244643
BmHel-8	DF090376:2244672-2244876
BmHel-8	DF090343:3854880-3855092
BmHel-8	DF090343:3845998-3846130
BmHel-8	DF090378:258294-258625
BmHel-8	DF090330:2230841-2230950
BmHel-8	DF090330:2230962-2231172

BmHel-8	DF090367:1030967-1031286
BmHel-8	DF090342:3810582-3810910
BmHel-8	DF090416:1244512-1244835
BmHel-8	DF090335:2230447-2230763
BmHel-8	DF090359:2345191-2345509
BmHel-8	DF090372:1305212-1305532
BmHel-8	DF090403:1098903-1099243
BmHel-8	DF090339:3252622-3252744
BmHel-8	DF090339:3252777-3252970
BmHel-8	DF090395:958103-958448
BmHel-8	DF090355:3451196-3451537
BmHel-8	DF090367:2744286-2744519
BmHel-8	DF090367:2744171-2744261
BmHel-8	DF090333:5883979-5884311
BmHel-8	DF090333:473362-473634
BmHel-8	DF090333:473648-473736
BmHel-8	DF090328:3462803-3463137
BmHel-8	DF090369:649236-649577
BmHel-8	DF090316:13241504-13241747
BmHel-8	DF090316:13244265-13244369
BmHel-8	DF090329:360872-360994
BmHel-8	DF090329:360650-360860
BmHel-8	DF090377:1351853-1352103
BmHel-8	DF090377:1350974-1351073
BmHel-8	DF090385:113870-114217
BmHel-8	DF090336:4682873-4683218
BmHel-8	DF090330:122968-123309
BmHel-8	DF090329:3624535-3624876
BmHel-8	DF090331:5205115-5205455
BmHel-8	DF090492:11153-11494
BmHel-8	DF090323:4507448-4507778
BmHel-8	DF090348:1192948-1193193
BmHel-8	DF090345:3818619-3818947
BmHel-8	DF090419:194004-194336
BmHel-8	DF090356:3424587-3424919
BmHel-8	DF090668:2754-3077
BmHel-8	DF090338:883879-884214
BmHel-8	DF090365:880747-880994
BmHel-8	DF090365:2407307-2407633
BmHel-8	DF090348:3306719-3306827
BmHel-8	DF090348:3306838-3307048
BmHel-8	DF090348:3307050-3307129
BmHel-8	DF090336:4836454-4836773
BmHel-8	DF090325:846520-846856
BmHel-8	DF090323:4797477-4797810
BmHel-8	DF090361:271038-271373
BmHel-8	DF090316:10584648-10584756
BmHel-8	DF090316:10584768-10584978

BmHel-8	DF090348:1904477-1904806
BmHel-8	DF090362:904747-905067
BmHel-8	DF090316:12972108-12972431
BmHel-8	DF090316:12972433-12972512
BmHel-8	DF090348:64667-64996
BmHel-8	DF090372:2356446-2356765
BmHel-8	DF090328:1001427-1001749
BmHel-8	DF090328:1001751-1001831
BmHel-8	BABH01049077:9-314
BmHel-8	DF090342:4465631-4465947
BmHel-8	DF090358:1885797-1886096
BmHel-8	DF090344:3808420-3808542
BmHel-8	DF090344:3808199-3808401
BmHel-8	DF090395:989542-989862
BmHel-8	DF090340:1649488-1649756
BmHel-8	DF090438:310042-310369
BmHel-8	DF090358:1932896-1933239
BmHel-8	DF090358:1720516-1720854
BmHel-8	DF090318:6769573-6769835
BmHel-8	DF090318:6769472-6769560
BmHel-8	DF090341:849540-849882
BmHel-8	DF090341:4086989-4087335
BmHel-8	DF090390:1227473-1227806
BmHel-8	DF090339:4841191-4841532
BmHel-8	BABH01045341:3143-3490
BmHel-8	DF090369:60323-60661
BmHel-8	DF090345:946140-946476
BmHel-8	DF090328:2105644-2105986
BmHel-8	DF090324:1460044-1460381
BmHel-8	DF090414:981652-981770
BmHel-8	DF090414:981434-981638
BmHel-8	DF090330:4465179-4465426
BmHel-8	DF090320:6853943-6854057
BmHel-8	DF090320:6853467-6853666
BmHel-8	DF090344:3471929-3472268
BmHel-8	DF090382:612694-612872
BmHel-8	DF090382:612561-612700
BmHel-8	DF090405:1430541-1430799
BmHel-8	DF090389:499204-499381
BmHel-8	DF090389:499065-499208
BmHel-8	DF090406:619853-620188
BmHel-8	DF090363:1044752-1045096
BmHel-8	DF090355:632184-632302
BmHel-8	DF090355:632315-632412
BmHel-8	DF090355:632429-632518
BmHel-8	DF090580:51740-52068
BmHel-8	DF090580:51619-51699
BmHel-8	DF090350:699530-699864

BmHel-8	DF090316:15993729-15994070
BmHel-8	DF090341:3954024-3954343
BmHel-8	DF090388:595160-595369
BmHel-8	DF090388:595367-595476
BmHel-8	DF090373:1693833-1694041
BmHel-8	DF090373:1693723-1693831
BmHel-8	DF090424:263683-264014
BmHel-8	DF090476:203937-204173
BmHel-8	DF090476:202181-202283
BmHel-8	DF090340:2427029-2427359
BmHel-8	DF090347:3124154-3124251
BmHel-8	DF090347:3123751-3123996
BmHel-8	DF090747:17722-18052
BmHel-8	DF090318:3068118-3068453
BmHel-8	DF090318:3340954-3341274
BmHel-8	DF090421:399111-399420
BmHel-8	DF090336:3634798-3635113
BmHel-8	DF090316:15110151-15110476
BmHel-8	BABH01083421:10-329
BmHel-8	DF090336:3675007-3675326
BmHel-8	DF090403:739285-739609
BmHel-8	DF090317:9549756-9550076
BmHel-8	DF090324:5255761-5256083
BmHel-8	BABH01045169:5521-5840
BmHel-8	DF090364:1653694-1654037
BmHel-8	DF090316:11137953-11138295
BmHel-8	DF090320:3081633-3081962
BmHel-8	DF090319:3121932-3122269
BmHel-8	DF090355:1461841-1462174
BmHel-8	DF090424:178827-179168
BmHel-8	DF090330:4272847-4273189
BmHel-8	DF090332:3052955-3053290
BmHel-8	DF090327:4313165-4313373
BmHel-8	DF090327:4313372-4313480
BmHel-8	DF090316:13439024-13439143
BmHel-8	DF090316:13438811-13438997
BmHel-8	DF090394:574257-574371
BmHel-8	DF090394:574043-574244
BmHel-8	DF090361:666791-667123
BmHel-8	DF090345:1084197-1084309
BmHel-8	DF090345:1084328-1084532
BmHel-8	DF090364:1396044-1396376
BmHel-8	DF090414:915472-915812
BmHel-8	DF090320:6262959-6263295
BmHel-8	DF090334:515219-515549
BmHel-8	DF090487:148962-149156
BmHel-8	DF090487:149159-149280
BmHel-8	DF090352:2117436-2117549

BmHel-8	DF090352:2117560-2117766
BmHel-8	DF090360:765634-765796
BmHel-8	DF090360:765823-765995
BmHel-8	DF090324:2657264-2657597
BmHel-8	DF090348:983235-983568
BmHel-8	DF090356:3411113-3411219
BmHel-8	DF090356:3411232-3411443
BmHel-8	DF090365:2266404-2266513
BmHel-8	DF090365:2266528-2266732
BmHel-8	DF090365:2266734-2266816
BmHel-8	BABH01087479:88-412
BmHel-8	DF090324:4697361-4697680
BmHel-8	DF090333:5784451-5784703
BmHel-8	DF090341:1683399-1683719
BmHel-8	BABH01081404:239-557
BmHel-8	BABH01045616:2024-2340
BmHel-8	DF090390:876870-877147
BmHel-8	DF090353:3356020-3356339
BmHel-8	DF090371:2071999-2072335
BmHel-8	DF090345:2612654-2612764
BmHel-8	DF090345:2612433-2612640
BmHel-8	DF090352:3001264-3001385
BmHel-8	DF090352:3001039-3001252
BmHel-8	DF090362:849176-849359
BmHel-8	DF090362:849358-849503
BmHel-8	DF090355:3330806-3331142
BmHel-8	DF090358:783089-783423
BmHel-8	DF090430:131257-131600
BmHel-8	DF090341:4072025-4072346
BmHel-8	DF090322:5654550-5654783
BmHel-8	DF090340:625768-626109
BmHel-8	DF090376:2042716-2043059
BmHel-8	DF090399:1496398-1496519
BmHel-8	DF090399:1496531-1496741
BmHel-8	DF090349:311026-311367
BmHel-8	BABH01086803:63-404
BmHel-8	DF090754:7897-8238
BmHel-8	DF090370:514699-515016
BmHel-8	DF090352:4096331-4096658
BmHel-8	DF090350:2614211-2614545
BmHel-8	DF090317:1433966-1434299
BmHel-8	DF090419:534817-535133
BmHel-8	BABH01044928:1955-2274
BmHel-8	DF090388:918310-918566
BmHel-8	DF090387:1673426-1673533
BmHel-8	DF090387:1673191-1673405
BmHel-8	DF090377:2045820-2046155
BmHel-8	DF090335:2104754-2105015

BmHel-8	DF090335:2104558-2104653
BmHel-8	DF090317:5186563-5186898
BmHel-8	DF090341:4011417-4011532
BmHel-8	DF090341:4011629-4011835
BmHel-8	DF090406:1029530-1029864
BmHel-8	DF091103:7863-8186
BmHel-8	DF090324:4720723-4721057
BmHel-8	DF090318:4145389-4145495
BmHel-8	DF090318:4145504-4145714
BmHel-8	DF090318:4145716-4145798
BmHel-8	DF090356:3423397-3423723
BmHel-8	DF090329:1609601-1609800
BmHel-8	DF090329:1611108-1611224
BmHel-8	DF090323:5861955-5862282
BmHel-8	DF090367:560825-561023
BmHel-8	DF090367:561049-561188
BmHel-8	DF090329:60417-60750
BmHel-8	DF090411:1157369-1157699
BmHel-8	DF090316:5785636-5785960
BmHel-8	DF090405:999196-999503
BmHel-8	DF090330:3678718-3679035
BmHel-8	DF090355:126898-127209
BmHel-8	DF090355:126814-126896
BmHel-8	DF090317:8565742-8566076
BmHel-8	DF090347:4090466-4090557
BmHel-8	DF090347:4090212-4090452
BmHel-8	DF090362:350486-350825
BmHel-8	DF090437:676307-676646
BmHel-8	DF090342:4929711-4930044
BmHel-8	DF090336:2337618-2337959
BmHel-8	DF090336:1146301-1146638
BmHel-8	DF090318:1939111-1939452
BmHel-8	DF090359:1423953-1424295
BmHel-8	DF090316:4901127-4901469
BmHel-8	DF090393:1674196-1674531
BmHel-8	DF090437:12037-12156
BmHel-8	DF090437:12182-12377
BmHel-8	DF090352:1715489-1715824
BmHel-8	DF090346:3162512-3162830
BmHel-8	DF090336:3429832-3430180
BmHel-8	DF090321:4225943-4226276
BmHel-8	DF090316:9928249-9928587
BmHel-8	DF090395:1643118-1643456
BmHel-8	DF090392:968379-968609
BmHel-8	DF090392:968292-968378
BmHel-8	DF090373:2301096-2301433
BmHel-8	DF090339:1717458-1717668
BmHel-8	DF090339:1717682-1717781

BmHel-8	DF090398:1311436-1311752
BmHel-8	DF090344:2750906-2751014
BmHel-8	DF090344:2751033-2751239
BmHel-8	DF090329:4413093-4413432
BmHel-8	DF090316:4358625-4358941
BmHel-8	DF090382:715529-715844
BmHel-8	DF090415:297951-298268
BmHel-8	DF090545:79775-80106
BmHel-8	DF090953:7234-7573
BmHel-8	BABH01055056:324-641
BmHel-8	DF090344:2792229-2792345
BmHel-8	DF090344:2792029-2792222
BmHel-8	DF090320:2001494-2001837
BmHel-8	DF090345:3817264-3817374
BmHel-8	DF090345:3817386-3817594
BmHel-8	DF090335:3183148-3183400
BmHel-8	DF090427:482212-482327
BmHel-8	DF090427:482006-482204
BmHel-8	DF090340:3736572-3736905
BmHel-8	DF090340:3625451-3625779
BmHel-8	BABH01045107:835-944
BmHel-8	BABH01045107:612-824
BmHel-8	DF090318:6951505-6951818
BmHel-8	DF090356:2286897-2287213
BmHel-8	DF090380:1645930-1646237
BmHel-8	DF090343:439372-439682
BmHel-8	DF090415:379570-379886
BmHel-8	DF090362:175351-175537
BmHel-8	DF090362:175184-175339
BmHel-8	DF090362:1339489-1339686
BmHel-8	DF090362:1339694-1339814
BmHel-8	DF090347:1515529-1515856
BmHel-8	DF090389:1393967-1394312
BmHel-8	DF090437:134295-134417
BmHel-8	DF090437:134437-134638
BmHel-8	DF090358:1022014-1022133
BmHel-8	DF090358:1021794-1022003
BmHel-8	DF090316:11201941-11202277
BmHel-8	DF090484:125670-126010
BmHel-8	DF090348:721434-721765
BmHel-8	DF090368:1913236-1913578
BmHel-8	DF090329:2574611-2574952
BmHel-8	DF090414:145575-145916
BmHel-8	DF090319:1795676-1795771
BmHel-8	DF090319:1795509-1795677
BmHel-8	DF090340:4156291-4156382
BmHel-8	DF090340:4156610-4156746
BmHel-8	DF090340:4156747-4156833

BmHel-8	DF090338:771184-771303
BmHel-8	DF090338:774921-775110
BmHel-8	DF090318:1824725-1825037
BmHel-8	DF090316:11600547-11600858
BmHel-8	DF090316:2684789-2685104
BmHel-8	DF090335:684261-684544
BmHel-8	DF090332:1305470-1305805
BmHel-8	DF090356:3001842-3002184
BmHel-8	DF090403:523701-524041
BmHel-8	DF090380:1721981-1722307
BmHel-8	DF090351:1675799-1676087
BmHel-8	DF090323:1305926-1306254
BmHel-8	DF090333:4250704-4250807
BmHel-8	DF090333:4250489-4250695
BmHel-8	DF090357:3766134-3766461
BmHel-8	DF090334:5863265-5863595
BmHel-8	BABH01044692:1393-1634
BmHel-8	BABH01044692:1735-1815
BmHel-8	DF090829:15661-15973
BmHel-8	DF090326:5539293-5539608
BmHel-8	DF090393:1363170-1363486
BmHel-8	DF090317:5765599-5765913
BmHel-8	BABH01080739:250-563
BmHel-8	DF090828:1052-1366
BmHel-8	DF090326:3090822-3091132
BmHel-8	DF090322:3517568-3517910
BmHel-8	DF090336:1524297-1524641
BmHel-8	DF090337:4842480-4842827
BmHel-8	DF090374:620666-620982
BmHel-8	DF090340:4552178-4552523
BmHel-8	DF090319:7525076-7525356
BmHel-8	BABH01045768:3322-3600
BmHel-8	DF090333:3733435-3733725
BmHel-8	DF090340:2823111-2823461
BmHel-8	DF090351:3753018-3753365
BmHel-8	DF090355:2945151-2945487
BmHel-8	DF090344:442301-442643
BmHel-8	DF090431:167255-167596
BmHel-8	BABH01055713:362-704
BmHel-8	DF090340:1900792-1901120
BmHel-8	DF090317:10889377-10889669
BmHel-8	DF090364:505180-505445
BmHel-8	DF090320:7223287-7223628
BmHel-8	DF090352:3623574-3623675
BmHel-8	DF090352:3623734-3623943
BmHel-8	DF090349:424748-425080
BmHel-8	DF090339:64713-65044
BmHel-8	DF090375:2002064-2002340

BmHel-8	DF090328:2037668-2038006
BmHel-8	DF090361:2948517-2948857
BmHel-8	DF090343:4464556-4464894
BmHel-8	DF090420:873800-874110
BmHel-8	DF090337:1550235-1550547
BmHel-8	DF090362:3195209-3195552
BmHel-8	DF090328:6413273-6413601
BmHel-8	DF090322:4987395-4987719
BmHel-8	DF090530:48384-48727
BmHel-8	DF090329:6099814-6099936
BmHel-8	DF090329:6099598-6099792
BmHel-8	DF090366:2086301-2086600
BmHel-8	DF090383:1920919-1921250
BmHel-8	DF090369:2263505-2263842
BmHel-8	DF090390:963775-964103
BmHel-8	DF090316:15519495-15519825
BmHel-8	DF090337:1032236-1032464
BmHel-8	DF090337:1031228-1031348
BmHel-8	DF090378:1773949-1774283
BmHel-8	DF090377:480589-480935
BmHel-8	DF090340:4900846-4901192
BmHel-8	DF090320:4957941-4958278
BmHel-8	DF090344:4690948-4691289
BmHel-8	DF090446:504197-504534
BmHel-8	DF090363:2729854-2730117
BmHel-8	DF090370:584696-585013
BmHel-8	DF090321:4387496-4387782
BmHel-8	DF090318:2643512-2643829
BmHel-8	BABH01054433:191-408
BmHel-8	DF090330:6335562-6335778
BmHel-8	DF090330:6335045-6335136
BmHel-8	DF090353:2086209-2086544
BmHel-8	DF090329:6378135-6378470
BmHel-8	BABH01049297:692-1027
BmHel-8	DF090386:8248-8558
BmHel-8	DF090338:3646938-3647250
BmHel-8	DF090392:1711059-1711401
BmHel-8	DF090319:4851011-4851344
BmHel-8	DF090423:916457-916790
BmHel-8	BABH01045800:3523-3850
BmHel-8	DF090350:1657804-1657906
BmHel-8	DF090350:1657568-1657786
BmHel-8	DF090322:8212178-8212520
BmHel-8	DF090781:89386-89713
BmHel-8	DF091657:838-1037
BmHel-8	DF091657:731-839
BmHel-8	DF090352:126115-126439
BmHel-8	DF090444:347931-348078

BmHel-8	DF090444:348087-348276
BmHel-8	DF090344:2862241-2862546
BmHel-8	DF090390:329369-329692
BmHel-8	DF090367:1760306-1760620
BmHel-8	DF090362:1320327-1320640
BmHel-8	DF090356:3638397-3638712
BmHel-8	DF090317:9830835-9831147
BmHel-8	DF090395:609983-610295
BmHel-8	BABH01081723:1-313
BmHel-8	DF090562:36564-36833
BmHel-8	DF090333:5565588-5565874
BmHel-8	DF090329:3752722-3753055
BmHel-8	DF090318:6676777-6676896
BmHel-8	DF090318:6676570-6676761
BmHel-8	DF090341:4747763-4748036
BmHel-8	DF090381:1738334-1738655
BmHel-8	DF090353:885012-885340
BmHel-8	DF090319:4625118-4625428
BmHel-8	DF090342:4311229-4311341
BmHel-8	DF090342:4311357-4311574
BmHel-8	DF090319:3325822-3326154
BmHel-8	DF090349:966917-967229
BmHel-8	DF090373:111067-111395
BmHel-8	DF090975:8012-8319
BmHel-8	DF090370:2675260-2675600
BmHel-8	DF090362:2978367-2978692
BmHel-8	DF090340:4844463-4844795
BmHel-8	DF090332:672674-672934
BmHel-8	DF090349:1822851-1822972
BmHel-8	DF090349:1822642-1822832
BmHel-8	DF090336:1978339-1978657
BmHel-8	DF090404:836383-836725
BmHel-8	DF090358:1962412-1962732
BmHel-8	DF090350:4186580-4186903
BmHel-8	DF090384:567767-568087
BmHel-8	DF090587:7359-7594
BmHel-8	DF090587:7253-7341
BmHel-8	DF090336:3973621-3973963
BmHel-8	DF090321:3798637-3798969
BmHel-8	DF090344:1170419-1170759
BmHel-8	DF090323:4390358-4390479
BmHel-8	DF090323:4390137-4390340
BmHel-8	DF090435:676474-676711
BmHel-8	DF090435:676736-676826
BmHel-8	DF090363:2911874-2912145
BmHel-8	DF090405:67899-68251
BmHel-8	DF090379:1603908-1604063
BmHel-8	DF090379:1603710-1603905

BmHel-8	DF090349:1229507-1229810
BmHel-8	DF090334:1942835-1943171
BmHel-8	DF090369:2615407-2615747
BmHel-8	DF090376:20057-20398
BmHel-8	DF090317:685170-685284
BmHel-8	DF090317:685032-685159
BmHel-8	DF090401:1115067-1115273
BmHel-8	DF090401:1115290-1115392
BmHel-8	DF090401:1364830-1365165
BmHel-8	DF090325:3288602-3288721
BmHel-8	DF090325:3288745-3288936
BmHel-8	DF090318:2601764-2602026
BmHel-8	DF090344:1086153-1086258
BmHel-8	DF090344:1086283-1086492
BmHel-8	DF090411:345077-345413
BmHel-8	BABH01082101:59-389
BmHel-8	DF090352:4064410-4064655
BmHel-8	DF090352:4063878-4063981
BmHel-8	DF090329:4984347-4984461
BmHel-8	DF090329:4984150-4984333
BmHel-8	DF090320:1951438-1951765
BmHel-8	DF090357:1702629-1702904
BmHel-8	DF090368:870148-870254
BmHel-8	DF090368:870276-870480
BmHel-8	DF090363:1743392-1743500
BmHel-8	DF090363:1743169-1743371
BmHel-8	DF090317:5007883-5008208
BmHel-8	DF090353:1030500-1030825
BmHel-8	DF090353:3796244-3796476
BmHel-8	DF090353:3795726-3795829
BmHel-8	DF090363:19832-20143
BmHel-8	DF090460:237882-238187
BmHel-8	DF090352:2157890-2158201
BmHel-8	DF090355:2948705-2949007
BmHel-8	DF090334:257169-257483
BmHel-8	DF090337:4591306-4591618
BmHel-8	DF090355:3494650-3494883
BmHel-8	DF090355:3494535-3494624
BmHel-8	DF090387:1339726-1339880
BmHel-8	DF090387:1341442-1341606
BmHel-8	DF090429:579954-580296
BmHel-8	DF090368:1708182-1708419
BmHel-8	DF090368:1708444-1708534
BmHel-8	DF090330:3784591-3784933
BmHel-8	DF090402:440868-441209
BmHel-8	DF090426:315965-316296
BmHel-8	DF090352:3348847-3349178
BmHel-8	DF090385:376197-376290

BmHel-8	DF090385:383129-383366
BmHel-8	DF090368:1379168-1379501
BmHel-8	DF090399:430261-430369
BmHel-8	DF090399:430397-430597
BmHel-8	DF090360:1972421-1972689
BmHel-8	DF090381:852217-852316
BmHel-8	DF090381:852342-852559
BmHel-8	DF090447:6977-7202
BmHel-8	DF090447:7216-7320
BmHel-8	DF090332:1435014-1435345
BmHel-8	DF090319:6900153-6900415
BmHel-8	DF090507:2002-2100
BmHel-8	DF090507:2117-2328
BmHel-8	DF090371:254290-254618
BmHel-8	DF090341:430815-431123
BmHel-8	DF090399:636262-636589
BmHel-8	DF090327:5929975-5930295
BmHel-8	DF090319:216932-217130
BmHel-8	DF090319:215402-215500
BmHel-8	DF090688:55727-55965
BmHel-8	DF090382:1833798-1834039
BmHel-8	BABH01047963:2-312
BmHel-8	BABH01085310:1-310
BmHel-8	DF090423:438271-438374
BmHel-8	DF090423:438050-438259
BmHel-8	BABH01042898:15073-15409
BmHel-8	DF090438:280971-281240
BmHel-8	DF090327:5257389-5257611
BmHel-8	DF090327:5257247-5257342
BmHel-8	DF090383:1221799-1221888
BmHel-8	DF090383:1221541-1221784
BmHel-8	DF090471:102900-103223
BmHel-8	DF090333:1993734-1993995
BmHel-8	DF090333:1993618-1993702
BmHel-8	DF090403:459989-460331
BmHel-8	DF090361:2313566-2313902
BmHel-8	DF090335:3254608-3254945
BmHel-8	BABH01081078:47-383
BmHel-8	BABH01085866:47-388
BmHel-8	DF090400:1167280-1167621
BmHel-8	DF090340:294450-294685
BmHel-8	DF090340:294342-294432
BmHel-8	DF090318:6159079-6159376
BmHel-8	DF090359:3014815-3015123
BmHel-8	DF090354:2026579-2026698
BmHel-8	DF090354:2026375-2026560
BmHel-8	DF090351:4322320-4322638
BmHel-8	DF090316:2460597-2460924

BmHel-8	DF090540:20245-20521
BmHel-8	DF090383:896598-896929
BmHel-8	DF090319:6198308-6198641
BmHel-8	DF090333:914170-914481
BmHel-8	DF090408:1277226-1277330
BmHel-8	DF090408:1277347-1277544
BmHel-8	DF090442:272349-272447
BmHel-8	DF090442:272130-272337
BmHel-8	DF090362:3150022-3150120
BmHel-8	DF090362:3149795-3150010
BmHel-8	DF090317:3045807-3046116
BmHel-8	BABH01049259:783-1089
BmHel-8	DF090540:84129-84435
BmHel-8	DF090355:683920-684020
BmHel-8	DF090355:684037-684245
BmHel-8	DF090332:1296415-1296564
BmHel-8	DF090332:1296590-1296769
BmHel-8	DF090317:9329841-9330175
BmHel-8	DF090319:1594248-1594413
BmHel-8	DF090319:1594096-1594248
BmHel-8	DF090326:2565642-2565963
BmHel-8	DF091000:12414-12735
BmHel-8	DF090388:1552606-1552954
BmHel-8	DF090327:2292184-2292512
BmHel-8	DF090373:1515263-1515378
BmHel-8	DF090373:1515392-1515606
BmHel-8	DF090336:543102-543443
BmHel-8	BABH01075679:46-387
BmHel-8	DF090348:1829171-1829506
BmHel-8	DF090335:2042492-2042833
BmHel-8	DF090364:913881-914219
BmHel-8	DF090349:1555585-1555910
BmHel-8	DF090385:243902-244241
BmHel-8	DF090346:3081915-3082232
BmHel-8	DF090344:3597597-3597933
BmHel-8	DF090317:3879958-3880068
BmHel-8	DF090317:3879736-3879938
BmHel-8	DF090321:3949747-3950070
BmHel-8	DF090344:1636557-1636869
BmHel-8	DF090349:2503108-2503221
BmHel-8	DF090349:2503232-2503443
BmHel-8	DF090390:1256076-1256372
BmHel-8	DF090425:655796-655910
BmHel-8	DF090425:655587-655785
BmHel-8	DF090387:652472-652797
BmHel-8	DF090376:1570556-1570876
BmHel-8	DF090355:240733-241040
BmHel-8	DF091850:1927-2227

BmHel-8	DF091430:2667-3008
BmHel-8	DF090325:1413625-1413809
BmHel-8	DF090325:1413834-1413994
BmHel-8	DF090401:758656-758892
BmHel-8	DF090401:758917-759003
BmHel-8	DF090339:688002-688119
BmHel-8	DF090339:687775-687985
BmHel-8	DF090580:38855-38960
BmHel-8	DF090580:38635-38803
BmHel-8	DF090316:2746186-2746433
BmHel-8	DF090316:2746843-2746946
BmHel-8	DF090320:1576491-1576828
BmHel-8	DF090326:180461-180796
BmHel-8	DF090412:713079-713346
BmHel-8	DF090400:1120141-1120319
BmHel-8	DF090400:1120344-1120497
BmHel-8	DF090365:1866273-1866607
BmHel-8	DF090334:4778805-4779018
BmHel-8	DF090378:787881-788140
BmHel-8	DF090429:222194-222292
BmHel-8	DF090429:221953-222159
BmHel-8	DF090429:221872-221951
BmHel-8	BABH01082830:1-234
BmHel-8	BABH01082830:259-349
BmHel-8	DF090383:1830030-1830333
BmHel-8	DF090318:9578751-9579000
BmHel-8	DF091031:1-309
BmHel-8	DF090573:4430-4730
BmHel-8	DF090551:19385-19716
BmHel-8	DF090317:9519258-9519376
BmHel-8	DF090317:9519396-9519606
BmHel-8	DF090365:2265342-2265628
BmHel-8	DF090343:3822428-3822740
BmHel-8	DF090324:3112639-3112984
BmHel-8	DF090342:1955454-1955690
BmHel-8	DF090342:1955738-1955826
BmHel-8	DF090345:3605733-3606080
BmHel-8	DF090357:1681532-1681873
BmHel-8	DF090321:517074-517337
BmHel-8	DF090320:3526960-3527201
BmHel-8	DF090320:3526826-3526961
BmHel-8	DF090334:5588850-5588945
BmHel-8	DF090334:5588949-5589158
BmHel-8	DF090324:6768205-6768405
BmHel-8	DF090324:6768410-6768531
BmHel-8	DF090349:1128891-1129230
BmHel-8	DF090345:3454271-3454606
BmHel-8	DF090324:3520135-3520217

BmHel-8	DF090324:3519691-3519915
BmHel-8	DF090389:1576356-1576585
BmHel-8	DF090389:1576611-1576703
BmHel-8	DF090368:1350696-1350953
BmHel-8	DF090386:1185552-1185845
BmHel-8	DF090390:1286771-1287077
BmHel-8	DF090350:2300732-2301037
BmHel-8	DF090372:2122016-2122350
BmHel-8	DF090348:709210-709478
BmHel-8	DF090357:729966-730296
BmHel-8	DF090325:6384908-6385017
BmHel-8	DF090325:6384687-6384886
BmHel-8	DF090415:385465-385741
BmHel-8	DF090330:5437054-5437361
BmHel-8	DF090316:5329788-5330118
BmHel-8	DF090396:177015-177244
BmHel-8	DF090396:176901-176989
BmHel-8	BABH01075885:296-600
BmHel-8	DF090358:1183376-1183722
BmHel-8	DF090349:2096471-2096694
BmHel-8	DF090349:2096737-2096827
BmHel-8	DF090475:139225-139444
BmHel-8	DF090475:139476-139563
BmHel-8	DF090439:124892-125179
BmHel-8	BABH01067640:269-555
BmHel-8	DF090720:10014-10197
BmHel-8	DF090720:10192-10329
BmHel-8	DF090322:7173911-7174029
BmHel-8	DF090322:7178468-7178673
BmHel-8	DF090328:2713072-2713406
BmHel-8	BABH01086681:46-387
BmHel-8	DF090317:11263898-11264011
BmHel-8	DF090317:11263680-11263882
BmHel-8	DF090348:2116565-2116869
BmHel-8	DF090354:2936896-2937203
BmHel-8	DF090412:793285-793403
BmHel-8	DF090412:793059-793244
BmHel-8	DF090321:2758719-2758952
BmHel-8	DF090321:2758589-2758677
BmHel-8	DF090319:3619378-3619646
BmHel-8	DF090375:470148-470483
BmHel-8	DF090427:72280-72391
BmHel-8	DF090427:72056-72257
BmHel-8	DF090383:1700164-1700259
BmHel-8	DF090383:1699937-1700142
BmHel-8	DF090339:4663302-4663603
BmHel-8	DF090353:1921592-1921701
BmHel-8	DF090353:1921733-1921928

BmHel-8	DF090357:3629210-3629319
BmHel-8	DF090357:3629349-3629545
BmHel-8	BABH01045180:5336-5445
BmHel-8	BABH01045180:5109-5304
BmHel-8	DF090342:2023453-2023566
BmHel-8	DF090342:2023215-2023406
BmHel-8	DF090443:404006-404332
BmHel-8	DF090354:987516-987660
BmHel-8	DF090354:987361-987520
BmHel-8	DF090483:171448-171774
BmHel-8	DF090401:536252-536503
BmHel-8	BABH01043098:2444-2595
BmHel-8	BABH01043098:2952-3137
BmHel-8	DF090342:137424-137729
BmHel-8	DF090316:2648482-2648782
BmHel-8	DF090321:7225801-7226136
BmHel-8	DF090329:5951315-5951436
BmHel-8	DF090329:5951450-5951658
BmHel-8	DF090319:2572617-2572957
BmHel-8	BABH01057972:346-682
BmHel-8	DF090351:1105989-1106224
BmHel-8	DF090351:1106259-1106352
BmHel-8	DF090340:4385991-4386333
BmHel-8	DF090326:5032779-5033119
BmHel-8	DF090379:1790502-1790622
BmHel-8	DF090379:1790284-1790476
BmHel-8	DF090352:2130228-2130346
BmHel-8	DF090352:2130014-2130201
BmHel-8	DF090328:4813065-4813368
BmHel-8	DF090390:841890-842008
BmHel-8	DF090390:834997-835197
BmHel-8	DF090376:1506758-1506976
BmHel-8	DF090376:1507008-1507101
BmHel-8	DF090325:5928965-5929263
BmHel-8	DF090387:61104-61288
BmHel-8	DF090387:61297-61416
BmHel-8	DF090357:3575582-3575912
BmHel-8	DF090338:3885041-3885377
BmHel-8	DF090348:1792886-1793190
BmHel-8	DF090348:1792798-1792878
BmHel-8	DF090398:183986-184268
BmHel-8	DF090375:2114839-2115153
BmHel-8	DF090399:1297887-1298191
BmHel-8	BABH01087077:121-445
BmHel-8	DF090485:130533-130847
BmHel-8	DF090316:15112367-15112565
BmHel-8	DF090316:15112220-15112338
BmHel-8	DF090455:388120-388357

BmHel-8	DF090324:1454877-1455219
BmHel-8	DF090359:2700974-2701247
BmHel-8	DF090763:3261-3383
BmHel-8	DF090763:3040-3235
BmHel-8	DF090333:307357-307527
BmHel-8	DF090333:307180-307341
BmHel-8	DF090328:6689322-6689661
BmHel-8	DF090340:995976-996318
BmHel-8	DF090411:70896-71241
BmHel-8	DF090421:13177-13289
BmHel-8	DF090421:12978-13167
BmHel-8	DF090345:2124239-2124352
BmHel-8	DF090345:2124381-2124585
BmHel-8	DF090335:1040619-1040710
BmHel-8	DF090335:1040382-1040592
BmHel-8	DF090335:1040298-1040380
BmHel-8	DF090324:4557290-4557390
BmHel-8	DF090324:4557412-4557626
BmHel-8	BABH01064800:289-626
BmHel-8	DF090320:5275778-5276035
BmHel-8	BABH01066708:46-377
BmHel-8	DF090335:5502856-5503155
BmHel-8	DF090340:3547969-3548267
BmHel-8	DF090332:6382952-6383214
BmHel-8	DF090411:1035687-1035989
BmHel-8	DF090321:2354549-2354639
BmHel-8	DF090321:2354831-2355034
BmHel-8	BABH01073222:1-304
BmHel-8	BABH01073222:306-386
BmHel-8	DF090374:1629349-1629569
BmHel-8	DF090374:1623458-1623549
BmHel-8	DF090351:1518739-1518843
BmHel-8	DF090351:1518379-1518574
BmHel-8	DF090332:4736948-4737268
BmHel-8	DF090326:244088-244179
BmHel-8	DF090326:243797-243885
BmHel-8	DF090326:243325-243481
BmHel-8	DF090335:4649530-4649646
BmHel-8	DF090335:4649676-4649873
BmHel-8	DF090339:877160-877377
BmHel-8	DF090339:877046-877146
BmHel-8	DF090442:154957-155077
BmHel-8	DF090442:155100-155297
BmHel-8	DF090352:1310291-1310407
BmHel-8	DF090352:1310063-1310271
BmHel-8	DF090364:139937-140188
BmHel-8	DF090352:3055281-3055622
BmHel-8	DF090402:935676-935933

BmHel-8	DF090358:320417-320613
BmHel-8	DF090358:320609-320731
BmHel-8	DF090344:1034256-1034594
BmHel-8	DF090330:6133303-6133640
BmHel-8	DF090344:1790920-1791221
BmHel-8	DF090369:628312-628488
BmHel-8	DF090369:628523-628650
BmHel-8	DF090325:2619473-2619563
BmHel-8	DF090325:2619594-2619804
BmHel-8	DF090325:3954216-3954446
BmHel-8	DF090325:3954467-3954555
BmHel-8	DF090343:299309-299583
BmHel-8	DF090343:4569141-4569374
BmHel-8	DF090424:690694-691015
BmHel-8	DF090319:2232337-2232663
BmHel-8	DF090456:279710-280026
BmHel-8	DF090316:2716385-2716683
BmHel-8	DF090347:3064840-3065142
BmHel-8	BABH01050093:4-304
BmHel-8	DF090357:346177-346480
BmHel-8	DF090377:2004116-2004320
BmHel-8	DF090377:2004341-2004463
BmHel-8	DF090362:3053229-3053351
BmHel-8	DF090362:3053370-3053567
BmHel-8	DF090377:2153917-2154253
BmHel-8	DF090359:2808102-2808440
BmHel-8	DF090350:3170942-3171055
BmHel-8	DF090350:3170722-3170924
BmHel-8	DF090398:1581000-1581097
BmHel-8	DF090398:1580762-1580974
BmHel-8	DF090328:1101022-1101137
BmHel-8	DF090328:1101145-1101324
BmHel-8	DF090336:1847565-1847882
BmHel-8	DF090325:2724517-2724827
BmHel-8	DF090838:6572-6674
BmHel-8	DF090838:6366-6557
BmHel-8	DF090332:2860713-2860968
BmHel-8	DF090337:954517-954846
BmHel-8	DF090321:3377727-3377937
BmHel-8	DF090321:3377618-3377712
BmHel-8	DF090443:427359-427669
BmHel-8	DF090316:7068036-7068330
BmHel-8	DF090334:133391-133698
BmHel-8	BABH01079924:259-566
BmHel-8	DF090360:264910-265207
BmHel-8	DF090405:889692-889993
BmHel-8	DF090325:6062523-6062824
BmHel-8	BABH01076709:1-301

BmHel-8	DF090474:299227-299410
BmHel-8	DF090474:299472-299559
BmHel-8	DF090352:269830-269967
BmHel-8	DF090352:269969-270126
BmHel-8	DF090351:4138799-4138905
BmHel-8	DF090351:4138929-4139148
BmHel-8	DF090411:80203-80325
BmHel-8	DF090411:80349-80546
BmHel-8	DF090360:2232206-2232491
BmHel-8	DF090325:5873533-5873840
BmHel-8	DF090453:453057-453397
BmHel-8	DF090436:173571-173814
BmHel-8	DF090436:173819-173902
BmHel-8	DF090335:6106-6223
BmHel-8	DF090335:5886-6082
BmHel-8	DF090471:199670-199852
BmHel-8	DF090471:199497-199624
BmHel-8	DF090996:4551-4782
BmHel-8	DF090996:4807-4897
BmHel-8	BABH01087638:1-333
BmHel-8	DF090361:1897102-1897425
BmHel-8	DF090391:857274-857482
BmHel-8	DF090391:857177-857271
BmHel-8	BABH01056527:363-691
BmHel-8	DF090374:1291956-1292202
BmHel-8	DF090358:327094-327275
BmHel-8	DF090358:326928-327050
BmHel-8	DF090360:1975243-1975590
BmHel-8	DF090340:3200223-3200341
BmHel-8	DF090340:3199997-3200210
BmHel-8	DF090441:114997-115108
BmHel-8	DF090441:114765-114974
BmHel-8	DF090346:1036276-1036512
BmHel-8	DF090357:1956994-1957116
BmHel-8	DF090357:1957143-1957338
BmHel-8	DF090377:266406-266736
BmHel-8	DF090361:1055796-1056132
BmHel-8	DF090319:5511667-5512002
BmHel-8	DF090316:9209099-9209200
BmHel-8	DF090316:9208687-9208883
BmHel-8	DF090396:1214105-1214404
BmHel-8	DF090585:58628-58927
BmHel-8	DF090316:14222448-14222535
BmHel-8	DF090316:14221882-14222128
BmHel-8	DF090400:493750-494063
BmHel-8	DF090449:426814-427149
BmHel-8	DF090446:275670-275849
BmHel-8	DF090446:275893-276017

BmHel-8	DF090354:739119-739217
BmHel-8	DF090354:739242-739452
BmHel-8	DF090349:2049688-2050027
BmHel-8	DF090345:2677771-2677955
BmHel-8	DF090345:2676159-2676278
BmHel-8	DF090326:36250-36453
BmHel-8	DF090326:36104-36227
BmHel-8	DF090359:602430-602649
BmHel-8	DF090359:602684-602779
BmHel-8	DF090317:2146307-2146635
BmHel-8	DF090430:821959-822174
BmHel-8	DF090430:822201-822296
BmHel-8	BABH01070249:37-372
BmHel-8	DF090353:3882749-3883040
BmHel-8	DF090366:2205338-2205638
BmHel-8	DF090366:2205915-2205997
BmHel-8	DF090364:2509695-2509814
BmHel-8	DF090364:2509836-2510011
BmHel-8	DF090317:1446366-1446660
BmHel-8	DF090349:74373-74670
BmHel-8	DF090324:7938009-7938112
BmHel-8	DF090324:7938146-7938345
BmHel-8	DF090368:1369524-1369735
BmHel-8	DF090368:1369406-1369512
BmHel-8	DF090355:522393-522707
BmHel-8	DF090549:62197-62504
BmHel-8	DF090320:6518524-6518817
BmHel-8	DF090388:1465323-1465625
BmHel-8	BABH01083334:238-535
BmHel-8	DF090338:4124018-4124233
BmHel-8	DF090338:4123893-4123979
BmHel-8	DF090342:3373264-3373599
BmHel-8	DF090378:1022208-1022426
BmHel-8	DF090378:1022084-1022175
BmHel-8	DF090348:1254100-1254412
BmHel-8	DF090352:357742-357961
BmHel-8	DF090352:358016-358106
BmHel-8	DF090355:3659359-3659578
BmHel-8	DF090355:3659230-3659320
BmHel-8	DF090317:7914782-7915001
BmHel-8	DF090317:7914649-7914739
BmHel-8	DF090331:2426807-2427030
BmHel-8	DF090331:2426674-2426764
BmHel-8	DF090333:389864-390082
BmHel-8	DF090333:389731-389821
BmHel-8	DF090469:36790-36912
BmHel-8	DF090469:36936-37120
BmHel-8	DF091074:7374-7597

BmHel-8	DF091074:7241-7331
BmHel-8	DF090365:1738822-1739164
BmHel-8	DF090316:1056115-1056444
BmHel-8	DF090362:764695-764930
BmHel-8	DF090407:148680-148857
BmHel-8	DF090407:148557-148687
BmHel-8	DF090338:4330518-4330603
BmHel-8	DF090338:4330286-4330502
BmHel-8	BABH01048211:979-1315
BmHel-8	DF090346:3517389-3517487
BmHel-8	DF090346:3517525-3517722
BmHel-8	DF090323:3256958-3257141
BmHel-8	DF090323:3256840-3256952
BmHel-8	DF090373:1061261-1061557
BmHel-8	DF090344:2147526-2147643
BmHel-8	DF090344:2147754-2147877
BmHel-8	DF090317:2993864-2994179
BmHel-8	DF090351:3704758-3704870
BmHel-8	DF090351:3704884-3705094
BmHel-8	DF090338:1652146-1652438
BmHel-8	DF090372:2271679-2272013
BmHel-8	DF090373:209960-210221
BmHel-8	DF090330:396051-396147
BmHel-8	DF090330:396160-396377
BmHel-8	DF090351:3052806-3053058
BmHel-8	DF090320:7632262-7632384
BmHel-8	DF090320:7632069-7632244
BmHel-8	DF090317:6532162-6532469
BmHel-8	DF090328:2674229-2674330
BmHel-8	DF090328:2674356-2674569
BmHel-8	DF090371:306688-306804
BmHel-8	DF090371:306834-307029
BmHel-8	DF090325:6998132-6998467
BmHel-8	DF090440:626833-627114
BmHel-8	BABH01082328:34-376
BmHel-8	DF090336:4283971-4284138
BmHel-8	DF090336:4283803-4283891
BmHel-8	DF090328:5512807-5513099
BmHel-8	DF090316:14843333-14843628
BmHel-8	DF090362:1286516-1286730
BmHel-8	DF090362:1286392-1286484
BmHel-8	DF090343:3133636-3133963
BmHel-8	DF090394:638733-638955
BmHel-8	DF090394:638618-638708
BmHel-8	DF090375:31860-32155
BmHel-8	BABH01071407:53-375
BmHel-8	DF090401:477134-477437
BmHel-8	DF090350:3715621-3715918

BmHel-8	DF090329:4674185-4674394
BmHel-8	DF090317:8923498-8923619
BmHel-8	DF090317:8923274-8923469
BmHel-8	DF090394:1425650-1425856
BmHel-8	DF090394:1425518-1425623
BmHel-8	DF090514:20080-20425
BmHel-8	DF090398:1392892-1393234
BmHel-8	DF090318:6815727-6816053
BmHel-8	DF090338:3452586-3452756
BmHel-8	DF090338:3452782-3452903
BmHel-8	DF090322:4100038-4100332
BmHel-8	DF090349:2291794-2292001
BmHel-8	DF090349:2291342-2291472
BmHel-8	DF090396:357741-357958
BmHel-8	DF090396:357610-357698
BmHel-8	DF090328:1191880-1192087
BmHel-8	DF090328:1192123-1192216
BmHel-8	BABH01045753:3588-3888
BmHel-8	DF090371:1265977-1266270
BmHel-8	BABH01049700:1-304
BmHel-8	DF090411:437217-437504
BmHel-8	DF090411:437511-437592
BmHel-8	DF090342:1097892-1098187
BmHel-8	DF090323:2364793-2365082
BmHel-8	DF090360:3157132-3157425
BmHel-8	DF090767:9766-10060
BmHel-8	DF090433:575021-575137
BmHel-8	DF090433:573226-573349
BmHel-8	DF090360:666572-666666
BmHel-8	DF090360:660633-660841
BmHel-8	DF090386:533339-533559
BmHel-8	DF090386:533221-533307
BmHel-8	DF090344:2404125-2404223
BmHel-8	DF090344:2404707-2404922
BmHel-8	DF090406:245053-245169
BmHel-8	DF090406:245203-245391
BmHel-8	DF090955:3354-3475
BmHel-8	DF090955:3488-3675
BmHel-8	DF090325:5484489-5484810
BmHel-8	BABH01083673:32-373
BmHel-8	DF091229:5816-6153
BmHel-8	DF090364:2541719-2541820
BmHel-8	DF090364:2541859-2542049
BmHel-8	DF090334:5699350-5699435
BmHel-8	DF090334:5699457-5699668
BmHel-8	BABH01049237:715-1052
BmHel-8	DF090362:626364-626572
BmHel-8	DF090362:626574-626656

BmHel-8	DF090384:468498-468791
BmHel-8	DF090318:3824690-3824799
BmHel-8	DF090318:3824468-3824670
BmHel-8	DF090344:43919-44020
BmHel-8	DF090344:44045-44245
BmHel-8	DF090322:4663288-4663616
BmHel-8	DF090350:1235090-1235195
BmHel-8	DF090350:1235210-1235425
BmHel-8	DF090318:4845402-4845695
BmHel-8	DF090329:3116775-3117110
BmHel-8	DF090366:69695-69777
BmHel-8	DF090366:70254-70465
BmHel-8	DF090384:368449-368733
BmHel-8	DF090317:10128749-10128857
BmHel-8	DF090317:10128534-10128736
BmHel-8	BABH01074714:1-313
BmHel-8	DF090343:4605965-4606262
BmHel-8	BABH01045443:4702-4999
BmHel-8	DF090360:1837221-1837510
BmHel-8	BABH01047693:11-313
BmHel-8	DF090359:1375732-1375920
BmHel-8	DF090359:1375927-1376035
BmHel-8	DF090398:1051960-1052264
BmHel-8	DF090330:4496556-4496885
BmHel-8	DF090386:439646-439763
BmHel-8	DF090386:439427-439633
BmHel-8	DF090338:4133126-4133345
BmHel-8	DF090338:4133392-4133478
BmHel-8	DF090322:3467857-3468038
BmHel-8	DF090322:3467651-3467769
BmHel-8	DF090316:3706275-3706496
BmHel-8	DF090316:3705983-3706071
BmHel-8	DF090354:3316591-3316809
BmHel-8	DF090354:3316864-3316951
BmHel-8	DF090455:264762-264884
BmHel-8	DF090455:264485-264589
BmHel-8	DF090455:264370-264460
BmHel-8	DF090325:2687171-2687273
BmHel-8	DF090325:2687300-2687514
BmHel-8	DF090316:13479711-13479928
BmHel-8	DF090316:13480000-13480090
BmHel-8	DF090360:1710293-1710509
BmHel-8	DF090360:1710551-1710645
BmHel-8	DF090337:4445990-4446112
BmHel-8	DF090337:4445772-4445963
BmHel-8	DF090366:328548-328800
BmHel-8	DF090408:1055043-1055357
BmHel-8	DF090317:7594657-7594994

BmHel-8	DF090371:2513466-2513805
BmHel-8	DF090370:2313662-2313975
BmHel-8	DF090352:2799463-2799747
BmHel-8	DF090332:5390453-5390563
BmHel-8	DF090332:5391535-5391742
BmHel-8	DF090350:3724282-3724617
BmHel-8	DF090327:3172084-3172421
BmHel-8	DF090323:2103443-2103561
BmHel-8	DF090323:2103577-2103755
BmHel-8	DF090390:1298478-1298752
BmHel-8	BABH01052667:466-802
BmHel-8	DF090707:2164-2276
BmHel-8	DF090707:2304-2498
BmHel-8	DF090383:1176936-1177251
BmHel-8	DF090324:7488249-7488345
BmHel-8	DF090324:7488371-7488593
BmHel-8	DF090400:963788-963901
BmHel-8	DF090400:963572-963764
BmHel-8	DF090338:4787217-4787447
BmHel-8	DF090407:865243-865569
BmHel-8	DF090348:4405085-4405340
BmHel-8	DF090358:1501276-1501568
BmHel-8	DF090367:2793423-2793710
BmHel-8	DF090401:5075-5365
BmHel-8	DF090365:2212551-2212658
BmHel-8	DF090365:2212321-2212536
BmHel-8	DF090344:816619-816788
BmHel-8	DF090344:816818-816940
BmHel-8	DF090513:12613-12724
BmHel-8	DF090513:12410-12595
BmHel-8	DF090351:1442798-1443127
BmHel-8	DF090372:1085387-1085659
BmHel-8	DF090369:322708-322811
BmHel-8	DF090369:322492-322696
BmHel-8	DF091156:4052-4357
BmHel-8	DF090341:1102455-1102556
BmHel-8	DF090341:1102220-1102430
BmHel-8	DF090361:2267532-2267832
BmHel-8	DF090357:2518782-2518895
BmHel-8	DF090357:2518541-2518742
BmHel-8	DF090352:2986965-2987141
BmHel-8	DF090352:2987143-2987277
BmHel-8	DF090317:7807658-7807985
BmHel-8	DF090319:3678298-3678471
BmHel-8	DF090319:3678471-3678619
BmHel-8	DF090364:2853162-2853435
BmHel-8	DF090320:3392390-3392616
BmHel-8	DF090320:3392236-3392343

BmHel-8	DF090327:6575098-6575310
BmHel-8	DF090327:6574961-6575053
BmHel-8	DF090409:1545856-1545937
BmHel-8	DF090409:1545390-1545594
BmHel-8	DF090322:4025443-4025545
BmHel-8	DF090322:4025222-4025428
BmHel-8	DF090349:361424-361512
BmHel-8	DF090349:361187-361396
BmHel-8	DF090484:237165-237365
BmHel-8	DF090484:237397-237491
BmHel-8	DF090329:3511177-3511505
BmHel-8	DF090338:3852818-3853109
BmHel-8	DF090401:1282082-1282193
BmHel-8	DF090401:1278682-1278868
BmHel-8	DF090366:702884-703175
BmHel-8	DF090348:4192961-4193263
BmHel-8	DF090343:1597739-1598035
BmHel-8	BABH01072345:331-623
BmHel-8	BABH01069130:1-81
BmHel-8	BABH01069130:93-303
BmHel-8	DF090365:625447-625654
BmHel-8	DF090365:625343-625439
BmHel-8	DF090349:2397406-2397693
BmHel-8	DF090389:966323-966616
BmHel-8	BABH01068221:356-648
BmHel-8	DF090368:2095689-2095949
BmHel-8	DF090335:5570161-5570283
BmHel-8	DF090335:5569942-5570135
BmHel-8	DF090381:292019-292257
BmHel-8	DF090480:76128-76342
BmHel-8	DF090480:76032-76118
BmHel-8	DF090387:1647669-1647780
BmHel-8	DF090387:1647814-1648014
BmHel-8	DF090343:159066-159157
BmHel-8	DF090343:158812-159017
BmHel-8	DF090398:825728-825902
BmHel-8	DF090398:825564-825685
BmHel-8	DF090351:2719284-2719523
BmHel-8	DF090370:2402269-2402482
BmHel-8	DF090370:2402521-2402611
BmHel-8	DF090370:2216812-2217145
BmHel-8	DF090365:840969-841055
BmHel-8	DF090365:840742-840948
BmHel-8	DF090328:2996032-2996128
BmHel-8	DF090328:2996149-2996357
BmHel-8	DF090349:2246860-2247181
BmHel-8	BABH01053043:579-811
BmHel-8	BABH01053043:61-164

BmHel-8	DF090448:90499-90701
BmHel-8	DF090448:90420-90502
BmHel-8	DF090354:3203782-3204074
BmHel-8	DF090322:6337462-6337566
BmHel-8	DF090322:6337966-6338152
BmHel-8	DF090383:1869548-1869662
BmHel-8	DF090383:1869696-1869795
BmHel-8	DF090383:1870215-1870313
BmHel-8	DF090358:2641895-2642194
BmHel-8	DF090336:4786976-4787160
BmHel-8	DF090336:4786813-4786938
BmHel-8	DF090316:2032749-2033064
BmHel-8	DF090857:14236-14420
BmHel-8	DF090567:37994-38104
BmHel-8	DF090567:37760-37959
BmHel-8	DF090383:376923-377102
BmHel-8	DF090383:376757-376880
BmHel-8	DF090335:4058418-4058615
BmHel-8	DF090335:4058277-4058365
BmHel-8	DF090367:2478344-2478521
BmHel-8	DF090367:2478180-2478304
BmHel-8	DF090318:6663915-6664248
BmHel-8	DF090319:5246127-5246229
BmHel-8	DF090319:5245897-5246089
BmHel-8	BABH01071543:40-374
BmHel-8	DF090331:2643395-2643503
BmHel-8	DF090331:2643179-2643384
BmHel-8	DF090394:718264-718593
BmHel-8	DF090429:333158-333482
BmHel-8	DF090351:2085756-2085847
BmHel-8	DF090351:2085543-2085743
BmHel-8	DF090325:1792056-1792380
BmHel-8	DF090340:1696319-1696629
BmHel-8	DF090342:276863-277145
BmHel-8	DF090343:3361121-3361405
BmHel-8	DF090339:1958426-1958524
BmHel-8	DF090339:1958568-1958758
BmHel-8	DF090323:6133482-6133664
BmHel-8	DF090323:6133284-6133408
BmHel-8	DF090412:1302751-1302844
BmHel-8	DF090412:1302878-1303095
BmHel-8	DF090342:4882455-4882669
BmHel-8	DF090342:4883368-4883454
BmHel-8	DF090341:1827067-1827167
BmHel-8	DF090341:1827198-1827403
BmHel-8	DF090339:2213010-2213120
BmHel-8	DF090339:2212771-2212981
BmHel-8	DF090384:1232448-1232567

BmHel-8	DF090384:1232582-1232717
BmHel-8	DF090356:3651224-3651425
BmHel-8	DF090356:3651076-3651204
BmHel-8	DF090365:523299-523513
BmHel-8	DF090365:523551-523637
BmHel-8	DF090319:7501603-7501817
BmHel-8	DF090319:7501476-7501563
BmHel-8	DF090401:214238-214332
BmHel-8	DF090401:213993-214195
BmHel-8	DF090386:164875-165201
BmHel-8	DF091011:9431-9720
BmHel-8	DF090349:600550-600859
BmHel-8	DF090578:28210-28505
BmHel-8	DF090393:1641887-1642009
BmHel-8	DF090393:1642112-1642279
BmHel-8	DF090323:2155000-2155297
BmHel-8	DF090466:153217-153328
BmHel-8	DF090466:153419-153610
BmHel-8	DF090340:416260-416441
BmHel-8	DF090340:416100-416229
BmHel-8	DF090347:842702-842917
BmHel-8	DF090347:842975-843055
BmHel-8	DF090342:4087780-4087995
BmHel-8	DF090342:4087652-4087739
BmHel-8	DF090345:4065198-4065309
BmHel-8	DF090345:4064962-4065159
BmHel-8	DF090361:632859-633196
BmHel-8	DF090316:8266877-8266969
BmHel-8	DF090316:8266640-8266851
BmHel-8	DF090336:3482356-3482472
BmHel-8	DF090336:3482135-3482330
BmHel-8	DF090328:3338493-3338596
BmHel-8	DF090328:3338331-3338480
BmHel-8	DF090323:60436-60537
BmHel-8	DF090323:60216-60408
BmHel-8	DF090343:4702283-4702581
BmHel-8	DF090322:5327077-5327169
BmHel-8	DF090322:5326834-5327051
BmHel-8	BABH01047327:320-424
BmHel-8	BABH01047327:95-296
BmHel-8	DF090394:1204150-1204254
BmHel-8	DF090394:1203953-1204129
BmHel-8	DF090330:3717642-3717929
BmHel-8	DF090317:7780978-7781143
BmHel-8	DF090317:7780813-7780948
BmHel-8	BABH01078552:6-294
BmHel-8	DF090345:889894-890180
BmHel-8	DF090376:730695-730789

BmHel-8	DF090376:730826-730987
BmHel-8	DF090336:2398162-2398431
BmHel-8	DF090360:2194881-2194971
BmHel-8	DF090360:2195003-2195218
BmHel-8	DF090389:1200988-1201090
BmHel-8	DF090389:1201125-1201330
BmHel-8	DF090325:971784-971889
BmHel-8	DF090325:971905-972125
BmHel-8	DF090317:855101-855350
BmHel-8	DF090340:1433380-1433697
BmHel-8	DF090336:1909798-1909913
BmHel-8	DF090336:1909577-1909768
BmHel-8	DF090367:1287059-1287258
BmHel-8	DF090367:1286919-1287013
BmHel-8	DF091334:2809-3093
BmHel-8	DF090341:4296457-4296567
BmHel-8	DF090341:4296600-4296775
BmHel-8	DF090322:1492740-1493030
BmHel-8	DF090321:3999673-3999843
BmHel-8	DF090321:3999240-3999395
BmHel-8	DF090422:950220-950407
BmHel-8	DF090422:950401-950572
BmHel-8	DF090422:950585-950665
BmHel-8	DF090326:5868778-5869088
BmHel-8	DF090362:2210542-2210661
BmHel-8	DF090362:2210771-2210895
BmHel-8	DF090390:909111-909202
BmHel-8	DF090390:908645-908846
BmHel-8	DF090354:1942457-1942551
BmHel-8	DF090354:1942217-1942430
BmHel-8	DF090338:1792103-1792199
BmHel-8	DF090338:1792230-1792438
BmHel-8	DF090338:4488741-4488951
BmHel-8	DF090338:4489000-4489090
BmHel-8	DF090348:3233714-3234019
BmHel-8	DF090356:2789015-2789137
BmHel-8	DF090356:2788798-2788991
BmHel-8	DF090322:757102-757433
BmHel-8	DF090318:8745486-8745604
BmHel-8	DF090318:8744937-8745103
BmHel-8	DF090323:6803331-6803445
BmHel-8	DF090323:6803457-6803619
BmHel-8	DF090319:8006965-8007065
BmHel-8	DF090319:8006731-8006920
BmHel-8	DF090790:19851-19967
BmHel-8	DF090790:19330-19527
BmHel-8	DF090317:4260843-4261063
BmHel-8	DF090317:4261067-4261153

BmHel-8	DF090317:1376664-1376956
BmHel-8	DF090328:4559252-4559491
BmHel-8	DF090317:909255-909543
BmHel-8	DF090407:281534-281731
BmHel-8	DF090363:2909053-2909192
BmHel-8	DF090363:2908832-2909043
BmHel-8	DF090323:5899156-5899449
BmHel-8	DF090323:4422014-4422298
BmHel-8	DF090481:170995-171267
BmHel-8	DF090319:3696954-3697238
BmHel-8	DF090340:1-286
BmHel-8	DF090372:231075-231362
BmHel-8	DF090342:4355613-4355818
BmHel-8	DF090342:4355529-4355612
BmHel-8	DF090330:1145296-1145517
BmHel-8	DF090375:445633-445849
BmHel-8	DF090375:441827-441926
BmHel-8	DF090342:1169114-1169226
BmHel-8	DF090342:1168857-1169072
BmHel-8	DF090335:3915191-3915378
BmHel-8	DF090335:3915407-3915573
BmHel-8	BABH01049379:746-852
BmHel-8	BABH01049379:544-744
BmHel-8	DF090344:1564509-1564793
BmHel-8	BABH01045330:5060-5339
BmHel-8	DF090394:1316413-1316517
BmHel-8	DF090394:1316557-1316755
BmHel-8	DF090368:1043121-1043421
BmHel-8	DF090415:941759-941878
BmHel-8	DF090415:941889-942071
BmHel-8	DF090339:587792-587913
BmHel-8	DF090339:587927-588134
BmHel-8	DF090378:460600-460786
BmHel-8	DF090386:1918584-1918766
BmHel-8	DF090386:1918810-1918924
BmHel-8	DF090441:233102-233218
BmHel-8	DF090441:232894-233084
BmHel-8	DF090487:138905-139237
BmHel-8	DF090318:939492-939687
BmHel-8	DF090318:939348-939441
BmHel-8	DF090373:44200-44310
BmHel-8	DF090373:43967-44170
BmHel-8	BABH01051275:1-285
BmHel-8	DF090316:5990032-5990351
BmHel-8	DF090370:1906680-1906960
BmHel-8	BABH01074517:1-284
BmHel-8	DF090347:566772-566946
BmHel-8	DF090347:566973-567097

BmHel-8	DF090359:2425810-2426024
BmHel-8	DF090397:908857-908973
BmHel-8	DF090397:905820-906013
BmHel-8	DF090330:5079909-5080020
BmHel-8	DF090330:5080036-5080222
BmHel-8	DF090353:2641991-2642095
BmHel-8	DF090353:2642137-2642324
BmHel-8	DF090346:2076723-2077024
BmHel-8	DF090397:618078-618335
BmHel-8	DF090327:1414687-1414896
BmHel-8	DF090327:1414579-1414662
BmHel-8	DF090319:1876051-1876349
BmHel-8	DF090461:270069-270366
BmHel-8	DF090319:823914-824020
BmHel-8	DF090319:823697-823879
BmHel-8	DF090386:1333518-1333842
BmHel-8	DF090370:791890-792082
BmHel-8	DF090370:791743-791837
BmHel-8	DF090407:586036-586339
BmHel-8	DF090330:6368731-6369021
BmHel-8	BABH01047753:1258-1550
BmHel-8	DF090410:389243-389525
BmHel-8	DF090449:256566-256847
BmHel-8	DF090378:2112623-2112906
BmHel-8	BABH01081257:275-558
BmHel-8	BABH01048618:1-283
BmHel-8	BABH01049423:229-510
BmHel-8	DF090466:300016-300225
BmHel-8	DF090466:300271-300357
BmHel-8	DF090353:1846707-1846907
BmHel-8	DF090353:1847505-1847629
BmHel-8	DF090327:6607558-6607753
BmHel-8	DF090327:6607800-6607880
BmHel-8	DF090384:1813580-1813692
BmHel-8	DF090384:1813400-1813569
BmHel-8	DF090349:109616-109901
BmHel-8	DF090346:1746876-1747041
BmHel-8	DF090346:1746711-1746834
BmHel-8	DF090373:934487-934697
BmHel-8	DF090324:7341933-7342130
BmHel-8	DF090324:7340866-7340945
BmHel-8	DF090324:7340781-7340860
BmHel-8	DF090382:886709-886986
BmHel-8	BABH01049648:3-284
BmHel-8	DF090333:3596737-3596853
BmHel-8	DF090333:3596893-3597077
BmHel-8	DF090358:1259450-1259668
BmHel-8	DF090358:1259354-1259433

BmHel-8	DF090316:6837141-6837440
BmHel-8	DF090322:3044888-3044996
BmHel-8	DF090322:3045031-3045222
BmHel-8	DF090322:1833994-1834088
BmHel-8	DF090322:1834570-1834757
BmHel-8	DF090419:142819-143132
BmHel-8	DF090330:2357212-2357325
BmHel-8	DF090330:2357026-2357193
BmHel-8	DF090330:5111711-5111795
BmHel-8	DF090330:5111482-5111696
BmHel-8	DF090342:1383196-1383312
BmHel-8	DF090342:1382991-1383167
BmHel-8	DF090319:3374822-3374925
BmHel-8	DF090319:3374953-3375053
BmHel-8	DF090319:3375078-3375169
BmHel-8	DF090488:126022-126324
BmHel-8	DF090412:544602-544872
BmHel-8	DF090350:3684244-3684553
BmHel-8	DF090343:3440473-3440570
BmHel-8	DF090343:3440609-3440802
BmHel-8	DF090425:882287-882402
BmHel-8	DF090425:882073-882189
BmHel-8	DF090350:1701861-1702115
BmHel-8	DF090332:2624118-2624394
BmHel-8	DF090396:863260-863534
BmHel-8	DF090374:2150557-2150830
BmHel-8	DF091235:1-273
BmHel-8	DF091235:275-355
BmHel-8	DF090356:1415168-1415441
BmHel-8	DF090344:3317941-3318052
BmHel-8	DF090344:3318073-3318156
BmHel-8	DF090344:3318194-3318281
BmHel-8	DF090322:3416811-3416911
BmHel-8	DF090322:3416943-3417050
BmHel-8	DF090322:3417072-3417157
BmHel-8	DF090412:280590-280711
BmHel-8	DF090412:280734-280899
BmHel-8	DF090346:626705-626802
BmHel-8	DF090346:626860-627052
BmHel-8	DF090344:4021261-4021347
BmHel-8	DF090344:4021046-4021255
BmHel-8	DF090358:516135-516312
BmHel-8	DF090412:736853-736966
BmHel-8	DF090412:736979-737190
BmHel-8	DF090398:255751-255841
BmHel-8	DF090398:256113-256271
BmHel-8	DF090347:207695-207796
BmHel-8	DF090347:207473-207671

BmHel-8	DF090363:543161-543448
BmHel-8	DF090321:1377162-1377309
BmHel-8	DF090360:3032098-3032209
BmHel-8	DF090360:3031604-3031790
BmHel-8	DF090329:3249683-3249767
BmHel-8	DF090329:3249448-3249647
BmHel-8	DF090418:906505-906584
BmHel-8	DF090418:906615-906832
BmHel-8	DF090460:196116-196413
BmHel-8	DF090395:1226349-1226645
BmHel-8	BABH01050131:677-972
BmHel-8	DF090319:1492491-1492701
BmHel-8	DF090338:3740320-3740620
BmHel-8	DF090330:2646620-2646735
BmHel-8	DF090330:2646746-2646916
BmHel-8	DF090358:2145813-2145924
BmHel-8	DF090358:2146064-2146178
BmHel-8	DF090350:4062101-4062190
BmHel-8	DF090350:4062230-4062375
BmHel-8	DF090347:1875428-1875668
BmHel-8	DF090363:1501409-1501514
BmHel-8	DF090363:1501177-1501382
BmHel-8	DF090421:395661-395778
BmHel-8	DF090421:395461-395631
BmHel-8	DF090324:260990-261108
BmHel-8	DF090324:260788-260961
BmHel-8	DF090340:1044654-1044937
BmHel-8	DF090401:1226739-1226947
BmHel-8	DF090401:1226603-1226698
BmHel-8	DF090362:1996011-1996189
BmHel-8	DF090362:1995837-1995956
BmHel-8	DF090373:1797936-1798046
BmHel-8	DF090373:1798091-1798279
BmHel-8	DF090362:2762614-2762924
BmHel-8	DF090358:2266643-2266744
BmHel-8	DF090358:2266767-2266960
BmHel-8	BABH01045090:5214-5345
BmHel-8	BABH01045090:5067-5214
BmHel-8	DF090562:2519-2752
BmHel-8	DF090356:3174832-3175135
BmHel-8	DF090317:2523531-2523702
BmHel-8	DF090317:2523706-2523827
BmHel-8	DF090422:819366-819643
BmHel-8	DF090329:3456505-3456775
BmHel-8	DF090342:424676-424787
BmHel-8	DF090342:432325-432535
BmHel-8	DF090359:3179875-3179985
BmHel-8	DF090359:3180012-3180196

BmHel-8	DF090345:886906-887110
BmHel-8	DF090345:887172-887262
BmHel-8	DF090353:3916939-3917227
BmHel-8	DF090316:3875385-3875484
BmHel-8	DF090316:3875160-3875357
BmHel-8	DF090316:4350334-4350522
BmHel-8	DF090316:4349955-4350055
BmHel-8	DF090400:1212713-1212903
BmHel-8	DF090400:1212627-1212709
BmHel-8	DF090400:1212335-1212414
BmHel-8	DF090355:1378784-1378866
BmHel-8	DF090355:1378576-1378771
BmHel-8	DF090339:1439713-1440018
BmHel-8	DF090396:488298-488565
BmHel-8	DF090386:49805-50081
BmHel-8	BABH01087215:158-435
BmHel-8	DF090443:535190-535393
BmHel-8	DF090319:2022117-2022439
BmHel-8	DF090339:4298657-4298862
BmHel-8	DF090339:4298922-4299011
BmHel-8	DF090375:45610-45788
BmHel-8	DF090375:45437-45532
BmHel-8	DF090402:248144-248341
BmHel-8	DF090402:249794-249888
BmHel-8	DF090316:4981733-4981972
BmHel-8	DF090354:2916602-2916885
BmHel-8	BABH01052172:472-780
BmHel-8	DF090408:28706-28967
BmHel-8	DF091073:7733-7942
BmHel-8	DF090318:5400494-5400769
BmHel-8	DF090359:479035-479132
BmHel-8	DF090359:479178-479269
BmHel-8	DF090359:479294-479383
BmHel-8	DF090319:6494789-6494899
BmHel-8	DF090319:6494556-6494746
BmHel-8	DF090510:36639-36740
BmHel-8	DF090510:36405-36608
BmHel-8	DF090319:1786080-1786264
BmHel-8	DF090319:1786305-1786394
BmHel-8	DF090320:1978995-1979271
BmHel-8	DF090339:2755680-2755928
BmHel-8	DF090339:2755978-2756060
BmHel-8	DF090396:1524132-1524329
BmHel-8	DF090396:1523981-1524071
BmHel-8	DF090400:1153556-1153833
BmHel-8	DF090354:2588070-2588173
BmHel-8	DF090354:2587881-2588051
BmHel-8	DF090316:16036899-16037208

BmHel-8	DF090327:3015597-3015890
BmHel-8	DF090416:832788-832907
BmHel-8	DF090416:832670-832771
BmHel-8	DF090388:1618906-1619199
BmHel-8	DF090367:1304579-1304747
BmHel-8	DF090367:1304798-1304922
BmHel-8	DF090392:1449085-1449264
BmHel-8	DF090392:1445123-1445240
BmHel-8	DF090354:2106724-2106841
BmHel-8	DF090354:2106487-2106607
BmHel-8	DF090358:53884-54170
BmHel-8	DF090340:3180365-3180664
BmHel-8	DF090348:4139167-4139370
BmHel-8	DF090348:4139031-4139116
BmHel-8	DF091129:2378-2674
BmHel-8	BABH01053649:2-222
BmHel-8	DF090343:3881404-3881605
BmHel-8	DF090343:3881633-3881727
BmHel-8	DF090345:1118707-1119004
BmHel-8	DF090324:3541178-3541463
BmHel-8	DF090744:5152-5240
BmHel-8	DF090744:5257-5361
BmHel-8	DF090744:5770-5873
BmHel-8	DF090744:5887-5966
BmHel-8	DF090418:300486-300574
BmHel-8	DF090418:300594-300798
BmHel-8	DF090353:3955300-3955574
BmHel-8	DF090329:3947727-3948020
BmHel-8	BABH01049907:735-1008
BmHel-8	DF090370:1403454-1403650
BmHel-8	DF090336:3246129-3246233
BmHel-8	DF090336:3245855-3245996
BmHel-8	DF090316:2077505-2077804
BmHel-8	DF090410:897269-897365
BmHel-8	DF090410:897530-897727
BmHel-8	DF090335:4074374-4074477
BmHel-8	DF090335:4074164-4074353
BmHel-8	DF090932:11260-11513
BmHel-8	DF090422:554969-555262
BmHel-8	DF090329:2806042-2806256
BmHel-8	DF090351:920445-920543
BmHel-8	DF090351:920575-920783
BmHel-8	DF090349:1574053-1574138
BmHel-8	DF090349:1574168-1574376
BmHel-8	DF090333:3883578-3883873
BmHel-8	DF090332:6334116-6334335
BmHel-8	BABH01082267:137-409
BmHel-8	DF090322:1794349-1794460

BmHel-8	DF090322:1794496-1794690
BmHel-8	DF090445:192700-192797
BmHel-8	DF090445:192504-192698
BmHel-8	DF090338:1222304-1222591
BmHel-8	DF090383:533441-533689
BmHel-8	DF090364:2200540-2200637
BmHel-8	DF090364:2200311-2200514
BmHel-8	DF090325:4259228-4259526
BmHel-8	DF090325:2191058-2191237
BmHel-8	DF090325:2190917-2191019
BmHel-8	DF090341:3145226-3145323
BmHel-8	DF090341:3145007-3145216
BmHel-8	DF090340:3299057-3299265
BmHel-8	DF090324:4573971-4574242
BmHel-8	DF090336:5333111-5333360
BmHel-8	DF090384:328008-328280
BmHel-8	DF090318:8911784-8912011
BmHel-8	DF090357:3205955-3206227
BmHel-8	DF090325:6288992-6289104
BmHel-8	DF090325:6289141-6289235
BmHel-8	DF090362:834842-835134
BmHel-8	DF090328:847090-847383
BmHel-8	DF090366:2711478-2711572
BmHel-8	DF090366:2711241-2711432
BmHel-8	DF090349:2518731-2518841
BmHel-8	DF090349:2518513-2518616
BmHel-8	DF090362:1854980-1855300
BmHel-8	DF090333:4077850-4078060
BmHel-8	DF090344:2169365-2169663
BmHel-8	DF090446:549621-549729
BmHel-8	DF090446:549469-549624
BmHel-8	DF090349:3103021-3103227
BmHel-8	DF090321:4085276-4085360
BmHel-8	DF090321:4084824-4085023
BmHel-8	DF090332:1767485-1767580
BmHel-8	DF090332:1767251-1767437
BmHel-8	DF090537:25839-26124
BmHel-8	DF090386:827254-827364
BmHel-8	DF090386:827080-827239
BmHel-8	DF090386:826996-827078
BmHel-8	DF090316:14736406-14736612
BmHel-8	DF090316:14736285-14736364
BmHel-8	DF090408:1451629-1451713
BmHel-8	DF090408:1451391-1451588
BmHel-8	DF090347:2373626-2373721
BmHel-8	DF090347:2373413-2373601
BmHel-8	DF090585:76271-76482
BmHel-8	BABH01074324:1-271

BmHel-8	DF090393:429784-429901
BmHel-8	DF090393:429974-430079
BmHel-8	DF090400:153198-153287
BmHel-8	DF090400:153472-153667
BmHel-8	DF090375:2290649-2290950
BmHel-8	DF090337:4894000-4894269
BmHel-8	DF090395:1413636-1413726
BmHel-8	DF090395:1413771-1413969
BmHel-8	DF090316:3826142-3826429
BmHel-8	DF090327:3321582-3321746
BmHel-8	DF090327:3321786-3321915
BmHel-8	DF090351:812222-812486
BmHel-8	DF090386:1913662-1913876
BmHel-8	DF090328:4742645-4742929
BmHel-8	DF090409:1081415-1081523
BmHel-8	DF090409:1081311-1081390
BmHel-8	DF090409:1081175-1081270
BmHel-8	DF090339:3824627-3824729
BmHel-8	DF090339:3824791-3824977
BmHel-8	DF090341:918764-918944
BmHel-8	DF090341:918594-918681
BmHel-8	DF090434:79164-79271
BmHel-8	DF090434:79303-79486
BmHel-8	DF090340:2808840-2809050
BmHel-8	DF090323:5116532-5116810
BmHel-8	DF090375:542518-542709
BmHel-8	DF090375:542741-542858
BmHel-8	DF090598:38660-38770
BmHel-8	DF090598:38505-38657
BmHel-8	DF090598:38420-38503
BmHel-8	DF090359:678802-678960
BmHel-8	DF090359:678959-679078
BmHel-8	DF090358:2517271-2517483
BmHel-8	DF090345:2406810-2407086
BmHel-8	DF090386:203517-203785
BmHel-8	DF090318:252253-252357
BmHel-8	DF090318:252410-252586
BmHel-8	DF090317:6572923-6573034
BmHel-8	DF090317:6573067-6573264
BmHel-8	DF090332:1899224-1899410
BmHel-8	DF090332:1899613-1899698
BmHel-8	DF090324:6154420-6154715
BmHel-8	DF090494:129824-129914
BmHel-8	DF090494:129941-130063
BmHel-8	DF090494:130074-130168
BmHel-8	DF090356:1969193-1969296
BmHel-8	DF090356:1969297-1969458
BmHel-8	DF090379:1857302-1857507

BmHel-8	DF090350:3955542-3955727
BmHel-8	DF090350:3963546-3963627
BmHel-8	BABH01087949:91-358
BmHel-8	DF090320:7016724-7016846
BmHel-8	DF090320:7016518-7016680
BmHel-8	DF090372:1706394-1706675
BmHel-8	DF090348:1948112-1948305
BmHel-8	DF090348:1948364-1948460
BmHel-8	DF090372:365014-365183
BmHel-8	DF090372:364898-364989
BmHel-8	DF090372:352370-352604
BmHel-8	DF090322:2664152-2664349
BmHel-8	DF090322:559812-559904
BmHel-8	DF090322:559916-560118
BmHel-8	DF090338:3680889-3681013
BmHel-8	DF090338:3681826-3681913
BmHel-8	DF090367:1060582-1060848
BmHel-8	BABH01060243:98-367
BmHel-8	DF090320:4626756-4626906
BmHel-8	DF090320:4627116-4627202
BmHel-8	DF090388:1446162-1446266
BmHel-8	DF090388:1445949-1446121
BmHel-8	DF090330:2427732-2427913
BmHel-8	DF090330:2427967-2428059
BmHel-8	DF090324:5004888-5004989
BmHel-8	DF090324:5004755-5004848
BmHel-8	DF090403:926564-926665
BmHel-8	DF090403:926329-926512
BmHel-8	DF090362:2938278-2938499
BmHel-8	DF090348:1472835-1472955
BmHel-8	DF090348:1472676-1472836
BmHel-8	DF090339:4080470-4080581
BmHel-8	DF090339:4080361-4080451
BmHel-8	DF090374:2193802-2193896
BmHel-8	DF090374:2194369-2194564
BmHel-8	DF090338:1721580-1721680
BmHel-8	DF090338:1721456-1721562
BmHel-8	DF090338:1721347-1721432
BmHel-8	DF090344:2566070-2566175
BmHel-8	DF090344:2566217-2566409
BmHel-8	DF090358:44181-44361
BmHel-8	DF090358:43336-43421
BmHel-8	DF090321:5030915-5031093
BmHel-8	DF090321:5031137-5031231
BmHel-8	DF090470:104845-105144
BmHel-8	DF090360:3100854-3101118
BmHel-8	DF090316:3245725-3245901
BmHel-8	DF090316:3245599-3245693

BmHel-8	BABH01045703:3906-4171
BmHel-8	DF090325:6088103-6088369
BmHel-8	DF090326:2372717-2372801
BmHel-8	DF090326:2372413-2372516
BmHel-8	DF090385:1880095-1880309
BmHel-8	DF090328:5964145-5964330
BmHel-8	DF090328:5964373-5964461
BmHel-8	DF090338:1541051-1541253
BmHel-8	DF090338:1540943-1541024
BmHel-8	DF090320:3572515-3572637
BmHel-8	DF090320:3572660-3572754
BmHel-8	DF090320:3572777-3572861
BmHel-8	DF090322:5515551-5515737
BmHel-8	DF090322:5515816-5515910
BmHel-8	DF090429:51345-51441
BmHel-8	DF090429:51486-51680
BmHel-8	DF090394:1299108-1299224
BmHel-8	DF090394:1298875-1299038
BmHel-8	DF090386:404993-405115
BmHel-8	DF090386:403109-403276
BmHel-8	DF090399:589116-589294
BmHel-8	DF090399:579411-579596
BmHel-8	DF090322:5743658-5743937
BmHel-8	DF090316:11671377-11671605
BmHel-8	DF090348:2469171-2469277
BmHel-8	DF090348:2469359-2469483
BmHel-8	DF090403:92277-92481
BmHel-8	DF090336:3350271-3350452
BmHel-8	DF090336:3350108-3350203
BmHel-8	DF090317:8896119-8896240
BmHel-8	DF090317:8896389-8896478
BmHel-8	DF090349:520319-520403
BmHel-8	DF090349:519858-520063
BmHel-8	DF090330:3088430-3088769
BmHel-8	DF090370:110888-111231
BmHel-8	DF090412:403088-403434
BmHel-8	DF090386:932814-933159
BmHel-8	DF090352:320706-321052
BmHel-8	DF090351:3941167-3941520
BmHel-8	DF090421:978931-979273
BmHel-8	DF090363:1556023-1556362
BmHel-8	DF090321:7647356-7647701
BmHel-8	DF090321:3978432-3978773
BmHel-8	DF090362:473857-474197
BmHel-8	DF090362:922965-923307
BmHel-8	DF090359:727139-727478
BmHel-8	DF090357:1857428-1857763
BmHel-8	DF090344:3526637-3526979

BmHel-8	DF090340:3313175-3313517
BmHel-8	DF090317:8185020-8185355
BmHel-8	DF090316:4566204-4566545
BmHel-8	DF090316:8757734-8758075
BmHel-8	DF090392:854588-854929
BmHel-8	DF090382:1138637-1138975
BmHel-8	DF090372:2061179-2061519
BmHel-8	DF090371:38196-38547
BmHel-8	DF090369:2003289-2003630
BmHel-8	DF090361:737810-738154
BmHel-8	DF090347:934657-935002
BmHel-8	DF090345:830914-831250
BmHel-8	DF090334:3290501-3290851
BmHel-8	DF090331:6000399-6000741
BmHel-8	DF090390:269105-269446
BmHel-8	DF090339:3736666-3737006
BmHel-8	DF090425:968062-968403
BmHel-8	DF090374:1171649-1171990
BmHel-8	DF090418:918013-918363
BmHel-8	BABH01087514:52-393
BmHel-8	BABH01049091:572-913
BmHel-8	DF090417:489774-490110
BmHel-8	DF090417:607697-608040
BmHel-8	BABH01048427:431-770
BmHel-8	BABH01051197:560-903
BmHel-8	DF090368:1386813-1387155
BmHel-8	DF090393:863724-864052
BmHel-8	DF091841:2470-2811
BmHel-8	BABH01082908:24-365
BmHel-8	BABH01050757:13-350
BmHel-8	BABH01071728:9-350
BmHel-8	DF090636:33276-33617
BmHel-8	DF090440:236012-236357
BmHel-8	BABH01049605:607-949
BmHel-8	DF091349:273-610
BmHel-8	BABH01044729:7204-7546
BmHel-8	BABH01050720:508-853
BmHel-8	DF090327:4444831-4445012
BmHel-8	DF090327:4444666-4444748
BmHel-8	DF090317:6301120-6301216
BmHel-8	DF090317:6300806-6300967
BmHel-8	DF090332:3443786-3443883
BmHel-8	DF090332:3443569-3443735
BmHel-8	DF090350:1519069-1519183
BmHel-8	DF090350:1519219-1519383
BmHel-8	DF090324:3975576-3975769
BmHel-8	DF090324:3975806-3975893
BmHel-8	DF090381:364054-364147

BmHel-8	DF090381:364188-364381
BmHel-8	DF090322:547122-547406
BmHel-8	DF090326:6175814-6176107
BmHel-8	DF090411:624449-624540
BmHel-8	DF090411:624569-624777
BmHel-8	DF090587:17289-17552
BmHel-8	DF090362:1404189-1404450
BmHel-8	DF090355:566830-567065
BmHel-8	DF090336:2813583-2813921
BmHel-8	DF090347:765218-765329
BmHel-8	DF090347:765005-765193
BmHel-8	DF090406:916231-916405
BmHel-8	DF090406:916070-916165
BmHel-8	DF090413:283398-283699
BmHel-8	DF090346:3375693-3376033
BmHel-8	DF090317:3478559-3478899
BmHel-8	DF090321:6354202-6354551
BmHel-8	DF090354:2005627-2005970
BmHel-8	DF090411:694366-694485
BmHel-8	DF090411:694140-694335
BmHel-8	DF090369:1790464-1790552
BmHel-8	DF090369:1790234-1790436
BmHel-8	DF090351:2030830-2031024
BmHel-8	DF090351:2023083-2023177
BmHel-8	DF090319:6918475-6918590
BmHel-8	DF090319:6918259-6918414
BmHel-8	DF090325:1989387-1989499
BmHel-8	DF090325:1989548-1989651
BmHel-8	DF090325:1990072-1990171
BmHel-8	DF090337:847365-847628
BmHel-8	DF090361:1545872-1546144
BmHel-8	DF090356:2143659-2143843
BmHel-8	DF090356:2143498-2143617
BmHel-8	DF090431:271629-271811
BmHel-8	DF090431:271887-271981
BmHel-8	DF090969:3024-3208
BmHel-8	DF090969:3255-3342
BmHel-8	DF090372:650876-651059
BmHel-8	DF090372:651104-651229
BmHel-8	DF090366:1725650-1725761
BmHel-8	DF090366:1725779-1725867
BmHel-8	DF090366:1725906-1725993
BmHel-8	DF090427:451752-452040
BmHel-8	DF090455:396678-396764
BmHel-8	DF090455:396264-396466
BmHel-8	DF090340:3454351-3454688
BmHel-8	DF090422:506521-506870
BmHel-8	DF090334:227671-228012

BmHel-8	DF090318:2563078-2563377
BmHel-8	DF091372:3052-3389
BmHel-8	DF090332:1710158-1710255
BmHel-8	DF090332:1710272-1710359
BmHel-8	DF090332:1716539-1716624
BmHel-8	DF090397:1212131-1212413
BmHel-8	DF090330:973618-973712
BmHel-8	DF090330:973448-973598
BmHel-8	DF090361:1077588-1077877
BmHel-8	DF090333:4319996-4320277
BmHel-8	DF090376:752609-752830
BmHel-8	BABH01069433:379-640
BmHel-8	DF090360:3286734-3286820
BmHel-8	DF090339:4773071-4773342
BmHel-8	DF090332:5692375-5692557
BmHel-8	DF090332:5692248-5692347
BmHel-8	DF090324:188121-188237
BmHel-8	DF090324:187926-188030
BmHel-8	DF090368:1002684-1002869
BmHel-8	DF090368:1002540-1002630
BmHel-8	DF090362:233846-233957
BmHel-8	DF090362:233979-234060
BmHel-8	DF090362:234100-234192
BmHel-8	DF090358:1763848-1764032
BmHel-8	DF090358:1766521-1766644
BmHel-8	DF090319:7243953-7244297
BmHel-8	DF090337:4355202-4355539
BmHel-8	DF090374:2275560-2275903
BmHel-8	BABH01086334:1-340
BmHel-8	BABH01081857:23-362
BmHel-8	BABH01049230:746-1085
BmHel-8	DF090422:820461-820730
BmHel-8	DF090402:344232-344570
BmHel-8	DF090360:2026148-2026482
BmHel-8	DF090335:5026066-5026404
BmHel-8	DF090414:1279919-1280258
BmHel-8	DF090364:715089-715427
BmHel-8	BABH01052090:418-750
BmHel-8	DF090433:540978-541175
BmHel-8	DF090433:540852-540931
BmHel-8	DF090339:2242230-2242431
BmHel-8	DF090339:2242470-2242564
BmHel-8	DF090322:4255251-4255427
BmHel-8	DF090322:4255114-4255231
BmHel-8	DF090459:330882-331184
BmHel-8	DF090431:400237-400446
BmHel-8	DF090357:2557453-2557548
BmHel-8	DF090357:2557226-2557416

BmHel-8	DF090316:6678616-6678922
BmHel-8	DF091365:2631-2882
BmHel-8	DF090628:8984-9240
BmHel-8	DF090368:1020661-1020844
BmHel-8	DF090368:1020500-1020592
BmHel-8	DF090354:1087703-1087882
BmHel-8	DF090354:1087536-1087630
BmHel-8	DF090340:3372217-3372338
BmHel-8	DF090340:3372368-3372523
BmHel-8	DF090321:2020077-2020199
BmHel-8	DF090321:2019922-2020047
BmHel-8	DF090341:2058118-2058240
BmHel-8	DF090341:2057946-2058099
BmHel-8	DF090373:476460-476571
BmHel-8	DF090373:476262-476441
BmHel-8	DF090383:1359293-1359634
BmHel-8	DF090317:1842842-1843182
BmHel-8	DF090369:2092477-2092821
BmHel-8	DF090374:2068956-2069298
BmHel-8	BABH01057798:357-697
BmHel-8	DF090324:3996362-3996704
BmHel-8	DF090787:15367-15708
BmHel-8	DF090349:1793500-1793779
BmHel-8	DF090331:4371151-4371484
BmHel-8	DF090344:3196653-3196943
BmHel-8	DF090353:2393271-2393502
BmHel-8	DF090401:19073-19169
BmHel-8	DF090401:18864-19032
BmHel-8	DF090349:1644360-1644614
BmHel-8	DF090466:130295-130568
BmHel-8	DF090319:273794-274057
BmHel-8	BABH01050350:705-966
BmHel-8	DF090319:2465520-2465781
BmHel-8	DF090347:1056827-1057126
BmHel-8	DF090369:986722-986841
BmHel-8	DF090369:986546-986695
BmHel-8	DF090405:455059-455242
BmHel-8	DF090405:454899-454990
BmHel-8	DF090325:3063808-3063907
BmHel-8	DF090325:3063953-3064138
BmHel-8	DF090330:2079912-2080035
BmHel-8	DF090330:2080083-2080247
BmHel-8	DF090573:48403-48557
BmHel-8	DF090573:48593-48779
BmHel-8	DF090398:984899-985235
BmHel-8	BABH01048207:409-741
BmHel-8	DF090352:247947-248288
BmHel-8	DF090355:431097-431279

BmHel-8	DF090355:430935-431023
BmHel-8	DF090369:2498431-2498529
BmHel-8	DF090369:2498193-2498373
BmHel-8	DF090377:510790-511126
BmHel-8	DF090322:6114776-6115113
BmHel-8	BABH01069258:179-515
BmHel-8	DF090354:3223617-3223728
BmHel-8	DF090354:3223798-3223900
BmHel-8	DF090329:6407207-6407380
BmHel-8	DF090329:6407063-6407154
BmHel-8	DF090619:37905-38108
BmHel-8	DF090328:6348935-6349108
BmHel-8	DF090328:6349166-6349261
BmHel-8	DF090319:5921623-5921879
BmHel-8	DF090401:1452622-1452744
BmHel-8	DF090401:1452870-1452994
BmHel-8	DF090316:3743564-3743658
BmHel-8	DF090316:3743878-3743966
BmHel-8	DF090316:3744044-3744136
BmHel-8	DF090413:999351-999625
BmHel-8	DF090351:3725599-3725935
BmHel-8	DF090342:287509-287844
BmHel-8	DF090384:904232-904558
BmHel-8	DF090345:2354292-2354552
BmHel-8	DF090347:1809173-1809268
BmHel-8	DF090347:1808970-1809143
BmHel-8	DF090393:773998-774336
BmHel-8	DF090579:28635-28748
BmHel-8	DF090579:28415-28597
BmHel-8	DF090349:3782591-3782888
BmHel-8	DF090340:3945757-3946101
BmHel-8	DF090390:1177018-1177352
BmHel-8	BABH01045581:4176-4510
BmHel-8	DF090414:679149-679422
BmHel-8	DF090336:4635246-4635418
BmHel-8	DF090336:4635473-4635572
BmHel-8	DF090316:2360114-2360376
BmHel-8	DF090428:674628-674709
BmHel-8	DF090428:674398-674590
BmHel-8	DF090369:1544244-1544504
BmHel-8	DF090547:31607-31865
BmHel-8	DF090332:4560584-4560764
BmHel-8	DF090332:4560833-4560919
BmHel-8	DF090410:217854-218130
BmHel-8	DF090363:475479-475659
BmHel-8	DF090363:475743-475839
BmHel-8	DF090334:2455779-2455963
BmHel-8	DF090332:5516030-5516354

BmHel-8	DF090367:2620269-2620609
BmHel-8	DF090359:3150858-3151192
BmHel-8	DF090341:3472004-3472333
BmHel-8	DF090331:231055-231389
BmHel-8	BABH01048213:1019-1352
BmHel-8	DF090339:992444-992770
BmHel-8	DF090833:10572-10834
BmHel-8	DF090411:610039-610300
BmHel-8	DF090380:1668338-1668519
BmHel-8	DF090380:1668164-1668258
BmHel-8	DF090390:1451540-1451874
BmHel-8	DF090343:112034-112122
BmHel-8	DF090343:111435-111626
BmHel-8	BABH01049215:29-365
BmHel-8	DF090382:109617-109780
BmHel-8	DF090382:109861-109953
BmHel-8	DF090344:1800172-1800380
BmHel-8	DF090362:2073184-2073269
BmHel-8	DF090362:2073299-2073404
BmHel-8	DF090362:2073438-2073527
BmHel-8	DF090486:141662-141853
BmHel-8	DF090317:6907588-6907844
BmHel-8	DF090351:426564-426660
BmHel-8	DF090351:427508-427680
BmHel-8	DF090321:538706-538822
BmHel-8	DF090321:538479-538565
BmHel-8	DF090324:5558961-5559201
BmHel-8	DF090324:2048385-2048662
BmHel-8	BABH01052587:126-353
BmHel-8	BABH01052587:1-122
BmHel-8	DF090351:3246455-3246780
BmHel-8	DF090324:2242566-2242900
BmHel-8	BABH01057545:393-727
BmHel-8	DF090741:8-335
BmHel-8	DF091237:2772-3100
BmHel-8	DF090333:3641427-3641725
BmHel-8	DF090316:1424666-1424763
BmHel-8	DF090316:1424799-1424962
BmHel-8	DF090360:1824300-1824562
BmHel-8	DF090376:170159-170497
BmHel-8	DF090378:506526-506636
BmHel-8	DF090378:506311-506470
BmHel-8	DF090332:703495-703701
BmHel-8	DF090389:1591629-1591764
BmHel-8	DF090349:3828878-3829128
BmHel-8	DF090716:52170-52425
BmHel-8	BABH01074921:351-606
BmHel-8	DF091813:1-255

BmHel-8	BABH01049618:1-251
BmHel-8	DF090347:1348795-1348981
BmHel-8	DF090347:1348628-1348716
BmHel-8	DF090431:114060-114243
BmHel-8	DF090431:114327-114411
BmHel-8	DF090393:1224069-1224252
BmHel-8	DF090393:1224321-1224406
BmHel-8	DF090446:41558-41664
BmHel-8	DF090446:41665-41828
BmHel-8	BABH01086624:1-334
BmHel-8	DF090345:2730670-2731004
BmHel-8	DF090775:15852-16185
BmHel-8	DF090342:1348536-1348718
BmHel-8	DF090316:6734834-6734950
BmHel-8	DF090316:6734997-6735137
BmHel-8	DF090381:713364-713536
BmHel-8	DF090381:713585-713666
BmHel-8	DF090339:1621206-1621315
BmHel-8	DF090339:1621320-1621459
BmHel-8	DF091416:1-335
BmHel-8	DF090362:2564199-2564530
BmHel-8	DF090365:830374-830591
BmHel-8	DF090328:283575-283840
BmHel-8	DF090354:784326-784590
BmHel-8	DF090448:37868-38067
BmHel-8	DF090448:38127-38217
BmHel-8	DF090318:4955246-4955525
BmHel-8	DF090347:3674551-3674645
BmHel-8	DF090347:3674688-3674879
BmHel-8	DF090369:41436-41647
BmHel-8	DF090369:40819-40954
BmHel-8	DF090336:2471053-2471386
BmHel-8	BABH01083326:205-535
BmHel-8	DF090351:586854-587049
BmHel-8	DF090326:2990200-2990301
BmHel-8	DF090326:2990334-2990431
BmHel-8	DF090389:1123881-1124210
BmHel-8	DF090327:5315806-5315894
BmHel-8	DF090327:5316027-5316153
BmHel-8	DF090332:2773816-2773931
BmHel-8	DF090332:2773989-2774146
BmHel-8	DF090431:95425-95603
BmHel-8	DF090431:95635-95728
BmHel-8	DF090365:2055891-2056065
BmHel-8	DF090365:2055722-2055816
BmHel-8	DF090408:1094518-1094629
BmHel-8	DF090408:1094275-1094363
BmHel-8	DF090386:1142179-1142512

BmHel-8	DF090342:2810753-2811076
BmHel-8	BABH01078687:219-551
BmHel-8	BABH01072063:10-342
BmHel-8	DF090338:3066931-3067213
BmHel-8	DF090336:3161981-3162234
BmHel-8	DF090342:2065989-2066242
BmHel-8	DF090317:4595926-4596174
BmHel-8	DF091087:4374-4575
BmHel-8	DF090331:4463647-4463773
BmHel-8	DF090330:5259234-5259416
BmHel-8	DF090330:5259074-5259153
BmHel-8	DF090318:1080413-1080529
BmHel-8	DF090318:1080541-1080624
BmHel-8	DF090388:609061-609177
BmHel-8	DF090388:608819-608914
BmHel-8	DF090319:5183007-5183123
BmHel-8	DF090319:5183257-5183347
BmHel-8	DF090349:3186003-3186104
BmHel-8	DF090349:3186138-3186260
BmHel-8	DF090434:299646-299830
BmHel-8	DF090434:299908-299998
BmHel-8	DF090329:6323676-6323861
BmHel-8	DF090329:6323526-6323620
BmHel-8	DF090317:169252-169537
BmHel-8	DF090340:1589586-1589917
BmHel-8	DF090373:1531850-1532176
BmHel-8	DF090332:4085202-4085316
BmHel-8	DF090332:4084963-4085057
BmHel-8	BABH01058691:387-716
BmHel-8	DF090323:1572490-1572675
BmHel-8	DF090543:19052-19382
BmHel-8	DF090342:4380042-4380154
BmHel-8	DF090342:4380204-4380367
BmHel-8	DF090328:6491443-6491553
BmHel-8	DF090328:6491891-6492060
BmHel-8	DF090330:1124946-1125229
BmHel-8	DF090317:4921377-4921614
BmHel-8	DF090337:2588466-2588582
BmHel-8	DF090337:2588243-2588458
BmHel-8	DF090402:1410476-1410806
BmHel-8	DF090336:1584742-1585069
BmHel-8	DF090357:1713104-1713285
BmHel-8	DF090357:1713355-1713444
BmHel-8	DF090332:2091152-2091264
BmHel-8	DF090332:2091295-2091449
BmHel-8	DF091544:2533-2733
BmHel-8	DF090418:281869-282039
BmHel-8	DF090418:281423-281510

BmHel-8	BABH01053274:10-332
BmHel-8	BABH01051898:7-335
BmHel-8	DF090397:1008056-1008255
BmHel-8	DF090377:1598643-1598902
BmHel-8	BABH01074478:1-251
BmHel-8	DF090377:1579019-1579214
BmHel-8	DF090326:4642829-4643014
BmHel-8	DF090326:4643079-4643166
BmHel-8	DF090398:1385364-1385482
BmHel-8	DF090398:1386280-1386364
BmHel-8	DF090316:13595996-13596328
BmHel-8	BABH01050689:1-318
BmHel-8	DF090358:2473237-2473374
BmHel-8	DF090358:2473066-2473189
BmHel-8	DF090412:25288-25618
BmHel-8	DF090361:1782040-1782368
BmHel-8	DF090363:497252-497460
BmHel-8	DF090374:335387-335590
BmHel-8	DF090341:2815197-2815368
BmHel-8	DF090341:2815433-2815527
BmHel-8	DF090350:1286313-1286641
BmHel-8	DF090518:5810-6073
BmHel-8	BABH01072624:371-621
BmHel-8	BABH01085263:254-504
BmHel-8	DF090363:943659-943846
BmHel-8	DF090363:943906-944007
BmHel-8	DF090413:89468-89599
BmHel-8	DF090413:89300-89387
BmHel-8	DF090410:267549-267650
BmHel-8	DF090410:267313-267498
BmHel-8	DF090382:1803357-1803691
BmHel-8	DF090404:1227662-1227984
BmHel-8	DF091620:2671-2997
BmHel-8	DF090385:1688794-1688972
BmHel-8	DF090385:1688681-1688763
BmHel-8	DF090377:1444057-1444306
BmHel-8	DF090379:178798-178877
BmHel-8	DF090379:178926-179022
BmHel-8	DF090332:1225653-1225740
BmHel-8	DF090332:1225399-1225491
BmHel-8	DF090339:4127681-4127770
BmHel-8	DF090339:4127430-4127527
BmHel-8	DF090380:1524611-1524789
BmHel-8	DF090378:902114-902294
BmHel-8	DF090378:902003-902082
BmHel-8	BABH01080644:308-561
BmHel-8	BABH01080644:227-306
BmHel-8	DF090411:54096-54346

BmHel-8	DF090327:6419795-6419917
BmHel-8	DF090327:6420051-6420142
BmHel-8	DF090331:3252467-3252578
BmHel-8	DF090331:3252349-3252433
BmHel-8	DF090341:1388128-1388300
BmHel-8	DF090341:1388398-1388479
BmHel-8	DF090349:3909594-3909833
BmHel-8	DF090349:3909858-3909948
BmHel-8	DF090345:4083332-4083657
BmHel-8	DF090450:396706-397035
BmHel-8	BABH01055206:1-321
BmHel-8	DF090335:5675417-5675620
BmHel-8	DF090476:145333-145587
BmHel-8	DF090332:1771413-1771671
BmHel-8	DF090349:3651189-3651439
BmHel-8	DF091843:862-1106
BmHel-8	DF090323:381282-381465
BmHel-8	DF090323:381117-381211
BmHel-8	DF090360:2469793-2469977
BmHel-8	DF090360:2469622-2469714
BmHel-8	DF090355:345686-345797
BmHel-8	DF090355:346568-346727
BmHel-8	DF090362:346563-346781
BmHel-8	DF090319:1580651-1580780
BmHel-8	DF090319:1580255-1580350
BmHel-8	DF090347:97878-97988
BmHel-8	DF090347:97647-97807
BmHel-8	DF090454:413324-413435
BmHel-8	DF090454:413509-413659
BmHel-8	DF091011:20784-20901
BmHel-8	DF091011:20556-20719
BmHel-8	DF090377:409985-410106
BmHel-8	DF090377:410257-410347
BmHel-8	DF090345:2636472-2636636
BmHel-8	DF090345:2636358-2636448
BmHel-8	DF090338:1993900-1994020
BmHel-8	DF090338:1994034-1994239
BmHel-8	BABH01045105:1276-1393
BmHel-8	BABH01045105:1404-1611
BmHel-8	BABH01051757:64-386
BmHel-8	DF090367:2611484-2611665
BmHel-8	DF090367:2611310-2611409
BmHel-8	DF090356:1085737-1085902
BmHel-8	DF090335:4262451-4262532
BmHel-8	DF090335:4262534-4262721
BmHel-8	DF090318:1933814-1933984
BmHel-8	DF090318:1933655-1933770
BmHel-8	DF090411:1239977-1240307

BmHel-8	DF090338:4115520-4115702
BmHel-8	DF090323:5847066-5847278
BmHel-8	DF090350:1677697-1677810
BmHel-8	DF090350:1677931-1678025
BmHel-8	DF090382:327946-328109
BmHel-8	DF090382:328200-328293
BmHel-8	DF090351:2250695-2250821
BmHel-8	DF090351:2250519-2250663
BmHel-8	DF090329:4049960-4050284
BmHel-8	DF090324:6767164-6767486
BmHel-8	DF090319:7859304-7859558
BmHel-8	DF090390:555997-556305
BmHel-8	BABH01087628:72-397
BmHel-8	DF090405:37132-37325
BmHel-8	DF090323:4587382-4587544
BmHel-8	DF090323:4587211-4587333
BmHel-8	DF090361:1913495-1913742
BmHel-8	DF090342:1204187-1204460
BmHel-8	DF090350:3932304-3932546
BmHel-8	DF090350:3932548-3932632
BmHel-8	DF090361:3189121-3189232
BmHel-8	DF090361:3188963-3189117
BmHel-8	DF090373:1604996-1605191
BmHel-8	DF090325:3160581-3160831
BmHel-8	DF090325:3160885-3160979
BmHel-8	DF090324:3171742-3172065
BmHel-8	BABH01080746:1-324
BmHel-8	DF090468:243809-244135
BmHel-8	DF090624:17112-17293
BmHel-8	DF090624:16941-17034
BmHel-8	DF090407:130355-130534
BmHel-8	DF090407:130612-130699
BmHel-8	DF090409:633047-633232
BmHel-8	DF090338:1150399-1150638
BmHel-8	BABH01086424:1-263
BmHel-8	DF090400:25113-25357
BmHel-8	DF090340:2583305-2583542
BmHel-8	DF090329:1666853-1667097
BmHel-8	DF090329:3828091-3828336
BmHel-8	DF090328:3707456-3707667
BmHel-8	DF090323:2693795-2693917
BmHel-8	DF090323:2693631-2693781
BmHel-8	DF090332:2014673-2014857
BmHel-8	DF090332:2014502-2014596
BmHel-8	DF090342:837159-837252
BmHel-8	DF090327:3047097-3047301
BmHel-8	DF090333:4945155-4945360
BmHel-8	DF090434:661197-661313

BmHel-8	DF090434:661034-661179
BmHel-8	DF090356:434553-434757
BmHel-8	DF090321:5257010-5257098
BmHel-8	DF090321:5256467-5256660
BmHel-8	DF090346:695655-695751
BmHel-8	DF090346:695819-695910
BmHel-8	DF090346:695924-696009
BmHel-8	DF090316:5584065-5584176
BmHel-8	DF090316:5583833-5584044
BmHel-8	DF090324:7044100-7044215
BmHel-8	DF090324:7043874-7044084
BmHel-8	DF090335:2635988-2636311
BmHel-8	DF090326:2727482-2727602
BmHel-8	DF090326:2727298-2727441
BmHel-8	DF090345:2950715-2950889
BmHel-8	DF090345:2950942-2951037
BmHel-8	DF090322:4962363-4962602
BmHel-8	DF090410:794259-794352
BmHel-8	DF090410:794002-794182
BmHel-8	DF090415:875602-875716
BmHel-8	DF090415:875789-875904
BmHel-8	DF090415:1156073-1156247
BmHel-8	DF090415:1156294-1156401
BmHel-8	DF090430:168368-168612
BmHel-8	DF090344:960937-961028
BmHel-8	DF090344:961064-961143
BmHel-8	DF090344:961175-961267
BmHel-8	BABH01085554:251-497
BmHel-8	BABH01085554:169-249
BmHel-8	BABH01044830:6968-7213
BmHel-8	DF090724:8362-8612
BmHel-8	DF090339:141859-142026
BmHel-8	DF090339:142042-142124
BmHel-8	DF090357:3174175-3174290
BmHel-8	DF090357:3173950-3174109
BmHel-8	DF090353:3225778-3225864
BmHel-8	DF090353:3226044-3226146
BmHel-8	DF090353:3226211-3226294
BmHel-8	DF090344:4450672-4450908
BmHel-8	DF090344:4450933-4451019
BmHel-8	DF090384:819005-819185
BmHel-8	DF090384:819181-819321
BmHel-8	BABH01045748:175-411
BmHel-8	BABH01045748:64-150
BmHel-8	DF090367:2505830-2506042
BmHel-8	DF090367:2505689-2505815
BmHel-8	DF090327:4620883-4621060
BmHel-8	DF090327:4621120-4621200

BmHel-8	DF090376:1853398-1853494
BmHel-8	DF090376:1853629-1853708
BmHel-8	DF090373:223326-223439
BmHel-8	DF090373:223163-223323
BmHel-8	DF090329:4709300-4709559
BmHel-8	DF090560:53244-53557
BmHel-8	DF090337:2141234-2141404
BmHel-8	DF090411:215445-215620
BmHel-8	DF090319:1183986-1184177
BmHel-8	DF090368:1137538-1137776
BmHel-8	DF090331:2816143-2816327
BmHel-8	DF090331:2815970-2816057
BmHel-8	DF090330:1630398-1630579
BmHel-8	DF090330:1630150-1630244
BmHel-8	DF090319:2993770-2993880
BmHel-8	DF090319:2993549-2993703
BmHel-8	DF090365:2316571-2316728
BmHel-8	DF090365:2316787-2316907
BmHel-8	DF090388:878895-879057
BmHel-8	DF090388:878753-878833
BmHel-8	DF090355:2220188-2220509
BmHel-8	BABH01087935:1-320
BmHel-8	DF090355:2199627-2199906
BmHel-8	DF090319:361535-361693
BmHel-8	DF090319:361774-361861
BmHel-8	BABH01042971:17830-18085
BmHel-8	DF090370:2663423-2663630
BmHel-8	DF090325:3339579-3339679
BmHel-8	DF090325:3339707-3339813
BmHel-8	DF090365:2951850-2952171
BmHel-8	BABH01080490:125-446
BmHel-8	DF090349:2804715-2804832
BmHel-8	DF090349:2804858-2805000
BmHel-8	DF090481:185157-185272
BmHel-8	DF090481:185286-185433
BmHel-8	DF090350:611732-611910
BmHel-8	DF090350:611589-611674
BmHel-8	DF090452:372420-372735
BmHel-8	DF090483:103858-104099
BmHel-8	DF090466:349108-349353
BmHel-8	BABH01048152:1-245
BmHel-8	DF090328:3874772-3874892
BmHel-8	DF090328:3874606-3874708
BmHel-8	DF090332:3307723-3307940
BmHel-8	DF090332:3307592-3307691
BmHel-8	DF090383:1258594-1258705
BmHel-8	DF090383:1258728-1258939
BmHel-8	DF090320:724778-724899

BmHel-8	DF090320:724561-724764
BmHel-8	DF090353:3934879-3935197
BmHel-8	DF090354:2843990-2844092
BmHel-8	DF090354:2844359-2844512
BmHel-8	DF090386:1983341-1983458
BmHel-8	DF090386:1983178-1983300
BmHel-8	DF090317:9898936-9899198
BmHel-8	DF090418:201138-201461
BmHel-8	DF090356:3729126-3729230
BmHel-8	DF090356:3728894-3729054
BmHel-8	DF090365:1355357-1355473
BmHel-8	DF090365:1355496-1355698
BmHel-8	DF090333:3294966-3295283
BmHel-8	DF090351:4011706-4012029
BmHel-8	DF090327:4710045-4710281
BmHel-8	BABH01060178:466-702
BmHel-8	DF090337:1378757-1378941
BmHel-8	DF090330:3818119-3818216
BmHel-8	DF090330:3818252-3818352
BmHel-8	DF090363:1704509-1704630
BmHel-8	DF090363:1704647-1704793
BmHel-8	DF090345:2391774-2391956
BmHel-8	DF090345:2390174-2390273
BmHel-8	DF090358:1557417-1557528
BmHel-8	DF090358:1557549-1557757
BmHel-8	DF090715:15734-15856
BmHel-8	DF090715:15881-16075
BmHel-8	DF090316:2939403-2939645
BmHel-8	DF090316:2939297-2939378
BmHel-8	BABH01045781:3581-3818
BmHel-8	BABH01045781:3843-3924
BmHel-8	BABH01083328:1-318
BmHel-8	DF090351:798428-798592
BmHel-8	DF090351:798594-798743
BmHel-8	DF090361:949621-949733
BmHel-8	DF090361:949502-949599
BmHel-8	DF090330:4175039-4175214
BmHel-8	DF090330:4175532-4175631
BmHel-8	DF090316:6914557-6914706
BmHel-8	DF090316:6914713-6914802
BmHel-8	DF090338:1150638-1150876
BmHel-8	DF090328:140894-140981
BmHel-8	DF090328:140669-140755
BmHel-8	DF090323:4809451-4809653
BmHel-8	DF090521:15406-15645
BmHel-8	DF090358:761373-761494
BmHel-8	DF090358:761145-761262
BmHel-8	DF090391:1174278-1174497

BmHel-8	DF090332:2629588-2629862
BmHel-8	DF090357:990660-990781
BmHel-8	DF090357:990431-990631
BmHel-8	DF090336:2873956-2874136
BmHel-8	DF090336:2873790-2873869
BmHel-8	DF090319:8260801-8260901
BmHel-8	DF090319:8260615-8260778
BmHel-8	DF090397:1473687-1473803
BmHel-8	DF090397:1473826-1473973
BmHel-8	DF090341:2214037-2214122
BmHel-8	DF090341:2214194-2214350
BmHel-8	DF090325:5210738-5210930
BmHel-8	DF090325:5210587-5210710
BmHel-8	DF090318:6641894-6642009
BmHel-8	DF090318:6641730-6641881
BmHel-8	DF090321:3835127-3835238
BmHel-8	DF090321:3834955-3835101
BmHel-8	DF090332:4303475-4303584
BmHel-8	DF090332:4303610-4303821
BmHel-8	DF090320:6191241-6191463
BmHel-8	DF090320:6191503-6191593
BmHel-8	DF090333:706527-706739
BmHel-8	DF090333:706765-706858
BmHel-8	DF090358:2367692-2367790
BmHel-8	DF090358:2367872-2368032
BmHel-8	DF090326:1169590-1169844
BmHel-8	DF090350:2514393-2514511
BmHel-8	DF090350:2514248-2514391
BmHel-8	DF090351:3116267-3116363
BmHel-8	DF090351:3116053-3116222
BmHel-8	DF090331:4118553-4118728
BmHel-8	DF090394:839366-839453
BmHel-8	DF090394:839179-839334
BmHel-8	DF090320:1065856-1065945
BmHel-8	DF090320:1065985-1066147
BmHel-8	DF090408:915887-916106
BmHel-8	DF090408:916543-916637
BmHel-8	BABH01048031:1184-1420
BmHel-8	DF090403:1195729-1195966
BmHel-8	DF090322:2927815-2927935
BmHel-8	DF090322:2928079-2928172
BmHel-8	DF090378:1547851-1547954
BmHel-8	DF090378:1547608-1547826
BmHel-8	DF090364:2361672-2361884
BmHel-8	DF090364:2361532-2361632
BmHel-8	DF090361:3238811-3238905
BmHel-8	DF090361:3238470-3238595
BmHel-8	DF090361:3238335-3238427

BmHel-8	BABH01049612:726-1040
BmHel-8	DF090318:6684005-6684290
BmHel-8	DF090347:882791-883011
BmHel-8	DF090347:883043-883137
BmHel-8	DF090368:2227568-2227754
BmHel-8	DF090368:2227366-2227499
BmHel-8	DF090342:68539-68646
BmHel-8	DF090342:68367-68500
BmHel-8	DF090357:2096694-2096773
BmHel-8	DF090357:2096611-2096701
BmHel-8	BABH01048411:955-1177
BmHel-8	BABH01048411:1188-1281
BmHel-8	DF090384:917096-917291
BmHel-8	DF090418:410023-410258
BmHel-8	DF090337:2413371-2413464
BmHel-8	DF090337:2413151-2413317
BmHel-8	DF090394:246855-246980
BmHel-8	DF090394:246635-246830
BmHel-8	BABH01074762:1-314
BmHel-8	DF090321:3247390-3247571
BmHel-8	DF090344:2632449-2632557
BmHel-8	DF090344:2631725-2631813
BmHel-8	DF090355:1342289-1342378
BmHel-8	DF090355:1342503-1342627
BmHel-8	DF090347:2599491-2599650
BmHel-8	DF090347:2599324-2599418
BmHel-8	BABH01045633:1-252
BmHel-8	DF090463:252944-253150
BmHel-8	DF090341:2144873-2144984
BmHel-8	DF090341:2144748-2144852
BmHel-8	DF090383:190367-190579
BmHel-8	DF090383:190604-190697
BmHel-8	DF090344:3777162-3777284
BmHel-8	DF090344:3776966-3777082
BmHel-8	DF090334:4238589-4238698
BmHel-8	DF090334:4238348-4238560
BmHel-8	DF090364:419068-419171
BmHel-8	DF090364:418870-419064
BmHel-8	BABH01076593:1-314
BmHel-8	BABH01045492:4540-4852
BmHel-8	DF090365:2084674-2084947
BmHel-8	DF090416:579681-579796
BmHel-8	DF090416:579798-579918
BmHel-8	DF090324:2266468-2266561
BmHel-8	DF090324:2266590-2266758
BmHel-8	DF090320:7579148-7579323
BmHel-8	DF090372:1728093-1728405
BmHel-8	DF090354:1463805-1463953

BmHel-8	DF090378:359648-359958
BmHel-8	DF090321:3722060-3722182
BmHel-8	DF090321:3721842-3722004
BmHel-8	DF090414:566912-567131
BmHel-8	DF090384:565258-565436
BmHel-8	DF090384:565093-565173
BmHel-8	DF090393:1552422-1552533
BmHel-8	DF090393:1552598-1552754
BmHel-8	DF090331:5091668-5091784
BmHel-8	DF090331:5091818-5092018
BmHel-8	DF090391:934500-934718
BmHel-8	DF090391:934404-934491
BmHel-8	DF090319:767651-767962
BmHel-8	DF090366:2111799-2112110
BmHel-8	DF090360:498122-498431
BmHel-8	DF090317:3299071-3299190
BmHel-8	DF090317:3299215-3299406
BmHel-8	DF090321:7076790-7077024
BmHel-8	DF090373:1638153-1638297
BmHel-8	DF090373:1638345-1638468
BmHel-8	DF090443:474989-475175
BmHel-8	DF090364:2355306-2355470
BmHel-8	DF090364:2360351-2360445
BmHel-8	DF090406:564228-564393
BmHel-8	DF090406:564053-564141
BmHel-8	DF090997:3859-3956
BmHel-8	DF090997:4114-4207
BmHel-8	DF090462:225335-225518
BmHel-8	DF090462:225169-225256
BmHel-8	DF090329:4859320-4859498
BmHel-8	DF090329:4859144-4859240
BmHel-8	DF090402:885911-886126
BmHel-8	DF090335:4178731-4179040
BmHel-8	DF090411:206749-207069
BmHel-8	DF090376:1486069-1486187
BmHel-8	DF090376:1479034-1479355
BmHel-8	DF090318:7785226-7785330
BmHel-8	DF090318:7785001-7785209
BmHel-8	DF090365:2792971-2793130
BmHel-8	DF090365:2792845-2792933
BmHel-8	DF090318:6820267-6820471
BmHel-8	DF090334:2621829-2622032
BmHel-8	DF090374:1123135-1123238
BmHel-8	DF090374:1122896-1123105
BmHel-8	DF090342:4748075-4748384
BmHel-8	DF090320:6152080-6152302
BmHel-8	DF090320:6152342-6152433
BmHel-8	DF090359:1617651-1617853

BmHel-8	DF090381:1720478-1720709
BmHel-8	DF090374:1532404-1532518
BmHel-8	DF090374:1532541-1532739
BmHel-8	DF090316:11798585-11798700
BmHel-8	DF090316:11798723-11798852
BmHel-8	DF090394:972490-972585
BmHel-8	DF090394:972609-972760
BmHel-8	DF090363:1181457-1181725
BmHel-8	BABH01047789:769-882
BmHel-8	BABH01047789:911-1028
BmHel-8	DF090318:9047964-9048043
BmHel-8	DF090318:9048081-9048165
BmHel-8	DF090318:9048196-9048279
BmHel-8	DF090317:6862416-6862590
BmHel-8	DF090371:688927-689025
BmHel-8	DF090371:689091-689241
BmHel-8	DF090329:1706066-1706248
BmHel-8	DF090319:7103437-7103584
BmHel-8	DF090319:7103623-7103702
BmHel-8	DF090330:6328872-6329140
BmHel-8	DF090361:1377323-1377535
BmHel-8	DF090361:1377201-1377296
BmHel-8	DF090338:2771112-2771371
BmHel-8	DF090340:3481186-3481486
BmHel-8	DF090364:1497237-1497545
BmHel-8	DF090343:2990136-2990243
BmHel-8	DF090460:249875-249956
BmHel-8	DF090460:249746-249865
BmHel-8	DF090320:7184772-7184874
BmHel-8	DF090320:7184574-7184654
BmHel-8	DF090317:4674614-4674693
BmHel-8	DF090317:4674178-4674418
BmHel-8	DF090348:3220496-3220675
BmHel-8	DF090380:1938946-1939209
BmHel-8	DF090324:1658904-1659013
BmHel-8	DF090324:1652952-1653143
BmHel-8	BABH01087098:1-310
BmHel-8	DF090402:424304-424534
BmHel-8	DF090437:90763-90968
BmHel-8	DF090365:1667181-1667282
BmHel-8	DF090365:1666993-1667148
BmHel-8	BABH01048171:750-939
BmHel-8	BABH01048171:531-661
BmHel-8	BABH01047155:1020-1214
BmHel-8	DF090328:674560-674673
BmHel-8	DF090328:674795-674879
BmHel-8	DF090356:2457301-2457478
BmHel-8	DF090356:2457132-2457219

BmHel-8	DF090403:1529802-1529970
BmHel-8	DF090322:4571610-4571845
BmHel-8	DF090319:1825784-1825963
BmHel-8	DF090319:1825616-1825707
BmHel-8	DF090393:1097633-1097744
BmHel-8	BABH01049965:1-311
BmHel-8	DF090316:15396820-15396997
BmHel-8	DF090316:15396674-15396760
BmHel-8	DF090319:8873711-8873822
BmHel-8	DF090319:8873846-8874044
BmHel-8	DF090324:520569-520839
BmHel-8	DF090325:6623762-6624072
BmHel-8	DF090353:2821101-2821261
BmHel-8	DF090321:5509991-5510112
BmHel-8	DF090321:5510129-5510236
BmHel-8	DF090388:1403296-1403408
BmHel-8	DF090388:1403427-1403569
BmHel-8	DF090320:6681486-6681608
BmHel-8	DF090320:6681272-6681460
BmHel-8	DF090422:46037-46354
BmHel-8	DF090342:1774654-1774762
BmHel-8	DF090342:1774863-1774985
BmHel-8	DF090537:9075-9189
BmHel-8	DF090537:9217-9416
BmHel-8	DF090349:788185-788300
BmHel-8	DF090349:787948-788072
BmHel-8	DF091006:9085-9297
BmHel-8	DF091006:8952-9042
BmHel-8	DF090359:998529-998840
BmHel-8	DF090322:3818746-3818938
BmHel-8	DF090336:5475330-5475563
BmHel-8	DF091075:3295-3416
BmHel-8	DF091075:3068-3190
BmHel-8	DF090358:1982067-1982165
BmHel-8	DF090358:1982309-1982421
BmHel-8	DF090362:410503-410686
BmHel-8	DF090362:410717-410838
BmHel-8	DF090496:58759-58964
BmHel-8	DF090496:58615-58713
BmHel-8	DF090417:891451-891567
BmHel-8	DF090350:3854042-3854305
BmHel-8	BABH01064719:361-670
BmHel-8	BABH01056710:435-739
BmHel-8	DF090367:1736691-1736867
BmHel-8	DF090334:3198888-3199008
BmHel-8	DF090334:3198658-3198782
BmHel-8	DF090389:1519039-1519276
BmHel-8	DF090423:895314-895436

BmHel-8	DF090423:895807-895901
BmHel-8	DF090541:32350-32554
BmHel-8	DF090323:17074-17292
BmHel-8	DF090323:17321-17400
BmHel-8	DF090382:2031504-2031610
BmHel-8	DF090382:2031256-2031464
BmHel-8	DF090366:2285063-2285273
BmHel-8	DF090366:2284924-2285005
BmHel-8	DF090480:151810-152017
BmHel-8	DF090480:151700-151792
BmHel-8	BABH01076798:243-361
BmHel-8	BABH01076798:388-575
BmHel-8	DF090365:1219987-1220083
BmHel-8	DF090365:1220143-1220301
BmHel-8	DF090319:4469374-4469490
BmHel-8	DF090319:4469602-4469717
BmHel-8	DF090323:1697728-1697819
BmHel-8	DF090323:1697863-1697948
BmHel-8	DF090382:1714503-1714612
BmHel-8	DF090382:1714640-1714841
BmHel-8	DF090318:1888474-1888648
BmHel-8	DF090338:552927-553097
BmHel-8	DF090439:370903-371090
BmHel-8	BABH01045578:553-740
BmHel-8	DF090338:671334-671515
BmHel-8	DF090338:671167-671291
BmHel-8	DF090321:7008670-7008963
BmHel-8	DF090387:640138-640438
BmHel-8	BABH01048248:8-309
BmHel-8	DF090360:2123901-2124016
BmHel-8	DF090360:2124089-2124199
BmHel-8	DF090324:4683089-4683318
BmHel-8	DF090354:822898-823110
BmHel-8	DF090354:822773-822859
BmHel-8	DF090354:2653174-2653479
BmHel-8	DF090316:13513142-13513366
BmHel-8	DF090374:1401422-1401663
BmHel-8	DF090342:4325866-4325960
BmHel-8	DF090342:4326317-4326533
BmHel-8	DF090344:2082229-2082330
BmHel-8	DF090344:2082012-2082207
BmHel-8	DF090320:3775391-3775696
BmHel-8	DF090390:208946-209247
BmHel-8	DF090424:924810-925111
BmHel-8	BABH01069844:21-313
BmHel-8	DF090377:1739518-1739636
BmHel-8	DF090377:1740311-1740641
BmHel-8	DF090336:2783424-2783521

BmHel-8	DF090336:2783559-2783717
BmHel-8	DF090339:2359413-2359640
BmHel-8	DF090316:5024120-5024317
BmHel-8	DF090430:658252-658346
BmHel-8	DF090430:658394-658486
BmHel-8	DF090323:4506131-4506317
BmHel-8	DF090339:3428905-3429088
BmHel-8	DF090475:122544-122648
BmHel-8	DF090475:122674-122830
BmHel-8	DF091266:3147-3331
BmHel-8	DF090386:65228-65325
BmHel-8	DF090386:65365-65576
BmHel-8	DF090409:1064514-1064619
BmHel-8	DF090409:1064274-1064471
BmHel-8	DF090416:1232838-1233057
BmHel-8	DF090352:381551-381845
BmHel-8	DF090625:38116-38416
BmHel-8	DF090409:1063674-1063788
BmHel-8	DF090409:1063796-1063902
BmHel-8	DF090319:6961285-6961384
BmHel-8	DF090319:6961092-6961240
BmHel-8	DF090317:6936482-6936682
BmHel-8	DF090336:2893546-2893712
BmHel-8	DF090336:2893382-2893476
BmHel-8	DF090384:787909-788117
BmHel-8	DF090336:3222789-3222877
BmHel-8	DF090336:3222603-3222761
BmHel-8	DF090371:1206375-1206575
BmHel-8	DF090371:1206249-1206343
BmHel-8	DF090346:454423-454660
BmHel-8	DF090338:431718-431955
BmHel-8	DF090316:8370771-8371009
BmHel-8	DF090324:4769750-4769987
BmHel-8	DF090408:7892-8128
BmHel-8	DF090355:3391791-3391907
BmHel-8	DF090355:3392016-3392119
BmHel-8	BABH01078610:1-300
BmHel-8	DF090447:208280-208580
BmHel-8	DF090357:2349940-2350120
BmHel-8	DF090320:2258090-2258206
BmHel-8	DF090319:1416129-1416432
BmHel-8	DF090416:1131537-1131698
BmHel-8	DF090416:1131360-1131446
BmHel-8	DF090328:1935820-1935939
BmHel-8	DF090328:1935527-1935651
BmHel-8	DF090499:73330-73445
BmHel-8	DF090499:73104-73224
BmHel-8	DF090374:1836659-1836770

BmHel-8	DF090374:1836882-1837004
BmHel-8	DF090320:2674997-2675210
BmHel-8	DF090325:6559215-6559303
BmHel-8	DF090325:6559011-6559148
BmHel-8	DF090368:527949-528084
BmHel-8	DF090367:699187-699290
BmHel-8	DF090367:699398-699523
BmHel-8	DF090350:1648390-1648478
BmHel-8	DF090350:1648740-1648891
BmHel-8	DF090383:857495-857681
BmHel-8	DF090383:857723-857841
BmHel-8	DF090343:2371298-2371482
BmHel-8	DF090343:2371524-2371641
BmHel-8	DF090357:2328134-2328426
BmHel-8	DF090345:3546961-3547259
BmHel-8	BABH01064748:1-299
BmHel-8	DF090677:11261-11560
BmHel-8	DF090330:396756-396955
BmHel-8	DF090330:396608-396700
BmHel-8	DF090321:3244007-3244125
BmHel-8	DF090321:3243784-3243867
BmHel-8	DF090320:916584-916785
BmHel-8	DF090326:5404710-5404823
BmHel-8	DF090326:5403181-5403311
BmHel-8	DF090324:7630213-7630386
BmHel-8	DF090752:19829-19988
BmHel-8	DF090410:976619-976733
BmHel-8	DF090410:976832-976955
BmHel-8	DF090408:845621-845802
BmHel-8	DF090329:5856461-5856554
BmHel-8	DF090329:5852531-5852629
BmHel-8	DF090339:3429568-3429870
BmHel-8	DF090340:1938770-1938876
BmHel-8	DF090340:1938912-1939064
BmHel-8	DF090343:911119-911215
BmHel-8	DF090343:910874-910959
BmHel-8	DF090340:2772388-2772492
BmHel-8	DF090340:2776161-2776285
BmHel-8	DF090426:442820-443085
BmHel-8	DF090321:6482646-6482826
BmHel-8	DF090418:86140-86324
BmHel-8	DF090341:4257876-4257987
BmHel-8	DF090341:4258021-4258167
BmHel-8	DF090628:26488-26793
BmHel-8	DF090363:1479142-1479230
BmHel-8	DF090363:1478602-1478809
BmHel-8	DF090322:32136-32312
BmHel-8	DF090355:2151797-2151877

BmHel-8	DF090355:2151998-2152157
BmHel-8	DF090373:1690036-1690238
BmHel-8	DF090373:1690291-1690383
BmHel-8	BABH01048355:930-1226
BmHel-8	DF090323:2293605-2293899
BmHel-8	DF090332:4105984-4106218
BmHel-8	DF090347:1443216-1443314
BmHel-8	DF090347:1443103-1443218
BmHel-8	DF090341:2643211-2643298
BmHel-8	DF090341:2643346-2643508
BmHel-8	DF090343:2649214-2649377
BmHel-8	DF090343:2649415-2649542
BmHel-8	BABH01048921:515-696
BmHel-8	DF090350:1666605-1666728
BmHel-8	DF090350:1667274-1667398
BmHel-8	DF090377:1949467-1949668
BmHel-8	DF090359:600108-600272
BmHel-8	DF090316:15959295-15959498
BmHel-8	DF090316:15959152-15959239
BmHel-8	DF090401:1265815-1266033
BmHel-8	DF090327:1442802-1442982
BmHel-8	DF090327:1443030-1443149
BmHel-8	BABH01085602:201-496
BmHel-8	DF090317:2538335-2538454
BmHel-8	DF090317:2538118-2538240
BmHel-8	DF090414:1345463-1345582
BmHel-8	DF090414:1345611-1345727
BmHel-8	DF090330:5640349-5640467
BmHel-8	DF090330:5640620-5640702
BmHel-8	DF090342:4886105-4886234
BmHel-8	BABH01076410:372-596
BmHel-8	DF090382:1864699-1864791
BmHel-8	DF090382:1864818-1865028
BmHel-8	DF090337:3743649-3743770
BmHel-8	DF090337:3743422-3743509
BmHel-8	DF090321:2418141-2418382
BmHel-8	DF090372:1731099-1731336
BmHel-8	DF090341:4175102-4175221
BmHel-8	DF090341:4175306-4175430
BmHel-8	DF090373:1256097-1256213
BmHel-8	DF090373:1255897-1256073
BmHel-8	DF090407:1061574-1061693
BmHel-8	DF090407:1061323-1061452
BmHel-8	BABH01064299:1-290
BmHel-8	DF090321:2937599-2937831
BmHel-8	DF090343:3681931-3682029
BmHel-8	DF090343:3681749-3681892
BmHel-8	DF090402:534776-534940

BmHel-8	DF090402:534345-534439
BmHel-8	DF090738:31186-31390
BmHel-8	DF090369:1572160-1572377
BmHel-8	DF090323:6511504-6511665
BmHel-8	DF090317:14652-14783
BmHel-8	DF090317:14435-14544
BmHel-8	DF090360:843156-843374
BmHel-8	DF090333:5017899-5018000
BmHel-8	DF090323:6437051-6437252
BmHel-8	DF090390:397077-397192
BmHel-8	DF090390:396891-397039
BmHel-8	DF090387:741122-741228
BmHel-8	DF090327:2889545-2889689
BmHel-8	DF090375:1125135-1125256
BmHel-8	DF090375:1125272-1125355
BmHel-8	DF090375:1125416-1125509
BmHel-8	DF090355:3521289-3521390
BmHel-8	DF090355:3521051-3521246
BmHel-8	DF090340:606646-606767
BmHel-8	DF090356:1418152-1418273
BmHel-8	DF090356:1418300-1418472
BmHel-8	DF090327:3681170-3681291
BmHel-8	DF090327:3681395-3681499
BmHel-8	DF090418:1102894-1103186
BmHel-8	DF090341:4537097-4537329
BmHel-8	DF090432:342592-342772
BmHel-8	DF090339:2464292-2464384
BmHel-8	DF090339:2464702-2464859
BmHel-8	DF090376:408651-408879
BmHel-8	DF090344:2268693-2268789
BmHel-8	DF090344:2268443-2268538
BmHel-8	DF090320:3105130-3105228
BmHel-8	DF090320:3105404-3105491
BmHel-8	DF090423:295056-295178
BmHel-8	BABH01075370:7-129
BmHel-8	BABH01075370:190-304
BmHel-8	DF090353:131310-131390
BmHel-8	DF090353:131477-131641
BmHel-8	DF090422:379254-379375
BmHel-8	DF090422:379508-379601
BmHel-8	DF090321:4205116-4205407
BmHel-8	DF090323:2121731-2122026
BmHel-8	DF090372:2245162-2245448
BmHel-8	DF090532:12137-12386
BmHel-8	DF090415:815948-816181
BmHel-8	DF090381:398062-398357
BmHel-8	DF090354:3420130-3420245
BmHel-8	DF090354:3420261-3420374

BmHel-8	DF090380:1998722-1998899
BmHel-8	DF090380:1998572-1998689
BmHel-8	DF090371:354004-354128
BmHel-8	DF090371:354242-354360
BmHel-8	DF090373:1645497-1645734
BmHel-8	DF090378:278224-278461
BmHel-8	DF090344:843737-843841
BmHel-8	DF090347:3861616-3861738
BmHel-8	DF090347:3861418-3861514
BmHel-8	DF090350:1227956-1228135
BmHel-8	DF090350:1227795-1227908
BmHel-8	DF090329:347564-347800
BmHel-8	DF090339:253576-253691
BmHel-8	DF090339:253776-253902
BmHel-8	DF090340:2927717-2927813
BmHel-8	DF090340:2927504-2927602
BmHel-8	BABH01044025:6185-6288
BmHel-8	DF090383:1415299-1415467
BmHel-8	DF090387:1721994-1722081
BmHel-8	DF090372:204175-204292
BmHel-8	DF090372:204358-204451
BmHel-8	DF090350:526532-526626
BmHel-8	DF090350:526296-526490
BmHel-8	DF090350:1727190-1727393
BmHel-8	DF090350:1727066-1727146
BmHel-8	DF090384:636774-637059
BmHel-8	BABH01084530:226-515
BmHel-8	DF090346:1872421-1872600
BmHel-8	DF090323:7674677-7674909
BmHel-8	DF091426:3367-3561
BmHel-8	DF090398:152596-152708
BmHel-8	DF090398:152241-152361
BmHel-8	DF090329:1258875-1258960
BmHel-8	DF090329:1259796-1259957
BmHel-8	DF090336:3251204-3251292
BmHel-8	DF090367:1352622-1352746
BmHel-8	DF090412:43202-43448
BmHel-8	DF090320:1342817-1343028
BmHel-8	DF090344:3047355-3047458
BmHel-8	DF090422:1003983-1004086
BmHel-8	DF090422:1004562-1004683
BmHel-8	DF090461:328579-328682
BmHel-8	DF090461:329065-329184
BmHel-8	DF090339:4722247-4722485
BmHel-8	DF090340:65654-65764
BmHel-8	DF090340:65294-65416
BmHel-8	DF090324:5157913-5158032
BmHel-8	DF090324:5158155-5158273

BmHel-8	DF090324:2270184-2270353
BmHel-8	DF090378:643885-644122
BmHel-8	DF090346:1781509-1781602
BmHel-8	DF090346:1780870-1781067
BmHel-8	DF090528:80717-80895
BmHel-8	DF090528:80545-80659
BmHel-8	DF090317:1349308-1349420
BmHel-8	DF090339:2708255-2708438
BmHel-8	DF090339:2708516-2708628
BmHel-8	DF090339:4596917-4597201
BmHel-8	DF090350:3232778-3232989
BmHel-8	DF090322:5841850-5841943
BmHel-8	DF090322:5841983-5842171
BmHel-8	DF090324:224652-224854
BmHel-8	DF090383:309294-309386
BmHel-8	DF090383:309694-309816
BmHel-8	BABH01066201:222-456
BmHel-8	DF090330:3872984-3873079
BmHel-8	DF090330:3872746-3872944
BmHel-8	DF090397:1379207-1379319
BmHel-8	DF090397:1378991-1379169
BmHel-8	DF090654:21824-21997
BmHel-8	DF090335:3969251-3969460
BmHel-8	BABH01052268:399-494
BmHel-8	BABH01052268:511-714
BmHel-8	DF090316:15180609-15180824
BmHel-8	DF090418:861055-861151
BmHel-8	DF090418:861256-861373
BmHel-8	DF090364:1545791-1545973
BmHel-8	DF090330:5572071-5572281
BmHel-8	DF090409:1132818-1133028
BmHel-8	DF090403:188246-188456
BmHel-8	DF090388:1026375-1026587
BmHel-8	DF090338:3555522-3555736
BmHel-8	DF090323:4651856-4652060
BmHel-8	DF090316:2957235-2957440
BmHel-8	DF090422:554381-554583
BmHel-8	DF090380:571115-571326
BmHel-8	DF090371:857364-857574
BmHel-8	DF090369:570176-570386
BmHel-8	DF090360:1999965-2000176
BmHel-8	BABH01075985:25-235
BmHel-8	BABH01069328:23-233
BmHel-8	BABH01067717:16-226
BmHel-8	BABH01056835:523-734
BmHel-8	DF091740:684-889
BmHel-8	BABH01052613:22-228
BmHel-8	DF090367:36726-36845

BmHel-8	DF090367:36951-37045
BmHel-8	DF090336:4451977-4452216
BmHel-8	DF090323:271878-271970
BmHel-8	DF090323:272016-272202
BmHel-8	DF090406:692662-692952
BmHel-8	DF090575:14199-14435
BmHel-8	DF090334:1998161-1998243
BmHel-8	DF090334:1998346-1998507
BmHel-8	DF090355:570037-570324
BmHel-8	DF090396:989338-989547
BmHel-8	DF090433:330515-330598
BmHel-8	DF090433:330256-330411
BmHel-8	DF090339:2045709-2045884
BmHel-8	DF090334:5036565-5036665
BmHel-8	DF090334:5036702-5036837
BmHel-8	DF090339:2691021-2691129
BmHel-8	DF090339:2681831-2682165
BmHel-8	DF090443:261285-261566
BmHel-8	DF090352:1274329-1274446
BmHel-8	DF090352:1274566-1274656
BmHel-8	DF090395:1297772-1297951
BmHel-8	DF090395:1297639-1297745
BmHel-8	DF091751:2613-2901
BmHel-8	DF090364:2450306-2450514
BmHel-8	BABH01047998:164-385
BmHel-8	DF090327:694718-694932
BmHel-8	DF090316:15565004-15565208
BmHel-8	DF090369:1372234-1372443
BmHel-8	DF090328:2072417-2072624
BmHel-8	DF090355:2494785-2495006
BmHel-8	DF090338:2894415-2894531
BmHel-8	DF090338:2894295-2894402
BmHel-8	DF090317:7725761-7725980
BmHel-8	DF090395:574905-575065
BmHel-8	BABH01061682:240-361
BmHel-8	BABH01061682:68-229
BmHel-8	DF090356:3127309-3127593
BmHel-8	DF090760:5281-5567
BmHel-8	DF090326:1801665-1801878
BmHel-8	DF090445:208114-208227
BmHel-8	DF090445:208243-208337
BmHel-8	DF090339:1533964-1534082
BmHel-8	DF090339:1533738-1533862
BmHel-8	DF091325:6110-6315
BmHel-8	DF090345:2457632-2457727
BmHel-8	DF090345:2457772-2457897
BmHel-8	DF090384:481216-481325
BmHel-8	DF090341:4312717-4312803

BmHel-8	DF090341:4311158-4311249
BmHel-8	DF090341:4311016-4311126
BmHel-8	DF090431:235683-235973
BmHel-8	DF090327:2129025-2129120
BmHel-8	DF090403:1383892-1384099
BmHel-8	DF090347:2247017-2247220
BmHel-8	BABH01049029:6-210
BmHel-8	DF090322:5804174-5804286
BmHel-8	DF090322:5800769-5800892
BmHel-8	DF090321:3814511-3814730
BmHel-8	DF090393:1400908-1401192
BmHel-8	DF090322:2908484-2908597
BmHel-8	DF090322:2908267-2908360
BmHel-8	DF090357:1641624-1641742
BmHel-8	DF090357:1641759-1641965
BmHel-8	DF090317:484174-484372
BmHel-8	DF090317:484044-484124
BmHel-8	DF090337:1768818-1769036
BmHel-8	DF090412:922146-922259
BmHel-8	DF090412:921930-922129
BmHel-8	DF090390:1364288-1364374
BmHel-8	DF090390:1354911-1355004
BmHel-8	DF090327:1783986-1784080
BmHel-8	DF090327:1784108-1784243
BmHel-8	DF090374:1860305-1860410
BmHel-8	DF090374:1860439-1860583
BmHel-8	BABH01047434:893-1079
BmHel-8	BABH01047434:1127-1221
BmHel-8	DF090838:1-211
BmHel-8	DF090362:1429928-1430134
BmHel-8	BABH01051692:426-632
BmHel-8	DF090402:646466-646563
BmHel-8	DF090402:646575-646659
BmHel-8	DF090402:646690-646785
BmHel-8	DF090387:1200702-1200818
BmHel-8	DF090387:1200475-1200596
BmHel-8	DF090328:3607898-3608071
BmHel-8	DF090328:3607359-3607479
BmHel-8	DF090373:1015896-1016178
BmHel-8	DF090368:557383-557669
BmHel-8	DF090317:1490422-1490509
BmHel-8	DF090317:1490202-1490411
BmHel-8	DF090319:5296294-5296412
BmHel-8	DF090319:5296545-5296632
BmHel-8	DF090328:338888-339062
BmHel-8	DF090475:95899-95995
BmHel-8	DF090475:96088-96225
BmHel-8	DF090331:2104276-2104383

BmHel-8	DF090331:2104067-2104242
BmHel-8	DF090320:4404956-4405066
BmHel-8	DF090320:4405194-4405317
BmHel-8	DF090330:675900-676021
BmHel-8	DF090330:675680-675885
BmHel-8	DF091612:2164-2286
BmHel-8	DF091612:1947-2059
BmHel-8	DF090328:5564503-5564785
BmHel-8	DF090444:334075-334357
BmHel-8	DF090323:7585995-7586166
BmHel-8	DF090317:9301877-9301979
BmHel-8	DF090374:1884120-1884229
BmHel-8	DF090322:780561-780659
BmHel-8	DF090322:781227-781320
BmHel-8	DF090322:783965-784054
BmHel-8	DF090372:2087209-2087493
BmHel-8	DF090336:2853639-2853856
BmHel-8	DF090316:16137879-16138093
BmHel-8	DF090316:16137789-16137868
BmHel-8	DF090387:1340904-1341026
BmHel-8	DF090379:1244765-1244984
BmHel-8	BABH01049639:451-560
BmHel-8	BABH01049639:247-418
BmHel-8	DF090378:1962009-1962214
BmHel-8	DF090349:4250246-4250462
BmHel-8	DF090324:5322994-5323121
BmHel-8	DF090388:1780678-1780886
BmHel-8	DF090453:377878-378097
BmHel-8	DF090324:383757-383879
BmHel-8	DF090324:383082-383416
BmHel-8	DF090328:66740-66865
BmHel-8	DF090328:66565-66723
BmHel-8	DF090437:74542-74661
BmHel-8	DF090437:74805-74888
BmHel-8	DF090330:2932913-2933031
BmHel-8	DF090330:2933137-2933260
BmHel-8	DF090440:529207-529314
BmHel-8	DF090440:529455-529549
BmHel-8	DF090336:998443-998659
BmHel-8	DF090412:701855-702135
BmHel-8	DF090377:1465793-1465996
BmHel-8	DF090332:1356512-1356675
BmHel-8	DF090382:1804532-1804627
BmHel-8	DF090321:4910732-4910942
BmHel-8	DF090374:55845-56054
BmHel-8	DF090329:6422812-6422934
BmHel-8	DF090329:6422613-6422708
BmHel-8	DF090369:2583746-2583930

BmHel-8	DF090369:2583573-2583667
BmHel-8	DF090369:613551-613827
BmHel-8	DF090344:102294-102402
BmHel-8	DF090344:102425-102511
BmHel-8	DF090334:4740808-4740899
BmHel-8	DF090334:4740648-4740766
BmHel-8	DF090328:377860-378041
BmHel-8	DF090436:252944-253048
BmHel-8	DF090436:253154-253278
BmHel-8	DF090356:3469799-3469882
BmHel-8	DF090356:3469502-3469594
BmHel-8	DF090321:3185448-3185532
BmHel-8	DF090321:3185331-3185422
BmHel-8	BABH01081961:277-551
BmHel-8	DF090354:2952593-2952709
BmHel-8	DF090399:88635-88756
BmHel-8	DF090399:84537-84659
BmHel-8	DF090325:7214126-7214248
BmHel-8	DF090325:7213982-7214106
BmHel-8	DF090357:287806-287923
BmHel-8	DF090357:288069-288152
BmHel-8	DF090697:7047-7149
BmHel-8	DF090697:6814-6998
BmHel-8	BABH01049224:815-1093
BmHel-8	BABH01046928:1462-1742
BmHel-8	DF090366:864035-864153
BmHel-8	DF090366:863808-863903
BmHel-8	BABH01079109:1-279
BmHel-8	DF090357:44474-44582
BmHel-8	DF090357:44243-44333
BmHel-8	DF090316:4653472-4653586
BmHel-8	DF090370:434435-434523
BmHel-8	DF090370:434141-434265
BmHel-8	DF090356:1493210-1493422
BmHel-8	DF090338:1405678-1405889
BmHel-8	DF090374:2331520-2331623
BmHel-8	DF090374:2331688-2331805
BmHel-8	DF090445:471690-471786
BmHel-8	DF090445:471455-471542
BmHel-8	BABH01087566:2-201
BmHel-8	DF090357:3767263-3767385
BmHel-8	DF090357:3767555-3767640
BmHel-8	DF090423:610466-610639
BmHel-8	DF090423:610715-610809
BmHel-8	DF090325:3569777-3569895
BmHel-8	DF090325:3569559-3569676
BmHel-8	DF090350:2106018-2106195
BmHel-8	DF090350:2105858-2105950

BmHel-8	DF090646:1-278
BmHel-8	DF090333:5294782-5294880
BmHel-8	DF090333:5295295-5295409
BmHel-8	DF090411:17492-17605
BmHel-8	DF090411:17277-17406
BmHel-8	DF090344:2351619-2351710
BmHel-8	DF090344:2351787-2351911
BmHel-8	DF090382:1444366-1444472
BmHel-8	DF090382:1444122-1444217
BmHel-8	DF090317:8054850-8054960
BmHel-8	DF090317:8055105-8055194
BmHel-8	DF090338:2586003-2586213
BmHel-8	DF090372:2253047-2253240
BmHel-8	BABH01053380:5-204
BmHel-8	DF090370:1787923-1788042
BmHel-8	DF090370:1787707-1787800
BmHel-8	DF090352:2581256-2581378
BmHel-8	DF090352:2581026-2581147
BmHel-8	DF090431:564733-564848
BmHel-8	DF090369:2541235-2541355
BmHel-8	DF090369:2540994-2541094
BmHel-8	DF090414:796577-796812
BmHel-8	DF090353:59730-59852
BmHel-8	DF090353:59982-60077
BmHel-8	DF090340:1007807-1007929
BmHel-8	DF090340:1007954-1008044
BmHel-8	DF090328:2254502-2254624
BmHel-8	DF090328:2254283-2254443
BmHel-8	DF090353:2192212-2192487
BmHel-8	DF090326:423918-424193
BmHel-8	DF090365:1468754-1469034
BmHel-8	DF092036:34-305
BmHel-8	DF090386:1580115-1580316
BmHel-8	DF090326:5704946-5705063
BmHel-8	DF090326:5704780-5704936
BmHel-8	DF090324:4117208-4117388
BmHel-8	DF090523:43148-43361
BmHel-8	DF090361:3211726-3211903
BmHel-8	DF090323:6893570-6893777
BmHel-8	DF090364:859849-859965
BmHel-8	DF090364:859614-859706
BmHel-8	DF090333:5772697-5772807
BmHel-8	DF090333:5772470-5772564
BmHel-8	DF090342:3324358-3324467
BmHel-8	DF090342:3324240-3324344
BmHel-8	DF090317:148242-148334
BmHel-8	DF090317:148003-148125
BmHel-8	DF090391:803042-803235

BmHel-8	DF090449:321743-321931
BmHel-8	DF090405:1255197-1255380
BmHel-8	DF090324:7576349-7576472
BmHel-8	DF090324:7576575-7576692
BmHel-8	DF090382:1582477-1582599
BmHel-8	DF090382:1582362-1582453
BmHel-8	DF090368:483838-484003
BmHel-8	DF090360:1906119-1906232
BmHel-8	DF090360:1905887-1906011
BmHel-8	DF090366:1119348-1119548
BmHel-8	DF090320:6243983-6244261
BmHel-8	DF090414:199262-199380
BmHel-8	DF090414:199064-199158
BmHel-8	BABH01048644:60-167
BmHel-8	BABH01048644:610-698
BmHel-8	DF090332:2690292-2690446
BmHel-8	DF090472:59626-59735
BmHel-8	DF090472:59874-59960
BmHel-8	DF090317:9294440-9294554
BmHel-8	DF090317:9294688-9294772
BmHel-8	DF090333:456717-456809
BmHel-8	DF090358:41312-41511
BmHel-8	DF090358:41513-41593
BmHel-8	DF090336:1023907-1024029
BmHel-8	DF090336:1024159-1024249
BmHel-8	DF090347:2331325-2331435
BmHel-8	DF090347:2331140-2331228
BmHel-8	DF090475:23838-23960
BmHel-8	DF090475:29652-29742
BmHel-8	DF090431:767706-767828
BmHel-8	DF090431:767478-767573
BmHel-8	DF090386:123144-123262
BmHel-8	DF090386:122926-123012
BmHel-8	DF090320:211465-211576
BmHel-8	DF090320:211024-211141
BmHel-8	DF090334:4768675-4768797
BmHel-8	DF090334:4768923-4769017
BmHel-8	DF090330:4232972-4233195
BmHel-8	DF090866:23585-23798
BmHel-8	DF090866:23798-23984
BmHel-8	DF090330:4679243-4679424
BmHel-8	DF090330:4679096-4679190
BmHel-8	DF090344:3895106-3895286
BmHel-8	DF090344:3895336-3895429
BmHel-8	DF090335:5182999-5183084
BmHel-8	DF090335:5182800-5182994
BmHel-8	DF090364:1926356-1926556
BmHel-8	DF090318:786064-786272

BmHel-8	DF090331:3313743-3313946
BmHel-8	DF090328:4514048-4514262
BmHel-8	DF090757:15402-15677
BmHel-8	BABH01053951:40-314
BmHel-8	DF090384:1970129-1970237
BmHel-8	DF090384:1969913-1970007
BmHel-8	DF090379:1841163-1841379
BmHel-8	DF090350:2833880-2834084
BmHel-8	DF090388:1737316-1737512
BmHel-8	DF090323:4587576-4587773
BmHel-8	DF090361:1311450-1311666
BmHel-8	DF090372:694202-694296
BmHel-8	DF090372:694038-694168
BmHel-8	DF090388:556024-556147
BmHel-8	DF090388:556272-556361
BmHel-8	DF090325:1885826-1885935
BmHel-8	DF090349:453486-453665
BmHel-8	DF090349:453330-453424
BmHel-8	DF090378:878586-878786
BmHel-8	DF090321:7113764-7114037
BmHel-8	DF090382:147991-148264
BmHel-8	DF090401:926106-926208
BmHel-8	DF090401:926322-926439
BmHel-8	DF090342:2587198-2587306
BmHel-8	DF090342:2587385-2587494
BmHel-8	DF090334:4192686-4192958
BmHel-8	DF090407:999074-999274
BmHel-8	DF091187:483-682
BmHel-8	DF090340:4419646-4419824
BmHel-8	DF090390:1421597-1421780
BmHel-8	DF090319:241186-241290
BmHel-8	DF090319:241754-241872
BmHel-8	DF090321:5492622-5492706
BmHel-8	DF090450:213087-213297
BmHel-8	DF090350:4079814-4080024
BmHel-8	DF090382:1032670-1032832
BmHel-8	BABH01048812:1-196
BmHel-8	DF090645:24766-24849
BmHel-8	DF090645:24560-24692
BmHel-8	DF090363:313484-313629
BmHel-8	DF090338:3061702-3061885
BmHel-8	DF090538:41983-42167
BmHel-8	DF090325:3415927-3416049
BmHel-8	DF090325:3415702-3415791
BmHel-8	DF090349:2671421-2671542
BmHel-8	DF090349:2672181-2672270
BmHel-8	DF090337:1664227-1664409
BmHel-8	DF090337:1664472-1664559

BmHel-8	DF090472:297380-297565
BmHel-8	DF090472:297637-297724
BmHel-8	DF090336:4549535-4549719
BmHel-8	DF090336:4549357-4549444
BmHel-8	DF090359:2521152-2521336
BmHel-8	DF090359:2521423-2521510
BmHel-8	DF090338:4174373-4174556
BmHel-8	DF090338:4174207-4174294
BmHel-8	DF090319:795061-795242
BmHel-8	DF090319:795321-795408
BmHel-8	DF090317:5652617-5652801
BmHel-8	DF090317:5652878-5652963
BmHel-8	DF090326:4557330-4557514
BmHel-8	DF090326:4557593-4557680
BmHel-8	DF090375:1192861-1193045
BmHel-8	DF090375:1192695-1192782
BmHel-8	DF090331:810137-810408
BmHel-8	DF090387:1525915-1526187
BmHel-8	DF090387:1277941-1278214
BmHel-8	DF090365:1722721-1722841
BmHel-8	DF090365:1722942-1723034
BmHel-8	DF090371:2416729-2416815
BmHel-8	DF090371:2416938-2417062
BmHel-8	DF090425:375899-375998
BmHel-8	DF090425:376079-376200
BmHel-8	DF090371:499590-499707
BmHel-8	DF090371:490994-491317
BmHel-8	DF090390:912936-913033
BmHel-8	DF090390:914328-914441
BmHel-8	DF090348:3033911-3034007
BmHel-8	DF090348:3034570-3034690
BmHel-8	DF090397:675037-675128
BmHel-8	DF090397:674793-674910
BmHel-8	DF090702:14843-15112
BmHel-8	DF090408:420207-420321
BmHel-8	DF090408:420099-420184
BmHel-8	DF090386:1578800-1578985
BmHel-8	DF090340:3625231-3625416
BmHel-8	DF090458:432343-432525
BmHel-8	DF090458:432049-432137
BmHel-8	DF090325:1909602-1909706
BmHel-8	DF090325:1909799-1909888
BmHel-8	DF090429:76333-76537
BmHel-8	DF090422:894948-895132
BmHel-8	DF090383:477586-477714
BmHel-8	DF090383:477804-477908
BmHel-8	DF090372:1614755-1614877
BmHel-8	DF090372:1614566-1614650

BmHel-8	DF091552:895-998
BmHel-8	DF091552:676-791
BmHel-8	DF090338:3277432-3277615
BmHel-8	DF090338:3277695-3277781
BmHel-8	DF090427:502011-502210
BmHel-8	BABH01069423:1-272
BmHel-8	DF090343:936009-936189
BmHel-8	DF090343:936250-936340
BmHel-8	DF090323:1285553-1285655
BmHel-8	DF090323:1285315-1285406
BmHel-8	DF090387:236802-236898
BmHel-8	DF090368:1399400-1399576
BmHel-8	DF090368:1403747-1403840
BmHel-8	DF090409:115036-115137
BmHel-8	DF090409:114878-115011
BmHel-8	DF090365:2076786-2076978
BmHel-8	DF090420:316080-316190
BmHel-8	DF090420:316202-316366
BmHel-8	DF090367:2022957-2023050
BmHel-8	DF090368:666905-667177
BmHel-8	DF090418:562344-562548
BmHel-8	DF090325:2691370-2691492
BmHel-8	DF090325:2691192-2691279
BmHel-8	DF090324:7941084-7941205
BmHel-8	DF090324:7940844-7940931
BmHel-8	DF090441:325217-325421
BmHel-8	DF090319:4570166-4570369
BmHel-8	DF090338:3662512-3662694
BmHel-8	DF090338:3662358-3662446
BmHel-8	DF090350:3763042-3763144
BmHel-8	DF090350:3763276-3763361
BmHel-8	DF090387:1449575-1449755
BmHel-8	DF090387:1449419-1449509
BmHel-8	DF090393:1642952-1643135
BmHel-8	DF090393:1637456-1637547
BmHel-8	DF090392:1591933-1592029
BmHel-8	DF090392:1592157-1592271
BmHel-8	DF090372:184612-184790
BmHel-8	DF090984:904-1015
BmHel-8	DF090984:673-762
BmHel-8	DF090324:7206602-7206712
BmHel-8	DF090324:7206368-7206458
BmHel-8	BABH01066111:369-639
BmHel-8	DF090319:7799776-7799884
BmHel-8	DF090319:7799546-7799638
BmHel-8	DF090373:388329-388489
BmHel-8	DF090337:1721509-1721714
BmHel-8	DF090317:5895346-5895446

BmHel-8	DF090327:2530296-2530392
BmHel-8	DF090327:2530106-2530248
BmHel-8	DF090325:1731246-1731449
BmHel-8	DF090354:1457633-1457837
BmHel-8	DF090397:1594888-1595057
BmHel-8	DF090468:148746-148949
BmHel-8	DF090408:170320-170442
BmHel-8	DF090408:170115-170236
BmHel-8	DF090929:6537-6624
BmHel-8	DF090929:6126-6216
BmHel-8	DF090929:5994-6082
BmHel-8	DF090334:4433472-4433660
BmHel-8	DF090387:1849545-1849690
BmHel-8	DF090387:1849394-1849486
BmHel-8	DF090404:879297-879413
BmHel-8	DF090404:879481-879643
BmHel-8	DF090354:1565246-1565426
BmHel-8	DF090354:1565078-1565166
BmHel-8	DF090321:5883324-5883440
BmHel-8	DF090321:5883142-5883294
BmHel-8	DF090322:4086859-4087131
BmHel-8	DF090326:2411837-2412116
BmHel-8	DF090360:1695470-1695739
BmHel-8	DF090328:1107910-1108024
BmHel-8	DF090328:1107677-1107772
BmHel-8	DF090443:254835-255017
BmHel-8	DF090443:254666-254753
BmHel-8	DF090376:2074270-2074547
BmHel-8	DF090375:2043357-2043537
BmHel-8	DF090375:2043194-2043281
BmHel-8	DF090354:2396726-2396904
BmHel-8	DF090330:6220236-6220448
BmHel-8	DF090734:2988-3183
BmHel-8	DF090338:1065605-1065717
BmHel-8	DF090338:1065398-1065490
BmHel-8	DF090422:94957-95163
BmHel-8	DF090418:347945-348055
BmHel-8	DF090317:5128975-5129081
BmHel-8	DF090317:5129104-5129265
BmHel-8	DF090531:27857-27958
BmHel-8	DF090456:139306-139429
BmHel-8	DF090456:139501-139594
BmHel-8	DF090319:5309157-5309365
BmHel-8	DF090327:5702679-5702893
BmHel-8	DF090346:1334554-1334657
BmHel-8	DF090346:1334735-1334856
BmHel-8	DF090316:1191333-1191446
BmHel-8	DF090316:1191585-1191680

BmHel-8	DF090439:355712-355817
BmHel-8	DF090439:355501-355607
BmHel-8	DF090426:512557-512665
BmHel-8	DF090426:512814-512909
BmHel-8	DF090328:3081413-3081590
BmHel-8	DF090379:1738358-1738464
BmHel-8	DF090379:1738527-1738699
BmHel-8	DF090388:168675-168855
BmHel-8	DF090388:168504-168592
BmHel-8	DF090359:2752962-2753160
BmHel-8	DF090330:1235680-1235948
BmHel-8	DF090325:7022558-7022826
BmHel-8	DF090683:10956-11069
BmHel-8	DF090683:9552-9709
BmHel-8	DF090330:3349886-3350011
BmHel-8	DF090330:3350038-3350191
BmHel-8	DF090395:919480-919598
BmHel-8	DF090395:919615-919767
BmHel-8	DF090415:604095-604213
BmHel-8	DF090415:604265-604358
BmHel-8	DF090352:618132-618216
BmHel-8	DF090352:618361-618472
BmHel-8	DF090322:6539553-6539667
BmHel-8	DF090322:6539737-6539895
BmHel-8	DF090356:1454065-1454161
BmHel-8	DF090356:1453861-1453955
BmHel-8	BABH01050372:9-213
BmHel-8	DF090384:1125589-1125772
BmHel-8	DF090342:2360410-2360614
BmHel-8	DF090316:1120100-1120283
BmHel-8	DF090316:1119954-1120037
BmHel-8	DF090334:2313906-2314085
BmHel-8	DF090334:2313745-2313830
BmHel-8	DF090357:870175-870383
BmHel-8	DF090348:2410677-2410949
BmHel-8	BABH01045735:3819-4086
BmHel-8	BABH01064498:402-671
BmHel-8	DF090578:42888-43107
BmHel-8	DF090326:1442967-1443070
BmHel-8	DF090326:1442787-1442942
BmHel-8	DF090789:13230-13424
BmHel-8	DF090554:65582-65691
BmHel-8	DF090554:65836-65929
BmHel-8	DF090317:8695532-8695734
BmHel-8	DF090349:495004-495220
BmHel-8	DF090326:5492716-5492929
BmHel-8	DF090322:6486815-6486926
BmHel-8	DF090322:6486607-6486700

BmHel-8	DF090395:870975-871098
BmHel-8	DF090395:870759-870849
BmHel-8	DF090441:534748-534867
BmHel-8	DF090441:535006-535088
BmHel-8	DF090338:594509-594630
BmHel-8	DF090338:594240-594333
BmHel-8	DF090323:4984355-4984528
BmHel-8	DF090323:4984206-4984298
BmHel-8	DF090324:4881245-4881349
BmHel-8	DF090324:4881381-4881541
BmHel-8	DF090330:218921-219040
BmHel-8	DF090317:2993318-2993532
BmHel-8	DF090345:11219-11485
BmHel-8	DF090319:7094188-7094301
BmHel-8	DF090376:1794252-1794430
BmHel-8	DF090376:1794533-1794620
BmHel-8	BABH01045151:3413-3591
BmHel-8	BABH01045151:3237-3327
BmHel-8	DF090379:250437-250629
BmHel-8	DF090459:284914-285123
BmHel-8	DF090350:3005760-3005960
BmHel-8	DF090377:1616240-1616433
BmHel-8	DF090410:889399-889593
BmHel-8	DF090370:454617-454754
BmHel-8	DF090373:1588988-1589129
BmHel-8	DF090326:3930129-3930338
BmHel-8	DF090339:1491280-1491485
BmHel-8	DF090412:518727-518932
BmHel-8	DF090341:4769586-4769778
BmHel-8	DF090362:3186356-3186546
BmHel-8	DF090344:340607-340794
BmHel-8	DF091204:8091-8275
BmHel-8	DF090379:916676-916794
BmHel-8	DF090379:917137-917218
BmHel-8	DF090468:271578-271700
BmHel-8	DF090468:271045-271138
BmHel-8	DF090418:150307-150511
BmHel-8	DF090316:4489242-4489363
BmHel-8	DF090316:4489054-4489152
BmHel-8	DF090453:306802-306983
BmHel-8	DF090453:307043-307123
BmHel-8	DF090318:9440256-9440440
BmHel-8	DF090318:9440090-9440170
BmHel-8	DF090320:3410151-3410336
BmHel-8	DF090320:3409986-3410066
BmHel-8	DF090362:529277-529461
BmHel-8	DF090362:529550-529630
BmHel-8	DF090365:1513866-1514051

BmHel-8	DF090365:1513692-1513772
BmHel-8	DF090345:3039016-3039200
BmHel-8	DF090345:3039289-3039369
BmHel-8	DF090328:868209-868392
BmHel-8	DF090328:868482-868562
BmHel-8	DF090442:104368-104551
BmHel-8	DF090442:104635-104715
BmHel-8	DF090334:2799972-2800237
BmHel-8	BABH01077918:1-266
BmHel-8	DF090585:31879-32144
BmHel-8	DF090317:10678512-10678631
BmHel-8	DF090317:10678312-10678391
BmHel-8	DF090321:2691101-2691208
BmHel-8	DF090321:2691750-2691869
BmHel-8	DF090348:1147050-1147244
BmHel-8	DF090393:1721955-1722154
BmHel-8	DF090323:2486349-2486541
BmHel-8	DF090326:174012-174204
BmHel-8	DF090318:7278166-7278263
BmHel-8	DF090318:7278604-7278716
BmHel-8	DF090379:74549-74634
BmHel-8	DF090379:74736-74818
BmHel-8	DF090354:3531706-3531931
BmHel-8	DF090399:226665-226782
BmHel-8	DF090328:1298675-1298884
BmHel-8	DF090336:4183155-4183343
BmHel-8	DF090447:212964-213151
BmHel-8	BABH01049354:886-1075
BmHel-8	DF090321:7041504-7041619
BmHel-8	DF090321:7041655-7041737
BmHel-8	DF090339:2771285-2771486
BmHel-8	DF090346:3706929-3707040
BmHel-8	DF090346:3707059-3707146
BmHel-8	DF090321:7656593-7656674
BmHel-8	DF090321:7656344-7656469
BmHel-8	DF090327:2836895-2837006
BmHel-8	DF090327:2837162-2837242
BmHel-8	DF090390:922963-923060
BmHel-8	DF090390:922761-922865
BmHel-8	DF090338:2197636-2197754
BmHel-8	DF090338:2197474-2197561
BmHel-8	DF090490:3622-3886
BmHel-8	DF090350:3601172-3601288
BmHel-8	DF090350:3601432-3601511
BmHel-8	DF090322:6459224-6459458
BmHel-8	DF090390:1222074-1222171
BmHel-8	DF090390:1221859-1221985
BmHel-8	DF090375:320734-320914

BmHel-8	DF090375:321009-321092
BmHel-8	DF090361:1458351-1458527
BmHel-8	DF090361:1458612-1458699
BmHel-8	DF090359:1407987-1408099
BmHel-8	DF090359:1408271-1408392
BmHel-8	DF090317:9911739-9911828
BmHel-8	DF090317:9910727-9910851
BmHel-8	DF090353:1076866-1076952
BmHel-8	DF090353:1076984-1077189
BmHel-8	DF090369:875487-875608
BmHel-8	DF090487:86003-86209
BmHel-8	DF090331:5406844-5406958
BmHel-8	DF090340:819166-819350
BmHel-8	DF090342:3867298-3867509
BmHel-8	DF090418:490021-490142
BmHel-8	DF090418:490290-490370
BmHel-8	DF090476:213593-213715
BmHel-8	DF090476:213798-213888
BmHel-8	DF090419:945112-945200
BmHel-8	DF090419:944865-945047
BmHel-8	DF091376:643-739
BmHel-8	DF091376:243-363
BmHel-8	DF090331:3467863-3467980
BmHel-8	DF090331:3467657-3467750
BmHel-8	DF090352:3751800-3751979
BmHel-8	DF090352:3752061-3752144
BmHel-8	DF090339:2528754-2528862
BmHel-8	DF090339:2528546-2528699
BmHel-8	DF090335:1619919-1620014
BmHel-8	DF090335:1620260-1620433
BmHel-8	DF090375:37523-37643
BmHel-8	DF090375:37671-37813
BmHel-8	DF090340:2545032-2545228
BmHel-8	DF090321:1476201-1476408
BmHel-8	DF090356:2737133-2737239
BmHel-8	DF090356:2736463-2736557
BmHel-8	DF090361:315534-315724
BmHel-8	DF090444:220575-220738
BmHel-8	DF090321:4471554-4471657
BmHel-8	DF090321:4471337-4471422
BmHel-8	DF090355:884005-884107
BmHel-8	DF090327:5385206-5385412
BmHel-8	DF090319:1806448-1806564
BmHel-8	DF090316:1598466-1598630
BmHel-8	DF090746:13397-13515
BmHel-8	DF090746:13161-13255
BmHel-8	BABH01054803:602-713
BmHel-8	BABH01054803:44-197

BmHel-8	DF090316:13787951-13788209
BmHel-8	DF090324:5636429-5636613
BmHel-8	DF090473:86483-86587
BmHel-8	DF090473:86730-86812
BmHel-8	DF091562:3691-3795
BmHel-8	DF091562:3466-3548
BmHel-8	DF090367:2088769-2088969
BmHel-8	DF090342:2228528-2228629
BmHel-8	DF090342:2228977-2229074
BmHel-8	DF090384:1415447-1415588
BmHel-8	DF090425:377473-377577
BmHel-8	DF090425:377246-377335
BmHel-8	DF090350:3708449-3708565
BmHel-8	DF090350:3708218-3708302
BmHel-8	DF090415:1008022-1008144
BmHel-8	DF090415:1007822-1007910
BmHel-8	DF090499:78293-78477
BmHel-8	DF090317:9944410-9944595
BmHel-8	DF090321:5681840-5682102
BmHel-8	DF090391:432371-432633
BmHel-8	DF090328:3088781-3088977
BmHel-8	DF090337:3826401-3826549
BmHel-8	DF090840:11063-11264
BmHel-8	DF090357:630532-630621
BmHel-8	DF090357:630768-630855
BmHel-8	DF090323:1072918-1073006
BmHel-8	DF090323:1073170-1073268
BmHel-8	DF090349:2879523-2879695
BmHel-8	DF090349:2879778-2879866
BmHel-8	DF090383:1616806-1616899
BmHel-8	DF090383:1616529-1616620
BmHel-8	DF090383:1616432-1616511
BmHel-8	DF090353:233825-234021
BmHel-8	DF090338:2332159-2332353
BmHel-8	DF090365:392004-392090
BmHel-8	DF090365:391603-391691
BmHel-8	DF090365:391437-391524
BmHel-8	DF090359:785982-786179
BmHel-8	DF090345:634512-634597
BmHel-8	DF090373:644567-644671
BmHel-8	DF090373:644736-644829
BmHel-8	DF090318:8660214-8660423
BmHel-8	DF090339:906427-906531
BmHel-8	DF090339:906685-906774
BmHel-8	DF090327:449817-449903
BmHel-8	DF090327:449590-449710
BmHel-8	DF090347:687254-687369
BmHel-8	DF090347:687471-687558

BmHel-8	DF090328:1391836-1391947
BmHel-8	DF090328:1392013-1392117
BmHel-8	DF090325:7053634-7053718
BmHel-8	DF090325:7053500-7053613
BmHel-8	DF090342:1724465-1724572
BmHel-8	DF090342:1724181-1724268
BmHel-8	DF090354:1148478-1148594
BmHel-8	DF090354:1148766-1148853
BmHel-8	DF090368:1165250-1165510
BmHel-8	DF090401:93533-93643
BmHel-8	DF090375:1530001-1530094
BmHel-8	DF090375:1529773-1529894
BmHel-8	DF090427:192320-192434
BmHel-8	DF090427:192156-192302
BmHel-8	DF090340:3468797-3468878
BmHel-8	DF090340:3469025-3469112
BmHel-8	DF090329:3088464-3088636
BmHel-8	DF090329:3088298-3088385
BmHel-8	DF090329:673993-674176
BmHel-8	DF090352:1579625-1579802
BmHel-8	DF090570:49766-49882
BmHel-8	DF090570:49912-50110
BmHel-8	DF090340:4319651-4319755
BmHel-8	DF090362:1807461-1807626
BmHel-8	DF090362:1807706-1807802
BmHel-8	DF090328:1143731-1143894
BmHel-8	DF090328:1143589-1143681
BmHel-8	DF090352:2967946-2968072
BmHel-8	DF090352:2968368-2968451
BmHel-8	DF090396:681491-681612
BmHel-8	DF090396:681298-681441
BmHel-8	DF090320:3760796-3760968
BmHel-8	DF090320:3761000-3761087
BmHel-8	DF090338:3919804-3919904
BmHel-8	DF090338:3920086-3920248
BmHel-8	DF090450:423637-423759
BmHel-8	DF090450:423476-423616
BmHel-8	BABH01047180:1-260
BmHel-8	DF090323:3045118-3045384
BmHel-8	DF090396:343704-343822
BmHel-8	DF090396:343222-343306
BmHel-8	DF090452:175953-176075
BmHel-8	DF090452:175727-175814
BmHel-8	DF090321:7259130-7259307
BmHel-8	DF090321:7258848-7258928
BmHel-8	DF090326:5166553-5166745
BmHel-8	DF090408:337666-337781
BmHel-8	DF090350:365225-365397

BmHel-8	DF090350:365098-365183
BmHel-8	BABH01056610:71-281
BmHel-8	DF090410:1121215-1121414
BmHel-8	DF090342:1572186-1572367
BmHel-8	DF090342:2978636-2978798
BmHel-8	DF090340:2296915-2297119
BmHel-8	DF090378:1600921-1601043
BmHel-8	DF090378:1604796-1604881
BmHel-8	DF090342:2253829-2253945
BmHel-8	DF090342:2253603-2253694
BmHel-8	DF090326:1488907-1489023
BmHel-8	DF090326:1488783-1488882
BmHel-8	DF090317:929378-929566
BmHel-8	DF090318:1099755-1100007
BmHel-8	DF090354:2704897-2705000
BmHel-8	DF090354:2704661-2704756
BmHel-8	DF090338:919082-919172
BmHel-8	DF090338:918855-918952
BmHel-8	DF090495:125950-126058
BmHel-8	DF090495:126285-126372
BmHel-8	DF090365:2036742-2036825
BmHel-8	DF090365:2036515-2036631
BmHel-8	DF090552:39550-39764
BmHel-8	BABH01049058:933-1118
BmHel-8	DF090325:4587981-4588161
BmHel-8	DF090325:4588163-4588246
BmHel-8	DF090354:481473-481595
BmHel-8	DF090354:481249-481332
BmHel-8	DF090352:4071025-4071227
BmHel-8	DF090413:717482-717686
BmHel-8	DF090332:2005706-2005827
BmHel-8	DF090332:2005853-2006046
BmHel-8	DF090322:1925300-1925481
BmHel-8	DF090329:4605204-4605294
BmHel-8	DF090329:4599240-4599479
BmHel-8	DF090343:2128344-2128609
BmHel-8	DF090670:20159-20380
BmHel-8	DF090449:652155-652331
BmHel-8	DF090366:2485314-2485401
BmHel-8	DF090366:2485075-2485193
BmHel-8	DF090317:7933333-7933532
BmHel-8	DF090328:272255-272365
BmHel-8	DF090368:1852025-1852216
BmHel-8	DF090327:970504-970612
BmHel-8	BABH01067309:33-138
BmHel-8	BABH01067309:163-253
BmHel-8	DF090316:5175590-5175770
BmHel-8	DF090346:188852-188974

BmHel-8	DF090346:188376-188458
BmHel-8	DF090337:3773117-3773221
BmHel-8	DF090337:3772916-3773020
BmHel-8	DF090413:385885-385983
BmHel-8	DF090413:386027-386189
BmHel-8	DF090369:600296-600403
BmHel-8	DF090370:1037271-1037356
BmHel-8	DF090316:13500292-13500495
BmHel-8	DF090322:7243081-7243227
BmHel-8	DF090322:2014156-2014337
BmHel-8	DF090411:34839-35017
BmHel-8	DF090343:255491-255661
BmHel-8	BABH01072465:448-622
BmHel-8	DF090317:7247808-7247912
BmHel-8	DF090396:1165551-1165735
BmHel-8	DF090360:1225169-1225290
BmHel-8	DF090598:30523-30707
BmHel-8	DF090319:8534182-8534299
BmHel-8	DF090340:263763-263874
BmHel-8	DF090340:263659-263744
BmHel-8	DF090449:455119-455325
BmHel-8	DF091020:2634-2740
BmHel-8	DF091020:2465-2613
BmHel-8	DF090413:134976-135175
BmHel-8	DF090352:964923-965030
BmHel-8	DF090352:965610-965762
BmHel-8	DF090319:1011192-1011309
BmHel-8	DF090319:1010720-1010801
BmHel-8	DF090340:3347386-3347566
BmHel-8	DF090365:1720499-1720609
BmHel-8	DF090365:1720311-1720407
BmHel-8	DF090354:712663-712782
BmHel-8	DF090354:712573-712656
BmHel-8	DF090350:254122-254206
BmHel-8	DF090350:254696-254786
BmHel-8	DF090329:4767770-4767892
BmHel-8	DF090429:635729-635870
BmHel-8	DF090435:152648-152831
BmHel-8	DF090319:7188925-7189047
BmHel-8	DF090319:7189076-7189167
BmHel-8	DF090327:4126521-4126619
BmHel-8	DF090327:4133025-4133149
BmHel-8	DF090336:4948660-4948843
BmHel-8	DF090388:74585-74769
BmHel-8	DF090442:190783-190971
BmHel-8	DF090378:55729-55847
BmHel-8	DF090378:55494-55589
BmHel-8	DF090368:1909860-1909972

BmHel-8	DF090340:3525194-3525306
BmHel-8	DF090340:3525419-3525498
BmHel-8	DF090335:3475111-3475200
BmHel-8	DF090331:5839512-5839596
BmHel-8	DF090331:5839260-5839476
BmHel-8	DF090418:433871-433989
BmHel-8	DF090585:3270-3446
BmHel-8	DF090416:655539-655661
BmHel-8	DF090413:310058-310242
BmHel-8	DF090322:3049389-3049506
BmHel-8	DF090322:3049262-3049350
BmHel-8	DF090321:2816145-2816249
BmHel-8	DF090449:787674-787796
BmHel-8	DF090449:787883-787967
BmHel-8	DF090354:2909439-2909549
BmHel-8	DF090354:2909171-2909263
BmHel-8	DF090358:1831314-1831416
BmHel-8	DF090358:1831077-1831239
BmHel-8	DF090352:333458-333633
BmHel-8	DF090341:4215695-4215797
BmHel-8	DF090341:4215532-4215683
BmHel-8	DF090403:1254980-1255083
BmHel-8	DF090456:121120-121220
BmHel-8	DF090320:2018345-2018449
BmHel-8	DF090320:2018460-2018635
BmHel-8	DF090436:463850-463942
BmHel-8	DF090332:820576-820769
BmHel-8	DF090354:501866-501954
BmHel-8	DF090354:501772-501859
BmHel-8	DF090321:4496603-4496787
BmHel-8	DF090324:6160288-6160490
BmHel-8	DF090457:74722-74817
BmHel-8	DF090336:4845313-4845414
BmHel-8	DF090336:4845130-4845287
BmHel-8	DF090323:5012594-5012716
BmHel-8	DF090323:5012745-5012874
BmHel-8	DF090440:234866-235121
BmHel-8	DF090332:907509-907707
BmHel-8	DF090340:2217377-2217487
BmHel-8	DF090340:2217624-2217708
BmHel-8	DF090322:7994980-7995070
BmHel-8	DF090322:7994743-7994834
BmHel-8	DF090352:3160431-3160512
BmHel-8	DF090352:3160649-3160743
BmHel-8	DF090390:57871-57957
BmHel-8	DF090390:57959-58050
BmHel-8	DF090370:1278808-1278926
BmHel-8	BABH01051466:703-892

BmHel-8	DF090323:5481689-5481874
BmHel-8	DF090336:4798633-4798721
BmHel-8	DF090368:1494497-1494612
BmHel-8	DF090368:1494277-1494467
BmHel-8	DF090387:1159011-1159115
BmHel-8	DF090387:1158771-1158865
BmHel-8	DF090320:5341838-5341929
BmHel-8	DF090320:5341972-5342082
BmHel-8	BABH01053130:1-252
BmHel-8	BABH01050192:729-980
BmHel-8	DF090336:607633-607750
BmHel-8	DF090317:7133386-7133585
BmHel-8	DF090366:1650968-1651156
BmHel-8	DF090461:325689-325785
BmHel-8	DF090461:325402-325489
BmHel-8	DF090316:3507488-3507581
BmHel-8	DF090316:3507768-3507848
BmHel-8	DF090345:679971-680141
BmHel-8	DF090345:680209-680288
BmHel-8	DF090332:1473788-1473963
BmHel-8	DF090361:2189834-2190003
BmHel-8	DF090391:1461996-1462118
BmHel-8	DF090391:1462235-1462324
BmHel-8	DF090389:601879-601982
BmHel-8	DF090389:601635-601722
BmHel-8	DF090350:1267937-1268128
BmHel-8	DF092040:2779-3023
BmHel-8	DF090421:490807-490988
BmHel-8	DF090342:4198219-4198400
BmHel-8	DF090403:333651-333832
BmHel-8	DF090316:1295907-1296087
BmHel-8	DF090411:142779-142961
BmHel-8	DF090404:1320703-1320884
BmHel-8	DF090415:428906-429087
BmHel-8	BABH01046138:1047-1228
BmHel-8	DF090340:1458381-1458499
BmHel-8	DF090373:805194-805306
BmHel-8	DF090373:805439-805533
BmHel-8	DF090339:4233093-4233199
BmHel-8	DF090339:4233222-4233306
BmHel-8	DF090629:45689-45785
BmHel-8	DF090629:45460-45559
BmHel-8	BABH01046982:1828-2033
BmHel-8	DF090328:1363384-1363497
BmHel-8	DF090328:1363628-1363713
BmHel-8	BABH01046727:276-378
BmHel-8	BABH01046727:86-236
BmHel-8	DF090347:3571286-3571395

BmHel-8	DF090347:3571054-3571141
BmHel-8	DF090417:637589-637711
BmHel-8	DF090373:430801-430985
BmHel-8	DF090365:1434549-1434750
BmHel-8	DF090359:2864859-2864960
BmHel-8	DF090359:2865120-2865212
BmHel-8	DF090374:2043091-2043195
BmHel-8	DF090374:2043299-2043392
BmHel-8	DF090342:4439081-4439265
BmHel-8	DF090316:9334935-9335117
BmHel-8	DF090330:1248463-1248643
BmHel-8	DF090321:5930080-5930259
BmHel-8	DF090388:577945-578126
BmHel-8	DF090382:1091998-1092178
BmHel-8	DF090383:844898-845109
BmHel-8	DF090380:1789351-1789445
BmHel-8	DF090380:1789113-1789205
BmHel-8	DF090324:204719-204917
BmHel-8	DF090329:1713111-1713206
BmHel-8	DF090329:1713338-1713425
BmHel-8	DF090401:864658-864854
BmHel-8	DF090433:84098-84293
BmHel-8	DF090319:2887538-2887625
BmHel-8	DF090319:2887920-2888087
BmHel-8	DF090394:172371-172533
BmHel-8	DF090394:172196-172281
BmHel-8	DF090360:2647588-2647681
BmHel-8	DF090360:2647808-2647902
BmHel-8	DF090317:2878963-2879135
BmHel-8	DF090518:76623-76794
BmHel-8	DF091163:4201-4373
BmHel-8	BABH01045038:533-709
BmHel-8	DF090592:16613-16815
BmHel-8	DF090323:2078414-2078513
BmHel-8	DF090323:2078171-2078260
BmHel-8	DF090383:2043121-2043304
BmHel-8	DF090374:246257-246368
BmHel-8	DF090374:246014-246110
BmHel-8	DF090549:51844-51945
BmHel-8	DF090549:51621-51715
BmHel-8	DF090337:1505041-1505235
BmHel-8	DF090322:6962090-6962291
BmHel-8	DF090317:4543188-4543304
BmHel-8	DF091604:1575-1778
BmHel-8	DF090366:2225101-2225202
BmHel-8	DF090366:2227840-2227933
BmHel-8	DF090427:114841-115045
BmHel-8	DF090355:1147289-1147383

BmHel-8	DF090355:1147415-1147574
BmHel-8	BABH01048289:756-1006
BmHel-8	DF091283:597-844
BmHel-8	DF090342:4840021-4840201
BmHel-8	DF090398:1068194-1068396
BmHel-8	DF090332:1065309-1065490
BmHel-8	DF090355:1480681-1480882
BmHel-8	DF090340:2268600-2268719
BmHel-8	DF090327:2708817-2709018
BmHel-8	DF090357:2572004-2572120
BmHel-8	DF090317:7623351-7623530
BmHel-8	DF090355:3027029-3027146
BmHel-8	DF090367:1115100-1115274
BmHel-8	DF090316:785177-785286
BmHel-8	DF090316:784974-785062
BmHel-8	DF090327:3640244-3640329
BmHel-8	DF090318:4237891-4238065
BmHel-8	BABH01075102:432-605
BmHel-8	BABH01075102:339-419
BmHel-8	DF090330:1539646-1539781
BmHel-8	DF090374:147037-147207
BmHel-8	BABH01067658:1-169
BmHel-8	DF090342:2504541-2504726
BmHel-8	DF090342:4080084-4080267
BmHel-8	DF090321:5868564-5868748
BmHel-8	DF090403:1289818-1290000
BmHel-8	DF090319:8296095-8296278
BmHel-8	DF090316:3284209-3284392
BmHel-8	DF090337:602117-602276
BmHel-8	DF090343:4244619-4244803
BmHel-8	DF090370:1169114-1169236
BmHel-8	DF090334:3723612-3723722
BmHel-8	DF090465:186997-187176
BmHel-8	DF090354:3856104-3856198
BmHel-8	DF090354:3856346-3856440
BmHel-8	DF090332:1522625-1522710
BmHel-8	DF090332:1522297-1522386
BmHel-8	DF090329:2544556-2544732
BmHel-8	DF090323:4166413-4166514
BmHel-8	DF090323:4166889-4166984
BmHel-8	DF090324:2686144-2686322
BmHel-8	DF090449:415150-415329
BmHel-8	DF090483:86398-86503
BmHel-8	DF090483:86185-86277
BmHel-8	BABH01052931:571-823
BmHel-8	DF090338:4300956-4301109
BmHel-8	BABH01088429:4-249
BmHel-8	DF090359:2004862-2005031

BmHel-8	DF090336:2575566-2575749
BmHel-8	DF090317:1152364-1152471
BmHel-8	DF090317:1152135-1152225
BmHel-8	DF090335:1851630-1851805
BmHel-8	DF090321:4433360-4433484
BmHel-8	DF090321:4433208-4433299
BmHel-8	DF090332:6144652-6144836
BmHel-8	DF090362:1443830-1444014
BmHel-8	DF090319:3699290-3699472
BmHel-8	DF090335:4105807-4105990
BmHel-8	DF090456:103861-103952
BmHel-8	DF090456:103632-103725
BmHel-8	DF090329:5839703-5839822
BmHel-8	DF090329:5839463-5839550
BmHel-8	DF090318:729427-729517
BmHel-8	DF090377:1440814-1440991
BmHel-8	DF090328:2372846-2373023
BmHel-8	DF090429:913175-913352
BmHel-8	DF090407:768965-769062
BmHel-8	DF090407:769211-769306
BmHel-8	DF090372:2024361-2024442
BmHel-8	DF090349:4118163-4118267
BmHel-8	DF090349:4117973-4118066
BmHel-8	DF090343:4379848-4380036
BmHel-8	DF090346:2902150-2902232
BmHel-8	DF090346:2902262-2902428
BmHel-8	DF091017:34933-35070
BmHel-8	DF090367:2690586-2690792
BmHel-8	DF090347:2036660-2036829
BmHel-8	BABH01051530:403-572
BmHel-8	DF090327:633383-633578
BmHel-8	DF090316:15577807-15577990
BmHel-8	DF090457:133296-133478
BmHel-8	DF090361:168673-168863
BmHel-8	DF090322:6277819-6277923
BmHel-8	DF090322:6277644-6277733
BmHel-8	DF090317:136268-136377
BmHel-8	DF090317:136524-136604
BmHel-8	DF090469:38593-38775
BmHel-8	DF090343:807884-807989
BmHel-8	DF090343:808134-808227
BmHel-8	DF090423:369123-369217
BmHel-8	DF090423:369299-369457
BmHel-8	DF090322:6043854-6043971
BmHel-8	DF090322:6043699-6043826
BmHel-8	BABH01071782:381-626
BmHel-8	DF090370:2681374-2681464
BmHel-8	DF090370:2681156-2681244

BmHel-8	DF090378:106762-106869
BmHel-8	DF090378:106655-106739
BmHel-8	DF090358:3365335-3365511
BmHel-8	DF090331:3459679-3459856
BmHel-8	DF090381:1590032-1590208
BmHel-8	DF090444:209400-209580
BmHel-8	BABH01050985:24-200
BmHel-8	DF090335:4057407-4057612
BmHel-8	DF090376:2244191-2244296
BmHel-8	BABH01048322:1147-1313
BmHel-8	DF090317:6694872-6695055
BmHel-8	DF090567:58885-59068
BmHel-8	DF090316:12978134-12978316
BmHel-8	DF090426:215646-215828
BmHel-8	DF090327:1701922-1702106
BmHel-8	DF090322:8037749-8037933
BmHel-8	DF090317:8512938-8513121
BmHel-8	DF090334:5643914-5644098
BmHel-8	DF090325:1780553-1780738
BmHel-8	DF090387:1490329-1490509
BmHel-8	DF090450:49282-49465
BmHel-8	BABH01051586:265-449
BmHel-8	DF090396:1022136-1022339
BmHel-8	DF090316:7315534-7315717
BmHel-8	DF090341:4525257-4525358
BmHel-8	DF090341:4525404-4525550
BmHel-8	DF090355:202660-202767
BmHel-8	DF090355:202553-202637
BmHel-8	DF090339:1623079-1623174
BmHel-8	DF090339:1622825-1622985
BmHel-8	DF090401:1235764-1235880
BmHel-8	DF090342:2067249-2067426
BmHel-8	DF090334:5146817-5146907
BmHel-8	DF090403:554713-554886
BmHel-8	DF090331:2468152-2468242
BmHel-8	DF090410:141441-141530
BmHel-8	DF090410:141561-141661
BmHel-8	DF090318:7106567-7106648
BmHel-8	DF090577:14853-15053
BmHel-8	DF090416:886699-886867
BmHel-8	DF090328:3697620-3697801
BmHel-8	DF090364:75971-76144
BmHel-8	DF090343:1955205-1955389
BmHel-8	DF090345:1266184-1266365
BmHel-8	DF090431:732760-732940
BmHel-8	DF090330:5873518-5873702
BmHel-8	DF090403:496265-496448
BmHel-8	DF090421:226500-226669

BmHel-8	DF090340:4857620-4857724
BmHel-8	DF090340:4857763-4857905
BmHel-8	DF090379:1039784-1040028
BmHel-8	DF090325:4645979-4646222
BmHel-8	DF090332:717301-717393
BmHel-8	DF090332:717223-717304
BmHel-8	DF090319:1505074-1505256
BmHel-8	DF090333:5053389-5053508
BmHel-8	DF090317:8994723-8994820
BmHel-8	DF090317:8994485-8994581
BmHel-8	DF090570:38404-38489
BmHel-8	DF090570:38142-38251
BmHel-8	DF090359:3221210-3221320
BmHel-8	DF090359:3221463-3221594
BmHel-8	DF090347:4249204-4249378
BmHel-8	BABH01052893:280-449
BmHel-8	DF090373:1470383-1470470
BmHel-8	DF090373:1470156-1470320
BmHel-8	DF090596:24755-24834
BmHel-8	DF090596:24520-24635
BmHel-8	BABH01049085:344-423
BmHel-8	BABH01049085:543-658
BmHel-8	DF090355:2186853-2187096
BmHel-8	DF090532:54596-54680
BmHel-8	DF090532:54381-54473
BmHel-8	DF090381:515243-515423
BmHel-8	DF090416:715581-715696
BmHel-8	DF090381:628910-629026
BmHel-8	DF090330:2459856-2459966
BmHel-8	DF090409:812931-813111
BmHel-8	DF090350:1196473-1196574
BmHel-8	DF090350:1196695-1196783
BmHel-8	DF090339:2949221-2949330
BmHel-8	DF090339:2944188-2944324
BmHel-8	BABH01055176:517-628
BmHel-8	BABH01055176:640-765
BmHel-8	DF090344:1223125-1223365
BmHel-8	DF090414:849598-849844
BmHel-8	DF091410:2738-2982
BmHel-8	DF090346:2329075-2329248
BmHel-8	DF090322:7097274-7097448
BmHel-8	DF090356:1332097-1332205
BmHel-8	DF090356:1332303-1332391
BmHel-8	DF090440:379358-379451
BmHel-8	DF090440:379612-379701
BmHel-8	DF090330:2064963-2065055
BmHel-8	DF090330:2064718-2064874
BmHel-8	DF090390:2891-3057

BmHel-8	DF090321:2878916-2879093
BmHel-8	DF090317:157216-157317
BmHel-8	DF090319:197539-197718
BmHel-8	DF090356:3509936-3510116
BmHel-8	DF090329:6309580-6309691
BmHel-8	DF090472:197496-197674
BmHel-8	DF090329:6571999-6572182
BmHel-8	DF090323:5662699-5662882
BmHel-8	DF090433:508661-508844
BmHel-8	DF090316:12805040-12805141
BmHel-8	DF090370:231099-231198
BmHel-8	DF090370:231308-231412
BmHel-8	DF090389:3182-3304
BmHel-8	DF090354:3134620-3134715
BmHel-8	DF090354:3134842-3134926
BmHel-8	DF090353:3358338-3358442
BmHel-8	DF090353:3358503-3358642
BmHel-8	DF090343:2633955-2634052
BmHel-8	DF090343:2633772-2633919
BmHel-8	BABH01047968:446-687
BmHel-8	DF090323:3689539-3689718
BmHel-8	DF090334:3215275-3215456
BmHel-8	DF090353:1892264-1892348
BmHel-8	DF090353:1891988-1892075
BmHel-8	DF090349:1444121-1444203
BmHel-8	DF090349:1444531-1444694
BmHel-8	DF090382:217542-217638
BmHel-8	DF090382:217371-217520
BmHel-8	DF090384:1106424-1106523
BmHel-8	DF090384:1106204-1106297
BmHel-8	DF090343:1138237-1138413
BmHel-8	DF090321:4800558-4800729
BmHel-8	DF090318:3648100-3648272
BmHel-8	DF090363:1357689-1357859
BmHel-8	DF090558:40765-40863
BmHel-8	DF090558:41000-41089
BmHel-8	DF090381:558547-558637
BmHel-8	DF091280:3766-3883
BmHel-8	DF090333:4169651-4169849
BmHel-8	DF090316:8428334-8428517
BmHel-8	DF090805:17000-17185
BmHel-8	DF090327:817283-817394
BmHel-8	DF090320:4766978-4767077
BmHel-8	DF090320:4767132-4767213
BmHel-8	DF090327:2952041-2952131
BmHel-8	DF090327:2952270-2952363
BmHel-8	DF090394:109959-110070
BmHel-8	DF090381:1906407-1906523

BmHel-8	DF090797:6042-6140
BmHel-8	DF090797:6270-6382
BmHel-8	DF090322:3704172-3704265
BmHel-8	DF090322:3703942-3704025
BmHel-8	DF090319:1062707-1062820
BmHel-8	DF090330:4118716-4118886
BmHel-8	DF090343:4082883-4082990
BmHel-8	DF090360:2595011-2595155
BmHel-8	DF090360:2594846-2594940
BmHel-8	DF090483:21890-22060
BmHel-8	DF090339:4380913-4381085
BmHel-8	DF090336:897632-897812
BmHel-8	DF090401:103427-103548
BmHel-8	DF090322:1323615-1323708
BmHel-8	DF090322:1323501-1323588
BmHel-8	DF090432:695660-695781
BmHel-8	DF090432:695439-695560
BmHel-8	DF090436:378963-379202
BmHel-8	BABH01087002:1-240
BmHel-8	BABH01049472:818-1057
BmHel-8	DF090325:3549098-3549276
BmHel-8	DF090318:1797976-1798153
BmHel-8	DF090416:393403-393579
BmHel-8	DF090390:806802-806890
BmHel-8	DF090390:805740-805898
BmHel-8	DF090353:3102966-3103055
BmHel-8	DF090353:3103215-3103302
BmHel-8	DF090454:510601-510767
BmHel-8	DF090382:2039298-2039464
BmHel-8	DF090318:7872787-7872947
BmHel-8	DF090482:44760-44921
BmHel-8	DF090828:7873-8035
BmHel-8	BABH01063960:403-565
BmHel-8	DF090356:1999390-1999506
BmHel-8	DF090469:195437-195614
BmHel-8	DF090342:2140671-2140855
BmHel-8	DF090402:734281-734465
BmHel-8	DF090350:1681018-1681200
BmHel-8	DF090344:1125325-1125425
BmHel-8	DF090344:1125472-1125667
BmHel-8	DF090332:3867219-3867399
BmHel-8	DF090319:8124464-8124657
BmHel-8	DF090393:1573450-1573651
BmHel-8	DF090317:351527-351643
BmHel-8	DF090329:4935435-4935536
BmHel-8	DF090344:893594-893763
BmHel-8	DF090360:3195326-3195429
BmHel-8	DF090324:6671695-6671777

BmHel-8	BABH01082354:1-234
BmHel-8	DF090468:278128-278229
BmHel-8	DF090331:836755-836935
BmHel-8	DF090405:619461-619646
BmHel-8	DF090341:3508327-3508510
BmHel-8	DF090341:4340029-4340144
BmHel-8	DF090341:4339799-4339880
BmHel-8	DF090319:5218900-5219019
BmHel-8	DF090319:5219127-5219249
BmHel-8	DF090354:912588-912776
BmHel-8	DF090346:1181726-1181964
BmHel-8	DF090326:5815484-5815721
BmHel-8	DF090391:1014447-1014690
BmHel-8	DF090360:1407156-1407382
BmHel-8	DF090393:1213583-1213820
BmHel-8	DF090332:280634-280755
BmHel-8	DF090316:13722518-13722695
BmHel-8	DF090382:1547939-1548120
BmHel-8	DF090316:15984000-15984112
BmHel-8	DF090316:15984254-15984341
BmHel-8	DF090335:4370808-4370896
BmHel-8	DF090341:1964384-1964552
BmHel-8	DF090359:230405-230516
BmHel-8	DF090319:93838-93922
BmHel-8	DF090319:93657-93742
BmHel-8	DF090397:451192-451346
BmHel-8	DF090343:656055-656228
BmHel-8	DF090334:6046810-6047009
BmHel-8	DF090333:1472504-1472592
BmHel-8	DF090333:1472969-1473059
BmHel-8	DF090356:1606844-1607025
BmHel-8	DF090323:2071093-2071215
BmHel-8	DF090407:1019445-1019557
BmHel-8	DF090407:1019197-1019276
BmHel-8	DF090511:68754-68940
BmHel-8	DF090352:4169916-4170154
BmHel-8	DF090340:4608441-4608619
BmHel-8	DF090316:621322-621481
BmHel-8	DF090345:4014625-4014809
BmHel-8	DF090358:182764-182940
BmHel-8	DF090421:599768-599939
BmHel-8	DF090322:8179651-8179740
BmHel-8	DF090325:6778840-6778996
BmHel-8	DF091287:2917-3073
BmHel-8	DF090357:958191-958309
BmHel-8	DF090462:84449-84570
BmHel-8	DF090384:582833-582947
BmHel-8	DF090347:2855330-2855421

BmHel-8	DF090347:2854802-2854889
BmHel-8	DF090429:580338-580456
BmHel-8	DF090429:580567-580689
BmHel-8	DF090332:938673-938770
BmHel-8	DF090332:938872-938952
BmHel-8	DF090340:1615910-1616087
BmHel-8	DF090431:689905-690019
BmHel-8	DF090431:690166-690246
BmHel-8	DF090318:5277240-5277352
BmHel-8	DF090328:3737603-3737698
BmHel-8	DF090409:1258164-1258360
BmHel-8	DF090317:8595839-8596035
BmHel-8	DF090390:1500034-1500145
BmHel-8	DF090390:1499850-1500008
BmHel-8	DF090528:22803-22912
BmHel-8	DF090528:18470-18558
BmHel-8	DF090318:5757634-5757722
BmHel-8	DF090364:161981-162156
BmHel-8	DF090316:233130-233310
BmHel-8	DF090320:2637079-2637164
BmHel-8	DF090320:2636838-2636926
BmHel-8	DF090360:389962-390119
BmHel-8	DF090317:4680863-4681031
BmHel-8	BABH01086939:294-451
BmHel-8	DF090329:5280963-5281147
BmHel-8	DF090321:5384459-5384572
BmHel-8	DF090321:5384321-5384407
BmHel-8	DF090346:1642942-1643143
BmHel-8	DF090332:2779504-2779626
BmHel-8	DF090332:3433206-3433290
BmHel-8	DF090332:3433428-3433516
BmHel-8	DF090338:1288238-1288358
BmHel-8	DF090487:106503-106606
BmHel-8	DF090319:3992538-3992653
BmHel-8	DF090397:1452701-1452789
BmHel-8	DF090397:1452809-1452894
BmHel-8	DF090394:1288947-1289064
BmHel-8	DF090394:1289162-1289280
BmHel-8	DF090331:3033848-3034021
BmHel-8	DF090366:436406-436576
BmHel-8	DF090380:557113-557291
BmHel-8	DF090901:5334-5427
BmHel-8	DF090369:1211958-1212061
BmHel-8	DF090331:2007087-2007178
BmHel-8	DF090327:6172298-6172388
BmHel-8	DF090327:6172053-6172147
BmHel-8	DF090402:65451-65560
BmHel-8	DF090402:65249-65330

BmHel-8	DF090377:424872-425044
BmHel-8	DF090373:212479-212559
BmHel-8	DF090339:3851732-3851901
BmHel-8	DF090341:2671269-2671464
BmHel-8	DF091693:43-203
BmHel-8	DF090375:1141375-1141530
BmHel-8	DF090319:5682899-5683054
BmHel-8	BABH01062338:423-580
BmHel-8	BABH01062338:582-664
BmHel-8	DF090323:6679069-6679185
BmHel-8	DF090392:1141053-1141236
BmHel-8	DF090368:1015858-1015957
BmHel-8	DF090391:1581862-1581980
BmHel-8	DF090390:414135-414256
BmHel-8	DF090367:2353217-2353400
BmHel-8	DF090335:4413658-4413753
BmHel-8	DF090366:369305-369483
BmHel-8	DF090343:3758966-3759145
BmHel-8	DF090342:4128242-4128425
BmHel-8	DF090349:1060251-1060366
BmHel-8	DF090361:756496-756591
BmHel-8	DF090361:756741-756828
BmHel-8	DF090394:1502684-1502767
BmHel-8	DF090394:1499874-1500203
BmHel-8	DF090358:2561876-2562052
BmHel-8	DF090344:2441651-2441754
BmHel-8	DF090344:2441907-2441993
BmHel-8	DF090338:3545122-3545298
BmHel-8	DF090355:2920621-2920769
BmHel-8	DF090355:2920773-2920929
BmHel-8	DF090355:2920931-2921013
BmHel-8	DF090371:2037798-2037881
BmHel-8	DF090371:2038081-2038264
BmHel-8	DF090413:240321-240420
BmHel-8	DF090413:240529-240611
BmHel-8	DF090355:1331173-1331261
BmHel-8	DF090355:1330919-1331015
BmHel-8	DF090344:3667953-3668116
BmHel-8	DF090317:8945118-8945306
BmHel-8	DF090981:732-892
BmHel-8	DF090386:1395526-1395725
BmHel-8	DF090377:1498164-1498283
BmHel-8	DF090342:4099722-4099906
BmHel-8	DF090320:4291997-4292182
BmHel-8	DF090348:14183-14368
BmHel-8	DF090436:323584-323768
BmHel-8	BABH01049397:495-680
BmHel-8	DF090333:3702517-3702628

BmHel-8	DF090317:11186864-11186965
BmHel-8	DF090317:11186643-11186731
BmHel-8	DF090327:2936963-2937075
BmHel-8	DF090327:2936736-2936860
BmHel-8	DF090383:412597-412718
BmHel-8	DF090383:412745-412855
BmHel-8	DF090357:2914993-2915177
BmHel-8	DF090383:42033-42145
BmHel-8	DF090324:957814-957931
BmHel-8	DF090320:6953724-6953900
BmHel-8	DF090324:4345156-4345263
BmHel-8	DF090488:50863-50975
BmHel-8	DF090327:1633257-1633428
BmHel-8	DF090414:1243019-1243179
BmHel-8	BABH01045690:2393-2491
BmHel-8	BABH01082254:5-85
BmHel-8	BABH01082254:157-318
BmHel-8	DF090323:5559388-5559554
BmHel-8	DF090468:177572-177653
BmHel-8	DF090319:6808808-6808958
BmHel-8	DF090319:1134650-1134767
BmHel-8	DF090381:121643-121764
BmHel-8	DF090321:1028080-1028263
BmHel-8	DF090317:5477401-5477585
BmHel-8	DF090366:292382-292566
BmHel-8	DF090396:1092940-1093114
BmHel-8	DF090397:1688405-1688588
BmHel-8	DF090319:3518212-3518305
BmHel-8	DF090319:3517970-3518052
BmHel-8	DF090554:61567-61800
BmHel-8	DF090350:2152530-2152647
BmHel-8	DF090335:2069247-2069364
BmHel-8	DF090320:2851554-2851634
BmHel-8	DF090320:2851081-2851241
BmHel-8	DF090336:4233097-4233181
BmHel-8	DF090336:4232851-4232943
BmHel-8	DF090324:7001401-7001572
BmHel-8	DF090344:478214-478367
BmHel-8	DF091014:6225-6304
BmHel-8	DF091014:6337-6421
BmHel-8	DF090342:1810293-1810458
BmHel-8	DF090323:4450658-4450778
BmHel-8	DF090343:2251950-2252066
BmHel-8	DF090721:3247-3369
BmHel-8	DF090317:10062246-10062428
BmHel-8	DF090490:47684-47869
BmHel-8	DF090334:4275658-4275779
BmHel-8	DF090321:3304227-3304310

BmHel-8	DF090321:3304447-3304541
BmHel-8	DF090318:4103247-4103358
BmHel-8	DF090318:4103011-4103129
BmHel-8	DF090794:15647-15766
BmHel-8	BABH01046290:493-722
BmHel-8	DF090362:2682847-2683023
BmHel-8	DF090320:3147620-3147739
BmHel-8	DF090382:387279-387397
BmHel-8	DF090326:3313612-3313728
BmHel-8	DF090326:3313447-3313568
BmHel-8	DF090341:18020-18135
BmHel-8	DF090344:188361-188472
BmHel-8	DF090344:188568-188690
BmHel-8	DF090340:1370787-1370961
BmHel-8	DF090335:3301166-3301277
BmHel-8	DF090322:4016712-4016884
BmHel-8	DF090316:11638180-11638279
BmHel-8	DF090349:1938638-1938740
BmHel-8	DF090333:5145574-5145666
BmHel-8	DF090352:3372553-3372637
BmHel-8	DF090352:3372416-3372515
BmHel-8	DF090319:2271758-2271882
BmHel-8	DF090421:784135-784287
BmHel-8	DF090316:5559630-5559752
BmHel-8	DF090355:3460365-3460543
BmHel-8	DF090326:3217453-3217636
BmHel-8	DF090345:2501804-2501987
BmHel-8	DF090321:733321-733421
BmHel-8	DF090321:733572-733662
BmHel-8	DF090382:1650074-1650162
BmHel-8	DF090382:1650557-1650647
BmHel-8	DF090316:2294200-2294288
BmHel-8	DF090316:2294888-2294980
BmHel-8	DF090337:1417953-1418069
BmHel-8	DF090387:1774960-1775142
BmHel-8	DF090369:1258201-1258296
BmHel-8	DF090369:1258083-1258166
BmHel-8	DF090324:2155365-2155545
BmHel-8	DF090413:631971-632082
BmHel-8	DF090338:4278559-4278646
BmHel-8	DF090338:4279203-4279294
BmHel-8	DF090332:6110672-6110763
BmHel-8	DF090332:6110913-6111005
BmHel-8	DF090599:40958-41057
BmHel-8	DF090599:40729-40816
BmHel-8	DF090387:1604108-1604278
BmHel-8	DF090456:127274-127374
BmHel-8	DF090456:127091-127186

BmHel-8	DF090388:916237-916399
BmHel-8	DF090383:1175547-1175738
BmHel-8	DF090324:3638560-3638743
BmHel-8	BABH01050413:468-649
BmHel-8	DF090359:2328394-2328579
BmHel-8	DF090441:637617-637737
BmHel-8	DF090441:637438-637551
BmHel-8	DF090343:3960127-3960357
BmHel-8	BABH01059779:478-706
BmHel-8	BABH01055296:3-230
BmHel-8	DF090321:1534287-1534464
BmHel-8	DF090357:3319561-3319679
BmHel-8	DF090324:1381819-1381929
BmHel-8	DF090403:794293-794380
BmHel-8	DF090403:794067-794154
BmHel-8	DF090333:3690532-3690619
BmHel-8	DF090333:3690299-3690383
BmHel-8	DF090483:49016-49131
BmHel-8	DF090374:2121771-2121965
BmHel-8	DF090368:506548-506656
BmHel-8	DF090368:506965-507089
BmHel-8	DF090463:125880-125965
BmHel-8	DF090463:125666-125747
BmHel-8	DF090316:6732980-6733098
BmHel-8	DF090329:6190856-6190972
BmHel-8	DF090417:784727-784816
BmHel-8	DF090338:3322656-3322832
BmHel-8	DF090340:3962556-3962660
BmHel-8	DF090338:4149156-4149334
BmHel-8	DF090404:318416-318597
BmHel-8	DF090331:5916846-5917029
BmHel-8	DF090351:3018173-3018356
BmHel-8	BABH01045777:966-1060
BmHel-8	DF090346:5329-5483
BmHel-8	DF090459:195907-196090
BmHel-8	DF090320:2543136-2543234
BmHel-8	DF090320:2542897-2542977
BmHel-8	BABH01086395:247-474
BmHel-8	DF090377:1832192-1832288
BmHel-8	DF090344:342185-342274
BmHel-8	DF090344:342313-342397
BmHel-8	DF090798:5799-5978
BmHel-8	DF090342:4119809-4119979
BmHel-8	DF090334:2884302-2884384
BmHel-8	DF090334:2883833-2883916
BmHel-8	DF090372:843111-843208
BmHel-8	DF090340:316518-316743
BmHel-8	DF090380:723009-723181

BmHel-8	DF090398:906921-907011
BmHel-8	DF090364:2086737-2086822
BmHel-8	DF090321:828618-828787
BmHel-8	DF090328:358582-358683
BmHel-8	DF090328:358353-358440
BmHel-8	DF090348:651668-651778
BmHel-8	DF090347:3848306-3848421
BmHel-8	DF090332:2136676-2136900
BmHel-8	DF090403:1712262-1712484
BmHel-8	DF090328:4849395-4849618
BmHel-8	BABH01055370:231-450
BmHel-8	DF090319:7214656-7214750
BmHel-8	DF090317:5742406-5742525
BmHel-8	DF090396:664453-664633
BmHel-8	DF090425:167884-168001
BmHel-8	DF090318:7062461-7062577
BmHel-8	DF090518:32001-32082
BmHel-8	DF090518:31735-31887
BmHel-8	DF090402:1477364-1477534
BmHel-8	DF090368:1865450-1865614
BmHel-8	DF090317:9792323-9792474
BmHel-8	BABH01062296:98-246
BmHel-8	DF090323:1495244-1495429
BmHel-8	DF090354:3737700-3737878
BmHel-8	DF090319:5681949-5682044
BmHel-8	DF090342:1068434-1068545
BmHel-8	DF090316:10162289-10162399
BmHel-8	DF090316:10162093-10162210
BmHel-8	DF090335:5740219-5740390
BmHel-8	DF090413:488299-488397
BmHel-8	DF090378:1127003-1127085
BmHel-8	DF090369:1393133-1393357
BmHel-8	DF090414:713133-713218
BmHel-8	DF090414:713917-713999
BmHel-8	DF090333:2692786-2692870
BmHel-8	DF090333:2692981-2693073
BmHel-8	DF090407:693411-693500
BmHel-8	DF090361:2646114-2646283
BmHel-8	BABH01053258:5-147
BmHel-8	DF090395:952243-952339
BmHel-8	DF090378:1884854-1884945
BmHel-8	DF090378:1884168-1884249
BmHel-8	DF090387:1396547-1396650
BmHel-8	DF090387:1396766-1396890
BmHel-8	DF090330:3579290-3579513
BmHel-8	DF090360:1106163-1106245
BmHel-8	DF090324:3586240-3586351
BmHel-8	BABH01058135:1-225

BmHel-8	DF090318:87570-87662
BmHel-8	BABH01049550:543-708
BmHel-8	DF090337:4128837-4128921
BmHel-8	DF090337:4128967-4129053
BmHel-8	DF090390:71606-71799
BmHel-8	DF090366:892967-893125
BmHel-8	DF090321:4560041-4560221
BmHel-8	DF090316:3091328-3091470
BmHel-8	DF090329:3210635-3210751
BmHel-8	DF090350:760550-760731
BmHel-8	DF090349:2489758-2489942
BmHel-8	DF090317:4737677-4737856
BmHel-8	DF090320:7031503-7031624
BmHel-8	DF090371:1305619-1305801
BmHel-8	DF090389:1412750-1412846
BmHel-8	DF090336:4007214-4007324
BmHel-8	DF090329:2436809-2437026
BmHel-8	DF090396:226684-226900
BmHel-8	DF090347:3865745-3865971
BmHel-8	DF090345:2517696-2517911
BmHel-8	DF090331:2536094-2536312
BmHel-8	DF090337:703637-703853
BmHel-8	DF090404:1384185-1384404
BmHel-8	DF090435:279422-279641
BmHel-8	DF090419:629788-630005
BmHel-8	BABH01064911:446-665
BmHel-8	DF090340:1737841-1737951
BmHel-8	DF090319:5941534-5941713
BmHel-8	DF090391:1599442-1599558
BmHel-8	DF090417:105172-105348
BmHel-8	DF090340:1335789-1335899
BmHel-8	DF090464:363316-363406
BmHel-8	DF090464:363079-363168
BmHel-8	DF090321:3482651-3482730
BmHel-8	DF090321:3482471-3482563
BmHel-8	DF090348:280144-280369
BmHel-8	DF090643:25572-25707
BmHel-8	BABH01051521:743-889
BmHel-8	BABH01075409:1-150
BmHel-8	DF090355:2356600-2356722
BmHel-8	DF090347:1593010-1593114
BmHel-8	DF090340:1012769-1012991
BmHel-8	DF090350:3427703-3427787
BmHel-8	DF090347:908315-908432
BmHel-8	DF090349:3501069-3501151
BmHel-8	DF090349:3500559-3500638
BmHel-8	DF090341:2886492-2886668
BmHel-8	DF090337:4644015-4644180

BmHel-8	DF090318:4092771-4092883
BmHel-8	DF090348:1548676-1548841
BmHel-8	DF090349:816108-816253
BmHel-8	DF090336:420189-420346
BmHel-8	DF090369:1720561-1720665
BmHel-8	DF090369:1720784-1720904
BmHel-8	BABH01059942:484-705
BmHel-8	DF091068:1-220
BmHel-8	DF090343:860628-860845
BmHel-8	DF090330:5595080-5595162
BmHel-8	DF090384:296818-296919
BmHel-8	DF090363:1361951-1362124
BmHel-8	DF090348:4215471-4215586
BmHel-8	DF090348:4215313-4215423
BmHel-8	DF090329:4447284-4447376
BmHel-8	DF090361:1558607-1558783
BmHel-8	DF090342:2451907-2452080
BmHel-8	DF090323:1995076-1995241
BmHel-8	DF090437:404140-404225
BmHel-8	DF090437:404366-404460
BmHel-8	DF090316:6129129-6129228
BmHel-8	DF090374:2228821-2228924
BmHel-8	DF090319:1228605-1228791
BmHel-8	DF090496:122476-122566
BmHel-8	DF090496:122234-122327
BmHel-8	DF090329:407077-407256
BmHel-8	DF090327:3515875-3516052
BmHel-8	DF090341:57160-57256
BmHel-8	DF090341:57396-57481
BmHel-8	DF090417:705694-705782
BmHel-8	DF090417:705959-706039
BmHel-8	DF090328:2531985-2532157
BmHel-8	DF090350:3159819-3159940
BmHel-8	DF090350:3159956-3160055
BmHel-8	DF091411:847-932
BmHel-8	DF091411:462-601
BmHel-8	DF090344:3131495-3131715
BmHel-8	DF090338:2219852-2220071
BmHel-8	DF090339:2877546-2877769
BmHel-8	DF090445:661278-661498
BmHel-8	BABH01060904:474-697
BmHel-8	DF090338:2376352-2376438
BmHel-8	DF090322:3774569-3774786
BmHel-8	DF090384:517543-517661
BmHel-8	DF090321:7766410-7766582
BmHel-8	DF090337:951991-952107
BmHel-8	DF090426:461637-461752
BmHel-8	DF090351:2708888-2708983

BmHel-8	DF090320:6350938-6351102
BmHel-8	DF090339:2075100-2075252
BmHel-8	DF090350:3834182-3834264
BmHel-8	DF090350:3834294-3834381
BmHel-8	DF090405:413894-413982
BmHel-8	DF090405:414202-414298
BmHel-8	DF090349:3353021-3353237
BmHel-8	DF090324:535240-535458
BmHel-8	DF090414:470215-470435
BmHel-8	BABH01059763:72-290
BmHel-8	BABH01050491:210-427
BmHel-8	BABH01077017:377-592
BmHel-8	DF090419:78076-78253
BmHel-8	DF090347:1070210-1070319
BmHel-8	DF090331:5967695-5967788
BmHel-8	DF090331:5967369-5967455
BmHel-8	DF090336:1808427-1808599
BmHel-8	DF090459:240716-240823
BmHel-8	DF090458:282064-282222
BmHel-8	DF090369:818987-819133
BmHel-8	BABH01049592:900-1041
BmHel-8	DF090319:8547203-8547310
BmHel-8	DF090347:744597-744704
BmHel-8	DF090389:51915-52009
BmHel-8	DF090389:51257-51338
BmHel-8	DF090320:4802573-4802690
BmHel-8	DF090317:9983454-9983576
BmHel-8	DF090332:6239717-6239804
BmHel-8	DF090479:88504-88614
BmHel-8	DF090479:88383-88488
BmHel-8	DF090347:2391891-2391988
BmHel-8	DF090345:1342295-1342458
BmHel-8	DF090336:4525859-4526071
BmHel-8	DF090327:835553-835726
BmHel-8	DF090434:724807-724986
BmHel-8	DF090417:439022-439199
BmHel-8	DF090421:233283-233378
BmHel-8	DF090421:233485-233611
BmHel-8	DF090699:17752-17831
BmHel-8	DF090699:17534-17698
BmHel-8	DF090344:2216099-2216209
BmHel-8	DF090336:5009032-5009117
BmHel-8	DF090329:2483540-2483634
BmHel-8	DF090328:5099801-5099976
BmHel-8	DF090363:2218796-2218987
BmHel-8	DF090320:5552447-5552545
BmHel-8	DF090327:4271611-4271719
BmHel-8	DF090359:2367173-2367356

BmHel-8	DF090372:1666657-1666779
BmHel-8	DF090328:5321494-5321678
BmHel-8	DF090363:2785448-2785551
BmHel-8	DF090329:3804989-3805100
BmHel-8	DF090370:277321-277469
BmHel-8	DF090409:249484-249610
BmHel-8	DF090409:249729-249828
BmHel-8	DF090342:4141309-4141431
BmHel-8	DF090342:4141116-4141210
BmHel-8	DF090431:571881-572058
BmHel-8	DF090435:196015-196137
BmHel-8	DF090435:195811-195904
BmHel-8	DF090318:4794316-4794526
BmHel-8	DF090316:7247891-7248103
BmHel-8	BABH01049574:343-560
BmHel-8	DF090346:1922864-1923022
BmHel-8	DF090317:99330-99425
BmHel-8	BABH01052659:1-219
BmHel-8	BABH01052816:610-827
BmHel-8	DF090362:2821075-2821286
BmHel-8	DF090317:1720549-1720642
BmHel-8	DF090316:9799420-9799592
BmHel-8	DF090361:2376284-2376375
BmHel-8	DF090361:2376509-2376607
BmHel-8	DF090531:47836-47915
BmHel-8	DF090378:389235-389386
BmHel-8	DF090358:978225-978326
BmHel-8	DF090356:2210164-2210285
BmHel-8	DF090356:2209967-2210061
BmHel-8	DF090361:2762672-2762793
BmHel-8	DF090361:2762897-2762991
BmHel-8	DF090339:1810709-1810830
BmHel-8	DF090339:1810967-1811061
BmHel-8	DF090436:336050-336172
BmHel-8	DF090436:336215-336309
BmHel-8	DF090317:10708155-10708256
BmHel-8	DF090317:10707916-10708034
BmHel-8	DF090375:994384-994520
BmHel-8	DF090317:10208265-10208476
BmHel-8	DF090321:4486683-4486897
BmHel-8	DF090420:877494-877672
BmHel-8	DF090329:4551211-4551389
BmHel-8	DF090461:195148-195232
BmHel-8	DF090481:224163-224336
BmHel-8	DF090324:2402921-2403137
BmHel-8	BABH01071300:357-573
BmHel-8	DF090343:2531686-2531858
BmHel-8	DF090380:266084-266179

BmHel-8	DF090409:672583-672683
BmHel-8	DF090322:1756644-1756749
BmHel-8	DF090600:4835-5018
BmHel-8	DF090411:416799-416879
BmHel-8	DF090349:824738-824834
BmHel-8	DF090349:824161-824285
BmHel-8	DF090316:3906353-3906475
BmHel-8	DF090316:3906611-3906705
BmHel-8	DF090406:650029-650203
BmHel-8	DF090350:2515492-2515665
BmHel-8	DF090350:2515660-2515798
BmHel-8	DF090350:2515801-2515882
BmHel-8	DF090350:2671983-2672196
BmHel-8	DF090341:3499007-3499101
BmHel-8	DF090338:1002412-1002552
BmHel-8	DF090324:5610814-5610947
BmHel-8	DF090357:2689361-2689472
BmHel-8	DF091707:517-703
BmHel-8	DF090355:660391-660568
BmHel-8	DF090322:6766667-6766793
BmHel-8	DF090322:6776278-6776371
BmHel-8	DF090350:3803363-3803484
BmHel-8	DF090350:3803626-3803718
BmHel-8	DF090365:2543176-2543298
BmHel-8	DF090365:2543425-2543519
BmHel-8	DF090361:2573955-2574078
BmHel-8	DF090361:2573771-2573858
BmHel-8	DF090321:2723529-2723744
BmHel-8	DF090318:1241882-1242096
BmHel-8	BABH01058159:491-705
BmHel-8	DF090323:6098057-6098176
BmHel-8	DF090323:6098324-6098418
BmHel-8	DF090374:1188510-1188691
BmHel-8	DF090367:1769490-1769612
BmHel-8	DF090367:1769271-1769374
BmHel-8	BABH01055428:1-214
BmHel-8	DF090322:7296229-7296333
BmHel-8	DF090352:3769299-3769389
BmHel-8	DF090352:3769545-3769626
BmHel-8	DF090794:1-121
BmHel-8	DF090329:2630075-2630167
BmHel-8	DF090375:176693-176829
BmHel-8	DF090356:3021945-3022066
BmHel-8	DF090327:4292833-4292949
BmHel-8	DF090319:9009186-9009282
BmHel-8	DF090349:2695571-2695784
BmHel-8	DF090348:3962589-3962804
BmHel-8	BABH01082735:305-518

BmHel-8	BABH01043618:5-218
BmHel-8	BABH01048195:420-633
BmHel-8	DF090322:1605866-1605964
BmHel-8	DF090422:397747-397842
BmHel-8	DF090422:397552-397670
BmHel-8	DF091809:96-270
BmHel-8	DF090386:1559321-1559501
BmHel-8	DF090320:2356216-2356329
BmHel-8	DF090362:2785607-2785779
BmHel-8	DF090376:2021080-2021192
BmHel-8	DF090376:2021231-2021334
BmHel-8	BABH01051378:539-625
BmHel-8	DF090319:8397872-8398024
BmHel-8	DF090480:90222-90335
BmHel-8	DF090421:205343-205460
BmHel-8	DF090422:455775-455902
BmHel-8	DF090422:455321-455413
BmHel-8	DF090318:7959751-7959852
BmHel-8	DF090318:7959965-7960079
BmHel-8	DF090369:1419122-1419233
BmHel-8	DF090369:1419352-1419458
BmHel-8	DF090317:2311403-2311525
BmHel-8	DF090317:2311302-2311389
BmHel-8	DF090344:985592-985711
BmHel-8	DF090344:985725-985812
BmHel-8	DF090680:31564-31776
BmHel-8	DF090359:1303172-1303379
BmHel-8	DF090357:1524779-1524986
BmHel-8	DF090410:919967-920172
BmHel-8	DF090366:1943432-1943638
BmHel-8	DF090374:1355794-1356001
BmHel-8	BABH01065350:398-610
BmHel-8	DF091069:219-431
BmHel-8	DF090340:4385021-4385108
BmHel-8	DF090475:186570-186676
BmHel-8	DF090434:676953-677065
BmHel-8	DF090344:3958924-3959024
BmHel-8	DF090360:2487288-2487399
BmHel-8	DF090400:1228001-1228213
BmHel-8	DF090340:2649344-2649515
BmHel-8	DF090564:55563-55735
BmHel-8	DF090356:3220165-3220252
BmHel-8	DF090342:450654-450735
BmHel-8	DF090342:450528-450618
BmHel-8	DF090380:244334-244421
BmHel-8	DF090324:1304001-1304112
BmHel-8	DF090343:2652124-2652218
BmHel-8	DF090330:5900182-5900291

BmHel-8	DF090340:3609507-3609623
BmHel-8	DF090340:3609286-3609381
BmHel-8	DF090406:781341-781457
BmHel-8	DF090406:781114-781210
BmHel-8	DF090383:558466-558570
BmHel-8	DF090371:2187774-2187860
BmHel-8	DF090335:1599524-1599648
BmHel-8	DF090342:605887-605991
BmHel-8	DF090327:3659621-3659730
BmHel-8	DF090352:3272964-3273141
BmHel-8	BABH01046475:1954-2063
BmHel-8	BABH01046475:1836-1937
BmHel-8	DF090359:1489628-1489719
BmHel-8	DF090353:1453312-1453475
BmHel-8	DF090360:405528-405651
BmHel-8	DF090360:405379-405466
BmHel-8	DF090409:500129-500267
BmHel-8	BABH01050864:403-576
BmHel-8	DF090348:2637709-2637829
BmHel-8	DF090326:4031444-4031566
BmHel-8	DF090326:4031240-4031327
BmHel-8	DF090337:2604052-2604140
BmHel-8	DF090337:2603821-2603943
BmHel-8	DF090356:863335-863457
BmHel-8	DF090356:863129-863220
BmHel-8	BABH01048108:969-1063
BmHel-8	BABH01048108:1282-1392
BmHel-8	DF090317:948762-948966
BmHel-8	DF090395:1319536-1319740
BmHel-8	DF090375:531830-532035
BmHel-8	DF090451:494837-495042
BmHel-8	DF090320:7405007-7405212
BmHel-8	DF090441:683341-683521
BmHel-8	DF090330:5302964-5303079
BmHel-8	DF090330:5303211-5303309
BmHel-8	DF090377:505297-505389
BmHel-8	DF090429:111474-111680
BmHel-8	DF090316:5700446-5700558
BmHel-8	DF090348:2642631-2642798
BmHel-8	BABH01088541:1-212
BmHel-8	DF090370:440892-441042
BmHel-8	BABH01086532:281-427
BmHel-8	DF090391:314322-314454
BmHel-8	BABH01088038:1-133
BmHel-8	DF090344:507773-507883
BmHel-8	DF090390:1416023-1416118
BmHel-8	DF090333:4496942-4497048
BmHel-8	DF090317:9776208-9776324

BmHel-8	DF090317:9776456-9776548
BmHel-8	DF090320:1753709-1753831
BmHel-8	DF090369:695198-695319
BmHel-8	DF090369:695335-695419
BmHel-8	DF090332:4270921-4271125
BmHel-8	BABH01074150:402-611
BmHel-8	DF090650:3771-3976
BmHel-8	DF090341:3846548-3846643
BmHel-8	BABH01048039:30-125
BmHel-8	DF090341:127820-127932
BmHel-8	DF090341:128044-128138
BmHel-8	DF090349:2397700-2397816
BmHel-8	DF090349:2397939-2398031
BmHel-8	DF090318:812593-812800
BmHel-8	DF090353:1755990-1756087
BmHel-8	DF090394:1235113-1235255
BmHel-8	DF090335:73803-73950
BmHel-8	DF090333:2756607-2756694
BmHel-8	DF090341:2430789-2430996
BmHel-8	DF090349:2683991-2684123
BmHel-8	DF091913:2259-2388
BmHel-8	DF090341:4427926-4428049
BmHel-8	DF090341:4427738-4427824
BmHel-8	DF090351:480843-481047
BmHel-8	DF090346:204488-204693
BmHel-8	DF090321:305460-305663
BmHel-8	DF090441:294797-295000
BmHel-8	DF090400:1306963-1307164
BmHel-8	DF090327:5440037-5440242
BmHel-8	DF090319:220743-220944
BmHel-8	DF090347:2169708-2169912
BmHel-8	DF090331:2859324-2859529
BmHel-8	DF090324:4460676-4460885
BmHel-8	DF090324:3076462-3076665
BmHel-8	DF090445:487323-487521
BmHel-8	DF090414:1316270-1316478
BmHel-8	DF090429:39570-39773
BmHel-8	DF090406:109494-109697
BmHel-8	DF090399:1431674-1431875
BmHel-8	BABH01086155:11-214
BmHel-8	BABH01053518:181-384
BmHel-8	DF090326:5094990-5095082
BmHel-8	DF090319:5821864-5821977
BmHel-8	DF090319:5821637-5821733
BmHel-8	DF090347:120394-120598
BmHel-8	DF090331:1264265-1264349
BmHel-8	DF090325:6303853-6303960
BmHel-8	DF090337:4316301-4316415

BmHel-8	DF090337:4316516-4316609
BmHel-8	DF090317:972857-972969
BmHel-8	DF090345:3428016-3428115
BmHel-8	DF090330:4353704-4353797
BmHel-8	DF090602:3647-3850
BmHel-8	DF090362:1461918-1462025
BmHel-8	DF090483:130995-131085
BmHel-8	DF090329:5834268-5834475
BmHel-8	BABH01048113:1060-1189
BmHel-8	DF090353:1015554-1015679
BmHel-8	DF090403:768417-768506
BmHel-8	DF090369:2649362-2649449
BmHel-8	DF090338:1926136-1926234
BmHel-8	DF090323:5777217-5777416
BmHel-8	DF090355:949617-949824
BmHel-8	DF090380:473478-473679
BmHel-8	DF090371:655848-656055
BmHel-8	BABH01082870:212-414
BmHel-8	BABH01053395:28-232
BmHel-8	DF090429:90304-90394
BmHel-8	DF090429:90724-90839
BmHel-8	DF090321:1788064-1788179
BmHel-8	DF090321:1787839-1787930
BmHel-8	DF090319:2698564-2698674
BmHel-8	DF090358:9189-9294
BmHel-8	DF090358:9355-9464
BmHel-8	DF090339:613409-613502
BmHel-8	DF090334:1522927-1523066
BmHel-8	DF090373:1396129-1396258
BmHel-8	BABH01045934:3402-3528
BmHel-8	DF090405:741159-741245
BmHel-8	DF090318:5409336-5409447
BmHel-8	DF090318:5409104-5409199
BmHel-8	DF090341:2801426-2801543
BmHel-8	DF090341:2801670-2801765
BmHel-8	DF090336:1071533-1071655
BmHel-8	DF090336:1071312-1071395
BmHel-8	BABH01048690:603-805
BmHel-8	DF090332:2706452-2706654
BmHel-8	DF090316:3965459-3965664
BmHel-8	DF090382:1698673-1698875
BmHel-8	DF090369:863289-863491
BmHel-8	DF090423:992850-993051
BmHel-8	DF090389:446053-446256
BmHel-8	BABH01045540:3478-3680
BmHel-8	DF090338:1317432-1317633
BmHel-8	DF090317:4313180-4313385
BmHel-8	DF090430:706399-706483

BmHel-8	DF090352:1446207-1446413
BmHel-8	DF090352:522827-522937
BmHel-8	DF090318:5781793-5782001
BmHel-8	DF090319:6996697-6996894
BmHel-8	DF090332:1398740-1398825
BmHel-8	BABH01046265:2081-2194
BmHel-8	DF090403:1477847-1477941
BmHel-8	DF090920:1-129
BmHel-8	DF090322:2644671-2644755
BmHel-8	DF090350:29069-29176
BmHel-8	DF090350:29748-29844
BmHel-8	DF090327:4636109-4636226
BmHel-8	DF090327:4636358-4636446
BmHel-8	DF090318:9300412-9300516
BmHel-8	DF090323:2968515-2968618
BmHel-8	DF090323:2968646-2968747
BmHel-8	DF090318:975002-975203
BmHel-8	DF090320:4378821-4378913
BmHel-8	DF090368:2262280-2262392
BmHel-8	DF090368:2262409-2262495
BmHel-8	DF090318:5248096-5248304
BmHel-8	DF090370:2111243-2111415
BmHel-8	DF090329:5306350-5306436
BmHel-8	BABH01050384:1-206
BmHel-8	DF090345:1028970-1029142
BmHel-8	DF090322:7156365-7156449
BmHel-8	DF090418:103956-104049
BmHel-8	DF090324:159816-159965
BmHel-8	DF090354:939265-939393
BmHel-8	DF090361:2365761-2365847
BmHel-8	BABH01080656:357-563
BmHel-8	DF090336:3572209-3572426
BmHel-8	DF090363:422757-422879
BmHel-8	DF090327:5980383-5980504
BmHel-8	DF090479:224168-224271
BmHel-8	BABH01049087:664-788
BmHel-8	DF090316:1009050-1009166
BmHel-8	DF090316:1009184-1009266
BmHel-8	DF090341:3255688-3255808
BmHel-8	DF090341:3255826-3255911
BmHel-8	DF090617:21022-21225
BmHel-8	DF090336:2849865-2849972
BmHel-8	DF090318:1073074-1073159
BmHel-8	DF090343:998177-998379
BmHel-8	DF090374:1306643-1306841
BmHel-8	DF090333:2253039-2253151
BmHel-8	DF090323:4997787-4997987
BmHel-8	DF090384:1385926-1386125

BmHel-8	DF090397:1509304-1509469
BmHel-8	DF090425:545007-545105
BmHel-8	BABH01066433:1-213
BmHel-8	DF090495:101141-101266
BmHel-8	DF090387:704398-704514
BmHel-8	BABH01045632:2513-2629
BmHel-8	DF090366:2354573-2354696
BmHel-8	DF090366:2354343-2354423
BmHel-8	DF090452:318473-318592
BmHel-8	DF090324:6176584-6176700
BmHel-8	DF090324:6176713-6176795
BmHel-8	DF090394:1013220-1013423
BmHel-8	DF090350:42939-43046
BmHel-8	DF090350:41859-41954
BmHel-8	DF090331:4919468-4919668
BmHel-8	DF090328:2609590-2609788
BmHel-8	DF090355:1004117-1004232
BmHel-8	DF090355:1004020-1004104
BmHel-8	DF090416:93293-93387
BmHel-8	DF090377:1015636-1015745
BmHel-8	DF090377:1015891-1015984
BmHel-8	DF090367:1682378-1682463
BmHel-8	DF090320:4585807-4585951
BmHel-8	BABH01050846:1-138
BmHel-8	DF090318:6580028-6580158
BmHel-8	DF090371:2526196-2526320
BmHel-8	DF090448:101955-102148
BmHel-8	BABH01050060:791-993
BmHel-8	BABH01084882:1-203
BmHel-8	DF090329:4874864-4874971
BmHel-8	DF090405:650399-650506
BmHel-8	DF090405:650650-650740
BmHel-8	DF090383:622738-622936
BmHel-8	DF090332:5324446-5324648
BmHel-8	DF090321:632342-632430
BmHel-8	DF090372:1351844-1351958
BmHel-8	DF090372:1351619-1351706
BmHel-8	DF090352:1832014-1832209
BmHel-8	DF090349:849646-849756
BmHel-8	DF090349:849869-849960
BmHel-8	DF090328:450933-451014
BmHel-8	DF090328:451112-451236
BmHel-8	DF090809:4508-4704
BmHel-8	DF090362:743983-744106
BmHel-8	DF090414:937842-937966
BmHel-8	BABH01070898:136-260
BmHel-8	BABH01049182:1-125
BmHel-8	DF090389:1094620-1094728

BmHel-8	DF090370:1880876-1880958
BmHel-8	DF090408:366657-366768
BmHel-8	DF090408:366880-366969
BmHel-8	DF090448:706926-707044
BmHel-8	DF090326:6276166-6276287
BmHel-8	DF090326:6276304-6276383
BmHel-8	DF090356:2028322-2028432
BmHel-8	DF090356:2028077-2028169
BmHel-8	DF090316:7531209-7531409
BmHel-8	DF090325:5395211-5395323
BmHel-8	DF090363:1982960-1983042
BmHel-8	DF090376:1348652-1348795
BmHel-8	DF090408:685073-685166
BmHel-8	DF090342:3088249-3088343
BmHel-8	DF090366:2300817-2300927
BmHel-8	DF090374:1014891-1014979
BmHel-8	DF090329:1060954-1061077
BmHel-8	DF090372:468636-468747
BmHel-8	DF090372:468896-468984
BmHel-8	DF090316:4470338-4470459
BmHel-8	DF090316:4470001-4470080
BmHel-8	DF090330:3096000-3096121
BmHel-8	DF090420:583877-583986
BmHel-8	DF090420:583751-583842
BmHel-8	DF090342:4625262-4625463
BmHel-8	DF090444:132456-132573
BmHel-8	DF090444:132718-132805
BmHel-8	DF090357:945030-945228
BmHel-8	DF090317:10856234-10856335
BmHel-8	DF090317:10856430-10856521
BmHel-8	DF090410:693596-693792
BmHel-8	BABH01074046:462-612
BmHel-8	DF090336:1406787-1406875
BmHel-8	DF090396:1290397-1290483
BmHel-8	DF090396:1289978-1290083
BmHel-8	DF090390:1105133-1105292
BmHel-8	BABH01047982:289-412
BmHel-8	DF090356:3396294-3396405
BmHel-8	DF090434:471044-471145
BmHel-8	DF090351:601170-601271
BmHel-8	DF090357:1702494-1702605
BmHel-8	DF090357:1702280-1702367
BmHel-8	DF090327:854853-854957
BmHel-8	DF090327:855083-855177
BmHel-8	DF090316:15191195-15191299
BmHel-8	DF090316:15191401-15191496
BmHel-8	DF090368:1072987-1073091
BmHel-8	DF090368:1073210-1073303

BmHel-8	DF090358:3190927-3191123
BmHel-8	DF090323:607056-607174
BmHel-8	DF090323:607491-607571
BmHel-8	DF090381:1241735-1241852
BmHel-8	DF090381:1242012-1242100
BmHel-8	DF090341:3526380-3526484
BmHel-8	DF090341:3526616-3526711
BmHel-8	DF090378:694987-695066
BmHel-8	DF090333:1005235-1005369
BmHel-8	BABH01087861:2-201
BmHel-8	DF090327:1685890-1686092
BmHel-8	BABH01045532:4183-4340
BmHel-8	BABH01072000:424-553
BmHel-8	DF090317:5404740-5404862
BmHel-8	DF090416:1270953-1271072
BmHel-8	BABH01047736:1184-1301
BmHel-8	DF090445:469478-469588
BmHel-8	DF090316:7875083-7875184
BmHel-8	DF090341:3772986-3773102
BmHel-8	DF090317:7610981-7611092
BmHel-8	DF090317:8478686-8478797
BmHel-8	DF090317:8478933-8479018
BmHel-8	DF090378:1524142-1524260
BmHel-8	DF090378:1524860-1524940
BmHel-8	DF090380:141965-142087
BmHel-8	DF090334:2609170-2609291
BmHel-8	DF090320:3333106-3333217
BmHel-8	DF090320:3332998-3333080
BmHel-8	DF090316:1891930-1892038
BmHel-8	DF090316:1892064-1892145
BmHel-8	DF090344:4030416-4030612
BmHel-8	DF090338:4229598-4229795
BmHel-8	DF091369:741-934
BmHel-8	DF090564:17766-17850
BmHel-8	DF090564:17464-17575
BmHel-8	DF090320:6356474-6356633
BmHel-8	BABH01059289:1-196
BmHel-8	BABH01087871:1-199
BmHel-8	BABH01088359:1-198
BmHel-8	BABH01045239:57-165
BmHel-8	DF091912:52-205
BmHel-8	DF090361:2803796-2803954
BmHel-8	DF090324:7262852-7262973
BmHel-8	BABH01066677:431-552
BmHel-8	BABH01042956:2-117
BmHel-8	DF090317:523764-523914
BmHel-8	DF090363:842167-842271
BmHel-8	DF090316:11327620-11327744

BmHel-8	DF090374:1496469-1496575
BmHel-8	DF090374:1496705-1496795
BmHel-8	DF090444:312632-312743
BmHel-8	DF090444:312424-312509
BmHel-8	DF090329:5488410-5488531
BmHel-8	DF090316:3493051-3493249
BmHel-8	DF090338:2139252-2139361
BmHel-8	DF090338:2139027-2139113
BmHel-8	DF090317:8419788-8419897
BmHel-8	DF090317:8419557-8419645
BmHel-8	DF090372:1817529-1817643
BmHel-8	DF090423:240418-240511
BmHel-8	DF090322:5598536-5598638
BmHel-8	DF090322:5598764-5598858
BmHel-8	DF090363:1145106-1145234
BmHel-8	DF090411:1341160-1341360
BmHel-8	DF090344:4312382-4312469
BmHel-8	DF090317:1959830-1959931
BmHel-8	DF090317:1959603-1959698
BmHel-8	DF090325:6705017-6705123
BmHel-8	DF090325:6704791-6704890
BmHel-8	DF090342:2037051-2037174
BmHel-8	DF090317:1788872-1789035
BmHel-8	DF090327:4372380-4372498
BmHel-8	DF090322:7681031-7681136
BmHel-8	DF090358:390420-390613
BmHel-8	DF090351:3133685-3133791
BmHel-8	DF090354:1975278-1975394
BmHel-8	DF090354:1975515-1975596
BmHel-8	BABH01080481:126-233
BmHel-8	DF090370:59149-59343
BmHel-8	DF090349:648406-648599
BmHel-8	DF090330:791753-791903
BmHel-8	DF090332:1875517-1875596
BmHel-8	DF090412:166627-166719
BmHel-8	DF090316:8592681-8592809
BmHel-8	DF090324:1474179-1474301
BmHel-8	DF090356:2632770-2632865
BmHel-8	DF090429:482357-482454
BmHel-8	DF090322:2707877-2707987
BmHel-8	DF090322:2708114-2708201
BmHel-8	DF090321:6777054-6777157
BmHel-8	DF090321:6776482-6776570
BmHel-8	DF090336:1435445-1435561
BmHel-8	DF090336:1435339-1435419
BmHel-8	DF090341:2637331-2637448
BmHel-8	DF090358:3127332-3127437
BmHel-8	DF090358:3127613-3127703

BmHel-8	DF090323:187043-187160
BmHel-8	DF090363:931504-931601
BmHel-8	DF090316:12311059-12311170
BmHel-8	DF090316:12311288-12311371
BmHel-8	DF090586:2800-2888
BmHel-8	DF090444:276245-276367
BmHel-8	DF090325:662114-662225
BmHel-8	DF091598:685-779
BmHel-8	DF090478:341079-341179
BmHel-8	DF090478:341305-341397
BmHel-8	DF090421:894091-894201
BmHel-8	DF090391:531712-531829
BmHel-8	DF090324:7345191-7345385
BmHel-8	BABH01056196:1-197
BmHel-8	DF090390:445835-445943
BmHel-8	BABH01050613:1-195
BmHel-8	DF090331:5288231-5288331
BmHel-8	DF090390:1020638-1020753
BmHel-8	DF090390:1020496-1020581
BmHel-8	DF090322:3090703-3090808
BmHel-8	DF090322:3090501-3090586
BmHel-8	DF090390:589975-590075
BmHel-8	DF090390:589745-589838
BmHel-8	DF090431:186685-186784
BmHel-8	DF090431:186492-186587
BmHel-8	DF090334:4054456-4054646
BmHel-8	DF090340:4057906-4058008
BmHel-8	BABH01045757:1510-1604
BmHel-8	DF090363:402144-402345
BmHel-8	DF090345:226940-227066
BmHel-8	DF092108:285-401
BmHel-8	DF090319:4909688-4909810
BmHel-8	DF090534:154752-154850
BmHel-8	DF090534:154515-154610
BmHel-8	DF090415:740531-740643
BmHel-8	DF090372:1043893-1044005
BmHel-8	BABH01056048:357-473
BmHel-8	DF090379:203045-203239
BmHel-8	BABH01058184:396-589
BmHel-8	DF090316:12513177-12513282
BmHel-8	DF090336:2514525-2514623
BmHel-8	DF090336:2514282-2514376
BmHel-8	DF090365:2096849-2096965
BmHel-8	DF090368:1425479-1425588
BmHel-8	DF090368:1425248-1425335
BmHel-8	DF090470:236703-236795
BmHel-8	DF090431:282825-282923
BmHel-8	DF090333:115773-115933

BmHel-8	DF090350:1588597-1588695
BmHel-8	DF090350:1589172-1589267
BmHel-8	DF090344:2700065-2700145
BmHel-8	DF090451:347826-348019
BmHel-8	DF090400:1131127-1131214
BmHel-8	DF090325:2582836-2582918
BmHel-8	DF090692:17138-17237
BmHel-8	DF090692:16872-16959
BmHel-8	DF090403:586655-586766
BmHel-8	DF090403:586801-586988
BmHel-8	DF090376:553440-553546
BmHel-8	DF090376:553563-553643
BmHel-8	DF090402:1019193-1019312
BmHel-8	DF090371:1932441-1932527
BmHel-8	DF090367:1519628-1519734
BmHel-8	DF090367:1519519-1519602
BmHel-8	DF090358:1789176-1789280
BmHel-8	DF090358:1789402-1789490
BmHel-8	DF090346:2178528-2178644
BmHel-8	DF090331:3401038-3401133
BmHel-8	DF090326:2229974-2230064
BmHel-8	DF090322:7817909-7818000
BmHel-8	DF090322:7817706-7817798
BmHel-8	DF090322:647465-647548
BmHel-8	BABH01079515:7-125
BmHel-8	BABH01079515:130-211
BmHel-8	DF091744:644-759
BmHel-8	DF090416:1174337-1174456
BmHel-8	DF090320:6787705-6787808
BmHel-8	DF090320:6787973-6788060
BmHel-8	DF090346:420549-420649
BmHel-8	DF090346:420314-420404
BmHel-8	DF090381:1207840-1207941
BmHel-8	DF090381:1207672-1207761
BmHel-8	DF090701:6182-6299
BmHel-8	DF090358:714896-715011
BmHel-8	DF090390:1363126-1363221
BmHel-8	DF090390:1363252-1363344
BmHel-8	DF090338:4428608-4428695
BmHel-8	DF090386:1433737-1433880
BmHel-8	DF090325:1067181-1067276
BmHel-8	DF090319:8684408-8684490
BmHel-8	DF090365:1879787-1879948
BmHel-8	DF090332:5985410-5985521
BmHel-8	BABH01054398:103-218
BmHel-8	DF090437:547658-547773
BmHel-8	DF090325:6048347-6048465
BmHel-8	DF090316:15969922-15970044

BmHel-8	DF090475:128248-128352
BmHel-8	DF090475:127526-127611
BmHel-8	DF090334:2968818-2968937
BmHel-8	DF090418:178847-178934
BmHel-8	DF090418:179250-179360
BmHel-8	DF090323:4520742-4520853
BmHel-8	DF090319:953213-953316
BmHel-8	DF090326:6609874-6609969
BmHel-8	DF090330:4066273-4066372
BmHel-8	DF090338:4864572-4864685
BmHel-8	DF090372:2413640-2413826
BmHel-8	DF090339:4190386-4190530
BmHel-8	DF090440:267971-268052
BmHel-8	DF090322:252695-252880
BmHel-8	BABH01049341:1-112
BmHel-8	DF090317:7545734-7545822
BmHel-8	DF090377:2032674-2032762
BmHel-8	DF090810:8209-8310
BmHel-8	DF090810:8465-8554
BmHel-8	DF090396:920679-920801
BmHel-8	DF090377:1276424-1276527
BmHel-8	DF090377:1276561-1276641
BmHel-8	DF090370:363852-363999
BmHel-8	DF090400:431122-431233
BmHel-8	DF090340:1544667-1544768
BmHel-8	DF090340:1544443-1544530
BmHel-8	DF090355:3590546-3590656
BmHel-8	DF090427:563746-563850
BmHel-8	DF090556:40357-40484
BmHel-8	DF090398:918522-918604
BmHel-8	DF090407:94366-94480
BmHel-8	DF090338:3969873-3969997
BmHel-8	DF090322:2231463-2231575
BmHel-8	DF090370:2059892-2059996
BmHel-8	DF090370:2060142-2060226
BmHel-8	DF090434:102423-102532
BmHel-8	BABH01072333:357-465
BmHel-8	BABH01072333:1-80
BmHel-8	DF090335:2188853-2188969
BmHel-8	DF090371:2404829-2404948
BmHel-8	DF090480:178058-178177
BmHel-8	DF090329:3282934-3283046
BmHel-8	DF090325:5209595-5209706
BmHel-8	DF090342:3061931-3062017
BmHel-8	DF090317:10617960-10618056
BmHel-8	DF090326:4859744-4859930
BmHel-8	DF090389:1667932-1668056
BmHel-8	DF090382:489505-489606

BmHel-8	DF090382:489761-489847
BmHel-8	DF090427:921374-921474
BmHel-8	DF090427:921146-921233
BmHel-8	DF090402:726229-726327
BmHel-8	DF090402:726474-726561
BmHel-8	DF090400:1488434-1488556
BmHel-8	DF090347:3790717-3790824
BmHel-8	DF090377:894963-895065
BmHel-8	DF090377:894747-894831
BmHel-8	DF090327:2956905-2956998
BmHel-8	DF090327:2956675-2956768
BmHel-8	DF090396:132616-132716
BmHel-8	DF090396:132734-132816
BmHel-8	DF090364:734466-734565
BmHel-8	DF090364:734264-734351
BmHel-8	DF090430:593358-593506
BmHel-8	DF090318:6792204-6792322
BmHel-8	BABH01050317:606-694
BmHel-8	BABH01050317:473-568
BmHel-8	BABH01045707:2160-2252
BmHel-8	BABH01045707:2412-2506
BmHel-8	DF090360:2603529-2603651
BmHel-8	DF090345:364243-364343
BmHel-8	DF090345:364447-364530
BmHel-8	DF090573:21946-22047
BmHel-8	DF090573:21541-21622
BmHel-8	DF090454:234610-234796
BmHel-8	DF090754:17301-17487
BmHel-8	DF090340:2979574-2979672
BmHel-8	DF090340:2979314-2979401
BmHel-8	DF090321:8005984-8006091
BmHel-8	DF090321:8005791-8005870
BmHel-8	DF090353:1388742-1388859
BmHel-8	DF090362:65068-65167
BmHel-8	DF090362:64585-64672
BmHel-8	DF090392:716628-716738
BmHel-8	DF090403:820398-820483
BmHel-8	DF090329:2930807-2930960
BmHel-8	DF090345:3666676-3666763
BmHel-8	DF090387:1147913-1148035
BmHel-8	DF090357:3366405-3366527
BmHel-8	DF090325:5753119-5753217
BmHel-8	DF090316:2490947-2491050
BmHel-8	DF090316:2490720-2490801
BmHel-8	DF090382:1754552-1754736
BmHel-8	BABH01048781:1-184
BmHel-8	DF090456:74456-74553
BmHel-8	DF090456:75877-75966

BmHel-8	DF091597:2840-2930
BmHel-8	DF091597:2423-2518
BmHel-8	DF090467:205753-205870
BmHel-8	DF090402:262980-263067
BmHel-8	DF090401:1300139-1300247
BmHel-8	DF090368:737391-737493
BmHel-8	DF090368:737156-737241
BmHel-8	DF090384:1266985-1267081
BmHel-8	DF090384:1267141-1267229
BmHel-8	BABH01071859:516-624
BmHel-8	DF090355:851476-851580
BmHel-8	DF090358:138440-138560
BmHel-8	DF090349:4028041-4028163
BmHel-8	DF090347:2539725-2539819
BmHel-8	DF090347:2538773-2538861
BmHel-8	DF090362:1814043-1814141
BmHel-8	DF090362:1813812-1813897
BmHel-8	DF090333:3732480-3732564
BmHel-8	DF090330:2979219-2979396
BmHel-8	DF090352:4136129-4136313
BmHel-8	DF090351:719716-719905
BmHel-8	DF090409:62969-63153
BmHel-8	DF090409:1559362-1559545
BmHel-8	DF090355:1216475-1216659
BmHel-8	DF090355:554124-554307
BmHel-8	DF090321:2598465-2598648
BmHel-8	DF090318:5823764-5823949
BmHel-8	DF090318:8466443-8466627
BmHel-8	DF090318:4856667-4856848
BmHel-8	DF090320:4973472-4973655
BmHel-8	DF090356:3333526-3333709
BmHel-8	DF090344:4512999-4513182
BmHel-8	DF090344:1545718-1545900
BmHel-8	DF090340:1332410-1332594
BmHel-8	DF090338:458805-458988
BmHel-8	DF090338:4032901-4033085
BmHel-8	DF090323:3819532-3819713
BmHel-8	DF090322:197873-198054
BmHel-8	DF090322:7571231-7571414
BmHel-8	DF090322:4592855-4593039
BmHel-8	DF090319:3795281-3795465
BmHel-8	DF090319:5396796-5396981
BmHel-8	DF090317:1457683-1457866
BmHel-8	DF090316:7816037-7816221
BmHel-8	DF090411:905517-905694
BmHel-8	DF090396:326073-326257
BmHel-8	DF090382:478581-478760
BmHel-8	DF090361:2168376-2168550

BmHel-8	DF090361:2984963-2985150
BmHel-8	DF090360:813305-813488
BmHel-8	DF090345:2022103-2022289
BmHel-8	DF090341:2549042-2549226
BmHel-8	DF090341:3766303-3766489
BmHel-8	DF090341:2713243-2713426
BmHel-8	DF090324:3740278-3740462
BmHel-8	DF090324:286064-286248
BmHel-8	DF090337:1706021-1706206
BmHel-8	DF090449:280588-280772
BmHel-8	DF090381:132567-132753
BmHel-8	DF090343:606635-606819
BmHel-8	DF090333:4060087-4060271
BmHel-8	DF090325:2718210-2718386
BmHel-8	DF090325:2173202-2173386
BmHel-8	DF090414:639322-639513
BmHel-8	DF090354:796328-796511
BmHel-8	DF090408:1170092-1170275
BmHel-8	DF090520:43909-44092
BmHel-8	DF090442:655354-655538
BmHel-8	BABH01047504:1130-1314
BmHel-8	BABH01081140:62-251
BmHel-8	BABH01050064:657-838
BmHel-8	BABH01047966:242-426
BmHel-8	BABH01042971:6236-6420
BmHel-8	BABH01045050:1575-1759
BmHel-8	DF090473:180626-180804
BmHel-8	DF090509:56492-56675
BmHel-8	DF090316:13455148-13455331
BmHel-8	DF091466:1528-1647
BmHel-8	DF090350:2267438-2267545
BmHel-8	DF090359:2462795-2462884
BmHel-8	DF090359:2462568-2462661
BmHel-8	BABH01061969:499-683
BmHel-8	BABH01059422:291-475
BmHel-8	BABH01059408:488-672
BmHel-8	BABH01068760:444-555
BmHel-8	BABH01071851:193-303
BmHel-8	BABH01061932:212-323
BmHel-8	BABH01058968:296-407
BmHel-8	DF090338:1850378-1850466
BmHel-8	DF090317:3042715-3042838
BmHel-8	DF090331:2986137-2986264
BmHel-8	DF090350:227785-227900
BmHel-8	DF090357:1898250-1898372
BmHel-8	DF090330:2825441-2825559
BmHel-8	DF090365:2198994-2199116
BmHel-8	DF090317:4392973-4393068

BmHel-8	DF090317:4392859-4392943
BmHel-8	DF090360:2407422-2407542
BmHel-8	DF090333:2842452-2842574
BmHel-8	DF090444:335614-335734
BmHel-8	DF090336:2060128-2060271
BmHel-8	DF090399:455510-455613
BmHel-8	DF090399:454369-454459
BmHel-8	DF090338:2178315-2178496
BmHel-8	DF090544:23074-23257
BmHel-8	DF090332:2419459-2419640
BmHel-8	DF090353:537740-537923
BmHel-8	DF090345:2572939-2573121
BmHel-8	DF090341:3879140-3879322
BmHel-8	DF090375:786601-786784
BmHel-8	DF090325:3760536-3760718
BmHel-8	DF090889:6179-6371
BmHel-8	DF090596:15810-15924
BmHel-8	DF090316:14058554-14058651
BmHel-8	DF090472:182926-183030
BmHel-8	DF091374:1939-2116
BmHel-8	BABH01060173:2-184
BmHel-8	DF090320:4133093-4133176
BmHel-8	DF090355:2596069-2596191
BmHel-8	DF090423:385339-385463
BmHel-8	DF090370:924597-924715
BmHel-8	BABH01048231:1-115
BmHel-8	DF090343:4187510-4187611
BmHel-8	DF090343:4187253-4187337
BmHel-8	DF090337:1253544-1253645
BmHel-8	DF090337:1253733-1253818
BmHel-8	DF090343:2225307-2225408
BmHel-8	DF090343:2225070-2225155
BmHel-8	DF090721:15254-15369
BmHel-8	DF090370:1189888-1190070
BmHel-8	DF090339:1072622-1072800
BmHel-8	DF090333:5291217-5291398
BmHel-8	DF090415:568481-568663
BmHel-8	DF090690:82-264
BmHel-8	BABH01073330:435-617
BmHel-8	DF090571:12405-12592
BmHel-8	DF090316:2171053-2171233
BmHel-8	DF090365:2966860-2967040
BmHel-8	DF090325:4361281-4361463
BmHel-8	DF090341:2083488-2083607
BmHel-8	DF090357:1118738-1118836
BmHel-8	DF090357:1118989-1119077
BmHel-8	DF090328:354157-354272
BmHel-8	DF090331:1629069-1629182

BmHel-8	DF090488:76847-76934
BmHel-8	DF090488:75926-76019
BmHel-8	DF090329:3067881-3068062
BmHel-8	DF090376:2003853-2003936
BmHel-8	DF090417:745796-745916
BmHel-8	BABH01078965:396-578
BmHel-8	DF090579:18575-18660
BmHel-8	DF090333:1194587-1194686
BmHel-8	DF090419:898189-898309
BmHel-8	DF090352:2014450-2014569
BmHel-8	DF090469:361513-361607
BmHel-8	DF090337:3576124-3576222
BmHel-8	DF090337:3575886-3575975
BmHel-8	DF090321:421466-421647
BmHel-8	DF090344:2397068-2397249
BmHel-8	DF090323:3128744-3128926
BmHel-8	DF090317:7188197-7188377
BmHel-8	DF090316:6181319-6181500
BmHel-8	DF090379:1310491-1310672
BmHel-8	DF090379:937726-937907
BmHel-8	DF090446:563110-563291
BmHel-8	DF090430:503843-504020
BmHel-8	DF090325:1429290-1429405
BmHel-8	DF090330:6418681-6418862
BmHel-8	DF090330:462434-462615
BmHel-8	DF090386:1300643-1300824
BmHel-8	DF090402:1307076-1307255
BmHel-8	DF090326:5359277-5359458
BmHel-8	DF090396:370956-371136
BmHel-8	BABH01049109:92-273
BmHel-8	BABH01044488:1506-1688
BmHel-8	DF090335:3934752-3934839
BmHel-8	DF090325:3341704-3341820
BmHel-8	DF090319:2487730-2487826
BmHel-8	DF090319:2487861-2487941
BmHel-8	DF090322:349470-349550
BmHel-8	DF090321:4375186-4375317
BmHel-8	DF090356:1600663-1600785
BmHel-8	DF090386:1510223-1510334
BmHel-8	DF090359:1077093-1077185
BmHel-8	DF090359:1076122-1076210
BmHel-8	DF090374:1380361-1380455
BmHel-8	DF090366:473408-473587
BmHel-8	DF090329:687759-687937
BmHel-8	DF090323:5646133-5646314
BmHel-8	DF090390:1566173-1566351
BmHel-8	DF090373:191393-191570
BmHel-8	DF090327:6507961-6508140

BmHel-8	DF090322:7006228-7006408
BmHel-8	DF090373:1785939-1786058
BmHel-8	DF090364:1177766-1177945
BmHel-8	DF090321:6144521-6144703
BmHel-8	DF090356:3827791-3827971
BmHel-8	DF090340:4425-4606
BmHel-8	DF090348:945514-945691
BmHel-8	DF090394:1311045-1311225
BmHel-8	BABH01063295:14-197
BmHel-8	DF090316:2088880-2089060
BmHel-8	DF090319:685192-685302
BmHel-8	DF090408:217422-217516
BmHel-8	DF090408:217651-217736
BmHel-8	DF090385:1791314-1791407
BmHel-8	DF090325:2636464-2636576
BmHel-8	DF090397:183271-183365
BmHel-8	DF090344:1935893-1936073
BmHel-8	BABH01088473:55-236
BmHel-8	DF090356:769174-769290
BmHel-8	DF090341:121501-121610
BmHel-8	BABH01084745:377-494
BmHel-8	DF090395:822597-822701
BmHel-8	DF090395:1475659-1475837
BmHel-8	DF090330:1943990-1944171
BmHel-8	DF090320:5548955-5549134
BmHel-8	DF090360:1886124-1886301
BmHel-8	DF090330:1849606-1849785
BmHel-8	DF090319:7682366-7682542
BmHel-8	DF090317:10124184-10124362
BmHel-8	BABH01042583:20486-20668
BmHel-8	DF090471:189029-189208
BmHel-8	DF090683:25766-25846
BmHel-8	DF090683:26800-26894
BmHel-8	DF090327:3258100-3258198
BmHel-8	DF090327:3258377-3258458
BmHel-8	DF090337:769708-769887
BmHel-8	DF090319:7112779-7112874
BmHel-8	DF090319:7112673-7112752
BmHel-8	DF090343:428587-428701
BmHel-8	DF090443:400245-400332
BmHel-8	DF090372:2226486-2226607
BmHel-8	DF090321:4402950-4403069
BmHel-8	DF090540:71072-71182
BmHel-8	DF090330:3326577-3326699
BmHel-8	DF090342:1856008-1856099
BmHel-8	DF090342:1856246-1856332
BmHel-8	DF090389:269606-269710
BmHel-8	DF090336:584772-584873

BmHel-8	DF090318:4280749-4280850
BmHel-8	DF090368:1398357-1398534
BmHel-8	DF090334:4563765-4563859
BmHel-8	DF090393:1540389-1540495
BmHel-8	DF090393:1540202-1540321
BmHel-8	DF090365:567709-567824
BmHel-8	DF090447:52722-52900
BmHel-8	BABH01074227:2-180
BmHel-8	DF090776:15418-15508
BmHel-8	DF090776:15838-15926
BmHel-8	DF090322:7342373-7342551
BmHel-8	DF090320:5536562-5536739
BmHel-8	DF090403:109660-109837
BmHel-8	DF090379:368827-369001
BmHel-8	DF090652:54354-54523
BmHel-8	BABH01072426:386-564
BmHel-8	DF090352:2210390-2210563
BmHel-8	BABH01087885:186-365
BmHel-8	DF090366:1395585-1395685
BmHel-8	DF090380:1910120-1910236
BmHel-8	DF090326:2414206-2414333
BmHel-8	BABH01068131:1-105
BmHel-8	DF090331:3845755-3845870
BmHel-8	DF090375:1911439-1911560
BmHel-8	DF090356:1099162-1099279
BmHel-8	DF090341:998254-998376
BmHel-8	DF090347:4073770-4073862
BmHel-8	DF090347:4073992-4074079
BmHel-8	DF090336:4011989-4012096
BmHel-8	DF090316:5575934-5576035
BmHel-8	DF090316:14470857-14470957
BmHel-8	DF090320:5142774-5142945
BmHel-8	DF090343:3684618-3684795
BmHel-8	DF090374:1229843-1230019
BmHel-8	DF090319:8526508-8526689
BmHel-8	DF090318:9774962-9775139
BmHel-8	DF090318:3571729-3571823
BmHel-8	DF090423:715913-716089
BmHel-8	DF090354:1675802-1675916
BmHel-8	BABH01058506:230-407
BmHel-8	DF090406:1121900-1122076
BmHel-8	DF090433:402368-402536
BmHel-8	DF090437:637636-637810
BmHel-8	DF090391:1424017-1424194
BmHel-8	DF090331:5661824-5662001
BmHel-8	DF090378:541539-541716
BmHel-8	DF090406:1022145-1022322
BmHel-8	DF090367:121212-121389

BmHel-8	DF090321:1555924-1556026
BmHel-8	DF090348:3704263-3704439
BmHel-8	BABH01051394:1-172
BmHel-8	BABH01049936:40-213
BmHel-8	BABH01074317:1-178
BmHel-8	DF090355:2458235-2458339
BmHel-8	DF090366:2181178-2181283
BmHel-8	DF090336:1887787-1887875
BmHel-8	DF090321:2473457-2473573
BmHel-8	DF090367:2024026-2024145
BmHel-8	DF090317:4603927-4604049
BmHel-8	DF090396:742201-742299
BmHel-8	DF090321:6433848-6433959
BmHel-8	DF090509:72229-72351
BmHel-8	BABH01088178:23-199
BmHel-8	DF090318:9198375-9198494
BmHel-8	DF090356:2393187-2393300
BmHel-8	DF090372:1578153-1578272
BmHel-8	DF090778:16903-17079
BmHel-8	DF090329:2090781-2090880
BmHel-8	DF090348:1437189-1437279
BmHel-8	DF090348:1435986-1436071
BmHel-8	BABH01076849:1-178
BmHel-8	BABH01062756:105-280
BmHel-8	DF090410:1195024-1195196
BmHel-8	DF090395:999410-999585
BmHel-8	DF090339:22747-22922
BmHel-8	DF090462:67081-67255
BmHel-8	DF090368:1160501-1160681
BmHel-8	DF090325:2947681-2947761
BmHel-8	DF090325:2947457-2947548
BmHel-8	DF090317:9184047-9184159
BmHel-8	DF090384:741633-741752
BmHel-8	DF090351:1774791-1774892
BmHel-8	DF090351:1776389-1776478
BmHel-8	DF090351:1776598-1776679
BmHel-8	DF090391:5289-5407
BmHel-8	DF090426:243462-243584
BmHel-8	DF090901:5069-5186
BmHel-8	DF090332:5770254-5770429
BmHel-8	DF090396:578363-578538
BmHel-8	DF090350:3779116-3779224
BmHel-8	DF090420:816206-816380
BmHel-8	DF090334:4130696-4130789
BmHel-8	DF090451:243-418
BmHel-8	DF090329:575341-575516
BmHel-8	DF090322:7153774-7153950
BmHel-8	DF090328:5846729-5846904

BmHel-8	DF090322:1165710-1165816
BmHel-8	BABH01046760:2040-2121
BmHel-8	BABH01070591:537-633
BmHel-8	DF090331:4963820-4963921
BmHel-8	DF090371:84536-84654
BmHel-8	DF090316:9236908-9237009
BmHel-8	DF090335:569487-569584
BmHel-8	DF090372:581758-581844
BmHel-8	DF090372:582032-582119
BmHel-8	DF090317:7310-7404
BmHel-8	DF090317:7561-7640
BmHel-8	DF090327:3421638-3421758
BmHel-8	DF090339:2388688-2388782
BmHel-8	DF090339:2389222-2389305
BmHel-8	DF090338:3699248-3699421
BmHel-8	DF090428:913545-913718
BmHel-8	BABH01056921:560-734
BmHel-8	DF090365:441217-441331
BmHel-8	DF090402:1353-1527
BmHel-8	DF090350:3890448-3890549
BmHel-8	DF090362:697510-697684
BmHel-8	DF090379:2092363-2092535
BmHel-8	DF090418:584679-584855
BmHel-8	DF090444:53274-53444
BmHel-8	DF090384:263247-263348
BmHel-8	DF090426:353894-354068
BmHel-8	BABH01056137:98-267
BmHel-8	BABH01076326:368-469
BmHel-8	BABH01074657:504-596
BmHel-8	BABH01070656:1-95
BmHel-8	BABH01070656:112-191
BmHel-8	BABH01064688:1-175
BmHel-8	DF090316:1544008-1544128
BmHel-8	DF090541:157440-157558
BmHel-8	DF090374:2338745-2338837
BmHel-8	DF090332:53660-53748
BmHel-8	DF090332:53937-54021
BmHel-8	DF090459:385954-386042
BmHel-8	DF090459:386225-386310
BmHel-8	DF091057:38351-38439
BmHel-8	DF091057:38083-38168
BmHel-8	DF090352:876676-876782
BmHel-8	DF090328:4070694-4070797
BmHel-8	DF090410:1047588-1047758
BmHel-8	DF090316:12660239-12660362
BmHel-8	DF090415:349379-349553
BmHel-8	DF090363:2625855-2625937
BmHel-8	DF090321:7153020-7153194

BmHel-8	DF090416:839211-839297
BmHel-8	DF091196:644-732
BmHel-8	DF091196:2632-2716
BmHel-8	DF090324:4762567-4762669
BmHel-8	DF090415:303949-304122
BmHel-8	DF090356:1682573-1682746
BmHel-8	BABH01045725:1002-1178
BmHel-8	DF090326:4268654-4268739
BmHel-8	DF090457:94699-94810
BmHel-8	BABH01075423:429-540
BmHel-8	DF091751:2980-3075
BmHel-8	DF090318:3445459-3445560
BmHel-8	DF090331:5972437-5972557
BmHel-8	DF090341:1905048-1905148
BmHel-8	DF090357:693581-693699
BmHel-8	DF090378:1754051-1754170
BmHel-8	DF090322:2072349-2072441
BmHel-8	DF090322:2066800-2066894
BmHel-8	DF090356:2606768-2606941
BmHel-8	DF090504:119632-119802
BmHel-8	DF090582:10916-11092
BmHel-8	DF090404:1181589-1181760
BmHel-8	DF090476:58241-58412
BmHel-8	DF090415:1275015-1275120
BmHel-8	DF090728:20796-20910
BmHel-8	DF090372:56716-56888
BmHel-8	DF090351:4280633-4280733
BmHel-8	DF090326:944775-944876
BmHel-8	DF090320:776741-776913
BmHel-8	DF090375:1776915-1777086
BmHel-8	DF090430:114863-115030
BmHel-8	DF090373:896157-896240
BmHel-8	BABH01088627:1-174
BmHel-8	DF090328:3291861-3291956
BmHel-8	DF090378:1423132-1423227
BmHel-8	DF090528:14572-14667
BmHel-8	BABH01049234:998-1092
BmHel-8	DF090352:4144775-4144896
BmHel-8	DF090351:400516-400631
BmHel-8	BABH01046082:983-1088
BmHel-8	DF090353:1285622-1285744
BmHel-8	DF090326:146013-146130
BmHel-8	DF090523:22632-22750
BmHel-8	DF090604:9778-9904
BmHel-8	DF090321:715375-715482
BmHel-8	DF090386:1751268-1751385
BmHel-8	DF090384:1056744-1056836
BmHel-8	DF090353:3131128-3131298

BmHel-8	DF090346:2616705-2616806
BmHel-8	DF090316:8570249-8570421
BmHel-8	DF090336:2081240-2081401
BmHel-8	DF091988:119-289
BmHel-8	DF090377:2020145-2020315
BmHel-8	DF090329:86907-87079
BmHel-8	DF090323:4748485-4748655
BmHel-8	DF090454:162629-162800
BmHel-8	DF090385:1590310-1590433
BmHel-8	BABH01045922:2344-2478
BmHel-8	BABH01079357:1-170
BmHel-8	DF090343:4274417-4274538
BmHel-8	DF090350:4102448-4102541
BmHel-8	DF090321:2335423-2335539
BmHel-8	DF090374:1902799-1902887
BmHel-8	DF090374:1902274-1902355
BmHel-8	DF090319:8648973-8649090
BmHel-8	DF090330:3002595-3002765
BmHel-8	DF090502:68840-68942
BmHel-8	DF090418:131060-131173
BmHel-8	DF090783:5322-5435
BmHel-8	DF090377:918973-919080
BmHel-8	DF090410:649558-649727
BmHel-8	DF091982:251-357
BmHel-8	BABH01087897:195-365
BmHel-8	DF090318:6122488-6122572
BmHel-8	DF090318:6122609-6122689
BmHel-8	DF090320:8566638-8566730
BmHel-8	BABH01070421:464-584
BmHel-8	DF090324:173287-173410
BmHel-8	DF090360:216801-216923
BmHel-8	DF090323:1827356-1827477
BmHel-8	DF090339:4750460-4750543
BmHel-8	DF090323:6310471-6310575
BmHel-8	DF090362:2829643-2829814
BmHel-8	DF090327:3196470-3196641
BmHel-8	BABH01052323:673-843
BmHel-8	BABH01069938:300-469
BmHel-8	BABH01048516:228-398
BmHel-8	BABH01046000:162-333
BmHel-8	DF090344:1830754-1830869
BmHel-8	DF091051:311-412
BmHel-8	DF090383:1989572-1989666
BmHel-8	DF090343:2845370-2845452
BmHel-8	DF090321:1801992-1802092
BmHel-8	DF090340:991718-991806
BmHel-8	DF090448:47288-47382
BmHel-8	DF090351:3326787-3326893

BmHel-8	DF090360:1481190-1481315
BmHel-8	DF090329:6044187-6044313
BmHel-8	DF090321:8237961-8238075
BmHel-8	DF090386:970563-970651
BmHel-8	DF090320:3688694-3688780
BmHel-8	DF090320:3688554-3688635
BmHel-8	BABH01051737:394-481
BmHel-8	DF090351:3552114-3552232
BmHel-8	DF090333:989101-989214
BmHel-8	DF090387:1794366-1794480
BmHel-8	DF090327:4746882-4747048
BmHel-8	DF090339:1698686-1698854
BmHel-8	DF090317:120278-120384
BmHel-8	DF090322:6020686-6020797
BmHel-8	DF090321:5977189-5977292
BmHel-8	DF090317:5664261-5664342
BmHel-8	DF090463:157188-157297
BmHel-8	BABH01052106:72-186
BmHel-8	DF090328:607936-608043
BmHel-8	DF090342:911275-911381
BmHel-8	DF090352:1240976-1241080
BmHel-8	DF090397:484547-484664
BmHel-8	DF090354:2014551-2014654
BmHel-8	DF090386:1785558-1785680
BmHel-8	DF090316:10510808-10510907
BmHel-8	DF090325:1294455-1294570
BmHel-8	DF090326:5227656-5227771
BmHel-8	DF090360:1486258-1486377
BmHel-8	DF090368:404585-404698
BmHel-8	DF090776:1231-1347
BmHel-8	DF090371:1299146-1299262
BmHel-8	DF090502:127428-127539
BmHel-8	DF090317:8665277-8665387
BmHel-8	DF090345:3690761-3690858
BmHel-8	DF090868:6631-6735
BmHel-8	DF090316:12154389-12154556
BmHel-8	DF090320:4532064-4532160
BmHel-8	DF090455:225128-225214
BmHel-8	DF090455:225343-225423
BmHel-8	DF090951:6830-6922
BmHel-8	DF090471:178975-179092
BmHel-8	DF090344:211419-211516
BmHel-8	BABH01080502:393-565
BmHel-8	BABH01082259:343-510
BmHel-8	DF090375:465594-465715
BmHel-8	DF090405:548178-548263
BmHel-8	DF090337:558223-558438
BmHel-8	DF090414:1358129-1358246

BmHel-8	DF090406:391520-391637
BmHel-8	DF090359:1732220-1732339
BmHel-8	DF090366:411447-411558
BmHel-8	DF090345:2365367-2365475
BmHel-8	DF090393:1379264-1379352
BmHel-8	DF090346:773051-773161
BmHel-8	DF090329:2759852-2759937
BmHel-8	DF091527:1599-1710
BmHel-8	DF090342:874233-874318
BmHel-8	DF090363:1337286-1337371
BmHel-8	DF090363:1337054-1337134
BmHel-8	BABH01073334:450-617
BmHel-8	DF090450:219526-219623
BmHel-8	DF090409:863001-863123
BmHel-8	DF090331:2381970-2382057
BmHel-8	DF090358:2820465-2820586
BmHel-8	DF090462:268978-269097
BmHel-8	DF091122:7459-7570
BmHel-8	DF090364:1353455-1353569
BmHel-8	DF090350:1372799-1372964
BmHel-8	DF090373:2170780-2170945
BmHel-8	BABH01066456:495-659
BmHel-8	BABH01070289:2-166
BmHel-8	DF090388:1228021-1228133
BmHel-8	DF090331:4146425-4146541
BmHel-8	DF090478:98469-98566
BmHel-8	DF090402:1090828-1090912
BmHel-8	DF090366:354355-354519
BmHel-8	DF091801:517-682
BmHel-8	DF090402:208169-208336
BmHel-8	DF090339:3072940-3073042
BmHel-8	DF090379:1986250-1986415
BmHel-8	DF090336:2539134-2539232
BmHel-8	DF090339:1769321-1769432
BmHel-8	BABH01068297:482-647
BmHel-8	BABH01059944:539-650
BmHel-8	DF090363:487702-487790
BmHel-8	BABH01069518:550-637
BmHel-8	DF090355:2681316-2681418
BmHel-8	DF090323:1217495-1217617
BmHel-8	DF090384:1202604-1202684
BmHel-8	DF090384:1202761-1202847
BmHel-8	DF090355:863545-863645
BmHel-8	DF090333:3999173-3999295
BmHel-8	DF090390:1284555-1284672
BmHel-8	DF090377:1871471-1871588
BmHel-8	DF090430:847829-847916
BmHel-8	DF090360:1859443-1859559

BmHel-8	DF090319:983614-983707
BmHel-8	DF090330:4569133-4569246
BmHel-8	DF090340:2090550-2090657
BmHel-8	DF090345:2931403-2931494
BmHel-8	DF090316:580430-580538
BmHel-8	DF090316:580664-580749
BmHel-8	DF090401:142310-142420
BmHel-8	DF090460:168479-168639
BmHel-8	DF090320:4254046-4254141
BmHel-8	DF090350:4102306-4102437
BmHel-8	BABH01048256:1249-1335
BmHel-8	DF090340:2122946-2123065
BmHel-8	DF090324:3723507-3723629
BmHel-8	DF090361:2250018-2250129
BmHel-8	DF090365:2693297-2693461
BmHel-8	BABH01049846:852-1014
BmHel-8	DF090340:3690940-3691044
BmHel-8	DF090337:2833593-2833706
BmHel-8	DF090381:1873839-1873946
BmHel-8	DF090377:1046359-1046473
BmHel-8	DF090447:107946-108063
BmHel-8	DF090425:1012533-1012648
BmHel-8	DF090425:1012349-1012443
BmHel-8	DF091471:501-610
BmHel-8	DF090316:15435801-15435911
BmHel-8	DF090349:3570405-3570492
BmHel-8	DF090322:6062569-6062651
BmHel-8	DF090369:799971-800071
BmHel-8	DF090350:590050-590171
BmHel-8	DF090356:1805060-1805151
BmHel-8	DF090318:6635685-6635766
BmHel-8	DF090383:1742805-1742916
BmHel-8	DF090390:205350-205449
BmHel-8	DF090401:656443-656538
BmHel-8	BABH01043953:3853-3975
BmHel-8	DF090384:1632856-1632972
BmHel-8	DF090332:2841-2993
BmHel-8	DF090318:3423448-3423557
BmHel-8	DF090374:191358-191474
BmHel-8	DF090344:2046842-2046954
BmHel-8	DF090414:865679-865782
BmHel-8	DF090411:607687-607784
BmHel-8	DF090339:4484283-4484368
BmHel-8	DF091582:541-641
BmHel-8	DF090337:5216574-5216660
BmHel-8	DF090316:4757495-4757601
BmHel-8	DF090460:4148-4246
BmHel-8	DF090330:5196673-5196834

BmHel-8	DF090411:652060-652175
BmHel-8	DF090345:160031-160131
BmHel-8	DF090329:3767104-3767198
BmHel-8	DF090361:2977002-2977097
BmHel-8	BABH01061171:285-386
BmHel-8	DF090618:17604-17688
BmHel-8	DF090439:619545-619666
BmHel-8	DF090341:4628040-4628162
BmHel-8	DF090329:710973-711094
BmHel-8	DF090339:4164769-4164870
BmHel-8	DF090318:4657466-4657574
BmHel-8	DF090358:2799892-2799976
BmHel-8	DF090331:3239204-3239323
BmHel-8	DF090370:413709-413821
BmHel-8	DF090322:7606974-7607078
BmHel-8	DF090338:1201508-1201601
BmHel-8	DF090321:501038-501130
BmHel-8	DF090374:2323291-2323395
BmHel-8	DF090351:2602784-2602951
BmHel-8	DF090335:3077686-3077845
BmHel-8	DF090372:1571209-1571369
BmHel-8	BABH01046211:2221-2314
BmHel-8	DF090327:4181855-4182013
BmHel-8	BABH01058165:29-190
BmHel-8	DF090376:1838924-1839041
BmHel-8	DF090347:3027976-3028086
BmHel-8	DF090318:4637530-4637611
BmHel-8	DF090318:6696385-6696504
BmHel-8	DF090418:785148-785271
BmHel-8	DF090332:4924826-4924927
BmHel-8	DF090499:43774-43893
BmHel-8	DF090391:1144315-1144414
BmHel-8	DF090426:673870-673968
BmHel-8	BABH01045989:1686-1785
BmHel-8	DF090404:1002412-1002567
BmHel-8	DF090423:268887-269002
BmHel-8	DF090324:1557353-1557456
BmHel-8	DF090350:1328679-1328789
BmHel-8	DF090382:1021275-1021378
BmHel-8	DF090360:3028995-3029108
BmHel-8	DF090345:3326081-3326163
BmHel-8	DF090429:647148-647231
BmHel-8	DF090329:4907456-4907567
BmHel-8	DF090330:938132-938232
BmHel-8	BABH01070699:530-628
BmHel-8	DF090331:1982740-1982852
BmHel-8	DF090366:802835-802950
BmHel-8	DF090402:1439660-1439782

BmHel-8	DF090340:474736-474857
BmHel-8	DF090440:460395-460516
BmHel-8	DF090447:90733-90892
BmHel-8	BABH01050471:1-160
BmHel-8	DF090316:13968168-13968270
BmHel-8	BABH01048610:632-720
BmHel-8	DF090319:8248885-8248966
BmHel-8	DF090403:1419019-1419119
BmHel-8	DF090316:695128-695225
BmHel-8	DF090328:6169708-6169811
BmHel-8	DF090327:824186-824277
BmHel-8	DF090330:4602235-4602393
BmHel-8	DF090348:899534-899624
BmHel-8	DF090361:1175881-1176040
BmHel-8	DF090379:972711-972877
BmHel-8	DF090396:416387-416509
BmHel-8	DF090350:3333025-3333126
BmHel-8	DF090329:346774-346873
BmHel-8	DF090352:3808582-3808701
BmHel-8	DF090380:1923862-1923981
BmHel-8	DF090335:1324612-1324720
BmHel-8	DF090337:2860061-2860169
BmHel-8	BABH01080280:402-560
BmHel-8	DF090378:2072143-2072233
BmHel-8	DF090432:181911-182007
BmHel-8	DF090374:546664-546778
BmHel-8	DF090425:642127-642210
BmHel-8	BABH01054477:417-535
BmHel-8	DF090389:25781-25942
BmHel-8	DF090405:999067-999163
BmHel-8	BABH01081441:465-554
BmHel-8	DF090316:15207500-15207580
BmHel-8	DF090330:5098552-5098649
BmHel-8	DF090320:6367189-6367306
BmHel-8	DF090325:2526647-2526745
BmHel-8	DF090360:3280887-3281003
BmHel-8	DF090320:6577801-6577895
BmHel-8	DF090344:247308-247411
BmHel-8	DF090349:419129-419286
BmHel-8	DF090380:555730-555837
BmHel-8	DF090508:105314-105405
BmHel-8	DF090397:643879-643996
BmHel-8	DF090431:323631-323784
BmHel-8	DF090352:2216648-2216748
BmHel-8	DF090316:7548627-7548711
BmHel-8	DF090319:355616-355739
BmHel-8	BABH01084653:322-412
BmHel-8	DF090330:2948927-2949046

BmHel-8	BABH01046870:1-80
BmHel-8	DF090336:3900638-3900739
BmHel-8	DF090324:6453934-6454050
BmHel-8	DF090335:5787522-5787630
BmHel-8	DF090417:218765-218883
BmHel-8	DF090341:376938-377057
BmHel-8	DF090317:10665894-10665998
BmHel-8	DF090386:865106-865218
BmHel-8	DF090387:1547522-1547638
BmHel-8	DF090387:1547304-1547461
BmHel-8	DF090418:894524-894618
BmHel-8	DF090325:824766-824878
BmHel-8	DF090317:7031976-7032075
BmHel-8	DF090329:2929565-2929717
BmHel-8	DF090319:1986637-1986792
BmHel-8	BABH01071899:538-625
BmHel-8	DF090330:3751597-3751762
BmHel-8	DF090472:48639-48735
BmHel-8	DF090321:5436063-5436144
BmHel-8	DF090400:1412238-1412333
BmHel-8	DF090392:1156774-1156874
BmHel-8	DF090454:390219-390323
BmHel-8	DF090384:935221-935343
BmHel-8	DF090357:2927143-2927264
BmHel-8	DF090332:5886539-5886641
BmHel-8	DF090386:360273-360395
BmHel-8	DF090342:1940059-1940180
BmHel-8	DF090337:4972136-4972257
BmHel-8	BABH01055755:377-498
BmHel-8	DF090324:5694301-5694455
BmHel-8	DF090378:2024609-2024710
BmHel-8	DF090322:3759564-3759654
BmHel-8	DF090323:4129007-4129119
BmHel-8	DF090316:6371564-6371679
BmHel-8	DF090340:1229184-1229272
BmHel-8	DF090318:8158141-8158240
BmHel-8	DF090412:507551-507705
BmHel-8	DF090350:2479955-2480073
BmHel-8	DF090323:290902-291005
BmHel-8	DF090321:4637952-4638048
BmHel-8	DF090394:914983-915067
BmHel-8	DF090318:6225860-6226014
BmHel-8	DF090403:768509-768663
BmHel-8	DF090400:1275672-1275826
BmHel-8	DF090318:9118675-9118764
BmHel-8	DF090373:1287898-1287997
BmHel-8	DF090350:4224129-4224236
BmHel-8	DF090350:4224028-4224116

BmHel-8	DF090360:1294119-1294202
BmHel-8	DF091058:2719-2883
BmHel-8	BABH01049414:599-683
BmHel-8	DF090351:2200702-2200799
BmHel-8	DF090388:1639105-1639221
BmHel-8	DF090376:1937832-1937947
BmHel-8	DF090347:4363440-4363541
BmHel-8	DF090404:505626-505742
BmHel-8	DF090391:20742-20828
BmHel-8	DF090356:3527994-3528090
BmHel-8	DF090321:7126510-7126600
BmHel-8	DF090325:5698071-5698166
BmHel-8	DF090509:97034-97150
BmHel-8	DF090318:5309781-5309895
BmHel-8	DF090375:832904-833013
BmHel-8	DF090505:161254-161349
BmHel-8	DF090376:830523-830632
BmHel-8	BABH01085549:346-498
BmHel-8	DF090323:1706104-1706198
BmHel-8	DF090411:965948-966035
BmHel-8	DF090324:47579-47677
BmHel-8	DF090322:3104434-3104514
BmHel-8	BABH01082492:1-152
BmHel-8	DF090368:2027006-2027154
BmHel-8	DF090801:19913-20001
BmHel-8	DF090326:2110480-2110594
BmHel-8	DF090427:701869-701976
BmHel-8	DF090483:142695-142845
BmHel-8	DF090511:107673-107756
BmHel-8	DF090377:1424500-1424605
BmHel-8	DF090353:3281727-3281844
BmHel-8	DF090374:2198499-2198607
BmHel-8	DF091718:1312-1406
BmHel-8	DF090334:5383348-5383458
BmHel-8	DF090329:3022191-3022300
BmHel-8	DF090356:2489795-2489890
BmHel-8	BABH01051708:305-410
BmHel-8	BABH01076119:1-151
BmHel-8	DF090396:1290088-1290169
BmHel-8	BABH01048871:374-495
BmHel-8	DF090321:3891027-3891121
BmHel-8	DF090330:5416769-5416863
BmHel-8	DF090433:635841-635934
BmHel-8	DF090430:100660-100761
BmHel-8	DF090517:96480-96573
BmHel-8	DF090316:6476198-6476299
BmHel-8	DF090338:4084856-4084954
BmHel-8	DF090318:6828070-6828162

BmHel-8	BABH01058454:315-409
BmHel-8	DF090334:3406816-3406902
BmHel-8	DF090362:1385493-1385580
BmHel-8	DF090338:4733771-4733863
BmHel-8	DF090387:429799-429887
BmHel-8	DF090374:1880818-1880901
BmHel-8	DF090324:2649756-2649848
BmHel-8	DF090405:1397493-1397594
BmHel-8	DF090345:1963686-1963798
BmHel-8	BABH01058661:104-198
BmHel-8	DF090400:508340-508451
BmHel-8	DF090354:2059495-2059590
BmHel-8	DF090396:103126-103239
BmHel-8	DF090740:9510-9589
BmHel-8	DF090363:600001-600100
BmHel-8	DF090321:6639615-6639704
BmHel-8	DF090420:887189-887295
BmHel-8	DF090343:503347-503435
BmHel-8	BABH01068062:5-152
BmHel-8	BABH01071008:484-631
BmHel-8	BABH01082594:7-155
BmHel-8	DF090372:1117322-1117415
BmHel-8	DF090392:346368-346447
BmHel-8	DF090444:576139-576250
BmHel-8	DF090380:372611-372705
BmHel-8	DF090339:2414071-2414172
BmHel-8	DF090321:7416545-7416642
BmHel-8	DF090468:104667-104765
BmHel-8	DF090364:2537026-2537111
BmHel-8	DF091029:8339-8453
BmHel-8	DF090450:132616-132705
BmHel-8	DF090317:3200674-3200816
BmHel-8	BABH01063158:1-143
BmHel-8	DF090361:3031787-3031875
BmHel-8	DF090366:1745219-1745331
BmHel-8	DF090373:820714-820811
BmHel-8	DF090405:435594-435691
BmHel-8	DF090321:146832-146937
BmHel-8	DF090413:839673-839787
BmHel-8	DF090345:804627-804720
BmHel-8	DF090576:3490-3632
BmHel-8	BABH01080066:39-185
BmHel-8	DF090354:3754571-3754686
BmHel-8	DF090353:1665047-1665158
BmHel-8	DF090316:13898152-13898256
BmHel-8	DF090356:1527400-1527498
BmHel-8	DF090331:5945894-5945985
BmHel-8	BABH01076445:2-147

BmHel-8	DF090348:4300101-4300185
BmHel-8	DF090318:2187627-2187772
BmHel-8	DF090397:288826-288972
BmHel-8	DF090317:2657445-2657528
BmHel-8	DF090375:1334616-1334760
BmHel-8	BABH01047401:773-866
BmHel-8	BABH01088649:1-148
BmHel-8	DF090654:21017-21111
BmHel-8	BABH01084998:370-509
BmHel-8	DF090337:5154628-5154719
BmHel-8	DF090361:3255045-3255130
BmHel-8	DF090387:1366947-1367057
BmHel-8	DF090320:4910441-4910530
BmHel-8	DF090374:1914507-1914595
BmHel-8	DF090329:689564-689653
BmHel-8	BABH01084269:96-175
BmHel-8	DF090419:308494-308639
BmHel-8	DF090350:2421455-2421542
BmHel-8	DF090316:11849041-11849138
BmHel-8	DF090710:22487-22575
BmHel-8	DF090319:3054135-3054238
BmHel-8	DF090333:4714333-4714424
BmHel-8	DF090323:561586-561692
BmHel-8	DF090332:3300692-3300772
BmHel-8	DF090327:5809218-5809318
BmHel-8	DF090337:47545-47629
BmHel-8	DF090394:663346-663434
BmHel-8	DF090369:1279298-1279381
BmHel-8	DF090397:1004601-1004688
BmHel-8	DF090345:3492054-3492136
BmHel-8	DF090345:3044594-3044685
BmHel-8	DF090373:1340464-1340668
BmHel-8	DF090373:1340324-1340415
BmHel-8	DF090348:1526277-1526377
BmHel-8	DF090398:875684-875772
BmHel-8	DF090337:3508949-3509050
BmHel-8	DF090351:1506870-1506962
BmHel-8	DF090357:2187214-2187320
BmHel-8	DF090335:5470504-5470592
BmHel-8	DF090412:892735-892836
BmHel-8	BABH01070559:1-141
BmHel-8	DF090322:1632471-1632557
BmHel-8	DF090331:5084264-5084349
BmHel-8	DF090325:3211407-3211494
BmHel-8	DF090324:409131-409216
BmHel-8	DF090458:239520-239615
BmHel-8	DF090328:2586109-2586188
BmHel-8	DF090360:194497-194596

BmHel-8	DF090393:1143887-1143966
BmHel-8	DF090377:1855087-1855187
BmHel-8	DF090324:1383715-1383855
BmHel-8	DF090322:3404019-3404106
BmHel-8	DF090781:103-195
BmHel-8	DF090316:14993517-14993600
BmHel-8	DF090835:3943-4047
BmHel-8	DF090361:2702892-2702989
BmHel-8	DF090412:360828-360921
BmHel-8	DF090368:1290144-1290233
BmHel-8	DF090327:5750701-5750804
BmHel-8	DF090361:133173-133258
BmHel-8	BABH01065933:525-662
BmHel-8	BABH01069456:1-143
BmHel-8	DF090380:1910004-1910113
BmHel-8	DF090374:1854950-1855048
BmHel-8	DF090347:3131030-3131114
BmHel-8	DF090357:3716108-3716211
BmHel-8	DF090324:2229745-2229848
BmHel-8	DF090386:1847985-1848087
BmHel-8	DF090350:1871591-1871680
BmHel-8	DF090339:1705288-1705369
BmHel-8	BABH01087702:1-139
BmHel-8	DF090410:787063-787153
BmHel-8	DF090342:1841811-1841899
BmHel-8	DF090383:697354-697452
BmHel-8	DF090455:356771-356867
BmHel-8	DF090346:2293984-2294082
BmHel-8	DF090316:6709113-6709209
BmHel-8	DF090322:5873857-5873941
BmHel-8	BABH01073181:47-132
BmHel-8	DF090492:37195-37279
BmHel-8	DF090377:688821-688908
BmHel-8	DF090388:892475-892557
BmHel-8	DF090317:9157840-9157937
BmHel-8	DF090324:5403418-5403505
BmHel-8	DF090414:901924-902010
BmHel-8	DF090324:7112344-7112444
BmHel-8	BABH01071835:490-626
BmHel-8	DF090365:988638-988733
BmHel-8	DF090348:4074560-4074652
BmHel-8	DF090356:3188343-3188423
BmHel-8	DF090340:429753-429848
BmHel-8	DF090326:6747867-6747967
BmHel-8	DF090397:142962-143068
BmHel-8	DF090327:6476915-6476995
BmHel-8	DF090414:660537-660623
BmHel-8	DF090363:2292719-2292804

BmHel-8	DF090348:4123796-4123879
BmHel-8	DF090451:147346-147442
BmHel-8	DF090364:2925465-2925556
BmHel-8	DF090327:4039359-4039440
BmHel-8	DF090407:218024-218119
BmHel-8	DF090322:6669047-6669146
BmHel-8	DF090349:2659888-2659984
BmHel-8	DF090341:1785702-1785807
BmHel-8	DF090335:5517825-5517905
BmHel-8	BABH01058593:584-717
BmHel-8	DF090418:545632-545714
BmHel-8	DF090365:2496467-2496571
BmHel-8	DF090415:115989-116079
BmHel-8	BABH01076910:1-134
BmHel-8	DF090317:760404-760490
BmHel-8	BABH01052683:318-412
BmHel-8	DF090673:24944-25046
BmHel-8	DF090430:798044-798123
BmHel-8	DF090350:3629714-3629850
BmHel-8	DF090408:601407-601494
BmHel-8	DF090374:2242932-2243024
BmHel-8	DF090348:92455-92539
BmHel-8	DF090367:1663165-1663260
BmHel-8	DF090352:559501-559594
BmHel-8	DF090322:62940-63034
BmHel-8	DF090321:2969569-2969662
BmHel-8	BABH01076414:1-132
BmHel-8	DF090380:484369-484463
BmHel-8	DF090346:1835488-1835584
BmHel-8	DF090322:6616036-6616128
BmHel-8	DF090372:2457639-2457742
BmHel-8	DF090372:2457899-2457992
BmHel-8	DF090343:2563162-2563241
BmHel-8	BABH01059157:1-131
BmHel-8	DF090321:2777536-2777618
BmHel-8	DF090352:3548173-3548273
BmHel-8	DF090339:2396514-2396605
BmHel-8	DF090357:3591077-3591164
BmHel-8	DF090321:1191133-1191214
BmHel-8	DF090406:271113-271204
BmHel-8	DF090406:994185-994279
BmHel-8	DF090317:2261477-2261557
BmHel-8	BABH01065901:4-133
BmHel-8	BABH01081956:423-551
BmHel-8	DF090361:785892-785980
BmHel-8	BABH01049179:19-111
BmHel-8	DF090368:1659062-1659146
BmHel-8	DF090349:1630976-1631057

BmHel-8	DF090343:4251622-4251705
BmHel-8	BABH01075430:14-142
BmHel-8	BABH01077408:462-589
BmHel-8	BABH01077002:464-592
BmHel-8	DF090419:641977-642073
BmHel-8	DF090334:5965635-5965726
BmHel-8	DF090324:2758031-2758116
BmHel-8	DF090454:235391-235515
BmHel-8	DF090329:4124655-4124738
BmHel-8	DF090354:1699137-1699230
BmHel-8	DF090387:617395-617479
BmHel-8	DF090565:96230-96316
BmHel-8	DF090341:1192132-1192220
BmHel-8	DF090323:6548756-6548853
BmHel-8	DF090322:2506101-2506226
BmHel-8	DF090401:519937-520030
BmHel-8	DF090398:654056-654144
BmHel-8	DF090374:2263974-2264056
BmHel-8	DF090346:564552-564632
BmHel-8	DF090364:568621-568700
BmHel-8	DF090320:4516517-4516611
BmHel-8	DF090418:367958-368077
BmHel-8	BABH01083029:392-516
BmHel-8	DF090486:176449-176534
BmHel-8	DF090380:2104247-2104327
BmHel-8	DF090370:540185-540268
BmHel-8	DF090358:1509088-1509210
BmHel-8	DF090358:2015373-2015495
BmHel-8	DF090352:2949678-2949799
BmHel-8	DF090332:6032296-6032424
BmHel-8	DF090332:6164918-6165040
BmHel-8	DF090332:3482271-3482393
BmHel-8	DF090355:320563-320685
BmHel-8	DF090353:2986945-2987067
BmHel-8	DF090350:2759795-2759917
BmHel-8	DF090350:3439158-3439280
BmHel-8	DF090350:2762864-2762986
BmHel-8	DF090342:2428251-2428373
BmHel-8	DF090342:2916609-2916730
BmHel-8	DF090342:2922279-2922401
BmHel-8	DF090336:2071750-2071872
BmHel-8	DF090321:7514532-7514654
BmHel-8	DF090321:8154462-8154583
BmHel-8	DF090318:9272894-9273016
BmHel-8	DF090320:650774-650896
BmHel-8	DF090403:1357719-1357841
BmHel-8	DF090357:823717-823839
BmHel-8	DF090344:2009894-2010016

BmHel-8	DF090344:1042575-1042699
BmHel-8	DF090340:4052176-4052297
BmHel-8	DF090340:4106894-4107016
BmHel-8	DF090340:1062181-1062303
BmHel-8	DF090329:2362108-2362227
BmHel-8	DF090327:493012-493135
BmHel-8	DF090323:6843430-6843552
BmHel-8	DF090322:4273580-4273692
BmHel-8	DF090319:1310488-1310608
BmHel-8	DF090316:14680106-14680232
BmHel-8	DF090410:206530-206652
BmHel-8	DF090395:390112-390234
BmHel-8	DF090326:3997971-3998093
BmHel-8	DF090433:304141-304263
BmHel-8	DF090433:451240-451362
BmHel-8	DF090439:113328-113450
BmHel-8	DF090411:886253-886374
BmHel-8	DF090397:942335-942457
BmHel-8	DF090396:617491-617613
BmHel-8	DF090394:1408031-1408153
BmHel-8	DF090382:1606365-1606487
BmHel-8	DF090372:882489-882612
BmHel-8	DF090372:2042593-2042715
BmHel-8	DF090372:2425453-2425575
BmHel-8	DF090371:1056033-1056155
BmHel-8	DF090347:2101276-2101398
BmHel-8	DF090345:4432488-4432610
BmHel-8	DF090331:945741-945863
BmHel-8	DF090337:2778825-2778946
BmHel-8	DF090337:4009839-4009961
BmHel-8	DF090390:534073-534195
BmHel-8	DF090373:2231638-2231760
BmHel-8	DF090339:2109690-2109812
BmHel-8	DF090339:4665727-4665849
BmHel-8	DF090418:335729-335851
BmHel-8	DF090418:258931-259053
BmHel-8	DF090431:711760-711884
BmHel-8	DF090364:1157325-1157447
BmHel-8	DF090406:1457238-1457357
BmHel-8	DF090407:1083701-1083826
BmHel-8	DF090447:68921-69043
BmHel-8	BABH01056710:24-146
BmHel-8	DF090757:1833-1955
BmHel-8	BABH01067477:515-637
BmHel-8	BABH01051124:427-549
BmHel-8	BABH01064222:14-136
BmHel-8	BABH01077662:13-135
BmHel-8	BABH01064461:6-127

BmHel-8	BABH01049303:224-346
BmHel-8	BABH01087399:29-155
BmHel-8	BABH01061203:29-151
BmHel-8	BABH01067849:504-632
BmHel-8	DF090843:5999-6127
BmHel-8	BABH01050777:52-174
BmHel-8	BABH01047601:1502-1624
BmHel-8	BABH01046007:2194-2316
BmHel-8	BABH01046159:631-753
BmHel-8	DF090761:481-603
BmHel-8	DF090326:1525188-1525275
BmHel-8	DF090377:1544775-1544897
BmHel-8	BABH01076064:4-127
BmHel-8	BABH01055797:641-723
BmHel-8	DF090342:2599706-2599824
BmHel-8	DF090358:2902368-2902489
BmHel-8	DF090386:480861-480982
BmHel-8	DF090332:884690-884815
BmHel-8	DF090362:2713179-2713300
BmHel-8	DF090340:4254373-4254494
BmHel-8	DF090329:2869525-2869646
BmHel-8	DF090327:3786548-3786669
BmHel-8	DF090316:7696475-7696596
BmHel-8	DF090316:13021277-13021397
BmHel-8	DF090401:1149554-1149675
BmHel-8	DF090394:1032103-1032227
BmHel-8	DF090394:382865-382986
BmHel-8	DF090347:1326691-1326813
BmHel-8	DF090341:719552-719673
BmHel-8	DF090343:4647625-4647748
BmHel-8	DF090427:521984-522105
BmHel-8	DF090835:43514-43635
BmHel-8	BABH01071230:25-146
BmHel-8	DF091277:4877-4998
BmHel-8	DF090713:6269-6390
BmHel-8	BABH01043558:13141-13262
BmHel-8	BABH01074770:465-585
BmHel-8	DF090335:3444097-3444180
BmHel-8	DF090322:6559787-6559909
BmHel-8	DF090332:207571-207691
BmHel-8	DF090415:783726-783846
BmHel-8	DF090332:2079419-2079539
BmHel-8	DF090318:9184961-9185081
BmHel-8	DF090327:1806722-1806843
BmHel-8	DF090365:2028191-2028311
BmHel-8	DF090376:958027-958147
BmHel-8	DF090483:212713-212833
BmHel-8	DF090454:526997-527113

BmHel-8	DF090351:2911912-2912032
BmHel-8	DF090332:2507244-2507363
BmHel-8	DF090344:1930645-1930765
BmHel-8	DF090317:464316-464407
BmHel-8	DF090377:1335631-1335741
BmHel-8	DF090342:3278661-3278780
BmHel-8	DF090318:358907-359027
BmHel-8	DF090344:2090667-2090781
BmHel-8	DF090319:7782417-7782536
BmHel-8	DF090316:8012144-8012262
BmHel-8	DF090392:1639301-1639420
BmHel-8	DF090337:2206945-2207064
BmHel-8	DF090337:4630232-4630351
BmHel-8	DF090494:331-450
BmHel-8	DF090417:818860-818977
BmHel-8	DF090368:1930442-1930562
BmHel-8	DF090567:224-343
BmHel-8	DF091122:6302-6421
BmHel-8	BABH01086146:195-314
BmHel-8	DF091627:3616-3735
BmHel-8	DF090335:2241399-2241518
BmHel-8	BABH01053064:656-772
BmHel-8	DF090342:3605735-3605854
BmHel-8	DF090327:4537848-4537969
BmHel-8	DF090365:1200781-1200899
BmHel-8	DF090348:1595016-1595135
BmHel-8	DF090381:275017-275136
BmHel-8	DF090481:43405-43522
BmHel-8	DF090448:257517-257636
BmHel-8	BABH01056613:608-727
BmHel-8	DF090318:9261885-9261966
BmHel-8	BABH01066726:538-657
BmHel-8	BABH01054364:313-432
BmHel-8	DF090355:362408-362526
BmHel-8	DF090609:14986-15104
BmHel-8	DF090429:646618-646736
BmHel-8	DF090476:246933-247051
BmHel-8	BABH01086246:29-147
BmHel-8	DF090318:1545810-1545928
BmHel-8	DF090319:7517551-7517669
BmHel-8	DF090397:602041-602160
BmHel-8	DF090442:216449-216567
BmHel-8	DF090355:2520284-2520402
BmHel-8	DF090355:2108070-2108188
BmHel-8	DF090320:3563728-3563846
BmHel-8	DF090344:3516113-3516231
BmHel-8	DF090319:6279144-6279262
BmHel-8	DF090411:748407-748529

BmHel-8	DF091000:1794-1913
BmHel-8	BABH01045771:483-564
BmHel-8	BABH01075834:430-548
BmHel-8	DF090358:1737928-1738012
BmHel-8	BABH01052369:491-609
BmHel-8	DF090330:5132176-5132293
BmHel-8	DF090352:2536179-2536296
BmHel-8	DF090357:834848-834965
BmHel-8	DF090371:1159764-1159881
BmHel-8	DF090379:706636-706752
BmHel-8	BABH01045218:33-151
BmHel-8	DF090321:5014480-5014588
BmHel-8	BABH01087359:305-422
BmHel-8	BABH01072988:493-610
BmHel-8	DF090344:1860256-1860370
BmHel-8	DF090365:2424558-2424675
BmHel-8	DF090374:1647061-1647178
BmHel-8	DF090459:179277-179394
BmHel-8	DF092076:15-132
BmHel-8	BABH01076505:13-130
BmHel-8	BABH01083140:1-118
BmHel-8	DF090410:1125767-1125884
BmHel-8	BABH01071363:1-119
BmHel-8	DF090330:6147765-6147881
BmHel-8	DF090367:2041934-2042050
BmHel-8	DF090353:3885537-3885654
BmHel-8	DF090350:164706-164822
BmHel-8	DF090359:857195-857311
BmHel-8	DF090327:2070525-2070642
BmHel-8	DF090323:22220-22336
BmHel-8	DF090319:6690972-6691087
BmHel-8	DF090348:347806-347922
BmHel-8	DF090391:1444251-1444367
BmHel-8	DF090341:861879-861995
BmHel-8	DF090375:750521-750637
BmHel-8	DF090463:55120-55233
BmHel-8	BABH01048268:891-1007
BmHel-8	DF090657:2805-2921
BmHel-8	DF090416:315594-315710
BmHel-8	DF090406:1554188-1554302
BmHel-8	DF090363:2842192-2842307
BmHel-8	DF090353:609356-609472
BmHel-8	DF090338:2349947-2350063
BmHel-8	DF090328:808230-808346
BmHel-8	DF090417:148859-148975
BmHel-8	DF090451:298288-298403
BmHel-8	DF090328:4722671-4722787
BmHel-8	DF090398:160155-160271

BmHel-8	BABH01074387:188-304
BmHel-8	DF090350:2301039-2301154
BmHel-8	DF090319:878626-878741
BmHel-8	DF090362:580828-580941
BmHel-8	DF090409:610044-610158
BmHel-8	DF090345:3003293-3003402
BmHel-8	DF090390:852040-852155
BmHel-8	DF090437:175001-175114
BmHel-8	DF090367:1004045-1004160
BmHel-8	DF090322:1945761-1945876
BmHel-8	DF090335:1345671-1345787
BmHel-8	DF090364:1228410-1228529
BmHel-8	BABH01064379:19-134
BmHel-8	DF090484:37006-37086
BmHel-8	DF090367:2579542-2579621
BmHel-8	DF090327:3173211-3173323
BmHel-8	DF090326:4453422-4453535
BmHel-8	DF090322:2262326-2262439
BmHel-8	DF090328:2741166-2741277
BmHel-8	DF090329:1559567-1559681
BmHel-8	DF090339:4105146-4105258
BmHel-8	DF090366:1113682-1113796
BmHel-8	DF090339:3772786-3772900
BmHel-8	DF090350:3587877-3587991
BmHel-8	DF090323:697243-697355
BmHel-8	DF090369:2693079-2693191
BmHel-8	DF090390:1052587-1052701
BmHel-8	DF090925:295-410
BmHel-8	DF090373:1432176-1432288
BmHel-8	DF090472:263072-263189
BmHel-8	BABH01044255:4935-5015
BmHel-8	DF090399:1626071-1626157
BmHel-8	DF090316:12572628-12572741
BmHel-8	DF090366:2138297-2138411
BmHel-8	DF090338:469955-470068
BmHel-8	DF090476:174919-175032
BmHel-8	DF090316:5672805-5672918
BmHel-8	DF090361:1299578-1299691
BmHel-8	DF090336:1115485-1115598
BmHel-8	DF090401:181092-181204
BmHel-8	DF090328:1503615-1503727
BmHel-8	DF090418:164258-164371
BmHel-8	DF090325:2288838-2288951
BmHel-8	DF090404:994591-994704
BmHel-8	DF090368:784725-784839
BmHel-8	DF090340:863260-863373
BmHel-8	DF090406:182676-182789
BmHel-8	DF090577:2091-2204

BmHel-8	DF090329:4760255-4760367
BmHel-8	DF090356:2932808-2932912
BmHel-8	DF090461:265210-265321
BmHel-8	DF090402:651255-651367
BmHel-8	DF090389:1001540-1001653
BmHel-8	DF090320:2156861-2156973
BmHel-8	DF090357:2523957-2524069
BmHel-8	DF090319:6887536-6887648
BmHel-8	DF090316:5981-6093
BmHel-8	DF090326:4096540-4096652
BmHel-8	DF090382:960205-960317
BmHel-8	DF090390:548433-548542
BmHel-8	DF090378:989453-989565
BmHel-8	DF090417:1089345-1089457
BmHel-8	BABH01068578:58-169
BmHel-8	BABH01049784:450-562
BmHel-8	BABH01055379:238-348
BmHel-8	DF091109:7453-7536
BmHel-8	BABH01046609:628-711
BmHel-8	BABH01050050:887-994
BmHel-8	DF090330:182929-183044
BmHel-8	DF090370:1571826-1571937
BmHel-8	DF090352:38623-38734
BmHel-8	DF090351:13053-13163
BmHel-8	DF090355:3734822-3734933
BmHel-8	DF090320:3550056-3550167
BmHel-8	DF090320:5043777-5043888
BmHel-8	DF090403:1729117-1729228
BmHel-8	DF090400:1073263-1073373
BmHel-8	DF090344:946338-946448
BmHel-8	DF090340:101690-101801
BmHel-8	DF090327:5302596-5302707
BmHel-8	DF090316:313612-313728
BmHel-8	DF090382:1867968-1868079
BmHel-8	DF090372:898389-898500
BmHel-8	DF090372:1067513-1067621
BmHel-8	DF090345:3232449-3232561
BmHel-8	DF090335:2712935-2713050
BmHel-8	DF090331:234215-234326
BmHel-8	DF090324:3343816-3343921
BmHel-8	DF090405:1208585-1208695
BmHel-8	DF090405:386034-386144
BmHel-8	DF090385:309753-309864
BmHel-8	DF090379:1508051-1508162
BmHel-8	DF090376:1653955-1654066
BmHel-8	DF090333:1777537-1777648
BmHel-8	DF090364:1025378-1025484
BmHel-8	DF090364:1686513-1686619

BmHel-8	DF090413:973604-973715
BmHel-8	DF090398:494500-494611
BmHel-8	DF090426:137581-137692
BmHel-8	BABH01069534:529-640
BmHel-8	DF090337:5076733-5076844
BmHel-8	DF090321:4995078-4995193
BmHel-8	DF090339:704867-704977
BmHel-8	DF090418:831696-831807
BmHel-8	DF090409:454870-454981
BmHel-8	DF090344:289192-289303
BmHel-8	DF090380:384739-384850
BmHel-8	BABH01075764:448-559
BmHel-8	BABH01054341:531-642
BmHel-8	DF090971:10631-10742
BmHel-8	BABH01067482:337-448
BmHel-8	BABH01082183:323-434
BmHel-8	BABH01060863:33-144
BmHel-8	BABH01049497:437-548
BmHel-8	DF090340:3097247-3097358
BmHel-8	DF090363:2043587-2043698
BmHel-8	DF090318:8437468-8437576
BmHel-8	DF090372:1503117-1503227
BmHel-8	DF090331:3325557-3325670
BmHel-8	DF090358:2158823-2158933
BmHel-8	DF090352:2398109-2398220
BmHel-8	DF090331:5233398-5233503
BmHel-8	BABH01046914:1593-1703
BmHel-8	DF090400:1453609-1453719
BmHel-8	DF090439:160095-160205
BmHel-8	DF090335:2126132-2126234
BmHel-8	DF090341:3821895-3822005
BmHel-8	DF090356:2777029-2777140
BmHel-8	DF090344:291337-291451
BmHel-8	DF090327:4694423-4694533
BmHel-8	DF090378:796616-796726
BmHel-8	DF090362:2103328-2103442
BmHel-8	DF090363:1304270-1304379
BmHel-8	DF090316:10697272-10697381
BmHel-8	DF090339:4563364-4563473
BmHel-8	DF090375:584507-584616
BmHel-8	DF090354:2446921-2447030
BmHel-8	BABH01056663:630-740
BmHel-8	DF090345:1318220-1318329
BmHel-8	DF090355:1316773-1316881
BmHel-8	DF090397:1543696-1543801
BmHel-8	DF090382:1942826-1942935
BmHel-8	DF090335:4611251-4611359
BmHel-8	DF090354:916625-916734

BmHel-8	DF090493:40057-40165
BmHel-8	DF090410:1199990-1200103
BmHel-8	DF090333:3451041-3451151
BmHel-8	DF090358:369812-369924
BmHel-8	DF090321:866386-866495
BmHel-8	DF090396:51860-51970
BmHel-8	DF090429:498423-498532
BmHel-8	BABH01079785:370-479
BmHel-8	DF090360:1534481-1534591
BmHel-8	DF090337:3218163-3218272
BmHel-8	DF090390:1471749-1471858
BmHel-8	DF090325:5365755-5365865
BmHel-8	DF090391:953745-953851
BmHel-8	DF090332:3105423-3105529
BmHel-8	DF090332:1142635-1142734
BmHel-8	DF090318:7698044-7698152
BmHel-8	DF090335:3431593-3431701
BmHel-8	DF090364:1114918-1115026
BmHel-8	BABH01084708:1-109
BmHel-8	BABH01057553:276-383
BmHel-8	BABH01047469:659-767
BmHel-8	DF090318:7444837-7444945
BmHel-8	DF090317:7809802-7809910
BmHel-8	DF090425:268121-268229
BmHel-8	BABH01084693:351-460
BmHel-8	DF090397:718322-718430
BmHel-8	DF090396:819432-819540
BmHel-8	DF090319:5324051-5324159
BmHel-8	BABH01083557:1-112
BmHel-8	DF091683:1612-1724
BmHel-8	BABH01069743:531-638
BmHel-8	BABH01047123:380-486
BmHel-8	BABH01065817:203-310
BmHel-8	DF090350:1717070-1717178
BmHel-8	DF090343:2592018-2592131
BmHel-8	BABH01063931:42-150
BmHel-8	DF090344:4491549-4491656
BmHel-8	DF090335:568089-568187
BmHel-8	DF090325:6552859-6552966
BmHel-8	DF090456:392846-392951
BmHel-8	DF090333:4612513-4612618
BmHel-8	DF090317:371678-371785
BmHel-8	DF090390:1631370-1631477
BmHel-8	DF090867:678-785
BmHel-8	DF090370:831706-831809
BmHel-8	DF090359:3273755-3273861
BmHel-8	DF090319:1951602-1951710
BmHel-8	DF090371:1092766-1092872

BmHel-8	DF090408:957313-957419
BmHel-8	DF090316:9372082-9372188
BmHel-8	DF090355:923082-923188
BmHel-8	DF090467:291851-291957
BmHel-8	DF090403:1299653-1299759
BmHel-8	DF090317:256249-256352
BmHel-8	DF090371:2122464-2122570
BmHel-8	DF090359:1904259-1904362
BmHel-8	DF090390:1465564-1465670
BmHel-8	DF090423:71640-71746
BmHel-8	DF090327:321442-321546
BmHel-8	DF090344:573695-573800
BmHel-8	DF090323:406813-406918
BmHel-8	DF090344:2252371-2252475
BmHel-8	DF090338:963590-963693
BmHel-8	DF090327:6445759-6445863
BmHel-8	DF090319:8045533-8045638
BmHel-8	DF090317:2415618-2415723
BmHel-8	DF090316:884386-884492
BmHel-8	DF090366:678730-678834
BmHel-8	DF090422:860021-860122
BmHel-8	DF090401:1453477-1453580
BmHel-8	DF090324:914079-914185
BmHel-8	DF090337:1367849-1367950
BmHel-8	DF090343:478304-478406
BmHel-8	DF090343:67539-67642
BmHel-8	DF090333:3187666-3187770
BmHel-8	DF090413:611235-611339
BmHel-8	BABH01047850:717-821
BmHel-8	DF091228:5440-5544
BmHel-8	BABH01049212:74-178
BmHel-8	DF090388:811484-811588
BmHel-8	DF090317:3495020-3495124
BmHel-8	DF090390:88428-88536
BmHel-8	DF090346:4190114-4190218
BmHel-8	BABH01081448:42-146
BmHel-8	DF090371:1535700-1535804
BmHel-8	BABH01056563:638-742
BmHel-8	DF090362:3094862-3094970
BmHel-8	DF090412:217372-217475
BmHel-8	DF090351:2355979-2356082
BmHel-8	DF090335:5820772-5820875
BmHel-8	DF090333:5033577-5033680
BmHel-8	DF090447:277570-277673
BmHel-8	DF090342:3421987-3422089
BmHel-8	DF090390:1189072-1189179
BmHel-8	DF090327:5652278-5652378
BmHel-8	DF090337:1450336-1450439

BmHel-8	DF090390:697217-697320
BmHel-8	DF090368:1358683-1358786
BmHel-8	BABH01049986:898-1000
BmHel-8	DF090349:690911-691015
BmHel-8	DF090320:2145502-2145605
BmHel-8	DF090379:1074367-1074470
BmHel-8	DF090357:3642080-3642187
BmHel-8	DF090502:115446-115553
BmHel-8	DF090332:6168235-6168338
BmHel-8	BABH01057305:626-729
BmHel-8	BABH01080799:456-562
BmHel-8	DF090444:568367-568469
BmHel-8	DF090438:495369-495473
BmHel-8	DF090352:1270203-1270304
BmHel-8	DF090355:745479-745581
BmHel-8	DF090344:2139754-2139856
BmHel-8	DF090316:1405406-1405508
BmHel-8	DF090369:1605749-1605851
BmHel-8	DF090328:1461487-1461593
BmHel-8	DF090324:989564-989667
BmHel-8	DF090974:384-486
BmHel-8	DF090349:2759295-2759397
BmHel-8	DF090364:2038263-2038365
BmHel-8	DF090319:8069618-8069721
BmHel-8	DF090346:1112839-1112935
BmHel-8	DF090332:1069878-1069979
BmHel-8	DF090353:1425757-1425858
BmHel-8	DF090350:3245662-3245762
BmHel-8	DF090321:6916740-6916841
BmHel-8	DF090318:6255140-6255242
BmHel-8	DF090318:8275459-8275561
BmHel-8	DF090338:855883-855984
BmHel-8	DF090317:10518540-10518631
BmHel-8	DF090317:7100977-7101079
BmHel-8	DF090349:2356322-2356423
BmHel-8	DF090394:1396288-1396389
BmHel-8	DF090372:1142756-1142853
BmHel-8	DF090335:5144091-5144194
BmHel-8	DF090337:3887400-3887501
BmHel-8	DF090343:2554776-2554877
BmHel-8	DF090376:537471-537572
BmHel-8	DF090333:2860744-2860845
BmHel-8	DF090475:215341-215442
BmHel-8	DF090445:646930-647031
BmHel-8	DF090389:296544-296645
BmHel-8	DF090387:401160-401262
BmHel-8	DF090384:763550-763651
BmHel-8	DF090364:2096290-2096391

BmHel-8	DF090415:538551-538652
BmHel-8	DF090598:1030-1131
BmHel-8	DF090947:11582-11684
BmHel-8	DF090552:18179-18279
BmHel-8	BABH01067303:332-433
BmHel-8	BABH01085856:83-184
BmHel-8	BABH01044709:6328-6429
BmHel-8	DF092053:119-220
BmHel-8	DF091361:2622-2722
BmHel-8	DF090418:611882-611983
BmHel-8	DF090321:7210851-7210947
BmHel-8	DF090403:712916-713017
BmHel-8	DF090356:2685632-2685733
BmHel-8	DF090382:1561752-1561854
BmHel-8	DF090402:753866-753967
BmHel-8	DF090317:7363763-7363864
BmHel-8	DF090416:1186439-1186539
BmHel-8	BABH01079476:100-201
BmHel-8	DF090657:6729-6831
BmHel-8	BABH01075345:317-418
BmHel-8	BABH01057095:470-571
BmHel-8	BABH01063695:518-619
BmHel-8	BABH01050227:874-975
BmHel-8	BABH01065313:555-656
BmHel-8	BABH01061084:5-106
BmHel-8	DF090409:723426-723526
BmHel-8	DF090355:1497882-1497983
BmHel-8	DF090353:2986657-2986757
BmHel-8	DF090350:1831541-1831641
BmHel-8	DF090388:1328535-1328635
BmHel-8	DF090345:4508441-4508542
BmHel-8	BABH01088213:1-98
BmHel-8	DF090409:429525-429625
BmHel-8	DF090326:1309471-1309572
BmHel-8	DF090353:371758-371858
BmHel-8	DF090323:5492897-5492995
BmHel-8	DF090375:679288-679388
BmHel-8	BABH01080070:269-369
BmHel-8	DF090335:1479479-1479579
BmHel-8	DF090398:1105394-1105494
BmHel-8	BABH01065962:14-115
BmHel-8	DF090368:1220324-1220423
BmHel-8	DF090329:4964236-4964335
BmHel-8	DF090323:6122133-6122231
BmHel-8	DF090334:3800971-3801070
BmHel-8	DF090524:75828-75929
BmHel-8	DF090476:185708-185806
BmHel-8	DF090337:543813-543912

BmHel-8	DF090358:948848-948947
BmHel-8	DF090359:2554048-2554147
BmHel-8	DF090327:4767237-4767336
BmHel-8	DF090423:880908-881007
BmHel-8	BABH01049307:549-648
BmHel-8	DF090355:1189339-1189437
BmHel-8	DF090353:2369731-2369830
BmHel-8	BABH01088140:201-299
BmHel-8	BABH01072825:520-620
BmHel-8	DF090370:1984106-1984203
BmHel-8	DF090437:47561-47659
BmHel-8	DF090362:1641845-1641943
BmHel-8	DF090338:4579278-4579376
BmHel-8	DF090425:251233-251329
BmHel-8	DF090379:781074-781172
BmHel-8	DF090378:36785-36884
BmHel-8	BABH01074120:509-606
BmHel-8	DF090332:3758040-3758138
BmHel-8	DF090483:8438-8537
BmHel-8	BABH01064253:575-673
BmHel-8	DF091318:1210-1307
BmHel-8	DF090356:466141-466240
BmHel-8	DF090328:4057529-4057627
BmHel-8	DF090742:21221-21319
BmHel-8	BABH01079185:31-129
BmHel-8	DF090334:2908228-2908326
BmHel-8	DF090367:294641-294739
BmHel-8	DF090358:3456533-3456631
BmHel-8	DF090467:6679-6778
BmHel-8	DF090348:4045392-4045489
BmHel-8	DF090401:617467-617564
BmHel-8	BABH01082135:452-549
BmHel-8	DF090334:5729133-5729230
BmHel-8	DF090420:213701-213798
BmHel-8	DF090320:7174931-7175025
BmHel-8	DF090422:915108-915205
BmHel-8	DF090343:2835622-2835719
BmHel-8	DF090343:1829242-1829335
BmHel-8	DF090317:6710294-6710391
BmHel-8	DF090344:475024-475121
BmHel-8	BABH01059559:610-708
BmHel-8	BABH01076014:75-171
BmHel-8	DF090356:3806089-3806185
BmHel-8	DF090380:769444-769540
BmHel-8	DF090423:45950-46046
BmHel-8	DF091127:6350-6446
BmHel-8	DF090343:1553157-1553253
BmHel-8	DF090351:4121420-4121516

BmHel-8	DF090348:3729020-3729116
BmHel-8	DF090422:241515-241615
BmHel-8	BABH01081743:457-553
BmHel-8	DF090328:948071-948167
BmHel-8	DF090355:3285647-3285743
BmHel-8	DF090356:1655015-1655111
BmHel-8	DF090317:9398475-9398570
BmHel-8	DF090369:1081911-1082006
BmHel-8	DF090390:1160788-1160880
BmHel-8	BABH01050570:855-950
BmHel-8	BABH01048457:689-784
BmHel-8	BABH01055283:1-96
BmHel-8	DF090350:2051533-2051628
BmHel-8	DF090644:1318-1413
BmHel-8	DF090351:2775808-2775901
BmHel-8	DF090323:6296297-6296392
BmHel-8	DF090349:3841808-3841903
BmHel-8	DF090328:1157188-1157283
BmHel-8	DF090324:4282728-4282823
BmHel-8	DF090378:1918071-1918172
BmHel-8	DF090389:1182560-1182655
BmHel-8	DF090350:2436435-2436532
BmHel-8	DF090322:7259457-7259552
BmHel-8	DF090338:4770333-4770428
BmHel-8	DF090936:11937-12032
BmHel-8	DF090322:8241408-8241503
BmHel-8	DF090356:3435577-3435672
BmHel-8	BABH01085491:405-499
BmHel-8	DF090412:778618-778712
BmHel-8	DF090350:3549531-3549626
BmHel-8	DF090342:1584252-1584346
BmHel-8	DF090362:1958113-1958208
BmHel-8	DF090356:3224115-3224209
BmHel-8	DF090344:1896418-1896512
BmHel-8	DF090323:5731750-5731844
BmHel-8	DF090322:1396978-1397072
BmHel-8	DF090319:5092843-5092937
BmHel-8	DF090326:281948-282042
BmHel-8	DF090341:3557165-3557260
BmHel-8	DF090334:150257-150351
BmHel-8	DF090354:2858955-2859050
BmHel-8	DF090398:387073-387167
BmHel-8	DF090454:458254-458348
BmHel-8	BABH01068208:461-555
BmHel-8	BABH01049502:350-444
BmHel-8	DF090661:19262-19356
BmHel-8	DF090342:1875940-1876036
BmHel-8	DF090354:2499574-2499668

BmHel-8	DF090317:1945306-1945400
BmHel-8	DF090390:991323-991417
BmHel-8	DF090340:1777328-1777422
BmHel-8	DF090355:2557187-2557281
BmHel-8	BABH01083515:17-111
BmHel-8	DF090367:973279-973374
BmHel-8	DF090338:2467717-2467811
BmHel-8	DF090322:4592230-4592324
BmHel-8	DF090316:14977104-14977199
BmHel-8	DF090411:521394-521491
BmHel-8	DF090468:57197-57291
BmHel-8	DF090613:73365-73459
BmHel-8	BABH01069976:522-621
BmHel-8	DF090317:7476006-7476098
BmHel-8	DF090316:15769930-15770022
BmHel-8	DF090348:3318199-3318292
BmHel-8	BABH01052860:23-116
BmHel-8	BABH01049035:1016-1110
BmHel-8	BABH01072180:3-93
BmHel-8	DF090349:1434986-1435079
BmHel-8	DF090318:848992-849085
BmHel-8	DF090377:77254-77347
BmHel-8	DF090328:1287840-1287933
BmHel-8	DF090387:1359634-1359725
BmHel-8	DF090339:1044562-1044654
BmHel-8	DF091086:7544-7637
BmHel-8	DF090321:2275584-2275675
BmHel-8	DF090316:1749798-1749890
BmHel-8	BABH01051162:1-93
BmHel-8	BABH01071369:10-102
BmHel-8	BABH01064568:396-488
BmHel-8	DF090367:1301853-1301945
BmHel-8	DF090320:2959190-2959280
BmHel-8	DF090336:2418155-2418247
BmHel-8	DF090344:2014728-2014820
BmHel-8	BABH01045476:561-653
BmHel-8	DF091884:51-143
BmHel-8	DF090338:1553859-1553951
BmHel-8	DF090398:873299-873391
BmHel-8	DF090342:4865313-4865405
BmHel-8	DF090334:2225322-2225414
BmHel-8	DF090383:1088839-1088931
BmHel-8	DF090321:1031712-1031800
BmHel-8	DF090360:1520783-1520871
BmHel-8	DF090341:2616016-2616107
BmHel-8	DF090398:1024656-1024749
BmHel-8	BABH01057057:644-735
BmHel-8	DF090736:22957-23048

BmHel-8	BABH01055925:17-105
BmHel-8	DF090397:1343138-1343222
BmHel-8	BABH01048065:1065-1156
BmHel-8	BABH01065715:171-262
BmHel-8	DF090419:667633-667726
BmHel-8	DF090393:1249496-1249588
BmHel-8	DF090317:999178-999268
BmHel-8	DF090319:5023749-5023840
BmHel-8	DF090381:1828438-1828529
BmHel-8	BABH01052206:558-649
BmHel-8	DF090366:1865706-1865797
BmHel-8	DF090370:2434228-2434319
BmHel-8	BABH01082678:1-92
BmHel-8	DF090413:333263-333355
BmHel-8	BABH01071612:275-367
BmHel-8	DF090336:726479-726569
BmHel-8	DF090316:3896172-3896261
BmHel-8	DF090375:62449-62544
BmHel-8	BABH01047971:901-991
BmHel-8	DF090316:13087745-13087834
BmHel-8	DF090347:4219004-4219094
BmHel-8	DF090370:2011990-2012080
BmHel-8	DF090323:176610-176700
BmHel-8	BABH01053549:1-91
BmHel-8	DF090390:457248-457338
BmHel-8	DF090372:163354-163444
BmHel-8	DF090355:1963776-1963868
BmHel-8	DF090386:1831639-1831722
BmHel-8	BABH01083003:1-91
BmHel-8	DF090340:125833-125923
BmHel-8	DF090333:5146038-5146129
BmHel-8	DF090318:2137716-2137805
BmHel-8	DF090327:4455995-4456084
BmHel-8	DF090319:2819211-2819300
BmHel-8	DF090345:2142209-2142298
BmHel-8	BABH01054863:1-89
BmHel-8	BABH01084397:230-319
BmHel-8	DF090335:4863074-4863162
BmHel-8	DF090321:4907353-4907442
BmHel-8	BABH01044633:5242-5329
BmHel-8	DF090394:1451985-1452073
BmHel-8	BABH01063733:583-671
BmHel-8	DF090317:386857-386946
BmHel-8	DF090348:2990271-2990360
BmHel-8	DF090357:1660698-1660786
BmHel-8	DF090454:420347-420436
BmHel-8	DF090319:8518586-8518676
BmHel-8	DF090366:2560591-2560680

BmHel-8	DF090469:71299-71388
BmHel-8	DF090343:2540850-2540937
BmHel-8	DF090367:830733-830822
BmHel-8	DF090441:522922-523010
BmHel-8	DF090362:1790108-1790193
BmHel-8	DF090340:4695139-4695226
BmHel-8	DF090365:1806817-1806905
BmHel-8	DF090324:5112874-5112962
BmHel-8	DF090428:832885-832973
BmHel-8	DF090484:63721-63808
BmHel-8	DF090336:3235064-3235152
BmHel-8	DF090375:856530-856618
BmHel-8	BABH01070604:1-89
BmHel-8	BABH01062375:598-686
BmHel-8	DF091281:5337-5425
BmHel-8	DF090338:3174575-3174663
BmHel-8	DF090365:1377609-1377697
BmHel-8	DF090763:10942-11030
BmHel-8	BABH01050524:609-697
BmHel-8	DF090342:3027446-3027534
BmHel-8	DF090378:204125-204213
BmHel-8	DF090512:8028-8117
BmHel-8	DF090336:3719853-3719941
BmHel-8	BABH01064134:586-674
BmHel-8	BABH01055173:606-695
BmHel-8	DF090381:1325098-1325187
BmHel-8	DF090555:58499-58585
BmHel-8	DF090346:4174702-4174790
BmHel-8	DF090320:7887192-7887279
BmHel-8	DF090323:1615210-1615295
BmHel-8	DF090317:2706823-2706910
BmHel-8	DF090317:234156-234243
BmHel-8	DF090845:13848-13934
BmHel-8	DF090341:4721527-4721614
BmHel-8	DF090359:1059949-1060036
BmHel-8	DF090379:1382056-1382143
BmHel-8	DF090426:260338-260425
BmHel-8	DF091382:2229-2315
BmHel-8	DF090364:2249342-2249430
BmHel-8	DF091155:4150-4237
BmHel-8	DF090355:3791965-3792052
BmHel-8	DF090326:2374113-2374200
BmHel-8	BABH01061906:586-674
BmHel-8	BABH01063600:46-133
BmHel-8	DF090358:1980284-1980369
BmHel-8	DF090344:3215099-3215179
BmHel-8	DF090319:3322706-3322793
BmHel-8	DF090382:2101862-2101948

BmHel-8	DF090324:2633379-2633466
BmHel-8	DF090418:714856-714942
BmHel-8	DF090351:2121119-2121205
BmHel-8	BABH01082141:1-87
BmHel-8	DF090343:141126-141212
BmHel-8	DF090429:271834-271920
BmHel-8	DF090330:1221358-1221444
BmHel-8	BABH01045813:401-485
BmHel-8	BABH01086934:354-440
BmHel-8	DF090349:1490924-1491011
BmHel-8	DF090393:227302-227388
BmHel-8	DF090442:204477-204563
BmHel-8	DF090320:1983006-1983093
BmHel-8	DF090328:3393372-3393459
BmHel-8	BABH01045295:1792-1876
BmHel-8	DF090360:775831-775916
BmHel-8	DF090405:789187-789273
BmHel-8	DF090353:2946562-2946647
BmHel-8	DF090319:6674198-6674283
BmHel-8	DF090327:3294719-3294808
BmHel-8	DF090417:529599-529678
BmHel-8	DF090367:2036738-2036824
BmHel-8	DF090365:1889683-1889769
BmHel-8	DF090329:3912394-3912478
BmHel-8	DF090344:15827-15912
BmHel-8	BABH01078532:480-565
BmHel-8	BABH01080174:483-568
BmHel-8	BABH01073581:1-86
BmHel-8	BABH01077062:1-81
BmHel-8	DF090350:1516178-1516263
BmHel-8	BABH01074523:512-596
BmHel-8	DF090328:6078839-6078927
BmHel-8	BABH01049751:701-789
BmHel-8	BABH01053650:400-488
BmHel-8	DF090404:422036-422119
BmHel-8	DF090377:1728338-1728423
BmHel-8	DF090374:205343-205424
BmHel-8	DF090322:7895724-7895808
BmHel-8	DF090435:73323-73407
BmHel-8	DF090344:3222616-3222700
BmHel-8	DF090338:2536832-2536916
BmHel-8	DF090341:612338-612422
BmHel-8	DF090327:4174981-4175065
BmHel-8	DF090342:2240866-2240950
BmHel-8	BABH01052984:1-85
BmHel-8	DF090352:1411564-1411648
BmHel-8	DF090346:1063717-1063801
BmHel-8	DF090382:747202-747286

BmHel-8	DF090379:1776153-1776237
BmHel-8	BABH01048470:949-1033
BmHel-8	BABH01086127:6-90
BmHel-8	BABH01068917:85-169
BmHel-8	DF090344:1600277-1600360
BmHel-8	DF090456:300915-300998
BmHel-8	DF090344:4339306-4339389
BmHel-8	DF090321:2220951-2221038
BmHel-8	DF090331:4869612-4869693
BmHel-8	DF090375:633178-633261
BmHel-8	DF090339:954253-954336
BmHel-8	DF090319:1315733-1315816
BmHel-8	BABH01071638:8-91
BmHel-8	BABH01086836:373-456
BmHel-8	DF091766:2250-2332
BmHel-8	DF090338:2495166-2495247
BmHel-8	DF090322:795067-795149
BmHel-8	DF090331:2485892-2485974
BmHel-8	DF090370:607253-607336
BmHel-8	DF090333:3355969-3356051
BmHel-8	DF090612:4161-4243
BmHel-8	DF090335:3278383-3278465
BmHel-8	DF090577:61584-61670
BmHel-8	BABH01067411:568-652
BmHel-8	DF090322:4806942-4807025
BmHel-8	DF090350:4162380-4162461
BmHel-8	DF090555:21124-21204
BmHel-8	BABH01072941:538-619
BmHel-8	DF090338:2640730-2640811
BmHel-8	DF090376:1923284-1923365
BmHel-8	BABH01050107:903-985
BmHel-8	BABH01048425:759-840
BmHel-8	DF090361:16681-16761
BmHel-8	DF090365:193750-193830
BmHel-8	DF090472:183945-184025
BmHel-8	DF090398:244931-245011
BmHel-8	DF090348:533083-533163
BmHel-8	BABH01063669:597-677
BmHel-8	DF090393:1307216-1307296
BmHel-8	BABH01077426:489-569
BmHel-8	DF090379:1548396-1548476
BmHel-8	DF090409:674561-674641
BmHel-8	DF090329:1742926-1743006
BmHel-8	DF090336:4353675-4353755
BmHel-8	BABH01065051:1-81
BmHel-8	BABH01083270:456-536
BmHel-8	DF090357:2498834-2498914
BmHel-8	DF090320:5692720-5692801

BmHel-8	DF090653:5650-5731
BmHel-8	DF090451:532978-533057
BmHel-8	DF090353:916019-916098
BmHel-8	DF090320:2671110-2671195
BmHel-8	DF090439:8298-8377
BmHel-8	DF090351:1806405-1806484
BmHel-8	BABH01075092:273-352
BmHel-8	BABH01080942:419-498
BmHel-8	BABH01067757:1-80
BmHel-11	DF090413:1094001-1094096
BmHel-11	DF090324:3454607-3454712
BmHel-11	DF090341:501422-501531
BmHel-11	DF090389:89602-89714
BmHel-11	DF090338:580120-580266
BmHel-11	DF090337:2366738-2366890
BmHel-11	DF090343:874489-874641
BmHel-11	DF090419:279258-279413
BmHel-11	DF090320:8094504-8094661
BmHel-11	DF090356:196561-196720
BmHel-11	DF090342:2620834-2620998
BmHel-11	BABH01052144:360-525
BmHel-11	DF090342:3318678-3318851
BmHel-11	DF090445:226440-226618
BmHel-11	DF090326:6539909-6540088
BmHel-11	DF090317:2136560-2136739
BmHel-11	DF090333:2513973-2514152
BmHel-11	DF090344:1826668-1826848
BmHel-11	DF090319:418950-419131
BmHel-11	DF090417:164796-164978
BmHel-11	DF090390:925703-925886
BmHel-11	DF090330:2145941-2146126
BmHel-11	DF090321:6491979-6492165
BmHel-11	DF090418:393405-393591
BmHel-11	DF090345:2665283-2665471
BmHel-11	DF090335:4568645-4568833
BmHel-11	DF090412:1059304-1059492
BmHel-11	DF090405:998663-998851
BmHel-11	DF090359:1725345-1725534
BmHel-11	DF090318:3209494-3209683
BmHel-11	DF090389:1635860-1636050
BmHel-11	DF090757:23343-23536
BmHel-11	DF090316:9098282-9098477
BmHel-11	DF090332:3532044-3532239
BmHel-11	DF090340:547257-547452
BmHel-11	DF090383:1889077-1889277
BmHel-11	DF090348:2552217-2552417
BmHel-11	DF090343:172857-173057

BmHel-11	DF090358:2973213-2973414
BmHel-11	DF090374:1292947-1293149
BmHel-11	BABH01047457:1418-1620
BmHel-11	DF091322:4434-4636
BmHel-11	DF090803:9297-9499
BmHel-11	DF090395:856970-857173
BmHel-11	DF090316:4196205-4196408
BmHel-11	DF090344:3471284-3471489
BmHel-11	DF090334:1982218-1982424
BmHel-11	DF090348:2706551-2706757
BmHel-11	DF090347:480144-480350
BmHel-11	DF090323:4385167-4385373
BmHel-11	DF090415:595936-596142
BmHel-11	DF090357:1449210-1449417
BmHel-11	DF090317:5017147-5017354
BmHel-11	DF090417:609787-609994
BmHel-11	DF090345:834951-835158
BmHel-11	DF090384:745600-745808
BmHel-11	DF090361:283202-283410
BmHel-11	DF090318:8743946-8744155
BmHel-11	DF090326:2374309-2374518
BmHel-11	DF090376:763595-763804
BmHel-11	DF090339:4504317-4504526
BmHel-11	DF090359:1351100-1351309
BmHel-11	DF090316:12477317-12477526
BmHel-11	DF090375:1170320-1170529
BmHel-11	DF090355:833324-833533
BmHel-11	DF090413:334065-334274
BmHel-11	DF090325:6363949-6364158
BmHel-11	DF090405:504390-504599
BmHel-11	DF090342:1788245-1788455
BmHel-11	DF090342:1961196-1961406
BmHel-11	DF090336:4982645-4982855
BmHel-11	DF090332:2773037-2773247
BmHel-11	DF090348:3561411-3561621
BmHel-11	DF090319:4171784-4171994
BmHel-11	DF090360:1010041-1010251
BmHel-11	DF090414:463917-464127
BmHel-11	DF090322:1163265-1163475
BmHel-11	DF090316:2202341-2202551
BmHel-11	DF090316:14707940-14708150
BmHel-11	DF090316:15156952-15157162
BmHel-11	DF090335:3368823-3369033
BmHel-11	DF090330:652997-653207
BmHel-11	DF090330:6248452-6248662
BmHel-11	DF090344:662467-662677
BmHel-11	DF090380:833513-833723
BmHel-11	DF090467:31980-32190

BmHel-11	DF090361:246486-246696
BmHel-11	DF090375:2089582-2089792
BmHel-11	DF090419:579337-579547
BmHel-11	DF090480:193276-193486
BmHel-11	DF090506:194125-194335
BmHel-11	BABH01049924:200-410
BmHel-11	DF090373:888106-888316
BmHel-11	DF090421:820586-820796
BmHel-11	DF090381:1697933-1698143
BmHel-11	DF090336:769774-769984
BmHel-11	DF090318:1376484-1376694
BmHel-11	DF090322:3901225-3901435
BmHel-11	DF090350:3036940-3037150
BmHel-11	DF090407:1044680-1044890
BmHel-11	DF090382:1797884-1798094
BmHel-11	DF090316:10867762-10867972
BmHel-11	DF090368:1148664-1148874
BmHel-11	DF090367:1897657-1897867
BmHel-11	DF090396:671736-671946
BmHel-11	DF090366:2025363-2025573
BmHel-11	DF090352:291528-291738
BmHel-11	DF090356:1747014-1747224
BmHel-11	DF090333:3695302-3695512
BmHel-11	DF090398:1282118-1282328
BmHel-11	DF090338:2618140-2618350
BmHel-11	DF090338:1121454-1121664
BmHel-11	DF090375:624503-624713
BmHel-11	DF090369:1028309-1028519
BmHel-11	DF090425:75700-75910
BmHel-11	DF090497:48933-49143
BmHel-11	DF090975:12059-12269
BmHel-11	DF090511:17-228
BmHel-11	DF090438:133651-133862
BmHel-11	DF090334:3804152-3804364
BmHel-11	DF090418:688938-689150
BmHel-11	DF090335:4152161-4152373
BmHel-11	DF090319:271137-271350
BmHel-11	DF090357:3380427-3380640
BmHel-11	DF090392:51188-51402
BmHel-11	DF090356:2044920-2045134
BmHel-11	BABH01045582:3426-3640
BmHel-11	DF090320:4175680-4175894
BmHel-11	DF090358:2776260-2776474
BmHel-11	DF090332:4361737-4361952
BmHel-11	DF090376:1428727-1428942
BmHel-11	DF090332:4541116-4541332
BmHel-11	DF090370:1017553-1017769
BmHel-11	DF090436:154125-154341

BmHel-11	DF090354:2907057-2907274
BmHel-11	DF090403:1408506-1408723
BmHel-11	DF090317:1527807-1528024
BmHel-11	DF090363:2126336-2126554
BmHel-11	DF090333:2577305-2577523
BmHel-11	DF090582:17521-17739
BmHel-11	DF090379:1284677-1284896
BmHel-11	DF090413:910942-911161
BmHel-11	DF090333:5014282-5014501
BmHel-11	DF090353:2152800-2153019
BmHel-11	DF090395:141829-142049
BmHel-11	DF090358:480081-480301
BmHel-11	DF090316:12162291-12162512
BmHel-11	DF090357:2881289-2881511
BmHel-11	DF090320:1826376-1826598
BmHel-11	DF090320:7717311-7717533
BmHel-11	BABH01087961:24-246
BmHel-11	DF090349:4057416-4057639
BmHel-11	DF090356:1485717-1485940
BmHel-11	DF090352:2229067-2229290
BmHel-11	DF090414:1365788-1366013
BmHel-11	DF090484:227259-227484
BmHel-11	DF090684:8462-8687
BmHel-11	DF090353:1464045-1464270
BmHel-11	DF090374:1060792-1061018
BmHel-11	DF090319:6498947-6499173
BmHel-11	DF090334:5979670-5979896
BmHel-11	DF090378:551738-551964
BmHel-11	DF090494:34304-34530
BmHel-11	DF090340:3947785-3948011
BmHel-11	DF090476:210731-210958
BmHel-11	DF090317:7568083-7568310
BmHel-11	DF090362:2507909-2508136
BmHel-11	DF090525:35402-35629
BmHel-11	DF090936:10996-11223
BmHel-11	DF090319:5099446-5099673
BmHel-11	DF090342:1749427-1749655
BmHel-11	DF090336:42560-42788
BmHel-11	DF090319:3050513-3050741
BmHel-11	DF090316:9302364-9302592
BmHel-11	DF090330:3068004-3068232
BmHel-11	DF090323:4198964-4199192
BmHel-11	DF090389:1523542-1523770
BmHel-11	DF090375:905137-905365
BmHel-11	DF090355:2172895-2173123
BmHel-11	DF090325:829342-829570
BmHel-11	DF090343:3349329-3349557
BmHel-11	DF090320:3073934-3074162

BmHel-11	DF090356:386967-387195
BmHel-11	DF090361:2005860-2006088
BmHel-11	DF090423:982260-982488
BmHel-11	DF090319:4500593-4500822
BmHel-11	DF090365:2986084-2986313
BmHel-11	DF090442:187094-187323
BmHel-11	DF090345:1990455-1990684
BmHel-11	BABH01051291:469-698
BmHel-11	DF090357:2482600-2482829
BmHel-11	DF090343:620649-620878
BmHel-11	DF090317:2311542-2311771
BmHel-11	DF091096:3863-4092
BmHel-11	BABH01050538:621-850
BmHel-11	DF090316:14783376-14783606
BmHel-11	DF090325:3414973-3415203
BmHel-11	DF090330:4200670-4200900
BmHel-11	DF090354:944731-944961
BmHel-11	DF090371:1136479-1136710
BmHel-11	DF090317:8074368-8074599
BmHel-11	DF090334:2815412-2815643
BmHel-11	DF090427:11192-11423
BmHel-11	DF090386:1291438-1291670
BmHel-11	DF090319:8140249-8140481
BmHel-11	DF090316:10604326-10604558
BmHel-11	DF090329:3919645-3919877
BmHel-11	DF090380:110411-110643
BmHel-11	DF090386:1375571-1375803
BmHel-11	DF090334:4191669-4191901
BmHel-11	DF090320:4037645-4037877
BmHel-11	DF090342:2070231-2070464
BmHel-11	DF090332:699623-699856
BmHel-11	DF090318:7170292-7170525
BmHel-11	DF090395:1411247-1411480
BmHel-11	DF090324:193794-194027
BmHel-11	DF090336:2588427-2588660
BmHel-11	DF090347:2189642-2189875
BmHel-11	DF090320:1367584-1367817
BmHel-11	DF090412:956493-956726
BmHel-11	DF090325:5014807-5015041
BmHel-11	DF090336:4044005-4044239
BmHel-11	DF090316:8107492-8107726
BmHel-11	DF090356:3646665-3646899
BmHel-11	DF090394:199082-199316
BmHel-11	DF090319:1506019-1506253
BmHel-11	DF090360:834520-834754
BmHel-11	DF090354:3431800-3432034
BmHel-11	DF091431:3591-3825
BmHel-11	DF090355:3753857-3754092

BmHel-11	DF090324:1040751-1040986
BmHel-11	DF090345:3022907-3023142
BmHel-11	DF090401:219802-220037
BmHel-11	DF090318:7379008-7379243
BmHel-11	DF090347:641339-641574
BmHel-11	DF090400:1559676-1559911
BmHel-11	DF090321:5866790-5867026
BmHel-11	DF090358:2739622-2739858
BmHel-11	DF090348:3228736-3228972
BmHel-11	DF090344:887057-887293
BmHel-11	DF090399:1190309-1190546
BmHel-11	DF090345:1590925-1591162
BmHel-11	DF090411:1340223-1340460
BmHel-11	DF090350:3340355-3340592
BmHel-11	DF090370:1361799-1362036
BmHel-11	DF090352:2648605-2648842
BmHel-11	DF090403:1513824-1514061
BmHel-11	DF091081:692-929
BmHel-11	DF091588:2159-2396
BmHel-11	DF090361:2450986-2451223
BmHel-11	DF090316:4779573-4779811
BmHel-11	DF090317:6346306-6346544
BmHel-11	DF090326:2905408-2905646
BmHel-11	DF090349:3527651-3527889
BmHel-11	DF090710:7044-7283
BmHel-11	DF090400:1405519-1405758
BmHel-11	DF090333:559591-559830
BmHel-11	DF090336:1343522-1343761
BmHel-11	DF090354:1704337-1704576
BmHel-11	DF090333:281675-281914
BmHel-11	DF090501:102148-102387
BmHel-11	DF090327:5979723-5979963
BmHel-11	DF090324:1784902-1785142
BmHel-11	DF090393:1040296-1040537
BmHel-11	DF090380:877524-877765
BmHel-11	DF090329:4248376-4248617
BmHel-11	DF090364:1591313-1591554
BmHel-11	DF090408:1410776-1411017
BmHel-11	DF090503:114248-114489
BmHel-11	DF090339:4867243-4867485
BmHel-11	DF090328:5882611-5882853
BmHel-11	DF090392:1757044-1757286
BmHel-11	DF090326:1512399-1512641
BmHel-11	DF090350:3583885-3584127
BmHel-11	DF090359:1013506-1013748
BmHel-11	DF090340:4496649-4496891
BmHel-11	DF090538:44414-44656
BmHel-11	BABH01048734:919-1161

BmHel-11	DF090319:81246-81488
BmHel-11	DF090326:624949-625191
BmHel-11	DF090323:4139333-4139575
BmHel-11	DF090320:5505733-5505975
BmHel-11	DF090354:1182639-1182881
BmHel-11	DF090317:5243339-5243581
BmHel-11	DF090324:4523981-4524223
BmHel-11	DF090355:3435518-3435761
BmHel-11	DF090421:25843-26086
BmHel-11	DF090386:1181142-1181385
BmHel-11	DF090342:1836358-1836601
BmHel-11	DF090332:3141386-3141629
BmHel-11	DF090318:2852108-2852351
BmHel-11	DF090328:5169276-5169519
BmHel-11	DF090395:1581420-1581663
BmHel-11	DF090377:1168356-1168599
BmHel-11	DF090347:1250765-1251008
BmHel-11	DF090347:3686631-3686874
BmHel-11	DF090343:1899674-1899917
BmHel-11	DF090334:3128502-3128745
BmHel-11	DF090368:475516-475759
BmHel-11	DF090335:2006667-2006910
BmHel-11	DF090330:4077117-4077360
BmHel-11	DF090370:1401471-1401714
BmHel-11	DF090384:1275687-1275930
BmHel-11	DF090364:718361-718604
BmHel-11	DF090317:1607570-1607813
BmHel-11	DF090435:442921-443164
BmHel-11	DF090358:568734-568977
BmHel-11	DF090358:1967150-1967393
BmHel-11	DF090416:522210-522453
BmHel-11	BABH01048282:139-382
BmHel-11	BABH01049208:694-937
BmHel-11	BABH01087819:8-251
BmHel-11	DF090318:5363937-5364180
BmHel-11	DF090328:5796042-5796285
BmHel-11	DF090365:2405693-2405936
BmHel-11	DF090321:5486518-5486761
BmHel-11	DF090321:4905262-4905505
BmHel-11	DF090393:1021702-1021945
BmHel-11	DF090359:2869763-2870006
BmHel-11	DF090331:5747328-5747571
BmHel-11	DF090331:1117600-1117843
BmHel-11	DF090316:1879831-1880074
BmHel-11	DF090330:3588615-3588858
BmHel-11	DF090329:2993348-2993591
BmHel-11	DF090320:5420451-5420694
BmHel-11	DF090366:1882288-1882531

BmHel-11	DF090352:2664504-2664747
BmHel-11	DF090435:526120-526363
BmHel-11	DF090356:1067246-1067489
BmHel-11	DF090327:3895984-3896227
BmHel-11	DF090477:2693-2936
BmHel-11	BABH01072144:183-426
BmHel-11	DF090332:4329658-4329902
BmHel-11	DF090357:857047-857291
BmHel-11	DF090331:524013-524257
BmHel-11	DF090400:1228799-1229043
BmHel-11	DF090330:3005408-3005652
BmHel-11	DF090320:2224166-2224410
BmHel-11	DF090540:57077-57321
BmHel-11	DF090363:973851-974095
BmHel-11	DF090340:2437653-2437897
BmHel-11	DF090442:308330-308574
BmHel-11	DF090317:9825953-9826197
BmHel-11	DF090317:335766-336010
BmHel-11	DF090389:586538-586782
BmHel-11	DF090397:721384-721628
BmHel-11	DF090446:513320-513564
BmHel-11	DF090419:645038-645282
BmHel-11	DF090431:66246-66490
BmHel-11	DF090381:659983-660228
BmHel-11	DF090357:159156-159401
BmHel-11	DF090360:2151810-2152055
BmHel-11	DF090316:12743364-12743609
BmHel-11	DF090316:15518819-15519064
BmHel-11	DF090337:892346-892591
BmHel-11	DF090323:6955944-6956189
BmHel-11	DF090367:731826-732071
BmHel-11	DF090416:660547-660792
BmHel-11	DF090374:1380898-1381143
BmHel-11	DF090448:54596-54841
BmHel-11	DF090419:772308-772553
BmHel-11	DF090512:21051-21296
BmHel-11	DF091005:8700-8945
BmHel-11	DF090409:78708-78953
BmHel-11	DF090317:8704251-8704496
BmHel-11	DF090318:5652821-5653066
BmHel-11	DF090383:507972-508217
BmHel-11	DF090322:7581321-7581566
BmHel-11	DF090322:852769-853014
BmHel-11	DF090349:2269013-2269258
BmHel-11	DF090331:4088423-4088668
BmHel-11	DF090316:15498724-15498969
BmHel-11	DF090316:6025907-6026152
BmHel-11	DF090351:303206-303451

BmHel-11	DF090323:3856497-3856742
BmHel-11	DF090379:1128370-1128615
BmHel-11	DF090378:856176-856421
BmHel-11	DF090404:167414-167659
BmHel-11	DF090358:35975-36220
BmHel-11	DF090327:4312422-4312667
BmHel-11	DF090346:225006-225251
BmHel-11	DF090415:870212-870457
BmHel-11	DF090413:165999-166244
BmHel-11	DF090562:8795-9040
BmHel-11	DF090369:574409-574654
BmHel-11	DF090324:2479575-2479820
BmHel-11	DF090370:2222833-2223078
BmHel-11	DF090369:2286759-2287004
BmHel-11	DF090325:3792901-3793147
BmHel-11	DF090362:1841573-1841819
BmHel-11	DF090324:790069-790315
BmHel-11	DF090324:5285943-5286189
BmHel-11	DF090386:1431905-1432151
BmHel-11	DF090328:2716875-2717121
BmHel-11	DF090357:2167492-2167738
BmHel-11	DF090414:1047790-1048036
BmHel-11	DF090365:181069-181315
BmHel-11	DF090326:2979458-2979704
BmHel-11	DF090326:5921138-5921384
BmHel-11	DF090326:6572485-6572731
BmHel-11	DF090322:2977166-2977412
BmHel-11	DF090343:974235-974481
BmHel-11	DF090341:3936280-3936526
BmHel-11	DF090340:2668367-2668613
BmHel-11	DF090316:3252517-3252763
BmHel-11	DF090316:12269284-12269530
BmHel-11	DF090388:1510664-1510910
BmHel-11	DF090351:402446-402692
BmHel-11	DF090337:1762124-1762370
BmHel-11	DF090330:3948161-3948407
BmHel-11	DF090329:3032859-3033105
BmHel-11	DF090367:2250957-2251203
BmHel-11	DF090344:2930258-2930504
BmHel-11	DF090380:227723-227969
BmHel-11	DF090379:1325286-1325532
BmHel-11	DF090378:1680200-1680446
BmHel-11	DF090354:1598743-1598989
BmHel-11	DF090317:5482107-5482353
BmHel-11	DF090435:571504-571750
BmHel-11	DF090372:1703584-1703830
BmHel-11	DF090361:1530570-1530816
BmHel-11	DF090361:2029292-2029538

BmHel-11	DF090462:205037-205283
BmHel-11	DF090375:1674781-1675027
BmHel-11	DF090446:277954-278200
BmHel-11	DF090408:833592-833838
BmHel-11	DF090447:453468-453714
BmHel-11	BABH01084233:269-515
BmHel-11	BABH01081448:166-412
BmHel-11	DF091020:426-672
BmHel-11	BABH01083948:184-430
BmHel-11	BABH01058700:5-251
BmHel-11	DF090322:7281543-7281789
BmHel-11	DF090353:664176-664422
BmHel-11	DF090339:1772430-1772676
BmHel-11	DF090318:1519660-1519906
BmHel-11	DF090383:1116113-1116359
BmHel-11	DF090319:5279117-5279363
BmHel-11	DF090357:166049-166295
BmHel-11	DF090377:1737026-1737272
BmHel-11	DF090360:2653391-2653637
BmHel-11	DF090414:1349903-1350149
BmHel-11	DF090365:2880775-2881021
BmHel-11	DF090321:5208140-5208386
BmHel-11	DF090363:2944417-2944663
BmHel-11	DF090363:2205843-2206089
BmHel-11	DF090363:1368478-1368724
BmHel-11	DF090349:4043400-4043646
BmHel-11	DF090341:398263-398509
BmHel-11	DF090340:3153042-3153288
BmHel-11	DF090334:3143314-3143560
BmHel-11	DF090331:2798818-2799064
BmHel-11	DF090331:878322-878568
BmHel-11	DF090316:6963130-6963376
BmHel-11	DF090400:1311073-1311319
BmHel-11	DF090388:1744039-1744285
BmHel-11	DF090351:2041636-2041882
BmHel-11	DF090335:4759622-4759868
BmHel-11	DF090320:304865-305111
BmHel-11	DF090490:87301-87547
BmHel-11	DF090384:3697-3943
BmHel-11	DF090317:5625950-5626196
BmHel-11	DF090317:3963726-3963972
BmHel-11	DF090317:132400-132646
BmHel-11	DF090438:507602-507848
BmHel-11	DF090372:2115069-2115315
BmHel-11	DF090372:1990557-1990803
BmHel-11	DF090372:1335437-1335683
BmHel-11	DF090358:2629618-2629864
BmHel-11	DF090356:917130-917376

BmHel-11	DF090345:2416838-2417084
BmHel-11	DF090333:413949-414195
BmHel-11	DF090346:1385817-1386063
BmHel-11	DF090375:1372312-1372558
BmHel-11	DF090460:153792-154038
BmHel-11	BABH01047515:572-818
BmHel-11	BABH01044729:537-783
BmHel-11	DF090380:1801329-1801575
BmHel-11	DF090324:398243-398490
BmHel-11	DF090336:2350821-2351068
BmHel-11	DF090336:5550212-5550459
BmHel-11	DF090347:674940-675187
BmHel-11	DF090347:2773809-2774056
BmHel-11	DF090326:6966722-6966969
BmHel-11	DF090322:7619661-7619908
BmHel-11	DF090340:4065059-4065306
BmHel-11	DF090330:1904336-1904583
BmHel-11	DF090330:3808818-3809065
BmHel-11	DF090396:472871-473118
BmHel-11	DF090364:2802598-2802845
BmHel-11	DF090352:3542343-3542590
BmHel-11	DF090398:786963-787210
BmHel-11	DF090541:115323-115570
BmHel-11	DF090336:517455-517702
BmHel-11	DF090328:5133536-5133783
BmHel-11	DF090392:108440-108687
BmHel-11	DF090365:1420475-1420722
BmHel-11	DF090326:5788529-5788776
BmHel-11	DF090326:216275-216522
BmHel-11	DF090322:2479929-2480176
BmHel-11	DF090321:4558056-4558303
BmHel-11	DF090350:2278069-2278316
BmHel-11	DF090363:2088107-2088354
BmHel-11	DF090359:3258742-3258989
BmHel-11	DF090316:6075954-6076201
BmHel-11	DF090400:687592-687839
BmHel-11	DF090373:989659-989906
BmHel-11	DF090335:5483717-5483964
BmHel-11	DF090329:2922811-2923058
BmHel-11	DF090329:249684-249931
BmHel-11	DF090320:1044532-1044779
BmHel-11	DF090467:262270-262517
BmHel-11	DF090327:5960720-5960967
BmHel-11	DF090413:259934-260181
BmHel-11	DF090556:10831-11078
BmHel-11	BABH01045064:1874-2121
BmHel-11	DF090405:1134943-1135190
BmHel-11	DF090318:6670793-6671040

BmHel-11	DF090331:626105-626352
BmHel-11	DF090336:2910693-2910941
BmHel-11	DF090318:4912673-4912921
BmHel-11	DF090322:5446726-5446974
BmHel-11	DF090316:1161535-1161783
BmHel-11	DF090316:15640994-15641242
BmHel-11	DF090373:180580-180828
BmHel-11	DF090337:3491114-3491362
BmHel-11	DF090406:117333-117581
BmHel-11	DF090454:295565-295813
BmHel-11	DF090324:7978393-7978641
BmHel-11	DF090328:3310925-3311173
BmHel-11	DF090328:2324786-2325034
BmHel-11	DF090322:1493869-1494117
BmHel-11	DF090407:238245-238493
BmHel-11	DF090334:1944349-1944597
BmHel-11	DF090330:188557-188805
BmHel-11	DF090346:1962914-1963162
BmHel-11	DF090622:4962-5210
BmHel-11	DF090431:565191-565439
BmHel-11	DF090318:7926685-7926934
BmHel-11	DF090348:1047472-1047721
BmHel-11	DF090322:2993345-2993594
BmHel-11	DF090350:2456808-2457057
BmHel-11	DF090334:1931365-1931614
BmHel-11	DF090367:1979640-1979889
BmHel-11	DF090378:2119199-2119448
BmHel-11	DF090333:1852283-1852532
BmHel-11	DF090386:1792852-1793101
BmHel-11	DF090317:6286209-6286458
BmHel-11	DF090386:2025408-2025657
BmHel-11	DF090342:1617853-1618102
BmHel-11	DF090326:3291860-3292109
BmHel-11	DF090469:262671-262920
BmHel-11	DF090337:4585634-4585883
BmHel-11	DF090335:488250-488499
BmHel-11	DF090330:3627729-3627978
BmHel-11	DF090329:3555244-3555493
BmHel-11	DF090452:161185-161434
BmHel-11	DF090366:1281039-1281288
BmHel-11	DF090345:2454740-2454989
BmHel-11	DF090443:576063-576312
BmHel-11	DF090426:537653-537902
BmHel-11	DF090330:6304908-6305157
BmHel-11	DF090405:818638-818887
BmHel-11	DF090492:216043-216292
BmHel-11	DF090325:3090176-3090426
BmHel-11	DF090328:1206573-1206823

BmHel-11	DF090322:1144449-1144699
BmHel-11	DF090344:916769-917019
BmHel-11	DF090379:987170-987420
BmHel-11	DF090356:2773714-2773964
BmHel-11	DF090361:2571261-2571511
BmHel-11	DF090408:640651-640901
BmHel-11	DF090369:1610697-1610947
BmHel-11	DF092094:295-545
BmHel-11	DF090325:2268226-2268476
BmHel-11	DF090324:6307051-6307301
BmHel-11	DF090332:3555424-3555674
BmHel-11	DF090319:5684184-5684434
BmHel-11	DF090323:7383320-7383570
BmHel-11	DF090364:2687945-2688195
BmHel-11	DF090422:548933-549183
BmHel-11	DF090369:1070728-1070978
BmHel-11	DF090325:1737523-1737774
BmHel-11	DF090339:3648784-3649035
BmHel-11	DF090342:4693557-4693808
BmHel-11	DF090332:1321318-1321569
BmHel-11	DF090365:2136201-2136452
BmHel-11	DF090463:271480-271731
BmHel-11	DF090317:3663054-3663305
BmHel-11	DF090346:2716298-2716549
BmHel-11	DF090338:3665482-3665733
BmHel-11	DF090432:415658-415909
BmHel-11	DF090367:2300983-2301234
BmHel-11	BABH01051272:351-602
BmHel-11	DF090324:3889097-3889349
BmHel-11	DF090326:2520060-2520312
BmHel-11	DF090317:2117057-2117309
BmHel-11	DF090371:1121584-1121836
BmHel-11	DF090361:1119095-1119347
BmHel-11	DF090587:23158-23410
BmHel-11	DF090325:1905069-1905321
BmHel-11	DF090400:1353089-1353341
BmHel-11	DF090344:1117204-1117456
BmHel-11	DF090384:1578461-1578713
BmHel-11	DF090363:495268-495521
BmHel-11	DF090316:7432627-7432880
BmHel-11	DF090320:1567079-1567332
BmHel-11	DF090317:10802563-10802816
BmHel-11	DF090356:2621729-2621982
BmHel-11	DF090394:142700-142953
BmHel-11	DF090385:1361955-1362208
BmHel-11	DF090371:2411977-2412230
BmHel-11	DF090325:5829656-5829910
BmHel-11	DF090336:2301029-2301283

BmHel-11	DF090332:5677061-5677315
BmHel-11	DF090334:4909447-4909701
BmHel-11	DF090316:11227833-11228087
BmHel-11	DF090344:4214606-4214860
BmHel-11	DF090320:2401915-2402169
BmHel-11	DF090361:3165532-3165786
BmHel-11	DF090420:98867-99121
BmHel-11	DF090415:944484-944738
BmHel-11	DF090413:984009-984263
BmHel-11	DF090339:4259946-4260200
BmHel-11	DF090336:4322634-4322888
BmHel-11	DF090332:2604031-2604285
BmHel-11	DF090357:1445287-1445541
BmHel-11	DF090326:5657337-5657591
BmHel-11	DF090354:1818339-1818593
BmHel-11	DF090415:504546-504800
BmHel-11	DF090429:324533-324787
BmHel-11	DF090425:168905-169159
BmHel-11	BABH01043618:11865-12119
BmHel-11	DF090940:13254-13508
BmHel-11	DF090319:117442-117696
BmHel-11	DF090366:1548914-1549168
BmHel-11	DF090325:4568118-4568373
BmHel-11	DF090349:1239766-1240021
BmHel-11	DF090412:102721-102976
BmHel-11	DF090354:491764-492019
BmHel-11	DF090318:3613031-3613286
BmHel-11	DF090377:1987253-1987508
BmHel-11	DF090382:347313-347568
BmHel-11	DF090351:1599387-1599642
BmHel-11	DF090320:4810076-4810331
BmHel-11	DF090412:351300-351555
BmHel-11	DF090354:2393837-2394092
BmHel-11	DF090372:1965902-1966157
BmHel-11	DF090337:4399478-4399734
BmHel-11	DF090330:2573031-2573287
BmHel-11	DF090431:165861-166117
BmHel-11	DF090343:3268396-3268652
BmHel-11	DF090328:6688750-6689006
BmHel-11	DF090326:5074265-5074521
BmHel-11	DF090321:3857994-3858250
BmHel-11	DF090352:4120736-4120992
BmHel-11	DF090327:3184490-3184746
BmHel-11	DF090335:2765578-2765834
BmHel-11	DF090369:1618538-1618794
BmHel-11	DF090402:636401-636658
BmHel-11	DF090348:3710758-3711015
BmHel-11	DF090357:34062-34319

BmHel-11	DF090363:2614777-2615034
BmHel-11	DF090316:3682970-3683227
BmHel-11	DF090388:1059621-1059878
BmHel-11	DF090329:5232561-5232818
BmHel-11	DF090380:272864-273121
BmHel-11	DF090317:4013800-4014057
BmHel-11	DF090358:1512537-1512794
BmHel-11	DF090327:2444536-2444793
BmHel-11	DF090444:595529-595786
BmHel-11	DF090570:51980-52237
BmHel-11	DF090325:1611653-1611910
BmHel-11	DF090342:3394593-3394850
BmHel-11	DF090319:5764459-5764716
BmHel-11	DF090319:3059677-3059934
BmHel-11	DF090411:233223-233480
BmHel-11	DF090391:1151706-1151963
BmHel-11	DF090337:3542504-3542761
BmHel-11	DF090354:3754058-3754315
BmHel-11	DF090356:3270461-3270718
BmHel-11	DF090333:2785348-2785605
BmHel-11	DF090371:2135072-2135329
BmHel-11	DF090375:1111144-1111401
BmHel-11	DF090549:61246-61503
BmHel-11	DF090342:2101159-2101416
BmHel-11	DF090319:4861078-4861335
BmHel-11	DF090378:946993-947250
BmHel-11	DF090346:2070840-2071097
BmHel-11	DF090319:6147562-6147819
BmHel-11	DF090355:511109-511367
BmHel-11	DF090357:3290239-3290497
BmHel-11	DF090350:556801-557059
BmHel-11	DF090334:1625020-1625278
BmHel-11	DF090334:3179988-3180246
BmHel-11	DF090331:2166398-2166656
BmHel-11	DF090388:165781-166039
BmHel-11	DF090330:4246570-4246828
BmHel-11	DF090329:4986753-4987011
BmHel-11	DF090318:3177639-3177897
BmHel-11	DF090336:3482609-3482867
BmHel-11	DF090340:304344-304602
BmHel-11	DF090331:2729863-2730121
BmHel-11	DF090316:12809303-12809561
BmHel-11	DF090316:2237401-2237659
BmHel-11	DF090335:2721061-2721319
BmHel-11	DF090379:1853627-1853885
BmHel-11	DF090438:357301-357559
BmHel-11	DF090358:1248732-1248990
BmHel-11	DF090356:1039761-1040019

BmHel-11	DF090398:633173-633431
BmHel-11	DF090408:593086-593344
BmHel-11	DF090353:1019017-1019276
BmHel-11	DF090328:6504536-6504795
BmHel-11	DF090319:573728-573987
BmHel-11	DF090482:43469-43728
BmHel-11	DF090322:5116029-5116288
BmHel-11	DF090321:1228624-1228883
BmHel-11	DF090316:2932154-2932413
BmHel-11	DF090417:373838-374097
BmHel-11	DF090441:262171-262430
BmHel-11	DF090492:34122-34381
BmHel-11	BABH01050312:271-530
BmHel-11	DF090348:1957044-1957303
BmHel-11	DF090316:8718314-8718573
BmHel-11	DF090332:568901-569160
BmHel-11	DF090377:386259-386518
BmHel-11	DF090359:580537-580796
BmHel-11	DF090424:260068-260327
BmHel-11	DF090323:3024063-3024322
BmHel-11	DF090320:2311986-2312245
BmHel-11	DF090317:6683831-6684090
BmHel-11	DF090317:2159893-2160152
BmHel-11	DF090665:796-1055
BmHel-11	BABH01045935:1649-1908
BmHel-11	DF090366:1391911-1392171
BmHel-11	DF090354:1528995-1529255
BmHel-11	DF090409:582652-582912
BmHel-11	DF090348:4008585-4008845
BmHel-11	DF090377:142671-142931
BmHel-11	DF090322:5830897-5831157
BmHel-11	DF090331:5111-5371
BmHel-11	DF090330:3697729-3697989
BmHel-11	DF090405:876448-876708
BmHel-11	DF090319:5126255-5126515
BmHel-11	DF090349:3260500-3260760
BmHel-11	DF090349:1753777-1754037
BmHel-11	DF090364:1506022-1506282
BmHel-11	DF090357:458727-458988
BmHel-11	DF090359:2600974-2601235
BmHel-11	DF090370:1769601-1769862
BmHel-11	DF090317:9796537-9796798
BmHel-11	DF090342:808960-809221
BmHel-11	DF090319:875189-875450
BmHel-11	DF090331:2824605-2824866
BmHel-11	DF090316:15649632-15649893
BmHel-11	DF090320:2482349-2482610
BmHel-11	DF090339:3139789-3140050

BmHel-11	DF090402:838398-838660
BmHel-11	DF090342:1180858-1181120
BmHel-11	DF090336:5003819-5004081
BmHel-11	DF090332:5903409-5903671
BmHel-11	DF090360:2063198-2063460
BmHel-11	DF090359:649328-649590
BmHel-11	DF090334:5618961-5619223
BmHel-11	DF090426:654062-654324
BmHel-11	DF090517:56979-57241
BmHel-11	DF090419:919541-919803
BmHel-11	DF090425:363666-363928
BmHel-11	DF090663:9719-9981
BmHel-11	DF090387:616083-616345
BmHel-11	DF090322:1275869-1276131
BmHel-11	DF090341:2851508-2851770
BmHel-11	DF090391:725609-725871
BmHel-11	DF090366:132214-132476
BmHel-11	DF090352:206812-207074
BmHel-11	DF090447:469175-469437
BmHel-11	BABH01047188:358-620
BmHel-11	DF090353:648195-648458
BmHel-11	DF090332:1488481-1488744
BmHel-11	DF090345:970569-970832
BmHel-11	DF090346:2850540-2850803
BmHel-11	DF090409:353509-353772
BmHel-11	DF090430:166804-167067
BmHel-11	DF090596:11486-11749
BmHel-11	DF090337:1699626-1699889
BmHel-11	DF090362:2097282-2097545
BmHel-11	DF090322:3721816-3722079
BmHel-11	DF090340:4685918-4686181
BmHel-11	DF090424:152251-152514
BmHel-11	DF090337:1730732-1730995
BmHel-11	DF090320:3730210-3730473
BmHel-11	DF090320:2533560-2533823
BmHel-11	DF090412:264993-265256
BmHel-11	DF090379:1423830-1424093
BmHel-11	DF090404:513895-514158
BmHel-11	DF090356:3566275-3566538
BmHel-11	DF090409:122264-122527
BmHel-11	DF090413:484414-484677
BmHel-11	DF090324:779724-779988
BmHel-11	DF090319:7004357-7004621
BmHel-11	DF090377:801893-802157
BmHel-11	DF090411:884634-884898
BmHel-11	DF090379:362686-362950
BmHel-11	DF090468:205572-205836
BmHel-11	DF090356:3195648-3195912

BmHel-11	DF090345:2105334-2105598
BmHel-11	DF090423:494870-495134
BmHel-11	DF090364:2059466-2059730
BmHel-11	DF090319:2977278-2977542
BmHel-11	DF090322:5784269-5784533
BmHel-11	DF090316:8191479-8191743
BmHel-11	DF090368:848465-848729
BmHel-11	DF090486:96354-96618
BmHel-11	DF090457:246490-246754
BmHel-11	DF090431:775708-775972
BmHel-11	DF090422:443837-444101
BmHel-11	DF090325:3616367-3616632
BmHel-11	DF090402:1172508-1172773
BmHel-11	DF090357:526832-527097
BmHel-11	DF090326:5107735-5108000
BmHel-11	DF090316:15087325-15087590
BmHel-11	DF090418:1150547-1150812
BmHel-11	DF090406:1150744-1151009
BmHel-11	DF090344:4148312-4148577
BmHel-11	DF090378:941061-941326
BmHel-11	DF090327:461118-461383
BmHel-11	DF090338:4545602-4545867
BmHel-11	DF090426:109732-109997
BmHel-11	DF090322:1454096-1454361
BmHel-11	DF090363:2249407-2249672
BmHel-11	DF090349:2041366-2041631
BmHel-11	DF090341:2704361-2704626
BmHel-11	DF090316:1456495-1456760
BmHel-11	DF090337:5218890-5219155
BmHel-11	DF090344:3344023-3344288
BmHel-11	DF090398:908038-908303
BmHel-11	DF090413:620806-621071
BmHel-11	BABH01048372:821-1086
BmHel-11	DF090355:446015-446281
BmHel-11	DF090325:6178301-6178567
BmHel-11	DF090339:167451-167717
BmHel-11	DF090386:547460-547726
BmHel-11	DF090342:1416506-1416772
BmHel-11	DF090383:1326896-1327162
BmHel-11	DF090328:1054798-1055064
BmHel-11	DF090319:1892039-1892305
BmHel-11	DF090382:1587104-1587370
BmHel-11	DF090316:12574269-12574535
BmHel-11	DF090424:383189-383455
BmHel-11	DF090330:2382597-2382863
BmHel-11	DF090327:1984048-1984314
BmHel-11	DF090558:46906-47172
BmHel-11	DF090367:818434-818700

BmHel-11	DF090591:47021-47287
BmHel-11	DF090336:2139528-2139794
BmHel-11	DF090347:179617-179883
BmHel-11	DF090326:5301857-5302123
BmHel-11	DF090322:1117868-1118134
BmHel-11	DF090354:1553371-1553637
BmHel-11	DF090317:844772-845038
BmHel-11	DF090334:5078152-5078418
BmHel-11	DF090421:465360-465627
BmHel-11	DF090318:6430904-6431171
BmHel-11	DF090328:121529-121796
BmHel-11	DF090360:2707565-2707832
BmHel-11	DF090365:1223166-1223433
BmHel-11	DF090321:7833232-7833499
BmHel-11	DF090340:4258197-4258464
BmHel-11	DF090316:11425752-11426019
BmHel-11	DF090351:3313737-3314004
BmHel-11	DF090335:2186878-2187145
BmHel-11	DF090330:3671903-3672170
BmHel-11	DF090462:94010-94277
BmHel-11	DF090421:788877-789144
BmHel-11	DF090353:2194792-2195059
BmHel-11	DF090332:2972239-2972506
BmHel-11	DF090318:4067656-4067923
BmHel-11	DF090367:976797-977064
BmHel-11	DF090354:778374-778641
BmHel-11	DF090317:5385488-5385755
BmHel-11	DF090317:2200432-2200699
BmHel-11	DF090376:1231307-1231574
BmHel-11	DF090333:3128825-3129092
BmHel-11	DF090338:4125841-4126108
BmHel-11	DF090441:637739-638006
BmHel-11	BABH01045299:5159-5426
BmHel-11	BABH01047565:725-992
BmHel-11	DF090353:1643541-1643809
BmHel-11	DF090326:5224464-5224732
BmHel-11	DF090446:24262-24530
BmHel-11	DF090520:895-1163
BmHel-11	DF090318:4631196-4631464
BmHel-11	DF090322:2073286-2073554
BmHel-11	DF090316:8287673-8287941
BmHel-11	DF090873:14236-14504
BmHel-11	DF090348:470896-471164
BmHel-11	DF090319:6161461-6161729
BmHel-11	DF090350:1523189-1523457
BmHel-11	DF090334:3819605-3819873
BmHel-11	DF090334:2181859-2182127
BmHel-11	DF090330:1093545-1093813

BmHel-11	DF090367:2667436-2667704
BmHel-11	DF090367:1146755-1147023
BmHel-11	DF090320:2352387-2352655
BmHel-11	DF090317:9169335-9169603
BmHel-11	DF090317:4870931-4871199
BmHel-11	DF090362:1742324-1742592
BmHel-11	DF090352:3065118-3065386
BmHel-11	DF090325:3523326-3523595
BmHel-11	DF090339:4989270-4989539
BmHel-11	DF090332:19947-20216
BmHel-11	DF090319:3610087-3610356
BmHel-11	DF090365:2089367-2089636
BmHel-11	DF090349:1495656-1495925
BmHel-11	DF090351:683533-683802
BmHel-11	DF090417:163161-163430
BmHel-11	DF090372:524645-524914
BmHel-11	DF091359:3790-4059
BmHel-11	DF090858:7159-7428
BmHel-11	DF091533:496-765
BmHel-11	DF090322:547419-547688
BmHel-11	DF090371:2260039-2260308
BmHel-11	DF090336:3595725-3595994
BmHel-11	DF090336:336222-336491
BmHel-11	DF090343:4634236-4634505
BmHel-11	DF090316:11853034-11853303
BmHel-11	DF090330:147999-148268
BmHel-11	DF090376:146854-147123
BmHel-11	DF090345:1997461-1997730
BmHel-11	DF090334:2045546-2045816
BmHel-11	DF090367:1369215-1369485
BmHel-11	DF090320:7748185-7748455
BmHel-11	DF090412:298324-298594
BmHel-11	DF090333:570766-571036
BmHel-11	DF090333:3998569-3998839
BmHel-11	DF090462:253256-253526
BmHel-11	DF090374:1539180-1539450
BmHel-11	DF090351:2234404-2234674
BmHel-11	DF091085:7422-7692
BmHel-11	DF090318:4836058-4836328
BmHel-11	DF090350:30297-30567
BmHel-11	DF090335:1960475-1960745
BmHel-11	DF090320:2177972-2178242
BmHel-11	DF090379:305312-305582
BmHel-11	DF090366:1973465-1973735
BmHel-11	DF091147:1173-1443
BmHel-11	DF090325:4366839-4367110
BmHel-11	DF090325:4732550-4732821
BmHel-11	DF090318:9828125-9828396

BmHel-11	DF090365:1825768-1826039
BmHel-11	DF090365:2414005-2414276
BmHel-11	DF090365:2492390-2492661
BmHel-11	DF090323:7561833-7562104
BmHel-11	DF090396:1654-1925
BmHel-11	DF090422:336322-336593
BmHel-11	DF090839:41194-41465
BmHel-11	DF090327:5649892-5650163
BmHel-11	DF090316:3863177-3863448
BmHel-11	DF090380:854065-854336
BmHel-11	DF090317:3007982-3008253
BmHel-11	DF090317:150939-151210
BmHel-11	DF090356:3331370-3331641
BmHel-11	DF090333:2258216-2258487
BmHel-11	DF090341:3931590-3931861
BmHel-11	DF090347:1746638-1746910
BmHel-11	DF090382:1867646-1867918
BmHel-11	DF090364:1080113-1080385
BmHel-11	DF090317:10362240-10362512
BmHel-11	DF090427:756163-756435
BmHel-11	DF090375:547383-547655
BmHel-11	DF090332:5031256-5031528
BmHel-11	DF090319:2656985-2657257
BmHel-11	DF090335:5794304-5794576
BmHel-11	DF090344:703507-703779
BmHel-11	DF090319:5209774-5210046
BmHel-11	DF090335:2047546-2047818
BmHel-11	DF090317:1744161-1744433
BmHel-11	DF090338:3629432-3629704
BmHel-11	DF090332:92708-92981
BmHel-11	DF090319:6548316-6548589
BmHel-11	DF090395:244311-244584
BmHel-11	DF090322:4718228-4718501
BmHel-11	DF090368:1732276-1732549
BmHel-11	DF090401:820268-820541
BmHel-11	DF090324:2721680-2721953
BmHel-11	DF090324:239344-239617
BmHel-11	DF090365:1367468-1367741
BmHel-11	DF090393:1634494-1634767
BmHel-11	DF090379:714152-714425
BmHel-11	DF090317:8839814-8840087
BmHel-11	DF090417:810844-811117
BmHel-11	DF090328:2730850-2731123
BmHel-11	DF090391:529632-529905
BmHel-11	DF090326:3186364-3186637
BmHel-11	DF090353:3921317-3921591
BmHel-11	DF090402:594784-595058
BmHel-11	DF090342:2910053-2910327

BmHel-11	DF090336:4517018-4517292
BmHel-11	DF090321:249000-249274
BmHel-11	DF090359:1383897-1384171
BmHel-11	DF090316:540242-540516
BmHel-11	DF090351:3978863-3979137
BmHel-11	DF090354:2847463-2847737
BmHel-11	DF090327:3604759-3605033
BmHel-11	DF090371:140227-140501
BmHel-11	DF090353:3407525-3407799
BmHel-11	DF090342:1402311-1402585
BmHel-11	DF090336:2516452-2516726
BmHel-11	DF090332:5125934-5126208
BmHel-11	DF090318:5072030-5072304
BmHel-11	DF090348:3985573-3985847
BmHel-11	DF090328:1778246-1778520
BmHel-11	DF090322:8223519-8223793
BmHel-11	DF090337:2876641-2876915
BmHel-11	DF090320:2927711-2927985
BmHel-11	DF090352:73217-73491
BmHel-11	DF090404:1477553-1477827
BmHel-11	DF090376:1971504-1971778
BmHel-11	DF090416:829591-829865
BmHel-11	DF090598:37316-37590
BmHel-11	DF090339:1283961-1284236
BmHel-11	DF090328:2740886-2741161
BmHel-11	DF090357:1292011-1292286
BmHel-11	DF090377:1565976-1566251
BmHel-11	DF090322:118170-118445
BmHel-11	DF090351:3580385-3580660
BmHel-11	DF090335:3447076-3447351
BmHel-11	DF090330:5460495-5460770
BmHel-11	DF090329:6547141-6547416
BmHel-11	DF090367:2235360-2235635
BmHel-11	DF090380:1719888-1720163
BmHel-11	DF090492:205587-205862
BmHel-11	DF090324:1726799-1727074
BmHel-11	DF090335:2548301-2548576
BmHel-11	DF090353:3516407-3516682
BmHel-11	DF090334:126176-126451
BmHel-11	DF090400:1077839-1078114
BmHel-11	DF090400:236867-237142
BmHel-11	DF090435:409160-409435
BmHel-11	DF090372:2467203-2467478
BmHel-11	DF090361:66904-67179
BmHel-11	DF090338:696214-696489
BmHel-11	DF090464:104598-104873
BmHel-11	DF090354:278129-278404
BmHel-11	DF090366:578391-578666

BmHel-11	BABH01048763:14-289
BmHel-11	DF090339:3144883-3145159
BmHel-11	DF090332:6275592-6275868
BmHel-11	DF090318:8256370-8256646
BmHel-11	DF090328:2706334-2706610
BmHel-11	DF090322:6162606-6162882
BmHel-11	DF090334:289510-289786
BmHel-11	DF090316:4285091-4285367
BmHel-11	DF090316:9709030-9709306
BmHel-11	DF090316:14525346-14525622
BmHel-11	DF090388:47293-47569
BmHel-11	DF090329:5814918-5815194
BmHel-11	DF090364:1904429-1904705
BmHel-11	DF090438:660422-660698
BmHel-11	DF090404:1044228-1044504
BmHel-11	DF090358:2399115-2399391
BmHel-11	DF090423:709652-709928
BmHel-11	DF090754:7176-7452
BmHel-11	DF090537:6895-7171
BmHel-11	DF090321:6584780-6585056
BmHel-11	DF090359:2476234-2476510
BmHel-11	DF090351:429598-429874
BmHel-11	DF090329:1176099-1176375
BmHel-11	DF090451:33146-33422
BmHel-11	DF090327:109379-109655
BmHel-11	DF090401:1111526-1111802
BmHel-11	DF090868:13836-14112
BmHel-11	DF090321:7849072-7849349
BmHel-11	DF090363:1155806-1156083
BmHel-11	DF090359:2085844-2086121
BmHel-11	DF090335:2913440-2913717
BmHel-11	DF090329:5313867-5314144
BmHel-11	DF090352:3488419-3488696
BmHel-11	DF090317:8580203-8580480
BmHel-11	DF090376:1486458-1486735
BmHel-11	DF090374:1468060-1468337
BmHel-11	DF090409:101516-101793
BmHel-11	DF090347:4053802-4054079
BmHel-11	DF090321:5993728-5994005
BmHel-11	DF090350:1630154-1630431
BmHel-11	DF090343:1268992-1269269
BmHel-11	DF090334:4259840-4260117
BmHel-11	DF090352:138645-138922
BmHel-11	DF090417:795598-795875
BmHel-11	DF090401:842734-843011
BmHel-11	DF090401:567708-567985
BmHel-11	DF090375:695557-695834
BmHel-11	DF090432:288727-289004

BmHel-11	DF090461:140151-140428
BmHel-11	DF090343:1115590-1115867
BmHel-11	DF090362:2493825-2494103
BmHel-11	DF090336:4591818-4592096
BmHel-11	DF090328:4080746-4081024
BmHel-11	DF090360:1153253-1153531
BmHel-11	DF090322:4243450-4243728
BmHel-11	DF090367:636297-636575
BmHel-11	DF090384:2014239-2014517
BmHel-11	DF090439:242046-242324
BmHel-11	DF090332:907713-907991
BmHel-11	DF090346:401740-402018
BmHel-11	DF090339:2755076-2755354
BmHel-11	DF090342:1449743-1450021
BmHel-11	DF090347:100501-100779
BmHel-11	DF090321:2249007-2249285
BmHel-11	DF090393:114941-115219
BmHel-11	DF090349:4012360-4012638
BmHel-11	DF090334:2774546-2774824
BmHel-11	DF090373:558027-558305
BmHel-11	DF090379:884766-885044
BmHel-11	DF090317:7258486-7258764
BmHel-11	DF090317:2173976-2174254
BmHel-11	DF090317:734281-734559
BmHel-11	DF090372:1684100-1684378
BmHel-11	DF090361:3106019-3106297
BmHel-11	DF090500:207040-207318
BmHel-11	DF090436:448560-448838
BmHel-11	DF090375:1606523-1606801
BmHel-11	DF090355:3508072-3508351
BmHel-11	DF090325:744777-745056
BmHel-11	DF090353:2776333-2776612
BmHel-11	DF090339:3110436-3110715
BmHel-11	DF090402:682549-682828
BmHel-11	DF090377:362976-363255
BmHel-11	DF090347:4361949-4362228
BmHel-11	DF090321:3925668-3925947
BmHel-11	DF090321:4237638-4237917
BmHel-11	DF090329:796361-796640
BmHel-11	DF090344:2356181-2356460
BmHel-11	DF090452:129928-130207
BmHel-11	DF090375:567963-568242
BmHel-11	DF090528:844-1123
BmHel-11	BABH01048053:340-619
BmHel-11	DF090319:6964505-6964784
BmHel-11	DF090349:388440-388719
BmHel-11	DF090330:867491-867770
BmHel-11	DF090330:4556700-4556979

BmHel-11	DF090317:5910102-5910381
BmHel-11	DF090357:2750393-2750672
BmHel-11	DF090409:115780-116059
BmHel-11	DF090332:2071827-2072106
BmHel-11	DF090322:7043734-7044013
BmHel-11	DF090355:2704131-2704410
BmHel-11	DF090324:181756-182035
BmHel-11	DF090381:1516125-1516404
BmHel-11	DF090433:649218-649497
BmHel-11	DF090360:229997-230276
BmHel-11	DF090347:3340126-3340405
BmHel-11	DF090326:5858669-5858948
BmHel-11	DF090343:3768019-3768298
BmHel-11	DF090340:4321157-4321436
BmHel-11	DF090370:721822-722101
BmHel-11	DF090320:668563-668842
BmHel-11	DF090416:1154813-1155092
BmHel-11	DF090423:917827-918106
BmHel-11	DF090526:62701-62980
BmHel-11	DF090330:6422172-6422451
BmHel-11	DF090320:4561820-4562099
BmHel-11	DF090352:1108450-1108729
BmHel-11	DF090333:2432543-2432822
BmHel-11	DF090318:2751856-2752136
BmHel-11	DF090363:212418-212698
BmHel-11	DF090331:5817163-5817443
BmHel-11	DF090418:707036-707316
BmHel-11	DF090391:1354041-1354321
BmHel-11	DF090323:7547874-7548154
BmHel-11	DF090320:4911140-4911420
BmHel-11	DF090366:2143273-2143553
BmHel-11	DF090364:64375-64655
BmHel-11	DF090364:1220872-1221152
BmHel-11	DF090468:124595-124875
BmHel-11	DF090357:2283117-2283397
BmHel-11	DF090326:4227765-4228045
BmHel-11	DF090375:1387240-1387520
BmHel-11	DF090355:1987314-1987594
BmHel-11	DF090336:1859319-1859599
BmHel-11	DF090319:6126105-6126385
BmHel-11	DF090377:523432-523712
BmHel-11	DF090347:2116074-2116354
BmHel-11	DF090326:4865188-4865468
BmHel-11	DF090469:315813-316093
BmHel-11	DF090349:3397549-3397829
BmHel-11	DF090340:2174259-2174539
BmHel-11	DF090388:1098780-1099060
BmHel-11	DF090344:3191979-3192259

BmHel-11	DF090320:2089734-2090014
BmHel-11	DF090427:896070-896350
BmHel-11	DF090375:523921-524201
BmHel-11	DF090446:578523-578803
BmHel-11	DF090394:1392049-1392329
BmHel-11	DF090413:875718-875998
BmHel-11	DF090425:926098-926378
BmHel-11	DF090318:2611380-2611660
BmHel-11	DF090414:63515-63795
BmHel-11	DF090416:413415-413695
BmHel-11	DF090428:854071-854351
BmHel-11	DF090383:1683119-1683400
BmHel-11	DF090433:569574-569855
BmHel-11	DF090322:2237769-2238050
BmHel-11	DF090316:4013797-4014078
BmHel-11	DF090335:5827969-5828250
BmHel-11	DF090330:6332359-6332640
BmHel-11	DF090323:1570147-1570428
BmHel-11	DF090344:2013998-2014279
BmHel-11	DF090354:3202651-3202932
BmHel-11	DF090376:2195958-2196239
BmHel-11	DF090361:2006555-2006836
BmHel-11	DF090338:3828978-3829259
BmHel-11	DF090338:4132010-4132291
BmHel-11	DF090408:1471372-1471653
BmHel-11	DF090339:119810-120091
BmHel-11	DF090326:4927758-4928039
BmHel-11	DF090361:1162811-1163092
BmHel-11	BABH01044028:8811-9092
BmHel-11	DF090353:1588975-1589256
BmHel-11	DF090328:2406829-2407110
BmHel-11	DF090357:1178094-1178375
BmHel-11	DF090340:4896613-4896894
BmHel-11	DF090316:13198659-13198940
BmHel-11	DF090368:1599470-1599751
BmHel-11	DF090335:927690-927971
BmHel-11	DF090330:3655260-3655541
BmHel-11	DF090345:269232-269513
BmHel-11	DF090403:1279438-1279719
BmHel-11	DF090423:233774-234055
BmHel-11	DF090375:1998021-1998302
BmHel-11	DF090447:93601-93882
BmHel-11	DF091245:4574-4855
BmHel-11	DF090325:3098299-3098580
BmHel-11	DF090335:309469-309750
BmHel-11	DF090370:2113709-2113990
BmHel-11	DF090352:871757-872038
BmHel-11	DF090336:1219295-1219577

BmHel-11	DF090332:1709869-1710151
BmHel-11	DF090348:517513-517795
BmHel-11	DF090328:3410982-3411264
BmHel-11	DF090347:1011747-1012029
BmHel-11	DF090414:283573-283855
BmHel-11	DF090322:7712183-7712465
BmHel-11	DF090322:7984909-7985191
BmHel-11	DF090321:1215096-1215378
BmHel-11	DF090321:1425478-1425760
BmHel-11	DF090393:1560627-1560909
BmHel-11	DF090331:2719936-2720218
BmHel-11	DF090351:3108553-3108835
BmHel-11	DF090344:4646189-4646471
BmHel-11	DF090320:5668591-5668873
BmHel-11	DF090399:1282281-1282563
BmHel-11	DF090364:136813-137095
BmHel-11	DF090364:1331467-1331749
BmHel-11	DF090317:8545040-8545322
BmHel-11	DF090372:383149-383431
BmHel-11	DF090401:1160171-1160453
BmHel-11	DF090441:436265-436547
BmHel-11	DF090492:223300-223582
BmHel-11	DF090699:10532-10814
BmHel-11	DF090450:30263-30545
BmHel-11	DF090384:293004-293286
BmHel-11	DF090325:1091782-1092064
BmHel-11	DF090339:2571008-2571290
BmHel-11	DF090362:2789646-2789928
BmHel-11	DF090436:3874-4156
BmHel-11	DF090348:3793472-3793754
BmHel-11	DF090319:2275887-2276169
BmHel-11	DF090357:1585255-1585537
BmHel-11	DF090365:2943129-2943411
BmHel-11	DF090326:1859875-1860157
BmHel-11	DF090321:7206662-7206944
BmHel-11	DF090341:687081-687363
BmHel-11	DF090340:905634-905916
BmHel-11	DF090334:5295101-5295383
BmHel-11	DF090316:2096898-2097180
BmHel-11	DF090396:514282-514564
BmHel-11	DF090417:218216-218498
BmHel-11	DF090345:3708819-3709101
BmHel-11	DF090327:1721076-1721358
BmHel-11	DF090374:69292-69574
BmHel-11	DF090420:749619-749901
BmHel-11	DF090369:1728405-1728687
BmHel-11	DF090425:970048-970330
BmHel-11	DF090528:70142-70424

BmHel-11	DF090437:57389-57671
BmHel-11	DF090413:1302107-1302389
BmHel-11	DF090444:590705-590987
BmHel-11	DF090429:425257-425539
BmHel-11	DF090339:2390416-2390699
BmHel-11	DF090324:1739510-1739793
BmHel-11	DF090342:3649933-3650216
BmHel-11	DF090360:995792-996075
BmHel-11	DF090343:115316-115599
BmHel-11	DF090341:2945293-2945576
BmHel-11	DF090320:7619689-7619972
BmHel-11	DF090358:734485-734768
BmHel-11	DF090361:2109840-2110123
BmHel-11	DF090338:3971737-3972020
BmHel-11	DF090413:960207-960490
BmHel-11	DF090481:180053-180336
BmHel-11	BABH01044635:4196-4479
BmHel-11	BABH01074640:315-598
BmHel-11	DF090386:278025-278308
BmHel-11	DF090418:259929-260212
BmHel-11	DF090353:1572898-1573181
BmHel-11	DF090342:2637824-2638107
BmHel-11	DF090342:494340-494623
BmHel-11	DF090336:4370083-4370366
BmHel-11	DF090318:7609258-7609541
BmHel-11	DF090328:3270340-3270623
BmHel-11	DF090392:787909-788192
BmHel-11	DF090407:668717-669000
BmHel-11	DF090407:175593-175876
BmHel-11	DF090363:106992-107275
BmHel-11	DF090368:1161883-1162166
BmHel-11	DF090320:737520-737803
BmHel-11	DF090376:1522557-1522840
BmHel-11	DF090405:319958-320241
BmHel-11	DF090346:3210122-3210405
BmHel-11	DF090441:404492-404775
BmHel-11	DF090328:2621389-2621672
BmHel-11	DF090330:3160228-3160511
BmHel-11	DF090325:1411011-1411295
BmHel-11	DF090325:6540846-6541130
BmHel-11	DF090325:7287102-7287386
BmHel-11	DF090377:448760-449044
BmHel-11	DF090360:2933261-2933545
BmHel-11	DF090347:1161800-1162084
BmHel-11	DF090321:2883494-2883778
BmHel-11	DF090407:1568128-1568412
BmHel-11	DF090331:597572-597856
BmHel-11	DF090388:972603-972887

BmHel-11	DF090388:1484140-1484424
BmHel-11	DF090368:1388720-1389004
BmHel-11	DF090330:2875692-2875976
BmHel-11	DF090330:5032828-5033112
BmHel-11	DF090329:4918556-4918840
BmHel-11	DF090344:1366474-1366758
BmHel-11	DF090344:4036029-4036313
BmHel-11	DF090399:284095-284379
BmHel-11	DF090379:1486270-1486554
BmHel-11	DF090404:1412315-1412599
BmHel-11	DF090392:600075-600359
BmHel-11	DF090342:4777978-4778262
BmHel-11	DF090336:1601294-1601578
BmHel-11	DF090332:3274873-3275157
BmHel-11	DF090328:6138055-6138339
BmHel-11	DF090319:8500113-8500397
BmHel-11	DF090357:925652-925936
BmHel-11	DF090357:811687-811971
BmHel-11	DF090411:397756-398040
BmHel-11	DF090326:3346735-3347019
BmHel-11	DF090321:1784902-1785186
BmHel-11	DF090363:2745890-2746174
BmHel-11	DF090334:3692933-3693217
BmHel-11	DF090316:4366777-4367061
BmHel-11	DF090335:2787697-2787981
BmHel-11	DF090323:4763553-4763837
BmHel-11	DF090323:1734979-1735263
BmHel-11	DF090344:1838794-1839078
BmHel-11	DF090380:2128318-2128602
BmHel-11	DF090370:1666415-1666699
BmHel-11	DF090396:784855-785139
BmHel-11	DF090317:8747668-8747952
BmHel-11	DF090317:4883739-4884023
BmHel-11	DF090372:880688-880972
BmHel-11	DF090361:1212735-1213019
BmHel-11	DF090394:1304073-1304357
BmHel-11	DF090321:1934039-1934323
BmHel-11	DF090316:9340559-9340843
BmHel-11	DF090388:1648465-1648749
BmHel-11	DF090333:4291491-4291775
BmHel-11	DF090320:818145-818429
BmHel-11	DF090339:4255790-4256075
BmHel-11	DF090332:1063133-1063418
BmHel-11	DF090332:2855086-2855371
BmHel-11	DF090332:5839300-5839585
BmHel-11	DF090318:3636863-3637148
BmHel-11	DF090318:8463984-8464269
BmHel-11	DF090348:1322127-1322412

BmHel-11	DF090319:7891421-7891706
BmHel-11	DF090357:2118817-2119102
BmHel-11	DF090347:1219013-1219298
BmHel-11	DF090321:4957723-4958008
BmHel-11	DF090363:653499-653784
BmHel-11	DF090359:1527639-1527924
BmHel-11	DF090341:3822852-3823137
BmHel-11	DF090331:5277327-5277612
BmHel-11	DF090316:4228300-4228585
BmHel-11	DF090400:688413-688698
BmHel-11	DF090388:951992-952277
BmHel-11	DF090406:99746-100031
BmHel-11	DF090344:1456403-1456688
BmHel-11	DF090344:1710734-1711019
BmHel-11	DF090320:1372617-1372902
BmHel-11	DF090320:2708622-2708907
BmHel-11	DF090354:3235043-3235328
BmHel-11	DF090592:4088-4373
BmHel-11	DF090443:448546-448831
BmHel-11	DF090429:92186-92471
BmHel-11	DF090429:463778-464063
BmHel-11	BABH01055439:242-527
BmHel-11	DF091006:6644-6929
BmHel-11	DF090880:10575-10860
BmHel-11	DF090389:1513218-1513503
BmHel-11	DF090328:6121709-6121994
BmHel-11	DF090339:3341316-3341601
BmHel-11	DF090362:265833-266118
BmHel-11	DF090336:3312784-3313069
BmHel-11	DF090336:945263-945548
BmHel-11	DF090318:6923347-6923632
BmHel-11	DF090348:2126724-2127009
BmHel-11	DF090328:1925205-1925490
BmHel-11	DF090319:5044140-5044425
BmHel-11	DF090321:6256735-6257020
BmHel-11	DF090321:796886-797171
BmHel-11	DF090476:197461-197746
BmHel-11	DF090476:125765-126050
BmHel-11	DF090393:55516-55801
BmHel-11	DF090331:1010137-1010422
BmHel-11	DF090316:816996-817281
BmHel-11	DF090391:934941-935226
BmHel-11	DF090368:1571787-1572072
BmHel-11	DF090367:787652-787937
BmHel-11	DF090412:132157-132442
BmHel-11	DF090358:51564-51849
BmHel-11	DF090346:3774079-3774364
BmHel-11	DF090346:2419322-2419607

BmHel-11	DF090415:480399-480684
BmHel-11	DF090409:1383798-1384083
BmHel-11	DF090441:183183-183468
BmHel-11	DF090427:654542-654827
BmHel-11	DF090353:1286919-1287205
BmHel-11	DF090342:689919-690205
BmHel-11	DF090318:2442412-2442698
BmHel-11	DF090383:1441019-1441305
BmHel-11	DF090348:3696201-3696487
BmHel-11	DF090328:4967649-4967935
BmHel-11	DF090319:5142249-5142535
BmHel-11	DF090365:280549-280835
BmHel-11	DF090365:1149682-1149968
BmHel-11	DF090321:4694392-4694678
BmHel-11	DF090382:67476-67762
BmHel-11	DF090316:12785265-12785551
BmHel-11	DF090388:1784017-1784303
BmHel-11	DF090373:1643957-1644243
BmHel-11	DF090351:3218368-3218654
BmHel-11	DF090330:3032175-3032461
BmHel-11	DF090329:5609419-5609705
BmHel-11	DF090323:1433093-1433379
BmHel-11	DF090367:1597330-1597616
BmHel-11	DF090320:6168118-6168404
BmHel-11	DF090320:7504447-7504733
BmHel-11	DF090396:232062-232348
BmHel-11	DF090317:5547051-5547337
BmHel-11	DF090317:8943791-8944077
BmHel-11	DF090358:2376153-2376439
BmHel-11	DF090345:2609856-2610142
BmHel-11	DF090405:966077-966363
BmHel-11	DF090346:108480-108766
BmHel-11	DF090361:137101-137387
BmHel-11	DF090432:435330-435616
BmHel-11	DF090430:679438-679724
BmHel-11	DF090419:987771-988057
BmHel-11	DF090475:369-655
BmHel-11	BABH01045757:3287-3573
BmHel-11	DF090350:1374084-1374370
BmHel-11	DF090364:372113-372399
BmHel-11	DF090352:3151152-3151438
BmHel-11	DF090548:26341-26627
BmHel-11	DF090325:4793702-4793988
BmHel-11	DF090325:4454281-4454567
BmHel-11	DF090339:1994538-1994824
BmHel-11	DF090362:14458-14744
BmHel-11	DF090326:5515486-5515772
BmHel-11	DF090321:7393186-7393472

BmHel-11	DF090321:3574775-3575061
BmHel-11	DF090340:2563389-2563675
BmHel-11	DF090334:5903752-5904038
BmHel-11	DF090334:2920895-2921181
BmHel-11	DF090385:966237-966523
BmHel-11	DF090368:2225776-2226062
BmHel-11	DF090337:4135875-4136161
BmHel-11	DF090330:3215589-3215875
BmHel-11	DF090323:6984078-6984364
BmHel-11	DF090323:3416349-3416635
BmHel-11	DF090320:1630580-1630866
BmHel-11	DF090364:2441087-2441373
BmHel-11	DF090352:164755-165041
BmHel-11	DF090317:6840071-6840357
BmHel-11	DF090317:5372289-5372575
BmHel-11	DF090317:4908062-4908348
BmHel-11	DF090486:39584-39870
BmHel-11	DF090333:400906-401192
BmHel-11	DF090346:3169952-3170238
BmHel-11	DF090427:930676-930962
BmHel-11	DF090339:2144973-2145259
BmHel-11	DF090324:4339274-4339560
BmHel-11	DF090381:87804-88090
BmHel-11	DF090433:244339-244625
BmHel-11	DF090359:2002696-2002982
BmHel-11	DF090331:2207013-2207299
BmHel-11	DF090316:3314171-3314457
BmHel-11	DF090323:7599257-7599543
BmHel-11	DF090344:3264300-3264586
BmHel-11	DF090355:913531-913818
BmHel-11	DF090325:6126991-6127278
BmHel-11	DF090353:1392086-1392373
BmHel-11	DF090353:3500609-3500896
BmHel-11	DF090339:2511132-2511419
BmHel-11	DF090362:1645432-1645719
BmHel-11	DF090324:3842044-3842331
BmHel-11	DF090324:5185497-5185784
BmHel-11	DF090386:873107-873394
BmHel-11	DF090332:133162-133449
BmHel-11	DF090332:2885610-2885897
BmHel-11	DF090348:535176-535463
BmHel-11	DF090328:889699-889986
BmHel-11	DF090319:300190-300477
BmHel-11	DF090319:5687689-5687976
BmHel-11	DF090377:1340805-1341092
BmHel-11	DF090377:1352426-1352713
BmHel-11	DF090360:309635-309922
BmHel-11	DF090322:6887105-6887392

BmHel-11	DF090382:1260818-1261105
BmHel-11	DF090359:2682264-2682551
BmHel-11	DF090343:4197903-4198190
BmHel-11	DF090343:4365684-4365971
BmHel-11	DF090316:14075111-14075398
BmHel-11	DF090351:2342986-2343273
BmHel-11	DF090330:4823366-4823653
BmHel-11	DF090330:6287454-6287741
BmHel-11	DF090384:146635-146922
BmHel-11	DF090384:1036568-1036855
BmHel-11	DF090384:1686209-1686496
BmHel-11	DF090366:287965-288252
BmHel-11	DF090364:109842-110129
BmHel-11	DF090364:921827-922114
BmHel-11	DF090352:1871487-1871774
BmHel-11	DF090372:1196695-1196982
BmHel-11	DF090338:2290802-2291089
BmHel-11	DF090437:284680-284967
BmHel-11	DF090449:787029-787316
BmHel-11	DF090458:12086-12373
BmHel-11	BABH01060459:389-676
BmHel-11	DF090355:683076-683363
BmHel-11	DF090325:722752-723039
BmHel-11	DF090319:2352764-2353051
BmHel-11	DF090414:1307334-1307621
BmHel-11	DF090370:783815-784102
BmHel-11	DF090396:1189266-1189553
BmHel-11	DF090440:505454-505741
BmHel-11	DF090333:3467125-3467412
BmHel-11	DF090521:69607-69894
BmHel-11	BABH01048151:461-748
BmHel-11	DF090377:1226035-1226322
BmHel-11	DF090355:3012935-3013222
BmHel-11	DF090355:203155-203442
BmHel-11	DF090325:1132856-1133143
BmHel-11	DF090402:474501-474788
BmHel-11	DF090390:465501-465788
BmHel-11	DF090386:765425-765712
BmHel-11	DF090381:327093-327380
BmHel-11	DF090342:978152-978439
BmHel-11	DF090348:2164705-2164992
BmHel-11	DF090348:215233-215520
BmHel-11	DF090319:5915806-5916093
BmHel-11	DF090357:2061238-2061525
BmHel-11	DF090365:2866087-2866374
BmHel-11	DF090326:5639249-5639536
BmHel-11	DF090321:2728461-2728748
BmHel-11	DF090321:2026375-2026662

BmHel-11	DF090407:820808-821095
BmHel-11	DF090340:4569970-4570257
BmHel-11	DF090340:813861-814148
BmHel-11	DF090331:4732102-4732389
BmHel-11	DF090316:10753036-10753323
BmHel-11	DF090316:9119162-9119449
BmHel-11	DF090388:683615-683902
BmHel-11	DF090351:3526718-3527005
BmHel-11	DF090323:1119886-1120173
BmHel-11	DF090344:4489448-4489735
BmHel-11	DF090320:8099028-8099315
BmHel-11	DF090320:174010-174297
BmHel-11	DF090378:279548-279835
BmHel-11	DF090317:190371-190658
BmHel-11	DF090389:771501-771788
BmHel-11	DF090389:37546-37833
BmHel-11	DF090356:3226213-3226500
BmHel-11	DF090345:2395162-2395449
BmHel-11	DF090333:5370236-5370523
BmHel-11	DF090333:5043021-5043308
BmHel-11	DF090327:2151890-2152177
BmHel-11	DF090371:1748621-1748908
BmHel-11	DF090346:941658-941945
BmHel-11	DF090361:1348410-1348697
BmHel-11	DF090426:941788-942075
BmHel-11	DF090408:1009793-1010080
BmHel-11	DF090480:108263-108550
BmHel-11	DF090355:1453147-1453434
BmHel-11	DF090348:3573144-3573431
BmHel-11	DF090344:522445-522732
BmHel-11	DF090417:919657-919944
BmHel-11	DF090353:3052613-3052901
BmHel-11	DF090387:802595-802883
BmHel-11	DF090336:4416615-4416903
BmHel-11	DF090332:4834258-4834546
BmHel-11	DF090318:3780676-3780964
BmHel-11	DF090328:2443623-2443911
BmHel-11	DF090328:4878329-4878617
BmHel-11	DF090360:1235776-1236064
BmHel-11	DF090365:1072475-1072763
BmHel-11	DF090349:4262333-4262621
BmHel-11	DF090343:3327984-3328272
BmHel-11	DF090323:2273093-2273381
BmHel-11	DF090344:2594752-2595040
BmHel-11	DF090344:4195282-4195570
BmHel-11	DF090384:188520-188808
BmHel-11	DF090384:1504902-1505190
BmHel-11	DF090378:76105-76393

BmHel-11	DF090378:308898-309186
BmHel-11	DF090354:3339304-3339592
BmHel-11	DF090352:436853-437141
BmHel-11	DF090352:1860264-1860552
BmHel-11	DF090372:1892839-1893127
BmHel-11	DF090345:4059333-4059621
BmHel-11	DF090327:1970143-1970431
BmHel-11	DF090346:1082126-1082414
BmHel-11	DF090427:248677-248965
BmHel-11	DF090361:1102508-1102796
BmHel-11	DF090477:228668-228956
BmHel-11	DF090439:337276-337564
BmHel-11	DF090724:11585-11873
BmHel-11	DF090571:5040-5328
BmHel-11	DF090456:329386-329674
BmHel-11	BABH01045341:1107-1395
BmHel-11	DF090499:103596-103884
BmHel-11	DF090348:2059225-2059513
BmHel-11	DF090433:409841-410129
BmHel-11	DF090329:2369475-2369763
BmHel-11	DF090364:651551-651839
BmHel-11	DF090317:9745406-9745694
BmHel-11	DF090398:727060-727348
BmHel-11	DF090415:432387-432675
BmHel-11	DF090426:501-789
BmHel-11	DF090318:8123022-8123310
BmHel-11	DF090352:2465576-2465864
BmHel-11	DF090353:71215-71503
BmHel-11	DF090339:4971169-4971457
BmHel-11	DF090387:868733-869021
BmHel-11	DF090324:4259429-4259717
BmHel-11	DF090402:1272680-1272968
BmHel-11	DF090390:609540-609828
BmHel-11	DF090342:433251-433539
BmHel-11	DF090336:4205960-4206248
BmHel-11	DF090332:4930687-4930975
BmHel-11	DF090318:520748-521036
BmHel-11	DF090383:980215-980503
BmHel-11	DF090319:521076-521364
BmHel-11	DF090357:345465-345753
BmHel-11	DF090322:6529851-6530139
BmHel-11	DF090382:1597116-1597404
BmHel-11	DF090382:474264-474552
BmHel-11	DF090334:5103087-5103375
BmHel-11	DF090329:4091607-4091895
BmHel-11	DF090367:364793-365081
BmHel-11	DF090344:910941-911229
BmHel-11	DF090344:473314-473602

BmHel-11	DF090452:9412-9700
BmHel-11	DF090379:1100248-1100536
BmHel-11	DF090317:10853829-10854117
BmHel-11	DF090317:5425218-5425506
BmHel-11	DF090317:1478590-1478878
BmHel-11	DF090333:4279471-4279759
BmHel-11	DF090333:510577-510865
BmHel-11	DF090327:1987608-1987896
BmHel-11	DF090346:2002488-2002776
BmHel-11	DF090346:268573-268861
BmHel-11	DF090375:1250323-1250611
BmHel-11	DF090460:45524-45812
BmHel-11	DF090448:30385-30673
BmHel-11	DF090369:2102088-2102376
BmHel-11	DF090520:151004-151292
BmHel-11	DF090355:3194518-3194806
BmHel-11	DF090324:2036880-2037168
BmHel-11	DF090342:791086-791374
BmHel-11	DF090395:1138581-1138869
BmHel-11	DF090322:7908991-7909279
BmHel-11	DF090343:636440-636728
BmHel-11	DF090340:1955757-1956045
BmHel-11	DF090416:537862-538150
BmHel-11	DF090413:1144682-1144970
BmHel-11	DF090441:295935-296223
BmHel-11	DF090335:4208168-4208456
BmHel-11	DF090325:2712646-2712935
BmHel-11	DF090325:5089266-5089555
BmHel-11	DF090353:1165483-1165772
BmHel-11	DF090353:4001172-4001461
BmHel-11	DF090362:707579-707868
BmHel-11	DF090324:6192357-6192646
BmHel-11	DF090386:494359-494648
BmHel-11	DF090386:1198160-1198449
BmHel-11	DF090336:2806046-2806335
BmHel-11	DF090332:5634305-5634594
BmHel-11	DF090332:6006954-6007243
BmHel-11	DF090318:1881024-1881313
BmHel-11	DF090383:31682-31971
BmHel-11	DF090357:1500223-1500512
BmHel-11	DF090360:2032464-2032753
BmHel-11	DF090347:988353-988642
BmHel-11	DF090326:1737246-1737535
BmHel-11	DF090326:2285756-2286045
BmHel-11	DF090326:6226998-6227287
BmHel-11	DF090321:873567-873856
BmHel-11	DF090382:930804-931093
BmHel-11	DF090349:4100607-4100896

BmHel-11	DF090331:1627218-1627507
BmHel-11	DF090331:2553089-2553378
BmHel-11	DF090388:563232-563521
BmHel-11	DF090351:2282408-2282697
BmHel-11	DF090351:2636144-2636433
BmHel-11	DF090337:1442611-1442900
BmHel-11	DF090330:1815776-1816065
BmHel-11	DF090330:4786049-4786338
BmHel-11	DF090329:1184552-1184841
BmHel-11	DF090329:4675917-4676206
BmHel-11	DF090329:6213917-6214206
BmHel-11	DF090323:84764-85053
BmHel-11	DF090323:1322016-1322305
BmHel-11	DF090323:5699375-5699664
BmHel-11	DF090450:474247-474536
BmHel-11	DF090354:978366-978655
BmHel-11	DF090354:2947744-2948033
BmHel-11	DF090438:541495-541784
BmHel-11	DF090346:3283202-3283491
BmHel-11	DF090338:2101346-2101635
BmHel-11	DF090338:4095244-4095533
BmHel-11	DF090375:866549-866838
BmHel-11	DF090397:652609-652898
BmHel-11	DF090397:1148987-1149276
BmHel-11	DF090443:423548-423837
BmHel-11	DF090413:1023789-1024078
BmHel-11	DF090492:83222-83511
BmHel-11	BABH01045180:3971-4260
BmHel-11	DF090342:1259350-1259639
BmHel-11	DF090318:808399-808688
BmHel-11	DF090348:148099-148388
BmHel-11	DF090347:2013979-2014268
BmHel-11	DF090365:1858711-1859000
BmHel-11	DF090316:4548988-4549277
BmHel-11	DF090351:1634917-1635206
BmHel-11	DF090330:1930932-1931221
BmHel-11	DF090366:2338134-2338423
BmHel-11	DF090317:2872269-2872558
BmHel-11	DF090358:195572-195861
BmHel-11	DF090573:10384-10673
BmHel-11	DF090420:625646-625935
BmHel-11	DF090399:23583-23872
BmHel-11	DF090318:2499834-2500123
BmHel-11	DF090353:1922777-1923066
BmHel-11	DF090362:2441206-2441495
BmHel-11	DF090387:1091099-1091388
BmHel-11	DF090324:973242-973531
BmHel-11	DF090386:733591-733880

BmHel-11	DF090348:1842547-1842836
BmHel-11	DF090328:4241576-4241865
BmHel-11	DF090328:3219952-3220241
BmHel-11	DF090319:8320271-8320560
BmHel-11	DF090319:4880620-4880909
BmHel-11	DF090319:3627630-3627919
BmHel-11	DF090319:2488627-2488916
BmHel-11	DF090377:1917572-1917861
BmHel-11	DF090347:228811-229100
BmHel-11	DF090322:1329587-1329876
BmHel-11	DF090321:4128008-4128297
BmHel-11	DF090343:862947-863236
BmHel-11	DF090334:355465-355754
BmHel-11	DF090316:12100612-12100901
BmHel-11	DF090316:6677371-6677660
BmHel-11	DF090316:6406940-6407229
BmHel-11	DF090316:1513572-1513861
BmHel-11	DF090388:1444124-1444413
BmHel-11	DF090385:1268622-1268911
BmHel-11	DF090373:429813-430102
BmHel-11	DF090368:1509563-1509852
BmHel-11	DF090351:1357587-1357876
BmHel-11	DF090337:1115527-1115816
BmHel-11	DF090335:4586803-4587092
BmHel-11	DF090330:4336481-4336770
BmHel-11	DF090330:1893372-1893661
BmHel-11	DF090323:3727456-3727745
BmHel-11	DF090323:1749630-1749919
BmHel-11	DF090367:2500429-2500718
BmHel-11	DF090344:4593122-4593411
BmHel-11	DF090320:2722647-2722936
BmHel-11	DF090320:1328743-1329032
BmHel-11	DF090442:268353-268642
BmHel-11	DF090412:885107-885396
BmHel-11	DF090399:1056620-1056909
BmHel-11	DF090364:305523-305812
BmHel-11	DF090317:7446747-7447036
BmHel-11	DF090376:2243057-2243346
BmHel-11	DF090327:6188135-6188424
BmHel-11	DF090398:187826-188115
BmHel-11	DF090346:2531298-2531587
BmHel-11	DF090346:2082367-2082656
BmHel-11	DF090501:91256-91545
BmHel-11	DF090416:115383-115672
BmHel-11	DF090403:937837-938126
BmHel-11	DF090374:666724-667013
BmHel-11	DF090394:1562027-1562316
BmHel-11	DF090944:366-655

BmHel-11	DF091273:561-850
BmHel-11	DF090324:7123184-7123473
BmHel-11	DF090332:5967793-5968082
BmHel-11	DF090326:6836760-6837049
BmHel-11	DF090326:5968559-5968848
BmHel-11	DF090326:5682789-5683078
BmHel-11	DF090326:1444455-1444744
BmHel-11	DF090349:222918-223207
BmHel-11	DF090331:3357912-3358201
BmHel-11	DF090351:2373990-2374279
BmHel-11	DF090329:1770322-1770611
BmHel-11	DF090323:4275363-4275652
BmHel-11	DF090384:1783517-1783806
BmHel-11	DF090325:7256897-7257186
BmHel-11	DF090348:4104141-4104430
BmHel-11	DF090326:2961163-2961452
BmHel-11	DF090409:381275-381564
BmHel-11	DF090355:2157464-2157754
BmHel-11	DF090325:5181131-5181421
BmHel-11	DF090353:580445-580735
BmHel-11	DF090362:325408-325698
BmHel-11	DF090362:1197919-1198209
BmHel-11	DF090362:1812873-1813163
BmHel-11	DF090324:3631195-3631485
BmHel-11	DF090324:5484087-5484377
BmHel-11	DF090386:1068543-1068833
BmHel-11	DF090342:2161836-2162126
BmHel-11	DF090342:2260712-2261002
BmHel-11	DF090336:1890126-1890416
BmHel-11	DF090332:311488-311778
BmHel-11	DF090332:872369-872659
BmHel-11	DF090332:4154335-4154625
BmHel-11	DF090332:4580380-4580670
BmHel-11	DF090318:2453518-2453808
BmHel-11	DF090383:1779523-1779813
BmHel-11	DF090348:4315761-4316051
BmHel-11	DF090328:3086418-3086708
BmHel-11	DF090319:3706527-3706817
BmHel-11	DF090319:4420252-4420542
BmHel-11	DF090319:8413409-8413699
BmHel-11	DF090357:1784005-1784295
BmHel-11	DF090377:895694-895984
BmHel-11	DF090377:1826200-1826490
BmHel-11	DF090414:596243-596533
BmHel-11	DF090392:134231-134521
BmHel-11	DF090392:1539846-1540136
BmHel-11	DF090326:4951964-4952254
BmHel-11	DF090326:6311575-6311865

BmHel-11	DF090322:1644649-1644939
BmHel-11	DF090321:3396331-3396621
BmHel-11	DF090321:7026839-7027129
BmHel-11	DF090410:892740-893030
BmHel-11	DF090350:1305619-1305909
BmHel-11	DF090407:14004-14294
BmHel-11	DF090382:1126116-1126406
BmHel-11	DF090349:890255-890545
BmHel-11	DF090349:1746730-1747020
BmHel-11	DF090349:2906752-2907042
BmHel-11	DF090343:1025516-1025806
BmHel-11	DF090341:4160009-4160299
BmHel-11	DF090341:4320595-4320885
BmHel-11	DF090334:1209958-1210248
BmHel-11	DF090316:4156045-4156335
BmHel-11	DF090316:4488097-4488387
BmHel-11	DF090800:5020-5310
BmHel-11	DF090424:197374-197664
BmHel-11	DF090424:964191-964481
BmHel-11	DF090368:2203867-2204157
BmHel-11	DF090368:2447206-2447496
BmHel-11	DF090351:1715602-1715892
BmHel-11	DF090337:1038485-1038775
BmHel-11	DF090337:2472073-2472363
BmHel-11	DF090337:3920077-3920367
BmHel-11	DF090337:4342865-4343155
BmHel-11	DF090335:2466783-2467073
BmHel-11	DF090335:3104574-3104864
BmHel-11	DF090335:5452816-5453106
BmHel-11	DF090330:2187075-2187365
BmHel-11	DF090330:3744466-3744756
BmHel-11	DF090329:421961-422251
BmHel-11	DF090329:2486397-2486687
BmHel-11	DF090329:3463946-3464236
BmHel-11	DF090344:3963364-3963654
BmHel-11	DF090320:495929-496219
BmHel-11	DF090320:3695692-3695982
BmHel-11	DF090320:4131703-4131993
BmHel-11	DF090412:789374-789664
BmHel-11	DF090379:842530-842820
BmHel-11	DF090366:987235-987525
BmHel-11	DF090366:1178504-1178794
BmHel-11	DF090364:1459174-1459464
BmHel-11	DF090352:3768768-3769058
BmHel-11	DF090317:53084-53374
BmHel-11	DF090317:3009779-3010069
BmHel-11	DF090435:46112-46402
BmHel-11	DF090404:963655-963945

BmHel-11	DF090389:431821-432111
BmHel-11	DF090389:982411-982701
BmHel-11	DF090389:1022207-1022497
BmHel-11	DF090358:1544722-1545012
BmHel-11	DF090356:1508549-1508839
BmHel-11	DF090356:2212418-2212708
BmHel-11	DF090356:2349850-2350140
BmHel-11	DF090434:374858-375148
BmHel-11	DF090345:1351251-1351541
BmHel-11	DF090333:4172780-4173070
BmHel-11	DF090333:5674719-5675009
BmHel-11	DF090327:5788968-5789258
BmHel-11	DF090401:1370898-1371188
BmHel-11	DF090371:1541186-1541476
BmHel-11	DF090371:1933754-1934044
BmHel-11	DF090371:2566932-2567222
BmHel-11	DF090361:368609-368899
BmHel-11	DF090423:650089-650379
BmHel-11	DF090374:336658-336948
BmHel-11	DF090409:393703-393993
BmHel-11	DF090432:343877-344167
BmHel-11	DF090413:1165207-1165497
BmHel-11	DF090756:8244-8534
BmHel-11	DF090425:903398-903688
BmHel-11	DF090703:25357-25647
BmHel-11	DF090456:290548-290838
BmHel-11	BABH01044460:4547-4837
BmHel-11	BABH01051860:411-701
BmHel-11	DF090905:6099-6389
BmHel-11	BABH01055020:332-622
BmHel-11	DF090325:6323387-6323677
BmHel-11	DF090353:3821975-3822265
BmHel-11	DF090339:603137-603427
BmHel-11	DF090339:722972-723262
BmHel-11	DF090339:1818825-1819115
BmHel-11	BABH01046693:2030-2320
BmHel-11	DF090402:89103-89393
BmHel-11	DF090386:1382809-1383099
BmHel-11	DF090386:1671703-1671993
BmHel-11	DF090383:2008803-2009093
BmHel-11	DF090348:3063684-3063974
BmHel-11	DF090328:2002137-2002427
BmHel-11	DF090319:2023340-2023630
BmHel-11	DF090319:3830206-3830496
BmHel-11	DF090357:3657916-3658206
BmHel-11	DF090377:642949-643239
BmHel-11	DF090347:1841387-1841677
BmHel-11	DF090347:4026137-4026427

BmHel-11	DF091137:33385-33675
BmHel-11	DF090322:138182-138472
BmHel-11	DF090322:3485900-3486190
BmHel-11	DF090407:514590-514880
BmHel-11	DF090349:3267782-3268072
BmHel-11	DF090343:12211-12501
BmHel-11	DF090388:1292549-1292839
BmHel-11	DF090351:163046-163336
BmHel-11	DF090337:1559581-1559871
BmHel-11	DF090335:5326726-5327016
BmHel-11	DF090330:1223111-1223401
BmHel-11	DF090323:2091185-2091475
BmHel-11	DF090450:323239-323529
BmHel-11	DF090406:791101-791391
BmHel-11	DF090367:2775689-2775979
BmHel-11	DF090380:1498034-1498324
BmHel-11	DF090320:7087257-7087547
BmHel-11	DF090399:192008-192298
BmHel-11	DF090366:1836608-1836898
BmHel-11	DF090364:339950-340240
BmHel-11	DF090352:2661074-2661364
BmHel-11	DF090352:2703217-2703507
BmHel-11	DF090317:1764616-1764906
BmHel-11	DF090356:167142-167432
BmHel-11	DF090333:2808366-2808656
BmHel-11	DF090333:3682478-3682768
BmHel-11	DF090327:441306-441596
BmHel-11	DF090401:1131684-1131974
BmHel-11	DF090346:1023480-1023770
BmHel-11	DF090403:1066038-1066328
BmHel-11	DF090374:1195438-1195728
BmHel-11	DF090409:1410928-1411218
BmHel-11	DF090394:1370075-1370365
BmHel-11	DF090496:59190-59480
BmHel-11	DF090413:1108691-1108981
BmHel-11	BABH01051688:562-852
BmHel-11	DF090425:193680-193970
BmHel-11	DF090425:436119-436409
BmHel-11	DF090582:42896-43186
BmHel-11	DF090395:974889-975179
BmHel-11	DF090347:524885-525175
BmHel-11	DF090343:4054057-4054347
BmHel-11	DF090345:799762-800052
BmHel-11	DF090338:3068170-3068460
BmHel-11	DF090355:2956287-2956577
BmHel-11	DF090325:4111888-4112178
BmHel-11	DF090325:2795134-2795424
BmHel-11	DF090353:3941847-3942137

BmHel-11	DF090339:2386864-2387154
BmHel-11	DF090362:3147029-3147319
BmHel-11	DF090362:1588700-1588990
BmHel-11	DF090362:302705-302995
BmHel-11	DF090445:34379-34669
BmHel-11	DF090387:439302-439592
BmHel-11	DF090324:4632522-4632812
BmHel-11	DF090324:3419083-3419373
BmHel-11	DF090324:3042129-3042419
BmHel-11	DF090324:703586-703876
BmHel-11	DF090421:919158-919448
BmHel-11	DF090390:1111366-1111656
BmHel-11	DF090390:636184-636474
BmHel-11	DF090342:4110298-4110588
BmHel-11	DF090342:3116324-3116614
BmHel-11	DF090342:2196859-2197149
BmHel-11	DF090336:2221340-2221630
BmHel-11	DF090336:200216-200506
BmHel-11	DF090332:5171929-5172219
BmHel-11	DF090332:5000917-5001207
BmHel-11	DF090318:6490266-6490556
BmHel-11	DF090318:5971520-5971810
BmHel-11	DF090318:4812545-4812835
BmHel-11	DF090318:3501475-3501765
BmHel-11	DF090318:1089709-1089999
BmHel-11	DF090348:3385532-3385822
BmHel-11	DF090348:2372710-2373000
BmHel-11	DF090348:1251799-1252089
BmHel-11	DF090319:5450055-5450345
BmHel-11	DF090357:2094756-2095046
BmHel-11	DF090395:1032676-1032966
BmHel-11	DF090395:657795-658085
BmHel-11	DF090347:992596-992886
BmHel-11	DF090414:1167584-1167874
BmHel-11	DF090365:2932468-2932758
BmHel-11	DF090326:6891205-6891495
BmHel-11	DF090321:257847-258137
BmHel-11	DF090382:2003093-2003383
BmHel-11	DF090359:2811038-2811328
BmHel-11	DF090359:1369694-1369984
BmHel-11	DF090359:773101-773391
BmHel-11	DF090349:1809350-1809640
BmHel-11	DF090343:3249359-3249649
BmHel-11	DF090340:4262704-4262994
BmHel-11	DF090334:5477730-5478020
BmHel-11	DF090334:3843643-3843933
BmHel-11	DF090331:4139637-4139927
BmHel-11	DF090331:1085069-1085359

BmHel-11	DF090316:14597246-14597536
BmHel-11	DF090316:13117417-13117707
BmHel-11	DF090479:290666-290956
BmHel-11	DF090368:1054636-1054926
BmHel-11	DF090335:4269496-4269786
BmHel-11	DF090335:3201109-3201399
BmHel-11	DF090335:445532-445822
BmHel-11	DF090329:2382303-2382593
BmHel-11	DF090329:821999-822289
BmHel-11	DF090323:1378367-1378657
BmHel-11	DF090323:1043345-1043635
BmHel-11	DF090344:4666356-4666646
BmHel-11	DF090344:3293196-3293486
BmHel-11	DF090344:702928-703218
BmHel-11	DF090320:3449103-3449393
BmHel-11	DF090320:1430258-1430548
BmHel-11	DF090379:1777071-1777361
BmHel-11	DF090378:659971-660261
BmHel-11	DF090366:290826-291116
BmHel-11	DF090317:7036057-7036347
BmHel-11	DF090317:6722134-6722424
BmHel-11	DF090459:289467-289757
BmHel-11	DF090435:565025-565315
BmHel-11	DF090372:617818-618108
BmHel-11	DF090356:3194582-3194872
BmHel-11	DF090356:1668439-1668729
BmHel-11	DF090345:4215712-4216002
BmHel-11	DF090345:1602446-1602736
BmHel-11	DF090333:3791331-3791621
BmHel-11	DF090327:2274994-2275284
BmHel-11	DF090398:110360-110650
BmHel-11	DF090371:473667-473957
BmHel-11	DF090416:628047-628337
BmHel-11	DF090403:1141603-1141893
BmHel-11	DF090361:433888-434178
BmHel-11	DF090338:1567874-1568164
BmHel-11	DF090422:339169-339459
BmHel-11	DF090375:2010280-2010570
BmHel-11	DF090415:1004640-1004930
BmHel-11	DF090394:1472671-1472961
BmHel-11	DF090466:156762-157052
BmHel-11	DF090466:113880-114170
BmHel-11	DF090457:218165-218455
BmHel-11	DF090413:1029274-1029564
BmHel-11	DF090408:985084-985374
BmHel-11	DF090510:29872-30162
BmHel-11	BABH01046480:2155-2445
BmHel-11	BABH01044232:10189-10479

BmHel-11	BABH01059727:160-450
BmHel-11	DF090355:3393118-3393408
BmHel-11	DF090353:3378770-3379060
BmHel-11	DF090336:1891220-1891510
BmHel-11	DF090336:1177001-1177291
BmHel-11	DF090318:9021634-9021924
BmHel-11	DF090348:1931146-1931436
BmHel-11	DF090348:899207-899497
BmHel-11	DF090348:237695-237985
BmHel-11	DF090328:3565487-3565777
BmHel-11	DF090319:272034-272324
BmHel-11	DF090377:319231-319521
BmHel-11	DF090360:1488565-1488855
BmHel-11	DF090347:2636934-2637224
BmHel-11	DF090347:343369-343659
BmHel-11	DF090411:1403956-1404246
BmHel-11	DF090411:15699-15989
BmHel-11	DF090392:1571404-1571694
BmHel-11	DF090321:3624572-3624862
BmHel-11	DF090382:847489-847779
BmHel-11	DF090343:4670653-4670943
BmHel-11	DF090343:1769325-1769615
BmHel-11	DF090341:3851581-3851871
BmHel-11	DF090340:3847186-3847476
BmHel-11	DF090340:3375933-3376223
BmHel-11	DF090340:1740467-1740757
BmHel-11	DF090334:2201747-2202037
BmHel-11	DF090331:5712870-5713160
BmHel-11	DF090331:2409301-2409591
BmHel-11	DF090331:1132585-1132875
BmHel-11	DF090316:12613688-12613978
BmHel-11	DF090335:4603597-4603887
BmHel-11	DF090335:2235888-2236178
BmHel-11	DF090330:3257632-3257922
BmHel-11	DF090323:3597290-3597580
BmHel-11	DF090406:705997-706287
BmHel-11	DF090367:2369942-2370232
BmHel-11	DF090344:557362-557652
BmHel-11	DF090320:4412364-4412654
BmHel-11	DF090364:1884275-1884565
BmHel-11	DF090317:7271319-7271609
BmHel-11	DF090317:1646137-1646427
BmHel-11	DF090317:1615473-1615763
BmHel-11	DF090372:1071682-1071972
BmHel-11	DF090358:511967-512257
BmHel-11	DF090327:876652-876942
BmHel-11	DF090401:424924-425214
BmHel-11	DF090361:566199-566489

BmHel-11	DF090338:1370928-1371218
BmHel-11	DF090375:132240-132530
BmHel-11	DF090374:910837-911127
BmHel-11	DF090374:574419-574709
BmHel-11	DF090374:335975-336265
BmHel-11	DF090374:300902-301192
BmHel-11	DF090639:15509-15799
BmHel-11	DF090431:511883-512173
BmHel-11	DF090347:1830372-1830662
BmHel-11	DF090326:2868835-2869125
BmHel-11	DF090316:3541117-3541407
BmHel-11	DF090351:3511675-3511965
BmHel-11	DF090384:1072166-1072456
BmHel-11	DF090417:857409-857699
BmHel-11	DF090510:40901-41191
BmHel-11	DF090355:203977-204268
BmHel-11	DF090355:1606676-1606967
BmHel-11	DF090355:2305882-2306173
BmHel-11	DF090355:2413567-2413858
BmHel-11	DF090355:3336364-3336655
BmHel-11	DF090325:3627924-3628215
BmHel-11	DF090325:4531266-4531557
BmHel-11	DF090325:4914746-4915037
BmHel-11	DF090325:6115096-6115387
BmHel-11	DF090353:29132-29423
BmHel-11	DF090353:974325-974616
BmHel-11	DF090353:1568472-1568763
BmHel-11	DF090353:3230442-3230733
BmHel-11	DF090339:514990-515281
BmHel-11	DF090339:1312947-1313238
BmHel-11	DF090339:1368789-1369080
BmHel-11	DF090339:1602698-1602989
BmHel-11	DF090339:1976612-1976903
BmHel-11	DF090339:2280718-2281009
BmHel-11	DF090339:2996112-2996403
BmHel-11	DF090339:3123865-3124156
BmHel-11	DF090339:3159677-3159968
BmHel-11	DF090362:157720-158011
BmHel-11	DF090362:492705-492996
BmHel-11	DF090387:1690867-1691158
BmHel-11	DF090324:551619-551910
BmHel-11	DF090324:1028774-1029065
BmHel-11	DF090324:2448238-2448529
BmHel-11	DF090324:2461484-2461775
BmHel-11	DF090324:3612512-3612803
BmHel-11	DF090324:5722773-5723064
BmHel-11	BABH01056570:290-581
BmHel-11	DF090421:591787-592078

BmHel-11	DF090421:872751-873042
BmHel-11	DF090421:1058835-1059126
BmHel-11	DF090402:310545-310836
BmHel-11	DF090402:370981-371272
BmHel-11	DF090402:517960-518251
BmHel-11	DF090390:792102-792393
BmHel-11	DF090390:1065487-1065778
BmHel-11	DF090386:140065-140356
BmHel-11	DF090386:831334-831625
BmHel-11	DF090386:1687105-1687396
BmHel-11	DF090381:163783-164074
BmHel-11	DF090381:813305-813596
BmHel-11	DF090381:1053510-1053801
BmHel-11	DF090381:1813524-1813815
BmHel-11	DF090342:987179-987470
BmHel-11	DF090342:1993733-1994024
BmHel-11	DF090342:3253444-3253735
BmHel-11	DF090336:627853-628144
BmHel-11	DF090336:1258540-1258831
BmHel-11	DF090336:1758929-1759220
BmHel-11	DF090336:2836966-2837257
BmHel-11	DF090332:2701861-2702152
BmHel-11	DF090332:2917354-2917645
BmHel-11	DF090332:4971071-4971362
BmHel-11	DF090332:6216400-6216691
BmHel-11	DF090318:315253-315544
BmHel-11	DF090318:2087586-2087877
BmHel-11	DF090318:2411005-2411296
BmHel-11	DF090318:3314644-3314935
BmHel-11	DF090318:3452669-3452960
BmHel-11	DF090318:3586592-3586883
BmHel-11	DF090318:4040471-4040762
BmHel-11	DF090318:4444289-4444580
BmHel-11	DF090318:5918868-5919159
BmHel-11	DF090318:8188947-8189238
BmHel-11	DF090318:9462769-9463060
BmHel-11	DF090383:498108-498399
BmHel-11	DF090383:1089651-1089942
BmHel-11	DF090383:1136990-1137281
BmHel-11	DF090348:1557156-1557447
BmHel-11	DF090348:2909086-2909377
BmHel-11	DF090348:4355825-4356116
BmHel-11	DF090328:1756906-1757197
BmHel-11	DF090328:3594889-3595180
BmHel-11	DF090328:4050170-4050461
BmHel-11	DF090328:4524968-4525259
BmHel-11	DF090328:4739186-4739477
BmHel-11	DF090319:1034803-1035094

BmHel-11	DF090319:1320429-1320720
BmHel-11	DF090319:3032316-3032607
BmHel-11	DF090319:3795636-3795927
BmHel-11	DF090319:7246857-7247148
BmHel-11	DF090483:64332-64623
BmHel-11	DF090433:431201-431492
BmHel-11	DF090693:3734-4025
BmHel-11	DF090395:1002382-1002673
BmHel-11	DF090395:1519432-1519723
BmHel-11	DF090377:462928-463219
BmHel-11	DF090377:1110150-1110441
BmHel-11	DF090377:1422851-1423142
BmHel-11	DF090360:204752-205043
BmHel-11	DF090360:706222-706513
BmHel-11	DF090360:1610003-1610294
BmHel-11	DF090360:2961342-2961633
BmHel-11	DF090347:891088-891379
BmHel-11	DF090414:338830-339121
BmHel-11	DF090411:1061776-1062067
BmHel-11	DF090365:61906-62197
BmHel-11	DF090365:2247098-2247389
BmHel-11	DF090365:2398377-2398668
BmHel-11	DF090326:110303-110594
BmHel-11	DF090326:4577044-4577335
BmHel-11	DF090326:5712559-5712850
BmHel-11	DF090326:6592051-6592342
BmHel-11	DF090322:1550436-1550727
BmHel-11	DF090322:3082654-3082945
BmHel-11	DF090322:3757624-3757915
BmHel-11	DF090322:5332259-5332550
BmHel-11	DF090322:6561845-6562136
BmHel-11	DF090322:8101334-8101625
BmHel-11	DF090321:2720275-2720566
BmHel-11	DF090321:2931841-2932132
BmHel-11	DF090321:3573311-3573602
BmHel-11	DF090321:3801873-3802164
BmHel-11	DF090321:5383214-5383505
BmHel-11	DF090321:6588644-6588935
BmHel-11	DF090350:280079-280370
BmHel-11	DF090350:325823-326114
BmHel-11	DF090350:1259663-1259954
BmHel-11	DF090350:1291094-1291385
BmHel-11	DF090407:92212-92503
BmHel-11	DF090407:272059-272350
BmHel-11	DF090407:964814-965105
BmHel-11	DF090363:1039435-1039726
BmHel-11	DF090363:1137555-1137846
BmHel-11	DF090363:2466769-2467060

BmHel-11	DF090363:2902906-2903197
BmHel-11	DF090359:1318157-1318448
BmHel-11	DF090359:1706551-1706842
BmHel-11	DF090359:2635298-2635589
BmHel-11	DF090359:3050688-3050979
BmHel-11	DF090349:9968-10259
BmHel-11	DF090349:1673624-1673915
BmHel-11	DF090349:2633674-2633965
BmHel-11	DF090349:4014636-4014927
BmHel-11	DF090343:4012239-4012530
BmHel-11	DF090343:4439972-4440263
BmHel-11	DF090341:1210436-1210727
BmHel-11	DF090341:3252168-3252459
BmHel-11	DF090340:1742746-1743037
BmHel-11	DF090340:2453968-2454259
BmHel-11	DF090340:3848251-3848542
BmHel-11	DF090334:906125-906416
BmHel-11	DF090334:1148664-1148955
BmHel-11	DF090334:2456487-2456778
BmHel-11	DF090334:3335043-3335334
BmHel-11	DF090334:4894682-4894973
BmHel-11	DF090334:5322777-5323068
BmHel-11	DF090331:5424728-5425019
BmHel-11	DF090316:725655-725946
BmHel-11	DF090316:751704-751995
BmHel-11	DF090316:1832357-1832648
BmHel-11	DF090316:2674946-2675237
BmHel-11	DF090316:4365960-4366251
BmHel-11	DF090316:4430179-4430470
BmHel-11	DF090316:9916090-9916381
BmHel-11	DF090316:10060189-10060480
BmHel-11	DF090316:10242609-10242900
BmHel-11	DF090316:10864826-10865117
BmHel-11	DF090316:10925322-10925613
BmHel-11	DF090316:11990841-11991132
BmHel-11	DF090316:12173548-12173839
BmHel-11	DF090316:12186326-12186617
BmHel-11	DF090316:12304736-12305027
BmHel-11	DF090806:5147-5438
BmHel-11	DF090525:73909-74200
BmHel-11	DF090502:111154-111445
BmHel-11	DF090479:246406-246697
BmHel-11	DF090424:336587-336878
BmHel-11	DF090424:657197-657488
BmHel-11	DF090400:810889-811180
BmHel-11	DF090400:884269-884560
BmHel-11	DF090391:761041-761332
BmHel-11	DF090388:492817-493108

BmHel-11	DF090388:542114-542405
BmHel-11	DF090388:1653161-1653452
BmHel-11	DF090385:99443-99734
BmHel-11	DF090385:189538-189829
BmHel-11	DF090373:415230-415521
BmHel-11	DF090373:1402468-1402759
BmHel-11	DF090373:1719218-1719509
BmHel-11	DF090373:2049225-2049516
BmHel-11	DF090368:176195-176486
BmHel-11	DF090368:1905546-1905837
BmHel-11	DF090351:1470364-1470655
BmHel-11	DF090351:1485485-1485776
BmHel-11	DF090351:1874548-1874839
BmHel-11	DF090351:2777209-2777500
BmHel-11	DF090337:1332305-1332596
BmHel-11	DF090337:4731491-4731782
BmHel-11	DF090335:936705-936996
BmHel-11	DF090335:1922811-1923102
BmHel-11	DF090335:2439477-2439768
BmHel-11	DF090335:3596672-3596963
BmHel-11	DF090330:466473-466764
BmHel-11	DF090330:1349131-1349422
BmHel-11	DF090330:2017685-2017976
BmHel-11	DF090330:3917664-3917955
BmHel-11	DF090330:3996950-3997241
BmHel-11	DF090330:5559532-5559823
BmHel-11	DF090329:1943022-1943313
BmHel-11	DF090329:5630067-5630358
BmHel-11	DF090329:6092826-6093117
BmHel-11	DF090323:1740202-1740493
BmHel-11	DF090323:5135167-5135458
BmHel-11	DF090450:512411-512702
BmHel-11	DF090367:1289464-1289755
BmHel-11	DF090344:1494475-1494766
BmHel-11	DF090344:2321930-2322221
BmHel-11	DF090344:2514144-2514435
BmHel-11	DF090344:2768786-2769077
BmHel-11	DF090344:3209995-3210286
BmHel-11	DF090380:2012410-2012701
BmHel-11	DF090370:528857-529148
BmHel-11	DF090370:2251216-2251507
BmHel-11	DF090370:2583772-2584063
BmHel-11	DF090320:1796161-1796452
BmHel-11	DF090320:2590683-2590974
BmHel-11	DF090320:2998025-2998316
BmHel-11	DF090320:6149244-6149535
BmHel-11	DF090320:6251771-6252062
BmHel-11	DF090412:174317-174608

BmHel-11	DF090412:1361019-1361310
BmHel-11	DF090399:1509531-1509822
BmHel-11	DF090396:571169-571460
BmHel-11	DF090384:845900-846191
BmHel-11	DF090384:1727927-1728218
BmHel-11	DF090379:237538-237829
BmHel-11	DF090379:386160-386451
BmHel-11	DF090378:283708-283999
BmHel-11	DF090378:352130-352421
BmHel-11	DF090366:303991-304282
BmHel-11	DF090366:875175-875466
BmHel-11	DF090366:1663916-1664207
BmHel-11	DF090366:1896811-1897102
BmHel-11	DF090364:1851715-1852006
BmHel-11	DF090364:2905442-2905733
BmHel-11	DF090364:2918217-2918508
BmHel-11	DF090354:2355453-2355744
BmHel-11	DF090354:2748767-2749058
BmHel-11	DF090352:1657295-1657586
BmHel-11	DF090317:952421-952712
BmHel-11	DF090317:1185164-1185455
BmHel-11	DF090317:2637952-2638243
BmHel-11	DF090317:4068541-4068832
BmHel-11	DF090317:4735926-4736217
BmHel-11	DF090317:7240201-7240492
BmHel-11	DF090317:7466600-7466891
BmHel-11	DF090317:9128745-9129036
BmHel-11	DF090317:9331337-9331628
BmHel-11	DF090317:9410561-9410852
BmHel-11	DF090498:153234-153525
BmHel-11	DF090486:150632-150923
BmHel-11	DF090438:496496-496787
BmHel-11	DF090435:362384-362675
BmHel-11	DF090417:868825-869116
BmHel-11	DF090404:1144619-1144910
BmHel-11	DF090389:1036274-1036565
BmHel-11	DF090372:73225-73516
BmHel-11	DF090372:586952-587243
BmHel-11	DF090372:1835190-1835481
BmHel-11	DF090358:2632380-2632671
BmHel-11	DF090358:3234790-3235081
BmHel-11	DF090356:483903-484194
BmHel-11	DF090434:427866-428157
BmHel-11	DF090376:1404760-1405051
BmHel-11	DF090345:584934-585225
BmHel-11	DF090345:1275813-1276104
BmHel-11	DF090345:2051289-2051580
BmHel-11	DF090333:1513254-1513545

BmHel-11	DF090333:3697268-3697559
BmHel-11	DF090333:4760533-4760824
BmHel-11	DF090333:5374973-5375264
BmHel-11	DF090333:5718756-5719047
BmHel-11	DF090327:2907432-2907723
BmHel-11	DF090327:2924203-2924494
BmHel-11	DF090327:4848801-4849092
BmHel-11	DF090494:101630-101921
BmHel-11	DF090405:699867-700158
BmHel-11	DF090405:1370876-1371167
BmHel-11	DF090405:1409362-1409653
BmHel-11	DF090398:270989-271280
BmHel-11	DF090371:863370-863661
BmHel-11	DF090346:240942-241233
BmHel-11	DF090346:1279061-1279352
BmHel-11	DF090346:3616508-3616799
BmHel-11	DF090346:3648529-3648820
BmHel-11	DF090622:815-1106
BmHel-11	DF090472:58945-59236
BmHel-11	DF090427:899886-900177
BmHel-11	DF090416:649155-649446
BmHel-11	DF090403:820487-820778
BmHel-11	DF090361:207201-207492
BmHel-11	DF090361:2188997-2189288
BmHel-11	DF090361:2432674-2432965
BmHel-11	DF090361:3067928-3068219
BmHel-11	DF090338:6775-7066
BmHel-11	DF090338:753564-753855
BmHel-11	DF090338:1599367-1599658
BmHel-11	DF090338:2264168-2264459
BmHel-11	DF090338:3942029-3942320
BmHel-11	BABH01043610:14232-14523
BmHel-11	DF090671:1907-2198
BmHel-11	DF090477:154491-154782
BmHel-11	DF090462:406020-406311
BmHel-11	DF090428:587191-587482
BmHel-11	DF090428:766833-767124
BmHel-11	DF090397:345052-345343
BmHel-11	DF090374:1025465-1025756
BmHel-11	DF090374:1155868-1156159
BmHel-11	DF090374:1564179-1564470
BmHel-11	DF090449:403102-403393
BmHel-11	DF090449:426209-426500
BmHel-11	DF090449:460779-461070
BmHel-11	DF090449:642386-642677
BmHel-11	DF090420:883547-883838
BmHel-11	DF090409:1484612-1484903
BmHel-11	BABH01048604:859-1150

BmHel-11	DF090439:47779-48070
BmHel-11	DF090394:1175231-1175522
BmHel-11	DF090592:31933-32224
BmHel-11	DF090466:222875-223166
BmHel-11	DF090457:143910-144201
BmHel-11	DF090413:245265-245556
BmHel-11	DF090408:1074631-1074922
BmHel-11	DF090571:37584-37875
BmHel-11	DF090460:147092-147383
BmHel-11	DF090448:684963-685254
BmHel-11	DF090419:686757-687048
BmHel-11	DF090419:838408-838699
BmHel-11	DF090369:1489948-1490239
BmHel-11	DF090369:1770043-1770334
BmHel-11	DF090369:1810512-1810803
BmHel-11	DF090369:2494130-2494421
BmHel-11	DF090447:325702-325993
BmHel-11	DF090518:82916-83207
BmHel-11	DF090461:268341-268632
BmHel-11	DF090461:324142-324433
BmHel-11	DF090455:197972-198263
BmHel-11	DF091903:103-394
BmHel-11	BABH01045079:1512-1803
BmHel-11	BABH01049118:437-728
BmHel-11	BABH01048951:412-703
BmHel-11	DF090528:17545-17836
BmHel-11	DF090383:1113048-1113339
BmHel-11	DF090395:1212397-1212688
BmHel-11	DF090359:1902700-1902991
BmHel-11	DF090330:3828195-3828486
BmHel-11	DF090330:6262411-6262702
BmHel-11	DF090320:2269155-2269446
BmHel-11	DF090317:8902871-8903162
BmHel-11	DF090333:5460322-5460613
BmHel-11	DF090338:659868-660159
BmHel-11	DF090338:4297514-4297805
BmHel-11	DF090504:100226-100517
BmHel-11	DF090394:1399357-1399648
BmHel-11	DF091225:12075-12366
BmHel-11	DF090808:8200-8491
BmHel-11	DF090355:1464891-1465182
BmHel-11	DF090333:3376439-3376730
BmHel-11	DF090375:1124565-1124856
BmHel-11	DF090355:2980629-2980920
BmHel-11	DF090325:5150511-5150802
BmHel-11	DF090325:4930607-4930898
BmHel-11	DF090325:4467458-4467749
BmHel-11	DF090325:4139001-4139292

BmHel-11	DF090325:2438342-2438633
BmHel-11	DF090353:3634623-3634914
BmHel-11	DF090353:3544694-3544985
BmHel-11	DF090353:2667881-2668172
BmHel-11	DF090353:1529745-1530036
BmHel-11	DF090353:1200848-1201139
BmHel-11	DF090353:501350-501641
BmHel-11	DF090339:4441149-4441440
BmHel-11	DF090339:2555568-2555859
BmHel-11	DF090339:1283273-1283564
BmHel-11	DF090362:2261781-2262072
BmHel-11	DF090362:2133014-2133305
BmHel-11	DF090362:1953510-1953801
BmHel-11	DF090362:782546-782837
BmHel-11	DF090362:494058-494349
BmHel-11	DF090387:998553-998844
BmHel-11	DF090324:7284792-7285083
BmHel-11	DF090324:1833010-1833301
BmHel-11	DF090421:43430-43721
BmHel-11	DF090402:1303591-1303882
BmHel-11	DF090402:1174917-1175208
BmHel-11	DF090402:952420-952711
BmHel-11	DF090390:1335949-1336240
BmHel-11	DF090386:1754743-1755034
BmHel-11	DF090381:1815759-1816050
BmHel-11	DF090381:1583398-1583689
BmHel-11	DF090381:936016-936307
BmHel-11	DF090381:726379-726670
BmHel-11	DF090381:686165-686456
BmHel-11	DF090342:3610411-3610702
BmHel-11	DF090342:3409641-3409932
BmHel-11	DF090342:1753877-1754168
BmHel-11	DF090342:1559209-1559500
BmHel-11	DF090342:1512214-1512505
BmHel-11	DF090342:1120367-1120658
BmHel-11	DF090342:408948-409239
BmHel-11	DF090336:4987895-4988186
BmHel-11	DF090336:4838865-4839156
BmHel-11	DF090336:2841850-2842141
BmHel-11	DF090332:2814782-2815073
BmHel-11	DF090318:8846686-8846977
BmHel-11	DF090318:5035564-5035855
BmHel-11	DF090318:3396613-3396904
BmHel-11	DF090318:732956-733247
BmHel-11	DF090318:138210-138501
BmHel-11	DF090348:1899427-1899718
BmHel-11	DF090328:4133111-4133402
BmHel-11	DF090328:3900944-3901235

BmHel-11	DF090328:2974798-2975089
BmHel-11	DF090328:1421946-1422237
BmHel-11	DF090328:693149-693440
BmHel-11	DF090328:88297-88588
BmHel-11	DF090319:7643934-7644225
BmHel-11	DF090319:5596545-5596836
BmHel-11	DF090319:3653561-3653852
BmHel-11	DF090319:2205858-2206149
BmHel-11	DF090319:1409113-1409404
BmHel-11	DF090357:3451161-3451452
BmHel-11	DF090357:2075854-2076145
BmHel-11	DF090487:146870-147161
BmHel-11	DF090487:75003-75294
BmHel-11	DF090395:468506-468797
BmHel-11	DF090377:1284204-1284495
BmHel-11	DF090377:1009521-1009812
BmHel-11	DF090377:692032-692323
BmHel-11	DF090360:469536-469827
BmHel-11	DF090347:2252178-2252469
BmHel-11	DF090347:1743258-1743549
BmHel-11	DF090347:1334078-1334369
BmHel-11	DF090347:1120974-1121265
BmHel-11	DF090411:930170-930461
BmHel-11	DF090392:1696381-1696672
BmHel-11	DF090392:356618-356909
BmHel-11	DF090365:2146206-2146497
BmHel-11	DF090365:1304140-1304431
BmHel-11	DF090365:677720-678011
BmHel-11	DF090365:649586-649877
BmHel-11	DF090326:6534144-6534435
BmHel-11	DF090326:5561544-5561835
BmHel-11	DF090326:5405242-5405533
BmHel-11	DF090326:4922420-4922711
BmHel-11	DF090326:3825909-3826200
BmHel-11	DF090326:2316912-2317203
BmHel-11	DF090326:1682813-1683104
BmHel-11	DF090326:436680-436971
BmHel-11	DF090322:5614752-5615043
BmHel-11	DF090322:5528295-5528586
BmHel-11	DF090322:5304625-5304916
BmHel-11	DF090322:3769718-3770009
BmHel-11	DF090322:3591117-3591408
BmHel-11	DF090322:1598982-1599273
BmHel-11	DF090321:6701638-6701929
BmHel-11	DF090321:5126012-5126303
BmHel-11	DF090321:4246759-4247050
BmHel-11	DF090321:3952993-3953284
BmHel-11	DF090321:838700-838991

BmHel-11	DF090410:408070-408361
BmHel-11	DF090363:2536464-2536755
BmHel-11	DF090363:591588-591879
BmHel-11	DF090363:204421-204712
BmHel-11	DF090359:1696375-1696666
BmHel-11	DF090359:1140029-1140320
BmHel-11	DF090359:266385-266676
BmHel-11	DF090349:2520746-2521037
BmHel-11	DF090349:2297704-2297995
BmHel-11	DF090349:1792297-1792588
BmHel-11	DF090349:1079653-1079944
BmHel-11	DF090349:204081-204372
BmHel-11	DF090343:4651724-4652015
BmHel-11	DF090343:4536100-4536391
BmHel-11	DF090343:2650064-2650355
BmHel-11	DF090343:1919046-1919337
BmHel-11	DF090343:763999-764290
BmHel-11	DF090341:2615188-2615479
BmHel-11	DF090341:345000-345291
BmHel-11	DF090340:3806540-3806831
BmHel-11	DF090340:3089388-3089679
BmHel-11	DF090340:2892492-2892783
BmHel-11	DF090340:1415348-1415639
BmHel-11	DF090340:1757-2048
BmHel-11	DF090334:4168751-4169042
BmHel-11	DF090331:4776044-4776335
BmHel-11	DF090331:4157253-4157544
BmHel-11	DF090331:3700932-3701223
BmHel-11	DF090331:2096031-2096322
BmHel-11	DF090316:15561306-15561597
BmHel-11	DF090316:14709892-14710183
BmHel-11	DF090316:14532981-14533272
BmHel-11	DF090316:9064310-9064601
BmHel-11	DF090316:7976948-7977239
BmHel-11	DF090316:4997479-4997770
BmHel-11	DF090316:4465322-4465613
BmHel-11	DF090316:3715387-3715678
BmHel-11	DF090316:2892160-2892451
BmHel-11	DF090316:2198791-2199082
BmHel-11	DF090418:1132770-1133061
BmHel-11	DF090418:978832-979123
BmHel-11	DF090400:1384121-1384412
BmHel-11	DF090400:1207552-1207843
BmHel-11	DF090400:780872-781163
BmHel-11	DF090400:288581-288872
BmHel-11	DF090391:250276-250567
BmHel-11	DF090391:171600-171891
BmHel-11	DF090388:1132571-1132862

BmHel-11	DF090388:517179-517470
BmHel-11	DF090385:339643-339934
BmHel-11	DF090373:1991569-1991860
BmHel-11	DF090373:659711-660002
BmHel-11	DF090368:2350575-2350866
BmHel-11	DF090368:2025820-2026111
BmHel-11	DF090368:1185522-1185813
BmHel-11	DF090351:4039371-4039662
BmHel-11	DF090351:2342028-2342319
BmHel-11	DF090337:3692115-3692406
BmHel-11	DF090335:4553003-4553294
BmHel-11	DF090335:3297603-3297894
BmHel-11	DF090330:5872803-5873094
BmHel-11	DF090330:3466153-3466444
BmHel-11	DF090330:1711108-1711399
BmHel-11	DF090330:604529-604820
BmHel-11	DF090329:6265323-6265614
BmHel-11	DF090329:5337487-5337778
BmHel-11	DF090329:4826737-4827028
BmHel-11	DF090329:4571591-4571882
BmHel-11	DF090329:4400755-4401046
BmHel-11	DF090329:4206580-4206871
BmHel-11	DF090329:3133557-3133848
BmHel-11	DF090329:2843516-2843807
BmHel-11	DF090329:2404895-2405186
BmHel-11	DF090329:84173-84464
BmHel-11	DF090323:8000185-8000476
BmHel-11	DF090323:7640924-7641215
BmHel-11	DF090323:7066588-7066879
BmHel-11	DF090323:2987617-2987908
BmHel-11	DF090323:2436644-2436935
BmHel-11	DF090323:1958500-1958791
BmHel-11	DF090323:1805325-1805616
BmHel-11	DF090323:1342217-1342508
BmHel-11	DF090323:437263-437554
BmHel-11	DF090323:392235-392526
BmHel-11	DF090450:247397-247688
BmHel-11	DF090367:923498-923789
BmHel-11	DF090367:670160-670451
BmHel-11	DF090367:376408-376699
BmHel-11	DF090344:2368956-2369247
BmHel-11	DF090380:1894651-1894942
BmHel-11	DF090380:1726121-1726412
BmHel-11	DF090380:419822-420113
BmHel-11	DF090370:2408579-2408870
BmHel-11	DF090370:1562845-1563136
BmHel-11	DF090370:1323758-1324049
BmHel-11	DF090370:269435-269726

BmHel-11	DF090320:7630464-7630755
BmHel-11	DF090320:7616896-7617187
BmHel-11	DF090320:6799149-6799440
BmHel-11	DF090320:6757489-6757780
BmHel-11	DF090320:5341014-5341305
BmHel-11	DF090320:1248355-1248646
BmHel-11	DF090442:257871-258162
BmHel-11	DF090412:317134-317425
BmHel-11	DF090396:1135920-1136211
BmHel-11	DF090384:582140-582431
BmHel-11	DF090379:1404343-1404634
BmHel-11	DF090379:1318399-1318690
BmHel-11	DF090379:632891-633182
BmHel-11	DF090378:1122676-1122967
BmHel-11	DF090366:1746168-1746459
BmHel-11	DF090366:1458942-1459233
BmHel-11	DF090366:884840-885131
BmHel-11	DF090366:631429-631720
BmHel-11	DF090366:268577-268868
BmHel-11	DF090366:241022-241313
BmHel-11	DF090364:2499432-2499723
BmHel-11	DF090364:836010-836301
BmHel-11	DF090354:534581-534872
BmHel-11	DF090352:3930864-3931155
BmHel-11	DF090352:641190-641481
BmHel-11	DF090317:11131496-11131787
BmHel-11	DF090317:7815507-7815798
BmHel-11	DF090317:7464420-7464711
BmHel-11	DF090317:7219728-7220019
BmHel-11	DF090317:5565159-5565450
BmHel-11	DF090317:4067671-4067962
BmHel-11	DF090317:2016918-2017209
BmHel-11	DF090317:1668099-1668390
BmHel-11	BABH01051867:125-416
BmHel-11	DF090486:160162-160453
BmHel-11	DF090468:265359-265650
BmHel-11	DF090468:84908-85199
BmHel-11	DF090459:331839-332130
BmHel-11	DF090438:674981-675272
BmHel-11	DF090404:546029-546320
BmHel-11	DF090389:996346-996637
BmHel-11	DF090372:2322026-2322317
BmHel-11	DF090372:2078263-2078554
BmHel-11	DF090372:1936270-1936561
BmHel-11	DF090372:947785-948076
BmHel-11	DF090358:3320181-3320472
BmHel-11	DF090358:997095-997386
BmHel-11	DF090358:385421-385712

BmHel-11	DF090573:21627-21918
BmHel-11	DF090376:2219536-2219827
BmHel-11	DF090345:3243739-3244030
BmHel-11	DF090333:5058649-5058940
BmHel-11	DF090333:4890047-4890338
BmHel-11	DF090333:1998780-1999071
BmHel-11	DF090333:1532568-1532859
BmHel-11	DF090333:1320961-1321252
BmHel-11	DF090333:38795-39086
BmHel-11	DF090327:5074019-5074310
BmHel-11	DF090327:832013-832304
BmHel-11	DF090327:123625-123916
BmHel-11	DF090398:1133591-1133882
BmHel-11	DF090398:817248-817539
BmHel-11	DF090371:2031396-2031687
BmHel-11	DF090371:1243222-1243513
BmHel-11	DF090371:682291-682582
BmHel-11	DF090371:308110-308401
BmHel-11	DF090371:175125-175416
BmHel-11	DF090346:4039659-4039950
BmHel-11	DF090346:3910327-3910618
BmHel-11	DF090346:1217339-1217630
BmHel-11	DF090427:565726-566017
BmHel-11	DF090427:444496-444787
BmHel-11	DF090427:294711-295002
BmHel-11	DF090416:854519-854810
BmHel-11	DF090403:1161596-1161887
BmHel-11	DF090361:1837431-1837722
BmHel-11	DF090361:772191-772482
BmHel-11	DF090338:2171369-2171660
BmHel-11	DF090338:2010024-2010315
BmHel-11	DF090338:1049982-1050273
BmHel-11	BABH01048401:715-1006
BmHel-11	DF090895:24407-24698
BmHel-11	DF090536:25113-25404
BmHel-11	DF090428:366997-367288
BmHel-11	DF090422:135959-136250
BmHel-11	DF090422:95911-96202
BmHel-11	DF090397:997519-997810
BmHel-11	DF090397:640296-640587
BmHel-11	DF090374:212688-212979
BmHel-11	DF090449:432001-432292
BmHel-11	DF090420:424724-425015
BmHel-11	DF090409:622808-623099
BmHel-11	BABH01059575:117-408
BmHel-11	DF090758:3667-3958
BmHel-11	DF090484:128054-128345
BmHel-11	DF090446:368885-369176

BmHel-11	DF090432:609806-610097
BmHel-11	DF090432:576081-576372
BmHel-11	DF090394:956155-956446
BmHel-11	DF090394:542740-543031
BmHel-11	DF090496:36711-37002
BmHel-11	DF090443:539194-539485
BmHel-11	DF090426:558176-558467
BmHel-11	DF090426:279212-279503
BmHel-11	DF090408:41639-41930
BmHel-11	DF090441:48738-49029
BmHel-11	DF090590:34908-35199
BmHel-11	DF090448:367131-367422
BmHel-11	DF090429:565157-565448
BmHel-11	DF090369:2317575-2317866
BmHel-11	DF090369:1699831-1700122
BmHel-11	DF090369:556503-556794
BmHel-11	DF090458:18246-18537
BmHel-11	DF090447:213948-214239
BmHel-11	DF090431:325850-326141
BmHel-11	DF090683:13520-13811
BmHel-11	DF090570:218-509
BmHel-11	DF091135:6158-6449
BmHel-11	DF090518:11026-11317
BmHel-11	DF090495:10431-10722
BmHel-11	DF090425:490201-490492
BmHel-11	DF090543:44270-44561
BmHel-11	DF090822:13231-13522
BmHel-11	BABH01045038:3715-4006
BmHel-11	DF090362:644128-644419
BmHel-11	DF090321:7655432-7655723
BmHel-11	DF090382:533429-533720
BmHel-11	DF090363:1485401-1485692
BmHel-11	DF090363:1441867-1442158
BmHel-11	DF090341:1705218-1705509
BmHel-11	DF090373:132637-132928
BmHel-11	DF090368:2428128-2428419
BmHel-11	DF090351:1413777-1414068
BmHel-11	DF090370:1771510-1771801
BmHel-11	DF090317:2336419-2336710
BmHel-11	DF090356:3426693-3426984
BmHel-11	DF090401:480608-480899
BmHel-11	DF090369:853665-853956
BmHel-11	DF091063:960-1251
BmHel-11	DF090353:2249394-2249685
BmHel-11	DF090340:1727224-1727515
BmHel-11	DF090316:13085312-13085603
BmHel-11	DF090351:1261938-1262229
BmHel-11	DF090353:118344-118636

BmHel-11	DF090353:1579253-1579545
BmHel-11	DF090339:989594-989886
BmHel-11	DF090362:2303092-2303384
BmHel-11	DF090387:1304590-1304882
BmHel-11	DF090324:7810170-7810462
BmHel-11	DF090390:1368203-1368495
BmHel-11	DF090390:1641132-1641424
BmHel-11	DF090386:313219-313511
BmHel-11	DF090342:422884-423176
BmHel-11	DF090342:4626313-4626605
BmHel-11	DF090336:3749197-3749489
BmHel-11	DF090332:1191073-1191365
BmHel-11	DF090332:1537714-1538006
BmHel-11	DF090332:1888903-1889195
BmHel-11	DF090332:4941068-4941360
BmHel-11	DF090318:1905392-1905684
BmHel-11	DF090318:6254464-6254756
BmHel-11	DF090318:7958469-7958761
BmHel-11	DF090348:991143-991435
BmHel-11	DF090348:2612799-2613091
BmHel-11	DF090328:419142-419434
BmHel-11	DF090319:882598-882890
BmHel-11	DF090319:7662533-7662825
BmHel-11	DF090357:178832-179124
BmHel-11	DF090357:608291-608583
BmHel-11	DF090357:1009324-1009616
BmHel-11	DF090347:102449-102741
BmHel-11	DF090347:3397122-3397414
BmHel-11	DF090347:4086883-4087175
BmHel-11	DF090411:840479-840771
BmHel-11	DF090411:1136317-1136609
BmHel-11	DF090326:2057245-2057537
BmHel-11	DF090322:2926664-2926956
BmHel-11	DF090322:4816788-4817080
BmHel-11	DF090322:8150097-8150389
BmHel-11	DF090350:1861314-1861606
BmHel-11	DF090463:240029-240321
BmHel-11	DF090382:1909358-1909650
BmHel-11	DF090359:1830534-1830826
BmHel-11	DF090349:1190702-1190994
BmHel-11	DF090349:1838782-1839074
BmHel-11	DF090349:3915711-3916003
BmHel-11	DF090349:3955965-3956257
BmHel-11	DF090343:494449-494741
BmHel-11	DF090341:3981967-3982259
BmHel-11	DF090340:483204-483496
BmHel-11	DF090340:1003155-1003447
BmHel-11	DF090331:4685439-4685731

BmHel-11	DF090316:6763257-6763549
BmHel-11	DF090316:8917091-8917383
BmHel-11	DF090316:9790731-9791023
BmHel-11	DF090391:278875-279167
BmHel-11	DF090388:615650-615942
BmHel-11	DF090388:1440267-1440559
BmHel-11	DF090373:726007-726299
BmHel-11	DF090351:3442827-3443119
BmHel-11	DF090351:4034115-4034407
BmHel-11	DF090335:4471645-4471937
BmHel-11	DF090330:3442314-3442606
BmHel-11	DF090330:5145024-5145316
BmHel-11	DF090344:500548-500840
BmHel-11	DF090344:863369-863661
BmHel-11	DF090344:2695467-2695759
BmHel-11	DF090370:2556442-2556734
BmHel-11	DF090320:3023095-3023387
BmHel-11	DF090320:3596684-3596976
BmHel-11	DF090577:34966-35258
BmHel-11	DF090451:313094-313386
BmHel-11	DF090396:87915-88207
BmHel-11	DF090396:1464603-1464895
BmHel-11	DF090384:333056-333348
BmHel-11	DF090384:478329-478621
BmHel-11	DF090384:1987846-1988138
BmHel-11	DF090379:334274-334566
BmHel-11	DF090364:2648583-2648875
BmHel-11	DF090364:2665901-2666193
BmHel-11	DF090354:1122106-1122398
BmHel-11	DF090354:2961069-2961361
BmHel-11	DF090352:343311-343603
BmHel-11	DF090352:565969-566261
BmHel-11	DF090352:1510301-1510593
BmHel-11	DF090352:2929980-2930272
BmHel-11	DF090352:3168649-3168941
BmHel-11	DF090317:7029131-7029423
BmHel-11	DF090438:1316-1608
BmHel-11	DF090438:446004-446296
BmHel-11	DF090356:2062557-2062849
BmHel-11	DF090333:3878316-3878608
BmHel-11	DF090333:4271368-4271660
BmHel-11	DF090333:5107412-5107704
BmHel-11	DF090327:5682082-5682374
BmHel-11	DF090371:886632-886924
BmHel-11	DF090472:88387-88679
BmHel-11	DF090361:2639654-2639946
BmHel-11	DF090422:865267-865559
BmHel-11	DF090375:1300622-1300914

BmHel-11	DF090375:1736824-1737116
BmHel-11	DF090437:493975-494267
BmHel-11	DF090415:206963-207255
BmHel-11	DF090898:1218-1510
BmHel-11	DF090503:49463-49755
BmHel-11	DF090441:683544-683836
BmHel-11	DF090539:18333-18625
BmHel-11	BABH01046158:55-347
BmHel-11	DF090473:156832-157124
BmHel-11	DF090473:237643-237935
BmHel-11	DF090473:352583-352875
BmHel-11	DF090425:763182-763474
BmHel-11	DF090425:854932-855224
BmHel-11	DF090716:8510-8802
BmHel-11	DF090336:4643802-4644094
BmHel-11	DF090318:5048016-5048308
BmHel-11	DF090360:3203200-3203492
BmHel-11	DF090411:1443679-1443971
BmHel-11	DF090340:4008038-4008330
BmHel-11	DF090340:4617367-4617659
BmHel-11	DF090340:4637000-4637292
BmHel-11	DF090334:5365990-5366282
BmHel-11	DF090335:5264371-5264663
BmHel-11	DF090358:2439956-2440248
BmHel-11	DF090355:110742-111034
BmHel-11	DF090325:5528833-5529125
BmHel-11	DF090325:4540131-4540423
BmHel-11	DF090325:3167571-3167863
BmHel-11	DF090325:230453-230745
BmHel-11	DF090353:1062369-1062661
BmHel-11	DF090339:2644624-2644916
BmHel-11	DF090324:4067512-4067804
BmHel-11	DF090324:3256593-3256885
BmHel-11	DF090386:56850-57142
BmHel-11	DF090342:1971479-1971771
BmHel-11	DF090342:658056-658348
BmHel-11	DF090332:3572519-3572811
BmHel-11	DF090318:7743285-7743577
BmHel-11	DF090318:1026795-1027087
BmHel-11	DF090348:2519064-2519356
BmHel-11	DF090348:130402-130694
BmHel-11	DF090328:5554150-5554442
BmHel-11	DF090328:5382789-5383081
BmHel-11	DF090328:5180476-5180768
BmHel-11	DF090328:1646681-1646973
BmHel-11	DF090328:708555-708847
BmHel-11	DF090319:6184010-6184302
BmHel-11	DF090357:629446-629738

BmHel-11	DF090357:549088-549380
BmHel-11	DF090377:1331685-1331977
BmHel-11	DF090347:1844806-1845098
BmHel-11	DF090365:460848-461140
BmHel-11	DF090326:3085182-3085474
BmHel-11	DF090322:7596629-7596921
BmHel-11	DF090322:6957359-6957651
BmHel-11	DF090322:4495279-4495571
BmHel-11	DF090322:940956-941248
BmHel-11	DF090350:2548781-2549073
BmHel-11	BABH01046700:1091-1383
BmHel-11	DF090469:353539-353831
BmHel-11	DF090407:26751-27043
BmHel-11	DF090382:1701614-1701906
BmHel-11	DF090382:1659659-1659951
BmHel-11	DF090363:2603548-2603840
BmHel-11	DF090363:2129608-2129900
BmHel-11	DF090349:3988935-3989227
BmHel-11	DF090349:3606568-3606860
BmHel-11	DF090349:3061649-3061941
BmHel-11	DF090349:2400540-2400832
BmHel-11	DF090349:400724-401016
BmHel-11	DF090343:3370761-3371053
BmHel-11	DF090343:3204154-3204446
BmHel-11	DF090343:1509912-1510204
BmHel-11	DF090341:1472902-1473194
BmHel-11	DF090340:4342971-4343263
BmHel-11	DF090316:13671267-13671559
BmHel-11	DF090316:13346008-13346300
BmHel-11	DF090316:10551283-10551575
BmHel-11	DF090373:362752-363044
BmHel-11	DF090337:4563681-4563973
BmHel-11	DF090329:5278922-5279214
BmHel-11	DF090329:685559-685851
BmHel-11	DF090344:3734199-3734491
BmHel-11	DF090370:627611-627903
BmHel-11	DF090320:6202241-6202533
BmHel-11	DF090320:5889555-5889847
BmHel-11	DF090396:1475232-1475524
BmHel-11	DF090384:1277917-1278209
BmHel-11	DF090366:104414-104706
BmHel-11	DF090364:2837765-2838057
BmHel-11	DF090364:736966-737258
BmHel-11	DF090354:2637130-2637422
BmHel-11	DF090354:2601167-2601459
BmHel-11	DF090354:1260393-1260685
BmHel-11	DF090352:616417-616709
BmHel-11	DF090317:10523469-10523761

BmHel-11	DF090317:9465081-9465373
BmHel-11	DF090435:92512-92804
BmHel-11	DF090389:23696-23988
BmHel-11	DF090358:116375-116667
BmHel-11	DF090356:3408119-3408411
BmHel-11	DF090434:133188-133480
BmHel-11	DF090345:2742643-2742935
BmHel-11	DF090333:3501619-3501911
BmHel-11	DF090333:855657-855949
BmHel-11	DF090405:622595-622887
BmHel-11	DF090371:2240714-2241006
BmHel-11	DF090346:3934685-3934977
BmHel-11	DF090346:1855192-1855484
BmHel-11	DF090346:4003-4295
BmHel-11	DF090416:672074-672366
BmHel-11	DF090361:3211435-3211727
BmHel-11	DF090423:50411-50703
BmHel-11	DF090422:392534-392826
BmHel-11	DF090375:291656-291948
BmHel-11	DF090375:105564-105856
BmHel-11	DF090397:1368390-1368682
BmHel-11	DF090374:975660-975952
BmHel-11	DF090449:453124-453416
BmHel-11	DF090420:322771-323063
BmHel-11	DF090415:312062-312354
BmHel-11	DF090439:500575-500867
BmHel-11	DF090517:44907-45199
BmHel-11	DF090644:90892-91184
BmHel-11	DF090728:19907-20199
BmHel-11	DF090447:490304-490596
BmHel-11	DF090478:58799-59091
BmHel-11	DF090579:53028-53320
BmHel-11	DF090325:3722880-3723172
BmHel-11	DF090339:1824563-1824855
BmHel-11	DF090342:4009283-4009575
BmHel-11	DF090318:6811831-6812123
BmHel-11	DF090319:2124913-2125205
BmHel-11	DF090316:372986-373278
BmHel-11	DF090378:534109-534401
BmHel-11	DF090317:4608348-4608640
BmHel-11	DF090358:2032575-2032867
BmHel-11	DF090467:248739-249031
BmHel-11	DF090454:451139-451431
BmHel-11	BABH01050185:238-530
BmHel-11	DF090355:3274167-3274460
BmHel-11	DF090325:3398946-3399239
BmHel-11	DF090325:4356536-4356829
BmHel-11	DF090339:4562544-4562837

BmHel-11	DF090339:4895982-4896275
BmHel-11	DF090324:2686962-2687255
BmHel-11	DF090324:3285621-3285914
BmHel-11	DF090421:321657-321950
BmHel-11	DF090342:1475029-1475322
BmHel-11	DF090342:1562645-1562938
BmHel-11	DF090342:2183122-2183415
BmHel-11	DF090336:1603800-1604093
BmHel-11	DF090336:2735060-2735353
BmHel-11	DF090332:5792693-5792986
BmHel-11	DF090318:3656422-3656715
BmHel-11	DF090383:152948-153241
BmHel-11	DF090348:10698-10991
BmHel-11	DF090348:1368444-1368737
BmHel-11	DF090319:4385818-4386111
BmHel-11	DF090414:711722-712015
BmHel-11	DF090321:1768856-1769149
BmHel-11	DF090407:715789-716082
BmHel-11	DF090349:3849075-3849368
BmHel-11	DF090343:3217000-3217293
BmHel-11	DF090340:801325-801618
BmHel-11	DF090340:3698767-3699060
BmHel-11	DF090334:4543973-4544266
BmHel-11	DF090331:5920145-5920438
BmHel-11	DF090316:8933897-8934190
BmHel-11	DF090316:9473244-9473537
BmHel-11	DF090418:1162931-1163224
BmHel-11	DF090351:1780998-1781291
BmHel-11	DF090351:1990211-1990504
BmHel-11	DF090337:3158878-3159171
BmHel-11	DF090337:4831767-4832060
BmHel-11	DF090330:1177126-1177419
BmHel-11	DF090330:4524368-4524661
BmHel-11	DF090329:2411407-2411700
BmHel-11	DF090329:6076966-6077259
BmHel-11	DF090367:2377952-2378245
BmHel-11	DF090344:1048140-1048433
BmHel-11	DF090344:4277683-4277976
BmHel-11	DF090370:197626-197919
BmHel-11	DF090320:3679630-3679923
BmHel-11	DF090320:5002740-5003033
BmHel-11	DF090396:667305-667598
BmHel-11	DF090384:416922-417215
BmHel-11	DF090378:483330-483623
BmHel-11	DF090354:382700-382993
BmHel-11	DF090354:2923267-2923560
BmHel-11	DF090317:6061459-6061752
BmHel-11	DF090317:7102725-7103018

BmHel-11	DF090404:1125063-1125356
BmHel-11	DF090389:1162438-1162731
BmHel-11	DF090356:1732354-1732647
BmHel-11	DF090467:282754-283047
BmHel-11	DF090327:4409169-4409462
BmHel-11	DF090361:531037-531330
BmHel-11	DF090361:2079168-2079461
BmHel-11	DF090361:2145339-2145632
BmHel-11	DF090338:1323882-1324175
BmHel-11	DF090369:693811-694104
BmHel-11	DF090580:43789-44082
BmHel-11	DF090699:21088-21381
BmHel-11	DF090431:772614-772907
BmHel-11	DF090579:48401-48694
BmHel-11	DF090334:4339059-4339352
BmHel-11	DF090355:2501864-2502157
BmHel-11	DF090325:1659778-1660071
BmHel-11	DF090362:2019121-2019414
BmHel-11	DF090324:7708237-7708530
BmHel-11	DF090324:6954201-6954494
BmHel-11	DF090421:746630-746923
BmHel-11	DF090386:1908842-1909135
BmHel-11	DF090386:937380-937673
BmHel-11	DF090381:1996727-1997020
BmHel-11	DF090336:5464902-5465195
BmHel-11	DF090336:3831862-3832155
BmHel-11	DF090336:729807-730100
BmHel-11	DF090332:1564382-1564675
BmHel-11	DF090383:1855117-1855410
BmHel-11	DF090383:452405-452698
BmHel-11	DF090348:973872-974165
BmHel-11	DF090319:3666161-3666454
BmHel-11	DF090483:94607-94900
BmHel-11	DF090357:2882855-2883148
BmHel-11	DF090357:860047-860340
BmHel-11	DF090395:1192201-1192494
BmHel-11	DF090365:2824699-2824992
BmHel-11	DF090365:787847-788140
BmHel-11	DF090326:4530785-4531078
BmHel-11	DF090322:8194885-8195178
BmHel-11	DF090322:6760679-6760972
BmHel-11	DF090322:6550106-6550399
BmHel-11	DF090321:5073369-5073662
BmHel-11	DF090321:4281247-4281540
BmHel-11	DF090321:4143775-4144068
BmHel-11	DF090349:4175801-4176094
BmHel-11	DF090340:1218060-1218353
BmHel-11	DF090334:2312592-2312885

BmHel-11	DF090331:5084637-5084930
BmHel-11	DF090316:12665366-12665659
BmHel-11	DF090316:2126757-2127050
BmHel-11	DF090316:1796005-1796298
BmHel-11	DF090316:1402566-1402859
BmHel-11	DF090418:1047406-1047699
BmHel-11	DF090400:933182-933475
BmHel-11	DF090400:480392-480685
BmHel-11	DF090385:316680-316973
BmHel-11	DF090351:640802-641095
BmHel-11	DF090337:4959687-4959980
BmHel-11	DF090330:2386252-2386545
BmHel-11	DF090329:5967183-5967476
BmHel-11	DF090323:6531392-6531685
BmHel-11	DF090367:1379558-1379851
BmHel-11	DF090367:151612-151905
BmHel-11	DF090380:867846-868139
BmHel-11	DF090320:1927941-1928234
BmHel-11	DF090354:1514212-1514505
BmHel-11	DF090317:9313576-9313869
BmHel-11	DF090358:3254016-3254309
BmHel-11	DF090358:2018940-2019233
BmHel-11	DF090333:5414265-5414558
BmHel-11	DF090333:4116801-4117094
BmHel-11	DF090405:301609-301902
BmHel-11	DF090346:3460589-3460882
BmHel-11	DF090338:2939346-2939639
BmHel-11	DF090439:108929-109222
BmHel-11	DF090432:445319-445612
BmHel-11	DF090426:923183-923476
BmHel-11	DF090431:359302-359595
BmHel-11	DF090425:323038-323331
BmHel-11	DF091269:3090-3383
BmHel-11	DF090519:27934-28227
BmHel-11	BABH01050537:146-439
BmHel-11	BABH01045809:756-1049
BmHel-11	DF090381:978813-979106
BmHel-11	DF090393:1275134-1275427
BmHel-11	DF090370:1908771-1909064
BmHel-11	DF090356:1132999-1133292
BmHel-11	DF090325:6054491-6054785
BmHel-11	DF090387:1570038-1570332
BmHel-11	DF090386:459012-459306
BmHel-11	DF090342:4254302-4254596
BmHel-11	DF090336:4383168-4383462
BmHel-11	DF090318:3366004-3366298
BmHel-11	DF090318:3816926-3817220
BmHel-11	DF090383:695538-695832

BmHel-11	DF090348:618138-618432
BmHel-11	DF090319:2742294-2742588
BmHel-11	DF090347:3832817-3833111
BmHel-11	DF090411:812438-812732
BmHel-11	DF090322:5419671-5419965
BmHel-11	DF090349:1428801-1429095
BmHel-11	DF090334:2889127-2889421
BmHel-11	DF090331:3126096-3126390
BmHel-11	DF090316:2178625-2178919
BmHel-11	DF090316:10573076-10573370
BmHel-11	DF090418:93910-94204
BmHel-11	DF090400:1490853-1491147
BmHel-11	DF090351:1669872-1670166
BmHel-11	DF090335:3171495-3171789
BmHel-11	DF090329:4075228-4075522
BmHel-11	DF090354:1706847-1707141
BmHel-11	DF090352:1802260-1802554
BmHel-11	DF090317:1294834-1295128
BmHel-11	DF090438:106468-106762
BmHel-11	DF090435:406326-406620
BmHel-11	DF090401:500449-500743
BmHel-11	DF090437:31355-31649
BmHel-11	DF090484:152442-152736
BmHel-11	DF090526:11480-11774
BmHel-11	DF091017:995-1289
BmHel-11	DF090324:462182-462476
BmHel-11	DF090340:2179059-2179353
BmHel-11	DF090331:5031204-5031498
BmHel-11	DF090418:124385-124679
BmHel-11	DF090323:5576560-5576854
BmHel-11	DF090358:110649-110943
BmHel-11	DF090327:5599812-5600106
BmHel-11	DF090329:1402255-1402549
BmHel-11	DF090434:104474-104768
BmHel-11	DF090339:3319818-3320112
BmHel-11	DF090339:1095203-1095497
BmHel-11	DF090324:2781654-2781948
BmHel-11	DF090342:4764367-4764661
BmHel-11	DF090336:4251792-4252086
BmHel-11	DF090336:2821099-2821393
BmHel-11	DF090332:4707317-4707611
BmHel-11	DF090332:17039-17333
BmHel-11	DF090318:5952888-5953182
BmHel-11	DF090318:5914553-5914847
BmHel-11	DF090318:5051654-5051948
BmHel-11	DF090348:949960-950254
BmHel-11	DF090347:3966791-3967085
BmHel-11	DF090321:5533232-5533526

BmHel-11	DF090476:31383-31677
BmHel-11	DF090410:754066-754360
BmHel-11	DF090363:1520255-1520549
BmHel-11	DF090349:3283537-3283831
BmHel-11	DF090343:2194805-2195099
BmHel-11	DF090334:367280-367574
BmHel-11	DF090316:8105515-8105809
BmHel-11	DF090316:7113095-7113389
BmHel-11	DF090385:497427-497721
BmHel-11	DF090330:5401462-5401756
BmHel-11	DF090330:3017744-3018038
BmHel-11	DF090329:2315740-2316034
BmHel-11	DF090380:1003600-1003894
BmHel-11	DF090320:4295915-4296209
BmHel-11	DF090320:4191526-4191820
BmHel-11	DF090452:372979-373273
BmHel-11	DF090399:652539-652833
BmHel-11	DF090378:1290711-1291005
BmHel-11	DF090378:1237186-1237480
BmHel-11	DF090354:1091622-1091916
BmHel-11	DF090354:1067229-1067523
BmHel-11	DF090352:3792341-3792635
BmHel-11	DF090389:673125-673419
BmHel-11	DF090356:3343716-3344010
BmHel-11	DF090346:3899301-3899595
BmHel-11	DF090472:175042-175336
BmHel-11	DF090427:476051-476345
BmHel-11	DF090416:511248-511542
BmHel-11	DF090416:326806-327100
BmHel-11	DF090397:492788-493082
BmHel-11	DF090408:74328-74622
BmHel-11	BABH01050603:165-459
BmHel-11	DF090429:886219-886513
BmHel-11	BABH01047257:336-630
BmHel-11	DF090746:19850-20144
BmHel-11	DF090328:6353441-6353735
BmHel-11	DF090392:537681-537975
BmHel-11	DF090316:4343096-4343390
BmHel-11	DF090417:879416-879710
BmHel-11	DF090361:2574132-2574426
BmHel-11	DF090521:88513-88807
BmHel-11	BABH01052004:496-790
BmHel-11	DF090321:4833173-4833467
BmHel-11	DF090353:510377-510672
BmHel-11	DF090362:1997523-1997818
BmHel-11	DF090402:738404-738699
BmHel-11	DF090386:698262-698557
BmHel-11	DF090342:1226859-1227154

BmHel-11	DF090336:2155516-2155811
BmHel-11	DF090436:221289-221584
BmHel-11	DF090319:2700111-2700406
BmHel-11	DF090319:3775165-3775460
BmHel-11	DF090322:1764051-1764346
BmHel-11	DF090322:5858543-5858838
BmHel-11	DF090322:6856476-6856771
BmHel-11	DF090349:1075981-1076276
BmHel-11	DF090316:10507932-10508227
BmHel-11	DF090316:11175441-11175736
BmHel-11	DF090368:1291246-1291541
BmHel-11	DF090337:5049824-5050119
BmHel-11	DF090330:2268424-2268719
BmHel-11	DF090367:366498-366793
BmHel-11	DF090354:475347-475642
BmHel-11	DF090354:1746410-1746705
BmHel-11	DF090345:3915360-3915655
BmHel-11	DF090327:1341371-1341666
BmHel-11	DF090472:42203-42498
BmHel-11	DF090416:572896-573191
BmHel-11	DF090338:845525-845820
BmHel-11	DF090429:679908-680203
BmHel-11	DF090557:54187-54482
BmHel-11	DF090325:5472565-5472860
BmHel-11	DF090365:2050910-2051205
BmHel-11	DF090374:1137955-1138250
BmHel-11	DF090355:3749104-3749399
BmHel-11	DF090325:92241-92536
BmHel-11	DF090339:4950767-4951062
BmHel-11	DF090324:4983139-4983434
BmHel-11	DF090342:4552065-4552360
BmHel-11	DF090342:4339329-4339624
BmHel-11	DF090318:6350690-6350985
BmHel-11	DF090319:2300505-2300800
BmHel-11	DF090347:1539198-1539493
BmHel-11	DF090411:257558-257853
BmHel-11	DF090365:154273-154568
BmHel-11	DF090322:210725-211020
BmHel-11	DF090350:2150823-2151118
BmHel-11	DF090343:3907880-3908175
BmHel-11	DF090343:1529448-1529743
BmHel-11	DF090341:1439776-1440071
BmHel-11	DF090334:4087972-4088267
BmHel-11	DF090331:3889801-3890096
BmHel-11	DF090316:13923512-13923807
BmHel-11	DF090316:13767896-13768191
BmHel-11	DF090316:10581627-10581922
BmHel-11	DF090368:1719120-1719415

BmHel-11	DF090367:2703318-2703613
BmHel-11	DF090367:1023890-1024185
BmHel-11	DF090399:551422-551717
BmHel-11	DF090364:1956583-1956878
BmHel-11	DF090317:10868053-10868348
BmHel-11	DF090317:10825998-10826293
BmHel-11	DF090358:1203176-1203471
BmHel-11	DF090376:409229-409524
BmHel-11	DF090371:2088523-2088818
BmHel-11	DF090346:4003671-4003966
BmHel-11	DF090346:2736168-2736463
BmHel-11	DF090403:68061-68356
BmHel-11	DF090361:2498886-2499181
BmHel-11	DF090361:1938999-1939294
BmHel-11	DF090423:867484-867779
BmHel-11	DF090409:992045-992340
BmHel-11	DF090408:1351962-1352257
BmHel-11	DF090460:26664-26959
BmHel-11	DF090369:948531-948826
BmHel-11	DF090692:20309-20604
BmHel-11	BABH01048038:853-1148
BmHel-11	DF090702:44859-45154
BmHel-11	DF090317:6949095-6949390
BmHel-11	DF090338:5080587-5080882
BmHel-11	DF090355:3692766-3693062
BmHel-11	DF090325:4985143-4985439
BmHel-11	DF090353:3245448-3245744
BmHel-11	DF090390:407987-408283
BmHel-11	DF090328:1377813-1378109
BmHel-11	DF090347:3296450-3296746
BmHel-11	DF090392:993685-993981
BmHel-11	DF090363:1580872-1581168
BmHel-11	DF090349:4223000-4223296
BmHel-11	DF090341:4727545-4727841
BmHel-11	DF090316:12079859-12080155
BmHel-11	DF090351:2154025-2154321
BmHel-11	DF090351:2961099-2961395
BmHel-11	DF090329:2674427-2674723
BmHel-11	DF090562:57636-57932
BmHel-11	DF090369:677535-677831
BmHel-11	DF090343:3280980-3281276
BmHel-11	DF090317:7533913-7534209
BmHel-11	DF090422:802837-803133
BmHel-11	DF090415:1142624-1142920
BmHel-11	DF090402:1327913-1328209
BmHel-11	DF090402:493925-494221
BmHel-11	DF090386:1716790-1717086
BmHel-11	DF090342:4037521-4037817

BmHel-11	DF090332:2435283-2435579
BmHel-11	DF090433:297749-298045
BmHel-11	DF090365:1759114-1759410
BmHel-11	DF090350:4138868-4139164
BmHel-11	DF090340:4718303-4718599
BmHel-11	DF090340:55206-55502
BmHel-11	DF090334:5218594-5218890
BmHel-11	DF090316:2482465-2482761
BmHel-11	DF090412:1100654-1100950
BmHel-11	DF090327:850605-850901
BmHel-11	DF090416:188911-189207
BmHel-11	DF090338:4650448-4650744
BmHel-11	DF090462:1718-2014
BmHel-11	DF090375:1439499-1439795
BmHel-11	DF090374:686349-686645
BmHel-11	DF090539:69984-70280
BmHel-11	BABH01047081:1652-1948
BmHel-11	DF090390:860667-860963
BmHel-11	DF090360:40297-40593
BmHel-11	DF090316:4846833-4847129
BmHel-11	DF090370:443431-443727
BmHel-11	DF090397:1254517-1254813
BmHel-11	DF090336:914777-915074
BmHel-11	DF090348:3819849-3820146
BmHel-11	DF090319:7145589-7145886
BmHel-11	DF090357:2706072-2706369
BmHel-11	DF090392:1600220-1600517
BmHel-11	DF090350:2805932-2806229
BmHel-11	DF090393:1320017-1320314
BmHel-11	DF090382:589313-589610
BmHel-11	DF090331:4709637-4709934
BmHel-11	DF090330:1445265-1445562
BmHel-11	DF090330:4434717-4435014
BmHel-11	DF090329:6126353-6126650
BmHel-11	DF090452:29882-30179
BmHel-11	DF090317:7141988-7142285
BmHel-11	DF090317:8761337-8761634
BmHel-11	DF090361:1624234-1624531
BmHel-11	DF090477:43525-43822
BmHel-11	DF090394:1136906-1137203
BmHel-11	DF090426:573775-574072
BmHel-11	DF090369:1365158-1365455
BmHel-11	DF091083:3072-3369
BmHel-11	DF090342:3379959-3380256
BmHel-11	DF090325:4864230-4864527
BmHel-11	DF090357:493457-493754
BmHel-11	DF090395:1281357-1281654
BmHel-11	DF090340:3581508-3581805

BmHel-11	DF090340:1082352-1082649
BmHel-11	DF090331:726800-727097
BmHel-11	DF090316:9670985-9671282
BmHel-11	DF090330:2097646-2097943
BmHel-11	DF090323:5034520-5034817
BmHel-11	DF090378:1384546-1384843
BmHel-11	DF090352:2020456-2020753
BmHel-11	DF090345:2519362-2519659
BmHel-11	DF090333:3144103-3144400
BmHel-11	DF090416:906764-907061
BmHel-11	DF090371:2265222-2265519
BmHel-11	DF090355:3129272-3129570
BmHel-11	DF090339:91882-92180
BmHel-11	DF090407:1066783-1067081
BmHel-11	DF090363:2284942-2285240
BmHel-11	DF090359:1037878-1038176
BmHel-11	DF090385:1806689-1806987
BmHel-11	DF090384:1091334-1091632
BmHel-11	DF090345:954850-955148
BmHel-11	DF090403:1565986-1566284
BmHel-11	DF090444:389862-390160
BmHel-11	DF090690:2414-2712
BmHel-11	DF091216:3619-3917
BmHel-11	DF090318:1377092-1377390
BmHel-11	DF090372:285988-286286
BmHel-11	DF090472:269401-269699
BmHel-11	DF090347:2904039-2904337
BmHel-11	DF090321:2793268-2793566
BmHel-11	DF090343:346615-346913
BmHel-11	DF090334:4037057-4037355
BmHel-11	DF090330:1151456-1151754
BmHel-11	DF090344:4020059-4020357
BmHel-11	DF090356:2895048-2895346
BmHel-11	DF090561:28099-28397
BmHel-11	DF090348:3344177-3344475
BmHel-11	DF090357:203472-203770
BmHel-11	DF090322:6283118-6283416
BmHel-11	DF090319:3979404-3979703
BmHel-11	DF090316:13403701-13404000
BmHel-11	DF090368:1940639-1940938
BmHel-11	DF090335:1417897-1418196
BmHel-11	DF090330:4030308-4030607
BmHel-11	DF090320:6888634-6888933
BmHel-11	DF090364:183159-183458
BmHel-11	DF090354:3497170-3497469
BmHel-11	DF090317:6811790-6812089
BmHel-11	DF090544:47339-47638
BmHel-11	BABH01047346:351-650

BmHel-11	DF090318:9033057-9033356
BmHel-11	DF090316:5046927-5047226
BmHel-11	DF090323:6568205-6568504
BmHel-11	DF090317:74580-74879
BmHel-11	DF090330:5604463-5604762
BmHel-11	DF090334:3204779-3205078
BmHel-11	DF090330:1760026-1760325
BmHel-11	DF090330:402012-402311
BmHel-11	DF090367:2645854-2646153
BmHel-11	DF090361:1006315-1006614
BmHel-11	DF090413:1196206-1196505
BmHel-11	DF090626:3033-3332
BmHel-11	BABH01051293:83-382
BmHel-11	DF090336:1183405-1183705
BmHel-11	DF090326:6442393-6442693
BmHel-11	DF090316:8515991-8516291
BmHel-11	DF090373:247019-247319
BmHel-11	DF090351:1090784-1091084
BmHel-11	DF090330:3180136-3180436
BmHel-11	DF090398:1294601-1294901
BmHel-11	DF090423:723019-723319
BmHel-11	DF090332:1469222-1469522
BmHel-11	BABH01042926:11001-11301
BmHel-11	DF090347:1667323-1667623
BmHel-11	DF090349:1549704-1550004
BmHel-11	DF090346:281725-282025
BmHel-11	DF091090:3812-4112
BmHel-11	DF090318:674-975
BmHel-11	DF090322:3637335-3637636
BmHel-11	DF090316:212156-212457
BmHel-11	DF090351:1288616-1288917
BmHel-11	DF090330:376167-376468
BmHel-11	DF090323:2751437-2751738
BmHel-11	DF090320:1856408-1856709
BmHel-11	DF090467:247359-247660
BmHel-11	DF090322:1755566-1755867
BmHel-11	DF090320:324257-324558
BmHel-11	DF090356:930966-931267
BmHel-11	DF090333:1769153-1769454
BmHel-11	DF090419:833040-833341
BmHel-11	DF090365:2283077-2283379
BmHel-11	DF090405:1153225-1153527
BmHel-11	DF090330:4152480-4152782
BmHel-11	DF090415:1163338-1163640
BmHel-11	DF090347:3894117-3894419
BmHel-11	DF090384:216507-216809
BmHel-11	DF090317:6134589-6134891
BmHel-11	DF090369:1172438-1172740

BmHel-11	DF090386:1616349-1616652
BmHel-11	DF090332:3339464-3339767
BmHel-11	DF090384:1244086-1244389
BmHel-11	DF090317:3873746-3874049
BmHel-11	DF090327:1226014-1226317
BmHel-11	DF090403:892212-892515
BmHel-11	DF090361:3173557-3173860
BmHel-11	DF090338:4161765-4162068
BmHel-11	DF090338:735005-735308
BmHel-11	DF090688:34102-34405
BmHel-11	DF090483:49590-49894
BmHel-11	DF090481:227745-228049
BmHel-11	DF090336:5084363-5084667
BmHel-11	DF090343:3075293-3075597
BmHel-11	DF090370:697267-697571
BmHel-11	DF090320:5014564-5014868
BmHel-11	DF090359:1799465-1799769
BmHel-11	DF090331:5373985-5374290
BmHel-11	DF090320:6542977-6543282
BmHel-11	DF090478:142269-142574
BmHel-11	DF090363:1194514-1194819
BmHel-11	DF090399:943540-943845
BmHel-11	DF090323:2206585-2206890
BmHel-11	DF090332:1259535-1259840
BmHel-11	DF090317:6863314-6863620
BmHel-11	DF090353:3165382-3165688
BmHel-11	DF090338:4919778-4920084
BmHel-11	DF090324:3463902-3464209
BmHel-11	DF090328:214900-215207
BmHel-11	DF090326:5551190-5551497
BmHel-11	DF090342:3572484-3572791
BmHel-11	DF090483:42160-42467
BmHel-11	DF090365:2553969-2554276
BmHel-11	DF090324:3493086-3493395
BmHel-11	DF090343:477652-477961
BmHel-11	DF090332:743772-744081
BmHel-11	DF090326:2242176-2242485
BmHel-11	DF090409:974963-975272
BmHel-11	DF090332:1820234-1820544
BmHel-11	DF090343:3345109-3345419
BmHel-11	DF090391:403440-403750
BmHel-11	DF090330:1294829-1295139
BmHel-11	DF090349:2534378-2534688
BmHel-11	DF090340:4067879-4068189
BmHel-11	DF090351:4198568-4198878
BmHel-11	DF090355:734527-734838
BmHel-11	DF090316:9492610-9492921
BmHel-11	DF090488:52214-52526

BmHel-11	DF090328:3279011-3279324
BmHel-11	DF090439:1704-2017
BmHel-11	DF090334:803986-804299
BmHel-11	DF090401:1419656-1419969
BmHel-11	DF090335:4980614-4980928
BmHel-11	DF090328:3622724-3623039
BmHel-11	DF090333:589288-589603
BmHel-11	DF090329:1912544-1912860
BmHel-11	DF090406:1101934-1102250
BmHel-11	DF090330:3108907-3109224
BmHel-11	DF090344:568169-568486
BmHel-11	DF090322:5479680-5479998
BmHel-11	DF090347:3333738-3334060
BmHel-11	DF090378:87309-87631
BmHel-11	DF090369:606374-606696
BmHel-11	DF090343:4392861-4393183
BmHel-11	DF090352:2855030-2855352
BmHel-11	DF090347:2757254-2757577
BmHel-11	DF090741:10719-11042
BmHel-11	DF090360:913179-913503
BmHel-11	DF090429:864699-865023
BmHel-11	DF090392:338931-339255
BmHel-11	DF090318:7969660-7969985
BmHel-11	DF090320:691696-692021
BmHel-11	DF090325:1071639-1071966
BmHel-11	DF090320:7046543-7046870
BmHel-11	DF090334:1398525-1398853
BmHel-11	DF090333:3724539-3724867
BmHel-11	DF090345:2081904-2082234
BmHel-11	DF090373:973519-973851
BmHel-11	DF090433:678198-678531
BmHel-11	DF090406:1073536-1073870
BmHel-11	DF090328:1957331-1957666
BmHel-11	DF090353:2970863-2971199
BmHel-11	DF090768:285-622
BmHel-11	DF090374:2225774-2226112
BmHel-11	DF090319:6851664-6852004
BmHel-11	DF090395:388659-388999
BmHel-11	DF090414:354082-354424
BmHel-11	DF090347:3624400-3624744
BmHel-11	DF090414:461068-461412
BmHel-11	DF090414:502834-503187
BmHel-11	DF090344:252851-253206
BmHel-11	DF090468:213508-213865
BmHel-11	DF090608:18999-19356
BmHel-11	DF090349:3626023-3626390
BmHel-11	DF090326:3007893-3008260
BmHel-11	DF090320:5809773-5810144

BmHel-11	DF090325:1014752-1015125
BmHel-11	DF090369:381083-381456
BmHel-11	DF090341:2765376-2765751
BmHel-11	DF090343:160735-161121
BmHel-11	DF090341:2716117-2716506
BmHel-11	DF090333:3552725-3553115
BmHel-11	DF090336:5509587-5509979
BmHel-11	DF090341:1997586-1997983
BmHel-11	DF090345:4305240-4305637
BmHel-11	DF090364:1715708-1716109
BmHel-11	DF090404:1358693-1359095
BmHel-11	DF090333:3182126-3182528
BmHel-11	DF090361:1593211-1593613
BmHel-11	DF090348:2481678-2482083
BmHel-11	DF090316:6418883-6419288
BmHel-11	DF090317:588532-588939
BmHel-11	DF090319:730251-730660
BmHel-11	DF090367:784530-784949
BmHel-11	DF090365:1225152-1225574
BmHel-11	DF090331:901003-901430
BmHel-11	DF090324:5655908-5656336
BmHel-11	DF090343:1619505-1619934
BmHel-11	DF090367:801457-801887
BmHel-11	DF090326:5470144-5470580
BmHel-11	DF090363:1817696-1818134
BmHel-11	DF090352:685189-685632
BmHel-11	DF090357:1062732-1063199
BmHel-11	DF090338:3992988-3993456
BmHel-11	DF090378:509299-509775
BmHel-11	DF090323:2961669-2962148
BmHel-11	DF090325:3225706-3226192
BmHel-11	DF090667:27887-28375
BmHel-11	DF090340:494849-495340
BmHel-11	DF090316:10555506-10555997
BmHel-11	DF090431:288256-288750
BmHel-11	DF090443:291766-292262
BmHel-11	DF090369:2453731-2454227
BmHel-11	DF090856:930-1427
BmHel-11	DF090390:546393-546890
BmHel-11	DF090327:4053917-4054415
BmHel-11	DF090339:3933958-3934462
BmHel-11	DF090360:1013607-1014113
BmHel-11	DF090316:6656036-6656543
BmHel-11	DF090395:230918-231427
BmHel-11	DF090350:3117703-3118213
BmHel-11	DF090415:536950-537460
BmHel-11	BABH01047413:963-1474
BmHel-11	DF090334:5369651-5370164

BmHel-11	DF090370:829219-829732
BmHel-11	DF090411:824339-824854
BmHel-11	DF090363:2062217-2062733
BmHel-11	DF090362:666888-667405
BmHel-11	DF090322:4558153-4558674
BmHel-11	DF090422:236434-236964
BmHel-11	DF090433:452170-452708
BmHel-11	DF090349:2562190-2562730
BmHel-11	DF090339:2692910-2693458
BmHel-11	DF090520:46518-47069
BmHel-11	DF090326:2268098-2268652
BmHel-11	DF090342:4473551-4474109
BmHel-11	DF090390:1463609-1464172
BmHel-11	DF090424:113066-113632
BmHel-11	DF090340:764671-765240
BmHel-11	DF090511:14114-14684
BmHel-11	DF090342:4399733-4400305
BmHel-11	DF090316:15015084-15015656
BmHel-11	DF090339:2529789-2530362
BmHel-11	DF090344:1232817-1233400
BmHel-11	DF090329:3167924-3168510
BmHel-11	DF090344:3970737-3971325
BmHel-11	DF090322:2655563-2656162
BmHel-11	DF090356:2907779-2908378
BmHel-11	DF090335:1469752-1470365
BmHel-11	DF090406:641684-642298
BmHel-11	DF090360:2914357-2914973
BmHel-11	DF090388:731955-732580
BmHel-11	DF090337:2758571-2759199
BmHel-11	DF090322:2534818-2535461
BmHel-11	DF090331:2120767-2121416
BmHel-11	DF090892:12242-12900
BmHel-11	DF090441:564481-565146
BmHel-11	DF090325:5760072-5760748
BmHel-11	DF090325:5365618-5366301
BmHel-11	DF090317:6571661-6572363
BmHel-11	DF090433:276189-276895
BmHel-11	DF090327:6164362-6165087
BmHel-11	DF090388:262693-263420
BmHel-11	DF090388:335024-335752
BmHel-11	DF090327:5518618-5519352
BmHel-11	DF090347:3790814-3791551
BmHel-11	DF090367:1415470-1416209
BmHel-11	DF090367:825854-826611
BmHel-11	DF090323:577383-578151
BmHel-11	DF090322:3690263-3691091
BmHel-11	DF090351:3635521-3636358
BmHel-11	DF090379:415435-416309

BmHel-11	DF090327:6119992-6120912
BmHel-11	DF090324:4778127-4779049
BmHel-11	DF090401:267564-268495
BmHel-11	DF090361:1406812-1407750
BmHel-11	DF090339:1357217-1358184
BmHel-11	DF090376:1282052-1283020
BmHel-11	DF090427:672928-673902
BmHel-11	DF090328:2918123-2919130
BmHel-11	DF090321:3292204-3293212
BmHel-11	DF091194:4758-5787
BmHel-11	DF090390:258031-259087
BmHel-11	DF090346:2372348-2373506
BmHel-11	DF090321:6533079-6534334
BmHel-11	DF090316:585767-587089
BmHel-11	DF090361:786670-788053
BmHel-11	DF090352:186294-187679
BmHel-11	DF090340:2135635-2137082
BmHel-11	DF090360:886947-888414
BmHel-11	DF090429:823736-825217
BmHel-11	DF090414:133298-134823
BmHel-11	DF090317:7060664-7062192
BmHel-11	DF090322:5036006-5037539
BmHel-11	DF090336:4449999-4451541
BmHel-11	DF090440:457462-459118
BmHel-11	DF090316:9382038-9383712
BmHel-11	DF090381:745321-747082
BmHel-11	DF090317:10839405-10841189
BmHel-11	DF090352:2482900-2484688
BmHel-11	DF090319:8203904-8205855
BmHel-11	DF090321:4308055-4310065
BmHel-11	DF090475:239002-241101
BmHel-11	DF090320:4673807-4676123
BmHel-11	DF090336:744302-747701
BmHel-11	DF090317:8386787-8390534
BmHel-11	DF090378:2072995-2076779
BmHel-11	DF090357:3621612-3625399
BmHel-11	DF090339:4360539-4364384
BmHel-11	DF090402:886876-890788
BmHel-11	DF090363:107743-111771
BmHel-11	DF090316:10376447-10380567
BmHel-11	DF090501:94208-98990
BmHel-11	DF090335:489477-494312
BmHel-11	DF090738:23882-29466
BmHel-11	DF090316:674387-680918
BmHel-11	DF090384:275439-282168
BmHel-11	DF090322:4614274-4621067
BmHel-11	DF090362:2397630-2405140
BmHel-11	DF090323:2862045-2870662

BmHel-11	DF090316:13250030-13259336
BmHel-11	DF090342:3678888-3688675
BmHel-11	DF090362:763984-764272
BmHel-11	DF090362:2538358-2538651
BmHel-11	DF090324:3221066-3221355
BmHel-11	DF090324:3818194-3818486
BmHel-11	DF090421:498697-498986
BmHel-11	DF090342:1288048-1288338
BmHel-11	DF090342:1530776-1531065
BmHel-11	DF090342:2378911-2379200
BmHel-11	DF090318:4722959-4723248
BmHel-11	DF090318:3272376-3272666
BmHel-11	DF090348:4202980-4203265
BmHel-11	DF090348:1599602-1599888
BmHel-11	DF090328:4615029-4615318
BmHel-11	DF090328:2308323-2308617
BmHel-11	DF090328:1847687-1847976
BmHel-11	DF090319:8699939-8700228
BmHel-11	DF090319:2403882-2404169
BmHel-11	DF090433:385112-385407
BmHel-11	DF090377:585222-585513
BmHel-11	DF090360:336249-336542
BmHel-11	DF090347:79126-79420
BmHel-11	DF090347:647306-647591
BmHel-11	DF090414:1257527-1257816
BmHel-11	DF090414:1089242-1089531
BmHel-11	DF090365:262658-262946
BmHel-11	DF090365:1815431-1815729
BmHel-11	DF090326:6106825-6107114
BmHel-11	DF090322:1027679-1027967
BmHel-11	DF090321:2550299-2550590
BmHel-11	DF090321:4427328-4427618
BmHel-11	DF090321:5218716-5219007
BmHel-11	DF090476:54998-55287
BmHel-11	DF090363:2628583-2628872
BmHel-11	DF090359:1957975-1958265
BmHel-11	DF090343:2170229-2170517
BmHel-11	DF090341:2017013-2017302
BmHel-11	DF090341:3880242-3880533
BmHel-11	DF090341:1847265-1847554
BmHel-11	DF090340:2717234-2717520
BmHel-11	DF090340:1663221-1663512
BmHel-11	DF090334:3323285-3323575
BmHel-11	DF090334:5552237-5552526
BmHel-11	DF090331:123933-124222
BmHel-11	DF090316:7446684-7446974
BmHel-11	DF090316:8047590-8047881
BmHel-11	DF090316:15129362-15129651

BmHel-11	DF090391:70887-71169
BmHel-11	DF090373:639298-639587
BmHel-11	DF090368:1938257-1938546
BmHel-11	DF090351:2751836-2752125
BmHel-11	DF090351:1314928-1315214
BmHel-11	DF090335:109233-109522
BmHel-11	DF090335:763349-763638
BmHel-11	DF090335:2784074-2784363
BmHel-11	DF090330:4687416-4687701
BmHel-11	DF090330:4265883-4266170
BmHel-11	DF090329:3525681-3525969
BmHel-11	DF090329:5150900-5151184
BmHel-11	DF090323:756003-756292
BmHel-11	DF090367:546849-547139
BmHel-11	DF090344:209797-210080
BmHel-11	DF090380:1635873-1636152
BmHel-11	DF090380:1920504-1920794
BmHel-11	DF090370:1189283-1189573
BmHel-11	DF090320:5148285-5148573
BmHel-11	DF090399:533003-533295
BmHel-11	DF090384:1021707-1021998
BmHel-11	DF090379:1447282-1447571
BmHel-11	DF090366:2128920-2129209
BmHel-11	DF090352:4168452-4168741
BmHel-11	DF090317:9626892-9627184
BmHel-11	DF090317:4785224-4785512
BmHel-11	DF090317:3346065-3346359
BmHel-11	DF090372:722976-723264
BmHel-11	DF090358:2606151-2606447
BmHel-11	DF090345:4170644-4170929
BmHel-11	DF090327:4399523-4399812
BmHel-11	DF090361:2484729-2485009
BmHel-11	DF090428:542038-542328
BmHel-11	DF090375:1267716-1267999
BmHel-11	DF090415:557803-558095
BmHel-11	BABH01044763:5278-5567
BmHel-11	DF090588:37582-37867
BmHel-11	DF090588:16998-17286
BmHel-11	DF090458:129094-129383
BmHel-11	DF090725:8215-8504
BmHel-11	DF090465:200105-200395
BmHel-11	DF090847:12024-12315
BmHel-11	DF091268:8892-9181
BmHel-11	DF090470:125467-125754
BmHel-11	BABH01048438:833-1122
BmHel-11	DF090771:8969-9256
BmHel-11	DF090560:1568-1856
BmHel-11	BABH01045310:3507-3793

BmHel-11	DF090325:4255836-4256121
BmHel-11	DF090339:4452131-4452422
BmHel-11	DF090339:1882816-1883105
BmHel-11	DF090339:1540233-1540522
BmHel-11	DF090362:1018706-1018995
BmHel-11	DF090387:1114733-1115022
BmHel-11	DF090386:629236-629525
BmHel-11	DF090342:1468116-1468405
BmHel-11	DF090328:3715137-3715426
BmHel-11	DF090328:4072799-4073090
BmHel-11	DF090411:747012-747301
BmHel-11	DF090350:322143-322432
BmHel-11	DF090340:688046-688333
BmHel-11	DF090334:925501-925790
BmHel-11	DF090316:462517-462806
BmHel-11	DF090316:14745981-14746265
BmHel-11	DF090400:1582620-1582910
BmHel-11	DF090351:3608644-3608938
BmHel-11	DF090335:3514574-3514857
BmHel-11	DF090335:3235364-3235653
BmHel-11	DF090344:3823466-3823755
BmHel-11	DF090320:1416536-1416825
BmHel-11	DF090320:3395986-3396275
BmHel-11	DF090366:154580-154868
BmHel-11	DF090372:1286005-1286295
BmHel-11	DF090467:39452-39732
BmHel-11	DF090376:890647-890934
BmHel-11	DF090345:3991218-3991508
BmHel-11	DF090327:2339419-2339701
BmHel-11	DF090494:125142-125430
BmHel-11	DF090346:3123196-3123482
BmHel-11	DF090427:116170-116459
BmHel-11	DF090338:158961-159255
BmHel-11	DF090338:916054-916343
BmHel-11	DF090338:4099854-4100143
BmHel-11	DF090338:2844231-2844520
BmHel-11	DF090375:964324-964617
BmHel-11	BABH01046757:1629-1918
BmHel-11	DF090466:189948-190244
BmHel-11	DF090426:746328-746614
BmHel-11	DF090523:77606-77894
BmHel-11	DF090465:110056-110345
BmHel-11	DF090570:53510-53800
BmHel-11	BABH01066824:311-604
BmHel-11	DF090362:784205-784418
BmHel-11	DF090400:1029775-1029993
BmHel-11	DF090441:206786-207026
BmHel-11	DF090324:7998403-7998694

BmHel-11	DF090395:712606-712894
BmHel-11	DF090382:272849-273137
BmHel-11	DF090331:2357991-2358279
BmHel-11	DF090316:579891-580179
BmHel-11	DF090391:1383468-1383759
BmHel-11	DF090388:1719938-1720224
BmHel-11	DF090366:634113-634401
BmHel-11	DF090354:290817-291105
BmHel-11	DF090352:1995242-1995522
BmHel-11	DF090358:3359229-3359517
BmHel-11	DF090356:1121534-1121822
BmHel-11	DF090369:897075-897372
BmHel-11	DF090492:23006-23295
BmHel-11	DF090781:80762-81053
BmHel-11	DF090325:2042196-2042483
BmHel-11	DF090325:5866663-5866945
BmHel-11	DF090325:1396964-1397255
BmHel-11	DF090339:2545927-2546215
BmHel-11	DF090342:2571370-2571658
BmHel-11	DF090342:1891129-1891416
BmHel-11	DF090342:1360705-1360993
BmHel-11	DF090336:2790523-2790811
BmHel-11	DF090318:2192758-2193049
BmHel-11	DF090318:7268057-7268345
BmHel-11	DF090319:7079600-7079889
BmHel-11	DF090433:565225-565513
BmHel-11	DF090322:1719037-1719324
BmHel-11	DF090322:2277650-2277940
BmHel-11	DF090322:7690472-7690756
BmHel-11	DF090350:1702991-1703278
BmHel-11	DF090359:3225303-3225591
BmHel-11	DF090331:3203551-3203839
BmHel-11	DF090331:6009745-6010038
BmHel-11	DF090331:5625213-5625504
BmHel-11	DF090316:1661116-1661389
BmHel-11	DF090316:10478385-10478676
BmHel-11	DF090316:13896714-13897003
BmHel-11	DF090391:727989-728278
BmHel-11	DF090351:1942160-1942447
BmHel-11	DF090329:6536344-6536629
BmHel-11	DF090320:4159682-4159973
BmHel-11	DF090364:2901882-2902166
BmHel-11	DF090317:289031-289317
BmHel-11	DF090438:465628-465909
BmHel-11	DF090404:885795-886080
BmHel-11	DF090372:1274145-1274433
BmHel-11	DF090358:3295855-3296144
BmHel-11	DF090345:1178461-1178749

BmHel-11	DF090371:2018475-2018757
BmHel-11	DF090371:1415375-1415666
BmHel-11	DF090346:4229806-4230094
BmHel-11	DF090416:1235715-1236004
BmHel-11	DF090361:1687703-1687990
BmHel-11	BABH01045735:2353-2642
BmHel-11	DF090685:13140-13428
BmHel-11	DF090453:323402-323688
BmHel-11	BABH01048180:294-583
BmHel-11	DF090324:391909-392197
BmHel-11	DF090317:9619903-9620073
BmHel-11	DF090317:9620719-9620836
BmHel-11	DF090346:4159932-4160142
BmHel-11	DF090355:593846-594134
BmHel-11	DF090355:852755-853044
BmHel-11	DF090355:2797815-2798103
BmHel-11	DF090355:3074076-3074360
BmHel-11	DF090355:2253930-2254217
BmHel-11	DF090355:2105526-2105817
BmHel-11	DF090355:1593555-1593843
BmHel-11	DF090355:1474709-1474994
BmHel-11	DF090325:4666543-4666821
BmHel-11	DF090325:3542678-3542966
BmHel-11	DF090325:2148345-2148637
BmHel-11	DF090325:925611-925900
BmHel-11	DF090325:404448-404742
BmHel-11	BABH01083504:5-293
BmHel-11	BABH01080528:37-325
BmHel-11	DF090353:354255-354543
BmHel-11	DF090353:1750314-1750602
BmHel-11	DF090353:1761672-1761958
BmHel-11	DF090353:3459868-3460156
BmHel-11	DF090353:2678703-2678990
BmHel-11	DF090353:2388916-2389204
BmHel-11	DF090339:1656074-1656360
BmHel-11	DF090339:2778949-2779236
BmHel-11	DF090339:4905660-4905945
BmHel-11	DF090339:4723064-4723347
BmHel-11	DF090362:2529915-2530204
BmHel-11	DF090362:1511460-1511747
BmHel-11	DF090362:699134-699422
BmHel-11	DF090445:583965-584253
BmHel-11	DF090387:9194-9482
BmHel-11	DF090387:1398149-1398422
BmHel-11	DF090387:1838092-1838380
BmHel-11	DF090387:1533606-1533893
BmHel-11	DF090324:1073-1353
BmHel-11	DF090324:1790862-1791151

BmHel-11	DF090324:2478299-2478578
BmHel-11	DF090324:7005053-7005341
BmHel-11	DF090324:7044748-7045028
BmHel-11	DF090324:8047564-8047852
BmHel-11	DF090324:6007674-6007960
BmHel-11	DF090324:4280249-4280537
BmHel-11	DF090324:4039806-4040095
BmHel-11	DF090402:706448-706736
BmHel-11	DF090390:174402-174692
BmHel-11	DF090390:588366-588654
BmHel-11	DF090390:439816-440102
BmHel-11	DF090390:314125-314407
BmHel-11	DF090390:105658-105946
BmHel-11	DF090386:1943708-1944000
BmHel-11	DF090386:803639-803932
BmHel-11	DF090386:422444-422726
BmHel-11	DF090386:274526-274814
BmHel-11	DF090386:186004-186292
BmHel-11	DF090381:1105601-1105887
BmHel-11	DF090381:2002155-2002439
BmHel-11	DF090381:804885-805170
BmHel-11	DF090342:1350524-1350812
BmHel-11	DF090342:1517297-1517573
BmHel-11	DF090342:4803173-4803447
BmHel-11	DF090342:4567503-4567791
BmHel-11	DF090342:2363890-2364176
BmHel-11	DF090336:121855-122139
BmHel-11	DF090336:203542-203842
BmHel-11	DF090336:261881-262162
BmHel-11	DF090336:1467422-1467715
BmHel-11	DF090336:1975651-1975938
BmHel-11	DF090336:3656062-3656350
BmHel-11	DF090336:5506754-5507045
BmHel-11	DF090336:3126805-3127086
BmHel-11	DF090332:1271879-1272164
BmHel-11	DF090332:2403839-2404128
BmHel-11	DF090332:2809104-2809391
BmHel-11	DF090332:3291701-3291990
BmHel-11	DF090332:5360579-5360860
BmHel-11	DF090332:6353892-6354180
BmHel-11	DF090332:3174105-3174392
BmHel-11	DF090332:1217870-1218155
BmHel-11	DF090332:50570-50852
BmHel-11	DF090318:3441830-3442118
BmHel-11	DF090318:3719280-3719568
BmHel-11	DF090318:5663690-5663984
BmHel-11	DF090318:6333983-6334274
BmHel-11	DF090318:6396070-6396356

BmHel-11	DF090318:8109595-8109883
BmHel-11	DF090318:9251401-9251688
BmHel-11	DF090318:8308259-8308535
BmHel-11	DF090318:7840545-7840836
BmHel-11	DF090318:7708161-7708439
BmHel-11	DF090318:5900343-5900627
BmHel-11	DF090318:4373453-4373741
BmHel-11	DF090318:2103114-2103402
BmHel-11	DF090318:1772565-1772857
BmHel-11	DF090436:532965-533253
BmHel-11	DF090436:137707-137995
BmHel-11	DF090436:123304-123594
BmHel-11	DF090383:1199060-1199348
BmHel-11	DF090383:1149117-1149405
BmHel-11	DF090383:127584-127870
BmHel-11	DF090348:2332670-2332958
BmHel-11	DF090348:2648679-2648966
BmHel-11	DF090348:3158207-3158482
BmHel-11	DF090348:2313980-2314271
BmHel-11	DF090328:527691-527980
BmHel-11	DF090328:1332357-1332645
BmHel-11	DF090328:4093613-4093898
BmHel-11	DF090328:6077287-6077569
BmHel-11	DF090328:5760175-5760459
BmHel-11	DF090328:1393964-1394252
BmHel-11	DF090328:1106068-1106362
BmHel-11	DF090328:350220-350508
BmHel-11	DF090319:84203-84501
BmHel-11	DF090319:110099-110387
BmHel-11	DF090319:2992558-2992845
BmHel-11	DF090319:3745064-3745357
BmHel-11	DF090319:5654300-5654588
BmHel-11	DF090319:6400338-6400626
BmHel-11	DF090319:6778319-6778608
BmHel-11	DF090319:7038249-7038537
BmHel-11	DF090319:7610226-7610518
BmHel-11	DF090319:7768044-7768331
BmHel-11	DF090319:8178501-8178790
BmHel-11	DF090319:7836383-7836673
BmHel-11	DF090319:4961362-4961650
BmHel-11	DF090319:3462740-3463027
BmHel-11	DF090319:3080719-3080999
BmHel-11	DF090319:2607745-2608033
BmHel-11	DF090319:2074900-2075187
BmHel-11	DF090319:1572577-1572865
BmHel-11	DF090319:1063919-1064208
BmHel-11	BABH01046749:1983-2271
BmHel-11	DF090483:173215-173497

BmHel-11	DF090433:421402-421682
BmHel-11	DF090357:1304979-1305267
BmHel-11	DF090357:3276422-3276714
BmHel-11	DF090357:3605469-3605757
BmHel-11	DF090357:2742753-2743040
BmHel-11	DF090357:2362607-2362893
BmHel-11	DF090395:1534362-1534650
BmHel-11	DF090377:545140-545428
BmHel-11	DF090377:1268258-1268546
BmHel-11	DF090377:2152228-2152521
BmHel-11	DF090377:2051400-2051688
BmHel-11	DF090377:270048-270336
BmHel-11	DF090360:1290451-1290736
BmHel-11	DF090360:1895322-1895610
BmHel-11	DF090360:2449207-2449496
BmHel-11	DF090347:2505644-2505929
BmHel-11	DF090347:2598444-2598732
BmHel-11	DF090347:3286038-3286326
BmHel-11	DF090347:3757753-3758034
BmHel-11	DF090347:4089146-4089431
BmHel-11	DF090347:3529092-3529380
BmHel-11	DF090347:3380172-3380459
BmHel-11	DF090347:3013008-3013291
BmHel-11	DF090347:2717146-2717437
BmHel-11	DF090347:2490727-2491015
BmHel-11	DF090347:926041-926329
BmHel-11	DF090347:135397-135692
BmHel-11	DF090414:410894-411182
BmHel-11	DF090414:804043-804331
BmHel-11	DF090392:1743211-1743501
BmHel-11	DF090392:961843-962130
BmHel-11	DF090392:756648-756938
BmHel-11	DF090365:37281-37571
BmHel-11	DF090365:748286-748574
BmHel-11	DF090365:1756062-1756354
BmHel-11	DF090365:2664835-2665123
BmHel-11	DF090326:2816087-2816376
BmHel-11	DF090326:5297102-5297390
BmHel-11	DF090326:5804801-5805089
BmHel-11	DF090326:6020569-6020849
BmHel-11	DF090326:6098575-6098863
BmHel-11	DF090326:6158014-6158302
BmHel-11	DF090326:6639256-6639539
BmHel-11	DF090326:4900018-4900302
BmHel-11	DF090326:4519016-4519297
BmHel-11	DF090326:4383573-4383861
BmHel-11	DF090326:3537612-3537895
BmHel-11	DF090322:3404259-3404546

BmHel-11	DF090322:4794458-4794737
BmHel-11	DF090322:6623226-6623514
BmHel-11	DF090322:6647782-6648071
BmHel-11	DF090322:7439501-7439786
BmHel-11	DF090322:7731861-7732154
BmHel-11	DF090322:8069201-8069491
BmHel-11	DF090322:6852776-6853064
BmHel-11	DF090322:6019255-6019544
BmHel-11	DF090322:5900210-5900498
BmHel-11	DF090322:5849054-5849340
BmHel-11	DF090322:4369447-4369737
BmHel-11	DF090322:2645460-2645747
BmHel-11	DF090322:2330233-2330520
BmHel-11	DF090322:810266-810559
BmHel-11	DF090321:2272472-2272760
BmHel-11	DF090321:3678803-3679090
BmHel-11	DF090321:5340257-5340542
BmHel-11	DF090321:5736475-5736763
BmHel-11	DF090321:5930263-5930551
BmHel-11	DF090321:7678006-7678296
BmHel-11	DF090321:6476091-6476371
BmHel-11	DF090321:6326801-6327090
BmHel-11	DF090321:4263254-4263542
BmHel-11	DF090321:4093481-4093769
BmHel-11	DF090321:1026584-1026872
BmHel-11	DF090321:969691-969978
BmHel-11	DF090321:928067-928348
BmHel-11	BABH01054882:478-769
BmHel-11	DF090476:13837-14128
BmHel-11	DF090350:2517164-2517452
BmHel-11	DF090350:1343233-1343521
BmHel-11	DF090350:644790-645079
BmHel-11	DF090471:116342-116638
BmHel-11	DF090469:43887-44175
BmHel-11	DF090463:72745-73032
BmHel-11	DF090463:267826-268118
BmHel-11	DF090407:499005-499283
BmHel-11	DF090407:1078551-1078839
BmHel-11	DF090407:844729-845015
BmHel-11	DF090407:327584-327872
BmHel-11	DF090407:213926-214214
BmHel-11	DF090393:116159-116447
BmHel-11	DF090393:677434-677721
BmHel-11	DF090393:1188453-1188748
BmHel-11	DF090393:1684998-1685285
BmHel-11	DF090393:569911-570200
BmHel-11	DF090382:52597-52887
BmHel-11	DF090382:516765-517044

BmHel-11	DF090382:986027-986313
BmHel-11	DF090382:1325364-1325653
BmHel-11	DF090382:1636049-1636333
BmHel-11	DF090382:1789837-1790117
BmHel-11	DF090363:2104924-2105215
BmHel-11	DF090363:1086000-1086288
BmHel-11	DF090363:955144-955432
BmHel-11	DF090363:746791-747088
BmHel-11	DF090363:7308-7595
BmHel-11	DF090359:576017-576304
BmHel-11	DF090359:2344301-2344580
BmHel-11	DF090359:2469252-2469540
BmHel-11	DF090359:2936622-2936910
BmHel-11	DF090359:2957549-2957834
BmHel-11	DF090359:3153131-3153421
BmHel-11	DF090359:2616388-2616672
BmHel-11	DF090359:2051158-2051446
BmHel-11	DF090359:2013845-2014134
BmHel-11	DF090359:1821965-1822253
BmHel-11	DF090349:334296-334579
BmHel-11	DF090349:3373552-3373840
BmHel-11	DF090349:4303813-4304101
BmHel-11	DF090349:4227410-4227699
BmHel-11	DF090349:3244219-3244500
BmHel-11	DF090349:2247874-2248162
BmHel-11	DF090343:1937409-1937690
BmHel-11	DF090343:2866821-2867105
BmHel-11	DF090343:2251476-2251766
BmHel-11	DF090343:480098-480386
BmHel-11	DF090343:144938-145226
BmHel-11	DF090343:102921-103209
BmHel-11	DF090341:724480-724768
BmHel-11	DF090341:1566932-1567221
BmHel-11	DF090341:4476982-4477263
BmHel-11	DF090341:3044884-3045172
BmHel-11	DF090341:892040-892328
BmHel-11	DF090341:334229-334517
BmHel-11	DF090340:1301072-1301362
BmHel-11	DF090340:2381527-2381804
BmHel-11	DF090340:2832018-2832306
BmHel-11	DF090340:3512784-3513072
BmHel-11	DF090340:4188431-4188719
BmHel-11	DF090340:3450666-3450954
BmHel-11	DF090334:338216-338501
BmHel-11	DF090334:1869595-1869885
BmHel-11	DF090334:2307660-2307947
BmHel-11	DF090334:4060048-4060336
BmHel-11	DF090334:5677540-5677833

BmHel-11	DF090334:5891649-5891921
BmHel-11	DF090334:5596553-5596841
BmHel-11	DF090334:4877255-4877543
BmHel-11	DF090334:3600231-3600519
BmHel-11	DF090334:3549752-3550040
BmHel-11	DF090334:1955827-1956115
BmHel-11	DF090331:3739065-3739353
BmHel-11	DF090331:5199190-5199478
BmHel-11	DF090331:4546546-4546834
BmHel-11	DF090331:2906908-2907197
BmHel-11	DF090316:375672-375960
BmHel-11	DF090316:2281619-2281906
BmHel-11	DF090316:2880570-2880858
BmHel-11	DF090316:4410064-4410355
BmHel-11	DF090316:6533340-6533628
BmHel-11	DF090316:7870946-7871233
BmHel-11	DF090316:11642350-11642638
BmHel-11	DF090316:11692231-11692517
BmHel-11	DF090316:11742545-11742825
BmHel-11	DF090316:11964482-11964769
BmHel-11	DF090316:12348835-12349117
BmHel-11	DF090316:13003627-13003915
BmHel-11	DF090316:13111077-13111373
BmHel-11	DF090316:13226082-13226372
BmHel-11	DF090316:15905939-15906229
BmHel-11	DF090316:15886675-15886964
BmHel-11	DF090316:15775063-15775351
BmHel-11	DF090316:14018625-14018909
BmHel-11	DF090316:13271089-13271377
BmHel-11	DF090316:10117643-10117931
BmHel-11	DF090316:9216678-9216966
BmHel-11	DF090316:9106330-9106602
BmHel-11	DF090316:8863737-8864025
BmHel-11	DF090316:8746935-8747229
BmHel-11	DF090316:8299643-8299924
BmHel-11	DF090316:8135615-8135903
BmHel-11	DF090316:7919982-7920270
BmHel-11	DF090316:7834168-7834458
BmHel-11	DF090316:5300799-5301101
BmHel-11	DF090316:3119319-3119607
BmHel-11	DF090316:2992679-2992963
BmHel-11	DF090316:1497146-1497429
BmHel-11	DF090316:436351-436641
BmHel-11	DF090424:458937-459225
BmHel-11	DF090418:981552-981842
BmHel-11	DF090418:1210504-1210791
BmHel-11	DF090418:1199592-1199880
BmHel-11	DF090400:732273-732552

BmHel-11	DF090391:365214-365496
BmHel-11	DF090391:629088-629376
BmHel-11	DF090391:1143192-1143474
BmHel-11	DF090391:844216-844502
BmHel-11	DF090391:511882-512169
BmHel-11	DF090388:277247-277535
BmHel-11	DF090388:815953-816240
BmHel-11	DF090388:849273-849562
BmHel-11	DF090388:1013739-1014027
BmHel-11	DF090388:1162515-1162806
BmHel-11	DF090388:944635-944919
BmHel-11	DF090373:1221032-1221320
BmHel-11	DF090373:1614334-1614624
BmHel-11	DF090373:460090-460386
BmHel-11	DF090373:72224-72512
BmHel-11	DF090368:1007023-1007313
BmHel-11	DF090368:1671925-1672221
BmHel-11	DF090368:1487074-1487370
BmHel-11	DF090368:711797-712087
BmHel-11	DF090368:339713-339994
BmHel-11	DF090351:290633-290923
BmHel-11	DF090351:1605115-1605403
BmHel-11	DF090351:1686131-1686427
BmHel-11	DF090351:1812482-1812771
BmHel-11	DF090351:2164965-2165259
BmHel-11	DF090351:2538232-2538514
BmHel-11	DF090351:2390085-2390373
BmHel-11	DF090351:1624098-1624384
BmHel-11	DF090351:340186-340473
BmHel-11	DF090337:3386414-3386705
BmHel-11	DF090337:3779104-3779392
BmHel-11	DF090337:1788652-1788937
BmHel-11	DF090337:1380876-1381168
BmHel-11	DF090335:2854194-2854479
BmHel-11	DF090335:4148687-4148975
BmHel-11	DF090335:4786568-4786858
BmHel-11	DF090335:4115272-4115560
BmHel-11	DF090335:2528063-2528351
BmHel-11	DF090330:2537915-2538202
BmHel-11	DF090330:3340638-3340926
BmHel-11	DF090330:3858699-3858821
BmHel-11	DF090330:3858837-3859001
BmHel-11	DF090330:5263259-5263552
BmHel-11	DF090330:5871877-5872165
BmHel-11	DF090330:6161354-6161639
BmHel-11	DF090330:5209410-5209696
BmHel-11	DF090330:4402491-4402782
BmHel-11	DF090330:3529403-3529690

BmHel-11	DF090329:400544-400833
BmHel-11	DF090329:2316336-2316624
BmHel-11	DF090329:5478820-5479108
BmHel-11	DF090329:5361807-5362093
BmHel-11	DF090329:4873899-4874195
BmHel-11	DF090329:4756598-4756884
BmHel-11	DF090329:4497020-4497305
BmHel-11	DF090323:1076573-1076861
BmHel-11	DF090323:2211034-2211326
BmHel-11	DF090323:2486813-2487099
BmHel-11	DF090323:3032310-3032598
BmHel-11	DF090323:5590762-5591060
BmHel-11	DF090323:7321854-7322150
BmHel-11	DF090323:7791262-7791552
BmHel-11	DF090323:6937608-6937891
BmHel-11	DF090323:6201032-6201320
BmHel-11	DF090323:2190306-2190597
BmHel-11	DF090323:531139-531427
BmHel-11	DF090323:96574-96862
BmHel-11	DF090406:141698-141989
BmHel-11	DF090406:1087651-1087934
BmHel-11	DF090367:136580-136868
BmHel-11	DF090367:1756333-1756626
BmHel-11	DF090344:2703435-2703715
BmHel-11	DF090344:1418349-1418638
BmHel-11	DF090344:1319120-1319399
BmHel-11	DF090344:486451-486738
BmHel-11	DF091283:2577-2868
BmHel-11	DF090380:696193-696481
BmHel-11	DF090380:849336-849626
BmHel-11	DF090380:2044454-2044733
BmHel-11	DF090380:1960859-1961146
BmHel-11	DF090380:1128347-1128637
BmHel-11	DF090380:694720-695003
BmHel-11	DF090380:75874-76162
BmHel-11	DF090370:621638-621929
BmHel-11	DF090370:1512852-1513143
BmHel-11	DF090370:2318027-2318316
BmHel-11	DF090370:1430739-1431020
BmHel-11	DF090320:4800290-4800565
BmHel-11	DF090320:6269442-6269729
BmHel-11	DF090320:6468288-6468576
BmHel-11	DF090320:6854778-6855067
BmHel-11	DF090320:7889236-7889525
BmHel-11	DF090320:8176258-8176548
BmHel-11	DF090320:8662117-8662400
BmHel-11	DF090320:4610267-4610553
BmHel-11	DF090320:4341388-4341677

BmHel-11	DF090320:999098-999388
BmHel-11	BABH01043665:2010-2297
BmHel-11	DF090452:69024-69312
BmHel-11	DF090451:409454-409744
BmHel-11	DF090442:538351-538639
BmHel-11	DF090442:290796-291084
BmHel-11	DF090396:259404-259694
BmHel-11	DF090396:634796-635084
BmHel-11	DF090396:1522400-1522688
BmHel-11	DF090379:1738948-1739236
BmHel-11	DF090379:1012006-1012293
BmHel-11	DF090378:1942748-1943036
BmHel-11	DF090378:473243-473532
BmHel-11	DF090378:401133-401421
BmHel-11	DF090378:67338-67624
BmHel-11	DF090366:1048704-1048991
BmHel-11	DF090366:1404118-1404413
BmHel-11	DF090366:171431-171719
BmHel-11	DF090364:254334-254621
BmHel-11	DF090364:464333-464622
BmHel-11	DF090364:1696643-1696936
BmHel-11	DF090364:1659140-1659428
BmHel-11	DF090364:1446649-1446937
BmHel-11	DF090354:3357975-3358260
BmHel-11	DF090354:849954-850239
BmHel-11	DF090352:3504370-3504658
BmHel-11	DF090352:1305568-1305856
BmHel-11	DF090352:560331-560619
BmHel-11	DF090352:401294-401582
BmHel-11	DF090317:896863-897151
BmHel-11	DF090317:1784644-1784932
BmHel-11	DF090317:1911722-1912013
BmHel-11	DF090317:2467069-2467358
BmHel-11	DF090317:4124011-4124299
BmHel-11	DF090317:4560683-4560974
BmHel-11	DF090317:6737505-6737786
BmHel-11	DF090317:7043877-7044168
BmHel-11	DF090317:8042310-8042598
BmHel-11	DF090317:8560620-8560908
BmHel-11	DF090317:9683041-9683330
BmHel-11	DF090317:9732988-9733274
BmHel-11	DF090317:10384273-10384561
BmHel-11	DF090317:10621397-10621533
BmHel-11	DF090317:10621545-10621696
BmHel-11	DF090317:10998117-10998402
BmHel-11	DF090317:7124714-7125004
BmHel-11	DF090317:6271147-6271431
BmHel-11	DF090317:5735052-5735331

BmHel-11	DF090317:4946804-4947100
BmHel-11	DF090317:4180902-4181190
BmHel-11	DF090317:1441043-1441327
BmHel-11	DF090317:23261-23551
BmHel-11	DF090468:170255-170557
BmHel-11	DF090459:35544-35837
BmHel-11	DF090438:480517-480802
BmHel-11	DF090438:313502-313780
BmHel-11	DF090435:24188-24475
BmHel-11	DF090417:61323-61611
BmHel-11	DF090417:387243-387535
BmHel-11	DF090417:981024-981312
BmHel-11	DF090417:532488-532775
BmHel-11	DF090404:363151-363435
BmHel-11	DF090389:717075-717363
BmHel-11	DF090389:521356-521650
BmHel-11	DF090372:1401901-1402188
BmHel-11	DF090358:473555-473842
BmHel-11	DF090358:1400511-1400808
BmHel-11	DF090358:1635662-1635953
BmHel-11	DF090358:2572477-2572755
BmHel-11	DF090358:1651896-1652190
BmHel-11	DF090358:1405886-1406175
BmHel-11	DF090358:1154416-1154703
BmHel-11	DF090358:1080008-1080296
BmHel-11	DF090356:2278423-2278711
BmHel-11	DF090356:3318315-3318603
BmHel-11	DF090356:3530361-3530646
BmHel-11	DF090356:1717535-1717822
BmHel-11	DF090356:1282045-1282334
BmHel-11	DF090467:99400-99688
BmHel-11	DF090434:578257-578546
BmHel-11	DF090376:682579-682867
BmHel-11	DF090376:1334954-1335242
BmHel-11	DF090376:1545841-1546117
BmHel-11	DF090345:4119130-4119418
BmHel-11	DF090333:3786404-3786686
BmHel-11	DF090333:5559163-5559450
BmHel-11	DF090333:1372618-1372907
BmHel-11	DF090333:101286-101581
BmHel-11	DF090327:784383-784666
BmHel-11	DF090327:4288570-4288858
BmHel-11	DF090327:4469927-4470201
BmHel-11	DF090327:4723144-4723431
BmHel-11	DF090327:3734752-3735044
BmHel-11	DF090327:2691089-2691376
BmHel-11	DF090327:1913685-1913971
BmHel-11	DF090398:1081957-1082242

BmHel-11	DF090398:1366472-1366760
BmHel-11	DF090371:353299-353588
BmHel-11	DF090371:686641-686932
BmHel-11	DF090371:1164622-1164909
BmHel-11	DF090371:1385697-1385991
BmHel-11	DF090346:2253354-2253642
BmHel-11	DF090346:3820306-3820590
BmHel-11	DF090346:3191637-3191918
BmHel-11	DF090346:1353980-1354268
BmHel-11	DF090346:682159-682440
BmHel-11	BABH01066512:323-611
BmHel-11	BABH01045849:2536-2823
BmHel-11	DF090472:325708-325996
BmHel-11	DF090472:301880-302169
BmHel-11	DF090427:237283-237568
BmHel-11	DF090416:608607-608894
BmHel-11	DF090416:1128507-1128794
BmHel-11	DF090403:757427-757714
BmHel-11	DF090403:1731523-1731810
BmHel-11	DF090403:213614-213902
BmHel-11	DF090361:548577-548865
BmHel-11	DF090361:1820091-1820375
BmHel-11	DF090361:2627504-2627796
BmHel-11	DF090338:4887262-4887550
BmHel-11	DF090338:4840429-4840719
BmHel-11	DF090338:4665299-4665594
BmHel-11	DF090338:4179633-4179924
BmHel-11	DF090338:3966853-3967143
BmHel-11	DF090671:15979-16267
BmHel-11	DF090462:358561-358849
BmHel-11	DF090462:292208-292488
BmHel-11	DF090423:64273-64563
BmHel-11	DF090422:662222-662511
BmHel-11	DF090422:981660-981947
BmHel-11	DF090375:1517901-1518189
BmHel-11	DF090375:2435714-2436000
BmHel-11	DF090375:1528409-1528697
BmHel-11	DF090397:1531545-1531832
BmHel-11	DF090374:202363-202656
BmHel-11	DF090374:1715894-1716182
BmHel-11	DF090437:547321-547608
BmHel-11	BABH01059970:153-441
BmHel-11	DF090449:751776-752064
BmHel-11	DF090415:1206421-1206709
BmHel-11	DF090415:372116-372404
BmHel-11	DF090409:466157-466445
BmHel-11	BABH01045090:2618-2910
BmHel-11	DF090446:294366-294654

BmHel-11	DF090432:300925-301214
BmHel-11	DF090394:648748-649036
BmHel-11	DF090394:1300571-1300861
BmHel-11	DF090394:86390-86679
BmHel-11	DF090457:269726-270017
BmHel-11	DF090426:451986-452276
BmHel-11	DF090426:202571-202856
BmHel-11	BABH01047571:1202-1490
BmHel-11	DF091597:987-1275
BmHel-11	DF091298:2271-2559
BmHel-11	DF091052:2872-3160
BmHel-11	DF090444:286182-286470
BmHel-11	DF090441:449366-449654
BmHel-11	DF090460:168758-169044
BmHel-11	DF090448:240863-241151
BmHel-11	DF090419:743342-743636
BmHel-11	DF090369:1206748-1207042
BmHel-11	DF090369:1381434-1381722
BmHel-11	DF090369:1690334-1690616
BmHel-11	DF090369:1472906-1473187
BmHel-11	DF090369:397775-398055
BmHel-11	DF090564:16756-17038
BmHel-11	BABH01081189:159-447
BmHel-11	DF091410:2068-2356
BmHel-11	DF090843:12176-12464
BmHel-11	DF090644:61780-62068
BmHel-11	DF090431:191617-191906
BmHel-11	DF090431:542029-542151
BmHel-11	DF090431:542176-542341
BmHel-11	DF090431:579847-580126
BmHel-11	DF091622:478-766
BmHel-11	DF090770:23937-24224
BmHel-11	DF090558:12757-13052
BmHel-11	DF090495:153541-153829
BmHel-11	DF090455:180208-180504
BmHel-11	DF090455:249864-250153
BmHel-11	DF090425:302503-302791
BmHel-11	DF090425:768937-769218
BmHel-11	DF090425:592002-592294
BmHel-11	BABH01050647:210-497
BmHel-11	BABH01047376:1048-1336
BmHel-11	DF090641:25199-25487
BmHel-11	DF090545:54315-54609
BmHel-11	BABH01056877:444-732
BmHel-11	DF091548:3278-3565
BmHel-11	BABH01045862:2645-2933
BmHel-11	DF090853:4210-4497
BmHel-11	DF090619:5441-5729

BmHel-11	DF090538:41597-41885
BmHel-11	DF090640:14010-14288
BmHel-11	DF090988:6825-7114
BmHel-11	DF090624:7945-8233
BmHel-11	DF090470:176263-176551
BmHel-11	DF090464:121698-121974
BmHel-11	DF090807:24352-24639
BmHel-11	BABH01051005:145-433
BmHel-11	BABH01043669:9737-10026
BmHel-11	BABH01048097:610-894
BmHel-11	BABH01049868:93-380
BmHel-11	DF090529:65733-66018
BmHel-11	BABH01053190:305-593
BmHel-11	DF091247:3790-4076
BmHel-11	DF090733:6915-7203
BmHel-11	BABH01045989:2626-2908
BmHel-11	DF090659:21341-21625
BmHel-11	DF090553:9307-9600
BmHel-11	DF090656:14275-14565
BmHel-11	DF090576:7543-7826
BmHel-11	DF091372:1128-1411
BmHel-11	DF090324:7544460-7544585
BmHel-11	DF090324:7544600-7544753
BmHel-11	DF090333:4393770-4393959
BmHel-11	DF090333:4393977-4394078
BmHel-11	DF090405:556074-556298
BmHel-11	DF090336:3886542-3886675
BmHel-11	DF090336:3886816-3886968
BmHel-11	DF090352:3834790-3835007
BmHel-11	DF090336:373696-373984
BmHel-11	DF090383:289646-289932
BmHel-11	DF090348:2698294-2698580
BmHel-11	DF090350:2934641-2934929
BmHel-11	DF090320:6062738-6063025
BmHel-11	DF090398:392252-392540
BmHel-11	DF090325:1750950-1751238
BmHel-11	DF090339:4853734-4854017
BmHel-11	DF090362:1035246-1035532
BmHel-11	DF090336:5573888-5574169
BmHel-11	DF090383:2066477-2066764
BmHel-11	DF090319:6804524-6804811
BmHel-11	DF090357:2809714-2809998
BmHel-11	DF090360:1795043-1795325
BmHel-11	DF090411:1232004-1232291
BmHel-11	DF090326:919193-919484
BmHel-11	DF090322:8033185-8033472
BmHel-11	DF090368:80163-80450
BmHel-11	DF090330:3620690-3620976

BmHel-11	DF090330:4869582-4869859
BmHel-11	DF090329:1972485-1972765
BmHel-11	DF090329:2540687-2540957
BmHel-11	DF090323:7649431-7649717
BmHel-11	DF090367:403065-403353
BmHel-11	DF090320:1880170-1880466
BmHel-11	DF090320:2758636-2758923
BmHel-11	DF090320:6294102-6294385
BmHel-11	DF090412:524732-525019
BmHel-11	DF090364:1412515-1412800
BmHel-11	DF090354:741920-742207
BmHel-11	DF090438:529783-530076
BmHel-11	DF090389:1187142-1187426
BmHel-11	DF090358:2536420-2536710
BmHel-11	DF090358:2797159-2797445
BmHel-11	DF090376:2106329-2106618
BmHel-11	DF090345:2975621-2975912
BmHel-11	DF090472:146583-146878
BmHel-11	DF090375:680153-680440
BmHel-11	DF090409:847317-847606
BmHel-11	DF090518:63733-64019
BmHel-11	DF090425:478612-478901
BmHel-11	DF090695:20350-20639
BmHel-11	BABH01045616:3667-3951
BmHel-11	DF090318:7573593-7573880
BmHel-11	DF090334:5453303-5453591
BmHel-11	DF090325:2776710-2776939
BmHel-11	DF090324:3484345-3484637
BmHel-11	DF090324:741648-741736
BmHel-11	DF090324:741000-741200
BmHel-11	DF090336:2548912-2549126
BmHel-11	DF090318:9434771-9434985
BmHel-11	DF090322:5874807-5875094
BmHel-11	DF090322:6702858-6703145
BmHel-11	DF090322:7608298-7608583
BmHel-11	DF090463:227819-228106
BmHel-11	DF090349:2115372-2115575
BmHel-11	DF090349:2111083-2111167
BmHel-11	DF090334:192915-193201
BmHel-11	DF090316:5189164-5189447
BmHel-11	DF090316:10375792-10376079
BmHel-11	DF090316:15935112-15935400
BmHel-11	DF090424:925449-925735
BmHel-11	DF090351:1176614-1176898
BmHel-11	DF090330:5376811-5377096
BmHel-11	DF090451:516176-516460
BmHel-11	DF090378:741343-741630
BmHel-11	DF090366:1701090-1701376

BmHel-11	DF090364:1142708-1142922
BmHel-11	DF090354:1733256-1733466
BmHel-11	DF090372:1221668-1221957
BmHel-11	DF090358:1580438-1580724
BmHel-11	DF090356:2352146-2352433
BmHel-11	DF090423:302244-302530
BmHel-11	DF090374:1023056-1023270
BmHel-11	DF090431:588907-589121
BmHel-11	DF090325:4485647-4485924
BmHel-11	DF090325:6472020-6472297
BmHel-11	DF090353:1251984-1252266
BmHel-11	DF090353:1981045-1981331
BmHel-11	DF090353:1744583-1744866
BmHel-11	DF090362:1259896-1260182
BmHel-11	DF090362:2906494-2906777
BmHel-11	DF090362:572220-572507
BmHel-11	DF090324:7912844-7913121
BmHel-11	DF090324:7259558-7259846
BmHel-11	DF090402:1424256-1424544
BmHel-11	DF090386:18145-18433
BmHel-11	DF090342:2196131-2196418
BmHel-11	DF090342:3072958-3073252
BmHel-11	DF090342:3930433-3930720
BmHel-11	DF090342:4728605-4728894
BmHel-11	DF090342:2233151-2233437
BmHel-11	DF090336:1041130-1041417
BmHel-11	DF090336:1902545-1902831
BmHel-11	DF090336:3478206-3478493
BmHel-11	DF090336:3670501-3670788
BmHel-11	DF090336:93170-93458
BmHel-11	DF090332:3882010-3882297
BmHel-11	DF090332:4129618-4129905
BmHel-11	DF090332:4316084-4316371
BmHel-11	DF090332:6286690-6286977
BmHel-11	DF090332:179044-179333
BmHel-11	DF090318:5791634-5791923
BmHel-11	DF090318:6570092-6570379
BmHel-11	DF090318:7630257-7630541
BmHel-11	DF090318:9720844-9721131
BmHel-11	DF090318:9110183-9110470
BmHel-11	DF090318:8798286-8798572
BmHel-11	DF090318:7972923-7973212
BmHel-11	DF090318:4396381-4396667
BmHel-11	DF090318:2941425-2941715
BmHel-11	DF090318:2654497-2654788
BmHel-11	DF090348:3763778-3764065
BmHel-11	DF090348:281376-281662
BmHel-11	DF090328:4635590-4635877

BmHel-11	DF090319:7824841-7825128
BmHel-11	DF090319:5797246-5797528
BmHel-11	DF090319:5432298-5432585
BmHel-11	DF090357:1609005-1609292
BmHel-11	DF090357:1854431-1854721
BmHel-11	DF090377:1302205-1302497
BmHel-11	DF090377:1380609-1380896
BmHel-11	DF090360:1464443-1464730
BmHel-11	DF090347:3522099-3522377
BmHel-11	DF090414:205161-205446
BmHel-11	DF090414:852216-852503
BmHel-11	DF090411:1243617-1243904
BmHel-11	DF090392:1239469-1239755
BmHel-11	DF090326:4633848-4634134
BmHel-11	DF090326:708696-708983
BmHel-11	DF090322:7007432-7007724
BmHel-11	DF090322:2233386-2233673
BmHel-11	DF090322:1554394-1554686
BmHel-11	DF090321:1288138-1288422
BmHel-11	DF090321:5060762-5061049
BmHel-11	DF090321:4767203-4767489
BmHel-11	DF090321:2147848-2148135
BmHel-11	DF090410:860258-860548
BmHel-11	DF090407:961292-961579
BmHel-11	DF090382:695769-696063
BmHel-11	DF090363:2510296-2510583
BmHel-11	DF090359:2570319-2570608
BmHel-11	DF090349:1130705-1130996
BmHel-11	DF090349:1877578-1877866
BmHel-11	DF090343:4490835-4491125
BmHel-11	DF090343:4084458-4084747
BmHel-11	DF090343:661794-662081
BmHel-11	DF090341:4614937-4615224
BmHel-11	DF090341:4784602-4784889
BmHel-11	DF090340:3892142-3892425
BmHel-11	DF090340:2384289-2384583
BmHel-11	DF090340:803435-803722
BmHel-11	DF090316:2155292-2155579
BmHel-11	DF090316:10360429-10360716
BmHel-11	DF090316:12675801-12676083
BmHel-11	DF090316:1421096-1421386
BmHel-11	BABH01065802:363-650
BmHel-11	DF090418:910503-910790
BmHel-11	DF090418:1060833-1061121
BmHel-11	DF090388:1726200-1726490
BmHel-11	DF090373:1906028-1906308
BmHel-11	DF090351:1392632-1392740
BmHel-11	DF090351:1392754-1392930

BmHel-11	DF090337:5137072-5137359
BmHel-11	DF090337:1278465-1278752
BmHel-11	DF090335:3915768-3916056
BmHel-11	DF090335:3467297-3467591
BmHel-11	DF090335:3030669-3030955
BmHel-11	DF090330:4240444-4240732
BmHel-11	DF090329:1534183-1534470
BmHel-11	DF090323:3110498-3110791
BmHel-11	DF090406:9283-9569
BmHel-11	DF090344:3900519-3900800
BmHel-11	DF090344:3018347-3018637
BmHel-11	DF090380:164215-164502
BmHel-11	DF090380:1535902-1536188
BmHel-11	DF090380:1814064-1814359
BmHel-11	DF090380:1983669-1983956
BmHel-11	DF090370:2569060-2569340
BmHel-11	DF090320:5363067-5363349
BmHel-11	DF090320:5631583-5631860
BmHel-11	DF090320:1275138-1275421
BmHel-11	DF090379:1023578-1023865
BmHel-11	DF090378:1393468-1393744
BmHel-11	DF090366:2470614-2470901
BmHel-11	DF090354:674990-675272
BmHel-11	DF090317:2520524-2520811
BmHel-11	DF090317:3272721-3273007
BmHel-11	DF090317:2769362-2769651
BmHel-11	DF090438:316611-316894
BmHel-11	DF090435:202636-202923
BmHel-11	DF090417:699362-699647
BmHel-11	DF090404:573929-574215
BmHel-11	DF090358:422847-423135
BmHel-11	DF090358:1912429-1912724
BmHel-11	DF090358:1827031-1827326
BmHel-11	DF090356:923789-924078
BmHel-11	DF090356:1459543-1459832
BmHel-11	DF090356:2649696-2649985
BmHel-11	DF090376:1460500-1460787
BmHel-11	DF090345:1080119-1080406
BmHel-11	DF090345:2371818-2372098
BmHel-11	DF090345:3599843-3600130
BmHel-11	DF090345:1098814-1099102
BmHel-11	DF090333:1621763-1622052
BmHel-11	DF090333:5286367-5286595
BmHel-11	DF090327:1789180-1789467
BmHel-11	DF090327:4983407-4983698
BmHel-11	DF090401:1040109-1040395
BmHel-11	DF090371:1793776-1794064
BmHel-11	DF090346:4207629-4207922

BmHel-11	DF090346:2596439-2596726
BmHel-11	DF090403:1106555-1106844
BmHel-11	DF090403:50490-50780
BmHel-11	DF090361:1732414-1732706
BmHel-11	DF090361:2502498-2502785
BmHel-11	DF090762:223-510
BmHel-11	DF090423:891870-892151
BmHel-11	DF090375:838424-838714
BmHel-11	DF090375:742532-742830
BmHel-11	DF090374:1575171-1575461
BmHel-11	DF090374:1450886-1451173
BmHel-11	DF090415:264710-264994
BmHel-11	BABH01077344:44-330
BmHel-11	DF090484:33861-34148
BmHel-11	DF090439:372088-372378
BmHel-11	DF090394:267296-267580
BmHel-11	DF090466:8578-8869
BmHel-11	DF090443:223722-223999
BmHel-11	DF090426:323828-324104
BmHel-11	DF090408:385333-385620
BmHel-11	DF090503:59156-59439
BmHel-11	DF090441:622151-622441
BmHel-11	DF090893:11137-11424
BmHel-11	DF090631:8653-8941
BmHel-11	DF090369:1375680-1375967
BmHel-11	DF090595:30783-31070
BmHel-11	DF090564:37068-37352
BmHel-11	BABH01047118:1359-1645
BmHel-11	DF090920:1996-2282
BmHel-11	BABH01044125:399-685
BmHel-11	DF090485:119953-120241
BmHel-11	BABH01045587:2352-2640
BmHel-11	DF090703:14156-14445
BmHel-11	DF090567:63046-63333
BmHel-11	BABH01048103:704-990
BmHel-11	BABH01047034:1046-1326
BmHel-11	DF090563:10016-10300
BmHel-11	DF090336:2689296-2689471
BmHel-11	DF090336:2689165-2689279
BmHel-11	DF090347:336417-336522
BmHel-11	DF090347:337775-337949
BmHel-11	DF090356:1598790-1599005
BmHel-11	DF090401:523943-524178
BmHel-11	DF090371:529955-530177
BmHel-11	DF090400:1444451-1444747
BmHel-11	DF090320:2389856-2390141
BmHel-11	DF090358:3183645-3183933
BmHel-11	DF090342:2526644-2526931

BmHel-11	DF090318:7752689-7752976
BmHel-11	DF090319:5842649-5842930
BmHel-11	DF090360:1069410-1069697
BmHel-11	DF090414:447000-447286
BmHel-11	DF090343:1544113-1544399
BmHel-11	DF090340:2283731-2284020
BmHel-11	DF090316:3299202-3299487
BmHel-11	DF090373:1058917-1059202
BmHel-11	DF090367:1561223-1561502
BmHel-11	DF090320:3802853-3803141
BmHel-11	DF090358:319793-320085
BmHel-11	DF090346:457500-457790
BmHel-11	DF090346:929361-929647
BmHel-11	DF090338:4474181-4474457
BmHel-11	DF090338:2350162-2350260
BmHel-11	DF090338:2342106-2342293
BmHel-11	DF090413:654702-654989
BmHel-11	BABH01059155:426-711
BmHel-11	DF090929:7884-8170
BmHel-11	DF090316:13780998-13781099
BmHel-11	DF090316:13780800-13780984
BmHel-11	DF090324:6276072-6276357
BmHel-11	DF090342:4460923-4461212
BmHel-11	DF090336:3606469-3606754
BmHel-11	DF090433:538606-538893
BmHel-11	DF090365:1893602-1893690
BmHel-11	DF090365:1893314-1893516
BmHel-11	DF090326:601887-602177
BmHel-11	DF090322:756497-756678
BmHel-11	DF090322:756690-756798
BmHel-11	DF090322:7080807-7081092
BmHel-11	DF090343:2550454-2550734
BmHel-11	DF090341:3491397-3491681
BmHel-11	DF090341:770687-770972
BmHel-11	DF090334:2953145-2953433
BmHel-11	DF090334:888221-888449
BmHel-11	DF090331:4763331-4763610
BmHel-11	DF090351:2593075-2593361
BmHel-11	DF090337:3005668-3005955
BmHel-11	DF090329:3146624-3146914
BmHel-11	DF090364:1670027-1670313
BmHel-11	DF090352:2830182-2830466
BmHel-11	DF090352:2892941-2893226
BmHel-11	DF090317:5344279-5344565
BmHel-11	DF090417:754170-754456
BmHel-11	DF090417:624165-624452
BmHel-11	DF090417:465177-465463
BmHel-11	DF090327:1469293-1469579

BmHel-11	DF090327:557771-558056
BmHel-11	DF090346:1605581-1605867
BmHel-11	DF090427:627941-628227
BmHel-11	DF090338:442732-443017
BmHel-11	DF090422:241159-241445
BmHel-11	DF090369:1952653-1952882
BmHel-11	DF090473:368408-368690
BmHel-11	BABH01046169:763-1052
BmHel-11	DF090690:24616-24734
BmHel-11	DF090690:24407-24572
BmHel-11	DF090357:1984228-1984441
BmHel-11	DF090323:1192380-1192661
BmHel-11	DF090384:1553541-1553827
BmHel-11	DF090426:134003-134203
BmHel-11	DF090426:133856-133944
BmHel-11	BABH01045967:2716-3009
BmHel-11	DF090355:1033046-1033336
BmHel-11	DF090355:1230758-1231044
BmHel-11	DF090355:1314177-1314462
BmHel-11	DF090355:2020751-2021041
BmHel-11	DF090355:3221942-3222230
BmHel-11	DF090355:940590-940873
BmHel-11	DF090325:2253327-2253623
BmHel-11	DF090325:2616636-2616921
BmHel-11	DF090325:3282625-3282911
BmHel-11	DF090325:2026640-2026926
BmHel-11	DF090325:1551925-1552210
BmHel-11	DF090353:3759373-3759655
BmHel-11	DF090353:3427918-3428204
BmHel-11	DF090353:3016163-3016452
BmHel-11	DF090353:2946263-2946551
BmHel-11	DF090353:800753-801039
BmHel-11	DF090339:142222-142504
BmHel-11	DF090339:490960-491245
BmHel-11	DF090339:1081948-1082230
BmHel-11	DF090339:1420134-1420420
BmHel-11	DF090339:4707218-4707504
BmHel-11	DF090339:4735927-4736213
BmHel-11	DF090339:1000676-1000964
BmHel-11	DF090339:854575-854863
BmHel-11	DF090362:1750332-1750618
BmHel-11	DF090362:3064750-3065036
BmHel-11	DF090362:2560277-2560563
BmHel-11	DF090362:2401350-2401632
BmHel-11	DF090362:1783985-1784270
BmHel-11	DF090362:1339121-1339410
BmHel-11	DF090362:909714-910005
BmHel-11	DF090362:808998-809282

BmHel-11	DF090362:399724-400010
BmHel-11	DF090445:177171-177456
BmHel-11	DF090387:1723097-1723383
BmHel-11	DF090324:1645839-1646128
BmHel-11	DF090324:2813958-2814244
BmHel-11	DF090324:6011383-6011666
BmHel-11	DF090324:6831069-6831357
BmHel-11	DF090324:3059817-3060103
BmHel-11	DF090421:12610-12896
BmHel-11	DF090402:908931-909220
BmHel-11	DF090402:424015-424302
BmHel-11	DF090390:1616991-1617277
BmHel-11	DF090390:1010076-1010362
BmHel-11	DF090390:178263-178556
BmHel-11	DF090381:625676-625958
BmHel-11	DF090381:131534-131822
BmHel-11	DF090342:18263-18544
BmHel-11	DF090342:590984-591270
BmHel-11	DF090342:2240217-2240503
BmHel-11	DF090342:2471842-2472132
BmHel-11	DF090342:2582775-2583062
BmHel-11	DF090342:2733288-2733574
BmHel-11	DF090342:2987395-2987686
BmHel-11	DF090342:4326582-4326868
BmHel-11	DF090342:4417139-4417425
BmHel-11	DF090342:3097046-3097333
BmHel-11	DF090336:436930-437225
BmHel-11	DF090336:2872434-2872720
BmHel-11	DF090336:3360661-3360948
BmHel-11	DF090336:3503053-3503333
BmHel-11	DF090336:4196340-4196629
BmHel-11	DF090336:5564980-5565267
BmHel-11	DF090336:4339132-4339424
BmHel-11	DF090336:1762288-1762575
BmHel-11	DF090332:505869-506154
BmHel-11	DF090332:1772617-1772903
BmHel-11	DF090332:3858725-3859011
BmHel-11	DF090332:3952500-3952785
BmHel-11	DF090332:2766068-2766369
BmHel-11	DF090332:2621254-2621543
BmHel-11	DF090332:2139795-2140078
BmHel-11	DF090332:354334-354620
BmHel-11	DF090318:1460430-1460716
BmHel-11	DF090318:3015134-3015422
BmHel-11	DF090318:6090057-6090340
BmHel-11	DF090318:8370364-8370657
BmHel-11	DF090318:8830117-8830408
BmHel-11	DF090318:8254363-8254648

BmHel-11	DF090318:7257534-7257827
BmHel-11	DF090318:7058754-7059040
BmHel-11	DF090318:2374820-2375106
BmHel-11	DF090318:1388295-1388581
BmHel-11	DF090436:253660-253943
BmHel-11	DF090436:666415-666701
BmHel-11	DF090383:1401748-1402034
BmHel-11	DF090383:1970869-1971155
BmHel-11	DF090383:643355-643642
BmHel-11	DF090348:4372090-4372375
BmHel-11	DF090348:3449301-3449587
BmHel-11	DF090348:3404226-3404511
BmHel-11	DF090348:3069671-3069953
BmHel-11	DF090348:2490264-2490550
BmHel-11	DF090348:292527-292812
BmHel-11	DF090328:24568-24853
BmHel-11	DF090328:4590702-4590988
BmHel-11	DF090328:4655968-4656253
BmHel-11	DF090328:3597048-3597343
BmHel-11	DF090328:2565774-2566060
BmHel-11	DF090328:971526-971812
BmHel-11	DF090319:1003940-1004219
BmHel-11	DF090319:4294723-4295008
BmHel-11	DF090319:4932693-4932979
BmHel-11	DF090319:5390766-5391051
BmHel-11	DF090319:7794811-7795097
BmHel-11	DF090319:8916981-8917266
BmHel-11	DF090319:8945084-8945361
BmHel-11	DF090319:6855231-6855517
BmHel-11	DF090319:5716876-5717155
BmHel-11	DF090319:4609987-4610265
BmHel-11	DF090357:1575151-1575437
BmHel-11	DF090357:2334187-2334474
BmHel-11	DF090357:3580111-3580396
BmHel-11	DF090357:3537913-3538199
BmHel-11	DF090395:1520529-1520815
BmHel-11	DF090360:649741-650027
BmHel-11	DF090360:3075658-3075950
BmHel-11	DF090360:3160324-3160610
BmHel-11	DF090360:673394-673674
BmHel-11	DF090347:3269596-3269881
BmHel-11	DF090347:3551783-3552073
BmHel-11	DF090347:4190568-4190858
BmHel-11	DF090347:2747789-2748073
BmHel-11	DF090347:2524963-2525242
BmHel-11	DF090482:32249-32535
BmHel-11	DF090482:160319-160605
BmHel-11	DF090414:613234-613519

BmHel-11	DF090414:1029026-1029310
BmHel-11	DF090414:1129094-1129380
BmHel-11	DF090414:109069-109349
BmHel-11	DF090411:129211-129493
BmHel-11	DF090392:1307064-1307350
BmHel-11	DF090392:1632917-1633205
BmHel-11	DF090392:1644669-1644951
BmHel-11	DF090365:569296-569583
BmHel-11	DF090365:701720-702006
BmHel-11	DF090365:1872536-1872824
BmHel-11	DF090365:1160465-1160750
BmHel-11	DF090326:6249200-6249486
BmHel-11	DF090326:6046261-6046553
BmHel-11	DF090326:3733574-3733852
BmHel-11	DF090326:666622-666904
BmHel-11	DF090322:1905130-1905417
BmHel-11	DF090322:5218555-5218841
BmHel-11	DF090322:6573804-6574090
BmHel-11	DF090322:3464585-3464876
BmHel-11	DF090322:3253240-3253524
BmHel-11	DF090322:2368321-2368608
BmHel-11	DF090322:27979-28263
BmHel-11	DF090321:342764-343054
BmHel-11	DF090321:3286864-3287149
BmHel-11	DF090321:5499925-5500212
BmHel-11	DF090321:5846302-5846581
BmHel-11	DF090321:5943299-5943585
BmHel-11	DF090321:8101809-8102095
BmHel-11	DF090321:6633411-6633695
BmHel-11	DF090321:2500410-2500694
BmHel-11	DF090410:502381-502668
BmHel-11	DF090410:831000-831287
BmHel-11	DF090410:167286-167572
BmHel-11	DF090350:3724619-3724905
BmHel-11	DF090407:477749-478035
BmHel-11	DF090393:1411877-1412162
BmHel-11	DF090393:442683-442973
BmHel-11	DF090382:865129-865412
BmHel-11	DF090382:1716117-1716403
BmHel-11	DF090382:2097394-2097680
BmHel-11	DF090363:2304421-2304716
BmHel-11	DF090363:2404973-2405258
BmHel-11	DF090363:2582353-2582639
BmHel-11	DF090363:228939-229224
BmHel-11	DF090359:339115-339400
BmHel-11	DF090349:116977-117263
BmHel-11	DF090349:533060-533350
BmHel-11	DF090349:1359145-1359431

BmHel-11	DF090349:4143563-4143849
BmHel-11	DF090349:4128735-4129023
BmHel-11	DF090349:2731312-2731589
BmHel-11	DF090349:61933-62219
BmHel-11	DF090343:2257226-2257511
BmHel-11	DF090343:3916962-3917248
BmHel-11	DF090343:2924237-2924521
BmHel-11	DF090343:1175-1461
BmHel-11	DF090341:1654034-1654326
BmHel-11	DF090341:2081460-2081746
BmHel-11	DF090341:4685802-4686088
BmHel-11	DF090341:2824312-2824597
BmHel-11	DF090340:2850212-2850504
BmHel-11	DF090340:3435699-3435981
BmHel-11	DF090340:4165508-4165795
BmHel-11	DF090340:3617587-3617879
BmHel-11	DF090340:3124074-3124363
BmHel-11	DF090340:872114-872400
BmHel-11	DF090340:627452-627738
BmHel-11	DF090340:435431-435717
BmHel-11	DF090334:3365303-3365592
BmHel-11	DF090334:3728313-3728588
BmHel-11	DF090334:3940775-3941061
BmHel-11	DF090334:4939457-4939743
BmHel-11	DF090334:5792788-5793074
BmHel-11	DF090331:3915406-3915694
BmHel-11	DF090331:3811551-3811838
BmHel-11	DF090331:3556006-3556292
BmHel-11	DF090331:2466583-2466866
BmHel-11	DF090331:161455-161742
BmHel-11	DF090316:110588-110871
BmHel-11	DF090316:2909284-2909568
BmHel-11	DF090316:6287009-6287295
BmHel-11	DF090316:9587913-9588199
BmHel-11	DF090316:10394268-10394562
BmHel-11	DF090316:11136396-11136682
BmHel-11	DF090316:14293362-14293485
BmHel-11	DF090316:14293516-14293686
BmHel-11	DF090316:16002408-16002696
BmHel-11	DF090316:15152273-15152559
BmHel-11	DF090316:14051364-14051650
BmHel-11	DF090316:13448904-13449190
BmHel-11	DF090316:11258185-11258472
BmHel-11	DF090316:5873655-5873942
BmHel-11	DF090316:4588072-4588358
BmHel-11	DF090316:4247385-4247671
BmHel-11	DF090316:1195999-1196279
BmHel-11	DF090502:52147-52434

BmHel-11	DF090424:236123-236409
BmHel-11	DF090424:682047-682334
BmHel-11	DF090418:742664-742946
BmHel-11	DF090400:656730-657017
BmHel-11	DF090400:1159039-1159326
BmHel-11	DF090400:549227-549509
BmHel-11	DF090391:1248451-1248651
BmHel-11	DF090391:1248668-1248753
BmHel-11	DF090391:1290871-1291159
BmHel-11	DF090391:1047798-1048083
BmHel-11	DF090391:545957-546240
BmHel-11	DF090388:1228465-1228750
BmHel-11	DF090385:342078-342364
BmHel-11	DF090385:1547199-1547484
BmHel-11	DF090385:1485022-1485308
BmHel-11	DF090373:529378-529664
BmHel-11	DF090373:786449-786733
BmHel-11	DF090373:1961457-1961743
BmHel-11	DF090373:2174067-2174352
BmHel-11	DF090373:920599-920885
BmHel-11	DF090368:261398-261684
BmHel-11	DF090368:2278161-2278448
BmHel-11	DF090368:459499-459785
BmHel-11	DF090351:1766719-1767006
BmHel-11	DF090351:2246488-2246774
BmHel-11	DF090351:861474-861760
BmHel-11	DF090351:839234-839519
BmHel-11	DF090337:10910-11187
BmHel-11	DF090337:5203114-5203394
BmHel-11	DF090337:4290756-4291042
BmHel-11	DF090337:3449665-3449951
BmHel-11	DF090337:833897-834185
BmHel-11	DF090335:3624238-3624526
BmHel-11	DF090335:4536692-4536977
BmHel-11	DF090335:4451978-4452264
BmHel-11	DF090335:3382218-3382504
BmHel-11	DF090335:2104194-2104479
BmHel-11	DF090335:1808928-1809214
BmHel-11	DF090330:3566810-3567093
BmHel-11	DF090330:4882672-4882956
BmHel-11	DF090330:4972887-4973176
BmHel-11	DF090330:6307630-6307916
BmHel-11	DF090330:6376945-6377238
BmHel-11	DF090330:6222946-6223231
BmHel-11	DF090330:6018779-6019064
BmHel-11	DF090330:3812208-3812492
BmHel-11	DF090330:237356-237642
BmHel-11	DF090329:1260552-1260838

BmHel-11	DF090329:1272128-1272410
BmHel-11	DF090329:3685637-3685926
BmHel-11	DF090329:4733352-4733638
BmHel-11	DF090329:5827405-5827693
BmHel-11	DF090329:4658426-4658714
BmHel-11	DF090329:4170697-4170979
BmHel-11	DF090329:1604123-1604406
BmHel-11	DF090323:3264168-3264454
BmHel-11	DF090323:4054964-4055248
BmHel-11	DF090323:6716086-6716367
BmHel-11	DF090323:6997192-6997473
BmHel-11	DF090323:7719208-7719494
BmHel-11	DF090323:4013035-4013313
BmHel-11	BABH01076723:268-554
BmHel-11	DF091572:1012-1298
BmHel-11	DF090450:394345-394633
BmHel-11	DF090406:1564227-1564513
BmHel-11	DF090406:1481101-1481388
BmHel-11	DF090367:1048602-1048888
BmHel-11	DF090367:1200607-1200893
BmHel-11	DF090367:2598181-2598469
BmHel-11	DF090367:1349001-1349283
BmHel-11	DF090344:36561-36848
BmHel-11	DF090344:1968191-1968478
BmHel-11	DF090344:2408378-2408664
BmHel-11	DF090344:2615241-2615528
BmHel-11	DF090344:3682074-3682360
BmHel-11	DF090344:4020512-4020799
BmHel-11	DF090344:4050934-4051216
BmHel-11	DF090344:4583116-4583400
BmHel-11	DF090344:4625076-4625360
BmHel-11	DF090344:3278153-3278441
BmHel-11	DF090344:833444-833730
BmHel-11	DF090380:658371-658657
BmHel-11	DF090380:743322-743610
BmHel-11	DF090370:1043658-1043944
BmHel-11	DF090320:573553-573839
BmHel-11	DF090320:993776-994062
BmHel-11	DF090320:1898756-1899044
BmHel-11	DF090320:6026265-6026551
BmHel-11	DF090320:4002758-4003045
BmHel-11	DF090320:3639197-3639483
BmHel-11	DF090320:1909223-1909507
BmHel-11	DF090451:14752-15039
BmHel-11	DF090399:588111-588396
BmHel-11	DF090396:406103-406393
BmHel-11	DF090384:823979-824265
BmHel-11	DF090384:599058-599345

BmHel-11	DF090379:528324-528610
BmHel-11	DF090379:1691727-1692013
BmHel-11	DF090379:1382659-1382947
BmHel-11	DF090379:558596-558877
BmHel-11	DF090378:1481501-1481787
BmHel-11	DF090378:1654952-1655237
BmHel-11	DF090378:1628357-1628653
BmHel-11	DF090378:1398242-1398528
BmHel-11	DF090366:144119-144406
BmHel-11	DF090366:497872-498158
BmHel-11	DF090364:1839260-1839543
BmHel-11	DF090364:1227202-1227491
BmHel-11	DF090364:188255-188541
BmHel-11	DF090364:56007-56293
BmHel-11	DF090354:1164569-1164853
BmHel-11	DF090354:2514321-2514600
BmHel-11	DF090354:3120600-3120886
BmHel-11	DF090354:3301033-3301319
BmHel-11	DF090354:3723036-3723322
BmHel-11	DF090354:3260293-3260579
BmHel-11	DF090354:2940466-2940749
BmHel-11	DF090354:605122-605412
BmHel-11	DF090352:278654-278940
BmHel-11	DF090352:1076687-1076970
BmHel-11	DF090352:1835409-1835693
BmHel-11	DF090352:3590942-3591228
BmHel-11	DF090352:2678115-2678403
BmHel-11	DF090352:2627780-2628064
BmHel-11	DF090352:1850161-1850446
BmHel-11	DF090352:1795325-1795610
BmHel-11	DF090352:535778-536059
BmHel-11	DF090317:3749629-3749915
BmHel-11	DF090317:4840843-4841129
BmHel-11	DF090317:6653319-6653606
BmHel-11	DF090317:10011576-10011862
BmHel-11	DF090317:10789541-10789827
BmHel-11	DF090317:8519549-8519836
BmHel-11	DF090317:8325819-8326106
BmHel-11	DF090317:6466187-6466473
BmHel-11	DF090317:4809193-4809479
BmHel-11	DF090317:4523528-4523806
BmHel-11	DF090317:3486219-3486505
BmHel-11	DF090317:3055796-3056077
BmHel-11	DF090317:2388868-2389145
BmHel-11	DF090317:1588162-1588443
BmHel-11	DF090317:1517468-1517750
BmHel-11	DF090486:92989-93275
BmHel-11	DF090459:266543-266830

BmHel-11	DF090438:331537-331824
BmHel-11	DF090435:599972-600258
BmHel-11	DF090417:505159-505439
BmHel-11	DF090404:291547-291839
BmHel-11	DF090389:792017-792299
BmHel-11	DF090372:2372428-2372713
BmHel-11	DF090372:711331-711616
BmHel-11	DF090372:422911-423198
BmHel-11	DF090358:1913230-1913521
BmHel-11	DF090358:2491184-2491467
BmHel-11	DF090358:1931369-1931658
BmHel-11	DF090358:279505-279795
BmHel-11	DF090356:3693958-3694243
BmHel-11	DF090356:2280856-2281143
BmHel-11	DF090356:1171647-1171933
BmHel-11	DF090356:166079-166365
BmHel-11	DF090573:54352-54638
BmHel-11	DF090440:552306-552592
BmHel-11	DF090434:660336-660621
BmHel-11	DF090434:168721-169007
BmHel-11	DF090376:1300621-1300913
BmHel-11	DF090376:1526918-1527204
BmHel-11	DF090376:1639088-1639372
BmHel-11	DF090376:190038-190324
BmHel-11	DF090345:531241-531531
BmHel-11	DF090345:2170280-2170567
BmHel-11	DF090345:4344926-4345212
BmHel-11	DF090345:1557087-1557371
BmHel-11	DF090333:5311315-5311601
BmHel-11	DF090333:5823062-5823348
BmHel-11	DF090333:4998049-4998340
BmHel-11	DF090333:3638700-3638992
BmHel-11	DF090333:3407787-3408073
BmHel-11	DF090333:1225159-1225445
BmHel-11	DF090333:1104345-1104633
BmHel-11	DF090333:1002002-1002288
BmHel-11	DF090327:2688240-2688531
BmHel-11	DF090327:6577810-6578097
BmHel-11	DF090327:5002517-5002800
BmHel-11	DF090327:4219009-4219285
BmHel-11	DF090327:2677280-2677569
BmHel-11	DF090327:1147625-1147913
BmHel-11	DF090327:1021574-1021857
BmHel-11	DF090327:384636-384917
BmHel-11	DF090405:298116-298407
BmHel-11	DF090405:493800-494086
BmHel-11	DF090405:571721-572007
BmHel-11	DF090405:774456-774742

BmHel-11	DF090405:1130837-1131127
BmHel-11	DF090405:1432574-1432860
BmHel-11	DF090405:1028320-1028613
BmHel-11	DF090405:411072-411358
BmHel-11	DF090401:1057185-1057471
BmHel-11	DF090398:544707-544996
BmHel-11	DF090371:2396722-2397009
BmHel-11	DF090346:1499137-1499427
BmHel-11	DF090346:2282938-2283231
BmHel-11	DF090501:36572-36859
BmHel-11	DF090427:287665-287948
BmHel-11	DF090416:40755-41043
BmHel-11	DF090403:468817-469103
BmHel-11	DF090403:1194729-1195015
BmHel-11	DF090403:658938-659224
BmHel-11	DF090361:1844664-1844951
BmHel-11	DF090361:2931801-2932082
BmHel-11	DF090361:538763-539053
BmHel-11	DF090338:740948-741234
BmHel-11	DF090338:5082401-5082686
BmHel-11	DF090338:831190-831475
BmHel-11	DF090462:360918-361206
BmHel-11	DF090428:621024-621301
BmHel-11	DF090422:660465-660754
BmHel-11	DF090375:233598-233883
BmHel-11	DF090375:626221-626502
BmHel-11	DF090375:2232730-2233016
BmHel-11	DF090374:1573574-1573869
BmHel-11	DF090374:275327-275607
BmHel-11	DF090449:825485-825771
BmHel-11	DF090449:486064-486347
BmHel-11	DF090420:865475-865760
BmHel-11	DF090484:191459-191738
BmHel-11	DF090439:509447-509732
BmHel-11	DF090439:605257-605543
BmHel-11	DF090439:303647-303935
BmHel-11	DF090394:654238-654526
BmHel-11	DF090592:21085-21371
BmHel-11	DF090466:306959-307245
BmHel-11	DF090466:254236-254522
BmHel-11	DF090443:243814-244100
BmHel-11	DF090413:819153-819444
BmHel-11	DF090503:29275-29561
BmHel-11	DF090444:116819-117100
BmHel-11	DF090441:717207-717495
BmHel-11	BABH01042898:10630-10916
BmHel-11	DF090590:4302-4588
BmHel-11	DF090430:336909-337190

BmHel-11	DF090430:322105-322383
BmHel-11	DF090698:20438-20723
BmHel-11	DF090561:6023-6309
BmHel-11	DF090429:818823-819109
BmHel-11	DF090429:756138-756424
BmHel-11	DF090419:642524-642810
BmHel-11	DF090369:942253-942535
BmHel-11	DF090369:1599610-1599892
BmHel-11	DF090369:1829235-1829521
BmHel-11	DF090369:907903-908188
BmHel-11	DF090369:787522-787808
BmHel-11	DF091669:1089-1378
BmHel-11	DF090431:626047-626332
BmHel-11	DF090465:2620-2906
BmHel-11	DF090455:371736-372018
BmHel-11	DF090455:410715-411001
BmHel-11	DF090838:290-576
BmHel-11	DF090772:14433-14719
BmHel-11	DF090425:541945-542231
BmHel-11	DF091264:1312-1600
BmHel-11	BABH01049592:533-821
BmHel-11	DF090475:202642-202925
BmHel-11	DF090530:40363-40649
BmHel-11	DF090740:9592-9875
BmHel-11	DF090531:65271-65556
BmHel-11	DF090925:1565-1853
BmHel-11	DF090481:217035-217326
BmHel-11	DF091051:711-1002
BmHel-11	DF090665:259-550
BmHel-11	DF090680:17928-18215
BmHel-11	DF090653:8536-8830
BmHel-11	BABH01068920:29-312
BmHel-11	DF090784:19885-20169
BmHel-11	BABH01046709:52-345
BmHel-11	DF090666:5374-5659
BmHel-11	DF090343:4471817-4472061
BmHel-11	DF090359:2561592-2561772
BmHel-11	DF090359:2561790-2561894
BmHel-11	DF090744:1586-1777
BmHel-11	DF090744:1790-1888
BmHel-11	DF090339:4172825-4173111
BmHel-11	DF090343:4159359-4159644
BmHel-11	DF090333:4167296-4167589
BmHel-11	DF090377:1617441-1617733
BmHel-11	DF090326:3927604-3927889
BmHel-11	DF090321:396374-396658
BmHel-11	DF090407:1000574-1000859
BmHel-11	DF090343:4148737-4149021

BmHel-11	DF090341:313934-314219
BmHel-11	DF090334:5387453-5387731
BmHel-11	DF090316:14722375-14722658
BmHel-11	DF090316:6379811-6380096
BmHel-11	DF090380:809963-810249
BmHel-11	DF090412:1154604-1154888
BmHel-11	DF090317:7773547-7773833
BmHel-11	DF090435:492473-492751
BmHel-11	DF090358:2045030-2045315
BmHel-11	DF090376:1766201-1766486
BmHel-11	DF090333:1137230-1137513
BmHel-11	DF090403:438167-438450
BmHel-11	DF090338:670499-670783
BmHel-11	DF090394:33691-33980
BmHel-11	DF090369:742365-742650
BmHel-11	DF090369:954674-954966
BmHel-11	DF090524:24352-24638
BmHel-11	DF090343:1063878-1064163
BmHel-11	DF090343:1176657-1176837
BmHel-11	DF090343:1176540-1176634
BmHel-11	DF090344:785048-785333
BmHel-11	DF090386:1547817-1548031
BmHel-11	DF090348:4334948-4335129
BmHel-11	DF090348:4335153-4335256
BmHel-11	DF090377:429101-429386
BmHel-11	DF090360:1072650-1072885
BmHel-11	DF090360:2574370-2574655
BmHel-11	DF090347:723538-723815
BmHel-11	DF090321:2568949-2569230
BmHel-11	DF090321:2706396-2706681
BmHel-11	DF090343:1622872-1623158
BmHel-11	DF090343:3228369-3228654
BmHel-11	DF090341:4576389-4576674
BmHel-11	DF090316:5844847-5845131
BmHel-11	DF090370:1466250-1466535
BmHel-11	DF090320:2935912-2936197
BmHel-11	DF090452:483731-484014
BmHel-11	DF090366:4164-4444
BmHel-11	DF090352:1290743-1291027
BmHel-11	DF090317:6743950-6744235
BmHel-11	DF090401:717747-718027
BmHel-11	DF090472:208518-208800
BmHel-11	DF090462:201783-202068
BmHel-11	DF090601:37476-37761
BmHel-11	DF090460:309853-310058
BmHel-11	DF090448:70619-70905
BmHel-11	DF090578:18077-18204
BmHel-11	DF090578:18218-18377

BmHel-11	DF090319:3955720-3956005
BmHel-11	DF090322:4195114-4195399
BmHel-11	DF090343:215168-215451
BmHel-11	DF090340:2553015-2553212
BmHel-11	DF090340:2554543-2554630
BmHel-11	DF090331:5675990-5676112
BmHel-11	DF090331:5674552-5674718
BmHel-11	DF090380:469903-470187
BmHel-11	DF090378:1138446-1138733
BmHel-11	DF090346:3217867-3218152
BmHel-11	DF090369:1500885-1501171
BmHel-11	DF090493:156923-157144
BmHel-11	DF090355:2467130-2467423
BmHel-11	DF090362:3190251-3190460
BmHel-11	DF090332:5311265-5311493
BmHel-11	DF090318:1846537-1846747
BmHel-11	DF090348:226256-226542
BmHel-11	DF090414:156224-156509
BmHel-11	DF090321:415124-415407
BmHel-11	DF090321:3224696-3224981
BmHel-11	DF090382:233490-233612
BmHel-11	DF090382:232920-233083
BmHel-11	DF090316:2227044-2227291
BmHel-11	DF090330:5938727-5938945
BmHel-11	DF090367:1522908-1523192
BmHel-11	DF090320:2963141-2963353
BmHel-11	DF090320:2436236-2436447
BmHel-11	DF090403:621788-622000
BmHel-11	DF090422:477765-478039
BmHel-11	DF090375:446351-446631
BmHel-11	DF090413:92513-92790
BmHel-11	DF090355:2123175-2123459
BmHel-11	DF090355:2257060-2257345
BmHel-11	DF090325:3007070-3007356
BmHel-11	DF090325:3484570-3484855
BmHel-11	DF090325:3872524-3872809
BmHel-11	DF090325:5378755-5379040
BmHel-11	DF090325:6100717-6101003
BmHel-11	DF090325:7199085-7199375
BmHel-11	DF090325:248105-248390
BmHel-11	DF090353:1943177-1943462
BmHel-11	DF090353:1316100-1316385
BmHel-11	DF090362:2283729-2284005
BmHel-11	DF090362:2970575-2970857
BmHel-11	DF090362:2542075-2542362
BmHel-11	DF090362:1914499-1914784
BmHel-11	DF090362:1839175-1839444
BmHel-11	DF090387:1859911-1860196

BmHel-11	DF090387:1605110-1605391
BmHel-11	DF090324:204030-204314
BmHel-11	DF090324:572803-573073
BmHel-11	DF090324:3239326-3239611
BmHel-11	DF090324:5557801-5558089
BmHel-11	DF090324:7818050-7818337
BmHel-11	DF090324:5597029-5597314
BmHel-11	DF090324:1257357-1257644
BmHel-11	DF090402:708227-708518
BmHel-11	DF090390:236308-236593
BmHel-11	DF090390:320681-320966
BmHel-11	DF090381:580150-580435
BmHel-11	DF090381:395216-395501
BmHel-11	DF090342:4370205-4370489
BmHel-11	DF090342:4123854-4124139
BmHel-11	DF090342:216439-216724
BmHel-11	DF090336:1394926-1395213
BmHel-11	DF090336:2564363-2564648
BmHel-11	DF090336:2943601-2943886
BmHel-11	DF090332:732979-733260
BmHel-11	DF090332:5331028-5331313
BmHel-11	DF090332:1646796-1647082
BmHel-11	DF090318:3601634-3601925
BmHel-11	DF090318:4705762-4706037
BmHel-11	DF090318:9506858-9507143
BmHel-11	DF090318:6558556-6558840
BmHel-11	DF090318:4277829-4278112
BmHel-11	DF090318:3459264-3459543
BmHel-11	DF090318:2718930-2719215
BmHel-11	DF090383:676419-676705
BmHel-11	DF090383:424969-425252
BmHel-11	DF090348:261663-261948
BmHel-11	DF090348:3835606-3835892
BmHel-11	DF090328:1472717-1472998
BmHel-11	DF090328:1524895-1525180
BmHel-11	DF090328:1462181-1462460
BmHel-11	DF090319:2149499-2149784
BmHel-11	DF090319:3192972-3193257
BmHel-11	DF090319:4008243-4008527
BmHel-11	DF090319:5127331-5127616
BmHel-11	DF090319:7386806-7387092
BmHel-11	DF090319:8451604-8451891
BmHel-11	DF090319:7465178-7465463
BmHel-11	DF090319:4017982-4018267
BmHel-11	DF090319:2846130-2846417
BmHel-11	DF090319:1427748-1428034
BmHel-11	DF090357:3798262-3798547
BmHel-11	DF090357:3304782-3305065

BmHel-11	DF090395:1415697-1415985
BmHel-11	DF090360:2663631-2663916
BmHel-11	DF090360:3331466-3331751
BmHel-11	DF090360:2383802-2384087
BmHel-11	DF090360:606585-606872
BmHel-11	DF090347:1890827-1891120
BmHel-11	DF090347:3148965-3149254
BmHel-11	DF090414:916387-916672
BmHel-11	DF090414:746209-746494
BmHel-11	DF090414:316295-316577
BmHel-11	DF090411:764622-764907
BmHel-11	DF090411:1110138-1110422
BmHel-11	DF090365:1000930-1001218
BmHel-11	DF090365:2437385-2437674
BmHel-11	DF090365:2591058-2591345
BmHel-11	DF090365:2954722-2955007
BmHel-11	DF090326:955966-956250
BmHel-11	DF090326:6116795-6117078
BmHel-11	DF090326:5346552-5346837
BmHel-11	DF090326:5152415-5152704
BmHel-11	DF090322:1960568-1960855
BmHel-11	DF090322:2743209-2743497
BmHel-11	DF090322:7293105-7293404
BmHel-11	DF090322:561064-561348
BmHel-11	DF090321:4378758-4379043
BmHel-11	DF090321:5753701-5753986
BmHel-11	DF090321:7992597-7992887
BmHel-11	DF090321:852499-852780
BmHel-11	DF090350:792022-792307
BmHel-11	DF090469:290644-290932
BmHel-11	DF090393:322705-322984
BmHel-11	DF090393:1180939-1181223
BmHel-11	DF090382:1280331-1280608
BmHel-11	DF090363:2240952-2241230
BmHel-11	DF090363:2334090-2334376
BmHel-11	DF090363:2689228-2689513
BmHel-11	DF090363:1048061-1048345
BmHel-11	DF090363:626879-627168
BmHel-11	DF090363:483877-484154
BmHel-11	DF090363:272906-273187
BmHel-11	DF090359:1487386-1487671
BmHel-11	DF090349:3791630-3791915
BmHel-11	DF090343:2595242-2595523
BmHel-11	DF090343:2800216-2800494
BmHel-11	DF090343:3421466-3421751
BmHel-11	DF090341:4475930-4476215
BmHel-11	DF090341:475748-476036
BmHel-11	DF090341:446087-446367

BmHel-11	DF090340:644407-644693
BmHel-11	DF090340:1210865-1211152
BmHel-11	DF090340:2510983-2511263
BmHel-11	DF090340:3901041-3901319
BmHel-11	DF090340:130859-131144
BmHel-11	DF090334:1674981-1675261
BmHel-11	DF090331:86435-86721
BmHel-11	DF090331:4351944-4352229
BmHel-11	DF090331:2158233-2158518
BmHel-11	DF090316:3707241-3707526
BmHel-11	DF090316:5673904-5674188
BmHel-11	DF090316:10173665-10173950
BmHel-11	DF090316:12375678-12375960
BmHel-11	DF090316:14133693-14133971
BmHel-11	DF090316:16127414-16127698
BmHel-11	DF090316:11215563-11215850
BmHel-11	DF090316:478693-478975
BmHel-11	DF090418:344192-344473
BmHel-11	DF090400:793935-794220
BmHel-11	DF090391:1480188-1480465
BmHel-11	DF090391:339817-340105
BmHel-11	DF090388:455838-456125
BmHel-11	DF090388:622099-622378
BmHel-11	DF090385:624708-624994
BmHel-11	DF090385:1504990-1505276
BmHel-11	DF090373:2030934-2031222
BmHel-11	DF090368:2093398-2093682
BmHel-11	DF090368:670064-670349
BmHel-11	DF090368:352485-352770
BmHel-11	DF090368:308710-308995
BmHel-11	DF090351:2649294-2649582
BmHel-11	DF090351:4266108-4266394
BmHel-11	DF090351:2996154-2996434
BmHel-11	DF090351:2934635-2934920
BmHel-11	DF090351:1339154-1339432
BmHel-11	DF090337:3629350-3629634
BmHel-11	DF090337:933102-933387
BmHel-11	DF090335:1317008-1317288
BmHel-11	DF090335:1491407-1491692
BmHel-11	DF090335:2874667-2874949
BmHel-11	DF090335:622532-622815
BmHel-11	DF090330:5269015-5269298
BmHel-11	DF090330:3104865-3105158
BmHel-11	DF090329:585780-586063
BmHel-11	DF090329:1388146-1388427
BmHel-11	DF090329:3284302-3284587
BmHel-11	DF090329:3908388-3908668
BmHel-11	DF090329:4872648-4872933

BmHel-11	DF090329:6011988-6012273
BmHel-11	DF090329:6704445-6704730
BmHel-11	DF090329:4855364-4855650
BmHel-11	DF090329:4389857-4390145
BmHel-11	DF090329:1234533-1234819
BmHel-11	DF090323:7912673-7912852
BmHel-11	DF090323:7912866-7912972
BmHel-11	DF090323:7664194-7664479
BmHel-11	DF090323:6106544-6106829
BmHel-11	DF090323:3695796-3696081
BmHel-11	DF090323:2616888-2617173
BmHel-11	DF090323:933848-934138
BmHel-11	DF090406:915513-915795
BmHel-11	DF090367:905822-906107
BmHel-11	DF090344:1722040-1722325
BmHel-11	DF090344:2149898-2150188
BmHel-11	DF090344:2397331-2397614
BmHel-11	DF090320:793296-793573
BmHel-11	DF090320:3291267-3291559
BmHel-11	DF090320:3544091-3544369
BmHel-11	DF090320:4776270-4776558
BmHel-11	DF090320:4508906-4509191
BmHel-11	DF090320:3881974-3882259
BmHel-11	DF090490:136749-137034
BmHel-11	DF090399:1219306-1219607
BmHel-11	DF090384:889469-889754
BmHel-11	DF090378:1746987-1747272
BmHel-11	DF090366:23081-23366
BmHel-11	DF090366:1216578-1216863
BmHel-11	DF090366:1850647-1850932
BmHel-11	DF090364:1055917-1056202
BmHel-11	DF090364:807657-807942
BmHel-11	DF090354:1246528-1246813
BmHel-11	DF090354:2194141-2194426
BmHel-11	DF090354:3280083-3280367
BmHel-11	DF090352:1182388-1182678
BmHel-11	DF090352:1557898-1558179
BmHel-11	DF090352:2553794-2554078
BmHel-11	DF090317:1305581-1305866
BmHel-11	DF090317:1503449-1503736
BmHel-11	DF090317:1524531-1524815
BmHel-11	DF090317:4611703-4611990
BmHel-11	DF090317:7495578-7495863
BmHel-11	DF090317:9256663-9256957
BmHel-11	DF090317:6555207-6555491
BmHel-11	DF090317:5179647-5179932
BmHel-11	DF090317:5082935-5083218
BmHel-11	DF090317:4974907-4975192

BmHel-11	DF090486:75013-75298
BmHel-11	DF090468:291973-292265
BmHel-11	DF090417:1160249-1160522
BmHel-11	DF090404:936117-936402
BmHel-11	DF090404:718635-718913
BmHel-11	DF090372:1811739-1812018
BmHel-11	DF090372:2301438-2301727
BmHel-11	DF090372:1834348-1834631
BmHel-11	DF090372:683671-683956
BmHel-11	DF090358:1496196-1496488
BmHel-11	DF090358:766037-766319
BmHel-11	DF090356:627786-628064
BmHel-11	DF090356:1917686-1917974
BmHel-11	DF090356:3499374-3499648
BmHel-11	DF090356:2925055-2925340
BmHel-11	DF090356:2331554-2331837
BmHel-11	DF090376:597753-598040
BmHel-11	DF090376:1359511-1359796
BmHel-11	DF090333:3515118-3515396
BmHel-11	DF090333:4494376-4494674
BmHel-11	DF090333:4058228-4058513
BmHel-11	DF090333:1969823-1970114
BmHel-11	DF090327:6475355-6475636
BmHel-11	DF090327:5936223-5936510
BmHel-11	DF090327:4941420-4941710
BmHel-11	DF090401:1441567-1441849
BmHel-11	DF090401:1309414-1309690
BmHel-11	DF090371:477980-478264
BmHel-11	DF090371:2021053-2021342
BmHel-11	DF090346:912215-912504
BmHel-11	DF090346:2865070-2865355
BmHel-11	DF090346:194674-194951
BmHel-11	DF090501:25188-25478
BmHel-11	DF090403:123767-124052
BmHel-11	DF090403:560897-561181
BmHel-11	DF090403:784779-785066
BmHel-11	DF090361:2275544-2275827
BmHel-11	DF090361:1304003-1304291
BmHel-11	DF090338:49370-49655
BmHel-11	DF090338:4083277-4083555
BmHel-11	DF090422:699497-699791
BmHel-11	DF090422:923589-923874
BmHel-11	DF090375:1318096-1318383
BmHel-11	DF090375:326856-327147
BmHel-11	DF090374:1238885-1239170
BmHel-11	DF090437:393660-393945
BmHel-11	DF090437:533785-534069
BmHel-11	DF090449:17391-17676

BmHel-11	DF090449:812905-813191
BmHel-11	DF090420:318265-318550
BmHel-11	DF090415:756610-756894
BmHel-11	DF090409:619107-619392
BmHel-11	DF090409:1316937-1317222
BmHel-11	DF090409:941241-941525
BmHel-11	DF090446:113952-114232
BmHel-11	DF090439:315451-315730
BmHel-11	DF090394:785653-785943
BmHel-11	DF090394:290657-290945
BmHel-11	DF090523:31682-31967
BmHel-11	DF090408:159532-159818
BmHel-11	DF090444:397267-397550
BmHel-11	DF090590:37969-38255
BmHel-11	DF090430:48983-49267
BmHel-11	DF090430:115868-116153
BmHel-11	DF090419:65004-65289
BmHel-11	DF090419:119581-119870
BmHel-11	DF090369:496186-496473
BmHel-11	DF090369:2682888-2683173
BmHel-11	DF090454:135173-135458
BmHel-11	DF090510:18989-19274
BmHel-11	DF090431:391226-391496
BmHel-11	BABH01049234:70-355
BmHel-11	DF090556:58612-58897
BmHel-11	DF090425:741064-741349
BmHel-11	DF090678:5007-5292
BmHel-11	DF090619:27749-28034
BmHel-11	DF090475:80867-81156
BmHel-11	BABH01049309:107-392
BmHel-11	DF090626:15538-15823
BmHel-11	DF091583:2936-3225
BmHel-11	DF090491:57012-57297
BmHel-11	DF090549:34212-34505
BmHel-11	BABH01045769:549-838
BmHel-11	BABH01053183:186-478
BmHel-11	DF090515:220214-220492
BmHel-11	DF090325:3451036-3451155
BmHel-11	DF090325:3451605-3451782
BmHel-11	DF090428:842766-842892
BmHel-11	DF090428:842464-842631
BmHel-11	DF090325:2986039-2986222
BmHel-11	DF090325:2985917-2986025
BmHel-11	DF090403:291336-291515
BmHel-11	DF090328:2350887-2351171
BmHel-11	DF090352:327227-327511
BmHel-11	BABH01047461:890-1173
BmHel-11	DF090342:139562-139839

BmHel-11	DF090319:5909964-5910249
BmHel-11	DF090393:1463966-1464250
BmHel-11	DF090363:1400601-1400885
BmHel-11	DF090334:4763570-4763861
BmHel-11	DF090373:2275161-2275445
BmHel-11	DF090370:827450-827734
BmHel-11	DF090412:944506-944791
BmHel-11	DF090372:1252696-1252976
BmHel-11	DF090338:584244-584518
BmHel-11	DF090428:505476-505760
BmHel-11	DF090353:1616353-1616581
BmHel-11	DF090393:616700-616984
BmHel-11	DF090335:1638177-1638462
BmHel-11	DF090339:1709167-1709451
BmHel-11	DF090336:4143055-4143188
BmHel-11	DF090336:4142772-4142931
BmHel-11	DF090332:5403312-5403596
BmHel-11	DF090318:5509781-5510064
BmHel-11	DF090326:6035050-6035287
BmHel-11	DF090321:822501-822782
BmHel-11	DF090410:1220581-1220865
BmHel-11	DF090359:3128565-3128729
BmHel-11	DF090359:3128428-3128548
BmHel-11	DF090388:994045-994223
BmHel-11	DF090388:993657-993763
BmHel-11	DF090373:1750304-1750556
BmHel-11	DF090337:2077784-2077979
BmHel-11	DF090337:2078086-2078182
BmHel-11	DF090323:2315107-2315402
BmHel-11	DF090367:1865814-1866002
BmHel-11	DF090367:1866015-1866109
BmHel-11	DF090344:746462-746681
BmHel-11	DF090320:1809629-1809877
BmHel-11	DF090417:139805-140089
BmHel-11	DF090358:6441-6726
BmHel-11	DF090440:161531-161812
BmHel-11	DF090345:4073188-4073470
BmHel-11	DF090333:3843190-3843309
BmHel-11	DF090333:3843001-3843163
BmHel-11	DF090422:802249-802499
BmHel-11	DF091004:3531-3816
BmHel-11	DF090786:25659-25837
BmHel-11	DF090786:25870-25975
BmHel-11	BABH01085090:82-334
BmHel-11	DF090833:6280-6464
BmHel-11	DF090833:6480-6580
BmHel-11	DF090343:1407482-1407631
BmHel-11	DF090343:1407322-1407455

BmHel-11	DF090351:87083-87368
BmHel-11	DF090330:4469448-4469732
BmHel-11	DF090486:49380-49606
BmHel-11	DF090355:2503352-2503637
BmHel-11	DF090355:3089697-3089913
BmHel-11	DF090324:926708-926992
BmHel-11	DF090402:379592-379876
BmHel-11	DF090383:1479023-1479307
BmHel-11	DF090377:1764428-1764715
BmHel-11	DF090347:1521534-1521818
BmHel-11	DF090469:285360-285646
BmHel-11	DF090393:1022214-1022497
BmHel-11	DF090349:649385-649668
BmHel-11	DF090331:820491-820775
BmHel-11	DF090316:15745809-15746093
BmHel-11	DF090316:3911378-3911659
BmHel-11	DF090316:2656910-2657195
BmHel-11	DF090316:515905-516199
BmHel-11	DF090388:408687-408894
BmHel-11	DF090335:3891801-3892014
BmHel-11	DF090370:1654286-1654442
BmHel-11	DF090370:1654458-1654588
BmHel-11	DF090354:1719814-1720097
BmHel-11	DF090317:2781144-2781431
BmHel-11	DF090317:5493435-5493719
BmHel-11	DF090372:2362376-2362650
BmHel-11	DF090358:3459434-3459708
BmHel-11	DF090356:3844002-3844287
BmHel-11	DF090401:872864-873146
BmHel-11	DF090375:2225810-2226094
BmHel-11	DF090426:311497-311780
BmHel-11	DF090618:25257-25541
BmHel-11	BABH01070556:350-631
BmHel-11	DF090355:640592-640873
BmHel-11	DF090386:1137292-1137504
BmHel-11	DF090342:1902291-1902571
BmHel-11	DF090348:105533-105817
BmHel-11	DF090328:6577058-6577333
BmHel-11	DF090347:2674434-2674713
BmHel-11	DF090326:2827163-2827380
BmHel-11	DF090321:6609872-6610024
BmHel-11	DF090321:6609399-6609518
BmHel-11	DF090331:2959423-2959564
BmHel-11	DF090331:2960019-2960160
BmHel-11	DF090320:6807334-6807617
BmHel-11	DF090317:9899544-9899819
BmHel-11	DF090327:837082-837229
BmHel-11	DF090327:837248-837338

BmHel-11	DF090346:1411146-1411356
BmHel-11	DF090427:372940-373150
BmHel-11	DF090419:488302-488590
BmHel-11	DF090458:99358-99568
BmHel-11	DF090518:46583-46803
BmHel-11	DF090832:2413-2694
BmHel-11	DF090355:1076570-1076856
BmHel-11	DF090355:1298426-1298710
BmHel-11	DF090355:2704826-2705104
BmHel-11	DF090355:3356408-3356679
BmHel-11	DF090355:1640132-1640422
BmHel-11	DF090355:795831-796108
BmHel-11	DF090325:2645544-2645828
BmHel-11	DF090325:4894126-4894404
BmHel-11	DF090353:3972088-3972372
BmHel-11	DF090353:2631471-2631753
BmHel-11	DF090353:1705291-1705579
BmHel-11	DF090353:1631027-1631310
BmHel-11	DF090339:2729486-2729772
BmHel-11	DF090339:2057865-2058149
BmHel-11	DF090339:633460-633745
BmHel-11	DF090339:528207-528499
BmHel-11	DF090339:212871-213153
BmHel-11	DF090362:871803-872087
BmHel-11	DF090362:2123308-2123592
BmHel-11	DF090362:2559604-2559889
BmHel-11	DF090362:1279203-1279487
BmHel-11	DF090362:727783-728060
BmHel-11	BABH01072502:338-622
BmHel-11	DF090534:127534-127818
BmHel-11	DF090445:533460-533744
BmHel-11	DF090324:1482795-1483072
BmHel-11	DF090324:1710205-1710489
BmHel-11	DF090324:1909653-1909946
BmHel-11	DF090324:3752305-3752591
BmHel-11	DF090324:6143478-6143762
BmHel-11	DF090324:7969503-7969789
BmHel-11	DF090324:7333588-7333870
BmHel-11	DF090324:5161639-5161923
BmHel-11	DF090324:4961495-4961770
BmHel-11	DF090324:1630959-1631248
BmHel-11	DF090324:1569255-1569545
BmHel-11	DF090402:214910-215194
BmHel-11	DF090390:113041-113329
BmHel-11	DF090390:382345-382631
BmHel-11	DF090390:692362-692641
BmHel-11	DF090390:1134314-1134600
BmHel-11	DF090386:1483567-1483851

BmHel-11	DF090386:1574256-1574547
BmHel-11	DF090381:135267-135551
BmHel-11	DF090381:1226495-1226783
BmHel-11	DF090381:1533992-1534277
BmHel-11	DF090381:1015424-1015709
BmHel-11	DF090342:3150325-3150617
BmHel-11	DF090342:3805983-3806267
BmHel-11	DF090342:4311835-4312122
BmHel-11	DF090342:4263320-4263605
BmHel-11	DF090342:3365749-3366025
BmHel-11	DF090342:1866401-1866685
BmHel-11	DF090342:1846478-1846761
BmHel-11	DF090336:586602-586892
BmHel-11	DF090336:1366063-1366348
BmHel-11	DF090336:4731255-4731544
BmHel-11	DF090336:5141280-5141560
BmHel-11	DF090336:4274338-4274621
BmHel-11	DF090336:2348695-2348978
BmHel-11	DF090336:1046977-1047261
BmHel-11	DF090332:5060478-5060764
BmHel-11	DF090332:4979602-4979886
BmHel-11	DF090332:3489448-3489733
BmHel-11	DF090332:1825618-1825901
BmHel-11	DF090332:1405517-1405801
BmHel-11	DF090318:1986289-1986573
BmHel-11	DF090318:2041976-2042260
BmHel-11	DF090318:6295563-6295843
BmHel-11	DF090318:8911477-8911756
BmHel-11	DF090318:8398118-8398404
BmHel-11	DF090318:7408053-7408339
BmHel-11	DF090318:7274469-7274745
BmHel-11	DF090318:3865010-3865295
BmHel-11	DF090318:493915-494200
BmHel-11	DF090318:468174-468459
BmHel-11	DF090318:50881-51164
BmHel-11	DF090436:188492-188767
BmHel-11	DF090436:284924-285204
BmHel-11	DF090436:503367-503651
BmHel-11	DF090383:923851-924130
BmHel-11	DF090383:995135-995419
BmHel-11	DF090383:1040337-1040617
BmHel-11	DF090383:1018490-1018776
BmHel-11	DF090348:3178697-3178983
BmHel-11	DF090328:4168282-4168561
BmHel-11	DF090328:5213590-5213874
BmHel-11	DF090328:5603303-5603587
BmHel-11	DF090328:5532943-5533225
BmHel-11	DF090328:3047006-3047294

BmHel-11	DF090328:1858113-1858411
BmHel-11	DF090328:1000230-1000515
BmHel-11	DF090319:248432-248709
BmHel-11	DF090319:3674587-3674874
BmHel-11	DF090319:5087822-5088105
BmHel-11	DF090319:6880616-6880894
BmHel-11	DF090319:7057655-7057942
BmHel-11	DF090319:8340314-8340592
BmHel-11	DF090319:8465048-8465332
BmHel-11	DF090319:5329162-5329454
BmHel-11	DF090319:4828356-4828640
BmHel-11	DF090319:3000467-3000751
BmHel-11	DF090319:2899170-2899450
BmHel-11	DF090433:640980-641261
BmHel-11	DF090357:1554213-1554492
BmHel-11	DF090395:843951-844235
BmHel-11	DF090377:316221-316505
BmHel-11	DF090377:659279-659556
BmHel-11	DF090360:2206563-2206847
BmHel-11	DF090360:3013640-3013926
BmHel-11	DF090360:1138120-1138404
BmHel-11	DF090347:2392542-2392829
BmHel-11	DF090347:1936716-1937000
BmHel-11	DF090347:1188188-1188472
BmHel-11	DF090347:1076114-1076406
BmHel-11	DF090414:733071-733355
BmHel-11	DF090411:1225933-1226213
BmHel-11	DF090411:637026-637312
BmHel-11	DF090392:1709205-1709489
BmHel-11	DF090365:1743842-1744128
BmHel-11	DF090365:2575860-2576134
BmHel-11	DF090365:2443388-2443677
BmHel-11	DF090326:130748-131031
BmHel-11	DF090326:357793-358072
BmHel-11	DF090326:1175481-1175766
BmHel-11	DF090326:4859944-4860214
BmHel-11	DF090326:6143999-6144283
BmHel-11	DF090326:6740234-6740519
BmHel-11	DF090326:6929030-6929314
BmHel-11	DF090326:6693534-6693818
BmHel-11	DF090326:5572959-5573241
BmHel-11	DF090326:3025916-3026200
BmHel-11	DF090326:1896008-1896293
BmHel-11	DF090322:3516937-3517221
BmHel-11	DF090322:6933638-6933923
BmHel-11	DF090322:4877213-4877499
BmHel-11	DF090322:2886610-2886891
BmHel-11	DF090321:4399843-4400127

BmHel-11	DF090321:4597187-4597471
BmHel-11	DF090321:5563059-5563342
BmHel-11	DF090321:5683536-5683820
BmHel-11	DF090321:7488593-7488870
BmHel-11	DF090321:5771730-5772010
BmHel-11	DF090321:4334057-4334340
BmHel-11	DF090321:572646-572922
BmHel-11	DF090410:34694-34971
BmHel-11	DF090410:822175-822459
BmHel-11	DF090410:99999-100283
BmHel-11	DF090350:367268-367553
BmHel-11	DF090471:191460-191743
BmHel-11	DF090469:218345-218630
BmHel-11	DF090469:201741-202026
BmHel-11	DF090393:805592-805877
BmHel-11	DF090393:1609651-1609938
BmHel-11	DF090393:1488055-1488339
BmHel-11	DF090393:1221302-1221586
BmHel-11	DF090382:1440827-1441111
BmHel-11	DF090363:1556979-1557263
BmHel-11	DF090363:2498140-2498424
BmHel-11	DF090363:792506-792790
BmHel-11	DF090363:284453-284740
BmHel-11	DF090363:56518-56803
BmHel-11	DF090359:1260837-1261121
BmHel-11	DF090359:2199130-2199414
BmHel-11	DF090359:825893-826179
BmHel-11	DF090359:753385-753661
BmHel-11	DF090359:148237-148517
BmHel-11	DF090349:33896-34180
BmHel-11	DF090349:2157250-2157536
BmHel-11	DF090349:2395151-2395435
BmHel-11	DF090349:3117840-3118129
BmHel-11	DF090349:3194022-3194306
BmHel-11	DF090349:3905375-3905659
BmHel-11	DF090349:144022-144308
BmHel-11	DF090343:1552601-1552890
BmHel-11	DF090343:3677517-3677800
BmHel-11	DF090343:3719444-3719732
BmHel-11	DF090343:3266851-3267135
BmHel-11	DF090341:299018-299301
BmHel-11	DF090341:921290-921574
BmHel-11	DF090341:2882889-2883173
BmHel-11	DF090341:2909675-2909959
BmHel-11	DF090341:4096470-4096753
BmHel-11	DF090341:199771-200056
BmHel-11	DF090340:1417862-1418148
BmHel-11	DF090340:2785300-2785580

BmHel-11	DF090340:3065230-3065516
BmHel-11	DF090340:83865-84143
BmHel-11	DF090334:1103042-1103323
BmHel-11	DF090334:1572086-1572368
BmHel-11	DF090334:2071611-2071894
BmHel-11	DF090334:3418473-3418753
BmHel-11	DF090334:4160417-4160699
BmHel-11	DF090334:4660667-4660951
BmHel-11	DF090334:5446198-5446481
BmHel-11	DF090334:3577772-3578060
BmHel-11	DF090334:2083012-2083297
BmHel-11	DF090334:1476120-1476404
BmHel-11	DF090331:27402-27688
BmHel-11	DF090331:5550372-5550650
BmHel-11	DF090331:3017878-3018156
BmHel-11	DF090331:271309-271605
BmHel-11	DF090316:1231802-1232087
BmHel-11	DF090316:5784898-5785182
BmHel-11	DF090316:9862881-9863165
BmHel-11	DF090316:10442155-10442439
BmHel-11	DF090316:15463984-15464267
BmHel-11	DF090316:15612224-15612508
BmHel-11	DF090316:16031795-16032075
BmHel-11	DF090316:15624721-15625004
BmHel-11	DF090316:14912142-14912426
BmHel-11	DF090316:13025798-13026082
BmHel-11	DF090316:12895440-12895713
BmHel-11	DF090316:12129279-12129564
BmHel-11	DF090316:9719976-9720259
BmHel-11	DF090316:9006492-9006774
BmHel-11	DF090316:8537648-8537926
BmHel-11	DF090316:4734699-4734983
BmHel-11	DF090316:4268260-4268544
BmHel-11	DF090316:1373059-1373344
BmHel-11	DF090418:1088742-1089026
BmHel-11	DF090418:587869-588153
BmHel-11	DF090400:661484-661783
BmHel-11	DF090400:28549-28837
BmHel-11	DF090391:1629024-1629303
BmHel-11	DF090391:1210740-1211024
BmHel-11	DF090388:709705-709989
BmHel-11	DF090388:919481-919769
BmHel-11	DF090388:1628053-1628337
BmHel-11	DF090388:826936-827223
BmHel-11	DF090388:779743-780027
BmHel-11	DF090388:457368-457659
BmHel-11	DF090373:1355336-1355614
BmHel-11	DF090373:1612601-1612888

BmHel-11	DF090351:4013451-4013739
BmHel-11	DF090351:1549995-1550286
BmHel-11	DF090337:4518175-4518454
BmHel-11	DF090337:1207206-1207488
BmHel-11	DF090335:1849133-1849418
BmHel-11	DF090335:1981585-1981869
BmHel-11	DF090335:2764913-2765195
BmHel-11	DF090335:3845047-3845339
BmHel-11	DF090335:4746374-4746658
BmHel-11	DF090335:4243420-4243693
BmHel-11	DF090335:3964209-3964493
BmHel-11	DF090335:3885842-3886126
BmHel-11	DF090335:707487-707769
BmHel-11	DF090330:688859-689138
BmHel-11	DF090330:2360438-2360724
BmHel-11	DF090330:5959738-5960022
BmHel-11	DF090330:6389848-6390132
BmHel-11	DF090330:6172091-6172374
BmHel-11	DF090330:5615230-5615515
BmHel-11	DF090330:3392035-3392325
BmHel-11	DF090330:3343326-3343607
BmHel-11	DF090330:2188381-2188664
BmHel-11	DF090330:1959483-1959766
BmHel-11	DF090330:765717-766002
BmHel-11	DF090330:297343-297627
BmHel-11	DF090329:232190-232474
BmHel-11	DF090329:604691-604979
BmHel-11	DF090329:773541-773824
BmHel-11	DF090329:1161835-1162124
BmHel-11	DF090329:1212635-1212920
BmHel-11	DF090329:4318418-4318702
BmHel-11	DF090329:5772211-5772498
BmHel-11	DF090329:4943476-4943762
BmHel-11	DF090329:4191639-4191924
BmHel-11	DF090329:3616329-3616616
BmHel-11	DF090329:448370-448654
BmHel-11	DF090329:409852-410136
BmHel-11	DF090323:2111410-2111694
BmHel-11	DF090323:3141306-3141589
BmHel-11	DF090323:5190035-5190320
BmHel-11	DF090323:7096626-7096912
BmHel-11	DF090323:5264886-5265168
BmHel-11	DF090406:217807-218090
BmHel-11	DF090406:425899-426181
BmHel-11	DF090406:754925-755204
BmHel-11	DF090367:322840-323125
BmHel-11	DF090367:1708521-1708806
BmHel-11	DF090344:2666087-2666370

BmHel-11	DF090344:2657124-2657402
BmHel-11	DF090344:977136-977420
BmHel-11	DF090344:869064-869348
BmHel-11	DF090344:67791-68074
BmHel-11	DF090370:949277-949568
BmHel-11	DF090320:1916568-1916852
BmHel-11	DF090320:2542221-2542502
BmHel-11	DF090320:5892807-5893095
BmHel-11	DF090320:6189722-6190001
BmHel-11	DF090320:7023652-7023939
BmHel-11	DF090320:8616394-8616672
BmHel-11	DF090320:6078216-6078498
BmHel-11	DF090320:4645653-4645937
BmHel-11	DF090320:625306-625597
BmHel-11	DF090490:128682-128967
BmHel-11	DF090490:100893-101177
BmHel-11	DF090451:423640-423925
BmHel-11	DF090442:230697-230975
BmHel-11	DF090412:616866-617150
BmHel-11	DF090412:1081194-1081478
BmHel-11	DF090399:602885-603169
BmHel-11	DF090399:131393-131678
BmHel-11	DF090396:862670-862954
BmHel-11	DF090384:245330-245617
BmHel-11	DF090384:1864631-1864915
BmHel-11	DF090384:1615233-1615517
BmHel-11	DF090384:904930-905215
BmHel-11	DF090384:742926-743204
BmHel-11	DF090379:221860-222144
BmHel-11	DF090379:1605853-1606137
BmHel-11	DF090379:582298-582583
BmHel-11	DF090379:418633-418920
BmHel-11	DF090378:1034286-1034570
BmHel-11	DF090366:249394-249677
BmHel-11	DF090366:2042872-2043156
BmHel-11	DF090366:219988-220268
BmHel-11	DF090364:408238-408520
BmHel-11	DF090364:750986-751270
BmHel-11	DF090364:1199340-1199620
BmHel-11	DF090364:1825835-1826115
BmHel-11	DF090354:430091-430374
BmHel-11	DF090354:726946-727229
BmHel-11	DF090354:1395329-1395613
BmHel-11	DF090354:2379232-2379502
BmHel-11	DF090354:2195667-2195951
BmHel-11	DF090354:1883738-1884022
BmHel-11	DF090352:370901-371185
BmHel-11	DF090352:423945-424225

BmHel-11	DF090352:1223822-1224096
BmHel-11	DF090317:2011350-2011634
BmHel-11	DF090317:4167723-4168003
BmHel-11	DF090317:6847333-6847616
BmHel-11	DF090317:9104394-9104682
BmHel-11	DF090317:9921456-9921740
BmHel-11	DF090317:10074514-10074796
BmHel-11	DF090317:9233990-9234274
BmHel-11	DF090317:8925124-8925407
BmHel-11	DF090317:5552504-5552788
BmHel-11	DF090317:5113489-5113773
BmHel-11	DF090317:3279195-3279481
BmHel-11	DF090317:71926-72214
BmHel-11	DF090498:51756-52038
BmHel-11	DF090498:72051-72337
BmHel-11	DF090459:279199-279480
BmHel-11	DF090438:516300-516584
BmHel-11	DF090417:237209-237490
BmHel-11	DF090404:911535-911819
BmHel-11	DF090389:612992-613280
BmHel-11	DF090389:945167-945449
BmHel-11	DF090372:1172653-1172939
BmHel-11	DF090358:1170500-1170774
BmHel-11	DF090358:2057883-2058172
BmHel-11	DF090358:2987129-2987413
BmHel-11	DF090358:2596943-2597227
BmHel-11	DF090358:893355-893633
BmHel-11	DF090356:2789507-2789791
BmHel-11	DF090356:3100685-3100969
BmHel-11	DF090356:3811368-3811656
BmHel-11	DF090356:1639922-1640206
BmHel-11	DF090440:41424-41706
BmHel-11	DF090434:293028-293311
BmHel-11	DF090376:30601-30887
BmHel-11	DF090345:1015550-1015834
BmHel-11	DF090345:2812333-2812607
BmHel-11	DF090345:2181638-2181922
BmHel-11	DF090333:5357645-5357928
BmHel-11	DF090327:3191887-3192171
BmHel-11	DF090327:3961671-3961961
BmHel-11	DF090327:1674099-1674383
BmHel-11	DF090327:1125334-1125620
BmHel-11	DF090494:53311-53594
BmHel-11	DF090405:656868-657145
BmHel-11	DF090405:836259-836538
BmHel-11	DF090405:874618-874905
BmHel-11	DF090405:704326-704605
BmHel-11	DF090401:1000768-1001044

BmHel-11	DF090401:62299-62585
BmHel-11	DF090398:1563870-1564145
BmHel-11	DF090398:1213030-1213314
BmHel-11	DF090346:2696824-2697110
BmHel-11	DF090346:2912978-2913264
BmHel-11	DF090346:4193744-4194026
BmHel-11	DF090346:3366966-3367252
BmHel-11	DF090346:160406-160691
BmHel-11	DF090403:396410-396694
BmHel-11	DF090403:1217491-1217783
BmHel-11	DF090403:159669-159945
BmHel-11	DF090361:1216749-1217034
BmHel-11	DF090361:1246915-1247200
BmHel-11	DF090361:3029289-3029573
BmHel-11	DF090338:1076374-1076664
BmHel-11	DF090338:1494743-1495029
BmHel-11	DF090338:2148895-2149183
BmHel-11	DF090338:4870149-4870434
BmHel-11	DF090338:3863759-3864047
BmHel-11	DF090338:1547804-1548088
BmHel-11	DF090338:661412-661686
BmHel-11	DF090477:69686-69970
BmHel-11	DF090428:357825-358112
BmHel-11	DF090423:254642-254926
BmHel-11	DF090423:675074-675357
BmHel-11	DF090422:490079-490366
BmHel-11	DF090375:1542202-1542487
BmHel-11	DF090375:562286-562562
BmHel-11	DF090375:549535-549818
BmHel-11	DF090375:91798-92077
BmHel-11	DF090397:958690-958968
BmHel-11	DF090437:405944-406224
BmHel-11	DF090437:330998-331281
BmHel-11	DF090437:109788-109902
BmHel-11	DF090437:109225-109387
BmHel-11	DF090449:498367-498651
BmHel-11	DF090415:1057815-1058099
BmHel-11	DF090409:1553432-1553707
BmHel-11	DF090409:484510-484800
BmHel-11	DF090446:344377-344661
BmHel-11	DF090432:578633-578917
BmHel-11	DF090394:798167-798452
BmHel-11	DF090394:950208-950492
BmHel-11	DF090394:1436367-1436654
BmHel-11	DF090443:561035-561317
BmHel-11	DF090426:553173-553458
BmHel-11	DF090426:711029-711311
BmHel-11	DF090413:310970-311246

BmHel-11	BABH01069753:197-480
BmHel-11	DF090971:4074-4358
BmHel-11	DF090732:21381-21665
BmHel-11	DF090444:295480-295764
BmHel-11	DF090444:313038-313323
BmHel-11	DF090441:655581-655862
BmHel-11	DF090441:584398-584675
BmHel-11	DF090429:300246-300524
BmHel-11	DF090419:496783-497068
BmHel-11	DF090419:455560-455838
BmHel-11	DF090369:2232950-2233234
BmHel-11	DF090369:1520487-1520770
BmHel-11	DF090369:1268568-1268846
BmHel-11	DF091573:1188-1470
BmHel-11	DF090580:12749-13040
BmHel-11	DF090704:22164-22443
BmHel-11	DF090473:217237-217521
BmHel-11	DF090431:688057-688345
BmHel-11	BABH01045702:2212-2496
BmHel-11	DF090657:7886-8170
BmHel-11	DF090425:54256-54545
BmHel-11	BABH01049900:652-948
BmHel-11	BABH01048266:225-509
BmHel-11	DF090661:21507-21791
BmHel-11	BABH01042583:12790-13074
BmHel-11	DF090545:74717-74993
BmHel-11	DF090919:10591-10875
BmHel-11	DF090475:257656-257940
BmHel-11	DF090530:33100-33388
BmHel-11	DF090530:47643-47924
BmHel-11	BABH01048238:379-667
BmHel-11	DF091066:7305-7588
BmHel-11	DF090705:840-1129
BmHel-11	DF090617:24100-24393
BmHel-11	DF090673:23930-24214
BmHel-11	DF090480:183922-184036
BmHel-11	DF090480:183739-183908
BmHel-11	DF090824:13523-13807
BmHel-11	DF090554:35985-36269
BmHel-11	DF090647:34081-34358
BmHel-11	BABH01047955:306-580
BmHel-11	DF090389:330264-330463
BmHel-11	DF090389:330543-330638
BmHel-11	DF090375:1183329-1183550
BmHel-11	DF090316:10815188-10815471
BmHel-11	DF090418:321951-322238
BmHel-11	DF090325:6206060-6206341
BmHel-11	DF090342:787581-787865

BmHel-11	DF090318:3960986-3961268
BmHel-11	DF090383:382955-383240
BmHel-11	DF090328:4484702-4484986
BmHel-11	DF090433:398540-398818
BmHel-11	DF090360:3030259-3030541
BmHel-11	DF090321:2476544-2476827
BmHel-11	DF090321:3196832-3197115
BmHel-11	DF090334:3779269-3779552
BmHel-11	DF090335:200765-201050
BmHel-11	DF090345:3791768-3792048
BmHel-11	DF090327:1352276-1352563
BmHel-11	DF090327:3560821-3561103
BmHel-11	BABH01079787:289-571
BmHel-11	DF090339:4736483-4736649
BmHel-11	DF090339:4736644-4736760
BmHel-11	DF090336:4532657-4532940
BmHel-11	DF090350:1530224-1530328
BmHel-11	DF090350:1530613-1530801
BmHel-11	DF090316:14792811-14792976
BmHel-11	DF090316:14792700-14792816
BmHel-11	DF090391:384745-384937
BmHel-11	DF090391:382918-383020
BmHel-11	DF090344:3180583-3180797
BmHel-11	DF090344:3180495-3180575
BmHel-11	DF090320:5983597-5983786
BmHel-11	DF090320:5983497-5983581
BmHel-11	DF090366:1023125-1023399
BmHel-11	DF090440:328413-328695
BmHel-11	DF090437:240483-240776
BmHel-11	DF090443:320291-320457
BmHel-11	DF090443:319661-319783
BmHel-11	BABH01059561:414-698
BmHel-11	DF090327:5620650-5620822
BmHel-11	DF090327:5620473-5620587
BmHel-11	DF090346:2933313-2933595
BmHel-11	DF090355:3121455-3121738
BmHel-11	DF090386:1125704-1125986
BmHel-11	DF090383:1063017-1063297
BmHel-11	DF090319:2215117-2215292
BmHel-11	DF090319:2215305-2215411
BmHel-11	DF090349:976494-976778
BmHel-11	DF090349:846023-846306
BmHel-11	DF090343:2444491-2444767
BmHel-11	DF090330:4325536-4325820
BmHel-11	DF090356:2584779-2584888
BmHel-11	DF090356:2584555-2584735
BmHel-11	DF090628:37118-37402
BmHel-11	DF090324:7613990-7614216

BmHel-11	DF090421:688956-689181
BmHel-11	DF090402:1057545-1057715
BmHel-11	DF090402:1058707-1058823
BmHel-11	DF090319:4659220-4659456
BmHel-11	DF090392:811061-811265
BmHel-11	DF090469:152388-152665
BmHel-11	DF090331:4501434-4501716
BmHel-11	DF090316:9149442-9149725
BmHel-11	DF090412:627179-627465
BmHel-11	DF090364:544274-544546
BmHel-11	DF090354:2717242-2717505
BmHel-11	DF090317:7329337-7329620
BmHel-11	DF090317:3852986-3853270
BmHel-11	DF090317:1915161-1915436
BmHel-11	DF090317:375499-375781
BmHel-11	DF090327:4994222-4994508
BmHel-11	DF090371:118325-118610
BmHel-11	DF090403:390156-390405
BmHel-11	DF090426:81419-81534
BmHel-11	DF090426:81233-81399
BmHel-11	DF090441:245034-245317
BmHel-11	DF090355:1519250-1519460
BmHel-11	DF090324:3516519-3516809
BmHel-11	DF090332:373742-373952
BmHel-11	DF090319:505327-505537
BmHel-11	DF090322:1734240-1734417
BmHel-11	DF090322:1734437-1734544
BmHel-11	DF090322:6195864-6196146
BmHel-11	DF090321:2682324-2682609
BmHel-11	DF090350:4081625-4081899
BmHel-11	DF090349:3752419-3752702
BmHel-11	DF090349:316734-317017
BmHel-11	DF090343:2506280-2506553
BmHel-11	DF090400:868421-868704
BmHel-11	DF090388:840686-840956
BmHel-11	DF090370:987525-987737
BmHel-11	DF090320:5500460-5500543
BmHel-11	DF090320:5501197-5501400
BmHel-11	DF090320:5451174-5451409
BmHel-11	DF090333:2419985-2420273
BmHel-11	DF090543:8468-8752
BmHel-11	DF090464:144471-144754
BmHel-11	DF090353:1799586-1799869
BmHel-11	DF090339:2321142-2321413
BmHel-11	DF090387:1345242-1345526
BmHel-11	DF090324:475109-475392
BmHel-11	DF090324:1809472-1809754
BmHel-11	DF090324:3077128-3077411

BmHel-11	DF090324:5627361-5627644
BmHel-11	DF090324:6180241-6180524
BmHel-11	DF090390:1540294-1540578
BmHel-11	DF090390:32135-32418
BmHel-11	DF090386:940511-940794
BmHel-11	DF090342:2333648-2333931
BmHel-11	DF090342:2424683-2424966
BmHel-11	DF090342:4493670-4493950
BmHel-11	DF090342:4750414-4750697
BmHel-11	DF090342:2990410-2990692
BmHel-11	DF090336:276991-277274
BmHel-11	DF090336:1928075-1928358
BmHel-11	DF090332:2443319-2443596
BmHel-11	DF090332:3090455-3090738
BmHel-11	DF090332:3644761-3645049
BmHel-11	DF090332:306602-306885
BmHel-11	DF090318:2711558-2711839
BmHel-11	DF090318:4162562-4162845
BmHel-11	DF090318:9148285-9148566
BmHel-11	DF090383:1606697-1606980
BmHel-11	DF090383:933236-933518
BmHel-11	DF090348:1764517-1764801
BmHel-11	DF090328:712273-712554
BmHel-11	DF090328:5703152-5703435
BmHel-11	DF090328:6397756-6398039
BmHel-11	DF090319:3527381-3527664
BmHel-11	DF090319:6053089-6053371
BmHel-11	DF090319:984413-984696
BmHel-11	DF090483:119886-120168
BmHel-11	DF090357:1195831-1196117
BmHel-11	DF090395:133963-134250
BmHel-11	DF090347:2797194-2797477
BmHel-11	DF090414:1196157-1196440
BmHel-11	DF090411:29971-30257
BmHel-11	DF090411:278249-278532
BmHel-11	DF090392:1268189-1268472
BmHel-11	DF090365:2477837-2478120
BmHel-11	DF090321:669520-669804
BmHel-11	DF090321:2692272-2692549
BmHel-11	DF090321:4274233-4274516
BmHel-11	DF090321:5366110-5366388
BmHel-11	DF090321:4021135-4021426
BmHel-11	DF090321:426521-426800
BmHel-11	DF090350:1809981-1810265
BmHel-11	DF090350:1738221-1738504
BmHel-11	DF090407:485303-485586
BmHel-11	DF090407:197890-198164
BmHel-11	DF090393:707800-708083

BmHel-11	DF090382:499690-499972
BmHel-11	DF090359:116502-116779
BmHel-11	DF090343:3905861-3906144
BmHel-11	DF090343:4081257-4081540
BmHel-11	DF090340:1076782-1077064
BmHel-11	DF090334:3937123-3937409
BmHel-11	DF090316:692373-692656
BmHel-11	DF090316:3882823-3883106
BmHel-11	DF090316:5599628-5599916
BmHel-11	DF090316:12704241-12704524
BmHel-11	DF090316:11658470-11658755
BmHel-11	DF090479:48065-48352
BmHel-11	DF090424:324662-324938
BmHel-11	DF090424:16021-16294
BmHel-11	DF090391:877240-877528
BmHel-11	DF090391:399326-399605
BmHel-11	DF090388:1573042-1573325
BmHel-11	DF090373:1651058-1651341
BmHel-11	DF090373:1513975-1514260
BmHel-11	DF090368:1919197-1919480
BmHel-11	DF090368:518879-519163
BmHel-11	DF090351:3955136-3955419
BmHel-11	DF090351:263409-263688
BmHel-11	DF090337:3702451-3702734
BmHel-11	DF090330:5307266-5307542
BmHel-11	DF090329:5118463-5118740
BmHel-11	DF090329:3864959-3865238
BmHel-11	DF090323:267933-268216
BmHel-11	DF090323:5602693-5602961
BmHel-11	DF090450:413413-413696
BmHel-11	DF090406:1054014-1054296
BmHel-11	DF090367:36259-36542
BmHel-11	DF090344:3491885-3492173
BmHel-11	DF090344:4386541-4386817
BmHel-11	DF090344:2456077-2456361
BmHel-11	DF090380:840643-840925
BmHel-11	DF090370:412067-412341
BmHel-11	DF090320:1113964-1114248
BmHel-11	DF090320:6705353-6705632
BmHel-11	DF090399:12549-12834
BmHel-11	DF090396:1046129-1046407
BmHel-11	DF090366:62587-62886
BmHel-11	DF090364:2113887-2114172
BmHel-11	DF090364:441443-441720
BmHel-11	DF090354:3072135-3072418
BmHel-11	DF090352:2807726-2808008
BmHel-11	DF090317:2800308-2800586
BmHel-11	DF090317:7879895-7880179

BmHel-11	DF090317:10942428-10942701
BmHel-11	DF090317:6479168-6479455
BmHel-11	DF090438:262812-263089
BmHel-11	DF090435:172279-172563
BmHel-11	DF090417:897568-897844
BmHel-11	DF090404:1230341-1230624
BmHel-11	DF090358:767224-767504
BmHel-11	DF090356:3475958-3476236
BmHel-11	DF090573:44926-45209
BmHel-11	DF090333:5776655-5776938
BmHel-11	DF090327:3320333-3320617
BmHel-11	DF090346:3064779-3065054
BmHel-11	DF090346:3708568-3708856
BmHel-11	DF090403:1231778-1232062
BmHel-11	DF090361:639910-640202
BmHel-11	DF090361:2702149-2702439
BmHel-11	DF090422:519250-519527
BmHel-11	DF090422:778417-778696
BmHel-11	DF090422:885063-885346
BmHel-11	DF090422:49165-49443
BmHel-11	DF090375:455019-455302
BmHel-11	DF090375:798122-798405
BmHel-11	DF090409:858071-858354
BmHel-11	DF090446:333805-334094
BmHel-11	DF090446:684289-684571
BmHel-11	DF090394:703861-704148
BmHel-11	DF090394:813811-814095
BmHel-11	DF090413:585579-585870
BmHel-11	DF090413:75173-75456
BmHel-11	DF091418:3146-3432
BmHel-11	DF090441:393292-393576
BmHel-11	BABH01048818:216-499
BmHel-11	DF090430:587163-587446
BmHel-11	DF090369:2316893-2317171
BmHel-11	DF090369:2565524-2565810
BmHel-11	DF090458:200069-200283
BmHel-11	BABH01064909:350-636
BmHel-11	BABH01048340:683-967
BmHel-11	DF090776:16769-17052
BmHel-11	DF090510:103723-103999
BmHel-11	DF090691:19738-20023
BmHel-11	DF090425:1003677-1003965
BmHel-11	DF090540:68905-69188
BmHel-11	DF090652:14455-14737
BmHel-11	DF090613:69365-69653
BmHel-11	DF090597:40534-40813
BmHel-11	DF090717:23707-23993
BmHel-11	BABH01048918:489-776

BmHel-11	DF091126:1638-1925
BmHel-11	DF090348:1386835-1387024
BmHel-11	DF090348:1392518-1392611
BmHel-11	DF090323:6735983-6736135
BmHel-11	DF090323:6735682-6735768
BmHel-11	DF090390:714637-714855
BmHel-11	DF090378:2120268-2120550
BmHel-11	DF090389:1384049-1384330
BmHel-11	DF090462:280549-280830
BmHel-11	DF090369:2221872-2222157
BmHel-11	DF090402:535519-535806
BmHel-11	DF090347:1919804-1920085
BmHel-11	DF090322:5637589-5637862
BmHel-11	DF090330:2421541-2421825
BmHel-11	DF090415:1195897-1196176
BmHel-11	DF090355:1395271-1395495
BmHel-11	DF090407:1530557-1530839
BmHel-11	DF090316:3459622-3459802
BmHel-11	DF090316:3459497-3459600
BmHel-11	DF090351:3457204-3457486
BmHel-11	DF090380:2022753-2022867
BmHel-11	DF090380:2022863-2023034
BmHel-11	DF090352:655805-655939
BmHel-11	DF090352:655540-655687
BmHel-11	DF090372:1873072-1873350
BmHel-11	DF090356:1766553-1766834
BmHel-11	DF090414:791620-791895
BmHel-11	DF090316:7998022-7998304
BmHel-11	DF090351:3965611-3965897
BmHel-11	DF090435:107441-107574
BmHel-11	DF090435:107270-107418
BmHel-11	DF090362:2069170-2069385
BmHel-11	DF090402:19854-20143
BmHel-11	DF090386:1033569-1033848
BmHel-11	DF090381:1099330-1099521
BmHel-11	DF090381:1094264-1094354
BmHel-11	DF090326:4886094-4886306
BmHel-11	DF090350:1159926-1160208
BmHel-11	DF090391:799591-799874
BmHel-11	DF090373:135436-135719
BmHel-11	DF090329:2812306-2812596
BmHel-11	DF090364:327687-327970
BmHel-11	DF090333:2091766-2091885
BmHel-11	DF090333:2091916-2092081
BmHel-11	DF090327:5145436-5145718
BmHel-11	DF090374:809194-809351
BmHel-11	DF090374:808706-808829
BmHel-11	DF090550:27040-27240

BmHel-11	DF090550:28180-28269
BmHel-11	DF090357:311404-311685
BmHel-11	DF090349:3030460-3030574
BmHel-11	DF090349:3030594-3030759
BmHel-11	DF090341:4257227-4257501
BmHel-11	DF090316:569180-569454
BmHel-11	DF090418:758631-758922
BmHel-11	DF090368:955359-955642
BmHel-11	DF090329:1929513-1929798
BmHel-11	DF090378:670758-671040
BmHel-11	DF090346:2341256-2341538
BmHel-11	DF090470:222908-223181
BmHel-11	DF090591:29889-30170
BmHel-11	DF090325:4308711-4308995
BmHel-11	DF090324:8060852-8061134
BmHel-11	DF090342:1132829-1133114
BmHel-11	DF090332:6041796-6042071
BmHel-11	DF090318:1197473-1197754
BmHel-11	DF090328:505223-505504
BmHel-11	DF090411:727047-727330
BmHel-11	DF090382:1203849-1204095
BmHel-11	DF090341:414128-414407
BmHel-11	DF090316:11794970-11795253
BmHel-11	DF090388:570079-570357
BmHel-11	DF090385:1780310-1780586
BmHel-11	DF090323:243771-244053
BmHel-11	DF090384:1896289-1896571
BmHel-11	DF090372:1457798-1458080
BmHel-11	DF090333:4935222-4935341
BmHel-11	DF090333:4935352-4935518
BmHel-11	DF090333:5402320-5402602
BmHel-11	DF090327:5045048-5045330
BmHel-11	DF090346:2015946-2016234
BmHel-11	DF090604:16953-17235
BmHel-11	DF090369:2249631-2249912
BmHel-11	DF090447:9174-9455
BmHel-11	BABH01079138:1-283
BmHel-11	BABH01047725:183-459
BmHel-11	DF091186:323-603
BmHel-11	DF090355:547833-548042
BmHel-11	DF090353:1170253-1170540
BmHel-11	DF090316:8581278-8581403
BmHel-11	DF090316:8580670-8580830
BmHel-11	DF090344:1405351-1405634
BmHel-11	DF090346:1265353-1265635
BmHel-11	DF090457:96404-96612
BmHel-11	DF090355:3108712-3108993
BmHel-11	DF090355:1927209-1927494

BmHel-11	DF090355:910094-910376
BmHel-11	DF090325:6259548-6259827
BmHel-11	DF090325:1634249-1634525
BmHel-11	DF090353:749131-749418
BmHel-11	DF090387:853039-853321
BmHel-11	DF090324:5881622-5881905
BmHel-11	DF090324:3581238-3581520
BmHel-11	DF090324:872134-872416
BmHel-11	DF090386:1816201-1816483
BmHel-11	DF090386:1985938-1986220
BmHel-11	DF090381:1662075-1662358
BmHel-11	DF090342:714947-715230
BmHel-11	DF090336:1583376-1583661
BmHel-11	DF090336:4568080-4568363
BmHel-11	DF090332:2098384-2098666
BmHel-11	DF090318:2967212-2967496
BmHel-11	DF090383:1794919-1795201
BmHel-11	DF090319:2066252-2066536
BmHel-11	DF090319:3456766-3457044
BmHel-11	DF090319:3949739-3950021
BmHel-11	DF090357:1761316-1761598
BmHel-11	DF090357:2832847-2833130
BmHel-11	DF090360:2149614-2149897
BmHel-11	DF090347:1777284-1777567
BmHel-11	DF090411:303790-304061
BmHel-11	DF090365:2271089-2271379
BmHel-11	DF090326:3797647-3797929
BmHel-11	DF090326:3362179-3362461
BmHel-11	DF090322:6197233-6197520
BmHel-11	DF090322:1833375-1833657
BmHel-11	DF090322:81909-82191
BmHel-11	DF090321:4327034-4327312
BmHel-11	DF090321:4876963-4877245
BmHel-11	DF090321:2743531-2743813
BmHel-11	DF090321:198952-199231
BmHel-11	DF090350:1726689-1726973
BmHel-11	DF090349:507797-508080
BmHel-11	DF090341:2205481-2205763
BmHel-11	DF090341:4184672-4184954
BmHel-11	DF090340:367806-368094
BmHel-11	DF090334:1233916-1234198
BmHel-11	DF090334:3795952-3796237
BmHel-11	DF090331:429169-429451
BmHel-11	DF090391:1341320-1341602
BmHel-11	DF090373:1861320-1861602
BmHel-11	DF090351:4349979-4350257
BmHel-11	DF090351:2672160-2672444
BmHel-11	DF090335:1904832-1905109

BmHel-11	DF090335:2562815-2563086
BmHel-11	DF090330:544083-544371
BmHel-11	DF090330:4144341-4144626
BmHel-11	DF090329:515880-516164
BmHel-11	DF090329:5542230-5542511
BmHel-11	DF090329:4993164-4993448
BmHel-11	DF090323:7863216-7863499
BmHel-11	DF090450:48742-49024
BmHel-11	DF090344:4063241-4063515
BmHel-11	DF090344:4100482-4100769
BmHel-11	DF090344:3433876-3434156
BmHel-11	DF090344:2621608-2621890
BmHel-11	DF090379:174450-174732
BmHel-11	DF090378:1594895-1595178
BmHel-11	DF090366:1928616-1928898
BmHel-11	DF090354:798701-798982
BmHel-11	DF090354:2669534-2669817
BmHel-11	DF090352:1428280-1428563
BmHel-11	DF090358:1757158-1757436
BmHel-11	DF090327:6409473-6409755
BmHel-11	DF090327:737225-737508
BmHel-11	DF090398:1040167-1040449
BmHel-11	DF090346:4015642-4015921
BmHel-11	DF090375:203937-204219
BmHel-11	DF090415:231046-231328
BmHel-11	DF090439:534658-534945
BmHel-11	DF090394:1001241-1001525
BmHel-11	DF090441:15092-15382
BmHel-11	DF090429:118234-118525
BmHel-11	DF090431:743568-743854
BmHel-11	DF090425:211324-211606
BmHel-11	DF090720:7516-7797
BmHel-11	DF090475:56879-57161
BmHel-11	DF090520:169789-170071
BmHel-11	BABH01046289:2197-2481
BmHel-11	DF090422:375137-375293
BmHel-11	DF090422:375552-375692
BmHel-11	DF090350:3462108-3462218
BmHel-11	DF090350:3461908-3462092
BmHel-11	DF090434:160842-161100
BmHel-11	DF090325:5447624-5447895
BmHel-11	DF090328:5749561-5749845
BmHel-11	DF090319:6463342-6463625
BmHel-11	DF090365:341614-341894
BmHel-11	DF090359:2396957-2397234
BmHel-11	DF090388:1688966-1689245
BmHel-11	DF090351:1284161-1284444
BmHel-11	DF090335:2249723-2250008

BmHel-11	DF090379:862312-862593
BmHel-11	DF090345:4203976-4204243
BmHel-11	DF090648:22421-22711
BmHel-11	DF090664:17619-17899
BmHel-11	DF090339:2200137-2200422
BmHel-11	DF090328:1496052-1496186
BmHel-11	DF090328:1496194-1496355
BmHel-11	DF090334:3444409-3444535
BmHel-11	DF090334:3444211-3444378
BmHel-11	DF090355:2523166-2523405
BmHel-11	DF090318:8873922-8874086
BmHel-11	DF090318:8873809-8873925
BmHel-11	DF090318:2552906-2553131
BmHel-11	DF090380:756398-756614
BmHel-11	DF090379:1330028-1330254
BmHel-11	DF090379:156650-156762
BmHel-11	DF090379:156487-156653
BmHel-11	DF090346:416960-417115
BmHel-11	DF090346:417143-417270
BmHel-11	DF090397:517219-517476
BmHel-11	DF090457:177597-177807
BmHel-11	DF090426:452844-453022
BmHel-11	DF090426:453038-453141
BmHel-11	DF090461:242022-242305
BmHel-11	DF090318:2589591-2589870
BmHel-11	DF090328:2503404-2503585
BmHel-11	DF090328:2503283-2503383
BmHel-11	DF090345:1378850-1379140
BmHel-11	DF090355:1564361-1564646
BmHel-11	DF090332:1118433-1118613
BmHel-11	DF090332:1118318-1118418
BmHel-11	DF090326:1530838-1531022
BmHel-11	DF090326:1530721-1530827
BmHel-11	DF090337:5158202-5158380
BmHel-11	DF090337:5158394-5158496
BmHel-11	DF090320:3086875-3087156
BmHel-11	DF090429:262265-262505
BmHel-11	DF090355:763712-763992
BmHel-11	DF090325:3969249-3969483
BmHel-11	DF090436:431456-431739
BmHel-11	DF090365:865988-866231
BmHel-11	DF090350:4123653-4123937
BmHel-11	DF090340:4822855-4823041
BmHel-11	DF090340:4823055-4823149
BmHel-11	DF090334:2605674-2605852
BmHel-11	DF090334:2605864-2605958
BmHel-11	DF090325:7198318-7198599
BmHel-11	DF090339:1244533-1244815

BmHel-11	DF090386:162786-163070
BmHel-11	DF090381:354269-354542
BmHel-11	DF090348:2228102-2228372
BmHel-11	DF090377:1932482-1932763
BmHel-11	DF090377:910003-910286
BmHel-11	DF090347:735065-735343
BmHel-11	DF090326:6192405-6192686
BmHel-11	DF090326:6955676-6955957
BmHel-11	DF090321:1571324-1571532
BmHel-11	DF090393:1282466-1282747
BmHel-11	DF090359:111476-111756
BmHel-11	DF090316:3225928-3226203
BmHel-11	DF090329:331366-331642
BmHel-11	DF090412:195407-195613
BmHel-11	DF090364:2286866-2286977
BmHel-11	DF090364:2286681-2286850
BmHel-11	DF090317:8916557-8916838
BmHel-11	DF090376:1872383-1872666
BmHel-11	DF090408:605672-605947
BmHel-11	DF090851:6891-7173
BmHel-11	DF090362:1820526-1820802
BmHel-11	DF090421:590499-590777
BmHel-11	DF090357:887280-887431
BmHel-11	DF090357:887446-887583
BmHel-11	DF090363:2962246-2962473
BmHel-11	DF090340:1925776-1926057
BmHel-11	DF090595:27049-27330
BmHel-11	DF090567:29938-30227
BmHel-11	DF090331:367850-368131
BmHel-11	DF090316:11817678-11817959
BmHel-11	DF090335:5351427-5351635
BmHel-11	DF090356:3458789-3458926
BmHel-11	DF090356:3459344-3459486
BmHel-11	DF090437:84296-84508
BmHel-11	BABH01046668:396-677
BmHel-11	DF090387:1037095-1037377
BmHel-11	DF090324:3265531-3265812
BmHel-11	DF090324:5638720-5639001
BmHel-11	DF090386:119053-119326
BmHel-11	DF090342:1897808-1898090
BmHel-11	DF090336:497857-498134
BmHel-11	DF090336:1641819-1642099
BmHel-11	DF090336:2395573-2395847
BmHel-11	DF090336:3402513-3402797
BmHel-11	DF090336:4900720-4901002
BmHel-11	DF090336:2414104-2414386
BmHel-11	DF090332:3259651-3259934
BmHel-11	DF090332:3520083-3520364

BmHel-11	DF090318:6926008-6926289
BmHel-11	DF090348:699630-699911
BmHel-11	DF090319:6471900-6472186
BmHel-11	DF090319:1210363-1210644
BmHel-11	DF090357:2938644-2938928
BmHel-11	DF090392:564392-564673
BmHel-11	DF090392:422961-423241
BmHel-11	DF090365:2224279-2224556
BmHel-11	DF090322:2022613-2022894
BmHel-11	DF090321:1877180-1877462
BmHel-11	DF090410:1332977-1333263
BmHel-11	DF090407:296795-297077
BmHel-11	DF090349:553741-554022
BmHel-11	DF090341:2623808-2624091
BmHel-11	DF090316:6362725-6363008
BmHel-11	DF090316:14580733-14581015
BmHel-11	DF090316:15809637-15809917
BmHel-11	DF090400:1244182-1244463
BmHel-11	DF090385:905114-905391
BmHel-11	DF090373:1780691-1780972
BmHel-11	DF090337:1608578-1608863
BmHel-11	DF090337:3204501-3204787
BmHel-11	DF090335:5595701-5595975
BmHel-11	DF090335:5131962-5132244
BmHel-11	DF090367:281877-282158
BmHel-11	DF090370:2155906-2156179
BmHel-11	DF090370:2371046-2371327
BmHel-11	DF090399:1254491-1254768
BmHel-11	DF090364:2177438-2177719
BmHel-11	DF090354:148838-149119
BmHel-11	DF090352:1098756-1099033
BmHel-11	DF090317:8300860-8301147
BmHel-11	DF090317:6865949-6866233
BmHel-11	DF090356:302384-302668
BmHel-11	DF090345:2678983-2679264
BmHel-11	DF090345:1812455-1812736
BmHel-11	DF090345:1258445-1258723
BmHel-11	DF090327:1482448-1482729
BmHel-11	DF090401:582899-583180
BmHel-11	DF090346:205344-205625
BmHel-11	DF090422:574165-574448
BmHel-11	DF090397:1166220-1166501
BmHel-11	DF090409:454572-454853
BmHel-11	DF090409:1247792-1248073
BmHel-11	DF090413:458904-459185
BmHel-11	DF090843:14127-14398
BmHel-11	DF090768:11825-12106
BmHel-11	DF090478:41479-41758

BmHel-11	BABH01045793:616-898
BmHel-11	DF090507:94666-94936
BmHel-11	DF090331:4557101-4557380
BmHel-11	DF090373:1242710-1242991
BmHel-11	DF090323:4620559-4620839
BmHel-11	DF090815:4911-5197
BmHel-11	DF090352:1944001-1944226
BmHel-11	DF090352:989726-989828
BmHel-11	DF090352:989547-989727
BmHel-11	DF090318:1699978-1700252
BmHel-11	DF090328:6433673-6433951
BmHel-11	DF090360:2369332-2369612
BmHel-11	DF090341:1722218-1722498
BmHel-11	DF090337:5108528-5108803
BmHel-11	DF090335:4328725-4328843
BmHel-11	DF090335:4328886-4329046
BmHel-11	DF090323:1652775-1652994
BmHel-11	DF090378:2157893-2158175
BmHel-11	DF090317:9587833-9588045
BmHel-11	DF090317:9588307-9588389
BmHel-11	DF090420:840141-840421
BmHel-11	DF090321:16825-17104
BmHel-11	DF090343:71394-71503
BmHel-11	DF090343:71185-71355
BmHel-11	DF090352:3905954-3906231
BmHel-11	DF090417:83481-83644
BmHel-11	DF090417:83368-83484
BmHel-11	DF090387:1608837-1609115
BmHel-11	DF090414:947029-947241
BmHel-11	DF090326:5502164-5502276
BmHel-11	DF090326:5502291-5502460
BmHel-11	DF090366:2219601-2219882
BmHel-11	DF090352:2064305-2064510
BmHel-11	DF090346:3469518-3469798
BmHel-11	DF090409:809235-809515
BmHel-11	DF090456:404450-404679
BmHel-11	DF090386:1405930-1406210
BmHel-11	DF090331:508556-508836
BmHel-11	DF090352:4130498-4130708
BmHel-11	DF090449:350677-350958
BmHel-11	DF090447:251096-251377
BmHel-11	DF090325:2272596-2272770
BmHel-11	DF090325:2272789-2272893
BmHel-11	DF090332:193763-193892
BmHel-11	DF090332:194205-194363
BmHel-11	DF090319:2571292-2571574
BmHel-11	DF090322:1934246-1934518
BmHel-11	DF090400:467190-467472

BmHel-11	DF090330:3054905-3055053
BmHel-11	DF090330:3054738-3054877
BmHel-11	DF090329:307127-307405
BmHel-11	DF090352:2136244-2136472
BmHel-11	DF090372:1349963-1350075
BmHel-11	DF090372:1350239-1350406
BmHel-11	DF090327:2067946-2068223
BmHel-11	DF090327:5759758-5759878
BmHel-11	DF090327:5759895-5760061
BmHel-11	DF090348:1087029-1087309
BmHel-11	DF090350:2866472-2866578
BmHel-11	DF090350:2866053-2866222
BmHel-11	DF090396:1499575-1499703
BmHel-11	DF090396:1499391-1499550
BmHel-11	DF090324:5974587-5974856
BmHel-11	DF090328:1144180-1144460
BmHel-11	DF090328:569232-569508
BmHel-11	DF090319:124848-124968
BmHel-11	DF090319:125245-125403
BmHel-11	DF090343:4623302-4623383
BmHel-11	DF090343:4623019-4623216
BmHel-11	DF090341:4540435-4540714
BmHel-11	DF090344:2190535-2190815
BmHel-11	DF090396:1074692-1074972
BmHel-11	DF090432:464675-464892
BmHel-11	DF090360:217427-217714
BmHel-11	DF090349:605202-605482
BmHel-11	DF090343:4379174-4379381
BmHel-11	DF090353:3680679-3680962
BmHel-11	DF090324:4450141-4450421
BmHel-11	DF090402:1032575-1032856
BmHel-11	DF090342:1454719-1455000
BmHel-11	DF090336:3448032-3448312
BmHel-11	DF090332:4846832-4847114
BmHel-11	DF090332:1870974-1871255
BmHel-11	DF090318:3353712-3353991
BmHel-11	DF090319:3418099-3418377
BmHel-11	DF090319:8933699-8933980
BmHel-11	DF090360:113069-113357
BmHel-11	DF090392:80041-80317
BmHel-11	DF090322:6810211-6810496
BmHel-11	DF090321:6429215-6429497
BmHel-11	DF090393:1107458-1107737
BmHel-11	DF090382:1411423-1411708
BmHel-11	DF090349:2660805-2661082
BmHel-11	DF090341:1245785-1246067
BmHel-11	DF090334:4574682-4574956
BmHel-11	DF090334:5560362-5560638

BmHel-11	DF090316:1024599-1024879
BmHel-11	DF090316:11744306-11744582
BmHel-11	DF090316:1159271-1159551
BmHel-11	DF090424:352338-352617
BmHel-11	DF090337:4705849-4706129
BmHel-11	DF090335:2820412-2820692
BmHel-11	DF090329:3829508-3829799
BmHel-11	DF090367:811402-811683
BmHel-11	DF090317:7142975-7143259
BmHel-11	DF090435:650515-650795
BmHel-11	DF090389:241789-242068
BmHel-11	DF090358:2767842-2768113
BmHel-11	DF090358:2561586-2561871
BmHel-11	DF090356:975377-975661
BmHel-11	DF090345:3779541-3779819
BmHel-11	DF090327:536786-537058
BmHel-11	DF090327:4630162-4630444
BmHel-11	DF090338:2711634-2711914
BmHel-11	DF091307:2142-2422
BmHel-11	DF090409:1348651-1348931
BmHel-11	DF090429:844744-845026
BmHel-11	DF090615:16502-16783
BmHel-11	DF090464:360406-360685
BmHel-11	DF090328:2481587-2481868
BmHel-11	DF090328:5360540-5360806
BmHel-11	DF090340:2757867-2758146
BmHel-11	DF090316:5062482-5062761
BmHel-11	DF090323:3562967-3563246
BmHel-11	DF090449:99052-99334
BmHel-11	DF090394:1541933-1542212
BmHel-11	BABH01054036:6-290
BmHel-11	DF090342:3171097-3171261
BmHel-11	DF090342:3171259-3171376
BmHel-11	DF090336:1941280-1941458
BmHel-11	DF090336:1941160-1941261
BmHel-11	DF090365:1853279-1853556
BmHel-11	DF090452:83984-84263
BmHel-11	DF090317:7435898-7436106
BmHel-11	DF090448:217104-217283
BmHel-11	DF090448:217315-217415
BmHel-11	DF090508:6679-6860
BmHel-11	DF090508:6878-6976
BmHel-11	DF090902:11245-11408
BmHel-11	DF090902:11406-11524
BmHel-11	DF090321:3026061-3026293
BmHel-11	DF090337:2737860-2738139
BmHel-11	DF090329:6270097-6270209
BmHel-11	DF090329:6270206-6270370

BmHel-11	DF090323:6526586-6526865
BmHel-11	DF090467:260941-261161
BmHel-11	DF090462:51032-51311
BmHel-11	DF090355:2191349-2191563
BmHel-11	DF090320:501069-501190
BmHel-11	DF090320:500674-500844
BmHel-11	DF090338:984109-984389
BmHel-11	DF090386:414117-414279
BmHel-11	DF090386:414274-414390
BmHel-11	DF090328:1897675-1897823
BmHel-11	DF090328:1897307-1897431
BmHel-11	DF090322:2730822-2731100
BmHel-11	DF090316:15585410-15585559
BmHel-11	DF090316:15585008-15585147
BmHel-11	DF090323:309693-309976
BmHel-11	DF090372:615890-616169
BmHel-11	DF090379:912218-912500
BmHel-11	DF090429:911903-912173
BmHel-11	DF090382:2040471-2040582
BmHel-11	DF090382:2040604-2040771
BmHel-11	DF090455:317480-317759
BmHel-11	DF090330:4716289-4716568
BmHel-11	DF090317:2607551-2607830
BmHel-11	DF090417:303368-303574
BmHel-11	DF090346:4158893-4159174
BmHel-11	DF090517:78975-79099
BmHel-11	DF090517:78691-78849
BmHel-11	DF090629:44840-45119
BmHel-11	DF090589:6371-6639
BmHel-11	DF090332:4004387-4004667
BmHel-11	DF090383:479384-479665
BmHel-11	DF090383:1948651-1948930
BmHel-11	DF090319:6505944-6506230
BmHel-11	DF090360:315369-315650
BmHel-11	DF090322:47735-48008
BmHel-11	DF090463:9044-9323
BmHel-11	DF090349:1588023-1588300
BmHel-11	DF090343:4069512-4069791
BmHel-11	DF090343:1604073-1604351
BmHel-11	DF090331:292205-292490
BmHel-11	DF090331:1174885-1175164
BmHel-11	DF090316:870828-871100
BmHel-11	DF090388:769050-769322
BmHel-11	DF090373:372406-372685
BmHel-11	DF090368:2322119-2322398
BmHel-11	DF090351:135142-135421
BmHel-11	DF090329:4720755-4721033
BmHel-11	DF090323:1169945-1170223

BmHel-11	DF090396:52387-52656
BmHel-11	DF090396:1356395-1356677
BmHel-11	DF090378:175430-175712
BmHel-11	DF090354:2867052-2867331
BmHel-11	DF090317:6179406-6179684
BmHel-11	DF090356:868560-868839
BmHel-11	DF090361:835798-836068
BmHel-11	DF090462:380060-380339
BmHel-11	DF090484:246415-246697
BmHel-11	DF090441:341180-341459
BmHel-11	DF090448:238963-239242
BmHel-11	DF090448:326334-326605
BmHel-11	DF090369:2063093-2063371
BmHel-11	DF090475:137001-137284
BmHel-11	DF090640:35454-35733
BmHel-11	DF091238:1532-1811
BmHel-11	DF090332:335674-335889
BmHel-11	DF090360:1723766-1723886
BmHel-11	DF090360:1723588-1723745
BmHel-11	DF090357:418188-418305
BmHel-11	DF090357:418028-418191
BmHel-11	DF090389:736391-736594
BmHel-11	DF090395:1671467-1671751
BmHel-11	BABH01081360:143-427
BmHel-11	DF090348:3286321-3286596
BmHel-11	DF090395:1311287-1311566
BmHel-11	DF090316:4134410-4134687
BmHel-11	DF090373:695330-695605
BmHel-11	DF090396:1058726-1059003
BmHel-11	DF090317:9863230-9863507
BmHel-11	DF090434:252782-253060
BmHel-11	DF090338:2088650-2088923
BmHel-11	DF090383:1069244-1069522
BmHel-11	DF090362:1974330-1974609
BmHel-11	DF090414:999096-999273
BmHel-11	DF090414:998996-999096
BmHel-11	DF090322:4895920-4896167
BmHel-11	DF090424:898318-898427
BmHel-11	DF090424:898125-898314
BmHel-11	DF090380:1865858-1866140
BmHel-11	DF090389:1040226-1040504
BmHel-11	DF090333:3486718-3486940
BmHel-11	DF090375:58358-58595
BmHel-11	DF090425:804566-804747
BmHel-11	DF090425:804441-804537
BmHel-11	DF090332:4860248-4860462
BmHel-11	DF090322:3206778-3206939
BmHel-11	DF090330:5252475-5252700

BmHel-11	DF090329:2104375-2104528
BmHel-11	DF090345:2558294-2558502
BmHel-11	DF090345:2558014-2558093
BmHel-11	DF090327:6414580-6414854
BmHel-11	BABH01073793:210-487
BmHel-11	BABH01067069:74-297
BmHel-11	BABH01045106:4606-4767
BmHel-11	DF090355:591868-592072
BmHel-11	DF090355:591772-591860
BmHel-11	DF090377:1167311-1167589
BmHel-11	DF090358:3064831-3064934
BmHel-11	DF090358:3064965-3065139
BmHel-11	DF090615:13196-13475
BmHel-11	DF090324:577878-578160
BmHel-11	DF090381:1679309-1679593
BmHel-11	DF090322:1965801-1966079
BmHel-11	DF090363:2043802-2044080
BmHel-11	DF090334:2656248-2656527
BmHel-11	DF090323:6855918-6856197
BmHel-11	DF090361:2817666-2817944
BmHel-11	DF090408:800974-801174
BmHel-11	DF090408:801304-801393
BmHel-11	DF090447:305832-306081
BmHel-11	DF090585:66339-66613
BmHel-11	DF090343:2826572-2826747
BmHel-11	DF090343:2826460-2826561
BmHel-11	DF090366:1380155-1380329
BmHel-11	DF090366:1380348-1380448
BmHel-11	DF090352:2601370-2601589
BmHel-11	DF090413:216252-216530
BmHel-11	DF090351:2430202-2430447
BmHel-11	DF090383:1266474-1266584
BmHel-11	DF090383:1266287-1266453
BmHel-11	DF090329:116351-116621
BmHel-11	DF090345:2334989-2335268
BmHel-11	DF090467:86773-87051
BmHel-11	DF090376:607290-607569
BmHel-11	DF090361:2361454-2361733
BmHel-11	DF090389:1369789-1369994
BmHel-11	DF090324:7700149-7700428
BmHel-11	DF090324:3887186-3887463
BmHel-11	DF090386:1891534-1891812
BmHel-11	DF090342:1924768-1925049
BmHel-11	DF090336:3115643-3115921
BmHel-11	DF090318:6774349-6774627
BmHel-11	DF090318:892176-892454
BmHel-11	DF090348:4305214-4305480
BmHel-11	DF090328:4113593-4113865

BmHel-11	DF090328:5371953-5372228
BmHel-11	DF090395:591062-591338
BmHel-11	DF090365:2938319-2938583
BmHel-11	DF090343:286863-287141
BmHel-11	DF090341:4062752-4063030
BmHel-11	DF090340:1682607-1682887
BmHel-11	DF090334:1728461-1728735
BmHel-11	DF090334:1870843-1871123
BmHel-11	DF090334:1404896-1405175
BmHel-11	DF090316:2854433-2854713
BmHel-11	DF090316:15580763-15581041
BmHel-11	DF090316:7354914-7355192
BmHel-11	DF090316:6580023-6580301
BmHel-11	DF090329:1020033-1020307
BmHel-11	DF090323:7248566-7248844
BmHel-11	DF090344:2977468-2977751
BmHel-11	DF090344:4640284-4640561
BmHel-11	DF090344:2830452-2830733
BmHel-11	DF090399:213937-214224
BmHel-11	DF090384:1679577-1679854
BmHel-11	DF090378:1537885-1538155
BmHel-11	DF090366:755612-755890
BmHel-11	DF090317:2210121-2210399
BmHel-11	DF090317:8406266-8406544
BmHel-11	DF090317:8817249-8817518
BmHel-11	DF090371:1599229-1599507
BmHel-11	DF090371:883275-883558
BmHel-11	DF090643:29280-29558
BmHel-11	DF090408:1073913-1074190
BmHel-11	DF090429:715289-715566
BmHel-11	DF090513:15048-15326
BmHel-11	BABH01045312:2062-2333
BmHel-11	DF090348:2345798-2346029
BmHel-11	DF090331:6160532-6160778
BmHel-11	DF090335:4004100-4004256
BmHel-11	DF090335:4004258-4004368
BmHel-11	DF090346:1009455-1009664
BmHel-11	DF090394:992698-992887
BmHel-11	DF090394:992595-992690
BmHel-11	DF090348:2100313-2100590
BmHel-11	DF090347:2470919-2471196
BmHel-11	DF090322:2679170-2679446
BmHel-11	DF090317:3307221-3307499
BmHel-11	DF090361:2747830-2748107
BmHel-11	DF090413:928358-928635
BmHel-11	DF090336:3502204-3502377
BmHel-11	DF090336:3502099-3502202
BmHel-11	DF090328:1888581-1888796

BmHel-11	DF090357:1603970-1604247
BmHel-11	DF090347:4207596-4207844
BmHel-11	DF090329:1389768-1390047
BmHel-11	DF090352:2735751-2735980
BmHel-11	DF090338:1495702-1495978
BmHel-11	DF090394:153658-153813
BmHel-11	DF090394:153532-153647
BmHel-11	BABH01064979:188-311
BmHel-11	BABH01064979:313-466
BmHel-11	BABH01060775:366-489
BmHel-11	BABH01060775:491-643
BmHel-11	DF090330:2948652-2948789
BmHel-11	DF090330:2948448-2948587
BmHel-11	DF090353:2811656-2811934
BmHel-11	DF090359:3116528-3116741
BmHel-11	DF090337:3808636-3808859
BmHel-11	DF090329:4554794-4554922
BmHel-11	DF090329:4553524-4553690
BmHel-11	DF090323:6508616-6508776
BmHel-11	DF090323:6508502-6508618
BmHel-11	DF090317:6714514-6714674
BmHel-11	DF090317:6714672-6714788
BmHel-11	DF090346:2187125-2187212
BmHel-11	DF090346:2187234-2187423
BmHel-11	DF090338:2309973-2310250
BmHel-11	DF090336:4857696-4857974
BmHel-11	DF090332:3203600-3203878
BmHel-11	DF090363:1107344-1107621
BmHel-11	DF090351:731657-731935
BmHel-11	DF090329:2391362-2391639
BmHel-11	DF090399:1120377-1120654
BmHel-11	DF090396:163097-163375
BmHel-11	DF090394:1257152-1257373
BmHel-11	BABH01044520:993-1271
BmHel-11	DF090516:14873-15144
BmHel-11	DF090325:5763091-5763369
BmHel-11	DF090337:5273331-5273442
BmHel-11	DF090337:5273145-5273310
BmHel-11	DF090380:1030397-1030611
BmHel-11	DF090432:128072-128231
BmHel-11	DF090432:127959-128075
BmHel-11	DF091348:3664-3875
BmHel-11	DF091348:2392-2473
BmHel-11	DF090318:8430723-8430999
BmHel-11	DF090366:2589619-2589793
BmHel-11	DF090366:2589504-2589606
BmHel-11	DF090317:1392755-1392871
BmHel-11	DF090317:1392443-1392613

BmHel-11	DF090365:2006744-2007024
BmHel-11	DF090335:673990-674267
BmHel-11	DF090323:1067222-1067500
BmHel-11	DF090327:624632-624910
BmHel-11	DF090401:427445-427563
BmHel-11	DF090401:427583-427741
BmHel-11	DF090402:1134317-1134594
BmHel-11	DF090317:1706966-1707250
BmHel-11	DF090346:1671072-1671349
BmHel-11	DF090325:4825861-4826133
BmHel-11	DF090336:47932-48214
BmHel-11	DF090364:680026-680303
BmHel-11	DF090333:2819360-2819637
BmHel-11	BABH01059904:370-647
BmHel-11	DF090355:1628259-1628464
BmHel-11	DF090355:2677855-2678134
BmHel-11	DF090355:2512690-2512968
BmHel-11	DF090355:2078328-2078605
BmHel-11	DF090353:3942555-3942832
BmHel-11	DF090387:1622522-1622798
BmHel-11	DF090324:5262139-5262417
BmHel-11	DF090342:3440198-3440473
BmHel-11	DF090342:2555043-2555320
BmHel-11	DF090332:2289402-2289681
BmHel-11	DF090318:6828861-6829138
BmHel-11	DF090318:149739-150017
BmHel-11	DF090348:878815-879091
BmHel-11	DF090328:1042079-1042355
BmHel-11	DF090319:5552829-5553106
BmHel-11	DF090319:8571135-8571411
BmHel-11	DF090347:2664523-2664805
BmHel-11	DF090411:980141-980420
BmHel-11	DF090326:2506333-2506610
BmHel-11	DF090326:6651014-6651288
BmHel-11	DF090321:4516295-4516572
BmHel-11	DF090350:4066368-4066645
BmHel-11	DF090350:2345393-2345670
BmHel-11	DF090363:1006873-1007151
BmHel-11	DF090341:3717891-3718167
BmHel-11	DF090334:5731883-5732156
BmHel-11	DF090331:5233019-5233296
BmHel-11	DF090316:14269984-14270262
BmHel-11	DF090316:9769443-9769720
BmHel-11	DF090388:470276-470553
BmHel-11	DF090388:1112441-1112721
BmHel-11	DF090373:492733-493010
BmHel-11	DF090351:2515717-2515993
BmHel-11	DF090351:4146778-4147055

BmHel-11	DF090351:1733581-1733859
BmHel-11	DF090329:3782504-3782781
BmHel-11	DF090323:2519594-2519864
BmHel-11	DF090320:3141441-3141719
BmHel-11	DF090379:542273-542550
BmHel-11	DF090352:3210524-3210800
BmHel-11	DF090317:150311-150585
BmHel-11	DF090317:10542513-10542785
BmHel-11	DF090356:3300820-3301096
BmHel-11	DF090356:277527-277803
BmHel-11	DF090376:1928465-1928741
BmHel-11	DF090345:4444415-4444692
BmHel-11	DF090345:1622171-1622449
BmHel-11	DF090333:2501987-2502264
BmHel-11	DF090346:2578759-2579034
BmHel-11	DF090397:98707-98976
BmHel-11	DF090449:316247-316524
BmHel-11	DF090439:153418-153696
BmHel-11	DF090454:332412-332695
BmHel-11	DF090619:9069-9352
BmHel-11	DF090456:95095-95370
BmHel-11	BABH01046609:1050-1327
BmHel-11	DF091109:6837-7114
BmHel-11	BABH01046915:1550-1816
BmHel-11	DF090707:21006-21296
BmHel-11	DF090418:542746-542898
BmHel-11	DF090418:542899-543021
BmHel-11	DF090321:8241530-8241721
BmHel-11	DF090321:8241720-8241806
BmHel-11	DF090327:6108887-6109011
BmHel-11	DF090327:6109459-6109633
BmHel-11	DF090373:1698860-1699137
BmHel-11	DF090322:5913595-5913875
BmHel-11	DF090366:1955291-1955567
BmHel-11	DF090327:5871047-5871324
BmHel-11	DF090425:4792-5071
BmHel-11	DF090383:1622235-1622437
BmHel-11	DF090355:3287468-3287577
BmHel-11	DF090355:3287258-3287427
BmHel-11	DF090325:3047640-3047739
BmHel-11	DF090325:3047473-3047639
BmHel-11	DF090353:2355722-2355921
BmHel-11	DF090386:1109760-1109990
BmHel-11	DF090328:2052498-2052774
BmHel-11	DF090319:7019327-7019608
BmHel-11	DF090487:61606-61860
BmHel-11	DF090367:1821167-1821375
BmHel-11	DF090466:176004-176227

BmHel-11	BABH01060409:355-637
BmHel-11	BABH01049346:655-937
BmHel-11	DF090355:2411308-2411446
BmHel-11	DF090355:2411170-2411307
BmHel-11	DF090351:2190338-2190571
BmHel-11	DF090351:2497181-2497280
BmHel-11	DF090351:2497003-2497180
BmHel-11	DF090358:3006451-3006713
BmHel-11	DF090325:1995812-1996044
BmHel-11	DF090339:1349978-1350196
BmHel-11	DF090321:3846572-3846808
BmHel-11	DF090382:1951748-1951931
BmHel-11	DF090382:1951931-1952028
BmHel-11	DF090984:10448-10723
BmHel-11	DF090362:2480505-2480650
BmHel-11	DF090362:2480377-2480507
BmHel-11	DF090317:1966108-1966329
BmHel-11	DF090321:3938878-3939163
BmHel-11	DF090340:2738690-2738965
BmHel-11	DF090329:6059345-6059540
BmHel-11	DF090329:6059245-6059331
BmHel-11	DF090371:1097680-1097813
BmHel-11	DF090371:1097833-1097982
BmHel-11	DF090338:3736060-3736349
BmHel-11	DF090351:2121251-2121525
BmHel-11	DF090381:104354-104632
BmHel-11	DF090344:3042155-3042311
BmHel-11	DF090344:3042022-3042141
BmHel-11	DF090319:5993952-5994159
BmHel-11	DF090326:3763705-3763981
BmHel-11	DF090367:2756247-2756494
BmHel-11	DF090352:2231246-2231482
BmHel-11	DF090331:2816641-2816852
BmHel-11	DF090358:2918978-2919255
BmHel-11	DF090338:3522698-3522973
BmHel-11	DF090317:5999044-5999330
BmHel-11	DF090333:3490378-3490653
BmHel-11	DF090392:232342-232616
BmHel-11	DF090468:264204-264480
BmHel-11	DF090325:6047242-6047516
BmHel-11	DF090319:3400328-3400603
BmHel-11	DF090319:5881698-5881970
BmHel-11	DF090392:674977-675253
BmHel-11	DF090321:2778215-2778491
BmHel-11	DF090334:4142759-4143036
BmHel-11	DF090316:4301057-4301338
BmHel-11	DF090424:884609-884884
BmHel-11	DF090335:4584729-4585004

BmHel-11	DF090367:2026498-2026773
BmHel-11	DF090317:2504753-2505023
BmHel-11	DF090317:890566-890838
BmHel-11	DF090358:1482816-1483099
BmHel-11	DF090422:560960-561237
BmHel-11	DF090409:537575-537843
BmHel-11	DF090441:3960-4237
BmHel-11	DF090465:169368-169638
BmHel-11	DF090319:9043507-9043666
BmHel-11	DF090319:9043390-9043506
BmHel-11	DF090339:4866487-4866758
BmHel-11	DF090394:685902-686177
BmHel-11	BABH01057768:302-582
BmHel-11	DF090332:5716448-5716721
BmHel-11	DF090318:91084-91359
BmHel-11	DF090348:3855980-3856254
BmHel-11	DF090316:8601299-8601574
BmHel-11	DF090316:560991-561262
BmHel-11	DF090457:85129-85404
BmHel-11	DF090631:36423-36697
BmHel-11	DF090369:2202698-2202973
BmHel-11	DF090475:183835-184110
BmHel-11	DF090367:1980941-1981216
BmHel-11	DF090339:1571013-1571289
BmHel-11	DF090362:3163900-3164176
BmHel-11	DF090350:2490781-2490974
BmHel-11	DF090350:2490694-2490775
BmHel-11	DF090351:2405834-2405977
BmHel-11	DF090351:2405707-2405837
BmHel-11	DF090323:7348645-7348759
BmHel-11	DF090323:7348481-7348643
BmHel-11	DF090370:2008666-2008822
BmHel-11	DF090370:2008442-2008560
BmHel-11	DF090354:2551683-2551782
BmHel-11	DF090354:2551786-2551953
BmHel-11	DF090354:644923-645054
BmHel-11	DF090354:644783-644925
BmHel-11	DF090352:2613875-2614013
BmHel-11	DF090352:2614014-2614150
BmHel-11	DF090317:2001085-2001293
BmHel-11	DF090317:9667643-9667802
BmHel-11	DF090317:9667811-9667926
BmHel-11	DF090356:2831907-2832074
BmHel-11	DF090356:2831800-2831907
BmHel-11	DF090333:2294905-2295031
BmHel-11	DF090333:2295034-2295182
BmHel-11	DF090447:558390-558534
BmHel-11	DF090447:558531-558660

BmHel-11	DF090456:356172-356357
BmHel-11	DF090456:356070-356158
BmHel-11	DF090521:28749-28949
BmHel-11	DF090624:32618-32748
BmHel-11	DF090624:32448-32592
BmHel-11	DF090417:1099832-1100060
BmHel-11	DF090344:483612-483888
BmHel-11	DF090412:681013-681192
BmHel-11	DF090412:681212-681307
BmHel-11	DF090358:1667707-1667829
BmHel-11	DF090358:1667479-1667576
BmHel-11	DF090356:1105125-1105298
BmHel-11	DF090356:1105308-1105409
BmHel-11	DF090333:3475356-3475509
BmHel-11	DF090333:3474559-3474686
BmHel-11	DF090403:1556231-1556339
BmHel-11	DF090403:1556066-1556231
BmHel-11	DF090485:171404-171674
BmHel-11	DF090325:1010204-1010438
BmHel-11	DF090349:3203432-3203566
BmHel-11	DF090349:3203291-3203432
BmHel-11	DF090344:4318904-4319046
BmHel-11	DF090344:4319046-4319178
BmHel-11	DF090425:312147-312422
BmHel-11	DF091531:1815-2091
BmHel-11	DF090359:1614260-1614418
BmHel-11	DF090359:1614146-1614262
BmHel-11	DF090379:131230-131396
BmHel-11	DF090379:131394-131501
BmHel-11	DF090354:2371604-2371877
BmHel-11	DF090434:412204-412352
BmHel-11	DF090434:412350-412466
BmHel-11	DF090346:2627952-2628100
BmHel-11	DF090346:2627793-2627935
BmHel-11	DF090374:1473447-1473661
BmHel-11	DF090394:1456462-1456572
BmHel-11	DF090394:1456569-1456733
BmHel-11	DF090408:988590-988699
BmHel-11	DF090408:988722-988890
BmHel-11	DF090360:1429194-1429415
BmHel-11	DF090523:55781-56042
BmHel-11	DF090321:7438824-7439032
BmHel-11	DF090399:1453034-1453315
BmHel-11	BABH01045786:162-272
BmHel-11	BABH01045786:292-464
BmHel-11	DF090363:2673841-2674117
BmHel-11	DF090371:1978277-1978552
BmHel-11	DF090424:413236-413506

BmHel-11	DF090329:6664451-6664726
BmHel-11	DF090321:6408174-6408441
BmHel-11	DF090325:5882125-5882398
BmHel-11	DF090353:2644220-2644497
BmHel-11	DF090387:1025822-1026096
BmHel-11	DF090342:4880376-4880652
BmHel-11	DF090336:1484269-1484543
BmHel-11	DF090348:1790637-1790922
BmHel-11	DF090328:629341-629616
BmHel-11	DF090319:1588793-1589070
BmHel-11	DF090319:5223308-5223585
BmHel-11	DF090357:1265693-1265968
BmHel-11	DF090357:1205820-1206089
BmHel-11	DF090360:15814-16089
BmHel-11	DF090411:536357-536637
BmHel-11	DF090326:554076-554351
BmHel-11	DF090349:820367-820637
BmHel-11	DF090349:2078095-2078370
BmHel-11	DF090340:2233520-2233794
BmHel-11	DF090316:14683008-14683282
BmHel-11	DF090316:14380023-14380298
BmHel-11	DF090385:1908127-1908392
BmHel-11	DF090337:2104953-2105229
BmHel-11	DF090329:5314963-5315240
BmHel-11	DF090323:4914199-4914473
BmHel-11	DF090367:2337467-2337742
BmHel-11	DF090344:1638108-1638384
BmHel-11	DF090320:8010320-8010595
BmHel-11	DF090320:2693523-2693805
BmHel-11	DF090378:153057-153331
BmHel-11	DF090366:1841792-1842065
BmHel-11	DF090364:2492947-2493222
BmHel-11	DF090317:8630875-8631153
BmHel-11	DF090389:1362447-1362721
BmHel-11	DF090401:317292-317563
BmHel-11	DF090403:47355-47630
BmHel-11	DF090361:2372127-2372403
BmHel-11	DF090338:958054-958329
BmHel-11	DF090419:104352-104627
BmHel-11	DF090818:2397-2672
BmHel-11	DF090709:17276-17550
BmHel-11	DF090323:6544802-6545016
BmHel-11	DF090378:1106328-1106547
BmHel-11	DF090396:1096896-1097167
BmHel-11	DF090456:109842-110120
BmHel-11	DF090763:22082-22356
BmHel-11	DF090355:2095112-2095386
BmHel-11	DF090347:2287015-2287289

BmHel-11	DF090330:4126099-4126372
BmHel-11	DF090327:1449340-1449621
BmHel-11	DF090361:673828-674095
BmHel-11	DF090328:1804709-1804978
BmHel-11	DF090377:2015841-2015968
BmHel-11	DF090377:2015969-2016115
BmHel-11	DF090322:3659299-3659426
BmHel-11	DF090322:3659425-3659560
BmHel-11	DF090333:2532495-2532698
BmHel-11	BABH01066134:387-661
BmHel-11	DF090365:430102-430287
BmHel-11	DF090365:430010-430098
BmHel-11	DF090434:290447-290572
BmHel-11	DF090434:290294-290442
BmHel-11	DF090416:366416-366616
BmHel-11	DF090416:366918-367017
BmHel-11	DF090325:4332263-4332541
BmHel-11	DF090336:1312854-1312976
BmHel-11	DF090336:1312979-1313130
BmHel-11	DF090319:7861219-7861493
BmHel-11	DF090414:82021-82191
BmHel-11	DF090414:81918-82022
BmHel-11	DF090411:1341477-1341677
BmHel-11	DF090359:856209-856414
BmHel-11	DF090316:185348-185474
BmHel-11	DF090316:184917-185064
BmHel-11	DF090388:1883447-1883655
BmHel-11	DF090320:3190475-3190708
BmHel-11	DF090352:3011784-3011873
BmHel-11	DF090352:3011603-3011784
BmHel-11	DF090333:2298270-2298491
BmHel-11	DF090401:23533-23807
BmHel-11	DF090338:247936-248210
BmHel-11	DF090437:475822-476085
BmHel-11	DF090426:16898-17093
BmHel-11	DF090343:574447-574683
BmHel-11	DF090323:4165098-4165256
BmHel-11	DF090323:4164972-4165087
BmHel-11	DF090442:129099-129301
BmHel-11	DF090442:129312-129403
BmHel-11	DF090332:5920333-5920508
BmHel-11	DF090332:5924283-5924401
BmHel-11	DF090328:6172395-6172518
BmHel-11	DF090328:6172518-6172672
BmHel-11	DF090375:5365-5489
BmHel-11	DF090375:5565-5726
BmHel-11	DF090397:397536-397809
BmHel-11	DF090496:63339-63613

BmHel-11	DF090630:38201-38331
BmHel-11	DF090630:38328-38471
BmHel-11	DF090390:1652414-1652539
BmHel-11	DF090390:1652185-1652328
BmHel-11	DF090342:4065721-4065995
BmHel-11	DF090410:285688-285962
BmHel-11	DF090327:4611529-4611803
BmHel-11	DF090353:2118490-2118762
BmHel-11	DF090425:91704-91848
BmHel-11	DF090425:92337-92476
BmHel-11	DF090319:1212829-1213105
BmHel-11	DF090347:2424788-2425059
BmHel-11	DF090319:7220632-7220855
BmHel-11	DF090353:2920076-2920357
BmHel-11	DF090362:1704133-1704401
BmHel-11	DF090324:1848234-1848506
BmHel-11	DF090332:944009-944280
BmHel-11	DF090350:2017506-2017780
BmHel-11	DF090335:3817174-3817451
BmHel-11	DF090367:1570796-1571071
BmHel-11	DF090333:1243439-1243710
BmHel-11	DF090409:748806-749078
BmHel-11	DF090431:491376-491638
BmHel-11	DF090339:1918519-1918748
BmHel-11	DF090326:6902746-6902983
BmHel-11	DF090386:1613614-1613895
BmHel-11	DF090321:1463695-1463968
BmHel-11	DF090336:183556-183830
BmHel-11	DF090347:2562773-2563045
BmHel-11	DF090423:919623-919890
BmHel-11	DF090355:382236-382461
BmHel-11	DF090336:900053-900325
BmHel-11	DF090322:1230288-1230514
BmHel-11	DF090359:2306952-2307195
BmHel-11	DF090340:319000-319226
BmHel-11	DF090323:347783-347928
BmHel-11	DF090323:347928-348061
BmHel-11	DF090366:1757741-1758014
BmHel-11	DF090366:1688352-1688581
BmHel-11	DF090345:2868546-2868766
BmHel-11	DF090537:32016-32232
BmHel-11	DF090766:1299-1465
BmHel-11	DF090766:1181-1287
BmHel-11	DF090319:8291170-8291442
BmHel-11	DF090337:970302-970541
BmHel-11	DF090335:4393675-4393847
BmHel-11	DF090335:4393575-4393675
BmHel-11	DF090470:309514-309688

BmHel-11	DF090470:309697-309795
BmHel-11	DF090328:2880152-2880309
BmHel-11	DF090328:2880037-2880150
BmHel-11	DF090322:3795103-3795250
BmHel-11	DF090322:3795251-3795376
BmHel-11	DF090322:2524470-2524704
BmHel-11	DF090410:1195444-1195539
BmHel-11	DF090410:1195541-1195718
BmHel-11	DF090316:6859811-6860042
BmHel-11	DF090337:3166628-3166868
BmHel-11	DF090398:919174-919392
BmHel-11	DF090622:21029-21229
BmHel-11	DF090458:366539-366812
BmHel-11	BABH01061020:422-696
BmHel-11	DF090670:16592-16768
BmHel-11	DF090670:16796-16892
BmHel-11	DF090373:1234116-1234383
BmHel-11	DF090355:2740864-2741099
BmHel-11	DF090318:5769645-5769856
BmHel-11	DF090341:3840596-3840861
BmHel-11	DF090330:1046888-1047050
BmHel-11	DF090330:1047048-1047159
BmHel-11	DF090536:86701-86980
BmHel-11	DF090461:291476-291749
BmHel-11	BABH01083070:1-273
BmHel-11	DF090354:503035-503308
BmHel-11	DF090376:1760677-1760950
BmHel-11	DF090338:1851108-1851375
BmHel-11	DF090436:508185-508340
BmHel-11	DF090436:508072-508188
BmHel-11	DF090408:526371-526644
BmHel-11	DF090336:1229736-1229921
BmHel-11	DF090336:1229366-1229453
BmHel-11	DF090368:570200-570473
BmHel-11	DF090374:145062-145260
BmHel-11	DF090337:943863-944132
BmHel-11	DF090325:2898829-2899102
BmHel-11	DF090325:6645922-6646193
BmHel-11	DF090335:243172-243445
BmHel-11	DF090320:2982319-2982593
BmHel-11	DF090317:379670-379945
BmHel-11	DF090389:387617-387893
BmHel-11	DF090425:799443-799711
BmHel-11	DF090345:2146193-2146324
BmHel-11	DF090345:2146324-2146464
BmHel-11	DF090418:947846-948118
BmHel-11	DF090325:4416285-4416557
BmHel-11	DF090356:3250460-3250727

BmHel-11	DF090655:24777-25048
BmHel-11	DF090362:2876751-2876899
BmHel-11	DF090362:2876209-2876296
BmHel-11	DF090390:567416-567537
BmHel-11	DF090390:567323-567415
BmHel-11	DF090487:179278-179550
BmHel-11	DF090316:6849557-6849646
BmHel-11	DF090316:6849373-6849556
BmHel-11	DF090329:5028737-5028829
BmHel-11	DF090329:5028556-5028735
BmHel-11	DF090352:2030298-2030502
BmHel-11	DF090361:2240728-2241001
BmHel-11	DF090448:172975-173179
BmHel-11	DF090321:1711122-1711329
BmHel-11	DF090349:1897267-1897539
BmHel-11	DF090340:291833-291949
BmHel-11	DF090340:291681-291833
BmHel-11	DF090327:6375477-6375656
BmHel-11	DF090327:6375656-6375747
BmHel-11	DF090397:368498-368718
BmHel-11	DF090408:1476008-1476207
BmHel-11	DF090324:5420690-5420841
BmHel-11	DF090348:2847524-2847794
BmHel-11	DF090319:4504822-4504993
BmHel-11	DF090319:4504722-4504822
BmHel-11	DF090320:6991501-6991622
BmHel-11	DF090320:6991333-6991477
BmHel-11	DF090384:305839-306111
BmHel-11	DF090333:5489702-5489974
BmHel-11	DF090343:820996-821268
BmHel-11	DF090323:6494320-6494414
BmHel-11	DF090323:6494151-6494322
BmHel-11	DF090317:9980831-9980946
BmHel-11	DF090317:9980643-9980799
BmHel-11	DF090485:135209-135459
BmHel-11	DF090575:29288-29508
BmHel-11	DF090353:2825609-2825747
BmHel-11	DF090353:2825779-2825902
BmHel-11	DF090359:2121197-2121423
BmHel-11	DF090316:11519728-11519996
BmHel-11	DF090320:2577471-2577745
BmHel-11	DF090327:1823704-1823861
BmHel-11	DF090327:1823579-1823692
BmHel-11	DF090437:643748-643889
BmHel-11	DF090437:644125-644256
BmHel-11	DF090415:53463-53745
BmHel-11	DF090376:458257-458498
BmHel-11	DF091844:190-300

BmHel-11	DF091844:1-170
BmHel-11	DF090380:53568-53777
BmHel-11	DF090369:2369958-2370063
BmHel-11	DF090369:2370088-2370263
BmHel-11	DF090374:1171317-1171424
BmHel-11	DF090374:1171127-1171307
BmHel-11	DF090348:1565520-1565612
BmHel-11	DF090348:1565404-1565505
BmHel-11	DF090327:595142-595412
BmHel-11	DF090338:1480916-1481118
BmHel-11	DF090384:868851-869123
BmHel-11	DF090353:2268738-2268963
BmHel-11	DF090599:19038-19176
BmHel-11	DF090599:19536-19672
BmHel-11	DF090353:3147614-3147886
BmHel-11	DF090342:1546989-1547258
BmHel-11	DF090336:3166896-3167168
BmHel-11	DF090332:3546667-3546938
BmHel-11	DF090318:6240379-6240650
BmHel-11	DF090360:1846048-1846322
BmHel-11	DF090347:1798842-1799113
BmHel-11	DF090322:2397569-2397839
BmHel-11	DF090410:977223-977495
BmHel-11	DF090359:3189194-3189468
BmHel-11	DF090349:3010690-3010961
BmHel-11	DF090316:1716480-1716754
BmHel-11	DF090316:4884979-4885250
BmHel-11	DF090316:11067429-11067697
BmHel-11	DF090316:14489935-14490200
BmHel-11	DF090330:2326815-2327090
BmHel-11	DF090344:2454220-2454492
BmHel-11	DF090344:1675952-1676228
BmHel-11	DF090352:1773662-1773926
BmHel-11	DF090346:1177096-1177366
BmHel-11	DF090504:12387-12643
BmHel-11	BABH01088069:1-272
BmHel-11	DF090509:33219-33495
BmHel-11	DF090747:16753-17026
BmHel-11	DF090318:5099807-5100001
BmHel-11	DF090327:3397529-3397734
BmHel-11	DF090426:184790-184898
BmHel-11	DF090426:185147-185253
BmHel-11	DF090319:3258529-3258798
BmHel-11	DF090326:6679397-6679668
BmHel-11	DF090393:724448-724717
BmHel-11	DF090373:1175151-1175426
BmHel-11	DF090378:1850605-1850877
BmHel-11	DF090361:1675375-1675656

BmHel-11	DF090338:1670801-1671062
BmHel-11	DF090322:7029462-7029636
BmHel-11	DF090322:7029644-7029739
BmHel-11	DF090319:629371-629583
BmHel-11	DF090341:4197395-4197578
BmHel-11	DF090341:4197578-4197669
BmHel-11	DF090316:7337473-7337731
BmHel-11	DF090331:5864087-5864291
BmHel-11	DF090451:113899-114058
BmHel-11	DF090451:113788-113899
BmHel-11	DF090376:1005192-1005458
BmHel-11	DF090386:654323-654439
BmHel-11	DF090386:654170-654322
BmHel-11	DF090365:828420-828691
BmHel-11	DF090321:1957582-1957803
BmHel-11	DF090316:13715661-13715882
BmHel-11	DF090316:12294733-12294935
BmHel-11	DF090316:10430269-10430382
BmHel-11	DF090316:10430186-10430270
BmHel-11	BABH01079517:1-269
BmHel-11	DF090319:4582587-4582793
BmHel-11	DF090347:4039186-4039449
BmHel-11	DF090469:273565-273822
BmHel-11	DF090407:54345-54523
BmHel-11	DF090407:54524-54615
BmHel-11	DF090331:3480707-3480848
BmHel-11	DF090331:3480525-3480653
BmHel-11	DF090354:2533571-2533796
BmHel-11	BABH01044357:1446-1652
BmHel-11	DF090339:1863232-1863511
BmHel-11	DF090318:7797225-7797360
BmHel-11	DF090318:7797083-7797220
BmHel-11	DF090352:3043675-3043915
BmHel-11	DF090468:54514-54615
BmHel-11	DF090468:54613-54782
BmHel-11	DF090401:897324-897464
BmHel-11	DF090401:897167-897294
BmHel-11	DF090361:2239807-2239893
BmHel-11	DF090361:2239897-2240081
BmHel-11	DF090416:21652-21853
BmHel-11	DF090353:177600-177871
BmHel-11	DF090318:3324310-3324580
BmHel-11	DF090322:2181428-2181508
BmHel-11	DF090322:2180601-2180809
BmHel-11	DF090329:5095946-5096216
BmHel-11	DF090361:2395683-2395877
BmHel-11	DF090324:7725058-7725330
BmHel-11	DF090358:155847-156113

BmHel-11	DF090504:85532-85803
BmHel-11	DF090362:1289488-1289764
BmHel-11	DF090379:704561-704833
BmHel-11	DF090319:4217036-4217306
BmHel-11	DF090345:3616513-3616785
BmHel-11	DF090394:1195128-1195399
BmHel-11	DF090324:7144122-7144392
BmHel-11	DF090332:4210155-4210424
BmHel-11	DF090348:4144693-4144964
BmHel-11	DF090328:3751887-3752158
BmHel-11	DF090382:359848-360119
BmHel-11	DF090316:12267258-12267530
BmHel-11	DF090330:6064-6341
BmHel-11	DF090344:1793289-1793560
BmHel-11	DF090317:6424850-6425125
BmHel-11	DF090389:851138-851399
BmHel-11	DF090356:497990-498261
BmHel-11	DF090415:822577-822851
BmHel-11	DF090409:1420079-1420350
BmHel-11	DF090394:701232-701503
BmHel-11	BABH01058278:416-690
BmHel-11	DF090346:1828173-1828296
BmHel-11	DF090346:1828306-1828454
BmHel-11	DF090373:480540-480717
BmHel-11	DF090373:480452-480542
BmHel-11	DF090389:577639-577907
BmHel-11	DF090332:2559275-2559505
BmHel-11	DF090319:3305816-3306090
BmHel-11	DF090360:1678369-1678598
BmHel-11	DF090365:2269748-2269965
BmHel-11	DF090350:1641947-1642215
BmHel-11	DF090368:1976458-1976688
BmHel-11	DF090335:46312-46540
BmHel-11	DF090330:3496608-3496716
BmHel-11	DF090330:3496751-3496912
BmHel-11	DF090370:1397723-1397837
BmHel-11	DF090370:1397574-1397685
BmHel-11	DF090317:831351-831582
BmHel-11	DF090462:218662-218892
BmHel-11	DF090375:971967-972161
BmHel-11	DF090415:1324483-1324690
BmHel-11	DF090571:41502-41732
BmHel-11	DF090319:1302016-1302166
BmHel-11	DF090319:1302171-1302289
BmHel-11	DF090395:1300062-1300273
BmHel-11	DF090354:2048735-2048875
BmHel-11	DF090354:2048606-2048735
BmHel-11	DF090444:306595-306834

BmHel-11	DF090325:3101520-3101632
BmHel-11	DF090325:3101630-3101787
BmHel-11	DF090340:1838735-1838954
BmHel-11	DF090351:3770707-3770931
BmHel-11	DF090323:4741264-4741534
BmHel-11	DF090389:88036-88308
BmHel-11	DF090428:601257-601453
BmHel-11	DF090446:321906-322041
BmHel-11	DF090446:322042-322176
BmHel-11	DF090429:216616-216846
BmHel-11	DF090324:6063425-6063695
BmHel-11	DF090332:5568681-5568812
BmHel-11	DF090332:5568543-5568685
BmHel-11	DF090357:1480379-1480580
BmHel-11	DF090322:2573766-2574032
BmHel-11	DF090316:291051-291181
BmHel-11	DF090316:291188-291327
BmHel-11	DF090316:4329558-4329828
BmHel-11	DF090355:501426-501699
BmHel-11	DF090353:3269533-3269742
BmHel-11	DF090357:1712722-1712874
BmHel-11	DF090357:1712883-1713000
BmHel-11	DF090395:1189794-1189961
BmHel-11	DF090395:1189960-1190057
BmHel-11	DF090411:1130992-1131236
BmHel-11	DF090341:4449704-4449943
BmHel-11	DF090380:1909040-1909204
BmHel-11	DF090380:1908886-1908991
BmHel-11	DF090352:1909426-1909537
BmHel-11	DF090352:1909254-1909420
BmHel-11	DF090389:1008740-1008842
BmHel-11	DF090389:1008573-1008740
BmHel-11	DF091339:4037-4188
BmHel-11	DF091339:4208-4326
BmHel-11	DF090319:8534660-8534814
BmHel-11	DF090319:8534546-8534660
BmHel-11	DF090418:369820-370090
BmHel-11	DF090326:5976268-5976383
BmHel-11	DF090326:5976386-5976534
BmHel-11	DF090379:2093357-2093539
BmHel-11	DF090379:2093270-2093357
BmHel-11	DF090361:1965686-1965879
BmHel-11	DF090395:350111-350268
BmHel-11	DF090395:350279-350394
BmHel-11	DF090344:3386665-3386847
BmHel-11	DF090344:3386852-3386939
BmHel-11	DF090459:323750-323912
BmHel-11	DF090459:323910-324017

BmHel-11	DF090349:1386647-1386749
BmHel-11	DF090349:1386780-1386949
BmHel-11	DF090412:907221-907494
BmHel-11	DF090389:557461-557580
BmHel-11	DF090389:557714-557860
BmHel-11	BABH01052671:567-836
BmHel-11	DF090354:1950699-1950937
BmHel-11	DF090319:3851980-3852183
BmHel-11	DF090348:2308167-2308432
BmHel-11	DF090411:68180-68452
BmHel-11	DF090365:1105355-1105625
BmHel-11	DF090340:2317029-2317298
BmHel-11	DF090337:2245954-2246224
BmHel-11	DF090366:857140-857410
BmHel-11	DF090415:268308-268578
BmHel-11	DF090409:1156605-1156878
BmHel-11	DF090413:1097093-1097363
BmHel-11	DF090721:14000-14270
BmHel-11	DF090324:2678167-2678400
BmHel-11	DF090349:3508119-3508353
BmHel-11	DF090351:2354947-2355127
BmHel-11	DF090351:2355125-2355213
BmHel-11	DF090446:551113-551344
BmHel-11	DF090359:1238705-1238881
BmHel-11	DF090360:421042-421311
BmHel-11	DF090338:2330988-2331259
BmHel-11	BABH01063390:67-335
BmHel-11	DF090339:1654370-1654603
BmHel-11	DF090348:1770252-1770374
BmHel-11	DF090348:1770106-1770253
BmHel-11	DF090343:4506489-4506707
BmHel-11	DF090418:68517-68743
BmHel-11	DF090317:4026893-4027162
BmHel-11	DF090317:561970-562073
BmHel-11	DF090317:561803-561968
BmHel-11	DF090403:369738-370007
BmHel-11	DF090342:1005001-1005274
BmHel-11	DF090328:3242406-3242646
BmHel-11	DF090365:1791241-1791513
BmHel-11	DF090321:8216260-8216491
BmHel-11	DF090363:2880803-2880990
BmHel-11	DF090363:2880708-2880802
BmHel-11	DF090330:1201285-1201378
BmHel-11	DF090330:1201410-1201584
BmHel-11	DF090406:695461-695630
BmHel-11	DF090406:695370-695461
BmHel-11	DF090354:1298927-1299018
BmHel-11	DF090354:1295457-1295668

BmHel-11	DF090317:5063366-5063470
BmHel-11	DF090317:5063469-5063632
BmHel-11	DF090591:24831-24977
BmHel-11	DF090591:24706-24828
BmHel-11	DF090345:1466072-1466345
BmHel-11	BABH01047915:1-261
BmHel-11	DF090328:5002435-5002660
BmHel-11	DF090319:3991517-3991683
BmHel-11	DF090357:2150608-2150736
BmHel-11	DF090357:2150476-2150612
BmHel-11	DF090326:1486064-1486289
BmHel-11	DF090359:208353-208587
BmHel-11	DF090334:2857041-2857167
BmHel-11	DF090334:2857408-2857549
BmHel-11	DF090316:15847035-15847263
BmHel-11	DF090337:1522069-1522281
BmHel-11	DF090323:4686888-4687160
BmHel-11	DF090390:88226-88320
BmHel-11	DF090390:87884-88068
BmHel-11	DF090319:7446458-7446629
BmHel-11	DF090319:7446641-7446738
BmHel-11	DF090334:727503-727730
BmHel-11	DF090410:679320-679463
BmHel-11	DF090410:679464-679589
BmHel-11	DF090319:5254685-5254956
BmHel-11	DF090352:266454-266650
BmHel-11	DF090352:3714547-3714816
BmHel-11	DF090319:1754165-1754382
BmHel-11	DF090389:161267-161361
BmHel-11	DF090389:161079-161251
BmHel-11	DF090322:799106-799375
BmHel-11	DF090322:633555-633813
BmHel-11	DF090379:207897-208163
BmHel-11	DF090333:431408-431608
BmHel-11	DF090363:2466108-2466378
BmHel-11	DF090317:6974962-6975229
BmHel-11	DF090319:527342-527611
BmHel-11	DF090360:2030324-2030594
BmHel-11	DF090326:2194752-2195011
BmHel-11	DF090340:3351511-3351783
BmHel-11	DF090368:2296414-2296683
BmHel-11	DF090329:1500042-1500305
BmHel-11	DF090323:6289069-6289341
BmHel-11	DF090367:1682888-1683157
BmHel-11	DF090356:2517774-2518041
BmHel-11	DF090376:2082935-2083206
BmHel-11	DF090347:2995331-2995458
BmHel-11	DF090347:2995492-2995632

BmHel-11	DF090316:9363926-9364194
BmHel-11	DF090356:587958-588226
BmHel-11	DF090336:4964409-4964677
BmHel-11	DF090378:341126-341396
BmHel-11	DF090374:1326278-1326546
BmHel-11	BABH01046820:1-271
BmHel-11	DF090339:3468123-3468231
BmHel-11	DF090339:3467960-3468119
BmHel-11	DF090342:2783040-2783217
BmHel-11	DF090342:2782942-2783032
BmHel-11	DF090332:2148256-2148458
BmHel-11	DF090328:3942137-3942327
BmHel-11	DF090319:6942542-6942743
BmHel-11	DF090344:2037311-2037540
BmHel-11	DF090426:610135-610342
BmHel-11	DF090430:130052-130194
BmHel-11	DF090430:129925-130050
BmHel-11	DF090321:8202036-8202249
BmHel-11	DF090351:2444388-2444504
BmHel-11	DF090351:2444236-2444387
BmHel-11	DF090337:1570481-1570704
BmHel-11	DF090335:4113774-4113997
BmHel-11	DF090318:3753932-3754155
BmHel-11	DF090399:1295455-1295719
BmHel-11	DF090371:1410734-1410850
BmHel-11	DF090371:1410856-1411007
BmHel-11	DF090423:514849-515019
BmHel-11	DF090423:515021-515117
BmHel-11	BABH01047909:775-970
BmHel-11	DF090339:564937-565049
BmHel-11	DF090339:564779-564934
BmHel-11	DF090334:2349900-2350023
BmHel-11	DF090334:2349765-2349905
BmHel-11	DF090358:315694-315831
BmHel-11	DF090346:1481568-1481683
BmHel-11	DF090346:1481396-1481548
BmHel-11	DF090504:73233-73460
BmHel-11	DF090343:3392074-3392230
BmHel-11	DF090343:3392234-3392345
BmHel-11	DF090447:70149-70355
BmHel-11	DF090349:300188-300456
BmHel-11	DF090355:2133564-2133834
BmHel-11	DF090367:2076681-2076947
BmHel-11	DF090409:654254-654334
BmHel-11	DF090409:654006-654193
BmHel-11	DF090353:3793975-3794236
BmHel-11	DF090336:1422657-1422925
BmHel-11	DF090343:3738200-3738468

BmHel-11	DF090320:6469577-6469844
BmHel-11	DF090490:2754-3024
BmHel-11	DF090327:6667940-6668211
BmHel-11	DF090360:1986723-1986889
BmHel-11	DF090360:1986890-1986990
BmHel-11	DF090375:1589876-1590092
BmHel-11	DF090337:427534-427637
BmHel-11	DF090337:427402-427536
BmHel-11	DF090339:3790726-3790918
BmHel-11	DF090391:825038-825166
BmHel-11	DF090391:824782-824909
BmHel-11	DF090317:2416605-2416872
BmHel-11	DF090715:7915-8182
BmHel-11	BABH01069246:139-404
BmHel-11	DF090325:6289933-6290203
BmHel-11	DF090362:1937527-1937797
BmHel-11	DF090377:282235-282505
BmHel-11	DF090343:2347247-2347514
BmHel-11	DF090324:1427232-1427358
BmHel-11	DF090324:1427062-1427202
BmHel-11	DF090343:1140734-1140861
BmHel-11	DF090343:1140864-1141004
BmHel-11	DF090351:258423-258588
BmHel-11	DF090351:258589-258691
BmHel-11	DF090352:4150557-4150650
BmHel-11	DF090352:4150377-4150550
BmHel-11	DF090317:1418335-1418432
BmHel-11	DF090317:1418431-1418604
BmHel-11	DF090316:3983401-3983528
BmHel-11	DF090316:3983261-3983388
BmHel-11	DF090391:672275-672526
BmHel-11	DF090346:2422652-2422795
BmHel-11	DF090346:2422794-2422917
BmHel-11	DF090351:1443434-1443661
BmHel-11	DF090323:4162407-4162633
BmHel-11	DF090404:61310-61578
BmHel-11	DF090346:1658131-1658278
BmHel-11	DF090346:1658012-1658131
BmHel-11	DF090346:306073-306296
BmHel-11	DF090402:1392152-1392297
BmHel-11	DF090402:1392028-1392151
BmHel-11	DF090328:908184-908274
BmHel-11	DF090328:908057-908164
BmHel-11	DF090414:1241669-1241781
BmHel-11	DF090414:1241784-1241938
BmHel-11	DF090471:222432-222637
BmHel-11	DF090319:1491215-1491436
BmHel-11	DF090321:749263-749486

BmHel-11	DF090388:1790443-1790679
BmHel-11	DF090385:834087-834325
BmHel-11	DF090367:1533804-1533975
BmHel-11	DF090367:1533994-1534088
BmHel-11	DF090372:1961990-1962258
BmHel-11	DF090356:2491810-2492028
BmHel-11	DF090356:2939771-2939898
BmHel-11	DF090356:2939904-2940037
BmHel-11	DF090338:1216336-1216603
BmHel-11	DF090413:618812-619005
BmHel-11	BABH01051797:1-263
BmHel-11	BABH01043991:7562-7659
BmHel-11	BABH01043991:7660-7821
BmHel-11	DF091341:5426-5525
BmHel-11	DF091341:5263-5424
BmHel-11	DF090353:2277492-2277677
BmHel-11	DF090353:2277678-2277759
BmHel-11	DF090421:1006397-1006659
BmHel-11	DF090331:5869502-5869769
BmHel-11	DF090317:6206972-6207201
BmHel-11	DF090323:13044-13264
BmHel-11	DF090388:657634-657808
BmHel-11	DF090388:657806-657896
BmHel-11	DF090452:502220-502488
BmHel-11	DF090317:11100971-11101121
BmHel-11	DF090317:11100846-11100957
BmHel-11	DF090358:2142306-2142569
BmHel-11	DF090358:69566-69677
BmHel-11	DF090358:69676-69831
BmHel-11	BABH01059191:1-268
BmHel-11	DF090319:1648787-1649062
BmHel-11	DF090347:2346041-2346311
BmHel-11	DF090341:1452770-1453039
BmHel-11	DF090316:420699-420966
BmHel-11	DF090417:147920-148191
BmHel-11	DF090345:1984213-1984481
BmHel-11	BABH01050946:650-923
BmHel-11	DF090324:8026166-8026374
BmHel-11	DF090886:13217-13356
BmHel-11	DF090886:13090-13216
BmHel-11	DF090373:1104333-1104589
BmHel-11	DF090355:2612888-2613062
BmHel-11	DF090355:2612803-2612894
BmHel-11	DF090381:1020578-1020752
BmHel-11	DF090381:1020746-1020836
BmHel-11	DF090360:712750-712972
BmHel-11	DF090321:7781128-7781302
BmHel-11	DF090321:7781043-7781134

BmHel-11	DF090343:1593157-1593298
BmHel-11	DF090343:1593028-1593156
BmHel-11	DF090316:5776980-5777193
BmHel-11	DF090391:1166759-1166870
BmHel-11	DF090391:1166870-1167024
BmHel-11	DF090320:1187507-1187655
BmHel-11	DF090320:1187656-1187773
BmHel-11	DF090358:2273178-2273403
BmHel-11	DF090327:1428792-1429062
BmHel-11	DF090536:5496-5597
BmHel-11	DF090536:5596-5788
BmHel-11	DF090375:331505-331771
BmHel-11	DF090374:1604574-1604710
BmHel-11	DF090374:1604711-1604840
BmHel-11	DF090443:535452-535626
BmHel-11	DF090443:535620-535708
BmHel-11	DF090317:2387779-2387892
BmHel-11	DF090317:2387889-2387972
BmHel-11	DF090376:396296-396420
BmHel-11	DF090414:780338-780548
BmHel-11	DF090331:5525565-5525650
BmHel-11	DF090331:5525372-5525551
BmHel-11	DF090317:2303080-2303178
BmHel-11	DF090317:2303667-2303836
BmHel-11	DF090317:5081211-5081443
BmHel-11	DF090317:2717936-2718164
BmHel-11	DF090376:1239653-1239763
BmHel-11	DF090376:1239765-1239920
BmHel-11	DF090327:6672427-6672659
BmHel-11	DF090331:230758-230973
BmHel-11	DF090424:105952-106132
BmHel-11	DF090424:105864-105952
BmHel-11	DF090342:2384597-2384823
BmHel-11	DF090347:1713958-1714181
BmHel-11	DF090380:641677-641907
BmHel-11	DF090464:92279-92419
BmHel-11	DF090464:92418-92543
BmHel-11	DF090343:372920-373097
BmHel-11	DF090343:373097-373185
BmHel-11	DF090352:3456303-3456569
BmHel-11	DF090321:4691586-4691733
BmHel-11	DF090321:4683283-4683400
BmHel-11	DF090330:5748865-5748999
BmHel-11	DF090330:5748761-5748864
BmHel-11	DF090330:733072-733250
BmHel-11	DF090330:732103-732190
BmHel-11	DF090329:4069324-4069528
BmHel-11	DF090325:3286625-3286866

BmHel-11	DF090342:298064-298330
BmHel-11	DF090364:2820550-2820809
BmHel-11	DF090421:663378-663471
BmHel-11	DF090421:662610-662783
BmHel-11	DF090339:3561404-3561666
BmHel-11	DF090387:1822799-1823065
BmHel-11	DF090342:1371042-1371308
BmHel-11	DF090377:2016296-2016562
BmHel-11	DF090322:838562-838828
BmHel-11	DF090382:148817-149083
BmHel-11	DF090330:5050805-5051072
BmHel-11	DF090379:1230635-1230903
BmHel-11	DF090354:889011-889277
BmHel-11	DF090317:9815151-9815415
BmHel-11	DF090317:7753633-7753897
BmHel-11	DF090398:382338-382605
BmHel-11	DF090346:2409076-2409342
BmHel-11	DF090415:230577-230843
BmHel-11	DF090448:11728-11996
BmHel-11	DF090613:55475-55745
BmHel-11	DF090340:3387409-3387590
BmHel-11	DF090554:42217-42306
BmHel-11	DF090554:42030-42194
BmHel-11	DF090316:10957577-10957752
BmHel-11	DF090316:10957751-10957838
BmHel-11	DF090414:637811-637893
BmHel-11	DF090414:637670-637812
BmHel-11	DF090323:314714-314814
BmHel-11	DF090323:314558-314716
BmHel-11	DF090352:2729268-2729532
BmHel-11	DF090384:18001-18183
BmHel-11	DF090347:3236447-3236712
BmHel-11	DF090322:4483495-4483629
BmHel-11	DF090322:4483358-4483497
BmHel-11	DF090321:3093930-3094189
BmHel-11	DF090321:1096429-1096691
BmHel-11	DF090343:3804118-3804262
BmHel-11	DF090343:3803997-3804118
BmHel-11	DF090388:1405856-1406125
BmHel-11	DF090352:1328626-1328890
BmHel-11	DF090369:2024039-2024136
BmHel-11	DF090369:2024135-2024300
BmHel-11	BABH01087805:99-368
BmHel-11	BABH01045002:268-537
BmHel-11	DF090343:575715-575946
BmHel-11	DF090546:6226-6399
BmHel-11	DF090546:6141-6232
BmHel-11	DF090342:1602518-1602780

BmHel-11	DF090348:490226-490370
BmHel-11	DF090348:490369-490492
BmHel-11	DF090319:4080146-4080261
BmHel-11	DF090319:4079998-4080147
BmHel-11	DF090360:2428491-2428694
BmHel-11	DF090389:695306-695421
BmHel-11	DF090389:695431-695543
BmHel-11	DF090413:784045-784237
BmHel-11	DF090355:698659-698875
BmHel-11	DF090321:303141-303371
BmHel-11	DF090316:15200395-15200477
BmHel-11	DF090316:15200197-15200379
BmHel-11	DF090355:874720-874948
BmHel-11	DF090324:4327587-4327851
BmHel-11	DF090391:1288116-1288344
BmHel-11	DF090329:6384763-6384915
BmHel-11	DF090329:6384651-6384763
BmHel-11	DF090367:1934503-1934680
BmHel-11	DF090367:1934415-1934502
BmHel-11	DF090371:37461-37729
BmHel-11	DF090361:1600372-1600538
BmHel-11	DF090397:249137-249331
BmHel-11	DF090336:2676184-2676304
BmHel-11	DF090336:2676306-2676450
BmHel-11	DF090322:6987231-6987389
BmHel-11	DF090322:6987393-6987498
BmHel-11	DF090332:6212152-6212310
BmHel-11	DF090332:6212049-6212155
BmHel-11	DF090397:1294666-1294871
BmHel-11	DF090664:37784-37955
BmHel-11	DF090664:37694-37786
BmHel-11	DF090340:4625953-4626218
BmHel-11	DF090387:1177488-1177568
BmHel-11	DF090387:1177568-1177753
BmHel-11	DF090405:1381432-1381640
BmHel-11	DF090346:4503879-4503981
BmHel-11	DF090346:4503761-4503863
BmHel-11	DF090357:2105296-2105505
BmHel-11	DF090391:1018073-1018341
BmHel-11	DF090355:3375612-3375877
BmHel-11	DF090319:1561846-1562107
BmHel-11	DF090322:2694316-2694583
BmHel-11	DF090400:1459517-1459780
BmHel-11	DF090367:1139471-1139741
BmHel-11	DF090417:965007-965272
BmHel-11	DF090345:868013-868278
BmHel-11	DF090345:4487994-4488263
BmHel-11	DF090403:874362-874630

BmHel-11	DF090478:132860-133128
BmHel-11	DF090488:74803-75071
BmHel-11	DF090332:4224183-4224399
BmHel-11	DF090323:7469998-7470216
BmHel-11	DF090358:810159-810323
BmHel-11	DF090358:810325-810424
BmHel-11	DF090342:2730779-2730983
BmHel-11	DF090365:1046668-1046877
BmHel-11	DF090323:27488-27605
BmHel-11	DF090323:27620-27775
BmHel-11	DF090323:4433417-4433631
BmHel-11	BABH01048896:358-564
BmHel-11	DF091305:3219-3425
BmHel-11	DF090630:52809-52901
BmHel-11	DF090630:52915-53088
BmHel-11	BABH01044930:694-829
BmHel-11	BABH01044930:832-962
BmHel-11	DF090324:498770-498931
BmHel-11	DF090324:498648-498750
BmHel-11	DF090534:148940-149104
BmHel-11	DF090328:1584851-1585021
BmHel-11	DF090328:1584770-1584857
BmHel-11	DF090321:2455768-2455940
BmHel-11	DF090321:2455683-2455774
BmHel-11	DF090382:1888964-1889220
BmHel-11	DF090317:2650012-2650276
BmHel-11	DF090460:371066-371238
BmHel-11	DF090460:370981-371072
BmHel-11	DF090532:80721-80893
BmHel-11	DF090532:80636-80727
BmHel-11	DF090377:55473-55614
BmHel-11	DF090377:55613-55735
BmHel-11	DF090336:258104-258298
BmHel-11	DF090343:3799898-3800122
BmHel-11	DF090409:1120255-1120422
BmHel-11	DF090409:1120446-1120540
BmHel-11	DF090798:20164-20340
BmHel-11	DF090341:1557517-1557774
BmHel-11	DF090344:2251409-2251570
BmHel-11	DF090344:2251302-2251404
BmHel-11	DF090352:263931-264154
BmHel-11	DF090345:4262670-4262928
BmHel-11	DF090333:1583166-1583314
BmHel-11	DF090333:1583323-1583437
BmHel-11	DF090346:581807-582071
BmHel-11	DF090361:407405-407511
BmHel-11	DF090361:407124-407281
BmHel-11	DF090365:2295886-2296116

BmHel-11	DF090375:466132-466393
BmHel-11	DF090320:1696415-1696618
BmHel-11	DF090338:4563884-4564148
BmHel-11	DF090373:295043-295307
BmHel-11	DF090320:2580602-2580866
BmHel-11	DF090356:1111086-1111350
BmHel-11	DF090342:4865610-4865870
BmHel-11	DF090336:4295436-4295700
BmHel-11	DF090322:1199466-1199734
BmHel-11	DF090334:1769119-1769383
BmHel-11	DF090337:3603191-3603456
BmHel-11	DF090366:2191982-2192249
BmHel-11	DF090401:759397-759660
BmHel-11	DF090416:581037-581295
BmHel-11	DF090397:1264514-1264782
BmHel-11	DF090415:844073-844327
BmHel-11	DF090409:1579622-1579888
BmHel-11	BABH01083242:93-357
BmHel-11	DF090360:270726-270903
BmHel-11	DF090360:270908-270993
BmHel-11	DF090407:1452816-1452993
BmHel-11	DF090407:1452992-1453077
BmHel-11	DF090525:63596-63775
BmHel-11	DF090525:63512-63597
BmHel-11	DF090333:1956052-1956218
BmHel-11	DF090333:1955954-1956050
BmHel-11	DF090344:978841-978977
BmHel-11	DF090344:978980-979106
BmHel-11	DF090328:3011415-3011678
BmHel-11	DF090404:1372594-1372859
BmHel-11	DF090350:3880693-3880857
BmHel-11	DF090350:3880592-3880691
BmHel-11	DF090349:3385377-3385548
BmHel-11	DF090349:3385545-3385636
BmHel-11	DF090337:1231267-1231477
BmHel-11	DF090337:725032-725128
BmHel-11	DF090337:724767-724933
BmHel-11	DF090344:1353646-1353807
BmHel-11	DF090344:1353817-1353918
BmHel-11	DF090556:7909-8088
BmHel-11	DF090556:8129-8210
BmHel-11	DF090668:29981-30117
BmHel-11	DF090668:30118-30244
BmHel-11	DF090342:1975804-1975977
BmHel-11	DF090342:1975973-1976062
BmHel-11	DF090360:281880-282124
BmHel-11	DF090316:13363756-13363956
BmHel-11	DF090361:236691-236961

BmHel-11	DF090426:243951-244174
BmHel-11	DF090462:17476-17654
BmHel-11	DF090462:17391-17476
BmHel-11	DF090335:283275-283540
BmHel-11	DF090376:982199-982359
BmHel-11	DF090376:982096-982198
BmHel-11	DF090327:2595233-2595361
BmHel-11	DF090327:2595089-2595223
BmHel-11	DF090316:3089350-3089471
BmHel-11	DF090316:3089467-3089608
BmHel-11	DF090424:775049-775208
BmHel-11	DF090424:774943-775045
BmHel-11	DF090330:2075866-2076041
BmHel-11	DF090330:2076041-2076128
BmHel-11	DF090412:1218787-1218950
BmHel-11	DF090412:1218684-1218783
BmHel-11	DF090403:455775-456038
BmHel-11	DF090752:23047-23310
BmHel-11	DF090353:1292512-1292699
BmHel-11	DF090335:2593181-2593319
BmHel-11	DF090335:2593542-2593666
BmHel-11	DF090348:3261922-3262033
BmHel-11	DF090348:3261352-3261498
BmHel-11	DF090336:546750-547013
BmHel-11	DF090373:747163-747426
BmHel-11	DF090383:1294730-1294984
BmHel-11	DF090326:1172185-1172292
BmHel-11	DF090326:1172011-1172174
BmHel-11	DF090364:2655284-2655549
BmHel-11	DF090431:83813-84028
BmHel-11	DF090334:5531014-5531277
BmHel-11	DF090323:6739190-6739453
BmHel-11	DF090320:8165263-8165535
BmHel-11	DF090333:5355713-5355976
BmHel-11	DF090422:1065138-1065403
BmHel-11	DF090661:11240-11503
BmHel-11	DF090327:6653161-6653327
BmHel-11	DF090338:4582134-4582223
BmHel-11	DF090338:4582237-4582345
BmHel-11	DF090447:273744-273876
BmHel-11	DF090447:273628-273728
BmHel-11	DF090352:2881178-2881395
BmHel-11	BABH01046455:589-806
BmHel-11	DF090322:7263661-7263911
BmHel-11	DF090388:1685453-1685717
BmHel-11	DF090358:1797882-1798143
BmHel-11	DF090443:318341-318602
BmHel-11	DF090324:4296842-4297067

BmHel-11	DF090319:695101-695282
BmHel-11	DF090319:695023-695103
BmHel-11	DF090395:184853-185039
BmHel-11	DF090411:1192044-1192219
BmHel-11	DF090411:1192222-1192309
BmHel-11	DF090341:2784268-2784380
BmHel-11	DF090341:2784418-2784563
BmHel-11	DF090331:4056882-4057137
BmHel-11	DF090323:3223860-3224041
BmHel-11	DF090323:3223780-3223860
BmHel-11	DF090345:3882086-3882217
BmHel-11	DF090345:3882234-3882364
BmHel-11	DF090338:2111406-2111580
BmHel-11	DF090338:2111322-2111407
BmHel-11	DF090428:831610-831788
BmHel-11	DF090428:831527-831610
BmHel-11	DF090328:4290614-4290876
BmHel-11	DF090335:1621709-1621871
BmHel-11	DF090335:1621872-1621971
BmHel-11	DF090396:920399-920562
BmHel-11	DF090396:920563-920672
BmHel-11	DF090434:394967-395084
BmHel-11	DF090434:394818-394962
BmHel-11	DF090427:917921-918158
BmHel-11	BABH01050226:708-973
BmHel-11	DF090333:2897695-2897866
BmHel-11	DF090333:2897861-2897951
BmHel-11	DF090348:1811028-1811198
BmHel-11	DF090348:1810944-1811034
BmHel-11	DF090341:3485953-3486210
BmHel-11	DF090316:10298181-10298387
BmHel-11	DF090316:14207726-14207941
BmHel-11	DF090316:10406597-10406729
BmHel-11	DF090316:10406467-10406596
BmHel-11	DF090352:961540-961710
BmHel-11	DF090352:961455-961546
BmHel-11	DF090347:1475593-1475755
BmHel-11	DF090347:1475754-1475849
BmHel-11	DF090343:456242-456437
BmHel-11	DF090334:4122960-4123222
BmHel-11	DF090412:1160026-1160144
BmHel-11	DF090412:1160140-1160282
BmHel-11	DF090483:36125-36282
BmHel-11	DF090483:36292-36401
BmHel-11	DF090434:620764-620893
BmHel-11	DF090434:620906-620999
BmHel-11	DF090397:1502237-1502499
BmHel-11	DF090368:2387783-2387933

BmHel-11	DF090368:2387662-2387773
BmHel-11	DF090340:534523-534781
BmHel-11	DF090421:134925-135075
BmHel-11	DF090421:135075-135186
BmHel-11	DF090369:1083516-1083668
BmHel-11	DF090369:1083411-1083515
BmHel-11	BABH01052670:227-322
BmHel-11	BABH01052670:53-223
BmHel-11	DF090391:954500-954616
BmHel-11	DF090391:954878-955023
BmHel-11	DF090365:217608-217870
BmHel-11	DF090420:494369-494514
BmHel-11	DF090420:494258-494374
BmHel-11	DF090368:754850-755110
BmHel-11	DF090327:4803899-4804104
BmHel-11	DF090336:5461654-5461918
BmHel-11	DF090344:2186506-2186768
BmHel-11	DF090501:48122-48380
BmHel-11	BABH01052897:564-826
BmHel-11	DF090383:75630-75769
BmHel-11	DF090383:75234-75354
BmHel-11	DF090358:672254-672420
BmHel-11	DF090358:672159-672252
BmHel-11	DF090361:1798021-1798125
BmHel-11	DF090361:1797799-1797978
BmHel-11	DF090342:373014-373228
BmHel-11	DF090316:12890659-12890915
BmHel-11	DF090379:1173552-1173805
BmHel-11	DF090351:919909-920070
BmHel-11	DF090351:920075-920174
BmHel-11	DF090365:1958075-1958282
BmHel-11	DF090349:359258-359372
BmHel-11	DF090349:359378-359524
BmHel-11	DF090316:12648420-12648597
BmHel-11	DF090316:12648601-12648684
BmHel-11	DF090352:840635-840769
BmHel-11	DF090352:840515-840635
BmHel-11	DF090369:1607316-1607545
BmHel-11	DF090456:159243-159398
BmHel-11	DF090456:159407-159514
BmHel-11	DF090359:2415057-2415245
BmHel-11	DF090324:4498753-4498924
BmHel-11	DF090324:4498918-4499006
BmHel-11	DF090354:2497320-2497443
BmHel-11	DF090354:2497178-2497316
BmHel-11	DF090316:12989020-12989271
BmHel-11	DF090412:79347-79516
BmHel-11	DF090412:79261-79352

BmHel-11	DF090354:3439448-3439672
BmHel-11	DF090338:3595422-3595598
BmHel-11	DF090338:3595598-3595683
BmHel-11	DF090466:129658-129744
BmHel-11	DF090466:129748-129922
BmHel-11	BABH01058948:457-714
BmHel-11	DF090575:53660-53791
BmHel-11	DF090575:53789-53921
BmHel-11	DF090325:6465928-6466084
BmHel-11	DF090325:6466103-6466206
BmHel-11	DF090322:1782143-1782312
BmHel-11	DF090322:1782306-1782396
BmHel-11	DF090321:3029474-3029735
BmHel-11	DF090361:3052308-3052439
BmHel-11	DF090361:3052440-3052568
BmHel-11	DF090320:200209-200470
BmHel-11	DF090378:190659-190820
BmHel-11	DF090378:190828-190927
BmHel-11	DF090323:7524764-7524989
BmHel-11	DF090316:15058267-15058535
BmHel-11	DF090372:2313720-2313949
BmHel-11	DF090328:5401054-5401317
BmHel-11	DF090316:5254526-5254793
BmHel-11	DF090353:28629-28890
BmHel-11	DF090336:319823-320087
BmHel-11	DF090319:7979367-7979628
BmHel-11	DF090343:2515494-2515758
BmHel-11	DF090334:5442674-5442935
BmHel-11	DF090331:4213272-4213523
BmHel-11	DF090418:178140-178405
BmHel-11	DF090467:317469-317724
BmHel-11	DF090374:1236889-1237148
BmHel-11	BABH01045859:1810-2071
BmHel-11	DF090343:434364-434488
BmHel-11	DF090353:2046956-2047141
BmHel-11	DF090322:2445432-2445603
BmHel-11	DF090322:2445613-2445700
BmHel-11	DF090321:2585197-2585336
BmHel-11	DF090321:2585337-2585457
BmHel-11	DF090420:87483-87743
BmHel-11	DF090332:4731566-4731663
BmHel-11	DF090332:4731686-4731838
BmHel-11	DF090392:1416285-1416448
BmHel-11	DF090392:1416184-1416280
BmHel-11	DF090322:2252239-2252448
BmHel-11	DF090321:2910497-2910722
BmHel-11	DF090321:2919993-2920147
BmHel-11	DF090321:2919888-2919993

BmHel-11	DF090341:2573568-2573687
BmHel-11	DF090341:2573430-2573569
BmHel-11	DF090334:5298538-5298746
BmHel-11	DF090344:3075713-3075881
BmHel-11	DF090344:3075619-3075710
BmHel-11	DF090396:1325742-1325965
BmHel-11	DF090369:2382745-2382897
BmHel-11	DF090369:2382638-2382743
BmHel-11	DF090960:846-1014
BmHel-11	DF090960:1017-1112
BmHel-11	DF090321:4924088-4924347
BmHel-11	DF090469:320401-320607
BmHel-11	DF090344:371057-371169
BmHel-11	DF090344:371177-371321
BmHel-11	DF090452:105130-105304
BmHel-11	DF090452:105307-105392
BmHel-11	DF090358:1844700-1844871
BmHel-11	DF090358:1844870-1844957
BmHel-11	DF090428:154348-154498
BmHel-11	DF090428:155092-155201
BmHel-11	DF090431:463159-463331
BmHel-11	DF090431:463330-463417
BmHel-11	DF090425:579110-579262
BmHel-11	DF090425:579262-579369
BmHel-11	DF090322:4597818-4597921
BmHel-11	DF090322:4597682-4597803
BmHel-11	DF090349:939799-939982
BmHel-11	DF090331:4282757-4282933
BmHel-11	DF090331:4282931-4283015
BmHel-11	DF090352:1821102-1821230
BmHel-11	DF090352:1821259-1821388
BmHel-11	DF090317:9029096-9029285
BmHel-11	DF090461:199344-199603
BmHel-11	DF090602:17344-17496
BmHel-11	DF090602:17501-17608
BmHel-11	DF090318:6534719-6534827
BmHel-11	DF090318:6534559-6534710
BmHel-11	DF090358:1737666-1737928
BmHel-11	DF090354:2269730-2269977
BmHel-11	DF090324:2972488-2972750
BmHel-11	DF090386:1912671-1912935
BmHel-11	DF090342:3918477-3918726
BmHel-11	DF090326:4720903-4721159
BmHel-11	DF090363:2253453-2253713
BmHel-11	DF090335:4417420-4417677
BmHel-11	DF090367:566712-566973
BmHel-11	DF090364:690374-690635
BmHel-11	DF090350:3531249-3531434

BmHel-11	DF090391:1222798-1222960
BmHel-11	DF090391:1222966-1223062
BmHel-11	DF090336:927295-927433
BmHel-11	DF090336:927452-927572
BmHel-11	DF090391:1497007-1497178
BmHel-11	DF090391:1497180-1497260
BmHel-11	DF090322:2947602-2947861
BmHel-11	DF090508:125565-125824
BmHel-11	DF090316:5177315-5177496
BmHel-11	DF090330:45452-45609
BmHel-11	DF090330:45609-45709
BmHel-11	DF090372:2229229-2229400
BmHel-11	DF090372:2229136-2229230
BmHel-11	DF090356:1816166-1816380
BmHel-11	DF090549:52449-52635
BmHel-11	DF090357:1281140-1281257
BmHel-11	DF090377:981702-981877
BmHel-11	DF090377:981888-981972
BmHel-11	DF090352:2446656-2446809
BmHel-11	DF090352:2446547-2446652
BmHel-11	DF090328:3550804-3550976
BmHel-11	DF090328:3550973-3551059
BmHel-11	DF090403:231234-231339
BmHel-11	DF090403:231342-231489
BmHel-11	DF090403:270482-270600
BmHel-11	DF090403:270616-270756
BmHel-11	DF090336:5216153-5216312
BmHel-11	DF090336:5216314-5216414
BmHel-11	DF090336:3093373-3093532
BmHel-11	DF090336:3093272-3093371
BmHel-11	DF090328:4156131-4156340
BmHel-11	DF090319:4431852-4431985
BmHel-11	DF090319:4431727-4431852
BmHel-11	DF090347:3806723-3806882
BmHel-11	DF090347:3806617-3806717
BmHel-11	DF090321:4538884-4539107
BmHel-11	DF090334:4791900-4792060
BmHel-11	DF090334:4791799-4791898
BmHel-11	DF090329:4642931-4643064
BmHel-11	DF090329:4642806-4642931
BmHel-11	DF090453:337118-337377
BmHel-11	DF090585:76524-76745
BmHel-11	DF090452:346211-346435
BmHel-11	DF090413:739445-739528
BmHel-11	DF090413:739267-739442
BmHel-11	DF090383:567718-567847
BmHel-11	DF090383:567848-567977
BmHel-11	DF090326:6683505-6683708

BmHel-11	DF090337:4051963-4052042
BmHel-11	DF090337:4051850-4051932
BmHel-11	DF090358:147435-147670
BmHel-11	DF090316:4378122-4378259
BmHel-11	DF090316:4378263-4378386
BmHel-11	DF090332:2376379-2376597
BmHel-11	DF090383:1547177-1547435
BmHel-11	DF090388:1237227-1237486
BmHel-11	BABH01058369:1-262
BmHel-11	DF090394:214163-214385
BmHel-11	DF090316:1649716-1649976
BmHel-11	DF090464:289790-290049
BmHel-11	DF090339:4818571-4818830
BmHel-11	DF090365:717011-717263
BmHel-11	DF090320:6537842-6538100
BmHel-11	DF090352:3271472-3271731
BmHel-11	DF090417:447713-447976
BmHel-11	DF090375:2187694-2187951
BmHel-11	DF090353:499848-500009
BmHel-11	DF090353:500046-500140
BmHel-11	DF090332:5791089-5791202
BmHel-11	DF090332:5790945-5791090
BmHel-11	DF090344:98421-98627
BmHel-11	DF090368:2598151-2598317
BmHel-11	DF090368:2598057-2598148
BmHel-11	DF090352:413832-414091
BmHel-11	DF090322:3066271-3066453
BmHel-11	DF090336:2088377-2088601
BmHel-11	DF090331:2976779-2976905
BmHel-11	DF090331:2976384-2976514
BmHel-11	DF090323:3434814-3434924
BmHel-11	DF090323:3434653-3434800
BmHel-11	DF090344:3154925-3155128
BmHel-11	DF090317:4310222-4310393
BmHel-11	DF090317:4310139-4310219
BmHel-11	DF090355:3779623-3779799
BmHel-11	DF090355:3779522-3779603
BmHel-11	DF090324:4915063-4915157
BmHel-11	DF090324:4915156-4915319
BmHel-11	DF090342:3143291-3143499
BmHel-11	DF090336:4182904-4183055
BmHel-11	DF090336:4182797-4182899
BmHel-11	DF090318:3245040-3245157
BmHel-11	DF090318:3244901-3245041
BmHel-11	DF090407:1363215-1363387
BmHel-11	DF090407:1363386-1363477
BmHel-11	DF090316:11969738-11969945
BmHel-11	DF090327:1758252-1758453

BmHel-11	DF090462:98393-98597
BmHel-11	DF090377:669768-669918
BmHel-11	DF090377:669939-670046
BmHel-11	DF090428:900036-900213
BmHel-11	DF090397:1035549-1035768
BmHel-11	DF090439:238774-238893
BmHel-11	DF090439:238602-238749
BmHel-11	DF090353:3470072-3470245
BmHel-11	DF090353:3469986-3470073
BmHel-11	DF090329:1214325-1214486
BmHel-11	DF090329:1214239-1214326
BmHel-11	DF090446:154826-154998
BmHel-11	DF090319:6549539-6549737
BmHel-11	DF090395:1050763-1050882
BmHel-11	DF090395:1050881-1051019
BmHel-11	DF090390:1478901-1479077
BmHel-11	DF090390:1479073-1479162
BmHel-11	DF090439:28378-28507
BmHel-11	DF090429:150045-150296
BmHel-11	DF090341:1735548-1735757
BmHel-11	DF090325:3559264-3559414
BmHel-11	DF090325:3559146-3559253
BmHel-11	DF090350:4247117-4247326
BmHel-11	DF090327:6252826-6252914
BmHel-11	DF090327:6253418-6253587
BmHel-11	DF090337:3182704-3182929
BmHel-11	DF090344:1889813-1889971
BmHel-11	DF090344:1889978-1890077
BmHel-11	DF090355:523253-523511
BmHel-11	DF090373:855757-855845
BmHel-11	DF090373:855565-855734
BmHel-11	DF090338:1799424-1799589
BmHel-11	DF090319:507692-507949
BmHel-11	DF090316:4986268-4986526
BmHel-11	DF090316:11371817-11372065
BmHel-11	DF090316:12508736-12508997
BmHel-11	DF090354:2218922-2219180
BmHel-11	DF090317:494239-494492
BmHel-11	DF090358:1019765-1020023
BmHel-11	DF090352:946167-946376
BmHel-11	DF090398:1160699-1160876
BmHel-11	DF090398:1160875-1160954
BmHel-11	DF090372:1901453-1901622
BmHel-11	DF090372:1901363-1901450
BmHel-11	DF090384:359052-359311
BmHel-11	DF090328:2410890-2411012
BmHel-11	DF090328:2411028-2411162
BmHel-11	DF090341:1776351-1776608

BmHel-11	DF090327:2773912-2774168
BmHel-11	DF090332:4169915-4170172
BmHel-11	DF090362:557293-557467
BmHel-11	DF090362:557209-557291
BmHel-11	DF090332:3712192-3712284
BmHel-11	DF090332:3711999-3712163
BmHel-11	DF090319:5351922-5352103
BmHel-11	DF090380:2137371-2137540
BmHel-11	DF090380:2137537-2137624
BmHel-11	DF090379:1143738-1143924
BmHel-11	DF090317:479020-479228
BmHel-11	DF090398:1149697-1149871
BmHel-11	DF090398:1149878-1149960
BmHel-11	DF090324:4570543-4570801
BmHel-11	DF090394:1276477-1276680
BmHel-11	DF090413:693377-693482
BmHel-11	DF090413:693483-693631
BmHel-11	DF090342:1646361-1646513
BmHel-11	DF090342:1646237-1646343
BmHel-11	DF090336:785574-785776
BmHel-11	DF090383:1527828-1528035
BmHel-11	DF090383:906410-906618
BmHel-11	DF090319:7189977-7190179
BmHel-11	DF090357:2957916-2958048
BmHel-11	DF090377:25893-26099
BmHel-11	DF090322:3020628-3020830
BmHel-11	DF090331:2490056-2490263
BmHel-11	DF090331:3000597-3000799
BmHel-11	DF090316:2975466-2975645
BmHel-11	DF090335:528641-528784
BmHel-11	DF090335:528785-528898
BmHel-11	DF090317:7513684-7513887
BmHel-11	DF091009:2848-3105
BmHel-11	DF090377:732754-732962
BmHel-11	DF090350:3824365-3824581
BmHel-11	DF090359:1924072-1924276
BmHel-11	DF090340:964924-965137
BmHel-11	DF090388:53709-53826
BmHel-11	DF090388:53536-53675
BmHel-11	DF090420:1059102-1059214
BmHel-11	DF090353:3924268-3924524
BmHel-11	DF090388:907404-907534
BmHel-11	DF090388:907278-907404
BmHel-11	DF090422:105636-105807
BmHel-11	DF090422:105807-105893
BmHel-11	DF090329:704680-704892
BmHel-11	DF090354:990295-990444
BmHel-11	DF090354:990444-990546

BmHel-11	DF090336:4684027-4684279
BmHel-11	DF090407:135999-136147
BmHel-11	DF090407:136147-136255
BmHel-11	DF090357:941849-942033
BmHel-11	DF090449:418601-418724
BmHel-11	DF090449:418459-418587
BmHel-11	DF090319:5221145-5221238
BmHel-11	DF090355:2666129-2666330
BmHel-11	BABH01044929:2625-2799
BmHel-11	DF090459:309470-309622
BmHel-11	DF090459:309623-309727
BmHel-11	DF090318:533906-534094
BmHel-11	DF090349:2361430-2361540
BmHel-11	DF090349:2361548-2361709
BmHel-11	DF090461:29098-29231
BmHel-11	DF090461:29249-29359
BmHel-11	DF090475:87897-88159
BmHel-11	DF090328:5980665-5980922
BmHel-11	DF090469:141375-141632
BmHel-11	DF090349:954975-955231
BmHel-11	DF090343:3862706-3862962
BmHel-11	DF090331:5943709-5943970
BmHel-11	DF090329:5684157-5684414
BmHel-11	DF090323:7113208-7113463
BmHel-11	DF090427:713188-713445
BmHel-11	DF090338:720754-721012
BmHel-11	DF090324:5856669-5856872
BmHel-11	DF090342:1203241-1203462
BmHel-11	DF090327:1746475-1746692
BmHel-11	DF090396:37710-37964
BmHel-11	DF090324:163313-163519
BmHel-11	DF090328:2296397-2296602
BmHel-11	DF090357:2572956-2573169
BmHel-11	DF090326:4467916-4468079
BmHel-11	DF090363:1798734-1798927
BmHel-11	DF090359:1538960-1539108
BmHel-11	DF090359:1539108-1539213
BmHel-11	DF090329:1834281-1834407
BmHel-11	DF090329:1834154-1834283
BmHel-11	DF090364:2517005-2517261
BmHel-11	DF090352:3760062-3760267
BmHel-11	DF090371:2052570-2052776
BmHel-11	DF090319:1045777-1045969
BmHel-11	DF090322:1257988-1258173
BmHel-11	DF090321:6689050-6689202
BmHel-11	DF090321:6689206-6689309
BmHel-11	DF090350:3376994-3377162
BmHel-11	DF090350:3376899-3376986

BmHel-11	DF090341:1902180-1902367
BmHel-11	DF090316:3235433-3235624
BmHel-11	DF090316:5905345-5905497
BmHel-11	DF090316:5905498-5905601
BmHel-11	DF090386:1231255-1231430
BmHel-11	DF090386:1231177-1231257
BmHel-11	DF090393:1385332-1385482
BmHel-11	DF090393:1385228-1385332
BmHel-11	DF090341:810701-810816
BmHel-11	DF090341:810815-810958
BmHel-11	DF090331:5898098-5898303
BmHel-11	DF090337:4420015-4420135
BmHel-11	DF090337:4420132-4420267
BmHel-11	DF090330:985364-985569
BmHel-11	DF090356:2377504-2377667
BmHel-11	DF090356:2377410-2377501
BmHel-11	DF090437:78769-78863
BmHel-11	DF090437:78871-79032
BmHel-11	DF090947:5282-5431
BmHel-11	DF090947:5432-5538
BmHel-11	DF090324:1230113-1230369
BmHel-11	DF090344:2034602-2034783
BmHel-11	DF090403:619489-619637
BmHel-11	DF090403:619637-619744
BmHel-11	DF090325:4781455-4781628
BmHel-11	DF090325:4781358-4781440
BmHel-11	DF090386:380437-380692
BmHel-11	DF090383:2089636-2089883
BmHel-11	DF090322:724638-724899
BmHel-11	DF090469:13867-14118
BmHel-11	DF090320:6392541-6392789
BmHel-11	DF090372:1884378-1884634
BmHel-11	DF090423:189772-190028
BmHel-11	DF090319:19106-19290
BmHel-11	DF090359:1214536-1214749
BmHel-11	DF090377:709519-709679
BmHel-11	DF090377:709426-709519
BmHel-11	DF090362:2462222-2462477
BmHel-11	DF090377:1092787-1093043
BmHel-11	DF090322:2946020-2946276
BmHel-11	DF090346:136901-137162
BmHel-11	DF090341:2185197-2185386
BmHel-11	DF090364:2888062-2888156
BmHel-11	DF090364:2887954-2888055
BmHel-11	DF090387:135595-135761
BmHel-11	DF090387:135754-135841
BmHel-11	DF090318:5793531-5793698
BmHel-11	DF090318:5793451-5793538

BmHel-11	DF090319:718461-718649
BmHel-11	DF090343:3587458-3587595
BmHel-11	DF090343:3587594-3587711
BmHel-11	DF090316:8254595-8254680
BmHel-11	DF090316:8254689-8254858
BmHel-11	DF090333:4339125-4339316
BmHel-11	DF090447:470430-470595
BmHel-11	DF090447:470597-470688
BmHel-11	DF090326:6483575-6483771
BmHel-11	DF090365:1342593-1342745
BmHel-11	DF090365:1342483-1342585
BmHel-11	DF090326:5385129-5385336
BmHel-11	DF090344:894735-894943
BmHel-11	DF090320:92048-92165
BmHel-11	DF090320:91900-92038
BmHel-11	DF090452:270045-270192
BmHel-11	DF090452:269943-270036
BmHel-11	DF090460:292172-292378
BmHel-11	DF090425:214998-215122
BmHel-11	DF090425:215120-215246
BmHel-11	DF090324:6221796-6221885
BmHel-11	DF090324:6221897-6222062
BmHel-11	DF090348:2866328-2866415
BmHel-11	DF090348:2866232-2866329
BmHel-11	DF090333:189744-189935
BmHel-11	DF090371:777829-778082
BmHel-11	DF090348:185364-185541
BmHel-11	DF090410:576677-576855
BmHel-11	DF090358:3020543-3020716
BmHel-11	DF090358:3020720-3020801
BmHel-11	DF090360:1557286-1557396
BmHel-11	DF090360:1557399-1557536
BmHel-11	DF090389:513515-513686
BmHel-11	DF090346:845870-846017
BmHel-11	DF090346:846018-846125
BmHel-11	DF090346:3092114-3092289
BmHel-11	DF090369:1644160-1644347
BmHel-11	DF090322:1159898-1160044
BmHel-11	DF090322:1159791-1159898
BmHel-11	DF090364:586972-587135
BmHel-11	DF090364:587129-587221
BmHel-11	DF090388:894573-894794
BmHel-11	DF090330:4493527-4493724
BmHel-11	DF090386:435200-435455
BmHel-11	DF090336:4963044-4963258
BmHel-11	DF090348:1674695-1674948
BmHel-11	DF090340:1485372-1485628
BmHel-11	DF090331:624298-624552

BmHel-11	DF090368:892888-893143
BmHel-11	DF090367:2427882-2428131
BmHel-11	DF090370:234145-234399
BmHel-11	DF090378:1200824-1201079
BmHel-11	DF090327:2394495-2394750
BmHel-11	DF090369:887120-887288
BmHel-11	DF090369:887040-887125
BmHel-11	DF090326:6433929-6434183
BmHel-11	DF090369:1035081-1035226
BmHel-11	DF090369:1035230-1035332
BmHel-11	DF090336:3941507-3941762
BmHel-11	DF090336:7653-7908
BmHel-11	DF090347:4046434-4046682
BmHel-11	DF090331:2004731-2004986
BmHel-11	DF090391:302707-302962
BmHel-11	DF090652:25380-25635
BmHel-11	BABH01047173:1509-1764
BmHel-11	DF090325:3690993-3691147
BmHel-11	DF090325:3690891-3690990
BmHel-11	DF090433:610049-610250
BmHel-11	DF090382:98845-99061
BmHel-11	DF090382:1244193-1244384
BmHel-11	DF090369:1873391-1873600
BmHel-11	DF090328:6611771-6611916
BmHel-11	DF090328:6611639-6611747
BmHel-11	DF090322:484142-484397
BmHel-11	DF090357:2837802-2837997
BmHel-11	DF090321:6343200-6343405
BmHel-11	DF090316:9942418-9942622
BmHel-11	DF090368:1567668-1567870
BmHel-11	DF090329:4833837-4834032
BmHel-11	DF090415:385978-386185
BmHel-11	DF090394:325449-325655
BmHel-11	DF090376:2089714-2089968
BmHel-11	DF090401:1171691-1171814
BmHel-11	DF090401:1171565-1171693
BmHel-11	DF090324:1373900-1374110
BmHel-11	DF090360:391054-391257
BmHel-11	DF090343:4025665-4025779
BmHel-11	DF090343:4025784-4025923
BmHel-11	DF090340:3832071-3832235
BmHel-11	DF090340:3832242-3832333
BmHel-11	DF090317:4046272-4046425
BmHel-11	DF090317:4046134-4046235
BmHel-11	DF090338:4601876-4602080
BmHel-11	DF090436:657182-657333
BmHel-11	DF090436:657334-657439
BmHel-11	DF091007:5419-5672

BmHel-11	DF090381:1712652-1712838
BmHel-11	DF090326:6716195-6716341
BmHel-11	DF090326:6716088-6716194
BmHel-11	DF090322:7823088-7823246
BmHel-11	DF090322:7822990-7823086
BmHel-11	DF090366:174713-174917
BmHel-11	DF090382:14160-14268
BmHel-11	DF090382:14395-14499
BmHel-11	DF090330:1636188-1636340
BmHel-11	DF090330:1636346-1636447
BmHel-11	DF090316:7694483-7694659
BmHel-11	DF090441:473728-473836
BmHel-11	DF090441:473835-473975
BmHel-11	DF090338:4709215-4709425
BmHel-11	DF090365:2742528-2742726
BmHel-11	DF090353:2073928-2074179
BmHel-11	DF090483:193250-193506
BmHel-11	DF090331:920889-921143
BmHel-11	DF090418:613224-613478
BmHel-11	DF090344:434216-434470
BmHel-11	DF090389:1143066-1143321
BmHel-11	DF090473:427670-427925
BmHel-11	BABH01049512:266-519
BmHel-11	DF090355:3204288-3204502
BmHel-11	DF090437:548264-548449
BmHel-11	DF090316:10663306-10663518
BmHel-11	DF090347:2095469-2095674
BmHel-11	DF090354:1445397-1445521
BmHel-11	DF090354:1445247-1445374
BmHel-11	DF090381:1242160-1242413
BmHel-11	DF090344:4116072-4116325
BmHel-11	DF090445:147134-147297
BmHel-11	DF090445:147040-147131
BmHel-11	DF090319:7591376-7591582
BmHel-11	DF090364:482873-483048
BmHel-11	DF090364:420068-420272
BmHel-11	DF090529:49633-49788
BmHel-11	DF090529:49793-49890
BmHel-11	DF090445:228746-228903
BmHel-11	DF090445:228633-228728
BmHel-11	DF090325:6893142-6893306
BmHel-11	DF090325:6893299-6893388
BmHel-11	DF090329:1559683-1559891
BmHel-11	DF090401:1038564-1038681
BmHel-11	DF090401:1038425-1038560
BmHel-11	DF090439:165519-165725
BmHel-11	DF090431:317380-317633
BmHel-11	DF090324:3559765-3559968

BmHel-11	DF090357:3029040-3029244
BmHel-11	DF090357:2988916-2989169
BmHel-11	DF090340:2893909-2894114
BmHel-11	DF090316:3422130-3422313
BmHel-11	DF090337:3661350-3661464
BmHel-11	DF090337:3661203-3661342
BmHel-11	DF090406:413301-413502
BmHel-11	DF090317:1855397-1855600
BmHel-11	DF090358:781988-782192
BmHel-11	DF090327:2650159-2650334
BmHel-11	DF090327:2650327-2650408
BmHel-11	DF090397:1288122-1288329
BmHel-11	DF090355:2316899-2317065
BmHel-11	DF090339:2686098-2686249
BmHel-11	DF090339:2686275-2686378
BmHel-11	DF090384:1135848-1136053
BmHel-11	DF090366:2347403-2347614
BmHel-11	BABH01074594:1-251
BmHel-11	DF090507:37629-37731
BmHel-11	DF090507:37752-37902
BmHel-11	DF090321:2363851-2363996
BmHel-11	DF090321:2363740-2363847
BmHel-11	DF090333:4875496-4875704
BmHel-11	DF090356:1379980-1380178
BmHel-11	DF090342:1162383-1162585
BmHel-11	DF090325:4273340-4273468
BmHel-11	DF090325:4273107-4273191
BmHel-11	DF090376:1446900-1447153
BmHel-11	DF090377:1520121-1520305
BmHel-11	DF090335:1885228-1885413
BmHel-11	DF091355:4060-4269
BmHel-11	DF090436:324544-324728
BmHel-11	DF090317:2091155-2091409
BmHel-11	DF090417:732222-732474
BmHel-11	DF090367:2486965-2487055
BmHel-11	DF090367:2486766-2486857
BmHel-11	BABH01046378:1773-2025
BmHel-11	DF090814:11958-12136
BmHel-11	DF090701:4383-4602
BmHel-11	DF090373:2112085-2112291
BmHel-11	DF090384:1955376-1955628
BmHel-11	DF090397:1347938-1348148
BmHel-11	DF090342:4791658-4791814
BmHel-11	DF090342:4791814-4791915
BmHel-11	DF090347:593395-593572
BmHel-11	DF090329:10576-10784
BmHel-11	DF090437:628213-628398
BmHel-11	BABH01084365:243-381

BmHel-11	BABH01084365:408-521
BmHel-11	DF090349:1972961-1973114
BmHel-11	DF090349:1973116-1973214
BmHel-11	DF090368:2171844-2171994
BmHel-11	DF090368:2171732-2171834
BmHel-11	DF090346:1224786-1224899
BmHel-11	DF090346:1224899-1225033
BmHel-11	DF090324:7480477-7480679
BmHel-11	DF090342:4236681-4236883
BmHel-11	DF090319:5622660-5622866
BmHel-11	DF090395:607099-607252
BmHel-11	DF090395:606966-607065
BmHel-11	DF090360:2636003-2636216
BmHel-11	DF090347:377474-377663
BmHel-11	DF090326:1366666-1366872
BmHel-11	DF090317:6528023-6528224
BmHel-11	DF090369:1290643-1290845
BmHel-11	DF090386:754395-754561
BmHel-11	DF090386:754563-754642
BmHel-11	DF090321:2365120-2365240
BmHel-11	DF090321:2365243-2365374
BmHel-11	DF090388:743855-744098
BmHel-11	DF090317:11135502-11135753
BmHel-11	DF090319:3909713-3909965
BmHel-11	DF090388:1773680-1773882
BmHel-11	DF090380:1436713-1436865
BmHel-11	DF090380:1436867-1436966
BmHel-11	DF090343:1746653-1746856
BmHel-11	DF090362:382280-382386
BmHel-11	DF090382:1275828-1276076
BmHel-11	DF090317:7780095-7780239
BmHel-11	DF090317:7779978-7780085
BmHel-11	DF090366:323735-323871
BmHel-11	DF090324:7505609-7505861
BmHel-11	DF090343:3103515-3103767
BmHel-11	DF090335:5368740-5368996
BmHel-11	DF090404:310001-310254
BmHel-11	DF090574:27549-27800
BmHel-11	DF090318:5149690-5149897
BmHel-11	DF090319:4647966-4648150
BmHel-11	DF090345:4500139-4500305
BmHel-11	DF090345:4500053-4500137
BmHel-11	DF090360:251884-252054
BmHel-11	DF090360:252047-252129
BmHel-11	DF090376:1818097-1818289
BmHel-11	DF090336:3833648-3833850
BmHel-11	DF090433:124206-124289
BmHel-11	DF090433:124293-124462

BmHel-11	DF090365:1576422-1576629
BmHel-11	DF090326:6008332-6008501
BmHel-11	DF090326:6008248-6008333
BmHel-11	DF090337:2095638-2095805
BmHel-11	DF090337:2095547-2095636
BmHel-11	DF090347:1235441-1235594
BmHel-11	DF090347:1235597-1235693
BmHel-11	DF090316:12452921-12453088
BmHel-11	DF090316:12452832-12452915
BmHel-11	DF090371:453687-453928
BmHel-11	DF090325:3506706-3506869
BmHel-11	DF090325:3506862-3506949
BmHel-11	DF090342:2818004-2818153
BmHel-11	DF090342:2818188-2818290
BmHel-11	DF090318:1587634-1587775
BmHel-11	DF090318:1587814-1587915
BmHel-11	DF090348:596984-597133
BmHel-11	DF090348:597159-597260
BmHel-11	DF090382:549738-549901
BmHel-11	DF090382:549658-549745
BmHel-11	DF090330:4164789-4164949
BmHel-11	DF090330:4164942-4165028
BmHel-11	DF090344:4187191-4187354
BmHel-11	DF090344:4187111-4187198
BmHel-11	DF090358:2343728-2343856
BmHel-11	DF090413:539842-540093
BmHel-11	DF090373:2083305-2083506
BmHel-11	DF090487:42891-43057
BmHel-11	DF090487:42797-42885
BmHel-11	DF090317:1744978-1745233
BmHel-11	DF090336:2620096-2620201
BmHel-11	DF090336:2619949-2620094
BmHel-11	DF090316:6907472-6907660
BmHel-11	DF090340:1916965-1917180
BmHel-11	BABH01049492:12-221
BmHel-11	DF090361:1424101-1424352
BmHel-11	DF090316:4389702-4389953
BmHel-11	DF090400:1140293-1140544
BmHel-11	DF090368:1886692-1886934
BmHel-11	DF090335:4376690-4376941
BmHel-11	DF091734:1-252
BmHel-11	DF090317:7254852-7255010
BmHel-11	DF090317:7255015-7255106
BmHel-11	DF090318:9500633-9500781
BmHel-11	DF090318:9500781-9500882
BmHel-11	DF090334:2400635-2400749
BmHel-11	DF090334:2400751-2400886
BmHel-11	DF090357:1351911-1352031

BmHel-11	DF090357:1351805-1351895
BmHel-11	DF090329:5449865-5450058
BmHel-11	DF090339:1755088-1755339
BmHel-11	DF090422:1091957-1092085
BmHel-11	DF090422:1091821-1091943
BmHel-11	DF090316:9873320-9873524
BmHel-11	DF090330:2681521-2681619
BmHel-11	DF090790:4306-4503
BmHel-11	DF090386:591812-591931
BmHel-11	DF090386:591431-591561
BmHel-11	DF090395:1322124-1322292
BmHel-11	DF090395:1322290-1322372
BmHel-11	DF090360:3349357-3349558
BmHel-11	DF090322:3265588-3265765
BmHel-11	DF090349:590346-590527
BmHel-11	DF090388:1239289-1239458
BmHel-11	DF090412:1196334-1196420
BmHel-11	DF090412:1196171-1196333
BmHel-11	DF090379:1343053-1343228
BmHel-11	DF090345:3675164-3675343
BmHel-11	DF090361:712024-712239
BmHel-11	DF090579:25518-25638
BmHel-11	DF090579:25637-25762
BmHel-11	DF090382:427849-428001
BmHel-11	DF090382:428013-428111
BmHel-11	DF090320:4759761-4759963
BmHel-11	DF090415:143127-143332
BmHel-11	DF090362:527905-528156
BmHel-11	DF090332:5453580-5453776
BmHel-11	DF090322:4749465-4749671
BmHel-11	DF090373:59988-60237
BmHel-11	DF090368:733439-733642
BmHel-11	DF090420:338622-338722
BmHel-11	DF090420:338411-338555
BmHel-11	DF090319:7109341-7109503
BmHel-11	DF090319:7109496-7109583
BmHel-11	DF090329:3251117-3251369
BmHel-11	DF090362:2727663-2727862
BmHel-11	DF090349:4112670-4112870
BmHel-11	DF090331:1732801-1732969
BmHel-11	DF090331:1732972-1733053
BmHel-11	DF090327:13293-13448
BmHel-11	DF090327:13459-13553
BmHel-11	DF090361:1488867-1489108
BmHel-11	BABH01045820:1601-1850
BmHel-11	DF090391:267661-267850
BmHel-11	DF090341:608686-608844
BmHel-11	DF090341:608838-608929

BmHel-11	DF090320:5961723-5961929
BmHel-11	DF090348:3523185-3523436
BmHel-11	DF090357:1789065-1789316
BmHel-11	DF090341:4631611-4631861
BmHel-11	DF090344:2240656-2240906
BmHel-11	DF090356:1226211-1226460
BmHel-11	DF092091:1531-1781
BmHel-11	DF090348:2121451-2121563
BmHel-11	DF090348:2121569-2121667
BmHel-11	DF090421:394597-394806
BmHel-11	DF090362:247454-247568
BmHel-11	DF090362:247373-247457
BmHel-11	DF090324:6324297-6324476
BmHel-11	DF090332:5655733-5655907
BmHel-11	DF090323:4847982-4848072
BmHel-11	DF090323:4848088-4848244
BmHel-11	DF090358:1624822-1625019
BmHel-11	DF090333:2858313-2858487
BmHel-11	DF090335:3597407-3597601
BmHel-11	DF090325:3446622-3446754
BmHel-11	DF090325:3446494-3446610
BmHel-11	DF090319:7304730-7304935
BmHel-11	DF090330:1741209-1741329
BmHel-11	DF090330:1741054-1741185
BmHel-11	DF090364:1165425-1165585
BmHel-11	DF090364:1165600-1165688
BmHel-11	DF090356:3244267-3244444
BmHel-11	DF090381:1769980-1770134
BmHel-11	DF090319:7533963-7534174
BmHel-11	DF090344:3566294-3566408
BmHel-11	DF090344:3566166-3566292
BmHel-11	DF090415:896681-896886
BmHel-11	DF090369:1304215-1304381
BmHel-11	DF090369:1304135-1304220
BmHel-11	DF091406:3747-3946
BmHel-11	DF090319:4773887-4774088
BmHel-11	DF090316:6074678-6074886
BmHel-11	DF090366:2573702-2573859
BmHel-11	DF090366:2573868-2573957
BmHel-11	DF090330:5293567-5293745
BmHel-11	BABH01085478:328-499
BmHel-11	DF090378:1362737-1362841
BmHel-11	DF090378:1362845-1362989
BmHel-11	DF090317:3223108-3223280
BmHel-11	DF090317:3223027-3223107
BmHel-11	DF090348:3482325-3482512
BmHel-11	DF090351:3292172-3292418
BmHel-11	DF090324:505622-505862

BmHel-11	DF090329:4513152-4513395
BmHel-11	DF090384:1563753-1564002
BmHel-11	DF090404:1356688-1356938
BmHel-11	DF090377:393381-393594
BmHel-11	DF090316:15332444-15332608
BmHel-11	DF090316:15332351-15332436
BmHel-11	DF090367:1954474-1954657
BmHel-11	DF090344:2986966-2987156
BmHel-11	DF090317:5910381-5910586
BmHel-11	DF090585:63146-63394
BmHel-11	DF090340:472527-472776
BmHel-11	DF090373:1896123-1896368
BmHel-11	DF090327:5985307-5985518
BmHel-11	DF090385:252508-252719
BmHel-11	DF090398:250135-250340
BmHel-11	BABH01048736:465-612
BmHel-11	BABH01048736:312-412
BmHel-11	DF090345:3486644-3486843
BmHel-11	DF090539:23741-23899
BmHel-11	DF090539:23647-23736
BmHel-11	DF090319:1010845-1011046
BmHel-11	DF090375:1628674-1628851
BmHel-11	DF090328:4890150-4890349
BmHel-11	DF090377:1480071-1480155
BmHel-11	DF090377:1480154-1480317
BmHel-11	DF090377:923366-923483
BmHel-11	DF090377:923206-923300
BmHel-11	DF090334:1528811-1528991
BmHel-11	DF090316:9281474-9281674
BmHel-11	DF090358:2478938-2479101
BmHel-11	DF090358:2479097-2479181
BmHel-11	DF090377:1043077-1043223
BmHel-11	DF090377:1043224-1043325
BmHel-11	DF090767:902-1103
BmHel-11	DF090350:3257893-3258094
BmHel-11	DF090367:1230606-1230779
BmHel-11	DF090352:485637-485837
BmHel-11	DF090322:8155894-8156032
BmHel-11	DF090366:1168272-1168427
BmHel-11	DF090366:1168180-1168271
BmHel-11	DF090349:3963129-3963209
BmHel-11	DF090349:3962639-3962823
BmHel-11	DF090628:10271-10523
BmHel-11	DF090316:243852-244091
BmHel-11	DF090402:580158-580328
BmHel-11	DF090323:6434828-6435076
BmHel-11	DF090332:3813569-3813750
BmHel-11	DF090368:1135131-1135320

BmHel-11	DF090356:1798894-1799016
BmHel-11	DF090356:1798769-1798893
BmHel-11	DF090343:556889-557018
BmHel-11	DF090343:556758-556875
BmHel-11	DF090355:2901853-2901999
BmHel-11	DF090355:2901749-2901850
BmHel-11	DF090322:2584665-2584912
BmHel-11	BABH01078533:8-255
BmHel-11	DF090318:5555705-5555891
BmHel-11	DF090328:5198349-5198535
BmHel-11	DF090334:3884371-3884557
BmHel-11	DF090316:6704869-6704958
BmHel-11	DF090316:6704713-6704814
BmHel-11	DF090380:1830861-1831063
BmHel-11	DF090352:3025183-3025353
BmHel-11	DF090317:7079427-7079571
BmHel-11	DF090317:7079324-7079426
BmHel-11	DF090409:1302247-1302363
BmHel-11	DF090409:1302109-1302239
BmHel-11	DF090460:320313-320499
BmHel-11	DF090429:612755-612918
BmHel-11	DF090429:612920-613003
BmHel-11	DF090358:75663-75907
BmHel-11	DF090376:1681426-1681568
BmHel-11	DF090376:1681589-1681693
BmHel-11	DF090318:9164155-9164296
BmHel-11	DF090318:9164297-9164399
BmHel-11	DF090376:534576-534755
BmHel-11	DF090405:600273-600466
BmHel-11	DF090321:3141165-3141377
BmHel-11	DF090316:6920487-6920642
BmHel-11	DF090316:6920650-6920741
BmHel-11	DF090388:1338830-1338976
BmHel-11	DF090388:1338985-1339084
BmHel-11	DF090324:631860-632013
BmHel-11	DF090324:632028-632115
BmHel-11	DF090333:1945332-1945504
BmHel-11	DF090358:696657-696904
BmHel-11	DF090412:947641-947888
BmHel-11	DF090384:1744722-1744968
BmHel-11	DF090358:2260043-2260296
BmHel-11	DF090351:4151481-4151602
BmHel-11	DF090351:4151613-4151736
BmHel-11	DF090484:18499-18588
BmHel-11	DF090484:18338-18493
BmHel-11	DF090316:7197293-7197541
BmHel-11	DF090372:287967-288213
BmHel-11	DF090329:4134940-4135097

BmHel-11	DF090329:4135099-4135190
BmHel-11	DF090339:5040696-5040883
BmHel-11	DF090321:2154515-2154699
BmHel-11	DF090342:3239980-3240126
BmHel-11	DF090342:3239857-3239959
BmHel-11	DF090336:4115780-4115948
BmHel-11	DF090328:777606-777853
BmHel-11	DF090391:860315-860466
BmHel-11	DF090391:860475-860569
BmHel-11	DF090323:5625977-5626182
BmHel-11	DF090403:263468-263719
BmHel-11	DF090319:4949543-4949754
BmHel-11	DF091944:1-201
BmHel-11	DF090327:4895402-4895571
BmHel-11	DF090321:4004303-4004516
BmHel-11	DF090469:295952-296145
BmHel-11	DF090343:3598951-3599197
BmHel-11	DF090367:2604471-2604641
BmHel-11	DF090392:1474954-1475116
BmHel-11	DF090318:641606-641723
BmHel-11	DF090318:641467-641597
BmHel-11	DF090353:1896750-1896833
BmHel-11	DF090353:1896617-1896749
BmHel-11	DF090320:2637897-2638092
BmHel-11	DF090444:493472-493572
BmHel-11	DF090444:493571-493716
BmHel-11	DF090425:429481-429654
BmHel-11	DF090402:305044-305247
BmHel-11	DF090367:98192-98439
BmHel-11	DF090356:955601-955812
BmHel-11	DF090332:2314578-2314714
BmHel-11	DF090332:2314720-2314828
BmHel-11	DF090365:1119734-1119882
BmHel-11	DF090365:1119630-1119730
BmHel-11	DF090329:2194790-2194969
BmHel-11	DF090388:331905-332151
BmHel-11	DF090316:3719279-3719401
BmHel-11	DF090316:3719417-3719541
BmHel-11	DF090340:119992-120093
BmHel-11	DF090340:120092-120234
BmHel-11	DF090338:3262312-3262456
BmHel-11	DF090338:3262473-3262574
BmHel-11	DF090347:1420023-1420207
BmHel-11	DF090350:360901-361078
BmHel-11	DF090334:3665026-3665144
BmHel-11	DF090334:3664898-3665026
BmHel-11	DF090412:1336411-1336517
BmHel-11	DF090412:1336522-1336660

BmHel-11	DF090397:1202374-1202612
BmHel-11	DF090336:5136331-5136474
BmHel-11	DF090336:5135662-5135755
BmHel-11	DF090383:2017966-2018141
BmHel-11	DF090627:14662-14768
BmHel-11	DF090627:14769-14905
BmHel-11	DF090386:1863204-1863398
BmHel-11	DF090322:7951752-7951997
BmHel-11	DF090331:5389374-5389618
BmHel-11	DF090333:5129397-5129554
BmHel-11	DF090333:5129561-5129649
BmHel-11	DF090324:3864894-3865072
BmHel-11	DF090328:586338-586504
BmHel-11	DF090323:6775121-6775232
BmHel-11	DF090323:6774931-6775062
BmHel-11	DF090323:5076042-5076138
BmHel-11	DF090499:108940-109106
BmHel-11	DF090317:9267581-9267831
BmHel-11	DF090425:69087-69332
BmHel-11	DF090528:60722-60867
BmHel-11	DF090528:60868-60967
BmHel-11	DF090341:882854-882971
BmHel-11	DF090341:882977-883058
BmHel-11	DF090320:4232954-4233123
BmHel-11	DF090342:1949124-1949337
BmHel-11	DF090542:12605-12849
BmHel-11	DF090388:930824-931076
BmHel-11	DF090329:1438306-1438547
BmHel-11	DF090354:1083891-1084136
BmHel-11	DF090372:2164615-2164864
BmHel-11	DF090348:1857428-1857635
BmHel-11	DF090319:5375070-5375279
BmHel-11	DF090828:7139-7383
BmHel-11	DF090409:747590-747840
BmHel-11	DF090344:1315467-1315618
BmHel-11	DF090344:1315634-1315724
BmHel-11	DF090319:5306307-5306505
BmHel-11	DF090364:616669-616823
BmHel-11	DF090364:616835-616924
BmHel-11	DF090364:2929183-2929336
BmHel-11	DF090364:2929086-2929176
BmHel-11	DF090448:116919-117052
BmHel-11	DF090448:117199-117308
BmHel-11	BABH01048679:585-738
BmHel-11	BABH01048679:745-835
BmHel-11	DF090339:715870-716037
BmHel-11	DF090349:1191890-1192073
BmHel-11	DF090330:3554124-3554256

BmHel-11	DF090404:1449543-1449726
BmHel-11	DF090324:3597924-3598076
BmHel-11	DF090324:3597822-3597912
BmHel-11	DF090326:3892649-3892779
BmHel-11	DF090326:3892514-3892627
BmHel-11	DF090350:2255715-2255850
BmHel-11	DF090350:2255611-2255714
BmHel-11	DF090351:1307926-1308019
BmHel-11	DF090351:1308073-1308175
BmHel-11	DF090416:871027-871142
BmHel-11	DF090416:871150-871281
BmHel-11	BABH01076042:208-338
BmHel-11	BABH01076042:73-186
BmHel-11	DF090331:2047057-2047168
BmHel-11	DF090331:2046907-2047039
BmHel-11	DF090316:7179380-7179581
BmHel-11	DF090330:5194343-5194538
BmHel-11	DF090504:30938-31095
BmHel-11	DF090504:31088-31175
BmHel-11	DF090446:469585-469690
BmHel-11	DF090446:469436-469575
BmHel-11	DF090382:1041660-1041838
BmHel-11	DF090330:474832-474988
BmHel-11	DF090330:474745-474832
BmHel-11	DF090316:11380419-11380604
BmHel-11	DF090367:1743985-1744066
BmHel-11	DF090367:1743822-1743978
BmHel-11	DF090323:7230101-7230187
BmHel-11	DF090323:7230205-7230367
BmHel-11	DF090322:2845950-2846193
BmHel-11	DF090433:208040-208234
BmHel-11	DF090729:12683-12877
BmHel-11	DF090316:2570358-2570503
BmHel-11	DF090316:2570262-2570357
BmHel-11	DF090343:4541094-4541257
BmHel-11	DF090343:4541262-4541342
BmHel-11	DF090324:5818859-5819106
BmHel-11	DF090319:6398991-6399237
BmHel-11	DF090382:1187628-1187871
BmHel-11	DF090441:362063-362255
BmHel-11	DF090319:199625-199731
BmHel-11	DF090319:199803-199888
BmHel-11	DF090475:75908-76117
BmHel-11	DF090333:5009247-5009457
BmHel-11	DF090324:1056369-1056472
BmHel-11	DF090347:2309678-2309839
BmHel-11	DF090347:2309453-2309535
BmHel-11	DF090319:4188101-4188231

BmHel-11	DF090319:4187974-4188084
BmHel-11	DF090378:1834189-1834299
BmHel-11	DF090347:138585-138768
BmHel-11	DF090350:1666355-1666522
BmHel-11	DF090363:419278-419461
BmHel-11	DF090427:197560-197722
BmHel-11	DF090329:1782837-1782993
BmHel-11	DF090329:1783007-1783093
BmHel-11	DF090320:874388-874549
BmHel-11	DF090530:21484-21727
BmHel-11	DF090320:6347997-6348166
BmHel-11	BABH01061358:574-693
BmHel-11	BABH01061358:441-564
BmHel-11	DF090340:3213140-3213284
BmHel-11	DF090352:3893828-3894020
BmHel-11	DF090356:2813600-2813789
BmHel-11	DF090504:15998-16191
BmHel-11	DF090316:15915567-15915770
BmHel-11	DF090381:1611926-1612082
BmHel-11	DF090369:2264690-2264935
BmHel-11	DF090319:6715930-6716125
BmHel-11	DF090365:2355705-2355857
BmHel-11	DF090378:1589712-1589870
BmHel-11	DF090378:1589632-1589711
BmHel-11	DF090443:397418-397611
BmHel-11	DF090329:6326589-6326806
BmHel-11	DF090382:973368-973484
BmHel-11	DF090382:973229-973354
BmHel-11	DF090341:729127-729322
BmHel-11	DF090320:1847069-1847306
BmHel-11	DF090412:394259-394440
BmHel-11	DF090358:2808469-2808644
BmHel-11	DF090644:73446-73535
BmHel-11	DF090644:73557-73707
BmHel-11	DF090328:1255923-1256165
BmHel-11	DF090370:376366-376445
BmHel-11	DF090370:376449-376600
BmHel-11	DF090440:446335-446515
BmHel-11	DF090427:148772-149015
BmHel-11	DF090428:881466-881549
BmHel-11	DF090428:881214-881373
BmHel-11	DF090375:2318822-2319000
BmHel-11	DF090328:402599-402762
BmHel-11	DF090398:1353809-1354048
BmHel-11	DF090323:412734-412927
BmHel-11	DF090342:3917317-3917496
BmHel-11	DF090334:2431863-2432105
BmHel-11	DF090323:291920-292162

BmHel-11	BABH01052382:594-838
BmHel-11	BABH01076599:1-239
BmHel-11	DF090317:7943699-7943893
BmHel-11	DF090361:144021-144156
BmHel-11	DF090335:2629752-2629993
BmHel-11	DF090396:1490091-1490333
BmHel-11	DF090352:1404169-1404399
BmHel-11	DF090317:1723893-1724128
BmHel-11	DF090344:3563671-3563790
BmHel-11	DF090344:3563803-3563924
BmHel-11	DF090339:4128342-4128453
BmHel-11	DF090316:11539573-11539763
BmHel-11	DF090379:23841-24035
BmHel-11	DF090338:1956069-1956231
BmHel-11	DF090322:5235999-5236243
BmHel-11	DF090316:3922636-3922747
BmHel-11	DF090333:3983282-3983463
BmHel-11	DF090398:1313649-1313822
BmHel-11	DF090328:4025718-4025861
BmHel-11	DF090328:4025617-4025716
BmHel-11	DF090463:364788-364956
BmHel-11	DF090343:2784856-2785038
BmHel-11	DF090347:1019916-1020030
BmHel-11	DF090347:1019775-1019900
BmHel-11	DF090472:193009-193140
BmHel-11	DF090472:193139-193248
BmHel-11	DF090426:899970-900085
BmHel-11	DF090426:899837-899956
BmHel-11	DF090340:1241629-1241820
BmHel-11	DF090351:4095820-4096012
BmHel-11	BABH01045277:4-245
BmHel-11	DF090324:1198027-1198237
BmHel-11	DF090331:6201475-6201717
BmHel-11	DF090331:1415550-1415791
BmHel-11	DF090394:426696-426937
BmHel-11	DF090518:96897-97089
BmHel-11	DF090445:456574-456805
BmHel-11	DF090326:6370362-6370596
BmHel-11	DF090335:2683338-2683582
BmHel-11	DF090355:3654848-3655129
BmHel-11	DF090353:1484521-1484701
BmHel-11	DF090319:5956704-5956948
BmHel-11	DF090359:1668772-1668901
BmHel-11	DF090359:1669047-1669157
BmHel-11	DF090344:2896124-2896330
BmHel-11	DF090366:408021-408118
BmHel-11	DF090366:407781-407920
BmHel-11	DF090317:5153881-5154119

BmHel-11	DF090345:4257056-4257303
BmHel-11	DF090509:75982-76222
BmHel-11	DF090325:3901859-3902050
BmHel-11	DF090324:4504105-4504294
BmHel-11	DF090381:752267-752441
BmHel-11	DF090326:6604594-6604783
BmHel-11	DF090321:3107721-3107872
BmHel-11	DF090321:3107880-3107970
BmHel-11	DF090331:644151-644292
BmHel-11	DF090331:644050-644144
BmHel-11	DF090316:7912978-7913168
BmHel-11	DF090400:1549649-1549836
BmHel-11	DF090351:4125809-4125998
BmHel-11	DF090320:6838671-6838861
BmHel-11	DF090564:38819-38987
BmHel-11	DF090542:62511-62699
BmHel-11	DF090402:1444861-1444944
BmHel-11	DF090402:1444948-1445105
BmHel-11	DF090351:3056615-3056849
BmHel-11	DF090452:241979-242218
BmHel-11	DF090333:4822644-4822854
BmHel-11	DF090411:5074-5256
BmHel-11	DF090316:10466481-10466574
BmHel-11	DF090316:10466275-10466421
BmHel-11	DF090358:855707-855947
BmHel-11	DF090326:2847357-2847457
BmHel-11	DF090326:2847455-2847583
BmHel-11	DF091006:573-816
BmHel-11	BABH01055042:279-521
BmHel-11	DF090360:815630-815867
BmHel-11	DF090363:535343-535581
BmHel-11	DF090317:515386-515623
BmHel-11	DF090358:611206-611443
BmHel-11	BABH01045439:3505-3743
BmHel-11	DF090339:439042-439228
BmHel-11	DF090370:2400176-2400358
BmHel-11	DF090337:1116856-1117048
BmHel-11	DF090373:509929-510086
BmHel-11	DF090373:510101-510182
BmHel-11	DF090356:1398538-1398629
BmHel-11	DF090356:1398636-1398783
BmHel-11	DF090360:3196259-3196486
BmHel-11	DF090420:124395-124632
BmHel-11	DF090318:1210562-1210801
BmHel-11	DF090414:682607-682800
BmHel-11	DF090349:485227-485415
BmHel-11	DF090370:2047121-2047331
BmHel-11	DF090320:3503731-3503876

BmHel-11	DF090352:3818784-3818973
BmHel-11	DF090441:176387-176569
BmHel-11	DF090452:150805-150918
BmHel-11	DF090452:150923-151039
BmHel-11	DF090379:1307231-1307336
BmHel-11	DF090379:1307341-1307465
BmHel-11	DF090348:3309119-3309307
BmHel-11	DF090360:1154449-1154640
BmHel-11	DF090359:3235936-3236049
BmHel-11	DF090359:3235791-3235916
BmHel-11	DF090316:2332189-2332389
BmHel-11	DF090345:1106610-1106691
BmHel-11	DF090345:1106691-1106780
BmHel-11	DF090339:773311-773477
BmHel-11	DF090348:353158-353361
BmHel-11	DF090359:1681320-1681501
BmHel-11	DF090331:1564960-1565165
BmHel-11	DF090344:1529425-1529593
BmHel-11	DF090333:1105653-1105817
BmHel-11	DF090520:19657-19836
BmHel-11	DF090427:411995-412237
BmHel-11	DF090352:1091470-1091598
BmHel-11	DF090352:1088474-1088668
BmHel-11	DF090383:1982070-1982168
BmHel-11	DF090411:859006-859085
BmHel-11	DF090411:863662-863769
BmHel-11	DF090335:1008879-1009049
BmHel-11	DF090362:389298-389437
BmHel-11	DF090362:389197-389298
BmHel-11	BABH01043650:7295-7527
BmHel-11	DF090442:118900-119073
BmHel-11	DF090355:1119993-1120233
BmHel-11	DF090355:3170131-3170370
BmHel-11	DF090325:2845249-2845488
BmHel-11	DF090325:4082334-4082567
BmHel-11	DF090353:3902588-3902827
BmHel-11	DF090339:3374759-3374999
BmHel-11	DF090362:2696322-2696561
BmHel-11	DF090324:6801440-6801679
BmHel-11	DF090421:772104-772343
BmHel-11	DF090421:141149-141389
BmHel-11	DF090390:1182513-1182753
BmHel-11	DF090342:510466-510705
BmHel-11	DF090332:947795-948033
BmHel-11	DF090318:3114817-3115057
BmHel-11	DF090318:7653744-7653983
BmHel-11	DF090383:522034-522273
BmHel-11	DF090348:3609028-3609269

BmHel-11	DF090319:1262060-1262299
BmHel-11	DF090319:1743747-1743986
BmHel-11	DF090319:6752230-6752470
BmHel-11	DF090433:389343-389582
BmHel-11	DF090357:2357715-2357954
BmHel-11	DF090357:3060623-3060863
BmHel-11	DF090357:1922191-1922430
BmHel-11	DF090377:1870697-1870936
BmHel-11	DF090360:1949488-1949727
BmHel-11	DF090360:458568-458807
BmHel-11	DF090347:3503132-3503371
BmHel-11	DF090365:1288803-1289042
BmHel-11	DF090326:762524-762763
BmHel-11	DF090350:978414-978653
BmHel-11	DF090359:2018965-2019210
BmHel-11	DF090349:2065010-2065249
BmHel-11	DF090349:1535761-1536002
BmHel-11	DF090343:837967-838207
BmHel-11	DF090340:1873050-1873289
BmHel-11	DF090340:2028261-2028506
BmHel-11	DF090334:5251435-5251675
BmHel-11	DF090316:9318845-9319084
BmHel-11	DF090316:608597-608836
BmHel-11	DF090479:206253-206492
BmHel-11	DF090391:302465-302704
BmHel-11	DF090373:1435503-1435745
BmHel-11	DF090373:1211580-1211820
BmHel-11	DF090351:272977-273215
BmHel-11	DF090351:1845671-1845910
BmHel-11	DF090337:4614374-4614613
BmHel-11	DF090337:4099388-4099628
BmHel-11	DF090335:4667751-4667990
BmHel-11	DF090330:1248183-1248416
BmHel-11	DF090406:332969-333199
BmHel-11	DF090344:1453830-1454069
BmHel-11	DF090370:1757736-1757975
BmHel-11	DF090320:2864468-2864708
BmHel-11	DF090320:3372932-3373172
BmHel-11	DF090352:2968503-2968742
BmHel-11	DF090317:3587576-3587814
BmHel-11	DF090317:6545816-6546057
BmHel-11	DF090317:11171572-11171811
BmHel-11	DF090317:5475013-5475252
BmHel-11	DF090438:171650-171889
BmHel-11	DF090358:2344796-2345035
BmHel-11	DF090358:2164431-2164670
BmHel-11	DF090356:3655925-3656164
BmHel-11	DF090345:1124336-1124574

BmHel-11	DF090345:1247292-1247531
BmHel-11	DF090327:3097338-3097577
BmHel-11	DF090327:1439252-1439491
BmHel-11	DF090416:406135-406380
BmHel-11	DF090361:2706739-2706978
BmHel-11	DF090338:2376610-2376850
BmHel-11	DF090428:696289-696521
BmHel-11	DF090423:461124-461364
BmHel-11	DF090444:409590-409777
BmHel-11	DF090369:678892-679132
BmHel-11	DF090369:234663-234902
BmHel-11	DF090425:778320-778558
BmHel-11	BABH01051019:171-410
BmHel-11	BABH01049352:723-963
BmHel-11	BABH01068634:175-414
BmHel-11	DF090867:5079-5319
BmHel-11	BABH01045975:707-946
BmHel-11	BABH01044805:3452-3691
BmHel-11	DF091056:1803-2044
BmHel-11	DF090373:1663083-1663206
BmHel-11	DF090332:5742018-5742166
BmHel-11	DF090357:3000933-3001120
BmHel-11	DF090494:74212-74406
BmHel-11	DF090403:850169-850286
BmHel-11	DF090403:850030-850150
BmHel-11	DF090416:1442-1554
BmHel-11	DF090416:1683-1805
BmHel-11	DF090322:7460367-7460610
BmHel-11	BABH01049912:6-244
BmHel-11	DF090321:7516176-7516375
BmHel-11	DF090358:2055161-2055402
BmHel-11	DF090316:2232782-2233019
BmHel-11	DF090458:210667-210837
BmHel-11	DF090516:23588-23703
BmHel-11	DF090351:3670919-3671025
BmHel-11	DF090351:3671028-3671109
BmHel-11	DF090333:3102226-3102342
BmHel-11	DF090333:3102088-3102209
BmHel-11	DF090348:161957-162136
BmHel-11	DF090326:4696688-4696926
BmHel-11	DF090321:7847317-7847504
BmHel-11	DF090410:223824-224011
BmHel-11	DF090334:1060387-1060574
BmHel-11	DF090334:4475440-4475625
BmHel-11	DF090352:3250974-3251167
BmHel-11	DF090403:495000-495166
BmHel-11	DF090360:3012427-3012577
BmHel-11	DF090360:3012335-3012424

BmHel-11	DF090337:3797661-3797899
BmHel-11	DF090330:4853204-4853381
BmHel-11	DF090345:4026972-4027124
BmHel-11	DF090391:1603794-1603978
BmHel-11	DF090341:3892245-3892433
BmHel-11	DF090373:1150668-1150855
BmHel-11	DF090414:1063013-1063181
BmHel-11	DF090325:2174744-2174887
BmHel-11	DF090325:2174645-2174743
BmHel-11	DF090390:958803-958998
BmHel-11	DF090435:225114-225284
BmHel-11	DF090325:6503924-6504089
BmHel-11	DF090342:3012660-3012898
BmHel-11	DF090344:3330468-3330708
BmHel-11	DF090333:5238502-5238745
BmHel-11	DF090394:1230294-1230531
BmHel-11	DF090421:286726-286885
BmHel-11	DF090324:5514388-5514625
BmHel-11	BABH01054619:546-777
BmHel-11	DF090362:749039-749231
BmHel-11	DF090322:4267723-4267960
BmHel-11	DF090323:2410779-2410958
BmHel-11	DF090323:3454159-3454336
BmHel-11	DF090347:1825904-1826092
BmHel-11	DF090410:735435-735623
BmHel-11	DF090364:668150-668234
BmHel-11	DF090364:668318-668408
BmHel-11	DF090358:1867474-1867664
BmHel-11	DF090356:1543641-1543818
BmHel-11	DF090444:251198-251381
BmHel-11	DF090362:2958718-2958824
BmHel-11	DF090362:2958579-2958664
BmHel-11	DF090324:7115862-7116057
BmHel-11	DF090343:1109712-1109907
BmHel-11	DF090320:8262096-8262208
BmHel-11	DF090320:8262236-8262361
BmHel-11	DF090322:6927413-6927618
BmHel-11	DF090437:601301-601504
BmHel-11	DF090431:434791-434946
BmHel-11	DF090323:174927-175058
BmHel-11	DF090323:175058-175158
BmHel-11	DF090470:186975-187215
BmHel-11	DF090321:6191257-6191500
BmHel-11	DF090431:141899-142061
BmHel-11	DF090339:4653675-4653912
BmHel-11	DF090336:5103167-5103404
BmHel-11	DF090319:161155-161392
BmHel-11	DF090340:1466771-1467008

BmHel-11	DF090327:4435928-4436165
BmHel-11	BABH01084625:1-238
BmHel-11	BABH01051113:1-238
BmHel-11	BABH01048131:576-813
BmHel-11	DF090343:1265722-1265911
BmHel-11	DF090330:3613016-3613204
BmHel-11	DF090405:589076-589264
BmHel-11	DF090397:1315391-1315581
BmHel-11	DF090405:846603-846697
BmHel-11	DF090405:846407-846489
BmHel-11	BABH01075708:328-447
BmHel-11	BABH01075708:485-601
BmHel-11	DF090360:914351-914529
BmHel-11	DF090350:1325386-1325622
BmHel-11	DF090321:7944454-7944690
BmHel-11	DF090349:3417095-3417333
BmHel-11	DF090345:3455109-3455346
BmHel-11	DF090470:129080-129315
BmHel-11	DF090324:2863486-2863673
BmHel-11	DF090342:1669760-1669953
BmHel-11	DF090316:1975296-1975474
BmHel-11	DF090332:5417735-5417976
BmHel-11	DF090328:4868667-4868855
BmHel-11	DF090379:1750255-1750421
BmHel-11	DF090352:1936780-1936909
BmHel-11	DF090317:212740-212977
BmHel-11	DF090338:1348694-1348883
BmHel-11	DF090433:189245-189430
BmHel-11	DF090340:4415431-4415661
BmHel-11	DF090428:649746-649871
BmHel-11	DF090387:1256447-1256562
BmHel-11	DF090387:1256335-1256422
BmHel-11	DF090359:2654573-2654706
BmHel-11	DF090359:2654707-2654808
BmHel-11	DF090334:5584393-5584526
BmHel-11	DF090334:5584290-5584392
BmHel-11	BABH01048625:642-876
BmHel-11	DF090330:673921-674062
BmHel-11	DF090326:6809297-6809533
BmHel-11	DF090316:7583897-7584134
BmHel-11	DF090412:863348-863584
BmHel-11	DF090333:1985097-1985327
BmHel-11	DF090375:1763745-1763983
BmHel-11	DF090374:1969566-1969802
BmHel-11	BABH01055092:1-237
BmHel-11	BABH01051658:353-592
BmHel-11	DF090366:1123789-1123977
BmHel-11	DF090384:203569-203758

BmHel-11	DF090356:3385421-3385609
BmHel-11	DF090615:28029-28222
BmHel-11	BABH01045516:1473-1662
BmHel-11	DF090328:434338-434496
BmHel-11	DF090318:5962347-5962582
BmHel-11	DF090417:525686-525923
BmHel-11	DF090375:1002926-1003161
BmHel-11	DF090372:563287-563462
BmHel-11	DF090387:118615-118801
BmHel-11	DF090324:7330090-7330274
BmHel-11	DF090421:1112465-1112700
BmHel-11	DF090342:2503884-2504071
BmHel-11	DF090318:991684-991863
BmHel-11	DF090321:2104271-2104457
BmHel-11	DF090469:241670-241855
BmHel-11	DF090388:1141635-1141820
BmHel-11	DF090344:825236-825422
BmHel-11	DF090344:1231381-1231567
BmHel-11	DF090378:2155069-2155255
BmHel-11	DF090317:10186509-10186695
BmHel-11	DF090356:3016970-3017156
BmHel-11	DF090434:158465-158652
BmHel-11	DF090345:2244026-2244213
BmHel-11	DF090327:5532057-5532160
BmHel-11	DF090327:5532160-5532291
BmHel-11	DF090327:6534071-6534260
BmHel-11	DF090327:651445-651584
BmHel-11	DF090416:341609-341796
BmHel-11	DF090375:1108945-1109131
BmHel-11	DF090408:502041-502175
BmHel-11	DF090408:502211-502312
BmHel-11	DF090653:5282-5444
BmHel-11	DF090904:23111-23297
BmHel-11	DF090320:3565057-3565241
BmHel-11	DF090361:2676814-2677002
BmHel-11	DF090325:5168454-5168628
BmHel-11	DF090362:1668747-1668846
BmHel-11	DF090362:1668850-1668978
BmHel-11	DF090365:251213-251444
BmHel-11	DF090343:2234500-2234734
BmHel-11	DF090359:1969925-1970109
BmHel-11	DF090324:7562107-7562288
BmHel-11	DF090402:936378-936479
BmHel-11	DF090343:2898698-2898887
BmHel-11	DF090345:3732752-3732927
BmHel-11	DF090394:44928-45043
BmHel-11	DF090377:1816948-1817183
BmHel-11	DF090333:1272936-1273119

BmHel-11	DF090369:100592-100778
BmHel-11	DF091229:4330-4489
BmHel-11	BABH01072727:385-488
BmHel-11	BABH01072727:486-620
BmHel-11	DF090358:3107827-3108062
BmHel-11	DF090328:5593354-5593588
BmHel-11	DF090395:676930-677164
BmHel-11	DF090400:1093935-1094123
BmHel-11	DF090362:3077709-3077898
BmHel-11	DF090322:6411546-6411717
BmHel-11	DF090427:617972-618081
BmHel-11	DF090427:618085-618209
BmHel-11	DF090357:1227386-1227549
BmHel-11	DF090325:5692119-5692251
BmHel-11	DF090325:5692271-5692373
BmHel-11	DF090336:4240017-4240198
BmHel-11	DF090360:1805584-1805819
BmHel-11	DF090326:5741625-5741797
BmHel-11	DF090322:5357838-5358015
BmHel-11	DF090322:2214548-2214717
BmHel-11	DF090322:2214282-2214445
BmHel-11	DF090455:408164-408299
BmHel-11	DF090455:408312-408411
BmHel-11	DF090636:13722-13903
BmHel-11	DF090376:633571-633756
BmHel-11	DF090330:4345247-4345481
BmHel-11	DF090379:1560501-1560678
BmHel-11	DF090409:1151792-1151887
BmHel-11	DF090409:1151889-1152032
BmHel-11	DF090336:978897-979065
BmHel-11	BABH01052105:304-487
BmHel-11	BABH01050957:657-840
BmHel-11	DF090381:1905001-1905229
BmHel-11	DF090381:454637-454800
BmHel-11	DF090352:3428741-3428911
BmHel-11	DF090567:11050-11190
BmHel-11	DF090567:10915-11008
BmHel-11	DF090319:6035995-6036129
BmHel-11	DF090319:6036129-6036228
BmHel-11	DF090332:2678037-2678268
BmHel-11	DF090466:163038-163272
BmHel-11	DF090386:960732-960969
BmHel-11	DF090348:847747-847981
BmHel-11	DF090357:2541814-2542051
BmHel-11	DF090468:139790-140021
BmHel-11	DF090328:738701-738887
BmHel-11	DF090350:1822068-1822254
BmHel-11	DF090327:1621432-1621619

BmHel-11	DF090522:88671-88860
BmHel-11	DF090430:541042-541148
BmHel-11	DF090430:540917-541039
BmHel-11	DF090332:2017057-2017292
BmHel-11	DF090339:805039-805228
BmHel-11	DF090319:181967-182149
BmHel-11	DF090330:4660552-4660664
BmHel-11	DF090330:4660382-4660503
BmHel-11	DF090346:647415-647598
BmHel-11	DF090319:8805019-8805121
BmHel-11	DF090319:8804870-8804999
BmHel-11	DF090377:2001364-2001526
BmHel-11	DF090355:180143-180324
BmHel-11	DF090362:1595230-1595414
BmHel-11	DF090336:2720976-2721161
BmHel-11	DF090383:1133207-1133391
BmHel-11	DF090383:211572-211756
BmHel-11	DF090348:2967148-2967333
BmHel-11	DF090328:4421648-4421832
BmHel-11	DF090328:3491554-3491738
BmHel-11	DF090319:28757-28942
BmHel-11	DF090395:1068542-1068724
BmHel-11	DF090347:253666-253850
BmHel-11	DF090326:1910270-1910453
BmHel-11	DF090321:3740638-3740822
BmHel-11	DF090321:4511302-4511494
BmHel-11	DF090382:373476-373661
BmHel-11	DF090363:1976317-1976501
BmHel-11	DF090349:3169596-3169781
BmHel-11	DF090343:1574151-1574335
BmHel-11	DF090331:5701472-5701657
BmHel-11	DF090502:19891-20075
BmHel-11	DF090330:5239188-5239372
BmHel-11	DF090329:1340351-1340536
BmHel-11	DF090344:18836-19024
BmHel-11	DF090396:211680-211866
BmHel-11	DF090327:6035402-6035588
BmHel-11	DF090327:5443711-5443944
BmHel-11	DF090401:173666-173850
BmHel-11	DF090403:87874-88058
BmHel-11	DF090375:280478-280662
BmHel-11	DF090394:1172446-1172628
BmHel-11	DF090330:809926-810072
BmHel-11	DF090339:2752936-2753103
BmHel-11	DF090339:2611087-2611258
BmHel-11	DF090363:764195-764324
BmHel-11	DF090363:764329-764431
BmHel-11	DF090341:4738414-4738544

BmHel-11	DF090341:4738549-4738651
BmHel-11	DF090316:2587665-2587776
BmHel-11	DF090356:2870092-2870203
BmHel-11	DF090356:2870251-2870331
BmHel-11	DF090402:1282149-1282258
BmHel-11	DF090402:1282310-1282433
BmHel-11	DF090351:2883030-2883263
BmHel-11	DF090421:481816-481998
BmHel-11	DF090401:874362-874544
BmHel-11	DF090332:360693-360797
BmHel-11	DF090332:360827-360955
BmHel-11	DF090349:2488145-2488326
BmHel-11	DF090363:1653791-1653984
BmHel-11	DF090321:3529589-3529717
BmHel-11	DF090321:3529718-3529822
BmHel-11	BABH01074395:215-374
BmHel-11	DF090377:1980218-1980416
BmHel-11	DF090337:1510665-1510902
BmHel-11	DF090367:262998-263231
BmHel-11	DF090353:3206955-3207140
BmHel-11	DF090486:109355-109540
BmHel-11	DF090346:3595277-3595465
BmHel-11	DF090322:2261123-2261355
BmHel-11	DF090321:8080726-8080957
BmHel-11	DF090424:757839-758075
BmHel-11	DF090380:1950220-1950403
BmHel-11	DF090411:1154004-1154236
BmHel-11	DF090368:2333422-2333652
BmHel-11	DF090330:2232447-2232619
BmHel-11	DF090317:6959880-6960063
BmHel-11	DF090427:338632-338796
BmHel-11	DF090422:836563-836748
BmHel-11	DF090422:214052-214170
BmHel-11	DF090369:1536265-1536447
BmHel-11	BABH01075250:1-233
BmHel-11	BABH01087331:58-290
BmHel-11	DF090318:169287-169419
BmHel-11	DF090318:168896-168994
BmHel-11	DF090319:5970519-5970671
BmHel-11	DF090319:5970678-5970757
BmHel-11	DF090326:4559201-4559385
BmHel-11	DF090373:270944-271176
BmHel-11	DF090337:919410-919593
BmHel-11	DF090484:170494-170678
BmHel-11	BABH01045616:347-528
BmHel-11	DF090362:1144677-1144859
BmHel-11	DF090324:3971186-3971369
BmHel-11	DF090316:15293591-15293770

BmHel-11	DF090351:115533-115716
BmHel-11	DF090366:2314991-2315174
BmHel-11	DF090364:885099-885284
BmHel-11	DF090317:367520-367702
BmHel-11	DF090358:529227-529416
BmHel-11	DF090333:4325365-4325548
BmHel-11	DF090441:130071-130257
BmHel-11	DF090418:143044-143222
BmHel-11	DF090367:2797475-2797656
BmHel-11	DF090352:1755207-1755356
BmHel-11	DF090352:1755107-1755189
BmHel-11	DF090347:33936-34018
BmHel-11	DF090347:33772-33926
BmHel-11	DF090418:492141-492322
BmHel-11	DF090333:1328755-1328932
BmHel-11	BABH01047654:940-1094
BmHel-11	BABH01047654:860-942
BmHel-11	DF090360:848678-848911
BmHel-11	DF090346:1566173-1566408
BmHel-11	DF090372:2434607-2434757
BmHel-11	BABH01073739:453-614
BmHel-11	DF090324:3505258-3505489
BmHel-11	DF090543:14004-14230
BmHel-11	DF090362:2114108-2114292
BmHel-11	DF090342:760306-760538
BmHel-11	DF090350:3173531-3173762
BmHel-11	DF090373:1456795-1456954
BmHel-11	DF090354:1364153-1364333
BmHel-11	BABH01063724:1-232
BmHel-11	DF090426:388770-388928
BmHel-11	DF090414:563518-563698
BmHel-11	DF090322:2299935-2300118
BmHel-11	DF090373:1709552-1709740
BmHel-11	DF090337:2680010-2680101
BmHel-11	DF090337:2680125-2680264
BmHel-11	DF090354:2469474-2469658
BmHel-11	BABH01064665:305-490
BmHel-11	BABH01064957:88-272
BmHel-11	DF090332:3126540-3126722
BmHel-11	DF090319:3167924-3168088
BmHel-11	DF090316:1522186-1522337
BmHel-11	DF090330:5899608-5899720
BmHel-11	DF090330:5896629-5896747
BmHel-11	DF090409:1013495-1013679
BmHel-11	DF090325:1511171-1511358
BmHel-11	DF090324:1888503-1888687
BmHel-11	DF090421:504017-504199
BmHel-11	DF090332:1621760-1621942

BmHel-11	DF090319:150035-150217
BmHel-11	DF090319:4911309-4911493
BmHel-11	DF090347:3971302-3971484
BmHel-11	DF090322:1259741-1259927
BmHel-11	DF090321:497130-497306
BmHel-11	DF090363:695713-695901
BmHel-11	DF090349:3661376-3661558
BmHel-11	DF090341:3428407-3428590
BmHel-11	DF090316:6355165-6355349
BmHel-11	DF090316:2846772-2846954
BmHel-11	DF090388:589493-589675
BmHel-11	DF090335:873268-873450
BmHel-11	DF090323:5021861-5022039
BmHel-11	DF090367:1913534-1913717
BmHel-11	DF090344:3611581-3611763
BmHel-11	DF090380:991076-991258
BmHel-11	DF090380:1265455-1265638
BmHel-11	DF090366:895821-896003
BmHel-11	DF090317:10372418-10372600
BmHel-11	DF090356:2002217-2002399
BmHel-11	DF090345:367632-367814
BmHel-11	DF090415:358111-358294
BmHel-11	DF090615:34605-34789
BmHel-11	DF090597:20027-20209
BmHel-11	DF090357:840777-840957
BmHel-11	DF090317:5949960-5950140
BmHel-11	DF090322:8161118-8161297
BmHel-11	DF090318:6940276-6940459
BmHel-11	DF090323:7195843-7196027
BmHel-11	DF090319:7780839-7781074
BmHel-11	BABH01085443:134-365
BmHel-11	BABH01051286:668-903
BmHel-11	DF090317:3418739-3418973
BmHel-11	DF090336:2983477-2983708
BmHel-11	DF090348:2386882-2387113
BmHel-11	DF090349:3871820-3872052
BmHel-11	DF090340:1578833-1579072
BmHel-11	DF090373:1578453-1578683
BmHel-11	DF090366:396208-396446
BmHel-11	DF090333:401498-401729
BmHel-11	DF090338:2494921-2495153
BmHel-11	BABH01072511:133-364
BmHel-11	DF090406:279524-279668
BmHel-11	DF090406:279434-279519
BmHel-11	DF090316:15859141-15859286
BmHel-11	DF090316:15859056-15859140
BmHel-11	DF090322:7363183-7363275
BmHel-11	DF090322:7363085-7363185

BmHel-11	DF090386:1662627-1662809
BmHel-11	DF090325:1871959-1872189
BmHel-11	DF090343:1000016-1000246
BmHel-11	DF090385:4366-4596
BmHel-11	DF090330:1132224-1132413
BmHel-11	DF090355:2363522-2363703
BmHel-11	DF090328:5322904-5323088
BmHel-11	DF090322:846207-846389
BmHel-11	DF090322:1402259-1402443
BmHel-11	DF090341:299640-299802
BmHel-11	DF090419:145900-146082
BmHel-11	BABH01079159:1-230
BmHel-11	BABH01077916:68-250
BmHel-11	DF090318:4246606-4246741
BmHel-11	DF090318:4246499-4246593
BmHel-11	DF090345:997380-997610
BmHel-11	DF090397:1130277-1130508
BmHel-11	DF090475:219829-220011
BmHel-11	DF090363:602139-602320
BmHel-11	DF090351:477426-477643
BmHel-11	DF090329:5188857-5189039
BmHel-11	DF090364:2733374-2733555
BmHel-11	DF090374:232447-232546
BmHel-11	DF090374:232563-232689
BmHel-11	DF090657:25757-25944
BmHel-11	DF090342:106601-106783
BmHel-11	DF090392:1671543-1671724
BmHel-11	DF090370:2197436-2197617
BmHel-11	DF090327:6592200-6592382
BmHel-11	DF090404:692624-692851
BmHel-11	BABH01074455:379-609
BmHel-11	DF090334:4906889-4907119
BmHel-11	DF090316:11308393-11308536
BmHel-11	DF090316:11308540-11308620
BmHel-11	DF090372:1482475-1482580
BmHel-11	DF090372:1482327-1482452
BmHel-11	DF090386:1055979-1056164
BmHel-11	DF090325:5199868-5200100
BmHel-11	DF090323:7520724-7520953
BmHel-11	DF090412:339304-339536
BmHel-11	DF090423:159068-159229
BmHel-11	DF090532:14711-14895
BmHel-11	DF090316:15331537-15331656
BmHel-11	DF090384:2026128-2026265
BmHel-11	DF090384:2026035-2026126
BmHel-11	DF090316:9894598-9894784
BmHel-11	DF090317:10330724-10330912
BmHel-11	DF090333:2011577-2011766

BmHel-11	DF090329:5044548-5044685
BmHel-11	DF090329:5044690-5044782
BmHel-11	DF090344:2587450-2587649
BmHel-11	DF090375:1746778-1746884
BmHel-11	DF090375:1746639-1746761
BmHel-11	DF090327:937863-938096
BmHel-11	DF090344:333013-333137
BmHel-11	DF090344:332886-332990
BmHel-11	DF090421:801753-801893
BmHel-11	DF090421:802696-802783
BmHel-11	DF090319:3876444-3876628
BmHel-11	DF090371:1605362-1605513
BmHel-11	DF090346:833887-834066
BmHel-11	DF090324:305408-305590
BmHel-11	DF090316:5700903-5701083
BmHel-11	DF090388:76292-76520
BmHel-11	DF090326:2175932-2176112
BmHel-11	DF090348:427730-427909
BmHel-11	DF090469:172331-172511
BmHel-11	DF090356:3262963-3263143
BmHel-11	DF090625:18451-18631
BmHel-11	DF090331:4491993-4492105
BmHel-11	DF090331:4492211-4492326
BmHel-11	DF090342:4854311-4854488
BmHel-11	DF090320:7767684-7767814
BmHel-11	DF090320:7767822-7767920
BmHel-11	DF090358:1283417-1283506
BmHel-11	DF090358:1283264-1283403
BmHel-11	DF090324:1973898-1974125
BmHel-11	DF090359:807964-808193
BmHel-11	DF090418:107273-107503
BmHel-11	DF090348:3052421-3052610
BmHel-11	DF090456:194800-194988
BmHel-11	DF090327:1870376-1870561
BmHel-11	DF090333:1801043-1801232
BmHel-11	DF090362:415214-415441
BmHel-11	DF090685:11251-11481
BmHel-11	DF090336:658940-659168
BmHel-11	DF090376:918208-918437
BmHel-11	DF090331:2450050-2450244
BmHel-11	DF090316:12022968-12023104
BmHel-11	DF090316:12022877-12022969
BmHel-11	DF090320:8034506-8034672
BmHel-11	DF090333:1393781-1393962
BmHel-11	DF090316:2810389-2810570
BmHel-11	DF090400:1421368-1421555
BmHel-11	DF090335:1174772-1174956
BmHel-11	DF092094:2115-2295

BmHel-11	BABH01051297:53-233
BmHel-11	DF090354:958856-959076
BmHel-11	DF090317:572406-572574
BmHel-11	DF090353:3543362-3543541
BmHel-11	DF090318:6700356-6700538
BmHel-11	DF090379:1774693-1774873
BmHel-11	DF090416:1076598-1076780
BmHel-11	DF091027:10511-10697
BmHel-11	BABH01047228:705-884
BmHel-11	BABH01045089:2136-2320
BmHel-11	DF090325:1534650-1534831
BmHel-11	DF090349:2928544-2928726
BmHel-11	DF090943:4007-4126
BmHel-11	DF090321:4066488-4066716
BmHel-11	DF090403:508089-508314
BmHel-11	DF090361:2872112-2872339
BmHel-11	DF090492:12607-12834
BmHel-11	BABH01045483:1742-1972
BmHel-11	DF090411:975731-975849
BmHel-11	DF090419:822920-823057
BmHel-11	DF090419:823064-823153
BmHel-11	DF090321:934454-934682
BmHel-11	DF090337:465890-466107
BmHel-11	DF090317:8156937-8157164
BmHel-11	DF090349:4074935-4075170
BmHel-11	DF090390:998735-998861
BmHel-11	DF090390:998309-998409
BmHel-11	DF090342:1497136-1497322
BmHel-11	DF090318:1956922-1957057
BmHel-11	DF090318:1957056-1957148
BmHel-11	DF090395:396465-396599
BmHel-11	DF090395:396598-396690
BmHel-11	DF090322:8044699-8044877
BmHel-11	DF090410:621085-621209
BmHel-11	DF090410:621208-621300
BmHel-11	DF090350:2702728-2702890
BmHel-11	DF090334:5763620-5763782
BmHel-11	DF090316:740220-740388
BmHel-11	DF090388:869601-869735
BmHel-11	DF090388:869734-869826
BmHel-11	DF090337:1145182-1145316
BmHel-11	DF090337:1145091-1145183
BmHel-11	DF090364:245142-245276
BmHel-11	DF090364:245051-245143
BmHel-11	DF090361:1286750-1286852
BmHel-11	DF090361:1288465-1288545
BmHel-11	DF090409:779126-779260
BmHel-11	DF090409:779035-779127

BmHel-11	DF090394:1068286-1068422
BmHel-11	DF090394:1068196-1068287
BmHel-11	DF090549:2063-2197
BmHel-11	DF090549:1972-2064
BmHel-11	DF090368:492269-492447
BmHel-11	DF090325:6903973-6904061
BmHel-11	DF090325:6903829-6903967
BmHel-11	DF090360:3106811-3106996
BmHel-11	DF090489:115751-115933
BmHel-11	DF090656:21608-21741
BmHel-11	DF090656:21512-21605
BmHel-11	DF090336:2859250-2859432
BmHel-11	DF090380:173152-173330
BmHel-11	DF090422:13651-13735
BmHel-11	DF090422:13259-13403
BmHel-11	DF090353:3228048-3228165
BmHel-11	DF090400:849190-849368
BmHel-11	DF090346:471779-471914
BmHel-11	DF090335:4639134-4639365
BmHel-11	DF090361:2333957-2334184
BmHel-11	DF090382:2111905-2112029
BmHel-11	DF090344:2422938-2423110
BmHel-11	DF090416:477128-477355
BmHel-11	DF090362:934258-934403
BmHel-11	DF090320:2607570-2607723
BmHel-11	DF090382:164323-164501
BmHel-11	DF090319:8400614-8400841
BmHel-11	DF090357:873650-873877
BmHel-11	DF090331:5095566-5095792
BmHel-11	DF090344:732934-733166
BmHel-11	DF090320:4211205-4211432
BmHel-11	DF090396:291949-292179
BmHel-11	DF090333:1381145-1381372
BmHel-11	DF090361:2817370-2817598
BmHel-11	DF090369:555240-555454
BmHel-11	DF090328:6487196-6487383
BmHel-11	DF090348:816545-816651
BmHel-11	DF090348:816676-816785
BmHel-11	DF090324:956067-956254
BmHel-11	DF090429:260141-260256
BmHel-11	DF090346:1991301-1991527
BmHel-11	DF090325:3465435-3465550
BmHel-11	DF090325:3465665-3465771
BmHel-11	DF090316:13681172-13681402
BmHel-11	DF090330:5895660-5895886
BmHel-11	DF090399:452432-452612
BmHel-11	DF091630:479-630
BmHel-11	DF090335:5713128-5713262

BmHel-11	DF090335:5713014-5713105
BmHel-11	DF090323:2424089-2424222
BmHel-11	DF090323:2423998-2424090
BmHel-11	DF090339:2843791-2843974
BmHel-11	DF090421:1076396-1076569
BmHel-11	DF090433:30254-30480
BmHel-11	DF090317:6130138-6130254
BmHel-11	DF090488:98210-98436
BmHel-11	DF090316:11856050-11856214
BmHel-11	DF090393:332429-332655
BmHel-11	DF090329:6024702-6024832
BmHel-11	DF090329:6024834-6024932
BmHel-11	DF090394:737576-737752
BmHel-11	DF090326:2860167-2860344
BmHel-11	DF090352:1604589-1604770
BmHel-11	DF090376:1904048-1904227
BmHel-11	DF090355:2432794-2432947
BmHel-11	DF090351:162454-162609
BmHel-11	DF090316:5932192-5932418
BmHel-11	DF090352:1926171-1926329
BmHel-11	DF090352:2254508-2254663
BmHel-11	DF090441:532708-532934
BmHel-11	DF090405:638330-638497
BmHel-11	DF090324:3108847-3109073
BmHel-11	DF090347:568348-568574
BmHel-11	DF090340:236359-236587
BmHel-11	DF090335:2396832-2397058
BmHel-11	DF090367:1689396-1689625
BmHel-11	DF090317:10000582-10000810
BmHel-11	DF090413:140623-140852
BmHel-11	DF090389:197632-197792
BmHel-11	DF090324:353613-353837
BmHel-11	DF090321:4207527-4207752
BmHel-11	DF090372:2076671-2076896
BmHel-11	DF090362:589646-589824
BmHel-11	DF090347:3891115-3891241
BmHel-11	DF090347:3891246-3891344
BmHel-11	DF090323:1143937-1144064
BmHel-11	DF090323:1144069-1144166
BmHel-11	DF090375:534379-534531
BmHel-11	DF090318:3748339-3748471
BmHel-11	DF090318:3748248-3748340
BmHel-11	DF090322:1695325-1695457
BmHel-11	DF090322:1695456-1695548
BmHel-11	DF090397:1243828-1243995
BmHel-11	DF090322:5982203-5982297
BmHel-11	DF090322:5982085-5982168
BmHel-11	DF090401:894594-894772

BmHel-11	DF090337:2199938-2200111
BmHel-11	DF090345:4251436-4251626
BmHel-11	DF090335:4438139-4438365
BmHel-11	DF090415:905910-906087
BmHel-11	DF090450:491701-491847
BmHel-11	DF090360:2050559-2050710
BmHel-11	DF090319:5182499-5182724
BmHel-11	DF090471:8395-8620
BmHel-11	DF090334:2885129-2885346
BmHel-11	DF090366:674780-675009
BmHel-11	DF090461:280478-280707
BmHel-11	DF090753:10971-11196
BmHel-11	DF091949:1-225
BmHel-11	DF090358:365937-366119
BmHel-11	DF090322:652746-652882
BmHel-11	DF090322:652881-652967
BmHel-11	DF090318:1929706-1929843
BmHel-11	DF090318:1929616-1929702
BmHel-11	DF090346:2787506-2787695
BmHel-11	DF091307:3904-4093
BmHel-11	DF090471:143282-143506
BmHel-11	DF090382:1680159-1680383
BmHel-11	DF090382:2037366-2037589
BmHel-11	DF090684:8232-8447
BmHel-11	DF090334:590388-590565
BmHel-11	DF090333:766568-766724
BmHel-11	DF090351:4007258-4007438
BmHel-11	DF090320:7593905-7594080
BmHel-11	BABH01086955:5-181
BmHel-11	BABH01083459:252-428
BmHel-11	DF090319:2436693-2436824
BmHel-11	DF090319:2436601-2436694
BmHel-11	DF090360:3282545-3282680
BmHel-11	DF090360:3282679-3282771
BmHel-11	DF090382:508738-508869
BmHel-11	DF090382:508648-508739
BmHel-11	DF090466:201332-201510
BmHel-11	BABH01045850:2792-2938
BmHel-11	DF090378:384182-384407
BmHel-11	DF090316:16157837-16158016
BmHel-11	DF090352:3589246-3589423
BmHel-11	DF090472:106855-107030
BmHel-11	DF090316:7078673-7078847
BmHel-11	DF090344:2082940-2083114
BmHel-11	DF090362:1118056-1118205
BmHel-11	DF090354:765144-765295
BmHel-11	DF090346:2434208-2434313
BmHel-11	DF090346:2434316-2434434

BmHel-11	DF090387:579019-579251
BmHel-11	BABH01066120:438-661
BmHel-11	DF090440:396387-396572
BmHel-11	DF090322:12753-12871
BmHel-11	DF090322:12874-12978
BmHel-11	DF090323:2679761-2679895
BmHel-11	DF090323:2679898-2679986
BmHel-11	DF090381:1979754-1979938
BmHel-11	DF090411:280063-280195
BmHel-11	DF090411:280197-280287
BmHel-11	DF090349:3545784-3545873
BmHel-11	DF090349:3545648-3545782
BmHel-11	DF090319:4236423-4236608
BmHel-11	DF090321:4949995-4950122
BmHel-11	DF090321:4949897-4949992
BmHel-11	DF090331:2469991-2470218
BmHel-11	DF090330:3710729-3710909
BmHel-11	DF090336:1939837-1940051
BmHel-11	DF090411:487894-488025
BmHel-11	DF090411:488024-488115
BmHel-11	DF090452:298221-298308
BmHel-11	DF090452:298141-298228
BmHel-11	DF090453:305180-305404
BmHel-11	DF090355:1305825-1305955
BmHel-11	DF090355:1305734-1305826
BmHel-11	DF090348:286759-286937
BmHel-11	DF090357:2610344-2610442
BmHel-11	DF090357:2610436-2610527
BmHel-11	DF090334:981243-981403
BmHel-11	DF090370:285899-286029
BmHel-11	DF090370:285808-285900
BmHel-11	DF090396:1220479-1220609
BmHel-11	DF090396:1220388-1220480
BmHel-11	DF090317:2686319-2686502
BmHel-11	DF090346:1246613-1246743
BmHel-11	DF090346:1246522-1246614
BmHel-11	DF090523:37907-38084
BmHel-11	DF090328:2637401-2637490
BmHel-11	DF090328:2637306-2637400
BmHel-11	DF090323:6746819-6746974
BmHel-11	DF090317:731673-731896
BmHel-11	DF090337:1293695-1293919
BmHel-11	DF090432:204410-204547
BmHel-11	DF090432:204546-204630
BmHel-11	DF090340:455332-455469
BmHel-11	DF090340:455475-455559
BmHel-11	DF090366:1152400-1152537
BmHel-11	DF090366:1152533-1152617

BmHel-11	DF090316:2871116-2871286
BmHel-11	DF090326:540050-540263
BmHel-11	DF090318:6045037-6045211
BmHel-11	DF090329:3755949-3756074
BmHel-11	DF090329:3756113-3756208
BmHel-11	DF090323:1057699-1057792
BmHel-11	DF090323:1057794-1057883
BmHel-11	DF090333:3315186-3315343
BmHel-11	DF090349:2032614-2032739
BmHel-11	DF090349:2032744-2032850
BmHel-11	DF090327:3572688-3572811
BmHel-11	DF090327:3572575-3572673
BmHel-11	DF090425:144702-144876
BmHel-11	DF090339:1267399-1267621
BmHel-11	DF090436:392732-392848
BmHel-11	DF090436:392611-392716
BmHel-11	DF090383:1074667-1074850
BmHel-11	DF090329:4426726-4426817
BmHel-11	DF090317:3986108-3986288
BmHel-11	DF090375:2243504-2243667
BmHel-11	DF090420:688505-688657
BmHel-11	DF090365:2974245-2974400
BmHel-11	DF090356:3525971-3526110
BmHel-11	DF090356:3525891-3525973
BmHel-11	DF090339:2878346-2878525
BmHel-11	DF090451:67052-67193
BmHel-11	DF090349:3829189-3829344
BmHel-11	DF090326:5697064-5697287
BmHel-11	DF090393:1420729-1420953
BmHel-11	DF090346:4521120-4521337
BmHel-11	DF090323:7376464-7376592
BmHel-11	DF090323:7376601-7376692
BmHel-11	DF090367:988279-988499
BmHel-11	DF090409:43444-43604
BmHel-11	BABH01048382:703-819
BmHel-11	DF090319:57083-57304
BmHel-11	DF090389:1574698-1574910
BmHel-11	DF091065:1664-1843
BmHel-11	DF090324:7922503-7922646
BmHel-11	DF090317:4589738-4589866
BmHel-11	DF090317:4589865-4589957
BmHel-11	DF090358:410702-410880
BmHel-11	DF090344:4530573-4530796
BmHel-11	DF090423:769046-769160
BmHel-11	DF090423:768919-769025
BmHel-11	DF090348:1836299-1836463
BmHel-11	DF090417:835354-835445
BmHel-11	DF090417:835451-835573

BmHel-11	DF090364:567896-567999
BmHel-11	DF090364:568010-568130
BmHel-11	DF090404:399849-400070
BmHel-11	DF090369:336994-337206
BmHel-11	DF090423:959314-959410
BmHel-11	DF090423:960315-960438
BmHel-11	DF090346:3006260-3006367
BmHel-11	DF090331:1974280-1974500
BmHel-11	DF090329:4448456-4448614
BmHel-11	DF090338:2696785-2696953
BmHel-11	BABH01069132:1-221
BmHel-11	DF090321:550891-551030
BmHel-11	DF090359:3131434-3131654
BmHel-11	DF090395:917544-917709
BmHel-11	DF090345:2777299-2777468
BmHel-11	DF090327:459667-459794
BmHel-11	DF090327:459575-459667
BmHel-11	DF090321:2324999-2325158
BmHel-11	DF090338:1646194-1646333
BmHel-11	DF090338:1646112-1646192
BmHel-11	DF090318:7021959-7022180
BmHel-11	DF090325:1150030-1150133
BmHel-11	DF090377:1454640-1454767
BmHel-11	DF090377:1454549-1454641
BmHel-11	DF090343:2424912-2425132
BmHel-11	DF090339:874875-875097
BmHel-11	DF090336:1401889-1402091
BmHel-11	DF090357:2437200-2437420
BmHel-11	DF090357:1325934-1326154
BmHel-11	BABH01044698:7650-7869
BmHel-11	DF090317:8094866-8095001
BmHel-11	DF090317:8095005-8095088
BmHel-11	DF090325:2959979-2960070
BmHel-11	DF090325:2959855-2959977
BmHel-11	DF090369:1006299-1006431
BmHel-11	DF090369:1006210-1006298
BmHel-11	DF090400:795789-795916
BmHel-11	DF090400:795696-795787
BmHel-11	DF090323:4421360-4421579
BmHel-11	DF090544:10987-11112
BmHel-11	DF090544:10892-10985
BmHel-11	DF090331:4621023-4621169
BmHel-11	DF090419:354245-354375
BmHel-11	DF090419:354376-354464
BmHel-11	DF090341:4110232-4110358
BmHel-11	DF090341:4110140-4110223
BmHel-11	DF090324:1596223-1596372
BmHel-11	DF090457:73369-73588

BmHel-11	DF090428:225661-225880
BmHel-11	DF090366:1059272-1059413
BmHel-11	DF090348:3559717-3559936
BmHel-11	DF090330:6094025-6094244
BmHel-11	DF090497:80570-80789
BmHel-11	DF090333:4861452-4861589
BmHel-11	DF090333:4861367-4861447
BmHel-11	DF090342:4077647-4077864
BmHel-11	DF090374:775011-775103
BmHel-11	DF090374:773665-773790
BmHel-11	DF090319:1107284-1107451
BmHel-11	DF090327:5800263-5800379
BmHel-11	DF090327:5800163-5800264
BmHel-11	DF090358:1585634-1585817
BmHel-11	DF090349:693932-694081
BmHel-11	DF090354:838652-838793
BmHel-11	DF090405:54561-54742
BmHel-11	DF090335:4423078-4423255
BmHel-11	DF090362:269421-269639
BmHel-11	DF090319:7912294-7912512
BmHel-11	DF090467:171928-172148
BmHel-11	DF091104:6340-6559
BmHel-11	DF090452:165479-165601
BmHel-11	DF090461:353300-353399
BmHel-11	DF090461:353156-353298
BmHel-11	DF090316:14869307-14869525
BmHel-11	DF090399:174625-174835
BmHel-11	DF090362:2587393-2587576
BmHel-11	DF090369:1641428-1641579
BmHel-11	DF090425:819471-819633
BmHel-11	BABH01046461:2337-2495
BmHel-11	DF090340:1002744-1002853
BmHel-11	DF090340:1001514-1001593
BmHel-11	DF090316:13867879-13867982
BmHel-11	BABH01049538:588-709
BmHel-11	BABH01049538:426-521
BmHel-11	DF090339:2129431-2129532
BmHel-11	DF090339:2129641-2129724
BmHel-11	DF090330:3366292-3366462
BmHel-11	DF090320:1298138-1298309
BmHel-11	DF090333:5631308-5631525
BmHel-11	DF090390:1086038-1086162
BmHel-11	DF090390:1085941-1086035
BmHel-11	DF090370:176181-176287
BmHel-11	DF090365:2121098-2121205
BmHel-11	DF090336:4637164-4637341
BmHel-11	DF090322:7519616-7519833
BmHel-11	DF090335:558912-559128

BmHel-11	DF090320:5824501-5824718
BmHel-11	DF090364:2528425-2528642
BmHel-11	DF090317:9608388-9608600
BmHel-11	DF090352:3347818-3347948
BmHel-11	DF090352:3347954-3348039
BmHel-11	DF090322:5462116-5462333
BmHel-11	DF090459:34198-34417
BmHel-11	DF090449:247426-247645
BmHel-11	DF090335:1075526-1075650
BmHel-11	DF090335:1075655-1075742
BmHel-11	DF090325:1489345-1489547
BmHel-11	DF090325:1489546-1489688
BmHel-11	DF090351:1169157-1169246
BmHel-11	DF090351:1169035-1169161
BmHel-11	DF090396:524972-525079
BmHel-11	DF090370:2236983-2237164
BmHel-11	DF090367:1512291-1512459
BmHel-11	DF090480:123290-123458
BmHel-11	DF090316:7167206-7167422
BmHel-11	DF090342:3872573-3872787
BmHel-11	DF090381:583023-583162
BmHel-11	DF090368:359983-360197
BmHel-11	DF090349:989457-989616
BmHel-11	DF090391:208595-208693
BmHel-11	DF090375:814344-814480
BmHel-11	DF090324:5679058-5679224
BmHel-11	DF090322:3743267-3743393
BmHel-11	DF090322:3743405-3743495
BmHel-11	DF090343:2414348-2414469
BmHel-11	DF090343:2414489-2414582
BmHel-11	DF090430:579583-579755
BmHel-11	DF090413:844670-844837
BmHel-11	DF090325:3225021-3225187
BmHel-11	DF090519:77813-78034
BmHel-11	BABH01044763:6263-6395
BmHel-11	DF090345:4287978-4288132
BmHel-11	DF090328:175045-175163
BmHel-11	DF090328:175165-175263
BmHel-11	DF090363:1872350-1872567
BmHel-11	DF090432:40920-41135
BmHel-11	DF090352:1565596-1565810
BmHel-11	DF090321:2481857-2481993
BmHel-11	DF090337:2283490-2283638
BmHel-11	DF090354:2334878-2334994
BmHel-11	DF090352:3236820-3236988
BmHel-11	DF091224:3364-3521
BmHel-11	DF090334:5954745-5954851
BmHel-11	DF090329:6189194-6189352

BmHel-11	DF090364:2950703-2950854
BmHel-11	DF090446:389508-389665
BmHel-11	DF090359:290310-290391
BmHel-11	DF090359:290175-290308
BmHel-11	DF090330:4479432-4479558
BmHel-11	DF090330:4479073-4479160
BmHel-11	DF090400:591364-591485
BmHel-11	DF090400:591484-591576
BmHel-11	DF090380:1073509-1073667
BmHel-11	DF090357:2461828-2462003
BmHel-11	DF090322:5511654-5511869
BmHel-11	DF090412:525074-525286
BmHel-11	DF090362:748781-748994
BmHel-11	DF090335:671433-671643
BmHel-11	DF090402:390450-390584
BmHel-11	DF090334:2097446-2097583
BmHel-11	DF090368:2423292-2423452
BmHel-11	DF090329:1536116-1536252
BmHel-11	DF090349:4126323-4126536
BmHel-11	DF090349:4313437-4313612
BmHel-11	DF090419:899590-899737
BmHel-11	DF090365:2683811-2684027
BmHel-11	DF091423:1555-1708
BmHel-11	DF090324:2621137-2621345
BmHel-11	DF090318:7088157-7088370
BmHel-11	DF090350:1943532-1943641
BmHel-11	DF090331:3869087-3869265
BmHel-11	DF090329:4813410-4813544
BmHel-11	DF090459:65541-65724
BmHel-11	DF090334:477812-477921
BmHel-11	DF090338:1302844-1303009
BmHel-11	DF090380:1792385-1792482
BmHel-11	DF090380:1792486-1792600
BmHel-11	DF090390:1630369-1630451
BmHel-11	DF090390:1630222-1630351
BmHel-11	BABH01049165:5-219
BmHel-11	DF090321:2407121-2407275
BmHel-11	DF090316:3495999-3496153
BmHel-11	DF090367:1659599-1659753
BmHel-11	DF090366:2664031-2664187
BmHel-11	DF090376:2174236-2174390
BmHel-11	DF090356:880852-881024
BmHel-11	DF091735:226-392
BmHel-11	DF090482:169691-169903
BmHel-11	DF090382:713507-713720
BmHel-11	DF090333:2270346-2270555
BmHel-11	BABH01049922:271-480
BmHel-11	DF091480:1273-1480

BmHel-11	DF090351:3386841-3386993
BmHel-11	DF090389:8233-8385
BmHel-11	DF090361:2536957-2537109
BmHel-11	DF090431:639834-640048
BmHel-11	DF090354:1777859-1777977
BmHel-11	DF090354:1777989-1778081
BmHel-11	DF090365:2707323-2707537
BmHel-11	DF090344:3863978-3864188
BmHel-11	BABH01058607:504-715
BmHel-11	DF090334:3791845-3791943
BmHel-11	DF090352:794457-794670
BmHel-11	DF090411:1026842-1026927
BmHel-11	DF090411:1026959-1027084
BmHel-11	DF090356:1893442-1893604
BmHel-11	DF090339:2738697-2738858
BmHel-11	DF090342:2004795-2005007
BmHel-11	DF090418:897671-897882
BmHel-11	DF090344:2161606-2161815
BmHel-11	DF090380:1709054-1709265
BmHel-11	DF090390:370649-370800
BmHel-11	DF090386:645694-645845
BmHel-11	DF090318:7519598-7519751
BmHel-11	DF090360:686413-686549
BmHel-11	DF090331:3593625-3593776
BmHel-11	DF090400:273283-273436
BmHel-11	DF090373:2092391-2092542
BmHel-11	DF090330:2790930-2791081
BmHel-11	DF090367:2537317-2537459
BmHel-11	DF090317:1308471-1308616
BmHel-11	DF090438:57874-58019
BmHel-11	DF090435:364667-364818
BmHel-11	DF090401:473359-473511
BmHel-11	DF090480:96943-97094
BmHel-11	DF090354:1994510-1994640
BmHel-11	DF090354:1994650-1994729
BmHel-11	DF090346:517018-517158
BmHel-11	BABH01075499:122-251
BmHel-11	BABH01075499:9-89
BmHel-11	DF090321:7733919-7734038
BmHel-11	DF090408:803499-803593
BmHel-11	DF090408:803384-803499
BmHel-11	DF090335:2305492-2305651
BmHel-11	DF090345:450905-451064
BmHel-11	DF090318:6593544-6593748
BmHel-11	DF090427:747752-747960
BmHel-11	DF090349:3898213-3898424
BmHel-11	DF090419:756120-756324
BmHel-11	DF090353:3166592-3166746

BmHel-11	DF090349:3936337-3936491
BmHel-11	DF090351:1917124-1917333
BmHel-11	DF090317:4082571-4082720
BmHel-11	DF090322:2979603-2979752
BmHel-11	DF090321:2815708-2815858
BmHel-11	DF090358:1439639-1439793
BmHel-11	DF090933:6464-6614
BmHel-11	DF090398:1262780-1262944
BmHel-11	DF090825:3383-3525
BmHel-11	DF090327:4822512-4822684
BmHel-11	DF090394:122209-122376
BmHel-11	DF090430:94841-95053
BmHel-11	DF090321:3457748-3457918
BmHel-11	DF090370:893644-893778
BmHel-11	DF090360:3224033-3224231
BmHel-11	DF090448:456894-457109
BmHel-11	DF090326:3968313-3968464
BmHel-11	DF090353:3474884-3475037
BmHel-11	DF090371:1686626-1686718
BmHel-11	DF090328:146335-146545
BmHel-11	DF090325:6602692-6602822
BmHel-11	DF090325:6602830-6602913
BmHel-11	DF090373:1938293-1938374
BmHel-11	DF090339:670364-670513
BmHel-11	DF090445:438214-438362
BmHel-11	DF090324:7855610-7855759
BmHel-11	DF090332:5544658-5544807
BmHel-11	DF090406:557323-557472
BmHel-11	DF090344:2826446-2826595
BmHel-11	DF090354:1235965-1236114
BmHel-11	DF090385:1392419-1392566
BmHel-11	DF090391:1332229-1332356
BmHel-11	DF090391:1332359-1332439
BmHel-11	DF090391:1519287-1519448
BmHel-11	DF090317:2821461-2821566
BmHel-11	DF090317:2821565-2821669
BmHel-11	DF090422:1002917-1003125
BmHel-11	DF090355:983922-984131
BmHel-11	DF090342:3627027-3627235
BmHel-11	DF090388:317777-317985
BmHel-11	DF090344:2024207-2024415
BmHel-11	DF090317:9705453-9705661
BmHel-11	BABH01045771:1654-1862
BmHel-11	DF090352:3505592-3505746
BmHel-11	DF090323:1594623-1594732
BmHel-11	DF090321:7360339-7360452
BmHel-11	DF090332:6123533-6123680
BmHel-11	DF090347:216774-216980

BmHel-11	DF090340:4563117-4563269
BmHel-11	DF090340:2124158-2124273
BmHel-11	DF090340:2124028-2124119
BmHel-11	DF090358:2278970-2279131
BmHel-11	DF090334:6035604-6035811
BmHel-11	DF090372:817938-818054
BmHel-11	DF090372:818069-818159
BmHel-11	DF090362:201013-201161
BmHel-11	DF090342:1313650-1313798
BmHel-11	DF090360:2700138-2700286
BmHel-11	DF090347:2423339-2423487
BmHel-11	DF090322:7217722-7217870
BmHel-11	DF090316:16171308-16171456
BmHel-11	DF090317:2588710-2588858
BmHel-11	DF090334:3471744-3471849
BmHel-11	DF090334:3471917-3472018
BmHel-11	DF090365:1740742-1740948
BmHel-11	DF090346:2462205-2462321
BmHel-11	DF090346:2462111-2462204
BmHel-11	DF090334:5562860-5563067
BmHel-11	DF090352:3911634-3911843
BmHel-11	BABH01050528:65-272
BmHel-11	DF090317:1323875-1324025
BmHel-11	DF090316:8674170-8674325
BmHel-11	DF090316:13064949-13065160
BmHel-11	DF090316:13549663-13549831
BmHel-11	DF090337:3030321-3030529
BmHel-11	DF090358:1732123-1732327
BmHel-11	BABH01065634:459-664
BmHel-11	DF090330:5350575-5350723
BmHel-11	DF090371:1923690-1923854
BmHel-11	DF090402:275522-275669
BmHel-11	DF090332:3224435-3224582
BmHel-11	DF090319:1729212-1729323
BmHel-11	DF090319:1729066-1729160
BmHel-11	DF090326:5781636-5781783
BmHel-11	DF090322:974948-975095
BmHel-11	DF090321:7780411-7780558
BmHel-11	DF090359:1473889-1474037
BmHel-11	DF090349:2053600-2053747
BmHel-11	DF090351:1872180-1872327
BmHel-11	DF090344:453882-454029
BmHel-11	DF090417:798717-798864
BmHel-11	DF090358:934869-935016
BmHel-11	DF090356:1191130-1191275
BmHel-11	BABH01047784:1328-1534
BmHel-11	BABH01077036:1-208
BmHel-11	DF090358:1706386-1706490

BmHel-11	DF090358:1706591-1706692
BmHel-11	DF090317:5175245-5175405
BmHel-11	DF090409:870169-870337
BmHel-11	DF090377:1681934-1682059
BmHel-11	DF090377:1682062-1682142
BmHel-11	DF090325:450110-450313
BmHel-11	DF090429:217646-217852
BmHel-11	DF090391:197632-197829
BmHel-11	DF090364:1129250-1129456
BmHel-11	DF090317:10188795-10188879
BmHel-11	DF090336:2113454-2113575
BmHel-11	DF090365:2685569-2685774
BmHel-11	BABH01069850:436-586
BmHel-11	DF090369:1358621-1358715
BmHel-11	DF090329:346354-346497
BmHel-11	DF090412:761345-761491
BmHel-11	DF090350:3761008-3761118
BmHel-11	DF090316:1010142-1010261
BmHel-11	DF090316:1010265-1010350
BmHel-11	DF090332:1491232-1491437
BmHel-11	DF090401:809461-809666
BmHel-11	DF090349:2129517-2129663
BmHel-11	DF090340:2217932-2218083
BmHel-11	DF090337:2145924-2146082
BmHel-11	DF090352:892172-892376
BmHel-11	DF090345:2425293-2425447
BmHel-11	DF090327:3342494-3342582
BmHel-11	DF090327:3342595-3342710
BmHel-11	DF090386:1831339-1831488
BmHel-11	DF090375:1992668-1992817
BmHel-11	DF090332:3106584-3106694
BmHel-11	DF090332:3106485-3106578
BmHel-11	DF090450:266881-266963
BmHel-11	DF090450:266684-266804
BmHel-11	DF090384:317487-317599
BmHel-11	DF090326:1741621-1741825
BmHel-11	DF090349:2159915-2160060
BmHel-11	DF090356:3782394-3782549
BmHel-11	DF090488:2913-3013
BmHel-11	DF090488:2809-2912
BmHel-11	DF090400:738018-738140
BmHel-11	DF090400:737931-738018
BmHel-11	DF090355:1139103-1139306
BmHel-11	DF090330:2737648-2737852
BmHel-11	DF090387:239858-239990
BmHel-11	DF090414:1320944-1321095
BmHel-11	DF090340:2350577-2350722
BmHel-11	DF090708:23786-23952

BmHel-11	BABH01042971:2615-2759
BmHel-11	DF090324:5929818-5929927
BmHel-11	DF090324:5929935-5930027
BmHel-11	DF091702:9370-9493
BmHel-11	DF090342:4684568-4684651
BmHel-11	DF090344:2062097-2062242
BmHel-11	DF090336:3142106-3142250
BmHel-11	DF090380:762683-762829
BmHel-11	DF090435:132245-132389
BmHel-11	DF090403:258602-258746
BmHel-11	DF090342:3839677-3839882
BmHel-11	DF090316:3344748-3344869
BmHel-11	DF090316:3345097-3345178
BmHel-11	DF090394:1347112-1347244
BmHel-11	DF090327:2545283-2545374
BmHel-11	BABH01051380:1-200
BmHel-11	DF090323:7687662-7687816
BmHel-11	DF090414:520762-520914
BmHel-11	DF090316:9706339-9706543
BmHel-11	DF090354:1673974-1674176
BmHel-11	DF090418:437805-437899
BmHel-11	DF090418:437898-438007
BmHel-11	DF090333:2190999-2191133
BmHel-11	DF090354:1963463-1963607
BmHel-11	DF090403:1051172-1051321
BmHel-11	DF090372:691741-691907
BmHel-11	DF090346:3405106-3405253
BmHel-11	DF090407:787878-788029
BmHel-11	DF090352:3994796-3994920
BmHel-11	DF090324:1832183-1832384
BmHel-11	DF090320:8306699-8306835
BmHel-11	DF090330:5674991-5675193
BmHel-11	DF090359:1808280-1808431
BmHel-11	DF090330:4587395-4587546
BmHel-11	DF090338:2462434-2462548
BmHel-11	DF090338:2462298-2462384
BmHel-11	DF090318:9005510-9005711
BmHel-11	DF090323:3641838-3641982
BmHel-11	DF090361:1646687-1646829
BmHel-11	DF090437:449011-449154
BmHel-11	BABH01048229:341-542
BmHel-11	DF090444:187706-187853
BmHel-11	DF090407:193777-193978
BmHel-11	BABH01043610:142-344
BmHel-11	DF090384:1220795-1220913
BmHel-11	DF090384:1220702-1220783
BmHel-11	DF090430:491239-491352
BmHel-11	DF090430:492320-492419

BmHel-11	DF090897:3598-3798
BmHel-11	DF090316:1811999-1812197
BmHel-11	DF090378:2088249-2088342
BmHel-11	DF090378:2088126-2088232
BmHel-11	DF090517:14644-14747
BmHel-11	DF090517:14538-14634
BmHel-11	DF090408:457297-457507
BmHel-11	DF090533:56063-56196
BmHel-11	BABH01072433:407-607
BmHel-11	DF090819:11051-11205
BmHel-11	DF090331:2920142-2920283
BmHel-11	DF090611:34608-34741
BmHel-11	DF090341:1385794-1385914
BmHel-11	DF090815:10505-10704
BmHel-11	BABH01066096:462-661
BmHel-11	DF090331:4237069-4237268
BmHel-11	DF090362:194936-195135
BmHel-11	DF090332:3508573-3508660
BmHel-11	DF090399:337218-337415
BmHel-11	DF090384:1295862-1296002
BmHel-11	DF090356:892709-892853
BmHel-11	DF090333:4950568-4950697
BmHel-11	DF090367:669736-669883
BmHel-11	DF090357:192227-192375
BmHel-11	DF090341:2162895-2163116
BmHel-11	DF090374:961406-961557
BmHel-11	DF090349:848761-848959
BmHel-11	DF090318:8809281-8809478
BmHel-11	DF090319:219056-219257
BmHel-11	DF090355:707610-707808
BmHel-11	DF090316:15103056-15103195
BmHel-11	DF090430:126565-126733
BmHel-11	DF090367:967098-967237
BmHel-11	DF090327:6466093-6466297
BmHel-11	DF090345:3295371-3295486
BmHel-11	DF090345:3295280-3295362
BmHel-11	DF090342:3440592-3440699
BmHel-11	DF090342:3440706-3440797
BmHel-11	DF090434:498547-498684
BmHel-11	DF090418:49575-49714
BmHel-11	BABH01062851:467-628
BmHel-11	DF090318:1595920-1596067
BmHel-11	DF090377:91832-91956
BmHel-11	DF090360:1855195-1855299
BmHel-11	DF090360:1855305-1855397
BmHel-11	DF090324:4464365-4464513
BmHel-11	DF090344:1438183-1438332
BmHel-11	DF090318:2661557-2661753

BmHel-11	DF090351:1990877-1991073
BmHel-11	DF090368:1395922-1396119
BmHel-11	BABH01048827:4-201
BmHel-11	BABH01070781:14-213
BmHel-11	DF090356:3515749-3515833
BmHel-11	DF090330:2623782-2623948
BmHel-11	DF090324:3431034-3431230
BmHel-11	DF090364:1111585-1111664
BmHel-11	DF090346:1962447-1962583
BmHel-11	DF090412:104544-104695
BmHel-11	BABH01079008:1-197
BmHel-11	DF090329:3975956-3976050
BmHel-11	DF090317:1330860-1330941
BmHel-11	DF090317:1330404-1330503
BmHel-11	DF090326:3665091-3665210
BmHel-11	DF090358:2332336-2332530
BmHel-11	DF090452:259472-259669
BmHel-11	DF090399:622843-623039
BmHel-11	DF090343:1059872-1060067
BmHel-11	DF090363:1738251-1738445
BmHel-11	DF090351:2354109-2354230
BmHel-11	BABH01086104:1-196
BmHel-11	DF090324:1769897-1770045
BmHel-11	DF090344:3021254-3021457
BmHel-11	DF090377:465616-465728
BmHel-11	DF090377:465512-465594
BmHel-11	BABH01054012:2-197
BmHel-11	DF090334:4314367-4314562
BmHel-11	DF090387:639297-639419
BmHel-11	DF090326:1943402-1943549
BmHel-11	BABH01064969:564-668
BmHel-11	BABH01064969:467-557
BmHel-11	DF090343:1666394-1666539
BmHel-11	BABH01050369:1-196
BmHel-11	DF090332:1591601-1591722
BmHel-11	DF090357:1819015-1819209
BmHel-11	DF090509:20733-20926
BmHel-11	DF090625:20385-20579
BmHel-11	DF090404:1286771-1286896
BmHel-11	DF090390:1340261-1340355
BmHel-11	DF090390:1340354-1340446
BmHel-11	DF090338:765961-766107
BmHel-11	DF090412:1192435-1192582
BmHel-11	BABH01061848:494-690
BmHel-11	DF090326:423539-423731
BmHel-11	DF090326:1807758-1807952
BmHel-11	DF090320:1223910-1224109
BmHel-11	BABH01048361:1-189

BmHel-11	DF090398:498226-498419
BmHel-11	DF091600:2738-2940
BmHel-11	DF090322:894349-894433
BmHel-11	DF090407:107019-107173
BmHel-11	DF090378:2023274-2023430
BmHel-11	DF090418:60885-61034
BmHel-11	DF090531:77110-77238
BmHel-11	DF090363:1710755-1710894
BmHel-11	DF090581:41212-41404
BmHel-11	DF090417:881235-881320
BmHel-11	DF090417:882725-882859
BmHel-11	DF090367:1479305-1479438
BmHel-11	DF090381:1488397-1488590
BmHel-11	DF090365:2030809-2030999
BmHel-11	DF090317:988982-989096
BmHel-11	DF090317:988758-988861
BmHel-11	DF090322:7065467-7065595
BmHel-11	DF090367:753090-753232
BmHel-11	DF090330:217134-217249
BmHel-11	DF090475:36532-36729
BmHel-11	DF090624:18741-18840
BmHel-11	DF090624:18642-18734
BmHel-11	BABH01061154:1-194
BmHel-11	DF091048:1527-1629
BmHel-11	DF090396:511221-511368
BmHel-11	DF090344:3305244-3305392
BmHel-11	DF090387:1779929-1780037
BmHel-11	DF090387:1785759-1786032
BmHel-11	DF090316:3000678-3000869
BmHel-11	DF090326:250321-250404
BmHel-11	DF090326:250178-250288
BmHel-11	DF090602:18824-18972
BmHel-11	DF090316:2276120-2276252
BmHel-11	DF090337:4199422-4199545
BmHel-11	BABH01053426:616-807
BmHel-11	DF090371:1715292-1715416
BmHel-11	DF090320:2237386-2237576
BmHel-11	DF090369:2004465-2004656
BmHel-11	DF090447:406128-406317
BmHel-11	DF090351:3094484-3094674
BmHel-11	DF090398:473784-473923
BmHel-11	DF090400:948062-948258
BmHel-11	DF090321:2485-2621
BmHel-11	DF090322:579317-579498
BmHel-11	DF090326:2228989-2229179
BmHel-11	DF090363:1902896-1903085
BmHel-11	BABH01086602:1-188
BmHel-11	DF090390:1440899-1441089

BmHel-11	DF090407:910050-910241
BmHel-11	DF090330:5524183-5524364
BmHel-11	DF090316:7251049-7251191
BmHel-11	DF090414:862842-863033
BmHel-11	DF090368:1107840-1107992
BmHel-11	BABH01078774:382-571
BmHel-11	DF090318:139770-139957
BmHel-11	DF090335:1957583-1957725
BmHel-11	DF090338:2773020-2773169
BmHel-11	DF090319:2340235-2340424
BmHel-11	DF090316:6613859-6613969
BmHel-11	DF090337:2927898-2928005
BmHel-11	DF090337:2927798-2927878
BmHel-11	BABH01052551:221-418
BmHel-11	DF090327:2120243-2120431
BmHel-11	DF090316:8218093-8218280
BmHel-11	DF090320:5920575-5920764
BmHel-11	DF090352:1716293-1716476
BmHel-11	DF090345:4227163-4227296
BmHel-11	BABH01067428:465-653
BmHel-11	DF090406:1118722-1118851
BmHel-11	DF090360:1551681-1551869
BmHel-11	DF090342:2088683-2088875
BmHel-11	DF090382:57194-57381
BmHel-11	DF090343:85996-86183
BmHel-11	DF090445:420442-420570
BmHel-11	DF090382:1079784-1079869
BmHel-11	DF090334:1965115-1965306
BmHel-11	DF090778:18855-18947
BmHel-11	DF090778:18758-18852
BmHel-11	DF090354:2825282-2825411
BmHel-11	DF090437:417281-417421
BmHel-11	BABH01084921:320-511
BmHel-11	DF090320:2465404-2465583
BmHel-11	DF090326:1458164-1458349
BmHel-11	DF090343:1371973-1372122
BmHel-11	DF090316:13770963-13771116
BmHel-11	DF090381:1616871-1616998
BmHel-11	DF090325:6656476-6656585
BmHel-11	BABH01045457:2096-2282
BmHel-11	DF091046:5758-5944
BmHel-11	DF090334:2381956-2382095
BmHel-11	BABH01045820:3231-3368
BmHel-11	BABH01072235:7-194
BmHel-11	DF090541:32670-32807
BmHel-11	DF090360:274420-274545
BmHel-11	BABH01053264:630-815
BmHel-11	DF090320:230265-230419

BmHel-11	DF090383:138049-138238
BmHel-11	DF090347:3076987-3077173
BmHel-11	DF090389:222106-222289
BmHel-11	DF090419:522165-522351
BmHel-11	DF090322:7881996-7882079
BmHel-11	DF090322:7881895-7881996
BmHel-11	DF090316:16058291-16058382
BmHel-11	DF090316:16058379-16058462
BmHel-11	DF090463:32433-32552
BmHel-11	DF090346:1009833-1009965
BmHel-11	DF090481:145702-145785
BmHel-11	DF090481:145415-145524
BmHel-11	DF090325:3727517-3727701
BmHel-11	DF090344:3449827-3450003
BmHel-11	DF090381:1858454-1858638
BmHel-11	DF090340:4731256-4731442
BmHel-11	DF090329:5136534-5136717
BmHel-11	DF090430:4872-5057
BmHel-11	BABH01059960:521-705
BmHel-11	DF090354:2006462-2006569
BmHel-11	DF090321:1984244-1984428
BmHel-11	DF090368:1122974-1123157
BmHel-11	DF090317:7537379-7537567
BmHel-11	DF090420:75635-75821
BmHel-11	BABH01087381:209-394
BmHel-11	BABH01081226:373-558
BmHel-11	DF090448:427250-427395
BmHel-11	DF090494:42756-42939
BmHel-11	DF090482:126636-126746
BmHel-11	DF090352:2390487-2390670
BmHel-11	DF090317:7090807-7090994
BmHel-11	DF090444:187870-188053
BmHel-11	DF090390:1155736-1155863
BmHel-11	DF090327:983664-983791
BmHel-11	DF090342:438317-438499
BmHel-11	DF090316:1120593-1120776
BmHel-11	DF090364:2638275-2638458
BmHel-11	DF090447:514893-515082
BmHel-11	DF090355:2551427-2551609
BmHel-11	DF090383:2041470-2041655
BmHel-11	DF090355:341188-341371
BmHel-11	DF090335:504605-504745
BmHel-11	DF090329:2623550-2623644
BmHel-11	DF090329:2623460-2623547
BmHel-11	BABH01044374:2256-2352
BmHel-11	BABH01044374:2160-2242
BmHel-11	DF090430:284621-284708
BmHel-11	DF090475:100914-101012

BmHel-11	DF090390:248362-248543
BmHel-11	DF090342:641618-641798
BmHel-11	DF090448:215928-216111
BmHel-11	DF090326:4243741-4243921
BmHel-11	DF090316:12028900-12029034
BmHel-11	DF090367:2723121-2723268
BmHel-11	BABH01046357:2587-2764
BmHel-11	DF090357:2185228-2185409
BmHel-11	BABH01046659:1517-1647
BmHel-11	BABH01072712:65-190
BmHel-11	DF090332:2614502-2614637
BmHel-11	DF090322:3987422-3987601
BmHel-11	DF090408:436730-436910
BmHel-11	DF090325:2670161-2670294
BmHel-11	DF090343:4198680-4198777
BmHel-11	DF090343:4198575-4198657
BmHel-11	DF090327:2343001-2343122
BmHel-11	BABH01081735:362-542
BmHel-11	DF090344:3410105-3410286
BmHel-11	DF090345:3643166-3643288
BmHel-11	DF090369:806968-807084
BmHel-11	DF090364:1409960-1410098
BmHel-11	DF090358:3119305-3119426
BmHel-11	BABH01083854:347-529
BmHel-11	DF090390:1148872-1149054
BmHel-11	BABH01087589:77-181
BmHel-11	DF090355:361169-361349
BmHel-11	DF090488:159417-159592
BmHel-11	DF090335:4863164-4863274
BmHel-11	DF090419:162860-162981
BmHel-11	DF090349:2216633-2216812
BmHel-11	DF090336:3846004-3846183
BmHel-11	DF090420:1126123-1126242
BmHel-11	DF090348:2904127-2904249
BmHel-11	DF090325:900518-900698
BmHel-11	DF090364:323342-323442
BmHel-11	DF090364:323441-323520
BmHel-11	BABH01079940:56-236
BmHel-11	DF090404:131351-131531
BmHel-11	DF090321:5279522-5279667
BmHel-11	BABH01056776:564-739
BmHel-11	DF090380:1165380-1165464
BmHel-11	DF090380:1165516-1165599
BmHel-11	BABH01088250:1-177
BmHel-11	DF090437:427267-427444
BmHel-11	BABH01084876:23-194
BmHel-11	DF090336:836046-836165
BmHel-11	DF090317:2696973-2697106

BmHel-11	DF090560:41518-41637
BmHel-11	DF090624:25505-25630
BmHel-11	DF090328:2171691-2171820
BmHel-11	DF090392:1432167-1432278
BmHel-11	BABH01050269:598-776
BmHel-11	DF090336:5287432-5287616
BmHel-11	DF090352:3803500-3803677
BmHel-11	DF090355:981342-981522
BmHel-11	DF090349:1860401-1860579
BmHel-11	DF090331:2478419-2478596
BmHel-11	DF090317:11041877-11042054
BmHel-11	DF090317:1880288-1880416
BmHel-11	DF090332:3613592-3613766
BmHel-11	DF090336:2068107-2068285
BmHel-11	DF090405:479006-479129
BmHel-11	DF090424:198965-199073
BmHel-11	DF090451:393606-393720
BmHel-11	DF090390:1518342-1518465
BmHel-11	DF090323:7603225-7603312
BmHel-11	DF090354:581971-582088
BmHel-11	BABH01074787:1-179
BmHel-11	BABH01054364:136-309
BmHel-11	BABH01086836:195-369
BmHel-11	BABH01066153:6-182
BmHel-11	DF090329:30043-30158
BmHel-11	DF090361:1554100-1554243
BmHel-11	DF090325:3460126-3460230
BmHel-11	DF090365:937519-937703
BmHel-11	DF090335:5070417-5070543
BmHel-11	DF090329:38768-38860
BmHel-11	DF090329:38856-38938
BmHel-11	DF090732:307-484
BmHel-11	BABH01071220:448-625
BmHel-11	DF090356:2382542-2382663
BmHel-11	DF090329:3384965-3385082
BmHel-11	BABH01088148:1-85
BmHel-11	BABH01088148:91-181
BmHel-11	DF090362:918823-918926
BmHel-11	DF090342:360820-360992
BmHel-11	BABH01070975:127-300
BmHel-11	DF090339:2243234-2243331
BmHel-11	DF090343:3885921-3886095
BmHel-11	BABH01080509:1-125
BmHel-11	DF090390:843112-843223
BmHel-11	DF090359:488118-488224
BmHel-11	DF090356:2191921-2192036
BmHel-11	BABH01059889:96-211
BmHel-11	DF090353:1040069-1040210

BmHel-11	BABH01068796:470-644
BmHel-11	BABH01047332:1602-1779
BmHel-11	DF090320:1342482-1342655
BmHel-11	BABH01047624:1196-1371
BmHel-11	BABH01060364:520-695
BmHel-11	DF090318:5998209-5998337
BmHel-11	DF090422:165958-166133
BmHel-11	DF090317:8777976-8778111
BmHel-11	DF090639:29088-29209
BmHel-11	DF090418:852821-852925
BmHel-11	DF090328:2222273-2222367
BmHel-11	DF090323:7331598-7331771
BmHel-11	DF090342:460383-460486
BmHel-11	BABH01075623:430-531
BmHel-11	DF090320:6483315-6483487
BmHel-11	DF090362:2736377-2736547
BmHel-11	DF090320:3108249-3108423
BmHel-11	DF090352:2709603-2709776
BmHel-11	BABH01079922:1-174
BmHel-11	DF090375:640607-640779
BmHel-11	DF090322:2096297-2096469
BmHel-11	DF090354:2332982-2333106
BmHel-11	DF090434:683496-683616
BmHel-11	BABH01070394:1-174
BmHel-11	DF090316:6035413-6035527
BmHel-11	DF090319:4805157-4805273
BmHel-11	DF090353:2613943-2614061
BmHel-11	DF090488:130514-130686
BmHel-11	DF090320:2501772-2501941
BmHel-11	BABH01046487:310-481
BmHel-11	DF090465:32740-32864
BmHel-11	BABH01078645:409-580
BmHel-11	DF090344:641975-642146
BmHel-11	DF090317:9518390-9518503
BmHel-11	DF090317:9518621-9518897
BmHel-11	DF090355:1615832-1616004
BmHel-11	BABH01055372:114-235
BmHel-11	DF090358:686035-686207
BmHel-11	DF090375:90472-90641
BmHel-11	BABH01051890:699-870
BmHel-11	DF090453:394686-394863
BmHel-11	BABH01048445:939-1108
BmHel-11	DF090317:9370491-9370588
BmHel-11	BABH01065560:484-608
BmHel-11	DF090401:440917-441037
BmHel-11	DF090328:2198793-2198888
BmHel-11	DF090336:4661445-4661607
BmHel-11	DF090321:6871955-6872125

BmHel-11	DF090316:5546641-5546811
BmHel-11	DF090337:4781484-4781654
BmHel-11	DF090329:4685987-4686155
BmHel-11	DF090317:9126282-9126456
BmHel-11	DF090372:1112385-1112555
BmHel-11	DF090346:2982944-2983113
BmHel-11	DF090338:2752299-2752470
BmHel-11	BABH01059398:533-702
BmHel-11	DF090330:1041265-1041416
BmHel-11	DF090379:592995-593164
BmHel-11	DF090324:319058-319180
BmHel-11	DF090319:827899-828023
BmHel-11	DF090329:570074-570243
BmHel-11	DF090364:2311428-2311597
BmHel-11	DF090464:185915-186086
BmHel-11	DF090398:199149-199317
BmHel-11	DF090341:2723630-2723798
BmHel-11	DF090405:1258306-1258393
BmHel-11	DF090415:128491-128600
BmHel-11	DF091354:2129-2296
BmHel-11	BABH01046129:1534-1702
BmHel-11	DF090377:1313218-1313389
BmHel-11	DF090340:4823703-4823823
BmHel-11	DF090371:1813305-1813472
BmHel-11	BABH01064809:502-669
BmHel-11	DF090364:790694-790808
BmHel-11	BABH01061027:1-168
BmHel-11	DF090465:291574-291741
BmHel-11	DF090354:3673005-3673120
BmHel-11	DF090431:117365-117473
BmHel-11	DF090361:1143940-1144050
BmHel-11	DF090370:2481574-2481744
BmHel-11	DF090319:1320220-1320387
BmHel-11	DF090334:519335-519502
BmHel-11	DF090319:7372380-7372502
BmHel-11	DF090374:1539542-1539707
BmHel-11	DF090565:956-1122
BmHel-11	BABH01074580:1-170
BmHel-11	DF090359:1768045-1768133
BmHel-11	DF090353:49279-49445
BmHel-11	DF090322:5727692-5727860
BmHel-11	BABH01081772:397-553
BmHel-11	DF090551:5111-5227
BmHel-11	DF090326:1398554-1398720
BmHel-11	DF090317:575647-575815
BmHel-11	DF090426:162197-162298
BmHel-11	DF090322:4224914-4225032
BmHel-11	DF091147:5548-5666

BmHel-11	DF090320:3153926-3154024
BmHel-11	DF090497:141304-141405
BmHel-11	DF090356:541179-541345
BmHel-11	DF090339:4544831-4544946
BmHel-11	DF090323:4519609-4519768
BmHel-11	DF090362:469818-469955
BmHel-11	DF090346:840965-841135
BmHel-11	DF090353:3080002-3080164
BmHel-11	DF090403:1385317-1385481
BmHel-11	DF090321:5215013-5215127
BmHel-11	DF090338:299895-300009
BmHel-11	DF090439:633920-634084
BmHel-11	BABH01083298:5-169
BmHel-11	DF090356:441048-441163
BmHel-11	DF090381:1155400-1155564
BmHel-11	DF090336:674212-674375
BmHel-11	DF090398:747025-747189
BmHel-11	DF090367:2242066-2242231
BmHel-11	DF090430:608726-608889
BmHel-11	DF091058:5704-5868
BmHel-11	DF090367:1315851-1315964
BmHel-11	DF090317:7184990-7185081
BmHel-11	DF090329:725784-725906
BmHel-11	DF090316:7834912-7835018
BmHel-11	DF090410:656923-657026
BmHel-11	DF090372:905537-905640
BmHel-11	DF090321:4286539-4286651
BmHel-11	DF090466:174021-174184
BmHel-11	DF090353:3419618-3419781
BmHel-11	DF090367:659195-659356
BmHel-11	BABH01088272:138-298
BmHel-11	DF090365:1138932-1139093
BmHel-11	DF090380:426873-426964
BmHel-11	DF090338:4988887-4989049
BmHel-11	BABH01071293:1-162
BmHel-11	DF090720:24444-24599
BmHel-11	DF090372:381600-381712
BmHel-11	DF090951:5929-6083
BmHel-11	DF090403:340554-340663
BmHel-11	DF090418:248714-248805
BmHel-11	BABH01073450:456-616
BmHel-11	BABH01080593:1-162
BmHel-11	DF090326:805552-805708
BmHel-11	DF090374:258636-258795
BmHel-11	BABH01046211:1005-1164
BmHel-11	DF090334:5315216-5315321
BmHel-11	DF090327:1064795-1064954
BmHel-11	DF090327:6165233-6165337

BmHel-11	DF090327:3298552-3298700
BmHel-11	DF090395:954269-954427
BmHel-11	DF090382:1127712-1127872
BmHel-11	DF090330:3863622-3863782
BmHel-11	DF090320:3397990-3398149
BmHel-11	BABH01045716:2817-2928
BmHel-11	DF090456:25456-25564
BmHel-11	DF090340:3993091-3993245
BmHel-11	DF090391:1035159-1035317
BmHel-11	DF090403:322169-322326
BmHel-11	BABH01088613:1-159
BmHel-11	DF090413:457696-457806
BmHel-11	DF090368:1659976-1660082
BmHel-11	DF090317:5190379-5190465
BmHel-11	DF090361:2302908-2302997
BmHel-11	DF090342:1045959-1046116
BmHel-11	DF090320:4378085-4378241
BmHel-11	DF090333:2256243-2256399
BmHel-11	DF090339:2713400-2713556
BmHel-11	DF090327:1694279-1694435
BmHel-11	BABH01058765:19-175
BmHel-11	DF090325:6952141-6952290
BmHel-11	DF090363:83037-83194
BmHel-11	BABH01076461:455-596
BmHel-11	DF090331:4479916-4480014
BmHel-11	DF090522:8994-9153
BmHel-11	DF090752:6132-6221
BmHel-11	DF090359:709774-709863
BmHel-11	DF090340:877603-877758
BmHel-11	DF090363:1637786-1637941
BmHel-11	DF090368:1240370-1240522
BmHel-11	DF090352:4143935-4144088
BmHel-11	DF090317:9841183-9841333
BmHel-11	DF090372:1322028-1322182
BmHel-11	DF090343:4494934-4495027
BmHel-11	BABH01088227:23-177
BmHel-11	DF090319:6677823-6677926
BmHel-11	DF090350:2503884-2504041
BmHel-11	DF090365:2340433-2340587
BmHel-11	DF090415:781553-781696
BmHel-11	DF090349:1517907-1518055
BmHel-11	DF090369:2137132-2137230
BmHel-11	DF090373:676471-676582
BmHel-11	DF090398:95545-95631
BmHel-11	BABH01062514:533-685
BmHel-11	DF090358:3423207-3423358
BmHel-11	DF090377:295478-295631
BmHel-11	DF090422:259597-259752

BmHel-11	DF090370:2277804-2277955
BmHel-11	BABH01081103:1-152
BmHel-11	DF090391:247964-248057
BmHel-11	DF090397:838056-838137
BmHel-11	DF090316:13290330-13290481
BmHel-11	DF090351:416744-416895
BmHel-11	DF090353:2684324-2684476
BmHel-11	DF090354:2579453-2579539
BmHel-11	DF090350:603-705
BmHel-11	BABH01071295:1-153
BmHel-11	DF090316:5997088-5997238
BmHel-11	DF090343:3624844-3624994
BmHel-11	DF090351:405374-405524
BmHel-11	DF090361:213253-213402
BmHel-11	DF090328:2279020-2279119
BmHel-11	DF090362:211281-211380
BmHel-11	DF090388:1161131-1161281
BmHel-11	DF090404:1404872-1405021
BmHel-11	DF090355:130509-130652
BmHel-11	DF090328:2388949-2389097
BmHel-11	DF090340:2974367-2974515
BmHel-11	DF090481:159074-159222
BmHel-11	DF090407:1226489-1226639
BmHel-11	BABH01061412:538-686
BmHel-11	DF090316:11643684-11643832
BmHel-11	DF090408:513642-513790
BmHel-11	BABH01080825:181-329
BmHel-11	DF090336:4936995-4937140
BmHel-11	BABH01066213:509-658
BmHel-11	DF090322:346822-346969
BmHel-11	DF090321:4457369-4457516
BmHel-11	BABH01065519:5-93
BmHel-11	DF090373:1224689-1224837
BmHel-11	DF090420:161572-161719
BmHel-11	DF090369:1105096-1105243
BmHel-11	DF091948:1734-1881
BmHel-11	DF090334:1290581-1290726
BmHel-11	DF090389:1401735-1401881
BmHel-11	DF091357:4540-4685
BmHel-11	BABH01049853:847-992
BmHel-11	DF090378:742335-742430
BmHel-11	BABH01080505:9-152
BmHel-11	BABH01049369:612-757
BmHel-11	BABH01078836:3-146
BmHel-11	DF090326:5024642-5024736
BmHel-11	DF090332:5592802-5592947
BmHel-11	DF090318:8534043-8534186
BmHel-11	BABH01078387:52-196

BmHel-11	DF090388:1675535-1675684
BmHel-11	DF090372:2015897-2016039
BmHel-11	DF090791:13789-13932
BmHel-11	DF090356:2427234-2427377
BmHel-11	DF090353:53146-53283
BmHel-11	DF090396:610218-610360
BmHel-11	DF090332:921936-922077
BmHel-11	DF090319:8347505-8347648
BmHel-11	DF090384:231418-231560
BmHel-11	DF090405:1394581-1394723
BmHel-11	BABH01077393:5-147
BmHel-11	BABH01075367:2-144
BmHel-11	BABH01058295:26-161
BmHel-11	BABH01044627:5972-6113
BmHel-11	DF090559:18529-18670
BmHel-11	BABH01071545:1-141
BmHel-11	DF090319:6244350-6244491
BmHel-11	DF090321:46759-46900
BmHel-11	DF090373:108482-108573
BmHel-11	DF090403:1367859-1367951
BmHel-11	DF090327:5314924-5315015
BmHel-11	DF090393:599187-599275
BmHel-11	DF090385:617532-617673
BmHel-11	DF090394:623923-624064
BmHel-11	DF090406:1417376-1417516
BmHel-11	DF090441:128172-128312
BmHel-11	DF090411:19722-19862
BmHel-11	BABH01076490:456-596
BmHel-11	DF090343:3255032-3255172
BmHel-11	DF090366:452899-453039
BmHel-11	DF090344:227685-227768
BmHel-11	DF090326:1611531-1611670
BmHel-11	DF090334:900679-900819
BmHel-11	DF090351:812493-812582
BmHel-11	BABH01070623:492-631
BmHel-11	DF090323:4049775-4049913
BmHel-11	BABH01076462:364-501
BmHel-11	BABH01071015:1-139
BmHel-11	DF090333:2162772-2162918
BmHel-11	DF090333:2163202-2163342
BmHel-11	DF090363:567916-568054
BmHel-11	DF090367:226262-226400
BmHel-11	DF090485:45833-45971
BmHel-11	DF090322:682840-682929
BmHel-11	DF090366:1313357-1313448
BmHel-11	DF090354:1369521-1369610
BmHel-11	DF090335:1275787-1275925
BmHel-11	DF090348:4077403-4077545

BmHel-11	DF090407:1186726-1186865
BmHel-11	DF090340:4918533-4918671
BmHel-11	DF090370:929102-929241
BmHel-11	DF090835:3258-3356
BmHel-11	DF090351:1472610-1472747
BmHel-11	DF090317:7490803-7490940
BmHel-11	DF090404:197564-197701
BmHel-11	DF090372:1425332-1425469
BmHel-11	DF090345:2352151-2352288
BmHel-11	BABH01052827:63-200
BmHel-11	DF090377:605676-605759
BmHel-11	BABH01067170:516-654
BmHel-11	DF090365:880144-880231
BmHel-11	DF090360:964122-964259
BmHel-11	DF090360:357465-357564
BmHel-11	BABH01068305:3-141
BmHel-11	DF090449:694130-694267
BmHel-11	DF090366:1520056-1520187
BmHel-11	DF090317:10866666-10866802
BmHel-11	BABH01081638:1-138
BmHel-11	DF090357:1375786-1375917
BmHel-11	DF090338:3037062-3037200
BmHel-11	DF090461:381909-382042
BmHel-11	DF090608:57405-57541
BmHel-11	DF090375:221754-221887
BmHel-11	BABH01073323:32-133
BmHel-11	DF090444:21325-21459
BmHel-11	BABH01051629:669-803
BmHel-11	DF091417:2283-2417
BmHel-11	BABH01082243:194-328
BmHel-11	BABH01063199:84-218
BmHel-11	BABH01057448:378-512
BmHel-11	BABH01083444:4-137
BmHel-11	BABH01049258:704-838
BmHel-11	BABH01047512:601-735
BmHel-11	BABH01083707:200-334
BmHel-11	BABH01083605:184-318
BmHel-11	BABH01082088:267-401
BmHel-11	BABH01053990:85-219
BmHel-11	BABH01087185:139-275
BmHel-11	BABH01062252:28-160
BmHel-11	BABH01069660:462-597
BmHel-11	DF090378:1509369-1509503
BmHel-11	BABH01086628:1-100
BmHel-11	BABH01054717:230-364
BmHel-11	DF090861:11733-11867
BmHel-11	DF090396:425111-425244
BmHel-11	DF090355:326646-326779

BmHel-11	DF090319:6426191-6426325
BmHel-11	DF090316:13539061-13539194
BmHel-11	DF090330:2547223-2547359
BmHel-11	DF090393:742622-742708
BmHel-11	DF090317:1256691-1256823
BmHel-11	DF090336:59209-59341
BmHel-11	BABH01072475:491-622
BmHel-11	DF090381:406477-406609
BmHel-11	DF090487:127588-127720
BmHel-11	DF090348:3548801-3548932
BmHel-11	DF090328:1551857-1551988
BmHel-11	DF090357:382430-382561
BmHel-11	DF090323:5509821-5509952
BmHel-11	BABH01072559:2-133
BmHel-11	BABH01049999:20-151
BmHel-11	DF090321:8057237-8057336
BmHel-11	DF090324:5024645-5024776
BmHel-11	BABH01076156:465-598
BmHel-11	DF091685:38-168
BmHel-11	BABH01061025:3-133
BmHel-11	DF090336:4883398-4883528
BmHel-11	BABH01081955:422-551
BmHel-11	BABH01051780:60-152
BmHel-11	DF090322:8212874-8212961
BmHel-11	DF090338:4989120-4989248
BmHel-11	BABH01067435:44-173
BmHel-11	DF090329:627171-627301
BmHel-11	DF090956:7586-7717
BmHel-11	DF091213:1324-1452
BmHel-11	BABH01088124:157-285
BmHel-11	DF090357:214237-214365
BmHel-11	DF090513:92910-93038
BmHel-11	DF090349:52747-52875
BmHel-11	DF090438:37145-37273
BmHel-11	BABH01068660:5-133
BmHel-11	DF090371:337589-337716
BmHel-11	DF091863:3-129
BmHel-11	DF090336:3642039-3642165
BmHel-11	DF090334:4300302-4300429
BmHel-11	DF090331:2528751-2528878
BmHel-11	DF090388:650390-650518
BmHel-11	BABH01046032:1-128
BmHel-11	DF090340:2283222-2283349
BmHel-11	BABH01083421:408-534
BmHel-11	DF090355:1664658-1664784
BmHel-11	BABH01054950:641-766
BmHel-11	BABH01083089:414-539
BmHel-11	DF090351:2568781-2568906

BmHel-11	DF090400:422594-422719
BmHel-11	DF090332:1734274-1734399
BmHel-11	DF090338:1999576-1999701
BmHel-11	DF090357:2022478-2022602
BmHel-11	DF090329:264723-264847
BmHel-11	DF090333:5208963-5209088
BmHel-11	BABH01054901:556-681
BmHel-11	DF090366:2186425-2186549
BmHel-11	DF090353:1541657-1541780
BmHel-11	DF090326:2623862-2623992
BmHel-11	DF090332:104224-104347
BmHel-11	BABH01076645:4-126
BmHel-11	DF090370:2337515-2337638
BmHel-11	BABH01049668:1-124
BmHel-11	BABH01073108:41-164
BmHel-11	DF090316:14038977-14039100
BmHel-11	DF090472:42575-42697
BmHel-11	BABH01048600:1100-1222
BmHel-11	DF090330:2964392-2964521
BmHel-11	DF090342:4766139-4766261
BmHel-11	DF090343:4337086-4337208
BmHel-11	DF090346:4269380-4269498
BmHel-11	DF090424:444152-444273
BmHel-11	DF091755:1701-1822
BmHel-11	DF090349:2515298-2515419
BmHel-11	DF090339:2904707-2904833
BmHel-11	DF090337:2634204-2634325
BmHel-11	DF090412:1021077-1021200
BmHel-11	DF090353:3704867-3704987
BmHel-11	BABH01082244:1-114
BmHel-11	BABH01083771:1-121
BmHel-11	DF090334:5917350-5917467
BmHel-11	DF090320:6846147-6846270
BmHel-11	DF090349:731859-731978
BmHel-11	DF090316:13922815-13922934
BmHel-11	DF090418:379012-379130
BmHel-11	DF090469:74658-74777
BmHel-11	DF090416:465320-465439
BmHel-11	DF090362:1781754-1781841
BmHel-11	DF090326:4257252-4257371
BmHel-11	DF090321:4979940-4980058
BmHel-11	DF090418:704473-704591
BmHel-11	DF090368:1991291-1991409
BmHel-11	DF090331:935684-935802
BmHel-11	DF090358:34037-34155
BmHel-11	BABH01069177:17-135
BmHel-11	BABH01066439:12-130
BmHel-11	BABH01073661:116-231

BmHel-11	DF090318:9550028-9550145
BmHel-11	DF090359:2230651-2230768
BmHel-11	DF090368:1200681-1200797
BmHel-11	BABH01083257:1-118
BmHel-11	BABH01075414:487-603
BmHel-11	DF090352:244054-244170
BmHel-11	DF090374:1684674-1684757
BmHel-11	DF090316:3882358-3882474
BmHel-11	DF090378:1020480-1020596
BmHel-11	DF090317:5637707-5637823
BmHel-11	BABH01079377:410-526
BmHel-11	DF090366:2086775-2086892
BmHel-11	DF090342:546328-546441
BmHel-11	DF090324:306944-307059
BmHel-11	DF090351:690539-690653
BmHel-11	DF091447:8330-8445
BmHel-11	DF090354:796145-796259
BmHel-11	BABH01081531:17-131
BmHel-11	BABH01051023:250-364
BmHel-11	BABH01077394:1-115
BmHel-11	DF090342:619824-619938
BmHel-11	BABH01081019:5-119
BmHel-11	DF090397:1562480-1562594
BmHel-11	DF090331:66012-66134
BmHel-11	DF090322:1586680-1586794
BmHel-11	DF090443:44206-44320
BmHel-11	BABH01054178:680-788
BmHel-11	DF090357:2569411-2569524
BmHel-11	BABH01081232:32-145
BmHel-11	DF090348:4048074-4048184
BmHel-11	DF090320:6517743-6517850
BmHel-11	DF090362:688433-688545
BmHel-11	DF090350:1017008-1017123
BmHel-11	DF090341:3055472-3055583
BmHel-11	DF090465:72483-72594
BmHel-11	BABH01073657:19-130
BmHel-11	DF090376:90793-90904
BmHel-11	DF090349:4159039-4159149
BmHel-11	DF090316:15068405-15068516
BmHel-11	DF090411:221962-222073
BmHel-11	BABH01080112:467-568
BmHel-11	DF090330:456515-456625
BmHel-11	BABH01085912:380-489
BmHel-11	DF090414:1015839-1015949
BmHel-11	DF090952:11421-11531
BmHel-11	DF090319:5464760-5464870
BmHel-11	DF090319:6303984-6304100
BmHel-11	DF090409:1493436-1493542

BmHel-11	BABH01063606:9-115
BmHel-11	DF090346:1713987-1714096
BmHel-11	BABH01061916:578-689
BmHel-11	DF090332:5691236-5691345
BmHel-11	BABH01072711:511-620
BmHel-11	DF090347:1664334-1664442
BmHel-11	BABH01079994:40-148
BmHel-11	BABH01074460:46-154
BmHel-11	BABH01070109:316-424
BmHel-11	BABH01049740:917-1025
BmHel-11	BABH01071077:522-630
BmHel-11	DF090414:649687-649795
BmHel-11	DF090317:8649248-8649355
BmHel-11	DF090320:7676765-7676874
BmHel-11	BABH01056706:359-466
BmHel-11	BABH01052961:450-557
BmHel-11	BABH01074705:461-568
BmHel-11	BABH01078592:1-108
BmHel-11	DF090342:3207260-3207366
BmHel-11	DF090384:1026028-1026135
BmHel-11	BABH01067301:518-624
BmHel-11	DF090343:1662635-1662741
BmHel-11	BABH01087044:1-107
BmHel-11	DF090325:4833241-4833347
BmHel-11	BABH01082124:6-112
BmHel-11	DF090395:316466-316572
BmHel-11	DF090341:2732624-2732725
BmHel-11	DF090414:582237-582342
BmHel-11	DF090383:2104875-2104979
BmHel-11	DF090348:1411428-1411533
BmHel-11	DF090327:6092539-6092644
BmHel-11	DF090864:3285-3386
BmHel-11	DF090317:1142950-1143054
BmHel-11	DF090341:1545022-1545126
BmHel-11	DF090363:2652017-2652120
BmHel-11	DF090338:2415029-2415132
BmHel-11	BABH01076421:7-110
BmHel-11	DF090348:4181955-4182058
BmHel-11	DF090316:3579349-3579451
BmHel-11	DF090384:1953285-1953387
BmHel-11	DF090317:8797999-8798101
BmHel-11	BABH01059807:1-103
BmHel-11	DF090549:37010-37112
BmHel-11	DF090358:3191415-3191517
BmHel-11	BABH01052152:469-570
BmHel-11	DF090327:5528727-5528829
BmHel-11	DF090489:108337-108442
BmHel-11	DF090375:1901381-1901483

BmHel-11	BABH01080668:462-563
BmHel-11	BABH01071802:1-102
BmHel-11	DF090322:5874131-5874238
BmHel-11	DF090364:1305884-1305985
BmHel-11	DF090678:90697-90798
BmHel-11	DF090354:2666743-2666843
BmHel-11	DF090387:286881-286980
BmHel-11	DF090318:932531-932629
BmHel-11	DF090500:115146-115243
BmHel-11	BABH01049111:1014-1112
BmHel-11	DF090321:1637504-1637602
BmHel-11	DF090627:11236-11334
BmHel-11	BABH01081241:11-109
BmHel-11	BABH01075184:1-98
BmHel-11	DF090340:716521-716621
BmHel-11	BABH01049560:16-114
BmHel-11	DF090319:4617551-4617648
BmHel-11	DF090331:1782224-1782321
BmHel-11	BABH01049349:1-98
BmHel-11	DF090390:281024-281120
BmHel-11	BABH01071685:532-627
BmHel-11	DF090334:1182523-1182620
BmHel-11	DF090330:1552546-1552643
BmHel-11	DF090316:1381056-1381154
BmHel-11	BABH01078850:479-573
BmHel-11	DF090514:96421-96517
BmHel-11	DF090366:1734369-1734465
BmHel-11	BABH01059821:1-97
BmHel-11	DF090344:346920-347015
BmHel-11	DF090350:938085-938180
BmHel-11	DF090359:2429286-2429381
BmHel-11	DF090434:463-558
BmHel-11	DF090390:942193-942288
BmHel-11	DF090318:6741177-6741275
BmHel-11	DF090354:3780291-3780385
BmHel-11	BABH01066106:567-661
BmHel-11	DF090329:5926474-5926568
BmHel-11	DF090317:11219952-11220046
BmHel-11	BABH01072878:4-97
BmHel-11	DF090358:1129886-1129979
BmHel-11	DF090347:2948281-2948374
BmHel-11	BABH01078256:10-103
BmHel-11	DF090351:1232705-1232798
BmHel-11	BABH01073488:523-616
BmHel-11	BABH01061454:91-182
BmHel-11	DF090334:5619610-5619702
BmHel-11	DF090356:1036916-1037007
BmHel-11	BABH01080566:1-93

BmHel-11	BABH01086392:1-92
BmHel-11	DF090361:2166760-2166849
BmHel-11	DF090326:3785882-3785973
BmHel-11	DF090316:15672831-15672922
BmHel-11	DF090391:754418-754514
BmHel-11	DF090368:2085926-2086017
BmHel-11	DF090363:764873-764963
BmHel-11	DF090361:1786063-1786153
BmHel-11	DF090344:131513-131603
BmHel-11	DF090346:1806477-1806567
BmHel-11	BABH01052873:737-827
BmHel-11	DF090316:12916685-12916774
BmHel-11	BABH01057899:1-90
BmHel-11	DF090349:161232-161319
BmHel-11	BABH01075235:517-604
BmHel-11	BABH01069714:1-90
BmHel-11	BABH01087706:1-90
BmHel-11	DF090348:4193818-4193907
BmHel-11	BABH01086912:1-90
BmHel-11	BABH01084737:3-91
BmHel-11	DF090342:4189021-4189109
BmHel-11	DF090321:1681806-1681894
BmHel-11	DF090358:2720055-2720142
BmHel-11	DF090339:2407327-2407414
BmHel-11	BABH01080051:482-569
BmHel-11	DF090672:27595-27682
BmHel-11	DF090340:676296-676383
BmHel-11	DF090370:194626-194713
BmHel-11	DF090437:355540-355627
BmHel-11	DF090394:663581-663668
BmHel-11	BABH01073850:1-88
BmHel-11	DF090422:926401-926487
BmHel-11	DF090316:14231330-14231416
BmHel-11	DF090355:3284985-3285074
BmHel-11	DF090322:2169635-2169721
BmHel-11	BABH01056779:654-739
BmHel-11	DF090317:1705749-1705834
BmHel-11	DF090316:1416131-1416218
BmHel-11	DF091737:1516-1601
BmHel-11	DF090344:3706035-3706118
BmHel-11	BABH01081005:1-85
BmHel-11	BABH01070493:550-634
BmHel-11	BABH01053667:717-801
BmHel-11	BABH01055678:673-756
BmHel-11	DF090433:137428-137510
BmHel-11	BABH01088046:250-333
BmHel-11	BABH01061252:577-660
BmHel-11	DF090317:250361-250444

BmHel-11	DF090432:395191-395274
BmHel-11	DF090325:6579242-6579324
BmHel-11	DF090433:59021-59103
BmHel-11	BABH01057521:647-729
BmHel-11	DF090393:135718-135799
BmHel-11	BABH01076140:513-594
BmHel-11	BABH01084982:428-509
BmHel-11	BABH01083926:447-528
BmHel-11	DF090342:142333-142414
BmHel-11	BABH01077390:502-583
BmHel-11	DF090485:159755-159836
BmHel-11	BABH01052603:758-838
BmHel-11	BABH01081447:476-556
BmHel-11	BABH01076774:515-594
BmHel-11	DF090319:7515359-7515441
BmHel-11	BABH01069549:506-585
BmHel-11	BABH01068961:388-467
BmHel-11	DF090378:1194943-1195022
BmHel-11	BABH01071055:551-630
BmHel-11	DF090340:1228451-1228530
BmHel-11	DF090374:2207034-2207113
BmHel-11	DF090363:1344012-1344091
BmHel-11	DF090424:182127-182206
BmHel-11	DF090366:523906-523985
BmHel-12	DF090324:1603365-1603516
BmHel-12	DF090354:3606643-3606808
BmHel-12	DF090330:241499-241687
BmHel-12	DF090365:881616-881805
BmHel-12	DF090372:512137-512329
BmHel-12	DF090319:5539267-5539467
BmHel-12	DF090323:7627583-7627801
BmHel-12	DF090423:650859-651079
BmHel-12	DF090360:306209-306441
BmHel-12	DF090329:4755629-4755863
BmHel-12	DF090318:6900423-6900664
BmHel-12	DF090321:3437802-3438046
BmHel-12	DF090398:594182-594430
BmHel-12	BABH01051435:611-864
BmHel-12	BABH01055118:25-278
BmHel-12	DF090932:9138-9391
BmHel-12	DF090336:919966-920231
BmHel-12	DF090326:2748153-2748419
BmHel-12	DF090338:1379143-1379415
BmHel-12	DF090352:1867892-1868165
BmHel-12	DF090447:112059-112332
BmHel-12	DF090420:624269-624546
BmHel-12	DF090346:2050102-2050382

BmHel-12	DF090340:723463-723746
BmHel-12	DF090362:3210601-3210885
BmHel-12	DF090327:3485279-3485563
BmHel-12	DF090399:1211568-1211852
BmHel-12	DF090389:1035467-1035752
BmHel-12	DF090391:1129879-1130164
BmHel-12	DF090446:47022-47308
BmHel-12	DF090374:885864-886150
BmHel-12	DF090417:827091-827378
BmHel-12	DF090338:4349485-4349773
BmHel-12	DF090337:3381857-3382146
BmHel-12	DF090331:4500687-4500976
BmHel-12	DF090319:35597-35887
BmHel-12	DF090356:2840497-2840787
BmHel-12	DF090335:290888-291180
BmHel-12	DF090341:444484-444776
BmHel-12	DF090408:141194-141486
BmHel-12	DF090384:531898-532191
BmHel-12	DF090364:514879-515172
BmHel-12	DF090411:649775-650069
BmHel-12	DF090363:912390-912685
BmHel-12	DF090342:2059007-2059303
BmHel-12	DF090498:134890-135187
BmHel-12	DF090316:15131895-15132192
BmHel-12	DF090340:3543541-3543839
BmHel-12	DF090326:3957516-3957814
BmHel-12	DF090323:3903561-3903859
BmHel-12	DF090936:7161-7459
BmHel-12	DF090322:1895960-1896258
BmHel-12	DF090322:842517-842815
BmHel-12	DF090351:1434265-1434563
BmHel-12	DF090357:1427978-1428277
BmHel-12	DF090367:1600466-1600765
BmHel-12	DF090341:4733960-4734259
BmHel-12	DF090360:2717751-2718052
BmHel-12	DF090329:4507284-4507588
BmHel-12	DF090317:7761365-7761670
BmHel-12	DF090344:774891-775197
BmHel-12	DF090339:1656787-1657094
BmHel-12	DF090386:1829676-1829985
BmHel-12	BABH01050534:280-589
BmHel-12	DF090327:4583023-4583333
BmHel-12	DF090350:239076-239386
BmHel-12	DF090361:1942861-1943172
BmHel-12	DF090316:999889-1000200
BmHel-12	DF090336:412124-412435
BmHel-12	DF090393:394960-395271
BmHel-12	DF090397:1573688-1573999

BmHel-12	DF090384:708852-709163
BmHel-12	DF090341:1388874-1389186
BmHel-12	DF090322:6902824-6903136
BmHel-12	DF090334:3059344-3059656
BmHel-12	DF090347:210362-210674
BmHel-12	DF090322:7005403-7005716
BmHel-12	DF091472:377-690
BmHel-12	DF090325:1657715-1658028
BmHel-12	DF090364:1454715-1455028
BmHel-12	DF090382:597762-598075
BmHel-12	DF090399:654568-654882
BmHel-12	DF090342:3619630-3619944
BmHel-12	DF090473:164339-164653
BmHel-12	DF090363:2882211-2882525
BmHel-12	DF090345:623103-623418
BmHel-12	DF090321:8134036-8134352
BmHel-12	DF090333:5403873-5404189
BmHel-12	DF090340:3671774-3672090
BmHel-12	DF090370:1460920-1461236
BmHel-12	DF090388:160693-161010
BmHel-12	DF090469:308130-308447
BmHel-12	DF090363:1677288-1677605
BmHel-12	DF090317:2027796-2028114
BmHel-12	DF090437:491600-491918
BmHel-12	DF090468:16498-16816
BmHel-12	DF090332:6085099-6085418
BmHel-12	DF090534:150146-150465
BmHel-12	DF090362:1875974-1876293
BmHel-12	DF090347:2125678-2125997
BmHel-12	DF090339:84338-84658
BmHel-12	DF090397:1478067-1478387
BmHel-12	DF090347:263958-264279
BmHel-12	DF090344:1390659-1390980
BmHel-12	DF090361:5214-5535
BmHel-12	DF090319:3835535-3835856
BmHel-12	DF090347:2193477-2193798
BmHel-12	DF090381:1936148-1936470
BmHel-12	DF090358:1334200-1334522
BmHel-12	DF090336:5593822-5594144
BmHel-12	DF090344:1833048-1833370
BmHel-12	DF090332:901743-902066
BmHel-12	DF090319:5481843-5482166
BmHel-12	DF090320:4679262-4679585
BmHel-12	DF090317:11205877-11206200
BmHel-12	DF090415:209008-209332
BmHel-12	DF090334:3194900-3195224
BmHel-12	DF090365:2317522-2317846
BmHel-12	DF090343:2219184-2219508

BmHel-12	DF090425:680003-680328
BmHel-12	DF090319:4138917-4139242
BmHel-12	DF090321:2543232-2543557
BmHel-12	DF090327:5128980-5129305
BmHel-12	DF090331:227382-227707
BmHel-12	DF090751:2747-3072
BmHel-12	DF090332:3246477-3246802
BmHel-12	DF090404:1173164-1173489
BmHel-12	DF090360:720056-720381
BmHel-12	DF090403:436966-437292
BmHel-12	DF090324:2121628-2121954
BmHel-12	DF090429:209932-210258
BmHel-12	DF090368:1033064-1033390
BmHel-12	DF090316:14140821-14141147
BmHel-12	DF090340:3389229-3389555
BmHel-12	DF090322:3261621-3261947
BmHel-12	DF090318:622378-622704
BmHel-12	DF090414:835532-835858
BmHel-12	DF090409:769966-770292
BmHel-12	DF090380:539718-540044
BmHel-12	DF090316:1638154-1638481
BmHel-12	DF090386:9650-9977
BmHel-12	DF090322:8263058-8263385
BmHel-12	DF090426:942683-943010
BmHel-12	DF090440:507386-507713
BmHel-12	DF090320:8184221-8184548
BmHel-12	DF090353:2030542-2030870
BmHel-12	DF090317:3071557-3071885
BmHel-12	DF090424:672403-672731
BmHel-12	DF090332:3233128-3233456
BmHel-12	DF090385:1182226-1182554
BmHel-12	DF090325:2499499-2499828
BmHel-12	DF090354:3234183-3234512
BmHel-12	DF090388:317425-317754
BmHel-12	DF090348:881645-881974
BmHel-12	DF090383:1774600-1774929
BmHel-12	DF090317:932627-932956
BmHel-12	DF090347:2945380-2945710
BmHel-12	DF090324:484983-485313
BmHel-12	DF090352:3755044-3755374
BmHel-12	DF090322:4882834-4883164
BmHel-12	DF090327:3949517-3949847
BmHel-12	DF090317:991342-991672
BmHel-12	DF090335:413073-413404
BmHel-12	DF090420:795621-795952
BmHel-12	DF090338:2146074-2146405
BmHel-12	DF090332:5169966-5170298
BmHel-12	DF090417:1053583-1053915

BmHel-12	DF090349:1107323-1107655
BmHel-12	DF090341:3834966-3835298
BmHel-12	DF090348:2926645-2926977
BmHel-12	DF090316:2309713-2310045
BmHel-12	DF090332:4335195-4335528
BmHel-12	DF090367:1949161-1949494
BmHel-12	DF090396:748650-748983
BmHel-12	DF090427:199432-199765
BmHel-12	DF090322:3635918-3636251
BmHel-12	DF090346:3089332-3089665
BmHel-12	DF090321:126530-126863
BmHel-12	DF090421:537722-538055
BmHel-12	DF090964:1027-1360
BmHel-12	DF090365:495974-496308
BmHel-12	DF090335:5325343-5325677
BmHel-12	DF090351:1661106-1661440
BmHel-12	DF090318:9446951-9447285
BmHel-12	DF090382:345627-345961
BmHel-12	DF090364:857731-858065
BmHel-12	DF090345:1670275-1670609
BmHel-12	DF090361:1676190-1676525
BmHel-12	DF090389:1315776-1316111
BmHel-12	DF090380:862165-862500
BmHel-12	DF090423:409279-409614
BmHel-12	DF090338:4949230-4949565
BmHel-12	DF090353:1906264-1906600
BmHel-12	DF090438:370469-370805
BmHel-12	DF090371:1734798-1735134
BmHel-12	DF090337:1832649-1832985
BmHel-12	DF090652:1056-1392
BmHel-12	DF090835:6788-7124
BmHel-12	DF090451:383169-383505
BmHel-12	DF090322:1863880-1864217
BmHel-12	DF090319:7827862-7828199
BmHel-12	DF090418:113109-113446
BmHel-12	DF090358:1772872-1773209
BmHel-12	DF090335:3131903-3132240
BmHel-12	DF090324:2025330-2025667
BmHel-12	DF090326:3107997-3108334
BmHel-12	DF090336:169820-170157
BmHel-12	DF090321:1036650-1036987
BmHel-12	DF090345:2716000-2716337
BmHel-12	DF090635:56677-57014
BmHel-12	DF090322:7859445-7859782
BmHel-12	DF090323:5944641-5944979
BmHel-12	DF090329:5063065-5063403
BmHel-12	DF090339:680285-680623
BmHel-12	DF090357:2282527-2282865

BmHel-12	DF090350:2932987-2933325
BmHel-12	DF090386:1111516-1111854
BmHel-12	DF090320:1140961-1141300
BmHel-12	DF090362:662838-663177
BmHel-12	DF090341:1260849-1261188
BmHel-12	DF090326:4686775-4687114
BmHel-12	DF090323:561050-561389
BmHel-12	DF090342:3472911-3473250
BmHel-12	DF090426:214918-215257
BmHel-12	DF090358:2563515-2563855
BmHel-12	DF090355:1008162-1008502
BmHel-12	DF090763:6659-6999
BmHel-12	DF090424:786988-787328
BmHel-12	DF090319:8319139-8319479
BmHel-12	DF090351:3017250-3017590
BmHel-12	DF090389:1261134-1261474
BmHel-12	DF090317:1006665-1007005
BmHel-12	DF090418:522892-523232
BmHel-12	DF090396:494326-494666
BmHel-12	DF090352:4072316-4072657
BmHel-12	DF090322:7948958-7949299
BmHel-12	DF090367:1466083-1466424
BmHel-12	DF090339:3592819-3593160
BmHel-12	DF090342:3738815-3739156
BmHel-12	DF090345:1609970-1610311
BmHel-12	DF090316:3355580-3355922
BmHel-12	DF090323:4132756-4133098
BmHel-12	DF090419:454294-454636
BmHel-12	DF090456:4094-4436
BmHel-12	DF090316:12621559-12621901
BmHel-12	DF090350:3505079-3505421
BmHel-12	DF090358:3261546-3261888
BmHel-12	DF090323:6193886-6194228
BmHel-12	DF090336:3755482-3755824
BmHel-12	DF090323:4667778-4668120
BmHel-12	DF090352:161536-161879
BmHel-12	DF090340:476741-477084
BmHel-12	DF090483:14400-14743
BmHel-12	DF090357:3751014-3751357
BmHel-12	DF090365:2104752-2105095
BmHel-12	DF090320:7035574-7035917
BmHel-12	DF090349:1732292-1732635
BmHel-12	DF090362:58394-58737
BmHel-12	DF090364:1337388-1337731
BmHel-12	DF090447:342322-342665
BmHel-12	DF090579:24767-25110
BmHel-12	DF090392:682473-682816
BmHel-12	DF090394:497527-497870

BmHel-12	DF090425:959229-959572
BmHel-12	DF090332:5841980-5842323
BmHel-12	DF090323:2873483-2873826
BmHel-12	DF090349:3326394-3326737
BmHel-12	DF090329:4659144-4659487
BmHel-12	DF090364:2352001-2352344
BmHel-12	DF090360:669265-669608
BmHel-12	DF090459:45482-45825
BmHel-12	DF090436:346282-346625
BmHel-12	DF090367:1888421-1888764
BmHel-12	DF090318:6288647-6288990
BmHel-12	DF090322:4983403-4983747
BmHel-12	DF090414:1364491-1364835
BmHel-12	DF090317:2819422-2819766
BmHel-12	DF090317:3768267-3768611
BmHel-12	DF090366:441069-441413
BmHel-12	DF090397:1201027-1201371
BmHel-12	DF090337:4576324-4576668
BmHel-12	DF090353:3249799-3250143
BmHel-12	DF090323:6673803-6674147
BmHel-12	DF090318:1326537-1326881
BmHel-12	DF090327:624167-624511
BmHel-12	DF090380:1433658-1434002
BmHel-12	DF090333:792421-792765
BmHel-12	DF090331:2413300-2413644
BmHel-12	DF090345:1532934-1533278
BmHel-12	DF090384:1343983-1344327
BmHel-12	DF090326:6210727-6211071
BmHel-12	DF090361:2049584-2049929
BmHel-12	DF090350:3553927-3554272
BmHel-12	DF090412:387872-388217
BmHel-12	DF090360:1965649-1965994
BmHel-12	DF090378:738236-738581
BmHel-12	DF090384:1475299-1475644
BmHel-12	DF090374:2245775-2246120
BmHel-12	DF090316:6224552-6224897
BmHel-12	DF090367:699850-700195
BmHel-12	DF090336:4216669-4217014
BmHel-12	DF090362:644419-644764
BmHel-12	DF090350:1676076-1676422
BmHel-12	DF090356:2461759-2462105
BmHel-12	DF090327:5060976-5061322
BmHel-12	DF090386:251588-251934
BmHel-12	DF090331:2768364-2768710
BmHel-12	DF090325:2714655-2715001
BmHel-12	DF090319:574225-574571
BmHel-12	DF090320:8093086-8093432
BmHel-12	DF090356:305333-305679

BmHel-12	DF090413:414756-415102
BmHel-12	DF090420:421629-421975
BmHel-12	DF090352:986218-986565
BmHel-12	DF090325:3775856-3776203
BmHel-12	DF090354:3098546-3098893
BmHel-12	DF090349:2240705-2241052
BmHel-12	DF090351:4323-4670
BmHel-12	DF090321:3836495-3836842
BmHel-12	DF090348:19842-20189
BmHel-12	DF090329:6142995-6143342
BmHel-12	DF090363:2404484-2404831
BmHel-12	DF090612:25741-26088
BmHel-12	DF090332:3700503-3700850
BmHel-12	DF090330:4540933-4541280
BmHel-12	DF090330:1814423-1814770
BmHel-12	DF090350:1972906-1973253
BmHel-12	DF090444:428729-429076
BmHel-12	DF090317:8429864-8430211
BmHel-12	DF090372:1280860-1281207
BmHel-12	DF090335:3076278-3076625
BmHel-12	DF090425:470316-470664
BmHel-12	DF090325:6692999-6693347
BmHel-12	DF090335:1410030-1410378
BmHel-12	DF090335:4956237-4956585
BmHel-12	DF090318:7000425-7000773
BmHel-12	DF090374:1558561-1558909
BmHel-12	DF090362:1998079-1998427
BmHel-12	DF090411:1304487-1304835
BmHel-12	DF090317:5701653-5702001
BmHel-12	DF090422:1077724-1078072
BmHel-12	DF090368:1805089-1805437
BmHel-12	DF090331:626797-627145
BmHel-12	DF090459:305811-306159
BmHel-12	DF090486:25104-25452
BmHel-12	DF090330:4300638-4300986
BmHel-12	DF090316:12334994-12335342
BmHel-12	DF090316:7443502-7443850
BmHel-12	DF090324:1014284-1014632
BmHel-12	DF090334:5367626-5367974
BmHel-12	DF090353:3673065-3673413
BmHel-12	DF090429:456609-456957
BmHel-12	DF090356:3085280-3085628
BmHel-12	DF090348:3934165-3934513
BmHel-12	DF090541:19953-20301
BmHel-12	DF090567:22788-23136
BmHel-12	DF090361:346352-346701
BmHel-12	DF090361:2804251-2804600
BmHel-12	DF090448:333781-334130

BmHel-12	DF090340:1687736-1688085
BmHel-12	DF090358:744616-744965
BmHel-12	DF090332:179874-180223
BmHel-12	DF090320:7766040-7766389
BmHel-12	DF090367:389539-389888
BmHel-12	DF090370:562600-562949
BmHel-12	DF090318:3963896-3964245
BmHel-12	DF090501:27629-27978
BmHel-12	DF090362:1891341-1891690
BmHel-12	DF090432:806443-806792
BmHel-12	DF090363:116775-117124
BmHel-12	DF090400:671421-671770
BmHel-12	DF090342:4419391-4419740
BmHel-12	DF090352:3627321-3627670
BmHel-12	DF090319:6730026-6730375
BmHel-12	DF090332:4892888-4893237
BmHel-12	DF090332:774512-774861
BmHel-12	DF090349:3557936-3558285
BmHel-12	DF090389:1178447-1178796
BmHel-12	DF090328:6257315-6257664
BmHel-12	DF090321:334715-335064
BmHel-12	DF090359:1964457-1964806
BmHel-12	DF090372:632918-633267
BmHel-12	DF090462:219659-220008
BmHel-12	DF090665:19238-19587
BmHel-12	DF090403:496955-497305
BmHel-12	DF090319:2463174-2463524
BmHel-12	DF090357:2985688-2986038
BmHel-12	DF090335:3436079-3436429
BmHel-12	DF090324:6366652-6367002
BmHel-12	DF090334:1328565-1328915
BmHel-12	DF090323:4462556-4462906
BmHel-12	DF090336:2568715-2569065
BmHel-12	DF090362:1495300-1495650
BmHel-12	DF090355:1578503-1578853
BmHel-12	DF090554:58999-59349
BmHel-12	DF090458:135359-135709
BmHel-12	DF090322:6174378-6174728
BmHel-12	DF090322:1640080-1640430
BmHel-12	DF090357:1866193-1866543
BmHel-12	DF090350:210077-210427
BmHel-12	DF090334:3357761-3358111
BmHel-12	DF090353:1464270-1464620
BmHel-12	DF090343:3600664-3601014
BmHel-12	DF090327:4813699-4814049
BmHel-12	DF090414:882525-882875
BmHel-12	DF090360:2220570-2220920
BmHel-12	DF090472:105549-105899

BmHel-12	BABH01055199:37-387
BmHel-12	DF090349:2635569-2635919
BmHel-12	DF090342:3004836-3005186
BmHel-12	DF090383:1891584-1891934
BmHel-12	DF090352:2450935-2451286
BmHel-12	DF090316:14308421-14308772
BmHel-12	DF090354:2909849-2910200
BmHel-12	DF090334:1878923-1879274
BmHel-12	DF090367:2470772-2471123
BmHel-12	DF090326:4772480-4772831
BmHel-12	DF090349:234243-234594
BmHel-12	DF090343:2559316-2559667
BmHel-12	DF090343:4496010-4496361
BmHel-12	DF090389:905476-905827
BmHel-12	DF090328:2856042-2856393
BmHel-12	DF090366:596791-597142
BmHel-12	DF090393:129375-129726
BmHel-12	DF090409:1378371-1378722
BmHel-12	DF090428:759988-760339
BmHel-12	DF090358:2739064-2739415
BmHel-12	DF090358:2048814-2049165
BmHel-12	DF090332:5662266-5662617
BmHel-12	DF090324:2473353-2473704
BmHel-12	DF090324:1277973-1278324
BmHel-12	DF090323:427034-427385
BmHel-12	DF090343:371200-371551
BmHel-12	DF090328:473103-473454
BmHel-12	DF090387:1054506-1054857
BmHel-12	DF090406:775002-775353
BmHel-12	DF090338:1702161-1702512
BmHel-12	DF090384:189275-189626
BmHel-12	DF090428:591907-592258
BmHel-12	DF090340:2888973-2889325
BmHel-12	DF090322:247166-247518
BmHel-12	DF090319:5165391-5165743
BmHel-12	DF090316:5680822-5681174
BmHel-12	DF090350:2328055-2328407
BmHel-12	DF090334:6067165-6067517
BmHel-12	DF090390:720237-720589
BmHel-12	DF090346:1604345-1604697
BmHel-12	DF090318:4066512-4066864
BmHel-12	DF090318:6103572-6103924
BmHel-12	BABH01056065:42-394
BmHel-12	DF090414:232264-232616
BmHel-12	DF090386:292362-292714
BmHel-12	DF090317:9865512-9865864
BmHel-12	DF090616:1563-1915
BmHel-12	DF090336:414556-414908

BmHel-12	DF090319:5206080-5206432
BmHel-12	DF090316:4533312-4533664
BmHel-12	DF090316:3504439-3504791
BmHel-12	DF090354:2922336-2922688
BmHel-12	DF090324:5055510-5055862
BmHel-12	DF090323:5010957-5011309
BmHel-12	DF090370:2013399-2013751
BmHel-12	DF090349:787017-787369
BmHel-12	DF090336:3632351-3632703
BmHel-12	DF090336:701339-701691
BmHel-12	DF090321:6631999-6632351
BmHel-12	DF090362:2326745-2327097
BmHel-12	DF090388:464091-464443
BmHel-12	DF090344:2140792-2141144
BmHel-12	DF090460:205977-206329
BmHel-12	DF090431:576860-577212
BmHel-12	DF090415:235541-235893
BmHel-12	DF090319:182276-182628
BmHel-12	DF090361:402718-403071
BmHel-12	DF090425:530563-530916
BmHel-12	DF090352:1655682-1656035
BmHel-12	DF090352:3454335-3454688
BmHel-12	DF090325:720817-721170
BmHel-12	DF090325:6857727-6858080
BmHel-12	DF090322:4087605-4087958
BmHel-12	DF090316:10080724-10081077
BmHel-12	DF090357:810867-811220
BmHel-12	DF090320:2428190-2428543
BmHel-12	DF090320:5766151-5766504
BmHel-12	DF090349:674484-674837
BmHel-12	DF090336:3313235-3313588
BmHel-12	DF090389:1787200-1787553
BmHel-12	DF090342:2380496-2380849
BmHel-12	DF090318:3521392-3521745
BmHel-12	DF090321:6756069-6756422
BmHel-12	DF090744:2282-2635
BmHel-12	DF090415:722501-722854
BmHel-12	DF090348:121718-122071
BmHel-12	DF090344:3964236-3964589
BmHel-12	DF090452:144695-145048
BmHel-12	DF090407:488658-489011
BmHel-12	DF090480:54408-54761
BmHel-12	DF090399:114698-115051
BmHel-12	DF090430:381451-381804
BmHel-12	BABH01045341:3795-4148
BmHel-12	DF090585:36044-36397
BmHel-12	BABH01080204:138-491
BmHel-12	DF090322:6620653-6621006

BmHel-12	DF090319:7289332-7289685
BmHel-12	DF090324:226435-226788
BmHel-12	DF090323:7323684-7324037
BmHel-12	DF090349:2720131-2720484
BmHel-12	DF090349:2094771-2095124
BmHel-12	DF090351:4091536-4091889
BmHel-12	DF090318:3929788-3930141
BmHel-12	DF090395:185913-186266
BmHel-12	DF090321:3837420-3837773
BmHel-12	DF090327:2165318-2165671
BmHel-12	DF090341:715973-716326
BmHel-12	DF090410:988962-989315
BmHel-12	DF090405:1216227-1216580
BmHel-12	DF090329:5140158-5140511
BmHel-12	DF090407:1143512-1143865
BmHel-12	DF090384:814670-815023
BmHel-12	DF090401:466189-466542
BmHel-12	DF090403:1106987-1107341
BmHel-12	DF090325:4677459-4677813
BmHel-12	DF090322:393918-394272
BmHel-12	DF090350:1465908-1466262
BmHel-12	DF090350:1562714-1563068
BmHel-12	DF090351:2917210-2917564
BmHel-12	DF090328:1737341-1737695
BmHel-12	DF090321:7739844-7740198
BmHel-12	DF090388:1807252-1807606
BmHel-12	DF090347:628544-628898
BmHel-12	DF090331:1200332-1200686
BmHel-12	DF090447:4017-4371
BmHel-12	DF090441:396853-397207
BmHel-12	DF090396:64483-64837
BmHel-12	DF091221:4234-4588
BmHel-12	DF090367:1289950-1290304
BmHel-12	DF090425:116385-116739
BmHel-12	DF090340:436140-436494
BmHel-12	DF090322:126780-127134
BmHel-12	DF090319:934056-934410
BmHel-12	DF090319:520024-520378
BmHel-12	DF090483:6494-6848
BmHel-12	DF090323:1446211-1446565
BmHel-12	DF090370:2491457-2491811
BmHel-12	DF090343:1754917-1755271
BmHel-12	DF090347:1008646-1009000
BmHel-12	DF090890:7303-7657
BmHel-12	DF090345:2461455-2461809
BmHel-12	DF090443:265717-266072
BmHel-12	DF090334:5902093-5902448
BmHel-12	DF090320:5499666-5500021

BmHel-12	DF090323:7505642-7505997
BmHel-12	DF090318:266223-266578
BmHel-12	DF090318:952730-953085
BmHel-12	DF090318:6332484-6332839
BmHel-12	DF090375:2356759-2357114
BmHel-12	DF090355:2076294-2076649
BmHel-12	DF090369:455829-456184
BmHel-12	DF090366:622768-623123
BmHel-12	DF090385:1031815-1032170
BmHel-12	DF090401:1255835-1256190
BmHel-12	DF090363:2301867-2302222
BmHel-12	DF090381:47398-47753
BmHel-12	DF090519:61566-61921
BmHel-12	DF090433:688972-689327
BmHel-12	DF090332:1528861-1529216
BmHel-12	DF090321:318696-319051
BmHel-12	DF090325:6686103-6686458
BmHel-12	DF090322:5414664-5415019
BmHel-12	DF090357:418621-418976
BmHel-12	DF090358:1592238-1592593
BmHel-12	DF090358:367357-367712
BmHel-12	DF090320:2683117-2683472
BmHel-12	DF090417:1091398-1091753
BmHel-12	DF090343:1970406-1970761
BmHel-12	DF090318:9253619-9253974
BmHel-12	DF090374:2139606-2139961
BmHel-12	DF090362:1808736-1809091
BmHel-12	DF090398:1481569-1481924
BmHel-12	DF090368:1404885-1405240
BmHel-12	DF090344:2068815-2069170
BmHel-12	DF090578:7439-7794
BmHel-12	DF090400:676024-676379
BmHel-12	DF090342:1412855-1413210
BmHel-12	DF090361:3015258-3015614
BmHel-12	DF090330:4614579-4614935
BmHel-12	DF090325:209293-209649
BmHel-12	DF090324:5854521-5854877
BmHel-12	DF090320:5347613-5347969
BmHel-12	DF090367:1766511-1766867
BmHel-12	DF090346:857164-857520
BmHel-12	DF090349:4144828-4145184
BmHel-12	DF090328:1067482-1067838
BmHel-12	DF090362:1412460-1412816
BmHel-12	DF090348:1553881-1554237
BmHel-12	DF090383:833475-833831
BmHel-12	DF090377:1327455-1327811
BmHel-12	DF090909:11312-11668
BmHel-12	DF090400:938608-938964

BmHel-12	DF090431:39069-39425
BmHel-12	DF090438:612471-612827
BmHel-12	DF090388:572315-572671
BmHel-12	DF090352:2461865-2462221
BmHel-12	DF090352:105067-105423
BmHel-12	DF090340:395758-396114
BmHel-12	DF090322:6198720-6199076
BmHel-12	DF090357:2714784-2715140
BmHel-12	DF090335:3794280-3794636
BmHel-12	DF090324:4006272-4006628
BmHel-12	DF090353:242175-242531
BmHel-12	DF090326:1543237-1543593
BmHel-12	DF090336:4317152-4317508
BmHel-12	DF090328:2652754-2653110
BmHel-12	DF090395:1210090-1210446
BmHel-12	DF090395:495160-495516
BmHel-12	DF090344:4308829-4309185
BmHel-12	DF090613:68673-69029
BmHel-12	DF090418:891937-892293
BmHel-12	DF090361:2300214-2300571
BmHel-12	DF090352:179505-179862
BmHel-12	DF090322:1400019-1400376
BmHel-12	DF090319:405269-405626
BmHel-12	DF090319:4209082-4209439
BmHel-12	DF090319:8442152-8442509
BmHel-12	DF090390:48810-49167
BmHel-12	DF090326:6926430-6926787
BmHel-12	DF090370:2014707-2015064
BmHel-12	DF090356:2803348-2803705
BmHel-12	DF090317:10674294-10674651
BmHel-12	DF090376:1423026-1423383
BmHel-12	DF090359:3193696-3194053
BmHel-12	DF090460:349338-349695
BmHel-12	DF090345:1230542-1230899
BmHel-12	DF090421:971577-971934
BmHel-12	DF090337:2595789-2596146
BmHel-12	DF090346:2993043-2993400
BmHel-12	DF090322:8166831-8167188
BmHel-12	DF090316:12494873-12495230
BmHel-12	DF090390:410722-411079
BmHel-12	DF090349:3217165-3217522
BmHel-12	DF090369:702559-702916
BmHel-12	DF090388:829102-829459
BmHel-12	DF090359:2202771-2203128
BmHel-12	DF090385:987930-988287
BmHel-12	DF090605:1657-2014
BmHel-12	DF090441:610659-611016
BmHel-12	DF090361:2865067-2865424

BmHel-12	DF090330:1665975-1666333
BmHel-12	DF090403:1500918-1501276
BmHel-12	DF090322:3119546-3119904
BmHel-12	DF090316:14026199-14026557
BmHel-12	DF090357:1366099-1366457
BmHel-12	DF090335:4348950-4349308
BmHel-12	DF090444:370991-371349
BmHel-12	DF090346:1156135-1156493
BmHel-12	DF090356:1289063-1289421
BmHel-12	DF090328:3237784-3238142
BmHel-12	DF090375:2090092-2090450
BmHel-12	DF090388:104222-104580
BmHel-12	DF090341:4247809-4248167
BmHel-12	DF090405:308652-309010
BmHel-12	DF090333:5218-5576
BmHel-12	DF090339:3501874-3502232
BmHel-12	DF090428:602715-603073
BmHel-12	DF090337:1243356-1243714
BmHel-12	DF090378:343048-343406
BmHel-12	DF090351:2761723-2762081
BmHel-12	DF090316:5395191-5395549
BmHel-12	DF090358:82457-82815
BmHel-12	DF090332:1217279-1217637
BmHel-12	DF090353:3072985-3073343
BmHel-12	DF090326:2012863-2013221
BmHel-12	DF090343:1095012-1095370
BmHel-12	DF090342:360252-360610
BmHel-12	DF090327:4043392-4043750
BmHel-12	DF090422:333329-333687
BmHel-12	DF090348:1214439-1214797
BmHel-12	DF090347:4302641-4302999
BmHel-12	DF090333:3540108-3540466
BmHel-12	DF090392:1251393-1251751
BmHel-12	DF090440:608798-609156
BmHel-12	DF090325:5314914-5315273
BmHel-12	DF090322:7298073-7298432
BmHel-12	DF090322:7688100-7688459
BmHel-12	DF090350:415186-415545
BmHel-12	DF090365:794514-794873
BmHel-12	DF090320:4478652-4479011
BmHel-12	DF090320:6495563-6495922
BmHel-12	DF090370:692188-692547
BmHel-12	DF090373:415853-416212
BmHel-12	DF090328:2205337-2205696
BmHel-12	DF090321:5242995-5243354
BmHel-12	DF090344:877222-877581
BmHel-12	DF090393:1247310-1247669
BmHel-12	DF090420:27294-27653

BmHel-12	DF090331:3729989-3730348
BmHel-12	DF090372:1225276-1225635
BmHel-12	DF090352:1052720-1053079
BmHel-12	DF090319:7445848-7446207
BmHel-12	DF090316:11628857-11629216
BmHel-12	DF090316:6940117-6940476
BmHel-12	DF090346:2270651-2271010
BmHel-12	DF090326:2639247-2639606
BmHel-12	DF090326:1525317-1525676
BmHel-12	DF090373:512180-512539
BmHel-12	DF090362:923906-924265
BmHel-12	DF090376:1454354-1454713
BmHel-12	DF090372:1201243-1201602
BmHel-12	DF090437:696415-696774
BmHel-12	DF090395:1006668-1007027
BmHel-12	DF090394:1455088-1455448
BmHel-12	DF090324:1240955-1241315
BmHel-12	DF090323:3443454-3443814
BmHel-12	DF090343:596125-596485
BmHel-12	DF090336:3124417-3124777
BmHel-12	DF090374:1261389-1261749
BmHel-12	DF090327:3946616-3946976
BmHel-12	DF090398:1089726-1090086
BmHel-12	DF090377:1516362-1516722
BmHel-12	DF090332:1354386-1354746
BmHel-12	DF090334:3090387-3090747
BmHel-12	DF090334:1985438-1985798
BmHel-12	DF090336:1466717-1467077
BmHel-12	DF090355:1251153-1251513
BmHel-12	DF090439:192405-192765
BmHel-12	DF090364:2766511-2766871
BmHel-12	DF090339:1604985-1605345
BmHel-12	DF090345:1379584-1379944
BmHel-12	DF090430:201938-202298
BmHel-12	DF090424:490584-490944
BmHel-12	DF090361:2648008-2648369
BmHel-12	DF090340:1954098-1954459
BmHel-12	DF090322:4500036-4500397
BmHel-12	DF090322:6960680-6961041
BmHel-12	DF090316:2757706-2758067
BmHel-12	DF090316:8605751-8606112
BmHel-12	DF090316:11678074-11678435
BmHel-12	DF090335:5171781-5172142
BmHel-12	DF090353:1531306-1531667
BmHel-12	DF090349:3226110-3226471
BmHel-12	DF090318:5734118-5734479
BmHel-12	DF090317:9496389-9496750
BmHel-12	DF090422:307704-308065

BmHel-12	DF090329:3450950-3451311
BmHel-12	DF090329:4904183-4904544
BmHel-12	DF090339:86635-86996
BmHel-12	DF090556:52225-52586
BmHel-12	DF090377:1172612-1172973
BmHel-12	DF090382:2100186-2100547
BmHel-12	DF090416:1199946-1200307
BmHel-12	DF090316:7544524-7544885
BmHel-12	DF090357:2947022-2947383
BmHel-12	DF090358:928914-929275
BmHel-12	DF090320:369913-370274
BmHel-12	DF090326:1716139-1716500
BmHel-12	DF090351:893472-893833
BmHel-12	DF090321:3905733-3906094
BmHel-12	DF090355:3146811-3147172
BmHel-12	DF090327:5395000-5395361
BmHel-12	DF090327:2353282-2353643
BmHel-12	DF090376:118313-118674
BmHel-12	DF090333:1940522-1940883
BmHel-12	DF090412:136217-136578
BmHel-12	DF090548:734-1095
BmHel-12	DF090429:113382-113744
BmHel-12	DF090356:1304866-1305228
BmHel-12	DF090318:3345198-3345560
BmHel-12	DF090317:9104916-9105278
BmHel-12	DF090439:584763-585125
BmHel-12	DF090344:4029147-4029509
BmHel-12	DF090357:3805267-3805629
BmHel-12	DF090358:3251009-3251371
BmHel-12	DF090335:5780395-5780757
BmHel-12	DF090324:2641557-2641919
BmHel-12	DF090334:812066-812428
BmHel-12	DF090320:3806298-3806660
BmHel-12	DF090353:3690356-3690718
BmHel-12	DF090373:942316-942678
BmHel-12	DF090321:289038-289400
BmHel-12	DF090422:800655-801017
BmHel-12	DF090366:176622-176984
BmHel-12	DF090348:3764604-3764966
BmHel-12	DF090347:1133633-1133995
BmHel-12	DF091119:4560-4922
BmHel-12	BABH01044605:6315-6677
BmHel-12	DF090325:7097488-7097851
BmHel-12	DF090316:14624703-14625066
BmHel-12	DF090483:176507-176870
BmHel-12	DF090323:6941768-6942131
BmHel-12	DF090349:3037750-3038113
BmHel-12	DF090328:224387-224750

BmHel-12	DF090380:2119988-2120351
BmHel-12	DF090366:97530-97893
BmHel-12	DF090397:385309-385672
BmHel-12	DF090472:40934-41297
BmHel-12	DF091007:8307-8670
BmHel-12	DF090357:2182079-2182442
BmHel-12	DF090335:2856234-2856597
BmHel-12	DF090334:1509398-1509761
BmHel-12	DF090326:5355607-5355970
BmHel-12	DF090323:2204553-2204916
BmHel-12	DF090351:2126967-2127330
BmHel-12	DF090318:94923-95286
BmHel-12	DF090438:342005-342368
BmHel-12	DF090317:4544090-4544453
BmHel-12	DF090366:651404-651767
BmHel-12	DF090447:461223-461586
BmHel-12	BABH01060125:261-624
BmHel-12	DF090323:365275-365639
BmHel-12	DF090343:4351632-4351996
BmHel-12	DF090356:2442420-2442784
BmHel-12	DF090318:7192850-7193214
BmHel-12	DF090375:205595-205959
BmHel-12	DF090317:8803394-8803758
BmHel-12	DF090415:807044-807408
BmHel-12	DF090408:1126971-1127335
BmHel-12	DF090368:624078-624442
BmHel-12	DF090392:212317-212681
BmHel-12	DF090320:1287896-1288260
BmHel-12	DF090349:2666616-2666980
BmHel-12	DF090321:6961872-6962236
BmHel-12	DF090317:3548821-3549185
BmHel-12	DF090344:3438031-3438395
BmHel-12	DF090407:724992-725356
BmHel-12	DF090400:752048-752412
BmHel-12	DF090338:3407183-3407547
BmHel-12	DF090434:703324-703688
BmHel-12	DF090384:388169-388533
BmHel-12	DF090335:4494204-4494568
BmHel-12	DF090341:801552-801916
BmHel-12	DF090361:218306-218671
BmHel-12	DF090335:2341839-2342204
BmHel-12	DF090324:6675282-6675647
BmHel-12	DF090327:611511-611876
BmHel-12	DF090411:139764-140129
BmHel-12	DF090348:435184-435549
BmHel-12	DF090348:1684662-1685027
BmHel-12	DF090347:3293163-3293528
BmHel-12	DF090405:870398-870763

BmHel-12	DF090340:1612875-1613240
BmHel-12	DF090354:3162178-3162543
BmHel-12	DF090318:8568552-8568917
BmHel-12	DF090321:2473667-2474032
BmHel-12	DF090321:1482857-1483222
BmHel-12	DF090329:6543489-6543854
BmHel-12	DF090344:1896512-1896877
BmHel-12	DF090344:453356-453721
BmHel-12	DF090345:3268718-3269083
BmHel-12	DF090391:668288-668653
BmHel-12	DF090396:1211026-1211391
BmHel-12	DF090352:3076510-3076876
BmHel-12	DF090340:1806901-1807267
BmHel-12	DF090316:430364-430730
BmHel-12	DF090332:989964-990330
BmHel-12	DF090332:5282818-5283184
BmHel-12	DF090367:1316845-1317211
BmHel-12	DF090342:1831770-1832136
BmHel-12	DF090372:1011898-1012264
BmHel-12	DF090345:1000914-1001280
BmHel-12	DF090400:437774-438140
BmHel-12	DF090711:15775-16141
BmHel-12	DF090728:12925-13291
BmHel-12	DF090358:2791405-2791771
BmHel-12	DF090358:788424-788790
BmHel-12	DF090356:2812086-2812452
BmHel-12	DF090328:2445750-2446116
BmHel-12	DF090375:119491-119857
BmHel-12	DF090327:4768913-4769279
BmHel-12	DF090462:189866-190232
BmHel-12	DF090362:3197090-3197457
BmHel-12	DF090355:912147-912514
BmHel-12	DF090317:4001323-4001690
BmHel-12	DF090363:375862-376229
BmHel-12	DF090384:607782-608149
BmHel-12	DF090387:1860804-1861171
BmHel-12	DF090368:731096-731463
BmHel-12	DF090319:7355202-7355569
BmHel-12	DF090429:207110-207477
BmHel-12	DF090318:3062781-3063148
BmHel-12	DF090345:874665-875032
BmHel-12	DF090378:835697-836064
BmHel-12	DF090379:1625286-1625653
BmHel-12	DF090344:3980398-3980765
BmHel-12	DF090319:7981723-7982091
BmHel-12	DF090320:6845635-6846003
BmHel-12	DF090339:3966426-3966794
BmHel-12	DF090378:1644377-1644745

BmHel-12	DF090494:9168-9536
BmHel-12	DF090327:1310067-1310435
BmHel-12	DF090418:424173-424541
BmHel-12	DF090339:4256426-4256794
BmHel-12	DF090378:1961255-1961623
BmHel-12	DF090320:74977-75345
BmHel-12	DF090317:9939649-9940018
BmHel-12	DF090405:626075-626444
BmHel-12	DF090463:314642-315011
BmHel-12	DF090363:1899951-1900320
BmHel-12	DF090420:879444-879813
BmHel-12	DF090551:7141-7510
BmHel-12	DF090316:14358697-14359066
BmHel-12	DF090321:1242255-1242624
BmHel-12	DF090387:1837341-1837710
BmHel-12	DF090387:1803523-1803892
BmHel-12	DF090329:2356104-2356473
BmHel-12	DF090339:615145-615514
BmHel-12	DF090340:2153876-2154245
BmHel-12	DF090319:4114185-4114555
BmHel-12	DF090375:2171188-2171558
BmHel-12	DF090361:134296-134666
BmHel-12	DF090354:2965163-2965533
BmHel-12	DF090326:2685008-2685378
BmHel-12	DF090318:5002994-5003364
BmHel-12	DF090359:2776550-2776920
BmHel-12	DF090321:1768298-1768669
BmHel-12	DF090317:1455287-1455658
BmHel-12	DF090412:231113-231484
BmHel-12	BABH01054118:110-481
BmHel-12	DF090330:4567103-4567474
BmHel-12	DF090323:2946687-2947058
BmHel-12	DF090418:579776-580147
BmHel-12	DF090377:2116515-2116886
BmHel-12	DF090431:70675-71046
BmHel-12	DF090525:68853-69224
BmHel-12	DF090369:1850865-1851237
BmHel-12	DF090320:3339712-3340084
BmHel-12	DF090357:619276-619648
BmHel-12	DF090324:5374997-5375369
BmHel-12	DF090353:3232440-3232812
BmHel-12	DF090344:1383649-1384021
BmHel-12	DF090396:487630-488003
BmHel-12	DF090436:283743-284116
BmHel-12	DF090423:109837-110210
BmHel-12	DF090930:7198-7571
BmHel-12	DF090374:2231035-2231408
BmHel-12	DF090336:3271668-3272041

BmHel-12	DF090353:3867010-3867384
BmHel-12	DF090370:1009521-1009895
BmHel-12	DF090336:4810524-4810898
BmHel-12	DF090321:4924369-4924743
BmHel-12	DF090379:725276-725650
BmHel-12	DF090321:2725550-2725924
BmHel-12	DF090372:643860-644234
BmHel-12	DF090328:1156615-1156990
BmHel-12	DF090362:1846309-1846684
BmHel-12	DF090321:7684846-7685221
BmHel-12	DF090415:614570-614945
BmHel-12	DF090446:86922-87298
BmHel-12	DF090495:3891-4267
BmHel-12	DF090325:3873611-3873987
BmHel-12	DF090318:3309772-3310148
BmHel-12	DF090356:3611488-3611865
BmHel-12	DF090380:1813092-1813469
BmHel-12	DF090348:3962807-3963184
BmHel-12	DF090318:8753693-8754070
BmHel-12	DF090380:561698-562075
BmHel-12	DF090452:143366-143743
BmHel-12	DF090316:8234860-8235238
BmHel-12	DF090433:356307-356685
BmHel-12	DF090332:1895756-1896134
BmHel-12	DF090458:133220-133598
BmHel-12	DF090324:5108529-5108908
BmHel-12	DF090318:7765534-7765913
BmHel-12	DF090331:5118992-5119371
BmHel-12	DF090340:3240421-3240800
BmHel-12	DF090326:5802840-5803219
BmHel-12	DF090321:508915-509295
BmHel-12	DF090330:2748684-2749064
BmHel-12	DF090324:7410527-7410907
BmHel-12	DF090407:1529403-1529783
BmHel-12	DF090950:6238-6618
BmHel-12	DF090349:1174346-1174727
BmHel-12	DF090328:3601729-3602110
BmHel-12	DF090324:1470677-1471058
BmHel-12	DF090368:496778-497159
BmHel-12	DF090338:1665738-1666120
BmHel-12	DF090338:3791522-3791904
BmHel-12	DF090316:12876670-12877053
BmHel-12	DF090316:5356395-5356779
BmHel-12	DF090364:497191-497576
BmHel-12	DF090324:552634-553021
BmHel-12	DF090515:203489-203876
BmHel-12	DF090349:1788918-1789307
BmHel-12	DF090316:1700249-1700641

BmHel-12	DF090356:54082-54474
BmHel-12	DF090378:420374-420766
BmHel-12	DF090350:3573678-3574074
BmHel-12	DF090686:18351-18749
BmHel-12	DF090320:5518336-5518735
BmHel-12	DF090339:560653-561052
BmHel-12	DF090336:692067-692467
BmHel-12	DF091108:6704-7104
BmHel-12	DF090325:3562839-3563240
BmHel-12	DF090327:3070954-3071357
BmHel-12	DF090431:274296-274700
BmHel-12	DF090317:8993659-8994064
BmHel-12	DF090320:4829049-4829455
BmHel-12	DF090328:633128-633538
BmHel-12	DF090372:1251656-1252069
BmHel-12	DF091019:3904-4317
BmHel-12	DF090352:1275198-1275614
BmHel-12	DF090338:844183-844599
BmHel-12	DF091641:533-952
BmHel-12	DF090427:280726-281147
BmHel-12	DF090319:6371615-6372042
BmHel-12	DF090385:120602-121034
BmHel-12	DF090439:220903-221344
BmHel-12	DF090395:856275-856723
BmHel-12	DF090398:70781-71235
BmHel-12	DF090324:4463243-4463715
BmHel-12	DF090434:28369-28845
BmHel-12	DF090370:335948-336424
BmHel-12	DF090322:7562011-7562499
BmHel-12	DF090365:2621456-2621945
BmHel-12	DF090379:262017-262519
BmHel-12	DF090316:16021457-16021960
BmHel-12	DF090528:12517-13026
BmHel-12	DF090347:2551280-2551813
BmHel-12	DF090400:1563324-1563861
BmHel-12	DF090344:3533294-3533837
BmHel-12	DF090371:1136215-1136760
BmHel-12	DF090353:3956251-3956798
BmHel-12	DF090338:676106-676653
BmHel-12	DF090564:44852-45401
BmHel-12	DF090401:1216329-1216879
BmHel-12	DF090411:219446-219997
BmHel-12	DF090333:4967817-4968372
BmHel-12	DF090344:916499-917069
BmHel-12	DF090334:5867866-5868438
BmHel-12	DF090326:6519318-6519894
BmHel-12	DF090398:1262687-1263270
BmHel-12	DF090400:1549349-1549935

BmHel-12	DF090337:2145812-2146408
BmHel-12	DF090319:3421409-3422006
BmHel-12	DF090361:2576825-2577425
BmHel-12	DF090365:233250-233857
BmHel-12	DF090316:4960407-4961019
BmHel-12	DF090342:3809076-3809690
BmHel-12	DF090346:468241-468856
BmHel-12	DF090328:1358247-1358864
BmHel-12	DF090380:568014-568634
BmHel-12	DF090329:5121916-5122536
BmHel-12	DF090396:1306047-1306668
BmHel-12	DF090372:1422278-1422899
BmHel-12	DF090431:775658-776282
BmHel-12	DF090347:1155258-1155887
BmHel-12	DF090375:1016771-1017401
BmHel-12	DF090323:2795291-2795925
BmHel-12	DF090364:357382-358027
BmHel-12	DF090405:802864-803513
BmHel-12	DF090520:156454-157109
BmHel-12	DF090319:7408763-7409419
BmHel-12	DF090392:1348352-1349013
BmHel-12	DF090462:145042-145725
BmHel-12	DF090369:1400543-1401240
BmHel-12	DF090353:3441178-3441882
BmHel-12	DF090427:523443-524171
BmHel-12	DF090411:436941-437674
BmHel-12	DF090386:121713-122452
BmHel-12	DF090369:1702413-1703162
BmHel-12	DF090352:1310755-1311520
BmHel-12	DF090332:1117068-1117836
BmHel-12	DF090319:5590664-5591436
BmHel-12	DF090346:2273398-2274179
BmHel-12	DF090683:25851-26642
BmHel-12	DF090351:3529933-3530732
BmHel-12	DF090345:2158068-2158871
BmHel-12	DF090444:172689-173499
BmHel-12	DF090359:1782439-1783255
BmHel-12	DF090599:42144-42962
BmHel-12	DF090644:87313-88139
BmHel-12	DF090316:7698585-7699428
BmHel-12	DF090420:980313-981167
BmHel-12	DF090388:1557630-1558489
BmHel-12	DF090317:7069422-7070306
BmHel-12	DF090401:1413899-1414833
BmHel-12	DF090331:2581933-2582875
BmHel-12	DF090342:756620-757587
BmHel-12	DF091821:975-1960
BmHel-12	DF090338:68607-69598

BmHel-12	DF090349:3376547-3377540
BmHel-12	DF090360:2370022-2371017
BmHel-12	DF090368:464230-465234
BmHel-12	DF090324:4240763-4241777
BmHel-12	DF090400:1469057-1470099
BmHel-12	DF090316:6034495-6035658
BmHel-12	DF090319:4587154-4588321
BmHel-12	DF090340:2086602-2087771
BmHel-12	DF090429:754359-755551
BmHel-12	DF090340:2165778-2166978
BmHel-12	DF090360:2604357-2605571
BmHel-12	DF090373:2156419-2157636
BmHel-12	DF090694:15808-17118
BmHel-12	DF090322:5411157-5412538
BmHel-12	DF090320:3605896-3607307
BmHel-12	DF090408:486903-488330
BmHel-12	DF090316:10127448-10128900
BmHel-12	DF090357:3131153-3132607
BmHel-12	DF090321:1086830-1088291
BmHel-12	DF090354:370113-371579
BmHel-12	DF090335:58768-60236
BmHel-12	DF090342:189372-190860
BmHel-12	DF090476:117698-119202
BmHel-12	DF090319:8022823-8024349
BmHel-12	DF090316:12994620-12996148
BmHel-12	DF090329:285268-286798
BmHel-12	DF090362:505054-506585
BmHel-12	DF090369:967530-969097
BmHel-12	DF090358:2064316-2065903
BmHel-12	DF090543:71292-72880
BmHel-12	DF090367:2380439-2382043
BmHel-12	DF090573:13113-14721
BmHel-12	DF090336:1883157-1884852
BmHel-12	DF090363:164145-165882
BmHel-12	DF090412:249340-251082
BmHel-12	DF090325:230403-232174
BmHel-12	DF090425:992639-994433
BmHel-12	DF090358:223749-225593
BmHel-12	DF090431:663520-665399
BmHel-12	DF090388:302841-304721
BmHel-12	DF090324:4462017-4463930
BmHel-12	DF090325:5648676-5650651
BmHel-12	DF090391:139484-141491
BmHel-12	DF090352:193590-195689
BmHel-12	DF090428:693073-695307
BmHel-12	DF090370:2327018-2329397
BmHel-12	DF090351:2493194-2495588
BmHel-12	DF090353:1694932-1697502

BmHel-12	DF090330:6364681-6367306
BmHel-12	DF090320:7088343-7091049
BmHel-12	DF090393:807619-810378
BmHel-12	DF090328:405745-408964
BmHel-12	DF090357:2186875-2190227
BmHel-12	DF090418:382323-386077
BmHel-12	DF090411:600977-604796
BmHel-12	DF090443:545445-549466
BmHel-12	DF090467:262991-267126
BmHel-12	DF090382:1408114-1412266
BmHel-12	DF090335:4632381-4636595
BmHel-12	DF090330:3795068-3799557
BmHel-12	DF090350:3282247-3286882
BmHel-12	DF090320:3783756-3788431
BmHel-12	DF090332:1967227-1971913
BmHel-12	DF090363:2888039-2892870
BmHel-12	DF090347:101200-106077
BmHel-12	DF090481:203319-208346
BmHel-12	DF090329:328060-333508
BmHel-12	DF090322:7816005-7821602
BmHel-12	DF090349:3304411-3310116
BmHel-12	DF090319:5737697-5743437
BmHel-12	DF090316:7492878-7498843
BmHel-12	DF090324:4635503-4641607
BmHel-12	DF090403:833730-839849
BmHel-12	DF090366:145031-151552
BmHel-12	DF090363:1552928-1559978
BmHel-12	DF090350:2499342-2506416
BmHel-12	DF090374:1162206-1169662
BmHel-12	DF090433:387110-395107
BmHel-12	DF090329:2739628-2747675
BmHel-12	DF090358:1697561-1705658
BmHel-12	DF090442:424638-432862
BmHel-12	DF090382:1929646-1938360
BmHel-12	DF090395:620827-629745
BmHel-12	DF090327:2594432-2603550
BmHel-12	DF090327:5496383-5505946
BmHel-12	DF090349:1990668-2000232
BmHel-12	DF090318:9088405-9099027
BmHel-12	DF090361:2464219-2464560
BmHel-12	DF090325:5063474-5063827
BmHel-12	DF090340:1046607-1046960
BmHel-12	DF090340:108380-108748
BmHel-12	DF090319:2699627-2699970
BmHel-12	DF090357:947968-948322
BmHel-12	DF090358:218963-219306
BmHel-12	DF090367:1364531-1364882
BmHel-12	DF090346:213893-214243

BmHel-12	DF090323:1648450-1648806
BmHel-12	DF090389:1027342-1027693
BmHel-12	DF090321:3982173-3982530
BmHel-12	BABH01048599:421-782
BmHel-12	DF090473:361411-361772
BmHel-12	DF090355:1550337-1550688
BmHel-12	DF090317:9775628-9775987
BmHel-12	DF090369:1998330-1998692
BmHel-12	DF090347:3590688-3591033
BmHel-12	DF090347:3680380-3680740
BmHel-12	DF090368:1602950-1603305
BmHel-12	DF090344:3035540-3035887
BmHel-12	DF090363:885821-886189
BmHel-12	DF090435:656971-657322
BmHel-12	DF090767:16932-17297
BmHel-12	DF090377:2054597-2054944
BmHel-12	DF090372:1816623-1816966
BmHel-12	DF090382:1607378-1607738
BmHel-12	DF090338:1957644-1958005
BmHel-12	BABH01050642:560-925
BmHel-12	DF090335:880772-881134
BmHel-12	DF090351:1104715-1105060
BmHel-12	DF090351:4249216-4249570
BmHel-12	DF090318:628803-629157
BmHel-12	DF090433:604912-605266
BmHel-12	DF090317:5933316-5933662
BmHel-12	DF090380:1898931-1899277
BmHel-12	DF090363:2694109-2694461
BmHel-12	DF090331:2762682-2763031
BmHel-12	DF091141:667-1026
BmHel-12	DF090472:41397-41745
BmHel-12	DF090391:870810-871151
BmHel-12	DF090468:111889-112241
BmHel-12	DF090369:1271583-1271937
BmHel-12	DF090340:2876167-2876522
BmHel-12	DF090324:7508882-7509232
BmHel-12	DF090320:2435751-2436097
BmHel-12	DF090467:198953-199309
BmHel-12	DF090411:1031766-1032118
BmHel-12	DF090386:616153-616507
BmHel-12	DF090366:986378-986726
BmHel-12	DF090344:965161-965517
BmHel-12	DF090363:1078666-1079020
BmHel-12	DF090437:5307-5650
BmHel-12	DF090361:1579426-1579775
BmHel-12	DF090319:2194372-2194714
BmHel-12	DF090316:12852339-12852691
BmHel-12	DF090316:11492204-11492554

BmHel-12	DF090357:1726073-1726430
BmHel-12	DF090350:2713154-2713508
BmHel-12	DF090335:5655700-5656052
BmHel-12	DF090335:2451513-2451866
BmHel-12	DF090324:701209-701558
BmHel-12	DF090320:6519208-6519548
BmHel-12	DF090390:182844-183194
BmHel-12	DF090367:1908801-1909146
BmHel-12	DF090370:582708-583057
BmHel-12	DF090429:652989-653342
BmHel-12	DF090336:246614-246968
BmHel-12	DF090351:1213061-1213418
BmHel-12	DF090328:5067826-5068173
BmHel-12	DF090328:5702376-5702723
BmHel-12	DF090328:3164926-3165281
BmHel-12	DF090318:2692469-2692820
BmHel-12	DF090374:1814484-1814839
BmHel-12	DF090327:2824632-2824988
BmHel-12	DF090386:726489-726845
BmHel-12	DF090386:1144383-1144736
BmHel-12	DF090432:78330-78683
BmHel-12	DF090329:1516677-1517031
BmHel-12	DF090368:594325-594679
BmHel-12	DF090359:2956816-2957155
BmHel-12	DF090344:4186100-4186453
BmHel-12	DF090344:4180969-4181317
BmHel-12	DF090588:30615-30958
BmHel-12	DF090333:5707362-5707711
BmHel-12	DF090420:71710-72061
BmHel-12	DF090437:594778-595118
BmHel-12	DF090532:54835-55190
BmHel-12	DF090377:1313547-1313890
BmHel-12	DF090428:389245-389599
BmHel-12	DF090391:1204563-1204913
BmHel-12	DF090419:91456-91800
BmHel-12	BABH01044459:6694-7050
BmHel-12	DF090858:8830-9195
BmHel-12	DF090343:3825019-3825299
BmHel-12	DF090411:293587-293862
BmHel-12	DF090349:423220-423575
BmHel-12	DF090322:5955086-5955431
BmHel-12	DF090319:2129309-2129667
BmHel-12	DF090332:3516705-3517066
BmHel-12	DF090417:735214-735565
BmHel-12	DF090417:287979-288328
BmHel-12	DF090576:10920-11269
BmHel-12	DF090356:1113402-1113747
BmHel-12	DF090369:1663996-1664345

BmHel-12	DF090341:1010546-1010904
BmHel-12	DF090366:2413152-2413507
BmHel-12	DF090348:982036-982395
BmHel-12	DF090359:3055550-3055906
BmHel-12	DF090331:3706487-3706845
BmHel-12	DF090391:1315143-1315473
BmHel-12	DF090463:3182-3533
BmHel-12	DF090330:4988516-4988865
BmHel-12	DF090319:6154303-6154652
BmHel-12	DF090358:861190-861539
BmHel-12	DF090323:1527906-1528258
BmHel-12	DF090343:4421821-4422157
BmHel-12	DF090356:543411-543764
BmHel-12	DF090374:1863103-1863453
BmHel-12	DF090374:1922707-1923064
BmHel-12	DF090327:6132216-6132558
BmHel-12	DF090369:2123773-2124129
BmHel-12	DF090398:959313-959666
BmHel-12	DF090347:1481393-1481748
BmHel-12	DF090401:1322828-1323184
BmHel-12	DF090331:1893177-1893532
BmHel-12	DF090331:3129399-3129739
BmHel-12	DF090360:652762-653132
BmHel-12	DF090413:793878-794223
BmHel-12	DF090356:2938381-2938483
BmHel-12	DF090356:2937879-2938139
BmHel-12	DF090350:2153753-2154102
BmHel-12	DF090410:128371-128730
BmHel-12	DF090363:301840-302198
BmHel-12	DF090335:1916782-1917127
BmHel-12	DF090353:2144743-2145086
BmHel-12	DF090342:4895336-4895420
BmHel-12	DF090342:4895022-4895294
BmHel-12	DF090318:6135410-6135755
BmHel-12	DF090386:671964-672295
BmHel-12	DF090405:280272-280628
BmHel-12	DF090319:716819-717167
BmHel-12	DF090316:2898468-2898810
BmHel-12	DF090334:5333051-5333393
BmHel-12	DF090328:2775523-2775875
BmHel-12	DF090329:732611-732961
BmHel-12	DF090601:20696-21042
BmHel-12	DF090406:1008732-1009079
BmHel-12	DF090382:50052-50415
BmHel-12	DF090608:50631-50984
BmHel-12	DF090416:665208-665560
BmHel-12	DF090358:1731771-1732121
BmHel-12	BABH01054631:36-394

BmHel-12	DF090327:2693899-2694246
BmHel-12	DF090333:3042514-3042868
BmHel-12	DF090340:1414377-1414732
BmHel-12	DF090362:1004208-1004561
BmHel-12	DF090327:6650279-6650638
BmHel-12	DF090317:6348987-6349347
BmHel-12	DF090796:15980-16317
BmHel-12	DF090329:5221750-5222093
BmHel-12	DF090391:1228583-1228667
BmHel-12	DF090391:1225402-1225675
BmHel-12	DF090360:2528568-2528835
BmHel-12	DF090330:4672195-4672545
BmHel-12	DF090316:15393293-15393637
BmHel-12	DF090365:2303025-2303368
BmHel-12	DF090335:3497661-3498020
BmHel-12	DF090351:474905-475263
BmHel-12	DF090375:789793-790126
BmHel-12	DF090387:1776927-1777281
BmHel-12	DF090326:1348697-1349043
BmHel-12	DF090360:1162903-1163247
BmHel-12	DF090360:3024162-3024493
BmHel-12	DF090418:150924-151266
BmHel-12	DF090421:1016388-1016746
BmHel-12	DF090928:6165-6522
BmHel-12	DF090356:2273513-2273816
BmHel-12	DF090355:2321905-2322159
BmHel-12	DF090355:2322153-2322250
BmHel-12	DF090413:240945-241240
BmHel-12	DF090330:4597959-4598300
BmHel-12	DF090352:2949913-2950256
BmHel-12	DF090340:2931955-2932313
BmHel-12	DF090332:3476675-3477026
BmHel-12	DF090338:1119473-1119824
BmHel-12	DF090322:6252320-6252664
BmHel-12	DF090357:1616755-1617105
BmHel-12	DF090526:64302-64651
BmHel-12	DF090444:101363-101700
BmHel-12	DF090323:7076957-7077308
BmHel-12	DF090348:381983-382337
BmHel-12	DF090368:1682582-1682935
BmHel-12	BABH01047873:1142-1491
BmHel-12	DF090359:317770-318130
BmHel-12	DF090363:2250069-2250421
BmHel-12	DF090472:124081-124437
BmHel-12	DF090459:93033-93377
BmHel-12	DF090378:2120560-2120865
BmHel-12	DF090372:2297481-2297777
BmHel-12	DF090319:6182925-6183265

BmHel-12	DF090341:4554205-4554546
BmHel-12	DF090330:2228673-2228887
BmHel-12	DF090330:2228333-2228460
BmHel-12	DF090413:861843-862086
BmHel-12	DF090413:861753-861848
BmHel-12	DF090352:3783999-3784344
BmHel-12	DF090390:1022629-1022950
BmHel-12	DF090435:317201-317454
BmHel-12	DF090435:317111-317207
BmHel-12	DF090328:1732641-1732727
BmHel-12	DF090328:1732496-1732626
BmHel-12	DF090328:1732354-1732481
BmHel-12	DF090514:40210-40482
BmHel-12	DF090357:1863238-1863588
BmHel-12	DF090354:1442532-1442810
BmHel-12	DF090365:2731273-2731612
BmHel-12	DF090356:1481534-1481882
BmHel-12	DF090473:215287-215637
BmHel-12	DF090380:705855-706196
BmHel-12	DF090329:5346274-5346610
BmHel-12	DF090404:1062578-1062921
BmHel-12	DF090437:515312-515662
BmHel-12	DF090371:229675-230021
BmHel-12	DF090413:742378-742725
BmHel-12	DF090606:44946-45299
BmHel-12	DF090352:1107175-1107507
BmHel-12	DF090325:6715103-6715434
BmHel-12	DF090346:4014486-4014833
BmHel-12	DF090352:1884577-1884879
BmHel-12	DF090328:167106-167258
BmHel-12	DF090328:167458-167638
BmHel-12	DF090373:430446-430802
BmHel-12	DF090356:1531981-1532329
BmHel-12	BABH01044692:4027-4375
BmHel-12	DF090376:2213497-2213720
BmHel-12	DF090376:2213376-2213498
BmHel-12	DF090324:2980763-2980886
BmHel-12	DF090324:2980528-2980747
BmHel-12	DF090380:320662-321000
BmHel-12	DF090398:41383-41662
BmHel-12	DF090405:419976-420318
BmHel-12	DF090317:3136888-3137238
BmHel-12	DF090344:4660238-4660557
BmHel-12	BABH01048147:692-823
BmHel-12	BABH01048147:585-674
BmHel-12	BABH01048147:424-543
BmHel-12	DF090378:1177587-1177933
BmHel-12	DF090350:3017715-3018046

BmHel-12	DF090332:6027912-6028193
BmHel-12	DF090326:5372351-5372615
BmHel-12	DF090317:6814556-6814678
BmHel-12	DF090317:6814676-6814898
BmHel-12	DF090352:3038998-3039127
BmHel-12	DF090352:3039132-3039359
BmHel-12	DF090443:26656-26934
BmHel-12	DF090342:2665589-2665929
BmHel-12	DF090323:7064292-7064422
BmHel-12	DF090323:7064424-7064660
BmHel-12	DF090386:943958-944221
BmHel-12	DF090328:5791201-5791283
BmHel-12	DF090328:5790881-5791155
BmHel-12	DF090330:3369217-3369564
BmHel-12	DF090351:597224-597570
BmHel-12	DF090459:17363-17717
BmHel-12	DF090418:115721-116067
BmHel-12	DF090431:694713-694841
BmHel-12	DF090431:694851-695077
BmHel-12	DF090332:5685734-5685864
BmHel-12	DF090332:5685514-5685724
BmHel-12	DF090337:4101379-4101670
BmHel-12	DF090319:132890-132992
BmHel-12	DF090319:133000-133246
BmHel-12	DF090364:774933-775147
BmHel-12	DF090340:1776323-1776479
BmHel-12	DF090340:1776502-1776689
BmHel-12	DF090319:7920935-7921119
BmHel-12	DF090319:7919978-7920129
BmHel-12	DF090397:704897-705121
BmHel-12	DF090397:704784-704896
BmHel-12	DF090325:7219315-7219601
BmHel-12	DF090348:3689909-3690246
BmHel-12	DF090333:1240432-1240781
BmHel-12	DF090383:1490586-1490924
BmHel-12	DF090377:1789651-1789988
BmHel-12	DF090317:2128281-2128552
BmHel-12	DF090383:1851283-1851621
BmHel-12	DF090520:9156-9455
BmHel-12	DF090322:212758-213107
BmHel-12	DF090356:2880297-2880564
BmHel-12	DF090356:2880581-2880661
BmHel-12	DF090416:1075208-1075472
BmHel-12	DF090319:6920816-6921079
BmHel-12	DF090338:638046-638290
BmHel-12	DF090338:638289-638386
BmHel-12	DF090362:1957196-1957468
BmHel-12	DF090340:14711-14926

BmHel-12	DF090444:469202-469455
BmHel-12	DF090361:3035673-3036016
BmHel-12	DF090533:48443-48784
BmHel-12	DF090371:60810-61144
BmHel-12	DF090340:1523361-1523490
BmHel-12	DF090340:1523494-1523728
BmHel-12	DF090322:306081-306299
BmHel-12	DF090376:2127429-2127776
BmHel-12	DF090350:4218529-4218841
BmHel-12	DF090351:3441693-3441999
BmHel-12	DF090364:90255-90363
BmHel-12	DF090364:90136-90234
BmHel-12	DF090364:89991-90118
BmHel-12	DF090354:3024611-3024875
BmHel-12	DF090322:1050389-1050741
BmHel-12	DF090413:194732-194953
BmHel-12	DF090340:4719546-4719742
BmHel-12	DF090340:4719738-4719883
BmHel-12	DF090350:1719177-1719524
BmHel-12	BABH01043176:3790-4120
BmHel-12	DF090370:618780-618910
BmHel-12	DF090370:618543-618776
BmHel-12	DF090411:976626-976885
BmHel-12	DF090333:5064629-5064890
BmHel-12	DF090483:47033-47172
BmHel-12	DF090483:46550-46778
BmHel-12	DF090318:2178593-2178745
BmHel-12	DF090318:2179071-2179287
BmHel-12	DF090407:470100-470405
BmHel-12	DF090615:36926-37114
BmHel-12	DF090615:36784-36911
BmHel-12	DF090369:894836-895096
BmHel-12	DF090348:1179068-1179292
BmHel-12	DF090348:1178948-1179071
BmHel-12	DF090344:2797030-2797308
BmHel-12	DF090431:81084-81331
BmHel-12	DF090431:81326-81421
BmHel-12	DF090350:3028473-3028814
BmHel-12	DF090348:3587795-3588128
BmHel-12	DF090647:20606-20948
BmHel-12	DF090633:9305-9438
BmHel-12	DF090633:9436-9663
BmHel-12	DF090328:4650568-4650698
BmHel-12	DF090328:4650698-4650929
BmHel-12	DF090348:998642-998770
BmHel-12	DF090348:998408-998637
BmHel-12	DF090377:97006-97088
BmHel-12	DF090377:97108-97384

BmHel-12	DF090373:1317394-1317737
BmHel-12	DF090347:4209277-4209538
BmHel-12	DF090365:37897-38065
BmHel-12	DF090365:38170-38335
BmHel-12	DF090378:216229-216368
BmHel-12	DF090378:215993-216225
BmHel-12	DF090363:163713-163861
BmHel-12	DF090363:162966-163182
BmHel-12	DF090318:9924863-9925166
BmHel-12	DF090383:2001697-2001984
BmHel-12	DF090340:2757553-2757853
BmHel-12	DF090353:3637359-3637689
BmHel-12	DF090362:1469480-1469812
BmHel-12	DF090318:1900582-1900715
BmHel-12	DF090318:1900773-1900989
BmHel-12	BABH01062372:238-371
BmHel-12	BABH01062372:1-232
BmHel-12	DF090382:358092-358348
BmHel-12	DF090347:2321153-2321293
BmHel-12	DF090347:2320935-2321149
BmHel-12	DF090343:444202-444485
BmHel-12	DF090348:2997002-2997132
BmHel-12	DF090348:2997142-2997375
BmHel-12	DF090468:235609-235873
BmHel-12	DF090468:235879-235959
BmHel-12	DF090356:2161927-2162250
BmHel-12	DF090326:1680753-1680884
BmHel-12	DF090326:1680519-1680744
BmHel-12	DF090317:2017508-2017776
BmHel-12	DF090344:2151186-2151450
BmHel-12	DF090347:1375809-1375934
BmHel-12	DF090347:1375940-1376174
BmHel-12	DF090339:3162638-3162860
BmHel-12	DF090339:3162116-3162241
BmHel-12	DF090324:1316144-1316485
BmHel-12	DF090345:3539349-3539608
BmHel-12	DF090420:835149-835413
BmHel-12	DF090400:1344158-1344287
BmHel-12	DF090400:1344303-1344527
BmHel-12	DF090394:1332170-1332493
BmHel-12	DF090333:4072514-4072801
BmHel-12	DF090383:806061-806265
BmHel-12	DF090383:806277-806357
BmHel-12	DF090328:2050754-2050883
BmHel-12	DF090328:2050517-2050752
BmHel-12	DF090411:1079665-1079999
BmHel-12	DF090431:735227-735493
BmHel-12	DF090341:3273769-3274096

BmHel-12	DF090390:1234288-1234555
BmHel-12	DF090426:922813-923149
BmHel-12	DF090352:1429726-1429977
BmHel-12	DF090418:959839-959969
BmHel-12	DF090418:959993-960223
BmHel-12	DF090322:5313923-5314181
BmHel-12	DF090320:6508222-6508348
BmHel-12	DF090320:6508354-6508592
BmHel-12	DF090389:102895-103216
BmHel-12	DF090412:119659-119781
BmHel-12	DF090412:119790-120023
BmHel-12	DF090319:8189876-8190099
BmHel-12	DF090348:149502-149626
BmHel-12	DF090348:149266-149482
BmHel-12	DF090431:677974-678234
BmHel-12	DF090367:737789-738022
BmHel-12	DF090367:737670-737784
BmHel-12	DF090358:466047-466172
BmHel-12	DF090358:466184-466402
BmHel-12	DF090342:4041642-4041918
BmHel-12	DF090630:12790-12924
BmHel-12	DF090630:12930-13123
BmHel-12	DF090359:2295001-2295124
BmHel-12	DF090359:2294766-2294997
BmHel-12	DF090359:3263453-3263746
BmHel-12	DF090325:5683834-5684081
BmHel-12	DF090352:2634553-2634822
BmHel-12	DF090369:1125391-1125665
BmHel-12	DF090334:3753053-3753319
BmHel-12	DF090318:5401369-5401636
BmHel-12	DF090337:623471-623733
BmHel-12	DF090337:623385-623464
BmHel-12	DF090318:3108734-3108924
BmHel-12	DF090317:4533830-4533963
BmHel-12	DF090317:4533971-4534192
BmHel-12	DF090352:2031742-2032006
BmHel-12	DF090422:588782-588995
BmHel-12	DF090422:588639-588760
BmHel-12	DF090338:1193720-1193999
BmHel-12	DF090327:4535169-4535373
BmHel-12	DF090327:4535372-4535520
BmHel-12	DF090328:649764-649988
BmHel-12	DF090328:649642-649746
BmHel-12	DF090326:1026873-1027210
BmHel-12	DF090357:1756752-1757011
BmHel-12	DF090350:1623981-1624168
BmHel-12	DF090343:1829820-1830100
BmHel-12	DF090465:111407-111544

BmHel-12	DF090465:111540-111765
BmHel-12	DF090318:5343872-5344001
BmHel-12	DF090318:5344003-5344232
BmHel-12	DF090317:3329397-3329591
BmHel-12	DF090350:3107754-3108008
BmHel-12	DF090429:579005-579270
BmHel-12	DF090319:326598-326855
BmHel-12	DF090328:4705068-4705323
BmHel-12	DF090344:3896468-3896590
BmHel-12	DF090344:3896233-3896464
BmHel-12	DF090424:854033-854155
BmHel-12	DF090424:853801-854029
BmHel-12	DF091451:36789-36927
BmHel-12	DF091451:36929-37153
BmHel-12	DF090460:52181-52449
BmHel-12	DF090326:3157581-3157790
BmHel-12	DF090326:3157429-3157553
BmHel-12	DF090326:6346239-6346491
BmHel-12	DF090330:4449156-4449254
BmHel-12	DF090330:4448785-4449054
BmHel-12	DF091121:7036-7127
BmHel-12	DF091121:6709-6943
BmHel-12	DF090338:4122739-4122937
BmHel-12	DF090353:886343-886608
BmHel-12	DF090336:1242069-1242210
BmHel-12	DF090336:1241849-1242071
BmHel-12	DF090423:526826-527093
BmHel-12	DF090332:6358171-6358434
BmHel-12	DF090415:314249-314510
BmHel-12	DF090345:3414542-3414811
BmHel-12	DF090364:1226162-1226403
BmHel-12	DF090364:1226415-1226495
BmHel-12	DF090327:4959908-4960231
BmHel-12	DF090331:6226210-6226549
BmHel-12	DF090399:541522-541857
BmHel-12	DF090384:638116-638210
BmHel-12	DF090384:637827-638054
BmHel-12	DF090407:1037379-1037717
BmHel-12	DF090358:1969130-1969377
BmHel-12	DF090354:2855448-2855774
BmHel-12	DF090316:8086672-8086797
BmHel-12	DF090316:8086420-8086654
BmHel-12	DF090317:58346-58671
BmHel-12	DF090317:1749756-1750033
BmHel-12	DF090778:1319-1567
BmHel-12	DF090331:2979145-2979288
BmHel-12	DF090331:2978925-2979149
BmHel-12	DF090362:2419318-2419499

BmHel-12	DF090362:2419509-2419651
BmHel-12	DF090358:1855392-1855665
BmHel-12	BABH01042583:23392-23492
BmHel-12	BABH01042583:23504-23734
BmHel-12	DF090323:1620578-1620850
BmHel-12	DF090324:7083927-7084056
BmHel-12	DF090324:7083708-7083923
BmHel-12	DF090362:1934821-1935141
BmHel-12	DF090324:5996716-5996963
BmHel-12	DF090353:180536-180800
BmHel-12	DF090358:3061790-3062050
BmHel-12	DF090366:1349352-1349623
BmHel-12	DF090400:457383-457708
BmHel-12	DF090343:4102394-4102527
BmHel-12	DF090343:4102598-4102748
BmHel-12	DF090446:70916-71045
BmHel-12	DF090446:70699-70918
BmHel-12	DF090338:3561039-3561297
BmHel-12	DF090319:5251200-5251291
BmHel-12	DF090319:5251011-5251181
BmHel-12	DF090316:7025437-7025658
BmHel-12	DF090356:3000294-3000458
BmHel-12	DF090356:3000197-3000277
BmHel-12	DF090362:1781899-1782107
BmHel-12	DF090355:2360222-2360551
BmHel-12	DF090316:7804956-7805232
BmHel-12	DF090370:1569815-1569944
BmHel-12	DF090370:1569661-1569815
BmHel-12	BABH01047762:953-1227
BmHel-12	DF090366:548992-549116
BmHel-12	DF090366:548846-548988
BmHel-12	DF090502:110832-111096
BmHel-12	DF090321:3513744-3513843
BmHel-12	DF090321:3513517-3513742
BmHel-12	DF090397:1191676-1191896
BmHel-12	DF090331:1420256-1420577
BmHel-12	DF090402:1293223-1293354
BmHel-12	DF090402:1293359-1293448
BmHel-12	DF090402:1293453-1293584
BmHel-12	DF090389:1242447-1242572
BmHel-12	DF090389:1242572-1242724
BmHel-12	DF090396:174473-174742
BmHel-12	DF090459:222306-222558
BmHel-12	DF090401:1135829-1136081
BmHel-12	DF090401:1136088-1136167
BmHel-12	DF090328:4676227-4676345
BmHel-12	DF090328:4676367-4676601
BmHel-12	DF090333:5082914-5083232

BmHel-12	DF090328:3997000-3997213
BmHel-12	DF090328:3996892-3997000
BmHel-12	DF090338:820599-820815
BmHel-12	DF090444:34418-34548
BmHel-12	DF090444:34218-34401
BmHel-12	DF090337:1499372-1499686
BmHel-12	DF090415:749169-749263
BmHel-12	DF090415:748996-749165
BmHel-12	DF090348:282545-282664
BmHel-12	DF090348:282673-282898
BmHel-12	DF090611:6650-6767
BmHel-12	DF090611:6781-7011
BmHel-12	DF090365:614061-614382
BmHel-12	DF090349:4073271-4073596
BmHel-12	DF090355:1125589-1125675
BmHel-12	DF090355:1125321-1125544
BmHel-12	DF090316:14907232-14907505
BmHel-12	DF090334:5805017-5805351
BmHel-12	DF090329:4434788-4434912
BmHel-12	DF090329:4434910-4435140
BmHel-12	DF090322:1969516-1969783
BmHel-12	DF090397:1273324-1273517
BmHel-12	DF090397:1271315-1271442
BmHel-12	DF090378:761284-761530
BmHel-12	DF090462:33343-33668
BmHel-12	DF090330:1881353-1881683
BmHel-12	DF090405:391491-391771
BmHel-12	DF090405:567822-568065
BmHel-12	DF090327:2054109-2054424
BmHel-12	DF090389:264012-264291
BmHel-12	DF090413:532292-532534
BmHel-12	DF090357:796905-797165
BmHel-12	DF090398:1005524-1005686
BmHel-12	DF090398:1005372-1005523
BmHel-12	DF090431:620977-621299
BmHel-12	DF090358:2754269-2754515
BmHel-12	DF090338:2777019-2777098
BmHel-12	DF090338:2776668-2776825
BmHel-12	DF090316:14949075-14949218
BmHel-12	DF090452:451126-451440
BmHel-12	DF090337:4096389-4096517
BmHel-12	DF090337:4096223-4096383
BmHel-12	DF090323:4801944-4802255
BmHel-12	DF090474:278016-278271
BmHel-12	DF090326:2160947-2161206
BmHel-12	DF090402:902389-902665
BmHel-12	DF090399:480235-480441
BmHel-12	DF090399:480113-480223

BmHel-12	DF090351:4276564-4276652
BmHel-12	DF090351:4276738-4276954
BmHel-12	DF090320:5538063-5538173
BmHel-12	DF090320:5537803-5538038
BmHel-12	DF090354:1864910-1865184
BmHel-12	DF090403:937228-937546
BmHel-12	DF090381:1614142-1614407
BmHel-12	DF090639:15804-16031
BmHel-12	DF090377:1406967-1407241
BmHel-12	DF090342:3868278-3868492
BmHel-12	DF090362:2396478-2396660
BmHel-12	DF090362:2396663-2396790
BmHel-12	DF090498:18603-18866
BmHel-12	DF090443:530276-530529
BmHel-12	DF090413:554417-554555
BmHel-12	DF090413:554626-554768
BmHel-12	DF090344:2166679-2166834
BmHel-12	DF090344:2166571-2166662
BmHel-12	DF090352:1953703-1953893
BmHel-12	DF090322:318142-318407
BmHel-12	DF090391:966437-966694
BmHel-12	DF090327:6053138-6053445
BmHel-12	DF090361:662327-662598
BmHel-12	DF090522:5992-6110
BmHel-12	DF090522:5765-5983
BmHel-12	DF090409:175847-176112
BmHel-12	DF090316:3078642-3078950
BmHel-12	DF091238:5410-5563
BmHel-12	DF091238:5566-5693
BmHel-12	DF090375:1217172-1217266
BmHel-12	DF090375:1216674-1216896
BmHel-12	DF090437:3439-3751
BmHel-12	DF090320:2114029-2114339
BmHel-12	DF090335:4236303-4236557
BmHel-12	DF090320:8365565-8365823
BmHel-12	DF090346:1628513-1628644
BmHel-12	DF090346:1628655-1628791
BmHel-12	DF090440:638709-638969
BmHel-12	DF090423:978489-978723
BmHel-12	DF090317:7932285-7932479
BmHel-12	BABH01049365:1-323
BmHel-12	DF090320:4655965-4656232
BmHel-12	DF090342:544158-544239
BmHel-12	DF090342:543993-544154
BmHel-12	DF090348:1070177-1070310
BmHel-12	DF090348:1069964-1070168
BmHel-12	DF090343:3621521-3621843
BmHel-12	DF090382:1445807-1445959

BmHel-12	DF090400:1522556-1522782
BmHel-12	DF090400:1522798-1522888
BmHel-12	DF090416:1271649-1271914
BmHel-12	DF090529:40004-40327
BmHel-12	DF090355:304081-304323
BmHel-12	DF090350:1522289-1522471
BmHel-12	DF090369:1968641-1968869
BmHel-12	DF090317:1401040-1401180
BmHel-12	DF090317:1399502-1399613
BmHel-12	DF090411:878795-879059
BmHel-12	DF090348:1411721-1411987
BmHel-12	DF090378:387289-387415
BmHel-12	DF090378:387053-387255
BmHel-12	DF090539:38807-38941
BmHel-12	DF090416:1245542-1245646
BmHel-12	DF090416:1245389-1245530
BmHel-12	DF090320:1479060-1479176
BmHel-12	DF090320:1479178-1479272
BmHel-12	DF090320:1479290-1479408
BmHel-12	DF090332:2753931-2754245
BmHel-12	DF090391:641440-641673
BmHel-12	DF090361:2358732-2358919
BmHel-12	DF090352:1121273-1121522
BmHel-12	DF090366:576439-576644
BmHel-12	DF090347:293828-294021
BmHel-12	DF090316:6305807-6306063
BmHel-12	DF090319:541166-541340
BmHel-12	DF090319:541355-541479
BmHel-12	DF090367:1069473-1069663
BmHel-12	DF090358:2135221-2135397
BmHel-12	DF090358:2135085-2135211
BmHel-12	DF090379:1055127-1055206
BmHel-12	DF090379:1054553-1054814
BmHel-12	DF090395:1082378-1082645
BmHel-12	DF090325:3273512-3273813
BmHel-12	DF090413:49471-49646
BmHel-12	DF090413:49666-49790
BmHel-12	DF090327:5842972-5843225
BmHel-12	DF090554:47137-47393
BmHel-12	DF090318:2823229-2823482
BmHel-12	DF090395:752968-753112
BmHel-12	DF090395:752771-752975
BmHel-12	DF090358:2493249-2493503
BmHel-12	DF090383:1340348-1340624
BmHel-12	DF090331:4944889-4945112
BmHel-12	DF090362:129567-129794
BmHel-12	DF090549:18186-18438
BmHel-12	DF090358:2398922-2399111

BmHel-12	DF090372:1560998-1561182
BmHel-12	DF090348:4180418-4180679
BmHel-12	DF090322:5579205-5579439
BmHel-12	DF090363:821664-821873
BmHel-12	DF090363:821550-821641
BmHel-12	DF090349:4176092-4176395
BmHel-12	DF090351:3604672-3604975
BmHel-12	DF090374:2300552-2300870
BmHel-12	DF090339:1596536-1596764
BmHel-12	DF090356:2374072-2374325
BmHel-12	DF090355:2970736-2970835
BmHel-12	DF090355:2970837-2970998
BmHel-12	DF090323:1206798-1207013
BmHel-12	DF090352:1844895-1845155
BmHel-12	DF090368:2310326-2310553
BmHel-12	DF090340:284116-284378
BmHel-12	DF090330:6313162-6313463
BmHel-12	DF090318:1589893-1590201
BmHel-12	DF090443:325213-325451
BmHel-12	DF090931:17711-17840
BmHel-12	DF090931:17524-17699
BmHel-12	DF090567:70871-71125
BmHel-12	DF090416:796992-797242
BmHel-12	DF090324:2105282-2105413
BmHel-12	DF090324:2105415-2105555
BmHel-12	DF090335:5578514-5578593
BmHel-12	DF090335:5577941-5578167
BmHel-12	DF090361:1844350-1844652
BmHel-12	DF090330:2057633-2057929
BmHel-12	DF090425:144882-145187
BmHel-12	DF090325:6630106-6630405
BmHel-12	DF090322:7928957-7929249
BmHel-12	DF090316:9250302-9250617
BmHel-12	DF090316:3888194-3888500
BmHel-12	DF090350:2628736-2629036
BmHel-12	DF090358:2587802-2588105
BmHel-12	DF090335:4942274-4942577
BmHel-12	DF090354:946670-946974
BmHel-12	DF090334:2914378-2914683
BmHel-12	DF090320:794659-794963
BmHel-12	DF090320:3164454-3164758
BmHel-12	DF090417:404422-404728
BmHel-12	DF090346:2139733-2140040
BmHel-12	DF090373:2122734-2123040
BmHel-12	DF090336:2926690-2926982
BmHel-12	DF090336:4664394-4664699
BmHel-12	DF090351:1910540-1910837
BmHel-12	DF090342:3676776-3677081

BmHel-12	DF090342:3230-3532
BmHel-12	DF090328:255120-255418
BmHel-12	DF090375:2190209-2190512
BmHel-12	DF090321:6866096-6866404
BmHel-12	DF090321:1763798-1764096
BmHel-12	DF090327:3386264-3386566
BmHel-12	DF090380:755184-755480
BmHel-12	DF090380:519437-519736
BmHel-12	DF090344:4464017-4464327
BmHel-12	DF090344:2274451-2274751
BmHel-12	DF090770:22905-23210
BmHel-12	DF090333:1202894-1203205
BmHel-12	DF090333:3734170-3734475
BmHel-12	DF090401:490452-490755
BmHel-12	DF090331:2796280-2796581
BmHel-12	DF090339:2462584-2462894
BmHel-12	DF090371:1045509-1045799
BmHel-12	DF090382:1449267-1449563
BmHel-12	DF090441:179405-179701
BmHel-12	DF090434:642731-643029
BmHel-12	DF090337:4717952-4718256
BmHel-12	BABH01044025:9319-9618
BmHel-12	DF090323:7539483-7539734
BmHel-12	DF090318:1938517-1938823
BmHel-12	DF090355:1125781-1126068
BmHel-12	DF090339:3669547-3669838
BmHel-12	DF090400:1518129-1518437
BmHel-12	DF090352:4120144-4120449
BmHel-12	DF090322:800633-800932
BmHel-12	DF090343:2328473-2328654
BmHel-12	DF090343:2328675-2328801
BmHel-12	DF090327:5042071-5042270
BmHel-12	DF090327:5041968-5042058
BmHel-12	DF090325:1719864-1720125
BmHel-12	DF090318:9884018-9884277
BmHel-12	DF090390:298626-298711
BmHel-12	DF090390:298431-298622
BmHel-12	DF090329:6593302-6593491
BmHel-12	DF090319:781413-781639
BmHel-12	DF090324:526334-526566
BmHel-12	DF090364:2304161-2304459
BmHel-12	DF090345:4413024-4413213
BmHel-12	DF090329:6046588-6046888
BmHel-12	DF090324:2941254-2941481
BmHel-12	DF090336:1297537-1297771
BmHel-12	DF090350:3261845-3262103
BmHel-12	DF090350:577407-577716
BmHel-12	DF090343:3022467-3022755

BmHel-12	DF090375:633785-634080
BmHel-12	DF090327:153761-154052
BmHel-12	DF090333:5049259-5049562
BmHel-12	DF090458:252333-252640
BmHel-12	DF090377:695001-695165
BmHel-12	DF090372:1780134-1780277
BmHel-12	DF090372:1780293-1780412
BmHel-12	DF090329:3177391-3177654
BmHel-12	BABH01047320:1473-1596
BmHel-12	BABH01047320:1598-1792
BmHel-12	BABH01045295:5183-5306
BmHel-12	BABH01045295:5308-5502
BmHel-12	DF090330:5112199-5112507
BmHel-12	DF090353:376643-376936
BmHel-12	DF090317:757280-757582
BmHel-12	DF090378:345844-346147
BmHel-12	BABH01063681:388-677
BmHel-12	DF090681:25570-25741
BmHel-12	DF090328:320508-320813
BmHel-12	DF090407:608333-608633
BmHel-12	BABH01079068:182-311
BmHel-12	BABH01079068:1-170
BmHel-12	DF090369:1322534-1322818
BmHel-12	DF090788:7643-7927
BmHel-12	DF090443:250758-251056
BmHel-12	DF090486:78427-78645
BmHel-12	DF090509:88914-89046
BmHel-12	DF090509:88770-88911
BmHel-12	DF090379:203933-204075
BmHel-12	DF090379:203816-203927
BmHel-12	DF090379:203724-203810
BmHel-12	DF090342:812197-812459
BmHel-12	DF090342:1859227-1859532
BmHel-12	DF090342:3219913-3220201
BmHel-12	DF090375:2434545-2434833
BmHel-12	DF090415:222779-223068
BmHel-12	DF090439:137102-137334
BmHel-12	DF090418:734414-734723
BmHel-12	DF090380:445655-445750
BmHel-12	DF090420:307306-307539
BmHel-12	DF090335:4428328-4428502
BmHel-12	DF090446:207331-207521
BmHel-12	DF090389:402062-402367
BmHel-12	DF090368:1523303-1523602
BmHel-12	DF090459:379597-379826
BmHel-12	DF090439:500265-500534
BmHel-12	DF090330:2132134-2132378
BmHel-12	DF090382:1283791-1284042

BmHel-12	DF090347:2534385-2534485
BmHel-12	DF090347:2534504-2534729
BmHel-12	DF090414:560649-560948
BmHel-12	DF090347:1986090-1986315
BmHel-12	DF090345:3211936-3212187
BmHel-12	BABH01043558:14455-14668
BmHel-12	BABH01043558:14710-14807
BmHel-12	DF090335:665739-665921
BmHel-12	DF090335:666052-666174
BmHel-12	DF090319:360517-360809
BmHel-12	DF090462:391995-392290
BmHel-12	DF090317:4620366-4620666
BmHel-12	DF090367:987653-987949
BmHel-12	DF090345:3529886-3530023
BmHel-12	DF090345:3530022-3530114
BmHel-12	DF090316:8359098-8359387
BmHel-12	DF090361:582837-582965
BmHel-12	DF090361:582967-583109
BmHel-12	DF090356:1006822-1007071
BmHel-12	DF090373:655067-655354
BmHel-12	DF090316:10413673-10413940
BmHel-12	DF090319:516745-516876
BmHel-12	DF090319:516903-517064
BmHel-12	DF090400:1108898-1109136
BmHel-12	DF090368:258345-258538
BmHel-12	DF090368:258562-258663
BmHel-12	DF090360:1699526-1699663
BmHel-12	DF090360:1699394-1699518
BmHel-12	DF090360:2508931-2509063
BmHel-12	DF090360:2508721-2508921
BmHel-12	DF090355:416491-416793
BmHel-12	DF090407:679196-679445
BmHel-12	DF090357:483208-483503
BmHel-12	DF090333:2901318-2901608
BmHel-12	DF090411:1216005-1216310
BmHel-12	DF090341:3881698-3881992
BmHel-12	DF090354:2086382-2086607
BmHel-12	DF090330:4120822-4120930
BmHel-12	DF090330:4120254-4120426
BmHel-12	DF090336:5110792-5111076
BmHel-12	DF090316:13414381-13414564
BmHel-12	DF090354:2989473-2989768
BmHel-12	DF090383:496296-496422
BmHel-12	DF090383:496447-496543
BmHel-12	DF090340:1643239-1643527
BmHel-12	DF090416:464869-465158
BmHel-12	DF090371:1330695-1330992
BmHel-12	DF090353:3113126-3113355

BmHel-12	DF090321:2638801-2638999
BmHel-12	DF090321:2639006-2639094
BmHel-12	DF090352:1333644-1333733
BmHel-12	DF090352:1333738-1333968
BmHel-12	DF090324:1553305-1553440
BmHel-12	DF090324:1553479-1553646
BmHel-12	DF090338:5087181-5087310
BmHel-12	DF090338:5086997-5087178
BmHel-12	DF090332:1228994-1229204
BmHel-12	DF090329:4926686-4926906
BmHel-12	DF090326:6802057-6802353
BmHel-12	DF090379:463872-464082
BmHel-12	DF090379:464089-464182
BmHel-12	DF090318:9770143-9770384
BmHel-12	DF090600:19015-19243
BmHel-12	DF090370:2404490-2404777
BmHel-12	DF090320:6076327-6076578
BmHel-12	DF090382:691947-692177
BmHel-12	DF090417:938175-938470
BmHel-12	DF090326:4184605-4184899
BmHel-12	DF090384:432540-432826
BmHel-12	DF090413:219422-219576
BmHel-12	DF090416:346259-346560
BmHel-12	DF090335:5563854-5564150
BmHel-12	DF090340:1776225-1776314
BmHel-12	DF090340:1776127-1776227
BmHel-12	DF090484:178229-178458
BmHel-12	DF091223:5239-5468
BmHel-12	DF090327:4977503-4977590
BmHel-12	DF090327:4977248-4977456
BmHel-12	DF090320:3316719-3316907
BmHel-12	DF090339:1576181-1576423
BmHel-12	DF090412:853628-853928
BmHel-12	DF090398:654168-654418
BmHel-12	DF090352:445140-445372
BmHel-12	DF090429:756475-756638
BmHel-12	DF090352:2155703-2155819
BmHel-12	DF090352:2145848-2145967
BmHel-12	DF090316:2631540-2631672
BmHel-12	DF090316:2631674-2631855
BmHel-12	DF090324:1485318-1485617
BmHel-12	DF090344:548787-549018
BmHel-12	DF090386:703034-703264
BmHel-12	DF090422:568904-569193
BmHel-12	DF090368:1123161-1123405
BmHel-12	DF090330:2327630-2327866
BmHel-12	DF090347:1349319-1349402
BmHel-12	DF090347:1349407-1349608

BmHel-12	DF090351:3480846-3481090
BmHel-12	DF090350:1997782-1998069
BmHel-12	DF090356:2914353-2914625
BmHel-12	DF090365:2519588-2519842
BmHel-12	DF090368:2249376-2249649
BmHel-12	DF090327:4822894-4822976
BmHel-12	DF090327:4823074-4823206
BmHel-12	DF090360:2575933-2576071
BmHel-12	DF090360:2576077-2576263
BmHel-12	DF090345:3041568-3041813
BmHel-12	DF090661:24592-24817
BmHel-12	DF090369:1548652-1548841
BmHel-12	DF090390:569948-570240
BmHel-12	DF090367:931331-931579
BmHel-12	DF090316:12643589-12643884
BmHel-12	DF090342:1407998-1408202
BmHel-12	DF090342:1408215-1408296
BmHel-12	DF090436:283623-283727
BmHel-12	DF090436:282230-282408
BmHel-12	DF090318:8939749-8939971
BmHel-12	DF090375:679515-679745
BmHel-12	DF090318:3640060-3640248
BmHel-12	DF090342:2307359-2307651
BmHel-12	DF090398:1193196-1193354
BmHel-12	DF090398:1193042-1193167
BmHel-12	DF090347:1218746-1218880
BmHel-12	DF090347:1218881-1218984
BmHel-12	DF090355:1514622-1514726
BmHel-12	DF090355:1513986-1514168
BmHel-12	DF090333:2863358-2863495
BmHel-12	DF090333:2863183-2863351
BmHel-12	DF090346:3825287-3825418
BmHel-12	DF090346:3825099-3825292
BmHel-12	DF090341:3546653-3546773
BmHel-12	DF090341:3546476-3546641
BmHel-12	DF090351:1807024-1807238
BmHel-12	DF090316:14855978-14856138
BmHel-12	DF090316:14856152-14856276
BmHel-12	DF090342:4195760-4195895
BmHel-12	DF090342:4195577-4195762
BmHel-12	DF090383:77280-77387
BmHel-12	DF090383:77154-77259
BmHel-12	DF090357:2070598-2070729
BmHel-12	DF090357:2070731-2070904
BmHel-12	DF090356:2433217-2433452
BmHel-12	DF090358:1979027-1979171
BmHel-12	DF090358:1979184-1979301
BmHel-12	DF090319:7996442-7996540

BmHel-12	DF090319:7996231-7996416
BmHel-12	DF090343:699141-699366
BmHel-12	DF090316:3303760-3303896
BmHel-12	DF090316:3303579-3303764
BmHel-12	DF090394:1530264-1530381
BmHel-12	DF090394:1530098-1530257
BmHel-12	DF090367:2324669-2324752
BmHel-12	DF090367:2324771-2324918
BmHel-12	DF090385:1404619-1404771
BmHel-12	DF090385:1404487-1404612
BmHel-12	DF090316:4110242-4110467
BmHel-12	DF090329:2864086-2864250
BmHel-12	DF090329:2864254-2864363
BmHel-12	DF090385:1697590-1697840
BmHel-12	DF090373:962473-962681
BmHel-12	DF090373:1824116-1824314
BmHel-12	DF090372:1730954-1731100
BmHel-12	DF090372:1730817-1730944
BmHel-12	DF090472:262193-262422
BmHel-12	DF090486:98833-98983
BmHel-12	DF090486:98686-98813
BmHel-12	DF090354:3104861-3105011
BmHel-12	DF090434:545420-545621
BmHel-12	DF090359:2526747-2526909
BmHel-12	DF090359:2526586-2526704
BmHel-12	DF090364:2025771-2025937
BmHel-12	DF090470:120916-121078
BmHel-12	DF090367:1907599-1907707
BmHel-12	DF090367:1907706-1907824
BmHel-12	DF090329:2799561-2799678
BmHel-12	DF090329:2799703-2799784
BmHel-12	DF090328:634466-634704
BmHel-12	DF090329:5595328-5595598
BmHel-12	BABH01047111:26-240
BmHel-12	DF090317:10568959-10569180
BmHel-12	DF090327:1545965-1546187
BmHel-12	DF090343:210216-210433
BmHel-12	DF090326:1993717-1993925
BmHel-12	BABH01051110:614-891
BmHel-12	DF090557:62254-62523
BmHel-12	DF090371:288611-288746
BmHel-12	DF090320:1538232-1538503
BmHel-12	DF090318:6208276-6208532
BmHel-12	DF090392:1592376-1592602
BmHel-12	DF090386:1414579-1414795
BmHel-12	DF090318:6319735-6319869
BmHel-12	DF090323:3749464-3749739
BmHel-12	DF090323:4333760-4333971

BmHel-12	DF090330:1984258-1984357
BmHel-12	DF090330:1984074-1984248
BmHel-12	DF090361:250422-250687
BmHel-12	DF090319:3387829-3388099
BmHel-12	DF090319:478021-478292
BmHel-12	DF090324:5323709-5323979
BmHel-12	DF090349:575388-575676
BmHel-12	DF090356:3505472-3505741
BmHel-12	DF090355:2358783-2359058
BmHel-12	DF090369:2293611-2293890
BmHel-12	DF090759:19146-19419
BmHel-12	DF090403:64609-64746
BmHel-12	DF090403:64509-64597
BmHel-12	DF090403:63967-64067
BmHel-12	DF090350:2594693-2594859
BmHel-12	DF090321:7652695-7652915
BmHel-12	DF090331:830698-830845
BmHel-12	DF090411:950620-950821
BmHel-12	DF090371:1187072-1187299
BmHel-12	DF090389:983855-984132
BmHel-12	DF090319:8586128-8586245
BmHel-12	DF090337:4736344-4736443
BmHel-12	DF090335:3395836-3396050
BmHel-12	DF090344:3357917-3358007
BmHel-12	DF090344:3357707-3357887
BmHel-12	DF090325:2189319-2189543
BmHel-12	DF090417:1028420-1028689
BmHel-12	DF090522:6402-6527
BmHel-12	DF090522:6598-6689
BmHel-12	DF090334:2527239-2527351
BmHel-12	DF090334:2527372-2527531
BmHel-12	DF090377:1518418-1518538
BmHel-12	DF090377:1518299-1518416
BmHel-12	DF090327:2443515-2443740
BmHel-12	DF090354:730251-730510
BmHel-12	DF090364:187611-187881
BmHel-12	DF090322:4745324-4745544
BmHel-12	DF090486:80817-81014
BmHel-12	DF091726:778-974
BmHel-12	DF090592:3567-3812
BmHel-12	DF090846:10230-10433
BmHel-12	DF090348:641190-641419
BmHel-12	DF090475:253867-254134
BmHel-12	DF090333:410193-410468
BmHel-12	DF090355:3740236-3740468
BmHel-12	DF090344:1439878-1440014
BmHel-12	DF090344:1440016-1440118
BmHel-12	DF090361:1127011-1127279

BmHel-12	DF090355:2578398-2578629
BmHel-12	DF090354:1436289-1436491
BmHel-12	DF090354:1436559-1436639
BmHel-12	DF090345:873102-873203
BmHel-12	DF090345:873208-873315
BmHel-12	DF090427:897549-897668
BmHel-12	DF090427:897362-897509
BmHel-12	DF090437:379583-379774
BmHel-12	DF090437:379789-379869
BmHel-12	DF090349:3525017-3525253
BmHel-12	DF090336:4773193-4773455
BmHel-12	DF090394:451287-451563
BmHel-12	DF090344:4275679-4275954
BmHel-12	DF090344:585777-585884
BmHel-12	DF090344:586183-586345
BmHel-12	DF090327:5484232-5484450
BmHel-12	DF090321:690933-691132
BmHel-12	DF090330:636323-636563
BmHel-12	DF090390:176559-176818
BmHel-12	DF090360:1259518-1259727
BmHel-12	DF090336:522501-522681
BmHel-12	DF090336:522403-522482
BmHel-12	DF090366:525519-525774
BmHel-12	DF090361:2701626-2701837
BmHel-12	DF090327:5541553-5541681
BmHel-12	DF090327:5541688-5541815
BmHel-12	DF090344:1806122-1806347
BmHel-12	DF090718:14428-14614
BmHel-12	DF090391:259818-260039
BmHel-12	DF090324:1574345-1574613
BmHel-12	DF090322:537569-537799
BmHel-12	DF090317:1854662-1854790
BmHel-12	DF090317:1854548-1854648
BmHel-12	DF090342:472999-473126
BmHel-12	DF090368:868213-868422
BmHel-12	DF090316:12884826-12884971
BmHel-12	DF090316:12884708-12884821
BmHel-12	DF090319:4615786-4616053
BmHel-12	DF090360:608382-608639
BmHel-12	DF090413:250943-251122
BmHel-12	DF090345:4363711-4363928
BmHel-12	DF090358:1157734-1157922
BmHel-12	DF090354:2887830-2887978
BmHel-12	DF090337:3823781-3823886
BmHel-12	DF090337:3823885-3824044
BmHel-12	DF091276:3951-4152
BmHel-12	DF090340:547483-547743
BmHel-12	DF090343:4055355-4055561

BmHel-12	DF090318:3818452-3818638
BmHel-12	DF090340:1476484-1476740
BmHel-12	DF090390:1164518-1164765
BmHel-12	DF090353:392099-392354
BmHel-12	DF090342:4217625-4217894
BmHel-12	DF090433:418406-418673
BmHel-12	DF090347:4247142-4247371
BmHel-12	DF090364:2586159-2586342
BmHel-12	DF090364:2585970-2586050
BmHel-12	BABH01060086:211-404
BmHel-12	DF090327:4824956-4825080
BmHel-12	DF090327:4824815-4824957
BmHel-12	DF090370:2572844-2573101
BmHel-12	DF090331:6204595-6204853
BmHel-12	DF090317:6007918-6008051
BmHel-12	DF090328:3270989-3271241
BmHel-12	DF090423:352848-352966
BmHel-12	DF090468:57924-58142
BmHel-12	DF090316:13574709-13574854
BmHel-12	DF090330:2397539-2397670
BmHel-12	DF090330:2397689-2397837
BmHel-12	DF090316:8374605-8374685
BmHel-12	DF090316:8374401-8374600
BmHel-12	DF090316:5361616-5361871
BmHel-12	DF090321:4073627-4073882
BmHel-12	DF090416:413888-413995
BmHel-12	DF090416:414010-414101
BmHel-12	DF090331:5880776-5880914
BmHel-12	DF090331:5880649-5880775
BmHel-12	DF090331:884541-884752
BmHel-12	BABH01063875:112-376
BmHel-12	DF090354:1259730-1259832
BmHel-12	DF090354:1259552-1259709
BmHel-12	DF090350:1765461-1765722
BmHel-12	DF090335:5271548-5271814
BmHel-12	DF090342:156147-156333
BmHel-12	DF090746:19192-19381
BmHel-12	DF090346:2317185-2317448
BmHel-12	DF090438:586468-586738
BmHel-12	DF090333:5714970-5715231
BmHel-12	DF090339:227395-227654
BmHel-12	DF090379:1720506-1720762
BmHel-12	DF090351:4132093-4132220
BmHel-12	DF090351:4132233-4132385
BmHel-12	DF090333:3541896-3542148
BmHel-12	DF090324:3252284-3252448
BmHel-12	DF090377:659596-659812
BmHel-12	DF090366:1851226-1851322

BmHel-12	DF090366:1851666-1851760
BmHel-12	DF090403:881306-881564
BmHel-12	BABH01046397:2017-2274
BmHel-12	DF090353:1010674-1010931
BmHel-12	DF090317:9214625-9214810
BmHel-12	DF090398:637727-637978
BmHel-12	DF090395:1152126-1152313
BmHel-12	BABH01051334:64-287
BmHel-12	DF090551:12654-12827
BmHel-12	DF090551:12565-12645
BmHel-12	DF090358:3237368-3237549
BmHel-12	DF090323:1800275-1800455
BmHel-12	DF090317:9272448-9272579
BmHel-12	DF090317:9272280-9272430
BmHel-12	DF090328:3603923-3604098
BmHel-12	DF090349:4165553-4165780
BmHel-12	DF090398:649649-649835
BmHel-12	DF090333:1479570-1479663
BmHel-12	DF090333:1479904-1479995
BmHel-12	DF090372:1441262-1441526
BmHel-12	DF090319:2301327-2301495
BmHel-12	DF090317:4051144-4051223
BmHel-12	DF090317:4051600-4051774
BmHel-12	DF090329:2846933-2847073
BmHel-12	DF090329:2847070-2847226
BmHel-12	DF090360:1782560-1782748
BmHel-12	DF090346:533271-533538
BmHel-12	DF090349:4156971-4157220
BmHel-12	DF090364:162856-162995
BmHel-12	DF090364:162993-163137
BmHel-12	DF090319:5614342-5614522
BmHel-12	DF090365:1962405-1962617
BmHel-12	DF090316:138468-138725
BmHel-12	DF090430:498892-499100
BmHel-12	DF090409:60432-60684
BmHel-12	DF090590:50931-51183
BmHel-12	DF090446:530324-530404
BmHel-12	DF090446:530088-530263
BmHel-12	DF090403:528622-528724
BmHel-12	DF090403:528434-528587
BmHel-12	DF090333:1967496-1967630
BmHel-12	DF090372:113676-113840
BmHel-12	DF090377:4862-5060
BmHel-12	DF090505:71491-71599
BmHel-12	DF090330:318061-318245
BmHel-12	DF090332:2902389-2902555
BmHel-12	DF090364:1679021-1679194
BmHel-12	DF090326:6085146-6085335

BmHel-12	DF090391:205141-205351
BmHel-12	DF090342:2456226-2456404
BmHel-12	DF090342:2456143-2456222
BmHel-12	DF090347:79914-80052
BmHel-12	DF090394:874752-874885
BmHel-12	DF090368:710159-710404
BmHel-12	DF090329:1627876-1628031
BmHel-12	DF090329:1627633-1627775
BmHel-12	DF090344:1914217-1914360
BmHel-12	DF090344:1914108-1914212
BmHel-12	DF090359:913019-913206
BmHel-12	DF090322:3604520-3604761
BmHel-12	DF090331:2572725-2572967
BmHel-12	DF090434:245897-246115
BmHel-12	BABH01074545:352-604
BmHel-12	DF090342:1091127-1091233
BmHel-12	DF090342:1090939-1091117
BmHel-12	DF090351:3278097-3278342
BmHel-12	DF090422:548080-548262
BmHel-12	BABH01045792:2554-2802
BmHel-12	DF090330:5219474-5219653
BmHel-12	DF090349:389521-389612
BmHel-12	DF090349:399356-399436
BmHel-12	DF090362:795107-795290
BmHel-12	DF090327:3265759-3265941
BmHel-12	DF090366:801022-801207
BmHel-12	DF090353:3782589-3782770
BmHel-12	DF090358:356484-356580
BmHel-12	DF090358:356307-356453
BmHel-12	DF090321:7444033-7444148
BmHel-12	DF090321:7444172-7444300
BmHel-12	DF090704:1056-1299
BmHel-12	DF090351:3954322-3954530
BmHel-12	DF090374:2106421-2106607
BmHel-12	DF090365:2813319-2813501
BmHel-12	DF090387:1370595-1370840
BmHel-12	DF090317:208735-208985
BmHel-12	DF090329:5421571-5421751
BmHel-12	DF090367:1518795-1519038
BmHel-12	DF090332:5787523-5787634
BmHel-12	DF090456:272493-272669
BmHel-12	DF090329:5878781-5878895
BmHel-12	DF090338:11376-11524
BmHel-12	DF090421:733050-733248
BmHel-12	DF090450:461791-461965
BmHel-12	DF090347:2663009-2663141
BmHel-12	DF090337:5207130-5207267
BmHel-12	DF090337:5207281-5207387

BmHel-12	DF090374:1902496-1902667
BmHel-12	DF090441:730251-730377
BmHel-12	DF090366:776394-776562
BmHel-12	DF090333:4870161-4870413
BmHel-12	DF090350:2517999-2518251
BmHel-12	DF090354:2744275-2744505
BmHel-12	DF090316:2329234-2329375
BmHel-12	DF090316:2329128-2329225
BmHel-12	DF090329:6526583-6526752
BmHel-12	DF090576:24677-24853
BmHel-12	DF090320:1696872-1697117
BmHel-12	DF090355:2557396-2557646
BmHel-12	DF090347:2563653-2563893
BmHel-12	DF090446:291663-291901
BmHel-12	DF090320:579637-579857
BmHel-12	DF090332:5012671-5012844
BmHel-12	DF090341:2732828-2733083
BmHel-12	DF090421:420680-420930
BmHel-12	DF090327:1446478-1446718
BmHel-12	DF090350:3598060-3598248
BmHel-12	DF090376:1339844-1340049
BmHel-12	DF090332:3207838-3207959
BmHel-12	DF090332:3207955-3208053
BmHel-12	DF090371:44438-44693
BmHel-12	DF090317:10295497-10295745
BmHel-12	DF090327:6602632-6602870
BmHel-12	DF090342:3060209-3060427
BmHel-12	DF090343:2851692-2851864
BmHel-12	DF090757:4061-4237
BmHel-12	DF090343:335446-335651
BmHel-12	DF090326:5221616-5221785
BmHel-12	DF090410:831442-831570
BmHel-12	DF090410:831298-831423
BmHel-12	BABH01067276:105-295
BmHel-12	DF090329:3072342-3072464
BmHel-12	DF090364:1953432-1953557
BmHel-12	DF090381:1715394-1715583
BmHel-12	DF090353:526050-526150
BmHel-12	DF090353:526216-526350
BmHel-12	DF090321:1596206-1596335
BmHel-12	DF090385:288354-288594
BmHel-12	DF090368:1303678-1303817
BmHel-12	DF090368:1304086-1304200
BmHel-12	DF090321:2443525-2443773
BmHel-12	DF090323:2969522-2969763
BmHel-12	DF090325:2388365-2388602
BmHel-12	BABH01044215:1881-2060
BmHel-12	DF090329:2280514-2280750

BmHel-12	DF090355:2639097-2639283
BmHel-12	DF090324:3259370-3259491
BmHel-12	DF090324:3259589-3259719
BmHel-12	DF090394:1261116-1261358
BmHel-12	DF090505:2482-2720
BmHel-12	DF090358:2442885-2443115
BmHel-12	DF090379:1650454-1650680
BmHel-12	DF090316:2363499-2363668
BmHel-12	DF090323:5197379-5197564
BmHel-12	DF090336:5051610-5051849
BmHel-12	DF090426:250519-250758
BmHel-12	DF090380:98766-98935
BmHel-12	DF090368:414679-414860
BmHel-12	DF090331:2922153-2922395
BmHel-12	DF090317:2886808-2886981
BmHel-12	DF090330:809250-809359
BmHel-12	DF090322:6920279-6920459
BmHel-12	DF090342:3781384-3781468
BmHel-12	DF090342:3781466-3781617
BmHel-12	DF090358:49558-49795
BmHel-12	DF090364:1843917-1844106
BmHel-12	DF090316:555546-555743
BmHel-12	DF090444:490139-490283
BmHel-12	DF090489:62118-62343
BmHel-12	DF090321:4926083-4926317
BmHel-12	DF090386:577948-578146
BmHel-12	DF090393:771998-772165
BmHel-12	DF090317:658590-658712
BmHel-12	DF090317:658726-658846
BmHel-12	DF090369:867983-868178
BmHel-12	DF090413:129770-129948
BmHel-12	DF090357:868230-868463
BmHel-12	DF090335:39707-39946
BmHel-12	DF090320:7405528-7405761
BmHel-12	DF090362:1684896-1685130
BmHel-12	DF090333:2527946-2528181
BmHel-12	DF090345:4150984-4151218
BmHel-12	DF090415:384644-384764
BmHel-12	DF090488:154364-154546
BmHel-12	DF090390:1064470-1064703
BmHel-12	DF090388:59307-59541
BmHel-12	DF090611:5006-5239
BmHel-12	DF090384:921358-921437
BmHel-12	DF090384:921199-921353
BmHel-12	DF090354:3591288-3591513
BmHel-12	DF090323:3017128-3017361
BmHel-12	DF090355:304968-305201
BmHel-12	DF090338:3682522-3682652

BmHel-12	DF090343:4384389-4384575
BmHel-12	DF090394:1426329-1426522
BmHel-12	DF090377:492962-493044
BmHel-12	DF090377:492832-492930
BmHel-12	DF090344:2736876-2737108
BmHel-12	DF090398:827362-827591
BmHel-12	DF090364:1635896-1636131
BmHel-12	DF090352:2887873-2888000
BmHel-12	DF090318:7329321-7329558
BmHel-12	DF090346:1214420-1214656
BmHel-12	DF090380:679568-679751
BmHel-12	DF090336:3375828-3376064
BmHel-12	BABH01044981:2586-2814
BmHel-12	DF090363:2459115-2459255
BmHel-12	DF090363:2459001-2459088
BmHel-12	DF090350:2768125-2768355
BmHel-12	DF090320:3033110-3033340
BmHel-12	DF090339:2345006-2345232
BmHel-12	DF090316:8922070-8922249
BmHel-12	DF090366:2136392-2136556
BmHel-12	DF090404:805718-805942
BmHel-12	DF090367:2535310-2535537
BmHel-12	DF090420:855563-855782
BmHel-12	BABH01044052:7274-7382
BmHel-12	BABH01044052:7134-7254
BmHel-12	DF090316:12348268-12348497
BmHel-12	DF090346:2829625-2829855
BmHel-12	DF090325:6149825-6149937
BmHel-12	DF091963:396-539
BmHel-12	DF090327:2773674-2773818
BmHel-12	DF090327:2773831-2773912
BmHel-12	DF090464:315142-315235
BmHel-12	DF090347:2073107-2073273
BmHel-12	DF090394:43439-43537
BmHel-12	DF090394:43569-43694
BmHel-12	DF090349:2184511-2184738
BmHel-12	DF090386:166228-166329
BmHel-12	DF090386:166327-166454
BmHel-12	DF090355:1714959-1715061
BmHel-12	DF090322:6779027-6779168
BmHel-12	DF090322:6779195-6779281
BmHel-12	DF090317:6423352-6423462
BmHel-12	DF090317:6423221-6423333
BmHel-12	DF090316:5964396-5964615
BmHel-12	DF090326:2965983-2966208
BmHel-12	DF090321:6799195-6799422
BmHel-12	DF090327:1519158-1519381
BmHel-12	DF090406:703022-703251

BmHel-12	DF090360:1184546-1184777
BmHel-12	DF090337:3377515-3377742
BmHel-12	DF090317:8641257-8641424
BmHel-12	DF090334:3474361-3474534
BmHel-12	DF090388:1139475-1139700
BmHel-12	DF090329:4598406-4598585
BmHel-12	DF090362:984506-984680
BmHel-12	DF090333:3043084-3043268
BmHel-12	DF090424:61001-61116
BmHel-12	DF090328:4450891-4451039
BmHel-12	DF090349:3126440-3126637
BmHel-12	DF090350:1748943-1749097
BmHel-12	DF090325:2149494-2149708
BmHel-12	DF090401:1301154-1301306
BmHel-12	DF090388:208807-208974
BmHel-12	DF090340:4833054-4833223
BmHel-12	DF090382:881689-881839
BmHel-12	DF090358:1893153-1893303
BmHel-12	DF090373:1136467-1136558
BmHel-12	DF090373:1136578-1136706
BmHel-12	DF090357:3800003-3800230
BmHel-12	DF090351:3380620-3380839
BmHel-12	DF090326:4974631-4974851
BmHel-12	DF090351:3206675-3206897
BmHel-12	DF090348:338359-338584
BmHel-12	DF090359:2863213-2863352
BmHel-12	DF090335:5363658-5363868
BmHel-12	DF090335:3110051-3110162
BmHel-12	DF090338:4960130-4960357
BmHel-12	DF090410:574677-574902
BmHel-12	DF090330:4514918-4515148
BmHel-12	DF090370:2121112-2121332
BmHel-12	DF090323:6549425-6549587
BmHel-12	DF090339:1482274-1482432
BmHel-12	DF090330:4673029-4673253
BmHel-12	DF090452:67484-67616
BmHel-12	DF090452:67626-67724
BmHel-12	DF090330:208626-208842
BmHel-12	DF090353:2580824-2581049
BmHel-12	DF090330:4463798-4464010
BmHel-12	DF090323:6595011-6595129
BmHel-12	DF090436:231725-231805
BmHel-12	DF090436:231629-231719
BmHel-12	BABH01067964:53-266
BmHel-12	DF090330:2809770-2809982
BmHel-12	DF090376:885815-886030
BmHel-12	DF090344:1046705-1046914
BmHel-12	BABH01057057:180-323

BmHel-12	DF090362:30549-30706
BmHel-12	DF090349:3192545-3192719
BmHel-12	DF090345:2236442-2236659
BmHel-12	DF090393:1148176-1148331
BmHel-12	DF090371:867193-867410
BmHel-12	DF090346:2606304-2606467
BmHel-12	DF090316:6846375-6846516
BmHel-12	DF090390:215243-215462
BmHel-12	DF090340:2089394-2089570
BmHel-12	DF090391:1197020-1197101
BmHel-12	DF090391:1198291-1198421
BmHel-12	DF090343:3211656-3211872
BmHel-12	DF090366:1868591-1868807
BmHel-12	DF090467:244228-244440
BmHel-12	DF090509:76252-76469
BmHel-12	DF090484:55583-55797
BmHel-12	DF090413:129400-129622
BmHel-12	BABH01070340:2-219
BmHel-12	DF090332:3542133-3542346
BmHel-12	DF090367:240023-240242
BmHel-12	DF090411:988774-988987
BmHel-12	DF090399:364020-364233
BmHel-12	BABH01075938:356-569
BmHel-12	BABH01061021:248-461
BmHel-12	BABH01060048:25-238
BmHel-12	BABH01059899:167-380
BmHel-12	BABH01057291:316-529
BmHel-12	DF090499:125140-125353
BmHel-12	BABH01062288:394-607
BmHel-12	BABH01061702:76-289
BmHel-12	BABH01050753:542-755
BmHel-12	BABH01064846:150-363
BmHel-12	DF090330:5679867-5680072
BmHel-12	DF090366:2004919-2005090
BmHel-12	DF090319:6398398-6398612
BmHel-12	DF090338:2610039-2610206
BmHel-12	DF090364:1854492-1854698
BmHel-12	DF090318:3429916-3430076
BmHel-12	DF090346:1473832-1474061
BmHel-12	DF090377:81829-82023
BmHel-12	DF090338:1089500-1089585
BmHel-12	DF090338:1089342-1089469
BmHel-12	DF090350:3158079-3158294
BmHel-12	DF090386:1499937-1500148
BmHel-12	DF090342:1508729-1508878
BmHel-12	DF090347:3994598-3994743
BmHel-12	DF090333:4289091-4289203
BmHel-12	DF090333:4288979-4289078

BmHel-12	DF090422:121102-121267
BmHel-12	BABH01083395:176-299
BmHel-12	BABH01083395:43-131
BmHel-12	DF090376:664920-665133
BmHel-12	DF090429:1345-1429
BmHel-12	DF090364:2252235-2252445
BmHel-12	DF090323:2172399-2172628
BmHel-12	DF090558:34490-34703
BmHel-12	DF090372:816875-817094
BmHel-12	DF090361:1019358-1019509
BmHel-12	DF090317:9159925-9160143
BmHel-12	BABH01044332:560-666
BmHel-12	BABH01044332:441-546
BmHel-12	DF090424:498164-498325
BmHel-12	DF090321:6181206-6181325
BmHel-12	DF090321:6181084-6181182
BmHel-12	DF090341:1916640-1916855
BmHel-12	DF090322:2445274-2445433
BmHel-12	DF090369:2570240-2570392
BmHel-12	DF090365:1537183-1537334
BmHel-12	DF090344:1787096-1787263
BmHel-12	DF090422:1102211-1102315
BmHel-12	DF090422:1102311-1102413
BmHel-12	DF090320:7924289-7924439
BmHel-12	DF090958:938-1151
BmHel-12	DF090396:371855-371989
BmHel-12	DF090316:9222590-9222796
BmHel-12	DF090320:4366852-4367020
BmHel-12	BABH01070087:10-215
BmHel-12	DF090389:342078-342210
BmHel-12	DF090343:2924536-2924737
BmHel-12	DF090369:2646709-2646803
BmHel-12	DF090369:2646821-2646931
BmHel-12	DF090316:12828090-12828292
BmHel-12	DF090335:2768284-2768441
BmHel-12	DF090340:4283535-4283678
BmHel-12	DF090317:52246-52344
BmHel-12	DF090317:51960-52061
BmHel-12	BABH01080045:104-232
BmHel-12	BABH01080045:5-102
BmHel-12	DF090344:1999318-1999515
BmHel-12	DF090382:920509-920634
BmHel-12	DF090382:920403-920511
BmHel-12	DF090358:1692614-1692701
BmHel-12	DF090358:1692373-1692493
BmHel-12	DF090325:5995375-5995583
BmHel-12	BABH01045315:2558-2769
BmHel-12	DF090328:2321050-2321248

BmHel-12	DF090346:577630-577838
BmHel-12	DF090324:885194-885291
BmHel-12	DF090324:885377-885480
BmHel-12	DF090336:2820666-2820746
BmHel-12	DF090336:2820538-2820663
BmHel-12	DF090330:1375726-1375932
BmHel-12	DF090380:1320938-1321144
BmHel-12	DF090349:4200764-4200921
BmHel-12	DF090345:285263-285459
BmHel-12	DF090386:441042-441153
BmHel-12	DF090386:441153-441241
BmHel-12	DF090368:1467142-1467275
BmHel-12	DF090316:2506504-2506705
BmHel-12	DF090384:4487-4584
BmHel-12	DF090384:4607-4717
BmHel-12	DF090348:2081056-2081290
BmHel-12	DF090338:2399120-2399316
BmHel-12	DF090441:587681-587888
BmHel-12	DF090362:3031617-3031820
BmHel-12	DF090316:6695238-6695402
BmHel-12	DF090358:2633227-2633352
BmHel-12	DF090342:1926864-1926951
BmHel-12	DF090496:51657-51753
BmHel-12	DF090496:51520-51620
BmHel-12	DF090330:4697774-4697980
BmHel-12	DF090411:371723-371914
BmHel-12	DF090387:1399193-1399317
BmHel-12	BABH01062650:297-421
BmHel-12	DF090330:4027175-4027315
BmHel-12	DF090406:193728-193927
BmHel-12	DF090338:1683079-1683279
BmHel-12	DF090403:512502-512630
BmHel-12	DF090390:142541-142663
BmHel-12	DF090435:391009-391128
BmHel-12	DF090332:242635-242839
BmHel-12	DF090342:3936209-3936408
BmHel-12	DF090638:16072-16193
BmHel-12	DF090377:1830852-1830996
BmHel-12	BABH01067099:389-533
BmHel-12	DF090363:2672209-2672338
BmHel-12	DF090339:4872300-4872501
BmHel-12	DF090444:99032-99182
BmHel-12	DF090329:4697894-4698023
BmHel-12	DF090329:4697798-4697893
BmHel-12	DF090347:501728-501871
BmHel-12	DF090352:410821-411017
BmHel-12	DF090348:1081057-1081248
BmHel-12	DF090349:240318-240433

BmHel-12	DF090555:55122-55245
BmHel-12	DF090555:55032-55120
BmHel-12	DF090365:2800827-2800967
BmHel-12	DF090339:1700428-1700557
BmHel-12	DF090327:3861944-3862148
BmHel-12	DF090361:579138-579334
BmHel-12	DF090390:1569577-1569770
BmHel-12	DF090351:744828-744932
BmHel-12	DF090402:1318048-1318164
BmHel-12	DF090477:187405-187542
BmHel-12	DF090477:187550-187750
BmHel-12	BABH01049853:241-373
BmHel-12	BABH01049853:150-238
BmHel-12	DF090319:5803160-5803359
BmHel-12	DF090347:2021035-2021142
BmHel-12	DF090347:2020899-2020982
BmHel-12	DF090343:1395894-1396082
BmHel-12	DF090322:7660863-7660957
BmHel-12	DF090322:7661000-7661091
BmHel-12	DF090319:353810-354014
BmHel-12	DF090367:1732046-1732228
BmHel-12	DF090356:2108500-2108702
BmHel-12	DF090317:313553-313677
BmHel-12	DF090330:3131584-3131714
BmHel-12	DF090331:4315549-4315666
BmHel-12	DF090383:339141-339230
BmHel-12	DF090383:339041-339139
BmHel-12	DF090438:621882-621980
BmHel-12	DF090438:621754-621840
BmHel-12	DF090358:368206-368391
BmHel-12	DF090328:6202745-6202854
BmHel-12	DF090335:2747632-2747760
BmHel-12	BABH01050606:752-946
BmHel-12	DF090324:5678253-5678391
BmHel-12	DF090330:5791177-5791361
BmHel-12	DF090342:2091456-2091644
BmHel-12	DF090391:762052-762241
BmHel-12	DF090590:6168-6300
BmHel-12	DF090590:6302-6384
BmHel-12	DF090336:4947315-4947498
BmHel-12	DF090342:794665-794789
BmHel-12	DF090316:7289606-7289793
BmHel-12	BABH01045683:4055-4242
BmHel-12	DF090756:13119-13238
BmHel-12	DF090332:952147-952227
BmHel-12	DF090332:952019-952118
BmHel-12	DF090357:3638969-3639160
BmHel-12	DF090357:1626696-1626884

BmHel-12	DF090319:3029676-3029862
BmHel-12	DF090376:26016-26202
BmHel-12	DF090442:189230-189341
BmHel-12	DF090566:35215-35334
BmHel-12	DF090321:3410273-3410465
BmHel-12	DF090336:1960819-1960931
BmHel-12	DF090430:294287-294392
BmHel-12	DF090430:294188-294285
BmHel-12	DF090434:333972-334151
BmHel-12	BABH01048731:672-805
BmHel-12	DF090382:285672-285825
BmHel-12	DF090339:2045025-2045212
BmHel-12	DF090331:1691956-1692107
BmHel-12	DF090343:3260203-3260340
BmHel-12	DF090370:1809736-1809825
BmHel-12	DF090370:1809623-1809714
BmHel-12	DF090662:1296-1480
BmHel-12	DF090338:3594473-3594608
BmHel-12	DF090362:3226-3413
BmHel-12	DF090317:6250820-6250944
BmHel-12	DF090467:314803-314939
BmHel-12	DF090328:1425334-1425517
BmHel-12	DF090387:1742171-1742351
BmHel-12	DF090365:1771132-1771315
BmHel-12	BABH01054923:588-770
BmHel-12	DF090373:1551797-1551930
BmHel-12	DF090344:897059-897255
BmHel-12	DF090316:8897065-8897248
BmHel-12	BABH01077361:263-435
BmHel-12	DF090370:1331842-1332019
BmHel-12	DF090360:2735591-2735771
BmHel-12	DF090352:851725-851851
BmHel-12	DF090383:438312-438493
BmHel-12	DF090403:1277625-1277747
BmHel-12	DF090356:1174628-1174758
BmHel-12	DF090336:3884129-3884307
BmHel-12	DF090594:40044-40224
BmHel-12	DF090459:9748-9924
BmHel-12	DF090340:4762175-4762354
BmHel-12	DF090322:2326401-2326580
BmHel-12	DF090331:3844992-3845172
BmHel-12	DF090318:7111137-7111312
BmHel-12	DF090326:1486649-1486761
BmHel-12	DF090340:3555296-3555472
BmHel-12	DF090337:3715864-3715999
BmHel-12	DF090333:5372283-5372389
BmHel-12	BABH01068560:73-203
BmHel-12	DF090317:4595178-4595363

BmHel-12	DF090328:4729372-4729548
BmHel-12	BABH01083393:1-168
BmHel-12	DF090355:72432-72533
BmHel-12	DF090451:522031-522211
BmHel-12	DF090317:1788164-1788341
BmHel-12	BABH01068588:148-321
BmHel-12	DF090344:3666567-3666660
BmHel-12	DF090344:3666456-3666535
BmHel-12	DF090322:410128-410305
BmHel-12	DF090355:1912434-1912611
BmHel-12	DF090319:6270515-6270610
BmHel-12	DF090414:1099007-1099114
BmHel-12	DF090352:1406304-1406458
BmHel-12	DF090367:1100891-1101027
BmHel-12	DF090344:3159648-3159824
BmHel-12	DF090402:1148734-1148828
BmHel-12	DF090365:2895596-2895697
BmHel-12	DF090503:90141-90302
BmHel-12	DF090340:1340021-1340194
BmHel-12	DF090472:286934-287108
BmHel-12	BABH01056525:554-731
BmHel-12	DF090320:5987673-5987841
BmHel-12	DF090411:1120362-1120535
BmHel-12	DF090435:224067-224240
BmHel-12	DF090419:310931-311057
BmHel-12	DF090373:740841-741009
BmHel-12	DF090319:5390421-5390537
BmHel-12	DF090370:924837-925009
BmHel-12	DF090383:105606-105778
BmHel-12	BABH01045807:2592-2759
BmHel-12	BABH01066584:2-169
BmHel-12	BABH01063296:511-679
BmHel-12	DF090441:550135-550308
BmHel-12	DF090344:3909438-3909608
BmHel-12	DF090318:2731461-2731563
BmHel-12	DF090317:3697085-3697203
BmHel-12	DF090316:10016676-10016767
BmHel-12	DF090363:1240943-1241099
BmHel-12	DF090444:600067-600235
BmHel-12	DF090382:1155546-1155625
BmHel-12	DF090382:1155660-1155746
BmHel-12	DF090358:1146635-1146758
BmHel-12	DF090323:5027829-5027953
BmHel-12	DF091797:703-869
BmHel-12	DF090328:4357230-4357324
BmHel-12	DF090365:2743691-2743786
BmHel-12	BABH01065323:503-666
BmHel-12	DF090320:5678114-5678281

BmHel-12	DF090335:4956660-4956828
BmHel-12	DF090316:13473641-13473753
BmHel-12	DF090336:1720019-1720181
BmHel-12	DF090357:1719532-1719640
BmHel-12	DF090342:3297154-3297267
BmHel-12	BABH01047936:1027-1137
BmHel-12	DF090414:82238-82320
BmHel-12	DF090414:82333-82413
BmHel-12	DF090357:3427858-3428018
BmHel-12	DF090431:298334-298461
BmHel-12	DF090364:495483-495584
BmHel-12	DF090393:425865-425953
BmHel-12	DF090339:4498485-4498568
BmHel-12	DF090329:2776105-2776257
BmHel-12	DF090417:855709-855801
BmHel-12	DF090351:3168516-3168670
BmHel-12	DF090370:2063157-2063237
BmHel-12	DF090358:2469744-2469824
BmHel-12	DF090336:1711165-1711247
BmHel-12	DF090331:1153298-1153455
BmHel-12	DF090361:515887-515999
BmHel-12	DF090350:2691081-2691163
BmHel-12	DF090347:1066393-1066520
BmHel-12	DF090344:2300801-2300966
BmHel-12	DF090408:30026-30141
BmHel-12	DF090388:824110-824218
BmHel-12	DF090338:2579284-2579437
BmHel-12	DF090389:1795692-1795816
BmHel-12	DF090348:1333900-1334056
BmHel-12	DF090344:1668909-1669012
BmHel-12	DF090384:207676-207775
BmHel-12	DF090334:3318158-3318272
BmHel-12	DF090338:2264585-2264733
BmHel-12	DF090384:1879455-1879565
BmHel-12	DF090379:491484-491621
BmHel-12	DF090327:3076303-3076456
BmHel-12	DF090554:12281-12401
BmHel-12	DF090316:4407666-4407819
BmHel-12	DF090345:3290530-3290640
BmHel-12	BABH01046596:1214-1368
BmHel-12	DF090352:4032751-4032853
BmHel-12	DF090371:598634-598749
BmHel-12	DF090413:47619-47764
BmHel-12	DF090465:175288-175404
BmHel-12	DF090356:1090761-1090911
BmHel-12	DF090356:3730294-3730417
BmHel-12	BABH01084588:348-494
BmHel-12	DF090333:3861623-3861771

BmHel-12	DF090388:937968-938072
BmHel-12	DF090402:515246-515351
BmHel-12	DF090390:882680-882759
BmHel-12	DF090324:607910-608056
BmHel-12	DF090338:3081746-3081880
BmHel-12	DF090362:1651730-1651872
BmHel-12	BABH01063020:541-681
BmHel-12	DF090327:4140654-4140793
BmHel-12	DF090364:716051-716141
BmHel-12	BABH01065925:492-632
BmHel-12	BABH01044974:6565-6707
BmHel-12	DF090372:2053131-2053272
BmHel-12	BABH01082286:405-547
BmHel-12	DF090331:5105365-5105490
BmHel-12	DF090327:3351330-3351469
BmHel-12	BABH01067142:11-150
BmHel-12	BABH01051757:707-850
BmHel-12	DF090375:2298000-2298079
BmHel-12	DF090373:257238-257370
BmHel-12	DF090363:2365283-2365415
BmHel-12	DF090391:1046434-1046573
BmHel-12	BABH01050956:1-133
BmHel-12	DF090342:4842025-4842126
BmHel-12	DF090316:11297061-11297197
BmHel-12	DF090364:2754269-2754403
BmHel-12	DF090361:2672735-2672871
BmHel-12	DF090405:1360741-1360873
BmHel-12	DF090329:3926921-3927001
BmHel-12	DF090350:3437476-3437607
BmHel-12	DF090425:932364-932452
BmHel-12	DF090352:1213687-1213819
BmHel-12	DF090332:32-174
BmHel-12	DF090430:864622-864715
BmHel-12	DF090330:3225234-3225358
BmHel-12	DF090350:2690929-2691060
BmHel-12	DF090388:944476-944600
BmHel-12	BABH01073201:489-617
BmHel-12	BABH01080825:41-164
BmHel-12	DF090316:12510702-12510783
BmHel-12	DF090346:2810804-2810931
BmHel-12	DF090324:260146-260273
BmHel-12	DF090450:362275-362401
BmHel-12	DF090339:4872502-4872630
BmHel-12	DF090391:1566194-1566323
BmHel-12	BABH01078980:444-578
BmHel-12	DF090330:4514700-4514827
BmHel-12	DF090325:2121569-2121700
BmHel-12	DF090365:151892-152023

BmHel-12	DF090374:2022102-2022229
BmHel-12	DF090363:1376129-1376252
BmHel-12	DF090480:231078-231211
BmHel-12	BABH01050792:153-280
BmHel-12	BABH01069069:490-621
BmHel-12	DF090344:345657-345737
BmHel-12	DF090433:216037-216152
BmHel-12	DF090574:22520-22645
BmHel-12	DF090396:1269756-1269882
BmHel-12	DF090344:211663-211788
BmHel-12	BABH01047435:585-674
BmHel-12	DF090339:2881662-2881788
BmHel-12	DF090370:2234399-2234523
BmHel-12	DF090333:4739853-4739976
BmHel-12	DF090324:6570799-6570911
BmHel-12	DF090411:6270-6393
BmHel-12	DF090462:354749-354869
BmHel-12	DF090488:157025-157146
BmHel-12	DF090385:1852676-1852798
BmHel-12	DF090377:438039-438120
BmHel-12	DF090319:4586134-4586256
BmHel-12	BABH01066147:534-661
BmHel-12	DF090388:1431739-1431861
BmHel-12	DF090433:468355-468476
BmHel-12	DF090328:4981614-4981734
BmHel-12	DF090316:11800362-11800484
BmHel-12	DF090350:4234368-4234488
BmHel-12	DF090331:918930-919044
BmHel-12	DF090378:1207179-1207298
BmHel-12	DF090350:3599031-3599149
BmHel-12	DF090374:488042-488158
BmHel-12	DF090342:2823950-2824072
BmHel-12	DF090447:415407-415525
BmHel-12	BABH01050549:841-951
BmHel-12	BABH01078686:120-236
BmHel-12	BABH01051638:1-117
BmHel-12	DF090390:1096633-1096747
BmHel-12	DF090325:2931675-2931787
BmHel-12	DF090319:4671216-4671331
BmHel-12	DF090332:663337-663452
BmHel-12	DF090334:2246025-2246140
BmHel-12	DF090327:5291267-5291382
BmHel-12	DF090345:2583376-2583488
BmHel-12	DF090343:4224615-4224730
BmHel-12	DF090323:617080-617192
BmHel-12	DF090360:2886820-2886930
BmHel-12	DF090365:459740-459851
BmHel-12	DF090356:3720814-3720923

BmHel-12	BABH01046305:2326-2443
BmHel-12	DF090334:4178889-4178998
BmHel-12	DF090419:9238-9351
BmHel-12	DF090343:70025-70134
BmHel-12	BABH01081341:447-556
BmHel-12	DF090413:254234-254341
BmHel-12	DF090376:1935268-1935386
BmHel-12	DF090626:23848-23955
BmHel-12	BABH01069616:1-107
BmHel-12	DF090342:3817394-3817496
BmHel-12	BABH01058261:483-585
BmHel-12	DF090370:2234287-2234394
BmHel-12	DF090316:2064961-2065068
BmHel-12	BABH01079949:26-130
BmHel-12	DF090410:493621-493725
BmHel-12	DF090327:1759089-1759194
BmHel-12	DF090417:892838-892941
BmHel-12	BABH01073453:13-116
BmHel-12	BABH01086083:344-446
BmHel-12	BABH01083120:364-466
BmHel-12	DF090464:199653-199755
BmHel-12	BABH01065286:1-101
BmHel-12	DF090319:246086-246190
BmHel-12	DF090349:2460288-2460393
BmHel-12	DF090377:707849-707949
BmHel-12	DF090414:391676-391776
BmHel-12	DF090386:1638951-1639054
BmHel-12	DF090325:2150270-2150371
BmHel-12	DF090319:416899-416999
BmHel-12	DF090330:382270-382369
BmHel-12	DF090367:2804497-2804596
BmHel-12	DF090358:1288923-1289025
BmHel-12	DF090358:2661999-2662098
BmHel-12	DF090397:363777-363875
BmHel-12	BABH01049182:503-601
BmHel-12	DF090344:4356581-4356679
BmHel-12	DF090443:57583-57689
BmHel-12	DF090348:2817304-2817402
BmHel-12	DF090344:2771219-2771318
BmHel-12	BABH01070375:31-131
BmHel-12	DF090344:2453771-2453870
BmHel-12	DF090410:320034-320130
BmHel-12	DF090342:1340551-1340647
BmHel-12	BABH01065997:450-546
BmHel-12	DF090366:457490-457587
BmHel-12	DF090517:15120-15216
BmHel-12	DF090395:1382858-1382954
BmHel-12	DF090322:6988975-6989067

BmHel-12	DF090403:939789-939883
BmHel-12	DF090319:8211981-8212077
BmHel-12	DF090339:2051576-2051669
BmHel-12	DF090382:1874298-1874389
BmHel-12	DF090609:5158-5250
BmHel-12	DF090345:2295303-2295393
BmHel-12	DF090339:3260305-3260396
BmHel-12	DF090350:3661241-3661335
BmHel-12	DF090368:1602704-1602793
BmHel-12	BABH01065080:580-668
BmHel-12	BABH01081259:417-505
BmHel-12	BABH01047764:384-472
BmHel-12	DF090377:1651988-1652077
BmHel-12	DF090320:2933608-2933691
BmHel-12	DF090316:3789891-3789978
BmHel-12	BABH01064497:584-671
BmHel-12	DF090354:1079822-1079913
BmHel-12	DF090405:564039-564127
BmHel-12	DF090350:2658079-2658165
BmHel-12	DF090345:1514962-1515049
BmHel-12	DF090349:2810615-2810700
BmHel-12	DF090327:1386914-1386999
BmHel-12	BABH01070256:1-86
BmHel-12	DF090382:1489124-1489209
BmHel-12	DF090345:1493621-1493707
BmHel-12	DF090359:205166-205250
BmHel-12	BABH01062046:549-633
BmHel-12	BABH01052689:7-90
BmHel-12	DF090318:93875-93959
BmHel-12	BABH01060171:326-412
BmHel-12	BABH01064740:1-86
BmHel-12	BABH01087474:331-413
BmHel-12	DF090357:1992815-1992896
BmHel-12	DF090353:3170239-3170319
BmHel-12	BABH01083204:306-386
BmHel-12	DF090341:1764451-1764531
BmHel-12	DF090352:663082-663161
BmHel-12	BABH01050527:638-717
BmHel-12	DF090349:4003321-4003403
BmHel-12	DF090438:331452-331531
BmHel-12	DF090438:460028-460107
BmHel-12	DF090450:304828-304907
BmHel-12	DF091774:551-630
BmHel-13	DF090384:185510-185794
BmHel-13	DF090409:101105-101389
BmHel-13	DF090317:4309242-4309532
BmHel-13	DF090358:3238923-3239217

BmHel-13	DF090366:1952646-1952941
BmHel-13	DF090325:4100153-4100450
BmHel-13	DF090328:6140913-6141210
BmHel-13	DF090321:3496984-3497281
BmHel-13	DF090384:1564576-1564873
BmHel-13	DF090429:486399-486697
BmHel-13	DF090359:107246-107545
BmHel-13	DF090351:617457-617756
BmHel-13	DF090325:6268410-6268709
BmHel-13	DF090383:440485-440787
BmHel-13	DF090361:790601-790904
BmHel-13	DF090322:5523673-5523976
BmHel-13	DF090345:2506830-2507134
BmHel-13	DF090367:218528-218832
BmHel-13	DF090316:10067938-10068244
BmHel-13	DF090366:1526158-1526464
BmHel-13	DF090426:764276-764584
BmHel-13	DF090401:989902-990210
BmHel-13	DF090342:4751752-4752060
BmHel-13	DF090326:3826614-3826922
BmHel-13	DF090348:418635-418944
BmHel-13	DF090326:5514573-5514882
BmHel-13	DF090331:3303819-3304128
BmHel-13	DF090329:6080051-6080360
BmHel-13	DF090328:5606525-5606834
BmHel-13	DF090422:1026509-1026818
BmHel-13	DF090353:3120518-3120828
BmHel-13	DF090344:155499-155810
BmHel-13	DF090360:3292165-3292476
BmHel-13	DF090352:848738-849051
BmHel-13	DF090487:109528-109841
BmHel-13	DF090389:931273-931588
BmHel-13	DF090321:5246387-5246726
BmHel-13	DF090324:39583-40069
BmHel-13	DF090328:3291740-3292261
BmHel-13	DF090388:1769477-1770006
BmHel-13	DF090342:2146867-2147437
BmHel-13	DF090348:3473443-3474022
BmHel-13	DF090335:1089760-1091810
BmHel-13	DF090329:6513078-6515200
BmHel-13	DF090375:772351-781314
BmHel-13	DF090359:506829-507085
BmHel-13	DF090317:11109411-11109662
BmHel-13	DF090372:1364090-1364213
BmHel-13	DF090372:1364213-1364329
BmHel-13	DF090339:2864293-2864540
BmHel-13	DF090318:4710971-4711229
BmHel-13	DF090318:5518855-5519113

BmHel-13	DF090375:479301-479559
BmHel-13	DF090931:10430-10688
BmHel-13	DF090324:729082-729334
BmHel-13	DF090347:2819353-2819602
BmHel-13	DF090343:942344-942602
BmHel-13	DF090337:2287381-2287639
BmHel-13	DF090329:1862534-1862790
BmHel-13	DF090329:4327545-4327803
BmHel-13	DF090329:3272005-3272263
BmHel-13	DF090326:1855852-1856106
BmHel-13	DF090371:24996-25253
BmHel-13	DF090338:76938-77197
BmHel-13	DF090400:1149234-1149492
BmHel-13	DF090364:145672-145930
BmHel-13	DF090356:3540853-3541111
BmHel-13	DF090336:2779006-2779272
BmHel-13	DF090336:1687970-1688228
BmHel-13	DF090335:765588-765836
BmHel-13	DF090373:983003-983260
BmHel-13	DF090327:1743408-1743666
BmHel-13	DF090327:6021928-6022186
BmHel-13	DF090334:5541551-5541808
BmHel-13	DF090394:1013873-1014131
BmHel-13	DF090385:1602482-1602740
BmHel-13	DF090415:550267-550525
BmHel-13	DF090429:659227-659486
BmHel-13	DF090316:9818442-9818697
BmHel-13	DF090330:1649518-1649780
BmHel-13	DF090439:586404-586660
BmHel-13	BABH01052210:161-417
BmHel-13	DF090318:8296057-8296309
BmHel-13	DF090334:2960982-2961236
BmHel-13	DF090334:2299706-2299834
BmHel-13	DF090334:2299556-2299684
BmHel-13	DF090389:1456865-1457078
BmHel-13	DF090316:9034739-9034994
BmHel-13	DF090341:4104263-4104515
BmHel-13	DF090384:1505549-1505804
BmHel-13	DF090356:3823526-3823671
BmHel-13	DF090356:3824985-3825101
BmHel-13	DF090319:2873789-2873953
BmHel-13	DF090319:2873388-2873487
BmHel-13	DF090324:5623638-5623853
BmHel-13	DF090323:4605859-4605987
BmHel-13	DF090323:4606009-4606137
BmHel-13	DF090318:5444508-5444759
BmHel-13	BABH01066863:278-499
BmHel-13	DF090461:211407-211637

BmHel-13	DF090461:184056-184277
BmHel-13	DF090355:1323570-1323791
BmHel-13	DF090354:3467894-3468115
BmHel-13	DF090354:2832763-2832984
BmHel-13	DF090354:2439881-2440102
BmHel-13	DF090354:1733502-1733720
BmHel-13	DF090339:99018-99239
BmHel-13	DF090339:1039538-1039762
BmHel-13	DF090339:1063866-1064087
BmHel-13	DF090339:1957565-1957786
BmHel-13	DF090339:3187244-3187465
BmHel-13	DF090339:3310669-3310890
BmHel-13	DF090339:1320103-1320324
BmHel-13	DF090339:525942-526163
BmHel-13	DF090323:3028209-3028427
BmHel-13	DF090323:4495043-4495273
BmHel-13	DF090323:6136039-6136260
BmHel-13	DF090323:6245345-6245570
BmHel-13	DF090323:4039333-4039554
BmHel-13	DF090323:3273418-3273641
BmHel-13	DF090323:2569703-2569924
BmHel-13	DF090323:1883671-1883890
BmHel-13	DF090323:1402864-1403085
BmHel-13	DF090323:823205-823426
BmHel-13	DF090323:774009-774230
BmHel-13	DF090318:6666928-6667148
BmHel-13	DF090318:9882853-9883073
BmHel-13	DF090318:2877365-2877586
BmHel-13	DF090318:1953873-1954094
BmHel-13	DF090318:330657-330878
BmHel-13	DF090342:1269772-1269993
BmHel-13	DF090342:2754183-2754404
BmHel-13	DF090342:3237483-3237704
BmHel-13	DF090342:4030972-4031201
BmHel-13	DF090342:4648027-4648248
BmHel-13	DF090342:2007945-2008166
BmHel-13	DF090342:786498-786719
BmHel-13	DF090342:149016-149238
BmHel-13	DF090316:3062714-3062939
BmHel-13	DF090316:4584675-4584896
BmHel-13	DF090316:6568306-6568527
BmHel-13	DF090316:7192794-7193015
BmHel-13	DF090316:9203358-9203578
BmHel-13	DF090316:9574353-9574577
BmHel-13	DF090316:15000362-15000583
BmHel-13	DF090316:16060352-16060573
BmHel-13	DF090316:11714141-11714359
BmHel-13	DF090316:7253268-7253486

BmHel-13	DF090316:5815141-5815366
BmHel-13	DF090316:3875770-3875990
BmHel-13	DF090368:478683-478904
BmHel-13	DF090412:753884-754105
BmHel-13	DF090412:935201-935422
BmHel-13	DF090412:895960-896184
BmHel-13	DF090365:1639085-1639307
BmHel-13	DF090341:4017753-4017885
BmHel-13	DF090341:4017908-4017996
BmHel-13	DF090341:4408393-4408614
BmHel-13	DF090341:3412271-3412496
BmHel-13	DF090330:2624582-2624803
BmHel-13	DF090330:3941836-3942055
BmHel-13	DF090330:6284346-6284567
BmHel-13	DF090330:5613465-5613686
BmHel-13	DF090330:3126245-3126466
BmHel-13	DF090330:676483-676704
BmHel-13	DF090345:2022901-2023119
BmHel-13	DF090345:2876977-2877199
BmHel-13	DF090345:3739479-3739699
BmHel-13	DF090345:4279196-4279417
BmHel-13	DF090345:4302198-4302419
BmHel-13	DF090345:4351825-4352046
BmHel-13	DF090345:4315760-4315980
BmHel-13	DF090345:3436145-3436366
BmHel-13	DF090345:2574172-2574393
BmHel-13	DF090345:1273553-1273775
BmHel-13	DF090344:81040-81259
BmHel-13	DF090344:2165856-2166077
BmHel-13	DF090344:4612483-4612704
BmHel-13	DF090344:2357152-2357373
BmHel-13	DF090344:1250086-1250308
BmHel-13	DF090324:757908-758129
BmHel-13	DF090324:278297-278518
BmHel-13	DF090319:8661196-8661417
BmHel-13	DF090319:7851933-7852154
BmHel-13	DF090319:5816581-5816801
BmHel-13	DF090319:4440120-4440341
BmHel-13	DF090319:2506726-2506947
BmHel-13	DF090319:1886101-1886322
BmHel-13	DF090319:1369989-1370214
BmHel-13	BABH01069824:363-584
BmHel-13	BABH01049760:245-466
BmHel-13	DF090391:1104122-1104338
BmHel-13	DF090388:294651-294873
BmHel-13	DF090388:1470005-1470226
BmHel-13	DF090380:1878976-1879198
BmHel-13	DF090380:1980233-1980454

BmHel-13	DF090380:2011537-2011758
BmHel-13	DF090362:26602-26824
BmHel-13	DF090362:1983270-1983491
BmHel-13	DF090362:2053741-2053962
BmHel-13	DF090362:3123580-3123811
BmHel-13	DF090362:2307397-2307618
BmHel-13	DF090362:1694508-1694729
BmHel-13	DF090362:1585078-1585299
BmHel-13	DF090362:1510618-1510839
BmHel-13	DF090362:845632-845855
BmHel-13	DF090351:1082368-1082593
BmHel-13	DF090351:3600526-3600747
BmHel-13	DF090348:1281677-1281899
BmHel-13	DF090348:1454420-1454641
BmHel-13	DF090348:2291614-2291835
BmHel-13	DF090348:3392510-3392731
BmHel-13	DF090347:553665-553886
BmHel-13	DF090347:784213-784434
BmHel-13	DF090347:3465882-3466103
BmHel-13	DF090347:3951290-3951511
BmHel-13	DF090347:4048058-4048279
BmHel-13	DF090347:3385687-3385908
BmHel-13	DF090347:416398-416619
BmHel-13	DF090346:1538601-1538822
BmHel-13	DF090346:1804651-1804873
BmHel-13	DF090346:2940276-2940497
BmHel-13	DF090346:3061438-3061659
BmHel-13	DF090343:1276322-1276543
BmHel-13	DF090343:4140307-4140528
BmHel-13	DF090343:3792315-3792536
BmHel-13	DF090343:3139433-3139653
BmHel-13	DF090343:2556525-2556746
BmHel-13	DF090337:882387-882608
BmHel-13	DF090337:4592453-4592674
BmHel-13	DF090337:4316032-4316251
BmHel-13	DF090329:3021728-3021956
BmHel-13	DF090329:4668312-4668530
BmHel-13	DF090329:5198723-5198946
BmHel-13	DF090329:4166141-4166364
BmHel-13	DF090329:2363534-2363755
BmHel-13	DF090329:336931-337152
BmHel-13	DF090328:1300019-1300240
BmHel-13	DF090328:3712744-3712965
BmHel-13	DF090326:606104-606322
BmHel-13	DF090326:1355465-1355686
BmHel-13	DF090326:1430340-1430551
BmHel-13	DF090326:2715309-2715530
BmHel-13	DF090326:6274454-6274675

BmHel-13	DF090326:6354739-6354958
BmHel-13	DF090326:6951138-6951359
BmHel-13	DF090326:6176220-6176441
BmHel-13	DF090326:6119577-6119798
BmHel-13	DF090326:2425245-2425466
BmHel-13	DF090326:530119-530339
BmHel-13	DF090321:5210897-5211118
BmHel-13	DF090321:8096651-8096865
BmHel-13	DF090321:6396370-6396591
BmHel-13	DF090321:6245868-6246091
BmHel-13	DF090321:4820157-4820375
BmHel-13	DF090321:3585828-3586049
BmHel-13	DF090321:7563-7784
BmHel-13	DF090317:70588-70809
BmHel-13	DF090317:10060453-10060674
BmHel-13	DF090317:9521444-9521662
BmHel-13	DF090317:4704789-4705010
BmHel-13	DF090317:3193894-3194114
BmHel-13	DF090317:1634967-1635188
BmHel-13	DF090460:297458-297679
BmHel-13	DF090371:2363735-2363956
BmHel-13	DF090371:1523200-1523422
BmHel-13	DF090360:2617549-2617770
BmHel-13	DF090360:643652-643873
BmHel-13	DF090338:2774943-2775164
BmHel-13	DF090338:2646458-2646678
BmHel-13	DF090332:6120783-6121003
BmHel-13	DF090332:6230471-6230697
BmHel-13	DF090332:1609742-1609963
BmHel-13	DF090325:6345197-6345418
BmHel-13	DF090325:3421507-3421730
BmHel-13	DF090361:298544-298763
BmHel-13	DF090361:397435-397654
BmHel-13	DF090361:460213-460434
BmHel-13	DF090361:1401422-1401646
BmHel-13	DF090361:2565764-2565985
BmHel-13	DF090361:2048198-2048419
BmHel-13	DF090381:1595985-1596206
BmHel-13	DF090381:46037-46258
BmHel-13	DF090352:372373-372598
BmHel-13	DF090352:3375005-3375226
BmHel-13	DF090322:1719842-1720063
BmHel-13	DF090322:2710474-2710696
BmHel-13	DF090322:7410589-7410809
BmHel-13	DF090322:6210880-6211102
BmHel-13	DF090322:4767896-4768114
BmHel-13	BABH01081304:200-421
BmHel-13	BABH01047827:36-257

BmHel-13	DF090886:11367-11588
BmHel-13	DF090600:34234-34455
BmHel-13	DF090444:275567-275788
BmHel-13	DF090444:105624-105843
BmHel-13	DF090435:2904-3125
BmHel-13	DF090435:643165-643386
BmHel-13	DF090435:24748-24969
BmHel-13	DF090433:27350-27571
BmHel-13	DF090426:921341-921562
BmHel-13	DF090403:257189-257410
BmHel-13	DF090396:248689-248910
BmHel-13	DF090386:371114-371337
BmHel-13	DF090386:457346-457567
BmHel-13	DF090384:251595-251810
BmHel-13	DF090364:429459-429680
BmHel-13	DF090364:275801-276022
BmHel-13	DF090359:3112424-3112645
BmHel-13	DF090359:1805958-1806179
BmHel-13	DF090358:1576616-1576837
BmHel-13	DF090358:816330-816551
BmHel-13	DF090356:323524-323744
BmHel-13	DF090356:3273646-3273867
BmHel-13	DF090349:1693066-1693287
BmHel-13	DF090340:1167342-1167563
BmHel-13	DF090340:2890260-2890481
BmHel-13	DF090340:3268770-3268993
BmHel-13	DF090336:1943558-1943779
BmHel-13	DF090336:5324091-5324312
BmHel-13	DF090335:643806-644027
BmHel-13	DF090335:1270646-1270868
BmHel-13	DF090335:2824494-2824715
BmHel-13	DF090335:3089327-3089545
BmHel-13	DF090335:4672064-4672285
BmHel-13	DF090335:5731286-5731511
BmHel-13	DF090335:4693284-4693505
BmHel-13	DF090335:3456307-3456516
BmHel-13	DF090335:582556-582777
BmHel-13	DF090331:1926801-1927023
BmHel-13	DF090331:2193484-2193706
BmHel-13	DF090331:2769955-2770176
BmHel-13	DF090331:3074502-3074729
BmHel-13	DF090331:3997462-3997683
BmHel-13	DF090331:3985340-3985561
BmHel-13	DF090331:3934278-3934499
BmHel-13	DF090331:748027-748248
BmHel-13	DF090420:364506-364727
BmHel-13	DF090390:686254-686475
BmHel-13	DF090382:1702328-1702549

BmHel-13	DF090373:1109177-1109398
BmHel-13	DF090373:1537363-1537584
BmHel-13	DF090373:1755300-1755521
BmHel-13	DF090373:804916-805137
BmHel-13	DF090372:1180619-1180841
BmHel-13	DF090372:841567-841788
BmHel-13	DF090350:3676976-3677197
BmHel-13	DF090350:3515885-3516109
BmHel-13	DF090350:2431940-2432161
BmHel-13	DF090422:1109575-1109799
BmHel-13	DF090401:1091120-1091341
BmHel-13	DF090405:417906-418127
BmHel-13	DF090363:861518-861739
BmHel-13	DF090363:336064-336285
BmHel-13	DF090577:25862-26083
BmHel-13	DF090484:185108-185329
BmHel-13	DF090457:88858-89079
BmHel-13	DF090431:173976-174197
BmHel-13	DF090411:704959-705180
BmHel-13	DF090411:608968-609185
BmHel-13	DF090411:234517-234738
BmHel-13	DF090395:943593-943814
BmHel-13	DF090393:1093722-1093943
BmHel-13	DF090378:1343729-1343950
BmHel-13	DF090378:2041159-2041382
BmHel-13	DF090378:265016-265238
BmHel-13	DF090367:2438995-2439220
BmHel-13	DF090367:1883467-1883688
BmHel-13	DF090333:574164-574385
BmHel-13	DF090333:4387779-4387997
BmHel-13	DF090333:3592561-3592775
BmHel-13	DF090333:431649-431870
BmHel-13	DF090327:2851095-2851316
BmHel-13	DF090327:5880079-5880300
BmHel-13	DF090327:1986013-1986234
BmHel-13	DF090418:679049-679269
BmHel-13	DF090398:190508-190725
BmHel-13	DF090334:2573519-2573742
BmHel-13	DF090334:3121638-3121854
BmHel-13	DF090334:5044725-5044943
BmHel-13	DF090334:3720120-3720342
BmHel-13	BABH01053060:357-579
BmHel-13	BABH01045814:314-535
BmHel-13	DF090802:2684-2905
BmHel-13	DF090607:2205-2426
BmHel-13	DF090472:270869-271090
BmHel-13	DF090466:235147-235371
BmHel-13	DF090421:484498-484719

BmHel-13	DF090421:932922-933143
BmHel-13	DF090408:787433-787654
BmHel-13	DF090394:1305665-1305886
BmHel-13	BABH01073754:40-262
BmHel-13	BABH01056119:305-524
BmHel-13	DF090383:307518-307736
BmHel-13	DF090383:360761-360982
BmHel-13	DF090383:1451176-1451397
BmHel-13	BABH01045017:5572-5793
BmHel-13	DF090612:13781-14002
BmHel-13	DF090481:188674-188895
BmHel-13	DF090448:352972-353192
BmHel-13	DF090442:538986-539215
BmHel-13	DF090407:760903-761123
BmHel-13	DF090402:496985-497206
BmHel-13	DF090385:1147261-1147485
BmHel-13	DF090441:684060-684281
BmHel-13	DF090415:85826-86048
BmHel-13	BABH01043741:7894-8115
BmHel-13	DF090570:41970-42191
BmHel-13	DF090539:13620-13840
BmHel-13	DF090468:199216-199437
BmHel-13	DF090449:754304-754526
BmHel-13	DF090617:34440-34653
BmHel-13	BABH01045502:2310-2531
BmHel-13	DF090522:3576-3802
BmHel-13	DF090423:904511-904733
BmHel-13	DF090496:139399-139619
BmHel-13	DF090640:14736-14957
BmHel-13	DF090410:286769-286990
BmHel-13	DF091042:9258-9477
BmHel-13	BABH01085299:118-334
BmHel-13	BABH01045685:2828-2957
BmHel-13	BABH01045685:2712-2803
BmHel-13	DF090330:400100-400227
BmHel-13	DF090330:400249-400377
BmHel-13	DF090342:3573767-3573899
BmHel-13	DF090342:3573925-3574012
BmHel-13	DF090342:591958-592178
BmHel-13	DF090346:1703762-1703979
BmHel-13	DF090332:5894980-5895108
BmHel-13	DF090332:5894863-5894954
BmHel-13	DF090361:2465406-2465489
BmHel-13	DF090361:2471219-2471355
BmHel-13	DF090358:945829-946049
BmHel-13	DF090355:2847484-2847704
BmHel-13	DF090354:2647702-2647921
BmHel-13	DF090339:4392263-4392485

BmHel-13	DF090339:3809319-3809539
BmHel-13	DF090339:1010085-1010305
BmHel-13	DF090339:478420-478640
BmHel-13	DF090323:2849061-2849281
BmHel-13	DF090342:1834111-1834333
BmHel-13	DF090316:4798084-4798304
BmHel-13	DF090316:9917394-9917614
BmHel-13	DF090316:3584846-3585066
BmHel-13	DF090316:1397187-1397407
BmHel-13	DF090368:2037313-2037533
BmHel-13	DF090375:1468103-1468323
BmHel-13	DF090341:432091-432310
BmHel-13	DF090341:2642950-2643165
BmHel-13	DF090357:822015-822235
BmHel-13	DF090357:2911905-2912125
BmHel-13	DF090357:1285425-1285645
BmHel-13	DF090345:3658943-3659163
BmHel-13	DF090345:1677228-1677452
BmHel-13	DF090345:1350216-1350436
BmHel-13	DF090324:2453540-2453756
BmHel-13	DF090319:3762817-3763037
BmHel-13	DF090319:2148373-2148594
BmHel-13	DF090319:229529-229749
BmHel-13	DF090629:12577-12797
BmHel-13	DF090388:188266-188487
BmHel-13	DF090388:876749-876969
BmHel-13	DF090370:1259078-1259293
BmHel-13	DF090362:3196650-3196870
BmHel-13	DF090362:2016218-2016438
BmHel-13	DF090353:993925-994144
BmHel-13	DF090353:1325615-1325836
BmHel-13	DF090353:3060973-3061194
BmHel-13	DF090343:3436023-3436243
BmHel-13	DF090328:3935720-3935940
BmHel-13	DF090328:4783661-4783885
BmHel-13	DF090328:3633436-3633656
BmHel-13	DF090326:3520493-3520713
BmHel-13	DF090326:801048-801268
BmHel-13	DF090317:9262392-9262615
BmHel-13	DF090317:3976190-3976410
BmHel-13	DF090460:172913-173133
BmHel-13	DF090371:393306-393526
BmHel-13	DF090360:1433735-1433957
BmHel-13	DF090360:2076233-2076452
BmHel-13	DF090360:550913-551133
BmHel-13	DF090332:4093661-4093884
BmHel-13	DF090332:1471696-1471916
BmHel-13	DF090325:1233947-1234165

BmHel-13	DF090325:3127511-3127734
BmHel-13	DF090325:3682049-3682271
BmHel-13	DF090381:1633759-1633979
BmHel-13	DF090352:1266184-1266404
BmHel-13	DF090352:1951268-1951488
BmHel-13	DF090352:3174412-3174631
BmHel-13	DF090352:221061-221285
BmHel-13	DF090403:872321-872541
BmHel-13	DF090400:559525-559745
BmHel-13	DF090396:483221-483441
BmHel-13	DF090396:1508918-1509146
BmHel-13	DF090364:2087092-2087321
BmHel-13	DF090364:1753608-1753828
BmHel-13	DF090358:747992-748212
BmHel-13	DF090358:1521715-1521936
BmHel-13	DF090358:1999738-1999956
BmHel-13	DF090349:2554970-2555198
BmHel-13	DF090349:263170-263389
BmHel-13	DF090340:3300032-3300252
BmHel-13	DF090340:4020723-4020950
BmHel-13	DF090340:4378653-4378873
BmHel-13	DF090340:4556965-4557185
BmHel-13	DF090340:4439593-4439822
BmHel-13	DF090336:2736853-2737073
BmHel-13	DF090336:4588060-4588277
BmHel-13	DF090336:4612245-4612464
BmHel-13	DF090335:3292251-3292471
BmHel-13	DF090420:468669-468889
BmHel-13	DF090420:816426-816646
BmHel-13	DF090382:32988-33208
BmHel-13	DF090320:3992555-3992775
BmHel-13	DF090405:568115-568335
BmHel-13	DF090363:941201-941416
BmHel-13	DF090367:1473147-1473367
BmHel-13	DF090327:2728496-2728716
BmHel-13	DF090541:132498-132718
BmHel-13	DF090418:1206862-1207082
BmHel-13	DF090399:320634-320854
BmHel-13	DF090377:1213521-1213744
BmHel-13	DF090334:5458480-5458700
BmHel-13	DF090421:541448-541668
BmHel-13	DF090394:829262-829482
BmHel-13	DF090379:125713-125930
BmHel-13	DF090376:1565790-1566010
BmHel-13	BABH01046120:2310-2530
BmHel-13	DF090414:1245701-1245830
BmHel-13	DF090414:1245586-1245676
BmHel-13	DF090383:287375-287595

BmHel-13	DF090383:532709-532929
BmHel-13	DF090476:271487-271707
BmHel-13	DF090442:199278-199497
BmHel-13	DF090712:18217-18436
BmHel-13	DF090409:768685-768905
BmHel-13	DF090548:35960-36180
BmHel-13	DF090489:136088-136308
BmHel-13	BABH01072502:51-275
BmHel-13	DF091299:1133-1358
BmHel-13	DF090460:181553-181671
BmHel-13	DF090323:534219-534351
BmHel-13	DF090323:534374-534461
BmHel-13	DF090318:1075853-1076015
BmHel-13	DF090391:722256-722480
BmHel-13	DF090351:2297424-2297555
BmHel-13	DF090351:2297578-2297672
BmHel-13	DF090329:3087382-3087601
BmHel-13	DF090321:7188007-7188226
BmHel-13	DF090317:2142767-2142986
BmHel-13	DF090317:3226457-3226545
BmHel-13	DF090349:2932992-2933211
BmHel-13	DF090363:2079008-2079136
BmHel-13	DF090363:2079163-2079252
BmHel-13	DF090438:525155-525374
BmHel-13	DF090344:2169126-2169348
BmHel-13	DF090329:4051520-4051651
BmHel-13	DF090329:4051406-4051496
BmHel-13	DF090323:4459959-4460178
BmHel-13	DF090318:2288492-2288710
BmHel-13	DF090368:1939569-1939792
BmHel-13	DF090330:3499755-3499884
BmHel-13	DF090330:3499641-3499730
BmHel-13	DF090319:7047420-7047639
BmHel-13	DF090328:1762552-1762771
BmHel-13	DF090326:1444767-1444986
BmHel-13	DF090360:2243172-2243388
BmHel-13	DF090322:5575194-5575413
BmHel-13	DF090336:955083-955302
BmHel-13	DF090414:883360-883579
BmHel-13	DF090355:2402154-2402286
BmHel-13	DF090355:2402308-2402399
BmHel-13	DF090339:3065996-3066127
BmHel-13	DF090339:3065883-3065974
BmHel-13	DF090318:6138959-6139091
BmHel-13	DF090318:6138846-6138937
BmHel-13	DF090342:4145796-4146014
BmHel-13	DF090316:11152271-11152404
BmHel-13	DF090316:11152426-11152514

BmHel-13	DF090369:445809-446028
BmHel-13	DF090369:2691278-2691496
BmHel-13	DF090341:1951029-1951247
BmHel-13	DF090341:2160598-2160730
BmHel-13	DF090341:2160752-2160843
BmHel-13	DF090341:3362377-3362599
BmHel-13	DF090330:4581326-4581544
BmHel-13	DF090324:6621776-6621994
BmHel-13	DF090343:1709625-1709757
BmHel-13	DF090343:1709779-1709870
BmHel-13	DF090343:4469876-4469988
BmHel-13	DF090343:4470221-4470332
BmHel-13	DF090329:2832932-2833074
BmHel-13	DF090317:6242991-6243123
BmHel-13	DF090317:6243145-6243236
BmHel-13	DF090317:7598769-7598901
BmHel-13	DF090317:7598923-7599014
BmHel-13	DF090317:10854667-10854839
BmHel-13	DF090360:1487958-1488176
BmHel-13	DF090386:1436741-1436959
BmHel-13	DF090340:1374764-1374893
BmHel-13	DF090340:1374915-1375006
BmHel-13	DF090382:1685093-1685311
BmHel-13	DF090401:648742-648965
BmHel-13	DF090320:2265705-2265837
BmHel-13	DF090320:2265859-2265950
BmHel-13	DF090320:6460812-6460945
BmHel-13	DF090320:6460699-6460790
BmHel-13	DF090938:7929-8147
BmHel-13	DF090363:911249-911381
BmHel-13	DF090363:911403-911494
BmHel-13	DF090446:425819-426037
BmHel-13	DF091102:3456-3565
BmHel-13	DF091102:3184-3298
BmHel-13	DF090328:1945619-1945837
BmHel-13	DF090381:506303-506521
BmHel-13	DF090374:523209-523376
BmHel-13	DF090369:1865922-1866140
BmHel-13	DF090344:3575547-3575769
BmHel-13	DF090348:4013918-4014141
BmHel-13	DF090317:2540004-2540224
BmHel-13	DF090371:1499030-1499248
BmHel-13	DF090338:2616619-2616846
BmHel-13	DF090325:3136402-3136617
BmHel-13	DF090322:4393722-4393940
BmHel-13	DF090437:567131-567350
BmHel-13	DF090437:196025-196244
BmHel-13	DF090364:2114463-2114681

BmHel-13	DF090487:138166-138384
BmHel-13	DF090387:1731180-1731398
BmHel-13	DF090316:8295841-8295986
BmHel-13	DF090351:1216883-1217096
BmHel-13	DF090346:645329-645457
BmHel-13	DF090346:645221-645309
BmHel-13	DF090332:1149168-1149299
BmHel-13	DF090332:1149321-1149412
BmHel-13	DF090358:3077618-3077828
BmHel-13	DF090358:237127-237344
BmHel-13	DF090378:2146458-2146675
BmHel-13	DF090334:2772862-2773077
BmHel-13	DF090387:1087236-1087363
BmHel-13	DF090387:1087397-1087486
BmHel-13	DF090449:712452-712669
BmHel-13	DF090355:2455013-2455145
BmHel-13	DF090355:2454902-2454991
BmHel-13	DF090318:8803556-8803773
BmHel-13	DF090342:2121370-2121586
BmHel-13	DF090316:6024858-6024990
BmHel-13	DF090316:6024746-6024836
BmHel-13	DF090316:4207027-4207244
BmHel-13	DF090412:782979-783112
BmHel-13	DF090412:783134-783224
BmHel-13	DF090412:320987-321119
BmHel-13	DF090412:320875-320965
BmHel-13	DF090357:3570785-3570917
BmHel-13	DF090357:3570673-3570763
BmHel-13	DF090319:2613048-2613265
BmHel-13	DF090353:486386-486518
BmHel-13	DF090353:486540-486630
BmHel-13	DF090351:3982464-3982596
BmHel-13	DF090351:3982352-3982442
BmHel-13	DF090337:1581195-1581412
BmHel-13	DF090329:2079805-2080022
BmHel-13	DF090321:1536570-1536698
BmHel-13	DF090321:1536461-1536550
BmHel-13	DF090317:869080-869212
BmHel-13	DF090317:869234-869324
BmHel-13	DF090360:3324804-3324937
BmHel-13	DF090360:3324959-3325049
BmHel-13	DF090361:2155861-2155995
BmHel-13	DF090361:2155747-2155839
BmHel-13	DF090352:3988476-3988610
BmHel-13	DF090352:3988632-3988722
BmHel-13	DF090426:670643-670860
BmHel-13	DF090386:645109-645241
BmHel-13	DF090386:644997-645087

BmHel-13	DF090335:900946-901077
BmHel-13	DF090335:901099-901189
BmHel-13	DF090390:812520-812652
BmHel-13	DF090390:812673-812763
BmHel-13	DF090363:2108865-2108998
BmHel-13	DF090363:2108753-2108843
BmHel-13	DF090363:518848-519065
BmHel-13	DF090377:169467-169599
BmHel-13	DF090377:169621-169711
BmHel-13	DF090448:148011-148143
BmHel-13	DF090448:147899-147989
BmHel-13	DF090402:1222538-1222670
BmHel-13	DF090402:1222426-1222516
BmHel-13	DF090339:487577-487798
BmHel-13	DF090328:5588577-5588798
BmHel-13	DF090321:3303571-3303788
BmHel-13	DF090322:5973701-5973918
BmHel-13	DF090359:2283855-2284072
BmHel-13	DF090320:2992417-2992634
BmHel-13	DF090316:12870359-12870574
BmHel-13	DF090316:5008160-5008376
BmHel-13	DF090403:533772-533988
BmHel-13	DF090316:5089977-5090102
BmHel-13	DF090316:5090119-5090209
BmHel-13	DF090330:5695830-5696046
BmHel-13	DF090324:1275242-1275458
BmHel-13	DF090331:5227113-5227323
BmHel-13	DF090469:278855-278985
BmHel-13	DF090469:279007-279097
BmHel-13	DF090391:393866-394086
BmHel-13	DF090357:3077665-3077881
BmHel-13	DF090321:949003-949214
BmHel-13	DF090317:4858170-4858394
BmHel-13	DF090317:8559812-8560028
BmHel-13	DF090338:751910-752124
BmHel-13	DF090518:61722-61937
BmHel-13	DF090361:3040964-3041208
BmHel-13	DF090344:609626-609752
BmHel-13	DF090344:609510-609601
BmHel-13	DF090346:3454144-3454265
BmHel-13	DF090346:3454280-3454379
BmHel-13	DF090343:1953947-1954158
BmHel-13	DF090326:6466582-6466797
BmHel-13	DF090408:107879-108008
BmHel-13	DF090408:108030-108121
BmHel-13	DF090383:1025993-1026208
BmHel-13	DF090344:3415751-3415966
BmHel-13	DF090355:1526931-1527146

BmHel-13	DF090380:720804-721019
BmHel-13	DF090403:1587929-1588141
BmHel-13	DF090430:520771-520986
BmHel-13	BABH01048663:994-1207
BmHel-13	DF090443:412690-412914
BmHel-13	DF090351:4175245-4175383
BmHel-13	BABH01045835:1114-1246
BmHel-13	BABH01045835:1266-1357
BmHel-13	DF090326:5316402-5316616
BmHel-13	DF090401:899379-899593
BmHel-13	DF090432:355999-356129
BmHel-13	DF090432:355890-355977
BmHel-13	DF090330:239203-239420
BmHel-13	DF090342:3604642-3604861
BmHel-13	DF090345:4188897-4189111
BmHel-13	DF090321:409535-409749
BmHel-13	DF090352:2816836-2817054
BmHel-13	DF090366:2118080-2118294
BmHel-13	DF090366:1651171-1651383
BmHel-13	DF090466:358509-358723
BmHel-13	BABH01042899:23376-23592
BmHel-13	DF090316:5632829-5633043
BmHel-13	DF090330:2153438-2153582
BmHel-13	DF090359:2478975-2479112
BmHel-13	DF090335:5497552-5497765
BmHel-13	DF090414:1079234-1079322
BmHel-13	DF090414:1078631-1078765
BmHel-13	DF090383:694888-695094
BmHel-13	DF090321:4449076-4449292
BmHel-13	DF090357:91889-92021
BmHel-13	DF090357:92043-92133
BmHel-13	DF090407:1352675-1352807
BmHel-13	DF090407:1352568-1352653
BmHel-13	DF090334:4965174-4965387
BmHel-13	DF090345:4412674-4412887
BmHel-13	DF090324:7932026-7932239
BmHel-13	DF090343:31834-32047
BmHel-13	DF090322:1279189-1279402
BmHel-13	DF090367:275291-275504
BmHel-13	DF090466:68513-68726
BmHel-13	DF090324:2652154-2652303
BmHel-13	DF090324:2652379-2652461
BmHel-13	DF090347:173509-173721
BmHel-13	DF090499:39463-39645
BmHel-13	DF090405:842853-842984
BmHel-13	DF090405:842743-842833
BmHel-13	DF090400:1011451-1011573
BmHel-13	DF090400:1011333-1011422

BmHel-13	DF090369:1432831-1433043
BmHel-13	DF090344:4253477-4253689
BmHel-13	DF090318:1923611-1923776
BmHel-13	DF090341:2174171-2174333
BmHel-13	DF090386:325863-325983
BmHel-13	DF090386:326001-326091
BmHel-13	DF090361:1360074-1360285
BmHel-13	DF090324:1619629-1619724
BmHel-13	DF090324:1619500-1619615
BmHel-13	DF090460:81117-81328
BmHel-13	DF090379:905135-905346
BmHel-13	DF090316:9831073-9831248
BmHel-13	DF090331:196102-196227
BmHel-13	DF090331:195972-196073
BmHel-13	DF090452:154192-154357
BmHel-13	DF090322:5674808-5674956
BmHel-13	DF090322:5674682-5674772
BmHel-13	DF090324:7786253-7786335
BmHel-13	DF090324:7786127-7786253
BmHel-13	DF090380:1221326-1221441
BmHel-13	DF090380:1221233-1221326
BmHel-13	DF090372:1818412-1818582
BmHel-13	DF090376:1867452-1867661
BmHel-13	DF090330:3739405-3739612
BmHel-13	DF090351:303849-304058
BmHel-13	DF090372:2203686-2203894
BmHel-13	DF090321:4044591-4044766
BmHel-13	DF090395:1079596-1079804
BmHel-13	DF090389:1413420-1413529
BmHel-13	DF090389:1412860-1412968
BmHel-13	DF090323:7419149-7419281
BmHel-13	DF090323:7419044-7419127
BmHel-13	DF090369:541278-541418
BmHel-13	DF090317:8893367-8893516
BmHel-13	DF090375:581616-581744
BmHel-13	DF090326:6033982-6034189
BmHel-13	DF090394:259940-260070
BmHel-13	DF090394:260075-260167
BmHel-13	DF090421:472793-472925
BmHel-13	DF090421:472947-473027
BmHel-13	DF090390:1647785-1647993
BmHel-13	DF090357:785441-785677
BmHel-13	DF090413:974223-974389
BmHel-13	DF090316:7516104-7516317
BmHel-13	DF090346:2848410-2848624
BmHel-13	DF090343:2819539-2819751
BmHel-13	DF090332:3168707-3168921
BmHel-13	DF090322:7250632-7250846

BmHel-13	DF090320:7597346-7597547
BmHel-13	DF090334:4677274-4677481
BmHel-13	DF090368:1628465-1628568
BmHel-13	DF090368:1628363-1628466
BmHel-13	DF090346:3718703-3718916
BmHel-13	DF090333:1556137-1556263
BmHel-13	DF090333:1556021-1556100
BmHel-13	DF090433:437648-437782
BmHel-13	DF090322:3125749-3125918
BmHel-13	DF090429:488874-488962
BmHel-13	DF090429:488732-488823
BmHel-13	DF090344:1675006-1675094
BmHel-13	DF090344:1675097-1675212
BmHel-13	DF090360:2923378-2923530
BmHel-13	DF090380:772709-772921
BmHel-13	DF090354:2308487-2308690
BmHel-13	DF090369:981550-981752
BmHel-13	DF090413:9402-9503
BmHel-13	DF090413:9503-9604
BmHel-13	DF090339:2152614-2152746
BmHel-13	DF090318:48473-48605
BmHel-13	DF090330:1991768-1991900
BmHel-13	DF090329:1460771-1460903
BmHel-13	DF090322:1263265-1263397
BmHel-13	DF090358:375566-375700
BmHel-13	DF090340:2194108-2194232
BmHel-13	DF090331:5876726-5876857
BmHel-13	DF090327:3552656-3552788
BmHel-13	DF090424:674366-674498
BmHel-13	DF090447:458701-458833
BmHel-13	DF090609:25205-25340
BmHel-13	DF090358:2487881-2488085
BmHel-13	DF090349:4197258-4197469
BmHel-13	DF090327:2971756-2971959
BmHel-13	DF090344:3010957-3011115
BmHel-13	DF090422:508385-508514
BmHel-13	DF090422:508184-508264
BmHel-13	DF090345:1744321-1744523
BmHel-13	DF090345:2497440-2497633
BmHel-13	DF090406:796117-796323
BmHel-13	DF090321:3338860-3338984
BmHel-13	DF090490:157886-158062
BmHel-13	DF090318:4962311-4962518
BmHel-13	DF090333:1275094-1275225
BmHel-13	DF090392:1479496-1479628
BmHel-13	DF090460:354457-354658
BmHel-13	DF090361:605909-606110
BmHel-13	DF090318:8069647-8069740

BmHel-13	DF090318:8069540-8069645
BmHel-13	DF090330:3786434-3786538
BmHel-13	DF090330:3786540-3786635
BmHel-13	DF090341:2289904-2290033
BmHel-13	DF090335:3566825-3567025
BmHel-13	DF090378:794135-794301
BmHel-13	DF090377:1155619-1155776
BmHel-13	DF090377:1155438-1155518
BmHel-13	DF090481:220188-220354
BmHel-13	DF090364:826445-826615
BmHel-13	DF090466:308766-308898
BmHel-13	DF090390:431404-431603
BmHel-13	DF090424:359565-359773
BmHel-13	DF090321:5891426-5891558
BmHel-13	DF090496:7823-7949
BmHel-13	DF090336:4556680-4556792
BmHel-13	DF090336:4556820-4556904
BmHel-13	DF090335:3410729-3410822
BmHel-13	DF090335:3410846-3410937
BmHel-13	DF090412:836990-837123
BmHel-13	DF090317:9734583-9734710
BmHel-13	DF090396:1194655-1194787
BmHel-13	DF090336:3298725-3298918
BmHel-13	DF090356:1769385-1769527
BmHel-13	BABH01054332:58-211
BmHel-13	DF090369:1483553-1483685
BmHel-13	DF090330:2065669-2065801
BmHel-13	DF090362:1305446-1305578
BmHel-13	DF090343:271483-271615
BmHel-13	DF090329:3587635-3587760
BmHel-13	DF090492:80208-80404
BmHel-13	DF090363:1161514-1161684
BmHel-13	DF090333:220729-220903
BmHel-13	DF090405:703608-703767
BmHel-13	DF090326:6485029-6485225
BmHel-13	DF090357:2549045-2549267
BmHel-13	DF090337:183628-183737
BmHel-13	DF090886:16969-17121
BmHel-13	DF090417:561191-561386
BmHel-13	DF090464:213832-214027
BmHel-13	DF090355:360591-360673
BmHel-13	DF090355:360476-360588
BmHel-13	DF090697:7540-7673
BmHel-13	DF090319:6052448-6052671
BmHel-13	DF090338:792911-793105
BmHel-13	DF090349:2370475-2370607
BmHel-13	DF090363:496866-496990
BmHel-13	DF090344:1750758-1750885

BmHel-13	DF090347:2038086-2038204
BmHel-13	DF090355:844340-844535
BmHel-13	DF090373:113908-114040
BmHel-13	DF090364:2531785-2531979
BmHel-13	DF090331:2237533-2237727
BmHel-13	DF090330:4519297-4519426
BmHel-13	DF090340:1577473-1577605
BmHel-13	DF090388:319745-319938
BmHel-13	DF090319:1058864-1059057
BmHel-13	DF090370:1269001-1269194
BmHel-13	DF090354:3105643-3105864
BmHel-13	DF090319:41224-41440
BmHel-13	DF090364:1143240-1143461
BmHel-13	DF090413:543945-544077
BmHel-13	DF090393:1707739-1707931
BmHel-13	BABH01083512:294-515
BmHel-13	BABH01063604:129-350
BmHel-13	DF090461:78386-78608
BmHel-13	DF090461:37829-38050
BmHel-13	DF090461:11692-11913
BmHel-13	DF090355:1423890-1424114
BmHel-13	DF090355:3448523-3448744
BmHel-13	DF090355:2406174-2406395
BmHel-13	DF090355:1623945-1624166
BmHel-13	DF090355:1312213-1312434
BmHel-13	DF090354:2348472-2348693
BmHel-13	DF090354:3371768-3371988
BmHel-13	DF090339:1076214-1076434
BmHel-13	DF090339:3463889-3464109
BmHel-13	DF090323:744875-745092
BmHel-13	DF090323:2143848-2144069
BmHel-13	DF090323:2567015-2567236
BmHel-13	DF090323:2824307-2824528
BmHel-13	DF090323:6744754-6744975
BmHel-13	DF090323:2773989-2774210
BmHel-13	DF090318:1600695-1600916
BmHel-13	DF090318:1825154-1825375
BmHel-13	DF090318:4640582-4640803
BmHel-13	DF090318:5630117-5630336
BmHel-13	DF090318:7858879-7859101
BmHel-13	DF090318:9204890-9205111
BmHel-13	DF090318:7151161-7151379
BmHel-13	DF090318:5824178-5824399
BmHel-13	DF090318:5690234-5690445
BmHel-13	DF090318:727574-727793
BmHel-13	DF090318:685637-685858
BmHel-13	DF090342:2079603-2079824
BmHel-13	DF090342:2199758-2199979

BmHel-13	DF090342:2457918-2458140
BmHel-13	DF090342:3361546-3361767
BmHel-13	DF090342:3378921-3379142
BmHel-13	DF090342:4056938-4057159
BmHel-13	DF090342:4497714-4497933
BmHel-13	DF090342:3503843-3504064
BmHel-13	DF090342:3368352-3368573
BmHel-13	DF090316:462824-463042
BmHel-13	DF090316:3529595-3529824
BmHel-13	DF090316:5496201-5496420
BmHel-13	DF090316:8909422-8909643
BmHel-13	DF090316:10007200-10007421
BmHel-13	DF090316:10034160-10034381
BmHel-13	DF090316:15321223-15321449
BmHel-13	DF090316:15750966-15751183
BmHel-13	DF090316:15618210-15618431
BmHel-13	DF090316:14309285-14309504
BmHel-13	DF090316:10492096-10492317
BmHel-13	DF090316:10157579-10157800
BmHel-13	DF090316:9338302-9338522
BmHel-13	DF090316:6547597-6547818
BmHel-13	DF090316:6356135-6356356
BmHel-13	DF090316:6106659-6106880
BmHel-13	DF090316:4273218-4273439
BmHel-13	DF090316:3741741-3741962
BmHel-13	DF090316:3224722-3224945
BmHel-13	DF090368:2211837-2212058
BmHel-13	DF090368:1962038-1962263
BmHel-13	DF090375:1345794-1346011
BmHel-13	DF090375:2191233-2191454
BmHel-13	DF091943:1827-2048
BmHel-13	DF090425:310254-310475
BmHel-13	DF090412:638400-638626
BmHel-13	DF090412:15279-15500
BmHel-13	DF090369:2633482-2633703
BmHel-13	DF090369:1637439-1637660
BmHel-13	DF090369:480954-481175
BmHel-13	DF090365:1381968-1382189
BmHel-13	DF090365:1611406-1611627
BmHel-13	DF090365:1774921-1775146
BmHel-13	DF090365:1190216-1190436
BmHel-13	DF090341:4312463-4312684
BmHel-13	DF090341:4365866-4366087
BmHel-13	DF090341:4283746-4283968
BmHel-13	DF090341:4177061-4177282
BmHel-13	DF090341:3826583-3826804
BmHel-13	DF090341:1978877-1979101
BmHel-13	DF090330:467179-467403

BmHel-13	DF090330:589737-589958
BmHel-13	DF090330:2042205-2042426
BmHel-13	DF090330:2348955-2349176
BmHel-13	DF090330:5097690-5097911
BmHel-13	DF090330:5869393-5869614
BmHel-13	DF090330:6228128-6228349
BmHel-13	DF090330:3022912-3023133
BmHel-13	DF090330:2351418-2351600
BmHel-13	DF090330:1321184-1321403
BmHel-13	DF090330:162041-162262
BmHel-13	DF090330:24653-24874
BmHel-13	DF090357:865045-865267
BmHel-13	DF090357:2179187-2179408
BmHel-13	DF090357:2886307-2886528
BmHel-13	DF090357:2753917-2754138
BmHel-13	DF090357:2447490-2447707
BmHel-13	DF090345:2310698-2310915
BmHel-13	DF090345:2800719-2800940
BmHel-13	DF090345:3724667-3724888
BmHel-13	DF090345:3888380-3888601
BmHel-13	DF090345:713388-713609
BmHel-13	DF090344:2389223-2389446
BmHel-13	DF090344:2589884-2590105
BmHel-13	DF090344:3045687-3045907
BmHel-13	DF090344:2249651-2249880
BmHel-13	DF090344:1925854-1926076
BmHel-13	DF090344:1303318-1303539
BmHel-13	DF090344:814652-814865
BmHel-13	DF090344:385624-385841
BmHel-13	DF090324:1086456-1086677
BmHel-13	DF090324:3719722-3719943
BmHel-13	DF090324:7320284-7320505
BmHel-13	DF090324:7622433-7622654
BmHel-13	DF090324:3867046-3867267
BmHel-13	DF090324:2968776-2968997
BmHel-13	DF090324:1321048-1321267
BmHel-13	DF090319:1295045-1295266
BmHel-13	DF090319:1635310-1635535
BmHel-13	DF090319:1718268-1718491
BmHel-13	DF090319:3040447-3040668
BmHel-13	DF090319:5212866-5213085
BmHel-13	DF090319:5959883-5960104
BmHel-13	DF090319:6121867-6122088
BmHel-13	DF090319:7550879-7551100
BmHel-13	DF090319:4899954-4900175
BmHel-13	DF090319:3151777-3152007
BmHel-13	BABH01085150:265-486
BmHel-13	BABH01058406:297-518

BmHel-13	BABH01050182:604-825
BmHel-13	BABH01048113:304-525
BmHel-13	BABH01047623:1378-1599
BmHel-13	BABH01044962:1598-1819
BmHel-13	DF090777:11284-11505
BmHel-13	DF090705:8236-8457
BmHel-13	DF090629:27617-27838
BmHel-13	DF090391:967944-968165
BmHel-13	DF090391:1511375-1511596
BmHel-13	DF090388:735868-736092
BmHel-13	DF090388:1610099-1610320
BmHel-13	DF090388:1666346-1666567
BmHel-13	DF090388:1168760-1168981
BmHel-13	DF090388:801886-802108
BmHel-13	DF090380:491201-491420
BmHel-13	DF090380:1297366-1297587
BmHel-13	DF090380:1632408-1632631
BmHel-13	DF090380:1708057-1708277
BmHel-13	DF090380:1853191-1853412
BmHel-13	DF090370:1418147-1418367
BmHel-13	DF090370:1732759-1732978
BmHel-13	DF090370:1486258-1486475
BmHel-13	DF090370:1177335-1177556
BmHel-13	DF090362:817462-817683
BmHel-13	DF090362:1077941-1078163
BmHel-13	DF090362:1135596-1135813
BmHel-13	DF090362:1164632-1164856
BmHel-13	DF090362:1373622-1373843
BmHel-13	DF090362:1463737-1463953
BmHel-13	DF090362:1626474-1626695
BmHel-13	DF090362:2632628-2632849
BmHel-13	DF090362:1423507-1423728
BmHel-13	DF090353:1686497-1686715
BmHel-13	DF090353:3702103-3702324
BmHel-13	DF090353:3928624-3928845
BmHel-13	DF090353:1680095-1680316
BmHel-13	DF090353:1538677-1538898
BmHel-13	DF090353:438802-439023
BmHel-13	DF090351:1981068-1981289
BmHel-13	DF090351:2235163-2235328
BmHel-13	DF090351:2398501-2398722
BmHel-13	DF090351:3579250-3579478
BmHel-13	DF090351:2834818-2835039
BmHel-13	DF090351:2702226-2702441
BmHel-13	DF090351:2177420-2177643
BmHel-13	DF090351:436928-437149
BmHel-13	DF090348:1867679-1867901
BmHel-13	DF090348:2643099-2643318

BmHel-13	DF090348:2953719-2953944
BmHel-13	DF090347:146860-147078
BmHel-13	DF090347:476063-476288
BmHel-13	DF090347:908435-908656
BmHel-13	DF090347:3829948-3830167
BmHel-13	DF090346:1105651-1105867
BmHel-13	DF090346:2725272-2725493
BmHel-13	DF090346:3020590-3020811
BmHel-13	DF090346:3617247-3617468
BmHel-13	DF090346:3751313-3751529
BmHel-13	DF090346:4531988-4532209
BmHel-13	DF090346:3641249-3641470
BmHel-13	DF090346:2518012-2518233
BmHel-13	DF090346:775737-775957
BmHel-13	DF090343:201473-201694
BmHel-13	DF090343:691949-692170
BmHel-13	DF090343:1298585-1298806
BmHel-13	DF090343:2167465-2167686
BmHel-13	DF090343:2643952-2644173
BmHel-13	DF090343:4202207-4202428
BmHel-13	DF090343:3594347-3594568
BmHel-13	DF090343:1567937-1568158
BmHel-13	DF090337:5140181-5140402
BmHel-13	DF090337:4868067-4868287
BmHel-13	DF090337:769445-769666
BmHel-13	DF090329:55266-55489
BmHel-13	DF090329:217846-218067
BmHel-13	DF090329:781687-781907
BmHel-13	DF090329:4245023-4245244
BmHel-13	DF090329:3949123-3949344
BmHel-13	DF090329:3502367-3502588
BmHel-13	DF090328:3493434-3493655
BmHel-13	DF090328:5053927-5054148
BmHel-13	DF090328:2962888-2963109
BmHel-13	DF090328:2326539-2326758
BmHel-13	DF090328:2219701-2219922
BmHel-13	DF090326:699322-699543
BmHel-13	DF090326:2208392-2208613
BmHel-13	DF090326:4638803-4639024
BmHel-13	DF090326:2997738-2997959
BmHel-13	DF090326:2633104-2633325
BmHel-13	DF090326:595905-596126
BmHel-13	DF090321:2496475-2496696
BmHel-13	DF090321:3839968-3840189
BmHel-13	DF090321:4117035-4117256
BmHel-13	DF090321:7887582-7887803
BmHel-13	DF090321:1186436-1186647
BmHel-13	DF090317:5218108-5218329

BmHel-13	DF090317:5627032-5627253
BmHel-13	DF090317:6592752-6592973
BmHel-13	DF090317:7855857-7856078
BmHel-13	DF090317:8516581-8516800
BmHel-13	DF090317:8788282-8788503
BmHel-13	DF090317:10526670-10526891
BmHel-13	DF090317:10083339-10083560
BmHel-13	DF090317:9384277-9384498
BmHel-13	DF090317:7906579-7906797
BmHel-13	DF090317:7081229-7081451
BmHel-13	DF090317:6873503-6873732
BmHel-13	DF090317:6184322-6184544
BmHel-13	DF090317:5245098-5245317
BmHel-13	DF090317:4520411-4520636
BmHel-13	DF090371:1961845-1962066
BmHel-13	DF090371:1979545-1979766
BmHel-13	DF090371:1713396-1713617
BmHel-13	DF090360:3259459-3259683
BmHel-13	DF090360:2372606-2372827
BmHel-13	DF090360:2231976-2232197
BmHel-13	DF090360:418348-418574
BmHel-13	DF090338:3297612-3297833
BmHel-13	DF090338:2706483-2706704
BmHel-13	DF090332:4045459-4045679
BmHel-13	DF090332:4077586-4077807
BmHel-13	DF090332:4650078-4650298
BmHel-13	DF090332:3209490-3209710
BmHel-13	DF090332:2951333-2951554
BmHel-13	DF090332:2039415-2039636
BmHel-13	DF090325:2341772-2341992
BmHel-13	DF090325:3820461-3820682
BmHel-13	DF090325:4629263-4629484
BmHel-13	DF090325:5804540-5804760
BmHel-13	DF090325:7065929-7066150
BmHel-13	DF090325:5343494-5343715
BmHel-13	DF090361:653191-653412
BmHel-13	DF090361:2491623-2491844
BmHel-13	DF090361:490840-491061
BmHel-13	DF090381:727777-727998
BmHel-13	DF090381:938684-938906
BmHel-13	DF090352:5849-6070
BmHel-13	DF090352:1515192-1515413
BmHel-13	DF090352:2010746-2010967
BmHel-13	DF090352:2275825-2276047
BmHel-13	DF090352:2379892-2380112
BmHel-13	DF090352:3458706-3458927
BmHel-13	DF090352:4037272-4037493
BmHel-13	DF090352:3078102-3078323

BmHel-13	DF090322:384630-384851
BmHel-13	DF090322:555852-556070
BmHel-13	DF090322:835159-835379
BmHel-13	DF090322:3230524-3230744
BmHel-13	DF090322:3753520-3753741
BmHel-13	DF090322:4640971-4641192
BmHel-13	DF090322:7209357-7209578
BmHel-13	DF090322:8183056-8183277
BmHel-13	DF090322:5491396-5491617
BmHel-13	DF090322:3420611-3420832
BmHel-13	DF090322:2683545-2683766
BmHel-13	DF090322:1061883-1062104
BmHel-13	DF091396:1225-1450
BmHel-13	DF091396:3415-3636
BmHel-13	DF090782:565-786
BmHel-13	DF090561:1367-1588
BmHel-13	DF090561:11830-12051
BmHel-13	DF090444:450417-450638
BmHel-13	DF090444:535280-535501
BmHel-13	DF090437:43653-43874
BmHel-13	DF090437:544435-544656
BmHel-13	DF090436:307041-307262
BmHel-13	DF090435:174346-174567
BmHel-13	DF090435:526703-526924
BmHel-13	DF090435:197466-197682
BmHel-13	DF090433:636937-637158
BmHel-13	DF090433:150323-150544
BmHel-13	DF090426:113382-113604
BmHel-13	DF090426:217018-217238
BmHel-13	DF090403:1248410-1248631
BmHel-13	DF090403:1155128-1155350
BmHel-13	DF090403:1081068-1081289
BmHel-13	DF090400:1515937-1516158
BmHel-13	DF090397:314676-314897
BmHel-13	DF090397:1500294-1500515
BmHel-13	DF090396:1279504-1279724
BmHel-13	DF090396:1397927-1398143
BmHel-13	DF090396:429714-429935
BmHel-13	DF090386:510798-511019
BmHel-13	DF090386:671122-671343
BmHel-13	DF090386:1951472-1951693
BmHel-13	DF090384:1100976-1101197
BmHel-13	DF090359:1300626-1300847
BmHel-13	DF090359:2293919-2294138
BmHel-13	DF090359:2359468-2359689
BmHel-13	DF090358:502658-502883
BmHel-13	DF090358:1169179-1169408
BmHel-13	DF090358:2872372-2872593

BmHel-13	DF090358:1155468-1155689
BmHel-13	DF090358:514699-514923
BmHel-13	DF090358:102300-102521
BmHel-13	DF090356:1788729-1788950
BmHel-13	DF090356:2214630-2214855
BmHel-13	DF090356:3177570-3177791
BmHel-13	DF090356:3081643-3081864
BmHel-13	DF090356:1587117-1587340
BmHel-13	DF090349:367646-367867
BmHel-13	DF090349:486400-486621
BmHel-13	DF090349:564226-564445
BmHel-13	DF090349:2924438-2924661
BmHel-13	DF090349:1799749-1799970
BmHel-13	DF090340:1437360-1437585
BmHel-13	DF090340:2038513-2038734
BmHel-13	DF090340:2114342-2114563
BmHel-13	DF090340:2957510-2957731
BmHel-13	DF090340:3238251-3238467
BmHel-13	DF090340:4665633-4665857
BmHel-13	DF090340:4201285-4201506
BmHel-13	DF090340:3572302-3572523
BmHel-13	DF090340:1524112-1524333
BmHel-13	DF090336:2135907-2136128
BmHel-13	DF090336:3483728-3483950
BmHel-13	DF090336:4771679-4771898
BmHel-13	DF090336:3058006-3058227
BmHel-13	DF090336:3044220-3044441
BmHel-13	DF090336:1505625-1505845
BmHel-13	DF090335:1180313-1180522
BmHel-13	DF090335:1900150-1900371
BmHel-13	DF090335:5641617-5641838
BmHel-13	DF090335:2397911-2398128
BmHel-13	DF090331:4110770-4110993
BmHel-13	DF090331:4707840-4708059
BmHel-13	DF090331:2822359-2822577
BmHel-13	DF090331:2422800-2423021
BmHel-13	DF090390:163361-163582
BmHel-13	DF090390:555222-555444
BmHel-13	DF090390:323519-323737
BmHel-13	DF090382:1751775-1751996
BmHel-13	DF090382:74333-74559
BmHel-13	DF090373:671124-671345
BmHel-13	DF090373:1807038-1807262
BmHel-13	DF090373:175326-175547
BmHel-13	DF090372:1448186-1448407
BmHel-13	DF090372:1941090-1941311
BmHel-13	DF090372:2154371-2154592
BmHel-13	DF090372:1971949-1972170

BmHel-13	DF090422:842835-843064
BmHel-13	DF090401:537360-537581
BmHel-13	DF090320:5052795-5053016
BmHel-13	DF090320:7033039-7033257
BmHel-13	DF090320:8561170-8561391
BmHel-13	DF090405:820957-821178
BmHel-13	DF090405:32528-32748
BmHel-13	DF090363:1568325-1568549
BmHel-13	DF090363:2098316-2098535
BmHel-13	DF090363:1272355-1272576
BmHel-13	DF090363:629215-629436
BmHel-13	BABH01045218:2758-2979
BmHel-13	DF091127:6479-6700
BmHel-13	DF091000:2102-2323
BmHel-13	DF090530:40937-41158
BmHel-13	DF090530:28519-28740
BmHel-13	DF090523:92100-92321
BmHel-13	DF090467:289781-290002
BmHel-13	DF090467:309272-309501
BmHel-13	DF090458:258238-258459
BmHel-13	DF090457:10460-10681
BmHel-13	DF090431:73561-73782
BmHel-13	DF090431:755234-755455
BmHel-13	DF090431:533030-533243
BmHel-13	DF090430:825891-826112
BmHel-13	DF090417:1057047-1057268
BmHel-13	DF090417:875297-875515
BmHel-13	DF090417:775771-775992
BmHel-13	DF090417:672563-672784
BmHel-13	DF090417:464274-464492
BmHel-13	DF090411:1243317-1243538
BmHel-13	DF090404:1295446-1295657
BmHel-13	DF090404:579224-579445
BmHel-13	DF090395:114619-114840
BmHel-13	DF090395:504316-504535
BmHel-13	DF090395:560693-560914
BmHel-13	DF090395:830806-831027
BmHel-13	DF090393:1252442-1252663
BmHel-13	DF090393:1602707-1602932
BmHel-13	DF090393:1675397-1675617
BmHel-13	DF090393:839410-839631
BmHel-13	DF090378:163045-163266
BmHel-13	DF090378:938629-938862
BmHel-13	DF090378:1424175-1424393
BmHel-13	DF090374:1383857-1384078
BmHel-13	DF090374:143189-143410
BmHel-13	DF090367:2220445-2220666
BmHel-13	DF090367:785892-786116

BmHel-13	DF090366:912803-913024
BmHel-13	DF090366:1163832-1164053
BmHel-13	DF090366:1182749-1182970
BmHel-13	DF090366:1731847-1732068
BmHel-13	DF090366:2309069-2309290
BmHel-13	DF090366:2037448-2037669
BmHel-13	DF090366:727090-727313
BmHel-13	DF090366:346020-346241
BmHel-13	DF090333:1232129-1232351
BmHel-13	DF090333:1577164-1577385
BmHel-13	DF090333:2511047-2511268
BmHel-13	DF090333:3879128-3879348
BmHel-13	DF090333:4889207-4889428
BmHel-13	DF090333:5358371-5358600
BmHel-13	DF090333:1593468-1593689
BmHel-13	DF090333:1132347-1132568
BmHel-13	DF090327:3931025-3931246
BmHel-13	DF090327:5831348-5831569
BmHel-13	DF090399:14123-14344
BmHel-13	DF090399:59732-59952
BmHel-13	DF090377:2021178-2021399
BmHel-13	DF090377:1737509-1737730
BmHel-13	DF090438:513034-513255
BmHel-13	DF090334:2457152-2457379
BmHel-13	DF090334:3828097-3828316
BmHel-13	DF090334:3903306-3903527
BmHel-13	DF090334:3755014-3755235
BmHel-13	DF090334:3595693-3595914
BmHel-13	BABH01049518:787-1008
BmHel-13	DF091199:3659-3880
BmHel-13	DF091037:6930-7151
BmHel-13	DF090802:6922-7143
BmHel-13	DF090498:16869-17089
BmHel-13	DF090498:43769-43990
BmHel-13	DF090446:386441-386662
BmHel-13	DF090421:644689-644907
BmHel-13	DF090421:118798-119019
BmHel-13	DF090419:703251-703472
BmHel-13	DF090419:671056-671277
BmHel-13	DF090408:7664-7884
BmHel-13	DF090394:1432720-1432941
BmHel-13	DF090376:1353695-1353916
BmHel-13	BABH01087170:50-275
BmHel-13	DF090508:147121-147343
BmHel-13	DF090383:875143-875365
BmHel-13	DF090383:1908583-1908804
BmHel-13	DF090383:1967448-1967670
BmHel-13	DF090383:793115-793333

BmHel-13	DF090528:54699-54920
BmHel-13	DF090476:50489-50710
BmHel-13	DF090448:241896-242126
BmHel-13	DF090442:443911-444132
BmHel-13	DF090442:192404-192625
BmHel-13	DF090428:353088-353312
BmHel-13	DF090428:332291-332512
BmHel-13	DF090413:871399-871618
BmHel-13	DF090407:513713-513933
BmHel-13	DF090407:1026752-1026968
BmHel-13	DF090407:884381-884602
BmHel-13	DF090407:170332-170553
BmHel-13	DF090402:180858-181079
BmHel-13	DF090385:1875035-1875256
BmHel-13	BABH01049929:307-532
BmHel-13	DF090441:350770-350993
BmHel-13	DF090409:801105-801326
BmHel-13	DF090409:841114-841343
BmHel-13	DF090409:485121-485350
BmHel-13	DF090609:19021-19242
BmHel-13	DF090563:33918-34139
BmHel-13	DF090551:50453-50674
BmHel-13	BABH01049654:805-1028
BmHel-13	DF090548:4006-4224
BmHel-13	BABH01044698:3112-3333
BmHel-13	DF090937:8804-9021
BmHel-13	DF090575:48738-48955
BmHel-13	DF090443:483550-483768
BmHel-13	BABH01069058:2-223
BmHel-13	BABH01047002:453-673
BmHel-13	DF090950:8508-8733
BmHel-13	DF090648:24420-24645
BmHel-13	DF091001:3598-3819
BmHel-13	DF090710:18360-18580
BmHel-13	DF090562:2915-3136
BmHel-13	DF090556:17897-18118
BmHel-13	DF091560:2228-2453
BmHel-13	BABH01044232:415-636
BmHel-13	DF090553:72620-72836
BmHel-13	BABH01056830:43-264
BmHel-13	DF090791:7360-7507
BmHel-13	DF090348:3028709-3028929
BmHel-13	DF090390:1705065-1705285
BmHel-13	DF090334:5076081-5076162
BmHel-13	DF090354:3123350-3123542
BmHel-13	DF090339:3295766-3295960
BmHel-13	DF090318:7649100-7649291
BmHel-13	DF090316:8227951-8228139

BmHel-13	DF090345:2620178-2620367
BmHel-13	DF090347:2110823-2111015
BmHel-13	DF090343:85108-85298
BmHel-13	DF090371:2522717-2522908
BmHel-13	DF090360:1903013-1903204
BmHel-13	DF090352:3028519-3028710
BmHel-13	DF090364:2034290-2034481
BmHel-13	DF090356:1451362-1451553
BmHel-13	DF090336:3398394-3398585
BmHel-13	DF090373:862855-863046
BmHel-13	DF090320:7952483-7952674
BmHel-13	DF090333:1304112-1304303
BmHel-13	DF090414:549499-549694
BmHel-13	DF090383:93127-93318
BmHel-13	DF091156:10714-10905
BmHel-13	DF090355:2606770-2606990
BmHel-13	DF090354:3362712-3362930
BmHel-13	DF090354:2552798-2553018
BmHel-13	DF090354:1914942-1915162
BmHel-13	DF090323:1496966-1497186
BmHel-13	DF090318:5990325-5990546
BmHel-13	DF090318:5661734-5661954
BmHel-13	DF090318:4093408-4093631
BmHel-13	DF090342:1417648-1417868
BmHel-13	DF090316:2275258-2275478
BmHel-13	DF090316:6589684-6589813
BmHel-13	DF090316:6589569-6589659
BmHel-13	DF090316:4066649-4066868
BmHel-13	DF090375:1170899-1171119
BmHel-13	DF090369:1148096-1148316
BmHel-13	DF090365:476013-476241
BmHel-13	DF090365:432343-432563
BmHel-13	DF090330:1627545-1627765
BmHel-13	DF090330:3113295-3113515
BmHel-13	DF090330:4687745-4687965
BmHel-13	DF090330:6378935-6379155
BmHel-13	DF090330:4373814-4374034
BmHel-13	DF090330:3254379-3254599
BmHel-13	DF090330:1028939-1029146
BmHel-13	DF090357:2481840-2482060
BmHel-13	DF090345:1244754-1244974
BmHel-13	DF090345:4104713-4104933
BmHel-13	DF090344:844505-844725
BmHel-13	DF090344:2258956-2259176
BmHel-13	DF090324:2261737-2261956
BmHel-13	DF090324:2105749-2105969
BmHel-13	DF090319:6087882-6088110
BmHel-13	DF090319:4819399-4819619

BmHel-13	DF090380:1421961-1422181
BmHel-13	DF090380:518533-518753
BmHel-13	DF090370:237357-237577
BmHel-13	DF090362:2214527-2214748
BmHel-13	DF090362:3041008-3041229
BmHel-13	DF090362:1334060-1334278
BmHel-13	DF090362:1085293-1085509
BmHel-13	DF090351:2111193-2111323
BmHel-13	DF090351:2111347-2111438
BmHel-13	DF090351:3067818-3068039
BmHel-13	DF090351:2073822-2074034
BmHel-13	DF090348:4194266-4194486
BmHel-13	DF090348:3193766-3193987
BmHel-13	DF090347:731973-732193
BmHel-13	DF090347:831296-831516
BmHel-13	DF090347:1815269-1815489
BmHel-13	DF090347:1944006-1944222
BmHel-13	DF090347:1823544-1823764
BmHel-13	DF090337:2943009-2943222
BmHel-13	DF090329:3006832-3006963
BmHel-13	DF090329:3006975-3007065
BmHel-13	DF090329:6714037-6714257
BmHel-13	DF090329:5684586-5684806
BmHel-13	DF090329:1964996-1965218
BmHel-13	DF090329:1577778-1577998
BmHel-13	DF090329:735373-735601
BmHel-13	DF090329:313551-313771
BmHel-13	DF090328:3115812-3116032
BmHel-13	DF090328:1083939-1084159
BmHel-13	DF090328:712600-712820
BmHel-13	DF090326:3091254-3091474
BmHel-13	DF090326:4549866-4550085
BmHel-13	DF090326:1417335-1417555
BmHel-13	DF090321:7103162-7103382
BmHel-13	DF090321:2464314-2464534
BmHel-13	DF090317:5306185-5306405
BmHel-13	DF090317:7752501-7752722
BmHel-13	DF090317:7825793-7825923
BmHel-13	DF090317:7825681-7825768
BmHel-13	DF090317:4538842-4539062
BmHel-13	DF090317:1215266-1215486
BmHel-13	DF090317:930557-930782
BmHel-13	DF090317:628091-628307
BmHel-13	BABH01069883:37-257
BmHel-13	DF090371:1060990-1061210
BmHel-13	DF090338:3235626-3235846
BmHel-13	DF090338:2026878-2027103
BmHel-13	DF090332:2096302-2096522

BmHel-13	DF090332:2394605-2394825
BmHel-13	DF090332:2802284-2802503
BmHel-13	DF090332:5062988-5063208
BmHel-13	DF090325:3964633-3964854
BmHel-13	DF090325:5201046-5201271
BmHel-13	DF090325:6053449-6053669
BmHel-13	DF090325:270110-270326
BmHel-13	DF090352:816698-816926
BmHel-13	DF090352:2415429-2415558
BmHel-13	DF090352:2415312-2415403
BmHel-13	DF090322:629550-629770
BmHel-13	DF090322:685431-685651
BmHel-13	DF090322:3361309-3361529
BmHel-13	DF090322:3902588-3902720
BmHel-13	DF090322:3902744-3902833
BmHel-13	DF090322:8017042-8017262
BmHel-13	DF090437:376636-376856
BmHel-13	DF090436:231027-231247
BmHel-13	DF090426:950897-951117
BmHel-13	DF090400:1287139-1287367
BmHel-13	DF090396:992458-992678
BmHel-13	DF090386:249894-250114
BmHel-13	DF090386:250789-251010
BmHel-13	DF090359:1674882-1675102
BmHel-13	DF090359:2786402-2786619
BmHel-13	DF090358:2853119-2853347
BmHel-13	DF090356:745778-745999
BmHel-13	DF090349:1322760-1322978
BmHel-13	DF090349:1363104-1363322
BmHel-13	DF090349:3929824-3930044
BmHel-13	DF090349:1714165-1714385
BmHel-13	DF090340:4420390-4420609
BmHel-13	DF090340:1659814-1660034
BmHel-13	DF090336:706215-706444
BmHel-13	DF090336:3092497-3092629
BmHel-13	DF090336:3092656-3092743
BmHel-13	DF090336:2941971-2942191
BmHel-13	DF090335:699522-699742
BmHel-13	DF090335:5814888-5815108
BmHel-13	DF090335:1523957-1524177
BmHel-13	DF090331:5807247-5807466
BmHel-13	DF090331:5715714-5715934
BmHel-13	DF090331:5244452-5244672
BmHel-13	BABH01054116:515-735
BmHel-13	DF090514:14047-14267
BmHel-13	DF090462:257200-257420
BmHel-13	DF090390:1547933-1548153
BmHel-13	DF090373:1600914-1601134

BmHel-13	DF090373:936447-936666
BmHel-13	DF090372:561310-561530
BmHel-13	DF090372:1963694-1963914
BmHel-13	DF090350:2932429-2932649
BmHel-13	DF090350:3833788-3834008
BmHel-13	DF090405:1347719-1347948
BmHel-13	DF090363:1243842-1244062
BmHel-13	DF090363:1991380-1991599
BmHel-13	DF090458:195438-195662
BmHel-13	DF090378:205623-205846
BmHel-13	DF090367:340772-340993
BmHel-13	DF090366:1698573-1698793
BmHel-13	DF090366:1784716-1784936
BmHel-13	DF090333:470644-470864
BmHel-13	DF090333:1385090-1385310
BmHel-13	DF090333:3213327-3213546
BmHel-13	DF090327:2363166-2363386
BmHel-13	BABH01081270:67-287
BmHel-13	DF090398:875426-875646
BmHel-13	DF090392:677554-677774
BmHel-13	DF090392:1237451-1237674
BmHel-13	DF090377:1784851-1785073
BmHel-13	DF090377:264625-264845
BmHel-13	DF090334:1826061-1826279
BmHel-13	DF090334:3596982-3597206
BmHel-13	DF090334:6095856-6096076
BmHel-13	DF090408:1066960-1067177
BmHel-13	DF090394:71991-72211
BmHel-13	DF090849:544-764
BmHel-13	DF090574:38212-38432
BmHel-13	DF090424:745657-745877
BmHel-13	DF090424:232356-232576
BmHel-13	DF090383:1653147-1653275
BmHel-13	DF090383:1653037-1653121
BmHel-13	DF090428:577389-577609
BmHel-13	DF090409:654796-655016
BmHel-13	DF090592:34698-34920
BmHel-13	DF091341:2995-3215
BmHel-13	DF090805:8075-8294
BmHel-13	DF090617:37367-37587
BmHel-13	DF090488:83148-83367
BmHel-13	BABH01044698:4401-4625
BmHel-13	DF091435:3799-4018
BmHel-13	DF090389:967187-967409
BmHel-13	DF090456:1475-1695
BmHel-13	DF090456:192959-193176
BmHel-13	DF090469:281142-281363
BmHel-13	DF090455:242108-242327

BmHel-13	DF090447:58253-58472
BmHel-13	BABH01075488:83-309
BmHel-13	DF090354:2450384-2450604
BmHel-13	DF090425:910661-910881
BmHel-13	DF090351:3972443-3972664
BmHel-13	DF090337:1550972-1551192
BmHel-13	DF090321:4994525-4994745
BmHel-13	DF090364:935839-936066
BmHel-13	DF090349:4401109-4401329
BmHel-13	DF090452:316210-316430
BmHel-13	DF090351:1912934-1913123
BmHel-13	DF090320:292669-292860
BmHel-13	DF090342:4853851-4854037
BmHel-13	DF090348:1738589-1738779
BmHel-13	DF090359:1488133-1488323
BmHel-13	DF090801:12412-12602
BmHel-13	DF090342:1183027-1183201
BmHel-13	DF090342:1127393-1127571
BmHel-13	DF090368:2292261-2292480
BmHel-13	DF090425:1006269-1006488
BmHel-13	DF090319:2625442-2625661
BmHel-13	DF090319:5317256-5317426
BmHel-13	DF090351:280192-280409
BmHel-13	DF090347:252179-252398
BmHel-13	DF090346:2646225-2646444
BmHel-13	DF090328:4494379-4494510
BmHel-13	DF090328:4494265-4494356
BmHel-13	DF090321:7967032-7967251
BmHel-13	DF090317:8489500-8489723
BmHel-13	DF090332:137138-137368
BmHel-13	DF090361:2276299-2276518
BmHel-13	DF090352:2204934-2205153
BmHel-13	DF090349:177225-177444
BmHel-13	DF090331:2142809-2143024
BmHel-13	DF090373:1412700-1412886
BmHel-13	DF090422:1057781-1058000
BmHel-13	DF090320:5385791-5385922
BmHel-13	DF090320:5385677-5385767
BmHel-13	DF090394:1334213-1334351
BmHel-13	DF090394:1334375-1334463
BmHel-13	DF090394:1400365-1400585
BmHel-13	BABH01044941:2056-2275
BmHel-13	DF090407:496494-496713
BmHel-13	DF090439:24054-24270
BmHel-13	BABH01045933:3256-3398
BmHel-13	DF090368:761760-761979
BmHel-13	DF090353:1908916-1909045
BmHel-13	DF090353:1908801-1908892

BmHel-13	DF090326:1609022-1609241
BmHel-13	DF090340:2983938-2984069
BmHel-13	DF090340:2983830-2983923
BmHel-13	DF090316:1093687-1093906
BmHel-13	DF090412:1303597-1303816
BmHel-13	DF090388:211143-211362
BmHel-13	DF090353:3857212-3857431
BmHel-13	DF090326:3330036-3330255
BmHel-13	DF090317:3039962-3040181
BmHel-13	DF090435:486272-486491
BmHel-13	DF090359:1504548-1504767
BmHel-13	DF090373:96667-96885
BmHel-13	DF090327:2641856-2642075
BmHel-13	DF090334:160474-160693
BmHel-13	DF090334:3277859-3278078
BmHel-13	DF090475:131628-131850
BmHel-13	DF090389:1517950-1518168
BmHel-13	DF090382:1689036-1689145
BmHel-13	DF090382:1688955-1689034
BmHel-13	DF090356:2366820-2366972
BmHel-13	DF090322:460467-460657
BmHel-13	DF090336:2153849-2154038
BmHel-13	DF090339:1969140-1969329
BmHel-13	DF090317:5724844-5725033
BmHel-13	DF090352:462361-462550
BmHel-13	DF090436:676048-676241
BmHel-13	DF090373:47529-47718
BmHel-13	DF090461:244524-244656
BmHel-13	DF090461:244414-244503
BmHel-13	DF090355:1245059-1245194
BmHel-13	DF090355:1244946-1245037
BmHel-13	DF090354:2625031-2625165
BmHel-13	DF090354:2624918-2625009
BmHel-13	DF090339:3106429-3106652
BmHel-13	DF090323:7676392-7676525
BmHel-13	DF090323:7676547-7676635
BmHel-13	DF090323:1565318-1565540
BmHel-13	DF090342:2552845-2552977
BmHel-13	DF090342:2552999-2553092
BmHel-13	DF090342:4326083-4326215
BmHel-13	DF090342:4325970-4326061
BmHel-13	DF090316:5375889-5376106
BmHel-13	DF090316:5993666-5993796
BmHel-13	DF090316:5993818-5993909
BmHel-13	DF090316:1003468-1003600
BmHel-13	DF090316:1003355-1003446
BmHel-13	DF090375:1558341-1558473
BmHel-13	DF090375:1558228-1558319

BmHel-13	DF090330:4239780-4239910
BmHel-13	DF090330:4239667-4239758
BmHel-13	DF090324:4777714-4777846
BmHel-13	DF090324:4777868-4777961
BmHel-13	BABH01085029:6-224
BmHel-13	DF090362:2860883-2861101
BmHel-13	DF090353:625813-626030
BmHel-13	DF090351:3558378-3558597
BmHel-13	DF090347:1074912-1075044
BmHel-13	DF090347:1075066-1075157
BmHel-13	DF090329:6559769-6559901
BmHel-13	DF090329:6559923-6560014
BmHel-13	DF090326:2626749-2626968
BmHel-13	DF090321:2618605-2618720
BmHel-13	DF090321:2618730-2618844
BmHel-13	DF090317:10739630-10739847
BmHel-13	DF090325:5403398-5403616
BmHel-13	DF090352:659210-659341
BmHel-13	DF090352:659363-659454
BmHel-13	DF090322:894828-895046
BmHel-13	DF090396:1493636-1493768
BmHel-13	DF090396:1493789-1493880
BmHel-13	DF090386:914166-914383
BmHel-13	DF090349:434402-434533
BmHel-13	DF090349:434556-434644
BmHel-13	DF090340:2065797-2065929
BmHel-13	DF090340:2065687-2065775
BmHel-13	DF090335:440827-440959
BmHel-13	DF090335:440714-440805
BmHel-13	DF090390:1061169-1061301
BmHel-13	DF090390:1061056-1061147
BmHel-13	DF090350:2819098-2819230
BmHel-13	DF090350:2819252-2819343
BmHel-13	DF090320:1732826-1732958
BmHel-13	DF090320:1732713-1732804
BmHel-13	DF090320:91758-91890
BmHel-13	DF090320:91645-91736
BmHel-13	DF090374:665113-665331
BmHel-13	DF090366:724533-724751
BmHel-13	DF090366:1363616-1363748
BmHel-13	DF090366:1363503-1363594
BmHel-13	DF090333:2720434-2720566
BmHel-13	DF090333:2720321-2720412
BmHel-13	DF090327:1856555-1856773
BmHel-13	DF090377:237994-238220
BmHel-13	DF090334:2386939-2387157
BmHel-13	DF090334:4001217-4001436
BmHel-13	DF090334:6004948-6005166

BmHel-13	DF090394:694198-694330
BmHel-13	DF090394:694352-694443
BmHel-13	DF090394:1062753-1062884
BmHel-13	DF090394:1062640-1062731
BmHel-13	DF090383:1728684-1728816
BmHel-13	DF090383:1728571-1728662
BmHel-13	DF090493:139449-139666
BmHel-13	DF090680:6833-7052
BmHel-13	DF090341:2931883-2932104
BmHel-13	DF090330:3481488-3481706
BmHel-13	DF090444:9807-10029
BmHel-13	DF090320:6970281-6970498
BmHel-13	BABH01056880:262-484
BmHel-13	DF091079:8911-9133
BmHel-13	DF090375:1941779-1941997
BmHel-13	DF090412:75056-75274
BmHel-13	DF090365:3007846-3008064
BmHel-13	DF090319:3601951-3602169
BmHel-13	BABH01075229:1-219
BmHel-13	DF090362:2657410-2657628
BmHel-13	DF090360:2094437-2094647
BmHel-13	DF090325:4798149-4798357
BmHel-13	BABH01080543:20-239
BmHel-13	DF090322:1492285-1492503
BmHel-13	DF090400:1252515-1252723
BmHel-13	DF090396:979104-979321
BmHel-13	DF090386:885701-885918
BmHel-13	DF090382:1497154-1497372
BmHel-13	DF090320:3820871-3821093
BmHel-13	DF090320:3822596-3822810
BmHel-13	BABH01055968:438-655
BmHel-13	DF090379:505340-505559
BmHel-13	DF090424:694358-694576
BmHel-13	DF090659:14855-15072
BmHel-13	DF090367:298237-298423
BmHel-13	DF090383:1608499-1608650
BmHel-13	DF090328:6491597-6491706
BmHel-13	DF090345:368423-368611
BmHel-13	DF090348:3315366-3315498
BmHel-13	DF090388:1605043-1605175
BmHel-13	DF090322:1836782-1836970
BmHel-13	DF090355:1223288-1223420
BmHel-13	DF090355:1223442-1223529
BmHel-13	DF090354:629016-629233
BmHel-13	DF090323:7306581-7306714
BmHel-13	DF090323:7306736-7306826
BmHel-13	DF090342:1676095-1676228
BmHel-13	DF090342:1676250-1676340

BmHel-13	DF090342:1293539-1293756
BmHel-13	DF090368:2352714-2352845
BmHel-13	DF090368:2352867-2352957
BmHel-13	DF090365:1960898-1961030
BmHel-13	DF090365:1961052-1961142
BmHel-13	DF090330:1834200-1834332
BmHel-13	DF090330:1834088-1834178
BmHel-13	DF090344:3854025-3854242
BmHel-13	DF090344:2084457-2084589
BmHel-13	DF090344:2084345-2084435
BmHel-13	DF090324:8016055-8016217
BmHel-13	DF090319:3673677-3673810
BmHel-13	DF090319:3673830-3673920
BmHel-13	DF090319:3062683-3062818
BmHel-13	DF090319:3062570-3062662
BmHel-13	DF090391:662482-662699
BmHel-13	DF090391:298888-299020
BmHel-13	DF090391:298776-298866
BmHel-13	DF090353:454215-454347
BmHel-13	DF090353:454103-454193
BmHel-13	DF090348:1151792-1151924
BmHel-13	DF090348:1151680-1151770
BmHel-13	DF090346:3079323-3079455
BmHel-13	DF090346:3079211-3079301
BmHel-13	DF090326:5638085-5638216
BmHel-13	DF090326:5637973-5638063
BmHel-13	DF090326:5545687-5545904
BmHel-13	DF090321:2775940-2776072
BmHel-13	DF090321:2776094-2776184
BmHel-13	DF090317:9362894-9363111
BmHel-13	DF090317:9812596-9812728
BmHel-13	DF090317:9812484-9812574
BmHel-13	DF090360:2046602-2046734
BmHel-13	DF090360:2046490-2046580
BmHel-13	DF090325:1749380-1749547
BmHel-13	DF090325:4847733-4847829
BmHel-13	DF090325:4847841-4847961
BmHel-13	DF090352:2994720-2994852
BmHel-13	DF090352:2994874-2994964
BmHel-13	DF090322:4990727-4990859
BmHel-13	DF090322:4990615-4990705
BmHel-13	DF090386:532886-533021
BmHel-13	DF090386:532774-532864
BmHel-13	DF090386:280422-280647
BmHel-13	DF090384:1728574-1728791
BmHel-13	DF090356:3545004-3545136
BmHel-13	DF090356:3544892-3544982
BmHel-13	DF090336:3969616-3969748

BmHel-13	DF090336:3969770-3969860
BmHel-13	DF090382:1713433-1713565
BmHel-13	DF090382:1713321-1713411
BmHel-13	DF090373:1164513-1164645
BmHel-13	DF090373:1164387-1164471
BmHel-13	DF090401:111516-111607
BmHel-13	DF090401:111237-111370
BmHel-13	DF090320:292124-292251
BmHel-13	DF090320:292012-292102
BmHel-13	DF090487:125552-125684
BmHel-13	DF090487:125706-125796
BmHel-13	DF090363:1855194-1855326
BmHel-13	DF090363:1855095-1855185
BmHel-13	DF090393:1251310-1251527
BmHel-13	DF090367:739593-739730
BmHel-13	DF090367:739752-739842
BmHel-13	DF090367:606725-606857
BmHel-13	DF090367:606612-606703
BmHel-13	DF090438:703197-703329
BmHel-13	DF090438:703085-703175
BmHel-13	DF090498:9094-9310
BmHel-13	DF090419:294987-295204
BmHel-13	DF090387:1573022-1573146
BmHel-13	DF090387:1573168-1573258
BmHel-13	DF090428:566439-566570
BmHel-13	DF090428:566326-566417
BmHel-13	DF090429:68717-68849
BmHel-13	DF090429:68871-68964
BmHel-13	DF090518:73097-73229
BmHel-13	DF090518:73251-73341
BmHel-13	DF090614:10009-10141
BmHel-13	DF090614:9897-9987
BmHel-13	DF090323:2141772-2141997
BmHel-13	DF090326:4402683-4402900
BmHel-13	DF090322:2269415-2269632
BmHel-13	DF090322:7106284-7106498
BmHel-13	DF090333:1920844-1921061
BmHel-13	DF090399:1148427-1148653
BmHel-13	DF090416:497376-497593
BmHel-13	DF090379:1134760-1134977
BmHel-13	DF090368:737837-738054
BmHel-13	DF090391:1090125-1090342
BmHel-13	DF090351:4058279-4058496
BmHel-13	DF090351:801995-802215
BmHel-13	DF090321:6730240-6730457
BmHel-13	DF090322:3895346-3895563
BmHel-13	DF090386:828870-829087
BmHel-13	DF090340:4723287-4723496

BmHel-13	DF090365:1445682-1445798
BmHel-13	DF090344:3318308-3318495
BmHel-13	DF090441:592267-592395
BmHel-13	DF090319:974904-975091
BmHel-13	DF090323:6567408-6567624
BmHel-13	DF090323:2583207-2583423
BmHel-13	DF090316:5883277-5883493
BmHel-13	DF090316:4923524-4923740
BmHel-13	DF090316:3139100-3139316
BmHel-13	DF090324:1079200-1079416
BmHel-13	DF090348:1646099-1646192
BmHel-13	DF090348:1646489-1646580
BmHel-13	DF090348:2514211-2514336
BmHel-13	DF090348:2514367-2514457
BmHel-13	DF090343:137705-137931
BmHel-13	DF090329:5910260-5910476
BmHel-13	DF090328:428908-429118
BmHel-13	DF090338:4586503-4586635
BmHel-13	DF090338:4586657-4586746
BmHel-13	DF090338:2241137-2241361
BmHel-13	DF090332:2134691-2134821
BmHel-13	DF090332:2134845-2134932
BmHel-13	DF090325:4331987-4332118
BmHel-13	DF090325:4332140-4332229
BmHel-13	DF090325:2535376-2535592
BmHel-13	DF090364:2672119-2672335
BmHel-13	DF090349:1131092-1131300
BmHel-13	DF090336:2970810-2970941
BmHel-13	DF090336:2970963-2971052
BmHel-13	DF090487:83385-83517
BmHel-13	DF090487:83270-83359
BmHel-13	DF090431:582901-583116
BmHel-13	DF090366:1942154-1942286
BmHel-13	DF090366:1942044-1942133
BmHel-13	DF090334:2360977-2361193
BmHel-13	DF090387:480710-480925
BmHel-13	DF090488:133217-133366
BmHel-13	DF090445:432822-432954
BmHel-13	DF090445:432709-432792
BmHel-13	DF091367:1055-1208
BmHel-13	BABH01073137:67-199
BmHel-13	BABH01073137:220-309
BmHel-13	BABH01044849:727-859
BmHel-13	BABH01044849:881-970
BmHel-13	DF090319:7469705-7469929
BmHel-13	DF090342:3563049-3563265
BmHel-13	DF090341:713281-713497
BmHel-13	DF090324:4249208-4249414

BmHel-13	DF090317:5327654-5327868
BmHel-13	DF090399:954255-954469
BmHel-13	DF090409:664543-664759
BmHel-13	DF090355:860084-860300
BmHel-13	DF090323:2502479-2502704
BmHel-13	DF090341:1558578-1558802
BmHel-13	DF090351:668244-668461
BmHel-13	DF090320:4440410-4440626
BmHel-13	DF090393:33919-34132
BmHel-13	DF090677:11713-11929
BmHel-13	BABH01042583:24988-25204
BmHel-13	DF090336:934765-934864
BmHel-13	DF090336:934888-934976
BmHel-13	DF090338:3523018-3523150
BmHel-13	DF090324:3792105-3792291
BmHel-13	DF090330:3101937-3102061
BmHel-13	DF090330:3102091-3102181
BmHel-13	DF090388:1903151-1903275
BmHel-13	DF090388:1903042-1903132
BmHel-13	DF090347:938058-938226
BmHel-13	DF090321:5015073-5015205
BmHel-13	DF090321:5014963-5015051
BmHel-13	DF090325:5558847-5558979
BmHel-13	DF090325:5558737-5558825
BmHel-13	DF090352:3511280-3511398
BmHel-13	DF090352:3511426-3511510
BmHel-13	DF090378:1473245-1473377
BmHel-13	DF090378:1473399-1473487
BmHel-13	DF090334:14785-15000
BmHel-13	DF090414:589884-590048
BmHel-13	DF090598:26247-26422
BmHel-13	DF090347:438961-439176
BmHel-13	DF090324:2711205-2711340
BmHel-13	DF090324:2711362-2711451
BmHel-13	DF090319:2991438-2991653
BmHel-13	DF090448:198960-199175
BmHel-13	DF090355:508482-508697
BmHel-13	DF090405:261166-261381
BmHel-13	DF090329:3601207-3601422
BmHel-13	DF090326:3035037-3035252
BmHel-13	DF090332:4433095-4433310
BmHel-13	DF090361:1468810-1469027
BmHel-13	DF090358:897644-897859
BmHel-13	DF090390:808527-808742
BmHel-13	DF090321:1150095-1150233
BmHel-13	DF090426:299073-299194
BmHel-13	DF090355:475463-475648
BmHel-13	DF090321:2423393-2423578

BmHel-13	DF090316:11892706-11892925
BmHel-13	DF090324:7100251-7100465
BmHel-13	DF090324:8069368-8069582
BmHel-13	DF090362:2725610-2725770
BmHel-13	DF090362:1406921-1407053
BmHel-13	DF090362:1406812-1406899
BmHel-13	DF090328:4757422-4757636
BmHel-13	DF090317:2523951-2524166
BmHel-13	DF090317:10328559-10328773
BmHel-13	DF090358:1237731-1237949
BmHel-13	DF090340:2556276-2556490
BmHel-13	DF090350:3662413-3662627
BmHel-13	DF090393:1346545-1346670
BmHel-13	DF090393:1346692-1346779
BmHel-13	BABH01069650:411-625
BmHel-13	DF090521:29744-29875
BmHel-13	DF090521:29897-29994
BmHel-13	DF090368:651462-651589
BmHel-13	DF090368:651352-651438
BmHel-13	DF090341:4550487-4550712
BmHel-13	DF090342:557999-558214
BmHel-13	DF090328:3407076-3407204
BmHel-13	DF090328:3407226-3407317
BmHel-13	DF090373:1985906-1986121
BmHel-13	DF090363:65490-65708
BmHel-13	DF090628:1232-1446
BmHel-13	DF090343:603791-603903
BmHel-13	DF090406:239898-240007
BmHel-13	DF090381:717830-718014
BmHel-13	DF090322:5917259-5917385
BmHel-13	DF090346:3734717-3734901
BmHel-13	DF090316:13068733-13068946
BmHel-13	DF090321:5364092-5364248
BmHel-13	DF090321:7201098-7201312
BmHel-13	DF090321:2710793-2711006
BmHel-13	DF090371:926706-926843
BmHel-13	DF090332:6042597-6042810
BmHel-13	DF090352:3897859-3898073
BmHel-13	DF090335:89154-89366
BmHel-13	DF090399:1134446-1134546
BmHel-13	DF090399:1135012-1135136
BmHel-13	DF090452:241127-241337
BmHel-13	DF090356:2676617-2676830
BmHel-13	DF090332:438508-438721
BmHel-13	DF090393:1336209-1336422
BmHel-13	DF090413:337478-337691
BmHel-13	DF090359:3254649-3254832
BmHel-13	DF090389:362107-362193

BmHel-13	DF090318:5387375-5387506
BmHel-13	DF090330:6329221-6329433
BmHel-13	DF090344:2059599-2059811
BmHel-13	DF090332:3874697-3874913
BmHel-13	DF090358:2826722-2826897
BmHel-13	DF090350:2571358-2571490
BmHel-13	DF090350:2571251-2571336
BmHel-13	DF090320:839644-839856
BmHel-13	DF090446:330233-330443
BmHel-13	DF090896:8871-9006
BmHel-13	DF090328:2774870-2775082
BmHel-13	DF090320:8202178-8202303
BmHel-13	DF090320:8202065-8202155
BmHel-13	DF090336:3850824-3851036
BmHel-13	DF090322:5147563-5147745
BmHel-13	DF090567:21058-21244
BmHel-13	DF090369:2682579-2682790
BmHel-13	DF090337:3999483-3999673
BmHel-13	DF090317:1515579-1515669
BmHel-13	DF090317:1515668-1515789
BmHel-13	DF090332:1143746-1143892
BmHel-13	DF090358:76926-77058
BmHel-13	DF090358:76820-76904
BmHel-13	DF090333:4008536-4008666
BmHel-13	DF090333:4008421-4008505
BmHel-13	DF090327:4595081-4595289
BmHel-13	DF090392:1623891-1624109
BmHel-13	DF090376:1880984-1881195
BmHel-13	DF090674:16922-17133
BmHel-13	DF090325:3506190-3506394
BmHel-13	DF090427:548844-549055
BmHel-13	DF090335:1653432-1653643
BmHel-13	DF090316:12255092-12255303
BmHel-13	DF090362:1059739-1059950
BmHel-13	DF090492:210128-210339
BmHel-13	BABH01070285:8-219
BmHel-13	DF090352:1535192-1535315
BmHel-13	DF090323:6164055-6164265
BmHel-13	DF090342:692599-692760
BmHel-13	DF090425:21516-21681
BmHel-13	DF090330:4862082-4862252
BmHel-13	DF090357:2540985-2541195
BmHel-13	DF090321:6285265-6285414
BmHel-13	DF090360:2708376-2708534
BmHel-13	DF090386:315003-315135
BmHel-13	DF090333:2162964-2163171
BmHel-13	DF090410:310919-311083
BmHel-13	DF090321:5965506-5965715

BmHel-13	DF090361:1629370-1629495
BmHel-13	DF090361:1629258-1629348
BmHel-13	DF090355:3246387-3246597
BmHel-13	DF090427:901443-901623
BmHel-13	DF090321:6126465-6126653
BmHel-13	DF090316:14860078-14860287
BmHel-13	DF090344:4542297-4542420
BmHel-13	DF090344:4542212-4542296
BmHel-13	DF090380:557509-557676
BmHel-13	DF090401:956175-956385
BmHel-13	DF090499:108385-108556
BmHel-13	DF090380:1889519-1889653
BmHel-13	DF090394:1534916-1535125
BmHel-13	DF090343:3708588-3708798
BmHel-13	DF090346:3869311-3869490
BmHel-13	DF090316:780403-780570
BmHel-13	DF090343:1436661-1436869
BmHel-13	DF090334:1868613-1868821
BmHel-13	DF090416:1239608-1239815
BmHel-13	BABH01048184:220-427
BmHel-13	DF090459:243497-243579
BmHel-13	DF090459:243587-243712
BmHel-13	DF090321:3307024-3307119
BmHel-13	DF090321:3307115-3307229
BmHel-13	DF090841:6325-6487
BmHel-13	DF090316:8569340-8569556
BmHel-13	DF090316:8151549-8151757
BmHel-13	DF090329:1917615-1917823
BmHel-13	DF090425:436479-436685
BmHel-13	DF090330:5211639-5211855
BmHel-13	DF090464:67440-67649
BmHel-13	DF090316:13162148-13162335
BmHel-13	DF090316:14095680-14095868
BmHel-13	DF090332:2780521-2780706
BmHel-13	DF090395:1308394-1308601
BmHel-13	DF090377:563833-564040
BmHel-13	DF090404:750379-750503
BmHel-13	DF090404:750270-750352
BmHel-13	DF090391:532960-533082
BmHel-13	DF090391:533104-533194
BmHel-13	DF090317:4086707-4086917
BmHel-13	DF090344:898091-898207
BmHel-13	DF090342:4410854-4410986
BmHel-13	DF090318:3933756-3933943
BmHel-13	DF090358:1197363-1197541
BmHel-13	DF090342:4435983-4436189
BmHel-13	DF090375:210601-210804
BmHel-13	DF090341:1736966-1737183

BmHel-13	DF090345:1275045-1275252
BmHel-13	DF090344:3160180-3160396
BmHel-13	DF090344:2312142-2312356
BmHel-13	DF090324:7249113-7249327
BmHel-13	DF090324:6766506-6766712
BmHel-13	DF090362:3138427-3138640
BmHel-13	DF090362:252912-253111
BmHel-13	DF090347:618372-618578
BmHel-13	DF090337:1710507-1710614
BmHel-13	DF090337:1710613-1710705
BmHel-13	DF090321:5326917-5327122
BmHel-13	DF090325:5490059-5490265
BmHel-13	DF090397:1370788-1371002
BmHel-13	DF090331:4880766-4880972
BmHel-13	DF090363:1129484-1129696
BmHel-13	DF090378:901209-901338
BmHel-13	DF090333:3001572-3001778
BmHel-13	DF090399:661606-661812
BmHel-13	DF090438:692301-692507
BmHel-13	DF090407:1148239-1148455
BmHel-13	DF090389:535801-536016
BmHel-13	BABH01047703:1245-1459
BmHel-13	BABH01075697:90-222
BmHel-13	DF090331:6007581-6007787
BmHel-13	DF090335:4729478-4729684
BmHel-13	DF090368:1539674-1539850
BmHel-13	DF090480:211570-211688
BmHel-13	DF090332:5135232-5135362
BmHel-13	DF090355:2859740-2859861
BmHel-13	DF090355:2859658-2859740
BmHel-13	DF090368:142937-143069
BmHel-13	DF090324:6136642-6136751
BmHel-13	DF090324:6136746-6136841
BmHel-13	DF090324:4485108-4485240
BmHel-13	DF090380:982972-983094
BmHel-13	DF090380:983094-983175
BmHel-13	DF090347:389825-389955
BmHel-13	DF090317:3425118-3425252
BmHel-13	DF090322:5887858-5887987
BmHel-13	DF090322:3115429-3115634
BmHel-13	DF090349:283231-283378
BmHel-13	DF090363:2563948-2564153
BmHel-13	DF090327:1272961-1273092
BmHel-13	DF090402:290877-291082
BmHel-13	BABH01073236:1-206
BmHel-13	DF090443:559070-559200
BmHel-13	DF090320:3377393-3377598
BmHel-13	DF090336:1465885-1466002

BmHel-13	DF090359:1798489-1798621
BmHel-13	DF090318:4838872-4839004
BmHel-13	DF090388:1075289-1075420
BmHel-13	DF090347:3523521-3523649
BmHel-13	DF090329:6646460-6646548
BmHel-13	DF090329:6646345-6646460
BmHel-13	DF090326:3967287-3967447
BmHel-13	DF090321:7355705-7355834
BmHel-13	DF090317:145346-145478
BmHel-13	DF090325:3146167-3146288
BmHel-13	DF090325:3146283-3146400
BmHel-13	DF090349:1859245-1859377
BmHel-13	DF090349:1859142-1859222
BmHel-13	DF090424:741158-741282
BmHel-13	DF090424:741281-741360
BmHel-13	BABH01045800:1-205
BmHel-13	DF090425:630051-630169
BmHel-13	DF090425:630191-630284
BmHel-13	DF090324:1461682-1461800
BmHel-13	DF090324:1461822-1461913
BmHel-13	DF090576:5667-5785
BmHel-13	DF090576:5807-5898
BmHel-13	DF090398:923755-923960
BmHel-13	DF090344:3595052-3595227
BmHel-13	DF090342:3800570-3800687
BmHel-13	DF090741:1849-1985
BmHel-13	DF090325:5991501-5991675
BmHel-13	DF090339:5038925-5039057
BmHel-13	DF090323:7484761-7484964
BmHel-13	DF090316:13366533-13366665
BmHel-13	DF090316:10544052-10544184
BmHel-13	DF090375:1430362-1430494
BmHel-13	DF090412:1107434-1107566
BmHel-13	DF090369:427550-427682
BmHel-13	DF090341:1789724-1789856
BmHel-13	DF090330:3766101-3766233
BmHel-13	DF090357:981197-981338
BmHel-13	DF090324:2174334-2174499
BmHel-13	DF090391:41662-41874
BmHel-13	DF090388:1177594-1177726
BmHel-13	DF090351:1657375-1657507
BmHel-13	DF090351:3036118-3036284
BmHel-13	DF090347:3324507-3324638
BmHel-13	DF090347:1468354-1468486
BmHel-13	DF090346:496267-496399
BmHel-13	DF090343:1221764-1221896
BmHel-13	DF090321:6355360-6355566
BmHel-13	DF090321:1110377-1110509

BmHel-13	DF090332:3135114-3135322
BmHel-13	DF090332:2865399-2865603
BmHel-13	DF090332:998043-998175
BmHel-13	DF090325:5531530-5531659
BmHel-13	DF090322:2478112-2478244
BmHel-13	DF090435:649718-649888
BmHel-13	DF090335:1044545-1044678
BmHel-13	DF090335:3973972-3974101
BmHel-13	DF090401:750371-750504
BmHel-13	DF090430:338908-339119
BmHel-13	DF090404:98912-99044
BmHel-13	DF090395:736818-736950
BmHel-13	DF090333:1437578-1437710
BmHel-13	DF090418:811627-811761
BmHel-13	DF090418:1131758-1131890
BmHel-13	DF090334:2397896-2398010
BmHel-13	DF090334:2398011-2398099
BmHel-13	DF090449:773759-773952
BmHel-13	DF090445:97726-97929
BmHel-13	BABH01050020:572-737
BmHel-13	DF090572:34215-34347
BmHel-13	DF090316:11640322-11640419
BmHel-13	DF090629:45804-46007
BmHel-13	DF090338:3683355-3683481
BmHel-13	DF090323:4360052-4360263
BmHel-13	DF090316:5848682-5848765
BmHel-13	DF090316:5848536-5848685
BmHel-13	DF090344:2478651-2478862
BmHel-13	DF090332:2197613-2197739
BmHel-13	DF090377:1422375-1422537
BmHel-13	DF090317:10842143-10842346
BmHel-13	DF090347:3318208-3318411
BmHel-13	DF090317:7631717-7631890
BmHel-13	DF090355:2417152-2417294
BmHel-13	DF090316:15653854-15653986
BmHel-13	DF090375:2046856-2047010
BmHel-13	DF090373:407249-407381
BmHel-13	DF090363:1866801-1866933
BmHel-13	BABH01053374:1-203
BmHel-13	DF090861:11931-12102
BmHel-13	DF090671:16802-16972
BmHel-13	DF090470:261924-262132
BmHel-13	DF090369:1571363-1571495
BmHel-13	DF090372:1794936-1795109
BmHel-13	DF090352:1811722-1811854
BmHel-13	DF090330:113803-113935
BmHel-13	DF090364:795805-795937
BmHel-13	DF090320:5333815-5333924

BmHel-13	DF090320:5333696-5333787
BmHel-13	DF090421:858662-858862
BmHel-13	DF090480:6769-6934
BmHel-13	DF090639:11841-11932
BmHel-13	DF090394:602531-602695
BmHel-13	DF090325:3801924-3802135
BmHel-13	DF090316:16063328-16063443
BmHel-13	DF090316:16063215-16063306
BmHel-13	DF090340:4284922-4285128
BmHel-13	DF090404:1366958-1367162
BmHel-13	DF090342:2630117-2630287
BmHel-13	DF090319:4838450-4838635
BmHel-13	DF090349:2048485-2048662
BmHel-13	DF090376:461975-462153
BmHel-13	DF090345:1432722-1432884
BmHel-13	DF090319:2222072-2222204
BmHel-13	DF090336:1734515-1734650
BmHel-13	DF090379:1262665-1262797
BmHel-13	DF090476:221796-221891
BmHel-13	DF090476:221697-221801
BmHel-13	DF090903:7149-7278
BmHel-13	DF090333:5442474-5442600
BmHel-13	DF090349:2810271-2810471
BmHel-13	DF090331:3425740-3425881
BmHel-13	DF090362:3015148-3015279
BmHel-13	DF090375:1841772-1841941
BmHel-13	DF090321:6618294-6618472
BmHel-13	DF090322:4747138-4747305
BmHel-13	DF090335:3444475-3444643
BmHel-13	DF090335:3118331-3118508
BmHel-13	DF090390:1423046-1423216
BmHel-13	DF090530:76590-76768
BmHel-13	DF090327:1336356-1336524
BmHel-13	DF090448:80884-81053
BmHel-13	BABH01045684:3821-3989
BmHel-13	DF090453:115353-115523
BmHel-13	DF090369:1858291-1858424
BmHel-13	DF090365:384645-384800
BmHel-13	DF090344:4482516-4482648
BmHel-13	DF090317:1439273-1439403
BmHel-13	DF090352:2229336-2229461
BmHel-13	DF090437:25467-25599
BmHel-13	DF090349:4070597-4070721
BmHel-13	DF090335:3913640-3913819
BmHel-13	DF090392:1291537-1291707
BmHel-13	DF090414:889785-889946
BmHel-13	DF090316:2783576-2783703
BmHel-13	DF090346:487337-487466

BmHel-13	DF090362:1236941-1237068
BmHel-13	DF090346:459434-459641
BmHel-13	DF090483:125876-126000
BmHel-13	DF090376:1485369-1485461
BmHel-13	DF090376:1485268-1485374
BmHel-13	DF090431:772366-772481
BmHel-13	DF090340:2314862-2314992
BmHel-13	DF090320:7880499-7880629
BmHel-13	DF090526:19557-19726
BmHel-13	DF090317:5060632-5060764
BmHel-13	DF090317:1687370-1687533
BmHel-13	DF090336:2846263-2846423
BmHel-13	DF090336:4730736-4730901
BmHel-13	DF090390:1548420-1548586
BmHel-13	DF090418:995872-996081
BmHel-13	DF090421:137716-137914
BmHel-13	DF090414:678431-678637
BmHel-13	DF090316:13432739-13432866
BmHel-13	DF090434:266617-266815
BmHel-13	DF090332:6060034-6060232
BmHel-13	DF090387:1058343-1058537
BmHel-13	DF090477:94552-94712
BmHel-13	DF090370:2292266-2292398
BmHel-13	DF090430:95918-96086
BmHel-13	DF090316:2877977-2878145
BmHel-13	DF090316:15736557-15736677
BmHel-13	DF090316:8514793-8514914
BmHel-13	DF090422:802692-802824
BmHel-13	DF090327:4859823-4859954
BmHel-13	DF090408:639929-640138
BmHel-13	DF090387:1110112-1110203
BmHel-13	DF090429:35188-35320
BmHel-13	DF090389:1828409-1828537
BmHel-13	DF090334:3053831-3053957
BmHel-13	DF090326:2409901-2410096
BmHel-13	DF090399:1033983-1034092
BmHel-13	DF090399:1033874-1033961
BmHel-13	DF090340:3784698-3784895
BmHel-13	DF090346:3958466-3958663
BmHel-13	DF090318:8623507-8623632
BmHel-13	DF090342:1429530-1429663
BmHel-13	DF090316:6247421-6247586
BmHel-13	DF090330:5622557-5622686
BmHel-13	DF090344:3646269-3646401
BmHel-13	DF090319:7692235-7692367
BmHel-13	DF090329:4246589-4246721
BmHel-13	DF090317:9517507-9517639
BmHel-13	DF090332:1248601-1248733

BmHel-13	DF090322:2257723-2257855
BmHel-13	DF090437:253193-253358
BmHel-13	DF090364:845676-845808
BmHel-13	DF090356:1724492-1724623
BmHel-13	DF090373:2222332-2222463
BmHel-13	DF090366:2677730-2677862
BmHel-13	DF090327:319122-319255
BmHel-13	DF090409:773810-773942
BmHel-13	DF090831:11089-11221
BmHel-13	DF090497:141970-142102
BmHel-13	DF090850:7351-7482
BmHel-13	DF090730:1241-1375
BmHel-13	DF090364:414182-414314
BmHel-13	DF090408:556502-556632
BmHel-13	DF090351:1540512-1540681
BmHel-13	DF090456:42823-42998
BmHel-13	DF090316:10665964-10666160
BmHel-13	DF090365:1253845-1253976
BmHel-13	DF090341:1685895-1686027
BmHel-13	DF090362:29211-29343
BmHel-13	DF090317:10401166-10401297
BmHel-13	DF090334:3026481-3026613
BmHel-13	DF090419:710082-710278
BmHel-13	DF090408:859079-859211
BmHel-13	DF090407:1329239-1329361
BmHel-13	DF090332:731477-731602
BmHel-13	DF090541:124295-124491
BmHel-13	DF090322:7334740-7334857
BmHel-13	DF090323:4911132-4911263
BmHel-13	DF090346:3882694-3882822
BmHel-13	DF090342:2658635-2658801
BmHel-13	DF090357:2865148-2865314
BmHel-13	DF090462:343467-343586
BmHel-13	DF090399:1224195-1224342
BmHel-13	DF090419:257144-257234
BmHel-13	DF090546:13011-13173
BmHel-13	DF090408:1470371-1470495
BmHel-13	DF090324:4806747-4806940
BmHel-13	DF090352:1440126-1440258
BmHel-13	DF090324:5217780-5217976
BmHel-13	DF090358:1036655-1036850
BmHel-13	DF090395:1485092-1485287
BmHel-13	DF090345:1034472-1034596
BmHel-13	DF090352:1731995-1732127
BmHel-13	DF090467:19842-19973
BmHel-13	DF090371:2463586-2463751
BmHel-13	DF090355:294412-294606
BmHel-13	DF090345:3632076-3632208

BmHel-13	DF090344:1623819-1624010
BmHel-13	DF090335:1657216-1657343
BmHel-13	BABH01074573:421-609
BmHel-13	DF090630:60215-60346
BmHel-13	DF090331:4343269-4343463
BmHel-13	DF090373:1307187-1307293
BmHel-13	DF090339:1221827-1221956
BmHel-13	DF090316:7717777-7717909
BmHel-13	DF090330:217868-218000
BmHel-13	DF090324:4317446-4317579
BmHel-13	DF090391:372899-373065
BmHel-13	DF090347:3485333-3485465
BmHel-13	DF090328:474278-474410
BmHel-13	DF090403:166724-166827
BmHel-13	DF090403:166535-166624
BmHel-13	DF090429:393100-393216
BmHel-13	DF090333:30356-30549
BmHel-13	DF090335:5315830-5315959
BmHel-13	DF090362:1578189-1578309
BmHel-13	DF090323:1853022-1853156
BmHel-13	DF090330:3337807-3337959
BmHel-13	DF090353:41722-41923
BmHel-13	DF090448:406048-406176
BmHel-13	DF090321:1352790-1352894
BmHel-13	DF090321:1352918-1353009
BmHel-13	DF090325:5170665-5170857
BmHel-13	DF090322:1414741-1414929
BmHel-13	DF090320:6105803-6105995
BmHel-13	DF090757:2223-2347
BmHel-13	DF090462:153806-153966
BmHel-13	DF090461:312854-312978
BmHel-13	DF090347:1992387-1992511
BmHel-13	DF090317:11250630-11250758
BmHel-13	DF090352:1321228-1321355
BmHel-13	DF090415:1247312-1247435
BmHel-13	DF090403:1539963-1540097
BmHel-13	DF090323:7721559-7721751
BmHel-13	DF090316:4753423-4753614
BmHel-13	DF090316:15656917-15657108
BmHel-13	DF090412:1260468-1260662
BmHel-13	DF090357:618038-618228
BmHel-13	DF090344:1600048-1600239
BmHel-13	DF090344:3212322-3212513
BmHel-13	DF090319:2508221-2508413
BmHel-13	DF090319:8050054-8050245
BmHel-13	DF090362:225860-226051
BmHel-13	DF090329:1372308-1372499
BmHel-13	DF090328:4740009-4740200

BmHel-13	DF090326:152842-153033
BmHel-13	DF090326:669155-669346
BmHel-13	DF090317:8505415-8505606
BmHel-13	DF090317:9806549-9806739
BmHel-13	DF090332:1044513-1044701
BmHel-13	DF090325:5817807-5817997
BmHel-13	DF090352:3437060-3437251
BmHel-13	DF090386:290833-291024
BmHel-13	DF090364:322398-322589
BmHel-13	DF090359:2612408-2612599
BmHel-13	DF090340:2699542-2699733
BmHel-13	DF090340:1966954-1967145
BmHel-13	DF090462:142034-142166
BmHel-13	DF090372:915962-916147
BmHel-13	DF090431:110896-111087
BmHel-13	DF090417:626710-626901
BmHel-13	DF090334:3920731-3920922
BmHel-13	DF090394:995049-995235
BmHel-13	DF090383:1270127-1270318
BmHel-13	DF090407:238977-239168
BmHel-13	DF090439:158869-159063
BmHel-13	DF090415:105439-105622
BmHel-13	BABH01053044:429-620
BmHel-13	BABH01043533:5382-5573
BmHel-13	DF091130:569-760
BmHel-13	BABH01052221:447-638
BmHel-13	BABH01045754:3294-3485
BmHel-13	BABH01047844:299-490
BmHel-13	DF090428:619818-619958
BmHel-13	DF090369:1665774-1665968
BmHel-13	DF090329:5818824-5818946
BmHel-13	DF090394:1558156-1558277
BmHel-13	DF090320:3807028-3807150
BmHel-13	DF090325:6113802-6113907
BmHel-13	DF090325:6113929-6114020
BmHel-13	DF090340:1768433-1768630
BmHel-13	DF090330:439843-440034
BmHel-13	DF090372:2420802-2420934
BmHel-13	DF090353:2835405-2835537
BmHel-13	DF090339:2086464-2086662
BmHel-13	DF090344:4661246-4661429
BmHel-13	DF090319:1067681-1067768
BmHel-13	DF090319:1067583-1067682
BmHel-13	DF090337:2063334-2063524
BmHel-13	DF090329:4190421-4190611
BmHel-13	DF090326:5054734-5054924
BmHel-13	DF090338:465646-465836
BmHel-13	DF090325:2823560-2823688

BmHel-13	DF090379:956502-956630
BmHel-13	DF090348:3754624-3754814
BmHel-13	DF090377:1831906-1832104
BmHel-13	DF090350:4031608-4031738
BmHel-13	DF090463:60687-60877
BmHel-13	DF090359:806564-806754
BmHel-13	DF090342:741857-742046
BmHel-13	DF090329:6423267-6423358
BmHel-13	DF090332:2407634-2407747
BmHel-13	DF090332:2407065-2407174
BmHel-13	DF090352:2616329-2616518
BmHel-13	DF090384:1315204-1315392
BmHel-13	DF090350:1167303-1167492
BmHel-13	DF090350:665435-665568
BmHel-13	DF090468:141318-141507
BmHel-13	DF090488:122259-122391
BmHel-13	BABH01081015:369-528
BmHel-13	DF090374:612652-612781
BmHel-13	DF090372:1314960-1315080
BmHel-13	DF090429:739853-740042
BmHel-13	DF090387:810857-810986
BmHel-13	DF090383:1326295-1326427
BmHel-13	DF090430:624151-624339
BmHel-13	DF090364:2099514-2099709
BmHel-13	DF090340:2527617-2527805
BmHel-13	DF090384:454199-454323
BmHel-13	DF090365:2205206-2205314
BmHel-13	DF090365:2205099-2205184
BmHel-13	DF090319:4392394-4392582
BmHel-13	DF090403:683330-683518
BmHel-13	DF090353:535779-535906
BmHel-13	DF090322:5599905-5600025
BmHel-13	DF090323:2022245-2022407
BmHel-13	DF090354:1583836-1584023
BmHel-13	DF090329:1690650-1690782
BmHel-13	DF090326:2565119-2565306
BmHel-13	DF090321:3037383-3037536
BmHel-13	DF090336:4089486-4089618
BmHel-13	DF090330:4865892-4866079
BmHel-13	DF090438:265822-266009
BmHel-13	BABH01049425:149-302
BmHel-13	DF090329:1454719-1454906
BmHel-13	DF090392:1169835-1169950
BmHel-13	DF090375:2167603-2167790
BmHel-13	DF090326:2952832-2952929
BmHel-13	DF090326:2952717-2952808
BmHel-13	DF090344:2841115-2841302
BmHel-13	DF090390:1564583-1564706

BmHel-13	DF090316:6787940-6788123
BmHel-13	DF090316:10475977-10476109
BmHel-13	DF090347:2239404-2239591
BmHel-13	DF090384:1670198-1670334
BmHel-13	DF090318:6487399-6487527
BmHel-13	DF090324:8040085-8040261
BmHel-13	DF090413:866298-866484
BmHel-13	DF090375:1485896-1486080
BmHel-13	DF090345:4238335-4238519
BmHel-13	DF090335:1409533-1409718
BmHel-13	DF090443:93890-94003
BmHel-13	DF090319:2467955-2468148
BmHel-13	DF090344:3707787-3707980
BmHel-13	BABH01079273:379-572
BmHel-13	DF090339:2635630-2635815
BmHel-13	DF090342:1034483-1034668
BmHel-13	DF090346:798262-798447
BmHel-13	DF090430:586155-586339
BmHel-13	DF090401:1272658-1272773
BmHel-13	DF090321:5307245-5307428
BmHel-13	DF090335:3577617-3577749
BmHel-13	DF090373:1255265-1255389
BmHel-13	DF090356:1629918-1630102
BmHel-13	DF090382:326692-326876
BmHel-13	DF090385:1527382-1527564
BmHel-13	DF090336:2583876-2584027
BmHel-13	DF090483:204810-204923
BmHel-13	DF090328:2799521-2799675
BmHel-13	DF090355:2795028-2795187
BmHel-13	DF090348:2099871-2100003
BmHel-13	DF090367:2366205-2366345
BmHel-13	BABH01074087:1-184
BmHel-13	DF090627:18798-18930
BmHel-13	DF090386:1570754-1570937
BmHel-13	DF090320:5156876-5157062
BmHel-13	DF090510:42854-43008
BmHel-13	DF090362:2121170-2121302
BmHel-13	DF090337:1418943-1419125
BmHel-13	DF090352:2049431-2049566
BmHel-13	DF090355:1710758-1710940
BmHel-13	DF090325:2710026-2710178
BmHel-13	DF090326:782248-782343
BmHel-13	DF090317:8574766-8574893
BmHel-13	DF090352:1695628-1695753
BmHel-13	DF090658:14307-14489
BmHel-13	DF090383:1350337-1350431
BmHel-13	DF090383:1350224-1350315
BmHel-13	DF090351:2222675-2222856

BmHel-13	DF090377:8324-8475
BmHel-13	DF090317:10999270-10999458
BmHel-13	DF090338:3027416-3027597
BmHel-13	DF090333:3717171-3717274
BmHel-13	DF090440:698258-698437
BmHel-13	DF090342:1767392-1767494
BmHel-13	BABH01049617:410-519
BmHel-13	DF090372:533272-533427
BmHel-13	DF090425:169721-169908
BmHel-13	DF090353:223477-223656
BmHel-13	DF090356:1415741-1415920
BmHel-13	BABH01047057:1058-1237
BmHel-13	DF091011:17554-17741
BmHel-13	DF090368:1338588-1338767
BmHel-13	DF090316:10579771-10579864
BmHel-13	DF090316:10579658-10579749
BmHel-13	DF090352:2478023-2478172
BmHel-13	DF090323:1152202-1152334
BmHel-13	DF090349:2124448-2124635
BmHel-13	BABH01068276:257-366
BmHel-13	DF090346:2113365-2113472
BmHel-13	DF090356:1230945-1231037
BmHel-13	DF090356:1231059-1231149
BmHel-13	DF090316:15561942-15562119
BmHel-13	DF090374:1704250-1704356
BmHel-13	DF090340:217660-217808
BmHel-13	DF090323:2971980-2972112
BmHel-13	DF090353:387912-388089
BmHel-13	DF090326:3807969-3808147
BmHel-13	DF090352:2801114-2801213
BmHel-13	DF090397:1093379-1093511
BmHel-13	DF090356:1009634-1009817
BmHel-13	DF090340:3857215-3857402
BmHel-13	DF090320:7054566-7054698
BmHel-13	DF090363:1220360-1220546
BmHel-13	DF090377:876328-876457
BmHel-13	DF090350:3943614-3943799
BmHel-13	DF090338:2833808-2833917
BmHel-13	DF090349:4118326-4118510
BmHel-13	DF090414:471398-471529
BmHel-13	BABH01072030:63-192
BmHel-13	DF090317:10020538-10020628
BmHel-13	DF090394:106155-106270
BmHel-13	DF090388:1139025-1139201
BmHel-13	DF090379:1526273-1526418
BmHel-13	DF090319:6271181-6271356
BmHel-13	DF090349:2357179-2357311
BmHel-13	DF090382:896891-897020

BmHel-13	DF090734:21686-21859
BmHel-13	DF090335:4380766-4380949
BmHel-13	DF090504:63874-64049
BmHel-13	DF090356:3057971-3058092
BmHel-13	DF090327:3105632-3105806
BmHel-13	DF090362:1913493-1913665
BmHel-13	BABH01052496:695-843
BmHel-13	DF090342:939848-940021
BmHel-13	DF090357:3215470-3215598
BmHel-13	DF090367:670729-670903
BmHel-13	DF090421:969227-969400
BmHel-13	DF090514:118202-118331
BmHel-13	DF090321:7742674-7742755
BmHel-13	DF090321:7742777-7742868
BmHel-13	DF090388:1414443-1414523
BmHel-13	DF090321:3438535-3438667
BmHel-13	DF090390:290621-290753
BmHel-13	DF090498:168251-168376
BmHel-13	DF090316:4752741-4752913
BmHel-13	DF090597:22121-22293
BmHel-13	DF090469:19335-19434
BmHel-13	DF090342:19357-19488
BmHel-13	DF090353:1475257-1475338
BmHel-13	DF090353:1475134-1475223
BmHel-13	DF090323:7004071-7004250
BmHel-13	DF090378:440121-440298
BmHel-13	DF090446:468538-468679
BmHel-13	DF090323:5543766-5543933
BmHel-13	DF090342:3482761-3482939
BmHel-13	DF090375:1072512-1072690
BmHel-13	DF090365:1772673-1772847
BmHel-13	DF090365:2570867-2570997
BmHel-13	DF090344:706382-706556
BmHel-13	DF090343:2144676-2144805
BmHel-13	DF090328:853832-853992
BmHel-13	DF090321:3437313-3437481
BmHel-13	DF090321:2851952-2852120
BmHel-13	DF090340:1248483-1248653
BmHel-13	DF090340:489111-489289
BmHel-13	DF090457:28928-29098
BmHel-13	DF090418:913290-913453
BmHel-13	DF090418:107798-107976
BmHel-13	DF090407:315312-315477
BmHel-13	DF090330:6041973-6042072
BmHel-13	DF090357:496042-496141
BmHel-13	BABH01044331:9147-9262
BmHel-13	DF090383:614352-614431
BmHel-13	DF090341:4702170-4702298

BmHel-13	DF090344:704318-704487
BmHel-13	DF090324:6793397-6793522
BmHel-13	DF090319:2031693-2031862
BmHel-13	DF090360:1409263-1409393
BmHel-13	DF090325:6046059-6046228
BmHel-13	DF090373:340125-340257
BmHel-13	DF090329:86186-86363
BmHel-13	DF090347:3913008-3913095
BmHel-13	DF090347:3913146-3913227
BmHel-13	DF090348:1957819-1957915
BmHel-13	DF090457:239866-240043
BmHel-13	DF090318:6228828-6228958
BmHel-13	DF090375:45083-45214
BmHel-13	DF090345:4260135-4260267
BmHel-13	DF090380:504535-504701
BmHel-13	DF090362:556877-557007
BmHel-13	DF090351:2984597-2984758
BmHel-13	DF090329:1999707-1999834
BmHel-13	DF090325:3012044-3012211
BmHel-13	DF090336:2494842-2494969
BmHel-13	DF090392:1251993-1252125
BmHel-13	DF090428:530584-530716
BmHel-13	DF090423:816904-817080
BmHel-13	DF090386:1078673-1078841
BmHel-13	DF090342:2790401-2790578
BmHel-13	DF090378:2071679-2071847
BmHel-13	DF090354:699223-699390
BmHel-13	DF090323:2325501-2325634
BmHel-13	DF090318:3906693-3906826
BmHel-13	DF090357:1466468-1466597
BmHel-13	DF090351:2491056-2491188
BmHel-13	DF090337:1683007-1683142
BmHel-13	DF090328:6588250-6588332
BmHel-13	DF090328:6588342-6588426
BmHel-13	DF090317:3835316-3835448
BmHel-13	DF090332:207822-207954
BmHel-13	DF090349:1812939-1813071
BmHel-13	DF090349:1422941-1423077
BmHel-13	DF090382:1883453-1883585
BmHel-13	DF090373:2082454-2082586
BmHel-13	DF090363:857131-857263
BmHel-13	DF090467:90803-90935
BmHel-13	DF090378:1857264-1857396
BmHel-13	DF090374:357241-357373
BmHel-13	DF090367:1252535-1252667
BmHel-13	DF090327:2875359-2875491
BmHel-13	DF090418:1183120-1183251
BmHel-13	DF090408:256498-256630

BmHel-13	DF090409:814576-814708
BmHel-13	DF090563:36773-36905
BmHel-13	DF090423:225805-225936
BmHel-13	DF090434:16381-16513
BmHel-13	DF090469:166325-166457
BmHel-13	DF090917:9303-9435
BmHel-13	BABH01064413:478-610
BmHel-13	DF090913:11715-11847
BmHel-13	DF090509:47502-47634
BmHel-13	BABH01050272:658-789
BmHel-13	DF090323:3893346-3893513
BmHel-13	BABH01075556:39-206
BmHel-13	BABH01075452:217-384
BmHel-13	DF090322:3322553-3322728
BmHel-13	DF090400:735973-736140
BmHel-13	DF090346:1357152-1357320
BmHel-13	DF090351:1453831-1453930
BmHel-13	DF090405:429374-429541
BmHel-13	DF090728:509-676
BmHel-13	DF090403:456233-456400
BmHel-13	DF090354:823724-823856
BmHel-13	DF090357:3569930-3570062
BmHel-13	DF090324:39695-39861
BmHel-13	DF090329:2882529-2882661
BmHel-13	DF090382:1589923-1590089
BmHel-13	DF090372:2151980-2152108
BmHel-13	DF090464:270407-270537
BmHel-13	DF090331:4241256-4241430
BmHel-13	DF090575:51915-52081
BmHel-13	DF090386:1974498-1974613
BmHel-13	DF090316:12370150-12370274
BmHel-13	DF090344:3388376-3388544
BmHel-13	DF090326:40457-40622
BmHel-13	DF090318:393561-393726
BmHel-13	DF090373:2084908-2085081
BmHel-13	DF090415:788511-788676
BmHel-13	BABH01046025:388-553
BmHel-13	DF090340:4407903-4408038
BmHel-13	DF090345:2056778-2056942
BmHel-13	DF090391:631497-631661
BmHel-13	DF090388:142461-142625
BmHel-13	DF090839:36654-36783
BmHel-13	DF090378:1391019-1391148
BmHel-13	BABH01063471:83-192
BmHel-13	DF090319:1293812-1293926
BmHel-13	DF090317:10005826-10005989
BmHel-13	DF090361:1294042-1294174
BmHel-13	DF090322:4003676-4003839

BmHel-13	DF090422:786329-786489
BmHel-13	DF090332:3420663-3420749
BmHel-13	DF090376:1765359-1765454
BmHel-13	DF090316:11909794-11909927
BmHel-13	DF090386:303860-303993
BmHel-13	DF090323:4970010-4970134
BmHel-13	DF090316:1920784-1920919
BmHel-13	DF090459:213793-213917
BmHel-13	DF090336:1142885-1143012
BmHel-13	DF090420:497065-497192
BmHel-13	DF090333:5523196-5523350
BmHel-13	DF090328:506164-506334
BmHel-13	DF090476:155554-155686
BmHel-13	DF090354:2102639-2102775
BmHel-13	DF090354:1149762-1149898
BmHel-13	DF090330:6298161-6298293
BmHel-13	DF090391:577757-577889
BmHel-13	DF090388:1292229-1292365
BmHel-13	DF090351:1287265-1287397
BmHel-13	DF090347:3616005-3616137
BmHel-13	DF090338:1929395-1929527
BmHel-13	DF090332:2726535-2726667
BmHel-13	DF090352:275823-275955
BmHel-13	DF090356:2070308-2070440
BmHel-13	DF090335:4457972-4458104
BmHel-13	DF090390:464088-464220
BmHel-13	DF090333:1235699-1235831
BmHel-13	DF090424:597267-597405
BmHel-13	DF090383:412215-412347
BmHel-13	DF090413:903953-904085
BmHel-13	DF090413:304061-304195
BmHel-13	BABH01045887:2-134
BmHel-13	DF090352:2514428-2514560
BmHel-13	DF090438:341307-341439
BmHel-13	DF090334:1481589-1481721
BmHel-13	DF090361:1364121-1364234
BmHel-13	DF090390:1371034-1371154
BmHel-13	DF090377:445963-446132
BmHel-13	DF090349:3386400-3386523
BmHel-13	DF090356:2909697-2909783
BmHel-13	DF090382:104571-104702
BmHel-13	DF090525:1871-2031
BmHel-13	DF090316:8853776-8853895
BmHel-13	DF090326:6916353-6916441
BmHel-13	DF090318:5067120-5067252
BmHel-13	DF090429:108095-108226
BmHel-13	DF090680:2331-2490
BmHel-13	DF090332:1695242-1695405

BmHel-13	DF090939:15202-15361
BmHel-13	DF090351:1291986-1292114
BmHel-13	DF090351:3097207-3097336
BmHel-13	DF090328:4107601-4107684
BmHel-13	DF090332:4223134-4223261
BmHel-13	DF090344:4326257-4326380
BmHel-13	DF090346:210096-210253
BmHel-13	DF090423:361615-361744
BmHel-13	DF090346:1034914-1035005
BmHel-13	DF090331:566263-566390
BmHel-13	DF090375:2152683-2152839
BmHel-13	DF090333:1616203-1616282
BmHel-13	DF090446:513087-513175
BmHel-13	DF090329:3833943-3834099
BmHel-13	DF090358:28839-28995
BmHel-13	DF090420:152026-152186
BmHel-13	DF090323:22483-22609
BmHel-13	DF090322:7474054-7474186
BmHel-13	DF090329:4405669-4405790
BmHel-13	DF090332:6189270-6189358
BmHel-13	DF090350:2011850-2011979
BmHel-13	DF090318:4267248-4267372
BmHel-13	DF090374:1229308-1229432
BmHel-13	DF090316:3645913-3646040
BmHel-13	DF090347:4042669-4042793
BmHel-13	DF090332:5485430-5485562
BmHel-13	DF090393:1156883-1156994
BmHel-13	DF090328:3856824-3856906
BmHel-13	BABH01079032:2-161
BmHel-13	DF090375:1779742-1779833
BmHel-13	BABH01048833:1010-1163
BmHel-13	DF090331:5906388-5906511
BmHel-13	DF090318:3318120-3318229
BmHel-13	DF090403:1519211-1519365
BmHel-13	DF090377:476450-476529
BmHel-13	DF090336:3281928-3282042
BmHel-13	DF090355:810842-810994
BmHel-13	DF090379:606798-606884
BmHel-13	DF090332:2886862-2887013
BmHel-13	DF090449:701704-701784
BmHel-13	DF090324:949984-950103
BmHel-13	DF090316:2333589-2333700
BmHel-13	DF090380:1880700-1880858
BmHel-13	DF090401:583718-583866
BmHel-13	DF091177:6356-6504
BmHel-13	DF090365:231664-231819
BmHel-13	DF090342:1538754-1538903
BmHel-13	DF090352:2126997-2127103

BmHel-13	DF090345:2685663-2685754
BmHel-13	DF090369:1411182-1411328
BmHel-13	DF090382:773495-773640
BmHel-13	DF090357:2041968-2042051
BmHel-13	DF090326:1564659-1564803
BmHel-13	DF090330:1047573-1047718
BmHel-13	DF090337:3073235-3073380
BmHel-13	DF090404:1095280-1095378
BmHel-13	DF090433:218370-218522
BmHel-13	DF090319:8742463-8742585
BmHel-13	DF090415:259874-260018
BmHel-13	DF090360:2724937-2725080
BmHel-13	DF090340:3815752-3815865
BmHel-13	DF090490:10140-10259
BmHel-13	DF090321:4879851-4879962
BmHel-13	DF090416:293096-293238
BmHel-13	DF090355:689950-690092
BmHel-13	DF090432:315502-315643
BmHel-13	BABH01052227:240-381
BmHel-13	BABH01056812:597-738
BmHel-13	DF090330:4569676-4569813
BmHel-13	BABH01088654:1-140
BmHel-13	DF090367:1899125-1899264
BmHel-13	DF090342:2563375-2563484
BmHel-13	DF090374:1616321-1616406
BmHel-13	DF090335:1479874-1480012
BmHel-13	DF090366:1442816-1442951
BmHel-13	DF090328:4157343-4157480
BmHel-13	DF090367:1294530-1294624
BmHel-13	DF090358:536741-536839
BmHel-13	DF090400:941598-941699
BmHel-13	DF090326:4908040-4908176
BmHel-13	DF090363:2634874-2634979
BmHel-13	DF091302:2795-2900
BmHel-13	DF090318:8885438-8885573
BmHel-13	DF090330:3017220-3017355
BmHel-13	DF090397:1655862-1655996
BmHel-13	DF090386:85334-85476
BmHel-13	DF090321:8246633-8246715
BmHel-13	DF090349:3779686-3779820
BmHel-13	DF090389:33079-33213
BmHel-13	DF090383:233241-233339
BmHel-13	DF090355:542509-542642
BmHel-13	DF090339:936120-936253
BmHel-13	DF090323:5975982-5976114
BmHel-13	DF090323:7985865-7985997
BmHel-13	DF090323:7869691-7869823
BmHel-13	DF090323:7059958-7060091

BmHel-13	DF090323:6331459-6331591
BmHel-13	DF090323:5224046-5224180
BmHel-13	DF090318:1005590-1005722
BmHel-13	DF090318:5979314-5979445
BmHel-13	DF090318:9186968-9187100
BmHel-13	DF090318:8327144-8327275
BmHel-13	DF090318:3523873-3524005
BmHel-13	DF090318:2479867-2480006
BmHel-13	DF090342:3265504-3265631
BmHel-13	DF090342:4465056-4465188
BmHel-13	DF090316:7625748-7625880
BmHel-13	DF090316:10536044-10536176
BmHel-13	DF090316:11328061-11328193
BmHel-13	DF090316:12410816-12410948
BmHel-13	DF090316:15288731-15288863
BmHel-13	DF090316:3988341-3988473
BmHel-13	DF090316:1844408-1844540
BmHel-13	DF090316:608449-608581
BmHel-13	DF090368:189282-189414
BmHel-13	DF090368:1923249-1923381
BmHel-13	DF090375:729247-729380
BmHel-13	DF090365:1866736-1866872
BmHel-13	DF090365:2135600-2135732
BmHel-13	DF090365:3027504-3027639
BmHel-13	DF090341:252853-252981
BmHel-13	DF090341:1346175-1346311
BmHel-13	DF090341:3771193-3771325
BmHel-13	DF090341:186472-186594
BmHel-13	DF090330:957074-957205
BmHel-13	DF090330:1188082-1188215
BmHel-13	DF090330:6015367-6015499
BmHel-13	DF090330:2173090-2173222
BmHel-13	DF090357:3204642-3204776
BmHel-13	DF090344:4563492-4563617
BmHel-13	DF090344:81587-81719
BmHel-13	DF090324:2490159-2490291
BmHel-13	DF090324:3079909-3080041
BmHel-13	DF090319:451312-451445
BmHel-13	DF090319:547796-547928
BmHel-13	DF090319:5596842-5596974
BmHel-13	DF090319:6686774-6686910
BmHel-13	DF090319:7283070-7283202
BmHel-13	DF090319:9062445-9062575
BmHel-13	DF090319:7566245-7566377
BmHel-13	DF090319:4194593-4194725
BmHel-13	DF090388:975364-975496
BmHel-13	DF090370:1326148-1326278
BmHel-13	DF090362:79333-79467

BmHel-13	DF090362:1154738-1154869
BmHel-13	DF090362:748297-748429
BmHel-13	DF090353:1707169-1707305
BmHel-13	DF090353:1749382-1749514
BmHel-13	DF090353:1184413-1184545
BmHel-13	DF090353:181293-181425
BmHel-13	DF090351:1944507-1944640
BmHel-13	DF090351:3690726-3690858
BmHel-13	DF090347:3495020-3495152
BmHel-13	DF090346:1107577-1107709
BmHel-13	DF090346:3031-3161
BmHel-13	DF090337:348038-348168
BmHel-13	DF090337:1682332-1682464
BmHel-13	DF090329:1506408-1506540
BmHel-13	DF090329:6448804-6448936
BmHel-13	DF090328:1503145-1503277
BmHel-13	DF090321:1439691-1439823
BmHel-13	DF090321:5222326-5222458
BmHel-13	DF090317:2007031-2007163
BmHel-13	DF090317:812322-812454
BmHel-13	DF090338:2627156-2627288
BmHel-13	DF090338:2051059-2051191
BmHel-13	DF090338:154872-155004
BmHel-13	DF090332:1603544-1603676
BmHel-13	DF090325:3067248-3067380
BmHel-13	DF090325:4219000-4219132
BmHel-13	DF090381:1654131-1654263
BmHel-13	DF090322:3954118-3954250
BmHel-13	DF090322:5122085-5122216
BmHel-13	DF090322:7358194-7358326
BmHel-13	DF090322:5307877-5308009
BmHel-13	DF090322:5009102-5009234
BmHel-13	DF090444:130668-130800
BmHel-13	DF090437:138202-138334
BmHel-13	DF090403:1565279-1565411
BmHel-13	DF090400:1284439-1284571
BmHel-13	DF090400:686712-686844
BmHel-13	DF090397:619704-619836
BmHel-13	DF090396:410426-410558
BmHel-13	DF090386:1889366-1889498
BmHel-13	DF090384:999601-999733
BmHel-13	DF090364:54322-54454
BmHel-13	DF090364:68713-68845
BmHel-13	DF090358:3278369-3278494
BmHel-13	DF090358:3457440-3457572
BmHel-13	DF090358:2128534-2128666
BmHel-13	DF090340:1507053-1507185
BmHel-13	DF090340:2263394-2263530

BmHel-13	DF090340:3126735-3126866
BmHel-13	DF090336:579659-579791
BmHel-13	DF090336:2687062-2687194
BmHel-13	DF090336:4250161-4250293
BmHel-13	DF090336:4771422-4771554
BmHel-13	DF090390:1352228-1352360
BmHel-13	DF090390:1601975-1602108
BmHel-13	DF090390:1454804-1454926
BmHel-13	DF090382:237195-237327
BmHel-13	DF090382:1325665-1325801
BmHel-13	DF090382:1966300-1966436
BmHel-13	DF090373:1668768-1668900
BmHel-13	DF090350:743782-743915
BmHel-13	DF090350:1884387-1884519
BmHel-13	DF090350:1470793-1470924
BmHel-13	DF090422:1112381-1112514
BmHel-13	DF090422:981507-981639
BmHel-13	DF090422:170069-170201
BmHel-13	DF090320:6903431-6903563
BmHel-13	DF090320:8151042-8151172
BmHel-13	DF090320:8568895-8569026
BmHel-13	DF090320:6135574-6135706
BmHel-13	DF090320:5154384-5154516
BmHel-13	DF090405:295854-295985
BmHel-13	DF090405:509321-509458
BmHel-13	DF090363:572614-572746
BmHel-13	DF090430:119063-119195
BmHel-13	DF090393:706512-706644
BmHel-13	DF090378:774728-774860
BmHel-13	DF090374:1907872-1908004
BmHel-13	DF090374:1219050-1219181
BmHel-13	DF090333:4376582-4376714
BmHel-13	DF090327:732722-732854
BmHel-13	DF090327:814422-814554
BmHel-13	DF090418:293488-293620
BmHel-13	DF090398:1282454-1282586
BmHel-13	DF090392:1520916-1521048
BmHel-13	DF090377:1673929-1674061
BmHel-13	DF090377:2122509-2122641
BmHel-13	DF090438:49206-49339
BmHel-13	DF090334:5286166-5286300
BmHel-13	DF090446:273099-273231
BmHel-13	DF090416:1211917-1212049
BmHel-13	DF090408:989542-989665
BmHel-13	DF090408:1188860-1188992
BmHel-13	DF090379:798682-798814
BmHel-13	DF090376:366094-366226
BmHel-13	DF090574:49731-49863

BmHel-13	DF090414:1339332-1339459
BmHel-13	DF090414:803690-803822
BmHel-13	DF090414:304241-304373
BmHel-13	DF090387:349247-349379
BmHel-13	DF090387:1655994-1656125
BmHel-13	DF090413:217306-217439
BmHel-13	DF090407:227370-227502
BmHel-13	DF090409:1183925-1184057
BmHel-13	DF090415:439446-439578
BmHel-13	DF090415:355167-355299
BmHel-13	DF090429:645837-645969
BmHel-13	DF090423:243588-243723
BmHel-13	DF090493:111478-111609
BmHel-13	DF090406:1146757-1146889
BmHel-13	DF090406:592708-592842
BmHel-13	DF090406:108743-108875
BmHel-13	DF090480:243635-243760
BmHel-13	DF091346:4455-4587
BmHel-13	DF090451:42567-42699
BmHel-13	DF091280:3212-3344
BmHel-13	DF090554:52502-52634
BmHel-13	BABH01083113:349-481
BmHel-13	BABH01081923:26-158
BmHel-13	DF090450:506686-506818
BmHel-13	BABH01045014:4603-4735
BmHel-13	DF090531:71672-71804
BmHel-13	DF090507:107680-107812
BmHel-13	BABH01043953:7052-7181
BmHel-13	DF091693:1889-2022
BmHel-13	DF090641:11400-11533
BmHel-13	DF090547:15659-15791
BmHel-13	DF090838:10996-11131
BmHel-13	DF090632:9677-9811
BmHel-13	DF090751:11881-12016
BmHel-13	DF090473:83491-83624
BmHel-13	DF090321:2478881-2479021
BmHel-13	DF090391:314468-314600
BmHel-13	DF090323:2299652-2299783
BmHel-13	DF090341:2666055-2666185
BmHel-13	DF090423:249948-250079
BmHel-13	DF090410:486925-487056
BmHel-13	DF090482:169112-169243
BmHel-13	BABH01048827:596-726
BmHel-13	DF090365:2823013-2823144
BmHel-13	DF090365:1309544-1309677
BmHel-13	DF090358:281106-281238
BmHel-13	DF090438:182632-182763
BmHel-13	BABH01048367:970-1102

BmHel-13	BABH01069994:1-135
BmHel-13	DF090858:5781-5913
BmHel-13	DF090441:520480-520567
BmHel-13	DF090318:7690471-7690572
BmHel-13	DF090339:2238518-2238656
BmHel-13	DF090339:890470-890600
BmHel-13	DF090318:9446330-9446460
BmHel-13	DF090316:13052803-13052924
BmHel-13	DF090369:1072881-1073012
BmHel-13	DF090391:579053-579182
BmHel-13	DF090348:3285443-3285573
BmHel-13	DF090346:1557053-1557183
BmHel-13	DF090371:1031668-1031766
BmHel-13	DF090338:4861780-4861910
BmHel-13	DF090325:2524630-2524760
BmHel-13	DF090331:947407-947537
BmHel-13	DF090422:573559-573689
BmHel-13	DF090419:442151-442282
BmHel-13	DF090423:560620-560701
BmHel-13	BABH01068706:5-135
BmHel-13	DF090330:2144573-2144703
BmHel-13	DF090327:5278064-5278198
BmHel-13	DF090365:1640727-1640811
BmHel-13	BABH01067544:59-189
BmHel-13	DF090349:2647621-2647721
BmHel-13	DF090318:1799449-1799577
BmHel-13	DF090353:3723659-3723787
BmHel-13	DF090321:520980-521109
BmHel-13	DF090335:2988564-2988693
BmHel-13	DF090366:502673-502802
BmHel-13	DF090699:20584-20713
BmHel-13	DF090317:7553047-7553176
BmHel-13	DF090323:6222479-6222608
BmHel-13	DF090316:9260742-9260867
BmHel-13	DF090412:195992-196121
BmHel-13	DF090357:2840904-2841033
BmHel-13	DF090357:2397889-2398014
BmHel-13	DF090353:1720438-1720567
BmHel-13	DF090328:2562019-2562152
BmHel-13	DF090397:1192095-1192224
BmHel-13	DF090331:2596280-2596407
BmHel-13	DF090408:911587-911715
BmHel-13	DF090427:315464-315593
BmHel-13	DF090621:32243-32372
BmHel-13	DF090454:23421-23554
BmHel-13	BABH01082627:46-173
BmHel-13	DF090360:6570-6700
BmHel-13	DF090397:363313-363439

BmHel-13	BABH01047502:515-644
BmHel-13	DF090354:2329499-2329627
BmHel-13	DF090354:3460111-3460238
BmHel-13	DF090318:5009944-5010072
BmHel-13	DF090326:719049-719177
BmHel-13	DF090356:1124313-1124432
BmHel-13	DF090383:486374-486501
BmHel-13	DF090329:1600395-1600523
BmHel-13	DF090340:708230-708358
BmHel-13	DF090319:5768738-5768866
BmHel-13	DF090318:3370756-3370883
BmHel-13	DF090352:3869686-3869814
BmHel-13	DF090322:4798685-4798814
BmHel-13	DF090372:2011535-2011663
BmHel-13	DF090374:1048284-1048411
BmHel-13	DF090414:292810-292938
BmHel-13	DF090432:779969-780097
BmHel-13	DF091552:1061-1189
BmHel-13	DF090596:33020-33148
BmHel-13	DF090321:1189076-1189213
BmHel-13	DF090342:2728827-2728953
BmHel-13	DF090316:12816296-12816423
BmHel-13	DF090319:2907889-2908016
BmHel-13	DF090361:2479905-2480032
BmHel-13	DF090418:807714-807841
BmHel-13	DF090387:323208-323325
BmHel-13	BABH01065207:1-128
BmHel-13	DF090318:7666290-7666418
BmHel-13	DF090348:3119983-3120110
BmHel-13	DF090343:4458326-4458452
BmHel-13	DF090374:1166031-1166114
BmHel-13	DF090355:1538836-1538963
BmHel-13	DF090322:5209457-5209584
BmHel-13	DF090327:3569598-3569723
BmHel-13	BABH01070627:331-446
BmHel-13	DF090320:1363999-1364096
BmHel-13	DF090370:1041968-1042094
BmHel-13	DF090321:5199909-5200039
BmHel-13	DF090367:2445588-2445715
BmHel-13	DF090318:2696704-2696795
BmHel-13	DF090391:967447-967572
BmHel-13	DF090321:115960-116087
BmHel-13	DF090430:297364-297455
BmHel-13	DF090466:9248-9373
BmHel-13	DF090787:14275-14402
BmHel-13	DF090423:622085-622166
BmHel-13	DF090342:2585165-2585290
BmHel-13	DF090336:3188306-3188431

BmHel-13	DF090465:110865-110960
BmHel-13	DF090365:2366160-2366284
BmHel-13	DF090335:5480976-5481100
BmHel-13	DF090366:368389-368513
BmHel-13	DF090327:2357360-2357484
BmHel-13	BABH01045354:743-867
BmHel-13	BABH01080020:1-123
BmHel-13	DF090331:317886-318009
BmHel-13	DF090351:2537069-2537196
BmHel-13	DF090316:16170898-16171022
BmHel-13	DF090357:1180268-1180355
BmHel-13	DF090332:6290959-6291077
BmHel-13	DF090443:236885-237008
BmHel-13	DF090329:5121429-5121560
BmHel-13	DF090322:888154-888277
BmHel-13	DF090365:2616787-2616909
BmHel-13	DF090359:3247274-3247396
BmHel-13	DF090349:1667244-1667337
BmHel-13	DF090328:3624242-3624368
BmHel-13	DF090355:1008515-1008635
BmHel-13	DF090333:5081311-5081432
BmHel-13	DF090354:785129-785250
BmHel-13	DF090317:8805206-8805327
BmHel-13	DF090346:997397-997526
BmHel-13	DF090999:8768-8889
BmHel-13	DF090359:772183-772305
BmHel-13	DF090330:5266116-5266236
BmHel-13	DF090382:1652728-1652848
BmHel-13	DF090406:125861-125982
BmHel-13	DF090349:3307518-3307638
BmHel-13	DF090326:6242614-6242702
BmHel-13	DF090327:1806971-1807095
BmHel-13	DF090365:2523361-2523480
BmHel-13	DF090341:445275-445397
BmHel-13	DF090317:8261125-8261231
BmHel-13	DF090346:277632-277751
BmHel-13	DF090332:2837516-2837634
BmHel-13	DF090390:156385-156503
BmHel-13	DF090418:1166288-1166368
BmHel-13	DF090961:4-113
BmHel-13	DF090349:3551254-3551371
BmHel-13	BABH01067997:1-117
BmHel-13	DF090378:969190-969307
BmHel-13	DF090468:127607-127693
BmHel-13	DF090342:3382021-3382138
BmHel-13	DF090325:4117087-4117204
BmHel-13	BABH01064226:1-117
BmHel-13	DF090388:1593892-1594002

BmHel-13	DF090617:8128-8214
BmHel-13	DF090712:30602-30718
BmHel-13	DF090407:694933-695031
BmHel-13	DF090340:1555284-1555399
BmHel-13	DF090365:236227-236312
BmHel-13	BABH01044598:633-747
BmHel-13	DF090340:4310897-4311010
BmHel-13	DF090348:1760907-1760990
BmHel-13	DF090866:1847-1959
BmHel-13	BABH01085565:385-497
BmHel-13	DF090381:1910387-1910507
BmHel-13	DF090354:971687-971798
BmHel-13	BABH01053531:2-84
BmHel-13	DF090316:1282070-1282173
BmHel-13	DF090331:1500513-1500624
BmHel-13	DF090403:1726234-1726344
BmHel-13	DF090367:648475-648588
BmHel-13	DF090383:1200395-1200505
BmHel-13	DF090365:1144197-1144307
BmHel-13	DF090316:15864912-15865026
BmHel-13	DF090321:1301373-1301483
BmHel-13	DF090323:922883-922992
BmHel-13	DF090358:943684-943793
BmHel-13	DF090374:1408849-1408958
BmHel-13	BABH01047971:157-266
BmHel-13	DF090579:14882-14992
BmHel-13	BABH01045444:306-415
BmHel-13	DF090615:36114-36223
BmHel-13	DF090461:272575-272684
BmHel-13	DF090316:11816687-11816766
BmHel-13	DF090369:2485844-2485923
BmHel-13	BABH01055020:685-768
BmHel-13	DF090355:3707195-3707303
BmHel-13	DF090336:2892093-2892201
BmHel-13	DF090382:275645-275753
BmHel-13	DF090363:2246203-2246310
BmHel-13	BABH01064023:1-107
BmHel-13	DF090342:3127702-3127808
BmHel-13	DF090410:1156694-1156800
BmHel-13	DF090632:36984-37090
BmHel-13	DF090336:5055375-5055485
BmHel-13	DF090319:7663398-7663504
BmHel-13	DF090348:4112806-4112912
BmHel-13	DF090352:160440-160545
BmHel-13	BABH01063851:571-676
BmHel-13	DF090318:8927745-8927850
BmHel-13	DF090344:1441897-1442001
BmHel-13	DF090325:4087348-4087444

BmHel-13	DF090325:2120640-2120744
BmHel-13	DF090355:1980588-1980691
BmHel-13	BABH01045401:1-104
BmHel-13	DF090347:1680882-1680984
BmHel-13	BABH01074328:508-610
BmHel-13	BABH01046609:365-467
BmHel-13	DF091109:7697-7799
BmHel-13	DF090453:266111-266207
BmHel-13	DF090448:708563-708665
BmHel-13	DF090369:792099-792201
BmHel-13	DF090344:4458401-4458503
BmHel-13	DF090456:139648-139750
BmHel-13	DF090358:1411554-1411656
BmHel-13	DF090342:1506117-1506218
BmHel-13	DF090332:2310510-2310611
BmHel-13	DF090359:1629209-1629310
BmHel-13	BABH01063651:577-677
BmHel-13	DF090925:3-101
BmHel-13	DF090405:64348-64447
BmHel-13	DF090377:375556-375656
BmHel-13	DF090329:2014784-2014883
BmHel-13	DF090327:4397903-4398002
BmHel-13	DF090340:2317299-2317395
BmHel-13	DF090368:1992941-1993037
BmHel-13	DF090340:2491325-2491421
BmHel-13	DF090365:1665765-1665860
BmHel-13	DF090329:1264246-1264340
BmHel-13	DF090340:3286156-3286250
BmHel-13	BABH01059759:1-94
BmHel-13	DF090361:820203-820295
BmHel-13	DF090352:1613328-1613423
BmHel-13	DF090356:3747805-3747893
BmHel-13	DF090391:942847-942938
BmHel-13	DF090320:7172597-7172687
BmHel-13	DF090398:797274-797364
BmHel-13	DF090394:476514-476605
BmHel-13	DF090330:3586760-3586850
BmHel-13	DF090324:7246158-7246248
BmHel-13	DF090338:3505392-3505481
BmHel-13	DF090398:1570631-1570720
BmHel-13	DF090354:762391-762479
BmHel-13	BABH01064767:1-94
BmHel-13	DF090318:6390689-6390781
BmHel-13	BABH01066053:574-661
BmHel-13	DF090327:1678952-1679039
BmHel-13	DF090334:4014396-4014483
BmHel-13	DF090347:3727989-3728076
BmHel-13	DF090316:8936565-8936652

BmHel-13	BABH01052837:160-247
BmHel-13	DF090337:2674402-2674488
BmHel-13	DF090360:840140-840225
BmHel-13	DF090338:2541842-2541922
BmHel-13	DF090345:2122173-2122255
BmHel-13	DF090384:1137673-1137754
BmHel-13	BABH01061349:483-564
BmHel-13	BABH01061537:357-438
BmHel-13	DF090644:290-371
BmHel-13	DF090332:2517588-2517669
BmHel-13	DF090385:527801-527881
BmHel-13	DF090443:34762-34842
BmHel-13	DF090473:329518-329597
BmHel-13	DF090319:5584705-5584784
BmHel-13	DF090316:12396815-12396894
BmHel-13	DF090414:1024841-1024920
BmHel-14	DF090367:1749776-1750033
BmHel-14	DF090391:577702-578048
BmHel-14	DF090333:1235505-1235878
BmHel-14	DF090332:2726341-2726716
BmHel-14	DF090383:412016-412394
BmHel-14	DF090318:48271-48651
BmHel-14	DF090413:903906-904302
BmHel-14	DF090332:2780341-2780753
BmHel-14	DF090316:13162101-13162536
BmHel-14	DF090433:437361-437828
BmHel-14	DF090322:7250371-7250887
BmHel-14	DF090322:5917212-5917737
BmHel-14	DF090327:3552609-3553142
BmHel-14	DF090392:1479449-1479986
BmHel-14	DF090329:1460728-1461268
BmHel-14	DF090374:1704204-1704744
BmHel-14	DF090322:1262898-1263442
BmHel-14	DF090318:8623140-8623685
BmHel-14	DF090496:7460-8075
BmHel-14	DF090358:2487738-2488359
BmHel-14	DF090413:543701-544331
BmHel-14	DF090341:2289862-2290589
BmHel-14	DF090466:308443-309440
BmHel-14	DF090362:3015105-3017827
BmHel-14	DF090316:7516182-7516317
BmHel-14	DF090316:7515883-7516195
BmHel-14	DF090334:4677274-4677403
BmHel-14	DF090334:4677390-4677692
BmHel-14	DF090330:1991768-1992209
BmHel-14	DF090447:458392-458833
BmHel-14	DF090323:7418839-7419281

BmHel-14	DF090373:113908-114345
BmHel-14	DF090346:2848488-2848624
BmHel-14	DF090346:2848201-2848501
BmHel-14	DF090424:674061-674498
BmHel-14	DF090396:1194655-1194898
BmHel-14	DF090396:1200256-1200478
BmHel-14	DF090332:997725-998175
BmHel-14	DF090322:2478112-2478568
BmHel-14	DF090418:1131438-1131890
BmHel-14	DF090369:1857971-1858424
BmHel-14	DF090395:736498-736950
BmHel-14	DF090379:1262665-1263117
BmHel-14	DF090347:3523521-3523972
BmHel-14	DF090322:5887537-5887987
BmHel-14	DF090388:1177594-1178043
BmHel-14	DF090357:981197-981648
BmHel-14	DF090349:1859038-1859377
BmHel-14	DF090349:1858709-1858825
BmHel-14	DF090351:1657375-1657825
BmHel-14	DF090369:427550-427999
BmHel-14	DF090378:901011-901338
BmHel-14	DF090378:900614-900737
BmHel-14	DF090414:678501-678637
BmHel-14	DF090414:678203-678514
BmHel-14	DF090572:34215-34663
BmHel-14	DF090316:15653538-15653986
BmHel-14	DF090418:995872-996008
BmHel-14	DF090418:995995-996308
BmHel-14	DF090344:3160258-3160396
BmHel-14	DF090344:3159959-3160271
BmHel-14	DF090391:41739-41874
BmHel-14	DF090391:41440-41752
BmHel-14	DF090316:11640322-11640419
BmHel-14	DF090316:11640449-11640795
BmHel-14	DF090443:558752-559200
BmHel-14	DF090392:1623891-1624026
BmHel-14	DF090392:1624013-1624324
BmHel-14	DF090340:2193822-2194232
BmHel-14	DF090321:1110180-1110509
BmHel-14	DF090321:1109842-1109963
BmHel-14	DF090338:3683355-3683802
BmHel-14	DF090347:1468110-1468486
BmHel-14	DF090347:1467631-1467714
BmHel-14	DF090335:3973972-3974241
BmHel-14	DF090335:3974258-3974431
BmHel-14	DF090356:2909697-2909856
BmHel-14	DF090356:2909863-2910090
BmHel-14	DF090341:1736966-1737105

BmHel-14	DF090341:1737092-1737398
BmHel-14	DF090407:1148318-1148455
BmHel-14	DF090407:1148061-1148331
BmHel-14	DF090332:2197294-2197739
BmHel-14	DF090470:262001-262132
BmHel-14	DF090470:261710-262012
BmHel-14	DF090375:1430247-1430494
BmHel-14	DF090375:1429837-1430013
BmHel-14	DF090408:1470050-1470495
BmHel-14	DF090418:811627-812080
BmHel-14	DF090318:4838872-4839306
BmHel-14	DF090379:956469-956630
BmHel-14	DF090379:955909-956190
BmHel-14	DF090437:25164-25599
BmHel-14	DF090397:1093081-1093511
BmHel-14	DF090363:1129562-1129696
BmHel-14	DF090363:1129479-1129575
BmHel-14	DF090363:1129156-1129382
BmHel-14	DF090408:556226-556632
BmHel-14	DF090319:2222072-2222510
BmHel-14	DF090342:1127449-1127571
BmHel-14	DF090342:1125720-1125820
BmHel-14	DF090342:1124753-1124970
BmHel-14	DF090346:459510-459641
BmHel-14	DF090346:459373-459523
BmHel-14	DF090346:458876-459040
BmHel-14	DF090326:2409978-2410096
BmHel-14	DF090326:2409679-2409991
BmHel-14	DF090361:1294042-1294232
BmHel-14	DF090361:1294233-1294482
BmHel-14	DF090344:4482439-4482648
BmHel-14	DF090344:4482343-4482440
BmHel-14	DF090344:4482025-4482158
BmHel-14	DF090344:2312220-2312356
BmHel-14	DF090344:2312090-2312233
BmHel-14	DF090344:2311934-2312090
BmHel-14	DF090352:2049354-2049566
BmHel-14	DF090352:2049126-2049355
BmHel-14	BABH01047703:1323-1459
BmHel-14	BABH01047703:1193-1336
BmHel-14	BABH01047703:1037-1193
BmHel-14	DF090389:1828409-1828848
BmHel-14	DF090372:1314960-1315390
BmHel-14	DF090320:3806705-3807150
BmHel-14	DF090341:1789724-1790038
BmHel-14	DF090341:1790041-1790164
BmHel-14	DF090316:13432739-13433175
BmHel-14	DF090333:5442163-5442600

BmHel-14	DF090362:2121093-2121302
BmHel-14	DF090362:2120871-2121094
BmHel-14	DF090391:372926-373065
BmHel-14	DF090391:372506-372790
BmHel-14	DF090316:15736246-15736677
BmHel-14	DF090356:3057659-3058092
BmHel-14	DF090319:2468030-2468148
BmHel-14	DF090319:2467737-2468043
BmHel-14	DF090347:389720-389955
BmHel-14	DF090347:389520-389712
BmHel-14	DF090365:2570867-2571065
BmHel-14	DF090365:2571078-2571309
BmHel-14	DF090332:731477-731697
BmHel-14	DF090332:731693-731900
BmHel-14	DF090404:98912-99180
BmHel-14	DF090404:99182-99339
BmHel-14	DF090488:122259-122449
BmHel-14	DF090488:122452-122712
BmHel-14	DF090425:169772-169908
BmHel-14	DF090425:169501-169716
BmHel-14	DF090339:2152419-2152746
BmHel-14	DF090324:7249191-7249327
BmHel-14	DF090324:7249044-7249204
BmHel-14	DF090324:7248823-7248914
BmHel-14	DF090389:535881-536016
BmHel-14	DF090389:535603-535892
BmHel-14	DF090367:1098334-1098662
BmHel-14	DF090351:1453829-1454259
BmHel-14	DF090388:1414349-1414742
BmHel-14	DF090390:1704685-1705010
BmHel-14	DF090390:1705312-1705409
BmHel-14	DF090368:142650-143069
BmHel-14	DF090316:2783576-2784001
BmHel-14	DF090357:496038-496449
BmHel-14	DF090325:3801924-3802057
BmHel-14	DF090325:3802044-3802327
BmHel-14	DF090394:1558156-1558568
BmHel-14	DF090420:496873-496985
BmHel-14	DF090420:496583-496886
BmHel-14	DF090349:4118326-4118433
BmHel-14	DF090349:4118420-4118724
BmHel-14	DF090903:7012-7278
BmHel-14	DF090903:6865-7013
BmHel-14	DF090343:1221482-1221896
BmHel-14	DF090361:615331-615686
BmHel-14	DF090323:7004071-7004179
BmHel-14	DF090323:7004166-7004250
BmHel-14	DF090323:7004278-7004510

BmHel-14	DF090325:5531530-5531936
BmHel-14	DF090323:4360052-4360184
BmHel-14	DF090323:4360164-4360361
BmHel-14	DF090323:4361742-4361834
BmHel-14	DF090397:1370788-1370924
BmHel-14	DF090397:1370911-1371173
BmHel-14	DF090521:29744-29956
BmHel-14	DF090521:29985-30094
BmHel-14	DF090521:30117-30204
BmHel-14	DF090368:651366-651589
BmHel-14	DF090368:649369-649548
BmHel-14	DF090346:487337-487741
BmHel-14	DF090329:5818824-5819042
BmHel-14	DF090329:5819040-5819223
BmHel-14	DF090340:4335867-4336003
BmHel-14	DF090340:4335620-4335833
BmHel-14	DF090449:701704-702104
BmHel-14	DF090326:782283-782566
BmHel-14	DF090352:1535104-1535315
BmHel-14	DF090352:1534946-1535105
BmHel-14	DF090330:3336484-3336837
BmHel-14	DF090326:595994-596126
BmHel-14	DF090326:585924-586004
BmHel-14	DF090326:583703-583784
BmHel-14	DF090316:10543884-10544184
BmHel-14	DF090386:85334-85514
BmHel-14	DF090386:85510-85733
BmHel-14	DF090316:8569428-8569556
BmHel-14	DF090316:8569180-8569437
BmHel-14	DF090320:7054566-7054772
BmHel-14	DF090320:7054831-7054948
BmHel-14	DF090380:772709-772843
BmHel-14	DF090380:772830-772971
BmHel-14	DF090380:772970-773051
BmHel-14	DF090317:3425118-3425508
BmHel-14	DF090319:3921041-3921424
BmHel-14	DF090321:5014975-5015205
BmHel-14	DF090321:5014821-5014972
BmHel-14	DF090359:1798489-1798691
BmHel-14	DF090359:1798751-1798833
BmHel-14	DF090412:1107287-1107566
BmHel-14	DF090412:1107195-1107288
BmHel-14	DF090347:3324507-3324785
BmHel-14	DF090347:3324784-3324874
BmHel-14	DF090328:4107601-4107684
BmHel-14	DF090328:4107694-4107980
BmHel-14	DF090356:1629990-1630102
BmHel-14	DF090356:1629818-1629992

BmHel-14	DF090356:1629737-1629817
BmHel-14	DF090344:3707787-3707902
BmHel-14	DF090344:3707889-3707989
BmHel-14	DF090344:3711047-3711208
BmHel-14	DF090332:5135232-5135565
BmHel-14	DF090358:375566-375900
BmHel-14	DF090741:1648-1985
BmHel-14	DF090429:108095-108280
BmHel-14	DF090429:108279-108453
BmHel-14	DF090354:823714-823856
BmHel-14	DF090354:823125-823241
BmHel-14	DF090358:3238960-3239092
BmHel-14	DF090358:3239217-3239341
BmHel-14	DF090328:3856676-3856934
BmHel-14	DF090328:3856584-3856677
BmHel-14	DF090321:2478938-2479021
BmHel-14	DF090321:2478675-2478882
BmHel-14	DF090360:6524-6700
BmHel-14	DF090360:6328-6511
BmHel-14	DF090403:1539963-1540154
BmHel-14	DF090403:1540202-1540294
BmHel-14	DF090362:3138427-3138562
BmHel-14	DF090362:3138551-3138690
BmHel-14	DF090362:3138692-3138778
BmHel-14	DF090359:2597404-2597760
BmHel-14	DF090333:1275094-1275329
BmHel-14	DF090333:1275357-1275448
BmHel-14	DF090363:1866801-1867073
BmHel-14	DF090363:1867080-1867161
BmHel-14	DF090398:47275-47500
BmHel-14	DF090398:47184-47273
BmHel-14	BABH01049617:410-701
BmHel-14	DF090347:3485245-3485465
BmHel-14	DF090347:3485119-3485212
BmHel-14	DF090324:4317446-4317667
BmHel-14	DF090324:4317700-4317793
BmHel-14	DF090379:233774-234015
BmHel-14	DF090379:234023-234145
BmHel-14	DF090429:393100-393216
BmHel-14	DF090429:393223-393365
BmHel-14	DF090429:393366-393454
BmHel-14	DF090339:2086526-2086662
BmHel-14	DF090339:2086330-2086539
BmHel-14	DF090346:997199-997398
BmHel-14	DF090466:358591-358723
BmHel-14	DF090466:355982-356085
BmHel-14	DF090332:3168707-3168843
BmHel-14	DF090332:3168830-3168968

BmHel-14	DF090457:239577-239677
BmHel-14	DF090457:240089-240248
BmHel-14	DF090388:1075084-1075420
BmHel-14	DF090339:5038925-5039261
BmHel-14	BABH01075697:90-426
BmHel-14	DF090382:896891-897226
BmHel-14	DF090335:1918375-1918709
BmHel-14	DF090335:3577542-3577749
BmHel-14	DF090335:3577420-3577543
BmHel-14	DF090346:496067-496399
BmHel-14	DF090330:217809-218000
BmHel-14	DF090330:217701-217808
BmHel-14	DF090340:990583-990907
BmHel-14	DF090424:597256-597405
BmHel-14	DF090424:597062-597152
BmHel-14	DF090353:2835349-2835537
BmHel-14	DF090353:2835238-2835345
BmHel-14	DF090317:1439087-1439403
BmHel-14	DF090373:340125-340321
BmHel-14	DF090373:340325-340452
BmHel-14	DF090328:506164-506256
BmHel-14	DF090328:506243-506384
BmHel-14	DF090352:1695628-1695841
BmHel-14	DF090352:1695843-1695954
BmHel-14	DF090316:7717777-7717968
BmHel-14	DF090316:7717969-7718071
BmHel-14	DF090346:3882694-3882876
BmHel-14	DF090346:3882877-3882976
BmHel-14	DF090390:464088-464280
BmHel-14	DF090390:464281-464380
BmHel-14	DF090349:2125946-2126082
BmHel-14	DF090554:43656-43835
BmHel-14	DF090348:1957671-1957915
BmHel-14	DF090332:5485430-5485614
BmHel-14	DF090483:125876-126199
BmHel-14	DF090317:11250630-11250812
BmHel-14	DF090322:5599885-5600025
BmHel-14	DF090322:5599737-5599844
BmHel-14	DF090461:312838-312978
BmHel-14	DF090461:312690-312797
BmHel-14	DF090403:947998-948315
BmHel-14	DF090347:1992371-1992511
BmHel-14	DF090347:1992223-1992328
BmHel-14	DF090350:1513201-1513306
BmHel-14	DF090350:1513054-1513202
BmHel-14	DF090366:368373-368513
BmHel-14	DF090366:367787-367894
BmHel-14	BABH01072030:63-279

BmHel-14	BABH01072030:310-401
BmHel-14	DF090377:476450-476529
BmHel-14	DF090377:476530-476674
BmHel-14	DF090377:476677-476766
BmHel-14	DF090398:501080-501247
BmHel-14	DF090327:134902-135214
BmHel-14	DF090356:3013183-3013397
BmHel-14	DF090356:3013396-3013489
BmHel-14	DF090414:471321-471529
BmHel-14	DF090350:3943614-3943738
BmHel-14	DF090350:3943725-3943867
BmHel-14	DF090351:1540512-1540647
BmHel-14	DF090351:1540682-1540781
BmHel-14	DF090414:1306589-1306886
BmHel-14	DF090331:5876591-5876857
BmHel-14	DF090333:1616203-1616282
BmHel-14	DF090333:1616298-1616424
BmHel-14	DF090333:1616423-1616516
BmHel-14	DF090374:221297-221500
BmHel-14	DF090374:221502-221588
BmHel-14	DF090330:3766003-3766233
BmHel-14	DF090333:1437478-1437710
BmHel-14	DF090839:36654-36842
BmHel-14	DF090839:36843-36950
BmHel-14	DF090347:3318297-3318411
BmHel-14	DF090347:3313381-3313488
BmHel-14	DF090374:1229308-1229568
BmHel-14	DF090415:1247296-1247435
BmHel-14	DF090321:1189122-1189263
BmHel-14	DF090321:1189262-1189353
BmHel-14	DF090324:4485092-4485240
BmHel-14	DF090324:4483258-4483337
BmHel-14	DF090373:867586-867780
BmHel-14	DF090388:1292202-1292365
BmHel-14	DF090388:1292098-1292201
BmHel-14	DF090334:3053731-3053957
BmHel-14	DF090459:213777-213917
BmHel-14	DF090459:213629-213736
BmHel-14	DF090330:5622557-5622748
BmHel-14	DF090320:7952483-7952615
BmHel-14	DF090319:4838490-4838635
BmHel-14	DF090319:4838378-4838489
BmHel-14	DF090318:3933802-3933943
BmHel-14	DF090318:3933690-3933801
BmHel-14	BABH01046182:256-542
BmHel-14	DF090372:2151980-2152162
BmHel-14	DF090372:2152168-2152261
BmHel-14	DF090334:1761334-1761618

BmHel-14	DF090353:535779-535923
BmHel-14	DF090323:4970010-4970146
BmHel-14	DF090323:4970191-4970308
BmHel-14	DF090354:2760550-2760672
BmHel-14	DF090354:2759990-2760071
BmHel-14	DF090354:2758114-2758210
BmHel-14	DF090316:13366533-13366814
BmHel-14	DF090357:1466468-1466659
BmHel-14	DF090357:1466660-1466749
BmHel-14	DF090321:115960-116087
BmHel-14	DF090321:115761-115847
BmHel-14	DF090467:19782-19973
BmHel-14	DF090339:3837173-3837357
BmHel-14	DF090339:3837065-3837159
BmHel-14	DF090336:3281912-3282042
BmHel-14	DF090336:3281764-3281871
BmHel-14	DF090336:4750835-4751063
BmHel-14	DF090316:3645913-3646056
BmHel-14	DF090316:3646100-3646207
BmHel-14	DF090352:2126889-2126996
BmHel-14	DF090373:438557-438804
BmHel-14	DF090326:461342-461617
BmHel-14	DF090388:142493-142625
BmHel-14	DF090388:141987-142093
BmHel-14	DF090401:956252-956385
BmHel-14	DF090362:252986-253111
BmHel-14	DF090362:252866-252997
BmHel-14	DF090342:4410778-4410986
BmHel-14	DF090327:1272961-1273227
BmHel-14	DF090321:7355578-7355834
BmHel-14	DF090364:1149872-1150139
BmHel-14	DF090337:5140181-5140312
BmHel-14	DF090320:7597416-7597547
BmHel-14	DF090320:7597305-7597410
BmHel-14	DF090433:218431-218626
BmHel-14	DF090322:6901956-6902223
BmHel-14	DF090373:407245-407381
BmHel-14	DF090373:407132-407258
BmHel-14	DF090363:1220411-1220546
BmHel-14	DF090363:1220290-1220423
BmHel-14	DF090456:42862-42998
BmHel-14	DF090329:1690511-1690782
BmHel-14	DF090362:1236941-1237203
BmHel-14	DF091388:3564-3830
BmHel-14	DF090335:4380816-4380949
BmHel-14	DF090335:4380700-4380828
BmHel-14	DF090609:25205-25445
BmHel-14	DF090372:2011535-2011710

BmHel-14	DF090372:2011737-2011826
BmHel-14	DF090464:270407-270601
BmHel-14	DF090407:1352575-1352807
BmHel-14	DF090346:1170436-1170582
BmHel-14	DF090346:1169852-1169967
BmHel-14	DF090323:1152134-1152334
BmHel-14	DF090530:40937-41069
BmHel-14	DF090849:632-764
BmHel-14	DF090323:751551-751812
BmHel-14	DF090349:2048530-2048662
BmHel-14	DF090349:2048413-2048506
BmHel-14	DF090332:5894878-5895108
BmHel-14	DF090351:2297424-2297657
BmHel-14	DF090351:3982366-3982596
BmHel-14	DF090316:11152271-11152499
BmHel-14	DF090316:6024760-6024990
BmHel-14	DF090330:3499654-3499884
BmHel-14	DF090318:6138861-6139091
BmHel-14	DF090343:1709625-1709855
BmHel-14	DF090323:534219-534446
BmHel-14	DF090357:91889-92123
BmHel-14	DF090357:3570687-3570917
BmHel-14	DF090346:645236-645457
BmHel-14	DF090320:2265705-2265935
BmHel-14	DF090320:6460714-6460945
BmHel-14	DF090341:4017753-4017981
BmHel-14	DF090339:3065898-3066127
BmHel-14	DF090321:1536474-1536698
BmHel-14	DF090390:812520-812749
BmHel-14	DF090335:900946-901175
BmHel-14	DF090334:2293713-2293944
BmHel-14	DF090317:869080-869310
BmHel-14	DF090317:7598769-7598999
BmHel-14	DF090363:911249-911479
BmHel-14	DF090363:2079008-2079237
BmHel-14	DF090414:1245600-1245830
BmHel-14	DF090412:782979-783210
BmHel-14	DF090412:320889-321119
BmHel-14	DF090355:2402154-2402384
BmHel-14	DF090355:2454916-2455145
BmHel-14	DF090352:3988476-3988708
BmHel-14	BABH01045685:2727-2957
BmHel-14	DF090421:472793-473023
BmHel-14	DF090377:169467-169697
BmHel-14	DF090353:486386-486616
BmHel-14	DF090402:1222440-1222670
BmHel-14	DF090342:3573767-3573997
BmHel-14	DF090448:147913-148143

BmHel-14	DF090432:355901-356129
BmHel-14	DF090387:1087236-1087471
BmHel-14	DF090332:1149168-1149397
BmHel-14	DF090329:3021728-3021862
BmHel-14	DF090329:3021848-3021941
BmHel-14	DF090329:4051421-4051651
BmHel-14	DF090317:6242991-6243220
BmHel-14	DF090340:4020723-4020858
BmHel-14	DF090340:4020845-4020936
BmHel-14	DF090340:4439686-4439822
BmHel-14	DF090340:4439607-4439699
BmHel-14	DF090317:3226377-3226530
BmHel-14	DF090317:4858170-4858306
BmHel-14	DF090317:4858293-4858384
BmHel-14	DF090364:2087184-2087321
BmHel-14	DF090364:2087106-2087197
BmHel-14	DF090362:3123675-3123811
BmHel-14	DF090362:3123595-3123688
BmHel-14	DF090396:1509010-1509146
BmHel-14	DF090396:1508932-1509023
BmHel-14	DF090342:4030972-4031108
BmHel-14	DF090342:4031095-4031186
BmHel-14	DF090442:539079-539215
BmHel-14	DF090442:539001-539092
BmHel-14	DF090332:3137087-3137228
BmHel-14	DF090332:3136975-3137086
BmHel-14	DF090361:534230-534441
BmHel-14	BABH01045684:3861-3989
BmHel-14	DF090408:107879-108106
BmHel-14	DF090344:609525-609752
BmHel-14	DF090400:1011346-1011573
BmHel-14	DF090405:842757-842984
BmHel-14	DF090469:278855-279081
BmHel-14	BABH01045835:1114-1341
BmHel-14	DF090386:645014-645241
BmHel-14	DF091392:2724-2970
BmHel-14	BABH01052746:314-564
BmHel-14	DF090340:3857215-3857356
BmHel-14	DF090340:3857357-3857466
BmHel-14	DF090338:2616619-2616756
BmHel-14	DF090338:2616736-2616834
BmHel-14	DF090323:4495043-4495180
BmHel-14	DF090323:4495160-4495258
BmHel-14	DF090349:4197333-4197469
BmHel-14	DF090349:4197258-4197346
BmHel-14	DF090341:2160598-2160823
BmHel-14	DF090395:504316-504448
BmHel-14	DF090393:1675487-1675617

BmHel-14	DF090437:253193-253318
BmHel-14	DF090443:412777-412914
BmHel-14	DF090443:412703-412790
BmHel-14	DF090336:4250161-4250304
BmHel-14	DF090336:4258490-4258599
BmHel-14	DF090375:1841772-1841901
BmHel-14	DF090318:4962311-4962447
BmHel-14	DF090318:4962448-4962559
BmHel-14	DF090333:1556035-1556263
BmHel-14	DF090316:1844351-1844540
BmHel-14	DF090337:1682286-1682464
BmHel-14	DF090365:861457-861549
BmHel-14	DF090365:861824-862005
BmHel-14	DF090346:2113365-2113616
BmHel-14	DF090356:1009634-1009764
BmHel-14	DF090356:1009772-1009882
BmHel-14	DF090326:3808014-3808147
BmHel-14	DF090326:3807903-3808016
BmHel-14	DF090317:10999322-10999458
BmHel-14	DF090317:10999227-10999335
BmHel-14	DF090374:2064595-2064744
BmHel-14	DF090374:2064745-2064841
BmHel-14	DF090327:1336356-1336484
BmHel-14	DF090427:896946-897092
BmHel-14	DF090418:1166288-1166388
BmHel-14	DF090418:1166429-1166541
BmHel-14	DF090361:2155775-2155995
BmHel-14	DF090323:2971980-2972188
BmHel-14	DF090473:83445-83624
BmHel-14	DF090329:4405669-4405850
BmHel-14	DF090335:3444475-3444603
BmHel-14	DF090367:68624-68870
BmHel-14	DF090429:488874-488962
BmHel-14	DF090429:488747-488874
BmHel-14	DF090446:512992-513175
BmHel-14	DF090375:210676-210804
BmHel-14	DF090375:210561-210676
BmHel-14	DF090352:1321228-1321355
BmHel-14	DF090358:1197417-1197541
BmHel-14	DF090358:1197336-1197421
BmHel-14	DF090348:3315366-3315580
BmHel-14	DF090482:169112-169253
BmHel-14	DF090482:169268-169365
BmHel-14	DF090316:5089977-5090102
BmHel-14	DF090316:5090105-5090192
BmHel-14	DF090388:1604962-1605175
BmHel-14	DF090352:2801114-2801213
BmHel-14	DF090352:2803332-2803413

BmHel-14	DF090343:31915-32047
BmHel-14	DF090343:31838-31917
BmHel-14	DF090323:1883760-1883890
BmHel-14	DF090323:1883683-1883762
BmHel-14	DF090346:1804651-1804784
BmHel-14	DF090346:1804782-1804861
BmHel-14	DF090341:432091-432223
BmHel-14	DF090411:234606-234738
BmHel-14	DF090411:234529-234608
BmHel-14	DF090336:2736853-2736985
BmHel-14	DF090336:2736983-2737062
BmHel-14	DF090331:3997462-3997594
BmHel-14	DF090331:3997592-3997671
BmHel-14	DF090350:3515977-3516109
BmHel-14	DF090350:3515897-3515979
BmHel-14	DF090371:393394-393526
BmHel-14	DF090371:393317-393396
BmHel-14	DF090426:921430-921562
BmHel-14	DF090426:921353-921432
BmHel-14	DF090346:3718703-3718823
BmHel-14	DF090346:3718810-3718901
BmHel-14	DF090401:648844-648965
BmHel-14	DF090401:648754-648846
BmHel-14	DF090401:750371-750612
BmHel-14	DF090324:6766585-6766712
BmHel-14	DF090324:6766468-6766579
BmHel-14	DF090340:1371302-1371430
BmHel-14	DF090320:3992555-3992689
BmHel-14	DF090336:4556680-4556889
BmHel-14	DF091299:1133-1257
BmHel-14	DF091299:1259-1344
BmHel-14	DF090391:631529-631661
BmHel-14	DF090391:631146-631235
BmHel-14	DF090320:7079945-7080126
BmHel-14	DF090332:6120783-6120914
BmHel-14	DF090332:4093752-4093884
BmHel-14	DF090332:4093675-4093754
BmHel-14	DF090332:1471784-1471916
BmHel-14	DF090322:1279189-1279321
BmHel-14	DF090322:1719842-1719974
BmHel-14	DF090322:2710474-2710607
BmHel-14	DF090322:6210969-6211102
BmHel-14	DF090322:5523799-5523931
BmHel-14	DF090351:303849-303981
BmHel-14	DF090351:1082368-1082504
BmHel-14	DF090351:3600615-3600747
BmHel-14	DF090319:7047420-7047552
BmHel-14	DF090319:8661196-8661328

BmHel-14	DF090319:7852022-7852154
BmHel-14	DF090319:4440209-4440341
BmHel-14	DF090319:3762905-3763037
BmHel-14	DF090319:2506815-2506947
BmHel-14	DF090319:229617-229749
BmHel-14	DF090316:4584675-4584807
BmHel-14	DF090316:4798084-4798216
BmHel-14	DF090316:7192794-7192929
BmHel-14	DF090316:9203358-9203489
BmHel-14	DF090316:9917394-9917526
BmHel-14	DF090316:15000362-15000494
BmHel-14	DF090316:16060352-16060484
BmHel-14	DF090316:11714230-11714359
BmHel-14	DF090316:10068064-10068196
BmHel-14	DF090316:7253354-7253486
BmHel-14	DF090316:1397275-1397407
BmHel-14	DF090418:679138-679269
BmHel-14	DF090329:6080177-6080309
BmHel-14	DF090329:5198812-5198946
BmHel-14	DF090329:4166232-4166364
BmHel-14	DF090329:2363623-2363755
BmHel-14	DF090369:1865922-1866054
BmHel-14	DF090330:2624582-2624714
BmHel-14	DF090330:6284346-6284478
BmHel-14	DF090330:3739481-3739612
BmHel-14	DF090330:3126334-3126466
BmHel-14	DF090330:676572-676704
BmHel-14	DF090318:9882853-9882984
BmHel-14	DF090318:2877454-2877586
BmHel-14	DF090318:2288579-2288710
BmHel-14	DF090318:1953962-1954094
BmHel-14	DF090318:330746-330878
BmHel-14	DF090368:2037313-2037445
BmHel-14	DF090338:2774943-2775075
BmHel-14	DF090338:2646547-2646678
BmHel-14	DF090343:1276322-1276454
BmHel-14	DF090343:3792404-3792536
BmHel-14	DF090343:3139522-3139653
BmHel-14	DF090343:2556614-2556746
BmHel-14	DF090408:787522-787654
BmHel-14	DF090395:943682-943814
BmHel-14	DF090372:2203762-2203894
BmHel-14	DF090372:1180708-1180841
BmHel-14	DF090372:841656-841788
BmHel-14	DF090379:905135-905267
BmHel-14	DF090388:294651-294784
BmHel-14	DF090388:1470094-1470226
BmHel-14	DF090388:876837-876969

BmHel-14	DF090323:6136039-6136171
BmHel-14	DF090323:6245438-6245570
BmHel-14	DF090323:6245360-6245440
BmHel-14	DF090323:4039422-4039554
BmHel-14	DF090323:2849149-2849281
BmHel-14	DF090323:1402953-1403085
BmHel-14	DF090323:823294-823426
BmHel-14	DF090340:3300032-3300164
BmHel-14	DF090340:4378653-4378785
BmHel-14	DF090449:754394-754526
BmHel-14	DF090373:1109177-1109309
BmHel-14	DF090373:1537363-1537495
BmHel-14	DF090373:1755300-1755432
BmHel-14	DF090333:4387779-4387911
BmHel-14	DF090437:567131-567264
BmHel-14	DF090437:196111-196244
BmHel-14	DF090357:822015-822147
BmHel-14	DF090357:1285513-1285645
BmHel-14	DF090394:1305665-1305797
BmHel-14	DF090394:829350-829482
BmHel-14	DF090346:1538601-1538733
BmHel-14	DF090346:2940276-2940408
BmHel-14	DF090325:3127511-3127643
BmHel-14	DF090325:3127641-3127720
BmHel-14	DF090325:4100192-4100324
BmHel-14	DF090325:3421598-3421730
BmHel-14	DF090356:3273735-3273867
BmHel-14	DF090359:3112513-3112645
BmHel-14	DF090359:2283940-2284072
BmHel-14	DF090359:1806047-1806179
BmHel-14	DF090347:553665-553797
BmHel-14	DF090347:784213-784345
BmHel-14	DF090347:3465882-3466014
BmHel-14	DF090347:3951290-3951422
BmHel-14	DF090347:4048058-4048190
BmHel-14	DF090347:416487-416619
BmHel-14	DF090320:2992502-2992634
BmHel-14	DF090401:989953-990085
BmHel-14	DF090401:1091120-1091252
BmHel-14	DF090341:3412360-3412496
BmHel-14	DF090327:2851095-2851227
BmHel-14	DF090327:2728584-2728716
BmHel-14	DF090327:1986102-1986234
BmHel-14	DF090361:298544-298674
BmHel-14	DF090361:2463588-2463720
BmHel-14	DF090361:2565764-2565896
BmHel-14	DF090361:2048287-2048419
BmHel-14	DF090466:68513-68645

BmHel-14	DF090466:235147-235282
BmHel-14	DF090339:99018-99150
BmHel-14	DF090339:1039538-1039671
BmHel-14	DF090339:1063866-1063998
BmHel-14	DF090339:1957565-1957697
BmHel-14	DF090339:3187244-3187376
BmHel-14	DF090339:4392353-4392485
BmHel-14	DF090339:3809407-3809539
BmHel-14	DF090339:1320192-1320324
BmHel-14	DF090339:1010173-1010305
BmHel-14	DF090339:526031-526163
BmHel-14	DF090321:8096651-8096776
BmHel-14	DF090321:6396459-6396591
BmHel-14	DF090321:3585917-3586049
BmHel-14	DF090321:3303656-3303788
BmHel-14	DF090321:7652-7784
BmHel-14	DF090367:2439084-2439220
BmHel-14	DF090367:1883556-1883688
BmHel-14	DF090367:1473235-1473367
BmHel-14	DF090367:275372-275504
BmHel-14	DF090349:2555058-2555198
BmHel-14	DF090349:263258-263389
BmHel-14	DF090344:155547-155684
BmHel-14	DF090344:2165856-2165988
BmHel-14	DF090344:3575547-3575683
BmHel-14	DF090344:4612572-4612704
BmHel-14	DF090344:4253557-4253689
BmHel-14	DF090420:468669-468801
BmHel-14	DF090390:686254-686386
BmHel-14	DF090390:1647785-1647916
BmHel-14	DF090328:1300019-1300151
BmHel-14	DF090328:1762552-1762684
BmHel-14	DF090328:3712744-3712876
BmHel-14	DF090328:5588577-5588713
BmHel-14	DF090403:872321-872453
BmHel-14	DF090403:1588009-1588141
BmHel-14	DF090391:1104122-1104249
BmHel-14	DF090335:643806-643938
BmHel-14	DF090335:1091553-1091684
BmHel-14	DF090335:1270646-1270778
BmHel-14	DF090335:2824494-2824626
BmHel-14	DF090335:4672064-4672196
BmHel-14	DF090335:4693373-4693505
BmHel-14	DF090335:3456396-3456516
BmHel-14	DF090335:3292339-3292471
BmHel-14	DF090335:582645-582777
BmHel-14	DF090358:747992-748124
BmHel-14	DF090358:1521715-1521848

BmHel-14	DF090358:1999826-1999956
BmHel-14	DF090358:1576705-1576837
BmHel-14	DF090334:2573519-2573650
BmHel-14	DF090334:2573648-2573727
BmHel-14	DF090334:5044725-5044854
BmHel-14	DF090334:5458568-5458700
BmHel-14	DF090334:3720209-3720342
BmHel-14	DF090378:1343729-1343861
BmHel-14	DF090378:2041248-2041382
BmHel-14	DF090326:606104-606236
BmHel-14	DF090326:1355465-1355597
BmHel-14	DF090326:5514624-5514756
BmHel-14	DF090326:6176309-6176441
BmHel-14	DF090326:6119666-6119798
BmHel-14	DF090326:3826739-3826871
BmHel-14	DF090326:3520581-3520713
BmHel-14	DF090326:2425334-2425466
BmHel-14	DF090326:1444854-1444986
BmHel-14	DF090326:801136-801268
BmHel-14	DF090317:8559812-8559944
BmHel-14	DF090317:9262392-9262527
BmHel-14	DF090317:10060453-10060585
BmHel-14	DF090317:11109530-11109662
BmHel-14	DF090317:9521530-9521662
BmHel-14	DF090317:3976278-3976410
BmHel-14	DF090317:1635056-1635188
BmHel-14	DF090382:32988-33120
BmHel-14	DF090382:1702417-1702549
BmHel-14	DF090364:2114549-2114681
BmHel-14	DF090364:1753696-1753828
BmHel-14	DF090364:429548-429680
BmHel-14	DF090364:275890-276022
BmHel-14	DF090363:861607-861739
BmHel-14	DF090363:336153-336285
BmHel-14	DF090414:883447-883579
BmHel-14	DF090412:753884-754016
BmHel-14	DF090412:896049-896184
BmHel-14	DF090348:1454420-1454552
BmHel-14	DF090348:2291614-2291746
BmHel-14	DF090348:3392510-3392642
BmHel-14	DF090348:3473569-3473701
BmHel-14	DF090348:3028797-3028929
BmHel-14	DF090433:27439-27571
BmHel-14	DF090411:705048-705180
BmHel-14	DF090411:609053-609185
BmHel-14	DF090355:1323659-1323791
BmHel-14	DF090352:372373-372505
BmHel-14	DF090352:372503-372583

BmHel-14	DF090352:848795-848927
BmHel-14	DF090352:1266184-1266316
BmHel-14	DF090352:2816836-2816968
BmHel-14	DF090352:2816966-2817046
BmHel-14	DF090352:3375005-3375137
BmHel-14	DF090352:3174500-3174631
BmHel-14	DF090375:781056-781188
BmHel-14	DF090375:1468191-1468323
BmHel-14	DF090362:1983270-1983402
BmHel-14	DF090362:2053741-2053873
BmHel-14	DF090362:2307486-2307618
BmHel-14	DF090362:2016306-2016438
BmHel-14	DF090362:1510707-1510839
BmHel-14	DF090362:845721-845855
BmHel-14	DF090336:1943558-1943690
BmHel-14	DF090336:4588060-4588192
BmHel-14	DF090336:5324180-5324312
BmHel-14	DF090336:955170-955302
BmHel-14	DF090396:483221-483353
BmHel-14	DF090396:248778-248910
BmHel-14	DF090331:1926801-1926934
BmHel-14	DF090331:2769955-2770087
BmHel-14	DF090331:3074502-3074640
BmHel-14	DF090331:3303870-3304002
BmHel-14	DF090331:3943367-3943499
BmHel-14	DF090331:748116-748248
BmHel-14	DF090398:190593-190725
BmHel-14	DF090366:2118080-2118212
BmHel-14	DF090421:484498-484630
BmHel-14	DF090421:933011-933143
BmHel-14	DF090421:541536-541668
BmHel-14	DF090383:440608-440740
BmHel-14	DF090377:1213521-1213656
BmHel-14	DF090360:2617549-2617681
BmHel-14	DF090360:643741-643873
BmHel-14	DF090353:993925-994056
BmHel-14	DF090353:1325615-1325748
BmHel-14	DF090353:3060973-3061106
BmHel-14	DF090353:3120644-3120775
BmHel-14	DF090400:559613-559745
BmHel-14	DF090386:371114-371246
BmHel-14	DF090518:61805-61937
BmHel-14	DF090405:411719-411851
BmHel-14	DF090405:568203-568335
BmHel-14	DF090350:3676976-3677108
BmHel-14	DF090350:2432029-2432161
BmHel-14	DF090342:2754183-2754315
BmHel-14	DF090342:3237483-3237615

BmHel-14	DF090342:4751878-4752011
BmHel-14	DF090342:3604724-3604861
BmHel-14	DF090342:2146993-2147125
BmHel-14	DF090342:2008034-2008166
BmHel-14	DF090342:786587-786719
BmHel-14	DF090342:149105-149238
BmHel-14	DF090448:353061-353192
BmHel-14	DF090381:1595985-1596117
BmHel-14	DF090381:1633759-1633891
BmHel-14	DF090381:46126-46258
BmHel-14	DF090354:2647702-2647834
BmHel-14	DF090354:3467894-3468026
BmHel-14	DF090354:2832852-2832984
BmHel-14	DF090354:2439970-2440102
BmHel-14	DF090324:2453625-2453756
BmHel-14	DF090324:278386-278518
BmHel-14	DF090422:1026635-1026767
BmHel-14	DF090387:1731180-1731312
BmHel-14	DF090461:184145-184277
BmHel-14	DF090345:2022901-2023030
BmHel-14	DF090345:2876977-2877110
BmHel-14	DF090345:4188897-4189029
BmHel-14	DF090345:4279196-4279328
BmHel-14	DF090345:4302198-4302330
BmHel-14	DF090345:4351825-4351957
BmHel-14	DF090345:4412755-4412887
BmHel-14	DF090345:3436234-3436366
BmHel-14	DF090345:1677316-1677452
BmHel-14	DF090345:1350304-1350436
BmHel-14	DF090345:1273642-1273775
BmHel-14	DF090384:1564702-1564834
BmHel-14	DF090337:882387-882519
BmHel-14	DF090337:4592453-4592585
BmHel-14	DF090399:320722-320854
BmHel-14	DF090457:88947-89079
BmHel-14	DF090460:172913-173045
BmHel-14	DF090380:1878976-1879108
BmHel-14	DF090380:1980233-1980365
BmHel-14	BABH01066863:278-410
BmHel-14	BABH01047827:125-257
BmHel-14	DF090629:12577-12709
BmHel-14	DF090371:1499030-1499162
BmHel-14	DF090371:1523290-1523422
BmHel-14	DF090886:11456-11588
BmHel-14	DF090484:185197-185329
BmHel-14	DF090444:105711-105843
BmHel-14	DF090442:199278-199409
BmHel-14	DF090415:85826-85959

BmHel-14	BABH01069824:452-584
BmHel-14	DF090612:13781-13913
BmHel-14	DF090541:132498-132630
BmHel-14	DF090435:2904-3036
BmHel-14	DF090435:643254-643386
BmHel-14	DF090435:24837-24969
BmHel-14	DF090426:764327-764459
BmHel-14	DF090430:520771-520903
BmHel-14	BABH01043741:7894-8026
BmHel-14	DF090577:25951-26083
BmHel-14	BABH01073754:129-262
BmHel-14	BABH01053060:357-490
BmHel-14	BABH01048663:994-1125
BmHel-14	BABH01046120:2398-2530
BmHel-14	DF090802:2773-2905
BmHel-14	DF090712:18217-18349
BmHel-14	DF090617:34529-34653
BmHel-14	DF090607:2205-2337
BmHel-14	DF090481:188763-188895
BmHel-14	BABH01045502:2310-2441
BmHel-14	BABH01042899:23376-23510
BmHel-14	DF090640:14825-14957
BmHel-14	DF090539:13709-13840
BmHel-14	DF091042:9347-9477
BmHel-14	DF090356:2070297-2070440
BmHel-14	DF090348:418690-418818
BmHel-14	DF090348:418816-418895
BmHel-14	DF090329:6513203-6513334
BmHel-14	DF090323:3028209-3028341
BmHel-14	DF090346:1703851-1703979
BmHel-14	DF090344:2169214-2169348
BmHel-14	DF090335:3410730-3410922
BmHel-14	DF090342:592047-592178
BmHel-14	DF090460:81197-81328
BmHel-14	DF090322:5973786-5973918
BmHel-14	DF090322:5575281-5575413
BmHel-14	DF090323:2569792-2569924
BmHel-14	DF090325:3136488-3136617
BmHel-14	DF090326:2715309-2715441
BmHel-14	DF090326:6354739-6354869
BmHel-14	DF090348:4014008-4014141
BmHel-14	DF090348:4013931-4014010
BmHel-14	DF090383:360761-360893
BmHel-14	DF090380:720887-721019
BmHel-14	DF090489:136088-136220
BmHel-14	DF090425:1010955-1011191
BmHel-14	DF090330:239203-239336
BmHel-14	DF090368:1939654-1939792

BmHel-14	DF090346:3061525-3061659
BmHel-14	DF090325:3682134-3682271
BmHel-14	DF090321:7188007-7188137
BmHel-14	DF090344:3415751-3415881
BmHel-14	DF090344:2357239-2357373
BmHel-14	DF090326:6274454-6274588
BmHel-14	DF090317:2142767-2142897
BmHel-14	DF090438:525155-525285
BmHel-14	DF090374:523209-523288
BmHel-14	DF090381:506391-506521
BmHel-14	DF090441:684060-684194
BmHel-14	BABH01081304:200-334
BmHel-14	DF090600:34321-34455
BmHel-14	DF090322:4393808-4393940
BmHel-14	DF090418:1206950-1207082
BmHel-14	DF090369:981550-981680
BmHel-14	DF090341:4408482-4408614
BmHel-14	DF090360:551001-551133
BmHel-14	DF090342:4648027-4648159
BmHel-14	DF090354:2308487-2308619
BmHel-14	DF090431:174065-174197
BmHel-14	DF090333:3717171-3717274
BmHel-14	DF090333:3717275-3717403
BmHel-14	DF090351:1291986-1292122
BmHel-14	DF090319:2613136-2613265
BmHel-14	DF090316:3062714-3062853
BmHel-14	DF090316:4207115-4207244
BmHel-14	DF090329:2079893-2080022
BmHel-14	DF090369:2691367-2691496
BmHel-14	DF090330:4581415-4581544
BmHel-14	DF090333:574164-574299
BmHel-14	DF090325:6268536-6268665
BmHel-14	DF090347:3385780-3385908
BmHel-14	DF090341:1951029-1951157
BmHel-14	DF090361:1360074-1360203
BmHel-14	DF090367:218653-218782
BmHel-14	DF090349:1693066-1693194
BmHel-14	DF090391:393957-394086
BmHel-14	DF090391:393879-393959
BmHel-14	DF090412:935201-935329
BmHel-14	DF090360:1487958-1488087
BmHel-14	DF090365:1639172-1639307
BmHel-14	DF090342:4145885-4146014
BmHel-14	DF090324:6621776-6621905
BmHel-14	DF090422:508385-508514
BmHel-14	DF090446:425819-425948
BmHel-14	DF090337:1581283-1581412
BmHel-14	DF090380:2011537-2011672

BmHel-14	BABH01049760:338-466
BmHel-14	DF090426:670643-670772
BmHel-14	DF090570:42063-42191
BmHel-14	DF090351:617583-617715
BmHel-14	DF090319:1886190-1886322
BmHel-14	DF090316:3875858-3875990
BmHel-14	DF090329:4668312-4668444
BmHel-14	DF090369:1432911-1433043
BmHel-14	DF090330:3941836-3941968
BmHel-14	DF090368:478772-478904
BmHel-14	DF090343:4140307-4140439
BmHel-14	DF090496:139399-139530
BmHel-14	DF090388:188266-188399
BmHel-14	DF090323:4460046-4460178
BmHel-14	DF090373:805005-805137
BmHel-14	DF090325:1233947-1234079
BmHel-14	DF090327:5880079-5880211
BmHel-14	DF090327:2971827-2971959
BmHel-14	DF090339:3310758-3310890
BmHel-14	DF090321:949087-949214
BmHel-14	DF090344:81040-81172
BmHel-14	DF090328:3935720-3935852
BmHel-14	DF090335:5731375-5731511
BmHel-14	DF090378:265106-265238
BmHel-14	DF090352:1951268-1951400
BmHel-14	DF090362:1694597-1694729
BmHel-14	DF090336:4612332-4612464
BmHel-14	DF090366:1526281-1526413
BmHel-14	DF090354:1734490-1734622
BmHel-14	DF090324:7932107-7932239
BmHel-14	DF090393:1093811-1093943
BmHel-14	DF090345:2506876-2507008
BmHel-14	DF090345:4315848-4315980
BmHel-14	DF090370:1259167-1259293
BmHel-14	DF090460:297547-297679
BmHel-14	BABH01045017:5661-5793
BmHel-14	BABH01056119:392-524
BmHel-14	DF090367:739593-739833
BmHel-14	DF090330:6298161-6298304
BmHel-14	DF090332:1609834-1609963
BmHel-14	DF090351:1216972-1217096
BmHel-14	DF090319:5816671-5816801
BmHel-14	DF090316:6568306-6568435
BmHel-14	DF090316:9574353-9574481
BmHel-14	DF090316:3584937-3585066
BmHel-14	DF090329:337024-337152
BmHel-14	DF090330:5695918-5696046
BmHel-14	DF090330:5613556-5613686

BmHel-14	DF090323:774100-774230
BmHel-14	DF090340:2890260-2890389
BmHel-14	DF090340:4557056-4557185
BmHel-14	DF090449:712541-712669
BmHel-14	DF090333:431741-431870
BmHel-14	DF090325:6345197-6345327
BmHel-14	DF090359:506953-507085
BmHel-14	DF090321:3497110-3497238
BmHel-14	DF090344:1250177-1250308
BmHel-14	DF090420:816518-816646
BmHel-14	DF090328:5606655-5606783
BmHel-14	DF090328:3633525-3633656
BmHel-14	DF090335:3089327-3089453
BmHel-14	DF090358:3077707-3077828
BmHel-14	DF090358:237216-237344
BmHel-14	DF090378:2146547-2146675
BmHel-14	DF090326:6951229-6951359
BmHel-14	DF090317:70588-70720
BmHel-14	DF090355:2847484-2847612
BmHel-14	DF090355:1527015-1527146
BmHel-14	DF090362:3196650-3196781
BmHel-14	DF090331:5227113-5227238
BmHel-14	DF090386:457436-457567
BmHel-14	DF090409:768685-768814
BmHel-14	DF090324:1275330-1275458
BmHel-14	DF090345:2574262-2574393
BmHel-14	DF090345:1744395-1744523
BmHel-14	DF090371:2363735-2363864
BmHel-14	DF090444:275567-275699
BmHel-14	BABH01045814:404-535
BmHel-14	DF090522:3576-3711
BmHel-14	DF090357:2911993-2912125
BmHel-14	DF090321:409535-409667
BmHel-14	DF090334:3121638-3121765
BmHel-14	DF090317:2540090-2540224
BmHel-14	DF090487:138252-138384
BmHel-14	DF090345:2497510-2497633
BmHel-14	DF090406:796187-796323
BmHel-14	DF090320:5156876-5156991
BmHel-14	DF090320:5156992-5157103
BmHel-14	DF090462:153806-153926
BmHel-14	DF090319:2148467-2148594
BmHel-14	DF090316:12870359-12870485
BmHel-14	DF090316:5008249-5008376
BmHel-14	DF090361:1401422-1401549
BmHel-14	DF090321:4820247-4820375
BmHel-14	DF090403:533772-533899
BmHel-14	DF090334:4965256-4965387

BmHel-14	DF090414:1079234-1079322
BmHel-14	DF090383:694971-695094
BmHel-14	DF090360:3292219-3292349
BmHel-14	DF090345:3739479-3739611
BmHel-14	DF090361:397435-397566
BmHel-14	DF090322:7410680-7410809
BmHel-14	DF090410:286862-286990
BmHel-14	DF090366:1651250-1651383
BmHel-14	DF090361:605978-606110
BmHel-14	DF090339:478508-478640
BmHel-14	DF090420:364595-364727
BmHel-14	DF090403:265994-266125
BmHel-14	DF090362:1585167-1585299
BmHel-14	DF090324:757908-758040
BmHel-14	DF090476:271575-271707
BmHel-14	DF090460:354526-354658
BmHel-14	DF090319:3062582-3062818
BmHel-14	DF090487:125552-125785
BmHel-14	DF090323:3273511-3273641
BmHel-14	DF090340:3268862-3268993
BmHel-14	DF090326:6466582-6466708
BmHel-14	DF090352:221151-221285
BmHel-14	DF090331:196102-196227
BmHel-14	DF090318:8803556-8803685
BmHel-14	DF090363:518936-519065
BmHel-14	DF090348:1281677-1281808
BmHel-14	DF090321:6126519-6126653
BmHel-14	DF090389:931410-931535
BmHel-14	DF090389:931324-931403
BmHel-14	DF090338:751992-752124
BmHel-14	DF090326:1430340-1430459
BmHel-14	DF090326:1430457-1430539
BmHel-14	DF090326:5316488-5316616
BmHel-14	DF090326:530214-530339
BmHel-14	DF090317:4704883-4705010
BmHel-14	DF090363:941293-941416
BmHel-14	DF090548:36053-36180
BmHel-14	BABH01085299:208-334
BmHel-14	DF090328:4783751-4783885
BmHel-14	DF090360:1433735-1433869
BmHel-14	BABH01072502:141-275
BmHel-14	DF090401:899379-899508
BmHel-14	DF090386:1436830-1436959
BmHel-14	DF090329:3087471-3087601
BmHel-14	DF090394:259940-260070
BmHel-14	DF090349:2933081-2933211
BmHel-14	DF090391:722256-722386
BmHel-14	DF090461:211507-211637

BmHel-14	DF090424:359565-359697
BmHel-14	DF090343:3436023-3436155
BmHel-14	DF090357:3077749-3077881
BmHel-14	DF090321:6245959-6246091
BmHel-14	DF090390:431404-431536
BmHel-14	DF090360:2243172-2243302
BmHel-14	DF090342:1269772-1269904
BmHel-14	DF090386:1078712-1078841
BmHel-14	DF090386:1078617-1078714
BmHel-14	DF090361:790733-790859
BmHel-14	DF090363:2108767-2108998
BmHel-14	DF090383:287375-287504
BmHel-14	DF090360:3324804-3325035
BmHel-14	DF090324:1619629-1619724
BmHel-14	DF090376:1565878-1566010
BmHel-14	DF090328:1945710-1945837
BmHel-14	DF090322:3902588-3902818
BmHel-14	DF090322:4990629-4990859
BmHel-14	DF090351:2111193-2111423
BmHel-14	DF090319:3673677-3673906
BmHel-14	DF090316:5993666-5993894
BmHel-14	DF090316:6589583-6589813
BmHel-14	DF090329:6559769-6559999
BmHel-14	DF090330:4239682-4239910
BmHel-14	DF090330:1834102-1834332
BmHel-14	DF090338:4586503-4586733
BmHel-14	DF090323:7306581-7306812
BmHel-14	DF090340:2065702-2065929
BmHel-14	DF090333:4008429-4008666
BmHel-14	DF090394:694198-694428
BmHel-14	DF090394:1334213-1334449
BmHel-14	DF090394:1062655-1062884
BmHel-14	DF090346:3079225-3079455
BmHel-14	DF090325:4331987-4332216
BmHel-14	DF090325:5558749-5558979
BmHel-14	DF090356:3544906-3545136
BmHel-14	DF090320:5385691-5385922
BmHel-14	DF090320:1732728-1732958
BmHel-14	DF090320:292026-292251
BmHel-14	DF090320:91660-91890
BmHel-14	DF090321:2775940-2776170
BmHel-14	DF090367:606626-606857
BmHel-14	DF090344:2084359-2084589
BmHel-14	DF090390:1061071-1061301
BmHel-14	DF090328:4494280-4494510
BmHel-14	DF090391:298790-299020
BmHel-14	DF090335:440729-440959
BmHel-14	DF090358:76828-77058

BmHel-14	DF090378:1473245-1473475
BmHel-14	DF090326:5637987-5638216
BmHel-14	DF090317:9812498-9812728
BmHel-14	DF090382:1713335-1713565
BmHel-14	DF090363:1855187-1855326
BmHel-14	DF090363:1855109-1855202
BmHel-14	DF090348:2514211-2514443
BmHel-14	DF090348:1151694-1151924
BmHel-14	DF090355:1223288-1223518
BmHel-14	DF090352:659210-659439
BmHel-14	DF090352:2994720-2994950
BmHel-14	DF090352:3511280-3511496
BmHel-14	DF090352:2415327-2415558
BmHel-14	DF090487:83283-83517
BmHel-14	DF090614:9911-10141
BmHel-14	DF090438:703099-703329
BmHel-14	DF090375:1558243-1558473
BmHel-14	DF090362:1406823-1407053
BmHel-14	DF090336:2970810-2971039
BmHel-14	DF090336:3969616-3969846
BmHel-14	DF090366:1941571-1941800
BmHel-14	BABH01044849:727-957
BmHel-14	DF090383:1728586-1728816
BmHel-14	DF090360:2046504-2046734
BmHel-14	DF090353:454117-454347
BmHel-14	DF090386:532788-533021
BmHel-14	DF090518:73097-73327
BmHel-14	DF090365:1960898-1961128
BmHel-14	DF090429:68717-68950
BmHel-14	BABH01073137:67-296
BmHel-14	DF090350:2819098-2819328
BmHel-14	DF090342:1676095-1676326
BmHel-14	DF090342:4325985-4326215
BmHel-14	DF090354:2624933-2625165
BmHel-14	DF090445:432724-432954
BmHel-14	DF090387:1573022-1573244
BmHel-14	DF090393:1346545-1346768
BmHel-14	DF090461:244428-244656
BmHel-14	DF090428:566340-566570
BmHel-14	DF090347:173598-173721
BmHel-14	DF090331:2193484-2193613
BmHel-14	DF090342:2121458-2121586
BmHel-14	DF090499:39463-39553
BmHel-14	DF090499:39551-39630
BmHel-14	DF090362:26602-26732
BmHel-14	DF090336:3298790-3298918
BmHel-14	DF090342:1834201-1834333
BmHel-14	DF090472:276581-276713

BmHel-14	DF090460:181605-181782
BmHel-14	DF090353:1908816-1909045
BmHel-14	DF090402:497072-497206
BmHel-14	DF090324:2652154-2652290
BmHel-14	DF090356:323524-323656
BmHel-14	DF090319:6087974-6088110
BmHel-14	DF090319:6087896-6087987
BmHel-14	DF090329:735465-735601
BmHel-14	DF090329:735387-735478
BmHel-14	DF090339:3106429-3106562
BmHel-14	DF090339:3106549-3106640
BmHel-14	DF090358:1169179-1169315
BmHel-14	DF090358:1169302-1169393
BmHel-14	DF090358:2853211-2853347
BmHel-14	DF090358:2853133-2853224
BmHel-14	DF090382:74426-74559
BmHel-14	DF090382:74348-74439
BmHel-14	DF090412:638400-638536
BmHel-14	DF090412:638523-638611
BmHel-14	DF090348:1646099-1646192
BmHel-14	DF090348:1646427-1646565
BmHel-14	DF090336:706215-706352
BmHel-14	DF090336:706339-706430
BmHel-14	DF090336:2779006-2779142
BmHel-14	DF090336:2779129-2779220
BmHel-14	DF090377:237994-238130
BmHel-14	DF090377:238117-238208
BmHel-14	DF090400:1287139-1287275
BmHel-14	DF090400:1287262-1287353
BmHel-14	DF090386:280511-280647
BmHel-14	DF090386:280433-280524
BmHel-14	DF090409:841114-841250
BmHel-14	DF090409:841237-841328
BmHel-14	DF090409:485214-485350
BmHel-14	DF090409:485136-485227
BmHel-14	DF090467:309272-309408
BmHel-14	DF090467:309395-309486
BmHel-14	DF090422:842928-843064
BmHel-14	DF090422:842850-842941
BmHel-14	DF090344:2478729-2478862
BmHel-14	DF090344:2478648-2478742
BmHel-14	DF090333:2783307-2783468
BmHel-14	DF090333:3592646-3592775
BmHel-14	DF090341:3362466-3362599
BmHel-14	DF090376:1867452-1867573
BmHel-14	DF090791:7372-7463
BmHel-14	DF090383:1451270-1451397
BmHel-14	DF090316:5815230-5815366

BmHel-14	DF090316:1003373-1003600
BmHel-14	DF090330:3101937-3102164
BmHel-14	DF090368:2352714-2352940
BmHel-14	DF090323:1853022-1853245
BmHel-14	DF090323:7676392-7676620
BmHel-14	DF090347:1074912-1075139
BmHel-14	DF090321:3437313-3437441
BmHel-14	DF090349:434402-434629
BmHel-14	DF090317:7825698-7825923
BmHel-14	DF090352:816698-816834
BmHel-14	DF090352:816822-816912
BmHel-14	DF090407:315352-315477
BmHel-14	DF090336:3092497-3092725
BmHel-14	DF090396:1493636-1493862
BmHel-14	DF090366:1363521-1363748
BmHel-14	DF090386:314910-315135
BmHel-14	DF090350:2571263-2571490
BmHel-14	DF090319:7469705-7469840
BmHel-14	DF090319:7469827-7469918
BmHel-14	DF090404:750276-750503
BmHel-14	DF090377:1155646-1155776
BmHel-14	DF090384:251690-251810
BmHel-14	DF090355:844340-844468
BmHel-14	DF090321:4449160-4449292
BmHel-14	DF090334:2772950-2773077
BmHel-14	DF090330:113708-113935
BmHel-14	DF090378:938629-938765
BmHel-14	DF090378:938746-938848
BmHel-14	DF090328:3407076-3407302
BmHel-14	DF090326:6034070-6034189
BmHel-14	DF090386:325863-325983
BmHel-14	DF090383:1026083-1026208
BmHel-14	DF090332:2134691-2134916
BmHel-14	DF090332:137229-137368
BmHel-14	DF090332:137154-137242
BmHel-14	DF090338:2241225-2241361
BmHel-14	DF090338:2241150-2241238
BmHel-14	DF090333:2720341-2720566
BmHel-14	DF090328:853832-853952
BmHel-14	DF090365:476105-476241
BmHel-14	DF090365:476030-476118
BmHel-14	DF090342:2552845-2553072
BmHel-14	DF090430:338983-339119
BmHel-14	DF090430:338908-338996
BmHel-14	DF090341:4550487-4550620
BmHel-14	DF090341:4550607-4550701
BmHel-14	DF090330:400100-400325
BmHel-14	DF090938:7929-8058

BmHel-14	DF090339:1969140-1969274
BmHel-14	DF090352:1811722-1811917
BmHel-14	DF090323:5543766-5543893
BmHel-14	DF090403:166724-166827
BmHel-14	DF090403:166548-166668
BmHel-14	DF090317:6873596-6873732
BmHel-14	DF090317:6873522-6873609
BmHel-14	DF090316:8295857-8295986
BmHel-14	DF090328:6141046-6141163
BmHel-14	DF090382:1685093-1685225
BmHel-14	DF090336:934767-934961
BmHel-14	DF090335:5497642-5497765
BmHel-14	DF090342:4853909-4854037
BmHel-14	DF090801:12470-12602
BmHel-14	DF090376:461975-462110
BmHel-14	DF090332:1248601-1248795
BmHel-14	DF090351:1287203-1287397
BmHel-14	DF090319:7692235-7692429
BmHel-14	DF090329:4246527-4246721
BmHel-14	DF090369:1483491-1483685
BmHel-14	DF090330:2065669-2065863
BmHel-14	DF090343:271483-271677
BmHel-14	DF090373:2222270-2222463
BmHel-14	DF090356:1724492-1724685
BmHel-14	DF090320:7880499-7880684
BmHel-14	DF090401:1272658-1272844
BmHel-14	DF090327:319060-319255
BmHel-14	DF090344:3646207-3646401
BmHel-14	DF090317:9517507-9517701
BmHel-14	DF090364:845614-845808
BmHel-14	DF090412:836990-837185
BmHel-14	DF090352:275823-275961
BmHel-14	DF090362:1305384-1305578
BmHel-14	DF090331:566196-566390
BmHel-14	DF090366:2677730-2677924
BmHel-14	DF090409:773748-773942
BmHel-14	DF090342:1429530-1429725
BmHel-14	DF090831:11089-11283
BmHel-14	DF090497:141908-142102
BmHel-14	DF090345:1034411-1034596
BmHel-14	DF090370:2292266-2292460
BmHel-14	DF090850:7351-7544
BmHel-14	DF090324:4777714-4777940
BmHel-14	DF090598:26247-26330
BmHel-14	DF090598:26317-26408
BmHel-14	DF090323:4605859-4606082
BmHel-14	DF090341:1558671-1558802
BmHel-14	DF090341:1558593-1558684

BmHel-14	DF090320:8202080-8202303
BmHel-14	DF090361:1629272-1629495
BmHel-14	DF090329:2832932-2833064
BmHel-14	DF090395:1079596-1079715
BmHel-14	DF090375:581616-581744
BmHel-14	DF090405:703608-703687
BmHel-14	DF090319:1370095-1370214
BmHel-14	DF090324:7786253-7786335
BmHel-14	DF090319:1058864-1058996
BmHel-14	DF090316:8227951-8228080
BmHel-14	DF090318:7649100-7649232
BmHel-14	DF090343:85166-85298
BmHel-14	DF090373:862914-863046
BmHel-14	DF090333:1304171-1304303
BmHel-14	DF090356:1451362-1451494
BmHel-14	DF090359:1488191-1488323
BmHel-14	DF090317:3193982-3194114
BmHel-14	DF090364:2034290-2034422
BmHel-14	DF090364:2531785-2531917
BmHel-14	DF090414:549558-549694
BmHel-14	DF090348:1738647-1738779
BmHel-14	DF090352:3028578-3028710
BmHel-14	DF090407:760991-761123
BmHel-14	DF090331:2237595-2237727
BmHel-14	DF090360:1903013-1903145
BmHel-14	DF090360:2076233-2076365
BmHel-14	DF090354:3123350-3123483
BmHel-14	DF090354:3109015-3109147
BmHel-14	DF090393:1707799-1707931
BmHel-14	DF090345:2620235-2620367
BmHel-14	DF090370:1269001-1269133
BmHel-14	DF090371:2522776-2522908
BmHel-14	DF091156:10714-10846
BmHel-14	DF090323:4911070-4911263
BmHel-14	DF090340:2314862-2314992
BmHel-14	DF090321:6618294-6618431
BmHel-14	DF090364:414182-414375
BmHel-14	DF090697:7479-7673
BmHel-14	DF090335:4457955-4458104
BmHel-14	DF090346:3454144-3454265
BmHel-14	DF090321:5210897-5211030
BmHel-14	DF090380:1221326-1221441
BmHel-14	DF090320:292669-292801
BmHel-14	DF090317:5724844-5724976
BmHel-14	DF090436:676048-676180
BmHel-14	DF090335:3118373-3118508
BmHel-14	DF090530:76590-76726
BmHel-14	DF090352:1731995-1732187

BmHel-14	DF090630:60122-60346
BmHel-14	DF090322:460526-460657
BmHel-14	DF090347:2110881-2111015
BmHel-14	DF090322:1836838-1836970
BmHel-14	DF090341:2648727-2648859
BmHel-14	DF090330:4519297-4519488
BmHel-14	DF090339:1221765-1221956
BmHel-14	DF090490:157928-158062
BmHel-14	DF090343:137705-137843
BmHel-14	DF090343:137823-137916
BmHel-14	DF090390:1548454-1548586
BmHel-14	DF090323:2502479-2502611
BmHel-14	DF090323:2502591-2502689
BmHel-14	DF090391:532960-533180
BmHel-14	DF090372:1364090-1364213
BmHel-14	DF090321:5246439-5246571
BmHel-14	DF090351:1912991-1913123
BmHel-14	DF090362:2725663-2725753
BmHel-14	DF090409:101231-101341
BmHel-14	DF090409:101154-101233
BmHel-14	DF090343:4469876-4469988
BmHel-14	DF090383:538796-538924
BmHel-14	DF090423:904511-904640
BmHel-14	DF090324:3792162-3792291
BmHel-14	DF090339:3295825-3295960
BmHel-14	DF090373:1164489-1164645
BmHel-14	DF090373:1164402-1164485
BmHel-14	DF090347:3913008-3913095
BmHel-14	DF090347:3913094-3913222
BmHel-14	DF090329:613127-613226
BmHel-14	DF090329:613010-613129
BmHel-14	DF090383:614352-614431
BmHel-14	DF090336:2153912-2154038
BmHel-14	DF090373:47586-47718
BmHel-14	DF090321:2423393-2423525
BmHel-14	DF090355:475516-475648
BmHel-14	DF090322:4747138-4747265
BmHel-14	DF090356:1769397-1769476
BmHel-14	DF090318:6487399-6487612
BmHel-14	DF090374:612565-612781
BmHel-14	DF090324:1461682-1461898
BmHel-14	DF090316:16063227-16063443
BmHel-14	BABH01079273:457-572
BmHel-14	BABH01079273:370-470
BmHel-14	BABH01050672:1-217
BmHel-14	DF090389:1413420-1413529
BmHel-14	DF090388:319745-319854
BmHel-14	DF090321:3338860-3338984

BmHel-14	DF090429:486462-486571
BmHel-14	DF090383:1326295-1326481
BmHel-14	DF090387:810857-811040
BmHel-14	DF090408:639929-640068
BmHel-14	DF090408:640059-640138
BmHel-14	DF090388:1903139-1903275
BmHel-14	DF090336:4089403-4089618
BmHel-14	DF090377:1673929-1674065
BmHel-14	DF090354:699223-699367
BmHel-14	DF090323:2141864-2141997
BmHel-14	DF090323:2141793-2141874
BmHel-14	DF090358:943524-943687
BmHel-14	DF090382:984148-984256
BmHel-14	DF090426:299073-299194
BmHel-14	DF090492:80208-80316
BmHel-14	DF090316:5632919-5633043
BmHel-14	DF090352:462361-462493
BmHel-14	DF090567:21058-21186
BmHel-14	BABH01050483:1-127
BmHel-14	DF090441:592210-592395
BmHel-14	DF090316:2275258-2275390
BmHel-14	DF090316:2275388-2275469
BmHel-14	DF090395:830895-831027
BmHel-14	DF090395:830816-830897
BmHel-14	DF090325:5201046-5201170
BmHel-14	DF090325:5201168-5201257
BmHel-14	DF090317:930650-930782
BmHel-14	DF090317:930566-930652
BmHel-14	DF090364:795778-795937
BmHel-14	DF090448:241985-242126
BmHel-14	DF090448:241906-241987
BmHel-14	DF090316:6247421-6247546
BmHel-14	DF090343:1954042-1954158
BmHel-14	DF091102:3456-3565
BmHel-14	DF090333:220729-220869
BmHel-14	DF090363:1161514-1161650
BmHel-14	DF090413:304009-304195
BmHel-14	DF090316:10475977-10476191
BmHel-14	DF090639:11856-12019
BmHel-14	DF090425:630051-630264
BmHel-14	DF090576:5667-5880
BmHel-14	DF090380:1880746-1880854
BmHel-14	DF090380:1880645-1880745
BmHel-14	DF090340:1167342-1167474
BmHel-14	DF090340:1167472-1167551
BmHel-14	DF090464:213832-213938
BmHel-14	DF090417:561280-561386
BmHel-14	DF090346:3734769-3734901

BmHel-14	DF090335:3566825-3566957
BmHel-14	DF090358:825711-825843
BmHel-14	DF090337:4316120-4316251
BmHel-14	DF090344:1750758-1750885
BmHel-14	DF090372:2420802-2420985
BmHel-14	DF090332:4650078-4650210
BmHel-14	DF090330:162130-162262
BmHel-14	DF090330:162053-162132
BmHel-14	DF090357:2886396-2886528
BmHel-14	DF090357:2886319-2886398
BmHel-14	DF090356:2214630-2214762
BmHel-14	DF090356:2214760-2214843
BmHel-14	DF090327:1856641-1856773
BmHel-14	DF090327:1856564-1856643
BmHel-14	DF090335:5641706-5641838
BmHel-14	DF090335:5641629-5641708
BmHel-14	DF090335:2398000-2398128
BmHel-14	DF090335:2397923-2398002
BmHel-14	DF090334:2457152-2457284
BmHel-14	DF090334:2457282-2457367
BmHel-14	DF090352:2204934-2205066
BmHel-14	DF090352:2205064-2205143
BmHel-14	DF090360:418437-418574
BmHel-14	DF090360:418360-418439
BmHel-14	DF090324:2261824-2261956
BmHel-14	DF090324:2261747-2261826
BmHel-14	DF090387:349247-349417
BmHel-14	DF090617:37367-37499
BmHel-14	DF090617:37497-37576
BmHel-14	DF090322:5147563-5147668
BmHel-14	DF090382:1689036-1689145
BmHel-14	DF090366:1952710-1952815
BmHel-14	DF090346:3869358-3869490
BmHel-14	DF090336:3398453-3398585
BmHel-14	DF090316:14095733-14095868
BmHel-14	DF090373:1255265-1255468
BmHel-14	DF090320:5333815-5333924
BmHel-14	DF090320:5333711-5333812
BmHel-14	DF090330:3786434-3786538
BmHel-14	DF090730:1227-1375
BmHel-14	DF090330:3017220-3017364
BmHel-14	DF090390:1601975-1602119
BmHel-14	DF090348:2099871-2100081
BmHel-14	DF090627:18720-18930
BmHel-14	DF090399:1033888-1034092
BmHel-14	DF090364:2778749-2778852
BmHel-14	DF090361:460213-460345
BmHel-14	DF090339:487577-487713

BmHel-14	DF090487:109654-109786
BmHel-14	DF090331:3985429-3985561
BmHel-14	DF090345:3659031-3659163
BmHel-14	DF090468:199305-199437
BmHel-14	DF090385:1147261-1147393
BmHel-14	DF090385:1147391-1147470
BmHel-14	DF090367:298324-298430
BmHel-14	DF090322:4767982-4768114
BmHel-14	DF090321:5891426-5891569
BmHel-14	DF090317:9734583-9734710
BmHel-14	DF090329:3587620-3587760
BmHel-14	DF090332:2096302-2096434
BmHel-14	DF090332:2394605-2394737
BmHel-14	DF090332:2802284-2802416
BmHel-14	DF090332:4045459-4045590
BmHel-14	DF090332:6042597-6042729
BmHel-14	DF090332:5063076-5063208
BmHel-14	DF090332:3874777-3874913
BmHel-14	DF090332:3209579-3209710
BmHel-14	DF090332:2951422-2951554
BmHel-14	DF090322:384630-384762
BmHel-14	DF090322:555852-555984
BmHel-14	DF090322:629550-629682
BmHel-14	DF090322:685431-685563
BmHel-14	DF090322:3361309-3361441
BmHel-14	DF090322:4640971-4641103
BmHel-14	DF090322:7209357-7209489
BmHel-14	DF090322:8017130-8017262
BmHel-14	DF090322:3420700-3420832
BmHel-14	DF090322:2683634-2683766
BmHel-14	DF090322:894914-895046
BmHel-14	DF090443:483636-483768
BmHel-14	DF090351:1981068-1981200
BmHel-14	DF090351:2398501-2398633
BmHel-14	DF090351:3067906-3068039
BmHel-14	DF090351:2834907-2835039
BmHel-14	DF090351:2073910-2074034
BmHel-14	DF090351:280279-280409
BmHel-14	DF090319:547796-547939
BmHel-14	DF090319:1295045-1295177
BmHel-14	DF090319:1635310-1635446
BmHel-14	DF090319:2625442-2625574
BmHel-14	DF090319:5959883-5960015
BmHel-14	DF090319:6121867-6121999
BmHel-14	DF090319:7550879-7551011
BmHel-14	DF090319:4900043-4900175
BmHel-14	DF090319:4194582-4194725
BmHel-14	DF090316:462824-462956

BmHel-14	DF090316:5375889-5376020
BmHel-14	DF090316:8909422-8909554
BmHel-14	DF090316:9818442-9818573
BmHel-14	DF090316:10007200-10007332
BmHel-14	DF090316:10034160-10034292
BmHel-14	DF090316:11892706-11892843
BmHel-14	DF090316:12816296-12816439
BmHel-14	DF090316:13052803-13052937
BmHel-14	DF090316:15750966-15751098
BmHel-14	DF090316:15618299-15618431
BmHel-14	DF090316:13068814-13068946
BmHel-14	DF090316:10492185-10492317
BmHel-14	DF090316:9338390-9338522
BmHel-14	DF090316:6547686-6547818
BmHel-14	DF090316:6356224-6356356
BmHel-14	DF090316:6106748-6106880
BmHel-14	DF090316:5883361-5883493
BmHel-14	DF090316:4923608-4923740
BmHel-14	DF090316:4066736-4066868
BmHel-14	DF090316:3741830-3741962
BmHel-14	DF090316:3224811-3224945
BmHel-14	DF090418:293477-293620
BmHel-14	DF090329:55266-55400
BmHel-14	DF090329:217846-217978
BmHel-14	DF090329:4245023-4245155
BmHel-14	DF090329:3949212-3949344
BmHel-14	DF090329:3502456-3502588
BmHel-14	DF090329:1965084-1965218
BmHel-14	DF090424:745745-745877
BmHel-14	DF090369:1637528-1637660
BmHel-14	DF090369:481043-481175
BmHel-14	DF090330:467179-467314
BmHel-14	DF090330:589737-589869
BmHel-14	DF090330:1627545-1627677
BmHel-14	DF090330:2042205-2042337
BmHel-14	DF090330:2348955-2349087
BmHel-14	DF090330:3113295-3113427
BmHel-14	DF090330:4687745-4687877
BmHel-14	DF090330:5097690-5097822
BmHel-14	DF090330:6329221-6329353
BmHel-14	DF090330:4373902-4374034
BmHel-14	DF090330:3254467-3254599
BmHel-14	DF090330:2351507-2351600
BmHel-14	DF090330:1649645-1649780
BmHel-14	DF090330:1649568-1649647
BmHel-14	DF090330:1321273-1321403
BmHel-14	DF090330:24742-24874
BmHel-14	DF090318:1600695-1600827

BmHel-14	DF090318:4640582-4640714
BmHel-14	DF090318:4710971-4711103
BmHel-14	DF090318:5990325-5990457
BmHel-14	DF090318:6658158-6658290
BmHel-14	DF090318:7858879-7859011
BmHel-14	DF090318:9204890-9205022
BmHel-14	DF090318:8296180-8296309
BmHel-14	DF090318:5824267-5824399
BmHel-14	DF090318:5661822-5661954
BmHel-14	DF090318:5518981-5519113
BmHel-14	DF090318:727663-727793
BmHel-14	DF090318:685726-685858
BmHel-14	DF090368:2211837-2211969
BmHel-14	DF090368:2292348-2292480
BmHel-14	DF090368:1962127-1962263
BmHel-14	DF090338:3235626-3235758
BmHel-14	DF090338:3297701-3297833
BmHel-14	DF090338:2706572-2706704
BmHel-14	DF090338:2051048-2051191
BmHel-14	DF090338:2026971-2027103
BmHel-14	DF090338:2026892-2026973
BmHel-14	DF090343:1298585-1298717
BmHel-14	DF090343:1436661-1436793
BmHel-14	DF090343:4202207-4202339
BmHel-14	DF090343:3594436-3594568
BmHel-14	DF090343:1568026-1568158
BmHel-14	DF090343:942470-942602
BmHel-14	DF090408:1067048-1067177
BmHel-14	DF090408:7753-7884
BmHel-14	DF090395:114619-114751
BmHel-14	DF090395:560693-560825
BmHel-14	DF090395:1308469-1308601
BmHel-14	DF090392:677554-677686
BmHel-14	DF090372:561310-561442
BmHel-14	DF090372:1448186-1448318
BmHel-14	DF090372:1941090-1941222
BmHel-14	DF090372:1963694-1963826
BmHel-14	DF090372:2154460-2154592
BmHel-14	DF090388:735868-736003
BmHel-14	DF090388:1610099-1610231
BmHel-14	DF090388:1666346-1666478
BmHel-14	DF090323:2143848-2143980
BmHel-14	DF090323:2824307-2824439
BmHel-14	DF090323:6567492-6567624
BmHel-14	DF090323:6164133-6164265
BmHel-14	DF090323:2774078-2774210
BmHel-14	DF090323:2583291-2583423
BmHel-14	DF090323:1565408-1565540

BmHel-14	DF090323:1565330-1565410
BmHel-14	DF090323:1497054-1497186
BmHel-14	DF090340:1437360-1437492
BmHel-14	DF090340:1437490-1437570
BmHel-14	DF090340:2114342-2114474
BmHel-14	DF090340:2957510-2957642
BmHel-14	DF090340:3238251-3238379
BmHel-14	DF090340:4420390-4420521
BmHel-14	DF090340:4665725-4665857
BmHel-14	DF090340:4665648-4665727
BmHel-14	DF090340:2556358-2556490
BmHel-14	DF090340:1659902-1660034
BmHel-14	DF090340:1533671-1533803
BmHel-14	DF090373:1600914-1601046
BmHel-14	DF090373:1807038-1807170
BmHel-14	DF090373:1807168-1807247
BmHel-14	DF090373:983129-983260
BmHel-14	DF090373:936534-936666
BmHel-14	DF090333:470644-470776
BmHel-14	DF090333:1232129-1232262
BmHel-14	DF090333:1577164-1577296
BmHel-14	DF090333:2162964-2163097
BmHel-14	DF090333:2511047-2511179
BmHel-14	DF090333:3879128-3879260
BmHel-14	DF090333:4889207-4889339
BmHel-14	DF090333:3001646-3001778
BmHel-14	DF090333:1593557-1593689
BmHel-14	DF090333:1132436-1132568
BmHel-14	DF090437:43653-43785
BmHel-14	DF090437:544435-544567
BmHel-14	DF090437:376724-376856
BmHel-14	DF090397:314676-314808
BmHel-14	DF090357:865045-865177
BmHel-14	DF090357:2179187-2179319
BmHel-14	DF090357:2540985-2541117
BmHel-14	DF090357:2754006-2754138
BmHel-14	DF090357:2552850-2552986
BmHel-14	DF090357:2481928-2482060
BmHel-14	DF090357:2447579-2447707
BmHel-14	DF090394:71991-72123
BmHel-14	DF090394:1013873-1014005
BmHel-14	DF090394:1432720-1432852
BmHel-14	DF090346:2646225-2646357
BmHel-14	DF090346:2725272-2725404
BmHel-14	DF090346:3020590-3020722
BmHel-14	DF090346:3617247-3617379
BmHel-14	DF090346:3751313-3751440
BmHel-14	DF090346:4531988-4532120

BmHel-14	DF090346:2518101-2518233
BmHel-14	DF090325:3820461-3820593
BmHel-14	DF090325:4629263-4629395
BmHel-14	DF090325:5490059-5490191
BmHel-14	DF090325:5804540-5804671
BmHel-14	DF090325:6053537-6053669
BmHel-14	DF090325:2535460-2535592
BmHel-14	DF090325:270194-270326
BmHel-14	DF090356:1788729-1788861
BmHel-14	DF090356:3540853-3540985
BmHel-14	DF090356:3177659-3177791
BmHel-14	DF090356:3081732-3081864
BmHel-14	DF090356:1587208-1587340
BmHel-14	DF090356:745867-745999
BmHel-14	DF090359:1300626-1300758
BmHel-14	DF090359:2359557-2359689
BmHel-14	DF090347:476063-476199
BmHel-14	DF090347:618372-618504
BmHel-14	DF090347:731973-732105
BmHel-14	DF090347:831296-831428
BmHel-14	DF090347:908435-908567
BmHel-14	DF090347:2819353-2819476
BmHel-14	DF090347:1823632-1823764
BmHel-14	DF090347:252266-252398
BmHel-14	DF090320:5052795-5052927
BmHel-14	DF090320:7033039-7033171
BmHel-14	DF090320:8561170-8561302
BmHel-14	DF090341:1346175-1346322
BmHel-14	DF090341:4365955-4366087
BmHel-14	DF090341:4283835-4283968
BmHel-14	DF090341:4177150-4177282
BmHel-14	DF090341:3826672-3826804
BmHel-14	DF090341:1978966-1979101
BmHel-14	DF090327:1743408-1743540
BmHel-14	DF090327:3931025-3931157
BmHel-14	DF090327:5831348-5831480
BmHel-14	DF090327:6022054-6022186
BmHel-14	DF090327:4595157-4595289
BmHel-14	DF090327:2363254-2363386
BmHel-14	DF090361:653191-653323
BmHel-14	DF090361:2491623-2491755
BmHel-14	DF090361:490929-491061
BmHel-14	DF090339:2864293-2864416
BmHel-14	DF090339:3463977-3464109
BmHel-14	DF090321:3839968-3840100
BmHel-14	DF090321:4117035-4117167
BmHel-14	DF090321:5222326-5222469
BmHel-14	DF090321:5326917-5327049

BmHel-14	DF090321:7887671-7887803
BmHel-14	DF090321:7201179-7201312
BmHel-14	DF090321:2464402-2464534
BmHel-14	DF090367:2220445-2220577
BmHel-14	DF090367:785985-786116
BmHel-14	DF090367:785907-785987
BmHel-14	DF090367:340860-340993
BmHel-14	DF090349:177225-177357
BmHel-14	DF090349:367646-367778
BmHel-14	DF090349:486400-486532
BmHel-14	DF090349:564226-564356
BmHel-14	DF090349:3929824-3929956
BmHel-14	DF090349:1799838-1799970
BmHel-14	DF090349:1714253-1714385
BmHel-14	DF090344:844505-844637
BmHel-14	DF090344:2059599-2059731
BmHel-14	DF090344:2258956-2259088
BmHel-14	DF090344:2389223-2389352
BmHel-14	DF090344:2389350-2389431
BmHel-14	DF090344:2589884-2590016
BmHel-14	DF090344:3854110-3854242
BmHel-14	DF090344:3045776-3045907
BmHel-14	DF090344:2249740-2249880
BmHel-14	DF090344:1925944-1926076
BmHel-14	DF090344:814741-814865
BmHel-14	DF090390:163361-163493
BmHel-14	DF090390:1547933-1548065
BmHel-14	DF090390:323608-323737
BmHel-14	DF090328:428908-429039
BmHel-14	DF090328:3493434-3493566
BmHel-14	DF090328:4757504-4757636
BmHel-14	DF090328:3115900-3116032
BmHel-14	DF090328:2219790-2219922
BmHel-14	DF090328:1084027-1084159
BmHel-14	DF090328:712688-712820
BmHel-14	DF090403:1248499-1248631
BmHel-14	DF090391:967944-968076
BmHel-14	DF090391:1511464-1511596
BmHel-14	DF090391:662567-662699
BmHel-14	DF090335:699522-699654
BmHel-14	DF090335:1900150-1900282
BmHel-14	DF090335:5480960-5481100
BmHel-14	DF090335:1524045-1524177
BmHel-14	DF090335:89235-89366
BmHel-14	DF090358:502658-502794
BmHel-14	DF090358:2872461-2872593
BmHel-14	DF090358:1155557-1155689
BmHel-14	DF090334:1868613-1868745

BmHel-14	DF090334:2386939-2387071
BmHel-14	DF090334:4001217-4001350
BmHel-14	DF090334:6095944-6096076
BmHel-14	DF090334:3755103-3755235
BmHel-14	DF090334:3595782-3595914
BmHel-14	DF090334:14868-15000
BmHel-14	DF090378:163045-163177
BmHel-14	DF090378:205623-205758
BmHel-14	DF090378:1424175-1424304
BmHel-14	DF090326:699322-699454
BmHel-14	DF090326:2208392-2208524
BmHel-14	DF090326:2626749-2626882
BmHel-14	DF090326:3091254-3091386
BmHel-14	DF090326:4638803-4638935
BmHel-14	DF090326:6484079-6484211
BmHel-14	DF090326:2633193-2633325
BmHel-14	DF090326:1417423-1417555
BmHel-14	DF090317:5218108-5218240
BmHel-14	DF090317:5306185-5306317
BmHel-14	DF090317:5627032-5627164
BmHel-14	DF090317:6592752-6592884
BmHel-14	DF090317:7752501-7752633
BmHel-14	DF090317:7855857-7855989
BmHel-14	DF090317:8516581-8516712
BmHel-14	DF090317:8788282-8788414
BmHel-14	DF090317:9362894-9363026
BmHel-14	DF090317:10328559-10328692
BmHel-14	DF090317:10526670-10526802
BmHel-14	DF090317:10739715-10739847
BmHel-14	DF090317:10083428-10083560
BmHel-14	DF090317:9384366-9384498
BmHel-14	DF090317:8489587-8489723
BmHel-14	DF090317:7906665-7906797
BmHel-14	DF090317:7081319-7081451
BmHel-14	DF090317:4538930-4539062
BmHel-14	DF090317:4520500-4520636
BmHel-14	DF090317:628179-628307
BmHel-14	DF090382:1751864-1751996
BmHel-14	DF090363:1243842-1243974
BmHel-14	DF090363:1568325-1568460
BmHel-14	DF090363:1991467-1991599
BmHel-14	DF090363:1272444-1272576
BmHel-14	DF090363:629304-629436
BmHel-14	DF090404:579313-579445
BmHel-14	DF090412:15368-15500
BmHel-14	DF090348:1867679-1867811
BmHel-14	DF090348:3193855-3193987
BmHel-14	DF090348:2953808-2953944

BmHel-14	DF090433:636937-637069
BmHel-14	DF090433:150412-150544
BmHel-14	DF090355:3448612-3448744
BmHel-14	DF090355:2406263-2406395
BmHel-14	DF090355:1624034-1624166
BmHel-14	DF090355:1312302-1312434
BmHel-14	DF090352:5849-5981
BmHel-14	DF090352:1515192-1515324
BmHel-14	DF090352:2010746-2010878
BmHel-14	DF090352:2275825-2275957
BmHel-14	DF090352:2379892-2380023
BmHel-14	DF090352:3458706-3458838
BmHel-14	DF090352:3897859-3897992
BmHel-14	DF090352:3078191-3078323
BmHel-14	DF090407:884470-884602
BmHel-14	DF090407:170421-170553
BmHel-14	DF090438:692375-692507
BmHel-14	DF090438:513123-513255
BmHel-14	DF090375:1345794-1345926
BmHel-14	DF090375:2191322-2191454
BmHel-14	DF090375:479427-479559
BmHel-14	DF090362:817462-817594
BmHel-14	DF090362:1135596-1135724
BmHel-14	DF090362:1373622-1373754
BmHel-14	DF090362:1463737-1463863
BmHel-14	DF090362:1626474-1626606
BmHel-14	DF090362:2632628-2632760
BmHel-14	DF090362:3041096-3041229
BmHel-14	DF090362:1423596-1423728
BmHel-14	DF090362:1085381-1085509
BmHel-14	DF090336:2135907-2136039
BmHel-14	DF090336:3483728-3483861
BmHel-14	DF090336:3058095-3058227
BmHel-14	DF090336:3044309-3044441
BmHel-14	DF090336:2942059-2942191
BmHel-14	DF090396:1279504-1279635
BmHel-14	DF090396:1397927-1398054
BmHel-14	DF090331:4880766-4880898
BmHel-14	DF090331:5715802-5715934
BmHel-14	DF090331:5244540-5244672
BmHel-14	DF090331:2822445-2822577
BmHel-14	DF090331:2422889-2423021
BmHel-14	DF090398:875426-875558
BmHel-14	DF090366:724533-724665
BmHel-14	DF090366:1163832-1163964
BmHel-14	DF090366:1182749-1182881
BmHel-14	DF090366:1731847-1731979
BmHel-14	DF090366:1784716-1784848

BmHel-14	DF090366:2309158-2309290
BmHel-14	DF090421:118887-119019
BmHel-14	DF090383:1908583-1908715
BmHel-14	DF090383:1967537-1967670
BmHel-14	DF090383:793201-793333
BmHel-14	DF090377:2021178-2021310
BmHel-14	DF090377:1784941-1785073
BmHel-14	DF090377:1737598-1737730
BmHel-14	DF090377:874608-874740
BmHel-14	DF090377:563908-564040
BmHel-14	DF090377:264713-264845
BmHel-14	DF090360:3259459-3259594
BmHel-14	DF090360:2372695-2372827
BmHel-14	DF090360:2232065-2232197
BmHel-14	DF090353:625813-625944
BmHel-14	DF090353:1686497-1686629
BmHel-14	DF090353:3928624-3928756
BmHel-14	DF090353:1680184-1680316
BmHel-14	DF090353:438891-439023
BmHel-14	DF090400:1515937-1516069
BmHel-14	DF090400:1149360-1149492
BmHel-14	DF090386:249894-250026
BmHel-14	DF090386:671122-671254
BmHel-14	DF090386:1951561-1951693
BmHel-14	DF090386:914252-914383
BmHel-14	DF090386:250877-251010
BmHel-14	DF090365:1381968-1382100
BmHel-14	DF090365:1611406-1611538
BmHel-14	DF090365:1775010-1775146
BmHel-14	DF090365:432431-432563
BmHel-14	DF090429:659227-659360
BmHel-14	DF090429:645826-645969
BmHel-14	DF090405:820957-821089
BmHel-14	DF090405:1347719-1347860
BmHel-14	DF090405:32617-32748
BmHel-14	DF090409:801105-801237
BmHel-14	DF090409:654884-655016
BmHel-14	DF090374:665113-665245
BmHel-14	DF090374:1383946-1384078
BmHel-14	DF090374:143278-143410
BmHel-14	DF090350:2932429-2932561
BmHel-14	DF090350:3833788-3833920
BmHel-14	DF090350:3662495-3662627
BmHel-14	DF090342:2199758-2199890
BmHel-14	DF090342:3265504-3265642
BmHel-14	DF090342:3361546-3361678
BmHel-14	DF090342:3378921-3379053
BmHel-14	DF090342:4056938-4057070

BmHel-14	DF090342:4436057-4436189
BmHel-14	DF090342:3503932-3504064
BmHel-14	DF090342:3368441-3368573
BmHel-14	DF090342:1417736-1417868
BmHel-14	DF090342:1293624-1293756
BmHel-14	DF090425:310254-310386
BmHel-14	DF090425:1006356-1006488
BmHel-14	DF090354:2348472-2348604
BmHel-14	DF090354:3371856-3371988
BmHel-14	DF090354:2552886-2553018
BmHel-14	DF090354:1915030-1915162
BmHel-14	DF090324:1086456-1086588
BmHel-14	DF090324:3719722-3719854
BmHel-14	DF090324:7100251-7100383
BmHel-14	DF090324:7622522-7622654
BmHel-14	DF090324:2968865-2968997
BmHel-14	DF090324:2105837-2105969
BmHel-14	DF090324:1079284-1079416
BmHel-14	DF090467:289781-289913
BmHel-14	DF090469:281231-281363
BmHel-14	DF090422:1057781-1057913
BmHel-14	DF090387:323208-323341
BmHel-14	DF090387:1655994-1656136
BmHel-14	DF090387:480793-480925
BmHel-14	DF090514:14135-14267
BmHel-14	DF090393:1252442-1252574
BmHel-14	DF090393:1602707-1602839
BmHel-14	DF090393:1602837-1602917
BmHel-14	DF090393:1251395-1251527
BmHel-14	DF090563:33918-34050
BmHel-14	DF090461:78386-78519
BmHel-14	DF090461:37918-38050
BmHel-14	DF090345:1244754-1244886
BmHel-14	DF090345:2800719-2800851
BmHel-14	DF090345:3724667-3724799
BmHel-14	DF090345:4102438-4102570
BmHel-14	DF090345:4261654-4261786
BmHel-14	DF090345:713477-713609
BmHel-14	DF090370:1418147-1418279
BmHel-14	DF090370:1732759-1732890
BmHel-14	DF090370:1486346-1486475
BmHel-14	DF090370:1177424-1177556
BmHel-14	DF090462:257288-257420
BmHel-14	DF090476:50489-50621
BmHel-14	DF090446:386441-386573
BmHel-14	DF090428:353176-353312
BmHel-14	DF090428:332380-332512
BmHel-14	DF090417:775860-775992

BmHel-14	DF090417:672652-672784
BmHel-14	DF090417:464360-464492
BmHel-14	DF090337:4868156-4868287
BmHel-14	DF090337:769534-769666
BmHel-14	DF090399:14123-14255
BmHel-14	DF090399:59732-59863
BmHel-14	DF090399:661606-661738
BmHel-14	DF090553:72709-72836
BmHel-14	DF090380:1297366-1297498
BmHel-14	DF090380:1632408-1632543
BmHel-14	DF090380:1853191-1853323
BmHel-14	DF090380:518621-518753
BmHel-14	BABH01085150:265-397
BmHel-14	BABH01083512:383-515
BmHel-14	BABH01063604:129-261
BmHel-14	BABH01058406:297-429
BmHel-14	BABH01050182:604-736
BmHel-14	BABH01048113:304-436
BmHel-14	DF090629:27706-27838
BmHel-14	DF090371:24996-25128
BmHel-14	DF090371:1060990-1061122
BmHel-14	DF090371:1961845-1961977
BmHel-14	BABH01054116:603-735
BmHel-14	DF091943:1827-1959
BmHel-14	BABH01045218:2847-2979
BmHel-14	BABH01044962:1598-1730
BmHel-14	DF091127:6568-6700
BmHel-14	DF090777:11373-11505
BmHel-14	DF090456:1475-1607
BmHel-14	DF090456:192959-193088
BmHel-14	DF090444:450417-450549
BmHel-14	DF090444:535369-535501
BmHel-14	DF090444:130657-130800
BmHel-14	DF090442:444000-444132
BmHel-14	DF090442:192493-192625
BmHel-14	DF090436:307130-307262
BmHel-14	DF090431:582901-583032
BmHel-14	DF090431:755323-755455
BmHel-14	DF090419:294987-295119
BmHel-14	DF090419:703251-703383
BmHel-14	DF090419:671145-671277
BmHel-14	DF090415:550267-550399
BmHel-14	DF090410:486925-487068
BmHel-14	DF090376:1881063-1881195
BmHel-14	DF090376:1353784-1353916
BmHel-14	DF090376:366083-366226
BmHel-14	DF090498:43858-43990
BmHel-14	DF090574:38300-38432

BmHel-14	BABH01047623:1378-1510
BmHel-14	DF091396:3504-3636
BmHel-14	DF091341:2995-3127
BmHel-14	DF090931:10430-10562
BmHel-14	DF090680:6833-6966
BmHel-14	DF090523:92189-92321
BmHel-14	DF090435:174346-174478
BmHel-14	DF090435:526792-526924
BmHel-14	DF090435:197550-197682
BmHel-14	DF090426:950985-951117
BmHel-14	DF090426:217107-217238
BmHel-14	DF090430:825980-826112
BmHel-14	BABH01081270:155-287
BmHel-14	DF091199:3748-3880
BmHel-14	DF090782:565-697
BmHel-14	DF090508:147121-147253
BmHel-14	DF090458:258238-258370
BmHel-14	DF090458:195526-195662
BmHel-14	DF090531:71661-71804
BmHel-14	BABH01069650:411-543
BmHel-14	BABH01049518:876-1008
BmHel-14	BABH01044941:2056-2188
BmHel-14	BABH01044232:504-636
BmHel-14	DF090805:8075-8207
BmHel-14	DF090802:6922-7054
BmHel-14	BABH01044698:3201-3333
BmHel-14	BABH01045354:743-883
BmHel-14	BABH01049929:396-532
BmHel-14	BABH01049654:894-1028
BmHel-14	DF091560:2228-2364
BmHel-14	DF090937:8893-9021
BmHel-14	BABH01056830:43-175
BmHel-14	DF091001:3598-3730
BmHel-14	DF090710:18360-18491
BmHel-14	BABH01047002:453-584
BmHel-14	DF091435:3887-4018
BmHel-14	BABH01048184:220-351
BmHel-14	DF090328:2774953-2775082
BmHel-14	DF090328:2774876-2774955
BmHel-14	DF090363:496866-496990
BmHel-14	DF090340:1577470-1577605
BmHel-14	DF090332:2310478-2310611
BmHel-14	DF090322:5307866-5308009
BmHel-14	DF090322:3115502-3115634
BmHel-14	DF090316:3529595-3529725
BmHel-14	DF090316:608438-608581
BmHel-14	DF090343:2167465-2167597
BmHel-14	DF090403:1081157-1081289

BmHel-14	DF090358:102389-102521
BmHel-14	DF090378:1857187-1857396
BmHel-14	DF090363:2564021-2564153
BmHel-14	DF090414:1339332-1339470
BmHel-14	DF090348:4194266-4194398
BmHel-14	DF090355:1423890-1424025
BmHel-14	DF090438:49195-49339
BmHel-14	DF090331:947395-947537
BmHel-14	DF090366:2037541-2037669
BmHel-14	DF090366:2037460-2037539
BmHel-14	DF090353:3702103-3702235
BmHel-14	DF090353:181282-181425
BmHel-14	DF090365:2366160-2366303
BmHel-14	DF090402:290950-291082
BmHel-14	DF090381:938684-938816
BmHel-14	DF090324:7320284-7320416
BmHel-14	DF090387:1110127-1110298
BmHel-14	DF090563:34857-34991
BmHel-14	DF090441:350770-350902
BmHel-14	DF090337:348038-348179
BmHel-14	DF090415:439436-439578
BmHel-14	DF091280:3202-3344
BmHel-14	BABH01073236:74-206
BmHel-14	DF090592:34788-34920
BmHel-14	DF090507:107670-107812
BmHel-14	DF091693:1878-2022
BmHel-14	DF090368:761760-761891
BmHel-14	DF090347:438961-439092
BmHel-14	DF090321:4994525-4994656
BmHel-14	DF090349:4401109-4401240
BmHel-14	DF090326:1609110-1609241
BmHel-14	DF090425:910661-910792
BmHel-14	DF090354:2450473-2450604
BmHel-14	DF090337:1551061-1551192
BmHel-14	DF090452:241127-241255
BmHel-14	DF090452:316299-316430
BmHel-14	DF090324:2711205-2711421
BmHel-14	DF090399:1148427-1148556
BmHel-14	DF090399:1148556-1148639
BmHel-14	DF090351:3097207-3097336
BmHel-14	DF090368:1628465-1628568
BmHel-14	DF090413:9402-9503
BmHel-14	DF090351:2177509-2177643
BmHel-14	DF090351:437017-437149
BmHel-14	DF090319:3040447-3040579
BmHel-14	DF090319:6686774-6686921
BmHel-14	DF090319:7566234-7566377
BmHel-14	DF090319:4819485-4819619

BmHel-14	DF090316:10157668-10157800
BmHel-14	DF090316:4273305-4273439
BmHel-14	DF090329:3006832-3006963
BmHel-14	DF090330:1029027-1029146
BmHel-14	DF090318:2479858-2480006
BmHel-14	DF090338:783676-783810
BmHel-14	DF090392:1520916-1521057
BmHel-14	DF090325:2341772-2341903
BmHel-14	DF090325:5403484-5403616
BmHel-14	DF090320:839722-839856
BmHel-14	DF090341:4312463-4312597
BmHel-14	DF090339:1076214-1076347
BmHel-14	DF090335:2988551-2988693
BmHel-14	DF090358:1237811-1237949
BmHel-14	DF090326:2997827-2997959
BmHel-14	DF090364:2672119-2672251
BmHel-14	DF090362:1164632-1164764
BmHel-14	DF090366:346109-346241
BmHel-14	DF090421:644776-644907
BmHel-14	DF090360:1415669-1415810
BmHel-14	DF090370:1041968-1042111
BmHel-14	DF090380:491201-491333
BmHel-14	DF090380:504573-504701
BmHel-14	BABH01085029:6-138
BmHel-14	BABH01069883:37-169
BmHel-14	DF090561:11830-11962
BmHel-14	DF090699:20571-20713
BmHel-14	BABH01075488:179-309
BmHel-14	BABH01069058:89-223
BmHel-14	DF090358:281106-281249
BmHel-14	DF090330:3481488-3481618
BmHel-14	DF090389:1518039-1518168
BmHel-14	DF090388:211232-211362
BmHel-14	DF090359:1504637-1504767
BmHel-14	DF090334:3277859-3277989
BmHel-14	DF090317:3039962-3040092
BmHel-14	DF090412:1303686-1303816
BmHel-14	DF090444:9899-10029
BmHel-14	DF090444:9821-9901
BmHel-14	DF090435:486361-486491
BmHel-14	BABH01056880:354-484
BmHel-14	BABH01056880:276-356
BmHel-14	BABH01052210:287-417
BmHel-14	DF091079:8911-9041
BmHel-14	DF091079:9039-9119
BmHel-14	DF090475:131717-131850
BmHel-14	DF090330:5211639-5211756
BmHel-14	DF090330:5211749-5211838

BmHel-14	DF090321:1150106-1150185
BmHel-14	DF090319:41224-41353
BmHel-14	DF090383:307518-307649
BmHel-14	DF090332:2865467-2865603
BmHel-14	DF090332:2039504-2039636
BmHel-14	DF090322:1061972-1062104
BmHel-14	DF090447:58340-58472
BmHel-14	DF090316:5496201-5496333
BmHel-14	DF090316:14860154-14860287
BmHel-14	DF090329:781687-781819
BmHel-14	DF090329:1862534-1862666
BmHel-14	DF090329:4327545-4327677
BmHel-14	DF090329:313635-313771
BmHel-14	DF090424:232444-232576
BmHel-14	DF090330:6378935-6379070
BmHel-14	DF090318:5630117-5630249
BmHel-14	DF090372:524282-524425
BmHel-14	DF090388:1168853-1168981
BmHel-14	DF090323:2567015-2567147
BmHel-14	DF090373:671124-671256
BmHel-14	DF090373:175419-175547
BmHel-14	DF090333:1385090-1385222
BmHel-14	DF090397:1500383-1500515
BmHel-14	DF090394:1400365-1400498
BmHel-14	DF090325:1749380-1749459
BmHel-14	DF090325:4847733-4847829
BmHel-14	DF090359:99519-99669
BmHel-14	DF090321:2496475-2496607
BmHel-14	DF090321:6355431-6355566
BmHel-14	DF090349:2924438-2924572
BmHel-14	DF090390:555311-555444
BmHel-14	DF090328:5054020-5054148
BmHel-14	DF090328:2962974-2963109
BmHel-14	DF090328:2326626-2326758
BmHel-14	DF090334:1826061-1826193
BmHel-14	DF090334:2361061-2361193
BmHel-14	DF090326:5545772-5545904
BmHel-14	DF090382:1325665-1325812
BmHel-14	DF090404:1295535-1295657
BmHel-14	DF090414:803679-803822
BmHel-14	DF090407:513713-513845
BmHel-14	DF090362:1334146-1334278
BmHel-14	DF090336:4771766-4771898
BmHel-14	DF090336:1505712-1505845
BmHel-14	DF090396:429803-429935
BmHel-14	DF090331:5807247-5807379
BmHel-14	DF090331:2142894-2143024
BmHel-14	DF090383:875143-875276

BmHel-14	DF090342:4497714-4497846
BmHel-14	DF090324:1321135-1321267
BmHel-14	DF090413:871486-871618
BmHel-14	DF090445:97726-97858
BmHel-14	DF090393:839499-839631
BmHel-14	DF090370:237357-237489
BmHel-14	DF090384:1100976-1101108
BmHel-14	DF090384:1728659-1728791
BmHel-14	DF090417:1057136-1057268
BmHel-14	DF090380:1422053-1422181
BmHel-14	DF090575:48738-48870
BmHel-14	DF090436:231115-231247
BmHel-14	DF090406:108735-108875
BmHel-14	DF090416:1239608-1239739
BmHel-14	DF090455:242108-242240
BmHel-14	DF090316:1093687-1093815
BmHel-14	DF090327:5278053-5278198
BmHel-14	DF090629:45877-46007
BmHel-14	DF090322:2269415-2269544
BmHel-14	DF090322:7106369-7106498
BmHel-14	DF090319:3602039-3602169
BmHel-14	DF090424:694358-694487
BmHel-14	DF090379:1134848-1134977
BmHel-14	DF090379:505430-505559
BmHel-14	DF090325:3506272-3506394
BmHel-14	DF090320:3822681-3822810
BmHel-14	DF090382:1497154-1497283
BmHel-14	DF090412:75145-75274
BmHel-14	DF090375:1941868-1941997
BmHel-14	DF090362:2657410-2657539
BmHel-14	DF090360:2094437-2094558
BmHel-14	DF090400:1252604-1252723
BmHel-14	DF090386:885701-885829
BmHel-14	DF090384:1505675-1505804
BmHel-14	DF090371:1974338-1974476
BmHel-14	BABH01075229:1-130
BmHel-14	BABH01080543:20-150
BmHel-14	DF090427:548926-549055
BmHel-14	DF090427:315453-315593
BmHel-14	DF090416:497464-497593
BmHel-14	BABH01055968:438-566
BmHel-14	DF090659:14943-15072
BmHel-14	DF090365:2205108-2205314
BmHel-14	DF090319:974989-975088
BmHel-14	DF090381:717914-718013
BmHel-14	DF090345:368509-368608
BmHel-14	DF090317:7631774-7631890
BmHel-14	DF090343:2813959-2814091

BmHel-14	DF090322:3753520-3753652
BmHel-14	DF090322:5491487-5491617
BmHel-14	DF090351:3558378-3558510
BmHel-14	DF090316:3139184-3139316
BmHel-14	DF090329:6714127-6714257
BmHel-14	DF090329:5910345-5910476
BmHel-14	DF090329:3272131-3272263
BmHel-14	DF090329:1577868-1577998
BmHel-14	DF090369:2633482-2633611
BmHel-14	DF090369:2682579-2682709
BmHel-14	DF090369:1148187-1148316
BmHel-14	DF090330:6015367-6015510
BmHel-14	DF090330:6228219-6228349
BmHel-14	DF090330:3023005-3023133
BmHel-14	DF090343:691949-692081
BmHel-14	DF090343:2643952-2644081
BmHel-14	DF090323:6744843-6744975
BmHel-14	DF090340:2038513-2038644
BmHel-14	DF090340:3572393-3572523
BmHel-14	DF090333:4376582-4376720
BmHel-14	DF090333:5358460-5358600
BmHel-14	DF090346:3641341-3641470
BmHel-14	DF090325:2524630-2524773
BmHel-14	DF090325:7065929-7066061
BmHel-14	DF090325:5343584-5343715
BmHel-14	DF090347:1944094-1944222
BmHel-14	DF090401:111516-111607
BmHel-14	DF090321:7103251-7103382
BmHel-14	DF090321:2710877-2711006
BmHel-14	DF090358:514789-514923
BmHel-14	DF090334:3596982-3597117
BmHel-14	DF090317:2523951-2524080
BmHel-14	DF090317:6184411-6184544
BmHel-14	DF090317:1215354-1215486
BmHel-14	DF090364:145800-145930
BmHel-14	DF090355:2606859-2606990
BmHel-14	DF090488:83148-83279
BmHel-14	DF090352:4037272-4037403
BmHel-14	DF090407:496494-496624
BmHel-14	DF090407:1026844-1026968
BmHel-14	DF090375:1170990-1171119
BmHel-14	DF090362:2860973-2861101
BmHel-14	DF090609:19112-19242
BmHel-14	DF090366:912803-912932
BmHel-14	DF090366:727181-727313
BmHel-14	DF090386:510798-510928
BmHel-14	DF090365:1190308-1190436
BmHel-14	DF090342:2079603-2079732

BmHel-14	DF090342:2457918-2458051
BmHel-14	DF090461:11781-11913
BmHel-14	DF090345:1275045-1275177
BmHel-14	DF090384:1670201-1670292
BmHel-14	DF090428:577389-577521
BmHel-14	DF090423:560609-560701
BmHel-14	DF090457:10460-10592
BmHel-14	BABH01053374:71-203
BmHel-14	DF090371:1713485-1713617
BmHel-14	DF090705:8236-8368
BmHel-14	DF090406:1146746-1146889
BmHel-14	DF090498:9180-9310
BmHel-14	DF090385:1875126-1875256
BmHel-14	DF090385:1602611-1602740
BmHel-14	BABH01087170:50-182
BmHel-14	DF091396:1225-1359
BmHel-14	DF090674:16922-17054
BmHel-14	DF090561:1367-1498
BmHel-14	DF090551:50453-50584
BmHel-14	DF090548:4095-4224
BmHel-14	DF091037:6930-7062
BmHel-14	DF090439:24143-24270
BmHel-14	BABH01044698:4401-4536
BmHel-14	DF090493:139449-139579
BmHel-14	DF090556:17897-18029
BmHel-14	DF090562:2915-3045
BmHel-14	DF090353:3857301-3857431
BmHel-14	DF090331:2596280-2596418
BmHel-14	DF090351:4058279-4058407
BmHel-14	DF090351:802084-802215
BmHel-14	DF090319:2991438-2991566
BmHel-14	DF090356:2676617-2676745
BmHel-14	DF090341:713369-713497
BmHel-14	DF090321:6730240-6730368
BmHel-14	DF090414:292810-292949
BmHel-14	DF090352:3869686-3869825
BmHel-14	DF090409:664543-664671
BmHel-14	DF090342:3563049-3563177
BmHel-14	DF090342:558086-558214
BmHel-14	DF090448:199047-199175
BmHel-14	DF090596:33020-33159
BmHel-14	DF090453:266111-266235
BmHel-14	DF090344:3318397-3318495
BmHel-14	DF090317:8893384-8893516
BmHel-14	DF090317:4309389-4309487
BmHel-14	DF090480:211570-211688
BmHel-14	DF090390:1564583-1564753
BmHel-14	DF090322:5009096-5009234

BmHel-14	DF090319:5212866-5212998
BmHel-14	DF090316:14309372-14309504
BmHel-14	DF090388:801976-802108
BmHel-14	DF090323:7869680-7869823
BmHel-14	DF090333:3213327-3213458
BmHel-14	DF090346:1105651-1105782
BmHel-14	DF090321:7967032-7967164
BmHel-14	DF090317:812311-812454
BmHel-14	DF090411:1243406-1243538
BmHel-14	DF090362:748286-748429
BmHel-14	DF090366:502673-502809
BmHel-14	DF090342:1183118-1183201
BmHel-14	DF090381:727777-727909
BmHel-14	DF090530:28608-28740
BmHel-14	DF090950:8508-8638
BmHel-14	DF090528:54789-54920
BmHel-14	DF090351:3972443-3972572
BmHel-14	DF090326:3330125-3330255
BmHel-14	DF090322:1492285-1492416
BmHel-14	DF090333:1920930-1921061
BmHel-14	DF090357:2397878-2398014
BmHel-14	DF090353:1720427-1720567
BmHel-14	DF090322:5209446-5209584
BmHel-14	DF090318:5444632-5444759
BmHel-14	DF090355:508482-508609
BmHel-14	DF090405:261254-261381
BmHel-14	DF090393:34008-34132
BmHel-14	DF090677:11713-11840
BmHel-14	DF090338:2833808-2834012
BmHel-14	DF090369:541296-541418
BmHel-14	DF090328:6491597-6491706
BmHel-14	DF090325:5991501-5991633
BmHel-14	DF090317:10854707-10854839
BmHel-14	DF090383:93127-93259
BmHel-14	DF090327:2301295-2301412
BmHel-14	DF090322:8183056-8183185
BmHel-14	DF090316:11328061-11328204
BmHel-14	DF090325:3964633-3964765
BmHel-14	DF090325:4087348-4087444
BmHel-14	DF090359:1674882-1675012
BmHel-14	DF090349:1322760-1322890
BmHel-14	DF090349:1363104-1363234
BmHel-14	DF090414:589884-590016
BmHel-14	DF090353:1538770-1538898
BmHel-14	DF090402:180858-180988
BmHel-14	DF090422:981499-981639
BmHel-14	DF090370:1326148-1326289
BmHel-14	DF090373:96759-96885

BmHel-14	DF090394:1534916-1535044
BmHel-14	DF090327:2641947-2642075
BmHel-14	DF090334:160474-160604
BmHel-14	DF090320:3820871-3821004
BmHel-14	DF090365:3007935-3008064
BmHel-14	DF090320:4440499-4440626
BmHel-14	DF090327:3569587-3569723
BmHel-14	DF090355:1538825-1538963
BmHel-14	DF090358:897733-897859
BmHel-14	DF090326:3035037-3035163
BmHel-14	DF090325:6113802-6114005
BmHel-14	DF090321:1352790-1352995
BmHel-14	DF090410:1156802-1156969
BmHel-14	DF090332:6230593-6230697
BmHel-14	DF090316:9831111-9831248
BmHel-14	DF090379:125713-125845
BmHel-14	DF090390:1423084-1423216
BmHel-14	DF090364:826483-826615
BmHel-14	DF090453:115391-115523
BmHel-14	DF090316:12410816-12410953
BmHel-14	DF090389:967187-967312
BmHel-14	DF090323:7484761-7484889
BmHel-14	DF090449:773831-773952
BmHel-14	DF090347:3829948-3830079
BmHel-14	DF090320:6135563-6135706
BmHel-14	DF090401:537449-537581
BmHel-14	DF090344:1303412-1303539
BmHel-14	DF090403:1155218-1155350
BmHel-14	DF090334:2960982-2961114
BmHel-14	DF090317:5245187-5245317
BmHel-14	DF090363:2098403-2098535
BmHel-14	DF090362:2214527-2214659
BmHel-14	DF090331:4110770-4110895
BmHel-14	DF090331:4707840-4707968
BmHel-14	DF090354:629016-629146
BmHel-14	DF090345:3888469-3888601
BmHel-14	DF090498:16869-16996
BmHel-14	DF091000:2102-2234
BmHel-14	DF090396:979104-979229
BmHel-14	DF090368:737837-737965
BmHel-14	DF090399:954255-954383
BmHel-14	DF090390:808615-808742
BmHel-14	DF090373:1985906-1986032
BmHel-14	DF090363:65490-65617
BmHel-14	DF090365:1445682-1445798
BmHel-14	DF090331:3425740-3425831
BmHel-14	DF090332:4077586-4077718
BmHel-14	DF090322:3954118-3954258

BmHel-14	DF090329:6423198-6423343
BmHel-14	DF090369:1072862-1073012
BmHel-14	DF090318:1825154-1825282
BmHel-14	DF090318:9446311-9446460
BmHel-14	DF090318:9186951-9187100
BmHel-14	DF090318:8885423-8885573
BmHel-14	DF090338:76938-77069
BmHel-14	DF090372:1972045-1972170
BmHel-14	DF090323:2299634-2299783
BmHel-14	DF090340:4201374-4201506
BmHel-14	DF090373:1668768-1668917
BmHel-14	DF090359:2293919-2294048
BmHel-14	DF090347:3495020-3495169
BmHel-14	DF090320:6903431-6903580
BmHel-14	DF090327:732722-732871
BmHel-14	DF090361:2276299-2276423
BmHel-14	DF090466:9224-9373
BmHel-14	DF090349:1667244-1667337
BmHel-14	DF090334:3903403-3903527
BmHel-14	DF090362:79333-79484
BmHel-14	DF090362:1077941-1078073
BmHel-14	DF090336:1688104-1688228
BmHel-14	DF090353:1707169-1707322
BmHel-14	DF090353:1749365-1749514
BmHel-14	DF090324:8069457-8069582
BmHel-14	DF090324:3867138-3867267
BmHel-14	DF090413:217289-217439
BmHel-14	DF090422:573559-573708
BmHel-14	DF090337:3999485-3999584
BmHel-14	DF090380:1708057-1708186
BmHel-14	DF091011:17605-17741
BmHel-14	DF090431:73561-73692
BmHel-14	DF090547:15659-15808
BmHel-14	DF090426:113382-113506
BmHel-14	DF090439:586536-586660
BmHel-14	DF090316:9034739-9034867
BmHel-14	DF090328:2562019-2562163
BmHel-14	DF090326:4402773-4402900
BmHel-14	DF090386:828961-829087
BmHel-14	DF090355:860173-860300
BmHel-14	DF090332:438508-438632
BmHel-14	DF090316:12255092-12255216
BmHel-14	DF090413:337478-337602
BmHel-14	DF090393:1336298-1336422
BmHel-14	DF090374:1408689-1408852
BmHel-14	DF090388:1769785-1769880
BmHel-14	DF090359:3254649-3254743
BmHel-14	DF090342:3800570-3800687

BmHel-14	DF090430:95918-96050
BmHel-14	DF090322:835159-835289
BmHel-14	DF090316:7625748-7625896
BmHel-14	DF090329:1506408-1506556
BmHel-14	DF090330:2173073-2173222
BmHel-14	DF090318:5979314-5979462
BmHel-14	DF090408:989542-989682
BmHel-14	DF090379:798682-798831
BmHel-14	DF090323:5975982-5976131
BmHel-14	DF090323:6331448-6331591
BmHel-14	DF090359:2786402-2786526
BmHel-14	DF090347:938147-938226
BmHel-14	DF090341:252853-252998
BmHel-14	DF090321:1439691-1439840
BmHel-14	DF090344:385715-385841
BmHel-14	DF090335:5814888-5815018
BmHel-14	DF090358:3278369-3278510
BmHel-14	DF090358:2128517-2128666
BmHel-14	DF090334:6005039-6005166
BmHel-14	DF090317:1515579-1515669
BmHel-14	DF090355:1246560-1246709
BmHel-14	DF090407:227370-227513
BmHel-14	DF090365:1866736-1866889
BmHel-14	DF090365:2135600-2135749
BmHel-14	DF090354:3362712-3362840
BmHel-14	DF090384:999584-999733
BmHel-14	DF090446:330233-330365
BmHel-14	DF090446:273082-273231
BmHel-14	DF090399:1134446-1134546
BmHel-14	DF090419:442133-442282
BmHel-14	DF090450:506675-506818
BmHel-14	BABH01068706:5-154
BmHel-14	DF090397:1192084-1192224
BmHel-14	DF090322:3895435-3895563
BmHel-14	DF090628:1321-1446
BmHel-14	DF090443:236874-237008
BmHel-14	DF090336:3850824-3850947
BmHel-14	DF090318:8069647-8069740
BmHel-14	DF090406:239898-240007
BmHel-14	DF090342:2630117-2630244
BmHel-14	DF090369:445809-445938
BmHel-14	DF090322:3125749-3125871
BmHel-14	DF090319:3151880-3152007
BmHel-14	DF090316:10665964-10666096
BmHel-14	DF090418:807698-807841
BmHel-14	DF090329:5684674-5684806
BmHel-14	DF090323:7059941-7060091
BmHel-14	DF090346:1557053-1557202

BmHel-14	DF090347:1815269-1815397
BmHel-14	DF090320:8151042-8151183
BmHel-14	DF090363:572597-572746
BmHel-14	DF090396:410426-410575
BmHel-14	DF090386:1889355-1889498
BmHel-14	DF090384:1137673-1137832
BmHel-14	DF090419:710146-710278
BmHel-14	DF090493:111461-111609
BmHel-14	DF090454:23416-23554
BmHel-14	DF090391:1090214-1090342
BmHel-14	DF090324:4249299-4249414
BmHel-14	DF090332:4433095-4433221
BmHel-14	DF090329:3601207-3601332
BmHel-14	DF090361:1468899-1469027
BmHel-14	DF090362:1059739-1059861
BmHel-14	BABH01045800:83-205
BmHel-14	DF090492:210217-210339
BmHel-14	BABH01070285:97-219
BmHel-14	DF090372:1818450-1818582
BmHel-14	DF090483:204810-204923
BmHel-14	DF090526:19591-19726
BmHel-14	DF090357:2865148-2865280
BmHel-14	DF090342:2658635-2658767
BmHel-14	DF090338:1929358-1929527
BmHel-14	DF090319:1718268-1718397
BmHel-14	DF090319:7283070-7283213
BmHel-14	DF090319:2907867-2908016
BmHel-14	DF090316:15321223-15321344
BmHel-14	DF090318:7151247-7151379
BmHel-14	DF090340:2263394-2263544
BmHel-14	DF090340:3126724-3126866
BmHel-14	DF090347:146860-146987
BmHel-14	DF090382:1966300-1966450
BmHel-14	DF090421:858736-858862
BmHel-14	DF090350:1470779-1470924
BmHel-14	DF090423:243573-243723
BmHel-14	DF090430:119052-119195
BmHel-14	DF090357:2840887-2841033
BmHel-14	DF090331:317886-318031
BmHel-14	DF090335:1653432-1653556
BmHel-14	DF090317:10020431-10020611
BmHel-14	DF090361:3041088-3041208
BmHel-14	DF090355:3246476-3246597
BmHel-14	DF090329:6448787-6448936
BmHel-14	DF090323:922883-923035
BmHel-14	DF090346:775834-775957
BmHel-14	DF090356:3823526-3823658
BmHel-14	DF090339:936109-936253

BmHel-14	DF090334:5541551-5541671
BmHel-14	DF090364:323154-323293
BmHel-14	DF090364:68700-68845
BmHel-14	DF090375:729230-729380
BmHel-14	DF090362:1154721-1154869
BmHel-14	DF090400:686701-686844
BmHel-14	DF090405:295854-296002
BmHel-14	DF090409:1183925-1184071
BmHel-14	DF090417:875393-875515
BmHel-14	DF090371:1031668-1031767
BmHel-14	DF090641:11386-11533
BmHel-14	DF090648:24523-24645
BmHel-14	DF090499:108474-108556
BmHel-14	DF090364:935839-935970
BmHel-14	BABH01048367:953-1102
BmHel-14	DF090318:3370739-3370883
BmHel-14	DF090351:668244-668371
BmHel-14	BABH01042583:24988-25113
BmHel-14	DF090316:8151637-8151757
BmHel-14	DF090329:1917615-1917735
BmHel-14	DF090343:3708677-3708798
BmHel-14	DF090326:2952732-2952929
BmHel-14	DF090318:1799449-1799597
BmHel-14	DF090325:3067248-3067397
BmHel-14	DF090341:3771176-3771325
BmHel-14	DF090358:2826722-2826809
BmHel-14	DF090364:54322-54471
BmHel-14	DF090459:243497-243579
BmHel-14	DF090320:6970368-6970498
BmHel-14	DF090331:6007581-6007703
BmHel-14	DF090355:1008515-1008654
BmHel-14	DF090317:5327740-5327868
BmHel-14	DF090317:8261114-8261231
BmHel-14	DF090425:436567-436685
BmHel-14	DF090464:67529-67649
BmHel-14	DF090379:666273-666469
BmHel-14	DF090321:4044634-4044766
BmHel-14	DF090378:794169-794301
BmHel-14	DF090351:3690726-3690869
BmHel-14	DF090319:9062445-9062579
BmHel-14	DF090318:5009923-5010072
BmHel-14	DF090343:201473-201605
BmHel-14	DF090388:975364-975507
BmHel-14	DF090340:1507053-1507196
BmHel-14	DF090341:2666055-2666185
BmHel-14	DF090335:765718-765836
BmHel-14	DF090353:3723659-3723787
BmHel-14	DF090400:1284424-1284571

BmHel-14	DF090428:530584-530772
BmHel-14	DF090480:243624-243760
BmHel-14	DF090316:9260731-9260867
BmHel-14	DF090333:5081286-5081432
BmHel-14	DF090383:1200361-1200505
BmHel-14	DF090321:5965506-5965633
BmHel-14	DF090390:156374-156503
BmHel-14	DF090335:4729566-4729684
BmHel-14	DF090317:4086799-4086917
BmHel-14	DF090317:4086722-4086801
BmHel-14	BABH01053435:4-200
BmHel-14	DF090344:1675006-1675094
BmHel-14	DF090322:2257723-2257917
BmHel-14	DF090476:155489-155686
BmHel-14	DF090362:1578189-1578309
BmHel-14	DF090332:207760-207954
BmHel-14	DF090322:7358194-7358337
BmHel-14	DF090351:2491056-2491250
BmHel-14	DF090316:12370150-12370335
BmHel-14	DF090316:15288731-15288867
BmHel-14	DF090418:1183120-1183313
BmHel-14	DF090329:2882529-2882723
BmHel-14	DF090329:4190421-4190553
BmHel-14	DF090318:3906693-3906888
BmHel-14	DF090343:2144611-2144805
BmHel-14	DF090408:859017-859211
BmHel-14	DF090408:256436-256630
BmHel-14	DF090392:1251931-1252125
BmHel-14	DF090323:2325501-2325696
BmHel-14	DF090373:2082454-2082648
BmHel-14	DF090325:3146167-3146288
BmHel-14	DF090341:4702104-4702298
BmHel-14	DF090341:1685833-1686027
BmHel-14	DF090327:2875295-2875491
BmHel-14	DF090321:1186530-1186647
BmHel-14	DF090367:1252473-1252667
BmHel-14	DF090349:1812877-1813071
BmHel-14	DF090349:1422880-1423077
BmHel-14	DF090344:4661246-4661371
BmHel-14	DF090344:4542297-4542420
BmHel-14	DF090335:1657146-1657343
BmHel-14	DF090334:1481589-1481783
BmHel-14	DF090334:3026481-3026675
BmHel-14	DF090334:5286166-5286316
BmHel-14	DF090317:3835316-3835510
BmHel-14	DF090317:10401104-10401297
BmHel-14	DF090317:5060570-5060764
BmHel-14	DF090382:1883453-1883647

BmHel-14	DF090363:857131-857325
BmHel-14	DF090348:2643099-2643233
BmHel-14	DF090352:2229336-2229519
BmHel-14	DF090352:2514376-2514560
BmHel-14	DF090407:1329177-1329361
BmHel-14	DF090362:29149-29343
BmHel-14	DF090421:137716-137848
BmHel-14	DF090383:1653081-1653275
BmHel-14	DF090353:1184348-1184545
BmHel-14	DF090365:1253845-1254038
BmHel-14	DF090429:35126-35320
BmHel-14	DF090409:814514-814708
BmHel-14	DF090374:357241-357435
BmHel-14	DF090342:4465056-4465205
BmHel-14	DF090434:16319-16513
BmHel-14	DF090381:1654120-1654263
BmHel-14	DF090917:9241-9435
BmHel-14	DF090324:6793397-6793587
BmHel-14	DF090467:90742-90935
BmHel-14	DF090469:166325-166519
BmHel-14	DF090422:1112381-1112515
BmHel-14	DF090422:802630-802824
BmHel-14	DF090345:3632076-3632265
BmHel-14	DF090509:47440-47634
BmHel-14	BABH01064413:478-672
BmHel-14	DF090913:11653-11847
BmHel-14	DF090337:1683007-1683204
BmHel-14	BABH01050272:596-789
BmHel-14	DF090423:225743-225936
BmHel-14	DF090342:2585165-2585309
BmHel-14	DF090356:3289636-3289776
BmHel-14	DF090345:927027-927210
BmHel-14	DF090368:1539674-1539761
BmHel-14	DF090452:154192-154321
BmHel-14	DF090371:2463631-2463751
BmHel-14	DF090322:3230524-3230657
BmHel-14	DF090319:8050113-8050245
BmHel-14	DF090316:4753423-4753555
BmHel-14	DF090316:15656917-15657049
BmHel-14	DF090329:1372367-1372499
BmHel-14	DF090329:1264246-1264340
BmHel-14	DF090318:6228828-6229021
BmHel-14	DF090368:189282-189425
BmHel-14	DF090338:465646-465778
BmHel-14	DF090323:7721559-7721691
BmHel-14	DF090340:2699601-2699733
BmHel-14	DF090340:1967013-1967145
BmHel-14	DF090357:3569930-3570123

BmHel-14	DF090357:618096-618228
BmHel-14	DF090394:995108-995235
BmHel-14	DF090325:5817866-5817997
BmHel-14	DF090344:1600048-1600180
BmHel-14	DF090344:1623819-1623951
BmHel-14	DF090344:3212381-3212513
BmHel-14	DF090328:4740009-4740141
BmHel-14	DF090334:3920731-3920863
BmHel-14	DF090326:152842-152974
BmHel-14	DF090326:5054734-5054866
BmHel-14	DF090317:8505415-8505547
BmHel-14	DF090317:9806549-9806680
BmHel-14	DF090355:294474-294606
BmHel-14	DF090352:3437119-3437251
BmHel-14	DF090407:238977-239109
BmHel-14	DF090375:45083-45277
BmHel-14	DF090362:556877-557070
BmHel-14	DF090362:225919-226051
BmHel-14	DF090383:1270186-1270318
BmHel-14	DF090386:290833-290965
BmHel-14	DF090405:509310-509458
BmHel-14	DF090417:626769-626901
BmHel-14	DF090337:2063392-2063524
BmHel-14	DF090451:42567-42706
BmHel-14	BABH01053044:429-561
BmHel-14	DF090431:110955-111087
BmHel-14	BABH01043533:5382-5514
BmHel-14	BABH01047844:358-490
BmHel-14	DF090323:6222468-6222608
BmHel-14	DF090398:923844-923960
BmHel-14	DF090383:1350239-1350431
BmHel-14	DF090356:1230945-1231137
BmHel-14	DF090332:2407634-2407747
BmHel-14	DF090418:107840-107976
BmHel-14	DF090318:3523856-3524005
BmHel-14	DF090392:1237451-1237581
BmHel-14	DF090340:489153-489289
BmHel-14	DF090359:772159-772305
BmHel-14	DF090390:1352228-1352372
BmHel-14	DF090403:1565279-1565416
BmHel-14	DF090326:1855974-1856106
BmHel-14	DF090348:3285443-3285592
BmHel-14	DF090352:2616386-2616518
BmHel-14	DF090350:1167303-1167435
BmHel-14	DF090342:741857-741989
BmHel-14	DF090342:3482803-3482939
BmHel-14	DF090340:2983924-2984069
BmHel-14	DF090348:3754683-3754814

BmHel-14	DF090430:624209-624339
BmHel-14	DF090514:118202-118394
BmHel-14	DF090317:10842143-10842258
BmHel-14	DF090344:3595142-3595227
BmHel-14	DF090344:81570-81719
BmHel-14	DF090328:474278-474469
BmHel-14	DF090350:1884376-1884519
BmHel-14	DF090324:3079898-3080041
BmHel-14	DF090422:170058-170201
BmHel-14	DF090329:86228-86363
BmHel-14	DF090378:1390957-1391148
BmHel-14	DF090320:3377393-3377509
BmHel-14	BABH01063471:1-192
BmHel-14	DF090332:4223072-4223261
BmHel-14	DF090332:1044569-1044701
BmHel-14	DF090318:4093518-4093631
BmHel-14	DF090368:1923232-1923381
BmHel-14	DF090397:619704-619853
BmHel-14	DF090326:2565119-2565251
BmHel-14	DF090317:1687402-1687533
BmHel-14	DF090355:2859740-2859861
BmHel-14	DF090354:1583836-1583968
BmHel-14	DF090337:1710507-1710614
BmHel-14	DF090423:249948-250090
BmHel-14	DF090380:982972-983094
BmHel-14	BABH01045014:4603-4752
BmHel-14	DF090416:1211917-1212066
BmHel-14	DF090423:816904-817038
BmHel-14	DF090364:2099573-2099709
BmHel-14	DF090374:1166031-1166125
BmHel-14	DF090390:1371034-1371215
BmHel-14	BABH01049425:216-302
BmHel-14	DF090317:8805206-8805344
BmHel-14	DF090340:4285007-4285128
BmHel-14	DF090316:10579674-10579864
BmHel-14	DF090372:1794936-1795020
BmHel-14	DF090351:2702315-2702441
BmHel-14	DF090316:6787940-6788069
BmHel-14	DF090316:1920727-1920919
BmHel-14	DF090424:741158-741282
BmHel-14	DF090330:957074-957222
BmHel-14	DF090408:1188849-1188992
BmHel-14	DF090347:2239458-2239591
BmHel-14	DF090326:669155-669287
BmHel-14	DF090345:2310698-2310830
BmHel-14	DF090462:142034-142223
BmHel-14	DF091130:628-760
BmHel-14	BABH01052221:506-638

BmHel-14	BABH01045754:3353-3485
BmHel-14	DF090439:158930-159063
BmHel-14	DF090322:3322595-3322728
BmHel-14	DF090438:265822-265950
BmHel-14	DF090336:1142885-1143073
BmHel-14	DF090384:454134-454323
BmHel-14	DF090406:125844-125982
BmHel-14	DF090349:2810271-2810383
BmHel-14	DF090404:1366958-1367071
BmHel-14	DF090449:757486-757592
BmHel-14	DF090355:360591-360673
BmHel-14	DF090319:5596842-5596985
BmHel-14	DF090316:2877977-2878109
BmHel-14	DF090335:1409586-1409718
BmHel-14	DF090334:2397896-2398010
BmHel-14	DF090375:1072512-1072648
BmHel-14	DF090345:4238388-4238519
BmHel-14	DF090384:1315204-1315335
BmHel-14	DF090415:355150-355299
BmHel-14	DF090644:290-371
BmHel-14	DF090349:3551225-3551371
BmHel-14	DF090316:5848682-5848765
BmHel-14	DF090341:4104263-4104392
BmHel-14	DF090331:4241298-4241430
BmHel-14	DF090350:4031546-4031738
BmHel-14	DF090341:445258-445397
BmHel-14	DF090365:2523354-2523480
BmHel-14	DF090329:1454795-1454906
BmHel-14	DF090316:1988089-1988200
BmHel-14	DF090347:3615979-3616137
BmHel-14	DF090321:2851992-2852120
BmHel-14	DF090336:1734518-1734650
BmHel-14	DF091367:1055-1184
BmHel-14	DF090318:7666271-7666418
BmHel-14	DF090373:2084950-2085081
BmHel-14	DF090354:785129-785263
BmHel-14	DF090316:15864901-15865026
BmHel-14	DF090357:785567-785677
BmHel-14	DF090434:266705-266815
BmHel-14	DF090318:1005590-1005739
BmHel-14	DF090318:5067066-5067252
BmHel-14	DF090338:2627103-2627288
BmHel-14	DF090359:2612408-2612533
BmHel-14	DF090339:890452-890600
BmHel-14	DF090321:2618605-2618720
BmHel-14	DF090344:4563492-4563634
BmHel-14	DF090391:967429-967572
BmHel-14	DF090335:1044545-1044676

BmHel-14	DF090324:6136642-6136751
BmHel-14	BABH01083113:349-535
BmHel-14	DF090440:698306-698437
BmHel-14	DF090340:2527617-2527744
BmHel-14	DF091552:1061-1200
BmHel-14	DF090386:1570811-1570937
BmHel-14	DF090378:969190-969324
BmHel-14	DF090332:6060123-6060232
BmHel-14	DF090340:3784698-3784807
BmHel-14	DF090324:4806833-4806940
BmHel-14	DF090317:4977834-4978027
BmHel-14	DF090325:4218999-4219132
BmHel-14	DF090349:4070597-4070721
BmHel-14	DF090336:2687062-2687212
BmHel-14	DF090398:1282443-1282586
BmHel-14	DF090330:2144573-2144720
BmHel-14	DF090319:5768726-5768866
BmHel-14	DF090344:4326257-4326440
BmHel-14	DF090346:3958555-3958663
BmHel-14	DF090346:1107576-1107709
BmHel-14	DF090334:3828097-3828204
BmHel-14	DF090488:133217-133349
BmHel-14	DF090336:579659-579808
BmHel-14	DF090377:1831906-1832034
BmHel-14	DF090341:2931971-2932104
BmHel-14	DF090658:14361-14489
BmHel-14	DF090388:1593875-1594002
BmHel-14	DF090397:363355-363439
BmHel-14	BABH01079032:2-83
BmHel-14	BABH01079032:70-170
BmHel-14	DF090322:7474003-7474186
BmHel-14	DF090316:8514793-8514914
BmHel-14	DF090329:1999707-1999834
BmHel-14	DF090338:3027464-3027597
BmHel-14	DF090335:1180313-1180419
BmHel-14	DF090355:542492-542642
BmHel-14	DF090336:2494842-2494969
BmHel-14	DF090337:1418993-1419125
BmHel-14	BABH01074087:52-184
BmHel-14	DF090377:445963-446090
BmHel-14	DF090429:739930-740042
BmHel-14	DF090387:1058432-1058537
BmHel-14	DF090541:124384-124491
BmHel-14	DF090395:1485181-1485287
BmHel-14	DF090324:5217869-5217976
BmHel-14	DF090332:1143760-1143892
BmHel-14	DF090323:4844146-4844229
BmHel-14	DF090327:4859823-4859954

BmHel-14	DF090391:579053-579182
BmHel-14	DF090353:41782-41923
BmHel-14	DF090374:1219039-1219181
BmHel-14	DF090340:1555277-1555399
BmHel-14	DF090368:1338588-1338693
BmHel-14	DF090363:2634878-2635030
BmHel-14	DF090326:6916353-6916463
BmHel-14	DF090325:4117087-4117204
BmHel-14	DF090349:1131092-1131196
BmHel-14	DF090349:2357130-2357311
BmHel-14	DF090396:1002321-1002454
BmHel-14	DF090406:592697-592842
BmHel-14	DF090322:4798685-4798815
BmHel-14	DF090321:4879817-4879962
BmHel-14	DF090332:2837494-2837634
BmHel-14	DF090318:8927731-8927850
BmHel-14	DF090340:217660-217780
BmHel-14	DF090367:2445588-2445715
BmHel-14	DF090335:3913676-3913819
BmHel-14	DF090326:719049-719177
BmHel-14	DF090353:387957-388089
BmHel-14	DF090468:141375-141507
BmHel-14	DF090431:533140-533243
BmHel-14	BABH01048827:596-726
BmHel-14	DF090321:7742674-7742755
BmHel-14	DF090321:7742755-7742853
BmHel-14	DF090351:3579250-3579382
BmHel-14	BABH01043953:7052-7192
BmHel-14	DF090330:4865951-4866079
BmHel-14	DF090428:619833-619912
BmHel-14	DF090369:1665844-1665968
BmHel-14	DF090382:1652728-1652859
BmHel-14	DF090333:30443-30549
BmHel-14	DF090348:4112795-4112912
BmHel-14	DF090354:2102622-2102775
BmHel-14	DF090354:1149745-1149898
BmHel-14	DF090318:5690344-5690445
BmHel-14	DF090323:7985854-7985997
BmHel-14	DF090325:2823560-2823688
BmHel-14	DF090353:223477-223606
BmHel-14	DF090342:2728805-2728953
BmHel-14	DF090448:406048-406176
BmHel-14	BABH01047057:1108-1237
BmHel-14	DF090329:1600395-1600523
BmHel-14	DF090412:195943-196121
BmHel-14	DF090504:63874-63993
BmHel-14	DF090325:2120640-2120755
BmHel-14	DF090319:4392483-4392584

BmHel-14	DF090420:896280-896458
BmHel-14	DF090448:80884-80999
BmHel-14	DF090390:1454793-1454926
BmHel-14	DF090326:4549866-4549966
BmHel-14	DF090331:4343358-4343463
BmHel-14	DF090316:3988330-3988473
BmHel-14	DF090321:5307296-5307428
BmHel-14	DF090382:237195-237328
BmHel-14	BABH01074573:421-548
BmHel-14	DF090351:2537063-2537196
BmHel-14	DF090322:1414745-1414840
BmHel-14	DF090330:439932-440031
BmHel-14	DF090325:5170669-5170768
BmHel-14	DF090320:6105892-6105991
BmHel-14	DF090375:2167603-2167702
BmHel-14	DF090463:60776-60875
BmHel-14	DF090320:1363999-1364096
BmHel-14	DF090438:341264-341439
BmHel-14	BABH01047971:157-266
BmHel-14	DF090367:648475-648588
BmHel-14	BABH01082627:1-173
BmHel-14	DF090365:1144197-1144307
BmHel-14	DF090316:8853776-8853895
BmHel-14	DF090319:7663398-7663521
BmHel-14	DF090403:683419-683521
BmHel-14	DF090319:6052573-6052671
BmHel-14	DF090344:2841115-2841213
BmHel-14	DF090342:2790401-2790488
BmHel-14	DF090342:2790472-2790562
BmHel-14	DF090319:6271181-6271313
BmHel-14	DF090367:670770-670903
BmHel-14	DF090421:969268-969400
BmHel-14	DF090342:939889-940021
BmHel-14	DF090861:11931-12063
BmHel-14	DF090340:708230-708371
BmHel-14	DF090332:6290959-6291077
BmHel-14	DF090340:4723287-4723387
BmHel-14	DF090340:4310897-4311021
BmHel-14	DF090358:1036655-1036761
BmHel-14	DF090340:1768529-1768626
BmHel-14	DF090346:798350-798447
BmHel-14	DF090339:2635630-2635727
BmHel-14	DF090342:1034483-1034580
BmHel-14	DF090413:866298-866395
BmHel-14	DF090319:2508221-2508353
BmHel-14	DF090319:5317294-5317426
BmHel-14	DF090418:913328-913453
BmHel-14	DF090392:1291537-1291669

BmHel-14	DF090340:1248521-1248653
BmHel-14	DF090373:1412700-1412832
BmHel-14	DF090356:1415741-1415873
BmHel-14	DF090344:706420-706556
BmHel-14	DF090355:1980588-1980691
BmHel-14	DF090365:1772673-1772809
BmHel-14	DF090350:743782-743916
BmHel-14	DF090342:692637-692760
BmHel-14	DF090457:28966-29098
BmHel-14	DF091346:4455-4588
BmHel-14	DF090365:1309533-1309677
BmHel-14	DF090432:779969-780108
BmHel-14	DF090369:792088-792198
BmHel-14	DF090361:820188-820295
BmHel-14	DF090325:6046059-6046191
BmHel-14	DF090344:704318-704450
BmHel-14	DF090375:1485896-1486028
BmHel-14	DF090476:221796-221891
BmHel-14	DF090337:2937424-2937558
BmHel-14	DF090336:3188259-3188431
BmHel-14	DF090351:2984633-2984758
BmHel-14	DF090382:326790-326876
BmHel-14	DF090382:326706-326790
BmHel-14	DF090369:1571363-1571504
BmHel-14	DF090319:2031740-2031862
BmHel-14	DF090354:2329499-2329627
BmHel-14	DF090321:3307024-3307119
BmHel-14	DF090323:3893346-3893476
BmHel-14	BABH01075556:76-206
BmHel-14	BABH01075452:217-347
BmHel-14	DF090400:735973-736102
BmHel-14	DF090389:1456991-1457078
BmHel-14	DF090355:1710758-1710851
BmHel-14	DF090347:4042653-4042793
BmHel-14	DF090351:3036152-3036284
BmHel-14	DF090330:4862082-4862213
BmHel-14	DF090325:3012077-3012211
BmHel-14	DF090382:1589923-1590055
BmHel-14	BABH01050020:606-737
BmHel-14	DF090575:51953-52081
BmHel-14	DF090346:1357152-1357279
BmHel-14	DF090376:1485369-1485461
BmHel-14	DF090337:183628-183737
BmHel-14	DF090318:1923644-1923776
BmHel-14	DF090336:4730769-4730901
BmHel-14	DF090480:6769-6901
BmHel-14	DF090734:21727-21859
BmHel-14	DF090326:40457-40588

BmHel-14	DF090546:13011-13139
BmHel-14	DF090318:393598-393726
BmHel-14	DF090378:774693-774860
BmHel-14	DF090342:1506117-1506218
BmHel-14	DF090345:2056778-2056910
BmHel-14	DF090325:4798228-4798357
BmHel-14	DF090340:2491325-2491440
BmHel-14	DF090351:2222675-2222769
BmHel-14	DF090346:1034832-1034990
BmHel-14	BABH01057285:18-184
BmHel-14	DF090349:2370470-2370607
BmHel-14	DF090317:10005857-10005989
BmHel-14	DF090422:786360-786489
BmHel-14	DF090866:1847-1959
BmHel-14	DF090316:15561942-15562031
BmHel-14	DF090324:5623638-5623727
BmHel-14	DF090344:3388376-3388507
BmHel-14	DF090324:8016055-8016187
BmHel-14	DF090324:39729-39861
BmHel-14	DF090405:64348-64447
BmHel-14	DF090336:5055400-5055499
BmHel-14	DF090375:1779656-1779818
BmHel-14	DF090414:889785-889917
BmHel-14	DF090324:7246158-7246256
BmHel-14	DF090388:1139114-1139201
BmHel-14	DF090379:1818790-1818920
BmHel-14	DF090510:42854-42989
BmHel-14	DF090525:1871-2003
BmHel-14	DF090377:375544-375657
BmHel-14	DF090340:3286139-3286250
BmHel-14	BABH01045887:1-134
BmHel-14	DF090329:6646460-6646548
BmHel-14	DF090346:277632-277751
BmHel-14	DF090680:2358-2490
BmHel-14	DF090368:1992941-1993037
BmHel-14	DF090423:361629-361717
BmHel-14	DF090357:2041879-2042041
BmHel-14	DF090334:3525443-3525605
BmHel-14	DF090325:2710026-2710158
BmHel-14	DF090341:2174171-2174303
BmHel-14	DF090383:1608518-1608650
BmHel-14	DF090386:303861-303993
BmHel-14	DF090400:941536-941697
BmHel-14	DF090330:3586755-3586850
BmHel-14	DF090327:3105720-3105806
BmHel-14	DF090403:55506-55613
BmHel-14	DF090382:104571-104702
BmHel-14	DF090383:233241-233401

BmHel-14	DF090346:210096-210179
BmHel-14	DF090597:22210-22293
BmHel-14	DF090417:502723-502892
BmHel-14	DF090323:2022245-2022379
BmHel-14	BABH01054332:58-188
BmHel-14	DF090886:16969-17099
BmHel-14	BABH01052496:695-825
BmHel-14	DF090362:1913493-1913578
BmHel-14	DF090446:468550-468679
BmHel-14	DF090325:3342264-3342382
BmHel-14	DF090337:2282399-2282503
BmHel-14	BABH01070627:359-463
BmHel-14	DF090316:4752829-4752913
BmHel-14	DF090335:5315830-5315916
BmHel-14	DF090328:2799521-2799649
BmHel-14	DF090363:2246203-2246310
BmHel-14	BABH01081923:1-158
BmHel-14	DF090386:1974498-1974613
BmHel-14	DF090366:1697321-1697401
BmHel-14	DF090353:1475257-1475338
BmHel-14	DF090456:139651-139766
BmHel-14	DF090405:429374-429453
BmHel-14	DF090728:597-676
BmHel-14	DF090323:22483-22609
BmHel-14	DF090423:622074-622166
BmHel-14	DF090332:2886915-2887013
BmHel-14	DF090435:649718-649800
BmHel-14	DF090321:8246639-8246793
BmHel-14	DF090757:2223-2347
BmHel-14	DF090365:231664-231777
BmHel-14	DF090318:2696704-2696857
BmHel-14	DF090378:2071768-2071847
BmHel-14	DF090331:5906388-5906511
BmHel-14	DF090333:5523196-5523278
BmHel-14	DF090316:1282070-1282173
BmHel-14	DF090357:3204642-3204794
BmHel-14	DF090324:949984-950103
BmHel-14	DF090332:1603544-1603693
BmHel-14	DF090351:1944507-1944657
BmHel-14	DF090319:451312-451462
BmHel-14	DF090318:8327127-8327275
BmHel-14	DF090338:4861761-4861910
BmHel-14	DF090338:154855-155004
BmHel-14	DF090323:5224029-5224180
BmHel-14	DF090327:814422-814571
BmHel-14	DF090339:2238518-2238675
BmHel-14	DF090328:1503145-1503294
BmHel-14	DF090358:3457440-3457589

BmHel-14	DF090375:2046895-2047010
BmHel-14	DF090336:4771422-4771571
BmHel-14	DF090383:486374-486522
BmHel-14	DF090365:3027487-3027639
BmHel-14	DF090324:2490159-2490308
BmHel-14	DF090838:10996-11148
BmHel-14	DF090787:14248-14402
BmHel-14	DF090751:11864-12016
BmHel-14	DF090322:5721106-5721256
BmHel-14	DF090367:1294457-1294609
BmHel-14	DF090365:1640654-1640803
BmHel-14	DF090422:1109680-1109799
BmHel-14	DF090354:3460091-3460238
BmHel-14	DF090858:5781-5930
BmHel-14	DF090398:1570631-1570720
BmHel-14	DF090369:1411182-1411269
BmHel-14	DF090316:12396798-12396894
BmHel-14	DF090343:4248834-4248982
BmHel-14	DF090393:706497-706644
BmHel-14	DF090382:275645-275754
BmHel-14	DF090408:911587-911732
BmHel-14	DF090329:5121429-5121519
BmHel-14	DF090374:1048267-1048411
BmHel-14	DF090319:5584705-5584784
BmHel-14	DF090350:2011886-2011967
BmHel-14	DF090322:5122085-5122228
BmHel-14	DF090379:606798-606884
BmHel-14	DF090341:186460-186594
BmHel-14	DF090385:1527382-1527477
BmHel-14	DF090378:440121-440204
BmHel-14	DF090416:293155-293238
BmHel-14	DF090331:3772577-3772716
BmHel-14	DF090420:152091-152171
BmHel-14	DF090320:8568884-8569026
BmHel-14	DF090361:2479889-2480032
BmHel-14	DF090321:5199909-5200056
BmHel-14	DF090414:304230-304373
BmHel-14	DF090632:9677-9822
BmHel-14	DF090340:3815752-3815865
BmHel-14	DF090490:10140-10259
BmHel-14	DF090437:138192-138334
BmHel-14	DF090328:6588250-6588332
BmHel-14	DF090317:7553047-7553189
BmHel-14	DF090438:182621-182763
BmHel-14	DF090365:2823002-2823144
BmHel-14	BABH01052227:290-381
BmHel-14	DF090397:1655862-1656003
BmHel-14	DF090320:5154375-5154516

BmHel-14	DF090316:16170881-16171022
BmHel-14	DF090330:5866355-5866448
BmHel-14	DF090338:3505392-3505481
BmHel-14	BABH01069994:1-144
BmHel-14	DF090343:4458313-4458452
BmHel-14	DF090348:3119983-3120123
BmHel-14	DF090621:32243-32383
BmHel-14	DF090357:1180216-1180355
BmHel-14	DF090342:2563375-2563484
BmHel-14	DF090374:1907872-1908010
BmHel-14	DF090321:731233-731371
BmHel-14	DF090318:6937684-6937821
BmHel-14	DF090346:3026-3161
BmHel-14	DF090377:2122504-2122641
BmHel-14	DF090321:6125562-6125657
BmHel-14	DF090327:2357360-2357496
BmHel-14	DF090319:2873818-2873953
BmHel-14	DF090324:8040164-8040261
BmHel-14	DF091302:2795-2900
BmHel-14	DF090468:127617-127751
BmHel-14	DF090316:10536044-10536177
BmHel-14	DF090330:1188082-1188216
BmHel-14	DF090317:2007031-2007164
BmHel-14	DF090362:2258514-2258594
BmHel-14	DF090574:49731-49864
BmHel-14	DF090327:1678952-1679039
BmHel-14	DF090352:3831600-3831681
BmHel-14	DF090322:5674824-5674956
BmHel-14	DF090322:4003707-4003839
BmHel-14	DF090372:915962-916094
BmHel-14	DF090321:5364092-5364224
BmHel-14	DF090382:773495-773627
BmHel-14	DF090336:2846263-2846395
BmHel-14	DF090360:2708376-2708500
BmHel-14	DF090360:2724937-2725069
BmHel-14	DF090432:315511-315643
BmHel-14	DF090324:729202-729334
BmHel-14	DF090345:1432722-1432854
BmHel-14	DF090399:1224211-1224342
BmHel-14	BABH01081015:369-501
BmHel-14	DF090415:105490-105622
BmHel-14	DF090671:16802-16939
BmHel-14	DF090407:694933-695031
BmHel-14	DF090318:6390689-6390781
BmHel-14	DF090355:2795028-2795159
BmHel-14	DF090394:602531-602662
BmHel-14	DF090318:7690471-7690572
BmHel-14	DF090381:1910387-1910475

BmHel-14	DF090349:2647621-2647721
BmHel-14	DF090321:520980-521109
BmHel-14	DF090412:1260468-1260597
BmHel-14	DF090375:2152710-2152839
BmHel-14	DF090356:1124313-1124432
BmHel-14	DF090498:168251-168330
BmHel-14	DF090429:61624-61752
BmHel-14	DF090317:8574766-8574893
BmHel-14	BABH01065207:1-128
BmHel-14	DF090403:1519211-1519338
BmHel-14	DF090366:1442851-1442938
BmHel-14	DF090349:3386436-3386523
BmHel-14	DF090465:110865-110960
BmHel-14	BABH01080020:1-123
BmHel-14	DF090359:3247274-3247396
BmHel-14	DF090319:1067681-1067768
BmHel-14	DF090328:3624242-3624368
BmHel-14	DF090335:1479874-1479995
BmHel-14	DF090377:460553-460674
BmHel-14	DF090330:5266116-5266236
BmHel-14	DF090615:36103-36223
BmHel-14	DF090327:1806971-1807095
BmHel-14	DF090347:3727946-3728065
BmHel-14	BABH01067997:1-117
BmHel-14	BABH01064226:1-117
BmHel-14	DF090712:30602-30718
BmHel-14	DF090398:797289-797403
BmHel-14	DF090332:3420663-3420749
BmHel-14	DF090365:236227-236312
BmHel-14	BABH01044598:633-747
BmHel-14	DF090344:4458404-4458518
BmHel-14	DF090348:1760907-1760990
BmHel-14	BABH01085565:385-497
BmHel-14	BABH01088654:28-140
BmHel-14	BABH01053531:2-84
BmHel-14	DF090331:1500513-1500624
BmHel-14	DF090403:1726234-1726344
BmHel-14	BABH01045444:305-415
BmHel-14	DF090358:943684-943793
BmHel-14	DF090374:1408849-1408958
BmHel-14	DF090579:14882-14992
BmHel-14	DF090372:1439975-1440080
BmHel-14	DF090316:11816687-11816766
BmHel-14	DF090369:2485844-2485923
BmHel-14	BABH01055020:685-768
BmHel-14	DF090355:3707195-3707303
BmHel-14	DF090336:2892093-2892201
BmHel-14	BABH01064023:1-107

BmHel-14	BABH01088041:1-108
BmHel-14	DF090410:1156694-1156800
BmHel-14	DF090632:36984-37090
BmHel-14	DF090352:160440-160545
BmHel-14	BABH01063851:571-676
BmHel-14	BABH01052837:175-280
BmHel-14	DF090344:1441897-1442001
BmHel-14	DF090337:2674402-2674505
BmHel-14	DF090347:1680882-1680984
BmHel-14	BABH01074328:508-610
BmHel-14	BABH01046609:365-467
BmHel-14	DF091109:7697-7799
BmHel-14	DF090448:708563-708665
BmHel-14	DF090359:1629209-1629310
BmHel-14	BABH01063651:577-677
BmHel-14	DF090925:3-101
BmHel-14	DF090358:536741-536839
BmHel-14	DF090334:4014396-4014494
BmHel-14	DF090340:2317299-2317395
BmHel-14	DF090316:2333604-2333700
BmHel-14	DF090360:840129-840225
BmHel-14	DF090365:1665765-1665860
BmHel-14	BABH01059759:1-94
BmHel-14	DF090394:476529-476620
BmHel-14	DF090356:3747805-3747893
BmHel-14	DF090352:1613328-1613423
BmHel-14	DF090342:3127729-3127819
BmHel-14	DF090319:6558705-6558794
BmHel-14	DF090354:762391-762479
BmHel-14	BABH01064767:1-94
BmHel-14	DF090391:942835-942923
BmHel-14	BABH01066053:574-661
BmHel-14	DF090340:3260604-3260690
BmHel-14	DF090338:2541842-2541922
BmHel-14	BABH01061349:483-564
BmHel-14	BABH01061537:357-438
BmHel-14	DF090443:34762-34842
BmHel-14	DF090385:527801-527881
BmHel-14	BABH01069511:18-98
BmHel-14	DF090473:329518-329597
BmHel-15	DF090327:5359478-5359619
BmHel-15	DF090340:2968129-2968323
BmHel-15	DF090354:1444132-1444334
BmHel-15	DF090364:1298259-1298463
BmHel-15	DF090336:4733594-4733800
BmHel-15	DF090353:778328-778536
BmHel-15	DF090352:3176112-3176321

BmHel-15	DF090344:1086647-1086857
BmHel-15	DF090375:910168-910378
BmHel-15	DF090392:24195-24407
BmHel-15	DF090351:612823-613035
BmHel-15	DF090413:1250155-1250388
BmHel-15	DF090354:1967759-1968001
BmHel-15	DF090439:338030-338274
BmHel-15	DF090369:1232262-1232509
BmHel-15	DF090526:29947-30198
BmHel-15	DF090320:1136308-1136560
BmHel-15	DF090358:28066-28318
BmHel-15	DF090389:363847-364099
BmHel-15	DF090323:1295285-1295541
BmHel-15	DF090324:775559-775816
BmHel-15	DF090317:3419597-3419858
BmHel-15	DF090334:3157687-3157950
BmHel-15	DF090362:1525185-1525454
BmHel-15	DF090336:4960224-4960496
BmHel-15	DF090449:641807-642091
BmHel-15	DF090319:4237216-4237500
BmHel-15	DF090316:10590504-10590794
BmHel-15	DF090369:1265369-1265663
BmHel-15	DF090319:6161960-6162255
BmHel-15	DF090403:478833-479130
BmHel-15	DF090352:2121566-2121864
BmHel-15	DF090328:937769-938070
BmHel-15	DF090340:3376223-3376524
BmHel-15	BABH01051110:312-614
BmHel-15	DF090426:310811-311114
BmHel-15	DF090433:374258-374561
BmHel-15	DF090323:7166632-7166943
BmHel-15	DF090345:3326895-3327207
BmHel-15	DF090353:3462721-3463036
BmHel-15	DF090374:1644857-1645173
BmHel-15	DF090352:2821911-2822229
BmHel-15	DF090367:272770-273090
BmHel-15	DF090355:3635667-3635988
BmHel-15	DF090326:5698272-5698594
BmHel-15	DF090345:2435797-2436120
BmHel-15	DF090412:1106437-1106760
BmHel-15	DF090319:8405019-8405342
BmHel-15	DF090335:2039737-2040060
BmHel-15	DF090332:3984292-3984616
BmHel-15	DF090325:4507031-4507358
BmHel-15	DF090336:5242983-5243312
BmHel-15	DF090323:56533-56863
BmHel-15	DF090318:5030368-5030701
BmHel-15	DF090406:1315994-1316328

BmHel-15	DF090351:80235-80574
BmHel-15	DF090484:182681-183027
BmHel-15	DF090319:2445006-2445353
BmHel-15	DF090403:1222108-1222456
BmHel-15	DF090347:2875635-2875985
BmHel-15	DF090415:78320-78671
BmHel-15	DF090345:2272245-2272597
BmHel-15	DF090323:4535135-4535488
BmHel-15	DF090405:479635-479991
BmHel-15	DF090323:7178344-7178701
BmHel-15	DF090328:4799132-4799490
BmHel-15	DF090436:541561-541919
BmHel-15	DF090328:3446658-3447016
BmHel-15	DF090344:1747069-1747428
BmHel-15	DF090804:2727-3086
BmHel-15	DF090320:836471-836830
BmHel-15	DF090366:159325-159684
BmHel-15	DF090320:1450594-1450954
BmHel-15	BABH01042956:5944-6305
BmHel-15	DF090398:1154154-1154516
BmHel-15	DF090356:1174853-1175215
BmHel-15	DF090337:3824085-3824447
BmHel-15	DF090358:3200930-3201292
BmHel-15	DF090364:1430999-1431363
BmHel-15	DF090410:874090-874454
BmHel-15	DF090475:168856-169221
BmHel-15	DF090318:6797206-6797572
BmHel-15	DF090555:44759-45125
BmHel-15	DF090329:5563017-5563384
BmHel-15	DF090328:3289055-3289423
BmHel-15	DF090340:1730484-1730855
BmHel-15	DF090321:5111379-5111751
BmHel-15	DF090372:2183189-2183562
BmHel-15	DF090415:859290-859663
BmHel-15	DF090576:8766-9139
BmHel-15	DF090379:1402683-1403056
BmHel-15	DF090375:2363321-2363695
BmHel-15	BABH01043095:3606-3980
BmHel-15	DF090361:3037813-3038187
BmHel-15	DF090356:2510272-2510647
BmHel-15	DF090319:6547428-6547804
BmHel-15	DF090437:316540-316917
BmHel-15	DF090404:1094035-1094413
BmHel-15	DF090399:11897-12275
BmHel-15	DF090322:1990025-1990405
BmHel-15	DF090361:864624-865004
BmHel-15	DF090436:199679-200060
BmHel-15	DF090316:15671515-15671897

BmHel-15	DF090330:5582264-5582646
BmHel-15	DF090380:1661744-1662126
BmHel-15	DF090324:5375946-5376328
BmHel-15	DF090438:90313-90696
BmHel-15	DF090336:5319369-5319753
BmHel-15	DF090351:1988777-1989161
BmHel-15	DF090370:2389039-2389424
BmHel-15	DF090711:16750-17135
BmHel-15	DF090336:4543029-4543415
BmHel-15	DF090350:1504866-1505252
BmHel-15	DF090319:4402909-4403296
BmHel-15	DF090421:90253-90640
BmHel-15	DF090355:3284399-3284787
BmHel-15	DF090324:7304472-7304860
BmHel-15	DF090361:1171526-1171915
BmHel-15	DF090323:2837423-2837812
BmHel-15	DF090355:3414057-3414446
BmHel-15	DF090334:793379-793769
BmHel-15	DF090359:1260336-1260726
BmHel-15	DF090359:2308198-2308588
BmHel-15	DF090325:6688219-6688610
BmHel-15	DF090334:3647933-3648324
BmHel-15	DF090362:2115432-2115823
BmHel-15	DF090349:2938796-2939188
BmHel-15	DF090378:52204-52596
BmHel-15	DF090328:3920989-3921381
BmHel-15	DF090406:595781-596174
BmHel-15	DF090322:4930123-4930516
BmHel-15	DF090330:593779-594172
BmHel-15	DF090324:6779129-6779522
BmHel-15	DF090341:1706006-1706399
BmHel-15	DF090318:5679110-5679504
BmHel-15	DF090412:696592-696986
BmHel-15	DF090621:1217-1612
BmHel-15	DF090378:1385335-1385730
BmHel-15	DF090335:2064147-2064542
BmHel-15	DF090316:12755796-12756192
BmHel-15	DF090327:2716068-2716464
BmHel-15	DF090415:42402-42798
BmHel-15	DF090323:6734092-6734489
BmHel-15	DF090331:2685967-2686364
BmHel-15	DF090456:271479-271876
BmHel-15	BABH01045793:2191-2588
BmHel-15	DF090406:1120296-1120693
BmHel-15	DF090388:210633-211030
BmHel-15	DF090339:3719721-3720119
BmHel-15	DF090425:542539-542937
BmHel-15	DF090336:4134751-4135150

BmHel-15	DF090424:219174-219573
BmHel-15	DF090377:89856-90255
BmHel-15	DF090330:1528329-1528728
BmHel-15	DF090382:1029550-1029949
BmHel-15	DF090373:1591428-1591828
BmHel-15	DF090361:321452-321852
BmHel-15	DF090341:4428051-4428451
BmHel-15	DF090343:766670-767071
BmHel-15	DF090384:372255-372656
BmHel-15	DF090318:5961945-5962347
BmHel-15	DF090378:286005-286407
BmHel-15	DF090360:1560742-1561144
BmHel-15	DF090407:1514485-1514888
BmHel-15	DF090351:1854653-1855056
BmHel-15	DF090363:2870083-2870486
BmHel-15	DF090316:13641361-13641764
BmHel-15	DF090342:4405381-4405784
BmHel-15	DF090353:560367-560770
BmHel-15	DF090328:633814-634217
BmHel-15	DF090356:1972410-1972813
BmHel-15	DF090349:2902511-2902916
BmHel-15	DF090323:8000738-8001143
BmHel-15	DF090351:209483-209888
BmHel-15	DF090317:10731563-10731968
BmHel-15	DF090354:1855851-1856256
BmHel-15	DF090326:4442562-4442967
BmHel-15	DF090348:4101143-4101548
BmHel-15	DF090340:2093223-2093628
BmHel-15	DF090322:2828416-2828822
BmHel-15	DF090496:75508-75914
BmHel-15	DF090316:11118562-11118968
BmHel-15	DF090331:2956203-2956609
BmHel-15	DF090345:4515409-4515815
BmHel-15	DF090388:1158269-1158675
BmHel-15	DF090369:1204552-1204958
BmHel-15	DF090316:15892091-15892498
BmHel-15	DF090338:2652987-2653394
BmHel-15	DF090374:72406-72813
BmHel-15	DF090328:2241811-2242218
BmHel-15	DF090365:2667117-2667524
BmHel-15	DF090339:1270501-1270909
BmHel-15	DF090358:2676097-2676505
BmHel-15	DF090319:4662-5070
BmHel-15	DF090318:5516469-5516877
BmHel-15	DF090373:524169-524578
BmHel-15	DF090361:3198073-3198482
BmHel-15	DF090316:3475901-3476311
BmHel-15	DF090317:8021145-8021555

BmHel-15	DF090334:5653834-5654244
BmHel-15	DF090343:3280345-3280755
BmHel-15	DF090407:613093-613503
BmHel-15	DF090355:3463858-3464268
BmHel-15	DF090339:5071097-5071507
BmHel-15	DF090335:3275583-3275993
BmHel-15	DF090321:5051395-5051805
BmHel-15	DF090389:762810-763220
BmHel-15	DF090322:3454236-3454646
BmHel-15	DF090328:4745954-4746365
BmHel-15	DF090316:12008528-12008939
BmHel-15	DF090354:1232815-1233226
BmHel-15	DF090396:1399859-1400270
BmHel-15	DF090363:417088-417499
BmHel-15	DF090349:3381304-3381715
BmHel-15	DF090363:1171934-1172345
BmHel-15	DF090374:1632697-1633108
BmHel-15	DF090388:357554-357965
BmHel-15	DF090453:434416-434827
BmHel-15	DF090432:608855-609267
BmHel-15	DF090337:4064801-4065213
BmHel-15	DF090375:82233-82645
BmHel-15	DF090316:5416711-5417124
BmHel-15	DF090411:435762-436175
BmHel-15	BABH01046000:1355-1768
BmHel-15	DF090325:1003753-1004166
BmHel-15	DF090327:1389702-1390115
BmHel-15	DF090396:1385246-1385659
BmHel-15	DF090316:14947946-14948359
BmHel-15	DF090327:3195035-3195448
BmHel-15	DF090340:4620316-4620730
BmHel-15	DF090337:469445-469859
BmHel-15	DF090413:745013-745427
BmHel-15	DF090355:1003047-1003461
BmHel-15	DF090396:681840-682254
BmHel-15	DF090363:328823-329237
BmHel-15	DF090414:742009-742423
BmHel-15	DF090362:3108971-3109386
BmHel-15	DF090329:5326003-5326418
BmHel-15	DF090317:9488899-9489314
BmHel-15	DF090402:1050837-1051252
BmHel-15	DF090362:1353098-1353513
BmHel-15	DF090333:3632158-3632573
BmHel-15	DF090331:176796-177211
BmHel-15	DF090358:1018243-1018658
BmHel-15	DF090328:2870286-2870701
BmHel-15	DF090437:463887-464302
BmHel-15	DF090438:482293-482708

BmHel-15	DF090535:39576-39991
BmHel-15	DF090486:37294-37709
BmHel-15	DF090507:90362-90777
BmHel-15	DF090342:4529207-4529623
BmHel-15	DF090359:2873305-2873721
BmHel-15	DF090377:176146-176562
BmHel-15	DF090326:6379071-6379487
BmHel-15	DF090359:2713229-2713645
BmHel-15	DF090318:3471548-3471964
BmHel-15	DF090368:2500049-2500465
BmHel-15	DF090364:230214-230630
BmHel-15	DF090343:74838-75255
BmHel-15	DF090319:8796061-8796478
BmHel-15	DF090317:10095264-10095681
BmHel-15	DF090397:319793-320210
BmHel-15	DF090368:1423818-1424235
BmHel-15	DF090456:307710-308127
BmHel-15	DF090316:6251339-6251756
BmHel-15	DF090367:187122-187539
BmHel-15	DF090355:2855868-2856286
BmHel-15	DF090328:4029781-4030199
BmHel-15	DF090335:5560768-5561186
BmHel-15	DF090332:262631-263049
BmHel-15	DF090344:1483146-1483565
BmHel-15	DF090317:2693355-2693774
BmHel-15	DF090357:404894-405313
BmHel-15	DF090342:2622312-2622731
BmHel-15	DF090342:514512-514932
BmHel-15	DF090347:4182971-4183391
BmHel-15	DF090322:3003725-3004145
BmHel-15	DF090334:4466780-4467200
BmHel-15	DF090627:25344-25764
BmHel-15	DF090354:2795552-2795972
BmHel-15	DF090365:1958719-1959139
BmHel-15	DF090459:373891-374311
BmHel-15	DF090443:545025-545445
BmHel-15	DF090316:4914265-4914686
BmHel-15	DF090360:794221-794642
BmHel-15	DF090368:535402-535823
BmHel-15	DF090321:1875859-1876280
BmHel-15	DF090482:45073-45494
BmHel-15	DF090359:769774-770195
BmHel-15	DF090333:2307213-2307634
BmHel-15	DF090335:2437046-2437468
BmHel-15	DF090328:5558815-5559237
BmHel-15	DF090391:372035-372457
BmHel-15	DF090396:345473-345895
BmHel-15	DF090360:2654553-2654975

BmHel-15	DF090317:1202616-1203038
BmHel-15	DF090358:2031079-2031501
BmHel-15	DF090404:463626-464049
BmHel-15	DF090329:5390725-5391148
BmHel-15	DF090466:139765-140188
BmHel-15	DF090348:1211821-1212244
BmHel-15	DF090366:1307334-1307757
BmHel-15	DF090368:924544-924967
BmHel-15	DF090321:4045322-4045745
BmHel-15	DF090373:1090558-1090981
BmHel-15	DF090351:1668223-1668646
BmHel-15	DF090320:7166153-7166577
BmHel-15	DF090403:565834-566258
BmHel-15	DF090323:4933492-4933916
BmHel-15	DF090329:5529812-5530236
BmHel-15	DF090452:503394-503818
BmHel-15	DF090327:1080343-1080767
BmHel-15	DF090318:7066635-7067059
BmHel-15	DF090347:3165120-3165544
BmHel-15	DF090385:129570-129994
BmHel-15	DF090352:2520250-2520674
BmHel-15	DF090325:2153707-2154131
BmHel-15	DF090339:219425-219849
BmHel-15	DF090381:1893084-1893508
BmHel-15	DF090335:3305122-3305546
BmHel-15	DF090332:2383348-2383772
BmHel-15	DF090366:723252-723677
BmHel-15	DF090328:2552821-2553246
BmHel-15	DF090324:5451436-5451861
BmHel-15	DF090318:3356909-3357334
BmHel-15	DF090362:243452-243877
BmHel-15	DF090360:954103-954528
BmHel-15	DF090363:2555544-2555969
BmHel-15	DF090316:1269078-1269503
BmHel-15	DF090333:5552417-5552842
BmHel-15	DF090329:4107116-4107541
BmHel-15	DF090376:545245-545670
BmHel-15	DF090316:14753252-14753678
BmHel-15	DF090361:2649768-2650194
BmHel-15	DF090386:523797-524223
BmHel-15	DF090374:882090-882516
BmHel-15	DF090665:10099-10525
BmHel-15	DF090344:2096688-2097114
BmHel-15	DF090332:4424987-4425413
BmHel-15	DF090333:4132194-4132620
BmHel-15	DF090443:28395-28821
BmHel-15	DF090317:5619789-5620215
BmHel-15	DF090325:4828592-4829018

BmHel-15	DF090347:2198348-2198774
BmHel-15	DF090330:3201757-3202183
BmHel-15	DF090367:2709711-2710138
BmHel-15	DF090349:3085835-3086262
BmHel-15	DF090377:342068-342495
BmHel-15	DF090346:976260-976687
BmHel-15	DF090329:5513416-5513843
BmHel-15	DF090397:1475437-1475864
BmHel-15	DF090383:1621472-1621899
BmHel-15	DF090573:41501-41928
BmHel-15	DF090319:16462-16889
BmHel-15	DF090332:4865133-4865560
BmHel-15	DF090326:3274350-3274777
BmHel-15	DF090318:5796732-5797159
BmHel-15	DF090364:255411-255838
BmHel-15	DF090332:4879611-4880038
BmHel-15	DF090317:8741732-8742159
BmHel-15	DF090369:869904-870331
BmHel-15	DF090433:635937-636364
BmHel-15	DF090363:2956183-2956610
BmHel-15	DF090349:1891270-1891697
BmHel-15	DF090356:3380066-3380493
BmHel-15	DF090333:2189094-2189521
BmHel-15	DF090351:2259246-2259673
BmHel-15	DF090317:4747239-4747666
BmHel-15	DF090348:3743430-3743857
BmHel-15	DF090447:484253-484680
BmHel-15	DF090318:2872981-2873409
BmHel-15	DF090350:1859758-1860186
BmHel-15	DF090326:1172637-1173065
BmHel-15	DF090338:1765372-1765800
BmHel-15	DF090330:4298516-4298944
BmHel-15	DF090317:353125-353553
BmHel-15	DF090355:1400232-1400660
BmHel-15	DF090458:255414-255842
BmHel-15	DF090344:2525923-2526351
BmHel-15	DF090325:4893235-4893663
BmHel-15	DF090362:1768072-1768500
BmHel-15	DF090323:5675069-5675497
BmHel-15	DF090418:469081-469509
BmHel-15	DF090334:1069311-1069739
BmHel-15	DF090352:1435848-1436276
BmHel-15	DF090352:144873-145301
BmHel-15	DF090416:810968-811396
BmHel-15	DF090324:6892995-6893423
BmHel-15	DF090337:4333560-4333988
BmHel-15	DF090360:2378093-2378521
BmHel-15	DF090393:1643410-1643838

BmHel-15	DF090434:296427-296855
BmHel-15	DF090325:2694607-2695036
BmHel-15	DF090404:1276148-1276577
BmHel-15	DF090362:1388438-1388867
BmHel-15	DF090345:2122886-2123315
BmHel-15	DF090337:2010252-2010681
BmHel-15	DF090323:1801249-1801678
BmHel-15	DF090323:1867836-1868265
BmHel-15	DF090317:9434288-9434717
BmHel-15	DF090428:724289-724718
BmHel-15	DF090368:1703650-1704079
BmHel-15	DF090439:292328-292757
BmHel-15	DF090543:81147-81576
BmHel-15	DF090343:4327785-4328214
BmHel-15	DF090329:3767217-3767646
BmHel-15	DF090317:3181429-3181858
BmHel-15	DF090422:1095820-1096249
BmHel-15	DF090321:3904731-3905160
BmHel-15	DF090359:1699061-1699490
BmHel-15	DF090350:3778312-3778741
BmHel-15	DF090371:2116220-2116649
BmHel-15	DF090326:3293966-3294395
BmHel-15	DF090360:2177971-2178400
BmHel-15	DF090317:8048347-8048776
BmHel-15	DF090366:2235420-2235849
BmHel-15	DF090321:651023-651452
BmHel-15	DF090420:928154-928583
BmHel-15	DF090441:587198-587627
BmHel-15	DF090360:1645496-1645925
BmHel-15	DF090344:660516-660946
BmHel-15	DF090388:1134997-1135427
BmHel-15	DF090324:1473316-1473746
BmHel-15	DF090318:1313984-1314414
BmHel-15	DF090362:2517966-2518396
BmHel-15	DF090351:1952393-1952823
BmHel-15	DF090331:1234523-1234953
BmHel-15	DF090330:711560-711990
BmHel-15	DF090414:1162715-1163145
BmHel-15	DF090341:708909-709339
BmHel-15	DF090637:8111-8541
BmHel-15	DF090316:2271577-2272007
BmHel-15	DF090318:2694590-2695020
BmHel-15	DF090368:1808155-1808585
BmHel-15	DF090321:3654210-3654640
BmHel-15	DF090404:1282599-1283029
BmHel-15	DF090354:3359795-3360225
BmHel-15	DF090342:434893-435323
BmHel-15	DF090340:3611994-3612424

BmHel-15	DF090323:1751680-1752110
BmHel-15	DF090476:206696-207126
BmHel-15	DF090317:10654313-10654743
BmHel-15	DF090397:1369102-1369532
BmHel-15	DF090334:5890357-5890787
BmHel-15	DF090352:3380394-3380824
BmHel-15	DF090435:305898-306328
BmHel-15	DF090580:13044-13474
BmHel-15	DF090408:389660-390090
BmHel-15	DF090383:892839-893269
BmHel-15	DF090396:1126987-1127417
BmHel-15	DF090353:3107783-3108213
BmHel-15	DF090317:4893581-4894011
BmHel-15	BABH01068220:66-496
BmHel-15	DF090356:3387607-3388038
BmHel-15	DF090323:3970326-3970757
BmHel-15	DF090323:7330562-7330993
BmHel-15	DF090412:814527-814958
BmHel-15	DF090355:1373535-1373966
BmHel-15	DF090420:297368-297799
BmHel-15	DF090398:821885-822316
BmHel-15	DF090336:4184085-4184516
BmHel-15	DF090335:1220335-1220766
BmHel-15	DF090334:3734751-3735182
BmHel-15	DF090409:616039-616470
BmHel-15	DF090443:470180-470611
BmHel-15	DF090436:477549-477980
BmHel-15	DF090316:12757463-12757894
BmHel-15	DF090316:11233174-11233605
BmHel-15	DF090339:3760742-3761173
BmHel-15	DF090328:749900-750331
BmHel-15	DF090342:3055865-3056296
BmHel-15	DF090362:1464414-1464845
BmHel-15	DF090406:320328-320759
BmHel-15	DF090323:2088576-2089007
BmHel-15	DF090366:1664510-1664941
BmHel-15	DF090437:277215-277646
BmHel-15	DF090339:2193691-2194122
BmHel-15	DF090319:1759654-1760085
BmHel-15	DF090353:2036710-2037141
BmHel-15	DF090334:4920597-4921028
BmHel-15	DF090444:127556-127987
BmHel-15	DF090859:13163-13594
BmHel-15	DF090321:3646336-3646767
BmHel-15	DF090357:2105832-2106264
BmHel-15	DF090357:2823619-2824051
BmHel-15	DF090322:2864294-2864726
BmHel-15	DF090349:2979297-2979729

BmHel-15	DF090327:424495-424927
BmHel-15	DF090359:1113950-1114382
BmHel-15	DF090403:1088979-1089411
BmHel-15	DF090337:1812269-1812701
BmHel-15	DF090360:1834075-1834507
BmHel-15	DF090317:9672151-9672583
BmHel-15	DF090348:3313378-3313810
BmHel-15	DF090387:559541-559973
BmHel-15	DF090363:1138814-1139246
BmHel-15	DF090321:3800753-3801185
BmHel-15	DF090434:669150-669582
BmHel-15	DF090391:1245383-1245815
BmHel-15	DF090323:6401771-6402203
BmHel-15	DF090333:2852317-2852749
BmHel-15	DF090338:4103465-4103897
BmHel-15	DF090397:1564824-1565256
BmHel-15	DF090348:1778581-1779013
BmHel-15	DF090379:1778562-1778994
BmHel-15	DF090366:2002729-2003161
BmHel-15	DF090363:895016-895448
BmHel-15	DF090321:1461037-1461469
BmHel-15	DF090392:1283331-1283763
BmHel-15	DF090316:15936269-15936701
BmHel-15	DF090318:4668561-4668993
BmHel-15	DF090367:105887-106319
BmHel-15	DF090349:3794627-3795059
BmHel-15	DF090327:6013485-6013917
BmHel-15	DF090319:5959077-5959509
BmHel-15	DF090319:1091316-1091748
BmHel-15	DF090340:4881907-4882339
BmHel-15	DF090419:137166-137598
BmHel-15	DF090418:1198835-1199267
BmHel-15	DF090326:4542664-4543096
BmHel-15	DF090351:241361-241793
BmHel-15	DF090334:1582926-1583358
BmHel-15	DF090358:148586-149018
BmHel-15	DF090387:856276-856708
BmHel-15	DF090389:859008-859440
BmHel-15	DF090344:42377-42809
BmHel-15	DF090318:2813472-2813904
BmHel-15	DF090367:2499576-2500008
BmHel-15	DF090332:5669545-5669977
BmHel-15	DF090340:1839833-1840265
BmHel-15	DF090412:368931-369363
BmHel-15	DF090422:956836-957268
BmHel-15	DF090382:874941-875373
BmHel-15	DF090374:1704962-1705394
BmHel-15	DF090384:1547970-1548403

BmHel-15	DF090354:2004837-2005270
BmHel-15	DF090413:726772-727205
BmHel-15	DF090405:683238-683671
BmHel-15	DF090483:15518-15951
BmHel-15	DF090578:44537-44970
BmHel-15	DF090325:4079340-4079773
BmHel-15	DF090425:912758-913191
BmHel-15	DF090342:1886606-1887039
BmHel-15	DF090390:1412550-1412983
BmHel-15	DF090323:2328898-2329331
BmHel-15	DF090334:887789-888222
BmHel-15	DF090358:3125884-3126317
BmHel-15	DF090492:73860-74293
BmHel-15	DF090357:275653-276086
BmHel-15	DF090343:3933354-3933787
BmHel-15	DF090324:2817249-2817682
BmHel-15	DF090391:596962-597395
BmHel-15	DF090322:4547925-4548358
BmHel-15	DF090319:7543949-7544382
BmHel-15	DF090372:693591-694024
BmHel-15	DF090365:1811083-1811516
BmHel-15	DF090340:3348318-3348751
BmHel-15	DF090346:111211-111644
BmHel-15	DF090330:3683986-3684419
BmHel-15	DF090397:651282-651715
BmHel-15	DF090369:579593-580026
BmHel-15	DF090334:3393071-3393504
BmHel-15	DF090435:362959-363392
BmHel-15	DF090351:2118855-2119288
BmHel-15	DF090330:3813178-3813611
BmHel-15	DF090330:3154033-3154466
BmHel-15	DF090317:227975-228408
BmHel-15	DF090358:1471880-1472313
BmHel-15	DF090647:20173-20606
BmHel-15	DF090316:14967652-14968086
BmHel-15	DF090343:3824328-3824762
BmHel-15	DF090322:3630272-3630706
BmHel-15	DF090351:630869-631303
BmHel-15	DF090330:1717056-1717490
BmHel-15	DF090317:1895543-1895977
BmHel-15	DF090427:408755-409189
BmHel-15	DF090437:399369-399803
BmHel-15	BABH01046737:1287-1721
BmHel-15	DF090488:121552-121986
BmHel-15	DF090316:1792981-1793415
BmHel-15	DF090320:6917462-6917896
BmHel-15	DF090788:15417-15851
BmHel-15	DF090398:317394-317828

BmHel-15	DF090322:772461-772895
BmHel-15	DF090327:6040215-6040649
BmHel-15	DF090319:8871617-8872051
BmHel-15	DF090403:592796-593230
BmHel-15	DF090377:73303-73737
BmHel-15	DF090353:3241270-3241704
BmHel-15	DF090329:4569956-4570390
BmHel-15	DF090317:5351597-5352031
BmHel-15	DF090369:991789-992223
BmHel-15	DF090352:2641202-2641636
BmHel-15	DF090368:1517765-1518199
BmHel-15	DF090387:816414-816848
BmHel-15	DF090341:4407246-4407680
BmHel-15	DF090321:2419301-2419735
BmHel-15	DF090321:539084-539518
BmHel-15	DF090349:3126919-3127353
BmHel-15	DF090320:2456857-2457291
BmHel-15	DF090396:125960-126394
BmHel-15	DF090330:1903801-1904235
BmHel-15	DF090334:3650877-3651311
BmHel-15	DF090395:920381-920815
BmHel-15	DF090316:5806335-5806770
BmHel-15	DF090339:3887017-3887452
BmHel-15	DF090320:3320955-3321390
BmHel-15	DF090340:2965934-2966369
BmHel-15	DF090377:2152682-2153117
BmHel-15	DF090361:1380910-1381345
BmHel-15	DF090317:11035777-11036212
BmHel-15	DF090369:422518-422953
BmHel-15	DF090394:1330266-1330701
BmHel-15	DF090388:1600496-1600931
BmHel-15	DF090328:6486029-6486464
BmHel-15	DF090417:568210-568645
BmHel-15	DF090322:2732850-2733285
BmHel-15	DF090404:538733-539168
BmHel-15	DF090354:2103545-2103980
BmHel-15	DF090396:1422225-1422660
BmHel-15	DF090422:855845-856280
BmHel-15	DF090368:1554278-1554713
BmHel-15	DF090755:3424-3859
BmHel-15	DF090721:2183-2618
BmHel-15	DF090459:145174-145609
BmHel-15	DF090316:11681098-11681533
BmHel-15	DF090325:4005992-4006427
BmHel-15	DF090425:939097-939532
BmHel-15	DF090349:3018989-3019424
BmHel-15	DF090319:2244124-2244559
BmHel-15	DF090403:803726-804161

BmHel-15	DF090323:7639344-7639779
BmHel-15	DF090317:570812-571247
BmHel-15	DF090409:1169270-1169705
BmHel-15	DF090348:3441325-3441760
BmHel-15	DF090479:237757-238192
BmHel-15	DF090475:252267-252702
BmHel-15	DF090339:4535098-4535533
BmHel-15	DF090318:6522670-6523105
BmHel-15	DF090367:2443164-2443599
BmHel-15	DF090340:1483429-1483864
BmHel-15	DF090331:4255314-4255749
BmHel-15	DF090428:503960-504395
BmHel-15	DF090389:265711-266146
BmHel-15	DF090560:39691-40126
BmHel-15	DF090316:4845224-4845660
BmHel-15	DF090316:10917424-10917860
BmHel-15	DF090316:12350507-12350943
BmHel-15	DF090343:3169893-3170329
BmHel-15	DF090324:165770-166206
BmHel-15	DF090324:3737927-3738363
BmHel-15	DF090324:7466382-7466818
BmHel-15	DF090407:254057-254493
BmHel-15	DF090349:4178239-4178675
BmHel-15	DF090327:24144-24580
BmHel-15	DF090327:4984930-4985366
BmHel-15	DF090319:357875-358311
BmHel-15	DF090332:3593863-3594299
BmHel-15	DF090396:1111581-1112017
BmHel-15	DF090602:12144-12580
BmHel-15	DF090335:1076071-1076507
BmHel-15	DF090413:550016-550452
BmHel-15	DF090353:1437517-1437953
BmHel-15	DF090326:50045-50481
BmHel-15	DF090346:532162-532598
BmHel-15	DF090329:818819-819255
BmHel-15	DF090416:967651-968087
BmHel-15	DF090363:2949884-2950320
BmHel-15	DF090415:632545-632981
BmHel-15	DF090411:1054615-1055051
BmHel-15	DF090364:2377608-2378044
BmHel-15	DF090322:868309-868745
BmHel-15	DF090319:7910696-7911132
BmHel-15	DF090342:3509212-3509648
BmHel-15	DF090340:1718611-1719047
BmHel-15	DF090390:1593446-1593882
BmHel-15	DF090346:575803-576239
BmHel-15	DF090346:4068269-4068705
BmHel-15	DF090376:1292165-1292601

BmHel-15	DF090382:101409-101845
BmHel-15	DF090321:780227-780663
BmHel-15	DF090316:13980055-13980491
BmHel-15	BABH01055541:89-525
BmHel-15	DF090344:2414065-2414501
BmHel-15	DF090339:438325-438761
BmHel-15	DF090411:944042-944478
BmHel-15	DF090343:96074-96510
BmHel-15	DF090324:4224720-4225156
BmHel-15	DF090318:8401087-8401523
BmHel-15	DF090417:1159522-1159958
BmHel-15	DF090367:1773277-1773713
BmHel-15	DF090349:2597229-2597665
BmHel-15	DF090327:2150660-2151096
BmHel-15	DF090319:552884-553320
BmHel-15	DF090362:2590623-2591059
BmHel-15	DF090320:4838986-4839422
BmHel-15	DF090359:1890038-1890474
BmHel-15	DF090359:971931-972367
BmHel-15	DF090345:1173255-1173691
BmHel-15	DF090323:7008554-7008990
BmHel-15	DF090333:4047465-4047901
BmHel-15	DF090360:3111397-3111833
BmHel-15	DF090338:4216709-4217145
BmHel-15	DF090317:300912-301348
BmHel-15	DF090348:4336185-4336621
BmHel-15	DF090348:4071726-4072162
BmHel-15	DF090358:685411-685847
BmHel-15	DF090321:290182-290618
BmHel-15	DF090357:2838765-2839201
BmHel-15	DF090350:1941224-1941660
BmHel-15	DF090342:4090681-4091117
BmHel-15	DF090320:685199-685635
BmHel-15	DF090359:809649-810085
BmHel-15	DF090340:782316-782752
BmHel-15	DF090410:216627-217063
BmHel-15	DF090351:3609764-3610200
BmHel-15	DF090329:4996914-4997350
BmHel-15	DF090329:2874065-2874501
BmHel-15	DF090317:9958243-9958679
BmHel-15	DF090334:2633888-2634324
BmHel-15	DF090422:501676-502112
BmHel-15	DF090505:4860-5296
BmHel-15	DF090440:514900-515336
BmHel-15	DF090436:246769-247205
BmHel-15	DF090339:2700484-2700921
BmHel-15	DF090425:974717-975154
BmHel-15	DF090357:2516792-2517229

BmHel-15	DF090328:3971191-3971628
BmHel-15	DF090318:8839166-8839603
BmHel-15	DF090407:220438-220875
BmHel-15	DF090350:4049963-4050400
BmHel-15	DF090349:2498709-2499146
BmHel-15	DF090319:2964988-2965425
BmHel-15	DF090319:7099855-7100292
BmHel-15	DF090342:271939-272376
BmHel-15	DF090336:1842393-1842830
BmHel-15	DF090373:1244229-1244666
BmHel-15	DF090373:2237044-2237481
BmHel-15	DF090332:45655-46092
BmHel-15	DF090365:1193378-1193815
BmHel-15	DF090406:876019-876456
BmHel-15	DF090337:767487-767924
BmHel-15	DF090347:1203716-1204153
BmHel-15	DF090351:1353103-1353540
BmHel-15	DF090331:4150627-4151064
BmHel-15	DF090352:2599196-2599633
BmHel-15	DF090446:299273-299710
BmHel-15	DF090321:4342478-4342915
BmHel-15	DF090344:2455309-2455746
BmHel-15	DF090322:1824678-1825115
BmHel-15	DF090319:3207709-3208146
BmHel-15	DF090342:4371948-4372385
BmHel-15	DF090361:606729-607166
BmHel-15	DF090402:993291-993728
BmHel-15	DF090444:589096-589533
BmHel-15	DF090420:608423-608860
BmHel-15	DF090316:9079581-9080018
BmHel-15	DF090325:1529389-1529826
BmHel-15	DF090339:4389747-4390184
BmHel-15	DF090398:210765-211202
BmHel-15	DF090425:133170-133607
BmHel-15	DF090411:1356274-1356711
BmHel-15	DF090357:793164-793601
BmHel-15	DF090328:4690739-4691176
BmHel-15	DF090423:110899-111336
BmHel-15	DF090319:1603256-1603693
BmHel-15	DF090319:992156-992593
BmHel-15	DF090340:4778115-4778552
BmHel-15	DF090377:1560594-1561031
BmHel-15	DF090361:372429-372866
BmHel-15	DF090337:43887-44324
BmHel-15	DF090371:887907-888344
BmHel-15	BABH01049522:393-830
BmHel-15	DF090370:58713-59150
BmHel-15	DF090351:1080718-1081155

BmHel-15	DF090346:2691023-2691460
BmHel-15	DF090338:4356134-4356571
BmHel-15	DF090329:1273984-1274421
BmHel-15	DF090317:172247-172684
BmHel-15	DF090369:109210-109647
BmHel-15	DF090429:277854-278291
BmHel-15	DF090358:2641441-2641878
BmHel-15	DF090389:1425576-1426013
BmHel-15	DF090325:3570342-3570779
BmHel-15	DF090325:2960074-2960511
BmHel-15	DF090339:1614601-1615038
BmHel-15	DF090398:252013-252450
BmHel-15	DF090318:5609330-5609767
BmHel-15	DF090318:487868-488305
BmHel-15	DF090320:5941805-5942242
BmHel-15	DF090340:2610260-2610697
BmHel-15	DF090323:7922375-7922812
BmHel-15	DF090323:4134887-4135324
BmHel-15	DF090346:254859-255296
BmHel-15	DF090334:2399566-2400003
BmHel-15	DF090401:532699-533136
BmHel-15	DF090328:2066850-2067288
BmHel-15	DF090343:1708637-1709075
BmHel-15	DF090322:3590263-3590701
BmHel-15	DF090342:208924-209362
BmHel-15	DF090340:2999298-2999736
BmHel-15	DF090377:97662-98100
BmHel-15	DF090347:718030-718468
BmHel-15	DF090405:498342-498780
BmHel-15	DF090452:332801-333239
BmHel-15	DF090363:1529226-1529664
BmHel-15	DF090341:409134-409572
BmHel-15	DF090316:10422664-10423102
BmHel-15	DF090344:45079-45517
BmHel-15	DF090328:1209632-1210070
BmHel-15	DF090318:2745098-2745536
BmHel-15	DF090384:409518-409956
BmHel-15	DF090349:126454-126892
BmHel-15	DF090335:5759507-5759945
BmHel-15	DF090333:5729362-5729800
BmHel-15	DF090317:10351586-10352024
BmHel-15	DF090316:14261537-14261975
BmHel-15	DF090343:3022757-3023195
BmHel-15	DF090349:1582117-1582555
BmHel-15	DF090332:312671-313109
BmHel-15	DF090323:7697924-7698362
BmHel-15	DF090323:7562479-7562917
BmHel-15	DF090418:97947-98385

BmHel-15	DF090347:2020188-2020626
BmHel-15	DF090326:6385378-6385816
BmHel-15	DF090360:882968-883406
BmHel-15	DF090358:864267-864705
BmHel-15	DF090321:1574729-1575167
BmHel-15	DF090355:906734-907172
BmHel-15	DF090424:804343-804781
BmHel-15	DF090408:1038343-1038781
BmHel-15	DF090344:3272896-3273334
BmHel-15	DF090509:56678-57116
BmHel-15	DF090332:2816599-2817037
BmHel-15	DF090320:6660952-6661390
BmHel-15	DF090340:23209-23647
BmHel-15	DF090351:692973-693411
BmHel-15	DF090369:1036235-1036673
BmHel-15	DF090366:293039-293477
BmHel-15	DF090341:2800594-2801032
BmHel-15	DF090392:725777-726215
BmHel-15	DF090449:794905-795343
BmHel-15	DF090325:5679775-5680214
BmHel-15	DF090417:89912-90351
BmHel-15	DF090322:764968-765407
BmHel-15	DF090349:3812936-3813375
BmHel-15	DF090319:4621027-4621466
BmHel-15	DF090336:1654555-1654994
BmHel-15	DF090410:856408-856847
BmHel-15	DF090330:2930652-2931091
BmHel-15	DF090317:174541-174980
BmHel-15	DF090341:372754-373193
BmHel-15	DF090490:30728-31167
BmHel-15	DF090520:45611-46050
BmHel-15	DF090380:448996-449435
BmHel-15	DF090316:8260401-8260840
BmHel-15	DF090339:3058119-3058558
BmHel-15	DF090384:1489542-1489981
BmHel-15	DF090347:3572333-3572772
BmHel-15	DF090473:316460-316899
BmHel-15	DF090350:1658315-1658754
BmHel-15	DF090534:66139-66578
BmHel-15	DF090359:3174011-3174450
BmHel-15	DF090340:1648077-1648516
BmHel-15	DF090346:422007-422446
BmHel-15	DF090329:5619742-5620181
BmHel-15	DF090409:970384-970823
BmHel-15	DF090396:882770-883209
BmHel-15	DF090340:867149-867588
BmHel-15	DF090329:2910461-2910900
BmHel-15	DF090435:66311-66750

BmHel-15	DF090502:122916-123355
BmHel-15	DF090357:1189677-1190117
BmHel-15	DF090319:5957302-5957742
BmHel-15	DF090317:1218234-1218674
BmHel-15	DF090348:3277721-3278161
BmHel-15	DF090358:875820-876260
BmHel-15	DF090355:3193749-3194189
BmHel-15	DF090322:1849655-1850095
BmHel-15	DF090337:688715-689155
BmHel-15	DF090323:7787153-7787593
BmHel-15	DF090333:3196032-3196472
BmHel-15	DF090316:10548114-10548554
BmHel-15	DF090325:6511466-6511906
BmHel-15	DF090343:1257308-1257748
BmHel-15	DF090318:788697-789137
BmHel-15	DF090391:1138067-1138507
BmHel-15	DF090349:3544584-3545024
BmHel-15	DF090396:1293118-1293558
BmHel-15	DF090340:2758416-2758856
BmHel-15	DF090805:13173-13613
BmHel-15	DF090403:1717324-1717764
BmHel-15	DF090403:631882-632322
BmHel-15	DF090347:1473307-1473747
BmHel-15	DF090326:5254928-5255368
BmHel-15	DF090317:3980814-3981254
BmHel-15	DF090483:9602-10042
BmHel-15	DF090427:207562-208002
BmHel-15	DF090395:1582510-1582950
BmHel-15	DF090374:1293656-1294096
BmHel-15	DF090474:276427-276867
BmHel-15	DF090364:2320964-2321404
BmHel-15	DF090349:1941419-1941859
BmHel-15	DF090390:1101066-1101506
BmHel-15	DF090358:3443270-3443710
BmHel-15	DF090435:648470-648910
BmHel-15	DF090384:1998674-1999115
BmHel-15	DF090419:819176-819617
BmHel-15	DF090421:996347-996788
BmHel-15	DF090360:122698-123139
BmHel-15	DF090334:4347247-4347688
BmHel-15	DF090400:455312-455753
BmHel-15	DF090318:3597060-3597501
BmHel-15	DF090381:1675645-1676086
BmHel-15	DF090361:2548938-2549379
BmHel-15	DF090355:3529101-3529542
BmHel-15	DF090316:12602906-12603347
BmHel-15	DF090332:3343860-3344301
BmHel-15	DF090332:1189014-1189455

BmHel-15	DF090320:1642011-1642452
BmHel-15	DF090353:3519010-3519451
BmHel-15	DF090352:620369-620810
BmHel-15	DF090348:3990309-3990750
BmHel-15	DF090386:855762-856203
BmHel-15	DF090384:615643-616084
BmHel-15	DF090369:852952-853393
BmHel-15	DF090358:2463476-2463917
BmHel-15	BABH01046305:330-771
BmHel-15	DF090371:784585-785027
BmHel-15	DF090421:449319-449761
BmHel-15	DF090319:3957976-3958418
BmHel-15	DF090356:2855020-2855462
BmHel-15	DF090468:188930-189372
BmHel-15	DF090343:2318708-2319150
BmHel-15	DF090326:6036069-6036511
BmHel-15	DF090317:4087393-4087835
BmHel-15	DF090366:69777-70219
BmHel-15	DF090349:3661880-3662323
BmHel-15	DF090319:582468-582911
BmHel-15	DF090390:1332196-1332639
BmHel-15	DF090326:264517-264960
BmHel-15	DF090432:742955-743398
BmHel-15	DF090423:523310-523753
BmHel-15	DF090373:255209-255652
BmHel-15	DF090320:7220448-7220891
BmHel-15	DF090355:1477576-1478019
BmHel-15	DF090526:64942-65385
BmHel-15	DF090333:4527721-4528164
BmHel-15	DF090412:1280803-1281247
BmHel-15	DF090412:1313184-1313628
BmHel-15	DF090444:499424-499868
BmHel-15	BABH01046907:715-1159
BmHel-15	DF090417:876124-876568
BmHel-15	DF090340:2360174-2360618
BmHel-15	DF090333:3104130-3104574
BmHel-15	DF090363:2234056-2234500
BmHel-15	DF090335:3788208-3788652
BmHel-15	DF090372:1843919-1844364
BmHel-15	DF090348:2474096-2474541
BmHel-15	DF090355:463510-463955
BmHel-15	DF090438:623038-623483
BmHel-15	DF090346:857520-857965
BmHel-15	DF090414:552452-552897
BmHel-15	DF090342:2187416-2187861
BmHel-15	DF090336:5381981-5382426
BmHel-15	DF090332:6095354-6095799
BmHel-15	DF090429:114307-114752

BmHel-15	DF090369:2276067-2276512
BmHel-15	DF090361:329447-329893
BmHel-15	DF090363:599240-599686
BmHel-15	DF090327:5837771-5838217
BmHel-15	DF090361:248167-248613
BmHel-15	DF090371:1572777-1573223
BmHel-15	DF090393:440192-440638
BmHel-15	DF090349:3778986-3779433
BmHel-15	DF090316:408031-408478
BmHel-15	DF090330:5170913-5171360
BmHel-15	DF090328:801107-801554
BmHel-15	DF090327:5467813-5468260
BmHel-15	DF090379:1194124-1194571
BmHel-15	DF090373:1346969-1347417
BmHel-15	DF090395:948703-949151
BmHel-15	DF090367:2559883-2560331
BmHel-15	DF090401:1027489-1027937
BmHel-15	DF090347:729039-729488
BmHel-15	DF090755:19749-20199
BmHel-15	DF090318:2617295-2617745
BmHel-15	DF090316:14113784-14114235
BmHel-15	DF090368:526051-526502
BmHel-15	DF090420:879821-880272
BmHel-15	DF090436:371607-372058
BmHel-15	DF090391:1040570-1041022
BmHel-15	DF090319:8141692-8142145
BmHel-15	DF090361:1511433-1511886
BmHel-15	DF090360:1792719-1793173
BmHel-15	DF090352:2812654-2813108
BmHel-15	DF090379:1167038-1167492
BmHel-15	DF090335:1441153-1441607
BmHel-15	DF090316:2194386-2194841
BmHel-15	DF090333:4070542-4070998
BmHel-15	DF090322:4484758-4485216
BmHel-15	DF090319:710044-710502
BmHel-15	DF090329:2473122-2473580
BmHel-15	DF090393:1377850-1378309
BmHel-15	DF090317:9779507-9779967
BmHel-15	DF090858:11832-12293
BmHel-15	DF090384:1929980-1930442
BmHel-15	DF090335:3988122-3988585
BmHel-15	DF090323:707278-707746
BmHel-15	DF090334:231775-232244
BmHel-15	DF090440:674837-675307
BmHel-15	DF090316:2847384-2847857
BmHel-15	DF090318:5730927-5731400
BmHel-15	DF090336:1400250-1400725
BmHel-15	DF090365:2319553-2320028

BmHel-15	DF090345:2123760-2124236
BmHel-15	DF090361:2167134-2167612
BmHel-15	DF090356:1827979-1828460
BmHel-15	DF090435:524414-524904
BmHel-15	DF090431:396417-396907
BmHel-15	DF090343:3180674-3181167
BmHel-15	DF090448:50436-50931
BmHel-15	DF090325:3887361-3887870
BmHel-15	DF090319:976490-976999
BmHel-15	DF090326:4274810-4275323
BmHel-15	DF090343:4674241-4674761
BmHel-15	DF090362:1607166-1607689
BmHel-15	DF090383:1752425-1752956
BmHel-15	DF090373:483308-483843
BmHel-15	DF090331:4066781-4067318
BmHel-15	DF090316:6571007-6571555
BmHel-15	DF090372:73973-74522
BmHel-15	DF090321:5785047-5785597
BmHel-15	DF090336:2984114-2984674
BmHel-15	DF090328:1637553-1638114
BmHel-15	DF090462:155916-156479
BmHel-15	DF090406:555188-555752
BmHel-15	DF090411:518389-518953
BmHel-15	DF090319:1950607-1951173
BmHel-15	DF090316:1576050-1576619
BmHel-15	DF090338:3855621-3856192
BmHel-15	DF090402:1110536-1111109
BmHel-15	DF090425:734316-734896
BmHel-15	DF090336:3643920-3644504
BmHel-15	DF090452:90397-90988
BmHel-15	DF090525:13176-13767
BmHel-15	DF090321:4381005-4381598
BmHel-15	DF090347:3585933-3586530
BmHel-15	DF090356:3571879-3572482
BmHel-15	DF090375:742843-743447
BmHel-15	DF090326:6790878-6791483
BmHel-15	DF090335:4034600-4035214
BmHel-15	DF090714:20247-20869
BmHel-15	DF090355:1270676-1271302
BmHel-15	DF090328:4599447-4600073
BmHel-15	DF090317:8672028-8672656
BmHel-15	DF090317:5323658-5324290
BmHel-15	DF090363:2892422-2893060
BmHel-15	DF090322:4500869-4501511
BmHel-15	DF090333:4167821-4168469
BmHel-15	DF090350:242508-243157
BmHel-15	DF090330:3067827-3068477
BmHel-15	DF090325:5783422-5784075

BmHel-15	DF090378:1098251-1098906
BmHel-15	DF090318:627532-628188
BmHel-15	DF090436:655252-655908
BmHel-15	DF090362:476581-477247
BmHel-15	DF090366:314285-314954
BmHel-15	DF090362:1211872-1212544
BmHel-15	DF090369:1667725-1668400
BmHel-15	DF090344:1411923-1412602
BmHel-15	DF090345:1106413-1107095
BmHel-15	DF090413:412133-412819
BmHel-15	DF090316:15163711-15164400
BmHel-15	DF090338:2357897-2358595
BmHel-15	DF090354:3393346-3394045
BmHel-15	DF090348:416161-416861
BmHel-15	DF090444:15416-16118
BmHel-15	DF090352:1112857-1113568
BmHel-15	DF090402:466256-466969
BmHel-15	DF090466:39287-40000
BmHel-15	DF090348:2494596-2495310
BmHel-15	DF090361:2122860-2123575
BmHel-15	DF090366:1712743-1713460
BmHel-15	DF090383:2087572-2088298
BmHel-15	DF090323:7021555-7022281
BmHel-15	DF090344:425904-426633
BmHel-15	DF090365:3003767-3004502
BmHel-15	DF090333:5799627-5800365
BmHel-15	DF090400:1036619-1037360
BmHel-15	DF090333:517119-517870
BmHel-15	DF090434:702571-703324
BmHel-15	DF090336:4594865-4595630
BmHel-15	DF090390:1234892-1235671
BmHel-15	DF090319:8359843-8360622
BmHel-15	DF090371:990641-991425
BmHel-15	DF090317:5796676-5797472
BmHel-15	DF090369:893401-894211
BmHel-15	DF090319:8519358-8520191
BmHel-15	DF090344:3906783-3907620
BmHel-15	DF090355:2670349-2671234
BmHel-15	DF090317:8757777-8758664
BmHel-15	DF090388:146140-147043
BmHel-15	DF090375:1373490-1374417
BmHel-15	DF090327:5532702-5533638
BmHel-15	DF090395:714942-715892
BmHel-15	DF090329:2012062-2013078
BmHel-15	DF090354:3162954-3163973
BmHel-15	DF090320:7919399-7920426
BmHel-15	DF090389:1803371-1804434
BmHel-15	DF090834:2703-3767

BmHel-15	DF090338:1786044-1787122
BmHel-15	DF090360:2666318-2667434
BmHel-15	DF090386:429453-430587
BmHel-15	DF090414:1100309-1101446
BmHel-15	DF090344:969983-971163
BmHel-15	DF090386:1955460-1956688
BmHel-15	DF090419:1227971-1229225
BmHel-15	DF090328:4799850-4801191
BmHel-15	DF090350:4364111-4365479
BmHel-15	DF090447:101549-102991
BmHel-15	DF090384:1521910-1523370
BmHel-15	DF090335:4843793-4845300
BmHel-15	DF090378:1510420-1512065
BmHel-15	DF090348:1025468-1027291
BmHel-15	DF090343:2055839-2057676
BmHel-15	DF090344:3643789-3645748
BmHel-15	DF090363:1958478-1960441
BmHel-15	DF090330:25428-27419
BmHel-15	DF090351:1295366-1297621
BmHel-15	DF090493:165795-168140
BmHel-15	DF090367:2247352-2249714
BmHel-15	DF090369:2439547-2442093
BmHel-15	DF090364:294001-296705
BmHel-15	DF090407:624979-627754
BmHel-15	DF090494:114117-117360
BmHel-15	DF090345:2615495-2619338
BmHel-15	DF090319:7461948-7466152
BmHel-15	DF090343:186284-190502
BmHel-15	DF090359:2539517-2544157
BmHel-15	DF090359:2418465-2423715
BmHel-15	DF090357:3761569-3767221
BmHel-15	DF090442:425262-431226
BmHel-15	DF090424:60633-66846
BmHel-15	DF090344:3415301-3421863
BmHel-15	DF090388:804008-810677
BmHel-15	DF090344:610750-617696
BmHel-15	DF090411:765560-772603
BmHel-15	DF090316:4640683-4647901
BmHel-15	DF090455:194077-202037
BmHel-15	DF090343:3632-12130
BmHel-15	DF090441:390251-398790
BmHel-15	DF090797:3256-11833
BmHel-15	DF090450:272878-281591
BmHel-15	DF090528:45671-54471
BmHel-15	DF090322:7030559-7040318
BmHel-15	DF090317:4783746-4793700
BmHel-15	DF090576:1509-11518
BmHel-15	DF090413:1179353-1189423

BmHel-15	DF090322:7715156-7725325
BmHel-15	DF090341:2811759-2822156
BmHel-15	DF090340:995106-1005573
BmHel-15	DF090316:9540243-9540680
BmHel-15	DF090343:511204-511643
BmHel-15	DF090343:251988-252419
BmHel-15	DF090324:702615-703045
BmHel-15	DF090318:6861175-6861610
BmHel-15	DF090322:1834086-1834524
BmHel-15	DF090322:1109469-1109910
BmHel-15	DF090327:6164430-6164864
BmHel-15	DF090319:5070339-5070778
BmHel-15	DF090342:4483398-4483835
BmHel-15	DF090342:3856671-3857103
BmHel-15	DF090340:760953-761387
BmHel-15	BABH01047836:134-569
BmHel-15	DF090356:1752685-1753117
BmHel-15	DF090370:2462852-2463275
BmHel-15	DF090351:16917-17352
BmHel-15	DF090330:2836439-2836883
BmHel-15	DF090334:4749784-4750220
BmHel-15	DF090366:2664375-2664821
BmHel-15	DF090446:494067-494501
BmHel-15	DF090386:1764642-1765075
BmHel-15	DF090355:3579122-3579553
BmHel-15	DF090472:120553-120989
BmHel-15	DF090395:1646329-1646756
BmHel-15	DF090415:542613-543044
BmHel-15	DF090316:9515138-9515573
BmHel-15	DF090425:879523-879955
BmHel-15	DF090411:1362043-1362475
BmHel-15	DF090328:5813700-5814140
BmHel-15	DF090318:3576894-3577312
BmHel-15	DF090407:903405-903840
BmHel-15	DF090349:1035499-1035933
BmHel-15	DF090319:6906738-6907171
BmHel-15	DF090377:192469-192905
BmHel-15	DF090333:4988478-4988911
BmHel-15	DF090348:3494971-3495416
BmHel-15	DF090366:640763-641201
BmHel-15	DF090376:1677796-1678230
BmHel-15	DF090321:5674099-5674532
BmHel-15	DF090321:5727974-5728405
BmHel-15	DF090375:1462232-1462661
BmHel-15	DF090317:10168898-10169162
BmHel-15	DF090317:10169259-10169436
BmHel-15	DF090316:8532490-8532932
BmHel-15	DF090339:2279670-2280105

BmHel-15	DF090388:670938-671375
BmHel-15	DF090388:1454466-1454911
BmHel-15	DF090357:2368105-2368539
BmHel-15	DF090324:2268367-2268802
BmHel-15	DF090318:159941-160381
BmHel-15	DF090319:1383249-1383677
BmHel-15	DF090354:3540765-3541188
BmHel-15	DF090320:6419658-6420095
BmHel-15	DF090347:3442883-3443319
BmHel-15	DF090317:2206603-2207040
BmHel-15	DF090366:1206882-1207307
BmHel-15	DF090414:1198342-1198770
BmHel-15	DF090394:1445352-1445789
BmHel-15	DF090427:439226-439652
BmHel-15	DF090389:843697-844125
BmHel-15	DF090316:2294287-2294726
BmHel-15	DF090316:15938049-15938486
BmHel-15	DF090316:4264407-4264835
BmHel-15	DF090325:6045155-6045594
BmHel-15	DF090411:560477-560913
BmHel-15	DF090411:784268-784701
BmHel-15	DF090357:2248600-2249031
BmHel-15	DF090343:3214718-3215152
BmHel-15	DF090324:4311504-4311938
BmHel-15	DF090324:2050190-2050622
BmHel-15	DF090324:1969987-1970422
BmHel-15	DF090318:6043471-6043900
BmHel-15	DF090350:3171063-3171507
BmHel-15	DF090384:1030945-1031380
BmHel-15	DF090384:1971855-1972290
BmHel-15	DF090322:5540624-5541058
BmHel-15	DF090322:3104824-3105256
BmHel-15	DF090349:3450041-3450474
BmHel-15	DF090349:3339746-3340175
BmHel-15	DF090598:2206-2639
BmHel-15	DF090327:620428-620852
BmHel-15	DF090319:865135-865575
BmHel-15	DF090404:939506-939939
BmHel-15	DF090381:1001335-1001769
BmHel-15	DF090372:480061-480495
BmHel-15	DF090342:1200765-1201203
BmHel-15	DF090342:3472362-3472796
BmHel-15	DF090342:4172959-4173384
BmHel-15	DF090342:4258709-4259149
BmHel-15	DF090342:2317295-2317729
BmHel-15	DF090336:3396431-3396865
BmHel-15	DF090362:242090-242523
BmHel-15	DF090362:2195207-2195638

BmHel-15	DF090332:2007185-2007613
BmHel-15	DF090332:4032776-4033206
BmHel-15	DF090320:6058495-6058929
BmHel-15	DF090320:4364364-4364797
BmHel-15	DF090396:327510-327944
BmHel-15	DF090365:693103-693537
BmHel-15	DF090359:1316835-1317273
BmHel-15	DF090359:3161101-3161535
BmHel-15	DF090359:2218951-2219384
BmHel-15	DF090340:1276668-1277101
BmHel-15	DF090340:3639376-3639803
BmHel-15	DF090805:4773-5212
BmHel-15	DF090378:410019-410456
BmHel-15	DF090378:1605518-1605952
BmHel-15	DF090377:1843233-1843667
BmHel-15	DF090377:1544332-1544765
BmHel-15	DF090345:1007855-1008293
BmHel-15	DF090337:2639127-2639554
BmHel-15	DF090356:3320878-3321320
BmHel-15	DF090356:1333055-1333489
BmHel-15	DF090323:5712086-5712522
BmHel-15	DF090323:3297647-3298077
BmHel-15	DF090413:311533-311964
BmHel-15	DF090412:1163489-1163917
BmHel-15	DF090353:1115025-1115458
BmHel-15	DF090353:3849784-3850223
BmHel-15	DF090333:991771-992196
BmHel-15	DF090347:73033-73461
BmHel-15	DF090326:1978536-1978966
BmHel-15	DF090326:1457210-1457639
BmHel-15	DF090326:999921-1000361
BmHel-15	DF090351:2849948-2850373
BmHel-15	DF090346:1223993-1224418
BmHel-15	DF090338:2949817-2950248
BmHel-15	DF090329:4792739-4793171
BmHel-15	DF090317:3715022-3715448
BmHel-15	DF090397:702412-702841
BmHel-15	DF090334:173604-174038
BmHel-15	DF090334:3173612-3174050
BmHel-15	DF090334:3200585-3201027
BmHel-15	DF090405:765822-766249
BmHel-15	DF090385:730616-731050
BmHel-15	DF090352:1048607-1049033
BmHel-15	DF090352:1169509-1169935
BmHel-15	DF090352:1664002-1664432
BmHel-15	DF090348:4363728-4364161
BmHel-15	DF090416:946491-946919
BmHel-15	DF090416:602832-603275

BmHel-15	DF090366:148355-148783
BmHel-15	DF090358:1195366-1195797
BmHel-15	DF090433:323509-323939
BmHel-15	DF090414:358316-358753
BmHel-15	DF090387:2003564-2003998
BmHel-15	DF090386:1435444-1435882
BmHel-15	DF090386:1320058-1320492
BmHel-15	DF090341:1951712-1952159
BmHel-15	DF090341:4585013-4585443
BmHel-15	DF090341:4651229-4651666
BmHel-15	DF090321:7663842-7664274
BmHel-15	DF090355:3092706-3093138
BmHel-15	DF090355:934977-935414
BmHel-15	DF090393:1188871-1189304
BmHel-15	DF090395:876750-877179
BmHel-15	DF090402:870149-870578
BmHel-15	DF090402:194671-195102
BmHel-15	DF090389:1511978-1512401
BmHel-15	DF090567:21484-21918
BmHel-15	DF090383:1276340-1276767
BmHel-15	BABH01061130:207-634
BmHel-15	DF090455:283212-283656
BmHel-15	DF090316:2179248-2179681
BmHel-15	DF090316:6128533-6128969
BmHel-15	DF090316:14234936-14235367
BmHel-15	DF090325:6548954-6549384
BmHel-15	DF090344:3771343-3771776
BmHel-15	DF090339:2714461-2714892
BmHel-15	DF090339:2388782-2389221
BmHel-15	DF090324:6894623-6895060
BmHel-15	DF090324:2181640-2182074
BmHel-15	DF090318:5310080-5310514
BmHel-15	DF090318:9309259-9309681
BmHel-15	DF090322:4579980-4580410
BmHel-15	DF090349:210267-210701
BmHel-15	DF090349:3767252-3767686
BmHel-15	DF090319:4898937-4899371
BmHel-15	DF090372:1828349-1828775
BmHel-15	DF090372:1501773-1502207
BmHel-15	DF090342:4343733-4344159
BmHel-15	DF090332:2123664-2124097
BmHel-15	DF090396:1151548-1151967
BmHel-15	DF090340:3094779-3095211
BmHel-15	DF090410:818941-819373
BmHel-15	DF090323:7206874-7207308
BmHel-15	DF090418:356477-356929
BmHel-15	DF090347:3672352-3672785
BmHel-15	DF090326:5393359-5393795

BmHel-15	DF090326:5303500-5303929
BmHel-15	DF090338:1217678-1218114
BmHel-15	DF090317:10557188-10557629
BmHel-15	DF090369:1968207-1968634
BmHel-15	DF090334:525174-525608
BmHel-15	DF090334:2764666-2765103
BmHel-15	DF091015:5769-6202
BmHel-15	DF090401:515447-515874
BmHel-15	DF090401:1325414-1325846
BmHel-15	DF090379:692463-692886
BmHel-15	DF090363:2180639-2181077
BmHel-15	DF090321:5537733-5538166
BmHel-15	DF090321:7697216-7697648
BmHel-15	DF090864:11054-11488
BmHel-15	DF090472:256725-257154
BmHel-15	DF090706:19551-19976
BmHel-15	DF090402:961347-961782
BmHel-15	DF090402:233782-234215
BmHel-15	DF090447:287654-288085
BmHel-15	DF090467:194495-194935
BmHel-15	DF090420:745085-745513
BmHel-15	DF090360:582412-582765
BmHel-15	DF090317:5436646-5436986
BmHel-15	DF090317:5436999-5437091
BmHel-15	DF090386:1950460-1950865
BmHel-15	DF090316:1816565-1816996
BmHel-15	DF090316:11025663-11026092
BmHel-15	DF090316:6116942-6117375
BmHel-15	DF090316:3102324-3102749
BmHel-15	DF090328:6622490-6622924
BmHel-15	DF090343:614865-615293
BmHel-15	DF090318:2726469-2726899
BmHel-15	DF090350:4174962-4175386
BmHel-15	DF090322:1594105-1594534
BmHel-15	DF090349:2118559-2118988
BmHel-15	DF090327:2714257-2714693
BmHel-15	DF090319:5190214-5190655
BmHel-15	DF090319:6274098-6274536
BmHel-15	DF090372:390720-391158
BmHel-15	DF090336:1631209-1631642
BmHel-15	DF090320:4226506-4226933
BmHel-15	DF090396:538224-538655
BmHel-15	DF090359:374890-375322
BmHel-15	DF090335:3495191-3495621
BmHel-15	BABH01054829:231-663
BmHel-15	DF090323:4621262-4621695
BmHel-15	DF090353:3404777-3405213
BmHel-15	DF090353:648522-648960

BmHel-15	DF090347:29457-29881
BmHel-15	DF090347:2533093-2533525
BmHel-15	DF090331:424306-424741
BmHel-15	DF090331:2076515-2076948
BmHel-15	DF090330:3950096-3950530
BmHel-15	DF090330:4450927-4451354
BmHel-15	DF090329:3643649-3644076
BmHel-15	DF090397:615165-615606
BmHel-15	DF090401:302391-302818
BmHel-15	DF090401:216223-216663
BmHel-15	DF090379:674299-674730
BmHel-15	DF090358:1217066-1217491
BmHel-15	DF090358:3381583-3382012
BmHel-15	DF090368:2438317-2438750
BmHel-15	DF090400:213035-213461
BmHel-15	DF090400:404094-404524
BmHel-15	DF090395:1193271-1193710
BmHel-15	DF090389:195752-196180
BmHel-15	DF090424:119510-119941
BmHel-15	DF090392:1321035-1321457
BmHel-15	BABH01045792:52-480
BmHel-15	DF090439:455985-456435
BmHel-15	DF090380:1449103-1449525
BmHel-15	DF090487:50530-50959
BmHel-15	DF090662:2150-2579
BmHel-15	DF091075:7416-7840
BmHel-15	DF090726:22688-23122
BmHel-15	DF090352:3836001-3836437
BmHel-15	DF090533:22919-23352
BmHel-15	BABH01068303:120-552
BmHel-15	DF090359:2201524-2201958
BmHel-15	DF090336:3790166-3790522
BmHel-15	DF090332:3694021-3694461
BmHel-15	DF090335:1313367-1313576
BmHel-15	DF090335:1313025-1313235
BmHel-15	DF090316:15566832-15567276
BmHel-15	DF090344:2226393-2226825
BmHel-15	DF090343:3906491-3906906
BmHel-15	DF090384:1844415-1844848
BmHel-15	DF090349:2226692-2227125
BmHel-15	DF090342:1261232-1261663
BmHel-15	DF090390:1761720-1762152
BmHel-15	DF090326:5552638-5553065
BmHel-15	DF090338:1298546-1298975
BmHel-15	DF090317:6038438-6038871
BmHel-15	DF090352:1424406-1424836
BmHel-15	DF090555:29367-29797
BmHel-15	DF090394:799995-800431

BmHel-15	DF090613:42355-42790
BmHel-15	DF090374:2193896-2194316
BmHel-15	DF090407:1093748-1094101
BmHel-15	DF090407:1093413-1093501
BmHel-15	DF090328:4900301-4900735
BmHel-15	DF090359:2797455-2797886
BmHel-15	DF090320:8181449-8181883
BmHel-15	DF090359:3258278-3258699
BmHel-15	DF090351:105519-105952
BmHel-15	DF090450:153097-153526
BmHel-15	DF090389:1175748-1176181
BmHel-15	DF090351:2953003-2953440
BmHel-15	DF090316:3424947-3425375
BmHel-15	DF090343:3223285-3223705
BmHel-15	DF090406:766364-766785
BmHel-15	DF090389:1550908-1551338
BmHel-15	DF090357:96028-96451
BmHel-15	DF090319:2480566-2480999
BmHel-15	DF090381:1191288-1191719
BmHel-15	DF090340:311896-312329
BmHel-15	DF090346:1170716-1171149
BmHel-15	DF090329:4952256-4952680
BmHel-15	DF090329:6317155-6317587
BmHel-15	DF090416:29650-30083
BmHel-15	DF090368:1146377-1146808
BmHel-15	DF090321:7267170-7267599
BmHel-15	DF090430:662292-662714
BmHel-15	DF090346:324180-324601
BmHel-15	DF090348:2339196-2339635
BmHel-15	DF090344:841025-841418
BmHel-15	DF090343:3965773-3966208
BmHel-15	DF090318:136645-137086
BmHel-15	DF090322:5229267-5229699
BmHel-15	DF090319:4905264-4905692
BmHel-15	DF090362:2702039-2702470
BmHel-15	DF090335:3844144-3844568
BmHel-15	DF090620:12889-13322
BmHel-15	DF090392:1703436-1703868
BmHel-15	DF090344:3319271-3319382
BmHel-15	DF090344:3319397-3319716
BmHel-15	DF090316:10899845-10900275
BmHel-15	DF090407:117763-118194
BmHel-15	DF090349:1441276-1441710
BmHel-15	DF090319:4599861-4600282
BmHel-15	DF090332:5371771-5372202
BmHel-15	DF090365:832938-833377
BmHel-15	DF090340:2160524-2160912
BmHel-15	DF090330:5310904-5311328

BmHel-15	DF090317:6787031-6787461
BmHel-15	DF090355:391246-391560
BmHel-15	DF090355:391127-391235
BmHel-15	DF090327:2432124-2432557
BmHel-15	DF090335:4241137-4241566
BmHel-15	DF090427:923764-924190
BmHel-15	DF090404:373540-373968
BmHel-15	DF090373:291074-291504
BmHel-15	DF090373:1495139-1495572
BmHel-15	DF090396:9921-10351
BmHel-15	DF090337:1336098-1336528
BmHel-15	DF090413:292562-292979
BmHel-15	DF090317:10805110-10805545
BmHel-15	DF090334:5198216-5198648
BmHel-15	DF090658:225-654
BmHel-15	DF091250:1389-1821
BmHel-15	DF090621:9854-10282
BmHel-15	DF090371:1124566-1124822
BmHel-15	DF090371:1123954-1124060
BmHel-15	DF090329:797624-797916
BmHel-15	DF090329:797022-797159
BmHel-15	DF090316:15754169-15754486
BmHel-15	DF090316:15754755-15754871
BmHel-15	DF090322:6346629-6346744
BmHel-15	DF090322:6346759-6347073
BmHel-15	DF090319:3626984-3627414
BmHel-15	DF090372:1956974-1957400
BmHel-15	DF090359:3132329-3132754
BmHel-15	DF090390:2144-2574
BmHel-15	DF090345:1209590-1210015
BmHel-15	DF090481:170213-170641
BmHel-15	DF090330:1430989-1431413
BmHel-15	BABH01045709:1049-1480
BmHel-15	DF090344:1615397-1615824
BmHel-15	DF090339:3031754-3032182
BmHel-15	DF090318:2081914-2082345
BmHel-15	DF090373:177047-177476
BmHel-15	DF090362:1001554-1001984
BmHel-15	DF090320:4826407-4826835
BmHel-15	DF090335:5541237-5541661
BmHel-15	DF090421:895630-896061
BmHel-15	DF090329:3248659-3249092
BmHel-15	DF090355:1682656-1683078
BmHel-15	DF090339:3170147-3170569
BmHel-15	DF090318:673031-673463
BmHel-15	DF090352:478533-478963
BmHel-15	DF090402:1463582-1464008
BmHel-15	DF090348:1761978-1762401

BmHel-15	DF090418:960232-960534
BmHel-15	DF090418:960745-960874
BmHel-15	DF090324:2436522-2436946
BmHel-15	DF090321:1024641-1025067
BmHel-15	DF090344:3823995-3824427
BmHel-15	DF090339:628053-628475
BmHel-15	DF090322:3618940-3619369
BmHel-15	DF090334:936782-937208
BmHel-15	DF090340:3453802-3454223
BmHel-15	DF090316:13982600-13983020
BmHel-15	DF090327:3945450-3945544
BmHel-15	DF090327:3945583-3945920
BmHel-15	DF090345:4059931-4060356
BmHel-15	DF090326:2861783-2862209
BmHel-15	BABH01045545:1350-1770
BmHel-15	DF090316:1749891-1750316
BmHel-15	DF090329:5306831-5307258
BmHel-15	DF090496:76781-77214
BmHel-15	DF090357:2295762-2296183
BmHel-15	DF090322:483047-483400
BmHel-15	BABH01043098:6621-7050
BmHel-15	DF090316:15028229-15028652
BmHel-15	DF090316:14807846-14808269
BmHel-15	DF090316:6186182-6186611
BmHel-15	DF090325:1426452-1426871
BmHel-15	DF090325:2370701-2371129
BmHel-15	DF090325:876464-876892
BmHel-15	DF090339:3389908-3390333
BmHel-15	DF090339:843868-844295
BmHel-15	DF090388:1152993-1153416
BmHel-15	DF090388:1655389-1655826
BmHel-15	DF090357:1727364-1727795
BmHel-15	DF090357:2140636-2141062
BmHel-15	DF090328:578644-579075
BmHel-15	DF090343:107500-107922
BmHel-15	DF090343:3338129-3338553
BmHel-15	DF090324:2812957-2813389
BmHel-15	DF090324:3441263-3441692
BmHel-15	DF090318:2887872-2888302
BmHel-15	DF090318:3662008-3662439
BmHel-15	DF090318:7521603-7522032
BmHel-15	DF090318:4335206-4335635
BmHel-15	DF090318:2955165-2955590
BmHel-15	DF090407:43294-43723
BmHel-15	DF090407:1554317-1554746
BmHel-15	DF090364:1506993-1507421
BmHel-15	DF090350:1878461-1878891
BmHel-15	DF090417:1162959-1163387

BmHel-15	DF090322:2252881-2253309
BmHel-15	DF090322:611503-611936
BmHel-15	DF090367:2194635-2195061
BmHel-15	DF090327:596277-596705
BmHel-15	DF090319:2472705-2473134
BmHel-15	DF090319:4506917-4507344
BmHel-15	DF090319:4607947-4608378
BmHel-15	DF090372:767382-767815
BmHel-15	DF090354:999322-999745
BmHel-15	DF090354:2641703-2642126
BmHel-15	DF090354:2859050-2859471
BmHel-15	DF090354:2424865-2425295
BmHel-15	DF090342:458078-458505
BmHel-15	DF090342:3163569-3163990
BmHel-15	DF090362:2593734-2594158
BmHel-15	DF090362:2745775-2746196
BmHel-15	DF090362:1985570-1986004
BmHel-15	DF090332:4826071-4826501
BmHel-15	DF090320:4114737-4115166
BmHel-15	DF090320:1896106-1896536
BmHel-15	DF090396:1474299-1474718
BmHel-15	DF090365:1145613-1146041
BmHel-15	DF090365:703936-704365
BmHel-15	DF090359:1772658-1773088
BmHel-15	DF090340:1151443-1151873
BmHel-15	DF090340:4207564-4207988
BmHel-15	DF090340:2492852-2493278
BmHel-15	DF090403:1588661-1589087
BmHel-15	DF090378:1900120-1900554
BmHel-15	DF090377:1155915-1156335
BmHel-15	DF090377:1707609-1708036
BmHel-15	DF090406:1550002-1550431
BmHel-15	DF090356:2809035-2809464
BmHel-15	DF090335:1448785-1449220
BmHel-15	DF090335:2968603-2969033
BmHel-15	DF090323:3100701-3101128
BmHel-15	DF090413:1128379-1128808
BmHel-15	DF090413:1015675-1016107
BmHel-15	DF090413:906250-906678
BmHel-15	DF090421:674419-674844
BmHel-15	DF090353:1426804-1427231
BmHel-15	DF090353:1562864-1563295
BmHel-15	DF090333:3044004-3044429
BmHel-15	DF090347:484275-484701
BmHel-15	DF090326:5485011-5485438
BmHel-15	DF090326:6678429-6678854
BmHel-15	DF090326:6508271-6508689
BmHel-15	DF090786:30717-31144

BmHel-15	DF090351:483965-484395
BmHel-15	DF090346:4168205-4168636
BmHel-15	DF090338:846641-847065
BmHel-15	DF090331:1047798-1048231
BmHel-15	DF090331:2269786-2270208
BmHel-15	DF090331:5426640-5427065
BmHel-15	DF090331:3108699-3109124
BmHel-15	DF090330:2979781-2980209
BmHel-15	DF090330:4061252-4061679
BmHel-15	DF090329:2990670-2991097
BmHel-15	DF090329:4928730-4929164
BmHel-15	DF090317:272255-272684
BmHel-15	DF090409:1109426-1109849
BmHel-15	DF090409:1409334-1409758
BmHel-15	DF090352:991892-992322
BmHel-15	DF090352:101073-101499
BmHel-15	DF090452:297445-297877
BmHel-15	DF090366:1522264-1522686
BmHel-15	DF090358:2825394-2825820
BmHel-15	DF090358:2428261-2428690
BmHel-15	DF090446:191334-191765
BmHel-15	DF090321:2711544-2711973
BmHel-15	BABH01046148:503-931
BmHel-15	DF090392:1268851-1269276
BmHel-15	BABH01080715:37-459
BmHel-15	DF090460:86918-87346
BmHel-15	DF090658:22714-23142
BmHel-15	DF090439:434443-434865
BmHel-15	DF090443:144403-144830
BmHel-15	DF090415:381044-381471
BmHel-15	DF090475:96910-97339
BmHel-15	DF090551:71046-71480
BmHel-15	DF090430:669173-669604
BmHel-15	DF090471:272642-273056
BmHel-15	DF090462:18667-18929
BmHel-15	DF090462:18493-18655
BmHel-15	DF090375:1344058-1344236
BmHel-15	DF090375:1343568-1343823
BmHel-15	DF090344:3485232-3485595
BmHel-15	DF090349:4158551-4158978
BmHel-15	DF090356:2022675-2023103
BmHel-15	DF090427:688143-688561
BmHel-15	DF090357:360090-360514
BmHel-15	DF090328:3809864-3810288
BmHel-15	DF090349:76912-77341
BmHel-15	DF090327:3722261-3722689
BmHel-15	DF090354:466746-467172
BmHel-15	DF090340:3689231-3689664

BmHel-15	DF090346:1308006-1308431
BmHel-15	DF090334:4103379-4103804
BmHel-15	DF090386:1549366-1549792
BmHel-15	DF090395:318796-319230
BmHel-15	DF090503:91716-92141
BmHel-15	DF090420:694755-695180
BmHel-15	DF090357:3603472-3603871
BmHel-15	DF090333:5043638-5044066
BmHel-15	DF090388:1320023-1320109
BmHel-15	DF090388:1319658-1320001
BmHel-15	DF090346:2237680-2238096
BmHel-15	DF090358:2904395-2904817
BmHel-15	DF090441:717502-717788
BmHel-15	DF090441:718074-718215
BmHel-15	DF090325:3154995-3155426
BmHel-15	DF090344:4278809-4279231
BmHel-15	DF090396:1168157-1168585
BmHel-15	DF090356:3585222-3585649
BmHel-15	DF090335:3452612-3453036
BmHel-15	DF090977:226-652
BmHel-15	DF090400:1048641-1049068
BmHel-15	DF090517:62594-63023
BmHel-15	DF090424:162988-163134
BmHel-15	DF090424:163147-163437
BmHel-15	DF090317:1869601-1869984
BmHel-15	DF090336:609175-609494
BmHel-15	DF090336:609053-609161
BmHel-15	DF090398:214646-215066
BmHel-15	DF090442:452308-452741
BmHel-15	DF090325:988885-989309
BmHel-15	DF090318:8760399-8760825
BmHel-15	DF090336:2354520-2354949
BmHel-15	DF090409:402700-403124
BmHel-15	DF090422:262293-262721
BmHel-15	DF090322:690219-690322
BmHel-15	DF090322:690354-690674
BmHel-15	DF090340:1161529-1161955
BmHel-15	DF090339:3497670-3498101
BmHel-15	DF090339:449912-450337
BmHel-15	DF090357:2656180-2656602
BmHel-15	DF090318:7018075-7018368
BmHel-15	DF090318:7018804-7018933
BmHel-15	DF090350:3460551-3460976
BmHel-15	DF090342:3101505-3101932
BmHel-15	DF090331:2356314-2356738
BmHel-15	DF090317:1582379-1582805
BmHel-15	DF090405:579819-580240
BmHel-15	DF090348:2061736-2062159

BmHel-15	DF090341:2085099-2085529
BmHel-15	DF090523:73071-73493
BmHel-15	DF090316:15482066-15482492
BmHel-15	DF090411:308523-308944
BmHel-15	DF090327:5383319-5383744
BmHel-15	DF090362:3003197-3003605
BmHel-15	DF090410:441681-442108
BmHel-15	DF090378:1590452-1590870
BmHel-15	DF090331:143035-143463
BmHel-15	DF090330:3968971-3969392
BmHel-15	DF090317:4566997-4567422
BmHel-15	DF090358:183449-183885
BmHel-15	DF090414:488306-488731
BmHel-15	DF090376:1240233-1240664
BmHel-15	DF090432:676228-676655
BmHel-15	DF090531:12963-13388
BmHel-15	DF090319:7256924-7257070
BmHel-15	DF090319:7257081-7257363
BmHel-15	DF090317:10230127-10230375
BmHel-15	DF090317:10229918-10230099
BmHel-15	DF090393:1328325-1328589
BmHel-15	DF090393:1328621-1328804
BmHel-15	DF090329:4178462-4178884
BmHel-15	DF090318:9332741-9333167
BmHel-15	DF090407:975309-975738
BmHel-15	DF090373:369184-369611
BmHel-15	DF090348:2322605-2323031
BmHel-15	DF090400:1307177-1307609
BmHel-15	DF090441:523009-523430
BmHel-15	DF090439:636831-637259
BmHel-15	DF090458:287150-287570
BmHel-15	DF090347:1299698-1300119
BmHel-15	DF090316:5298048-5298238
BmHel-15	DF090316:5297800-5298036
BmHel-15	DF090336:2340911-2341010
BmHel-15	DF090336:2341037-2341378
BmHel-15	DF090421:967291-967717
BmHel-15	DF090338:1637685-1637824
BmHel-15	DF090338:1637387-1637674
BmHel-15	DF090317:4056267-4056581
BmHel-15	DF090317:4056608-4056738
BmHel-15	DF090342:3886156-3886475
BmHel-15	DF090342:3886896-3887012
BmHel-15	DF090316:14187590-14188016
BmHel-15	DF090325:2186130-2186551
BmHel-15	DF090398:872437-872866
BmHel-15	DF090388:537386-537812
BmHel-15	DF090324:5521768-5522199

BmHel-15	DF090318:539943-540356
BmHel-15	DF090318:5993148-5993577
BmHel-15	DF090318:1213565-1213990
BmHel-15	DF090364:1521448-1521877
BmHel-15	DF090322:7614716-7615141
BmHel-15	DF090404:683390-683813
BmHel-15	DF090342:2456725-2457144
BmHel-15	DF090342:2969563-2969984
BmHel-15	DF090362:223251-223677
BmHel-15	DF090362:2881033-2881457
BmHel-15	DF090332:5565017-5565444
BmHel-15	DF090340:1912463-1912880
BmHel-15	DF090337:258501-258926
BmHel-15	DF090335:5552445-5552870
BmHel-15	DF090412:134760-135187
BmHel-15	DF090331:1876006-1876434
BmHel-15	DF090330:2222251-2222674
BmHel-15	DF090330:4381405-4381832
BmHel-15	DF090329:4881375-4881796
BmHel-15	DF090317:4533061-4533483
BmHel-15	DF090369:1770939-1771376
BmHel-15	DF090352:3383727-3384153
BmHel-15	DF090452:432982-433397
BmHel-15	DF090452:424041-424465
BmHel-15	DF090379:272025-272440
BmHel-15	DF090433:561317-561743
BmHel-15	DF090363:2055170-2055596
BmHel-15	DF090355:416058-416483
BmHel-15	DF090427:410799-411224
BmHel-15	DF090400:1298255-1298681
BmHel-15	DF090466:177338-177762
BmHel-15	DF090389:75430-75853
BmHel-15	DF090707:9938-10364
BmHel-15	DF090374:1547410-1547830
BmHel-15	DF090375:965757-966177
BmHel-15	DF090339:2737489-2737838
BmHel-15	DF090329:1261842-1262263
BmHel-15	DF090348:290661-291086
BmHel-15	DF090316:15913098-15913524
BmHel-15	DF090325:5180252-5180667
BmHel-15	DF090344:1150415-1150830
BmHel-15	DF090350:1236057-1236478
BmHel-15	DF090336:5049599-5050023
BmHel-15	DF090373:1579712-1580135
BmHel-15	DF090337:4575455-4575882
BmHel-15	DF090335:2827107-2827531
BmHel-15	DF090355:2234241-2234662
BmHel-15	DF090355:1595726-1596104

BmHel-15	DF090317:5756027-5756205
BmHel-15	DF090317:5756462-5756730
BmHel-15	DF090317:2291411-2291551
BmHel-15	DF090317:2291563-2291839
BmHel-15	DF090358:3012337-3012475
BmHel-15	DF090358:3012487-3012768
BmHel-15	DF090327:4013460-4013856
BmHel-15	DF090319:8238591-8239012
BmHel-15	DF090342:2786125-2786545
BmHel-15	DF090377:1197276-1197699
BmHel-15	DF090389:438048-438478
BmHel-15	DF090427:155432-155789
BmHel-15	DF090330:4693815-4694077
BmHel-15	DF090330:4693656-4693818
BmHel-15	DF090386:733892-734323
BmHel-15	DF090352:1541974-1542391
BmHel-15	DF090344:1550153-1550582
BmHel-15	DF090356:1779534-1779959
BmHel-15	DF090318:3634244-3634668
BmHel-15	DF090396:62314-62637
BmHel-15	DF090396:62189-62295
BmHel-15	DF090370:736801-737227
BmHel-15	DF090355:1889740-1890073
BmHel-15	DF090355:1891553-1891643
BmHel-15	DF090393:437274-437578
BmHel-15	DF090393:438006-438136
BmHel-15	DF090336:647562-647985
BmHel-15	DF090361:3241403-3241826
BmHel-15	DF090330:2628764-2629191
BmHel-15	DF090392:1043783-1044202
BmHel-15	DF090345:1811665-1812093
BmHel-15	DF090330:3747983-3748407
BmHel-15	DF090344:876449-876873
BmHel-15	DF090388:1206648-1207063
BmHel-15	DF090343:4263358-4263778
BmHel-15	DF090343:3707640-3708063
BmHel-15	DF090350:4120133-4120560
BmHel-15	DF090322:625633-626057
BmHel-15	DF090322:2704019-2704435
BmHel-15	DF090349:3673258-3673683
BmHel-15	DF090342:1705460-1705887
BmHel-15	DF090342:1405861-1406286
BmHel-15	DF090396:867367-867791
BmHel-15	DF090346:3959330-3959754
BmHel-15	DF090369:679183-679609
BmHel-15	DF090348:2519687-2520114
BmHel-15	DF090368:2077807-2078237
BmHel-15	DF090363:1038481-1038902

BmHel-15	DF090451:107379-107803
BmHel-15	DF090336:966586-966784
BmHel-15	DF090336:966805-967032
BmHel-15	DF090338:4698930-4699207
BmHel-15	DF090338:4699207-4699347
BmHel-15	DF090343:2018604-2018999
BmHel-15	DF090341:4395402-4395518
BmHel-15	DF090341:4396385-4396711
BmHel-15	DF090443:297937-298163
BmHel-15	DF090443:298196-298387
BmHel-15	DF090320:3414339-3414596
BmHel-15	DF090320:3414653-3414805
BmHel-15	DF090676:5018-5447
BmHel-15	DF090358:2350982-2351124
BmHel-15	DF090358:2351135-2351411
BmHel-15	DF090364:1135784-1136139
BmHel-15	DF090329:5128475-5128660
BmHel-15	DF090329:5128223-5128456
BmHel-15	BABH01045133:2333-2688
BmHel-15	DF090404:1305732-1306157
BmHel-15	DF090348:2545457-2545879
BmHel-15	DF090318:4925445-4925869
BmHel-15	DF090354:3233403-3233655
BmHel-15	DF090354:3231748-3231927
BmHel-15	DF090428:821779-822207
BmHel-15	DF090344:3476419-3476841
BmHel-15	DF090324:5529965-5530389
BmHel-15	DF090362:769894-770127
BmHel-15	DF090362:769679-769874
BmHel-15	DF090479:231479-231908
BmHel-15	DF090411:738427-738854
BmHel-15	DF090345:1140649-1141078
BmHel-15	DF090332:6387137-6387561
BmHel-15	DF090326:6971060-6971484
BmHel-15	DF090360:1625118-1625533
BmHel-15	DF090330:3618736-3618878
BmHel-15	DF090330:3618890-3619167
BmHel-15	DF090325:5468481-5468623
BmHel-15	DF090325:5468634-5468807
BmHel-15	DF090325:5469958-5470066
BmHel-15	DF090370:2633428-2633784
BmHel-15	DF090330:37351-37739
BmHel-15	DF091262:3098-3240
BmHel-15	DF091262:3254-3533
BmHel-15	DF090364:2464522-2464665
BmHel-15	DF090364:2464232-2464511
BmHel-15	DF090718:11186-11510
BmHel-15	DF090718:10791-10887

BmHel-15	DF090316:2994985-2995406
BmHel-15	DF090382:138731-139147
BmHel-15	DF090827:8404-8544
BmHel-15	DF090827:8115-8398
BmHel-15	DF090317:5625260-5625677
BmHel-15	DF090321:3365123-3365548
BmHel-15	DF090362:2675411-2675799
BmHel-15	DF090336:4801083-4801502
BmHel-15	DF090337:4051348-4051773
BmHel-15	DF090397:32933-33354
BmHel-15	DF090327:5819309-5819719
BmHel-15	DF090339:4280004-4280273
BmHel-15	DF090339:4279829-4279981
BmHel-15	DF090322:6182182-6182603
BmHel-15	DF090362:3181724-3182146
BmHel-15	DF090332:579115-579539
BmHel-15	DF090320:4627590-4627893
BmHel-15	DF090320:4628028-4628156
BmHel-15	DF090365:2524287-2524709
BmHel-15	DF090413:142017-142439
BmHel-15	DF090355:3441080-3441504
BmHel-15	DF090400:1366796-1367220
BmHel-15	DF090415:253211-253636
BmHel-15	DF090388:978980-979405
BmHel-15	DF090354:2966476-2966904
BmHel-15	DF090345:2288271-2288685
BmHel-15	DF090351:398871-399289
BmHel-15	DF090369:658939-659358
BmHel-15	DF090358:2525594-2526018
BmHel-15	DF090321:1707421-1707847
BmHel-15	DF090339:5094210-5094397
BmHel-15	DF090339:5094409-5094639
BmHel-15	DF090320:5996302-5996440
BmHel-15	DF090320:5996458-5996725
BmHel-15	DF090396:1054679-1054861
BmHel-15	DF090396:1054875-1055117
BmHel-15	DF090344:4311806-4311945
BmHel-15	DF090344:4311524-4311793
BmHel-15	DF090322:6865768-6866183
BmHel-15	DF090352:735286-735696
BmHel-15	DF090717:19480-19908
BmHel-15	DF090391:594734-595151
BmHel-15	DF090317:4104309-4104735
BmHel-15	DF090806:17010-17434
BmHel-15	DF090368:2001366-2001781
BmHel-15	DF090355:169850-170262
BmHel-15	DF090344:1563742-1564054
BmHel-15	DF090344:1564056-1564162

BmHel-15	DF090322:2883369-2883684
BmHel-15	DF090322:2883685-2883788
BmHel-15	DF090345:3082748-3083171
BmHel-15	DF090574:45549-45971
BmHel-15	DF090379:800690-800830
BmHel-15	DF090379:800398-800679
BmHel-15	BABH01043610:1208-1556
BmHel-15	DF090321:6839971-6840384
BmHel-15	DF090344:3241656-3242078
BmHel-15	DF090332:696016-696322
BmHel-15	DF090332:696354-696470
BmHel-15	DF090365:2827680-2828102
BmHel-15	DF090365:2232498-2232726
BmHel-15	DF090365:2232273-2232464
BmHel-15	DF090323:5150954-5151368
BmHel-15	DF090317:8535314-8535747
BmHel-15	DF090348:1406433-1406620
BmHel-15	DF090348:1406632-1406867
BmHel-15	DF090394:1135623-1135970
BmHel-15	DF090363:2321409-2321828
BmHel-15	DF090374:436734-437145
BmHel-15	DF090423:353140-353561
BmHel-15	DF090351:4269708-4270130
BmHel-15	DF090334:2349340-2349759
BmHel-15	DF090348:1528680-1529098
BmHel-15	DF090517:40804-41222
BmHel-15	DF090336:1669543-1669974
BmHel-15	DF090362:1338351-1338773
BmHel-15	DF090360:3088090-3088511
BmHel-15	DF090329:5180331-5180633
BmHel-15	DF090328:6157818-6158012
BmHel-15	DF090328:6158026-6158253
BmHel-15	DF090349:1534239-1534381
BmHel-15	DF090349:1534398-1534683
BmHel-15	DF090420:126985-127338
BmHel-15	DF090377:1107032-1107225
BmHel-15	DF090377:1107244-1107473
BmHel-15	DF090355:2557722-2558080
BmHel-15	DF090386:2004206-2004511
BmHel-15	DF090386:2004081-2004197
BmHel-15	DF090339:514286-514424
BmHel-15	DF090339:514440-514717
BmHel-15	DF090440:156795-157212
BmHel-15	DF090537:1159-1584
BmHel-15	BABH01046347:242-650
BmHel-15	DF090414:10580-10896
BmHel-15	DF090414:10477-10579
BmHel-15	DF090350:1819270-1819622

BmHel-15	DF090319:3057723-3058135
BmHel-15	DF090372:1419642-1419777
BmHel-15	DF090372:1419337-1419626
BmHel-15	DF090331:5898353-5898592
BmHel-15	DF090331:5897912-5898093
BmHel-15	DF090329:366255-366386
BmHel-15	DF090329:365961-366242
BmHel-15	DF090341:4091738-4092155
BmHel-15	DF090420:1160441-1160812
BmHel-15	DF090431:382763-382893
BmHel-15	DF090431:382910-383192
BmHel-15	DF090321:2667303-2667465
BmHel-15	DF090321:2667473-2667743
BmHel-15	DF090318:3336331-3336754
BmHel-15	DF090392:1355095-1355508
BmHel-15	DF090319:4542442-4542753
BmHel-15	DF090319:4542754-4542852
BmHel-15	DF090333:908803-909071
BmHel-15	DF090333:908627-908738
BmHel-15	DF090330:5136213-5136355
BmHel-15	DF090330:5136369-5136644
BmHel-15	DF090358:2756583-2756681
BmHel-15	DF090358:2756365-2756535
BmHel-15	DF090358:2756212-2756368
BmHel-15	DF090862:2609-2912
BmHel-15	DF090862:2496-2608
BmHel-15	DF090352:2174295-2174487
BmHel-15	DF090352:2174067-2174293
BmHel-15	DF090602:5986-6183
BmHel-15	DF090602:5746-5974
BmHel-15	DF090325:938468-938884
BmHel-15	DF090377:1224003-1224426
BmHel-15	DF090325:3076564-3076982
BmHel-15	DF090473:9027-9440
BmHel-15	DF090321:1431641-1432058
BmHel-15	DF090322:972399-972726
BmHel-15	DF090322:965767-965876
BmHel-15	DF090361:829600-829733
BmHel-15	DF090361:829307-829588
BmHel-15	DF090337:5223795-5224212
BmHel-15	DF090335:1717278-1717554
BmHel-15	DF090335:1717034-1717188
BmHel-15	DF090338:754954-755372
BmHel-15	DF090317:116212-116562
BmHel-15	DF090363:1176749-1177171
BmHel-15	DF090385:656205-656619
BmHel-15	DF090349:2334420-2334835
BmHel-15	DF090404:1340793-1341211

BmHel-15	DF090360:1900487-1900906
BmHel-15	DF090339:862815-862958
BmHel-15	DF090339:862969-863179
BmHel-15	DF090322:2839295-2839658
BmHel-15	DF090321:4137583-4137718
BmHel-15	DF090321:4137749-4138031
BmHel-15	DF090355:2875621-2875983
BmHel-15	DF090412:332109-332299
BmHel-15	DF090412:332784-333012
BmHel-15	DF090423:125453-125872
BmHel-15	DF090353:3277204-3277622
BmHel-15	DF090321:2082350-2082535
BmHel-15	DF090321:2082668-2082907
BmHel-15	DF090400:724307-724660
BmHel-15	DF090422:404747-405107
BmHel-15	DF090422:404356-404448
BmHel-15	DF090388:575118-575296
BmHel-15	DF090388:574870-575105
BmHel-15	DF090322:6535748-6536168
BmHel-15	DF090354:2903112-2903238
BmHel-15	DF090354:2902829-2903112
BmHel-15	DF090320:3023957-3024377
BmHel-15	DF090365:149736-150064
BmHel-15	DF090365:149379-149490
BmHel-15	DF090335:2952301-2952436
BmHel-15	DF090335:2952004-2952280
BmHel-15	DF090333:3545021-3545372
BmHel-15	DF090318:5416064-5416474
BmHel-15	DF090387:1527134-1527553
BmHel-15	DF090400:782235-782422
BmHel-15	DF090400:782004-782231
BmHel-15	DF090387:759519-759821
BmHel-15	DF090387:758997-759110
BmHel-15	DF090342:4166400-4166791
BmHel-15	DF090362:826418-826553
BmHel-15	DF090362:826565-826841
BmHel-15	DF090350:689221-689403
BmHel-15	DF090350:689416-689643
BmHel-15	DF090422:993193-993325
BmHel-15	DF090422:992888-993174
BmHel-15	DF090340:1661994-1662131
BmHel-15	DF090340:1662145-1662421
BmHel-15	DF090390:747312-747661
BmHel-15	DF090348:1785755-1786112
BmHel-15	DF090327:2139849-2139979
BmHel-15	DF090327:2139994-2140273
BmHel-15	DF090328:3113959-3114373
BmHel-15	DF090318:1984690-1985052

BmHel-15	DF090384:1315847-1316266
BmHel-15	DF090319:8638183-8638594
BmHel-15	DF090372:1858887-1859231
BmHel-15	DF090337:2798330-2798629
BmHel-15	DF090337:2798215-2798333
BmHel-15	DF090331:3039113-3039248
BmHel-15	DF090331:3039259-3039542
BmHel-15	DF090334:3437622-3438040
BmHel-15	DF090422:750981-751408
BmHel-15	DF090326:1464-1876
BmHel-15	DF090363:709918-710277
BmHel-15	DF090322:1959167-1959585
BmHel-15	DF090426:869533-869951
BmHel-15	DF090329:1315959-1316375
BmHel-15	DF090344:3693820-3694232
BmHel-15	DF090412:1352062-1352195
BmHel-15	DF090412:1351762-1352045
BmHel-15	DF090372:2333443-2333577
BmHel-15	DF090372:2333596-2333878
BmHel-15	DF090320:3151014-3151194
BmHel-15	DF090320:3151199-3151427
BmHel-15	DF090386:150848-150979
BmHel-15	DF090386:150998-151324
BmHel-15	DF090582:34205-34398
BmHel-15	DF090582:34409-34562
BmHel-15	DF090328:4540021-4540308
BmHel-15	DF090328:4540361-4540460
BmHel-15	DF090323:2750759-2751064
BmHel-15	DF090323:2750650-2750758
BmHel-15	DF090335:1466293-1466433
BmHel-15	DF090335:1466017-1466282
BmHel-15	DF090351:420910-421090
BmHel-15	DF090351:421096-421328
BmHel-15	DF090429:105462-105880
BmHel-15	DF090460:106026-106156
BmHel-15	DF090460:108773-109064
BmHel-15	DF090316:4337510-4337802
BmHel-15	DF090316:4337386-4337510
BmHel-15	DF090343:1219807-1219987
BmHel-15	DF090343:1219999-1220239
BmHel-15	DF090324:7617451-7617583
BmHel-15	DF090324:7617597-7617880
BmHel-15	DF090332:5632173-5632591
BmHel-15	DF090320:5428578-5428994
BmHel-15	DF090378:218033-218218
BmHel-15	DF090378:218232-218467
BmHel-15	DF090378:1971800-1972031
BmHel-15	DF090378:1971580-1971773

BmHel-15	DF090330:5100002-5100088
BmHel-15	DF090330:5100085-5100416
BmHel-15	DF090317:7504783-7504915
BmHel-15	DF090317:7504486-7504774
BmHel-15	DF090383:615254-615480
BmHel-15	DF090383:612814-613031
BmHel-15	DF090324:5652708-5653022
BmHel-15	DF090324:5652608-5652709
BmHel-15	DF090390:1355146-1355555
BmHel-15	DF090316:15919650-15919792
BmHel-15	DF090316:15919356-15919629
BmHel-15	DF090336:5477608-5477790
BmHel-15	DF090336:5477373-5477605
BmHel-15	DF090410:990063-990201
BmHel-15	DF090410:989762-990033
BmHel-15	DF090378:642073-642447
BmHel-15	DF090433:152492-152634
BmHel-15	DF090433:152195-152473
BmHel-15	DF090324:3167424-3167662
BmHel-15	DF090324:3167668-3167863
BmHel-15	DF090395:1219488-1219901
BmHel-15	DF090477:218168-218580
BmHel-15	DF090388:235733-235868
BmHel-15	DF090388:235890-236166
BmHel-15	DF090327:5006960-5007308
BmHel-15	DF090327:5007309-5007405
BmHel-15	DF090387:1093662-1093796
BmHel-15	DF090387:1093809-1094083
BmHel-15	DF090316:7598200-7598544
BmHel-15	DF090364:1850256-1850439
BmHel-15	DF090364:1850035-1850252
BmHel-15	DF090342:3891831-3891962
BmHel-15	DF090342:3891535-3891819
BmHel-15	DF090383:871299-871428
BmHel-15	DF090383:871439-871724
BmHel-15	DF090339:509522-509938
BmHel-15	DF090360:2571438-2571855
BmHel-15	DF090354:2946392-2946806
BmHel-15	DF090355:3244027-3244379
BmHel-15	DF090353:1471133-1471272
BmHel-15	DF090353:1471278-1471548
BmHel-15	DF090352:857691-858046
BmHel-15	DF090321:8209910-8210045
BmHel-15	DF090321:8210069-8210346
BmHel-15	DF090355:604819-605168
BmHel-15	DF090322:2172922-2173109
BmHel-15	DF090322:2173115-2173347
BmHel-15	DF090334:1300051-1300371

BmHel-15	DF090334:1300373-1300463
BmHel-15	DF090321:580273-580685
BmHel-15	DF090365:2780837-2781258
BmHel-15	DF090361:1154913-1155328
BmHel-15	DF090321:1182547-1182967
BmHel-15	DF090318:1242214-1242574
BmHel-15	DF090320:1953444-1953796
BmHel-15	DF090319:177058-177348
BmHel-15	DF090319:177355-177475
BmHel-15	DF090342:482567-482679
BmHel-15	DF090342:483702-484027
BmHel-15	DF090329:438953-439088
BmHel-15	DF090329:438762-438921
BmHel-15	DF090329:438631-438750
BmHel-15	DF090402:793634-793769
BmHel-15	DF090402:793339-793618
BmHel-15	DF090450:523048-523182
BmHel-15	DF090450:522731-523013
BmHel-15	DF090319:212221-212399
BmHel-15	DF090319:212726-212959
BmHel-15	DF090347:3521052-3521469
BmHel-15	DF090353:911577-911721
BmHel-15	DF090353:911731-911938
BmHel-15	DF090328:837149-837281
BmHel-15	DF090328:837295-837582
BmHel-15	DF090372:1326731-1326861
BmHel-15	DF090372:1326421-1326704
BmHel-15	DF090356:3757976-3758280
BmHel-15	DF090356:3758283-3758392
BmHel-15	DF090346:3636136-3636496
BmHel-15	DF090321:3979285-3979420
BmHel-15	DF090321:3979422-3979708
BmHel-15	DF090362:1769327-1769514
BmHel-15	DF090362:1769532-1769752
BmHel-15	DF090347:2879234-2879650
BmHel-15	DF090359:2833583-2833997
BmHel-15	DF090330:4865117-4865257
BmHel-15	DF090330:4864913-4865104
BmHel-15	DF090330:4864698-4864797
BmHel-15	DF090374:1025756-1026000
BmHel-15	DF090374:1025355-1025467
BmHel-15	DF090320:3290598-3290737
BmHel-15	DF090320:3290311-3290586
BmHel-15	DF090427:934278-934401
BmHel-15	DF090427:933983-934264
BmHel-15	DF090331:707107-707291
BmHel-15	DF090331:706859-707085
BmHel-15	DF091070:7466-7650

BmHel-15	DF091070:7672-7898
BmHel-15	DF090340:3204958-3205367
BmHel-15	DF090377:1941686-1941813
BmHel-15	DF090377:1941386-1941673
BmHel-15	DF090333:1549725-1549858
BmHel-15	DF090333:1549878-1550160
BmHel-15	DF090333:3976897-3977248
BmHel-15	DF090330:1356858-1357270
BmHel-15	DF090383:1208809-1209158
BmHel-15	DF091366:3-417
BmHel-15	DF090320:707511-707862
BmHel-15	DF090342:72538-72671
BmHel-15	DF090342:72251-72523
BmHel-15	DF090326:6435030-6435213
BmHel-15	DF090326:6434791-6435017
BmHel-15	DF090320:5726281-5726627
BmHel-15	DF090459:38052-38183
BmHel-15	DF090459:37761-38040
BmHel-15	DF090349:231401-231721
BmHel-15	DF090349:232137-232242
BmHel-15	DF090317:3257485-3257760
BmHel-15	DF090317:3257328-3257469
BmHel-15	DF090316:12875274-12875541
BmHel-15	DF090316:12875568-12875674
BmHel-15	DF090339:3354730-3355041
BmHel-15	DF090339:3355042-3355144
BmHel-15	DF090324:7807361-7807496
BmHel-15	DF090324:7807522-7807799
BmHel-15	DF090336:835821-836010
BmHel-15	DF090336:835694-835801
BmHel-15	DF090336:835585-835697
BmHel-15	DF090409:605673-606024
BmHel-15	DF090400:1382296-1382545
BmHel-15	DF090400:1382544-1382707
BmHel-15	DF090334:4277636-4277954
BmHel-15	DF090360:1757463-1757597
BmHel-15	DF090360:1757168-1757442
BmHel-15	DF090316:12622865-12623040
BmHel-15	DF090316:12623067-12623298
BmHel-15	DF090318:9084667-9084798
BmHel-15	DF090318:9084374-9084653
BmHel-15	BABH01077516:426-557
BmHel-15	BABH01077516:133-412
BmHel-15	DF090316:7311890-7311983
BmHel-15	DF090316:7311537-7311861
BmHel-15	DF090338:4064967-4065063
BmHel-15	DF090338:4064624-4064946
BmHel-15	DF090321:2858752-2859167

BmHel-15	DF090345:1995055-1995303
BmHel-15	DF090345:1995460-1995625
BmHel-15	DF090322:7853381-7853797
BmHel-15	DF090332:916525-916929
BmHel-15	DF090357:1669464-1669599
BmHel-15	DF090357:1669624-1669898
BmHel-15	DF090342:3753239-3753366
BmHel-15	DF090342:3753387-3753664
BmHel-15	DF090356:654883-655013
BmHel-15	DF090356:654577-654861
BmHel-15	DF090368:1279842-1280243
BmHel-15	DF090408:960098-960498
BmHel-15	DF090330:70487-70753
BmHel-15	DF090330:70341-70485
BmHel-15	DF090413:329998-330330
BmHel-15	DF090326:4283856-4284196
BmHel-15	DF090351:1104264-1104613
BmHel-15	DF090336:3300069-3300197
BmHel-15	DF090336:3300222-3300507
BmHel-15	DF090404:1352139-1352274
BmHel-15	DF090404:1352285-1352559
BmHel-15	DF090344:313195-313325
BmHel-15	DF090344:312908-313183
BmHel-15	DF090364:2698216-2698568
BmHel-15	DF090365:1830876-1831003
BmHel-15	DF090365:1831021-1831305
BmHel-15	DF090329:1457261-1457494
BmHel-15	DF090329:1457083-1457257
BmHel-15	DF090399:1629286-1629537
BmHel-15	DF090399:1629134-1629285
BmHel-15	DF090791:11786-12127
BmHel-15	DF090323:4067699-4068042
BmHel-15	DF090319:1335458-1335870
BmHel-15	DF090354:1972528-1972651
BmHel-15	DF090354:1972228-1972510
BmHel-15	DF090390:1507431-1507559
BmHel-15	DF090390:1507126-1507405
BmHel-15	DF090346:3167455-3167592
BmHel-15	DF090346:3167631-3167911
BmHel-15	DF090336:1699000-1699420
BmHel-15	DF090327:2594231-2594408
BmHel-15	DF090327:2593958-2594192
BmHel-15	DF090339:2527762-2527886
BmHel-15	DF090339:2527883-2528160
BmHel-15	DF090335:5828253-5828442
BmHel-15	DF090335:5828470-5828689
BmHel-15	BABH01049801:384-727
BmHel-15	DF090329:3132253-3132577

BmHel-15	DF090329:3132578-3132662
BmHel-15	DF090340:2737402-2737529
BmHel-15	DF090340:2737547-2737829
BmHel-15	DF090368:294018-294364
BmHel-15	DF090363:2223018-2223141
BmHel-15	DF090363:2222703-2222987
BmHel-15	DF090363:1136953-1137294
BmHel-15	DF090375:1762100-1762282
BmHel-15	DF090375:1762297-1762484
BmHel-15	DF090344:1420790-1420971
BmHel-15	DF090344:1420542-1420775
BmHel-15	DF090388:558704-559047
BmHel-15	DF090351:2577892-2578301
BmHel-15	DF090381:356604-356947
BmHel-15	DF090342:4152462-4152805
BmHel-15	DF090339:3708626-3708768
BmHel-15	DF090339:3708800-3709062
BmHel-15	DF090361:931601-931740
BmHel-15	DF090361:931754-931843
BmHel-15	DF090361:931841-932019
BmHel-15	DF090369:1171870-1172062
BmHel-15	DF090369:1169690-1169906
BmHel-15	DF090385:1348650-1348966
BmHel-15	DF090385:1348541-1348631
BmHel-15	DF090343:1488968-1489321
BmHel-15	DF090357:2853936-2854069
BmHel-15	DF090357:2854103-2854383
BmHel-15	DF090368:1030806-1031168
BmHel-15	DF090366:253826-254015
BmHel-15	DF090366:254032-254244
BmHel-15	DF090343:47186-47604
BmHel-15	DF090396:1574585-1574999
BmHel-15	DF090431:192627-192972
BmHel-15	DF090349:881835-882200
BmHel-15	DF090390:1491159-1491284
BmHel-15	DF090390:1490830-1491114
BmHel-15	DF090544:21710-22061
BmHel-15	DF090321:520000-520345
BmHel-15	DF090407:600059-600317
BmHel-15	DF090407:600755-600907
BmHel-15	DF090340:2709229-2709636
BmHel-15	DF090329:4259473-4259826
BmHel-15	DF090334:4039064-4039199
BmHel-15	DF090334:4038762-4039042
BmHel-15	DF090368:571410-571533
BmHel-15	DF090368:571540-571829
BmHel-15	DF090441:158815-159168
BmHel-15	DF090342:151837-152243

BmHel-15	DF090346:381345-381472
BmHel-15	DF090346:381052-381321
BmHel-15	DF090320:3660444-3660848
BmHel-15	DF090317:8327070-8327478
BmHel-15	DF090348:2503558-2503968
BmHel-15	DF090329:5009412-5009808
BmHel-15	BABH01053050:402-820
BmHel-15	DF090480:154689-154814
BmHel-15	DF090480:154386-154668
BmHel-15	DF090319:645008-645129
BmHel-15	DF090319:644716-644998
BmHel-15	DF090324:5599844-5599979
BmHel-15	DF090324:5599617-5599826
BmHel-15	DF090365:1440932-1441246
BmHel-15	DF090365:1441247-1441345
BmHel-15	DF090328:2419457-2419871
BmHel-15	DF090340:2039607-2039731
BmHel-15	DF090340:2039762-2040045
BmHel-15	DF090363:1688549-1688671
BmHel-15	DF090363:1688242-1688531
BmHel-15	DF090334:3442543-3442807
BmHel-15	DF090334:3442807-3442957
BmHel-15	DF090324:1817751-1817886
BmHel-15	DF090324:1817467-1817740
BmHel-15	DF090340:412803-413157
BmHel-15	DF090379:1856812-1856937
BmHel-15	DF090379:1856977-1857260
BmHel-15	DF090368:1059152-1059283
BmHel-15	DF090368:1058853-1059124
BmHel-15	DF090341:2453284-2453471
BmHel-15	DF090341:2453494-2453728
BmHel-15	DF090316:11765853-11766027
BmHel-15	DF090316:11766046-11766272
BmHel-15	DF090396:1113776-1114184
BmHel-15	DF090370:1474972-1475382
BmHel-15	DF090322:5820825-5820950
BmHel-15	DF090322:5820962-5821244
BmHel-15	DF090317:2649627-2649757
BmHel-15	DF090317:2649319-2649597
BmHel-15	DF090415:599310-599664
BmHel-15	DF090325:2028848-2028973
BmHel-15	DF090325:2029006-2029289
BmHel-15	DF090376:1529649-1529953
BmHel-15	DF090376:1529531-1529635
BmHel-15	DF090424:862638-862766
BmHel-15	DF090424:862340-862617
BmHel-15	DF090321:4075953-4076247
BmHel-15	DF090321:4075823-4075932

BmHel-15	DF090357:3244926-3245334
BmHel-15	DF090326:5252775-5253179
BmHel-15	DF090318:5906844-5907246
BmHel-15	DF090319:1721663-1722067
BmHel-15	DF090326:3103336-3103424
BmHel-15	DF090326:3097178-3097271
BmHel-15	DF090326:3096922-3097147
BmHel-15	DF090341:597452-597810
BmHel-15	BABH01075539:97-461
BmHel-15	DF090375:1320525-1320657
BmHel-15	DF090375:1320229-1320500
BmHel-15	DF090454:288489-288841
BmHel-15	DF090354:2954920-2955045
BmHel-15	DF090354:2955063-2955346
BmHel-15	DF090333:1596439-1596563
BmHel-15	DF090333:1596569-1596846
BmHel-15	DF090347:334895-335239
BmHel-15	DF090326:6525759-6525939
BmHel-15	DF090326:6523356-6523582
BmHel-15	DF090405:1154461-1154860
BmHel-15	DF090375:98628-98817
BmHel-15	DF090375:98832-99054
BmHel-15	DF090327:2748364-2748667
BmHel-15	DF090327:2748201-2748322
BmHel-15	DF090419:280198-280323
BmHel-15	DF090419:279903-280182
BmHel-15	DF090445:428979-429342
BmHel-15	DF090319:5606085-5606489
BmHel-15	DF090792:14747-15148
BmHel-15	DF090317:9055100-9055293
BmHel-15	DF090317:9054995-9055095
BmHel-15	DF090317:9054884-9054994
BmHel-15	DF090401:928501-928628
BmHel-15	DF090401:928653-928901
BmHel-15	DF090424:100201-100557
BmHel-15	DF090360:2741702-2741831
BmHel-15	DF090360:2741402-2741676
BmHel-15	DF090356:3204736-3204914
BmHel-15	DF090356:3204495-3204723
BmHel-15	DF090388:495596-495789
BmHel-15	DF090388:495364-495580
BmHel-15	DF090320:4268353-4268712
BmHel-15	DF090386:1511914-1512273
BmHel-15	DF090447:304347-304473
BmHel-15	DF090447:304522-304801
BmHel-15	DF090324:4545049-4545390
BmHel-15	DF090393:1079078-1079417
BmHel-15	DF090322:5497384-5497729

BmHel-15	DF090319:33398-33525
BmHel-15	DF090319:33104-33379
BmHel-15	DF090337:4236005-4236345
BmHel-15	DF090317:10976525-10976853
BmHel-15	DF090317:10976858-10976966
BmHel-15	DF090394:785943-786253
BmHel-15	DF090394:786349-786443
BmHel-15	DF090341:4293439-4293574
BmHel-15	DF090341:4293154-4293424
BmHel-15	DF090354:3277814-3278161
BmHel-15	DF090338:4596287-4596408
BmHel-15	DF090338:4596426-4596708
BmHel-15	DF090338:3084148-3084510
BmHel-15	DF090331:1634655-1634950
BmHel-15	DF090331:1634391-1634554
BmHel-15	DF090481:113177-113368
BmHel-15	DF090481:112959-113172
BmHel-15	DF090329:6651590-6651717
BmHel-15	DF090329:6651735-6651899
BmHel-15	DF090329:6651909-6652026
BmHel-15	DF090358:3420441-3420615
BmHel-15	DF090358:3420197-3420425
BmHel-15	DF090412:308388-308717
BmHel-15	DF090325:7289679-7290041
BmHel-15	DF090459:370978-371382
BmHel-15	DF090437:373199-373299
BmHel-15	DF090437:371978-372063
BmHel-15	DF090437:371718-371954
BmHel-15	DF090362:3124699-3124820
BmHel-15	DF090362:3124397-3124683
BmHel-15	DF090347:272830-273162
BmHel-15	DF090352:2011259-2011563
BmHel-15	DF090352:2011562-2011660
BmHel-15	DF090352:2424296-2424653
BmHel-15	DF090340:169821-170227
BmHel-15	DF090370:891256-891663
BmHel-15	DF090404:1472726-1472934
BmHel-15	DF090404:1472535-1472727
BmHel-15	DF090421:822003-822351
BmHel-15	DF090363:510699-510884
BmHel-15	DF090363:510904-511015
BmHel-15	DF090363:514983-515105
BmHel-15	DF090630:53096-53225
BmHel-15	DF090630:53251-53526
BmHel-15	DF090359:979508-979915
BmHel-15	DF090350:3491262-3491433
BmHel-15	DF090350:3491450-3491683
BmHel-15	DF090338:1679052-1679425

BmHel-15	DF090369:1555715-1555944
BmHel-15	DF090369:1555947-1556125
BmHel-15	DF090387:1412908-1413050
BmHel-15	DF090387:1413077-1413196
BmHel-15	DF090387:1414565-1414707
BmHel-15	DF090880:5992-6117
BmHel-15	DF090880:6133-6412
BmHel-15	DF090360:2900396-2900483
BmHel-15	DF090360:2900077-2900397
BmHel-15	DF090326:6529665-6530064
BmHel-15	BABH01048642:1-404
BmHel-15	DF090356:2756676-2756796
BmHel-15	DF090356:2756826-2757109
BmHel-15	DF090319:1908626-1908746
BmHel-15	DF090319:1908760-1909045
BmHel-15	DF090408:1147210-1147328
BmHel-15	DF090408:1146908-1147191
BmHel-15	DF090425:1001304-1001658
BmHel-15	DF090322:2969257-2969606
BmHel-15	DF090367:142845-142963
BmHel-15	DF090367:142983-143265
BmHel-15	DF090354:3151499-3151631
BmHel-15	DF090354:3151654-3151924
BmHel-15	DF090323:5317491-5317832
BmHel-15	DF090348:419601-419950
BmHel-15	DF090434:108366-108712
BmHel-15	DF090431:342100-342226
BmHel-15	DF090431:341894-342089
BmHel-15	DF090431:341582-341674
BmHel-15	DF090365:2247403-2247810
BmHel-15	DF090351:162629-162967
BmHel-15	DF090384:1470216-1470465
BmHel-15	DF090384:1470063-1470216
BmHel-15	DF090319:7220493-7220627
BmHel-15	DF090319:7220224-7220490
BmHel-15	DF090393:599793-599914
BmHel-15	DF090393:599939-600219
BmHel-15	DF090391:1062459-1062601
BmHel-15	DF090391:1062243-1062448
BmHel-15	DF090353:3069021-3069152
BmHel-15	DF090353:3069185-3069457
BmHel-15	DF090411:1190971-1191113
BmHel-15	DF090411:1191128-1191387
BmHel-15	DF090321:2389895-2390145
BmHel-15	DF090321:2389696-2389867
BmHel-15	DF090378:1016749-1017140
BmHel-15	DF090378:980770-981185
BmHel-15	DF090340:2629210-2629614

BmHel-15	DF090329:5411636-5411755
BmHel-15	DF090329:5411342-5411623
BmHel-15	DF090579:29007-29371
BmHel-15	DF090321:1102612-1103018
BmHel-15	DF090508:115443-115566
BmHel-15	DF090508:115133-115413
BmHel-15	DF090344:3775184-3775319
BmHel-15	DF090344:3775338-3775620
BmHel-15	DF090339:748869-749144
BmHel-15	DF090339:749150-749278
BmHel-15	DF090342:1391087-1391264
BmHel-15	DF090342:1391452-1391683
BmHel-15	DF090323:2291556-2291679
BmHel-15	DF090323:2291259-2291533
BmHel-15	DF090427:947237-947589
BmHel-15	DF090437:341927-342272
BmHel-15	DF090356:1019571-1019973
BmHel-15	DF090400:87925-88043
BmHel-15	DF090400:88093-88374
BmHel-15	DF090323:1664135-1664264
BmHel-15	DF090323:1664275-1664485
BmHel-15	DF090483:99767-99942
BmHel-15	DF090483:99520-99749
BmHel-15	DF090340:4355153-4355550
BmHel-15	DF090388:122479-122879
BmHel-15	DF090388:1308704-1309114
BmHel-15	DF090373:449665-450063
BmHel-15	DF090340:2829984-2830388
BmHel-15	DF090340:227631-228035
BmHel-15	BABH01066976:12-415
BmHel-15	DF090319:933703-934056
BmHel-15	DF090320:6494445-6494564
BmHel-15	DF090320:6494141-6494424
BmHel-15	DF090410:1387491-1387850
BmHel-15	DF090366:1361741-1361919
BmHel-15	DF090366:1361519-1361744
BmHel-15	DF090480:28884-29023
BmHel-15	DF090480:28650-28859
BmHel-15	DF090464:269478-269834
BmHel-15	DF090909:11668-11802
BmHel-15	DF090909:11872-12103
BmHel-15	DF090365:2361527-2361817
BmHel-15	DF090365:2361401-2361513
BmHel-15	DF090622:24711-24830
BmHel-15	DF090622:24408-24688
BmHel-15	DF090317:8898730-8898812
BmHel-15	DF090317:8898821-8898968
BmHel-15	DF090317:8899010-8899182

BmHel-15	DF090420:746164-746494
BmHel-15	DF091005:3399-3527
BmHel-15	DF091005:3067-3338
BmHel-15	DF090316:5037445-5037579
BmHel-15	DF090316:5037596-5037862
BmHel-15	DF090318:6047623-6048016
BmHel-15	DF090372:521969-522325
BmHel-15	DF090377:127912-128273
BmHel-15	DF090418:322568-322753
BmHel-15	DF090418:322441-322552
BmHel-15	DF090418:322240-322357
BmHel-15	DF090363:916190-916399
BmHel-15	DF090363:916003-916191
BmHel-15	DF090358:555842-556180
BmHel-15	DF090334:4053991-4054395
BmHel-15	DF090323:2130331-2130731
BmHel-15	DF090597:25037-25172
BmHel-15	DF090597:24738-24973
BmHel-15	DF090318:962057-962199
BmHel-15	DF090318:962262-962479
BmHel-15	DF090318:6646460-6646598
BmHel-15	DF090318:6646222-6646455
BmHel-15	DF090353:141125-141244
BmHel-15	DF090353:140802-141090
BmHel-15	DF090322:6093573-6093698
BmHel-15	DF090322:6093447-6093553
BmHel-15	DF090322:6092865-6093037
BmHel-15	DF090353:377459-377591
BmHel-15	DF090353:377615-377883
BmHel-15	DF090439:495503-495861
BmHel-15	DF090415:166220-166574
BmHel-15	DF090368:2046356-2046760
BmHel-15	DF090330:853818-853923
BmHel-15	DF090330:853633-853820
BmHel-15	DF090330:853522-853632
BmHel-15	DF090351:2094528-2094933
BmHel-15	DF090394:1309723-1309959
BmHel-15	DF090394:1309502-1309658
BmHel-15	DF090347:507250-507603
BmHel-15	DF090334:4981472-4981823
BmHel-15	DF090460:129927-130042
BmHel-15	DF090460:129626-129912
BmHel-15	DF090316:9241842-9241964
BmHel-15	DF090316:9241612-9241822
BmHel-15	DF090335:197841-197966
BmHel-15	DF090335:196936-197059
BmHel-15	DF090335:196790-196935
BmHel-15	DF090573:5034-5163

BmHel-15	DF090573:4816-5023
BmHel-15	DF090415:472867-473006
BmHel-15	DF090415:473037-473298
BmHel-15	DF090331:5403863-5404264
BmHel-15	DF090448:88192-88592
BmHel-15	DF090335:4170403-4170635
BmHel-15	DF090335:4170634-4170801
BmHel-15	DF090339:4504953-4505084
BmHel-15	DF090339:4505119-4505403
BmHel-15	DF090388:795061-795185
BmHel-15	DF090388:795211-795490
BmHel-15	DF090357:2894552-2894843
BmHel-15	DF090357:2894884-2894978
BmHel-15	DF090401:333999-334317
BmHel-15	DF090392:411138-411330
BmHel-15	DF090392:411347-411497
BmHel-15	DF091913:267-566
BmHel-15	DF091913:568-669
BmHel-15	DF090364:2042332-2042462
BmHel-15	DF090364:2042504-2042773
BmHel-15	DF090336:554364-554760
BmHel-15	DF090324:7060621-7061021
BmHel-15	DF090347:1219440-1219561
BmHel-15	DF090347:1219594-1219772
BmHel-15	DF090347:1220919-1221026
BmHel-15	DF090316:1547649-1548055
BmHel-15	DF090372:1177992-1178338
BmHel-15	DF090330:3482025-3482375
BmHel-15	DF090368:1483885-1484239
BmHel-15	DF090483:32013-32366
BmHel-15	DF090447:110779-111126
BmHel-15	DF090396:601023-601282
BmHel-15	DF090396:601285-601421
BmHel-15	DF090543:45232-45578
BmHel-15	DF090358:1569466-1569585
BmHel-15	DF090358:1569610-1569892
BmHel-15	DF090319:1782321-1782720
BmHel-15	DF090411:504321-504722
BmHel-15	DF090388:114914-115315
BmHel-15	DF090326:2541185-2541588
BmHel-15	DF090351:2126279-2126678
BmHel-15	DF090488:167113-167517
BmHel-15	DF090326:4379840-4380244
BmHel-15	DF090386:1165767-1166173
BmHel-15	BABH01045073:4709-5115
BmHel-15	DF090452:11531-11711
BmHel-15	DF090452:11735-11954
BmHel-15	DF090330:385009-385188

BmHel-15	DF090330:385201-385412
BmHel-15	DF090316:4224065-4224465
BmHel-15	DF090322:7352169-7352576
BmHel-15	DF090365:339604-340005
BmHel-15	DF090367:507254-507598
BmHel-15	DF090316:3411150-3411510
BmHel-15	DF090382:375812-376002
BmHel-15	DF090382:375582-375784
BmHel-15	DF090564:31252-31611
BmHel-15	DF090351:4350837-4350955
BmHel-15	DF090351:4350542-4350827
BmHel-15	DF090480:110305-110555
BmHel-15	DF090480:110556-110700
BmHel-15	DF090354:3552567-3552909
BmHel-15	DF090378:1217275-1217443
BmHel-15	DF090378:1217468-1217699
BmHel-15	DF090370:770948-771069
BmHel-15	DF090370:770644-770919
BmHel-15	DF090346:1881376-1881729
BmHel-15	DF090436:140425-140683
BmHel-15	DF090436:140712-140852
BmHel-15	DF090363:1243687-1243801
BmHel-15	DF090363:1243376-1243661
BmHel-15	DF090325:5565418-5565749
BmHel-15	DF090340:2312534-2312697
BmHel-15	DF090340:2312287-2312521
BmHel-15	DF090424:238639-239047
BmHel-15	DF090400:972221-972613
BmHel-15	DF090316:3433866-3434265
BmHel-15	DF090324:4043141-4043549
BmHel-15	DF090352:1749061-1749459
BmHel-15	DF090352:3065386-3065790
BmHel-15	DF090358:2047114-2047511
BmHel-15	DF090355:2726570-2726969
BmHel-15	DF090392:111446-111833
BmHel-15	DF090374:1489986-1490378
BmHel-15	DF090416:728450-728566
BmHel-15	DF090416:728608-728886
BmHel-15	DF090318:7213538-7213894
BmHel-15	DF090347:2318631-2318920
BmHel-15	DF090347:2318513-2318621
BmHel-15	DF090363:243698-243882
BmHel-15	DF090363:243467-243677
BmHel-15	DF090317:8651753-8651885
BmHel-15	DF090317:8649728-8649999
BmHel-15	DF090324:1830969-1831087
BmHel-15	DF090324:1831137-1831414
BmHel-15	DF090486:163881-164064

BmHel-15	DF090486:164085-164300
BmHel-15	DF090339:1844129-1844485
BmHel-15	DF090339:1593017-1593128
BmHel-15	DF090339:1592703-1592985
BmHel-15	DF090333:4002721-4002995
BmHel-15	DF090333:4002596-4002719
BmHel-15	DF090334:5344355-5344467
BmHel-15	DF090334:5344041-5344326
BmHel-15	DF090335:5316992-5317349
BmHel-15	DF090355:3345182-3345533
BmHel-15	DF090447:435116-435474
BmHel-15	DF090317:6610966-6611298
BmHel-15	DF090344:2138206-2138324
BmHel-15	DF090344:2137886-2138165
BmHel-15	DF090339:4527227-4527477
BmHel-15	DF090339:4527484-4527626
BmHel-15	DF090326:3497080-3497420
BmHel-15	DF090366:2405003-2405300
BmHel-15	DF090366:2404898-2404998
BmHel-15	DF090392:1585931-1586284
BmHel-15	DF090464:131628-131752
BmHel-15	DF090464:131346-131613
BmHel-15	DF090361:1721189-1721582
BmHel-15	DF090317:9067861-9068201
BmHel-15	DF090526:12332-12610
BmHel-15	DF090526:12217-12334
BmHel-15	DF090332:5723523-5723734
BmHel-15	DF090332:5723348-5723502
BmHel-15	BABH01065529:198-319
BmHel-15	BABH01065529:336-607
BmHel-15	DF090326:3820181-3820355
BmHel-15	DF090326:3819948-3820167
BmHel-15	DF090316:2549363-2549482
BmHel-15	DF090316:2549523-2549799
BmHel-15	DF090342:596292-596625
BmHel-15	DF090377:1761609-1761943
BmHel-15	DF090319:4271172-4271289
BmHel-15	DF090319:4270894-4271165
BmHel-15	DF090337:728580-728848
BmHel-15	DF090337:728875-728992
BmHel-15	DF090324:1691812-1691940
BmHel-15	DF090324:1691965-1692243
BmHel-15	DF090328:5822170-5822493
BmHel-15	DF090385:643356-643705
BmHel-15	DF090324:6376895-6377013
BmHel-15	DF090324:6376595-6376870
BmHel-15	DF090327:289057-289189
BmHel-15	DF090327:289208-289419

BmHel-15	DF090385:368015-368197
BmHel-15	DF090385:368219-368430
BmHel-15	DF090389:242950-243240
BmHel-15	DF090389:242841-242949
BmHel-15	DF090402:833023-833201
BmHel-15	DF090402:833217-833434
BmHel-15	DF090358:1172114-1172223
BmHel-15	DF090358:1171813-1172096
BmHel-15	DF090323:1409751-1409928
BmHel-15	DF090323:1409930-1410149
BmHel-15	DF090483:78562-78961
BmHel-15	DF090342:2028-2426
BmHel-15	DF090364:904136-904248
BmHel-15	DF090364:903832-904119
BmHel-15	DF090338:2495246-2495385
BmHel-15	DF090338:2495396-2495596
BmHel-15	DF090324:4959025-4959154
BmHel-15	DF090324:4958734-4959005
BmHel-15	DF090381:1725442-1725838
BmHel-15	DF090333:5268801-5269210
BmHel-15	DF090339:2730465-2730736
BmHel-15	DF090339:2730739-2730862
BmHel-15	DF090342:3971318-3971604
BmHel-15	DF090342:3971620-3971733
BmHel-15	DF090396:1230155-1230385
BmHel-15	DF090396:1230386-1230557
BmHel-15	DF090422:813959-814350
BmHel-15	DF090321:106287-106420
BmHel-15	DF090321:106447-106712
BmHel-15	DF090342:2243804-2244080
BmHel-15	DF090342:2243658-2243779
BmHel-15	DF090359:2199009-2199115
BmHel-15	DF090359:2198712-2198992
BmHel-15	DF090325:4518310-4518705
BmHel-15	DF090356:3068126-3068358
BmHel-15	DF090356:3068364-3068522
BmHel-15	DF090360:590994-591134
BmHel-15	DF090360:591155-591355
BmHel-15	DF090371:154642-155037
BmHel-15	DF090317:1439459-1439855
BmHel-15	DF090316:15517179-15517539
BmHel-15	DF090316:11289018-11289149
BmHel-15	DF090316:11288844-11288997
BmHel-15	DF090316:11288740-11288847
BmHel-15	DF090370:2126661-2126780
BmHel-15	DF090370:2126810-2127085
BmHel-15	DF090456:141168-141361
BmHel-15	DF090456:140942-141149

BmHel-15	DF090363:1885644-1885928
BmHel-15	DF090328:2107840-2108168
BmHel-15	DF090319:5229029-5229167
BmHel-15	DF090319:5228805-5229017
BmHel-15	DF090406:1069642-1069992
BmHel-15	DF090466:104577-104694
BmHel-15	DF090466:104268-104551
BmHel-15	DF090416:505623-505735
BmHel-15	DF090416:505764-506044
BmHel-15	DF090333:4447311-4447661
BmHel-15	DF090357:1777945-1778333
BmHel-15	DF090327:3573107-3573223
BmHel-15	DF090327:3572815-3573084
BmHel-15	DF090316:11519273-11519668
BmHel-15	DF090319:8381266-8381662
BmHel-15	DF090342:4689533-4689928
BmHel-15	DF090323:5601100-5601194
BmHel-15	DF090323:5600792-5601003
BmHel-15	DF090321:4610426-4610823
BmHel-15	DF090408:473624-474024
BmHel-15	DF090367:1694419-1694823
BmHel-15	DF090387:315034-315292
BmHel-15	DF090387:315292-315389
BmHel-15	DF090326:5624079-5624209
BmHel-15	DF090326:5624310-5624545
BmHel-15	DF090344:1186255-1186589
BmHel-15	DF090318:4740557-4740881
BmHel-15	DF090322:6922765-6922984
BmHel-15	DF090322:6922596-6922766
BmHel-15	DF090356:2245970-2246295
BmHel-15	DF090355:2632739-2632925
BmHel-15	DF090355:2632946-2633164
BmHel-15	DF090418:367195-367442
BmHel-15	DF090418:367701-367851
BmHel-15	DF090366:1386083-1386192
BmHel-15	DF090366:1385769-1386053
BmHel-15	DF090359:1796556-1796839
BmHel-15	DF090359:1796442-1796556
BmHel-15	DF090344:1804229-1804425
BmHel-15	DF090344:1804010-1804214
BmHel-15	DF090427:917267-917378
BmHel-15	DF090427:916948-917232
BmHel-15	DF090329:1619506-1619635
BmHel-15	DF090329:1619720-1619809
BmHel-15	DF090329:1620059-1620199
BmHel-15	DF090326:1395016-1395360
BmHel-15	DF090326:4984181-4984572
BmHel-15	DF090340:3112090-3112434

BmHel-15	DF090343:267836-267963
BmHel-15	DF090343:268001-268234
BmHel-15	DF090345:964896-965255
BmHel-15	DF090323:7399874-7400212
BmHel-15	DF090408:734574-734908
BmHel-15	DF090324:2637198-2637509
BmHel-15	DF090355:596706-596945
BmHel-15	DF090355:596553-596706
BmHel-15	DF090360:1785419-1785764
BmHel-15	DF090330:1998635-1998969
BmHel-15	DF090316:15733222-15733468
BmHel-15	DF090316:15733571-15733738
BmHel-15	DF090353:3082823-3082933
BmHel-15	DF090353:3082958-3083241
BmHel-15	DF090435:338064-338173
BmHel-15	DF090435:337766-338049
BmHel-15	DF090487:100058-100201
BmHel-15	DF090487:99862-100046
BmHel-15	DF090325:274473-274586
BmHel-15	DF090325:274623-274777
BmHel-15	DF090325:274800-274924
BmHel-15	DF091579:410-692
BmHel-15	DF091579:775-883
BmHel-15	DF090380:1981134-1981272
BmHel-15	DF090380:1980908-1981115
BmHel-15	DF090348:3340486-3340705
BmHel-15	DF090348:3340719-3340893
BmHel-15	DF090333:3789489-3789872
BmHel-15	DF090330:2187837-2188190
BmHel-15	DF090447:96745-97083
BmHel-15	DF090391:448108-448452
BmHel-15	DF090335:4455028-4455191
BmHel-15	DF090335:4454800-4455026
BmHel-15	DF090356:34012-34406
BmHel-15	DF090412:241759-242152
BmHel-15	DF090422:316641-316869
BmHel-15	DF090422:320196-320376
BmHel-15	DF090348:480154-480545
BmHel-15	DF090319:3410716-3411111
BmHel-15	DF090429:146921-147313
BmHel-15	DF090406:200307-200629
BmHel-15	DF090316:60522-60742
BmHel-15	DF090316:60762-60926
BmHel-15	DF090342:1829075-1829187
BmHel-15	DF090342:1829216-1829423
BmHel-15	DF090403:183117-183461
BmHel-15	DF090382:1192341-1192661
BmHel-15	DF090347:3418508-3418857

BmHel-15	DF090317:9760114-9760230
BmHel-15	DF090317:9757003-9757269
BmHel-15	DF090344:1001763-1002163
BmHel-15	DF090368:1266660-1266984
BmHel-15	DF090330:2900844-2900949
BmHel-15	DF090330:2900535-2900822
BmHel-15	DF090324:4898949-4899054
BmHel-15	DF090324:4899077-4899365
BmHel-15	DF090319:2256166-2256564
BmHel-15	DF090320:1251456-1251639
BmHel-15	DF090320:1251644-1251851
BmHel-15	DF090365:1544916-1545197
BmHel-15	DF090423:88655-88784
BmHel-15	DF090423:88802-89061
BmHel-15	DF090322:8159183-8159311
BmHel-15	DF090322:8158900-8159158
BmHel-15	DF090444:584640-585031
BmHel-15	BABH01046137:910-1303
BmHel-15	DF090339:2093462-2093848
BmHel-15	DF090316:9505626-9505739
BmHel-15	DF090316:9505302-9505573
BmHel-15	DF090463:257935-258212
BmHel-15	DF090463:258218-258324
BmHel-15	DF090332:4075615-4075792
BmHel-15	DF090332:4075824-4076030
BmHel-15	DF090424:141284-141388
BmHel-15	DF090424:141415-141699
BmHel-15	DF090449:368724-368837
BmHel-15	DF090449:368399-368680
BmHel-15	DF090401:559878-559992
BmHel-15	DF090401:566751-566957
BmHel-15	DF090328:3937624-3937966
BmHel-15	BABH01046134:894-1049
BmHel-15	BABH01046134:1063-1302
BmHel-15	DF090472:278816-279209
BmHel-15	DF090347:3838141-3838523
BmHel-15	DF090356:3620544-3620665
BmHel-15	DF090356:3620263-3620490
BmHel-15	DF090468:95289-95402
BmHel-15	DF090468:95439-95715
BmHel-15	DF090332:5021820-5022212
BmHel-15	DF090475:237796-238182
BmHel-15	DF090346:1742764-1743155
BmHel-15	DF090393:1043276-1043556
BmHel-15	DF090393:1043161-1043262
BmHel-15	DF090383:1518616-1518889
BmHel-15	DF090383:1518490-1518602
BmHel-15	DF090340:3201196-3201317

BmHel-15	DF090340:3200928-3201196
BmHel-15	DF090344:1782491-1782597
BmHel-15	DF090344:1782182-1782464
BmHel-15	DF090419:192778-192885
BmHel-15	DF090419:192467-192752
BmHel-15	DF090318:9491501-9491792
BmHel-15	DF090318:9491396-9491500
BmHel-15	DF090318:6915061-6915441
BmHel-15	DF091515:2444-2835
BmHel-15	BABH01047397:1082-1221
BmHel-15	BABH01047397:1235-1387
BmHel-15	BABH01047397:1391-1487
BmHel-15	DF090317:2405699-2405892
BmHel-15	DF090317:2405900-2406090
BmHel-15	DF090338:2314277-2314406
BmHel-15	DF090338:2315073-2315287
BmHel-15	DF090316:12535603-12535847
BmHel-15	DF090316:12535891-12536035
BmHel-15	DF090316:4944493-4944613
BmHel-15	DF090316:4944638-4944839
BmHel-15	DF090327:1781801-1781947
BmHel-15	DF090327:1781701-1781786
BmHel-15	DF090327:1781605-1781701
BmHel-15	DF090325:2987009-2987125
BmHel-15	DF090325:2987148-2987363
BmHel-15	DF090355:2418995-2419125
BmHel-15	DF090355:2419189-2419404
BmHel-15	DF090339:3016733-3017055
BmHel-15	DF090357:825015-825197
BmHel-15	DF090357:825203-825331
BmHel-15	DF090317:1703102-1703403
BmHel-15	DF090317:1702999-1703090
BmHel-15	DF090402:1169942-1170335
BmHel-15	DF090319:5357959-5358041
BmHel-15	DF090319:5357645-5357958
BmHel-15	DF090357:1021581-1021931
BmHel-15	DF090354:2433281-2433561
BmHel-15	DF090354:2433573-2433680
BmHel-15	DF090343:1568201-1568377
BmHel-15	DF090343:1568393-1568603
BmHel-15	DF090339:3329158-3329488
BmHel-15	DF090360:781705-781812
BmHel-15	DF090360:781824-782104
BmHel-15	DF090350:358755-359144
BmHel-15	DF090321:5071219-5071606
BmHel-15	DF090319:3320667-3321058
BmHel-15	DF090390:684912-685304
BmHel-15	DF090394:1290528-1290922

BmHel-15	DF090327:6490664-6490843
BmHel-15	DF090327:6490860-6491063
BmHel-15	DF090316:14230331-14230662
BmHel-15	DF090316:4356058-4356168
BmHel-15	DF090316:4355721-4355895
BmHel-15	DF090362:1591478-1591796
BmHel-15	DF090335:3809846-3810171
BmHel-15	DF090388:223026-223191
BmHel-15	DF090388:222789-223011
BmHel-15	DF090334:3764217-3764490
BmHel-15	DF090334:3764489-3764600
BmHel-15	BABH01046012:2734-2854
BmHel-15	BABH01046012:2462-2734
BmHel-15	DF090345:1379175-1379332
BmHel-15	DF090345:1379336-1379566
BmHel-15	DF090480:2975-3317
BmHel-15	DF090375:1955147-1955254
BmHel-15	DF090375:1955305-1955579
BmHel-15	DF090323:2499093-2499218
BmHel-15	DF090323:2498821-2499076
BmHel-15	DF090352:145669-145792
BmHel-15	DF090352:145939-146203
BmHel-15	DF090365:2404812-2404902
BmHel-15	DF090365:2404549-2404761
BmHel-15	DF090392:1355872-1356223
BmHel-15	DF090316:14307902-14308183
BmHel-15	DF090316:14307797-14307901
BmHel-15	DF090441:674322-674439
BmHel-15	DF090441:674466-674733
BmHel-15	DF090649:31301-31400
BmHel-15	DF090649:30727-31000
BmHel-15	DF090357:217954-218197
BmHel-15	DF090357:217818-217959
BmHel-15	DF090347:3735639-3736031
BmHel-15	DF090362:3122159-3122290
BmHel-15	DF090362:3122304-3122514
BmHel-15	DF090531:49191-49492
BmHel-15	DF090411:862991-863110
BmHel-15	DF090411:863112-863376
BmHel-15	DF090320:3625910-3626193
BmHel-15	DF090320:3626212-3626311
BmHel-15	DF090387:450996-451124
BmHel-15	DF090387:451140-451351
BmHel-15	DF090391:153812-154199
BmHel-15	DF090316:16107614-16107951
BmHel-15	DF090455:24179-24279
BmHel-15	DF090455:23869-24158
BmHel-15	DF090339:4715357-4715676

BmHel-15	DF090324:2081462-2081634
BmHel-15	DF090324:2081280-2081440
BmHel-15	DF090327:2474507-2474798
BmHel-15	DF090324:4274104-4274224
BmHel-15	DF090324:4274305-4274526
BmHel-15	DF090415:922015-922371
BmHel-15	DF090415:922414-922524
BmHel-15	DF090335:3260710-3260837
BmHel-15	DF090335:3260866-3260979
BmHel-15	DF090335:3260976-3261127
BmHel-15	DF090328:6012398-6012525
BmHel-15	DF090328:6012565-6012772
BmHel-15	DF090369:1740017-1740393
BmHel-15	DF090345:3659578-3659761
BmHel-15	DF090345:3659320-3659524
BmHel-15	DF090319:2738090-2738331
BmHel-15	DF090319:2738350-2738442
BmHel-15	DF090323:6801912-6802041
BmHel-15	DF090323:6802074-6802261
BmHel-15	DF090353:1150908-1151038
BmHel-15	DF090353:1151042-1151255
BmHel-15	DF090353:3013127-3013369
BmHel-15	DF090353:3005085-3005327
BmHel-15	DF090354:2663783-2663941
BmHel-15	DF090354:2663951-2664137
BmHel-15	DF090324:3715938-3716034
BmHel-15	DF090324:3716055-3716339
BmHel-15	DF090319:5571099-5571377
BmHel-15	DF090319:5571420-5571524
BmHel-15	DF090350:324795-325112
BmHel-15	DF090329:3426315-3426497
BmHel-15	DF090329:3426095-3426287
BmHel-15	DF090362:2372157-2372283
BmHel-15	DF090362:2371859-2372073
BmHel-15	DF090406:904591-904703
BmHel-15	DF090406:904732-905006
BmHel-15	DF090318:6099818-6099918
BmHel-15	DF090318:6099946-6100214
BmHel-15	DF090396:354492-354758
BmHel-15	DF090396:354773-354885
BmHel-15	DF090362:615817-615926
BmHel-15	DF090362:615608-615804
BmHel-15	DF090362:615418-615514
BmHel-15	DF090726:17695-17840
BmHel-15	DF090726:17442-17681
BmHel-15	DF090346:3528861-3529019
BmHel-15	DF090346:3529034-3529258
BmHel-15	DF090328:3240290-3240426

BmHel-15	DF090328:3240013-3240215
BmHel-15	DF090324:3483521-3483610
BmHel-15	DF090324:3483286-3483509
BmHel-15	DF090352:608573-608705
BmHel-15	DF090352:611977-612186
BmHel-15	DF090430:595988-596072
BmHel-15	DF090430:595515-595770
BmHel-15	DF090327:4039048-4039339
BmHel-15	DF090327:4038921-4039001
BmHel-15	DF090318:5465756-5465861
BmHel-15	DF090318:5465885-5466160
BmHel-15	DF090319:8666115-8666222
BmHel-15	DF090319:8666228-8666501
BmHel-15	DF090323:5759211-5759311
BmHel-15	DF090323:5758928-5759199
BmHel-15	DF090347:1189050-1189425
BmHel-15	DF090317:5377294-5377508
BmHel-15	DF090317:5377512-5377675
BmHel-15	DF090375:77961-78089
BmHel-15	DF090375:78094-78289
BmHel-15	DF090350:1357055-1357157
BmHel-15	DF090350:1357187-1357465
BmHel-15	DF090349:280856-281050
BmHel-15	DF090349:280668-280849
BmHel-15	DF090378:1052675-1052813
BmHel-15	DF090378:1051924-1052127
BmHel-15	DF090383:1671193-1671328
BmHel-15	DF090383:1670915-1671158
BmHel-15	DF090412:1217975-1218076
BmHel-15	DF090412:1217651-1217930
BmHel-15	DF090386:1994447-1994577
BmHel-15	DF090386:1994604-1994803
BmHel-15	DF090327:1466667-1466988
BmHel-15	DF090354:3608244-3608550
BmHel-15	DF090319:742425-742517
BmHel-15	DF090319:742085-742380
BmHel-15	DF090378:11405-11530
BmHel-15	DF090378:11185-11394
BmHel-15	DF090441:134508-134607
BmHel-15	DF090441:133896-134175
BmHel-15	DF090466:232035-232361
BmHel-15	DF090316:7442836-7443215
BmHel-15	DF090344:2628566-2628845
BmHel-15	DF090344:2625036-2625152
BmHel-15	DF090364:117579-117753
BmHel-15	DF090364:117382-117578
BmHel-15	DF090358:2392260-2392371
BmHel-15	DF090358:2392089-2392240

BmHel-15	DF090358:2391976-2392089
BmHel-15	DF090316:11496263-11496396
BmHel-15	DF090316:11496053-11496248
BmHel-15	DF090336:5157504-5157639
BmHel-15	DF090336:5157274-5157492
BmHel-15	DF090429:48230-48611
BmHel-15	DF090327:2678714-2679033
BmHel-15	DF090323:3101730-3102115
BmHel-15	DF090348:520979-521112
BmHel-15	DF090348:521121-521233
BmHel-15	DF090348:521232-521321
BmHel-15	DF090382:1964986-1965098
BmHel-15	DF090382:1965205-1965474
BmHel-15	DF090417:977540-977683
BmHel-15	DF090417:977695-977828
BmHel-15	DF090417:977827-977927
BmHel-15	DF090447:4371-4495
BmHel-15	DF090447:4570-4787
BmHel-15	DF090334:5095152-5095284
BmHel-15	DF090334:5095301-5095517
BmHel-15	DF090344:1641043-1641197
BmHel-15	DF090344:1640562-1640701
BmHel-15	DF090407:1124280-1124389
BmHel-15	DF090407:1123147-1123366
BmHel-15	DF090317:2733752-2733876
BmHel-15	DF090317:2733551-2733734
BmHel-15	DF090464:366136-366506
BmHel-15	DF090430:334249-334353
BmHel-15	DF090430:334020-334227
BmHel-15	DF090573:20380-20709
BmHel-15	DF090343:1795024-1795346
BmHel-15	DF090395:1142841-1142933
BmHel-15	DF090395:1142557-1142834
BmHel-15	DF090353:3325819-3326008
BmHel-15	DF090353:3326048-3326235
BmHel-15	DF090360:2047752-2047894
BmHel-15	DF090360:2047456-2047694
BmHel-15	DF090348:65934-66242
BmHel-15	DF090392:986926-987067
BmHel-15	DF090392:987088-987322
BmHel-15	DF090362:2083984-2084107
BmHel-15	DF090362:2084115-2084322
BmHel-15	DF090371:2550683-2550824
BmHel-15	DF090371:2550425-2550662
BmHel-15	DF090535:69028-69150
BmHel-15	DF090535:68802-69006
BmHel-15	DF090388:1541068-1541171
BmHel-15	DF090388:1541182-1541454

BmHel-15	BABH01047467:946-1135
BmHel-15	BABH01047467:1241-1354
BmHel-15	DF090721:11956-12050
BmHel-15	DF090721:11648-11921
BmHel-15	DF090334:299712-300043
BmHel-15	DF090318:6139428-6139532
BmHel-15	DF090318:6139601-6139836
BmHel-15	DF090429:513097-513474
BmHel-15	DF090330:3361682-3361866
BmHel-15	DF090330:3361899-3361983
BmHel-15	DF090330:3361985-3362088
BmHel-15	DF090320:7611214-7611522
BmHel-15	DF090411:177757-178046
BmHel-15	DF090411:178075-178158
BmHel-15	DF090325:5950433-5950649
BmHel-15	DF090325:5950645-5950796
BmHel-15	DF090321:5131296-5131415
BmHel-15	DF090321:5131445-5131654
BmHel-15	DF090375:568672-568799
BmHel-15	DF090375:568520-568633
BmHel-15	DF090375:568437-568523
BmHel-15	DF090360:1172415-1172744
BmHel-15	DF090332:395112-395422
BmHel-15	DF090322:301168-301539
BmHel-15	DF090374:1169273-1169364
BmHel-15	DF090374:1168972-1169255
BmHel-15	DF090408:265024-265349
BmHel-15	DF090347:2075069-2075450
BmHel-15	DF090353:1425447-1425591
BmHel-15	DF090353:1424925-1425097
BmHel-15	DF090936:11518-11658
BmHel-15	DF090936:11385-11507
BmHel-15	DF090936:11257-11365
BmHel-15	DF090425:813258-813395
BmHel-15	DF090425:812971-813204
BmHel-15	DF090317:6975253-6975391
BmHel-15	DF090317:6975411-6975646
BmHel-15	DF090583:27997-28084
BmHel-15	DF090583:27682-27969
BmHel-15	DF090319:6858194-6858393
BmHel-15	DF090316:5105097-5105202
BmHel-15	DF090316:5105261-5105526
BmHel-15	DF090454:437561-437744
BmHel-15	DF090454:437371-437558
BmHel-15	DF091189:5706-5818
BmHel-15	DF091189:4873-5071
BmHel-15	DF090601:34961-35086
BmHel-15	DF090601:34804-34938

BmHel-15	DF090601:34697-34803
BmHel-15	DF090318:2592697-2592956
BmHel-15	DF090318:2592962-2593072
BmHel-15	DF090355:3789525-3789701
BmHel-15	DF090355:3789296-3789488
BmHel-15	DF090378:972331-972418
BmHel-15	DF090378:972019-972306
BmHel-15	DF090354:902979-903070
BmHel-15	DF090354:902680-902958
BmHel-15	BABH01079551:2-375
BmHel-15	DF090352:1615415-1615664
BmHel-15	DF090352:1615682-1615798
BmHel-15	DF090322:5569974-5570151
BmHel-15	DF090322:5569807-5569954
BmHel-15	DF090325:5042947-5043256
BmHel-15	DF090349:4266203-4266330
BmHel-15	DF090349:4266358-4266491
BmHel-15	DF090349:4266492-4266603
BmHel-15	DF090377:1298369-1298542
BmHel-15	DF090377:1298169-1298368
BmHel-15	DF090358:121828-122133
BmHel-15	DF090355:1213709-1214084
BmHel-15	DF090364:1222007-1222150
BmHel-15	DF090364:1221721-1221951
BmHel-15	DF090370:2338152-2338307
BmHel-15	DF090370:2338050-2338150
BmHel-15	DF090357:1378557-1378655
BmHel-15	DF090357:1378296-1378558
BmHel-15	DF090337:2823841-2823980
BmHel-15	DF090337:2823647-2823824
BmHel-15	DF090339:1020881-1021016
BmHel-15	DF090339:1020615-1020844
BmHel-15	DF090358:345172-345307
BmHel-15	DF090358:345324-345560
BmHel-15	DF090347:1010603-1010975
BmHel-15	DF090586:22199-22573
BmHel-15	DF090355:2335715-2335813
BmHel-15	DF090355:2335825-2336101
BmHel-15	DF090511:1618-1803
BmHel-15	DF090511:845-984
BmHel-15	DF090316:14856288-14856591
BmHel-15	DF090349:2055985-2056103
BmHel-15	DF090349:2055748-2055952
BmHel-15	DF090390:1278617-1278802
BmHel-15	DF090390:1278254-1278371
BmHel-15	DF090377:160897-161028
BmHel-15	DF090377:160652-160887
BmHel-15	DF090322:655279-655482

BmHel-15	DF090322:655483-655577
BmHel-15	BABH01052544:290-593
BmHel-15	DF090352:4176911-4177214
BmHel-15	DF090407:847626-847991
BmHel-15	DF090319:7061584-7061956
BmHel-15	DF090377:1471872-1472177
BmHel-15	DF090409:1157186-1157325
BmHel-15	DF090409:1157409-1157632
BmHel-15	DF090361:1428308-1428431
BmHel-15	DF090361:1428101-1428282
BmHel-15	DF090422:297472-297660
BmHel-15	DF090422:297213-297337
BmHel-15	DF090316:7282047-7282348
BmHel-15	DF090316:7282518-7282699
BmHel-15	DF090376:1454212-1454354
BmHel-15	DF090376:1453951-1454188
BmHel-15	DF090335:5701801-5702086
BmHel-15	DF090335:5704484-5704567
BmHel-15	DF091783:505-790
BmHel-15	DF091783:358-441
BmHel-15	DF090562:62399-62509
BmHel-15	DF090562:62271-62363
BmHel-15	DF090562:62099-62265
BmHel-15	DF091163:5390-5525
BmHel-15	DF091163:5128-5362
BmHel-15	DF090341:4372347-4372439
BmHel-15	DF090341:4372044-4372320
BmHel-15	DF090317:5673882-5674066
BmHel-15	DF090317:5673744-5673879
BmHel-15	DF090611:1564-1706
BmHel-15	DF090611:1721-1950
BmHel-15	DF090411:494689-494998
BmHel-15	DF090325:2635682-2635810
BmHel-15	DF090325:2635856-2636085
BmHel-15	DF090335:2828924-2829006
BmHel-15	DF090335:2828603-2828888
BmHel-15	DF090407:350264-350504
BmHel-15	DF090407:350141-350265
BmHel-15	DF090360:524488-524620
BmHel-15	DF090360:524633-524868
BmHel-15	DF090349:1992173-1992276
BmHel-15	DF090349:1991897-1992131
BmHel-15	DF090397:280380-280511
BmHel-15	DF090397:280597-280829
BmHel-15	DF090331:4109874-4110014
BmHel-15	DF090331:4110029-4110256
BmHel-15	DF090329:596336-596422
BmHel-15	DF090329:602123-602388

BmHel-15	DF090329:602393-602531
BmHel-15	DF090380:1781093-1781306
BmHel-15	DF090380:1781307-1781463
BmHel-15	DF090358:1648143-1648269
BmHel-15	DF090358:1647866-1647996
BmHel-15	DF090316:9927223-9927477
BmHel-15	DF090316:9927066-9927184
BmHel-15	DF090317:5809868-5810137
BmHel-15	DF090382:631780-631907
BmHel-15	DF090382:631946-632175
BmHel-15	DF090488:75384-75519
BmHel-15	DF090488:75563-75795
BmHel-15	DF090331:3417336-3417466
BmHel-15	DF090331:3417513-3417733
BmHel-15	DF090317:6136746-6136875
BmHel-15	DF090317:6136452-6136687
BmHel-15	DF090443:284721-284898
BmHel-15	DF090443:284918-285107
BmHel-15	DF090410:902624-902934
BmHel-15	DF090321:5076138-5076277
BmHel-15	DF090321:5076316-5076543
BmHel-15	DF090349:3903512-3903641
BmHel-15	DF090349:3903257-3903493
BmHel-15	BABH01043665:6049-6132
BmHel-15	BABH01043665:5372-5603
BmHel-15	DF090350:207427-207556
BmHel-15	DF090350:207614-207847
BmHel-15	DF090432:337711-337791
BmHel-15	DF090432:337429-337703
BmHel-15	DF090349:3632485-3632565
BmHel-15	DF090349:3632578-3632867
BmHel-15	DF090411:42608-42975
BmHel-15	DF090411:346725-346848
BmHel-15	DF090411:345977-346156
BmHel-15	DF090321:7418667-7418801
BmHel-15	DF090321:7418474-7418641
BmHel-15	DF090319:2173767-2174030
BmHel-15	DF090319:2173666-2173763
BmHel-15	DF090403:522125-522318
BmHel-15	DF090403:521877-522047
BmHel-15	DF090326:1945063-1945200
BmHel-15	DF090326:1945235-1945464
BmHel-15	DF090323:736658-736793
BmHel-15	DF090323:736518-736635
BmHel-15	DF090323:736405-736516
BmHel-15	DF090353:665241-665441
BmHel-15	DF090353:671366-671489
BmHel-15	DF090463:98415-98520

BmHel-15	DF090463:98149-98410
BmHel-15	DF090348:998921-999145
BmHel-15	DF090348:998772-998911
BmHel-15	DF090413:1138329-1138461
BmHel-15	DF090413:1146164-1146313
BmHel-15	DF090361:635177-635303
BmHel-15	DF090361:634974-635147
BmHel-15	DF090350:3832809-3832891
BmHel-15	DF090350:3832488-3832759
BmHel-15	DF090357:2282280-2282523
BmHel-15	DF090357:2282156-2282279
BmHel-15	DF090547:70592-70716
BmHel-15	DF090547:70778-71010
BmHel-15	DF090320:7282233-7282518
BmHel-15	DF090355:2190579-2190892
BmHel-15	DF090417:1062457-1062599
BmHel-15	DF090417:1062341-1062450
BmHel-15	DF090417:1062206-1062318
BmHel-15	DF090322:3485023-3485347
BmHel-15	DF090368:1979424-1979616
BmHel-15	DF090368:1979329-1979421
BmHel-15	DF090340:4694985-4695117
BmHel-15	DF090340:4694669-4694900
BmHel-15	DF090328:3078150-3078333
BmHel-15	DF090328:3078371-3078493
BmHel-15	DF090362:1319450-1319625
BmHel-15	DF090362:1319643-1319726
BmHel-15	DF090362:1319726-1319836
BmHel-15	DF090362:151009-151134
BmHel-15	DF090362:149748-149891
BmHel-15	DF090362:149654-149747
BmHel-15	DF090455:556935-557122
BmHel-15	DF090455:556746-556864
BmHel-15	DF090384:177187-177435
BmHel-15	DF090384:185349-185463
BmHel-15	DF090415:1333641-1333968
BmHel-15	DF090319:8616658-8616794
BmHel-15	DF090319:8616806-8616973
BmHel-15	DF090457:114781-114954
BmHel-15	DF090457:114573-114761
BmHel-15	DF090319:5180896-5180978
BmHel-15	DF090319:5179308-5179587
BmHel-15	DF090331:5707376-5707738
BmHel-15	DF090325:384940-385306
BmHel-15	DF090390:1746307-1746672
BmHel-15	DF090334:6062489-6062631
BmHel-15	DF090334:6062339-6062445
BmHel-15	DF090334:6062226-6062339

BmHel-15	DF090420:61464-61652
BmHel-15	DF090420:61272-61445
BmHel-15	DF090336:4469346-4469492
BmHel-15	DF090336:4469508-4469676
BmHel-15	DF090316:15150264-15150626
BmHel-15	DF090325:5275453-5275819
BmHel-15	DF090344:71306-71663
BmHel-15	DF090388:1768261-1768622
BmHel-15	DF090318:4665046-4665399
BmHel-15	DF090322:1608718-1609080
BmHel-15	DF090322:3021824-3022182
BmHel-15	DF090354:1914245-1914603
BmHel-15	DF090362:320485-320841
BmHel-15	DF090365:1806904-1807267
BmHel-15	DF090340:3904704-3905066
BmHel-15	DF090406:457117-457471
BmHel-15	DF090345:3555287-3555645
BmHel-15	DF090345:1594706-1595064
BmHel-15	DF090337:671346-671709
BmHel-15	DF090335:2226125-2226488
BmHel-15	DF090323:4558189-4558549
BmHel-15	DF090329:4897437-4897792
BmHel-15	DF090329:5431248-5431609
BmHel-15	DF090317:6635008-6635366
BmHel-15	DF090317:1757533-1757889
BmHel-15	DF090334:5929211-5929572
BmHel-15	DF090429:632928-633292
BmHel-15	DF090385:378447-378803
BmHel-15	DF090368:1110620-1110982
BmHel-15	DF090450:342981-343342
BmHel-15	DF090456:130857-131219
BmHel-15	BABH01060103:326-684
BmHel-15	BABH01066093:162-525
BmHel-15	BABH01070361:7-361
BmHel-15	BABH01050165:357-711
BmHel-15	BABH01051163:318-672
BmHel-15	DF090399:170027-170388
BmHel-15	DF090672:27087-27448
BmHel-15	DF090377:1301200-1301379
BmHel-15	DF090377:1301399-1301512
BmHel-15	DF090330:3742-3868
BmHel-15	DF090330:3366-3596
BmHel-15	DF090317:1346170-1346295
BmHel-15	DF090317:1346348-1346590
BmHel-15	DF090322:787417-787730
BmHel-15	DF090413:685596-685728
BmHel-15	DF090413:685774-686003
BmHel-15	DF090318:1054961-1055083

BmHel-15	DF090318:1055150-1055318
BmHel-15	DF090340:3473047-3473407
BmHel-15	DF090390:566896-567255
BmHel-15	DF090347:600565-600927
BmHel-15	DF090715:7517-7881
BmHel-15	DF090339:942271-942632
BmHel-15	DF090393:738319-738675
BmHel-15	DF090377:2030088-2030217
BmHel-15	DF090377:2030216-2030297
BmHel-15	DF090377:2030451-2030599
BmHel-15	DF090322:1211630-1211712
BmHel-15	DF090322:1220422-1220703
BmHel-15	DF090334:3216302-3216485
BmHel-15	DF090334:3216505-3216675
BmHel-15	DF091174:2412-2714
BmHel-15	DF090322:3814114-3814421
BmHel-15	DF090410:934943-935090
BmHel-15	DF090410:934796-934932
BmHel-15	DF090334:2020537-2020895
BmHel-15	DF090436:73100-73464
BmHel-15	DF090317:2027438-2027797
BmHel-15	DF090336:1715458-1715813
BmHel-15	DF090337:3609010-3609370
BmHel-15	DF090344:3051929-3052056
BmHel-15	DF090344:3052122-3052241
BmHel-15	DF090344:3052598-3052705
BmHel-15	DF090331:5182243-5182376
BmHel-15	DF090331:5182433-5182663
BmHel-15	DF090366:2121437-2121723
BmHel-15	DF090569:41963-42251
BmHel-15	DF090362:2449487-2449612
BmHel-15	DF090362:2449184-2449416
BmHel-15	BABH01085384:8-361
BmHel-15	DF090366:842485-842852
BmHel-15	DF090319:234886-235000
BmHel-15	DF090319:234639-234838
BmHel-15	DF090359:286834-287193
BmHel-15	DF090341:3139794-3140159
BmHel-15	BABH01043176:13758-13883
BmHel-15	BABH01043176:13476-13706
BmHel-15	DF090325:415571-415695
BmHel-15	DF090325:415720-415952
BmHel-15	DF090375:1944127-1944243
BmHel-15	DF090375:1943622-1943826
BmHel-15	DF090330:5370825-5371192
BmHel-15	DF090329:1312947-1313305
BmHel-15	DF090324:1235761-1236119
BmHel-15	DF090316:6372847-6373020

BmHel-15	DF090316:6372688-6372822
BmHel-15	DF090361:2201411-2201544
BmHel-15	DF090361:2201596-2201780
BmHel-15	DF090415:325299-325423
BmHel-15	DF090415:324997-325220
BmHel-15	DF090321:4298279-4298531
BmHel-15	DF090321:4298536-4298640
BmHel-15	DF090345:1268921-1269187
BmHel-15	DF090345:1268796-1268887
BmHel-15	DF090360:408020-408347
BmHel-15	DF090402:4627-4985
BmHel-15	DF090357:1434232-1434590
BmHel-15	DF090319:290466-290832
BmHel-15	DF090323:7200501-7200622
BmHel-15	DF090323:7200687-7200925
BmHel-15	DF090325:6264247-6264374
BmHel-15	DF090325:6265102-6265232
BmHel-15	DF090319:3783210-3783331
BmHel-15	DF090319:3782924-3783160
BmHel-15	DF090338:4753916-4754037
BmHel-15	DF090338:4754089-4754315
BmHel-15	BABH01047187:73-194
BmHel-15	BABH01047187:246-472
BmHel-15	DF090340:1627137-1627443
BmHel-15	DF090370:1743903-1744055
BmHel-15	DF090370:1737254-1737391
BmHel-15	DF090362:1146098-1146180
BmHel-15	DF090362:1145873-1146085
BmHel-15	DF090317:6127265-6127398
BmHel-15	DF090317:6127462-6127689
BmHel-15	DF090454:222431-222619
BmHel-15	DF090454:222654-222817
BmHel-15	DF090334:6028976-6029330
BmHel-15	DF090358:3087654-3088015
BmHel-15	DF090396:1021091-1021456
BmHel-15	DF090317:8426026-8426375
BmHel-15	DF090406:981082-981216
BmHel-15	DF090406:981272-981494
BmHel-15	DF090326:5460154-5460279
BmHel-15	DF090326:5460317-5460544
BmHel-15	DF090336:2281701-2282054
BmHel-15	DF090343:2122602-2122914
BmHel-15	DF090316:11962737-11963094
BmHel-15	DF090338:4554768-4555126
BmHel-15	DF090367:2050998-2051359
BmHel-15	DF090355:1968641-1968939
BmHel-15	DF090351:849781-850072
BmHel-15	DF090355:728689-729043

BmHel-15	DF090388:1506104-1506458
BmHel-15	DF090350:3150494-3150855
BmHel-15	DF090358:2810749-2811110
BmHel-15	DF090402:133173-133527
BmHel-15	BABH01050102:624-978
BmHel-15	DF090446:243589-243722
BmHel-15	DF090446:243403-243579
BmHel-15	DF090320:2962316-2962668
BmHel-15	DF090322:7026906-7027224
BmHel-15	DF090376:1222268-1222411
BmHel-15	DF090376:1222422-1222633
BmHel-15	DF090428:585782-586136
BmHel-15	BABH01083878:178-528
BmHel-15	DF090322:2646218-2646350
BmHel-15	DF090322:2645940-2646161
BmHel-15	DF090335:1643598-1643721
BmHel-15	DF090335:1643767-1643988
BmHel-15	DF090398:890857-891211
BmHel-15	DF090407:515197-515545
BmHel-15	DF090381:80501-80709
BmHel-15	DF090381:80772-80864
BmHel-15	DF090365:1589365-1589488
BmHel-15	DF090365:1589119-1589349
BmHel-15	DF090330:6306675-6307028
BmHel-15	DF090394:635033-635386
BmHel-15	BABH01070236:1-355
BmHel-15	DF090838:8722-9082
BmHel-15	DF090342:1865827-1865927
BmHel-15	DF090342:1865547-1865759
BmHel-15	DF090437:709225-709313
BmHel-15	DF090437:709373-709595
BmHel-15	DF090322:2374661-2375015
BmHel-15	DF090402:525052-525200
BmHel-15	DF090402:524838-524985
BmHel-15	DF090321:549186-549479
BmHel-15	DF090386:1416117-1416247
BmHel-15	DF090386:1415813-1416034
BmHel-15	DF090321:413561-413699
BmHel-15	DF090321:413758-413941
BmHel-15	DF090482:152576-152928
BmHel-15	DF090339:1332821-1332952
BmHel-15	DF090339:1333032-1333163
BmHel-15	DF090322:5477563-5477747
BmHel-15	DF090322:5477759-5477869
BmHel-15	DF090418:1095641-1095991
BmHel-15	DF090375:2204833-2204952
BmHel-15	DF090375:2204382-2204618
BmHel-15	DF090348:230021-230374

BmHel-15	DF090983:8978-9096
BmHel-15	DF090983:8744-8964
BmHel-15	DF090322:498764-499114
BmHel-15	DF090316:10736836-10737189
BmHel-15	DF090344:1465796-1465888
BmHel-15	DF090344:1465907-1466097
BmHel-15	DF090319:3687711-3687900
BmHel-15	DF090319:3687912-3688069
BmHel-15	DF090320:4732543-4732735
BmHel-15	DF090320:4732747-4732904
BmHel-15	DF090352:466025-466214
BmHel-15	DF090352:465856-466013
BmHel-15	DF091789:108-301
BmHel-15	DF091789:313-470
BmHel-15	DF090360:180058-180411
BmHel-15	DF090316:10599664-10599977
BmHel-15	DF090389:606864-607216
BmHel-15	DF090451:532570-532744
BmHel-15	DF090451:532395-532569
BmHel-15	DF090434:302334-302682
BmHel-15	DF090343:2447527-2447656
BmHel-15	DF090343:2447683-2447904
BmHel-15	DF090365:2312370-2312494
BmHel-15	DF090365:2312509-2312739
BmHel-15	DF090319:3048443-3048586
BmHel-15	DF090319:3048190-3048397
BmHel-15	DF090401:1425412-1425723
BmHel-15	DF090403:404285-404642
BmHel-15	DF090345:565506-565626
BmHel-15	DF090345:565321-565499
BmHel-15	DF090390:295552-295741
BmHel-15	DF090390:295434-295530
BmHel-15	DF090370:863434-863604
BmHel-15	DF090370:863232-863415
BmHel-15	DF090334:1830250-1830361
BmHel-15	DF090334:1829991-1830228
BmHel-15	DF090403:951927-952281
BmHel-15	DF090419:264064-264174
BmHel-15	DF090419:263553-263733
BmHel-15	DF090333:2053059-2053410
BmHel-15	DF091413:1838-2191
BmHel-15	DF090335:1433556-1433673
BmHel-15	DF090335:1433759-1434003
BmHel-15	DF090369:1649249-1649391
BmHel-15	DF090369:1648980-1649150
BmHel-15	DF090318:4612099-4612288
BmHel-15	DF090318:4611963-4612079
BmHel-15	DF090461:198168-198305

BmHel-15	DF090461:198329-198483
BmHel-15	DF090334:34731-34918
BmHel-15	DF090334:34932-35089
BmHel-15	DF090323:1817117-1817232
BmHel-15	DF090323:1817329-1817464
BmHel-15	DF090347:3074900-3075031
BmHel-15	DF090347:3075062-3075215
BmHel-15	DF090430:429540-429885
BmHel-15	DF090316:2049844-2050025
BmHel-15	DF090316:2050060-2050164
BmHel-15	DF090319:6114919-6115267
BmHel-15	DF090357:2842607-2842865
BmHel-15	BABH01045068:1086-1211
BmHel-15	BABH01045068:1245-1341
BmHel-15	BABH01045068:1341-1466
BmHel-15	DF090465:18634-18878
BmHel-15	DF090465:18886-19015
BmHel-15	DF090338:3378611-3378927
BmHel-15	DF090325:6825981-6826328
BmHel-15	DF090327:6122279-6122377
BmHel-15	DF090327:6122631-6122881
BmHel-15	DF090514:54595-54704
BmHel-15	DF090514:54367-54574
BmHel-15	DF090365:36738-36984
BmHel-15	DF090365:36630-36733
BmHel-15	DF090349:681291-681421
BmHel-15	DF090349:681463-681622
BmHel-15	DF090334:3551105-3551358
BmHel-15	BABH01044634:4691-5032
BmHel-15	DF090348:1547392-1547736
BmHel-15	DF090373:2063108-2063296
BmHel-15	DF090373:2062989-2063101
BmHel-15	DF090351:2017557-2017780
BmHel-15	DF090351:2017423-2017547
BmHel-15	DF090374:1348850-1349035
BmHel-15	DF090374:1349073-1349240
BmHel-15	DF090489:3577-3698
BmHel-15	DF090489:3797-4021
BmHel-15	DF090355:2605412-2605759
BmHel-15	DF090393:899894-900020
BmHel-15	DF090393:900019-900234
BmHel-15	DF090379:537937-538059
BmHel-15	DF090379:537802-537904
BmHel-15	DF090379:537683-537803
BmHel-15	DF090405:1147304-1147389
BmHel-15	DF090405:1151887-1152147
BmHel-15	DF090338:1351835-1351974
BmHel-15	DF090338:1351608-1351815

BmHel-15	DF090436:621012-621097
BmHel-15	DF090436:617035-617346
BmHel-15	DF090436:616909-617017
BmHel-15	DF090363:877135-877270
BmHel-15	DF090363:877279-877490
BmHel-15	DF090389:39491-39785
BmHel-15	DF090392:1135850-1136188
BmHel-15	DF090370:1887263-1887565
BmHel-15	DF090386:2024909-2025100
BmHel-15	DF090386:2025147-2025257
BmHel-15	DF090443:172033-172380
BmHel-15	DF090384:1108875-1108998
BmHel-15	DF090384:1108996-1109211
BmHel-15	DF090316:717447-717559
BmHel-15	DF090316:717192-717425
BmHel-15	DF091258:480-586
BmHel-15	DF091258:217-451
BmHel-15	DF090349:4146319-4146442
BmHel-15	DF090349:4146054-4146268
BmHel-15	DF090390:1127322-1127466
BmHel-15	DF090390:1126910-1127072
BmHel-15	DF090363:1869157-1869293
BmHel-15	DF090363:1869020-1869124
BmHel-15	DF090363:1868901-1869005
BmHel-15	DF090349:2022259-2022603
BmHel-15	DF090363:1577051-1577392
BmHel-15	DF090319:6771041-6771135
BmHel-15	DF090319:6770761-6771007
BmHel-15	BABH01086653:123-465
BmHel-15	DF090336:4280916-4281215
BmHel-15	DF090363:1788061-1788406
BmHel-15	DF090384:1672186-1672421
BmHel-15	DF090345:4347828-4347950
BmHel-15	DF090345:4347982-4348150
BmHel-15	DF091314:2431-2626
BmHel-15	DF091314:2324-2410
BmHel-15	DF090374:1558924-1559028
BmHel-15	DF090374:1559033-1559268
BmHel-15	DF090467:11371-11711
BmHel-15	DF090372:1758153-1758286
BmHel-15	DF090372:1758348-1758498
BmHel-15	DF090371:635173-635287
BmHel-15	DF090371:635344-635577
BmHel-15	DF090330:4310882-4311063
BmHel-15	DF090330:4310472-4310570
BmHel-15	DF090439:261481-261817
BmHel-15	DF090429:929161-929405
BmHel-15	DF090429:929404-929503

BmHel-15	DF090340:3089816-3090129
BmHel-15	DF090444:496524-496654
BmHel-15	DF090444:496288-496498
BmHel-15	BABH01056608:396-740
BmHel-15	DF090316:12584462-12584577
BmHel-15	DF090316:12584211-12584440
BmHel-15	DF090366:997624-997922
BmHel-15	DF090380:387693-387975
BmHel-15	DF090354:851489-851591
[truncated: 427,233 more chars]
