# Supplementary material for: Identification and Evolution of the Silkworm Helitrons and their Contribution to Transcripts
Source: DNA Res. 2013 Jun 14;20(5):471–84. doi: 10.1093/dnares/dst024 (PMC3789558; doi:10.1093/dnares/dst024)
Supplement: Supplementary Data [file supp_dst024_dst024supp_table2.pdf]

TableS2 Positions of helitrons in Heliconius melpomene genome

Family	Accession numbers
HmHel-1	CAEZ01003019:101905-102768
HmHel-1	CAEZ01003019:131494-132199
HmHel-1	CAEZ01009407:10023-10853
HmHel-1	CAEZ01009407:2411-2733
HmHel-1	CAEZ01011354:234-1062
HmHel-1	CAEZ01006866:13283-13495
HmHel-1	CAEZ01006866:33748-33947
HmHel-1	CAEZ01006866:11621-11987
HmHel-1	CAEZ01002673:19272-20102
HmHel-1	CAEZ01004505:12357-13186
HmHel-1	CAEZ01008735:3629-3716
HmHel-1	CAEZ01008735:42019-42845
HmHel-1	CAEZ01008735:29352-29567
HmHel-1	CAEZ01003151:41210-41555
HmHel-1	CAEZ01003151:64555-65393
HmHel-1	CAEZ01003151:83660-83999
HmHel-1	CAEZ01011462:3983-4808
HmHel-1	CAEZ01009614:16518-16933
HmHel-1	CAEZ01009614:46571-47396
HmHel-1	CAEZ01009614:31786-32116
HmHel-1	CAEZ01005043:104194-104578
HmHel-1	CAEZ01005043:157570-159265
HmHel-1	CAEZ01005043:212872-213190
HmHel-1	CAEZ01005043:183734-184140
HmHel-1	CAEZ01005043:158002-158809
HmHel-1	CAEZ01005043:52564-53229
HmHel-1	CAEZ01010740:63392-64222
HmHel-1	CAEZ01008916:46048-46878
HmHel-1	CAEZ01008916:2203-2752
HmHel-1	CAEZ01002396:1647-2477
HmHel-1	CAEZ01000879:167750-168004
HmHel-1	CAEZ01000879:122998-123827
HmHel-1	CAEZ01000879:60385-60986
HmHel-1	CAEZ01009070:31011-31238
HmHel-1	CAEZ01009070:21321-21660
HmHel-1	CAEZ01009701:10079-10904
HmHel-1	CAEZ01007413:92700-92919
HmHel-1	CAEZ01007413:21231-22054
HmHel-1	CAEZ01004232:5102-5933
HmHel-1	CAEZ01008258:156024-156363
HmHel-1	CAEZ01008258:155594-156024
HmHel-1	CAEZ01008258:18903-19643
HmHel-1	CAEZ01007584:107885-108227
HmHel-1	CAEZ01007584:129756-130439
HmHel-1	CAEZ01007584:193692-194520
HmHel-1	CAEZ01007584:68599-69265

HmHel-1	CAEZ01007584:48593-49418
HmHel-1	CAEZ01007584:24646-24956
HmHel-1	CAEZ01006560:2926-3758
HmHel-1	CAEZ01006560:101535-101874
HmHel-1	CAEZ01006560:68321-68986
HmHel-1	CAEZ01006001:9749-10577
HmHel-1	CAEZ01006001:8130-8829
HmHel-1	CAEZ01004533:1244-2077
HmHel-1	CAEZ01001775:192-435
HmHel-1	CAEZ01001775:32566-33309
HmHel-1	CAEZ01010918:73658-74914
HmHel-1	CAEZ01010918:91230-92055
HmHel-1	CAEZ01010918:74013-74448
HmHel-1	CAEZ01010918:40496-40585
HmHel-1	CAEZ01010827:11583-12239
HmHel-1	CAEZ01010827:124809-125016
HmHel-1	CAEZ01010827:163106-163930
HmHel-1	CAEZ01010827:282582-282676
HmHel-1	CAEZ01010827:423250-423884
HmHel-1	CAEZ01010827:98507-98842
HmHel-1	CAEZ01010724:1488-1703
HmHel-1	CAEZ01010724:25206-26030
HmHel-1	CAEZ01004973:18937-19761
HmHel-1	CAEZ01003034:3036-3861
HmHel-1	CAEZ01002719:4315-5140
HmHel-1	CAEZ01002719:29009-29336
HmHel-1	CAEZ01001577:71351-71985
HmHel-1	CAEZ01001577:51326-52151
HmHel-1	CAEZ01001577:26530-26871
HmHel-1	CAEZ01010752:22268-23094
HmHel-1	CAEZ01001362:3165-3996
HmHel-1	CAEZ01001362:7315-7543
HmHel-1	CAEZ01008821:14814-15646
HmHel-1	CAEZ01002665:7883-8691
HmHel-1	CAEZ01002665:40995-41328
HmHel-1	CAEZ01009774:17415-18239
HmHel-1	CAEZ01008567:1461-2284
HmHel-1	CAEZ01006660:12199-13024
HmHel-1	CAEZ01004535:23924-24339
HmHel-1	CAEZ01007941:192892-193721
HmHel-1	CAEZ01007941:34117-34456
HmHel-1	CAEZ01007941:19606-19885
HmHel-1	CAEZ01004437:3717-4525
HmHel-1	CAEZ01004437:5669-6491
HmHel-1	CAEZ01006124:11391-11623
HmHel-1	CAEZ01006124:37273-38104
HmHel-1	CAEZ01006124:20285-20369
HmHel-1	CAEZ01006124:7234-7573

HmHel-1	CAEZ01009456:172426-173098
HmHel-1	CAEZ01009456:114186-115012
HmHel-1	CAEZ01006328:60814-61624
HmHel-1	CAEZ01003407:66703-67377
HmHel-1	CAEZ01010007:11033-11857
HmHel-1	CAEZ01009443:40905-41642
HmHel-1	CAEZ01009443:63383-64207
HmHel-1	CAEZ01005108:64151-64976
HmHel-1	CAEZ01004633:43472-43704
HmHel-1	CAEZ01004633:24026-24851
HmHel-1	CAEZ01001639:178-999
HmHel-1	CAEZ01001203:2552-3372
HmHel-1	CAEZ01005433:70243-70702
HmHel-1	CAEZ01006718:361-1191
HmHel-1	CAEZ01009871:10384-10616
HmHel-1	CAEZ01009871:39343-40172
HmHel-1	CAEZ01009698:10232-10501
HmHel-1	CAEZ01008742:5211-6050
HmHel-1	CAEZ01008182:18991-19330
HmHel-1	CAEZ01008182:55650-56378
HmHel-1	CAEZ01008182:43815-43903
HmHel-1	CAEZ01008182:25723-25967
HmHel-1	CAEZ01008182:4295-4668
HmHel-1	CAEZ01011306:12113-12947
HmHel-1	CAEZ01009723:15109-15934
HmHel-1	CAEZ01009723:16170-16389
HmHel-1	CAEZ01006738:137-348
HmHel-1	CAEZ01006738:12911-13291
HmHel-1	CAEZ01003070:85349-86174
HmHel-1	CAEZ01003070:146373-146606
HmHel-1	CAEZ01003070:208886-209642
HmHel-1	CAEZ01003070:111112-111919
HmHel-1	CAEZ01003070:40504-40843
HmHel-1	CAEZ01005515:20916-21255
HmHel-1	CAEZ01005515:52191-52996
HmHel-1	CAEZ01005515:3856-4078
HmHel-1	CAEZ01008723:23481-24319
HmHel-1	CAEZ01008723:5399-5758
HmHel-1	CAEZ01007865:29775-30200
HmHel-1	CAEZ01007865:42678-43509
HmHel-1	CAEZ01010616:595-1436
HmHel-1	CAEZ01004814:3951-4779
HmHel-1	CAEZ01004814:51545-51821
HmHel-1	CAEZ01004814:1041-1304
HmHel-1	CAEZ01001650:45237-46063
HmHel-1	CAEZ01009896:30521-30771
HmHel-1	CAEZ01009896:146852-147677
HmHel-1	CAEZ01008926:21-300

HmHel-1	CAEZ01008926:16292-17116
HmHel-1	CAEZ01008550:16815-17639
HmHel-1	CAEZ01008550:10336-10673
HmHel-1	CAEZ01008414:18419-19243
HmHel-1	CAEZ01008270:67128-67467
HmHel-1	CAEZ01008270:100925-101749
HmHel-1	CAEZ01008270:63954-64587
HmHel-1	CAEZ01007975:76781-77122
HmHel-1	CAEZ01007975:71423-72126
HmHel-1	CAEZ01005272:1-684
HmHel-1	CAEZ01001668:9860-10889
HmHel-1	CAEZ01001668:26053-26891
HmHel-1	CAEZ01008951:1455-2290
HmHel-1	CAEZ01005493:1-606
HmHel-1	CAEZ01004829:7-346
HmHel-1	CAEZ01004829:12019-12841
HmHel-1	CAEZ01000876:22870-23705
HmHel-1	CAEZ01008769:15023-15805
HmHel-1	CAEZ01008769:90716-91055
HmHel-1	CAEZ01008769:44608-45268
HmHel-1	CAEZ01005798:16419-17217
HmHel-1	CAEZ01004991:1-825
HmHel-1	CAEZ01004762:13323-13547
HmHel-1	CAEZ01004762:29771-30595
HmHel-1	CAEZ01004762:25038-25376
HmHel-1	CAEZ01004762:12075-12309
HmHel-1	CAEZ01001521:14849-15678
HmHel-1	CAEZ01007273:51842-52536
HmHel-1	CAEZ01007273:94845-95019
HmHel-1	CAEZ01007273:157243-157462
HmHel-1	CAEZ01007273:131963-132790
HmHel-1	CAEZ01007273:110904-111213
HmHel-1	CAEZ01007273:55845-56662
HmHel-1	CAEZ01007273:44988-45793
HmHel-1	CAEZ01007273:25936-26813
HmHel-1	CAEZ01011318:4888-5216
HmHel-1	CAEZ01011318:42429-42768
HmHel-1	CAEZ01011318:19575-20410
HmHel-1	CAEZ01008883:717-1541
HmHel-1	CAEZ01007256:19048-19482
HmHel-1	CAEZ01007256:62513-63274
HmHel-1	CAEZ01007256:77284-77623
HmHel-1	CAEZ01007256:39766-40103
HmHel-1	CAEZ01007256:1-599
HmHel-1	CAEZ01009312:519-1336
HmHel-1	CAEZ01006534:43513-44343
HmHel-1	CAEZ01006534:85847-86488
HmHel-1	CAEZ01006534:121211-121412

HmHel-1	CAEZ01006534:49620-49822
HmHel-1	CAEZ01001125:7610-8437
HmHel-1	CAEZ01010972:7603-7697
HmHel-1	CAEZ01010972:411551-412362
HmHel-1	CAEZ01010972:472631-472870
HmHel-1	CAEZ01010972:418508-418923
HmHel-1	CAEZ01010972:270587-271007
HmHel-1	CAEZ01010972:202413-203192
HmHel-1	CAEZ01010972:175199-175431
HmHel-1	CAEZ01010972:137851-138658
HmHel-1	CAEZ01010972:38150-38242
HmHel-1	CAEZ01010424:73716-74304
HmHel-1	CAEZ01010424:169546-169875
HmHel-1	CAEZ01010424:112913-113118
HmHel-1	CAEZ01010424:101296-101537
HmHel-1	CAEZ01009071:5478-6305
HmHel-1	CAEZ01009071:6495-9892
HmHel-1	CAEZ01004496:58765-59540
HmHel-1	CAEZ01004496:3478-4314
HmHel-1	CAEZ01001338:98489-99319
HmHel-1	CAEZ01001338:60430-60769
HmHel-1	CAEZ01007836:912-1734
HmHel-1	CAEZ01005714:21476-22318
HmHel-1	CAEZ01002797:2528-3352
HmHel-1	CAEZ01001538:21043-21853
HmHel-1	CAEZ01001538:52186-53010
HmHel-1	CAEZ01008960:14474-14693
HmHel-1	CAEZ01008960:42205-42543
HmHel-1	CAEZ01008960:97183-101707
HmHel-1	CAEZ01008960:120450-121041
HmHel-1	CAEZ01008960:132713-133129
HmHel-1	CAEZ01008960:160638-160878
HmHel-1	CAEZ01008960:155315-155953
HmHel-1	CAEZ01008960:100839-101175
HmHel-1	CAEZ01008960:29822-30950
HmHel-1	CAEZ01007519:8306-8640
HmHel-1	CAEZ01007519:55-869
HmHel-1	CAEZ01005757:3911-4736
HmHel-1	CAEZ01007272:610-949
HmHel-1	CAEZ01007272:49417-49756
HmHel-1	CAEZ01007272:390-1068
HmHel-1	CAEZ01002839:16353-17178
HmHel-1	CAEZ01001103:19976-20315
HmHel-1	CAEZ01001103:33716-34539
HmHel-1	CAEZ01010140:22685-23018
HmHel-1	CAEZ01009136:27408-29131
HmHel-1	CAEZ01009136:113492-113831
HmHel-1	CAEZ01009136:2283-2370

HmHel-1	CAEZ01007254:18514-19341
HmHel-1	CAEZ01007108:106334-107158
HmHel-1	CAEZ01004291:14204-15024
HmHel-1	CAEZ01007974:75071-75879
HmHel-1	CAEZ01007974:55182-55998
HmHel-1	CAEZ01007974:31826-32640
HmHel-1	CAEZ01007974:9-340
HmHel-1	CAEZ01010621:21705-22529
HmHel-1	CAEZ01010517:57727-58387
HmHel-1	CAEZ01010517:16766-17578
HmHel-1	CAEZ01008101:13821-14243
HmHel-1	CAEZ01007232:57833-58198
HmHel-1	CAEZ01007232:67787-68601
HmHel-1	CAEZ01003335:48113-48717
HmHel-1	CAEZ01003335:23865-24345
HmHel-1	CAEZ01009503:7836-8662
HmHel-1	CAEZ01007709:3387-3482
HmHel-1	CAEZ01007709:31259-31762
HmHel-1	CAEZ01007709:42669-43497
HmHel-1	CAEZ01007709:43728-44070
HmHel-1	CAEZ01007545:47074-47902
HmHel-1	CAEZ01000912:11260-11624
HmHel-1	CAEZ01000912:17155-17398
HmHel-1	CAEZ01009754:3034-3860
HmHel-1	CAEZ01008578:40803-41235
HmHel-1	CAEZ01008578:91540-92373
HmHel-1	CAEZ01005690:7931-8272
HmHel-1	CAEZ01004679:20173-20977
HmHel-1	CAEZ01005442:151689-152506
HmHel-1	CAEZ01009179:7941-8760
HmHel-1	CAEZ01006445:89731-89935
HmHel-1	CAEZ01006445:232753-233579
HmHel-1	CAEZ01006445:222547-222635
HmHel-1	CAEZ01006445:189051-189474
HmHel-1	CAEZ01006445:85061-85727
HmHel-1	CAEZ01003061:27725-28550
HmHel-1	CAEZ01002767:2069-2408
HmHel-1	CAEZ01002767:27082-27314
HmHel-1	CAEZ01002767:40350-42332
HmHel-1	CAEZ01002767:2653-3498
HmHel-1	CAEZ01003548:16381-17204
HmHel-1	CAEZ01008284:3-771
HmHel-1	CAEZ01005468:1-813
HmHel-1	CAEZ01001464:6741-7567
HmHel-1	CAEZ01001464:30230-30434
HmHel-1	CAEZ01001464:23800-23888
HmHel-1	CAEZ01010886:1-307
HmHel-1	CAEZ01010886:11147-14408

HmHel-1	CAEZ01008606:46311-47138
HmHel-1	CAEZ01008606:91193-91430
HmHel-1	CAEZ01004252:10529-10676
HmHel-1	CAEZ01004252:49538-50375
HmHel-1	CAEZ01009971:13130-13496
HmHel-1	CAEZ01009971:32145-32486
HmHel-1	CAEZ01009971:84497-84906
HmHel-1	CAEZ01009971:50211-50844
HmHel-1	CAEZ01009971:12596-12832
HmHel-1	CAEZ01009610:14281-14513
HmHel-1	CAEZ01009610:67742-67959
HmHel-1	CAEZ01009610:30692-31508
HmHel-1	CAEZ01006494:34498-35313
HmHel-1	CAEZ01006494:35407-35737
HmHel-1	CAEZ01004661:22589-23421
HmHel-1	CAEZ01002231:15720-16548
HmHel-1	CAEZ01011133:26749-27592
HmHel-1	CAEZ01007624:5473-6300
HmHel-1	CAEZ01007624:53546-54185
HmHel-1	CAEZ01005743:15107-15308
HmHel-1	CAEZ01005743:51609-51842
HmHel-1	CAEZ01005743:71966-72054
HmHel-1	CAEZ01005743:48604-49430
HmHel-1	CAEZ01002721:37916-38729
HmHel-1	CAEZ01011245:40083-40425
HmHel-1	CAEZ01011245:75868-76201
HmHel-1	CAEZ01011245:57890-59222
HmHel-1	CAEZ01006760:12325-13114
HmHel-1	CAEZ01006760:51088-51508
HmHel-1	CAEZ01007252:52114-52318
HmHel-1	CAEZ01007252:156358-156840
HmHel-1	CAEZ01007252:211062-211399
HmHel-1	CAEZ01007252:233689-234028
HmHel-1	CAEZ01007252:244590-244929
HmHel-1	CAEZ01007252:120518-120852
HmHel-1	CAEZ01007252:18152-18980
HmHel-1	CAEZ01007252:6399-6487
HmHel-1	CAEZ01010462:11906-12720
HmHel-1	CAEZ01008365:11350-12162
HmHel-1	CAEZ01004383:11236-11579
HmHel-1	CAEZ01003647:41290-42104
HmHel-1	CAEZ01003647:6870-7209
HmHel-1	CAEZ01009750:38288-39119
HmHel-1	CAEZ01005711:15390-15812
HmHel-1	CAEZ01005711:153036-153168
HmHel-1	CAEZ01005711:167332-167554
HmHel-1	CAEZ01005711:182904-183325
HmHel-1	CAEZ01005711:143056-143871

HmHel-1	CAEZ01005711:98008-98794
HmHel-1	CAEZ01005711:2846-3066
HmHel-1	CAEZ01005333:4463-5267
HmHel-1	CAEZ01005333:48089-48747
HmHel-1	CAEZ01011416:49952-50802
HmHel-1	CAEZ01011416:45871-46559
HmHel-1	CAEZ01011416:21698-22505
HmHel-1	CAEZ01002210:2662-3523
HmHel-1	CAEZ01009404:58105-58915
HmHel-1	CAEZ01010501:2154-2986
HmHel-1	CAEZ01010501:35731-35819
HmHel-1	CAEZ01006785:17857-19138
HmHel-1	CAEZ01006785:3561-4380
HmHel-1	CAEZ01009827:17243-17458
HmHel-1	CAEZ01009827:5074-5896
HmHel-1	CAEZ01008512:99796-100620
HmHel-1	CAEZ01007870:37493-37749
HmHel-1	CAEZ01007870:82259-83107
HmHel-1	CAEZ01010318:6943-7277
HmHel-1	CAEZ01010318:53531-54362
HmHel-1	CAEZ01010318:55626-56468
HmHel-1	CAEZ01004735:27046-27853
HmHel-1	CAEZ01009883:51054-51720
HmHel-1	CAEZ01009883:63624-64033
HmHel-1	CAEZ01009883:120444-121273
HmHel-1	CAEZ01009883:73035-73414
HmHel-1	CAEZ01009883:17232-17571
HmHel-1	CAEZ01005126:1763-2594
HmHel-1	CAEZ01001868:1283-1562
HmHel-1	CAEZ01001868:17865-18204
HmHel-1	CAEZ01001868:29207-30032
HmHel-1	CAEZ01011323:19973-20467
HmHel-1	CAEZ01011323:55887-56706
HmHel-1	CAEZ01009270:26163-26502
HmHel-1	CAEZ01009481:52791-53377
HmHel-1	CAEZ01007926:8880-9707
HmHel-1	CAEZ01004740:37972-38801
HmHel-1	CAEZ01004740:50000-50335
HmHel-1	CAEZ01001871:807-1638
HmHel-1	CAEZ01006279:19809-20614
HmHel-1	CAEZ01010127:83613-84398
HmHel-1	CAEZ01010127:98941-99282
HmHel-1	CAEZ01010025:31130-31942
HmHel-1	CAEZ01009120:29384-29626
HmHel-1	CAEZ01009120:39654-39858
HmHel-1	CAEZ01009120:77316-77738
HmHel-1	CAEZ01009120:22740-23502
HmHel-1	CAEZ01000909:25755-26077

HmHel-1	CAEZ01000909:155626-156048
HmHel-1	CAEZ01000909:139305-140107
HmHel-1	CAEZ01000909:107236-107458
HmHel-1	CAEZ01000909:81634-81867
HmHel-1	CAEZ01007765:4194-5290
HmHel-1	CAEZ01007765:112458-112799
HmHel-1	CAEZ01007765:409161-409982
HmHel-1	CAEZ01007765:493771-494579
HmHel-1	CAEZ01007765:435441-435780
HmHel-1	CAEZ01007765:384727-385398
HmHel-1	CAEZ01007765:202608-202960
HmHel-1	CAEZ01007765:4518-4857
HmHel-1	CAEZ01003307:5505-5973
HmHel-1	CAEZ01003307:1057-1396
HmHel-1	CAEZ01009748:98440-99241
HmHel-1	CAEZ01005321:50679-51490
HmHel-1	CAEZ01004962:1882-2864
HmHel-1	CAEZ01004962:2512-2624
HmHel-1	CAEZ01001286:4183-4921
HmHel-1	CAEZ01003422:8419-12037
HmHel-1	CAEZ01003422:31007-31609
HmHel-1	CAEZ01008882:14403-14825
HmHel-1	CAEZ01008882:1248-3281
HmHel-1	CAEZ01008486:10444-10821
HmHel-1	CAEZ01008486:32075-32898
HmHel-1	CAEZ01008486:10004-11181
HmHel-1	CAEZ01009996:12140-12978
HmHel-1	CAEZ01009996:33668-33893
HmHel-1	CAEZ01009996:57566-57798
HmHel-1	CAEZ01009996:83066-83268
HmHel-1	CAEZ01009996:73974-74404
HmHel-1	CAEZ01009996:49193-49393
HmHel-1	CAEZ01007062:19859-20720
HmHel-1	CAEZ01007062:38757-39087
HmHel-1	CAEZ01007062:53322-53588
HmHel-1	CAEZ01007062:14391-14694
HmHel-1	CAEZ01004038:41806-42631
HmHel-1	CAEZ01007207:120821-120955
HmHel-1	CAEZ01007207:174762-175106
HmHel-1	CAEZ01007207:236411-236636
HmHel-1	CAEZ01007207:267033-267248
HmHel-1	CAEZ01007207:236703-237505
HmHel-1	CAEZ01007207:222189-222996
HmHel-1	CAEZ01007207:158152-158491
HmHel-1	CAEZ01007207:43931-44272
HmHel-1	CAEZ01007207:23015-23430
HmHel-1	CAEZ01005417:8943-9751
HmHel-1	CAEZ01005417:2262-3088

HmHel-1	CAEZ01003849:7773-8610
HmHel-1	CAEZ01001984:14842-15670
HmHel-1	CAEZ01010864:28048-28737
HmHel-1	CAEZ01010864:113491-114294
HmHel-1	CAEZ01010864:66503-67146
HmHel-1	CAEZ01009729:16020-16843
HmHel-1	CAEZ01006594:12818-13146
HmHel-1	CAEZ01006594:47505-48330
HmHel-1	CAEZ01010528:8299-9124
HmHel-1	CAEZ01010528:35880-36329
HmHel-1	CAEZ01006773:78411-78750
HmHel-1	CAEZ01006773:110023-110367
HmHel-1	CAEZ01006773:125784-126647
HmHel-1	CAEZ01006773:77412-77603
HmHel-1	CAEZ01005430:55202-56482
HmHel-1	CAEZ01005430:67565-67797
HmHel-1	CAEZ01005430:352-690
HmHel-1	CAEZ01003275:23679-23991
HmHel-1	CAEZ01003275:17501-18317
HmHel-1	CAEZ01005235:116543-116882
HmHel-1	CAEZ01005235:252471-252821
HmHel-1	CAEZ01005235:275684-276023
HmHel-1	CAEZ01005235:134237-135057
HmHel-1	CAEZ01005044:59703-60467
HmHel-1	CAEZ01005044:163085-163420
HmHel-1	CAEZ01003505:288-1120
HmHel-1	CAEZ01009373:62525-63316
HmHel-1	CAEZ01009373:81766-82607
HmHel-1	CAEZ01009373:49240-49891
HmHel-1	CAEZ01002875:50255-51084
HmHel-1	CAEZ01002875:202037-202152
HmHel-1	CAEZ01002875:258260-258698
HmHel-1	CAEZ01002875:110671-110759
HmHel-1	CAEZ01002875:76900-77564
HmHel-1	CAEZ01002875:20559-21290
HmHel-1	CAEZ01009205:5502-6279
HmHel-1	CAEZ01003326:18901-18989
HmHel-1	CAEZ01003326:34868-35677
HmHel-1	CAEZ01003326:53342-53546
HmHel-1	CAEZ01003326:34631-35820
HmHel-1	CAEZ01001892:21832-22653
HmHel-1	CAEZ01010243:63693-63902
HmHel-1	CAEZ01010243:34305-35129
HmHel-1	CAEZ01003063:13285-13719
HmHel-1	CAEZ01003063:8675-9493
HmHel-1	CAEZ01010954:7903-8735
HmHel-1	CAEZ01003762:6450-6629
HmHel-1	CAEZ01003762:16632-17442

HmHel-1	CAEZ01003762:5412-5500
HmHel-1	CAEZ01009042:32451-42664
HmHel-1	CAEZ01007938:30446-30781
HmHel-1	CAEZ01007938:43907-44338
HmHel-1	CAEZ01007938:9606-10419
HmHel-1	CAEZ01004544:26002-26809
HmHel-1	CAEZ01004544:16483-17209
HmHel-1	CAEZ01001815:1-802
HmHel-1	CAEZ01008721:1-781
HmHel-1	CAEZ01008721:36411-37210
HmHel-1	CAEZ01008721:36222-37696
HmHel-1	CAEZ01008721:20011-20333
HmHel-1	CAEZ01008305:99434-103808
HmHel-1	CAEZ01011519:12-835
HmHel-1	CAEZ01011519:55981-56320
HmHel-1	CAEZ01011519:165133-165474
HmHel-1	CAEZ01011519:168327-168749
HmHel-1	CAEZ01011519:116452-117418
HmHel-1	CAEZ01011519:103386-104055
HmHel-1	CAEZ01011519:28439-28778
HmHel-1	CAEZ01007147:338-1123
HmHel-1	CAEZ01006724:3791-4615
HmHel-1	CAEZ01009656:34567-34976
HmHel-1	CAEZ01009656:48524-48760
HmHel-1	CAEZ01009656:19285-19701
HmHel-1	CAEZ01009044:133823-134641
HmHel-1	CAEZ01008910:5441-5645
HmHel-1	CAEZ01008910:20018-20357
HmHel-1	CAEZ01008910:20565-21381
HmHel-1	CAEZ01011083:74978-75632
HmHel-1	CAEZ01011083:217478-217817
HmHel-1	CAEZ01011083:187088-187891
HmHel-1	CAEZ01011538:1850-2672
HmHel-1	CAEZ01006366:16803-17162
HmHel-1	CAEZ01006366:20642-21452
HmHel-1	CAEZ01006366:26-365
HmHel-1	CAEZ01003387:1-692
HmHel-1	CAEZ01010753:969-1177
HmHel-1	CAEZ01010753:2479-3297
HmHel-1	CAEZ01006348:31082-31904
HmHel-1	CAEZ01005274:95561-95984
HmHel-1	CAEZ01005274:117422-118246
HmHel-1	CAEZ01005274:119710-120049
HmHel-1	CAEZ01005274:96871-97222
HmHel-1	CAEZ01001964:1-532
HmHel-1	CAEZ01000960:3218-4042
HmHel-1	CAEZ01000960:15615-15956
HmHel-1	CAEZ01011101:71272-71570

HmHel-1	CAEZ01011101:53565-54375
HmHel-1	CAEZ01010117:63349-64197
HmHel-1	CAEZ01010117:1-529
HmHel-1	CAEZ01004408:14244-15089
HmHel-1	CAEZ01003827:57836-58177
HmHel-1	CAEZ01003827:71723-72560
HmHel-1	CAEZ01009633:43629-44454
HmHel-1	CAEZ01009633:103004-103425
HmHel-1	CAEZ01007140:5499-5855
HmHel-1	CAEZ01007140:44342-44681
HmHel-1	CAEZ01007140:55941-56772
HmHel-1	CAEZ01009622:6700-7535
HmHel-1	CAEZ01011465:27721-28543
HmHel-1	CAEZ01006249:21451-21873
HmHel-1	CAEZ01006249:41780-42645
HmHel-1	CAEZ01006249:62495-63137
HmHel-1	CAEZ01002785:1-431
HmHel-1	CAEZ01003392:9065-9887
HmHel-1	CAEZ01011144:1045-1848
HmHel-1	CAEZ01011144:36968-37777
HmHel-1	CAEZ01009232:22317-23173
HmHel-1	CAEZ01009232:8534-9222
HmHel-1	CAEZ01002815:95891-96230
HmHel-1	CAEZ01002815:181778-181979
HmHel-1	CAEZ01002815:193482-193821
HmHel-1	CAEZ01002815:192741-193958
HmHel-1	CAEZ01002815:101867-102206
HmHel-1	CAEZ01002815:54749-55088
HmHel-1	CAEZ01011285:44273-45095
HmHel-1	CAEZ01007721:15527-15869
HmHel-1	CAEZ01007721:30725-31534
HmHel-1	CAEZ01010927:76605-77270
HmHel-1	CAEZ01010927:108141-108229
HmHel-1	CAEZ01010927:130882-131690
HmHel-1	CAEZ01010927:61528-62212
HmHel-1	CAEZ01007603:31356-32167
HmHel-1	CAEZ01007266:46609-47437
HmHel-1	CAEZ01008234:20371-21196
HmHel-1	CAEZ01008234:50885-51588
HmHel-1	CAEZ01006231:578-2105
HmHel-1	CAEZ01006231:2367-2680
HmHel-1	CAEZ01011049:65702-66337
HmHel-1	CAEZ01010801:6834-7082
HmHel-1	CAEZ01010801:24745-25574
HmHel-1	CAEZ01003890:13489-14337
HmHel-1	CAEZ01003890:5494-5847
HmHel-1	CAEZ01003207:65436-66145
HmHel-1	CAEZ01010217:36695-37488

HmHel-1	CAEZ01010217:51616-51859
HmHel-1	CAEZ01010217:122601-122803
HmHel-1	CAEZ01007072:21815-22228
HmHel-1	CAEZ01004582:31918-32789
HmHel-1	CAEZ01010340:6927-7354
HmHel-1	CAEZ01004857:3622-4300
HmHel-1	CAEZ01009085:17116-17391
HmHel-1	CAEZ01009085:49640-50390
HmHel-1	CAEZ01009085:72918-73333
HmHel-1	CAEZ01009085:111545-111957
HmHel-1	CAEZ01009085:99872-100098
HmHel-1	CAEZ01009085:50754-51047
HmHel-1	CAEZ01009249:3191-3530
HmHel-1	CAEZ01009249:25149-25949
HmHel-1	CAEZ01005770:22314-23121
HmHel-1	CAEZ01001854:8364-9187
HmHel-1	CAEZ01011197:8357-10445
HmHel-1	CAEZ01011197:39256-40080
HmHel-1	CAEZ01011197:140979-141787
HmHel-1	CAEZ01011197:103213-104031
HmHel-1	CAEZ01010677:45135-45943
HmHel-1	CAEZ01010677:69704-69802
HmHel-1	CAEZ01010677:172968-173181
HmHel-1	CAEZ01010677:213690-213778
HmHel-1	CAEZ01010677:285273-285450
HmHel-1	CAEZ01010677:253574-253790
HmHel-1	CAEZ01010677:127442-127781
HmHel-1	CAEZ01010677:107383-107606
HmHel-1	CAEZ01010677:69994-70239
HmHel-1	CAEZ01010017:10398-11219
HmHel-1	CAEZ01008261:86761-87575
HmHel-1	CAEZ01008261:110047-110384
HmHel-1	CAEZ01008261:165066-165296
HmHel-1	CAEZ01008261:190902-191241
HmHel-1	CAEZ01008261:188067-188758
HmHel-1	CAEZ01008261:148067-148301
HmHel-1	CAEZ01008261:86677-88049
HmHel-1	CAEZ01008261:23241-24956
HmHel-1	CAEZ01005439:100708-101514
HmHel-1	CAEZ01005439:174841-175248
HmHel-1	CAEZ01005439:2988-3327
HmHel-1	CAEZ01001441:40672-41006
HmHel-1	CAEZ01001441:90070-90292
HmHel-1	CAEZ01001441:40221-40672
HmHel-1	CAEZ01009168:11645-12075
HmHel-1	CAEZ01008001:41334-42116
HmHel-1	CAEZ01008001:23699-23942
HmHel-1	CAEZ01009124:13629-15510

HmHel-1	CAEZ01009124:25611-26302
HmHel-1	CAEZ01009124:65376-65781
HmHel-1	CAEZ01009124:1952-2758
HmHel-1	CAEZ01010907:124621-124960
HmHel-1	CAEZ01010907:25350-26166
HmHel-1	CAEZ01010239:2595-3399
HmHel-1	CAEZ01010130:68107-78173
HmHel-1	CAEZ01010130:131897-132129
HmHel-1	CAEZ01010130:83504-83713
HmHel-1	CAEZ01009975:25198-26006
HmHel-1	CAEZ01009975:104525-104857
HmHel-1	CAEZ01009975:135294-136102
HmHel-1	CAEZ01009975:116996-117200
HmHel-1	CAEZ01009975:14857-15446
HmHel-1	CAEZ01009887:169609-170435
HmHel-1	CAEZ01009887:183971-184059
HmHel-1	CAEZ01009887:43108-43287
HmHel-1	CAEZ01009887:19561-20373
HmHel-1	CAEZ01009884:4414-5222
HmHel-1	CAEZ01009458:67596-68402
HmHel-1	CAEZ01009458:79353-80024
HmHel-1	CAEZ01009458:175783-176124
HmHel-1	CAEZ01009458:155481-155681
HmHel-1	CAEZ01009450:650-1470
HmHel-1	CAEZ01009450:29703-34653
HmHel-1	CAEZ01009217:11617-12440
HmHel-1	CAEZ01009123:2764-3572
HmHel-1	CAEZ01008734:11754-12566
HmHel-1	CAEZ01008393:42699-42911
HmHel-1	CAEZ01008393:132373-132787
HmHel-1	CAEZ01008393:115227-115463
HmHel-1	CAEZ01008393:89433-90247
HmHel-1	CAEZ01008117:13025-13819
HmHel-1	CAEZ01008117:138-222
HmHel-1	CAEZ01007752:26908-27185
HmHel-1	CAEZ01007752:42220-43005
HmHel-1	CAEZ01007752:102438-102653
HmHel-1	CAEZ01007752:80576-80920
HmHel-1	CAEZ01007102:5371-5820
HmHel-1	CAEZ01006995:1972-2780
HmHel-1	CAEZ01006117:993-1788
HmHel-1	CAEZ01005840:3500-3733
HmHel-1	CAEZ01005840:7673-8454
HmHel-1	CAEZ01005286:4701-5511
HmHel-1	CAEZ01005286:803-1142
HmHel-1	CAEZ01004506:7595-8399
HmHel-1	CAEZ01004482:2761-3181
HmHel-1	CAEZ01002864:10543-11351

HmHel-1	CAEZ01002776:5692-6907
HmHel-1	CAEZ01002776:5815-6623
HmHel-1	CAEZ01000063:13014-17766
HmHel-1	CAEZ01000063:56121-56696
HmHel-1	CAEZ01000063:4730-5735
HmHel-1	CAEZ01010124:4142-4953
HmHel-1	CAEZ01010124:385-1651
HmHel-1	CAEZ01006317:13932-14271
HmHel-1	CAEZ01006317:13194-14413
HmHel-1	CAEZ01003202:108703-109521
HmHel-1	CAEZ01003202:117245-118050
HmHel-1	CAEZ01003202:97816-98617
HmHel-1	CAEZ01011282:23235-23907
HmHel-1	CAEZ01011282:110784-111592
HmHel-1	CAEZ01011282:448-869
HmHel-1	CAEZ01010030:872-1213
HmHel-1	CAEZ01010030:97446-98071
HmHel-1	CAEZ01010030:187191-187592
HmHel-1	CAEZ01010030:261247-261676
HmHel-1	CAEZ01010030:222664-223472
HmHel-1	CAEZ01010030:138055-138273
HmHel-1	CAEZ01010030:12942-13280
HmHel-1	CAEZ01009576:7505-8321
HmHel-1	CAEZ01009576:41178-41434
HmHel-1	CAEZ01009576:25659-26738
HmHel-1	CAEZ01009131:17572-18348
HmHel-1	CAEZ01009131:15557-15645
HmHel-1	CAEZ01009131:2393-2481
HmHel-1	CAEZ01008743:111807-112164
HmHel-1	CAEZ01008743:147321-148127
HmHel-1	CAEZ01008743:100309-101117
HmHel-1	CAEZ01008743:50443-50782
HmHel-1	CAEZ01007849:37527-38335
HmHel-1	CAEZ01007830:13303-13707
HmHel-1	CAEZ01006810:28789-29618
HmHel-1	CAEZ01006154:11602-11691
HmHel-1	CAEZ01006154:26247-26476
HmHel-1	CAEZ01006154:113537-113875
HmHel-1	CAEZ01006154:173717-173932
HmHel-1	CAEZ01006154:251027-251357
HmHel-1	CAEZ01006154:285547-285775
HmHel-1	CAEZ01006154:90639-91748
HmHel-1	CAEZ01005495:39123-39346
HmHel-1	CAEZ01005495:126-934
HmHel-1	CAEZ01005049:1627-2435
HmHel-1	CAEZ01004993:9260-10074
HmHel-1	CAEZ01004975:6033-6838
HmHel-1	CAEZ01004975:44265-44902

HmHel-1	CAEZ01004486:21894-22702
HmHel-1	CAEZ01003971:1829-2639
HmHel-1	CAEZ01001832:39820-40159
HmHel-1	CAEZ01001832:59939-60766
HmHel-1	CAEZ01001832:51773-52590
HmHel-1	CAEZ01001510:5780-6102
HmHel-1	CAEZ01001510:31559-32367
HmHel-1	CAEZ01001446:67231-67919
HmHel-1	CAEZ01001446:149277-149643
HmHel-1	CAEZ01001446:94761-94966
HmHel-1	CAEZ01001446:50654-51464
HmHel-1	CAEZ01000800:14680-15488
HmHel-1	CAEZ01009264:49886-50753
HmHel-1	CAEZ01009264:22730-23532
HmHel-1	CAEZ01005729:263-1081
HmHel-1	CAEZ01005219:184862-185552
HmHel-1	CAEZ01005219:66413-67223
HmHel-1	CAEZ01005219:47967-48676
HmHel-1	CAEZ01010730:16596-17285
HmHel-1	CAEZ01010730:29774-30579
HmHel-1	CAEZ01009844:174485-175312
HmHel-1	CAEZ01009844:185607-185840
HmHel-1	CAEZ01009844:165948-166750
HmHel-1	CAEZ01009844:26726-26954
HmHel-1	CAEZ01011395:18350-19170
HmHel-1	CAEZ01011395:1994-2198
HmHel-1	CAEZ01011296:18203-22392
HmHel-1	CAEZ01011296:67556-68420
HmHel-1	CAEZ01011296:109593-109934
HmHel-1	CAEZ01011296:151226-151332
HmHel-1	CAEZ01011296:173074-173409
HmHel-1	CAEZ01011296:110156-110501
HmHel-1	CAEZ01011296:21816-22160
HmHel-1	CAEZ01010746:5076-5884
HmHel-1	CAEZ01010746:42397-42736
HmHel-1	CAEZ01010746:22950-23376
HmHel-1	CAEZ01010583:5866-6097
HmHel-1	CAEZ01010583:44733-45457
HmHel-1	CAEZ01010583:39815-39902
HmHel-1	CAEZ01010583:23345-24153
HmHel-1	CAEZ01010209:16353-17161
HmHel-1	CAEZ01009897:3992-4800
HmHel-1	CAEZ01009897:5475-5897
HmHel-1	CAEZ01009888:28819-29623
HmHel-1	CAEZ01009888:83326-83667
HmHel-1	CAEZ01009888:5457-5832
HmHel-1	CAEZ01009846:1505-1924
HmHel-1	CAEZ01009138:52606-53414

HmHel-1	CAEZ01009138:42354-42968
HmHel-1	CAEZ01009056:8871-9678
HmHel-1	CAEZ01008739:14597-15405
HmHel-1	CAEZ01008620:133313-134136
HmHel-1	CAEZ01008620:145745-146440
HmHel-1	CAEZ01008620:115546-116207
HmHel-1	CAEZ01008620:79307-79646
HmHel-1	CAEZ01008401:16126-16936
HmHel-1	CAEZ01008315:135984-136310
HmHel-1	CAEZ01008315:267394-268191
HmHel-1	CAEZ01008315:357290-357629
HmHel-1	CAEZ01008315:55565-56374
HmHel-1	CAEZ01008315:13221-14031
HmHel-1	CAEZ01008097:4194-4998
HmHel-1	CAEZ01007505:14933-15741
HmHel-1	CAEZ01007431:16677-17483
HmHel-1	CAEZ01007431:35646-36331
HmHel-1	CAEZ01007184:68084-68288
HmHel-1	CAEZ01007184:86515-86859
HmHel-1	CAEZ01007184:167386-167602
HmHel-1	CAEZ01007184:137840-138062
HmHel-1	CAEZ01007184:51358-52172
HmHel-1	CAEZ01007184:36301-36661
HmHel-1	CAEZ01006863:19197-20011
HmHel-1	CAEZ01006863:81678-82003
HmHel-1	CAEZ01006863:7610-8018
HmHel-1	CAEZ01006781:7550-7882
HmHel-1	CAEZ01006781:24991-25807
HmHel-1	CAEZ01006542:67969-68315
HmHel-1	CAEZ01006542:119293-120072
HmHel-1	CAEZ01006542:232498-233302
HmHel-1	CAEZ01006542:226230-226660
HmHel-1	CAEZ01006287:21512-21600
HmHel-1	CAEZ01006287:55788-56127
HmHel-1	CAEZ01006287:57176-57419
HmHel-1	CAEZ01005975:96990-97385
HmHel-1	CAEZ01005975:152895-153204
HmHel-1	CAEZ01005975:163793-164675
HmHel-1	CAEZ01005975:99299-99506
HmHel-1	CAEZ01005946:24319-24896
HmHel-1	CAEZ01005946:163672-164477
HmHel-1	CAEZ01005946:9307-9526
HmHel-1	CAEZ01005864:18675-19415
HmHel-1	CAEZ01005427:993-1801
HmHel-1	CAEZ01005427:84795-85615
HmHel-1	CAEZ01005293:33-636
HmHel-1	CAEZ01005203:86999-87660
HmHel-1	CAEZ01005203:9302-10110

HmHel-1	CAEZ01005141:1960-2769
HmHel-1	CAEZ01004894:5708-6523
HmHel-1	CAEZ01003750:167701-167818
HmHel-1	CAEZ01003750:9046-9853
HmHel-1	CAEZ01003673:9306-10115
HmHel-1	CAEZ01003069:190725-191069
HmHel-1	CAEZ01003069:288742-289081
HmHel-1	CAEZ01003069:302283-303088
HmHel-1	CAEZ01003069:248544-248632
HmHel-1	CAEZ01003069:130972-131782
HmHel-1	CAEZ01003069:12680-13369
HmHel-1	CAEZ01002891:8720-8942
HmHel-1	CAEZ01002891:14741-15553
HmHel-1	CAEZ01002724:52794-53602
HmHel-1	CAEZ01002724:109963-110165
HmHel-1	CAEZ01002724:78071-78288
HmHel-1	CAEZ01002724:6805-7494
HmHel-1	CAEZ01002629:41700-41903
HmHel-1	CAEZ01002629:31940-32748
HmHel-1	CAEZ01001765:45120-45898
HmHel-1	CAEZ01001765:62612-63420
HmHel-1	CAEZ01010620:72417-72758
HmHel-1	CAEZ01010620:96356-97164
HmHel-1	CAEZ01010096:9090-9898
HmHel-1	CAEZ01007949:14322-15122
HmHel-1	CAEZ01007686:28793-29602
HmHel-1	CAEZ01003884:821-1160
HmHel-1	CAEZ01011523:20501-21309
HmHel-1	CAEZ01011523:68492-68735
HmHel-1	CAEZ01011523:73682-74104
HmHel-1	CAEZ01010817:2726-3534
HmHel-1	CAEZ01010226:22-410
HmHel-1	CAEZ01010033:1816-2157
HmHel-1	CAEZ01010033:17958-18766
HmHel-1	CAEZ01009322:4525-5334
HmHel-1	CAEZ01009304:23595-24395
HmHel-1	CAEZ01009281:28734-29542
HmHel-1	CAEZ01009084:16677-17031
HmHel-1	CAEZ01009084:75292-75621
HmHel-1	CAEZ01009084:74090-74448
HmHel-1	CAEZ01009084:12657-13468
HmHel-1	CAEZ01008231:917-1699
HmHel-1	CAEZ01008231:37900-37988
HmHel-1	CAEZ01008231:24521-26582
HmHel-1	CAEZ01007940:25383-25612
HmHel-1	CAEZ01007940:481-1289
HmHel-1	CAEZ01007781:4365-5169
HmHel-1	CAEZ01007115:25-413

HmHel-1	CAEZ01006128:66466-66885
HmHel-1	CAEZ01006128:55862-56667
HmHel-1	CAEZ01006128:40261-40624
HmHel-1	CAEZ01005508:31180-31988
HmHel-1	CAEZ01005447:8340-9151
HmHel-1	CAEZ01005279:65494-66129
HmHel-1	CAEZ01005279:49830-50636
HmHel-1	CAEZ01005279:26357-27165
HmHel-1	CAEZ01005175:30332-31140
HmHel-1	CAEZ01005175:32079-32828
HmHel-1	CAEZ01004303:11431-12245
HmHel-1	CAEZ01003250:56268-56472
HmHel-1	CAEZ01003250:78229-79022
HmHel-1	CAEZ01003250:161837-162033
HmHel-1	CAEZ01002754:51305-52113
HmHel-1	CAEZ01002353:847-1278
HmHel-1	CAEZ01001089:19411-20219
HmHel-1	CAEZ01001089:51249-51936
HmHel-1	CAEZ01001089:12158-12497
HmHel-1	CAEZ01000074:19316-20123
HmHel-1	CAEZ01009475:17806-18615
HmHel-1	CAEZ01008124:17840-18515
HmHel-1	CAEZ01008124:125640-126450
HmHel-1	CAEZ01008124:239194-239600
HmHel-1	CAEZ01008124:89225-89872
HmHel-1	CAEZ01008124:7812-8114
HmHel-1	CAEZ01004826:64840-65652
HmHel-1	CAEZ01004792:29902-30243
HmHel-1	CAEZ01004792:105736-106389
HmHel-1	CAEZ01004792:69487-70296
HmHel-1	CAEZ01001909:5146-5965
HmHel-1	CAEZ01001909:7693-7888
HmHel-1	CAEZ01009300:27531-28337
HmHel-1	CAEZ01009300:12521-12942
HmHel-1	CAEZ01008944:1657-2462
HmHel-1	CAEZ01008944:67520-67934
HmHel-1	CAEZ01008944:51160-51582
HmHel-1	CAEZ01007281:5260-6059
HmHel-1	CAEZ01007281:39901-40657
HmHel-1	CAEZ01004119:1814-2566
HmHel-1	CAEZ01004119:9085-9891
HmHel-1	CAEZ01003996:3870-4675
HmHel-1	CAEZ01003926:7741-8082
HmHel-1	CAEZ01003926:12791-13596
HmHel-1	CAEZ01008402:45897-46713
HmHel-1	CAEZ01008402:72893-73132
HmHel-1	CAEZ01008402:81663-81994
HmHel-1	CAEZ01008402:26163-26502

HmHel-1	CAEZ01008211:125248-126111
HmHel-1	CAEZ01008211:171335-171562
HmHel-1	CAEZ01008211:238499-238911
HmHel-1	CAEZ01008211:231100-231439
HmHel-1	CAEZ01008211:198930-199269
HmHel-1	CAEZ01008211:27337-27570
HmHel-1	CAEZ01008175:13803-14609
HmHel-1	CAEZ01007341:6352-7158
HmHel-1	CAEZ01007101:15369-15708
HmHel-1	CAEZ01007101:95631-96438
HmHel-1	CAEZ01004852:9946-10764
HmHel-1	CAEZ01004852:12044-12490
HmHel-1	CAEZ01002229:28283-29100
HmHel-1	CAEZ01010288:167169-167410
HmHel-1	CAEZ01010288:282513-290595
HmHel-1	CAEZ01010288:248075-248414
HmHel-1	CAEZ01010288:165470-166278
HmHel-1	CAEZ01010288:121307-121675
HmHel-1	CAEZ01009892:46556-46875
HmHel-1	CAEZ01009892:94753-95555
HmHel-1	CAEZ01009892:17586-18392
HmHel-1	CAEZ01009501:60-864
HmHel-1	CAEZ01009501:13168-13345
HmHel-1	CAEZ01009501:83575-84329
HmHel-1	CAEZ01009501:131096-131188
HmHel-1	CAEZ01009501:32015-32447
HmHel-1	CAEZ01009426:6826-7274
HmHel-1	CAEZ01009422:96748-96970
HmHel-1	CAEZ01009422:118886-119694
HmHel-1	CAEZ01009422:132035-132278
HmHel-1	CAEZ01009422:107842-108620
HmHel-1	CAEZ01009422:88348-88800
HmHel-1	CAEZ01009422:24085-24445
HmHel-1	CAEZ01009422:7741-7950
HmHel-1	CAEZ01008889:20822-21623
HmHel-1	CAEZ01008489:25678-26460
HmHel-1	CAEZ01008310:4378-5188
HmHel-1	CAEZ01008203:3022-3821
HmHel-1	CAEZ01007753:15-706
HmHel-1	CAEZ01007753:31199-32007
HmHel-1	CAEZ01007735:2466-3285
HmHel-1	CAEZ01007201:44332-44420
HmHel-1	CAEZ01007201:94821-94909
HmHel-1	CAEZ01007201:9285-10092
HmHel-1	CAEZ01006526:3026-3834
HmHel-1	CAEZ01006496:5247-6057
HmHel-1	CAEZ01006324:54411-55218
HmHel-1	CAEZ01005973:16974-17309

HmHel-1	CAEZ01005973:17606-18414
HmHel-1	CAEZ01005076:7717-8130
HmHel-1	CAEZ01005070:13128-13939
HmHel-1	CAEZ01002680:8868-9672
HmHel-1	CAEZ01002680:20537-20625
HmHel-1	CAEZ01001347:9372-10179
HmHel-1	CAEZ01001347:9065-9304
HmHel-1	CAEZ01010701:43249-44050
HmHel-1	CAEZ01010701:146955-147174
HmHel-1	CAEZ01010701:175391-175735
HmHel-1	CAEZ01010701:175208-175833
HmHel-1	CAEZ01010701:158231-158405
HmHel-1	CAEZ01010701:61999-62649
HmHel-1	CAEZ01010701:538-1207
HmHel-1	CAEZ01009685:16122-16918
HmHel-1	CAEZ01001266:106493-106842
HmHel-1	CAEZ01001266:75363-76171
HmHel-1	CAEZ01001266:9835-10017
HmHel-1	CAEZ01010320:34046-34749
HmHel-1	CAEZ01010320:73720-74538
HmHel-1	CAEZ01010320:43575-43916
HmHel-1	CAEZ01010320:25393-26059
HmHel-1	CAEZ01010115:1-358
HmHel-1	CAEZ01009016:18355-18677
HmHel-1	CAEZ01009016:48424-49230
HmHel-1	CAEZ01005451:28510-29310
HmHel-1	CAEZ01005451:121245-121584
HmHel-1	CAEZ01005451:83787-84096
HmHel-1	CAEZ01005451:50102-50890
HmHel-1	CAEZ01005242:3912-4483
HmHel-1	CAEZ01009460:7556-8040
HmHel-1	CAEZ01009460:31091-31899
HmHel-1	CAEZ01009460:11262-11592
HmHel-1	CAEZ01008726:100697-101503
HmHel-1	CAEZ01008726:145384-145705
HmHel-1	CAEZ01007666:11588-12396
HmHel-1	CAEZ01006347:96988-97797
HmHel-1	CAEZ01006347:53989-54321
HmHel-1	CAEZ01006181:6001-6803
HmHel-1	CAEZ01003254:18947-19757
HmHel-1	CAEZ01003076:362-1168
HmHel-1	CAEZ01002641:2409-3223
HmHel-1	CAEZ01007987:4128-4934
HmHel-1	CAEZ01003264:1-806
HmHel-1	CAEZ01010521:13420-13796
HmHel-1	CAEZ01008514:25628-25957
HmHel-1	CAEZ01008514:176-983
HmHel-1	CAEZ01007365:8978-9788

HmHel-1	CAEZ01007365:29454-29798
HmHel-1	CAEZ01006618:63216-64025
HmHel-1	CAEZ01006618:147803-148492
HmHel-1	CAEZ01006618:228188-228525
HmHel-1	CAEZ01000055:11537-12329
HmHel-1	CAEZ01011137:101894-102991
HmHel-1	CAEZ01011137:117321-117474
HmHel-1	CAEZ01011137:55715-56522
HmHel-1	CAEZ01011137:43578-43779
HmHel-1	CAEZ01010602:37719-38527
HmHel-1	CAEZ01010602:28814-29236
HmHel-1	CAEZ01010534:33458-33875
HmHel-1	CAEZ01010534:57636-57876
HmHel-1	CAEZ01010534:79362-79581
HmHel-1	CAEZ01010494:4991-5752
HmHel-1	CAEZ01009716:8527-9334
HmHel-1	CAEZ01009716:60913-61166
HmHel-1	CAEZ01009716:74487-75090
HmHel-1	CAEZ01009716:99203-99625
HmHel-1	CAEZ01009716:217032-217615
HmHel-1	CAEZ01009673:52796-53600
HmHel-1	CAEZ01005984:19612-20420
HmHel-1	CAEZ01004403:603-1417
HmHel-1	CAEZ01003636:10322-11179
HmHel-1	CAEZ01002097:41009-41213
HmHel-1	CAEZ01002097:23534-23741
HmHel-1	CAEZ01002097:2902-3710
HmHel-1	CAEZ01000968:32275-33083
HmHel-1	CAEZ01009391:8844-9648
HmHel-1	CAEZ01009272:23504-24320
HmHel-1	CAEZ01008510:19001-19811
HmHel-1	CAEZ01007298:6-388
HmHel-1	CAEZ01002428:4639-6390
HmHel-1	CAEZ01002428:4739-5548
HmHel-1	CAEZ01010926:17481-17887
HmHel-1	CAEZ01010926:83036-83626
HmHel-1	CAEZ01010926:96100-96906
HmHel-1	CAEZ01010926:72056-72460
HmHel-1	CAEZ01008989:158600-159408
HmHel-1	CAEZ01008989:95507-95739
HmHel-1	CAEZ01008989:25906-26249
HmHel-1	CAEZ01006406:25619-25858
HmHel-1	CAEZ01006406:38744-39568
HmHel-1	CAEZ01005481:13009-17960
HmHel-1	CAEZ01005481:38069-38878
HmHel-1	CAEZ01005481:89866-90288
HmHel-1	CAEZ01005225:40799-41046
HmHel-1	CAEZ01005225:17798-18605

HmHel-1	CAEZ01002684:7501-8369
HmHel-1	CAEZ01002684:8400-8937
HmHel-1	CAEZ01005683:1765-2475
HmHel-1	CAEZ01009150:2760-3084
HmHel-1	CAEZ01009150:24007-24812
HmHel-1	CAEZ01005402:2010-2813
HmHel-1	CAEZ01005402:16514-16918
HmHel-1	CAEZ01005402:35343-35591
HmHel-1	CAEZ01005402:58909-59565
HmHel-1	CAEZ01005402:37803-38542
HmHel-1	CAEZ01005402:20947-21736
HmHel-1	CAEZ01011492:2227-2422
HmHel-1	CAEZ01011492:699-997
HmHel-1	CAEZ01007250:1-373
HmHel-1	CAEZ01008614:4743-5082
HmHel-1	CAEZ01008614:40362-40703
HmHel-1	CAEZ01008614:24572-25243
HmHel-1	CAEZ01001754:1-792
HmHel-1	CAEZ01010535:2088-2508
HmHel-1	CAEZ01010535:59109-59593
HmHel-1	CAEZ01010535:8589-9011
HmHel-1	CAEZ01009680:33570-34382
HmHel-1	CAEZ01009680:65703-66044
HmHel-1	CAEZ01009680:20815-21015
HmHel-1	CAEZ01009680:2267-3109
HmHel-1	CAEZ01008152:1-371
HmHel-1	CAEZ01005143:1914-2731
HmHel-1	CAEZ01008851:3238-3607
HmHel-1	CAEZ01008504:3988-4778
HmHel-1	CAEZ01008425:5366-5701
HmHel-1	CAEZ01008425:35593-35932
HmHel-1	CAEZ01008425:83812-84618
HmHel-1	CAEZ01008425:107475-108275
HmHel-1	CAEZ01008425:59207-59419
HmHel-1	CAEZ01005015:499-2021
HmHel-1	CAEZ01005015:20646-21023
HmHel-1	CAEZ01005015:33589-33684
HmHel-1	CAEZ01005015:132151-132492
HmHel-1	CAEZ01005015:238566-238765
HmHel-1	CAEZ01005015:235339-235427
HmHel-1	CAEZ01005015:218943-219693
HmHel-1	CAEZ01005015:145285-146091
HmHel-1	CAEZ01004793:87061-87477
HmHel-1	CAEZ01004793:30423-30622
HmHel-1	CAEZ01004793:12-459
HmHel-1	CAEZ01003575:838-1637
HmHel-1	CAEZ01002886:1-598
HmHel-1	CAEZ01009396:1254-2047

HmHel-1	CAEZ01009339:1-588
HmHel-1	CAEZ01006157:1114-1480
HmHel-1	CAEZ01005241:6936-7761
HmHel-1	CAEZ01001932:4492-5285
HmHel-1	CAEZ01009355:61168-61987
HmHel-1	CAEZ01009355:82857-83520
HmHel-1	CAEZ01009355:19897-20294
HmHel-1	CAEZ01004107:1-518
HmHel-1	CAEZ01008649:10220-11032
HmHel-1	CAEZ01004751:35432-36238
HmHel-1	CAEZ01000834:38657-39459
HmHel-1	CAEZ01000834:2103-2294
HmHel-1	CAEZ01010815:2888-3255
HmHel-1	CAEZ01009800:16387-17814
HmHel-1	CAEZ01009800:19730-20140
HmHel-1	CAEZ01008123:32271-32961
HmHel-1	CAEZ01008123:47731-47938
HmHel-1	CAEZ01008123:33551-34354
HmHel-1	CAEZ01001894:4038-4370
HmHel-1	CAEZ01001894:3599-4801
HmHel-1	CAEZ01011046:128214-129022
HmHel-1	CAEZ01011046:161151-161490
HmHel-1	CAEZ01009901:75913-76576
HmHel-1	CAEZ01009901:46172-46400
HmHel-1	CAEZ01009901:1-337
HmHel-1	CAEZ01001886:1-801
HmHel-1	CAEZ01001059:476-744
HmHel-1	CAEZ01011569:4-353
HmHel-1	CAEZ01007708:72035-72267
HmHel-1	CAEZ01007708:28802-30979
HmHel-1	CAEZ01004720:7-385
HmHel-1	CAEZ01004251:717-1523
HmHel-1	CAEZ01009961:29385-29726
HmHel-1	CAEZ01003624:2894-3660
HmHel-1	CAEZ01010066:12192-12580
HmHel-1	CAEZ01008316:42710-42950
HmHel-1	CAEZ01008316:17631-18450
HmHel-1	CAEZ01005884:18092-19466
HmHel-1	CAEZ01005884:18392-19217
HmHel-1	CAEZ01010455:49208-49547
HmHel-1	CAEZ01010455:59652-59992
HmHel-1	CAEZ01010455:116365-116721
HmHel-1	CAEZ01010455:73578-74208
HmHel-1	CAEZ01004545:50151-50569
HmHel-1	CAEZ01004545:41441-42234
HmHel-1	CAEZ01010227:40874-41216
HmHel-1	CAEZ01010227:99577-100000
HmHel-1	CAEZ01010227:124036-124360

HmHel-1	CAEZ01010227:110771-111335
HmHel-1	CAEZ01010227:41941-42414
HmHel-1	CAEZ01007912:64740-65588
HmHel-1	CAEZ01007912:81885-82107
HmHel-1	CAEZ01007912:102096-102527
HmHel-1	CAEZ01003828:60066-60702
HmHel-1	CAEZ01003828:43428-43850
HmHel-1	CAEZ01003828:20809-21148
HmHel-1	CAEZ01003828:1-329
HmHel-1	CAEZ01010005:2669-3534
HmHel-1	CAEZ01008372:138668-139328
HmHel-1	CAEZ01008372:229258-230079
HmHel-1	CAEZ01008372:267029-267453
HmHel-1	CAEZ01008372:286162-286362
HmHel-1	CAEZ01008372:19169-19658
HmHel-1	CAEZ01008372:2-303
HmHel-1	CAEZ01005151:27-732
HmHel-1	CAEZ01004741:53719-54334
HmHel-1	CAEZ01004741:559-1366
HmHel-1	CAEZ01009511:47953-48418
HmHel-1	CAEZ01003985:403-734
HmHel-1	CAEZ01005501:12400-13250
HmHel-1	CAEZ01008737:24880-25084
HmHel-1	CAEZ01008737:67822-68613
HmHel-1	CAEZ01008737:80781-81048
HmHel-1	CAEZ01007878:8024-8363
HmHel-1	CAEZ01006233:4725-5064
HmHel-1	CAEZ01006233:15070-15157
HmHel-1	CAEZ01006233:25386-26221
HmHel-1	CAEZ01002013:573-1346
HmHel-1	CAEZ01008329:1-741
HmHel-1	CAEZ01003695:13-377
HmHel-1	CAEZ01009435:46088-46488
HmHel-1	CAEZ01009435:73718-74299
HmHel-1	CAEZ01009435:21345-21684
HmHel-1	CAEZ01010858:22985-23324
HmHel-1	CAEZ01010858:38536-39365
HmHel-1	CAEZ01005083:92703-93408
HmHel-1	CAEZ01005083:72177-72406
HmHel-1	CAEZ01005083:35533-35908
HmHel-1	CAEZ01002866:122619-123427
HmHel-1	CAEZ01010216:56473-57300
HmHel-1	CAEZ01004738:169777-170055
HmHel-1	CAEZ01004738:221779-222550
HmHel-1	CAEZ01004738:298637-299505
HmHel-1	CAEZ01004738:269077-269809
HmHel-1	CAEZ01004738:241346-241682
HmHel-1	CAEZ01004738:42927-43416

HmHel-1	CAEZ01004738:13896-14730
HmHel-1	CAEZ01007857:6513-6933
HmHel-1	CAEZ01007857:39330-39669
HmHel-1	CAEZ01007857:53946-54776
HmHel-1	CAEZ01007857:14780-14994
HmHel-1	CAEZ01007255:7098-7824
HmHel-1	CAEZ01010093:5411-6199
HmHel-1	CAEZ01006804:158602-159358
HmHel-1	CAEZ01006804:195740-195953
HmHel-1	CAEZ01006590:26600-27430
HmHel-1	CAEZ01006590:43805-44150
HmHel-1	CAEZ01006590:44986-45318
HmHel-1	CAEZ01004203:1-300
HmHel-1	CAEZ01005342:5600-6366
HmHel-1	CAEZ01005400:15056-15630
HmHel-1	CAEZ01005400:7077-7525
HmHel-1	CAEZ01005304:13258-14046
HmHel-1	CAEZ01003923:23915-24706
HmHel-1	CAEZ01007785:7273-9352
HmHel-1	CAEZ01007114:6049-6476
HmHel-1	CAEZ01007114:394-715
HmHel-1	CAEZ01000031:449-1011
HmHel-1	CAEZ01008286:3367-4130
HmHel-1	CAEZ01002603:1434-2212
HmHel-1	CAEZ01001004:125079-125310
HmHel-1	CAEZ01001004:154465-154703
HmHel-1	CAEZ01001004:127081-127504
HmHel-1	CAEZ01001004:6729-7483
HmHel-1	CAEZ01009647:331-670
HmHel-1	CAEZ01008719:67817-68648
HmHel-1	CAEZ01008719:7775-8104
HmHel-1	CAEZ01010272:108946-109285
HmHel-1	CAEZ01010272:110219-110441
HmHel-1	CAEZ01010272:61477-61692
HmHel-1	CAEZ01010272:47250-48028
HmHel-1	CAEZ01005418:68979-69639
HmHel-1	CAEZ01005418:194803-195233
HmHel-1	CAEZ01005418:260544-261391
HmHel-1	CAEZ01005418:289328-289535
HmHel-1	CAEZ01005418:294006-294190
HmHel-1	CAEZ01005418:273993-274354
HmHel-1	CAEZ01005418:246279-246933
HmHel-1	CAEZ01005418:94071-94407
HmHel-1	CAEZ01000949:293-1125
HmHel-1	CAEZ01006943:11799-12138
HmHel-1	CAEZ01006943:339-680
HmHel-1	CAEZ01006299:8671-10930
HmHel-1	CAEZ01006299:24913-25252

HmHel-1	CAEZ01006299:55411-55828
HmHel-1	CAEZ01003263:2878-2966
HmHel-1	CAEZ01003263:4079-4874
HmHel-1	CAEZ01011107:62345-63179
HmHel-1	CAEZ01011107:103487-103835
HmHel-1	CAEZ01008420:10944-11787
HmHel-1	CAEZ01008420:77065-77406
HmHel-1	CAEZ01008420:40986-41325
HmHel-1	CAEZ01008420:7793-8012
HmHel-1	CAEZ01004484:6410-7171
HmHel-1	CAEZ01004484:1988-2327
HmHel-1	CAEZ01007691:17465-18046
HmHel-1	CAEZ01007749:21153-21561
HmHel-1	CAEZ01007749:83691-83896
HmHel-1	CAEZ01007749:90477-91329
HmHel-1	CAEZ01009204:1011-1723
HmHel-1	CAEZ01007848:29639-30023
HmHel-1	CAEZ01008925:10225-10563
HmHel-1	CAEZ01007777:49644-50039
HmHel-1	CAEZ01007777:36359-36591
HmHel-1	CAEZ01007777:11871-12538
HmHel-1	CAEZ01005712:16407-16685
HmHel-1	CAEZ01000942:22310-22651
HmHel-1	CAEZ01000942:6-484
HmHel-1	CAEZ01005549:1-283
HmHel-1	CAEZ01005399:1-637
HmHel-1	CAEZ01005399:29003-29486
HmHel-1	CAEZ01007112:38054-38157
HmHel-1	CAEZ01007112:37895-38755
HmHel-1	CAEZ01006853:25435-26204
HmHel-1	CAEZ01006853:26752-27085
HmHel-1	CAEZ01005114:5364-5764
HmHel-1	CAEZ01010692:9965-10761
HmHel-1	CAEZ01010692:11306-11635
HmHel-1	CAEZ01009328:2193-2846
HmHel-1	CAEZ01009328:722-981
HmHel-1	CAEZ01009311:33945-34149
HmHel-1	CAEZ01009311:32520-33321
HmHel-1	CAEZ01007782:1-263
HmHel-1	CAEZ01009428:5192-5996
HmHel-1	CAEZ01007737:3773-4628
HmHel-1	CAEZ01007737:52721-53059
HmHel-1	CAEZ01007737:3591-4742
HmHel-1	CAEZ01003385:29642-30351
HmHel-1	CAEZ01003420:13277-14088
HmHel-1	CAEZ01006961:15012-15842
HmHel-1	CAEZ01006961:49259-49347
HmHel-1	CAEZ01006961:15884-16014

HmHel-1	CAEZ01006961:3067-3286
HmHel-1	CAEZ01008210:64054-64266
HmHel-1	CAEZ01008210:51982-52199
HmHel-1	CAEZ01008210:40688-41465
HmHel-1	CAEZ01008147:11530-12225
HmHel-1	CAEZ01008147:27531-27794
HmHel-1	CAEZ01008335:2214-2873
HmHel-1	CAEZ01008335:16866-17392
HmHel-1	CAEZ01011598:33047-33251
HmHel-1	CAEZ01011598:110088-110912
HmHel-1	CAEZ01011598:37754-39706
HmHel-1	CAEZ01007410:22-789
HmHel-1	CAEZ01006283:8934-9268
HmHel-1	CAEZ01006283:41165-41619
HmHel-1	CAEZ01006283:51921-52003
HmHel-1	CAEZ01006283:15458-16203
HmHel-1	CAEZ01009833:15687-16050
HmHel-1	CAEZ01009833:32847-37995
HmHel-1	CAEZ01009833:880-1621
HmHel-1	CAEZ01011544:1-406
HmHel-1	CAEZ01006136:1008-1339
HmHel-1	CAEZ01008771:96173-98133
HmHel-1	CAEZ01008771:166213-166301
HmHel-1	CAEZ01008771:118794-119462
HmHel-1	CAEZ01008771:96590-97344
HmHel-1	CAEZ01008771:29787-30126
HmHel-1	CAEZ01008771:15319-15658
HmHel-1	CAEZ01001965:9540-9911
HmHel-1	CAEZ01000817:29511-29850
HmHel-1	CAEZ01000817:26453-27221
HmHel-1	CAEZ01010260:52253-52467
HmHel-1	CAEZ01010260:108137-108928
HmHel-1	CAEZ01010260:111142-111654
HmHel-1	CAEZ01010739:18062-18625
HmHel-1	CAEZ01010739:37610-37949
HmHel-1	CAEZ01010739:6296-6875
HmHel-1	CAEZ01006711:11504-12383
HmHel-1	CAEZ01006711:81541-81873
HmHel-1	CAEZ01003478:16867-17206
HmHel-1	CAEZ01003478:1189-1458
HmHel-1	CAEZ01000818:414-1208
HmHel-1	CAEZ01004067:8712-9174
HmHel-1	CAEZ01004619:4977-5856
HmHel-1	CAEZ01006885:1536-2324
HmHel-1	CAEZ01006885:1077-1416
HmHel-1	CAEZ01002675:39202-40052
HmHel-1	CAEZ01002675:20639-21482
HmHel-1	CAEZ01002223:4213-4688

HmHel-1	CAEZ01000839:4937-5807
HmHel-1	CAEZ01010242:42227-42566
HmHel-1	CAEZ01010242:40044-40799
HmHel-1	CAEZ01002951:806-1648
HmHel-1	CAEZ01005740:10788-11464
HmHel-1	CAEZ01005740:1-394
HmHel-1	CAEZ01003272:10331-10670
HmHel-1	CAEZ01003272:973-1304
HmHel-1	CAEZ01005424:11699-12525
HmHel-1	CAEZ01001594:8515-9308
HmHel-1	CAEZ01000076:914-1280
HmHel-1	CAEZ01001736:97279-97501
HmHel-1	CAEZ01001736:34758-35564
HmHel-1	CAEZ01004306:231-8163
HmHel-1	CAEZ01004306:10907-11161
HmHel-1	CAEZ01000799:13080-13331
HmHel-1	CAEZ01000200:38-377
HmHel-1	CAEZ01002668:75839-76178
HmHel-1	CAEZ01002668:73282-73621
HmHel-1	CAEZ01002668:32391-32813
HmHel-1	CAEZ01006510:1-229
HmHel-1	CAEZ01005600:1-596
HmHel-1	CAEZ01000873:10956-11301
HmHel-1	CAEZ01008764:4762-5512
HmHel-1	CAEZ01008435:79476-80152
HmHel-1	CAEZ01008435:97035-97213
HmHel-1	CAEZ01008435:158456-158850
HmHel-1	CAEZ01008435:230862-232367
HmHel-1	CAEZ01008435:240393-241061
HmHel-1	CAEZ01008435:107091-107308
HmHel-1	CAEZ01008435:85894-86233
HmHel-1	CAEZ01008435:73715-74017
HmHel-1	CAEZ01008435:5071-5493
HmHel-1	CAEZ01008046:51-324
HmHel-1	CAEZ01007537:1-412
HmHel-1	CAEZ01006835:564-979
HmHel-1	CAEZ01006632:23661-24410
HmHel-1	CAEZ01006632:52680-53012
HmHel-1	CAEZ01006276:8155-8374
HmHel-1	CAEZ01006276:18502-19361
HmHel-1	CAEZ01004427:2256-2674
HmHel-1	CAEZ01004271:14010-14426
HmHel-1	CAEZ01003763:491-1240
HmHel-1	CAEZ01001742:5405-5746
HmHel-1	CAEZ01001742:17876-18629
HmHel-1	CAEZ01001742:7005-7346
HmHel-1	CAEZ01001020:602-810
HmHel-1	CAEZ01005311:1-639

HmHel-1	CAEZ01002946:2386-2697
HmHel-1	CAEZ01002946:1-207
HmHel-1	CAEZ01011208:4-656
HmHel-1	CAEZ01008138:163182-163424
HmHel-1	CAEZ01008138:176958-177297
HmHel-1	CAEZ01008138:200641-201331
HmHel-1	CAEZ01008138:318108-318530
HmHel-1	CAEZ01008138:339381-340021
HmHel-1	CAEZ01008138:351033-356030
HmHel-1	CAEZ01008138:58528-59266
HmHel-1	CAEZ01001785:6987-7640
HmHel-1	CAEZ01011452:25678-26104
HmHel-1	CAEZ01011452:4951-5639
HmHel-1	CAEZ01008266:5712-5909
HmHel-1	CAEZ01008266:22221-22879
HmHel-1	CAEZ01008266:1534-2362
HmHel-1	CAEZ01011050:1-276
HmHel-1	CAEZ01011050:14076-14747
HmHel-1	CAEZ01009962:35-230
HmHel-1	CAEZ01009962:38621-39037
HmHel-1	CAEZ01009449:159-570
HmHel-1	CAEZ01009372:1351-1781
HmHel-1	CAEZ01009332:16425-17171
HmHel-1	CAEZ01009332:27608-27947
HmHel-1	CAEZ01009332:6126-6335
HmHel-1	CAEZ01008874:33717-34456
HmHel-1	CAEZ01008874:46986-48119
HmHel-1	CAEZ01008874:88563-88795
HmHel-1	CAEZ01008874:124585-124908
HmHel-1	CAEZ01008729:57049-57253
HmHel-1	CAEZ01008729:30511-30599
HmHel-1	CAEZ01008729:13280-13898
HmHel-1	CAEZ01008609:6455-6868
HmHel-1	CAEZ01007804:72605-72944
HmHel-1	CAEZ01007804:28041-28795
HmHel-1	CAEZ01007568:21751-21953
HmHel-1	CAEZ01007568:51911-59612
HmHel-1	CAEZ01007568:80687-81109
HmHel-1	CAEZ01007568:101021-101370
HmHel-1	CAEZ01007568:113493-113983
HmHel-1	CAEZ01007568:62240-62441
HmHel-1	CAEZ01007568:34737-35152
HmHel-1	CAEZ01007463:2934-3274
HmHel-1	CAEZ01007463:13889-14361
HmHel-1	CAEZ01007463:587-673
HmHel-1	CAEZ01006778:67094-67782
HmHel-1	CAEZ01006778:25167-25852
HmHel-1	CAEZ01006241:729-1155

HmHel-1	CAEZ01006241:35819-36228
HmHel-1	CAEZ01005739:30465-30660
HmHel-1	CAEZ01005721:10005-10389
HmHel-1	CAEZ01005721:66799-67003
HmHel-1	CAEZ01005721:113888-113975
HmHel-1	CAEZ01005721:124292-124635
HmHel-1	CAEZ01005721:97287-97627
HmHel-1	CAEZ01005721:80037-80254
HmHel-1	CAEZ01005721:63575-64239
HmHel-1	CAEZ01005721:3489-4198
HmHel-1	CAEZ01005617:3385-3840
HmHel-1	CAEZ01005529:1-569
HmHel-1	CAEZ01005529:13641-14132
HmHel-1	CAEZ01005307:3706-4413
HmHel-1	CAEZ01005307:3158-5743
HmHel-1	CAEZ01005252:36324-36679
HmHel-1	CAEZ01005196:33793-34543
HmHel-1	CAEZ01004811:169879-170567
HmHel-1	CAEZ01004811:358738-358981
HmHel-1	CAEZ01004811:425165-425504
HmHel-1	CAEZ01004811:76903-77319
HmHel-1	CAEZ01004406:894-1317
HmHel-1	CAEZ01003262:20963-21305
HmHel-1	CAEZ01003197:45400-45739
HmHel-1	CAEZ01003197:12262-12880
HmHel-1	CAEZ01002939:853-1161
HmHel-1	CAEZ01002666:27498-27920
HmHel-1	CAEZ01002666:54673-54905
HmHel-1	CAEZ01002666:81657-82306
HmHel-1	CAEZ01002666:37853-38603
HmHel-1	CAEZ01000905:32350-32771
HmHel-1	CAEZ01001213:532-958
HmHel-1	CAEZ01009414:16850-17093
HmHel-1	CAEZ01008667:357-1105
HmHel-1	CAEZ01003578:855-1608
HmHel-1	CAEZ01002620:34914-35118
HmHel-1	CAEZ01002620:69360-69689
HmHel-1	CAEZ01002620:70015-70387
HmHel-1	CAEZ01002620:21486-21828
HmHel-1	CAEZ01002154:4213-4951
HmHel-1	CAEZ01010608:6064-6453
HmHel-1	CAEZ01009835:24613-25128
HmHel-1	CAEZ01009484:13979-14399
HmHel-1	CAEZ01009306:80836-81256
HmHel-1	CAEZ01009306:65247-65616
HmHel-1	CAEZ01009306:28620-29113
HmHel-1	CAEZ01006273:15826-16052
HmHel-1	CAEZ01003678:9320-10095

HmHel-1	CAEZ01001648:26306-26771
HmHel-1	CAEZ01011488:6972-7665
HmHel-1	CAEZ01010917:26576-26791
HmHel-1	CAEZ01010917:32161-32827
HmHel-1	CAEZ01010917:10432-10773
HmHel-1	CAEZ01010816:17-212
HmHel-1	CAEZ01010816:37871-38210
HmHel-1	CAEZ01010816:66433-66772
HmHel-1	CAEZ01010816:12130-12550
HmHel-1	CAEZ01010749:8482-8841
HmHel-1	CAEZ01010749:9470-9665
HmHel-1	CAEZ01010731:10114-10535
HmHel-1	CAEZ01010728:70113-70452
HmHel-1	CAEZ01010728:67001-68039
HmHel-1	CAEZ01010728:13744-14005
HmHel-1	CAEZ01010540:5272-5694
HmHel-1	CAEZ01009759:7247-7663
HmHel-1	CAEZ01009589:4548-4962
HmHel-1	CAEZ01009589:790-1222
HmHel-1	CAEZ01009077:1-287
HmHel-1	CAEZ01009077:52599-52958
HmHel-1	CAEZ01009077:225974-226333
HmHel-1	CAEZ01009077:143926-144675
HmHel-1	CAEZ01009077:119587-119943
HmHel-1	CAEZ01008915:84694-85033
HmHel-1	CAEZ01008915:140917-141376
HmHel-1	CAEZ01008915:257522-257860
HmHel-1	CAEZ01008915:307760-308437
HmHel-1	CAEZ01008915:256192-256322
HmHel-1	CAEZ01008915:129891-129979
HmHel-1	CAEZ01008915:106216-106905
HmHel-1	CAEZ01008684:1380-1805
HmHel-1	CAEZ01008500:107914-108606
HmHel-1	CAEZ01008500:139376-139571
HmHel-1	CAEZ01008500:57023-57361
HmHel-1	CAEZ01008349:144898-145330
HmHel-1	CAEZ01008349:44667-45023
HmHel-1	CAEZ01007939:66077-66874
HmHel-1	CAEZ01007939:110924-111152
HmHel-1	CAEZ01007939:127326-127947
HmHel-1	CAEZ01007939:304857-305173
HmHel-1	CAEZ01007939:200362-200718
HmHel-1	CAEZ01007713:5-355
HmHel-1	CAEZ01007713:65839-66253
HmHel-1	CAEZ01007713:32097-32495
HmHel-1	CAEZ01007704:15547-15963
HmHel-1	CAEZ01007499:97372-98097
HmHel-1	CAEZ01007478:79597-79826

HmHel-1	CAEZ01007478:107812-108236
HmHel-1	CAEZ01007216:6611-7320
HmHel-1	CAEZ01007074:1154-1554
HmHel-1	CAEZ01006960:36211-36928
HmHel-1	CAEZ01006884:12689-13030
HmHel-1	CAEZ01006646:7-286
HmHel-1	CAEZ01006493:20493-20915
HmHel-1	CAEZ01006419:2-298
HmHel-1	CAEZ01006340:34964-35390
HmHel-1	CAEZ01006326:3946-4386
HmHel-1	CAEZ01005967:18453-18864
HmHel-1	CAEZ01005967:5990-6340
HmHel-1	CAEZ01005912:4189-4601
HmHel-1	CAEZ01005841:1-540
HmHel-1	CAEZ01005841:51301-51501
HmHel-1	CAEZ01005682:9550-10293
HmHel-1	CAEZ01005682:10704-11406
HmHel-1	CAEZ01005478:3746-4091
HmHel-1	CAEZ01005478:18800-19000
HmHel-1	CAEZ01005478:82243-82663
HmHel-1	CAEZ01005478:71843-72058
HmHel-1	CAEZ01005478:1683-1898
HmHel-1	CAEZ01005428:96890-97564
HmHel-1	CAEZ01005428:176-901
HmHel-1	CAEZ01005275:12263-12683
HmHel-1	CAEZ01005208:27711-28067
HmHel-1	CAEZ01004737:1-339
HmHel-1	CAEZ01004659:975-1395
HmHel-1	CAEZ01004030:9707-10398
HmHel-1	CAEZ01003547:2758-3099
HmHel-1	CAEZ01003020:77-1736
HmHel-1	CAEZ01002798:23124-23339
HmHel-1	CAEZ01002798:28930-29656
HmHel-1	CAEZ01002732:25908-26254
HmHel-1	CAEZ01001479:993-1717
HmHel-1	CAEZ01001447:1-429
HmHel-1	CAEZ01001447:30251-30583
HmHel-1	CAEZ01001447:47579-47918
HmHel-1	CAEZ01001408:186575-186916
HmHel-1	CAEZ01001408:75203-75629
HmHel-1	CAEZ01000993:1543-1907
HmHel-1	CAEZ01000791:12-207
HmHel-1	CAEZ01007204:45320-45661
HmHel-1	CAEZ01007204:63817-64137
HmHel-1	CAEZ01007204:52470-52692
HmHel-1	CAEZ01007204:2147-2483
HmHel-1	CAEZ01008254:10819-11565
HmHel-1	CAEZ01008034:2938-3674

HmHel-1	CAEZ01008471:1-547
HmHel-1	CAEZ01011263:359-1083
HmHel-1	CAEZ01010868:28958-29679
HmHel-1	CAEZ01010832:19-214
HmHel-1	CAEZ01010809:61879-62222
HmHel-1	CAEZ01010206:15656-16414
HmHel-1	CAEZ01010026:228736-229160
HmHel-1	CAEZ01010026:49283-49699
HmHel-1	CAEZ01010001:25047-25469
HmHel-1	CAEZ01009681:1-358
HmHel-1	CAEZ01009681:44108-44530
HmHel-1	CAEZ01009681:72632-72970
HmHel-1	CAEZ01009681:73639-73972
HmHel-1	CAEZ01009271:1-330
HmHel-1	CAEZ01009271:169088-169718
HmHel-1	CAEZ01009271:69248-69604
HmHel-1	CAEZ01009143:1-245
HmHel-1	CAEZ01008928:23089-23293
HmHel-1	CAEZ01008928:7550-7909
HmHel-1	CAEZ01008919:3440-3799
HmHel-1	CAEZ01008919:55759-55974
HmHel-1	CAEZ01008276:20-296
HmHel-1	CAEZ01008233:10216-10438
HmHel-1	CAEZ01008233:91265-91624
HmHel-1	CAEZ01008233:20148-20489
HmHel-1	CAEZ01007954:4615-4954
HmHel-1	CAEZ01007875:4055-4414
HmHel-1	CAEZ01007875:245-960
HmHel-1	CAEZ01007812:1346-1687
HmHel-1	CAEZ01007812:199731-199977
HmHel-1	CAEZ01007812:179851-180379
HmHel-1	CAEZ01007764:16392-16730
HmHel-1	CAEZ01007390:20451-20792
HmHel-1	CAEZ01007249:20107-20536
HmHel-1	CAEZ01007157:12631-13054
HmHel-1	CAEZ01007069:7132-7473
HmHel-1	CAEZ01006897:9550-9962
HmHel-1	CAEZ01006897:21732-21937
HmHel-1	CAEZ01006897:77-411
HmHel-1	CAEZ01006772:226844-227264
HmHel-1	CAEZ01006772:183759-184098
HmHel-1	CAEZ01006772:72843-73507
HmHel-1	CAEZ01006675:73544-73766
HmHel-1	CAEZ01006675:95487-95826
HmHel-1	CAEZ01006675:118039-118398
HmHel-1	CAEZ01006675:88904-89263
HmHel-1	CAEZ01006675:52644-52848
HmHel-1	CAEZ01006562:29671-29998

HmHel-1	CAEZ01006562:40634-41056
HmHel-1	CAEZ01006562:170621-170754
HmHel-1	CAEZ01006562:41562-41984
HmHel-1	CAEZ01006562:30811-31150
HmHel-1	CAEZ01006548:3867-5118
HmHel-1	CAEZ01006548:95387-95809
HmHel-1	CAEZ01006548:14777-15472
HmHel-1	CAEZ01006449:4141-4563
HmHel-1	CAEZ01006171:113129-113540
HmHel-1	CAEZ01006171:113727-114067
HmHel-1	CAEZ01006171:69272-69504
HmHel-1	CAEZ01006153:12506-12846
HmHel-1	CAEZ01006153:23309-24084
HmHel-1	CAEZ01005962:8936-9404
HmHel-1	CAEZ01005858:575-990
HmHel-1	CAEZ01005803:7222-7944
HmHel-1	CAEZ01005796:4-340
HmHel-1	CAEZ01005746:38057-38428
HmHel-1	CAEZ01005746:67620-68392
HmHel-1	CAEZ01005654:995-1417
HmHel-1	CAEZ01005426:11559-11971
HmHel-1	CAEZ01005211:118333-119018
HmHel-1	CAEZ01005211:12977-13372
HmHel-1	CAEZ01005180:2848-3207
HmHel-1	CAEZ01005180:14019-14438
HmHel-1	CAEZ01005152:71859-72061
HmHel-1	CAEZ01005152:33610-34305
HmHel-1	CAEZ01005131:1759-2452
HmHel-1	CAEZ01005131:19290-19624
HmHel-1	CAEZ01005077:1-421
HmHel-1	CAEZ01004733:2153-2537
HmHel-1	CAEZ01004733:22120-22594
HmHel-1	CAEZ01004733:40117-40439
HmHel-1	CAEZ01004733:26207-26841
HmHel-1	CAEZ01004562:84615-84954
HmHel-1	CAEZ01004562:222158-222613
HmHel-1	CAEZ01004562:34783-35140
HmHel-1	CAEZ01004368:30864-31223
HmHel-1	CAEZ01004196:21737-22107
HmHel-1	CAEZ01004025:4102-4503
HmHel-1	CAEZ01004014:18233-18525
HmHel-1	CAEZ01004014:51531-51953
HmHel-1	CAEZ01003621:3692-11138
HmHel-1	CAEZ01003621:13459-13794
HmHel-1	CAEZ01003194:43453-44097
HmHel-1	CAEZ01003194:60884-61306
HmHel-1	CAEZ01003194:2849-3603
HmHel-1	CAEZ01003007:57485-57907

HmHel-1	CAEZ01002630:20731-21052
HmHel-1	CAEZ01001281:445-804
HmHel-1	CAEZ01001249:18613-19339
HmHel-1	CAEZ01000878:106-330
HmHel-1	CAEZ01000878:36080-36770
HmHel-1	CAEZ01000878:84037-84793
HmHel-1	CAEZ01000878:47982-48306
HmHel-1	CAEZ01000878:30215-30303
HmHel-1	CAEZ01011527:26984-27320
HmHel-1	CAEZ01011527:21715-24798
HmHel-1	CAEZ01008674:39338-39677
HmHel-1	CAEZ01008674:78873-79297
HmHel-1	CAEZ01003711:6505-6706
HmHel-1	CAEZ01003711:19609-19948
HmHel-1	CAEZ01003711:22948-23360
HmHel-1	CAEZ01009893:36533-36870
HmHel-1	CAEZ01009893:18219-19122
HmHel-1	CAEZ01007259:58234-59323
HmHel-1	CAEZ01007259:91946-92288
HmHel-1	CAEZ01007259:24459-25027
HmHel-1	CAEZ01011540:1-407
HmHel-1	CAEZ01011485:9-584
HmHel-1	CAEZ01009374:60598-61335
HmHel-1	CAEZ01008878:37956-38758
HmHel-1	CAEZ01006509:4120-4316
HmHel-1	CAEZ01002941:22459-22867
HmHel-1	CAEZ01002889:1062-1700
HmHel-1	CAEZ01002729:11863-12602
HmHel-1	CAEZ01011572:12812-13234
HmHel-1	CAEZ01011489:77023-77651
HmHel-1	CAEZ01011489:33562-34286
HmHel-1	CAEZ01010879:5108-5901
HmHel-1	CAEZ01010732:4858-5277
HmHel-1	CAEZ01010641:3630-3995
HmHel-1	CAEZ01010457:16144-16560
HmHel-1	CAEZ01010457:20095-20811
HmHel-1	CAEZ01010407:592-1333
HmHel-1	CAEZ01010285:44558-44897
HmHel-1	CAEZ01010285:122864-123223
HmHel-1	CAEZ01010285:181630-181968
HmHel-1	CAEZ01010285:109295-109608
HmHel-1	CAEZ01010285:63775-64210
HmHel-1	CAEZ01010184:26183-26605
HmHel-1	CAEZ01010076:6199-6306
HmHel-1	CAEZ01010076:2951-3324
HmHel-1	CAEZ01009832:11438-11785
HmHel-1	CAEZ01009832:29743-30070
HmHel-1	CAEZ01009832:19625-19952

HmHel-1	CAEZ01009696:18887-21904
HmHel-1	CAEZ01009465:18559-18984
HmHel-1	CAEZ01009359:30823-31163
HmHel-1	CAEZ01009223:38836-39257
HmHel-1	CAEZ01009223:49750-50176
HmHel-1	CAEZ01009223:73837-74176
HmHel-1	CAEZ01009223:53798-54037
HmHel-1	CAEZ01009039:4515-5228
HmHel-1	CAEZ01008707:115387-115610
HmHel-1	CAEZ01008707:123630-123969
HmHel-1	CAEZ01008707:100161-100500
HmHel-1	CAEZ01008707:66161-69890
HmHel-1	CAEZ01008707:24221-24975
HmHel-1	CAEZ01008707:379-776
HmHel-1	CAEZ01008677:85319-85660
HmHel-1	CAEZ01008675:13235-13657
HmHel-1	CAEZ01008404:18267-18689
HmHel-1	CAEZ01008404:182401-183065
HmHel-1	CAEZ01008404:74670-74902
HmHel-1	CAEZ01008404:44581-44669
HmHel-1	CAEZ01008238:24811-24995
HmHel-1	CAEZ01008238:52360-52775
HmHel-1	CAEZ01007858:169236-169479
HmHel-1	CAEZ01007858:15603-15944
HmHel-1	CAEZ01007706:31043-31464
HmHel-1	CAEZ01007706:45578-55136
HmHel-1	CAEZ01007706:139472-139729
HmHel-1	CAEZ01007706:159087-159512
HmHel-1	CAEZ01007706:253199-253291
HmHel-1	CAEZ01007706:111058-111397
HmHel-1	CAEZ01007465:19797-20144
HmHel-1	CAEZ01007192:2272-2688
HmHel-1	CAEZ01007192:6896-7111
HmHel-1	CAEZ01007090:10062-10225
HmHel-1	CAEZ01007090:5939-6361
HmHel-1	CAEZ01006974:9263-9622
HmHel-1	CAEZ01006827:10856-11215
HmHel-1	CAEZ01006335:3408-3824
HmHel-1	CAEZ01006131:38434-38773
HmHel-1	CAEZ01006131:161073-161807
HmHel-1	CAEZ01005923:20944-21283
HmHel-1	CAEZ01005923:20009-20206
HmHel-1	CAEZ01005923:8086-8502
HmHel-1	CAEZ01005866:6334-6674
HmHel-1	CAEZ01005756:22975-23204
HmHel-1	CAEZ01005756:5126-5548
HmHel-1	CAEZ01005717:48876-49215
HmHel-1	CAEZ01005717:41734-42153

HmHel-1	CAEZ01005695:8256-8655
HmHel-1	CAEZ01005676:1-470
HmHel-1	CAEZ01005487:1-399
HmHel-1	CAEZ01005487:1575-1914
HmHel-1	CAEZ01005466:13364-13723
HmHel-1	CAEZ01005385:3849-4607
HmHel-1	CAEZ01005356:405-746
HmHel-1	CAEZ01004816:77256-77575
HmHel-1	CAEZ01004816:34513-34872
HmHel-1	CAEZ01004519:26427-26950
HmHel-1	CAEZ01004000:6228-6529
HmHel-1	CAEZ01003927:2703-3956
HmHel-1	CAEZ01003345:25360-25775
HmHel-1	CAEZ01003191:929-1288
HmHel-1	CAEZ01003163:2060-2148
HmHel-1	CAEZ01003163:70684-71060
HmHel-1	CAEZ01003163:29127-29818
HmHel-1	CAEZ01002802:12175-12597
HmHel-1	CAEZ01002762:37305-37717
HmHel-1	CAEZ01002695:2853-3605
HmHel-1	CAEZ01002579:2196-3362
HmHel-1	CAEZ01002579:2507-2904
HmHel-1	CAEZ01002244:5427-5849
HmHel-1	CAEZ01002184:21287-22788
HmHel-1	CAEZ01002184:21470-22220
HmHel-1	CAEZ01002184:7760-7989
HmHel-1	CAEZ01001623:10-681
HmHel-1	CAEZ01000921:15990-16685
HmHel-1	CAEZ01000921:35257-35345
HmHel-1	CAEZ01000831:12932-13351
HmHel-1	CAEZ01000795:7491-7764
HmHel-1	CAEZ01010828:29153-29494
HmHel-1	CAEZ01004923:66509-66845
HmHel-1	CAEZ01004923:80363-80587
HmHel-1	CAEZ01011507:10089-12084
HmHel-1	CAEZ01009186:19553-20378
HmHel-1	CAEZ01007146:5210-5624
HmHel-1	CAEZ01005898:2474-3224
HmHel-1	CAEZ01011514:1444-1775
HmHel-1	CAEZ01011338:1793-1988
HmHel-1	CAEZ01010228:132-403
HmHel-1	CAEZ01010228:30633-30855
HmHel-1	CAEZ01009424:29320-29659
HmHel-1	CAEZ01008403:34902-35237
HmHel-1	CAEZ01008403:91259-91598
HmHel-1	CAEZ01008403:9015-9357
HmHel-1	CAEZ01005410:19319-19732
HmHel-1	CAEZ01005409:36630-36969

HmHel-1	CAEZ01005409:38478-38817
HmHel-1	CAEZ01004656:17473-17781
HmHel-1	CAEZ01004027:6339-9083
HmHel-1	CAEZ01003811:582-1337
HmHel-1	CAEZ01003811:18510-19000
HmHel-1	CAEZ01002912:1288-1711
HmHel-1	CAEZ01002645:3035-3392
HmHel-1	CAEZ01002607:141634-141973
HmHel-1	CAEZ01002607:200278-200871
HmHel-1	CAEZ01002607:121129-121451
HmHel-1	CAEZ01011625:5133-5555
HmHel-1	CAEZ01011558:14446-15089
HmHel-1	CAEZ01011558:1188-1610
HmHel-1	CAEZ01011388:4063-4351
HmHel-1	CAEZ01011330:20584-21006
HmHel-1	CAEZ01011054:48552-48893
HmHel-1	CAEZ01011054:24813-25155
HmHel-1	CAEZ01010943:11119-11542
HmHel-1	CAEZ01010942:17771-18191
HmHel-1	CAEZ01010667:6530-7660
HmHel-1	CAEZ01010667:29377-31004
HmHel-1	CAEZ01010477:1206-1807
HmHel-1	CAEZ01010118:30046-30387
HmHel-1	CAEZ01010114:13905-14283
HmHel-1	CAEZ01009900:96492-96783
HmHel-1	CAEZ01009669:603-1018
HmHel-1	CAEZ01009427:1-396
HmHel-1	CAEZ01009338:14788-14967
HmHel-1	CAEZ01008168:21268-21605
HmHel-1	CAEZ01007693:1171-1499
HmHel-1	CAEZ01007634:13052-13537
HmHel-1	CAEZ01007450:26472-26883
HmHel-1	CAEZ01007389:90992-91406
HmHel-1	CAEZ01007389:176475-176565
HmHel-1	CAEZ01007389:150785-159196
HmHel-1	CAEZ01007389:132567-133233
HmHel-1	CAEZ01007389:87309-87555
HmHel-1	CAEZ01007288:26977-27172
HmHel-1	CAEZ01006958:17400-17759
HmHel-1	CAEZ01006937:35961-36383
HmHel-1	CAEZ01006937:35437-35885
HmHel-1	CAEZ01006900:6999-7415
HmHel-1	CAEZ01006702:1851-4073
HmHel-1	CAEZ01006530:34522-34861
HmHel-1	CAEZ01006530:76607-77372
HmHel-1	CAEZ01006530:262683-263344
HmHel-1	CAEZ01006530:412-905
HmHel-1	CAEZ01006422:36721-36921

HmHel-1	CAEZ01006422:80350-80766
HmHel-1	CAEZ01006422:42833-43289
HmHel-1	CAEZ01006422:15180-15521
HmHel-1	CAEZ01006422:41-476
HmHel-1	CAEZ01006164:10800-11268
HmHel-1	CAEZ01006164:24142-24478
HmHel-1	CAEZ01006164:40829-41178
HmHel-1	CAEZ01006164:55194-55372
HmHel-1	CAEZ01006164:39092-39431
HmHel-1	CAEZ01006164:21831-22019
HmHel-1	CAEZ01006164:9908-10112
HmHel-1	CAEZ01006161:56194-56416
HmHel-1	CAEZ01006161:90836-91583
HmHel-1	CAEZ01006161:107203-107289
HmHel-1	CAEZ01006161:75808-76013
HmHel-1	CAEZ01006161:53997-54243
HmHel-1	CAEZ01006023:26334-27398
HmHel-1	CAEZ01006023:9132-9350
HmHel-1	CAEZ01005269:85167-85384
HmHel-1	CAEZ01005269:103909-104113
HmHel-1	CAEZ01005269:131070-131492
HmHel-1	CAEZ01005269:82192-82630
HmHel-1	CAEZ01005269:61945-62149
HmHel-1	CAEZ01005105:8149-8490
HmHel-1	CAEZ01005105:37498-37919
HmHel-1	CAEZ01005105:13138-13479
HmHel-1	CAEZ01004983:5404-5637
HmHel-1	CAEZ01004939:1182-1458
HmHel-1	CAEZ01004629:34155-34567
HmHel-1	CAEZ01004629:70528-71207
HmHel-1	CAEZ01004629:6950-7290
HmHel-1	CAEZ01004590:4-299
HmHel-1	CAEZ01004325:17999-18221
HmHel-1	CAEZ01004325:18748-19086
HmHel-1	CAEZ01004094:107986-108719
HmHel-1	CAEZ01003143:22457-22707
HmHel-1	CAEZ01003143:29012-29353
HmHel-1	CAEZ01003106:13930-14247
HmHel-1	CAEZ01003106:18623-19039
HmHel-1	CAEZ01003106:7994-8396
HmHel-1	CAEZ01002840:1369-1793
HmHel-1	CAEZ01002701:805-1055
HmHel-1	CAEZ01002701:1807-2218
HmHel-1	CAEZ01002691:11609-12196
HmHel-1	CAEZ01002182:78979-79179
HmHel-1	CAEZ01002182:99979-100064
HmHel-1	CAEZ01002182:2316-2668
HmHel-1	CAEZ01002087:75979-76395

HmHel-1	CAEZ01002087:28130-28341
HmHel-1	CAEZ01001794:1134-1604
HmHel-1	CAEZ01001028:909-1331
HmHel-1	CAEZ01008807:2548-2948
HmHel-1	CAEZ01006470:22872-23187
HmHel-1	CAEZ01006470:21887-22309
HmHel-1	CAEZ01006238:14659-14909
HmHel-1	CAEZ01003400:1782-2156
HmHel-1	CAEZ01003400:22196-22535
HmHel-1	CAEZ01003400:5032-5365
HmHel-1	CAEZ01001571:1066-1487
HmHel-1	CAEZ01001571:2709-3092
HmHel-1	CAEZ01011559:1-597
HmHel-1	CAEZ01010803:123469-123810
HmHel-1	CAEZ01010803:60989-61656
HmHel-1	CAEZ01008126:25220-25888
HmHel-1	CAEZ01008126:50000-50679
HmHel-1	CAEZ01008126:59881-60149
HmHel-1	CAEZ01005620:9894-10180
HmHel-1	CAEZ01005160:4145-4543
HmHel-1	CAEZ01003756:23096-23760
HmHel-1	CAEZ01001998:320-688
HmHel-1	CAEZ01001802:17747-18228
HmHel-1	CAEZ01000965:2190-2610
HmHel-1	CAEZ01011410:50172-50397
HmHel-1	CAEZ01011410:30043-30382
HmHel-1	CAEZ01010969:60986-61325
HmHel-1	CAEZ01010786:1485-1899
HmHel-1	CAEZ01010727:52959-53379
HmHel-1	CAEZ01010727:38400-38842
HmHel-1	CAEZ01010355:34483-34858
HmHel-1	CAEZ01009919:771-1110
HmHel-1	CAEZ01009778:166615-167303
HmHel-1	CAEZ01009778:382603-383325
HmHel-1	CAEZ01009778:404881-405243
HmHel-1	CAEZ01009778:153699-154038
HmHel-1	CAEZ01009219:8860-9284
HmHel-1	CAEZ01009012:16949-17288
HmHel-1	CAEZ01008621:43384-43662
HmHel-1	CAEZ01008381:9165-10080
HmHel-1	CAEZ01008381:33307-33721
HmHel-1	CAEZ01008381:18070-18462
HmHel-1	CAEZ01007659:12640-13061
HmHel-1	CAEZ01006830:9786-10137
HmHel-1	CAEZ01006537:13742-14081
HmHel-1	CAEZ01006525:181-511
HmHel-1	CAEZ01005949:40525-40864
HmHel-1	CAEZ01005150:30321-30660

HmHel-1	CAEZ01005150:28590-28819
HmHel-1	CAEZ01004990:19125-19471
HmHel-1	CAEZ01004990:71712-72134
HmHel-1	CAEZ01004591:1126-1336
HmHel-1	CAEZ01004591:19569-19908
HmHel-1	CAEZ01004591:14653-21211
HmHel-1	CAEZ01004034:44960-45299
HmHel-1	CAEZ01003839:4710-5049
HmHel-1	CAEZ01003532:2546-2885
HmHel-1	CAEZ01001349:6368-6742
HmHel-1	CAEZ01001349:47103-47333
HmHel-1	CAEZ01001349:11105-11444
HmHel-1	CAEZ01011531:1-296
HmHel-1	CAEZ01011339:6-422
HmHel-1	CAEZ01011284:34038-34377
HmHel-1	CAEZ01011284:911-1332
HmHel-1	CAEZ01011190:31496-31797
HmHel-1	CAEZ01011190:19730-19938
HmHel-1	CAEZ01011015:2422-2712
HmHel-1	CAEZ01010880:167-382
HmHel-1	CAEZ01010880:28947-29204
HmHel-1	CAEZ01010880:17891-18233
HmHel-1	CAEZ01010584:204-544
HmHel-1	CAEZ01010584:15786-16125
HmHel-1	CAEZ01010584:91802-91890
HmHel-1	CAEZ01010584:177903-178564
HmHel-1	CAEZ01010584:191016-191354
HmHel-1	CAEZ01010584:288284-288773
HmHel-1	CAEZ01010584:47300-48004
HmHel-1	CAEZ01010584:23361-23824
HmHel-1	CAEZ01010495:3-182
HmHel-1	CAEZ01010428:16541-16957
HmHel-1	CAEZ01010428:42056-44683
HmHel-1	CAEZ01010428:120058-120397
HmHel-1	CAEZ01010428:106164-106580
HmHel-1	CAEZ01010356:60030-60458
HmHel-1	CAEZ01010351:20712-21134
HmHel-1	CAEZ01010351:62372-62819
HmHel-1	CAEZ01009979:15567-15906
HmHel-1	CAEZ01009943:1-313
HmHel-1	CAEZ01009943:2042-2221
HmHel-1	CAEZ01009802:138743-139092
HmHel-1	CAEZ01009802:259384-259662
HmHel-1	CAEZ01009243:1-305
HmHel-1	CAEZ01008924:51324-51659
HmHel-1	CAEZ01008924:19857-20198
HmHel-1	CAEZ01008713:112312-112734
HmHel-1	CAEZ01008713:98915-99628

HmHel-1	CAEZ01008713:81618-82176
HmHel-1	CAEZ01008713:6057-6394
HmHel-1	CAEZ01008630:4776-5115
HmHel-1	CAEZ01008518:35748-35989
HmHel-1	CAEZ01008208:51716-52132
HmHel-1	CAEZ01008208:96855-97276
HmHel-1	CAEZ01008208:142333-142553
HmHel-1	CAEZ01008208:188973-189302
HmHel-1	CAEZ01008208:205695-207397
HmHel-1	CAEZ01008208:148779-148957
HmHel-1	CAEZ01008151:3665-4055
HmHel-1	CAEZ01008096:2341-2772
HmHel-1	CAEZ01007863:13942-14264
HmHel-1	CAEZ01007863:105812-106252
HmHel-1	CAEZ01007828:282-762
HmHel-1	CAEZ01007828:1-282
HmHel-1	CAEZ01006876:6780-7220
HmHel-1	CAEZ01006342:1936-2277
HmHel-1	CAEZ01006130:54556-54887
HmHel-1	CAEZ01006130:179068-179489
HmHel-1	CAEZ01006130:156152-156746
HmHel-1	CAEZ01006130:22173-22356
HmHel-1	CAEZ01005954:3648-4397
HmHel-1	CAEZ01005800:1775-2198
HmHel-1	CAEZ01005532:2582-2969
HmHel-1	CAEZ01005116:2489-2910
HmHel-1	CAEZ01004774:707-1025
HmHel-1	CAEZ01004495:4493-4830
HmHel-1	CAEZ01004003:16855-17281
HmHel-1	CAEZ01003976:4528-4869
HmHel-1	CAEZ01003933:32158-32497
HmHel-1	CAEZ01003236:15140-15558
HmHel-1	CAEZ01003208:3-506
HmHel-1	CAEZ01003080:16052-16274
HmHel-1	CAEZ01003080:47859-48237
HmHel-1	CAEZ01003080:120502-120843
HmHel-1	CAEZ01003080:196294-196511
HmHel-1	CAEZ01003080:25762-26177
HmHel-1	CAEZ01003080:1104-1445
HmHel-1	CAEZ01002444:997-1369
HmHel-1	CAEZ01002138:1-700
HmHel-1	CAEZ01002138:23686-23995
HmHel-1	CAEZ01011167:569-981
HmHel-1	CAEZ01009947:83-504
HmHel-1	CAEZ01009631:16299-16638
HmHel-1	CAEZ01009631:88094-88278
HmHel-1	CAEZ01009631:43117-43543
HmHel-1	CAEZ01009631:4470-5511

HmHel-1	CAEZ01007215:1119-1544
HmHel-1	CAEZ01006638:15094-15508
HmHel-1	CAEZ01005877:7861-8279
HmHel-1	CAEZ01005667:2024-2381
HmHel-1	CAEZ01003122:1026-1417
HmHel-1	CAEZ01002804:29973-30332
HmHel-1	CAEZ01010417:4336-4525
HmHel-1	CAEZ01009502:4384-4557
HmHel-1	CAEZ01006177:196225-196567
HmHel-1	CAEZ01006177:126078-126290
HmHel-1	CAEZ01005162:48623-49037
HmHel-1	CAEZ01004941:3785-3976
HmHel-1	CAEZ01004941:34186-34860
HmHel-1	CAEZ01004941:119378-119656
HmHel-1	CAEZ01004941:21231-21572
HmHel-1	CAEZ01004808:672-1009
HmHel-1	CAEZ01011526:22950-23289
HmHel-1	CAEZ01011179:2950-3289
HmHel-1	CAEZ01011051:22413-22752
HmHel-1	CAEZ01010897:8619-8958
HmHel-1	CAEZ01010270:12811-13242
HmHel-1	CAEZ01010270:162525-162864
HmHel-1	CAEZ01010270:276431-276654
HmHel-1	CAEZ01010270:203686-204030
HmHel-1	CAEZ01010176:1948-2287
HmHel-1	CAEZ01009231:10970-11309
HmHel-1	CAEZ01009154:10215-10554
HmHel-1	CAEZ01008556:1925-2264
HmHel-1	CAEZ01008517:21422-21761
HmHel-1	CAEZ01008469:13278-13617
HmHel-1	CAEZ01008427:51830-52171
HmHel-1	CAEZ01008427:64607-65265
HmHel-1	CAEZ01008427:51035-51266
HmHel-1	CAEZ01008419:10065-10406
HmHel-1	CAEZ01008419:23294-23471
HmHel-1	CAEZ01007707:28711-29228
HmHel-1	CAEZ01007707:98640-98863
HmHel-1	CAEZ01007707:110459-110800
HmHel-1	CAEZ01007629:3215-3441
HmHel-1	CAEZ01007629:16248-16587
HmHel-1	CAEZ01007231:23-332
HmHel-1	CAEZ01006896:7658-7986
HmHel-1	CAEZ01006842:1169-1512
HmHel-1	CAEZ01006842:13958-14157
HmHel-1	CAEZ01006701:5-345
HmHel-1	CAEZ01006701:2845-3761
HmHel-1	CAEZ01006567:72159-72489
HmHel-1	CAEZ01006559:16438-16760

HmHel-1	CAEZ01006423:214559-214886
HmHel-1	CAEZ01006423:331565-331780
HmHel-1	CAEZ01006423:78263-78913
HmHel-1	CAEZ01006423:28293-28597
HmHel-1	CAEZ01006010:3251-3460
HmHel-1	CAEZ01006010:33038-33377
HmHel-1	CAEZ01006004:6569-6878
HmHel-1	CAEZ01005831:10955-11294
HmHel-1	CAEZ01005678:980-1319
HmHel-1	CAEZ01005632:20651-21002
HmHel-1	CAEZ01005590:34176-34294
HmHel-1	CAEZ01005590:6679-7792
HmHel-1	CAEZ01005454:20348-20687
HmHel-1	CAEZ01005392:5368-5611
HmHel-1	CAEZ01005392:72589-72928
HmHel-1	CAEZ01005392:25163-25523
HmHel-1	CAEZ01005169:17-226
HmHel-1	CAEZ01005169:723-1001
HmHel-1	CAEZ01004858:21557-21961
HmHel-1	CAEZ01004707:8744-9090
HmHel-1	CAEZ01004707:10331-10675
HmHel-1	CAEZ01004417:14339-14678
HmHel-1	CAEZ01004387:12571-12910
HmHel-1	CAEZ01004376:2072-2493
HmHel-1	CAEZ01004218:11041-11380
HmHel-1	CAEZ01004121:4519-4858
HmHel-1	CAEZ01003781:15586-15925
HmHel-1	CAEZ01003781:7228-7468
HmHel-1	CAEZ01003545:3396-3735
HmHel-1	CAEZ01003107:4129-4468
HmHel-1	CAEZ01003107:13776-14115
HmHel-1	CAEZ01003015:19-357
HmHel-1	CAEZ01003015:20844-21183
HmHel-1	CAEZ01002872:16681-16855
HmHel-1	CAEZ01002872:31815-32034
HmHel-1	CAEZ01002872:16247-16586
HmHel-1	CAEZ01002727:14419-14758
HmHel-1	CAEZ01002727:5321-5521
HmHel-1	CAEZ01002275:1374-1606
HmHel-1	CAEZ01002275:2033-2372
HmHel-1	CAEZ01001766:45687-46352
HmHel-1	CAEZ01001766:152974-153228
HmHel-1	CAEZ01001766:184899-185238
HmHel-1	CAEZ01001450:43775-44114
HmHel-1	CAEZ01001449:11477-11811
HmHel-1	CAEZ01001011:1-320
HmHel-1	CAEZ01000988:19309-19648
HmHel-1	CAEZ01000847:3914-4246

HmHel-1	CAEZ01000808:1047-1312
HmHel-1	CAEZ01011577:8404-8822
HmHel-1	CAEZ01011529:29024-29267
HmHel-1	CAEZ01011529:50276-50931
HmHel-1	CAEZ01011529:35072-35478
HmHel-1	CAEZ01011529:22311-22723
HmHel-1	CAEZ01011529:2881-3159
HmHel-1	CAEZ01011520:4528-5644
HmHel-1	CAEZ01011491:8701-8880
HmHel-1	CAEZ01011132:29801-30118
HmHel-1	CAEZ01011132:28285-28745
HmHel-1	CAEZ01011132:4742-5177
HmHel-1	CAEZ01010626:4233-4491
HmHel-1	CAEZ01008888:2504-2926
HmHel-1	CAEZ01008888:24029-24368
HmHel-1	CAEZ01008513:7212-7578
HmHel-1	CAEZ01008513:2741-2833
HmHel-1	CAEZ01008002:1-416
HmHel-1	CAEZ01007716:645-986
HmHel-1	CAEZ01007572:15-431
HmHel-1	CAEZ01007466:3321-3747
HmHel-1	CAEZ01007395:38326-38667
HmHel-1	CAEZ01007395:17874-26473
HmHel-1	CAEZ01007263:54030-54273
HmHel-1	CAEZ01007263:71051-71438
HmHel-1	CAEZ01007263:89060-89401
HmHel-1	CAEZ01007263:12983-13213
HmHel-1	CAEZ01007260:13303-13642
HmHel-1	CAEZ01007219:18615-19668
HmHel-1	CAEZ01007219:28227-28566
HmHel-1	CAEZ01006392:72767-73106
HmHel-1	CAEZ01006392:94899-95240
HmHel-1	CAEZ01006392:91835-92068
HmHel-1	CAEZ01006392:73316-73645
HmHel-1	CAEZ01006162:74385-74724
HmHel-1	CAEZ01006162:112978-113571
HmHel-1	CAEZ01006162:30585-30926
HmHel-1	CAEZ01004794:10776-11107
HmHel-1	CAEZ01004745:5877-6218
HmHel-1	CAEZ01004097:241004-241760
HmHel-1	CAEZ01004097:231289-231824
HmHel-1	CAEZ01004097:67076-67289
HmHel-1	CAEZ01002962:3217-3556
HmHel-1	CAEZ01002211:8366-9032
HmHel-1	CAEZ01002211:51052-51135
HmHel-1	CAEZ01002207:1-385
HmHel-1	CAEZ01002203:9438-9860
HmHel-1	CAEZ01001715:6791-7150

HmHel-1	CAEZ01010669:903-1237
HmHel-1	CAEZ01009843:6821-7160
HmHel-1	CAEZ01009843:65472-65896
HmHel-1	CAEZ01006095:10876-11291
HmHel-1	CAEZ01006081:17863-18198
HmHel-1	CAEZ01011642:16512-17261
HmHel-1	CAEZ01009000:3648-4131
HmHel-1	CAEZ01008671:20223-21251
HmHel-1	CAEZ01006572:2371-3523
HmHel-1	CAEZ01006557:2700-3098
HmHel-1	CAEZ01003680:13284-13491
HmHel-1	CAEZ01003452:24122-24541
HmHel-1	CAEZ01002725:1-299
HmHel-1	CAEZ01000824:141957-142295
HmHel-1	CAEZ01000824:107440-107982
HmHel-1	CAEZ01011619:26-304
HmHel-1	CAEZ01011596:3272-3611
HmHel-1	CAEZ01011478:17151-17490
HmHel-1	CAEZ01010829:237028-237367
HmHel-1	CAEZ01010829:263825-264490
HmHel-1	CAEZ01010829:160292-160798
HmHel-1	CAEZ01010829:119676-119887
HmHel-1	CAEZ01010829:91434-91636
HmHel-1	CAEZ01010829:51006-51186
HmHel-1	CAEZ01010813:3711-4050
HmHel-1	CAEZ01010807:79863-80185
HmHel-1	CAEZ01010807:189233-189463
HmHel-1	CAEZ01010807:5288-6068
HmHel-1	CAEZ01010794:11756-11952
HmHel-1	CAEZ01010666:63498-63817
HmHel-1	CAEZ01010632:2396-2735
HmHel-1	CAEZ01010632:22842-23183
HmHel-1	CAEZ01010612:989-1328
HmHel-1	CAEZ01010419:3815-4154
HmHel-1	CAEZ01010343:8012-8443
HmHel-1	CAEZ01010281:13656-13994
HmHel-1	CAEZ01010281:159-493
HmHel-1	CAEZ01010275:49817-50156
HmHel-1	CAEZ01010275:24971-25059
HmHel-1	CAEZ01010237:147064-147403
HmHel-1	CAEZ01010219:73962-74625
HmHel-1	CAEZ01010219:43385-43724
HmHel-1	CAEZ01010193:15755-16094
HmHel-1	CAEZ01010187:18-354
HmHel-1	CAEZ01010187:683-1022
HmHel-1	CAEZ01010150:1391-2048
HmHel-1	CAEZ01010041:27667-27999
HmHel-1	CAEZ01010041:2306-2645

HmHel-1	CAEZ01010004:5476-5815
HmHel-1	CAEZ01009974:32194-32881
HmHel-1	CAEZ01009974:34192-34422
HmHel-1	CAEZ01009917:5286-5619
HmHel-1	CAEZ01009841:13457-13793
HmHel-1	CAEZ01009841:34730-34960
HmHel-1	CAEZ01009841:60035-60374
HmHel-1	CAEZ01009437:36480-36819
HmHel-1	CAEZ01009248:13928-14263
HmHel-1	CAEZ01009248:56093-56340
HmHel-1	CAEZ01009025:3052-3141
HmHel-1	CAEZ01009025:38921-39260
HmHel-1	CAEZ01009025:67-406
HmHel-1	CAEZ01008746:3346-3685
HmHel-1	CAEZ01008746:193784-194123
HmHel-1	CAEZ01008746:196265-196955
HmHel-1	CAEZ01008746:115007-115611
HmHel-1	CAEZ01008568:5885-6163
HmHel-1	CAEZ01008398:35121-35453
HmHel-1	CAEZ01008398:58454-58793
HmHel-1	CAEZ01008398:43057-43396
HmHel-1	CAEZ01008359:182498-182837
HmHel-1	CAEZ01008359:89126-89465
HmHel-1	CAEZ01008303:38452-38791
HmHel-1	CAEZ01008294:27542-28207
HmHel-1	CAEZ01008294:170944-171069
HmHel-1	CAEZ01008294:181657-182312
HmHel-1	CAEZ01008294:200500-201764
HmHel-1	CAEZ01008294:144668-144946
HmHel-1	CAEZ01008294:69322-69631
HmHel-1	CAEZ01008294:50036-50279
HmHel-1	CAEZ01008224:30478-30817
HmHel-1	CAEZ01008202:50097-50240
HmHel-1	CAEZ01008202:44335-44681
HmHel-1	CAEZ01008202:32004-32222
HmHel-1	CAEZ01008122:10132-10338
HmHel-1	CAEZ01008122:22438-22666
HmHel-1	CAEZ01008122:83708-83929
HmHel-1	CAEZ01008122:44399-44738
HmHel-1	CAEZ01008122:14703-15042
HmHel-1	CAEZ01008063:14441-14780
HmHel-1	CAEZ01007443:3884-4223
HmHel-1	CAEZ01007439:14005-14350
HmHel-1	CAEZ01007374:29619-29958
HmHel-1	CAEZ01006759:15001-15340
HmHel-1	CAEZ01006759:82619-82819
HmHel-1	CAEZ01006755:10868-11207
HmHel-1	CAEZ01006561:37819-38487

HmHel-1	CAEZ01006561:67320-67659
HmHel-1	CAEZ01006479:12251-12590
HmHel-1	CAEZ01006479:1366-1575
HmHel-1	CAEZ01006438:7733-8072
HmHel-1	CAEZ01006319:5274-5613
HmHel-1	CAEZ01006319:9874-10091
HmHel-1	CAEZ01006306:2534-2895
HmHel-1	CAEZ01006278:34043-34733
HmHel-1	CAEZ01006278:53275-53597
HmHel-1	CAEZ01006185:24178-24513
HmHel-1	CAEZ01006066:625-965
HmHel-1	CAEZ01006059:12794-13133
HmHel-1	CAEZ01006046:15888-16223
HmHel-1	CAEZ01005977:2006-2345
HmHel-1	CAEZ01005944:8762-9104
HmHel-1	CAEZ01005944:12289-12709
HmHel-1	CAEZ01005596:7158-7497
HmHel-1	CAEZ01005500:5059-5398
HmHel-1	CAEZ01005386:16434-16712
HmHel-1	CAEZ01005386:7806-7928
HmHel-1	CAEZ01005373:57694-58106
HmHel-1	CAEZ01005373:76221-76560
HmHel-1	CAEZ01005373:43347-43590
HmHel-1	CAEZ01005276:11683-12022
HmHel-1	CAEZ01005248:4785-5204
HmHel-1	CAEZ01005247:21269-21604
HmHel-1	CAEZ01005207:52044-52383
HmHel-1	CAEZ01005207:92121-92460
HmHel-1	CAEZ01005207:46187-46529
HmHel-1	CAEZ01005088:16173-16512
HmHel-1	CAEZ01005088:29567-29655
HmHel-1	CAEZ01005088:61427-61759
HmHel-1	CAEZ01005088:23505-23844
HmHel-1	CAEZ01004905:6895-7429
HmHel-1	CAEZ01004905:48114-48296
HmHel-1	CAEZ01004870:2262-2599
HmHel-1	CAEZ01004688:12745-13084
HmHel-1	CAEZ01004684:3119-3783
HmHel-1	CAEZ01004684:5256-5591
HmHel-1	CAEZ01004541:129742-130081
HmHel-1	CAEZ01004541:74655-74997
HmHel-1	CAEZ01004356:5370-5709
HmHel-1	CAEZ01004139:11728-12379
HmHel-1	CAEZ01004011:10049-10753
HmHel-1	CAEZ01004011:2561-2976
HmHel-1	CAEZ01003929:5881-6220
HmHel-1	CAEZ01003854:21792-22198
HmHel-1	CAEZ01003747:10870-11207

HmHel-1	CAEZ01003739:16437-16775
HmHel-1	CAEZ01003689:30214-30553
HmHel-1	CAEZ01003675:3749-4088
HmHel-1	CAEZ01003077:12635-12894
HmHel-1	CAEZ01003077:7607-7942
HmHel-1	CAEZ01002947:2603-2948
HmHel-1	CAEZ01002947:14344-14568
HmHel-1	CAEZ01002926:1192-1531
HmHel-1	CAEZ01002881:8719-9058
HmHel-1	CAEZ01002870:93414-93753
HmHel-1	CAEZ01002863:61068-61407
HmHel-1	CAEZ01002826:34332-34586
HmHel-1	CAEZ01002826:113773-114011
HmHel-1	CAEZ01002779:25187-25528
HmHel-1	CAEZ01002779:80643-80876
HmHel-1	CAEZ01002779:78248-78587
HmHel-1	CAEZ01002742:122683-123022
HmHel-1	CAEZ01002742:85840-86502
HmHel-1	CAEZ01002640:2103-2442
HmHel-1	CAEZ01001831:58180-58519
HmHel-1	CAEZ01001649:1-345
HmHel-1	CAEZ01001572:30728-31067
HmHel-1	CAEZ01001572:47644-47987
HmHel-1	CAEZ01001572:26809-27013
HmHel-1	CAEZ01001435:122616-122821
HmHel-1	CAEZ01001435:157465-157804
HmHel-1	CAEZ01001435:10871-11540
HmHel-1	CAEZ01000958:14125-14464
HmHel-1	CAEZ01000529:410-749
HmHel-1	CAEZ01000083:14353-14692
HmHel-1	CAEZ01000083:1-231
HmHel-1	CAEZ01011207:6453-6873
HmHel-1	CAEZ01008277:34148-34446
HmHel-1	CAEZ01008087:15754-16085
HmHel-1	CAEZ01008087:13271-16309
HmHel-1	CAEZ01007518:3323-3498
HmHel-1	CAEZ01007234:39708-40482
HmHel-1	CAEZ01006799:74318-74657
HmHel-1	CAEZ01006126:20254-20458
HmHel-1	CAEZ01006126:21416-23860
HmHel-1	CAEZ01006126:10408-10699
HmHel-1	CAEZ01005751:4845-5186
HmHel-1	CAEZ01005243:8742-9417
HmHel-1	CAEZ01004734:37641-38063
HmHel-1	CAEZ01004734:75131-75360
HmHel-1	CAEZ01003081:747-1161
HmHel-1	CAEZ01002963:31336-31567
HmHel-1	CAEZ01002963:214-543

HmHel-1	CAEZ01000934:7754-8112
HmHel-1	CAEZ01007559:4252-4666
HmHel-1	CAEZ01003595:25165-25502
HmHel-1	CAEZ01003099:21756-22073
HmHel-1	CAEZ01002159:482-820
HmHel-1	CAEZ01000853:4930-5269
HmHel-1	CAEZ01011274:14515-14851
HmHel-1	CAEZ01011193:81299-81527
HmHel-1	CAEZ01011193:99797-100471
HmHel-1	CAEZ01010024:3299-3719
HmHel-1	CAEZ01009925:245-431
HmHel-1	CAEZ01009925:29466-29670
HmHel-1	CAEZ01009925:5636-5908
HmHel-1	CAEZ01009686:16716-17044
HmHel-1	CAEZ01009676:15941-16361
HmHel-1	CAEZ01009275:29-232
HmHel-1	CAEZ01008640:17924-18482
HmHel-1	CAEZ01008640:30931-31341
HmHel-1	CAEZ01008640:8030-8625
HmHel-1	CAEZ01006234:1-337
HmHel-1	CAEZ01006196:24072-24413
HmHel-1	CAEZ01006196:59679-59767
HmHel-1	CAEZ01006196:234170-234842
HmHel-1	CAEZ01005456:5469-6143
HmHel-1	CAEZ01003328:113206-113545
HmHel-1	CAEZ01002982:17730-17973
HmHel-1	CAEZ01002608:42087-43328
HmHel-1	CAEZ01011616:16990-17329
HmHel-1	CAEZ01011616:19246-19497
HmHel-1	CAEZ01011500:13036-13375
HmHel-1	CAEZ01011500:7894-13724
HmHel-1	CAEZ01011366:2010-2349
HmHel-1	CAEZ01011362:35066-35405
HmHel-1	CAEZ01011293:1103-2721
HmHel-1	CAEZ01011271:3111-3531
HmHel-1	CAEZ01011232:2899-4182
HmHel-1	CAEZ01011139:41541-41819
HmHel-1	CAEZ01011091:9130-10130
HmHel-1	CAEZ01011091:9258-9597
HmHel-1	CAEZ01011067:17756-18095
HmHel-1	CAEZ01011057:6740-7068
HmHel-1	CAEZ01011048:45395-45734
HmHel-1	CAEZ01010896:159976-160396
HmHel-1	CAEZ01010896:212000-212339
HmHel-1	CAEZ01010808:148734-149068
HmHel-1	CAEZ01010808:207826-208494
HmHel-1	CAEZ01010808:173144-173483
HmHel-1	CAEZ01010808:141625-141964

HmHel-1	CAEZ01010787:8733-9072
HmHel-1	CAEZ01010748:3265-3604
HmHel-1	CAEZ01010748:18465-18885
HmHel-1	CAEZ01010707:137254-137606
HmHel-1	CAEZ01010695:59509-59837
HmHel-1	CAEZ01010617:51700-52039
HmHel-1	CAEZ01010614:8147-8486
HmHel-1	CAEZ01010614:71075-71418
HmHel-1	CAEZ01010591:1235-1574
HmHel-1	CAEZ01010591:12546-15493
HmHel-1	CAEZ01010529:24-204
HmHel-1	CAEZ01010277:49836-50175
HmHel-1	CAEZ01010277:3864-4115
HmHel-1	CAEZ01010218:41809-42148
HmHel-1	CAEZ01010218:57331-57415
HmHel-1	CAEZ01010218:43133-43551
HmHel-1	CAEZ01010036:265-604
HmHel-1	CAEZ01009994:14512-14851
HmHel-1	CAEZ01009903:131-447
HmHel-1	CAEZ01009903:6291-6630
HmHel-1	CAEZ01009902:8586-8925
HmHel-1	CAEZ01009894:36461-36799
HmHel-1	CAEZ01009894:53291-53509
HmHel-1	CAEZ01009894:25320-25974
HmHel-1	CAEZ01009879:5187-5522
HmHel-1	CAEZ01009879:16565-17290
HmHel-1	CAEZ01009845:24517-24856
HmHel-1	CAEZ01009781:38823-39162
HmHel-1	CAEZ01009777:40716-41055
HmHel-1	CAEZ01009585:22105-32091
HmHel-1	CAEZ01009509:120512-120716
HmHel-1	CAEZ01009509:135767-136106
HmHel-1	CAEZ01009507:42989-43294
HmHel-1	CAEZ01009507:44679-45788
HmHel-1	CAEZ01009507:20303-20525
HmHel-1	CAEZ01009462:12181-12520
HmHel-1	CAEZ01009462:13979-14318
HmHel-1	CAEZ01009425:3377-3716
HmHel-1	CAEZ01009425:15032-15272
HmHel-1	CAEZ01009387:10055-10394
HmHel-1	CAEZ01009308:315-654
HmHel-1	CAEZ01009209:3335-3674
HmHel-1	CAEZ01009055:2423-2767
HmHel-1	CAEZ01009018:2115-2459
HmHel-1	CAEZ01008963:72709-72797
HmHel-1	CAEZ01008963:128111-128357
HmHel-1	CAEZ01008963:206672-207011
HmHel-1	CAEZ01008963:146708-147047

HmHel-1	CAEZ01008963:13101-13766
HmHel-1	CAEZ01008939:17240-17579
HmHel-1	CAEZ01008931:3566-3905
HmHel-1	CAEZ01008879:88688-89027
HmHel-1	CAEZ01008879:84537-84882
HmHel-1	CAEZ01008879:64467-64710
HmHel-1	CAEZ01008879:50849-51116
HmHel-1	CAEZ01008850:119-454
HmHel-1	CAEZ01008733:1469-1778
HmHel-1	CAEZ01008668:2356-2695
HmHel-1	CAEZ01008613:3920-4259
HmHel-1	CAEZ01008482:3-241
HmHel-1	CAEZ01008472:1845-2175
HmHel-1	CAEZ01008413:48729-48942
HmHel-1	CAEZ01008413:7606-7945
HmHel-1	CAEZ01008242:3221-3530
HmHel-1	CAEZ01008236:137949-138287
HmHel-1	CAEZ01008236:123681-124013
HmHel-1	CAEZ01008236:10262-10596
HmHel-1	CAEZ01008209:1618-1837
HmHel-1	CAEZ01008209:58722-59000
HmHel-1	CAEZ01008209:98379-98592
HmHel-1	CAEZ01008105:60860-61199
HmHel-1	CAEZ01008100:6220-6559
HmHel-1	CAEZ01008100:63957-64288
HmHel-1	CAEZ01008100:70248-70583
HmHel-1	CAEZ01008021:26790-27129
HmHel-1	CAEZ01007960:24440-25102
HmHel-1	CAEZ01007960:52574-52998
HmHel-1	CAEZ01007960:57927-58145
HmHel-1	CAEZ01007881:435-774
HmHel-1	CAEZ01007802:31455-31794
HmHel-1	CAEZ01007802:46259-46479
HmHel-1	CAEZ01007771:19-358
HmHel-1	CAEZ01007761:38291-38630
HmHel-1	CAEZ01007711:20077-20348
HmHel-1	CAEZ01007711:35322-35652
HmHel-1	CAEZ01007711:18691-19615
HmHel-1	CAEZ01007700:1730-2069
HmHel-1	CAEZ01007696:22438-22777
HmHel-1	CAEZ01007678:41647-41890
HmHel-1	CAEZ01007678:27322-27662
HmHel-1	CAEZ01007676:203668-203813
HmHel-1	CAEZ01007676:161791-162125
HmHel-1	CAEZ01007676:146455-146653
HmHel-1	CAEZ01007676:29953-30174
HmHel-1	CAEZ01007405:17013-17340
HmHel-1	CAEZ01007373:3312-3651

HmHel-1	CAEZ01007339:25114-25320
HmHel-1	CAEZ01007268:33263-33614
HmHel-1	CAEZ01007268:6853-7087
HmHel-1	CAEZ01007133:1658-1997
HmHel-1	CAEZ01007051:15840-16677
HmHel-1	CAEZ01006936:4271-4607
HmHel-1	CAEZ01006911:7048-7711
HmHel-1	CAEZ01006911:10521-10860
HmHel-1	CAEZ01006893:391-713
HmHel-1	CAEZ01006881:33683-34060
HmHel-1	CAEZ01006833:7426-7764
HmHel-1	CAEZ01006767:12277-12616
HmHel-1	CAEZ01006616:3335-3674
HmHel-1	CAEZ01006616:34238-34579
HmHel-1	CAEZ01006616:75839-76182
HmHel-1	CAEZ01006616:92347-92605
HmHel-1	CAEZ01006616:171029-171369
HmHel-1	CAEZ01006616:185699-185903
HmHel-1	CAEZ01006616:181496-181728
HmHel-1	CAEZ01006515:21003-21342
HmHel-1	CAEZ01006495:46014-46357
HmHel-1	CAEZ01006468:5206-5545
HmHel-1	CAEZ01006450:6661-6996
HmHel-1	CAEZ01006450:2280-2484
HmHel-1	CAEZ01006417:196-535
HmHel-1	CAEZ01006417:27430-27769
HmHel-1	CAEZ01006417:53489-54131
HmHel-1	CAEZ01006400:40438-40659
HmHel-1	CAEZ01006400:50681-51020
HmHel-1	CAEZ01006395:48197-48538
HmHel-1	CAEZ01006367:83856-84195
HmHel-1	CAEZ01006227:13699-14038
HmHel-1	CAEZ01006144:1188-1527
HmHel-1	CAEZ01005792:17039-17378
HmHel-1	CAEZ01005645:22030-22366
HmHel-1	CAEZ01005618:11189-11528
HmHel-1	CAEZ01005612:31393-31732
HmHel-1	CAEZ01005595:7196-7465
HmHel-1	CAEZ01005498:13724-13956
HmHel-1	CAEZ01005498:5952-6291
HmHel-1	CAEZ01005477:43144-43483
HmHel-1	CAEZ01005460:26415-26754
HmHel-1	CAEZ01005393:12890-13223
HmHel-1	CAEZ01005393:96954-97191
HmHel-1	CAEZ01005309:56817-57156
HmHel-1	CAEZ01005308:11241-11580
HmHel-1	CAEZ01005308:41333-41423
HmHel-1	CAEZ01005259:3861-4200

HmHel-1	CAEZ01005250:52654-52993
HmHel-1	CAEZ01005250:90538-91171
HmHel-1	CAEZ01005035:35573-35912
HmHel-1	CAEZ01004997:16053-16392
HmHel-1	CAEZ01004895:10819-11170
HmHel-1	CAEZ01004727:7741-8080
HmHel-1	CAEZ01004631:199950-200301
HmHel-1	CAEZ01004631:318846-320036
HmHel-1	CAEZ01004631:210474-210820
HmHel-1	CAEZ01004588:401-740
HmHel-1	CAEZ01004569:2472-2811
HmHel-1	CAEZ01004432:49995-50333
HmHel-1	CAEZ01004432:39026-39258
HmHel-1	CAEZ01004432:28272-28731
HmHel-1	CAEZ01004381:8392-8747
HmHel-1	CAEZ01004292:5531-5870
HmHel-1	CAEZ01004292:2305-2644
HmHel-1	CAEZ01004239:17222-17561
HmHel-1	CAEZ01004239:34385-34620
HmHel-1	CAEZ01004239:10108-10312
HmHel-1	CAEZ01004195:10815-11154
HmHel-1	CAEZ01004172:13197-13536
HmHel-1	CAEZ01004141:17866-18167
HmHel-1	CAEZ01004033:64871-65210
HmHel-1	CAEZ01004033:103976-104315
HmHel-1	CAEZ01004033:65368-65709
HmHel-1	CAEZ01004033:24644-24831
HmHel-1	CAEZ01004017:11685-12024
HmHel-1	CAEZ01003991:580-858
HmHel-1	CAEZ01003869:6670-7002
HmHel-1	CAEZ01003830:29763-30102
HmHel-1	CAEZ01003814:7587-8212
HmHel-1	CAEZ01003814:27041-27221
HmHel-1	CAEZ01003769:7441-7780
HmHel-1	CAEZ01003759:24111-24452
HmHel-1	CAEZ01003759:25163-25301
HmHel-1	CAEZ01003507:6282-6615
HmHel-1	CAEZ01003506:8913-9252
HmHel-1	CAEZ01003415:56744-57083
HmHel-1	CAEZ01003410:15540-15879
HmHel-1	CAEZ01003406:13730-14069
HmHel-1	CAEZ01003283:29-307
HmHel-1	CAEZ01003243:2624-2963
HmHel-1	CAEZ01002916:1312-1650
HmHel-1	CAEZ01002763:2853-3193
HmHel-1	CAEZ01002759:18088-18398
HmHel-1	CAEZ01002759:16026-16369
HmHel-1	CAEZ01002704:30782-31059

HmHel-1	CAEZ01002634:12941-13029
HmHel-1	CAEZ01002634:24606-24945
HmHel-1	CAEZ01002383:25591-25930
HmHel-1	CAEZ01002362:12746-13087
HmHel-1	CAEZ01002323:38663-39328
HmHel-1	CAEZ01002323:57499-57833
HmHel-1	CAEZ01002320:23020-23359
HmHel-1	CAEZ01002285:306-502
HmHel-1	CAEZ01002285:17151-17489
HmHel-1	CAEZ01002285:1152-1491
HmHel-1	CAEZ01001740:91353-91631
HmHel-1	CAEZ01001740:73288-73620
HmHel-1	CAEZ01001537:5463-5802
HmHel-1	CAEZ01001517:6996-7335
HmHel-1	CAEZ01001207:977-1750
HmHel-1	CAEZ01001207:12164-12500
HmHel-1	CAEZ01001152:4944-5266
HmHel-1	CAEZ01001152:8237-8577
HmHel-1	CAEZ01000871:555-980
HmHel-1	CAEZ01000828:1762-2186
HmHel-1	CAEZ01000828:1103-1442
HmHel-1	CAEZ01000811:14054-14395
HmHel-1	CAEZ01000071:14289-14633
HmHel-1	CAEZ01011313:10036-10765
HmHel-1	CAEZ01010825:18241-18580
HmHel-1	CAEZ01010484:8059-8398
HmHel-1	CAEZ01010399:28888-29166
HmHel-1	CAEZ01009859:19110-19450
HmHel-1	CAEZ01009403:46519-46937
HmHel-1	CAEZ01009403:39963-40198
HmHel-1	CAEZ01008873:24314-24634
HmHel-1	CAEZ01008608:46602-46843
HmHel-1	CAEZ01008608:37464-37884
HmHel-1	CAEZ01006930:2197-2477
HmHel-1	CAEZ01006339:44176-44598
HmHel-1	CAEZ01006339:47569-47905
HmHel-1	CAEZ01006094:40707-41046
HmHel-1	CAEZ01005778:3609-4335
HmHel-1	CAEZ01004878:1-164
HmHel-1	CAEZ01004878:5628-6110
HmHel-1	CAEZ01004429:2475-2653
HmHel-1	CAEZ01004429:16595-16774
HmHel-1	CAEZ01003627:3744-11725
HmHel-1	CAEZ01003334:13193-13505
HmHel-1	CAEZ01003304:31000-31325
HmHel-1	CAEZ01003304:30602-30941
HmHel-1	CAEZ01001265:94466-95137
HmHel-1	CAEZ01001265:124593-125157

HmHel-1	CAEZ01001265:62942-63030
HmHel-1	CAEZ01000854:77998-78411
HmHel-1	CAEZ01008994:7249-7665
HmHel-1	CAEZ01007811:2716-3410
HmHel-1	CAEZ01007497:9248-9706
HmHel-1	CAEZ01005817:37954-38656
HmHel-1	CAEZ01005817:20413-20766
HmHel-1	CAEZ01005416:28513-28852
HmHel-1	CAEZ01002705:24327-24663
HmHel-1	CAEZ01002047:11865-12596
HmHel-1	CAEZ01009239:3696-4034
HmHel-1	CAEZ01006086:28085-28453
HmHel-1	CAEZ01006011:10148-10476
HmHel-1	CAEZ01005671:11886-12236
HmHel-1	CAEZ01005403:4966-11393
HmHel-1	CAEZ01005403:22470-22888
HmHel-1	CAEZ01005403:38904-39133
HmHel-1	CAEZ01005403:6626-6967
HmHel-1	CAEZ01005371:4-628
HmHel-1	CAEZ01004925:2447-2775
HmHel-1	CAEZ01003435:1910-2246
HmHel-1	CAEZ01002100:752-1057
HmHel-1	CAEZ01001235:2018-2354
HmHel-1	CAEZ01011626:48302-48646
HmHel-1	CAEZ01011626:119110-119198
HmHel-1	CAEZ01011626:15728-15991
HmHel-1	CAEZ01011560:21460-21799
HmHel-1	CAEZ01011522:33986-34325
HmHel-1	CAEZ01011417:10137-10788
HmHel-1	CAEZ01011399:4189-4609
HmHel-1	CAEZ01011289:3763-4102
HmHel-1	CAEZ01011022:19063-19402
HmHel-1	CAEZ01010925:27421-27690
HmHel-1	CAEZ01010925:33935-34279
HmHel-1	CAEZ01010925:347-859
HmHel-1	CAEZ01010855:6949-7290
HmHel-1	CAEZ01010820:54341-54574
HmHel-1	CAEZ01010820:102344-102682
HmHel-1	CAEZ01010820:64517-64903
HmHel-1	CAEZ01010802:29126-29465
HmHel-1	CAEZ01010737:55669-55872
HmHel-1	CAEZ01010737:131148-131568
HmHel-1	CAEZ01010737:179580-180070
HmHel-1	CAEZ01010737:156860-156948
HmHel-1	CAEZ01010737:115584-115788
HmHel-1	CAEZ01010622:15740-16081
HmHel-1	CAEZ01010622:22-361
HmHel-1	CAEZ01010512:3520-3841

HmHel-1	CAEZ01010451:5444-5783
HmHel-1	CAEZ01010451:48263-48566
HmHel-1	CAEZ01010451:105029-105233
HmHel-1	CAEZ01010451:113044-113383
HmHel-1	CAEZ01010451:47358-47697
HmHel-1	CAEZ01010365:9009-9232
HmHel-1	CAEZ01010365:58619-59585
HmHel-1	CAEZ01010365:26506-26845
HmHel-1	CAEZ01010264:3231-3570
HmHel-1	CAEZ01010244:50939-51367
HmHel-1	CAEZ01010244:50505-50834
HmHel-1	CAEZ01009926:11123-11462
HmHel-1	CAEZ01009915:789-1128
HmHel-1	CAEZ01009858:5740-6057
HmHel-1	CAEZ01009770:28316-28655
HmHel-1	CAEZ01009770:73100-73441
HmHel-1	CAEZ01009609:66198-66537
HmHel-1	CAEZ01009609:95528-95624
HmHel-1	CAEZ01009173:6046-6324
HmHel-1	CAEZ01009030:51673-51861
HmHel-1	CAEZ01009030:73206-73425
HmHel-1	CAEZ01009030:77186-77437
HmHel-1	CAEZ01009030:30869-31208
HmHel-1	CAEZ01008901:2272-2455
HmHel-1	CAEZ01008901:17813-18152
HmHel-1	CAEZ01008860:17339-17678
HmHel-1	CAEZ01008822:19716-20044
HmHel-1	CAEZ01008430:24633-24972
HmHel-1	CAEZ01008410:30843-31077
HmHel-1	CAEZ01008410:82268-82937
HmHel-1	CAEZ01008410:38981-39320
HmHel-1	CAEZ01008358:29252-29474
HmHel-1	CAEZ01008358:46632-46945
HmHel-1	CAEZ01008358:33278-33617
HmHel-1	CAEZ01008358:14980-15671
HmHel-1	CAEZ01008327:2671-3010
HmHel-1	CAEZ01008159:7315-7648
HmHel-1	CAEZ01008089:43549-43888
HmHel-1	CAEZ01008089:18799-18887
HmHel-1	CAEZ01007897:2745-3084
HmHel-1	CAEZ01007855:43100-43441
HmHel-1	CAEZ01007853:34849-35188
HmHel-1	CAEZ01007853:110895-111159
HmHel-1	CAEZ01007831:66-403
HmHel-1	CAEZ01007736:11600-11861
HmHel-1	CAEZ01007736:17925-18349
HmHel-1	CAEZ01007703:30571-30814
HmHel-1	CAEZ01007703:22452-22791

HmHel-1	CAEZ01007585:31294-31633
HmHel-1	CAEZ01007325:92033-92372
HmHel-1	CAEZ01007311:13701-14033
HmHel-1	CAEZ01007223:2283-2622
HmHel-1	CAEZ01007193:6109-6448
HmHel-1	CAEZ01007176:28509-28850
HmHel-1	CAEZ01007061:42954-43293
HmHel-1	CAEZ01007061:23760-24430
HmHel-1	CAEZ01007026:11128-11467
HmHel-1	CAEZ01006672:9727-10059
HmHel-1	CAEZ01006596:919-1258
HmHel-1	CAEZ01006588:3775-4114
HmHel-1	CAEZ01006372:39589-39928
HmHel-1	CAEZ01006372:45637-45852
HmHel-1	CAEZ01006262:34999-35489
HmHel-1	CAEZ01006262:28332-28672
HmHel-1	CAEZ01006252:16878-17213
HmHel-1	CAEZ01006252:154773-155110
HmHel-1	CAEZ01006228:29300-31552
HmHel-1	CAEZ01006228:1233-1452
HmHel-1	CAEZ01006121:129224-129467
HmHel-1	CAEZ01006121:226479-232718
HmHel-1	CAEZ01006121:240429-240637
HmHel-1	CAEZ01006121:182273-182612
HmHel-1	CAEZ01006121:163041-163272
HmHel-1	CAEZ01006121:127973-128194
HmHel-1	CAEZ01006021:23299-23638
HmHel-1	CAEZ01006007:23258-23473
HmHel-1	CAEZ01006007:36904-37243
HmHel-1	CAEZ01005913:12720-13059
HmHel-1	CAEZ01005820:2880-3221
HmHel-1	CAEZ01005716:111213-111552
HmHel-1	CAEZ01005675:259-598
HmHel-1	CAEZ01005665:37221-37499
HmHel-1	CAEZ01005637:6538-6921
HmHel-1	CAEZ01005628:9896-10218
HmHel-1	CAEZ01005472:8541-8880
HmHel-1	CAEZ01005319:25390-25668
HmHel-1	CAEZ01005287:677-1016
HmHel-1	CAEZ01005186:23450-23789
HmHel-1	CAEZ01004930:19019-19223
HmHel-1	CAEZ01004930:7053-7392
HmHel-1	CAEZ01004864:342-429
HmHel-1	CAEZ01004864:880-1219
HmHel-1	CAEZ01004838:5311-5650
HmHel-1	CAEZ01004755:17891-18230
HmHel-1	CAEZ01004736:355-694
HmHel-1	CAEZ01004730:14350-14556

HmHel-1	CAEZ01004077:468-807
HmHel-1	CAEZ01004068:17814-18071
HmHel-1	CAEZ01003974:4697-5053
HmHel-1	CAEZ01003943:23783-24122
HmHel-1	CAEZ01003807:610-949
HmHel-1	CAEZ01003717:89107-89451
HmHel-1	CAEZ01003717:48957-49296
HmHel-1	CAEZ01003484:16357-16696
HmHel-1	CAEZ01003413:8542-8843
HmHel-1	CAEZ01003413:34258-34594
HmHel-1	CAEZ01003349:8576-8717
HmHel-1	CAEZ01003349:1453-1792
HmHel-1	CAEZ01003291:3620-3959
HmHel-1	CAEZ01003279:25980-26318
HmHel-1	CAEZ01003165:36098-36437
HmHel-1	CAEZ01003165:28149-28481
HmHel-1	CAEZ01003135:4243-4582
HmHel-1	CAEZ01003111:30882-31109
HmHel-1	CAEZ01003111:60204-60543
HmHel-1	CAEZ01002964:51466-51805
HmHel-1	CAEZ01002936:722-1000
HmHel-1	CAEZ01002904:4121-4460
HmHel-1	CAEZ01002777:149-427
HmHel-1	CAEZ01002777:1-143
HmHel-1	CAEZ01002731:28388-28727
HmHel-1	CAEZ01002709:20983-21322
HmHel-1	CAEZ01002709:1793-2463
HmHel-1	CAEZ01002678:7381-7659
HmHel-1	CAEZ01002393:1941-2276
HmHel-1	CAEZ01001899:2830-3169
HmHel-1	CAEZ01001697:333-672
HmHel-1	CAEZ01001578:28661-28749
HmHel-1	CAEZ01001578:74258-74597
HmHel-1	CAEZ01001578:86737-86823
HmHel-1	CAEZ01001223:768-1107
HmHel-1	CAEZ01001022:33530-33869
HmHel-1	CAEZ01001022:32846-33165
HmHel-1	CAEZ01000936:345-779
HmHel-1	CAEZ01000931:25031-25352
HmHel-1	CAEZ01000867:48774-49113
HmHel-1	CAEZ01000867:77165-77504
HmHel-1	CAEZ01000867:61519-61607
HmHel-1	CAEZ01000867:15830-16197
HmHel-1	CAEZ01011013:8082-8757
HmHel-1	CAEZ01010364:26780-27469
HmHel-1	CAEZ01010183:2254-2593
HmHel-1	CAEZ01010183:32144-32366
HmHel-1	CAEZ01010107:9525-10213

HmHel-1	CAEZ01009804:14991-15330
HmHel-1	CAEZ01009444:1-269
HmHel-1	CAEZ01008076:16673-16880
HmHel-1	CAEZ01008076:32-478
HmHel-1	CAEZ01007271:17023-17362
HmHel-1	CAEZ01007271:4290-4522
HmHel-1	CAEZ01006820:6836-7532
HmHel-1	CAEZ01005619:26050-26540
HmHel-1	CAEZ01005045:39832-40171
HmHel-1	CAEZ01004835:4892-5557
HmHel-1	CAEZ01004835:17095-17434
HmHel-1	CAEZ01003608:494-835
HmHel-1	CAEZ01002095:1-163
HmHel-1	CAEZ01011270:8772-9044
HmHel-1	CAEZ01010104:414-6355
HmHel-1	CAEZ01008872:12473-12882
HmHel-1	CAEZ01007625:66221-66541
HmHel-1	CAEZ01007625:92100-92305
HmHel-1	CAEZ01006467:5731-6073
HmHel-1	CAEZ01005999:11636-12176
HmHel-1	CAEZ01005999:25-513
HmHel-1	CAEZ01005229:33013-33352
HmHel-1	CAEZ01005144:14093-14559
HmHel-1	CAEZ01003173:42914-43143
HmHel-1	CAEZ01003173:62659-64206
HmHel-1	CAEZ01009646:23219-23502
HmHel-1	CAEZ01009555:11203-11540
HmHel-1	CAEZ01009533:8393-8809
HmHel-1	CAEZ01009533:9476-9564
HmHel-1	CAEZ01009104:12030-13037
HmHel-1	CAEZ01009104:12404-12713
HmHel-1	CAEZ01008911:27351-27682
HmHel-1	CAEZ01008603:63062-63319
HmHel-1	CAEZ01008603:2596-3284
HmHel-1	CAEZ01006931:3841-4076
HmHel-1	CAEZ01006816:11719-12058
HmHel-1	CAEZ01006761:2693-3044
HmHel-1	CAEZ01006336:19134-19464
HmHel-1	CAEZ01006336:38266-38605
HmHel-1	CAEZ01006336:25996-26230
HmHel-1	CAEZ01005881:28396-28729
HmHel-1	CAEZ01005780:12547-12962
HmHel-1	CAEZ01005170:26-366
HmHel-1	CAEZ01004708:1124-1552
HmHel-1	CAEZ01004708:18069-18310
HmHel-1	CAEZ01004257:1851-2189
HmHel-1	CAEZ01003560:1100-1518
HmHel-1	CAEZ01002376:584-1258

HmHel-1	CAEZ01001403:24909-25250
HmHel-1	CAEZ01001403:46834-47175
HmHel-1	CAEZ01001403:26901-27350
HmHel-1	CAEZ01001302:5018-5352
HmHel-1	CAEZ01011495:91080-91407
HmHel-1	CAEZ01011495:37895-38096
HmHel-1	CAEZ01011408:2756-2939
HmHel-1	CAEZ01011408:19321-19660
HmHel-1	CAEZ01011408:1179-6977
HmHel-1	CAEZ01011131:632-971
HmHel-1	CAEZ01011104:35154-35493
HmHel-1	CAEZ01011104:98063-98546
HmHel-1	CAEZ01011008:6160-6499
HmHel-1	CAEZ01010974:11528-11867
HmHel-1	CAEZ01010908:12623-12939
HmHel-1	CAEZ01010823:8909-9248
HmHel-1	CAEZ01010805:96199-96538
HmHel-1	CAEZ01010805:131776-131986
HmHel-1	CAEZ01010805:107880-108135
HmHel-1	CAEZ01010805:76304-76529
HmHel-1	CAEZ01010541:169744-169930
HmHel-1	CAEZ01010541:189215-189554
HmHel-1	CAEZ01010309:3026-3359
HmHel-1	CAEZ01010301:7258-8035
HmHel-1	CAEZ01010301:37240-37402
HmHel-1	CAEZ01010301:91181-91520
HmHel-1	CAEZ01010301:15174-15415
HmHel-1	CAEZ01010039:100559-100898
HmHel-1	CAEZ01010039:170704-170936
HmHel-1	CAEZ01010039:140950-141528
HmHel-1	CAEZ01009906:3822-4155
HmHel-1	CAEZ01009873:52763-53358
HmHel-1	CAEZ01009873:52143-52482
HmHel-1	CAEZ01009863:2238-2577
HmHel-1	CAEZ01009787:1-242
HmHel-1	CAEZ01009603:494-833
HmHel-1	CAEZ01009583:77879-78218
HmHel-1	CAEZ01009474:62104-62446
HmHel-1	CAEZ01009474:41879-42091
HmHel-1	CAEZ01009361:409-751
HmHel-1	CAEZ01009234:2627-2968
HmHel-1	CAEZ01009210:21238-21579
HmHel-1	CAEZ01009122:1494-1835
HmHel-1	CAEZ01008990:14074-14415
HmHel-1	CAEZ01008966:47506-47900
HmHel-1	CAEZ01008966:13069-13489
HmHel-1	CAEZ01008945:69795-70134
HmHel-1	CAEZ01008732:59900-60239

HmHel-1	CAEZ01008388:9941-10280
HmHel-1	CAEZ01008384:5381-5720
HmHel-1	CAEZ01008361:20805-21136
HmHel-1	CAEZ01008361:7755-8100
HmHel-1	CAEZ01008251:8826-9165
HmHel-1	CAEZ01008251:30536-30875
HmHel-1	CAEZ01008167:7210-7544
HmHel-1	CAEZ01008098:33751-34090
HmHel-1	CAEZ01007758:18849-19184
HmHel-1	CAEZ01007576:23800-24139
HmHel-1	CAEZ01007529:5929-6349
HmHel-1	CAEZ01007529:702-5520
HmHel-1	CAEZ01007507:7917-8195
HmHel-1	CAEZ01007402:5555-5887
HmHel-1	CAEZ01007340:8344-8683
HmHel-1	CAEZ01007078:3779-4120
HmHel-1	CAEZ01007008:2947-3220
HmHel-1	CAEZ01006919:33484-33824
HmHel-1	CAEZ01006919:33134-33349
HmHel-1	CAEZ01006869:21676-22015
HmHel-1	CAEZ01006628:40337-40674
HmHel-1	CAEZ01006442:27134-27467
HmHel-1	CAEZ01006433:5-338
HmHel-1	CAEZ01006409:32310-32651
HmHel-1	CAEZ01006402:7822-8157
HmHel-1	CAEZ01006402:29623-29962
HmHel-1	CAEZ01006346:4280-8838
HmHel-1	CAEZ01006346:2561-2900
HmHel-1	CAEZ01006280:10933-11267
HmHel-1	CAEZ01006151:2574-2662
HmHel-1	CAEZ01006151:16207-16439
HmHel-1	CAEZ01006151:77627-78298
HmHel-1	CAEZ01006151:68826-69165
HmHel-1	CAEZ01006151:18763-18964
HmHel-1	CAEZ01006054:20588-20927
HmHel-1	CAEZ01005972:32604-32943
HmHel-1	CAEZ01005964:56080-56424
HmHel-1	CAEZ01005911:50667-51329
HmHel-1	CAEZ01005911:89159-89391
HmHel-1	CAEZ01005911:94252-94472
HmHel-1	CAEZ01005911:18218-18557
HmHel-1	CAEZ01005875:8564-8905
HmHel-1	CAEZ01005839:16843-17182
HmHel-1	CAEZ01005772:19804-20169
HmHel-1	CAEZ01005758:4857-5288
HmHel-1	CAEZ01005539:476-815
HmHel-1	CAEZ01005476:24440-25035
HmHel-1	CAEZ01005476:67579-67915

HmHel-1	CAEZ01005267:100072-100382
HmHel-1	CAEZ01005267:62545-62884
HmHel-1	CAEZ01005221:67511-68383
HmHel-1	CAEZ01005221:47850-48189
HmHel-1	CAEZ01005221:3468-3702
HmHel-1	CAEZ01005174:31128-31467
HmHel-1	CAEZ01005153:35131-35754
HmHel-1	CAEZ01005153:67308-67671
HmHel-1	CAEZ01005153:40867-41055
HmHel-1	CAEZ01005153:27928-28224
HmHel-1	CAEZ01005005:5774-6115
HmHel-1	CAEZ01004909:84917-85001
HmHel-1	CAEZ01004909:29026-29365
HmHel-1	CAEZ01004716:2386-2725
HmHel-1	CAEZ01004531:5720-6059
HmHel-1	CAEZ01004531:12855-13484
HmHel-1	CAEZ01004469:13592-13931
HmHel-1	CAEZ01004362:4188-4775
HmHel-1	CAEZ01004362:13526-13833
HmHel-1	CAEZ01004244:144412-144751
HmHel-1	CAEZ01004244:11075-11163
HmHel-1	CAEZ01004182:12382-12721
HmHel-1	CAEZ01004104:32345-32754
HmHel-1	CAEZ01004007:25028-25367
HmHel-1	CAEZ01003959:2215-2554
HmHel-1	CAEZ01003959:12645-12816
HmHel-1	CAEZ01003414:14690-15006
HmHel-1	CAEZ01003414:25167-25506
HmHel-1	CAEZ01003414:36630-36845
HmHel-1	CAEZ01003192:82061-82400
HmHel-1	CAEZ01003186:11217-11495
HmHel-1	CAEZ01003060:7123-7462
HmHel-1	CAEZ01003060:34682-34770
HmHel-1	CAEZ01002973:109-448
HmHel-1	CAEZ01002896:3276-3493
HmHel-1	CAEZ01002896:28744-28994
HmHel-1	CAEZ01002896:8407-9151
HmHel-1	CAEZ01002720:8965-9274
HmHel-1	CAEZ01002667:1050-1389
HmHel-1	CAEZ01002667:17252-17473
HmHel-1	CAEZ01002568:24721-25141
HmHel-1	CAEZ01002051:9685-10520
HmHel-1	CAEZ01002020:414-736
HmHel-1	CAEZ01001963:8175-9290
HmHel-1	CAEZ01001940:3829-4170
HmHel-1	CAEZ01001826:421-762
HmHel-1	CAEZ01001797:614-953
HmHel-1	CAEZ01001457:5421-5760

HmHel-1	CAEZ01001386:27648-27987
HmHel-1	CAEZ01001215:12-351
HmHel-1	CAEZ01001150:4071-4410
HmHel-1	CAEZ01001073:19143-19482
HmHel-1	CAEZ01001042:3162-4067
HmHel-1	CAEZ01001042:16026-16259
HmHel-1	CAEZ01001042:11341-11682
HmHel-1	CAEZ01011494:20221-20443
HmHel-1	CAEZ01011494:63617-63825
HmHel-1	CAEZ01011494:30552-30785
HmHel-1	CAEZ01011494:15263-15951
HmHel-1	CAEZ01009786:20269-20448
HmHel-1	CAEZ01009786:34661-35000
HmHel-1	CAEZ01009227:48330-49014
HmHel-1	CAEZ01008829:27411-27749
HmHel-1	CAEZ01008829:13776-14009
HmHel-1	CAEZ01008547:91-581
HmHel-1	CAEZ01008532:25821-26470
HmHel-1	CAEZ01008532:62853-63056
HmHel-1	CAEZ01008497:28247-31479
HmHel-1	CAEZ01008449:7234-7573
HmHel-1	CAEZ01008405:11-449
HmHel-1	CAEZ01008405:19591-20021
HmHel-1	CAEZ01007854:6274-6684
HmHel-1	CAEZ01007854:3985-4324
HmHel-1	CAEZ01007770:9336-9529
HmHel-1	CAEZ01007122:67471-67925
HmHel-1	CAEZ01006864:19658-20113
HmHel-1	CAEZ01006635:3651-4343
HmHel-1	CAEZ01006549:4322-4661
HmHel-1	CAEZ01006477:11473-12150
HmHel-1	CAEZ01006149:31322-31821
HmHel-1	CAEZ01005922:24474-24962
HmHel-1	CAEZ01005507:12329-12676
HmHel-1	CAEZ01005166:5931-6141
HmHel-1	CAEZ01005166:10832-11355
HmHel-1	CAEZ01004810:2435-3106
HmHel-1	CAEZ01004370:74774-75229
HmHel-1	CAEZ01004233:32858-33166
HmHel-1	CAEZ01003650:4969-5197
HmHel-1	CAEZ01003650:16294-26796
HmHel-1	CAEZ01003095:2737-3076
HmHel-1	CAEZ01001981:38848-39263
HmHel-1	CAEZ01001970:5091-5780
HmHel-1	CAEZ01008921:8-229
HmHel-1	CAEZ01008921:71598-71699
HmHel-1	CAEZ01008921:108741-109464
HmHel-1	CAEZ01008921:88771-90570

HmHel-1	CAEZ01008921:44790-45030
HmHel-1	CAEZ01008921:15680-24689
HmHel-1	CAEZ01007494:21929-22267
HmHel-1	CAEZ01006698:62486-64512
HmHel-1	CAEZ01004153:13293-13573
HmHel-1	CAEZ01004127:52548-52997
HmHel-1	CAEZ01003634:849-1193
HmHel-1	CAEZ01001458:356-770
HmHel-1	CAEZ01010673:5219-5563
HmHel-1	CAEZ01009380:41340-41679
HmHel-1	CAEZ01009380:1722-1943
HmHel-1	CAEZ01007943:5458-5950
HmHel-1	CAEZ01007943:8163-8408
HmHel-1	CAEZ01006566:84798-85020
HmHel-1	CAEZ01006566:114963-115300
HmHel-1	CAEZ01006566:14938-15026
HmHel-1	CAEZ01006370:53727-54063
HmHel-1	CAEZ01006370:50317-50551
HmHel-1	CAEZ01006370:16844-17063
HmHel-1	CAEZ01006145:1-280
HmHel-1	CAEZ01006092:60681-61105
HmHel-1	CAEZ01005106:2404-2743
HmHel-1	CAEZ01003765:16535-16875
HmHel-1	CAEZ01003757:201-533
HmHel-1	CAEZ01003616:2246-2522
HmHel-1	CAEZ01003222:66004-66676
HmHel-1	CAEZ01011315:5660-5999
HmHel-1	CAEZ01011269:12414-12753
HmHel-1	CAEZ01011256:7548-7889
HmHel-1	CAEZ01011127:11190-11529
HmHel-1	CAEZ01010936:26153-26494
HmHel-1	CAEZ01010771:11508-11847
HmHel-1	CAEZ01010709:68269-68608
HmHel-1	CAEZ01010141:12705-13045
HmHel-1	CAEZ01009710:2225-2566
HmHel-1	CAEZ01009442:21441-21780
HmHel-1	CAEZ01009133:22282-22623
HmHel-1	CAEZ01009133:14-364
HmHel-1	CAEZ01008849:37846-38185
HmHel-1	CAEZ01008629:9228-9569
HmHel-1	CAEZ01008475:24114-24452
HmHel-1	CAEZ01008351:1-272
HmHel-1	CAEZ01008351:133135-133789
HmHel-1	CAEZ01008351:95784-96125
HmHel-1	CAEZ01008137:766-1105
HmHel-1	CAEZ01008119:6284-6606
HmHel-1	CAEZ01007983:94830-95174
HmHel-1	CAEZ01007983:34495-34583

HmHel-1	CAEZ01007981:32079-32517
HmHel-1	CAEZ01007922:374-713
HmHel-1	CAEZ01007462:6099-6431
HmHel-1	CAEZ01007404:1-320
HmHel-1	CAEZ01007274:2193-2782
HmHel-1	CAEZ01007166:918-1259
HmHel-1	CAEZ01007160:18447-18786
HmHel-1	CAEZ01006861:30593-30928
HmHel-1	CAEZ01006838:2892-3233
HmHel-1	CAEZ01006806:3566-3901
HmHel-1	CAEZ01006739:3684-4022
HmHel-1	CAEZ01006595:22349-22686
HmHel-1	CAEZ01006592:36889-37123
HmHel-1	CAEZ01006592:120102-120443
HmHel-1	CAEZ01006492:6706-7374
HmHel-1	CAEZ01006492:13210-13549
HmHel-1	CAEZ01006069:2301-2640
HmHel-1	CAEZ01006044:46344-46685
HmHel-1	CAEZ01005869:7389-7728
HmHel-1	CAEZ01005850:16511-16843
HmHel-1	CAEZ01005827:22072-22305
HmHel-1	CAEZ01005827:35679-36351
HmHel-1	CAEZ01005698:3430-3768
HmHel-1	CAEZ01005606:49403-49675
HmHel-1	CAEZ01005606:73974-74064
HmHel-1	CAEZ01005543:8512-8853
HmHel-1	CAEZ01005494:50547-50886
HmHel-1	CAEZ01004940:3499-3828
HmHel-1	CAEZ01004888:6715-7054
HmHel-1	CAEZ01004855:17758-17870
HmHel-1	CAEZ01004855:7490-7822
HmHel-1	CAEZ01004815:9229-9583
HmHel-1	CAEZ01004815:19350-19967
HmHel-1	CAEZ01004330:9833-10172
HmHel-1	CAEZ01004248:9608-9947
HmHel-1	CAEZ01004173:14136-14455
HmHel-1	CAEZ01003921:17283-17624
HmHel-1	CAEZ01003896:8039-8378
HmHel-1	CAEZ01003896:14821-15163
HmHel-1	CAEZ01003707:3124-3463
HmHel-1	CAEZ01002414:124013-124345
HmHel-1	CAEZ01002293:55-392
HmHel-1	CAEZ01001982:18663-19167
HmHel-1	CAEZ01001980:26560-26901
HmHel-1	CAEZ01001969:19821-20160
HmHel-1	CAEZ01001954:41309-41645
HmHel-1	CAEZ01001769:494-833
HmHel-1	CAEZ01001557:23110-23413

HmHel-1	CAEZ01001455:3138-3477
HmHel-1	CAEZ01001315:12183-12514
HmHel-1	CAEZ01001260:7260-7599
HmHel-1	CAEZ01000043:8349-12426
HmHel-1	CAEZ01011414:69013-69702
HmHel-1	CAEZ01010788:4824-5485
HmHel-1	CAEZ01010321:4493-4759
HmHel-1	CAEZ01009970:47057-47473
HmHel-1	CAEZ01009970:59194-59534
HmHel-1	CAEZ01009648:7933-8356
HmHel-1	CAEZ01009224:22179-29882
HmHel-1	CAEZ01009224:55545-55767
HmHel-1	CAEZ01009224:21993-23299
HmHel-1	CAEZ01009126:5700-6104
HmHel-1	CAEZ01009096:1018-1217
HmHel-1	CAEZ01009096:9928-10268
HmHel-1	CAEZ01009065:6444-7118
HmHel-1	CAEZ01008396:10617-11305
HmHel-1	CAEZ01007780:462-1151
HmHel-1	CAEZ01007751:47847-48152
HmHel-1	CAEZ01007751:59059-59748
HmHel-1	CAEZ01007745:35632-36319
HmHel-1	CAEZ01007372:188-444
HmHel-1	CAEZ01006415:18342-27798
HmHel-1	CAEZ01006415:14563-15234
HmHel-1	CAEZ01006261:3804-4493
HmHel-1	CAEZ01005830:13658-14351
HmHel-1	CAEZ01005830:159448-160245
HmHel-1	CAEZ01005320:7140-7479
HmHel-1	CAEZ01005089:49723-49945
HmHel-1	CAEZ01005089:16378-17067
HmHel-1	CAEZ01004818:29906-30162
HmHel-1	CAEZ01004818:117752-118441
HmHel-1	CAEZ01004818:92504-92737
HmHel-1	CAEZ01004818:71797-72033
HmHel-1	CAEZ01004818:9837-10150
HmHel-1	CAEZ01004528:9134-9551
HmHel-1	CAEZ01004312:15521-16010
HmHel-1	CAEZ01003108:408-821
HmHel-1	CAEZ01002876:23827-24051
HmHel-1	CAEZ01002876:122114-122798
HmHel-1	CAEZ01002876:137575-137807
HmHel-1	CAEZ01002755:46768-47213
HmHel-1	CAEZ01002685:1837-2511
HmHel-1	CAEZ01002643:34169-34858
HmHel-1	CAEZ01002281:1842-2185
HmHel-1	CAEZ01001452:6558-6998
HmHel-1	CAEZ01001452:6141-6482

HmHel-1	CAEZ01000920:9625-10312
HmHel-1	CAEZ01000033:12159-12499
HmHel-1	CAEZ01010662:9710-10908
HmHel-1	CAEZ01007258:89587-89930
HmHel-1	CAEZ01006989:15607-16022
HmHel-1	CAEZ01004234:320-2183
HmHel-1	CAEZ01003761:15988-16330
HmHel-1	CAEZ01001703:15990-16332
HmHel-1	CAEZ01010900:61732-61820
HmHel-1	CAEZ01010900:23041-23435
HmHel-1	CAEZ01007498:8649-9336
HmHel-1	CAEZ01007498:16167-16839
HmHel-1	CAEZ01007257:20335-20671
HmHel-1	CAEZ01007009:1-195
HmHel-1	CAEZ01007009:18363-18704
HmHel-1	CAEZ01007009:17512-17726
HmHel-1	CAEZ01006490:4317-4654
HmHel-1	CAEZ01005821:8367-8631
HmHel-1	CAEZ01005696:11287-11624
HmHel-1	CAEZ01005576:19273-19611
HmHel-1	CAEZ01005576:18316-18554
HmHel-1	CAEZ01005202:12029-12510
HmHel-1	CAEZ01005202:12866-13193
HmHel-1	CAEZ01002638:14758-15100
HmHel-1	CAEZ01001801:12938-16881
HmHel-1	CAEZ01000903:1914-2349
HmHel-1	CAEZ01011539:11915-12253
HmHel-1	CAEZ01011528:1279-1557
HmHel-1	CAEZ01011528:39647-40200
HmHel-1	CAEZ01011528:32317-33083
HmHel-1	CAEZ01011528:18851-19192
HmHel-1	CAEZ01011528:6794-6882
HmHel-1	CAEZ01011461:23136-23470
HmHel-1	CAEZ01011211:4914-5255
HmHel-1	CAEZ01010624:23219-23560
HmHel-1	CAEZ01010527:19045-19386
HmHel-1	CAEZ01010527:27755-27843
HmHel-1	CAEZ01010511:26018-26912
HmHel-1	CAEZ01010280:259-1782
HmHel-1	CAEZ01010160:7472-7814
HmHel-1	CAEZ01009733:53399-53628
HmHel-1	CAEZ01009733:67957-68184
HmHel-1	CAEZ01009733:76953-77318
HmHel-1	CAEZ01009720:981-1322
HmHel-1	CAEZ01009436:29335-29675
HmHel-1	CAEZ01009076:31543-31882
HmHel-1	CAEZ01008691:37459-37737
HmHel-1	CAEZ01008273:4882-5221

HmHel-1	CAEZ01008181:5549-5888
HmHel-1	CAEZ01008181:33786-34122
HmHel-1	CAEZ01007986:14336-14677
HmHel-1	CAEZ01007851:91080-91337
HmHel-1	CAEZ01007851:120508-120997
HmHel-1	CAEZ01007851:24752-25093
HmHel-1	CAEZ01007809:83831-83919
HmHel-1	CAEZ01007809:104716-105056
HmHel-1	CAEZ01007809:252526-252755
HmHel-1	CAEZ01007809:41017-41358
HmHel-1	CAEZ01007809:9375-9618
HmHel-1	CAEZ01007759:3075-5059
HmHel-1	CAEZ01007590:12176-12517
HmHel-1	CAEZ01007264:3061-3402
HmHel-1	CAEZ01007222:144130-144342
HmHel-1	CAEZ01007222:208157-208490
HmHel-1	CAEZ01007222:278910-279504
HmHel-1	CAEZ01007222:222556-222788
HmHel-1	CAEZ01007020:17728-18067
HmHel-1	CAEZ01006983:3505-3846
HmHel-1	CAEZ01006686:47983-48337
HmHel-1	CAEZ01006666:48280-48621
HmHel-1	CAEZ01006666:44549-45208
HmHel-1	CAEZ01006517:1333-1674
HmHel-1	CAEZ01006478:11245-11584
HmHel-1	CAEZ01005822:112-453
HmHel-1	CAEZ01005548:7434-7869
HmHel-1	CAEZ01005355:951-1273
HmHel-1	CAEZ01005335:32731-32922
HmHel-1	CAEZ01005335:73243-73584
HmHel-1	CAEZ01005335:92497-92714
HmHel-1	CAEZ01005066:51251-51915
HmHel-1	CAEZ01005066:94192-94394
HmHel-1	CAEZ01005066:63792-64131
HmHel-1	CAEZ01004986:7136-7477
HmHel-1	CAEZ01004901:20988-21322
HmHel-1	CAEZ01004605:5369-5571
HmHel-1	CAEZ01004605:561-902
HmHel-1	CAEZ01004474:16488-16827
HmHel-1	CAEZ01004422:1963-2304
HmHel-1	CAEZ01003777:6495-6860
HmHel-1	CAEZ01003346:87923-88379
HmHel-1	CAEZ01003346:67983-68650
HmHel-1	CAEZ01003346:49620-49705
HmHel-1	CAEZ01003316:15634-15973
HmHel-1	CAEZ01003130:19574-19912
HmHel-1	CAEZ01003130:2339-3004
HmHel-1	CAEZ01003110:3-195

HmHel-1	CAEZ01003110:69849-70188
HmHel-1	CAEZ01003110:4516-4852
HmHel-1	CAEZ01002851:6388-6727
HmHel-1	CAEZ01002811:23611-23815
HmHel-1	CAEZ01002811:167393-167828
HmHel-1	CAEZ01002811:151054-151690
HmHel-1	CAEZ01002811:75302-77434
HmHel-1	CAEZ01002765:62780-63420
HmHel-1	CAEZ01002765:8409-8620
HmHel-1	CAEZ01002429:26120-26335
HmHel-1	CAEZ01002429:18081-18423
HmHel-1	CAEZ01002429:2478-2890
HmHel-1	CAEZ01002318:24291-24628
HmHel-1	CAEZ01002199:684-1023
HmHel-1	CAEZ01002120:27657-27997
HmHel-1	CAEZ01001900:10795-11107
HmHel-1	CAEZ01001887:1171-1512
HmHel-1	CAEZ01001750:9906-10247
HmHel-1	CAEZ01001506:8344-8684
HmHel-1	CAEZ01000990:13210-13551
HmHel-1	CAEZ01000805:26116-26389
HmHel-1	CAEZ01000805:20915-21333
HmHel-1	CAEZ01011320:569-1239
HmHel-1	CAEZ01010738:83612-83953
HmHel-1	CAEZ01010738:231028-231268
HmHel-1	CAEZ01010738:208276-208965
HmHel-1	CAEZ01010738:184300-184502
HmHel-1	CAEZ01010738:162372-162458
HmHel-1	CAEZ01010738:138844-139523
HmHel-1	CAEZ01010738:92847-93068
HmHel-1	CAEZ01010738:36223-36652
HmHel-1	CAEZ01010720:14687-15376
HmHel-1	CAEZ01009801:73942-74650
HmHel-1	CAEZ01009682:74-417
HmHel-1	CAEZ01009221:19060-19756
HmHel-1	CAEZ01009221:20632-21347
HmHel-1	CAEZ01009221:7294-7391
HmHel-1	CAEZ01008470:21142-21355
HmHel-1	CAEZ01008470:18074-18408
HmHel-1	CAEZ01007070:16743-17198
HmHel-1	CAEZ01006771:3572-4109
HmHel-1	CAEZ01006771:4109-4386
HmHel-1	CAEZ01005933:36555-37227
HmHel-1	CAEZ01005413:5831-6496
HmHel-1	CAEZ01004830:10860-11549
HmHel-1	CAEZ01004259:100-438
HmHel-1	CAEZ01004174:11744-12318
HmHel-1	CAEZ01004174:56-451

HmHel-1	CAEZ01003815:13236-13727
HmHel-1	CAEZ01003233:9819-10058
HmHel-1	CAEZ01003233:53893-54231
HmHel-1	CAEZ01003038:8803-9021
HmHel-1	CAEZ01003038:17781-18111
HmHel-1	CAEZ01002417:589-1079
HmHel-1	CAEZ01001276:21093-21547
HmHel-1	CAEZ01011038:5980-6312
HmHel-1	CAEZ01010799:39852-40249
HmHel-1	CAEZ01008704:488-968
HmHel-1	CAEZ01007727:54647-55335
HmHel-1	CAEZ01006056:17064-17396
HmHel-1	CAEZ01005924:33250-33589
HmHel-1	CAEZ01004904:11000-11335
HmHel-1	CAEZ01003803:3892-4627
HmHel-1	CAEZ01001250:200-542
HmHel-1	CAEZ01010876:18377-18701
HmHel-1	CAEZ01010086:5837-6182
HmHel-1	CAEZ01009867:20234-20668
HmHel-1	CAEZ01008818:21386-21711
HmHel-1	CAEZ01007663:32492-32816
HmHel-1	CAEZ01006689:23498-23916
HmHel-1	CAEZ01006689:8571-8897
HmHel-1	CAEZ01005173:2478-2566
HmHel-1	CAEZ01005173:99469-99742
HmHel-1	CAEZ01005173:130258-130462
HmHel-1	CAEZ01002943:14129-14461
HmHel-1	CAEZ01011518:5240-5577
HmHel-1	CAEZ01011423:2373-3039
HmHel-1	CAEZ01011092:11397-11738
HmHel-1	CAEZ01010910:935-1273
HmHel-1	CAEZ01010725:31991-32231
HmHel-1	CAEZ01010587:20397-20817
HmHel-1	CAEZ01010444:10892-11233
HmHel-1	CAEZ01010078:60304-60712
HmHel-1	CAEZ01009978:4971-5213
HmHel-1	CAEZ01009978:25940-26281
HmHel-1	CAEZ01009978:9370-9614
HmHel-1	CAEZ01009488:23761-24102
HmHel-1	CAEZ01009092:10821-11160
HmHel-1	CAEZ01008585:12585-12926
HmHel-1	CAEZ01008585:3274-3373
HmHel-1	CAEZ01008298:4373-4714
HmHel-1	CAEZ01008298:4211-4373
HmHel-1	CAEZ01008215:3187-3528
HmHel-1	CAEZ01007911:18077-18418
HmHel-1	CAEZ01007801:15369-15513
HmHel-1	CAEZ01007728:5680-5903

HmHel-1	CAEZ01007728:22426-22769
HmHel-1	CAEZ01007362:12088-12387
HmHel-1	CAEZ01007362:3954-5512
HmHel-1	CAEZ01007248:370-568
HmHel-1	CAEZ01007248:7966-8726
HmHel-1	CAEZ01006934:13846-14186
HmHel-1	CAEZ01006721:1464-1805
HmHel-1	CAEZ01006188:19948-20610
HmHel-1	CAEZ01005980:17402-17708
HmHel-1	CAEZ01005980:45503-45842
HmHel-1	CAEZ01005383:1404-1745
HmHel-1	CAEZ01005334:19072-19413
HmHel-1	CAEZ01005251:10251-10813
HmHel-1	CAEZ01005251:25015-25354
HmHel-1	CAEZ01005230:23308-23649
HmHel-1	CAEZ01005120:474-813
HmHel-1	CAEZ01004225:14681-15022
HmHel-1	CAEZ01003819:11839-12329
HmHel-1	CAEZ01003819:27931-28162
HmHel-1	CAEZ01003732:2893-13563
HmHel-1	CAEZ01003457:2412-2589
HmHel-1	CAEZ01003457:3365-3566
HmHel-1	CAEZ01003226:143491-144143
HmHel-1	CAEZ01003226:14304-14642
HmHel-1	CAEZ01003001:14-356
HmHel-1	CAEZ01002906:7236-7928
HmHel-1	CAEZ01002837:8-347
HmHel-1	CAEZ01002266:2182-2524
HmHel-1	CAEZ01002140:12454-12730
HmHel-1	CAEZ01001692:883-1056
HmHel-1	CAEZ01001581:14388-14722
HmHel-1	CAEZ01001419:5697-13344
HmHel-1	CAEZ01001166:1237-10193
HmHel-1	CAEZ01000962:1-239
HmHel-1	CAEZ01000890:1414-1709
HmHel-1	CAEZ01000819:1077-1364
HmHel-1	CAEZ01000061:11478-11818
HmHel-1	CAEZ01000061:23614-23702
HmHel-1	CAEZ01011297:147658-148347
HmHel-1	CAEZ01011204:5876-6217
HmHel-1	CAEZ01011095:76-531
HmHel-1	CAEZ01011095:28888-29069
HmHel-1	CAEZ01010700:98632-99305
HmHel-1	CAEZ01010085:1589-2277
HmHel-1	CAEZ01010050:9039-9725
HmHel-1	CAEZ01009506:14661-15350
HmHel-1	CAEZ01009153:1908-2397
HmHel-1	CAEZ01009153:5972-6312

HmHel-1	CAEZ01008710:62653-63341
HmHel-1	CAEZ01008710:170-486
HmHel-1	CAEZ01008632:7692-8380
HmHel-1	CAEZ01008590:125513-125749
HmHel-1	CAEZ01008590:62504-65719
HmHel-1	CAEZ01008590:27419-28069
HmHel-1	CAEZ01007988:133-336
HmHel-1	CAEZ01007427:13536-14223
HmHel-1	CAEZ01006083:18631-19319
HmHel-1	CAEZ01005458:15150-15606
HmHel-1	CAEZ01004510:4688-5028
HmHel-1	CAEZ01003954:34932-39546
HmHel-1	CAEZ01003727:1977-2317
HmHel-1	CAEZ01003639:13738-14083
HmHel-1	CAEZ01003142:4178-4522
HmHel-1	CAEZ01001803:1-633
HmHel-1	CAEZ01011248:13808-14096
HmHel-1	CAEZ01010967:20085-20386
HmHel-1	CAEZ01009364:4104-4187
HmHel-1	CAEZ01009364:26949-27601
HmHel-1	CAEZ01009141:13669-14322
HmHel-1	CAEZ01008525:5113-5445
HmHel-1	CAEZ01006691:916-1248
HmHel-1	CAEZ01004660:1-172
HmHel-1	CAEZ01003292:95888-96094
HmHel-1	CAEZ01003292:75572-76232
HmHel-1	CAEZ01003292:62569-62793
HmHel-1	CAEZ01002852:4301-4648
HmHel-1	CAEZ01002358:3173-3470
HmHel-1	CAEZ01001824:5097-5768
HmHel-1	CAEZ01009830:45-419
HmHel-1	CAEZ01009830:7485-8152
HmHel-1	CAEZ01009829:930-1303
HmHel-1	CAEZ01009336:6150-6496
HmHel-1	CAEZ01009336:22884-23116
HmHel-1	CAEZ01009336:4560-4782
HmHel-1	CAEZ01006979:1875-2126
HmHel-1	CAEZ01006403:1722-1965
HmHel-1	CAEZ01006403:115499-115600
HmHel-1	CAEZ01006403:88089-88316
HmHel-1	CAEZ01006403:73125-73369
HmHel-1	CAEZ01006403:62274-62625
HmHel-1	CAEZ01004921:10638-12186
HmHel-1	CAEZ01004921:51369-52039
HmHel-1	CAEZ01002183:9040-10318
HmHel-1	CAEZ01002004:15705-15982
HmHel-1	CAEZ01011212:13708-14008
HmHel-1	CAEZ01010770:21889-22295

HmHel-1	CAEZ01010629:6361-6776
HmHel-1	CAEZ01010420:9016-9355
HmHel-1	CAEZ01009504:2313-2681
HmHel-1	CAEZ01008828:8298-8638
HmHel-1	CAEZ01007739:7195-7857
HmHel-1	CAEZ01007739:59377-59559
HmHel-1	CAEZ01007739:61535-61872
HmHel-1	CAEZ01007739:28530-28745
HmHel-1	CAEZ01006749:28511-28849
HmHel-1	CAEZ01006250:17160-17597
HmHel-1	CAEZ01006012:39817-40491
HmHel-1	CAEZ01005707:1322-1663
HmHel-1	CAEZ01005605:77008-77381
HmHel-1	CAEZ01005558:6729-7070
HmHel-1	CAEZ01005054:183-873
HmHel-1	CAEZ01004294:3604-3805
HmHel-1	CAEZ01004294:11583-11752
HmHel-1	CAEZ01003347:6192-6503
HmHel-1	CAEZ01003137:21400-21724
HmHel-1	CAEZ01003137:21285-21400
HmHel-1	CAEZ01002907:7259-7598
HmHel-1	CAEZ01001996:3152-3563
HmHel-1	CAEZ01001991:3-443
HmHel-1	CAEZ01001327:8971-9312
HmHel-1	CAEZ01001327:5203-5530
HmHel-1	CAEZ01001291:18221-18562
HmHel-1	CAEZ01010810:38507-38961
HmHel-1	CAEZ01009890:4882-5532
HmHel-1	CAEZ01006489:32480-32822
HmHel-1	CAEZ01004648:15123-15468
HmHel-1	CAEZ01003408:698-1369
HmHel-1	CAEZ01003312:28883-29228
HmHel-1	CAEZ01001437:28119-28454
HmHel-1	CAEZ01011348:6872-7190
HmHel-1	CAEZ01010915:21862-21947
HmHel-1	CAEZ01010915:49913-50674
HmHel-1	CAEZ01010915:22179-22474
HmHel-1	CAEZ01009639:12905-13238
HmHel-1	CAEZ01006047:13-181
HmHel-1	CAEZ01004051:12209-12554
HmHel-1	CAEZ01003861:14501-14803
HmHel-1	CAEZ01002308:12-321
HmHel-1	CAEZ01001085:4968-5293
HmHel-1	CAEZ01011484:6581-6722
HmHel-1	CAEZ01011196:9336-9695
HmHel-1	CAEZ01009515:414-735
HmHel-1	CAEZ01009255:5034-5188
HmHel-1	CAEZ01005726:6444-6777

HmHel-1	CAEZ01004507:23864-24549
HmHel-1	CAEZ01003947:18309-18649
HmHel-1	CAEZ01010798:12921-13262
HmHel-1	CAEZ01010798:9809-9950
HmHel-1	CAEZ01009717:42767-43108
HmHel-1	CAEZ01009608:27795-28114
HmHel-1	CAEZ01008374:25055-25378
HmHel-1	CAEZ01008374:42080-42397
HmHel-1	CAEZ01008244:25087-25430
HmHel-1	CAEZ01007657:982-1281
HmHel-1	CAEZ01006947:6255-7434
HmHel-1	CAEZ01006471:2420-3007
HmHel-1	CAEZ01006269:13951-14108
HmHel-1	CAEZ01006150:43868-44200
HmHel-1	CAEZ01006150:35559-35790
HmHel-1	CAEZ01005155:2748-2940
HmHel-1	CAEZ01005155:16060-16401
HmHel-1	CAEZ01004572:1073-1408
HmHel-1	CAEZ01004004:623-899
HmHel-1	CAEZ01003185:968-1352
HmHel-1	CAEZ01002910:1318-1658
HmHel-1	CAEZ01002770:15772-16110
HmHel-1	CAEZ01001891:29853-30262
HmHel-1	CAEZ01001345:4076-4419
HmHel-1	CAEZ01009455:6207-7962
HmHel-1	CAEZ01009455:26122-26465
HmHel-1	CAEZ01009455:19127-19343
HmHel-1	CAEZ01005224:17862-18212
HmHel-1	CAEZ01004942:3793-3997
HmHel-1	CAEZ01004942:1-325
HmHel-1	CAEZ01002436:10270-10558
HmHel-1	CAEZ01011533:20253-20978
HmHel-1	CAEZ01009965:14585-15070
HmHel-1	CAEZ01009146:16823-17479
HmHel-1	CAEZ01009146:1-252
HmHel-1	CAEZ01008437:103156-103586
HmHel-1	CAEZ01008437:144727-145384
HmHel-1	CAEZ01008437:31906-32149
HmHel-1	CAEZ01008437:1526-1758
HmHel-1	CAEZ01007879:1-362
HmHel-1	CAEZ01006416:24060-24385
HmHel-1	CAEZ01005414:39009-39213
HmHel-1	CAEZ01005414:39760-40429
HmHel-1	CAEZ01005137:879-1193
HmHel-1	CAEZ01002651:8941-9611
HmHel-1	CAEZ01010215:26-435
HmHel-1	CAEZ01010215:12853-13085
HmHel-1	CAEZ01010145:4797-5169

HmHel-1	CAEZ01009002:27250-27548
HmHel-1	CAEZ01008767:24934-25273
HmHel-1	CAEZ01008115:14222-14477
HmHel-1	CAEZ01008104:11007-13458
HmHel-1	CAEZ01007164:306-464
HmHel-1	CAEZ01007164:10813-11017
HmHel-1	CAEZ01007164:21945-22169
HmHel-1	CAEZ01007100:23889-24121
HmHel-1	CAEZ01007100:65857-66505
HmHel-1	CAEZ01006281:728-991
HmHel-1	CAEZ01005844:18904-19041
HmHel-1	CAEZ01005228:35921-36164
HmHel-1	CAEZ01005228:52956-53624
HmHel-1	CAEZ01011173:221-736
HmHel-1	CAEZ01010449:32125-32609
HmHel-1	CAEZ01010449:81145-81486
HmHel-1	CAEZ01010090:2257-2599
HmHel-1	CAEZ01009905:40-527
HmHel-1	CAEZ01009864:1713-2367
HmHel-1	CAEZ01009864:6086-6411
HmHel-1	CAEZ01009401:473-856
HmHel-1	CAEZ01008952:33754-34094
HmHel-1	CAEZ01008773:9765-10251
HmHel-1	CAEZ01008408:10801-11213
HmHel-1	CAEZ01007969:13-327
HmHel-1	CAEZ01005795:10499-10832
HmHel-1	CAEZ01005087:19813-20122
HmHel-1	CAEZ01002924:78495-78738
HmHel-1	CAEZ01002924:20889-21577
HmHel-1	CAEZ01002669:8855-9199
HmHel-1	CAEZ01002143:12775-13116
HmHel-1	CAEZ01002064:29403-29744
HmHel-1	CAEZ01009081:23204-23891
HmHel-1	CAEZ01009081:45250-45459
HmHel-1	CAEZ01007111:1-1056
HmHel-1	CAEZ01004585:11204-16475
HmHel-1	CAEZ01004585:31891-31972
HmHel-1	CAEZ01003365:32826-32965
HmHel-1	CAEZ01011283:5088-5884
HmHel-1	CAEZ01011283:5391-5523
HmHel-1	CAEZ01010110:3466-4103
HmHel-1	CAEZ01010072:8015-8347
HmHel-1	CAEZ01009727:11113-11444
HmHel-1	CAEZ01006573:9160-9339
HmHel-1	CAEZ01006541:33508-33831
HmHel-1	CAEZ01005900:24178-24848
HmHel-1	CAEZ01002807:39208-39526
HmHel-1	CAEZ01002706:86171-86503

HmHel-1	CAEZ01002706:113200-113421
HmHel-1	CAEZ01002706:59481-59709
HmHel-1	CAEZ01010287:8935-9276
HmHel-1	CAEZ01010079:20645-20877
HmHel-1	CAEZ01010079:91628-91967
HmHel-1	CAEZ01010079:23555-23788
HmHel-1	CAEZ01008336:1-301
HmHel-1	CAEZ01007712:13980-14247
HmHel-1	CAEZ01007712:6940-7267
HmHel-1	CAEZ01005808:1127-1471
HmHel-1	CAEZ01004328:6101-6725
HmHel-1	CAEZ01003690:20603-20822
HmHel-1	CAEZ01002653:11704-11866
HmHel-1	CAEZ01002160:5009-5338
HmHel-1	CAEZ01001463:44932-45185
HmHel-1	CAEZ01001463:5109-5799
HmHel-1	CAEZ01011600:3965-4365
HmHel-1	CAEZ01010461:7805-8446
HmHel-1	CAEZ01010461:22645-22862
HmHel-1	CAEZ01007024:2318-2675
HmHel-1	CAEZ01004819:18670-19294
HmHel-1	CAEZ01004819:30803-31042
HmHel-1	CAEZ01004819:19029-19202
HmHel-1	CAEZ01003510:8641-8977
HmHel-1	CAEZ01002036:9771-10104
HmHel-1	CAEZ01000895:11688-12364
HmHel-1	CAEZ01000895:10901-11105
HmHel-1	CAEZ01000806:374-539
HmHel-1	CAEZ01010008:32075-32514
HmHel-1	CAEZ01009242:17755-18086
HmHel-1	CAEZ01009242:65601-65830
HmHel-1	CAEZ01009242:80551-80641
HmHel-1	CAEZ01003681:172-494
HmHel-1	CAEZ01002581:12060-12464
HmHel-1	CAEZ01009982:112607-113281
HmHel-1	CAEZ01009982:61604-61818
HmHel-1	CAEZ01009383:7818-16308
HmHel-1	CAEZ01009263:27927-34206
HmHel-1	CAEZ01006867:10639-10932
HmHel-1	CAEZ01005725:4979-5411
HmHel-1	CAEZ01005556:8852-9158
HmHel-1	CAEZ01005136:7816-8035
HmHel-1	CAEZ01005136:37159-37476
HmHel-1	CAEZ01004597:5697-6365
HmHel-1	CAEZ01004542:3246-3805
HmHel-1	CAEZ01004542:201-335
HmHel-1	CAEZ01003487:22814-23030
HmHel-1	CAEZ01002929:4076-4231

HmHel-1	CAEZ01000846:18651-19563
HmHel-1	CAEZ01011425:1-122
HmHel-1	CAEZ01011425:12239-12829
HmHel-1	CAEZ01010293:62304-62906
HmHel-1	CAEZ01008664:7921-8263
HmHel-1	CAEZ01006440:111531-111763
HmHel-1	CAEZ01005425:20780-20930
HmHel-1	CAEZ01004289:13233-13748
HmHel-1	CAEZ01003823:14298-14666
HmHel-1	CAEZ01001431:12195-12466
HmHel-1	CAEZ01009741:469-1138
HmHel-1	CAEZ01007684:3971-4378
HmHel-1	CAEZ01004680:36499-36841
HmHel-1	CAEZ01003053:2420-2786
HmHel-1	CAEZ01001772:6527-6982
HmHel-1	CAEZ01005485:56225-56671
HmHel-1	CAEZ01002049:103924-104332
HmHel-1	CAEZ01000049:2-381
HmHel-1	CAEZ01010898:15360-16027
HmHel-1	CAEZ01010292:23636-24226
HmHel-1	CAEZ01010292:8311-8761
HmHel-1	CAEZ01009137:107514-107960
HmHel-1	CAEZ01008352:4677-4901
HmHel-1	CAEZ01008352:110021-110459
HmHel-1	CAEZ01008352:126361-127010
HmHel-1	CAEZ01008125:188177-188838
HmHel-1	CAEZ01008125:44555-44689
HmHel-1	CAEZ01007121:29852-30231
HmHel-1	CAEZ01007063:5-380
HmHel-1	CAEZ01006669:11177-11856
HmHel-1	CAEZ01006669:9507-9674
HmHel-1	CAEZ01006365:15104-15772
HmHel-1	CAEZ01006237:730-929
HmHel-1	CAEZ01006237:2295-2783
HmHel-1	CAEZ01005981:12-449
HmHel-1	CAEZ01005981:4895-5110
HmHel-1	CAEZ01005909:28171-28384
HmHel-1	CAEZ01005909:40627-41136
HmHel-1	CAEZ01005541:9198-10338
HmHel-1	CAEZ01004477:16738-17170
HmHel-1	CAEZ01003979:18-1212
HmHel-1	CAEZ01003783:52879-53575
HmHel-1	CAEZ01003546:186-1414
HmHel-1	CAEZ01001046:999-1362
HmHel-1	CAEZ01009342:18879-19543
HmHel-1	CAEZ01009206:7834-8084
HmHel-1	CAEZ01009206:1-145
HmHel-1	CAEZ01008979:8191-8537

HmHel-1	CAEZ01008495:17177-17863
HmHel-1	CAEZ01008350:52850-53262
HmHel-1	CAEZ01008350:125193-125459
HmHel-1	CAEZ01008263:29281-29879
HmHel-1	CAEZ01008263:46749-47433
HmHel-1	CAEZ01008263:29109-29985
HmHel-1	CAEZ01006734:10875-11218
HmHel-1	CAEZ01005825:4153-4556
HmHel-1	CAEZ01003380:316-636
HmHel-1	CAEZ01011605:51272-51928
HmHel-1	CAEZ01011605:32975-33306
HmHel-1	CAEZ01010922:47420-47963
HmHel-1	CAEZ01010543:43409-43659
HmHel-1	CAEZ01010464:118780-119441
HmHel-1	CAEZ01010464:116884-117549
HmHel-1	CAEZ01009765:10641-11306
HmHel-1	CAEZ01009541:4450-4863
HmHel-1	CAEZ01009288:24797-25024
HmHel-1	CAEZ01009288:9062-9492
HmHel-1	CAEZ01009099:22422-22629
HmHel-1	CAEZ01009099:5380-5618
HmHel-1	CAEZ01008796:15603-15841
HmHel-1	CAEZ01007746:123906-124266
HmHel-1	CAEZ01007495:26299-26542
HmHel-1	CAEZ01007495:28183-36227
HmHel-1	CAEZ01007099:1829-2285
HmHel-1	CAEZ01006591:32959-33047
HmHel-1	CAEZ01006591:6119-6785
HmHel-1	CAEZ01006351:8321-8987
HmHel-1	CAEZ01006313:476-1054
HmHel-1	CAEZ01005361:8775-9259
HmHel-1	CAEZ01005013:41873-42463
HmHel-1	CAEZ01004845:70222-70538
HmHel-1	CAEZ01004677:50640-50890
HmHel-1	CAEZ01004527:29559-29872
HmHel-1	CAEZ01003562:23178-23849
HmHel-1	CAEZ01003265:13898-14349
HmHel-1	CAEZ01003006:8693-9379
HmHel-1	CAEZ01002224:27712-28836
HmHel-1	CAEZ01001865:917-3087
HmHel-1	CAEZ01001218:1013-1326
HmHel-1	CAEZ01005677:19552-19868
HmHel-1	CAEZ01010139:80926-81435
HmHel-1	CAEZ01009513:6126-6442
HmHel-1	CAEZ01009356:4625-4941
HmHel-1	CAEZ01008709:30873-31382
HmHel-1	CAEZ01007874:3161-3477
HmHel-1	CAEZ01006352:24658-24880

HmHel-1	CAEZ01006352:157935-158596
HmHel-1	CAEZ01006352:108345-109004
HmHel-1	CAEZ01006352:34817-35051
HmHel-1	CAEZ01006028:6483-7163
HmHel-1	CAEZ01004596:14975-15671
HmHel-1	CAEZ01003705:4027-4714
HmHel-1	CAEZ01002025:3321-3812
HmHel-1	CAEZ01001076:31146-31336
HmHel-1	CAEZ01001076:5607-6258
HmHel-1	CAEZ01008012:12533-13198
HmHel-1	CAEZ01007185:2249-2671
HmHel-1	CAEZ01007066:95429-95830
HmHel-1	CAEZ01007066:75397-75826
HmHel-1	CAEZ01003301:18160-18851
HmHel-1	CAEZ01003301:9936-10219
HmHel-1	CAEZ01001943:459-1178
HmHel-1	CAEZ01011525:33092-33522
HmHel-1	CAEZ01011093:27873-28302
HmHel-1	CAEZ01011093:43295-43517
HmHel-1	CAEZ01011093:131725-132376
HmHel-1	CAEZ01011093:179636-181176
HmHel-1	CAEZ01011093:129155-129387
HmHel-1	CAEZ01010839:10116-10758
HmHel-1	CAEZ01010605:4289-4954
HmHel-1	CAEZ01010401:6983-7652
HmHel-1	CAEZ01009688:20916-24037
HmHel-1	CAEZ01009441:9010-9434
HmHel-1	CAEZ01009094:3357-3993
HmHel-1	CAEZ01008784:1087-1519
HmHel-1	CAEZ01008383:9846-10511
HmHel-1	CAEZ01008033:5191-5621
HmHel-1	CAEZ01007434:13160-13592
HmHel-1	CAEZ01007037:489-1153
HmHel-1	CAEZ01006152:9335-9766
HmHel-1	CAEZ01006098:36371-36577
HmHel-1	CAEZ01005148:27475-28134
HmHel-1	CAEZ01005102:3753-4493
HmHel-1	CAEZ01004787:13940-14497
HmHel-1	CAEZ01003894:25653-26309
HmHel-1	CAEZ01003794:2595-7293
HmHel-1	CAEZ01003646:3911-4042
HmHel-1	CAEZ01003646:4873-4961
HmHel-1	CAEZ01003606:2649-3000
HmHel-1	CAEZ01003582:68053-68488
HmHel-1	CAEZ01003404:20761-22276
HmHel-1	CAEZ01003404:58891-59133
HmHel-1	CAEZ01003404:21097-21651
HmHel-1	CAEZ01003211:14416-14776

HmHel-1	CAEZ01002648:10153-10698
HmHel-1	CAEZ01002589:26859-27149
HmHel-1	CAEZ01002001:17586-18243
HmHel-1	CAEZ01001466:56829-57481
HmHel-1	CAEZ01001466:103344-103469
HmHel-1	CAEZ01001466:54498-54738
HmHel-1	CAEZ01001414:1578-2247
HmHel-1	CAEZ01001009:1535-1993
HmHel-1	CAEZ01010619:46335-47012
HmHel-1	CAEZ01009471:5529-6244
HmHel-1	CAEZ01006428:2103-2787
HmHel-1	CAEZ01006428:4404-5094
HmHel-1	CAEZ01005828:20733-21398
HmHel-1	CAEZ01011324:1-165
HmHel-1	CAEZ01010257:3847-4509
HmHel-1	CAEZ01008639:5720-6110
HmHel-1	CAEZ01008433:16568-17233
HmHel-1	CAEZ01007985:7806-18156
HmHel-1	CAEZ01007985:20973-21188
HmHel-1	CAEZ01007985:10180-10365
HmHel-1	CAEZ01007212:1088-1750
HmHel-1	CAEZ01007080:15807-16456
HmHel-1	CAEZ01006802:14541-15450
HmHel-1	CAEZ01003723:24136-24805
HmHel-1	CAEZ01001959:167-599
HmHel-1	CAEZ01001847:483-1077
HmHel-1	CAEZ01001822:540-1181
HmHel-1	CAEZ01009714:11450-12112
HmHel-1	CAEZ01009491:8883-9214
HmHel-1	CAEZ01008842:1-328
HmHel-1	CAEZ01006985:18216-18702
HmHel-1	CAEZ01006195:2074-2498
HmHel-1	CAEZ01004862:7789-8447
HmHel-1	CAEZ01003784:55314-55431
HmHel-1	CAEZ01001426:9671-10198
HmHel-1	CAEZ01001212:1-173
HmHel-1	CAEZ01001212:1427-1670
HmHel-1	CAEZ01000970:37353-37930
HmHel-1	CAEZ01010877:1363-2001
HmHel-1	CAEZ01010830:6026-6743
HmHel-1	CAEZ01010830:29786-30018
HmHel-1	CAEZ01010251:148-813
HmHel-1	CAEZ01009898:27742-27862
HmHel-1	CAEZ01009898:25482-26456
HmHel-1	CAEZ01008959:4980-5294
HmHel-1	CAEZ01008728:36557-37162
HmHel-1	CAEZ01008643:34906-35619
HmHel-1	CAEZ01008026:18632-19270

HmHel-1	CAEZ01008003:9765-10095
HmHel-1	CAEZ01007928:47026-47708
HmHel-1	CAEZ01007928:71998-72086
HmHel-1	CAEZ01007740:59955-60634
HmHel-1	CAEZ01007586:73761-74744
HmHel-1	CAEZ01007275:89542-90159
HmHel-1	CAEZ01007275:110809-111026
HmHel-1	CAEZ01007067:109-538
HmHel-1	CAEZ01006622:12540-13208
HmHel-1	CAEZ01005771:335-1000
HmHel-1	CAEZ01003940:1932-2527
HmHel-1	CAEZ01003332:951-3646
HmHel-1	CAEZ01003247:23567-23757
HmHel-1	CAEZ01003247:30291-30865
HmHel-1	CAEZ01002890:13464-14157
HmHel-1	CAEZ01002868:544-919
HmHel-1	CAEZ01002843:1465-1573
HmHel-1	CAEZ01002245:4173-4762
HmHel-1	CAEZ01001248:169-402
HmHel-1	CAEZ01001062:32397-32485
HmHel-1	CAEZ01001062:51830-52039
HmHel-1	CAEZ01001062:47188-47441
HmHel-1	CAEZ01000206:430-805
HmHel-1	CAEZ01011047:969-1460
HmHel-1	CAEZ01011027:1390-1720
HmHel-1	CAEZ01008795:10284-10715
HmHel-1	CAEZ01005227:16643-17150
HmHel-1	CAEZ01010250:13509-14168
HmHel-1	CAEZ01009822:3117-5199
HmHel-1	CAEZ01008839:10146-10814
HmHel-1	CAEZ01008400:78071-78497
HmHel-1	CAEZ01008400:46189-46878
HmHel-1	CAEZ01008371:61114-61442
HmHel-1	CAEZ01008371:37623-38279
HmHel-1	CAEZ01008371:11820-12052
HmHel-1	CAEZ01006690:16584-17050
HmHel-1	CAEZ01006073:4834-5093
HmHel-1	CAEZ01006022:4737-5365
HmHel-1	CAEZ01005745:9736-10271
HmHel-1	CAEZ01004584:20180-20643
HmHel-1	CAEZ01002079:5513-6175
HmHel-1	CAEZ01010405:6974-7131
HmHel-1	CAEZ01010405:9399-9562
HmHel-1	CAEZ01009510:19481-19598
HmHel-1	CAEZ01009366:60507-61169
HmHel-1	CAEZ01006670:12018-12670
HmHel-1	CAEZ01005976:36223-36883
HmHel-1	CAEZ01003127:13775-14447

HmHel-1	CAEZ01002416:66447-66663
HmHel-1	CAEZ01002416:55288-55998
HmHel-1	CAEZ01001100:491-829
HmHel-1	CAEZ01011659:16630-17295
HmHel-1	CAEZ01011300:5659-6325
HmHel-1	CAEZ01011178:1679-1787
HmHel-1	CAEZ01010269:5317-5979
HmHel-1	CAEZ01009826:25623-26238
HmHel-1	CAEZ01009793:1864-2524
HmHel-1	CAEZ01009145:24496-24822
HmHel-1	CAEZ01009032:6147-6822
HmHel-1	CAEZ01008922:2992-3415
HmHel-1	CAEZ01008754:2076-2726
HmHel-1	CAEZ01008604:3104-3613
HmHel-1	CAEZ01008091:70116-70584
HmHel-1	CAEZ01007725:108726-109157
HmHel-1	CAEZ01007060:10554-10752
HmHel-1	CAEZ01007060:6947-7187
HmHel-1	CAEZ01007011:1-212
HmHel-1	CAEZ01006798:9549-10211
HmHel-1	CAEZ01006531:741-1272
HmHel-1	CAEZ01006129:4491-5154
HmHel-1	CAEZ01005625:958-1360
HmHel-1	CAEZ01005491:8883-10604
HmHel-1	CAEZ01005082:10105-10761
HmHel-1	CAEZ01005082:40444-40789
HmHel-1	CAEZ01003825:1425-2010
HmHel-1	CAEZ01003280:162-589
HmHel-1	CAEZ01002944:71144-71348
HmHel-1	CAEZ01002944:5739-6404
HmHel-1	CAEZ01002736:17456-26339
HmHel-1	CAEZ01000060:18101-18376
HmHel-1	CAEZ01011442:11702-11852
HmHel-1	CAEZ01009562:25922-26144
HmHel-1	CAEZ01009562:23541-24212
HmHel-1	CAEZ01001793:40215-40455
HmHel-1	CAEZ01001793:18453-18719
HmHel-1	CAEZ01000390:1-108
HmHel-1	CAEZ01011134:20219-20809
HmHel-1	CAEZ01007302:7-129
HmHel-1	CAEZ01006218:43228-43896
HmHel-1	CAEZ01003984:1421-1632
HmHel-1	CAEZ01003984:2023-2640
HmHel-1	CAEZ01003558:34919-35274
HmHel-1	CAEZ01003558:603-1245
HmHel-1	CAEZ01007903:2984-3454
HmHel-1	CAEZ01007903:17818-18389
HmHel-1	CAEZ01006216:3433-4091

HmHel-1	CAEZ01006067:261-549
HmHel-1	CAEZ01006067:21262-21539
HmHel-1	CAEZ01006067:9053-9137
HmHel-1	CAEZ01003720:1921-2432
HmHel-1	CAEZ01011521:3241-3868
HmHel-1	CAEZ01010797:12866-13090
HmHel-1	CAEZ01010797:87318-87740
HmHel-1	CAEZ01010797:104387-104616
HmHel-1	CAEZ01010797:115329-115544
HmHel-1	CAEZ01010797:144670-144761
HmHel-1	CAEZ01010797:120690-121365
HmHel-1	CAEZ01009650:11551-12030
HmHel-1	CAEZ01009497:17110-17590
HmHel-1	CAEZ01009370:103491-104071
HmHel-1	CAEZ01009370:57705-57946
HmHel-1	CAEZ01007208:43778-45533
HmHel-1	CAEZ01007124:114426-114669
HmHel-1	CAEZ01007124:68205-73303
HmHel-1	CAEZ01006501:38483-38913
HmHel-1	CAEZ01006501:24455-28398
HmHel-1	CAEZ01006338:38033-38689
HmHel-1	CAEZ01006270:2771-3434
HmHel-1	CAEZ01006242:7965-8595
HmHel-1	CAEZ01004436:1391-1875
HmHel-1	CAEZ01003057:309-970
HmHel-1	CAEZ01002792:10455-10698
HmHel-1	CAEZ01002792:26178-26828
HmHel-1	CAEZ01002286:42963-43626
HmHel-1	CAEZ01001817:8682-9341
HmHel-1	CAEZ01009721:9626-10307
HmHel-1	CAEZ01008499:1024-1692
HmHel-1	CAEZ01006895:8475-8889
HmHel-1	CAEZ01006539:18275-18940
HmHel-1	CAEZ01004761:1-301
HmHel-1	CAEZ01004571:5910-6347
HmHel-1	CAEZ01003366:1-123
HmHel-1	CAEZ01001247:1378-1485
HmHel-1	CAEZ01011171:562-1043
HmHel-1	CAEZ01010893:27644-30207
HmHel-1	CAEZ01010149:24078-24369
HmHel-1	CAEZ01009453:56086-56196
HmHel-1	CAEZ01007984:38553-39218
HmHel-1	CAEZ01007392:12088-12449
HmHel-1	CAEZ01006747:14234-14553
HmHel-1	CAEZ01003922:9155-9818
HmHel-1	CAEZ01010938:13952-18851
HmHel-1	CAEZ01010249:7384-7736
HmHel-1	CAEZ01009922:34150-34825

HmHel-1	CAEZ01008028:8902-9781
HmHel-1	CAEZ01007680:1281-1915
HmHel-1	CAEZ01007538:8630-8955
HmHel-1	CAEZ01007538:15733-16326
HmHel-1	CAEZ01007538:4542-4746
HmHel-1	CAEZ01003887:3064-3414
HmHel-1	CAEZ01001496:853-962
HmHel-1	CAEZ01011517:4619-5250
HmHel-1	CAEZ01009470:68928-69588
HmHel-1	CAEZ01009470:9065-9319
HmHel-1	CAEZ01008184:458-1130
HmHel-1	CAEZ01005041:4903-5097
HmHel-1	CAEZ01004958:30469-31451
HmHel-1	CAEZ01004169:11964-12523
HmHel-1	CAEZ01001902:1294-2024
HmHel-1	CAEZ01001862:883-1548
HmHel-1	CAEZ01001372:8-424
HmHel-1	CAEZ01000062:19198-19834
HmHel-1	CAEZ01009314:26010-26461
HmHel-1	CAEZ01004072:5707-6080
HmHel-1	CAEZ01011562:23-269
HmHel-1	CAEZ01009637:1921-2267
HmHel-1	CAEZ01008031:20551-20673
HmHel-1	CAEZ01007230:4420-4730
HmHel-1	CAEZ01006987:7732-8426
HmHel-1	CAEZ01006987:6297-6512
HmHel-1	CAEZ01006388:1-126
HmHel-1	CAEZ01004515:14351-14751
HmHel-1	CAEZ01003348:34437-34735
HmHel-1	CAEZ01011630:4552-5017
HmHel-1	CAEZ01010792:11705-12656
HmHel-1	CAEZ01009872:122689-123347
HmHel-1	CAEZ01009176:2608-3287
HmHel-1	CAEZ01008544:4-304
HmHel-1	CAEZ01008112:164-475
HmHel-1	CAEZ01006558:34-242
HmHel-1	CAEZ01006357:9031-9436
HmHel-1	CAEZ01006223:838-1301
HmHel-1	CAEZ01005901:1-284
HmHel-1	CAEZ01002269:107-768
HmHel-1	CAEZ01011242:12282-12967
HmHel-1	CAEZ01010192:12645-13254
HmHel-1	CAEZ01008502:3857-4503
HmHel-1	CAEZ01008324:3490-3787
HmHel-1	CAEZ01007754:1178-2409
HmHel-1	CAEZ01006889:6054-6714
HmHel-1	CAEZ01005163:33515-34094
HmHel-1	CAEZ01005163:35262-35496

HmHel-1	CAEZ01005094:9144-10026
HmHel-1	CAEZ01005025:151801-152466
HmHel-1	CAEZ01005025:112150-112389
HmHel-1	CAEZ01002687:1316-1977
HmHel-1	CAEZ01011309:1384-1705
HmHel-1	CAEZ01010437:72386-72814
HmHel-1	CAEZ01010437:45641-46312
HmHel-1	CAEZ01009842:13963-14344
HmHel-1	CAEZ01009689:189-568
HmHel-1	CAEZ01009489:311-594
HmHel-1	CAEZ01008178:6992-7898
HmHel-1	CAEZ01006248:536-871
HmHel-1	CAEZ01006202:36464-36822
HmHel-1	CAEZ01005857:3435-3999
HmHel-1	CAEZ01003138:426-529
HmHel-1	CAEZ01003138:580-799
HmHel-1	CAEZ01010358:4260-5138
HmHel-1	CAEZ01002332:26220-26895
HmHel-1	CAEZ01002053:4618-4900
HmHel-1	CAEZ01001738:4003-4604
HmHel-1	CAEZ01001738:46366-48282
HmHel-1	CAEZ01008800:5278-6984
HmHel-1	CAEZ01007446:20645-20894
HmHel-1	CAEZ01005732:20331-21023
HmHel-1	CAEZ01010312:12976-13573
HmHel-1	CAEZ01009415:3-453
HmHel-1	CAEZ01007825:2264-4609
HmHel-1	CAEZ01007289:2953-3295
HmHel-1	CAEZ01006288:2548-3204
HmHel-1	CAEZ01005656:1461-2115
HmHel-1	CAEZ01005572:10642-10984
HmHel-1	CAEZ01003527:2121-2317
HmHel-1	CAEZ01003527:3171-3833
HmHel-1	CAEZ01002612:13128-13792
HmHel-1	CAEZ01001321:2470-2578
HmHel-1	CAEZ01005438:8836-9523
HmHel-1	CAEZ01005419:24442-24643
HmHel-1	CAEZ01005419:18696-18822
HmHel-1	CAEZ01005419:1828-2048
HmHel-1	CAEZ01005053:13880-14534
HmHel-1	CAEZ01004371:1991-2407
HmHel-1	CAEZ01003235:77316-77914
HmHel-1	CAEZ01002181:27062-27159
HmHel-1	CAEZ01002181:23470-24110
HmHel-1	CAEZ01002119:12615-12825
HmHel-1	CAEZ01002119:1965-2321
HmHel-1	CAEZ01000996:433-1112
HmHel-1	CAEZ01008120:3030-3286

HmHel-1	CAEZ01007599:29002-29614
HmHel-1	CAEZ01004756:7843-8505
HmHel-1	CAEZ01003571:388-494
HmHel-1	CAEZ01003401:60981-61520
HmHel-1	CAEZ01008356:29251-29682
HmHel-1	CAEZ01008356:59235-59403
HmHel-1	CAEZ01008256:17436-18386
HmHel-1	CAEZ01007313:39033-39655
HmHel-1	CAEZ01006529:178285-179516
HmHel-1	CAEZ01008843:5553-5998
HmHel-1	CAEZ01008736:22596-32311
HmHel-1	CAEZ01008283:5319-5431
HmHel-1	CAEZ01007047:1395-1503
HmHel-1	CAEZ01006846:14778-15099
HmHel-1	CAEZ01005863:7162-7832
HmHel-1	CAEZ01005588:7379-7734
HmHel-1	CAEZ01010290:10536-10945
HmHel-1	CAEZ01010212:30572-32700
HmHel-1	CAEZ01001629:1-170
HmHel-1	CAEZ01010924:13769-13957
HmHel-1	CAEZ01009602:5401-5777
HmHel-1	CAEZ01003570:11156-11351
HmHel-1	CAEZ01005783:15826-16306
HmHel-1	CAEZ01009413:3745-4228
HmHel-1	CAEZ01002711:10836-11410
HmHel-1	CAEZ01009305:7597-7812
HmHel-1	CAEZ01008421:31998-32349
HmHel-1	CAEZ01011221:6764-7086
HmHel-1	CAEZ01001289:20109-20207
HmHel-1	CAEZ01011475:36836-37105
HmHel-1	CAEZ01007500:64104-64381
HmHel-1	CAEZ01005231:91258-91471
HmHel-1	CAEZ01005231:8556-9176
HmHel-1	CAEZ01004264:4012-4582
HmHel-1	CAEZ01011036:12845-13059
HmHel-1	CAEZ01010336:15356-15588
HmHel-1	CAEZ01010291:1-225
HmHel-1	CAEZ01010291:35996-36141
HmHel-1	CAEZ01010291:25963-26164
HmHel-1	CAEZ01010091:161-241
HmHel-1	CAEZ01008431:15651-15878
HmHel-1	CAEZ01008367:89408-89637
HmHel-1	CAEZ01008094:23075-23167
HmHel-1	CAEZ01007861:183837-184193
HmHel-1	CAEZ01007861:148279-148755
HmHel-1	CAEZ01007822:26605-26827
HmHel-1	CAEZ01006570:4843-5047
HmHel-1	CAEZ01005843:924-1724

HmHel-1	CAEZ01005486:16319-16407
HmHel-1	CAEZ01003897:5643-5865
HmHel-1	CAEZ01003744:17098-17313
HmHel-1	CAEZ01002602:18209-18412
HmHel-1	CAEZ01001038:1953-2614
HmHel-1	CAEZ01011094:9031-9437
HmHel-1	CAEZ01006284:6039-6162
HmHel-1	CAEZ01010349:5262-5843
HmHel-1	CAEZ01008904:6700-7024
HmHel-1	CAEZ01007705:8230-13819
HmHel-1	CAEZ01007705:85149-85390
HmHel-1	CAEZ01006865:51091-51411
HmHel-1	CAEZ01006434:16055-16242
HmHel-1	CAEZ01006434:9-399
HmHel-1	CAEZ01004181:2810-3468
HmHel-1	CAEZ01002993:3433-3666
HmHel-1	CAEZ01011502:4888-4985
HmHel-1	CAEZ01010979:850-1563
HmHel-1	CAEZ01010585:167660-167877
HmHel-1	CAEZ01010585:73384-73613
HmHel-1	CAEZ01008622:1-93
HmHel-1	CAEZ01007968:15111-15330
HmHel-1	CAEZ01007333:11300-11552
HmHel-1	CAEZ01006955:10533-11644
HmHel-1	CAEZ01006097:71502-71667
HmHel-1	CAEZ01005010:1718-2014
HmHel-1	CAEZ01011466:404-492
HmHel-1	CAEZ01011155:2379-2551
HmHel-1	CAEZ01011155:20110-20317
HmHel-1	CAEZ01010555:420-648
HmHel-1	CAEZ01010510:437-525
HmHel-1	CAEZ01010460:61817-62049
HmHel-1	CAEZ01010427:19434-19666
HmHel-1	CAEZ01010276:34591-34808
HmHel-1	CAEZ01010259:5191-5279
HmHel-1	CAEZ01010202:2882-3086
HmHel-1	CAEZ01010181:50371-50575
HmHel-1	CAEZ01010012:12012-18351
HmHel-1	CAEZ01009973:3087-3175
HmHel-1	CAEZ01009973:8129-8215
HmHel-1	CAEZ01009891:79250-79483
HmHel-1	CAEZ01009891:28458-29118
HmHel-1	CAEZ01009706:40739-40943
HmHel-1	CAEZ01009514:1208-1451
HmHel-1	CAEZ01009500:2193-2423
HmHel-1	CAEZ01009287:1182-1386
HmHel-1	CAEZ01009285:2935-3146
HmHel-1	CAEZ01009159:4321-4549

HmHel-1	CAEZ01009024:6095-6330
HmHel-1	CAEZ01008935:5465-5694
HmHel-1	CAEZ01008701:2666-2754
HmHel-1	CAEZ01008616:3438-3670
HmHel-1	CAEZ01008589:2618-2833
HmHel-1	CAEZ01008589:14500-14739
HmHel-1	CAEZ01008131:7317-7545
HmHel-1	CAEZ01008084:3030-3234
HmHel-1	CAEZ01007789:1-424
HmHel-1	CAEZ01007722:60582-60814
HmHel-1	CAEZ01007722:145906-146129
HmHel-1	CAEZ01007722:161715-161939
HmHel-1	CAEZ01007651:12915-13003
HmHel-1	CAEZ01007597:13987-14230
HmHel-1	CAEZ01007571:15686-15890
HmHel-1	CAEZ01007426:2963-3051
HmHel-1	CAEZ01007382:3543-3747
HmHel-1	CAEZ01007267:25281-25365
HmHel-1	CAEZ01007265:1060-1276
HmHel-1	CAEZ01007265:27602-27831
HmHel-1	CAEZ01007265:18333-18445
HmHel-1	CAEZ01006722:421-633
HmHel-1	CAEZ01006643:28047-28135
HmHel-1	CAEZ01006619:10516-10740
HmHel-1	CAEZ01006619:30254-30483
HmHel-1	CAEZ01006619:33903-34146
HmHel-1	CAEZ01006603:11373-11597
HmHel-1	CAEZ01006528:5128-5414
HmHel-1	CAEZ01006369:36694-36909
HmHel-1	CAEZ01006369:26901-28447
HmHel-1	CAEZ01005774:8455-8659
HmHel-1	CAEZ01005639:4990-5193
HmHel-1	CAEZ01005611:2292-2528
HmHel-1	CAEZ01005555:4326-4509
HmHel-1	CAEZ01005534:981-1205
HmHel-1	CAEZ01005455:3841-4045
HmHel-1	CAEZ01005218:922-1132
HmHel-1	CAEZ01005204:9612-9854
HmHel-1	CAEZ01004994:281-523
HmHel-1	CAEZ01004883:1302-1509
HmHel-1	CAEZ01004832:32892-33116
HmHel-1	CAEZ01004714:4257-4486
HmHel-1	CAEZ01004641:4364-4452
HmHel-1	CAEZ01004452:44704-44926
HmHel-1	CAEZ01004093:18062-18266
HmHel-1	CAEZ01004093:21386-21552
HmHel-1	CAEZ01003993:3971-4203
HmHel-1	CAEZ01003952:33006-33209

HmHel-1	CAEZ01003809:729-933
HmHel-1	CAEZ01003067:82159-82374
HmHel-1	CAEZ01003067:98877-99083
HmHel-1	CAEZ01003067:34176-34409
HmHel-1	CAEZ01002987:4276-4487
HmHel-1	CAEZ01002901:9383-9598
HmHel-1	CAEZ01002821:36219-36441
HmHel-1	CAEZ01002814:5905-6120
HmHel-1	CAEZ01002627:608-802
HmHel-1	CAEZ01002601:137-370
HmHel-1	CAEZ01002398:2078-2280
HmHel-1	CAEZ01002268:2023-2227
HmHel-1	CAEZ01002157:926-1148
HmHel-1	CAEZ01002144:7804-7892
HmHel-1	CAEZ01002134:14184-14391
HmHel-1	CAEZ01002134:26436-26651
HmHel-1	CAEZ01002072:4113-4321
HmHel-1	CAEZ01001879:798-1002
HmHel-1	CAEZ01001828:8579-8779
HmHel-1	CAEZ01001828:6560-6648
HmHel-1	CAEZ01001773:3360-3552
HmHel-1	CAEZ01001417:4743-4992
HmHel-1	CAEZ01001417:30126-30345
HmHel-1	CAEZ01001417:22936-23024
HmHel-1	CAEZ01001238:8924-9131
HmHel-1	CAEZ01001208:2682-2890
HmHel-1	CAEZ01001082:3393-3617
HmHel-1	CAEZ01001051:790-982
HmHel-1	CAEZ01000850:21962-22177
HmHel-1	CAEZ01000850:85482-92288
HmHel-1	CAEZ01000850:79249-79464
HmHel-1	CAEZ01000850:20190-20278
HmHel-1	CAEZ01000849:40578-40782
HmHel-1	CAEZ01000849:71479-72006
HmHel-1	CAEZ01000849:20146-20348
HmHel-1	CAEZ01000040:3484-3706
HmHel-1	CAEZ01011146:1-194
HmHel-1	CAEZ01011082:4882-5285
HmHel-1	CAEZ01010445:14-280
HmHel-1	CAEZ01010445:27639-31128
HmHel-1	CAEZ01009824:32457-32690
HmHel-1	CAEZ01009824:74635-75624
HmHel-1	CAEZ01009824:3027-3115
HmHel-1	CAEZ01009479:44803-45014
HmHel-1	CAEZ01009463:42-174
HmHel-1	CAEZ01009307:3063-3142
HmHel-1	CAEZ01007902:19022-19109
HmHel-1	CAEZ01006665:787-1998

HmHel-1	CAEZ01006300:20505-20584
HmHel-1	CAEZ01005652:6162-6245
HmHel-1	CAEZ01004307:4160-4352
HmHel-1	CAEZ01004307:18751-18831
HmHel-1	CAEZ01003704:15608-15740
HmHel-1	CAEZ01003704:1203-1407
HmHel-1	CAEZ01002150:414-608
HmHel-1	CAEZ01001494:21918-22137
HmHel-1	CAEZ01011154:2960-3116
HmHel-1	CAEZ01008824:25656-25988
HmHel-1	CAEZ01008824:47762-47852
HmHel-1	CAEZ01008423:3749-3831
HmHel-1	CAEZ01007665:119095-119181
HmHel-1	CAEZ01007665:81255-81443
HmHel-1	CAEZ01006745:31662-31904
HmHel-1	CAEZ01006120:7231-7474
HmHel-1	CAEZ01006120:43006-43092
HmHel-1	CAEZ01005885:4629-4848
HmHel-1	CAEZ01005781:6865-7716
HmHel-1	CAEZ01005190:16808-17044
HmHel-1	CAEZ01010172:58-147
HmHel-1	CAEZ01007890:11575-11767
HmHel-1	CAEZ01007816:15832-16048
HmHel-1	CAEZ01007403:29696-29901
HmHel-1	CAEZ01007403:23340-23552
HmHel-1	CAEZ01006637:1721-1810
HmHel-1	CAEZ01006412:37105-37228
HmHel-1	CAEZ01006412:65523-65745
HmHel-1	CAEZ01005391:5163-5392
HmHel-1	CAEZ01003620:7023-7242
HmHel-1	CAEZ01002284:3179-3460
HmHel-1	CAEZ01002015:10585-10674
HmHel-1	CAEZ01011501:15558-15782
HmHel-1	CAEZ01011498:1-203
HmHel-1	CAEZ01011430:5158-5384
HmHel-1	CAEZ01011351:14-242
HmHel-1	CAEZ01010933:274-478
HmHel-1	CAEZ01010777:781-862
HmHel-1	CAEZ01010734:26596-26825
HmHel-1	CAEZ01010734:127540-127770
HmHel-1	CAEZ01010682:1329-1580
HmHel-1	CAEZ01010589:5599-5815
HmHel-1	CAEZ01010509:7298-7530
HmHel-1	CAEZ01010500:9282-9498
HmHel-1	CAEZ01010489:8670-8902
HmHel-1	CAEZ01010489:29665-29898
HmHel-1	CAEZ01010421:9221-9436
HmHel-1	CAEZ01010282:16519-16719

HmHel-1	CAEZ01010171:26967-27210
HmHel-1	CAEZ01010119:7914-8120
HmHel-1	CAEZ01010059:5237-5325
HmHel-1	CAEZ01010013:3856-3944
HmHel-1	CAEZ01009999:49505-49727
HmHel-1	CAEZ01009885:34250-34450
HmHel-1	CAEZ01009869:11283-11522
HmHel-1	CAEZ01009862:485-714
HmHel-1	CAEZ01009737:8176-8398
HmHel-1	CAEZ01009705:51181-51424
HmHel-1	CAEZ01009421:55422-55644
HmHel-1	CAEZ01009417:35327-35573
HmHel-1	CAEZ01009417:22062-30471
HmHel-1	CAEZ01009196:15468-15690
HmHel-1	CAEZ01009023:90793-91037
HmHel-1	CAEZ01008885:1920-2142
HmHel-1	CAEZ01008852:27048-27136
HmHel-1	CAEZ01008740:79550-79772
HmHel-1	CAEZ01008627:8639-8872
HmHel-1	CAEZ01008588:2484-3119
HmHel-1	CAEZ01008588:41298-41512
HmHel-1	CAEZ01008561:1132-1336
HmHel-1	CAEZ01008560:4905-5138
HmHel-1	CAEZ01008524:39-267
HmHel-1	CAEZ01008508:10831-10919
HmHel-1	CAEZ01008508:26694-26782
HmHel-1	CAEZ01008508:24507-24735
HmHel-1	CAEZ01008477:8835-9050
HmHel-1	CAEZ01008448:11407-11613
HmHel-1	CAEZ01008360:8167-8391
HmHel-1	CAEZ01008334:1623-1711
HmHel-1	CAEZ01008313:10286-10517
HmHel-1	CAEZ01008248:42780-42868
HmHel-1	CAEZ01008082:9935-10139
HmHel-1	CAEZ01008023:9643-9876
HmHel-1	CAEZ01008023:140-252
HmHel-1	CAEZ01008022:11257-11500
HmHel-1	CAEZ01007978:2602-2804
HmHel-1	CAEZ01007832:7380-7602
HmHel-1	CAEZ01007741:74178-74393
HmHel-1	CAEZ01007741:102125-102357
HmHel-1	CAEZ01007741:96291-96519
HmHel-1	CAEZ01007741:3458-3653
HmHel-1	CAEZ01007631:23246-23489
HmHel-1	CAEZ01007565:4644-4861
HmHel-1	CAEZ01007480:16626-16869
HmHel-1	CAEZ01007460:17337-17542
HmHel-1	CAEZ01007398:4259-4477

HmHel-1	CAEZ01007361:3187-3419
HmHel-1	CAEZ01006957:2063-2285
HmHel-1	CAEZ01006836:12897-13116
HmHel-1	CAEZ01006633:14962-15194
HmHel-1	CAEZ01006615:944-1166
HmHel-1	CAEZ01006602:10045-10133
HmHel-1	CAEZ01006508:6698-6920
HmHel-1	CAEZ01006389:12204-12439
HmHel-1	CAEZ01006322:6664-6888
HmHel-1	CAEZ01006191:3349-3581
HmHel-1	CAEZ01006179:21570-21803
HmHel-1	CAEZ01006096:1811-2015
HmHel-1	CAEZ01006085:3065-3153
HmHel-1	CAEZ01006079:3137-3343
HmHel-1	CAEZ01005929:2753-2968
HmHel-1	CAEZ01005845:13008-13230
HmHel-1	CAEZ01005773:1333-1513
HmHel-1	CAEZ01005773:5420-5649
HmHel-1	CAEZ01005722:25310-25539
HmHel-1	CAEZ01005705:2795-3003
HmHel-1	CAEZ01005580:5804-6035
HmHel-1	CAEZ01005580:6929-7157
HmHel-1	CAEZ01005421:29212-29424
HmHel-1	CAEZ01005042:1479-1703
HmHel-1	CAEZ01004987:9536-9751
HmHel-1	CAEZ01004964:1282-1497
HmHel-1	CAEZ01004936:5848-6081
HmHel-1	CAEZ01004934:22641-22729
HmHel-1	CAEZ01004920:28766-28977
HmHel-1	CAEZ01004843:7158-7391
HmHel-1	CAEZ01004843:28022-28270
HmHel-1	CAEZ01004822:8330-8410
HmHel-1	CAEZ01004796:40252-40474
HmHel-1	CAEZ01004789:5062-5261
HmHel-1	CAEZ01004703:20856-21060
HmHel-1	CAEZ01004653:17980-18208
HmHel-1	CAEZ01004551:22689-22915
HmHel-1	CAEZ01004529:12-370
HmHel-1	CAEZ01004492:17378-17617
HmHel-1	CAEZ01004492:31752-31984
HmHel-1	CAEZ01004464:6543-6746
HmHel-1	CAEZ01004413:1515-1747
HmHel-1	CAEZ01004314:2196-2387
HmHel-1	CAEZ01004314:13366-13588
HmHel-1	CAEZ01004285:29235-29323
HmHel-1	CAEZ01004249:17310-17553
HmHel-1	CAEZ01004249:58-153
HmHel-1	CAEZ01004247:11796-12000

HmHel-1	CAEZ01004189:1534-1766
HmHel-1	CAEZ01004112:9317-9521
HmHel-1	CAEZ01004096:119579-119811
HmHel-1	CAEZ01004081:44742-45257
HmHel-1	CAEZ01004045:27336-27569
HmHel-1	CAEZ01004008:64980-65068
HmHel-1	CAEZ01004001:4058-4146
HmHel-1	CAEZ01003994:12454-12686
HmHel-1	CAEZ01003912:4511-4599
HmHel-1	CAEZ01003863:4659-4863
HmHel-1	CAEZ01003842:28542-28746
HmHel-1	CAEZ01003842:27868-28083
HmHel-1	CAEZ01003722:3138-3348
HmHel-1	CAEZ01003719:23581-23809
HmHel-1	CAEZ01003718:22095-27810
HmHel-1	CAEZ01003718:23873-24116
HmHel-1	CAEZ01003686:17310-17398
HmHel-1	CAEZ01003622:17319-17549
HmHel-1	CAEZ01003610:17564-17807
HmHel-1	CAEZ01003476:89-177
HmHel-1	CAEZ01003363:9060-9265
HmHel-1	CAEZ01003293:1822-1978
HmHel-1	CAEZ01003200:20549-20773
HmHel-1	CAEZ01003195:25506-25738
HmHel-1	CAEZ01003195:96916-97142
HmHel-1	CAEZ01003102:7166-7254
HmHel-1	CAEZ01003071:22827-23059
HmHel-1	CAEZ01002966:2795-3025
HmHel-1	CAEZ01002957:296-518
HmHel-1	CAEZ01002810:39526-39698
HmHel-1	CAEZ01002810:68503-68706
HmHel-1	CAEZ01002769:66936-67150
HmHel-1	CAEZ01002726:3473-3705
HmHel-1	CAEZ01002689:17888-18130
HmHel-1	CAEZ01002499:3907-4139
HmHel-1	CAEZ01002451:239-327
HmHel-1	CAEZ01002331:24162-24409
HmHel-1	CAEZ01002170:8384-8472
HmHel-1	CAEZ01002113:2151-2389
HmHel-1	CAEZ01002039:17039-17280
HmHel-1	CAEZ01001936:4921-5148
HmHel-1	CAEZ01001850:25869-26097
HmHel-1	CAEZ01001759:5413-5638
HmHel-1	CAEZ01001559:10894-11120
HmHel-1	CAEZ01001508:1546-1746
HmHel-1	CAEZ01001416:1983-2071
HmHel-1	CAEZ01001415:21764-22007
HmHel-1	CAEZ01001142:527-770

HmHel-1	CAEZ01001077:722-916
HmHel-1	CAEZ01001048:1208-1409
HmHel-1	CAEZ01001021:11374-11617
HmHel-1	CAEZ01000959:10894-11114
HmHel-1	CAEZ01000084:663-900
HmHel-1	CAEZ01000070:366-454
HmHel-1	CAEZ01011443:8-123
HmHel-1	CAEZ01010912:5165-5407
HmHel-1	CAEZ01009954:1799-1984
HmHel-1	CAEZ01009642:23173-23395
HmHel-1	CAEZ01009420:6628-6722
HmHel-1	CAEZ01009420:41557-41656
HmHel-1	CAEZ01008971:35173-35260
HmHel-1	CAEZ01008570:14362-14594
HmHel-1	CAEZ01008272:22550-22772
HmHel-1	CAEZ01007998:3989-4077
HmHel-1	CAEZ01007998:1-141
HmHel-1	CAEZ01006374:8511-8740
HmHel-1	CAEZ01005201:27242-27374
HmHel-1	CAEZ01003629:1-191
HmHel-1	CAEZ01003351:45-209
HmHel-1	CAEZ01002633:35662-35745
HmHel-1	CAEZ01010607:4597-4679
HmHel-1	CAEZ01009788:11129-11215
HmHel-1	CAEZ01007296:6635-8159
HmHel-1	CAEZ01007296:12434-12663
HmHel-1	CAEZ01006657:4707-4945
HmHel-1	CAEZ01006080:14632-14865
HmHel-1	CAEZ01006019:4619-4720
HmHel-1	CAEZ01005861:772-1013
HmHel-1	CAEZ01004336:3177-3386
HmHel-1	CAEZ01003948:1441-2817
HmHel-1	CAEZ01003938:3151-3348
HmHel-1	CAEZ01003881:4987-5212
HmHel-1	CAEZ01003715:2646-2865
HmHel-1	CAEZ01002255:42801-43008
HmHel-1	CAEZ01002048:21038-21718
HmHel-1	CAEZ01001134:8380-8605
HmHel-1	CAEZ01011411:10742-10982
HmHel-1	CAEZ01011039:8362-8594
HmHel-1	CAEZ01011009:36676-36919
HmHel-1	CAEZ01010824:21668-21757
HmHel-1	CAEZ01008950:12733-12988
HmHel-1	CAEZ01008658:1-381
HmHel-1	CAEZ01008341:56539-57117
HmHel-1	CAEZ01008260:18085-18320
HmHel-1	CAEZ01008146:1161-1394
HmHel-1	CAEZ01007475:72989-73117

HmHel-1	CAEZ01007475:873-1096
HmHel-1	CAEZ01007094:5965-6058
HmHel-1	CAEZ01006343:23099-23425
HmHel-1	CAEZ01005215:8542-8757
HmHel-1	CAEZ01004513:21312-21965
HmHel-1	CAEZ01004300:20691-20913
HmHel-1	CAEZ01002723:25242-25471
HmHel-1	CAEZ01001951:1552-1763
HmHel-1	CAEZ01011584:1894-2100
HmHel-1	CAEZ01011548:3554-3790
HmHel-1	CAEZ01011463:6096-6184
HmHel-1	CAEZ01011463:17936-18179
HmHel-1	CAEZ01011275:11668-12256
HmHel-1	CAEZ01011071:9539-9627
HmHel-1	CAEZ01011010:1512-1727
HmHel-1	CAEZ01010719:15915-16158
HmHel-1	CAEZ01010659:7621-7825
HmHel-1	CAEZ01010453:101014-101229
HmHel-1	CAEZ01010453:59830-60043
HmHel-1	CAEZ01010415:22949-23171
HmHel-1	CAEZ01010346:7825-8057
HmHel-1	CAEZ01010341:371-614
HmHel-1	CAEZ01010211:11799-11887
HmHel-1	CAEZ01010190:21428-21632
HmHel-1	CAEZ01010052:7649-7871
HmHel-1	CAEZ01010015:11728-11973
HmHel-1	CAEZ01009823:37261-37443
HmHel-1	CAEZ01009813:78710-78953
HmHel-1	CAEZ01009672:8894-9136
HmHel-1	CAEZ01009521:7160-7392
HmHel-1	CAEZ01009476:25640-25840
HmHel-1	CAEZ01009452:81589-81677
HmHel-1	CAEZ01009395:695-914
HmHel-1	CAEZ01009395:11695-11910
HmHel-1	CAEZ01009379:22567-22799
HmHel-1	CAEZ01009265:32382-32614
HmHel-1	CAEZ01009250:7-242
HmHel-1	CAEZ01009250:15043-15286
HmHel-1	CAEZ01009114:21384-21472
HmHel-1	CAEZ01008918:8058-8326
HmHel-1	CAEZ01008837:11562-11773
HmHel-1	CAEZ01008833:5890-6108
HmHel-1	CAEZ01008791:2271-2477
HmHel-1	CAEZ01008686:3058-3140
HmHel-1	CAEZ01008686:2087-2175
HmHel-1	CAEZ01008665:5093-5336
HmHel-1	CAEZ01008587:654-868
HmHel-1	CAEZ01008574:12288-12383

HmHel-1	CAEZ01008574:105224-105428
HmHel-1	CAEZ01008492:4015-4256
HmHel-1	CAEZ01008390:54779-54983
HmHel-1	CAEZ01008289:21184-21416
HmHel-1	CAEZ01008271:1386-1608
HmHel-1	CAEZ01008213:5229-5462
HmHel-1	CAEZ01008212:13437-13678
HmHel-1	CAEZ01007967:6931-7019
HmHel-1	CAEZ01007950:14532-14749
HmHel-1	CAEZ01007731:110391-110594
HmHel-1	CAEZ01007731:101089-101293
HmHel-1	CAEZ01007637:14207-14409
HmHel-1	CAEZ01007598:229-474
HmHel-1	CAEZ01007567:3971-4188
HmHel-1	CAEZ01007552:8945-9033
HmHel-1	CAEZ01007522:3561-3769
HmHel-1	CAEZ01007429:59721-59945
HmHel-1	CAEZ01007429:86673-86908
HmHel-1	CAEZ01007342:12427-12515
HmHel-1	CAEZ01007304:3920-4142
HmHel-1	CAEZ01007245:9062-9150
HmHel-1	CAEZ01007151:2717-2886
HmHel-1	CAEZ01007151:23006-23239
HmHel-1	CAEZ01007151:25165-25397
HmHel-1	CAEZ01007137:17591-17679
HmHel-1	CAEZ01007137:2548-2749
HmHel-1	CAEZ01007076:7403-7491
HmHel-1	CAEZ01007053:6182-6379
HmHel-1	CAEZ01007001:11560-11793
HmHel-1	CAEZ01006992:15087-15263
HmHel-1	CAEZ01006992:1938-2132
HmHel-1	CAEZ01006945:923-1104
HmHel-1	CAEZ01006847:111878-112093
HmHel-1	CAEZ01006621:2329-2545
HmHel-1	CAEZ01006621:7739-7982
HmHel-1	CAEZ01006294:3050-3250
HmHel-1	CAEZ01006125:4274-4486
HmHel-1	CAEZ01006061:9562-9736
HmHel-1	CAEZ01006057:3428-3661
HmHel-1	CAEZ01006030:4590-4793
HmHel-1	CAEZ01005982:8312-8527
HmHel-1	CAEZ01005955:2416-2650
HmHel-1	CAEZ01005938:15766-15969
HmHel-1	CAEZ01005927:14471-14686
HmHel-1	CAEZ01005760:543-771
HmHel-1	CAEZ01005744:5214-5448
HmHel-1	CAEZ01005660:22568-22783
HmHel-1	CAEZ01005516:19774-19862

HmHel-1	CAEZ01005499:28529-28743
HmHel-1	CAEZ01005475:1232-1429
HmHel-1	CAEZ01005264:140-228
HmHel-1	CAEZ01005161:16326-16558
HmHel-1	CAEZ01004722:6332-6558
HmHel-1	CAEZ01004625:8023-8226
HmHel-1	CAEZ01004589:1695-1899
HmHel-1	CAEZ01004574:30175-30399
HmHel-1	CAEZ01004222:66389-66621
HmHel-1	CAEZ01004185:25163-25378
HmHel-1	CAEZ01004158:4765-4987
HmHel-1	CAEZ01004133:14608-14851
HmHel-1	CAEZ01004026:543-1145
HmHel-1	CAEZ01003939:15698-15930
HmHel-1	CAEZ01003904:1781-1975
HmHel-1	CAEZ01003554:3694-3909
HmHel-1	CAEZ01003543:3107-3324
HmHel-1	CAEZ01003358:1007-1239
HmHel-1	CAEZ01003229:278-493
HmHel-1	CAEZ01003228:3138-3353
HmHel-1	CAEZ01003217:20472-20702
HmHel-1	CAEZ01003196:86335-86552
HmHel-1	CAEZ01003196:34277-34365
HmHel-1	CAEZ01003175:4753-4984
HmHel-1	CAEZ01003023:1196-1413
HmHel-1	CAEZ01002838:86312-86524
HmHel-1	CAEZ01002803:8960-9048
HmHel-1	CAEZ01002744:34147-34227
HmHel-1	CAEZ01002744:21055-21143
HmHel-1	CAEZ01002737:1314-1538
HmHel-1	CAEZ01002586:367-600
HmHel-1	CAEZ01002379:4412-4661
HmHel-1	CAEZ01002379:2687-2772
HmHel-1	CAEZ01002363:57420-57508
HmHel-1	CAEZ01002363:50232-50430
HmHel-1	CAEZ01002132:1534-1736
HmHel-1	CAEZ01002066:13007-13196
HmHel-1	CAEZ01001914:8567-8782
HmHel-1	CAEZ01001771:14515-14718
HmHel-1	CAEZ01001634:5810-6053
HmHel-1	CAEZ01001595:124236-124437
HmHel-1	CAEZ01001579:17876-18045
HmHel-1	CAEZ01001579:60814-61057
HmHel-1	CAEZ01001564:35821-36049
HmHel-1	CAEZ01001564:16653-16861
HmHel-1	CAEZ01001564:1-353
HmHel-1	CAEZ01001465:3647-3890
HmHel-1	CAEZ01001465:81656-81886

HmHel-1	CAEZ01001301:16260-16482
HmHel-1	CAEZ01001222:6931-7160
HmHel-1	CAEZ01001174:11437-11652
HmHel-1	CAEZ01001140:4433-4669
HmHel-1	CAEZ01001129:25937-26141
HmHel-1	CAEZ01000835:13866-14109
HmHel-1	CAEZ01000681:1831-2046
HmHel-1	CAEZ01010857:3077-3295
HmHel-1	CAEZ01009828:5343-5575
HmHel-1	CAEZ01007977:8270-8502
HmHel-1	CAEZ01007747:167912-168133
HmHel-1	CAEZ01007747:29040-29284
HmHel-1	CAEZ01007556:3048-3270
HmHel-1	CAEZ01004570:5522-5705
HmHel-1	CAEZ01003797:31944-32144
HmHel-1	CAEZ01003472:7344-7565
HmHel-1	CAEZ01003353:1343-1570
HmHel-1	CAEZ01003353:9459-9655
HmHel-1	CAEZ01003212:2545-2768
HmHel-1	CAEZ01001043:1475-1718
HmHel-1	CAEZ01011126:34395-34595
HmHel-1	CAEZ01010256:35467-35553
HmHel-1	CAEZ01010144:74240-74410
HmHel-1	CAEZ01010144:78337-78539
HmHel-1	CAEZ01009799:2385-2973
HmHel-1	CAEZ01009440:61933-62137
HmHel-1	CAEZ01008428:2452-2655
HmHel-1	CAEZ01007886:3245-3488
HmHel-1	CAEZ01007630:933-1161
HmHel-1	CAEZ01005566:3-180
HmHel-1	CAEZ01005467:8439-8659
HmHel-1	CAEZ01003941:10170-10399
HmHel-1	CAEZ01003851:3186-3395
HmHel-1	CAEZ01002800:13116-13494
HmHel-1	CAEZ01001512:16011-16213
HmHel-1	CAEZ01000886:1094-1176
HmHel-1	CAEZ01000820:58053-58147
HmHel-1	CAEZ01011437:12327-12560
HmHel-1	CAEZ01010578:17023-17224
HmHel-1	CAEZ01010289:138141-138342
HmHel-1	CAEZ01009347:9646-9735
HmHel-1	CAEZ01008355:13023-13225
HmHel-1	CAEZ01007573:6439-6662
HmHel-1	CAEZ01007564:13461-13704
HmHel-1	CAEZ01007458:8379-8468
HmHel-1	CAEZ01007218:10705-10786
HmHel-1	CAEZ01005624:14173-14370
HmHel-1	CAEZ01002836:1450-1680

HmHel-1	CAEZ01001427:39-140
HmHel-1	CAEZ01001402:24292-24378
HmHel-1	CAEZ01000913:3135-3224
HmHel-1	CAEZ01000542:546-635
HmHel-1	CAEZ01000080:8523-8767
HmHel-1	CAEZ01011493:58643-58849
HmHel-1	CAEZ01011493:1691-1906
HmHel-1	CAEZ01011162:3189-3387
HmHel-1	CAEZ01011160:4232-4466
HmHel-1	CAEZ01010971:56718-56933
HmHel-1	CAEZ01010931:13992-14196
HmHel-1	CAEZ01010671:16174-16376
HmHel-1	CAEZ01010627:16328-16570
HmHel-1	CAEZ01010425:1-360
HmHel-1	CAEZ01010362:35800-35888
HmHel-1	CAEZ01010304:17166-17362
HmHel-1	CAEZ01010048:974-1206
HmHel-1	CAEZ01009586:24966-25194
HmHel-1	CAEZ01009586:52000-52224
HmHel-1	CAEZ01009528:2444-2532
HmHel-1	CAEZ01009482:7014-7289
HmHel-1	CAEZ01009291:19577-19665
HmHel-1	CAEZ01008846:7952-8169
HmHel-1	CAEZ01008610:7777-8020
HmHel-1	CAEZ01008610:4620-4841
HmHel-1	CAEZ01008446:961-1165
HmHel-1	CAEZ01008386:2052-2140
HmHel-1	CAEZ01008337:11744-11932
HmHel-1	CAEZ01008337:38402-38634
HmHel-1	CAEZ01008240:26818-27050
HmHel-1	CAEZ01007820:4669-4881
HmHel-1	CAEZ01007715:1625-1831
HmHel-1	CAEZ01007627:7804-7892
HmHel-1	CAEZ01007419:992-1080
HmHel-1	CAEZ01007226:1722-1931
HmHel-1	CAEZ01007059:1011-1205
HmHel-1	CAEZ01006912:7569-7772
HmHel-1	CAEZ01006843:509-728
HmHel-1	CAEZ01006780:8289-8525
HmHel-1	CAEZ01006664:14423-14627
HmHel-1	CAEZ01006650:19-206
HmHel-1	CAEZ01006569:9644-9732
HmHel-1	CAEZ01006398:35961-36164
HmHel-1	CAEZ01006308:29177-29265
HmHel-1	CAEZ01006199:877-1102
HmHel-1	CAEZ01006158:1788-2003
HmHel-1	CAEZ01006053:2532-2736
HmHel-1	CAEZ01006042:2105-2193

HmHel-1	CAEZ01006018:4295-4515
HmHel-1	CAEZ01006009:7941-8182
HmHel-1	CAEZ01005942:4899-5103
HmHel-1	CAEZ01005669:34869-34957
HmHel-1	CAEZ01005504:424-639
HmHel-1	CAEZ01005394:19743-19831
HmHel-1	CAEZ01005142:7267-7509
HmHel-1	CAEZ01004601:1721-1933
HmHel-1	CAEZ01004536:5187-5391
HmHel-1	CAEZ01004359:21404-21636
HmHel-1	CAEZ01004316:541-629
HmHel-1	CAEZ01004309:11385-11606
HmHel-1	CAEZ01003642:4330-4534
HmHel-1	CAEZ01003468:1762-1967
HmHel-1	CAEZ01003434:3167-3380
HmHel-1	CAEZ01003152:4213-4419
HmHel-1	CAEZ01002950:32811-33026
HmHel-1	CAEZ01002825:13621-13709
HmHel-1	CAEZ01002670:11462-11712
HmHel-1	CAEZ01002650:44158-44401
HmHel-1	CAEZ01002360:9656-9744
HmHel-1	CAEZ01001616:7729-7948
HmHel-1	CAEZ01001556:9638-9855
HmHel-1	CAEZ01001473:933-1021
HmHel-1	CAEZ01001351:11072-11328
HmHel-1	CAEZ01001351:4957-5170
HmHel-1	CAEZ01001300:2947-3035
HmHel-1	CAEZ01000830:8932-9162
HmHel-1	CAEZ01000682:6550-6695
HmHel-1	CAEZ01000124:1587-1805
HmHel-1	CAEZ01011473:10915-11115
HmHel-1	CAEZ01010772:3083-3325
HmHel-1	CAEZ01008688:20631-20839
HmHel-1	CAEZ01008183:1113-1349
HmHel-1	CAEZ01007893:1545-1754
HmHel-1	CAEZ01006659:8356-8599
HmHel-1	CAEZ01006624:14553-14907
HmHel-1	CAEZ01006421:13261-13487
HmHel-1	CAEZ01006027:26072-26650
HmHel-1	CAEZ01003079:12168-12815
HmHel-1	CAEZ01011650:4559-4783
HmHel-1	CAEZ01011356:178-379
HmHel-1	CAEZ01008857:6350-6567
HmHel-1	CAEZ01008543:8798-9155
HmHel-1	CAEZ01008516:10120-10351
HmHel-1	CAEZ01008067:11397-11574
HmHel-1	CAEZ01005210:3828-3914
HmHel-1	CAEZ01003496:4548-4781

HmHel-1	CAEZ01001472:5009-5100
HmHel-1	CAEZ01000035:2386-2590
HmHel-1	CAEZ01010765:7509-7598
HmHel-1	CAEZ01007420:4227-4445
HmHel-1	CAEZ01007035:881-1121
HmHel-1	CAEZ01005986:101107-101309
HmHel-1	CAEZ01005848:2435-2636
HmHel-1	CAEZ01005198:26409-26598
HmHel-1	CAEZ01004046:9-239
HmHel-1	CAEZ01003907:18718-18908
HmHel-1	CAEZ01002570:948-1144
HmHel-1	CAEZ01001672:10-404
HmHel-1	CAEZ01001375:2473-2708
HmHel-1	CAEZ01001123:9045-9231
HmHel-1	CAEZ01011653:28-140
HmHel-1	CAEZ01011217:29191-29405
HmHel-1	CAEZ01011006:1584-1816
HmHel-1	CAEZ01010423:20437-20665
HmHel-1	CAEZ01010208:11913-12001
HmHel-1	CAEZ01010195:8810-9362
HmHel-1	CAEZ01010189:14519-14762
HmHel-1	CAEZ01010116:2460-3065
HmHel-1	CAEZ01010106:2649-2895
HmHel-1	CAEZ01009776:37819-37907
HmHel-1	CAEZ01009742:23070-23285
HmHel-1	CAEZ01009556:13360-13594
HmHel-1	CAEZ01009310:16513-16625
HmHel-1	CAEZ01009274:8069-8290
HmHel-1	CAEZ01008933:13939-14027
HmHel-1	CAEZ01008342:33283-33609
HmHel-1	CAEZ01007995:7091-7291
HmHel-1	CAEZ01007944:2045-2283
HmHel-1	CAEZ01007921:11483-11670
HmHel-1	CAEZ01007636:2310-2514
HmHel-1	CAEZ01007504:7619-7707
HmHel-1	CAEZ01007238:3997-4212
HmHel-1	CAEZ01006461:16887-16975
HmHel-1	CAEZ01006427:493-708
HmHel-1	CAEZ01006068:8566-8765
HmHel-1	CAEZ01005834:19865-20064
HmHel-1	CAEZ01005811:5242-5456
HmHel-1	CAEZ01005807:1060-1277
HmHel-1	CAEZ01005710:1842-1954
HmHel-1	CAEZ01005703:5358-5539
HmHel-1	CAEZ01005519:5085-5289
HmHel-1	CAEZ01005370:8695-8779
HmHel-1	CAEZ01005205:39484-39680
HmHel-1	CAEZ01005113:5767-5855

HmHel-1	CAEZ01004972:23318-31200
HmHel-1	CAEZ01004955:8373-8577
HmHel-1	CAEZ01004634:2378-2611
HmHel-1	CAEZ01004198:36569-36773
HmHel-1	CAEZ01003931:3764-3978
HmHel-1	CAEZ01003801:5834-5922
HmHel-1	CAEZ01003059:27178-27407
HmHel-1	CAEZ01003056:24412-24524
HmHel-1	CAEZ01003056:3062-3286
HmHel-1	CAEZ01003041:5856-6097
HmHel-1	CAEZ01002971:13373-13461
HmHel-1	CAEZ01002788:34612-34843
HmHel-1	CAEZ01002635:50709-50943
HmHel-1	CAEZ01001990:2147-2259
HmHel-1	CAEZ01001805:19605-19820
HmHel-1	CAEZ01001768:1842-2046
HmHel-1	CAEZ01001747:10331-10549
HmHel-1	CAEZ01001018:6316-6570
HmHel-1	CAEZ01010609:9739-10484
HmHel-1	CAEZ01008468:409-621
HmHel-1	CAEZ01007195:18215-18605
HmHel-1	CAEZ01006617:5004-5247
HmHel-1	CAEZ01005996:8988-9195
HmHel-1	CAEZ01005704:8789-8981
HmHel-1	CAEZ01005704:10358-10580
HmHel-1	CAEZ01004215:5988-6196
HmHel-1	CAEZ01003852:25935-26168
HmHel-1	CAEZ01003139:2856-3101
HmHel-1	CAEZ01002361:11639-11846
HmHel-1	CAEZ01011340:12675-12995
HmHel-1	CAEZ01008761:4736-4964
HmHel-1	CAEZ01007073:6776-7010
HmHel-1	CAEZ01005474:23558-23780
HmHel-1	CAEZ01004731:2921-3146
HmHel-1	CAEZ01004054:6669-8261
HmHel-1	CAEZ01004053:3009-3099
HmHel-1	CAEZ01003875:14143-14585
HmHel-1	CAEZ01003209:8978-10028
HmHel-1	CAEZ01002708:25491-25695
HmHel-1	CAEZ01002341:7973-8395
HmHel-1	CAEZ01011511:1-264
HmHel-1	CAEZ01011361:5146-5352
HmHel-1	CAEZ01010560:4656-4877
HmHel-1	CAEZ01010019:518-759
HmHel-1	CAEZ01010016:10434-10523
HmHel-1	CAEZ01009623:10425-10506
HmHel-1	CAEZ01008794:10028-10238
HmHel-1	CAEZ01008772:13005-13098

HmHel-1	CAEZ01008417:34936-35179
HmHel-1	CAEZ01008331:11957-12078
HmHel-1	CAEZ01008128:725-855
HmHel-1	CAEZ01007930:34097-34327
HmHel-1	CAEZ01007077:4111-4339
HmHel-1	CAEZ01004632:13127-13350
HmHel-1	CAEZ01004632:71547-71648
HmHel-1	CAEZ01004632:20686-20934
HmHel-1	CAEZ01004587:3991-4130
HmHel-1	CAEZ01004487:1967-2072
HmHel-1	CAEZ01003416:51942-52125
HmHel-1	CAEZ01001999:860-1166
HmHel-1	CAEZ01001582:1031-1136
HmHel-1	CAEZ01010580:14009-14121
HmHel-1	CAEZ01009772:48062-48286
HmHel-1	CAEZ01009736:3126-3320
HmHel-1	CAEZ01009636:7050-7211
HmHel-1	CAEZ01009487:45498-45730
HmHel-1	CAEZ01008705:10718-10932
HmHel-1	CAEZ01008434:7514-7626
HmHel-1	CAEZ01007640:587-809
HmHel-1	CAEZ01007442:8214-8406
HmHel-1	CAEZ01007262:23-353
HmHel-1	CAEZ01006332:68901-69101
HmHel-1	CAEZ01005470:14637-14914
HmHel-1	CAEZ01005047:10486-10820
HmHel-1	CAEZ01004508:3006-3253
HmHel-1	CAEZ01004319:629-717
HmHel-1	CAEZ01004128:9589-9814
HmHel-1	CAEZ01003932:10024-10112
HmHel-1	CAEZ01003322:7215-7416
HmHel-1	CAEZ01002730:5344-5545
HmHel-1	CAEZ01002730:1078-1254
HmHel-1	CAEZ01002307:324-523
HmHel-1	CAEZ01001124:625-840
HmHel-1	CAEZ01011421:16193-16409
HmHel-1	CAEZ01010266:7458-7662
HmHel-1	CAEZ01008683:8205-8345
HmHel-1	CAEZ01005396:11225-11412
HmHel-1	CAEZ01010161:2920-3136
HmHel-1	CAEZ01010049:13294-13489
HmHel-1	CAEZ01009302:790-1206
HmHel-1	CAEZ01007129:4998-5241
HmHel-1	CAEZ01005953:167-372
HmHel-1	CAEZ01005805:2313-2510
HmHel-1	CAEZ01005524:2401-2503
HmHel-1	CAEZ01002712:8890-9120
HmHel-1	CAEZ01002449:219-326

HmHel-1	CAEZ01002122:3517-3725
HmHel-1	CAEZ01002074:6808-7012
HmHel-1	CAEZ01001576:10767-10982
HmHel-1	CAEZ01000716:9498-9683
HmHel-1	CAEZ01010486:4379-4607
HmHel-1	CAEZ01008389:8002-8211
HmHel-1	CAEZ01007551:12444-12682
HmHel-1	CAEZ01007416:5389-5561
HmHel-1	CAEZ01005939:14588-14781
HmHel-1	CAEZ01004120:3712-3802
HmHel-1	CAEZ01003730:15285-15462
HmHel-1	CAEZ01001159:9637-9860
HmHel-1	CAEZ01010439:41607-41822
HmHel-1	CAEZ01009876:24074-24162
HmHel-1	CAEZ01009542:25-137
HmHel-1	CAEZ01009269:59296-59483
HmHel-1	CAEZ01008948:3504-3702
HmHel-1	CAEZ01008455:20232-20421
HmHel-1	CAEZ01007979:106-372
HmHel-1	CAEZ01007626:115-227
HmHel-1	CAEZ01007560:6850-7083
HmHel-1	CAEZ01007381:4376-4569
HmHel-1	CAEZ01006286:104909-105086
HmHel-1	CAEZ01005344:5150-5362
HmHel-1	CAEZ01005282:15939-16172
HmHel-1	CAEZ01004949:7468-7686
HmHel-1	CAEZ01004212:4569-4907
HmHel-1	CAEZ01004126:40544-40910
HmHel-1	CAEZ01003075:24288-24507
HmHel-1	CAEZ01002152:3787-4140
HmHel-1	CAEZ01011195:50771-50988
HmHel-1	CAEZ01010869:73278-73499
HmHel-1	CAEZ01010863:14469-14818
HmHel-1	CAEZ01010757:10512-10731
HmHel-1	CAEZ01009976:4879-5070
HmHel-1	CAEZ01009557:9413-9632
HmHel-1	CAEZ01009432:5348-5539
HmHel-1	CAEZ01008695:12009-12217
HmHel-1	CAEZ01007539:6683-6913
HmHel-1	CAEZ01007235:37655-37862
HmHel-1	CAEZ01006235:582-1101
HmHel-1	CAEZ01005584:2988-3224
HmHel-1	CAEZ01004137:11152-11363
HmHel-1	CAEZ01003177:3169-3374
HmHel-1	CAEZ01000963:22598-22784
HmHel-1	CAEZ01009775:15188-15281
HmHel-1	CAEZ01009767:8575-8779
HmHel-1	CAEZ01007327:7779-7957

HmHel-1	CAEZ01006413:15198-15324
HmHel-1	CAEZ01003300:29118-29228
HmHel-1	CAEZ01002501:38138-38363
HmHel-1	CAEZ01002060:1290-1790
HmHel-1	CAEZ01001838:4984-5460
HmHel-1	CAEZ01001251:1451-1629
HmHel-1	CAEZ01001193:7891-8106
HmHel-1	CAEZ01011227:16134-18122
HmHel-1	CAEZ01010963:4416-4629
HmHel-1	CAEZ01010182:16563-16807
HmHel-1	CAEZ01008278:8182-8414
HmHel-1	CAEZ01006839:15324-15521
HmHel-1	CAEZ01005463:1238-1453
HmHel-1	CAEZ01005075:757-976
HmHel-1	CAEZ01005007:4729-4909
HmHel-1	CAEZ01003945:6172-6402
HmHel-1	CAEZ01003455:1583-1830
HmHel-1	CAEZ01002986:15591-15789
HmHel-1	CAEZ01011032:24695-24911
HmHel-1	CAEZ01010546:1-189
HmHel-1	CAEZ01010342:11334-11567
HmHel-1	CAEZ01008887:2831-2967
HmHel-1	CAEZ01008628:3011-3217
HmHel-1	CAEZ01008412:7934-8165
HmHel-1	CAEZ01007089:19618-19845
HmHel-1	CAEZ01006969:854-946
HmHel-1	CAEZ01006382:4234-4425
HmHel-1	CAEZ01005855:7781-7989
HmHel-1	CAEZ01005090:2904-3135
HmHel-1	CAEZ01005061:9291-9383
HmHel-1	CAEZ01004457:10363-10593
HmHel-1	CAEZ01001764:20194-20424
HmHel-1	CAEZ01001075:1032-1238
HmHel-1	CAEZ01011373:14160-14388
HmHel-1	CAEZ01011063:19082-19275
HmHel-1	CAEZ01010503:4710-4912
HmHel-1	CAEZ01009957:12931-13147
HmHel-1	CAEZ01007594:2263-2353
HmHel-1	CAEZ01006973:8027-8247
HmHel-1	CAEZ01006521:4288-4370
HmHel-1	CAEZ01004617:13649-13847
HmHel-1	CAEZ01004402:817-1008
HmHel-1	CAEZ01003555:193-413
HmHel-1	CAEZ01001843:9298-9486
HmHel-1	CAEZ01001529:507-636
HmHel-1	CAEZ01001364:1397-1649
HmHel-1	CAEZ01009592:11359-12829
HmHel-1	CAEZ01008977:7872-8081

HmHel-1	CAEZ01006857:385-581
HmHel-1	CAEZ01005067:37773-37982
HmHel-1	CAEZ01001074:21519-21718
HmHel-1	CAEZ01011241:7278-7482
HmHel-1	CAEZ01010214:10223-10320
HmHel-1	CAEZ01008461:1824-2025
HmHel-1	CAEZ01008399:12754-12907
HmHel-1	CAEZ01007654:2359-2456
HmHel-1	CAEZ01003855:20365-20592
HmHel-1	CAEZ01003526:3548-4068
HmHel-1	CAEZ01001599:6472-6568
HmHel-1	CAEZ01000746:509-692
HmHel-1	CAEZ01000069:3014-3102
HmHel-1	CAEZ01000048:42494-42591
HmHel-1	CAEZ01011277:5711-5807
HmHel-1	CAEZ01011184:6846-7066
HmHel-1	CAEZ01008264:6588-6787
HmHel-1	CAEZ01007655:1477-1573
HmHel-1	CAEZ01006320:21002-21087
HmHel-1	CAEZ01005565:6541-6919
HmHel-1	CAEZ01005464:20-355
HmHel-1	CAEZ01005343:19057-19240
HmHel-1	CAEZ01004441:824-1064
HmHel-1	CAEZ01000877:3380-3604
HmHel-1	CAEZ01005322:8194-8395
HmHel-1	CAEZ01003826:37443-37526
HmHel-1	CAEZ01003403:6223-6414
HmHel-1	CAEZ01001755:1688-1876
HmHel-1	CAEZ01001729:9153-10091
HmHel-1	CAEZ01011590:9329-9514
HmHel-1	CAEZ01009495:27494-27698
HmHel-1	CAEZ01005503:8653-8855
HmHel-1	CAEZ01003031:5793-5985
HmHel-1	CAEZ01001146:404-796
HmHel-1	CAEZ01011058:11408-11627
HmHel-1	CAEZ01000601:5148-5234
HmHel-1	CAEZ01010995:28782-28894
HmHel-1	CAEZ01009061:7498-7582
HmHel-1	CAEZ01008174:9-93
HmHel-1	CAEZ01006726:1750-1834
HmHel-1	CAEZ01006609:14-352
HmHel-1	CAEZ01004509:123-207
HmHel-1	CAEZ01003113:3281-3365
HmHel-1	CAEZ01001322:56-140
HmHel-1	CAEZ01001199:10445-10529
HmHel-1	CAEZ01000900:964-1205
HmHel-1	CAEZ01000646:840-919
HmHel-1	CAEZ01007819:2909-2988

HmHel-1	CAEZ01003617:24459-24544
HmHel-1	CAEZ01003564:4530-4743
HmHel-1	CAEZ01011472:13306-13386
HmHel-1	CAEZ01005117:568-663
HmHel-1	CAEZ01004999:9145-9247
	:-
HmHel-2	CAEZ01007112:7224-7819
HmHel-2	CAEZ01011282:7959-8554
HmHel-2	CAEZ01011282:112757-113213
HmHel-2	CAEZ01011282:168704-169262
HmHel-2	CAEZ01009627:20824-21418
HmHel-2	CAEZ01005946:156616-157201
HmHel-2	CAEZ01005946:120682-120955
HmHel-2	CAEZ01005946:108910-109252
HmHel-2	CAEZ01011137:102148-102739
HmHel-2	CAEZ01011137:177940-178157
HmHel-2	CAEZ01011137:88866-89325
HmHel-2	CAEZ01004495:3045-3639
HmHel-2	CAEZ01009154:63346-63940
HmHel-2	CAEZ01009154:2513-2834
HmHel-2	CAEZ01011489:62015-62610
HmHel-2	CAEZ01011489:38499-39094
HmHel-2	CAEZ01009804:65497-66092
HmHel-2	CAEZ01009804:106068-106251
HmHel-2	CAEZ01009804:94931-95030
HmHel-2	CAEZ01009778:101735-102330
HmHel-2	CAEZ01009778:116384-116842
HmHel-2	CAEZ01009778:172306-172901
HmHel-2	CAEZ01009778:231615-232178
HmHel-2	CAEZ01009778:244099-244698
HmHel-2	CAEZ01009778:298777-299070
HmHel-2	CAEZ01009778:369643-369873
HmHel-2	CAEZ01009778:391824-392419
HmHel-2	CAEZ01009778:378808-379267
HmHel-2	CAEZ01009778:266115-267038
HmHel-2	CAEZ01009778:202529-202987
HmHel-2	CAEZ01009778:169773-170233
HmHel-2	CAEZ01009778:113126-113220
HmHel-2	CAEZ01009778:46393-46571
HmHel-2	CAEZ01007911:29343-29622
HmHel-2	CAEZ01007911:11582-12177
HmHel-2	CAEZ01006804:137225-137662
HmHel-2	CAEZ01006804:130770-131357
HmHel-2	CAEZ01005830:160258-160716
HmHel-2	CAEZ01005830:12971-13566
HmHel-2	CAEZ01004735:18093-18687
HmHel-2	CAEZ01004735:26591-27046
HmHel-2	CAEZ01004735:7438-8032

HmHel-2	CAEZ01003784:1639-2232
HmHel-2	CAEZ01003784:47681-48269
HmHel-2	CAEZ01003717:23839-30255
HmHel-2	CAEZ01003717:78558-79147
HmHel-2	CAEZ01003717:95741-96331
HmHel-2	CAEZ01003717:66820-67386
HmHel-2	CAEZ01001398:1661-2256
HmHel-2	CAEZ01010030:38754-39206
HmHel-2	CAEZ01010030:89198-89666
HmHel-2	CAEZ01010030:112098-112556
HmHel-2	CAEZ01010030:261678-262145
HmHel-2	CAEZ01010030:297985-298299
HmHel-2	CAEZ01010030:197788-198379
HmHel-2	CAEZ01010972:100544-100996
HmHel-2	CAEZ01010972:422728-423083
HmHel-2	CAEZ01010972:458259-458572
HmHel-2	CAEZ01010972:429010-429288
HmHel-2	CAEZ01010972:315680-316275
HmHel-2	CAEZ01010972:3228-3417
HmHel-2	CAEZ01004261:17725-18320
HmHel-2	CAEZ01003338:28357-28952
HmHel-2	CAEZ01003338:33584-34134
HmHel-2	CAEZ01001517:8062-8657
HmHel-2	CAEZ01008710:81450-81888
HmHel-2	CAEZ01008710:101205-101799
HmHel-2	CAEZ01008710:46812-52080
HmHel-2	CAEZ01007427:17572-17718
HmHel-2	CAEZ01007427:22777-22986
HmHel-2	CAEZ01006202:19872-20466
HmHel-2	CAEZ01005150:2037-2597
HmHel-2	CAEZ01009138:355-951
HmHel-2	CAEZ01009138:25172-26445
HmHel-2	CAEZ01005087:18618-19209
HmHel-2	CAEZ01002744:13477-14072
HmHel-2	CAEZ01002744:33590-34143
HmHel-2	CAEZ01002744:6080-6577
HmHel-2	CAEZ01000045:13883-14476
HmHel-2	CAEZ01011567:5334-5928
HmHel-2	CAEZ01011567:4757-5058
HmHel-2	CAEZ01011506:2551-3145
HmHel-2	CAEZ01010484:31373-31968
HmHel-2	CAEZ01009974:26971-27566
HmHel-2	CAEZ01009974:1565-2080
HmHel-2	CAEZ01009020:17698-18285
HmHel-2	CAEZ01008886:32681-32967
HmHel-2	CAEZ01008886:16129-16724
HmHel-2	CAEZ01008466:1694-2289
HmHel-2	CAEZ01008466:377-972

HmHel-2	CAEZ01006541:28306-28901
HmHel-2	CAEZ01006392:41146-41706
HmHel-2	CAEZ01006392:77791-78386
HmHel-2	CAEZ01006392:107317-107912
HmHel-2	CAEZ01001431:2312-2907
HmHel-2	CAEZ01009025:47091-47412
HmHel-2	CAEZ01005269:1620-1808
HmHel-2	CAEZ01005269:42210-43095
HmHel-2	CAEZ01005269:144038-144480
HmHel-2	CAEZ01005269:126320-126883
HmHel-2	CAEZ01002838:40703-41152
HmHel-2	CAEZ01002838:81931-82525
HmHel-2	CAEZ01002838:38154-38470
HmHel-2	CAEZ01002838:23149-23709
HmHel-2	CAEZ01002838:7720-8317
HmHel-2	CAEZ01007706:65396-65826
HmHel-2	CAEZ01007706:119939-120178
HmHel-2	CAEZ01007706:159849-160177
HmHel-2	CAEZ01007706:200727-201323
HmHel-2	CAEZ01007706:235382-235839
HmHel-2	CAEZ01007706:277548-277658
HmHel-2	CAEZ01007706:273658-274106
HmHel-2	CAEZ01007706:161540-161969
HmHel-2	CAEZ01007706:112295-112848
HmHel-2	CAEZ01007706:84654-84880
HmHel-2	CAEZ01005439:177590-178182
HmHel-2	CAEZ01005439:101594-102011
HmHel-2	CAEZ01005439:76128-76717
HmHel-2	CAEZ01005409:14234-14677
HmHel-2	CAEZ01005409:46824-47416
HmHel-2	CAEZ01005409:31509-31965
HmHel-2	CAEZ01003080:119424-120019
HmHel-2	CAEZ01003080:160434-160890
HmHel-2	CAEZ01003080:49165-49757
HmHel-2	CAEZ01009691:70339-70934
HmHel-2	CAEZ01009691:57060-57657
HmHel-2	CAEZ01009609:19070-19665
HmHel-2	CAEZ01008596:1562-2157
HmHel-2	CAEZ01008263:34834-35293
HmHel-2	CAEZ01008091:29724-30319
HmHel-2	CAEZ01008091:65241-65699
HmHel-2	CAEZ01008034:8148-8725
HmHel-2	CAEZ01008034:24267-24862
HmHel-2	CAEZ01007222:6703-6983
HmHel-2	CAEZ01007222:17947-18540
HmHel-2	CAEZ01007222:199193-199513
HmHel-2	CAEZ01007222:234752-234957
HmHel-2	CAEZ01007222:262971-263429

HmHel-2	CAEZ01007222:246576-246854
HmHel-2	CAEZ01007222:234419-234725
HmHel-2	CAEZ01007222:50098-50419
HmHel-2	CAEZ01006283:35905-36364
HmHel-2	CAEZ01006283:62071-62492
HmHel-2	CAEZ01006283:27913-28508
HmHel-2	CAEZ01006063:19717-20312
HmHel-2	CAEZ01005659:13738-14332
HmHel-2	CAEZ01005659:9769-10340
HmHel-2	CAEZ01005272:24943-25090
HmHel-2	CAEZ01005067:7319-7754
HmHel-2	CAEZ01005067:139112-139706
HmHel-2	CAEZ01004088:1130-1725
HmHel-2	CAEZ01004088:3317-4054
HmHel-2	CAEZ01003033:1818-2412
HmHel-2	CAEZ01009929:15101-15689
HmHel-2	CAEZ01009990:18077-18530
HmHel-2	CAEZ01009990:44294-44887
HmHel-2	CAEZ01009990:31765-32048
HmHel-2	CAEZ01009982:71071-71528
HmHel-2	CAEZ01009982:85169-85763
HmHel-2	CAEZ01009982:102225-102813
HmHel-2	CAEZ01009982:30717-31310
HmHel-2	CAEZ01009982:14318-14910
HmHel-2	CAEZ01008359:163163-163759
HmHel-2	CAEZ01008359:107151-107740
HmHel-2	CAEZ01005587:1188-1784
HmHel-2	CAEZ01005451:64740-64840
HmHel-2	CAEZ01005451:115494-116047
HmHel-2	CAEZ01005451:99497-100089
HmHel-2	CAEZ01002413:563-1020
HmHel-2	CAEZ01002413:20071-20671
HmHel-2	CAEZ01002413:92038-92288
HmHel-2	CAEZ01002413:63736-64334
HmHel-2	CAEZ01002413:49628-50085
HmHel-2	CAEZ01010270:1958-2551
HmHel-2	CAEZ01010270:164833-165428
HmHel-2	CAEZ01010270:214357-214909
HmHel-2	CAEZ01010270:193209-193804
HmHel-2	CAEZ01010270:121895-122487
HmHel-2	CAEZ01010270:12258-12715
HmHel-2	CAEZ01009673:9254-9849
HmHel-2	CAEZ01008427:106853-107443
HmHel-2	CAEZ01008427:96785-97246
HmHel-2	CAEZ01008427:78666-79214
HmHel-2	CAEZ01008427:35639-36216
HmHel-2	CAEZ01008427:16396-16854
HmHel-2	CAEZ01006660:3704-4298

HmHel-2	CAEZ01005270:35427-35886
HmHel-2	CAEZ01005270:22463-22916
HmHel-2	CAEZ01005270:6722-7179
HmHel-2	CAEZ01002876:25976-26603
HmHel-2	CAEZ01002876:135398-135856
HmHel-2	CAEZ01002876:20834-21429
HmHel-2	CAEZ01002658:48673-49268
HmHel-2	CAEZ01002658:12436-13028
HmHel-2	CAEZ01006675:73243-73544
HmHel-2	CAEZ01006675:47296-47890
HmHel-2	CAEZ01006367:167436-167888
HmHel-2	CAEZ01006367:93175-93295
HmHel-2	CAEZ01007808:10659-11252
HmHel-2	CAEZ01009017:78755-79351
HmHel-2	CAEZ01009017:59653-60015
HmHel-2	CAEZ01009017:45969-46529
HmHel-2	CAEZ01005239:10545-11137
HmHel-2	CAEZ01010621:8460-9055
HmHel-2	CAEZ01008718:30140-30735
HmHel-2	CAEZ01008718:5026-5373
HmHel-2	CAEZ01007751:55961-56556
HmHel-2	CAEZ01007751:113952-114400
HmHel-2	CAEZ01007751:54756-55210
HmHel-2	CAEZ01007751:14789-15214
HmHel-2	CAEZ01007624:8698-8873
HmHel-2	CAEZ01007624:55599-56058
HmHel-2	CAEZ01007624:4881-5473
HmHel-2	CAEZ01006576:678-1273
HmHel-2	CAEZ01006566:41624-42219
HmHel-2	CAEZ01006150:143-738
HmHel-2	CAEZ01005868:13892-14487
HmHel-2	CAEZ01005868:1677-2116
HmHel-2	CAEZ01005827:18653-19248
HmHel-2	CAEZ01005605:32691-33286
HmHel-2	CAEZ01005605:11677-12233
HmHel-2	CAEZ01005431:7952-8547
HmHel-2	CAEZ01004097:10673-11130
HmHel-2	CAEZ01004097:238403-239161
HmHel-2	CAEZ01004097:191866-192427
HmHel-2	CAEZ01004097:32877-33472
HmHel-2	CAEZ01003595:1333-1928
HmHel-2	CAEZ01002231:12449-13044
HmHel-2	CAEZ01000850:6603-6687
HmHel-2	CAEZ01000850:32215-32672
HmHel-2	CAEZ01000850:90069-90651
HmHel-2	CAEZ01000850:21365-21550
HmHel-2	CAEZ01007108:7358-7815
HmHel-2	CAEZ01007108:87884-88478

HmHel-2	CAEZ01007108:111258-111999
HmHel-2	CAEZ01007108:134973-135568
HmHel-2	CAEZ01007108:80367-84578
HmHel-2	CAEZ01003192:107289-107540
HmHel-2	CAEZ01003192:125469-125707
HmHel-2	CAEZ01003192:100916-101510
HmHel-2	CAEZ01003192:10938-11535
HmHel-2	CAEZ01010617:36616-37213
HmHel-2	CAEZ01010617:51380-51698
HmHel-2	CAEZ01010617:15815-16411
HmHel-2	CAEZ01008169:914-999
HmHel-2	CAEZ01008169:37681-38271
HmHel-2	CAEZ01004826:28412-28520
HmHel-2	CAEZ01004826:69365-69956
HmHel-2	CAEZ01004631:137840-138433
HmHel-2	CAEZ01004631:257103-257696
HmHel-2	CAEZ01004631:297301-297759
HmHel-2	CAEZ01004631:267749-268207
HmHel-2	CAEZ01004631:220679-221130
HmHel-2	CAEZ01004631:44663-45094
HmHel-2	CAEZ01003276:34919-35515
HmHel-2	CAEZ01003276:56167-56292
HmHel-2	CAEZ01011207:19-474
HmHel-2	CAEZ01011207:16726-17320
HmHel-2	CAEZ01010894:105605-106165
HmHel-2	CAEZ01010894:172812-173275
HmHel-2	CAEZ01010894:129065-129660
HmHel-2	CAEZ01010894:22867-23325
HmHel-2	CAEZ01010740:45549-45896
HmHel-2	CAEZ01010740:29448-30042
HmHel-2	CAEZ01010392:5706-6301
HmHel-2	CAEZ01010285:2380-2975
HmHel-2	CAEZ01010144:107675-108270
HmHel-2	CAEZ01010144:71634-71757
HmHel-2	CAEZ01010144:34860-35316
HmHel-2	CAEZ01010130:151849-152395
HmHel-2	CAEZ01010130:130424-130851
HmHel-2	CAEZ01010130:93555-94004
HmHel-2	CAEZ01010130:42760-43355
HmHel-2	CAEZ01009540:8967-9562
HmHel-2	CAEZ01009484:78420-79015
HmHel-2	CAEZ01009484:92764-92943
HmHel-2	CAEZ01009484:59909-60230
HmHel-2	CAEZ01009484:45801-46245
HmHel-2	CAEZ01008732:38942-39387
HmHel-2	CAEZ01008732:62364-62453
HmHel-2	CAEZ01008732:122359-122953
HmHel-2	CAEZ01008732:102749-103207

HmHel-2	CAEZ01008732:85009-85467
HmHel-2	CAEZ01008732:5688-6283
HmHel-2	CAEZ01006483:85-680
HmHel-2	CAEZ01006483:4273-4828
HmHel-2	CAEZ01005493:7416-8011
HmHel-2	CAEZ01004432:48622-49217
HmHel-2	CAEZ01004315:2086-2680
HmHel-2	CAEZ01003668:766-1361
HmHel-2	CAEZ01003409:22332-22927
HmHel-2	CAEZ01003409:34712-35138
HmHel-2	CAEZ01003409:39927-40517
HmHel-2	CAEZ01003401:174100-174289
HmHel-2	CAEZ01003401:199144-199601
HmHel-2	CAEZ01003401:211588-212183
HmHel-2	CAEZ01003401:37370-37799
HmHel-2	CAEZ01003091:803-1266
HmHel-2	CAEZ01003091:14453-15048
HmHel-2	CAEZ01003091:18639-19194
HmHel-2	CAEZ01002049:92885-93205
HmHel-2	CAEZ01002049:20492-21039
HmHel-2	CAEZ01002049:6710-7297
HmHel-2	CAEZ01001564:33873-34468
HmHel-2	CAEZ01001356:26563-27158
HmHel-2	CAEZ01001356:23896-24228
HmHel-2	CAEZ01008393:115794-116379
HmHel-2	CAEZ01008393:151724-152320
HmHel-2	CAEZ01008393:88778-89355
HmHel-2	CAEZ01008393:36321-36590
HmHel-2	CAEZ01007246:1203-1797
HmHel-2	CAEZ01007234:2721-3304
HmHel-2	CAEZ01007234:51102-51697
HmHel-2	CAEZ01007234:43199-43648
HmHel-2	CAEZ01007234:25567-26162
HmHel-2	CAEZ01005745:1304-1898
HmHel-2	CAEZ01004122:13029-13625
HmHel-2	CAEZ01000878:28923-29517
HmHel-2	CAEZ01008966:14435-15012
HmHel-2	CAEZ01008966:44598-45056
HmHel-2	CAEZ01008966:11973-12429
HmHel-2	CAEZ01008960:13548-13848
HmHel-2	CAEZ01008960:180597-181187
HmHel-2	CAEZ01008960:138362-138684
HmHel-2	CAEZ01007469:53312-53904
HmHel-2	CAEZ01007469:12999-13317
HmHel-2	CAEZ01006394:6710-7301
HmHel-2	CAEZ01005316:23884-24341
HmHel-2	CAEZ01005316:5361-5950
HmHel-2	CAEZ01004368:78488-79048

HmHel-2	CAEZ01004368:54138-54731
HmHel-2	CAEZ01004368:35233-35537
HmHel-2	CAEZ01003997:6332-6929
HmHel-2	CAEZ01002218:1418-2014
HmHel-2	CAEZ01011471:6002-6597
HmHel-2	CAEZ01011163:3529-4124
HmHel-2	CAEZ01011069:1-239
HmHel-2	CAEZ01011069:11327-11922
HmHel-2	CAEZ01010734:97844-98400
HmHel-2	CAEZ01010594:25579-26173
HmHel-2	CAEZ01009077:121027-121487
HmHel-2	CAEZ01009077:204941-205535
HmHel-2	CAEZ01009077:167996-168458
HmHel-2	CAEZ01009077:44487-44937
HmHel-2	CAEZ01009077:19314-19915
HmHel-2	CAEZ01009064:5363-5958
HmHel-2	CAEZ01008287:4451-5046
HmHel-2	CAEZ01008208:66268-66857
HmHel-2	CAEZ01008208:157418-157876
HmHel-2	CAEZ01008208:223046-223504
HmHel-2	CAEZ01008208:224995-225554
HmHel-2	CAEZ01008208:198632-199090
HmHel-2	CAEZ01008208:153531-153781
HmHel-2	CAEZ01008208:109875-110326
HmHel-2	CAEZ01008149:4402-4996
HmHel-2	CAEZ01007974:126743-127201
HmHel-2	CAEZ01007974:162030-162431
HmHel-2	CAEZ01007974:147465-148060
HmHel-2	CAEZ01007974:74294-74754
HmHel-2	CAEZ01007974:13202-13798
HmHel-2	CAEZ01007189:8704-9299
HmHel-2	CAEZ01007127:4019-4613
HmHel-2	CAEZ01006032:95-689
HmHel-2	CAEZ01004355:8903-9498
HmHel-2	CAEZ01003487:6-134
HmHel-2	CAEZ01003487:11014-11604
HmHel-2	CAEZ01003487:23024-23158
HmHel-2	CAEZ01003348:1422-1711
HmHel-2	CAEZ01003348:83821-84416
HmHel-2	CAEZ01003348:80311-80769
HmHel-2	CAEZ01003348:49039-49587
HmHel-2	CAEZ01003171:22818-23413
HmHel-2	CAEZ01002870:24423-25018
HmHel-2	CAEZ01002501:5890-6485
HmHel-2	CAEZ01002139:2577-3172
HmHel-2	CAEZ01002083:2305-2900
HmHel-2	CAEZ01001741:11738-12333
HmHel-2	CAEZ01011179:24332-24919

HmHel-2	CAEZ01009887:33346-33909
HmHel-2	CAEZ01009887:228440-229043
HmHel-2	CAEZ01009887:227042-227513
HmHel-2	CAEZ01009887:206911-207505
HmHel-2	CAEZ01009887:132433-132864
HmHel-2	CAEZ01009887:26658-27204
HmHel-2	CAEZ01007184:114566-115024
HmHel-2	CAEZ01007184:149039-149367
HmHel-2	CAEZ01007184:218382-218703
HmHel-2	CAEZ01007184:102160-102750
HmHel-2	CAEZ01007184:58034-58127
HmHel-2	CAEZ01007184:43007-43565
HmHel-2	CAEZ01003420:3865-4425
HmHel-2	CAEZ01003420:2449-3042
HmHel-2	CAEZ01009836:4628-5220
HmHel-2	CAEZ01009177:9550-10142
HmHel-2	CAEZ01008282:8730-9314
HmHel-2	CAEZ01007329:7625-7846
HmHel-2	CAEZ01007329:45113-45695
HmHel-2	CAEZ01005025:26495-27088
HmHel-2	CAEZ01005025:111251-111703
HmHel-2	CAEZ01005025:128557-129142
HmHel-2	CAEZ01005025:155422-155880
HmHel-2	CAEZ01005025:9610-10044
HmHel-2	CAEZ01004259:438-895
HmHel-2	CAEZ01004259:44174-44745
HmHel-2	CAEZ01004259:17797-18386
HmHel-2	CAEZ01002878:1199-1788
HmHel-2	CAEZ01011296:43377-43973
HmHel-2	CAEZ01011296:189474-189932
HmHel-2	CAEZ01011296:163503-166197
HmHel-2	CAEZ01011296:147414-147539
HmHel-2	CAEZ01007752:21134-21730
HmHel-2	CAEZ01007752:120761-121122
HmHel-2	CAEZ01007752:85561-86156
HmHel-2	CAEZ01002706:19592-20164
HmHel-2	CAEZ01002706:71999-72604
HmHel-2	CAEZ01002706:103959-104517
HmHel-2	CAEZ01002706:3973-4567
HmHel-2	CAEZ01001062:4808-5366
HmHel-2	CAEZ01001062:6336-6928
HmHel-2	CAEZ01010967:24351-25172
HmHel-2	CAEZ01010967:316-911
HmHel-2	CAEZ01010277:36554-37190
HmHel-2	CAEZ01010277:104542-105137
HmHel-2	CAEZ01009403:35602-36160
HmHel-2	CAEZ01009403:5938-6533
HmHel-2	CAEZ01009373:83174-83757

HmHel-2	CAEZ01009373:21383-21615
HmHel-2	CAEZ01009373:2225-2627
HmHel-2	CAEZ01009282:36204-36792
HmHel-2	CAEZ01009282:23067-23528
HmHel-2	CAEZ01009242:38598-39192
HmHel-2	CAEZ01009023:12843-13274
HmHel-2	CAEZ01009023:88222-88402
HmHel-2	CAEZ01009023:45072-45667
HmHel-2	CAEZ01008946:5261-5819
HmHel-2	CAEZ01007972:23270-23864
HmHel-2	CAEZ01007941:147897-148492
HmHel-2	CAEZ01005834:32471-33063
HmHel-2	CAEZ01005437:10631-11225
HmHel-2	CAEZ01005236:6785-7315
HmHel-2	CAEZ01003860:19110-19705
HmHel-2	CAEZ01003260:1291-7889
HmHel-2	CAEZ01003260:126831-127378
HmHel-2	CAEZ01003260:82277-82871
HmHel-2	CAEZ01002754:47578-48164
HmHel-2	CAEZ01002754:5588-6040
HmHel-2	CAEZ01002640:914-1508
HmHel-2	CAEZ01001160:993-1587
HmHel-2	CAEZ01010343:7419-8012
HmHel-2	CAEZ01008578:106236-106509
HmHel-2	CAEZ01008578:137740-138197
HmHel-2	CAEZ01008578:79453-80047
HmHel-2	CAEZ01008578:12071-12666
HmHel-2	CAEZ01005366:5338-5935
HmHel-2	CAEZ01009893:36870-37456
HmHel-2	CAEZ01010928:40114-40356
HmHel-2	CAEZ01010928:3117-3581
HmHel-2	CAEZ01009229:1432-2019
HmHel-2	CAEZ01008410:42695-43147
HmHel-2	CAEZ01008410:67367-67959
HmHel-2	CAEZ01011564:2602-3196
HmHel-2	CAEZ01011564:45315-45865
HmHel-2	CAEZ01011495:16334-16787
HmHel-2	CAEZ01011495:68428-68882
HmHel-2	CAEZ01011495:83190-83784
HmHel-2	CAEZ01011495:56921-57382
HmHel-2	CAEZ01011495:15180-15289
HmHel-2	CAEZ01011134:47631-48363
HmHel-2	CAEZ01011134:108439-108990
HmHel-2	CAEZ01011134:85284-85375
HmHel-2	CAEZ01011134:68595-69190
HmHel-2	CAEZ01010362:13440-14035
HmHel-2	CAEZ01009939:10554-11148
HmHel-2	CAEZ01009716:25558-25640

HmHel-2	CAEZ01009716:123051-123644
HmHel-2	CAEZ01009580:8114-8708
HmHel-2	CAEZ01009399:6040-6580
HmHel-2	CAEZ01009399:19895-20489
HmHel-2	CAEZ01009399:16-336
HmHel-2	CAEZ01009252:8334-8929
HmHel-2	CAEZ01009252:17884-18479
HmHel-2	CAEZ01009233:1602-1711
HmHel-2	CAEZ01009233:17071-17662
HmHel-2	CAEZ01008942:10784-11379
HmHel-2	CAEZ01008942:1198-1649
HmHel-2	CAEZ01008651:9268-9850
HmHel-2	CAEZ01008236:29880-30637
HmHel-2	CAEZ01008236:95840-96435
HmHel-2	CAEZ01008236:35965-36423
HmHel-2	CAEZ01007855:34242-34834
HmHel-2	CAEZ01007269:21722-22187
HmHel-2	CAEZ01007269:39175-39633
HmHel-2	CAEZ01007269:46059-46616
HmHel-2	CAEZ01006802:19100-19695
HmHel-2	CAEZ01006404:15376-15473
HmHel-2	CAEZ01006404:13378-13973
HmHel-2	CAEZ01006130:25945-26540
HmHel-2	CAEZ01006130:40456-40754
HmHel-2	CAEZ01006130:131650-132244
HmHel-2	CAEZ01006130:68413-68728
HmHel-2	CAEZ01006130:9121-9557
HmHel-2	CAEZ01005336:969-1564
HmHel-2	CAEZ01004738:43503-44094
HmHel-2	CAEZ01004738:134987-135579
HmHel-2	CAEZ01004738:173427-173612
HmHel-2	CAEZ01004738:216984-217442
HmHel-2	CAEZ01004738:283744-283987
HmHel-2	CAEZ01004738:312154-312611
HmHel-2	CAEZ01004738:172833-173097
HmHel-2	CAEZ01002792:2621-3031
HmHel-2	CAEZ01002792:49619-50214
HmHel-2	CAEZ01002792:8497-8690
HmHel-2	CAEZ01002376:5998-6593
HmHel-2	CAEZ01001092:4353-4948
HmHel-2	CAEZ01009827:20804-21262
HmHel-2	CAEZ01009827:16002-16282
HmHel-2	CAEZ01006749:24278-24806
HmHel-2	CAEZ01006749:1-571
HmHel-2	CAEZ01000874:393-985
HmHel-2	CAEZ01007939:66136-66596
HmHel-2	CAEZ01007939:124960-125553
HmHel-2	CAEZ01007939:193935-194396

HmHel-2	CAEZ01007939:269688-269820
HmHel-2	CAEZ01007939:159678-160270
HmHel-2	CAEZ01007939:30886-31343
HmHel-2	CAEZ01007939:2484-3079
HmHel-2	CAEZ01007386:10807-11398
HmHel-2	CAEZ01010221:1211-1804
HmHel-2	CAEZ01010221:75927-76486
HmHel-2	CAEZ01009988:6085-6329
HmHel-2	CAEZ01009988:16773-17395
HmHel-2	CAEZ01008279:3184-3778
HmHel-2	CAEZ01008279:28115-28358
HmHel-2	CAEZ01008279:46991-56537
HmHel-2	CAEZ01008279:83149-83744
HmHel-2	CAEZ01008279:247734-248327
HmHel-2	CAEZ01008279:233826-233987
HmHel-2	CAEZ01008279:86373-86827
HmHel-2	CAEZ01008279:7330-7611
HmHel-2	CAEZ01008024:16665-17256
HmHel-2	CAEZ01007275:8985-9572
HmHel-2	CAEZ01007275:44000-44096
HmHel-2	CAEZ01007275:131848-132443
HmHel-2	CAEZ01007275:146661-147252
HmHel-2	CAEZ01007275:166698-167294
HmHel-2	CAEZ01007275:88834-89428
HmHel-2	CAEZ01007275:58288-58555
HmHel-2	CAEZ01007275:17343-17486
HmHel-2	CAEZ01006454:22634-23230
HmHel-2	CAEZ01006422:16551-17039
HmHel-2	CAEZ01006422:29754-30348
HmHel-2	CAEZ01006422:43609-44203
HmHel-2	CAEZ01006422:155841-156142
HmHel-2	CAEZ01006422:12862-12993
HmHel-2	CAEZ01006351:25359-25673
HmHel-2	CAEZ01006351:1551-2147
HmHel-2	CAEZ01004653:8392-8858
HmHel-2	CAEZ01004653:26554-27142
HmHel-2	CAEZ01004081:43064-43521
HmHel-2	CAEZ01004081:28067-28660
HmHel-2	CAEZ01003592:2121-2713
HmHel-2	CAEZ01003059:4488-5079
HmHel-2	CAEZ01003059:17226-17684
HmHel-2	CAEZ01001888:16191-16647
HmHel-2	CAEZ01001888:38792-39379
HmHel-2	CAEZ01001888:9699-10157
HmHel-2	CAEZ01001162:7143-7735
HmHel-2	CAEZ01010952:1079-1371
HmHel-2	CAEZ01010952:53165-53760
HmHel-2	CAEZ01010952:88797-89360

HmHel-2	CAEZ01010952:1-463
HmHel-2	CAEZ01010462:9835-10430
HmHel-2	CAEZ01010462:16276-16869
HmHel-2	CAEZ01010455:51085-51500
HmHel-2	CAEZ01010455:62176-62771
HmHel-2	CAEZ01010455:89880-90433
HmHel-2	CAEZ01010455:114398-114713
HmHel-2	CAEZ01010181:3171-3730
HmHel-2	CAEZ01010181:51744-52288
HmHel-2	CAEZ01010181:58670-59265
HmHel-2	CAEZ01009556:6996-7591
HmHel-2	CAEZ01009308:15319-15914
HmHel-2	CAEZ01008752:2385-2980
HmHel-2	CAEZ01008752:16352-16808
HmHel-2	CAEZ01008374:26600-27193
HmHel-2	CAEZ01008103:2073-2668
HmHel-2	CAEZ01006396:8977-9578
HmHel-2	CAEZ01006097:36622-37032
HmHel-2	CAEZ01006097:95562-96157
HmHel-2	CAEZ01006097:6481-6804
HmHel-2	CAEZ01006073:30902-31354
HmHel-2	CAEZ01006073:57907-58365
HmHel-2	CAEZ01006073:42146-42839
HmHel-2	CAEZ01005508:40143-40319
HmHel-2	CAEZ01005508:83137-83734
HmHel-2	CAEZ01005508:72666-73261
HmHel-2	CAEZ01005508:16447-17042
HmHel-2	CAEZ01005014:20980-21575
HmHel-2	CAEZ01004972:30988-31138
HmHel-2	CAEZ01004972:44711-45305
HmHel-2	CAEZ01004972:22884-23112
HmHel-2	CAEZ01004496:69515-70110
HmHel-2	CAEZ01004496:46853-47449
HmHel-2	CAEZ01004083:32610-33205
HmHel-2	CAEZ01003662:253-848
HmHel-2	CAEZ01001890:9760-10354
HmHel-2	CAEZ01000909:191628-191944
HmHel-2	CAEZ01000909:121866-122331
HmHel-2	CAEZ01000909:30792-31394
HmHel-2	CAEZ01000879:109627-110220
HmHel-2	CAEZ01000879:176797-177392
HmHel-2	CAEZ01000879:168589-169017
HmHel-2	CAEZ01000879:51026-51267
HmHel-2	CAEZ01007571:45045-45599
HmHel-2	CAEZ01007571:49775-50332
HmHel-2	CAEZ01007571:8614-9153
HmHel-2	CAEZ01005198:35271-35830
HmHel-2	CAEZ01005198:6703-7314

HmHel-2	CAEZ01008771:90314-90910
HmHel-2	CAEZ01008771:185724-186261
HmHel-2	CAEZ01008771:146538-146967
HmHel-2	CAEZ01008771:29163-29706
HmHel-2	CAEZ01008138:9947-10373
HmHel-2	CAEZ01008138:194010-194432
HmHel-2	CAEZ01008138:244553-245145
HmHel-2	CAEZ01008138:383543-384001
HmHel-2	CAEZ01008138:366763-366860
HmHel-2	CAEZ01008138:341780-342235
HmHel-2	CAEZ01007975:7787-8384
HmHel-2	CAEZ01007975:51139-51322
HmHel-2	CAEZ01007975:47821-47974
HmHel-2	CAEZ01007628:4306-4895
HmHel-2	CAEZ01005483:8082-8671
HmHel-2	CAEZ01005483:47954-48257
HmHel-2	CAEZ01005483:26946-27537
HmHel-2	CAEZ01005483:11491-11603
HmHel-2	CAEZ01005282:11943-12533
HmHel-2	CAEZ01005282:2963-3169
HmHel-2	CAEZ01011596:436-1028
HmHel-2	CAEZ01010829:15272-15865
HmHel-2	CAEZ01010829:26961-27419
HmHel-2	CAEZ01010829:91640-92229
HmHel-2	CAEZ01010829:179048-179629
HmHel-2	CAEZ01010829:129701-130259
HmHel-2	CAEZ01010829:3381-3846
HmHel-2	CAEZ01010084:49187-49785
HmHel-2	CAEZ01009650:14188-14784
HmHel-2	CAEZ01009011:4639-5235
HmHel-2	CAEZ01008762:60832-61390
HmHel-2	CAEZ01008762:69477-70070
HmHel-2	CAEZ01008762:16644-17230
HmHel-2	CAEZ01008762:3160-3748
HmHel-2	CAEZ01008517:20378-20972
HmHel-2	CAEZ01007429:42521-52901
HmHel-2	CAEZ01007429:34653-34908
HmHel-2	CAEZ01007429:2107-2305
HmHel-2	CAEZ01007286:7710-8303
HmHel-2	CAEZ01007252:143750-144338
HmHel-2	CAEZ01006970:3218-3655
HmHel-2	CAEZ01005430:8916-9471
HmHel-2	CAEZ01005430:26282-26711
HmHel-2	CAEZ01005430:55251-55706
HmHel-2	CAEZ01005430:70654-71238
HmHel-2	CAEZ01005430:10052-10647
HmHel-2	CAEZ01004997:9418-9876
HmHel-2	CAEZ01004997:7337-7933

HmHel-2	CAEZ01004744:7491-8083
HmHel-2	CAEZ01004020:31468-32064
HmHel-2	CAEZ01002250:28459-29050
HmHel-2	CAEZ01010827:149574-149744
HmHel-2	CAEZ01010827:176889-177480
HmHel-2	CAEZ01010827:257010-257589
HmHel-2	CAEZ01010827:445025-445134
HmHel-2	CAEZ01010827:332746-333038
HmHel-2	CAEZ01010827:96886-97480
HmHel-2	CAEZ01010827:69643-70237
HmHel-2	CAEZ01010120:17955-18550
HmHel-2	CAEZ01008939:425-1016
HmHel-2	CAEZ01008939:26639-27097
HmHel-2	CAEZ01008806:30621-31215
HmHel-2	CAEZ01008806:14862-15203
HmHel-2	CAEZ01008726:43414-43748
HmHel-2	CAEZ01008726:48745-49340
HmHel-2	CAEZ01008726:20400-20858
HmHel-2	CAEZ01008688:24861-25455
HmHel-2	CAEZ01008677:104197-104792
HmHel-2	CAEZ01008677:134030-135013
HmHel-2	CAEZ01007716:28232-28827
HmHel-2	CAEZ01007609:473-1055
HmHel-2	CAEZ01007609:21231-21790
HmHel-2	CAEZ01007609:46388-46982
HmHel-2	CAEZ01007176:39357-39952
HmHel-2	CAEZ01006196:35267-35655
HmHel-2	CAEZ01006196:52243-52694
HmHel-2	CAEZ01006196:71719-72314
HmHel-2	CAEZ01006196:130570-131129
HmHel-2	CAEZ01006196:199118-205329
HmHel-2	CAEZ01006196:190776-191322
HmHel-2	CAEZ01006196:38907-39182
HmHel-2	CAEZ01005746:62820-63415
HmHel-2	CAEZ01005746:52546-53003
HmHel-2	CAEZ01004811:21754-22049
HmHel-2	CAEZ01004811:70603-71058
HmHel-2	CAEZ01004811:100613-101202
HmHel-2	CAEZ01004811:142909-144531
HmHel-2	CAEZ01004811:232718-233312
HmHel-2	CAEZ01004811:282673-283217
HmHel-2	CAEZ01004811:259088-259546
HmHel-2	CAEZ01004811:209406-215575
HmHel-2	CAEZ01004370:51610-52205
HmHel-2	CAEZ01004304:8998-9593
HmHel-2	CAEZ01003346:43737-44195
HmHel-2	CAEZ01003346:18893-19204
HmHel-2	CAEZ01003346:3077-3668

HmHel-2	CAEZ01002210:191-786
HmHel-2	CAEZ01002097:36435-36994
HmHel-2	CAEZ01002097:60776-61097
HmHel-2	CAEZ01002097:65061-65292
HmHel-2	CAEZ01001965:1002-1597
HmHel-2	CAEZ01001784:1084-1678
HmHel-2	CAEZ01001742:11382-11833
HmHel-2	CAEZ01001736:13786-14335
HmHel-2	CAEZ01001736:27125-27718
HmHel-2	CAEZ01001736:73621-74216
HmHel-2	CAEZ01001736:11444-12040
HmHel-2	CAEZ01001436:33683-34278
HmHel-2	CAEZ01000931:15241-15836
HmHel-2	CAEZ01010535:32609-33075
HmHel-2	CAEZ01010535:58207-58800
HmHel-2	CAEZ01010189:18363-18966
HmHel-2	CAEZ01008183:26999-27593
HmHel-2	CAEZ01008183:59291-59884
HmHel-2	CAEZ01007662:19153-19735
HmHel-2	CAEZ01005899:3112-3567
HmHel-2	CAEZ01005899:16912-17471
HmHel-2	CAEZ01002814:202790-203248
HmHel-2	CAEZ01002814:231948-232236
HmHel-2	CAEZ01002814:356478-356936
HmHel-2	CAEZ01002814:345244-345796
HmHel-2	CAEZ01002814:307042-307217
HmHel-2	CAEZ01002814:266717-267275
HmHel-2	CAEZ01002814:245207-245663
HmHel-2	CAEZ01002814:219421-219981
HmHel-2	CAEZ01002814:180544-181000
HmHel-2	CAEZ01010731:3726-4315
HmHel-2	CAEZ01010731:6739-7055
HmHel-2	CAEZ01003865:1276-1869
HmHel-2	CAEZ01003336:8781-9366
HmHel-2	CAEZ01001435:78591-79049
HmHel-2	CAEZ01001435:206646-207236
HmHel-2	CAEZ01001435:221938-222360
HmHel-2	CAEZ01001435:213309-213899
HmHel-2	CAEZ01001435:31192-31650
HmHel-2	CAEZ01010584:28993-29549
HmHel-2	CAEZ01010584:156572-157132
HmHel-2	CAEZ01010584:263256-263852
HmHel-2	CAEZ01010584:322411-322571
HmHel-2	CAEZ01010584:269944-270191
HmHel-2	CAEZ01010584:90627-91221
HmHel-2	CAEZ01010127:96413-97008
HmHel-2	CAEZ01010127:21257-21817
HmHel-2	CAEZ01009463:9062-9655

HmHel-2	CAEZ01009084:42054-42642
HmHel-2	CAEZ01009084:69380-69838
HmHel-2	CAEZ01009021:15402-15998
HmHel-2	CAEZ01007809:25738-26330
HmHel-2	CAEZ01007809:57745-58289
HmHel-2	CAEZ01007809:112496-112954
HmHel-2	CAEZ01007809:289675-290217
HmHel-2	CAEZ01007809:312072-312640
HmHel-2	CAEZ01007809:177339-177933
HmHel-2	CAEZ01007533:3355-3950
HmHel-2	CAEZ01005434:497-1085
HmHel-2	CAEZ01003109:6655-7292
HmHel-2	CAEZ01003109:60540-61099
HmHel-2	CAEZ01003109:18300-18891
HmHel-2	CAEZ01002704:17280-17872
HmHel-2	CAEZ01002704:27281-27730
HmHel-2	CAEZ01002182:142112-142698
HmHel-2	CAEZ01002182:131401-131988
HmHel-2	CAEZ01000970:42028-42624
HmHel-2	CAEZ01000895:41445-41810
HmHel-2	CAEZ01000895:61259-61704
HmHel-2	CAEZ01000895:77228-77796
HmHel-2	CAEZ01000895:41303-41438
HmHel-2	CAEZ01010460:21959-22374
HmHel-2	CAEZ01010460:33623-34010
HmHel-2	CAEZ01010460:113403-113991
HmHel-2	CAEZ01010460:123824-124419
HmHel-2	CAEZ01010460:19000-19458
HmHel-2	CAEZ01009824:27257-27714
HmHel-2	CAEZ01009824:87669-88263
HmHel-2	CAEZ01009768:1046-1641
HmHel-2	CAEZ01009281:12463-13052
HmHel-2	CAEZ01008663:5514-6108
HmHel-2	CAEZ01008552:29166-29760
HmHel-2	CAEZ01008516:13025-13620
HmHel-2	CAEZ01007523:5144-5737
HmHel-2	CAEZ01007121:27041-27623
HmHel-2	CAEZ01007121:46917-47511
HmHel-2	CAEZ01006294:21295-21890
HmHel-2	CAEZ01005407:17693-18288
HmHel-2	CAEZ01005152:9554-10043
HmHel-2	CAEZ01005152:72902-73457
HmHel-2	CAEZ01005152:6567-7161
HmHel-2	CAEZ01004198:21108-21433
HmHel-2	CAEZ01004198:31100-31499
HmHel-2	CAEZ01004198:18010-18605
HmHel-2	CAEZ01003919:5947-6541
HmHel-2	CAEZ01003113:1851-2446

HmHel-2	CAEZ01002736:1220-1742
HmHel-2	CAEZ01002736:15468-15907
HmHel-2	CAEZ01002369:372-904
HmHel-2	CAEZ01002369:50506-51101
HmHel-2	CAEZ01002369:65651-66109
HmHel-2	CAEZ01001766:69-527
HmHel-2	CAEZ01001766:75570-76129
HmHel-2	CAEZ01001766:106170-106313
HmHel-2	CAEZ01001766:191841-192435
HmHel-2	CAEZ01001766:157240-157795
HmHel-2	CAEZ01001766:115007-115562
HmHel-2	CAEZ01001766:45133-45588
HmHel-2	CAEZ01009898:605-1207
HmHel-2	CAEZ01009898:26007-26328
HmHel-2	CAEZ01009723:9449-10039
HmHel-2	CAEZ01008040:1921-2510
HmHel-2	CAEZ01007764:27573-28129
HmHel-2	CAEZ01007764:13701-14260
HmHel-2	CAEZ01007764:2065-2659
HmHel-2	CAEZ01007605:7371-7829
HmHel-2	CAEZ01007605:23184-23742
HmHel-2	CAEZ01007964:3027-3623
HmHel-2	CAEZ01007848:12168-12758
HmHel-2	CAEZ01006560:29007-29444
HmHel-2	CAEZ01006560:40498-41088
HmHel-2	CAEZ01006560:84991-85257
HmHel-2	CAEZ01006560:9310-9599
HmHel-2	CAEZ01005083:30617-31213
HmHel-2	CAEZ01005083:47010-57145
HmHel-2	CAEZ01005083:8173-8520
HmHel-2	CAEZ01010228:2463-3007
HmHel-2	CAEZ01010228:88124-88367
HmHel-2	CAEZ01010228:104233-104555
HmHel-2	CAEZ01010228:55514-56109
HmHel-2	CAEZ01009706:10965-11554
HmHel-2	CAEZ01009706:82066-82641
HmHel-2	CAEZ01009706:79920-80377
HmHel-2	CAEZ01009706:41463-41990
HmHel-2	CAEZ01009659:3893-4485
HmHel-2	CAEZ01008246:8638-9228
HmHel-2	CAEZ01008246:23552-24010
HmHel-2	CAEZ01008213:2352-2945
HmHel-2	CAEZ01007493:7943-8402
HmHel-2	CAEZ01007493:19697-20289
HmHel-2	CAEZ01002964:6512-6968
HmHel-2	CAEZ01002964:40978-41566
HmHel-2	CAEZ01002964:25199-25699
HmHel-2	CAEZ01002729:8475-9067

HmHel-2	CAEZ01002729:42339-42466
HmHel-2	CAEZ01002729:33140-33723
HmHel-2	CAEZ01002675:36505-37080
HmHel-2	CAEZ01009844:165611-174468
HmHel-2	CAEZ01009844:115757-116058
HmHel-2	CAEZ01009844:24309-24904
HmHel-2	CAEZ01009841:18032-18371
HmHel-2	CAEZ01009841:83439-83877
HmHel-2	CAEZ01009841:107597-108191
HmHel-2	CAEZ01009841:82556-83014
HmHel-2	CAEZ01009841:65409-65867
HmHel-2	CAEZ01009001:1597-2192
HmHel-2	CAEZ01008315:274381-274975
HmHel-2	CAEZ01008315:311253-311711
HmHel-2	CAEZ01008315:431086-431544
HmHel-2	CAEZ01008315:363481-364072
HmHel-2	CAEZ01008315:135683-135817
HmHel-2	CAEZ01007803:1747-2342
HmHel-2	CAEZ01007612:8323-8917
HmHel-2	CAEZ01007232:14054-14648
HmHel-2	CAEZ01007232:34220-34682
HmHel-2	CAEZ01007232:38940-39407
HmHel-2	CAEZ01007200:6821-7416
HmHel-2	CAEZ01007144:2164-2758
HmHel-2	CAEZ01006799:16732-17286
HmHel-2	CAEZ01006799:59953-60407
HmHel-2	CAEZ01006799:58251-58548
HmHel-2	CAEZ01006799:28904-29496
HmHel-2	CAEZ01006799:13817-14275
HmHel-2	CAEZ01006698:16856-17450
HmHel-2	CAEZ01006698:64703-64843
HmHel-2	CAEZ01006529:11229-11787
HmHel-2	CAEZ01006529:51343-51488
HmHel-2	CAEZ01006529:125542-133263
HmHel-2	CAEZ01006529:178853-179311
HmHel-2	CAEZ01006529:215376-215826
HmHel-2	CAEZ01006529:65899-66493
HmHel-2	CAEZ01006319:47298-47891
HmHel-2	CAEZ01006319:8750-9342
HmHel-2	CAEZ01006252:49606-50201
HmHel-2	CAEZ01006131:228287-228744
HmHel-2	CAEZ01006131:217673-218268
HmHel-2	CAEZ01005844:4940-5534
HmHel-2	CAEZ01005649:27361-27955
HmHel-2	CAEZ01005649:14832-15289
HmHel-2	CAEZ01004792:14181-14776
HmHel-2	CAEZ01004792:93264-93728
HmHel-2	CAEZ01004309:19476-19929

HmHel-2	CAEZ01004309:8483-9078
HmHel-2	CAEZ01003709:16984-17578
HmHel-2	CAEZ01003082:18262-18857
HmHel-2	CAEZ01003082:19544-19971
HmHel-2	CAEZ01002800:6843-7437
HmHel-2	CAEZ01002657:45502-46097
HmHel-2	CAEZ01002183:10407-10808
HmHel-2	CAEZ01002183:9568-10163
HmHel-2	CAEZ01001891:22221-22816
HmHel-2	CAEZ01001891:46731-47326
HmHel-2	CAEZ01001891:17080-17527
HmHel-2	CAEZ01001465:75093-75420
HmHel-2	CAEZ01001465:19966-20561
HmHel-2	CAEZ01001216:10021-10596
HmHel-2	CAEZ01011394:5667-6257
HmHel-2	CAEZ01009772:33953-34409
HmHel-2	CAEZ01009772:3625-4019
HmHel-2	CAEZ01008105:4843-5294
HmHel-2	CAEZ01008105:35851-36309
HmHel-2	CAEZ01008105:47888-48477
HmHel-2	CAEZ01011539:5269-5857
HmHel-2	CAEZ01006878:1968-2564
HmHel-2	CAEZ01005739:10701-11293
HmHel-2	CAEZ01005485:1901-2359
HmHel-2	CAEZ01005485:17471-18065
HmHel-2	CAEZ01005485:1-493
HmHel-2	CAEZ01002982:2043-2632
HmHel-2	CAEZ01001249:3316-3908
HmHel-2	CAEZ01011392:15626-16222
HmHel-2	CAEZ01011037:9414-10006
HmHel-2	CAEZ01010702:1901-2361
HmHel-2	CAEZ01010702:8249-8845
HmHel-2	CAEZ01010289:146150-146770
HmHel-2	CAEZ01010289:137682-138134
HmHel-2	CAEZ01010289:48057-48652
HmHel-2	CAEZ01009802:7512-7970
HmHel-2	CAEZ01009802:101425-101574
HmHel-2	CAEZ01009802:152036-152631
HmHel-2	CAEZ01009802:276681-276977
HmHel-2	CAEZ01009802:271527-272120
HmHel-2	CAEZ01009802:238431-238715
HmHel-2	CAEZ01009789:5811-6403
HmHel-2	CAEZ01009251:4121-4551
HmHel-2	CAEZ01009251:16111-16705
HmHel-2	CAEZ01009044:31776-32369
HmHel-2	CAEZ01009044:139101-139697
HmHel-2	CAEZ01009044:156702-156821
HmHel-2	CAEZ01009044:88231-88814

HmHel-2	CAEZ01008122:82406-82998
HmHel-2	CAEZ01008122:124064-124403
HmHel-2	CAEZ01008122:30323-30761
HmHel-2	CAEZ01008122:162-755
HmHel-2	CAEZ01006670:10920-11512
HmHel-2	CAEZ01004566:27846-28438
HmHel-2	CAEZ01004566:19664-20096
HmHel-2	CAEZ01002607:199607-199806
HmHel-2	CAEZ01002607:190301-190421
HmHel-2	CAEZ01002607:134788-135384
HmHel-2	CAEZ01002607:79691-80286
HmHel-2	CAEZ01002607:58351-58805
HmHel-2	CAEZ01001294:977-1570
HmHel-2	CAEZ01011367:22719-23314
HmHel-2	CAEZ01011009:9943-10490
HmHel-2	CAEZ01011009:28564-29157
HmHel-2	CAEZ01011009:37635-38212
HmHel-2	CAEZ01010021:26332-26899
HmHel-2	CAEZ01008055:14374-14968
HmHel-2	CAEZ01007066:104832-105009
HmHel-2	CAEZ01007066:62488-63083
HmHel-2	CAEZ01007066:40754-41120
HmHel-2	CAEZ01007066:24713-25308
HmHel-2	CAEZ01006772:20281-20876
HmHel-2	CAEZ01006772:53527-53984
HmHel-2	CAEZ01006772:251886-252482
HmHel-2	CAEZ01006772:269054-269646
HmHel-2	CAEZ01006772:276502-277097
HmHel-2	CAEZ01006772:239952-240366
HmHel-2	CAEZ01006772:18599-19193
HmHel-2	CAEZ01006701:18267-18354
HmHel-2	CAEZ01006701:8174-8727
HmHel-2	CAEZ01006641:7972-8547
HmHel-2	CAEZ01006007:5350-5945
HmHel-2	CAEZ01006007:20479-20985
HmHel-2	CAEZ01006007:17517-17975
HmHel-2	CAEZ01006006:16-611
HmHel-2	CAEZ01004656:31264-31858
HmHel-2	CAEZ01004656:11060-11365
HmHel-2	CAEZ01004079:23040-23634
HmHel-2	CAEZ01004079:8204-8678
HmHel-2	CAEZ01003946:8420-9014
HmHel-2	CAEZ01002875:15242-15698
HmHel-2	CAEZ01002875:47449-47986
HmHel-2	CAEZ01002875:86393-86682
HmHel-2	CAEZ01002875:143880-144115
HmHel-2	CAEZ01002875:225549-226092
HmHel-2	CAEZ01002875:150712-151304

HmHel-2	CAEZ01002415:84519-85114
HmHel-2	CAEZ01001975:28-555
HmHel-2	CAEZ01010751:13163-13756
HmHel-2	CAEZ01009663:9884-10480
HmHel-2	CAEZ01009415:7279-7870
HmHel-2	CAEZ01005956:65-622
HmHel-2	CAEZ01004204:3102-3688
HmHel-2	CAEZ01003715:517-1107
HmHel-2	CAEZ01003673:8644-9243
HmHel-2	CAEZ01001185:1936-2486
HmHel-2	CAEZ01011461:26370-26963
HmHel-2	CAEZ01009124:32345-41395
HmHel-2	CAEZ01009124:59746-60205
HmHel-2	CAEZ01009124:64743-65331
HmHel-2	CAEZ01009124:50462-51056
HmHel-2	CAEZ01009124:38933-39093
HmHel-2	CAEZ01005007:2312-2909
HmHel-2	CAEZ01004055:14540-14645
HmHel-2	CAEZ01002363:12137-12734
HmHel-2	CAEZ01001933:936-1493
HmHel-2	CAEZ01001572:43552-43808
HmHel-2	CAEZ01001572:27747-28335
HmHel-2	CAEZ01001450:27616-28203
HmHel-2	CAEZ01010666:62127-62722
HmHel-2	CAEZ01010666:78296-78892
HmHel-2	CAEZ01010666:4415-4859
HmHel-2	CAEZ01010454:1550-2146
HmHel-2	CAEZ01009539:1878-2472
HmHel-2	CAEZ01009359:1628-2184
HmHel-2	CAEZ01009359:27091-27236
HmHel-2	CAEZ01009359:14224-14546
HmHel-2	CAEZ01008989:63423-63858
HmHel-2	CAEZ01008989:188303-188896
HmHel-2	CAEZ01008989:7803-8373
HmHel-2	CAEZ01008175:6293-6885
HmHel-2	CAEZ01008175:22525-23810
HmHel-2	CAEZ01007952:8010-8585
HmHel-2	CAEZ01007952:10177-10770
HmHel-2	CAEZ01007679:9621-10217
HmHel-2	CAEZ01007604:30525-30983
HmHel-2	CAEZ01006540:3260-3817
HmHel-2	CAEZ01006540:60998-61404
HmHel-2	CAEZ01006540:42201-42797
HmHel-2	CAEZ01005436:742-1198
HmHel-2	CAEZ01004730:10181-10773
HmHel-2	CAEZ01004224:5007-5599
HmHel-2	CAEZ01001101:16092-16690
HmHel-2	CAEZ01000990:12020-12616

HmHel-2	CAEZ01010858:5731-6326
HmHel-2	CAEZ01010858:39413-39972
HmHel-2	CAEZ01010851:3236-3831
HmHel-2	CAEZ01010851:40818-41377
HmHel-2	CAEZ01010133:11795-12385
HmHel-2	CAEZ01010133:39878-40472
HmHel-2	CAEZ01010133:42337-42923
HmHel-2	CAEZ01009806:8229-8819
HmHel-2	CAEZ01009075:786-1376
HmHel-2	CAEZ01008590:121013-121329
HmHel-2	CAEZ01008590:41841-42162
HmHel-2	CAEZ01008590:9063-9658
HmHel-2	CAEZ01008176:50485-51035
HmHel-2	CAEZ01008176:7550-8140
HmHel-2	CAEZ01008162:7646-8236
HmHel-2	CAEZ01007500:35937-36530
HmHel-2	CAEZ01007500:46881-47437
HmHel-2	CAEZ01007500:58802-59229
HmHel-2	CAEZ01007500:81908-82503
HmHel-2	CAEZ01007500:12906-13370
HmHel-2	CAEZ01007487:11868-12462
HmHel-2	CAEZ01007287:566-1161
HmHel-2	CAEZ01007124:60365-60959
HmHel-2	CAEZ01007124:54617-55211
HmHel-2	CAEZ01006891:2356-2814
HmHel-2	CAEZ01006891:7348-7756
HmHel-2	CAEZ01006128:69265-69849
HmHel-2	CAEZ01006128:54997-55556
HmHel-2	CAEZ01005924:11851-12446
HmHel-2	CAEZ01005711:121061-121655
HmHel-2	CAEZ01005711:177711-177908
HmHel-2	CAEZ01005711:119256-119493
HmHel-2	CAEZ01005711:85685-87020
HmHel-2	CAEZ01005711:63324-63906
HmHel-2	CAEZ01005711:20246-20962
HmHel-2	CAEZ01004813:41645-41872
HmHel-2	CAEZ01004813:65242-65835
HmHel-2	CAEZ01003746:93153-93747
HmHel-2	CAEZ01002194:38216-38810
HmHel-2	CAEZ01002184:22800-23394
HmHel-2	CAEZ01002184:24930-25523
HmHel-2	CAEZ01001338:29596-30190
HmHel-2	CAEZ01011107:80327-80783
HmHel-2	CAEZ01011107:97864-98423
HmHel-2	CAEZ01011107:109039-109600
HmHel-2	CAEZ01010653:8669-9268
HmHel-2	CAEZ01008662:12134-12718
HmHel-2	CAEZ01007700:14671-15129

HmHel-2	CAEZ01007389:10744-11294
HmHel-2	CAEZ01007389:83571-84165
HmHel-2	CAEZ01007389:74933-75373
HmHel-2	CAEZ01007389:14286-14409
HmHel-2	CAEZ01006435:51002-51594
HmHel-2	CAEZ01004740:1662-2119
HmHel-2	CAEZ01004740:13736-14049
HmHel-2	CAEZ01004740:4002-4595
HmHel-2	CAEZ01004698:11674-12262
HmHel-2	CAEZ01002902:2399-3000
HmHel-2	CAEZ01002830:20132-20572
HmHel-2	CAEZ01009370:126650-127245
HmHel-2	CAEZ01009370:102808-103404
HmHel-2	CAEZ01009370:28710-28970
HmHel-2	CAEZ01006168:12095-12409
HmHel-2	CAEZ01006168:30433-31030
HmHel-2	CAEZ01005173:1-220
HmHel-2	CAEZ01005173:97265-98183
HmHel-2	CAEZ01005173:137578-138172
HmHel-2	CAEZ01005173:161465-161922
HmHel-2	CAEZ01005173:57105-57223
HmHel-2	CAEZ01011561:1183-1778
HmHel-2	CAEZ01010451:21459-21917
HmHel-2	CAEZ01010451:62441-62894
HmHel-2	CAEZ01010451:115915-116486
HmHel-2	CAEZ01010451:43839-44432
HmHel-2	CAEZ01009992:4921-5378
HmHel-2	CAEZ01009380:54975-55559
HmHel-2	CAEZ01008507:60-655
HmHel-2	CAEZ01008125:80199-80626
HmHel-2	CAEZ01008125:102437-102653
HmHel-2	CAEZ01008125:146256-150785
HmHel-2	CAEZ01008125:182491-183079
HmHel-2	CAEZ01008125:108783-108950
HmHel-2	CAEZ01008125:61626-62082
HmHel-2	CAEZ01008125:50161-50478
HmHel-2	CAEZ01008124:34121-34440
HmHel-2	CAEZ01008124:133704-133821
HmHel-2	CAEZ01008124:81715-82273
HmHel-2	CAEZ01008124:64769-65362
HmHel-2	CAEZ01008124:52012-52423
HmHel-2	CAEZ01006812:554-1149
HmHel-2	CAEZ01006095:14219-14808
HmHel-2	CAEZ01006095:37176-37738
HmHel-2	CAEZ01005186:28062-28647
HmHel-2	CAEZ01004934:18348-18941
HmHel-2	CAEZ01004545:6500-7092
HmHel-2	CAEZ01004545:21633-22091

HmHel-2	CAEZ01004033:23427-23886
HmHel-2	CAEZ01004033:200865-201051
HmHel-2	CAEZ01004033:107753-108345
HmHel-2	CAEZ01002797:13358-13951
HmHel-2	CAEZ01002797:1926-2528
HmHel-2	CAEZ01011242:6210-6804
HmHel-2	CAEZ01011242:16930-17517
HmHel-2	CAEZ01010962:4490-5084
HmHel-2	CAEZ01010697:30104-30706
HmHel-2	CAEZ01010697:24264-24859
HmHel-2	CAEZ01010223:1835-2422
HmHel-2	CAEZ01010223:8299-9022
HmHel-2	CAEZ01009801:122-709
HmHel-2	CAEZ01009801:29976-30060
HmHel-2	CAEZ01009801:74740-75197
HmHel-2	CAEZ01009801:22548-23142
HmHel-2	CAEZ01009767:15670-16265
HmHel-2	CAEZ01008919:23693-24014
HmHel-2	CAEZ01008919:71011-71606
HmHel-2	CAEZ01007461:39656-40250
HmHel-2	CAEZ01007306:7832-8426
HmHel-2	CAEZ01007306:10717-11280
HmHel-2	CAEZ01007279:851-1446
HmHel-2	CAEZ01006690:7915-8508
HmHel-2	CAEZ01006230:13958-14553
HmHel-2	CAEZ01006230:12516-12987
HmHel-2	CAEZ01005174:24454-25049
HmHel-2	CAEZ01005174:32718-33169
HmHel-2	CAEZ01005108:123729-124309
HmHel-2	CAEZ01004823:15071-15666
HmHel-2	CAEZ01004236:13014-13607
HmHel-2	CAEZ01004236:13636-13779
HmHel-2	CAEZ01004165:16752-17207
HmHel-2	CAEZ01004165:9892-10487
HmHel-2	CAEZ01003762:20030-20586
HmHel-2	CAEZ01003364:403-997
HmHel-2	CAEZ01002684:15589-16148
HmHel-2	CAEZ01001224:1725-2320
HmHel-2	CAEZ01008316:22306-22903
HmHel-2	CAEZ01008316:37762-38355
HmHel-2	CAEZ01005026:15294-15502
HmHel-2	CAEZ01005026:1591-2184
HmHel-2	CAEZ01004627:2458-3041
HmHel-2	CAEZ01004535:38726-39311
HmHel-2	CAEZ01004535:6843-7389
HmHel-2	CAEZ01010458:9648-10106
HmHel-2	CAEZ01010458:28558-29010
HmHel-2	CAEZ01010458:84482-85075

HmHel-2	CAEZ01010458:77680-78139
HmHel-2	CAEZ01010046:1018-1612
HmHel-2	CAEZ01006061:14771-15343
HmHel-2	CAEZ01003252:26891-27213
HmHel-2	CAEZ01003196:35831-36423
HmHel-2	CAEZ01003196:42053-42502
HmHel-2	CAEZ01002639:3888-4480
HmHel-2	CAEZ01001645:8097-8549
HmHel-2	CAEZ01011598:25709-26166
HmHel-2	CAEZ01011598:137812-138405
HmHel-2	CAEZ01011598:5842-6114
HmHel-2	CAEZ01011091:6971-7564
HmHel-2	CAEZ01010915:24654-25247
HmHel-2	CAEZ01010587:7309-7902
HmHel-2	CAEZ01010587:26660-27118
HmHel-2	CAEZ01008857:65332-65811
HmHel-2	CAEZ01008857:9550-10145
HmHel-2	CAEZ01008743:1182-1770
HmHel-2	CAEZ01008743:152571-152707
HmHel-2	CAEZ01008743:70036-70495
HmHel-2	CAEZ01008294:16882-17029
HmHel-2	CAEZ01008294:128551-129009
HmHel-2	CAEZ01008294:146299-146890
HmHel-2	CAEZ01008294:201033-201624
HmHel-2	CAEZ01008294:195690-196148
HmHel-2	CAEZ01008294:64738-65326
HmHel-2	CAEZ01007295:1783-2375
HmHel-2	CAEZ01007205:2974-3570
HmHel-2	CAEZ01007205:14679-15251
HmHel-2	CAEZ01006820:15365-15956
HmHel-2	CAEZ01006820:8965-9286
HmHel-2	CAEZ01005953:7915-8511
HmHel-2	CAEZ01005953:3757-4346
HmHel-2	CAEZ01005207:41794-42392
HmHel-2	CAEZ01005207:20813-21409
HmHel-2	CAEZ01005048:9930-10523
HmHel-2	CAEZ01004478:222-813
HmHel-2	CAEZ01004383:2466-2787
HmHel-2	CAEZ01004383:7982-8578
HmHel-2	CAEZ01003823:3174-3765
HmHel-2	CAEZ01003778:10272-10868
HmHel-2	CAEZ01003644:7251-7843
HmHel-2	CAEZ01003177:3390-3942
HmHel-2	CAEZ01003051:2623-3082
HmHel-2	CAEZ01002947:4532-5128
HmHel-2	CAEZ01002947:1900-2596
HmHel-2	CAEZ01002882:2339-2931
HmHel-2	CAEZ01000867:1366-1929

HmHel-2	CAEZ01000867:34232-34507
HmHel-2	CAEZ01000867:54261-54566
HmHel-2	CAEZ01000867:27635-28177
HmHel-2	CAEZ01000523:823-1041
HmHel-2	CAEZ01000041:17879-18474
HmHel-2	CAEZ01011231:15923-16517
HmHel-2	CAEZ01011121:1325-1919
HmHel-2	CAEZ01011095:47090-47645
HmHel-2	CAEZ01011095:56078-56671
HmHel-2	CAEZ01011095:11698-12301
HmHel-2	CAEZ01010887:11733-12315
HmHel-2	CAEZ01010810:2963-3554
HmHel-2	CAEZ01010810:25800-26257
HmHel-2	CAEZ01010659:12453-13047
HmHel-2	CAEZ01010489:28998-29456
HmHel-2	CAEZ01010489:888-1483
HmHel-2	CAEZ01009892:166814-166982
HmHel-2	CAEZ01009892:195794-196388
HmHel-2	CAEZ01009892:123265-123859
HmHel-2	CAEZ01009437:19245-19701
HmHel-2	CAEZ01009437:48895-49352
HmHel-2	CAEZ01009437:65152-65603
HmHel-2	CAEZ01009437:33288-33849
HmHel-2	CAEZ01009437:16912-17505
HmHel-2	CAEZ01008423:44854-45448
HmHel-2	CAEZ01008423:54947-55504
HmHel-2	CAEZ01008423:44247-44700
HmHel-2	CAEZ01008258:8097-8382
HmHel-2	CAEZ01008258:34718-35312
HmHel-2	CAEZ01008258:128581-129046
HmHel-2	CAEZ01008258:144721-145179
HmHel-2	CAEZ01008258:159705-159990
HmHel-2	CAEZ01008258:193525-194119
HmHel-2	CAEZ01008258:106829-107352
HmHel-2	CAEZ01008258:90107-90547
HmHel-2	CAEZ01008258:36708-37302
HmHel-2	CAEZ01007567:24960-25554
HmHel-2	CAEZ01007194:7168-7763
HmHel-2	CAEZ01007105:7670-8264
HmHel-2	CAEZ01006978:15859-16454
HmHel-2	CAEZ01006758:6003-6595
HmHel-2	CAEZ01006185:9090-9683
HmHel-2	CAEZ01006012:24979-25573
HmHel-2	CAEZ01006012:41648-42237
HmHel-2	CAEZ01006012:59457-60008
HmHel-2	CAEZ01005930:3050-3816
HmHel-2	CAEZ01005268:9042-9633
HmHel-2	CAEZ01005061:10144-10742

HmHel-2	CAEZ01004873:7391-7985
HmHel-2	CAEZ01004629:74181-74776
HmHel-2	CAEZ01004501:13029-13623
HmHel-2	CAEZ01002786:551-1146
HmHel-2	CAEZ01002119:6505-7100
HmHel-2	CAEZ01001403:33259-33854
HmHel-2	CAEZ01001343:20259-20851
HmHel-2	CAEZ01000820:51105-51425
HmHel-2	CAEZ01000820:58214-58445
HmHel-2	CAEZ01000820:46830-47041
HmHel-2	CAEZ01000820:14694-15288
HmHel-2	CAEZ01011519:95017-95609
HmHel-2	CAEZ01011519:113351-113640
HmHel-2	CAEZ01011519:163108-163703
HmHel-2	CAEZ01011519:166023-166514
HmHel-2	CAEZ01011519:149512-149756
HmHel-2	CAEZ01011519:93281-93830
HmHel-2	CAEZ01010332:34403-34996
HmHel-2	CAEZ01010117:72267-72866
HmHel-2	CAEZ01010117:72115-72208
HmHel-2	CAEZ01006177:87256-87699
HmHel-2	CAEZ01006177:218677-219206
HmHel-2	CAEZ01006177:195550-196137
HmHel-2	CAEZ01003682:2366-2909
HmHel-2	CAEZ01002670:8756-9350
HmHel-2	CAEZ01002670:45623-46216
HmHel-2	CAEZ01010275:15023-15479
HmHel-2	CAEZ01010275:51562-51861
HmHel-2	CAEZ01010275:19719-20313
HmHel-2	CAEZ01009595:34-435
HmHel-2	CAEZ01009362:4307-4867
HmHel-2	CAEZ01008167:16610-17208
HmHel-2	CAEZ01008167:2913-3473
HmHel-2	CAEZ01007851:118654-119247
HmHel-2	CAEZ01007274:7980-8541
HmHel-2	CAEZ01006863:32244-32456
HmHel-2	CAEZ01006863:61647-62237
HmHel-2	CAEZ01006863:82080-82274
HmHel-2	CAEZ01006863:105057-105506
HmHel-2	CAEZ01006863:94545-94736
HmHel-2	CAEZ01004632:39273-39866
HmHel-2	CAEZ01004632:71054-78296
HmHel-2	CAEZ01004632:109577-109995
HmHel-2	CAEZ01004632:106140-106598
HmHel-2	CAEZ01004454:154-751
HmHel-2	CAEZ01003777:975-1563
HmHel-2	CAEZ01011083:18675-19133
HmHel-2	CAEZ01011083:126553-127011

HmHel-2	CAEZ01011083:190938-191395
HmHel-2	CAEZ01011083:144853-145309
HmHel-2	CAEZ01011083:8706-8899
HmHel-2	CAEZ01010987:7941-8527
HmHel-2	CAEZ01010882:30848-31424
HmHel-2	CAEZ01010796:20800-21390
HmHel-2	CAEZ01010796:7438-8033
HmHel-2	CAEZ01010622:813-1268
HmHel-2	CAEZ01010622:16264-16857
HmHel-2	CAEZ01009970:46334-46894
HmHel-2	CAEZ01009970:72173-72756
HmHel-2	CAEZ01009970:68919-69377
HmHel-2	CAEZ01008598:10173-10719
HmHel-2	CAEZ01008442:12480-13074
HmHel-2	CAEZ01008199:9238-9814
HmHel-2	CAEZ01008199:6993-7585
HmHel-2	CAEZ01007849:69695-70290
HmHel-2	CAEZ01007849:18987-19446
HmHel-2	CAEZ01007562:11479-12072
HmHel-2	CAEZ01007070:4129-4724
HmHel-2	CAEZ01006825:4262-4855
HmHel-2	CAEZ01004118:5417-6011
HmHel-2	CAEZ01002707:6755-7347
HmHel-2	CAEZ01002663:12690-13283
HmHel-2	CAEZ01001983:13680-14276
HmHel-2	CAEZ01001775:1597-2192
HmHel-2	CAEZ01011361:21451-21909
HmHel-2	CAEZ01011361:42515-43072
HmHel-2	CAEZ01011361:68831-69426
HmHel-2	CAEZ01011361:32881-33310
HmHel-2	CAEZ01010784:15029-15621
HmHel-2	CAEZ01010762:16178-16768
HmHel-2	CAEZ01010701:218519-218977
HmHel-2	CAEZ01010701:139560-140155
HmHel-2	CAEZ01010701:89402-89720
HmHel-2	CAEZ01010556:466-1060
HmHel-2	CAEZ01010470:5332-5927
HmHel-2	CAEZ01010281:2810-3144
HmHel-2	CAEZ01010281:10794-11389
HmHel-2	CAEZ01009975:83174-83610
HmHel-2	CAEZ01009975:106521-106844
HmHel-2	CAEZ01009975:124640-125185
HmHel-2	CAEZ01009975:111385-111538
HmHel-2	CAEZ01009975:70296-71011
HmHel-2	CAEZ01009465:81999-82592
HmHel-2	CAEZ01009078:31515-31830
HmHel-2	CAEZ01009078:46015-46609
HmHel-2	CAEZ01007812:78710-79305

HmHel-2	CAEZ01007812:209674-209992
HmHel-2	CAEZ01007812:74338-74899
HmHel-2	CAEZ01007056:9877-10472
HmHel-2	CAEZ01006727:1020-1614
HmHel-2	CAEZ01006352:14551-14855
HmHel-2	CAEZ01006352:25711-26305
HmHel-2	CAEZ01006352:44686-45271
HmHel-2	CAEZ01006352:59429-59599
HmHel-2	CAEZ01006352:140254-140504
HmHel-2	CAEZ01006352:117491-117631
HmHel-2	CAEZ01006352:43641-43887
HmHel-2	CAEZ01006352:497-955
HmHel-2	CAEZ01005851:5190-5785
HmHel-2	CAEZ01005851:26825-27425
HmHel-2	CAEZ01005403:1629-2205
HmHel-2	CAEZ01005403:32647-33241
HmHel-2	CAEZ01005241:27284-27878
HmHel-2	CAEZ01005241:16149-17750
HmHel-2	CAEZ01005237:437-1029
HmHel-2	CAEZ01004562:193502-194036
HmHel-2	CAEZ01004562:212073-212667
HmHel-2	CAEZ01004562:183042-183601
HmHel-2	CAEZ01004562:88514-89095
HmHel-2	CAEZ01004546:289-881
HmHel-2	CAEZ01004505:9809-10404
HmHel-2	CAEZ01004505:28125-28346
HmHel-2	CAEZ01003718:11011-11604
HmHel-2	CAEZ01003718:8932-9511
HmHel-2	CAEZ01003135:2283-2877
HmHel-2	CAEZ01003035:1920-2512
HmHel-2	CAEZ01002047:12740-13334
HmHel-2	CAEZ01001446:18452-19046
HmHel-2	CAEZ01011104:49212-49669
HmHel-2	CAEZ01011104:136839-137294
HmHel-2	CAEZ01011104:136240-136831
HmHel-2	CAEZ01007499:99448-100042
HmHel-2	CAEZ01006489:63840-64156
HmHel-2	CAEZ01006489:13429-14027
HmHel-2	CAEZ01003439:10439-11032
HmHel-2	CAEZ01011486:25215-25812
HmHel-2	CAEZ01011486:51920-52480
HmHel-2	CAEZ01010086:17145-17740
HmHel-2	CAEZ01009965:19227-19784
HmHel-2	CAEZ01009965:20375-20620
HmHel-2	CAEZ01008205:19841-20426
HmHel-2	CAEZ01005722:295-872
HmHel-2	CAEZ01005722:14030-14463
HmHel-2	CAEZ01005707:1663-2264

HmHel-2	CAEZ01004981:22699-23301
HmHel-2	CAEZ01004981:19977-20564
HmHel-2	CAEZ01001715:7194-7780
HmHel-2	CAEZ01009928:30682-31274
HmHel-2	CAEZ01009928:39834-40136
HmHel-2	CAEZ01009886:6675-6786
HmHel-2	CAEZ01009886:141308-141466
HmHel-2	CAEZ01009886:1149-1741
HmHel-2	CAEZ01009717:1994-2587
HmHel-2	CAEZ01009717:43753-44073
HmHel-2	CAEZ01009079:29027-29619
HmHel-2	CAEZ01008576:132695-133286
HmHel-2	CAEZ01008576:92962-93095
HmHel-2	CAEZ01007951:15204-15796
HmHel-2	CAEZ01006559:15369-15929
HmHel-2	CAEZ01006559:134-585
HmHel-2	CAEZ01006417:1407-1859
HmHel-2	CAEZ01006417:47307-47696
HmHel-2	CAEZ01006417:73069-73658
HmHel-2	CAEZ01006417:141647-142202
HmHel-2	CAEZ01005885:7389-7938
HmHel-2	CAEZ01005382:32993-33582
HmHel-2	CAEZ01005140:3411-4007
HmHel-2	CAEZ01004914:7763-8356
HmHel-2	CAEZ01004786:12726-12913
HmHel-2	CAEZ01004786:59820-60064
HmHel-2	CAEZ01004786:53977-54536
HmHel-2	CAEZ01004786:21801-22393
HmHel-2	CAEZ01004558:15961-16548
HmHel-2	CAEZ01004186:1-87
HmHel-2	CAEZ01004186:6988-7580
HmHel-2	CAEZ01004067:4863-5289
HmHel-2	CAEZ01004067:48139-48731
HmHel-2	CAEZ01003300:17417-18008
HmHel-2	CAEZ01003167:29342-29793
HmHel-2	CAEZ01003167:22636-23232
HmHel-2	CAEZ01003110:54751-55346
HmHel-2	CAEZ01003110:73479-73627
HmHel-2	CAEZ01003110:75509-75704
HmHel-2	CAEZ01003110:6263-6854
HmHel-2	CAEZ01001831:49519-50067
HmHel-2	CAEZ01001831:32830-41409
HmHel-2	CAEZ01010776:24182-24572
HmHel-2	CAEZ01010776:3117-3706
HmHel-2	CAEZ01010507:2424-3018
HmHel-2	CAEZ01009931:9771-10334
HmHel-2	CAEZ01009808:8523-9117
HmHel-2	CAEZ01008305:73289-73883

HmHel-2	CAEZ01008305:108931-109036
HmHel-2	CAEZ01008197:1-185
HmHel-2	CAEZ01008189:449-1041
HmHel-2	CAEZ01008185:12739-13324
HmHel-2	CAEZ01007861:179034-179489
HmHel-2	CAEZ01007861:204041-204519
HmHel-2	CAEZ01007861:117504-118099
HmHel-2	CAEZ01007861:73121-73579
HmHel-2	CAEZ01007722:4195-4653
HmHel-2	CAEZ01007722:37035-37361
HmHel-2	CAEZ01007722:94823-97514
HmHel-2	CAEZ01007722:155480-159382
HmHel-2	CAEZ01007722:128829-129150
HmHel-2	CAEZ01007722:44536-44993
HmHel-2	CAEZ01007568:114199-114793
HmHel-2	CAEZ01007568:26810-27014
HmHel-2	CAEZ01006400:14605-15198
HmHel-2	CAEZ01005033:673-1268
HmHel-2	CAEZ01004379:1383-1977
HmHel-2	CAEZ01003422:13923-14482
HmHel-2	CAEZ01003422:74242-74836
HmHel-2	CAEZ01002260:4166-4760
HmHel-2	CAEZ01001484:1302-1896
HmHel-2	CAEZ01001463:103283-103872
HmHel-2	CAEZ01001463:111435-112028
HmHel-2	CAEZ01010918:70384-70975
HmHel-2	CAEZ01010918:33759-34088
HmHel-2	CAEZ01010450:55041-55633
HmHel-2	CAEZ01010129:12232-12791
HmHel-2	CAEZ01010129:81579-82163
HmHel-2	CAEZ01007646:10856-11454
HmHel-2	CAEZ01007285:5467-6060
HmHel-2	CAEZ01006347:14650-15241
HmHel-2	CAEZ01006347:44774-45574
HmHel-2	CAEZ01006347:119682-119993
HmHel-2	CAEZ01006347:176481-176902
HmHel-2	CAEZ01006347:210040-210188
HmHel-2	CAEZ01006347:34151-34303
HmHel-2	CAEZ01005997:1464-1730
HmHel-2	CAEZ01003069:62326-62784
HmHel-2	CAEZ01003069:211024-211620
HmHel-2	CAEZ01003069:312757-313357
HmHel-2	CAEZ01003069:265821-266403
HmHel-2	CAEZ01003069:40254-40848
HmHel-2	CAEZ01000992:28121-28714
HmHel-2	CAEZ01010364:20843-21431
HmHel-2	CAEZ01010260:66827-67399
HmHel-2	CAEZ01010260:94485-94719

HmHel-2	CAEZ01010260:127913-128377
HmHel-2	CAEZ01010260:117645-118236
HmHel-2	CAEZ01010260:102889-103457
HmHel-2	CAEZ01010260:79497-79955
HmHel-2	CAEZ01010260:44999-45559
HmHel-2	CAEZ01008621:37609-38155
HmHel-2	CAEZ01008621:30533-30943
HmHel-2	CAEZ01008352:155114-155705
HmHel-2	CAEZ01008206:9347-9941
HmHel-2	CAEZ01005615:34544-35113
HmHel-2	CAEZ01005235:14684-15005
HmHel-2	CAEZ01005235:233367-233646
HmHel-2	CAEZ01005235:188834-188930
HmHel-2	CAEZ01005235:81072-81210
HmHel-2	CAEZ01005235:51104-51697
HmHel-2	CAEZ01004822:17789-18381
HmHel-2	CAEZ01004822:90030-90200
HmHel-2	CAEZ01004822:24083-24368
HmHel-2	CAEZ01003660:5869-6319
HmHel-2	CAEZ01003660:22430-22751
HmHel-2	CAEZ01010629:22513-23100
HmHel-2	CAEZ01010629:11573-12035
HmHel-2	CAEZ01009357:8919-9511
HmHel-2	CAEZ01009145:1373-1916
HmHel-2	CAEZ01009145:18483-18747
HmHel-2	CAEZ01009145:12679-13137
HmHel-2	CAEZ01008879:82744-83336
HmHel-2	CAEZ01008879:105238-105345
HmHel-2	CAEZ01008879:108164-108722
HmHel-2	CAEZ01008879:49306-49989
HmHel-2	CAEZ01008345:33467-35792
HmHel-2	CAEZ01008345:33970-34565
HmHel-2	CAEZ01008106:12313-12904
HmHel-2	CAEZ01008106:742-1337
HmHel-2	CAEZ01007324:10848-11440
HmHel-2	CAEZ01006056:21680-21947
HmHel-2	CAEZ01005813:2840-3385
HmHel-2	CAEZ01004751:21882-22463
HmHel-2	CAEZ01004751:56574-57147
HmHel-2	CAEZ01004531:9393-9986
HmHel-2	CAEZ01003075:32824-33384
HmHel-2	CAEZ01003007:9492-10050
HmHel-2	CAEZ01003007:60733-61292
HmHel-2	CAEZ01002654:3160-3708
HmHel-2	CAEZ01001934:1548-2143
HmHel-2	CAEZ01001934:45075-45662
HmHel-2	CAEZ01001934:8188-8733
HmHel-2	CAEZ01001836:3783-4371

HmHel-2	CAEZ01001087:6836-7428
HmHel-2	CAEZ01001078:1696-2280
HmHel-2	CAEZ01010863:9142-9736
HmHel-2	CAEZ01010863:13540-14095
HmHel-2	CAEZ01010725:13617-14211
HmHel-2	CAEZ01008381:2263-2856
HmHel-2	CAEZ01008101:11519-11951
HmHel-2	CAEZ01008101:28283-28877
HmHel-2	CAEZ01007740:33569-34019
HmHel-2	CAEZ01007740:19162-19745
HmHel-2	CAEZ01007730:16863-17441
HmHel-2	CAEZ01007730:91347-91940
HmHel-2	CAEZ01007730:100471-100657
HmHel-2	CAEZ01006984:8344-8484
HmHel-2	CAEZ01006377:7935-8528
HmHel-2	CAEZ01006249:53559-54150
HmHel-2	CAEZ01006249:20098-20565
HmHel-2	CAEZ01005897:3101-3665
HmHel-2	CAEZ01004682:59541-60135
HmHel-2	CAEZ01004682:42176-42627
HmHel-2	CAEZ01003681:6052-6647
HmHel-2	CAEZ01003413:15364-15958
HmHel-2	CAEZ01002103:995-1589
HmHel-2	CAEZ01001327:20545-21140
HmHel-2	CAEZ01008889:2891-3481
HmHel-2	CAEZ01008889:30195-30652
HmHel-2	CAEZ01008889:18581-20456
HmHel-2	CAEZ01008351:187673-188267
HmHel-2	CAEZ01008331:156-749
HmHel-2	CAEZ01001375:1880-2473
HmHel-2	CAEZ01006666:46859-47400
HmHel-2	CAEZ01006504:10856-11448
HmHel-2	CAEZ01006504:19615-19739
HmHel-2	CAEZ01004148:12410-13008
HmHel-2	CAEZ01003827:30576-31164
HmHel-2	CAEZ01003827:47298-47696
HmHel-2	CAEZ01002620:3068-3630
HmHel-2	CAEZ01002620:33667-34125
HmHel-2	CAEZ01002620:55574-56129
HmHel-2	CAEZ01002620:76389-76845
HmHel-2	CAEZ01002620:42740-43301
HmHel-2	CAEZ01001253:216-806
HmHel-2	CAEZ01011043:5288-5847
HmHel-2	CAEZ01008963:2388-2865
HmHel-2	CAEZ01008963:202016-202577
HmHel-2	CAEZ01008963:101940-102067
HmHel-2	CAEZ01008963:5665-6122
HmHel-2	CAEZ01008937:2245-2838

HmHel-2	CAEZ01008674:99445-99903
HmHel-2	CAEZ01008674:60230-60822
HmHel-2	CAEZ01008674:27857-28184
HmHel-2	CAEZ01007664:103-663
HmHel-2	CAEZ01007550:29491-30082
HmHel-2	CAEZ01006374:5-598
HmHel-2	CAEZ01005911:5891-6479
HmHel-2	CAEZ01005911:76790-77226
HmHel-2	CAEZ01005886:5299-5891
HmHel-2	CAEZ01005247:29496-30086
HmHel-2	CAEZ01004986:11513-12105
HmHel-2	CAEZ01002653:8076-8669
HmHel-2	CAEZ01011169:1967-2561
HmHel-2	CAEZ01011169:26157-26478
HmHel-2	CAEZ01011169:6619-7049
HmHel-2	CAEZ01009969:15356-15921
HmHel-2	CAEZ01009630:960-1553
HmHel-2	CAEZ01009150:47401-47992
HmHel-2	CAEZ01008849:35958-36402
HmHel-2	CAEZ01008531:14419-14877
HmHel-2	CAEZ01008531:25586-26179
HmHel-2	CAEZ01008063:13846-14441
HmHel-2	CAEZ01007626:108143-108702
HmHel-2	CAEZ01007626:129847-130097
HmHel-2	CAEZ01007626:60257-60816
HmHel-2	CAEZ01007626:45853-46443
HmHel-2	CAEZ01007579:1844-2438
HmHel-2	CAEZ01007101:38389-38843
HmHel-2	CAEZ01007101:63931-64054
HmHel-2	CAEZ01007101:93521-94115
HmHel-2	CAEZ01007101:67166-67529
HmHel-2	CAEZ01007101:24154-24713
HmHel-2	CAEZ01006981:11961-12555
HmHel-2	CAEZ01006440:84501-85111
HmHel-2	CAEZ01006440:125114-125603
HmHel-2	CAEZ01006440:159398-159992
HmHel-2	CAEZ01006440:15575-16115
HmHel-2	CAEZ01005889:6076-6671
HmHel-2	CAEZ01005257:2133-2727
HmHel-2	CAEZ01004349:10028-10612
HmHel-2	CAEZ01003975:9006-9128
HmHel-2	CAEZ01003975:497-1044
HmHel-2	CAEZ01003883:47250-47649
HmHel-2	CAEZ01003883:57080-57289
HmHel-2	CAEZ01003883:46059-46651
HmHel-2	CAEZ01010080:224-802
HmHel-2	CAEZ01008715:9998-10557
HmHel-2	CAEZ01007100:34637-35239

HmHel-2	CAEZ01006242:43162-43745
HmHel-2	CAEZ01004138:1573-2166
HmHel-2	CAEZ01000263:428-1009
HmHel-2	CAEZ01008620:41038-41483
HmHel-2	CAEZ01008620:124739-125300
HmHel-2	CAEZ01008620:166742-167029
HmHel-2	CAEZ01008620:197027-197485
HmHel-2	CAEZ01008356:37437-37895
HmHel-2	CAEZ01008356:50953-51531
HmHel-2	CAEZ01008356:5502-6060
HmHel-2	CAEZ01007731:51237-51804
HmHel-2	CAEZ01007731:106060-106295
HmHel-2	CAEZ01007731:119571-120029
HmHel-2	CAEZ01007731:114303-114894
HmHel-2	CAEZ01007731:17227-17333
HmHel-2	CAEZ01007727:58987-59545
HmHel-2	CAEZ01006618:1602-2189
HmHel-2	CAEZ01006618:105313-105623
HmHel-2	CAEZ01006618:239075-239215
HmHel-2	CAEZ01006618:258353-258811
HmHel-2	CAEZ01006618:214457-214609
HmHel-2	CAEZ01006618:44013-44600
HmHel-2	CAEZ01006434:3699-4153
HmHel-2	CAEZ01006434:71551-72144
HmHel-2	CAEZ01005462:23343-23943
HmHel-2	CAEZ01005462:20774-21231
HmHel-2	CAEZ01005267:50248-50700
HmHel-2	CAEZ01005267:127530-128091
HmHel-2	CAEZ01005267:4275-4733
HmHel-2	CAEZ01004919:6002-6587
HmHel-2	CAEZ01010690:10094-10552
HmHel-2	CAEZ01010453:20663-21260
HmHel-2	CAEZ01010453:62060-62518
HmHel-2	CAEZ01010453:116661-126559
HmHel-2	CAEZ01010453:56038-56721
HmHel-2	CAEZ01010453:37989-38442
HmHel-2	CAEZ01009024:33003-33596
HmHel-2	CAEZ01009024:43905-44364
HmHel-2	CAEZ01009024:29750-30208
HmHel-2	CAEZ01008721:118413-118871
HmHel-2	CAEZ01008721:194720-195315
HmHel-2	CAEZ01008721:164101-164644
HmHel-2	CAEZ01008721:77813-78269
HmHel-2	CAEZ01008721:66371-66962
HmHel-2	CAEZ01007597:11560-12155
HmHel-2	CAEZ01007240:2099-2690
HmHel-2	CAEZ01006149:3214-3802
HmHel-2	CAEZ01004863:466-1058

HmHel-2	CAEZ01004030:19614-20203
HmHel-2	CAEZ01004030:18492-18575
HmHel-2	CAEZ01003994:11711-12271
HmHel-2	CAEZ01003994:11265-11719
HmHel-2	CAEZ01003943:13964-14555
HmHel-2	CAEZ01002938:7250-7702
HmHel-2	CAEZ01002769:15682-16275
HmHel-2	CAEZ01002765:31048-31639
HmHel-2	CAEZ01001235:239-803
HmHel-2	CAEZ01001235:13654-14099
HmHel-2	CAEZ01000833:3635-3902
HmHel-2	CAEZ01000833:62463-63023
HmHel-2	CAEZ01009996:53319-53886
HmHel-2	CAEZ01009996:39351-39764
HmHel-2	CAEZ01009748:43691-43977
HmHel-2	CAEZ01009748:108249-108808
HmHel-2	CAEZ01008915:8956-9092
HmHel-2	CAEZ01008915:59499-59766
HmHel-2	CAEZ01008915:105797-106014
HmHel-2	CAEZ01008915:265797-266118
HmHel-2	CAEZ01008915:261737-261910
HmHel-2	CAEZ01008915:177862-178440
HmHel-2	CAEZ01008915:141574-141780
HmHel-2	CAEZ01008915:130596-131152
HmHel-2	CAEZ01008915:95692-96036
HmHel-2	CAEZ01008915:52495-53084
HmHel-2	CAEZ01008421:19923-20493
HmHel-2	CAEZ01008421:3525-4120
HmHel-2	CAEZ01007985:52101-52694
HmHel-2	CAEZ01007985:31009-31289
HmHel-2	CAEZ01007985:16770-17366
HmHel-2	CAEZ01007690:960-1545
HmHel-2	CAEZ01007259:79387-79674
HmHel-2	CAEZ01007259:97716-98274
HmHel-2	CAEZ01007259:28096-28699
HmHel-2	CAEZ01007259:9856-10421
HmHel-2	CAEZ01006923:8565-9022
HmHel-2	CAEZ01006923:15871-16465
HmHel-2	CAEZ01005211:55487-56082
HmHel-2	CAEZ01005211:134682-135090
HmHel-2	CAEZ01005203:137213-137807
HmHel-2	CAEZ01005162:28554-29013
HmHel-2	CAEZ01005162:75017-75611
HmHel-2	CAEZ01004835:14834-15423
HmHel-2	CAEZ01004260:18443-19018
HmHel-2	CAEZ01000081:20110-20704
HmHel-2	CAEZ01000062:1046-1640
HmHel-2	CAEZ01000062:41350-41936

HmHel-2	CAEZ01009773:11216-11798
HmHel-2	CAEZ01008761:422-1012
HmHel-2	CAEZ01008761:23873-24195
HmHel-2	CAEZ01008238:12790-13324
HmHel-2	CAEZ01004130:1-520
HmHel-2	CAEZ01003896:15163-15754
HmHel-2	CAEZ01003896:1619-2074
HmHel-2	CAEZ01010818:21383-21832
HmHel-2	CAEZ01009576:62802-63176
HmHel-2	CAEZ01009576:74754-75304
HmHel-2	CAEZ01009576:52212-52673
HmHel-2	CAEZ01009576:25991-26582
HmHel-2	CAEZ01009576:6643-7158
HmHel-2	CAEZ01007870:86037-86635
HmHel-2	CAEZ01007870:74259-74441
HmHel-2	CAEZ01005435:1136-1737
HmHel-2	CAEZ01002866:156325-156782
HmHel-2	CAEZ01002866:157225-157770
HmHel-2	CAEZ01002866:116634-117142
HmHel-2	CAEZ01002866:68834-71779
HmHel-2	CAEZ01011639:11606-12198
HmHel-2	CAEZ01011602:12735-13328
HmHel-2	CAEZ01010892:7112-7705
HmHel-2	CAEZ01010816:40397-40993
HmHel-2	CAEZ01010026:89823-90417
HmHel-2	CAEZ01010026:136832-137628
HmHel-2	CAEZ01009470:50266-50724
HmHel-2	CAEZ01009470:76492-76848
HmHel-2	CAEZ01009470:79599-79680
HmHel-2	CAEZ01009470:55664-56255
HmHel-2	CAEZ01009435:34116-34708
HmHel-2	CAEZ01009054:3220-3818
HmHel-2	CAEZ01007898:6598-7157
HmHel-2	CAEZ01007294:5741-6328
HmHel-2	CAEZ01007273:15451-16041
HmHel-2	CAEZ01007273:39454-39762
HmHel-2	CAEZ01007273:123621-123959
HmHel-2	CAEZ01007273:160389-160973
HmHel-2	CAEZ01007273:130697-131114
HmHel-2	CAEZ01007273:28842-29281
HmHel-2	CAEZ01007273:3343-3797
HmHel-2	CAEZ01007061:33466-33763
HmHel-2	CAEZ01007061:27300-27892
HmHel-2	CAEZ01007061:1411-1685
HmHel-2	CAEZ01006616:166655-167215
HmHel-2	CAEZ01006616:263234-263826
HmHel-2	CAEZ01006616:128327-128888
HmHel-2	CAEZ01006216:7105-7664

HmHel-2	CAEZ01005624:11711-12299
HmHel-2	CAEZ01005391:13920-14517
HmHel-2	CAEZ01004913:8634-9229
HmHel-2	CAEZ01004800:8355-8950
HmHel-2	CAEZ01003979:463-1056
HmHel-2	CAEZ01003193:27046-27640
HmHel-2	CAEZ01003193:39459-40047
HmHel-2	CAEZ01003193:34061-34499
HmHel-2	CAEZ01002709:11495-11792
HmHel-2	CAEZ01002709:5329-5921
HmHel-2	CAEZ01002680:50677-51135
HmHel-2	CAEZ01002680:32752-32973
HmHel-2	CAEZ01002433:318-911
HmHel-2	CAEZ01002332:30251-30847
HmHel-2	CAEZ01000961:19641-20237
HmHel-2	CAEZ01011513:4893-5485
HmHel-2	CAEZ01011033:1-455
HmHel-2	CAEZ01011033:9283-9774
HmHel-2	CAEZ01010716:8108-8665
HmHel-2	CAEZ01010561:14421-15007
HmHel-2	CAEZ01010474:18729-19357
HmHel-2	CAEZ01010430:7153-7654
HmHel-2	CAEZ01009775:25768-26226
HmHel-2	CAEZ01009775:3424-4006
HmHel-2	CAEZ01009136:16434-17028
HmHel-2	CAEZ01009136:21496-21875
HmHel-2	CAEZ01009076:25193-25782
HmHel-2	CAEZ01008414:10800-11395
HmHel-2	CAEZ01008190:3333-3775
HmHel-2	CAEZ01008190:11285-11845
HmHel-2	CAEZ01007707:16787-16942
HmHel-2	CAEZ01007707:80821-81102
HmHel-2	CAEZ01007707:127895-138039
HmHel-2	CAEZ01007707:153852-154033
HmHel-2	CAEZ01007707:100845-101144
HmHel-2	CAEZ01007707:53607-54201
HmHel-2	CAEZ01007707:22930-23184
HmHel-2	CAEZ01005764:408-1002
HmHel-2	CAEZ01005392:16240-16835
HmHel-2	CAEZ01005392:4652-5080
HmHel-2	CAEZ01005016:10715-11156
HmHel-2	CAEZ01004674:5065-5614
HmHel-2	CAEZ01004571:5205-5748
HmHel-2	CAEZ01002812:25372-25966
HmHel-2	CAEZ01001220:7778-8341
HmHel-2	CAEZ01001147:38027-38619
HmHel-2	CAEZ01001147:20182-23647
HmHel-2	CAEZ01011459:3595-4178

HmHel-2	CAEZ01011324:12310-12896
HmHel-2	CAEZ01009036:5534-6129
HmHel-2	CAEZ01007831:11002-11597
HmHel-2	CAEZ01003034:1-258
HmHel-2	CAEZ01003034:8584-9174
HmHel-2	CAEZ01003031:2636-3227
HmHel-2	CAEZ01008949:265-1675
HmHel-2	CAEZ01007721:2237-2695
HmHel-2	CAEZ01007721:28139-28728
HmHel-2	CAEZ01007496:53597-54189
HmHel-2	CAEZ01001805:52472-53065
HmHel-2	CAEZ01001805:83754-84355
HmHel-2	CAEZ01001805:68074-68530
HmHel-2	CAEZ01001805:33652-34208
HmHel-2	CAEZ01010737:40250-40718
HmHel-2	CAEZ01010737:68735-69191
HmHel-2	CAEZ01010737:135721-136023
HmHel-2	CAEZ01010737:95293-95469
HmHel-2	CAEZ01010737:73500-74035
HmHel-2	CAEZ01010737:61143-61591
HmHel-2	CAEZ01010367:6850-7410
HmHel-2	CAEZ01010367:19040-19455
HmHel-2	CAEZ01010367:9919-10512
HmHel-2	CAEZ01009436:19300-19702
HmHel-2	CAEZ01009436:92002-92582
HmHel-2	CAEZ01009311:9973-10532
HmHel-2	CAEZ01009196:11401-11960
HmHel-2	CAEZ01009196:72206-72480
HmHel-2	CAEZ01009196:32233-32550
HmHel-2	CAEZ01008234:44212-44760
HmHel-2	CAEZ01007863:29124-29662
HmHel-2	CAEZ01007863:112988-113573
HmHel-2	CAEZ01007863:85370-85962
HmHel-2	CAEZ01006988:1547-2148
HmHel-2	CAEZ01005696:6627-7219
HmHel-2	CAEZ01005219:1357-1952
HmHel-2	CAEZ01004995:13740-14343
HmHel-2	CAEZ01004743:4814-5373
HmHel-2	CAEZ01004497:24034-24456
HmHel-2	CAEZ01004497:1784-2344
HmHel-2	CAEZ01003265:64431-65529
HmHel-2	CAEZ01003265:68853-69117
HmHel-2	CAEZ01002568:11626-12210
HmHel-2	CAEZ01002216:1192-1532
HmHel-2	CAEZ01002087:15215-15811
HmHel-2	CAEZ01001932:51727-52319
HmHel-2	CAEZ01001932:26185-26282
HmHel-2	CAEZ01001921:638-1218

HmHel-2	CAEZ01001765:42978-43571
HmHel-2	CAEZ01001765:6866-7101
HmHel-2	CAEZ01001265:44652-45046
HmHel-2	CAEZ01001265:108884-109247
HmHel-2	CAEZ01001265:122080-122368
HmHel-2	CAEZ01001265:138053-138511
HmHel-2	CAEZ01001265:108571-108846
HmHel-2	CAEZ01001265:8813-9408
HmHel-2	CAEZ01001065:7699-8013
HmHel-2	CAEZ01001065:6341-6796
HmHel-2	CAEZ01010802:31172-31734
HmHel-2	CAEZ01010662:8482-9006
HmHel-2	CAEZ01010662:8194-8483
HmHel-2	CAEZ01008661:17585-18179
HmHel-2	CAEZ01007940:26558-27117
HmHel-2	CAEZ01007940:67679-68272
HmHel-2	CAEZ01005867:2422-3016
HmHel-2	CAEZ01005556:4506-5079
HmHel-2	CAEZ01005226:100-686
HmHel-2	CAEZ01005226:7127-7721
HmHel-2	CAEZ01005051:18839-19434
HmHel-2	CAEZ01005051:2095-2552
HmHel-2	CAEZ01004075:19504-20098
HmHel-2	CAEZ01003329:389-983
HmHel-2	CAEZ01002636:501-1030
HmHel-2	CAEZ01002636:1686-1962
HmHel-2	CAEZ01002329:3576-4144
HmHel-2	CAEZ01011238:11094-11684
HmHel-2	CAEZ01010745:7668-8256
HmHel-2	CAEZ01007785:20269-20864
HmHel-2	CAEZ01004651:1199-1744
HmHel-2	CAEZ01011197:137960-138521
HmHel-2	CAEZ01011197:4794-5252
HmHel-2	CAEZ01011174:1007-1604
HmHel-2	CAEZ01010797:68484-68873
HmHel-2	CAEZ01010797:167961-168558
HmHel-2	CAEZ01010797:156613-157046
HmHel-2	CAEZ01010797:85560-86936
HmHel-2	CAEZ01010670:5937-6532
HmHel-2	CAEZ01010254:2881-3468
HmHel-2	CAEZ01007648:533-1123
HmHel-2	CAEZ01005393:75255-75816
HmHel-2	CAEZ01005393:27434-27886
HmHel-2	CAEZ01003582:1016-1474
HmHel-2	CAEZ01003582:74253-74710
HmHel-2	CAEZ01003582:78779-79229
HmHel-2	CAEZ01003582:29078-29675
HmHel-2	CAEZ01011343:4850-5409

HmHel-2	CAEZ01010736:57114-57625
HmHel-2	CAEZ01010736:20100-20696
HmHel-2	CAEZ01010728:73741-74312
HmHel-2	CAEZ01010728:57031-57184
HmHel-2	CAEZ01010728:35061-35363
HmHel-2	CAEZ01010728:23487-24079
HmHel-2	CAEZ01010431:17090-17650
HmHel-2	CAEZ01010431:13329-13920
HmHel-2	CAEZ01010219:99905-100162
HmHel-2	CAEZ01010219:159272-159831
HmHel-2	CAEZ01010219:172875-173237
HmHel-2	CAEZ01010219:18516-21878
HmHel-2	CAEZ01009458:29462-29923
HmHel-2	CAEZ01009458:119229-119550
HmHel-2	CAEZ01009458:139022-139614
HmHel-2	CAEZ01009458:123477-123923
HmHel-2	CAEZ01009458:35843-36189
HmHel-2	CAEZ01009104:11160-11433
HmHel-2	CAEZ01008435:18947-19681
HmHel-2	CAEZ01008435:45984-46442
HmHel-2	CAEZ01008435:74383-74944
HmHel-2	CAEZ01008435:107308-107734
HmHel-2	CAEZ01008435:171372-171827
HmHel-2	CAEZ01008435:219950-220408
HmHel-2	CAEZ01008435:229423-229880
HmHel-2	CAEZ01008435:134650-135109
HmHel-2	CAEZ01008435:104129-104394
HmHel-2	CAEZ01008435:60912-61370
HmHel-2	CAEZ01008415:18034-18622
HmHel-2	CAEZ01008100:10518-10976
HmHel-2	CAEZ01008100:80165-80622
HmHel-2	CAEZ01008100:56113-56375
HmHel-2	CAEZ01008100:39797-40092
HmHel-2	CAEZ01007280:293-751
HmHel-2	CAEZ01005039:4976-5435
HmHel-2	CAEZ01004486:48538-49131
HmHel-2	CAEZ01004486:20275-20702
HmHel-2	CAEZ01003316:11682-12274
HmHel-2	CAEZ01003248:9883-10443
HmHel-2	CAEZ01003248:73114-73562
HmHel-2	CAEZ01003248:59613-60071
HmHel-2	CAEZ01003006:24736-25328
HmHel-2	CAEZ01008555:3188-3792
HmHel-2	CAEZ01008409:10513-11133
HmHel-2	CAEZ01008409:29298-29853
HmHel-2	CAEZ01006961:48669-49249
HmHel-2	CAEZ01006961:44309-44871
HmHel-2	CAEZ01006581:3415-4006

HmHel-2	CAEZ01006403:42542-43115
HmHel-2	CAEZ01006403:115002-115441
HmHel-2	CAEZ01006403:87561-88077
HmHel-2	CAEZ01003831:8260-8854
HmHel-2	CAEZ01003292:63976-64564
HmHel-2	CAEZ01003292:98875-99333
HmHel-2	CAEZ01011210:2803-3405
HmHel-2	CAEZ01008763:3422-4007
HmHel-2	CAEZ01007665:1237-1558
HmHel-2	CAEZ01007665:57289-57878
HmHel-2	CAEZ01006624:858-1454
HmHel-2	CAEZ01005475:79887-80341
HmHel-2	CAEZ01005475:139427-139980
HmHel-2	CAEZ01005475:155301-155494
HmHel-2	CAEZ01005475:140957-141221
HmHel-2	CAEZ01005475:40338-40614
HmHel-2	CAEZ01004014:3-355
HmHel-2	CAEZ01004014:60586-61119
HmHel-2	CAEZ01004014:39379-40138
HmHel-2	CAEZ01004014:2509-2965
HmHel-2	CAEZ01002825:963-1552
HmHel-2	CAEZ01002825:23131-23573
HmHel-2	CAEZ01001846:2123-2724
HmHel-2	CAEZ01001799:5005-5575
HmHel-2	CAEZ01001799:7735-8324
HmHel-2	CAEZ01011093:19818-20354
HmHel-2	CAEZ01011093:180168-180726
HmHel-2	CAEZ01011093:187472-187790
HmHel-2	CAEZ01011093:71684-71812
HmHel-2	CAEZ01011093:21649-22107
HmHel-2	CAEZ01003111:32088-32644
HmHel-2	CAEZ01003111:6760-6877
HmHel-2	CAEZ01011051:17774-19008
HmHel-2	CAEZ01010965:2433-2532
HmHel-2	CAEZ01010965:19382-19937
HmHel-2	CAEZ01010667:6579-7138
HmHel-2	CAEZ01010667:32974-33434
HmHel-2	CAEZ01010667:46244-46803
HmHel-2	CAEZ01010667:47539-48135
HmHel-2	CAEZ01010667:19027-19479
HmHel-2	CAEZ01010072:659-1215
HmHel-2	CAEZ01010041:24398-24796
HmHel-2	CAEZ01010041:23420-24008
HmHel-2	CAEZ01008437:71475-72043
HmHel-2	CAEZ01008437:58810-59097
HmHel-2	CAEZ01007396:5556-6116
HmHel-2	CAEZ01006220:40037-40597
HmHel-2	CAEZ01006220:11260-11996

HmHel-2	CAEZ01005732:11267-11724
HmHel-2	CAEZ01005196:27354-27947
HmHel-2	CAEZ01005196:8389-8983
HmHel-2	CAEZ01004929:489-1082
HmHel-2	CAEZ01004819:41411-41999
HmHel-2	CAEZ01003968:2647-3207
HmHel-2	CAEZ01003488:19775-20335
HmHel-2	CAEZ01003286:13501-14060
HmHel-2	CAEZ01002341:9148-9707
HmHel-2	CAEZ01002323:3499-4058
HmHel-2	CAEZ01002323:54333-54880
HmHel-2	CAEZ01002323:65978-66431
HmHel-2	CAEZ01002323:51142-51595
HmHel-2	CAEZ01002323:39665-40121
HmHel-2	CAEZ01002323:35-299
HmHel-2	CAEZ01008803:34954-43016
HmHel-2	CAEZ01008500:90756-91316
HmHel-2	CAEZ01008500:54427-54884
HmHel-2	CAEZ01008500:16662-17077
HmHel-2	CAEZ01008500:2478-2854
HmHel-2	CAEZ01007705:60279-60553
HmHel-2	CAEZ01007705:79759-80205
HmHel-2	CAEZ01007705:73384-73920
HmHel-2	CAEZ01006423:54263-54865
HmHel-2	CAEZ01006423:262496-262637
HmHel-2	CAEZ01006423:349181-349626
HmHel-2	CAEZ01006423:189835-190266
HmHel-2	CAEZ01006423:160916-160995
HmHel-2	CAEZ01006423:5645-5935
HmHel-2	CAEZ01006285:884-2306
HmHel-2	CAEZ01006285:7904-8498
HmHel-2	CAEZ01006162:129638-130233
HmHel-2	CAEZ01006162:56589-57047
HmHel-2	CAEZ01006162:29603-29827
HmHel-2	CAEZ01005955:36581-37086
HmHel-2	CAEZ01005955:15355-15902
HmHel-2	CAEZ01011423:7134-7732
HmHel-2	CAEZ01011414:65425-66018
HmHel-2	CAEZ01007827:946-1495
HmHel-2	CAEZ01004643:6685-7394
HmHel-2	CAEZ01004643:43205-43661
HmHel-2	CAEZ01004643:1-555
HmHel-2	CAEZ01004077:5049-5645
HmHel-2	CAEZ01004077:3436-3897
HmHel-2	CAEZ01002301:5982-6562
HmHel-2	CAEZ01009800:13006-13578
HmHel-2	CAEZ01008168:13567-14023
HmHel-2	CAEZ01008168:28718-29148

HmHel-2	CAEZ01008021:27129-27683
HmHel-2	CAEZ01007925:2487-3045
HmHel-2	CAEZ01004677:10873-11329
HmHel-2	CAEZ01004677:35029-35452
HmHel-2	CAEZ01004677:75279-75533
HmHel-2	CAEZ01004677:60278-62357
HmHel-2	CAEZ01004677:1506-1964
HmHel-2	CAEZ01002713:3391-3935
HmHel-2	CAEZ01002015:15146-15707
HmHel-2	CAEZ01011421:8925-9509
HmHel-2	CAEZ01009510:7134-7693
HmHel-2	CAEZ01009510:9670-9825
HmHel-2	CAEZ01009014:1853-2411
HmHel-2	CAEZ01006530:17157-17242
HmHel-2	CAEZ01006530:83380-83458
HmHel-2	CAEZ01006530:52274-52830
HmHel-2	CAEZ01006121:214440-214839
HmHel-2	CAEZ01006121:244537-244978
HmHel-2	CAEZ01006121:225251-225847
HmHel-2	CAEZ01006121:83429-83988
HmHel-2	CAEZ01005975:93941-94529
HmHel-2	CAEZ01005975:127988-128713
HmHel-2	CAEZ01005975:120891-121342
HmHel-2	CAEZ01005606:12543-12975
HmHel-2	CAEZ01005606:65988-66547
HmHel-2	CAEZ01005606:138865-139325
HmHel-2	CAEZ01005416:35796-36255
HmHel-2	CAEZ01005416:57012-57142
HmHel-2	CAEZ01005416:43666-44226
HmHel-2	CAEZ01005416:29538-30112
HmHel-2	CAEZ01005086:3697-4257
HmHel-2	CAEZ01004515:39881-40574
HmHel-2	CAEZ01003802:22765-23118
HmHel-2	CAEZ01003802:4937-5389
HmHel-2	CAEZ01000812:549-1142
HmHel-2	CAEZ01010808:701-1021
HmHel-2	CAEZ01010808:120883-121341
HmHel-2	CAEZ01010808:219529-219973
HmHel-2	CAEZ01010808:193678-194232
HmHel-2	CAEZ01010808:146522-146842
HmHel-2	CAEZ01010795:13426-13571
HmHel-2	CAEZ01010795:26326-26921
HmHel-2	CAEZ01010673:4661-5219
HmHel-2	CAEZ01008460:940-1398
HmHel-2	CAEZ01006534:66948-67406
HmHel-2	CAEZ01006534:42955-43513
HmHel-2	CAEZ01006260:8228-8823
HmHel-2	CAEZ01003868:2231-2825

HmHel-2	CAEZ01003722:13036-13629
HmHel-2	CAEZ01003722:10406-10951
HmHel-2	CAEZ01000969:19094-19656
HmHel-2	CAEZ01011258:5652-6210
HmHel-2	CAEZ01011048:127438-128016
HmHel-2	CAEZ01011048:94983-95250
HmHel-2	CAEZ01011048:12845-13295
HmHel-2	CAEZ01010183:1-545
HmHel-2	CAEZ01010183:12290-12850
HmHel-2	CAEZ01009871:13430-13984
HmHel-2	CAEZ01009306:83868-84346
HmHel-2	CAEZ01009306:130688-130945
HmHel-2	CAEZ01009306:111415-111873
HmHel-2	CAEZ01009306:63500-64099
HmHel-2	CAEZ01007272:40401-40834
HmHel-2	CAEZ01007272:85292-85888
HmHel-2	CAEZ01007272:27774-28232
HmHel-2	CAEZ01005399:17791-18341
HmHel-2	CAEZ01003516:4618-5076
HmHel-2	CAEZ01003516:1-411
HmHel-2	CAEZ01003165:14803-15333
HmHel-2	CAEZ01007920:4453-5042
HmHel-2	CAEZ01007478:121018-121384
HmHel-2	CAEZ01007478:118140-118696
HmHel-2	CAEZ01007478:107354-107812
HmHel-2	CAEZ01007383:183-676
HmHel-2	CAEZ01010665:4491-5050
HmHel-2	CAEZ01010665:40436-40995
HmHel-2	CAEZ01009424:30354-30913
HmHel-2	CAEZ01008632:21674-22234
HmHel-2	CAEZ01007373:7455-8050
HmHel-2	CAEZ01007065:748-1343
HmHel-2	CAEZ01006671:9339-9931
HmHel-2	CAEZ01005521:1675-2234
HmHel-2	CAEZ01005402:5426-5694
HmHel-2	CAEZ01005402:39572-39795
HmHel-2	CAEZ01005402:65795-66378
HmHel-2	CAEZ01005402:4652-4798
HmHel-2	CAEZ01004373:5430-5989
HmHel-2	CAEZ01003824:1030-1589
HmHel-2	CAEZ01003625:1752-2312
HmHel-2	CAEZ01002911:1690-2284
HmHel-2	CAEZ01011289:7210-7819
HmHel-2	CAEZ01011082:4202-4791
HmHel-2	CAEZ01009063:15982-16577
HmHel-2	CAEZ01009063:39729-39822
HmHel-2	CAEZ01009063:3870-4425
HmHel-2	CAEZ01008673:535-777

HmHel-2	CAEZ01008673:26693-27251
HmHel-2	CAEZ01008673:30755-36548
HmHel-2	CAEZ01007002:11748-12306
HmHel-2	CAEZ01004815:47090-47685
HmHel-2	CAEZ01003909:4172-4762
HmHel-2	CAEZ01003238:2192-2780
HmHel-2	CAEZ01002906:2446-3000
HmHel-2	CAEZ01001892:21274-21832
HmHel-2	CAEZ01001823:1717-2311
HmHel-2	CAEZ01001556:6463-7057
HmHel-2	CAEZ01006057:7009-7598
HmHel-2	CAEZ01005496:93990-94548
HmHel-2	CAEZ01005145:50784-51120
HmHel-2	CAEZ01003349:9236-9675
HmHel-2	CAEZ01003349:54143-54600
HmHel-2	CAEZ01003349:44211-44796
HmHel-2	CAEZ01003349:6095-6293
HmHel-2	CAEZ01003143:21863-22426
HmHel-2	CAEZ01003143:1442-1997
HmHel-2	CAEZ01000912:46410-46868
HmHel-2	CAEZ01000912:7138-7732
HmHel-2	CAEZ01009933:2980-3536
HmHel-2	CAEZ01007413:31167-31696
HmHel-2	CAEZ01007413:33179-33776
HmHel-2	CAEZ01007413:19073-19659
HmHel-2	CAEZ01006689:62528-66189
HmHel-2	CAEZ01002910:297-881
HmHel-2	CAEZ01001326:3650-4206
HmHel-2	CAEZ01011101:26569-27112
HmHel-2	CAEZ01011101:72448-73975
HmHel-2	CAEZ01011101:77885-78445
HmHel-2	CAEZ01011101:56455-56732
HmHel-2	CAEZ01011101:25877-26256
HmHel-2	CAEZ01011050:33363-33922
HmHel-2	CAEZ01009676:25969-26525
HmHel-2	CAEZ01008574:111320-111773
HmHel-2	CAEZ01008574:87521-88081
HmHel-2	CAEZ01006055:2829-3433
HmHel-2	CAEZ01006055:13980-14440
HmHel-2	CAEZ01009883:16286-16744
HmHel-2	CAEZ01009883:114242-114789
HmHel-2	CAEZ01009379:54082-54538
HmHel-2	CAEZ01007267:7-256
HmHel-2	CAEZ01007267:37923-38486
HmHel-2	CAEZ01006562:32237-32831
HmHel-2	CAEZ01006562:148472-148929
HmHel-2	CAEZ01005729:1144-1698
HmHel-2	CAEZ01005729:30097-30501

HmHel-2	CAEZ01003067:57329-57892
HmHel-2	CAEZ01003067:10641-10957
HmHel-2	CAEZ01001102:54933-55524
HmHel-2	CAEZ01011640:1486-2043
HmHel-2	CAEZ01010139:83821-84274
HmHel-2	CAEZ01010139:47548-48100
HmHel-2	CAEZ01009012:2931-3456
HmHel-2	CAEZ01009012:14551-15148
HmHel-2	CAEZ01007708:1-211
HmHel-2	CAEZ01007708:61694-62126
HmHel-2	CAEZ01007708:78680-79233
HmHel-2	CAEZ01007708:41784-41987
HmHel-2	CAEZ01007708:1697-2008
HmHel-2	CAEZ01004222:66670-67213
HmHel-2	CAEZ01004222:32879-33469
HmHel-2	CAEZ01003663:12751-13315
HmHel-2	CAEZ01002787:5305-5867
HmHel-2	CAEZ01011607:6856-7410
HmHel-2	CAEZ01009140:3481-4093
HmHel-2	CAEZ01009120:13669-13919
HmHel-2	CAEZ01009120:71150-71705
HmHel-2	CAEZ01009120:61799-62387
HmHel-2	CAEZ01007584:158152-158610
HmHel-2	CAEZ01007584:114567-115127
HmHel-2	CAEZ01007067:8890-9346
HmHel-2	CAEZ01005080:3783-4336
HmHel-2	CAEZ01005080:1-425
HmHel-2	CAEZ01004817:1337-1895
HmHel-2	CAEZ01003250:60445-60876
HmHel-2	CAEZ01003250:144193-144751
HmHel-2	CAEZ01003250:162058-162515
HmHel-2	CAEZ01003250:196108-196426
HmHel-2	CAEZ01003152:25328-25889
HmHel-2	CAEZ01011139:460-1020
HmHel-2	CAEZ01011139:44352-44673
HmHel-2	CAEZ01010896:31101-31335
HmHel-2	CAEZ01010896:107669-108130
HmHel-2	CAEZ01010896:118864-119322
HmHel-2	CAEZ01010896:334169-334728
HmHel-2	CAEZ01010674:17976-18535
HmHel-2	CAEZ01009842:2386-2945
HmHel-2	CAEZ01009842:15377-16000
HmHel-2	CAEZ01009239:2254-2844
HmHel-2	CAEZ01008588:19771-20196
HmHel-2	CAEZ01008588:4256-4815
HmHel-2	CAEZ01008275:1-509
HmHel-2	CAEZ01006632:7276-7860
HmHel-2	CAEZ01006302:16031-16748

HmHel-2	CAEZ01005586:41382-41783
HmHel-2	CAEZ01005586:47263-47822
HmHel-2	CAEZ01004699:11732-12340
HmHel-2	CAEZ01004408:12500-13108
HmHel-2	CAEZ01004202:24518-24974
HmHel-2	CAEZ01004202:39918-40258
HmHel-2	CAEZ01004202:20974-21533
HmHel-2	CAEZ01002676:4286-4845
HmHel-2	CAEZ01009971:4192-4750
HmHel-2	CAEZ01009971:58724-59182
HmHel-2	CAEZ01009971:8425-8772
HmHel-2	CAEZ01009428:15-469
HmHel-2	CAEZ01005848:17897-18487
HmHel-2	CAEZ01005148:3626-3799
HmHel-2	CAEZ01005148:19987-20539
HmHel-2	CAEZ01005148:55836-56463
HmHel-2	CAEZ01005148:59249-59350
HmHel-2	CAEZ01003517:36198-36756
HmHel-2	CAEZ01003050:11121-11721
HmHel-2	CAEZ01001858:22945-23383
HmHel-2	CAEZ01001825:14456-15014
HmHel-2	CAEZ01001825:27761-28219
HmHel-2	CAEZ01001643:562-1156
HmHel-2	CAEZ01009514:1-569
HmHel-2	CAEZ01006634:26862-27015
HmHel-2	CAEZ01006634:33990-34588
HmHel-2	CAEZ01006634:394-942
HmHel-2	CAEZ01005593:2300-2868
HmHel-2	CAEZ01004858:9431-10020
HmHel-2	CAEZ01004493:473-1057
HmHel-2	CAEZ01010322:2235-2831
HmHel-2	CAEZ01009426:6269-6825
HmHel-2	CAEZ01008229:4627-5339
HmHel-2	CAEZ01007743:1833-2376
HmHel-2	CAEZ01006475:2563-3124
HmHel-2	CAEZ01005385:35614-36046
HmHel-2	CAEZ01005385:10278-10847
HmHel-2	CAEZ01005315:112-670
HmHel-2	CAEZ01003388:2604-3164
HmHel-2	CAEZ01002863:22716-23165
HmHel-2	CAEZ01002863:70884-71119
HmHel-2	CAEZ01002863:5851-6408
HmHel-2	CAEZ01002352:1979-2563
HmHel-2	CAEZ01002215:213-758
HmHel-2	CAEZ01001755:3105-3663
HmHel-2	CAEZ01001432:1-545
HmHel-2	CAEZ01000068:25965-26515
HmHel-2	CAEZ01011054:38040-38599

HmHel-2	CAEZ01010888:8644-9204
HmHel-2	CAEZ01010590:151-710
HmHel-2	CAEZ01010423:1196-1738
HmHel-2	CAEZ01010357:126-686
HmHel-2	CAEZ01009930:25669-26121
HmHel-2	CAEZ01009930:2372-2968
HmHel-2	CAEZ01009147:27342-27953
HmHel-2	CAEZ01008944:67974-68561
HmHel-2	CAEZ01008944:25893-26345
HmHel-2	CAEZ01008944:3-551
HmHel-2	CAEZ01008470:28282-28875
HmHel-2	CAEZ01008327:1010-1520
HmHel-2	CAEZ01008327:18110-18669
HmHel-2	CAEZ01007853:13702-14004
HmHel-2	CAEZ01007853:114056-114642
HmHel-2	CAEZ01007853:89072-89624
HmHel-2	CAEZ01007853:75199-75758
HmHel-2	CAEZ01007502:9824-10568
HmHel-2	CAEZ01007336:3895-4454
HmHel-2	CAEZ01006039:1875-2430
HmHel-2	CAEZ01004519:13008-13601
HmHel-2	CAEZ01004519:33083-33353
HmHel-2	CAEZ01004519:6333-6791
HmHel-2	CAEZ01004128:8824-9384
HmHel-2	CAEZ01002148:5603-6162
HmHel-2	CAEZ01001911:757-1316
HmHel-2	CAEZ01001842:12324-12884
HmHel-2	CAEZ01010968:10316-10773
HmHel-2	CAEZ01010968:33293-33887
HmHel-2	CAEZ01010926:22080-22412
HmHel-2	CAEZ01010926:141464-141984
HmHel-2	CAEZ01010926:123004-123439
HmHel-2	CAEZ01010926:107844-108396
HmHel-2	CAEZ01010926:22423-23017
HmHel-2	CAEZ01009422:45467-45923
HmHel-2	CAEZ01009422:91740-92333
HmHel-2	CAEZ01009422:111763-111934
HmHel-2	CAEZ01009422:86614-87071
HmHel-2	CAEZ01009422:70071-70391
HmHel-2	CAEZ01004818:118528-119404
HmHel-2	CAEZ01004818:140804-141085
HmHel-2	CAEZ01004818:43887-44453
HmHel-2	CAEZ01004803:8332-8882
HmHel-2	CAEZ01003174:8077-8684
HmHel-2	CAEZ01000807:7592-8178
HmHel-2	CAEZ01009834:1502-2095
HmHel-2	CAEZ01009473:8611-9200
HmHel-2	CAEZ01009473:5167-5722

HmHel-2	CAEZ01007165:11719-12314
HmHel-2	CAEZ01007019:3805-4430
HmHel-2	CAEZ01006899:2480-3041
HmHel-2	CAEZ01006776:25253-25812
HmHel-2	CAEZ01006776:4307-4759
HmHel-2	CAEZ01002732:97406-97862
HmHel-2	CAEZ01002732:80645-81103
HmHel-2	CAEZ01011606:121-679
HmHel-2	CAEZ01010971:7151-7727
HmHel-2	CAEZ01010361:11023-11586
HmHel-2	CAEZ01008489:20674-21218
HmHel-2	CAEZ01008085:2383-2983
HmHel-2	CAEZ01008085:17333-17877
HmHel-2	CAEZ01007563:3034-3596
HmHel-2	CAEZ01005992:23199-23794
HmHel-2	CAEZ01005228:33857-34411
HmHel-2	CAEZ01004120:2544-3112
HmHel-2	CAEZ01003650:2886-3446
HmHel-2	CAEZ01003650:17777-18176
HmHel-2	CAEZ01003650:29854-30194
HmHel-2	CAEZ01002383:7354-7497
HmHel-2	CAEZ01002383:27130-27686
HmHel-2	CAEZ01001772:8610-9075
HmHel-2	CAEZ01001331:88343-88895
HmHel-2	CAEZ01001331:103469-103927
HmHel-2	CAEZ01011627:39533-40092
HmHel-2	CAEZ01011627:82073-82360
HmHel-2	CAEZ01011049:31633-32191
HmHel-2	CAEZ01011049:58772-59331
HmHel-2	CAEZ01010534:94074-94633
HmHel-2	CAEZ01009989:25314-25874
HmHel-2	CAEZ01009989:18885-19140
HmHel-2	CAEZ01009885:78123-78202
HmHel-2	CAEZ01009885:36173-36609
HmHel-2	CAEZ01009885:21123-21713
HmHel-2	CAEZ01009133:13179-13768
HmHel-2	CAEZ01009046:24252-24800
HmHel-2	CAEZ01009028:432-978
HmHel-2	CAEZ01006332:36694-37253
HmHel-2	CAEZ01006199:4419-4978
HmHel-2	CAEZ01005841:40855-41413
HmHel-2	CAEZ01005474:34203-34762
HmHel-2	CAEZ01004578:1905-2464
HmHel-2	CAEZ01002715:5018-5575
HmHel-2	CAEZ01002689:4604-5162
HmHel-2	CAEZ01011105:37526-38120
HmHel-2	CAEZ01010150:1-452
HmHel-2	CAEZ01010150:38808-39366

HmHel-2	CAEZ01010150:46995-47451
HmHel-2	CAEZ01010039:153811-154376
HmHel-2	CAEZ01009506:9588-10012
HmHel-2	CAEZ01009506:8147-8605
HmHel-2	CAEZ01008931:12956-13514
HmHel-2	CAEZ01002772:11377-11624
HmHel-2	CAEZ01002772:35645-36208
HmHel-2	CAEZ01000831:7082-7629
HmHel-2	CAEZ01008811:1498-2059
HmHel-2	CAEZ01005418:62711-63302
HmHel-2	CAEZ01005418:107239-107693
HmHel-2	CAEZ01005418:324319-324777
HmHel-2	CAEZ01005418:297314-305049
HmHel-2	CAEZ01005418:259016-259470
HmHel-2	CAEZ01005418:217069-217299
HmHel-2	CAEZ01005418:163649-164107
HmHel-2	CAEZ01005418:125120-127292
HmHel-2	CAEZ01005418:105211-105418
HmHel-2	CAEZ01005418:45278-45726
HmHel-2	CAEZ01005418:8627-8929
HmHel-2	CAEZ01003190:5757-6349
HmHel-2	CAEZ01003190:11843-12075
HmHel-2	CAEZ01002606:4799-5386
HmHel-2	CAEZ01010073:9280-9838
HmHel-2	CAEZ01008376:10115-10675
HmHel-2	CAEZ01007030:4352-4910
HmHel-2	CAEZ01006849:107-705
HmHel-2	CAEZ01006836:13602-14200
HmHel-2	CAEZ01006765:3901-4459
HmHel-2	CAEZ01006619:1003-1461
HmHel-2	CAEZ01006619:27901-28489
HmHel-2	CAEZ01006287:39627-40179
HmHel-2	CAEZ01006287:55084-55538
HmHel-2	CAEZ01005502:7220-7765
HmHel-2	CAEZ01005427:15952-16197
HmHel-2	CAEZ01005427:6608-7160
HmHel-2	CAEZ01003233:13266-13660
HmHel-2	CAEZ01003233:33278-33581
HmHel-2	CAEZ01003233:58620-59178
HmHel-2	CAEZ01001004:63412-64008
HmHel-2	CAEZ01001004:152371-152928
HmHel-2	CAEZ01001004:69516-69975
HmHel-2	CAEZ01010729:16438-16865
HmHel-2	CAEZ01010729:72098-72386
HmHel-2	CAEZ01010729:45365-45954
HmHel-2	CAEZ01010190:28711-29270
HmHel-2	CAEZ01009413:10478-11037
HmHel-2	CAEZ01008413:60857-61416

HmHel-2	CAEZ01007983:38871-39430
HmHel-2	CAEZ01007983:174663-175256
HmHel-2	CAEZ01007983:139439-139900
HmHel-2	CAEZ01007983:93809-94209
HmHel-2	CAEZ01007960:55604-56192
HmHel-2	CAEZ01007960:51996-52574
HmHel-2	CAEZ01007960:15926-16383
HmHel-2	CAEZ01007361:9999-10560
HmHel-2	CAEZ01007339:21438-21997
HmHel-2	CAEZ01006224:3687-4271
HmHel-2	CAEZ01005977:92281-92736
HmHel-2	CAEZ01005977:24554-25005
HmHel-2	CAEZ01005977:13474-14022
HmHel-2	CAEZ01003602:2521-3116
HmHel-2	CAEZ01003437:3459-4014
HmHel-2	CAEZ01003132:22283-22842
HmHel-2	CAEZ01002943:6154-6713
HmHel-2	CAEZ01001663:326-873
HmHel-2	CAEZ01001153:16638-17197
HmHel-2	CAEZ01009932:4639-5206
HmHel-2	CAEZ01009932:81232-81691
HmHel-2	CAEZ01009932:64021-64491
HmHel-2	CAEZ01009932:25091-25547
HmHel-2	CAEZ01009932:12889-13237
HmHel-2	CAEZ01009932:1-410
HmHel-2	CAEZ01008270:11722-12267
HmHel-2	CAEZ01008270:44210-44943
HmHel-2	CAEZ01008270:91926-92027
HmHel-2	CAEZ01008270:34752-35264
HmHel-2	CAEZ01007198:391-946
HmHel-2	CAEZ01006847:112793-113346
HmHel-2	CAEZ01006847:85076-85515
HmHel-2	CAEZ01006847:22949-23400
HmHel-2	CAEZ01005015:95145-95700
HmHel-2	CAEZ01005015:228087-228326
HmHel-2	CAEZ01005015:12548-12832
HmHel-2	CAEZ01003768:6892-7450
HmHel-2	CAEZ01003178:32102-32422
HmHel-2	CAEZ01003178:8146-8700
HmHel-2	CAEZ01001740:48072-48529
HmHel-2	CAEZ01001740:139582-140154
HmHel-2	CAEZ01001740:163382-163668
HmHel-2	CAEZ01001740:120555-120876
HmHel-2	CAEZ01001740:102473-103026
HmHel-2	CAEZ01010898:16290-16843
HmHel-2	CAEZ01008643:28308-28896
HmHel-2	CAEZ01008643:52767-53215
HmHel-2	CAEZ01008643:42078-42588

HmHel-2	CAEZ01008425:66612-66933
HmHel-2	CAEZ01008425:113252-113850
HmHel-2	CAEZ01005695:9931-10484
HmHel-2	CAEZ01005361:16476-17029
HmHel-2	CAEZ01005230:120639-121034
HmHel-2	CAEZ01005230:102230-104298
HmHel-2	CAEZ01005230:90396-90992
HmHel-2	CAEZ01005230:18419-18861
HmHel-2	CAEZ01005156:1-531
HmHel-2	CAEZ01005099:1-478
HmHel-2	CAEZ01002608:2633-3089
HmHel-2	CAEZ01002608:42223-42795
HmHel-2	CAEZ01002608:27567-28058
HmHel-2	CAEZ01010428:37961-38529
HmHel-2	CAEZ01010428:1-123
HmHel-2	CAEZ01010268:33229-33785
HmHel-2	CAEZ01010163:17260-17817
HmHel-2	CAEZ01009729:20612-21156
HmHel-2	CAEZ01009729:42672-43134
HmHel-2	CAEZ01009420:1893-2174
HmHel-2	CAEZ01009420:23052-23413
HmHel-2	CAEZ01009420:31437-31687
HmHel-2	CAEZ01009420:16445-16863
HmHel-2	CAEZ01008104:28163-28739
HmHel-2	CAEZ01007914:69-551
HmHel-2	CAEZ01007498:43742-44203
HmHel-2	CAEZ01007419:396-992
HmHel-2	CAEZ01006773:108488-108805
HmHel-2	CAEZ01006773:191079-191639
HmHel-2	CAEZ01006773:36445-36901
HmHel-2	CAEZ01005438:11831-12389
HmHel-2	CAEZ01004770:13794-14348
HmHel-2	CAEZ01003450:221-767
HmHel-2	CAEZ01002414:34624-34771
HmHel-2	CAEZ01002414:134747-135304
HmHel-2	CAEZ01002414:136538-137150
HmHel-2	CAEZ01002414:71794-72355
HmHel-2	CAEZ01011132:22160-22719
HmHel-2	CAEZ01011132:46691-47149
HmHel-2	CAEZ01011132:34978-35417
HmHel-2	CAEZ01010750:987-1546
HmHel-2	CAEZ01010677:86407-86923
HmHel-2	CAEZ01010677:178056-178513
HmHel-2	CAEZ01010677:237662-237941
HmHel-2	CAEZ01010677:303593-304051
HmHel-2	CAEZ01010677:280706-281266
HmHel-2	CAEZ01010677:263072-263522
HmHel-2	CAEZ01010677:166287-166690

HmHel-2	CAEZ01010677:152697-153037
HmHel-2	CAEZ01010387:11089-15032
HmHel-2	CAEZ01010141:5299-5745
HmHel-2	CAEZ01010141:7592-17909
HmHel-2	CAEZ01010054:1632-2192
HmHel-2	CAEZ01009891:35683-36242
HmHel-2	CAEZ01009891:51437-51895
HmHel-2	CAEZ01009891:64985-65337
HmHel-2	CAEZ01009891:34712-35079
HmHel-2	CAEZ01009071:4920-5379
HmHel-2	CAEZ01009071:6763-7322
HmHel-2	CAEZ01006745:28447-29026
HmHel-2	CAEZ01005396:26920-27473
HmHel-2	CAEZ01005175:52354-52913
HmHel-2	CAEZ01005031:2422-3008
HmHel-2	CAEZ01003884:2490-3088
HmHel-2	CAEZ01003647:2976-3433
HmHel-2	CAEZ01003647:83943-84494
HmHel-2	CAEZ01003647:68559-69009
HmHel-2	CAEZ01003417:13534-14075
HmHel-2	CAEZ01002799:4411-5068
HmHel-2	CAEZ01002719:11118-11576
HmHel-2	CAEZ01002719:10061-10203
HmHel-2	CAEZ01002616:1826-3346
HmHel-2	CAEZ01002616:6063-6519
HmHel-2	CAEZ01011215:209864-210323
HmHel-2	CAEZ01011215:160168-160626
HmHel-2	CAEZ01010620:203704-204296
HmHel-2	CAEZ01010620:68567-68692
HmHel-2	CAEZ01009417:13514-13699
HmHel-2	CAEZ01009417:16291-16845
HmHel-2	CAEZ01008987:25503-26098
HmHel-2	CAEZ01008987:3287-3832
HmHel-2	CAEZ01007799:18483-19041
HmHel-2	CAEZ01005082:19849-20186
HmHel-2	CAEZ01005082:52368-52963
HmHel-2	CAEZ01004664:806-1387
HmHel-2	CAEZ01001773:14065-14619
HmHel-2	CAEZ01001773:47-599
HmHel-2	CAEZ01010266:6219-6436
HmHel-2	CAEZ01010266:8875-9464
HmHel-2	CAEZ01008211:218802-218984
HmHel-2	CAEZ01008211:229577-230027
HmHel-2	CAEZ01008211:166614-166733
HmHel-2	CAEZ01008211:138903-139494
HmHel-2	CAEZ01003514:8453-9057
HmHel-2	CAEZ01003102:14557-14790
HmHel-2	CAEZ01011418:3891-4493

HmHel-2	CAEZ01011100:20118-20742
HmHel-2	CAEZ01006278:1-162
HmHel-2	CAEZ01006278:54792-55239
HmHel-2	CAEZ01005720:22526-22788
HmHel-2	CAEZ01003328:74072-74646
HmHel-2	CAEZ01002891:12486-13038
HmHel-2	CAEZ01011626:117860-118313
HmHel-2	CAEZ01011626:63796-64355
HmHel-2	CAEZ01011281:422-1125
HmHel-2	CAEZ01010252:4350-4440
HmHel-2	CAEZ01008480:13177-13775
HmHel-2	CAEZ01007747:127719-128278
HmHel-2	CAEZ01007747:205636-205761
HmHel-2	CAEZ01007747:249647-249755
HmHel-2	CAEZ01006890:3048-3639
HmHel-2	CAEZ01006890:127-442
HmHel-2	CAEZ01006778:142903-143462
HmHel-2	CAEZ01004551:16055-16509
HmHel-2	CAEZ01004551:7329-7888
HmHel-2	CAEZ01004433:2245-2805
HmHel-2	CAEZ01003667:4137-4695
HmHel-2	CAEZ01003667:2799-3212
HmHel-2	CAEZ01001368:1769-2329
HmHel-2	CAEZ01001196:10718-11168
HmHel-2	CAEZ01001196:52513-53071
HmHel-2	CAEZ01001196:43696-43952
HmHel-2	CAEZ01000839:20022-20479
HmHel-2	CAEZ01000839:26065-26623
HmHel-2	CAEZ01008602:17868-18422
HmHel-2	CAEZ01007676:73579-73900
HmHel-2	CAEZ01007676:209915-210490
HmHel-2	CAEZ01007676:65828-66060
HmHel-2	CAEZ01007569:33529-34095
HmHel-2	CAEZ01007569:614-1027
HmHel-2	CAEZ01006098:38420-39010
HmHel-2	CAEZ01005384:683-1277
HmHel-2	CAEZ01005293:10781-11340
HmHel-2	CAEZ01003564:3176-3734
HmHel-2	CAEZ01001557:33085-33643
HmHel-2	CAEZ01010592:2023-2157
HmHel-2	CAEZ01010272:90665-90874
HmHel-2	CAEZ01010272:86537-87119
HmHel-2	CAEZ01005986:44239-44796
HmHel-2	CAEZ01005986:112830-113283
HmHel-2	CAEZ01005986:130905-131107
HmHel-2	CAEZ01005986:106653-106873
HmHel-2	CAEZ01005957:14016-14580
HmHel-2	CAEZ01002034:1106-1664

HmHel-2	CAEZ01002034:455-739
HmHel-2	CAEZ01010792:21656-22216
HmHel-2	CAEZ01010787:1-521
HmHel-2	CAEZ01010730:3723-4551
HmHel-2	CAEZ01010730:18335-18784
HmHel-2	CAEZ01010730:70764-70864
HmHel-2	CAEZ01010730:57615-57924
HmHel-2	CAEZ01010730:22113-22667
HmHel-2	CAEZ01010457:31494-32085
HmHel-2	CAEZ01009940:22-423
HmHel-2	CAEZ01007366:5302-5811
HmHel-2	CAEZ01006412:96071-96319
HmHel-2	CAEZ01006412:46658-47215
HmHel-2	CAEZ01006393:136-689
HmHel-2	CAEZ01004733:15762-16072
HmHel-2	CAEZ01004733:59267-59577
HmHel-2	CAEZ01004733:80055-80138
HmHel-2	CAEZ01004733:106645-107323
HmHel-2	CAEZ01004094:77280-77731
HmHel-2	CAEZ01004094:93325-93587
HmHel-2	CAEZ01001213:5403-5963
HmHel-2	CAEZ01000876:85569-86130
HmHel-2	CAEZ01000876:4612-4904
HmHel-2	CAEZ01010956:1325-2403
HmHel-2	CAEZ01010292:31149-31708
HmHel-2	CAEZ01009460:33033-33591
HmHel-2	CAEZ01009344:3509-4069
HmHel-2	CAEZ01009291:62-621
HmHel-2	CAEZ01009249:9267-9699
HmHel-2	CAEZ01008401:11835-12408
HmHel-2	CAEZ01006019:1205-1401
HmHel-2	CAEZ01005509:52850-53140
HmHel-2	CAEZ01005509:64861-65419
HmHel-2	CAEZ01004923:50550-51109
HmHel-2	CAEZ01004923:92842-93163
HmHel-2	CAEZ01003974:5054-5613
HmHel-2	CAEZ01003493:1160-1706
HmHel-2	CAEZ01002798:503-807
HmHel-2	CAEZ01002798:51257-51815
HmHel-2	CAEZ01011075:1707-2262
HmHel-2	CAEZ01010237:114754-115191
HmHel-2	CAEZ01010237:151777-152271
HmHel-2	CAEZ01010237:110462-110916
HmHel-2	CAEZ01009364:54-1073
HmHel-2	CAEZ01009364:28401-28965
HmHel-2	CAEZ01008982:20624-20737
HmHel-2	CAEZ01007937:60784-61242
HmHel-2	CAEZ01006974:558-1122

HmHel-2	CAEZ01004661:17863-18451
HmHel-2	CAEZ01004442:7613-8170
HmHel-2	CAEZ01004045:13070-13624
HmHel-2	CAEZ01003791:16908-17019
HmHel-2	CAEZ01003791:143-696
HmHel-2	CAEZ01003119:10338-10896
HmHel-2	CAEZ01010604:7448-7968
HmHel-2	CAEZ01010464:58061-58519
HmHel-2	CAEZ01010464:83187-83789
HmHel-2	CAEZ01010464:118233-118691
HmHel-2	CAEZ01010464:102550-103132
HmHel-2	CAEZ01010464:59581-60123
HmHel-2	CAEZ01010464:8085-8166
HmHel-2	CAEZ01007063:32305-32864
HmHel-2	CAEZ01007063:8350-8909
HmHel-2	CAEZ01006194:6262-6720
HmHel-2	CAEZ01006194:31450-31877
HmHel-2	CAEZ01006194:13-493
HmHel-2	CAEZ01004541:107540-110324
HmHel-2	CAEZ01004541:152318-152897
HmHel-2	CAEZ01004541:123575-123896
HmHel-2	CAEZ01004541:37288-37589
HmHel-2	CAEZ01004541:21294-21897
HmHel-2	CAEZ01001305:9295-9856
HmHel-2	CAEZ01007913:17434-18055
HmHel-2	CAEZ01004936:48528-48830
HmHel-2	CAEZ01004936:39939-40563
HmHel-2	CAEZ01004936:8901-9359
HmHel-2	CAEZ01004330:11550-12140
HmHel-2	CAEZ01001251:549-1107
HmHel-2	CAEZ01011307:1647-2105
HmHel-2	CAEZ01011307:14851-15513
HmHel-2	CAEZ01009805:1-190
HmHel-2	CAEZ01009805:25248-25806
HmHel-2	CAEZ01009022:7445-7996
HmHel-2	CAEZ01007631:14092-14652
HmHel-2	CAEZ01007631:76170-76611
HmHel-2	CAEZ01007631:75465-75876
HmHel-2	CAEZ01007631:20964-21522
HmHel-2	CAEZ01006478:435-994
HmHel-2	CAEZ01006028:584-1570
HmHel-2	CAEZ01005531:5478-6036
HmHel-2	CAEZ01002368:42785-43375
HmHel-2	CAEZ01009381:25653-26212
HmHel-2	CAEZ01007299:35297-35755
HmHel-2	CAEZ01003194:1198-1763
HmHel-2	CAEZ01003194:7461-7919
HmHel-2	CAEZ01003123:15490-15626

HmHel-2	CAEZ01003123:871-1450
HmHel-2	CAEZ01002567:178-731
HmHel-2	CAEZ01011572:19575-20104
HmHel-2	CAEZ01011572:19464-19572
HmHel-2	CAEZ01009327:1-459
HmHel-2	CAEZ01006151:35365-35632
HmHel-2	CAEZ01006151:81187-81581
HmHel-2	CAEZ01006151:93991-94562
HmHel-2	CAEZ01006151:8990-9435
HmHel-2	CAEZ01002831:751-1347
HmHel-2	CAEZ01002810:70983-71548
HmHel-2	CAEZ01002810:84077-84535
HmHel-2	CAEZ01002810:41664-42117
HmHel-2	CAEZ01004958:29924-30296
HmHel-2	CAEZ01004958:39287-39745
HmHel-2	CAEZ01004958:17310-17918
HmHel-2	CAEZ01004555:15370-15943
HmHel-2	CAEZ01004340:2072-2632
HmHel-2	CAEZ01004340:5154-5474
HmHel-2	CAEZ01001172:274-823
HmHel-2	CAEZ01010092:3361-3921
HmHel-2	CAEZ01008737:73023-81591
HmHel-2	CAEZ01008737:85624-86206
HmHel-2	CAEZ01008737:46298-46606
HmHel-2	CAEZ01007204:28627-28850
HmHel-2	CAEZ01007204:42047-42154
HmHel-2	CAEZ01007204:55380-55515
HmHel-2	CAEZ01007204:21088-21677
HmHel-2	CAEZ01007204:857-1405
HmHel-2	CAEZ01006072:808-1244
HmHel-2	CAEZ01005373:76607-77065
HmHel-2	CAEZ01005373:96352-96810
HmHel-2	CAEZ01005373:81275-81411
HmHel-2	CAEZ01005373:62110-62317
HmHel-2	CAEZ01004544:4358-4917
HmHel-2	CAEZ01004544:2788-2924
HmHel-2	CAEZ01004034:2314-2879
HmHel-2	CAEZ01002217:29462-29612
HmHel-2	CAEZ01002217:52488-52811
HmHel-2	CAEZ01002217:55800-56272
HmHel-2	CAEZ01001771:3401-3859
HmHel-2	CAEZ01001771:19669-20168
HmHel-2	CAEZ01001771:11949-12509
HmHel-2	CAEZ01009521:1520-2062
HmHel-2	CAEZ01008510:5778-8638
HmHel-2	CAEZ01008329:18525-19078
HmHel-2	CAEZ01006090:4235-4799
HmHel-2	CAEZ01006090:20331-20415

HmHel-2	CAEZ01004178:5525-6077
HmHel-2	CAEZ01000866:732-837
HmHel-2	CAEZ01011586:1777-2333
HmHel-2	CAEZ01010958:1305-1886
HmHel-2	CAEZ01010439:3078-3192
HmHel-2	CAEZ01010439:50154-50742
HmHel-2	CAEZ01008689:32309-32767
HmHel-2	CAEZ01008689:795-6270
HmHel-2	CAEZ01008623:10676-11231
HmHel-2	CAEZ01007663:693-1006
HmHel-2	CAEZ01007663:49416-49854
HmHel-2	CAEZ01005797:545-1139
HmHel-2	CAEZ01005503:1746-2300
HmHel-2	CAEZ01004435:548-1113
HmHel-2	CAEZ01004113:1-462
HmHel-2	CAEZ01002894:16746-17035
HmHel-2	CAEZ01002894:10105-13460
HmHel-2	CAEZ01010070:3-518
HmHel-2	CAEZ01009784:6199-6804
HmHel-2	CAEZ01008615:19595-20179
HmHel-2	CAEZ01008615:14007-14473
HmHel-2	CAEZ01004816:105618-106076
HmHel-2	CAEZ01004816:26404-26862
HmHel-2	CAEZ01001793:32562-33107
HmHel-2	CAEZ01009614:35652-36208
HmHel-2	CAEZ01009614:42673-42770
HmHel-2	CAEZ01008337:40473-40930
HmHel-2	CAEZ01008337:25446-26006
HmHel-2	CAEZ01006723:7057-7600
HmHel-2	CAEZ01006647:1-484
HmHel-2	CAEZ01005738:11148-12019
HmHel-2	CAEZ01004252:47915-48475
HmHel-2	CAEZ01010700:31305-31884
HmHel-2	CAEZ01010700:91215-91768
HmHel-2	CAEZ01010700:6917-7375
HmHel-2	CAEZ01008945:41338-41930
HmHel-2	CAEZ01008945:30562-30864
HmHel-2	CAEZ01003262:27-415
HmHel-2	CAEZ01003262:8575-9033
HmHel-2	CAEZ01003195:15679-16137
HmHel-2	CAEZ01003195:55946-56339
HmHel-2	CAEZ01000843:12553-13011
HmHel-2	CAEZ01011617:5-532
HmHel-2	CAEZ01009121:4625-5078
HmHel-2	CAEZ01008868:16619-17176
HmHel-2	CAEZ01008868:289-743
HmHel-2	CAEZ01008580:18351-18892
HmHel-2	CAEZ01007210:2839-3295

HmHel-2	CAEZ01007130:446-1003
HmHel-2	CAEZ01006897:26372-26930
HmHel-2	CAEZ01005231:10414-10802
HmHel-2	CAEZ01005231:44741-45312
HmHel-2	CAEZ01005231:58013-58562
HmHel-2	CAEZ01005231:15763-16238
HmHel-2	CAEZ01002314:1658-2241
HmHel-2	CAEZ01001140:3000-3585
HmHel-2	CAEZ01011206:84-677
HmHel-2	CAEZ01009032:251-807
HmHel-2	CAEZ01007149:1-478
HmHel-2	CAEZ01005381:10377-10966
HmHel-2	CAEZ01004050:7686-8242
HmHel-2	CAEZ01010907:3353-3912
HmHel-2	CAEZ01009978:32106-32662
HmHel-2	CAEZ01008202:31080-31640
HmHel-2	CAEZ01008202:26399-26540
HmHel-2	CAEZ01008047:1-497
HmHel-2	CAEZ01007298:4371-4970
HmHel-2	CAEZ01007163:2947-3543
HmHel-2	CAEZ01002669:16603-17201
HmHel-2	CAEZ01010071:1299-1757
HmHel-2	CAEZ01009125:14857-15068
HmHel-2	CAEZ01009125:34881-35459
HmHel-2	CAEZ01009125:2812-3144
HmHel-2	CAEZ01007603:36253-36711
HmHel-2	CAEZ01007603:19951-20039
HmHel-2	CAEZ01006277:1745-2291
HmHel-2	CAEZ01000050:636-1094
HmHel-2	CAEZ01000042:33301-33846
HmHel-2	CAEZ01011245:35610-36162
HmHel-2	CAEZ01010035:11351-11920
HmHel-2	CAEZ01008209:12777-13235
HmHel-2	CAEZ01008209:94494-94943
HmHel-2	CAEZ01008194:21511-22059
HmHel-2	CAEZ01008141:884-1440
HmHel-2	CAEZ01008141:33042-33499
HmHel-2	CAEZ01008141:1-237
HmHel-2	CAEZ01007893:8047-8505
HmHel-2	CAEZ01007399:57302-57843
HmHel-2	CAEZ01003771:6301-6932
HmHel-2	CAEZ01002811:3255-3837
HmHel-2	CAEZ01002811:46039-46628
HmHel-2	CAEZ01002811:135533-135665
HmHel-2	CAEZ01002811:168030-168141
HmHel-2	CAEZ01002811:149486-149810
HmHel-2	CAEZ01011570:22-423
HmHel-2	CAEZ01010619:47099-47658

HmHel-2	CAEZ01010619:60666-60799
HmHel-2	CAEZ01010320:59168-59484
HmHel-2	CAEZ01010320:957-1513
HmHel-2	CAEZ01009655:31709-32297
HmHel-2	CAEZ01007266:31913-32374
HmHel-2	CAEZ01007266:53374-53827
HmHel-2	CAEZ01007266:13784-14336
HmHel-2	CAEZ01004749:11753-12211
HmHel-2	CAEZ01004749:35256-35713
HmHel-2	CAEZ01004589:1900-2468
HmHel-2	CAEZ01004279:1567-2120
HmHel-2	CAEZ01002836:3759-4308
HmHel-2	CAEZ01010178:11795-12220
HmHel-2	CAEZ01007207:126821-127414
HmHel-2	CAEZ01007207:102403-102846
HmHel-2	CAEZ01006092:33285-33843
HmHel-2	CAEZ01004182:21019-21575
HmHel-2	CAEZ01008416:3999-4558
HmHel-2	CAEZ01008335:4899-5500
HmHel-2	CAEZ01007995:11209-11794
HmHel-2	CAEZ01007995:24186-24636
HmHel-2	CAEZ01007965:1-520
HmHel-2	CAEZ01006841:2782-3240
HmHel-2	CAEZ01005669:33061-33519
HmHel-2	CAEZ01005669:5939-6234
HmHel-2	CAEZ01005404:1215-1303
HmHel-2	CAEZ01005404:37526-38087
HmHel-2	CAEZ01004441:5032-5491
HmHel-2	CAEZ01003291:106734-107050
HmHel-2	CAEZ01003291:38852-39310
HmHel-2	CAEZ01002635:35603-36060
HmHel-2	CAEZ01002635:47259-47726
HmHel-2	CAEZ01002635:59900-60129
HmHel-2	CAEZ01001039:2-513
HmHel-2	CAEZ01011449:10784-11358
HmHel-2	CAEZ01009495:18965-19231
HmHel-2	CAEZ01009495:56234-56815
HmHel-2	CAEZ01008509:29366-29823
HmHel-2	CAEZ01007815:20662-21273
HmHel-2	CAEZ01002705:18008-18596
HmHel-2	CAEZ01011410:5-175
HmHel-2	CAEZ01011410:18984-19298
HmHel-2	CAEZ01011410:21072-21650
HmHel-2	CAEZ01011410:10380-10938
HmHel-2	CAEZ01005153:26205-26373
HmHel-2	CAEZ01005153:78755-79056
HmHel-2	CAEZ01005153:89077-89637
HmHel-2	CAEZ01003287:3275-3668

HmHel-2	CAEZ01002629:1-489
HmHel-2	CAEZ01001981:47716-48174
HmHel-2	CAEZ01000918:494-950
HmHel-2	CAEZ01000864:11712-12169
HmHel-2	CAEZ01011217:95289-95677
HmHel-2	CAEZ01011217:146561-147122
HmHel-2	CAEZ01010969:13638-14095
HmHel-2	CAEZ01010969:118635-119131
HmHel-2	CAEZ01010969:231909-232456
HmHel-2	CAEZ01010969:247907-248039
HmHel-2	CAEZ01010969:38681-39130
HmHel-2	CAEZ01010079:31584-32143
HmHel-2	CAEZ01010079:25771-26217
HmHel-2	CAEZ01010066:10181-10740
HmHel-2	CAEZ01010029:18051-18478
HmHel-2	CAEZ01010029:93237-93834
HmHel-2	CAEZ01010029:83953-84410
HmHel-2	CAEZ01009770:2723-3111
HmHel-2	CAEZ01009770:29643-30101
HmHel-2	CAEZ01009770:52247-52676
HmHel-2	CAEZ01009512:10904-11458
HmHel-2	CAEZ01008386:9756-10346
HmHel-2	CAEZ01008239:42400-42998
HmHel-2	CAEZ01006769:43037-43156
HmHel-2	CAEZ01001457:3498-4058
HmHel-2	CAEZ01011178:1222-1680
HmHel-2	CAEZ01011142:2122-2579
HmHel-2	CAEZ01010724:22637-23096
HmHel-2	CAEZ01010521:8895-9353
HmHel-2	CAEZ01010300:24073-24664
HmHel-2	CAEZ01008729:28860-29318
HmHel-2	CAEZ01008161:18799-19399
HmHel-2	CAEZ01007943:16552-17010
HmHel-2	CAEZ01007943:17586-17809
HmHel-2	CAEZ01007449:11668-12126
HmHel-2	CAEZ01006171:98674-99216
HmHel-2	CAEZ01006171:85984-86442
HmHel-2	CAEZ01005163:32987-33422
HmHel-2	CAEZ01005163:16802-17259
HmHel-2	CAEZ01004868:14078-14641
HmHel-2	CAEZ01004429:15693-16214
HmHel-2	CAEZ01001221:16825-17372
HmHel-2	CAEZ01000940:1086-1545
HmHel-2	CAEZ01009185:6948-7406
HmHel-2	CAEZ01007950:14749-15037
HmHel-2	CAEZ01007950:2861-3318
HmHel-2	CAEZ01007804:118267-118722
HmHel-2	CAEZ01007804:99233-99694

HmHel-2	CAEZ01007040:7836-18413
HmHel-2	CAEZ01001756:517-975
HmHel-2	CAEZ01010718:3231-3816
HmHel-2	CAEZ01010542:38838-39311
HmHel-2	CAEZ01009156:9154-9618
HmHel-2	CAEZ01007247:4305-4863
HmHel-2	CAEZ01007157:9520-10115
HmHel-2	CAEZ01003944:990-1449
HmHel-2	CAEZ01003511:3929-4454
HmHel-2	CAEZ01002711:23299-23836
HmHel-2	CAEZ01001477:5618-6104
HmHel-2	CAEZ01001477:8296-8840
HmHel-2	CAEZ01009686:26746-27295
HmHel-2	CAEZ01009179:24277-24810
HmHel-2	CAEZ01008709:19958-20412
HmHel-2	CAEZ01008089:4395-4854
HmHel-2	CAEZ01005643:43895-44112
HmHel-2	CAEZ01003202:53553-54091
HmHel-2	CAEZ01002632:2095-2650
HmHel-2	CAEZ01002208:119678-120136
HmHel-2	CAEZ01002208:50438-50994
HmHel-2	CAEZ01001562:3896-4457
HmHel-2	CAEZ01011599:18545-18997
HmHel-2	CAEZ01011599:104-326
HmHel-2	CAEZ01011023:26548-27006
HmHel-2	CAEZ01010871:5545-6000
HmHel-2	CAEZ01010871:13879-14829
HmHel-2	CAEZ01009285:452-1015
HmHel-2	CAEZ01009057:8866-9325
HmHel-2	CAEZ01008289:6258-6717
HmHel-2	CAEZ01007869:16715-17173
HmHel-2	CAEZ01007723:4839-5297
HmHel-2	CAEZ01007723:5769-5960
HmHel-2	CAEZ01007007:1412-1870
HmHel-2	CAEZ01006907:1-484
HmHel-2	CAEZ01006681:15017-15560
HmHel-2	CAEZ01005809:16311-16769
HmHel-2	CAEZ01004273:4411-4869
HmHel-2	CAEZ01004027:7714-8172
HmHel-2	CAEZ01002072:4607-5065
HmHel-2	CAEZ01001571:11615-12149
HmHel-2	CAEZ01001455:4-447
HmHel-2	CAEZ01000872:1346-1936
HmHel-2	CAEZ01011344:1674-2140
HmHel-2	CAEZ01011344:7616-8074
HmHel-2	CAEZ01010720:24285-24735
HmHel-2	CAEZ01010193:482-1040
HmHel-2	CAEZ01009264:73231-73530

HmHel-2	CAEZ01009264:6885-7337
HmHel-2	CAEZ01009201:3977-4435
HmHel-2	CAEZ01009031:7077-7659
HmHel-2	CAEZ01008722:30011-30265
HmHel-2	CAEZ01008722:10886-11341
HmHel-2	CAEZ01007938:5857-6315
HmHel-2	CAEZ01007938:88380-88837
HmHel-2	CAEZ01007938:4339-4797
HmHel-2	CAEZ01004542:28468-28926
HmHel-2	CAEZ01004489:11763-12221
HmHel-2	CAEZ01004041:14408-14866
HmHel-2	CAEZ01001729:9315-9875
HmHel-2	CAEZ01010605:3107-3578
HmHel-2	CAEZ01009531:4053-4506
HmHel-2	CAEZ01008257:33332-33914
HmHel-2	CAEZ01008257:8107-8564
HmHel-2	CAEZ01008120:12314-12808
HmHel-2	CAEZ01008120:2573-3026
HmHel-2	CAEZ01007536:10075-10649
HmHel-2	CAEZ01007395:18115-18432
HmHel-2	CAEZ01007395:29915-30659
HmHel-2	CAEZ01006801:3392-3950
HmHel-2	CAEZ01006801:1-362
HmHel-2	CAEZ01001807:57152-57750
HmHel-2	CAEZ01001452:2053-2510
HmHel-2	CAEZ01003586:5155-5705
HmHel-2	CAEZ01002851:29748-30306
HmHel-2	CAEZ01002851:5075-5530
HmHel-2	CAEZ01002374:367-926
HmHel-2	CAEZ01011472:12064-12522
HmHel-2	CAEZ01009016:14354-14909
HmHel-2	CAEZ01009016:8274-8529
HmHel-2	CAEZ01007749:38223-38458
HmHel-2	CAEZ01007749:8838-9296
HmHel-2	CAEZ01007483:3861-4317
HmHel-2	CAEZ01007453:10711-11169
HmHel-2	CAEZ01006617:12861-13319
HmHel-2	CAEZ01006617:58571-58892
HmHel-2	CAEZ01006617:26680-26955
HmHel-2	CAEZ01005693:28036-28621
HmHel-2	CAEZ01005415:470-927
HmHel-2	CAEZ01003832:11359-11817
HmHel-2	CAEZ01003398:40061-40522
HmHel-2	CAEZ01002985:8370-8764
HmHel-2	CAEZ01002977:143-598
HmHel-2	CAEZ01011483:3917-4345
HmHel-2	CAEZ01010738:166223-166639
HmHel-2	CAEZ01010738:205210-205329

HmHel-2	CAEZ01010738:228767-229224
HmHel-2	CAEZ01010738:136815-137272
HmHel-2	CAEZ01008898:3946-4404
HmHel-2	CAEZ01008504:1607-2065
HmHel-2	CAEZ01006992:28035-28607
HmHel-2	CAEZ01006992:25597-26068
HmHel-2	CAEZ01006401:8863-9318
HmHel-2	CAEZ01006348:41913-42109
HmHel-2	CAEZ01006348:54330-54787
HmHel-2	CAEZ01006002:4349-4618
HmHel-2	CAEZ01006002:18238-18692
HmHel-2	CAEZ01005944:7056-7618
HmHel-2	CAEZ01004732:735-1153
HmHel-2	CAEZ01004722:5278-5740
HmHel-2	CAEZ01003956:14196-14654
HmHel-2	CAEZ01002865:5776-6233
HmHel-2	CAEZ01009280:34142-34590
HmHel-2	CAEZ01008219:9700-10171
HmHel-2	CAEZ01006286:25954-26395
HmHel-2	CAEZ01006286:133137-133645
HmHel-2	CAEZ01005721:12882-13208
HmHel-2	CAEZ01005721:83649-84106
HmHel-2	CAEZ01005721:112209-115837
HmHel-2	CAEZ01005721:144530-144962
HmHel-2	CAEZ01005721:147658-148244
HmHel-2	CAEZ01005721:121272-121426
HmHel-2	CAEZ01003604:396-941
HmHel-2	CAEZ01001979:93-564
HmHel-2	CAEZ01008933:6244-6697
HmHel-2	CAEZ01007854:241-800
HmHel-2	CAEZ01005966:3473-4033
HmHel-2	CAEZ01005089:87865-88316
HmHel-2	CAEZ01005089:29780-30228
HmHel-2	CAEZ01004626:1-550
HmHel-2	CAEZ01004527:427-885
HmHel-2	CAEZ01004347:5348-5768
HmHel-2	CAEZ01003721:2577-3035
HmHel-2	CAEZ01001229:350-806
HmHel-2	CAEZ01010755:17278-17839
HmHel-2	CAEZ01010585:42512-42970
HmHel-2	CAEZ01010585:30581-30734
HmHel-2	CAEZ01010425:6052-6511
HmHel-2	CAEZ01010425:18955-19410
HmHel-2	CAEZ01010217:123714-124172
HmHel-2	CAEZ01010217:32535-32993
HmHel-2	CAEZ01009999:23280-23685
HmHel-2	CAEZ01009994:42590-43048
HmHel-2	CAEZ01009962:57295-57753

HmHel-2	CAEZ01009700:2170-2629
HmHel-2	CAEZ01008917:2061-2515
HmHel-2	CAEZ01008084:11484-12038
HmHel-2	CAEZ01007666:19287-19795
HmHel-2	CAEZ01006744:1-546
HmHel-2	CAEZ01006744:2064-2411
HmHel-2	CAEZ01005646:14483-14941
HmHel-2	CAEZ01005476:10723-11181
HmHel-2	CAEZ01005476:83355-83803
HmHel-2	CAEZ01005476:536-1172
HmHel-2	CAEZ01004741:10711-11295
HmHel-2	CAEZ01004741:51749-52208
HmHel-2	CAEZ01004313:15550-15976
HmHel-2	CAEZ01003862:6866-7429
HmHel-2	CAEZ01003785:25760-26275
HmHel-2	CAEZ01003503:10023-10481
HmHel-2	CAEZ01002880:19288-19747
HmHel-2	CAEZ01000056:37881-38342
HmHel-2	CAEZ01010427:22117-22556
HmHel-2	CAEZ01010427:31703-32133
HmHel-2	CAEZ01010427:7479-7934
HmHel-2	CAEZ01009976:14785-15392
HmHel-2	CAEZ01008935:8-296
HmHel-2	CAEZ01008420:66314-66456
HmHel-2	CAEZ01008420:173802-174116
HmHel-2	CAEZ01008420:54649-54921
HmHel-2	CAEZ01008420:18935-19393
HmHel-2	CAEZ01007454:41124-41582
HmHel-2	CAEZ01007454:35703-36153
HmHel-2	CAEZ01006535:1168-1681
HmHel-2	CAEZ01006535:15457-15914
HmHel-2	CAEZ01006362:8322-8904
HmHel-2	CAEZ01004980:19528-19985
HmHel-2	CAEZ01004254:27002-27460
HmHel-2	CAEZ01003416:115756-116213
HmHel-2	CAEZ01001658:5383-5841
HmHel-2	CAEZ01001408:191184-191642
HmHel-2	CAEZ01001408:49661-49981
HmHel-2	CAEZ01001228:558-1016
HmHel-2	CAEZ01000968:27623-28081
HmHel-2	CAEZ01000968:31080-31401
HmHel-2	CAEZ01010847:645-1191
HmHel-2	CAEZ01010078:41916-42370
HmHel-2	CAEZ01010078:22261-22372
HmHel-2	CAEZ01009822:15056-15638
HmHel-2	CAEZ01009822:27234-27689
HmHel-2	CAEZ01009624:7677-8260
HmHel-2	CAEZ01009570:6911-7469

HmHel-2	CAEZ01009553:18657-19234
HmHel-2	CAEZ01007984:25387-25915
HmHel-2	CAEZ01006686:77196-77650
HmHel-2	CAEZ01006686:41296-41892
HmHel-2	CAEZ01006156:1-255
HmHel-2	CAEZ01006156:4448-4885
HmHel-2	CAEZ01003765:8652-9085
HmHel-2	CAEZ01003765:12015-12419
HmHel-2	CAEZ01003226:48977-49267
HmHel-2	CAEZ01003226:138995-139453
HmHel-2	CAEZ01003226:90995-91452
HmHel-2	CAEZ01003127:4724-5202
HmHel-2	CAEZ01002650:4392-4847
HmHel-2	CAEZ01001207:1200-1639
HmHel-2	CAEZ01000888:786-1240
HmHel-2	CAEZ01011529:20659-20891
HmHel-2	CAEZ01011529:43991-44449
HmHel-2	CAEZ01011529:17318-17723
HmHel-2	CAEZ01009917:5627-6083
HmHel-2	CAEZ01007615:5858-6416
HmHel-2	CAEZ01005541:3943-4404
HmHel-2	CAEZ01005541:9634-10182
HmHel-2	CAEZ01005255:9612-10069
HmHel-2	CAEZ01005147:124193-124651
HmHel-2	CAEZ01000959:8595-9070
HmHel-2	CAEZ01000815:3139-3578
HmHel-2	CAEZ01000815:2171-2629
HmHel-2	CAEZ01011138:11877-12335
HmHel-2	CAEZ01010785:16040-16496
HmHel-2	CAEZ01010435:9733-10191
HmHel-2	CAEZ01009979:8599-9056
HmHel-2	CAEZ01009896:190005-190431
HmHel-2	CAEZ01009896:118529-118947
HmHel-2	CAEZ01008746:37110-37568
HmHel-2	CAEZ01008746:59450-59753
HmHel-2	CAEZ01008098:42943-43527
HmHel-2	CAEZ01007790:1787-2245
HmHel-2	CAEZ01007725:138081-138540
HmHel-2	CAEZ01007546:339-1078
HmHel-2	CAEZ01007546:13616-14069
HmHel-2	CAEZ01007330:4792-5250
HmHel-2	CAEZ01007059:14364-14822
HmHel-2	CAEZ01006426:12569-13027
HmHel-2	CAEZ01006402:35055-35513
HmHel-2	CAEZ01005962:8395-8852
HmHel-2	CAEZ01005455:54884-55342
HmHel-2	CAEZ01004833:2389-2838
HmHel-2	CAEZ01004833:13328-13422

HmHel-2	CAEZ01004833:3909-4367
HmHel-2	CAEZ01004745:6719-7176
HmHel-2	CAEZ01004559:6821-7277
HmHel-2	CAEZ01004267:1366-1826
HmHel-2	CAEZ01003967:5689-6146
HmHel-2	CAEZ01003510:78407-78864
HmHel-2	CAEZ01002643:49419-49877
HmHel-2	CAEZ01002617:1305-1763
HmHel-2	CAEZ01002336:5668-6127
HmHel-2	CAEZ01001758:1730-2187
HmHel-2	CAEZ01000053:22198-22656
HmHel-2	CAEZ01010648:11294-11727
HmHel-2	CAEZ01009300:28392-28993
HmHel-2	CAEZ01008403:81886-82479
HmHel-2	CAEZ01006893:18403-18945
HmHel-2	CAEZ01005010:586-1044
HmHel-2	CAEZ01004199:8118-8576
HmHel-2	CAEZ01002846:31750-32209
HmHel-2	CAEZ01001801:9946-10404
HmHel-2	CAEZ01001801:5678-5767
HmHel-2	CAEZ01001279:2505-2963
HmHel-2	CAEZ01011221:9007-9467
HmHel-2	CAEZ01010424:20554-21006
HmHel-2	CAEZ01010424:137434-137579
HmHel-2	CAEZ01010424:149284-149753
HmHel-2	CAEZ01010424:124144-124603
HmHel-2	CAEZ01010424:44342-44786
HmHel-2	CAEZ01009957:1-488
HmHel-2	CAEZ01009856:4175-4719
HmHel-2	CAEZ01009509:106297-106718
HmHel-2	CAEZ01009509:112240-112698
HmHel-2	CAEZ01006943:14561-15017
HmHel-2	CAEZ01005517:1059-1526
HmHel-2	CAEZ01004353:7099-7556
HmHel-2	CAEZ01001811:775-1233
HmHel-2	CAEZ01010912:9811-10270
HmHel-2	CAEZ01008719:57617-58075
HmHel-2	CAEZ01008719:69973-70422
HmHel-2	CAEZ01008260:18733-18930
HmHel-2	CAEZ01008260:28692-29131
HmHel-2	CAEZ01008126:59192-59650
HmHel-2	CAEZ01008126:38469-38578
HmHel-2	CAEZ01007745:104295-104933
HmHel-2	CAEZ01007745:50742-51200
HmHel-2	CAEZ01006413:19700-20275
HmHel-2	CAEZ01005495:9045-9239
HmHel-2	CAEZ01005495:30133-30537
HmHel-2	CAEZ01005495:36483-36939

HmHel-2	CAEZ01005105:45849-46305
HmHel-2	CAEZ01004479:19236-19677
HmHel-2	CAEZ01004479:53699-54150
HmHel-2	CAEZ01004302:4099-4553
HmHel-2	CAEZ01003712:13170-13629
HmHel-2	CAEZ01001853:3173-3609
HmHel-2	CAEZ01010815:439-884
HmHel-2	CAEZ01010310:21632-22078
HmHel-2	CAEZ01010310:20611-21069
HmHel-2	CAEZ01010195:1466-1973
HmHel-2	CAEZ01010107:7397-7852
HmHel-2	CAEZ01009583:2438-2896
HmHel-2	CAEZ01009274:8786-9592
HmHel-2	CAEZ01008961:45127-45585
HmHel-2	CAEZ01008961:46987-47444
HmHel-2	CAEZ01008298:470-913
HmHel-2	CAEZ01008261:194006-194464
HmHel-2	CAEZ01008261:101437-101889
HmHel-2	CAEZ01008261:7602-7689
HmHel-2	CAEZ01008012:8096-8554
HmHel-2	CAEZ01007987:3673-4122
HmHel-2	CAEZ01007738:92656-93112
HmHel-2	CAEZ01007531:6171-6629
HmHel-2	CAEZ01007531:652-1058
HmHel-2	CAEZ01006326:116769-117176
HmHel-2	CAEZ01006326:3283-6970
HmHel-2	CAEZ01006321:6116-6382
HmHel-2	CAEZ01006079:3513-3968
HmHel-2	CAEZ01005724:10172-10643
HmHel-2	CAEZ01004845:65200-65474
HmHel-2	CAEZ01004845:31890-32348
HmHel-2	CAEZ01001822:55384-55839
HmHel-2	CAEZ01001822:22768-23110
HmHel-2	CAEZ01001089:23781-24239
HmHel-2	CAEZ01000841:478-938
HmHel-2	CAEZ01009813:75594-76031
HmHel-2	CAEZ01009813:70798-71072
HmHel-2	CAEZ01009813:33874-34388
HmHel-2	CAEZ01009318:7836-8291
HmHel-2	CAEZ01007342:19250-19863
HmHel-2	CAEZ01006803:33181-33638
HmHel-2	CAEZ01005692:28879-29337
HmHel-2	CAEZ01005688:21453-21911
HmHel-2	CAEZ01005190:6515-6971
HmHel-2	CAEZ01004899:9988-10606
HmHel-2	CAEZ01004899:322-760
HmHel-2	CAEZ01002898:3931-4388
HmHel-2	CAEZ01001903:708-1276

HmHel-2	CAEZ01001802:1-547
HmHel-2	CAEZ01001802:6760-7213
HmHel-2	CAEZ01001538:43477-43935
HmHel-2	CAEZ01000071:10-372
HmHel-2	CAEZ01009954:14770-15227
HmHel-2	CAEZ01009954:45189-45652
HmHel-2	CAEZ01009667:9829-10391
HmHel-2	CAEZ01009325:12318-12772
HmHel-2	CAEZ01008839:7508-7966
HmHel-2	CAEZ01008839:21437-21578
HmHel-2	CAEZ01008707:51309-51765
HmHel-2	CAEZ01008707:55006-55274
HmHel-2	CAEZ01004372:1961-2558
HmHel-2	CAEZ01002970:1307-1696
HmHel-2	CAEZ01002008:921-1385
HmHel-2	CAEZ01010868:20991-21090
HmHel-2	CAEZ01010868:2865-3320
HmHel-2	CAEZ01010811:1-452
HmHel-2	CAEZ01009430:9762-10222
HmHel-2	CAEZ01009192:12643-13171
HmHel-2	CAEZ01008824:40311-40768
HmHel-2	CAEZ01008824:1509-2086
HmHel-2	CAEZ01007010:1-473
HmHel-2	CAEZ01007010:13986-14405
HmHel-2	CAEZ01007010:9973-10430
HmHel-2	CAEZ01006711:77046-77599
HmHel-2	CAEZ01006711:57364-57495
HmHel-2	CAEZ01006182:2574-3032
HmHel-2	CAEZ01005511:25377-25947
HmHel-2	CAEZ01005511:32562-32648
HmHel-2	CAEZ01003811:28680-29137
HmHel-2	CAEZ01003163:62106-62509
HmHel-2	CAEZ01011416:98563-99148
HmHel-2	CAEZ01010541:155690-155865
HmHel-2	CAEZ01010541:311959-312553
HmHel-2	CAEZ01010499:1537-1996
HmHel-2	CAEZ01010437:78283-78402
HmHel-2	CAEZ01010437:60121-60579
HmHel-2	CAEZ01010437:33146-33328
HmHel-2	CAEZ01010437:17924-18382
HmHel-2	CAEZ01009589:1318-1776
HmHel-2	CAEZ01009546:1272-1728
HmHel-2	CAEZ01009546:18068-18323
HmHel-2	CAEZ01009301:10574-11033
HmHel-2	CAEZ01008192:2429-2765
HmHel-2	CAEZ01008142:384-840
HmHel-2	CAEZ01006638:14250-14711
HmHel-2	CAEZ01004921:14801-15062

HmHel-2	CAEZ01004921:101412-101503
HmHel-2	CAEZ01004921:24327-24800
HmHel-2	CAEZ01004574:21945-22401
HmHel-2	CAEZ01004574:40404-40857
HmHel-2	CAEZ01004574:18074-18532
HmHel-2	CAEZ01003750:79911-80326
HmHel-2	CAEZ01003750:99289-99977
HmHel-2	CAEZ01003750:111207-111329
HmHel-2	CAEZ01003750:94038-94597
HmHel-2	CAEZ01003750:59929-60388
HmHel-2	CAEZ01002890:5506-5964
HmHel-2	CAEZ01002872:96937-97395
HmHel-2	CAEZ01002872:70897-71144
HmHel-2	CAEZ01002203:6423-6804
HmHel-2	CAEZ01002203:26059-26515
HmHel-2	CAEZ01002203:12663-12857
HmHel-2	CAEZ01002180:46189-46647
HmHel-2	CAEZ01010772:10310-10757
HmHel-2	CAEZ01009843:40687-40827
HmHel-2	CAEZ01009843:52956-53414
HmHel-2	CAEZ01009843:87269-87558
HmHel-2	CAEZ01009843:77369-77805
HmHel-2	CAEZ01009843:58292-58760
HmHel-2	CAEZ01009843:23782-24240
HmHel-2	CAEZ01009843:12279-12603
HmHel-2	CAEZ01009455:26636-26764
HmHel-2	CAEZ01009455:45721-46007
HmHel-2	CAEZ01009455:54629-55086
HmHel-2	CAEZ01009248:14-416
HmHel-2	CAEZ01009248:12348-12553
HmHel-2	CAEZ01009248:34156-34749
HmHel-2	CAEZ01009248:44503-44779
HmHel-2	CAEZ01009248:12621-12768
HmHel-2	CAEZ01007947:8127-8576
HmHel-2	CAEZ01007765:221022-221478
HmHel-2	CAEZ01007765:606350-606593
HmHel-2	CAEZ01007765:524072-524267
HmHel-2	CAEZ01007765:28084-28555
HmHel-2	CAEZ01007592:1267-1719
HmHel-2	CAEZ01005274:80553-90557
HmHel-2	CAEZ01004774:1139-1545
HmHel-2	CAEZ01003507:25023-25482
HmHel-2	CAEZ01003236:27719-28177
HmHel-2	CAEZ01003236:11783-12399
HmHel-2	CAEZ01001441:1516-2073
HmHel-2	CAEZ01001441:36375-36829
HmHel-2	CAEZ01001441:54407-54726
HmHel-2	CAEZ01000849:70095-70400

HmHel-2	CAEZ01000849:114733-115191
HmHel-2	CAEZ01000849:79557-79656
HmHel-2	CAEZ01000849:62641-62955
HmHel-2	CAEZ01010243:45892-46407
HmHel-2	CAEZ01009816:5763-6207
HmHel-2	CAEZ01009230:270-820
HmHel-2	CAEZ01008001:32401-32856
HmHel-2	CAEZ01008001:28602-29030
HmHel-2	CAEZ01007076:35-492
HmHel-2	CAEZ01005804:6130-6531
HmHel-2	CAEZ01005583:6850-7251
HmHel-2	CAEZ01005463:4106-4522
HmHel-2	CAEZ01005298:1464-1841
HmHel-2	CAEZ01005298:993-1448
HmHel-2	CAEZ01003475:11543-12096
HmHel-2	CAEZ01003231:3141-3580
HmHel-2	CAEZ01002815:102433-102890
HmHel-2	CAEZ01002815:186216-186741
HmHel-2	CAEZ01002776:16-416
HmHel-2	CAEZ01001649:5627-6085
HmHel-2	CAEZ01008888:18845-19296
HmHel-2	CAEZ01008888:7116-7296
HmHel-2	CAEZ01008606:57385-57846
HmHel-2	CAEZ01008606:41756-42209
HmHel-2	CAEZ01008523:24125-24563
HmHel-2	CAEZ01006418:2460-2937
HmHel-2	CAEZ01006418:40911-41367
HmHel-2	CAEZ01003990:2277-2732
HmHel-2	CAEZ01003261:18250-18686
HmHel-2	CAEZ01002360:20935-21390
HmHel-2	CAEZ01002360:4533-5080
HmHel-2	CAEZ01011605:25867-26325
HmHel-2	CAEZ01011605:31618-32085
HmHel-2	CAEZ01008885:5709-6325
HmHel-2	CAEZ01008029:12251-12500
HmHel-2	CAEZ01008029:2865-3322
HmHel-2	CAEZ01007976:7390-7894
HmHel-2	CAEZ01007976:1-282
HmHel-2	CAEZ01007845:4703-5161
HmHel-2	CAEZ01007599:8663-9113
HmHel-2	CAEZ01007585:12784-13242
HmHel-2	CAEZ01006787:11467-19751
HmHel-2	CAEZ01006477:15143-15601
HmHel-2	CAEZ01006069:685-1277
HmHel-2	CAEZ01005817:12064-12513
HmHel-2	CAEZ01005817:2-460
HmHel-2	CAEZ01005636:1-408
HmHel-2	CAEZ01005442:111663-112121

HmHel-2	CAEZ01005442:65414-65635
HmHel-2	CAEZ01004911:10808-11261
HmHel-2	CAEZ01004832:6291-6749
HmHel-2	CAEZ01004832:21614-21940
HmHel-2	CAEZ01004832:74417-74863
HmHel-2	CAEZ01003797:1802-10205
HmHel-2	CAEZ01003797:20493-20574
HmHel-2	CAEZ01003216:2995-3453
HmHel-2	CAEZ01003147:9101-9559
HmHel-2	CAEZ01002762:554-1012
HmHel-2	CAEZ01002759:34396-34827
HmHel-2	CAEZ01001868:10748-11206
HmHel-2	CAEZ01001806:90929-91161
HmHel-2	CAEZ01001806:72087-72545
HmHel-2	CAEZ01001123:2460-2915
HmHel-2	CAEZ01000048:7256-7714
HmHel-2	CAEZ01000048:38509-38715
HmHel-2	CAEZ01010927:257-715
HmHel-2	CAEZ01010927:108358-108832
HmHel-2	CAEZ01010301:87232-87639
HmHel-2	CAEZ01010301:51755-52327
HmHel-2	CAEZ01010301:6052-6620
HmHel-2	CAEZ01009610:74637-75189
HmHel-2	CAEZ01007600:22920-23511
HmHel-2	CAEZ01006406:27778-28236
HmHel-2	CAEZ01006406:77996-78234
HmHel-2	CAEZ01006406:60113-60475
HmHel-2	CAEZ01006240:1919-2377
HmHel-2	CAEZ01005815:26135-26593
HmHel-2	CAEZ01005043:177441-177971
HmHel-2	CAEZ01005043:114203-114661
HmHel-2	CAEZ01005043:28890-29215
HmHel-2	CAEZ01004056:7608-8067
HmHel-2	CAEZ01004056:26633-27091
HmHel-2	CAEZ01004056:28375-28623
HmHel-2	CAEZ01004056:8069-8443
HmHel-2	CAEZ01003563:14309-14543
HmHel-2	CAEZ01003275:28103-28424
HmHel-2	CAEZ01003275:25565-26022
HmHel-2	CAEZ01003179:2663-3120
HmHel-2	CAEZ01001247:1-441
HmHel-2	CAEZ01011141:1372-1816
HmHel-2	CAEZ01011129:13051-13504
HmHel-2	CAEZ01010401:19573-20029
HmHel-2	CAEZ01010401:4851-5313
HmHel-2	CAEZ01009740:1-445
HmHel-2	CAEZ01009474:12267-12726
HmHel-2	CAEZ01009474:43739-43834

HmHel-2	CAEZ01009224:2859-3316
HmHel-2	CAEZ01009224:40672-40846
HmHel-2	CAEZ01009224:47172-47626
HmHel-2	CAEZ01007106:2240-2540
HmHel-2	CAEZ01006949:10703-11100
HmHel-2	CAEZ01005473:5482-5934
HmHel-2	CAEZ01005473:19805-20261
HmHel-2	CAEZ01003210:19374-19828
HmHel-2	CAEZ01002696:4868-5269
HmHel-2	CAEZ01000054:59-515
HmHel-2	CAEZ01011283:11619-12077
HmHel-2	CAEZ01010805:111591-112030
HmHel-2	CAEZ01009605:37248-37706
HmHel-2	CAEZ01009605:3573-4032
HmHel-2	CAEZ01009321:30381-30760
HmHel-2	CAEZ01009321:9374-9798
HmHel-2	CAEZ01008600:3173-3646
HmHel-2	CAEZ01008278:5886-6341
HmHel-2	CAEZ01008231:34608-35203
HmHel-2	CAEZ01008231:21224-21685
HmHel-2	CAEZ01008087:13544-14005
HmHel-2	CAEZ01007989:22279-22736
HmHel-2	CAEZ01007741:27764-28215
HmHel-2	CAEZ01007741:76344-76798
HmHel-2	CAEZ01006445:85826-86365
HmHel-2	CAEZ01006445:171393-171985
HmHel-2	CAEZ01006445:160991-161284
HmHel-2	CAEZ01006187:1863-2322
HmHel-2	CAEZ01006137:46182-46637
HmHel-2	CAEZ01005334:26034-26489
HmHel-2	CAEZ01005318:14403-14969
HmHel-2	CAEZ01004904:9248-9703
HmHel-2	CAEZ01004528:6920-7319
HmHel-2	CAEZ01003901:18015-18395
HmHel-2	CAEZ01003742:7103-7558
HmHel-2	CAEZ01003597:1-363
HmHel-2	CAEZ01003597:9234-9630
HmHel-2	CAEZ01002724:19149-19303
HmHel-2	CAEZ01002724:38561-39078
HmHel-2	CAEZ01002724:121840-122299
HmHel-2	CAEZ01002724:35917-36375
HmHel-2	CAEZ01001854:628-956
HmHel-2	CAEZ01001854:2506-2962
HmHel-2	CAEZ01000828:2660-2819
HmHel-2	CAEZ01000828:4686-5274
HmHel-2	CAEZ01011144:16220-16814
HmHel-2	CAEZ01011144:43128-43554
HmHel-2	CAEZ01011144:38723-39180

HmHel-2	CAEZ01010780:1871-2329
HmHel-2	CAEZ01010494:2000-2458
HmHel-2	CAEZ01010420:1039-1192
HmHel-2	CAEZ01010420:14376-14834
HmHel-2	CAEZ01009766:60850-61308
HmHel-2	CAEZ01009701:27296-27752
HmHel-2	CAEZ01009336:21147-21604
HmHel-2	CAEZ01007362:8932-9390
HmHel-2	CAEZ01007213:9070-9528
HmHel-2	CAEZ01006525:28885-29343
HmHel-2	CAEZ01006268:5620-6076
HmHel-2	CAEZ01006135:38273-38747
HmHel-2	CAEZ01005524:2537-2995
HmHel-2	CAEZ01004185:22282-22740
HmHel-2	CAEZ01004008:18519-18976
HmHel-2	CAEZ01003548:11925-12379
HmHel-2	CAEZ01002758:17250-17609
HmHel-2	CAEZ01002758:21709-22030
HmHel-2	CAEZ01002337:2822-3280
HmHel-2	CAEZ01011255:7-464
HmHel-2	CAEZ01010242:32275-32733
HmHel-2	CAEZ01010206:5635-6206
HmHel-2	CAEZ01010033:6675-7257
HmHel-2	CAEZ01009042:11525-11982
HmHel-2	CAEZ01008203:12919-13470
HmHel-2	CAEZ01008203:22433-22720
HmHel-2	CAEZ01006567:65642-66099
HmHel-2	CAEZ01006567:28104-28452
HmHel-2	CAEZ01006164:4035-4459
HmHel-2	CAEZ01006164:22886-23226
HmHel-2	CAEZ01006164:60428-60885
HmHel-2	CAEZ01006164:58387-58804
HmHel-2	CAEZ01005713:38916-39372
HmHel-2	CAEZ01005713:7435-7892
HmHel-2	CAEZ01004772:11524-11980
HmHel-2	CAEZ01004540:66-496
HmHel-2	CAEZ01003492:250-713
HmHel-2	CAEZ01003359:1383-1841
HmHel-2	CAEZ01001648:13665-14074
HmHel-2	CAEZ01010999:1092-1511
HmHel-2	CAEZ01010011:2441-2555
HmHel-2	CAEZ01010011:20180-20752
HmHel-2	CAEZ01009725:9443-9895
HmHel-2	CAEZ01008253:7307-7623
HmHel-2	CAEZ01008253:9490-10086
HmHel-2	CAEZ01007140:52402-52868
HmHel-2	CAEZ01007140:53903-54366
HmHel-2	CAEZ01007140:12518-13104

HmHel-2	CAEZ01006070:5-445
HmHel-2	CAEZ01005842:3180-3788
HmHel-2	CAEZ01004678:18201-18655
HmHel-2	CAEZ01004678:1306-1386
HmHel-2	CAEZ01002742:137768-138226
HmHel-2	CAEZ01002742:213619-214077
HmHel-2	CAEZ01002742:123886-124347
HmHel-2	CAEZ01002742:84811-85272
HmHel-2	CAEZ01001453:14740-15281
HmHel-2	CAEZ01009511:10320-10771
HmHel-2	CAEZ01008793:33499-33954
HmHel-2	CAEZ01008201:1625-2083
HmHel-2	CAEZ01008201:18970-19291
HmHel-2	CAEZ01007928:30347-30546
HmHel-2	CAEZ01007928:56620-57098
HmHel-2	CAEZ01007928:29219-29742
HmHel-2	CAEZ01006732:8092-8550
HmHel-2	CAEZ01004862:7243-7698
HmHel-2	CAEZ01004681:26580-27032
HmHel-2	CAEZ01004572:4336-4642
HmHel-2	CAEZ01004572:12935-13358
HmHel-2	CAEZ01003246:27735-28185
HmHel-2	CAEZ01003019:35793-36206
HmHel-2	CAEZ01003019:69891-70327
HmHel-2	CAEZ01002255:64219-64678
HmHel-2	CAEZ01011346:7166-7624
HmHel-2	CAEZ01011295:44372-44830
HmHel-2	CAEZ01010287:37842-38158
HmHel-2	CAEZ01010287:50998-51456
HmHel-2	CAEZ01010125:20809-21267
HmHel-2	CAEZ01009991:7094-7544
HmHel-2	CAEZ01009991:44637-45095
HmHel-2	CAEZ01009184:3935-4393
HmHel-2	CAEZ01008890:16872-17329
HmHel-2	CAEZ01008816:4442-4900
HmHel-2	CAEZ01008724:21717-22171
HmHel-2	CAEZ01008181:28242-28697
HmHel-2	CAEZ01008030:4421-4880
HmHel-2	CAEZ01007871:1469-1927
HmHel-2	CAEZ01007772:19875-19996
HmHel-2	CAEZ01007772:49442-49657
HmHel-2	CAEZ01007772:49246-49389
HmHel-2	CAEZ01007772:17982-18440
HmHel-2	CAEZ01007164:5-306
HmHel-2	CAEZ01007164:17036-17427
HmHel-2	CAEZ01006571:787-1246
HmHel-2	CAEZ01006548:12406-12864
HmHel-2	CAEZ01006548:89892-91303

HmHel-2	CAEZ01006476:5526-5984
HmHel-2	CAEZ01006447:3447-3905
HmHel-2	CAEZ01005779:34139-34597
HmHel-2	CAEZ01005779:6726-6878
HmHel-2	CAEZ01005595:1-376
HmHel-2	CAEZ01005595:7068-7193
HmHel-2	CAEZ01005572:9048-9502
HmHel-2	CAEZ01005339:1734-2193
HmHel-2	CAEZ01005070:21856-22314
HmHel-2	CAEZ01005070:20149-20470
HmHel-2	CAEZ01003885:518-1061
HmHel-2	CAEZ01003885:35996-36454
HmHel-2	CAEZ01003600:11476-11568
HmHel-2	CAEZ01003600:56932-57252
HmHel-2	CAEZ01003600:44567-45025
HmHel-2	CAEZ01002954:2398-2856
HmHel-2	CAEZ01002110:513-971
HmHel-2	CAEZ01001917:7506-7962
HmHel-2	CAEZ01010807:97245-97559
HmHel-2	CAEZ01010807:5556-6014
HmHel-2	CAEZ01010616:31284-31996
HmHel-2	CAEZ01009600:1-411
HmHel-2	CAEZ01009383:1602-2059
HmHel-2	CAEZ01008687:1-414
HmHel-2	CAEZ01007265:12178-12636
HmHel-2	CAEZ01007208:16747-17265
HmHel-2	CAEZ01004869:8950-9128
HmHel-2	CAEZ01004869:31068-31523
HmHel-2	CAEZ01003646:9696-10149
HmHel-2	CAEZ01001957:431-889
HmHel-2	CAEZ01001304:4776-5359
HmHel-2	CAEZ01011046:19359-19818
HmHel-2	CAEZ01009067:362-820
HmHel-2	CAEZ01007954:2909-3347
HmHel-2	CAEZ01007822:14563-15023
HmHel-2	CAEZ01007440:1041-1498
HmHel-2	CAEZ01006672:1562-2120
HmHel-2	CAEZ01006672:13885-14157
HmHel-2	CAEZ01005999:600-1153
HmHel-2	CAEZ01003305:9785-10221
HmHel-2	CAEZ01001826:1309-1767
HmHel-2	CAEZ01010132:5043-5501
HmHel-2	CAEZ01010112:28506-29072
HmHel-2	CAEZ01010049:16781-17236
HmHel-2	CAEZ01009993:15033-15635
HmHel-2	CAEZ01009859:14916-15193
HmHel-2	CAEZ01009402:267-723
HmHel-2	CAEZ01009126:14980-15400

HmHel-2	CAEZ01007858:19660-20099
HmHel-2	CAEZ01007394:422-875
HmHel-2	CAEZ01006989:23742-24201
HmHel-2	CAEZ01006860:2721-3121
HmHel-2	CAEZ01006860:10425-10883
HmHel-2	CAEZ01005603:3966-4287
HmHel-2	CAEZ01005603:24045-24503
HmHel-2	CAEZ01005240:29-431
HmHel-2	CAEZ01004790:5102-5556
HmHel-2	CAEZ01004403:1417-1811
HmHel-2	CAEZ01004112:17980-18365
HmHel-2	CAEZ01011601:3248-3674
HmHel-2	CAEZ01011122:16414-16889
HmHel-2	CAEZ01009756:4507-4965
HmHel-2	CAEZ01009756:7310-7646
HmHel-2	CAEZ01009527:15851-16309
HmHel-2	CAEZ01009298:4712-5191
HmHel-2	CAEZ01008943:7459-7917
HmHel-2	CAEZ01008499:8307-8388
HmHel-2	CAEZ01008499:21349-21808
HmHel-2	CAEZ01008499:8390-8637
HmHel-2	CAEZ01007736:24145-24603
HmHel-2	CAEZ01006415:60772-61267
HmHel-2	CAEZ01006415:21129-21587
HmHel-2	CAEZ01006378:20137-20595
HmHel-2	CAEZ01006241:22718-23176
HmHel-2	CAEZ01006043:2-1079
HmHel-2	CAEZ01005682:32627-33085
HmHel-2	CAEZ01005505:2567-3025
HmHel-2	CAEZ01005044:48978-49437
HmHel-2	CAEZ01005044:117814-118266
HmHel-2	CAEZ01005044:53364-53734
HmHel-2	CAEZ01004249:30433-30891
HmHel-2	CAEZ01002793:754-1323
HmHel-2	CAEZ01002430:19479-19932
HmHel-2	CAEZ01001749:408-863
HmHel-2	CAEZ01010434:5402-6027
HmHel-2	CAEZ01009257:982-1439
HmHel-2	CAEZ01008805:411-882
HmHel-2	CAEZ01008508:36629-37079
HmHel-2	CAEZ01008508:5058-5292
HmHel-2	CAEZ01008248:25401-25818
HmHel-2	CAEZ01008248:10957-11415
HmHel-2	CAEZ01008218:24873-25014
HmHel-2	CAEZ01008218:9516-9978
HmHel-2	CAEZ01006031:2239-2689
HmHel-2	CAEZ01004762:19922-20375
HmHel-2	CAEZ01004431:1647-2232

HmHel-2	CAEZ01003524:13730-14188
HmHel-2	CAEZ01002922:6839-7295
HmHel-2	CAEZ01001333:682-1137
HmHel-2	CAEZ01001152:6322-6779
HmHel-2	CAEZ01000766:2016-2418
HmHel-2	CAEZ01010803:95371-95961
HmHel-2	CAEZ01008741:1-423
HmHel-2	CAEZ01008350:22730-23187
HmHel-2	CAEZ01008350:45964-46416
HmHel-2	CAEZ01007538:14133-14590
HmHel-2	CAEZ01007538:14703-14839
HmHel-2	CAEZ01006945:12992-13393
HmHel-2	CAEZ01006877:31473-31802
HmHel-2	CAEZ01006877:2557-3113
HmHel-2	CAEZ01006405:12145-12576
HmHel-2	CAEZ01006271:8251-8709
HmHel-2	CAEZ01006166:11591-12049
HmHel-2	CAEZ01006166:19911-20317
HmHel-2	CAEZ01002335:2484-2941
HmHel-2	CAEZ01010821:19875-20333
HmHel-2	CAEZ01010821:48994-49452
HmHel-2	CAEZ01010634:13668-14323
HmHel-2	CAEZ01010067:4700-7356
HmHel-2	CAEZ01010002:3488-3942
HmHel-2	CAEZ01009476:4107-4552
HmHel-2	CAEZ01009476:74050-74508
HmHel-2	CAEZ01008767:73791-74201
HmHel-2	CAEZ01008532:21969-22086
HmHel-2	CAEZ01008532:63799-64253
HmHel-2	CAEZ01008532:87432-87749
HmHel-2	CAEZ01007697:14550-15005
HmHel-2	CAEZ01007148:14566-14939
HmHel-2	CAEZ01006920:5521-5960
HmHel-2	CAEZ01006721:17034-17498
HmHel-2	CAEZ01006154:102798-103064
HmHel-2	CAEZ01006154:255694-255960
HmHel-2	CAEZ01006154:273032-273489
HmHel-2	CAEZ01004650:4221-7967
HmHel-2	CAEZ01004597:12509-13101
HmHel-2	CAEZ01004597:33362-33636
HmHel-2	CAEZ01004241:11106-11501
HmHel-2	CAEZ01003386:5206-5662
HmHel-2	CAEZ01002934:2382-2841
HmHel-2	CAEZ01002934:5408-5841
HmHel-2	CAEZ01002925:31751-32207
HmHel-2	CAEZ01010118:30402-30857
HmHel-2	CAEZ01010004:29613-29992
HmHel-2	CAEZ01009501:1741-2196

HmHel-2	CAEZ01008922:26158-26723
HmHel-2	CAEZ01008377:12445-12903
HmHel-2	CAEZ01008159:13604-14062
HmHel-2	CAEZ01007902:16156-16617
HmHel-2	CAEZ01007902:18077-18515
HmHel-2	CAEZ01006866:9279-9737
HmHel-2	CAEZ01005627:12958-13380
HmHel-2	CAEZ01004687:302-760
HmHel-2	CAEZ01004508:2319-2774
HmHel-2	CAEZ01004360:6403-6861
HmHel-2	CAEZ01003686:5129-5587
HmHel-2	CAEZ01003363:1844-2301
HmHel-2	CAEZ01002924:67891-68349
HmHel-2	CAEZ01002652:19140-19571
HmHel-2	CAEZ01002652:18857-19142
HmHel-2	CAEZ01001878:3219-3679
HmHel-2	CAEZ01001873:5299-5756
HmHel-2	CAEZ01010917:25652-26480
HmHel-2	CAEZ01010917:5835-6029
HmHel-2	CAEZ01010527:10337-10795
HmHel-2	CAEZ01009633:94883-95191
HmHel-2	CAEZ01009633:14968-15269
HmHel-2	CAEZ01009080:7835-8369
HmHel-2	CAEZ01008579:49342-49794
HmHel-2	CAEZ01007228:1027-1516
HmHel-2	CAEZ01006808:8312-8702
HmHel-2	CAEZ01006343:8741-9193
HmHel-2	CAEZ01006343:41815-42272
HmHel-2	CAEZ01006126:24185-24644
HmHel-2	CAEZ01006126:63239-63539
HmHel-2	CAEZ01006042:1374-1509
HmHel-2	CAEZ01006042:5768-6125
HmHel-2	CAEZ01005847:22785-22877
HmHel-2	CAEZ01005847:22317-22783
HmHel-2	CAEZ01004802:9430-9886
HmHel-2	CAEZ01004795:19041-19465
HmHel-2	CAEZ01003875:9224-9681
HmHel-2	CAEZ01003852:9861-10423
HmHel-2	CAEZ01002576:7945-8402
HmHel-2	CAEZ01011062:5775-6230
HmHel-2	CAEZ01010278:23472-23924
HmHel-2	CAEZ01010175:7481-7878
HmHel-2	CAEZ01008684:8173-8621
HmHel-2	CAEZ01007840:12-506
HmHel-2	CAEZ01007761:24111-24704
HmHel-2	CAEZ01004623:10127-10589
HmHel-2	CAEZ01004123:1-354
HmHel-2	CAEZ01003855:16614-17068

HmHel-2	CAEZ01003855:28607-29216
HmHel-2	CAEZ01001926:3843-4301
HmHel-2	CAEZ01001433:1969-2424
HmHel-2	CAEZ01000952:734-1193
HmHel-2	CAEZ01010864:91906-92337
HmHel-2	CAEZ01010864:106815-107242
HmHel-2	CAEZ01010864:220293-220798
HmHel-2	CAEZ01010864:152015-152494
HmHel-2	CAEZ01010864:75807-75943
HmHel-2	CAEZ01009874:19194-19703
HmHel-2	CAEZ01008284:10693-11100
HmHel-2	CAEZ01007795:4716-5194
HmHel-2	CAEZ01007795:72451-72594
HmHel-2	CAEZ01007795:57015-57453
HmHel-2	CAEZ01007048:7073-7545
HmHel-2	CAEZ01004723:6461-7003
HmHel-2	CAEZ01004512:544-984
HmHel-2	CAEZ01001956:586-1024
HmHel-2	CAEZ01011393:1545-2005
HmHel-2	CAEZ01010630:18-478
HmHel-2	CAEZ01010220:2433-2568
HmHel-2	CAEZ01009689:654-1112
HmHel-2	CAEZ01008418:31816-32267
HmHel-2	CAEZ01008418:90455-90911
HmHel-2	CAEZ01007758:5392-5806
HmHel-2	CAEZ01007401:39304-39762
HmHel-2	CAEZ01007209:8595-8942
HmHel-2	CAEZ01006511:3381-3839
HmHel-2	CAEZ01006040:8887-9345
HmHel-2	CAEZ01005069:9742-10218
HmHel-2	CAEZ01004799:4614-5067
HmHel-2	CAEZ01004357:6861-7318
HmHel-2	CAEZ01004196:15021-15479
HmHel-2	CAEZ01003817:6877-7335
HmHel-2	CAEZ01003499:7-440
HmHel-2	CAEZ01003112:60576-61035
HmHel-2	CAEZ01002017:1998-2456
HmHel-2	CAEZ01001845:3873-4334
HmHel-2	CAEZ01001750:11736-11914
HmHel-2	CAEZ01010901:941-1398
HmHel-2	CAEZ01010874:8144-8601
HmHel-2	CAEZ01010358:3713-4170
HmHel-2	CAEZ01009709:88013-88405
HmHel-2	CAEZ01009586:65737-66135
HmHel-2	CAEZ01008296:3245-3703
HmHel-2	CAEZ01007873:591-1043
HmHel-2	CAEZ01007094:5044-5485
HmHel-2	CAEZ01004901:28241-28698

HmHel-2	CAEZ01004901:5030-5195
HmHel-2	CAEZ01004326:6321-6778
HmHel-2	CAEZ01003253:2458-2914
HmHel-2	CAEZ01003212:11569-12026
HmHel-2	CAEZ01000902:6891-7444
HmHel-2	CAEZ01009765:3079-3513
HmHel-2	CAEZ01009371:3545-4000
HmHel-2	CAEZ01008832:6577-7154
HmHel-2	CAEZ01006531:23-217
HmHel-2	CAEZ01006491:10133-10589
HmHel-2	CAEZ01004570:1716-2174
HmHel-2	CAEZ01001954:23360-23944
HmHel-2	CAEZ01011359:16169-16621
HmHel-2	CAEZ01011359:17597-18060
HmHel-2	CAEZ01010923:9361-9772
HmHel-2	CAEZ01010398:16801-17108
HmHel-2	CAEZ01010398:13914-14002
HmHel-2	CAEZ01010398:1542-2093
HmHel-2	CAEZ01006620:1608-2067
HmHel-2	CAEZ01006115:447-667
HmHel-2	CAEZ01005744:6127-6664
HmHel-2	CAEZ01005466:2306-2701
HmHel-2	CAEZ01005466:1788-2313
HmHel-2	CAEZ01005387:1-439
HmHel-2	CAEZ01004724:5817-6398
HmHel-2	CAEZ01004581:4075-4516
HmHel-2	CAEZ01003729:2259-2715
HmHel-2	CAEZ01003714:5266-5722
HmHel-2	CAEZ01002036:8068-8239
HmHel-2	CAEZ01011527:34695-35153
HmHel-2	CAEZ01011478:22728-23035
HmHel-2	CAEZ01010503:22472-22930
HmHel-2	CAEZ01009419:16585-17203
HmHel-2	CAEZ01009286:69434-69892
HmHel-2	CAEZ01009085:51190-51291
HmHel-2	CAEZ01009085:109613-109834
HmHel-2	CAEZ01009085:109846-110004
HmHel-2	CAEZ01009085:58064-61779
HmHel-2	CAEZ01009085:13519-13994
HmHel-2	CAEZ01008815:1150-5660
HmHel-2	CAEZ01007978:3924-4381
HmHel-2	CAEZ01007704:11490-11948
HmHel-2	CAEZ01007686:10940-11134
HmHel-2	CAEZ01007686:43710-44169
HmHel-2	CAEZ01007625:14581-15090
HmHel-2	CAEZ01007625:51998-52117
HmHel-2	CAEZ01007625:23-574
HmHel-2	CAEZ01006623:6381-6803

HmHel-2	CAEZ01006337:102812-103250
HmHel-2	CAEZ01006337:78833-79291
HmHel-2	CAEZ01006261:11463-11921
HmHel-2	CAEZ01005836:2366-2825
HmHel-2	CAEZ01005803:5953-6403
HmHel-2	CAEZ01005342:541-999
HmHel-2	CAEZ01004752:57730-58465
HmHel-2	CAEZ01004457:7242-7697
HmHel-2	CAEZ01004213:21719-22178
HmHel-2	CAEZ01004213:54428-54886
HmHel-2	CAEZ01004197:1606-2062
HmHel-2	CAEZ01002667:2243-2698
HmHel-2	CAEZ01002040:15248-15706
HmHel-2	CAEZ01001439:39275-39733
HmHel-2	CAEZ01001381:172-625
HmHel-2	CAEZ01008723:27594-28058
HmHel-2	CAEZ01007618:5134-5593
HmHel-2	CAEZ01006462:987-1438
HmHel-2	CAEZ01006462:1-365
HmHel-2	CAEZ01006102:28010-28467
HmHel-2	CAEZ01004942:74096-74532
HmHel-2	CAEZ01004809:15892-16339
HmHel-2	CAEZ01003741:4278-4550
HmHel-2	CAEZ01001402:34960-35057
HmHel-2	CAEZ01001402:15221-15812
HmHel-2	CAEZ01001266:29520-29980
HmHel-2	CAEZ01011408:9312-9855
HmHel-2	CAEZ01011408:21697-22335
HmHel-2	CAEZ01011166:6548-7003
HmHel-2	CAEZ01009987:23226-23745
HmHel-2	CAEZ01008226:12817-13277
HmHel-2	CAEZ01008193:18-394
HmHel-2	CAEZ01006407:11264-11722
HmHel-2	CAEZ01006324:25177-25663
HmHel-2	CAEZ01005639:2894-3352
HmHel-2	CAEZ01005480:8057-8512
HmHel-2	CAEZ01010981:11250-11409
HmHel-2	CAEZ01010508:9259-9698
HmHel-2	CAEZ01008367:9316-9746
HmHel-2	CAEZ01008367:168983-169439
HmHel-2	CAEZ01008367:110070-110255
HmHel-2	CAEZ01008367:31050-31515
HmHel-2	CAEZ01008349:138656-138978
HmHel-2	CAEZ01008349:145995-146589
HmHel-2	CAEZ01008349:53514-53970
HmHel-2	CAEZ01007028:2846-3296
HmHel-2	CAEZ01006228:53731-54190
HmHel-2	CAEZ01006086:8470-8631

HmHel-2	CAEZ01006086:41619-42192
HmHel-2	CAEZ01006086:15458-15909
HmHel-2	CAEZ01005215:2347-2795
HmHel-2	CAEZ01005215:24716-24961
HmHel-2	CAEZ01004821:35425-35745
HmHel-2	CAEZ01004821:51934-52390
HmHel-2	CAEZ01004821:42821-43277
HmHel-2	CAEZ01003878:960-1416
HmHel-2	CAEZ01003699:1-364
HmHel-2	CAEZ01002971:10494-10950
HmHel-2	CAEZ01001071:4197-4557
HmHel-2	CAEZ01000854:71715-72033
HmHel-2	CAEZ01000854:87750-88189
HmHel-2	CAEZ01000854:4466-4622
HmHel-2	CAEZ01011227:44163-44621
HmHel-2	CAEZ01009711:801-1262
HmHel-2	CAEZ01008916:48728-48977
HmHel-2	CAEZ01008916:25242-25699
HmHel-2	CAEZ01008882:14866-15324
HmHel-2	CAEZ01007414:13282-13458
HmHel-2	CAEZ01007414:7038-7475
HmHel-2	CAEZ01007258:124137-124600
HmHel-2	CAEZ01006431:11364-11822
HmHel-2	CAEZ01003674:6159-6617
HmHel-2	CAEZ01003173:2214-2815
HmHel-2	CAEZ01003173:17021-17481
HmHel-2	CAEZ01001919:3461-3917
HmHel-2	CAEZ01001902:328-517
HmHel-2	CAEZ01001902:1480-1938
HmHel-2	CAEZ01001539:406-864
HmHel-2	CAEZ01001386:20446-20536
HmHel-2	CAEZ01001386:1235-1586
HmHel-2	CAEZ01011228:1443-1900
HmHel-2	CAEZ01010350:1607-1774
HmHel-2	CAEZ01010350:11704-12158
HmHel-2	CAEZ01009625:764-1329
HmHel-2	CAEZ01007886:17374-17798
HmHel-2	CAEZ01007095:10331-11052
HmHel-2	CAEZ01006165:1-388
HmHel-2	CAEZ01005728:10687-11057
HmHel-2	CAEZ01005644:406-862
HmHel-2	CAEZ01004754:4962-5419
HmHel-2	CAEZ01004592:30058-30501
HmHel-2	CAEZ01004592:12864-13324
HmHel-2	CAEZ01004018:1507-1718
HmHel-2	CAEZ01004018:47262-47727
HmHel-2	CAEZ01002984:1110-1562
HmHel-2	CAEZ01002409:794-1128

HmHel-2	CAEZ01010354:8881-9339
HmHel-2	CAEZ01009888:34083-34550
HmHel-2	CAEZ01008398:18267-18683
HmHel-2	CAEZ01008398:73885-74206
HmHel-2	CAEZ01008398:55072-55527
HmHel-2	CAEZ01008398:4329-4505
HmHel-2	CAEZ01006935:1900-2442
HmHel-2	CAEZ01006370:2133-2584
HmHel-2	CAEZ01006370:20283-20630
HmHel-2	CAEZ01006142:1-560
HmHel-2	CAEZ01006142:789-973
HmHel-2	CAEZ01005146:11-387
HmHel-2	CAEZ01005006:2101-2557
HmHel-2	CAEZ01004905:30166-30602
HmHel-2	CAEZ01004582:31456-31875
HmHel-2	CAEZ01004582:13826-14271
HmHel-2	CAEZ01010801:5171-5622
HmHel-2	CAEZ01009880:25642-26098
HmHel-2	CAEZ01009819:6797-7253
HmHel-2	CAEZ01009444:2657-3105
HmHel-2	CAEZ01009367:19812-20267
HmHel-2	CAEZ01007909:10302-10736
HmHel-2	CAEZ01006892:441-1020
HmHel-2	CAEZ01006116:3191-3503
HmHel-2	CAEZ01004939:2307-2761
HmHel-2	CAEZ01004726:2324-2783
HmHel-2	CAEZ01001510:45486-45948
HmHel-2	CAEZ01011188:5668-6271
HmHel-2	CAEZ01011160:12670-13114
HmHel-2	CAEZ01010947:2822-3557
HmHel-2	CAEZ01010475:4-363
HmHel-2	CAEZ01009479:48155-48603
HmHel-2	CAEZ01009479:56933-57402
HmHel-2	CAEZ01007541:1480-2075
HmHel-2	CAEZ01007177:2497-3197
HmHel-2	CAEZ01004917:12399-12857
HmHel-2	CAEZ01003237:46587-47176
HmHel-2	CAEZ01002634:61711-62165
HmHel-2	CAEZ01002634:36074-36532
HmHel-2	CAEZ01010828:28706-29151
HmHel-2	CAEZ01009681:9472-10031
HmHel-2	CAEZ01007374:23269-23784
HmHel-2	CAEZ01005528:5771-6229
HmHel-2	CAEZ01005428:72761-73065
HmHel-2	CAEZ01005428:92234-92674
HmHel-2	CAEZ01005428:100041-100594
HmHel-2	CAEZ01005362:9674-10122
HmHel-2	CAEZ01004771:7302-7750

HmHel-2	CAEZ01004771:5273-5406
HmHel-2	CAEZ01004759:10026-10478
HmHel-2	CAEZ01003980:4784-5238
HmHel-2	CAEZ01002952:963-1400
HmHel-2	CAEZ01002118:11230-11687
HmHel-2	CAEZ01001234:1124-1800
HmHel-2	CAEZ01001064:29329-29778
HmHel-2	CAEZ01010385:7144-7593
HmHel-2	CAEZ01010191:4302-4920
HmHel-2	CAEZ01009983:5708-6127
HmHel-2	CAEZ01009850:2113-2536
HmHel-2	CAEZ01009414:9396-9932
HmHel-2	CAEZ01009414:5970-6431
HmHel-2	CAEZ01008330:1065-1960
HmHel-2	CAEZ01008297:2986-3440
HmHel-2	CAEZ01008237:62708-63175
HmHel-2	CAEZ01007480:1119-1576
HmHel-2	CAEZ01007218:13891-14457
HmHel-2	CAEZ01005564:1179-1630
HmHel-2	CAEZ01004703:19466-19914
HmHel-2	CAEZ01004468:6437-7089
HmHel-2	CAEZ01002416:22494-22649
HmHel-2	CAEZ01002416:67327-67587
HmHel-2	CAEZ01002416:5510-5950
HmHel-2	CAEZ01002195:13669-14064
HmHel-2	CAEZ01002195:11883-12161
HmHel-2	CAEZ01002079:7334-7928
HmHel-2	CAEZ01011220:1-328
HmHel-2	CAEZ01011146:22993-23449
HmHel-2	CAEZ01008400:39464-39938
HmHel-2	CAEZ01008400:87580-87705
HmHel-2	CAEZ01008400:70915-71444
HmHel-2	CAEZ01008400:26158-26450
HmHel-2	CAEZ01007713:35427-35962
HmHel-2	CAEZ01007713:53564-53758
HmHel-2	CAEZ01007713:60945-61205
HmHel-2	CAEZ01007713:9974-10432
HmHel-2	CAEZ01007271:20025-20481
HmHel-2	CAEZ01005440:32-356
HmHel-2	CAEZ01004093:13474-21973
HmHel-2	CAEZ01003954:12682-13139
HmHel-2	CAEZ01003794:13748-14228
HmHel-2	CAEZ01003794:6813-7070
HmHel-2	CAEZ01000939:1241-1696
HmHel-2	CAEZ01000903:19657-20111
HmHel-2	CAEZ01011496:27-446
HmHel-2	CAEZ01009832:22972-23405
HmHel-2	CAEZ01008184:2320-2768

HmHel-2	CAEZ01008054:8169-8709
HmHel-2	CAEZ01005890:23346-23780
HmHel-2	CAEZ01005111:24494-24949
HmHel-2	CAEZ01005111:15538-15757
HmHel-2	CAEZ01004721:7933-8391
HmHel-2	CAEZ01003891:4868-5341
HmHel-2	CAEZ01002583:1-347
HmHel-2	CAEZ01002090:677-1138
HmHel-2	CAEZ01002090:1686-1816
HmHel-2	CAEZ01000852:479-937
HmHel-2	CAEZ01010273:21444-21810
HmHel-2	CAEZ01010273:77788-78134
HmHel-2	CAEZ01008843:1607-2066
HmHel-2	CAEZ01007363:948-1361
HmHel-2	CAEZ01007351:1973-2430
HmHel-2	CAEZ01006759:6075-6342
HmHel-2	CAEZ01006759:80170-80552
HmHel-2	CAEZ01006759:36542-37125
HmHel-2	CAEZ01005353:2-446
HmHel-2	CAEZ01003854:6951-7394
HmHel-2	CAEZ01000040:8082-8499
HmHel-2	CAEZ01010723:475-914
HmHel-2	CAEZ01010663:24451-24772
HmHel-2	CAEZ01010663:28171-29221
HmHel-2	CAEZ01010173:9359-9538
HmHel-2	CAEZ01008372:296946-297398
HmHel-2	CAEZ01008372:113496-113810
HmHel-2	CAEZ01008077:7917-8365
HmHel-2	CAEZ01008045:15832-16290
HmHel-2	CAEZ01007961:6454-6911
HmHel-2	CAEZ01006592:104632-104954
HmHel-2	CAEZ01006592:44727-45064
HmHel-2	CAEZ01005131:20527-20792
HmHel-2	CAEZ01005131:16605-16896
HmHel-2	CAEZ01004397:13872-14400
HmHel-2	CAEZ01003745:13982-14433
HmHel-2	CAEZ01003418:3529-4143
HmHel-2	CAEZ01002166:16057-16446
HmHel-2	CAEZ01002035:1592-2028
HmHel-2	CAEZ01009944:1-333
HmHel-2	CAEZ01009798:13636-14091
HmHel-2	CAEZ01009552:9-581
HmHel-2	CAEZ01007046:9907-10363
HmHel-2	CAEZ01006962:6-318
HmHel-2	CAEZ01006763:2934-3897
HmHel-2	CAEZ01005251:26471-26919
HmHel-2	CAEZ01004828:8266-8677
HmHel-2	CAEZ01003234:14-433

HmHel-2	CAEZ01002973:1337-1794
HmHel-2	CAEZ01011194:469-923
HmHel-2	CAEZ01009431:75979-76400
HmHel-2	CAEZ01009190:42-393
HmHel-2	CAEZ01008765:27454-27895
HmHel-2	CAEZ01008068:7176-7626
HmHel-2	CAEZ01007732:3418-3836
HmHel-2	CAEZ01007278:12691-13058
HmHel-2	CAEZ01006593:8330-8906
HmHel-2	CAEZ01006561:88065-88523
HmHel-2	CAEZ01006561:123921-124379
HmHel-2	CAEZ01006561:110604-110925
HmHel-2	CAEZ01005302:7096-7398
HmHel-2	CAEZ01005302:2495-2959
HmHel-2	CAEZ01004689:25428-25775
HmHel-2	CAEZ01004169:7908-8439
HmHel-2	CAEZ01003535:10415-10873
HmHel-2	CAEZ01001054:2-466
HmHel-2	CAEZ01000322:1-366
HmHel-2	CAEZ01011313:1695-2200
HmHel-2	CAEZ01010900:44986-45446
HmHel-2	CAEZ01010900:15755-16312
HmHel-2	CAEZ01010758:2345-2893
HmHel-2	CAEZ01009215:2225-2682
HmHel-2	CAEZ01008738:8992-9449
HmHel-2	CAEZ01007688:6506-6963
HmHel-2	CAEZ01004665:2570-4416
HmHel-2	CAEZ01004099:5640-6103
HmHel-2	CAEZ01003020:27091-27787
HmHel-2	CAEZ01003020:19859-20221
HmHel-2	CAEZ01002582:1250-1596
HmHel-2	CAEZ01009481:6662-7107
HmHel-2	CAEZ01009481:42507-42967
HmHel-2	CAEZ01009271:154319-154602
HmHel-2	CAEZ01009271:133184-133636
HmHel-2	CAEZ01009271:42509-42968
HmHel-2	CAEZ01008450:76067-76388
HmHel-2	CAEZ01008450:30896-31312
HmHel-2	CAEZ01007967:7083-7597
HmHel-2	CAEZ01007823:2034-2360
HmHel-2	CAEZ01007689:36567-37027
HmHel-2	CAEZ01006001:11624-12049
HmHel-2	CAEZ01002128:8200-8617
HmHel-2	CAEZ01001006:734-1185
HmHel-2	CAEZ01011652:980-1435
HmHel-2	CAEZ01011111:7467-7918
HmHel-2	CAEZ01009349:2409-2857
HmHel-2	CAEZ01007771:7138-7450

HmHel-2	CAEZ01006496:11142-11601
HmHel-2	CAEZ01006496:9903-10145
HmHel-2	CAEZ01006461:797-1254
HmHel-2	CAEZ01005958:3878-4334
HmHel-2	CAEZ01004464:14675-15157
HmHel-2	CAEZ01001777:11994-12453
HmHel-2	CAEZ01001777:27195-27477
HmHel-2	CAEZ01000809:9955-10539
HmHel-2	CAEZ01010865:1-376
HmHel-2	CAEZ01010212:840-1159
HmHel-2	CAEZ01010212:112247-112698
HmHel-2	CAEZ01010212:39555-39874
HmHel-2	CAEZ01009043:17-368
HmHel-2	CAEZ01008502:1712-2161
HmHel-2	CAEZ01007793:19144-19553
HmHel-2	CAEZ01007658:7241-7695
HmHel-2	CAEZ01006700:7818-8078
HmHel-2	CAEZ01006700:19157-19690
HmHel-2	CAEZ01005425:13536-13991
HmHel-2	CAEZ01005294:660-1109
HmHel-2	CAEZ01001113:602-1020
HmHel-2	CAEZ01000059:25631-26102
HmHel-2	CAEZ01000059:16115-16426
HmHel-2	CAEZ01011526:27159-27614
HmHel-2	CAEZ01011526:21184-21462
HmHel-2	CAEZ01011405:10181-10643
HmHel-2	CAEZ01009529:8333-8790
HmHel-2	CAEZ01008389:15645-16064
HmHel-2	CAEZ01007451:12905-13363
HmHel-2	CAEZ01006922:9-383
HmHel-2	CAEZ01005735:2550-3007
HmHel-2	CAEZ01005589:1-409
HmHel-2	CAEZ01005072:5790-6371
HmHel-2	CAEZ01004938:16459-16690
HmHel-2	CAEZ01004938:20929-21833
HmHel-2	CAEZ01004852:9495-9941
HmHel-2	CAEZ01003732:15806-16268
HmHel-2	CAEZ01002857:1497-1928
HmHel-2	CAEZ01002857:1309-1505
HmHel-2	CAEZ01001033:3495-3683
HmHel-2	CAEZ01010081:18022-18475
HmHel-2	CAEZ01009710:3759-4214
HmHel-2	CAEZ01009070:25498-25954
HmHel-2	CAEZ01008407:15430-15866
HmHel-2	CAEZ01008346:34223-34682
HmHel-2	CAEZ01008019:4734-5169
HmHel-2	CAEZ01006946:8049-8618
HmHel-2	CAEZ01005964:113295-113734

HmHel-2	CAEZ01004082:13093-13547
HmHel-2	CAEZ01003207:2610-2903
HmHel-2	CAEZ01003207:52598-53052
HmHel-2	CAEZ01002322:9157-9605
HmHel-2	CAEZ01002322:10441-10732
HmHel-2	CAEZ01002147:10038-10597
HmHel-2	CAEZ01011488:10597-11043
HmHel-2	CAEZ01009757:8-327
HmHel-2	CAEZ01009750:27769-28323
HmHel-2	CAEZ01009418:1-425
HmHel-2	CAEZ01006365:6079-6230
HmHel-2	CAEZ01006365:17874-18331
HmHel-2	CAEZ01006365:14576-14730
HmHel-2	CAEZ01005807:26703-27154
HmHel-2	CAEZ01005807:36843-37300
HmHel-2	CAEZ01005725:7458-7818
HmHel-2	CAEZ01004362:14594-15050
HmHel-2	CAEZ01003257:4418-4874
HmHel-2	CAEZ01003257:7826-8287
HmHel-2	CAEZ01002644:16216-16665
HmHel-2	CAEZ01001738:22325-22646
HmHel-2	CAEZ01001738:28120-28578
HmHel-2	CAEZ01001474:23-383
HmHel-2	CAEZ01000927:1415-1790
HmHel-2	CAEZ01010778:627-974
HmHel-2	CAEZ01010171:27210-27665
HmHel-2	CAEZ01009448:15560-16018
HmHel-2	CAEZ01009255:6818-7165
HmHel-2	CAEZ01008819:9704-10173
HmHel-2	CAEZ01007283:965-1152
HmHel-2	CAEZ01006633:37780-38212
HmHel-2	CAEZ01003166:3938-4387
HmHel-2	CAEZ01002664:6294-6621
HmHel-2	CAEZ01011088:482-939
HmHel-2	CAEZ01010536:1-364
HmHel-2	CAEZ01007970:3157-3749
HmHel-2	CAEZ01006564:19578-20035
HmHel-2	CAEZ01006384:5222-5776
HmHel-2	CAEZ01006384:24711-25008
HmHel-2	CAEZ01006384:19049-19632
HmHel-2	CAEZ01005846:9773-10235
HmHel-2	CAEZ01002950:57593-58058
HmHel-2	CAEZ01002950:25669-26127
HmHel-2	CAEZ01002927:14203-14898
HmHel-2	CAEZ01010766:2742-3195
HmHel-2	CAEZ01009737:483-937
HmHel-2	CAEZ01009664:7625-8081
HmHel-2	CAEZ01009637:7717-8134

HmHel-2	CAEZ01008725:14719-15201
HmHel-2	CAEZ01007619:749-1202
HmHel-2	CAEZ01007537:1112-1562
HmHel-2	CAEZ01006788:11653-12181
HmHel-2	CAEZ01006084:21381-21701
HmHel-2	CAEZ01006084:58486-58578
HmHel-2	CAEZ01006084:45259-45526
HmHel-2	CAEZ01006084:33370-33818
HmHel-2	CAEZ01006084:19539-19701
HmHel-2	CAEZ01006047:26854-27411
HmHel-2	CAEZ01005453:8657-9112
HmHel-2	CAEZ01003556:453-904
HmHel-2	CAEZ01010210:11728-12057
HmHel-2	CAEZ01009350:5244-5703
HmHel-2	CAEZ01007488:1-341
HmHel-2	CAEZ01007153:886-1246
HmHel-2	CAEZ01004646:29-349
HmHel-2	CAEZ01002785:2131-2431
HmHel-2	CAEZ01001176:45-365
HmHel-2	CAEZ01010465:2417-2875
HmHel-2	CAEZ01010060:5510-5889
HmHel-2	CAEZ01006848:12171-12518
HmHel-2	CAEZ01006490:70897-71519
HmHel-2	CAEZ01009828:11-383
HmHel-2	CAEZ01009828:20655-27883
HmHel-2	CAEZ01009828:1986-2310
HmHel-2	CAEZ01006735:8493-9065
HmHel-2	CAEZ01005020:362-814
HmHel-2	CAEZ01004059:22218-22332
HmHel-2	CAEZ01004059:24418-24861
HmHel-2	CAEZ01004038:11714-12246
HmHel-2	CAEZ01011285:11328-11751
HmHel-2	CAEZ01011285:44095-45451
HmHel-2	CAEZ01010447:25542-25994
HmHel-2	CAEZ01009569:7825-8280
HmHel-2	CAEZ01009322:25994-26445
HmHel-2	CAEZ01009186:5319-5751
HmHel-2	CAEZ01006965:6523-6923
HmHel-2	CAEZ01005408:7972-8289
HmHel-2	CAEZ01005066:24257-24708
HmHel-2	CAEZ01005021:4326-4771
HmHel-2	CAEZ01011528:19600-20039
HmHel-2	CAEZ01010770:8333-8789
HmHel-2	CAEZ01009755:487-922
HmHel-2	CAEZ01008990:10-326
HmHel-2	CAEZ01007495:36643-37139
HmHel-2	CAEZ01007495:32-351
HmHel-2	CAEZ01007434:12069-12508

HmHel-2	CAEZ01006138:60620-60773
HmHel-2	CAEZ01006138:80106-80572
HmHel-2	CAEZ01005799:33-352
HmHel-2	CAEZ01004245:8879-9337
HmHel-2	CAEZ01009013:22812-23176
HmHel-2	CAEZ01009013:1350-1807
HmHel-2	CAEZ01007442:14406-14863
HmHel-2	CAEZ01006239:5653-6110
HmHel-2	CAEZ01005637:7953-8422
HmHel-2	CAEZ01005481:27005-27460
HmHel-2	CAEZ01005481:18981-19220
HmHel-2	CAEZ01004907:530-988
HmHel-2	CAEZ01004756:2391-2832
HmHel-2	CAEZ01003279:47155-47606
HmHel-2	CAEZ01011476:3929-4377
HmHel-2	CAEZ01011476:20517-20635
HmHel-2	CAEZ01011176:1-393
HmHel-2	CAEZ01010752:36289-36767
HmHel-2	CAEZ01010752:1764-2333
HmHel-2	CAEZ01009781:22604-22966
HmHel-2	CAEZ01007607:37318-37758
HmHel-2	CAEZ01007151:26665-26867
HmHel-2	CAEZ01007151:1-459
HmHel-2	CAEZ01003321:4289-4721
HmHel-2	CAEZ01000905:7-450
HmHel-2	CAEZ01000858:23149-23590
HmHel-2	CAEZ01000858:1007-1394
HmHel-2	CAEZ01009937:4483-5046
HmHel-2	CAEZ01007379:6794-7247
HmHel-2	CAEZ01007376:37207-37583
HmHel-2	CAEZ01006372:70822-71143
HmHel-2	CAEZ01006372:47301-47622
HmHel-2	CAEZ01006372:8730-9003
HmHel-2	CAEZ01005845:3015-3463
HmHel-2	CAEZ01002140:13132-13468
HmHel-2	CAEZ01001323:2-311
HmHel-2	CAEZ01001254:327-913
HmHel-2	CAEZ01010746:14207-14668
HmHel-2	CAEZ01009945:22045-22507
HmHel-2	CAEZ01008678:797-1171
HmHel-2	CAEZ01007973:25189-25622
HmHel-2	CAEZ01006605:4635-4951
HmHel-2	CAEZ01005578:7071-7616
HmHel-2	CAEZ01005309:58205-58891
HmHel-2	CAEZ01003494:20557-20901
HmHel-2	CAEZ01003206:11335-11624
HmHel-2	CAEZ01001074:14603-15058
HmHel-2	CAEZ01000967:7490-7962

HmHel-2	CAEZ01009894:37179-37655
HmHel-2	CAEZ01008733:11777-12222
HmHel-2	CAEZ01005923:20486-20944
HmHel-2	CAEZ01005177:89326-89722
HmHel-2	CAEZ01004086:8605-8951
HmHel-2	CAEZ01003847:42-505
HmHel-2	CAEZ01002779:25801-26160
HmHel-2	CAEZ01010540:22746-23344
HmHel-2	CAEZ01008753:2679-3062
HmHel-2	CAEZ01008057:9146-9576
HmHel-2	CAEZ01007981:30170-30568
HmHel-2	CAEZ01007093:2303-2860
HmHel-2	CAEZ01006474:10582-11018
HmHel-2	CAEZ01011375:4389-4841
HmHel-2	CAEZ01007173:2871-3339
HmHel-2	CAEZ01006736:28194-28491
HmHel-2	CAEZ01005685:16-432
HmHel-2	CAEZ01005421:29437-29791
HmHel-2	CAEZ01005421:9236-9671
HmHel-2	CAEZ01004663:2903-3336
HmHel-2	CAEZ01007901:12081-12491
HmHel-2	CAEZ01006113:1588-2033
HmHel-2	CAEZ01004367:2566-3297
HmHel-2	CAEZ01008501:861-1139
HmHel-2	CAEZ01006556:4859-5317
HmHel-2	CAEZ01004492:2271-2879
HmHel-2	CAEZ01004492:36160-36598
HmHel-2	CAEZ01003381:1649-2219
HmHel-2	CAEZ01008928:7040-7495
HmHel-2	CAEZ01008119:3024-3279
HmHel-2	CAEZ01008119:12716-13273
HmHel-2	CAEZ01004282:7984-8437
HmHel-2	CAEZ01002881:21785-22083
HmHel-2	CAEZ01002843:904-1417
HmHel-2	CAEZ01009872:98587-99037
HmHel-2	CAEZ01009872:122166-122602
HmHel-2	CAEZ01009575:2070-6857
HmHel-2	CAEZ01006414:10502-10981
HmHel-2	CAEZ01004011:53547-53999
HmHel-2	CAEZ01010822:18704-19041
HmHel-2	CAEZ01006702:9679-10017
HmHel-2	CAEZ01005216:2643-3081
HmHel-2	CAEZ01003343:2820-3208
HmHel-2	CAEZ01003282:1302-1749
HmHel-2	CAEZ01001751:1-361
HmHel-2	CAEZ01001559:10448-10892
HmHel-2	CAEZ01011064:13769-14040
HmHel-2	CAEZ01010111:3401-3812

HmHel-2	CAEZ01009721:31793-32251
HmHel-2	CAEZ01008170:32314-32772
HmHel-2	CAEZ01006658:270-573
HmHel-2	CAEZ01006516:13842-14375
HmHel-2	CAEZ01005567:182-485
HmHel-2	CAEZ01004159:6512-7078
HmHel-2	CAEZ01002867:4-275
HmHel-2	CAEZ01009475:6922-7377
HmHel-2	CAEZ01009256:11795-12221
HmHel-2	CAEZ01009256:8827-9190
HmHel-2	CAEZ01008835:1776-2381
HmHel-2	CAEZ01005214:8786-9061
HmHel-2	CAEZ01004404:555-1001
HmHel-2	CAEZ01002813:38903-39458
HmHel-2	CAEZ01001809:1076-2794
HmHel-2	CAEZ01011336:3530-3976
HmHel-2	CAEZ01011167:1694-2155
HmHel-2	CAEZ01007709:42518-42612
HmHel-2	CAEZ01005157:15487-16051
HmHel-2	CAEZ01003977:2600-2985
HmHel-2	CAEZ01011342:1-407
HmHel-2	CAEZ01010951:21553-21833
HmHel-2	CAEZ01009694:36023-36470
HmHel-2	CAEZ01008094:13138-13494
HmHel-2	CAEZ01007582:1-277
HmHel-2	CAEZ01005596:1385-1468
HmHel-2	CAEZ01005596:2147-2699
HmHel-2	CAEZ01005406:331-778
HmHel-2	CAEZ01010940:5956-6407
HmHel-2	CAEZ01009427:23051-23188
HmHel-2	CAEZ01008614:22752-23079
HmHel-2	CAEZ01005029:687-1245
HmHel-2	CAEZ01001574:1299-1598
HmHel-2	CAEZ01010288:261381-261683
HmHel-2	CAEZ01008911:120342-120522
HmHel-2	CAEZ01008911:1781-2145
HmHel-2	CAEZ01007714:1-293
HmHel-2	CAEZ01007353:1-392
HmHel-2	CAEZ01006231:7433-7814
HmHel-2	CAEZ01005727:3579-4011
HmHel-2	CAEZ01005097:9827-10294
HmHel-2	CAEZ01003819:31376-31671
HmHel-2	CAEZ01003819:1096-1526
HmHel-2	CAEZ01002771:30515-30816
HmHel-2	CAEZ01002275:8549-8998
HmHel-2	CAEZ01002028:1-240
HmHel-2	CAEZ01010982:11806-12252
HmHel-2	CAEZ01009769:17723-18169

HmHel-2	CAEZ01009551:4628-4925
HmHel-2	CAEZ01007553:21-342
HmHel-2	CAEZ01006859:11161-11623
HmHel-2	CAEZ01005250:67990-68455
HmHel-2	CAEZ01000439:326-903
HmHel-2	CAEZ01009180:1-382
HmHel-2	CAEZ01006160:4219-4764
HmHel-2	CAEZ01004937:4872-5301
HmHel-2	CAEZ01004888:13153-13744
HmHel-2	CAEZ01009496:5079-5362
HmHel-2	CAEZ01008608:72443-72790
HmHel-2	CAEZ01008608:14-194
HmHel-2	CAEZ01008127:24703-24824
HmHel-2	CAEZ01008127:2929-3499
HmHel-2	CAEZ01003303:14802-15247
HmHel-2	CAEZ01002694:24874-25405
HmHel-2	CAEZ01002694:20224-20344
HmHel-2	CAEZ01001429:3791-4248
HmHel-2	CAEZ01010744:6294-6576
HmHel-2	CAEZ01008388:19656-20033
HmHel-2	CAEZ01008363:6892-7443
HmHel-2	CAEZ01006395:72358-73068
HmHel-2	CAEZ01006395:57178-57505
HmHel-2	CAEZ01005479:11540-11996
HmHel-2	CAEZ01003781:42687-43154
HmHel-2	CAEZ01002102:1281-1721
HmHel-2	CAEZ01002030:1160-1555
HmHel-2	CAEZ01005141:3-292
HmHel-2	CAEZ01008015:2258-2652
HmHel-2	CAEZ01006667:24318-24895
HmHel-2	CAEZ01004614:5153-5601
HmHel-2	CAEZ01004006:16968-17299
HmHel-2	CAEZ01004006:9225-9657
HmHel-2	CAEZ01000991:6455-6999
HmHel-2	CAEZ01000910:1-307
HmHel-2	CAEZ01010707:197220-197822
HmHel-2	CAEZ01010707:103719-104037
HmHel-2	CAEZ01004503:5578-6040
HmHel-2	CAEZ01004031:792-1383
HmHel-2	CAEZ01011011:1140-1566
HmHel-2	CAEZ01010658:10909-11477
HmHel-2	CAEZ01009408:10106-10445
HmHel-2	CAEZ01008740:99693-99807
HmHel-2	CAEZ01008740:94910-95518
HmHel-2	CAEZ01006492:26592-26709
HmHel-2	CAEZ01006492:12580-13146
HmHel-2	CAEZ01004127:38080-38320
HmHel-2	CAEZ01004127:85435-85702

HmHel-2	CAEZ01001602:6978-7428
HmHel-2	CAEZ01011159:1-372
HmHel-2	CAEZ01005743:12604-12719
HmHel-2	CAEZ01009594:8416-8724
HmHel-2	CAEZ01007616:2822-3261
HmHel-2	CAEZ01007501:4903-5350
HmHel-2	CAEZ01005335:87755-88204
HmHel-2	CAEZ01005335:10101-10422
HmHel-2	CAEZ01003826:9336-9779
HmHel-2	CAEZ01003826:35110-35274
HmHel-2	CAEZ01003251:1896-2358
HmHel-2	CAEZ01002681:2757-3212
HmHel-2	CAEZ01008488:120-432
HmHel-2	CAEZ01004843:9326-9795
HmHel-2	CAEZ01009003:16759-17147
HmHel-2	CAEZ01005242:2821-3178
HmHel-2	CAEZ01003074:460-845
HmHel-2	CAEZ01011234:6357-6820
HmHel-2	CAEZ01008616:4399-4842
HmHel-2	CAEZ01006155:9008-9391
HmHel-2	CAEZ01006153:18954-19425
HmHel-2	CAEZ01004365:5781-6118
HmHel-2	CAEZ01002211:73564-74009
HmHel-2	CAEZ01000474:486-925
HmHel-2	CAEZ01009513:5240-5720
HmHel-2	CAEZ01008075:18210-18486
HmHel-2	CAEZ01008075:3353-3503
HmHel-2	CAEZ01006265:4224-4687
HmHel-2	CAEZ01003107:2-444
HmHel-2	CAEZ01001205:5766-6201
HmHel-2	CAEZ01010890:3710-4201
HmHel-2	CAEZ01010735:9550-10022
HmHel-2	CAEZ01009656:59928-60558
HmHel-2	CAEZ01008716:13156-13541
HmHel-2	CAEZ01008697:1-283
HmHel-2	CAEZ01003427:1549-1826
HmHel-2	CAEZ01003045:12783-13092
HmHel-2	CAEZ01001119:5275-5861
HmHel-2	CAEZ01010623:4599-5051
HmHel-2	CAEZ01008967:877-961
HmHel-2	CAEZ01008967:13960-14147
HmHel-2	CAEZ01008967:431-792
HmHel-2	CAEZ01006957:11484-11769
HmHel-2	CAEZ01003515:19-296
HmHel-2	CAEZ01003326:36627-37203
HmHel-2	CAEZ01002177:1-301
HmHel-2	CAEZ01001563:20174-20546
HmHel-2	CAEZ01001563:31180-31586

HmHel-2	CAEZ01009692:8426-8861
HmHel-2	CAEZ01005273:18894-19332
HmHel-2	CAEZ01004354:7599-8025
HmHel-2	CAEZ01005928:9621-10041
HmHel-2	CAEZ01001951:1-324
HmHel-2	CAEZ01001466:36451-36874
HmHel-2	CAEZ01007548:4-285
HmHel-2	CAEZ01006884:13030-13485
HmHel-2	CAEZ01006884:17642-17952
HmHel-2	CAEZ01006790:3068-3429
HmHel-2	CAEZ01005299:33379-33831
HmHel-2	CAEZ01000834:37618-38009
HmHel-2	CAEZ01010748:1152-1531
HmHel-2	CAEZ01006780:15990-16275
HmHel-2	CAEZ01005585:10562-10886
HmHel-2	CAEZ01004244:150580-151997
HmHel-2	CAEZ01002429:9405-10009
HmHel-2	CAEZ01002429:37354-37671
HmHel-2	CAEZ01010809:20901-21324
HmHel-2	CAEZ01010809:66452-67023
HmHel-2	CAEZ01009491:7542-8009
HmHel-2	CAEZ01009151:15587-16025
HmHel-2	CAEZ01009151:5333-5698
HmHel-2	CAEZ01007957:59514-59915
HmHel-2	CAEZ01007957:221-758
HmHel-2	CAEZ01007530:1544-1957
HmHel-2	CAEZ01006245:5587-6034
HmHel-2	CAEZ01001700:515-965
HmHel-2	CAEZ01007687:2-272
HmHel-2	CAEZ01004352:5883-6327
HmHel-2	CAEZ01003077:107533-107922
HmHel-2	CAEZ01003029:12242-12586
HmHel-2	CAEZ01002321:52441-52871
HmHel-2	CAEZ01000810:2572-2971
HmHel-2	CAEZ01010994:3050-3474
HmHel-2	CAEZ01010994:7623-7976
HmHel-2	CAEZ01010869:28784-29256
HmHel-2	CAEZ01009807:22695-23281
HmHel-2	CAEZ01009566:6071-6421
HmHel-2	CAEZ01007314:11-436
HmHel-2	CAEZ01006236:13083-13527
HmHel-2	CAEZ01003092:5206-5460
HmHel-2	CAEZ01001015:891-1212
HmHel-2	CAEZ01010975:5569-6180
HmHel-2	CAEZ01008921:98622-98705
HmHel-2	CAEZ01008921:148657-149074
HmHel-2	CAEZ01008838:8444-8872
HmHel-2	CAEZ01007215:10081-10465

HmHel-2	CAEZ01007215:3294-4261
HmHel-2	CAEZ01004791:1687-2144
HmHel-2	CAEZ01002561:5-312
HmHel-2	CAEZ01001731:13250-13511
HmHel-2	CAEZ01007766:6092-6490
HmHel-2	CAEZ01002586:4-236
HmHel-2	CAEZ01010234:17368-17627
HmHel-2	CAEZ01009607:28665-29130
HmHel-2	CAEZ01009342:19746-20156
HmHel-2	CAEZ01006125:9788-10236
HmHel-2	CAEZ01002176:462-725
HmHel-2	CAEZ01001984:1-312
HmHel-2	CAEZ01001984:15670-16145
HmHel-2	CAEZ01001984:24802-25231
HmHel-2	CAEZ01009489:5887-6170
HmHel-2	CAEZ01007411:20198-20519
HmHel-2	CAEZ01007292:2607-3194
HmHel-2	CAEZ01003840:255-842
HmHel-2	CAEZ01003735:16547-16868
HmHel-2	CAEZ01003735:2495-2712
HmHel-2	CAEZ01003254:14822-15080
HmHel-2	CAEZ01003254:17339-17793
HmHel-2	CAEZ01010656:12260-12724
HmHel-2	CAEZ01008099:5259-5766
HmHel-2	CAEZ01006914:3990-4300
HmHel-2	CAEZ01005866:38681-39090
HmHel-2	CAEZ01005858:13460-13812
HmHel-2	CAEZ01005488:1397-1685
HmHel-2	CAEZ01004019:6595-6747
HmHel-2	CAEZ01004019:1045-1358
HmHel-2	CAEZ01001867:366-947
HmHel-2	CAEZ01010977:1001-1447
HmHel-2	CAEZ01010586:19579-19880
HmHel-2	CAEZ01006506:16906-17367
HmHel-2	CAEZ01006506:19517-19786
HmHel-2	CAEZ01006276:19740-20329
HmHel-2	CAEZ01005862:7422-7949
HmHel-2	CAEZ01004718:1057-1312
HmHel-2	CAEZ01003933:10-130
HmHel-2	CAEZ01003933:24102-24551
HmHel-2	CAEZ01003933:36029-36167
HmHel-2	CAEZ01010013:38330-38453
HmHel-2	CAEZ01010013:20670-21111
HmHel-2	CAEZ01005861:5514-5781
HmHel-2	CAEZ01005071:461-2177
HmHel-2	CAEZ01004212:4210-4560
HmHel-2	CAEZ01003208:31721-32167
HmHel-2	CAEZ01001428:7-318

HmHel-2	CAEZ01001083:459-781
HmHel-2	CAEZ01000679:5233-5492
HmHel-2	CAEZ01011387:7929-8250
HmHel-2	CAEZ01010618:5482-5803
HmHel-2	CAEZ01010140:16012-16333
HmHel-2	CAEZ01009833:33813-34399
HmHel-2	CAEZ01009490:100-674
HmHel-2	CAEZ01009487:15987-16308
HmHel-2	CAEZ01009115:24273-24719
HmHel-2	CAEZ01008918:6263-6584
HmHel-2	CAEZ01007930:1549-2135
HmHel-2	CAEZ01007930:21783-21936
HmHel-2	CAEZ01007572:22848-23434
HmHel-2	CAEZ01007572:9764-9927
HmHel-2	CAEZ01007358:15143-15544
HmHel-2	CAEZ01002595:1368-1645
HmHel-2	CAEZ01001501:4467-4788
HmHel-2	CAEZ01001501:40594-40715
HmHel-2	CAEZ01000951:574-987
HmHel-2	CAEZ01010502:4011-4454
HmHel-2	CAEZ01008064:12136-12445
HmHel-2	CAEZ01005684:9462-9760
HmHel-2	CAEZ01004951:5-254
HmHel-2	CAEZ01001451:1-354
HmHel-2	CAEZ01005854:1141-1484
HmHel-2	CAEZ01005854:10389-10755
HmHel-2	CAEZ01005176:148-380
HmHel-2	CAEZ01003046:1-269
HmHel-2	CAEZ01002160:9405-9694
HmHel-2	CAEZ01000069:1-273
HmHel-2	CAEZ01011154:7-258
HmHel-2	CAEZ01010136:10166-10748
HmHel-2	CAEZ01008370:23582-24162
HmHel-2	CAEZ01009823:9274-9712
HmHel-2	CAEZ01009210:8692-9015
HmHel-2	CAEZ01007460:19783-20370
HmHel-2	CAEZ01005506:1396-1717
HmHel-2	CAEZ01005233:3388-3988
HmHel-2	CAEZ01003318:10763-11084
HmHel-2	CAEZ01003076:2136-2500
HmHel-2	CAEZ01002821:68160-68482
HmHel-2	CAEZ01002821:6294-6751
HmHel-2	CAEZ01002132:12881-13201
HmHel-2	CAEZ01001269:499-820
HmHel-2	CAEZ01007877:1-302
HmHel-2	CAEZ01007678:1229-1591
HmHel-2	CAEZ01007678:27700-27961
HmHel-2	CAEZ01006781:2192-2501

HmHel-2	CAEZ01005825:41800-42233
HmHel-2	CAEZ01005460:21001-21286
HmHel-2	CAEZ01003873:1712-2028
HmHel-2	CAEZ01002271:1048-1335
HmHel-2	CAEZ01002271:604-1043
HmHel-2	CAEZ01002066:23157-23422
HmHel-2	CAEZ01000935:4688-5154
HmHel-2	CAEZ01010061:1-245
HmHel-2	CAEZ01007784:7312-7747
HmHel-2	CAEZ01007519:4045-4366
HmHel-2	CAEZ01001877:14090-14478
HmHel-2	CAEZ01011025:14479-15056
HmHel-2	CAEZ01009341:3098-3490
HmHel-2	CAEZ01007865:17964-18286
HmHel-2	CAEZ01007494:42078-42341
HmHel-2	CAEZ01004644:8485-8941
HmHel-2	CAEZ01002630:2168-2423
HmHel-2	CAEZ01002630:5226-5525
HmHel-2	CAEZ01002318:2828-3266
HmHel-2	CAEZ01002002:123-407
HmHel-2	CAEZ01001703:17928-18193
HmHel-2	CAEZ01001703:9480-9782
HmHel-2	CAEZ01001134:2691-3184
HmHel-2	CAEZ01011233:2761-3279
HmHel-2	CAEZ01010936:8603-9189
HmHel-2	CAEZ01010765:8428-8701
HmHel-2	CAEZ01009158:5565-6020
HmHel-2	CAEZ01007045:9-538
HmHel-2	CAEZ01006775:7578-8048
HmHel-2	CAEZ01006210:530-850
HmHel-2	CAEZ01006119:1668-2788
HmHel-2	CAEZ01005225:41951-42237
HmHel-2	CAEZ01004797:12031-12345
HmHel-2	CAEZ01004157:2722-3042
HmHel-2	CAEZ01002591:3267-3626
HmHel-2	CAEZ01011065:1460-1780
HmHel-2	CAEZ01010176:1-214
HmHel-2	CAEZ01002619:209-587
HmHel-2	CAEZ01002334:436-976
HmHel-2	CAEZ01010823:50-301
HmHel-2	CAEZ01004583:8364-8648
HmHel-2	CAEZ01002596:1-228
HmHel-2	CAEZ01001109:613-1045
HmHel-2	CAEZ01008339:1731-2304
HmHel-2	CAEZ01007492:29731-30212
HmHel-2	CAEZ01005860:16593-17183
HmHel-2	CAEZ01005079:5708-6016
HmHel-2	CAEZ01005054:959-1286

HmHel-2	CAEZ01004834:717-1074
HmHel-2	CAEZ01002983:5710-5948
HmHel-2	CAEZ01001889:2948-3264
HmHel-2	CAEZ01006243:1646-2088
HmHel-2	CAEZ01000966:29090-29677
HmHel-2	CAEZ01010235:1-253
HmHel-2	CAEZ01007197:1707-2028
HmHel-2	CAEZ01005030:208-481
HmHel-2	CAEZ01004779:617-1055
HmHel-2	CAEZ01003829:6426-6747
HmHel-2	CAEZ01003122:64-338
HmHel-2	CAEZ01010169:12001-12239
HmHel-2	CAEZ01009451:15561-16135
HmHel-2	CAEZ01008411:2472-3058
HmHel-2	CAEZ01006826:7646-8110
HmHel-2	CAEZ01004584:15497-15952
HmHel-2	CAEZ01004328:3244-3748
HmHel-2	CAEZ01004183:10997-11264
HmHel-2	CAEZ01002944:86620-86928
HmHel-2	CAEZ01002052:1691-2069
HmHel-2	CAEZ01010985:6953-7274
HmHel-2	CAEZ01010571:20-307
HmHel-2	CAEZ01009273:8217-8801
HmHel-2	CAEZ01009143:3608-3929
HmHel-2	CAEZ01008751:80149-80326
HmHel-2	CAEZ01008751:52652-53252
HmHel-2	CAEZ01008264:1-334
HmHel-2	CAEZ01007656:6076-6426
HmHel-2	CAEZ01006858:681-1268
HmHel-2	CAEZ01006553:410-804
HmHel-2	CAEZ01006470:6440-6756
HmHel-2	CAEZ01005300:3599-3865
HmHel-2	CAEZ01002100:2940-3523
HmHel-2	CAEZ01001099:827-1151
HmHel-2	CAEZ01010376:5070-5643
HmHel-2	CAEZ01008404:17305-17614
HmHel-2	CAEZ01008174:5789-6261
HmHel-2	CAEZ01006720:4270-4591
HmHel-2	CAEZ01005694:2807-3043
HmHel-2	CAEZ01004409:8493-8725
HmHel-2	CAEZ01003164:24013-24461
HmHel-2	CAEZ01001473:1-296
HmHel-2	CAEZ01008313:5128-5569
HmHel-2	CAEZ01008313:41707-41904
HmHel-2	CAEZ01007557:8708-9024
HmHel-2	CAEZ01005951:19771-20239
HmHel-2	CAEZ01004890:4063-4378
HmHel-2	CAEZ01004294:9919-10241

HmHel-2	CAEZ01008884:53749-54202
HmHel-2	CAEZ01008884:11799-12104
HmHel-2	CAEZ01007403:15072-15388
HmHel-2	CAEZ01007226:3656-4144
HmHel-2	CAEZ01007055:4307-4623
HmHel-2	CAEZ01006409:19282-19579
HmHel-2	CAEZ01005819:1-212
HmHel-2	CAEZ01004908:4088-4647
HmHel-2	CAEZ01002626:1130-1451
HmHel-2	CAEZ01002333:3323-3625
HmHel-2	CAEZ01001912:1356-1675
HmHel-2	CAEZ01001796:1867-2542
HmHel-2	CAEZ01011014:9624-10045
HmHel-2	CAEZ01011014:5299-5615
HmHel-2	CAEZ01010633:8978-9374
HmHel-2	CAEZ01009649:2759-3079
HmHel-2	CAEZ01009122:2427-2727
HmHel-2	CAEZ01008675:3541-3998
HmHel-2	CAEZ01007720:2199-2640
HmHel-2	CAEZ01004903:3263-3584
HmHel-2	CAEZ01003966:236-550
HmHel-2	CAEZ01003099:16849-17170
HmHel-2	CAEZ01001884:2015-2336
HmHel-2	CAEZ01009440:4874-13958
HmHel-2	CAEZ01007539:1949-2312
HmHel-2	CAEZ01006342:8846-9235
HmHel-2	CAEZ01006342:15486-15741
HmHel-2	CAEZ01004248:1083-1403
HmHel-2	CAEZ01003992:624-1082
HmHel-2	CAEZ01011484:3-207
HmHel-2	CAEZ01006027:11161-17498
HmHel-2	CAEZ01003197:47915-48180
HmHel-2	CAEZ01010764:2715-3034
HmHel-2	CAEZ01008955:17536-17823
HmHel-2	CAEZ01008609:5399-5872
HmHel-2	CAEZ01008524:9924-10241
HmHel-2	CAEZ01005671:5208-5529
HmHel-2	CAEZ01002981:8294-8736
HmHel-2	CAEZ01002757:18080-18381
HmHel-2	CAEZ01011460:6784-7094
HmHel-2	CAEZ01010493:879-1200
HmHel-2	CAEZ01010282:1-130
HmHel-2	CAEZ01010282:13788-14109
HmHel-2	CAEZ01010197:9326-9647
HmHel-2	CAEZ01008382:3459-3780
HmHel-2	CAEZ01005949:105907-106222
HmHel-2	CAEZ01003385:10343-10569
HmHel-2	CAEZ01002638:11079-11400

HmHel-2	CAEZ01009528:30078-30612
HmHel-2	CAEZ01005419:15211-15658
HmHel-2	CAEZ01004871:6513-6830
HmHel-2	CAEZ01009015:11962-12261
HmHel-2	CAEZ01005168:13182-13432
HmHel-2	CAEZ01003431:896-1115
HmHel-2	CAEZ01011278:7736-8054
HmHel-2	CAEZ01009304:31917-32377
HmHel-2	CAEZ01007470:29913-30231
HmHel-2	CAEZ01006867:14113-14580
HmHel-2	CAEZ01006867:16081-16204
HmHel-2	CAEZ01005208:6954-7256
HmHel-2	CAEZ01011580:2924-3245
HmHel-2	CAEZ01010817:325-543
HmHel-2	CAEZ01010628:2476-2797
HmHel-2	CAEZ01010192:4167-4696
HmHel-2	CAEZ01009171:12679-12999
HmHel-2	CAEZ01008842:19849-20210
HmHel-2	CAEZ01007935:661-979
HmHel-2	CAEZ01005288:8051-8350
HmHel-2	CAEZ01004648:4297-4742
HmHel-2	CAEZ01003335:27957-28073
HmHel-2	CAEZ01003335:47515-48026
HmHel-2	CAEZ01009029:7-326
HmHel-2	CAEZ01004827:22413-22825
HmHel-2	CAEZ01002594:2-210
HmHel-2	CAEZ01011535:9109-9593
HmHel-2	CAEZ01008441:5939-6255
HmHel-2	CAEZ01007447:3563-3884
HmHel-2	CAEZ01007253:36351-36682
HmHel-2	CAEZ01006948:917-1238
HmHel-2	CAEZ01003148:5407-5679
HmHel-2	CAEZ01009247:3540-3811
HmHel-2	CAEZ01007268:20647-21086
HmHel-2	CAEZ01007268:32800-33120
HmHel-2	CAEZ01005290:24341-24695
HmHel-2	CAEZ01005122:14790-15944
HmHel-2	CAEZ01000908:920-1253
HmHel-2	CAEZ01000908:7458-7971
HmHel-2	CAEZ01010028:69294-69516
HmHel-2	CAEZ01009567:8550-8912
HmHel-2	CAEZ01008402:46767-47178
HmHel-2	CAEZ01008402:58616-58756
HmHel-2	CAEZ01008402:28394-28634
HmHel-2	CAEZ01007291:6380-6863
HmHel-2	CAEZ01004855:7133-7454
HmHel-2	CAEZ01004190:1312-1570
HmHel-2	CAEZ01003056:19621-20050

HmHel-2	CAEZ01000960:2369-2690
HmHel-2	CAEZ01009835:13366-13934
HmHel-2	CAEZ01008446:34582-34865
HmHel-2	CAEZ01006346:12811-13177
HmHel-2	CAEZ01004554:8428-8899
HmHel-2	CAEZ01004324:16982-17285
HmHel-2	CAEZ01004238:1158-1478
HmHel-2	CAEZ01002835:7568-7957
HmHel-2	CAEZ01001167:1062-1382
HmHel-2	CAEZ01000174:2949-3235
HmHel-2	CAEZ01007301:12992-13101
HmHel-2	CAEZ01007301:11744-12058
HmHel-2	CAEZ01006004:77063-77479
HmHel-2	CAEZ01006004:69383-69633
HmHel-2	CAEZ01006004:26967-27082
HmHel-2	CAEZ01004184:1-225
HmHel-2	CAEZ01003519:13123-13359
HmHel-2	CAEZ01000963:31753-32219
HmHel-2	CAEZ01000588:3051-3433
HmHel-2	CAEZ01005321:19253-19666
HmHel-2	CAEZ01005204:3376-3618
HmHel-2	CAEZ01004940:1146-1471
HmHel-2	CAEZ01004474:14903-15314
HmHel-2	CAEZ01003733:517-809
HmHel-2	CAEZ01002371:94636-95086
HmHel-2	CAEZ01002304:4050-4508
HmHel-2	CAEZ01000921:29628-29906
HmHel-2	CAEZ01009378:8042-8351
HmHel-2	CAEZ01008511:15247-15695
HmHel-2	CAEZ01006320:6883-7181
HmHel-2	CAEZ01003479:605-1187
HmHel-2	CAEZ01003055:647-989
HmHel-2	CAEZ01001349:882-1196
HmHel-2	CAEZ01001349:45526-46000
HmHel-2	CAEZ01010375:23603-23903
HmHel-2	CAEZ01008844:15613-16037
HmHel-2	CAEZ01006865:56635-57158
HmHel-2	CAEZ01006129:22161-22555
HmHel-2	CAEZ01005838:9502-9723
HmHel-2	CAEZ01005657:1118-1351
HmHel-2	CAEZ01004280:3245-3524
HmHel-2	CAEZ01003596:1-504
HmHel-2	CAEZ01003038:3941-4376
HmHel-2	CAEZ01000846:9619-9929
HmHel-2	CAEZ01009599:4182-4382
HmHel-2	CAEZ01008443:7578-8007
HmHel-2	CAEZ01007551:26767-26924
HmHel-2	CAEZ01007551:25458-25787

HmHel-2	CAEZ01003277:10713-11021
HmHel-2	CAEZ01002056:320-903
HmHel-2	CAEZ01009788:80167-80415
HmHel-2	CAEZ01008846:9618-9789
HmHel-2	CAEZ01007365:16909-17228
HmHel-2	CAEZ01006067:32881-33129
HmHel-2	CAEZ01002847:28697-28915
HmHel-2	CAEZ01011184:9712-10025
HmHel-2	CAEZ01011019:6994-7287
HmHel-2	CAEZ01008681:10060-10373
HmHel-2	CAEZ01008286:759-1070
HmHel-2	CAEZ01008196:37622-37856
HmHel-2	CAEZ01007338:4596-4917
HmHel-2	CAEZ01006831:23027-23346
HmHel-2	CAEZ01005013:39822-40251
HmHel-2	CAEZ01000184:100-394
HmHel-2	CAEZ01011412:390-991
HmHel-2	CAEZ01011323:19357-19840
HmHel-2	CAEZ01010501:13077-13394
HmHel-2	CAEZ01007872:18770-19137
HmHel-2	CAEZ01007872:84140-84469
HmHel-2	CAEZ01007872:54913-55092
HmHel-2	CAEZ01007596:9896-10289
HmHel-2	CAEZ01007263:50422-57444
HmHel-2	CAEZ01006364:13782-14210
HmHel-2	CAEZ01005794:7802-8253
HmHel-2	CAEZ01004841:32416-32801
HmHel-2	CAEZ01004841:13162-13483
HmHel-2	CAEZ01004539:517-1023
HmHel-2	CAEZ01011362:34552-34869
HmHel-2	CAEZ01008583:8778-9101
HmHel-2	CAEZ01006411:9467-9784
HmHel-2	CAEZ01003509:13848-14072
HmHel-2	CAEZ01001142:3464-3784
HmHel-2	CAEZ01011482:9586-9863
HmHel-2	CAEZ01008081:1439-1909
HmHel-2	CAEZ01005394:14650-14913
HmHel-2	CAEZ01003089:2513-2823
HmHel-2	CAEZ01002695:21252-21604
HmHel-2	CAEZ01001764:3382-3523
HmHel-2	CAEZ01001764:10548-10877
HmHel-2	CAEZ01011354:1570-1978
HmHel-2	CAEZ01010644:1-208
HmHel-2	CAEZ01001887:813-1114
HmHel-2	CAEZ01001788:9601-9916
HmHel-2	CAEZ01011120:50997-51239
HmHel-2	CAEZ01008200:2621-3071
HmHel-2	CAEZ01007322:11746-12008

HmHel-2	CAEZ01007322:1567-1679
HmHel-2	CAEZ01007199:1-186
HmHel-2	CAEZ01006637:2246-2554
HmHel-2	CAEZ01006495:19515-19826
HmHel-2	CAEZ01006493:3903-4305
HmHel-2	CAEZ01005931:2-191
HmHel-2	CAEZ01004407:7500-7717
HmHel-2	CAEZ01003070:108776-109156
HmHel-2	CAEZ01002330:12742-13136
HmHel-2	CAEZ01001138:8-213
HmHel-2	CAEZ01011494:64465-64702
HmHel-2	CAEZ01009747:7332-7607
HmHel-2	CAEZ01009747:9903-10203
HmHel-2	CAEZ01008497:6245-6568
HmHel-2	CAEZ01007879:14101-14525
HmHel-2	CAEZ01007879:13853-14089
HmHel-2	CAEZ01005909:24021-24416
HmHel-2	CAEZ01004158:2077-2361
HmHel-2	CAEZ01002361:20067-20384
HmHel-2	CAEZ01011314:22501-22872
HmHel-2	CAEZ01009384:5221-5673
HmHel-2	CAEZ01006730:1-292
HmHel-2	CAEZ01005700:602-918
HmHel-2	CAEZ01005205:57265-57563
HmHel-2	CAEZ01002579:12591-12911
HmHel-2	CAEZ01010461:6018-6413
HmHel-2	CAEZ01008171:13508-14112
HmHel-2	CAEZ01007504:6-398
HmHel-2	CAEZ01002723:6896-7192
HmHel-2	CAEZ01010998:9707-10108
HmHel-2	CAEZ01010238:120666-120986
HmHel-2	CAEZ01005906:17430-17691
HmHel-2	CAEZ01005243:8221-8635
HmHel-2	CAEZ01003702:7585-7770
HmHel-2	CAEZ01003093:5-190
HmHel-2	CAEZ01001941:1845-2122
HmHel-2	CAEZ01001424:1595-2034
HmHel-2	CAEZ01010593:40-255
HmHel-2	CAEZ01010485:10784-11056
HmHel-2	CAEZ01009683:848-1024
HmHel-2	CAEZ01008430:47510-47780
HmHel-2	CAEZ01008290:9983-10098
HmHel-2	CAEZ01008290:71274-71600
HmHel-2	CAEZ01007640:9874-10066
HmHel-2	CAEZ01007640:9507-9856
HmHel-2	CAEZ01007201:95257-95492
HmHel-2	CAEZ01007201:110001-110272
HmHel-2	CAEZ01007201:84997-85296

HmHel-2	CAEZ01004647:2512-2966
HmHel-2	CAEZ01004016:41983-42078
HmHel-2	CAEZ01004016:31212-31427
HmHel-2	CAEZ01004016:10508-10937
HmHel-2	CAEZ01003229:1957-2319
HmHel-2	CAEZ01011631:1023-1297
HmHel-2	CAEZ01010308:7373-7673
HmHel-2	CAEZ01007904:307-724
HmHel-2	CAEZ01006960:16727-17030
HmHel-2	CAEZ01005980:45965-46320
HmHel-2	CAEZ01005560:5023-5322
HmHel-2	CAEZ01001527:3858-4177
HmHel-2	CAEZ01007768:26041-26564
HmHel-2	CAEZ01006204:5510-5820
HmHel-2	CAEZ01011583:1634-1879
HmHel-2	CAEZ01009571:10828-11264
HmHel-2	CAEZ01008108:1015-1328
HmHel-2	CAEZ01007754:33348-33609
HmHel-2	CAEZ01005772:5561-5950
HmHel-2	CAEZ01005158:1570-1783
HmHel-2	CAEZ01004676:2314-2784
HmHel-2	CAEZ01003861:10392-10707
HmHel-2	CAEZ01002303:1148-1403
HmHel-2	CAEZ01000662:7347-7686
HmHel-2	CAEZ01010922:2237-2548
HmHel-2	CAEZ01009998:13739-14061
HmHel-2	CAEZ01008512:38863-39158
HmHel-2	CAEZ01008182:48320-48636
HmHel-2	CAEZ01007435:23734-24038
HmHel-2	CAEZ01007260:15366-15660
HmHel-2	CAEZ01003484:11787-12106
HmHel-2	CAEZ01002997:2890-3287
HmHel-2	CAEZ01008093:1-160
HmHel-2	CAEZ01007034:428-742
HmHel-2	CAEZ01003304:29314-29716
HmHel-2	CAEZ01002243:7012-7319
HmHel-2	CAEZ01011589:9199-9496
HmHel-2	CAEZ01003209:33594-33844
HmHel-2	CAEZ01009631:47333-47518
HmHel-2	CAEZ01008847:15312-15511
HmHel-2	CAEZ01006218:40093-40404
HmHel-2	CAEZ01006049:245-674
HmHel-2	CAEZ01004796:33028-33268
HmHel-2	CAEZ01003634:8241-8593
HmHel-2	CAEZ01001081:11317-11617
HmHel-2	CAEZ01011565:6977-7204
HmHel-2	CAEZ01010759:1-176
HmHel-2	CAEZ01008666:656-958

HmHel-2	CAEZ01006190:7196-7644
HmHel-2	CAEZ01005400:8931-9493
HmHel-2	CAEZ01005400:14659-15056
HmHel-2	CAEZ01003345:75528-75721
HmHel-2	CAEZ01003345:5967-8080
HmHel-2	CAEZ01008448:7646-8087
HmHel-2	CAEZ01004720:13124-13438
HmHel-2	CAEZ01004200:1740-2021
HmHel-2	CAEZ01002585:1195-1505
HmHel-2	CAEZ01002585:979-1179
HmHel-2	CAEZ01009526:3965-4190
HmHel-2	CAEZ01007114:21414-21782
HmHel-2	CAEZ01005433:2258-2483
HmHel-2	CAEZ01005433:21295-21700
HmHel-2	CAEZ01005123:9769-9947
HmHel-2	CAEZ01004750:243-405
HmHel-2	CAEZ01001518:18929-19141
HmHel-2	CAEZ01001351:16126-16585
HmHel-2	CAEZ01001351:13001-13147
HmHel-2	CAEZ01008304:12407-12864
HmHel-2	CAEZ01005925:1238-1646
HmHel-2	CAEZ01005510:6404-6828
HmHel-2	CAEZ01004814:42388-42740
HmHel-2	CAEZ01002366:9212-9365
HmHel-2	CAEZ01010615:21170-21422
HmHel-2	CAEZ01010199:29084-29403
HmHel-2	CAEZ01010199:1739-7117
HmHel-2	CAEZ01009395:10014-10308
HmHel-2	CAEZ01008638:1346-1601
HmHel-2	CAEZ01007677:10277-10582
HmHel-2	CAEZ01007332:1-181
HmHel-2	CAEZ01004765:4454-4782
HmHel-2	CAEZ01002320:16445-16827
HmHel-2	CAEZ01001856:5655-5988
HmHel-2	CAEZ01001761:3666-3910
HmHel-2	CAEZ01000984:1094-1383
HmHel-2	CAEZ01005717:35551-45814
HmHel-2	CAEZ01003804:7898-8201
HmHel-2	CAEZ01010978:7321-8417
HmHel-2	CAEZ01010093:4215-4521
HmHel-2	CAEZ01008585:6967-7292
HmHel-2	CAEZ01008023:5482-5872
HmHel-2	CAEZ01008023:12889-13141
HmHel-2	CAEZ01005057:8374-8706
HmHel-2	CAEZ01004297:1-183
HmHel-2	CAEZ01003513:7343-7501
HmHel-2	CAEZ01008440:6288-6473
HmHel-2	CAEZ01006408:1-354

HmHel-2	CAEZ01008328:8272-8722
HmHel-2	CAEZ01006565:19180-19561
HmHel-2	CAEZ01008028:16089-16512
HmHel-2	CAEZ01006839:17099-17539
HmHel-2	CAEZ01002940:5392-5708
HmHel-2	CAEZ01002022:14137-14567
HmHel-2	CAEZ01001683:1-223
HmHel-2	CAEZ01010727:42788-43218
HmHel-2	CAEZ01010647:2379-2723
HmHel-2	CAEZ01009095:1665-1993
HmHel-2	CAEZ01007346:5220-5676
HmHel-2	CAEZ01006051:636-821
HmHel-2	CAEZ01011251:4336-4702
HmHel-2	CAEZ01008429:20962-21217
HmHel-2	CAEZ01010509:17561-17731
HmHel-2	CAEZ01010509:3965-4233
HmHel-2	CAEZ01006226:12783-13239
HmHel-2	CAEZ01006136:1-140
HmHel-2	CAEZ01001743:51905-52377
HmHel-2	CAEZ01009340:7053-7203
HmHel-2	CAEZ01009340:8898-9322
HmHel-2	CAEZ01008631:21725-22013
HmHel-2	CAEZ01006603:9023-9337
HmHel-2	CAEZ01005397:128-270
HmHel-2	CAEZ01005073:6463-6629
HmHel-2	CAEZ01004878:6237-6562
HmHel-2	CAEZ01010247:14551-15117
HmHel-2	CAEZ01007486:1139-1566
HmHel-2	CAEZ01006229:7-192
HmHel-2	CAEZ01003180:1-384
HmHel-2	CAEZ01008635:230-413
HmHel-2	CAEZ01008635:13104-13187
HmHel-2	CAEZ01005618:3560-3985
HmHel-2	CAEZ01002588:1-169
HmHel-2	CAEZ01001817:4013-4344
HmHel-2	CAEZ01008706:9157-9485
HmHel-2	CAEZ01007182:17220-17528
HmHel-2	CAEZ01004909:8841-11135
HmHel-2	CAEZ01003850:38337-38511
HmHel-2	CAEZ01002580:1033-1460
HmHel-2	CAEZ01002204:1019-1375
HmHel-2	CAEZ01005101:10915-11340
HmHel-2	CAEZ01001293:380-705
HmHel-2	CAEZ01010351:4046-4507
HmHel-2	CAEZ01010351:44101-44305
HmHel-2	CAEZ01010180:18411-18592
HmHel-2	CAEZ01008836:2186-2372
HmHel-2	CAEZ01006939:8394-8551

HmHel-2	CAEZ01006482:18121-18274
HmHel-2	CAEZ01006026:1-150
HmHel-2	CAEZ01003527:1-313
HmHel-2	CAEZ01001025:20115-20395
HmHel-2	CAEZ01011431:1-239
HmHel-2	CAEZ01010833:10145-10478
HmHel-2	CAEZ01009153:19195-19512
HmHel-2	CAEZ01006768:21899-22326
HmHel-2	CAEZ01006096:16560-16747
HmHel-2	CAEZ01006053:4039-4366
HmHel-2	CAEZ01009517:8405-8628
HmHel-2	CAEZ01005859:295-540
HmHel-2	CAEZ01002224:19037-19420
HmHel-2	CAEZ01009980:7326-7491
HmHel-2	CAEZ01009980:7500-7601
HmHel-2	CAEZ01005119:27516-27678
HmHel-2	CAEZ01005119:10740-10845
HmHel-2	CAEZ01002285:18747-19012
HmHel-2	CAEZ01002199:409-681
HmHel-2	CAEZ01009116:5019-5322
HmHel-2	CAEZ01005193:1126-1406
HmHel-2	CAEZ01004345:6125-6380
HmHel-2	CAEZ01001850:37247-37400
HmHel-2	CAEZ01011195:53190-53377
HmHel-2	CAEZ01002055:80772-81069
HmHel-2	CAEZ01002055:69750-69841
HmHel-2	CAEZ01008639:20043-20421
HmHel-2	CAEZ01006699:613-973
HmHel-2	CAEZ01002099:23804-24203
HmHel-2	CAEZ01002099:13310-13549
HmHel-2	CAEZ01005584:23570-23812
HmHel-2	CAEZ01011067:18110-18346
HmHel-2	CAEZ01008271:38166-38558
HmHel-2	CAEZ01000803:459-755
HmHel-2	CAEZ01009628:9144-9335
HmHel-2	CAEZ01006481:392-614
HmHel-2	CAEZ01002338:9788-10106
HmHel-2	CAEZ01001442:11911-12169
HmHel-2	CAEZ01001442:39186-39444
HmHel-2	CAEZ01001442:1902-1983
HmHel-2	CAEZ01001352:4497-4760
HmHel-2	CAEZ01010613:748-8522
HmHel-2	CAEZ01007576:25709-26230
HmHel-2	CAEZ01005761:24092-24404
HmHel-2	CAEZ01005287:6955-7243
HmHel-2	CAEZ01005287:10449-10598
HmHel-2	CAEZ01010614:88886-89115
HmHel-2	CAEZ01010614:1807-1919

HmHel-2	CAEZ01009786:32827-33124
HmHel-2	CAEZ01009786:20010-20259
HmHel-2	CAEZ01009331:91692-91978
HmHel-2	CAEZ01009228:25952-26071
HmHel-2	CAEZ01009228:59136-59370
HmHel-2	CAEZ01009221:9813-10173
HmHel-2	CAEZ01009128:1480-1711
HmHel-2	CAEZ01006861:18012-18213
HmHel-2	CAEZ01002615:126-357
HmHel-2	CAEZ01001828:3296-3541
HmHel-2	CAEZ01001722:161-392
HmHel-2	CAEZ01001637:20-251
HmHel-2	CAEZ01000517:555-786
HmHel-2	CAEZ01000516:815-1046
HmHel-2	CAEZ01011376:10682-11024
HmHel-2	CAEZ01007231:3849-4111
HmHel-2	CAEZ01005307:5948-6196
HmHel-2	CAEZ01003339:19577-19869
HmHel-2	CAEZ01000860:7569-7877
HmHel-2	CAEZ01009870:1433-1632
HmHel-2	CAEZ01005477:10133-10252
HmHel-2	CAEZ01005477:37600-37719
HmHel-2	CAEZ01005472:17755-17998
HmHel-2	CAEZ01008548:3175-3420
HmHel-2	CAEZ01005220:7333-9268
HmHel-2	CAEZ01005220:44180-44310
HmHel-2	CAEZ01007131:1-129
HmHel-2	CAEZ01005610:20601-20908
HmHel-2	CAEZ01010741:15922-16074
HmHel-2	CAEZ01003331:14181-14362
HmHel-2	CAEZ01003331:6556-6911
HmHel-2	CAEZ01000145:935-1100
HmHel-2	CAEZ01009374:21571-21951
HmHel-2	CAEZ01001952:33-194
HmHel-2	CAEZ01001627:716-948
HmHel-2	CAEZ01001174:1683-1941
HmHel-2	CAEZ01000790:3-130
HmHel-2	CAEZ01000266:658-793
HmHel-2	CAEZ01008601:272-398
HmHel-2	CAEZ01006308:32880-33041
HmHel-2	CAEZ01004149:569-881
HmHel-2	CAEZ01005829:6742-6951
HmHel-2	CAEZ01005486:39491-39645
HmHel-2	CAEZ01009439:5209-5504
HmHel-2	CAEZ01011276:34959-35160
HmHel-2	CAEZ01003063:3782-4042
HmHel-2	CAEZ01001723:1-221
HmHel-2	CAEZ01000244:1-124

HmHel-2	CAEZ01008031:19068-19454
HmHel-2	CAEZ01003971:1140-1455
HmHel-2	CAEZ01011345:5343-5496
HmHel-2	CAEZ01009356:3811-3968
HmHel-2	CAEZ01007588:23595-23902
HmHel-2	CAEZ01011380:6198-6338
HmHel-2	CAEZ01010573:1913-2146
HmHel-2	CAEZ01010299:4585-4773
HmHel-2	CAEZ01009617:19754-19977
HmHel-2	CAEZ01005843:6827-6999
HmHel-2	CAEZ01002677:797-1031
HmHel-2	CAEZ01010305:70258-70677
HmHel-2	CAEZ01010218:13219-13593
HmHel-2	CAEZ01009680:66232-66497
HmHel-2	CAEZ01005824:5076-5344
HmHel-2	CAEZ01010318:35340-44122
HmHel-2	CAEZ01007711:6858-7149
HmHel-2	CAEZ01007711:45041-45183
HmHel-2	CAEZ01007711:20365-20538
HmHel-2	CAEZ01002584:4947-5238
HmHel-2	CAEZ01007248:3286-3656
HmHel-2	CAEZ01004073:20688-20933
HmHel-2	CAEZ01002141:1-184
HmHel-2	CAEZ01008273:7429-7636
HmHel-2	CAEZ01007586:74136-74277
HmHel-2	CAEZ01007586:88214-88542
HmHel-2	CAEZ01005623:1-157
HmHel-2	CAEZ01005623:58593-59110
HmHel-2	CAEZ01004881:2473-2619
HmHel-2	CAEZ01010906:3648-3816
HmHel-2	CAEZ01010906:28609-28699
HmHel-2	CAEZ01010906:22016-22194
HmHel-2	CAEZ01010356:38681-39086
HmHel-2	CAEZ01009585:11182-11467
HmHel-2	CAEZ01009585:33019-33098
HmHel-2	CAEZ01007051:14814-15063
HmHel-2	CAEZ01005283:3375-3490
HmHel-2	CAEZ01002009:5931-6034
HmHel-2	CAEZ01006876:11664-11865
HmHel-2	CAEZ01003302:30189-30339
HmHel-2	CAEZ01010578:22728-22862
HmHel-2	CAEZ01009830:32313-32518
HmHel-2	CAEZ01006366:13325-13459
HmHel-2	CAEZ01005645:1426-1560
HmHel-2	CAEZ01005159:10352-10493
HmHel-2	CAEZ01001385:3676-3785
HmHel-2	CAEZ01010681:937-1061
HmHel-2	CAEZ01007757:2187-2513

HmHel-2	CAEZ01006044:50317-50501
HmHel-2	CAEZ01003562:50393-50487
HmHel-2	CAEZ01003562:14383-14550
HmHel-2	CAEZ01001779:16496-16765
HmHel-2	CAEZ01007426:13176-13580
HmHel-2	CAEZ01004338:11650-11901
HmHel-2	CAEZ01008604:33472-33650
HmHel-2	CAEZ01010498:9815-9924
HmHel-2	CAEZ01008877:17930-18238
HmHel-2	CAEZ01007062:13850-14003
HmHel-2	CAEZ01006760:87650-87935
HmHel-2	CAEZ01005183:17851-18054
HmHel-2	CAEZ01001313:13759-13911
HmHel-2	CAEZ01011309:1752-2040
HmHel-2	CAEZ01010406:1852-1980
HmHel-2	CAEZ01009404:24735-24888
HmHel-2	CAEZ01008788:4376-4678
HmHel-2	CAEZ01005210:90031-90171
HmHel-2	CAEZ01004587:17244-17468
HmHel-2	CAEZ01003186:5679-6027
HmHel-2	CAEZ01000033:15370-15621
HmHel-2	CAEZ01011219:29688-29910
HmHel-2	CAEZ01009869:17539-17666
HmHel-2	CAEZ01009456:52426-52716
HmHel-2	CAEZ01009456:161152-161317
HmHel-2	CAEZ01009456:142088-142174
HmHel-2	CAEZ01006152:5017-5171
HmHel-2	CAEZ01003557:14334-14518
HmHel-2	CAEZ01002172:2973-3157
HmHel-2	CAEZ01006959:8273-8423
HmHel-2	CAEZ01003463:912-1192
HmHel-2	CAEZ01001048:4677-4820
HmHel-2	CAEZ01010992:14859-14992
HmHel-2	CAEZ01010227:121491-121600
HmHel-2	CAEZ01009738:2305-2575
HmHel-2	CAEZ01008784:19046-19179
HmHel-2	CAEZ01008452:4884-5027
HmHel-2	CAEZ01007245:1285-1637
HmHel-2	CAEZ01005716:73303-73446
HmHel-2	CAEZ01000001:481-634
HmHel-2	CAEZ01010008:35591-36159
HmHel-2	CAEZ01009876:22971-23226
HmHel-2	CAEZ01007219:43903-44068
HmHel-2	CAEZ01007096:2281-2444
HmHel-2	CAEZ01006416:3837-10174
HmHel-2	CAEZ01003473:6537-6832
HmHel-2	CAEZ01011320:1455-1701
HmHel-2	CAEZ01007734:8297-8463

HmHel-2	CAEZ01004317:1807-2081
HmHel-2	CAEZ01009223:18041-18193
HmHel-2	CAEZ01009223:7551-7673
HmHel-2	CAEZ01009194:15447-15605
HmHel-2	CAEZ01003370:396-623
HmHel-2	CAEZ01001420:8682-8808
HmHel-2	CAEZ01009519:3979-4209
HmHel-2	CAEZ01009516:3-127
HmHel-2	CAEZ01008607:18232-18557
HmHel-2	CAEZ01008496:6934-7086
HmHel-2	CAEZ01006009:9827-9970
HmHel-2	CAEZ01004385:7521-7819
HmHel-2	CAEZ01004230:30403-30556
HmHel-2	CAEZ01001097:26470-26619
HmHel-2	CAEZ01011420:9952-10090
HmHel-2	CAEZ01008972:14051-14201
HmHel-2	CAEZ01006985:1-182
HmHel-2	CAEZ01006819:11281-11433
HmHel-2	CAEZ01003593:3114-3366
HmHel-2	CAEZ01001550:12769-12922
HmHel-2	CAEZ01006233:1523-1610
HmHel-2	CAEZ01005609:13020-13251
HmHel-2	CAEZ01001416:8607-8754
HmHel-2	CAEZ01000715:14737-14989
HmHel-2	CAEZ01010569:7969-8351
HmHel-2	CAEZ01008641:18961-19218
HmHel-2	CAEZ01008079:1233-1378
HmHel-2	CAEZ01004124:31608-31878
HmHel-2	CAEZ01003627:3854-3984
HmHel-2	CAEZ01010505:11075-11218
HmHel-2	CAEZ01007545:83083-83395
HmHel-2	CAEZ01007545:2112-2410
HmHel-2	CAEZ01005810:17463-17606
HmHel-2	CAEZ01005059:4474-4600
HmHel-2	CAEZ01004348:780-933
HmHel-2	CAEZ01001301:10582-10804
HmHel-2	CAEZ01011659:20984-21137
HmHel-2	CAEZ01009707:16591-16730
HmHel-2	CAEZ01008354:22065-22196
HmHel-2	CAEZ01006498:2860-2996
HmHel-2	CAEZ01005833:15130-18021
HmHel-2	CAEZ01005826:160-313
HmHel-2	CAEZ01004990:33512-33624
HmHel-2	CAEZ01002065:10158-10285
HmHel-2	CAEZ01009733:71047-71486
HmHel-2	CAEZ01007966:903-1020
HmHel-2	CAEZ01007446:14494-14769
HmHel-2	CAEZ01006600:6060-6248

HmHel-2	CAEZ01006076:8630-8810
HmHel-2	CAEZ01001747:9736-9843
HmHel-2	CAEZ01007836:10724-10857
HmHel-2	CAEZ01000234:631-757
HmHel-2	CAEZ01010884:6223-6399
HmHel-2	CAEZ01009443:3531-3672
HmHel-2	CAEZ01003929:7180-7309
HmHel-2	CAEZ01003834:14110-14248
HmHel-2	CAEZ01001530:987-1134
HmHel-2	CAEZ01001506:1955-2056
HmHel-2	CAEZ01001112:779-864
HmHel-2	CAEZ01011030:1435-1549
HmHel-2	CAEZ01008952:696-828
HmHel-2	CAEZ01005172:7982-8108
HmHel-2	CAEZ01005172:4302-4438
HmHel-2	CAEZ01003424:26264-26500
HmHel-2	CAEZ01006555:6272-6383
HmHel-2	CAEZ01006368:9317-9591
HmHel-2	CAEZ01003761:1883-2127
HmHel-2	CAEZ01001783:809-2547
HmHel-2	CAEZ01005941:15285-15438
HmHel-2	CAEZ01004807:995-1094
HmHel-2	CAEZ01004321:1075-1188
HmHel-2	CAEZ01002354:2308-2450
HmHel-2	CAEZ01010449:98379-98491
HmHel-2	CAEZ01009881:38505-38598
HmHel-2	CAEZ01008624:15248-15370
HmHel-2	CAEZ01006771:520-661
HmHel-2	CAEZ01006443:2007-2128
HmHel-2	CAEZ01006093:8330-8605
HmHel-2	CAEZ01005631:4222-4318
HmHel-2	CAEZ01005619:8737-8853
HmHel-2	CAEZ01004205:15619-15759
HmHel-2	CAEZ01000934:2850-2974
HmHel-2	CAEZ01009102:7848-8091
HmHel-2	CAEZ01006306:608-810
HmHel-2	CAEZ01006157:4-95
HmHel-2	CAEZ01004146:1-92
HmHel-2	CAEZ01003142:16977-17076
HmHel-2	CAEZ01001504:1115-1239
HmHel-2	CAEZ01011422:1-126
HmHel-2	CAEZ01008874:104375-104469
HmHel-2	CAEZ01008874:88247-88467
HmHel-2	CAEZ01006464:21998-22114
HmHel-2	CAEZ01004691:22835-22972
HmHel-2	CAEZ01000933:14116-14202
HmHel-2	CAEZ01008948:2270-2420
HmHel-2	CAEZ01007412:7914-8025

HmHel-2	CAEZ01005783:12331-12433
HmHel-2	CAEZ01005783:20250-23155
HmHel-2	CAEZ01005783:645-794
HmHel-2	CAEZ01000421:597-800
HmHel-2	CAEZ01003332:3877-4003
HmHel-2	CAEZ01000063:47037-47156
HmHel-2	CAEZ01005452:14780-14955
HmHel-2	CAEZ01000403:1-172
HmHel-2	CAEZ01000180:236-695
HmHel-2	CAEZ01000091:910-993
HmHel-2	CAEZ01007013:33734-33848
HmHel-2	CAEZ01006937:33296-33494
HmHel-2	CAEZ01006862:22056-22190
HmHel-2	CAEZ01006862:14404-14635
HmHel-2	CAEZ01004039:27855-27941
HmHel-2	CAEZ01003683:2690-2829
HmHel-2	CAEZ01001011:417-692
HmHel-2	CAEZ01009838:16768-17088
HmHel-2	CAEZ01009698:8111-8210
HmHel-2	CAEZ01006682:2773-3040
HmHel-2	CAEZ01004502:9801-9917
HmHel-2	CAEZ01008978:905-1065
HmHel-2	CAEZ01008570:2531-2778
HmHel-2	CAEZ01008210:44432-44543
HmHel-2	CAEZ01005161:28155-28306
HmHel-2	CAEZ01004830:3647-3877
HmHel-2	CAEZ01003925:9742-9861
HmHel-2	CAEZ01003027:1211-1381
HmHel-2	CAEZ01010600:40424-40570
HmHel-2	CAEZ01008905:759-912
HmHel-2	CAEZ01008272:12532-12644
HmHel-2	CAEZ01008132:4217-4299
HmHel-2	CAEZ01006133:2701-2801
HmHel-2	CAEZ01003957:13491-13706
HmHel-2	CAEZ01009560:20727-20815
HmHel-2	CAEZ01009183:8517-8749
HmHel-2	CAEZ01009103:871-1116
HmHel-2	CAEZ01006991:1452-1559
HmHel-2	CAEZ01005608:690-776
HmHel-2	CAEZ01004335:2907-3193
HmHel-2	CAEZ01002951:18828-19070
HmHel-2	CAEZ01002936:3107-3269
HmHel-2	CAEZ01000480:701-881
HmHel-2	CAEZ01009642:24306-24403
HmHel-2	CAEZ01009469:1210-1313
HmHel-2	CAEZ01006502:8770-8894
HmHel-2	CAEZ01004948:1027-1203
HmHel-2	CAEZ01003871:783-909

HmHel-2	CAEZ01011537:9524-9649
HmHel-2	CAEZ01010244:30319-30512
HmHel-2	CAEZ01008971:19072-19198
HmHel-2	CAEZ01004825:8177-8273
HmHel-2	CAEZ01003676:1343-1460
HmHel-2	CAEZ01011172:9143-9227
HmHel-2	CAEZ01010407:3917-4036
HmHel-2	CAEZ01008654:1027-1154
HmHel-2	CAEZ01007069:14808-14968
HmHel-2	CAEZ01004530:16243-16466
HmHel-2	CAEZ01002904:11958-12037
HmHel-2	CAEZ01002131:1159-1326
HmHel-2	CAEZ01000118:522-681
HmHel-2	CAEZ01011173:11392-11559
HmHel-2	CAEZ01008568:8283-8384
HmHel-2	CAEZ01005974:18430-18665
HmHel-2	CAEZ01003633:54643-54749
HmHel-2	CAEZ01001348:2-103
HmHel-2	CAEZ01004975:14720-14812
HmHel-2	CAEZ01004975:48994-49277
HmHel-2	CAEZ01010516:29801-29885
HmHel-2	CAEZ01008818:4119-4236
HmHel-2	CAEZ01005774:9023-9111
HmHel-2	CAEZ01004320:18608-18713
HmHel-2	CAEZ01011559:10892-10978
HmHel-2	CAEZ01010726:15537-15657
HmHel-2	CAEZ01010317:6035-6131
HmHel-2	CAEZ01010157:15078-15269
HmHel-2	CAEZ01010003:12280-12391
HmHel-2	CAEZ01009127:8622-8764
HmHel-2	CAEZ01006338:42403-42597
HmHel-2	CAEZ01002686:485-572
HmHel-2	CAEZ01000168:1080-1312
HmHel-2	CAEZ01009446:39098-39213
HmHel-2	CAEZ01007344:3541-3676
HmHel-2	CAEZ01006533:19171-19347
HmHel-2	CAEZ01003798:13673-13942
HmHel-2	CAEZ01008770:4228-4370
HmHel-2	CAEZ01004126:31443-31674
HmHel-2	CAEZ01003939:1567-1694
HmHel-2	CAEZ01007660:21782-21877
HmHel-2	CAEZ01006952:7532-7665
HmHel-2	CAEZ01004715:27355-27567
HmHel-2	CAEZ01000853:1076-1196
HmHel-2	CAEZ01010313:3154-3300
HmHel-2	CAEZ01007926:14350-14435
HmHel-2	CAEZ01004306:1889-1976
HmHel-2	CAEZ01004235:704-792

HmHel-2	CAEZ01005971:12434-12536
HmHel-2	CAEZ01002051:8852-9050
HmHel-2	CAEZ01006873:23403-23533
HmHel-2	CAEZ01006864:7919-8108
HmHel-2	CAEZ01001579:58975-59097
HmHel-2	CAEZ01011612:29528-29610
HmHel-2	CAEZ01006838:10211-10372
HmHel-2	CAEZ01004514:4615-4713
HmHel-2	CAEZ01008050:23949-24216
HmHel-2	CAEZ01005206:67296-67405
HmHel-2	CAEZ01005206:21162-21345
HmHel-2	CAEZ01001166:8949-9217
HmHel-2	CAEZ01003228:2290-2483
HmHel-2	CAEZ01006299:62256-62819
HmHel-2	CAEZ01005500:17492-17601
HmHel-2	CAEZ01005331:18639-18725
HmHel-2	CAEZ01005331:14662-14865
HmHel-2	CAEZ01007659:9782-9967
HmHel-2	CAEZ01007544:26451-26616
HmHel-2	CAEZ01006995:6035-6205
HmHel-2	CAEZ01006659:10660-10758
HmHel-2	CAEZ01005581:11767-11818
HmHel-2	CAEZ01004203:1740-1824
HmHel-2	CAEZ01001136:592-673
HmHel-2	CAEZ01006706:9380-9488
HmHel-2	CAEZ01010276:8231-8342
HmHel-2	CAEZ01008826:5551-5679
HmHel-2	CAEZ01008640:30546-30636
HmHel-2	CAEZ01005933:26091-26230
HmHel-2	CAEZ01004601:3749-3928
HmHel-2	CAEZ01002151:507-683
HmHel-2	CAEZ01007325:87345-87457
HmHel-2	CAEZ01000229:163-250
HmHel-2	CAEZ01006487:4977-5096
HmHel-2	CAEZ01005714:35482-35618
HmHel-2	CAEZ01005454:14612-14717
HmHel-2	CAEZ01007996:14147-14262
HmHel-2	CAEZ01006766:22293-22392
HmHel-2	CAEZ01004234:3162-3251
HmHel-2	CAEZ01011292:2371-2493
HmHel-2	CAEZ01006433:27924-28073
HmHel-2	CAEZ01001347:10728-10839
HmHel-2	CAEZ01010942:64221-64302
HmHel-2	CAEZ01010657:7011-7093
HmHel-2	CAEZ01004513:20101-20187
	:-
HmHel-3	CAEZ01010427:19434-19835
HmHel-3	CAEZ01011039:8194-8594

HmHel-3	CAEZ01005774:8258-8659
HmHel-3	CAEZ01010808:160188-161768
HmHel-3	CAEZ01010808:208372-208705
HmHel-3	CAEZ01010808:144089-144498
HmHel-3	CAEZ01010130:68107-68519
HmHel-3	CAEZ01010130:131897-132302
HmHel-3	CAEZ01010130:81682-83713
HmHel-3	CAEZ01008729:57049-57454
HmHel-3	CAEZ01008729:30199-30599
HmHel-3	CAEZ01008729:13397-13898
HmHel-3	CAEZ01003200:20549-20956
HmHel-3	CAEZ01003994:3209-3621
HmHel-3	CAEZ01004413:1342-1747
HmHel-3	CAEZ01001736:110733-110905
HmHel-3	CAEZ01001736:97096-97501
HmHel-3	CAEZ01001736:34893-35564
HmHel-3	CAEZ01010144:74240-74606
HmHel-3	CAEZ01010144:78140-78539
HmHel-3	CAEZ01008183:940-1349
HmHel-3	CAEZ01003071:22827-23223
HmHel-3	CAEZ01010289:138141-138539
HmHel-3	CAEZ01006319:9689-10091
HmHel-3	CAEZ01010460:61648-62049
HmHel-3	CAEZ01010336:15356-15757
HmHel-3	CAEZ01007296:6635-8367
HmHel-3	CAEZ01007296:12261-12663
HmHel-3	CAEZ01005208:18859-19226
HmHel-3	CAEZ01005208:25220-25296
HmHel-3	CAEZ01010737:55669-56065
HmHel-3	CAEZ01010737:121146-124183
HmHel-3	CAEZ01010737:180147-180477
HmHel-3	CAEZ01010737:156788-156948
HmHel-3	CAEZ01010737:115393-115788
HmHel-3	CAEZ01009421:55239-55644
HmHel-3	CAEZ01008532:26355-26673
HmHel-3	CAEZ01008532:62853-63252
HmHel-3	CAEZ01008435:80030-80325
HmHel-3	CAEZ01008435:97041-97419
HmHel-3	CAEZ01008435:158456-158745
HmHel-3	CAEZ01008435:230862-232587
HmHel-3	CAEZ01008435:240194-240510
HmHel-3	CAEZ01008435:117630-118000
HmHel-3	CAEZ01008435:106903-107308
HmHel-3	CAEZ01008435:4740-5493
HmHel-3	CAEZ01008352:4677-5079
HmHel-3	CAEZ01008352:110342-110666
HmHel-3	CAEZ01008352:126150-126483
HmHel-3	CAEZ01003067:82159-82555

HmHel-3	CAEZ01003067:98877-99279
HmHel-3	CAEZ01003067:34010-34409
HmHel-3	CAEZ01003067:8486-8759
HmHel-3	CAEZ01001512:16011-16410
HmHel-3	CAEZ01009287:1182-1584
HmHel-3	CAEZ01005066:61446-61847
HmHel-3	CAEZ01005066:94192-94592
HmHel-3	CAEZ01010215:391-650
HmHel-3	CAEZ01010215:12684-13085
HmHel-3	CAEZ01009509:120512-120913
HmHel-3	CAEZ01008208:51716-51997
HmHel-3	CAEZ01008208:96855-97141
HmHel-3	CAEZ01008208:152510-152911
HmHel-3	CAEZ01008208:188973-189200
HmHel-3	CAEZ01008208:206986-207397
HmHel-3	CAEZ01007571:18791-19083
HmHel-3	CAEZ01005711:15390-15707
HmHel-3	CAEZ01005711:167332-167748
HmHel-3	CAEZ01005711:143191-143871
HmHel-3	CAEZ01005711:98143-98794
HmHel-3	CAEZ01005711:2668-3066
HmHel-3	CAEZ01004536:4990-5391
HmHel-3	CAEZ01007741:74178-74557
HmHel-3	CAEZ01007741:102125-102530
HmHel-3	CAEZ01007741:96123-96519
HmHel-3	CAEZ01007741:3285-3653
HmHel-3	CAEZ01003353:1343-1739
HmHel-3	CAEZ01003353:9258-9655
HmHel-3	CAEZ01001301:16260-16660
HmHel-3	CAEZ01009925:245-604
HmHel-3	CAEZ01009925:29466-29879
HmHel-3	CAEZ01009925:5742-5825
HmHel-3	CAEZ01009610:14281-14677
HmHel-3	CAEZ01009610:67742-68135
HmHel-3	CAEZ01009610:30827-31508
HmHel-3	CAEZ01008410:30843-31245
HmHel-3	CAEZ01008410:82815-83032
HmHel-3	CAEZ01008209:1618-2015
HmHel-3	CAEZ01008209:98650-98739
HmHel-3	CAEZ01008209:98213-98592
HmHel-3	CAEZ01003897:5468-5865
HmHel-3	CAEZ01005986:101107-101506
HmHel-3	CAEZ01001238:1656-2054
HmHel-3	CAEZ01001129:25747-26141
HmHel-3	CAEZ01008616:3438-3851
HmHel-3	CAEZ01008561:938-1336
HmHel-3	CAEZ01007630:933-1340
HmHel-3	CAEZ01007389:90992-91271

HmHel-3	CAEZ01007389:172255-172625
HmHel-3	CAEZ01007389:158795-159196
HmHel-3	CAEZ01007389:135023-135411
HmHel-3	CAEZ01007389:87137-87555
HmHel-3	CAEZ01006389:12204-12607
HmHel-3	CAEZ01005760:543-940
HmHel-3	CAEZ01003217:20305-20702
HmHel-3	CAEZ01001464:6741-7432
HmHel-3	CAEZ01001464:30230-30631
HmHel-3	CAEZ01001464:23492-23888
HmHel-3	CAEZ01009586:24966-25364
HmHel-3	CAEZ01009586:51816-52224
HmHel-3	CAEZ01008261:86761-87440
HmHel-3	CAEZ01008261:165066-165432
HmHel-3	CAEZ01008261:187860-188189
HmHel-3	CAEZ01008261:147898-148301
HmHel-3	CAEZ01008261:87704-88049
HmHel-3	CAEZ01008261:38638-38837
HmHel-3	CAEZ01008261:24550-24956
HmHel-3	CAEZ01007822:26605-27014
HmHel-3	CAEZ01007382:3543-3948
HmHel-3	CAEZ01002499:3742-4139
HmHel-3	CAEZ01011494:20221-20625
HmHel-3	CAEZ01011494:63346-63825
HmHel-3	CAEZ01011494:30374-30785
HmHel-3	CAEZ01011494:15057-15385
HmHel-3	CAEZ01009196:15468-15870
HmHel-3	CAEZ01007268:33377-33614
HmHel-3	CAEZ01007268:6685-7087
HmHel-3	CAEZ01005955:26386-26532
HmHel-3	CAEZ01005955:2243-2650
HmHel-3	CAEZ01011527:24388-24798
HmHel-3	CAEZ01006171:113129-113435
HmHel-3	CAEZ01006171:113832-114067
HmHel-3	CAEZ01006171:69090-69504
HmHel-3	CAEZ01011492:2227-2629
HmHel-3	CAEZ01011492:393-481
HmHel-3	CAEZ01007998:3989-4390
HmHel-3	CAEZ01005639:4990-5391
HmHel-3	CAEZ01004432:38864-39258
HmHel-3	CAEZ01004432:28377-28731
HmHel-3	CAEZ01004093:18062-18462
HmHel-3	CAEZ01004093:21184-21552
HmHel-3	CAEZ01011093:28182-28504
HmHel-3	CAEZ01011093:43295-43703
HmHel-3	CAEZ01011093:132254-132625
HmHel-3	CAEZ01011093:179449-179766
HmHel-3	CAEZ01011093:128981-129387

HmHel-3	CAEZ01010030:872-1108
HmHel-3	CAEZ01010030:97956-98282
HmHel-3	CAEZ01010030:139202-139595
HmHel-3	CAEZ01010030:187191-187475
HmHel-3	CAEZ01010030:261382-261676
HmHel-3	CAEZ01010030:222799-223472
HmHel-3	CAEZ01010030:137874-138273
HmHel-3	CAEZ01008574:105019-105428
HmHel-3	CAEZ01005221:67511-68584
HmHel-3	CAEZ01005221:3299-3702
HmHel-3	CAEZ01004314:2196-2597
HmHel-3	CAEZ01004314:13366-13775
HmHel-3	CAEZ01003993:3794-4203
HmHel-3	CAEZ01009975:20494-20773
HmHel-3	CAEZ01009975:104525-104740
HmHel-3	CAEZ01009975:135294-135967
HmHel-3	CAEZ01009975:116807-117200
HmHel-3	CAEZ01009975:14583-14904
HmHel-3	CAEZ01009871:10384-10788
HmHel-3	CAEZ01009871:42891-43295
HmHel-3	CAEZ01008240:26818-27222
HmHel-3	CAEZ01006124:11391-11795
HmHel-3	CAEZ01006124:36898-38104
HmHel-3	CAEZ01006124:19977-20369
HmHel-3	CAEZ01005175:30332-34909
HmHel-3	CAEZ01005175:32977-33213
HmHel-3	CAEZ01005163:33977-34352
HmHel-3	CAEZ01005163:35085-35496
HmHel-3	CAEZ01002944:70946-71348
HmHel-3	CAEZ01002944:5554-5861
HmHel-3	CAEZ01009696:21503-21904
HmHel-3	CAEZ01008606:46311-47003
HmHel-3	CAEZ01008606:91193-91603
HmHel-3	CAEZ01006897:9550-9825
HmHel-3	CAEZ01006897:21732-22138
HmHel-3	CAEZ01006897:181-260
HmHel-3	CAEZ01006287:21512-21913
HmHel-3	CAEZ01006287:65157-65245
HmHel-3	CAEZ01006287:57015-57419
HmHel-3	CAEZ01006164:40829-41070
HmHel-3	CAEZ01006164:54994-55372
HmHel-3	CAEZ01006164:21645-22019
HmHel-3	CAEZ01006164:9706-10112
HmHel-3	CAEZ01005498:13724-14125
HmHel-3	CAEZ01010931:13992-14397
HmHel-3	CAEZ01010039:109422-109747
HmHel-3	CAEZ01010039:170531-170936
HmHel-3	CAEZ01010039:140753-141072

HmHel-3	CAEZ01008477:8651-9050
HmHel-3	CAEZ01008272:22369-22772
HmHel-3	CAEZ01006780:8121-8525
HmHel-3	CAEZ01006493:20610-20915
HmHel-3	CAEZ01006191:3349-3754
HmHel-3	CAEZ01004818:117549-117874
HmHel-3	CAEZ01004818:92335-92737
HmHel-3	CAEZ01004818:71683-72033
HmHel-3	CAEZ01004189:1361-1766
HmHel-3	CAEZ01003711:6505-6909
HmHel-3	CAEZ01003711:23061-23360
HmHel-3	CAEZ01002726:3473-3866
HmHel-3	CAEZ01000909:28043-28455
HmHel-3	CAEZ01000909:152074-152471
HmHel-3	CAEZ01000909:139422-140107
HmHel-3	CAEZ01000909:107058-107458
HmHel-3	CAEZ01000909:81450-81867
HmHel-3	CAEZ01010583:5866-6269
HmHel-3	CAEZ01010583:44733-45353
HmHel-3	CAEZ01010583:39505-39902
HmHel-3	CAEZ01010583:23480-24153
HmHel-3	CAEZ01008885:1920-2315
HmHel-3	CAEZ01004574:29993-30399
HmHel-3	CAEZ01001559:10894-11289
HmHel-3	CAEZ01007265:1060-1483
HmHel-3	CAEZ01007265:27602-28004
HmHel-3	CAEZ01001134:8380-8773
HmHel-3	CAEZ01010079:28145-28550
HmHel-3	CAEZ01010079:91628-91862
HmHel-3	CAEZ01010079:23406-23788
HmHel-3	CAEZ01008260:18085-18489
HmHel-3	CAEZ01007568:21751-22154
HmHel-3	CAEZ01007568:51911-52351
HmHel-3	CAEZ01007568:80687-80992
HmHel-3	CAEZ01007568:109407-109799
HmHel-3	CAEZ01007568:113084-113416
HmHel-3	CAEZ01007568:62044-62441
HmHel-3	CAEZ01007568:25218-35152
HmHel-3	CAEZ01006616:34238-34474
HmHel-3	CAEZ01006616:75839-76063
HmHel-3	CAEZ01006616:92485-92803
HmHel-3	CAEZ01006616:171029-171264
HmHel-3	CAEZ01006616:185699-186128
HmHel-3	CAEZ01006616:258166-258484
HmHel-3	CAEZ01006616:181287-181728
HmHel-3	CAEZ01004864:4014-4415
HmHel-3	CAEZ01003527:2121-2511
HmHel-3	CAEZ01003527:2968-3293

HmHel-3	CAEZ01010276:34403-34808
HmHel-3	CAEZ01009562:25922-26331
HmHel-3	CAEZ01009562:23339-23663
HmHel-3	CAEZ01009425:15032-15433
HmHel-3	CAEZ01009311:33945-34348
HmHel-3	CAEZ01009311:32637-33321
HmHel-3	CAEZ01007252:52114-52518
HmHel-3	CAEZ01007252:156723-157051
HmHel-3	CAEZ01007252:21764-22167
HmHel-3	CAEZ01007252:6080-6487
HmHel-3	CAEZ01006508:6698-7103
HmHel-3	CAEZ01004796:40252-40657
HmHel-3	CAEZ01004703:20856-21261
HmHel-3	CAEZ01004452:44704-45101
HmHel-3	CAEZ01003809:528-933
HmHel-3	CAEZ01002821:117513-117905
HmHel-3	CAEZ01002821:36032-36441
HmHel-3	CAEZ01010578:16825-17224
HmHel-3	CAEZ01009224:29649-30048
HmHel-3	CAEZ01009224:55545-55949
HmHel-3	CAEZ01007960:19783-25329
HmHel-3	CAEZ01007960:52574-53303
HmHel-3	CAEZ01007960:57744-58145
HmHel-3	CAEZ01007731:110391-110774
HmHel-3	CAEZ01007731:128470-128787
HmHel-3	CAEZ01007731:100897-101293
HmHel-3	CAEZ01007708:72035-72442
HmHel-3	CAEZ01007708:36761-37489
HmHel-3	CAEZ01007304:3920-4328
HmHel-3	CAEZ01007137:17297-17679
HmHel-3	CAEZ01007137:2351-2749
HmHel-3	CAEZ01006615:758-1166
HmHel-3	CAEZ01005403:5515-5833
HmHel-3	CAEZ01005403:22470-22774
HmHel-3	CAEZ01005403:38737-39133
HmHel-3	CAEZ01005403:6732-6967
HmHel-3	CAEZ01005042:1292-1703
HmHel-3	CAEZ01004046:9-411
HmHel-3	CAEZ01003939:15534-15930
HmHel-3	CAEZ01001349:46927-47333
HmHel-3	CAEZ01000895:12245-12588
HmHel-3	CAEZ01000895:10703-11105
HmHel-3	CAEZ01010451:48263-48552
HmHel-3	CAEZ01010451:105029-105431
HmHel-3	CAEZ01009841:13457-13684
HmHel-3	CAEZ01009841:34730-35130
HmHel-3	CAEZ01007539:6506-6913
HmHel-3	CAEZ01004734:37641-37946

HmHel-3	CAEZ01004734:75131-75533
HmHel-3	CAEZ01001082:3211-3617
HmHel-3	CAEZ01011598:33047-33448
HmHel-3	CAEZ01011598:110088-110807
HmHel-3	CAEZ01011598:37570-37887
HmHel-3	CAEZ01009824:32457-32866
HmHel-3	CAEZ01009824:74635-75789
HmHel-3	CAEZ01009824:2855-3115
HmHel-3	CAEZ01008393:42699-43098
HmHel-3	CAEZ01008393:128692-132787
HmHel-3	CAEZ01008393:115061-115463
HmHel-3	CAEZ01008393:89568-90247
HmHel-3	CAEZ01008337:11744-12133
HmHel-3	CAEZ01008337:38402-38827
HmHel-3	CAEZ01008211:125248-125977
HmHel-3	CAEZ01008211:171335-171730
HmHel-3	CAEZ01008211:189429-189662
HmHel-3	CAEZ01008211:238499-238804
HmHel-3	CAEZ01008211:237051-237445
HmHel-3	CAEZ01008211:199035-199269
HmHel-3	CAEZ01008211:27155-27570
HmHel-3	CAEZ01007977:8101-8502
HmHel-3	CAEZ01006370:53874-53958
HmHel-3	CAEZ01006370:50153-50551
HmHel-3	CAEZ01006370:16662-17063
HmHel-3	CAEZ01005467:8262-8659
HmHel-3	CAEZ01005455:3841-4254
HmHel-3	CAEZ01002638:14758-16917
HmHel-3	CAEZ01010926:17481-17782
HmHel-3	CAEZ01010926:83570-83712
HmHel-3	CAEZ01010926:96217-96906
HmHel-3	CAEZ01010926:72173-72460
HmHel-3	CAEZ01010059:5237-5642
HmHel-3	CAEZ01010052:7475-7871
HmHel-3	CAEZ01009380:1507-1943
HmHel-3	CAEZ01008740:79550-79954
HmHel-3	CAEZ01008248:42463-42868
HmHel-3	CAEZ01007944:1876-2283
HmHel-3	CAEZ01007930:33926-34327
HmHel-3	CAEZ01007722:60582-60994
HmHel-3	CAEZ01007722:145906-146312
HmHel-3	CAEZ01007722:161715-162130
HmHel-3	CAEZ01007640:404-809
HmHel-3	CAEZ01007164:306-646
HmHel-3	CAEZ01007164:10813-11218
HmHel-3	CAEZ01006154:26247-26642
HmHel-3	CAEZ01006154:173717-174105
HmHel-3	CAEZ01006154:285547-285945

HmHel-3	CAEZ01006154:90744-91748
HmHel-3	CAEZ01005474:23558-23963
HmHel-3	CAEZ01004633:43299-43704
HmHel-3	CAEZ01004633:24131-24851
HmHel-3	CAEZ01002730:5344-5746
HmHel-3	CAEZ01002730:881-1257
HmHel-3	CAEZ01002620:34914-35319
HmHel-3	CAEZ01002620:69498-69584
HmHel-3	CAEZ01002620:70299-70387
HmHel-3	CAEZ01002620:21593-21828
HmHel-3	CAEZ01000061:23297-23702
HmHel-3	CAEZ01009336:22884-23280
HmHel-3	CAEZ01009336:4400-4782
HmHel-3	CAEZ01008289:21184-21580
HmHel-3	CAEZ01006057:3257-3661
HmHel-3	CAEZ01005501:20065-20475
HmHel-3	CAEZ01004239:34248-34620
HmHel-3	CAEZ01004239:9908-10312
HmHel-3	CAEZ01002361:11639-12052
HmHel-3	CAEZ01010585:191605-191702
HmHel-3	CAEZ01010585:167458-167877
HmHel-3	CAEZ01010585:73216-73613
HmHel-3	CAEZ01009962:867-1260
HmHel-3	CAEZ01008707:115387-115800
HmHel-3	CAEZ01008707:137024-137397
HmHel-3	CAEZ01008707:65911-66074
HmHel-3	CAEZ01008707:24328-24975
HmHel-3	CAEZ01008707:496-776
HmHel-3	CAEZ01008688:20439-20839
HmHel-3	CAEZ01008590:125513-125914
HmHel-3	CAEZ01008590:62297-62607
HmHel-3	CAEZ01008590:27214-27531
HmHel-3	CAEZ01011528:32545-40409
HmHel-3	CAEZ01011528:41854-42255
HmHel-3	CAEZ01011528:18957-19192
HmHel-3	CAEZ01011528:6495-6882
HmHel-3	CAEZ01010509:22362-22601
HmHel-3	CAEZ01010509:7129-7530
HmHel-3	CAEZ01008313:10117-10517
HmHel-3	CAEZ01007429:59721-60122
HmHel-3	CAEZ01007429:86673-88399
HmHel-3	CAEZ01007053:5982-6379
HmHel-3	CAEZ01003842:28542-28955
HmHel-3	CAEZ01003842:27664-28083
HmHel-3	CAEZ01010738:33-254
HmHel-3	CAEZ01010738:83612-83848
HmHel-3	CAEZ01010738:230860-231268
HmHel-3	CAEZ01010738:208077-208398

HmHel-3	CAEZ01010738:184118-184504
HmHel-3	CAEZ01010738:162064-162458
HmHel-3	CAEZ01010738:138633-138966
HmHel-3	CAEZ01010738:92677-93068
HmHel-3	CAEZ01010738:36002-36345
HmHel-3	CAEZ01009642:22990-23395
HmHel-3	CAEZ01009422:96748-97153
HmHel-3	CAEZ01009422:118886-119559
HmHel-3	CAEZ01009422:131676-132278
HmHel-3	CAEZ01009422:107667-108620
HmHel-3	CAEZ01009422:88712-88800
HmHel-3	CAEZ01009422:24190-24445
HmHel-3	CAEZ01009422:7572-7950
HmHel-3	CAEZ01009096:1018-1412
HmHel-3	CAEZ01008360:7982-8391
HmHel-3	CAEZ01007912:64369-64598
HmHel-3	CAEZ01007912:81885-82282
HmHel-3	CAEZ01007912:101861-102216
HmHel-3	CAEZ01007100:23889-24298
HmHel-3	CAEZ01007100:65650-65965
HmHel-3	CAEZ01006332:68701-69101
HmHel-3	CAEZ01004941:8674-9088
HmHel-3	CAEZ01004941:34738-35084
HmHel-3	CAEZ01004941:89085-89467
HmHel-3	CAEZ01004941:21345-21572
HmHel-3	CAEZ01004589:1490-1899
HmHel-3	CAEZ01004441:824-1233
HmHel-3	CAEZ01004096:119579-119984
HmHel-3	CAEZ01003622:17140-17549
HmHel-3	CAEZ01003326:18901-19301
HmHel-3	CAEZ01003326:34868-35547
HmHel-3	CAEZ01003326:53342-53754
HmHel-3	CAEZ01003326:33331-34594
HmHel-3	CAEZ01002589:18037-18438
HmHel-3	CAEZ01009265:32382-32790
HmHel-3	CAEZ01009024:6095-6516
HmHel-3	CAEZ01008468:413-800
HmHel-3	CAEZ01007928:47586-47943
HmHel-3	CAEZ01007928:71998-72394
HmHel-3	CAEZ01007928:32394-32775
HmHel-3	CAEZ01007747:167727-168133
HmHel-3	CAEZ01007747:28886-29284
HmHel-3	CAEZ01006352:24658-25067
HmHel-3	CAEZ01006352:158474-158799
HmHel-3	CAEZ01006352:108138-108467
HmHel-3	CAEZ01006352:34613-35051
HmHel-3	CAEZ01006352:2936-3313
HmHel-3	CAEZ01003416:51942-52326

HmHel-3	CAEZ01003250:56268-56676
HmHel-3	CAEZ01003250:78229-78890
HmHel-3	CAEZ01003250:161837-162677
HmHel-3	CAEZ01002957:296-705
HmHel-3	CAEZ01010765:7197-7598
HmHel-3	CAEZ01009733:53399-53796
HmHel-3	CAEZ01009733:67957-68354
HmHel-3	CAEZ01009733:77058-77318
HmHel-3	CAEZ01008791:2271-2684
HmHel-3	CAEZ01006961:14631-14865
HmHel-3	CAEZ01006961:49259-49506
HmHel-3	CAEZ01006961:2884-3286
HmHel-3	CAEZ01005756:22975-23372
HmHel-3	CAEZ01005756:10671-11086
HmHel-3	CAEZ01010807:79996-80080
HmHel-3	CAEZ01010807:189060-189463
HmHel-3	CAEZ01010807:4864-6068
HmHel-3	CAEZ01010671:16174-16571
HmHel-3	CAEZ01009223:38560-38658
HmHel-3	CAEZ01009223:49750-50041
HmHel-3	CAEZ01009223:70262-71951
HmHel-3	CAEZ01009223:81222-81635
HmHel-3	CAEZ01009223:53625-54037
HmHel-3	CAEZ01007978:2400-2804
HmHel-3	CAEZ01006445:89731-90144
HmHel-3	CAEZ01006445:232753-239818
HmHel-3	CAEZ01006445:222222-222635
HmHel-3	CAEZ01006445:189168-189474
HmHel-3	CAEZ01006445:84860-85178
HmHel-3	CAEZ01005721:10005-10523
HmHel-3	CAEZ01005721:66799-67155
HmHel-3	CAEZ01005721:124425-124635
HmHel-3	CAEZ01005721:79817-80254
HmHel-3	CAEZ01005721:63369-63697
HmHel-3	CAEZ01005721:4741-5001
HmHel-3	CAEZ01005624:13980-14370
HmHel-3	CAEZ01004045:27336-28138
HmHel-3	CAEZ01002966:4489-4708
HmHel-3	CAEZ01002966:2622-3025
HmHel-3	CAEZ01002724:52794-53467
HmHel-3	CAEZ01002724:109963-110366
HmHel-3	CAEZ01002724:77992-78288
HmHel-3	CAEZ01002724:6562-6927
HmHel-3	CAEZ01002712:8717-9120
HmHel-3	CAEZ01002601:1-370
HmHel-3	CAEZ01001508:1351-1746
HmHel-3	CAEZ01008389:7809-8211
HmHel-3	CAEZ01007184:68084-68481

HmHel-3	CAEZ01007184:86515-86754
HmHel-3	CAEZ01007184:167383-167768
HmHel-3	CAEZ01007184:137649-138062
HmHel-3	CAEZ01007184:51493-52172
HmHel-3	CAEZ01007184:36406-36661
HmHel-3	CAEZ01006843:333-728
HmHel-3	CAEZ01006675:75747-76165
HmHel-3	CAEZ01006675:118039-118281
HmHel-3	CAEZ01006675:89021-89263
HmHel-3	CAEZ01006675:52539-52848
HmHel-3	CAEZ01006619:10516-10919
HmHel-3	CAEZ01006619:30254-30612
HmHel-3	CAEZ01006619:33743-34146
HmHel-3	CAEZ01005089:49540-49945
HmHel-3	CAEZ01005089:16175-16500
HmHel-3	CAEZ01004923:80363-80786
HmHel-3	CAEZ01004738:169866-169950
HmHel-3	CAEZ01004738:221779-222433
HmHel-3	CAEZ01004738:298637-299391
HmHel-3	CAEZ01004738:269194-269809
HmHel-3	CAEZ01004738:240981-241217
HmHel-3	CAEZ01004738:42529-42850
HmHel-3	CAEZ01004738:15216-15628
HmHel-3	CAEZ01004677:41451-41856
HmHel-3	CAEZ01003546:999-1414
HmHel-3	CAEZ01002838:86119-86524
HmHel-3	CAEZ01001203:8175-8584
HmHel-3	CAEZ01010272:108946-109180
HmHel-3	CAEZ01010272:110044-110441
HmHel-3	CAEZ01010272:61289-61692
HmHel-3	CAEZ01010272:48177-48413
HmHel-3	CAEZ01008915:140917-141259
HmHel-3	CAEZ01008915:129655-129979
HmHel-3	CAEZ01008915:96198-96595
HmHel-3	CAEZ01008437:103466-103793
HmHel-3	CAEZ01008437:145262-145637
HmHel-3	CAEZ01008437:31737-32149
HmHel-3	CAEZ01008437:1354-1758
HmHel-3	CAEZ01007522:3561-3966
HmHel-3	CAEZ01007263:54030-54437
HmHel-3	CAEZ01007263:71051-71303
HmHel-3	CAEZ01007263:89060-89287
HmHel-3	CAEZ01007263:12819-13213
HmHel-3	CAEZ01006570:4634-5047
HmHel-3	CAEZ01005481:13009-13385
HmHel-3	CAEZ01005481:38069-38743
HmHel-3	CAEZ01005481:80024-80432
HmHel-3	CAEZ01005198:26218-26614

HmHel-3	CAEZ01001879:798-1194
HmHel-3	CAEZ01010734:26596-26986
HmHel-3	CAEZ01010734:127540-127937
HmHel-3	CAEZ01007151:2717-3071
HmHel-3	CAEZ01007151:23006-23376
HmHel-3	CAEZ01007151:24982-25397
HmHel-3	CAEZ01007124:114426-114838
HmHel-3	CAEZ01007124:72891-73303
HmHel-3	CAEZ01006068:8367-8765
HmHel-3	CAEZ01002767:27082-27481
HmHel-3	CAEZ01002767:39870-40472
HmHel-3	CAEZ01002767:2779-3498
HmHel-3	CAEZ01002097:11742-12105
HmHel-3	CAEZ01002097:40806-41213
HmHel-3	CAEZ01002097:23534-23741
HmHel-3	CAEZ01002097:3037-3710
HmHel-3	CAEZ01011395:17851-19170
HmHel-3	CAEZ01011395:1788-2198
HmHel-3	CAEZ01011006:1403-1816
HmHel-3	CAEZ01010589:5599-6002
HmHel-3	CAEZ01010346:7825-8234
HmHel-3	CAEZ01008971:35173-35580
HmHel-3	CAEZ01008963:72709-73130
HmHel-3	CAEZ01008963:128111-128531
HmHel-3	CAEZ01008963:12902-13223
HmHel-3	CAEZ01008675:8575-8973
HmHel-3	CAEZ01006912:7382-7772
HmHel-3	CAEZ01004158:4583-4987
HmHel-3	CAEZ01003472:7344-7745
HmHel-3	CAEZ01002891:8720-9161
HmHel-3	CAEZ01002891:14846-15553
HmHel-3	CAEZ01002826:113773-114183
HmHel-3	CAEZ01000040:3484-3892
HmHel-3	CAEZ01011095:409-738
HmHel-3	CAEZ01011095:28679-29069
HmHel-3	CAEZ01010797:12866-13305
HmHel-3	CAEZ01010797:87618-87934
HmHel-3	CAEZ01010797:104387-104808
HmHel-3	CAEZ01010797:115329-115741
HmHel-3	CAEZ01010797:144566-144761
HmHel-3	CAEZ01010797:120517-120835
HmHel-3	CAEZ01009288:24797-25197
HmHel-3	CAEZ01009288:8856-9182
HmHel-3	CAEZ01008404:18267-18572
HmHel-3	CAEZ01008404:182948-183276
HmHel-3	CAEZ01008404:74511-74902
HmHel-3	CAEZ01008404:44260-44669
HmHel-3	CAEZ01007809:83831-84236

HmHel-3	CAEZ01007809:104716-104951
HmHel-3	CAEZ01007809:252526-252940
HmHel-3	CAEZ01007809:41122-41358
HmHel-3	CAEZ01007809:9206-9618
HmHel-3	CAEZ01005269:85167-85537
HmHel-3	CAEZ01005269:103909-104314
HmHel-3	CAEZ01005269:131070-131375
HmHel-3	CAEZ01005269:81993-82314
HmHel-3	CAEZ01005269:61750-62149
HmHel-3	CAEZ01004714:4077-4486
HmHel-3	CAEZ01004325:17999-18404
HmHel-3	CAEZ01003732:2893-3319
HmHel-3	CAEZ01003715:2469-2865
HmHel-3	CAEZ01003543:2919-3324
HmHel-3	CAEZ01003059:27178-27577
HmHel-3	CAEZ01003056:2878-3286
HmHel-3	CAEZ01001771:14326-14718
HmHel-3	CAEZ01011584:1894-2302
HmHel-3	CAEZ01010486:4214-4607
HmHel-3	CAEZ01006398:35765-36164
HmHel-3	CAEZ01005722:29308-29703
HmHel-3	CAEZ01005430:55202-56347
HmHel-3	CAEZ01005430:67384-67797
HmHel-3	CAEZ01005430:454-690
HmHel-3	CAEZ01010217:36695-37371
HmHel-3	CAEZ01010217:51616-52031
HmHel-3	CAEZ01010217:122397-122803
HmHel-3	CAEZ01009458:67596-68271
HmHel-3	CAEZ01009458:79902-80223
HmHel-3	CAEZ01009458:175783-176019
HmHel-3	CAEZ01009458:155282-155681
HmHel-3	CAEZ01008123:32839-33192
HmHel-3	CAEZ01008123:47731-48151
HmHel-3	CAEZ01008123:33686-34354
HmHel-3	CAEZ01008123:23173-23586
HmHel-3	CAEZ01008122:10132-10538
HmHel-3	CAEZ01008122:22438-22810
HmHel-3	CAEZ01008122:83550-83929
HmHel-3	CAEZ01007140:5499-5738
HmHel-3	CAEZ01007140:38600-39008
HmHel-3	CAEZ01007140:55941-56637
HmHel-3	CAEZ01005534:981-1391
HmHel-3	CAEZ01001742:17876-19563
HmHel-3	CAEZ01001742:7110-7346
HmHel-3	CAEZ01007739:7740-8060
HmHel-3	CAEZ01007739:59301-59738
HmHel-3	CAEZ01007739:52178-52555
HmHel-3	CAEZ01007739:28388-28745

HmHel-3	CAEZ01005885:4443-4848
HmHel-3	CAEZ01003060:34367-34770
HmHel-3	CAEZ01002708:25491-25911
HmHel-3	CAEZ01002630:14902-21052
HmHel-3	CAEZ01001572:47644-47882
HmHel-3	CAEZ01001572:26620-27013
HmHel-3	CAEZ01010972:410277-410986
HmHel-3	CAEZ01010972:472472-472870
HmHel-3	CAEZ01010972:418605-418923
HmHel-3	CAEZ01010972:270692-271007
HmHel-3	CAEZ01010972:202548-203192
HmHel-3	CAEZ01010972:175034-175431
HmHel-3	CAEZ01010972:137984-138658
HmHel-3	CAEZ01010546:1-362
HmHel-3	CAEZ01009521:6980-7392
HmHel-3	CAEZ01009474:41697-42091
HmHel-3	CAEZ01007361:3187-3584
HmHel-3	CAEZ01003863:4440-4863
HmHel-3	CAEZ01003212:2365-2768
HmHel-3	CAEZ01003152:4213-4624
HmHel-3	CAEZ01003080:16052-16456
HmHel-3	CAEZ01003080:48115-48436
HmHel-3	CAEZ01003080:120502-120729
HmHel-3	CAEZ01003080:196294-196700
HmHel-3	CAEZ01003080:25091-26177
HmHel-3	CAEZ01003080:1159-1445
HmHel-3	CAEZ01002398:1875-2280
HmHel-3	CAEZ01002119:16193-16592
HmHel-3	CAEZ01009832:3870-4198
HmHel-3	CAEZ01009832:29743-29976
HmHel-3	CAEZ01009832:19271-19562
HmHel-3	CAEZ01009770:67002-67404
HmHel-3	CAEZ01008736:31898-32311
HmHel-3	CAEZ01007556:3048-3452
HmHel-3	CAEZ01006603:11175-11597
HmHel-3	CAEZ01005136:7816-8215
HmHel-3	CAEZ01003620:7023-7428
HmHel-3	CAEZ01002665:7883-8556
HmHel-3	CAEZ01002665:40275-40963
HmHel-3	CAEZ01001914:8567-8989
HmHel-3	CAEZ01011190:31613-31797
HmHel-3	CAEZ01011190:19555-19946
HmHel-3	CAEZ01008989:158600-159291
HmHel-3	CAEZ01008989:95357-95739
HmHel-3	CAEZ01007560:6665-7083
HmHel-3	CAEZ01005773:1332-1703
HmHel-3	CAEZ01005773:5255-5649
HmHel-3	CAEZ01005190:16808-17212

HmHel-3	CAEZ01004643:31068-31466
HmHel-3	CAEZ01003686:15860-16262
HmHel-3	CAEZ01002689:17888-18294
HmHel-3	CAEZ01002666:27498-27803
HmHel-3	CAEZ01002666:54673-55088
HmHel-3	CAEZ01002666:81494-81779
HmHel-3	CAEZ01002666:37958-38603
HmHel-3	CAEZ01001048:1012-1409
HmHel-3	CAEZ01010824:21352-21757
HmHel-3	CAEZ01010048:821-1206
HmHel-3	CAEZ01008761:4736-5129
HmHel-3	CAEZ01007460:17125-17542
HmHel-3	CAEZ01006400:40438-40830
HmHel-3	CAEZ01005519:5085-5542
HmHel-3	CAEZ01005495:39123-39524
HmHel-3	CAEZ01005495:261-934
HmHel-3	CAEZ01005218:922-1339
HmHel-3	CAEZ01005067:37594-37982
HmHel-3	CAEZ01004492:17378-17790
HmHel-3	CAEZ01004492:31563-31984
HmHel-3	CAEZ01004001:3753-4146
HmHel-3	CAEZ01003938:3151-3557
HmHel-3	CAEZ01003496:4376-4781
HmHel-3	CAEZ01001419:12934-13344
HmHel-3	CAEZ01001222:6931-12229
HmHel-3	CAEZ01009737:8176-8569
HmHel-3	CAEZ01009487:45333-45730
HmHel-3	CAEZ01008921:8-411
HmHel-3	CAEZ01008921:108741-109325
HmHel-3	CAEZ01008921:88664-88888
HmHel-3	CAEZ01008921:44614-45030
HmHel-3	CAEZ01008921:15483-15905
HmHel-3	CAEZ01006421:13261-13634
HmHel-3	CAEZ01006421:43937-44282
HmHel-3	CAEZ01005043:104194-104461
HmHel-3	CAEZ01005043:155753-155989
HmHel-3	CAEZ01005043:212872-213085
HmHel-3	CAEZ01005043:183824-184140
HmHel-3	CAEZ01005043:163157-163571
HmHel-3	CAEZ01005043:52350-52684
HmHel-3	CAEZ01004319:312-717
HmHel-3	CAEZ01003952:32801-33209
HmHel-3	CAEZ01002627:2573-2982
HmHel-3	CAEZ01010415:22949-23354
HmHel-3	CAEZ01009828:5180-5575
HmHel-3	CAEZ01009291:19577-19985
HmHel-3	CAEZ01009120:29384-29753
HmHel-3	CAEZ01009120:77433-77738

HmHel-3	CAEZ01009120:22857-23502
HmHel-3	CAEZ01007551:12276-12682
HmHel-3	CAEZ01006804:158602-159229
HmHel-3	CAEZ01006804:195561-195972
HmHel-3	CAEZ01005396:11024-11412
HmHel-3	CAEZ01005173:2478-2820
HmHel-3	CAEZ01005173:18867-18999
HmHel-3	CAEZ01005173:99553-99637
HmHel-3	CAEZ01005173:130258-130654
HmHel-3	CAEZ01004921:10638-12355
HmHel-3	CAEZ01004921:51171-51491
HmHel-3	CAEZ01004402:651-1011
HmHel-3	CAEZ01010829:264368-264689
HmHel-3	CAEZ01010829:160098-160412
HmHel-3	CAEZ01010829:119449-119887
HmHel-3	CAEZ01010829:91185-91636
HmHel-3	CAEZ01010829:50802-51186
HmHel-3	CAEZ01006369:36694-37084
HmHel-3	CAEZ01006369:26728-28447
HmHel-3	CAEZ01006151:2574-2735
HmHel-3	CAEZ01006151:16207-16579
HmHel-3	CAEZ01006151:78176-78581
HmHel-3	CAEZ01006151:18564-18964
HmHel-3	CAEZ01004198:36569-36963
HmHel-3	CAEZ01004104:32345-39898
HmHel-3	CAEZ01002737:1127-1538
HmHel-3	CAEZ01002634:12941-13347
HmHel-3	CAEZ01002134:14184-14600
HmHel-3	CAEZ01002134:26229-26651
HmHel-3	CAEZ01009974:32759-33116
HmHel-3	CAEZ01009974:34032-34422
HmHel-3	CAEZ01008960:14474-14868
HmHel-3	CAEZ01008960:97183-97536
HmHel-3	CAEZ01008960:120450-120538
HmHel-3	CAEZ01008960:132713-133012
HmHel-3	CAEZ01008960:160638-161039
HmHel-3	CAEZ01008960:155095-155433
HmHel-3	CAEZ01008960:20367-20804
HmHel-3	CAEZ01008935:5465-5868
HmHel-3	CAEZ01008910:5441-5829
HmHel-3	CAEZ01008910:20670-21381
HmHel-3	CAEZ01008367:89408-89801
HmHel-3	CAEZ01007707:28711-29113
HmHel-3	CAEZ01007707:98640-99037
HmHel-3	CAEZ01007707:124504-128249
HmHel-3	CAEZ01007207:120820-121129
HmHel-3	CAEZ01007207:174762-175001
HmHel-3	CAEZ01007207:236411-237685

HmHel-3	CAEZ01007207:266833-267248
HmHel-3	CAEZ01007207:236820-237505
HmHel-3	CAEZ01007207:222324-222996
HmHel-3	CAEZ01007207:44036-44272
HmHel-3	CAEZ01007207:23121-23430
HmHel-3	CAEZ01009982:113148-113478
HmHel-3	CAEZ01009982:61406-61818
HmHel-3	CAEZ01008431:15468-15878
HmHel-3	CAEZ01004359:21404-21768
HmHel-3	CAEZ01003912:4194-4599
HmHel-3	CAEZ01003730:15094-15461
HmHel-3	CAEZ01003070:81170-81561
HmHel-3	CAEZ01003070:146373-146796
HmHel-3	CAEZ01003070:208553-208685
HmHel-3	CAEZ01003070:111247-111919
HmHel-3	CAEZ01002810:39526-39699
HmHel-3	CAEZ01002810:68303-68706
HmHel-3	CAEZ01002810:46139-46477
HmHel-3	CAEZ01002727:5128-5521
HmHel-3	CAEZ01001564:35821-36220
HmHel-3	CAEZ01001564:16492-16861
HmHel-3	CAEZ01009124:9623-9940
HmHel-3	CAEZ01009124:26182-26500
HmHel-3	CAEZ01009124:44722-45140
HmHel-3	CAEZ01009124:2921-3219
HmHel-3	CAEZ01007737:3773-4511
HmHel-3	CAEZ01007737:52721-55366
HmHel-3	CAEZ01007737:3415-4742
HmHel-3	CAEZ01006374:8511-8917
HmHel-3	CAEZ01006010:3251-3657
HmHel-3	CAEZ01006010:30527-30935
HmHel-3	CAEZ01005515:25001-25708
HmHel-3	CAEZ01005515:52191-52879
HmHel-3	CAEZ01005515:3674-4078
HmHel-3	CAEZ01005211:117766-119225
HmHel-3	CAEZ01005211:13094-13372
HmHel-3	CAEZ01004789:5062-7230
HmHel-3	CAEZ01001595:27545-27788
HmHel-3	CAEZ01001595:123963-124437
HmHel-3	CAEZ01007985:7806-8211
HmHel-3	CAEZ01007985:20809-21188
HmHel-3	CAEZ01007651:12604-13003
HmHel-3	CAEZ01002429:26120-26514
HmHel-3	CAEZ01002429:18186-18423
HmHel-3	CAEZ01000084:501-900
HmHel-3	CAEZ01009557:9413-9801
HmHel-3	CAEZ01009274:8069-8417
HmHel-3	CAEZ01009274:24011-26023

HmHel-3	CAEZ01005478:3746-3986
HmHel-3	CAEZ01005478:18800-19189
HmHel-3	CAEZ01005478:82243-82560
HmHel-3	CAEZ01005478:71650-72058
HmHel-3	CAEZ01005478:1494-1898
HmHel-3	CAEZ01011473:10709-11115
HmHel-3	CAEZ01007413:92700-93103
HmHel-3	CAEZ01007413:21334-22054
HmHel-3	CAEZ01006299:8719-10862
HmHel-3	CAEZ01003704:6720-7027
HmHel-3	CAEZ01003704:988-1407
HmHel-3	CAEZ01002268:1813-2227
HmHel-3	CAEZ01010304:17166-17590
HmHel-3	CAEZ01010086:11042-11432
HmHel-3	CAEZ01009978:4971-5378
HmHel-3	CAEZ01009978:26045-26281
HmHel-3	CAEZ01009978:9195-9614
HmHel-3	CAEZ01008124:18393-18741
HmHel-3	CAEZ01008124:125640-126315
HmHel-3	CAEZ01008124:239300-239600
HmHel-3	CAEZ01008124:97973-98389
HmHel-3	CAEZ01008124:4174-4580
HmHel-3	CAEZ01006346:213-538
HmHel-3	CAEZ01005953:3-372
HmHel-3	CAEZ01004832:32702-33116
HmHel-3	CAEZ01003292:95769-96094
HmHel-3	CAEZ01003292:75677-75837
HmHel-3	CAEZ01003292:62404-62793
HmHel-3	CAEZ01009999:49320-49727
HmHel-3	CAEZ01009263:33784-34206
HmHel-3	CAEZ01007395:38431-38667
HmHel-3	CAEZ01007395:26060-26473
HmHel-3	CAEZ01004653:17980-18383
HmHel-3	CAEZ01003335:56343-56743
HmHel-3	CAEZ01003335:23486-23786
HmHel-3	CAEZ01002570:747-1144
HmHel-3	CAEZ01011162:3189-3592
HmHel-3	CAEZ01010827:12117-12438
HmHel-3	CAEZ01010827:124809-125183
HmHel-3	CAEZ01010827:163106-163813
HmHel-3	CAEZ01010827:423762-424105
HmHel-3	CAEZ01005946:24788-25089
HmHel-3	CAEZ01005946:163672-164373
HmHel-3	CAEZ01005946:9166-9526
HmHel-3	CAEZ01005335:32731-33127
HmHel-3	CAEZ01005335:73243-73479
HmHel-3	CAEZ01005335:87160-87477
HmHel-3	CAEZ01002122:3326-3725

HmHel-3	CAEZ01010805:131566-131986
HmHel-3	CAEZ01010805:107677-108135
HmHel-3	CAEZ01010805:76131-76529
HmHel-3	CAEZ01010677:45135-45808
HmHel-3	CAEZ01010677:172968-173181
HmHel-3	CAEZ01010677:213690-214053
HmHel-3	CAEZ01010677:283459-283664
HmHel-3	CAEZ01010677:285087-285450
HmHel-3	CAEZ01010677:253375-253790
HmHel-3	CAEZ01010677:69801-70116
HmHel-3	CAEZ01009778:167181-167506
HmHel-3	CAEZ01009778:243202-243543
HmHel-3	CAEZ01009778:404658-405003
HmHel-3	CAEZ01006633:14962-15349
HmHel-3	CAEZ01006096:1651-2015
HmHel-3	CAEZ01008794:10028-10414
HmHel-3	CAEZ01008455:20231-20617
HmHel-3	CAEZ01005911:51207-51535
HmHel-3	CAEZ01005911:89159-89680
HmHel-3	CAEZ01005911:94171-94472
HmHel-3	CAEZ01005419:24442-24854
HmHel-3	CAEZ01005419:1766-2048
HmHel-3	CAEZ01005205:39286-39680
HmHel-3	CAEZ01003322:7023-7416
HmHel-3	CAEZ01003110:1-386
HmHel-3	CAEZ01003110:69849-70068
HmHel-3	CAEZ01003110:4621-4852
HmHel-3	CAEZ01003069:302381-303088
HmHel-3	CAEZ01003069:248228-248632
HmHel-3	CAEZ01003069:147069-147418
HmHel-3	CAEZ01003069:131107-131782
HmHel-3	CAEZ01003069:12466-12802
HmHel-3	CAEZ01010365:9009-9406
HmHel-3	CAEZ01010365:58619-59480
HmHel-3	CAEZ01006479:7242-7554
HmHel-3	CAEZ01006406:25619-26009
HmHel-3	CAEZ01006406:38744-39433
HmHel-3	CAEZ01004591:1126-1502
HmHel-3	CAEZ01004591:20788-21211
HmHel-3	CAEZ01003722:3138-3548
HmHel-3	CAEZ01003468:1762-2154
HmHel-3	CAEZ01003041:5856-6263
HmHel-3	CAEZ01002680:8868-9537
HmHel-3	CAEZ01002680:20537-20939
HmHel-3	CAEZ01006415:18342-18745
HmHel-3	CAEZ01006415:14335-23566
HmHel-3	CAEZ01005841:51088-51501
HmHel-3	CAEZ01003272:1724-2136

HmHel-3	CAEZ01000741:4073-4475
HmHel-3	CAEZ01009885:34250-34636
HmHel-3	CAEZ01009556:13360-13783
HmHel-3	CAEZ01011430:4975-5384
HmHel-3	CAEZ01009901:75719-76035
HmHel-3	CAEZ01009901:45999-46400
HmHel-3	CAEZ01009901:249-337
HmHel-3	CAEZ01009030:32849-32975
HmHel-3	CAEZ01009030:51673-52018
HmHel-3	CAEZ01009030:73206-73604
HmHel-3	CAEZ01009030:90104-90400
HmHel-3	CAEZ01009030:77018-77437
HmHel-3	CAEZ01009030:27020-27404
HmHel-3	CAEZ01008842:206-539
HmHel-3	CAEZ01008420:10944-11670
HmHel-3	CAEZ01008420:77173-77406
HmHel-3	CAEZ01008420:7608-8012
HmHel-3	CAEZ01005938:15569-15969
HmHel-3	CAEZ01008355:12800-13225
HmHel-3	CAEZ01005082:10639-10959
HmHel-3	CAEZ01005082:40271-40789
HmHel-3	CAEZ01002876:23827-24212
HmHel-3	CAEZ01002876:122754-123009
HmHel-3	CAEZ01002876:137575-137933
HmHel-3	CAEZ01001074:21339-21719
HmHel-3	CAEZ01010534:33994-34663
HmHel-3	CAEZ01010534:57636-58045
HmHel-3	CAEZ01010534:79362-79758
HmHel-3	CAEZ01009379:22567-22980
HmHel-3	CAEZ01006322:6473-6888
HmHel-3	CAEZ01005996:8775-9195
HmHel-3	CAEZ01004464:6328-6746
HmHel-3	CAEZ01002706:113012-113421
HmHel-3	CAEZ01002706:59292-59709
HmHel-3	CAEZ01001159:9637-10047
HmHel-3	CAEZ01010405:6974-7058
HmHel-3	CAEZ01010405:9197-9566
HmHel-3	CAEZ01009099:22215-22629
HmHel-3	CAEZ01009099:5099-5422
HmHel-3	CAEZ01006759:82394-82819
HmHel-3	CAEZ01006501:43119-43602
HmHel-3	CAEZ01006501:24286-24609
HmHel-3	CAEZ01006308:29177-29564
HmHel-3	CAEZ01005775:9025-9402
HmHel-3	CAEZ01002307:118-523
HmHel-3	CAEZ01010877:1879-2218
HmHel-3	CAEZ01005845:12818-13230
HmHel-3	CAEZ01003718:27581-28028

HmHel-3	CAEZ01003718:23703-24116
HmHel-3	CAEZ01006534:43513-44208
HmHel-3	CAEZ01006534:86366-86682
HmHel-3	CAEZ01006534:120998-121412
HmHel-3	CAEZ01006534:49427-49822
HmHel-3	CAEZ01005855:7781-8194
HmHel-3	CAEZ01004300:20691-21074
HmHel-3	CAEZ01011241:7278-7665
HmHel-3	CAEZ01007832:7212-7602
HmHel-3	CAEZ01007475:72820-73117
HmHel-3	CAEZ01007475:686-1096
HmHel-3	CAEZ01004955:8373-8737
HmHel-3	CAEZ01004953:10562-10978
HmHel-3	CAEZ01009776:37501-37907
HmHel-3	CAEZ01005155:2748-3161
HmHel-3	CAEZ01005155:16060-16296
HmHel-3	CAEZ01004632:13127-13544
HmHel-3	CAEZ01004632:20524-20934
HmHel-3	CAEZ01011408:2756-3126
HmHel-3	CAEZ01011408:6654-6977
HmHel-3	CAEZ01011126:34395-34799
HmHel-3	CAEZ01010266:7458-7853
HmHel-3	CAEZ01007258:17769-18092
HmHel-3	CAEZ01002157:926-1286
HmHel-3	CAEZ01011361:5146-5600
HmHel-3	CAEZ01009846:1505-1807
HmHel-3	CAEZ01009846:8094-8482
HmHel-3	CAEZ01008386:1729-2140
HmHel-3	CAEZ01006030:4590-4931
HmHel-3	CAEZ01005463:1074-1453
HmHel-3	CAEZ01001351:11072-11504
HmHel-3	CAEZ01001351:4774-5170
HmHel-3	CAEZ01009584:2426-2822
HmHel-3	CAEZ01008084:2821-3234
HmHel-3	CAEZ01007567:3771-4188
HmHel-3	CAEZ01007059:819-1205
HmHel-3	CAEZ01006023:26650-27036
HmHel-3	CAEZ01006023:8957-9350
HmHel-3	CAEZ01003196:86335-86699
HmHel-3	CAEZ01003196:33976-34365
HmHel-3	CAEZ01000800:5041-5785
HmHel-3	CAEZ01010291:103-435
HmHel-3	CAEZ01010291:25754-26164
HmHel-3	CAEZ01002908:6192-6575
HmHel-3	CAEZ01008695:12009-12395
HmHel-3	CAEZ01007820:4669-5069
HmHel-3	CAEZ01004336:3177-3585
HmHel-3	CAEZ01001364:1397-1821

HmHel-3	CAEZ01008508:10831-11012
HmHel-3	CAEZ01008508:26694-27099
HmHel-3	CAEZ01008508:24331-24735
HmHel-3	CAEZ01007754:1178-2595
HmHel-3	CAEZ01006235:582-1287
HmHel-3	CAEZ01005503:8653-9035
HmHel-3	CAEZ01011184:6846-7269
HmHel-3	CAEZ01008928:23089-23504
HmHel-3	CAEZ01008928:7667-7909
HmHel-3	CAEZ01005923:20944-21178
HmHel-3	CAEZ01005923:19813-20206
HmHel-3	CAEZ01005923:1908-2234
HmHel-3	CAEZ01003855:20182-20592
HmHel-3	CAEZ01002947:14344-14763
HmHel-3	CAEZ01002836:1289-1680
HmHel-3	CAEZ01002765:62885-63420
HmHel-3	CAEZ01002765:8222-8620
HmHel-3	CAEZ01010560:4471-4877
HmHel-3	CAEZ01009894:53291-53677
HmHel-3	CAEZ01009894:25109-25442
HmHel-3	CAEZ01005710:1256-1594
HmHel-3	CAEZ01001936:4921-5317
HmHel-3	CAEZ01009976:4879-5351
HmHel-3	CAEZ01003363:9060-9478
HmHel-3	CAEZ01002815:96044-96125
HmHel-3	CAEZ01002815:181778-182163
HmHel-3	CAEZ01002815:192876-193958
HmHel-3	CAEZ01001062:32397-32566
HmHel-3	CAEZ01001062:51830-52258
HmHel-3	CAEZ01001062:46818-47132
HmHel-3	CAEZ01010243:63693-64097
HmHel-3	CAEZ01010243:34410-35129
HmHel-3	CAEZ01009887:169609-170288
HmHel-3	CAEZ01009887:183672-184059
HmHel-3	CAEZ01009887:42943-43291
HmHel-3	CAEZ01009887:19696-20373
HmHel-3	CAEZ01009085:59474-59870
HmHel-3	CAEZ01009085:71162-71579
HmHel-3	CAEZ01009085:111545-111840
HmHel-3	CAEZ01009085:99694-100098
HmHel-3	CAEZ01009085:50811-51047
HmHel-3	CAEZ01006839:15324-15709
HmHel-3	CAEZ01006351:4739-5125
HmHel-3	CAEZ01003403:6027-6414
HmHel-3	CAEZ01010161:2747-3136
HmHel-3	CAEZ01005228:35921-36333
HmHel-3	CAEZ01005228:53491-53810
HmHel-3	CAEZ01002255:42801-43173

HmHel-3	CAEZ01011501:15444-15782
HmHel-3	CAEZ01007235:37478-37862
HmHel-3	CAEZ01004112:9102-9521
HmHel-3	CAEZ01010228:30453-30855
HmHel-3	CAEZ01010202:2882-3411
HmHel-3	CAEZ01009455:6207-6591
HmHel-3	CAEZ01009455:26122-26358
HmHel-3	CAEZ01009455:18965-19343
HmHel-3	CAEZ01002629:41700-42085
HmHel-3	CAEZ01002629:32075-32748
HmHel-3	CAEZ01001208:2481-2890
HmHel-3	CAEZ01008399:12557-12967
HmHel-3	CAEZ01011626:118911-119198
HmHel-3	CAEZ01011626:15558-15991
HmHel-3	CAEZ01003739:1916-2252
HmHel-3	CAEZ01003038:8803-9201
HmHel-3	CAEZ01003038:17883-17967
HmHel-3	CAEZ01007271:4162-4522
HmHel-3	CAEZ01005848:2287-2636
HmHel-3	CAEZ01003031:5793-6170
HmHel-3	CAEZ01002635:50709-51054
HmHel-3	CAEZ01009495:27294-27698
HmHel-3	CAEZ01006669:11723-12047
HmHel-3	CAEZ01006669:9316-9689
HmHel-3	CAEZ01001843:9298-9685
HmHel-3	CAEZ01008977:7872-8260
HmHel-3	CAEZ01008428:2273-2655
HmHel-3	CAEZ01008358:29252-29658
HmHel-3	CAEZ01008358:46785-46869
HmHel-3	CAEZ01008358:14761-15080
HmHel-3	CAEZ01005235:252471-254885
HmHel-3	CAEZ01005235:134354-135057
HmHel-3	CAEZ01004930:18810-19223
HmHel-3	CAEZ01006286:104708-105086
HmHel-3	CAEZ01004605:5369-5770
HmHel-3	CAEZ01004605:666-902
HmHel-3	CAEZ01002808:182952-183302
HmHel-3	CAEZ01002792:10455-10863
HmHel-3	CAEZ01002792:25967-26298
HmHel-3	CAEZ01000746:439-856
HmHel-3	CAEZ01009585:31719-32091
HmHel-3	CAEZ01007598:229-646
HmHel-3	CAEZ01001768:1842-2154
HmHel-3	CAEZ01001022:32639-33165
HmHel-3	CAEZ01008263:46554-46871
HmHel-3	CAEZ01008263:28925-29985
HmHel-3	CAEZ01001042:3162-4264
HmHel-3	CAEZ01001042:11446-11682

HmHel-3	CAEZ01000850:21962-22367
HmHel-3	CAEZ01000850:85482-85823
HmHel-3	CAEZ01000850:82307-82646
HmHel-3	CAEZ01000850:19921-20278
HmHel-3	CAEZ01007222:144130-144480
HmHel-3	CAEZ01007222:208157-208385
HmHel-3	CAEZ01007222:279459-279764
HmHel-3	CAEZ01007222:222377-222788
HmHel-3	CAEZ01006773:110023-110259
HmHel-3	CAEZ01006773:125889-126647
HmHel-3	CAEZ01006773:77247-77603
HmHel-3	CAEZ01010541:169744-170131
HmHel-3	CAEZ01008128:556-907
HmHel-3	CAEZ01005771:9840-10234
HmHel-3	CAEZ01004371:1362-1757
HmHel-3	CAEZ01008425:83812-84483
HmHel-3	CAEZ01008425:107475-108140
HmHel-3	CAEZ01008425:59031-59419
HmHel-3	CAEZ01008182:55784-56378
HmHel-3	CAEZ01008182:43588-43903
HmHel-3	CAEZ01008182:8-699
HmHel-3	CAEZ01009042:38741-39165
HmHel-3	CAEZ01009042:41741-42098
HmHel-3	CAEZ01006018:4295-4661
HmHel-3	CAEZ01004617:13468-13847
HmHel-3	CAEZ01002736:17456-26535
HmHel-3	CAEZ01006161:56194-56588
HmHel-3	CAEZ01006161:90836-91466
HmHel-3	CAEZ01006161:106902-107289
HmHel-3	CAEZ01006161:75629-76013
HmHel-3	CAEZ01006161:53839-54243
HmHel-3	CAEZ01006028:6305-6613
HmHel-3	CAEZ01010181:50187-50575
HmHel-3	CAEZ01008686:3058-3366
HmHel-3	CAEZ01008686:1819-2175
HmHel-3	CAEZ01007736:11600-12028
HmHel-3	CAEZ01007736:18030-18349
HmHel-3	CAEZ01005830:14229-14546
HmHel-3	CAEZ01005830:159268-160245
HmHel-3	CAEZ01005231:91258-91654
HmHel-3	CAEZ01005231:8962-9176
HmHel-3	CAEZ01003526:3342-4068
HmHel-3	CAEZ01010963:4222-4629
HmHel-3	CAEZ01008818:18481-18877
HmHel-3	CAEZ01009592:11359-12959
HmHel-3	CAEZ01007218:10408-10786
HmHel-3	CAEZ01007077:3948-4339
HmHel-3	CAEZ01005391:5163-5543

HmHel-3	CAEZ01005250:23241-23572
HmHel-3	CAEZ01005250:90335-90655
HmHel-3	CAEZ01005225:40629-40921
HmHel-3	CAEZ01005225:25619-25985
HmHel-3	CAEZ01002755:47456-47606
HmHel-3	CAEZ01002755:46589-46901
HmHel-3	CAEZ01002723:25085-25471
HmHel-3	CAEZ01010301:7258-8586
HmHel-3	CAEZ01010301:37240-37482
HmHel-3	CAEZ01010301:15008-15415
HmHel-3	CAEZ01010757:10512-10923
HmHel-3	CAEZ01007706:31043-31327
HmHel-3	CAEZ01007706:45579-45953
HmHel-3	CAEZ01007706:139617-139926
HmHel-3	CAEZ01007706:159087-165448
HmHel-3	CAEZ01007706:84290-84371
HmHel-3	CAEZ01009417:35327-35741
HmHel-3	CAEZ01009417:30056-30471
HmHel-3	CAEZ01008281:14540-14872
HmHel-3	CAEZ01008082:9935-10178
HmHel-3	CAEZ01003173:42914-43295
HmHel-3	CAEZ01003173:62659-63275
HmHel-3	CAEZ01002066:13007-19816
HmHel-3	CAEZ01008067:11196-11574
HmHel-3	CAEZ01004762:13323-13727
HmHel-3	CAEZ01004762:29771-30490
HmHel-3	CAEZ01004762:11765-12309
HmHel-3	CAEZ01004385:13084-13386
HmHel-3	CAEZ01003896:4720-4970
HmHel-3	CAEZ01003896:14926-15163
HmHel-3	CAEZ01001124:625-933
HmHel-3	CAEZ01009507:42989-43189
HmHel-3	CAEZ01009507:44472-44801
HmHel-3	CAEZ01009507:20103-20525
HmHel-3	CAEZ01008371:60907-61231
HmHel-3	CAEZ01008371:37425-37734
HmHel-3	CAEZ01008371:11635-12052
HmHel-3	CAEZ01007273:52414-52730
HmHel-3	CAEZ01007273:94845-95234
HmHel-3	CAEZ01007273:157089-157462
HmHel-3	CAEZ01007273:132082-132790
HmHel-3	CAEZ01007273:55950-56662
HmHel-3	CAEZ01007273:45105-45793
HmHel-3	CAEZ01007273:26073-26813
HmHel-3	CAEZ01006126:17789-18164
HmHel-3	CAEZ01006126:23347-23860
HmHel-3	CAEZ01006126:9064-10699
HmHel-3	CAEZ01005161:16326-16732

HmHel-3	CAEZ01003175:4753-5137
HmHel-3	CAEZ01002127:4370-4694
HmHel-3	CAEZ01010190:21428-21817
HmHel-3	CAEZ01006973:7840-8247
HmHel-3	CAEZ01006413:15026-15341
HmHel-3	CAEZ01005414:39009-39329
HmHel-3	CAEZ01005414:39557-39882
HmHel-3	CAEZ01006529:178285-179685
HmHel-3	CAEZ01001828:8579-8814
HmHel-3	CAEZ01001828:6306-6648
HmHel-3	CAEZ01010869:73108-73499
HmHel-3	CAEZ01007715:1446-1831
HmHel-3	CAEZ01006450:2089-2484
HmHel-3	CAEZ01006199:877-1275
HmHel-3	CAEZ01003358:843-1239
HmHel-3	CAEZ01003195:25506-25822
HmHel-3	CAEZ01003195:56394-56479
HmHel-3	CAEZ01003195:96916-97309
HmHel-3	CAEZ01007636:2116-2514
HmHel-3	CAEZ01005584:2988-3427
HmHel-3	CAEZ01004081:44541-44854
HmHel-3	CAEZ01010810:32717-33128
HmHel-3	CAEZ01009631:15654-16068
HmHel-3	CAEZ01009631:87911-88278
HmHel-3	CAEZ01009631:42902-43239
HmHel-3	CAEZ01007538:8630-8852
HmHel-3	CAEZ01007538:15562-15881
HmHel-3	CAEZ01007538:4330-4746
HmHel-3	CAEZ01005744:5214-5464
HmHel-3	CAEZ01009772:48062-48477
HmHel-3	CAEZ01009432:5347-5722
HmHel-3	CAEZ01011519:12-730
HmHel-3	CAEZ01011519:58485-58724
HmHel-3	CAEZ01011519:165133-165369
HmHel-3	CAEZ01011519:168444-168749
HmHel-3	CAEZ01011519:103203-103519
HmHel-3	CAEZ01009996:12140-12843
HmHel-3	CAEZ01009996:33668-34076
HmHel-3	CAEZ01009996:57566-57961
HmHel-3	CAEZ01009996:83066-83463
HmHel-3	CAEZ01009996:73762-74094
HmHel-3	CAEZ01009996:49005-49393
HmHel-3	CAEZ01008413:48520-48942
HmHel-3	CAEZ01008266:5712-6130
HmHel-3	CAEZ01008266:22043-22346
HmHel-3	CAEZ01008266:1644-2362
HmHel-3	CAEZ01008229:2245-2595
HmHel-3	CAEZ01004905:7192-7324

HmHel-3	CAEZ01004905:48114-48487
HmHel-3	CAEZ01009081:23769-24097
HmHel-3	CAEZ01009081:45077-45459
HmHel-3	CAEZ01008271:1210-1608
HmHel-3	CAEZ01006566:84798-85217
HmHel-3	CAEZ01006566:14677-15026
HmHel-3	CAEZ01006244:10715-11015
HmHel-3	CAEZ01009242:17755-17994
HmHel-3	CAEZ01009242:65601-65989
HmHel-3	CAEZ01001793:40051-40455
HmHel-3	CAEZ01001793:9354-9768
HmHel-3	CAEZ01001466:57364-57675
HmHel-3	CAEZ01001466:103167-103469
HmHel-3	CAEZ01001466:54371-54738
HmHel-3	CAEZ01009723:15109-15829
HmHel-3	CAEZ01009723:15981-16389
HmHel-3	CAEZ01009533:8393-8704
HmHel-3	CAEZ01009533:9163-9564
HmHel-3	CAEZ01002711:10571-10883
HmHel-3	CAEZ01001417:4743-5168
HmHel-3	CAEZ01001417:30126-30531
HmHel-3	CAEZ01001417:22906-23024
HmHel-3	CAEZ01007716:4667-4941
HmHel-3	CAEZ01007705:8230-9058
HmHel-3	CAEZ01007705:85149-85555
HmHel-3	CAEZ01007565:4467-4861
HmHel-3	CAEZ01001218:4050-4441
HmHel-3	CAEZ01009243:2019-2247
HmHel-3	CAEZ01008427:64412-64729
HmHel-3	CAEZ01008427:50839-51266
HmHel-3	CAEZ01009896:30521-30953
HmHel-3	CAEZ01009896:146852-147572
HmHel-3	CAEZ01009383:7818-16704
HmHel-3	CAEZ01008270:67515-67751
HmHel-3	CAEZ01008270:95924-101749
HmHel-3	CAEZ01008270:63763-64074
HmHel-3	CAEZ01007327:7779-8143
HmHel-3	CAEZ01007967:6627-7019
HmHel-3	CAEZ01006957:1843-2285
HmHel-3	CAEZ01006664:14374-14627
HmHel-3	CAEZ01005942:4689-5103
HmHel-3	CAEZ01003414:14884-15200
HmHel-3	CAEZ01003414:36441-36845
HmHel-3	CAEZ01003414:14129-15245
HmHel-3	CAEZ01008518:28125-28475
HmHel-3	CAEZ01008372:139206-139354
HmHel-3	CAEZ01008372:229258-229944
HmHel-3	CAEZ01008372:267029-267348

HmHel-3	CAEZ01008372:286040-286362
HmHel-3	CAEZ01008372:159656-159991
HmHel-3	CAEZ01008372:18776-19092
HmHel-3	CAEZ01007442:8049-8406
HmHel-3	CAEZ01005732:20138-20427
HmHel-3	CAEZ01009767:8422-8779
HmHel-3	CAEZ01011373:14160-14565
HmHel-3	CAEZ01008721:1-646
HmHel-3	CAEZ01008721:36411-37489
HmHel-3	CAEZ01008721:113783-114187
HmHel-3	CAEZ01008721:36049-37696
HmHel-3	CAEZ01008721:20482-20683
HmHel-3	CAEZ01003642:4129-4534
HmHel-3	CAEZ01002416:66447-66862
HmHel-3	CAEZ01002416:61887-62121
HmHel-3	CAEZ01011155:2379-2721
HmHel-3	CAEZ01011155:20110-20530
HmHel-3	CAEZ01009440:61741-62137
HmHel-3	CAEZ01008178:6992-8090
HmHel-3	CAEZ01007073:6607-7010
HmHel-3	CAEZ01006560:2926-4781
HmHel-3	CAEZ01006560:68127-68443
HmHel-3	CAEZ01006417:54009-54325
HmHel-3	CAEZ01006121:129224-129635
HmHel-3	CAEZ01006121:226479-226851
HmHel-3	CAEZ01006121:240252-240637
HmHel-3	CAEZ01006121:162839-163272
HmHel-3	CAEZ01006121:127794-128194
HmHel-3	CAEZ01005251:10691-11007
HmHel-3	CAEZ01001775:192-604
HmHel-3	CAEZ01001775:32116-32431
HmHel-3	CAEZ01000849:40578-40965
HmHel-3	CAEZ01000849:71479-72850
HmHel-3	CAEZ01000849:19943-20348
HmHel-3	CAEZ01010270:13147-13433
HmHel-3	CAEZ01010270:276431-276746
HmHel-3	CAEZ01009137:107323-107647
HmHel-3	CAEZ01008400:78383-78700
HmHel-3	CAEZ01008400:46017-46339
HmHel-3	CAEZ01007680:1782-2097
HmHel-3	CAEZ01006836:12897-13279
HmHel-3	CAEZ01006152:9141-9449
HmHel-3	CAEZ01000897:4-319
HmHel-3	CAEZ01008956:668-967
HmHel-3	CAEZ01008524:18-267
HmHel-3	CAEZ01007204:13606-13788
HmHel-3	CAEZ01007204:45320-45556
HmHel-3	CAEZ01007204:63922-64137

HmHel-3	CAEZ01007204:51910-52692
HmHel-3	CAEZ01006294:2868-3250
HmHel-3	CAEZ01004247:11728-12000
HmHel-3	CAEZ01000846:18651-19759
HmHel-3	CAEZ01010494:3192-3578
HmHel-3	CAEZ01008833:5709-6108
HmHel-3	CAEZ01007723:9318-9681
HmHel-3	CAEZ01004878:5433-5750
HmHel-3	CAEZ01004793:87175-87477
HmHel-3	CAEZ01004793:30254-30622
HmHel-3	CAEZ01002132:1534-1946
HmHel-3	CAEZ01011300:5465-5781
HmHel-3	CAEZ01010933:274-697
HmHel-3	CAEZ01009682:1522-1921
HmHel-3	CAEZ01008356:29560-29893
HmHel-3	CAEZ01008356:59235-59580
HmHel-3	CAEZ01004938:31085-31419
HmHel-3	CAEZ01004811:170445-170770
HmHel-3	CAEZ01004811:358738-359150
HmHel-3	CAEZ01004811:77020-77319
HmHel-3	CAEZ01003881:4842-5212
HmHel-3	CAEZ01003228:2941-3353
HmHel-3	CAEZ01001951:1552-1961
HmHel-3	CAEZ01001416:1105-2071
HmHel-3	CAEZ01001043:1306-1718
HmHel-3	CAEZ01005975:96990-97293
HmHel-3	CAEZ01005975:163927-164675
HmHel-3	CAEZ01005975:99090-99506
HmHel-3	CAEZ01004975:6033-6703
HmHel-3	CAEZ01004975:44072-44387
HmHel-3	CAEZ01004942:3793-4187
HmHel-3	CAEZ01004942:89-325
HmHel-3	CAEZ01004920:28766-29118
HmHel-3	CAEZ01004920:30115-30194
HmHel-3	CAEZ01006698:62486-64701
HmHel-3	CAEZ01006548:3831-4932
HmHel-3	CAEZ01006548:95387-103716
HmHel-3	CAEZ01006548:14894-15472
HmHel-3	CAEZ01005939:14582-14973
HmHel-3	CAEZ01005939:10897-11257
HmHel-3	CAEZ01004815:19163-19477
HmHel-3	CAEZ01003959:12645-13011
HmHel-3	CAEZ01003762:6450-6805
HmHel-3	CAEZ01003762:16737-17442
HmHel-3	CAEZ01003762:5121-5500
HmHel-3	CAEZ01002039:17039-17439
HmHel-3	CAEZ01008824:25656-25873
HmHel-3	CAEZ01008824:35966-36368

HmHel-3	CAEZ01008213:5042-5462
HmHel-3	CAEZ01005210:3828-4202
HmHel-3	CAEZ01004843:7158-7550
HmHel-3	CAEZ01004843:27855-28270
HmHel-3	CAEZ01003954:35474-35791
HmHel-3	CAEZ01011529:29024-29441
HmHel-3	CAEZ01011529:50096-50409
HmHel-3	CAEZ01011529:35187-35478
HmHel-3	CAEZ01011529:22425-22723
HmHel-3	CAEZ01011283:5837-6153
HmHel-3	CAEZ01010119:7723-8120
HmHel-3	CAEZ01009023:90793-91206
HmHel-3	CAEZ01008104:11007-11336
HmHel-3	CAEZ01007950:9221-14749
HmHel-3	CAEZ01006423:223607-223910
HmHel-3	CAEZ01006423:331565-331977
HmHel-3	CAEZ01006423:78056-78385
HmHel-3	CAEZ01005499:28529-28941
HmHel-3	CAEZ01003404:22162-22470
HmHel-3	CAEZ01003404:58737-59133
HmHel-3	CAEZ01003404:20887-21219
HmHel-3	CAEZ01003229:82-493
HmHel-3	CAEZ01002890:14033-14376
HmHel-3	CAEZ01002811:23611-24022
HmHel-3	CAEZ01002811:167203-167515
HmHel-3	CAEZ01002811:150804-151171
HmHel-3	CAEZ01002811:75407-77436
HmHel-3	CAEZ01001465:3647-4045
HmHel-3	CAEZ01001465:96062-96359
HmHel-3	CAEZ01001465:81473-81886
HmHel-3	CAEZ01001075:835-1238
HmHel-3	CAEZ01011091:10001-10310
HmHel-3	CAEZ01010627:16157-16570
HmHel-3	CAEZ01009476:25507-25840
HmHel-3	CAEZ01009342:19421-19741
HmHel-3	CAEZ01007275:90123-90367
HmHel-3	CAEZ01007275:110809-111163
HmHel-3	CAEZ01007275:127864-127976
HmHel-3	CAEZ01007275:67321-67679
HmHel-3	CAEZ01007238:3997-4413
HmHel-3	CAEZ01007208:43584-45533
HmHel-3	CAEZ01006392:72767-73001
HmHel-3	CAEZ01006392:94899-95135
HmHel-3	CAEZ01006392:91668-92068
HmHel-3	CAEZ01006392:78462-78782
HmHel-3	CAEZ01003434:2971-3380
HmHel-3	CAEZ01003262:16910-17322
HmHel-3	CAEZ01008919:3440-3682

HmHel-3	CAEZ01008919:55560-55974
HmHel-3	CAEZ01002651:8743-9058
HmHel-3	CAEZ01010927:77153-77484
HmHel-3	CAEZ01010927:108141-109015
HmHel-3	CAEZ01010927:130882-131555
HmHel-3	CAEZ01010927:61321-61645
HmHel-3	CAEZ01009736:3125-4814
HmHel-3	CAEZ01009370:103949-104272
HmHel-3	CAEZ01009370:57535-57946
HmHel-3	CAEZ01009306:74-390
HmHel-3	CAEZ01009306:65382-65616
HmHel-3	CAEZ01009306:28431-28536
HmHel-3	CAEZ01006179:21570-21969
HmHel-3	CAEZ01003422:8419-11915
HmHel-3	CAEZ01003422:30812-31129
HmHel-3	CAEZ01001414:2125-2442
HmHel-3	CAEZ01010445:14-233
HmHel-3	CAEZ01010445:27639-28031
HmHel-3	CAEZ01008944:1657-11168
HmHel-3	CAEZ01008944:43034-43455
HmHel-3	CAEZ01007943:6010-6331
HmHel-3	CAEZ01007943:7994-8408
HmHel-3	CAEZ01005590:34161-34463
HmHel-3	CAEZ01001766:46230-46546
HmHel-3	CAEZ01001766:152974-153396
HmHel-3	CAEZ01001576:10583-10982
HmHel-3	CAEZ01000959:10326-11114
HmHel-3	CAEZ01011195:50593-50988
HmHel-3	CAEZ01010830:6625-6959
HmHel-3	CAEZ01010830:22778-23194
HmHel-3	CAEZ01009879:16397-17290
HmHel-3	CAEZ01008879:84348-84659
HmHel-3	CAEZ01008879:64298-64710
HmHel-3	CAEZ01004244:138491-138902
HmHel-3	CAEZ01004244:10811-11163
HmHel-3	CAEZ01003940:1738-2045
HmHel-3	CAEZ01003139:2683-3101
HmHel-3	CAEZ01010753:969-1381
HmHel-3	CAEZ01010753:2584-3297
HmHel-3	CAEZ01010527:19045-19281
HmHel-3	CAEZ01010527:27431-27843
HmHel-3	CAEZ01009514:1036-1451
HmHel-3	CAEZ01008843:4615-5025
HmHel-3	CAEZ01008746:196062-196387
HmHel-3	CAEZ01008746:114805-115129
HmHel-3	CAEZ01007495:26299-26711
HmHel-3	CAEZ01007495:35819-36227
HmHel-3	CAEZ01007035:881-1312

HmHel-3	CAEZ01006802:14368-15450
HmHel-3	CAEZ01006403:1722-2109
HmHel-3	CAEZ01006403:87217-88316
HmHel-3	CAEZ01006403:72949-73369
HmHel-3	CAEZ01006249:21451-21756
HmHel-3	CAEZ01006249:41780-42510
HmHel-3	CAEZ01006249:62301-62539
HmHel-3	CAEZ01011137:103068-103383
HmHel-3	CAEZ01011137:127553-127894
HmHel-3	CAEZ01011137:117132-117474
HmHel-3	CAEZ01011137:55850-56522
HmHel-3	CAEZ01011137:43398-43781
HmHel-3	CAEZ01009822:4227-7833
HmHel-3	CAEZ01009500:2193-2594
HmHel-3	CAEZ01009332:16425-17054
HmHel-3	CAEZ01009332:5958-6335
HmHel-3	CAEZ01006592:36889-37292
HmHel-3	CAEZ01006592:120207-120443
HmHel-3	CAEZ01006242:8481-8790
HmHel-3	CAEZ01006188:19753-20070
HmHel-3	CAEZ01004531:12637-12967
HmHel-3	CAEZ01003907:18537-18908
HmHel-3	CAEZ01001455:1415-1732
HmHel-3	CAEZ01001004:125079-125488
HmHel-3	CAEZ01001004:154303-154703
HmHel-3	CAEZ01001004:127653-127889
HmHel-3	CAEZ01001004:15532-16232
HmHel-3	CAEZ01011523:20501-21174
HmHel-3	CAEZ01011523:68492-68904
HmHel-3	CAEZ01011523:73787-74104
HmHel-3	CAEZ01011521:3048-3363
HmHel-3	CAEZ01009706:40526-40943
HmHel-3	CAEZ01009441:8816-9132
HmHel-3	CAEZ01007825:2264-2660
HmHel-3	CAEZ01005807:1060-1442
HmHel-3	CAEZ01000124:1587-2002
HmHel-3	CAEZ01011548:3554-3973
HmHel-3	CAEZ01008588:3002-3313
HmHel-3	CAEZ01008588:41097-41512
HmHel-3	CAEZ01008448:11407-11786
HmHel-3	CAEZ01008349:145213-145523
HmHel-3	CAEZ01008349:44784-45023
HmHel-3	CAEZ01008294:21510-21838
HmHel-3	CAEZ01008294:171252-171417
HmHel-3	CAEZ01008294:182190-182510
HmHel-3	CAEZ01008294:200298-200622
HmHel-3	CAEZ01008294:49863-50279
HmHel-3	CAEZ01007903:3211-3619

HmHel-3	CAEZ01007903:18342-18668
HmHel-3	CAEZ01006621:2329-4076
HmHel-3	CAEZ01006621:7670-7982
HmHel-3	CAEZ01006079:2937-3343
HmHel-3	CAEZ01005476:24988-25303
HmHel-3	CAEZ01004684:3661-3981
HmHel-3	CAEZ01004307:4160-4549
HmHel-3	CAEZ01002868:271-591
HmHel-3	CAEZ01011256:4615-7889
HmHel-3	CAEZ01010772:2911-3325
HmHel-3	CAEZ01010292:24179-24507
HmHel-3	CAEZ01010292:8113-8428
HmHel-3	CAEZ01009355:61168-66290
HmHel-3	CAEZ01009355:82657-82979
HmHel-3	CAEZ01008665:4929-5336
HmHel-3	CAEZ01008578:41113-41437
HmHel-3	CAEZ01008578:91540-92256
HmHel-3	CAEZ01005153:35634-35953
HmHel-3	CAEZ01005153:67308-67566
HmHel-3	CAEZ01005153:40701-41055
HmHel-3	CAEZ01003582:67851-68170
HmHel-3	CAEZ01003209:8978-10193
HmHel-3	CAEZ01002788:34422-34843
HmHel-3	CAEZ01002769:66739-67150
HmHel-3	CAEZ01001805:19411-19820
HmHel-3	CAEZ01001738:4521-4795
HmHel-3	CAEZ01001738:48160-48471
HmHel-3	CAEZ01011410:50172-50572
HmHel-3	CAEZ01011410:30148-30382
HmHel-3	CAEZ01009971:32145-32381
HmHel-3	CAEZ01009971:84497-84789
HmHel-3	CAEZ01009971:50016-50333
HmHel-3	CAEZ01009971:12395-12832
HmHel-3	CAEZ01009138:52606-53279
HmHel-3	CAEZ01009138:42144-42467
HmHel-3	CAEZ01008640:18360-18677
HmHel-3	CAEZ01008640:31050-31341
HmHel-3	CAEZ01008640:7893-8092
HmHel-3	CAEZ01008570:14362-14763
HmHel-3	CAEZ01008516:9944-10351
HmHel-3	CAEZ01007637:14038-14409
HmHel-3	CAEZ01007398:4091-4477
HmHel-3	CAEZ01003797:31730-32144
HmHel-3	CAEZ01002803:8658-9048
HmHel-3	CAEZ01001212:1262-1670
HmHel-3	CAEZ01010701:43249-43915
HmHel-3	CAEZ01010701:146955-147326
HmHel-3	CAEZ01010701:175033-175833

HmHel-3	CAEZ01010701:158027-158408
HmHel-3	CAEZ01010701:61792-62121
HmHel-3	CAEZ01010701:344-660
HmHel-3	CAEZ01010183:32144-32373
HmHel-3	CAEZ01008620:133313-134001
HmHel-3	CAEZ01008620:145551-145867
HmHel-3	CAEZ01008620:115339-115668
HmHel-3	CAEZ01008335:2753-3075
HmHel-3	CAEZ01007886:3245-3662
HmHel-3	CAEZ01006842:13958-14326
HmHel-3	CAEZ01005418:69517-69833
HmHel-3	CAEZ01005418:183383-183723
HmHel-3	CAEZ01005418:195113-195448
HmHel-3	CAEZ01005418:260544-261274
HmHel-3	CAEZ01005418:289328-289703
HmHel-3	CAEZ01005418:294103-294190
HmHel-3	CAEZ01005418:274266-274354
HmHel-3	CAEZ01005418:246076-246398
HmHel-3	CAEZ01003713:6691-7018
HmHel-3	CAEZ01003233:9819-10227
HmHel-3	CAEZ01001265:95015-95336
HmHel-3	CAEZ01001265:125035-125359
HmHel-3	CAEZ01001265:62604-63030
HmHel-3	CAEZ01001265:35846-36190
HmHel-3	CAEZ01010461:8324-8644
HmHel-3	CAEZ01010461:22647-23021
HmHel-3	CAEZ01009395:695-1101
HmHel-3	CAEZ01007753:584-908
HmHel-3	CAEZ01007753:31199-36851
HmHel-3	CAEZ01007629:3215-3624
HmHel-3	CAEZ01007573:6274-6662
HmHel-3	CAEZ01005827:22072-22488
HmHel-3	CAEZ01005827:35265-35801
HmHel-3	CAEZ01005281:7762-8167
HmHel-3	CAEZ01004661:20102-20506
HmHel-3	CAEZ01003852:25755-26168
HmHel-3	CAEZ01003077:12691-12789
HmHel-3	CAEZ01003077:17153-17568
HmHel-3	CAEZ01002612:12926-13250
HmHel-3	CAEZ01002182:78979-79377
HmHel-3	CAEZ01002182:99665-100064
HmHel-3	CAEZ01002182:2433-2668
HmHel-3	CAEZ01001850:25869-26270
HmHel-3	CAEZ01000080:8523-8939
HmHel-3	CAEZ01010682:1329-1752
HmHel-3	CAEZ01010659:7413-7825
HmHel-3	CAEZ01010619:46148-46462
HmHel-3	CAEZ01009813:78533-78953

HmHel-3	CAEZ01007995:5754-7291
HmHel-3	CAEZ01007192:2272-2571
HmHel-3	CAEZ01007192:6701-7111
HmHel-3	CAEZ01006866:13283-13676
HmHel-3	CAEZ01006866:8380-11576
HmHel-3	CAEZ01006426:9561-9811
HmHel-3	CAEZ01006130:54556-54782
HmHel-3	CAEZ01006130:179204-179489
HmHel-3	CAEZ01006130:155941-156274
HmHel-3	CAEZ01006130:31203-31531
HmHel-3	CAEZ01004819:30630-31042
HmHel-3	CAEZ01004249:17310-17718
HmHel-3	CAEZ01002670:11462-11884
HmHel-3	CAEZ01002602:18079-18412
HmHel-3	CAEZ01010857:3077-3457
HmHel-3	CAEZ01008298:4373-4609
HmHel-3	CAEZ01008298:4031-4820
HmHel-3	CAEZ01008012:12338-12655
HmHel-3	CAEZ01007746:123636-123953
HmHel-3	CAEZ01006847:89304-89538
HmHel-3	CAEZ01006847:111878-112285
HmHel-3	CAEZ01005933:37105-37422
HmHel-3	CAEZ01003555:193-594
HmHel-3	CAEZ01003332:3524-3842
HmHel-3	CAEZ01002183:8845-9162
HmHel-3	CAEZ01002113:1971-2389
HmHel-3	CAEZ01011605:51813-52147
HmHel-3	CAEZ01010918:74811-75120
HmHel-3	CAEZ01010918:91230-91938
HmHel-3	CAEZ01010918:73814-74135
HmHel-3	CAEZ01010918:40222-40585
HmHel-3	CAEZ01010511:25809-26912
HmHel-3	CAEZ01010453:101014-101372
HmHel-3	CAEZ01010453:59631-60043
HmHel-3	CAEZ01010288:167169-167579
HmHel-3	CAEZ01010288:289749-290087
HmHel-3	CAEZ01010288:179827-180243
HmHel-3	CAEZ01010288:165605-166278
HmHel-3	CAEZ01010288:121444-121675
HmHel-3	CAEZ01010257:1204-1574
HmHel-3	CAEZ01009903:325-646
HmHel-3	CAEZ01009891:79250-79662
HmHel-3	CAEZ01009891:28282-28607
HmHel-3	CAEZ01008604:3491-3812
HmHel-3	CAEZ01008324:3665-3986
HmHel-3	CAEZ01007597:13818-14230
HmHel-3	CAEZ01007419:992-1383
HmHel-3	CAEZ01007009:18363-18599

HmHel-3	CAEZ01007009:17312-17726
HmHel-3	CAEZ01007001:11381-11793
HmHel-3	CAEZ01006945:923-1308
HmHel-3	CAEZ01006747:6601-8379
HmHel-3	CAEZ01005981:10811-11162
HmHel-3	CAEZ01005981:4698-5110
HmHel-3	CAEZ01005015:8488-8724
HmHel-3	CAEZ01005015:20646-20916
HmHel-3	CAEZ01005015:132151-132387
HmHel-3	CAEZ01005015:238550-238962
HmHel-3	CAEZ01005015:235012-235427
HmHel-3	CAEZ01005015:219060-219693
HmHel-3	CAEZ01005015:145420-146091
HmHel-3	CAEZ01003484:3-249
HmHel-3	CAEZ01002963:31336-31751
HmHel-3	CAEZ01002701:805-1196
HmHel-3	CAEZ01002701:1942-2218
HmHel-3	CAEZ01011507:11456-11772
HmHel-3	CAEZ01011463:6096-6196
HmHel-3	CAEZ01011463:17771-18179
HmHel-3	CAEZ01010858:28434-28806
HmHel-3	CAEZ01010803:62642-62986
HmHel-3	CAEZ01010803:60790-61106
HmHel-3	CAEZ01010801:6834-7255
HmHel-3	CAEZ01010801:24745-25470
HmHel-3	CAEZ01008643:29821-30133
HmHel-3	CAEZ01008587:654-1061
HmHel-3	CAEZ01008264:6552-6952
HmHel-3	CAEZ01007678:41647-42063
HmHel-3	CAEZ01006820:6638-6958
HmHel-3	CAEZ01006218:43024-43350
HmHel-3	CAEZ01005400:15056-15590
HmHel-3	CAEZ01005400:4895-5215
HmHel-3	CAEZ01005393:8280-8534
HmHel-3	CAEZ01005393:96954-97364
HmHel-3	CAEZ01005102:3568-3876
HmHel-3	CAEZ01003794:2397-7293
HmHel-3	CAEZ01003211:14149-14463
HmHel-3	CAEZ01002363:57420-57820
HmHel-3	CAEZ01002363:50038-50430
HmHel-3	CAEZ01002001:18121-18441
HmHel-3	CAEZ01001021:11374-11782
HmHel-3	CAEZ01001009:1876-2192
HmHel-3	CAEZ01000878:106-503
HmHel-3	CAEZ01000878:36648-36973
HmHel-3	CAEZ01000878:84037-84676
HmHel-3	CAEZ01000878:29952-30303
HmHel-3	CAEZ01010189:14347-14762

HmHel-3	CAEZ01009328:2724-3043
HmHel-3	CAEZ01009328:893-981
HmHel-3	CAEZ01008771:96173-98016
HmHel-3	CAEZ01008771:165924-166301
HmHel-3	CAEZ01008771:118596-118916
HmHel-3	CAEZ01008771:96725-97344
HmHel-3	CAEZ01008771:29893-29976
HmHel-3	CAEZ01008771:15424-15658
HmHel-3	CAEZ01008560:4905-5325
HmHel-3	CAEZ01006542:67969-68205
HmHel-3	CAEZ01006542:119293-119937
HmHel-3	CAEZ01006542:232498-233167
HmHel-3	CAEZ01006542:226031-226350
HmHel-3	CAEZ01005743:15094-15494
HmHel-3	CAEZ01005743:51609-51986
HmHel-3	CAEZ01005743:71662-72054
HmHel-3	CAEZ01005743:48739-49430
HmHel-3	CAEZ01005279:65292-65616
HmHel-3	CAEZ01005279:49947-50636
HmHel-3	CAEZ01005279:26492-27165
HmHel-3	CAEZ01004596:14806-15125
HmHel-3	CAEZ01004370:75109-75428
HmHel-3	CAEZ01003610:17392-17807
HmHel-3	CAEZ01003558:1-394
HmHel-3	CAEZ01003558:34625-34964
HmHel-3	CAEZ01003558:407-725
HmHel-3	CAEZ01003194:43975-44299
HmHel-3	CAEZ01003194:60884-61201
HmHel-3	CAEZ01003194:15207-15301
HmHel-3	CAEZ01003194:2966-3603
HmHel-3	CAEZ01001251:1269-1629
HmHel-3	CAEZ01001115:22401-22774
HmHel-3	CAEZ01009844:174485-175177
HmHel-3	CAEZ01009844:185432-185840
HmHel-3	CAEZ01009844:166076-166750
HmHel-3	CAEZ01009844:26559-26954
HmHel-3	CAEZ01009470:30170-30258
HmHel-3	CAEZ01009470:68725-69050
HmHel-3	CAEZ01009470:8900-9319
HmHel-3	CAEZ01008614:8810-9228
HmHel-3	CAEZ01008614:40362-40598
HmHel-3	CAEZ01008614:24362-24694
HmHel-3	CAEZ01008022:11103-11500
HmHel-3	CAEZ01006991:15700-15982
HmHel-3	CAEZ01004008:64698-65068
HmHel-3	CAEZ01011525:32893-33214
HmHel-3	CAEZ01011466:404-755
HmHel-3	CAEZ01010788:4625-4946

HmHel-3	CAEZ01009714:11990-12338
HmHel-3	CAEZ01008610:7777-8186
HmHel-3	CAEZ01008610:4425-4841
HmHel-3	CAEZ01007874:2967-3283
HmHel-3	CAEZ01007812:1346-1582
HmHel-3	CAEZ01007812:199731-200141
HmHel-3	CAEZ01007812:179656-179973
HmHel-3	CAEZ01007212:1628-1949
HmHel-3	CAEZ01005927:14471-14885
HmHel-3	CAEZ01005456:6021-6353
HmHel-3	CAEZ01004835:5441-5759
HmHel-3	CAEZ01004542:3683-4004
HmHel-3	CAEZ01004542:3304-3420
HmHel-3	CAEZ01004137:11152-11501
HmHel-3	CAEZ01004137:15873-16214
HmHel-3	CAEZ01003301:18729-19057
HmHel-3	CAEZ01003301:9763-10219
HmHel-3	CAEZ01002798:23124-23536
HmHel-3	CAEZ01002798:34583-34911
HmHel-3	CAEZ01002275:1377-1775
HmHel-3	CAEZ01002274:12061-12456
HmHel-3	CAEZ01001077:558-916
HmHel-3	CAEZ01000035:2378-2787
HmHel-3	CAEZ01011193:81299-81715
HmHel-3	CAEZ01011193:99579-99919
HmHel-3	CAEZ01010971:56525-56933
HmHel-3	CAEZ01010489:8670-9077
HmHel-3	CAEZ01010489:29665-30119
HmHel-3	CAEZ01010342:11151-11567
HmHel-3	CAEZ01009765:11184-11504
HmHel-3	CAEZ01008796:15333-15650
HmHel-3	CAEZ01008585:12585-12821
HmHel-3	CAEZ01008585:3077-3373
HmHel-3	CAEZ01008390:54779-54987
HmHel-3	CAEZ01008341:56341-56661
HmHel-3	CAEZ01007748:3377-3637
HmHel-3	CAEZ01007480:16626-17042
HmHel-3	CAEZ01006270:2573-2893
HmHel-3	CAEZ01004810:2984-3304
HmHel-3	CAEZ01002074:6304-7012
HmHel-3	CAEZ01002048:20865-21186
HmHel-3	CAEZ01010139:81313-81624
HmHel-3	CAEZ01008278:8012-8414
HmHel-3	CAEZ01008146:1161-1568
HmHel-3	CAEZ01007811:3260-3595
HmHel-3	CAEZ01007802:46259-46653
HmHel-3	CAEZ01007752:42220-42882
HmHel-3	CAEZ01007752:102438-102845

HmHel-3	CAEZ01007416:5189-5560
HmHel-3	CAEZ01007392:12407-12722
HmHel-3	CAEZ01006276:8155-8542
HmHel-3	CAEZ01006276:19510-19740
HmHel-3	CAEZ01005439:100708-101397
HmHel-3	CAEZ01005439:175132-175451
HmHel-3	CAEZ01005075:586-975
HmHel-3	CAEZ01004648:537-781
HmHel-3	CAEZ01004648:15123-15331
HmHel-3	CAEZ01003828:58905-59305
HmHel-3	CAEZ01003828:43545-43850
HmHel-3	CAEZ01003828:241-329
HmHel-3	CAEZ01003705:3858-4177
HmHel-3	CAEZ01003646:4102-4431
HmHel-3	CAEZ01003646:13786-14082
HmHel-3	CAEZ01002184:22666-23599
HmHel-3	CAEZ01002184:21587-22220
HmHel-3	CAEZ01002184:7596-7993
HmHel-3	CAEZ01001824:4895-5219
HmHel-3	CAEZ01000835:13866-14274
HmHel-3	CAEZ01009993:14303-14682
HmHel-3	CAEZ01009589:4082-4488
HmHel-3	CAEZ01009589:592-912
HmHel-3	CAEZ01008901:2272-2611
HmHel-3	CAEZ01008846:7952-8369
HmHel-3	CAEZ01008461:1824-2225
HmHel-3	CAEZ01008233:10216-10614
HmHel-3	CAEZ01008233:91265-91507
HmHel-3	CAEZ01008233:20253-20489
HmHel-3	CAEZ01007749:21153-21450
HmHel-3	CAEZ01007749:83691-84107
HmHel-3	CAEZ01007749:90582-91329
HmHel-3	CAEZ01006992:15087-15434
HmHel-3	CAEZ01006992:1914-2132
HmHel-3	CAEZ01006778:67211-67782
HmHel-3	CAEZ01006778:24966-25289
HmHel-3	CAEZ01006569:9362-9732
HmHel-3	CAEZ01006530:76607-77237
HmHel-3	CAEZ01006530:262476-262805
HmHel-3	CAEZ01006158:1788-2203
HmHel-3	CAEZ01005576:18141-18554
HmHel-3	CAEZ01005025:151592-151923
HmHel-3	CAEZ01005025:111980-112389
HmHel-3	CAEZ01002586:367-760
HmHel-3	CAEZ01001838:4660-4947
HmHel-3	CAEZ01011475:36983-37304
HmHel-3	CAEZ01011083:9-233
HmHel-3	CAEZ01011083:75510-75823

HmHel-3	CAEZ01011083:187223-187891
HmHel-3	CAEZ01009965:14234-14482
HmHel-3	CAEZ01009872:123225-123546
HmHel-3	CAEZ01009541:4247-4568
HmHel-3	CAEZ01008728:37040-37384
HmHel-3	CAEZ01008710:63219-63551
HmHel-3	CAEZ01008710:8-292
HmHel-3	CAEZ01007434:12961-13282
HmHel-3	CAEZ01006702:15235-15517
HmHel-3	CAEZ01006702:3196-3528
HmHel-3	CAEZ01006659:8190-8599
HmHel-3	CAEZ01006365:14893-15221
HmHel-3	CAEZ01006148:10148-10447
HmHel-3	CAEZ01005834:19865-20241
HmHel-3	CAEZ01005152:10329-10768
HmHel-3	CAEZ01005152:71859-72256
HmHel-3	CAEZ01005152:33727-34305
HmHel-3	CAEZ01004929:2302-2535
HmHel-3	CAEZ01004634:2378-2795
HmHel-3	CAEZ01003825:1963-2272
HmHel-3	CAEZ01002286:43504-43840
HmHel-3	CAEZ01010886:11692-12019
HmHel-3	CAEZ01010886:21997-22142
HmHel-3	CAEZ01010428:16541-16823
HmHel-3	CAEZ01010428:42056-44577
HmHel-3	CAEZ01010428:105951-106286
HmHel-3	CAEZ01010421:9025-9436
HmHel-3	CAEZ01010269:5857-6182
HmHel-3	CAEZ01010212:30370-30694
HmHel-3	CAEZ01007858:169064-169479
HmHel-3	CAEZ01007478:79595-79990
HmHel-3	CAEZ01007478:107812-109164
HmHel-3	CAEZ01007201:44332-44743
HmHel-3	CAEZ01007201:94516-94909
HmHel-3	CAEZ01007201:9420-10092
HmHel-3	CAEZ01006061:9562-9898
HmHel-3	CAEZ01005982:8111-8527
HmHel-3	CAEZ01002501:37920-38255
HmHel-3	CAEZ01000071:7442-7860
HmHel-3	CAEZ01011498:1-203
HmHel-3	CAEZ01011495:37753-38096
HmHel-3	CAEZ01011411:10576-10982
HmHel-3	CAEZ01010864:28617-28971
HmHel-3	CAEZ01010864:113608-114294
HmHel-3	CAEZ01010864:66296-66547
HmHel-3	CAEZ01007816:15659-16048
HmHel-3	CAEZ01004883:1136-1509
HmHel-3	CAEZ01004741:53509-53835

HmHel-3	CAEZ01004741:352-1366
HmHel-3	CAEZ01004215:5797-6196
HmHel-3	CAEZ01001759:5413-5814
HmHel-3	CAEZ01010309:27269-27667
HmHel-3	CAEZ01010026:231122-231358
HmHel-3	CAEZ01010026:40811-49699
HmHel-3	CAEZ01010015:11728-12139
HmHel-3	CAEZ01008589:2618-3008
HmHel-3	CAEZ01008589:14330-14739
HmHel-3	CAEZ01007676:146260-146671
HmHel-3	CAEZ01007676:29407-30174
HmHel-3	CAEZ01006525:2321-2715
HmHel-3	CAEZ01004587:3991-4375
HmHel-3	CAEZ01004587:19977-20380
HmHel-3	CAEZ01004570:5522-5905
HmHel-3	CAEZ01002814:5905-6327
HmHel-3	CAEZ01002323:39208-39531
HmHel-3	CAEZ01001450:4663-5068
HmHel-3	CAEZ01000760:231-465
HmHel-3	CAEZ01011013:8635-8956
HmHel-3	CAEZ01010358:5018-5340
HmHel-3	CAEZ01009888:28819-29488
HmHel-3	CAEZ01009888:83431-83667
HmHel-3	CAEZ01009888:5251-5579
HmHel-3	CAEZ01008639:6877-7198
HmHel-3	CAEZ01008412:7761-8165
HmHel-3	CAEZ01007975:82315-82740
HmHel-3	CAEZ01007975:71558-72126
HmHel-3	CAEZ01007940:25383-25793
HmHel-3	CAEZ01007940:616-1289
HmHel-3	CAEZ01007498:9214-9543
HmHel-3	CAEZ01007498:51190-51338
HmHel-3	CAEZ01007498:15966-16287
HmHel-3	CAEZ01006781:24991-26634
HmHel-3	CAEZ01006666:48280-48516
HmHel-3	CAEZ01006666:44343-44671
HmHel-3	CAEZ01006602:10045-10465
HmHel-3	CAEZ01006602:26846-27254
HmHel-3	CAEZ01006236:24070-24385
HmHel-3	CAEZ01005611:2134-2528
HmHel-3	CAEZ01005045:33616-34028
HmHel-3	CAEZ01004814:71-399
HmHel-3	CAEZ01004814:50268-50504
HmHel-3	CAEZ01004814:1146-1278
HmHel-3	CAEZ01004513:21106-21432
HmHel-3	CAEZ01003948:1242-1563
HmHel-3	CAEZ01003673:8064-10115
HmHel-3	CAEZ01002875:50255-50979

HmHel-3	CAEZ01002875:258576-258904
HmHel-3	CAEZ01002875:110372-110759
HmHel-3	CAEZ01002650:44158-44605
HmHel-3	CAEZ01002643:33970-34291
HmHel-3	CAEZ01011058:11408-11785
HmHel-3	CAEZ01011046:128214-128887
HmHel-3	CAEZ01011046:178668-178993
HmHel-3	CAEZ01010351:20712-21017
HmHel-3	CAEZ01010351:62079-62311
HmHel-3	CAEZ01009898:25279-25604
HmHel-3	CAEZ01009898:4549-4752
HmHel-3	CAEZ01009890:4666-4987
HmHel-3	CAEZ01009793:1665-1985
HmHel-3	CAEZ01009742:22873-23285
HmHel-3	CAEZ01009742:2268-2508
HmHel-3	CAEZ01008784:1397-1722
HmHel-3	CAEZ01008305:101356-101668
HmHel-3	CAEZ01008028:8711-9781
HmHel-3	CAEZ01007599:29497-29828
HmHel-3	CAEZ01006004:52587-52929
HmHel-3	CAEZ01002950:32811-33206
HmHel-3	CAEZ01002950:15174-15511
HmHel-3	CAEZ01001248:462-782
HmHel-3	CAEZ01000921:15990-16568
HmHel-3	CAEZ01000921:35257-35664
HmHel-3	CAEZ01011266:2359-2743
HmHel-3	CAEZ01010250:14046-14374
HmHel-3	CAEZ01010250:34046-34367
HmHel-3	CAEZ01008737:24878-25248
HmHel-3	CAEZ01008737:67822-68478
HmHel-3	CAEZ01007777:49644-49732
HmHel-3	CAEZ01007777:36182-36591
HmHel-3	CAEZ01007777:11976-12538
HmHel-3	CAEZ01007765:5168-5521
HmHel-3	CAEZ01007765:112458-112694
HmHel-3	CAEZ01007765:409161-409847
HmHel-3	CAEZ01007765:493906-494579
HmHel-3	CAEZ01007765:384521-384849
HmHel-3	CAEZ01007765:202745-202960
HmHel-3	CAEZ01007129:4828-5241
HmHel-3	CAEZ01006531:1230-1550
HmHel-3	CAEZ01006129:1664-3125
HmHel-3	CAEZ01006129:4289-4613
HmHel-3	CAEZ01005840:3500-3909
HmHel-3	CAEZ01005840:7808-8454
HmHel-3	CAEZ01005504:424-843
HmHel-3	CAEZ01005421:29212-29984
HmHel-3	CAEZ01004285:29235-29638

HmHel-3	CAEZ01004174:12196-12520
HmHel-3	CAEZ01004174:153-451
HmHel-3	CAEZ01004033:65473-65709
HmHel-3	CAEZ01004033:24465-24831
HmHel-3	CAEZ01003904:1781-2161
HmHel-3	CAEZ01002779:25187-25423
HmHel-3	CAEZ01002779:80643-81055
HmHel-3	CAEZ01002687:1855-2224
HmHel-3	CAEZ01001773:3360-3713
HmHel-3	CAEZ01000062:18996-19320
HmHel-3	CAEZ01010591:15108-15493
HmHel-3	CAEZ01010446:42095-42467
HmHel-3	CAEZ01010437:72183-72508
HmHel-3	CAEZ01010437:45440-45749
HmHel-3	CAEZ01010423:20437-20846
HmHel-3	CAEZ01010320:34046-34632
HmHel-3	CAEZ01010320:73855-74538
HmHel-3	CAEZ01010320:43680-43916
HmHel-3	CAEZ01010320:25188-25515
HmHel-3	CAEZ01008922:3293-3622
HmHel-3	CAEZ01008500:108484-108821
HmHel-3	CAEZ01008500:49791-50028
HmHel-3	CAEZ01007759:4941-5269
HmHel-3	CAEZ01007665:118767-119181
HmHel-3	CAEZ01007665:81076-81443
HmHel-3	CAEZ01006738:137-543
HmHel-3	CAEZ01006738:13016-13291
HmHel-3	CAEZ01006067:8744-9137
HmHel-3	CAEZ01005704:8789-9152
HmHel-3	CAEZ01005704:10185-10580
HmHel-3	CAEZ01004026:316-665
HmHel-3	CAEZ01000063:13014-13427
HmHel-3	CAEZ01000063:56121-56209
HmHel-3	CAEZ01000063:4516-5735
HmHel-3	CAEZ01010275:49967-50051
HmHel-3	CAEZ01010275:24694-25059
HmHel-3	CAEZ01009435:46088-46371
HmHel-3	CAEZ01009435:68518-74299
HmHel-3	CAEZ01009435:15911-16310
HmHel-3	CAEZ01009403:46519-46832
HmHel-3	CAEZ01009403:39582-39903
HmHel-3	CAEZ01008961:50092-51882
HmHel-3	CAEZ01008961:28030-28276
HmHel-3	CAEZ01008502:3651-3977
HmHel-3	CAEZ01008125:187967-188291
HmHel-3	CAEZ01008125:44167-44495
HmHel-3	CAEZ01007942:1-358
HmHel-3	CAEZ01007226:1722-2130

HmHel-3	CAEZ01006422:36721-37102
HmHel-3	CAEZ01006422:80467-80766
HmHel-3	CAEZ01006422:42611-42955
HmHel-3	CAEZ01006422:15285-15521
HmHel-3	CAEZ01006073:5150-5476
HmHel-3	CAEZ01006009:7770-8182
HmHel-3	CAEZ01005857:3277-3557
HmHel-3	CAEZ01005717:44575-44994
HmHel-3	CAEZ01005475:1129-1429
HmHel-3	CAEZ01005344:5150-5491
HmHel-3	CAEZ01004699:1468-1738
HmHel-3	CAEZ01002742:85626-85962
HmHel-3	CAEZ01002369:50296-51327
HmHel-3	CAEZ01010724:1488-1906
HmHel-3	CAEZ01010724:25206-25925
HmHel-3	CAEZ01010424:74184-74503
HmHel-3	CAEZ01010424:178852-179181
HmHel-3	CAEZ01010424:112719-113119
HmHel-3	CAEZ01010424:101132-101537
HmHel-3	CAEZ01009482:7014-7452
HmHel-3	CAEZ01008705:10718-11121
HmHel-3	CAEZ01008514:25429-25745
HmHel-3	CAEZ01008514:311-983
HmHel-3	CAEZ01007939:66077-66772
HmHel-3	CAEZ01007939:110924-111337
HmHel-3	CAEZ01007939:127825-128150
HmHel-3	CAEZ01007939:304941-305173
HmHel-3	CAEZ01007939:200479-200718
HmHel-3	CAEZ01006911:7597-7914
HmHel-3	CAEZ01006591:32959-33292
HmHel-3	CAEZ01006591:5916-6241
HmHel-3	CAEZ01005909:28171-28562
HmHel-3	CAEZ01005909:40429-40749
HmHel-3	CAEZ01005166:356-604
HmHel-3	CAEZ01005166:10558-10864
HmHel-3	CAEZ01005053:13694-14002
HmHel-3	CAEZ01004708:1124-1424
HmHel-3	CAEZ01004708:17887-18310
HmHel-3	CAEZ01004597:5494-5819
HmHel-3	CAEZ01003280:469-804
HmHel-3	CAEZ01003177:2986-3375
HmHel-3	CAEZ01003023:1014-1413
HmHel-3	CAEZ01002987:4071-4487
HmHel-3	CAEZ01001435:122616-123007
HmHel-3	CAEZ01001435:10685-10993
HmHel-3	CAEZ01001051:790-1134
HmHel-3	CAEZ01000920:9435-9747
HmHel-3	CAEZ01009269:59097-59491

HmHel-3	CAEZ01008950:12525-12853
HmHel-3	CAEZ01006427:289-708
HmHel-3	CAEZ01006382:4054-4425
HmHel-3	CAEZ01006198:141-384
HmHel-3	CAEZ01005215:8358-8757
HmHel-3	CAEZ01004722:6332-6753
HmHel-3	CAEZ01004185:24971-25378
HmHel-3	CAEZ01003664:14886-15271
HmHel-3	CAEZ01002954:4573-4896
HmHel-3	CAEZ01001375:2473-2872
HmHel-3	CAEZ01000578:70-394
HmHel-3	CAEZ01011171:367-684
HmHel-3	CAEZ01011032:24537-24911
HmHel-3	CAEZ01010605:4078-4411
HmHel-3	CAEZ01010116:2229-2582
HmHel-3	CAEZ01009741:1022-1328
HmHel-3	CAEZ01009513:6322-6649
HmHel-3	CAEZ01008231:917-1564
HmHel-3	CAEZ01008231:37900-38299
HmHel-3	CAEZ01008231:26494-26582
HmHel-3	CAEZ01006660:12199-15181
HmHel-3	CAEZ01006196:24072-24308
HmHel-3	CAEZ01006196:59679-62950
HmHel-3	CAEZ01006196:233963-234292
HmHel-3	CAEZ01004936:5848-6240
HmHel-3	CAEZ01004128:9589-9997
HmHel-3	CAEZ01002901:9184-9598
HmHel-3	CAEZ01002709:1622-1915
HmHel-3	CAEZ01001981:38848-39158
HmHel-3	CAEZ01001801:13485-13814
HmHel-3	CAEZ01001747:10331-10740
HmHel-3	CAEZ01001205:8606-9012
HmHel-3	CAEZ01000065:24232-24572
HmHel-3	CAEZ01000049:7501-7908
HmHel-3	CAEZ01011511:142-455
HmHel-3	CAEZ01011242:12845-13189
HmHel-3	CAEZ01010792:12536-12861
HmHel-3	CAEZ01010584:204-427
HmHel-3	CAEZ01010584:91802-92033
HmHel-3	CAEZ01010584:178442-178751
HmHel-3	CAEZ01010584:287895-288224
HmHel-3	CAEZ01010584:47417-48004
HmHel-3	CAEZ01010584:23466-23824
HmHel-3	CAEZ01009905:604-925
HmHel-3	CAEZ01009826:25419-25740
HmHel-3	CAEZ01008701:2403-2754
HmHel-3	CAEZ01008405:327-663
HmHel-3	CAEZ01008405:19392-19713

HmHel-3	CAEZ01007857:14607-14994
HmHel-3	CAEZ01007713:233-561
HmHel-3	CAEZ01007713:32214-32495
HmHel-3	CAEZ01007061:23591-23882
HmHel-3	CAEZ01006549:71-396
HmHel-3	CAEZ01005485:56549-56870
HmHel-3	CAEZ01005361:9336-9657
HmHel-3	CAEZ01005361:28865-29202
HmHel-3	CAEZ01005196:40778-41206
HmHel-3	CAEZ01004584:20493-20820
HmHel-3	CAEZ01009302:583-912
HmHel-3	CAEZ01009159:4321-4736
HmHel-3	CAEZ01008433:17111-17438
HmHel-3	CAEZ01007519:1265-1676
HmHel-3	CAEZ01006618:63216-63890
HmHel-3	CAEZ01006618:148370-148695
HmHel-3	CAEZ01006434:15871-16242
HmHel-3	CAEZ01006177:204604-204998
HmHel-3	CAEZ01006177:125878-126290
HmHel-3	CAEZ01005491:10482-10813
HmHel-3	CAEZ01005013:41599-41915
HmHel-3	CAEZ01004934:22641-22824
HmHel-3	CAEZ01003984:1421-1792
HmHel-3	CAEZ01003984:1804-2131
HmHel-3	CAEZ01003226:144021-144350
HmHel-3	CAEZ01002376:377-706
HmHel-3	CAEZ01001426:9490-9812
HmHel-3	CAEZ01011297:148225-148549
HmHel-3	CAEZ01011009:36509-36919
HmHel-3	CAEZ01010260:20818-20934
HmHel-3	CAEZ01010260:52253-52658
HmHel-3	CAEZ01010260:108137-108793
HmHel-3	CAEZ01010260:110969-111214
HmHel-3	CAEZ01010013:3856-4205
HmHel-3	CAEZ01009957:12716-13147
HmHel-3	CAEZ01009827:17243-17644
HmHel-3	CAEZ01009827:5209-5896
HmHel-3	CAEZ01008234:20371-21091
HmHel-3	CAEZ01008234:51466-51790
HmHel-3	CAEZ01008138:163182-163596
HmHel-3	CAEZ01008138:201209-201534
HmHel-3	CAEZ01008138:318108-318425
HmHel-3	CAEZ01008138:339901-340215
HmHel-3	CAEZ01008138:351150-356030
HmHel-3	CAEZ01008138:58644-59266
HmHel-3	CAEZ01005725:5289-5612
HmHel-3	CAEZ01005625:2000-2149
HmHel-3	CAEZ01005625:756-1047

HmHel-3	CAEZ01005580:5804-6214
HmHel-3	CAEZ01005580:6750-7157
HmHel-3	CAEZ01004508:2804-3128
HmHel-3	CAEZ01003247:23567-25313
HmHel-3	CAEZ01003247:29998-30338
HmHel-3	CAEZ01002924:78495-78926
HmHel-3	CAEZ01002924:20686-21011
HmHel-3	CAEZ01002084:403-689
HmHel-3	CAEZ01001817:8457-8804
HmHel-3	CAEZ01001563:56227-56551
HmHel-3	CAEZ01010728:66833-68039
HmHel-3	CAEZ01010728:13596-14005
HmHel-3	CAEZ01010464:119319-119648
HmHel-3	CAEZ01010464:116678-117006
HmHel-3	CAEZ01009672:8708-9136
HmHel-3	CAEZ01009366:60301-60629
HmHel-3	CAEZ01009150:34736-35154
HmHel-3	CAEZ01008713:112451-112734
HmHel-3	CAEZ01008713:99050-99628
HmHel-3	CAEZ01008713:81419-81730
HmHel-3	CAEZ01007624:5473-6178
HmHel-3	CAEZ01007624:53327-53668
HmHel-3	CAEZ01006477:12028-12357
HmHel-3	CAEZ01006120:7231-7605
HmHel-3	CAEZ01006120:42931-43092
HmHel-3	CAEZ01006080:14447-14865
HmHel-3	CAEZ01005998:15823-16126
HmHel-3	CAEZ01005402:2010-2678
HmHel-3	CAEZ01005402:16514-16791
HmHel-3	CAEZ01005402:35343-35717
HmHel-3	CAEZ01005402:58697-59031
HmHel-3	CAEZ01005402:37935-38542
HmHel-3	CAEZ01005402:21052-21736
HmHel-3	CAEZ01004252:10529-10879
HmHel-3	CAEZ01004252:49673-50375
HmHel-3	CAEZ01003650:4969-5389
HmHel-3	CAEZ01003650:16860-17202
HmHel-3	CAEZ01003562:22975-23298
HmHel-3	CAEZ01003130:19574-19779
HmHel-3	CAEZ01003130:2129-2461
HmHel-3	CAEZ01003063:17770-18107
HmHel-3	CAEZ01003063:8809-9493
HmHel-3	CAEZ01002986:15591-15951
HmHel-3	CAEZ01002675:38994-40050
HmHel-3	CAEZ01002675:20774-21482
HmHel-3	CAEZ01002607:200001-200325
HmHel-3	CAEZ01001822:1059-1440
HmHel-3	CAEZ01001441:89892-90292

HmHel-3	CAEZ01001441:53862-54929
HmHel-3	CAEZ01001441:39456-40180
HmHel-3	CAEZ01001123:9045-9420
HmHel-3	CAEZ01000830:8932-9340
HmHel-3	CAEZ01011558:14228-14568
HmHel-3	CAEZ01011558:1305-1610
HmHel-3	CAEZ01011296:19128-19492
HmHel-3	CAEZ01011296:67556-68315
HmHel-3	CAEZ01011296:109593-109817
HmHel-3	CAEZ01011296:151226-151314
HmHel-3	CAEZ01010555:416-648
HmHel-3	CAEZ01010209:11273-17161
HmHel-3	CAEZ01009786:20269-20658
HmHel-3	CAEZ01009285:2935-3349
HmHel-3	CAEZ01008628:3008-3386
HmHel-3	CAEZ01007984:39096-39440
HmHel-3	CAEZ01007751:47847-48073
HmHel-3	CAEZ01007751:58841-59181
HmHel-3	CAEZ01006318:48-286
HmHel-3	CAEZ01005828:20521-20853
HmHel-3	CAEZ01005471:807-1181
HmHel-3	CAEZ01005307:2952-3204
HmHel-3	CAEZ01004830:2828-3202
HmHel-3	CAEZ01004761:179-575
HmHel-3	CAEZ01004429:2475-2862
HmHel-3	CAEZ01004316:391-629
HmHel-3	CAEZ01003819:12406-12731
HmHel-3	CAEZ01003819:27931-28337
HmHel-3	CAEZ01003102:7166-7577
HmHel-3	CAEZ01002648:10651-10991
HmHel-3	CAEZ01002211:8915-9239
HmHel-3	CAEZ01002211:51052-51203
HmHel-3	CAEZ01001970:5658-6066
HmHel-3	CAEZ01000996:990-1330
HmHel-3	CAEZ01000877:3380-3754
HmHel-3	CAEZ01011517:4417-4740
HmHel-3	CAEZ01011351:14-421
HmHel-3	CAEZ01011340:12503-12995
HmHel-3	CAEZ01010912:5165-5884
HmHel-3	CAEZ01010662:10260-10580
HmHel-3	CAEZ01010044:105-539
HmHel-3	CAEZ01009404:58105-61356
HmHel-3	CAEZ01009356:4819-5162
HmHel-3	CAEZ01009176:2405-2730
HmHel-3	CAEZ01008544:182-507
HmHel-3	CAEZ01008334:1297-1711
HmHel-3	CAEZ01007727:55213-55538
HmHel-3	CAEZ01007451:16557-16918

HmHel-3	CAEZ01007067:416-745
HmHel-3	CAEZ01006357:8824-9153
HmHel-3	CAEZ01006027:26528-26811
HmHel-3	CAEZ01004541:125951-126374
HmHel-3	CAEZ01004126:40342-40666
HmHel-3	CAEZ01003346:88257-88586
HmHel-3	CAEZ01003346:67773-68105
HmHel-3	CAEZ01001939:30-355
HmHel-3	CAEZ01001463:45245-45575
HmHel-3	CAEZ01001463:4906-5231
HmHel-3	CAEZ01001276:20890-21215
HmHel-3	CAEZ01000879:123115-123827
HmHel-3	CAEZ01000879:60103-60432
HmHel-3	CAEZ01011650:4385-4783
HmHel-3	CAEZ01011323:20345-20669
HmHel-3	CAEZ01011323:55887-56601
HmHel-3	CAEZ01010839:10643-10949
HmHel-3	CAEZ01010110:3260-3582
HmHel-3	CAEZ01010016:10434-10680
HmHel-3	CAEZ01010016:22566-22870
HmHel-3	CAEZ01010016:12011-12413
HmHel-3	CAEZ01009271:169674-169909
HmHel-3	CAEZ01009271:69365-69604
HmHel-3	CAEZ01008497:22066-22652
HmHel-3	CAEZ01007988:299-546
HmHel-3	CAEZ01006022:5258-5572
HmHel-3	CAEZ01004625:7832-8226
HmHel-3	CAEZ01003945:5977-6402
HmHel-3	CAEZ01003305:25750-25993
HmHel-3	CAEZ01002087:75979-76278
HmHel-3	CAEZ01002087:27922-28341
HmHel-3	CAEZ01002079:6053-6395
HmHel-3	CAEZ01011659:16411-16752
HmHel-3	CAEZ01011134:20762-21095
HmHel-3	CAEZ01010401:7530-7863
HmHel-3	CAEZ01010312:13459-13780
HmHel-3	CAEZ01010150:1926-2259
HmHel-3	CAEZ01009721:10185-10513
HmHel-3	CAEZ01009131:17023-17241
HmHel-3	CAEZ01009131:15280-15645
HmHel-3	CAEZ01009131:2060-2481
HmHel-3	CAEZ01008315:268251-268579
HmHel-3	CAEZ01008315:55700-56374
HmHel-3	CAEZ01008315:8792-14031
HmHel-3	CAEZ01006798:10088-10405
HmHel-3	CAEZ01005901:162-447
HmHel-3	CAEZ01005884:19528-19849
HmHel-3	CAEZ01005884:18508-19217

HmHel-3	CAEZ01005656:1238-1583
HmHel-3	CAEZ01005566:135-462
HmHel-3	CAEZ01005227:17028-17358
HmHel-3	CAEZ01004169:11682-12011
HmHel-3	CAEZ01004139:12264-12589
HmHel-3	CAEZ01004097:241004-241643
HmHel-3	CAEZ01004097:231023-231317
HmHel-3	CAEZ01004097:66904-67289
HmHel-3	CAEZ01003941:10015-10401
HmHel-3	CAEZ01003781:7066-7468
HmHel-3	CAEZ01003720:1710-2043
HmHel-3	CAEZ01003606:2972-3274
HmHel-3	CAEZ01003111:30882-31296
HmHel-3	CAEZ01003006:8487-8815
HmHel-3	CAEZ01002269:646-975
HmHel-3	CAEZ01002267:2-334
HmHel-3	CAEZ01001579:17876-18231
HmHel-3	CAEZ01001579:60686-61057
HmHel-3	CAEZ01001237:852-1181
HmHel-3	CAEZ01000903:1714-2036
HmHel-3	CAEZ01011423:2155-2495
HmHel-3	CAEZ01011421:16193-16604
HmHel-3	CAEZ01011197:8685-9009
HmHel-3	CAEZ01011197:140979-141652
HmHel-3	CAEZ01011197:103348-104031
HmHel-3	CAEZ01010922:47135-47467
HmHel-3	CAEZ01010719:15757-16158
HmHel-3	CAEZ01010171:26805-27210
HmHel-3	CAEZ01010012:17940-18351
HmHel-3	CAEZ01009823:37261-37669
HmHel-3	CAEZ01009705:51000-51424
HmHel-3	CAEZ01009650:12097-12421
HmHel-3	CAEZ01009456:172976-173301
HmHel-3	CAEZ01009456:114291-115012
HmHel-3	CAEZ01009094:3871-4227
HmHel-3	CAEZ01009065:6206-6566
HmHel-3	CAEZ01008381:9165-10308
HmHel-3	CAEZ01008381:33413-33721
HmHel-3	CAEZ01008381:18205-18462
HmHel-3	CAEZ01008026:19226-19471
HmHel-3	CAEZ01008001:41334-41979
HmHel-3	CAEZ01008001:23582-23942
HmHel-3	CAEZ01007685:33261-33584
HmHel-3	CAEZ01007426:2602-3051
HmHel-3	CAEZ01007420:4055-4453
HmHel-3	CAEZ01007403:29696-30091
HmHel-3	CAEZ01007403:23174-23552
HmHel-3	CAEZ01007066:95529-95830

HmHel-3	CAEZ01007066:75270-75516
HmHel-3	CAEZ01006433:82490-82818
HmHel-3	CAEZ01006085:2931-3153
HmHel-3	CAEZ01005556:8645-8959
HmHel-3	CAEZ01005486:16319-16634
HmHel-3	CAEZ01005433:70580-70920
HmHel-3	CAEZ01005282:15939-16367
HmHel-3	CAEZ01004585:11204-11403
HmHel-3	CAEZ01004585:9366-10040
HmHel-3	CAEZ01004527:29351-29679
HmHel-3	CAEZ01003877:1418-1754
HmHel-3	CAEZ01003554:3694-3910
HmHel-3	CAEZ01002144:7804-8169
HmHel-3	CAEZ01001529:492-815
HmHel-3	CAEZ01001437:28275-36969
HmHel-3	CAEZ01001415:21420-22007
HmHel-3	CAEZ01001193:7891-8285
HmHel-3	CAEZ01011630:4897-5228
HmHel-3	CAEZ01010282:16332-16719
HmHel-3	CAEZ01010182:16360-16685
HmHel-3	CAEZ01009232:22317-23068
HmHel-3	CAEZ01009232:8331-8656
HmHel-3	CAEZ01008933:13644-14027
HmHel-3	CAEZ01008839:9943-10268
HmHel-3	CAEZ01008795:10065-10404
HmHel-3	CAEZ01008351:150-475
HmHel-3	CAEZ01008351:132928-133218
HmHel-3	CAEZ01008351:95889-96125
HmHel-3	CAEZ01007851:121057-121382
HmHel-3	CAEZ01007851:24857-25093
HmHel-3	CAEZ01007631:23078-23489
HmHel-3	CAEZ01007381:4376-4755
HmHel-3	CAEZ01007261:2983-3308
HmHel-3	CAEZ01007070:17076-17401
HmHel-3	CAEZ01006478:8329-8519
HmHel-3	CAEZ01006223:635-960
HmHel-3	CAEZ01005438:8633-8958
HmHel-3	CAEZ01005204:9431-9854
HmHel-3	CAEZ01004551:22689-23095
HmHel-3	CAEZ01002245:3895-4220
HmHel-3	CAEZ01001959:477-802
HmHel-3	CAEZ01001372:302-627
HmHel-3	CAEZ01009680:33570-34247
HmHel-3	CAEZ01009680:20744-21015
HmHel-3	CAEZ01009680:2384-3109
HmHel-3	CAEZ01009497:17650-17917
HmHel-3	CAEZ01009497:2923-3338
HmHel-3	CAEZ01009221:19634-19962

HmHel-3	CAEZ01009221:20447-20741
HmHel-3	CAEZ01009221:7108-7200
HmHel-3	CAEZ01008837:11362-11773
HmHel-3	CAEZ01008383:10394-10731
HmHel-3	CAEZ01006888:17697-18084
HmHel-3	CAEZ01006338:37826-38136
HmHel-3	CAEZ01006168:6665-7035
HmHel-3	CAEZ01005746:38384-38619
HmHel-3	CAEZ01005746:67755-68392
HmHel-3	CAEZ01004362:4733-5049
HmHel-3	CAEZ01003851:2994-3395
HmHel-3	CAEZ01000059:16425-16830
HmHel-3	CAEZ01010917:11345-11631
HmHel-3	CAEZ01010917:26576-26963
HmHel-3	CAEZ01010917:31958-32277
HmHel-3	CAEZ01010917:10549-10773
HmHel-3	CAEZ01010730:17163-17500
HmHel-3	CAEZ01010730:29879-30579
HmHel-3	CAEZ01010449:32669-32998
HmHel-3	CAEZ01010449:81278-81486
HmHel-3	CAEZ01010364:26569-26902
HmHel-3	CAEZ01010349:5796-6120
HmHel-3	CAEZ01010107:9302-9647
HmHel-3	CAEZ01009695:99-440
HmHel-3	CAEZ01009146:17357-17682
HmHel-3	CAEZ01008396:11183-11532
HmHel-3	CAEZ01007983:34242-34583
HmHel-3	CAEZ01007745:35425-35754
HmHel-3	CAEZ01007586:74622-74974
HmHel-3	CAEZ01006622:12333-12662
HmHel-3	CAEZ01006492:7252-7581
HmHel-3	CAEZ01006336:25851-26230
HmHel-3	CAEZ01006172:66-392
HmHel-3	CAEZ01005825:42227-42453
HmHel-3	CAEZ01005131:1759-2373
HmHel-3	CAEZ01005131:19290-19507
HmHel-3	CAEZ01004973:9231-19761
HmHel-3	CAEZ01002872:31815-32203
HmHel-3	CAEZ01002606:25-354
HmHel-3	CAEZ01002060:1290-2000
HmHel-3	CAEZ01001135:888-1229
HmHel-3	CAEZ01010227:99577-100270
HmHel-3	CAEZ01010227:124036-124124
HmHel-3	CAEZ01010227:110566-110893
HmHel-3	CAEZ01010227:42046-42133
HmHel-3	CAEZ01010085:1386-1711
HmHel-3	CAEZ01009788:11129-11521
HmHel-3	CAEZ01009788:73454-73712

HmHel-3	CAEZ01009471:5323-5651
HmHel-3	CAEZ01009361:5013-5337
HmHel-3	CAEZ01009321:666-1041
HmHel-3	CAEZ01009032:6690-7038
HmHel-3	CAEZ01008829:13588-14009
HmHel-3	CAEZ01008343:41-336
HmHel-3	CAEZ01008112:2-286
HmHel-3	CAEZ01007890:11575-11927
HmHel-3	CAEZ01007725:108515-108847
HmHel-3	CAEZ01007552:8945-9106
HmHel-3	CAEZ01007230:4239-4730
HmHel-3	CAEZ01006986:1-320
HmHel-3	CAEZ01006947:3149-6645
HmHel-3	CAEZ01006724:1260-1650
HmHel-3	CAEZ01006722:421-810
HmHel-3	CAEZ01006657:4707-5132
HmHel-3	CAEZ01006643:28047-28224
HmHel-3	CAEZ01006539:18021-18397
HmHel-3	CAEZ01006367:86340-86701
HmHel-3	CAEZ01006125:4102-4486
HmHel-3	CAEZ01005839:12778-14167
HmHel-3	CAEZ01005817:37792-38076
HmHel-3	CAEZ01005817:20518-20766
HmHel-3	CAEZ01004986:14408-14764
HmHel-3	CAEZ01003185:3243-3515
HmHel-3	CAEZ01003185:2980-3209
HmHel-3	CAEZ01003163:2060-2217
HmHel-3	CAEZ01003163:70684-70925
HmHel-3	CAEZ01003163:28917-29249
HmHel-3	CAEZ01001403:25059-25146
HmHel-3	CAEZ01001403:46939-47175
HmHel-3	CAEZ01001403:21729-27350
HmHel-3	CAEZ01001140:4433-4758
HmHel-3	CAEZ01011227:16134-16451
HmHel-3	CAEZ01010050:8836-9161
HmHel-3	CAEZ01009506:14458-14783
HmHel-3	CAEZ01008632:7489-7814
HmHel-3	CAEZ01008495:16974-17299
HmHel-3	CAEZ01008470:21142-21548
HmHel-3	CAEZ01008470:18181-18408
HmHel-3	CAEZ01008359:183775-184121
HmHel-3	CAEZ01008131:7118-7545
HmHel-3	CAEZ01006261:3601-3926
HmHel-3	CAEZ01005669:34869-35280
HmHel-3	CAEZ01004994:81-523
HmHel-3	CAEZ01004312:15119-15444
HmHel-3	CAEZ01004133:14608-15007
HmHel-3	CAEZ01002952:7-322

HmHel-3	CAEZ01002633:35662-35818
HmHel-3	CAEZ01001991:321-646
HmHel-3	CAEZ01001446:67797-68122
HmHel-3	CAEZ01001446:149277-149365
HmHel-3	CAEZ01001446:94619-94966
HmHel-3	CAEZ01001446:50789-51464
HmHel-3	CAEZ01011320:1117-1428
HmHel-3	CAEZ01010820:54341-54748
HmHel-3	CAEZ01010820:102864-103100
HmHel-3	CAEZ01010820:64603-64903
HmHel-3	CAEZ01010517:58271-58634
HmHel-3	CAEZ01010517:16899-17578
HmHel-3	CAEZ01010256:35212-35553
HmHel-3	CAEZ01009145:24290-24618
HmHel-3	CAEZ01007768:5185-5648
HmHel-3	CAEZ01006864:19456-19780
HmHel-3	CAEZ01006202:39070-39491
HmHel-3	CAEZ01005861:772-1140
HmHel-3	CAEZ01005740:10585-10914
HmHel-3	CAEZ01005740:306-394
HmHel-3	CAEZ01002241:1442-1707
HmHel-3	CAEZ01002072:4113-4321
HmHel-3	CAEZ01000970:37075-37395
HmHel-3	CAEZ01011010:1512-1926
HmHel-3	CAEZ01010149:23859-24200
HmHel-3	CAEZ01009374:53017-53342
HmHel-3	CAEZ01009153:2474-2803
HmHel-3	CAEZ01008754:2606-2933
HmHel-3	CAEZ01007780:247-584
HmHel-3	CAEZ01006867:3216-3635
HmHel-3	CAEZ01006637:1721-2144
HmHel-3	CAEZ01006216:3975-4295
HmHel-3	CAEZ01006149:30800-31245
HmHel-3	CAEZ01005428:97442-97771
HmHel-3	CAEZ01005428:281-901
HmHel-3	CAEZ01005369:6361-6706
HmHel-3	CAEZ01004507:23653-23986
HmHel-3	CAEZ01003815:12814-13159
HmHel-3	CAEZ01003801:5834-6166
HmHel-3	CAEZ01003235:77792-78065
HmHel-3	CAEZ01002332:26005-26342
HmHel-3	CAEZ01002150:414-817
HmHel-3	CAEZ01002061:14-261
HmHel-3	CAEZ01001616:7729-8074
HmHel-3	CAEZ01001577:71852-72616
HmHel-3	CAEZ01001577:51443-52151
HmHel-3	CAEZ01001577:26649-26871
HmHel-3	CAEZ01001556:9447-9855

HmHel-3	CAEZ01000039:11843-12090
HmHel-3	CAEZ01011590:9329-9746
HmHel-3	CAEZ01010915:49913-50569
HmHel-3	CAEZ01010915:20887-21249
HmHel-3	CAEZ01010700:98402-98754
HmHel-3	CAEZ01010455:116365-116453
HmHel-3	CAEZ01010455:73370-73658
HmHel-3	CAEZ01010341:371-791
HmHel-3	CAEZ01009364:4104-4491
HmHel-3	CAEZ01009364:35551-35974
HmHel-3	CAEZ01009300:27535-28232
HmHel-3	CAEZ01009300:17295-17696
HmHel-3	CAEZ01009250:120-369
HmHel-3	CAEZ01009250:14893-15286
HmHel-3	CAEZ01008089:18738-18887
HmHel-3	CAEZ01007564:13294-13704
HmHel-3	CAEZ01007098:1290-1622
HmHel-3	CAEZ01006919:32947-33349
HmHel-3	CAEZ01006007:23258-24048
HmHel-3	CAEZ01005705:2795-3406
HmHel-3	CAEZ01005660:22364-22783
HmHel-3	CAEZ01005373:57694-58001
HmHel-3	CAEZ01005373:43222-43590
HmHel-3	CAEZ01004987:9536-9942
HmHel-3	CAEZ01003932:10024-10452
HmHel-3	CAEZ01003922:9696-10023
HmHel-3	CAEZ01003814:8099-8392
HmHel-3	CAEZ01003719:23548-23809
HmHel-3	CAEZ01001634:5652-6053
HmHel-3	CAEZ01011616:19043-19497
HmHel-3	CAEZ01011533:20828-21115
HmHel-3	CAEZ01010501:2154-2869
HmHel-3	CAEZ01010501:35667-35819
HmHel-3	CAEZ01009576:7505-8186
HmHel-3	CAEZ01009576:41511-41833
HmHel-3	CAEZ01009576:25257-25498
HmHel-3	CAEZ01008065:99-424
HmHel-3	CAEZ01006690:16356-16706
HmHel-3	CAEZ01006428:2647-2984
HmHel-3	CAEZ01006428:4225-4443
HmHel-3	CAEZ01006195:1835-2182
HmHel-3	CAEZ01006162:112661-113025
HmHel-3	CAEZ01005976:36812-37056
HmHel-3	CAEZ01005652:5846-6245
HmHel-3	CAEZ01005308:41333-41684
HmHel-3	CAEZ01004011:10631-10956
HmHel-3	CAEZ01004011:2678-2976
HmHel-3	CAEZ01003744:17098-17516

HmHel-3	CAEZ01002025:2960-3257
HmHel-3	CAEZ01009862:485-913
HmHel-3	CAEZ01006241:729-1038
HmHel-3	CAEZ01006241:35585-35941
HmHel-3	CAEZ01006053:2347-2736
HmHel-3	CAEZ01005458:14924-15194
HmHel-3	CAEZ01005264:73-228
HmHel-3	CAEZ01000886:1094-1379
HmHel-3	CAEZ01011275:12107-12436
HmHel-3	CAEZ01008258:155395-155715
HmHel-3	CAEZ01008258:19020-19643
HmHel-3	CAEZ01007740:60484-60982
HmHel-3	CAEZ01007625:91703-92040
HmHel-3	CAEZ01007529:5929-6244
HmHel-3	CAEZ01007529:519-5520
HmHel-3	CAEZ01007462:6099-6326
HmHel-3	CAEZ01007462:7270-7561
HmHel-3	CAEZ01006772:226844-227159
HmHel-3	CAEZ01006772:72616-72965
HmHel-3	CAEZ01006635:4221-4594
HmHel-3	CAEZ01006313:273-520
HmHel-3	CAEZ01006083:34174-34451
HmHel-3	CAEZ01006083:18425-18753
HmHel-3	CAEZ01005219:184647-184984
HmHel-3	CAEZ01005219:67376-67612
HmHel-3	CAEZ01005219:48084-48676
HmHel-3	CAEZ01004528:16896-17225
HmHel-3	CAEZ01003407:66492-66825
HmHel-3	CAEZ01003263:2878-3177
HmHel-3	CAEZ01003263:4184-4874
HmHel-3	CAEZ01002971:13091-13461
HmHel-3	CAEZ01002719:4315-5521
HmHel-3	CAEZ01002170:8384-8775
HmHel-3	CAEZ01011437:12327-12723
HmHel-3	CAEZ01011217:29191-29534
HmHel-3	CAEZ01010900:61732-61893
HmHel-3	CAEZ01010900:23170-23435
HmHel-3	CAEZ01008882:7625-8002
HmHel-3	CAEZ01008882:2986-3281
HmHel-3	CAEZ01008210:63840-64266
HmHel-3	CAEZ01008210:51889-52199
HmHel-3	CAEZ01008210:40823-41465
HmHel-3	CAEZ01008091:69726-69969
HmHel-3	CAEZ01007861:183837-184073
HmHel-3	CAEZ01007861:147913-148208
HmHel-3	CAEZ01007060:6756-7187
HmHel-3	CAEZ01006987:8318-8579
HmHel-3	CAEZ01006987:6081-6512

HmHel-3	CAEZ01006228:29300-29692
HmHel-3	CAEZ01006042:2105-2475
HmHel-3	CAEZ01005142:7105-7509
HmHel-3	CAEZ01004964:1115-1497
HmHel-3	CAEZ01004949:7466-7860
HmHel-3	CAEZ01003943:28177-28585
HmHel-3	CAEZ01003476:16-177
HmHel-3	CAEZ01002181:23255-23583
HmHel-3	CAEZ01001473:933-1094
HmHel-3	CAEZ01001362:3165-3879
HmHel-3	CAEZ01001362:7175-7543
HmHel-3	CAEZ01000912:11668-12379
HmHel-3	CAEZ01000912:17011-17398
HmHel-3	CAEZ01000681:1831-2084
HmHel-3	CAEZ01011050:14625-14950
HmHel-3	CAEZ01010554:3990-4175
HmHel-3	CAEZ01009801:73735-73986
HmHel-3	CAEZ01008023:9643-10042
HmHel-3	CAEZ01007875:4055-4297
HmHel-3	CAEZ01007875:84-367
HmHel-3	CAEZ01007627:7804-8135
HmHel-3	CAEZ01006372:39589-39823
HmHel-3	CAEZ01006372:45515-45852
HmHel-3	CAEZ01006278:34625-34952
HmHel-3	CAEZ01006262:35566-35891
HmHel-3	CAEZ01005924:20172-20417
HmHel-3	CAEZ01005546:5090-5338
HmHel-3	CAEZ01003811:582-1234
HmHel-3	CAEZ01003811:19060-19389
HmHel-3	CAEZ01003408:491-820
HmHel-3	CAEZ01001888:3303-3711
HmHel-3	CAEZ01001452:6881-7148
HmHel-3	CAEZ01000900:753-1085
HmHel-3	CAEZ01010551:26480-26855
HmHel-3	CAEZ01008948:3332-3702
HmHel-3	CAEZ01008033:4982-5311
HmHel-3	CAEZ01007245:9062-9209
HmHel-3	CAEZ01007021:4389-4735
HmHel-3	CAEZ01005425:19220-19515
HmHel-3	CAEZ01005392:5368-5788
HmHel-3	CAEZ01005392:72589-72823
HmHel-3	CAEZ01005392:25248-25523
HmHel-3	CAEZ01005094:9144-10233
HmHel-3	CAEZ01003933:37921-38292
HmHel-3	CAEZ01011416:49952-50667
HmHel-3	CAEZ01011416:45644-45993
HmHel-3	CAEZ01011416:21834-22505
HmHel-3	CAEZ01011356:175-552

HmHel-3	CAEZ01010251:1-270
HmHel-3	CAEZ01010172:1-147
HmHel-3	CAEZ01009973:3087-3194
HmHel-3	CAEZ01009973:7799-8215
HmHel-3	CAEZ01008126:25766-26098
HmHel-3	CAEZ01008126:50000-50544
HmHel-3	CAEZ01007427:14101-14454
HmHel-3	CAEZ01007063:336-592
HmHel-3	CAEZ01006561:38365-38659
HmHel-3	CAEZ01006150:43868-44090
HmHel-3	CAEZ01006150:35326-35790
HmHel-3	CAEZ01006012:39590-39939
HmHel-3	CAEZ01005470:14831-15158
HmHel-3	CAEZ01004641:4364-4759
HmHel-3	CAEZ01003457:2388-2484
HmHel-3	CAEZ01003457:3192-3569
HmHel-3	CAEZ01002758:23950-24300
HmHel-3	CAEZ01011493:58643-59030
HmHel-3	CAEZ01011493:1533-1906
HmHel-3	CAEZ01010412:438-779
HmHel-3	CAEZ01010208:11732-12001
HmHel-3	CAEZ01010049:13294-13630
HmHel-3	CAEZ01009114:21384-21792
HmHel-3	CAEZ01008627:8639-9038
HmHel-3	CAEZ01007728:5680-6092
HmHel-3	CAEZ01007728:22426-22664
HmHel-3	CAEZ01006617:5004-5416
HmHel-3	CAEZ01005811:5242-5644
HmHel-3	CAEZ01005150:28404-28819
HmHel-3	CAEZ01001100:312-639
HmHel-3	CAEZ01000070:366-641
HmHel-3	CAEZ01008603:70242-70493
HmHel-3	CAEZ01008603:2701-3284
HmHel-3	CAEZ01007076:7403-7551
HmHel-3	CAEZ01006128:66466-66765
HmHel-3	CAEZ01006128:55979-56667
HmHel-3	CAEZ01006128:39976-40308
HmHel-3	CAEZ01008694:3-251
HmHel-3	CAEZ01007267:25078-25365
HmHel-3	CAEZ01005922:25039-25390
HmHel-3	CAEZ01005516:19715-19862
HmHel-3	CAEZ01004309:11177-11606
HmHel-3	CAEZ01001420:2718-3103
HmHel-3	CAEZ01000716:9303-9685
HmHel-3	CAEZ01011146:18415-18647
HmHel-3	CAEZ01010211:11799-12108
HmHel-3	CAEZ01006384:32623-32960
HmHel-3	CAEZ01002331:24162-24498

HmHel-3	CAEZ01001578:28661-29013
HmHel-3	CAEZ01001578:74408-74492
HmHel-3	CAEZ01001578:86737-87065
HmHel-3	CAEZ01001076:31090-31336
HmHel-3	CAEZ01001076:5380-5723
HmHel-3	CAEZ01000913:2902-3224
HmHel-3	CAEZ01010259:5191-5476
HmHel-3	CAEZ01009623:10120-10506
HmHel-3	CAEZ01007703:30571-30984
HmHel-3	CAEZ01005293:8775-8974
HmHel-3	CAEZ01005090:2728-3135
HmHel-3	CAEZ01004181:2601-2921
HmHel-3	CAEZ01011160:4059-4466
HmHel-3	CAEZ01010318:53531-54245
HmHel-3	CAEZ01010318:55731-56468
HmHel-3	CAEZ01010318:38095-38322
HmHel-3	CAEZ01009528:2214-2532
HmHel-3	CAEZ01007893:1370-1754
HmHel-3	CAEZ01007870:37848-38145
HmHel-3	CAEZ01007870:82398-83107
HmHel-3	CAEZ01005597:13795-14087
HmHel-3	CAEZ01005384:1-221
HmHel-3	CAEZ01003460:3799-4012
HmHel-3	CAEZ01003334:13328-13511
HmHel-3	CAEZ01010019:351-759
HmHel-3	CAEZ01009883:51600-51903
HmHel-3	CAEZ01009883:63624-63927
HmHel-3	CAEZ01009883:120579-121273
HmHel-3	CAEZ01009688:21464-21787
HmHel-3	CAEZ01008609:4487-4821
HmHel-3	CAEZ01007504:7619-7976
HmHel-3	CAEZ01007289:3379-3682
HmHel-3	CAEZ01006670:11837-12167
HmHel-3	CAEZ01004792:14886-15025
HmHel-3	CAEZ01004792:29902-30138
HmHel-3	CAEZ01004792:69622-70296
HmHel-3	CAEZ01003875:13936-14180
HmHel-3	CAEZ01001300:2947-3179
HmHel-3	CAEZ01009638:10702-11028
HmHel-3	CAEZ01008417:34936-35326
HmHel-3	CAEZ01008212:13261-13678
HmHel-3	CAEZ01007122:67243-67571
HmHel-3	CAEZ01006785:17887-18247
HmHel-3	CAEZ01006785:3696-4380
HmHel-3	CAEZ01005805:2157-2521
HmHel-3	CAEZ01003783:53430-53804
HmHel-3	CAEZ01011036:12660-13059
HmHel-3	CAEZ01010218:57331-57674

HmHel-3	CAEZ01010218:43238-43551
HmHel-3	CAEZ01008492:4015-4419
HmHel-3	CAEZ01008391:2604-2777
HmHel-3	CAEZ01007902:19022-19244
HmHel-3	CAEZ01005203:86789-87121
HmHel-3	CAEZ01005203:9419-10110
HmHel-3	CAEZ01002015:10585-10922
HmHel-3	CAEZ01001174:11437-11854
HmHel-3	CAEZ01001142:446-770
HmHel-3	CAEZ01011489:76818-77145
HmHel-3	CAEZ01010500:9092-9498
HmHel-3	CAEZ01010184:793-970
HmHel-3	CAEZ01010184:26288-26605
HmHel-3	CAEZ01007446:20858-21095
HmHel-3	CAEZ01006388:291-554
HmHel-3	CAEZ01004306:245-8046
HmHel-3	CAEZ01004306:10779-11161
HmHel-3	CAEZ01003931:3745-3978
HmHel-3	CAEZ01003348:34231-34544
HmHel-3	CAEZ01003254:2336-2632
HmHel-3	CAEZ01003254:19052-19757
HmHel-3	CAEZ01003088:1-182
HmHel-3	CAEZ01002360:9656-10052
HmHel-3	CAEZ01000867:48774-49008
HmHel-3	CAEZ01000867:61359-61607
HmHel-3	CAEZ01009922:34676-35006
HmHel-3	CAEZ01006855:7904-8220
HmHel-3	CAEZ01002797:2528-11901
HmHel-3	CAEZ01000542:348-635
HmHel-3	CAEZ01011071:9219-9627
HmHel-3	CAEZ01010880:167-596
HmHel-3	CAEZ01010439:41606-41967
HmHel-3	CAEZ01009833:15687-15774
HmHel-3	CAEZ01009833:32847-37891
HmHel-3	CAEZ01009833:498-1621
HmHel-3	CAEZ01007342:12427-12627
HmHel-3	CAEZ01007342:11327-11538
HmHel-3	CAEZ01005273:22156-22290
HmHel-3	CAEZ01004756:7644-7965
HmHel-3	CAEZ01002379:4412-4609
HmHel-3	CAEZ01002379:2483-2772
HmHel-3	CAEZ01001755:1688-1987
HmHel-3	CAEZ01001290:1587-1832
HmHel-3	CAEZ01007333:10900-11552
HmHel-3	CAEZ01007259:58234-58590
HmHel-3	CAEZ01007259:91946-92186
HmHel-3	CAEZ01007259:24564-25027
HmHel-3	CAEZ01005343:19057-19397

HmHel-3	CAEZ01003894:25456-25766
HmHel-3	CAEZ01002744:17628-21143
HmHel-3	CAEZ01002685:1654-1959
HmHel-3	CAEZ01002112:23-168
HmHel-3	CAEZ01001765:45120-45783
HmHel-3	CAEZ01001765:62612-73982
HmHel-3	CAEZ01009869:11283-11752
HmHel-3	CAEZ01008202:44440-44681
HmHel-3	CAEZ01008202:31812-32222
HmHel-3	CAEZ01003980:1481-1809
HmHel-3	CAEZ01003455:1583-1976
HmHel-3	CAEZ01011263:359-8056
HmHel-3	CAEZ01009104:12030-13187
HmHel-3	CAEZ01008769:15023-15735
HmHel-3	CAEZ01008769:44417-44725
HmHel-3	CAEZ01005083:84275-84562
HmHel-3	CAEZ01005083:72007-72406
HmHel-3	CAEZ01005083:28177-28492
HmHel-3	CAEZ01001038:2492-2786
HmHel-3	CAEZ01005665:26847-27143
HmHel-3	CAEZ01004862:8306-8652
HmHel-3	CAEZ01010609:10367-10591
HmHel-3	CAEZ01010226:21028-21162
HmHel-3	CAEZ01010226:322-410
HmHel-3	CAEZ01009025:3052-3250
HmHel-3	CAEZ01007638:8517-8881
HmHel-3	CAEZ01007350:695-873
HmHel-3	CAEZ01007089:19434-19845
HmHel-3	CAEZ01005555:4326-4680
HmHel-3	CAEZ01001668:9868-11051
HmHel-3	CAEZ01001668:26170-26891
HmHel-3	CAEZ01001453:25-265
HmHel-3	CAEZ01010362:34066-35888
HmHel-3	CAEZ01010106:2649-3056
HmHel-3	CAEZ01008402:45897-46596
HmHel-3	CAEZ01008402:72892-73295
HmHel-3	CAEZ01008402:81769-81994
HmHel-3	CAEZ01006856:7748-7890
HmHel-3	CAEZ01005333:4463-5132
HmHel-3	CAEZ01005333:47882-48133
HmHel-3	CAEZ01005007:4729-5085
HmHel-3	CAEZ01002451:239-523
HmHel-3	CAEZ01008857:6187-6567
HmHel-3	CAEZ01006461:16887-17213
HmHel-3	CAEZ01010249:7125-7431
HmHel-3	CAEZ01010195:8810-9509
HmHel-3	CAEZ01008076:720-910
HmHel-3	CAEZ01008076:1-165

HmHel-3	CAEZ01007248:359-769
HmHel-3	CAEZ01007248:8075-8726
HmHel-3	CAEZ01004457:10363-10748
HmHel-3	CAEZ01003204:703-841
HmHel-3	CAEZ01010192:10980-12760
HmHel-3	CAEZ01009264:49700-50753
HmHel-3	CAEZ01009264:22835-23532
HmHel-3	CAEZ01008419:10065-10301
HmHel-3	CAEZ01008419:23294-23656
HmHel-3	CAEZ01005929:2602-2968
HmHel-3	CAEZ01000963:22416-22784
HmHel-3	CAEZ01005394:19743-19959
HmHel-3	CAEZ01001729:9007-10090
HmHel-3	CAEZ01011406:1-168
HmHel-3	CAEZ01010798:12921-13157
HmHel-3	CAEZ01010798:9627-9950
HmHel-3	CAEZ01005047:10297-10416
HmHel-3	CAEZ01004631:318846-320222
HmHel-3	CAEZ01004601:1721-2148
HmHel-3	CAEZ01008446:961-1374
HmHel-3	CAEZ01005868:15811-16009
HmHel-3	CAEZ01002639:3232-3375
HmHel-3	CAEZ01002038:299-501
HmHel-3	CAEZ01004629:34155-34432
HmHel-3	CAEZ01004629:62832-62982
HmHel-3	CAEZ01009246:14931-15127
HmHel-3	CAEZ01008852:26901-27136
HmHel-3	CAEZ01008734:69193-69537
HmHel-3	CAEZ01008734:11889-12566
HmHel-3	CAEZ01005745:10227-10537
HmHel-3	CAEZ01004731:2921-3328
HmHel-3	CAEZ01007921:11317-11670
HmHel-3	CAEZ01003564:4325-4574
HmHel-3	CAEZ01001080:11413-11548
HmHel-3	CAEZ01009501:60-721
HmHel-3	CAEZ01009501:83575-84212
HmHel-3	CAEZ01009501:131096-131392
HmHel-3	CAEZ01009501:32128-32447
HmHel-3	CAEZ01003075:24113-24507
HmHel-3	CAEZ01009343:317-499
HmHel-3	CAEZ01006624:14385-14610
HmHel-3	CAEZ01006572:2175-3523
HmHel-3	CAEZ01004086:8054-8326
HmHel-3	CAEZ01002896:3276-3702
HmHel-3	CAEZ01002896:8684-8920
HmHel-3	CAEZ01011520:544-925
HmHel-3	CAEZ01008925:15501-15716
HmHel-3	CAEZ01008925:190-278

HmHel-3	CAEZ01005117:247-436
HmHel-3	CAEZ01004972:23111-31202
HmHel-3	CAEZ01004499:37868-38052
HmHel-3	CAEZ01010091:15-162
HmHel-3	CAEZ01005703:5358-5725
HmHel-3	CAEZ01001862:1426-1653
HmHel-3	CAEZ01011063:19079-19391
HmHel-3	CAEZ01010244:51257-51562
HmHel-3	CAEZ01005479:10424-10712
HmHel-3	CAEZ01003384:74-269
HmHel-3	CAEZ01002341:7772-8395
HmHel-3	CAEZ01009248:56089-56715
HmHel-3	CAEZ01009248:27626-27739
HmHel-3	CAEZ01004328:6617-6862
HmHel-3	CAEZ01011461:4220-4589
HmHel-3	CAEZ01010285:122864-123106
HmHel-3	CAEZ01010285:63568-63897
HmHel-3	CAEZ01007772:45525-45747
HmHel-3	CAEZ01001266:106628-106842
HmHel-3	CAEZ01001266:75498-76171
HmHel-3	CAEZ01001266:9649-10017
HmHel-3	CAEZ01005464:442-680
HmHel-3	CAEZ01005399:28620-28945
HmHel-3	CAEZ01002309:1-214
HmHel-3	CAEZ01001853:1503-3165
HmHel-3	CAEZ01009227:48122-48452
HmHel-3	CAEZ01008709:30638-30995
HmHel-3	CAEZ01007528:4036-4191
HmHel-3	CAEZ01007371:24279-24474
HmHel-3	CAEZ01005783:15566-16306
HmHel-3	CAEZ01010510:427-525
HmHel-3	CAEZ01010293:62083-62424
HmHel-3	CAEZ01010219:52718-53100
HmHel-3	CAEZ01010219:73794-74084
HmHel-3	CAEZ01009864:2245-2550
HmHel-3	CAEZ01009864:6175-6411
HmHel-3	CAEZ01006608:21677-21855
HmHel-3	CAEZ01005413:6374-6715
HmHel-3	CAEZ01002417:1156-1397
HmHel-3	CAEZ01001089:19411-20084
HmHel-3	CAEZ01001089:51004-51371
HmHel-3	CAEZ01010739:18062-18842
HmHel-3	CAEZ01009830:45-314
HmHel-3	CAEZ01009830:7292-7607
HmHel-3	CAEZ01009084:75292-75515
HmHel-3	CAEZ01009084:74212-74448
HmHel-3	CAEZ01009084:3473-13468
HmHel-3	CAEZ01005863:7712-8040

HmHel-3	CAEZ01004787:14450-14780
HmHel-3	CAEZ01004294:3604-3834
HmHel-3	CAEZ01000043:12145-12426
HmHel-3	CAEZ01006326:3946-4269
HmHel-3	CAEZ01006326:106559-106684
HmHel-3	CAEZ01011500:7753-8058
HmHel-3	CAEZ01010503:8-187
HmHel-3	CAEZ01010503:4560-4912
HmHel-3	CAEZ01009452:81574-81677
HmHel-3	CAEZ01006895:8259-8595
HmHel-3	CAEZ01005595:14183-14303
HmHel-3	CAEZ01004515:14351-14922
HmHel-3	CAEZ01002984:14710-14825
HmHel-3	CAEZ01002825:13621-13854
HmHel-3	CAEZ01008812:7311-7548
HmHel-3	CAEZ01010863:14838-14987
HmHel-3	CAEZ01010720:15254-15604
HmHel-3	CAEZ01007080:15558-15929
HmHel-3	CAEZ01006099:28060-28221
HmHel-3	CAEZ01005558:6729-6965
HmHel-3	CAEZ01005322:8027-8395
HmHel-3	CAEZ01002996:1-115
HmHel-3	CAEZ01001906:23-204
HmHel-3	CAEZ01011104:98634-98832
HmHel-3	CAEZ01006665:1867-2208
HmHel-3	CAEZ01006002:18190-24095
HmHel-3	CAEZ01005088:29567-29667
HmHel-3	CAEZ01001825:12788-13120
HmHel-3	CAEZ01008874:33717-34629
HmHel-3	CAEZ01008874:46986-48002
HmHel-3	CAEZ01008874:99142-99373
HmHel-3	CAEZ01003222:66554-66897
HmHel-3	CAEZ01001764:20194-20577
HmHel-3	CAEZ01005541:8942-9245
HmHel-3	CAEZ01005054:18-305
HmHel-3	CAEZ01004264:4479-4680
HmHel-3	CAEZ01006233:15070-15294
HmHel-3	CAEZ01006233:25521-26221
HmHel-3	CAEZ01001018:6121-6438
HmHel-3	CAEZ01010043:5683-5970
HmHel-3	CAEZ01007463:573-673
HmHel-3	CAEZ01005151:9023-9177
HmHel-3	CAEZ01005148:28014-28303
HmHel-3	CAEZ01008918:7930-8180
HmHel-3	CAEZ01008499:978-1146
HmHel-3	CAEZ01007365:8978-9653
HmHel-3	CAEZ01007365:29454-29693
HmHel-3	CAEZ01006937:35961-36266

HmHel-3	CAEZ01006937:35231-35557
HmHel-3	CAEZ01004224:4456-4952
HmHel-3	CAEZ01003723:24683-24851
HmHel-3	CAEZ01001166:9360-9648
HmHel-3	CAEZ01008262:4662-4797
HmHel-3	CAEZ01010762:3470-3684
HmHel-3	CAEZ01007458:8379-8473
HmHel-3	CAEZ01006288:2328-2669
HmHel-3	CAEZ01004241:6202-6580
HmHel-3	CAEZ01002608:43211-43530
HmHel-3	CAEZ01009799:3814-4023
HmHel-3	CAEZ01007821:852-1060
HmHel-3	CAEZ01006955:10533-11690
HmHel-3	CAEZ01006889:5557-6168
HmHel-3	CAEZ01003057:848-1016
HmHel-3	CAEZ01011414:68768-69135
HmHel-3	CAEZ01006739:3789-4022
HmHel-3	CAEZ01002568:24624-25401
HmHel-3	CAEZ01002568:24835-25141
HmHel-3	CAEZ01000601:5148-5376
HmHel-3	CAEZ01010970:62261-62501
HmHel-3	CAEZ01009716:8527-9199
HmHel-3	CAEZ01009716:61226-61603
HmHel-3	CAEZ01009716:74487-74977
HmHel-3	CAEZ01009716:99203-99510
HmHel-3	CAEZ01009716:217032-217336
HmHel-3	CAEZ01008423:3498-3831
HmHel-3	CAEZ01010938:13952-14175
HmHel-3	CAEZ01008460:12064-12212
HmHel-3	CAEZ01004733:2153-2432
HmHel-3	CAEZ01004733:22120-22477
HmHel-3	CAEZ01004733:26004-26845
HmHel-3	CAEZ01002684:7501-8234
HmHel-3	CAEZ01010187:117-249
HmHel-3	CAEZ01009710:2330-2566
HmHel-3	CAEZ01009391:17358-17593
HmHel-3	CAEZ01005900:24726-24894
HmHel-3	CAEZ01003890:13489-13577
HmHel-3	CAEZ01003890:5599-5845
HmHel-3	CAEZ01001039:1739-1907
HmHel-3	CAEZ01000942:22310-22546
HmHel-3	CAEZ01000942:398-484
HmHel-3	CAEZ01011462:4100-4808
HmHel-3	CAEZ01009511:47953-48041
HmHel-3	CAEZ01008329:89-741
HmHel-3	CAEZ01007062:19859-20616
HmHel-3	CAEZ01007062:38757-38982
HmHel-3	CAEZ01007051:15840-16889

HmHel-3	CAEZ01007037:1036-1199
HmHel-3	CAEZ01001892:21832-22518
HmHel-3	CAEZ01011221:6853-7086
HmHel-3	CAEZ01001152:8342-8577
HmHel-3	CAEZ01011348:6872-7099
HmHel-3	CAEZ01010936:26153-26389
HmHel-3	CAEZ01010141:12810-13045
HmHel-3	CAEZ01010127:83613-84263
HmHel-3	CAEZ01010127:98941-102309
HmHel-3	CAEZ01010104:516-6355
HmHel-3	CAEZ01009488:23761-23997
HmHel-3	CAEZ01009234:2732-2968
HmHel-3	CAEZ01008677:93948-94184
HmHel-3	CAEZ01008100:70365-70583
HmHel-3	CAEZ01008100:35584-35878
HmHel-3	CAEZ01007497:9352-9706
HmHel-3	CAEZ01007232:57833-58093
HmHel-3	CAEZ01007232:67904-68601
HmHel-3	CAEZ01007166:918-1154
HmHel-3	CAEZ01006857:364-581
HmHel-3	CAEZ01005850:16616-16843
HmHel-3	CAEZ01005772:19804-20064
HmHel-3	CAEZ01005267:100177-100382
HmHel-3	CAEZ01005105:8149-8385
HmHel-3	CAEZ01005105:13243-13479
HmHel-3	CAEZ01005005:5879-6115
HmHel-3	CAEZ01004904:11099-11335
HmHel-3	CAEZ01004417:6985-7190
HmHel-3	CAEZ01003547:2758-2994
HmHel-3	CAEZ01003478:11617-12008
HmHel-3	CAEZ01003478:282-370
HmHel-3	CAEZ01003312:28883-29119
HmHel-3	CAEZ01002759:18442-18678
HmHel-3	CAEZ01002759:16131-16369
HmHel-3	CAEZ01002414:124013-124240
HmHel-3	CAEZ01002362:12746-12982
HmHel-3	CAEZ01001887:1276-1512
HmHel-3	CAEZ01000990:13315-13551
HmHel-3	CAEZ01000811:14159-14395
HmHel-3	CAEZ01011101:71336-74040
HmHel-3	CAEZ01011101:53700-54375
HmHel-3	CAEZ01010005:2774-3534
HmHel-3	CAEZ01008513:7212-7473
HmHel-3	CAEZ01008120:3030-3118
HmHel-3	CAEZ01008031:20551-20761
HmHel-3	CAEZ01006153:23422-24084
HmHel-3	CAEZ01004940:3499-3723
HmHel-3	CAEZ01004510:4795-5028

HmHel-3	CAEZ01001291:18221-18448
HmHel-3	CAEZ01010041:27667-27902
HmHel-3	CAEZ01009681:44108-44425
HmHel-3	CAEZ01009681:72632-72856
HmHel-3	CAEZ01009681:73746-73972
HmHel-3	CAEZ01007584:107885-108122
HmHel-3	CAEZ01007584:129756-130334
HmHel-3	CAEZ01007584:193809-194520
HmHel-3	CAEZ01007584:68599-69265
HmHel-3	CAEZ01007584:48698-49418
HmHel-3	CAEZ01007584:24749-24956
HmHel-3	CAEZ01006366:16803-17027
HmHel-3	CAEZ01006366:20747-21452
HmHel-3	CAEZ01003727:1977-2213
HmHel-3	CAEZ01003165:28254-28481
HmHel-3	CAEZ01011284:34143-34377
HmHel-3	CAEZ01011284:1028-1332
HmHel-3	CAEZ01010855:6949-7185
HmHel-3	CAEZ01009717:42872-43108
HmHel-3	CAEZ01009133:22282-22518
HmHel-3	CAEZ01009133:129-364
HmHel-3	CAEZ01008629:9333-9569
HmHel-3	CAEZ01007260:5939-13537
HmHel-3	CAEZ01006711:11504-12247
HmHel-3	CAEZ01006711:74600-74836
HmHel-3	CAEZ01006402:7822-8052
HmHel-3	CAEZ01005543:8617-8853
HmHel-3	CAEZ01004422:1963-2199
HmHel-3	CAEZ01003510:8741-8977
HmHel-3	CAEZ01001900:10795-11002
HmHel-3	CAEZ01001594:8143-8379
HmHel-3	CAEZ01011518:5341-5577
HmHel-3	CAEZ01011211:4914-5150
HmHel-3	CAEZ01011133:26866-27592
HmHel-3	CAEZ01010620:72417-72653
HmHel-3	CAEZ01010620:96356-97029
HmHel-3	CAEZ01010444:10892-11128
HmHel-3	CAEZ01010287:9040-9276
HmHel-3	CAEZ01010078:54091-55207
HmHel-3	CAEZ01010033:1816-2052
HmHel-3	CAEZ01010033:17958-18661
HmHel-3	CAEZ01009906:3822-4050
HmHel-3	CAEZ01009892:94753-95450
HmHel-3	CAEZ01009892:17733-18392
HmHel-3	CAEZ01009720:1086-1322
HmHel-3	CAEZ01009491:8883-9121
HmHel-3	CAEZ01008990:14179-14415
HmHel-3	CAEZ01008926:63-195

HmHel-3	CAEZ01008926:16397-17116
HmHel-3	CAEZ01008924:19962-20198
HmHel-3	CAEZ01008743:74350-74464
HmHel-3	CAEZ01008743:111807-112104
HmHel-3	CAEZ01008743:146940-147179
HmHel-3	CAEZ01008743:100444-101117
HmHel-3	CAEZ01008486:10444-10716
HmHel-3	CAEZ01008486:32210-32898
HmHel-3	CAEZ01008486:11330-11566
HmHel-3	CAEZ01008244:25192-25430
HmHel-3	CAEZ01008236:138072-138287
HmHel-3	CAEZ01008236:10360-10596
HmHel-3	CAEZ01008147:11087-11323
HmHel-3	CAEZ01008147:27531-27617
HmHel-3	CAEZ01007264:3166-3402
HmHel-3	CAEZ01006983:3610-3846
HmHel-3	CAEZ01006934:13846-14082
HmHel-3	CAEZ01006838:2997-3233
HmHel-3	CAEZ01006721:1464-1700
HmHel-3	CAEZ01006395:48302-48538
HmHel-3	CAEZ01006231:578-1988
HmHel-3	CAEZ01006231:2444-2680
HmHel-3	CAEZ01006066:999-1235
HmHel-3	CAEZ01006044:46344-46580
HmHel-3	CAEZ01005334:19072-19308
HmHel-3	CAEZ01004233:32858-33061
HmHel-3	CAEZ01003921:17283-17519
HmHel-3	CAEZ01003839:5292-5528
HmHel-3	CAEZ01003757:306-533
HmHel-3	CAEZ01003400:2196-2432
HmHel-3	CAEZ01003400:5137-5221
HmHel-3	CAEZ01003079:12697-12812
HmHel-3	CAEZ01003019:101905-102669
HmHel-3	CAEZ01003019:131494-132065
HmHel-3	CAEZ01003001:119-356
HmHel-3	CAEZ01002120:27657-27892
HmHel-3	CAEZ01001750:9906-10142
HmHel-3	CAEZ01001408:186680-186916
HmHel-3	CAEZ01001408:75320-75629
HmHel-3	CAEZ01001250:200-437
HmHel-3	CAEZ01000962:2-134
HmHel-3	CAEZ01010898:15247-15477
HmHel-3	CAEZ01007986:14441-14677
HmHel-3	CAEZ01005468:133-813
HmHel-3	CAEZ01005274:95561-95846
HmHel-3	CAEZ01005274:117422-118141
HmHel-3	CAEZ01005274:97134-97222
HmHel-3	CAEZ01003624:2894-12243

HmHel-3	CAEZ01002866:122724-123427
HmHel-3	CAEZ01002051:9685-10415
HmHel-3	CAEZ01001965:9540-9620
HmHel-3	CAEZ01000890:1528-1709
HmHel-3	CAEZ01010355:34899-35135
HmHel-3	CAEZ01010160:7577-7814
HmHel-3	CAEZ01009413:3476-3655
HmHel-3	CAEZ01010457:16144-16425
HmHel-3	CAEZ01010457:21095-21322
HmHel-3	CAEZ01009622:6817-7535
HmHel-3	CAEZ01008904:6790-7024
HmHel-3	CAEZ01008449:14433-14653
HmHel-3	CAEZ01008215:3292-3528
HmHel-3	CAEZ01007711:18573-19599
HmHel-3	CAEZ01007405:18953-19189
HmHel-3	CAEZ01006686:47983-48232
HmHel-3	CAEZ01006590:43805-44047
HmHel-3	CAEZ01006489:32480-32718
HmHel-3	CAEZ01006409:32415-32651
HmHel-3	CAEZ01004680:36603-36841
HmHel-3	CAEZ01004054:6651-6813
HmHel-3	CAEZ01003929:10771-11031
HmHel-3	CAEZ01003926:7741-7978
HmHel-3	CAEZ01003926:12908-13596
HmHel-3	CAEZ01003616:2319-2417
HmHel-3	CAEZ01001345:4076-4314
HmHel-3	CAEZ01011092:11397-11633
HmHel-3	CAEZ01010673:5219-5458
HmHel-3	CAEZ01010624:23219-23455
HmHel-3	CAEZ01010622:15740-15976
HmHel-3	CAEZ01009122:1599-1835
HmHel-3	CAEZ01008828:8298-8534
HmHel-3	CAEZ01008475:24219-24452
HmHel-3	CAEZ01007590:12281-12517
HmHel-3	CAEZ01007008:2983-3115
HmHel-3	CAEZ01006541:33508-33744
HmHel-3	CAEZ01006517:1438-1674
HmHel-3	CAEZ01005822:112-348
HmHel-3	CAEZ01005707:1427-1663
HmHel-3	CAEZ01005677:19654-19868
HmHel-3	CAEZ01005230:23413-23649
HmHel-3	CAEZ01005144:14198-14330
HmHel-3	CAEZ01004958:30473-31593
HmHel-3	CAEZ01003827:57836-58072
HmHel-3	CAEZ01003827:71858-72560
HmHel-3	CAEZ01003777:6495-6755
HmHel-3	CAEZ01003415:56036-56272
HmHel-3	CAEZ01002951:911-1648

HmHel-3	CAEZ01002906:7190-7358
HmHel-3	CAEZ01002293:160-392
HmHel-3	CAEZ01002143:12880-13116
HmHel-3	CAEZ01001996:3152-3458
HmHel-3	CAEZ01001982:18768-19004
HmHel-3	CAEZ01001940:3934-4170
HmHel-3	CAEZ01001327:8971-9207
HmHel-3	CAEZ01011465:27856-28543
HmHel-3	CAEZ01011318:19710-20410
HmHel-3	CAEZ01011313:10036-10975
HmHel-3	CAEZ01011306:12113-12812
HmHel-3	CAEZ01011245:58025-59222
HmHel-3	CAEZ01010879:5108-5784
HmHel-3	CAEZ01010815:2888-2976
HmHel-3	CAEZ01010752:22268-22959
HmHel-3	CAEZ01010616:730-1436
HmHel-3	CAEZ01010462:11906-12585
HmHel-3	CAEZ01010140:23070-23773
HmHel-3	CAEZ01010124:4142-4818
HmHel-3	CAEZ01010124:520-1651
HmHel-3	CAEZ01010066:12192-12280
HmHel-3	CAEZ01009961:29385-29473
HmHel-3	CAEZ01009774:17537-18239
HmHel-3	CAEZ01009748:98561-99241
HmHel-3	CAEZ01009698:10232-10501
HmHel-3	CAEZ01009647:197-285
HmHel-3	CAEZ01009614:16518-16816
HmHel-3	CAEZ01009614:46571-47291
HmHel-3	CAEZ01009475:17941-18615
HmHel-3	CAEZ01009044:133823-134524
HmHel-3	CAEZ01008951:1455-2155
HmHel-3	CAEZ01008821:14814-15529
HmHel-3	CAEZ01008742:5211-5917
HmHel-3	CAEZ01008550:16815-17504
HmHel-3	CAEZ01008504:4101-4778
HmHel-3	CAEZ01008284:75-771
HmHel-3	CAEZ01007974:75071-75744
HmHel-3	CAEZ01007974:55287-55998
HmHel-3	CAEZ01007974:31961-32640
HmHel-3	CAEZ01007865:29775-30095
HmHel-3	CAEZ01007865:42795-43509
HmHel-3	CAEZ01007545:47074-47785
HmHel-3	CAEZ01007272:2088-2778
HmHel-3	CAEZ01007250:285-373
HmHel-3	CAEZ01006559:16571-16655
HmHel-3	CAEZ01006494:34498-35196
HmHel-3	CAEZ01006279:19944-20614
HmHel-3	CAEZ01006001:9749-10455

HmHel-3	CAEZ01006001:8235-8829
HmHel-3	CAEZ01005714:21611-22318
HmHel-3	CAEZ01005493:518-606
HmHel-3	CAEZ01005427:993-1666
HmHel-3	CAEZ01005427:84900-85615
HmHel-3	CAEZ01005386:7840-7928
HmHel-3	CAEZ01005076:7717-7805
HmHel-3	CAEZ01004894:5843-6523
HmHel-3	CAEZ01004888:6820-7054
HmHel-3	CAEZ01004794:10776-10864
HmHel-3	CAEZ01004720:297-385
HmHel-3	CAEZ01004582:32023-32789
HmHel-3	CAEZ01004496:58882-59540
HmHel-3	CAEZ01004496:3613-4314
HmHel-3	CAEZ01004437:3717-4390
HmHel-3	CAEZ01004437:5804-6491
HmHel-3	CAEZ01003923:23530-23766
HmHel-3	CAEZ01003849:7773-8475
HmHel-3	CAEZ01003707:3124-3358
HmHel-3	CAEZ01003695:289-377
HmHel-3	CAEZ01003636:9910-10146
HmHel-3	CAEZ01003307:5505-5593
HmHel-3	CAEZ01003293:1861-1984
HmHel-3	CAEZ01003202:108703-109404
HmHel-3	CAEZ01003202:117380-118050
HmHel-3	CAEZ01003202:97933-98617
HmHel-3	CAEZ01003151:64555-65258
HmHel-3	CAEZ01002721:37916-38594
HmHel-3	CAEZ01002673:19272-19985
HmHel-3	CAEZ01002396:1647-2360
HmHel-3	CAEZ01002064:29403-29630
HmHel-3	CAEZ01000876:22870-23570
HmHel-3	CAEZ01000083:143-231
HmHel-3	CAEZ01010017:10533-11219
HmHel-3	CAEZ01009602:5401-5640
HmHel-3	CAEZ01008003:9863-10095
HmHel-3	CAEZ01004572:1073-1289
HmHel-3	CAEZ01002266:2287-2524
HmHel-3	CAEZ01000076:1190-1280
HmHel-3	CAEZ01000069:3014-3108
HmHel-3	CAEZ01000033:12159-12366
HmHel-3	CAEZ01011309:1384-1620
HmHel-3	CAEZ01010632:22947-23183
HmHel-3	CAEZ01009804:14715-14951
HmHel-3	CAEZ01008101:13938-14243
HmHel-3	CAEZ01006865:51187-51411
HmHel-3	CAEZ01006339:44176-44493
HmHel-3	CAEZ01006339:47674-47905

HmHel-3	CAEZ01005778:30830-31047
HmHel-3	CAEZ01005778:3714-4335
HmHel-3	CAEZ01005248:4398-4634
HmHel-3	CAEZ01004562:222491-222660
HmHel-3	CAEZ01004477:16837-17170
HmHel-3	CAEZ01003974:4417-4653
HmHel-3	CAEZ01003265:13898-14244
HmHel-3	CAEZ01002318:24291-24527
HmHel-3	CAEZ01001980:26665-26901
HmHel-3	CAEZ01010007:11033-11752
HmHel-3	CAEZ01009979:15307-15515
HmHel-3	CAEZ01007949:13514-13750
HmHel-3	CAEZ01007911:18182-18418
HmHel-3	CAEZ01007078:3884-4120
HmHel-3	CAEZ01006943:444-680
HmHel-3	CAEZ01005821:8675-8883
HmHel-3	CAEZ01005383:1404-1640
HmHel-3	CAEZ01003236:21124-21351
HmHel-3	CAEZ01002140:12559-12691
HmHel-3	CAEZ01001697:486-567
HmHel-3	CAEZ01011569:265-353
HmHel-3	CAEZ01011538:1850-2537
HmHel-3	CAEZ01011484:6581-6669
HmHel-3	CAEZ01011338:1793-1881
HmHel-3	CAEZ01011285:44408-45095
HmHel-3	CAEZ01011282:23235-23792
HmHel-3	CAEZ01011282:110784-120457
HmHel-3	CAEZ01011282:553-869
HmHel-3	CAEZ01011204:5995-6217
HmHel-3	CAEZ01011144:1045-1743
HmHel-3	CAEZ01011144:36968-37642
HmHel-3	CAEZ01011107:62345-63062
HmHel-3	CAEZ01011107:103602-103835
HmHel-3	CAEZ01010954:7903-8630
HmHel-3	CAEZ01010907:25485-26166
HmHel-3	CAEZ01010746:5076-5749
HmHel-3	CAEZ01010746:23082-23376
HmHel-3	CAEZ01010740:61990-64222
HmHel-3	CAEZ01010692:9965-10656
HmHel-3	CAEZ01010667:6530-7346
HmHel-3	CAEZ01010667:29377-30942
HmHel-3	CAEZ01010621:21705-22424
HmHel-3	CAEZ01010608:6369-6453
HmHel-3	CAEZ01010528:7452-7682
HmHel-3	CAEZ01010528:35880-35968
HmHel-3	CAEZ01010340:6927-7015
HmHel-3	CAEZ01010242:40149-40799
HmHel-3	CAEZ01010239:2595-3264

HmHel-3	CAEZ01010117:63349-64080
HmHel-3	CAEZ01010117:441-529
HmHel-3	CAEZ01010115:270-358
HmHel-3	CAEZ01010093:5516-6199
HmHel-3	CAEZ01009919:876-960
HmHel-3	CAEZ01009884:4414-5087
HmHel-3	CAEZ01009754:3034-3743
HmHel-3	CAEZ01009750:38288-38984
HmHel-3	CAEZ01009729:16155-16843
HmHel-3	CAEZ01009685:16122-16783
HmHel-3	CAEZ01009656:34567-34861
HmHel-3	CAEZ01009656:19422-19701
HmHel-3	CAEZ01009633:43629-44340
HmHel-3	CAEZ01009633:103004-103308
HmHel-3	CAEZ01009503:7953-8662
HmHel-3	CAEZ01009481:52791-52879
HmHel-3	CAEZ01009450:650-1335
HmHel-3	CAEZ01009443:40905-41525
HmHel-3	CAEZ01009443:63383-64102
HmHel-3	CAEZ01009428:5192-5891
HmHel-3	CAEZ01009407:10023-10736
HmHel-3	CAEZ01009396:1254-1942
HmHel-3	CAEZ01009373:60070-60283
HmHel-3	CAEZ01009373:81902-82607
HmHel-3	CAEZ01009373:49357-49891
HmHel-3	CAEZ01009339:500-588
HmHel-3	CAEZ01009304:23595-30950
HmHel-3	CAEZ01009281:28734-29407
HmHel-3	CAEZ01009272:23609-24320
HmHel-3	CAEZ01009270:26539-26627
HmHel-3	CAEZ01009249:25149-25853
HmHel-3	CAEZ01009136:21882-22555
HmHel-3	CAEZ01009123:2764-3437
HmHel-3	CAEZ01009071:5478-6170
HmHel-3	CAEZ01009056:8871-9543
HmHel-3	CAEZ01008916:46170-46878
HmHel-3	CAEZ01008916:2086-2752
HmHel-3	CAEZ01008767:24934-25168
HmHel-3	CAEZ01008735:42019-42710
HmHel-3	CAEZ01008726:100697-101368
HmHel-3	CAEZ01008723:23616-24319
HmHel-3	CAEZ01008723:5516-5758
HmHel-3	CAEZ01008649:10220-10915
HmHel-3	CAEZ01008567:1461-2179
HmHel-3	CAEZ01008512:99796-100503
HmHel-3	CAEZ01008489:25813-26460
HmHel-3	CAEZ01008414:18419-19126
HmHel-3	CAEZ01008316:17766-18450

HmHel-3	CAEZ01008310:11662-11953
HmHel-3	CAEZ01008286:4279-4925
HmHel-3	CAEZ01008175:13938-14609
HmHel-3	CAEZ01008152:283-371
HmHel-3	CAEZ01008117:13025-13682
HmHel-3	CAEZ01007987:4128-4817
HmHel-3	CAEZ01007941:192892-193599
HmHel-3	CAEZ01007849:37662-38335
HmHel-3	CAEZ01007848:29935-30023
HmHel-3	CAEZ01007836:1047-1734
HmHel-3	CAEZ01007785:9264-9352
HmHel-3	CAEZ01007782:175-263
HmHel-3	CAEZ01007735:2601-3285
HmHel-3	CAEZ01007721:30725-31399
HmHel-3	CAEZ01007709:42669-43381
HmHel-3	CAEZ01007666:11588-12261
HmHel-3	CAEZ01007603:31356-32062
HmHel-3	CAEZ01007505:15068-15741
HmHel-3	CAEZ01007431:16677-17378
HmHel-3	CAEZ01007431:35646-36224
HmHel-3	CAEZ01007410:127-789
HmHel-3	CAEZ01007341:6352-7023
HmHel-3	CAEZ01007298:300-388
HmHel-3	CAEZ01007281:5260-5924
HmHel-3	CAEZ01007281:39901-40540
HmHel-3	CAEZ01007266:46732-47437
HmHel-3	CAEZ01007256:62513-63139
HmHel-3	CAEZ01007256:39871-40103
HmHel-3	CAEZ01007256:511-599
HmHel-3	CAEZ01007254:18649-19341
HmHel-3	CAEZ01007101:95748-96438
HmHel-3	CAEZ01007047:1395-1483
HmHel-3	CAEZ01006995:1972-2645
HmHel-3	CAEZ01006885:1536-2189
HmHel-3	CAEZ01006885:1182-1266
HmHel-3	CAEZ01006863:19197-19876
HmHel-3	CAEZ01006863:81783-82003
HmHel-3	CAEZ01006863:7715-8018
HmHel-3	CAEZ01006810:28906-29618
HmHel-3	CAEZ01006769:42955-43035
HmHel-3	CAEZ01006760:12325-12979
HmHel-3	CAEZ01006760:51202-51508
HmHel-3	CAEZ01006718:361-1086
HmHel-3	CAEZ01006594:12818-13041
HmHel-3	CAEZ01006594:47641-48330
HmHel-3	CAEZ01006510:141-229
HmHel-3	CAEZ01006347:97093-97797
HmHel-3	CAEZ01006328:60929-61624

HmHel-3	CAEZ01006324:54411-55083
HmHel-3	CAEZ01006273:15826-15914
HmHel-3	CAEZ01006181:6136-6803
HmHel-3	CAEZ01006157:1392-1480
HmHel-3	CAEZ01006136:495-796
HmHel-3	CAEZ01006117:993-1653
HmHel-3	CAEZ01005973:17741-18414
HmHel-3	CAEZ01005864:18675-19375
HmHel-3	CAEZ01005729:263-946
HmHel-3	CAEZ01005690:884-1609
HmHel-3	CAEZ01005678:1085-1169
HmHel-3	CAEZ01005549:85-177
HmHel-3	CAEZ01005548:7434-7764
HmHel-3	CAEZ01005508:31180-31853
HmHel-3	CAEZ01005451:28510-33220
HmHel-3	CAEZ01005451:50237-50890
HmHel-3	CAEZ01005447:8340-9016
HmHel-3	CAEZ01005442:151824-152506
HmHel-3	CAEZ01005417:8943-9616
HmHel-3	CAEZ01005417:2397-3088
HmHel-3	CAEZ01005342:5705-6366
HmHel-3	CAEZ01005321:50679-51373
HmHel-3	CAEZ01005304:13363-14046
HmHel-3	CAEZ01005286:4701-5376
HmHel-3	CAEZ01005242:4527-5270
HmHel-3	CAEZ01005143:2049-2731
HmHel-3	CAEZ01005141:2065-2769
HmHel-3	CAEZ01005049:1627-2300
HmHel-3	CAEZ01005044:59703-59791
HmHel-3	CAEZ01004852:9946-10617
HmHel-3	CAEZ01004829:12019-12706
HmHel-3	CAEZ01004826:64840-65517
HmHel-3	CAEZ01004679:21142-21411
HmHel-3	CAEZ01004545:50151-50455
HmHel-3	CAEZ01004545:41576-42234
HmHel-3	CAEZ01004544:26002-26674
HmHel-3	CAEZ01004533:1244-1972
HmHel-3	CAEZ01004506:7724-8399
HmHel-3	CAEZ01004505:12357-13081
HmHel-3	CAEZ01004486:21894-22567
HmHel-3	CAEZ01004484:6410-7054
HmHel-3	CAEZ01004482:2761-2849
HmHel-3	CAEZ01004383:11341-11579
HmHel-3	CAEZ01004251:717-1388
HmHel-3	CAEZ01004232:5102-5828
HmHel-3	CAEZ01004225:14786-15022
HmHel-3	CAEZ01004203:212-300
HmHel-3	CAEZ01004119:1814-2461

HmHel-3	CAEZ01004119:9190-9891
HmHel-3	CAEZ01004038:41544-42631
HmHel-3	CAEZ01003996:3870-4570
HmHel-3	CAEZ01003971:1829-2504
HmHel-3	CAEZ01003884:357-445
HmHel-3	CAEZ01003763:596-1240
HmHel-3	CAEZ01003647:41425-42104
HmHel-3	CAEZ01003548:16503-17204
HmHel-3	CAEZ01003275:23679-23889
HmHel-3	CAEZ01003275:17606-18317
HmHel-3	CAEZ01003264:132-806
HmHel-3	CAEZ01003034:3036-3756
HmHel-3	CAEZ01002982:17784-17868
HmHel-3	CAEZ01002946:2387-2609
HmHel-3	CAEZ01002946:119-207
HmHel-3	CAEZ01002864:10648-11351
HmHel-3	CAEZ01002776:5950-6623
HmHel-3	CAEZ01002603:2362-2596
HmHel-3	CAEZ01002231:15720-16431
HmHel-3	CAEZ01002229:28418-29100
HmHel-3	CAEZ01002210:3435-3523
HmHel-3	CAEZ01002013:690-1346
HmHel-3	CAEZ01001964:444-532
HmHel-3	CAEZ01001932:4597-5285
HmHel-3	CAEZ01001854:8499-9187
HmHel-3	CAEZ01001832:59939-60631
HmHel-3	CAEZ01001832:51908-52590
HmHel-3	CAEZ01001639:178-868
HmHel-3	CAEZ01001521:14849-15573
HmHel-3	CAEZ01001510:31559-32232
HmHel-3	CAEZ01001125:7610-8302
HmHel-3	CAEZ01001103:33716-34433
HmHel-3	CAEZ01001059:796-884
HmHel-3	CAEZ01000960:3218-3907
HmHel-3	CAEZ01000839:5054-5807
HmHel-3	CAEZ01000834:38657-39354
HmHel-3	CAEZ01000833:27480-27585
HmHel-3	CAEZ01000817:26588-27221
HmHel-3	CAEZ01000055:11537-12194
HmHel-3	CAEZ01010727:53074-53379
HmHel-3	CAEZ01010727:38505-38842
HmHel-3	CAEZ01008889:20824-21509
HmHel-3	CAEZ01007500:64145-64381
HmHel-3	CAEZ01006896:7762-7841
HmHel-3	CAEZ01003823:14298-14558
HmHel-3	CAEZ01003304:30707-30790
HmHel-3	CAEZ01011642:16617-17261
HmHel-3	CAEZ01010910:935-1601

HmHel-3	CAEZ01010264:3231-3465
HmHel-3	CAEZ01009835:24708-24806
HmHel-3	CAEZ01008184:455-593
HmHel-3	CAEZ01008087:17569-17793
HmHel-3	CAEZ01008087:13376-16309
HmHel-3	CAEZ01007981:32079-32412
HmHel-3	CAEZ01006689:23610-23916
HmHel-3	CAEZ01006689:8670-8897
HmHel-3	CAEZ01006537:13742-13976
HmHel-3	CAEZ01005898:2579-3224
HmHel-3	CAEZ01004845:70222-70457
HmHel-3	CAEZ01003678:9425-10095
HmHel-3	CAEZ01002579:2196-3257
HmHel-3	CAEZ01002579:2612-2904
HmHel-3	CAEZ01010817:2861-3534
HmHel-3	CAEZ01010407:691-1333
HmHel-3	CAEZ01008408:10902-11213
HmHel-3	CAEZ01007340:134-361
HmHel-3	CAEZ01006317:13329-14413
HmHel-3	CAEZ01005843:699-1724
HmHel-3	CAEZ01001797:767-848
HmHel-3	CAEZ01000871:660-980
HmHel-3	CAEZ01011544:318-406
HmHel-3	CAEZ01011539:11915-12148
HmHel-3	CAEZ01011452:25783-26104
HmHel-3	CAEZ01011452:5056-5639
HmHel-3	CAEZ01011354:338-1062
HmHel-3	CAEZ01010748:3415-3499
HmHel-3	CAEZ01010748:18564-18885
HmHel-3	CAEZ01010602:37719-38392
HmHel-3	CAEZ01010602:28931-29236
HmHel-3	CAEZ01010535:2088-2403
HmHel-3	CAEZ01010535:8726-9011
HmHel-3	CAEZ01010521:13420-13508
HmHel-3	CAEZ01010024:3299-3614
HmHel-3	CAEZ01009897:3992-4665
HmHel-3	CAEZ01009897:5592-5897
HmHel-3	CAEZ01009893:18358-19122
HmHel-3	CAEZ01009701:10184-10904
HmHel-3	CAEZ01009460:31226-31899
HmHel-3	CAEZ01009426:6826-6914
HmHel-3	CAEZ01009322:4660-5334
HmHel-3	CAEZ01009312:519-1231
HmHel-3	CAEZ01009204:1121-1723
HmHel-3	CAEZ01009186:19553-20243
HmHel-3	CAEZ01009168:11645-11729
HmHel-3	CAEZ01009070:19462-20184
HmHel-3	CAEZ01009016:18355-18572

HmHel-3	CAEZ01009016:48424-49095
HmHel-3	CAEZ01008883:852-1541
HmHel-3	CAEZ01008510:19136-19811
HmHel-3	CAEZ01008401:16261-16936
HmHel-3	CAEZ01008203:3157-3821
HmHel-3	CAEZ01008097:4194-4863
HmHel-3	CAEZ01007926:8997-9707
HmHel-3	CAEZ01007830:13303-13391
HmHel-3	CAEZ01007781:4500-5169
HmHel-3	CAEZ01007114:6049-6358
HmHel-3	CAEZ01007114:627-715
HmHel-3	CAEZ01007108:106439-107158
HmHel-3	CAEZ01007011:124-212
HmHel-3	CAEZ01006672:11171-11259
HmHel-3	CAEZ01006567:72300-72384
HmHel-3	CAEZ01006526:3161-3834
HmHel-3	CAEZ01006496:5247-5952
HmHel-3	CAEZ01006348:31199-31904
HmHel-3	CAEZ01005984:19612-20285
HmHel-3	CAEZ01005770:22314-23016
HmHel-3	CAEZ01005757:3911-4631
HmHel-3	CAEZ01005424:10965-12762
HmHel-3	CAEZ01005241:7070-7761
HmHel-3	CAEZ01005114:5676-5764
HmHel-3	CAEZ01005108:64151-65270
HmHel-3	CAEZ01005070:13128-13834
HmHel-3	CAEZ01004993:9395-10074
HmHel-3	CAEZ01004991:1-720
HmHel-3	CAEZ01004990:19125-19366
HmHel-3	CAEZ01004990:71829-72134
HmHel-3	CAEZ01004751:35567-36238
HmHel-3	CAEZ01004740:37972-38666
HmHel-3	CAEZ01004735:27046-27718
HmHel-3	CAEZ01004707:10436-10520
HmHel-3	CAEZ01004619:5082-5856
HmHel-3	CAEZ01004535:23924-24222
HmHel-3	CAEZ01004303:11431-12110
HmHel-3	CAEZ01004291:14309-15024
HmHel-3	CAEZ01003750:9151-9853
HmHel-3	CAEZ01003575:838-1502
HmHel-3	CAEZ01003505:393-1120
HmHel-3	CAEZ01003385:29642-30246
HmHel-3	CAEZ01003365:32826-32914
HmHel-3	CAEZ01003207:65436-66028
HmHel-3	CAEZ01003076:362-4023
HmHel-3	CAEZ01003061:27725-28415
HmHel-3	CAEZ01002839:16487-17178
HmHel-3	CAEZ01002785:343-431

HmHel-3	CAEZ01002754:51305-51978
HmHel-3	CAEZ01002428:4874-5548
HmHel-3	CAEZ01001894:3704-4801
HmHel-3	CAEZ01001868:29324-30032
HmHel-3	CAEZ01001650:45237-46341
HmHel-3	CAEZ01001538:21043-21718
HmHel-3	CAEZ01001538:52303-53010
HmHel-3	CAEZ01001347:9372-10044
HmHel-3	CAEZ01001347:9200-10882
HmHel-3	CAEZ01001338:98489-99214
HmHel-3	CAEZ01001286:4288-4921
HmHel-3	CAEZ01000818:531-1208
HmHel-3	CAEZ01000799:13080-13168
HmHel-3	CAEZ01009829:930-1198
HmHel-3	CAEZ01007185:2355-2671
HmHel-3	CAEZ01006097:71502-71588
HmHel-3	CAEZ01005712:16732-16815
HmHel-3	CAEZ01003119:4982-5076
HmHel-3	CAEZ01001479:10867-11135
HmHel-3	CAEZ01010816:12247-12550
HmHel-3	CAEZ01010587:20503-20817
HmHel-3	CAEZ01010281:13761-13994
HmHel-3	CAEZ01010216:56608-57294
HmHel-3	CAEZ01009219:8860-9179
HmHel-3	CAEZ01008888:2504-2821
HmHel-3	CAEZ01008664:8026-8263
HmHel-3	CAEZ01007758:18849-19083
HmHel-3	CAEZ01007657:982-1216
HmHel-3	CAEZ01007219:18615-19519
HmHel-3	CAEZ01007072:21815-21901
HmHel-3	CAEZ01006701:2651-3927
HmHel-3	CAEZ01006468:5311-5545
HmHel-3	CAEZ01006131:161073-161690
HmHel-3	CAEZ01006092:60681-61000
HmHel-3	CAEZ01005944:12396-12709
HmHel-3	CAEZ01005758:4962-5288
HmHel-3	CAEZ01005600:64-596
HmHel-3	CAEZ01005410:19319-19615
HmHel-3	CAEZ01005145:37255-37383
HmHel-3	CAEZ01004997:16053-16287
HmHel-3	CAEZ01004376:2177-2493
HmHel-3	CAEZ01004027:6339-8978
HmHel-3	CAEZ01004014:51531-52239
HmHel-3	CAEZ01004004:696-794
HmHel-3	CAEZ01003639:13849-14083
HmHel-3	CAEZ01003621:3692-11021
HmHel-3	CAEZ01003106:18740-19039
HmHel-3	CAEZ01002668:32496-32813

HmHel-3	CAEZ01001963:8175-9564
HmHel-3	CAEZ01001909:5146-5830
HmHel-3	CAEZ01001207:977-1197
HmHel-3	CAEZ01000828:1762-2081
HmHel-3	CAEZ01011271:3219-3531
HmHel-3	CAEZ01011132:29801-30013
HmHel-3	CAEZ01011132:28458-28745
HmHel-3	CAEZ01011132:4859-5177
HmHel-3	CAEZ01009843:65598-65896
HmHel-3	CAEZ01006412:37105-37449
HmHel-3	CAEZ01005637:6538-6816
HmHel-3	CAEZ01001128:1311-1388
HmHel-3	CAEZ01010786:1485-1794
HmHel-3	CAEZ01010512:3624-3708
HmHel-3	CAEZ01010176:2098-2182
HmHel-3	CAEZ01009800:16387-17679
HmHel-3	CAEZ01009800:19847-20140
HmHel-3	CAEZ01009673:52931-53600
HmHel-3	CAEZ01008851:3238-3326
HmHel-3	CAEZ01008739:14732-15405
HmHel-3	CAEZ01008608:37569-37884
HmHel-3	CAEZ01007115:325-413
HmHel-3	CAEZ01007112:38667-38755
HmHel-3	CAEZ01006471:2817-2901
HmHel-3	CAEZ01006343:55936-56054
HmHel-3	CAEZ01006284:6039-6127
HmHel-3	CAEZ01005683:1822-2475
HmHel-3	CAEZ01005606:73974-74054
HmHel-3	CAEZ01004962:1882-2729
HmHel-3	CAEZ01004855:17758-17846
HmHel-3	CAEZ01004744:12366-12584
HmHel-3	CAEZ01003985:266-354
HmHel-3	CAEZ01003765:16685-16769
HmHel-3	CAEZ01003420:13394-14088
HmHel-3	CAEZ01003392:9065-9782
HmHel-3	CAEZ01002777:55-143
HmHel-3	CAEZ01002645:3035-3287
HmHel-3	CAEZ01002641:2409-3088
HmHel-3	CAEZ01002353:847-935
HmHel-3	CAEZ01002047:11865-12491
HmHel-3	CAEZ01001984:14961-15670
HmHel-3	CAEZ01001517:6996-7230
HmHel-3	CAEZ01000074:19316-19988
HmHel-3	CAEZ01010096:9225-9898
HmHel-3	CAEZ01005605:77113-77381
HmHel-3	CAEZ01003137:21400-21705
HmHel-3	CAEZ01011526:23055-23289
HmHel-3	CAEZ01010896:159976-160291

HmHel-3	CAEZ01009970:47057-47357
HmHel-3	CAEZ01009873:52763-53063
HmHel-3	CAEZ01008674:78873-79192
HmHel-3	CAEZ01008273:4882-5116
HmHel-3	CAEZ01008171:16758-16877
HmHel-3	CAEZ01007863:105812-106130
HmHel-3	CAEZ01007804:28158-28795
HmHel-3	CAEZ01007362:5231-5512
HmHel-3	CAEZ01006853:25435-26087
HmHel-3	CAEZ01006632:23661-24293
HmHel-3	CAEZ01006628:40337-40569
HmHel-3	CAEZ01006562:40634-40939
HmHel-3	CAEZ01006562:41667-41984
HmHel-3	CAEZ01006250:17265-17597
HmHel-3	CAEZ01005596:1335-7392
HmHel-3	CAEZ01005588:7379-7613
HmHel-3	CAEZ01004858:21557-22250
HmHel-3	CAEZ01004127:52653-52997
HmHel-3	CAEZ01003197:21290-21607
HmHel-3	CAEZ01002910:1318-1537
HmHel-3	CAEZ01001150:4071-4305
HmHel-3	CAEZ01009648:8042-8356
HmHel-3	CAEZ01009217:11752-12440
HmHel-3	CAEZ01008952:33754-33989
HmHel-3	CAEZ01007854:6274-6565
HmHel-3	CAEZ01006011:10390-10476
HmHel-3	CAEZ01005869:7389-7614
HmHel-3	CAEZ01005682:9550-10176
HmHel-3	CAEZ01005682:10821-11406
HmHel-3	CAEZ01005180:2848-3090
HmHel-3	CAEZ01005180:14142-14438
HmHel-3	CAEZ01004519:26530-26916
HmHel-3	CAEZ01004094:107986-108602
HmHel-3	CAEZ01002199:837-918
HmHel-3	CAEZ01000936:345-674
HmHel-3	CAEZ01011577:8404-8705
HmHel-3	CAEZ01011207:6453-6768
HmHel-3	CAEZ01011027:1390-1609
HmHel-3	CAEZ01010770:21985-22295
HmHel-3	CAEZ01009484:14084-14399
HmHel-3	CAEZ01009179:7941-8625
HmHel-3	CAEZ01007938:44028-44338
HmHel-3	CAEZ01007938:9711-10419
HmHel-3	CAEZ01007274:1538-2376
HmHel-3	CAEZ01005272:1-684
HmHel-3	CAEZ01004816:34630-34872
HmHel-3	CAEZ01004408:14244-14972
HmHel-3	CAEZ01004403:738-1417

HmHel-3	CAEZ01003560:1205-1518
HmHel-3	CAEZ01002224:27754-27912
HmHel-3	CAEZ01001886:1-696
HmHel-3	CAEZ01001871:807-1503
HmHel-3	CAEZ01001815:1-667
HmHel-3	CAEZ01000968:32410-33083
HmHel-3	CAEZ01009676:16055-16361
HmHel-3	CAEZ01008622:3-93
HmHel-3	CAEZ01007157:12736-13054
HmHel-3	CAEZ01004107:428-518
HmHel-3	CAEZ01003634:954-1193
HmHel-3	CAEZ01001020:707-793
HmHel-3	CAEZ01011488:7089-7665
HmHel-3	CAEZ01010206:15773-16414
HmHel-3	CAEZ01009359:30940-31163
HmHel-3	CAEZ01009239:3816-4034
HmHel-3	CAEZ01007576:23800-24034
HmHel-3	CAEZ01007499:97372-97980
HmHel-3	CAEZ01007465:19914-20144
HmHel-3	CAEZ01007313:39033-39640
HmHel-3	CAEZ01007216:6728-7320
HmHel-3	CAEZ01007147:342-1006
HmHel-3	CAEZ01006989:15607-16292
HmHel-3	CAEZ01005803:7339-7944
HmHel-3	CAEZ01005798:16554-17215
HmHel-3	CAEZ01004007:25028-25262
HmHel-3	CAEZ01003328:113700-113942
HmHel-3	CAEZ01003020:2093-2326
HmHel-3	CAEZ01002729:11863-12485
HmHel-3	CAEZ01001754:4-657
HmHel-3	CAEZ01001740:70176-73464
HmHel-3	CAEZ01001703:16095-16332
HmHel-3	CAEZ01001458:78-770
HmHel-3	CAEZ01001249:18613-19222
HmHel-3	CAEZ01000873:10956-11179
HmHel-3	CAEZ01011208:826-1123
HmHel-3	CAEZ01010868:29075-29679
HmHel-3	CAEZ01008667:357-970
HmHel-3	CAEZ01008034:2938-3557
HmHel-3	CAEZ01007663:32492-32711
HmHel-3	CAEZ01007146:5210-5519
HmHel-3	CAEZ01005385:3984-4607
HmHel-3	CAEZ01004234:320-2078
HmHel-3	CAEZ01004072:5813-6080
HmHel-3	CAEZ01004025:4102-4386
HmHel-3	CAEZ01004003:16960-17281
HmHel-3	CAEZ01003380:316-526
HmHel-3	CAEZ01002695:2853-3488

HmHel-3	CAEZ01002154:4213-4834
HmHel-3	CAEZ01001943:459-1072
HmHel-3	CAEZ01001865:1022-3087
HmHel-3	CAEZ01001803:4-633
HmHel-3	CAEZ01001785:7104-7640
HmHel-3	CAEZ01009802:138743-138957
HmHel-3	CAEZ01009802:259473-259557
HmHel-3	CAEZ01009637:1921-2194
HmHel-3	CAEZ01008807:2548-2831
HmHel-3	CAEZ01008671:20223-20837
HmHel-3	CAEZ01008350:52850-53167
HmHel-3	CAEZ01007234:39708-40357
HmHel-3	CAEZ01006881:33788-34060
HmHel-3	CAEZ01006830:9891-10137
HmHel-3	CAEZ01006771:4144-4386
HmHel-3	CAEZ01005632:20756-21002
HmHel-3	CAEZ01005371:22-628
HmHel-3	CAEZ01005126:1763-2477
HmHel-3	CAEZ01003627:3732-11603
HmHel-3	CAEZ01002444:1114-1369
HmHel-3	CAEZ01011625:5133-5450
HmHel-3	CAEZ01010731:10114-10430
HmHel-3	CAEZ01010008:32075-32392
HmHel-3	CAEZ01009465:18664-18984
HmHel-3	CAEZ01009372:1351-1676
HmHel-3	CAEZ01008966:47583-47900
HmHel-3	CAEZ01008966:13174-13489
HmHel-3	CAEZ01006979:1875-1950
HmHel-3	CAEZ01006086:28085-28349
HmHel-3	CAEZ01005275:12368-12683
HmHel-3	CAEZ01001571:1066-1381
HmHel-3	CAEZ01001571:2826-3092
HmHel-3	CAEZ01000905:32350-32666
HmHel-3	CAEZ01000200:285-377
HmHel-3	CAEZ01011572:12929-13234
HmHel-3	CAEZ01011399:4189-4504
HmHel-3	CAEZ01011330:20701-21006
HmHel-3	CAEZ01010749:8482-8724
HmHel-3	CAEZ01010732:4858-5160
HmHel-3	CAEZ01010641:3747-3995
HmHel-3	CAEZ01010025:31130-31825
HmHel-3	CAEZ01009947:200-504
HmHel-3	CAEZ01009759:7364-7663
HmHel-3	CAEZ01009449:159-453
HmHel-3	CAEZ01009077:52599-52841
HmHel-3	CAEZ01009077:225974-226216
HmHel-3	CAEZ01009077:144043-144675
HmHel-3	CAEZ01009077:119728-119943

HmHel-3	CAEZ01008764:4879-5512
HmHel-3	CAEZ01008732:60005-62519
HmHel-3	CAEZ01008365:11350-12045
HmHel-3	CAEZ01008277:26379-34373
HmHel-3	CAEZ01007704:15547-15846
HmHel-3	CAEZ01006974:9380-9622
HmHel-3	CAEZ01006960:36328-36928
HmHel-3	CAEZ01006958:8654-17759
HmHel-3	CAEZ01006835:681-979
HmHel-3	CAEZ01006827:10973-11215
HmHel-3	CAEZ01006638:15211-15508
HmHel-3	CAEZ01006557:2803-3098
HmHel-3	CAEZ01006470:22004-22309
HmHel-3	CAEZ01006449:4258-4563
HmHel-3	CAEZ01005877:7980-8279
HmHel-3	CAEZ01005781:6844-7837
HmHel-3	CAEZ01005780:12547-12852
HmHel-3	CAEZ01005695:8373-8655
HmHel-3	CAEZ01005667:2024-2264
HmHel-3	CAEZ01005466:13481-13723
HmHel-3	CAEZ01005426:11559-11854
HmHel-3	CAEZ01005243:8877-9417
HmHel-3	CAEZ01005162:48728-49037
HmHel-3	CAEZ01004406:894-1200
HmHel-3	CAEZ01004368:30864-31106
HmHel-3	CAEZ01004271:14127-14426
HmHel-3	CAEZ01004000:6345-6529
HmHel-3	CAEZ01003803:3892-4536
HmHel-3	CAEZ01003345:25477-25775
HmHel-3	CAEZ01003081:747-1026
HmHel-3	CAEZ01002941:22569-22867
HmHel-3	CAEZ01002436:10270-10505
HmHel-3	CAEZ01002036:9799-10104
HmHel-3	CAEZ01001998:320-572
HmHel-3	CAEZ01001281:562-804
HmHel-3	CAEZ01001213:532-841
HmHel-3	CAEZ01000206:505-805
HmHel-3	CAEZ01003578:855-1473
HmHel-3	CAEZ01003347:6192-6398
HmHel-3	CAEZ01010540:5272-5559
HmHel-3	CAEZ01010001:25047-25334
HmHel-3	CAEZ01009039:4650-5228
HmHel-3	CAEZ01008684:1380-1670
HmHel-3	CAEZ01008238:52477-52775
HmHel-3	CAEZ01006283:41165-41504
HmHel-3	CAEZ01006283:15563-16202
HmHel-3	CAEZ01005532:2687-2969
HmHel-3	CAEZ01003191:929-1153

HmHel-3	CAEZ01003122:1161-1417
HmHel-3	CAEZ01002912:1288-1575
HmHel-3	CAEZ01002581:12060-12367
HmHel-3	CAEZ01000831:12932-13234
HmHel-3	CAEZ01006335:3408-3719
HmHel-3	CAEZ01005967:18453-18748
HmHel-3	CAEZ01005967:6107-6340
HmHel-3	CAEZ01005160:4145-4452
HmHel-3	CAEZ01003127:13775-14379
HmHel-3	CAEZ01003007:57485-57790
HmHel-3	CAEZ01002802:12280-12597
HmHel-3	CAEZ01010943:11119-11425
HmHel-3	CAEZ01010343:8012-8321
HmHel-3	CAEZ01008872:12473-12765
HmHel-3	CAEZ01008254:10819-11448
HmHel-3	CAEZ01008096:2478-2772
HmHel-3	CAEZ01007466:3321-3630
HmHel-3	CAEZ01007090:6056-6361
HmHel-3	CAEZ01007074:1154-1417
HmHel-3	CAEZ01006876:6780-7103
HmHel-3	CAEZ01005954:3648-4280
HmHel-3	CAEZ01005912:4326-4601
HmHel-3	CAEZ01005858:692-990
HmHel-3	CAEZ01004196:21737-21990
HmHel-3	CAEZ01002840:1485-1793
HmHel-3	CAEZ01002804:29973-30215
HmHel-3	CAEZ01002203:9575-9860
HmHel-3	CAEZ01001715:6791-7033
HmHel-3	CAEZ01001623:127-681
HmHel-3	CAEZ01001028:909-1214
HmHel-3	CAEZ01000993:1660-1907
HmHel-3	CAEZ01000934:7754-7977
HmHel-3	CAEZ01000854:77998-78299
HmHel-3	CAEZ01010035:16066-19391
HmHel-3	CAEZ01008773:10328-10437
HmHel-3	CAEZ01005252:36446-36679
HmHel-3	CAEZ01011392:10241-10360
HmHel-3	CAEZ01010725:31991-32130
HmHel-3	CAEZ01010280:364-1782
HmHel-3	CAEZ01009867:20338-20668
HmHel-3	CAEZ01009000:3648-3992
HmHel-3	CAEZ01008994:7387-7665
HmHel-3	CAEZ01008979:8296-8495
HmHel-3	CAEZ01007450:26472-26766
HmHel-3	CAEZ01007215:1119-1422
HmHel-3	CAEZ01005800:1892-2198
HmHel-3	CAEZ01005671:11886-12101
HmHel-3	CAEZ01002244:5427-5732

HmHel-3	CAEZ01000965:2323-2610
HmHel-3	CAEZ01003681:300-494
HmHel-3	CAEZ01009401:473-753
HmHel-3	CAEZ01006900:6999-7278
HmHel-3	CAEZ01005654:1112-1417
HmHel-3	CAEZ01005116:2489-2793
HmHel-3	CAEZ01003452:24122-24427
HmHel-3	CAEZ01003014:2752-2822
HmHel-3	CAEZ01010942:17771-18074
HmHel-3	CAEZ01010799:39969-40249
HmHel-3	CAEZ01010629:6472-6776
HmHel-3	CAEZ01006655:359-481
HmHel-3	CAEZ01009126:5700-5969
HmHel-3	CAEZ01002049:103924-104199
HmHel-3	CAEZ01011472:13306-13386
	:-
HmHel-4	CAEZ01011095:42151-42451
HmHel-4	CAEZ01011095:10292-10598
HmHel-4	CAEZ01010911:3116-3416
HmHel-4	CAEZ01008621:15058-15358
HmHel-4	CAEZ01008621:26935-27256
HmHel-4	CAEZ01008404:87543-87843
HmHel-4	CAEZ01008404:104222-104522
HmHel-4	CAEZ01008404:169828-170128
HmHel-4	CAEZ01001465:131686-131983
HmHel-4	CAEZ01011506:3599-3903
HmHel-4	CAEZ01011506:34279-34603
HmHel-4	CAEZ01010930:5483-5783
HmHel-4	CAEZ01010930:45666-45934
HmHel-4	CAEZ01010896:179076-179381
HmHel-4	CAEZ01010896:285321-285617
HmHel-4	CAEZ01010896:379421-379728
HmHel-4	CAEZ01010896:268018-268324
HmHel-4	CAEZ01010896:252043-252341
HmHel-4	CAEZ01010896:133849-134149
HmHel-4	CAEZ01009835:37293-37593
HmHel-4	CAEZ01009835:34062-34344
HmHel-4	CAEZ01008126:6483-6790
HmHel-4	CAEZ01008126:47125-47425
HmHel-4	CAEZ01008126:67577-67842
HmHel-4	CAEZ01007484:1521-1821
HmHel-4	CAEZ01007266:31069-31369
HmHel-4	CAEZ01004104:17098-17398
HmHel-4	CAEZ01003738:2383-2683
HmHel-4	CAEZ01001738:30522-30822
HmHel-4	CAEZ01001738:15116-15416
HmHel-4	CAEZ01001464:9612-9909
HmHel-4	CAEZ01001464:21878-22178

HmHel-4	CAEZ01001422:7595-7895
HmHel-4	CAEZ01010935:36496-36749
HmHel-4	CAEZ01010935:24669-24983
HmHel-4	CAEZ01009802:105058-105358
HmHel-4	CAEZ01009802:218732-219029
HmHel-4	CAEZ01009802:179535-179842
HmHel-4	CAEZ01009802:103009-103309
HmHel-4	CAEZ01011022:13042-13342
HmHel-4	CAEZ01011022:18523-18820
HmHel-4	CAEZ01009783:753-1050
HmHel-4	CAEZ01001904:10280-10577
HmHel-4	CAEZ01011651:9735-10035
HmHel-4	CAEZ01011518:877-1177
HmHel-4	CAEZ01011137:9859-10159
HmHel-4	CAEZ01011137:56943-57243
HmHel-4	CAEZ01011137:70008-70201
HmHel-4	CAEZ01011137:175345-175645
HmHel-4	CAEZ01010864:15800-16100
HmHel-4	CAEZ01010864:188903-189203
HmHel-4	CAEZ01010738:281961-282270
HmHel-4	CAEZ01010738:261192-261376
HmHel-4	CAEZ01010738:198284-198584
HmHel-4	CAEZ01010738:172486-172760
HmHel-4	CAEZ01010738:81206-81505
HmHel-4	CAEZ01010711:1847-2147
HmHel-4	CAEZ01010418:380-680
HmHel-4	CAEZ01010299:3377-3677
HmHel-4	CAEZ01010266:5858-6158
HmHel-4	CAEZ01010144:3521-3821
HmHel-4	CAEZ01010144:42314-42597
HmHel-4	CAEZ01010144:60722-61508
HmHel-4	CAEZ01010144:83056-83361
HmHel-4	CAEZ01010144:109801-110104
HmHel-4	CAEZ01010129:10415-10687
HmHel-4	CAEZ01010129:67169-67469
HmHel-4	CAEZ01010129:85626-85939
HmHel-4	CAEZ01010129:49375-49675
HmHel-4	CAEZ01010087:11801-12101
HmHel-4	CAEZ01010039:107988-108275
HmHel-4	CAEZ01010039:159501-159801
HmHel-4	CAEZ01010039:198043-198350
HmHel-4	CAEZ01010039:182024-182183
HmHel-4	CAEZ01010039:115527-115692
HmHel-4	CAEZ01010039:12667-12967
HmHel-4	CAEZ01009999:20056-20283
HmHel-4	CAEZ01009999:64334-64653
HmHel-4	CAEZ01009999:47513-47619
HmHel-4	CAEZ01009999:3582-3882

HmHel-4	CAEZ01009842:9504-9804
HmHel-4	CAEZ01009725:752-1052
HmHel-4	CAEZ01009725:12488-12788
HmHel-4	CAEZ01009484:107664-107964
HmHel-4	CAEZ01009484:123905-124204
HmHel-4	CAEZ01009484:20706-20874
HmHel-4	CAEZ01009473:6424-6724
HmHel-4	CAEZ01009448:4010-4310
HmHel-4	CAEZ01009448:36830-37130
HmHel-4	CAEZ01008966:38328-38628
HmHel-4	CAEZ01008962:472-745
HmHel-4	CAEZ01008574:128312-128612
HmHel-4	CAEZ01008574:143587-143887
HmHel-4	CAEZ01008574:139447-139670
HmHel-4	CAEZ01008574:95497-95797
HmHel-4	CAEZ01008574:38483-38786
HmHel-4	CAEZ01008393:167440-167766
HmHel-4	CAEZ01008393:138728-139039
HmHel-4	CAEZ01008393:111183-111492
HmHel-4	CAEZ01008393:16316-16621
HmHel-4	CAEZ01008345:54830-55137
HmHel-4	CAEZ01008345:87711-87922
HmHel-4	CAEZ01008345:34567-34867
HmHel-4	CAEZ01008284:1586-1885
HmHel-4	CAEZ01008210:16757-17057
HmHel-4	CAEZ01008210:44327-44467
HmHel-4	CAEZ01008210:59503-59803
HmHel-4	CAEZ01008210:47071-47371
HmHel-4	CAEZ01008176:35858-36158
HmHel-4	CAEZ01007747:36986-37293
HmHel-4	CAEZ01007747:119770-120070
HmHel-4	CAEZ01007747:221537-221835
HmHel-4	CAEZ01007747:254289-254578
HmHel-4	CAEZ01007747:167105-167264
HmHel-4	CAEZ01007747:84659-84964
HmHel-4	CAEZ01007722:17235-17535
HmHel-4	CAEZ01007722:41564-41787
HmHel-4	CAEZ01007722:77531-77751
HmHel-4	CAEZ01007722:100042-100353
HmHel-4	CAEZ01007722:126672-126972
HmHel-4	CAEZ01007722:160289-160512
HmHel-4	CAEZ01007722:122354-122577
HmHel-4	CAEZ01007722:70137-70444
HmHel-4	CAEZ01007722:28199-28492
HmHel-4	CAEZ01007499:20781-21081
HmHel-4	CAEZ01007499:123924-124224
HmHel-4	CAEZ01007499:183058-183340
HmHel-4	CAEZ01007499:25758-26057

HmHel-4	CAEZ01007353:710-1010
HmHel-4	CAEZ01007268:3981-4308
HmHel-4	CAEZ01007268:32501-32800
HmHel-4	CAEZ01007268:45227-45527
HmHel-4	CAEZ01006988:11890-12217
HmHel-4	CAEZ01006988:8692-8992
HmHel-4	CAEZ01006681:10486-10786
HmHel-4	CAEZ01006529:135251-135551
HmHel-4	CAEZ01006529:196963-197263
HmHel-4	CAEZ01006326:22319-22619
HmHel-4	CAEZ01006326:85216-85516
HmHel-4	CAEZ01006326:103541-103849
HmHel-4	CAEZ01006326:79271-79578
HmHel-4	CAEZ01006308:44611-44776
HmHel-4	CAEZ01006308:14738-15038
HmHel-4	CAEZ01006262:51006-51306
HmHel-4	CAEZ01006185:23274-23574
HmHel-4	CAEZ01005482:69537-69834
HmHel-4	CAEZ01005482:17307-17607
HmHel-4	CAEZ01005393:16871-17171
HmHel-4	CAEZ01005393:102216-102495
HmHel-4	CAEZ01005393:133079-133298
HmHel-4	CAEZ01005393:69801-70090
HmHel-4	CAEZ01005393:15496-15796
HmHel-4	CAEZ01005393:2401-2686
HmHel-4	CAEZ01005261:9483-9785
HmHel-4	CAEZ01005261:11962-12262
HmHel-4	CAEZ01005088:7573-7900
HmHel-4	CAEZ01005088:11853-12153
HmHel-4	CAEZ01004917:14625-14925
HmHel-4	CAEZ01004869:28453-28753
HmHel-4	CAEZ01004811:138726-139027
HmHel-4	CAEZ01004811:158289-158588
HmHel-4	CAEZ01004811:236625-236895
HmHel-4	CAEZ01004811:257206-257506
HmHel-4	CAEZ01004811:327456-327767
HmHel-4	CAEZ01004811:427999-428097
HmHel-4	CAEZ01004811:368527-368817
HmHel-4	CAEZ01004811:272727-273027
HmHel-4	CAEZ01004811:235934-236234
HmHel-4	CAEZ01004811:175777-175968
HmHel-4	CAEZ01004811:108677-108971
HmHel-4	CAEZ01004811:92675-92983
HmHel-4	CAEZ01004616:4622-4922
HmHel-4	CAEZ01004481:10002-10302
HmHel-4	CAEZ01004481:27460-27768
HmHel-4	CAEZ01004239:30864-31152
HmHel-4	CAEZ01004239:7296-7594

HmHel-4	CAEZ01004222:10113-10413
HmHel-4	CAEZ01004094:87942-88246
HmHel-4	CAEZ01004094:92080-92380
HmHel-4	CAEZ01003695:5744-6044
HmHel-4	CAEZ01003416:46983-47283
HmHel-4	CAEZ01003300:18000-18308
HmHel-4	CAEZ01003300:26349-26649
HmHel-4	CAEZ01003070:59779-60084
HmHel-4	CAEZ01003070:133690-133989
HmHel-4	CAEZ01003070:63367-63652
HmHel-4	CAEZ01003070:27804-28104
HmHel-4	CAEZ01003050:3939-4239
HmHel-4	CAEZ01002950:43186-43486
HmHel-4	CAEZ01002950:62669-62934
HmHel-4	CAEZ01002950:31717-32017
HmHel-4	CAEZ01002501:25840-26140
HmHel-4	CAEZ01002501:10909-11204
HmHel-4	CAEZ01002332:3808-4090
HmHel-4	CAEZ01002332:14371-14671
HmHel-4	CAEZ01002213:1297-1597
HmHel-4	CAEZ01001842:1486-1786
HmHel-4	CAEZ01001805:71278-71555
HmHel-4	CAEZ01001805:87390-87677
HmHel-4	CAEZ01001805:34315-34615
HmHel-4	CAEZ01001736:104199-104499
HmHel-4	CAEZ01001510:15064-15364
HmHel-4	CAEZ01001510:34821-35128
HmHel-4	CAEZ01000907:1027-1327
HmHel-4	CAEZ01008909:3574-3873
HmHel-4	CAEZ01008035:3881-4174
HmHel-4	CAEZ01005972:36693-36992
HmHel-4	CAEZ01005972:16774-17074
HmHel-4	CAEZ01005928:874-1173
HmHel-4	CAEZ01005928:6960-7259
HmHel-4	CAEZ01001766:17994-18292
HmHel-4	CAEZ01001766:96670-96981
HmHel-4	CAEZ01001766:127647-127896
HmHel-4	CAEZ01001766:143528-143835
HmHel-4	CAEZ01001766:107519-107818
HmHel-4	CAEZ01001766:14207-14506
HmHel-4	CAEZ01001147:29598-29897
HmHel-4	CAEZ01009332:17236-17534
HmHel-4	CAEZ01006337:71076-71391
HmHel-4	CAEZ01006337:75553-75851
HmHel-4	CAEZ01004126:33116-33414
HmHel-4	CAEZ01009786:33494-33791
HmHel-4	CAEZ01009126:30-327
HmHel-4	CAEZ01008866:9732-10038

HmHel-4	CAEZ01008866:927-1227
HmHel-4	CAEZ01007985:8906-9203
HmHel-4	CAEZ01007985:36977-37274
HmHel-4	CAEZ01007985:43076-43375
HmHel-4	CAEZ01007940:46560-46858
HmHel-4	CAEZ01006530:54971-55271
HmHel-4	CAEZ01006530:115653-115950
HmHel-4	CAEZ01006530:219773-220069
HmHel-4	CAEZ01006530:120335-120621
HmHel-4	CAEZ01006530:108725-109025
HmHel-4	CAEZ01006530:93558-93855
HmHel-4	CAEZ01006186:8919-9245
HmHel-4	CAEZ01003647:8458-8758
HmHel-4	CAEZ01003647:70647-70960
HmHel-4	CAEZ01003647:26499-26796
HmHel-4	CAEZ01002815:81759-82056
HmHel-4	CAEZ01002815:49910-50210
HmHel-4	CAEZ01011616:14438-14738
HmHel-4	CAEZ01011554:22293-22593
HmHel-4	CAEZ01011149:5360-5555
HmHel-4	CAEZ01011149:3668-3968
HmHel-4	CAEZ01011134:75352-75652
HmHel-4	CAEZ01011134:98701-99001
HmHel-4	CAEZ01011134:85431-85579
HmHel-4	CAEZ01011057:4390-4690
HmHel-4	CAEZ01011025:15058-15358
HmHel-4	CAEZ01010876:17033-17333
HmHel-4	CAEZ01010843:13390-13690
HmHel-4	CAEZ01010827:123908-124092
HmHel-4	CAEZ01010827:368464-368776
HmHel-4	CAEZ01010827:430284-430508
HmHel-4	CAEZ01010827:390343-390643
HmHel-4	CAEZ01010827:213325-213625
HmHel-4	CAEZ01010799:2935-3162
HmHel-4	CAEZ01010799:34281-34581
HmHel-4	CAEZ01010757:10207-10507
HmHel-4	CAEZ01010708:14028-14328
HmHel-4	CAEZ01010700:13802-14102
HmHel-4	CAEZ01010682:8601-8901
HmHel-4	CAEZ01010628:5655-5827
HmHel-4	CAEZ01010628:40426-40726
HmHel-4	CAEZ01010628:1529-1836
HmHel-4	CAEZ01010428:11667-11989
HmHel-4	CAEZ01010428:52947-53211
HmHel-4	CAEZ01010428:117018-117240
HmHel-4	CAEZ01010428:58434-58734
HmHel-4	CAEZ01010428:36363-36544
HmHel-4	CAEZ01010428:5968-6268

HmHel-4	CAEZ01010139:87984-88284
HmHel-4	CAEZ01010139:120912-121210
HmHel-4	CAEZ01010139:107453-107734
HmHel-4	CAEZ01010139:42144-42444
HmHel-4	CAEZ01009935:4295-4595
HmHel-4	CAEZ01009891:29863-30149
HmHel-4	CAEZ01009891:76187-76487
HmHel-4	CAEZ01009891:23669-23969
HmHel-4	CAEZ01009891:10458-10758
HmHel-4	CAEZ01009886:50174-50474
HmHel-4	CAEZ01009886:36249-36532
HmHel-4	CAEZ01009872:2992-3273
HmHel-4	CAEZ01009872:119008-119179
HmHel-4	CAEZ01009872:124419-124719
HmHel-4	CAEZ01009872:70445-70745
HmHel-4	CAEZ01009872:6970-7267
HmHel-4	CAEZ01009609:7068-7368
HmHel-4	CAEZ01009608:17961-18164
HmHel-4	CAEZ01009608:22471-22771
HmHel-4	CAEZ01009511:24855-25155
HmHel-4	CAEZ01009511:7431-7725
HmHel-4	CAEZ01009452:24614-24914
HmHel-4	CAEZ01009442:1249-1549
HmHel-4	CAEZ01009325:24084-24384
HmHel-4	CAEZ01009313:1327-1627
HmHel-4	CAEZ01009021:5327-5623
HmHel-4	CAEZ01009013:30154-30454
HmHel-4	CAEZ01008857:8423-8723
HmHel-4	CAEZ01008857:39834-40139
HmHel-4	CAEZ01008857:21125-21405
HmHel-4	CAEZ01008847:7357-7657
HmHel-4	CAEZ01008847:20415-20715
HmHel-4	CAEZ01008740:13287-13545
HmHel-4	CAEZ01008740:95652-95932
HmHel-4	CAEZ01008740:77713-78013
HmHel-4	CAEZ01008740:66810-67110
HmHel-4	CAEZ01008499:24313-24601
HmHel-4	CAEZ01008499:1945-2245
HmHel-4	CAEZ01008427:82355-82655
HmHel-4	CAEZ01008427:33862-34041
HmHel-4	CAEZ01008367:85593-85859
HmHel-4	CAEZ01008367:14414-14714
HmHel-4	CAEZ01008294:6057-6357
HmHel-4	CAEZ01008294:16726-16901
HmHel-4	CAEZ01008294:62701-63000
HmHel-4	CAEZ01008294:80580-80873
HmHel-4	CAEZ01008294:130578-130878
HmHel-4	CAEZ01008294:139703-140003

HmHel-4	CAEZ01008234:184381-184681
HmHel-4	CAEZ01007968:9919-10219
HmHel-4	CAEZ01007923:24505-24805
HmHel-4	CAEZ01007730:50105-50356
HmHel-4	CAEZ01007730:83366-83666
HmHel-4	CAEZ01007716:38474-38774
HmHel-4	CAEZ01007662:12795-13095
HmHel-4	CAEZ01007389:7359-7660
HmHel-4	CAEZ01007389:29707-29993
HmHel-4	CAEZ01007389:117551-117844
HmHel-4	CAEZ01007389:171404-171704
HmHel-4	CAEZ01007389:132032-132323
HmHel-4	CAEZ01007272:50686-50931
HmHel-4	CAEZ01007272:50136-50431
HmHel-4	CAEZ01007272:24565-24865
HmHel-4	CAEZ01007267:12152-12452
HmHel-4	CAEZ01007267:22296-22529
HmHel-4	CAEZ01007259:39031-39251
HmHel-4	CAEZ01007259:109655-109955
HmHel-4	CAEZ01007259:108952-109230
HmHel-4	CAEZ01007259:88482-88782
HmHel-4	CAEZ01007259:50588-50847
HmHel-4	CAEZ01007156:4407-4707
HmHel-4	CAEZ01007096:6692-6801
HmHel-4	CAEZ01007096:7689-7989
HmHel-4	CAEZ01007066:73841-74134
HmHel-4	CAEZ01007066:109363-109656
HmHel-4	CAEZ01006803:35055-35379
HmHel-4	CAEZ01006803:43838-44138
HmHel-4	CAEZ01006803:7522-7822
HmHel-4	CAEZ01006772:16713-17011
HmHel-4	CAEZ01006772:103854-104154
HmHel-4	CAEZ01006772:126996-127303
HmHel-4	CAEZ01006772:173470-173770
HmHel-4	CAEZ01006772:205139-205441
HmHel-4	CAEZ01006772:229739-230046
HmHel-4	CAEZ01006772:248902-249202
HmHel-4	CAEZ01006772:211885-212179
HmHel-4	CAEZ01006772:198527-198854
HmHel-4	CAEZ01006772:121791-122090
HmHel-4	CAEZ01006772:10050-10355
HmHel-4	CAEZ01006761:13591-13891
HmHel-4	CAEZ01006695:5526-5826
HmHel-4	CAEZ01006487:13723-14023
HmHel-4	CAEZ01006346:14414-14707
HmHel-4	CAEZ01006346:6018-6318
HmHel-4	CAEZ01006317:3731-4031
HmHel-4	CAEZ01006317:22264-22491

HmHel-4	CAEZ01006285:5719-6038
HmHel-4	CAEZ01006285:16903-17203
HmHel-4	CAEZ01006285:8498-8797
HmHel-4	CAEZ01006228:36110-36356
HmHel-4	CAEZ01006228:45128-45428
HmHel-4	CAEZ01006228:8392-8687
HmHel-4	CAEZ01006162:45232-45532
HmHel-4	CAEZ01006162:64224-64519
HmHel-4	CAEZ01006162:114136-114435
HmHel-4	CAEZ01006162:78297-78598
HmHel-4	CAEZ01006162:63469-63724
HmHel-4	CAEZ01006162:13549-13850
HmHel-4	CAEZ01006161:5516-5816
HmHel-4	CAEZ01006161:97305-97605
HmHel-4	CAEZ01006161:73729-73912
HmHel-4	CAEZ01006150:18500-18803
HmHel-4	CAEZ01006150:31428-31719
HmHel-4	CAEZ01006150:45068-45331
HmHel-4	CAEZ01006150:58280-58580
HmHel-4	CAEZ01006150:43138-43438
HmHel-4	CAEZ01006042:1238-1382
HmHel-4	CAEZ01006042:5423-5723
HmHel-4	CAEZ01005919:17054-17353
HmHel-4	CAEZ01005919:12286-12586
HmHel-4	CAEZ01005690:9412-9712
HmHel-4	CAEZ01005615:34242-34542
HmHel-4	CAEZ01005615:1941-2241
HmHel-4	CAEZ01005581:18171-18471
HmHel-4	CAEZ01005581:4926-5235
HmHel-4	CAEZ01005392:68957-69257
HmHel-4	CAEZ01005235:125329-125629
HmHel-4	CAEZ01005235:89149-89456
HmHel-4	CAEZ01005235:55614-61953
HmHel-4	CAEZ01005108:83892-84192
HmHel-4	CAEZ01005108:48029-48329
HmHel-4	CAEZ01005105:7972-8132
HmHel-4	CAEZ01005105:35855-36156
HmHel-4	CAEZ01005066:27933-28195
HmHel-4	CAEZ01005066:80016-80316
HmHel-4	CAEZ01005066:12918-13211
HmHel-4	CAEZ01004795:24196-24496
HmHel-4	CAEZ01004795:3383-3694
HmHel-4	CAEZ01004786:43431-43638
HmHel-4	CAEZ01004786:20900-21200
HmHel-4	CAEZ01004535:1333-1599
HmHel-4	CAEZ01004535:21801-22108
HmHel-4	CAEZ01004535:33350-33650
HmHel-4	CAEZ01004389:8064-8364

HmHel-4	CAEZ01004125:17253-17553
HmHel-4	CAEZ01003882:25938-26238
HmHel-4	CAEZ01003844:2106-2406
HmHel-4	CAEZ01003844:21601-21902
HmHel-4	CAEZ01003842:24323-24623
HmHel-4	CAEZ01003800:5950-6250
HmHel-4	CAEZ01003750:137894-138193
HmHel-4	CAEZ01003750:115456-115746
HmHel-4	CAEZ01003750:79967-80267
HmHel-4	CAEZ01003750:39093-39346
HmHel-4	CAEZ01003710:3555-3855
HmHel-4	CAEZ01003422:50156-50463
HmHel-4	CAEZ01003422:72774-73073
HmHel-4	CAEZ01003422:60209-60509
HmHel-4	CAEZ01003420:7881-8181
HmHel-4	CAEZ01003328:60548-60860
HmHel-4	CAEZ01003328:109358-109656
HmHel-4	CAEZ01003328:118700-119000
HmHel-4	CAEZ01003328:84375-84675
HmHel-4	CAEZ01003328:28987-29287
HmHel-4	CAEZ01003260:103882-104190
HmHel-4	CAEZ01003260:126527-126827
HmHel-4	CAEZ01003260:97819-98119
HmHel-4	CAEZ01003260:27814-28121
HmHel-4	CAEZ01003112:15800-16100
HmHel-4	CAEZ01002808:13986-14286
HmHel-4	CAEZ01002808:45903-46203
HmHel-4	CAEZ01002765:63052-63352
HmHel-4	CAEZ01002765:43379-43587
HmHel-4	CAEZ01002729:39515-39815
HmHel-4	CAEZ01002729:38434-38734
HmHel-4	CAEZ01002729:11417-11717
HmHel-4	CAEZ01002680:12848-13138
HmHel-4	CAEZ01002680:25517-25817
HmHel-4	CAEZ01002643:45456-45756
HmHel-4	CAEZ01002633:24957-25267
HmHel-4	CAEZ01002633:4198-4497
HmHel-4	CAEZ01002074:22061-22361
HmHel-4	CAEZ01002074:6493-6793
HmHel-4	CAEZ01001595:37684-37801
HmHel-4	CAEZ01001595:94068-94232
HmHel-4	CAEZ01001595:115323-115513
HmHel-4	CAEZ01001595:79939-80239
HmHel-4	CAEZ01001595:20064-20364
HmHel-4	CAEZ01001443:43545-43845
HmHel-4	CAEZ01001238:875-1175
HmHel-4	CAEZ01001114:3386-3686
HmHel-4	CAEZ01001023:8877-9177

HmHel-4	CAEZ01000849:71528-71828
HmHel-4	CAEZ01000849:93298-93621
HmHel-4	CAEZ01000849:109314-109614
HmHel-4	CAEZ01000849:48550-48854
HmHel-4	CAEZ01010083:5302-5601
HmHel-4	CAEZ01009507:15229-15528
HmHel-4	CAEZ01009507:45875-46174
HmHel-4	CAEZ01009507:39827-39972
HmHel-4	CAEZ01009441:20897-21197
HmHel-4	CAEZ01009441:2406-2705
HmHel-4	CAEZ01006416:6752-7051
HmHel-4	CAEZ01006416:10189-10315
HmHel-4	CAEZ01002915:1966-2265
HmHel-4	CAEZ01001265:119464-119763
HmHel-4	CAEZ01001265:61433-61740
HmHel-4	CAEZ01001265:4042-4310
HmHel-4	CAEZ01001217:9355-9657
HmHel-4	CAEZ01005402:34278-34578
HmHel-4	CAEZ01005402:62575-62876
HmHel-4	CAEZ01005402:18374-18672
HmHel-4	CAEZ01005402:5124-5423
HmHel-4	CAEZ01001499:1460-1758
HmHel-4	CAEZ01010221:13562-13863
HmHel-4	CAEZ01010221:55675-55972
HmHel-4	CAEZ01010221:8299-8599
HmHel-4	CAEZ01008677:16890-17187
HmHel-4	CAEZ01008677:28516-28816
HmHel-4	CAEZ01008677:98672-98972
HmHel-4	CAEZ01008677:27702-28009
HmHel-4	CAEZ01008270:12479-12784
HmHel-4	CAEZ01008270:93264-93561
HmHel-4	CAEZ01008270:106305-106448
HmHel-4	CAEZ01007726:12439-12736
HmHel-4	CAEZ01007726:13709-14010
HmHel-4	CAEZ01006202:22407-22708
HmHel-4	CAEZ01004096:120643-120943
HmHel-4	CAEZ01004096:31755-32055
HmHel-4	CAEZ01004096:4878-5175
HmHel-4	CAEZ01002360:19996-20293
HmHel-4	CAEZ01002360:2782-3081
HmHel-4	CAEZ01001869:23786-24083
HmHel-4	CAEZ01011495:15310-15433
HmHel-4	CAEZ01011330:4981-5281
HmHel-4	CAEZ01011330:22394-22694
HmHel-4	CAEZ01011330:8729-9022
HmHel-4	CAEZ01011239:14574-14874
HmHel-4	CAEZ01011227:25853-26087
HmHel-4	CAEZ01011227:28623-28923

HmHel-4	CAEZ01011227:4758-5053
HmHel-4	CAEZ01011038:7508-7812
HmHel-4	CAEZ01011038:1911-2211
HmHel-4	CAEZ01010963:2331-2631
HmHel-4	CAEZ01010932:5156-5452
HmHel-4	CAEZ01010932:52991-53291
HmHel-4	CAEZ01010932:54264-54483
HmHel-4	CAEZ01010859:290-590
HmHel-4	CAEZ01010850:7130-7430
HmHel-4	CAEZ01010841:5420-5720
HmHel-4	CAEZ01010770:6015-6325
HmHel-4	CAEZ01010770:15268-15568
HmHel-4	CAEZ01010753:17131-17446
HmHel-4	CAEZ01010753:13161-13461
HmHel-4	CAEZ01010701:48127-48427
HmHel-4	CAEZ01010701:88844-89144
HmHel-4	CAEZ01010701:180669-180969
HmHel-4	CAEZ01010701:198781-199081
HmHel-4	CAEZ01010701:170270-170522
HmHel-4	CAEZ01010701:9365-9660
HmHel-4	CAEZ01010666:61825-62125
HmHel-4	CAEZ01010666:74453-74782
HmHel-4	CAEZ01010666:48495-48802
HmHel-4	CAEZ01010558:7873-8173
HmHel-4	CAEZ01010446:69939-70239
HmHel-4	CAEZ01010301:49006-49306
HmHel-4	CAEZ01010301:87658-87928
HmHel-4	CAEZ01010301:91602-91812
HmHel-4	CAEZ01010301:72801-73105
HmHel-4	CAEZ01010084:45475-45772
HmHel-4	CAEZ01010084:5575-5875
HmHel-4	CAEZ01010026:114901-115201
HmHel-4	CAEZ01010026:93998-94272
HmHel-4	CAEZ01009937:1137-1437
HmHel-4	CAEZ01009885:37422-37714
HmHel-4	CAEZ01009885:4043-4343
HmHel-4	CAEZ01009871:1050-1336
HmHel-4	CAEZ01009871:24711-25011
HmHel-4	CAEZ01009859:14608-14910
HmHel-4	CAEZ01009859:21113-21413
HmHel-4	CAEZ01009844:8767-9228
HmHel-4	CAEZ01009844:48168-48468
HmHel-4	CAEZ01009844:138228-138450
HmHel-4	CAEZ01009844:145243-145542
HmHel-4	CAEZ01009844:133526-133826
HmHel-4	CAEZ01009844:115001-115301
HmHel-4	CAEZ01009832:1-288
HmHel-4	CAEZ01009832:20348-20648

HmHel-4	CAEZ01009832:13608-13740
HmHel-4	CAEZ01009807:11998-12298
HmHel-4	CAEZ01009806:6183-6483
HmHel-4	CAEZ01009796:1331-1631
HmHel-4	CAEZ01009703:10879-11179
HmHel-4	CAEZ01009681:61523-61823
HmHel-4	CAEZ01009681:33643-33943
HmHel-4	CAEZ01009643:26388-26688
HmHel-4	CAEZ01009643:898-1205
HmHel-4	CAEZ01009583:15234-15534
HmHel-4	CAEZ01009583:72566-72866
HmHel-4	CAEZ01009583:47886-48175
HmHel-4	CAEZ01009583:17748-18048
HmHel-4	CAEZ01009564:10278-10578
HmHel-4	CAEZ01009481:45482-45782
HmHel-4	CAEZ01009481:47742-48042
HmHel-4	CAEZ01009476:1941-2245
HmHel-4	CAEZ01009476:18154-18461
HmHel-4	CAEZ01009476:68855-69152
HmHel-4	CAEZ01009476:48465-48697
HmHel-4	CAEZ01009476:23276-23576
HmHel-4	CAEZ01009470:78980-79159
HmHel-4	CAEZ01009470:99090-99390
HmHel-4	CAEZ01009470:62323-62615
HmHel-4	CAEZ01009470:38557-38857
HmHel-4	CAEZ01009470:5127-5424
HmHel-4	CAEZ01009263:32464-32768
HmHel-4	CAEZ01009263:19974-20274
HmHel-4	CAEZ01009223:20853-21160
HmHel-4	CAEZ01009223:41381-41704
HmHel-4	CAEZ01009223:63801-64101
HmHel-4	CAEZ01009223:11487-18440
HmHel-4	CAEZ01009115:12350-12650
HmHel-4	CAEZ01009115:2031-2134
HmHel-4	CAEZ01009107:3955-4255
HmHel-4	CAEZ01009044:86165-86465
HmHel-4	CAEZ01009044:43735-43984
HmHel-4	CAEZ01008977:15256-15563
HmHel-4	CAEZ01008977:2033-2338
HmHel-4	CAEZ01008883:5240-5540
HmHel-4	CAEZ01008879:1-256
HmHel-4	CAEZ01008879:56022-56321
HmHel-4	CAEZ01008879:115731-116038
HmHel-4	CAEZ01008879:96970-97082
HmHel-4	CAEZ01008879:61729-62029
HmHel-4	CAEZ01008879:21974-22274
HmHel-4	CAEZ01008763:19436-19736
HmHel-4	CAEZ01008760:8794-9094

HmHel-4	CAEZ01008746:11986-12267
HmHel-4	CAEZ01008746:23644-23941
HmHel-4	CAEZ01008746:115698-116024
HmHel-4	CAEZ01008746:194182-194485
HmHel-4	CAEZ01008746:162796-163096
HmHel-4	CAEZ01008734:61736-62036
HmHel-4	CAEZ01008673:13941-14241
HmHel-4	CAEZ01008673:25471-25771
HmHel-4	CAEZ01008673:5601-5899
HmHel-4	CAEZ01008620:161672-161972
HmHel-4	CAEZ01008620:189122-189422
HmHel-4	CAEZ01008435:5493-5790
HmHel-4	CAEZ01008435:89004-89311
HmHel-4	CAEZ01008435:143615-143959
HmHel-4	CAEZ01008435:202073-202380
HmHel-4	CAEZ01008435:244828-245135
HmHel-4	CAEZ01008435:126166-126466
HmHel-4	CAEZ01008435:80647-80946
HmHel-4	CAEZ01008435:51535-51682
HmHel-4	CAEZ01008435:27760-27945
HmHel-4	CAEZ01008419:13530-13830
HmHel-4	CAEZ01008280:9819-10121
HmHel-4	CAEZ01008279:8069-8376
HmHel-4	CAEZ01008279:80325-80625
HmHel-4	CAEZ01008279:263514-263821
HmHel-4	CAEZ01008279:253514-253824
HmHel-4	CAEZ01008279:228627-228927
HmHel-4	CAEZ01008279:154804-155078
HmHel-4	CAEZ01008279:64608-64932
HmHel-4	CAEZ01008279:31697-31997
HmHel-4	CAEZ01008279:10651-10925
HmHel-4	CAEZ01008261:61080-61387
HmHel-4	CAEZ01008261:75427-75727
HmHel-4	CAEZ01008261:97393-97558
HmHel-4	CAEZ01008261:114417-114717
HmHel-4	CAEZ01008261:130079-130383
HmHel-4	CAEZ01008261:171030-171330
HmHel-4	CAEZ01008261:167553-167848
HmHel-4	CAEZ01008261:92775-93075
HmHel-4	CAEZ01008260:2028-2328
HmHel-4	CAEZ01008237:23213-23513
HmHel-4	CAEZ01008237:14875-15102
HmHel-4	CAEZ01008208:11028-11264
HmHel-4	CAEZ01008208:106287-106594
HmHel-4	CAEZ01008208:198121-198416
HmHel-4	CAEZ01008208:223675-223805
HmHel-4	CAEZ01008208:210485-210676
HmHel-4	CAEZ01008208:135453-135747

HmHel-4	CAEZ01008208:103641-103969
HmHel-4	CAEZ01008208:59147-59457
HmHel-4	CAEZ01008139:33455-33755
HmHel-4	CAEZ01008104:14277-14577
HmHel-4	CAEZ01008089:40530-40674
HmHel-4	CAEZ01008089:11414-11713
HmHel-4	CAEZ01008028:16586-16886
HmHel-4	CAEZ01008028:8044-8344
HmHel-4	CAEZ01008017:3438-3738
HmHel-4	CAEZ01007983:35897-36190
HmHel-4	CAEZ01007983:91712-92012
HmHel-4	CAEZ01007983:193160-193436
HmHel-4	CAEZ01007983:101590-101924
HmHel-4	CAEZ01007983:40365-40669
HmHel-4	CAEZ01007983:20572-20872
HmHel-4	CAEZ01007849:38338-38635
HmHel-4	CAEZ01007849:44258-44558
HmHel-4	CAEZ01007849:12493-12793
HmHel-4	CAEZ01007773:880-1180
HmHel-4	CAEZ01007765:165671-165970
HmHel-4	CAEZ01007765:544440-544740
HmHel-4	CAEZ01007765:503393-503693
HmHel-4	CAEZ01007765:309518-309815
HmHel-4	CAEZ01007765:261193-261493
HmHel-4	CAEZ01007765:197571-197916
HmHel-4	CAEZ01007556:19672-19972
HmHel-4	CAEZ01007509:468-768
HmHel-4	CAEZ01007480:19420-19635
HmHel-4	CAEZ01007480:35648-35948
HmHel-4	CAEZ01007480:30510-30813
HmHel-4	CAEZ01007413:3079-3208
HmHel-4	CAEZ01007413:24108-24369
HmHel-4	CAEZ01007413:45664-45782
HmHel-4	CAEZ01007413:84155-84456
HmHel-4	CAEZ01007413:34109-34411
HmHel-4	CAEZ01007413:16210-16510
HmHel-4	CAEZ01007371:23686-23986
HmHel-4	CAEZ01007371:7150-7437
HmHel-4	CAEZ01007213:7434-7734
HmHel-4	CAEZ01007207:11841-12165
HmHel-4	CAEZ01007207:62043-62322
HmHel-4	CAEZ01007207:138945-139168
HmHel-4	CAEZ01007207:276724-277024
HmHel-4	CAEZ01007207:166470-166632
HmHel-4	CAEZ01007207:126000-126300
HmHel-4	CAEZ01007207:51430-51723
HmHel-4	CAEZ01007184:21190-21490
HmHel-4	CAEZ01007184:65855-66147

HmHel-4	CAEZ01007184:285143-285450
HmHel-4	CAEZ01007184:138640-138898
HmHel-4	CAEZ01007137:4020-4320
HmHel-4	CAEZ01007137:22106-22406
HmHel-4	CAEZ01007124:64404-64704
HmHel-4	CAEZ01007080:12798-13098
HmHel-4	CAEZ01007079:1361-1661
HmHel-4	CAEZ01007078:4648-4948
HmHel-4	CAEZ01007062:14014-14162
HmHel-4	CAEZ01007062:2266-2566
HmHel-4	CAEZ01006991:13189-13516
HmHel-4	CAEZ01006950:497-797
HmHel-4	CAEZ01006716:3068-3368
HmHel-4	CAEZ01006628:13133-13433
HmHel-4	CAEZ01006628:39580-39873
HmHel-4	CAEZ01006618:25114-25414
HmHel-4	CAEZ01006618:117376-117678
HmHel-4	CAEZ01006618:145839-146134
HmHel-4	CAEZ01006618:195719-196019
HmHel-4	CAEZ01006618:60878-61177
HmHel-4	CAEZ01006592:12801-13093
HmHel-4	CAEZ01006592:24414-24713
HmHel-4	CAEZ01006592:73376-73683
HmHel-4	CAEZ01006592:103104-103406
HmHel-4	CAEZ01006592:31148-31448
HmHel-4	CAEZ01006592:8320-8620
HmHel-4	CAEZ01006562:57700-58000
HmHel-4	CAEZ01006562:108324-108640
HmHel-4	CAEZ01006562:259861-260161
HmHel-4	CAEZ01006562:150953-151255
HmHel-4	CAEZ01006468:6380-6680
HmHel-4	CAEZ01006403:24787-25087
HmHel-4	CAEZ01006403:84399-84695
HmHel-4	CAEZ01006286:8537-8837
HmHel-4	CAEZ01006286:42606-42908
HmHel-4	CAEZ01006286:99312-99611
HmHel-4	CAEZ01006286:131685-131985
HmHel-4	CAEZ01006286:111846-112065
HmHel-4	CAEZ01006286:18347-18647
HmHel-4	CAEZ01006230:20765-21065
HmHel-4	CAEZ01006212:8416-8716
HmHel-4	CAEZ01006166:10834-11134
HmHel-4	CAEZ01006166:21830-22126
HmHel-4	CAEZ01006154:44258-44557
HmHel-4	CAEZ01006154:62599-62904
HmHel-4	CAEZ01006154:123281-123608
HmHel-4	CAEZ01006154:280784-281084
HmHel-4	CAEZ01006154:150798-151098

HmHel-4	CAEZ01006154:139930-140230
HmHel-4	CAEZ01006154:56930-57230
HmHel-4	CAEZ01005951:27670-27970
HmHel-4	CAEZ01005721:9030-9330
HmHel-4	CAEZ01005721:22036-22192
HmHel-4	CAEZ01005721:53035-53258
HmHel-4	CAEZ01005721:69019-69348
HmHel-4	CAEZ01005721:105800-106100
HmHel-4	CAEZ01005721:115238-115538
HmHel-4	CAEZ01005721:57416-57719
HmHel-4	CAEZ01005721:20931-21228
HmHel-4	CAEZ01005711:48935-49262
HmHel-4	CAEZ01005711:71985-72285
HmHel-4	CAEZ01005711:145908-146203
HmHel-4	CAEZ01005711:169189-169402
HmHel-4	CAEZ01005711:122402-122731
HmHel-4	CAEZ01005711:95554-95853
HmHel-4	CAEZ01005711:64208-64535
HmHel-4	CAEZ01005669:17313-17613
HmHel-4	CAEZ01005669:26170-26264
HmHel-4	CAEZ01005660:53462-53754
HmHel-4	CAEZ01005660:33419-33719
HmHel-4	CAEZ01005660:1856-2156
HmHel-4	CAEZ01005605:27163-27391
HmHel-4	CAEZ01005605:57234-57534
HmHel-4	CAEZ01005605:91994-92294
HmHel-4	CAEZ01005605:116633-116940
HmHel-4	CAEZ01005557:5941-6242
HmHel-4	CAEZ01005557:14148-14448
HmHel-4	CAEZ01005527:30758-31058
HmHel-4	CAEZ01005527:16392-16692
HmHel-4	CAEZ01005439:146419-146719
HmHel-4	CAEZ01005439:163033-163291
HmHel-4	CAEZ01005373:45958-46285
HmHel-4	CAEZ01005373:99025-99200
HmHel-4	CAEZ01005373:126480-126775
HmHel-4	CAEZ01005373:118935-119230
HmHel-4	CAEZ01005373:81419-81571
HmHel-4	CAEZ01005373:49807-50107
HmHel-4	CAEZ01005334:23971-24271
HmHel-4	CAEZ01005269:3195-3502
HmHel-4	CAEZ01005269:19865-20158
HmHel-4	CAEZ01005269:74493-74820
HmHel-4	CAEZ01005269:109336-109663
HmHel-4	CAEZ01005269:110858-111158
HmHel-4	CAEZ01005269:9585-9885
HmHel-4	CAEZ01005266:2771-3071
HmHel-4	CAEZ01005152:69495-69795

HmHel-4	CAEZ01004841:20182-20482
HmHel-4	CAEZ01004841:9294-9593
HmHel-4	CAEZ01004754:1582-1882
HmHel-4	CAEZ01004735:15685-15985
HmHel-4	CAEZ01004631:80054-80378
HmHel-4	CAEZ01004631:115434-115733
HmHel-4	CAEZ01004631:295388-295688
HmHel-4	CAEZ01004631:311767-312094
HmHel-4	CAEZ01004631:255004-255309
HmHel-4	CAEZ01004631:152194-152501
HmHel-4	CAEZ01004631:35761-36068
HmHel-4	CAEZ01004622:8087-8387
HmHel-4	CAEZ01004545:7313-7634
HmHel-4	CAEZ01004545:44722-45020
HmHel-4	CAEZ01004545:30178-30478
HmHel-4	CAEZ01004271:33642-33946
HmHel-4	CAEZ01004271:6102-6402
HmHel-4	CAEZ01004259:33604-33904
HmHel-4	CAEZ01004259:54674-54974
HmHel-4	CAEZ01004259:24766-25066
HmHel-4	CAEZ01004252:50974-51274
HmHel-4	CAEZ01004056:9731-10031
HmHel-4	CAEZ01004011:54486-54734
HmHel-4	CAEZ01004011:133549-133849
HmHel-4	CAEZ01004011:51370-51542
HmHel-4	CAEZ01003945:28279-28579
HmHel-4	CAEZ01003945:37654-37954
HmHel-4	CAEZ01003746:45454-45754
HmHel-4	CAEZ01003746:75836-76143
HmHel-4	CAEZ01003746:30369-30676
HmHel-4	CAEZ01003746:11775-12087
HmHel-4	CAEZ01003517:43489-43805
HmHel-4	CAEZ01003517:18357-18657
HmHel-4	CAEZ01003466:5170-5470
HmHel-4	CAEZ01003447:302-602
HmHel-4	CAEZ01003247:6132-6431
HmHel-4	CAEZ01003222:8044-8344
HmHel-4	CAEZ01003222:65605-65900
HmHel-4	CAEZ01003222:51636-51936
HmHel-4	CAEZ01003175:10810-11114
HmHel-4	CAEZ01003175:32468-32768
HmHel-4	CAEZ01003173:1069-1293
HmHel-4	CAEZ01003173:42332-42609
HmHel-4	CAEZ01003173:68711-68977
HmHel-4	CAEZ01003173:53192-53511
HmHel-4	CAEZ01003173:24933-25232
HmHel-4	CAEZ01003110:48292-48599
HmHel-4	CAEZ01003110:59794-60101

HmHel-4	CAEZ01003110:70675-70975
HmHel-4	CAEZ01003110:91903-92178
HmHel-4	CAEZ01003110:74334-74634
HmHel-4	CAEZ01003110:39122-39400
HmHel-4	CAEZ01003110:16600-16907
HmHel-4	CAEZ01002964:46675-46975
HmHel-4	CAEZ01002964:4001-4301
HmHel-4	CAEZ01002944:19465-19767
HmHel-4	CAEZ01002944:79172-79473
HmHel-4	CAEZ01002944:66731-67031
HmHel-4	CAEZ01002936:16222-16522
HmHel-4	CAEZ01002936:26585-26822
HmHel-4	CAEZ01002838:64013-64313
HmHel-4	CAEZ01002838:9663-9970
HmHel-4	CAEZ01002713:6720-6954
HmHel-4	CAEZ01002713:7299-7599
HmHel-4	CAEZ01002706:105869-106164
HmHel-4	CAEZ01002706:77729-78025
HmHel-4	CAEZ01002706:66039-66339
HmHel-4	CAEZ01002629:35663-35861
HmHel-4	CAEZ01002629:14975-15275
HmHel-4	CAEZ01002289:21311-21610
HmHel-4	CAEZ01002221:1229-1529
HmHel-4	CAEZ01002195:10336-10636
HmHel-4	CAEZ01002182:35277-35576
HmHel-4	CAEZ01002182:100863-101187
HmHel-4	CAEZ01002182:125267-125567
HmHel-4	CAEZ01002182:98092-98393
HmHel-4	CAEZ01002182:11404-11704
HmHel-4	CAEZ01002064:21023-21323
HmHel-4	CAEZ01002064:26201-26512
HmHel-4	CAEZ01001947:8955-9255
HmHel-4	CAEZ01001850:29295-29595
HmHel-4	CAEZ01001850:42051-42350
HmHel-4	CAEZ01001777:23300-23599
HmHel-4	CAEZ01001466:87808-88108
HmHel-4	CAEZ01001466:49639-49881
HmHel-4	CAEZ01001466:4374-4670
HmHel-4	CAEZ01001403:38212-44054
HmHel-4	CAEZ01001403:4125-4425
HmHel-4	CAEZ01001033:2116-2416
HmHel-4	CAEZ01001004:15883-16183
HmHel-4	CAEZ01000923:1605-1905
HmHel-4	CAEZ01000912:31202-31502
HmHel-4	CAEZ01000912:57556-57867
HmHel-4	CAEZ01000912:18085-18386
HmHel-4	CAEZ01000056:25427-25727
HmHel-4	CAEZ01000056:41924-42224

HmHel-4	CAEZ01000052:1414-1714
HmHel-4	CAEZ01011083:130517-130807
HmHel-4	CAEZ01011083:192372-192672
HmHel-4	CAEZ01011083:140614-140914
HmHel-4	CAEZ01011083:110483-110782
HmHel-4	CAEZ01010927:17424-17722
HmHel-4	CAEZ01010927:54565-54850
HmHel-4	CAEZ01010927:166986-167085
HmHel-4	CAEZ01010927:100467-100776
HmHel-4	CAEZ01010927:32993-33158
HmHel-4	CAEZ01010796:63744-64044
HmHel-4	CAEZ01010796:62616-62843
HmHel-4	CAEZ01010796:34158-34457
HmHel-4	CAEZ01010796:16310-16609
HmHel-4	CAEZ01010540:10756-11055
HmHel-4	CAEZ01009769:18172-18471
HmHel-4	CAEZ01009435:30543-30827
HmHel-4	CAEZ01009435:1484-1786
HmHel-4	CAEZ01009120:55458-55650
HmHel-4	CAEZ01009120:55136-55431
HmHel-4	CAEZ01007297:12334-12633
HmHel-4	CAEZ01006237:2870-3169
HmHel-4	CAEZ01005184:13229-13528
HmHel-4	CAEZ01004958:48597-48896
HmHel-4	CAEZ01004822:7151-7466
HmHel-4	CAEZ01003233:78946-79247
HmHel-4	CAEZ01003233:48592-48891
HmHel-4	CAEZ01002897:447-742
HmHel-4	CAEZ01002036:10104-10403
HmHel-4	CAEZ01002036:18660-18967
HmHel-4	CAEZ01002036:2019-2304
HmHel-4	CAEZ01001362:1757-2056
HmHel-4	CAEZ01001362:2871-3163
HmHel-4	CAEZ01010758:13716-14010
HmHel-4	CAEZ01010130:49063-49367
HmHel-4	CAEZ01010130:91012-91116
HmHel-4	CAEZ01010130:127543-127843
HmHel-4	CAEZ01010130:99635-99840
HmHel-4	CAEZ01010130:50408-50706
HmHel-4	CAEZ01005535:3653-3951
HmHel-4	CAEZ01000974:1054-1348
HmHel-4	CAEZ01000049:1307-1608
HmHel-4	CAEZ01000049:15675-16002
HmHel-4	CAEZ01010677:7329-7576
HmHel-4	CAEZ01010677:57213-57510
HmHel-4	CAEZ01010677:108405-108697
HmHel-4	CAEZ01010677:168791-169092
HmHel-4	CAEZ01010677:258448-258741

HmHel-4	CAEZ01010677:168091-168391
HmHel-4	CAEZ01010677:121694-121994
HmHel-4	CAEZ01010677:91722-92029
HmHel-4	CAEZ01010219:3180-3479
HmHel-4	CAEZ01010219:24766-25063
HmHel-4	CAEZ01010219:137379-137652
HmHel-4	CAEZ01010219:158660-158985
HmHel-4	CAEZ01010219:21888-22031
HmHel-4	CAEZ01010030:70348-70648
HmHel-4	CAEZ01010030:103273-103573
HmHel-4	CAEZ01010030:168408-168677
HmHel-4	CAEZ01010030:258139-258419
HmHel-4	CAEZ01010030:274152-274452
HmHel-4	CAEZ01010030:142527-142827
HmHel-4	CAEZ01010030:132249-132404
HmHel-4	CAEZ01010030:86631-86928
HmHel-4	CAEZ01010030:43346-43646
HmHel-4	CAEZ01009619:1810-2107
HmHel-4	CAEZ01009501:70245-70547
HmHel-4	CAEZ01009501:108602-108899
HmHel-4	CAEZ01009456:41154-41454
HmHel-4	CAEZ01009456:85691-85863
HmHel-4	CAEZ01009456:142214-142472
HmHel-4	CAEZ01009456:118441-118668
HmHel-4	CAEZ01009456:9586-9884
HmHel-4	CAEZ01008967:37-281
HmHel-4	CAEZ01008967:17393-17690
HmHel-4	CAEZ01008961:1778-1992
HmHel-4	CAEZ01008961:48075-48250
HmHel-4	CAEZ01008961:60923-61230
HmHel-4	CAEZ01008961:26904-27156
HmHel-4	CAEZ01008912:14684-14953
HmHel-4	CAEZ01008912:35942-36241
HmHel-4	CAEZ01008849:45-346
HmHel-4	CAEZ01008849:33662-33959
HmHel-4	CAEZ01008849:38596-38896
HmHel-4	CAEZ01008849:26002-26297
HmHel-4	CAEZ01008301:14276-14572
HmHel-4	CAEZ01006296:15290-15587
HmHel-4	CAEZ01006279:16479-16776
HmHel-4	CAEZ01006279:13853-14153
HmHel-4	CAEZ01006249:13102-13399
HmHel-4	CAEZ01006177:188786-189052
HmHel-4	CAEZ01006177:4470-4763
HmHel-4	CAEZ01006137:1027-1142
HmHel-4	CAEZ01006137:25616-25913
HmHel-4	CAEZ01005773:10835-11136
HmHel-4	CAEZ01005133:14201-14502

HmHel-4	CAEZ01005043:37504-37807
HmHel-4	CAEZ01005043:61522-61716
HmHel-4	CAEZ01005043:162381-162688
HmHel-4	CAEZ01005043:247979-248286
HmHel-4	CAEZ01005043:186621-186921
HmHel-4	CAEZ01005043:133407-133572
HmHel-4	CAEZ01005043:113326-113609
HmHel-4	CAEZ01004633:42668-42965
HmHel-4	CAEZ01004633:47668-47968
HmHel-4	CAEZ01004549:17374-17671
HmHel-4	CAEZ01003410:4416-4713
HmHel-4	CAEZ01002621:22242-22538
HmHel-4	CAEZ01001963:223-520
HmHel-4	CAEZ01001739:10627-10924
HmHel-4	CAEZ01001420:8951-9248
HmHel-4	CAEZ01000043:3712-4012
HmHel-4	CAEZ01000043:17287-17580
HmHel-4	CAEZ01011626:91386-91693
HmHel-4	CAEZ01011626:125377-125684
HmHel-4	CAEZ01011626:154819-155114
HmHel-4	CAEZ01011626:87252-87556
HmHel-4	CAEZ01011626:33091-33420
HmHel-4	CAEZ01011583:537-837
HmHel-4	CAEZ01011512:1601-1901
HmHel-4	CAEZ01011500:29574-29874
HmHel-4	CAEZ01011387:6547-6846
HmHel-4	CAEZ01011387:590-891
HmHel-4	CAEZ01011317:6285-6585
HmHel-4	CAEZ01011283:53407-53707
HmHel-4	CAEZ01011258:896-1196
HmHel-4	CAEZ01011086:3957-4257
HmHel-4	CAEZ01011074:10490-10790
HmHel-4	CAEZ01011000:6678-6979
HmHel-4	CAEZ01011000:35947-36247
HmHel-4	CAEZ01010893:74821-75121
HmHel-4	CAEZ01010802:917-1217
HmHel-4	CAEZ01010802:43471-43766
HmHel-4	CAEZ01010802:3408-3708
HmHel-4	CAEZ01010787:10354-10654
HmHel-4	CAEZ01010787:14334-14634
HmHel-4	CAEZ01010787:3820-4120
HmHel-4	CAEZ01010737:59661-59964
HmHel-4	CAEZ01010737:119002-119302
HmHel-4	CAEZ01010737:162376-162676
HmHel-4	CAEZ01010737:67847-68136
HmHel-4	CAEZ01010737:34206-34511
HmHel-4	CAEZ01010678:4298-4598
HmHel-4	CAEZ01010661:4984-5284

HmHel-4	CAEZ01010649:6125-6425
HmHel-4	CAEZ01010390:4984-5284
HmHel-4	CAEZ01010312:42594-42894
HmHel-4	CAEZ01010260:41653-41953
HmHel-4	CAEZ01010260:96722-97029
HmHel-4	CAEZ01010260:87718-88017
HmHel-4	CAEZ01010260:59853-60159
HmHel-4	CAEZ01010260:48288-48425
HmHel-4	CAEZ01010260:30583-30883
HmHel-4	CAEZ01010252:34830-35126
HmHel-4	CAEZ01010252:1347-1607
HmHel-4	CAEZ01010237:72765-73061
HmHel-4	CAEZ01010237:45829-46129
HmHel-4	CAEZ01010229:13134-13434
HmHel-4	CAEZ01010216:7215-7515
HmHel-4	CAEZ01010216:45679-46006
HmHel-4	CAEZ01010216:57819-58119
HmHel-4	CAEZ01010181:33565-33865
HmHel-4	CAEZ01010181:64589-64889
HmHel-4	CAEZ01010118:42850-43150
HmHel-4	CAEZ01010032:19414-19713
HmHel-4	CAEZ01010032:31637-31927
HmHel-4	CAEZ01010032:27448-27763
HmHel-4	CAEZ01010032:2128-2428
HmHel-4	CAEZ01009876:26381-26681
HmHel-4	CAEZ01009776:55755-56055
HmHel-4	CAEZ01009748:53975-54302
HmHel-4	CAEZ01009748:157734-158037
HmHel-4	CAEZ01009748:102434-102734
HmHel-4	CAEZ01009733:5282-5579
HmHel-4	CAEZ01009733:64661-64961
HmHel-4	CAEZ01009733:66708-67008
HmHel-4	CAEZ01009694:40459-40749
HmHel-4	CAEZ01009694:20963-21262
HmHel-4	CAEZ01009631:62361-62637
HmHel-4	CAEZ01009631:59167-59467
HmHel-4	CAEZ01009631:32193-32358
HmHel-4	CAEZ01009631:20696-21285
HmHel-4	CAEZ01009478:757-1057
HmHel-4	CAEZ01009432:40136-40436
HmHel-4	CAEZ01009432:4014-4314
HmHel-4	CAEZ01009422:5901-6198
HmHel-4	CAEZ01009422:26035-26344
HmHel-4	CAEZ01009422:48355-48662
HmHel-4	CAEZ01009422:83606-83911
HmHel-4	CAEZ01009422:114785-115085
HmHel-4	CAEZ01009422:129780-129970
HmHel-4	CAEZ01009422:93781-94098

HmHel-4	CAEZ01009375:76115-76415
HmHel-4	CAEZ01009228:44659-44957
HmHel-4	CAEZ01009228:57806-58110
HmHel-4	CAEZ01009228:22811-23118
HmHel-4	CAEZ01009150:43778-44078
HmHel-4	CAEZ01009150:28467-28767
HmHel-4	CAEZ01009079:28705-29026
HmHel-4	CAEZ01009079:9852-10152
HmHel-4	CAEZ01009077:18343-18508
HmHel-4	CAEZ01009077:35105-35405
HmHel-4	CAEZ01009077:127867-128145
HmHel-4	CAEZ01009077:202389-202687
HmHel-4	CAEZ01009030:79715-80042
HmHel-4	CAEZ01009030:66035-66335
HmHel-4	CAEZ01009030:47229-47502
HmHel-4	CAEZ01009030:23164-23451
HmHel-4	CAEZ01008989:8640-8921
HmHel-4	CAEZ01008989:31963-32264
HmHel-4	CAEZ01008989:45966-46273
HmHel-4	CAEZ01008989:80279-80578
HmHel-4	CAEZ01008989:141941-142240
HmHel-4	CAEZ01008989:187030-187329
HmHel-4	CAEZ01008989:183853-184157
HmHel-4	CAEZ01008989:143656-143985
HmHel-4	CAEZ01008989:16094-16388
HmHel-4	CAEZ01008982:37149-37449
HmHel-4	CAEZ01008915:46378-46678
HmHel-4	CAEZ01008915:92426-92733
HmHel-4	CAEZ01008915:129979-130273
HmHel-4	CAEZ01008915:308610-308903
HmHel-4	CAEZ01008915:177559-177859
HmHel-4	CAEZ01008888:908-1208
HmHel-4	CAEZ01008888:17476-17776
HmHel-4	CAEZ01008888:10647-10954
HmHel-4	CAEZ01008824:14535-14836
HmHel-4	CAEZ01008824:63338-63638
HmHel-4	CAEZ01008796:1617-1917
HmHel-4	CAEZ01008632:39978-40278
HmHel-4	CAEZ01008632:16775-17096
HmHel-4	CAEZ01008515:13469-13748
HmHel-4	CAEZ01008515:26808-27108
HmHel-4	CAEZ01008399:44872-45172
HmHel-4	CAEZ01008398:61402-61702
HmHel-4	CAEZ01008398:64924-65224
HmHel-4	CAEZ01008372:4084-4249
HmHel-4	CAEZ01008372:38893-39193
HmHel-4	CAEZ01008372:295003-295302
HmHel-4	CAEZ01008372:319199-319499

HmHel-4	CAEZ01008372:276574-276874
HmHel-4	CAEZ01008372:245264-245562
HmHel-4	CAEZ01008372:90398-90698
HmHel-4	CAEZ01008372:11251-11469
HmHel-4	CAEZ01008340:10911-11211
HmHel-4	CAEZ01008262:34712-34940
HmHel-4	CAEZ01008262:56315-56615
HmHel-4	CAEZ01008262:25804-26104
HmHel-4	CAEZ01008236:64194-64501
HmHel-4	CAEZ01008236:7267-7601
HmHel-4	CAEZ01008233:86808-87108
HmHel-4	CAEZ01008233:11398-11698
HmHel-4	CAEZ01008087:3193-3493
HmHel-4	CAEZ01008087:31721-32016
HmHel-4	CAEZ01008087:20458-20758
HmHel-4	CAEZ01008046:12315-12614
HmHel-4	CAEZ01008026:15693-15993
HmHel-4	CAEZ01007939:156432-156638
HmHel-4	CAEZ01007939:73429-73789
HmHel-4	CAEZ01007939:3082-3382
HmHel-4	CAEZ01007928:75474-75774
HmHel-4	CAEZ01007928:31934-32234
HmHel-4	CAEZ01007919:6428-6728
HmHel-4	CAEZ01007855:13988-14288
HmHel-4	CAEZ01007802:11839-12120
HmHel-4	CAEZ01007802:35645-35989
HmHel-4	CAEZ01007741:42314-42589
HmHel-4	CAEZ01007741:80905-81219
HmHel-4	CAEZ01007741:89041-89348
HmHel-4	CAEZ01007741:64415-64715
HmHel-4	CAEZ01007741:53738-53948
HmHel-4	CAEZ01007741:37587-37908
HmHel-4	CAEZ01007737:67173-67337
HmHel-4	CAEZ01007711:1919-2080
HmHel-4	CAEZ01007711:25224-25524
HmHel-4	CAEZ01007711:53868-54167
HmHel-4	CAEZ01007711:49593-49890
HmHel-4	CAEZ01007711:10028-10328
HmHel-4	CAEZ01007682:5454-5754
HmHel-4	CAEZ01007676:48483-48710
HmHel-4	CAEZ01007676:98317-98622
HmHel-4	CAEZ01007676:168219-168554
HmHel-4	CAEZ01007676:192577-192683
HmHel-4	CAEZ01007676:208187-208487
HmHel-4	CAEZ01007676:115826-116123
HmHel-4	CAEZ01007625:57104-57276
HmHel-4	CAEZ01007625:43049-43349
HmHel-4	CAEZ01007568:96205-96512

HmHel-4	CAEZ01007568:29333-29633
HmHel-4	CAEZ01007545:118512-118812
HmHel-4	CAEZ01007494:1492-1792
HmHel-4	CAEZ01007475:33819-34119
HmHel-4	CAEZ01007419:5188-5488
HmHel-4	CAEZ01007376:30530-30827
HmHel-4	CAEZ01007376:9686-9985
HmHel-4	CAEZ01007307:8190-8490
HmHel-4	CAEZ01007304:5016-5316
HmHel-4	CAEZ01007299:501-691
HmHel-4	CAEZ01007299:15574-15874
HmHel-4	CAEZ01007164:18581-18881
HmHel-4	CAEZ01007121:23812-24117
HmHel-4	CAEZ01007121:37413-37713
HmHel-4	CAEZ01007121:11744-12042
HmHel-4	CAEZ01006962:25985-26212
HmHel-4	CAEZ01006962:10668-10968
HmHel-4	CAEZ01006880:11300-11600
HmHel-4	CAEZ01006773:20-461
HmHel-4	CAEZ01006773:52139-52439
HmHel-4	CAEZ01006773:169416-169538
HmHel-4	CAEZ01006773:179769-180069
HmHel-4	CAEZ01006773:36960-37066
HmHel-4	CAEZ01006749:33558-33855
HmHel-4	CAEZ01006749:34294-34594
HmHel-4	CAEZ01006717:10165-10465
HmHel-4	CAEZ01006710:995-1295
HmHel-4	CAEZ01006661:3566-3866
HmHel-4	CAEZ01006596:60964-61264
HmHel-4	CAEZ01006596:17349-17649
HmHel-4	CAEZ01006567:80204-80504
HmHel-4	CAEZ01006567:50392-50697
HmHel-4	CAEZ01006567:27919-28068
HmHel-4	CAEZ01006567:8255-8559
HmHel-4	CAEZ01006566:52335-54219
HmHel-4	CAEZ01006566:81900-82203
HmHel-4	CAEZ01006566:122525-122825
HmHel-4	CAEZ01006566:29050-29358
HmHel-4	CAEZ01006501:4973-5273
HmHel-4	CAEZ01006445:276482-276782
HmHel-4	CAEZ01006443:2431-2731
HmHel-4	CAEZ01006418:61002-61302
HmHel-4	CAEZ01006343:60648-60948
HmHel-4	CAEZ01006149:24351-24651
HmHel-4	CAEZ01006096:48941-49235
HmHel-4	CAEZ01006096:47899-48199
HmHel-4	CAEZ01006054:3707-4007
HmHel-4	CAEZ01006054:1661-1956

HmHel-4	CAEZ01005977:51223-51452
HmHel-4	CAEZ01005977:93907-94207
HmHel-4	CAEZ01005869:7106-7278
HmHel-4	CAEZ01005869:24-324
HmHel-4	CAEZ01005841:57440-57716
HmHel-4	CAEZ01005841:4108-4408
HmHel-4	CAEZ01005839:13520-13820
HmHel-4	CAEZ01005839:14543-14845
HmHel-4	CAEZ01005792:7329-7629
HmHel-4	CAEZ01005679:591-891
HmHel-4	CAEZ01005679:28516-28822
HmHel-4	CAEZ01005569:6430-6730
HmHel-4	CAEZ01005504:20825-21125
HmHel-4	CAEZ01005442:44027-44329
HmHel-4	CAEZ01005442:109488-109785
HmHel-4	CAEZ01005442:150799-151096
HmHel-4	CAEZ01005442:79520-79820
HmHel-4	CAEZ01005415:38548-38696
HmHel-4	CAEZ01005415:35952-36281
HmHel-4	CAEZ01005415:10994-11290
HmHel-4	CAEZ01005343:7414-7714
HmHel-4	CAEZ01005310:1747-2047
HmHel-4	CAEZ01005039:727-1027
HmHel-4	CAEZ01005015:220615-220914
HmHel-4	CAEZ01005015:213221-213521
HmHel-4	CAEZ01005008:1945-2245
HmHel-4	CAEZ01004974:6641-6941
HmHel-4	CAEZ01004952:12035-12334
HmHel-4	CAEZ01004952:28788-29087
HmHel-4	CAEZ01004952:4267-4569
HmHel-4	CAEZ01004941:21574-21851
HmHel-4	CAEZ01004941:116933-117236
HmHel-4	CAEZ01004941:105931-106231
HmHel-4	CAEZ01004941:84177-84500
HmHel-4	CAEZ01004814:821-967
HmHel-4	CAEZ01004814:43277-43577
HmHel-4	CAEZ01004814:77795-78073
HmHel-4	CAEZ01004814:32237-32537
HmHel-4	CAEZ01004812:2448-2748
HmHel-4	CAEZ01004566:8781-9110
HmHel-4	CAEZ01004566:38618-38915
HmHel-4	CAEZ01004566:40208-40508
HmHel-4	CAEZ01004566:18699-19006
HmHel-4	CAEZ01004507:1753-2053
HmHel-4	CAEZ01004499:8693-8993
HmHel-4	CAEZ01004475:17128-17428
HmHel-4	CAEZ01004352:45-345
HmHel-4	CAEZ01004352:9802-10102

HmHel-4	CAEZ01004345:5697-5993
HmHel-4	CAEZ01004304:5354-5654
HmHel-4	CAEZ01004260:10773-11073
HmHel-4	CAEZ01004174:16945-17245
HmHel-4	CAEZ01004140:7946-8246
HmHel-4	CAEZ01003891:15831-16131
HmHel-4	CAEZ01003808:8191-8491
HmHel-4	CAEZ01003774:6807-7107
HmHel-4	CAEZ01003717:120859-121159
HmHel-4	CAEZ01003717:55279-55579
HmHel-4	CAEZ01003472:5714-6014
HmHel-4	CAEZ01003401:34226-34477
HmHel-4	CAEZ01003401:148013-148311
HmHel-4	CAEZ01003401:224231-224392
HmHel-4	CAEZ01003401:134037-134340
HmHel-4	CAEZ01003401:108599-108899
HmHel-4	CAEZ01003353:4314-4597
HmHel-4	CAEZ01003353:481-781
HmHel-4	CAEZ01003291:108261-108561
HmHel-4	CAEZ01003291:64846-65147
HmHel-4	CAEZ01003277:7977-8277
HmHel-4	CAEZ01003145:460-782
HmHel-4	CAEZ01003145:4355-4655
HmHel-4	CAEZ01003055:18-318
HmHel-4	CAEZ01002987:5352-5652
HmHel-4	CAEZ01002869:971-1271
HmHel-4	CAEZ01002828:12428-12728
HmHel-4	CAEZ01002817:4468-4768
HmHel-4	CAEZ01002811:17681-17978
HmHel-4	CAEZ01002811:32116-32410
HmHel-4	CAEZ01002811:50058-50358
HmHel-4	CAEZ01002810:20474-20781
HmHel-4	CAEZ01002810:37070-37370
HmHel-4	CAEZ01002810:74339-74654
HmHel-4	CAEZ01002810:26247-26547
HmHel-4	CAEZ01002810:1571-1878
HmHel-4	CAEZ01002780:24741-25041
HmHel-4	CAEZ01002779:693-1068
HmHel-4	CAEZ01002779:37943-38242
HmHel-4	CAEZ01002779:1707-2007
HmHel-4	CAEZ01002732:112767-113016
HmHel-4	CAEZ01002732:60762-61062
HmHel-4	CAEZ01002732:47852-48162
HmHel-4	CAEZ01002663:1436-1763
HmHel-4	CAEZ01002663:9218-9518
HmHel-4	CAEZ01002631:17783-18083
HmHel-4	CAEZ01002631:25554-25854
HmHel-4	CAEZ01002620:15596-15896

HmHel-4	CAEZ01002368:998-1298
HmHel-4	CAEZ01002323:42547-42863
HmHel-4	CAEZ01002323:67299-67617
HmHel-4	CAEZ01002323:35521-35828
HmHel-4	CAEZ01002320:18891-19191
HmHel-4	CAEZ01002320:40809-41109
HmHel-4	CAEZ01002211:20677-20977
HmHel-4	CAEZ01002208:46729-47029
HmHel-4	CAEZ01002208:58251-58388
HmHel-4	CAEZ01002048:30905-31205
HmHel-4	CAEZ01002026:12993-13293
HmHel-4	CAEZ01001944:303-603
HmHel-4	CAEZ01001908:845-1145
HmHel-4	CAEZ01001891:48933-49233
HmHel-4	CAEZ01001889:10784-11084
HmHel-4	CAEZ01001868:43786-44086
HmHel-4	CAEZ01001868:34246-34546
HmHel-4	CAEZ01001835:4755-5055
HmHel-4	CAEZ01001775:1174-1474
HmHel-4	CAEZ01001579:42106-42405
HmHel-4	CAEZ01001533:6360-6660
HmHel-4	CAEZ01001521:3904-4204
HmHel-4	CAEZ01001521:13830-14137
HmHel-4	CAEZ01001463:28282-28581
HmHel-4	CAEZ01001463:130468-130590
HmHel-4	CAEZ01001463:124435-124599
HmHel-4	CAEZ01001463:71670-71969
HmHel-4	CAEZ01001463:3732-4031
HmHel-4	CAEZ01001437:16783-17083
HmHel-4	CAEZ01001437:27376-27670
HmHel-4	CAEZ01001301:42141-42441
HmHel-4	CAEZ01001301:28864-29164
HmHel-4	CAEZ01001244:275-575
HmHel-4	CAEZ01001233:2757-3060
HmHel-4	CAEZ01001233:8360-8660
HmHel-4	CAEZ01001102:55523-55823
HmHel-4	CAEZ01001058:339-639
HmHel-4	CAEZ01000853:331-631
HmHel-4	CAEZ01000850:88695-88995
HmHel-4	CAEZ01000850:58858-59050
HmHel-4	CAEZ01000850:38123-38423
HmHel-4	CAEZ01000850:19399-19535
HmHel-4	CAEZ01000828:2248-2548
HmHel-4	CAEZ01011141:3956-4254
HmHel-4	CAEZ01011141:8307-8613
HmHel-4	CAEZ01010918:7314-7613
HmHel-4	CAEZ01010918:43365-43662
HmHel-4	CAEZ01010918:90349-90642

HmHel-4	CAEZ01010918:27993-28293
HmHel-4	CAEZ01010918:9978-10277
HmHel-4	CAEZ01010364:38682-38981
HmHel-4	CAEZ01010364:36496-36800
HmHel-4	CAEZ01007974:37775-38077
HmHel-4	CAEZ01007974:130832-131049
HmHel-4	CAEZ01007974:143216-143517
HmHel-4	CAEZ01007247:3942-4242
HmHel-4	CAEZ01006974:10073-10375
HmHel-4	CAEZ01006974:5667-5968
HmHel-4	CAEZ01006865:148-427
HmHel-4	CAEZ01006865:41644-41944
HmHel-4	CAEZ01006865:36138-36437
HmHel-4	CAEZ01006865:24231-24536
HmHel-4	CAEZ01006865:10839-11116
HmHel-4	CAEZ01006804:188611-188918
HmHel-4	CAEZ01006804:158372-158543
HmHel-4	CAEZ01006804:65504-65811
HmHel-4	CAEZ01006804:13103-13402
HmHel-4	CAEZ01006423:323112-323439
HmHel-4	CAEZ01006423:289978-290277
HmHel-4	CAEZ01006423:266191-266312
HmHel-4	CAEZ01006423:160994-161120
HmHel-4	CAEZ01006423:104435-104733
HmHel-4	CAEZ01006127:2996-3163
HmHel-4	CAEZ01006127:4139-4443
HmHel-4	CAEZ01005253:33884-34183
HmHel-4	CAEZ01003799:3417-3708
HmHel-4	CAEZ01003510:30256-30551
HmHel-4	CAEZ01002416:51467-51771
HmHel-4	CAEZ01002416:66105-66410
HmHel-4	CAEZ01002416:24564-24817
HmHel-4	CAEZ01002416:11430-11730
HmHel-4	CAEZ01002414:17639-17939
HmHel-4	CAEZ01002414:154018-154317
HmHel-4	CAEZ01002285:17492-17791
HmHel-4	CAEZ01001230:1107-1406
HmHel-4	CAEZ01010828:35823-36120
HmHel-4	CAEZ01008861:18192-18490
HmHel-4	CAEZ01007845:2440-2738
HmHel-4	CAEZ01004510:3492-3790
HmHel-4	CAEZ01004038:18773-19043
HmHel-4	CAEZ01000916:785-1079
HmHel-4	CAEZ01010936:1469-1766
HmHel-4	CAEZ01010395:15595-15903
HmHel-4	CAEZ01010395:2534-2832
HmHel-4	CAEZ01010275:8575-8882
HmHel-4	CAEZ01010275:4815-5112

HmHel-4	CAEZ01009794:1960-2256
HmHel-4	CAEZ01009275:2458-2755
HmHel-4	CAEZ01009080:9183-9486
HmHel-4	CAEZ01008970:24099-24393
HmHel-4	CAEZ01008767:4201-4508
HmHel-4	CAEZ01008767:23415-23684
HmHel-4	CAEZ01008767:39278-39571
HmHel-4	CAEZ01008767:41712-41926
HmHel-4	CAEZ01008765:37162-37455
HmHel-4	CAEZ01008661:2636-2946
HmHel-4	CAEZ01008661:24217-24514
HmHel-4	CAEZ01008424:20442-20742
HmHel-4	CAEZ01008352:87792-88092
HmHel-4	CAEZ01008352:134801-135104
HmHel-4	CAEZ01008352:121263-121597
HmHel-4	CAEZ01008135:135-432
HmHel-4	CAEZ01008135:12567-12866
HmHel-4	CAEZ01008100:84276-84577
HmHel-4	CAEZ01007705:2571-2798
HmHel-4	CAEZ01007705:81233-81531
HmHel-4	CAEZ01006561:1545-1856
HmHel-4	CAEZ01006561:39828-40113
HmHel-4	CAEZ01006561:81316-81622
HmHel-4	CAEZ01006561:121908-122207
HmHel-4	CAEZ01006097:14408-14735
HmHel-4	CAEZ01005458:4837-5129
HmHel-4	CAEZ01005350:2819-3116
HmHel-4	CAEZ01005196:64221-64514
HmHel-4	CAEZ01005196:44077-44374
HmHel-4	CAEZ01004118:8699-8997
HmHel-4	CAEZ01003764:2743-2866
HmHel-4	CAEZ01003764:9386-9678
HmHel-4	CAEZ01003759:4873-5170
HmHel-4	CAEZ01003630:5245-5542
HmHel-4	CAEZ01003582:1533-1839
HmHel-4	CAEZ01003582:46473-46772
HmHel-4	CAEZ01003582:63341-63645
HmHel-4	CAEZ01003582:69873-70170
HmHel-4	CAEZ01003582:49822-50122
HmHel-4	CAEZ01003582:36916-37223
HmHel-4	CAEZ01003345:16670-16967
HmHel-4	CAEZ01003215:5082-5379
HmHel-4	CAEZ01003196:4649-4946
HmHel-4	CAEZ01003196:37343-37649
HmHel-4	CAEZ01003196:62843-63150
HmHel-4	CAEZ01003196:97569-97869
HmHel-4	CAEZ01003196:99685-99938
HmHel-4	CAEZ01003196:38812-39117

HmHel-4	CAEZ01003196:1308-1608
HmHel-4	CAEZ01003190:6732-7029
HmHel-4	CAEZ01002818:48-194
HmHel-4	CAEZ01002818:10495-10792
HmHel-4	CAEZ01002725:636-936
HmHel-4	CAEZ01002724:113973-114270
HmHel-4	CAEZ01002724:93460-93763
HmHel-4	CAEZ01001807:2569-2866
HmHel-4	CAEZ01001807:39896-40199
HmHel-4	CAEZ01001807:6159-6479
HmHel-4	CAEZ01001765:97446-97739
HmHel-4	CAEZ01001765:75548-75820
HmHel-4	CAEZ01011494:65593-65893
HmHel-4	CAEZ01011494:58602-58902
HmHel-4	CAEZ01011494:37733-38004
HmHel-4	CAEZ01011493:13728-14027
HmHel-4	CAEZ01011493:21254-21364
HmHel-4	CAEZ01011476:1473-1773
HmHel-4	CAEZ01011476:20713-20930
HmHel-4	CAEZ01011318:10615-10915
HmHel-4	CAEZ01011314:49992-50291
HmHel-4	CAEZ01011314:25026-25207
HmHel-4	CAEZ01011276:8009-8298
HmHel-4	CAEZ01011276:6646-6946
HmHel-4	CAEZ01011249:10667-10967
HmHel-4	CAEZ01010931:17293-17598
HmHel-4	CAEZ01010931:13446-13746
HmHel-4	CAEZ01010880:12386-12686
HmHel-4	CAEZ01010808:141084-141384
HmHel-4	CAEZ01010808:113331-113630
HmHel-4	CAEZ01010808:89648-89947
HmHel-4	CAEZ01010660:2803-3103
HmHel-4	CAEZ01010583:4486-4718
HmHel-4	CAEZ01010583:18764-19052
HmHel-4	CAEZ01010583:41329-41629
HmHel-4	CAEZ01010541:39300-39460
HmHel-4	CAEZ01010541:138393-138692
HmHel-4	CAEZ01010541:202019-202325
HmHel-4	CAEZ01010541:338459-338759
HmHel-4	CAEZ01010541:209707-209997
HmHel-4	CAEZ01010470:2660-2960
HmHel-4	CAEZ01010453:60057-60357
HmHel-4	CAEZ01010453:108205-108504
HmHel-4	CAEZ01010438:1672-1972
HmHel-4	CAEZ01010361:8567-8874
HmHel-4	CAEZ01010361:30874-31169
HmHel-4	CAEZ01010357:11880-12180
HmHel-4	CAEZ01010326:33671-33971

HmHel-4	CAEZ01010300:28738-29038
HmHel-4	CAEZ01010212:9298-9598
HmHel-4	CAEZ01010212:70172-70452
HmHel-4	CAEZ01010212:111024-111327
HmHel-4	CAEZ01010212:125258-125565
HmHel-4	CAEZ01010212:36867-37200
HmHel-4	CAEZ01010127:37347-37654
HmHel-4	CAEZ01010127:58424-58724
HmHel-4	CAEZ01009982:91074-91351
HmHel-4	CAEZ01009982:102829-103028
HmHel-4	CAEZ01009982:90812-90980
HmHel-4	CAEZ01009982:67909-68209
HmHel-4	CAEZ01009982:333-636
HmHel-4	CAEZ01009971:75785-76085
HmHel-4	CAEZ01009932:27692-27992
HmHel-4	CAEZ01009922:10898-11198
HmHel-4	CAEZ01009841:105941-106241
HmHel-4	CAEZ01009841:86244-86551
HmHel-4	CAEZ01009841:56408-56712
HmHel-4	CAEZ01009841:25945-26245
HmHel-4	CAEZ01009841:11201-11501
HmHel-4	CAEZ01009813:70213-70512
HmHel-4	CAEZ01009813:52038-52338
HmHel-4	CAEZ01009813:29295-29602
HmHel-4	CAEZ01009731:20104-20404
HmHel-4	CAEZ01009560:20927-21227
HmHel-4	CAEZ01009560:22339-22508
HmHel-4	CAEZ01009560:8911-9211
HmHel-4	CAEZ01009553:21392-21692
HmHel-4	CAEZ01009446:2294-2592
HmHel-4	CAEZ01009446:39559-39859
HmHel-4	CAEZ01009379:46118-46423
HmHel-4	CAEZ01009379:27417-27717
HmHel-4	CAEZ01009370:174767-175067
HmHel-4	CAEZ01009370:94519-94819
HmHel-4	CAEZ01009342:1618-1918
HmHel-4	CAEZ01009339:11499-11799
HmHel-4	CAEZ01009322:28541-28847
HmHel-4	CAEZ01009322:39209-39509
HmHel-4	CAEZ01009322:1349-1666
HmHel-4	CAEZ01009268:75510-75810
HmHel-4	CAEZ01009268:23580-23887
HmHel-4	CAEZ01009227:27950-28250
HmHel-4	CAEZ01009213:3373-3673
HmHel-4	CAEZ01009182:22243-22535
HmHel-4	CAEZ01009182:13898-14203
HmHel-4	CAEZ01009137:55375-55675
HmHel-4	CAEZ01009099:25339-25639

HmHel-4	CAEZ01009070:8382-8689
HmHel-4	CAEZ01009070:24884-25188
HmHel-4	CAEZ01009070:18289-18589
HmHel-4	CAEZ01008960:7919-8184
HmHel-4	CAEZ01008960:29075-29363
HmHel-4	CAEZ01008960:79024-79322
HmHel-4	CAEZ01008960:104892-105180
HmHel-4	CAEZ01008960:117050-117195
HmHel-4	CAEZ01008960:250086-250393
HmHel-4	CAEZ01008960:255406-255635
HmHel-4	CAEZ01008960:223958-224263
HmHel-4	CAEZ01008960:159597-159900
HmHel-4	CAEZ01008960:122260-122566
HmHel-4	CAEZ01008960:87080-87379
HmHel-4	CAEZ01008910:23903-24203
HmHel-4	CAEZ01008910:6334-6633
HmHel-4	CAEZ01008848:906-1206
HmHel-4	CAEZ01008812:7542-7842
HmHel-4	CAEZ01008784:19171-19319
HmHel-4	CAEZ01008784:1861-2161
HmHel-4	CAEZ01008781:8940-9240
HmHel-4	CAEZ01008769:8796-9096
HmHel-4	CAEZ01008769:46012-46307
HmHel-4	CAEZ01008722:23480-23791
HmHel-4	CAEZ01008722:9653-9953
HmHel-4	CAEZ01008721:126058-126358
HmHel-4	CAEZ01008721:97895-98193
HmHel-4	CAEZ01008649:12320-12620
HmHel-4	CAEZ01008619:15712-15939
HmHel-4	CAEZ01008619:8920-9220
HmHel-4	CAEZ01008589:6769-11865
HmHel-4	CAEZ01008589:24921-25182
HmHel-4	CAEZ01008589:4912-5212
HmHel-4	CAEZ01008576:6734-7034
HmHel-4	CAEZ01008576:109182-109424
HmHel-4	CAEZ01008576:93087-93235
HmHel-4	CAEZ01008576:5499-5724
HmHel-4	CAEZ01008472:32854-33147
HmHel-4	CAEZ01008472:63098-63398
HmHel-4	CAEZ01008223:13117-13417
HmHel-4	CAEZ01008215:4968-5268
HmHel-4	CAEZ01008211:70880-71158
HmHel-4	CAEZ01008211:216911-217210
HmHel-4	CAEZ01008211:236052-236302
HmHel-4	CAEZ01008211:230309-230609
HmHel-4	CAEZ01008211:166714-166992
HmHel-4	CAEZ01008211:126533-126833
HmHel-4	CAEZ01008211:67288-67591

HmHel-4	CAEZ01008211:38576-38848
HmHel-4	CAEZ01008182:72168-72473
HmHel-4	CAEZ01008182:20227-20527
HmHel-4	CAEZ01008182:3792-4092
HmHel-4	CAEZ01008124:93188-93488
HmHel-4	CAEZ01008124:260038-260335
HmHel-4	CAEZ01008124:186333-186616
HmHel-4	CAEZ01008124:146832-147123
HmHel-4	CAEZ01008124:17470-17753
HmHel-4	CAEZ01008123:31314-31614
HmHel-4	CAEZ01008123:50494-50794
HmHel-4	CAEZ01008123:28840-29160
HmHel-4	CAEZ01008105:118174-118473
HmHel-4	CAEZ01008105:46573-46869
HmHel-4	CAEZ01007902:11904-12204
HmHel-4	CAEZ01007817:12669-12969
HmHel-4	CAEZ01007804:41338-41585
HmHel-4	CAEZ01007804:112907-113207
HmHel-4	CAEZ01007804:22776-23103
HmHel-4	CAEZ01007798:1242-1542
HmHel-4	CAEZ01007749:56774-57078
HmHel-4	CAEZ01007749:112031-112336
HmHel-4	CAEZ01007749:65354-65654
HmHel-4	CAEZ01007707:77682-77982
HmHel-4	CAEZ01007707:125667-125975
HmHel-4	CAEZ01007707:131925-132385
HmHel-4	CAEZ01007706:149207-149507
HmHel-4	CAEZ01007706:177001-177299
HmHel-4	CAEZ01007706:277256-277467
HmHel-4	CAEZ01007706:238753-239053
HmHel-4	CAEZ01007706:196424-196724
HmHel-4	CAEZ01007706:139011-139300
HmHel-4	CAEZ01007706:105140-105420
HmHel-4	CAEZ01007683:2827-3127
HmHel-4	CAEZ01007642:10567-10867
HmHel-4	CAEZ01007449:14156-14456
HmHel-4	CAEZ01007449:26664-26961
HmHel-4	CAEZ01007414:17312-17612
HmHel-4	CAEZ01007412:24480-24780
HmHel-4	CAEZ01007412:19878-20178
HmHel-4	CAEZ01007367:9199-9499
HmHel-4	CAEZ01007356:7679-7955
HmHel-4	CAEZ01007324:8632-8932
HmHel-4	CAEZ01007306:14685-14985
HmHel-4	CAEZ01007263:8838-9138
HmHel-4	CAEZ01007263:15711-15992
HmHel-4	CAEZ01007230:843-1143
HmHel-4	CAEZ01007230:15126-15426

HmHel-4	CAEZ01007150:2382-2682
HmHel-4	CAEZ01007039:6023-6323
HmHel-4	CAEZ01007020:10404-10704
HmHel-4	CAEZ01007015:20241-20541
HmHel-4	CAEZ01007009:11844-12144
HmHel-4	CAEZ01006847:10730-11030
HmHel-4	CAEZ01006847:93937-94191
HmHel-4	CAEZ01006698:59300-59600
HmHel-4	CAEZ01006698:66773-67073
HmHel-4	CAEZ01006689:84939-85239
HmHel-4	CAEZ01006616:9512-9826
HmHel-4	CAEZ01006616:265796-265971
HmHel-4	CAEZ01006616:252553-252847
HmHel-4	CAEZ01006616:235874-236170
HmHel-4	CAEZ01006616:209676-209976
HmHel-4	CAEZ01006616:184818-185115
HmHel-4	CAEZ01006590:43081-43381
HmHel-4	CAEZ01006587:29305-29605
HmHel-4	CAEZ01006509:656-956
HmHel-4	CAEZ01006474:2426-2725
HmHel-4	CAEZ01006417:107530-107856
HmHel-4	CAEZ01006417:168867-169167
HmHel-4	CAEZ01006400:1575-1875
HmHel-4	CAEZ01006400:58610-58914
HmHel-4	CAEZ01006395:5673-5973
HmHel-4	CAEZ01006347:9227-9536
HmHel-4	CAEZ01006347:53207-53506
HmHel-4	CAEZ01006347:221324-221674
HmHel-4	CAEZ01006347:140745-141052
HmHel-4	CAEZ01006347:114087-114373
HmHel-4	CAEZ01006347:98767-99067
HmHel-4	CAEZ01006347:34269-34444
HmHel-4	CAEZ01006347:13027-13327
HmHel-4	CAEZ01006171:112143-112443
HmHel-4	CAEZ01006171:46736-46918
HmHel-4	CAEZ01006153:4795-5095
HmHel-4	CAEZ01006085:10163-10456
HmHel-4	CAEZ01006085:67634-67930
HmHel-4	CAEZ01006085:50866-51132
HmHel-4	CAEZ01006085:33796-34096
HmHel-4	CAEZ01006004:95229-95529
HmHel-4	CAEZ01006004:27206-27343
HmHel-4	CAEZ01005975:44393-44727
HmHel-4	CAEZ01005975:152678-152889
HmHel-4	CAEZ01005975:7476-7776
HmHel-4	CAEZ01005964:31328-31628
HmHel-4	CAEZ01005911:28129-28381
HmHel-4	CAEZ01005911:56063-56331

HmHel-4	CAEZ01005911:93383-93694
HmHel-4	CAEZ01005911:121923-122223
HmHel-4	CAEZ01005911:81059-81363
HmHel-4	CAEZ01005911:64073-64366
HmHel-4	CAEZ01005911:51864-52164
HmHel-4	CAEZ01005898:19175-19475
HmHel-4	CAEZ01005851:18864-19164
HmHel-4	CAEZ01005851:1495-1795
HmHel-4	CAEZ01005706:8559-8866
HmHel-4	CAEZ01005684:1066-1366
HmHel-4	CAEZ01005584:9531-9843
HmHel-4	CAEZ01005584:36420-36720
HmHel-4	CAEZ01005495:33381-33681
HmHel-4	CAEZ01005495:25349-25521
HmHel-4	CAEZ01005495:1606-1906
HmHel-4	CAEZ01005485:22741-23034
HmHel-4	CAEZ01005485:52996-53296
HmHel-4	CAEZ01005455:14902-15202
HmHel-4	CAEZ01005429:65209-65509
HmHel-4	CAEZ01005429:49475-49772
HmHel-4	CAEZ01005418:51459-51759
HmHel-4	CAEZ01005418:104430-104727
HmHel-4	CAEZ01005418:178845-179149
HmHel-4	CAEZ01005418:191489-191796
HmHel-4	CAEZ01005418:236902-237125
HmHel-4	CAEZ01005418:273607-273913
HmHel-4	CAEZ01005418:190345-190650
HmHel-4	CAEZ01005418:164108-164408
HmHel-4	CAEZ01005418:35218-35511
HmHel-4	CAEZ01005409:76078-76378
HmHel-4	CAEZ01005221:20165-20465
HmHel-4	CAEZ01005221:37278-37575
HmHel-4	CAEZ01005221:56224-56551
HmHel-4	CAEZ01005221:37010-37169
HmHel-4	CAEZ01005210:39165-39467
HmHel-4	CAEZ01005210:57412-57688
HmHel-4	CAEZ01005210:80586-90305
HmHel-4	CAEZ01005210:33110-33417
HmHel-4	CAEZ01005210:3012-3191
HmHel-4	CAEZ01005163:26309-26609
HmHel-4	CAEZ01005157:12389-12689
HmHel-4	CAEZ01005145:37407-37666
HmHel-4	CAEZ01005145:21924-22224
HmHel-4	CAEZ01005145:6190-6487
HmHel-4	CAEZ01005144:7026-7326
HmHel-4	CAEZ01005134:2699-2999
HmHel-4	CAEZ01005119:38985-39284
HmHel-4	CAEZ01005112:559-859

HmHel-4	CAEZ01005025:118746-119036
HmHel-4	CAEZ01005025:59400-59700
HmHel-4	CAEZ01004885:2194-2494
HmHel-4	CAEZ01004873:19523-19823
HmHel-4	CAEZ01004873:2584-2806
HmHel-4	CAEZ01004757:21924-22224
HmHel-4	CAEZ01004757:6190-6487
HmHel-4	CAEZ01004740:16450-16751
HmHel-4	CAEZ01004740:4595-4895
HmHel-4	CAEZ01004738:173118-173425
HmHel-4	CAEZ01004738:189365-189669
HmHel-4	CAEZ01004738:246777-247039
HmHel-4	CAEZ01004738:317199-317499
HmHel-4	CAEZ01004738:139645-139901
HmHel-4	CAEZ01004677:69353-69558
HmHel-4	CAEZ01004677:90293-90593
HmHel-4	CAEZ01004592:7726-8018
HmHel-4	CAEZ01004592:27851-28068
HmHel-4	CAEZ01004562:115302-115602
HmHel-4	CAEZ01004562:194570-194876
HmHel-4	CAEZ01004562:218811-219111
HmHel-4	CAEZ01004562:185480-185777
HmHel-4	CAEZ01004562:122813-123092
HmHel-4	CAEZ01004533:941-1241
HmHel-4	CAEZ01004457:17200-17500
HmHel-4	CAEZ01004457:22572-22773
HmHel-4	CAEZ01004297:17833-18133
HmHel-4	CAEZ01004295:6284-6584
HmHel-4	CAEZ01004291:9228-9528
HmHel-4	CAEZ01004155:2224-2524
HmHel-4	CAEZ01003885:33300-33600
HmHel-4	CAEZ01003885:1945-2272
HmHel-4	CAEZ01003747:30823-31126
HmHel-4	CAEZ01003747:8746-9046
HmHel-4	CAEZ01003503:2259-2559
HmHel-4	CAEZ01003437:9275-9575
HmHel-4	CAEZ01003316:12695-12995
HmHel-4	CAEZ01003144:28291-28591
HmHel-4	CAEZ01002963:29905-30205
HmHel-4	CAEZ01002924:54253-54559
HmHel-4	CAEZ01002924:93075-93371
HmHel-4	CAEZ01002924:14686-14986
HmHel-4	CAEZ01002876:118928-119228
HmHel-4	CAEZ01002876:140179-140477
HmHel-4	CAEZ01002876:86300-86558
HmHel-4	CAEZ01002876:64634-64933
HmHel-4	CAEZ01002866:46288-46603
HmHel-4	CAEZ01002866:150518-150818

HmHel-4	CAEZ01002826:7326-7626
HmHel-4	CAEZ01002712:1969-2268
HmHel-4	CAEZ01002597:1121-1421
HmHel-4	CAEZ01002431:87-394
HmHel-4	CAEZ01002431:60230-60530
HmHel-4	CAEZ01002361:26908-27208
HmHel-4	CAEZ01002361:46958-47179
HmHel-4	CAEZ01002268:2627-2927
HmHel-4	CAEZ01002240:967-1267
HmHel-4	CAEZ01002181:20210-20510
HmHel-4	CAEZ01002181:21597-21897
HmHel-4	CAEZ01002181:98-382
HmHel-4	CAEZ01002098:6403-6706
HmHel-4	CAEZ01001931:7015-7315
HmHel-4	CAEZ01001888:2756-3089
HmHel-4	CAEZ01001888:29009-29309
HmHel-4	CAEZ01001216:3300-3599
HmHel-4	CAEZ01001216:13694-13993
HmHel-4	CAEZ01001216:18944-19244
HmHel-4	CAEZ01001089:34803-35102
HmHel-4	CAEZ01000975:715-1015
HmHel-4	CAEZ01000935:7396-7696
HmHel-4	CAEZ01000878:39839-40139
HmHel-4	CAEZ01000867:2211-2518
HmHel-4	CAEZ01000867:25766-26036
HmHel-4	CAEZ01000867:55461-55760
HmHel-4	CAEZ01000867:67219-67528
HmHel-4	CAEZ01000867:32924-33221
HmHel-4	CAEZ01000867:12464-12733
HmHel-4	CAEZ01000162:349-649
HmHel-4	CAEZ01000039:4951-5250
HmHel-4	CAEZ01011419:15610-15909
HmHel-4	CAEZ01010965:22990-23290
HmHel-4	CAEZ01010965:20999-21295
HmHel-4	CAEZ01009717:10811-11115
HmHel-4	CAEZ01009717:29120-29422
HmHel-4	CAEZ01009717:41982-42291
HmHel-4	CAEZ01009242:51005-51343
HmHel-4	CAEZ01009136:23322-23626
HmHel-4	CAEZ01009136:131100-131407
HmHel-4	CAEZ01008743:77107-77406
HmHel-4	CAEZ01008743:135282-135582
HmHel-4	CAEZ01008743:60251-60502
HmHel-4	CAEZ01008396:12651-12950
HmHel-4	CAEZ01008396:2523-2830
HmHel-4	CAEZ01008349:45083-45388
HmHel-4	CAEZ01008349:139943-140230
HmHel-4	CAEZ01008349:51522-51823

HmHel-4	CAEZ01008253:12369-12667
HmHel-4	CAEZ01008045:11948-12247
HmHel-4	CAEZ01007898:25519-25819
HmHel-4	CAEZ01007898:16675-16926
HmHel-4	CAEZ01007898:5606-5905
HmHel-4	CAEZ01007788:3849-4148
HmHel-4	CAEZ01007649:395-695
HmHel-4	CAEZ01007649:27269-27454
HmHel-4	CAEZ01007649:12469-12767
HmHel-4	CAEZ01006943:24502-24801
HmHel-4	CAEZ01006759:99130-99392
HmHel-4	CAEZ01006759:2925-3224
HmHel-4	CAEZ01006583:7416-7711
HmHel-4	CAEZ01006422:44203-44503
HmHel-4	CAEZ01006422:60725-61032
HmHel-4	CAEZ01006422:121542-121842
HmHel-4	CAEZ01006422:113328-113627
HmHel-4	CAEZ01006422:56737-57036
HmHel-4	CAEZ01006422:23358-23667
HmHel-4	CAEZ01006422:12968-13137
HmHel-4	CAEZ01006342:4947-5251
HmHel-4	CAEZ01006342:17498-17805
HmHel-4	CAEZ01006205:6901-7200
HmHel-4	CAEZ01006205:11901-12198
HmHel-4	CAEZ01006101:1570-1872
HmHel-4	CAEZ01006084:24743-25043
HmHel-4	CAEZ01006084:58717-59014
HmHel-4	CAEZ01006084:14873-15172
HmHel-4	CAEZ01005190:779-1078
HmHel-4	CAEZ01005173:25762-26056
HmHel-4	CAEZ01005173:100371-100676
HmHel-4	CAEZ01005173:138172-138471
HmHel-4	CAEZ01005173:157860-158164
HmHel-4	CAEZ01005173:108912-109219
HmHel-4	CAEZ01004542:27559-27859
HmHel-4	CAEZ01004542:21226-21525
HmHel-4	CAEZ01004460:8478-8777
HmHel-4	CAEZ01004188:15846-16143
HmHel-4	CAEZ01004188:3797-4088
HmHel-4	CAEZ01003749:597-896
HmHel-4	CAEZ01003238:3644-3944
HmHel-4	CAEZ01003056:2078-2377
HmHel-4	CAEZ01003019:116311-116610
HmHel-4	CAEZ01003019:93483-93703
HmHel-4	CAEZ01003019:32289-32594
HmHel-4	CAEZ01003015:22190-22492
HmHel-4	CAEZ01002703:7451-7750
HmHel-4	CAEZ01002274:4782-5082

HmHel-4	CAEZ01001538:37187-37494
HmHel-4	CAEZ01001538:49959-50254
HmHel-4	CAEZ01001436:26558-26860
HmHel-4	CAEZ01001199:3968-4267
HmHel-4	CAEZ01011284:45932-46234
HmHel-4	CAEZ01010695:20645-20942
HmHel-4	CAEZ01010228:63921-64218
HmHel-4	CAEZ01010228:101045-101276
HmHel-4	CAEZ01010228:5705-6004
HmHel-4	CAEZ01010210:19963-20257
HmHel-4	CAEZ01009281:42497-42795
HmHel-4	CAEZ01008578:11756-12056
HmHel-4	CAEZ01008578:125363-125603
HmHel-4	CAEZ01008578:107562-107860
HmHel-4	CAEZ01008578:45167-45474
HmHel-4	CAEZ01008578:3610-3912
HmHel-4	CAEZ01007286:2948-3219
HmHel-4	CAEZ01007286:18630-18925
HmHel-4	CAEZ01007286:14371-14656
HmHel-4	CAEZ01006504:20393-20687
HmHel-4	CAEZ01002180:120005-120298
HmHel-4	CAEZ01002041:17434-17741
HmHel-4	CAEZ01002041:73382-73692
HmHel-4	CAEZ01011416:21099-21397
HmHel-4	CAEZ01011416:63655-63882
HmHel-4	CAEZ01011122:8820-9117
HmHel-4	CAEZ01011093:200223-200523
HmHel-4	CAEZ01011093:68170-68468
HmHel-4	CAEZ01011093:39332-39559
HmHel-4	CAEZ01010969:59057-59356
HmHel-4	CAEZ01010969:88449-88749
HmHel-4	CAEZ01010969:199002-199299
HmHel-4	CAEZ01010969:125448-125746
HmHel-4	CAEZ01010969:91285-91582
HmHel-4	CAEZ01010969:9833-10091
HmHel-4	CAEZ01010810:17387-17684
HmHel-4	CAEZ01010704:131893-132191
HmHel-4	CAEZ01010620:4844-5173
HmHel-4	CAEZ01010620:152134-152453
HmHel-4	CAEZ01010620:182090-182383
HmHel-4	CAEZ01010415:571-872
HmHel-4	CAEZ01010277:125757-126055
HmHel-4	CAEZ01010277:18703-19004
HmHel-4	CAEZ01010033:4678-4975
HmHel-4	CAEZ01009893:6949-7242
HmHel-4	CAEZ01009893:19505-19781
HmHel-4	CAEZ01009887:56865-57082
HmHel-4	CAEZ01009887:137326-137623

HmHel-4	CAEZ01009887:193045-193345
HmHel-4	CAEZ01009887:211135-211440
HmHel-4	CAEZ01009887:244379-244662
HmHel-4	CAEZ01009887:233782-234086
HmHel-4	CAEZ01009887:205778-206078
HmHel-4	CAEZ01009887:138058-138359
HmHel-4	CAEZ01009887:108817-109126
HmHel-4	CAEZ01009887:27462-27740
HmHel-4	CAEZ01009887:12789-13055
HmHel-4	CAEZ01009614:5880-6177
HmHel-4	CAEZ01009482:2518-2816
HmHel-4	CAEZ01009084:3792-4031
HmHel-4	CAEZ01009084:86149-86450
HmHel-4	CAEZ01009084:79779-80081
HmHel-4	CAEZ01009020:2529-2822
HmHel-4	CAEZ01009020:12318-12619
HmHel-4	CAEZ01008662:21113-21414
HmHel-4	CAEZ01008662:1992-2301
HmHel-4	CAEZ01008269:7903-8199
HmHel-4	CAEZ01008269:30663-30960
HmHel-4	CAEZ01007731:63311-63619
HmHel-4	CAEZ01007128:3699-4000
HmHel-4	CAEZ01006863:67054-67363
HmHel-4	CAEZ01006863:103082-103389
HmHel-4	CAEZ01006863:101663-101948
HmHel-4	CAEZ01006863:21488-21770
HmHel-4	CAEZ01006863:9776-9882
HmHel-4	CAEZ01006352:41720-42017
HmHel-4	CAEZ01006352:84035-86287
HmHel-4	CAEZ01006352:5331-5558
HmHel-4	CAEZ01006164:7947-8227
HmHel-4	CAEZ01006164:22023-22320
HmHel-4	CAEZ01006164:56961-57258
HmHel-4	CAEZ01005475:44060-44358
HmHel-4	CAEZ01005475:112361-112668
HmHel-4	CAEZ01005475:98467-98749
HmHel-4	CAEZ01005475:64188-64495
HmHel-4	CAEZ01004802:41470-41771
HmHel-4	CAEZ01004792:5048-5203
HmHel-4	CAEZ01004792:20522-20823
HmHel-4	CAEZ01004792:104567-104832
HmHel-4	CAEZ01004792:73941-74239
HmHel-4	CAEZ01004792:58084-58391
HmHel-4	CAEZ01004640:1258-1556
HmHel-4	CAEZ01004632:113265-113410
HmHel-4	CAEZ01004632:115636-115723
HmHel-4	CAEZ01004632:55911-56204
HmHel-4	CAEZ01003069:154591-154899

HmHel-4	CAEZ01003069:172574-172867
HmHel-4	CAEZ01003069:212118-212445
HmHel-4	CAEZ01003069:297579-297883
HmHel-4	CAEZ01003069:281751-282051
HmHel-4	CAEZ01003033:6945-7243
HmHel-4	CAEZ01001494:17026-17324
HmHel-4	CAEZ01001327:6497-6794
HmHel-4	CAEZ01001201:7190-7486
HmHel-4	CAEZ01000971:15649-15945
HmHel-4	CAEZ01000971:14074-14371
HmHel-4	CAEZ01000824:58609-58910
HmHel-4	CAEZ01000044:10816-11109
HmHel-4	CAEZ01011598:37192-37449
HmHel-4	CAEZ01011598:115638-115960
HmHel-4	CAEZ01011598:24713-25013
HmHel-4	CAEZ01011521:6459-6761
HmHel-4	CAEZ01011521:21845-22145
HmHel-4	CAEZ01011386:16900-17200
HmHel-4	CAEZ01011386:13838-14145
HmHel-4	CAEZ01011362:19119-19346
HmHel-4	CAEZ01011362:34173-34473
HmHel-4	CAEZ01011307:26146-26446
HmHel-4	CAEZ01011275:4591-4891
HmHel-4	CAEZ01011214:3377-3677
HmHel-4	CAEZ01011158:1281-1581
HmHel-4	CAEZ01011139:21298-21609
HmHel-4	CAEZ01011139:37316-37622
HmHel-4	CAEZ01011139:94127-94431
HmHel-4	CAEZ01011139:135985-136226
HmHel-4	CAEZ01011139:68790-69090
HmHel-4	CAEZ01011113:3827-4126
HmHel-4	CAEZ01010990:4053-4353
HmHel-4	CAEZ01010920:53824-54129
HmHel-4	CAEZ01010920:1756-2056
HmHel-4	CAEZ01010909:6453-6752
HmHel-4	CAEZ01010892:4645-4945
HmHel-4	CAEZ01010836:3454-3698
HmHel-4	CAEZ01010728:15045-15345
HmHel-4	CAEZ01010728:57149-57324
HmHel-4	CAEZ01010728:41342-41642
HmHel-4	CAEZ01010728:27624-27920
HmHel-4	CAEZ01010728:406-669
HmHel-4	CAEZ01010707:163785-164086
HmHel-4	CAEZ01010707:145749-146039
HmHel-4	CAEZ01010707:114839-115135
HmHel-4	CAEZ01010707:48626-48926
HmHel-4	CAEZ01010689:2506-2804
HmHel-4	CAEZ01010676:11247-11547

HmHel-4	CAEZ01010667:3440-3732
HmHel-4	CAEZ01010667:21510-21810
HmHel-4	CAEZ01010667:36196-36488
HmHel-4	CAEZ01010667:6122-6429
HmHel-4	CAEZ01010665:29595-29895
HmHel-4	CAEZ01010642:441-737
HmHel-4	CAEZ01010534:85927-86227
HmHel-4	CAEZ01010423:57998-58298
HmHel-4	CAEZ01010423:25377-25676
HmHel-4	CAEZ01010318:43326-43626
HmHel-4	CAEZ01010318:54418-54715
HmHel-4	CAEZ01010289:153352-153652
HmHel-4	CAEZ01010288:48204-48508
HmHel-4	CAEZ01010288:91739-92039
HmHel-4	CAEZ01010288:173985-174284
HmHel-4	CAEZ01010288:26583-26755
HmHel-4	CAEZ01010241:13676-13975
HmHel-4	CAEZ01009978:26987-27282
HmHel-4	CAEZ01009978:53949-54256
HmHel-4	CAEZ01009978:42344-42643
HmHel-4	CAEZ01009978:23651-23838
HmHel-4	CAEZ01009978:8322-8622
HmHel-4	CAEZ01009894:56136-56436
HmHel-4	CAEZ01009894:34247-34554
HmHel-4	CAEZ01009843:42824-43110
HmHel-4	CAEZ01009843:61289-61585
HmHel-4	CAEZ01009746:2440-2742
HmHel-4	CAEZ01009746:18427-18727
HmHel-4	CAEZ01009671:6693-6993
HmHel-4	CAEZ01009582:12526-12824
HmHel-4	CAEZ01009582:9816-10115
HmHel-4	CAEZ01009559:1197-1497
HmHel-4	CAEZ01009558:660-960
HmHel-4	CAEZ01009538:3311-3611
HmHel-4	CAEZ01009538:13615-13915
HmHel-4	CAEZ01009533:9885-10177
HmHel-4	CAEZ01009497:18410-18696
HmHel-4	CAEZ01009497:24327-24554
HmHel-4	CAEZ01009497:9068-9368
HmHel-4	CAEZ01009488:19867-20167
HmHel-4	CAEZ01009440:15277-15577
HmHel-4	CAEZ01009431:43427-43513
HmHel-4	CAEZ01009431:41175-41472
HmHel-4	CAEZ01009431:29415-29715
HmHel-4	CAEZ01009289:6582-6882
HmHel-4	CAEZ01009265:15280-15579
HmHel-4	CAEZ01009224:30127-30230
HmHel-4	CAEZ01009224:45857-46170

HmHel-4	CAEZ01009224:53497-53798
HmHel-4	CAEZ01009224:31844-32140
HmHel-4	CAEZ01009048:36517-36817
HmHel-4	CAEZ01009022:5276-5576
HmHel-4	CAEZ01008945:25292-25592
HmHel-4	CAEZ01008945:72922-73216
HmHel-4	CAEZ01008934:9121-9421
HmHel-4	CAEZ01008921:64039-64339
HmHel-4	CAEZ01008921:146988-147296
HmHel-4	CAEZ01008921:115763-121667
HmHel-4	CAEZ01008921:91352-91659
HmHel-4	CAEZ01008921:39926-40230
HmHel-4	CAEZ01008921:2015-2289
HmHel-4	CAEZ01008911:31429-33657
HmHel-4	CAEZ01008911:85839-86153
HmHel-4	CAEZ01008911:139226-139519
HmHel-4	CAEZ01008911:42837-43137
HmHel-4	CAEZ01008911:15113-15249
HmHel-4	CAEZ01008843:633-933
HmHel-4	CAEZ01008688:13457-13756
HmHel-4	CAEZ01008675:12064-12348
HmHel-4	CAEZ01008675:20429-20729
HmHel-4	CAEZ01008675:765-1064
HmHel-4	CAEZ01008529:2162-2462
HmHel-4	CAEZ01008388:3289-3589
HmHel-4	CAEZ01008333:4820-5119
HmHel-4	CAEZ01008333:5900-6200
HmHel-4	CAEZ01008315:50833-51121
HmHel-4	CAEZ01008315:254578-254866
HmHel-4	CAEZ01008315:267661-267961
HmHel-4	CAEZ01008315:407459-407766
HmHel-4	CAEZ01008315:369872-370171
HmHel-4	CAEZ01008315:136371-136667
HmHel-4	CAEZ01008315:89046-89351
HmHel-4	CAEZ01008315:19070-19397
HmHel-4	CAEZ01008303:77718-78016
HmHel-4	CAEZ01008303:16645-16942
HmHel-4	CAEZ01008303:3743-4043
HmHel-4	CAEZ01008295:11636-11932
HmHel-4	CAEZ01008293:53891-54190
HmHel-4	CAEZ01008258:189056-189356
HmHel-4	CAEZ01008258:174590-174897
HmHel-4	CAEZ01008258:66021-66348
HmHel-4	CAEZ01008258:54229-54529
HmHel-4	CAEZ01008239:8272-8572
HmHel-4	CAEZ01008171:4142-4442
HmHel-4	CAEZ01008167:3475-3775
HmHel-4	CAEZ01008118:7183-7483

HmHel-4	CAEZ01007978:5234-5534
HmHel-4	CAEZ01007812:200650-200950
HmHel-4	CAEZ01007812:267557-267864
HmHel-4	CAEZ01007812:233852-234149
HmHel-4	CAEZ01007812:66288-66580
HmHel-4	CAEZ01007812:7490-7782
HmHel-4	CAEZ01007808:33400-33525
HmHel-4	CAEZ01007808:88279-88579
HmHel-4	CAEZ01007797:5265-5565
HmHel-4	CAEZ01007777:27140-27440
HmHel-4	CAEZ01007739:13361-13661
HmHel-4	CAEZ01007739:46332-46614
HmHel-4	CAEZ01007739:70734-71034
HmHel-4	CAEZ01007739:16835-17142
HmHel-4	CAEZ01007739:4093-4396
HmHel-4	CAEZ01007704:861-1161
HmHel-4	CAEZ01007704:17555-17841
HmHel-4	CAEZ01007584:127618-127917
HmHel-4	CAEZ01007584:167515-167824
HmHel-4	CAEZ01007584:134352-134652
HmHel-4	CAEZ01007584:99123-99397
HmHel-4	CAEZ01007578:4207-4507
HmHel-4	CAEZ01007535:3467-3767
HmHel-4	CAEZ01007498:35613-35913
HmHel-4	CAEZ01007498:25230-25530
HmHel-4	CAEZ01007492:33343-33623
HmHel-4	CAEZ01007492:46612-46912
HmHel-4	CAEZ01007492:26817-27113
HmHel-4	CAEZ01007360:9371-9671
HmHel-4	CAEZ01007352:4540-4840
HmHel-4	CAEZ01007232:43093-43393
HmHel-4	CAEZ01007232:15367-15674
HmHel-4	CAEZ01006914:8756-9056
HmHel-4	CAEZ01006914:13712-14014
HmHel-4	CAEZ01006894:192-492
HmHel-4	CAEZ01006787:22201-22494
HmHel-4	CAEZ01006675:23254-23554
HmHel-4	CAEZ01006675:69922-70220
HmHel-4	CAEZ01006675:81353-81611
HmHel-4	CAEZ01006675:65250-65516
HmHel-4	CAEZ01006657:4014-4316
HmHel-4	CAEZ01006638:13627-13927
HmHel-4	CAEZ01006318:14754-15054
HmHel-4	CAEZ01006315:9092-9392
HmHel-4	CAEZ01006281:5468-5760
HmHel-4	CAEZ01006281:24983-25279
HmHel-4	CAEZ01006281:36438-36748
HmHel-4	CAEZ01006281:11128-11314

HmHel-4	CAEZ01006242:16314-16614
HmHel-4	CAEZ01006196:23106-23406
HmHel-4	CAEZ01006196:54378-54678
HmHel-4	CAEZ01006196:175016-175318
HmHel-4	CAEZ01005716:24967-25100
HmHel-4	CAEZ01005716:73153-73328
HmHel-4	CAEZ01005716:74989-75289
HmHel-4	CAEZ01005716:35974-36273
HmHel-4	CAEZ01005716:3949-4270
HmHel-4	CAEZ01005657:15567-15867
HmHel-4	CAEZ01005628:308-608
HmHel-4	CAEZ01005616:278-578
HmHel-4	CAEZ01005576:17486-17786
HmHel-4	CAEZ01005568:1464-1764
HmHel-4	CAEZ01005321:36700-37000
HmHel-4	CAEZ01005313:6649-6949
HmHel-4	CAEZ01005262:3718-4018
HmHel-4	CAEZ01005229:31169-31410
HmHel-4	CAEZ01005229:45355-45658
HmHel-4	CAEZ01005226:7765-8065
HmHel-4	CAEZ01005177:4861-5088
HmHel-4	CAEZ01005177:36837-37137
HmHel-4	CAEZ01005177:62342-62642
HmHel-4	CAEZ01005177:82969-83276
HmHel-4	CAEZ01005131:16930-17230
HmHel-4	CAEZ01005048:22457-22757
HmHel-4	CAEZ01005047:11293-11592
HmHel-4	CAEZ01004996:23469-23769
HmHel-4	CAEZ01004996:2547-2858
HmHel-4	CAEZ01004850:7682-7982
HmHel-4	CAEZ01004759:4528-4828
HmHel-4	CAEZ01004591:27211-27511
HmHel-4	CAEZ01004469:3777-4077
HmHel-4	CAEZ01004386:1249-1549
HmHel-4	CAEZ01003913:12164-12464
HmHel-4	CAEZ01003761:17983-18283
HmHel-4	CAEZ01003722:33039-33339
HmHel-4	CAEZ01003722:50588-50887
HmHel-4	CAEZ01003620:9100-9400
HmHel-4	CAEZ01003545:12677-12977
HmHel-4	CAEZ01003508:5550-5850
HmHel-4	CAEZ01003231:11818-12118
HmHel-4	CAEZ01003195:3807-4026
HmHel-4	CAEZ01003195:48967-49266
HmHel-4	CAEZ01003171:3368-3668
HmHel-4	CAEZ01003134:5127-5427
HmHel-4	CAEZ01003063:25403-25619
HmHel-4	CAEZ01003063:20514-20813

HmHel-4	CAEZ01003030:2194-2492
HmHel-4	CAEZ01002957:1-293
HmHel-4	CAEZ01002890:26459-26759
HmHel-4	CAEZ01002823:10-310
HmHel-4	CAEZ01002670:20553-20853
HmHel-4	CAEZ01002667:28990-29297
HmHel-4	CAEZ01002667:26966-27265
HmHel-4	CAEZ01002429:38279-38593
HmHel-4	CAEZ01002429:63872-63960
HmHel-4	CAEZ01002429:40288-40588
HmHel-4	CAEZ01002429:8789-9088
HmHel-4	CAEZ01002255:53970-54270
HmHel-4	CAEZ01002255:53032-53339
HmHel-4	CAEZ01002250:2423-2723
HmHel-4	CAEZ01002125:733-1033
HmHel-4	CAEZ01002066:16484-16783
HmHel-4	CAEZ01002060:1339-1639
HmHel-4	CAEZ01002039:24906-25217
HmHel-4	CAEZ01002039:6424-6724
HmHel-4	CAEZ01001787:11065-11365
HmHel-4	CAEZ01001650:10433-10733
HmHel-4	CAEZ01001650:110106-110406
HmHel-4	CAEZ01001556:13680-13979
HmHel-4	CAEZ01001522:547-846
HmHel-4	CAEZ01001496:19673-19972
HmHel-4	CAEZ01001435:9705-10005
HmHel-4	CAEZ01001435:97456-97761
HmHel-4	CAEZ01001435:228047-228326
HmHel-4	CAEZ01001435:225242-225558
HmHel-4	CAEZ01001435:53133-53467
HmHel-4	CAEZ01001435:42334-42618
HmHel-4	CAEZ01001141:853-1153
HmHel-4	CAEZ01000879:115130-115414
HmHel-4	CAEZ01000879:182621-182838
HmHel-4	CAEZ01000879:164887-165198
HmHel-4	CAEZ01000879:133126-133426
HmHel-4	CAEZ01000879:111554-111674
HmHel-4	CAEZ01000879:54871-55171
HmHel-4	CAEZ01000879:4382-4689
HmHel-4	CAEZ01000067:8910-9210
HmHel-4	CAEZ01000063:1718-2018
HmHel-4	CAEZ01000063:54825-55125
HmHel-4	CAEZ01000063:1110-1420
HmHel-4	CAEZ01000059:1281-1608
HmHel-4	CAEZ01000059:14191-14490
HmHel-4	CAEZ01011519:154205-154448
HmHel-4	CAEZ01011519:175336-175643
HmHel-4	CAEZ01011519:160123-160422

HmHel-4	CAEZ01011519:34354-34652
HmHel-4	CAEZ01011215:28468-28763
HmHel-4	CAEZ01011215:166008-166316
HmHel-4	CAEZ01011215:177562-177869
HmHel-4	CAEZ01011215:60624-60947
HmHel-4	CAEZ01010746:38223-38440
HmHel-4	CAEZ01010746:2252-2553
HmHel-4	CAEZ01010509:7916-8215
HmHel-4	CAEZ01009684:19167-19466
HmHel-4	CAEZ01009135:15345-15644
HmHel-4	CAEZ01008944:36818-37117
HmHel-4	CAEZ01008944:52850-53044
HmHel-4	CAEZ01008944:1244-1414
HmHel-4	CAEZ01008674:21187-21414
HmHel-4	CAEZ01008674:48795-49087
HmHel-4	CAEZ01008674:69973-70268
HmHel-4	CAEZ01008674:82988-83295
HmHel-4	CAEZ01008674:89753-90051
HmHel-4	CAEZ01008674:60065-60227
HmHel-4	CAEZ01008674:24599-24898
HmHel-4	CAEZ01008147:14929-15228
HmHel-4	CAEZ01007275:94777-95084
HmHel-4	CAEZ01007275:140179-140488
HmHel-4	CAEZ01007275:158946-159281
HmHel-4	CAEZ01007275:75816-76112
HmHel-4	CAEZ01007275:17488-17624
HmHel-4	CAEZ01006333:7309-7611
HmHel-4	CAEZ01006081:25113-25412
HmHel-4	CAEZ01005447:5049-5344
HmHel-4	CAEZ01004733:59577-59876
HmHel-4	CAEZ01004733:101906-102208
HmHel-4	CAEZ01004244:27438-27720
HmHel-4	CAEZ01004244:105165-105442
HmHel-4	CAEZ01004244:136027-136293
HmHel-4	CAEZ01004244:156185-156489
HmHel-4	CAEZ01004244:110086-110385
HmHel-4	CAEZ01002365:3023-3319
HmHel-4	CAEZ01002097:20960-21109
HmHel-4	CAEZ01002097:64590-64881
HmHel-4	CAEZ01002097:14591-14898
HmHel-4	CAEZ01001741:9983-10282
HmHel-4	CAEZ01000990:32144-32435
HmHel-4	CAEZ01000909:45999-46161
HmHel-4	CAEZ01000909:152996-153165
HmHel-4	CAEZ01000909:1637-1920
HmHel-4	CAEZ01000812:1142-1440
HmHel-4	CAEZ01011348:1690-1987
HmHel-4	CAEZ01010803:10532-10839

HmHel-4	CAEZ01010803:28273-28548
HmHel-4	CAEZ01010803:61856-62150
HmHel-4	CAEZ01010803:83047-83335
HmHel-4	CAEZ01010244:49519-49817
HmHel-4	CAEZ01010244:34347-34641
HmHel-4	CAEZ01010141:1129-1427
HmHel-4	CAEZ01010141:16488-16660
HmHel-4	CAEZ01009386:17187-17481
HmHel-4	CAEZ01009386:5853-6111
HmHel-4	CAEZ01008951:2937-3235
HmHel-4	CAEZ01007861:95495-95821
HmHel-4	CAEZ01007861:4447-4733
HmHel-4	CAEZ01007378:4398-4697
HmHel-4	CAEZ01005183:5780-6074
HmHel-4	CAEZ01005183:22437-22737
HmHel-4	CAEZ01004224:4606-4903
HmHel-4	CAEZ01011564:2279-2599
HmHel-4	CAEZ01011564:42950-43253
HmHel-4	CAEZ01011529:1124-1424
HmHel-4	CAEZ01011529:18517-18817
HmHel-4	CAEZ01011529:46185-46474
HmHel-4	CAEZ01011529:27507-27810
HmHel-4	CAEZ01011430:10983-11286
HmHel-4	CAEZ01011073:843-1141
HmHel-4	CAEZ01010972:95590-95897
HmHel-4	CAEZ01010972:402610-402907
HmHel-4	CAEZ01010972:452157-452456
HmHel-4	CAEZ01010972:473560-473846
HmHel-4	CAEZ01010972:345195-345497
HmHel-4	CAEZ01010972:331247-331544
HmHel-4	CAEZ01010818:11347-11646
HmHel-4	CAEZ01010818:60865-61166
HmHel-4	CAEZ01010741:3844-4142
HmHel-4	CAEZ01009394:637-936
HmHel-4	CAEZ01008930:437-738
HmHel-4	CAEZ01008275:9145-9445
HmHel-4	CAEZ01008247:15-308
HmHel-4	CAEZ01007887:11206-11507
HmHel-4	CAEZ01007590:74733-75034
HmHel-4	CAEZ01007590:70865-71165
HmHel-4	CAEZ01007590:35391-35676
HmHel-4	CAEZ01007222:68977-69284
HmHel-4	CAEZ01007222:244602-244909
HmHel-4	CAEZ01007222:193582-193744
HmHel-4	CAEZ01007222:179245-179521
HmHel-4	CAEZ01007222:134644-134906
HmHel-4	CAEZ01007201:17623-17898
HmHel-4	CAEZ01007201:98312-98615

HmHel-4	CAEZ01007201:110510-110761
HmHel-4	CAEZ01007201:2282-2579
HmHel-4	CAEZ01006522:11986-12291
HmHel-4	CAEZ01006435:52444-52741
HmHel-4	CAEZ01006401:3315-3613
HmHel-4	CAEZ01006336:7682-7979
HmHel-4	CAEZ01006336:21728-22024
HmHel-4	CAEZ01006235:631-929
HmHel-4	CAEZ01006092:9386-9682
HmHel-4	CAEZ01006092:42591-42895
HmHel-4	CAEZ01006092:50120-50403
HmHel-4	CAEZ01005825:8005-8303
HmHel-4	CAEZ01005825:34398-34698
HmHel-4	CAEZ01005825:17972-18248
HmHel-4	CAEZ01005803:6873-7170
HmHel-4	CAEZ01005720:16333-16630
HmHel-4	CAEZ01005697:9703-10000
HmHel-4	CAEZ01005499:38238-38530
HmHel-4	CAEZ01005499:74857-75156
HmHel-4	CAEZ01005499:35871-36175
HmHel-4	CAEZ01005499:40-345
HmHel-4	CAEZ01005193:4028-4322
HmHel-4	CAEZ01004682:68653-68949
HmHel-4	CAEZ01004682:33964-34261
HmHel-4	CAEZ01004179:5367-5665
HmHel-4	CAEZ01003778:4822-5129
HmHel-4	CAEZ01003699:8257-8554
HmHel-4	CAEZ01003605:1507-1803
HmHel-4	CAEZ01003339:4832-5124
HmHel-4	CAEZ01003209:22733-23030
HmHel-4	CAEZ01003192:99878-100176
HmHel-4	CAEZ01003192:107588-107745
HmHel-4	CAEZ01003192:84633-84913
HmHel-4	CAEZ01003192:53289-53596
HmHel-4	CAEZ01003150:3054-3381
HmHel-4	CAEZ01003150:4081-4378
HmHel-4	CAEZ01002792:22350-22648
HmHel-4	CAEZ01002792:2416-2616
HmHel-4	CAEZ01002226:1216-1517
HmHel-4	CAEZ01001935:959-1248
HmHel-4	CAEZ01001894:2651-2949
HmHel-4	CAEZ01001535:2577-2875
HmHel-4	CAEZ01001111:1452-1744
HmHel-4	CAEZ01001111:1-297
HmHel-4	CAEZ01011659:14370-14669
HmHel-4	CAEZ01011454:4603-4902
HmHel-4	CAEZ01011424:1519-1805
HmHel-4	CAEZ01011424:568-868

HmHel-4	CAEZ01011337:1096-1395
HmHel-4	CAEZ01011008:8693-8993
HmHel-4	CAEZ01010974:18318-18618
HmHel-4	CAEZ01010894:165221-165403
HmHel-4	CAEZ01010894:153246-153539
HmHel-4	CAEZ01010894:19626-19926
HmHel-4	CAEZ01010868:65485-65784
HmHel-4	CAEZ01010764:1476-1784
HmHel-4	CAEZ01010734:69711-70010
HmHel-4	CAEZ01010734:107991-108295
HmHel-4	CAEZ01010674:7526-7826
HmHel-4	CAEZ01010489:21536-21828
HmHel-4	CAEZ01010489:5791-6125
HmHel-4	CAEZ01010435:29126-29426
HmHel-4	CAEZ01010276:51601-51900
HmHel-4	CAEZ01010276:14257-14501
HmHel-4	CAEZ01010269:10730-11030
HmHel-4	CAEZ01010227:87786-88084
HmHel-4	CAEZ01010227:121620-121918
HmHel-4	CAEZ01010086:16361-16661
HmHel-4	CAEZ01010029:4554-4787
HmHel-4	CAEZ01010029:85800-86106
HmHel-4	CAEZ01010024:916-1216
HmHel-4	CAEZ01009992:10410-10710
HmHel-4	CAEZ01009900:83439-83766
HmHel-4	CAEZ01009900:57576-57880
HmHel-4	CAEZ01009881:19431-19731
HmHel-4	CAEZ01009881:51327-51631
HmHel-4	CAEZ01009788:8159-8465
HmHel-4	CAEZ01009716:37460-37760
HmHel-4	CAEZ01009607:20297-20624
HmHel-4	CAEZ01009607:24298-24598
HmHel-4	CAEZ01009602:1808-2092
HmHel-4	CAEZ01009602:4538-4838
HmHel-4	CAEZ01009525:2322-2622
HmHel-4	CAEZ01009506:35065-35365
HmHel-4	CAEZ01009424:19326-19550
HmHel-4	CAEZ01009424:36322-36622
HmHel-4	CAEZ01009424:3793-4088
HmHel-4	CAEZ01009229:7571-7871
HmHel-4	CAEZ01009229:39423-39715
HmHel-4	CAEZ01009229:44913-45019
HmHel-4	CAEZ01009196:6858-7044
HmHel-4	CAEZ01009196:59563-59862
HmHel-4	CAEZ01009165:9040-9339
HmHel-4	CAEZ01009096:18120-18420
HmHel-4	CAEZ01009086:7693-7993
HmHel-4	CAEZ01008922:58096-58396

HmHel-4	CAEZ01008894:10693-10995
HmHel-4	CAEZ01008894:30512-30812
HmHel-4	CAEZ01008889:8951-9251
HmHel-4	CAEZ01008882:35377-35677
HmHel-4	CAEZ01008723:26146-26446
HmHel-4	CAEZ01008657:2249-2549
HmHel-4	CAEZ01008518:2139-2413
HmHel-4	CAEZ01008518:12009-12308
HmHel-4	CAEZ01008450:3598-3898
HmHel-4	CAEZ01008209:6471-6771
HmHel-4	CAEZ01008209:67016-67205
HmHel-4	CAEZ01008202:10484-10749
HmHel-4	CAEZ01008202:48187-48482
HmHel-4	CAEZ01008175:5991-6291
HmHel-4	CAEZ01008019:2697-2997
HmHel-4	CAEZ01007938:102371-102675
HmHel-4	CAEZ01007938:134823-135123
HmHel-4	CAEZ01007927:5937-6237
HmHel-4	CAEZ01007851:20112-20412
HmHel-4	CAEZ01007851:99950-100250
HmHel-4	CAEZ01007851:65036-65331
HmHel-4	CAEZ01007809:207870-208170
HmHel-4	CAEZ01007809:322061-322332
HmHel-4	CAEZ01007809:253717-253988
HmHel-4	CAEZ01007774:839-1139
HmHel-4	CAEZ01007721:32705-33005
HmHel-4	CAEZ01007721:24864-24968
HmHel-4	CAEZ01007721:13859-14157
HmHel-4	CAEZ01007599:12120-12424
HmHel-4	CAEZ01007599:23509-23809
HmHel-4	CAEZ01007586:34077-34376
HmHel-4	CAEZ01007586:73996-74171
HmHel-4	CAEZ01007586:21751-22058
HmHel-4	CAEZ01007557:9025-9325
HmHel-4	CAEZ01007537:11510-11810
HmHel-4	CAEZ01007431:6146-6446
HmHel-4	CAEZ01007398:5142-5442
HmHel-4	CAEZ01007258:13505-13835
HmHel-4	CAEZ01007258:44225-44532
HmHel-4	CAEZ01007258:112209-112513
HmHel-4	CAEZ01007258:101979-102279
HmHel-4	CAEZ01007050:2101-2401
HmHel-4	CAEZ01006874:9291-9591
HmHel-4	CAEZ01006795:9239-9531
HmHel-4	CAEZ01006778:92235-92535
HmHel-4	CAEZ01006669:12435-12735
HmHel-4	CAEZ01006560:38195-38495
HmHel-4	CAEZ01006560:131585-131884

HmHel-4	CAEZ01006560:146529-146828
HmHel-4	CAEZ01006560:94277-94562
HmHel-4	CAEZ01006560:67094-67365
HmHel-4	CAEZ01006560:50044-50344
HmHel-4	CAEZ01006534:88897-89197
HmHel-4	CAEZ01006506:11015-11315
HmHel-4	CAEZ01006370:30677-30978
HmHel-4	CAEZ01006370:59740-60055
HmHel-4	CAEZ01006370:34090-34397
HmHel-4	CAEZ01006370:18543-18850
HmHel-4	CAEZ01006319:38079-38373
HmHel-4	CAEZ01006319:23478-23766
HmHel-4	CAEZ01006234:13775-14075
HmHel-4	CAEZ01006234:24961-25268
HmHel-4	CAEZ01006234:30282-30620
HmHel-4	CAEZ01006224:18242-18542
HmHel-4	CAEZ01006214:19207-19507
HmHel-4	CAEZ01006062:2103-2403
HmHel-4	CAEZ01006026:2157-2456
HmHel-4	CAEZ01005980:30232-30532
HmHel-4	CAEZ01005850:12148-12448
HmHel-4	CAEZ01005848:8202-8502
HmHel-4	CAEZ01005802:22359-22659
HmHel-4	CAEZ01005717:35422-35508
HmHel-4	CAEZ01005717:48567-48867
HmHel-4	CAEZ01005717:36610-36912
HmHel-4	CAEZ01005666:4794-5125
HmHel-4	CAEZ01005633:2034-2334
HmHel-4	CAEZ01005624:15788-16088
HmHel-4	CAEZ01005411:4434-4734
HmHel-4	CAEZ01005410:20934-21233
HmHel-4	CAEZ01005279:35477-35777
HmHel-4	CAEZ01005279:103879-104179
HmHel-4	CAEZ01005279:75147-75367
HmHel-4	CAEZ01005279:41685-41929
HmHel-4	CAEZ01005230:46621-46912
HmHel-4	CAEZ01005230:84713-85013
HmHel-4	CAEZ01005230:64034-64312
HmHel-4	CAEZ01005213:8575-8875
HmHel-4	CAEZ01005211:3312-3575
HmHel-4	CAEZ01005211:36223-36499
HmHel-4	CAEZ01005211:51154-51460
HmHel-4	CAEZ01005211:71903-72202
HmHel-4	CAEZ01005211:93929-94152
HmHel-4	CAEZ01005211:124990-125290
HmHel-4	CAEZ01005211:123258-123558
HmHel-4	CAEZ01005207:89722-90022
HmHel-4	CAEZ01005207:55170-55497

HmHel-4	CAEZ01005186:6663-6963
HmHel-4	CAEZ01004920:30194-30494
HmHel-4	CAEZ01004793:56290-56597
HmHel-4	CAEZ01004793:71929-72212
HmHel-4	CAEZ01004793:91865-92170
HmHel-4	CAEZ01004793:44057-44360
HmHel-4	CAEZ01004793:33082-33382
HmHel-4	CAEZ01004667:3323-3623
HmHel-4	CAEZ01004626:3568-3868
HmHel-4	CAEZ01004484:49-349
HmHel-4	CAEZ01004418:608-908
HmHel-4	CAEZ01004311:244-544
HmHel-4	CAEZ01004198:7343-7636
HmHel-4	CAEZ01004198:30339-30639
HmHel-4	CAEZ01004198:1077-1377
HmHel-4	CAEZ01003940:3887-4187
HmHel-4	CAEZ01003875:29079-29379
HmHel-4	CAEZ01003850:21128-21428
HmHel-4	CAEZ01003729:946-1245
HmHel-4	CAEZ01003691:3699-3999
HmHel-4	CAEZ01003668:253-553
HmHel-4	CAEZ01003595:4910-5209
HmHel-4	CAEZ01003584:860-1159
HmHel-4	CAEZ01003326:39182-39482
HmHel-4	CAEZ01003321:2197-2497
HmHel-4	CAEZ01003235:106841-107141
HmHel-4	CAEZ01003235:82147-82443
HmHel-4	CAEZ01003099:6634-6935
HmHel-4	CAEZ01003099:8846-9144
HmHel-4	CAEZ01003045:3918-4216
HmHel-4	CAEZ01002951:22130-22430
HmHel-4	CAEZ01002951:45512-45845
HmHel-4	CAEZ01002906:26469-26769
HmHel-4	CAEZ01002906:26859-26981
HmHel-4	CAEZ01002870:19009-19309
HmHel-4	CAEZ01002870:3794-4141
HmHel-4	CAEZ01002863:71267-71567
HmHel-4	CAEZ01002863:19492-19787
HmHel-4	CAEZ01002822:10334-10634
HmHel-4	CAEZ01002742:36004-36309
HmHel-4	CAEZ01002742:178454-178750
HmHel-4	CAEZ01002742:211420-211727
HmHel-4	CAEZ01002742:215390-215607
HmHel-4	CAEZ01002742:105293-105600
HmHel-4	CAEZ01002742:85278-85578
HmHel-4	CAEZ01002675:30048-30348
HmHel-4	CAEZ01002599:284-584
HmHel-4	CAEZ01002143:5262-5562

HmHel-4	CAEZ01001773:2654-2954
HmHel-4	CAEZ01001753:1391-1691
HmHel-4	CAEZ01001441:93642-93941
HmHel-4	CAEZ01001441:106157-106454
HmHel-4	CAEZ01001441:69829-70136
HmHel-4	CAEZ01001441:25243-25515
HmHel-4	CAEZ01001375:15776-16073
HmHel-4	CAEZ01001375:9904-10204
HmHel-4	CAEZ01001094:767-1067
HmHel-4	CAEZ01001070:24730-25029
HmHel-4	CAEZ01001018:4616-4916
HmHel-4	CAEZ01010807:148509-148830
HmHel-4	CAEZ01010807:126240-126531
HmHel-4	CAEZ01010201:565-861
HmHel-4	CAEZ01009892:1305-1602
HmHel-4	CAEZ01009892:83369-83527
HmHel-4	CAEZ01009892:119365-119645
HmHel-4	CAEZ01009892:191602-191896
HmHel-4	CAEZ01009892:94442-94737
HmHel-4	CAEZ01009775:8981-9285
HmHel-4	CAEZ01009679:15057-15356
HmHel-4	CAEZ01009282:14338-14645
HmHel-4	CAEZ01009282:260965-261271
HmHel-4	CAEZ01009282:90757-91051
HmHel-4	CAEZ01008766:8997-9293
HmHel-4	CAEZ01008458:8338-8642
HmHel-4	CAEZ01008110:23153-23457
HmHel-4	CAEZ01007551:20668-20893
HmHel-4	CAEZ01007551:31567-31873
HmHel-4	CAEZ01006975:5081-5379
HmHel-4	CAEZ01006960:45034-45333
HmHel-4	CAEZ01006189:13653-13944
HmHel-4	CAEZ01005921:8042-8321
HmHel-4	CAEZ01005515:61230-61526
HmHel-4	CAEZ01005396:2749-3048
HmHel-4	CAEZ01005155:5548-5853
HmHel-4	CAEZ01005155:14290-14585
HmHel-4	CAEZ01005002:682-977
HmHel-4	CAEZ01004849:5000-5302
HmHel-4	CAEZ01004685:256-558
HmHel-4	CAEZ01004685:14482-14777
HmHel-4	CAEZ01004387:4622-4917
HmHel-4	CAEZ01004248:5562-5868
HmHel-4	CAEZ01004018:40354-40650
HmHel-4	CAEZ01004018:100484-100750
HmHel-4	CAEZ01004018:577-887
HmHel-4	CAEZ01003189:9324-9646
HmHel-4	CAEZ01003189:369-665

HmHel-4	CAEZ01002898:2575-2874
HmHel-4	CAEZ01000864:12169-12471
HmHel-4	CAEZ01010041:12252-12550
HmHel-4	CAEZ01009509:84784-85083
HmHel-4	CAEZ01009509:122563-122861
HmHel-4	CAEZ01006958:1236-1534
HmHel-4	CAEZ01006958:19487-19793
HmHel-4	CAEZ01006958:5809-6116
HmHel-4	CAEZ01005988:1196-1493
HmHel-4	CAEZ01004824:10753-11043
HmHel-4	CAEZ01002577:1047-1268
HmHel-4	CAEZ01002577:23975-24272
HmHel-4	CAEZ01011565:10167-10470
HmHel-4	CAEZ01010449:33876-34173
HmHel-4	CAEZ01010449:83076-83403
HmHel-4	CAEZ01010449:82513-82840
HmHel-4	CAEZ01010189:12932-13228
HmHel-4	CAEZ01010079:11267-11565
HmHel-4	CAEZ01010079:80578-80905
HmHel-4	CAEZ01010079:20063-20361
HmHel-4	CAEZ01010079:7950-8244
HmHel-4	CAEZ01009500:15039-15340
HmHel-4	CAEZ01009471:1872-2168
HmHel-4	CAEZ01009373:30489-30786
HmHel-4	CAEZ01009271:168550-168853
HmHel-4	CAEZ01009251:10640-10938
HmHel-4	CAEZ01009125:5440-5741
HmHel-4	CAEZ01009125:34611-34882
HmHel-4	CAEZ01008359:76160-76460
HmHel-4	CAEZ01008359:188764-188929
HmHel-4	CAEZ01008359:168448-168749
HmHel-4	CAEZ01006322:3950-4247
HmHel-4	CAEZ01006316:3053-3350
HmHel-4	CAEZ01006302:6246-6650
HmHel-4	CAEZ01006302:16752-17049
HmHel-4	CAEZ01006302:17664-17987
HmHel-4	CAEZ01006278:1513-1810
HmHel-4	CAEZ01006278:33671-33918
HmHel-4	CAEZ01006270:3866-4163
HmHel-4	CAEZ01006151:21984-22269
HmHel-4	CAEZ01006151:35032-35339
HmHel-4	CAEZ01005394:15506-15800
HmHel-4	CAEZ01005265:12576-12874
HmHel-4	CAEZ01005265:16056-16350
HmHel-4	CAEZ01004955:2824-3121
HmHel-4	CAEZ01004761:25985-26282
HmHel-4	CAEZ01004097:119504-119811
HmHel-4	CAEZ01004097:140914-141211

HmHel-4	CAEZ01004097:161393-161690
HmHel-4	CAEZ01004097:215833-216132
HmHel-4	CAEZ01004097:198283-198411
HmHel-4	CAEZ01004097:133998-134176
HmHel-4	CAEZ01004097:29312-29619
HmHel-4	CAEZ01004034:61676-61937
HmHel-4	CAEZ01004034:84348-84645
HmHel-4	CAEZ01003881:15540-15829
HmHel-4	CAEZ01003514:8157-8451
HmHel-4	CAEZ01003344:29401-29698
HmHel-4	CAEZ01002788:30265-30562
HmHel-4	CAEZ01002788:3546-3872
HmHel-4	CAEZ01002576:16447-16619
HmHel-4	CAEZ01001331:99115-99411
HmHel-4	CAEZ01000231:183-477
HmHel-4	CAEZ01011513:463-760
HmHel-4	CAEZ01011513:3252-3544
HmHel-4	CAEZ01010853:2656-2956
HmHel-4	CAEZ01010838:11227-11523
HmHel-4	CAEZ01010825:29958-30250
HmHel-4	CAEZ01010805:7609-7908
HmHel-4	CAEZ01010805:78963-79235
HmHel-4	CAEZ01010805:83290-83548
HmHel-4	CAEZ01010797:2421-2721
HmHel-4	CAEZ01010797:101466-101745
HmHel-4	CAEZ01010797:145676-145878
HmHel-4	CAEZ01010797:161607-161834
HmHel-4	CAEZ01010797:110906-111206
HmHel-4	CAEZ01010797:35207-35371
HmHel-4	CAEZ01010777:5916-6216
HmHel-4	CAEZ01010727:47299-47596
HmHel-4	CAEZ01010727:42247-42546
HmHel-4	CAEZ01010539:3475-3775
HmHel-4	CAEZ01010481:1039-2661
HmHel-4	CAEZ01010481:9564-9864
HmHel-4	CAEZ01010190:30391-30699
HmHel-4	CAEZ01010184:31164-31464
HmHel-4	CAEZ01009988:13145-13445
HmHel-4	CAEZ01009966:4190-4490
HmHel-4	CAEZ01009884:22598-22898
HmHel-4	CAEZ01009884:28769-29070
HmHel-4	CAEZ01009780:1741-2041
HmHel-4	CAEZ01009723:16832-17132
HmHel-4	CAEZ01009718:17253-17553
HmHel-4	CAEZ01009566:825-1124
HmHel-4	CAEZ01009566:3366-3693
HmHel-4	CAEZ01009415:2244-2544
HmHel-4	CAEZ01009415:10452-10756

HmHel-4	CAEZ01009320:10623-10838
HmHel-4	CAEZ01009320:4395-4695
HmHel-4	CAEZ01009113:8175-8474
HmHel-4	CAEZ01009085:153-452
HmHel-4	CAEZ01009085:57630-57949
HmHel-4	CAEZ01009085:37433-37734
HmHel-4	CAEZ01009023:57685-57985
HmHel-4	CAEZ01009023:94003-94268
HmHel-4	CAEZ01009023:40853-41157
HmHel-4	CAEZ01009023:6323-6619
HmHel-4	CAEZ01008919:63424-63723
HmHel-4	CAEZ01008873:2882-3182
HmHel-4	CAEZ01008724:24165-24461
HmHel-4	CAEZ01008550:20443-20762
HmHel-4	CAEZ01008526:10022-10322
HmHel-4	CAEZ01008475:407-707
HmHel-4	CAEZ01008392:41769-42069
HmHel-4	CAEZ01008392:16279-16545
HmHel-4	CAEZ01008392:1112-1338
HmHel-4	CAEZ01008083:1553-1849
HmHel-4	CAEZ01008002:2075-2375
HmHel-4	CAEZ01007865:32434-32734
HmHel-4	CAEZ01007865:34489-34616
HmHel-4	CAEZ01007772:44799-45099
HmHel-4	CAEZ01007751:56970-57278
HmHel-4	CAEZ01007751:128531-128823
HmHel-4	CAEZ01007751:65338-65510
HmHel-4	CAEZ01007736:13827-14134
HmHel-4	CAEZ01007736:32574-32874
HmHel-4	CAEZ01007736:45055-45306
HmHel-4	CAEZ01007665:129795-130095
HmHel-4	CAEZ01007626:66999-67299
HmHel-4	CAEZ01007626:20914-21214
HmHel-4	CAEZ01007600:10845-11145
HmHel-4	CAEZ01007600:11754-12037
HmHel-4	CAEZ01007576:35880-36180
HmHel-4	CAEZ01007508:3593-3893
HmHel-4	CAEZ01007265:22313-22613
HmHel-4	CAEZ01007185:9384-9684
HmHel-4	CAEZ01007072:18953-19252
HmHel-4	CAEZ01006968:198-493
HmHel-4	CAEZ01006968:11915-12195
HmHel-4	CAEZ01006938:4809-5109
HmHel-4	CAEZ01006937:7260-7559
HmHel-4	CAEZ01006760:7911-8193
HmHel-4	CAEZ01006760:103497-103797
HmHel-4	CAEZ01006760:101993-102262
HmHel-4	CAEZ01006760:8212-8439

HmHel-4	CAEZ01006711:80835-81135
HmHel-4	CAEZ01006653:1479-1778
HmHel-4	CAEZ01006490:63494-63794
HmHel-4	CAEZ01006124:32307-32607
HmHel-4	CAEZ01006100:5419-5719
HmHel-4	CAEZ01006007:1695-1995
HmHel-4	CAEZ01005997:7055-7348
HmHel-4	CAEZ01005997:34589-34884
HmHel-4	CAEZ01005997:9292-9592
HmHel-4	CAEZ01005966:3166-3473
HmHel-4	CAEZ01005949:15685-15992
HmHel-4	CAEZ01005949:89835-90142
HmHel-4	CAEZ01005949:56198-56498
HmHel-4	CAEZ01005924:21402-21701
HmHel-4	CAEZ01005817:14661-14961
HmHel-4	CAEZ01005688:18481-18781
HmHel-4	CAEZ01005688:1684-1959
HmHel-4	CAEZ01005656:12214-12514
HmHel-4	CAEZ01005590:32144-32443
HmHel-4	CAEZ01005562:5615-5915
HmHel-4	CAEZ01005498:7156-7456
HmHel-4	CAEZ01005480:37972-38272
HmHel-4	CAEZ01005478:54525-54825
HmHel-4	CAEZ01005478:79334-79621
HmHel-4	CAEZ01005478:67704-68011
HmHel-4	CAEZ01005478:13283-13606
HmHel-4	CAEZ01005362:17119-17414
HmHel-4	CAEZ01005314:6076-6375
HmHel-4	CAEZ01005150:27686-27985
HmHel-4	CAEZ01005071:27567-27862
HmHel-4	CAEZ01004832:33118-33451
HmHel-4	CAEZ01004832:83980-84205
HmHel-4	CAEZ01004832:74111-74417
HmHel-4	CAEZ01004832:53588-53887
HmHel-4	CAEZ01004832:8497-8788
HmHel-4	CAEZ01004745:22749-22919
HmHel-4	CAEZ01004745:10168-10468
HmHel-4	CAEZ01004570:2805-3105
HmHel-4	CAEZ01004429:4293-4593
HmHel-4	CAEZ01004359:5240-5491
HmHel-4	CAEZ01004359:22644-22907
HmHel-4	CAEZ01004359:1032-1332
HmHel-4	CAEZ01004302:14929-15229
HmHel-4	CAEZ01004016:35236-35536
HmHel-4	CAEZ01004016:35994-36300
HmHel-4	CAEZ01004016:2809-3115
HmHel-4	CAEZ01003830:19486-19786
HmHel-4	CAEZ01003811:10183-10483

HmHel-4	CAEZ01003740:19072-19372
HmHel-4	CAEZ01003740:17904-18211
HmHel-4	CAEZ01003504:3917-4208
HmHel-4	CAEZ01003234:13531-13827
HmHel-4	CAEZ01003194:62527-62802
HmHel-4	CAEZ01003194:49238-49545
HmHel-4	CAEZ01003194:30164-30471
HmHel-4	CAEZ01003194:9547-9847
HmHel-4	CAEZ01003184:6399-6699
HmHel-4	CAEZ01003106:21641-21938
HmHel-4	CAEZ01003106:1084-1384
HmHel-4	CAEZ01003102:1657-1957
HmHel-4	CAEZ01003080:199736-200043
HmHel-4	CAEZ01003080:47547-47860
HmHel-4	CAEZ01003080:20433-20738
HmHel-4	CAEZ01002972:1429-1729
HmHel-4	CAEZ01002965:28284-28584
HmHel-4	CAEZ01002922:1406-1705
HmHel-4	CAEZ01002684:16928-17197
HmHel-4	CAEZ01002684:25291-25598
HmHel-4	CAEZ01002658:24939-25239
HmHel-4	CAEZ01002257:18307-18607
HmHel-4	CAEZ01002257:44344-44643
HmHel-4	CAEZ01002032:691-991
HmHel-4	CAEZ01001813:207-506
HmHel-4	CAEZ01001444:27292-27592
HmHel-4	CAEZ01001444:33672-33899
HmHel-4	CAEZ01001125:9517-9817
HmHel-4	CAEZ01001095:6130-6430
HmHel-4	CAEZ01000970:27306-27605
HmHel-4	CAEZ01000970:6211-6539
HmHel-4	CAEZ01000834:21709-22002
HmHel-4	CAEZ01000834:33835-34135
HmHel-4	CAEZ01000806:1236-1536
HmHel-4	CAEZ01011114:8177-8476
HmHel-4	CAEZ01011084:13302-13608
HmHel-4	CAEZ01011084:1-301
HmHel-4	CAEZ01010725:9297-9599
HmHel-4	CAEZ01010725:42527-42799
HmHel-4	CAEZ01009888:49523-49829
HmHel-4	CAEZ01009888:62916-64592
HmHel-4	CAEZ01009778:195152-195452
HmHel-4	CAEZ01009778:367875-368174
HmHel-4	CAEZ01009316:7863-8171
HmHel-4	CAEZ01008494:10449-10751
HmHel-4	CAEZ01008337:3874-4165
HmHel-4	CAEZ01008257:7247-7549
HmHel-4	CAEZ01008244:9757-9929

HmHel-4	CAEZ01008244:24655-24957
HmHel-4	CAEZ01008020:1927-2226
HmHel-4	CAEZ01007958:9351-9653
HmHel-4	CAEZ01007958:25043-25342
HmHel-4	CAEZ01006884:3050-3356
HmHel-4	CAEZ01006884:1369-1668
HmHel-4	CAEZ01006434:68533-68829
HmHel-4	CAEZ01006384:7164-7462
HmHel-4	CAEZ01005845:5529-5832
HmHel-4	CAEZ01005732:23384-23688
HmHel-4	CAEZ01005199:10863-11165
HmHel-4	CAEZ01004994:2344-2638
HmHel-4	CAEZ01004829:8023-8286
HmHel-4	CAEZ01004829:50336-50639
HmHel-4	CAEZ01004674:11411-11708
HmHel-4	CAEZ01004674:7537-7725
HmHel-4	CAEZ01004672:908-1203
HmHel-4	CAEZ01004651:4344-4643
HmHel-4	CAEZ01003810:20085-20384
HmHel-4	CAEZ01002363:31116-31412
HmHel-4	CAEZ01009637:5466-5756
HmHel-4	CAEZ01009463:174-475
HmHel-4	CAEZ01008904:1-301
HmHel-4	CAEZ01008904:9090-9364
HmHel-4	CAEZ01008478:2718-3021
HmHel-4	CAEZ01007478:89786-90086
HmHel-4	CAEZ01007478:116728-117020
HmHel-4	CAEZ01007478:132387-132687
HmHel-4	CAEZ01007478:109830-110133
HmHel-4	CAEZ01007478:95013-95303
HmHel-4	CAEZ01007478:80331-80627
HmHel-4	CAEZ01007478:61113-61402
HmHel-4	CAEZ01007273:55371-55628
HmHel-4	CAEZ01007273:153008-153310
HmHel-4	CAEZ01007273:96754-97058
HmHel-4	CAEZ01006339:62330-62623
HmHel-4	CAEZ01006021:36530-36820
HmHel-4	CAEZ01005201:3316-3610
HmHel-4	CAEZ01005147:73997-74291
HmHel-4	CAEZ01005147:116061-116358
HmHel-4	CAEZ01005140:11869-12166
HmHel-4	CAEZ01004193:7147-7449
HmHel-4	CAEZ01004020:15691-15991
HmHel-4	CAEZ01003972:43-340
HmHel-4	CAEZ01003697:5010-5295
HmHel-4	CAEZ01003635:8244-8540
HmHel-4	CAEZ01003125:5192-5478
HmHel-4	CAEZ01001497:2560-2857

HmHel-4	CAEZ01001137:1578-1875
HmHel-4	CAEZ01011605:34197-34500
HmHel-4	CAEZ01011605:64127-64354
HmHel-4	CAEZ01011605:32095-32409
HmHel-4	CAEZ01011528:31548-31875
HmHel-4	CAEZ01011528:3187-3497
HmHel-4	CAEZ01011527:11756-12059
HmHel-4	CAEZ01011527:26395-26695
HmHel-4	CAEZ01011527:36146-36448
HmHel-4	CAEZ01011361:22175-22481
HmHel-4	CAEZ01011361:6711-7004
HmHel-4	CAEZ01011282:104769-105019
HmHel-4	CAEZ01011282:81677-81974
HmHel-4	CAEZ01011282:13133-13437
HmHel-4	CAEZ01010854:1465-1768
HmHel-4	CAEZ01010597:15764-16062
HmHel-4	CAEZ01010076:5831-6134
HmHel-4	CAEZ01009833:16666-16967
HmHel-4	CAEZ01009830:14637-14931
HmHel-4	CAEZ01009308:18225-18522
HmHel-4	CAEZ01009274:8422-8553
HmHel-4	CAEZ01009274:36410-36687
HmHel-4	CAEZ01009108:7374-7672
HmHel-4	CAEZ01008953:2-299
HmHel-4	CAEZ01008900:6968-7269
HmHel-4	CAEZ01008900:1679-1988
HmHel-4	CAEZ01008710:12818-13118
HmHel-4	CAEZ01008710:41484-41806
HmHel-4	CAEZ01008710:97906-98211
HmHel-4	CAEZ01008500:803-1100
HmHel-4	CAEZ01008500:42490-42655
HmHel-4	CAEZ01008500:36084-36411
HmHel-4	CAEZ01008391:5067-5374
HmHel-4	CAEZ01008391:7467-7769
HmHel-4	CAEZ01008076:18173-18469
HmHel-4	CAEZ01008076:6503-6764
HmHel-4	CAEZ01007799:20084-20377
HmHel-4	CAEZ01006755:1050-1348
HmHel-4	CAEZ01006664:13535-13837
HmHel-4	CAEZ01006497:3157-3450
HmHel-4	CAEZ01006121:140790-141070
HmHel-4	CAEZ01006121:238959-239263
HmHel-4	CAEZ01006121:165576-165887
HmHel-4	CAEZ01006121:147153-147456
HmHel-4	CAEZ01006121:94200-94483
HmHel-4	CAEZ01005630:2914-3215
HmHel-4	CAEZ01005630:15641-15949
HmHel-4	CAEZ01005264:767-1065

HmHel-4	CAEZ01005251:4763-5056
HmHel-4	CAEZ01005223:9781-10077
HmHel-4	CAEZ01004956:453-750
HmHel-4	CAEZ01004942:7658-7923
HmHel-4	CAEZ01004942:23290-23587
HmHel-4	CAEZ01004942:67959-68260
HmHel-4	CAEZ01004942:95700-95981
HmHel-4	CAEZ01004942:66298-66598
HmHel-4	CAEZ01004942:30353-30580
HmHel-4	CAEZ01004942:12392-12557
HmHel-4	CAEZ01004022:9253-9551
HmHel-4	CAEZ01003216:8410-8705
HmHel-4	CAEZ01003216:26978-27275
HmHel-4	CAEZ01003216:10085-10394
HmHel-4	CAEZ01003147:2927-3230
HmHel-4	CAEZ01002892:16947-17244
HmHel-4	CAEZ01002194:41974-42282
HmHel-4	CAEZ01002194:60955-61252
HmHel-4	CAEZ01001133:343-640
HmHel-4	CAEZ01000889:1136-1429
HmHel-4	CAEZ01011423:10239-10539
HmHel-4	CAEZ01011409:3958-4258
HmHel-4	CAEZ01011219:3937-4237
HmHel-4	CAEZ01011197:78283-78583
HmHel-4	CAEZ01011197:160193-160493
HmHel-4	CAEZ01011197:143934-144224
HmHel-4	CAEZ01011197:51768-52075
HmHel-4	CAEZ01011197:15415-15688
HmHel-4	CAEZ01010948:510-810
HmHel-4	CAEZ01010915:30937-31233
HmHel-4	CAEZ01010915:18962-19262
HmHel-4	CAEZ01010800:9678-9953
HmHel-4	CAEZ01010800:51993-52293
HmHel-4	CAEZ01010800:25695-25995
HmHel-4	CAEZ01010584:35385-35708
HmHel-4	CAEZ01010584:257190-257417
HmHel-4	CAEZ01010584:270239-270530
HmHel-4	CAEZ01010584:316335-316639
HmHel-4	CAEZ01010584:292683-292993
HmHel-4	CAEZ01010584:259791-260091
HmHel-4	CAEZ01010584:115651-115964
HmHel-4	CAEZ01010544:27878-28178
HmHel-4	CAEZ01010400:3484-3784
HmHel-4	CAEZ01010199:36385-36683
HmHel-4	CAEZ01010168:5684-5984
HmHel-4	CAEZ01009954:3690-3990
HmHel-4	CAEZ01009954:27825-28111
HmHel-4	CAEZ01009928:25913-26213

HmHel-4	CAEZ01009896:61814-62114
HmHel-4	CAEZ01009896:62237-62544
HmHel-4	CAEZ01009761:13937-14237
HmHel-4	CAEZ01009752:3656-3956
HmHel-4	CAEZ01009648:8542-8842
HmHel-4	CAEZ01009493:4499-4799
HmHel-4	CAEZ01009455:11175-11475
HmHel-4	CAEZ01009162:10458-10758
HmHel-4	CAEZ01009112:1999-2300
HmHel-4	CAEZ01008957:981-1281
HmHel-4	CAEZ01008943:8177-8476
HmHel-4	CAEZ01008751:76772-77072
HmHel-4	CAEZ01008751:80291-80466
HmHel-4	CAEZ01008751:48019-48326
HmHel-4	CAEZ01008606:98771-99071
HmHel-4	CAEZ01008606:74130-74426
HmHel-4	CAEZ01008606:54723-55023
HmHel-4	CAEZ01008405:13780-14080
HmHel-4	CAEZ01008400:11000-11300
HmHel-4	CAEZ01008318:401-701
HmHel-4	CAEZ01008305:85536-85811
HmHel-4	CAEZ01008305:52792-53096
HmHel-4	CAEZ01008305:21143-21443
HmHel-4	CAEZ01008198:13188-13488
HmHel-4	CAEZ01007740:68276-68576
HmHel-4	CAEZ01007539:8699-8947
HmHel-4	CAEZ01007539:5051-5351
HmHel-4	CAEZ01007208:29760-30060
HmHel-4	CAEZ01006985:4151-4451
HmHel-4	CAEZ01006881:5800-6100
HmHel-4	CAEZ01006881:36150-36377
HmHel-4	CAEZ01006868:6272-6564
HmHel-4	CAEZ01006862:16735-17035
HmHel-4	CAEZ01006862:36464-36570
HmHel-4	CAEZ01006862:6887-7073
HmHel-4	CAEZ01006763:14284-14591
HmHel-4	CAEZ01006763:11827-12127
HmHel-4	CAEZ01006505:13151-13451
HmHel-4	CAEZ01006421:6855-7155
HmHel-4	CAEZ01005981:453-753
HmHel-4	CAEZ01005981:11651-11954
HmHel-4	CAEZ01005831:31532-31832
HmHel-4	CAEZ01005831:14667-14938
HmHel-4	CAEZ01005299:41546-41846
HmHel-4	CAEZ01005299:28262-28486
HmHel-4	CAEZ01005172:7842-8017
HmHel-4	CAEZ01005172:27405-27592
HmHel-4	CAEZ01005172:20127-20425

HmHel-4	CAEZ01004500:1032-1332
HmHel-4	CAEZ01004070:560-860
HmHel-4	CAEZ01003946:31671-34852
HmHel-4	CAEZ01003946:25014-25315
HmHel-4	CAEZ01003797:51879-52179
HmHel-4	CAEZ01003797:25138-25445
HmHel-4	CAEZ01003714:3140-3439
HmHel-4	CAEZ01003617:3232-3532
HmHel-4	CAEZ01003430:2225-2525
HmHel-4	CAEZ01003409:18788-19088
HmHel-4	CAEZ01003037:1365-1665
HmHel-4	CAEZ01002968:669-969
HmHel-4	CAEZ01002947:17005-17301
HmHel-4	CAEZ01002630:11184-11472
HmHel-4	CAEZ01002217:38650-38943
HmHel-4	CAEZ01002087:59945-60239
HmHel-4	CAEZ01002087:40243-40546
HmHel-4	CAEZ01001861:647-947
HmHel-4	CAEZ01001550:2360-2660
HmHel-4	CAEZ01001550:12887-13059
HmHel-4	CAEZ01001415:15642-15925
HmHel-4	CAEZ01011418:330-629
HmHel-4	CAEZ01011059:5494-5769
HmHel-4	CAEZ01009989:46426-46730
HmHel-4	CAEZ01009989:23365-23608
HmHel-4	CAEZ01009180:579-862
HmHel-4	CAEZ01008569:621-904
HmHel-4	CAEZ01008164:29278-29580
HmHel-4	CAEZ01006866:21969-22268
HmHel-4	CAEZ01006866:6733-7030
HmHel-4	CAEZ01006785:31997-32305
HmHel-4	CAEZ01006008:5940-6234
HmHel-4	CAEZ01005868:8833-9131
HmHel-4	CAEZ01005586:15853-16145
HmHel-4	CAEZ01004726:3061-3360
HmHel-4	CAEZ01004370:61266-61577
HmHel-4	CAEZ01004370:41891-42189
HmHel-4	CAEZ01004310:4937-5228
HmHel-4	CAEZ01004218:25807-25937
HmHel-4	CAEZ01004218:10541-10824
HmHel-4	CAEZ01002986:16152-16310
HmHel-4	CAEZ01002986:960-1259
HmHel-4	CAEZ01001408:20201-20497
HmHel-4	CAEZ01001408:92302-92606
HmHel-4	CAEZ01001399:11269-11544
HmHel-4	CAEZ01000932:592-888
HmHel-4	CAEZ01000839:2767-2906
HmHel-4	CAEZ01000839:22437-22741

HmHel-4	CAEZ01010049:1977-2251
HmHel-4	CAEZ01009587:8461-8759
HmHel-4	CAEZ01008868:5391-5689
HmHel-4	CAEZ01008726:105236-105530
HmHel-4	CAEZ01008726:52895-53177
HmHel-4	CAEZ01008288:6465-6759
HmHel-4	CAEZ01006553:3892-4189
HmHel-4	CAEZ01006136:4087-4382
HmHel-4	CAEZ01005984:20589-20886
HmHel-4	CAEZ01005335:60337-60631
HmHel-4	CAEZ01004938:33918-34218
HmHel-4	CAEZ01004938:9998-10298
HmHel-4	CAEZ01004375:13612-13906
HmHel-4	CAEZ01011190:3289-3581
HmHel-4	CAEZ01011050:9214-9512
HmHel-4	CAEZ01010740:33933-34217
HmHel-4	CAEZ01010740:10360-10657
HmHel-4	CAEZ01010663:25831-26137
HmHel-4	CAEZ01010663:3560-3857
HmHel-4	CAEZ01010281:16906-17202
HmHel-4	CAEZ01009402:17197-17493
HmHel-4	CAEZ01009280:1194-1498
HmHel-4	CAEZ01009017:69165-69463
HmHel-4	CAEZ01008762:17230-17531
HmHel-4	CAEZ01008350:5723-6024
HmHel-4	CAEZ01008350:31055-31355
HmHel-4	CAEZ01008191:7858-8156
HmHel-4	CAEZ01006495:44647-44944
HmHel-4	CAEZ01006376:7804-8096
HmHel-4	CAEZ01006340:33664-33962
HmHel-4	CAEZ01006299:7114-7419
HmHel-4	CAEZ01005939:1360-1657
HmHel-4	CAEZ01005925:20916-21228
HmHel-4	CAEZ01005925:17094-17422
HmHel-4	CAEZ01005249:12098-12396
HmHel-4	CAEZ01004803:9006-9299
HmHel-4	CAEZ01004032:10954-11261
HmHel-4	CAEZ01004032:1167-1633
HmHel-4	CAEZ01003304:9769-10066
HmHel-4	CAEZ01000641:4778-5050
HmHel-4	CAEZ01011634:6177-6501
HmHel-4	CAEZ01011634:6854-7154
HmHel-4	CAEZ01011489:13031-13275
HmHel-4	CAEZ01011489:79342-79513
HmHel-4	CAEZ01011489:86728-87023
HmHel-4	CAEZ01011273:18268-18568
HmHel-4	CAEZ01010980:3500-3800
HmHel-4	CAEZ01010895:12946-13246

HmHel-4	CAEZ01010816:26843-27143
HmHel-4	CAEZ01010784:16065-16365
HmHel-4	CAEZ01010270:83304-83611
HmHel-4	CAEZ01010270:165854-166161
HmHel-4	CAEZ01010270:248708-249015
HmHel-4	CAEZ01010270:250939-251242
HmHel-4	CAEZ01010270:146192-146486
HmHel-4	CAEZ01010270:24303-24601
HmHel-4	CAEZ01010254:3477-3775
HmHel-4	CAEZ01010242:35852-36152
HmHel-4	CAEZ01010242:33510-33803
HmHel-4	CAEZ01010197:7426-7726
HmHel-4	CAEZ01010106:8865-9164
HmHel-4	CAEZ01009816:1267-1559
HmHel-4	CAEZ01009404:30735-31030
HmHel-4	CAEZ01009404:51317-51624
HmHel-4	CAEZ01009003:8143-8443
HmHel-4	CAEZ01008884:48412-48706
HmHel-4	CAEZ01008884:50026-50326
HmHel-4	CAEZ01008418:2858-3166
HmHel-4	CAEZ01008298:6879-7179
HmHel-4	CAEZ01008218:25030-25123
HmHel-4	CAEZ01008218:14220-14519
HmHel-4	CAEZ01007950:12803-13103
HmHel-4	CAEZ01007822:8369-8664
HmHel-4	CAEZ01007785:22376-22676
HmHel-4	CAEZ01007619:19050-19349
HmHel-4	CAEZ01007588:7489-7789
HmHel-4	CAEZ01006786:2340-2639
HmHel-4	CAEZ01006396:5841-6140
HmHel-4	CAEZ01006311:2665-2965
HmHel-4	CAEZ01006311:959-1248
HmHel-4	CAEZ01005958:4987-5287
HmHel-4	CAEZ01005618:3242-3557
HmHel-4	CAEZ01005618:20787-21079
HmHel-4	CAEZ01005618:18315-18619
HmHel-4	CAEZ01005481:78425-78723
HmHel-4	CAEZ01005400:3143-3442
HmHel-4	CAEZ01005311:6565-6865
HmHel-4	CAEZ01005067:112168-112444
HmHel-4	CAEZ01005067:58726-59022
HmHel-4	CAEZ01005067:38707-39007
HmHel-4	CAEZ01004686:2369-2669
HmHel-4	CAEZ01004331:3738-4040
HmHel-4	CAEZ01004085:8840-9139
HmHel-4	CAEZ01003088:645-945
HmHel-4	CAEZ01002719:22372-22600
HmHel-4	CAEZ01002719:8577-8877

HmHel-4	CAEZ01002091:446-745
HmHel-4	CAEZ01000071:3104-3401
HmHel-4	CAEZ01000071:21108-21407
HmHel-4	CAEZ01011436:570-874
HmHel-4	CAEZ01010820:133786-134061
HmHel-4	CAEZ01010477:4670-4972
HmHel-4	CAEZ01010069:7470-7769
HmHel-4	CAEZ01009140:5483-5787
HmHel-4	CAEZ01008403:74186-74484
HmHel-4	CAEZ01008403:4412-4720
HmHel-4	CAEZ01008138:42493-42776
HmHel-4	CAEZ01008138:24232-24536
HmHel-4	CAEZ01007500:28565-28867
HmHel-4	CAEZ01007108:122421-122717
HmHel-4	CAEZ01007108:5656-5961
HmHel-4	CAEZ01006824:7119-7406
HmHel-4	CAEZ01006312:1095-1391
HmHel-4	CAEZ01006130:12370-12664
HmHel-4	CAEZ01006130:81579-81843
HmHel-4	CAEZ01006130:194574-194873
HmHel-4	CAEZ01006130:176896-177201
HmHel-4	CAEZ01006130:56310-56602
HmHel-4	CAEZ01006130:18062-18330
HmHel-4	CAEZ01005339:1098-1388
HmHel-4	CAEZ01005148:3251-3547
HmHel-4	CAEZ01004541:124254-124542
HmHel-4	CAEZ01004541:106068-106339
HmHel-4	CAEZ01003618:418-717
HmHel-4	CAEZ01009516:12617-12911
HmHel-4	CAEZ01008980:5720-6007
HmHel-4	CAEZ01008412:4176-4474
HmHel-4	CAEZ01008125:31113-31278
HmHel-4	CAEZ01008125:65304-65602
HmHel-4	CAEZ01008125:173051-173359
HmHel-4	CAEZ01008125:159509-159692
HmHel-4	CAEZ01008125:35286-35498
HmHel-4	CAEZ01008125:7459-7759
HmHel-4	CAEZ01007636:1510-1808
HmHel-4	CAEZ01007104:2798-3080
HmHel-4	CAEZ01007104:116-412
HmHel-4	CAEZ01006000:15518-15792
HmHel-4	CAEZ01005643:71284-71583
HmHel-4	CAEZ01005603:2567-2867
HmHel-4	CAEZ01005603:7132-7398
HmHel-4	CAEZ01004452:2242-2431
HmHel-4	CAEZ01004452:24596-24895
HmHel-4	CAEZ01004452:26505-26810
HmHel-4	CAEZ01004452:14808-15101

HmHel-4	CAEZ01004132:7410-7707
HmHel-4	CAEZ01010455:9672-9977
HmHel-4	CAEZ01010414:1167-1464
HmHel-4	CAEZ01009487:659-928
HmHel-4	CAEZ01009248:34044-34153
HmHel-4	CAEZ01009248:38334-38634
HmHel-4	CAEZ01009027:4240-4537
HmHel-4	CAEZ01008650:11257-11550
HmHel-4	CAEZ01008614:35547-35844
HmHel-4	CAEZ01008532:17217-17490
HmHel-4	CAEZ01008068:764-1060
HmHel-4	CAEZ01006939:3797-4092
HmHel-4	CAEZ01006731:10012-10313
HmHel-4	CAEZ01006593:1205-1510
HmHel-4	CAEZ01006541:60206-60498
HmHel-4	CAEZ01006488:40045-40346
HmHel-4	CAEZ01006314:27170-27467
HmHel-4	CAEZ01005833:38381-38589
HmHel-4	CAEZ01005833:15248-15420
HmHel-4	CAEZ01005450:635-933
HmHel-4	CAEZ01005391:10602-10898
HmHel-4	CAEZ01003408:6218-6502
HmHel-4	CAEZ01003182:8560-8854
HmHel-4	CAEZ01002022:11023-11321
HmHel-4	CAEZ01001941:12731-13027
HmHel-4	CAEZ01001343:13904-14202
HmHel-4	CAEZ01011652:2274-2574
HmHel-4	CAEZ01011652:6460-6696
HmHel-4	CAEZ01010891:40441-40737
HmHel-4	CAEZ01010794:10960-11260
HmHel-4	CAEZ01010783:4379-4679
HmHel-4	CAEZ01010613:15153-15448
HmHel-4	CAEZ01010356:66585-66888
HmHel-4	CAEZ01010356:86672-86933
HmHel-4	CAEZ01010356:6941-7241
HmHel-4	CAEZ01010355:35308-35608
HmHel-4	CAEZ01009706:67952-68250
HmHel-4	CAEZ01009706:1470-1774
HmHel-4	CAEZ01009656:54455-55590
HmHel-4	CAEZ01009656:52682-52987
HmHel-4	CAEZ01009656:29124-29430
HmHel-4	CAEZ01009425:1773-2073
HmHel-4	CAEZ01009057:19817-20117
HmHel-4	CAEZ01008482:5344-5647
HmHel-4	CAEZ01007743:555-854
HmHel-4	CAEZ01007689:37924-38219
HmHel-4	CAEZ01007182:14298-14589
HmHel-4	CAEZ01007097:12651-12951

HmHel-4	CAEZ01006845:5646-5943
HmHel-4	CAEZ01006845:26348-26647
HmHel-4	CAEZ01006517:3527-3827
HmHel-4	CAEZ01006387:9774-10069
HmHel-4	CAEZ01005986:91766-92065
HmHel-4	CAEZ01005830:11852-12152
HmHel-4	CAEZ01005693:1-269
HmHel-4	CAEZ01005089:29437-29744
HmHel-4	CAEZ01005087:18334-18610
HmHel-4	CAEZ01005012:10664-10964
HmHel-4	CAEZ01004921:29176-29383
HmHel-4	CAEZ01004921:45780-45993
HmHel-4	CAEZ01004921:64261-64435
HmHel-4	CAEZ01004921:94364-94676
HmHel-4	CAEZ01004921:101598-101779
HmHel-4	CAEZ01004921:57866-58385
HmHel-4	CAEZ01004921:46754-47053
HmHel-4	CAEZ01004921:13719-14026
HmHel-4	CAEZ01004852:13596-13891
HmHel-4	CAEZ01004182:12031-12322
HmHel-4	CAEZ01004101:15772-16065
HmHel-4	CAEZ01003792:3669-3969
HmHel-4	CAEZ01003207:34398-34698
HmHel-4	CAEZ01003111:2576-2876
HmHel-4	CAEZ01003111:28278-28506
HmHel-4	CAEZ01003111:49391-49679
HmHel-4	CAEZ01002272:1170-1467
HmHel-4	CAEZ01001740:76977-77191
HmHel-4	CAEZ01001740:130142-130416
HmHel-4	CAEZ01001740:179625-179924
HmHel-4	CAEZ01001740:92529-92836
HmHel-4	CAEZ01010925:14379-14674
HmHel-4	CAEZ01010925:23579-23876
HmHel-4	CAEZ01010925:2926-3228
HmHel-4	CAEZ01010751:4474-4782
HmHel-4	CAEZ01010751:24641-24904
HmHel-4	CAEZ01009738:1998-2289
HmHel-4	CAEZ01009331:26164-26471
HmHel-4	CAEZ01009331:27275-27578
HmHel-4	CAEZ01009331:6618-6920
HmHel-4	CAEZ01009326:17089-17381
HmHel-4	CAEZ01008409:14445-14736
HmHel-4	CAEZ01008409:26469-26772
HmHel-4	CAEZ01008316:25872-26176
HmHel-4	CAEZ01004006:17496-17792
HmHel-4	CAEZ01003964:4040-4344
HmHel-4	CAEZ01001492:999-1310
HmHel-4	CAEZ01011599:22470-22769

HmHel-4	CAEZ01011171:6934-7182
HmHel-4	CAEZ01011171:5058-5358
HmHel-4	CAEZ01010530:485-763
HmHel-4	CAEZ01010208:15567-15852
HmHel-4	CAEZ01010208:33913-34215
HmHel-4	CAEZ01010146:14538-14832
HmHel-4	CAEZ01009993:14686-14981
HmHel-4	CAEZ01007341:6041-6331
HmHel-4	CAEZ01005776:2924-3221
HmHel-4	CAEZ01005606:124418-124727
HmHel-4	CAEZ01005606:62789-63071
HmHel-4	CAEZ01005417:8643-8940
HmHel-4	CAEZ01005017:6221-6519
HmHel-4	CAEZ01004836:6955-7253
HmHel-4	CAEZ01002634:28894-29182
HmHel-4	CAEZ01002634:14122-14418
HmHel-4	CAEZ01001822:5289-5592
HmHel-4	CAEZ01001703:19290-19589
HmHel-4	CAEZ01011183:3067-3364
HmHel-4	CAEZ01009594:7662-7943
HmHel-4	CAEZ01009495:32105-32401
HmHel-4	CAEZ01009436:33125-33414
HmHel-4	CAEZ01009436:50557-50863
HmHel-4	CAEZ01009436:7601-7908
HmHel-4	CAEZ01008872:36556-36859
HmHel-4	CAEZ01008446:26176-26473
HmHel-4	CAEZ01007912:100235-100536
HmHel-4	CAEZ01007912:41419-41726
HmHel-4	CAEZ01007532:572-870
HmHel-4	CAEZ01006736:3893-4189
HmHel-4	CAEZ01006736:8936-9249
HmHel-4	CAEZ01006174:31950-32247
HmHel-4	CAEZ01006129:2685-2977
HmHel-4	CAEZ01005454:20009-20305
HmHel-4	CAEZ01005077:3297-3602
HmHel-4	CAEZ01005051:8118-8419
HmHel-4	CAEZ01003515:4029-4326
HmHel-4	CAEZ01002444:583-884
HmHel-4	CAEZ01001743:16579-16876
HmHel-4	CAEZ01001661:995-1298
HmHel-4	CAEZ01011488:22721-23015
HmHel-4	CAEZ01011124:2275-2535
HmHel-4	CAEZ01010206:13028-13327
HmHel-4	CAEZ01010206:8029-8336
HmHel-4	CAEZ01008735:21021-21321
HmHel-4	CAEZ01008213:12028-12323
HmHel-4	CAEZ01007725:28804-29031
HmHel-4	CAEZ01007725:85513-85813

HmHel-4	CAEZ01007725:138843-139019
HmHel-4	CAEZ01007725:16006-16306
HmHel-4	CAEZ01007672:3050-3350
HmHel-4	CAEZ01007672:20537-20817
HmHel-4	CAEZ01007325:80897-81192
HmHel-4	CAEZ01006595:9435-9735
HmHel-4	CAEZ01006548:15766-16073
HmHel-4	CAEZ01006548:97606-97905
HmHel-4	CAEZ01006548:18019-18339
HmHel-4	CAEZ01006548:3524-3796
HmHel-4	CAEZ01006108:3604-3903
HmHel-4	CAEZ01006039:16684-16960
HmHel-4	CAEZ01005385:40585-40811
HmHel-4	CAEZ01005385:28461-28761
HmHel-4	CAEZ01005274:27406-27536
HmHel-4	CAEZ01005274:106190-107024
HmHel-4	CAEZ01005274:120049-120344
HmHel-4	CAEZ01005274:133723-134024
HmHel-4	CAEZ01005274:104110-104410
HmHel-4	CAEZ01004092:1503-1797
HmHel-4	CAEZ01003730:12969-13266
HmHel-4	CAEZ01003337:13881-14181
HmHel-4	CAEZ01003279:45882-46182
HmHel-4	CAEZ01003082:7827-8126
HmHel-4	CAEZ01001857:2173-2460
HmHel-4	CAEZ01001825:2281-2576
HmHel-4	CAEZ01000037:36988-37287
HmHel-4	CAEZ01011421:4882-5184
HmHel-4	CAEZ01011155:1798-2101
HmHel-4	CAEZ01011155:21235-21476
HmHel-4	CAEZ01010225:1116-1410
HmHel-4	CAEZ01009465:20960-21231
HmHel-4	CAEZ01009270:24764-25060
HmHel-4	CAEZ01006604:4447-4737
HmHel-4	CAEZ01006392:117783-118080
HmHel-4	CAEZ01006392:69011-69319
HmHel-4	CAEZ01006392:25380-25677
HmHel-4	CAEZ01006090:18062-18361
HmHel-4	CAEZ01005231:58967-59294
HmHel-4	CAEZ01005231:8655-8951
HmHel-4	CAEZ01004914:8656-8924
HmHel-4	CAEZ01004913:1870-2161
HmHel-4	CAEZ01004432:3839-4131
HmHel-4	CAEZ01004432:54565-54842
HmHel-4	CAEZ01004249:48905-49201
HmHel-4	CAEZ01003689:13339-13635
HmHel-4	CAEZ01003307:4045-4312
HmHel-4	CAEZ01003219:12490-12761

HmHel-4	CAEZ01001572:10661-10951
HmHel-4	CAEZ01001159:930-1234
HmHel-4	CAEZ01001016:1275-1571
HmHel-4	CAEZ01011541:619-925
HmHel-4	CAEZ01010464:34378-34678
HmHel-4	CAEZ01010464:107547-107848
HmHel-4	CAEZ01010464:109937-110230
HmHel-4	CAEZ01006040:4167-4486
HmHel-4	CAEZ01006040:6719-7016
HmHel-4	CAEZ01005811:3063-3360
HmHel-4	CAEZ01004720:12824-13121
HmHel-4	CAEZ01003735:10740-11033
HmHel-4	CAEZ01003211:48132-48432
HmHel-4	CAEZ01003138:21685-21978
HmHel-4	CAEZ01002694:26380-26683
HmHel-4	CAEZ01002694:20468-20626
HmHel-4	CAEZ01000876:52493-52782
HmHel-4	CAEZ01000846:12016-12312
HmHel-4	CAEZ01011535:1-277
HmHel-4	CAEZ01011261:2307-2471
HmHel-4	CAEZ01010484:31066-31370
HmHel-4	CAEZ01010484:8791-9091
HmHel-4	CAEZ01009344:4914-5215
HmHel-4	CAEZ01009288:33355-33653
HmHel-4	CAEZ01009183:7998-8267
HmHel-4	CAEZ01008634:7553-7855
HmHel-4	CAEZ01007018:10949-11243
HmHel-4	CAEZ01005228:34435-34733
HmHel-4	CAEZ01005060:1214-1512
HmHel-4	CAEZ01004089:1008-1302
HmHel-4	CAEZ01004089:14427-14734
HmHel-4	CAEZ01004044:35577-35884
HmHel-4	CAEZ01003771:9375-9680
HmHel-4	CAEZ01003739:13449-13722
HmHel-4	CAEZ01003202:66346-66650
HmHel-4	CAEZ01002666:38604-38899
HmHel-4	CAEZ01001364:967-1265
HmHel-4	CAEZ01001324:2382-2679
HmHel-4	CAEZ01011543:9121-9421
HmHel-4	CAEZ01010739:11214-11514
HmHel-4	CAEZ01010739:17597-17733
HmHel-4	CAEZ01009996:78798-79105
HmHel-4	CAEZ01009996:61405-61714
HmHel-4	CAEZ01009996:16616-16843
HmHel-4	CAEZ01009334:3973-4268
HmHel-4	CAEZ01008925:28202-28507
HmHel-4	CAEZ01008925:16180-16480
HmHel-4	CAEZ01008512:58102-58402

HmHel-4	CAEZ01008031:19461-19766
HmHel-4	CAEZ01007857:55509-55797
HmHel-4	CAEZ01007769:327-634
HmHel-4	CAEZ01006635:1755-2062
HmHel-4	CAEZ01006635:3250-3554
HmHel-4	CAEZ01006321:4353-4639
HmHel-4	CAEZ01005556:5093-5393
HmHel-4	CAEZ01005250:44908-45215
HmHel-4	CAEZ01004978:1863-2131
HmHel-4	CAEZ01004964:34-332
HmHel-4	CAEZ01004596:10747-11047
HmHel-4	CAEZ01004112:12751-13058
HmHel-4	CAEZ01003798:8584-8861
HmHel-4	CAEZ01002984:18849-19109
HmHel-4	CAEZ01011153:10573-10861
HmHel-4	CAEZ01009754:10684-10986
HmHel-4	CAEZ01008954:14496-14799
HmHel-4	CAEZ01007872:32486-32782
HmHel-4	CAEZ01007106:15325-15624
HmHel-4	CAEZ01006823:11345-11652
HmHel-4	CAEZ01006252:125646-125929
HmHel-4	CAEZ01003993:2077-2383
HmHel-4	CAEZ01001501:19328-19621
HmHel-4	CAEZ01001501:40433-40581
HmHel-4	CAEZ01001257:693-1018
HmHel-4	CAEZ01001189:511-803
HmHel-4	CAEZ01011269:24067-24365
HmHel-4	CAEZ01011142:21456-21748
HmHel-4	CAEZ01010249:8796-9095
HmHel-4	CAEZ01009737:5725-5807
HmHel-4	CAEZ01008623:5122-5428
HmHel-4	CAEZ01005595:2086-2390
HmHel-4	CAEZ01005283:14368-14671
HmHel-4	CAEZ01005283:4447-4744
HmHel-4	CAEZ01003809:7170-7475
HmHel-4	CAEZ01003801:5264-5555
HmHel-4	CAEZ01001645:25854-26144
HmHel-4	CAEZ01001645:4955-5256
HmHel-4	CAEZ01011163:5336-5632
HmHel-4	CAEZ01010829:58854-59081
HmHel-4	CAEZ01010829:189107-189414
HmHel-4	CAEZ01010720:17068-17364
HmHel-4	CAEZ01010372:10330-10623
HmHel-4	CAEZ01009498:4855-4966
HmHel-4	CAEZ01009498:1-254
HmHel-4	CAEZ01009396:10484-10781
HmHel-4	CAEZ01008821:10483-10743
HmHel-4	CAEZ01007758:20693-21000

HmHel-4	CAEZ01006861:14209-14506
HmHel-4	CAEZ01005992:21544-21845
HmHel-4	CAEZ01004826:56760-57025
HmHel-4	CAEZ01003613:2982-3283
HmHel-4	CAEZ01002806:1062-1357
HmHel-4	CAEZ01001020:34978-35283
HmHel-4	CAEZ01011496:3444-3751
HmHel-4	CAEZ01011026:4625-4932
HmHel-4	CAEZ01010063:5540-5840
HmHel-4	CAEZ01009458:28126-28421
HmHel-4	CAEZ01009403:34713-35013
HmHel-4	CAEZ01009403:22047-22323
HmHel-4	CAEZ01007873:11157-11464
HmHel-4	CAEZ01007863:35475-35782
HmHel-4	CAEZ01006614:7065-7337
HmHel-4	CAEZ01006271:301-600
HmHel-4	CAEZ01005743:79862-80132
HmHel-4	CAEZ01005743:54904-55211
HmHel-4	CAEZ01004816:69149-69417
HmHel-4	CAEZ01004816:107345-107652
HmHel-4	CAEZ01004816:79755-80040
HmHel-4	CAEZ01004202:34525-34832
HmHel-4	CAEZ01004202:14531-14818
HmHel-4	CAEZ01003893:2544-2851
HmHel-4	CAEZ01003782:4975-5269
HmHel-4	CAEZ01003349:43658-43962
HmHel-4	CAEZ01002872:21302-21545
HmHel-4	CAEZ01002872:56175-56466
HmHel-4	CAEZ01002872:81166-81446
HmHel-4	CAEZ01002830:19283-19543
HmHel-4	CAEZ01000833:3905-4176
HmHel-4	CAEZ01000833:36861-37062
HmHel-4	CAEZ01000833:50600-50907
HmHel-4	CAEZ01000833:78247-78574
HmHel-4	CAEZ01011640:12712-13040
HmHel-4	CAEZ01010358:11935-12231
HmHel-4	CAEZ01010116:1079-1375
HmHel-4	CAEZ01009412:5991-6274
HmHel-4	CAEZ01009381:2991-3279
HmHel-4	CAEZ01008926:17342-17638
HmHel-4	CAEZ01008101:969-1271
HmHel-4	CAEZ01007238:10254-10556
HmHel-4	CAEZ01006045:10779-11085
HmHel-4	CAEZ01005388:2512-2808
HmHel-4	CAEZ01004749:24621-24923
HmHel-4	CAEZ01004409:103-405
HmHel-4	CAEZ01001776:11395-11701
HmHel-4	CAEZ01001776:15071-15379

HmHel-4	CAEZ01008511:14136-14422
HmHel-4	CAEZ01007734:9971-10273
HmHel-4	CAEZ01006413:32712-32974
HmHel-4	CAEZ01003726:2557-2852
HmHel-4	CAEZ01000913:3-253
HmHel-4	CAEZ01011425:9756-10062
HmHel-4	CAEZ01010238:33687-33990
HmHel-4	CAEZ01010238:107285-107511
HmHel-4	CAEZ01010158:7073-7346
HmHel-4	CAEZ01009383:14909-15212
HmHel-4	CAEZ01008748:5286-5851
HmHel-4	CAEZ01007663:18578-18905
HmHel-4	CAEZ01006357:19879-20159
HmHel-4	CAEZ01004033:34595-34860
HmHel-4	CAEZ01004033:48164-48498
HmHel-4	CAEZ01003159:3779-4070
HmHel-4	CAEZ01010188:6356-6493
HmHel-4	CAEZ01010188:21065-21372
HmHel-4	CAEZ01009586:27632-27930
HmHel-4	CAEZ01009586:39160-39467
HmHel-4	CAEZ01009586:7574-7881
HmHel-4	CAEZ01009443:3087-3394
HmHel-4	CAEZ01009443:60566-60873
HmHel-4	CAEZ01009384:2913-3220
HmHel-4	CAEZ01009321:23908-24215
HmHel-4	CAEZ01009287:21366-21638
HmHel-4	CAEZ01009287:34820-35127
HmHel-4	CAEZ01008874:64116-64416
HmHel-4	CAEZ01008770:22430-22539
HmHel-4	CAEZ01008770:38866-39126
HmHel-4	CAEZ01008425:6874-7181
HmHel-4	CAEZ01008425:64347-64645
HmHel-4	CAEZ01008425:3386-3686
HmHel-4	CAEZ01006831:27535-27842
HmHel-4	CAEZ01006083:34890-35197
HmHel-4	CAEZ01005770:11687-11994
HmHel-4	CAEZ01005398:3322-3629
HmHel-4	CAEZ01005398:14580-14880
HmHel-4	CAEZ01004489:12938-13245
HmHel-4	CAEZ01004014:45021-45319
HmHel-4	CAEZ01003826:35276-35583
HmHel-4	CAEZ01003641:2375-2682
HmHel-4	CAEZ01011516:3878-4177
HmHel-4	CAEZ01011487:15059-15357
HmHel-4	CAEZ01010209:7833-8142
HmHel-4	CAEZ01008509:4464-4759
HmHel-4	CAEZ01004196:5870-6177
HmHel-4	CAEZ01004196:21305-21611

HmHel-4	CAEZ01003834:10905-11191
HmHel-4	CAEZ01002718:4049-4341
HmHel-4	CAEZ01000910:5523-5666
HmHel-4	CAEZ01000910:24533-24837
HmHel-4	CAEZ01000910:20128-20416
HmHel-4	CAEZ01011627:42521-42827
HmHel-4	CAEZ01011627:75423-75749
HmHel-4	CAEZ01009532:398-696
HmHel-4	CAEZ01009508:5378-5685
HmHel-4	CAEZ01009508:1-271
HmHel-4	CAEZ01007329:6392-6650
HmHel-4	CAEZ01007329:44863-45111
HmHel-4	CAEZ01007329:18143-18560
HmHel-4	CAEZ01002622:9971-10268
HmHel-4	CAEZ01002001:10132-10419
HmHel-4	CAEZ01001841:5554-5880
HmHel-4	CAEZ01001841:11805-12067
HmHel-4	CAEZ01001356:29039-29313
HmHel-4	CAEZ01010878:12538-12828
HmHel-4	CAEZ01008905:5463-5756
HmHel-4	CAEZ01006181:6964-7449
HmHel-4	CAEZ01005459:10183-10481
HmHel-4	CAEZ01004516:2690-2991
HmHel-4	CAEZ01003794:9632-9922
HmHel-4	CAEZ01003794:62701-62927
HmHel-4	CAEZ01000942:22006-22310
HmHel-4	CAEZ01011380:5754-6054
HmHel-4	CAEZ01011373:8627-8934
HmHel-4	CAEZ01011098:8589-8856
HmHel-4	CAEZ01011098:32934-33171
HmHel-4	CAEZ01011098:21588-21895
HmHel-4	CAEZ01010851:3861-4150
HmHel-4	CAEZ01010606:19133-19424
HmHel-4	CAEZ01010236:1256-1563
HmHel-4	CAEZ01009890:378-662
HmHel-4	CAEZ01009836:3514-3820
HmHel-4	CAEZ01008707:32275-32450
HmHel-4	CAEZ01008707:126830-127137
HmHel-4	CAEZ01008640:21048-21354
HmHel-4	CAEZ01008490:14447-14754
HmHel-4	CAEZ01008448:3089-3396
HmHel-4	CAEZ01008229:10495-10794
HmHel-4	CAEZ01007960:62595-62887
HmHel-4	CAEZ01007869:18063-18370
HmHel-4	CAEZ01007438:4345-4652
HmHel-4	CAEZ01007271:21496-21803
HmHel-4	CAEZ01007218:12770-13077
HmHel-4	CAEZ01007173:31318-31620

HmHel-4	CAEZ01007173:4610-4817
HmHel-4	CAEZ01006563:29774-30081
HmHel-4	CAEZ01006563:18871-19144
HmHel-4	CAEZ01006246:16241-16501
HmHel-4	CAEZ01006013:61845-62140
HmHel-4	CAEZ01006013:30149-30456
HmHel-4	CAEZ01005696:5470-5777
HmHel-4	CAEZ01005551:13074-13381
HmHel-4	CAEZ01004768:1054-1145
HmHel-4	CAEZ01004768:405-693
HmHel-4	CAEZ01003737:425-720
HmHel-4	CAEZ01003301:32415-32722
HmHel-4	CAEZ01003067:99660-99958
HmHel-4	CAEZ01002730:4739-5046
HmHel-4	CAEZ01002608:44867-45194
HmHel-4	CAEZ01002118:10975-11728
HmHel-4	CAEZ01001577:61031-61320
HmHel-4	CAEZ01001221:16529-16824
HmHel-4	CAEZ01001021:12762-13061
HmHel-4	CAEZ01011340:9970-10124
HmHel-4	CAEZ01011340:21870-22157
HmHel-4	CAEZ01007562:1605-1912
HmHel-4	CAEZ01007562:12846-13081
HmHel-4	CAEZ01007462:2757-3046
HmHel-4	CAEZ01006776:11549-11854
HmHel-4	CAEZ01006640:26136-26442
HmHel-4	CAEZ01006556:11613-11891
HmHel-4	CAEZ01006320:6143-6441
HmHel-4	CAEZ01005982:11145-11420
HmHel-4	CAEZ01004965:8510-8812
HmHel-4	CAEZ01004365:5025-5327
HmHel-4	CAEZ01000731:1131-1433
HmHel-4	CAEZ01010285:56288-56594
HmHel-4	CAEZ01010012:20773-21064
HmHel-4	CAEZ01009430:22456-22761
HmHel-4	CAEZ01009430:20047-20319
HmHel-4	CAEZ01008027:11025-11317
HmHel-4	CAEZ01006967:9802-10044
HmHel-4	CAEZ01005188:237-552
HmHel-4	CAEZ01004765:6009-6255
HmHel-4	CAEZ01002879:7294-7556
HmHel-4	CAEZ01001413:1334-1639
HmHel-4	CAEZ01010073:10619-10923
HmHel-4	CAEZ01009479:29200-29508
HmHel-4	CAEZ01009479:17701-17999
HmHel-4	CAEZ01008963:31409-31709
HmHel-4	CAEZ01008963:60876-61203
HmHel-4	CAEZ01006398:53960-54264

HmHel-4	CAEZ01006366:19642-19951
HmHel-4	CAEZ01004233:8224-8531
HmHel-4	CAEZ01004205:15279-15579
HmHel-4	CAEZ01000038:5814-6094
HmHel-4	CAEZ01000038:19852-20161
HmHel-4	CAEZ01011132:36768-37008
HmHel-4	CAEZ01011132:17441-17748
HmHel-4	CAEZ01011049:24637-24944
HmHel-4	CAEZ01008732:19924-20231
HmHel-4	CAEZ01008732:125982-126289
HmHel-4	CAEZ01008430:40666-40973
HmHel-4	CAEZ01008174:208-284
HmHel-4	CAEZ01008174:315-622
HmHel-4	CAEZ01005771:6724-7031
HmHel-4	CAEZ01005277:11110-11417
HmHel-4	CAEZ01005153:66244-66548
HmHel-4	CAEZ01005153:33293-33599
HmHel-4	CAEZ01004734:8769-9076
HmHel-4	CAEZ01003649:7478-7775
HmHel-4	CAEZ01003059:15393-15700
HmHel-4	CAEZ01002755:30442-30749
HmHel-4	CAEZ01002681:3212-3519
HmHel-4	CAEZ01001953:4938-5245
HmHel-4	CAEZ01001235:7791-8098
HmHel-4	CAEZ01001235:14531-14838
HmHel-4	CAEZ01000956:366-673
HmHel-4	CAEZ01000859:4674-4981
HmHel-4	CAEZ01010424:102395-102680
HmHel-4	CAEZ01010424:88632-88919
HmHel-4	CAEZ01010424:61358-61463
HmHel-4	CAEZ01009392:5104-5408
HmHel-4	CAEZ01008625:6664-6971
HmHel-4	CAEZ01008625:2917-3204
HmHel-4	CAEZ01007417:14098-14378
HmHel-4	CAEZ01005613:439-741
HmHel-4	CAEZ01004845:57601-57873
HmHel-4	CAEZ01003085:28306-28561
HmHel-4	CAEZ01000758:1-292
HmHel-4	CAEZ01009824:8364-8657
HmHel-4	CAEZ01009824:17506-17813
HmHel-4	CAEZ01009217:9456-9749
HmHel-4	CAEZ01008878:36238-36546
HmHel-4	CAEZ01003526:3710-4011
HmHel-4	CAEZ01002971:12261-12540
HmHel-4	CAEZ01002672:1-263
HmHel-4	CAEZ01001304:5801-6117
HmHel-4	CAEZ01000046:2611-2906
HmHel-4	CAEZ01011410:3575-3855

HmHel-4	CAEZ01008731:51032-51336
HmHel-4	CAEZ01008731:7923-8160
HmHel-4	CAEZ01007651:5801-6074
HmHel-4	CAEZ01007429:75548-75852
HmHel-4	CAEZ01007429:27554-27861
HmHel-4	CAEZ01007269:35248-35555
HmHel-4	CAEZ01007011:220-510
HmHel-4	CAEZ01006672:26003-26286
HmHel-4	CAEZ01006672:1078-1405
HmHel-4	CAEZ01005973:24504-24745
HmHel-4	CAEZ01005973:58602-58787
HmHel-4	CAEZ01003077:68414-68717
HmHel-4	CAEZ01001076:17054-17358
HmHel-4	CAEZ01001076:4334-4623
HmHel-4	CAEZ01009676:20232-20539
HmHel-4	CAEZ01009413:1418-1712
HmHel-4	CAEZ01008232:13346-13642
HmHel-4	CAEZ01008098:82985-83278
HmHel-4	CAEZ01008098:56594-56901
HmHel-4	CAEZ01008023:4513-4820
HmHel-4	CAEZ01007615:10900-11207
HmHel-4	CAEZ01006989:31250-31577
HmHel-4	CAEZ01006989:19682-19972
HmHel-4	CAEZ01006799:78668-78833
HmHel-4	CAEZ01006799:5197-5504
HmHel-4	CAEZ01006172:5884-6191
HmHel-4	CAEZ01006172:20254-20559
HmHel-4	CAEZ01006152:4081-4388
HmHel-4	CAEZ01006135:26697-27005
HmHel-4	CAEZ01006029:1105-1412
HmHel-4	CAEZ01005428:114836-114997
HmHel-4	CAEZ01005428:64830-65137
HmHel-4	CAEZ01005428:29574-29876
HmHel-4	CAEZ01005309:35515-35821
HmHel-4	CAEZ01005222:16183-16490
HmHel-4	CAEZ01005222:12817-13070
HmHel-4	CAEZ01004363:2964-3271
HmHel-4	CAEZ01004230:6203-6510
HmHel-4	CAEZ01004230:30521-30696
HmHel-4	CAEZ01003886:7417-7724
HmHel-4	CAEZ01003524:5571-5878
HmHel-4	CAEZ01001924:2124-2431
HmHel-4	CAEZ01001196:55957-56264
HmHel-4	CAEZ01010585:62609-62885
HmHel-4	CAEZ01010585:147411-147722
HmHel-4	CAEZ01010585:199237-199534
HmHel-4	CAEZ01010585:159945-160248
HmHel-4	CAEZ01010585:44223-44512

HmHel-4	CAEZ01010585:30726-30874
HmHel-4	CAEZ01009975:15515-15821
HmHel-4	CAEZ01009975:40658-41011
HmHel-4	CAEZ01009970:18918-19389
HmHel-4	CAEZ01009970:83215-83502
HmHel-4	CAEZ01009970:29715-29910
HmHel-4	CAEZ01007403:26638-26944
HmHel-4	CAEZ01006741:601-903
HmHel-4	CAEZ01006667:30518-30847
HmHel-4	CAEZ01006667:61619-61925
HmHel-4	CAEZ01006667:27187-27469
HmHel-4	CAEZ01006629:4723-5043
HmHel-4	CAEZ01006605:2518-2824
HmHel-4	CAEZ01006411:13312-13547
HmHel-4	CAEZ01004797:3705-4011
HmHel-4	CAEZ01003214:724-1028
HmHel-4	CAEZ01002168:27361-27637
HmHel-4	CAEZ01002023:816-1108
HmHel-4	CAEZ01001491:420-647
HmHel-4	CAEZ01010257:31623-31936
HmHel-4	CAEZ01010257:5567-5875
HmHel-4	CAEZ01009264:2169-2471
HmHel-4	CAEZ01006412:60897-61174
HmHel-4	CAEZ01005479:19157-19463
HmHel-4	CAEZ01002679:26637-26879
HmHel-4	CAEZ01001154:1325-1563
HmHel-4	CAEZ01010954:7313-7617
HmHel-4	CAEZ01009646:12194-12474
HmHel-4	CAEZ01009646:15601-15867
HmHel-4	CAEZ01008410:17475-17779
HmHel-4	CAEZ01008410:52682-52909
HmHel-4	CAEZ01008122:4564-4872
HmHel-4	CAEZ01008122:41931-42242
HmHel-4	CAEZ01008122:126401-126725
HmHel-4	CAEZ01008122:45027-45320
HmHel-4	CAEZ01006660:1178-1446
HmHel-4	CAEZ01006660:10824-11134
HmHel-4	CAEZ01005441:6832-7122
HmHel-4	CAEZ01005244:1791-1947
HmHel-4	CAEZ01005244:13977-14287
HmHel-4	CAEZ01005208:13302-13572
HmHel-4	CAEZ01005208:77446-77748
HmHel-4	CAEZ01005208:62431-62720
HmHel-4	CAEZ01005205:1458-1738
HmHel-4	CAEZ01005205:53135-53439
HmHel-4	CAEZ01004751:55400-55704
HmHel-4	CAEZ01004751:47713-48039
HmHel-4	CAEZ01004066:1793-2050

HmHel-4	CAEZ01002358:2308-2569
HmHel-4	CAEZ01001910:4810-5110
HmHel-4	CAEZ01001078:2768-3075
HmHel-4	CAEZ01011222:1289-1595
HmHel-4	CAEZ01010712:1741-2048
HmHel-4	CAEZ01010580:7304-7581
HmHel-4	CAEZ01010580:12023-12330
HmHel-4	CAEZ01010383:2323-2630
HmHel-4	CAEZ01009449:3660-3967
HmHel-4	CAEZ01009166:3230-3537
HmHel-4	CAEZ01009114:3110-3417
HmHel-4	CAEZ01008718:6334-6641
HmHel-4	CAEZ01008603:28501-28780
HmHel-4	CAEZ01008510:819-1126
HmHel-4	CAEZ01008510:29534-29843
HmHel-4	CAEZ01008510:5552-5775
HmHel-4	CAEZ01008483:3379-3686
HmHel-4	CAEZ01008437:26399-26566
HmHel-4	CAEZ01008437:83624-83931
HmHel-4	CAEZ01008437:87442-87740
HmHel-4	CAEZ01008271:3655-3962
HmHel-4	CAEZ01008271:9-336
HmHel-4	CAEZ01007998:7814-8081
HmHel-4	CAEZ01007761:23545-23852
HmHel-4	CAEZ01007339:15585-15892
HmHel-4	CAEZ01006348:49920-50224
HmHel-4	CAEZ01006348:39090-39393
HmHel-4	CAEZ01005483:29866-30173
HmHel-4	CAEZ01005139:13-314
HmHel-4	CAEZ01005139:5673-5980
HmHel-4	CAEZ01005044:164659-164966
HmHel-4	CAEZ01004805:585-884
HmHel-4	CAEZ01004595:1307-1614
HmHel-4	CAEZ01004400:19437-19744
HmHel-4	CAEZ01004285:16255-16562
HmHel-4	CAEZ01004203:1051-1337
HmHel-4	CAEZ01003923:16493-16800
HmHel-4	CAEZ01003276:31245-31544
HmHel-4	CAEZ01003276:9301-9608
HmHel-4	CAEZ01003075:77885-78410
HmHel-4	CAEZ01003075:6147-6450
HmHel-4	CAEZ01002942:1007-1314
HmHel-4	CAEZ01002821:106545-106872
HmHel-4	CAEZ01002821:83776-84107
HmHel-4	CAEZ01002814:179341-179634
HmHel-4	CAEZ01002814:340515-340796
HmHel-4	CAEZ01001876:675-982
HmHel-4	CAEZ01001772:7068-7365

HmHel-4	CAEZ01001129:30542-30848
HmHel-4	CAEZ01001129:231-528
HmHel-4	CAEZ01001062:55306-55606
HmHel-4	CAEZ01001062:23472-23773
HmHel-4	CAEZ01000041:29601-29891
HmHel-4	CAEZ01010516:29404-29710
HmHel-4	CAEZ01009025:20885-21210
HmHel-4	CAEZ01008423:84284-84511
HmHel-4	CAEZ01008423:53999-54229
HmHel-4	CAEZ01007678:28294-28596
HmHel-4	CAEZ01002654:8089-8395
HmHel-4	CAEZ01001778:1181-1416
HmHel-4	CAEZ01001245:376-682
HmHel-4	CAEZ01010926:39913-40206
HmHel-4	CAEZ01010917:8184-8490
HmHel-4	CAEZ01010917:3024-3330
HmHel-4	CAEZ01010113:4705-5023
HmHel-4	CAEZ01009284:13820-14108
HmHel-4	CAEZ01007120:11556-11802
HmHel-4	CAEZ01002749:1-243
HmHel-4	CAEZ01002749:1062-1359
HmHel-4	CAEZ01002665:6558-6840
HmHel-4	CAEZ01002665:35937-36229
HmHel-4	CAEZ01011537:3294-3598
HmHel-4	CAEZ01010795:24417-24685
HmHel-4	CAEZ01010419:179-483
HmHel-4	CAEZ01010419:11831-11978
HmHel-4	CAEZ01010417:12614-12894
HmHel-4	CAEZ01010078:1399-1699
HmHel-4	CAEZ01010078:22552-22853
HmHel-4	CAEZ01008739:4-308
HmHel-4	CAEZ01008579:72222-72549
HmHel-4	CAEZ01008579:21694-21980
HmHel-4	CAEZ01008416:17664-17964
HmHel-4	CAEZ01006961:37817-38121
HmHel-4	CAEZ01006932:4801-5081
HmHel-4	CAEZ01006163:8175-8467
HmHel-4	CAEZ01003097:17329-17605
HmHel-4	CAEZ01002969:9827-10131
HmHel-4	CAEZ01002695:19145-19449
HmHel-4	CAEZ01000908:17185-17492
HmHel-4	CAEZ01000908:3844-4151
HmHel-4	CAEZ01011292:15212-15519
HmHel-4	CAEZ01010887:4266-4569
HmHel-4	CAEZ01010557:4044-4347
HmHel-4	CAEZ01010382:17692-17998
HmHel-4	CAEZ01009983:19034-19341
HmHel-4	CAEZ01008588:3312-3615

HmHel-4	CAEZ01008588:24219-24523
HmHel-4	CAEZ01008588:10328-10554
HmHel-4	CAEZ01008434:7127-7427
HmHel-4	CAEZ01008395:7802-8050
HmHel-4	CAEZ01007984:37833-38132
HmHel-4	CAEZ01007004:3377-3684
HmHel-4	CAEZ01006844:8255-8562
HmHel-4	CAEZ01006630:12909-13207
HmHel-4	CAEZ01006440:91295-91590
HmHel-4	CAEZ01005725:317-624
HmHel-4	CAEZ01005667:19811-20038
HmHel-4	CAEZ01005667:33026-33332
HmHel-4	CAEZ01005667:9766-9924
HmHel-4	CAEZ01005267:115560-115838
HmHel-4	CAEZ01005267:7517-7828
HmHel-4	CAEZ01004760:2868-3167
HmHel-4	CAEZ01004574:36089-36283
HmHel-4	CAEZ01004574:46099-46406
HmHel-4	CAEZ01004428:271-577
HmHel-4	CAEZ01004146:1758-2062
HmHel-4	CAEZ01004075:13640-13947
HmHel-4	CAEZ01003262:19033-19332
HmHel-4	CAEZ01003181:52882-53189
HmHel-4	CAEZ01003130:6123-6430
HmHel-4	CAEZ01003130:21057-21364
HmHel-4	CAEZ01002004:1283-1590
HmHel-4	CAEZ01001386:12437-12744
HmHel-4	CAEZ01010387:32763-33066
HmHel-4	CAEZ01010015:8154-8445
HmHel-4	CAEZ01009895:36805-37114
HmHel-4	CAEZ01009617:18526-18835
HmHel-4	CAEZ01009028:3522-3825
HmHel-4	CAEZ01009005:10102-10406
HmHel-4	CAEZ01008822:55558-55876
HmHel-4	CAEZ01008681:3854-4089
HmHel-4	CAEZ01006406:2093-2379
HmHel-4	CAEZ01004923:3330-3638
HmHel-4	CAEZ01004923:67318-67621
HmHel-4	CAEZ01004729:3173-3475
HmHel-4	CAEZ01003413:15088-15362
HmHel-4	CAEZ01003376:1380-1660
HmHel-4	CAEZ01001523:351-653
HmHel-4	CAEZ01001300:2235-2546
HmHel-4	CAEZ01009078:46609-46914
HmHel-4	CAEZ01008590:111854-111964
HmHel-4	CAEZ01008590:61161-61387
HmHel-4	CAEZ01008527:28587-28893
HmHel-4	CAEZ01008470:25895-26187

HmHel-4	CAEZ01008022:12688-12993
HmHel-4	CAEZ01005587:32853-33157
HmHel-4	CAEZ01005587:19118-19425
HmHel-4	CAEZ01005272:18905-19067
HmHel-4	CAEZ01005272:13242-13546
HmHel-4	CAEZ01003954:42707-42965
HmHel-4	CAEZ01010762:15792-16095
HmHel-4	CAEZ01010627:12199-12503
HmHel-4	CAEZ01010362:41315-41552
HmHel-4	CAEZ01009690:14090-14395
HmHel-4	CAEZ01009286:71514-71818
HmHel-4	CAEZ01009286:63620-63907
HmHel-4	CAEZ01009286:43098-43406
HmHel-4	CAEZ01008181:22562-22886
HmHel-4	CAEZ01007922:15439-15729
HmHel-4	CAEZ01007922:993-1170
HmHel-4	CAEZ01007340:11062-11354
HmHel-4	CAEZ01007024:19012-19312
HmHel-4	CAEZ01006833:15331-15635
HmHel-4	CAEZ01006009:10001-10305
HmHel-4	CAEZ01006009:11948-12188
HmHel-4	CAEZ01005042:10489-10718
HmHel-4	CAEZ01004896:13262-13564
HmHel-4	CAEZ01004896:5171-5434
HmHel-4	CAEZ01004831:1311-1616
HmHel-4	CAEZ01004368:75838-76142
HmHel-4	CAEZ01004127:7907-8215
HmHel-4	CAEZ01003914:1029-1333
HmHel-4	CAEZ01003146:4751-5061
HmHel-4	CAEZ01003096:18351-18619
HmHel-4	CAEZ01002635:48551-48855
HmHel-4	CAEZ01001457:11087-11375
HmHel-4	CAEZ01011019:8391-8560
HmHel-4	CAEZ01011019:2832-3139
HmHel-4	CAEZ01010697:7777-8066
HmHel-4	CAEZ01010697:2568-2875
HmHel-4	CAEZ01010243:40489-40798
HmHel-4	CAEZ01010013:22427-22729
HmHel-4	CAEZ01010013:38052-38242
HmHel-4	CAEZ01009930:26383-26690
HmHel-4	CAEZ01009925:6029-6298
HmHel-4	CAEZ01009319:1576-1883
HmHel-4	CAEZ01008771:136705-137011
HmHel-4	CAEZ01008497:25915-26202
HmHel-4	CAEZ01008497:2736-3043
HmHel-4	CAEZ01007979:7379-7603
HmHel-4	CAEZ01007979:12270-12577
HmHel-4	CAEZ01007826:7406-7713

HmHel-4	CAEZ01007631:4214-4521
HmHel-4	CAEZ01007631:34028-34331
HmHel-4	CAEZ01006886:5710-6013
HmHel-4	CAEZ01006886:7334-7641
HmHel-4	CAEZ01006739:4630-4937
HmHel-4	CAEZ01006633:38581-38893
HmHel-4	CAEZ01006633:49892-50170
HmHel-4	CAEZ01006535:8315-8622
HmHel-4	CAEZ01006525:28545-28851
HmHel-4	CAEZ01006512:13913-14220
HmHel-4	CAEZ01006405:42256-42562
HmHel-4	CAEZ01006405:1348-1654
HmHel-4	CAEZ01006088:6765-7080
HmHel-4	CAEZ01006088:29767-30074
HmHel-4	CAEZ01005936:18708-19015
HmHel-4	CAEZ01005887:338-645
HmHel-4	CAEZ01005496:42122-42449
HmHel-4	CAEZ01005496:49028-49335
HmHel-4	CAEZ01004770:4611-4918
HmHel-4	CAEZ01004629:88596-88903
HmHel-4	CAEZ01004165:1668-1975
HmHel-4	CAEZ01003790:7-142
HmHel-4	CAEZ01003790:27846-28010
HmHel-4	CAEZ01003548:15377-15683
HmHel-4	CAEZ01002106:274-581
HmHel-4	CAEZ01001896:64-371
HmHel-4	CAEZ01001488:3126-3432
HmHel-4	CAEZ01001292:2782-3089
HmHel-4	CAEZ01001292:7111-7233
HmHel-4	CAEZ01001104:260-567
HmHel-4	CAEZ01001054:1060-1367
HmHel-4	CAEZ01010821:5525-5831
HmHel-4	CAEZ01010359:1193-1498
HmHel-4	CAEZ01010067:2697-2920
HmHel-4	CAEZ01008715:9461-9750
HmHel-4	CAEZ01008536:6040-6352
HmHel-4	CAEZ01006369:37405-37725
HmHel-4	CAEZ01005694:199-504
HmHel-4	CAEZ01005462:10278-10600
HmHel-4	CAEZ01004349:23398-23625
HmHel-4	CAEZ01004332:11523-11809
HmHel-4	CAEZ01003650:19820-20119
HmHel-4	CAEZ01003650:15150-15447
HmHel-4	CAEZ01003590:349-660
HmHel-4	CAEZ01003377:7787-8111
HmHel-4	CAEZ01003377:6688-6969
HmHel-4	CAEZ01003100:6082-6385
HmHel-4	CAEZ01001291:8923-9182

HmHel-4	CAEZ01011014:12157-12430
HmHel-4	CAEZ01007059:12796-13101
HmHel-4	CAEZ01006449:19249-19557
HmHel-4	CAEZ01006126:25571-25877
HmHel-4	CAEZ01006126:37201-37410
HmHel-4	CAEZ01006126:3204-3501
HmHel-4	CAEZ01005772:10429-10726
HmHel-4	CAEZ01004889:11385-11685
HmHel-4	CAEZ01003745:363-664
HmHel-4	CAEZ01003226:82483-82787
HmHel-4	CAEZ01003226:69327-69620
HmHel-4	CAEZ01002764:2038-2256
HmHel-4	CAEZ01001965:31951-32248
HmHel-4	CAEZ01010466:4616-4920
HmHel-4	CAEZ01010460:78637-78964
HmHel-4	CAEZ01010460:132307-132614
HmHel-4	CAEZ01010460:131823-132103
HmHel-4	CAEZ01009380:81184-81473
HmHel-4	CAEZ01009380:64458-64787
HmHel-4	CAEZ01007550:16278-16582
HmHel-4	CAEZ01007402:19345-19625
HmHel-4	CAEZ01006043:4344-4652
HmHel-4	CAEZ01005874:9531-9839
HmHel-4	CAEZ01005714:34814-35094
HmHel-4	CAEZ01005670:53456-53736
HmHel-4	CAEZ01005670:3710-3935
HmHel-4	CAEZ01005351:12302-12606
HmHel-4	CAEZ01004972:49845-50150
HmHel-4	CAEZ01004835:19504-19794
HmHel-4	CAEZ01004216:9734-10014
HmHel-4	CAEZ01004073:1290-1589
HmHel-4	CAEZ01002415:70878-71182
HmHel-4	CAEZ01002152:183-487
HmHel-4	CAEZ01001206:4343-4560
HmHel-4	CAEZ01011021:3468-3762
HmHel-4	CAEZ01010517:6910-7217
HmHel-4	CAEZ01009883:51950-52257
HmHel-4	CAEZ01009883:86551-86778
HmHel-4	CAEZ01009883:24891-25076
HmHel-4	CAEZ01009774:16471-16776
HmHel-4	CAEZ01009192:7138-7445
HmHel-4	CAEZ01007129:1075-1382
HmHel-4	CAEZ01007129:4171-4467
HmHel-4	CAEZ01006765:8136-8439
HmHel-4	CAEZ01006571:19772-20079
HmHel-4	CAEZ01006414:10188-10495
HmHel-4	CAEZ01006025:2079-2386
HmHel-4	CAEZ01005472:407-688

HmHel-4	CAEZ01005472:21408-21699
HmHel-4	CAEZ01005472:10684-10991
HmHel-4	CAEZ01005076:6696-7003
HmHel-4	CAEZ01004995:10844-11151
HmHel-4	CAEZ01004823:22144-22451
HmHel-4	CAEZ01004540:2241-2548
HmHel-4	CAEZ01004306:7222-7497
HmHel-4	CAEZ01003509:12571-12880
HmHel-4	CAEZ01003509:9892-10186
HmHel-4	CAEZ01002875:7163-7470
HmHel-4	CAEZ01002793:2751-3058
HmHel-4	CAEZ01002611:1405-1712
HmHel-4	CAEZ01002203:10808-11105
HmHel-4	CAEZ01001971:4235-4541
HmHel-4	CAEZ01001887:3350-3657
HmHel-4	CAEZ01001458:1023-1329
HmHel-4	CAEZ01000811:946-1237
HmHel-4	CAEZ01011408:20959-21198
HmHel-4	CAEZ01010971:25242-25516
HmHel-4	CAEZ01010971:102679-102982
HmHel-4	CAEZ01010427:13095-13400
HmHel-4	CAEZ01010173:4355-4650
HmHel-4	CAEZ01008678:45410-45685
HmHel-4	CAEZ01008678:2162-2456
HmHel-4	CAEZ01008487:424-651
HmHel-4	CAEZ01007738:101488-101795
HmHel-4	CAEZ01007700:9816-10128
HmHel-4	CAEZ01007506:1855-2161
HmHel-4	CAEZ01006829:5183-5488
HmHel-4	CAEZ01006493:21043-21346
HmHel-4	CAEZ01005808:8247-8549
HmHel-4	CAEZ01004999:1644-1959
HmHel-4	CAEZ01004678:12494-12805
HmHel-4	CAEZ01004643:21939-22201
HmHel-4	CAEZ01004385:11238-11545
HmHel-4	CAEZ01003861:9510-9798
HmHel-4	CAEZ01003137:18556-18830
HmHel-4	CAEZ01002012:461-744
HmHel-4	CAEZ01001816:556-864
HmHel-4	CAEZ01001316:454-763
HmHel-4	CAEZ01000989:5484-5787
HmHel-4	CAEZ01010735:2-220
HmHel-4	CAEZ01009071:2253-2559
HmHel-4	CAEZ01008523:13251-13551
HmHel-4	CAEZ01008341:64447-64749
HmHel-4	CAEZ01008064:4239-4512
HmHel-4	CAEZ01006287:4583-4849
HmHel-4	CAEZ01005224:27685-27985

HmHel-4	CAEZ01004899:13851-14150
HmHel-4	CAEZ01004818:5233-5539
HmHel-4	CAEZ01004818:118991-129058
HmHel-4	CAEZ01004818:96517-98344
HmHel-4	CAEZ01004818:4114-4441
HmHel-4	CAEZ01004511:34632-34862
HmHel-4	CAEZ01003895:25035-25366
HmHel-4	CAEZ01003895:23073-23377
HmHel-4	CAEZ01003151:31073-31353
HmHel-4	CAEZ01003151:44274-44574
HmHel-4	CAEZ01003151:51758-51993
HmHel-4	CAEZ01002100:3523-3808
HmHel-4	CAEZ01001266:54950-55164
HmHel-4	CAEZ01001266:67482-67775
HmHel-4	CAEZ01001266:76336-76593
HmHel-4	CAEZ01010919:8983-9291
HmHel-4	CAEZ01009154:55000-55328
HmHel-4	CAEZ01009154:17923-18213
HmHel-4	CAEZ01009131:6641-6940
HmHel-4	CAEZ01008859:514-826
HmHel-4	CAEZ01008183:25884-26105
HmHel-4	CAEZ01007930:32516-32824
HmHel-4	CAEZ01007930:28982-29289
HmHel-4	CAEZ01006820:16907-17207
HmHel-4	CAEZ01006472:22038-22346
HmHel-4	CAEZ01006273:10090-10390
HmHel-4	CAEZ01005781:15939-16148
HmHel-4	CAEZ01005767:3772-4076
HmHel-4	CAEZ01005421:38973-39260
HmHel-4	CAEZ01005141:13079-13384
HmHel-4	CAEZ01003888:14995-15297
HmHel-4	CAEZ01003346:51609-51912
HmHel-4	CAEZ01002849:4640-4910
HmHel-4	CAEZ01002581:21998-22306
HmHel-4	CAEZ01000968:39509-39797
HmHel-4	CAEZ01000968:3367-3672
HmHel-4	CAEZ01011452:22574-22881
HmHel-4	CAEZ01011452:19131-19355
HmHel-4	CAEZ01011304:2937-3243
HmHel-4	CAEZ01010914:6126-6429
HmHel-4	CAEZ01010729:1-253
HmHel-4	CAEZ01010729:46051-46357
HmHel-4	CAEZ01010729:75007-75309
HmHel-4	CAEZ01010396:1-308
HmHel-4	CAEZ01009063:13516-13790
HmHel-4	CAEZ01008917:4917-5262
HmHel-4	CAEZ01007967:10631-10938
HmHel-4	CAEZ01007634:17194-17450

HmHel-4	CAEZ01007617:930-1237
HmHel-4	CAEZ01007467:5706-6012
HmHel-4	CAEZ01007461:34152-34376
HmHel-4	CAEZ01007461:33324-33611
HmHel-4	CAEZ01007461:9912-10219
HmHel-4	CAEZ01007303:12722-13029
HmHel-4	CAEZ01007303:1208-1542
HmHel-4	CAEZ01006623:19612-19919
HmHel-4	CAEZ01005836:22850-23157
HmHel-4	CAEZ01005125:9002-9309
HmHel-4	CAEZ01005027:14633-14931
HmHel-4	CAEZ01004513:19294-19601
HmHel-4	CAEZ01004474:3119-3426
HmHel-4	CAEZ01004413:3628-3937
HmHel-4	CAEZ01004209:11133-11305
HmHel-4	CAEZ01004209:12116-12423
HmHel-4	CAEZ01003475:4329-4636
HmHel-4	CAEZ01003338:18647-18954
HmHel-4	CAEZ01003133:13665-13972
HmHel-4	CAEZ01002771:10884-11191
HmHel-4	CAEZ01001878:4634-4940
HmHel-4	CAEZ01001307:1296-1584
HmHel-4	CAEZ01000738:426-721
HmHel-4	CAEZ01010308:30398-30705
HmHel-4	CAEZ01010308:9465-9682
HmHel-4	CAEZ01009898:18748-19054
HmHel-4	CAEZ01009867:2325-2552
HmHel-4	CAEZ01009867:2000-2331
HmHel-4	CAEZ01008612:16304-16534
HmHel-4	CAEZ01008356:32036-32332
HmHel-4	CAEZ01008296:8411-8638
HmHel-4	CAEZ01007571:55283-55494
HmHel-4	CAEZ01007571:5236-5407
HmHel-4	CAEZ01006888:23871-24177
HmHel-4	CAEZ01006888:9124-9424
HmHel-4	CAEZ01006194:45113-45340
HmHel-4	CAEZ01005695:1033-1322
HmHel-4	CAEZ01005695:14255-14420
HmHel-4	CAEZ01004707:5075-5378
HmHel-4	CAEZ01004496:7-234
HmHel-4	CAEZ01004496:27998-28298
HmHel-4	CAEZ01002067:1783-2010
HmHel-4	CAEZ01001097:26066-26368
HmHel-4	CAEZ01010528:2966-3242
HmHel-4	CAEZ01010528:32599-32832
HmHel-4	CAEZ01009901:1832-2109
HmHel-4	CAEZ01009124:34846-35145
HmHel-4	CAEZ01008263:5032-5259

HmHel-4	CAEZ01008263:16790-17103
HmHel-4	CAEZ01008263:13203-13618
HmHel-4	CAEZ01007157:12093-12394
HmHel-4	CAEZ01007157:30395-30677
HmHel-4	CAEZ01007112:28194-28438
HmHel-4	CAEZ01006332:47725-48014
HmHel-4	CAEZ01006283:16216-16495
HmHel-4	CAEZ01005944:6708-6972
HmHel-4	CAEZ01003076:49-359
HmHel-4	CAEZ01002948:634-935
HmHel-4	CAEZ01002784:1018-1299
HmHel-4	CAEZ01002728:10699-10956
HmHel-4	CAEZ01002723:1348-1632
HmHel-4	CAEZ01009961:18784-19048
HmHel-4	CAEZ01008794:17740-18048
HmHel-4	CAEZ01007708:11936-12019
HmHel-4	CAEZ01007708:100175-100322
HmHel-4	CAEZ01007708:7804-8013
HmHel-4	CAEZ01006973:159-469
HmHel-4	CAEZ01006565:17436-17740
HmHel-4	CAEZ01006402:37274-37578
HmHel-4	CAEZ01006377:24581-24884
HmHel-4	CAEZ01006377:35242-35529
HmHel-4	CAEZ01005416:40078-40382
HmHel-4	CAEZ01003661:4886-5183
HmHel-4	CAEZ01003633:43799-44082
HmHel-4	CAEZ01003633:27590-28303
HmHel-4	CAEZ01003633:3704-3964
HmHel-4	CAEZ01001207:5117-5426
HmHel-4	CAEZ01001207:694-970
HmHel-4	CAEZ01000841:1316-1533
HmHel-4	CAEZ01000208:272-481
HmHel-4	CAEZ01011523:50361-50688
HmHel-4	CAEZ01011523:61517-61685
HmHel-4	CAEZ01011523:38723-39030
HmHel-4	CAEZ01011523:11180-11656
HmHel-4	CAEZ01011417:2152-2599
HmHel-4	CAEZ01011101:71808-72051
HmHel-4	CAEZ01011101:39343-39544
HmHel-4	CAEZ01011032:2332-2634
HmHel-4	CAEZ01011032:13408-13714
HmHel-4	CAEZ01010726:2615-2922
HmHel-4	CAEZ01010034:65083-65386
HmHel-4	CAEZ01008582:5163-5466
HmHel-4	CAEZ01008582:6772-7079
HmHel-4	CAEZ01007470:28317-28624
HmHel-4	CAEZ01007260:3846-4151
HmHel-4	CAEZ01007204:54806-55017

HmHel-4	CAEZ01007204:71239-71430
HmHel-4	CAEZ01007204:49747-50039
HmHel-4	CAEZ01007204:36020-36235
HmHel-4	CAEZ01007142:17200-17507
HmHel-4	CAEZ01006929:3856-4159
HmHel-4	CAEZ01006690:26543-26843
HmHel-4	CAEZ01006549:21400-21692
HmHel-4	CAEZ01006549:11044-11350
HmHel-4	CAEZ01005754:23666-23973
HmHel-4	CAEZ01005677:7350-7657
HmHel-4	CAEZ01005387:1172-1479
HmHel-4	CAEZ01004819:21345-21652
HmHel-4	CAEZ01004819:42155-42482
HmHel-4	CAEZ01004671:3323-3606
HmHel-4	CAEZ01003417:16588-16865
HmHel-4	CAEZ01003087:2644-2950
HmHel-4	CAEZ01003034:7717-8024
HmHel-4	CAEZ01002293:2799-3106
HmHel-4	CAEZ01000895:61874-62102
HmHel-4	CAEZ01000895:11394-11598
HmHel-4	CAEZ01010703:41491-41806
HmHel-4	CAEZ01010671:1466-1763
HmHel-4	CAEZ01010439:44396-44702
HmHel-4	CAEZ01009756:2079-2354
HmHel-4	CAEZ01009678:1328-1555
HmHel-4	CAEZ01009433:3961-4188
HmHel-4	CAEZ01009188:1001-1228
HmHel-4	CAEZ01008916:74948-75175
HmHel-4	CAEZ01008916:13903-14181
HmHel-4	CAEZ01008243:5604-5831
HmHel-4	CAEZ01007563:4959-5245
HmHel-4	CAEZ01007161:4834-5137
HmHel-4	CAEZ01007109:14029-14339
HmHel-4	CAEZ01006659:8898-9210
HmHel-4	CAEZ01006573:6016-6302
HmHel-4	CAEZ01005976:44117-44422
HmHel-4	CAEZ01005955:25774-26061
HmHel-4	CAEZ01005955:14247-14558
HmHel-4	CAEZ01005215:11839-12147
HmHel-4	CAEZ01003896:11522-11748
HmHel-4	CAEZ01003704:4603-4902
HmHel-4	CAEZ01003183:18320-18547
HmHel-4	CAEZ01002669:3681-3908
HmHel-4	CAEZ01002371:77870-78156
HmHel-4	CAEZ01002371:24697-24924
HmHel-4	CAEZ01002052:226-453
HmHel-4	CAEZ01002015:1759-2053
HmHel-4	CAEZ01011581:2041-2334

HmHel-4	CAEZ01010019:2343-2643
HmHel-4	CAEZ01009589:1974-2279
HmHel-4	CAEZ01008737:67119-67356
HmHel-4	CAEZ01008390:36778-37087
HmHel-4	CAEZ01008390:43311-43645
HmHel-4	CAEZ01007365:27008-27294
HmHel-4	CAEZ01007365:9974-10149
HmHel-4	CAEZ01006367:64053-64355
HmHel-4	CAEZ01006367:128313-128620
HmHel-4	CAEZ01006367:93344-93451
HmHel-4	CAEZ01006328:1987-2271
HmHel-4	CAEZ01005806:14974-15255
HmHel-4	CAEZ01005783:6086-6391
HmHel-4	CAEZ01005175:49818-50111
HmHel-4	CAEZ01004902:16404-16679
HmHel-4	CAEZ01004552:2707-2973
HmHel-4	CAEZ01003975:10276-10545
HmHel-4	CAEZ01003919:5380-5685
HmHel-4	CAEZ01002430:24961-25272
HmHel-4	CAEZ01001750:4149-4425
HmHel-4	CAEZ01000938:1532-1839
HmHel-4	CAEZ01000065:10534-10833
HmHel-4	CAEZ01011549:1922-2225
HmHel-4	CAEZ01011313:10-267
HmHel-4	CAEZ01010402:775-1058
HmHel-4	CAEZ01010217:31625-31917
HmHel-4	CAEZ01010217:118399-118719
HmHel-4	CAEZ01008635:23912-24216
HmHel-4	CAEZ01008168:18480-18780
HmHel-4	CAEZ01007982:11933-12228
HmHel-4	CAEZ01007982:4037-4341
HmHel-4	CAEZ01006349:37411-37673
HmHel-4	CAEZ01005722:9954-10254
HmHel-4	CAEZ01005722:30377-30656
HmHel-4	CAEZ01005574:4473-4781
HmHel-4	CAEZ01005338:422-730
HmHel-4	CAEZ01005243:15434-15710
HmHel-4	CAEZ01004497:3193-3394
HmHel-4	CAEZ01004486:27113-27374
HmHel-4	CAEZ01004486:23537-23676
HmHel-4	CAEZ01004172:558-826
HmHel-4	CAEZ01004055:15596-15904
HmHel-4	CAEZ01003845:5635-5903
HmHel-4	CAEZ01002739:2089-2394
HmHel-4	CAEZ01011144:2829-3114
HmHel-4	CAEZ01011144:25051-25355
HmHel-4	CAEZ01011031:11655-11923
HmHel-4	CAEZ01010968:40066-40376

HmHel-4	CAEZ01010615:18226-18449
HmHel-4	CAEZ01008354:24447-24754
HmHel-4	CAEZ01008186:3270-3577
HmHel-4	CAEZ01007987:22417-22724
HmHel-4	CAEZ01007987:1671-1978
HmHel-4	CAEZ01007328:5462-5662
HmHel-4	CAEZ01007100:29488-29795
HmHel-4	CAEZ01003706:2153-2464
HmHel-4	CAEZ01003177:631-937
HmHel-4	CAEZ01001892:24319-24543
HmHel-4	CAEZ01001892:22720-23098
HmHel-4	CAEZ01011407:122-349
HmHel-4	CAEZ01011245:29067-29218
HmHel-4	CAEZ01011245:7689-8000
HmHel-4	CAEZ01010998:1172-1475
HmHel-4	CAEZ01010264:33428-33742
HmHel-4	CAEZ01008981:147-371
HmHel-4	CAEZ01008974:4099-4410
HmHel-4	CAEZ01006882:3787-4089
HmHel-4	CAEZ01006821:38966-39193
HmHel-4	CAEZ01006095:8770-9073
HmHel-4	CAEZ01006095:21360-21665
HmHel-4	CAEZ01004647:8762-9065
HmHel-4	CAEZ01007973:17657-17959
HmHel-4	CAEZ01007597:2624-2830
HmHel-4	CAEZ01006836:8319-8629
HmHel-4	CAEZ01006515:14026-14312
HmHel-4	CAEZ01004853:36235-36538
HmHel-4	CAEZ01004623:13239-13525
HmHel-4	CAEZ01004441:2626-2924
HmHel-4	CAEZ01004079:9754-10050
HmHel-4	CAEZ01003598:21340-21641
HmHel-4	CAEZ01003435:1276-1582
HmHel-4	CAEZ01011538:7042-7317
HmHel-4	CAEZ01011444:3924-4224
HmHel-4	CAEZ01011043:49931-50191
HmHel-4	CAEZ01010497:12188-12503
HmHel-4	CAEZ01010497:4573-4805
HmHel-4	CAEZ01010432:8520-8737
HmHel-4	CAEZ01009946:5493-5798
HmHel-4	CAEZ01008947:2115-2419
HmHel-4	CAEZ01008685:1-304
HmHel-4	CAEZ01008685:11934-12233
HmHel-4	CAEZ01007894:2075-2296
HmHel-4	CAEZ01007681:199-498
HmHel-4	CAEZ01007458:11573-11833
HmHel-4	CAEZ01006632:37819-38123
HmHel-4	CAEZ01005525:1655-1897

HmHel-4	CAEZ01004918:58739-59052
HmHel-4	CAEZ01004843:10155-10424
HmHel-4	CAEZ01004843:15845-16042
HmHel-4	CAEZ01004124:8356-8657
HmHel-4	CAEZ01003688:5-230
HmHel-4	CAEZ01003645:10528-10833
HmHel-4	CAEZ01003266:1649-1948
HmHel-4	CAEZ01001506:5962-6262
HmHel-4	CAEZ01000992:29792-30095
HmHel-4	CAEZ01011186:24487-24793
HmHel-4	CAEZ01010992:5001-5307
HmHel-4	CAEZ01010897:50809-51136
HmHel-4	CAEZ01010897:39100-39316
HmHel-4	CAEZ01010589:5080-5374
HmHel-4	CAEZ01010310:16229-16517
HmHel-4	CAEZ01009304:41550-41857
HmHel-4	CAEZ01008224:27711-28018
HmHel-4	CAEZ01008050:86808-87089
HmHel-4	CAEZ01006999:11632-11907
HmHel-4	CAEZ01006350:5954-6177
HmHel-4	CAEZ01005164:9500-9804
HmHel-4	CAEZ01004975:45018-45298
HmHel-4	CAEZ01004261:6823-7118
HmHel-4	CAEZ01002102:3657-3964
HmHel-4	CAEZ01001195:4874-5178
HmHel-4	CAEZ01000809:22509-22717
HmHel-4	CAEZ01000066:7676-7903
HmHel-4	CAEZ01000066:23342-23645
HmHel-4	CAEZ01011435:5087-5314
HmHel-4	CAEZ01011173:24336-24623
HmHel-4	CAEZ01010793:55608-55915
HmHel-4	CAEZ01010793:48933-49066
HmHel-4	CAEZ01009969:24453-24755
HmHel-4	CAEZ01009421:90-317
HmHel-4	CAEZ01008631:2958-3185
HmHel-4	CAEZ01008106:17230-17543
HmHel-4	CAEZ01007319:20322-20600
HmHel-4	CAEZ01007319:7222-7536
HmHel-4	CAEZ01005827:17441-17751
HmHel-4	CAEZ01004950:6278-6505
HmHel-4	CAEZ01004661:5145-5462
HmHel-4	CAEZ01003533:11374-11681
HmHel-4	CAEZ01002613:2329-2630
HmHel-4	CAEZ01001450:31910-32209
HmHel-4	CAEZ01001450:28441-28768
HmHel-4	CAEZ01010723:3535-3846
HmHel-4	CAEZ01010440:63748-64052
HmHel-4	CAEZ01009826:10587-10813

HmHel-4	CAEZ01009742:17417-22288
HmHel-4	CAEZ01009673:78233-78534
HmHel-4	CAEZ01008327:28083-28352
HmHel-4	CAEZ01008327:69735-69959
HmHel-4	CAEZ01007405:2512-2817
HmHel-4	CAEZ01005282:757-1033
HmHel-4	CAEZ01005209:6391-6711
HmHel-4	CAEZ01011298:2937-3225
HmHel-4	CAEZ01010906:2416-2712
HmHel-4	CAEZ01010554:449-750
HmHel-4	CAEZ01009933:5099-5400
HmHel-4	CAEZ01009827:14198-14483
HmHel-4	CAEZ01009827:19600-19908
HmHel-4	CAEZ01007002:14956-15244
HmHel-4	CAEZ01006027:19046-19346
HmHel-4	CAEZ01005913:3004-3237
HmHel-4	CAEZ01004925:7498-7798
HmHel-4	CAEZ01004378:351-621
HmHel-4	CAEZ01004378:7387-7714
HmHel-4	CAEZ01003957:647-944
HmHel-4	CAEZ01003250:164622-164909
HmHel-4	CAEZ01010907:61426-61729
HmHel-4	CAEZ01010016:15958-16265
HmHel-4	CAEZ01009828:23441-23661
HmHel-4	CAEZ01009633:85917-86224
HmHel-4	CAEZ01007745:15576-15861
HmHel-4	CAEZ01007564:21935-22242
HmHel-4	CAEZ01007564:12562-12890
HmHel-4	CAEZ01007395:16159-16373
HmHel-4	CAEZ01007395:48932-49207
HmHel-4	CAEZ01005789:3238-3545
HmHel-4	CAEZ01003925:18191-18455
HmHel-4	CAEZ01003460:1805-2112
HmHel-4	CAEZ01003265:17339-17602
HmHel-4	CAEZ01003265:20908-21188
HmHel-4	CAEZ01003117:4307-4664
HmHel-4	CAEZ01002704:23830-24137
HmHel-4	CAEZ01002642:32794-33018
HmHel-4	CAEZ01002112:11440-11739
HmHel-4	CAEZ01011133:8976-9250
HmHel-4	CAEZ01011133:27592-27916
HmHel-4	CAEZ01009635:426-660
HmHel-4	CAEZ01008736:6135-6437
HmHel-4	CAEZ01008091:32467-32793
HmHel-4	CAEZ01006692:11359-11675
HmHel-4	CAEZ01005305:43-356
HmHel-4	CAEZ01005273:9797-10109
HmHel-4	CAEZ01005273:21162-21350

HmHel-4	CAEZ01004825:3801-4114
HmHel-4	CAEZ01004708:22993-23290
HmHel-4	CAEZ01004381:24413-24640
HmHel-4	CAEZ01003124:20493-20828
HmHel-4	CAEZ01003103:2567-2880
HmHel-4	CAEZ01002720:2630-2938
HmHel-4	CAEZ01000633:1688-1915
HmHel-4	CAEZ01000057:3756-4050
HmHel-4	CAEZ01011414:16906-17100
HmHel-4	CAEZ01010399:23955-24230
HmHel-4	CAEZ01010399:760-1070
HmHel-4	CAEZ01008939:18596-18873
HmHel-4	CAEZ01008443:4369-4663
HmHel-4	CAEZ01008077:15413-15685
HmHel-4	CAEZ01005281:2169-2473
HmHel-4	CAEZ01005217:8576-8841
HmHel-4	CAEZ01004936:36117-36410
HmHel-4	CAEZ01004936:18371-18663
HmHel-4	CAEZ01004564:11295-11564
HmHel-4	CAEZ01004187:7088-7412
HmHel-4	CAEZ01003051:5455-5760
HmHel-4	CAEZ01011611:3375-3667
HmHel-4	CAEZ01010492:230-535
HmHel-4	CAEZ01009705:50400-50680
HmHel-4	CAEZ01007972:22625-22842
HmHel-4	CAEZ01006742:8395-8695
HmHel-4	CAEZ01006654:5758-5982
HmHel-4	CAEZ01006558:244-449
HmHel-4	CAEZ01005337:1520-1854
HmHel-4	CAEZ01004279:1137-1438
HmHel-4	CAEZ01004225:13107-13421
HmHel-4	CAEZ01004225:23797-23962
HmHel-4	CAEZ01004225:102-3313
HmHel-4	CAEZ01003719:26727-26932
HmHel-4	CAEZ01010398:10684-10979
HmHel-4	CAEZ01010294:1084-1308
HmHel-4	CAEZ01009696:15622-15926
HmHel-4	CAEZ01009337:6367-6643
HmHel-4	CAEZ01008730:5315-5539
HmHel-4	CAEZ01008626:23-251
HmHel-4	CAEZ01008420:66161-66311
HmHel-4	CAEZ01008420:195787-196048
HmHel-4	CAEZ01008273:8807-9114
HmHel-4	CAEZ01007970:2133-2442
HmHel-4	CAEZ01007746:56498-56801
HmHel-4	CAEZ01006069:5089-5379
HmHel-4	CAEZ01005909:22833-23097
HmHel-4	CAEZ01002568:18305-18612

HmHel-4	CAEZ01001326:40457-40763
HmHel-4	CAEZ01000639:1009-1313
HmHel-4	CAEZ01011628:2941-3202
HmHel-4	CAEZ01009926:6175-6462
HmHel-4	CAEZ01009686:24849-25132
HmHel-4	CAEZ01009686:37979-38282
HmHel-4	CAEZ01009686:50392-50474
HmHel-4	CAEZ01009179:4360-4666
HmHel-4	CAEZ01009116:6368-6595
HmHel-4	CAEZ01008656:3063-3366
HmHel-4	CAEZ01006201:469-780
HmHel-4	CAEZ01005333:21078-21305
HmHel-4	CAEZ01004519:10821-11110
HmHel-4	CAEZ01010693:8051-8334
HmHel-4	CAEZ01005929:7232-7570
HmHel-4	CAEZ01005252:50772-50987
HmHel-4	CAEZ01002900:810-1099
HmHel-4	CAEZ01002362:43661-43921
HmHel-4	CAEZ01002362:35824-36083
HmHel-4	CAEZ01002362:15120-15421
HmHel-4	CAEZ01001367:2464-2737
HmHel-4	CAEZ01010824:27636-27829
HmHel-4	CAEZ01010824:5193-5512
HmHel-4	CAEZ01010268:22138-22466
HmHel-4	CAEZ01010268:9294-9587
HmHel-4	CAEZ01009543:5546-5826
HmHel-4	CAEZ01007981:2039-2288
HmHel-4	CAEZ01007981:34536-34846
HmHel-4	CAEZ01006686:67971-68160
HmHel-4	CAEZ01006218:25097-25424
HmHel-4	CAEZ01005777:7937-8264
HmHel-4	CAEZ01005161:24074-24396
HmHel-4	CAEZ01004870:277-590
HmHel-4	CAEZ01004544:3444-3728
HmHel-4	CAEZ01004277:16540-16874
HmHel-4	CAEZ01003795:13039-13366
HmHel-4	CAEZ01003388:6937-7158
HmHel-4	CAEZ01003187:2560-2887
HmHel-4	CAEZ01001439:37870-38190
HmHel-4	CAEZ01009962:6765-6992
HmHel-4	CAEZ01009962:36394-36682
HmHel-4	CAEZ01009076:29005-29310
HmHel-4	CAEZ01005443:18786-19086
HmHel-4	CAEZ01004634:7428-7735
HmHel-4	CAEZ01003629:18299-18527
HmHel-4	CAEZ01001737:15369-15676
HmHel-4	CAEZ01000848:12156-12278
HmHel-4	CAEZ01000848:25727-25997

HmHel-4	CAEZ01011287:10182-10469
HmHel-4	CAEZ01011135:23567-23860
HmHel-4	CAEZ01010730:15790-16095
HmHel-4	CAEZ01010730:25548-25807
HmHel-4	CAEZ01008654:771-953
HmHel-4	CAEZ01008654:1973-2270
HmHel-4	CAEZ01008248:36352-36658
HmHel-4	CAEZ01007453:8176-8479
HmHel-4	CAEZ01006982:3915-4141
HmHel-4	CAEZ01005463:6407-6702
HmHel-4	CAEZ01011006:278-553
HmHel-4	CAEZ01010385:18248-18446
HmHel-4	CAEZ01010385:8799-9094
HmHel-4	CAEZ01010191:8132-8426
HmHel-4	CAEZ01009102:375-657
HmHel-4	CAEZ01008639:23189-23492
HmHel-4	CAEZ01005192:10771-10997
HmHel-4	CAEZ01003071:6721-7019
HmHel-4	CAEZ01002229:15043-15328
HmHel-4	CAEZ01002132:5353-5646
HmHel-4	CAEZ01011576:22455-22749
HmHel-4	CAEZ01011576:10802-11086
HmHel-4	CAEZ01011296:18750-19110
HmHel-4	CAEZ01011254:17211-17400
HmHel-4	CAEZ01010952:20836-21157
HmHel-4	CAEZ01010898:31254-31532
HmHel-4	CAEZ01010898:30580-30713
HmHel-4	CAEZ01010658:1072-1399
HmHel-4	CAEZ01010503:21883-22096
HmHel-4	CAEZ01009889:23432-23759
HmHel-4	CAEZ01009889:86167-86579
HmHel-4	CAEZ01009655:29899-30166
HmHel-4	CAEZ01009655:33094-33419
HmHel-4	CAEZ01007397:6727-6912
HmHel-4	CAEZ01006324:106371-106668
HmHel-4	CAEZ01005813:7208-7509
HmHel-4	CAEZ01005316:38098-38279
HmHel-4	CAEZ01003210:11027-11327
HmHel-4	CAEZ01002846:16225-16424
HmHel-4	CAEZ01002359:606-940
HmHel-4	CAEZ01001838:457-678
HmHel-4	CAEZ01001155:5724-5905
HmHel-4	CAEZ01001022:8485-8812
HmHel-4	CAEZ01011020:11590-11884
HmHel-4	CAEZ01008220:25595-25896
HmHel-4	CAEZ01007495:3542-3695
HmHel-4	CAEZ01007495:1505-1822
HmHel-4	CAEZ01006438:19895-20179

HmHel-4	CAEZ01005815:19372-19678
HmHel-4	CAEZ01005061:13235-13508
HmHel-4	CAEZ01003912:1998-2252
HmHel-4	CAEZ01003643:5831-6127
HmHel-4	CAEZ01000832:2149-2372
HmHel-4	CAEZ01011508:6556-6826
HmHel-4	CAEZ01010681:653-935
HmHel-4	CAEZ01008655:2847-3161
HmHel-4	CAEZ01008643:49727-49950
HmHel-4	CAEZ01008034:7170-7464
HmHel-4	CAEZ01007989:18113-18877
HmHel-4	CAEZ01007953:28672-28899
HmHel-4	CAEZ01006732:2104-3717
HmHel-4	CAEZ01006295:14530-14842
HmHel-4	CAEZ01005805:1703-1996
HmHel-4	CAEZ01003788:3393-3659
HmHel-4	CAEZ01002790:7517-7825
HmHel-4	CAEZ01002188:5805-6101
HmHel-4	CAEZ01002188:1-261
HmHel-4	CAEZ01010983:12645-12827
HmHel-4	CAEZ01008277:21585-21865
HmHel-4	CAEZ01008246:17243-17534
HmHel-4	CAEZ01007920:2756-3080
HmHel-4	CAEZ01007001:4935-5222
HmHel-4	CAEZ01007001:19128-19297
HmHel-4	CAEZ01007001:14100-14426
HmHel-4	CAEZ01006666:50934-51191
HmHel-4	CAEZ01006666:25736-26004
HmHel-4	CAEZ01005917:9001-9283
HmHel-4	CAEZ01005652:8342-8657
HmHel-4	CAEZ01005519:4804-5085
HmHel-4	CAEZ01004008:17496-17876
HmHel-4	CAEZ01003532:11810-12004
HmHel-4	CAEZ01003415:19940-20161
HmHel-4	CAEZ01002671:1-199
HmHel-4	CAEZ01001338:71596-71932
HmHel-4	CAEZ01001338:49462-49787
HmHel-4	CAEZ01000966:25282-25590
HmHel-4	CAEZ01011210:4193-4493
HmHel-4	CAEZ01010957:8757-9084
HmHel-4	CAEZ01009994:21822-22149
HmHel-4	CAEZ01009211:30535-30862
HmHel-4	CAEZ01009181:10216-10509
HmHel-4	CAEZ01008460:2482-2749
HmHel-4	CAEZ01008436:4023-4352
HmHel-4	CAEZ01008194:19828-20155
HmHel-4	CAEZ01007992:10479-10753
HmHel-4	CAEZ01007517:6882-7207

HmHel-4	CAEZ01006651:10727-11051
HmHel-4	CAEZ01006645:4212-4542
HmHel-4	CAEZ01005933:14401-14712
HmHel-4	CAEZ01005403:13861-14188
HmHel-4	CAEZ01004815:22352-22612
HmHel-4	CAEZ01004815:48560-49004
HmHel-4	CAEZ01004815:86827-87108
HmHel-4	CAEZ01004815:58621-58725
HmHel-4	CAEZ01004662:74907-75184
HmHel-4	CAEZ01004108:4080-4407
HmHel-4	CAEZ01003802:18658-18970
HmHel-4	CAEZ01003802:41322-41649
HmHel-4	CAEZ01003047:8434-8631
HmHel-4	CAEZ01000800:5090-5417
HmHel-4	CAEZ01010929:6858-7145
HmHel-4	CAEZ01010508:923-1188
HmHel-4	CAEZ01010317:13842-14140
HmHel-4	CAEZ01009766:63021-63316
HmHel-4	CAEZ01009306:55386-55692
HmHel-4	CAEZ01009004:1361-1668
HmHel-4	CAEZ01008449:6129-6429
HmHel-4	CAEZ01007358:3706-4011
HmHel-4	CAEZ01007171:4952-5250
HmHel-4	CAEZ01006619:5-1003
HmHel-4	CAEZ01006023:17995-18219
HmHel-4	CAEZ01005592:7891-8216
HmHel-4	CAEZ01005330:491-769
HmHel-4	CAEZ01005049:2491-2793
HmHel-4	CAEZ01004820:64681-64892
HmHel-4	CAEZ01003665:1818-2123
HmHel-4	CAEZ01003367:5115-5379
HmHel-4	CAEZ01003367:2162-2452
HmHel-4	CAEZ01002051:8489-8759
HmHel-4	CAEZ01001447:29618-29786
HmHel-4	CAEZ01001447:8752-9029
HmHel-4	CAEZ01010578:15338-15653
HmHel-4	CAEZ01009528:17631-17917
HmHel-4	CAEZ01009510:21311-21583
HmHel-4	CAEZ01008846:1920-2224
HmHel-4	CAEZ01008162:10736-11015
HmHel-4	CAEZ01005746:23041-23343
HmHel-4	CAEZ01005665:30960-31269
HmHel-4	CAEZ01005665:15699-15972
HmHel-4	CAEZ01004821:20671-20962
HmHel-4	CAEZ01004821:69365-69665
HmHel-4	CAEZ01004796:25334-25627
HmHel-4	CAEZ01003621:4628-4823
HmHel-4	CAEZ01002119:5032-5346

HmHel-4	CAEZ01002119:15951-16190
HmHel-4	CAEZ01001243:1237-1547
HmHel-4	CAEZ01010309:14751-14974
HmHel-4	CAEZ01009095:2807-3107
HmHel-4	CAEZ01008842:10650-10907
HmHel-4	CAEZ01007252:206920-207206
HmHel-4	CAEZ01007252:50510-50795
HmHel-4	CAEZ01006873:8674-8868
HmHel-4	CAEZ01006086:14668-14949
HmHel-4	CAEZ01004168:3682-3971
HmHel-4	CAEZ01003372:4172-4499
HmHel-4	CAEZ01010718:9376-9630
HmHel-4	CAEZ01009345:2884-3097
HmHel-4	CAEZ01009220:1025-1352
HmHel-4	CAEZ01009153:3655-3952
HmHel-4	CAEZ01007858:135562-135889
HmHel-4	CAEZ01007823:172-471
HmHel-4	CAEZ01007771:929-1227
HmHel-4	CAEZ01007582:364-691
HmHel-4	CAEZ01006415:51937-52207
HmHel-4	CAEZ01006415:33253-33574
HmHel-4	CAEZ01006325:12538-12865
HmHel-4	CAEZ01006098:2309-2492
HmHel-4	CAEZ01006098:28682-29009
HmHel-4	CAEZ01005111:11028-11355
HmHel-4	CAEZ01004919:5675-6002
HmHel-4	CAEZ01004649:8990-9295
HmHel-4	CAEZ01004526:3-266
HmHel-4	CAEZ01004258:4871-5193
HmHel-4	CAEZ01002072:336-584
HmHel-4	CAEZ01001479:8357-8562
HmHel-4	CAEZ01010320:67170-67494
HmHel-4	CAEZ01007140:6654-6830
HmHel-4	CAEZ01007140:58415-58718
HmHel-4	CAEZ01007140:18363-18654
HmHel-4	CAEZ01006701:18004-18231
HmHel-4	CAEZ01006514:4380-4757
HmHel-4	CAEZ01010193:2933-3242
HmHel-4	CAEZ01009169:7033-7328
HmHel-4	CAEZ01007211:7290-7429
HmHel-4	CAEZ01007211:3858-4146
HmHel-4	CAEZ01003634:5938-6265
HmHel-4	CAEZ01010437:55942-56266
HmHel-4	CAEZ01010365:38628-38952
HmHel-4	CAEZ01007027:9897-10221
HmHel-4	CAEZ01007027:14250-14527
HmHel-4	CAEZ01006910:29172-29469
HmHel-4	CAEZ01005861:4165-4443

HmHel-4	CAEZ01003519:8858-9135
HmHel-4	CAEZ01003407:1011-1253
HmHel-4	CAEZ01002149:436-764
HmHel-4	CAEZ01001514:448-646
HmHel-4	CAEZ01000061:57752-58019
HmHel-4	CAEZ01011621:5296-5623
HmHel-4	CAEZ01011498:554-881
HmHel-4	CAEZ01010624:4051-4274
HmHel-4	CAEZ01010624:9219-9546
HmHel-4	CAEZ01009634:14834-15161
HmHel-4	CAEZ01008439:2733-3060
HmHel-4	CAEZ01006106:7466-7754
HmHel-4	CAEZ01006030:6272-6595
HmHel-4	CAEZ01005884:21074-21401
HmHel-4	CAEZ01005564:1635-1956
HmHel-4	CAEZ01004903:17781-18115
HmHel-4	CAEZ01004654:4710-5037
HmHel-4	CAEZ01004281:464-792
HmHel-4	CAEZ01004142:24219-24463
HmHel-4	CAEZ01003335:17131-17408
HmHel-4	CAEZ01003335:54394-54695
HmHel-4	CAEZ01003335:1748-1993
HmHel-4	CAEZ01001989:5632-5966
HmHel-4	CAEZ01001761:4003-4330
HmHel-4	CAEZ01011636:6047-6286
HmHel-4	CAEZ01011196:5597-5925
HmHel-4	CAEZ01010900:53595-53857
HmHel-4	CAEZ01010447:14854-15134
HmHel-4	CAEZ01009521:4293-4628
HmHel-4	CAEZ01009521:6030-6236
HmHel-4	CAEZ01008918:12444-12723
HmHel-4	CAEZ01004665:2228-2533
HmHel-4	CAEZ01004581:3805-4070
HmHel-4	CAEZ01003819:24592-24918
HmHel-4	CAEZ01003819:32076-32410
HmHel-4	CAEZ01003023:109-397
HmHel-4	CAEZ01000902:1035-1271
HmHel-4	CAEZ01011184:7919-8216
HmHel-4	CAEZ01010282:22405-22699
HmHel-4	CAEZ01009374:58603-58929
HmHel-4	CAEZ01008146:2648-2914
HmHel-4	CAEZ01006774:64955-65267
HmHel-4	CAEZ01006774:116102-116359
HmHel-4	CAEZ01006471:1489-1729
HmHel-4	CAEZ01003152:19162-19473
HmHel-4	CAEZ01001065:7286-7592
HmHel-4	CAEZ01009965:10620-10934
HmHel-4	CAEZ01009158:7325-7556

HmHel-4	CAEZ01007657:1671-1953
HmHel-4	CAEZ01007628:14736-15063
HmHel-4	CAEZ01005999:12802-13268
HmHel-4	CAEZ01004211:4298-4446
HmHel-4	CAEZ01004211:34021-34195
HmHel-4	CAEZ01003197:35906-44946
HmHel-4	CAEZ01002406:1-309
HmHel-4	CAEZ01002096:3357-3649
HmHel-4	CAEZ01000858:19273-19566
HmHel-4	CAEZ01000062:4369-4978
HmHel-4	CAEZ01011460:5646-5945
HmHel-4	CAEZ01011432:3827-4154
HmHel-4	CAEZ01011400:1-322
HmHel-4	CAEZ01009033:5226-5558
HmHel-4	CAEZ01008997:6040-6336
HmHel-4	CAEZ01008346:42770-43066
HmHel-4	CAEZ01008272:12244-12424
HmHel-4	CAEZ01008272:21570-21878
HmHel-4	CAEZ01007637:3591-3884
HmHel-4	CAEZ01006617:26955-27282
HmHel-4	CAEZ01006540:122797-123001
HmHel-4	CAEZ01006470:20957-21138
HmHel-4	CAEZ01006372:11057-11384
HmHel-4	CAEZ01006122:931-1240
HmHel-4	CAEZ01005889:6864-7147
HmHel-4	CAEZ01005877:5634-7574
HmHel-4	CAEZ01004360:18604-18931
HmHel-4	CAEZ01004100:1258-1585
HmHel-4	CAEZ01003568:41223-41550
HmHel-4	CAEZ01003538:836-1080
HmHel-4	CAEZ01002799:1901-2228
HmHel-4	CAEZ01002065:9863-10075
HmHel-4	CAEZ01002055:71915-72242
HmHel-4	CAEZ01011526:29040-29369
HmHel-4	CAEZ01007647:30873-31084
HmHel-4	CAEZ01007190:7486-7812
HmHel-4	CAEZ01005460:14692-14985
HmHel-4	CAEZ01004698:11395-11674
HmHel-4	CAEZ01003358:2496-2822
HmHel-4	CAEZ01003122:3823-4097
HmHel-4	CAEZ01001553:2290-2579
HmHel-4	CAEZ01011540:26056-26346
HmHel-4	CAEZ01011193:85135-85416
HmHel-4	CAEZ01011193:9680-10025
HmHel-4	CAEZ01010461:36563-36876
HmHel-4	CAEZ01010461:32050-32306
HmHel-4	CAEZ01007435:20397-20568
HmHel-4	CAEZ01006380:1731-6351

HmHel-4	CAEZ01005804:4856-5158
HmHel-4	CAEZ01005572:7570-7869
HmHel-4	CAEZ01003863:9332-9646
HmHel-4	CAEZ01011120:8867-9036
HmHel-4	CAEZ01008351:83588-83916
HmHel-4	CAEZ01006564:4629-4960
HmHel-4	CAEZ01006073:37523-37847
HmHel-4	CAEZ01004741:14135-14430
HmHel-4	CAEZ01004739:18766-19079
HmHel-4	CAEZ01002758:12789-13026
HmHel-4	CAEZ01001073:5280-5568
HmHel-4	CAEZ01010801:7683-8017
HmHel-4	CAEZ01009364:39580-39846
HmHel-4	CAEZ01009364:24130-24405
HmHel-4	CAEZ01007891:570-897
HmHel-4	CAEZ01007446:27160-27387
HmHel-4	CAEZ01001858:10317-10610
HmHel-4	CAEZ01010498:9244-9531
HmHel-4	CAEZ01007454:801-1076
HmHel-4	CAEZ01005647:22538-22869
HmHel-4	CAEZ01005308:33819-34075
HmHel-4	CAEZ01004940:7914-8141
HmHel-4	CAEZ01004289:7987-8318
HmHel-4	CAEZ01000136:821-1005
HmHel-4	CAEZ01010025:37513-37855
HmHel-4	CAEZ01009957:11026-11342
HmHel-4	CAEZ01009123:3758-3933
HmHel-4	CAEZ01005775:3051-3333
HmHel-4	CAEZ01005733:1466-1742
HmHel-4	CAEZ01004534:5085-5391
HmHel-4	CAEZ01004492:10268-10572
HmHel-4	CAEZ01003153:20978-21287
HmHel-4	CAEZ01002736:17624-17799
HmHel-4	CAEZ01000934:2956-3131
HmHel-4	CAEZ01010381:13867-14076
HmHel-4	CAEZ01007870:50700-50980
HmHel-4	CAEZ01004641:7457-7766
HmHel-4	CAEZ01004137:24300-24614
HmHel-4	CAEZ01004137:12351-12602
HmHel-4	CAEZ01001742:14584-14909
HmHel-4	CAEZ01009979:14369-14639
HmHel-4	CAEZ01009979:1116-1351
HmHel-4	CAEZ01009630:39003-39331
HmHel-4	CAEZ01009557:5555-5830
HmHel-4	CAEZ01009243:38372-38654
HmHel-4	CAEZ01008127:24551-24695
HmHel-4	CAEZ01008127:21848-22182
HmHel-4	CAEZ01005280:314-640

HmHel-4	CAEZ01005189:195-494
HmHel-4	CAEZ01003765:4285-4619
HmHel-4	CAEZ01010486:1027-1358
HmHel-4	CAEZ01010028:49158-49493
HmHel-4	CAEZ01009193:9384-9572
HmHel-4	CAEZ01005005:15523-15735
HmHel-4	CAEZ01002928:14286-14513
HmHel-4	CAEZ01001764:3101-3324
HmHel-4	CAEZ01011463:12919-13139
HmHel-4	CAEZ01009439:23179-23462
HmHel-4	CAEZ01008624:15091-15266
HmHel-4	CAEZ01007975:47939-48114
HmHel-4	CAEZ01007905:13550-13873
HmHel-4	CAEZ01006959:8389-8564
HmHel-4	CAEZ01004362:6779-7048
HmHel-4	CAEZ01003683:2794-2969
HmHel-4	CAEZ01008738:8174-8505
HmHel-4	CAEZ01007723:10442-10674
HmHel-4	CAEZ01007723:8468-8574
HmHel-4	CAEZ01006182:16398-16690
HmHel-4	CAEZ01005623:30387-30711
HmHel-4	CAEZ01003303:7617-7895
HmHel-4	CAEZ01011402:1057-1320
HmHel-4	CAEZ01002769:69268-69602
HmHel-4	CAEZ01002321:62298-62625
HmHel-4	CAEZ01002263:3984-4298
HmHel-4	CAEZ01001873:5002-5296
HmHel-4	CAEZ01010662:4106-4450
HmHel-4	CAEZ01009212:35311-35602
HmHel-4	CAEZ01009074:5840-6070
HmHel-4	CAEZ01008950:807-1080
HmHel-4	CAEZ01005768:53-209
HmHel-4	CAEZ01001351:8506-8790
HmHel-4	CAEZ01007795:72301-72448
HmHel-4	CAEZ01006634:26722-26897
HmHel-4	CAEZ01005430:25533-26282
HmHel-4	CAEZ01005430:8251-8445
HmHel-4	CAEZ01004348:640-815
HmHel-4	CAEZ01004309:26284-26595
HmHel-4	CAEZ01002277:1-174
HmHel-4	CAEZ01000982:980-1273
HmHel-4	CAEZ01007205:7772-8054
HmHel-4	CAEZ01006334:24659-24805
HmHel-4	CAEZ01002049:21309-21580
HmHel-4	CAEZ01007937:11634-11925
HmHel-4	CAEZ01007633:17392-17701
HmHel-4	CAEZ01001814:1328-1605
HmHel-4	CAEZ01001285:495-795

HmHel-4	CAEZ01000947:9793-10120
HmHel-4	CAEZ01007468:1-168
HmHel-4	CAEZ01006160:2278-2592
HmHel-4	CAEZ01004890:4761-5010
HmHel-4	CAEZ01004372:3100-3425
HmHel-4	CAEZ01001081:15458-15721
HmHel-4	CAEZ01007162:3713-3995
HmHel-4	CAEZ01006133:2525-2664
HmHel-4	CAEZ01003624:8301-8581
HmHel-4	CAEZ01003193:6406-6694
HmHel-4	CAEZ01002727:1584-1882
HmHel-4	CAEZ01011364:5145-5487
HmHel-4	CAEZ01007322:15736-16024
HmHel-4	CAEZ01003286:12780-13092
HmHel-4	CAEZ01002731:19414-19684
HmHel-4	CAEZ01009698:7946-8091
HmHel-4	CAEZ01009576:18663-18942
HmHel-4	CAEZ01007803:2968-3198
HmHel-4	CAEZ01007177:3683-3900
HmHel-4	CAEZ01008568:8349-8521
HmHel-4	CAEZ01008311:7909-8215
HmHel-4	CAEZ01008254:3307-3472
HmHel-4	CAEZ01008254:13053-13232
HmHel-4	CAEZ01007596:8380-8677
HmHel-4	CAEZ01007411:13286-13548
HmHel-4	CAEZ01006878:6669-6868
HmHel-4	CAEZ01005779:6871-7019
HmHel-4	CAEZ01005296:6984-7277
HmHel-4	CAEZ01004688:11244-11392
HmHel-4	CAEZ01001856:4155-4391
HmHel-4	CAEZ01010420:900-1047
HmHel-4	CAEZ01009366:11929-12104
HmHel-4	CAEZ01008972:13911-14086
HmHel-4	CAEZ01008948:2130-2305
HmHel-4	CAEZ01008496:7051-7226
HmHel-4	CAEZ01008452:4738-4909
HmHel-4	CAEZ01006622:2431-2723
HmHel-4	CAEZ01006138:60481-60628
HmHel-4	CAEZ01005082:6513-6780
HmHel-4	CAEZ01004590:1312-1479
HmHel-4	CAEZ01011570:7374-7516
HmHel-4	CAEZ01001562:11808-12082
HmHel-4	CAEZ01001440:8348-8556
HmHel-4	CAEZ01007847:643-824
HmHel-4	CAEZ01006338:41061-41346
HmHel-4	CAEZ01003237:46275-46502
HmHel-4	CAEZ01011654:10-158
HmHel-4	CAEZ01008586:4492-4808

HmHel-4	CAEZ01007670:3734-4063
HmHel-4	CAEZ01006771:380-528
HmHel-4	CAEZ01002800:12477-12790
HmHel-4	CAEZ01002762:35963-36251
HmHel-4	CAEZ01002377:3298-3628
HmHel-4	CAEZ01009823:23854-24100
HmHel-4	CAEZ01009278:8841-9178
HmHel-4	CAEZ01008952:1555-1810
HmHel-4	CAEZ01008952:800-975
HmHel-4	CAEZ01007977:4774-5085
HmHel-4	CAEZ01007390:3893-4147
HmHel-4	CAEZ01006404:15236-15411
HmHel-4	CAEZ01005381:1318-1548
HmHel-4	CAEZ01004054:8352-8648
HmHel-4	CAEZ01003264:2521-2791
HmHel-4	CAEZ01001658:4464-4678
HmHel-4	CAEZ01011242:16641-16925
HmHel-4	CAEZ01011236:7903-8204
HmHel-4	CAEZ01009758:2924-3219
HmHel-4	CAEZ01007115:3422-3729
HmHel-4	CAEZ01006533:14734-15039
HmHel-4	CAEZ01005159:1320-1517
HmHel-4	CAEZ01010003:12399-12544
HmHel-4	CAEZ01008304:5478-5781
HmHel-4	CAEZ01004897:8667-8961
HmHel-4	CAEZ01002885:3711-4010
HmHel-4	CAEZ01001417:28172-28436
HmHel-4	CAEZ01007836:10869-11017
HmHel-4	CAEZ01005427:19539-19810
HmHel-4	CAEZ01003292:90575-90742
HmHel-4	CAEZ01000001:341-489
HmHel-4	CAEZ01009104:10107-10314
HmHel-4	CAEZ01008141:6595-6890
HmHel-4	CAEZ01005826:1125-1312
HmHel-4	CAEZ01005826:278-446
HmHel-4	CAEZ01005368:5811-6138
HmHel-4	CAEZ01005301:10636-10852
HmHel-4	CAEZ01003221:9294-9576
HmHel-4	CAEZ01001806:10641-10863
HmHel-4	CAEZ01009170:20406-20559
HmHel-4	CAEZ01007911:22657-22930
HmHel-4	CAEZ01006203:2889-3035
HmHel-4	CAEZ01003768:56898-57174
HmHel-4	CAEZ01010505:11223-11368
HmHel-4	CAEZ01009437:67412-67652
HmHel-4	CAEZ01007709:41964-42181
HmHel-4	CAEZ01007709:25745-25856
HmHel-4	CAEZ01006846:2711-2947

HmHel-4	CAEZ01005631:4049-4194
HmHel-4	CAEZ01005179:20680-20821
HmHel-4	CAEZ01007614:1-156
HmHel-4	CAEZ01004691:22937-23105
HmHel-4	CAEZ01003933:35848-35996
HmHel-4	CAEZ01002689:19288-19603
HmHel-4	CAEZ01008542:1247-1569
HmHel-4	CAEZ01008480:17699-17866
HmHel-4	CAEZ01008402:58722-58897
HmHel-4	CAEZ01007778:12727-12992
HmHel-4	CAEZ01004019:6455-6630
HmHel-4	CAEZ01004019:471-787
HmHel-4	CAEZ01002373:926-1108
HmHel-4	CAEZ01002090:1797-1972
HmHel-4	CAEZ01007081:8867-9048
HmHel-4	CAEZ01001136:452-630
HmHel-4	CAEZ01000999:9307-9589
HmHel-4	CAEZ01010511:33699-33824
HmHel-4	CAEZ01009800:13980-14261
HmHel-4	CAEZ01006140:6701-7004
HmHel-4	CAEZ01004987:8935-9229
HmHel-4	CAEZ01010871:25201-25414
HmHel-4	CAEZ01008815:5383-5531
HmHel-4	CAEZ01008717:62972-63180
HmHel-4	CAEZ01007118:18238-18437
HmHel-4	CAEZ01006791:31023-31258
HmHel-4	CAEZ01005227:12886-13211
HmHel-4	CAEZ01005198:96799-97070
HmHel-4	CAEZ01004939:7685-7833
HmHel-4	CAEZ01003690:14705-15009
HmHel-4	CAEZ01003123:15634-15782
HmHel-4	CAEZ01001048:4825-4961
HmHel-4	CAEZ01008383:17961-18243
HmHel-4	CAEZ01006498:2978-3153
HmHel-4	CAEZ01001442:25704-25956
HmHel-4	CAEZ01006557:3100-3230
HmHel-4	CAEZ01003791:17097-17316
HmHel-4	CAEZ01007685:16201-16342
HmHel-4	CAEZ01007443:2713-2929
HmHel-4	CAEZ01003929:7283-7467
HmHel-4	CAEZ01010036:13248-13377
HmHel-4	CAEZ01001785:9533-9847
HmHel-4	CAEZ01008689:1095-1419
HmHel-4	CAEZ01003994:23260-23457
HmHel-4	CAEZ01003994:10135-10376
HmHel-4	CAEZ01002678:1290-1405
HmHel-4	CAEZ01001504:975-1150
HmHel-4	CAEZ01000951:1168-1487

HmHel-4	CAEZ01011510:16347-16492
HmHel-4	CAEZ01011306:8717-8902
HmHel-4	CAEZ01010961:15292-15605
HmHel-4	CAEZ01008941:3129-3324
HmHel-4	CAEZ01008890:17842-17976
HmHel-4	CAEZ01008741:1797-2105
HmHel-4	CAEZ01006276:5141-5326
HmHel-4	CAEZ01006010:25142-25309
HmHel-4	CAEZ01010051:490-762
HmHel-4	CAEZ01009399:6758-7061
HmHel-4	CAEZ01007926:4689-4887
HmHel-4	CAEZ01004398:15961-16078
HmHel-4	CAEZ01006080:3817-4089
HmHel-4	CAEZ01005863:2749-3032
HmHel-4	CAEZ01004341:1285-1560
HmHel-4	CAEZ01004236:13771-13919
HmHel-4	CAEZ01010058:1638-1761
HmHel-4	CAEZ01007401:28377-28512
HmHel-4	CAEZ01008819:14864-15105
HmHel-4	CAEZ01005142:1237-1496
HmHel-4	CAEZ01001385:2048-2182
HmHel-4	CAEZ01011448:2544-2709
HmHel-4	CAEZ01006464:5001-5225
HmHel-4	CAEZ01006464:22116-22265
HmHel-4	CAEZ01005867:4768-5060
HmHel-4	CAEZ01005287:21297-21501
HmHel-4	CAEZ01004771:5408-5545
HmHel-4	CAEZ01000036:29571-29680
HmHel-4	CAEZ01009585:5445-5651
HmHel-4	CAEZ01009249:18806-19008
HmHel-4	CAEZ01007310:9447-9726
HmHel-4	CAEZ01006893:21003-21258
HmHel-4	CAEZ01004099:2160-2369
HmHel-4	CAEZ01003732:187-478
HmHel-4	CAEZ01002941:20944-21157
HmHel-4	CAEZ01001302:5934-6186
HmHel-4	CAEZ01010180:5692-5867
HmHel-4	CAEZ01009934:936-1101
HmHel-4	CAEZ01006508:6425-6695
HmHel-4	CAEZ01005923:1443-1608
HmHel-4	CAEZ01005083:30455-30617
HmHel-4	CAEZ01008928:27357-27525
HmHel-4	CAEZ01003676:1467-1584
HmHel-4	CAEZ01001240:4117-4285
HmHel-4	CAEZ01001112:1005-1275
HmHel-4	CAEZ01005953:2791-3082
HmHel-4	CAEZ01011018:4454-4747
HmHel-4	CAEZ01008818:3962-4137

HmHel-4	CAEZ01005617:744-1042
HmHel-4	CAEZ01011179:11791-12086
HmHel-4	CAEZ01010494:4550-4840
HmHel-4	CAEZ01010397:5813-5947
HmHel-4	CAEZ01007256:45221-45382
HmHel-4	CAEZ01006424:10105-10270
HmHel-4	CAEZ01004548:486-658
HmHel-4	CAEZ01011502:31401-31506
HmHel-4	CAEZ01009968:1760-2042
HmHel-4	CAEZ01009045:17002-17279
HmHel-4	CAEZ01011189:13606-13860
HmHel-4	CAEZ01008456:22289-22582
HmHel-4	CAEZ01006465:6616-6862
HmHel-4	CAEZ01005906:22022-22135
HmHel-4	CAEZ01004762:18744-19190
HmHel-4	CAEZ01011405:3459-3752
HmHel-4	CAEZ01007406:6169-6457
HmHel-4	CAEZ01004072:7320-7567
HmHel-4	CAEZ01002082:998-1105
HmHel-4	CAEZ01009680:55650-55821
HmHel-4	CAEZ01009024:12728-12893
HmHel-4	CAEZ01008374:43179-43344
HmHel-4	CAEZ01006454:15549-15787
HmHel-4	CAEZ01005899:6173-6414
HmHel-4	CAEZ01005698:2925-3152
HmHel-4	CAEZ01004081:18623-18788
HmHel-4	CAEZ01003943:27159-27324
HmHel-4	CAEZ01007885:2742-2851
HmHel-4	CAEZ01005580:9671-9928
HmHel-4	CAEZ01005347:6932-7103
HmHel-4	CAEZ01004683:3489-3749
HmHel-4	CAEZ01003780:372-502
HmHel-4	CAEZ01007047:1551-1718
HmHel-4	CAEZ01001446:13819-14134
HmHel-4	CAEZ01006941:3427-3609
HmHel-4	CAEZ01005941:15144-15271
HmHel-4	CAEZ01011486:20372-20537
HmHel-4	CAEZ01011126:20979-21151
HmHel-4	CAEZ01010133:43418-43528
HmHel-4	CAEZ01008201:20748-21020
HmHel-4	CAEZ01007048:24823-24985
HmHel-4	CAEZ01006899:1176-1341
HmHel-4	CAEZ01006852:7348-7486
HmHel-4	CAEZ01006191:897-1059
HmHel-4	CAEZ01005057:9941-10105
HmHel-4	CAEZ01003766:3906-4071
HmHel-4	CAEZ01008946:6648-6868
HmHel-4	CAEZ01006948:21291-21698

HmHel-4	CAEZ01006694:8124-8298
HmHel-4	CAEZ01006531:4735-4844
HmHel-4	CAEZ01003931:6149-6377
HmHel-4	CAEZ01000829:577-722
HmHel-4	CAEZ01000829:1-98
HmHel-4	CAEZ01000064:2859-3137
HmHel-4	CAEZ01011033:4818-4949
HmHel-4	CAEZ01009273:726-996
HmHel-4	CAEZ01000547:2909-3005
HmHel-4	CAEZ01010211:5041-5209
HmHel-4	CAEZ01004017:9265-9511
HmHel-4	CAEZ01002862:1968-2063
HmHel-4	CAEZ01001352:8095-8213
HmHel-4	CAEZ01007660:4161-4445
HmHel-4	CAEZ01006247:3953-4209
HmHel-4	CAEZ01006005:560-725
HmHel-4	CAEZ01004905:32445-32617
HmHel-4	CAEZ01001032:5-103
HmHel-4	CAEZ01011632:10-268
HmHel-4	CAEZ01006867:16213-16354
HmHel-4	CAEZ01003711:26019-26311
HmHel-4	CAEZ01009719:15229-15481
HmHel-4	CAEZ01005303:1164-1329
HmHel-4	CAEZ01005219:162697-162931
HmHel-4	CAEZ01010619:60558-60685
HmHel-4	CAEZ01009246:17942-18134
HmHel-4	CAEZ01003432:19995-20189
HmHel-4	CAEZ01001222:9053-9218
HmHel-4	CAEZ01000485:357-464
HmHel-4	CAEZ01009378:11614-11752
HmHel-4	CAEZ01007130:2962-3072
HmHel-4	CAEZ01006003:1-153
HmHel-4	CAEZ01005886:37926-38084
HmHel-4	CAEZ01005886:22383-22552
HmHel-4	CAEZ01003060:8751-8923
HmHel-4	CAEZ01000854:77508-77673
HmHel-4	CAEZ01009998:4372-4527
HmHel-4	CAEZ01009647:14439-14634
HmHel-4	CAEZ01006168:11124-11381
HmHel-4	CAEZ01003814:446-690
HmHel-4	CAEZ01001306:1-102
HmHel-4	CAEZ01006951:1285-1401
HmHel-4	CAEZ01004354:9756-9901
HmHel-4	CAEZ01003709:23038-23148
HmHel-4	CAEZ01011601:23194-23354
HmHel-4	CAEZ01008920:9717-9815
HmHel-4	CAEZ01008268:4-94
HmHel-4	CAEZ01006700:17771-17958

HmHel-4	CAEZ01002847:14351-14516
HmHel-4	CAEZ01002058:1410-1578
HmHel-4	CAEZ01001580:11822-11994
HmHel-4	CAEZ01001580:12792-12893
HmHel-4	CAEZ01008469:20939-21107
HmHel-4	CAEZ01004934:19945-20116
HmHel-4	CAEZ01003248:61441-61603
HmHel-4	CAEZ01010353:70-198
HmHel-4	CAEZ01007194:5516-5685
HmHel-4	CAEZ01002309:946-1053
HmHel-4	CAEZ01009831:1170-1268
HmHel-4	CAEZ01008525:10654-10953
HmHel-4	CAEZ01005097:5497-5787
HmHel-4	CAEZ01003825:1-170
HmHel-4	CAEZ01003334:33963-34117
HmHel-4	CAEZ01000630:1467-1630
HmHel-4	CAEZ01011618:1415-1589
HmHel-4	CAEZ01011347:11498-11590
HmHel-4	CAEZ01009318:7689-7835
HmHel-4	CAEZ01005622:767-864
HmHel-4	CAEZ01006491:20781-20908
HmHel-4	CAEZ01002383:7240-7379
HmHel-4	CAEZ01003562:11486-11739
HmHel-4	CAEZ01002142:6981-7153
HmHel-4	CAEZ01000725:5326-5498
HmHel-4	CAEZ01007632:2258-2359
HmHel-4	CAEZ01006972:20536-20710
HmHel-4	CAEZ01007301:12804-12964
HmHel-4	CAEZ01004083:3584-3823
HmHel-4	CAEZ01010099:9633-9716
HmHel-4	CAEZ01010001:5349-5681
HmHel-4	CAEZ01007101:63713-63860
HmHel-4	CAEZ01007101:42061-42230
HmHel-4	CAEZ01003642:5291-5460
HmHel-4	CAEZ01002668:63772-63937
HmHel-4	CAEZ01011199:3588-3755
HmHel-4	CAEZ01009197:31938-32170
HmHel-4	CAEZ01005162:1300-1945
HmHel-4	CAEZ01004997:1518-1660
HmHel-4	CAEZ01008531:23472-23615
HmHel-4	CAEZ01010403:3237-3320
HmHel-4	CAEZ01005619:8876-8985
HmHel-4	CAEZ01004676:11075-11247
HmHel-4	CAEZ01004664:7489-7681
HmHel-4	CAEZ01002798:12073-12325
HmHel-4	CAEZ01008117:9130-9435
HmHel-4	CAEZ01006502:9052-9136
HmHel-4	CAEZ01004477:19507-19676

HmHel-4	CAEZ01008545:10795-10926
HmHel-4	CAEZ01006890:6834-7084
HmHel-4	CAEZ01010736:387-571
HmHel-4	CAEZ01010351:89351-89495
HmHel-4	CAEZ01004502:9930-10045
HmHel-4	CAEZ01010581:8628-8768
HmHel-4	CAEZ01011009:22402-22563
HmHel-4	CAEZ01004161:6645-6726
HmHel-4	CAEZ01006713:2988-3184
HmHel-4	CAEZ01005703:8379-8527
HmHel-4	CAEZ01011588:1835-1993
HmHel-4	CAEZ01004714:10089-10190
HmHel-4	CAEZ01011571:4708-4796
HmHel-4	CAEZ01009642:32550-32665
HmHel-4	CAEZ01008001:41043-41186
HmHel-4	CAEZ01002638:1-111
HmHel-4	CAEZ01003257:5724-5839
	:-
HmHel-5	CAEZ01004815:102552-103918
HmHel-5	CAEZ01004632:118914-119781
HmHel-5	CAEZ01004632:109995-111274
HmHel-5	CAEZ01004632:85887-87094
HmHel-5	CAEZ01007576:133-503
HmHel-5	CAEZ01007576:16268-17639
HmHel-5	CAEZ01002194:39620-40923
HmHel-5	CAEZ01002194:61252-62602
HmHel-5	CAEZ01006406:73039-74315
HmHel-5	CAEZ01006406:83213-84574
HmHel-5	CAEZ01006406:54499-55821
HmHel-5	CAEZ01006177:224026-225404
HmHel-5	CAEZ01010668:686-2045
HmHel-5	CAEZ01010668:15432-16571
HmHel-5	CAEZ01006799:58782-59926
HmHel-5	CAEZ01006799:97830-99135
HmHel-5	CAEZ01006799:58552-58782
HmHel-5	CAEZ01007518:1812-3153
HmHel-5	CAEZ01007518:40-371
HmHel-5	CAEZ01007100:27401-28683
HmHel-5	CAEZ01004068:1906-3252
HmHel-5	CAEZ01010301:13693-14976
HmHel-5	CAEZ01010301:66685-68014
HmHel-5	CAEZ01010301:62788-63502
HmHel-5	CAEZ01009843:1-369
HmHel-5	CAEZ01009843:27023-28143
HmHel-5	CAEZ01009843:39099-40381
HmHel-5	CAEZ01002847:388-1671
HmHel-5	CAEZ01002847:1810-3163
HmHel-5	CAEZ01010828:4779-6059

HmHel-5	CAEZ01010460:104172-105390
HmHel-5	CAEZ01010460:46955-48225
HmHel-5	CAEZ01010424:97290-98656
HmHel-5	CAEZ01010424:163553-164822
HmHel-5	CAEZ01004033:116037-117220
HmHel-5	CAEZ01004033:201117-202379
HmHel-5	CAEZ01004033:3869-5154
HmHel-5	CAEZ01010677:31437-32719
HmHel-5	CAEZ01010677:232609-234291
HmHel-5	CAEZ01007706:120627-121909
HmHel-5	CAEZ01001457:1-1276
HmHel-5	CAEZ01009875:1-1128
HmHel-5	CAEZ01009607:25558-26848
HmHel-5	CAEZ01002825:7308-8590
HmHel-5	CAEZ01008427:62038-63372
HmHel-5	CAEZ01008427:75902-77183
HmHel-5	CAEZ01007269:54576-55860
HmHel-5	CAEZ01011107:75929-77265
HmHel-5	CAEZ01011107:41067-42348
HmHel-5	CAEZ01010129:68171-69516
HmHel-5	CAEZ01008233:94556-95725
HmHel-5	CAEZ01008233:42624-43906
HmHel-5	CAEZ01007453:35352-36632
HmHel-5	CAEZ01008164:43424-44707
HmHel-5	CAEZ01006778:133866-135135
HmHel-5	CAEZ01002712:18249-19531
HmHel-5	CAEZ01002134:2792-4071
HmHel-5	CAEZ01006347:25350-26708
HmHel-5	CAEZ01006347:41957-43228
HmHel-5	CAEZ01006347:235961-236307
HmHel-5	CAEZ01006347:61702-62134
HmHel-5	CAEZ01006347:10641-11922
HmHel-5	CAEZ01002211:47392-48653
HmHel-5	CAEZ01008248:32-967
HmHel-5	CAEZ01008248:43405-45624
HmHel-5	CAEZ01008248:35002-36352
HmHel-5	CAEZ01009733:37334-38618
HmHel-5	CAEZ01009506:51475-52681
HmHel-5	CAEZ01005886:26411-27777
HmHel-5	CAEZ01005410:28937-30219
HmHel-5	CAEZ01005410:53244-54582
HmHel-5	CAEZ01009431:15246-16525
HmHel-5	CAEZ01009229:43172-44451
HmHel-5	CAEZ01008112:5806-7081
HmHel-5	CAEZ01008112:561-1902
HmHel-5	CAEZ01007047:5506-6786
HmHel-5	CAEZ01003123:4215-5501
HmHel-5	CAEZ01002616:475-1762

HmHel-5	CAEZ01009246:6988-8268
HmHel-5	CAEZ01010730:32868-34151
HmHel-5	CAEZ01002203:29152-30435
HmHel-5	CAEZ01010920:38086-39370
HmHel-5	CAEZ01010920:1-549
HmHel-5	CAEZ01010700:88831-90112
HmHel-5	CAEZ01009078:11986-13324
HmHel-5	CAEZ01005572:1580-2862
HmHel-5	CAEZ01004741:30623-31905
HmHel-5	CAEZ01010219:1897-3181
HmHel-5	CAEZ01010219:70918-72274
HmHel-5	CAEZ01010219:151319-152673
HmHel-5	CAEZ01009321:9799-11081
HmHel-5	CAEZ01009120:80385-81579
HmHel-5	CAEZ01007695:13770-15056
HmHel-5	CAEZ01007695:1-409
HmHel-5	CAEZ01007660:20005-21368
HmHel-5	CAEZ01009592:11419-12661
HmHel-5	CAEZ01010918:115263-115680
HmHel-5	CAEZ01010918:17625-18912
HmHel-5	CAEZ01009302:22138-23423
HmHel-5	CAEZ01008178:11366-12639
HmHel-5	CAEZ01004972:47277-48560
HmHel-5	CAEZ01008616:4846-6137
HmHel-5	CAEZ01003962:295-1576
HmHel-5	CAEZ01007960:19910-21191
HmHel-5	CAEZ01007063:23103-24382
HmHel-5	CAEZ01005015:157814-159118
HmHel-5	CAEZ01005015:230862-232153
HmHel-5	CAEZ01005015:170290-171631
HmHel-5	CAEZ01003111:45249-46530
HmHel-5	CAEZ01001463:49421-50699
HmHel-5	CAEZ01009742:15773-17051
HmHel-5	CAEZ01000968:6488-7585
HmHel-5	CAEZ01000968:40796-41647
HmHel-5	CAEZ01011139:63727-65077
HmHel-5	CAEZ01011139:87048-88429
HmHel-5	CAEZ01011139:32625-33905
HmHel-5	CAEZ01010250:5590-6870
HmHel-5	CAEZ01007275:74176-75524
HmHel-5	CAEZ01007275:68763-70045
HmHel-5	CAEZ01007271:1955-3314
HmHel-5	CAEZ01007271:17770-19069
HmHel-5	CAEZ01006131:40124-41480
HmHel-5	CAEZ01006131:226934-228287
HmHel-5	CAEZ01006131:9982-10642
HmHel-5	CAEZ01004829:40225-41507
HmHel-5	CAEZ01010216:8592-9953

HmHel-5	CAEZ01001466:79482-80724
HmHel-5	CAEZ01001466:8814-10100
HmHel-5	CAEZ01011120:44705-45986
HmHel-5	CAEZ01008247:10984-12089
HmHel-5	CAEZ01008247:12091-12502
HmHel-5	CAEZ01006908:7446-8726
HmHel-5	CAEZ01004903:17475-19113
HmHel-5	CAEZ01010900:53885-55236
HmHel-5	CAEZ01007232:24613-25836
HmHel-5	CAEZ01007232:48001-49352
HmHel-5	CAEZ01007066:64882-66014
HmHel-5	CAEZ01007066:77620-78989
HmHel-5	CAEZ01007066:25760-27021
HmHel-5	CAEZ01002892:19409-20692
HmHel-5	CAEZ01001064:28047-29323
HmHel-5	CAEZ01005250:27221-28568
HmHel-5	CAEZ01005250:88582-89853
HmHel-5	CAEZ01005250:45552-46871
HmHel-5	CAEZ01001888:19035-20159
HmHel-5	CAEZ01009674:1-1327
HmHel-5	CAEZ01003401:24697-25978
HmHel-5	CAEZ01003235:87701-88945
HmHel-5	CAEZ01003235:99102-100430
HmHel-5	CAEZ01010300:3261-4479
HmHel-5	CAEZ01010272:90955-92236
HmHel-5	CAEZ01010272:1-1231
HmHel-5	CAEZ01009085:13204-14984
HmHel-5	CAEZ01009085:76779-78128
HmHel-5	CAEZ01009085:97698-98925
HmHel-5	CAEZ01009085:88403-89454
HmHel-5	CAEZ01008191:1-932
HmHel-5	CAEZ01009730:3160-4439
HmHel-5	CAEZ01009379:7532-8811
HmHel-5	CAEZ01009077:160014-161322
HmHel-5	CAEZ01007785:1-385
HmHel-5	CAEZ01007785:18379-19661
HmHel-5	CAEZ01005418:259491-263986
HmHel-5	CAEZ01005418:257625-258907
HmHel-5	CAEZ01009844:36403-37759
HmHel-5	CAEZ01006252:18676-19954
HmHel-5	CAEZ01006252:63099-64443
HmHel-5	CAEZ01006252:128988-130370
HmHel-5	CAEZ01005606:41614-42896
HmHel-5	CAEZ01005606:86750-88057
HmHel-5	CAEZ01005606:70328-71554
HmHel-5	CAEZ01005606:47848-49134
HmHel-5	CAEZ01005393:128481-129764
HmHel-5	CAEZ01005393:70089-71393

HmHel-5	CAEZ01005393:1-330
HmHel-5	CAEZ01004816:47747-49026
HmHel-5	CAEZ01001166:2235-3517
HmHel-5	CAEZ01008968:347-1622
HmHel-5	CAEZ01007385:10048-11337
HmHel-5	CAEZ01003635:34403-35663
HmHel-5	CAEZ01003192:115777-117058
HmHel-5	CAEZ01003192:121908-123254
HmHel-5	CAEZ01003192:34000-35356
HmHel-5	CAEZ01002301:460-1738
HmHel-5	CAEZ01001891:51593-52819
HmHel-5	CAEZ01001891:578-1853
HmHel-5	CAEZ01008246:7348-8638
HmHel-5	CAEZ01007845:43-977
HmHel-5	CAEZ01001556:11680-13030
HmHel-5	CAEZ01001556:3994-5343
HmHel-5	CAEZ01007721:17766-18733
HmHel-5	CAEZ01007721:25476-26842
HmHel-5	CAEZ01005183:9116-10398
HmHel-5	CAEZ01003079:4849-6115
HmHel-5	CAEZ01002646:8375-9549
HmHel-5	CAEZ01002646:19888-21142
HmHel-5	CAEZ01001496:24111-25423
HmHel-5	CAEZ01010232:5552-6814
HmHel-5	CAEZ01009975:4803-6148
HmHel-5	CAEZ01009975:71011-72355
HmHel-5	CAEZ01009975:113258-114589
HmHel-5	CAEZ01009975:136179-137536
HmHel-5	CAEZ01009975:111542-112243
HmHel-5	CAEZ01009975:56613-57895
HmHel-5	CAEZ01005489:8865-10230
HmHel-5	CAEZ01002754:15835-17114
HmHel-5	CAEZ01005221:1-920
HmHel-5	CAEZ01005221:37742-38957
HmHel-5	CAEZ01005175:1181-2395
HmHel-5	CAEZ01005175:42165-43413
HmHel-5	CAEZ01001738:12401-13763
HmHel-5	CAEZ01001738:44876-46222
HmHel-5	CAEZ01008638:3649-4654
HmHel-5	CAEZ01006350:12974-14328
HmHel-5	CAEZ01006350:37072-38002
HmHel-5	CAEZ01003137:7786-9030
HmHel-5	CAEZ01011093:134793-136069
HmHel-5	CAEZ01011093:193594-194965
HmHel-5	CAEZ01011093:22106-23463
HmHel-5	CAEZ01010270:83834-85114
HmHel-5	CAEZ01010270:146885-148250
HmHel-5	CAEZ01010270:290678-291251

HmHel-5	CAEZ01010270:182399-183747
HmHel-5	CAEZ01010270:67484-68827
HmHel-5	CAEZ01008460:10251-11537
HmHel-5	CAEZ01002713:23137-24417
HmHel-5	CAEZ01002184:1-1213
HmHel-5	CAEZ01002184:28573-29903
HmHel-5	CAEZ01008287:15752-17043
HmHel-5	CAEZ01010287:68180-69446
HmHel-5	CAEZ01004814:8172-9451
HmHel-5	CAEZ01010706:59469-60751
HmHel-5	CAEZ01010706:4006-5358
HmHel-5	CAEZ01009030:67753-68341
HmHel-5	CAEZ01009030:100950-101530
HmHel-5	CAEZ01009030:163-1445
HmHel-5	CAEZ01000942:2367-3648
HmHel-5	CAEZ01010820:13669-14977
HmHel-5	CAEZ01010820:3-793
HmHel-5	CAEZ01010461:6417-7692
HmHel-5	CAEZ01009929:42301-43520
HmHel-5	CAEZ01009929:39462-40817
HmHel-5	CAEZ01008597:12700-13979
HmHel-5	CAEZ01007951:3186-4577
HmHel-5	CAEZ01007446:27418-28696
HmHel-5	CAEZ01007446:9-696
HmHel-5	CAEZ01006823:6422-7702
HmHel-5	CAEZ01006249:45554-46837
HmHel-5	CAEZ01006249:64765-66020
HmHel-5	CAEZ01006249:25590-26922
HmHel-5	CAEZ01005097:10766-12045
HmHel-5	CAEZ01003385:16858-18139
HmHel-5	CAEZ01010285:105520-106801
HmHel-5	CAEZ01010285:51323-52663
HmHel-5	CAEZ01007372:3912-5198
HmHel-5	CAEZ01006202:20466-21750
HmHel-5	CAEZ01006202:34031-35380
HmHel-5	CAEZ01011522:34734-36017
HmHel-5	CAEZ01011230:6991-8266
HmHel-5	CAEZ01008602:6550-7803
HmHel-5	CAEZ01008602:11199-13529
HmHel-5	CAEZ01005255:1-1072
HmHel-5	CAEZ01004833:21031-22269
HmHel-5	CAEZ01010132:8881-9811
HmHel-5	CAEZ01010952:106831-107737
HmHel-5	CAEZ01010952:93228-94502
HmHel-5	CAEZ01010952:21991-23336
HmHel-5	CAEZ01010952:6536-7769
HmHel-5	CAEZ01010166:15407-16406
HmHel-5	CAEZ01006399:7690-9023

HmHel-5	CAEZ01006399:14381-15744
HmHel-5	CAEZ01005355:1309-2590
HmHel-5	CAEZ01004662:810-2161
HmHel-5	CAEZ01004662:75478-76791
HmHel-5	CAEZ01004662:48497-49811
HmHel-5	CAEZ01004662:29520-30849
HmHel-5	CAEZ01003717:113340-114694
HmHel-5	CAEZ01003717:52827-54186
HmHel-5	CAEZ01003717:32163-33441
HmHel-5	CAEZ01011495:74683-75959
HmHel-5	CAEZ01011495:93572-94445
HmHel-5	CAEZ01010450:31673-33043
HmHel-5	CAEZ01010450:6733-8011
HmHel-5	CAEZ01011259:8431-9554
HmHel-5	CAEZ01010094:16076-17001
HmHel-5	CAEZ01005025:117397-118746
HmHel-5	CAEZ01005025:109891-111244
HmHel-5	CAEZ01005025:55776-57115
HmHel-5	CAEZ01005025:2761-4051
HmHel-5	CAEZ01008182:67468-68836
HmHel-5	CAEZ01007603:23441-24748
HmHel-5	CAEZ01007603:52746-54081
HmHel-5	CAEZ01005088:37314-38624
HmHel-5	CAEZ01005088:26639-28163
HmHel-5	CAEZ01009716:203942-205102
HmHel-5	CAEZ01005827:73259-74525
HmHel-5	CAEZ01005827:76236-77330
HmHel-5	CAEZ01001898:2864-4129
HmHel-5	CAEZ01001898:6574-7858
HmHel-5	CAEZ01011410:57035-57426
HmHel-5	CAEZ01011410:37639-38920
HmHel-5	CAEZ01010455:100317-101575
HmHel-5	CAEZ01010071:15-1299
HmHel-5	CAEZ01010734:7284-8568
HmHel-5	CAEZ01007772:52260-53593
HmHel-5	CAEZ01004752:2372-3726
HmHel-5	CAEZ01004752:117792-119074
HmHel-5	CAEZ01004752:20643-21332
HmHel-5	CAEZ01003349:35068-36409
HmHel-5	CAEZ01003349:59607-60890
HmHel-5	CAEZ01001919:3955-4887
HmHel-5	CAEZ01011171:11120-12410
HmHel-5	CAEZ01011171:1130-2482
HmHel-5	CAEZ01010221:94838-96114
HmHel-5	CAEZ01008391:24280-25569
HmHel-5	CAEZ01006750:6622-7709
HmHel-5	CAEZ01006750:8536-9802
HmHel-5	CAEZ01010212:22393-23683

HmHel-5	CAEZ01009458:3592-4864
HmHel-5	CAEZ01009458:87041-87139
HmHel-5	CAEZ01009458:188590-189182
HmHel-5	CAEZ01009458:182923-183929
HmHel-5	CAEZ01010851:45305-46608
HmHel-5	CAEZ01010027:12954-14316
HmHel-5	CAEZ01009788:9837-11120
HmHel-5	CAEZ01009788:30487-31832
HmHel-5	CAEZ01009788:80663-81727
HmHel-5	CAEZ01006643:8298-9579
HmHel-5	CAEZ01001829:377-1627
HmHel-5	CAEZ01010907:114838-116199
HmHel-5	CAEZ01010335:3153-4229
HmHel-5	CAEZ01006196:61492-62767
HmHel-5	CAEZ01006196:4733-6544
HmHel-5	CAEZ01002105:1442-2801
HmHel-5	CAEZ01009223:70307-71590
HmHel-5	CAEZ01008258:76816-78124
HmHel-5	CAEZ01008258:104516-105869
HmHel-5	CAEZ01007809:1-288
HmHel-5	CAEZ01007809:279270-281596
HmHel-5	CAEZ01007380:9416-10670
HmHel-5	CAEZ01001778:1-1169
HmHel-5	CAEZ01004079:7966-9754
HmHel-5	CAEZ01003407:65244-66457
HmHel-5	CAEZ01002875:87922-89229
HmHel-5	CAEZ01002875:116605-117868
HmHel-5	CAEZ01002875:51148-52435
HmHel-5	CAEZ01006372:62714-64040
HmHel-5	CAEZ01006372:40745-43703
HmHel-5	CAEZ01005429:42908-44176
HmHel-5	CAEZ01005429:2-922
HmHel-5	CAEZ01001743:55495-56773
HmHel-5	CAEZ01009609:56571-57850
HmHel-5	CAEZ01008435:260084-261011
HmHel-5	CAEZ01008435:230916-232200
HmHel-5	CAEZ01008435:163179-164517
HmHel-5	CAEZ01001879:1257-2188
HmHel-5	CAEZ01001444:10532-11775
HmHel-5	CAEZ01001444:14012-15354
HmHel-5	CAEZ01009300:61312-62592
HmHel-5	CAEZ01009300:4864-6183
HmHel-5	CAEZ01010864:137570-138805
HmHel-5	CAEZ01008269:3595-4871
HmHel-5	CAEZ01010157:17532-18874
HmHel-5	CAEZ01009823:10750-12043
HmHel-5	CAEZ01008515:46232-47515
HmHel-5	CAEZ01001419:616-1989

HmHel-5	CAEZ01003338:10481-11843
HmHel-5	CAEZ01004983:1-1154
HmHel-5	CAEZ01011020:17591-18691
HmHel-5	CAEZ01006255:3951-5288
HmHel-5	CAEZ01008606:5766-7122
HmHel-5	CAEZ01010199:339-1623
HmHel-5	CAEZ01009996:31837-33201
HmHel-5	CAEZ01009996:66997-68348
HmHel-5	CAEZ01009996:40135-40904
HmHel-5	CAEZ01004407:1-1352
HmHel-5	CAEZ01003963:1101-2380
HmHel-5	CAEZ01007565:1057-2345
HmHel-5	CAEZ01005145:4-905
HmHel-5	CAEZ01004757:4-905
HmHel-5	CAEZ01011616:35-1374
HmHel-5	CAEZ01010493:4030-4962
HmHel-5	CAEZ01008186:9163-10509
HmHel-5	CAEZ01003854:26904-28246
HmHel-5	CAEZ01010138:509-1837
HmHel-5	CAEZ01010916:44-1420
HmHel-5	CAEZ01007224:3054-4332
HmHel-5	CAEZ01006228:12255-13460
HmHel-5	CAEZ01006228:62655-63995
HmHel-5	CAEZ01005139:6111-7369
HmHel-5	CAEZ01001550:1-1314
HmHel-5	CAEZ01007978:14941-16286
HmHel-5	CAEZ01005696:1679-2963
HmHel-5	CAEZ01006835:28321-29610
HmHel-5	CAEZ01009192:1-889
HmHel-5	CAEZ01009192:15611-15972
HmHel-5	CAEZ01004515:12977-14322
HmHel-5	CAEZ01002850:1-1338
HmHel-5	CAEZ01006218:44164-45011
HmHel-5	CAEZ01001133:6915-8180
HmHel-5	CAEZ01008356:7944-9285
HmHel-5	CAEZ01009994:4570-5914
HmHel-5	CAEZ01008525:523-1798
HmHel-5	CAEZ01008162:19212-20496
HmHel-5	CAEZ01000046:6665-8023
HmHel-5	CAEZ01007336:6697-7909
HmHel-5	CAEZ01006634:41062-42419
HmHel-5	CAEZ01003750:89065-90436
HmHel-5	CAEZ01003750:173546-174904
HmHel-5	CAEZ01003750:113344-114700
HmHel-5	CAEZ01004934:8018-9407
HmHel-5	CAEZ01007456:486-1341
HmHel-5	CAEZ01006824:8948-10194
HmHel-5	CAEZ01001775:35387-36763

HmHel-5	CAEZ01006853:22381-24042
HmHel-5	CAEZ01011292:27075-28338
HmHel-5	CAEZ01007958:18599-19903
HmHel-5	CAEZ01002742:219532-220850
HmHel-5	CAEZ01002742:154011-155221
HmHel-5	CAEZ01002742:1-563
HmHel-5	CAEZ01011411:1-1308
HmHel-5	CAEZ01007784:10887-12203
HmHel-5	CAEZ01003548:32183-32976
HmHel-5	CAEZ01003413:9323-10660
HmHel-5	CAEZ01008405:10202-11578
HmHel-5	CAEZ01007749:64204-65354
HmHel-5	CAEZ01007429:86915-88296
HmHel-5	CAEZ01007429:339-548
HmHel-5	CAEZ01008621:51804-54948
HmHel-5	CAEZ01008621:18329-19666
HmHel-5	CAEZ01005949:66334-67688
HmHel-5	CAEZ01004634:10563-11906
HmHel-5	CAEZ01009736:3327-4632
HmHel-5	CAEZ01006926:1-822
HmHel-5	CAEZ01003828:26142-27490
HmHel-5	CAEZ01003828:95781-97123
HmHel-5	CAEZ01003828:329-1630
HmHel-5	CAEZ01003557:15719-16728
HmHel-5	CAEZ01008490:938-1953
HmHel-5	CAEZ01002258:15117-16473
HmHel-5	CAEZ01006352:61680-63028
HmHel-5	CAEZ01006352:35327-36675
HmHel-5	CAEZ01005746:65827-67206
HmHel-5	CAEZ01002849:17355-18717
HmHel-5	CAEZ01001871:1-807
HmHel-5	CAEZ01003760:25608-26919
HmHel-5	CAEZ01003760:8120-9619
HmHel-5	CAEZ01010707:722-2064
HmHel-5	CAEZ01002341:6020-7345
HmHel-5	CAEZ01002341:15930-16608
HmHel-5	CAEZ01009916:2-847
HmHel-5	CAEZ01009404:40056-41403
HmHel-5	CAEZ01008921:141284-142638
HmHel-5	CAEZ01008921:94119-96446
HmHel-5	CAEZ01006638:905-2178
HmHel-5	CAEZ01006286:65434-66727
HmHel-5	CAEZ01006286:79758-81098
HmHel-5	CAEZ01006286:39921-41276
HmHel-5	CAEZ01011135:47453-48803
HmHel-5	CAEZ01011135:1399-2708
HmHel-5	CAEZ01008800:5464-6812
HmHel-5	CAEZ01005674:219-1571

HmHel-5	CAEZ01003719:23823-25173
HmHel-5	CAEZ01002924:13339-14686
HmHel-5	CAEZ01002924:140531-141890
HmHel-5	CAEZ01002924:112555-113908
HmHel-5	CAEZ01009859:10065-11389
HmHel-5	CAEZ01008179:23-750
HmHel-5	CAEZ01008179:9210-9772
HmHel-5	CAEZ01011169:3793-5145
HmHel-5	CAEZ01005496:110316-110810
HmHel-5	CAEZ01005496:68986-70333
HmHel-5	CAEZ01005496:7842-8722
HmHel-5	CAEZ01008100:80952-82273
HmHel-5	CAEZ01007061:12437-13792
HmHel-5	CAEZ01006562:226977-228309
HmHel-5	CAEZ01004273:3-806
HmHel-5	CAEZ01001454:3296-4570
HmHel-5	CAEZ01008620:175-1355
HmHel-5	CAEZ01008620:94031-95342
HmHel-5	CAEZ01008620:213717-215006
HmHel-5	CAEZ01008620:44750-46104
HmHel-5	CAEZ01005152:18614-19932
HmHel-5	CAEZ01005152:13732-14876
HmHel-5	CAEZ01004097:60104-61466
HmHel-5	CAEZ01002729:34067-35442
HmHel-5	CAEZ01002729:29839-31181
HmHel-5	CAEZ01010737:129155-130506
HmHel-5	CAEZ01010737:65172-66510
HmHel-5	CAEZ01010737:1-943
HmHel-5	CAEZ01010026:161927-163246
HmHel-5	CAEZ01010026:101921-103278
HmHel-5	CAEZ01009359:11245-12594
HmHel-5	CAEZ01008263:15346-16687
HmHel-5	CAEZ01006845:45478-46835
HmHel-5	CAEZ01005474:46329-47673
HmHel-5	CAEZ01005320:5578-6944
HmHel-5	CAEZ01003019:24325-25657
HmHel-5	CAEZ01003019:104378-105664
HmHel-5	CAEZ01003019:78676-80029
HmHel-5	CAEZ01003019:1-1285
HmHel-5	CAEZ01010974:9129-10462
HmHel-5	CAEZ01010974:14765-16032
HmHel-5	CAEZ01010915:1347-2131
HmHel-5	CAEZ01010915:17624-18962
HmHel-5	CAEZ01010915:51761-53104
HmHel-5	CAEZ01009381:10940-12108
HmHel-5	CAEZ01009381:3278-4631
HmHel-5	CAEZ01008946:7602-8896
HmHel-5	CAEZ01008230:8-928

HmHel-5	CAEZ01005850:711-2076
HmHel-5	CAEZ01004038:15456-16788
HmHel-5	CAEZ01004038:27868-29203
HmHel-5	CAEZ01010496:1-1141
HmHel-5	CAEZ01008183:50247-51612
HmHel-5	CAEZ01008183:71973-73321
HmHel-5	CAEZ01002991:70046-71262
HmHel-5	CAEZ01002780:10666-12019
HmHel-5	CAEZ01002736:21162-22518
HmHel-5	CAEZ01008423:49470-50820
HmHel-5	CAEZ01008106:1-442
HmHel-5	CAEZ01008106:19477-20843
HmHel-5	CAEZ01007018:6059-7401
HmHel-5	CAEZ01006400:27699-28985
HmHel-5	CAEZ01006400:22063-23379
HmHel-5	CAEZ01006400:5354-6671
HmHel-5	CAEZ01003761:63-1381
HmHel-5	CAEZ01009984:10831-12187
HmHel-5	CAEZ01009186:20914-22269
HmHel-5	CAEZ01008499:5900-7253
HmHel-5	CAEZ01008499:28732-30077
HmHel-5	CAEZ01008443:16263-17611
HmHel-5	CAEZ01008418:82413-83741
HmHel-5	CAEZ01005810:20330-21039
HmHel-5	CAEZ01004811:165608-166966
HmHel-5	CAEZ01004811:434393-434773
HmHel-5	CAEZ01004811:19068-19976
HmHel-5	CAEZ01004042:3498-4418
HmHel-5	CAEZ01003006:11457-12808
HmHel-5	CAEZ01000820:33705-35042
HmHel-5	CAEZ01011307:552-2332
HmHel-5	CAEZ01009258:14279-15626
HmHel-5	CAEZ01008260:29645-30974
HmHel-5	CAEZ01007975:37790-39082
HmHel-5	CAEZ01007975:45072-46428
HmHel-5	CAEZ01002772:34125-35395
HmHel-5	CAEZ01002383:21982-23323
HmHel-5	CAEZ01000802:2631-3986
HmHel-5	CAEZ01011651:17334-18607
HmHel-5	CAEZ01007329:11064-12416
HmHel-5	CAEZ01007329:40770-42108
HmHel-5	CAEZ01005475:145702-147047
HmHel-5	CAEZ01002814:186230-187593
HmHel-5	CAEZ01001510:32891-33759
HmHel-5	CAEZ01001510:10710-12064
HmHel-5	CAEZ01001025:16016-17193
HmHel-5	CAEZ01000070:5091-6198
HmHel-5	CAEZ01011050:3403-4754

HmHel-5	CAEZ01010885:1246-2592
HmHel-5	CAEZ01008615:1402-2743
HmHel-5	CAEZ01008614:49336-50142
HmHel-5	CAEZ01008614:2-1004
HmHel-5	CAEZ01007662:1-1152
HmHel-5	CAEZ01005373:120290-121612
HmHel-5	CAEZ01003345:25823-27161
HmHel-5	CAEZ01003345:49100-50454
HmHel-5	CAEZ01010507:5062-6392
HmHel-5	CAEZ01010507:6890-7446
HmHel-5	CAEZ01008425:126875-127645
HmHel-5	CAEZ01008425:99244-100597
HmHel-5	CAEZ01005465:6186-7367
HmHel-5	CAEZ01004226:23459-24550
HmHel-5	CAEZ01011137:18715-20113
HmHel-5	CAEZ01010361:8874-10193
HmHel-5	CAEZ01010237:17123-18475
HmHel-5	CAEZ01010237:59553-60912
HmHel-5	CAEZ01010174:12955-14304
HmHel-5	CAEZ01008920:11400-12753
HmHel-5	CAEZ01004524:7460-8189
HmHel-5	CAEZ01004096:25397-26745
HmHel-5	CAEZ01004096:41896-43023
HmHel-5	CAEZ01002238:1585-2944
HmHel-5	CAEZ01011417:2904-4258
HmHel-5	CAEZ01009170:11999-13266
HmHel-5	CAEZ01005154:592-1903
HmHel-5	CAEZ01004117:1-797
HmHel-5	CAEZ01011179:27594-28934
HmHel-5	CAEZ01008404:90174-91466
HmHel-5	CAEZ01007993:2958-4251
HmHel-5	CAEZ01006098:31159-32511
HmHel-5	CAEZ01006098:21100-22448
HmHel-5	CAEZ01005392:83453-84334
HmHel-5	CAEZ01005392:1-1376
HmHel-5	CAEZ01002589:1307-1744
HmHel-5	CAEZ01002589:19870-21225
HmHel-5	CAEZ01001890:1-866
HmHel-5	CAEZ01001890:10862-12081
HmHel-5	CAEZ01001403:36663-37949
HmHel-5	CAEZ01010703:14154-15340
HmHel-5	CAEZ01010703:56759-58098
HmHel-5	CAEZ01009982:30022-32375
HmHel-5	CAEZ01009982:65167-66519
HmHel-5	CAEZ01009982:107843-109162
HmHel-5	CAEZ01009209:10248-11602
HmHel-5	CAEZ01008367:149563-150912
HmHel-5	CAEZ01008367:125633-126994

HmHel-5	CAEZ01008367:28724-29857
HmHel-5	CAEZ01007982:4876-6220
HmHel-5	CAEZ01007982:16253-17563
HmHel-5	CAEZ01007475:83573-84901
HmHel-5	CAEZ01005388:558-1825
HmHel-5	CAEZ01005388:1-326
HmHel-5	CAEZ01004734:1-1342
HmHel-5	CAEZ01003747:25915-27265
HmHel-5	CAEZ01000907:2061-3395
HmHel-5	CAEZ01009274:20499-21777
HmHel-5	CAEZ01008122:1-162
HmHel-5	CAEZ01008122:68575-69086
HmHel-5	CAEZ01008122:11396-12750
HmHel-5	CAEZ01006577:2892-4222
HmHel-5	CAEZ01006346:11081-12212
HmHel-5	CAEZ01006148:6160-7636
HmHel-5	CAEZ01006061:7397-8732
HmHel-5	CAEZ01004534:8446-9429
HmHel-5	CAEZ01003863:12030-13376
HmHel-5	CAEZ01003610:17827-19183
HmHel-5	CAEZ01003496:14705-16002
HmHel-5	CAEZ01003247:23772-25119
HmHel-5	CAEZ01003247:12142-13103
HmHel-5	CAEZ01002767:40870-42196
HmHel-5	CAEZ01009417:33300-34659
HmHel-5	CAEZ01008234:20145-20371
HmHel-5	CAEZ01008234:37316-38666
HmHel-5	CAEZ01008234:153858-155210
HmHel-5	CAEZ01008234:99259-100410
HmHel-5	CAEZ01008234:635-1578
HmHel-5	CAEZ01007373:565-1904
HmHel-5	CAEZ01006693:504-1772
HmHel-5	CAEZ01005416:48078-50607
HmHel-5	CAEZ01005416:52410-53762
HmHel-5	CAEZ01005163:26618-27697
HmHel-5	CAEZ01004387:26-754
HmHel-5	CAEZ01000839:10985-12318
HmHel-5	CAEZ01000839:23358-24696
HmHel-5	CAEZ01011529:35786-37142
HmHel-5	CAEZ01009894:32525-33882
HmHel-5	CAEZ01008915:73041-74381
HmHel-5	CAEZ01008721:44910-46262
HmHel-5	CAEZ01008721:205903-207221
HmHel-5	CAEZ01007441:5717-6416
HmHel-5	CAEZ01006149:25867-27219
HmHel-5	CAEZ01005981:20958-22198
HmHel-5	CAEZ01005981:9143-10407
HmHel-5	CAEZ01005942:22304-23662

HmHel-5	CAEZ01004631:260178-261051
HmHel-5	CAEZ01004631:320627-321984
HmHel-5	CAEZ01003363:712-2518
HmHel-5	CAEZ01002361:52228-53481
HmHel-5	CAEZ01002361:16719-18064
HmHel-5	CAEZ01010667:53843-53990
HmHel-5	CAEZ01010667:29467-30807
HmHel-5	CAEZ01010278:22113-23463
HmHel-5	CAEZ01009845:15456-16664
HmHel-5	CAEZ01009845:22679-23997
HmHel-5	CAEZ01009767:2959-4292
HmHel-5	CAEZ01009766:63343-63922
HmHel-5	CAEZ01009766:59487-60827
HmHel-5	CAEZ01009766:42864-44207
HmHel-5	CAEZ01008383:5707-7064
HmHel-5	CAEZ01007754:28947-30297
HmHel-5	CAEZ01007222:230577-231891
HmHel-5	CAEZ01007222:266102-267406
HmHel-5	CAEZ01007222:208677-209811
HmHel-5	CAEZ01006227:1737-3071
HmHel-5	CAEZ01005669:37022-37906
HmHel-5	CAEZ01005400:1-1212
HmHel-5	CAEZ01004740:34559-35893
HmHel-5	CAEZ01004724:11659-13004
HmHel-5	CAEZ01002663:11415-12632
HmHel-5	CAEZ01001416:1-1069
HmHel-5	CAEZ01010580:4241-5522
HmHel-5	CAEZ01008784:13275-14630
HmHel-5	CAEZ01008398:27605-28955
HmHel-5	CAEZ01008398:78248-79644
HmHel-5	CAEZ01005934:623-1727
HmHel-5	CAEZ01005716:69120-70472
HmHel-5	CAEZ01005438:20061-21402
HmHel-5	CAEZ01004656:31857-32984
HmHel-5	CAEZ01003322:4826-6214
HmHel-5	CAEZ01002634:3283-4555
HmHel-5	CAEZ01002634:17067-18387
HmHel-5	CAEZ01008437:53746-54780
HmHel-5	CAEZ01008437:74798-76115
HmHel-5	CAEZ01008099:2464-3580
HmHel-5	CAEZ01007937:28383-29731
HmHel-5	CAEZ01007609:5087-6771
HmHel-5	CAEZ01007609:45431-47216
HmHel-5	CAEZ01007609:18387-19654
HmHel-5	CAEZ01006970:9009-10323
HmHel-5	CAEZ01006618:40045-41319
HmHel-5	CAEZ01006618:275354-276703
HmHel-5	CAEZ01006618:213110-214383

HmHel-5	CAEZ01005425:20980-22314
HmHel-5	CAEZ01009807:15495-16853
HmHel-5	CAEZ01009807:20262-20793
HmHel-5	CAEZ01009791:562-1918
HmHel-5	CAEZ01009336:4803-6150
HmHel-5	CAEZ01007428:7176-8313
HmHel-5	CAEZ01006169:10311-11645
HmHel-5	CAEZ01005894:9107-10147
HmHel-5	CAEZ01005840:6081-7435
HmHel-5	CAEZ01002662:541-1798
HmHel-5	CAEZ01001326:43344-44548
HmHel-5	CAEZ01001326:11516-12851
HmHel-5	CAEZ01008176:8144-10386
HmHel-5	CAEZ01008043:8937-9730
HmHel-5	CAEZ01004793:12095-13373
HmHel-5	CAEZ01004793:123951-125121
HmHel-5	CAEZ01001349:1-745
HmHel-5	CAEZ01001349:71798-72155
HmHel-5	CAEZ01001349:1511-2888
HmHel-5	CAEZ01011272:4683-6052
HmHel-5	CAEZ01008950:35-786
HmHel-5	CAEZ01008884:54465-55772
HmHel-5	CAEZ01008331:4487-5852
HmHel-5	CAEZ01006272:5-495
HmHel-5	CAEZ01006272:1319-2040
HmHel-5	CAEZ01005269:76684-78108
HmHel-5	CAEZ01005269:36362-37540
HmHel-5	CAEZ01004136:9464-10825
HmHel-5	CAEZ01003894:4464-5786
HmHel-5	CAEZ01003746:43173-44513
HmHel-5	CAEZ01002904:13236-14586
HmHel-5	CAEZ01002904:2-550
HmHel-5	CAEZ01002108:116-984
HmHel-5	CAEZ01010926:126369-127703
HmHel-5	CAEZ01010819:7675-8980
HmHel-5	CAEZ01010745:1732-3053
HmHel-5	CAEZ01008961:50141-51492
HmHel-5	CAEZ01006749:39328-40650
HmHel-5	CAEZ01006632:15780-17119
HmHel-5	CAEZ01005491:8950-10057
HmHel-5	CAEZ01005443:15082-17636
HmHel-5	CAEZ01005426:18620-19895
HmHel-5	CAEZ01001007:1-719
HmHel-5	CAEZ01011132:43255-44590
HmHel-5	CAEZ01011132:26-440
HmHel-5	CAEZ01008091:84036-85318
HmHel-5	CAEZ01008091:34108-35440
HmHel-5	CAEZ01003081:6474-7291

HmHel-5	CAEZ01001449:14915-16136
HmHel-5	CAEZ01010581:11123-12479
HmHel-5	CAEZ01008431:25573-26929
HmHel-5	CAEZ01007631:50078-51422
HmHel-5	CAEZ01004449:2405-3741
HmHel-5	CAEZ01011278:183-1493
HmHel-5	CAEZ01010755:13938-15053
HmHel-5	CAEZ01010704:44448-45770
HmHel-5	CAEZ01010370:6955-8310
HmHel-5	CAEZ01010218:51077-52455
HmHel-5	CAEZ01010218:66717-67248
HmHel-5	CAEZ01010218:37359-38664
HmHel-5	CAEZ01010130:44836-46189
HmHel-5	CAEZ01010130:20759-21958
HmHel-5	CAEZ01009033:13420-14767
HmHel-5	CAEZ01008194:34387-35706
HmHel-5	CAEZ01008194:20158-21511
HmHel-5	CAEZ01006491:4-476
HmHel-5	CAEZ01006491:10589-13296
HmHel-5	CAEZ01006491:26205-26343
HmHel-5	CAEZ01006491:10807-12151
HmHel-5	CAEZ01005964:24089-25405
HmHel-5	CAEZ01005964:1-1048
HmHel-5	CAEZ01005750:131-1054
HmHel-5	CAEZ01005270:24896-26254
HmHel-5	CAEZ01005270:10762-11581
HmHel-5	CAEZ01003645:10837-12167
HmHel-5	CAEZ01003346:44197-45534
HmHel-5	CAEZ01003346:53751-55082
HmHel-5	CAEZ01003231:10489-11818
HmHel-5	CAEZ01010702:10070-12111
HmHel-5	CAEZ01009145:18273-18484
HmHel-5	CAEZ01009145:8230-9559
HmHel-5	CAEZ01009023:95711-96636
HmHel-5	CAEZ01009023:82791-83473
HmHel-5	CAEZ01005038:3467-4997
HmHel-5	CAEZ01005038:585-948
HmHel-5	CAEZ01004150:6442-6580
HmHel-5	CAEZ01004150:1431-2778
HmHel-5	CAEZ01002866:160132-161477
HmHel-5	CAEZ01002866:82168-82969
HmHel-5	CAEZ01009456:152811-154137
HmHel-5	CAEZ01009456:55357-56710
HmHel-5	CAEZ01008919:72625-73775
HmHel-5	CAEZ01008919:83565-83799
HmHel-5	CAEZ01008619:98193-98342
HmHel-5	CAEZ01008619:4040-5391
HmHel-5	CAEZ01006617:51269-52575

HmHel-5	CAEZ01006617:28201-29536
HmHel-5	CAEZ01006283:46483-47371
HmHel-5	CAEZ01004930:17380-18724
HmHel-5	CAEZ01004771:5544-6881
HmHel-5	CAEZ01004259:56670-57917
HmHel-5	CAEZ01004259:18413-19767
HmHel-5	CAEZ01008245:5577-6928
HmHel-5	CAEZ01007606:1-717
HmHel-5	CAEZ01005511:847-2182
HmHel-5	CAEZ01001526:6584-7934
HmHel-5	CAEZ01010691:2696-4042
HmHel-5	CAEZ01008598:13-1368
HmHel-5	CAEZ01008018:3200-4488
HmHel-5	CAEZ01006616:240148-241499
HmHel-5	CAEZ01006616:78339-79572
HmHel-5	CAEZ01006416:10391-11729
HmHel-5	CAEZ01006073:63270-64620
HmHel-5	CAEZ01005473:26565-27911
HmHel-5	CAEZ01005198:76381-77699
HmHel-5	CAEZ01005198:22666-24002
HmHel-5	CAEZ01003626:8125-9468
HmHel-5	CAEZ01003105:4710-6059
HmHel-5	CAEZ01010725:1199-2538
HmHel-5	CAEZ01010127:89459-90810
HmHel-5	CAEZ01009891:70083-71436
HmHel-5	CAEZ01009891:7983-9335
HmHel-5	CAEZ01009011:7952-9159
HmHel-5	CAEZ01004832:28999-30314
HmHel-5	CAEZ01000823:1-1316
HmHel-5	CAEZ01010437:61535-62889
HmHel-5	CAEZ01008963:181687-183029
HmHel-5	CAEZ01008963:120788-123767
HmHel-5	CAEZ01008963:105220-106569
HmHel-5	CAEZ01008963:62174-63259
HmHel-5	CAEZ01006176:5331-7804
HmHel-5	CAEZ01006053:4897-6243
HmHel-5	CAEZ01004011:55746-58205
HmHel-5	CAEZ01004011:148754-150077
HmHel-5	CAEZ01004011:99136-100417
HmHel-5	CAEZ01004011:26037-27411
HmHel-5	CAEZ01002147:1-466
HmHel-5	CAEZ01002147:5600-6927
HmHel-5	CAEZ01001850:33527-34861
HmHel-5	CAEZ01001239:1578-2860
HmHel-5	CAEZ01001239:6186-6699
HmHel-5	CAEZ01009435:65735-66980
HmHel-5	CAEZ01009435:43122-44464
HmHel-5	CAEZ01008294:52571-55120

HmHel-5	CAEZ01008294:152626-153744
HmHel-5	CAEZ01008294:194082-195383
HmHel-5	CAEZ01003881:13709-15056
HmHel-5	CAEZ01003881:40248-41511
HmHel-5	CAEZ01003881:806-2285
HmHel-5	CAEZ01003208:31503-33301
HmHel-5	CAEZ01002289:449-1570
HmHel-5	CAEZ01001908:6921-8267
HmHel-5	CAEZ01011027:8007-9300
HmHel-5	CAEZ01011007:6695-8078
HmHel-5	CAEZ01009969:29384-31866
HmHel-5	CAEZ01009828:28193-29548
HmHel-5	CAEZ01009460:8557-9909
HmHel-5	CAEZ01009460:34910-35615
HmHel-5	CAEZ01009279:1632-3358
HmHel-5	CAEZ01007730:75245-76550
HmHel-5	CAEZ01007730:89414-90762
HmHel-5	CAEZ01007730:1318-2665
HmHel-5	CAEZ01007498:17037-18165
HmHel-5	CAEZ01007498:38329-39658
HmHel-5	CAEZ01007498:64224-65596
HmHel-5	CAEZ01007463:12575-13829
HmHel-5	CAEZ01006479:4645-6000
HmHel-5	CAEZ01005711:163149-164498
HmHel-5	CAEZ01005478:70332-71649
HmHel-5	CAEZ01005478:24338-25645
HmHel-5	CAEZ01004452:55256-56596
HmHel-5	CAEZ01004452:22384-23693
HmHel-5	CAEZ01004213:1308-2639
HmHel-5	CAEZ01002321:64306-64999
HmHel-5	CAEZ01002321:48296-48772
HmHel-5	CAEZ01011293:1148-2418
HmHel-5	CAEZ01011149:6180-6968
HmHel-5	CAEZ01009928:42495-43828
HmHel-5	CAEZ01009806:2357-3647
HmHel-5	CAEZ01008751:86202-87489
HmHel-5	CAEZ01008719:54440-55793
HmHel-5	CAEZ01008603:25240-26551
HmHel-5	CAEZ01007731:92762-92889
HmHel-5	CAEZ01007731:72785-74120
HmHel-5	CAEZ01007090:11416-12570
HmHel-5	CAEZ01006548:99537-102023
HmHel-5	CAEZ01006548:64485-65790
HmHel-5	CAEZ01005458:15935-17283
HmHel-5	CAEZ01004867:1-824
HmHel-5	CAEZ01003305:21-740
HmHel-5	CAEZ01003260:82893-84128
HmHel-5	CAEZ01003171:3668-5019

HmHel-5	CAEZ01001965:18806-20158
HmHel-5	CAEZ01001965:26935-28285
HmHel-5	CAEZ01011494:35481-36745
HmHel-5	CAEZ01011143:740-1921
HmHel-5	CAEZ01009583:20975-22347
HmHel-5	CAEZ01009583:89837-90582
HmHel-5	CAEZ01009583:4400-5529
HmHel-5	CAEZ01008576:4066-5425
HmHel-5	CAEZ01008576:70580-71931
HmHel-5	CAEZ01007765:262652-263955
HmHel-5	CAEZ01006369:27083-28398
HmHel-5	CAEZ01005514:2478-3751
HmHel-5	CAEZ01003827:1115-2172
HmHel-5	CAEZ01011285:48525-49872
HmHel-5	CAEZ01006772:17026-18363
HmHel-5	CAEZ01006130:198537-199836
HmHel-5	CAEZ01006130:62634-63963
HmHel-5	CAEZ01005396:6371-7727
HmHel-5	CAEZ01005396:20449-21814
HmHel-5	CAEZ01001033:4409-5677
HmHel-5	CAEZ01009586:4086-6533
HmHel-5	CAEZ01009586:58630-59948
HmHel-5	CAEZ01009380:24155-25475
HmHel-5	CAEZ01007621:7409-8747
HmHel-5	CAEZ01006534:15693-17050
HmHel-5	CAEZ01006300:30-948
HmHel-5	CAEZ01005268:11547-12829
HmHel-5	CAEZ01005268:9647-11005
HmHel-5	CAEZ01003987:1989-3349
HmHel-5	CAEZ01003933:26681-28007
HmHel-5	CAEZ01003933:11189-12473
HmHel-5	CAEZ01003722:47823-49160
HmHel-5	CAEZ01003722:20548-21882
HmHel-5	CAEZ01003718:1211-2561
HmHel-5	CAEZ01001877:1-793
HmHel-5	CAEZ01011055:726-1947
HmHel-5	CAEZ01010795:26109-28043
HmHel-5	CAEZ01009044:135357-137688
HmHel-5	CAEZ01009044:35989-37365
HmHel-5	CAEZ01008879:58539-61716
HmHel-5	CAEZ01007769:14675-16013
HmHel-5	CAEZ01006503:7479-8837
HmHel-5	CAEZ01006434:30160-31416
HmHel-5	CAEZ01003222:73786-75101
HmHel-5	CAEZ01000967:4460-5640
HmHel-5	CAEZ01011631:2-979
HmHel-5	CAEZ01010139:84280-85625
HmHel-5	CAEZ01010139:111192-111953

HmHel-5	CAEZ01010139:61400-62736
HmHel-5	CAEZ01010139:30805-32156
HmHel-5	CAEZ01008994:1-1059
HmHel-5	CAEZ01011088:1636-2970
HmHel-5	CAEZ01009801:60126-61457
HmHel-5	CAEZ01009801:44583-45919
HmHel-5	CAEZ01008713:17731-18969
HmHel-5	CAEZ01008713:134138-135405
HmHel-5	CAEZ01007812:153159-154522
HmHel-5	CAEZ01007812:243218-244543
HmHel-5	CAEZ01007812:98058-99400
HmHel-5	CAEZ01007747:77192-78525
HmHel-5	CAEZ01007747:241002-242272
HmHel-5	CAEZ01006433:43854-45156
HmHel-5	CAEZ01006433:21882-23241
HmHel-5	CAEZ01004179:1-1271
HmHel-5	CAEZ01003875:4859-6206
HmHel-5	CAEZ01003875:14672-15996
HmHel-5	CAEZ01009638:19662-20997
HmHel-5	CAEZ01006423:292743-294054
HmHel-5	CAEZ01006423:334027-335335
HmHel-5	CAEZ01006423:274830-276069
HmHel-5	CAEZ01006423:225539-226864
HmHel-5	CAEZ01006230:25254-26570
HmHel-5	CAEZ01006007:42826-44117
HmHel-5	CAEZ01006007:13326-14616
HmHel-5	CAEZ01005093:11951-13101
HmHel-5	CAEZ01003149:13427-14308
HmHel-5	CAEZ01001579:32489-33809
HmHel-5	CAEZ01001579:55238-57122
HmHel-5	CAEZ01001579:10742-12121
HmHel-5	CAEZ01001214:1-697
HmHel-5	CAEZ01011547:2096-3407
HmHel-5	CAEZ01010701:154064-155400
HmHel-5	CAEZ01010252:9050-10385
HmHel-5	CAEZ01009629:1-842
HmHel-5	CAEZ01008771:19110-25402
HmHel-5	CAEZ01008771:108347-109678
HmHel-5	CAEZ01007272:6626-7968
HmHel-5	CAEZ01007272:38851-40178
HmHel-5	CAEZ01005977:114497-117036
HmHel-5	CAEZ01005430:16344-17696
HmHel-5	CAEZ01005428:100811-101964
HmHel-5	CAEZ01005428:126366-128744
HmHel-5	CAEZ01002813:2-843
HmHel-5	CAEZ01001765:72496-73789
HmHel-5	CAEZ01001765:27814-29161
HmHel-5	CAEZ01000935:1525-2884

HmHel-5	CAEZ01010451:71855-73178
HmHel-5	CAEZ01010451:89372-90671
HmHel-5	CAEZ01009153:20399-21150
HmHel-5	CAEZ01009153:19533-20250
HmHel-5	CAEZ01007638:1-1009
HmHel-5	CAEZ01005722:21176-22303
HmHel-5	CAEZ01004538:4758-6067
HmHel-5	CAEZ01004270:24666-26003
HmHel-5	CAEZ01004076:2637-3993
HmHel-5	CAEZ01003575:8248-9407
HmHel-5	CAEZ01010535:9-1286
HmHel-5	CAEZ01010291:2513-3836
HmHel-5	CAEZ01009899:16640-17841
HmHel-5	CAEZ01009224:56508-56918
HmHel-5	CAEZ01009224:1527-2859
HmHel-5	CAEZ01008105:25732-27092
HmHel-5	CAEZ01008105:129887-130708
HmHel-5	CAEZ01007297:1439-2661
HmHel-5	CAEZ01006529:1-424
HmHel-5	CAEZ01006529:7484-8825
HmHel-5	CAEZ01006184:34-1043
HmHel-5	CAEZ01003415:8860-10217
HmHel-5	CAEZ01003415:67031-68391
HmHel-5	CAEZ01002401:1147-1929
HmHel-5	CAEZ01002363:9377-10732
HmHel-5	CAEZ01011554:11001-12386
HmHel-5	CAEZ01010444:6628-7962
HmHel-5	CAEZ01009999:55810-57002
HmHel-5	CAEZ01009974:13318-14654
HmHel-5	CAEZ01009484:8380-9747
HmHel-5	CAEZ01009212:47496-48411
HmHel-5	CAEZ01008261:107941-109289
HmHel-5	CAEZ01007184:257296-258632
HmHel-5	CAEZ01007184:3736-5057
HmHel-5	CAEZ01007177:8595-9959
HmHel-5	CAEZ01005319:28530-29839
HmHel-5	CAEZ01003647:5016-6371
HmHel-5	CAEZ01003075:28224-29579
HmHel-5	CAEZ01002097:38756-40113
HmHel-5	CAEZ01002097:62953-64247
HmHel-5	CAEZ01001233:6892-8237
HmHel-5	CAEZ01001233:11-417
HmHel-5	CAEZ01001196:46159-47479
HmHel-5	CAEZ01001147:25912-27248
HmHel-5	CAEZ01010257:18118-19454
HmHel-5	CAEZ01010039:52224-52308
HmHel-5	CAEZ01010039:101157-102490
HmHel-5	CAEZ01010039:191519-192851

HmHel-5	CAEZ01008420:162218-163548
HmHel-5	CAEZ01008420:8219-9463
HmHel-5	CAEZ01006370:7621-8993
HmHel-5	CAEZ01006370:40367-41706
HmHel-5	CAEZ01006008:10458-11794
HmHel-5	CAEZ01006008:1-305
HmHel-5	CAEZ01004822:86926-89370
HmHel-5	CAEZ01003099:7343-8683
HmHel-5	CAEZ01002984:19141-21659
HmHel-5	CAEZ01001330:7377-8723
HmHel-5	CAEZ01010868:50543-51858
HmHel-5	CAEZ01010161:465-1589
HmHel-5	CAEZ01009481:34530-35551
HmHel-5	CAEZ01009481:48115-48198
HmHel-5	CAEZ01009481:8317-9656
HmHel-5	CAEZ01006495:1-719
HmHel-5	CAEZ01006495:25423-25950
HmHel-5	CAEZ01003929:1085-2421
HmHel-5	CAEZ01003265:4092-5414
HmHel-5	CAEZ01003248:70056-71404
HmHel-5	CAEZ01010770:30472-31827
HmHel-5	CAEZ01010347:3167-4407
HmHel-5	CAEZ01008734:58994-60333
HmHel-5	CAEZ01008121:371-1461
HmHel-5	CAEZ01008121:21730-22705
HmHel-5	CAEZ01007207:36379-37723
HmHel-5	CAEZ01005259:6342-7697
HmHel-5	CAEZ01005219:36996-38343
HmHel-5	CAEZ01004810:1-1027
HmHel-5	CAEZ01002811:118077-119337
HmHel-5	CAEZ01002811:153968-155256
HmHel-5	CAEZ01002172:4938-7191
HmHel-5	CAEZ01010028:68186-69290
HmHel-5	CAEZ01009770:103473-104791
HmHel-5	CAEZ01009770:119753-120925
HmHel-5	CAEZ01009025:24837-26169
HmHel-5	CAEZ01007939:40-959
HmHel-5	CAEZ01007939:56822-57927
HmHel-5	CAEZ01007499:131055-132398
HmHel-5	CAEZ01007499:158023-159316
HmHel-5	CAEZ01006101:34-945
HmHel-5	CAEZ01005924:25510-26844
HmHel-5	CAEZ01005721:33562-34897
HmHel-5	CAEZ01005721:81221-82546
HmHel-5	CAEZ01002255:2307-3667
HmHel-5	CAEZ01002255:10-568
HmHel-5	CAEZ01002180:92309-93645
HmHel-5	CAEZ01001446:144455-145671

HmHel-5	CAEZ01001446:126511-127798
HmHel-5	CAEZ01000067:3404-4740
HmHel-5	CAEZ01009066:4245-5577
HmHel-5	CAEZ01009066:16-855
HmHel-5	CAEZ01007700:3384-4727
HmHel-5	CAEZ01003077:60129-61151
HmHel-5	CAEZ01002620:15896-18046
HmHel-5	CAEZ01002620:1954-5533
HmHel-5	CAEZ01002015:24219-25534
HmHel-5	CAEZ01011399:30052-30976
HmHel-5	CAEZ01010827:8251-9626
HmHel-5	CAEZ01010827:384402-385676
HmHel-5	CAEZ01009887:225570-227731
HmHel-5	CAEZ01009887:167661-168995
HmHel-5	CAEZ01009887:4428-5792
HmHel-5	CAEZ01008360:37502-38725
HmHel-5	CAEZ01008360:43129-43817
HmHel-5	CAEZ01008360:30001-31327
HmHel-5	CAEZ01007611:1108-2447
HmHel-5	CAEZ01006299:27363-27497
HmHel-5	CAEZ01006299:47252-48602
HmHel-5	CAEZ01006030:8459-9264
HmHel-5	CAEZ01005954:4473-5808
HmHel-5	CAEZ01005290:27395-28752
HmHel-5	CAEZ01004633:724-2066
HmHel-5	CAEZ01001968:933-2242
HmHel-5	CAEZ01001665:802-2107
HmHel-5	CAEZ01011636:12908-13695
HmHel-5	CAEZ01009346:19898-21209
HmHel-5	CAEZ01009306:86077-87402
HmHel-5	CAEZ01007530:8039-9044
HmHel-5	CAEZ01006992:2967-4301
HmHel-5	CAEZ01005910:23114-24470
HmHel-5	CAEZ01005910:27686-28417
HmHel-5	CAEZ01002332:32742-34085
HmHel-5	CAEZ01002098:15502-16849
HmHel-5	CAEZ01000858:20198-21181
HmHel-5	CAEZ01010893:68770-70087
HmHel-5	CAEZ01009762:4753-6040
HmHel-5	CAEZ01008655:8-698
HmHel-5	CAEZ01007941:154800-156130
HmHel-5	CAEZ01007620:24531-25586
HmHel-5	CAEZ01007325:58147-59500
HmHel-5	CAEZ01006758:6595-7929
HmHel-5	CAEZ01006417:149976-151332
HmHel-5	CAEZ01004362:10895-12230
HmHel-5	CAEZ01003653:5242-6550
HmHel-5	CAEZ01003629:16346-17731

HmHel-5	CAEZ01003629:9017-10326
HmHel-5	CAEZ01001802:5624-9342
HmHel-5	CAEZ01011437:6142-7469
HmHel-5	CAEZ01011414:95319-95729
HmHel-5	CAEZ01011414:19044-21522
HmHel-5	CAEZ01010584:24434-25591
HmHel-5	CAEZ01007370:18522-19443
HmHel-5	CAEZ01007245:1821-3132
HmHel-5	CAEZ01005556:9589-10960
HmHel-5	CAEZ01003138:21977-23309
HmHel-5	CAEZ01002603:6222-7571
HmHel-5	CAEZ01001940:467-1770
HmHel-5	CAEZ01009495:13358-14558
HmHel-5	CAEZ01008327:65751-67071
HmHel-5	CAEZ01008327:13430-14727
HmHel-5	CAEZ01007259:84107-85464
HmHel-5	CAEZ01000849:86724-88059
HmHel-5	CAEZ01011545:6571-7929
HmHel-5	CAEZ01010865:8074-9424
HmHel-5	CAEZ01009268:52182-53540
HmHel-5	CAEZ01009233:3-386
HmHel-5	CAEZ01009233:15018-16404
HmHel-5	CAEZ01009068:260-1621
HmHel-5	CAEZ01007426:4074-5431
HmHel-5	CAEZ01007140:30270-31544
HmHel-5	CAEZ01006129:5244-6588
HmHel-5	CAEZ01006002:15535-16741
HmHel-5	CAEZ01005741:413-1691
HmHel-5	CAEZ01005693:17285-18630
HmHel-5	CAEZ01005114:13616-14958
HmHel-5	CAEZ01004796:14480-15817
HmHel-5	CAEZ01003175:32768-34119
HmHel-5	CAEZ01010969:242594-243323
HmHel-5	CAEZ01010969:106718-108080
HmHel-5	CAEZ01010969:17728-20303
HmHel-5	CAEZ01010735:14997-16343
HmHel-5	CAEZ01009989:68549-69837
HmHel-5	CAEZ01007983:120550-120910
HmHel-5	CAEZ01007983:6690-8034
HmHel-5	CAEZ01007728:11650-12986
HmHel-5	CAEZ01007728:4053-5392
HmHel-5	CAEZ01007601:8509-9366
HmHel-5	CAEZ01007601:21884-23228
HmHel-5	CAEZ01007601:69-648
HmHel-5	CAEZ01007413:14539-15827
HmHel-5	CAEZ01007413:74251-75871
HmHel-5	CAEZ01006883:52-566
HmHel-5	CAEZ01006883:1731-3054

HmHel-5	CAEZ01006014:2643-3995
HmHel-5	CAEZ01005860:1-458
HmHel-5	CAEZ01005860:30225-31554
HmHel-5	CAEZ01005083:53384-53947
HmHel-5	CAEZ01005083:94805-95390
HmHel-5	CAEZ01005083:62256-63610
HmHel-5	CAEZ01005083:29130-30455
HmHel-5	CAEZ01004755:14142-15474
HmHel-5	CAEZ01004755:329-1343
HmHel-5	CAEZ01002170:1-1284
HmHel-5	CAEZ01001435:168657-169985
HmHel-5	CAEZ01001088:312-1040
HmHel-5	CAEZ01010863:38218-39590
HmHel-5	CAEZ01010863:18182-20802
HmHel-5	CAEZ01010112:1494-2776
HmHel-5	CAEZ01009150:44081-44300
HmHel-5	CAEZ01009150:44304-44963
HmHel-5	CAEZ01009150:13486-14820
HmHel-5	CAEZ01008926:26056-27359
HmHel-5	CAEZ01008691:1443-2812
HmHel-5	CAEZ01008532:86047-87420
HmHel-5	CAEZ01006675:47890-49206
HmHel-5	CAEZ01006675:62988-64333
HmHel-5	CAEZ01005558:190-1298
HmHel-5	CAEZ01005451:114176-115490
HmHel-5	CAEZ01005451:99173-102042
HmHel-5	CAEZ01005215:6715-8064
HmHel-5	CAEZ01003690:12475-13811
HmHel-5	CAEZ01003187:1209-2560
HmHel-5	CAEZ01003005:4158-5445
HmHel-5	CAEZ01002771:7729-9080
HmHel-5	CAEZ01002771:25355-26412
HmHel-5	CAEZ01001805:88806-90141
HmHel-5	CAEZ01001805:8678-10031
HmHel-5	CAEZ01001557:31369-32654
HmHel-5	CAEZ01001557:14186-14402
HmHel-5	CAEZ01000850:104926-106179
HmHel-5	CAEZ01010405:1-1196
HmHel-5	CAEZ01009631:53374-55919
HmHel-5	CAEZ01008159:7648-10135
HmHel-5	CAEZ01007395:14581-15751
HmHel-5	CAEZ01007395:46674-47997
HmHel-5	CAEZ01002642:22384-23726
HmHel-5	CAEZ01011245:59222-60539
HmHel-5	CAEZ01010888:1-586
HmHel-5	CAEZ01010888:9203-10257
HmHel-5	CAEZ01010018:643-1978
HmHel-5	CAEZ01007264:13197-14551

HmHel-5	CAEZ01006747:6968-8323
HmHel-5	CAEZ01004044:20031-21381
HmHel-5	CAEZ01003420:6066-6995
HmHel-5	CAEZ01009978:18719-20067
HmHel-5	CAEZ01009763:355-1646
HmHel-5	CAEZ01008918:2044-3406
HmHel-5	CAEZ01008677:19639-20978
HmHel-5	CAEZ01008021:18732-20012
HmHel-5	CAEZ01006126:4058-4604
HmHel-5	CAEZ01006126:44584-45931
HmHel-5	CAEZ01006126:10553-10644
HmHel-5	CAEZ01005876:5049-6383
HmHel-5	CAEZ01005149:20712-21926
HmHel-5	CAEZ01003695:18526-19838
HmHel-5	CAEZ01003211:25243-26501
HmHel-5	CAEZ01003211:45948-47208
HmHel-5	CAEZ01002318:1550-2828
HmHel-5	CAEZ01006803:44432-45763
HmHel-5	CAEZ01006200:1-710
HmHel-5	CAEZ01005233:9693-11040
HmHel-5	CAEZ01001062:64154-65482
HmHel-5	CAEZ01011283:77214-78572
HmHel-5	CAEZ01011283:79913-81254
HmHel-5	CAEZ01008372:295573-296912
HmHel-5	CAEZ01007708:29566-30930
HmHel-5	CAEZ01007708:52334-52902
HmHel-5	CAEZ01007708:91977-93189
HmHel-5	CAEZ01007708:62127-63442
HmHel-5	CAEZ01005044:30646-31795
HmHel-5	CAEZ01004083:25452-26821
HmHel-5	CAEZ01009005:10742-12050
HmHel-5	CAEZ01008886:15895-17496
HmHel-5	CAEZ01008886:10554-11907
HmHel-5	CAEZ01008436:1-1094
HmHel-5	CAEZ01007624:18993-26409
HmHel-5	CAEZ01007273:108334-109566
HmHel-5	CAEZ01007273:54163-55368
HmHel-5	CAEZ01007273:16041-17234
HmHel-5	CAEZ01007267:16886-19209
HmHel-5	CAEZ01006020:4837-6193
HmHel-5	CAEZ01005402:56873-58232
HmHel-5	CAEZ01005402:13352-13979
HmHel-5	CAEZ01003762:26291-27631
HmHel-5	CAEZ01003682:3677-5018
HmHel-5	CAEZ01003151:69026-70401
HmHel-5	CAEZ01001595:131966-132821
HmHel-5	CAEZ01001450:56616-57927
HmHel-5	CAEZ01001441:32992-34347

HmHel-5	CAEZ01000969:15275-16604
HmHel-5	CAEZ01000969:83-651
HmHel-5	CAEZ01010965:1-416
HmHel-5	CAEZ01010965:13082-14384
HmHel-5	CAEZ01010805:74095-75404
HmHel-5	CAEZ01010719:6770-8148
HmHel-5	CAEZ01010206:17084-18084
HmHel-5	CAEZ01009945:1355-2703
HmHel-5	CAEZ01008175:19690-21042
HmHel-5	CAEZ01007758:5806-7163
HmHel-5	CAEZ01007374:1736-3020
HmHel-5	CAEZ01007374:3146-4512
HmHel-5	CAEZ01007225:795-2129
HmHel-5	CAEZ01006396:18446-19782
HmHel-5	CAEZ01005122:19702-21057
HmHel-5	CAEZ01004792:5771-7107
HmHel-5	CAEZ01003245:1784-2638
HmHel-5	CAEZ01002286:16997-18530
HmHel-5	CAEZ01001779:4248-5516
HmHel-5	CAEZ01011489:90-1440
HmHel-5	CAEZ01011022:4917-6214
HmHel-5	CAEZ01008835:3531-4858
HmHel-5	CAEZ01008756:1-709
HmHel-5	CAEZ01008604:13130-15629
HmHel-5	CAEZ01008352:121873-123219
HmHel-5	CAEZ01007985:41937-43062
HmHel-5	CAEZ01007751:76404-78932
HmHel-5	CAEZ01007647:2944-4277
HmHel-5	CAEZ01006967:1-699
HmHel-5	CAEZ01006302:10722-12053
HmHel-5	CAEZ01005676:7941-9038
HmHel-5	CAEZ01005508:87474-88506
HmHel-5	CAEZ01005508:122486-123692
HmHel-5	CAEZ01005271:1-722
HmHel-5	CAEZ01004823:19241-20585
HmHel-5	CAEZ01004733:102211-103023
HmHel-5	CAEZ01004733:20418-21724
HmHel-5	CAEZ01004562:87380-89308
HmHel-5	CAEZ01003818:4171-6708
HmHel-5	CAEZ01002208:1104-2248
HmHel-5	CAEZ01001777:12453-13814
HmHel-5	CAEZ01001501:1-787
HmHel-5	CAEZ01010103:297-1613
HmHel-5	CAEZ01008280:783-3066
HmHel-5	CAEZ01007263:1297-3778
HmHel-5	CAEZ01007263:75734-76578
HmHel-5	CAEZ01002636:1962-3310
HmHel-5	CAEZ01002038:4584-5842

HmHel-5	CAEZ01000991:1-788
HmHel-5	CAEZ01010803:1463-2825
HmHel-5	CAEZ01010803:13860-15196
HmHel-5	CAEZ01010242:14481-15813
HmHel-5	CAEZ01007464:3736-5089
HmHel-5	CAEZ01006303:9661-10674
HmHel-5	CAEZ01005735:31689-32694
HmHel-5	CAEZ01005603:14191-15545
HmHel-5	CAEZ01004408:10120-11476
HmHel-5	CAEZ01003686:10087-11345
HmHel-5	CAEZ01003202:74744-76076
HmHel-5	CAEZ01002833:2016-5229
HmHel-5	CAEZ01001070:37986-39333
HmHel-5	CAEZ01001070:59245-60593
HmHel-5	CAEZ01009939:12333-13673
HmHel-5	CAEZ01009010:11785-11982
HmHel-5	CAEZ01009010:12241-13593
HmHel-5	CAEZ01008680:7353-8883
HmHel-5	CAEZ01008211:164810-166162
HmHel-5	CAEZ01005660:72004-73360
HmHel-5	CAEZ01005449:14850-15773
HmHel-5	CAEZ01004284:4356-5724
HmHel-5	CAEZ01003940:42-958
HmHel-5	CAEZ01003510:4989-6319
HmHel-5	CAEZ01003510:84094-85090
HmHel-5	CAEZ01003416:29704-30965
HmHel-5	CAEZ01002230:12701-13618
HmHel-5	CAEZ01001408:38603-39935
HmHel-5	CAEZ01000970:23820-25089
HmHel-5	CAEZ01000864:17243-18579
HmHel-5	CAEZ01009138:10464-11726
HmHel-5	CAEZ01009138:30958-32305
HmHel-5	CAEZ01007964:12122-14651
HmHel-5	CAEZ01007449:25373-26662
HmHel-5	CAEZ01006880:19901-20576
HmHel-5	CAEZ01006660:17590-20073
HmHel-5	CAEZ01006062:11375-13614
HmHel-5	CAEZ01005498:1437-2718
HmHel-5	CAEZ01003678:7553-8653
HmHel-5	CAEZ01002749:2254-3577
HmHel-5	CAEZ01007587:27007-28368
HmHel-5	CAEZ01005016:7108-8401
HmHel-5	CAEZ01003041:2888-4230
HmHel-5	CAEZ01011207:1150-2461
HmHel-5	CAEZ01010636:7247-8582
HmHel-5	CAEZ01008783:3499-4821
HmHel-5	CAEZ01007528:645-2010
HmHel-5	CAEZ01007492:59047-60407

HmHel-5	CAEZ01007317:20190-21490
HmHel-5	CAEZ01006974:2336-3691
HmHel-5	CAEZ01006521:11056-11985
HmHel-5	CAEZ01003595:11817-13132
HmHel-5	CAEZ01003513:6009-7343
HmHel-5	CAEZ01003250:159083-160424
HmHel-5	CAEZ01003250:24844-26196
HmHel-5	CAEZ01001598:779-1986
HmHel-5	CAEZ01001598:2220-3353
HmHel-5	CAEZ01001458:4750-5975
HmHel-5	CAEZ01011640:56085-57439
HmHel-5	CAEZ01011625:9522-10850
HmHel-5	CAEZ01007121:44735-46086
HmHel-5	CAEZ01007121:25704-27040
HmHel-5	CAEZ01005504:13083-14462
HmHel-5	CAEZ01004195:5772-7124
HmHel-5	CAEZ01000986:2546-3875
HmHel-5	CAEZ01011577:3009-4117
HmHel-5	CAEZ01011577:4141-5059
HmHel-5	CAEZ01011046:89414-90765
HmHel-5	CAEZ01011046:123066-124413
HmHel-5	CAEZ01011046:740-2070
HmHel-5	CAEZ01010886:22163-23382
HmHel-5	CAEZ01008323:851-2163
HmHel-5	CAEZ01008168:19214-20440
HmHel-5	CAEZ01008168:14473-15638
HmHel-5	CAEZ01007168:1-1300
HmHel-5	CAEZ01005086:5071-6415
HmHel-5	CAEZ01004261:388-1795
HmHel-5	CAEZ01003879:9376-10377
HmHel-5	CAEZ01003365:1-679
HmHel-5	CAEZ01010466:24209-25544
HmHel-5	CAEZ01009946:822-2192
HmHel-5	CAEZ01009056:1-1132
HmHel-5	CAEZ01007777:12855-14186
HmHel-5	CAEZ01007292:1648-3513
HmHel-5	CAEZ01006220:67840-68723
HmHel-5	CAEZ01002792:19136-20515
HmHel-5	CAEZ01009465:18984-19866
HmHel-5	CAEZ01008947:6149-7506
HmHel-5	CAEZ01008290:99394-100724
HmHel-5	CAEZ01007804:199752-201106
HmHel-5	CAEZ01007402:949-2311
HmHel-5	CAEZ01005883:7699-9023
HmHel-5	CAEZ01005407:10793-12032
HmHel-5	CAEZ01004986:1756-3113
HmHel-5	CAEZ01004684:15278-16629
HmHel-5	CAEZ01003114:23-747

HmHel-5	CAEZ01002368:35442-36483
HmHel-5	CAEZ01010981:38092-39447
HmHel-5	CAEZ01010190:44657-45984
HmHel-5	CAEZ01009627:21418-22817
HmHel-5	CAEZ01008119:310-1666
HmHel-5	CAEZ01008119:9951-11266
HmHel-5	CAEZ01007473:20092-20752
HmHel-5	CAEZ01006621:2552-3884
HmHel-5	CAEZ01004014:52466-53773
HmHel-5	CAEZ01002821:9080-11613
HmHel-5	CAEZ01002821:119748-121050
HmHel-5	CAEZ01001114:14158-15313
HmHel-5	CAEZ01001114:3686-4972
HmHel-5	CAEZ01007759:3178-4526
HmHel-5	CAEZ01002974:539-1653
HmHel-5	CAEZ01009842:1-676
HmHel-5	CAEZ01009842:21197-21806
HmHel-5	CAEZ01009232:32425-33783
HmHel-5	CAEZ01008116:3659-4999
HmHel-5	CAEZ01007902:6983-8340
HmHel-5	CAEZ01007495:34054-35377
HmHel-5	CAEZ01007358:5941-7253
HmHel-5	CAEZ01005611:13739-15098
HmHel-5	CAEZ01002674:5828-7097
HmHel-5	CAEZ01011413:36335-37696
HmHel-5	CAEZ01011413:282-1661
HmHel-5	CAEZ01011158:8100-9382
HmHel-5	CAEZ01010030:53748-55149
HmHel-5	CAEZ01009691:70934-72136
HmHel-5	CAEZ01008799:5-873
HmHel-5	CAEZ01008104:38180-39515
HmHel-5	CAEZ01007093:980-2288
HmHel-5	CAEZ01007093:8896-10220
HmHel-5	CAEZ01005244:8846-10061
HmHel-5	CAEZ01004941:4738-6052
HmHel-5	CAEZ01004236:10649-11998
HmHel-5	CAEZ01002049:57798-59159
HmHel-5	CAEZ01000937:19-1240
HmHel-5	CAEZ01009617:43263-44439
HmHel-5	CAEZ01009017:84797-86184
HmHel-5	CAEZ01008110:19717-21348
HmHel-5	CAEZ01007986:2554-3714
HmHel-5	CAEZ01007912:116026-117374
HmHel-5	CAEZ01005495:35257-36443
HmHel-5	CAEZ01005239:6242-7562
HmHel-5	CAEZ01005239:3751-5082
HmHel-5	CAEZ01003767:2289-2949
HmHel-5	CAEZ01009124:24250-25548

HmHel-5	CAEZ01009124:66155-66389
HmHel-5	CAEZ01009124:62809-63985
HmHel-5	CAEZ01008857:12479-13706
HmHel-5	CAEZ01007626:43796-45142
HmHel-5	CAEZ01004914:9039-10396
HmHel-5	CAEZ01004677:32034-33343
HmHel-5	CAEZ01004677:55441-56503
HmHel-5	CAEZ01004370:64995-66106
HmHel-5	CAEZ01003904:9871-10950
HmHel-5	CAEZ01002638:8098-9287
HmHel-5	CAEZ01001668:1817-3183
HmHel-5	CAEZ01010927:89393-90755
HmHel-5	CAEZ01008899:4791-6101
HmHel-5	CAEZ01002769:21760-23005
HmHel-5	CAEZ01002769:29121-30396
HmHel-5	CAEZ01001249:1-519
HmHel-5	CAEZ01001249:20929-21846
HmHel-5	CAEZ01001157:11885-13223
HmHel-5	CAEZ01009992:1-692
HmHel-5	CAEZ01009746:5650-6747
HmHel-5	CAEZ01008838:250-1415
HmHel-5	CAEZ01006530:269278-270539
HmHel-5	CAEZ01006335:1-910
HmHel-5	CAEZ01005230:1641-2982
HmHel-5	CAEZ01003511:83-944
HmHel-5	CAEZ01001703:24242-25453
HmHel-5	CAEZ01010666:6052-7382
HmHel-5	CAEZ01009032:2370-3746
HmHel-5	CAEZ01007029:3265-4613
HmHel-5	CAEZ01006907:21031-22363
HmHel-5	CAEZ01005053:24505-25870
HmHel-5	CAEZ01004855:16245-17503
HmHel-5	CAEZ01001660:1-693
HmHel-5	CAEZ01008353:52465-53825
HmHel-5	CAEZ01002870:21950-23146
HmHel-5	CAEZ01008632:20145-21458
HmHel-5	CAEZ01008329:2131-3460
HmHel-5	CAEZ01008329:19251-20528
HmHel-5	CAEZ01006339:71672-73013
HmHel-5	CAEZ01004804:12362-13429
HmHel-5	CAEZ01010288:291081-292243
HmHel-5	CAEZ01010288:203926-205245
HmHel-5	CAEZ01003641:4097-4983
HmHel-5	CAEZ01010699:3759-5105
HmHel-5	CAEZ01010348:4973-6290
HmHel-5	CAEZ01008500:1-804
HmHel-5	CAEZ01008500:34815-36084
HmHel-5	CAEZ01008500:129870-131148

HmHel-5	CAEZ01006558:4918-6230
HmHel-5	CAEZ01006540:63512-64865
HmHel-5	CAEZ01004681:31744-32270
HmHel-5	CAEZ01004681:1800-3161
HmHel-5	CAEZ01003989:3669-4354
HmHel-5	CAEZ01003989:350-1706
HmHel-5	CAEZ01001417:7361-8685
HmHel-5	CAEZ01011134:126085-126989
HmHel-5	CAEZ01011134:86644-87968
HmHel-5	CAEZ01011048:62806-63733
HmHel-5	CAEZ01009473:16547-17937
HmHel-5	CAEZ01008315:82822-84164
HmHel-5	CAEZ01008315:370564-370685
HmHel-5	CAEZ01008315:66542-67872
HmHel-5	CAEZ01007458:1-179
HmHel-5	CAEZ01007458:12452-13597
HmHel-5	CAEZ01006343:1003-2345
HmHel-5	CAEZ01002650:55919-57223
HmHel-5	CAEZ01002635:33737-35065
HmHel-5	CAEZ01002635:63118-64414
HmHel-5	CAEZ01002635:60167-61527
HmHel-5	CAEZ01011269:5715-6985
HmHel-5	CAEZ01009134:8-650
HmHel-5	CAEZ01007917:6344-7697
HmHel-5	CAEZ01006640:7436-8774
HmHel-5	CAEZ01005578:11296-12647
HmHel-5	CAEZ01003964:4384-5055
HmHel-5	CAEZ01003102:9416-10774
HmHel-5	CAEZ01011032:19019-20095
HmHel-5	CAEZ01010771:31897-32062
HmHel-5	CAEZ01010771:4105-5464
HmHel-5	CAEZ01003109:13693-15051
HmHel-5	CAEZ01008607:8568-9872
HmHel-5	CAEZ01007863:83212-84233
HmHel-5	CAEZ01006833:3043-4316
HmHel-5	CAEZ01006492:5303-6620
HmHel-5	CAEZ01006365:11086-12435
HmHel-5	CAEZ01005196:10325-11616
HmHel-5	CAEZ01008724:3786-5057
HmHel-5	CAEZ01007722:9260-10382
HmHel-5	CAEZ01007722:26003-27305
HmHel-5	CAEZ01007722:148443-149793
HmHel-5	CAEZ01007722:39264-40359
HmHel-5	CAEZ01005437:31387-31800
HmHel-5	CAEZ01005437:22651-23922
HmHel-5	CAEZ01005437:1-889
HmHel-5	CAEZ01003714:11429-12736
HmHel-5	CAEZ01007704:18447-19149

HmHel-5	CAEZ01006145:18749-19429
HmHel-5	CAEZ01004334:3589-4929
HmHel-5	CAEZ01004119:4393-5641
HmHel-5	CAEZ01003667:6882-8227
HmHel-5	CAEZ01001673:1-1179
HmHel-5	CAEZ01011195:14575-15919
HmHel-5	CAEZ01009470:72747-74078
HmHel-5	CAEZ01009470:118495-119848
HmHel-5	CAEZ01009470:79884-81236
HmHel-5	CAEZ01009470:1074-1836
HmHel-5	CAEZ01008361:3680-4476
HmHel-5	CAEZ01007433:414-1613
HmHel-5	CAEZ01002961:7646-8872
HmHel-5	CAEZ01001658:687-2030
HmHel-5	CAEZ01007012:908-2191
HmHel-5	CAEZ01004981:9422-10799
HmHel-5	CAEZ01010619:32394-33489
HmHel-5	CAEZ01010453:35839-37111
HmHel-5	CAEZ01010453:124651-125981
HmHel-5	CAEZ01010453:60357-61461
HmHel-5	CAEZ01008381:1150-3090
HmHel-5	CAEZ01007727:7945-9281
HmHel-5	CAEZ01007727:26899-27927
HmHel-5	CAEZ01010428:42136-43473
HmHel-5	CAEZ01010428:38529-39852
HmHel-5	CAEZ01009822:19761-20308
HmHel-5	CAEZ01009822:13253-14556
HmHel-5	CAEZ01009556:8399-9741
HmHel-5	CAEZ01008416:970-2318
HmHel-5	CAEZ01007205:9020-10337
HmHel-5	CAEZ01011204:348-1680
HmHel-5	CAEZ01009213:1-1302
HmHel-5	CAEZ01004927:18853-20098
HmHel-5	CAEZ01004826:70320-71675
HmHel-5	CAEZ01004492:24488-25832
HmHel-5	CAEZ01003868:9355-10698
HmHel-5	CAEZ01003549:1496-2381
HmHel-5	CAEZ01003439:11032-12357
HmHel-5	CAEZ01008069:15355-16036
HmHel-5	CAEZ01007457:196-1536
HmHel-5	CAEZ01005210:21590-22931
HmHel-5	CAEZ01002919:4911-5975
HmHel-5	CAEZ01002415:1745-3082
HmHel-5	CAEZ01002415:29200-30623
HmHel-5	CAEZ01011063:8530-9782
HmHel-5	CAEZ01010972:8744-10092
HmHel-5	CAEZ01010318:33911-35222
HmHel-5	CAEZ01009178:5196-6523

HmHel-5	CAEZ01003517:27512-28857
HmHel-5	CAEZ01001518:12899-14233
HmHel-5	CAEZ01000994:761-1401
HmHel-5	CAEZ01010411:39154-40067
HmHel-5	CAEZ01010351:90389-91737
HmHel-5	CAEZ01003070:199436-200773
HmHel-5	CAEZ01003070:42879-45450
HmHel-5	CAEZ01002759:4820-6137
HmHel-5	CAEZ01010799:30289-31614
HmHel-5	CAEZ01010697:858-2196
HmHel-5	CAEZ01010505:14755-16116
HmHel-5	CAEZ01010505:13-399
HmHel-5	CAEZ01006010:13165-14477
HmHel-5	CAEZ01003701:224-1508
HmHel-5	CAEZ01000983:189-1364
HmHel-5	CAEZ01009872:15514-16875
HmHel-5	CAEZ01009064:6968-8177
HmHel-5	CAEZ01008707:55999-56899
HmHel-5	CAEZ01008707:85238-86545
HmHel-5	CAEZ01008707:89012-90172
HmHel-5	CAEZ01004745:18589-19710
HmHel-5	CAEZ01003735:62-1339
HmHel-5	CAEZ01001184:853-2008
HmHel-5	CAEZ01010431:5-3088
HmHel-5	CAEZ01010260:8625-9899
HmHel-5	CAEZ01010260:25406-26770
HmHel-5	CAEZ01008204:9443-11829
HmHel-5	CAEZ01006454:11082-12442
HmHel-5	CAEZ01010808:195015-196098
HmHel-5	CAEZ01008330:8116-8721
HmHel-5	CAEZ01007571:41951-43276
HmHel-5	CAEZ01007571:45770-46994
HmHel-5	CAEZ01010960:7308-8629
HmHel-5	CAEZ01005274:122627-123867
HmHel-5	CAEZ01000879:181388-182618
HmHel-5	CAEZ01000838:4780-6115
HmHel-5	CAEZ01000019:1-705
HmHel-5	CAEZ01011504:4798-6100
HmHel-5	CAEZ01006866:20643-21969
HmHel-5	CAEZ01002724:7581-8818
HmHel-5	CAEZ01007517:19526-20155
HmHel-5	CAEZ01005623:12537-13643
HmHel-5	CAEZ01002867:282-1163
HmHel-5	CAEZ01010993:3000-4326
HmHel-5	CAEZ01004574:18543-19886
HmHel-5	CAEZ01000527:356-1028
HmHel-5	CAEZ01003323:1-950
HmHel-5	CAEZ01010643:24257-25623

HmHel-5	CAEZ01003785:18636-19955
HmHel-5	CAEZ01003785:24873-25366
HmHel-5	CAEZ01005195:8605-9913
HmHel-5	CAEZ01003097:13591-14918
HmHel-5	CAEZ01006840:84-1409
HmHel-5	CAEZ01006389:4975-6322
HmHel-5	CAEZ01005084:1-643
HmHel-5	CAEZ01011104:10828-11662
HmHel-5	CAEZ01011104:115130-116476
HmHel-5	CAEZ01011104:52941-53930
HmHel-5	CAEZ01007950:13103-14377
HmHel-5	CAEZ01003320:390-1440
HmHel-5	CAEZ01000904:1-584
HmHel-5	CAEZ01011394:11171-12379
HmHel-5	CAEZ01005624:17932-19259
HmHel-5	CAEZ01004385:9892-11238
HmHel-5	CAEZ01010029:28876-30119
HmHel-5	CAEZ01010029:74557-75520
HmHel-5	CAEZ01010029:53993-55285
HmHel-5	CAEZ01005177:28-1046
HmHel-5	CAEZ01007431:22818-24155
HmHel-5	CAEZ01007431:46593-46725
HmHel-5	CAEZ01005515:1-392
HmHel-5	CAEZ01005515:32637-33946
HmHel-5	CAEZ01006318:3294-4473
HmHel-5	CAEZ01006318:19257-19882
HmHel-5	CAEZ01004274:4604-5943
HmHel-5	CAEZ01004274:15310-15715
HmHel-5	CAEZ01007661:18068-19268
HmHel-5	CAEZ01004921:10687-12004
HmHel-5	CAEZ01009293:1-628
HmHel-5	CAEZ01008957:16206-16885
HmHel-5	CAEZ01006901:12300-13597
HmHel-5	CAEZ01003977:1279-2399
HmHel-5	CAEZ01011314:18357-21196
HmHel-5	CAEZ01006877:3119-4450
HmHel-5	CAEZ01008841:1-1142
HmHel-5	CAEZ01003823:15499-15825
HmHel-5	CAEZ01003823:3783-5007
HmHel-5	CAEZ01003491:964-2017
HmHel-5	CAEZ01008498:1-604
HmHel-5	CAEZ01007348:3129-4478
HmHel-5	CAEZ01002181:21900-23187
HmHel-5	CAEZ01002243:190-1550
HmHel-5	CAEZ01009970:1-609
HmHel-5	CAEZ01009776:11054-12362
HmHel-5	CAEZ01005628:5479-6822
HmHel-5	CAEZ01001572:1-410

HmHel-5	CAEZ01001572:29406-30728
HmHel-5	CAEZ01011598:38139-39412
HmHel-5	CAEZ01011598:1-1084
HmHel-5	CAEZ01001336:1567-2932
HmHel-5	CAEZ01002982:3625-4939
HmHel-5	CAEZ01004542:5447-6679
HmHel-5	CAEZ01004542:23290-24619
HmHel-5	CAEZ01004351:8-1230
HmHel-5	CAEZ01010423:48680-49859
HmHel-5	CAEZ01008410:93936-94845
HmHel-5	CAEZ01005134:1-554
HmHel-5	CAEZ01009694:40753-41701
HmHel-5	CAEZ01009255:9-569
HmHel-5	CAEZ01005586:17414-18688
HmHel-5	CAEZ01005586:1-948
HmHel-5	CAEZ01003507:31-1263
HmHel-5	CAEZ01008508:823-2129
HmHel-5	CAEZ01006056:6572-7861
HmHel-5	CAEZ01004693:18166-19410
HmHel-5	CAEZ01010740:9073-10351
HmHel-5	CAEZ01010740:76713-77298
HmHel-5	CAEZ01007817:1-1201
HmHel-5	CAEZ01007568:44619-46042
HmHel-5	CAEZ01010137:1919-3206
HmHel-5	CAEZ01008392:18382-19725
HmHel-5	CAEZ01007633:21527-22888
HmHel-5	CAEZ01004649:9299-9859
HmHel-5	CAEZ01003766:8-1223
HmHel-5	CAEZ01001210:1345-2694
HmHel-5	CAEZ01011415:13715-14310
HmHel-5	CAEZ01011415:1-822
HmHel-5	CAEZ01011219:18359-19653
HmHel-5	CAEZ01005582:2544-3876
HmHel-5	CAEZ01004377:5270-6565
HmHel-5	CAEZ01011197:75309-76596
HmHel-5	CAEZ01010831:4151-5525
HmHel-5	CAEZ01007740:3409-4718
HmHel-5	CAEZ01007740:89400-90706
HmHel-5	CAEZ01007740:18332-18457
HmHel-5	CAEZ01006649:11859-12490
HmHel-5	CAEZ01005911:160068-161447
HmHel-5	CAEZ01010930:4554-5461
HmHel-5	CAEZ01009181:3533-4428
HmHel-5	CAEZ01004198:10-576
HmHel-5	CAEZ01010575:7-568
HmHel-5	CAEZ01009135:7-568
HmHel-5	CAEZ01007191:2845-3779
HmHel-5	CAEZ01001317:4439-5645

HmHel-5	CAEZ01010541:224246-225514
HmHel-5	CAEZ01009459:1-576
HmHel-5	CAEZ01008238:23270-24705
HmHel-5	CAEZ01008061:1-597
HmHel-5	CAEZ01005299:60799-61386
HmHel-5	CAEZ01003334:17470-18808
HmHel-5	CAEZ01001234:1-899
HmHel-5	CAEZ01004511:34903-35773
HmHel-5	CAEZ01000953:346-1015
HmHel-5	CAEZ01010133:22284-23671
HmHel-5	CAEZ01010133:9-314
HmHel-5	CAEZ01009452:101540-102086
HmHel-5	CAEZ01008413:21345-22848
HmHel-5	CAEZ01002118:15971-16545
HmHel-5	CAEZ01010274:71-664
HmHel-5	CAEZ01008904:7453-8767
HmHel-5	CAEZ01006661:4688-5988
HmHel-5	CAEZ01009271:101755-103058
HmHel-5	CAEZ01008617:5744-7085
HmHel-5	CAEZ01008617:1-1079
HmHel-5	CAEZ01006863:98616-99958
HmHel-5	CAEZ01005199:30058-31399
HmHel-5	CAEZ01002535:1-667
HmHel-5	CAEZ01002338:11550-12412
HmHel-5	CAEZ01010617:3-577
HmHel-5	CAEZ01004923:46362-47627
HmHel-5	CAEZ01002633:32693-33989
HmHel-5	CAEZ01010034:28747-29870
HmHel-5	CAEZ01008636:19769-21087
HmHel-5	CAEZ01003715:1107-2458
HmHel-5	CAEZ01002656:6382-7669
HmHel-5	CAEZ01002656:322-1031
HmHel-5	CAEZ01010414:16255-16845
HmHel-5	CAEZ01010036:21140-22414
HmHel-5	CAEZ01007163:14743-15619
HmHel-5	CAEZ01005296:9591-10142
HmHel-5	CAEZ01004404:14044-14782
HmHel-5	CAEZ01001645:30446-31759
HmHel-5	CAEZ01005846:6234-7466
HmHel-5	CAEZ01005321:19666-20984
HmHel-5	CAEZ01009003:1-601
HmHel-5	CAEZ01006287:22633-24044
HmHel-5	CAEZ01009197:23-1352
HmHel-5	CAEZ01006125:15939-17274
HmHel-5	CAEZ01006045:1-667
HmHel-5	CAEZ01000895:41810-43120
HmHel-5	CAEZ01008527:1-1036
HmHel-5	CAEZ01005204:10431-11017

HmHel-5	CAEZ01003210:22742-24078
HmHel-5	CAEZ01010325:1-859
HmHel-5	CAEZ01009709:99142-100408
HmHel-5	CAEZ01004786:38645-39980
HmHel-5	CAEZ01010100:26173-26870
HmHel-5	CAEZ01008656:394-1718
HmHel-5	CAEZ01004465:1484-2752
HmHel-5	CAEZ01001278:4385-5651
HmHel-5	CAEZ01008629:6079-7417
HmHel-5	CAEZ01008629:20837-21226
HmHel-5	CAEZ01008130:7861-9106
HmHel-5	CAEZ01002841:13978-14565
HmHel-5	CAEZ01001369:1-1019
HmHel-5	CAEZ01011193:108571-109845
HmHel-5	CAEZ01010420:1-900
HmHel-5	CAEZ01006279:9818-11153
HmHel-5	CAEZ01004995:14391-15501
HmHel-5	CAEZ01004034:20610-23419
HmHel-5	CAEZ01006972:21098-22317
HmHel-5	CAEZ01005433:80017-80554
HmHel-5	CAEZ01002732:43566-44946
HmHel-5	CAEZ01002459:1-1172
HmHel-5	CAEZ01011474:6504-7417
HmHel-5	CAEZ01008477:31433-32543
HmHel-5	CAEZ01006143:4804-6013
HmHel-5	CAEZ01006611:9463-10021
HmHel-5	CAEZ01004975:37518-38759
HmHel-5	CAEZ01003738:13826-14442
HmHel-5	CAEZ01006322:1-594
HmHel-5	CAEZ01006322:21512-21836
HmHel-5	CAEZ01004663:1607-2903
HmHel-5	CAEZ01004500:4669-5677
HmHel-5	CAEZ01009444:32244-35636
HmHel-5	CAEZ01011559:4195-5520
HmHel-5	CAEZ01009029:1725-2252
HmHel-5	CAEZ01008101:32754-33345
HmHel-5	CAEZ01008055:16606-17945
HmHel-5	CAEZ01008128:1-531
HmHel-5	CAEZ01006198:2079-2908
HmHel-5	CAEZ01008654:3060-4257
HmHel-5	CAEZ01007676:137641-139019
HmHel-5	CAEZ01007053:643-1983
HmHel-5	CAEZ01005068:1-882
HmHel-5	CAEZ01004468:15905-16429
HmHel-5	CAEZ01003148:351-1650
HmHel-5	CAEZ01001806:120123-121228
HmHel-5	CAEZ01001776:1-996
HmHel-5	CAEZ01011493:15583-16694

HmHel-5	CAEZ01009331:14175-15479
HmHel-5	CAEZ01007233:6608-7138
HmHel-5	CAEZ01005569:9990-11310
HmHel-5	CAEZ01004495:574-1444
HmHel-5	CAEZ01003938:5969-7179
HmHel-5	CAEZ01009531:1-669
HmHel-5	CAEZ01004697:3465-4000
HmHel-5	CAEZ01001857:2906-3761
HmHel-5	CAEZ01006567:66099-68534
HmHel-5	CAEZ01002818:16918-18247
HmHel-5	CAEZ01011392:58-640
HmHel-5	CAEZ01008678:46557-47119
HmHel-5	CAEZ01005180:15070-15632
HmHel-5	CAEZ01004635:1-1224
HmHel-5	CAEZ01006323:15-898
HmHel-5	CAEZ01009196:10555-12501
HmHel-5	CAEZ01008212:16030-17364
HmHel-5	CAEZ01006555:4363-6116
HmHel-5	CAEZ01002992:22528-23088
HmHel-5	CAEZ01009824:93204-94988
HmHel-5	CAEZ01002654:9932-10536
HmHel-5	CAEZ01009422:134767-135002
HmHel-5	CAEZ01009422:101330-102667
HmHel-5	CAEZ01004020:9483-11165
HmHel-5	CAEZ01002711:31712-32238
HmHel-5	CAEZ01001972:1876-3143
HmHel-5	CAEZ01010814:307-1666
HmHel-5	CAEZ01005486:1-529
HmHel-5	CAEZ01004794:11732-12257
HmHel-5	CAEZ01001740:33073-34368
HmHel-5	CAEZ01000020:1-550
HmHel-5	CAEZ01009463:17313-18661
HmHel-5	CAEZ01008279:1-537
HmHel-5	CAEZ01008166:1-571
HmHel-5	CAEZ01008166:2811-2940
HmHel-5	CAEZ01004644:12503-13830
HmHel-5	CAEZ01008278:4-1133
HmHel-5	CAEZ01007359:21016-22348
HmHel-5	CAEZ01008031:24174-25519
HmHel-5	CAEZ01006761:548-1937
HmHel-5	CAEZ01006761:17045-17607
HmHel-5	CAEZ01010111:36643-37305
HmHel-5	CAEZ01001799:9622-10163
HmHel-5	CAEZ01005173:149022-150124
HmHel-5	CAEZ01001868:1-581
HmHel-5	CAEZ01007204:24328-25637
HmHel-5	CAEZ01004820:67090-68389
HmHel-5	CAEZ01003453:5075-5621

HmHel-5	CAEZ01011110:498-1464
HmHel-5	CAEZ01009364:32992-34132
HmHel-5	CAEZ01005366:29280-30255
HmHel-5	CAEZ01002948:1-635
HmHel-5	CAEZ01002886:1866-2979
HmHel-5	CAEZ01001933:2223-2784
HmHel-5	CAEZ01008170:24510-26997
HmHel-5	CAEZ01008170:41746-42911
HmHel-5	CAEZ01011296:42135-44114
HmHel-5	CAEZ01010264:1117-2420
HmHel-5	CAEZ01009384:7534-8868
HmHel-5	CAEZ01008967:20450-20970
HmHel-5	CAEZ01007228:1-1023
HmHel-5	CAEZ01007008:1-528
HmHel-5	CAEZ01005159:9248-10363
HmHel-5	CAEZ01001566:712-1462
HmHel-5	CAEZ01008257:1549-2868
HmHel-5	CAEZ01007157:36746-38042
HmHel-5	CAEZ01011412:13295-13776
HmHel-5	CAEZ01009931:6315-7016
HmHel-5	CAEZ01008140:1-514
HmHel-5	CAEZ01002644:1-523
HmHel-5	CAEZ01001571:30430-30837
HmHel-5	CAEZ01001571:23466-24546
HmHel-5	CAEZ01007705:37237-37991
HmHel-5	CAEZ01007238:10556-11812
HmHel-5	CAEZ01005585:13292-14037
HmHel-5	CAEZ01004118:1-539
HmHel-5	CAEZ01003741:2178-3509
HmHel-5	CAEZ01000061:32386-33550
HmHel-5	CAEZ01010615:43448-45712
HmHel-5	CAEZ01008966:3658-4686
HmHel-5	CAEZ01008960:45156-46483
HmHel-5	CAEZ01002838:11909-13260
HmHel-5	CAEZ01001789:12103-12594
HmHel-5	CAEZ01004949:805-2166
HmHel-5	CAEZ01004678:24322-25194
HmHel-5	CAEZ01003633:59554-60205
HmHel-5	CAEZ01003173:18406-19692
HmHel-5	CAEZ01010202:9090-10369
HmHel-5	CAEZ01002860:379-995
HmHel-5	CAEZ01008295:5322-6650
HmHel-5	CAEZ01006948:18477-19766
HmHel-5	CAEZ01004738:62445-63766
HmHel-5	CAEZ01004738:5-369
HmHel-5	CAEZ01008169:22250-25565
HmHel-5	CAEZ01005241:8205-9547
HmHel-5	CAEZ01005040:1-604

HmHel-5	CAEZ01003592:813-2110
HmHel-5	CAEZ01010113:9915-12006
HmHel-5	CAEZ01009060:1-693
HmHel-5	CAEZ01004996:6358-7656
HmHel-5	CAEZ01003506:1-1147
HmHel-5	CAEZ01006947:1-889
HmHel-5	CAEZ01002608:51336-51811
HmHel-5	CAEZ01001825:13121-14444
HmHel-5	CAEZ01009462:243-777
HmHel-5	CAEZ01008546:831-1889
HmHel-5	CAEZ01006502:10862-11357
HmHel-5	CAEZ01001739:9328-10627
HmHel-5	CAEZ01001060:4-569
HmHel-5	CAEZ01010635:16566-17477
HmHel-5	CAEZ01009342:7878-9276
HmHel-5	CAEZ01008419:40445-41549
HmHel-5	CAEZ01005307:6198-7622
HmHel-5	CAEZ01005153:70024-71349
HmHel-5	CAEZ01011371:15836-16363
HmHel-5	CAEZ01007716:5468-6807
HmHel-5	CAEZ01006850:446-1708
HmHel-5	CAEZ01004859:1-877
HmHel-5	CAEZ01002678:13823-15076
HmHel-5	CAEZ01001922:2-997
HmHel-5	CAEZ01006231:1-574
HmHel-5	CAEZ01005082:2165-3258
HmHel-5	CAEZ01005082:52965-54064
HmHel-5	CAEZ01003325:4-542
HmHel-5	CAEZ01003325:588-3153
HmHel-5	CAEZ01002836:1-1128
HmHel-5	CAEZ01008350:62075-63635
HmHel-5	CAEZ01005806:13545-14921
HmHel-5	CAEZ01005806:34022-34771
HmHel-5	CAEZ01005806:15255-16324
HmHel-5	CAEZ01009676:28131-29345
HmHel-5	CAEZ01007567:735-2039
HmHel-5	CAEZ01006490:111490-112189
HmHel-5	CAEZ01006490:107441-107752
HmHel-5	CAEZ01010266:11085-12383
HmHel-5	CAEZ01003071:31743-32232
HmHel-5	CAEZ01000038:506-1847
HmHel-5	CAEZ01001811:1929-2480
HmHel-5	CAEZ01009685:1-491
HmHel-5	CAEZ01008497:33943-34803
HmHel-5	CAEZ01005573:1-513
HmHel-5	CAEZ01010164:8567-9916
HmHel-5	CAEZ01008063:1-487
HmHel-5	CAEZ01006348:1-862

HmHel-5	CAEZ01002583:1284-1783
HmHel-5	CAEZ01010283:407-1116
HmHel-5	CAEZ01006405:35710-37046
HmHel-5	CAEZ01009374:34807-36157
HmHel-5	CAEZ01008184:1-456
HmHel-5	CAEZ01005424:471-1844
HmHel-5	CAEZ01004381:19988-21314
HmHel-5	CAEZ01011325:6213-7415
HmHel-5	CAEZ01011310:16-1272
HmHel-5	CAEZ01006088:16910-19148
HmHel-5	CAEZ01002883:2-508
HmHel-5	CAEZ01002675:7759-9096
HmHel-5	CAEZ01010185:12675-13144
HmHel-5	CAEZ01003733:1-518
HmHel-5	CAEZ01010435:30998-31527
HmHel-5	CAEZ01010509:15761-17014
HmHel-5	CAEZ01003321:4730-6042
HmHel-5	CAEZ01002625:3822-4996
HmHel-5	CAEZ01001681:1-1072
HmHel-5	CAEZ01011649:10227-11537
HmHel-5	CAEZ01005098:1463-2794
HmHel-5	CAEZ01010953:1-466
HmHel-5	CAEZ01006773:113013-114353
HmHel-5	CAEZ01005176:9815-10662
HmHel-5	CAEZ01008905:26653-27416
HmHel-5	CAEZ01005513:1-466
HmHel-5	CAEZ01005513:2473-2929
HmHel-5	CAEZ01004723:15883-16355
HmHel-5	CAEZ01004565:6-1325
HmHel-5	CAEZ01009210:1-492
HmHel-5	CAEZ01004754:6077-6603
HmHel-5	CAEZ01003189:14907-15406
HmHel-5	CAEZ01003337:1-504
HmHel-5	CAEZ01008935:16559-17565
HmHel-5	CAEZ01008935:3389-4692
HmHel-5	CAEZ01002112:3743-4678
HmHel-5	CAEZ01004389:1-496
HmHel-5	CAEZ01004139:19257-20617
HmHel-5	CAEZ01004254:24977-26297
HmHel-5	CAEZ01001158:1-472
HmHel-5	CAEZ01010944:16046-16524
HmHel-5	CAEZ01010695:13173-14249
HmHel-5	CAEZ01008190:1067-3332
HmHel-5	CAEZ01008190:19020-19841
HmHel-5	CAEZ01008190:26-611
HmHel-5	CAEZ01007389:119775-121084
HmHel-5	CAEZ01007389:30441-30531
HmHel-5	CAEZ01010430:24574-25670

HmHel-5	CAEZ01009826:4725-5581
HmHel-5	CAEZ01009826:27-121
HmHel-5	CAEZ01009225:1-520
HmHel-5	CAEZ01008943:4-524
HmHel-5	CAEZ01008943:21731-23026
HmHel-5	CAEZ01008943:1082-1982
HmHel-5	CAEZ01008595:13826-14271
HmHel-5	CAEZ01004809:16578-17636
HmHel-5	CAEZ01003392:5437-6721
HmHel-5	CAEZ01002254:31003-31494
HmHel-5	CAEZ01006776:4763-5649
HmHel-5	CAEZ01005935:1045-2176
HmHel-5	CAEZ01005626:1-492
HmHel-5	CAEZ01003885:1234-1945
HmHel-5	CAEZ01010582:4747-5221
HmHel-5	CAEZ01003155:1-447
HmHel-5	CAEZ01003155:12109-12940
HmHel-5	CAEZ01002300:698-1180
HmHel-5	CAEZ01010547:6774-7207
HmHel-5	CAEZ01011256:11710-12182
HmHel-5	CAEZ01008458:2272-3655
HmHel-5	CAEZ01010922:36468-37609
HmHel-5	CAEZ01001347:14640-15435
HmHel-5	CAEZ01007725:1-385
HmHel-5	CAEZ01007725:133358-134258
HmHel-5	CAEZ01005382:28973-30242
HmHel-5	CAEZ01004496:8069-9287
HmHel-5	CAEZ01002062:1143-2255
HmHel-5	CAEZ01000999:39824-41173
HmHel-5	CAEZ01010448:3315-3906
HmHel-5	CAEZ01009386:80-218
HmHel-5	CAEZ01009386:1-1126
HmHel-5	CAEZ01005361:16-266
HmHel-5	CAEZ01005361:5851-7211
HmHel-5	CAEZ01010522:7870-8650
HmHel-5	CAEZ01009936:12300-13543
HmHel-5	CAEZ01007600:1-441
HmHel-5	CAEZ01004795:1-771
HmHel-5	CAEZ01001679:601-1094
HmHel-5	CAEZ01009573:1-935
HmHel-5	CAEZ01006153:83002-83834
HmHel-5	CAEZ01003529:1221-2529
HmHel-5	CAEZ01001624:1-459
HmHel-5	CAEZ01010449:1-440
HmHel-5	CAEZ01010449:25366-25756
HmHel-5	CAEZ01006652:31217-32482
HmHel-5	CAEZ01006071:10933-11376
HmHel-5	CAEZ01005203:14196-14801

HmHel-5	CAEZ01005203:2013-2754
HmHel-5	CAEZ01010001:1-1013
HmHel-5	CAEZ01008177:4514-4921
HmHel-5	CAEZ01005220:57656-58426
HmHel-5	CAEZ01002609:17-857
HmHel-5	CAEZ01000339:1-458
HmHel-5	CAEZ01011528:51468-52267
HmHel-5	CAEZ01011528:6885-8075
HmHel-5	CAEZ01009684:27834-28404
HmHel-5	CAEZ01009315:1-847
HmHel-5	CAEZ01004936:23999-25172
HmHel-5	CAEZ01002262:1523-2821
HmHel-5	CAEZ01001366:7568-8084
HmHel-5	CAEZ01001056:930-2253
HmHel-5	CAEZ01005334:12858-14207
HmHel-5	CAEZ01002041:76523-77210
HmHel-5	CAEZ01001764:20616-22220
HmHel-5	CAEZ01010095:1-831
HmHel-5	CAEZ01010938:11681-12689
HmHel-5	CAEZ01009511:2615-3713
HmHel-5	CAEZ01003263:1-1025
HmHel-5	CAEZ01002146:12801-13318
HmHel-5	CAEZ01011519:64722-65936
HmHel-5	CAEZ01010605:875-1657
HmHel-5	CAEZ01008647:4013-4478
HmHel-5	CAEZ01003223:1-830
HmHel-5	CAEZ01011373:1-817
HmHel-5	CAEZ01011105:44362-45165
HmHel-5	CAEZ01008757:1886-2299
HmHel-5	CAEZ01007724:80458-81520
HmHel-5	CAEZ01005902:13957-14368
HmHel-5	CAEZ01002416:87035-87518
HmHel-5	CAEZ01001577:7310-7898
HmHel-5	CAEZ01007768:2217-2623
HmHel-5	CAEZ01007057:1687-2126
HmHel-5	CAEZ01003819:6934-8219
HmHel-5	CAEZ01000926:12321-13413
HmHel-5	CAEZ01011163:11175-11678
HmHel-5	CAEZ01003091:8555-9143
HmHel-5	CAEZ01003091:1-9437
HmHel-5	CAEZ01009133:33068-33600
HmHel-5	CAEZ01003562:35604-36923
HmHel-5	CAEZ01010462:100063-101377
HmHel-5	CAEZ01010247:17014-17476
HmHel-5	CAEZ01005085:12836-14199
HmHel-5	CAEZ01004165:7505-8876
HmHel-5	CAEZ01004165:16475-18142
HmHel-5	CAEZ01004165:1-269

HmHel-5	CAEZ01011313:8709-10027
HmHel-5	CAEZ01010434:4-490
HmHel-5	CAEZ01008328:11264-11941
HmHel-5	CAEZ01008328:1-478
HmHel-5	CAEZ01008082:10182-11217
HmHel-5	CAEZ01004940:1-522
HmHel-5	CAEZ01003035:8-852
HmHel-5	CAEZ01002322:11-555
HmHel-5	CAEZ01009679:13724-15057
HmHel-5	CAEZ01009560:21227-22305
HmHel-5	CAEZ01005995:4315-5656
HmHel-5	CAEZ01011182:8-421
HmHel-5	CAEZ01010338:1-223
HmHel-5	CAEZ01010338:10763-11389
HmHel-5	CAEZ01008492:1884-3267
HmHel-5	CAEZ01003640:6613-7932
HmHel-5	CAEZ01000840:1-460
HmHel-5	CAEZ01000021:1-460
HmHel-5	CAEZ01010743:1244-2628
HmHel-5	CAEZ01010736:57674-57960
HmHel-5	CAEZ01010736:1-385
HmHel-5	CAEZ01010534:114297-115051
HmHel-5	CAEZ01010534:20493-21885
HmHel-5	CAEZ01007929:10528-11839
HmHel-5	CAEZ01007893:8-877
HmHel-5	CAEZ01004682:1-937
HmHel-5	CAEZ01003454:59-465
HmHel-5	CAEZ01003388:4575-5907
HmHel-5	CAEZ01001172:5630-15565
HmHel-5	CAEZ01011615:9546-10557
HmHel-5	CAEZ01010903:1405-2013
HmHel-5	CAEZ01009389:13487-14265
HmHel-5	CAEZ01009254:14644-15058
HmHel-5	CAEZ01008070:1-494
HmHel-5	CAEZ01007736:52388-53409
HmHel-5	CAEZ01006349:22789-23876
HmHel-5	CAEZ01004958:28282-29617
HmHel-5	CAEZ01010289:9015-10391
HmHel-5	CAEZ01009073:1-468
HmHel-5	CAEZ01006745:1204-2227
HmHel-5	CAEZ01006354:1451-2042
HmHel-5	CAEZ01005427:1-434
HmHel-5	CAEZ01005360:56536-57828
HmHel-5	CAEZ01004405:1-471
HmHel-5	CAEZ01000995:436-1537
HmHel-5	CAEZ01003283:609-1919
HmHel-5	CAEZ01002075:696-1720
HmHel-5	CAEZ01011065:1-377

HmHel-5	CAEZ01009681:50013-51354
HmHel-5	CAEZ01007501:6595-7306
HmHel-5	CAEZ01005304:942-2275
HmHel-5	CAEZ01004904:14471-15725
HmHel-5	CAEZ01004360:22549-23893
HmHel-5	CAEZ01003945:1741-2970
HmHel-5	CAEZ01002615:3559-4007
HmHel-5	CAEZ01000990:39009-39416
HmHel-5	CAEZ01009841:109264-109675
HmHel-5	CAEZ01006004:3-529
HmHel-5	CAEZ01005061:1-1062
HmHel-5	CAEZ01004650:14-412
HmHel-5	CAEZ01004618:1403-1814
HmHel-5	CAEZ01003853:2095-2787
HmHel-5	CAEZ01009986:27-440
HmHel-5	CAEZ01008123:57026-57834
HmHel-5	CAEZ01007227:5677-6063
HmHel-5	CAEZ01005205:3-528
HmHel-5	CAEZ01003303:1-468
HmHel-5	CAEZ01010070:3559-4953
HmHel-5	CAEZ01009898:24846-27314
HmHel-5	CAEZ01009535:10351-11704
HmHel-5	CAEZ01008062:10128-10529
HmHel-5	CAEZ01007816:8493-14214
HmHel-5	CAEZ01004637:1-443
HmHel-5	CAEZ01003082:1-384
HmHel-5	CAEZ01002414:145875-147272
HmHel-5	CAEZ01010440:53186-54514
HmHel-5	CAEZ01009971:23431-24771
HmHel-5	CAEZ01009803:1-511
HmHel-5	CAEZ01006221:35-446
HmHel-5	CAEZ01005059:1-452
HmHel-5	CAEZ01003700:11139-11550
HmHel-5	CAEZ01003364:4476-4889
HmHel-5	CAEZ01001402:26531-27859
HmHel-5	CAEZ01004501:1-380
HmHel-5	CAEZ01003944:3822-5086
HmHel-5	CAEZ01010141:3367-4453
HmHel-5	CAEZ01006100:5921-6828
HmHel-5	CAEZ01005058:12649-13324
HmHel-5	CAEZ01003588:5-394
HmHel-5	CAEZ01007579:10113-11190
HmHel-5	CAEZ01007362:4063-5206
HmHel-5	CAEZ01007035:1-417
HmHel-5	CAEZ01005479:7175-7687
HmHel-5	CAEZ01005267:16367-17668
HmHel-5	CAEZ01002040:3606-4133
HmHel-5	CAEZ01001971:12660-13026

HmHel-5	CAEZ01009253:1-371
HmHel-5	CAEZ01006003:879-1274
HmHel-5	CAEZ01003332:1925-3290
HmHel-5	CAEZ01007067:5072-6037
HmHel-5	CAEZ01006816:10366-11718
HmHel-5	CAEZ01005743:84325-85086
HmHel-5	CAEZ01005743:45351-45618
HmHel-5	CAEZ01002249:24827-25790
HmHel-5	CAEZ01008939:6664-7527
HmHel-5	CAEZ01007713:26166-26900
HmHel-5	CAEZ01007713:55197-56335
HmHel-5	CAEZ01005137:2576-3229
HmHel-5	CAEZ01003324:3-483
HmHel-5	CAEZ01003227:4172-4607
HmHel-5	CAEZ01002706:54781-55326
HmHel-5	CAEZ01002658:52759-53127
HmHel-5	CAEZ01001203:8786-9516
HmHel-5	CAEZ01010614:21123-21598
HmHel-5	CAEZ01009350:17079-18429
HmHel-5	CAEZ01008354:71254-72214
HmHel-5	CAEZ01008041:6389-7792
HmHel-5	CAEZ01002990:2231-3192
HmHel-5	CAEZ01001880:1-899
HmHel-5	CAEZ01007105:1-568
HmHel-5	CAEZ01003249:2320-3597
HmHel-5	CAEZ01002051:6973-8329
HmHel-5	CAEZ01001939:856-1515
HmHel-5	CAEZ01010800:1-395
HmHel-5	CAEZ01009576:43128-44200
HmHel-5	CAEZ01009474:59460-60610
HmHel-5	CAEZ01008506:9464-10451
HmHel-5	CAEZ01007349:4989-5430
HmHel-5	CAEZ01004737:7001-7403
HmHel-5	CAEZ01003936:1-440
HmHel-5	CAEZ01001782:4-525
HmHel-5	CAEZ01011282:75255-76597
HmHel-5	CAEZ01010413:12498-12934
HmHel-5	CAEZ01009944:741-1755
HmHel-5	CAEZ01002906:1-1016
HmHel-5	CAEZ01001849:153-621
HmHel-5	CAEZ01010053:12453-13758
HmHel-5	CAEZ01005895:23300-23712
HmHel-5	CAEZ01005895:13-113
HmHel-5	CAEZ01002267:1786-2810
HmHel-5	CAEZ01001226:985-1595
HmHel-5	CAEZ01011421:17366-17741
HmHel-5	CAEZ01010764:3034-6078
HmHel-5	CAEZ01010628:14334-15381

HmHel-5	CAEZ01008888:1-420
HmHel-5	CAEZ01008888:697-833
HmHel-5	CAEZ01008671:4079-5368
HmHel-5	CAEZ01008231:33216-33631
HmHel-5	CAEZ01008231:24732-25953
HmHel-5	CAEZ01008254:16745-17183
HmHel-5	CAEZ01004446:46-458
HmHel-5	CAEZ01003373:10-467
HmHel-5	CAEZ01003162:2030-3399
HmHel-5	CAEZ01001945:3-835
HmHel-5	CAEZ01010924:74-1140
HmHel-5	CAEZ01009991:53668-54343
HmHel-5	CAEZ01006072:1-380
HmHel-5	CAEZ01005896:1-468
HmHel-5	CAEZ01005181:1-827
HmHel-5	CAEZ01003603:9-416
HmHel-5	CAEZ01002890:12064-13401
HmHel-5	CAEZ01010356:27-648
HmHel-5	CAEZ01008798:6628-7015
HmHel-5	CAEZ01007602:1-836
HmHel-5	CAEZ01003784:19731-21084
HmHel-5	CAEZ01002019:1-594
HmHel-5	CAEZ01001925:1149-1894
HmHel-5	CAEZ01010923:26777-27027
HmHel-5	CAEZ01010923:3038-6255
HmHel-5	CAEZ01006956:1-379
HmHel-5	CAEZ01006450:8011-8478
HmHel-5	CAEZ01002607:135516-141244
HmHel-5	CAEZ01007366:6855-8214
HmHel-5	CAEZ01007118:23622-24986
HmHel-5	CAEZ01006841:6542-6926
HmHel-5	CAEZ01004679:1-408
HmHel-5	CAEZ01008188:1-530
HmHel-5	CAEZ01006549:14326-15731
HmHel-5	CAEZ01002354:3928-5083
HmHel-5	CAEZ01011372:9411-9811
HmHel-5	CAEZ01007185:13808-16140
HmHel-5	CAEZ01005460:1-434
HmHel-5	CAEZ01008882:8188-8807
HmHel-5	CAEZ01008882:45361-46613
HmHel-5	CAEZ01005855:1-371
HmHel-5	CAEZ01005641:31627-32138
HmHel-5	CAEZ01005574:5840-6614
HmHel-5	CAEZ01010271:9088-9950
HmHel-5	CAEZ01005117:7009-7955
HmHel-5	CAEZ01001926:1-350
HmHel-5	CAEZ01010273:126475-126851
HmHel-5	CAEZ01005836:9-394

HmHel-5	CAEZ01005710:1-825
HmHel-5	CAEZ01004953:43-802
HmHel-5	CAEZ01003661:11041-11466
HmHel-5	CAEZ01010102:2036-3136
HmHel-5	CAEZ01008355:1-417
HmHel-5	CAEZ01001892:37531-37717
HmHel-5	CAEZ01001892:1-352
HmHel-5	CAEZ01011280:4821-5184
HmHel-5	CAEZ01008124:1-384
HmHel-5	CAEZ01008124:75839-76963
HmHel-5	CAEZ01006154:1-360
HmHel-5	CAEZ01005521:1-387
HmHel-5	CAEZ01004906:10233-11241
HmHel-5	CAEZ01001465:98129-98502
HmHel-5	CAEZ01001465:110107-110984
HmHel-5	CAEZ01001465:1-831
HmHel-5	CAEZ01010511:40676-41265
HmHel-5	CAEZ01006796:31612-32023
HmHel-5	CAEZ01006326:87177-88363
HmHel-5	CAEZ01005661:620-1002
HmHel-5	CAEZ01002374:1-367
HmHel-5	CAEZ01011142:2-444
HmHel-5	CAEZ01011142:35737-35888
HmHel-5	CAEZ01002717:4749-6005
HmHel-5	CAEZ01002064:40506-40868
HmHel-5	CAEZ01010203:4-410
HmHel-5	CAEZ01008493:6-433
HmHel-5	CAEZ01007938:136911-137302
HmHel-5	CAEZ01007938:40911-41969
HmHel-5	CAEZ01005890:20370-20922
HmHel-5	CAEZ01004939:13259-13673
HmHel-5	CAEZ01001041:404-1512
HmHel-5	CAEZ01009063:27689-29873
HmHel-5	CAEZ01006699:25799-27082
HmHel-5	CAEZ01001189:1-371
HmHel-5	CAEZ01007908:2701-3747
HmHel-5	CAEZ01005026:10810-11411
HmHel-5	CAEZ01001026:12701-14000
HmHel-5	CAEZ01003731:6195-6573
HmHel-5	CAEZ01005662:1-362
HmHel-5	CAEZ01005108:116105-117430
HmHel-5	CAEZ01003737:4349-5707
HmHel-5	CAEZ01011058:11785-12778
HmHel-5	CAEZ01010506:22-417
HmHel-5	CAEZ01008987:2-829
HmHel-5	CAEZ01008255:1-508
HmHel-5	CAEZ01008102:1-356
HmHel-5	CAEZ01006152:148-1094

HmHel-5	CAEZ01005280:5851-6452
HmHel-5	CAEZ01004412:1-733
HmHel-5	CAEZ01004232:9849-10428
HmHel-5	CAEZ01001867:15398-15753
HmHel-5	CAEZ01001512:22-417
HmHel-5	CAEZ01000027:1965-2329
HmHel-5	CAEZ01010769:1225-1596
HmHel-5	CAEZ01009959:883-2160
HmHel-5	CAEZ01009193:1-536
HmHel-5	CAEZ01005385:1927-2971
HmHel-5	CAEZ01011404:744-1110
HmHel-5	CAEZ01009252:11462-12723
HmHel-5	CAEZ01009034:5-528
HmHel-5	CAEZ01006812:12027-12512
HmHel-5	CAEZ01002840:424-1286
HmHel-5	CAEZ01011190:1-735
HmHel-5	CAEZ01009155:7-661
HmHel-5	CAEZ01008161:1-717
HmHel-5	CAEZ01006903:32-355
HmHel-5	CAEZ01006672:12637-13869
HmHel-5	CAEZ01006494:50562-51061
HmHel-5	CAEZ01006009:1-499
HmHel-5	CAEZ01004717:10027-10995
HmHel-5	CAEZ01002843:1572-1976
HmHel-5	CAEZ01002798:27834-28617
HmHel-5	CAEZ01002661:7-409
HmHel-5	CAEZ01001046:1-477
HmHel-5	CAEZ01001162:10182-10905
HmHel-5	CAEZ01007434:14581-17189
HmHel-5	CAEZ01007048:9038-9758
HmHel-5	CAEZ01009618:1-366
HmHel-5	CAEZ01008871:1-369
HmHel-5	CAEZ01002288:3362-3843
HmHel-5	CAEZ01006775:5638-6503
HmHel-5	CAEZ01006351:4-763
HmHel-5	CAEZ01005391:16158-16576
HmHel-5	CAEZ01002250:1-364
HmHel-5	CAEZ01006813:3-739
HmHel-5	CAEZ01005436:28344-28752
HmHel-5	CAEZ01002183:16225-17179
HmHel-5	CAEZ01008823:1-858
HmHel-5	CAEZ01006762:14338-14611
HmHel-5	CAEZ01006762:1-473
HmHel-5	CAEZ01010248:1-744
HmHel-5	CAEZ01008643:53940-55211
HmHel-5	CAEZ01008167:62-875
HmHel-5	CAEZ01005459:51515-52011
HmHel-5	CAEZ01001180:1-711

HmHel-5	CAEZ01001094:1067-1546
HmHel-5	CAEZ01004519:5-374
HmHel-5	CAEZ01001523:2753-3170
HmHel-5	CAEZ01011630:6913-7236
HmHel-5	CAEZ01007038:1527-1938
HmHel-5	CAEZ01010201:1-352
HmHel-5	CAEZ01009659:43896-46866
HmHel-5	CAEZ01006921:13109-13891
HmHel-5	CAEZ01002252:738-1730
HmHel-5	CAEZ01011488:27505-28044
HmHel-5	CAEZ01010687:5999-6450
HmHel-5	CAEZ01001363:1-350
HmHel-5	CAEZ01000018:44-526
HmHel-5	CAEZ01010852:19426-20127
HmHel-5	CAEZ01010182:1365-2154
HmHel-5	CAEZ01001318:1-1066
HmHel-5	CAEZ01009469:3443-3838
HmHel-5	CAEZ01008276:600-1046
HmHel-5	CAEZ01005368:7691-8750
HmHel-5	CAEZ01004673:1-364
HmHel-5	CAEZ01000263:3-424
HmHel-5	CAEZ01008997:5392-6037
HmHel-5	CAEZ01001353:1-343
HmHel-5	CAEZ01009453:1-747
HmHel-5	CAEZ01007703:32763-33714
HmHel-5	CAEZ01001630:657-1013
HmHel-5	CAEZ01011618:897-1415
HmHel-5	CAEZ01001339:5790-6153
HmHel-5	CAEZ01010468:1-700
HmHel-5	CAEZ01005891:1-937
HmHel-5	CAEZ01003994:3-387
HmHel-5	CAEZ01011593:379-1592
HmHel-5	CAEZ01008887:16712-17329
HmHel-5	CAEZ01008648:1-353
HmHel-5	CAEZ01010772:1-926
HmHel-5	CAEZ01006769:32474-33290
HmHel-5	CAEZ01003237:49910-50291
HmHel-5	CAEZ01001704:37-431
HmHel-5	CAEZ01008623:18457-19505
HmHel-5	CAEZ01003196:58385-59636
HmHel-5	CAEZ01011595:1633-2406
HmHel-5	CAEZ01011523:82-468
HmHel-5	CAEZ01011523:67318-68492
HmHel-5	CAEZ01009087:2141-2449
HmHel-5	CAEZ01008758:3121-3874
HmHel-5	CAEZ01008127:13445-14910
HmHel-5	CAEZ01010101:1-304
HmHel-5	CAEZ01007811:26770-27077

HmHel-5	CAEZ01005757:5455-6368
HmHel-5	CAEZ01010583:1344-2284
HmHel-5	CAEZ01009075:5857-6240
HmHel-5	CAEZ01007251:34725-35278
HmHel-5	CAEZ01007502:1-335
HmHel-5	CAEZ01007037:17-402
HmHel-5	CAEZ01008722:36109-37397
HmHel-5	CAEZ01010107:1-296
HmHel-5	CAEZ01009088:1-911
HmHel-5	CAEZ01003398:46420-47526
HmHel-5	CAEZ01001439:39068-40536
HmHel-5	CAEZ01001164:1-339
HmHel-5	CAEZ01009156:884-1212
HmHel-5	CAEZ01008630:1-919
HmHel-5	CAEZ01003602:3116-3469
HmHel-5	CAEZ01010887:18102-18893
HmHel-5	CAEZ01006651:28720-30285
HmHel-5	CAEZ01006637:1-340
HmHel-5	CAEZ01004003:4-512
HmHel-5	CAEZ01008822:72326-72712
HmHel-5	CAEZ01002910:1-297
HmHel-5	CAEZ01000964:1-911
HmHel-5	CAEZ01007234:1649-2544
HmHel-5	CAEZ01004145:3518-4677
HmHel-5	CAEZ01003544:1-1188
HmHel-5	CAEZ01001038:3522-3823
HmHel-5	CAEZ01004121:2-355
HmHel-5	CAEZ01003577:1-362
HmHel-5	CAEZ01001467:755-1071
HmHel-5	CAEZ01005417:1-316
HmHel-5	CAEZ01004168:6094-7172
HmHel-5	CAEZ01002366:445-746
HmHel-5	CAEZ01009628:12100-12392
HmHel-5	CAEZ01004692:3955-4280
HmHel-5	CAEZ01004399:44659-45033
HmHel-5	CAEZ01002065:1-432
HmHel-5	CAEZ01007445:3357-3576
HmHel-5	CAEZ01007445:3580-3880
HmHel-5	CAEZ01004812:1-870
HmHel-5	CAEZ01001760:1-777
HmHel-5	CAEZ01005920:756-1067
HmHel-5	CAEZ01003167:29919-30216
HmHel-5	CAEZ01011010:1-339
HmHel-5	CAEZ01006345:1599-1933
HmHel-5	CAEZ01001085:1-339
HmHel-5	CAEZ01007092:5376-5875
HmHel-5	CAEZ01006142:1436-1783
HmHel-5	CAEZ01004339:25609-25879

HmHel-5	CAEZ01006151:100722-101069
HmHel-5	CAEZ01005737:3-284
HmHel-5	CAEZ01003426:1-311
HmHel-5	CAEZ01001245:723-1226
HmHel-5	CAEZ01009912:2377-2734
HmHel-5	CAEZ01006946:8781-9106
HmHel-5	CAEZ01002812:45244-45636
HmHel-5	CAEZ01008311:1-268
HmHel-5	CAEZ01006744:11341-11623
HmHel-5	CAEZ01011349:6707-7051
HmHel-5	CAEZ01009084:311-912
HmHel-5	CAEZ01004672:2032-2563
HmHel-5	CAEZ01006910:31023-31314
HmHel-5	CAEZ01003913:1-349
HmHel-5	CAEZ01002386:1222-1853
HmHel-5	CAEZ01001858:1-305
HmHel-5	CAEZ01009012:11852-13197
HmHel-5	CAEZ01009012:1173-1305
HmHel-5	CAEZ01000821:9666-10178
HmHel-5	CAEZ01011551:1-731
HmHel-5	CAEZ01011389:24-350
HmHel-5	CAEZ01008341:45135-45838
HmHel-5	CAEZ01005485:79109-79674
HmHel-5	CAEZ01002629:42456-43756
HmHel-5	CAEZ01001443:57367-57626
HmHel-5	CAEZ01009365:25-275
HmHel-5	CAEZ01003880:1938-2833
HmHel-5	CAEZ01011059:1-335
HmHel-5	CAEZ01006113:40-366
HmHel-5	CAEZ01004116:5196-5522
HmHel-5	CAEZ01003623:5442-5747
HmHel-5	CAEZ01000987:633-959
HmHel-5	CAEZ01011167:3499-3805
HmHel-5	CAEZ01008165:2212-2877
HmHel-5	CAEZ01007277:1-696
HmHel-5	CAEZ01008995:3131-3391
HmHel-5	CAEZ01008129:1-533
HmHel-5	CAEZ01007808:114768-115192
HmHel-5	CAEZ01006578:1760-2086
HmHel-5	CAEZ01005921:11-270
HmHel-5	CAEZ01001303:1-436
HmHel-5	CAEZ01008545:24757-25043
HmHel-5	CAEZ01008531:845-1575
HmHel-5	CAEZ01007605:23742-24068
HmHel-5	CAEZ01011597:2808-3068
HmHel-5	CAEZ01007829:1-506
HmHel-5	CAEZ01007482:11349-11906
HmHel-5	CAEZ01006178:23-349

HmHel-5	CAEZ01002723:834-1102
HmHel-5	CAEZ01001117:32-358
HmHel-5	CAEZ01009390:1-285
HmHel-5	CAEZ01011150:2-252
HmHel-5	CAEZ01008782:7966-8357
HmHel-5	CAEZ01007918:2-252
HmHel-5	CAEZ01007470:21670-22248
HmHel-5	CAEZ01007058:1-735
HmHel-5	CAEZ01007031:23-273
HmHel-5	CAEZ01005123:2-277
HmHel-5	CAEZ01004463:1-534
HmHel-5	CAEZ01004124:3-386
HmHel-5	CAEZ01001516:301-627
HmHel-5	CAEZ01001131:4376-4626
HmHel-5	CAEZ01010779:1-583
HmHel-5	CAEZ01010527:32692-33132
HmHel-5	CAEZ01009507:1-383
HmHel-5	CAEZ01009182:24572-25086
HmHel-5	CAEZ01009031:7-339
HmHel-5	CAEZ01006219:1-492
HmHel-5	CAEZ01005383:13268-14311
HmHel-5	CAEZ01003304:47656-48264
HmHel-5	CAEZ01002844:1-435
HmHel-5	CAEZ01005420:29909-33834
HmHel-5	CAEZ01011416:16-406
HmHel-5	CAEZ01010616:52799-53176
HmHel-5	CAEZ01009697:3895-4436
HmHel-5	CAEZ01007838:4846-5096
HmHel-5	CAEZ01004233:1-275
HmHel-5	CAEZ01001672:594-1323
HmHel-5	CAEZ01001563:8852-9552
HmHel-5	CAEZ01005928:20874-21143
HmHel-5	CAEZ01002187:787-1582
HmHel-5	CAEZ01000003:1-407
HmHel-5	CAEZ01008316:49884-50215
HmHel-5	CAEZ01008044:8-538
HmHel-5	CAEZ01007219:4323-4583
HmHel-5	CAEZ01003215:1-478
HmHel-5	CAEZ01000004:1-389
HmHel-5	CAEZ01003576:4543-4778
HmHel-5	CAEZ01007762:1-334
HmHel-5	CAEZ01003302:33122-33596
HmHel-5	CAEZ01002362:3-532
HmHel-5	CAEZ01000071:372-606
HmHel-5	CAEZ01010690:9876-11637
HmHel-5	CAEZ01003435:6861-7097
HmHel-5	CAEZ01010579:10805-10903
HmHel-5	CAEZ01010579:7852-8989

HmHel-5	CAEZ01009764:9-336
HmHel-5	CAEZ01005654:3-329
HmHel-5	CAEZ01001901:6-569
HmHel-5	CAEZ01001817:14732-15317
HmHel-5	CAEZ01001229:958-1253
HmHel-5	CAEZ01008986:20220-20520
HmHel-5	CAEZ01005399:29816-30156
HmHel-5	CAEZ01003861:16871-17171
HmHel-5	CAEZ01003106:1-281
HmHel-5	CAEZ01001087:4298-5081
HmHel-5	CAEZ01010531:1116-1888
HmHel-5	CAEZ01010355:49803-50041
HmHel-5	CAEZ01008202:10856-11578
HmHel-5	CAEZ01007821:96-852
HmHel-5	CAEZ01006784:21285-21789
HmHel-5	CAEZ01003765:1-510
HmHel-5	CAEZ01003274:1-591
HmHel-5	CAEZ01003246:1-227
HmHel-5	CAEZ01001368:15-1024
HmHel-5	CAEZ01000032:22-272
HmHel-5	CAEZ01010945:1-479
HmHel-5	CAEZ01010853:2346-2656
HmHel-5	CAEZ01003072:1-825
HmHel-5	CAEZ01002708:9280-9617
HmHel-5	CAEZ01008870:2518-2770
HmHel-5	CAEZ01010186:1-736
HmHel-5	CAEZ01008115:37195-37429
HmHel-5	CAEZ01003966:1-236
HmHel-5	CAEZ01011076:1-441
HmHel-5	CAEZ01007520:4576-5142
HmHel-5	CAEZ01005590:6972-7747
HmHel-5	CAEZ01001361:9-530
HmHel-5	CAEZ01000227:1-227
HmHel-5	CAEZ01002901:1-535
HmHel-5	CAEZ01001437:36228-36758
HmHel-5	CAEZ01008936:11-263
HmHel-5	CAEZ01005614:1-479
HmHel-5	CAEZ01005471:1-377
HmHel-5	CAEZ01011627:26870-27521
HmHel-5	CAEZ01008806:38965-39480
HmHel-5	CAEZ01004636:3377-4315
HmHel-5	CAEZ01003038:18303-18823
HmHel-5	CAEZ01002920:414-659
HmHel-5	CAEZ01002697:6199-6476
HmHel-5	CAEZ01001251:1629-1890
HmHel-5	CAEZ01011432:441-954
HmHel-5	CAEZ01002673:26-277
HmHel-5	CAEZ01001766:101531-101766

HmHel-5	CAEZ01010436:2-444
HmHel-5	CAEZ01009698:5-493
HmHel-5	CAEZ01009146:381-615
HmHel-5	CAEZ01005369:1-488
HmHel-5	CAEZ01005372:14-263
HmHel-5	CAEZ01005136:38839-39088
HmHel-5	CAEZ01003432:13030-14348
HmHel-5	CAEZ01010313:4553-5553
HmHel-5	CAEZ01009111:1-237
HmHel-5	CAEZ01010982:7607-7842
HmHel-5	CAEZ01005655:6567-6794
HmHel-5	CAEZ01004137:22036-23658
HmHel-5	CAEZ01007282:20126-21791
HmHel-5	CAEZ01005231:9828-10062
HmHel-5	CAEZ01009249:1-538
HmHel-5	CAEZ01011475:1-311
HmHel-5	CAEZ01006859:1-229
HmHel-5	CAEZ01001351:1-539
HmHel-5	CAEZ01003935:1680-2227
HmHel-5	CAEZ01000628:28-279
HmHel-5	CAEZ01008160:1-227
HmHel-5	CAEZ01004836:34916-35214
HmHel-5	CAEZ01008189:25-348
HmHel-5	CAEZ01001790:1-763
HmHel-5	CAEZ01000846:877-1100
HmHel-5	CAEZ01000677:1-282
HmHel-5	CAEZ01005892:1-203
HmHel-5	CAEZ01001464:34244-34494
HmHel-5	CAEZ01007588:222-503
HmHel-5	CAEZ01005512:23645-23964
HmHel-5	CAEZ01010149:10610-10985
HmHel-5	CAEZ01005520:9976-10490
HmHel-5	CAEZ01011281:1299-1770
HmHel-5	CAEZ01009510:26120-26362
HmHel-5	CAEZ01003624:1022-1556
HmHel-5	CAEZ01001787:1-207
HmHel-5	CAEZ01002876:48244-48642
HmHel-5	CAEZ01009154:27-477
HmHel-5	CAEZ01007618:4910-5787
HmHel-5	CAEZ01002189:1056-1838
HmHel-5	CAEZ01001544:1050-1260
HmHel-5	CAEZ01001773:601-818
HmHel-5	CAEZ01006358:1-295
HmHel-5	CAEZ01005243:16164-18345
HmHel-5	CAEZ01005125:1-267
HmHel-5	CAEZ01009624:1330-1553
HmHel-5	CAEZ01005967:15230-16289
HmHel-5	CAEZ01000768:1-591

HmHel-5	CAEZ01003135:2047-2283
HmHel-5	CAEZ01000405:1-202
HmHel-5	CAEZ01003732:5444-5656
HmHel-5	CAEZ01004406:14665-14900
HmHel-5	CAEZ01011255:2384-2971
HmHel-5	CAEZ01004055:6424-6956
HmHel-5	CAEZ01008249:474-1078
HmHel-5	CAEZ01006199:5632-6056
HmHel-5	CAEZ01000321:56-254
HmHel-5	CAEZ01006882:10901-11276
HmHel-5	CAEZ01003939:30794-31047
HmHel-5	CAEZ01002643:57403-57900
HmHel-5	CAEZ01011144:1-202
HmHel-5	CAEZ01008958:1222-1566
HmHel-5	CAEZ01007656:4718-5053
HmHel-5	CAEZ01011623:16187-16609
HmHel-5	CAEZ01006610:15243-15566
HmHel-5	CAEZ01009881:22584-23972
HmHel-5	CAEZ01004244:111878-112793
HmHel-5	CAEZ01006112:1800-1982
HmHel-5	CAEZ01001876:1834-1997
HmHel-5	CAEZ01005670:12-264
HmHel-5	CAEZ01011214:5278-5442
HmHel-5	CAEZ01004469:25-494
HmHel-5	CAEZ01003953:7354-7521
HmHel-5	CAEZ01000176:5102-5317
HmHel-5	CAEZ01008380:2-196
HmHel-5	CAEZ01010167:1-171
HmHel-5	CAEZ01009957:25603-25800
HmHel-5	CAEZ01007350:1-153
HmHel-5	CAEZ01006273:2-174
HmHel-5	CAEZ01004509:8348-8936
HmHel-5	CAEZ01003943:3885-4750
HmHel-5	CAEZ01010658:9542-9750
HmHel-5	CAEZ01001096:1-555
HmHel-5	CAEZ01007737:6-194
HmHel-5	CAEZ01005027:1-187
HmHel-5	CAEZ01010548:1-441
HmHel-5	CAEZ01007322:11-1553
HmHel-5	CAEZ01002718:4-566
HmHel-5	CAEZ01003912:9280-9682
HmHel-5	CAEZ01007262:5238-5389
HmHel-5	CAEZ01009900:2109-2236
HmHel-5	CAEZ01008644:595-721
HmHel-5	CAEZ01007252:96579-96729
HmHel-5	CAEZ01004597:21737-21887
HmHel-5	CAEZ01004597:21587-21676
HmHel-5	CAEZ01010890:8042-8170

HmHel-5	CAEZ01008471:609-773
HmHel-5	CAEZ01005792:2-408
HmHel-5	CAEZ01004942:82114-82265
HmHel-5	CAEZ01002963:3954-4105
HmHel-5	CAEZ01010608:5713-5982
HmHel-5	CAEZ01006182:818-954
HmHel-5	CAEZ01009593:10968-11591
HmHel-5	CAEZ01004521:936-1054
HmHel-5	CAEZ01008056:19-130
HmHel-5	CAEZ01001505:3-125
HmHel-5	CAEZ01007208:37861-37997
HmHel-5	CAEZ01006161:18309-18444
HmHel-5	CAEZ01007519:5298-5413
HmHel-5	CAEZ01006422:15949-16085
HmHel-5	CAEZ01003826:20396-20531
HmHel-5	CAEZ01009227:7423-7557
HmHel-5	CAEZ01011555:1-138
HmHel-5	CAEZ01001286:5581-5696
HmHel-5	CAEZ01008524:22883-22985
HmHel-5	CAEZ01010539:9356-9485
HmHel-5	CAEZ01000773:2379-2487
HmHel-5	CAEZ01000394:1-92
HmHel-5	CAEZ01009024:1190-1337
HmHel-5	CAEZ01004561:1-104
HmHel-5	CAEZ01006559:19835-20086
HmHel-5	CAEZ01002641:1916-2023
HmHel-5	CAEZ01005987:22869-22970
HmHel-5	CAEZ01006277:55-322
HmHel-5	CAEZ01009074:816-925
HmHel-5	CAEZ01000400:4-92
HmHel-5	CAEZ01000387:1-85
HmHel-5	CAEZ01001287:1-84
HmHel-5	CAEZ01009475:23637-23808
HmHel-5	CAEZ01006005:14628-14716
HmHel-5	CAEZ01009437:22713-22813
HmHel-5	CAEZ01004848:1215-1294
	:-
HmHel-6	CAEZ01004321:16494-16747
HmHel-6	CAEZ01006154:23981-24104
HmHel-6	CAEZ01006154:45235-45472
HmHel-6	CAEZ01006154:58384-66783
HmHel-6	CAEZ01006154:91817-92028
HmHel-6	CAEZ01006154:134207-134329
HmHel-6	CAEZ01006154:171910-172091
HmHel-6	CAEZ01006154:201784-209594
HmHel-6	CAEZ01006154:231385-234112
HmHel-6	CAEZ01006154:261771-266924
HmHel-6	CAEZ01006154:284981-285188

HmHel-6	CAEZ01006154:260965-261197
HmHel-6	CAEZ01006154:220842-221080
HmHel-6	CAEZ01006154:198038-198170
HmHel-6	CAEZ01006154:173077-173279
HmHel-6	CAEZ01006154:149517-149685
HmHel-6	CAEZ01006154:74246-74482
HmHel-6	CAEZ01006154:51680-51940
HmHel-6	CAEZ01006154:30544-30751
HmHel-6	CAEZ01003194:6429-6539
HmHel-6	CAEZ01003194:62960-65441
HmHel-6	CAEZ01003194:64403-72064
HmHel-6	CAEZ01003194:26631-26791
HmHel-6	CAEZ01006295:6797-6912
HmHel-6	CAEZ01006295:39327-39435
HmHel-6	CAEZ01006295:22278-22522
HmHel-6	CAEZ01005633:8059-8312
HmHel-6	CAEZ01005633:14716-14967
HmHel-6	CAEZ01008921:3775-4043
HmHel-6	CAEZ01008921:38186-38301
HmHel-6	CAEZ01008921:112887-113081
HmHel-6	CAEZ01008921:154329-154571
HmHel-6	CAEZ01008921:125443-132001
HmHel-6	CAEZ01008921:70568-70679
HmHel-6	CAEZ01008921:46905-55247
HmHel-6	CAEZ01008921:10354-10499
HmHel-6	CAEZ01007730:6038-6222
HmHel-6	CAEZ01007730:79197-83063
HmHel-6	CAEZ01007730:95898-103026
HmHel-6	CAEZ01007730:118983-119078
HmHel-6	CAEZ01007730:95300-95482
HmHel-6	CAEZ01007730:54208-54296
HmHel-6	CAEZ01007730:35462-35605
HmHel-6	CAEZ01003413:13128-13249
HmHel-6	CAEZ01003413:28337-28584
HmHel-6	CAEZ01003413:37194-44579
HmHel-6	CAEZ01005711:39809-39982
HmHel-6	CAEZ01005711:85028-95104
HmHel-6	CAEZ01005711:134588-144473
HmHel-6	CAEZ01005711:179603-179717
HmHel-6	CAEZ01005711:175867-175991
HmHel-6	CAEZ01005711:90845-91068
HmHel-6	CAEZ01005711:37324-37577
HmHel-6	CAEZ01005711:19612-19870
HmHel-6	CAEZ01005711:2405-2655
HmHel-6	CAEZ01002870:57139-58701
HmHel-6	CAEZ01002870:76883-77097
HmHel-6	CAEZ01002870:105657-105801
HmHel-6	CAEZ01002870:104708-104801

HmHel-6	CAEZ01002870:89311-89536
HmHel-6	CAEZ01002870:46726-52401
HmHel-6	CAEZ01002870:13230-13378
HmHel-6	CAEZ01003753:11949-12192
HmHel-6	CAEZ01003753:12686-12848
HmHel-6	CAEZ01008941:5098-5349
HmHel-6	CAEZ01008941:7935-9944
HmHel-6	CAEZ01006863:1661-1904
HmHel-6	CAEZ01006863:25983-27073
HmHel-6	CAEZ01006863:96662-103534
HmHel-6	CAEZ01006863:105978-106199
HmHel-6	CAEZ01006863:61112-61309
HmHel-6	CAEZ01006863:22533-22770
HmHel-6	CAEZ01001740:34576-34759
HmHel-6	CAEZ01001740:50167-55117
HmHel-6	CAEZ01001740:90026-90272
HmHel-6	CAEZ01001740:108038-113644
HmHel-6	CAEZ01001740:142478-145605
HmHel-6	CAEZ01001740:178692-178833
HmHel-6	CAEZ01001740:138541-138677
HmHel-6	CAEZ01001740:72202-72416
HmHel-6	CAEZ01001740:59558-59683
HmHel-6	CAEZ01001740:38001-38194
HmHel-6	CAEZ01011171:4281-4534
HmHel-6	CAEZ01010720:2452-2638
HmHel-6	CAEZ01010720:28791-28960
HmHel-6	CAEZ01008523:824-986
HmHel-6	CAEZ01008523:27552-27689
HmHel-6	CAEZ01008523:9124-9377
HmHel-6	CAEZ01007722:3355-6367
HmHel-6	CAEZ01007722:28569-28697
HmHel-6	CAEZ01007722:54943-55141
HmHel-6	CAEZ01007722:153949-154918
HmHel-6	CAEZ01007722:163902-164118
HmHel-6	CAEZ01007722:45385-46119
HmHel-6	CAEZ01007722:17080-17590
HmHel-6	CAEZ01005044:6646-6743
HmHel-6	CAEZ01005044:28995-29093
HmHel-6	CAEZ01005044:46740-46937
HmHel-6	CAEZ01005044:82692-84298
HmHel-6	CAEZ01005044:145781-146606
HmHel-6	CAEZ01005044:173030-173207
HmHel-6	CAEZ01005044:150193-153046
HmHel-6	CAEZ01005044:130563-130725
HmHel-6	CAEZ01005044:100723-100947
HmHel-6	CAEZ01005044:55258-55411
HmHel-6	CAEZ01005044:29845-30043
HmHel-6	CAEZ01005044:7347-7469

HmHel-6	CAEZ01002798:5355-5604
HmHel-6	CAEZ01002798:40551-40714
HmHel-6	CAEZ01002182:20-247
HmHel-6	CAEZ01002182:29181-36003
HmHel-6	CAEZ01002182:68260-73630
HmHel-6	CAEZ01002182:85079-85332
HmHel-6	CAEZ01002182:124561-124734
HmHel-6	CAEZ01002182:129097-139070
HmHel-6	CAEZ01002182:106877-107098
HmHel-6	CAEZ01002182:41472-43759
HmHel-6	CAEZ01002182:12185-12346
HmHel-6	CAEZ01005862:10458-10694
HmHel-6	CAEZ01010729:6413-6659
HmHel-6	CAEZ01010729:23531-23780
HmHel-6	CAEZ01010729:52665-52816
HmHel-6	CAEZ01010729:66067-66318
HmHel-6	CAEZ01010729:99071-103296
HmHel-6	CAEZ01010729:102490-102657
HmHel-6	CAEZ01010729:80518-84206
HmHel-6	CAEZ01010729:54451-54639
HmHel-6	CAEZ01010729:29980-30174
HmHel-6	CAEZ01009701:14814-15061
HmHel-6	CAEZ01009701:9861-12171
HmHel-6	CAEZ01006817:13361-13525
HmHel-6	CAEZ01010364:2882-6085
HmHel-6	CAEZ01010364:19163-19280
HmHel-6	CAEZ01010364:13007-20020
HmHel-6	CAEZ01009351:5723-8997
HmHel-6	CAEZ01007870:1502-1583
HmHel-6	CAEZ01007870:100013-100264
HmHel-6	CAEZ01007870:104109-104217
HmHel-6	CAEZ01007870:85579-85762
HmHel-6	CAEZ01007870:44078-52331
HmHel-6	CAEZ01011626:14856-15109
HmHel-6	CAEZ01011626:50811-50910
HmHel-6	CAEZ01011626:71651-71884
HmHel-6	CAEZ01011626:93414-93603
HmHel-6	CAEZ01011626:121512-133654
HmHel-6	CAEZ01011626:157120-157260
HmHel-6	CAEZ01011626:141531-145723
HmHel-6	CAEZ01011626:117549-117693
HmHel-6	CAEZ01011626:85956-92080
HmHel-6	CAEZ01011626:74087-75216
HmHel-6	CAEZ01011626:14265-29456
HmHel-6	CAEZ01009862:5046-5298
HmHel-6	CAEZ01007273:14196-14336
HmHel-6	CAEZ01007273:57311-62250
HmHel-6	CAEZ01007273:93384-93508

HmHel-6	CAEZ01007273:105584-105693
HmHel-6	CAEZ01007273:162160-162374
HmHel-6	CAEZ01007273:77427-80202
HmHel-6	CAEZ01007273:58487-58619
HmHel-6	CAEZ01007273:30756-30877
HmHel-6	CAEZ01007273:11290-11408
HmHel-6	CAEZ01006888:3816-8615
HmHel-6	CAEZ01006888:34563-34810
HmHel-6	CAEZ01006888:42926-43010
HmHel-6	CAEZ01006888:6609-6800
HmHel-6	CAEZ01006328:6975-7068
HmHel-6	CAEZ01006328:20696-20919
HmHel-6	CAEZ01006328:61641-61853
HmHel-6	CAEZ01006328:4139-5724
HmHel-6	CAEZ01005646:9932-10125
HmHel-6	CAEZ01005646:19130-19383
HmHel-6	CAEZ01005175:4002-4237
HmHel-6	CAEZ01005175:49031-49284
HmHel-6	CAEZ01005175:35431-35624
HmHel-6	CAEZ01002322:7383-7636
HmHel-6	CAEZ01001742:10715-10849
HmHel-6	CAEZ01001742:17596-17849
HmHel-6	CAEZ01008977:18419-18649
HmHel-6	CAEZ01008977:4164-4415
HmHel-6	CAEZ01008210:6006-6155
HmHel-6	CAEZ01008210:16357-16615
HmHel-6	CAEZ01008210:28211-29548
HmHel-6	CAEZ01008210:62867-63119
HmHel-6	CAEZ01008210:35298-35542
HmHel-6	CAEZ01008210:3173-3327
HmHel-6	CAEZ01010792:18260-18459
HmHel-6	CAEZ01009447:5711-5954
HmHel-6	CAEZ01008741:5120-5338
HmHel-6	CAEZ01008741:26675-26922
HmHel-6	CAEZ01008741:10854-11939
HmHel-6	CAEZ01005594:602-802
HmHel-6	CAEZ01005594:4267-4522
HmHel-6	CAEZ01003815:5503-5758
HmHel-6	CAEZ01011361:9278-9481
HmHel-6	CAEZ01011361:42041-42217
HmHel-6	CAEZ01011361:73352-73443
HmHel-6	CAEZ01011361:34133-34383
HmHel-6	CAEZ01009525:8620-8874
HmHel-6	CAEZ01009525:10011-10134
HmHel-6	CAEZ01009326:2791-3047
HmHel-6	CAEZ01009326:20805-21016
HmHel-6	CAEZ01009326:1486-1788
HmHel-6	CAEZ01002964:16136-16311

HmHel-6	CAEZ01002964:43527-46213
HmHel-6	CAEZ01002964:31091-31203
HmHel-6	CAEZ01002964:13305-13514
HmHel-6	CAEZ01002732:90302-90440
HmHel-6	CAEZ01002732:76711-76864
HmHel-6	CAEZ01002732:58977-59130
HmHel-6	CAEZ01002732:34051-34305
HmHel-6	CAEZ01002732:19907-20011
HmHel-6	CAEZ01010498:8682-8923
HmHel-6	CAEZ01010498:6007-6116
HmHel-6	CAEZ01009871:4565-4693
HmHel-6	CAEZ01009871:29935-30015
HmHel-6	CAEZ01009871:18871-19106
HmHel-6	CAEZ01008960:28262-28471
HmHel-6	CAEZ01008960:56899-57048
HmHel-6	CAEZ01008960:93869-94602
HmHel-6	CAEZ01008960:115918-116115
HmHel-6	CAEZ01008960:130813-130992
HmHel-6	CAEZ01008960:157064-158859
HmHel-6	CAEZ01008960:253575-253756
HmHel-6	CAEZ01008960:195731-195983
HmHel-6	CAEZ01008960:152429-152682
HmHel-6	CAEZ01008960:130147-130373
HmHel-6	CAEZ01008960:107980-108130
HmHel-6	CAEZ01008960:71484-76123
HmHel-6	CAEZ01008527:3262-3503
HmHel-6	CAEZ01008527:12866-12989
HmHel-6	CAEZ01008456:11798-11961
HmHel-6	CAEZ01006968:16556-16809
HmHel-6	CAEZ01006968:17-150
HmHel-6	CAEZ01006590:35660-35912
HmHel-6	CAEZ01006590:2582-2785
HmHel-6	CAEZ01005721:2147-3068
HmHel-6	CAEZ01005721:90588-90803
HmHel-6	CAEZ01005721:109931-110094
HmHel-6	CAEZ01005721:141922-142156
HmHel-6	CAEZ01005721:80903-81058
HmHel-6	CAEZ01005721:61045-69636
HmHel-6	CAEZ01005721:42180-42359
HmHel-6	CAEZ01005721:22896-23074
HmHel-6	CAEZ01005136:31943-32109
HmHel-6	CAEZ01005136:17994-18121
HmHel-6	CAEZ01004896:3043-4663
HmHel-6	CAEZ01004896:7324-7576
HmHel-6	CAEZ01003798:1973-2179
HmHel-6	CAEZ01002887:3450-3665
HmHel-6	CAEZ01001210:4576-4825
HmHel-6	CAEZ01001210:297-430

HmHel-6	CAEZ01010301:4722-12843
HmHel-6	CAEZ01010301:60071-60222
HmHel-6	CAEZ01010301:76476-83426
HmHel-6	CAEZ01010301:80130-80368
HmHel-6	CAEZ01010301:24852-32955
HmHel-6	CAEZ01010301:2053-2158
HmHel-6	CAEZ01009586:2527-2754
HmHel-6	CAEZ01009586:52292-52508
HmHel-6	CAEZ01009586:66768-66983
HmHel-6	CAEZ01009586:70324-70579
HmHel-6	CAEZ01007898:26199-26325
HmHel-6	CAEZ01007898:23181-23421
HmHel-6	CAEZ01006440:67843-68017
HmHel-6	CAEZ01006440:90455-90603
HmHel-6	CAEZ01006440:109594-109728
HmHel-6	CAEZ01006440:135759-135890
HmHel-6	CAEZ01006440:115526-115624
HmHel-6	CAEZ01006440:89618-89862
HmHel-6	CAEZ01006440:59121-59202
HmHel-6	CAEZ01006440:3634-3735
HmHel-6	CAEZ01005418:5640-5796
HmHel-6	CAEZ01005418:26235-26376
HmHel-6	CAEZ01005418:46921-47163
HmHel-6	CAEZ01005418:81305-81551
HmHel-6	CAEZ01005418:112874-112955
HmHel-6	CAEZ01005418:130033-130251
HmHel-6	CAEZ01005418:147241-147443
HmHel-6	CAEZ01005418:194059-194256
HmHel-6	CAEZ01005418:208560-208667
HmHel-6	CAEZ01005418:226009-230775
HmHel-6	CAEZ01005418:295984-303223
HmHel-6	CAEZ01005418:351512-351752
HmHel-6	CAEZ01005418:315796-315978
HmHel-6	CAEZ01005418:231636-231840
HmHel-6	CAEZ01005418:211099-211289
HmHel-6	CAEZ01005418:181363-190920
HmHel-6	CAEZ01005418:160336-160548
HmHel-6	CAEZ01005418:132064-132314
HmHel-6	CAEZ01005418:110153-110399
HmHel-6	CAEZ01005418:91562-91675
HmHel-6	CAEZ01005418:60179-60382
HmHel-6	CAEZ01003825:7368-7491
HmHel-6	CAEZ01003825:3791-4039
HmHel-6	CAEZ01002429:11831-11924
HmHel-6	CAEZ01002429:73556-73722
HmHel-6	CAEZ01002429:64673-68214
HmHel-6	CAEZ01002028:684-924
HmHel-6	CAEZ01002028:3695-3837

HmHel-6	CAEZ01009969:12932-13160
HmHel-6	CAEZ01009969:27988-28239
HmHel-6	CAEZ01009969:2963-3766
HmHel-6	CAEZ01006666:18267-20460
HmHel-6	CAEZ01006666:51563-51779
HmHel-6	CAEZ01006666:45504-55375
HmHel-6	CAEZ01006666:9194-9319
HmHel-6	CAEZ01006036:3752-4003
HmHel-6	CAEZ01006036:7319-7526
HmHel-6	CAEZ01005499:2193-2316
HmHel-6	CAEZ01005499:48591-48704
HmHel-6	CAEZ01005499:73309-73551
HmHel-6	CAEZ01005499:75637-75851
HmHel-6	CAEZ01005499:9996-11805
HmHel-6	CAEZ01002331:708-959
HmHel-6	CAEZ01002331:26896-27113
HmHel-6	CAEZ01009801:30574-30711
HmHel-6	CAEZ01009801:71337-71430
HmHel-6	CAEZ01009801:65865-66113
HmHel-6	CAEZ01009801:33609-33827
HmHel-6	CAEZ01008449:12976-13057
HmHel-6	CAEZ01008449:16214-16316
HmHel-6	CAEZ01006398:5945-6061
HmHel-6	CAEZ01006398:17621-17795
HmHel-6	CAEZ01006398:39189-39435
HmHel-6	CAEZ01006398:47613-52816
HmHel-6	CAEZ01006398:36432-36594
HmHel-6	CAEZ01005827:36721-36851
HmHel-6	CAEZ01005827:61851-61955
HmHel-6	CAEZ01005827:15639-15812
HmHel-6	CAEZ01005827:3515-3765
HmHel-6	CAEZ01005208:6292-6479
HmHel-6	CAEZ01005208:75637-75884
HmHel-6	CAEZ01005208:82678-82764
HmHel-6	CAEZ01005208:65138-65353
HmHel-6	CAEZ01005208:32121-32279
HmHel-6	CAEZ01005208:11527-11651
HmHel-6	CAEZ01005206:39401-46039
HmHel-6	CAEZ01005206:43458-45120
HmHel-6	CAEZ01005206:22462-22716
HmHel-6	CAEZ01003026:5314-5552
HmHel-6	CAEZ01003020:8485-8619
HmHel-6	CAEZ01003020:24871-25083
HmHel-6	CAEZ01003020:4439-4584
HmHel-6	CAEZ01000910:14588-18770
HmHel-6	CAEZ01000910:40871-41121
HmHel-6	CAEZ01011492:11541-11744
HmHel-6	CAEZ01011492:9659-9900

HmHel-6	CAEZ01010751:18576-18741
HmHel-6	CAEZ01010751:3972-4225
HmHel-6	CAEZ01010217:12208-12344
HmHel-6	CAEZ01010217:34782-40238
HmHel-6	CAEZ01010217:77081-77168
HmHel-6	CAEZ01010217:94363-94575
HmHel-6	CAEZ01010217:113969-114173
HmHel-6	CAEZ01010217:120672-120797
HmHel-6	CAEZ01010217:81403-81595
HmHel-6	CAEZ01010217:62014-62267
HmHel-6	CAEZ01010217:44473-44579
HmHel-6	CAEZ01010217:8070-8154
HmHel-6	CAEZ01009392:2447-2651
HmHel-6	CAEZ01009392:3949-4126
HmHel-6	CAEZ01008915:3652-6085
HmHel-6	CAEZ01008915:26253-33897
HmHel-6	CAEZ01008915:69731-69816
HmHel-6	CAEZ01008915:81081-85759
HmHel-6	CAEZ01008915:119958-120092
HmHel-6	CAEZ01008915:137555-137694
HmHel-6	CAEZ01008915:151199-160302
HmHel-6	CAEZ01008915:248422-248594
HmHel-6	CAEZ01008915:262253-262334
HmHel-6	CAEZ01008915:295532-295685
HmHel-6	CAEZ01008915:316365-316576
HmHel-6	CAEZ01008915:276498-276748
HmHel-6	CAEZ01008915:223415-223670
HmHel-6	CAEZ01008915:199233-199405
HmHel-6	CAEZ01008915:135291-135400
HmHel-6	CAEZ01008915:115424-115585
HmHel-6	CAEZ01008915:83236-83442
HmHel-6	CAEZ01008915:51932-52073
HmHel-6	CAEZ01008915:4822-11224
HmHel-6	CAEZ01008546:1923-2172
HmHel-6	CAEZ01007809:10466-10662
HmHel-6	CAEZ01007809:34297-34457
HmHel-6	CAEZ01007809:86409-86539
HmHel-6	CAEZ01007809:108571-109233
HmHel-6	CAEZ01007809:154102-154231
HmHel-6	CAEZ01007809:210140-217624
HmHel-6	CAEZ01007809:264475-264710
HmHel-6	CAEZ01007809:288945-295274
HmHel-6	CAEZ01007809:323304-323437
HmHel-6	CAEZ01007809:324470-324567
HmHel-6	CAEZ01007809:310134-310345
HmHel-6	CAEZ01007809:285447-285582
HmHel-6	CAEZ01007809:263648-263735
HmHel-6	CAEZ01007809:252114-253536

HmHel-6	CAEZ01007809:236043-236219
HmHel-6	CAEZ01007809:209046-209259
HmHel-6	CAEZ01007809:187828-187956
HmHel-6	CAEZ01007809:166081-166275
HmHel-6	CAEZ01007809:147797-148049
HmHel-6	CAEZ01007809:97322-97407
HmHel-6	CAEZ01007809:34218-37745
HmHel-6	CAEZ01007809:8134-8217
HmHel-6	CAEZ01007778:17510-17763
HmHel-6	CAEZ01007747:46515-46602
HmHel-6	CAEZ01007747:61224-61408
HmHel-6	CAEZ01007747:125435-125675
HmHel-6	CAEZ01007747:216719-216931
HmHel-6	CAEZ01007747:256468-256604
HmHel-6	CAEZ01007747:259687-263278
HmHel-6	CAEZ01007747:242480-242652
HmHel-6	CAEZ01007747:187476-199888
HmHel-6	CAEZ01007747:160541-160666
HmHel-6	CAEZ01007747:90075-90168
HmHel-6	CAEZ01007747:31774-31993
HmHel-6	CAEZ01007731:23712-23800
HmHel-6	CAEZ01007731:40421-40567
HmHel-6	CAEZ01007731:74906-75091
HmHel-6	CAEZ01007731:95688-95776
HmHel-6	CAEZ01007731:115505-115754
HmHel-6	CAEZ01007731:90384-90515
HmHel-6	CAEZ01007731:21430-21630
HmHel-6	CAEZ01007080:5620-12527
HmHel-6	CAEZ01007080:22079-22197
HmHel-6	CAEZ01007080:4964-5114
HmHel-6	CAEZ01006897:7893-8599
HmHel-6	CAEZ01006897:35193-35281
HmHel-6	CAEZ01006897:10760-11009
HmHel-6	CAEZ01006196:11759-21023
HmHel-6	CAEZ01006196:67195-67311
HmHel-6	CAEZ01006196:129495-129589
HmHel-6	CAEZ01006196:142477-142635
HmHel-6	CAEZ01006196:187539-187660
HmHel-6	CAEZ01006196:212918-213010
HmHel-6	CAEZ01006196:252173-252258
HmHel-6	CAEZ01006196:233384-233543
HmHel-6	CAEZ01006196:194360-196440
HmHel-6	CAEZ01006196:176915-177102
HmHel-6	CAEZ01006196:131734-131917
HmHel-6	CAEZ01006196:105191-105307
HmHel-6	CAEZ01006196:79175-79357
HmHel-6	CAEZ01006196:56166-56376
HmHel-6	CAEZ01005394:6413-6666

HmHel-6	CAEZ01005394:35946-36942
HmHel-6	CAEZ01011272:7546-7793
HmHel-6	CAEZ01010805:49053-49228
HmHel-6	CAEZ01010805:72049-72180
HmHel-6	CAEZ01010805:109993-113023
HmHel-6	CAEZ01010805:113962-118035
HmHel-6	CAEZ01010805:54022-54265
HmHel-6	CAEZ01010805:35177-35338
HmHel-6	CAEZ01010805:18847-19061
HmHel-6	CAEZ01010542:41712-41960
HmHel-6	CAEZ01010542:50804-50981
HmHel-6	CAEZ01009085:41229-41364
HmHel-6	CAEZ01009085:52928-53178
HmHel-6	CAEZ01009085:87068-96260
HmHel-6	CAEZ01009085:108587-108667
HmHel-6	CAEZ01009085:79951-80210
HmHel-6	CAEZ01009085:48913-49061
HmHel-6	CAEZ01009085:36641-36840
HmHel-6	CAEZ01009085:17426-17674
HmHel-6	CAEZ01008026:4560-4808
HmHel-6	CAEZ01006864:3526-3771
HmHel-6	CAEZ01006864:16624-16838
HmHel-6	CAEZ01004019:7667-7919
HmHel-6	CAEZ01003861:13250-13475
HmHel-6	CAEZ01003861:22523-22652
HmHel-6	CAEZ01011487:2277-2409
HmHel-6	CAEZ01011487:12542-12667
HmHel-6	CAEZ01010824:7190-7437
HmHel-6	CAEZ01010824:14607-24491
HmHel-6	CAEZ01010288:38011-44694
HmHel-6	CAEZ01010288:87124-87210
HmHel-6	CAEZ01010288:101627-102743
HmHel-6	CAEZ01010288:122057-122173
HmHel-6	CAEZ01010288:183454-183640
HmHel-6	CAEZ01010288:206735-206870
HmHel-6	CAEZ01010288:271502-271672
HmHel-6	CAEZ01010288:283405-283619
HmHel-6	CAEZ01010288:263219-263448
HmHel-6	CAEZ01010288:198271-198367
HmHel-6	CAEZ01010288:176371-176533
HmHel-6	CAEZ01010288:146159-146331
HmHel-6	CAEZ01010288:99867-104393
HmHel-6	CAEZ01010288:59666-59877
HmHel-6	CAEZ01010288:46274-46435
HmHel-6	CAEZ01010288:21817-27861
HmHel-6	CAEZ01007858:31276-31527
HmHel-6	CAEZ01007858:89620-89863
HmHel-6	CAEZ01007858:117635-122041

HmHel-6	CAEZ01007858:138919-139130
HmHel-6	CAEZ01007858:174271-174439
HmHel-6	CAEZ01007858:167021-173725
HmHel-6	CAEZ01007858:64096-64260
HmHel-6	CAEZ01007858:53257-53358
HmHel-6	CAEZ01007858:19076-23677
HmHel-6	CAEZ01007480:5129-12002
HmHel-6	CAEZ01007480:32770-32958
HmHel-6	CAEZ01006348:1023-1278
HmHel-6	CAEZ01006348:35235-35500
HmHel-6	CAEZ01006348:54058-54191
HmHel-6	CAEZ01006348:7006-7090
HmHel-6	CAEZ01005660:1235-1332
HmHel-6	CAEZ01005660:23912-24158
HmHel-6	CAEZ01005660:70655-76823
HmHel-6	CAEZ01005660:50460-50662
HmHel-6	CAEZ01005660:19574-19782
HmHel-6	CAEZ01005147:16189-16350
HmHel-6	CAEZ01005147:66428-66584
HmHel-6	CAEZ01005147:100318-100424
HmHel-6	CAEZ01005147:129915-130134
HmHel-6	CAEZ01005147:85287-85401
HmHel-6	CAEZ01005147:44730-44899
HmHel-6	CAEZ01002341:13228-15866
HmHel-6	CAEZ01002341:12249-12390
HmHel-6	CAEZ01011519:2595-2846
HmHel-6	CAEZ01011519:31684-31805
HmHel-6	CAEZ01011519:41834-41937
HmHel-6	CAEZ01011519:100714-106032
HmHel-6	CAEZ01011519:134853-134955
HmHel-6	CAEZ01011519:145425-145660
HmHel-6	CAEZ01011519:166956-167151
HmHel-6	CAEZ01011519:124153-124390
HmHel-6	CAEZ01011519:80663-83604
HmHel-6	CAEZ01011519:45930-46139
HmHel-6	CAEZ01011519:23418-23612
HmHel-6	CAEZ01009048:20785-20939
HmHel-6	CAEZ01009048:38196-38293
HmHel-6	CAEZ01009048:22496-31164
HmHel-6	CAEZ01009048:11466-11600
HmHel-6	CAEZ01007626:883-1009
HmHel-6	CAEZ01007626:78719-78972
HmHel-6	CAEZ01007626:124777-124889
HmHel-6	CAEZ01007626:52130-52260
HmHel-6	CAEZ01007059:2067-2278
HmHel-6	CAEZ01007059:9942-10191
HmHel-6	CAEZ01006853:10040-10155
HmHel-6	CAEZ01006853:31683-31816

HmHel-6	CAEZ01006853:35576-35830
HmHel-6	CAEZ01006853:16011-16124
HmHel-6	CAEZ01006395:5577-5659
HmHel-6	CAEZ01006395:41279-41399
HmHel-6	CAEZ01006395:60155-60838
HmHel-6	CAEZ01006395:73901-74050
HmHel-6	CAEZ01006395:32182-32327
HmHel-6	CAEZ01006395:1833-11282
HmHel-6	CAEZ01006012:18202-23654
HmHel-6	CAEZ01006012:48892-49039
HmHel-6	CAEZ01006012:58414-58591
HmHel-6	CAEZ01006012:28417-28544
HmHel-6	CAEZ01005235:5239-5415
HmHel-6	CAEZ01005235:127742-127873
HmHel-6	CAEZ01005235:195096-195218
HmHel-6	CAEZ01005235:253443-258200
HmHel-6	CAEZ01005235:302461-302657
HmHel-6	CAEZ01005235:248782-249024
HmHel-6	CAEZ01005235:231192-231425
HmHel-6	CAEZ01005235:146781-146957
HmHel-6	CAEZ01005235:133440-133657
HmHel-6	CAEZ01005235:114114-119360
HmHel-6	CAEZ01005235:62092-64720
HmHel-6	CAEZ01005235:28611-28775
HmHel-6	CAEZ01005235:2519-2610
HmHel-6	CAEZ01004734:3228-3317
HmHel-6	CAEZ01004734:15700-15850
HmHel-6	CAEZ01004734:45248-47349
HmHel-6	CAEZ01004734:9422-9515
HmHel-6	CAEZ01003682:1767-2018
HmHel-6	CAEZ01003528:4271-4517
HmHel-6	CAEZ01011538:1316-1569
HmHel-6	CAEZ01011538:197-305
HmHel-6	CAEZ01010701:9792-10045
HmHel-6	CAEZ01010701:91085-92759
HmHel-6	CAEZ01010701:133787-133994
HmHel-6	CAEZ01010701:176139-176307
HmHel-6	CAEZ01010701:198526-198625
HmHel-6	CAEZ01010701:257390-257499
HmHel-6	CAEZ01010701:239329-239430
HmHel-6	CAEZ01010701:211760-211845
HmHel-6	CAEZ01010701:197590-197677
HmHel-6	CAEZ01010701:174416-174669
HmHel-6	CAEZ01010701:90465-93634
HmHel-6	CAEZ01010701:44429-44565
HmHel-6	CAEZ01010701:25461-25561
HmHel-6	CAEZ01010701:13088-14955
HmHel-6	CAEZ01010039:25-170

HmHel-6	CAEZ01010039:14873-14953
HmHel-6	CAEZ01010039:27132-27385
HmHel-6	CAEZ01010039:53774-53996
HmHel-6	CAEZ01010039:90124-90234
HmHel-6	CAEZ01010039:110524-114979
HmHel-6	CAEZ01010039:146021-146276
HmHel-6	CAEZ01010039:165173-165256
HmHel-6	CAEZ01010039:182937-183118
HmHel-6	CAEZ01010039:195307-195389
HmHel-6	CAEZ01010039:206086-206208
HmHel-6	CAEZ01010039:149556-149694
HmHel-6	CAEZ01010039:20802-32299
HmHel-6	CAEZ01009538:6401-6638
HmHel-6	CAEZ01009421:7417-7534
HmHel-6	CAEZ01009421:31398-31488
HmHel-6	CAEZ01009421:51603-53955
HmHel-6	CAEZ01009030:5272-5509
HmHel-6	CAEZ01009030:70461-70709
HmHel-6	CAEZ01009030:50916-51075
HmHel-6	CAEZ01008737:3916-4168
HmHel-6	CAEZ01008737:50014-50151
HmHel-6	CAEZ01008737:90987-91188
HmHel-6	CAEZ01008737:115738-119320
HmHel-6	CAEZ01008737:116374-116627
HmHel-6	CAEZ01008737:48596-57094
HmHel-6	CAEZ01008737:26722-26968
HmHel-6	CAEZ01008091:1441-1587
HmHel-6	CAEZ01008091:24774-26576
HmHel-6	CAEZ01008091:50203-50456
HmHel-6	CAEZ01008091:37349-37515
HmHel-6	CAEZ01008091:22638-22725
HmHel-6	CAEZ01007975:6169-6394
HmHel-6	CAEZ01007975:18886-19057
HmHel-6	CAEZ01007975:66469-66677
HmHel-6	CAEZ01007975:79871-80027
HmHel-6	CAEZ01007975:58856-58974
HmHel-6	CAEZ01007975:21444-21694
HmHel-6	CAEZ01007765:86488-86587
HmHel-6	CAEZ01007765:100041-100176
HmHel-6	CAEZ01007765:118594-118740
HmHel-6	CAEZ01007765:194703-194880
HmHel-6	CAEZ01007765:262209-262317
HmHel-6	CAEZ01007765:288567-291930
HmHel-6	CAEZ01007765:351267-351396
HmHel-6	CAEZ01007765:374766-374931
HmHel-6	CAEZ01007765:423272-423465
HmHel-6	CAEZ01007765:486498-486711
HmHel-6	CAEZ01007765:506362-506512

HmHel-6	CAEZ01007765:553807-553905
HmHel-6	CAEZ01007765:585317-585484
HmHel-6	CAEZ01007765:547328-550498
HmHel-6	CAEZ01007765:524333-524465
HmHel-6	CAEZ01007765:362614-363707
HmHel-6	CAEZ01007765:224704-224915
HmHel-6	CAEZ01007765:139405-139534
HmHel-6	CAEZ01007765:97786-98027
HmHel-6	CAEZ01007765:74969-75222
HmHel-6	CAEZ01007765:55734-64785
HmHel-6	CAEZ01007765:33100-33304
HmHel-6	CAEZ01006294:15370-15598
HmHel-6	CAEZ01006177:123596-123769
HmHel-6	CAEZ01006177:160716-160956
HmHel-6	CAEZ01006177:172032-172187
HmHel-6	CAEZ01006177:192122-192238
HmHel-6	CAEZ01006177:210674-215347
HmHel-6	CAEZ01006177:188541-197804
HmHel-6	CAEZ01006177:169044-169190
HmHel-6	CAEZ01006177:8698-8782
HmHel-6	CAEZ01005501:1829-2074
HmHel-6	CAEZ01005501:22997-23083
HmHel-6	CAEZ01005501:8430-8535
HmHel-6	CAEZ01005263:6757-6963
HmHel-6	CAEZ01005263:22867-23401
HmHel-6	CAEZ01005263:19663-24903
HmHel-6	CAEZ01005043:90219-90461
HmHel-6	CAEZ01005043:129137-131546
HmHel-6	CAEZ01005043:167401-173956
HmHel-6	CAEZ01005043:217168-217402
HmHel-6	CAEZ01005043:239539-245412
HmHel-6	CAEZ01005043:248760-257568
HmHel-6	CAEZ01005043:222308-222559
HmHel-6	CAEZ01005043:179147-179327
HmHel-6	CAEZ01005043:148338-148450
HmHel-6	CAEZ01005043:108085-108196
HmHel-6	CAEZ01005043:82283-82512
HmHel-6	CAEZ01003205:11086-11338
HmHel-6	CAEZ01003205:579-683
HmHel-6	CAEZ01002863:93968-94092
HmHel-6	CAEZ01002863:117116-117308
HmHel-6	CAEZ01002863:113297-113550
HmHel-6	CAEZ01002863:69105-69333
HmHel-6	CAEZ01002863:37774-41843
HmHel-6	CAEZ01011500:2015-6310
HmHel-6	CAEZ01010981:217-465
HmHel-6	CAEZ01010981:43208-43304
HmHel-6	CAEZ01010981:16171-16275

HmHel-6	CAEZ01009470:4370-4619
HmHel-6	CAEZ01009470:83696-83792
HmHel-6	CAEZ01009470:121634-121749
HmHel-6	CAEZ01009470:112581-116857
HmHel-6	CAEZ01009470:90684-90783
HmHel-6	CAEZ01009470:79709-79868
HmHel-6	CAEZ01009470:63576-63669
HmHel-6	CAEZ01009470:51287-51406
HmHel-6	CAEZ01009470:37504-37598
HmHel-6	CAEZ01009470:3720-3807
HmHel-6	CAEZ01009322:409-522
HmHel-6	CAEZ01009322:12327-14804
HmHel-6	CAEZ01009322:35104-35356
HmHel-6	CAEZ01008123:6843-7004
HmHel-6	CAEZ01008123:34766-34849
HmHel-6	CAEZ01008123:56650-56762
HmHel-6	CAEZ01008123:45641-45782
HmHel-6	CAEZ01008123:30348-30531
HmHel-6	CAEZ01008123:13159-13411
HmHel-6	CAEZ01005926:1337-1565
HmHel-6	CAEZ01005926:22266-22349
HmHel-6	CAEZ01005337:9110-9357
HmHel-6	CAEZ01005337:11840-16255
HmHel-6	CAEZ01005337:1271-1358
HmHel-6	CAEZ01005183:876-1116
HmHel-6	CAEZ01005183:31337-31540
HmHel-6	CAEZ01005183:20857-29280
HmHel-6	CAEZ01003790:17147-17243
HmHel-6	CAEZ01003790:28055-28186
HmHel-6	CAEZ01003111:3740-3991
HmHel-6	CAEZ01003111:54432-63940
HmHel-6	CAEZ01003111:37284-37508
HmHel-6	CAEZ01000849:84255-92182
HmHel-6	CAEZ01000849:118738-125720
HmHel-6	CAEZ01000849:129796-130040
HmHel-6	CAEZ01000849:68422-68581
HmHel-6	CAEZ01000849:46788-46924
HmHel-6	CAEZ01010715:1186-1277
HmHel-6	CAEZ01010715:3094-3281
HmHel-6	CAEZ01009583:12365-12474
HmHel-6	CAEZ01009583:24827-25007
HmHel-6	CAEZ01009583:51745-51834
HmHel-6	CAEZ01009583:67162-67402
HmHel-6	CAEZ01009583:70787-72433
HmHel-6	CAEZ01009583:16708-34399
HmHel-6	CAEZ01007976:14617-14864
HmHel-6	CAEZ01007976:5765-5944
HmHel-6	CAEZ01007298:11118-11340

HmHel-6	CAEZ01005475:45722-45810
HmHel-6	CAEZ01005475:125818-128642
HmHel-6	CAEZ01005475:140597-145229
HmHel-6	CAEZ01005475:111884-112094
HmHel-6	CAEZ01005475:87944-95286
HmHel-6	CAEZ01005475:43201-52753
HmHel-6	CAEZ01005475:12848-12943
HmHel-6	CAEZ01004397:379-3313
HmHel-6	CAEZ01001832:3451-3630
HmHel-6	CAEZ01001832:17702-17949
HmHel-6	CAEZ01001832:37612-37697
HmHel-6	CAEZ01001832:55207-55419
HmHel-6	CAEZ01001832:36784-36929
HmHel-6	CAEZ01001832:24340-24432
HmHel-6	CAEZ01001832:5994-6147
HmHel-6	CAEZ01011343:150-400
HmHel-6	CAEZ01010872:6168-6413
HmHel-6	CAEZ01009891:5038-5189
HmHel-6	CAEZ01009891:65849-66039
HmHel-6	CAEZ01009891:77408-77536
HmHel-6	CAEZ01009891:72116-72199
HmHel-6	CAEZ01009891:53706-53918
HmHel-6	CAEZ01009891:4028-4166
HmHel-6	CAEZ01009154:7056-7136
HmHel-6	CAEZ01008857:18423-18583
HmHel-6	CAEZ01008857:41847-41965
HmHel-6	CAEZ01008857:30334-30546
HmHel-6	CAEZ01008857:8156-8263
HmHel-6	CAEZ01008360:4961-5831
HmHel-6	CAEZ01008360:41316-41570
HmHel-6	CAEZ01008360:6120-6321
HmHel-6	CAEZ01008208:1215-7282
HmHel-6	CAEZ01008208:79218-79309
HmHel-6	CAEZ01008208:89856-90095
HmHel-6	CAEZ01008208:109312-109541
HmHel-6	CAEZ01008208:128479-129991
HmHel-6	CAEZ01008208:155217-155469
HmHel-6	CAEZ01008208:175502-175756
HmHel-6	CAEZ01008208:235906-238185
HmHel-6	CAEZ01008208:204115-212289
HmHel-6	CAEZ01008208:191502-191675
HmHel-6	CAEZ01008208:174340-174570
HmHel-6	CAEZ01008208:150001-157339
HmHel-6	CAEZ01008208:130460-135175
HmHel-6	CAEZ01008208:118958-119203
HmHel-6	CAEZ01008208:88445-88584
HmHel-6	CAEZ01008208:22064-22173
HmHel-6	CAEZ01006381:4627-4798

HmHel-6	CAEZ01006381:4002-4253
HmHel-6	CAEZ01006010:3111-3190
HmHel-6	CAEZ01006010:14813-15848
HmHel-6	CAEZ01006010:43129-43246
HmHel-6	CAEZ01006010:26320-26570
HmHel-6	CAEZ01006010:11041-11130
HmHel-6	CAEZ01005496:1783-2032
HmHel-6	CAEZ01005496:36270-36483
HmHel-6	CAEZ01005496:63712-65120
HmHel-6	CAEZ01005496:92570-95432
HmHel-6	CAEZ01005496:107290-107392
HmHel-6	CAEZ01005496:88188-88410
HmHel-6	CAEZ01005496:65343-65498
HmHel-6	CAEZ01005496:6454-6938
HmHel-6	CAEZ01005378:13208-13454
HmHel-6	CAEZ01005378:7952-16896
HmHel-6	CAEZ01005153:17289-17444
HmHel-6	CAEZ01005153:43486-43617
HmHel-6	CAEZ01005153:108840-113014
HmHel-6	CAEZ01005153:71368-71606
HmHel-6	CAEZ01005153:30142-39148
HmHel-6	CAEZ01005153:11915-12061
HmHel-6	CAEZ01003610:14413-14654
HmHel-6	CAEZ01002618:2362-2608
HmHel-6	CAEZ01000988:6320-6573
HmHel-6	CAEZ01000745:2808-3054
HmHel-6	CAEZ01000745:3913-4049
HmHel-6	CAEZ01011134:52013-52126
HmHel-6	CAEZ01011134:119522-122941
HmHel-6	CAEZ01011134:83884-84133
HmHel-6	CAEZ01010896:11897-12001
HmHel-6	CAEZ01010896:44891-45090
HmHel-6	CAEZ01010896:89674-90670
HmHel-6	CAEZ01010896:101893-102020
HmHel-6	CAEZ01010896:246839-246923
HmHel-6	CAEZ01010896:289875-290003
HmHel-6	CAEZ01010896:309816-309921
HmHel-6	CAEZ01010896:324107-324251
HmHel-6	CAEZ01010896:338093-338309
HmHel-6	CAEZ01010896:388168-388421
HmHel-6	CAEZ01010896:378894-384320
HmHel-6	CAEZ01010896:342467-342655
HmHel-6	CAEZ01010896:309104-309214
HmHel-6	CAEZ01010896:294170-294290
HmHel-6	CAEZ01010896:203325-210146
HmHel-6	CAEZ01010896:95787-95898
HmHel-6	CAEZ01010896:66864-68642
HmHel-6	CAEZ01010896:52288-52492

HmHel-6	CAEZ01010896:34465-34599
HmHel-6	CAEZ01009894:9514-9715
HmHel-6	CAEZ01009894:49100-49428
HmHel-6	CAEZ01009894:64752-64841
HmHel-6	CAEZ01009894:47751-47856
HmHel-6	CAEZ01009894:31062-31301
HmHel-6	CAEZ01009894:2250-2543
HmHel-6	CAEZ01008678:4768-4889
HmHel-6	CAEZ01008678:24746-24877
HmHel-6	CAEZ01008678:43384-44558
HmHel-6	CAEZ01008678:15110-15197
HmHel-6	CAEZ01008678:2468-2674
HmHel-6	CAEZ01008418:46255-46384
HmHel-6	CAEZ01008418:59750-66073
HmHel-6	CAEZ01008418:65364-65555
HmHel-6	CAEZ01008418:29380-29527
HmHel-6	CAEZ01008418:6601-14559
HmHel-6	CAEZ01008234:11129-11243
HmHel-6	CAEZ01008234:88495-88748
HmHel-6	CAEZ01008234:107953-115038
HmHel-6	CAEZ01008234:130477-130569
HmHel-6	CAEZ01008234:178660-178740
HmHel-6	CAEZ01008234:218230-218483
HmHel-6	CAEZ01008234:158245-158368
HmHel-6	CAEZ01008234:47033-47205
HmHel-6	CAEZ01008036:2504-2756
HmHel-6	CAEZ01007983:8609-8747
HmHel-6	CAEZ01007983:31697-31812
HmHel-6	CAEZ01007983:91092-91179
HmHel-6	CAEZ01007983:136628-136732
HmHel-6	CAEZ01007983:162027-162211
HmHel-6	CAEZ01007983:179389-179496
HmHel-6	CAEZ01007983:195100-196892
HmHel-6	CAEZ01007983:142921-143070
HmHel-6	CAEZ01007983:113808-114061
HmHel-6	CAEZ01007983:10623-10710
HmHel-6	CAEZ01007571:6687-7339
HmHel-6	CAEZ01007571:28330-28570
HmHel-6	CAEZ01007571:36012-36393
HmHel-6	CAEZ01007313:8658-8911
HmHel-6	CAEZ01007313:5209-5306
HmHel-6	CAEZ01006675:39834-39961
HmHel-6	CAEZ01006675:82438-82627
HmHel-6	CAEZ01006675:103896-104101
HmHel-6	CAEZ01006675:78321-78574
HmHel-6	CAEZ01006675:62417-64513
HmHel-6	CAEZ01006675:4470-4634
HmHel-6	CAEZ01006603:2245-2342

HmHel-6	CAEZ01006603:10555-10808
HmHel-6	CAEZ01006405:6325-6579
HmHel-6	CAEZ01006405:56477-56641
HmHel-6	CAEZ01006405:46925-55841
HmHel-6	CAEZ01006405:10262-10511
HmHel-6	CAEZ01006403:6309-6481
HmHel-6	CAEZ01006403:97736-97913
HmHel-6	CAEZ01006403:125556-125695
HmHel-6	CAEZ01006403:95141-97309
HmHel-6	CAEZ01006403:67665-76305
HmHel-6	CAEZ01006403:35685-35932
HmHel-6	CAEZ01005343:17815-17895
HmHel-6	CAEZ01004762:7680-7872
HmHel-6	CAEZ01004762:30924-31177
HmHel-6	CAEZ01004762:1323-1533
HmHel-6	CAEZ01004492:10837-14937
HmHel-6	CAEZ01004492:37682-37820
HmHel-6	CAEZ01004492:18235-18408
HmHel-6	CAEZ01004442:16405-16658
HmHel-6	CAEZ01003759:4312-4525
HmHel-6	CAEZ01003759:21679-21795
HmHel-6	CAEZ01003759:18929-19182
HmHel-6	CAEZ01003403:7803-8056
HmHel-6	CAEZ01002568:10226-10414
HmHel-6	CAEZ01002568:23732-23861
HmHel-6	CAEZ01002352:1557-1810
HmHel-6	CAEZ01001331:8081-8208
HmHel-6	CAEZ01001331:89129-89215
HmHel-6	CAEZ01001331:30410-31413
HmHel-6	CAEZ01001308:3116-3223
HmHel-6	CAEZ01001308:2350-2603
HmHel-6	CAEZ01000589:1906-2119
HmHel-6	CAEZ01000589:3684-3937
HmHel-6	CAEZ01011545:1935-2092
HmHel-6	CAEZ01011545:8394-8638
HmHel-6	CAEZ01011170:3310-3565
HmHel-6	CAEZ01008912:3960-4212
HmHel-6	CAEZ01008912:49761-49928
HmHel-6	CAEZ01008912:19185-19399
HmHel-6	CAEZ01008396:1262-1509
HmHel-6	CAEZ01008396:15770-15880
HmHel-6	CAEZ01008396:17499-17662
HmHel-6	CAEZ01007573:2672-2870
HmHel-6	CAEZ01007573:8702-8866
HmHel-6	CAEZ01006564:1957-2135
HmHel-6	CAEZ01006564:12419-17331
HmHel-6	CAEZ01006564:12901-13141
HmHel-6	CAEZ01006088:7658-7739

HmHel-6	CAEZ01006088:29359-29524
HmHel-6	CAEZ01006088:49868-50023
HmHel-6	CAEZ01006088:49230-49388
HmHel-6	CAEZ01006088:34055-34305
HmHel-6	CAEZ01006088:23621-23872
HmHel-6	CAEZ01005579:5025-5280
HmHel-6	CAEZ01004591:13903-13994
HmHel-6	CAEZ01004591:29071-29205
HmHel-6	CAEZ01004591:33207-33338
HmHel-6	CAEZ01004591:4810-5069
HmHel-6	CAEZ01002821:22760-23004
HmHel-6	CAEZ01002821:39493-39705
HmHel-6	CAEZ01002821:84215-92390
HmHel-6	CAEZ01002821:114976-115228
HmHel-6	CAEZ01002821:121494-121613
HmHel-6	CAEZ01002821:69781-69964
HmHel-6	CAEZ01002821:7183-7382
HmHel-6	CAEZ01001974:2202-2426
HmHel-6	CAEZ01001974:12888-12993
HmHel-6	CAEZ01010666:20497-20708
HmHel-6	CAEZ01010666:40477-40658
HmHel-6	CAEZ01010666:63882-64130
HmHel-6	CAEZ01010666:66697-66926
HmHel-6	CAEZ01010666:42223-50576
HmHel-6	CAEZ01010666:8711-8890
HmHel-6	CAEZ01010281:511-758
HmHel-6	CAEZ01010281:15591-15752
HmHel-6	CAEZ01010281:10562-10733
HmHel-6	CAEZ01009976:15468-15715
HmHel-6	CAEZ01009976:16652-16743
HmHel-6	CAEZ01009976:3324-3499
HmHel-6	CAEZ01009900:29450-29680
HmHel-6	CAEZ01009900:55314-55439
HmHel-6	CAEZ01009900:66291-66461
HmHel-6	CAEZ01009900:58761-58975
HmHel-6	CAEZ01008313:24789-25028
HmHel-6	CAEZ01008313:41934-42185
HmHel-6	CAEZ01008313:45119-45283
HmHel-6	CAEZ01004850:2402-2654
HmHel-6	CAEZ01004850:6262-6363
HmHel-6	CAEZ01004793:1652-7005
HmHel-6	CAEZ01004793:58465-58609
HmHel-6	CAEZ01004793:84300-84412
HmHel-6	CAEZ01004793:98814-98913
HmHel-6	CAEZ01004793:135902-136053
HmHel-6	CAEZ01004793:133232-133387
HmHel-6	CAEZ01004793:97179-97298
HmHel-6	CAEZ01004793:48610-48710

HmHel-6	CAEZ01004793:20219-20439
HmHel-6	CAEZ01004252:16909-17123
HmHel-6	CAEZ01004252:38392-38478
HmHel-6	CAEZ01004252:65926-66026
HmHel-6	CAEZ01004252:46570-49371
HmHel-6	CAEZ01004252:14225-14452
HmHel-6	CAEZ01004127:16130-16302
HmHel-6	CAEZ01004127:35367-35452
HmHel-6	CAEZ01004127:70548-70664
HmHel-6	CAEZ01004127:85756-85989
HmHel-6	CAEZ01004127:48083-49024
HmHel-6	CAEZ01004127:15461-15708
HmHel-6	CAEZ01003197:4293-4400
HmHel-6	CAEZ01003197:26497-34529
HmHel-6	CAEZ01003197:18475-18712
HmHel-6	CAEZ01001081:13385-13497
HmHel-6	CAEZ01011318:38454-44313
HmHel-6	CAEZ01011318:21920-22138
HmHel-6	CAEZ01011318:5435-5669
HmHel-6	CAEZ01010969:11207-11305
HmHel-6	CAEZ01010969:35403-35522
HmHel-6	CAEZ01010969:72495-72592
HmHel-6	CAEZ01010969:114210-114372
HmHel-6	CAEZ01010969:227997-228234
HmHel-6	CAEZ01010969:241585-248463
HmHel-6	CAEZ01010969:226229-226852
HmHel-6	CAEZ01010969:213350-213454
HmHel-6	CAEZ01010969:145250-153855
HmHel-6	CAEZ01010969:102227-102372
HmHel-6	CAEZ01010969:65811-66010
HmHel-6	CAEZ01010969:3394-3644
HmHel-6	CAEZ01010677:5398-10264
HmHel-6	CAEZ01010677:36814-36939
HmHel-6	CAEZ01010677:52434-55731
HmHel-6	CAEZ01010677:96826-102764
HmHel-6	CAEZ01010677:153701-153784
HmHel-6	CAEZ01010677:180797-180954
HmHel-6	CAEZ01010677:214066-214231
HmHel-6	CAEZ01010677:310093-310222
HmHel-6	CAEZ01010677:273152-276768
HmHel-6	CAEZ01010677:216551-216694
HmHel-6	CAEZ01010677:188808-189021
HmHel-6	CAEZ01010677:167611-167711
HmHel-6	CAEZ01010677:116812-120254
HmHel-6	CAEZ01010677:100744-100980
HmHel-6	CAEZ01010677:88163-88250
HmHel-6	CAEZ01010677:62797-62907
HmHel-6	CAEZ01010677:52242-52343

HmHel-6	CAEZ01010677:1674-1827
HmHel-6	CAEZ01010285:31209-31355
HmHel-6	CAEZ01010285:46792-46944
HmHel-6	CAEZ01010285:67923-74160
HmHel-6	CAEZ01010285:111479-111644
HmHel-6	CAEZ01010285:164615-164762
HmHel-6	CAEZ01010285:180027-180239
HmHel-6	CAEZ01010285:142137-142229
HmHel-6	CAEZ01010285:36208-36299
HmHel-6	CAEZ01010285:13445-13615
HmHel-6	CAEZ01009716:106929-107109
HmHel-6	CAEZ01009716:136459-136631
HmHel-6	CAEZ01009716:146881-147095
HmHel-6	CAEZ01009716:177947-178115
HmHel-6	CAEZ01009716:210072-210154
HmHel-6	CAEZ01009716:223024-223211
HmHel-6	CAEZ01009716:218005-218196
HmHel-6	CAEZ01009716:153779-153991
HmHel-6	CAEZ01009716:123907-124140
HmHel-6	CAEZ01009716:98840-101080
HmHel-6	CAEZ01009716:64881-64998
HmHel-6	CAEZ01009716:24673-24923
HmHel-6	CAEZ01009368:6277-6433
HmHel-6	CAEZ01009368:25447-25598
HmHel-6	CAEZ01009368:70568-70694
HmHel-6	CAEZ01009368:480-723
HmHel-6	CAEZ01008989:3966-11951
HmHel-6	CAEZ01008989:57290-60061
HmHel-6	CAEZ01008989:102705-102843
HmHel-6	CAEZ01008989:143116-143320
HmHel-6	CAEZ01008989:180981-187471
HmHel-6	CAEZ01008989:203140-203273
HmHel-6	CAEZ01008989:166613-167317
HmHel-6	CAEZ01008989:107880-114757
HmHel-6	CAEZ01008989:48898-49007
HmHel-6	CAEZ01008989:27250-27483
HmHel-6	CAEZ01008989:1319-1490
HmHel-6	CAEZ01008677:7165-16527
HmHel-6	CAEZ01008677:83422-83507
HmHel-6	CAEZ01008677:99767-100058
HmHel-6	CAEZ01008677:130073-130308
HmHel-6	CAEZ01008677:122557-122780
HmHel-6	CAEZ01008677:86479-86630
HmHel-6	CAEZ01008677:23408-23658
HmHel-6	CAEZ01007707:15610-15849
HmHel-6	CAEZ01007707:60843-61057
HmHel-6	CAEZ01007707:89254-89385
HmHel-6	CAEZ01007707:102746-102893

HmHel-6	CAEZ01007707:145063-148238
HmHel-6	CAEZ01007707:165674-165804
HmHel-6	CAEZ01007707:171732-171925
HmHel-6	CAEZ01007707:137535-137716
HmHel-6	CAEZ01007707:94491-94730
HmHel-6	CAEZ01007707:80138-80352
HmHel-6	CAEZ01007707:37800-37891
HmHel-6	CAEZ01007707:22610-26539
HmHel-6	CAEZ01007662:3905-4159
HmHel-6	CAEZ01007662:16145-16243
HmHel-6	CAEZ01007396:2358-2443
HmHel-6	CAEZ01006862:16475-16693
HmHel-6	CAEZ01006862:37733-37919
HmHel-6	CAEZ01006862:49838-49953
HmHel-6	CAEZ01006862:34824-34949
HmHel-6	CAEZ01006862:20787-20909
HmHel-6	CAEZ01006286:24307-24390
HmHel-6	CAEZ01006286:70461-70610
HmHel-6	CAEZ01006286:134856-134936
HmHel-6	CAEZ01006286:107733-107896
HmHel-6	CAEZ01006286:5268-5942
HmHel-6	CAEZ01005911:9274-9388
HmHel-6	CAEZ01005911:73333-73468
HmHel-6	CAEZ01005911:95696-95908
HmHel-6	CAEZ01005911:139470-139683
HmHel-6	CAEZ01005911:153168-153342
HmHel-6	CAEZ01005911:133094-133294
HmHel-6	CAEZ01005911:111005-111177
HmHel-6	CAEZ01005911:589-724
HmHel-6	CAEZ01005772:350-789
HmHel-6	CAEZ01005772:15875-23166
HmHel-6	CAEZ01005650:4656-4912
HmHel-6	CAEZ01005099:34579-34830
HmHel-6	CAEZ01005099:39935-40038
HmHel-6	CAEZ01005099:13938-14025
HmHel-6	CAEZ01004602:1480-1730
HmHel-6	CAEZ01001456:3558-4937
HmHel-6	CAEZ01001456:3269-3396
HmHel-6	CAEZ01000066:2171-2421
HmHel-6	CAEZ01000066:14235-22366
HmHel-6	CAEZ01011494:25945-26152
HmHel-6	CAEZ01011494:46603-54132
HmHel-6	CAEZ01011494:18497-18588
HmHel-6	CAEZ01011494:1250-1503
HmHel-6	CAEZ01010362:15137-15347
HmHel-6	CAEZ01010362:41787-42442
HmHel-6	CAEZ01010362:59299-59424
HmHel-6	CAEZ01010362:34831-35045

HmHel-6	CAEZ01010362:6116-6304
HmHel-6	CAEZ01008583:2348-2595
HmHel-6	CAEZ01008583:21348-21554
HmHel-6	CAEZ01008404:50416-50549
HmHel-6	CAEZ01008404:74159-78842
HmHel-6	CAEZ01008404:102238-102491
HmHel-6	CAEZ01008404:117464-117596
HmHel-6	CAEZ01008404:136012-136140
HmHel-6	CAEZ01008404:160538-164308
HmHel-6	CAEZ01008404:147146-147330
HmHel-6	CAEZ01008404:89050-89281
HmHel-6	CAEZ01008404:69441-69576
HmHel-6	CAEZ01008404:56606-56709
HmHel-6	CAEZ01008404:42737-42920
HmHel-6	CAEZ01008346:11929-12959
HmHel-6	CAEZ01008346:39274-41402
HmHel-6	CAEZ01008346:36159-36412
HmHel-6	CAEZ01007984:13556-13797
HmHel-6	CAEZ01007984:59472-59671
HmHel-6	CAEZ01007984:60771-69849
HmHel-6	CAEZ01007974:5807-5938
HmHel-6	CAEZ01007974:47601-47803
HmHel-6	CAEZ01007974:139387-139502
HmHel-6	CAEZ01007974:131647-131751
HmHel-6	CAEZ01007974:93181-93434
HmHel-6	CAEZ01007974:68227-70872
HmHel-6	CAEZ01007974:46400-46608
HmHel-6	CAEZ01007802:7263-7463
HmHel-6	CAEZ01007802:27261-27465
HmHel-6	CAEZ01007802:41052-47292
HmHel-6	CAEZ01006372:13647-18965
HmHel-6	CAEZ01006372:34452-35750
HmHel-6	CAEZ01006372:65407-69430
HmHel-6	CAEZ01006372:66154-66289
HmHel-6	CAEZ01006372:31261-31514
HmHel-6	CAEZ01006194:2501-2657
HmHel-6	CAEZ01006194:34477-34730
HmHel-6	CAEZ01006194:45890-52893
HmHel-6	CAEZ01006194:34154-44158
HmHel-6	CAEZ01005776:1090-1273
HmHel-6	CAEZ01005776:6012-6123
HmHel-6	CAEZ01005623:19390-19643
HmHel-6	CAEZ01005623:31560-31662
HmHel-6	CAEZ01005623:58671-58836
HmHel-6	CAEZ01005623:56675-58006
HmHel-6	CAEZ01005623:22174-22415
HmHel-6	CAEZ01005440:18010-18232
HmHel-6	CAEZ01005440:29096-29206

HmHel-6	CAEZ01005399:25129-25216
HmHel-6	CAEZ01005399:26948-27171
HmHel-6	CAEZ01005399:9877-10074
HmHel-6	CAEZ01005111:20405-20657
HmHel-6	CAEZ01005111:11483-11591
HmHel-6	CAEZ01005047:817-7645
HmHel-6	CAEZ01005047:10036-10289
HmHel-6	CAEZ01004195:9585-9723
HmHel-6	CAEZ01004195:11302-11539
HmHel-6	CAEZ01003791:21525-21778
HmHel-6	CAEZ01003791:48137-48217
HmHel-6	CAEZ01003791:23450-26930
HmHel-6	CAEZ01003791:2605-2693
HmHel-6	CAEZ01003190:1825-2073
HmHel-6	CAEZ01002882:206-455
HmHel-6	CAEZ01002726:4949-5198
HmHel-6	CAEZ01002669:312-433
HmHel-6	CAEZ01002669:32352-32555
HmHel-6	CAEZ01002669:25209-25458
HmHel-6	CAEZ01001842:6582-6830
HmHel-6	CAEZ01001392:441-694
HmHel-6	CAEZ01011647:1402-1650
HmHel-6	CAEZ01011647:10083-10179
HmHel-6	CAEZ01010818:14880-15082
HmHel-6	CAEZ01010818:45467-45674
HmHel-6	CAEZ01009706:23310-23427
HmHel-6	CAEZ01009706:51088-51331
HmHel-6	CAEZ01009706:118875-119045
HmHel-6	CAEZ01009706:101829-101940
HmHel-6	CAEZ01009706:68461-76650
HmHel-6	CAEZ01009706:32920-33052
HmHel-6	CAEZ01009706:15461-15596
HmHel-6	CAEZ01009576:75572-78885
HmHel-6	CAEZ01009576:55366-55613
HmHel-6	CAEZ01009576:19385-19554
HmHel-6	CAEZ01009576:4931-5010
HmHel-6	CAEZ01009435:20359-20568
HmHel-6	CAEZ01009435:34772-34949
HmHel-6	CAEZ01009435:50506-50621
HmHel-6	CAEZ01009435:67826-68012
HmHel-6	CAEZ01008981:2541-2785
HmHel-6	CAEZ01008981:26070-26246
HmHel-6	CAEZ01008981:23682-23931
HmHel-6	CAEZ01008713:58564-58660
HmHel-6	CAEZ01008713:71604-71760
HmHel-6	CAEZ01008713:103026-106266
HmHel-6	CAEZ01008713:128733-128852
HmHel-6	CAEZ01008713:158896-159078

HmHel-6	CAEZ01008713:124726-124808
HmHel-6	CAEZ01008713:104908-105156
HmHel-6	CAEZ01008713:68056-68242
HmHel-6	CAEZ01008713:43793-43958
HmHel-6	CAEZ01008713:26038-26199
HmHel-6	CAEZ01007857:7471-8901
HmHel-6	CAEZ01007857:22874-22961
HmHel-6	CAEZ01007857:43221-46413
HmHel-6	CAEZ01007857:30069-30185
HmHel-6	CAEZ01007857:5812-5923
HmHel-6	CAEZ01007804:19000-19253
HmHel-6	CAEZ01007804:34888-34988
HmHel-6	CAEZ01007804:76246-76462
HmHel-6	CAEZ01007804:104250-105605
HmHel-6	CAEZ01007804:121634-121757
HmHel-6	CAEZ01007804:144752-144877
HmHel-6	CAEZ01007804:163678-163797
HmHel-6	CAEZ01007804:190847-191059
HmHel-6	CAEZ01007804:221247-221483
HmHel-6	CAEZ01007804:236558-236712
HmHel-6	CAEZ01007804:198802-198951
HmHel-6	CAEZ01007804:183451-183530
HmHel-6	CAEZ01007804:127960-129339
HmHel-6	CAEZ01007804:79744-79985
HmHel-6	CAEZ01007804:58513-58646
HmHel-6	CAEZ01007499:38204-38456
HmHel-6	CAEZ01007499:86739-86836
HmHel-6	CAEZ01007499:115461-115672
HmHel-6	CAEZ01007499:176909-181921
HmHel-6	CAEZ01007499:167621-167759
HmHel-6	CAEZ01007499:40589-40827
HmHel-6	CAEZ01006783:7112-7362
HmHel-6	CAEZ01006571:7723-7943
HmHel-6	CAEZ01006571:41254-41362
HmHel-6	CAEZ01006571:41582-41801
HmHel-6	CAEZ01006347:27066-27234
HmHel-6	CAEZ01006347:95515-100284
HmHel-6	CAEZ01006347:152048-156270
HmHel-6	CAEZ01006347:167200-167451
HmHel-6	CAEZ01006347:191592-191820
HmHel-6	CAEZ01006347:229105-229330
HmHel-6	CAEZ01006347:179972-180051
HmHel-6	CAEZ01006347:138273-138457
HmHel-6	CAEZ01006347:106140-108588
HmHel-6	CAEZ01006347:93343-93464
HmHel-6	CAEZ01006347:43352-43503
HmHel-6	CAEZ01005066:923-1027
HmHel-6	CAEZ01005066:31121-34096

HmHel-6	CAEZ01005066:90864-91075
HmHel-6	CAEZ01005066:89324-89463
HmHel-6	CAEZ01005066:72189-72313
HmHel-6	CAEZ01005066:58630-58884
HmHel-6	CAEZ01005066:30374-30462
HmHel-6	CAEZ01004244:499-611
HmHel-6	CAEZ01004244:12654-12892
HmHel-6	CAEZ01004244:37706-37908
HmHel-6	CAEZ01004244:61763-61889
HmHel-6	CAEZ01004244:97218-106495
HmHel-6	CAEZ01004244:139016-145393
HmHel-6	CAEZ01004244:131257-131392
HmHel-6	CAEZ01004244:109337-109508
HmHel-6	CAEZ01004244:49508-50387
HmHel-6	CAEZ01004244:25959-26829
HmHel-6	CAEZ01003781:15414-15506
HmHel-6	CAEZ01003781:45246-45426
HmHel-6	CAEZ01003781:35837-36078
HmHel-6	CAEZ01002363:19183-19371
HmHel-6	CAEZ01002363:35373-38142
HmHel-6	CAEZ01002363:60595-61336
HmHel-6	CAEZ01002363:45587-45700
HmHel-6	CAEZ01001578:7452-7589
HmHel-6	CAEZ01001578:20587-24394
HmHel-6	CAEZ01001578:78145-78376
HmHel-6	CAEZ01001578:93932-94012
HmHel-6	CAEZ01001578:71742-71994
HmHel-6	CAEZ01001578:51157-58165
HmHel-6	CAEZ01001578:11255-11336
HmHel-6	CAEZ01011481:4282-4404
HmHel-6	CAEZ01010464:33095-33211
HmHel-6	CAEZ01010464:44208-44459
HmHel-6	CAEZ01010464:77684-77914
HmHel-6	CAEZ01010464:120195-120309
HmHel-6	CAEZ01010464:63849-63999
HmHel-6	CAEZ01010464:41075-41290
HmHel-6	CAEZ01010457:32180-32431
HmHel-6	CAEZ01010457:21464-21594
HmHel-6	CAEZ01009954:13815-14023
HmHel-6	CAEZ01009954:41269-44143
HmHel-6	CAEZ01009954:46881-47003
HmHel-6	CAEZ01009954:23854-30594
HmHel-6	CAEZ01009892:9996-10149
HmHel-6	CAEZ01009892:72409-73018
HmHel-6	CAEZ01009892:105270-105401
HmHel-6	CAEZ01009892:153160-153410
HmHel-6	CAEZ01009892:196907-199266
HmHel-6	CAEZ01009892:205729-205914

HmHel-6	CAEZ01009892:174941-175039
HmHel-6	CAEZ01009892:115715-115883
HmHel-6	CAEZ01009892:96495-96641
HmHel-6	CAEZ01009892:62015-62238
HmHel-6	CAEZ01009892:33336-38080
HmHel-6	CAEZ01009888:10969-16272
HmHel-6	CAEZ01009888:47934-57842
HmHel-6	CAEZ01009888:111643-111733
HmHel-6	CAEZ01009888:147459-147705
HmHel-6	CAEZ01009888:94121-94321
HmHel-6	CAEZ01009888:53628-53876
HmHel-6	CAEZ01009888:3029-3121
HmHel-6	CAEZ01009617:3886-6489
HmHel-6	CAEZ01009617:22002-22216
HmHel-6	CAEZ01009321:13310-13557
HmHel-6	CAEZ01009321:33273-33365
HmHel-6	CAEZ01009321:23064-31269
HmHel-6	CAEZ01008726:2868-2972
HmHel-6	CAEZ01008726:44651-44889
HmHel-6	CAEZ01008726:65316-75301
HmHel-6	CAEZ01008726:121901-122036
HmHel-6	CAEZ01008726:143770-143980
HmHel-6	CAEZ01008726:109275-110035
HmHel-6	CAEZ01008726:71599-79389
HmHel-6	CAEZ01008726:45194-54046
HmHel-6	CAEZ01008726:15608-15823
HmHel-6	CAEZ01008588:11284-11383
HmHel-6	CAEZ01008588:37146-37329
HmHel-6	CAEZ01008588:38617-40699
HmHel-6	CAEZ01008578:15588-15744
HmHel-6	CAEZ01008578:63396-63644
HmHel-6	CAEZ01008578:104313-117627
HmHel-6	CAEZ01008578:159140-159265
HmHel-6	CAEZ01008578:133580-133712
HmHel-6	CAEZ01008578:95370-103809
HmHel-6	CAEZ01008578:81492-81728
HmHel-6	CAEZ01008578:47621-47762
HmHel-6	CAEZ01008578:18141-18357
HmHel-6	CAEZ01007720:3047-3298
HmHel-6	CAEZ01007352:445-668
HmHel-6	CAEZ01007352:14084-14240
HmHel-6	CAEZ01007352:3273-3527
HmHel-6	CAEZ01006847:490-715
HmHel-6	CAEZ01006847:33728-33956
HmHel-6	CAEZ01006847:64848-68186
HmHel-6	CAEZ01006847:84892-95516
HmHel-6	CAEZ01006847:105160-105307
HmHel-6	CAEZ01006847:75132-75346

HmHel-6	CAEZ01006847:38847-40449
HmHel-6	CAEZ01006839:9443-9678
HmHel-6	CAEZ01006839:2178-2382
HmHel-6	CAEZ01006788:9612-9859
HmHel-6	CAEZ01005991:1128-1252
HmHel-6	CAEZ01005991:7336-7591
HmHel-6	CAEZ01005733:3030-3110
HmHel-6	CAEZ01005733:11856-12103
HmHel-6	CAEZ01004733:3114-3216
HmHel-6	CAEZ01004733:25318-25573
HmHel-6	CAEZ01004733:71640-76432
HmHel-6	CAEZ01004733:104199-104448
HmHel-6	CAEZ01004733:119945-120061
HmHel-6	CAEZ01004733:74616-74712
HmHel-6	CAEZ01004733:28791-28956
HmHel-6	CAEZ01004733:12017-12233
HmHel-6	CAEZ01003719:30546-30760
HmHel-6	CAEZ01003233:48491-53647
HmHel-6	CAEZ01003233:70989-71214
HmHel-6	CAEZ01002611:865-1120
HmHel-6	CAEZ01001323:7169-7413
HmHel-6	CAEZ01001260:1965-2120
HmHel-6	CAEZ01001260:12372-12601
HmHel-6	CAEZ01000521:878-985
HmHel-6	CAEZ01000521:1-247
HmHel-6	CAEZ01010972:53426-53659
HmHel-6	CAEZ01010972:64418-64576
HmHel-6	CAEZ01010972:83220-83470
HmHel-6	CAEZ01010972:93514-93755
HmHel-6	CAEZ01010972:105114-105351
HmHel-6	CAEZ01010972:120705-120869
HmHel-6	CAEZ01010972:148981-149202
HmHel-6	CAEZ01010972:207703-207939
HmHel-6	CAEZ01010972:219687-222978
HmHel-6	CAEZ01010972:295976-296120
HmHel-6	CAEZ01010972:344233-352908
HmHel-6	CAEZ01010972:388036-388137
HmHel-6	CAEZ01010972:400780-401000
HmHel-6	CAEZ01010972:418995-419087
HmHel-6	CAEZ01010972:465691-465799
HmHel-6	CAEZ01010972:419401-419583
HmHel-6	CAEZ01010972:345628-345850
HmHel-6	CAEZ01010972:328582-328703
HmHel-6	CAEZ01010972:271092-271233
HmHel-6	CAEZ01010972:250301-250554
HmHel-6	CAEZ01010972:235586-235812
HmHel-6	CAEZ01010972:191489-191653
HmHel-6	CAEZ01010972:174033-174193

HmHel-6	CAEZ01010972:94502-94642
HmHel-6	CAEZ01010972:65642-65800
HmHel-6	CAEZ01010972:18405-18557
HmHel-6	CAEZ01010907:21687-21936
HmHel-6	CAEZ01010907:37745-37971
HmHel-6	CAEZ01010907:64402-64502
HmHel-6	CAEZ01010907:85855-86088
HmHel-6	CAEZ01010907:112441-112602
HmHel-6	CAEZ01010907:126930-127036
HmHel-6	CAEZ01010907:118906-118994
HmHel-6	CAEZ01010907:42108-42301
HmHel-6	CAEZ01010907:11094-11325
HmHel-6	CAEZ01009439:3546-3667
HmHel-6	CAEZ01009439:26237-26387
HmHel-6	CAEZ01009439:12165-21601
HmHel-6	CAEZ01009271:4185-4394
HmHel-6	CAEZ01009271:39065-39152
HmHel-6	CAEZ01009271:55101-55253
HmHel-6	CAEZ01009271:65446-65526
HmHel-6	CAEZ01009271:94176-94289
HmHel-6	CAEZ01009271:113083-113704
HmHel-6	CAEZ01009271:140189-140326
HmHel-6	CAEZ01009271:153793-153977
HmHel-6	CAEZ01009271:56296-56459
HmHel-6	CAEZ01009271:6113-6246
HmHel-6	CAEZ01008970:55968-66098
HmHel-6	CAEZ01008970:79701-79781
HmHel-6	CAEZ01008970:120056-122912
HmHel-6	CAEZ01008970:125613-125807
HmHel-6	CAEZ01007478:21968-22167
HmHel-6	CAEZ01007478:60561-60774
HmHel-6	CAEZ01007478:115847-115950
HmHel-6	CAEZ01007478:149701-150026
HmHel-6	CAEZ01007478:147170-147264
HmHel-6	CAEZ01007478:124649-129207
HmHel-6	CAEZ01007417:5285-8103
HmHel-6	CAEZ01007417:12301-12425
HmHel-6	CAEZ01006985:1216-1336
HmHel-6	CAEZ01006985:12001-12162
HmHel-6	CAEZ01006985:13016-13107
HmHel-6	CAEZ01005008:938-1091
HmHel-6	CAEZ01005008:1690-1928
HmHel-6	CAEZ01004661:13838-14092
HmHel-6	CAEZ01004661:2052-2202
HmHel-6	CAEZ01004533:3089-3337
HmHel-6	CAEZ01003558:12194-12311
HmHel-6	CAEZ01003558:28836-28917
HmHel-6	CAEZ01003558:31895-32149

HmHel-6	CAEZ01001403:30820-31006
HmHel-6	CAEZ01001403:46350-46600
HmHel-6	CAEZ01000068:2133-2314
HmHel-6	CAEZ01011307:25865-26118
HmHel-6	CAEZ01011193:23473-23638
HmHel-6	CAEZ01011193:71016-71251
HmHel-6	CAEZ01011193:92427-94130
HmHel-6	CAEZ01011193:79600-89685
HmHel-6	CAEZ01011193:30299-40470
HmHel-6	CAEZ01011193:15591-15824
HmHel-6	CAEZ01010144:22946-23112
HmHel-6	CAEZ01010144:62217-62324
HmHel-6	CAEZ01010144:108894-109150
HmHel-6	CAEZ01010144:129783-129903
HmHel-6	CAEZ01010144:149010-149263
HmHel-6	CAEZ01010144:50975-56392
HmHel-6	CAEZ01009579:9315-9564
HmHel-6	CAEZ01009431:18808-19017
HmHel-6	CAEZ01009431:50872-52992
HmHel-6	CAEZ01009431:69229-69404
HmHel-6	CAEZ01009431:70421-70633
HmHel-6	CAEZ01009431:47470-47714
HmHel-6	CAEZ01009431:18277-18421
HmHel-6	CAEZ01009079:23682-23770
HmHel-6	CAEZ01009079:48487-48567
HmHel-6	CAEZ01009079:69764-69958
HmHel-6	CAEZ01009079:44122-44371
HmHel-6	CAEZ01009079:16652-16846
HmHel-6	CAEZ01008874:11090-11342
HmHel-6	CAEZ01008874:40769-41019
HmHel-6	CAEZ01008874:67446-67662
HmHel-6	CAEZ01008874:85378-85532
HmHel-6	CAEZ01008874:127566-127781
HmHel-6	CAEZ01008874:105236-105340
HmHel-6	CAEZ01008874:62998-63092
HmHel-6	CAEZ01008874:37436-37611
HmHel-6	CAEZ01008874:18801-18965
HmHel-6	CAEZ01008820:3010-3258
HmHel-6	CAEZ01008289:5808-6060
HmHel-6	CAEZ01008289:23604-23717
HmHel-6	CAEZ01008289:3348-3574
HmHel-6	CAEZ01008279:12833-12982
HmHel-6	CAEZ01008279:111506-121520
HmHel-6	CAEZ01008279:149462-149573
HmHel-6	CAEZ01008279:199783-199996
HmHel-6	CAEZ01008279:222225-222321
HmHel-6	CAEZ01008279:260485-260710
HmHel-6	CAEZ01008279:253976-254191

HmHel-6	CAEZ01008279:199209-211680
HmHel-6	CAEZ01008279:147103-150030
HmHel-6	CAEZ01008279:124556-124736
HmHel-6	CAEZ01008279:60861-65685
HmHel-6	CAEZ01008279:43591-43813
HmHel-6	CAEZ01008279:9327-9527
HmHel-6	CAEZ01008209:2028-2278
HmHel-6	CAEZ01008209:25073-25174
HmHel-6	CAEZ01008209:38645-38895
HmHel-6	CAEZ01008209:68846-76255
HmHel-6	CAEZ01008209:97803-98052
HmHel-6	CAEZ01008209:104106-104228
HmHel-6	CAEZ01008209:90346-90460
HmHel-6	CAEZ01008209:39869-40083
HmHel-6	CAEZ01008209:24469-24549
HmHel-6	CAEZ01006689:28680-28793
HmHel-6	CAEZ01006689:87252-87505
HmHel-6	CAEZ01006689:87139-87218
HmHel-6	CAEZ01006689:51373-58450
HmHel-6	CAEZ01006337:63762-64003
HmHel-6	CAEZ01006337:110066-110231
HmHel-6	CAEZ01006337:48754-50709
HmHel-6	CAEZ01006337:32088-32317
HmHel-6	CAEZ01006337:21009-21157
HmHel-6	CAEZ01005859:562-814
HmHel-6	CAEZ01005859:1868-2066
HmHel-6	CAEZ01005688:5309-5562
HmHel-6	CAEZ01005688:39404-39626
HmHel-6	CAEZ01005688:3975-4079
HmHel-6	CAEZ01005334:9821-15096
HmHel-6	CAEZ01005334:28434-28648
HmHel-6	CAEZ01005334:31524-31701
HmHel-6	CAEZ01005334:16763-16909
HmHel-6	CAEZ01004955:10230-10459
HmHel-6	CAEZ01004955:9265-9407
HmHel-6	CAEZ01004649:5702-5802
HmHel-6	CAEZ01004649:228-481
HmHel-6	CAEZ01003660:15963-16077
HmHel-6	CAEZ01003660:3110-3363
HmHel-6	CAEZ01002742:12799-16529
HmHel-6	CAEZ01002742:45291-45397
HmHel-6	CAEZ01002742:75669-75830
HmHel-6	CAEZ01002742:126830-127083
HmHel-6	CAEZ01002742:182384-190523
HmHel-6	CAEZ01002742:218289-218496
HmHel-6	CAEZ01002742:197745-197998
HmHel-6	CAEZ01002742:133321-133506
HmHel-6	CAEZ01002742:74124-84306

HmHel-6	CAEZ01002742:37737-38932
HmHel-6	CAEZ01002742:2255-2348
HmHel-6	CAEZ01002097:18183-18412
HmHel-6	CAEZ01002097:48636-48877
HmHel-6	CAEZ01002097:62554-62697
HmHel-6	CAEZ01002097:9445-9671
HmHel-6	CAEZ01001917:2204-2429
HmHel-6	CAEZ01001087:9902-10155
HmHel-6	CAEZ01001087:13573-15580
HmHel-6	CAEZ01011524:8154-8402
HmHel-6	CAEZ01010880:12936-13185
HmHel-6	CAEZ01010880:27622-27801
HmHel-6	CAEZ01010880:1157-1266
HmHel-6	CAEZ01010659:7039-7279
HmHel-6	CAEZ01010659:65137-65340
HmHel-6	CAEZ01010659:52196-52447
HmHel-6	CAEZ01010501:228-1762
HmHel-6	CAEZ01010501:35103-35213
HmHel-6	CAEZ01010501:34197-36593
HmHel-6	CAEZ01010501:14086-14168
HmHel-6	CAEZ01010437:47336-47591
HmHel-6	CAEZ01010437:65961-66053
HmHel-6	CAEZ01010437:50850-50980
HmHel-6	CAEZ01010437:20459-20599
HmHel-6	CAEZ01010437:6183-6393
HmHel-6	CAEZ01010005:1591-1691
HmHel-6	CAEZ01010005:21049-24469
HmHel-6	CAEZ01010005:16657-16881
HmHel-6	CAEZ01009694:2610-2787
HmHel-6	CAEZ01009694:27659-27899
HmHel-6	CAEZ01009694:12252-12470
HmHel-6	CAEZ01009611:7810-8062
HmHel-6	CAEZ01009611:3221-6897
HmHel-6	CAEZ01008705:1582-1818
HmHel-6	CAEZ01007380:1203-1332
HmHel-6	CAEZ01007380:23666-23914
HmHel-6	CAEZ01007380:13482-13634
HmHel-6	CAEZ01006228:46180-46358
HmHel-6	CAEZ01006092:10123-10309
HmHel-6	CAEZ01006092:22588-22670
HmHel-6	CAEZ01006092:46444-53697
HmHel-6	CAEZ01006092:14309-14406
HmHel-6	CAEZ01005495:7592-7739
HmHel-6	CAEZ01005495:32288-32458
HmHel-6	CAEZ01005495:43080-43182
HmHel-6	CAEZ01005495:47112-47278
HmHel-6	CAEZ01005495:29085-29321
HmHel-6	CAEZ01005495:13026-13273

HmHel-6	CAEZ01005181:1592-12573
HmHel-6	CAEZ01004389:8829-8941
HmHel-6	CAEZ01004389:11136-11376
HmHel-6	CAEZ01003341:7879-7962
HmHel-6	CAEZ01003341:5565-5808
HmHel-6	CAEZ01001062:7820-7921
HmHel-6	CAEZ01001062:48395-48582
HmHel-6	CAEZ01001062:51474-58876
HmHel-6	CAEZ01001062:4203-4328
HmHel-6	CAEZ01010597:10868-11023
HmHel-6	CAEZ01010597:39621-39748
HmHel-6	CAEZ01010466:2201-8546
HmHel-6	CAEZ01010466:19430-19676
HmHel-6	CAEZ01010466:18334-18514
HmHel-6	CAEZ01010309:23181-23785
HmHel-6	CAEZ01010309:22221-22375
HmHel-6	CAEZ01009057:11353-11470
HmHel-6	CAEZ01009057:33249-33496
HmHel-6	CAEZ01009057:19264-19382
HmHel-6	CAEZ01008884:10480-10727
HmHel-6	CAEZ01008884:32403-32544
HmHel-6	CAEZ01008884:54235-54453
HmHel-6	CAEZ01008884:61737-61991
HmHel-6	CAEZ01008884:45545-45667
HmHel-6	CAEZ01008884:31550-31655
HmHel-6	CAEZ01008884:18414-18518
HmHel-6	CAEZ01008884:8025-8245
HmHel-6	CAEZ01008488:13920-14034
HmHel-6	CAEZ01008488:759-990
HmHel-6	CAEZ01008211:14469-17676
HmHel-6	CAEZ01008211:60402-60614
HmHel-6	CAEZ01008211:168032-168229
HmHel-6	CAEZ01008211:207373-208273
HmHel-6	CAEZ01008211:224828-225082
HmHel-6	CAEZ01008211:215796-215981
HmHel-6	CAEZ01008211:182461-184988
HmHel-6	CAEZ01008211:90181-90300
HmHel-6	CAEZ01008211:74135-74360
HmHel-6	CAEZ01008211:39180-39365
HmHel-6	CAEZ01008211:12562-12777
HmHel-6	CAEZ01008126:183-376
HmHel-6	CAEZ01008126:30861-32056
HmHel-6	CAEZ01008126:46099-46346
HmHel-6	CAEZ01008126:68531-71660
HmHel-6	CAEZ01008126:66002-66113
HmHel-6	CAEZ01008126:29024-29111
HmHel-6	CAEZ01007389:2894-3015
HmHel-6	CAEZ01007389:28858-29005

HmHel-6	CAEZ01007389:63331-63545
HmHel-6	CAEZ01007389:128929-129172
HmHel-6	CAEZ01007389:150231-153398
HmHel-6	CAEZ01007389:80637-82870
HmHel-6	CAEZ01007389:48806-51057
HmHel-6	CAEZ01007389:33722-33803
HmHel-6	CAEZ01006956:4221-4326
HmHel-6	CAEZ01006635:16132-16379
HmHel-6	CAEZ01006635:40319-49061
HmHel-6	CAEZ01006635:17605-17836
HmHel-6	CAEZ01006470:416-3035
HmHel-6	CAEZ01006470:10769-13443
HmHel-6	CAEZ01005402:22425-22522
HmHel-6	CAEZ01005402:46173-46339
HmHel-6	CAEZ01005402:63250-63459
HmHel-6	CAEZ01005402:41613-41864
HmHel-6	CAEZ01005402:14419-14509
HmHel-6	CAEZ01005335:6487-6665
HmHel-6	CAEZ01005335:53639-53739
HmHel-6	CAEZ01005335:97980-102895
HmHel-6	CAEZ01005335:101862-107046
HmHel-6	CAEZ01005335:51316-51422
HmHel-6	CAEZ01004796:3367-3585
HmHel-6	CAEZ01004796:24508-24759
HmHel-6	CAEZ01004796:7549-7682
HmHel-6	CAEZ01004018:64091-64302
HmHel-6	CAEZ01004018:92719-92905
HmHel-6	CAEZ01004018:85720-85971
HmHel-6	CAEZ01004018:69092-69302
HmHel-6	CAEZ01004018:37847-39635
HmHel-6	CAEZ01003248:17560-17811
HmHel-6	CAEZ01003248:60093-60283
HmHel-6	CAEZ01003248:19318-19399
HmHel-6	CAEZ01003248:6054-6191
HmHel-6	CAEZ01000858:31384-31478
HmHel-6	CAEZ01000858:40669-40919
HmHel-6	CAEZ01011493:22704-22885
HmHel-6	CAEZ01011493:36401-36502
HmHel-6	CAEZ01011493:51135-51319
HmHel-6	CAEZ01011493:44349-44584
HmHel-6	CAEZ01011493:13006-15510
HmHel-6	CAEZ01011128:2482-2614
HmHel-6	CAEZ01011128:11916-12165
HmHel-6	CAEZ01010028:50268-50416
HmHel-6	CAEZ01010028:79187-79356
HmHel-6	CAEZ01010028:90071-90227
HmHel-6	CAEZ01010028:111591-111713
HmHel-6	CAEZ01010028:92049-92142

HmHel-6	CAEZ01010028:59229-59314
HmHel-6	CAEZ01010028:36814-36901
HmHel-6	CAEZ01010028:20631-20881
HmHel-6	CAEZ01010016:2985-3235
HmHel-6	CAEZ01010016:17774-17953
HmHel-6	CAEZ01010016:13356-15493
HmHel-6	CAEZ01010016:513-673
HmHel-6	CAEZ01009473:12256-12499
HmHel-6	CAEZ01009473:4796-4935
HmHel-6	CAEZ01008894:10175-10292
HmHel-6	CAEZ01008894:29906-30151
HmHel-6	CAEZ01007938:5321-5438
HmHel-6	CAEZ01007938:42034-46517
HmHel-6	CAEZ01007938:99360-99516
HmHel-6	CAEZ01007938:123218-123450
HmHel-6	CAEZ01007938:112048-112149
HmHel-6	CAEZ01007938:94319-94403
HmHel-6	CAEZ01007938:69739-72255
HmHel-6	CAEZ01007938:55146-56501
HmHel-6	CAEZ01007938:22181-28810
HmHel-6	CAEZ01007712:5113-5366
HmHel-6	CAEZ01006796:23128-23272
HmHel-6	CAEZ01006796:22445-22698
HmHel-6	CAEZ01006352:1569-1694
HmHel-6	CAEZ01006352:61339-61444
HmHel-6	CAEZ01006352:109113-109338
HmHel-6	CAEZ01006352:138097-138241
HmHel-6	CAEZ01006352:151842-151995
HmHel-6	CAEZ01006352:162717-162883
HmHel-6	CAEZ01006352:117682-117818
HmHel-6	CAEZ01006352:104946-105072
HmHel-6	CAEZ01006352:81922-82110
HmHel-6	CAEZ01006352:4247-5875
HmHel-6	CAEZ01005930:5362-5615
HmHel-6	CAEZ01005930:32320-32496
HmHel-6	CAEZ01005930:36059-36152
HmHel-6	CAEZ01005310:18855-23041
HmHel-6	CAEZ01005310:50380-50630
HmHel-6	CAEZ01005310:32593-32838
HmHel-6	CAEZ01005310:6474-6577
HmHel-6	CAEZ01004921:14450-14582
HmHel-6	CAEZ01004921:56745-56873
HmHel-6	CAEZ01004921:69213-76844
HmHel-6	CAEZ01004921:96488-96734
HmHel-6	CAEZ01004921:103909-104088
HmHel-6	CAEZ01004921:76102-76356
HmHel-6	CAEZ01004921:47641-47817
HmHel-6	CAEZ01004921:29782-29941

HmHel-6	CAEZ01004080:116-366
HmHel-6	CAEZ01003717:5120-5208
HmHel-6	CAEZ01003717:24431-24518
HmHel-6	CAEZ01003717:37009-37197
HmHel-6	CAEZ01003717:64910-65016
HmHel-6	CAEZ01003717:105357-105611
HmHel-6	CAEZ01003717:125072-125215
HmHel-6	CAEZ01003717:111245-120480
HmHel-6	CAEZ01003717:78295-78497
HmHel-6	CAEZ01003717:63648-63797
HmHel-6	CAEZ01003422:14944-15136
HmHel-6	CAEZ01003422:29831-29998
HmHel-6	CAEZ01003422:64496-70713
HmHel-6	CAEZ01003422:71800-73473
HmHel-6	CAEZ01003422:32978-39700
HmHel-6	CAEZ01003422:6705-6954
HmHel-6	CAEZ01011283:44950-46315
HmHel-6	CAEZ01011283:92525-92746
HmHel-6	CAEZ01010460:44726-44814
HmHel-6	CAEZ01010460:83141-92111
HmHel-6	CAEZ01010460:139353-139445
HmHel-6	CAEZ01010460:134343-138453
HmHel-6	CAEZ01010460:111093-114142
HmHel-6	CAEZ01010460:97270-97378
HmHel-6	CAEZ01010460:73291-73481
HmHel-6	CAEZ01010460:55590-55785
HmHel-6	CAEZ01010460:39436-39643
HmHel-6	CAEZ01010316:3308-3398
HmHel-6	CAEZ01010316:1108-1361
HmHel-6	CAEZ01010277:56576-56782
HmHel-6	CAEZ01010277:82750-83003
HmHel-6	CAEZ01010277:118781-118876
HmHel-6	CAEZ01010277:102656-102774
HmHel-6	CAEZ01010277:74509-74749
HmHel-6	CAEZ01010277:48770-48986
HmHel-6	CAEZ01010277:12020-12110
HmHel-6	CAEZ01010026:124250-124371
HmHel-6	CAEZ01010026:135383-135540
HmHel-6	CAEZ01010026:216274-216526
HmHel-6	CAEZ01010026:272135-272230
HmHel-6	CAEZ01010026:110587-110669
HmHel-6	CAEZ01010026:78726-88905
HmHel-6	CAEZ01010026:67908-68007
HmHel-6	CAEZ01010026:30702-30826
HmHel-6	CAEZ01010026:10324-18546
HmHel-6	CAEZ01008911:74183-77427
HmHel-6	CAEZ01008911:115427-115831
HmHel-6	CAEZ01008911:120901-121381

HmHel-6	CAEZ01008911:106785-106991
HmHel-6	CAEZ01008911:59011-67948
HmHel-6	CAEZ01008911:34978-42453
HmHel-6	CAEZ01008711:9999-10254
HmHel-6	CAEZ01008711:4242-4386
HmHel-6	CAEZ01007985:11690-11902
HmHel-6	CAEZ01007985:25482-25707
HmHel-6	CAEZ01007985:56699-56942
HmHel-6	CAEZ01007985:39788-39877
HmHel-6	CAEZ01007985:14060-14232
HmHel-6	CAEZ01007982:58883-61745
HmHel-6	CAEZ01007982:52831-53002
HmHel-6	CAEZ01007982:19453-28713
HmHel-6	CAEZ01007982:1625-1705
HmHel-6	CAEZ01007258:14541-14628
HmHel-6	CAEZ01007258:99071-104223
HmHel-6	CAEZ01007258:117541-117743
HmHel-6	CAEZ01007258:123184-123300
HmHel-6	CAEZ01007258:91051-91300
HmHel-6	CAEZ01007258:45054-45169
HmHel-6	CAEZ01006422:8808-8921
HmHel-6	CAEZ01006422:20001-20170
HmHel-6	CAEZ01006422:51127-51210
HmHel-6	CAEZ01006422:95340-95458
HmHel-6	CAEZ01006422:128412-128661
HmHel-6	CAEZ01006422:93396-99044
HmHel-6	CAEZ01006422:75686-75803
HmHel-6	CAEZ01006422:56512-56678
HmHel-6	CAEZ01006422:18337-18491
HmHel-6	CAEZ01005808:22223-22358
HmHel-6	CAEZ01005808:13014-22816
HmHel-6	CAEZ01005771:3830-4024
HmHel-6	CAEZ01005771:24370-24581
HmHel-6	CAEZ01004815:195-2268
HmHel-6	CAEZ01004815:24271-24442
HmHel-6	CAEZ01004815:75300-77584
HmHel-6	CAEZ01004815:91722-97582
HmHel-6	CAEZ01004815:89824-89956
HmHel-6	CAEZ01004815:52312-52495
HmHel-6	CAEZ01004815:22006-22190
HmHel-6	CAEZ01004429:1249-1368
HmHel-6	CAEZ01004429:10273-10519
HmHel-6	CAEZ01004406:9245-9490
HmHel-6	CAEZ01004406:3521-3613
HmHel-6	CAEZ01004233:2086-2315
HmHel-6	CAEZ01004233:4281-13201
HmHel-6	CAEZ01004107:2461-2599
HmHel-6	CAEZ01004107:2112-11663

HmHel-6	CAEZ01004011:36193-42416
HmHel-6	CAEZ01004011:72666-72747
HmHel-6	CAEZ01004011:136605-136804
HmHel-6	CAEZ01004011:147728-147973
HmHel-6	CAEZ01004011:129558-133168
HmHel-6	CAEZ01004011:113535-116085
HmHel-6	CAEZ01004011:97728-97808
HmHel-6	CAEZ01004011:77719-77806
HmHel-6	CAEZ01004011:64363-64457
HmHel-6	CAEZ01004011:37280-37459
HmHel-6	CAEZ01004011:20388-20515
HmHel-6	CAEZ01004011:8499-8712
HmHel-6	CAEZ01003139:9083-9205
HmHel-6	CAEZ01003139:4266-4486
HmHel-6	CAEZ01011575:4528-4701
HmHel-6	CAEZ01011575:6653-6897
HmHel-6	CAEZ01011359:12485-12717
HmHel-6	CAEZ01011359:28998-29098
HmHel-6	CAEZ01011296:5652-5755
HmHel-6	CAEZ01011296:17854-25073
HmHel-6	CAEZ01011296:79083-84974
HmHel-6	CAEZ01011296:132570-132743
HmHel-6	CAEZ01011296:151679-151927
HmHel-6	CAEZ01011296:172458-172594
HmHel-6	CAEZ01011296:194436-195237
HmHel-6	CAEZ01011296:164324-164577
HmHel-6	CAEZ01011296:144067-144285
HmHel-6	CAEZ01011296:126909-127138
HmHel-6	CAEZ01011296:81231-81439
HmHel-6	CAEZ01011296:34990-35084
HmHel-6	CAEZ01011288:8428-8673
HmHel-6	CAEZ01010029:3148-3403
HmHel-6	CAEZ01010029:85000-85146
HmHel-6	CAEZ01010029:83138-87869
HmHel-6	CAEZ01010029:49821-50053
HmHel-6	CAEZ01010029:12830-12935
HmHel-6	CAEZ01009873:14379-14631
HmHel-6	CAEZ01009873:46941-51964
HmHel-6	CAEZ01009873:34652-34883
HmHel-6	CAEZ01009788:59215-62953
HmHel-6	CAEZ01009788:64624-67282
HmHel-6	CAEZ01009788:48468-54075
HmHel-6	CAEZ01009788:20813-21013
HmHel-6	CAEZ01009788:7842-8090
HmHel-6	CAEZ01009021:823-963
HmHel-6	CAEZ01009021:21192-21765
HmHel-6	CAEZ01008517:19584-19836
HmHel-6	CAEZ01008517:15148-15265

HmHel-6	CAEZ01007855:21848-22093
HmHel-6	CAEZ01007855:53356-53565
HmHel-6	CAEZ01007855:18789-19038
HmHel-6	CAEZ01007812:30018-30153
HmHel-6	CAEZ01007812:92790-92883
HmHel-6	CAEZ01007812:201405-201613
HmHel-6	CAEZ01007812:216806-218235
HmHel-6	CAEZ01007812:236563-236776
HmHel-6	CAEZ01007812:274275-274436
HmHel-6	CAEZ01007812:286027-286144
HmHel-6	CAEZ01007812:266463-266598
HmHel-6	CAEZ01007812:245091-245227
HmHel-6	CAEZ01007812:224392-224510
HmHel-6	CAEZ01007812:188757-194552
HmHel-6	CAEZ01007812:136904-140020
HmHel-6	CAEZ01007812:123876-124124
HmHel-6	CAEZ01007812:61824-62028
HmHel-6	CAEZ01007812:20504-20724
HmHel-6	CAEZ01007586:9621-19744
HmHel-6	CAEZ01007586:56290-56446
HmHel-6	CAEZ01007586:59581-59699
HmHel-6	CAEZ01007586:39395-39499
HmHel-6	CAEZ01007586:16686-16938
HmHel-6	CAEZ01007586:538-780
HmHel-6	CAEZ01006361:10730-10982
HmHel-6	CAEZ01005373:14620-14755
HmHel-6	CAEZ01005373:31980-32082
HmHel-6	CAEZ01005373:69636-69792
HmHel-6	CAEZ01005373:123422-123667
HmHel-6	CAEZ01005373:126120-126207
HmHel-6	CAEZ01005373:99704-99853
HmHel-6	CAEZ01005373:65509-65725
HmHel-6	CAEZ01005373:9921-10480
HmHel-6	CAEZ01005211:4149-4228
HmHel-6	CAEZ01005211:15670-22216
HmHel-6	CAEZ01005211:93000-93187
HmHel-6	CAEZ01005211:133519-133771
HmHel-6	CAEZ01005211:95438-95535
HmHel-6	CAEZ01005211:64390-64539
HmHel-6	CAEZ01005211:48335-48541
HmHel-6	CAEZ01005205:10530-10663
HmHel-6	CAEZ01005205:29176-29423
HmHel-6	CAEZ01005205:42880-42960
HmHel-6	CAEZ01005205:62328-62567
HmHel-6	CAEZ01005205:45530-54684
HmHel-6	CAEZ01005205:17470-22493
HmHel-6	CAEZ01005205:2483-2609
HmHel-6	CAEZ01002826:33491-33571

HmHel-6	CAEZ01002826:60001-68713
HmHel-6	CAEZ01002826:89146-89375
HmHel-6	CAEZ01002826:107808-108013
HmHel-6	CAEZ01002826:126946-127166
HmHel-6	CAEZ01002826:135204-135448
HmHel-6	CAEZ01002826:98022-98231
HmHel-6	CAEZ01002826:64952-65098
HmHel-6	CAEZ01002826:33128-33249
HmHel-6	CAEZ01002826:8124-8248
HmHel-6	CAEZ01002368:7774-7903
HmHel-6	CAEZ01002368:11045-11285
HmHel-6	CAEZ01001743:4202-4296
HmHel-6	CAEZ01001743:50132-50302
HmHel-6	CAEZ01001743:31716-31970
HmHel-6	CAEZ01001743:20625-20726
HmHel-6	CAEZ01000048:33578-33830
HmHel-6	CAEZ01000048:22534-22655
HmHel-6	CAEZ01010942:18654-18755
HmHel-6	CAEZ01010942:63069-64722
HmHel-6	CAEZ01010942:69050-69187
HmHel-6	CAEZ01010942:35055-35229
HmHel-6	CAEZ01010730:5014-5179
HmHel-6	CAEZ01010730:62685-67099
HmHel-6	CAEZ01010730:80483-80694
HmHel-6	CAEZ01010730:75631-75726
HmHel-6	CAEZ01010730:42955-43107
HmHel-6	CAEZ01010730:16169-16370
HmHel-6	CAEZ01009422:13860-14057
HmHel-6	CAEZ01009422:88018-88206
HmHel-6	CAEZ01009422:101267-105217
HmHel-6	CAEZ01009422:133943-134034
HmHel-6	CAEZ01009422:120909-121052
HmHel-6	CAEZ01009422:67403-67540
HmHel-6	CAEZ01009422:49075-53338
HmHel-6	CAEZ01005455:12191-12288
HmHel-6	CAEZ01005455:48918-49109
HmHel-6	CAEZ01005455:81511-85249
HmHel-6	CAEZ01005455:41911-49886
HmHel-6	CAEZ01004038:4929-5068
HmHel-6	CAEZ01004038:32857-40805
HmHel-6	CAEZ01004038:37984-38102
HmHel-6	CAEZ01002589:5648-5807
HmHel-6	CAEZ01002589:27418-27539
HmHel-6	CAEZ01002589:35281-37488
HmHel-6	CAEZ01002589:1811-4477
HmHel-6	CAEZ01002046:1701-1829
HmHel-6	CAEZ01002046:31455-31562
HmHel-6	CAEZ01001910:3342-3573

HmHel-6	CAEZ01010930:4355-4478
HmHel-6	CAEZ01010930:20909-21155
HmHel-6	CAEZ01010930:40856-45179
HmHel-6	CAEZ01010930:38466-38617
HmHel-6	CAEZ01010797:1251-1428
HmHel-6	CAEZ01010797:30263-30440
HmHel-6	CAEZ01010797:63276-63394
HmHel-6	CAEZ01010797:88581-88703
HmHel-6	CAEZ01010797:106495-106596
HmHel-6	CAEZ01010797:120339-128350
HmHel-6	CAEZ01010797:157412-165048
HmHel-6	CAEZ01010797:177398-177516
HmHel-6	CAEZ01010797:153981-154122
HmHel-6	CAEZ01010797:129134-129384
HmHel-6	CAEZ01010797:83588-83805
HmHel-6	CAEZ01010797:5748-5853
HmHel-6	CAEZ01010722:2725-2976
HmHel-6	CAEZ01010556:1112-1362
HmHel-6	CAEZ01010511:14358-21035
HmHel-6	CAEZ01010511:23648-23851
HmHel-6	CAEZ01010188:8893-8983
HmHel-6	CAEZ01010188:21725-21961
HmHel-6	CAEZ01010188:30216-30303
HmHel-6	CAEZ01008919:11709-11876
HmHel-6	CAEZ01008919:31964-33080
HmHel-6	CAEZ01008919:43600-43700
HmHel-6	CAEZ01008919:55071-55167
HmHel-6	CAEZ01008919:77961-78204
HmHel-6	CAEZ01008919:46428-48456
HmHel-6	CAEZ01008444:21301-21551
HmHel-6	CAEZ01008444:51252-51371
HmHel-6	CAEZ01008315:1531-3571
HmHel-6	CAEZ01008315:61597-70217
HmHel-6	CAEZ01008315:127177-127260
HmHel-6	CAEZ01008315:164090-164327
HmHel-6	CAEZ01008315:200329-200474
HmHel-6	CAEZ01008315:217757-226124
HmHel-6	CAEZ01008315:256057-263929
HmHel-6	CAEZ01008315:323665-323763
HmHel-6	CAEZ01008315:434871-435080
HmHel-6	CAEZ01008315:446576-446705
HmHel-6	CAEZ01008315:427025-427266
HmHel-6	CAEZ01008315:409243-409359
HmHel-6	CAEZ01008315:367219-367447
HmHel-6	CAEZ01008315:316063-316289
HmHel-6	CAEZ01008315:284964-290108
HmHel-6	CAEZ01008315:258645-260942
HmHel-6	CAEZ01008315:238583-245694

HmHel-6	CAEZ01008315:225125-225228
HmHel-6	CAEZ01008315:204365-204446
HmHel-6	CAEZ01008315:171189-179775
HmHel-6	CAEZ01008315:153907-160443
HmHel-6	CAEZ01008239:8008-8182
HmHel-6	CAEZ01008239:47275-47355
HmHel-6	CAEZ01008239:37499-37745
HmHel-6	CAEZ01007637:6086-6336
HmHel-6	CAEZ01007637:12419-12504
HmHel-6	CAEZ01007637:229-310
HmHel-6	CAEZ01007291:5383-5576
HmHel-6	CAEZ01006367:2846-2974
HmHel-6	CAEZ01006367:67377-67539
HmHel-6	CAEZ01006367:131205-131442
HmHel-6	CAEZ01006367:147798-147901
HmHel-6	CAEZ01006367:156585-156726
HmHel-6	CAEZ01006367:79512-79747
HmHel-6	CAEZ01006367:38226-38417
HmHel-6	CAEZ01006131:10991-11125
HmHel-6	CAEZ01006131:60903-61024
HmHel-6	CAEZ01006131:71625-71863
HmHel-6	CAEZ01006131:88983-89141
HmHel-6	CAEZ01006131:108545-108756
HmHel-6	CAEZ01006131:127233-128268
HmHel-6	CAEZ01006131:145854-153988
HmHel-6	CAEZ01006131:226741-226918
HmHel-6	CAEZ01006131:195033-205051
HmHel-6	CAEZ01006131:136639-136857
HmHel-6	CAEZ01006131:97350-97498
HmHel-6	CAEZ01005015:2313-2469
HmHel-6	CAEZ01005015:58079-58291
HmHel-6	CAEZ01005015:85787-92216
HmHel-6	CAEZ01005015:131187-131426
HmHel-6	CAEZ01005015:161707-166425
HmHel-6	CAEZ01005015:185187-185290
HmHel-6	CAEZ01005015:199173-199352
HmHel-6	CAEZ01005015:232189-232432
HmHel-6	CAEZ01005015:256222-256319
HmHel-6	CAEZ01005015:211569-211712
HmHel-6	CAEZ01005015:197186-197436
HmHel-6	CAEZ01005015:168385-168551
HmHel-6	CAEZ01005015:102547-102796
HmHel-6	CAEZ01005015:81805-81903
HmHel-6	CAEZ01005015:67011-67237
HmHel-6	CAEZ01005015:31405-31653
HmHel-6	CAEZ01004925:405-544
HmHel-6	CAEZ01004925:10087-10322
HmHel-6	CAEZ01004811:30698-30860

HmHel-6	CAEZ01004811:48703-48887
HmHel-6	CAEZ01004811:77751-77965
HmHel-6	CAEZ01004811:122478-122578
HmHel-6	CAEZ01004811:159785-159956
HmHel-6	CAEZ01004811:178232-178328
HmHel-6	CAEZ01004811:194081-194235
HmHel-6	CAEZ01004811:241719-241913
HmHel-6	CAEZ01004811:262109-262337
HmHel-6	CAEZ01004811:274544-274686
HmHel-6	CAEZ01004811:289044-289260
HmHel-6	CAEZ01004811:359507-359644
HmHel-6	CAEZ01004811:405474-405659
HmHel-6	CAEZ01004811:418427-418544
HmHel-6	CAEZ01004811:427403-428957
HmHel-6	CAEZ01004811:402710-402813
HmHel-6	CAEZ01004811:376143-382955
HmHel-6	CAEZ01004811:329491-331625
HmHel-6	CAEZ01004811:313150-313276
HmHel-6	CAEZ01004811:278648-280523
HmHel-6	CAEZ01004811:218143-224523
HmHel-6	CAEZ01004811:140629-140813
HmHel-6	CAEZ01004811:101306-101396
HmHel-6	CAEZ01004811:74629-74854
HmHel-6	CAEZ01004811:38167-38351
HmHel-6	CAEZ01004648:3249-3384
HmHel-6	CAEZ01004457:23686-23784
HmHel-6	CAEZ01003679:1513-1763
HmHel-6	CAEZ01002981:1223-1302
HmHel-6	CAEZ01002962:1182-1400
HmHel-6	CAEZ01001326:10110-10351
HmHel-6	CAEZ01001326:24880-24985
HmHel-6	CAEZ01001326:62031-62114
HmHel-6	CAEZ01001326:74269-76790
HmHel-6	CAEZ01001326:110156-110402
HmHel-6	CAEZ01001326:96643-97212
HmHel-6	CAEZ01001326:75172-75277
HmHel-6	CAEZ01001326:64080-64165
HmHel-6	CAEZ01001326:46059-46935
HmHel-6	CAEZ01001326:13042-13174
HmHel-6	CAEZ01000908:2103-2335
HmHel-6	CAEZ01000908:9163-18469
HmHel-6	CAEZ01011410:11847-14444
HmHel-6	CAEZ01011410:48122-48209
HmHel-6	CAEZ01011410:30990-31213
HmHel-6	CAEZ01011410:15877-15998
HmHel-6	CAEZ01010295:21090-21337
HmHel-6	CAEZ01010295:15879-16082
HmHel-6	CAEZ01009748:27097-27256

HmHel-6	CAEZ01009748:48232-48496
HmHel-6	CAEZ01009748:66252-66335
HmHel-6	CAEZ01009748:85530-85641
HmHel-6	CAEZ01009748:155161-155379
HmHel-6	CAEZ01009748:124380-124558
HmHel-6	CAEZ01009748:58256-58438
HmHel-6	CAEZ01009748:40778-40969
HmHel-6	CAEZ01009613:5431-5680
HmHel-6	CAEZ01009613:4256-4397
HmHel-6	CAEZ01009075:1788-2033
HmHel-6	CAEZ01009075:23373-23481
HmHel-6	CAEZ01009075:24045-24291
HmHel-6	CAEZ01009075:8576-9689
HmHel-6	CAEZ01008838:12407-12510
HmHel-6	CAEZ01008838:8173-11886
HmHel-6	CAEZ01008808:1360-2236
HmHel-6	CAEZ01008808:10480-10709
HmHel-6	CAEZ01008766:31448-40219
HmHel-6	CAEZ01008766:51668-51758
HmHel-6	CAEZ01008766:53792-54040
HmHel-6	CAEZ01008632:30854-37571
HmHel-6	CAEZ01008632:36504-44520
HmHel-6	CAEZ01008356:20680-25618
HmHel-6	CAEZ01008356:39817-40015
HmHel-6	CAEZ01008356:51697-51785
HmHel-6	CAEZ01008356:71914-72135
HmHel-6	CAEZ01008356:42133-42287
HmHel-6	CAEZ01008356:17334-17451
HmHel-6	CAEZ01008281:12559-12796
HmHel-6	CAEZ01008281:35964-36121
HmHel-6	CAEZ01008281:50064-50250
HmHel-6	CAEZ01008281:10376-10515
HmHel-6	CAEZ01008189:15249-15485
HmHel-6	CAEZ01008144:7246-7490
HmHel-6	CAEZ01008020:14708-14813
HmHel-6	CAEZ01008020:9733-9973
HmHel-6	CAEZ01007880:5268-5424
HmHel-6	CAEZ01007880:6604-6841
HmHel-6	CAEZ01007801:5351-5604
HmHel-6	CAEZ01007801:9586-9673
HmHel-6	CAEZ01007796:458-711
HmHel-6	CAEZ01006861:7453-7705
HmHel-6	CAEZ01006861:22899-29761
HmHel-6	CAEZ01006861:4293-4536
HmHel-6	CAEZ01006696:8368-8486
HmHel-6	CAEZ01005805:1294-1439
HmHel-6	CAEZ01005805:3330-3582
HmHel-6	CAEZ01005649:23854-29893

HmHel-6	CAEZ01005649:35228-35397
HmHel-6	CAEZ01005144:4975-5231
HmHel-6	CAEZ01003947:500-968
HmHel-6	CAEZ01003401:11223-11456
HmHel-6	CAEZ01003401:26383-26530
HmHel-6	CAEZ01003401:46945-47038
HmHel-6	CAEZ01003401:157038-160875
HmHel-6	CAEZ01003401:227242-227402
HmHel-6	CAEZ01003401:238492-242426
HmHel-6	CAEZ01003401:187780-192006
HmHel-6	CAEZ01003401:85106-85289
HmHel-6	CAEZ01003401:43210-43455
HmHel-6	CAEZ01003380:1068-1301
HmHel-6	CAEZ01003143:4502-4602
HmHel-6	CAEZ01003143:21728-21861
HmHel-6	CAEZ01003143:50891-50975
HmHel-6	CAEZ01002607:19797-24073
HmHel-6	CAEZ01002607:96213-96325
HmHel-6	CAEZ01002607:120198-120423
HmHel-6	CAEZ01002607:176193-176302
HmHel-6	CAEZ01002607:104736-111396
HmHel-6	CAEZ01002607:73160-73238
HmHel-6	CAEZ01002607:43267-52825
HmHel-6	CAEZ01002607:29897-30142
HmHel-6	CAEZ01002607:16518-16604
HmHel-6	CAEZ01001345:2732-2983
HmHel-6	CAEZ01001345:9723-9939
HmHel-6	CAEZ01011495:6792-6919
HmHel-6	CAEZ01011495:64322-64539
HmHel-6	CAEZ01011495:90206-93145
HmHel-6	CAEZ01011495:86701-86937
HmHel-6	CAEZ01011495:34786-38625
HmHel-6	CAEZ01011323:27258-27377
HmHel-6	CAEZ01011323:52675-52883
HmHel-6	CAEZ01010727:13099-13346
HmHel-6	CAEZ01010727:33248-33363
HmHel-6	CAEZ01010727:57412-57657
HmHel-6	CAEZ01010305:36463-36685
HmHel-6	CAEZ01010305:58052-58190
HmHel-6	CAEZ01010305:69295-69442
HmHel-6	CAEZ01010305:77476-80606
HmHel-6	CAEZ01009802:9464-9602
HmHel-6	CAEZ01009802:63654-63745
HmHel-6	CAEZ01009802:112677-112930
HmHel-6	CAEZ01009802:125270-125483
HmHel-6	CAEZ01009802:146370-146568
HmHel-6	CAEZ01009802:202110-202231
HmHel-6	CAEZ01009802:237839-238001

HmHel-6	CAEZ01009802:276470-285669
HmHel-6	CAEZ01009802:253589-253866
HmHel-6	CAEZ01009802:241255-241507
HmHel-6	CAEZ01009802:224761-224893
HmHel-6	CAEZ01009802:210135-210244
HmHel-6	CAEZ01009802:194114-194371
HmHel-6	CAEZ01009802:164819-164956
HmHel-6	CAEZ01009802:118318-127544
HmHel-6	CAEZ01009802:98570-98725
HmHel-6	CAEZ01009802:29032-37848
HmHel-6	CAEZ01008710:34171-39950
HmHel-6	CAEZ01008710:96723-99029
HmHel-6	CAEZ01008710:45165-45395
HmHel-6	CAEZ01008674:23387-33189
HmHel-6	CAEZ01008674:94210-94432
HmHel-6	CAEZ01008674:81833-82043
HmHel-6	CAEZ01008674:42826-46698
HmHel-6	CAEZ01008619:2648-2881
HmHel-6	CAEZ01008619:25462-25676
HmHel-6	CAEZ01008619:38600-38763
HmHel-6	CAEZ01008619:64577-64801
HmHel-6	CAEZ01008619:67025-72641
HmHel-6	CAEZ01008619:35581-47516
HmHel-6	CAEZ01008619:19342-19593
HmHel-6	CAEZ01008603:3326-3447
HmHel-6	CAEZ01008603:16861-17012
HmHel-6	CAEZ01008603:48062-51976
HmHel-6	CAEZ01008603:69390-72393
HmHel-6	CAEZ01008603:62833-66182
HmHel-6	CAEZ01007727:23993-24093
HmHel-6	CAEZ01007727:89629-89811
HmHel-6	CAEZ01007727:81484-81574
HmHel-6	CAEZ01007727:52791-61460
HmHel-6	CAEZ01007727:39245-39432
HmHel-6	CAEZ01007727:28510-28623
HmHel-6	CAEZ01007408:271-511
HmHel-6	CAEZ01007408:14922-15085
HmHel-6	CAEZ01007408:12659-12877
HmHel-6	CAEZ01007401:9071-9315
HmHel-6	CAEZ01007401:27541-27646
HmHel-6	CAEZ01007401:54710-54953
HmHel-6	CAEZ01007401:46624-46729
HmHel-6	CAEZ01007401:20595-20724
HmHel-6	CAEZ01006772:29480-29673
HmHel-6	CAEZ01006772:57299-57437
HmHel-6	CAEZ01006772:86097-86230
HmHel-6	CAEZ01006772:107305-107531
HmHel-6	CAEZ01006772:201545-201756

HmHel-6	CAEZ01006772:249805-250049
HmHel-6	CAEZ01006772:270202-270316
HmHel-6	CAEZ01006772:237046-239536
HmHel-6	CAEZ01006772:200753-200912
HmHel-6	CAEZ01006772:133784-133875
HmHel-6	CAEZ01006318:900-5701
HmHel-6	CAEZ01006318:15439-15687
HmHel-6	CAEZ01006214:10375-10492
HmHel-6	CAEZ01006214:18836-18970
HmHel-6	CAEZ01006162:19064-19158
HmHel-6	CAEZ01006162:40092-40230
HmHel-6	CAEZ01006162:65137-65237
HmHel-6	CAEZ01006162:76398-76554
HmHel-6	CAEZ01006162:90974-91210
HmHel-6	CAEZ01006162:119417-119597
HmHel-6	CAEZ01006162:122781-122932
HmHel-6	CAEZ01006162:103949-104192
HmHel-6	CAEZ01006162:85511-85601
HmHel-6	CAEZ01006162:47870-48045
HmHel-6	CAEZ01006162:29071-29180
HmHel-6	CAEZ01005839:1426-7178
HmHel-6	CAEZ01005665:13605-15558
HmHel-6	CAEZ01004751:22798-22929
HmHel-6	CAEZ01004751:59300-59457
HmHel-6	CAEZ01004751:17343-17563
HmHel-6	CAEZ01003612:2414-2546
HmHel-6	CAEZ01003612:4284-4471
HmHel-6	CAEZ01002724:23659-23906
HmHel-6	CAEZ01002724:87909-88115
HmHel-6	CAEZ01002724:100271-106208
HmHel-6	CAEZ01002724:126366-126505
HmHel-6	CAEZ01002724:103992-104206
HmHel-6	CAEZ01002724:70395-70541
HmHel-6	CAEZ01002724:3295-11923
HmHel-6	CAEZ01002690:5413-5672
HmHel-6	CAEZ01002690:4154-4376
HmHel-6	CAEZ01000912:10744-10863
HmHel-6	CAEZ01000912:40378-43189
HmHel-6	CAEZ01000912:56255-58492
HmHel-6	CAEZ01000912:42098-42344
HmHel-6	CAEZ01000912:15695-15904
HmHel-6	CAEZ01000912:5431-5637
HmHel-6	CAEZ01010965:9360-9476
HmHel-6	CAEZ01010965:19060-19307
HmHel-6	CAEZ01010332:1582-4149
HmHel-6	CAEZ01010332:26318-26430
HmHel-6	CAEZ01010332:4496-4747
HmHel-6	CAEZ01010023:13352-13582

HmHel-6	CAEZ01009882:9371-9553
HmHel-6	CAEZ01009882:16187-17601
HmHel-6	CAEZ01009881:2522-2646
HmHel-6	CAEZ01009881:17902-18081
HmHel-6	CAEZ01008293:20178-20311
HmHel-6	CAEZ01008293:61112-61569
HmHel-6	CAEZ01008293:73141-73250
HmHel-6	CAEZ01008293:54650-54743
HmHel-6	CAEZ01008293:25069-25199
HmHel-6	CAEZ01007686:34848-34961
HmHel-6	CAEZ01007686:14970-15220
HmHel-6	CAEZ01006069:2815-3042
HmHel-6	CAEZ01006069:7667-7759
HmHel-6	CAEZ01005992:17723-17974
HmHel-6	CAEZ01005438:34192-34436
HmHel-6	CAEZ01005438:23564-23670
HmHel-6	CAEZ01005220:14114-14246
HmHel-6	CAEZ01005220:28434-28651
HmHel-6	CAEZ01005220:43323-43480
HmHel-6	CAEZ01005220:56561-56718
HmHel-6	CAEZ01005220:39829-39980
HmHel-6	CAEZ01005220:10444-10620
HmHel-6	CAEZ01004225:2674-2809
HmHel-6	CAEZ01004225:26409-26538
HmHel-6	CAEZ01004207:2972-3214
HmHel-6	CAEZ01004207:3723-3805
HmHel-6	CAEZ01003879:2492-2585
HmHel-6	CAEZ01003879:23971-24086
HmHel-6	CAEZ01003879:12447-12697
HmHel-6	CAEZ01003879:1632-1757
HmHel-6	CAEZ01003681:2544-2775
HmHel-6	CAEZ01002828:6440-11624
HmHel-6	CAEZ01002828:8850-9085
HmHel-6	CAEZ01002400:290-526
HmHel-6	CAEZ01002203:25594-25786
HmHel-6	CAEZ01002041:32019-35968
HmHel-6	CAEZ01002041:72368-72589
HmHel-6	CAEZ01002041:60824-61071
HmHel-6	CAEZ01002041:17862-18025
HmHel-6	CAEZ01001284:1132-10115
HmHel-6	CAEZ01001284:20901-22406
HmHel-6	CAEZ01011525:19349-19510
HmHel-6	CAEZ01011525:15615-15869
HmHel-6	CAEZ01011208:2387-2636
HmHel-6	CAEZ01011208:1846-2001
HmHel-6	CAEZ01010774:5129-5370
HmHel-6	CAEZ01010774:4806-4962
HmHel-6	CAEZ01010497:8296-8456

HmHel-6	CAEZ01010497:9642-13719
HmHel-6	CAEZ01010030:5165-5315
HmHel-6	CAEZ01010030:30171-30321
HmHel-6	CAEZ01010030:47654-47869
HmHel-6	CAEZ01010030:73553-73760
HmHel-6	CAEZ01010030:104987-105177
HmHel-6	CAEZ01010030:149522-159486
HmHel-6	CAEZ01010030:178359-178442
HmHel-6	CAEZ01010030:191635-191863
HmHel-6	CAEZ01010030:229127-229356
HmHel-6	CAEZ01010030:240025-240231
HmHel-6	CAEZ01010030:297724-297917
HmHel-6	CAEZ01010030:308861-309004
HmHel-6	CAEZ01010030:279676-279796
HmHel-6	CAEZ01010030:232344-232540
HmHel-6	CAEZ01010030:220149-220354
HmHel-6	CAEZ01010030:174527-174745
HmHel-6	CAEZ01010030:119127-119319
HmHel-6	CAEZ01010030:107713-107897
HmHel-6	CAEZ01010030:64633-66100
HmHel-6	CAEZ01010030:40065-40237
HmHel-6	CAEZ01009714:1838-2019
HmHel-6	CAEZ01009714:16446-25859
HmHel-6	CAEZ01009714:25242-25348
HmHel-6	CAEZ01009448:3081-3208
HmHel-6	CAEZ01009448:25159-25262
HmHel-6	CAEZ01009448:43985-44188
HmHel-6	CAEZ01009023:18293-18423
HmHel-6	CAEZ01009023:33886-34090
HmHel-6	CAEZ01009023:67745-67922
HmHel-6	CAEZ01009023:82557-87030
HmHel-6	CAEZ01009023:76562-80602
HmHel-6	CAEZ01009023:66127-66217
HmHel-6	CAEZ01009023:25760-25998
HmHel-6	CAEZ01008420:6418-6591
HmHel-6	CAEZ01008420:17061-17223
HmHel-6	CAEZ01008420:30107-34440
HmHel-6	CAEZ01008420:125693-125956
HmHel-6	CAEZ01008420:138563-142410
HmHel-6	CAEZ01008420:196525-196670
HmHel-6	CAEZ01008420:192895-193145
HmHel-6	CAEZ01008420:161936-162148
HmHel-6	CAEZ01008420:141198-141449
HmHel-6	CAEZ01008420:118463-118570
HmHel-6	CAEZ01007413:1802-1912
HmHel-6	CAEZ01007413:17207-17315
HmHel-6	CAEZ01007413:57963-58217
HmHel-6	CAEZ01007413:80612-80821

HmHel-6	CAEZ01007413:20991-21096
HmHel-6	CAEZ01007000:4858-8285
HmHel-6	CAEZ01007000:6039-6245
HmHel-6	CAEZ01006633:1076-3854
HmHel-6	CAEZ01006633:50859-51081
HmHel-6	CAEZ01006203:1211-1442
HmHel-6	CAEZ01006203:14232-14327
HmHel-6	CAEZ01006181:2174-2306
HmHel-6	CAEZ01005736:2283-2535
HmHel-6	CAEZ01005736:1545-1792
HmHel-6	CAEZ01005482:1577-1806
HmHel-6	CAEZ01005482:52651-52771
HmHel-6	CAEZ01005482:76037-76207
HmHel-6	CAEZ01005482:109759-115441
HmHel-6	CAEZ01005482:122076-122218
HmHel-6	CAEZ01005482:83490-87373
HmHel-6	CAEZ01005482:54529-61695
HmHel-6	CAEZ01005482:31854-31979
HmHel-6	CAEZ01005482:10486-10568
HmHel-6	CAEZ01004343:14570-14820
HmHel-6	CAEZ01004343:6255-6406
HmHel-6	CAEZ01003583:12126-12376
HmHel-6	CAEZ01003478:8061-8700
HmHel-6	CAEZ01003478:13129-13245
HmHel-6	CAEZ01003478:1813-2003
HmHel-6	CAEZ01002132:8209-8340
HmHel-6	CAEZ01002132:8077-9443
HmHel-6	CAEZ01001466:23049-23128
HmHel-6	CAEZ01001466:53893-53981
HmHel-6	CAEZ01001466:68657-68783
HmHel-6	CAEZ01001466:90566-90650
HmHel-6	CAEZ01001466:56583-57761
HmHel-6	CAEZ01001466:24200-24446
HmHel-6	CAEZ01011219:9315-9434
HmHel-6	CAEZ01011219:21487-21704
HmHel-6	CAEZ01011219:46472-46580
HmHel-6	CAEZ01011219:62695-62904
HmHel-6	CAEZ01011219:40745-47029
HmHel-6	CAEZ01011219:27056-27190
HmHel-6	CAEZ01011022:55-249
HmHel-6	CAEZ01011022:20133-20383
HmHel-6	CAEZ01011022:890-8784
HmHel-6	CAEZ01010614:12979-13129
HmHel-6	CAEZ01010614:91537-91628
HmHel-6	CAEZ01010614:89422-89538
HmHel-6	CAEZ01010614:76268-76405
HmHel-6	CAEZ01010614:1351-1454
HmHel-6	CAEZ01010503:20022-20275

HmHel-6	CAEZ01010503:7765-8016
HmHel-6	CAEZ01009633:12339-12480
HmHel-6	CAEZ01009633:35812-38960
HmHel-6	CAEZ01009633:87468-87741
HmHel-6	CAEZ01009633:102588-102776
HmHel-6	CAEZ01009633:89622-90616
HmHel-6	CAEZ01009633:6122-6201
HmHel-6	CAEZ01009610:20731-22449
HmHel-6	CAEZ01009610:59171-59424
HmHel-6	CAEZ01009610:60350-60508
HmHel-6	CAEZ01009610:26645-26783
HmHel-6	CAEZ01009500:10697-10947
HmHel-6	CAEZ01009500:5870-6018
HmHel-6	CAEZ01007674:22700-22948
HmHel-6	CAEZ01007674:19743-19831
HmHel-6	CAEZ01007674:9334-9445
HmHel-6	CAEZ01006632:9504-9718
HmHel-6	CAEZ01006632:47772-47973
HmHel-6	CAEZ01006632:63350-63510
HmHel-6	CAEZ01006632:65881-67757
HmHel-6	CAEZ01006632:43310-43524
HmHel-6	CAEZ01006566:13280-13453
HmHel-6	CAEZ01006566:71908-72000
HmHel-6	CAEZ01006566:99162-99350
HmHel-6	CAEZ01006566:116115-116367
HmHel-6	CAEZ01006566:80704-80910
HmHel-6	CAEZ01006566:47706-48532
HmHel-6	CAEZ01006566:11522-11674
HmHel-6	CAEZ01006522:10248-10500
HmHel-6	CAEZ01006522:14168-14355
HmHel-6	CAEZ01006399:9765-10001
HmHel-6	CAEZ01005479:3952-4036
HmHel-6	CAEZ01005479:45605-45768
HmHel-6	CAEZ01005479:27468-27605
HmHel-6	CAEZ01005219:47571-47808
HmHel-6	CAEZ01005219:106483-106732
HmHel-6	CAEZ01005219:170023-178868
HmHel-6	CAEZ01005219:199732-199884
HmHel-6	CAEZ01005219:216350-216471
HmHel-6	CAEZ01005219:162075-162233
HmHel-6	CAEZ01005219:149185-150852
HmHel-6	CAEZ01005219:122665-122786
HmHel-6	CAEZ01005219:85797-85907
HmHel-6	CAEZ01005219:43030-43224
HmHel-6	CAEZ01005219:8428-8654
HmHel-6	CAEZ01004691:7866-8103
HmHel-6	CAEZ01002224:5611-5856
HmHel-6	CAEZ01002224:26524-26605

HmHel-6	CAEZ01002224:25127-25301
HmHel-6	CAEZ01002224:4509-4695
HmHel-6	CAEZ01011398:5922-6169
HmHel-6	CAEZ01010275:6026-8249
HmHel-6	CAEZ01010275:36745-44344
HmHel-6	CAEZ01010275:76682-76920
HmHel-6	CAEZ01010275:20386-20476
HmHel-6	CAEZ01010259:11936-12148
HmHel-6	CAEZ01010094:450-702
HmHel-6	CAEZ01010094:12187-15602
HmHel-6	CAEZ01010094:9624-9782
HmHel-6	CAEZ01009656:8096-8325
HmHel-6	CAEZ01009656:48039-48141
HmHel-6	CAEZ01009656:68448-68547
HmHel-6	CAEZ01009656:69658-69873
HmHel-6	CAEZ01009656:37639-37891
HmHel-6	CAEZ01009325:2678-2824
HmHel-6	CAEZ01009325:3544-3767
HmHel-6	CAEZ01009196:3918-4105
HmHel-6	CAEZ01009196:34419-34505
HmHel-6	CAEZ01009196:65466-66246
HmHel-6	CAEZ01009196:56809-57001
HmHel-6	CAEZ01009196:10018-10247
HmHel-6	CAEZ01008982:3380-3529
HmHel-6	CAEZ01008982:16276-16517
HmHel-6	CAEZ01008982:45668-45862
HmHel-6	CAEZ01008982:27301-27432
HmHel-6	CAEZ01008982:12590-12807
HmHel-6	CAEZ01008933:1735-1981
HmHel-6	CAEZ01008918:765-954
HmHel-6	CAEZ01008918:4672-4912
HmHel-6	CAEZ01008828:4608-4832
HmHel-6	CAEZ01008654:2398-2621
HmHel-6	CAEZ01008585:3997-6713
HmHel-6	CAEZ01008585:15163-15377
HmHel-6	CAEZ01008585:2130-2367
HmHel-6	CAEZ01007503:3050-3298
HmHel-6	CAEZ01007461:33623-35467
HmHel-6	CAEZ01007461:28433-28647
HmHel-6	CAEZ01007461:826-13285
HmHel-6	CAEZ01006749:27100-27299
HmHel-6	CAEZ01006749:64036-64148
HmHel-6	CAEZ01006749:66249-66485
HmHel-6	CAEZ01006749:7689-11464
HmHel-6	CAEZ01006340:606-1344
HmHel-6	CAEZ01006340:26838-27028
HmHel-6	CAEZ01006340:37457-37543
HmHel-6	CAEZ01006340:33557-41046

HmHel-6	CAEZ01006340:12708-12897
HmHel-6	CAEZ01006300:12142-12393
HmHel-6	CAEZ01006300:16932-17112
HmHel-6	CAEZ01006067:4035-4167
HmHel-6	CAEZ01006067:31143-31347
HmHel-6	CAEZ01006067:24857-31469
HmHel-6	CAEZ01005987:2607-2714
HmHel-6	CAEZ01005987:13177-13372
HmHel-6	CAEZ01005987:16324-16556
HmHel-6	CAEZ01005987:1080-1230
HmHel-6	CAEZ01005691:418-655
HmHel-6	CAEZ01005691:5398-5683
HmHel-6	CAEZ01005351:10851-11091
HmHel-6	CAEZ01005351:4536-8701
HmHel-6	CAEZ01004544:2145-2373
HmHel-6	CAEZ01004544:25162-25321
HmHel-6	CAEZ01004003:3484-3695
HmHel-6	CAEZ01004003:18671-22633
HmHel-6	CAEZ01003676:3007-3231
HmHel-6	CAEZ01003676:7578-7676
HmHel-6	CAEZ01003433:2055-2213
HmHel-6	CAEZ01003433:7259-7471
HmHel-6	CAEZ01003187:3457-3677
HmHel-6	CAEZ01000059:15373-15609
HmHel-6	CAEZ01000059:45535-45655
HmHel-6	CAEZ01000059:30100-35317
HmHel-6	CAEZ01000059:17230-17425
HmHel-6	CAEZ01011417:345-478
HmHel-6	CAEZ01011417:4910-5001
HmHel-6	CAEZ01010739:67356-67605
HmHel-6	CAEZ01010739:85678-85911
HmHel-6	CAEZ01010739:109519-109683
HmHel-6	CAEZ01010739:130914-130996
HmHel-6	CAEZ01010739:143562-145101
HmHel-6	CAEZ01010739:168294-168381
HmHel-6	CAEZ01010739:140161-140382
HmHel-6	CAEZ01010739:116095-124511
HmHel-6	CAEZ01010739:79804-87538
HmHel-6	CAEZ01010739:45007-48939
HmHel-6	CAEZ01010739:31039-31157
HmHel-6	CAEZ01010737:22798-30203
HmHel-6	CAEZ01010737:71768-81815
HmHel-6	CAEZ01010737:98806-98956
HmHel-6	CAEZ01010737:138072-138205
HmHel-6	CAEZ01010737:154499-156227
HmHel-6	CAEZ01010737:147834-148035
HmHel-6	CAEZ01010737:68172-68336
HmHel-6	CAEZ01010737:33599-33691

HmHel-6	CAEZ01010737:14741-21555
HmHel-6	CAEZ01010208:911-1146
HmHel-6	CAEZ01010208:16260-18361
HmHel-6	CAEZ01010208:38793-38928
HmHel-6	CAEZ01010208:42705-43361
HmHel-6	CAEZ01010208:25919-26111
HmHel-6	CAEZ01010140:15659-15881
HmHel-6	CAEZ01010140:27189-27275
HmHel-6	CAEZ01010140:18708-18948
HmHel-6	CAEZ01009815:9754-10000
HmHel-6	CAEZ01008940:3856-4106
HmHel-6	CAEZ01008689:22164-22247
HmHel-6	CAEZ01008689:5761-6012
HmHel-6	CAEZ01008675:30798-30997
HmHel-6	CAEZ01008675:15211-16987
HmHel-6	CAEZ01008675:1869-5062
HmHel-6	CAEZ01008649:7514-7763
HmHel-6	CAEZ01008124:33150-33255
HmHel-6	CAEZ01008124:86446-86593
HmHel-6	CAEZ01008124:147963-148071
HmHel-6	CAEZ01008124:180251-184861
HmHel-6	CAEZ01008124:241183-241341
HmHel-6	CAEZ01008124:255665-258055
HmHel-6	CAEZ01008124:234476-234714
HmHel-6	CAEZ01008124:207566-207817
HmHel-6	CAEZ01008124:167137-167234
HmHel-6	CAEZ01008124:155945-156043
HmHel-6	CAEZ01008124:136681-136906
HmHel-6	CAEZ01008124:73488-73662
HmHel-6	CAEZ01008124:29284-29499
HmHel-6	CAEZ01007426:1525-1693
HmHel-6	CAEZ01007426:14008-14142
HmHel-6	CAEZ01007407:27552-27796
HmHel-6	CAEZ01007407:21168-21330
HmHel-6	CAEZ01007222:8677-9492
HmHel-6	CAEZ01007222:22362-22592
HmHel-6	CAEZ01007222:36400-36581
HmHel-6	CAEZ01007222:72735-72945
HmHel-6	CAEZ01007222:90607-90822
HmHel-6	CAEZ01007222:132186-132281
HmHel-6	CAEZ01007222:147515-151310
HmHel-6	CAEZ01007222:172868-180364
HmHel-6	CAEZ01007222:210619-210821
HmHel-6	CAEZ01007222:228525-228635
HmHel-6	CAEZ01007222:258288-258378
HmHel-6	CAEZ01007222:275349-275531
HmHel-6	CAEZ01007222:244114-244343
HmHel-6	CAEZ01007222:226884-227116

HmHel-6	CAEZ01007222:164042-166189
HmHel-6	CAEZ01007222:134055-134172
HmHel-6	CAEZ01007222:110651-110845
HmHel-6	CAEZ01007222:85590-85721
HmHel-6	CAEZ01007222:71775-72007
HmHel-6	CAEZ01007222:15708-15862
HmHel-6	CAEZ01007017:657-863
HmHel-6	CAEZ01007017:18759-24128
HmHel-6	CAEZ01007017:27206-27328
HmHel-6	CAEZ01006320:5887-6135
HmHel-6	CAEZ01006320:23874-23975
HmHel-6	CAEZ01005613:11982-12230
HmHel-6	CAEZ01005613:2294-2485
HmHel-6	CAEZ01004580:39671-39898
HmHel-6	CAEZ01004362:6536-6772
HmHel-6	CAEZ01004089:19212-30700
HmHel-6	CAEZ01003591:5264-5498
HmHel-6	CAEZ01002416:6277-6474
HmHel-6	CAEZ01002416:21304-24287
HmHel-6	CAEZ01002416:60096-60295
HmHel-6	CAEZ01002416:81854-86935
HmHel-6	CAEZ01002416:41958-42211
HmHel-6	CAEZ01002416:23661-25706
HmHel-6	CAEZ01001572:27195-27433
HmHel-6	CAEZ01001572:52429-52646
HmHel-6	CAEZ01001436:12429-12510
HmHel-6	CAEZ01001436:32991-37363
HmHel-6	CAEZ01001266:2534-2635
HmHel-6	CAEZ01001266:20518-22742
HmHel-6	CAEZ01001266:77519-77624
HmHel-6	CAEZ01001266:99775-109794
HmHel-6	CAEZ01001266:72504-72607
HmHel-6	CAEZ01001266:30074-30235
HmHel-6	CAEZ01000833:35121-35319
HmHel-6	CAEZ01000833:66468-66652
HmHel-6	CAEZ01011190:2435-2568
HmHel-6	CAEZ01011190:20491-24891
HmHel-6	CAEZ01011190:45696-45863
HmHel-6	CAEZ01011190:19111-19204
HmHel-6	CAEZ01010807:13466-13555
HmHel-6	CAEZ01010807:49956-50051
HmHel-6	CAEZ01010807:143742-143825
HmHel-6	CAEZ01010807:180672-180875
HmHel-6	CAEZ01010807:202327-202498
HmHel-6	CAEZ01010807:150892-151094
HmHel-6	CAEZ01010807:95367-103643
HmHel-6	CAEZ01010807:42005-42091
HmHel-6	CAEZ01010807:27164-27346

HmHel-6	CAEZ01010800:4611-4824
HmHel-6	CAEZ01010800:35695-35796
HmHel-6	CAEZ01010800:59147-59278
HmHel-6	CAEZ01010800:22746-22956
HmHel-6	CAEZ01010297:7784-8034
HmHel-6	CAEZ01010296:2573-2824
HmHel-6	CAEZ01010296:20558-20775
HmHel-6	CAEZ01010296:15396-15512
HmHel-6	CAEZ01010229:3707-3812
HmHel-6	CAEZ01010229:39137-39339
HmHel-6	CAEZ01010229:21122-21220
HmHel-6	CAEZ01010117:31616-31854
HmHel-6	CAEZ01010117:61736-63276
HmHel-6	CAEZ01010117:56510-56680
HmHel-6	CAEZ01010117:39524-39708
HmHel-6	CAEZ01010117:3971-4224
HmHel-6	CAEZ01009228:6260-6384
HmHel-6	CAEZ01009228:37552-37663
HmHel-6	CAEZ01009228:83848-84061
HmHel-6	CAEZ01009228:94236-94897
HmHel-6	CAEZ01009228:95955-96147
HmHel-6	CAEZ01009228:72170-72262
HmHel-6	CAEZ01009228:59795-60029
HmHel-6	CAEZ01009228:32686-32946
HmHel-6	CAEZ01008916:6430-7145
HmHel-6	CAEZ01008916:61006-61179
HmHel-6	CAEZ01008916:37444-37579
HmHel-6	CAEZ01008916:21852-22099
HmHel-6	CAEZ01008635:11920-12005
HmHel-6	CAEZ01008635:25842-25922
HmHel-6	CAEZ01008635:8289-8534
HmHel-6	CAEZ01008413:12145-12235
HmHel-6	CAEZ01008413:51896-52142
HmHel-6	CAEZ01008413:65702-65822
HmHel-6	CAEZ01008413:49409-49517
HmHel-6	CAEZ01008194:15718-23064
HmHel-6	CAEZ01008194:18670-18859
HmHel-6	CAEZ01007939:13814-15443
HmHel-6	CAEZ01007939:37822-37961
HmHel-6	CAEZ01007939:70381-70585
HmHel-6	CAEZ01007939:121039-121203
HmHel-6	CAEZ01007939:153963-158112
HmHel-6	CAEZ01007939:184319-191777
HmHel-6	CAEZ01007939:215944-216197
HmHel-6	CAEZ01007939:235321-244733
HmHel-6	CAEZ01007939:275704-275803
HmHel-6	CAEZ01007939:305909-306132
HmHel-6	CAEZ01007939:279927-280129

HmHel-6	CAEZ01007939:260534-260661
HmHel-6	CAEZ01007939:215027-215194
HmHel-6	CAEZ01007939:195137-195247
HmHel-6	CAEZ01007939:142758-142995
HmHel-6	CAEZ01007939:77837-78001
HmHel-6	CAEZ01007939:64218-64322
HmHel-6	CAEZ01007939:24804-24891
HmHel-6	CAEZ01007736:2375-4330
HmHel-6	CAEZ01007736:36920-40768
HmHel-6	CAEZ01007736:43326-43445
HmHel-6	CAEZ01006972:8901-9135
HmHel-6	CAEZ01006972:13214-13448
HmHel-6	CAEZ01006821:2988-3205
HmHel-6	CAEZ01006821:22748-31233
HmHel-6	CAEZ01006821:40221-40301
HmHel-6	CAEZ01005573:627-845
HmHel-6	CAEZ01005573:4856-4967
HmHel-6	CAEZ01005338:2239-2488
HmHel-6	CAEZ01005338:1060-1160
HmHel-6	CAEZ01005193:2357-2571
HmHel-6	CAEZ01005193:5534-5620
HmHel-6	CAEZ01004912:22417-22511
HmHel-6	CAEZ01004912:2652-2796
HmHel-6	CAEZ01004155:4171-4404
HmHel-6	CAEZ01004155:5735-5965
HmHel-6	CAEZ01003714:5790-6040
HmHel-6	CAEZ01003714:14282-14362
HmHel-6	CAEZ01003714:2851-2961
HmHel-6	CAEZ01002800:3946-4196
HmHel-6	CAEZ01002188:2270-2509
HmHel-6	CAEZ01002188:5022-6787
HmHel-6	CAEZ01002134:8964-9062
HmHel-6	CAEZ01002134:26940-27142
HmHel-6	CAEZ01002134:4966-5105
HmHel-6	CAEZ01002057:9865-9986
HmHel-6	CAEZ01002057:590-844
HmHel-6	CAEZ01001205:4417-4662
HmHel-6	CAEZ01001205:10500-10658
HmHel-6	CAEZ01011634:8289-8410
HmHel-6	CAEZ01011634:15971-16204
HmHel-6	CAEZ01011421:508-594
HmHel-6	CAEZ01011251:5542-5793
HmHel-6	CAEZ01010966:636-715
HmHel-6	CAEZ01010966:2757-3012
HmHel-6	CAEZ01010952:44718-44809
HmHel-6	CAEZ01010952:57273-57410
HmHel-6	CAEZ01010952:85298-85512
HmHel-6	CAEZ01010952:96998-97083

HmHel-6	CAEZ01010952:35791-35950
HmHel-6	CAEZ01010799:22517-22665
HmHel-6	CAEZ01010799:37879-45314
HmHel-6	CAEZ01010799:42087-48834
HmHel-6	CAEZ01010528:18164-18288
HmHel-6	CAEZ01010528:11426-11615
HmHel-6	CAEZ01010286:2049-2151
HmHel-6	CAEZ01010286:13109-13299
HmHel-6	CAEZ01010286:10274-10526
HmHel-6	CAEZ01009999:12743-16714
HmHel-6	CAEZ01009999:43497-59237
HmHel-6	CAEZ01009999:30866-31082
HmHel-6	CAEZ01009778:27789-27949
HmHel-6	CAEZ01009778:43876-47263
HmHel-6	CAEZ01009778:107435-107592
HmHel-6	CAEZ01009778:131014-131262
HmHel-6	CAEZ01009778:185702-194452
HmHel-6	CAEZ01009778:221246-221376
HmHel-6	CAEZ01009778:334986-335164
HmHel-6	CAEZ01009778:345383-345505
HmHel-6	CAEZ01009778:365303-365399
HmHel-6	CAEZ01009778:403343-403470
HmHel-6	CAEZ01009778:303709-303847
HmHel-6	CAEZ01009778:288782-288935
HmHel-6	CAEZ01009778:236183-236268
HmHel-6	CAEZ01009778:225045-225224
HmHel-6	CAEZ01009778:211334-211551
HmHel-6	CAEZ01009778:160731-160812
HmHel-6	CAEZ01009778:141771-141862
HmHel-6	CAEZ01009778:121637-121749
HmHel-6	CAEZ01009778:105109-105194
HmHel-6	CAEZ01009778:84893-85010
HmHel-6	CAEZ01009778:9279-9469
HmHel-6	CAEZ01008943:15643-15889
HmHel-6	CAEZ01008943:33022-35649
HmHel-6	CAEZ01008469:20171-20383
HmHel-6	CAEZ01008469:24116-24308
HmHel-6	CAEZ01008371:20799-21046
HmHel-6	CAEZ01008371:60436-60616
HmHel-6	CAEZ01008371:55177-60082
HmHel-6	CAEZ01007872:4548-4759
HmHel-6	CAEZ01007872:78757-78893
HmHel-6	CAEZ01007872:77917-78002
HmHel-6	CAEZ01007872:31768-32009
HmHel-6	CAEZ01007872:18125-19437
HmHel-6	CAEZ01007823:7872-8097
HmHel-6	CAEZ01007823:3579-3761
HmHel-6	CAEZ01007758:10451-10605

HmHel-6	CAEZ01007758:30419-30672
HmHel-6	CAEZ01007562:8141-8381
HmHel-6	CAEZ01007562:7541-7651
HmHel-6	CAEZ01007374:18941-22703
HmHel-6	CAEZ01007374:34110-34338
HmHel-6	CAEZ01007374:35851-35973
HmHel-6	CAEZ01007374:21247-21370
HmHel-6	CAEZ01007374:801-1038
HmHel-6	CAEZ01007270:3532-3777
HmHel-6	CAEZ01007202:1515-1630
HmHel-6	CAEZ01007202:6376-10362
HmHel-6	CAEZ01007140:11232-11347
HmHel-6	CAEZ01007140:26794-26878
HmHel-6	CAEZ01007140:55619-60803
HmHel-6	CAEZ01007140:103569-103696
HmHel-6	CAEZ01007140:95589-95727
HmHel-6	CAEZ01007140:22967-24089
HmHel-6	CAEZ01007140:6000-6245
HmHel-6	CAEZ01007129:1856-9020
HmHel-6	CAEZ01007129:2244-2492
HmHel-6	CAEZ01006578:381-594
HmHel-6	CAEZ01006230:20003-20152
HmHel-6	CAEZ01006230:10602-13205
HmHel-6	CAEZ01006161:25570-25754
HmHel-6	CAEZ01006161:64752-64914
HmHel-6	CAEZ01006161:81149-81230
HmHel-6	CAEZ01006161:101823-103007
HmHel-6	CAEZ01006161:115463-115670
HmHel-6	CAEZ01006161:69114-69304
HmHel-6	CAEZ01006161:30276-34006
HmHel-6	CAEZ01006161:1881-2003
HmHel-6	CAEZ01005419:3972-4222
HmHel-6	CAEZ01005419:32276-39728
HmHel-6	CAEZ01005419:32169-32274
HmHel-6	CAEZ01005108:95973-96115
HmHel-6	CAEZ01005108:117516-119636
HmHel-6	CAEZ01005108:111682-111795
HmHel-6	CAEZ01005108:89987-97007
HmHel-6	CAEZ01005108:73547-73651
HmHel-6	CAEZ01005108:41071-41279
HmHel-6	CAEZ01004903:1304-1462
HmHel-6	CAEZ01004876:18668-21146
HmHel-6	CAEZ01004876:28040-28281
HmHel-6	CAEZ01004821:32393-33104
HmHel-6	CAEZ01004821:74532-74646
HmHel-6	CAEZ01004821:60284-60506
HmHel-6	CAEZ01004821:38479-38579
HmHel-6	CAEZ01004821:23100-23301

HmHel-6	CAEZ01004821:3743-3956
HmHel-6	CAEZ01004137:5823-6071
HmHel-6	CAEZ01004137:34594-34744
HmHel-6	CAEZ01004081:15708-15940
HmHel-6	CAEZ01004081:33695-33848
HmHel-6	CAEZ01004081:39373-39590
HmHel-6	CAEZ01004081:21047-21293
HmHel-6	CAEZ01003973:8638-12497
HmHel-6	CAEZ01003973:3207-3444
HmHel-6	CAEZ01003291:4685-4843
HmHel-6	CAEZ01003291:30330-30451
HmHel-6	CAEZ01003291:127744-127824
HmHel-6	CAEZ01003291:111212-111294
HmHel-6	CAEZ01003069:48099-48312
HmHel-6	CAEZ01003069:83308-83446
HmHel-6	CAEZ01003069:106508-106590
HmHel-6	CAEZ01003069:148017-152961
HmHel-6	CAEZ01003069:204787-204943
HmHel-6	CAEZ01003069:261979-270571
HmHel-6	CAEZ01003069:329143-335628
HmHel-6	CAEZ01003069:234646-234764
HmHel-6	CAEZ01003069:169404-169551
HmHel-6	CAEZ01003069:143892-143975
HmHel-6	CAEZ01003069:97969-98148
HmHel-6	CAEZ01003069:49406-54873
HmHel-6	CAEZ01003069:32457-32542
HmHel-6	CAEZ01002913:205-418
HmHel-6	CAEZ01001595:9234-15523
HmHel-6	CAEZ01001595:51897-52048
HmHel-6	CAEZ01001595:78704-78893
HmHel-6	CAEZ01001595:131632-131882
HmHel-6	CAEZ01001595:46168-46370
HmHel-6	CAEZ01001595:9935-13727
HmHel-6	CAEZ01001447:29460-38918
HmHel-6	CAEZ01001447:43630-43719
HmHel-6	CAEZ01001447:16293-16534
HmHel-6	CAEZ01000921:10091-18464
HmHel-6	CAEZ01000921:31422-31666
HmHel-6	CAEZ01011542:4594-4829
HmHel-6	CAEZ01010405:1291-1532
HmHel-6	CAEZ01009968:4667-4883
HmHel-6	CAEZ01009887:52640-52816
HmHel-6	CAEZ01009887:139114-139274
HmHel-6	CAEZ01009887:153381-162032
HmHel-6	CAEZ01009887:206094-207737
HmHel-6	CAEZ01009887:245466-245680
HmHel-6	CAEZ01009887:207990-208208
HmHel-6	CAEZ01009887:142868-152690

HmHel-6	CAEZ01009887:114811-114909
HmHel-6	CAEZ01009887:83673-83769
HmHel-6	CAEZ01009887:62393-62499
HmHel-6	CAEZ01009887:49105-49223
HmHel-6	CAEZ01009887:15697-15894
HmHel-6	CAEZ01008771:3450-3702
HmHel-6	CAEZ01008771:14898-14982
HmHel-6	CAEZ01008771:33395-33632
HmHel-6	CAEZ01008771:94200-94303
HmHel-6	CAEZ01008771:112539-122396
HmHel-6	CAEZ01008771:152064-152251
HmHel-6	CAEZ01008771:165140-165333
HmHel-6	CAEZ01008771:164117-164247
HmHel-6	CAEZ01008771:125674-125822
HmHel-6	CAEZ01008771:94892-95088
HmHel-6	CAEZ01008771:5015-5256
HmHel-6	CAEZ01008590:16896-17001
HmHel-6	CAEZ01008590:32121-32254
HmHel-6	CAEZ01008590:56321-56453
HmHel-6	CAEZ01008590:98879-102147
HmHel-6	CAEZ01008590:133858-134108
HmHel-6	CAEZ01008590:115667-115758
HmHel-6	CAEZ01008590:80540-80714
HmHel-6	CAEZ01008590:69614-69720
HmHel-6	CAEZ01008590:50960-52590
HmHel-6	CAEZ01008590:39087-39270
HmHel-6	CAEZ01008590:16328-17763
HmHel-6	CAEZ01008266:306-5355
HmHel-6	CAEZ01008266:26151-26375
HmHel-6	CAEZ01008266:28075-28162
HmHel-6	CAEZ01007418:635-750
HmHel-6	CAEZ01007418:2211-2450
HmHel-6	CAEZ01007326:23990-24117
HmHel-6	CAEZ01007326:49756-49984
HmHel-6	CAEZ01007326:18585-22192
HmHel-6	CAEZ01007184:33770-39636
HmHel-6	CAEZ01007184:65586-65809
HmHel-6	CAEZ01007184:90863-91021
HmHel-6	CAEZ01007184:111165-111329
HmHel-6	CAEZ01007184:135523-135664
HmHel-6	CAEZ01007184:157081-159681
HmHel-6	CAEZ01007184:218859-219077
HmHel-6	CAEZ01007184:273759-273866
HmHel-6	CAEZ01007184:293271-293464
HmHel-6	CAEZ01007184:318330-325941
HmHel-6	CAEZ01007184:325129-325382
HmHel-6	CAEZ01007184:275680-275806
HmHel-6	CAEZ01007184:235805-236204

HmHel-6	CAEZ01007184:178995-183466
HmHel-6	CAEZ01007184:154799-155051
HmHel-6	CAEZ01007184:85087-85237
HmHel-6	CAEZ01007184:8574-8725
HmHel-6	CAEZ01005114:9273-15834
HmHel-6	CAEZ01005114:11192-11297
HmHel-6	CAEZ01005013:723-832
HmHel-6	CAEZ01005013:30066-30279
HmHel-6	CAEZ01005013:38884-40552
HmHel-6	CAEZ01005013:26035-26173
HmHel-6	CAEZ01004545:21357-21606
HmHel-6	CAEZ01004545:31822-32030
HmHel-6	CAEZ01004545:14937-15069
HmHel-6	CAEZ01004368:32885-33043
HmHel-6	CAEZ01004368:57790-58041
HmHel-6	CAEZ01004368:53666-53819
HmHel-6	CAEZ01004368:3296-3486
HmHel-6	CAEZ01002685:1401-1646
HmHel-6	CAEZ01002685:4714-4829
HmHel-6	CAEZ01002578:8244-8330
HmHel-6	CAEZ01002578:5509-5703
HmHel-6	CAEZ01001004:13375-13610
HmHel-6	CAEZ01001004:32304-32456
HmHel-6	CAEZ01001004:94610-98716
HmHel-6	CAEZ01001004:121970-122095
HmHel-6	CAEZ01001004:141730-141853
HmHel-6	CAEZ01001004:135062-135258
HmHel-6	CAEZ01001004:116716-116801
HmHel-6	CAEZ01001004:73423-78025
HmHel-6	CAEZ01001004:55849-56088
HmHel-6	CAEZ01001004:44334-44419
HmHel-6	CAEZ01001004:2947-3197
HmHel-6	CAEZ01010189:6927-7187
HmHel-6	CAEZ01010189:30100-30230
HmHel-6	CAEZ01009509:23156-23281
HmHel-6	CAEZ01009509:163146-163390
HmHel-6	CAEZ01009509:159390-159505
HmHel-6	CAEZ01009509:107552-107764
HmHel-6	CAEZ01009509:65836-66064
HmHel-6	CAEZ01009509:24446-30179
HmHel-6	CAEZ01009476:2551-2634
HmHel-6	CAEZ01009476:18759-18970
HmHel-6	CAEZ01009476:49981-50084
HmHel-6	CAEZ01009476:54516-59900
HmHel-6	CAEZ01009476:17894-18019
HmHel-6	CAEZ01009373:54718-58437
HmHel-6	CAEZ01009373:79932-80188
HmHel-6	CAEZ01008986:4635-4794

HmHel-6	CAEZ01008986:10023-10270
HmHel-6	CAEZ01008261:18249-24345
HmHel-6	CAEZ01008261:71369-71587
HmHel-6	CAEZ01008261:89517-89770
HmHel-6	CAEZ01008261:125919-132850
HmHel-6	CAEZ01008261:191403-191501
HmHel-6	CAEZ01008261:194626-194860
HmHel-6	CAEZ01008261:144377-144557
HmHel-6	CAEZ01008261:127690-127939
HmHel-6	CAEZ01008261:91749-91935
HmHel-6	CAEZ01008261:25654-25902
HmHel-6	CAEZ01008261:2239-2342
HmHel-6	CAEZ01007895:3808-3889
HmHel-6	CAEZ01007895:7759-8005
HmHel-6	CAEZ01007595:10840-11059
HmHel-6	CAEZ01007595:21404-21569
HmHel-6	CAEZ01007204:2746-4059
HmHel-6	CAEZ01007204:17751-17848
HmHel-6	CAEZ01007204:36378-36490
HmHel-6	CAEZ01007204:50893-50987
HmHel-6	CAEZ01007204:70108-70339
HmHel-6	CAEZ01007204:55033-55136
HmHel-6	CAEZ01007204:35063-35194
HmHel-6	CAEZ01007204:13076-19336
HmHel-6	CAEZ01007204:542-632
HmHel-6	CAEZ01006865:11665-13681
HmHel-6	CAEZ01006865:37892-38159
HmHel-6	CAEZ01006865:55452-55576
HmHel-6	CAEZ01006865:52188-52391
HmHel-6	CAEZ01006865:40918-41144
HmHel-6	CAEZ01006865:17328-23667
HmHel-6	CAEZ01006569:6402-6624
HmHel-6	CAEZ01006569:17371-17489
HmHel-6	CAEZ01006548:9178-9297
HmHel-6	CAEZ01006548:78323-78515
HmHel-6	CAEZ01006548:98402-98602
HmHel-6	CAEZ01006548:50035-50229
HmHel-6	CAEZ01006548:14269-14446
HmHel-6	CAEZ01006441:3037-3134
HmHel-6	CAEZ01006121:10356-10571
HmHel-6	CAEZ01006121:51943-52101
HmHel-6	CAEZ01006121:66580-66760
HmHel-6	CAEZ01006121:84034-85610
HmHel-6	CAEZ01006121:104418-104505
HmHel-6	CAEZ01006121:116249-116355
HmHel-6	CAEZ01006121:131595-131845
HmHel-6	CAEZ01006121:180400-180668
HmHel-6	CAEZ01006121:207128-212976

HmHel-6	CAEZ01006121:243323-243539
HmHel-6	CAEZ01006121:241235-242755
HmHel-6	CAEZ01006121:182069-182229
HmHel-6	CAEZ01006121:151489-159445
HmHel-6	CAEZ01006121:88476-88711
HmHel-6	CAEZ01005830:35852-41577
HmHel-6	CAEZ01005830:81336-81551
HmHel-6	CAEZ01005830:62084-62183
HmHel-6	CAEZ01005830:32882-33082
HmHel-6	CAEZ01005830:4086-4234
HmHel-6	CAEZ01005803:8574-8822
HmHel-6	CAEZ01005331:2004-9976
HmHel-6	CAEZ01005331:34691-34926
HmHel-6	CAEZ01005331:39122-39253
HmHel-6	CAEZ01005313:1578-1820
HmHel-6	CAEZ01005313:6033-6262
HmHel-6	CAEZ01004381:8146-8295
HmHel-6	CAEZ01004381:25948-26159
HmHel-6	CAEZ01003317:3940-4082
HmHel-6	CAEZ01003216:9136-9383
HmHel-6	CAEZ01003080:12157-12384
HmHel-6	CAEZ01003080:45146-45287
HmHel-6	CAEZ01003080:114514-114647
HmHel-6	CAEZ01003080:148507-148667
HmHel-6	CAEZ01003080:182884-182968
HmHel-6	CAEZ01003080:201091-201298
HmHel-6	CAEZ01003080:184851-185004
HmHel-6	CAEZ01003080:150477-156790
HmHel-6	CAEZ01003080:112202-112374
HmHel-6	CAEZ01003080:77143-80979
HmHel-6	CAEZ01003080:51956-52189
HmHel-6	CAEZ01003080:15436-15551
HmHel-6	CAEZ01002684:17894-18145
HmHel-6	CAEZ01002684:26702-26789
HmHel-6	CAEZ01010918:10942-11645
HmHel-6	CAEZ01010918:52454-52542
HmHel-6	CAEZ01010918:110537-110661
HmHel-6	CAEZ01010918:12903-13078
HmHel-6	CAEZ01010555:2343-2593
HmHel-6	CAEZ01010541:20134-25146
HmHel-6	CAEZ01010541:36057-36181
HmHel-6	CAEZ01010541:55417-55557
HmHel-6	CAEZ01010541:86247-86394
HmHel-6	CAEZ01010541:111867-116314
HmHel-6	CAEZ01010541:146486-146605
HmHel-6	CAEZ01010541:202379-202493
HmHel-6	CAEZ01010541:220412-220516
HmHel-6	CAEZ01010541:252095-252179

HmHel-6	CAEZ01010541:286814-286944
HmHel-6	CAEZ01010541:309908-316800
HmHel-6	CAEZ01010541:348031-348170
HmHel-6	CAEZ01010541:237733-237892
HmHel-6	CAEZ01010541:204257-204362
HmHel-6	CAEZ01010541:164755-171226
HmHel-6	CAEZ01010541:59340-60206
HmHel-6	CAEZ01008721:18979-19214
HmHel-6	CAEZ01008721:54402-54620
HmHel-6	CAEZ01008721:92415-92562
HmHel-6	CAEZ01008721:138662-143860
HmHel-6	CAEZ01008721:165608-165821
HmHel-6	CAEZ01008721:211344-211455
HmHel-6	CAEZ01008721:169464-175310
HmHel-6	CAEZ01008721:140399-140644
HmHel-6	CAEZ01008721:98205-98419
HmHel-6	CAEZ01008721:51123-51284
HmHel-6	CAEZ01008721:32379-32593
HmHel-6	CAEZ01008721:10708-10833
HmHel-6	CAEZ01007881:3234-3484
HmHel-6	CAEZ01007881:5336-12318
HmHel-6	CAEZ01007861:1723-1917
HmHel-6	CAEZ01007861:32668-37989
HmHel-6	CAEZ01007861:48455-48535
HmHel-6	CAEZ01007861:88221-88311
HmHel-6	CAEZ01007861:114110-116633
HmHel-6	CAEZ01007861:142045-142170
HmHel-6	CAEZ01007861:173110-173355
HmHel-6	CAEZ01007861:112407-112620
HmHel-6	CAEZ01007861:71828-71955
HmHel-6	CAEZ01007861:60532-60666
HmHel-6	CAEZ01007861:21387-22836
HmHel-6	CAEZ01007861:896-5857
HmHel-6	CAEZ01007633:18359-18596
HmHel-6	CAEZ01007633:1179-1423
HmHel-6	CAEZ01007297:17878-21588
HmHel-6	CAEZ01007297:9310-9516
HmHel-6	CAEZ01006242:2717-6363
HmHel-6	CAEZ01006242:19906-20124
HmHel-6	CAEZ01006242:32516-32672
HmHel-6	CAEZ01006242:48720-48841
HmHel-6	CAEZ01006242:10026-10230
HmHel-6	CAEZ01006084:1352-1443
HmHel-6	CAEZ01006084:32704-32928
HmHel-6	CAEZ01006084:5-129
HmHel-6	CAEZ01005936:15579-15668
HmHel-6	CAEZ01005371:18467-18648
HmHel-6	CAEZ01005371:30349-30429

HmHel-6	CAEZ01005371:7266-7454
HmHel-6	CAEZ01004975:28476-28601
HmHel-6	CAEZ01004975:25791-25905
HmHel-6	CAEZ01004975:5033-5165
HmHel-6	CAEZ01004920:30815-30900
HmHel-6	CAEZ01004920:28532-28680
HmHel-6	CAEZ01003567:317-571
HmHel-6	CAEZ01003178:16371-16502
HmHel-6	CAEZ01003178:38800-38972
HmHel-6	CAEZ01003178:27849-28098
HmHel-6	CAEZ01003178:3283-3405
HmHel-6	CAEZ01003019:60207-60447
HmHel-6	CAEZ01003019:105704-105783
HmHel-6	CAEZ01003019:122256-122455
HmHel-6	CAEZ01003019:101095-101216
HmHel-6	CAEZ01003019:67586-67669
HmHel-6	CAEZ01003019:46764-46968
HmHel-6	CAEZ01003019:14924-15126
HmHel-6	CAEZ01002951:3076-3256
HmHel-6	CAEZ01002951:15143-15252
HmHel-6	CAEZ01002951:41021-50720
HmHel-6	CAEZ01002951:43633-49715
HmHel-6	CAEZ01002885:2317-2559
HmHel-6	CAEZ01002885:4653-4779
HmHel-6	CAEZ01002838:24166-33490
HmHel-6	CAEZ01002838:46563-46647
HmHel-6	CAEZ01002838:65207-73210
HmHel-6	CAEZ01000824:79047-79257
HmHel-6	CAEZ01000824:182838-183071
HmHel-6	CAEZ01000824:153703-153925
HmHel-6	CAEZ01000824:119781-119889
HmHel-6	CAEZ01000824:92812-95594
HmHel-6	CAEZ01000824:70363-70527
HmHel-6	CAEZ01000824:30572-30784
HmHel-6	CAEZ01000824:16749-16931
HmHel-6	CAEZ01011566:4674-4755
HmHel-6	CAEZ01011566:2292-2501
HmHel-6	CAEZ01010922:9718-17520
HmHel-6	CAEZ01010922:84273-84526
HmHel-6	CAEZ01010922:50875-51021
HmHel-6	CAEZ01010916:8564-8816
HmHel-6	CAEZ01010495:348-500
HmHel-6	CAEZ01010495:6312-8225
HmHel-6	CAEZ01010238:19480-19730
HmHel-6	CAEZ01010238:30873-30976
HmHel-6	CAEZ01010238:54591-61423
HmHel-6	CAEZ01010238:78337-78586
HmHel-6	CAEZ01010238:126208-126340

HmHel-6	CAEZ01010238:154722-154804
HmHel-6	CAEZ01010238:23736-31955
HmHel-6	CAEZ01010238:10014-10179
HmHel-6	CAEZ01010036:54551-54685
HmHel-6	CAEZ01010036:18056-18228
HmHel-6	CAEZ01009883:3456-3661
HmHel-6	CAEZ01009883:38210-38322
HmHel-6	CAEZ01009883:58567-74705
HmHel-6	CAEZ01009883:104131-104379
HmHel-6	CAEZ01009883:151638-151738
HmHel-6	CAEZ01009883:153242-153436
HmHel-6	CAEZ01009883:141892-141979
HmHel-6	CAEZ01009883:112248-112341
HmHel-6	CAEZ01009883:54373-54609
HmHel-6	CAEZ01009883:30235-30431
HmHel-6	CAEZ01009883:14150-14228
HmHel-6	CAEZ01009832:17902-18059
HmHel-6	CAEZ01009832:32944-33186
HmHel-6	CAEZ01009832:31225-36547
HmHel-6	CAEZ01009481:33353-33592
HmHel-6	CAEZ01009481:19392-19513
HmHel-6	CAEZ01009339:3182-3435
HmHel-6	CAEZ01008574:908-1068
HmHel-6	CAEZ01008574:50634-50716
HmHel-6	CAEZ01008574:151178-151283
HmHel-6	CAEZ01008574:139071-139317
HmHel-6	CAEZ01008574:91548-91766
HmHel-6	CAEZ01008574:57992-58080
HmHel-6	CAEZ01008574:32723-32959
HmHel-6	CAEZ01008467:32119-32240
HmHel-6	CAEZ01008467:9473-9591
HmHel-6	CAEZ01008435:6289-6510
HmHel-6	CAEZ01008435:44075-54174
HmHel-6	CAEZ01008435:101960-102197
HmHel-6	CAEZ01008435:156465-162522
HmHel-6	CAEZ01008435:185094-187617
HmHel-6	CAEZ01008435:215425-215551
HmHel-6	CAEZ01008435:230295-230417
HmHel-6	CAEZ01008435:241465-242217
HmHel-6	CAEZ01008435:254807-254953
HmHel-6	CAEZ01008435:237509-242851
HmHel-6	CAEZ01008435:222537-223385
HmHel-6	CAEZ01008435:164559-164649
HmHel-6	CAEZ01008435:154367-154521
HmHel-6	CAEZ01008435:134172-134415
HmHel-6	CAEZ01008435:87808-87949
HmHel-6	CAEZ01008435:10321-16144
HmHel-6	CAEZ01007987:22294-22409

HmHel-6	CAEZ01007987:1109-1317
HmHel-6	CAEZ01007741:3954-11870
HmHel-6	CAEZ01007741:25057-25702
HmHel-6	CAEZ01007741:58974-59211
HmHel-6	CAEZ01007741:97097-102760
HmHel-6	CAEZ01007741:61560-61660
HmHel-6	CAEZ01007741:30183-30281
HmHel-6	CAEZ01006866:8694-8908
HmHel-6	CAEZ01006866:31465-31662
HmHel-6	CAEZ01006233:5238-5319
HmHel-6	CAEZ01005860:7883-8110
HmHel-6	CAEZ01005860:15842-15992
HmHel-6	CAEZ01005793:15809-15911
HmHel-6	CAEZ01005793:31823-31940
HmHel-6	CAEZ01005793:53726-53877
HmHel-6	CAEZ01005793:29131-29384
HmHel-6	CAEZ01005793:7049-7147
HmHel-6	CAEZ01005763:6954-7165
HmHel-6	CAEZ01005763:5384-5553
HmHel-6	CAEZ01005274:468-682
HmHel-6	CAEZ01005274:35377-35500
HmHel-6	CAEZ01005274:52531-55049
HmHel-6	CAEZ01005274:65773-66008
HmHel-6	CAEZ01005274:97636-97882
HmHel-6	CAEZ01005274:109468-109699
HmHel-6	CAEZ01005274:151041-156373
HmHel-6	CAEZ01005274:166665-166850
HmHel-6	CAEZ01005274:153458-153673
HmHel-6	CAEZ01005274:89730-89837
HmHel-6	CAEZ01005274:58955-59078
HmHel-6	CAEZ01005274:38143-38258
HmHel-6	CAEZ01005274:14239-14371
HmHel-6	CAEZ01005231:32083-32904
HmHel-6	CAEZ01005231:54617-54697
HmHel-6	CAEZ01005231:66571-67538
HmHel-6	CAEZ01005231:92160-92413
HmHel-6	CAEZ01005231:40849-40940
HmHel-6	CAEZ01005231:2456-7209
HmHel-6	CAEZ01004249:6044-6155
HmHel-6	CAEZ01003737:3489-3710
HmHel-6	CAEZ01003452:9779-9998
HmHel-6	CAEZ01003452:15506-15755
HmHel-6	CAEZ01001873:3308-3529
HmHel-6	CAEZ01000909:31749-31868
HmHel-6	CAEZ01000909:65207-65369
HmHel-6	CAEZ01000909:90810-90948
HmHel-6	CAEZ01000909:110796-111043
HmHel-6	CAEZ01000909:142423-145474

HmHel-6	CAEZ01000909:158814-158902
HmHel-6	CAEZ01000909:181943-182147
HmHel-6	CAEZ01000909:192241-192408
HmHel-6	CAEZ01000909:173067-173186
HmHel-6	CAEZ01000909:154089-154267
HmHel-6	CAEZ01000909:106317-106437
HmHel-6	CAEZ01011486:2874-3119
HmHel-6	CAEZ01011486:24000-24228
HmHel-6	CAEZ01011486:57707-57819
HmHel-6	CAEZ01011486:45412-45498
HmHel-6	CAEZ01011486:29285-29466
HmHel-6	CAEZ01011486:4564-4723
HmHel-6	CAEZ01011056:1861-2081
HmHel-6	CAEZ01010820:8705-8820
HmHel-6	CAEZ01010820:97288-97403
HmHel-6	CAEZ01010820:131452-131697
HmHel-6	CAEZ01010820:144745-144875
HmHel-6	CAEZ01010820:141356-141593
HmHel-6	CAEZ01010820:107789-107903
HmHel-6	CAEZ01010820:76102-76259
HmHel-6	CAEZ01010820:29542-29625
HmHel-6	CAEZ01010633:5690-5938
HmHel-6	CAEZ01009889:34069-34285
HmHel-6	CAEZ01009889:54189-54321
HmHel-6	CAEZ01009889:75509-75761
HmHel-6	CAEZ01009889:82745-82899
HmHel-6	CAEZ01009889:21801-31829
HmHel-6	CAEZ01008430:3639-3740
HmHel-6	CAEZ01008430:14295-14512
HmHel-6	CAEZ01008430:34986-42328
HmHel-6	CAEZ01008430:37441-37638
HmHel-6	CAEZ01008410:2017-2132
HmHel-6	CAEZ01008410:18244-18486
HmHel-6	CAEZ01008410:53846-53966
HmHel-6	CAEZ01008410:67154-67242
HmHel-6	CAEZ01008410:77652-77900
HmHel-6	CAEZ01008410:92657-92854
HmHel-6	CAEZ01008410:64817-64905
HmHel-6	CAEZ01008410:34894-41003
HmHel-6	CAEZ01008270:3442-3585
HmHel-6	CAEZ01008270:36102-36286
HmHel-6	CAEZ01008270:85958-86190
HmHel-6	CAEZ01008270:101960-104901
HmHel-6	CAEZ01008270:89094-89253
HmHel-6	CAEZ01008270:57766-57951
HmHel-6	CAEZ01008270:13801-14047
HmHel-6	CAEZ01007714:7686-7898
HmHel-6	CAEZ01007588:3113-7208

HmHel-6	CAEZ01007588:19663-25545
HmHel-6	CAEZ01007588:24664-24900
HmHel-6	CAEZ01007281:6873-12855
HmHel-6	CAEZ01007281:34172-34360
HmHel-6	CAEZ01007281:23342-23570
HmHel-6	CAEZ01007077:3077-3309
HmHel-6	CAEZ01007077:10034-10156
HmHel-6	CAEZ01006672:6591-6794
HmHel-6	CAEZ01006672:17169-17417
HmHel-6	CAEZ01006672:25668-25878
HmHel-6	CAEZ01006672:2606-2692
HmHel-6	CAEZ01006329:741-961
HmHel-6	CAEZ01006213:1616-1856
HmHel-6	CAEZ01006213:11391-11493
HmHel-6	CAEZ01006164:1422-10359
HmHel-6	CAEZ01006164:39579-39714
HmHel-6	CAEZ01006164:65389-65594
HmHel-6	CAEZ01006164:59772-59904
HmHel-6	CAEZ01006164:35982-36092
HmHel-6	CAEZ01006164:7614-7746
HmHel-6	CAEZ01005767:2989-3233
HmHel-6	CAEZ01004795:5800-5945
HmHel-6	CAEZ01004795:24061-28950
HmHel-6	CAEZ01004795:7133-7341
HmHel-6	CAEZ01004727:15802-16054
HmHel-6	CAEZ01004549:18232-18396
HmHel-6	CAEZ01004549:12072-13589
HmHel-6	CAEZ01004230:24330-24474
HmHel-6	CAEZ01004230:15504-15678
HmHel-6	CAEZ01003110:34072-39775
HmHel-6	CAEZ01003110:74954-75079
HmHel-6	CAEZ01003110:76555-84889
HmHel-6	CAEZ01003110:50405-50623
HmHel-6	CAEZ01003110:39506-39735
HmHel-6	CAEZ01002630:19018-19230
HmHel-6	CAEZ01002630:26034-26153
HmHel-6	CAEZ01002630:3230-3941
HmHel-6	CAEZ01001616:5287-8751
HmHel-6	CAEZ01000035:4320-4551
HmHel-6	CAEZ01011648:5325-5544
HmHel-6	CAEZ01011513:2576-2736
HmHel-6	CAEZ01011513:13401-13552
HmHel-6	CAEZ01011513:22793-22995
HmHel-6	CAEZ01011315:14236-14462
HmHel-6	CAEZ01011021:4902-5029
HmHel-6	CAEZ01011021:1758-2009
HmHel-6	CAEZ01010365:3877-3996
HmHel-6	CAEZ01010365:46031-46129

HmHel-6	CAEZ01010365:57814-57904
HmHel-6	CAEZ01010365:31215-31368
HmHel-6	CAEZ01010318:6055-6262
HmHel-6	CAEZ01010318:38356-45631
HmHel-6	CAEZ01010318:87945-88196
HmHel-6	CAEZ01010318:71139-71342
HmHel-6	CAEZ01010318:22130-22283
HmHel-6	CAEZ01009872:10849-10981
HmHel-6	CAEZ01009872:26216-26388
HmHel-6	CAEZ01009872:53741-53958
HmHel-6	CAEZ01009872:114772-114995
HmHel-6	CAEZ01009872:100812-100931
HmHel-6	CAEZ01009872:75284-82249
HmHel-6	CAEZ01009872:64446-64606
HmHel-6	CAEZ01009872:21456-21567
HmHel-6	CAEZ01009224:11946-25484
HmHel-6	CAEZ01009224:36447-36678
HmHel-6	CAEZ01009224:47753-50009
HmHel-6	CAEZ01009224:14197-14323
HmHel-6	CAEZ01008545:993-1109
HmHel-6	CAEZ01008545:16436-16693
HmHel-6	CAEZ01008545:21897-22035
HmHel-6	CAEZ01007360:6948-7196
HmHel-6	CAEZ01007360:3162-10339
HmHel-6	CAEZ01006791:17373-28731
HmHel-6	CAEZ01006791:40340-40486
HmHel-6	CAEZ01006791:16288-16499
HmHel-6	CAEZ01006475:4192-4420
HmHel-6	CAEZ01006252:1757-1902
HmHel-6	CAEZ01006252:31595-31702
HmHel-6	CAEZ01006252:72591-72774
HmHel-6	CAEZ01006252:131266-131429
HmHel-6	CAEZ01006252:178746-178853
HmHel-6	CAEZ01006252:114597-114702
HmHel-6	CAEZ01006252:93308-96593
HmHel-6	CAEZ01006252:70902-71071
HmHel-6	CAEZ01006252:58922-59074
HmHel-6	CAEZ01006252:23920-24163
HmHel-6	CAEZ01006252:6176-6286
HmHel-6	CAEZ01006226:6349-6563
HmHel-6	CAEZ01006013:20458-20588
HmHel-6	CAEZ01006013:40172-40379
HmHel-6	CAEZ01006013:54076-56195
HmHel-6	CAEZ01006013:15698-15817
HmHel-6	CAEZ01005360:34723-34919
HmHel-6	CAEZ01005360:24293-24379
HmHel-6	CAEZ01005025:6008-12388
HmHel-6	CAEZ01005025:68867-68949

HmHel-6	CAEZ01005025:116690-125214
HmHel-6	CAEZ01005025:151173-154486
HmHel-6	CAEZ01005025:156913-157159
HmHel-6	CAEZ01005025:79754-79849
HmHel-6	CAEZ01005025:40915-41134
HmHel-6	CAEZ01003797:618-703
HmHel-6	CAEZ01003797:51398-51612
HmHel-6	CAEZ01003797:49676-49791
HmHel-6	CAEZ01003797:23059-28049
HmHel-6	CAEZ01003797:997-1173
HmHel-6	CAEZ01003633:10176-10419
HmHel-6	CAEZ01003633:22798-25919
HmHel-6	CAEZ01003633:51716-51808
HmHel-6	CAEZ01003633:12670-12914
HmHel-6	CAEZ01003091:6194-6428
HmHel-6	CAEZ01003091:10813-12819
HmHel-6	CAEZ01003089:14093-14334
HmHel-6	CAEZ01002812:24499-24747
HmHel-6	CAEZ01002812:18376-20819
HmHel-6	CAEZ01002074:15942-16192
HmHel-6	CAEZ01002065:3025-3276
HmHel-6	CAEZ01001019:2487-2732
HmHel-6	CAEZ01010915:2387-2649
HmHel-6	CAEZ01010915:33425-33577
HmHel-6	CAEZ01010915:56923-57006
HmHel-6	CAEZ01010915:32526-32743
HmHel-6	CAEZ01010803:478-676
HmHel-6	CAEZ01010803:22882-23012
HmHel-6	CAEZ01010803:63162-63404
HmHel-6	CAEZ01010803:84270-84514
HmHel-6	CAEZ01010803:114628-114769
HmHel-6	CAEZ01010803:122841-122993
HmHel-6	CAEZ01010803:69570-69805
HmHel-6	CAEZ01010803:42824-42985
HmHel-6	CAEZ01010803:13196-13407
HmHel-6	CAEZ01010624:205-7167
HmHel-6	CAEZ01010216:3198-3319
HmHel-6	CAEZ01010216:68686-68900
HmHel-6	CAEZ01010216:55695-55803
HmHel-6	CAEZ01009399:21826-22072
HmHel-6	CAEZ01009399:2410-11686
HmHel-6	CAEZ01009024:21850-21981
HmHel-6	CAEZ01009024:29273-29357
HmHel-6	CAEZ01009024:10739-10912
HmHel-6	CAEZ01008510:13490-15907
HmHel-6	CAEZ01008510:39653-39763
HmHel-6	CAEZ01008510:68589-68833
HmHel-6	CAEZ01008510:46792-51629

HmHel-6	CAEZ01008510:12729-12965
HmHel-6	CAEZ01008398:10363-10573
HmHel-6	CAEZ01008398:75441-75524
HmHel-6	CAEZ01008398:86996-88224
HmHel-6	CAEZ01008398:75783-87325
HmHel-6	CAEZ01008398:56724-56825
HmHel-6	CAEZ01008141:8034-8118
HmHel-6	CAEZ01008141:33934-34118
HmHel-6	CAEZ01008141:3408-3655
HmHel-6	CAEZ01008104:10398-10554
HmHel-6	CAEZ01008104:7308-17144
HmHel-6	CAEZ01005689:1203-1434
HmHel-6	CAEZ01005669:10314-10509
HmHel-6	CAEZ01005669:17061-26740
HmHel-6	CAEZ01005669:3035-3222
HmHel-6	CAEZ01005627:1295-1521
HmHel-6	CAEZ01005627:12743-12928
HmHel-6	CAEZ01005627:2112-2368
HmHel-6	CAEZ01005266:5627-5716
HmHel-6	CAEZ01005266:9090-9339
HmHel-6	CAEZ01004239:12765-12999
HmHel-6	CAEZ01004239:33790-33947
HmHel-6	CAEZ01004096:4347-4478
HmHel-6	CAEZ01004096:31575-31697
HmHel-6	CAEZ01004096:53537-53711
HmHel-6	CAEZ01004096:70832-74829
HmHel-6	CAEZ01004096:109248-111273
HmHel-6	CAEZ01004096:133159-133338
HmHel-6	CAEZ01004096:107710-117861
HmHel-6	CAEZ01004096:92070-92197
HmHel-6	CAEZ01004096:78128-78222
HmHel-6	CAEZ01004096:47392-53129
HmHel-6	CAEZ01004096:30856-31027
HmHel-6	CAEZ01004096:17225-17346
HmHel-6	CAEZ01003740:13481-13731
HmHel-6	CAEZ01003740:3605-8763
HmHel-6	CAEZ01002596:13473-13723
HmHel-6	CAEZ01010489:133-6466
HmHel-6	CAEZ01010489:37667-37897
HmHel-6	CAEZ01010489:36232-36358
HmHel-6	CAEZ01010440:18511-18749
HmHel-6	CAEZ01010440:34858-35038
HmHel-6	CAEZ01010440:64476-64638
HmHel-6	CAEZ01010440:35566-35789
HmHel-6	CAEZ01010440:15332-19309
HmHel-6	CAEZ01008892:6804-11693
HmHel-6	CAEZ01008892:2566-6493
HmHel-6	CAEZ01008271:2671-2758

HmHel-6	CAEZ01008271:25288-25440
HmHel-6	CAEZ01008271:33249-33450
HmHel-6	CAEZ01008271:6591-6674
HmHel-6	CAEZ01008258:25354-26935
HmHel-6	CAEZ01008258:61953-62076
HmHel-6	CAEZ01008258:89607-89816
HmHel-6	CAEZ01008258:108456-108543
HmHel-6	CAEZ01008258:153725-153939
HmHel-6	CAEZ01008258:168170-168291
HmHel-6	CAEZ01008258:179590-179691
HmHel-6	CAEZ01008258:121237-121467
HmHel-6	CAEZ01008258:102202-102338
HmHel-6	CAEZ01008258:64968-65132
HmHel-6	CAEZ01008258:9505-9658
HmHel-6	CAEZ01007941:671-790
HmHel-6	CAEZ01007941:14237-14479
HmHel-6	CAEZ01007941:38043-38222
HmHel-6	CAEZ01007941:49782-52265
HmHel-6	CAEZ01007941:96787-96880
HmHel-6	CAEZ01007941:151664-151917
HmHel-6	CAEZ01007941:176554-176683
HmHel-6	CAEZ01007941:139100-146393
HmHel-6	CAEZ01007941:124214-124292
HmHel-6	CAEZ01007941:60887-61006
HmHel-6	CAEZ01007941:30410-30614
HmHel-6	CAEZ01007941:9824-9949
HmHel-6	CAEZ01007865:52792-55127
HmHel-6	CAEZ01007411:6037-6290
HmHel-6	CAEZ01007411:5172-5262
HmHel-6	CAEZ01007268:4338-4535
HmHel-6	CAEZ01007268:23509-23712
HmHel-6	CAEZ01007268:54393-54558
HmHel-6	CAEZ01007268:16786-16911
HmHel-6	CAEZ01006763:11215-11389
HmHel-6	CAEZ01006763:16767-17013
HmHel-6	CAEZ01006401:6332-6558
HmHel-6	CAEZ01006401:23961-24119
HmHel-6	CAEZ01006401:5125-8429
HmHel-6	CAEZ01006343:10775-10863
HmHel-6	CAEZ01006343:24133-24344
HmHel-6	CAEZ01006343:36463-36570
HmHel-6	CAEZ01006343:65251-65432
HmHel-6	CAEZ01006343:54311-54455
HmHel-6	CAEZ01006343:9431-14149
HmHel-6	CAEZ01006137:11134-15094
HmHel-6	CAEZ01006137:35738-35977
HmHel-6	CAEZ01006137:25931-26085
HmHel-6	CAEZ01006137:6383-6541

HmHel-6	CAEZ01006029:527-782
HmHel-6	CAEZ01005692:19275-19423
HmHel-6	CAEZ01005692:32097-32350
HmHel-6	CAEZ01005692:22675-32806
HmHel-6	CAEZ01005606:10557-11536
HmHel-6	CAEZ01005606:68290-68531
HmHel-6	CAEZ01005606:99055-102082
HmHel-6	CAEZ01005606:136046-141157
HmHel-6	CAEZ01005606:144894-145032
HmHel-6	CAEZ01005606:131908-132122
HmHel-6	CAEZ01005606:75872-75997
HmHel-6	CAEZ01005606:40987-47817
HmHel-6	CAEZ01005606:25762-28710
HmHel-6	CAEZ01005606:15246-15463
HmHel-6	CAEZ01005067:9575-9667
HmHel-6	CAEZ01005067:31969-32072
HmHel-6	CAEZ01005067:49092-49228
HmHel-6	CAEZ01005067:68546-68786
HmHel-6	CAEZ01005067:83256-83507
HmHel-6	CAEZ01005067:103276-103368
HmHel-6	CAEZ01005067:126378-126534
HmHel-6	CAEZ01005067:137602-137769
HmHel-6	CAEZ01005067:106341-113479
HmHel-6	CAEZ01005067:18820-19012
HmHel-6	CAEZ01004395:7785-7876
HmHel-6	CAEZ01004395:28062-28297
HmHel-6	CAEZ01004395:1975-2215
HmHel-6	CAEZ01004097:11376-11468
HmHel-6	CAEZ01004097:28348-29864
HmHel-6	CAEZ01004097:64621-64773
HmHel-6	CAEZ01004097:124770-124964
HmHel-6	CAEZ01004097:135016-135241
HmHel-6	CAEZ01004097:199650-204582
HmHel-6	CAEZ01004097:238074-246383
HmHel-6	CAEZ01004097:228419-228592
HmHel-6	CAEZ01004097:207160-208091
HmHel-6	CAEZ01004097:145274-145474
HmHel-6	CAEZ01004097:101878-102120
HmHel-6	CAEZ01004097:57190-59460
HmHel-6	CAEZ01004097:30728-39069
HmHel-6	CAEZ01004097:11995-12088
HmHel-6	CAEZ01004016:21009-21133
HmHel-6	CAEZ01004016:39752-39999
HmHel-6	CAEZ01004016:7587-7693
HmHel-6	CAEZ01003647:599-724
HmHel-6	CAEZ01003647:54255-59737
HmHel-6	CAEZ01003647:75977-85598
HmHel-6	CAEZ01003647:8775-8965

HmHel-6	CAEZ01002036:10948-19769
HmHel-6	CAEZ01002036:20648-25010
HmHel-6	CAEZ01000539:1443-1700
HmHel-6	CAEZ01011234:4716-4824
HmHel-6	CAEZ01011234:19561-19773
HmHel-6	CAEZ01011234:8497-8713
HmHel-6	CAEZ01008866:6003-6255
HmHel-6	CAEZ01008130:4682-4898
HmHel-6	CAEZ01008130:14077-14161
HmHel-6	CAEZ01007981:4647-4782
HmHel-6	CAEZ01007981:17587-17666
HmHel-6	CAEZ01007981:28071-28273
HmHel-6	CAEZ01007981:34322-34429
HmHel-6	CAEZ01007981:5770-5945
HmHel-6	CAEZ01007738:7821-8040
HmHel-6	CAEZ01007738:72241-80446
HmHel-6	CAEZ01007738:116632-116775
HmHel-6	CAEZ01007738:100462-101371
HmHel-6	CAEZ01007738:75607-75771
HmHel-6	CAEZ01007738:51950-52039
HmHel-6	CAEZ01007738:37089-40023
HmHel-6	CAEZ01007738:12284-12408
HmHel-6	CAEZ01007267:9199-9398
HmHel-6	CAEZ01007267:20241-20347
HmHel-6	CAEZ01007267:52592-52808
HmHel-6	CAEZ01007267:65510-65741
HmHel-6	CAEZ01007267:37148-37268
HmHel-6	CAEZ01007267:2315-2563
HmHel-6	CAEZ01007201:4013-15077
HmHel-6	CAEZ01007201:26292-26504
HmHel-6	CAEZ01007201:43003-43226
HmHel-6	CAEZ01007201:54853-55089
HmHel-6	CAEZ01007201:81116-81351
HmHel-6	CAEZ01007201:104761-104965
HmHel-6	CAEZ01007201:108436-108670
HmHel-6	CAEZ01007201:84175-84798
HmHel-6	CAEZ01007201:56428-56553
HmHel-6	CAEZ01006133:16229-16337
HmHel-6	CAEZ01006133:4789-4914
HmHel-6	CAEZ01005997:5899-6119
HmHel-6	CAEZ01005997:23497-23692
HmHel-6	CAEZ01005997:32248-33526
HmHel-6	CAEZ01005997:13234-13398
HmHel-6	CAEZ01005714:21140-21247
HmHel-6	CAEZ01005714:39330-39531
HmHel-6	CAEZ01005714:34057-34139
HmHel-6	CAEZ01005714:13972-14212
HmHel-6	CAEZ01005299:1765-9598

HmHel-6	CAEZ01005299:35683-35929
HmHel-6	CAEZ01005299:36498-40169
HmHel-6	CAEZ01005299:9170-9397
HmHel-6	CAEZ01004792:7662-7894
HmHel-6	CAEZ01004792:29795-29880
HmHel-6	CAEZ01004792:56099-56186
HmHel-6	CAEZ01004792:66200-66306
HmHel-6	CAEZ01004792:92156-101278
HmHel-6	CAEZ01004792:96833-97130
HmHel-6	CAEZ01004792:29185-29271
HmHel-6	CAEZ01004792:3035-11674
HmHel-6	CAEZ01004654:17412-17642
HmHel-6	CAEZ01003718:24921-25106
HmHel-6	CAEZ01003718:18531-18759
HmHel-6	CAEZ01001349:19471-19679
HmHel-6	CAEZ01001349:70796-70898
HmHel-6	CAEZ01001349:54307-54481
HmHel-6	CAEZ01001349:6076-6229
HmHel-6	CAEZ01011101:66982-67238
HmHel-6	CAEZ01011101:91379-91464
HmHel-6	CAEZ01011101:70416-70667
HmHel-6	CAEZ01011101:32565-32745
HmHel-6	CAEZ01011101:10597-10708
HmHel-6	CAEZ01010322:12147-14335
HmHel-6	CAEZ01010322:14755-14864
HmHel-6	CAEZ01009725:4224-4475
HmHel-6	CAEZ01009688:7679-7789
HmHel-6	CAEZ01009688:34614-34787
HmHel-6	CAEZ01009688:50272-50391
HmHel-6	CAEZ01009688:89945-90133
HmHel-6	CAEZ01009688:60172-66735
HmHel-6	CAEZ01009688:37810-37978
HmHel-6	CAEZ01009688:24545-24649
HmHel-6	CAEZ01009623:11318-11554
HmHel-6	CAEZ01009269:28503-28678
HmHel-6	CAEZ01009269:46505-46732
HmHel-6	CAEZ01008833:9229-9342
HmHel-6	CAEZ01008833:14023-14256
HmHel-6	CAEZ01008833:2758-2969
HmHel-6	CAEZ01008440:1836-2078
HmHel-6	CAEZ01007568:8684-8859
HmHel-6	CAEZ01007568:64059-64286
HmHel-6	CAEZ01007568:100100-100312
HmHel-6	CAEZ01007568:109005-109136
HmHel-6	CAEZ01007568:9288-9369
HmHel-6	CAEZ01007070:14426-21404
HmHel-6	CAEZ01007070:23032-23118
HmHel-6	CAEZ01007070:11914-12141

HmHel-6	CAEZ01006618:193-289
HmHel-6	CAEZ01006618:14195-24467
HmHel-6	CAEZ01006618:47978-49655
HmHel-6	CAEZ01006618:134334-134961
HmHel-6	CAEZ01006618:149300-149392
HmHel-6	CAEZ01006618:177061-177192
HmHel-6	CAEZ01006618:218306-220234
HmHel-6	CAEZ01006618:248754-248846
HmHel-6	CAEZ01006618:286868-286957
HmHel-6	CAEZ01006618:274018-274151
HmHel-6	CAEZ01006618:196459-196638
HmHel-6	CAEZ01006618:149635-151586
HmHel-6	CAEZ01006618:103295-103523
HmHel-6	CAEZ01006618:80951-86592
HmHel-6	CAEZ01006618:54843-64856
HmHel-6	CAEZ01006618:32200-32415
HmHel-6	CAEZ01006618:11783-11914
HmHel-6	CAEZ01006204:6837-7077
HmHel-6	CAEZ01006086:3864-3997
HmHel-6	CAEZ01006086:38298-38524
HmHel-6	CAEZ01005478:7228-7473
HmHel-6	CAEZ01005478:37304-37518
HmHel-6	CAEZ01005478:66462-67367
HmHel-6	CAEZ01005478:40888-41030
HmHel-6	CAEZ01005117:3141-3241
HmHel-6	CAEZ01005117:26639-26863
HmHel-6	CAEZ01005117:1929-2027
HmHel-6	CAEZ01004631:50156-50344
HmHel-6	CAEZ01004631:99470-99607
HmHel-6	CAEZ01004631:121510-121696
HmHel-6	CAEZ01004631:182898-183008
HmHel-6	CAEZ01004631:205640-205776
HmHel-6	CAEZ01004631:316456-316609
HmHel-6	CAEZ01004631:300935-301045
HmHel-6	CAEZ01004631:290031-290130
HmHel-6	CAEZ01004631:273352-273547
HmHel-6	CAEZ01004631:216777-216927
HmHel-6	CAEZ01004631:200809-200932
HmHel-6	CAEZ01004631:145417-145572
HmHel-6	CAEZ01004631:94407-94547
HmHel-6	CAEZ01004415:1706-1949
HmHel-6	CAEZ01004382:8713-8835
HmHel-6	CAEZ01004382:19180-19391
HmHel-6	CAEZ01004382:20238-20325
HmHel-6	CAEZ01004382:9461-9656
HmHel-6	CAEZ01004131:1346-1595
HmHel-6	CAEZ01003946:1217-9158
HmHel-6	CAEZ01003946:27533-27806

HmHel-6	CAEZ01003946:22501-22591
HmHel-6	CAEZ01003568:965-1104
HmHel-6	CAEZ01003568:47061-47291
HmHel-6	CAEZ01003568:59098-59246
HmHel-6	CAEZ01003568:61010-61218
HmHel-6	CAEZ01003568:46400-46496
HmHel-6	CAEZ01002719:3932-4039
HmHel-6	CAEZ01002719:14145-19194
HmHel-6	CAEZ01002719:1334-1547
HmHel-6	CAEZ01001446:29945-30151
HmHel-6	CAEZ01001446:73880-73994
HmHel-6	CAEZ01001446:100578-100746
HmHel-6	CAEZ01001446:128655-128741
HmHel-6	CAEZ01001446:147190-147387
HmHel-6	CAEZ01001446:108035-114389
HmHel-6	CAEZ01001446:84831-84932
HmHel-6	CAEZ01001446:65724-65932
HmHel-6	CAEZ01001446:14788-17912
HmHel-6	CAEZ01000978:8450-8697
HmHel-6	CAEZ01011215:86845-86958
HmHel-6	CAEZ01011215:113274-113357
HmHel-6	CAEZ01011215:137051-137269
HmHel-6	CAEZ01011215:199845-201046
HmHel-6	CAEZ01011215:219914-223526
HmHel-6	CAEZ01011215:167230-167377
HmHel-6	CAEZ01011215:156336-156569
HmHel-6	CAEZ01011215:124969-129192
HmHel-6	CAEZ01011215:112702-112956
HmHel-6	CAEZ01011215:85816-85974
HmHel-6	CAEZ01011215:58403-58503
HmHel-6	CAEZ01010867:12751-12993
HmHel-6	CAEZ01010867:21788-21942
HmHel-6	CAEZ01010867:2878-2961
HmHel-6	CAEZ01009886:14776-14867
HmHel-6	CAEZ01009886:33434-33625
HmHel-6	CAEZ01009886:66496-70572
HmHel-6	CAEZ01009886:136976-137150
HmHel-6	CAEZ01009886:155333-155592
HmHel-6	CAEZ01009886:146910-147066
HmHel-6	CAEZ01009886:127278-136157
HmHel-6	CAEZ01009886:65802-66058
HmHel-6	CAEZ01009886:27837-27945
HmHel-6	CAEZ01009886:2045-2213
HmHel-6	CAEZ01009729:13747-21648
HmHel-6	CAEZ01009729:41157-41255
HmHel-6	CAEZ01009729:51396-51602
HmHel-6	CAEZ01009729:32506-32617
HmHel-6	CAEZ01009646:13365-13579

HmHel-6	CAEZ01009442:1770-1865
HmHel-6	CAEZ01009442:24171-24378
HmHel-6	CAEZ01009442:5929-6156
HmHel-6	CAEZ01008978:26456-26703
HmHel-6	CAEZ01008978:7528-7696
HmHel-6	CAEZ01008409:15143-15331
HmHel-6	CAEZ01008409:26229-26404
HmHel-6	CAEZ01008409:24969-25090
HmHel-6	CAEZ01008409:4106-4312
HmHel-6	CAEZ01008280:35287-35522
HmHel-6	CAEZ01008280:33117-33244
HmHel-6	CAEZ01008223:661-744
HmHel-6	CAEZ01008223:7717-7959
HmHel-6	CAEZ01007979:5355-5582
HmHel-6	CAEZ01007819:15489-15771
HmHel-6	CAEZ01007819:12781-13024
HmHel-6	CAEZ01006487:10929-11144
HmHel-6	CAEZ01006487:16791-17001
HmHel-6	CAEZ01006370:1317-1422
HmHel-6	CAEZ01006370:47666-47873
HmHel-6	CAEZ01006370:26109-26188
HmHel-6	CAEZ01006370:6071-6294
HmHel-6	CAEZ01005618:17463-20056
HmHel-6	CAEZ01005618:3058-3180
HmHel-6	CAEZ01005333:9335-9562
HmHel-6	CAEZ01005333:23315-29526
HmHel-6	CAEZ01005149:12639-12852
HmHel-6	CAEZ01005149:10861-11010
HmHel-6	CAEZ01004527:5010-5117
HmHel-6	CAEZ01004527:24355-30741
HmHel-6	CAEZ01004527:56386-56519
HmHel-6	CAEZ01004527:1406-1656
HmHel-6	CAEZ01003750:4152-4342
HmHel-6	CAEZ01003750:58726-62790
HmHel-6	CAEZ01003750:96158-96308
HmHel-6	CAEZ01003750:184540-184707
HmHel-6	CAEZ01003750:132714-135810
HmHel-6	CAEZ01003750:98911-99069
HmHel-6	CAEZ01003750:83570-83763
HmHel-6	CAEZ01003750:44744-44957
HmHel-6	CAEZ01003750:24924-25054
HmHel-6	CAEZ01003725:1361-1607
HmHel-6	CAEZ01003526:1766-1897
HmHel-6	CAEZ01003526:1124-1375
HmHel-6	CAEZ01003277:5049-5889
HmHel-6	CAEZ01003277:25842-29647
HmHel-6	CAEZ01003277:9483-9610
HmHel-6	CAEZ01002851:5840-6054

HmHel-6	CAEZ01002851:30896-31145
HmHel-6	CAEZ01002851:3656-3797
HmHel-6	CAEZ01002576:20659-20857
HmHel-6	CAEZ01002576:17938-18124
HmHel-6	CAEZ01001336:6408-7314
HmHel-6	CAEZ01001336:4905-5003
HmHel-6	CAEZ01000873:7357-16792
HmHel-6	CAEZ01000873:13214-13428
HmHel-6	CAEZ01010951:19603-19721
HmHel-6	CAEZ01010951:1217-1453
HmHel-6	CAEZ01010251:16007-16231
HmHel-6	CAEZ01010251:16874-17114
HmHel-6	CAEZ01009807:12837-23983
HmHel-6	CAEZ01009807:24601-24706
HmHel-6	CAEZ01009807:6578-6807
HmHel-6	CAEZ01009703:14004-20226
HmHel-6	CAEZ01008275:4287-4539
HmHel-6	CAEZ01008275:1357-1535
HmHel-6	CAEZ01006464:17172-17253
HmHel-6	CAEZ01006464:7840-16410
HmHel-6	CAEZ01006130:13248-13427
HmHel-6	CAEZ01006130:28168-28253
HmHel-6	CAEZ01006130:80738-80864
HmHel-6	CAEZ01006130:92072-101588
HmHel-6	CAEZ01006130:152301-152468
HmHel-6	CAEZ01006130:192761-192864
HmHel-6	CAEZ01006130:110698-110821
HmHel-6	CAEZ01006130:66186-66311
HmHel-6	CAEZ01006130:24393-24595
HmHel-6	CAEZ01005850:2855-5530
HmHel-6	CAEZ01005850:19452-19654
HmHel-6	CAEZ01005850:13351-13583
HmHel-6	CAEZ01005597:5669-5898
HmHel-6	CAEZ01005597:16894-17165
HmHel-6	CAEZ01005597:12624-12824
HmHel-6	CAEZ01005179:11871-12096
HmHel-6	CAEZ01005179:3755-11168
HmHel-6	CAEZ01005140:935-1096
HmHel-6	CAEZ01005140:25633-25866
HmHel-6	CAEZ01004787:9674-9789
HmHel-6	CAEZ01004787:2216-2457
HmHel-6	CAEZ01004745:16179-16384
HmHel-6	CAEZ01004745:8450-17767
HmHel-6	CAEZ01004193:4676-5913
HmHel-6	CAEZ01004193:18280-18489
HmHel-6	CAEZ01003856:1648-7748
HmHel-6	CAEZ01003856:2191-2420
HmHel-6	CAEZ01003488:13928-14144

HmHel-6	CAEZ01003488:22892-22991
HmHel-6	CAEZ01002950:17054-17205
HmHel-6	CAEZ01002950:35225-35432
HmHel-6	CAEZ01002950:58954-59504
HmHel-6	CAEZ01002950:17952-18079
HmHel-6	CAEZ01002193:478-650
HmHel-6	CAEZ01002193:7787-7891
HmHel-6	CAEZ01001948:2757-2994
HmHel-6	CAEZ01001806:33060-33293
HmHel-6	CAEZ01001806:73071-73209
HmHel-6	CAEZ01001806:101940-102031
HmHel-6	CAEZ01001806:126115-126331
HmHel-6	CAEZ01001806:61602-61739
HmHel-6	CAEZ01001806:24088-24293
HmHel-6	CAEZ01001327:3449-3694
HmHel-6	CAEZ01001327:21719-30609
HmHel-6	CAEZ01011606:4931-5058
HmHel-6	CAEZ01011606:2053-2278
HmHel-6	CAEZ01010740:1286-11071
HmHel-6	CAEZ01010740:54381-54609
HmHel-6	CAEZ01010740:76181-76363
HmHel-6	CAEZ01010740:48126-56256
HmHel-6	CAEZ01010740:18731-25961
HmHel-6	CAEZ01010328:1482-1723
HmHel-6	CAEZ01010088:3233-3452
HmHel-6	CAEZ01009120:16797-17034
HmHel-6	CAEZ01009120:29853-31435
HmHel-6	CAEZ01009120:77805-78044
HmHel-6	CAEZ01009120:66197-72091
HmHel-6	CAEZ01009120:42426-42618
HmHel-6	CAEZ01008886:9664-9893
HmHel-6	CAEZ01008886:26592-30090
HmHel-6	CAEZ01008886:4661-4801
HmHel-6	CAEZ01008278:19521-19759
HmHel-6	CAEZ01008232:9271-9431
HmHel-6	CAEZ01008232:4783-12013
HmHel-6	CAEZ01005527:11114-11373
HmHel-6	CAEZ01005388:5720-5816
HmHel-6	CAEZ01005388:2859-3027
HmHel-6	CAEZ01005269:10580-12370
HmHel-6	CAEZ01005269:32499-32678
HmHel-6	CAEZ01005269:68753-68899
HmHel-6	CAEZ01005269:118621-118715
HmHel-6	CAEZ01005269:133007-138115
HmHel-6	CAEZ01005269:98063-98207
HmHel-6	CAEZ01005269:73627-73721
HmHel-6	CAEZ01005269:56165-56296
HmHel-6	CAEZ01005269:45558-45765

HmHel-6	CAEZ01005269:20266-24615
HmHel-6	CAEZ01005163:7638-7825
HmHel-6	CAEZ01005163:40631-40890
HmHel-6	CAEZ01005163:13975-14080
HmHel-6	CAEZ01004907:3651-3884
HmHel-6	CAEZ01004853:1977-2189
HmHel-6	CAEZ01004853:29605-29837
HmHel-6	CAEZ01004853:24152-24320
HmHel-6	CAEZ01004752:5205-12743
HmHel-6	CAEZ01004752:139736-139859
HmHel-6	CAEZ01004752:132074-132172
HmHel-6	CAEZ01004752:115285-115427
HmHel-6	CAEZ01004752:93848-94088
HmHel-6	CAEZ01004752:67542-67622
HmHel-6	CAEZ01004752:52371-52516
HmHel-6	CAEZ01004752:25705-25886
HmHel-6	CAEZ01004614:2382-2597
HmHel-6	CAEZ01004614:1152-5023
HmHel-6	CAEZ01004399:23927-24138
HmHel-6	CAEZ01004399:31337-31584
HmHel-6	CAEZ01004043:6419-6626
HmHel-6	CAEZ01004043:2005-2166
HmHel-6	CAEZ01002678:9175-9274
HmHel-6	CAEZ01002678:10487-10739
HmHel-6	CAEZ01011640:13338-13463
HmHel-6	CAEZ01011640:36130-36358
HmHel-6	CAEZ01011640:55964-64049
HmHel-6	CAEZ01011640:4449-6213
HmHel-6	CAEZ01011527:33952-34203
HmHel-6	CAEZ01011527:22916-23107
HmHel-6	CAEZ01011265:12588-15238
HmHel-6	CAEZ01011265:18888-18989
HmHel-6	CAEZ01010738:13393-13625
HmHel-6	CAEZ01010738:70943-71420
HmHel-6	CAEZ01010738:81883-82125
HmHel-6	CAEZ01010738:112849-113041
HmHel-6	CAEZ01010738:175523-175623
HmHel-6	CAEZ01010738:196492-196668
HmHel-6	CAEZ01010738:228193-228291
HmHel-6	CAEZ01010738:243998-244193
HmHel-6	CAEZ01010738:270742-270832
HmHel-6	CAEZ01010738:282817-282917
HmHel-6	CAEZ01010738:273550-273636
HmHel-6	CAEZ01010738:261657-261808
HmHel-6	CAEZ01010738:242807-243022
HmHel-6	CAEZ01010738:122800-122911
HmHel-6	CAEZ01010738:93516-93696
HmHel-6	CAEZ01010738:82341-83158

HmHel-6	CAEZ01010738:49917-50024
HmHel-6	CAEZ01010481:5491-5689
HmHel-6	CAEZ01010481:21713-21945
HmHel-6	CAEZ01010481:3947-4052
HmHel-6	CAEZ01010228:38394-38499
HmHel-6	CAEZ01010228:85048-85240
HmHel-6	CAEZ01010228:108668-108846
HmHel-6	CAEZ01010228:43951-44049
HmHel-6	CAEZ01009876:4478-4684
HmHel-6	CAEZ01009876:25469-25723
HmHel-6	CAEZ01009876:21586-21701
HmHel-6	CAEZ01009876:5450-5607
HmHel-6	CAEZ01009828:15279-15418
HmHel-6	CAEZ01009828:49743-49967
HmHel-6	CAEZ01009828:9749-9941
HmHel-6	CAEZ01009659:16169-16338
HmHel-6	CAEZ01009659:52103-52305
HmHel-6	CAEZ01009659:14798-19578
HmHel-6	CAEZ01009638:2310-5256
HmHel-6	CAEZ01009638:16146-16318
HmHel-6	CAEZ01009272:7110-7308
HmHel-6	CAEZ01009272:40806-40920
HmHel-6	CAEZ01009272:29888-30135
HmHel-6	CAEZ01009272:606-696
HmHel-6	CAEZ01009084:19029-19133
HmHel-6	CAEZ01009084:76394-76587
HmHel-6	CAEZ01009084:88747-88956
HmHel-6	CAEZ01009084:68111-68358
HmHel-6	CAEZ01009084:43371-45662
HmHel-6	CAEZ01009084:32328-32581
HmHel-6	CAEZ01009084:7749-8878
HmHel-6	CAEZ01008744:25693-25825
HmHel-6	CAEZ01008744:40552-40674
HmHel-6	CAEZ01008744:6516-6764
HmHel-6	CAEZ01008367:7250-13908
HmHel-6	CAEZ01008367:129219-129442
HmHel-6	CAEZ01008367:143389-143581
HmHel-6	CAEZ01008367:162285-162404
HmHel-6	CAEZ01008367:137082-137240
HmHel-6	CAEZ01008367:111199-111391
HmHel-6	CAEZ01008367:62988-70138
HmHel-6	CAEZ01008367:51351-51449
HmHel-6	CAEZ01008367:40686-40772
HmHel-6	CAEZ01008367:27869-28022
HmHel-6	CAEZ01007207:58458-58673
HmHel-6	CAEZ01007207:77544-81178
HmHel-6	CAEZ01007207:156819-156917
HmHel-6	CAEZ01007207:224734-224932

HmHel-6	CAEZ01007207:250876-257237
HmHel-6	CAEZ01007207:278720-278827
HmHel-6	CAEZ01007207:271281-276190
HmHel-6	CAEZ01007207:232511-232738
HmHel-6	CAEZ01007207:164037-164121
HmHel-6	CAEZ01007207:144264-146083
HmHel-6	CAEZ01007207:110036-110202
HmHel-6	CAEZ01007207:78670-78874
HmHel-6	CAEZ01007207:43049-49526
HmHel-6	CAEZ01007207:10398-13867
HmHel-6	CAEZ01006941:70-770
HmHel-6	CAEZ01006941:5001-5133
HmHel-6	CAEZ01006805:15492-15712
HmHel-6	CAEZ01006805:20959-21107
HmHel-6	CAEZ01006465:3461-3695
HmHel-6	CAEZ01005071:8573-12371
HmHel-6	CAEZ01005071:31791-31890
HmHel-6	CAEZ01005071:13011-13261
HmHel-6	CAEZ01004940:30632-30814
HmHel-6	CAEZ01004940:78071-83171
HmHel-6	CAEZ01004940:82287-82514
HmHel-6	CAEZ01004940:57643-57763
HmHel-6	CAEZ01004940:23811-39883
HmHel-6	CAEZ01004940:4430-9324
HmHel-6	CAEZ01004738:7643-7855
HmHel-6	CAEZ01004738:26433-26555
HmHel-6	CAEZ01004738:46271-46396
HmHel-6	CAEZ01004738:92177-95172
HmHel-6	CAEZ01004738:154948-155191
HmHel-6	CAEZ01004738:184660-184876
HmHel-6	CAEZ01004738:224434-224518
HmHel-6	CAEZ01004738:248953-249049
HmHel-6	CAEZ01004738:266182-275113
HmHel-6	CAEZ01004738:289546-289694
HmHel-6	CAEZ01004738:306944-315509
HmHel-6	CAEZ01004738:278525-278624
HmHel-6	CAEZ01004738:261071-261160
HmHel-6	CAEZ01004738:188859-188949
HmHel-6	CAEZ01004738:140490-141341
HmHel-6	CAEZ01004738:113513-114988
HmHel-6	CAEZ01004738:88095-88184
HmHel-6	CAEZ01004738:50735-50820
HmHel-6	CAEZ01004738:517-714
HmHel-6	CAEZ01003699:4266-4512
HmHel-6	CAEZ01003699:2294-2407
HmHel-6	CAEZ01003292:2244-2350
HmHel-6	CAEZ01003292:23419-33348
HmHel-6	CAEZ01003292:98483-98702

HmHel-6	CAEZ01003292:65053-68122
HmHel-6	CAEZ01003292:38411-38589
HmHel-6	CAEZ01001576:6569-6669
HmHel-6	CAEZ01001576:11154-11361
HmHel-6	CAEZ01001415:2593-2820
HmHel-6	CAEZ01001415:9899-10008
HmHel-6	CAEZ01000056:28920-29167
HmHel-6	CAEZ01000056:42233-42462
HmHel-6	CAEZ01000056:30723-30866
HmHel-6	CAEZ01000056:17535-17645
HmHel-6	CAEZ01000038:2353-4894
HmHel-6	CAEZ01000038:53708-65740
HmHel-6	CAEZ01000038:69660-69853
HmHel-6	CAEZ01000038:24549-27543
HmHel-6	CAEZ01011601:8166-8417
HmHel-6	CAEZ01011601:34915-34998
HmHel-6	CAEZ01011601:21347-21529
HmHel-6	CAEZ01011601:309-1453
HmHel-6	CAEZ01010970:56733-56958
HmHel-6	CAEZ01010970:78960-79157
HmHel-6	CAEZ01010970:65620-65778
HmHel-6	CAEZ01010970:37614-37860
HmHel-6	CAEZ01010629:11445-11552
HmHel-6	CAEZ01010629:12855-15754
HmHel-6	CAEZ01010113:410-574
HmHel-6	CAEZ01010113:25519-25706
HmHel-6	CAEZ01010113:17017-17232
HmHel-6	CAEZ01010079:8538-8775
HmHel-6	CAEZ01010079:64735-64832
HmHel-6	CAEZ01010079:78566-78694
HmHel-6	CAEZ01010079:102232-102332
HmHel-6	CAEZ01010079:93086-93340
HmHel-6	CAEZ01010079:73878-73972
HmHel-6	CAEZ01010079:33449-36835
HmHel-6	CAEZ01010025:9681-15608
HmHel-6	CAEZ01010025:36875-36975
HmHel-6	CAEZ01010025:7887-7992
HmHel-6	CAEZ01009606:718-2565
HmHel-6	CAEZ01009243:2863-2964
HmHel-6	CAEZ01009243:31288-31393
HmHel-6	CAEZ01009243:46608-46810
HmHel-6	CAEZ01009243:28697-28778
HmHel-6	CAEZ01009227:32358-32938
HmHel-6	CAEZ01009227:75864-76435
HmHel-6	CAEZ01009227:77997-78140
HmHel-6	CAEZ01009227:38588-42906
HmHel-6	CAEZ01009099:232-343
HmHel-6	CAEZ01009099:20755-20997

HmHel-6	CAEZ01009099:23349-23585
HmHel-6	CAEZ01009099:8511-8595
HmHel-6	CAEZ01008913:4766-5020
HmHel-6	CAEZ01008913:38294-38418
HmHel-6	CAEZ01008913:45908-46047
HmHel-6	CAEZ01008872:13882-13994
HmHel-6	CAEZ01008872:25167-25388
HmHel-6	CAEZ01008872:32670-32852
HmHel-6	CAEZ01008872:10773-10992
HmHel-6	CAEZ01008767:7236-7366
HmHel-6	CAEZ01008767:44663-44841
HmHel-6	CAEZ01008767:65200-65421
HmHel-6	CAEZ01008767:63618-72361
HmHel-6	CAEZ01008767:10415-10508
HmHel-6	CAEZ01008358:9068-9161
HmHel-6	CAEZ01008358:30637-30719
HmHel-6	CAEZ01008358:49852-49941
HmHel-6	CAEZ01008358:52431-52637
HmHel-6	CAEZ01008358:17488-23985
HmHel-6	CAEZ01008351:3675-3773
HmHel-6	CAEZ01008351:20522-20695
HmHel-6	CAEZ01008351:44465-44580
HmHel-6	CAEZ01008351:66135-66381
HmHel-6	CAEZ01008351:97909-98133
HmHel-6	CAEZ01008351:190285-190421
HmHel-6	CAEZ01008351:205600-205849
HmHel-6	CAEZ01008351:131738-131881
HmHel-6	CAEZ01008351:91124-91301
HmHel-6	CAEZ01008351:10815-10984
HmHel-6	CAEZ01007533:1679-1925
HmHel-6	CAEZ01007533:15331-16955
HmHel-6	CAEZ01006844:605-709
HmHel-6	CAEZ01006844:3487-3734
HmHel-6	CAEZ01006835:36261-36504
HmHel-6	CAEZ01006835:10094-10186
HmHel-6	CAEZ01006326:6318-6523
HmHel-6	CAEZ01006326:66748-74631
HmHel-6	CAEZ01006326:92218-92424
HmHel-6	CAEZ01006326:83350-83464
HmHel-6	CAEZ01006326:46586-46837
HmHel-6	CAEZ01006326:26025-31619
HmHel-6	CAEZ01006326:15569-15701
HmHel-6	CAEZ01006262:25711-25917
HmHel-6	CAEZ01006262:17462-17680
HmHel-6	CAEZ01006262:6567-6752
HmHel-6	CAEZ01006057:9119-9836
HmHel-6	CAEZ01006057:16267-16371
HmHel-6	CAEZ01005841:39744-39890

HmHel-6	CAEZ01005841:50610-50860
HmHel-6	CAEZ01005682:11800-16144
HmHel-6	CAEZ01005682:30025-30233
HmHel-6	CAEZ01005682:16595-16702
HmHel-6	CAEZ01005682:1781-2005
HmHel-6	CAEZ01005494:18263-18415
HmHel-6	CAEZ01005494:46811-47024
HmHel-6	CAEZ01003859:2932-3164
HmHel-6	CAEZ01002810:17314-17422
HmHel-6	CAEZ01002810:60939-61152
HmHel-6	CAEZ01002810:84018-93952
HmHel-6	CAEZ01002810:93785-93877
HmHel-6	CAEZ01002810:50911-58267
HmHel-6	CAEZ01002810:29139-29301
HmHel-6	CAEZ01002810:8947-9173
HmHel-6	CAEZ01002695:1672-1787
HmHel-6	CAEZ01002695:17588-17836
HmHel-6	CAEZ01002695:26518-26723
HmHel-6	CAEZ01002695:9613-10728
HmHel-6	CAEZ01001703:3919-4029
HmHel-6	CAEZ01001703:14077-17718
HmHel-6	CAEZ01001464:5607-5723
HmHel-6	CAEZ01011608:68-321
HmHel-6	CAEZ01011510:7089-7309
HmHel-6	CAEZ01011510:17642-17742
HmHel-6	CAEZ01011510:9623-12285
HmHel-6	CAEZ01011273:3095-3341
HmHel-6	CAEZ01011273:20528-20647
HmHel-6	CAEZ01010793:21328-21581
HmHel-6	CAEZ01009046:10863-10949
HmHel-6	CAEZ01009046:10190-10395
HmHel-6	CAEZ01006562:39385-39637
HmHel-6	CAEZ01006562:50070-50208
HmHel-6	CAEZ01006562:109882-109970
HmHel-6	CAEZ01006562:144315-144477
HmHel-6	CAEZ01006562:171288-171405
HmHel-6	CAEZ01006562:183492-183636
HmHel-6	CAEZ01006562:197923-198010
HmHel-6	CAEZ01006562:228341-228534
HmHel-6	CAEZ01006562:193678-193913
HmHel-6	CAEZ01006562:90308-90424
HmHel-6	CAEZ01006562:67425-73862
HmHel-6	CAEZ01006562:33461-35541
HmHel-6	CAEZ01006151:4232-4491
HmHel-6	CAEZ01006151:43892-44014
HmHel-6	CAEZ01006151:86986-87220
HmHel-6	CAEZ01006151:19243-26403
HmHel-6	CAEZ01006056:12107-12200

HmHel-6	CAEZ01006056:16457-16704
HmHel-6	CAEZ01006056:5607-5811
HmHel-6	CAEZ01005603:6969-9604
HmHel-6	CAEZ01005603:11002-11251
HmHel-6	CAEZ01005230:107957-108146
HmHel-6	CAEZ01005230:112331-112541
HmHel-6	CAEZ01005230:92804-93020
HmHel-6	CAEZ01005152:3168-3249
HmHel-6	CAEZ01005152:45154-45343
HmHel-6	CAEZ01005152:76791-76905
HmHel-6	CAEZ01005152:41682-61262
HmHel-6	CAEZ01005152:7196-7389
HmHel-6	CAEZ01004324:19784-20021
HmHel-6	CAEZ01004037:8199-8420
HmHel-6	CAEZ01002997:1205-1458
HmHel-6	CAEZ01002823:5025-5251
HmHel-6	CAEZ01002718:5036-5280
HmHel-6	CAEZ01002718:11936-12151
HmHel-6	CAEZ01002635:28672-28842
HmHel-6	CAEZ01002635:53100-53343
HmHel-6	CAEZ01002635:18276-27566
HmHel-6	CAEZ01002592:7853-8096
HmHel-6	CAEZ01002026:413-546
HmHel-6	CAEZ01002026:12032-12240
HmHel-6	CAEZ01001175:720-937
HmHel-6	CAEZ01011132:9653-9777
HmHel-6	CAEZ01011132:36594-36678
HmHel-6	CAEZ01011132:10551-10803
HmHel-6	CAEZ01010260:37796-37972
HmHel-6	CAEZ01010260:52908-53128
HmHel-6	CAEZ01010260:93645-93785
HmHel-6	CAEZ01010260:128388-128536
HmHel-6	CAEZ01010260:103522-103694
HmHel-6	CAEZ01009621:10655-10870
HmHel-6	CAEZ01009621:11480-13627
HmHel-6	CAEZ01009608:8542-8797
HmHel-6	CAEZ01009608:11863-11977
HmHel-6	CAEZ01009488:10239-10492
HmHel-6	CAEZ01009488:8711-8890
HmHel-6	CAEZ01008525:6852-6935
HmHel-6	CAEZ01008525:30723-33772
HmHel-6	CAEZ01008236:24838-24947
HmHel-6	CAEZ01008236:50925-51034
HmHel-6	CAEZ01008236:104514-108995
HmHel-6	CAEZ01008236:82782-82931
HmHel-6	CAEZ01008236:62010-62162
HmHel-6	CAEZ01008236:33701-37036
HmHel-6	CAEZ01008236:4872-5107

HmHel-6	CAEZ01007669:289-481
HmHel-6	CAEZ01007492:19586-20976
HmHel-6	CAEZ01007492:62744-62983
HmHel-6	CAEZ01007492:28034-28161
HmHel-6	CAEZ01007492:3513-3727
HmHel-6	CAEZ01007219:3879-4256
HmHel-6	CAEZ01007219:27869-29577
HmHel-6	CAEZ01007219:38119-38241
HmHel-6	CAEZ01006875:902-1129
HmHel-6	CAEZ01006875:6600-6834
HmHel-6	CAEZ01006096:7782-7905
HmHel-6	CAEZ01006096:39327-39515
HmHel-6	CAEZ01006096:48813-48921
HmHel-6	CAEZ01005951:6774-6966
HmHel-6	CAEZ01005951:21969-22060
HmHel-6	CAEZ01005951:12102-12192
HmHel-6	CAEZ01005393:111111-111311
HmHel-6	CAEZ01005393:128025-128174
HmHel-6	CAEZ01005393:117733-117985
HmHel-6	CAEZ01005393:97469-97557
HmHel-6	CAEZ01005393:37915-38018
HmHel-6	CAEZ01005393:10880-11071
HmHel-6	CAEZ01004870:4768-5009
HmHel-6	CAEZ01004870:5870-5952
HmHel-6	CAEZ01004179:1891-2143
HmHel-6	CAEZ01004130:3061-3143
HmHel-6	CAEZ01004130:22996-23243
HmHel-6	CAEZ01004130:14747-14943
HmHel-6	CAEZ01003151:5525-5662
HmHel-6	CAEZ01003151:37086-37222
HmHel-6	CAEZ01003151:71016-71212
HmHel-6	CAEZ01003151:73816-73947
HmHel-6	CAEZ01003151:40116-40270
HmHel-6	CAEZ01003151:17906-18045
HmHel-6	CAEZ01003151:3026-3263
HmHel-6	CAEZ01002990:3551-3653
HmHel-6	CAEZ01002990:22719-22930
HmHel-6	CAEZ01002923:25550-25783
HmHel-6	CAEZ01002866:12852-15879
HmHel-6	CAEZ01002866:59064-67736
HmHel-6	CAEZ01002866:108231-108355
HmHel-6	CAEZ01002866:119998-120176
HmHel-6	CAEZ01002866:140370-149735
HmHel-6	CAEZ01002866:172205-172289
HmHel-6	CAEZ01002866:145147-156854
HmHel-6	CAEZ01002866:117251-117356
HmHel-6	CAEZ01002866:43397-43532
HmHel-6	CAEZ01002866:9274-9386

HmHel-6	CAEZ01000123:8-196
HmHel-6	CAEZ01011475:3372-3454
HmHel-6	CAEZ01011475:17667-17798
HmHel-6	CAEZ01011475:10849-11082
HmHel-6	CAEZ01011023:22700-22808
HmHel-6	CAEZ01011023:4699-4792
HmHel-6	CAEZ01009676:3198-3336
HmHel-6	CAEZ01009676:20044-20160
HmHel-6	CAEZ01009676:10371-10554
HmHel-6	CAEZ01009671:9634-9719
HmHel-6	CAEZ01009332:53438-53621
HmHel-6	CAEZ01009332:700-790
HmHel-6	CAEZ01008890:2902-3132
HmHel-6	CAEZ01008500:8973-9061
HmHel-6	CAEZ01008500:27082-27279
HmHel-6	CAEZ01008500:62717-62941
HmHel-6	CAEZ01008500:79615-79742
HmHel-6	CAEZ01008500:111879-112086
HmHel-6	CAEZ01008500:83322-86800
HmHel-6	CAEZ01008500:50842-51061
HmHel-6	CAEZ01008500:24446-24547
HmHel-6	CAEZ01008500:9559-14246
HmHel-6	CAEZ01007591:14095-14306
HmHel-6	CAEZ01007591:11441-15601
HmHel-6	CAEZ01007193:12682-12913
HmHel-6	CAEZ01006671:11970-12056
HmHel-6	CAEZ01006319:8324-8560
HmHel-6	CAEZ01006319:39879-40531
HmHel-6	CAEZ01006319:47224-51860
HmHel-6	CAEZ01006319:28192-28394
HmHel-6	CAEZ01006319:4043-4161
HmHel-6	CAEZ01005834:27196-27368
HmHel-6	CAEZ01005834:31847-34951
HmHel-6	CAEZ01005834:2476-2686
HmHel-6	CAEZ01005476:26070-26169
HmHel-6	CAEZ01005476:70626-70871
HmHel-6	CAEZ01005476:87638-87788
HmHel-6	CAEZ01005476:38159-38261
HmHel-6	CAEZ01005476:7681-7919
HmHel-6	CAEZ01004535:1193-1332
HmHel-6	CAEZ01004535:38260-48473
HmHel-6	CAEZ01004535:40334-47701
HmHel-6	CAEZ01004059:9673-9822
HmHel-6	CAEZ01004059:18259-18492
HmHel-6	CAEZ01004059:1835-3450
HmHel-6	CAEZ01002814:2991-3213
HmHel-6	CAEZ01002814:17747-17947
HmHel-6	CAEZ01002814:43524-46169

HmHel-6	CAEZ01002814:97907-98068
HmHel-6	CAEZ01002814:110243-112721
HmHel-6	CAEZ01002814:170992-171083
HmHel-6	CAEZ01002814:197561-197645
HmHel-6	CAEZ01002814:262702-262937
HmHel-6	CAEZ01002814:289331-289555
HmHel-6	CAEZ01002814:339308-339526
HmHel-6	CAEZ01002814:353107-353348
HmHel-6	CAEZ01002814:328425-328611
HmHel-6	CAEZ01002814:311267-311424
HmHel-6	CAEZ01002814:237561-246944
HmHel-6	CAEZ01002814:190033-190228
HmHel-6	CAEZ01002814:152394-155857
HmHel-6	CAEZ01002814:131416-134993
HmHel-6	CAEZ01002814:117463-120985
HmHel-6	CAEZ01001199:3052-3157
HmHel-6	CAEZ01001199:306-477
HmHel-6	CAEZ01011197:13033-19226
HmHel-6	CAEZ01011197:49506-49708
HmHel-6	CAEZ01011197:84576-84678
HmHel-6	CAEZ01011197:104641-104835
HmHel-6	CAEZ01011197:145754-146010
HmHel-6	CAEZ01011197:48359-60925
HmHel-6	CAEZ01011197:7075-7712
HmHel-6	CAEZ01010853:6898-7112
HmHel-6	CAEZ01010853:8829-8923
HmHel-6	CAEZ01010724:4790-4872
HmHel-6	CAEZ01010724:18027-18193
HmHel-6	CAEZ01010724:26635-26783
HmHel-6	CAEZ01009446:2947-3045
HmHel-6	CAEZ01009446:30304-30403
HmHel-6	CAEZ01009446:37636-37825
HmHel-6	CAEZ01009446:19894-21738
HmHel-6	CAEZ01009077:5068-5152
HmHel-6	CAEZ01009077:38378-44205
HmHel-6	CAEZ01009077:98347-99536
HmHel-6	CAEZ01009077:114091-114206
HmHel-6	CAEZ01009077:139294-145402
HmHel-6	CAEZ01009077:214098-214202
HmHel-6	CAEZ01009077:245715-245949
HmHel-6	CAEZ01009077:189312-189512
HmHel-6	CAEZ01009077:171326-171421
HmHel-6	CAEZ01009077:116787-126448
HmHel-6	CAEZ01009077:87580-87746
HmHel-6	CAEZ01008883:2730-2985
HmHel-6	CAEZ01008824:4136-11898
HmHel-6	CAEZ01008824:72155-72423
HmHel-6	CAEZ01008824:66286-66381

HmHel-6	CAEZ01008824:43745-43947
HmHel-6	CAEZ01008824:29321-29563
HmHel-6	CAEZ01008824:10256-10448
HmHel-6	CAEZ01008769:16302-17447
HmHel-6	CAEZ01008769:38779-38877
HmHel-6	CAEZ01008769:73318-73551
HmHel-6	CAEZ01008769:64258-64464
HmHel-6	CAEZ01008769:16721-16887
HmHel-6	CAEZ01008486:7972-8104
HmHel-6	CAEZ01008486:35054-35302
HmHel-6	CAEZ01008353:49920-50142
HmHel-6	CAEZ01008353:82288-82518
HmHel-6	CAEZ01008353:37852-38058
HmHel-6	CAEZ01008353:19937-20080
HmHel-6	CAEZ01008353:7508-7671
HmHel-6	CAEZ01007427:28068-28157
HmHel-6	CAEZ01007427:3264-3501
HmHel-6	CAEZ01006746:427-533
HmHel-6	CAEZ01006746:3637-3895
HmHel-6	CAEZ01006688:3914-4150
HmHel-6	CAEZ01006283:3894-7554
HmHel-6	CAEZ01006283:30899-37613
HmHel-6	CAEZ01006283:51415-51624
HmHel-6	CAEZ01006283:15232-15385
HmHel-6	CAEZ01006129:14412-14624
HmHel-6	CAEZ01005416:12047-12269
HmHel-6	CAEZ01005416:25702-25823
HmHel-6	CAEZ01005416:56273-56369
HmHel-6	CAEZ01005416:38000-38115
HmHel-6	CAEZ01005300:7086-11668
HmHel-6	CAEZ01003814:2571-2672
HmHel-6	CAEZ01003814:15003-15246
HmHel-6	CAEZ01001738:16168-16251
HmHel-6	CAEZ01001738:59425-59584
HmHel-6	CAEZ01001738:30013-36997
HmHel-6	CAEZ01001738:6246-6337
HmHel-6	CAEZ01001650:11414-11553
HmHel-6	CAEZ01001650:35720-40803
HmHel-6	CAEZ01001650:58787-58894
HmHel-6	CAEZ01001650:85484-94061
HmHel-6	CAEZ01001650:110979-111094
HmHel-6	CAEZ01001650:49392-51894
HmHel-6	CAEZ01001443:3346-3593
HmHel-6	CAEZ01001443:37007-37133
HmHel-6	CAEZ01000878:31115-31309
HmHel-6	CAEZ01000878:48781-48935
HmHel-6	CAEZ01000878:71094-71221
HmHel-6	CAEZ01000878:81234-81343

HmHel-6	CAEZ01000878:42662-42788
HmHel-6	CAEZ01000878:26825-27016
HmHel-6	CAEZ01000580:144-394
HmHel-6	CAEZ01000095:2208-2430
HmHel-6	CAEZ01011528:24457-24588
HmHel-6	CAEZ01011528:43130-43350
HmHel-6	CAEZ01011528:50885-51135
HmHel-6	CAEZ01011528:15819-16317
HmHel-6	CAEZ01011501:1691-1779
HmHel-6	CAEZ01011501:14366-14598
HmHel-6	CAEZ01011314:25283-25500
HmHel-6	CAEZ01011314:544-653
HmHel-6	CAEZ01011133:59849-59990
HmHel-6	CAEZ01011133:50439-50658
HmHel-6	CAEZ01011133:4731-4979
HmHel-6	CAEZ01010369:4879-5087
HmHel-6	CAEZ01009458:4944-5171
HmHel-6	CAEZ01009458:26392-26637
HmHel-6	CAEZ01009458:37349-37449
HmHel-6	CAEZ01009458:68512-69516
HmHel-6	CAEZ01009458:95139-95313
HmHel-6	CAEZ01009458:110145-117526
HmHel-6	CAEZ01009458:135089-135289
HmHel-6	CAEZ01009458:169141-170747
HmHel-6	CAEZ01009458:180365-180521
HmHel-6	CAEZ01009458:138765-138973
HmHel-6	CAEZ01009458:111359-115024
HmHel-6	CAEZ01009458:81959-82154
HmHel-6	CAEZ01009458:46821-46978
HmHel-6	CAEZ01009458:7667-9664
HmHel-6	CAEZ01009440:35632-44775
HmHel-6	CAEZ01009440:68242-68423
HmHel-6	CAEZ01009440:59594-59702
HmHel-6	CAEZ01009440:37344-37557
HmHel-6	CAEZ01009440:27157-27250
HmHel-6	CAEZ01009027:8779-8899
HmHel-6	CAEZ01009027:20104-20256
HmHel-6	CAEZ01009027:3232-3368
HmHel-6	CAEZ01008871:7397-7645
HmHel-6	CAEZ01008821:14111-14299
HmHel-6	CAEZ01008821:12666-12911
HmHel-6	CAEZ01008532:40555-40792
HmHel-6	CAEZ01008532:62769-63486
HmHel-6	CAEZ01008532:62152-70159
HmHel-6	CAEZ01007795:7477-7720
HmHel-6	CAEZ01007795:33861-34070
HmHel-6	CAEZ01007795:61392-61645
HmHel-6	CAEZ01007795:79387-79539

HmHel-6	CAEZ01007795:74846-78228
HmHel-6	CAEZ01007795:54856-54938
HmHel-6	CAEZ01007795:3006-3209
HmHel-6	CAEZ01007625:25804-27075
HmHel-6	CAEZ01007625:47631-47828
HmHel-6	CAEZ01007625:86994-87344
HmHel-6	CAEZ01007625:84427-84579
HmHel-6	CAEZ01007625:15177-15281
HmHel-6	CAEZ01006910:11374-11540
HmHel-6	CAEZ01006910:35224-35421
HmHel-6	CAEZ01006910:25335-25494
HmHel-6	CAEZ01006910:13448-13666
HmHel-6	CAEZ01006649:8056-8261
HmHel-6	CAEZ01006338:4620-6207
HmHel-6	CAEZ01006338:26117-26322
HmHel-6	CAEZ01006338:40781-41034
HmHel-6	CAEZ01006322:881-1085
HmHel-6	CAEZ01006322:13816-14045
HmHel-6	CAEZ01006218:8833-9165
HmHel-6	CAEZ01006218:38677-38922
HmHel-6	CAEZ01006218:33961-34110
HmHel-6	CAEZ01005632:541-5645
HmHel-6	CAEZ01005632:18716-18920
HmHel-6	CAEZ01005632:5007-5134
HmHel-6	CAEZ01005433:4512-4760
HmHel-6	CAEZ01005433:23521-23696
HmHel-6	CAEZ01005433:43932-44142
HmHel-6	CAEZ01005433:60085-60213
HmHel-6	CAEZ01005433:77454-77535
HmHel-6	CAEZ01005433:62554-62655
HmHel-6	CAEZ01005433:31383-31560
HmHel-6	CAEZ01005433:12410-12624
HmHel-6	CAEZ01005392:2023-8799
HmHel-6	CAEZ01005392:74959-75210
HmHel-6	CAEZ01005392:20753-20840
HmHel-6	CAEZ01005194:6956-7178
HmHel-6	CAEZ01004387:1164-5730
HmHel-6	CAEZ01004387:10615-12106
HmHel-6	CAEZ01004070:4924-5186
HmHel-6	CAEZ01004070:1118-1295
HmHel-6	CAEZ01004025:4621-4784
HmHel-6	CAEZ01004025:6254-6498
HmHel-6	CAEZ01003348:4387-4530
HmHel-6	CAEZ01003348:77621-77756
HmHel-6	CAEZ01002811:15011-20754
HmHel-6	CAEZ01002811:56993-57128
HmHel-6	CAEZ01002811:84369-84606
HmHel-6	CAEZ01002811:110196-110321

HmHel-6	CAEZ01002811:180474-180695
HmHel-6	CAEZ01002811:162579-162699
HmHel-6	CAEZ01002811:150205-150379
HmHel-6	CAEZ01002811:94528-94701
HmHel-6	CAEZ01002811:19571-19805
HmHel-6	CAEZ01002165:1723-1822
HmHel-6	CAEZ01000965:2956-3137
HmHel-6	CAEZ01000879:698-3871
HmHel-6	CAEZ01000879:25649-35712
HmHel-6	CAEZ01000879:68161-68372
HmHel-6	CAEZ01000879:91934-92100
HmHel-6	CAEZ01000879:110628-110801
HmHel-6	CAEZ01000879:148176-149027
HmHel-6	CAEZ01000879:165869-165999
HmHel-6	CAEZ01000879:180734-180870
HmHel-6	CAEZ01000879:161583-161835
HmHel-6	CAEZ01000879:145884-145969
HmHel-6	CAEZ01000879:133522-133722
HmHel-6	CAEZ01000879:114305-114468
HmHel-6	CAEZ01000879:83470-83684
HmHel-6	CAEZ01000879:49480-62403
HmHel-6	CAEZ01000879:11888-12015
HmHel-6	CAEZ01010709:9531-9670
HmHel-6	CAEZ01010709:80830-80926
HmHel-6	CAEZ01010709:92430-92586
HmHel-6	CAEZ01010709:30665-30877
HmHel-6	CAEZ01010709:168-389
HmHel-6	CAEZ01010423:19135-19221
HmHel-6	CAEZ01010423:46792-47024
HmHel-6	CAEZ01010423:22340-22536
HmHel-6	CAEZ01010423:8319-8444
HmHel-6	CAEZ01009982:42166-42377
HmHel-6	CAEZ01009982:112328-112453
HmHel-6	CAEZ01009982:91387-91935
HmHel-6	CAEZ01009982:29094-29317
HmHel-6	CAEZ01009895:840-922
HmHel-6	CAEZ01009895:17721-17888
HmHel-6	CAEZ01009895:32737-32976
HmHel-6	CAEZ01009895:43052-43193
HmHel-6	CAEZ01009585:2858-4001
HmHel-6	CAEZ01009585:32596-34253
HmHel-6	CAEZ01008922:19714-19826
HmHel-6	CAEZ01008922:32371-32607
HmHel-6	CAEZ01008922:63585-69894
HmHel-6	CAEZ01008922:44415-44599
HmHel-6	CAEZ01008922:27997-28151
HmHel-6	CAEZ01008922:8797-8912
HmHel-6	CAEZ01008470:4278-4389

HmHel-6	CAEZ01008470:35765-35883
HmHel-6	CAEZ01008446:2337-2447
HmHel-6	CAEZ01008446:31151-31370
HmHel-6	CAEZ01008100:9310-9428
HmHel-6	CAEZ01008100:65504-65594
HmHel-6	CAEZ01008100:72165-72392
HmHel-6	CAEZ01007968:6225-6376
HmHel-6	CAEZ01007968:14418-14659
HmHel-6	CAEZ01007709:60306-60530
HmHel-6	CAEZ01007709:42275-42492
HmHel-6	CAEZ01007709:14728-14892
HmHel-6	CAEZ01007275:19522-22455
HmHel-6	CAEZ01007275:51404-51508
HmHel-6	CAEZ01007275:90589-90837
HmHel-6	CAEZ01007275:127221-127330
HmHel-6	CAEZ01007275:159292-159504
HmHel-6	CAEZ01007275:134480-134700
HmHel-6	CAEZ01007275:96346-96459
HmHel-6	CAEZ01007275:66282-66370
HmHel-6	CAEZ01007275:13562-13730
HmHel-6	CAEZ01007115:606-725
HmHel-6	CAEZ01007115:5710-5802
HmHel-6	CAEZ01006553:9243-9393
HmHel-6	CAEZ01006553:4276-4525
HmHel-6	CAEZ01006366:8188-8430
HmHel-6	CAEZ01005133:2736-2977
HmHel-6	CAEZ01005133:7565-7699
HmHel-6	CAEZ01004676:2869-2957
HmHel-6	CAEZ01004676:12255-12356
HmHel-6	CAEZ01004029:1784-1972
HmHel-6	CAEZ01003056:9097-9178
HmHel-6	CAEZ01003056:21209-21417
HmHel-6	CAEZ01002710:14960-15111
HmHel-6	CAEZ01002710:45925-46169
HmHel-6	CAEZ01002710:10904-11019
HmHel-6	CAEZ01002668:31-7350
HmHel-6	CAEZ01002668:27447-35442
HmHel-6	CAEZ01002668:77991-78198
HmHel-6	CAEZ01002668:38176-44415
HmHel-6	CAEZ01002637:2808-3046
HmHel-6	CAEZ01002039:4503-4602
HmHel-6	CAEZ01002039:10096-19874
HmHel-6	CAEZ01001571:3287-10778
HmHel-6	CAEZ01001076:7651-7776
HmHel-6	CAEZ01001076:4234-10668
HmHel-6	CAEZ01011067:23109-23368
HmHel-6	CAEZ01011067:3867-3981
HmHel-6	CAEZ01011025:683-4905

HmHel-6	CAEZ01011025:16003-16201
HmHel-6	CAEZ01011025:12956-13175
HmHel-6	CAEZ01010979:5380-5606
HmHel-6	CAEZ01010954:4320-4571
HmHel-6	CAEZ01010954:23810-29928
HmHel-6	CAEZ01010783:2560-2781
HmHel-6	CAEZ01010783:4798-5010
HmHel-6	CAEZ01010089:5983-6100
HmHel-6	CAEZ01010089:6894-6975
HmHel-6	CAEZ01009846:6886-7088
HmHel-6	CAEZ01009589:10437-15227
HmHel-6	CAEZ01009589:5987-6139
HmHel-6	CAEZ01009101:2142-2356
HmHel-6	CAEZ01008829:35522-35724
HmHel-6	CAEZ01008829:33261-33511
HmHel-6	CAEZ01008751:11398-15422
HmHel-6	CAEZ01008751:81513-82875
HmHel-6	CAEZ01008751:66401-66551
HmHel-6	CAEZ01008751:39012-47929
HmHel-6	CAEZ01007121:4101-4199
HmHel-6	CAEZ01007121:27883-28122
HmHel-6	CAEZ01007121:32258-32369
HmHel-6	CAEZ01007121:12268-12968
HmHel-6	CAEZ01005977:2551-2765
HmHel-6	CAEZ01005977:43781-52796
HmHel-6	CAEZ01005977:86019-86202
HmHel-6	CAEZ01005977:106227-106333
HmHel-6	CAEZ01005977:90183-94459
HmHel-6	CAEZ01005977:74624-78807
HmHel-6	CAEZ01005977:3679-3831
HmHel-6	CAEZ01005795:12681-12895
HmHel-6	CAEZ01005224:32322-32537
HmHel-6	CAEZ01005224:12989-15928
HmHel-6	CAEZ01005216:4725-4870
HmHel-6	CAEZ01005216:11744-11944
HmHel-6	CAEZ01003739:6549-6681
HmHel-6	CAEZ01003739:12895-13131
HmHel-6	CAEZ01003577:17987-18204
HmHel-6	CAEZ01002944:16623-16706
HmHel-6	CAEZ01002944:32125-32250
HmHel-6	CAEZ01002944:50625-50753
HmHel-6	CAEZ01002944:77217-78614
HmHel-6	CAEZ01002944:93034-93161
HmHel-6	CAEZ01002944:91230-91420
HmHel-6	CAEZ01002944:37128-37247
HmHel-6	CAEZ01002744:10302-10510
HmHel-6	CAEZ01002744:25911-26031
HmHel-6	CAEZ01002706:8830-8914

HmHel-6	CAEZ01002706:47444-50055
HmHel-6	CAEZ01002706:98410-98522
HmHel-6	CAEZ01002706:109211-109298
HmHel-6	CAEZ01002706:106238-106445
HmHel-6	CAEZ01002706:74799-76461
HmHel-6	CAEZ01002706:51533-51634
HmHel-6	CAEZ01002217:1016-1114
HmHel-6	CAEZ01002217:28472-28707
HmHel-6	CAEZ01002217:42620-42797
HmHel-6	CAEZ01002217:46019-46115
HmHel-6	CAEZ01002217:22406-29452
HmHel-6	CAEZ01001594:9939-10130
HmHel-6	CAEZ01001594:7501-7705
HmHel-6	CAEZ01001557:9288-9417
HmHel-6	CAEZ01001557:25168-25261
HmHel-6	CAEZ01001557:30249-30424
HmHel-6	CAEZ01001557:11786-11976
HmHel-6	CAEZ01001022:1197-1324
HmHel-6	CAEZ01001022:17549-17768
HmHel-6	CAEZ01010925:50024-50106
HmHel-6	CAEZ01010925:58163-58402
HmHel-6	CAEZ01010925:13213-13293
HmHel-6	CAEZ01010874:12288-12381
HmHel-6	CAEZ01010874:7126-7249
HmHel-6	CAEZ01009822:10526-13009
HmHel-6	CAEZ01009822:34176-34362
HmHel-6	CAEZ01009117:942-1162
HmHel-6	CAEZ01009071:12791-13008
HmHel-6	CAEZ01009071:4742-4903
HmHel-6	CAEZ01008305:9715-9866
HmHel-6	CAEZ01008305:63855-64101
HmHel-6	CAEZ01008305:53448-53526
HmHel-6	CAEZ01008305:37459-37544
HmHel-6	CAEZ01008305:10901-20124
HmHel-6	CAEZ01008169:45450-45593
HmHel-6	CAEZ01008169:44712-44906
HmHel-6	CAEZ01008169:33973-34056
HmHel-6	CAEZ01007963:8082-8325
HmHel-6	CAEZ01007963:4553-4660
HmHel-6	CAEZ01007655:191-444
HmHel-6	CAEZ01007654:1546-1799
HmHel-6	CAEZ01006774:23608-23760
HmHel-6	CAEZ01006774:46531-46746
HmHel-6	CAEZ01006774:56785-56968
HmHel-6	CAEZ01006774:91913-92084
HmHel-6	CAEZ01006774:118917-123752
HmHel-6	CAEZ01006774:135946-140225
HmHel-6	CAEZ01006774:113589-113670

HmHel-6	CAEZ01006774:7791-14723
HmHel-6	CAEZ01006447:6946-7167
HmHel-6	CAEZ01006145:8555-8792
HmHel-6	CAEZ01006145:18235-18412
HmHel-6	CAEZ01006128:7317-7403
HmHel-6	CAEZ01006128:47269-47375
HmHel-6	CAEZ01006128:64623-64842
HmHel-6	CAEZ01006128:68638-77518
HmHel-6	CAEZ01006128:50372-50546
HmHel-6	CAEZ01006128:24362-26327
HmHel-6	CAEZ01005261:692-876
HmHel-6	CAEZ01005261:16887-17558
HmHel-6	CAEZ01004942:17565-24684
HmHel-6	CAEZ01004942:64450-64541
HmHel-6	CAEZ01004942:97519-97606
HmHel-6	CAEZ01004942:68697-76826
HmHel-6	CAEZ01004942:10554-10733
HmHel-6	CAEZ01004584:20882-21102
HmHel-6	CAEZ01004584:19299-19548
HmHel-6	CAEZ01004007:1315-1532
HmHel-6	CAEZ01004007:5512-15678
HmHel-6	CAEZ01001386:18563-23944
HmHel-6	CAEZ01001386:30561-30750
HmHel-6	CAEZ01001386:8953-18182
HmHel-6	CAEZ01011523:14053-14145
HmHel-6	CAEZ01011523:36789-36971
HmHel-6	CAEZ01011523:63021-63227
HmHel-6	CAEZ01011523:74937-75169
HmHel-6	CAEZ01011523:72683-72869
HmHel-6	CAEZ01011523:501-699
HmHel-6	CAEZ01011095:52376-52552
HmHel-6	CAEZ01011095:62941-63045
HmHel-6	CAEZ01011095:63527-68993
HmHel-6	CAEZ01011095:32979-33146
HmHel-6	CAEZ01010594:7554-7735
HmHel-6	CAEZ01010594:28571-28678
HmHel-6	CAEZ01010594:56737-65437
HmHel-6	CAEZ01010594:80237-85379
HmHel-6	CAEZ01010594:78487-78597
HmHel-6	CAEZ01010594:41822-50075
HmHel-6	CAEZ01010594:24210-24327
HmHel-6	CAEZ01009474:7382-7618
HmHel-6	CAEZ01009474:57345-65463
HmHel-6	CAEZ01009474:37536-43542
HmHel-6	CAEZ01009282:78351-78442
HmHel-6	CAEZ01009282:169243-169420
HmHel-6	CAEZ01009282:206546-214339
HmHel-6	CAEZ01009282:270793-270942

HmHel-6	CAEZ01009282:267766-267888
HmHel-6	CAEZ01009282:219757-219878
HmHel-6	CAEZ01009282:114545-114780
HmHel-6	CAEZ01009282:42716-42843
HmHel-6	CAEZ01009282:27411-27514
HmHel-6	CAEZ01008003:14353-14520
HmHel-6	CAEZ01008003:6141-6338
HmHel-6	CAEZ01007725:11138-11239
HmHel-6	CAEZ01007725:30067-30262
HmHel-6	CAEZ01007725:43253-43381
HmHel-6	CAEZ01007725:81108-87408
HmHel-6	CAEZ01007725:120960-121089
HmHel-6	CAEZ01007725:113452-114725
HmHel-6	CAEZ01007725:83043-86706
HmHel-6	CAEZ01007725:49981-50091
HmHel-6	CAEZ01006700:8466-8717
HmHel-6	CAEZ01006638:2983-3224
HmHel-6	CAEZ01006638:8728-18376
HmHel-6	CAEZ01006184:1840-2084
HmHel-6	CAEZ01005885:16721-16939
HmHel-6	CAEZ01005885:26706-34647
HmHel-6	CAEZ01005584:38904-44202
HmHel-6	CAEZ01005584:7651-7884
HmHel-6	CAEZ01004740:5823-6056
HmHel-6	CAEZ01004740:37264-37405
HmHel-6	CAEZ01004740:55054-55690
HmHel-6	CAEZ01004740:7335-7436
HmHel-6	CAEZ01003994:24536-27107
HmHel-6	CAEZ01003994:23888-24044
HmHel-6	CAEZ01003935:586-830
HmHel-6	CAEZ01003774:23421-23581
HmHel-6	CAEZ01003774:5303-5520
HmHel-6	CAEZ01003646:3244-3482
HmHel-6	CAEZ01003646:14100-14204
HmHel-6	CAEZ01003646:1531-2740
HmHel-6	CAEZ01003600:10188-10400
HmHel-6	CAEZ01003600:57366-57587
HmHel-6	CAEZ01003600:69435-69631
HmHel-6	CAEZ01003600:48504-50892
HmHel-6	CAEZ01003428:804-1800
HmHel-6	CAEZ01003428:9016-9197
HmHel-6	CAEZ01003212:14296-14508
HmHel-6	CAEZ01003212:4506-16572
HmHel-6	CAEZ01003100:14978-15076
HmHel-6	CAEZ01002759:2262-2371
HmHel-6	CAEZ01002759:30215-30340
HmHel-6	CAEZ01002759:10102-19755
HmHel-6	CAEZ01002338:3640-3887

HmHel-6	CAEZ01001002:7703-7875
HmHel-6	CAEZ01001002:6016-6236
HmHel-6	CAEZ01000536:53-277
HmHel-6	CAEZ01011579:4184-4429
HmHel-6	CAEZ01010864:2667-2868
HmHel-6	CAEZ01010864:46462-46623
HmHel-6	CAEZ01010864:87610-87831
HmHel-6	CAEZ01010864:140107-143703
HmHel-6	CAEZ01010864:175475-175560
HmHel-6	CAEZ01010864:188590-188694
HmHel-6	CAEZ01010864:218850-219355
HmHel-6	CAEZ01010864:204654-204818
HmHel-6	CAEZ01010864:156142-156230
HmHel-6	CAEZ01010864:139060-140377
HmHel-6	CAEZ01010864:64435-64627
HmHel-6	CAEZ01010864:14143-14239
HmHel-6	CAEZ01010663:1891-2097
HmHel-6	CAEZ01010663:23930-24170
HmHel-6	CAEZ01010584:39704-41755
HmHel-6	CAEZ01010584:61428-61542
HmHel-6	CAEZ01010584:78897-78978
HmHel-6	CAEZ01010584:114394-114651
HmHel-6	CAEZ01010584:150920-151001
HmHel-6	CAEZ01010584:215649-215748
HmHel-6	CAEZ01010584:232908-233159
HmHel-6	CAEZ01010584:261769-261906
HmHel-6	CAEZ01010584:284528-284709
HmHel-6	CAEZ01010584:303729-313401
HmHel-6	CAEZ01010584:294368-294530
HmHel-6	CAEZ01010584:272063-272274
HmHel-6	CAEZ01010584:232000-232183
HmHel-6	CAEZ01010584:216372-216586
HmHel-6	CAEZ01010584:144487-144610
HmHel-6	CAEZ01010584:132720-132816
HmHel-6	CAEZ01010584:78441-78538
HmHel-6	CAEZ01010584:42178-42281
HmHel-6	CAEZ01010584:4695-7033
HmHel-6	CAEZ01010361:21840-21966
HmHel-6	CAEZ01010361:32918-33158
HmHel-6	CAEZ01010361:67931-68182
HmHel-6	CAEZ01010361:74736-74840
HmHel-6	CAEZ01010361:57955-60093
HmHel-6	CAEZ01010361:40433-40638
HmHel-6	CAEZ01010150:971-2585
HmHel-6	CAEZ01010150:23678-23826
HmHel-6	CAEZ01010150:36861-37063
HmHel-6	CAEZ01010150:16799-17007
HmHel-6	CAEZ01010083:2107-4609

HmHel-6	CAEZ01010083:6371-6629
HmHel-6	CAEZ01009975:24060-24260
HmHel-6	CAEZ01009975:43643-44353
HmHel-6	CAEZ01009975:58009-58993
HmHel-6	CAEZ01009975:95319-95429
HmHel-6	CAEZ01009975:115071-115208
HmHel-6	CAEZ01009975:132385-132597
HmHel-6	CAEZ01009975:98575-98664
HmHel-6	CAEZ01009975:78650-78782
HmHel-6	CAEZ01009975:43076-50678
HmHel-6	CAEZ01009455:29665-29896
HmHel-6	CAEZ01009455:47952-48084
HmHel-6	CAEZ01009455:28209-31445
HmHel-6	CAEZ01009419:24404-24493
HmHel-6	CAEZ01009318:2114-2337
HmHel-6	CAEZ01009136:93232-93339
HmHel-6	CAEZ01009136:139129-142286
HmHel-6	CAEZ01009136:170220-170420
HmHel-6	CAEZ01009136:159981-160088
HmHel-6	CAEZ01009136:135929-136140
HmHel-6	CAEZ01009136:117372-117461
HmHel-6	CAEZ01009136:95728-104527
HmHel-6	CAEZ01009136:66127-66310
HmHel-6	CAEZ01009136:40377-40509
HmHel-6	CAEZ01008867:6120-6284
HmHel-6	CAEZ01008867:12972-13101
HmHel-6	CAEZ01008508:4522-5552
HmHel-6	CAEZ01008508:28538-28668
HmHel-6	CAEZ01008508:21886-21998
HmHel-6	CAEZ01008508:7293-7502
HmHel-6	CAEZ01008292:1895-2094
HmHel-6	CAEZ01008292:24065-27919
HmHel-6	CAEZ01008292:38834-42067
HmHel-6	CAEZ01008292:2963-3099
HmHel-6	CAEZ01008264:3148-3357
HmHel-6	CAEZ01008264:13417-13538
HmHel-6	CAEZ01008200:4599-4823
HmHel-6	CAEZ01007676:15989-18068
HmHel-6	CAEZ01007676:34523-34610
HmHel-6	CAEZ01007676:49492-49687
HmHel-6	CAEZ01007676:94905-95026
HmHel-6	CAEZ01007676:118500-118759
HmHel-6	CAEZ01007676:181871-182007
HmHel-6	CAEZ01007676:150711-150946
HmHel-6	CAEZ01007676:95348-98257
HmHel-6	CAEZ01007676:39738-39978
HmHel-6	CAEZ01006959:7242-7490
HmHel-6	CAEZ01006959:12154-12295

HmHel-6	CAEZ01006898:6680-6917
HmHel-6	CAEZ01006898:1473-1670
HmHel-6	CAEZ01006692:3824-4044
HmHel-6	CAEZ01006692:16574-16814
HmHel-6	CAEZ01006659:641-3527
HmHel-6	CAEZ01006659:7864-10606
HmHel-6	CAEZ01006402:9341-9557
HmHel-6	CAEZ01006402:36833-36926
HmHel-6	CAEZ01006402:28826-29017
HmHel-6	CAEZ01006402:11314-11549
HmHel-6	CAEZ01005946:2340-2582
HmHel-6	CAEZ01005946:17287-17417
HmHel-6	CAEZ01005946:45493-45573
HmHel-6	CAEZ01005946:64900-65042
HmHel-6	CAEZ01005946:110114-114849
HmHel-6	CAEZ01005946:146051-146140
HmHel-6	CAEZ01005946:107181-107361
HmHel-6	CAEZ01005946:83406-84760
HmHel-6	CAEZ01005946:52336-52527
HmHel-6	CAEZ01005946:19182-19320
HmHel-6	CAEZ01005946:6946-7167
HmHel-6	CAEZ01005538:10851-10939
HmHel-6	CAEZ01005538:6470-7997
HmHel-6	CAEZ01004813:515-5576
HmHel-6	CAEZ01004813:22446-22657
HmHel-6	CAEZ01004813:49352-49529
HmHel-6	CAEZ01004813:69464-69678
HmHel-6	CAEZ01004813:70089-73140
HmHel-6	CAEZ01004596:3551-3793
HmHel-6	CAEZ01004240:6206-6440
HmHel-6	CAEZ01004240:5740-5945
HmHel-6	CAEZ01002803:23267-23491
HmHel-6	CAEZ01002310:498-742
HmHel-6	CAEZ01001309:21677-21860
HmHel-6	CAEZ01001309:27567-28897
HmHel-6	CAEZ01001309:2152-2364
HmHel-6	CAEZ01000821:143-383
HmHel-6	CAEZ01000821:3910-4048
HmHel-6	CAEZ01011276:6015-6201
HmHel-6	CAEZ01011276:37073-37181
HmHel-6	CAEZ01011276:22206-22334
HmHel-6	CAEZ01011276:11643-11761
HmHel-6	CAEZ01011227:18439-18572
HmHel-6	CAEZ01011227:33842-34021
HmHel-6	CAEZ01011227:28286-28469
HmHel-6	CAEZ01011227:7075-7283
HmHel-6	CAEZ01011130:3660-3862
HmHel-6	CAEZ01011130:2378-2587

HmHel-6	CAEZ01011033:5042-5249
HmHel-6	CAEZ01011033:7675-7773
HmHel-6	CAEZ01010714:7026-7119
HmHel-6	CAEZ01010714:6046-6241
HmHel-6	CAEZ01010142:7300-7516
HmHel-6	CAEZ01010142:2903-3119
HmHel-6	CAEZ01010013:20464-20640
HmHel-6	CAEZ01010013:23027-25233
HmHel-6	CAEZ01009880:7006-7240
HmHel-6	CAEZ01009396:487-670
HmHel-6	CAEZ01009396:9194-9392
HmHel-6	CAEZ01008961:8287-17243
HmHel-6	CAEZ01008961:61381-61594
HmHel-6	CAEZ01008961:24590-33122
HmHel-6	CAEZ01008961:11739-11966
HmHel-6	CAEZ01008718:24-222
HmHel-6	CAEZ01008718:21849-24319
HmHel-6	CAEZ01008718:39088-39259
HmHel-6	CAEZ01007420:2588-2779
HmHel-6	CAEZ01006960:6930-7176
HmHel-6	CAEZ01006960:55968-56128
HmHel-6	CAEZ01006960:32730-32919
HmHel-6	CAEZ01006960:11899-12109
HmHel-6	CAEZ01006826:6333-9905
HmHel-6	CAEZ01006720:6541-6712
HmHel-6	CAEZ01006146:4610-4865
HmHel-6	CAEZ01006146:954-1047
HmHel-6	CAEZ01006021:16022-16126
HmHel-6	CAEZ01006021:37187-37375
HmHel-6	CAEZ01006021:38881-39092
HmHel-6	CAEZ01006021:5456-5577
HmHel-6	CAEZ01005695:14701-14779
HmHel-6	CAEZ01005695:9474-9660
HmHel-6	CAEZ01005364:5377-5582
HmHel-6	CAEZ01005364:3607-3814
HmHel-6	CAEZ01005321:11206-11444
HmHel-6	CAEZ01005321:34453-34676
HmHel-6	CAEZ01005321:22033-22175
HmHel-6	CAEZ01005321:8280-8365
HmHel-6	CAEZ01004688:11425-11678
HmHel-6	CAEZ01004688:8645-10382
HmHel-6	CAEZ01003783:31125-35276
HmHel-6	CAEZ01003783:41714-41793
HmHel-6	CAEZ01003783:21398-21613
HmHel-6	CAEZ01003236:15841-16024
HmHel-6	CAEZ01003236:658-908
HmHel-6	CAEZ01000867:257-965
HmHel-6	CAEZ01000867:47937-48104

HmHel-6	CAEZ01000867:96850-97049
HmHel-6	CAEZ01000867:36441-36615
HmHel-6	CAEZ01000867:17227-17363
HmHel-6	CAEZ01000867:2570-2715
HmHel-6	CAEZ01010875:6915-9314
HmHel-6	CAEZ01010875:31789-31951
HmHel-6	CAEZ01010875:8537-8747
HmHel-6	CAEZ01010695:39345-39548
HmHel-6	CAEZ01010695:49067-49268
HmHel-6	CAEZ01010695:25722-25839
HmHel-6	CAEZ01010695:14570-14813
HmHel-6	CAEZ01010648:9947-10205
HmHel-6	CAEZ01010648:10506-10617
HmHel-6	CAEZ01010449:55823-56039
HmHel-6	CAEZ01010449:92488-92585
HmHel-6	CAEZ01010449:74683-77490
HmHel-6	CAEZ01009925:6610-6853
HmHel-6	CAEZ01009925:25469-25553
HmHel-6	CAEZ01009925:11330-11428
HmHel-6	CAEZ01009769:9915-19112
HmHel-6	CAEZ01009769:40937-41119
HmHel-6	CAEZ01009769:45219-45345
HmHel-6	CAEZ01009631:18691-18895
HmHel-6	CAEZ01009631:43973-44100
HmHel-6	CAEZ01009631:59069-65305
HmHel-6	CAEZ01009631:62062-70458
HmHel-6	CAEZ01009631:24676-24869
HmHel-6	CAEZ01009631:4702-4919
HmHel-6	CAEZ01009425:12946-13699
HmHel-6	CAEZ01008723:4141-4278
HmHel-6	CAEZ01008723:35862-35983
HmHel-6	CAEZ01008547:2852-2932
HmHel-6	CAEZ01008547:2036-2276
HmHel-6	CAEZ01007912:38814-38959
HmHel-6	CAEZ01007912:129505-129740
HmHel-6	CAEZ01007912:111415-111519
HmHel-6	CAEZ01007912:68697-68875
HmHel-6	CAEZ01007912:27212-32002
HmHel-6	CAEZ01006779:18571-18813
HmHel-6	CAEZ01004938:36271-36477
HmHel-6	CAEZ01004562:52827-53014
HmHel-6	CAEZ01004562:83664-85426
HmHel-6	CAEZ01004562:97225-97471
HmHel-6	CAEZ01004562:120094-120206
HmHel-6	CAEZ01004562:158827-158913
HmHel-6	CAEZ01004562:213250-213360
HmHel-6	CAEZ01004562:208393-208533
HmHel-6	CAEZ01004562:145676-145848

HmHel-6	CAEZ01004562:121237-121364
HmHel-6	CAEZ01004562:90440-90542
HmHel-6	CAEZ01004562:49400-49533
HmHel-6	CAEZ01004562:23339-23446
HmHel-6	CAEZ01003009:1229-1475
HmHel-6	CAEZ01002414:3315-3439
HmHel-6	CAEZ01002414:34908-34995
HmHel-6	CAEZ01002414:65340-65437
HmHel-6	CAEZ01002414:104454-104565
HmHel-6	CAEZ01002414:132162-132364
HmHel-6	CAEZ01002414:155168-155253
HmHel-6	CAEZ01002414:138221-138463
HmHel-6	CAEZ01002414:101168-110264
HmHel-6	CAEZ01002414:89880-90007
HmHel-6	CAEZ01002414:46799-46913
HmHel-6	CAEZ01002414:10032-17494
HmHel-6	CAEZ01001599:1449-1632
HmHel-6	CAEZ01001599:8961-9129
HmHel-6	CAEZ01001435:39388-39604
HmHel-6	CAEZ01001435:60852-61065
HmHel-6	CAEZ01001435:135437-135551
HmHel-6	CAEZ01001435:155405-164312
HmHel-6	CAEZ01001435:211065-211282
HmHel-6	CAEZ01001435:224717-224831
HmHel-6	CAEZ01001435:198713-198915
HmHel-6	CAEZ01001435:176050-176209
HmHel-6	CAEZ01001435:157065-157188
HmHel-6	CAEZ01001435:129587-129746
HmHel-6	CAEZ01001435:85541-85640
HmHel-6	CAEZ01001435:61719-61858
HmHel-6	CAEZ01001435:24256-24396
HmHel-6	CAEZ01001211:942-1224
HmHel-6	CAEZ01000854:14422-14665
HmHel-6	CAEZ01000854:27864-28125
HmHel-6	CAEZ01000854:83314-84463
HmHel-6	CAEZ01000854:90111-90284
HmHel-6	CAEZ01000854:70186-70308
HmHel-6	CAEZ01000854:34545-34628
HmHel-6	CAEZ01011521:10692-16396
HmHel-6	CAEZ01011521:4854-5079
HmHel-6	CAEZ01010939:3041-3222
HmHel-6	CAEZ01010057:2492-2739
HmHel-6	CAEZ01010057:5995-6187
HmHel-6	CAEZ01009824:2305-2475
HmHel-6	CAEZ01009824:49624-49730
HmHel-6	CAEZ01009824:69825-70066
HmHel-6	CAEZ01009824:31183-33813
HmHel-6	CAEZ01009717:35288-35521

HmHel-6	CAEZ01009717:33365-33623
HmHel-6	CAEZ01009717:19860-19939
HmHel-6	CAEZ01009334:19133-19237
HmHel-6	CAEZ01009334:4741-4950
HmHel-6	CAEZ01009262:11453-11671
HmHel-6	CAEZ01009262:19129-19389
HmHel-6	CAEZ01009162:1388-1585
HmHel-6	CAEZ01008920:8060-8288
HmHel-6	CAEZ01008361:2774-2924
HmHel-6	CAEZ01008361:14751-14976
HmHel-6	CAEZ01008016:569-804
HmHel-6	CAEZ01007620:23191-23433
HmHel-6	CAEZ01007545:25132-32602
HmHel-6	CAEZ01007545:52883-52980
HmHel-6	CAEZ01007545:80019-80250
HmHel-6	CAEZ01007545:121187-121274
HmHel-6	CAEZ01007545:124204-124340
HmHel-6	CAEZ01007545:99725-99809
HmHel-6	CAEZ01007545:59501-62403
HmHel-6	CAEZ01007545:22121-22352
HmHel-6	CAEZ01006725:3020-3230
HmHel-6	CAEZ01006542:98209-98302
HmHel-6	CAEZ01006542:120796-123738
HmHel-6	CAEZ01006542:177708-177857
HmHel-6	CAEZ01006542:199861-200114
HmHel-6	CAEZ01006542:147118-147252
HmHel-6	CAEZ01006542:108993-109075
HmHel-6	CAEZ01006542:63220-64569
HmHel-6	CAEZ01006542:17489-17597
HmHel-6	CAEZ01005982:4551-4661
HmHel-6	CAEZ01005982:10884-11104
HmHel-6	CAEZ01005854:5028-5120
HmHel-6	CAEZ01005854:3472-3705
HmHel-6	CAEZ01005809:2221-5002
HmHel-6	CAEZ01005809:7373-7726
HmHel-6	CAEZ01004918:29170-29392
HmHel-6	CAEZ01004918:70503-70734
HmHel-6	CAEZ01004918:37829-37948
HmHel-6	CAEZ01004918:25456-25641
HmHel-6	CAEZ01004359:12010-13667
HmHel-6	CAEZ01004359:9801-10019
HmHel-6	CAEZ01003834:11550-11637
HmHel-6	CAEZ01003834:18906-19151
HmHel-6	CAEZ01003279:13358-13483
HmHel-6	CAEZ01003279:45130-45261
HmHel-6	CAEZ01003279:30306-30429
HmHel-6	CAEZ01003204:11604-11848
HmHel-6	CAEZ01001714:8266-8412

HmHel-6	CAEZ01001714:2125-2334
HmHel-6	CAEZ01001265:83155-83290
HmHel-6	CAEZ01001265:103897-104034
HmHel-6	CAEZ01001265:114856-115024
HmHel-6	CAEZ01001265:68948-69704
HmHel-6	CAEZ01001265:3806-3888
HmHel-6	CAEZ01000717:8116-8363
HmHel-6	CAEZ01000717:6009-6180
HmHel-6	CAEZ01010811:8840-9062
HmHel-6	CAEZ01010811:14686-14863
HmHel-6	CAEZ01010725:3853-4067
HmHel-6	CAEZ01010725:23145-23333
HmHel-6	CAEZ01010725:43709-43795
HmHel-6	CAEZ01010725:33219-33422
HmHel-6	CAEZ01010725:12033-12119
HmHel-6	CAEZ01010543:13143-13391
HmHel-6	CAEZ01010414:3151-3331
HmHel-6	CAEZ01010389:7838-8714
HmHel-6	CAEZ01010389:7577-7822
HmHel-6	CAEZ01010078:12401-12499
HmHel-6	CAEZ01010078:23830-23960
HmHel-6	CAEZ01010078:40835-41068
HmHel-6	CAEZ01009893:11812-15211
HmHel-6	CAEZ01009893:14591-20748
HmHel-6	CAEZ01009598:6302-6467
HmHel-6	CAEZ01009598:10775-11007
HmHel-6	CAEZ01009115:3051-6044
HmHel-6	CAEZ01009115:23990-24074
HmHel-6	CAEZ01009115:998-1238
HmHel-6	CAEZ01009082:1349-1470
HmHel-6	CAEZ01009082:14450-14662
HmHel-6	CAEZ01008779:19240-19332
HmHel-6	CAEZ01008779:3883-4040
HmHel-6	CAEZ01008516:1605-7051
HmHel-6	CAEZ01008516:25485-25686
HmHel-6	CAEZ01008516:3661-4508
HmHel-6	CAEZ01008427:4749-4843
HmHel-6	CAEZ01008427:28110-28233
HmHel-6	CAEZ01008427:57601-57805
HmHel-6	CAEZ01008427:79261-79447
HmHel-6	CAEZ01008427:112557-112768
HmHel-6	CAEZ01008427:116365-116499
HmHel-6	CAEZ01008427:34667-34928
HmHel-6	CAEZ01007779:184-7298
HmHel-6	CAEZ01007779:3284-3503
HmHel-6	CAEZ01007584:7706-7827
HmHel-6	CAEZ01007584:53319-53546
HmHel-6	CAEZ01007584:93690-98846

HmHel-6	CAEZ01007584:187969-188098
HmHel-6	CAEZ01007584:199442-199633
HmHel-6	CAEZ01007584:128849-132318
HmHel-6	CAEZ01007584:40340-40419
HmHel-6	CAEZ01006989:12963-13098
HmHel-6	CAEZ01006989:27038-33174
HmHel-6	CAEZ01006669:8265-8506
HmHel-6	CAEZ01006669:10530-10746
HmHel-6	CAEZ01006019:4735-4941
HmHel-6	CAEZ01006019:17242-17393
HmHel-6	CAEZ01005174:30681-30928
HmHel-6	CAEZ01005174:11917-12040
HmHel-6	CAEZ01004827:21530-21706
HmHel-6	CAEZ01004452:1634-1818
HmHel-6	CAEZ01004452:27579-27712
HmHel-6	CAEZ01004452:45905-45986
HmHel-6	CAEZ01004452:36727-36929
HmHel-6	CAEZ01004142:3625-3761
HmHel-6	CAEZ01004142:22983-23236
HmHel-6	CAEZ01004142:16725-16854
HmHel-6	CAEZ01004142:5524-5639
HmHel-6	CAEZ01003932:5132-5370
HmHel-6	CAEZ01003932:1636-8595
HmHel-6	CAEZ01003134:10273-10526
HmHel-6	CAEZ01002875:28431-37514
HmHel-6	CAEZ01002875:72594-72703
HmHel-6	CAEZ01002875:92497-92659
HmHel-6	CAEZ01002875:196926-197174
HmHel-6	CAEZ01002875:241639-241850
HmHel-6	CAEZ01002875:228223-228438
HmHel-6	CAEZ01002875:217508-217726
HmHel-6	CAEZ01002875:172159-172288
HmHel-6	CAEZ01002875:103337-103519
HmHel-6	CAEZ01002875:68261-76789
HmHel-6	CAEZ01002875:40814-40922
HmHel-6	CAEZ01002875:10804-12217
HmHel-6	CAEZ01000883:2052-2289
HmHel-6	CAEZ01000754:6852-7096
HmHel-6	CAEZ01011617:11469-11552
HmHel-6	CAEZ01011617:14409-14527
HmHel-6	CAEZ01011540:12287-12448
HmHel-6	CAEZ01011540:17084-17173
HmHel-6	CAEZ01010917:20828-22774
HmHel-6	CAEZ01010917:28975-29184
HmHel-6	CAEZ01009843:816-3157
HmHel-6	CAEZ01009843:47225-47471
HmHel-6	CAEZ01009843:61650-71439
HmHel-6	CAEZ01009843:61813-61898

HmHel-6	CAEZ01009843:35789-42720
HmHel-6	CAEZ01009681:13419-19445
HmHel-6	CAEZ01009681:16406-16593
HmHel-6	CAEZ01009420:21411-21594
HmHel-6	CAEZ01009420:33580-37578
HmHel-6	CAEZ01009420:34556-34713
HmHel-6	CAEZ01009359:12998-15697
HmHel-6	CAEZ01009359:37592-37818
HmHel-6	CAEZ01009359:7301-7412
HmHel-6	CAEZ01009349:1316-1559
HmHel-6	CAEZ01008598:25061-25299
HmHel-6	CAEZ01008598:5161-5373
HmHel-6	CAEZ01008345:1682-1837
HmHel-6	CAEZ01008345:25821-29543
HmHel-6	CAEZ01008345:40402-44899
HmHel-6	CAEZ01008345:89997-90205
HmHel-6	CAEZ01008345:88813-88980
HmHel-6	CAEZ01008345:71825-71934
HmHel-6	CAEZ01008345:35230-35376
HmHel-6	CAEZ01007998:14770-14869
HmHel-6	CAEZ01006877:17817-17931
HmHel-6	CAEZ01006877:27007-27092
HmHel-6	CAEZ01006754:7736-7982
HmHel-6	CAEZ01006413:1663-1887
HmHel-6	CAEZ01006413:84189-84450
HmHel-6	CAEZ01006413:71393-72698
HmHel-6	CAEZ01006413:38006-42432
HmHel-6	CAEZ01006413:11491-11672
HmHel-6	CAEZ01006332:40126-40265
HmHel-6	CAEZ01006332:62902-63022
HmHel-6	CAEZ01006332:86384-86487
HmHel-6	CAEZ01006332:74581-74819
HmHel-6	CAEZ01006332:9098-23928
HmHel-6	CAEZ01006095:11708-18776
HmHel-6	CAEZ01006095:50111-50340
HmHel-6	CAEZ01006095:12645-12815
HmHel-6	CAEZ01005976:59119-59329
HmHel-6	CAEZ01005976:96112-96274
HmHel-6	CAEZ01005976:81341-81552
HmHel-6	CAEZ01005976:56244-56347
HmHel-6	CAEZ01005976:26734-28744
HmHel-6	CAEZ01005748:6945-7061
HmHel-6	CAEZ01005748:14238-14413
HmHel-6	CAEZ01005425:11159-15604
HmHel-6	CAEZ01005425:15687-15940
HmHel-6	CAEZ01004588:3131-3337
HmHel-6	CAEZ01004588:3961-4133
HmHel-6	CAEZ01004218:23153-23290

HmHel-6	CAEZ01004218:25192-32281
HmHel-6	CAEZ01004218:12189-12385
HmHel-6	CAEZ01003708:4555-4746
HmHel-6	CAEZ01003708:193-324
HmHel-6	CAEZ01003074:884-991
HmHel-6	CAEZ01003074:20428-20636
HmHel-6	CAEZ01003074:6058-6147
HmHel-6	CAEZ01002792:18576-24485
HmHel-6	CAEZ01002792:42745-44023
HmHel-6	CAEZ01002680:40090-40268
HmHel-6	CAEZ01002680:51182-51396
HmHel-6	CAEZ01002680:13295-23379
HmHel-6	CAEZ01001771:9840-9925
HmHel-6	CAEZ01001103:2359-5917
HmHel-6	CAEZ01001103:29299-29406
HmHel-6	CAEZ01001103:26301-26418
HmHel-6	CAEZ01001103:10400-10661
HmHel-6	CAEZ01010181:24624-27386
HmHel-6	CAEZ01010181:56566-56718
HmHel-6	CAEZ01010181:69348-76121
HmHel-6	CAEZ01010181:80982-81062
HmHel-6	CAEZ01010181:62263-62445
HmHel-6	CAEZ01010181:38579-44609
HmHel-6	CAEZ01009983:16688-16791
HmHel-6	CAEZ01009436:4329-14445
HmHel-6	CAEZ01009436:33878-34062
HmHel-6	CAEZ01009436:86128-86347
HmHel-6	CAEZ01009436:87887-97003
HmHel-6	CAEZ01009436:61354-61587
HmHel-6	CAEZ01009436:21702-24050
HmHel-6	CAEZ01009376:3154-8352
HmHel-6	CAEZ01008392:11537-11787
HmHel-6	CAEZ01008392:34400-34511
HmHel-6	CAEZ01008392:27195-42249
HmHel-6	CAEZ01008350:19875-20046
HmHel-6	CAEZ01008350:48143-48251
HmHel-6	CAEZ01008350:71124-71329
HmHel-6	CAEZ01008350:104357-105366
HmHel-6	CAEZ01008350:87418-87670
HmHel-6	CAEZ01007752:26063-26270
HmHel-6	CAEZ01007752:55267-55472
HmHel-6	CAEZ01007752:81681-81762
HmHel-6	CAEZ01007752:104614-104785
HmHel-6	CAEZ01007752:117346-117481
HmHel-6	CAEZ01007752:136814-136978
HmHel-6	CAEZ01007752:127113-133606
HmHel-6	CAEZ01007752:111042-111126
HmHel-6	CAEZ01007752:82462-83560

HmHel-6	CAEZ01007752:47071-47191
HmHel-6	CAEZ01007463:5721-5928
HmHel-6	CAEZ01007463:1807-9013
HmHel-6	CAEZ01006310:950-1200
HmHel-6	CAEZ01006308:17376-17531
HmHel-6	CAEZ01006308:8588-14485
HmHel-6	CAEZ01006222:2741-2979
HmHel-6	CAEZ01005192:5382-5589
HmHel-6	CAEZ01004923:8083-8183
HmHel-6	CAEZ01004923:56098-56306
HmHel-6	CAEZ01004923:83787-93379
HmHel-6	CAEZ01004923:60227-67224
HmHel-6	CAEZ01004818:10999-19139
HmHel-6	CAEZ01004818:46629-46833
HmHel-6	CAEZ01004818:81805-81905
HmHel-6	CAEZ01004818:97385-97529
HmHel-6	CAEZ01004818:134339-134543
HmHel-6	CAEZ01004818:153436-153646
HmHel-6	CAEZ01004818:160403-160512
HmHel-6	CAEZ01004818:129769-131397
HmHel-6	CAEZ01004818:92754-92935
HmHel-6	CAEZ01004818:70087-72415
HmHel-6	CAEZ01004818:9543-9757
HmHel-6	CAEZ01003928:7052-7255
HmHel-6	CAEZ01003475:3424-3508
HmHel-6	CAEZ01002330:6534-6665
HmHel-6	CAEZ01002330:24149-24355
HmHel-6	CAEZ01002330:25068-25240
HmHel-6	CAEZ01002330:6728-6918
HmHel-6	CAEZ01001353:8970-9140
HmHel-6	CAEZ01000846:5368-6115
HmHel-6	CAEZ01000846:26301-26476
HmHel-6	CAEZ01000061:34172-34534
HmHel-6	CAEZ01000061:57220-57323
HmHel-6	CAEZ01000061:7762-8002
HmHel-6	CAEZ01010139:26764-27015
HmHel-6	CAEZ01010139:56993-57214
HmHel-6	CAEZ01010139:80355-80470
HmHel-6	CAEZ01010139:120128-120308
HmHel-6	CAEZ01010139:85877-86116
HmHel-6	CAEZ01010139:72904-73024
HmHel-6	CAEZ01010139:55996-56185
HmHel-6	CAEZ01010139:42811-42950
HmHel-6	CAEZ01009562:20144-21960
HmHel-6	CAEZ01009562:27606-31539
HmHel-6	CAEZ01009294:2807-3020
HmHel-6	CAEZ01009291:7289-7399
HmHel-6	CAEZ01009291:21051-21300

HmHel-6	CAEZ01009291:28920-29078
HmHel-6	CAEZ01009291:5704-5812
HmHel-6	CAEZ01009268:3869-4066
HmHel-6	CAEZ01009268:31461-31720
HmHel-6	CAEZ01009268:70373-70451
HmHel-6	CAEZ01009268:84758-84996
HmHel-6	CAEZ01009268:66759-66857
HmHel-6	CAEZ01009268:27689-27950
HmHel-6	CAEZ01009237:7718-7907
HmHel-6	CAEZ01009237:15641-15855
HmHel-6	CAEZ01009237:396-602
HmHel-6	CAEZ01008437:11007-11087
HmHel-6	CAEZ01008437:42370-42547
HmHel-6	CAEZ01008437:71162-71304
HmHel-6	CAEZ01008437:89519-89645
HmHel-6	CAEZ01008437:144382-144555
HmHel-6	CAEZ01008437:132340-132591
HmHel-6	CAEZ01008437:97568-97741
HmHel-6	CAEZ01008437:49700-49896
HmHel-6	CAEZ01008437:7961-12143
HmHel-6	CAEZ01008263:4677-4798
HmHel-6	CAEZ01008263:17196-17343
HmHel-6	CAEZ01008263:31211-31323
HmHel-6	CAEZ01008263:21184-21379
HmHel-6	CAEZ01008202:801-2898
HmHel-6	CAEZ01008202:15921-19491
HmHel-6	CAEZ01008202:47174-47267
HmHel-6	CAEZ01008202:26838-27040
HmHel-6	CAEZ01008035:15959-16212
HmHel-6	CAEZ01007450:36753-36950
HmHel-6	CAEZ01007450:25510-25628
HmHel-6	CAEZ01007450:6191-10856
HmHel-6	CAEZ01007001:12263-12472
HmHel-6	CAEZ01007001:21072-21262
HmHel-6	CAEZ01006874:4444-10201
HmHel-6	CAEZ01006874:5324-5445
HmHel-6	CAEZ01006312:63-292
HmHel-6	CAEZ01006312:14982-15128
HmHel-6	CAEZ01005240:1374-1579
HmHel-6	CAEZ01005240:3183-3268
HmHel-6	CAEZ01004344:442-616
HmHel-6	CAEZ01004344:14737-14877
HmHel-6	CAEZ01003260:562-697
HmHel-6	CAEZ01003260:38578-38708
HmHel-6	CAEZ01003260:86192-86442
HmHel-6	CAEZ01003260:113790-113994
HmHel-6	CAEZ01003260:95824-101224
HmHel-6	CAEZ01003260:70526-70745

HmHel-6	CAEZ01003260:28195-28315
HmHel-6	CAEZ01003260:4976-10161
HmHel-6	CAEZ01001789:2296-2530
HmHel-6	CAEZ01001070:4693-11961
HmHel-6	CAEZ01001070:51949-52114
HmHel-6	CAEZ01001070:69489-69583
HmHel-6	CAEZ01001070:94423-94536
HmHel-6	CAEZ01001070:129496-137491
HmHel-6	CAEZ01001070:156973-157061
HmHel-6	CAEZ01001070:229573-229749
HmHel-6	CAEZ01001070:244588-248602
HmHel-6	CAEZ01001070:136759-136903
HmHel-6	CAEZ01001070:122187-122372
HmHel-6	CAEZ01001070:95042-95174
HmHel-6	CAEZ01001070:68862-69042
HmHel-6	CAEZ01001070:9386-9466
HmHel-6	CAEZ01010796:13803-13892
HmHel-6	CAEZ01010796:86253-86461
HmHel-6	CAEZ01010796:110717-110911
HmHel-6	CAEZ01010796:100024-100135
HmHel-6	CAEZ01010796:84764-85003
HmHel-6	CAEZ01010796:59851-60003
HmHel-6	CAEZ01010796:11097-11318
HmHel-6	CAEZ01010273:1150-1400
HmHel-6	CAEZ01010273:39911-39993
HmHel-6	CAEZ01010273:50918-51105
HmHel-6	CAEZ01010273:70938-71153
HmHel-6	CAEZ01010273:91934-92136
HmHel-6	CAEZ01010273:82821-83254
HmHel-6	CAEZ01010273:51111-53744
HmHel-6	CAEZ01010273:16827-17039
HmHel-6	CAEZ01010008:17231-17408
HmHel-6	CAEZ01010008:35778-35991
HmHel-6	CAEZ01010008:10063-10220
HmHel-6	CAEZ01009940:1568-1756
HmHel-6	CAEZ01009926:1210-10053
HmHel-6	CAEZ01009926:3158-3283
HmHel-6	CAEZ01009816:27563-27791
HmHel-6	CAEZ01009816:1015-1222
HmHel-6	CAEZ01008184:1361-1591
HmHel-6	CAEZ01007810:1672-1759
HmHel-6	CAEZ01007810:56703-56863
HmHel-6	CAEZ01007810:70530-70726
HmHel-6	CAEZ01007810:109869-109949
HmHel-6	CAEZ01007810:107470-107630
HmHel-6	CAEZ01007810:52163-60607
HmHel-6	CAEZ01007810:30660-30773
HmHel-6	CAEZ01007713:58473-58685

HmHel-6	CAEZ01007713:33840-33975
HmHel-6	CAEZ01007706:83358-83496
HmHel-6	CAEZ01007706:106691-106901
HmHel-6	CAEZ01007706:130602-130723
HmHel-6	CAEZ01007706:148844-148962
HmHel-6	CAEZ01007706:270164-279464
HmHel-6	CAEZ01007706:280113-280211
HmHel-6	CAEZ01007706:255379-263999
HmHel-6	CAEZ01007706:230839-231061
HmHel-6	CAEZ01007706:191092-191190
HmHel-6	CAEZ01007706:134178-140320
HmHel-6	CAEZ01007706:110711-110904
HmHel-6	CAEZ01007706:82667-82809
HmHel-6	CAEZ01007706:51460-51688
HmHel-6	CAEZ01007706:3652-11115
HmHel-6	CAEZ01007464:9093-10991
HmHel-6	CAEZ01007464:6340-6569
HmHel-6	CAEZ01007260:2434-2674
HmHel-6	CAEZ01007260:10626-15231
HmHel-6	CAEZ01006732:4515-4740
HmHel-6	CAEZ01006038:93-273
HmHel-6	CAEZ01005228:12428-12608
HmHel-6	CAEZ01005228:814-923
HmHel-6	CAEZ01005085:18241-18448
HmHel-6	CAEZ01005053:11172-11298
HmHel-6	CAEZ01005053:28654-28863
HmHel-6	CAEZ01003871:1548-1797
HmHel-6	CAEZ01003871:28019-28144
HmHel-6	CAEZ01003871:30488-30609
HmHel-6	CAEZ01003871:7362-7450
HmHel-6	CAEZ01003038:1450-1686
HmHel-6	CAEZ01003038:16868-16988
HmHel-6	CAEZ01001766:23768-23995
HmHel-6	CAEZ01001766:70132-77115
HmHel-6	CAEZ01001766:107155-107266
HmHel-6	CAEZ01001766:192548-192728
HmHel-6	CAEZ01001766:198605-201764
HmHel-6	CAEZ01001766:183497-183596
HmHel-6	CAEZ01001766:126728-130600
HmHel-6	CAEZ01001766:95056-95211
HmHel-6	CAEZ01001766:70949-71081
HmHel-6	CAEZ01001766:38448-38655
HmHel-6	CAEZ01001766:24935-25179
HmHel-6	CAEZ01000074:12018-12193
HmHel-6	CAEZ01000074:8925-9104
HmHel-6	CAEZ01011209:151-358
HmHel-6	CAEZ01010453:50801-51008
HmHel-6	CAEZ01010453:92028-95937

HmHel-6	CAEZ01010453:141981-142122
HmHel-6	CAEZ01010453:97136-98978
HmHel-6	CAEZ01010453:27006-27171
HmHel-6	CAEZ01009437:20240-20456
HmHel-6	CAEZ01009437:64573-64766
HmHel-6	CAEZ01009437:50260-54903
HmHel-6	CAEZ01009437:30764-31660
HmHel-6	CAEZ01009338:14110-14245
HmHel-6	CAEZ01009338:9674-9898
HmHel-6	CAEZ01009337:5368-5576
HmHel-6	CAEZ01008627:780-1024
HmHel-6	CAEZ01008586:1546-1762
HmHel-6	CAEZ01008586:6571-6792
HmHel-6	CAEZ01007925:37932-38052
HmHel-6	CAEZ01007925:53908-54099
HmHel-6	CAEZ01007925:54964-55174
HmHel-6	CAEZ01007925:28223-32720
HmHel-6	CAEZ01007925:4261-4448
HmHel-6	CAEZ01007636:6058-11361
HmHel-6	CAEZ01006392:5999-13843
HmHel-6	CAEZ01006392:66834-67439
HmHel-6	CAEZ01006392:77474-77557
HmHel-6	CAEZ01006392:95582-95810
HmHel-6	CAEZ01006392:103554-103749
HmHel-6	CAEZ01006392:76808-76965
HmHel-6	CAEZ01006392:59556-59667
HmHel-6	CAEZ01006325:11738-11985
HmHel-6	CAEZ01006325:27758-28002
HmHel-6	CAEZ01005897:5675-5836
HmHel-6	CAEZ01005897:17384-17565
HmHel-6	CAEZ01005287:1103-9185
HmHel-6	CAEZ01004826:9866-10109
HmHel-6	CAEZ01004826:87794-87995
HmHel-6	CAEZ01004826:96293-96476
HmHel-6	CAEZ01004826:7671-7827
HmHel-6	CAEZ01003934:980-1236
HmHel-6	CAEZ01003365:3434-3645
HmHel-6	CAEZ01003365:8402-8498
HmHel-6	CAEZ01002805:6313-6527
HmHel-6	CAEZ01000850:3402-3603
HmHel-6	CAEZ01000850:104464-104582
HmHel-6	CAEZ01000850:68098-68300
HmHel-6	CAEZ01010598:20448-20658
HmHel-6	CAEZ01010138:11123-19845
HmHel-6	CAEZ01010138:23918-24104
HmHel-6	CAEZ01010032:17782-17947
HmHel-6	CAEZ01010032:30881-31091
HmHel-6	CAEZ01009614:6780-6990

HmHel-6	CAEZ01009614:43298-44995
HmHel-6	CAEZ01009614:59624-60980
HmHel-6	CAEZ01009614:25911-26079
HmHel-6	CAEZ01008602:8968-9193
HmHel-6	CAEZ01008502:7441-7697
HmHel-6	CAEZ01007879:2186-2374
HmHel-6	CAEZ01007879:15023-22263
HmHel-6	CAEZ01007879:22306-22445
HmHel-6	CAEZ01007863:15876-15999
HmHel-6	CAEZ01007863:36619-39115
HmHel-6	CAEZ01007863:59661-59836
HmHel-6	CAEZ01007863:109236-109351
HmHel-6	CAEZ01007863:131732-131823
HmHel-6	CAEZ01007863:169760-169889
HmHel-6	CAEZ01007863:120714-124022
HmHel-6	CAEZ01007863:106703-110405
HmHel-6	CAEZ01007863:76587-76790
HmHel-6	CAEZ01007863:54238-54404
HmHel-6	CAEZ01007863:22759-22841
HmHel-6	CAEZ01007828:1035-1257
HmHel-6	CAEZ01007814:12059-12281
HmHel-6	CAEZ01007395:9106-9288
HmHel-6	CAEZ01007395:37708-37917
HmHel-6	CAEZ01007395:50098-50218
HmHel-6	CAEZ01007395:312-491
HmHel-6	CAEZ01006619:12698-12905
HmHel-6	CAEZ01006619:9018-9167
HmHel-6	CAEZ01006561:33093-35167
HmHel-6	CAEZ01006561:78082-78290
HmHel-6	CAEZ01006561:95031-95119
HmHel-6	CAEZ01006561:114079-114194
HmHel-6	CAEZ01006561:105154-105280
HmHel-6	CAEZ01006561:86688-86841
HmHel-6	CAEZ01006561:75335-75514
HmHel-6	CAEZ01006561:34290-39450
HmHel-6	CAEZ01006561:2776-7545
HmHel-6	CAEZ01004541:83965-84173
HmHel-6	CAEZ01004541:133766-133897
HmHel-6	CAEZ01004541:112815-112995
HmHel-6	CAEZ01004541:49233-49457
HmHel-6	CAEZ01004541:37718-37976
HmHel-6	CAEZ01004541:14090-14198
HmHel-6	CAEZ01004027:13033-13139
HmHel-6	CAEZ01004027:9320-9530
HmHel-6	CAEZ01003923:2852-3064
HmHel-6	CAEZ01003923:19017-19265
HmHel-6	CAEZ01003842:926-1023
HmHel-6	CAEZ01003842:41964-42186

HmHel-6	CAEZ01003842:40607-40745
HmHel-6	CAEZ01002888:856-1057
HmHel-6	CAEZ01002736:23869-24100
HmHel-6	CAEZ01002624:11672-11803
HmHel-6	CAEZ01002624:954-1132
HmHel-6	CAEZ01001868:2590-2827
HmHel-6	CAEZ01001868:26734-26993
HmHel-6	CAEZ01001868:40591-40804
HmHel-6	CAEZ01001868:28578-28673
HmHel-6	CAEZ01001868:10604-10730
HmHel-6	CAEZ01001216:6808-7009
HmHel-6	CAEZ01001216:18708-18869
HmHel-6	CAEZ01001216:8702-8867
HmHel-6	CAEZ01000072:14538-14695
HmHel-6	CAEZ01000072:18086-18486
HmHel-6	CAEZ01011369:5184-5440
HmHel-6	CAEZ01011369:16061-16189
HmHel-6	CAEZ01011268:4709-4870
HmHel-6	CAEZ01011260:5648-5765
HmHel-6	CAEZ01010827:53298-53568
HmHel-6	CAEZ01010827:76513-82980
HmHel-6	CAEZ01010827:128722-128813
HmHel-6	CAEZ01010827:165581-165810
HmHel-6	CAEZ01010827:220409-220566
HmHel-6	CAEZ01010827:300395-306496
HmHel-6	CAEZ01010827:336829-341712
HmHel-6	CAEZ01010827:361841-362024
HmHel-6	CAEZ01010827:397160-397305
HmHel-6	CAEZ01010827:469754-469877
HmHel-6	CAEZ01010827:441113-445259
HmHel-6	CAEZ01010827:356812-356978
HmHel-6	CAEZ01010827:302981-303080
HmHel-6	CAEZ01010827:285902-285992
HmHel-6	CAEZ01010827:221669-221896
HmHel-6	CAEZ01010827:195651-195765
HmHel-6	CAEZ01010827:153745-153834
HmHel-6	CAEZ01010827:125608-125774
HmHel-6	CAEZ01010827:98878-99010
HmHel-6	CAEZ01010827:33144-33261
HmHel-6	CAEZ01010822:13204-13340
HmHel-6	CAEZ01010822:14201-14414
HmHel-6	CAEZ01010601:7899-8115
HmHel-6	CAEZ01010601:6944-7027
HmHel-6	CAEZ01009567:3812-4035
HmHel-6	CAEZ01009567:10338-10444
HmHel-6	CAEZ01009452:330-500
HmHel-6	CAEZ01009452:57040-57226
HmHel-6	CAEZ01009452:98499-98694

HmHel-6	CAEZ01009452:83404-91374
HmHel-6	CAEZ01009452:1279-4105
HmHel-6	CAEZ01009317:8332-8521
HmHel-6	CAEZ01009288:311-528
HmHel-6	CAEZ01009288:48771-51387
HmHel-6	CAEZ01008403:9889-15127
HmHel-6	CAEZ01008403:76089-76297
HmHel-6	CAEZ01008403:91686-91895
HmHel-6	CAEZ01007678:771-1000
HmHel-6	CAEZ01007678:22376-30007
HmHel-6	CAEZ01007678:15914-24065
HmHel-6	CAEZ01007312:5489-5696
HmHel-6	CAEZ01007312:8890-8971
HmHel-6	CAEZ01006400:10100-10564
HmHel-6	CAEZ01006400:24616-24791
HmHel-6	CAEZ01006400:38621-38718
HmHel-6	CAEZ01006400:63046-71147
HmHel-6	CAEZ01006400:68231-68358
HmHel-6	CAEZ01006400:42906-43170
HmHel-6	CAEZ01006400:32401-32560
HmHel-6	CAEZ01006400:11775-11856
HmHel-6	CAEZ01006400:402-483
HmHel-6	CAEZ01006152:5639-5837
HmHel-6	CAEZ01006152:4392-4484
HmHel-6	CAEZ01006126:916-1061
HmHel-6	CAEZ01006126:20960-21062
HmHel-6	CAEZ01006126:35304-35404
HmHel-6	CAEZ01005927:1772-1929
HmHel-6	CAEZ01005927:19534-19627
HmHel-6	CAEZ01005927:31764-31920
HmHel-6	CAEZ01005927:12416-16219
HmHel-6	CAEZ01005833:11853-11993
HmHel-6	CAEZ01005833:27564-27751
HmHel-6	CAEZ01005833:30627-30755
HmHel-6	CAEZ01005833:18567-18808
HmHel-6	CAEZ01005833:3970-4089
HmHel-6	CAEZ01005825:7293-12896
HmHel-6	CAEZ01005825:49931-50096
HmHel-6	CAEZ01005825:9176-17615
HmHel-6	CAEZ01005198:80550-80664
HmHel-6	CAEZ01005198:118960-119049
HmHel-6	CAEZ01005198:58204-58432
HmHel-6	CAEZ01005198:41010-41183
HmHel-6	CAEZ01005198:1709-1822
HmHel-6	CAEZ01005191:2119-2367
HmHel-6	CAEZ01004479:42702-42919
HmHel-6	CAEZ01004479:55242-55387
HmHel-6	CAEZ01004314:4112-4324

HmHel-6	CAEZ01004122:1491-5804
HmHel-6	CAEZ01004122:15897-16149
HmHel-6	CAEZ01003738:13326-13512
HmHel-6	CAEZ01003485:11115-11372
HmHel-6	CAEZ01003485:3476-9618
HmHel-6	CAEZ01002729:56788-56906
HmHel-6	CAEZ01002729:37085-37179
HmHel-6	CAEZ01002099:17145-17393
HmHel-6	CAEZ01002099:39396-39602
HmHel-6	CAEZ01001512:11539-11752
HmHel-6	CAEZ01001457:7627-7791
HmHel-6	CAEZ01001457:10555-10688
HmHel-6	CAEZ01001338:25475-25559
HmHel-6	CAEZ01001338:60892-62719
HmHel-6	CAEZ01001338:123091-124368
HmHel-6	CAEZ01001338:131691-131895
HmHel-6	CAEZ01001338:100123-100334
HmHel-6	CAEZ01001338:53897-56934
HmHel-6	CAEZ01001338:16155-16363
HmHel-6	CAEZ01000037:652-3527
HmHel-6	CAEZ01000037:29248-29346
HmHel-6	CAEZ01000037:47557-47814
HmHel-6	CAEZ01000037:68012-68120
HmHel-6	CAEZ01000037:69447-73870
HmHel-6	CAEZ01000037:21976-22063
HmHel-6	CAEZ01011298:921-1028
HmHel-6	CAEZ01011298:5403-5528
HmHel-6	CAEZ01010863:35959-36042
HmHel-6	CAEZ01010863:47364-47516
HmHel-6	CAEZ01010863:36524-36682
HmHel-6	CAEZ01010863:7298-7468
HmHel-6	CAEZ01010752:15923-16036
HmHel-6	CAEZ01010752:34703-34825
HmHel-6	CAEZ01010752:10449-19581
HmHel-6	CAEZ01010499:10469-10722
HmHel-6	CAEZ01010499:5558-5669
HmHel-6	CAEZ01009813:32766-32892
HmHel-6	CAEZ01009813:45538-59767
HmHel-6	CAEZ01009813:79905-80085
HmHel-6	CAEZ01009813:51821-52000
HmHel-6	CAEZ01009813:19863-20066
HmHel-6	CAEZ01009813:4138-4250
HmHel-6	CAEZ01009503:64555-64798
HmHel-6	CAEZ01008963:61664-61810
HmHel-6	CAEZ01008963:91822-98233
HmHel-6	CAEZ01008963:141347-149000
HmHel-6	CAEZ01008963:169185-169305
HmHel-6	CAEZ01008963:197322-197439

HmHel-6	CAEZ01008963:196353-204313
HmHel-6	CAEZ01008963:161574-161766
HmHel-6	CAEZ01008963:128875-129008
HmHel-6	CAEZ01008963:116591-116704
HmHel-6	CAEZ01008963:103009-103128
HmHel-6	CAEZ01008963:85823-85984
HmHel-6	CAEZ01008963:72418-72610
HmHel-6	CAEZ01008963:19815-20030
HmHel-6	CAEZ01008849:352-478
HmHel-6	CAEZ01008849:26462-31934
HmHel-6	CAEZ01008849:32890-40748
HmHel-6	CAEZ01008849:20059-20143
HmHel-6	CAEZ01008706:4236-4420
HmHel-6	CAEZ01008706:11146-11352
HmHel-6	CAEZ01008327:40523-40725
HmHel-6	CAEZ01008327:55726-55978
HmHel-6	CAEZ01008327:65075-65193
HmHel-6	CAEZ01008327:11379-11498
HmHel-6	CAEZ01007739:29828-30005
HmHel-6	CAEZ01007739:60980-61114
HmHel-6	CAEZ01007739:89637-92546
HmHel-6	CAEZ01007739:83709-96336
HmHel-6	CAEZ01007739:25649-25760
HmHel-6	CAEZ01007572:8234-8342
HmHel-6	CAEZ01007572:20643-20829
HmHel-6	CAEZ01007208:8983-9116
HmHel-6	CAEZ01007208:24804-34005
HmHel-6	CAEZ01007208:26173-26394
HmHel-6	CAEZ01007208:6145-6384
HmHel-6	CAEZ01006529:36968-37201
HmHel-6	CAEZ01006529:65094-65254
HmHel-6	CAEZ01006529:98649-98801
HmHel-6	CAEZ01006529:131833-131936
HmHel-6	CAEZ01006529:165994-166147
HmHel-6	CAEZ01006529:190715-190941
HmHel-6	CAEZ01006529:216211-216312
HmHel-6	CAEZ01006529:226979-227126
HmHel-6	CAEZ01006529:108913-109054
HmHel-6	CAEZ01006529:90176-90375
HmHel-6	CAEZ01006529:58684-58828
HmHel-6	CAEZ01006183:1821-2048
HmHel-6	CAEZ01005929:3996-4091
HmHel-6	CAEZ01005929:5710-5951
HmHel-6	CAEZ01005732:8555-8783
HmHel-6	CAEZ01005732:30901-31111
HmHel-6	CAEZ01005732:23063-23289
HmHel-6	CAEZ01005706:13879-20705
HmHel-6	CAEZ01004392:225-393

HmHel-6	CAEZ01003944:2030-2265
HmHel-6	CAEZ01003768:280-3023
HmHel-6	CAEZ01003768:19090-19281
HmHel-6	CAEZ01003768:55540-55656
HmHel-6	CAEZ01003768:67797-67995
HmHel-6	CAEZ01003768:85583-85677
HmHel-6	CAEZ01003768:69610-69733
HmHel-6	CAEZ01003109:7399-10339
HmHel-6	CAEZ01003109:30013-30223
HmHel-6	CAEZ01003109:66758-73066
HmHel-6	CAEZ01003109:85120-85231
HmHel-6	CAEZ01003109:61192-70764
HmHel-6	CAEZ01003109:9223-9455
HmHel-6	CAEZ01002779:9328-9411
HmHel-6	CAEZ01002779:33790-33946
HmHel-6	CAEZ01002779:49996-50230
HmHel-6	CAEZ01002779:71743-71954
HmHel-6	CAEZ01002779:86416-86508
HmHel-6	CAEZ01002779:52760-52977
HmHel-6	CAEZ01002779:36441-36522
HmHel-6	CAEZ01002779:22363-24150
HmHel-6	CAEZ01002158:3764-3928
HmHel-6	CAEZ01001537:4004-4249
HmHel-6	CAEZ01001537:17786-17876
HmHel-6	CAEZ01001537:14700-14931
HmHel-6	CAEZ01011169:2590-2830
HmHel-6	CAEZ01010870:30501-30683
HmHel-6	CAEZ01010870:27873-28103
HmHel-6	CAEZ01010617:3351-3553
HmHel-6	CAEZ01010617:14263-14457
HmHel-6	CAEZ01010617:48302-48464
HmHel-6	CAEZ01010617:30329-30525
HmHel-6	CAEZ01010487:3504-3699
HmHel-6	CAEZ01010487:4834-4961
HmHel-6	CAEZ01010484:1416-1618
HmHel-6	CAEZ01010484:30726-30816
HmHel-6	CAEZ01010256:6030-10447
HmHel-6	CAEZ01010256:25910-30687
HmHel-6	CAEZ01010256:15077-15185
HmHel-6	CAEZ01010050:5567-5766
HmHel-6	CAEZ01010050:1293-4590
HmHel-6	CAEZ01009367:3690-9034
HmHel-6	CAEZ01009367:20547-20736
HmHel-6	CAEZ01009081:5857-6037
HmHel-6	CAEZ01009081:52270-55482
HmHel-6	CAEZ01009081:44560-44708
HmHel-6	CAEZ01009081:19168-19386
HmHel-6	CAEZ01009015:12702-12938

HmHel-6	CAEZ01008826:16533-16740
HmHel-6	CAEZ01008226:17798-17891
HmHel-6	CAEZ01008125:28539-28662
HmHel-6	CAEZ01008125:50966-51216
HmHel-6	CAEZ01008125:103244-103454
HmHel-6	CAEZ01008125:119539-124030
HmHel-6	CAEZ01008125:136978-137114
HmHel-6	CAEZ01008125:185788-185871
HmHel-6	CAEZ01008125:181490-181570
HmHel-6	CAEZ01008125:156495-156700
HmHel-6	CAEZ01008125:77144-77223
HmHel-6	CAEZ01008125:66259-66479
HmHel-6	CAEZ01008125:23309-23428
HmHel-6	CAEZ01007582:2529-2698
HmHel-6	CAEZ01007582:18994-19081
HmHel-6	CAEZ01007494:5429-5625
HmHel-6	CAEZ01007494:33143-40740
HmHel-6	CAEZ01007494:33089-35947
HmHel-6	CAEZ01007494:14591-14779
HmHel-6	CAEZ01007118:1376-1470
HmHel-6	CAEZ01007118:19435-19689
HmHel-6	CAEZ01006919:25103-25391
HmHel-6	CAEZ01006919:35873-36124
HmHel-6	CAEZ01006804:73030-73152
HmHel-6	CAEZ01006804:132240-132479
HmHel-6	CAEZ01006804:146502-146717
HmHel-6	CAEZ01006804:206242-206338
HmHel-6	CAEZ01006804:150067-150171
HmHel-6	CAEZ01006804:125241-125340
HmHel-6	CAEZ01006804:57407-58316
HmHel-6	CAEZ01006804:19004-19124
HmHel-6	CAEZ01006804:4743-4898
HmHel-6	CAEZ01006534:25972-26468
HmHel-6	CAEZ01006534:44639-44825
HmHel-6	CAEZ01006534:61322-61430
HmHel-6	CAEZ01006534:102377-105261
HmHel-6	CAEZ01006534:74200-74376
HmHel-6	CAEZ01006534:45423-45674
HmHel-6	CAEZ01006534:17313-17415
HmHel-6	CAEZ01006185:25017-26721
HmHel-6	CAEZ01006185:21612-21811
HmHel-6	CAEZ01006073:2324-2532
HmHel-6	CAEZ01006073:50677-50897
HmHel-6	CAEZ01006073:64968-65174
HmHel-6	CAEZ01006073:69601-69833
HmHel-6	CAEZ01004829:7769-8820
HmHel-6	CAEZ01004829:44497-44641
HmHel-6	CAEZ01004829:55018-55227

HmHel-6	CAEZ01004829:11547-15922
HmHel-6	CAEZ01004828:5308-5527
HmHel-6	CAEZ01004741:9302-9407
HmHel-6	CAEZ01004741:25436-25538
HmHel-6	CAEZ01004741:10007-14704
HmHel-6	CAEZ01004561:18694-18940
HmHel-6	CAEZ01003710:6625-6824
HmHel-6	CAEZ01003664:5169-5350
HmHel-6	CAEZ01003664:3918-4137
HmHel-6	CAEZ01003548:11704-11835
HmHel-6	CAEZ01003548:26774-28747
HmHel-6	CAEZ01003548:13073-13181
HmHel-6	CAEZ01003370:8255-8446
HmHel-6	CAEZ01003370:4169-4275
HmHel-6	CAEZ01003276:26504-26691
HmHel-6	CAEZ01003276:52228-52395
HmHel-6	CAEZ01002413:5393-8945
HmHel-6	CAEZ01002413:81697-81944
HmHel-6	CAEZ01002413:53068-55868
HmHel-6	CAEZ01002413:13641-16890
HmHel-6	CAEZ01002286:10175-10341
HmHel-6	CAEZ01002286:26164-26412
HmHel-6	CAEZ01002286:13274-16565
HmHel-6	CAEZ01001799:1966-2125
HmHel-6	CAEZ01011612:10185-10436
HmHel-6	CAEZ01011612:25544-25712
HmHel-6	CAEZ01011612:30695-30897
HmHel-6	CAEZ01011612:20115-20257
HmHel-6	CAEZ01011612:1026-1208
HmHel-6	CAEZ01010262:4842-5037
HmHel-6	CAEZ01009814:500-662
HmHel-6	CAEZ01009465:14570-14688
HmHel-6	CAEZ01009465:47204-47342
HmHel-6	CAEZ01009465:90607-90731
HmHel-6	CAEZ01009465:101242-101346
HmHel-6	CAEZ01009465:61308-71273
HmHel-6	CAEZ01009465:50834-50988
HmHel-6	CAEZ01009465:33936-34127
HmHel-6	CAEZ01009465:6652-6803
HmHel-6	CAEZ01007223:2041-2214
HmHel-6	CAEZ01007143:4384-4618
HmHel-6	CAEZ01006992:4445-14011
HmHel-6	CAEZ01006889:2966-7733
HmHel-6	CAEZ01006889:9677-11063
HmHel-6	CAEZ01006873:21961-22167
HmHel-6	CAEZ01006656:8251-8411
HmHel-6	CAEZ01006656:13164-13416
HmHel-6	CAEZ01005964:10252-10454

HmHel-6	CAEZ01005964:91932-92080
HmHel-6	CAEZ01005964:106970-107206
HmHel-6	CAEZ01005964:104436-104516
HmHel-6	CAEZ01005964:75750-75850
HmHel-6	CAEZ01005964:21178-26039
HmHel-6	CAEZ01005962:6319-6549
HmHel-6	CAEZ01005962:2080-2245
HmHel-6	CAEZ01005925:661-871
HmHel-6	CAEZ01005925:18723-18808
HmHel-6	CAEZ01005925:20436-20639
HmHel-6	CAEZ01005877:21932-22181
HmHel-6	CAEZ01005638:9287-9473
HmHel-6	CAEZ01005638:3058-11513
HmHel-6	CAEZ01005451:20017-20188
HmHel-6	CAEZ01005451:54778-54918
HmHel-6	CAEZ01005451:90281-90362
HmHel-6	CAEZ01005451:40911-42895
HmHel-6	CAEZ01005451:16955-17362
HmHel-6	CAEZ01005451:2548-2639
HmHel-6	CAEZ01005204:7087-7261
HmHel-6	CAEZ01004400:9925-10069
HmHel-6	CAEZ01004400:20982-21111
HmHel-6	CAEZ01003954:24395-24544
HmHel-6	CAEZ01003954:43451-46977
HmHel-6	CAEZ01003954:38279-38443
HmHel-6	CAEZ01003954:16175-16373
HmHel-6	CAEZ01003754:13693-13924
HmHel-6	CAEZ01003754:4274-5069
HmHel-6	CAEZ01003746:24163-24331
HmHel-6	CAEZ01003746:66195-75170
HmHel-6	CAEZ01003746:86290-86550
HmHel-6	CAEZ01003746:75716-75830
HmHel-6	CAEZ01003746:40394-40596
HmHel-6	CAEZ01003593:3403-3632
HmHel-6	CAEZ01003593:2453-5274
HmHel-6	CAEZ01003195:9984-10064
HmHel-6	CAEZ01003195:22679-22806
HmHel-6	CAEZ01003195:89333-94919
HmHel-6	CAEZ01003195:60880-61096
HmHel-6	CAEZ01003195:19765-19853
HmHel-6	CAEZ01003167:8853-11503
HmHel-6	CAEZ01003167:29043-29168
HmHel-6	CAEZ01000971:1472-1596
HmHel-6	CAEZ01000971:26586-26799
HmHel-6	CAEZ01000971:2576-2769
HmHel-6	CAEZ01000847:3155-3357
HmHel-6	CAEZ01011020:1551-1683
HmHel-6	CAEZ01011020:5982-6186

HmHel-6	CAEZ01010270:9468-9559
HmHel-6	CAEZ01010270:27591-27723
HmHel-6	CAEZ01010270:65690-65967
HmHel-6	CAEZ01010270:151560-151667
HmHel-6	CAEZ01010270:188876-192279
HmHel-6	CAEZ01010270:225015-229589
HmHel-6	CAEZ01010270:265842-266016
HmHel-6	CAEZ01010270:278031-278140
HmHel-6	CAEZ01010270:289846-289933
HmHel-6	CAEZ01010270:277236-281248
HmHel-6	CAEZ01010270:245671-249538
HmHel-6	CAEZ01010270:206544-206628
HmHel-6	CAEZ01010270:188366-188469
HmHel-6	CAEZ01010270:168934-169061
HmHel-6	CAEZ01010270:135001-135231
HmHel-6	CAEZ01010270:74292-74433
HmHel-6	CAEZ01010270:50956-51068
HmHel-6	CAEZ01010270:38987-39144
HmHel-6	CAEZ01010059:4208-4314
HmHel-6	CAEZ01010059:7731-7929
HmHel-6	CAEZ01008277:1279-17254
HmHel-6	CAEZ01008277:29787-29987
HmHel-6	CAEZ01008277:36443-36654
HmHel-6	CAEZ01007390:9639-9767
HmHel-6	CAEZ01007390:19295-19400
HmHel-6	CAEZ01006733:1007-1163
HmHel-6	CAEZ01006733:2522-7189
HmHel-6	CAEZ01006710:4402-4623
HmHel-6	CAEZ01006710:6367-6600
HmHel-6	CAEZ01006068:10342-10503
HmHel-6	CAEZ01006068:9760-12615
HmHel-6	CAEZ01005670:52732-52916
HmHel-6	CAEZ01005670:51557-55175
HmHel-6	CAEZ01005578:9333-9514
HmHel-6	CAEZ01005578:28437-28562
HmHel-6	CAEZ01005511:34866-34989
HmHel-6	CAEZ01005511:21485-21694
HmHel-6	CAEZ01005511:7297-8972
HmHel-6	CAEZ01005449:7094-7259
HmHel-6	CAEZ01005262:12437-12647
HmHel-6	CAEZ01005262:24888-25831
HmHel-6	CAEZ01005262:11596-11736
HmHel-6	CAEZ01004832:28068-28189
HmHel-6	CAEZ01004832:61413-64936
HmHel-6	CAEZ01004832:97916-98037
HmHel-6	CAEZ01004832:52516-52601
HmHel-6	CAEZ01004832:40787-40984
HmHel-6	CAEZ01001564:1620-1766

HmHel-6	CAEZ01001564:17609-27532
HmHel-6	CAEZ01001564:58226-58458
HmHel-6	CAEZ01001564:37276-37404
HmHel-6	CAEZ01001564:8844-9056
HmHel-6	CAEZ01001301:30244-30328
HmHel-6	CAEZ01001301:40862-41095
HmHel-6	CAEZ01001301:15737-15827
HmHel-6	CAEZ01001089:3288-3390
HmHel-6	CAEZ01001089:22102-22212
HmHel-6	CAEZ01001089:40838-40992
HmHel-6	CAEZ01001089:43126-43338
HmHel-6	CAEZ01001089:14082-14203
HmHel-6	CAEZ01011269:1192-1279
HmHel-6	CAEZ01011269:18169-18275
HmHel-6	CAEZ01011269:20142-26204
HmHel-6	CAEZ01010329:10009-10205
HmHel-6	CAEZ01010329:14434-14526
HmHel-6	CAEZ01010199:14991-15159
HmHel-6	CAEZ01010199:33785-34001
HmHel-6	CAEZ01010199:12103-12196
HmHel-6	CAEZ01009867:5450-13001
HmHel-6	CAEZ01009867:20752-24373
HmHel-6	CAEZ01009242:7882-8055
HmHel-6	CAEZ01009242:46775-46912
HmHel-6	CAEZ01009242:67419-67648
HmHel-6	CAEZ01009242:89383-89605
HmHel-6	CAEZ01009242:31517-34616
HmHel-6	CAEZ01009242:2650-2843
HmHel-6	CAEZ01008736:4212-4395
HmHel-6	CAEZ01008736:61728-63312
HmHel-6	CAEZ01008736:59449-62680
HmHel-6	CAEZ01008736:30526-30709
HmHel-6	CAEZ01007817:2445-2618
HmHel-6	CAEZ01007817:2174-7746
HmHel-6	CAEZ01007557:11455-11701
HmHel-6	CAEZ01007557:9620-9795
HmHel-6	CAEZ01007446:14950-15039
HmHel-6	CAEZ01007446:36245-36406
HmHel-6	CAEZ01007446:36869-36979
HmHel-6	CAEZ01007353:1266-1466
HmHel-6	CAEZ01007230:1257-2846
HmHel-6	CAEZ01007230:14941-19605
HmHel-6	CAEZ01006598:4548-4706
HmHel-6	CAEZ01006598:15854-16021
HmHel-6	CAEZ01006417:592-689
HmHel-6	CAEZ01006417:22118-22230
HmHel-6	CAEZ01006417:35555-39321
HmHel-6	CAEZ01006417:110251-114986

HmHel-6	CAEZ01006417:155251-155340
HmHel-6	CAEZ01006417:170237-170323
HmHel-6	CAEZ01006417:177870-178017
HmHel-6	CAEZ01006417:160932-161061
HmHel-6	CAEZ01006417:99567-103098
HmHel-6	CAEZ01006417:31705-36941
HmHel-6	CAEZ01006417:867-1081
HmHel-6	CAEZ01006309:6008-7410
HmHel-6	CAEZ01006125:12919-13004
HmHel-6	CAEZ01006125:26327-26573
HmHel-6	CAEZ01006125:11235-11324
HmHel-6	CAEZ01005941:11155-11381
HmHel-6	CAEZ01005941:4276-4420
HmHel-6	CAEZ01005580:24461-24564
HmHel-6	CAEZ01005580:22438-22650
HmHel-6	CAEZ01005580:3924-4019
HmHel-6	CAEZ01004877:1036-1208
HmHel-6	CAEZ01004271:3099-3308
HmHel-6	CAEZ01004271:53679-53904
HmHel-6	CAEZ01004271:57321-58158
HmHel-6	CAEZ01002876:4934-5032
HmHel-6	CAEZ01002876:44457-44721
HmHel-6	CAEZ01002876:86967-87052
HmHel-6	CAEZ01002876:140632-140791
HmHel-6	CAEZ01002876:130608-136054
HmHel-6	CAEZ01002876:68236-68319
HmHel-6	CAEZ01002876:11213-11422
HmHel-6	CAEZ01002430:6515-6698
HmHel-6	CAEZ01002430:18995-21086
HmHel-6	CAEZ01002430:28995-29167
HmHel-6	CAEZ01002323:9887-10115
HmHel-6	CAEZ01002323:44842-44932
HmHel-6	CAEZ01002323:66511-66669
HmHel-6	CAEZ01002323:44061-52692
HmHel-6	CAEZ01002323:24704-28084
HmHel-6	CAEZ01002323:6605-6806
HmHel-6	CAEZ01002138:3676-7576
HmHel-6	CAEZ01002138:12025-12252
HmHel-6	CAEZ01010406:6805-6894
HmHel-6	CAEZ01010406:8205-8431
HmHel-6	CAEZ01010387:10510-10595
HmHel-6	CAEZ01010387:28755-29839
HmHel-6	CAEZ01010387:4586-4790
HmHel-6	CAEZ01009996:7541-7707
HmHel-6	CAEZ01009996:20523-20651
HmHel-6	CAEZ01009996:38847-44733
HmHel-6	CAEZ01009996:38222-38460
HmHel-6	CAEZ01009823:6085-7870

HmHel-6	CAEZ01009823:23064-23184
HmHel-6	CAEZ01009823:36087-43107
HmHel-6	CAEZ01009678:1213-1294
HmHel-6	CAEZ01009678:5436-5636
HmHel-6	CAEZ01009487:50339-50531
HmHel-6	CAEZ01009487:83017-83234
HmHel-6	CAEZ01009487:79368-79461
HmHel-6	CAEZ01009487:46277-46365
HmHel-6	CAEZ01009487:22905-29083
HmHel-6	CAEZ01007922:3989-4195
HmHel-6	CAEZ01007922:15801-15947
HmHel-6	CAEZ01007853:3502-18526
HmHel-6	CAEZ01007853:53440-53561
HmHel-6	CAEZ01007853:72373-72476
HmHel-6	CAEZ01007853:105635-112757
HmHel-6	CAEZ01007853:104385-104557
HmHel-6	CAEZ01007853:48172-48281
HmHel-6	CAEZ01007853:18036-18205
HmHel-6	CAEZ01007342:7186-7428
HmHel-6	CAEZ01007272:4495-6370
HmHel-6	CAEZ01007272:32443-41327
HmHel-6	CAEZ01007272:68907-69008
HmHel-6	CAEZ01007272:84354-84521
HmHel-6	CAEZ01007272:65605-66956
HmHel-6	CAEZ01007272:52016-52262
HmHel-6	CAEZ01007272:4995-12542
HmHel-6	CAEZ01006755:8315-8493
HmHel-6	CAEZ01006755:10212-10379
HmHel-6	CAEZ01006533:28391-28642
HmHel-6	CAEZ01006533:24819-24939
HmHel-6	CAEZ01006533:13467-13665
HmHel-6	CAEZ01004889:12939-13091
HmHel-6	CAEZ01004889:13760-13943
HmHel-6	CAEZ01004094:13460-13578
HmHel-6	CAEZ01004094:32493-32573
HmHel-6	CAEZ01004094:48299-48410
HmHel-6	CAEZ01004094:101819-102027
HmHel-6	CAEZ01004094:102773-102872
HmHel-6	CAEZ01004094:87063-87298
HmHel-6	CAEZ01004094:67962-68209
HmHel-6	CAEZ01004094:39028-43466
HmHel-6	CAEZ01004094:16055-16214
HmHel-6	CAEZ01004094:5802-5921
HmHel-6	CAEZ01003189:2202-2413
HmHel-6	CAEZ01003189:14200-14410
HmHel-6	CAEZ01002806:17578-17809
HmHel-6	CAEZ01001645:5976-6198
HmHel-6	CAEZ01001645:20533-20765

HmHel-6	CAEZ01001385:2921-3154
HmHel-6	CAEZ01001383:549-752
HmHel-6	CAEZ01000930:3143-3334
HmHel-6	CAEZ01011394:8572-8815
HmHel-6	CAEZ01011394:10912-11115
HmHel-6	CAEZ01010603:13541-13755
HmHel-6	CAEZ01010396:7348-7594
HmHel-6	CAEZ01010396:6575-8516
HmHel-6	CAEZ01010130:7858-7961
HmHel-6	CAEZ01010130:23571-23717
HmHel-6	CAEZ01010130:39942-42708
HmHel-6	CAEZ01010130:61247-61403
HmHel-6	CAEZ01010130:124941-125131
HmHel-6	CAEZ01010130:158846-159013
HmHel-6	CAEZ01010130:114299-114451
HmHel-6	CAEZ01010130:89607-95380
HmHel-6	CAEZ01010130:73010-73181
HmHel-6	CAEZ01010130:46858-46998
HmHel-6	CAEZ01010130:8803-8910
HmHel-6	CAEZ01009853:534-755
HmHel-6	CAEZ01009025:18064-18143
HmHel-6	CAEZ01009025:33615-33829
HmHel-6	CAEZ01009025:44799-46032
HmHel-6	CAEZ01009025:50638-50847
HmHel-6	CAEZ01009025:31438-31520
HmHel-6	CAEZ01008896:6319-6480
HmHel-6	CAEZ01008896:4999-5225
HmHel-6	CAEZ01008822:1399-1614
HmHel-6	CAEZ01008822:35870-36057
HmHel-6	CAEZ01008822:46318-54456
HmHel-6	CAEZ01008822:60973-61191
HmHel-6	CAEZ01008719:36170-36267
HmHel-6	CAEZ01008719:69145-69238
HmHel-6	CAEZ01008719:7233-7405
HmHel-6	CAEZ01008570:8685-18122
HmHel-6	CAEZ01008570:12996-19353
HmHel-6	CAEZ01008311:632-865
HmHel-6	CAEZ01007271:34112-34350
HmHel-6	CAEZ01007271:9782-15882
HmHel-6	CAEZ01007209:1839-8199
HmHel-6	CAEZ01007209:5765-5996
HmHel-6	CAEZ01006881:4839-5125
HmHel-6	CAEZ01006881:25896-26615
HmHel-6	CAEZ01006881:3808-4022
HmHel-6	CAEZ01006567:17689-25513
HmHel-6	CAEZ01006567:56208-56390
HmHel-6	CAEZ01006567:8579-9428
HmHel-6	CAEZ01006540:7340-7423

HmHel-6	CAEZ01006540:61972-67389
HmHel-6	CAEZ01006540:135531-135613
HmHel-6	CAEZ01006540:119523-119769
HmHel-6	CAEZ01006540:81984-82111
HmHel-6	CAEZ01006540:49430-56389
HmHel-6	CAEZ01006540:28931-33560
HmHel-6	CAEZ01006540:2792-11163
HmHel-6	CAEZ01006349:14464-14646
HmHel-6	CAEZ01006349:41099-41247
HmHel-6	CAEZ01006349:19038-19192
HmHel-6	CAEZ01006124:27844-28314
HmHel-6	CAEZ01006124:33089-33271
HmHel-6	CAEZ01006124:1418-1630
HmHel-6	CAEZ01005664:8336-8550
HmHel-6	CAEZ01005664:9478-9616
HmHel-6	CAEZ01004911:13461-13667
HmHel-6	CAEZ01004517:988-7336
HmHel-6	CAEZ01004471:248-426
HmHel-6	CAEZ01004471:13026-13228
HmHel-6	CAEZ01004471:8053-8280
HmHel-6	CAEZ01004217:5956-6155
HmHel-6	CAEZ01004169:1813-2032
HmHel-6	CAEZ01004169:8714-8922
HmHel-6	CAEZ01003385:7744-7851
HmHel-6	CAEZ01003385:13830-14020
HmHel-6	CAEZ01003192:8727-8820
HmHel-6	CAEZ01003192:25125-25208
HmHel-6	CAEZ01003192:48346-48547
HmHel-6	CAEZ01003192:123933-124076
HmHel-6	CAEZ01003192:105573-105793
HmHel-6	CAEZ01003192:93714-93820
HmHel-6	CAEZ01003192:78593-78714
HmHel-6	CAEZ01003192:55120-58904
HmHel-6	CAEZ01002288:1686-1864
HmHel-6	CAEZ01000069:3991-4208
HmHel-6	CAEZ01011659:1744-2543
HmHel-6	CAEZ01011659:15292-15501
HmHel-6	CAEZ01011092:7136-7310
HmHel-6	CAEZ01011092:687-910
HmHel-6	CAEZ01010855:5438-5688
HmHel-6	CAEZ01010312:1466-1616
HmHel-6	CAEZ01010312:18778-27474
HmHel-6	CAEZ01010312:58392-58742
HmHel-6	CAEZ01010312:59868-59974
HmHel-6	CAEZ01010312:18271-18430
HmHel-6	CAEZ01009710:1662-1867
HmHel-6	CAEZ01009456:34759-34880
HmHel-6	CAEZ01009456:151573-156581

HmHel-6	CAEZ01009456:155017-155273
HmHel-6	CAEZ01009456:144416-144629
HmHel-6	CAEZ01009456:129847-129999
HmHel-6	CAEZ01009456:61898-61980
HmHel-6	CAEZ01009456:44968-45118
HmHel-6	CAEZ01009456:17195-17279
HmHel-6	CAEZ01009032:4668-4879
HmHel-6	CAEZ01008254:6109-6263
HmHel-6	CAEZ01008185:8429-8649
HmHel-6	CAEZ01008153:2585-2814
HmHel-6	CAEZ01007946:532-733
HmHel-6	CAEZ01007946:6008-6141
HmHel-6	CAEZ01007746:24503-24732
HmHel-6	CAEZ01007746:41011-41136
HmHel-6	CAEZ01007746:55012-63100
HmHel-6	CAEZ01007746:99097-99311
HmHel-6	CAEZ01007746:126375-126522
HmHel-6	CAEZ01007746:129887-130058
HmHel-6	CAEZ01007746:110703-110864
HmHel-6	CAEZ01007746:69878-70067
HmHel-6	CAEZ01007746:37602-37837
HmHel-6	CAEZ01007536:8698-8879
HmHel-6	CAEZ01006304:2056-2253
HmHel-6	CAEZ01006304:2644-2736
HmHel-6	CAEZ01005869:4220-4361
HmHel-6	CAEZ01005869:20416-20523
HmHel-6	CAEZ01005869:10235-10339
HmHel-6	CAEZ01005857:3097-3208
HmHel-6	CAEZ01005857:2115-2332
HmHel-6	CAEZ01005739:19027-19129
HmHel-6	CAEZ01005739:27379-27594
HmHel-6	CAEZ01005739:6159-6325
HmHel-6	CAEZ01005720:11317-12982
HmHel-6	CAEZ01005720:13448-13540
HmHel-6	CAEZ01005430:3982-4084
HmHel-6	CAEZ01005430:18783-29671
HmHel-6	CAEZ01005430:45149-45282
HmHel-6	CAEZ01005430:87180-89308
HmHel-6	CAEZ01005430:68787-68963
HmHel-6	CAEZ01005430:54682-54893
HmHel-6	CAEZ01005430:35015-35229
HmHel-6	CAEZ01005430:14422-14683
HmHel-6	CAEZ01005409:22816-22904
HmHel-6	CAEZ01005409:43894-44096
HmHel-6	CAEZ01005409:60708-60789
HmHel-6	CAEZ01005409:78096-78272
HmHel-6	CAEZ01005409:69273-69352
HmHel-6	CAEZ01004973:29326-29539

HmHel-6	CAEZ01004936:45549-45628
HmHel-6	CAEZ01004936:35478-35607
HmHel-6	CAEZ01004909:2456-2648
HmHel-6	CAEZ01004909:48867-48961
HmHel-6	CAEZ01004909:71077-78795
HmHel-6	CAEZ01004909:77262-77384
HmHel-6	CAEZ01004909:64595-64705
HmHel-6	CAEZ01004909:23337-23438
HmHel-6	CAEZ01003883:3611-3783
HmHel-6	CAEZ01003883:64341-65015
HmHel-6	CAEZ01003883:24622-24718
HmHel-6	CAEZ01002670:1657-1857
HmHel-6	CAEZ01002670:61630-62741
HmHel-6	CAEZ01002670:31888-32120
HmHel-6	CAEZ01002670:3274-11071
HmHel-6	CAEZ01001501:4814-4937
HmHel-6	CAEZ01001501:48627-48712
HmHel-6	CAEZ01001501:61793-62028
HmHel-6	CAEZ01001501:21613-21822
HmHel-6	CAEZ01001501:6924-7100
HmHel-6	CAEZ01001437:8123-11292
HmHel-6	CAEZ01001437:35255-35467
HmHel-6	CAEZ01001437:56750-56889
HmHel-6	CAEZ01001437:17404-17584
HmHel-6	CAEZ01011362:5351-8058
HmHel-6	CAEZ01011362:33775-33987
HmHel-6	CAEZ01011362:12562-21042
HmHel-6	CAEZ01010782:2044-2247
HmHel-6	CAEZ01010561:454-637
HmHel-6	CAEZ01010561:10929-11010
HmHel-6	CAEZ01009747:1658-1866
HmHel-6	CAEZ01009747:14455-14695
HmHel-6	CAEZ01009747:12693-12855
HmHel-6	CAEZ01009454:6601-6688
HmHel-6	CAEZ01009454:22358-23516
HmHel-6	CAEZ01009454:17888-18132
HmHel-6	CAEZ01009454:5560-5662
HmHel-6	CAEZ01009044:8296-13566
HmHel-6	CAEZ01009044:45564-45746
HmHel-6	CAEZ01009044:151380-151481
HmHel-6	CAEZ01009044:170893-170976
HmHel-6	CAEZ01009044:115495-117011
HmHel-6	CAEZ01009044:98826-98921
HmHel-6	CAEZ01009044:40689-49246
HmHel-6	CAEZ01009044:14541-14746
HmHel-6	CAEZ01008861:7894-8143
HmHel-6	CAEZ01008861:23497-23723
HmHel-6	CAEZ01008861:9459-9582

HmHel-6	CAEZ01008858:1563-1793
HmHel-6	CAEZ01008742:49686-49829
HmHel-6	CAEZ01008742:31845-37863
HmHel-6	CAEZ01008742:6688-6782
HmHel-6	CAEZ01008641:19693-19909
HmHel-6	CAEZ01008641:8987-9115
HmHel-6	CAEZ01008407:63-8379
HmHel-6	CAEZ01008407:11552-11681
HmHel-6	CAEZ01007913:9940-10148
HmHel-6	CAEZ01007667:4912-5127
HmHel-6	CAEZ01007505:3547-3711
HmHel-6	CAEZ01007505:32619-32848
HmHel-6	CAEZ01007505:1151-1306
HmHel-6	CAEZ01006530:36779-36970
HmHel-6	CAEZ01006530:84650-84762
HmHel-6	CAEZ01006530:132060-132227
HmHel-6	CAEZ01006530:232886-233043
HmHel-6	CAEZ01006530:272000-272098
HmHel-6	CAEZ01006530:296286-296529
HmHel-6	CAEZ01006530:287663-287804
HmHel-6	CAEZ01006530:254310-254442
HmHel-6	CAEZ01006530:196274-196374
HmHel-6	CAEZ01006530:145911-150211
HmHel-6	CAEZ01006530:113934-114024
HmHel-6	CAEZ01006530:98145-98230
HmHel-6	CAEZ01006530:50453-50615
HmHel-6	CAEZ01006433:28374-28541
HmHel-6	CAEZ01006433:56748-56870
HmHel-6	CAEZ01006150:4840-4981
HmHel-6	CAEZ01006150:23726-23919
HmHel-6	CAEZ01006150:43486-43720
HmHel-6	CAEZ01006150:54889-55097
HmHel-6	CAEZ01006150:27297-27439
HmHel-6	CAEZ01006150:14009-14106
HmHel-6	CAEZ01005871:5061-5310
HmHel-6	CAEZ01005836:6677-6871
HmHel-6	CAEZ01005836:21754-21859
HmHel-6	CAEZ01005836:11796-11980
HmHel-6	CAEZ01004671:7792-7915
HmHel-6	CAEZ01004671:134-357
HmHel-6	CAEZ01004227:1-181
HmHel-6	CAEZ01004198:19154-26014
HmHel-6	CAEZ01004198:35576-35794
HmHel-6	CAEZ01003353:7443-7622
HmHel-6	CAEZ01003063:1309-1521
HmHel-6	CAEZ01003063:12231-12440
HmHel-6	CAEZ01002665:7669-9936
HmHel-6	CAEZ01002665:46768-46987

HmHel-6	CAEZ01002320:7502-7709
HmHel-6	CAEZ01002320:18560-18659
HmHel-6	CAEZ01002320:28808-31310
HmHel-6	CAEZ01002320:44951-45163
HmHel-6	CAEZ01002320:27883-27995
HmHel-6	CAEZ01002320:1950-2153
HmHel-6	CAEZ01001932:1763-1961
HmHel-6	CAEZ01001932:29911-30111
HmHel-6	CAEZ01001932:52908-53186
HmHel-6	CAEZ01001932:22572-22773
HmHel-6	CAEZ01001932:6028-6185
HmHel-6	CAEZ01000969:5206-5373
HmHel-6	CAEZ01000969:7287-7482
HmHel-6	CAEZ01009804:19118-19210
HmHel-6	CAEZ01009804:49163-49273
HmHel-6	CAEZ01009804:79538-79790
HmHel-6	CAEZ01009804:94547-94669
HmHel-6	CAEZ01009804:89189-89376
HmHel-6	CAEZ01009804:9379-9520
HmHel-6	CAEZ01009284:49813-61610
HmHel-6	CAEZ01009284:64753-64846
HmHel-6	CAEZ01009284:45195-45288
HmHel-6	CAEZ01009284:15985-16081
HmHel-6	CAEZ01009241:2167-2427
HmHel-6	CAEZ01008997:9145-9355
HmHel-6	CAEZ01008997:13148-13366
HmHel-6	CAEZ01008997:763-1216
HmHel-6	CAEZ01008971:5825-6002
HmHel-6	CAEZ01008971:38507-38709
HmHel-6	CAEZ01008971:38054-46632
HmHel-6	CAEZ01008971:5270-5459
HmHel-6	CAEZ01008885:1516-11117
HmHel-6	CAEZ01008885:27480-27588
HmHel-6	CAEZ01008885:17229-24741
HmHel-6	CAEZ01008885:3443-3600
HmHel-6	CAEZ01008416:10499-16299
HmHel-6	CAEZ01008416:49926-50007
HmHel-6	CAEZ01008416:12728-12945
HmHel-6	CAEZ01008359:14717-14845
HmHel-6	CAEZ01008359:50649-50732
HmHel-6	CAEZ01008359:69167-69283
HmHel-6	CAEZ01008359:83067-86409
HmHel-6	CAEZ01008359:154987-155075
HmHel-6	CAEZ01008359:165153-165305
HmHel-6	CAEZ01008359:169181-169398
HmHel-6	CAEZ01008359:117459-117559
HmHel-6	CAEZ01008359:72672-72822
HmHel-6	CAEZ01008359:51846-52057

HmHel-6	CAEZ01008359:22789-22941
HmHel-6	CAEZ01008138:7995-8136
HmHel-6	CAEZ01008138:51344-55584
HmHel-6	CAEZ01008138:74980-75094
HmHel-6	CAEZ01008138:130738-134108
HmHel-6	CAEZ01008138:156232-156316
HmHel-6	CAEZ01008138:178127-182310
HmHel-6	CAEZ01008138:226294-228117
HmHel-6	CAEZ01008138:247247-247373
HmHel-6	CAEZ01008138:260108-260246
HmHel-6	CAEZ01008138:315855-315952
HmHel-6	CAEZ01008138:385478-385591
HmHel-6	CAEZ01008138:349723-349950
HmHel-6	CAEZ01008138:334472-334617
HmHel-6	CAEZ01008138:320435-320618
HmHel-6	CAEZ01008138:288139-288229
HmHel-6	CAEZ01008138:263392-263485
HmHel-6	CAEZ01008138:248397-248643
HmHel-6	CAEZ01008138:205257-205411
HmHel-6	CAEZ01008138:163825-163931
HmHel-6	CAEZ01008138:141805-142012
HmHel-6	CAEZ01008138:120927-121040
HmHel-6	CAEZ01008138:43120-43242
HmHel-6	CAEZ01008138:9490-9578
HmHel-6	CAEZ01008071:10527-16849
HmHel-6	CAEZ01008071:9749-9993
HmHel-6	CAEZ01007883:3610-3821
HmHel-6	CAEZ01007883:19892-20001
HmHel-6	CAEZ01007689:52497-52594
HmHel-6	CAEZ01007689:51929-52173
HmHel-6	CAEZ01007689:17595-17703
HmHel-6	CAEZ01007370:7638-8052
HmHel-6	CAEZ01007370:17999-18213
HmHel-6	CAEZ01007319:16219-16324
HmHel-6	CAEZ01007253:21752-21977
HmHel-6	CAEZ01007253:38584-38723
HmHel-6	CAEZ01007253:8407-8507
HmHel-6	CAEZ01006501:11170-11377
HmHel-6	CAEZ01006063:10100-10307
HmHel-6	CAEZ01006063:16414-16550
HmHel-6	CAEZ01005536:404-645
HmHel-6	CAEZ01005510:14060-14174
HmHel-6	CAEZ01005500:21592-21831
HmHel-6	CAEZ01005500:24554-24746
HmHel-6	CAEZ01004764:8554-8793
HmHel-6	CAEZ01004380:5378-5580
HmHel-6	CAEZ01002361:18131-18411
HmHel-6	CAEZ01002361:30442-30584

HmHel-6	CAEZ01002361:50961-51181
HmHel-6	CAEZ01002361:3836-3937
HmHel-6	CAEZ01002098:168-335
HmHel-6	CAEZ01002098:4867-4975
HmHel-6	CAEZ01000968:2978-3157
HmHel-6	CAEZ01000968:36913-37116
HmHel-6	CAEZ01010573:1645-1836
HmHel-6	CAEZ01010461:8942-9104
HmHel-6	CAEZ01010461:24125-25426
HmHel-6	CAEZ01010461:30500-30666
HmHel-6	CAEZ01010393:6741-6947
HmHel-6	CAEZ01009691:9082-9199
HmHel-6	CAEZ01009691:19471-19598
HmHel-6	CAEZ01009691:81548-81749
HmHel-6	CAEZ01009691:36704-38071
HmHel-6	CAEZ01009691:22649-22757
HmHel-6	CAEZ01009411:1959-2165
HmHel-6	CAEZ01009408:5904-5991
HmHel-6	CAEZ01009408:868-1073
HmHel-6	CAEZ01008955:2339-2423
HmHel-6	CAEZ01008955:16526-16739
HmHel-6	CAEZ01008955:2760-4460
HmHel-6	CAEZ01008746:13826-15190
HmHel-6	CAEZ01008746:59761-59906
HmHel-6	CAEZ01008746:135172-135289
HmHel-6	CAEZ01008746:163589-163676
HmHel-6	CAEZ01008746:182927-185536
HmHel-6	CAEZ01008746:186928-187087
HmHel-6	CAEZ01008746:158780-158862
HmHel-6	CAEZ01008746:89079-93288
HmHel-6	CAEZ01008746:54881-58553
HmHel-6	CAEZ01007911:2759-2921
HmHel-6	CAEZ01007911:6482-6584
HmHel-6	CAEZ01007894:1320-8578
HmHel-6	CAEZ01007894:14608-14748
HmHel-6	CAEZ01007278:2572-2814
HmHel-6	CAEZ01007002:10434-10672
HmHel-6	CAEZ01006660:53-274
HmHel-6	CAEZ01006660:15464-16109
HmHel-6	CAEZ01006660:8987-9077
HmHel-6	CAEZ01006423:68061-68167
HmHel-6	CAEZ01006423:143690-143916
HmHel-6	CAEZ01006423:218477-218582
HmHel-6	CAEZ01006423:271782-271877
HmHel-6	CAEZ01006423:311436-311559
HmHel-6	CAEZ01006423:343949-344169
HmHel-6	CAEZ01006423:314927-319780
HmHel-6	CAEZ01006423:224791-224914

HmHel-6	CAEZ01006423:156020-156247
HmHel-6	CAEZ01006423:107033-107125
HmHel-6	CAEZ01006423:93871-93957
HmHel-6	CAEZ01006423:76433-76595
HmHel-6	CAEZ01006085:4631-4834
HmHel-6	CAEZ01006085:24311-24479
HmHel-6	CAEZ01006085:47956-48936
HmHel-6	CAEZ01006085:47465-47548
HmHel-6	CAEZ01006085:12916-13038
HmHel-6	CAEZ01005520:7021-8707
HmHel-6	CAEZ01005520:241-451
HmHel-6	CAEZ01003484:13912-14017
HmHel-6	CAEZ01003484:48479-48694
HmHel-6	CAEZ01003484:27275-27384
HmHel-6	CAEZ01003484:8625-8716
HmHel-6	CAEZ01003226:8515-9493
HmHel-6	CAEZ01003226:21308-21519
HmHel-6	CAEZ01003226:59923-60018
HmHel-6	CAEZ01003226:73856-74023
HmHel-6	CAEZ01003226:84675-84924
HmHel-6	CAEZ01003226:129846-129983
HmHel-6	CAEZ01003226:145629-145724
HmHel-6	CAEZ01003226:113078-113247
HmHel-6	CAEZ01003226:85970-86122
HmHel-6	CAEZ01003226:47673-47774
HmHel-6	CAEZ01003226:6227-6401
HmHel-6	CAEZ01001747:5407-5569
HmHel-6	CAEZ01001747:1594-3134
HmHel-6	CAEZ01001442:17491-17685
HmHel-6	CAEZ01001442:40974-41130
HmHel-6	CAEZ01001442:34089-34173
HmHel-6	CAEZ01001442:13503-13590
HmHel-6	CAEZ01000966:22267-30384
HmHel-6	CAEZ01000966:23111-23251
HmHel-6	CAEZ01000966:12672-12837
HmHel-6	CAEZ01010953:4348-18075
HmHel-6	CAEZ01010953:30393-30555
HmHel-6	CAEZ01010953:19637-19744
HmHel-6	CAEZ01010953:6630-6750
HmHel-6	CAEZ01010802:624-774
HmHel-6	CAEZ01010802:29839-30008
HmHel-6	CAEZ01010802:40406-40502
HmHel-6	CAEZ01010007:848-1044
HmHel-6	CAEZ01010007:15370-15579
HmHel-6	CAEZ01010007:3125-3328
HmHel-6	CAEZ01009845:9323-9520
HmHel-6	CAEZ01009845:26125-26312
HmHel-6	CAEZ01009845:28238-28452

HmHel-6	CAEZ01009844:3575-13827
HmHel-6	CAEZ01009844:74744-74837
HmHel-6	CAEZ01009844:153607-153783
HmHel-6	CAEZ01009844:181790-181926
HmHel-6	CAEZ01009844:181093-181285
HmHel-6	CAEZ01009844:81479-81569
HmHel-6	CAEZ01009844:33936-34148
HmHel-6	CAEZ01009844:5453-5548
HmHel-6	CAEZ01009391:8600-8698
HmHel-6	CAEZ01009391:18803-19050
HmHel-6	CAEZ01008889:1015-1167
HmHel-6	CAEZ01008889:25237-25335
HmHel-6	CAEZ01008889:11008-11245
HmHel-6	CAEZ01008390:40343-40459
HmHel-6	CAEZ01008390:50555-50739
HmHel-6	CAEZ01008390:4154-4240
HmHel-6	CAEZ01008294:1988-2153
HmHel-6	CAEZ01008294:32706-32905
HmHel-6	CAEZ01008294:44452-49048
HmHel-6	CAEZ01008294:102304-104839
HmHel-6	CAEZ01008294:119531-119661
HmHel-6	CAEZ01008294:171781-172689
HmHel-6	CAEZ01008294:183921-187720
HmHel-6	CAEZ01008294:169489-170549
HmHel-6	CAEZ01008294:111709-111903
HmHel-6	CAEZ01008294:68679-79945
HmHel-6	CAEZ01008294:35058-40288
HmHel-6	CAEZ01008206:7852-8035
HmHel-6	CAEZ01007751:42497-42693
HmHel-6	CAEZ01007751:84037-84125
HmHel-6	CAEZ01007751:165823-165905
HmHel-6	CAEZ01007751:47595-47800
HmHel-6	CAEZ01007748:32608-32686
HmHel-6	CAEZ01007748:57699-57837
HmHel-6	CAEZ01007748:63388-63548
HmHel-6	CAEZ01007748:45173-46635
HmHel-6	CAEZ01007748:15701-19439
HmHel-6	CAEZ01007320:538-730
HmHel-6	CAEZ01007296:1372-1527
HmHel-6	CAEZ01007296:11653-21652
HmHel-6	CAEZ01007296:19557-19669
HmHel-6	CAEZ01006711:9306-18262
HmHel-6	CAEZ01006711:55587-55784
HmHel-6	CAEZ01006711:16236-16375
HmHel-6	CAEZ01006624:15034-15231
HmHel-6	CAEZ01006624:1879-2026
HmHel-6	CAEZ01005702:1990-2167
HmHel-6	CAEZ01005101:7264-7357

HmHel-6	CAEZ01005101:30840-31093
HmHel-6	CAEZ01005101:41160-41311
HmHel-6	CAEZ01005101:6644-6876
HmHel-6	CAEZ01004463:3797-4035
HmHel-6	CAEZ01003852:772-891
HmHel-6	CAEZ01003852:29673-29889
HmHel-6	CAEZ01003852:4611-4766
HmHel-6	CAEZ01003302:11966-16483
HmHel-6	CAEZ01003302:16904-17105
HmHel-6	CAEZ01003044:1954-2119
HmHel-6	CAEZ01003044:13216-13313
HmHel-6	CAEZ01002629:11638-11785
HmHel-6	CAEZ01002629:37364-37498
HmHel-6	CAEZ01002629:43851-43949
HmHel-6	CAEZ01002629:12315-12965
HmHel-6	CAEZ01002629:953-1154
HmHel-6	CAEZ01002289:6114-16254
HmHel-6	CAEZ01002289:25412-25540
HmHel-6	CAEZ01002289:11781-11907
HmHel-6	CAEZ01001065:5173-5431
HmHel-6	CAEZ01001020:55022-55107
HmHel-6	CAEZ01001020:37162-37300
HmHel-6	CAEZ01001020:18127-19620
HmHel-6	CAEZ01011144:12880-13023
HmHel-6	CAEZ01011144:20662-24939
HmHel-6	CAEZ01010736:16201-16298
HmHel-6	CAEZ01010736:33243-33466
HmHel-6	CAEZ01010736:19208-19321
HmHel-6	CAEZ01010410:5124-5361
HmHel-6	CAEZ01009835:26947-27041
HmHel-6	CAEZ01009835:38649-39880
HmHel-6	CAEZ01009835:28152-28390
HmHel-6	CAEZ01009835:17022-17104
HmHel-6	CAEZ01008117:15355-15503
HmHel-6	CAEZ01008117:10492-10690
HmHel-6	CAEZ01008089:10458-15907
HmHel-6	CAEZ01008089:37369-37561
HmHel-6	CAEZ01007388:348-543
HmHel-6	CAEZ01007388:3639-3748
HmHel-6	CAEZ01007168:5990-6086
HmHel-6	CAEZ01007168:24368-24465
HmHel-6	CAEZ01007168:16175-16391
HmHel-6	CAEZ01007168:2154-2236
HmHel-6	CAEZ01007083:6971-8154
HmHel-6	CAEZ01006823:11991-19582
HmHel-6	CAEZ01006823:13076-13205
HmHel-6	CAEZ01006616:51300-51422
HmHel-6	CAEZ01006616:108424-109635

HmHel-6	CAEZ01006616:146333-146465
HmHel-6	CAEZ01006616:179748-179917
HmHel-6	CAEZ01006616:219445-219595
HmHel-6	CAEZ01006616:246553-246776
HmHel-6	CAEZ01006616:191600-191819
HmHel-6	CAEZ01006616:134762-141705
HmHel-6	CAEZ01006616:111199-111281
HmHel-6	CAEZ01006616:93525-93654
HmHel-6	CAEZ01006616:82811-82930
HmHel-6	CAEZ01006616:60337-63174
HmHel-6	CAEZ01006616:41976-42171
HmHel-6	CAEZ01006616:13031-13118
HmHel-6	CAEZ01006061:13863-14100
HmHel-6	CAEZ01005605:27004-31832
HmHel-6	CAEZ01005605:89506-89673
HmHel-6	CAEZ01005605:108910-109037
HmHel-6	CAEZ01005605:96917-97115
HmHel-6	CAEZ01005555:9754-9965
HmHel-6	CAEZ01005508:5391-5580
HmHel-6	CAEZ01005508:40535-44415
HmHel-6	CAEZ01005508:65359-73902
HmHel-6	CAEZ01005508:105502-105588
HmHel-6	CAEZ01005508:89962-90101
HmHel-6	CAEZ01005508:72294-72473
HmHel-6	CAEZ01005148:17336-17500
HmHel-6	CAEZ01005148:29939-30099
HmHel-6	CAEZ01004546:7073-7303
HmHel-6	CAEZ01004546:7838-8040
HmHel-6	CAEZ01003726:425-568
HmHel-6	CAEZ01003726:2399-2903
HmHel-6	CAEZ01003672:7471-7725
HmHel-6	CAEZ01003672:4448-4667
HmHel-6	CAEZ01003265:9769-11181
HmHel-6	CAEZ01003265:31555-33392
HmHel-6	CAEZ01003265:45237-45437
HmHel-6	CAEZ01003265:80799-80978
HmHel-6	CAEZ01003265:36553-36770
HmHel-6	CAEZ01002905:7078-7250
HmHel-6	CAEZ01002830:8867-9096
HmHel-6	CAEZ01000583:392-588
HmHel-6	CAEZ01011489:5669-12059
HmHel-6	CAEZ01011489:37802-42671
HmHel-6	CAEZ01011489:75268-75459
HmHel-6	CAEZ01011489:88898-88998
HmHel-6	CAEZ01011489:61431-61513
HmHel-6	CAEZ01011489:16711-16804
HmHel-6	CAEZ01011393:11399-11635
HmHel-6	CAEZ01011393:10592-10743

HmHel-6	CAEZ01011083:41031-41154
HmHel-6	CAEZ01011083:123783-128597
HmHel-6	CAEZ01011083:141462-141541
HmHel-6	CAEZ01011083:161772-170311
HmHel-6	CAEZ01011083:204395-204483
HmHel-6	CAEZ01011083:227010-227124
HmHel-6	CAEZ01011083:226826-226905
HmHel-6	CAEZ01011083:196260-196485
HmHel-6	CAEZ01011083:162163-165780
HmHel-6	CAEZ01011083:118439-120905
HmHel-6	CAEZ01011083:104764-104919
HmHel-6	CAEZ01011083:57255-60572
HmHel-6	CAEZ01011083:30708-39591
HmHel-6	CAEZ01011068:1482-1709
HmHel-6	CAEZ01011046:34200-34316
HmHel-6	CAEZ01011046:49787-51313
HmHel-6	CAEZ01011046:64994-65155
HmHel-6	CAEZ01011046:104261-105452
HmHel-6	CAEZ01011046:126504-126588
HmHel-6	CAEZ01011046:160468-160679
HmHel-6	CAEZ01011046:98617-99057
HmHel-6	CAEZ01011046:59804-68729
HmHel-6	CAEZ01010871:6942-7150
HmHel-6	CAEZ01010661:2658-9771
HmHel-6	CAEZ01010661:860-2074
HmHel-6	CAEZ01010424:57513-57659
HmHel-6	CAEZ01010424:83594-83785
HmHel-6	CAEZ01010424:133137-133246
HmHel-6	CAEZ01010424:143986-151801
HmHel-6	CAEZ01010424:138999-139078
HmHel-6	CAEZ01010424:126251-126385
HmHel-6	CAEZ01010424:90882-90968
HmHel-6	CAEZ01010424:42290-42408
HmHel-6	CAEZ01010351:4537-4669
HmHel-6	CAEZ01010351:89901-90144
HmHel-6	CAEZ01010351:94086-94215
HmHel-6	CAEZ01010351:34311-42069
HmHel-6	CAEZ01010351:9911-10028
HmHel-6	CAEZ01009962:20328-20423
HmHel-6	CAEZ01009962:48576-48784
HmHel-6	CAEZ01009962:34200-34458
HmHel-6	CAEZ01009931:1463-1544
HmHel-6	CAEZ01009931:13226-13446
HmHel-6	CAEZ01009680:4057-8325
HmHel-6	CAEZ01009680:30411-30532
HmHel-6	CAEZ01009680:58520-58668
HmHel-6	CAEZ01009680:40043-40141
HmHel-6	CAEZ01009191:408-504

HmHel-6	CAEZ01009191:682-897
HmHel-6	CAEZ01009183:2776-2946
HmHel-6	CAEZ01009183:7677-7854
HmHel-6	CAEZ01008975:2053-2216
HmHel-6	CAEZ01008806:37435-37565
HmHel-6	CAEZ01008806:9326-9552
HmHel-6	CAEZ01008671:18889-19052
HmHel-6	CAEZ01008671:12672-12762
HmHel-6	CAEZ01008640:10021-10193
HmHel-6	CAEZ01008640:42271-42508
HmHel-6	CAEZ01008291:34576-43528
HmHel-6	CAEZ01008291:50463-50595
HmHel-6	CAEZ01008291:20396-20553
HmHel-6	CAEZ01006588:8583-10696
HmHel-6	CAEZ01006588:23404-23554
HmHel-6	CAEZ01006588:41431-41642
HmHel-6	CAEZ01006588:14435-15431
HmHel-6	CAEZ01005738:9629-9828
HmHel-6	CAEZ01005738:1382-1625
HmHel-6	CAEZ01005354:16655-16835
HmHel-6	CAEZ01005354:28684-28922
HmHel-6	CAEZ01005354:33704-33823
HmHel-6	CAEZ01004320:7765-7959
HmHel-6	CAEZ01004254:6490-6688
HmHel-6	CAEZ01004254:32581-32670
HmHel-6	CAEZ01004124:6990-7152
HmHel-6	CAEZ01004124:19669-20033
HmHel-6	CAEZ01000609:3114-3236
HmHel-6	CAEZ01000609:900-1134
HmHel-6	CAEZ01011210:7783-9553
HmHel-6	CAEZ01011210:8160-9157
HmHel-6	CAEZ01010723:1788-1947
HmHel-6	CAEZ01010723:9122-9383
HmHel-6	CAEZ01010586:12707-12894
HmHel-6	CAEZ01010586:10832-22944
HmHel-6	CAEZ01010411:9428-9508
HmHel-6	CAEZ01010411:45304-45388
HmHel-6	CAEZ01010411:35038-35296
HmHel-6	CAEZ01010411:8606-8798
HmHel-6	CAEZ01010356:16274-16377
HmHel-6	CAEZ01010356:80503-88202
HmHel-6	CAEZ01010356:52973-59975
HmHel-6	CAEZ01010356:31440-31564
HmHel-6	CAEZ01009404:8614-8852
HmHel-6	CAEZ01009404:64141-64228
HmHel-6	CAEZ01009404:21489-21723
HmHel-6	CAEZ01009404:7981-11384
HmHel-6	CAEZ01009240:4759-4953

HmHel-6	CAEZ01009240:12520-12669
HmHel-6	CAEZ01009230:17675-17767
HmHel-6	CAEZ01009230:6711-6892
HmHel-6	CAEZ01008374:11768-11944
HmHel-6	CAEZ01008374:29215-29411
HmHel-6	CAEZ01008374:44648-47642
HmHel-6	CAEZ01008374:58392-66642
HmHel-6	CAEZ01008374:18394-18626
HmHel-6	CAEZ01008290:38553-38665
HmHel-6	CAEZ01008290:84779-94412
HmHel-6	CAEZ01008290:53474-53679
HmHel-6	CAEZ01008290:35591-35735
HmHel-6	CAEZ01008290:16282-16398
HmHel-6	CAEZ01008231:2776-10727
HmHel-6	CAEZ01008231:36541-36636
HmHel-6	CAEZ01008231:20476-20648
HmHel-6	CAEZ01007897:2225-2419
HmHel-6	CAEZ01007897:16716-16942
HmHel-6	CAEZ01007564:16419-16539
HmHel-6	CAEZ01007564:4664-4862
HmHel-6	CAEZ01007489:1926-2132
HmHel-6	CAEZ01007453:18493-18626
HmHel-6	CAEZ01007453:42771-42929
HmHel-6	CAEZ01007453:11870-18131
HmHel-6	CAEZ01007156:4771-5006
HmHel-6	CAEZ01007156:5481-5570
HmHel-6	CAEZ01006622:8474-14199
HmHel-6	CAEZ01006622:11814-12050
HmHel-6	CAEZ01006560:6253-6467
HmHel-6	CAEZ01006560:37445-37630
HmHel-6	CAEZ01006560:62348-62529
HmHel-6	CAEZ01006560:75334-75482
HmHel-6	CAEZ01006560:93770-94950
HmHel-6	CAEZ01006560:116957-117148
HmHel-6	CAEZ01006560:138600-138696
HmHel-6	CAEZ01006560:101975-111572
HmHel-6	CAEZ01006560:42395-51545
HmHel-6	CAEZ01006560:17769-17904
HmHel-6	CAEZ01006136:1502-1687
HmHel-6	CAEZ01005696:1-164
HmHel-6	CAEZ01004706:5014-5243
HmHel-6	CAEZ01004569:5342-5592
HmHel-6	CAEZ01004497:3701-3814
HmHel-6	CAEZ01004497:41825-41938
HmHel-6	CAEZ01004497:30358-30439
HmHel-6	CAEZ01004497:6175-6427
HmHel-6	CAEZ01004222:9332-9469
HmHel-6	CAEZ01004222:39797-40014

HmHel-6	CAEZ01004222:67795-67925
HmHel-6	CAEZ01002833:707-889
HmHel-6	CAEZ01002833:28505-28723
HmHel-6	CAEZ01002833:51432-52103
HmHel-6	CAEZ01002087:24723-24936
HmHel-6	CAEZ01002087:50899-51039
HmHel-6	CAEZ01002087:25774-25926
HmHel-6	CAEZ01002087:14014-14127
HmHel-6	CAEZ01001222:1641-9780
HmHel-6	CAEZ01001222:7733-8030
HmHel-6	CAEZ01000838:1257-1345
HmHel-6	CAEZ01000838:6207-6400
HmHel-6	CAEZ01000049:3562-5873
HmHel-6	CAEZ01000049:17464-17617
HmHel-6	CAEZ01000049:25775-25934
HmHel-6	CAEZ01000049:13331-13520
HmHel-6	CAEZ01010733:61-249
HmHel-6	CAEZ01010733:29672-29829
HmHel-6	CAEZ01010073:10377-10566
HmHel-6	CAEZ01010017:3088-3301
HmHel-6	CAEZ01009634:8908-9124
HmHel-6	CAEZ01009634:12490-12674
HmHel-6	CAEZ01009223:51254-53044
HmHel-6	CAEZ01009223:73567-80623
HmHel-6	CAEZ01009223:101638-101825
HmHel-6	CAEZ01009223:98586-98735
HmHel-6	CAEZ01009223:20587-20780
HmHel-6	CAEZ01009210:7535-7764
HmHel-6	CAEZ01008996:787-940
HmHel-6	CAEZ01008996:1305-1499
HmHel-6	CAEZ01008101:23726-31320
HmHel-6	CAEZ01008101:21828-21978
HmHel-6	CAEZ01007101:11164-11763
HmHel-6	CAEZ01007101:24850-25039
HmHel-6	CAEZ01007101:68134-68239
HmHel-6	CAEZ01007101:85831-85942
HmHel-6	CAEZ01007101:65097-65230
HmHel-6	CAEZ01007101:29339-39210
HmHel-6	CAEZ01006667:51702-51953
HmHel-6	CAEZ01006667:76981-78904
HmHel-6	CAEZ01006667:71221-74603
HmHel-6	CAEZ01006667:24002-24174
HmHel-6	CAEZ01006339:4248-4473
HmHel-6	CAEZ01006339:58463-58585
HmHel-6	CAEZ01006339:46165-53668
HmHel-6	CAEZ01006339:10344-12180
HmHel-6	CAEZ01006174:17461-17676
HmHel-6	CAEZ01006174:30644-33354

HmHel-6	CAEZ01006174:60743-64358
HmHel-6	CAEZ01006174:26377-34878
HmHel-6	CAEZ01005383:2010-8879
HmHel-6	CAEZ01005383:10501-10730
HmHel-6	CAEZ01005166:9542-9689
HmHel-6	CAEZ01005166:30380-30593
HmHel-6	CAEZ01004228:4858-5068
HmHel-6	CAEZ01004228:378-506
HmHel-6	CAEZ01003855:9965-10198
HmHel-6	CAEZ01003855:29377-29457
HmHel-6	CAEZ01003855:17590-17673
HmHel-6	CAEZ01003732:7818-8074
HmHel-6	CAEZ01002711:4033-4260
HmHel-6	CAEZ01002711:24524-24631
HmHel-6	CAEZ01002711:13716-13948
HmHel-6	CAEZ01002210:12755-12995
HmHel-6	CAEZ01002210:15418-15580
HmHel-6	CAEZ01001431:1753-1858
HmHel-6	CAEZ01001431:2028-2241
HmHel-6	CAEZ01011408:19708-19864
HmHel-6	CAEZ01011408:14360-20227
HmHel-6	CAEZ01011322:3400-3638
HmHel-6	CAEZ01011091:5477-5616
HmHel-6	CAEZ01011091:23121-23279
HmHel-6	CAEZ01011091:35445-35626
HmHel-6	CAEZ01011091:30460-30643
HmHel-6	CAEZ01011091:10568-15585
HmHel-6	CAEZ01011035:6780-6951
HmHel-6	CAEZ01010898:27650-27858
HmHel-6	CAEZ01010898:5107-5264
HmHel-6	CAEZ01010772:5657-12203
HmHel-6	CAEZ01010772:13761-13969
HmHel-6	CAEZ01010728:2428-2579
HmHel-6	CAEZ01010728:26244-26453
HmHel-6	CAEZ01010728:38115-38325
HmHel-6	CAEZ01010728:9777-10690
HmHel-6	CAEZ01010485:17815-17990
HmHel-6	CAEZ01010485:6932-7053
HmHel-6	CAEZ01010304:26299-26499
HmHel-6	CAEZ01010304:7719-7885
HmHel-6	CAEZ01009841:11816-11945
HmHel-6	CAEZ01009841:27913-27998
HmHel-6	CAEZ01009841:57143-65270
HmHel-6	CAEZ01009841:95383-104936
HmHel-6	CAEZ01009841:62405-62577
HmHel-6	CAEZ01009841:32718-41810
HmHel-6	CAEZ01008839:27458-27718
HmHel-6	CAEZ01008839:16094-16315

HmHel-6	CAEZ01008662:14699-14935
HmHel-6	CAEZ01008662:10044-10155
HmHel-6	CAEZ01008342:19680-19924
HmHel-6	CAEZ01008342:24947-25142
HmHel-6	CAEZ01008342:2470-2658
HmHel-6	CAEZ01008295:8759-8977
HmHel-6	CAEZ01008147:2265-2486
HmHel-6	CAEZ01007902:8657-8895
HmHel-6	CAEZ01007902:10717-12870
HmHel-6	CAEZ01007768:13176-13322
HmHel-6	CAEZ01007768:17549-17709
HmHel-6	CAEZ01007768:2897-4999
HmHel-6	CAEZ01007726:11785-11887
HmHel-6	CAEZ01007726:38691-38782
HmHel-6	CAEZ01007726:37023-37237
HmHel-6	CAEZ01007726:6684-6885
HmHel-6	CAEZ01007661:8018-8265
HmHel-6	CAEZ01007537:9080-11377
HmHel-6	CAEZ01007537:9749-9963
HmHel-6	CAEZ01007449:12889-13084
HmHel-6	CAEZ01007449:18697-18818
HmHel-6	CAEZ01007322:6716-6909
HmHel-6	CAEZ01007322:14317-14396
HmHel-6	CAEZ01006929:17482-17705
HmHel-6	CAEZ01006929:14118-14266
HmHel-6	CAEZ01006929:158-262
HmHel-6	CAEZ01006220:7085-7258
HmHel-6	CAEZ01006220:18287-18413
HmHel-6	CAEZ01006220:53642-53804
HmHel-6	CAEZ01006220:31441-31598
HmHel-6	CAEZ01005807:34565-34773
HmHel-6	CAEZ01005676:3557-3773
HmHel-6	CAEZ01005676:805-1029
HmHel-6	CAEZ01005387:1505-1747
HmHel-6	CAEZ01004313:16768-16856
HmHel-6	CAEZ01004313:8572-8736
HmHel-6	CAEZ01002254:9403-9612
HmHel-6	CAEZ01001805:7714-7837
HmHel-6	CAEZ01001805:44910-50862
HmHel-6	CAEZ01001805:81427-82117
HmHel-6	CAEZ01001805:97758-97921
HmHel-6	CAEZ01001805:59714-59802
HmHel-6	CAEZ01001805:37939-38101
HmHel-6	CAEZ01001805:1423-1545
HmHel-6	CAEZ01000060:16314-16487
HmHel-6	CAEZ01000060:13559-13656
HmHel-6	CAEZ01011465:3387-11916
HmHel-6	CAEZ01011414:37213-37293

HmHel-6	CAEZ01011414:88890-89081
HmHel-6	CAEZ01011414:44824-44965
HmHel-6	CAEZ01011414:25312-25545
HmHel-6	CAEZ01011258:267-7923
HmHel-6	CAEZ01011258:13710-13803
HmHel-6	CAEZ01011192:3337-3573
HmHel-6	CAEZ01011137:54730-54918
HmHel-6	CAEZ01011137:70666-70819
HmHel-6	CAEZ01011137:172277-172400
HmHel-6	CAEZ01011137:193592-193694
HmHel-6	CAEZ01011137:175685-175771
HmHel-6	CAEZ01011137:138507-138732
HmHel-6	CAEZ01011137:51048-51223
HmHel-6	CAEZ01010698:3705-3904
HmHel-6	CAEZ01010212:29003-29185
HmHel-6	CAEZ01010212:94164-94341
HmHel-6	CAEZ01010212:225-376
HmHel-6	CAEZ01010190:46792-47035
HmHel-6	CAEZ01010190:42623-42730
HmHel-6	CAEZ01010190:28277-28509
HmHel-6	CAEZ01010190:17494-17638
HmHel-6	CAEZ01010141:6059-6266
HmHel-6	CAEZ01010141:10633-14622
HmHel-6	CAEZ01009896:2453-7826
HmHel-6	CAEZ01009896:31015-31618
HmHel-6	CAEZ01009896:46966-47048
HmHel-6	CAEZ01009896:79796-79930
HmHel-6	CAEZ01009896:100337-100589
HmHel-6	CAEZ01009896:140047-145652
HmHel-6	CAEZ01009896:180195-180301
HmHel-6	CAEZ01009896:188187-188302
HmHel-6	CAEZ01009896:176607-176816
HmHel-6	CAEZ01009896:101727-109026
HmHel-6	CAEZ01009896:91160-91239
HmHel-6	CAEZ01009896:66744-66840
HmHel-6	CAEZ01009896:44803-44975
HmHel-6	CAEZ01009896:14836-20343
HmHel-6	CAEZ01009874:8263-8477
HmHel-6	CAEZ01009874:9472-9624
HmHel-6	CAEZ01009696:7077-7296
HmHel-6	CAEZ01009696:21055-21309
HmHel-6	CAEZ01009696:6004-6118
HmHel-6	CAEZ01009547:4590-4833
HmHel-6	CAEZ01009132:6379-6534
HmHel-6	CAEZ01009042:464-622
HmHel-6	CAEZ01009042:21232-21330
HmHel-6	CAEZ01009042:41445-41652
HmHel-6	CAEZ01009042:42829-42934

HmHel-6	CAEZ01009042:17408-21586
HmHel-6	CAEZ01008369:36088-37078
HmHel-6	CAEZ01008369:22679-22883
HmHel-6	CAEZ01008369:7840-7995
HmHel-6	CAEZ01008349:10768-10891
HmHel-6	CAEZ01008349:31576-31772
HmHel-6	CAEZ01008349:61115-61219
HmHel-6	CAEZ01008349:113933-114115
HmHel-6	CAEZ01008349:129364-129542
HmHel-6	CAEZ01008349:143397-143621
HmHel-6	CAEZ01008349:189683-196692
HmHel-6	CAEZ01008349:206334-206435
HmHel-6	CAEZ01008349:180208-180384
HmHel-6	CAEZ01008349:169081-169218
HmHel-6	CAEZ01008349:152709-152934
HmHel-6	CAEZ01008349:107496-107653
HmHel-6	CAEZ01008349:75612-85436
HmHel-6	CAEZ01008349:29523-37234
HmHel-6	CAEZ01008246:2608-2753
HmHel-6	CAEZ01008246:16503-24832
HmHel-6	CAEZ01008246:25008-25215
HmHel-6	CAEZ01007851:52729-52861
HmHel-6	CAEZ01007851:90422-90632
HmHel-6	CAEZ01007851:107123-107224
HmHel-6	CAEZ01007851:111476-111558
HmHel-6	CAEZ01007851:89737-89921
HmHel-6	CAEZ01007851:75868-76015
HmHel-6	CAEZ01007851:3176-3308
HmHel-6	CAEZ01007325:4940-5079
HmHel-6	CAEZ01007325:49794-49972
HmHel-6	CAEZ01007325:81497-89475
HmHel-6	CAEZ01007325:90486-99482
HmHel-6	CAEZ01007325:76311-76434
HmHel-6	CAEZ01007325:57830-60040
HmHel-6	CAEZ01007325:32701-32873
HmHel-6	CAEZ01007325:12279-12360
HmHel-6	CAEZ01007234:5430-5634
HmHel-6	CAEZ01007234:65159-65244
HmHel-6	CAEZ01007234:11305-11462
HmHel-6	CAEZ01007097:3024-3220
HmHel-6	CAEZ01006586:6572-6717
HmHel-6	CAEZ01006586:21568-21784
HmHel-6	CAEZ01006586:18093-18240
HmHel-6	CAEZ01006415:4068-12987
HmHel-6	CAEZ01006415:23729-30278
HmHel-6	CAEZ01006415:43396-43575
HmHel-6	CAEZ01006415:54020-54157
HmHel-6	CAEZ01006415:56472-59213

HmHel-6	CAEZ01006415:28380-28496
HmHel-6	CAEZ01006415:5779-5964
HmHel-6	CAEZ01006394:12652-12864
HmHel-6	CAEZ01006394:4950-9402
HmHel-6	CAEZ01006118:18658-20177
HmHel-6	CAEZ01006118:7948-8111
HmHel-6	CAEZ01006097:7589-7805
HmHel-6	CAEZ01006097:76440-76525
HmHel-6	CAEZ01006097:89853-90026
HmHel-6	CAEZ01006097:62324-62416
HmHel-6	CAEZ01006097:45578-45777
HmHel-6	CAEZ01005975:33433-34332
HmHel-6	CAEZ01005975:64421-64649
HmHel-6	CAEZ01005975:109589-109724
HmHel-6	CAEZ01005975:148059-148234
HmHel-6	CAEZ01005975:166884-166966
HmHel-6	CAEZ01005975:178800-178890
HmHel-6	CAEZ01005975:155442-158103
HmHel-6	CAEZ01005975:138364-138503
HmHel-6	CAEZ01005975:119096-119332
HmHel-6	CAEZ01005975:35519-35605
HmHel-6	CAEZ01005325:10516-10687
HmHel-6	CAEZ01005325:7013-7229
HmHel-6	CAEZ01004852:13912-14114
HmHel-6	CAEZ01003795:3997-4230
HmHel-6	CAEZ01003760:11441-17428
HmHel-6	CAEZ01003760:17041-17251
HmHel-6	CAEZ01003339:1009-1111
HmHel-6	CAEZ01003339:34077-34228
HmHel-6	CAEZ01003339:8661-8821
HmHel-6	CAEZ01002265:651-756
HmHel-6	CAEZ01002265:924-1131
HmHel-6	CAEZ01002160:2817-4894
HmHel-6	CAEZ01001943:1764-1931
HmHel-6	CAEZ01001556:10232-10467
HmHel-6	CAEZ01001556:7515-7726
HmHel-6	CAEZ01001152:19158-19237
HmHel-6	CAEZ01001152:4400-4488
HmHel-6	CAEZ01000931:17205-17368
HmHel-6	CAEZ01000931:16028-16170
HmHel-6	CAEZ01000763:3016-3252
HmHel-6	CAEZ01000082:4587-4842
HmHel-6	CAEZ01011179:6146-6352
HmHel-6	CAEZ01011179:31048-31179
HmHel-6	CAEZ01011179:16508-16709
HmHel-6	CAEZ01010932:52675-52842
HmHel-6	CAEZ01010932:18757-18867
HmHel-6	CAEZ01010893:4425-4627

HmHel-6	CAEZ01010893:17493-17584
HmHel-6	CAEZ01010893:44391-44539
HmHel-6	CAEZ01010893:105614-105711
HmHel-6	CAEZ01010893:71672-71881
HmHel-6	CAEZ01010893:54313-54440
HmHel-6	CAEZ01010893:17035-17237
HmHel-6	CAEZ01010218:11811-11896
HmHel-6	CAEZ01010218:30629-30723
HmHel-6	CAEZ01010218:64369-64605
HmHel-6	CAEZ01010218:44214-44414
HmHel-6	CAEZ01010145:7374-7551
HmHel-6	CAEZ01010145:24289-24557
HmHel-6	CAEZ01010145:986-1087
HmHel-6	CAEZ01009264:19148-19339
HmHel-6	CAEZ01009264:38227-38339
HmHel-6	CAEZ01009264:61154-61295
HmHel-6	CAEZ01009264:78644-80390
HmHel-6	CAEZ01009264:65963-66120
HmHel-6	CAEZ01009264:5402-12183
HmHel-6	CAEZ01008722:1679-1821
HmHel-6	CAEZ01008722:29019-36055
HmHel-6	CAEZ01008722:45093-45339
HmHel-6	CAEZ01008722:28647-28780
HmHel-6	CAEZ01008722:9375-16415
HmHel-6	CAEZ01008673:2466-3962
HmHel-6	CAEZ01008673:15116-15739
HmHel-6	CAEZ01008673:41764-41890
HmHel-6	CAEZ01008673:15764-15926
HmHel-6	CAEZ01008082:6979-7173
HmHel-6	CAEZ01008082:2511-2654
HmHel-6	CAEZ01007929:2951-3137
HmHel-6	CAEZ01007672:6407-6647
HmHel-6	CAEZ01007672:46162-48091
HmHel-6	CAEZ01007672:26884-27015
HmHel-6	CAEZ01007590:9827-10071
HmHel-6	CAEZ01007590:51550-51685
HmHel-6	CAEZ01007590:63955-64069
HmHel-6	CAEZ01007590:64603-64750
HmHel-6	CAEZ01007590:53953-54181
HmHel-6	CAEZ01007590:30425-33811
HmHel-6	CAEZ01007142:19658-20962
HmHel-6	CAEZ01007142:74523-74712
HmHel-6	CAEZ01007142:73265-73462
HmHel-6	CAEZ01007142:52250-52348
HmHel-6	CAEZ01007142:37853-40122
HmHel-6	CAEZ01006911:3343-3520
HmHel-6	CAEZ01006911:230-345
HmHel-6	CAEZ01006778:28834-29005

HmHel-6	CAEZ01006778:50679-50870
HmHel-6	CAEZ01006778:73628-77712
HmHel-6	CAEZ01006778:112064-119722
HmHel-6	CAEZ01006778:160596-160700
HmHel-6	CAEZ01006778:197869-197996
HmHel-6	CAEZ01006778:187460-187674
HmHel-6	CAEZ01006778:167053-167234
HmHel-6	CAEZ01006778:150556-150644
HmHel-6	CAEZ01006778:136358-136501
HmHel-6	CAEZ01006778:94590-94689
HmHel-6	CAEZ01006778:53499-53672
HmHel-6	CAEZ01006778:15141-18067
HmHel-6	CAEZ01005769:6811-7015
HmHel-6	CAEZ01005769:1895-1976
HmHel-6	CAEZ01005745:8562-8776
HmHel-6	CAEZ01005207:39267-39385
HmHel-6	CAEZ01005207:50330-50446
HmHel-6	CAEZ01005207:90691-90824
HmHel-6	CAEZ01005207:97734-97931
HmHel-6	CAEZ01005207:68148-68253
HmHel-6	CAEZ01005207:45717-45939
HmHel-6	CAEZ01004990:24408-24660
HmHel-6	CAEZ01004990:71385-71636
HmHel-6	CAEZ01004990:37025-44517
HmHel-6	CAEZ01004990:14277-21378
HmHel-6	CAEZ01004814:1953-5304
HmHel-6	CAEZ01004814:26350-30092
HmHel-6	CAEZ01004814:68114-68322
HmHel-6	CAEZ01004814:55584-55683
HmHel-6	CAEZ01004814:29400-30358
HmHel-6	CAEZ01004814:2463-2607
HmHel-6	CAEZ01004597:25017-30044
HmHel-6	CAEZ01003843:1625-1841
HmHel-6	CAEZ01003843:329-546
HmHel-6	CAEZ01003826:12371-12609
HmHel-6	CAEZ01003826:27177-31395
HmHel-6	CAEZ01003745:5109-5361
HmHel-6	CAEZ01003745:19297-19423
HmHel-6	CAEZ01003745:4025-4107
HmHel-6	CAEZ01003209:18127-18362
HmHel-6	CAEZ01003209:15004-15217
HmHel-6	CAEZ01003070:31167-31392
HmHel-6	CAEZ01003070:73570-76257
HmHel-6	CAEZ01003070:221403-221613
HmHel-6	CAEZ01003070:191919-192166
HmHel-6	CAEZ01003070:177156-177242
HmHel-6	CAEZ01003070:148309-148394
HmHel-6	CAEZ01003070:125027-125150

HmHel-6	CAEZ01003070:91376-91498
HmHel-6	CAEZ01003070:33242-38552
HmHel-6	CAEZ01003070:8865-8950
HmHel-6	CAEZ01002965:6706-6823
HmHel-6	CAEZ01002965:27240-30639
HmHel-6	CAEZ01002965:22023-22239
HmHel-6	CAEZ01002211:2264-2490
HmHel-6	CAEZ01002211:27607-33599
HmHel-6	CAEZ01002211:51238-51335
HmHel-6	CAEZ01002211:77603-77776
HmHel-6	CAEZ01002211:65076-65155
HmHel-6	CAEZ01002211:50463-54748
HmHel-6	CAEZ01001463:13361-14122
HmHel-6	CAEZ01001463:88245-88334
HmHel-6	CAEZ01001463:129516-129692
HmHel-6	CAEZ01001463:147029-147167
HmHel-6	CAEZ01001463:140705-141475
HmHel-6	CAEZ01001463:55127-62774
HmHel-6	CAEZ01001463:26692-33894
HmHel-6	CAEZ01001463:2129-2313
HmHel-6	CAEZ01000934:4695-4930
HmHel-6	CAEZ01010927:21438-21634
HmHel-6	CAEZ01010927:127365-128098
HmHel-6	CAEZ01010927:200571-200720
HmHel-6	CAEZ01010927:182046-183591
HmHel-6	CAEZ01010927:111461-111555
HmHel-6	CAEZ01010927:88100-89199
HmHel-6	CAEZ01010927:34533-34701
HmHel-6	CAEZ01010836:10982-11190
HmHel-6	CAEZ01010755:18067-18247
HmHel-6	CAEZ01010358:9910-12439
HmHel-6	CAEZ01010358:16176-16382
HmHel-6	CAEZ01010310:10754-10901
HmHel-6	CAEZ01010116:871-7492
HmHel-6	CAEZ01009609:38409-38534
HmHel-6	CAEZ01009609:74958-78306
HmHel-6	CAEZ01009609:68510-68691
HmHel-6	CAEZ01009609:41780-42034
HmHel-6	CAEZ01009609:24900-25013
HmHel-6	CAEZ01009170:7324-7444
HmHel-6	CAEZ01009170:11599-11820
HmHel-6	CAEZ01008770:13399-13599
HmHel-6	CAEZ01008770:48446-48583
HmHel-6	CAEZ01008770:68102-75434
HmHel-6	CAEZ01008770:51344-51561
HmHel-6	CAEZ01008770:14060-14213
HmHel-6	CAEZ01008576:94626-94774
HmHel-6	CAEZ01008576:136696-136777

HmHel-6	CAEZ01008576:86375-86587
HmHel-6	CAEZ01008576:32451-32676
HmHel-6	CAEZ01008539:1175-2712
HmHel-6	CAEZ01008539:16303-16552
HmHel-6	CAEZ01008468:3855-4055
HmHel-6	CAEZ01008468:257-1410
HmHel-6	CAEZ01008399:38902-47288
HmHel-6	CAEZ01008399:21376-21579
HmHel-6	CAEZ01008383:3088-3296
HmHel-6	CAEZ01008383:21716-21951
HmHel-6	CAEZ01007659:6062-10372
HmHel-6	CAEZ01007659:12237-12434
HmHel-6	CAEZ01006102:37605-42552
HmHel-6	CAEZ01006102:670-867
HmHel-6	CAEZ01006041:5444-5617
HmHel-6	CAEZ01005986:45750-46337
HmHel-6	CAEZ01005986:104889-105109
HmHel-6	CAEZ01005986:125453-125643
HmHel-6	CAEZ01005986:128426-128648
HmHel-6	CAEZ01005986:99460-103527
HmHel-6	CAEZ01005986:88742-88821
HmHel-6	CAEZ01005986:48810-48909
HmHel-6	CAEZ01005986:27018-27179
HmHel-6	CAEZ01005986:7297-7428
HmHel-6	CAEZ01004689:4504-4665
HmHel-6	CAEZ01004689:15024-15120
HmHel-6	CAEZ01004689:1206-1397
HmHel-6	CAEZ01004216:5860-6042
HmHel-6	CAEZ01004174:9271-9479
HmHel-6	CAEZ01004174:14682-14784
HmHel-6	CAEZ01004128:1344-1551
HmHel-6	CAEZ01004128:26097-26293
HmHel-6	CAEZ01003346:81796-81965
HmHel-6	CAEZ01003346:84250-89383
HmHel-6	CAEZ01003346:61284-65202
HmHel-6	CAEZ01003346:37584-40872
HmHel-6	CAEZ01003346:8214-8314
HmHel-6	CAEZ01003338:7768-7872
HmHel-6	CAEZ01003338:18594-24365
HmHel-6	CAEZ01003338:34815-34979
HmHel-6	CAEZ01003235:22611-22808
HmHel-6	CAEZ01003235:48464-48605
HmHel-6	CAEZ01003235:97760-97846
HmHel-6	CAEZ01003235:107194-107286
HmHel-6	CAEZ01003235:82865-82987
HmHel-6	CAEZ01003235:62693-64600
HmHel-6	CAEZ01003235:50696-50906
HmHel-6	CAEZ01003235:3543-3791

HmHel-6	CAEZ01002601:2607-2815
HmHel-6	CAEZ01001454:1678-1871
HmHel-6	CAEZ01000885:14170-14399
HmHel-6	CAEZ01000885:16596-16804
HmHel-6	CAEZ01011399:9002-9156
HmHel-6	CAEZ01011399:19737-29311
HmHel-6	CAEZ01011399:20199-20355
HmHel-6	CAEZ01010579:10162-10369
HmHel-6	CAEZ01010462:10851-15143
HmHel-6	CAEZ01010462:30936-31026
HmHel-6	CAEZ01010462:99649-99781
HmHel-6	CAEZ01010462:68863-73847
HmHel-6	CAEZ01010462:25820-25968
HmHel-6	CAEZ01010462:3954-4133
HmHel-6	CAEZ01009293:7666-7847
HmHel-6	CAEZ01008910:12721-12905
HmHel-6	CAEZ01008910:24393-32808
HmHel-6	CAEZ01008910:47642-47736
HmHel-6	CAEZ01008910:36944-37107
HmHel-6	CAEZ01008910:3243-3366
HmHel-6	CAEZ01008887:1667-7533
HmHel-6	CAEZ01008034:393-594
HmHel-6	CAEZ01008034:22831-23038
HmHel-6	CAEZ01008034:6467-6680
HmHel-6	CAEZ01007616:8450-8662
HmHel-6	CAEZ01007500:4134-4314
HmHel-6	CAEZ01007500:33001-39096
HmHel-6	CAEZ01007500:73348-75658
HmHel-6	CAEZ01007500:73595-80659
HmHel-6	CAEZ01007500:37075-39771
HmHel-6	CAEZ01007500:1517-11060
HmHel-6	CAEZ01006759:35840-35978
HmHel-6	CAEZ01006759:47566-47718
HmHel-6	CAEZ01006759:68654-71439
HmHel-6	CAEZ01006759:99476-100540
HmHel-6	CAEZ01006759:89863-90071
HmHel-6	CAEZ01006759:76193-76279
HmHel-6	CAEZ01006759:19196-19395
HmHel-6	CAEZ01006759:6959-7140
HmHel-6	CAEZ01005781:19739-19927
HmHel-6	CAEZ01005781:495-10627
HmHel-6	CAEZ01005746:47862-48074
HmHel-6	CAEZ01005746:15510-15610
HmHel-6	CAEZ01005221:14792-14969
HmHel-6	CAEZ01005221:39499-48841
HmHel-6	CAEZ01005221:55954-58006
HmHel-6	CAEZ01005221:35342-35450
HmHel-6	CAEZ01004507:20159-20412

HmHel-6	CAEZ01004507:11223-11446
HmHel-6	CAEZ01004226:33762-33926
HmHel-6	CAEZ01004161:7703-7907
HmHel-6	CAEZ01004161:14531-14821
HmHel-6	CAEZ01003945:14726-20476
HmHel-6	CAEZ01003945:26838-27043
HmHel-6	CAEZ01003945:566-5360
HmHel-6	CAEZ01003171:20808-21000
HmHel-6	CAEZ01003171:17252-17408
HmHel-6	CAEZ01002636:6932-7171
HmHel-6	CAEZ01002208:17592-17698
HmHel-6	CAEZ01002208:67673-69528
HmHel-6	CAEZ01002208:97160-97292
HmHel-6	CAEZ01002208:113705-113787
HmHel-6	CAEZ01002208:98404-107401
HmHel-6	CAEZ01002208:73763-76347
HmHel-6	CAEZ01002208:52033-52132
HmHel-6	CAEZ01010906:17295-17505
HmHel-6	CAEZ01010906:23760-23940
HmHel-6	CAEZ01010771:10952-11163
HmHel-6	CAEZ01010505:11936-12053
HmHel-6	CAEZ01010505:12833-13035
HmHel-6	CAEZ01009261:13467-15029
HmHel-6	CAEZ01009050:1569-1807
HmHel-6	CAEZ01009050:23818-23961
HmHel-6	CAEZ01009050:6874-14796
HmHel-6	CAEZ01009031:12420-12627
HmHel-6	CAEZ01009031:12795-12900
HmHel-6	CAEZ01008827:5052-5246
HmHel-6	CAEZ01008827:886-3838
HmHel-6	CAEZ01008259:527-734
HmHel-6	CAEZ01008098:3920-4054
HmHel-6	CAEZ01008098:92827-92935
HmHel-6	CAEZ01008098:48696-48894
HmHel-6	CAEZ01008098:15230-15341
HmHel-6	CAEZ01007282:11026-11246
HmHel-6	CAEZ01007282:1168-1325
HmHel-6	CAEZ01007073:10929-11147
HmHel-6	CAEZ01007040:12043-12175
HmHel-6	CAEZ01007040:17856-17977
HmHel-6	CAEZ01007014:3195-3378
HmHel-6	CAEZ01006765:7726-7909
HmHel-6	CAEZ01006634:43188-43355
HmHel-6	CAEZ01006634:31736-31845
HmHel-6	CAEZ01006634:8016-8248
HmHel-6	CAEZ01006525:1871-1964
HmHel-6	CAEZ01006525:952-1167
HmHel-6	CAEZ01006420:3330-3541

HmHel-6	CAEZ01006195:13755-13977
HmHel-6	CAEZ01006195:9419-9556
HmHel-6	CAEZ01006168:8399-8521
HmHel-6	CAEZ01006168:22979-23068
HmHel-6	CAEZ01006168:18729-18944
HmHel-6	CAEZ01006168:2637-2831
HmHel-6	CAEZ01005246:4346-4437
HmHel-6	CAEZ01005246:1229-1444
HmHel-6	CAEZ01004629:63923-64042
HmHel-6	CAEZ01004629:93312-93457
HmHel-6	CAEZ01004629:129360-129564
HmHel-6	CAEZ01004629:92547-92753
HmHel-6	CAEZ01004629:52811-53001
HmHel-6	CAEZ01004629:23863-24075
HmHel-6	CAEZ01004540:2632-6234
HmHel-6	CAEZ01004540:4867-4950
HmHel-6	CAEZ01004484:970-1180
HmHel-6	CAEZ01004460:11522-12722
HmHel-6	CAEZ01004460:10963-11082
HmHel-6	CAEZ01004460:421-631
HmHel-6	CAEZ01004036:1416-1627
HmHel-6	CAEZ01003369:3935-4043
HmHel-6	CAEZ01003369:27041-27143
HmHel-6	CAEZ01003369:23822-24029
HmHel-6	CAEZ01003369:4615-12549
HmHel-6	CAEZ01003316:10923-11066
HmHel-6	CAEZ01003316:17077-17203
HmHel-6	CAEZ01003303:750-917
HmHel-6	CAEZ01003303:15492-15707
HmHel-6	CAEZ01003261:8227-8465
HmHel-6	CAEZ01003261:12614-12738
HmHel-6	CAEZ01003246:11866-12080
HmHel-6	CAEZ01003246:32165-33819
HmHel-6	CAEZ01003246:20002-20087
HmHel-6	CAEZ01003246:3259-3470
HmHel-6	CAEZ01003112:6069-6770
HmHel-6	CAEZ01003112:27073-32724
HmHel-6	CAEZ01003112:1019-1170
HmHel-6	CAEZ01002723:19715-19892
HmHel-6	CAEZ01002723:41255-41383
HmHel-6	CAEZ01002723:15271-15466
HmHel-6	CAEZ01002643:11948-12091
HmHel-6	CAEZ01002643:51957-55650
HmHel-6	CAEZ01002643:57259-57364
HmHel-6	CAEZ01002643:38793-39784
HmHel-6	CAEZ01002643:7194-7312
HmHel-6	CAEZ01002501:9118-9325
HmHel-6	CAEZ01002501:13577-13760

HmHel-6	CAEZ01002271:92-307
HmHel-6	CAEZ01001465:8398-8534
HmHel-6	CAEZ01001465:23230-23370
HmHel-6	CAEZ01001465:40824-40910
HmHel-6	CAEZ01001465:73889-75986
HmHel-6	CAEZ01001465:98564-98660
HmHel-6	CAEZ01001465:44411-44607
HmHel-6	CAEZ01001465:16352-16576
HmHel-6	CAEZ01001332:79-294
HmHel-6	CAEZ01001228:1-85
HmHel-6	CAEZ01001228:259-466
HmHel-6	CAEZ01011572:973-1079
HmHel-6	CAEZ01011572:11296-11392
HmHel-6	CAEZ01010850:2540-2721
HmHel-6	CAEZ01010129:21674-21765
HmHel-6	CAEZ01010129:42774-52385
HmHel-6	CAEZ01010129:63267-63356
HmHel-6	CAEZ01010129:86280-86429
HmHel-6	CAEZ01010129:86916-88921
HmHel-6	CAEZ01010129:22839-22987
HmHel-6	CAEZ01010129:2816-2986
HmHel-6	CAEZ01010103:3187-9546
HmHel-6	CAEZ01010103:8853-9067
HmHel-6	CAEZ01009217:20694-20880
HmHel-6	CAEZ01009124:49672-49898
HmHel-6	CAEZ01009124:61120-61209
HmHel-6	CAEZ01009124:48622-48799
HmHel-6	CAEZ01008743:2883-2981
HmHel-6	CAEZ01008743:14464-14680
HmHel-6	CAEZ01008743:25177-25357
HmHel-6	CAEZ01008743:71048-76775
HmHel-6	CAEZ01008743:104555-104682
HmHel-6	CAEZ01008743:130183-130377
HmHel-6	CAEZ01008743:129835-136780
HmHel-6	CAEZ01008743:70538-79621
HmHel-6	CAEZ01008743:23045-23173
HmHel-6	CAEZ01008423:4253-4364
HmHel-6	CAEZ01008423:40658-40794
HmHel-6	CAEZ01008423:66729-66851
HmHel-6	CAEZ01008423:85565-85760
HmHel-6	CAEZ01008423:55612-55862
HmHel-6	CAEZ01008423:36262-36471
HmHel-6	CAEZ01007716:2396-3809
HmHel-6	CAEZ01007716:29402-35922
HmHel-6	CAEZ01007716:43025-43157
HmHel-6	CAEZ01007716:21598-26770
HmHel-6	CAEZ01007658:15320-15452
HmHel-6	CAEZ01007658:13283-13438

HmHel-6	CAEZ01007630:6190-6671
HmHel-6	CAEZ01007569:3669-3869
HmHel-6	CAEZ01007569:31585-31694
HmHel-6	CAEZ01007569:35109-35253
HmHel-6	CAEZ01007569:17145-17251
HmHel-6	CAEZ01007203:2032-2217
HmHel-6	CAEZ01007203:1814-1944
HmHel-6	CAEZ01007036:8573-8771
HmHel-6	CAEZ01007006:5929-6043
HmHel-6	CAEZ01007006:26312-26393
HmHel-6	CAEZ01007006:69322-69453
HmHel-6	CAEZ01007006:38445-38556
HmHel-6	CAEZ01007006:18984-19202
HmHel-6	CAEZ01006974:4509-4657
HmHel-6	CAEZ01006974:9907-10456
HmHel-6	CAEZ01006953:1375-1617
HmHel-6	CAEZ01006953:8473-8667
HmHel-6	CAEZ01006752:3930-4096
HmHel-6	CAEZ01006651:8302-8398
HmHel-6	CAEZ01006651:45876-46008
HmHel-6	CAEZ01006651:57856-57994
HmHel-6	CAEZ01006651:61321-61464
HmHel-6	CAEZ01006651:20564-20781
HmHel-6	CAEZ01006640:9563-9792
HmHel-6	CAEZ01006640:10155-10350
HmHel-6	CAEZ01006377:35978-36069
HmHel-6	CAEZ01006377:1710-1934
HmHel-6	CAEZ01005719:14863-15042
HmHel-6	CAEZ01005381:14869-15085
HmHel-6	CAEZ01005381:14441-14540
HmHel-6	CAEZ01005381:1103-1285
HmHel-6	CAEZ01005283:6664-6917
HmHel-6	CAEZ01005264:5688-5949
HmHel-6	CAEZ01005264:26685-26790
HmHel-6	CAEZ01005264:14226-14414
HmHel-6	CAEZ01005217:10066-10229
HmHel-6	CAEZ01005217:11085-11305
HmHel-6	CAEZ01004650:3684-3786
HmHel-6	CAEZ01004650:1164-1354
HmHel-6	CAEZ01003914:19609-19919
HmHel-6	CAEZ01003914:219-383
HmHel-6	CAEZ01003607:22918-23124
HmHel-6	CAEZ01003607:15345-15424
HmHel-6	CAEZ01002671:3072-3296
HmHel-6	CAEZ01001941:11150-11308
HmHel-6	CAEZ01001941:13790-13910
HmHel-6	CAEZ01001202:4626-4780
HmHel-6	CAEZ01000831:11325-11426

HmHel-6	CAEZ01000831:12312-16486
HmHel-6	CAEZ01010776:10041-10308
HmHel-6	CAEZ01010776:26232-26438
HmHel-6	CAEZ01010776:23577-24091
HmHel-6	CAEZ01010320:8017-8176
HmHel-6	CAEZ01010320:50578-50756
HmHel-6	CAEZ01010320:74904-78806
HmHel-6	CAEZ01010320:80746-80853
HmHel-6	CAEZ01010320:33085-33226
HmHel-6	CAEZ01010221:22620-22789
HmHel-6	CAEZ01010221:45153-45330
HmHel-6	CAEZ01010221:61150-61248
HmHel-6	CAEZ01010221:75493-75646
HmHel-6	CAEZ01010221:117300-124313
HmHel-6	CAEZ01010184:22184-22316
HmHel-6	CAEZ01010184:34531-34724
HmHel-6	CAEZ01010184:19112-19297
HmHel-6	CAEZ01010014:7312-7487
HmHel-6	CAEZ01010014:5144-7285
HmHel-6	CAEZ01009884:2720-6851
HmHel-6	CAEZ01009884:67005-67187
HmHel-6	CAEZ01009884:31516-31699
HmHel-6	CAEZ01009630:3413-3570
HmHel-6	CAEZ01009630:18287-18448
HmHel-6	CAEZ01009630:28504-28697
HmHel-6	CAEZ01009630:33781-33901
HmHel-6	CAEZ01009630:11259-11473
HmHel-6	CAEZ01009565:9721-9850
HmHel-6	CAEZ01009432:6142-6342
HmHel-6	CAEZ01009432:18943-19075
HmHel-6	CAEZ01009432:32800-33047
HmHel-6	CAEZ01009432:40724-40882
HmHel-6	CAEZ01009383:10191-19079
HmHel-6	CAEZ01009248:39144-39405
HmHel-6	CAEZ01009248:54491-54584
HmHel-6	CAEZ01008864:659-877
HmHel-6	CAEZ01008864:1323-1447
HmHel-6	CAEZ01008110:9745-9998
HmHel-6	CAEZ01008110:4758-4990
HmHel-6	CAEZ01006948:2065-2144
HmHel-6	CAEZ01006852:7280-7660
HmHel-6	CAEZ01006852:10309-10537
HmHel-6	CAEZ01006486:3273-3450
HmHel-6	CAEZ01005737:3331-3554
HmHel-6	CAEZ01005737:1623-7217
HmHel-6	CAEZ01005250:33100-33228
HmHel-6	CAEZ01005250:55965-59377
HmHel-6	CAEZ01005250:115441-115562

HmHel-6	CAEZ01005250:112479-112665
HmHel-6	CAEZ01005250:81482-81638
HmHel-6	CAEZ01004759:6101-11024
HmHel-6	CAEZ01004759:5891-6036
HmHel-6	CAEZ01004684:603-688
HmHel-6	CAEZ01004684:14076-14209
HmHel-6	CAEZ01004530:3580-12548
HmHel-6	CAEZ01004530:1655-1841
HmHel-6	CAEZ01003986:17710-17825
HmHel-6	CAEZ01003986:13117-17271
HmHel-6	CAEZ01003986:1712-1845
HmHel-6	CAEZ01003827:36093-36318
HmHel-6	CAEZ01003827:68007-71121
HmHel-6	CAEZ01003827:19455-19553
HmHel-6	CAEZ01003196:1978-2076
HmHel-6	CAEZ01003196:18679-18850
HmHel-6	CAEZ01003196:49425-49550
HmHel-6	CAEZ01003196:115464-115592
HmHel-6	CAEZ01003196:111296-116229
HmHel-6	CAEZ01003196:88788-88924
HmHel-6	CAEZ01003196:71205-71308
HmHel-6	CAEZ01003196:14694-20966
HmHel-6	CAEZ01003196:4313-4404
HmHel-6	CAEZ01003107:751-912
HmHel-6	CAEZ01003107:12264-12465
HmHel-6	CAEZ01003107:27516-29577
HmHel-6	CAEZ01003107:33649-35543
HmHel-6	CAEZ01003107:17711-18981
HmHel-6	CAEZ01003071:11495-11674
HmHel-6	CAEZ01003071:21620-21746
HmHel-6	CAEZ01003071:6139-6379
HmHel-6	CAEZ01001535:3284-3523
HmHel-6	CAEZ01001523:1729-1970
HmHel-6	CAEZ01001208:6297-6465
HmHel-6	CAEZ01000848:3842-13377
HmHel-6	CAEZ01000848:22560-26376
HmHel-6	CAEZ01011469:224-429
HmHel-6	CAEZ01010777:7174-7347
HmHel-6	CAEZ01010311:1052-1270
HmHel-6	CAEZ01010311:12267-12417
HmHel-6	CAEZ01009965:15378-15511
HmHel-6	CAEZ01009965:944-1064
HmHel-6	CAEZ01008863:6800-6911
HmHel-6	CAEZ01008863:8157-8318
HmHel-6	CAEZ01008092:1726-1971
HmHel-6	CAEZ01007967:1790-8558
HmHel-6	CAEZ01007967:3373-3546
HmHel-6	CAEZ01007847:6968-7171

HmHel-6	CAEZ01007847:5807-6032
HmHel-6	CAEZ01007749:28772-31837
HmHel-6	CAEZ01007749:46883-47039
HmHel-6	CAEZ01007368:7794-8062
HmHel-6	CAEZ01007368:7624-7737
HmHel-6	CAEZ01007108:22466-29162
HmHel-6	CAEZ01007108:63701-63796
HmHel-6	CAEZ01007108:79420-86361
HmHel-6	CAEZ01007108:131550-131769
HmHel-6	CAEZ01007108:93736-93917
HmHel-6	CAEZ01007108:50847-50982
HmHel-6	CAEZ01007108:36406-36497
HmHel-6	CAEZ01006648:1673-1780
HmHel-6	CAEZ01006648:13983-14163
HmHel-6	CAEZ01006412:5982-7403
HmHel-6	CAEZ01006412:36252-37843
HmHel-6	CAEZ01006412:55347-59032
HmHel-6	CAEZ01006412:102215-110539
HmHel-6	CAEZ01006412:108276-108476
HmHel-6	CAEZ01006412:13474-13627
HmHel-6	CAEZ01005666:7778-7954
HmHel-6	CAEZ01005666:11463-11598
HmHel-6	CAEZ01005323:3098-3246
HmHel-6	CAEZ01005260:11831-12036
HmHel-6	CAEZ01005072:10885-11092
HmHel-6	CAEZ01005072:4830-4948
HmHel-6	CAEZ01004845:7815-12502
HmHel-6	CAEZ01004845:55009-55220
HmHel-6	CAEZ01004845:18625-18826
HmHel-6	CAEZ01004093:17088-17279
HmHel-6	CAEZ01004093:41081-41279
HmHel-6	CAEZ01004093:38759-43652
HmHel-6	CAEZ01003497:5195-7729
HmHel-6	CAEZ01002943:11478-11691
HmHel-6	CAEZ01002943:758-909
HmHel-6	CAEZ01002872:18120-18275
HmHel-6	CAEZ01002872:55829-60682
HmHel-6	CAEZ01002872:56758-61912
HmHel-6	CAEZ01002872:32812-32901
HmHel-6	CAEZ01002332:29727-29942
HmHel-6	CAEZ01002180:23756-30413
HmHel-6	CAEZ01002180:59239-59324
HmHel-6	CAEZ01002180:71627-71832
HmHel-6	CAEZ01002180:103406-112528
HmHel-6	CAEZ01002180:60118-60301
HmHel-6	CAEZ01002180:24719-33360
HmHel-6	CAEZ01000957:14175-14324
HmHel-6	CAEZ01000957:11564-11813

HmHel-6	CAEZ01011611:1288-1462
HmHel-6	CAEZ01011611:27425-27579
HmHel-6	CAEZ01011611:1018-8163
HmHel-6	CAEZ01011598:8873-9090
HmHel-6	CAEZ01011598:21702-21917
HmHel-6	CAEZ01011598:61976-62113
HmHel-6	CAEZ01011598:85419-85577
HmHel-6	CAEZ01011598:137413-137616
HmHel-6	CAEZ01011598:84993-85101
HmHel-6	CAEZ01011598:42594-42715
HmHel-6	CAEZ01011598:22087-31982
HmHel-6	CAEZ01011435:3505-3748
HmHel-6	CAEZ01011418:1488-1639
HmHel-6	CAEZ01011016:2974-3101
HmHel-6	CAEZ01010923:10875-11002
HmHel-6	CAEZ01010923:7781-8024
HmHel-6	CAEZ01010151:2133-2260
HmHel-6	CAEZ01010151:12746-12831
HmHel-6	CAEZ01010151:4015-4112
HmHel-6	CAEZ01010049:6246-8921
HmHel-6	CAEZ01010049:15787-15874
HmHel-6	CAEZ01010049:957-1113
HmHel-6	CAEZ01010048:9591-9747
HmHel-6	CAEZ01009673:31600-35024
HmHel-6	CAEZ01009673:69473-69687
HmHel-6	CAEZ01009673:60028-60194
HmHel-6	CAEZ01009673:36109-45193
HmHel-6	CAEZ01009355:85157-85321
HmHel-6	CAEZ01009355:41246-41452
HmHel-6	CAEZ01009355:27370-27560
HmHel-6	CAEZ01009355:16064-16186
HmHel-6	CAEZ01008987:13582-13783
HmHel-6	CAEZ01008987:24422-26265
HmHel-6	CAEZ01008948:5943-13950
HmHel-6	CAEZ01008939:3047-3131
HmHel-6	CAEZ01008939:8881-9052
HmHel-6	CAEZ01008882:5274-11037
HmHel-6	CAEZ01008882:40418-40626
HmHel-6	CAEZ01008774:27613-27865
HmHel-6	CAEZ01008339:276-483
HmHel-6	CAEZ01008339:649-754
HmHel-6	CAEZ01008134:1161-1404
HmHel-6	CAEZ01007527:1528-1735
HmHel-6	CAEZ01007416:517-709
HmHel-6	CAEZ01007354:1333-1580
HmHel-6	CAEZ01007354:3895-4005
HmHel-6	CAEZ01006999:2881-3091
HmHel-6	CAEZ01006999:9738-9961

HmHel-6	CAEZ01006494:16593-16685
HmHel-6	CAEZ01006494:29034-29248
HmHel-6	CAEZ01006494:45119-45333
HmHel-6	CAEZ01006494:34124-34290
HmHel-6	CAEZ01006493:1934-2015
HmHel-6	CAEZ01006493:6141-6332
HmHel-6	CAEZ01006153:429-4532
HmHel-6	CAEZ01006153:16819-17045
HmHel-6	CAEZ01006153:60208-60297
HmHel-6	CAEZ01006153:46230-48699
HmHel-6	CAEZ01006153:14901-15060
HmHel-6	CAEZ01006153:2344-2548
HmHel-6	CAEZ01006077:38236-38449
HmHel-6	CAEZ01005906:12810-13003
HmHel-6	CAEZ01005906:21815-21980
HmHel-6	CAEZ01005906:255-2191
HmHel-6	CAEZ01005658:1855-3044
HmHel-6	CAEZ01005504:7900-8055
HmHel-6	CAEZ01005504:17322-24065
HmHel-6	CAEZ01005309:5906-6007
HmHel-6	CAEZ01005309:29118-29241
HmHel-6	CAEZ01005074:1153-1368
HmHel-6	CAEZ01005068:1867-2082
HmHel-6	CAEZ01004820:17327-17419
HmHel-6	CAEZ01004820:34479-34662
HmHel-6	CAEZ01004820:58664-58877
HmHel-6	CAEZ01004820:68965-69072
HmHel-6	CAEZ01004820:63626-69979
HmHel-6	CAEZ01004820:42505-42613
HmHel-6	CAEZ01004820:20463-20724
HmHel-6	CAEZ01004446:2067-2327
HmHel-6	CAEZ01004304:11648-11855
HmHel-6	CAEZ01004104:5967-6134
HmHel-6	CAEZ01004104:40287-40396
HmHel-6	CAEZ01004104:3233-3390
HmHel-6	CAEZ01004032:5858-5994
HmHel-6	CAEZ01004032:22255-22490
HmHel-6	CAEZ01004032:20971-23812
HmHel-6	CAEZ01004032:5329-5447
HmHel-6	CAEZ01003981:6009-9330
HmHel-6	CAEZ01003981:8236-8367
HmHel-6	CAEZ01003565:580-756
HmHel-6	CAEZ01003211:5112-5203
HmHel-6	CAEZ01003211:17416-17529
HmHel-6	CAEZ01003211:53063-55493
HmHel-6	CAEZ01003211:51708-51913
HmHel-6	CAEZ01003211:11480-12372
HmHel-6	CAEZ01002701:2661-4258

HmHel-6	CAEZ01002701:5977-6128
HmHel-6	CAEZ01002620:6655-6780
HmHel-6	CAEZ01002620:45235-45433
HmHel-6	CAEZ01002620:57178-57283
HmHel-6	CAEZ01002620:91097-98923
HmHel-6	CAEZ01002620:87868-88089
HmHel-6	CAEZ01002620:14752-14842
HmHel-6	CAEZ01002301:3173-3392
HmHel-6	CAEZ01002120:19935-20142
HmHel-6	CAEZ01001914:13346-13480
HmHel-6	CAEZ01001914:33575-33750
HmHel-6	CAEZ01001914:52791-52983
HmHel-6	CAEZ01001563:27616-35508
HmHel-6	CAEZ01001563:41644-46458
HmHel-6	CAEZ01001174:4700-8337
HmHel-6	CAEZ01011045:3821-4050
HmHel-6	CAEZ01011045:11489-11587
HmHel-6	CAEZ01010668:17047-17247
HmHel-6	CAEZ01010033:745-967
HmHel-6	CAEZ01010033:7316-15147
HmHel-6	CAEZ01009989:35622-35749
HmHel-6	CAEZ01009989:61954-62130
HmHel-6	CAEZ01009989:75756-75990
HmHel-6	CAEZ01009989:57658-59019
HmHel-6	CAEZ01009989:36673-36774
HmHel-6	CAEZ01009901:18160-18363
HmHel-6	CAEZ01009901:56423-56580
HmHel-6	CAEZ01009901:71994-72148
HmHel-6	CAEZ01009901:84165-84274
HmHel-6	CAEZ01009901:103005-103089
HmHel-6	CAEZ01009901:83030-83174
HmHel-6	CAEZ01009901:54553-54644
HmHel-6	CAEZ01009901:32023-32185
HmHel-6	CAEZ01009790:987-1173
HmHel-6	CAEZ01009790:3563-3655
HmHel-6	CAEZ01008734:5678-6309
HmHel-6	CAEZ01008734:62919-63059
HmHel-6	CAEZ01008734:76491-76572
HmHel-6	CAEZ01008734:68846-69039
HmHel-6	CAEZ01008543:112-214
HmHel-6	CAEZ01008543:5118-5252
HmHel-6	CAEZ01008375:16643-16850
HmHel-6	CAEZ01008375:28983-29140
HmHel-6	CAEZ01008375:28032-28141
HmHel-6	CAEZ01008158:5771-5879
HmHel-6	CAEZ01008158:32270-32376
HmHel-6	CAEZ01008158:43665-43870
HmHel-6	CAEZ01008158:21454-21535

HmHel-6	CAEZ01007995:18973-19137
HmHel-6	CAEZ01007995:26049-26219
HmHel-6	CAEZ01007995:12733-12895
HmHel-6	CAEZ01007960:16471-16603
HmHel-6	CAEZ01007960:63265-70606
HmHel-6	CAEZ01007960:105829-105937
HmHel-6	CAEZ01007960:77561-77640
HmHel-6	CAEZ01007960:30973-31075
HmHel-6	CAEZ01007429:22148-22298
HmHel-6	CAEZ01007429:69359-69549
HmHel-6	CAEZ01007429:76364-81644
HmHel-6	CAEZ01007429:48442-51322
HmHel-6	CAEZ01007429:35864-36023
HmHel-6	CAEZ01007429:8478-8595
HmHel-6	CAEZ01007137:4582-4725
HmHel-6	CAEZ01007137:3691-3853
HmHel-6	CAEZ01007056:2612-3408
HmHel-6	CAEZ01006594:2276-3047
HmHel-6	CAEZ01006594:21847-31804
HmHel-6	CAEZ01006594:46760-47008
HmHel-6	CAEZ01006594:64990-65077
HmHel-6	CAEZ01006281:8305-8446
HmHel-6	CAEZ01006281:30837-31135
HmHel-6	CAEZ01006281:14924-15081
HmHel-6	CAEZ01005949:41798-41922
HmHel-6	CAEZ01005949:58585-59001
HmHel-6	CAEZ01005949:101804-101998
HmHel-6	CAEZ01005949:114627-122481
HmHel-6	CAEZ01005949:78570-78672
HmHel-6	CAEZ01005949:55472-56808
HmHel-6	CAEZ01005949:28792-34744
HmHel-6	CAEZ01005861:5347-5492
HmHel-6	CAEZ01005253:7485-7995
HmHel-6	CAEZ01005253:38630-38708
HmHel-6	CAEZ01005253:41405-41565
HmHel-6	CAEZ01005253:22481-22619
HmHel-6	CAEZ01004575:31510-31752
HmHel-6	CAEZ01004296:13215-13441
HmHel-6	CAEZ01004296:10351-10586
HmHel-6	CAEZ01004190:6268-6371
HmHel-6	CAEZ01004190:3797-6161
HmHel-6	CAEZ01003955:6398-6538
HmHel-6	CAEZ01003955:5141-5374
HmHel-6	CAEZ01003895:26357-26519
HmHel-6	CAEZ01003895:8797-8960
HmHel-6	CAEZ01003829:4849-5088
HmHel-6	CAEZ01003208:1001-1229
HmHel-6	CAEZ01003208:22808-23013

HmHel-6	CAEZ01003208:35445-35554
HmHel-6	CAEZ01003208:18443-18627
HmHel-6	CAEZ01002720:12160-12272
HmHel-6	CAEZ01002720:3650-3862
HmHel-6	CAEZ01001807:3965-4146
HmHel-6	CAEZ01001807:48436-49684
HmHel-6	CAEZ01001807:20572-20726
HmHel-6	CAEZ01000704:2209-2403
HmHel-6	CAEZ01000585:1303-1544
HmHel-6	CAEZ01010829:29284-29378
HmHel-6	CAEZ01010829:54803-60536
HmHel-6	CAEZ01010829:135027-135184
HmHel-6	CAEZ01010829:154904-155133
HmHel-6	CAEZ01010829:172194-172395
HmHel-6	CAEZ01010829:185992-190936
HmHel-6	CAEZ01010829:200989-201129
HmHel-6	CAEZ01010829:221083-225016
HmHel-6	CAEZ01010829:239456-239669
HmHel-6	CAEZ01010829:265113-266206
HmHel-6	CAEZ01010829:206186-206423
HmHel-6	CAEZ01010829:177676-177791
HmHel-6	CAEZ01010829:159720-159898
HmHel-6	CAEZ01010829:111254-111395
HmHel-6	CAEZ01010829:67883-68001
HmHel-6	CAEZ01010566:5854-5941
HmHel-6	CAEZ01010566:24257-34153
HmHel-6	CAEZ01010566:29259-29349
HmHel-6	CAEZ01010566:15728-15970
HmHel-6	CAEZ01010566:2456-2553
HmHel-6	CAEZ01010244:8798-9007
HmHel-6	CAEZ01010244:47897-48016
HmHel-6	CAEZ01010244:18780-18929
HmHel-6	CAEZ01010075:5689-5875
HmHel-6	CAEZ01009808:2722-2812
HmHel-6	CAEZ01009808:13618-13770
HmHel-6	CAEZ01009808:10457-13060
HmHel-6	CAEZ01009654:2051-2176
HmHel-6	CAEZ01009654:15401-15484
HmHel-6	CAEZ01009654:1337-1425
HmHel-6	CAEZ01009593:3917-8616
HmHel-6	CAEZ01009582:922-1004
HmHel-6	CAEZ01009582:16148-17972
HmHel-6	CAEZ01009582:17167-17367
HmHel-6	CAEZ01009582:5435-5643
HmHel-6	CAEZ01009569:4694-4851
HmHel-6	CAEZ01009569:6875-7120
HmHel-6	CAEZ01009462:2352-5481
HmHel-6	CAEZ01009462:9345-9538

HmHel-6	CAEZ01009370:152645-152818
HmHel-6	CAEZ01009370:166068-166192
HmHel-6	CAEZ01009370:153260-153371
HmHel-6	CAEZ01009370:69750-69919
HmHel-6	CAEZ01009370:4608-4705
HmHel-6	CAEZ01008691:13659-13858
HmHel-6	CAEZ01008262:4054-4252
HmHel-6	CAEZ01008262:33717-35407
HmHel-6	CAEZ01008262:46416-47381
HmHel-6	CAEZ01008262:63238-63458
HmHel-6	CAEZ01008262:23736-23853
HmHel-6	CAEZ01008143:7407-7586
HmHel-6	CAEZ01008127:30611-30821
HmHel-6	CAEZ01008127:15647-15814
HmHel-6	CAEZ01007928:23242-23548
HmHel-6	CAEZ01007928:39173-41374
HmHel-6	CAEZ01007928:8787-8888
HmHel-6	CAEZ01007387:3250-3418
HmHel-6	CAEZ01007387:4013-4099
HmHel-6	CAEZ01007256:44489-44751
HmHel-6	CAEZ01007256:63790-69170
HmHel-6	CAEZ01007256:48274-53389
HmHel-6	CAEZ01007256:871-962
HmHel-6	CAEZ01007191:2412-2593
HmHel-6	CAEZ01007151:21858-21999
HmHel-6	CAEZ01007151:22426-22572
HmHel-6	CAEZ01006988:347-4677
HmHel-6	CAEZ01006988:14816-23273
HmHel-6	CAEZ01006798:7767-8004
HmHel-6	CAEZ01006579:1818-1943
HmHel-6	CAEZ01006579:8193-8339
HmHel-6	CAEZ01006030:11781-11904
HmHel-6	CAEZ01006030:6914-7130
HmHel-6	CAEZ01006023:933-1063
HmHel-6	CAEZ01006023:32162-33734
HmHel-6	CAEZ01006023:27699-27880
HmHel-6	CAEZ01006023:13172-13320
HmHel-6	CAEZ01005831:1772-6664
HmHel-6	CAEZ01005831:27336-27429
HmHel-6	CAEZ01005762:7021-7253
HmHel-6	CAEZ01005514:5679-5885
HmHel-6	CAEZ01005442:20190-20288
HmHel-6	CAEZ01005442:114633-114881
HmHel-6	CAEZ01005442:136100-136312
HmHel-6	CAEZ01005199:29714-34211
HmHel-6	CAEZ01005199:60286-60494
HmHel-6	CAEZ01005199:26696-28364
HmHel-6	CAEZ01004518:4294-4467

HmHel-6	CAEZ01004430:9592-9674
HmHel-6	CAEZ01004430:26857-27066
HmHel-6	CAEZ01002966:845-3567
HmHel-6	CAEZ01002113:2968-3109
HmHel-6	CAEZ01002113:252-362
HmHel-6	CAEZ01001412:742-819
HmHel-6	CAEZ01001412:3201-3427
HmHel-6	CAEZ01000999:22746-22956
HmHel-6	CAEZ01000999:46760-46915
HmHel-6	CAEZ01000999:65769-65862
HmHel-6	CAEZ01000999:51112-51280
HmHel-6	CAEZ01011488:7692-14636
HmHel-6	CAEZ01011488:25453-25613
HmHel-6	CAEZ01011488:11126-11227
HmHel-6	CAEZ01011217:2859-11068
HmHel-6	CAEZ01011217:45521-45745
HmHel-6	CAEZ01011217:65440-65588
HmHel-6	CAEZ01011217:76030-76240
HmHel-6	CAEZ01011217:104194-104300
HmHel-6	CAEZ01011217:155651-155755
HmHel-6	CAEZ01011217:101635-101743
HmHel-6	CAEZ01011217:70865-71027
HmHel-6	CAEZ01011217:31672-31775
HmHel-6	CAEZ01011217:11800-11934
HmHel-6	CAEZ01011122:12036-12253
HmHel-6	CAEZ01011122:14272-17453
HmHel-6	CAEZ01009970:1011-1264
HmHel-6	CAEZ01009970:20466-26312
HmHel-6	CAEZ01009970:77261-77352
HmHel-6	CAEZ01009970:79055-82540
HmHel-6	CAEZ01009970:62647-62830
HmHel-6	CAEZ01009970:47522-47649
HmHel-6	CAEZ01009970:17530-25475
HmHel-6	CAEZ01009709:58944-59114
HmHel-6	CAEZ01009709:102318-102439
HmHel-6	CAEZ01009709:69575-77298
HmHel-6	CAEZ01009709:16186-16323
HmHel-6	CAEZ01009707:20990-21210
HmHel-6	CAEZ01009605:5540-11405
HmHel-6	CAEZ01009605:22748-22842
HmHel-6	CAEZ01009605:30111-30245
HmHel-6	CAEZ01009113:27157-27377
HmHel-6	CAEZ01008923:22187-22397
HmHel-6	CAEZ01008923:20987-21205
HmHel-6	CAEZ01008923:6742-6948
HmHel-6	CAEZ01008823:5643-5864
HmHel-6	CAEZ01008761:1315-1517
HmHel-6	CAEZ01008761:12028-12228

HmHel-6	CAEZ01008761:8527-8753
HmHel-6	CAEZ01008341:17131-17233
HmHel-6	CAEZ01008341:59543-59715
HmHel-6	CAEZ01008341:86240-86475
HmHel-6	CAEZ01008341:108865-109100
HmHel-6	CAEZ01008341:73852-77577
HmHel-6	CAEZ01008341:47193-47348
HmHel-6	CAEZ01008341:22822-22946
HmHel-6	CAEZ01008341:9838-9964
HmHel-6	CAEZ01008240:19648-19808
HmHel-6	CAEZ01008029:9617-9765
HmHel-6	CAEZ01007708:3157-3369
HmHel-6	CAEZ01007708:31837-32065
HmHel-6	CAEZ01007708:54853-55196
HmHel-6	CAEZ01007708:74616-74775
HmHel-6	CAEZ01007708:88192-88301
HmHel-6	CAEZ01007708:12991-13095
HmHel-6	CAEZ01007265:10780-11013
HmHel-6	CAEZ01007265:29051-31226
HmHel-6	CAEZ01007265:4173-4378
HmHel-6	CAEZ01007247:1280-1513
HmHel-6	CAEZ01007109:3940-4032
HmHel-6	CAEZ01007109:15915-18990
HmHel-6	CAEZ01007109:19155-19261
HmHel-6	CAEZ01007069:4193-4424
HmHel-6	CAEZ01006773:37987-38147
HmHel-6	CAEZ01006773:82900-83011
HmHel-6	CAEZ01006773:93046-97073
HmHel-6	CAEZ01006773:180130-189891
HmHel-6	CAEZ01006773:190781-190893
HmHel-6	CAEZ01006773:135238-135355
HmHel-6	CAEZ01006773:95252-99936
HmHel-6	CAEZ01006773:69301-69464
HmHel-6	CAEZ01006490:32079-32280
HmHel-6	CAEZ01006490:57201-57430
HmHel-6	CAEZ01006490:85801-85900
HmHel-6	CAEZ01006490:91286-91434
HmHel-6	CAEZ01006490:49081-49297
HmHel-6	CAEZ01006490:5399-10609
HmHel-6	CAEZ01006048:3921-4006
HmHel-6	CAEZ01006048:13208-13306
HmHel-6	CAEZ01005799:388-7022
HmHel-6	CAEZ01005799:6270-12614
HmHel-6	CAEZ01005469:4757-4934
HmHel-6	CAEZ01005469:11932-12110
HmHel-6	CAEZ01005035:14934-15013
HmHel-6	CAEZ01005035:29258-29419
HmHel-6	CAEZ01005035:39951-45580

HmHel-6	CAEZ01005035:7559-7745
HmHel-6	CAEZ01004156:7165-7371
HmHel-6	CAEZ01003802:13169-13284
HmHel-6	CAEZ01003802:21663-21815
HmHel-6	CAEZ01003802:566-758
HmHel-6	CAEZ01003207:47856-47990
HmHel-6	CAEZ01003207:25783-25987
HmHel-6	CAEZ01002815:27360-42307
HmHel-6	CAEZ01002815:137151-139233
HmHel-6	CAEZ01002815:176439-176616
HmHel-6	CAEZ01002815:149969-150071
HmHel-6	CAEZ01002815:111002-111143
HmHel-6	CAEZ01002815:72788-72954
HmHel-6	CAEZ01002815:55635-55751
HmHel-6	CAEZ01002815:18890-19007
HmHel-6	CAEZ01001441:3115-10126
HmHel-6	CAEZ01001441:30028-30257
HmHel-6	CAEZ01001441:101439-101548
HmHel-6	CAEZ01001441:57005-57213
HmHel-6	CAEZ01001441:43306-43490
HmHel-6	CAEZ01001441:18963-19219
HmHel-6	CAEZ01001281:4428-4645
HmHel-6	CAEZ01001281:2301-2400
HmHel-6	CAEZ01001181:4890-5013
HmHel-6	CAEZ01011473:9524-9632
HmHel-6	CAEZ01011473:2294-2444
HmHel-6	CAEZ01011117:15580-15775
HmHel-6	CAEZ01010652:15686-15767
HmHel-6	CAEZ01010652:18339-18546
HmHel-6	CAEZ01010123:6723-6940
HmHel-6	CAEZ01010123:27020-27149
HmHel-6	CAEZ01010123:21844-29494
HmHel-6	CAEZ01010123:11526-11677
HmHel-6	CAEZ01010085:492-698
HmHel-6	CAEZ01009781:8932-9150
HmHel-6	CAEZ01009781:33450-33619
HmHel-6	CAEZ01009537:5156-9437
HmHel-6	CAEZ01009537:27306-27398
HmHel-6	CAEZ01009537:16838-17021
HmHel-6	CAEZ01009512:29427-29516
HmHel-6	CAEZ01009512:26984-30226
HmHel-6	CAEZ01009403:29576-29699
HmHel-6	CAEZ01009403:44435-44570
HmHel-6	CAEZ01009403:11623-11789
HmHel-6	CAEZ01009137:13975-14147
HmHel-6	CAEZ01009137:52806-52905
HmHel-6	CAEZ01009137:101361-101525
HmHel-6	CAEZ01009137:56331-56565

HmHel-6	CAEZ01009137:10701-13443
HmHel-6	CAEZ01008956:8190-17165
HmHel-6	CAEZ01008956:44335-44565
HmHel-6	CAEZ01008688:10287-10376
HmHel-6	CAEZ01008688:13987-14172
HmHel-6	CAEZ01008611:23951-24148
HmHel-6	CAEZ01008611:428-532
HmHel-6	CAEZ01008425:8121-8248
HmHel-6	CAEZ01008425:67627-67818
HmHel-6	CAEZ01008425:106539-106660
HmHel-6	CAEZ01008425:128449-128559
HmHel-6	CAEZ01008425:75869-76176
HmHel-6	CAEZ01008425:36716-36899
HmHel-6	CAEZ01008425:2922-3086
HmHel-6	CAEZ01008190:4074-4289
HmHel-6	CAEZ01008190:13940-14105
HmHel-6	CAEZ01007745:4956-10993
HmHel-6	CAEZ01007745:49122-49219
HmHel-6	CAEZ01007745:73182-73397
HmHel-6	CAEZ01007745:106262-106372
HmHel-6	CAEZ01007745:63663-63794
HmHel-6	CAEZ01007745:33773-40493
HmHel-6	CAEZ01007745:6294-9801
HmHel-6	CAEZ01007559:7707-7928
HmHel-6	CAEZ01007229:2005-2187
HmHel-6	CAEZ01006317:1848-2077
HmHel-6	CAEZ01006317:11822-11925
HmHel-6	CAEZ01006285:66-281
HmHel-6	CAEZ01005729:24340-28464
HmHel-6	CAEZ01005729:26403-26570
HmHel-6	CAEZ01005590:25061-25256
HmHel-6	CAEZ01005590:6128-12683
HmHel-6	CAEZ01005583:1185-1348
HmHel-6	CAEZ01005443:12613-12765
HmHel-6	CAEZ01005443:18095-18292
HmHel-6	CAEZ01005267:30336-30475
HmHel-6	CAEZ01005267:86514-86624
HmHel-6	CAEZ01005267:105427-105543
HmHel-6	CAEZ01005267:120425-120536
HmHel-6	CAEZ01005267:150992-151174
HmHel-6	CAEZ01005267:148071-148217
HmHel-6	CAEZ01005267:135060-135195
HmHel-6	CAEZ01005267:81216-89212
HmHel-6	CAEZ01005267:58328-58457
HmHel-6	CAEZ01005141:12535-14732
HmHel-6	CAEZ01004864:7721-7859
HmHel-6	CAEZ01004864:7132-7213
HmHel-6	CAEZ01004501:1192-1415

HmHel-6	CAEZ01004261:21194-21361
HmHel-6	CAEZ01003796:7025-7143
HmHel-6	CAEZ01003796:10876-11128
HmHel-6	CAEZ01003635:5750-5977
HmHel-6	CAEZ01003635:19091-19194
HmHel-6	CAEZ01003375:9297-9508
HmHel-6	CAEZ01003181:42182-42325
HmHel-6	CAEZ01003181:38354-38587
HmHel-6	CAEZ01003181:4501-12401
HmHel-6	CAEZ01003085:22019-22206
HmHel-6	CAEZ01003085:2235-2471
HmHel-6	CAEZ01002360:8847-8974
HmHel-6	CAEZ01002360:1327-1570
HmHel-6	CAEZ01001420:3212-3378
HmHel-6	CAEZ01001114:13112-13323
HmHel-6	CAEZ01001114:15528-15620
HmHel-6	CAEZ01011228:4865-5075
HmHel-6	CAEZ01008944:1429-1538
HmHel-6	CAEZ01008944:65311-65491
HmHel-6	CAEZ01008944:46496-50522
HmHel-6	CAEZ01008944:10366-15678
HmHel-6	CAEZ01008656:5273-5475
HmHel-6	CAEZ01008352:6769-6893
HmHel-6	CAEZ01008352:30368-30500
HmHel-6	CAEZ01008352:40791-40892
HmHel-6	CAEZ01008352:104311-104524
HmHel-6	CAEZ01008352:123833-124001
HmHel-6	CAEZ01008352:7401-7491
HmHel-6	CAEZ01008288:10483-10704
HmHel-6	CAEZ01008288:30177-30329
HmHel-6	CAEZ01007951:6798-7067
HmHel-6	CAEZ01007808:4666-4850
HmHel-6	CAEZ01007808:24719-24897
HmHel-6	CAEZ01007808:95751-95842
HmHel-6	CAEZ01007808:63076-63157
HmHel-6	CAEZ01007808:19876-20047
HmHel-6	CAEZ01006699:29349-29563
HmHel-6	CAEZ01006301:16501-16742
HmHel-6	CAEZ01006260:4803-4985
HmHel-6	CAEZ01006205:10959-11198
HmHel-6	CAEZ01006205:6282-6485
HmHel-6	CAEZ01006005:9736-9914
HmHel-6	CAEZ01005157:12949-13069
HmHel-6	CAEZ01005157:697-911
HmHel-6	CAEZ01005057:3595-3690
HmHel-6	CAEZ01004993:11-173
HmHel-6	CAEZ01004993:2316-7090
HmHel-6	CAEZ01004836:10468-18558

HmHel-6	CAEZ01004836:31290-31494
HmHel-6	CAEZ01004836:1782-5262
HmHel-6	CAEZ01004585:36226-36378
HmHel-6	CAEZ01004585:30833-30924
HmHel-6	CAEZ01004585:11730-11920
HmHel-6	CAEZ01004519:15637-15763
HmHel-6	CAEZ01004322:218-470
HmHel-6	CAEZ01003922:6014-6182
HmHel-6	CAEZ01003922:7266-7506
HmHel-6	CAEZ01003073:9156-9290
HmHel-6	CAEZ01003073:3909-12935
HmHel-6	CAEZ01002697:4511-4657
HmHel-6	CAEZ01002697:3818-3976
HmHel-6	CAEZ01001538:17482-17610
HmHel-6	CAEZ01001538:50694-50831
HmHel-6	CAEZ01001078:1284-1470
HmHel-6	CAEZ01001034:2469-2725
HmHel-6	CAEZ01001034:7158-7363
HmHel-6	CAEZ01011605:2156-2305
HmHel-6	CAEZ01011605:24573-24693
HmHel-6	CAEZ01011605:68271-68365
HmHel-6	CAEZ01011605:62991-63186
HmHel-6	CAEZ01011605:9926-10056
HmHel-6	CAEZ01011380:1649-1800
HmHel-6	CAEZ01011380:4611-4759
HmHel-6	CAEZ01011031:11055-11227
HmHel-6	CAEZ01011031:7355-7472
HmHel-6	CAEZ01010436:856-1079
HmHel-6	CAEZ01010436:11118-11212
HmHel-6	CAEZ01010436:15575-15787
HmHel-6	CAEZ01010381:8925-9124
HmHel-6	CAEZ01010381:19134-19383
HmHel-6	CAEZ01009750:25590-25727
HmHel-6	CAEZ01009750:5007-5220
HmHel-6	CAEZ01008950:2078-3352
HmHel-6	CAEZ01008878:730-923
HmHel-6	CAEZ01008878:48252-48362
HmHel-6	CAEZ01008878:39090-41079
HmHel-6	CAEZ01008684:3331-3553
HmHel-6	CAEZ01008610:2617-2836
HmHel-6	CAEZ01008610:5825-6035
HmHel-6	CAEZ01008503:740-951
HmHel-6	CAEZ01008303:4463-4611
HmHel-6	CAEZ01008303:15710-15804
HmHel-6	CAEZ01008303:72896-82680
HmHel-6	CAEZ01008303:73619-73772
HmHel-6	CAEZ01008303:7176-7400
HmHel-6	CAEZ01007705:3850-3984

HmHel-6	CAEZ01007705:48145-48322
HmHel-6	CAEZ01007705:61862-62021
HmHel-6	CAEZ01007705:75698-75849
HmHel-6	CAEZ01007705:38734-38938
HmHel-6	CAEZ01007705:2220-2402
HmHel-6	CAEZ01007612:4515-4752
HmHel-6	CAEZ01007593:5206-5361
HmHel-6	CAEZ01007593:7056-7229
HmHel-6	CAEZ01007232:12612-12816
HmHel-6	CAEZ01007232:35031-42512
HmHel-6	CAEZ01007232:57282-57414
HmHel-6	CAEZ01007232:33151-38409
HmHel-6	CAEZ01007066:1897-4181
HmHel-6	CAEZ01007066:34643-34851
HmHel-6	CAEZ01007066:86011-86206
HmHel-6	CAEZ01007066:64609-64763
HmHel-6	CAEZ01007066:36712-36952
HmHel-6	CAEZ01007066:13363-13553
HmHel-6	CAEZ01006802:7764-7967
HmHel-6	CAEZ01006802:10049-11915
HmHel-6	CAEZ01006572:3929-4118
HmHel-6	CAEZ01006541:56814-56945
HmHel-6	CAEZ01006541:80655-90170
HmHel-6	CAEZ01006541:9230-15927
HmHel-6	CAEZ01006156:2059-10112
HmHel-6	CAEZ01005918:12924-13057
HmHel-6	CAEZ01005918:20746-20931
HmHel-6	CAEZ01005899:5694-11289
HmHel-6	CAEZ01005899:5443-5599
HmHel-6	CAEZ01005761:1587-1673
HmHel-6	CAEZ01005761:29213-29818
HmHel-6	CAEZ01005761:7022-7172
HmHel-6	CAEZ01005461:16693-16798
HmHel-6	CAEZ01005461:4677-4861
HmHel-6	CAEZ01005304:10048-10253
HmHel-6	CAEZ01005233:1929-2034
HmHel-6	CAEZ01005233:34232-35344
HmHel-6	CAEZ01005233:36230-36346
HmHel-6	CAEZ01004898:3528-6800
HmHel-6	CAEZ01004898:1431-1616
HmHel-6	CAEZ01004623:5413-13086
HmHel-6	CAEZ01004623:3540-3673
HmHel-6	CAEZ01004208:12540-12734
HmHel-6	CAEZ01004144:6895-7071
HmHel-6	CAEZ01004144:7776-7920
HmHel-6	CAEZ01004140:12530-12750
HmHel-6	CAEZ01004140:4841-4955
HmHel-6	CAEZ01003806:3794-4014

HmHel-6	CAEZ01003806:11879-11973
HmHel-6	CAEZ01001900:2433-9550
HmHel-6	CAEZ01001900:7787-7979
HmHel-6	CAEZ01001814:1653-1782
HmHel-6	CAEZ01001814:13608-15913
HmHel-6	CAEZ01000933:5548-5697
HmHel-6	CAEZ01000933:3985-4195
HmHel-6	CAEZ01000429:5-142
HmHel-6	CAEZ01011478:787-935
HmHel-6	CAEZ01011478:21030-21245
HmHel-6	CAEZ01011452:7023-15882
HmHel-6	CAEZ01011452:15611-23748
HmHel-6	CAEZ01011250:999-1189
HmHel-6	CAEZ01011250:4165-4268
HmHel-6	CAEZ01011173:5770-5901
HmHel-6	CAEZ01011173:3247-3477
HmHel-6	CAEZ01010830:21845-21984
HmHel-6	CAEZ01010830:27390-28713
HmHel-6	CAEZ01010830:5617-5742
HmHel-6	CAEZ01010821:1117-1259
HmHel-6	CAEZ01010821:58222-58315
HmHel-6	CAEZ01010821:52056-56491
HmHel-6	CAEZ01010816:4273-13003
HmHel-6	CAEZ01010816:60650-60805
HmHel-6	CAEZ01010816:63454-63588
HmHel-6	CAEZ01010816:653-751
HmHel-6	CAEZ01010367:8998-9126
HmHel-6	CAEZ01009859:20102-21031
HmHel-6	CAEZ01009091:13350-13474
HmHel-6	CAEZ01009091:1327-1537
HmHel-6	CAEZ01009016:34899-35120
HmHel-6	CAEZ01009016:7708-9898
HmHel-6	CAEZ01008628:5019-7610
HmHel-6	CAEZ01007969:2447-2688
HmHel-6	CAEZ01007969:4457-4597
HmHel-6	CAEZ01007934:20848-21062
HmHel-6	CAEZ01007793:1242-1333
HmHel-6	CAEZ01007793:23320-23471
HmHel-6	CAEZ01007793:20418-20569
HmHel-6	CAEZ01007519:2444-2692
HmHel-6	CAEZ01007341:8023-8173
HmHel-6	CAEZ01007266:20522-26748
HmHel-6	CAEZ01007266:49020-49238
HmHel-6	CAEZ01007266:50788-50951
HmHel-6	CAEZ01007266:30793-31016
HmHel-6	CAEZ01007266:18876-19020
HmHel-6	CAEZ01007248:2487-2662
HmHel-6	CAEZ01006563:33404-33625

HmHel-6	CAEZ01006563:29463-29584
HmHel-6	CAEZ01006563:16137-16320
HmHel-6	CAEZ01006435:6288-6454
HmHel-6	CAEZ01006435:33373-36817
HmHel-6	CAEZ01006435:53781-55149
HmHel-6	CAEZ01006435:66937-67025
HmHel-6	CAEZ01006435:48414-48561
HmHel-6	CAEZ01006435:26520-26650
HmHel-6	CAEZ01006406:52434-52691
HmHel-6	CAEZ01006406:77205-77285
HmHel-6	CAEZ01006406:47020-47163
HmHel-6	CAEZ01006406:21040-21166
HmHel-6	CAEZ01006180:660-808
HmHel-6	CAEZ01005715:1003-1224
HmHel-6	CAEZ01005515:1970-11617
HmHel-6	CAEZ01005515:31326-31530
HmHel-6	CAEZ01005515:54311-54516
HmHel-6	CAEZ01005515:72829-74203
HmHel-6	CAEZ01005515:73233-73381
HmHel-6	CAEZ01004986:12713-12950
HmHel-6	CAEZ01004885:1871-2031
HmHel-6	CAEZ01004885:13588-13834
HmHel-6	CAEZ01004885:5161-5363
HmHel-6	CAEZ01004260:10582-10660
HmHel-6	CAEZ01004260:2988-12072
HmHel-6	CAEZ01004158:3600-3789
HmHel-6	CAEZ01004048:4368-4516
HmHel-6	CAEZ01003812:860-1088
HmHel-6	CAEZ01003812:23248-23358
HmHel-6	CAEZ01003812:18227-18355
HmHel-6	CAEZ01003652:3248-3435
HmHel-6	CAEZ01003652:5809-5953
HmHel-6	CAEZ01003415:4496-4647
HmHel-6	CAEZ01003415:59179-59274
HmHel-6	CAEZ01003415:70443-70619
HmHel-6	CAEZ01002318:17981-18105
HmHel-6	CAEZ01002318:23115-23263
HmHel-6	CAEZ01002318:12152-12270
HmHel-6	CAEZ01001884:899-1101
HmHel-6	CAEZ01001413:1652-1817
HmHel-6	CAEZ01001233:1677-1868
HmHel-6	CAEZ01001233:3869-3973
HmHel-6	CAEZ01001172:13156-13372
HmHel-6	CAEZ01001140:7279-7500
HmHel-6	CAEZ01000579:2411-2575
HmHel-6	CAEZ01000479:1-196
HmHel-6	CAEZ01011282:10988-12396
HmHel-6	CAEZ01011282:61217-61310

HmHel-6	CAEZ01011282:83882-83968
HmHel-6	CAEZ01011282:140867-140982
HmHel-6	CAEZ01011282:171660-171758
HmHel-6	CAEZ01011282:59505-59589
HmHel-6	CAEZ01011282:30029-30135
HmHel-6	CAEZ01011182:7827-8010
HmHel-6	CAEZ01011182:2729-2856
HmHel-6	CAEZ01010902:4428-8399
HmHel-6	CAEZ01010839:7522-9935
HmHel-6	CAEZ01010839:675-822
HmHel-6	CAEZ01010619:4142-4353
HmHel-6	CAEZ01010619:45007-45088
HmHel-6	CAEZ01010533:8773-8931
HmHel-6	CAEZ01010533:4097-4224
HmHel-6	CAEZ01010015:2410-2594
HmHel-6	CAEZ01010015:7248-15434
HmHel-6	CAEZ01009731:4865-5075
HmHel-6	CAEZ01009731:19809-20019
HmHel-6	CAEZ01009731:2072-2200
HmHel-6	CAEZ01009301:21274-21496
HmHel-6	CAEZ01008658:6401-6490
HmHel-6	CAEZ01008658:2141-2312
HmHel-6	CAEZ01008251:1988-7443
HmHel-6	CAEZ01008251:26149-26260
HmHel-6	CAEZ01008251:37180-37395
HmHel-6	CAEZ01008251:8384-8602
HmHel-6	CAEZ01008247:4529-4649
HmHel-6	CAEZ01008167:15439-15621
HmHel-6	CAEZ01008167:13115-13899
HmHel-6	CAEZ01008043:3146-3290
HmHel-6	CAEZ01007867:109-3585
HmHel-6	CAEZ01007663:34222-34407
HmHel-6	CAEZ01007651:16350-16473
HmHel-6	CAEZ01007185:7717-7948
HmHel-6	CAEZ01007185:54589-54682
HmHel-6	CAEZ01007185:26171-26359
HmHel-6	CAEZ01007183:10027-10221
HmHel-6	CAEZ01007183:7544-7644
HmHel-6	CAEZ01007093:4996-5218
HmHel-6	CAEZ01006947:9559-9726
HmHel-6	CAEZ01006384:1520-1658
HmHel-6	CAEZ01006384:31392-31616
HmHel-6	CAEZ01006384:28855-29119
HmHel-6	CAEZ01006384:1-10466
HmHel-6	CAEZ01006182:13335-13609
HmHel-6	CAEZ01006182:25450-26295
HmHel-6	CAEZ01006182:4576-4678
HmHel-6	CAEZ01005747:4390-4601

HmHel-6	CAEZ01005352:402-768
HmHel-6	CAEZ01005196:7246-7350
HmHel-6	CAEZ01005196:56133-56299
HmHel-6	CAEZ01005196:66671-66779
HmHel-6	CAEZ01005196:60911-61015
HmHel-6	CAEZ01005196:3517-3601
HmHel-6	CAEZ01004960:4542-4653
HmHel-6	CAEZ01004960:2947-3106
HmHel-6	CAEZ01004300:16726-16885
HmHel-6	CAEZ01003355:14407-14494
HmHel-6	CAEZ01003355:12578-12783
HmHel-6	CAEZ01003345:12832-12957
HmHel-6	CAEZ01003345:69772-71141
HmHel-6	CAEZ01003345:73355-77728
HmHel-6	CAEZ01003299:23634-23751
HmHel-6	CAEZ01003299:10426-10664
HmHel-6	CAEZ01003119:8888-8968
HmHel-6	CAEZ01003119:2043-2254
HmHel-6	CAEZ01002880:11609-11729
HmHel-6	CAEZ01002880:14237-14420
HmHel-6	CAEZ01002774:5809-8162
HmHel-6	CAEZ01002774:3173-3276
HmHel-6	CAEZ01002772:5761-7051
HmHel-6	CAEZ01002772:38985-39162
HmHel-6	CAEZ01002772:13010-13168
HmHel-6	CAEZ01002679:22249-24525
HmHel-6	CAEZ01002679:35133-35267
HmHel-6	CAEZ01002679:9037-9189
HmHel-6	CAEZ01002255:60670-60864
HmHel-6	CAEZ01002229:12325-12411
HmHel-6	CAEZ01002229:12062-21244
HmHel-6	CAEZ01001798:3382-3585
HmHel-6	CAEZ01000970:29778-29937
HmHel-6	CAEZ01000970:45496-48459
HmHel-6	CAEZ01000970:39084-39261
HmHel-6	CAEZ01000970:14539-18520
HmHel-6	CAEZ01000902:1889-2125
HmHel-6	CAEZ01000902:6104-6240
HmHel-6	CAEZ01000652:776-978
HmHel-6	CAEZ01011123:4923-5039
HmHel-6	CAEZ01011123:7136-7234
HmHel-6	CAEZ01010613:16626-16842
HmHel-6	CAEZ01010399:30102-30246
HmHel-6	CAEZ01010241:1793-6941
HmHel-6	CAEZ01010241:28186-28398
HmHel-6	CAEZ01010107:44893-45038
HmHel-6	CAEZ01010107:10341-17323
HmHel-6	CAEZ01009831:1684-1897

HmHel-6	CAEZ01009831:3481-3572
HmHel-6	CAEZ01009772:5449-5662
HmHel-6	CAEZ01009772:42206-51220
HmHel-6	CAEZ01009772:49492-49654
HmHel-6	CAEZ01009772:38775-38900
HmHel-6	CAEZ01009772:4747-4897
HmHel-6	CAEZ01009737:4513-4695
HmHel-6	CAEZ01009181:3014-3144
HmHel-6	CAEZ01009181:8709-8906
HmHel-6	CAEZ01008579:6197-14179
HmHel-6	CAEZ01008579:31819-31943
HmHel-6	CAEZ01008579:59727-59961
HmHel-6	CAEZ01008579:63774-63902
HmHel-6	CAEZ01008414:13161-15626
HmHel-6	CAEZ01008414:30742-31006
HmHel-6	CAEZ01008414:18227-19660
HmHel-6	CAEZ01008414:4155-7578
HmHel-6	CAEZ01008354:12910-13143
HmHel-6	CAEZ01008354:40606-40722
HmHel-6	CAEZ01008354:55247-55413
HmHel-6	CAEZ01008354:68928-69148
HmHel-6	CAEZ01008354:10057-10271
HmHel-6	CAEZ01008285:3372-3530
HmHel-6	CAEZ01008285:6069-6207
HmHel-6	CAEZ01008039:12089-12303
HmHel-6	CAEZ01007991:1108-1261
HmHel-6	CAEZ01007991:11988-13937
HmHel-6	CAEZ01007884:785-892
HmHel-6	CAEZ01007884:1241-1440
HmHel-6	CAEZ01007441:5138-5274
HmHel-6	CAEZ01007441:2033-2231
HmHel-6	CAEZ01007399:28099-28313
HmHel-6	CAEZ01007399:67942-68029
HmHel-6	CAEZ01007399:59154-66039
HmHel-6	CAEZ01007399:30603-38457
HmHel-6	CAEZ01006787:8595-8757
HmHel-6	CAEZ01006702:10182-10361
HmHel-6	CAEZ01006443:5049-5263
HmHel-6	CAEZ01006127:7890-8151
HmHel-6	CAEZ01006127:8747-8915
HmHel-6	CAEZ01005886:24895-25089
HmHel-6	CAEZ01005886:23280-23417
HmHel-6	CAEZ01005070:1857-2401
HmHel-6	CAEZ01005070:14896-15038
HmHel-6	CAEZ01004971:8764-9000
HmHel-6	CAEZ01004843:8682-8910
HmHel-6	CAEZ01004843:21208-27725
HmHel-6	CAEZ01004843:3923-4061

HmHel-6	CAEZ01004732:11810-11956
HmHel-6	CAEZ01004643:4268-5406
HmHel-6	CAEZ01004643:5899-10848
HmHel-6	CAEZ01004116:1566-1775
HmHel-6	CAEZ01003849:12527-12749
HmHel-6	CAEZ01003849:13325-13511
HmHel-6	CAEZ01003823:13333-13527
HmHel-6	CAEZ01002776:4634-4840
HmHel-6	CAEZ01002776:3017-3143
HmHel-6	CAEZ01002642:10588-15967
HmHel-6	CAEZ01002642:26689-26908
HmHel-6	CAEZ01001938:1129-1343
HmHel-6	CAEZ01001938:206-328
HmHel-6	CAEZ01001765:15139-19224
HmHel-6	CAEZ01001765:44008-44221
HmHel-6	CAEZ01001765:85298-92276
HmHel-6	CAEZ01001765:108433-108638
HmHel-6	CAEZ01001765:97979-98098
HmHel-6	CAEZ01001510:3-256
HmHel-6	CAEZ01001510:43919-44115
HmHel-6	CAEZ01001510:18516-18618
HmHel-6	CAEZ01001438:6377-6591
HmHel-6	CAEZ01001438:13203-13309
HmHel-6	CAEZ01001193:2380-2514
HmHel-6	CAEZ01001193:15247-15400
HmHel-6	CAEZ01001193:13255-13444
HmHel-6	CAEZ01011556:11337-11471
HmHel-6	CAEZ01011556:8813-9064
HmHel-6	CAEZ01011555:21350-21500
HmHel-6	CAEZ01011555:9324-9533
HmHel-6	CAEZ01011285:39527-42138
HmHel-6	CAEZ01011285:67126-67235
HmHel-6	CAEZ01011285:20827-21015
HmHel-6	CAEZ01011278:8117-8211
HmHel-6	CAEZ01011278:4569-4714
HmHel-6	CAEZ01011218:6886-7071
HmHel-6	CAEZ01011218:43150-43247
HmHel-6	CAEZ01011218:39684-45855
HmHel-6	CAEZ01011218:9610-9710
HmHel-6	CAEZ01011011:10067-10274
HmHel-6	CAEZ01010257:36804-37056
HmHel-6	CAEZ01010257:22853-25023
HmHel-6	CAEZ01010257:8781-9675
HmHel-6	CAEZ01010227:2265-2351
HmHel-6	CAEZ01010227:51301-51514
HmHel-6	CAEZ01010227:79939-80072
HmHel-6	CAEZ01010227:105084-111579
HmHel-6	CAEZ01010227:70533-79340

HmHel-6	CAEZ01009776:10897-16684
HmHel-6	CAEZ01009776:41765-42025
HmHel-6	CAEZ01009776:62780-63013
HmHel-6	CAEZ01009776:45352-47985
HmHel-6	CAEZ01009776:32351-32556
HmHel-6	CAEZ01009776:6010-13500
HmHel-6	CAEZ01009641:3483-9965
HmHel-6	CAEZ01009641:8700-8866
HmHel-6	CAEZ01008739:6208-6295
HmHel-6	CAEZ01008739:22972-23097
HmHel-6	CAEZ01008544:8359-8610
HmHel-6	CAEZ01008544:3970-4100
HmHel-6	CAEZ01008326:205-310
HmHel-6	CAEZ01008326:636-858
HmHel-6	CAEZ01008122:93386-97914
HmHel-6	CAEZ01008122:129423-133449
HmHel-6	CAEZ01008122:109095-109227
HmHel-6	CAEZ01008122:62241-62347
HmHel-6	CAEZ01008122:20751-20980
HmHel-6	CAEZ01007816:3660-7769
HmHel-6	CAEZ01007816:2154-4915
HmHel-6	CAEZ01007803:2344-2497
HmHel-6	CAEZ01007803:13561-13689
HmHel-6	CAEZ01007090:3044-3233
HmHel-6	CAEZ01006987:2870-11134
HmHel-6	CAEZ01006987:9905-10060
HmHel-6	CAEZ01006776:29367-29548
HmHel-6	CAEZ01006776:51428-52344
HmHel-6	CAEZ01006776:45688-45914
HmHel-6	CAEZ01006734:1832-1953
HmHel-6	CAEZ01006734:1393-3798
HmHel-6	CAEZ01006698:16232-16416
HmHel-6	CAEZ01006698:51264-51477
HmHel-6	CAEZ01006141:1720-1882
HmHel-6	CAEZ01006074:1196-4177
HmHel-6	CAEZ01006074:602-777
HmHel-6	CAEZ01005612:1641-1850
HmHel-6	CAEZ01005612:44295-44469
HmHel-6	CAEZ01005612:51038-51218
HmHel-6	CAEZ01005612:20101-20304
HmHel-6	CAEZ01005612:1100-4309
HmHel-6	CAEZ01005444:246-4710
HmHel-6	CAEZ01005203:12746-12843
HmHel-6	CAEZ01005203:50403-55836
HmHel-6	CAEZ01005203:116049-116241
HmHel-6	CAEZ01005203:129724-129900
HmHel-6	CAEZ01005203:152552-154693
HmHel-6	CAEZ01005203:165579-165717

HmHel-6	CAEZ01005203:103325-105434
HmHel-6	CAEZ01005203:29087-36693
HmHel-6	CAEZ01005203:2803-3031
HmHel-6	CAEZ01005105:19992-20240
HmHel-6	CAEZ01004486:49466-49591
HmHel-6	CAEZ01004486:17660-17821
HmHel-6	CAEZ01004419:5-157
HmHel-6	CAEZ01004126:1044-1253
HmHel-6	CAEZ01004126:22960-23053
HmHel-6	CAEZ01004126:44949-45132
HmHel-6	CAEZ01004126:29924-30049
HmHel-6	CAEZ01004126:18514-18699
HmHel-6	CAEZ01004071:17288-17427
HmHel-6	CAEZ01003881:11762-11842
HmHel-6	CAEZ01003881:25207-25414
HmHel-6	CAEZ01003537:10027-10184
HmHel-6	CAEZ01003537:339-549
HmHel-6	CAEZ01003234:10049-10210
HmHel-6	CAEZ01003077:39152-39272
HmHel-6	CAEZ01003077:109273-109372
HmHel-6	CAEZ01003077:116770-116909
HmHel-6	CAEZ01003077:82376-90793
HmHel-6	CAEZ01001965:1700-6979
HmHel-6	CAEZ01001965:17381-17463
HmHel-6	CAEZ01001965:25717-25919
HmHel-6	CAEZ01001092:1480-1665
HmHel-6	CAEZ01001092:3604-3783
HmHel-6	CAEZ01000834:8334-8425
HmHel-6	CAEZ01000834:28397-28577
HmHel-6	CAEZ01000834:13049-13169
HmHel-6	CAEZ01011652:7329-7547
HmHel-6	CAEZ01011652:28729-28902
HmHel-6	CAEZ01011571:12805-12999
HmHel-6	CAEZ01011571:1056-1160
HmHel-6	CAEZ01011189:19108-19310
HmHel-6	CAEZ01011008:5443-5561
HmHel-6	CAEZ01011008:1598-1796
HmHel-6	CAEZ01010881:20457-20673
HmHel-6	CAEZ01010704:64576-64789
HmHel-6	CAEZ01010704:95009-95118
HmHel-6	CAEZ01010704:109208-112168
HmHel-6	CAEZ01010704:137212-138828
HmHel-6	CAEZ01010704:60454-62823
HmHel-6	CAEZ01010704:47604-47700
HmHel-6	CAEZ01010458:9488-9613
HmHel-6	CAEZ01010458:33421-33545
HmHel-6	CAEZ01010458:68218-68326
HmHel-6	CAEZ01010458:83078-92526

HmHel-6	CAEZ01010458:87965-89227
HmHel-6	CAEZ01010439:38547-38758
HmHel-6	CAEZ01010439:50947-51170
HmHel-6	CAEZ01010439:29844-30039
HmHel-6	CAEZ01010272:33110-33292
HmHel-6	CAEZ01010272:96306-105265
HmHel-6	CAEZ01010272:78175-78354
HmHel-6	CAEZ01010272:67648-67774
HmHel-6	CAEZ01010272:45965-46165
HmHel-6	CAEZ01010272:25401-25516
HmHel-6	CAEZ01010272:10052-10264
HmHel-6	CAEZ01010087:11599-11727
HmHel-6	CAEZ01010087:17172-17297
HmHel-6	CAEZ01009977:14440-14649
HmHel-6	CAEZ01009840:13-234
HmHel-6	CAEZ01009718:24459-24559
HmHel-6	CAEZ01009718:26314-26482
HmHel-6	CAEZ01009495:22146-22238
HmHel-6	CAEZ01009495:61125-61206
HmHel-6	CAEZ01009495:40178-40333
HmHel-6	CAEZ01009495:1645-1735
HmHel-6	CAEZ01009231:8929-9044
HmHel-6	CAEZ01009231:6308-13084
HmHel-6	CAEZ01008170:2111-2295
HmHel-6	CAEZ01008170:22232-22325
HmHel-6	CAEZ01008161:12785-12968
HmHel-6	CAEZ01007753:35273-35422
HmHel-6	CAEZ01007753:4432-4645
HmHel-6	CAEZ01007264:5513-5734
HmHel-6	CAEZ01007264:22520-22603
HmHel-6	CAEZ01007264:23113-23314
HmHel-6	CAEZ01007176:3010-3214
HmHel-6	CAEZ01007176:25955-26051
HmHel-6	CAEZ01007176:18755-18955
HmHel-6	CAEZ01007063:27111-27327
HmHel-6	CAEZ01007063:14395-14530
HmHel-6	CAEZ01007029:5969-8042
HmHel-6	CAEZ01007029:21775-21982
HmHel-6	CAEZ01006549:19489-19613
HmHel-6	CAEZ01006549:29610-29728
HmHel-6	CAEZ01006549:11813-12013
HmHel-6	CAEZ01006547:1577-1807
HmHel-6	CAEZ01006346:13463-13552
HmHel-6	CAEZ01006346:22567-22722
HmHel-6	CAEZ01006083:4636-5076
HmHel-6	CAEZ01006083:20925-27943
HmHel-6	CAEZ01006083:32513-33515
HmHel-6	CAEZ01006083:14925-16154

HmHel-6	CAEZ01005878:2203-2447
HmHel-6	CAEZ01005878:15042-15151
HmHel-6	CAEZ01005878:16979-17058
HmHel-6	CAEZ01005878:3686-3818
HmHel-6	CAEZ01005717:1491-1696
HmHel-6	CAEZ01005717:38199-38406
HmHel-6	CAEZ01005717:21202-21370
HmHel-6	CAEZ01005717:5444-5563
HmHel-6	CAEZ01005716:298-8951
HmHel-6	CAEZ01005716:25369-25502
HmHel-6	CAEZ01005716:61425-61505
HmHel-6	CAEZ01005716:82440-91582
HmHel-6	CAEZ01005716:117953-118136
HmHel-6	CAEZ01005716:112058-121926
HmHel-6	CAEZ01005716:16375-16550
HmHel-6	CAEZ01005699:5553-5745
HmHel-6	CAEZ01005687:5672-13900
HmHel-6	CAEZ01005687:50829-50982
HmHel-6	CAEZ01005687:38607-38806
HmHel-6	CAEZ01005687:2964-3110
HmHel-6	CAEZ01005184:2591-4730
HmHel-6	CAEZ01005184:10979-11174
HmHel-6	CAEZ01005184:245-417
HmHel-6	CAEZ01005055:945-1164
HmHel-6	CAEZ01005031:8861-8989
HmHel-6	CAEZ01004761:28232-28321
HmHel-6	CAEZ01004761:25478-25674
HmHel-6	CAEZ01004513:3108-5466
HmHel-6	CAEZ01004513:32772-32874
HmHel-6	CAEZ01004513:23835-23998
HmHel-6	CAEZ01004303:1839-8434
HmHel-6	CAEZ01004303:26309-26491
HmHel-6	CAEZ01004267:11178-11362
HmHel-6	CAEZ01003801:4031-4231
HmHel-6	CAEZ01003801:5041-5167
HmHel-6	CAEZ01003724:13523-13725
HmHel-6	CAEZ01003724:30029-30112
HmHel-6	CAEZ01003724:11543-12827
HmHel-6	CAEZ01003400:18263-18360
HmHel-6	CAEZ01003400:24846-25056
HmHel-6	CAEZ01002581:20897-21017
HmHel-6	CAEZ01002581:28537-28734
HmHel-6	CAEZ01002110:3528-3740
HmHel-6	CAEZ01001793:46716-46962
HmHel-6	CAEZ01001577:9743-9983
HmHel-6	CAEZ01001577:39746-39945
HmHel-6	CAEZ01001577:72645-72876
HmHel-6	CAEZ01001577:25167-30635

HmHel-6	CAEZ01001329:944-1120
HmHel-6	CAEZ01001123:12670-12811
HmHel-6	CAEZ01001123:19284-19508
HmHel-6	CAEZ01000990:33765-33903
HmHel-6	CAEZ01000990:22700-22898
HmHel-6	CAEZ01000963:15564-15648
HmHel-6	CAEZ01000963:28008-28131
HmHel-6	CAEZ01000963:9704-9866
HmHel-6	CAEZ01000780:105-326
HmHel-6	CAEZ01011616:17863-18046
HmHel-6	CAEZ01011616:7709-7825
HmHel-6	CAEZ01011334:1619-1754
HmHel-6	CAEZ01011195:8666-8746
HmHel-6	CAEZ01011195:24604-24748
HmHel-6	CAEZ01011195:39902-39990
HmHel-6	CAEZ01011195:37104-37291
HmHel-6	CAEZ01011195:3516-10111
HmHel-6	CAEZ01010938:1283-1520
HmHel-6	CAEZ01010938:21374-21464
HmHel-6	CAEZ01010935:1909-11818
HmHel-6	CAEZ01010935:38332-38565
HmHel-6	CAEZ01010665:674-830
HmHel-6	CAEZ01010665:22683-22770
HmHel-6	CAEZ01010665:39357-39440
HmHel-6	CAEZ01010665:13046-13250
HmHel-6	CAEZ01010482:1399-1542
HmHel-6	CAEZ01010425:8106-8345
HmHel-6	CAEZ01010363:12138-12327
HmHel-6	CAEZ01010363:32685-34541
HmHel-6	CAEZ01010363:30718-30824
HmHel-6	CAEZ01010363:11076-11290
HmHel-6	CAEZ01010360:6620-6775
HmHel-6	CAEZ01010268:28949-29092
HmHel-6	CAEZ01010268:5508-5707
HmHel-6	CAEZ01010133:1728-1829
HmHel-6	CAEZ01010133:29388-31948
HmHel-6	CAEZ01010133:45296-45429
HmHel-6	CAEZ01010133:29983-30092
HmHel-6	CAEZ01010128:3373-3552
HmHel-6	CAEZ01010128:21660-31561
HmHel-6	CAEZ01010128:24507-26561
HmHel-6	CAEZ01010042:310-552
HmHel-6	CAEZ01009577:7481-7691
HmHel-6	CAEZ01009550:6190-6405
HmHel-6	CAEZ01009550:17100-19630
HmHel-6	CAEZ01009274:10627-10757
HmHel-6	CAEZ01009274:41384-41499
HmHel-6	CAEZ01009274:13708-13801

HmHel-6	CAEZ01008707:13920-14038
HmHel-6	CAEZ01008707:38125-46678
HmHel-6	CAEZ01008707:133217-134720
HmHel-6	CAEZ01008707:65402-65525
HmHel-6	CAEZ01008707:11037-11287
HmHel-6	CAEZ01008607:3605-3690
HmHel-6	CAEZ01008607:19286-19369
HmHel-6	CAEZ01008607:33936-34022
HmHel-6	CAEZ01008607:38314-38410
HmHel-6	CAEZ01008273:9577-13358
HmHel-6	CAEZ01008273:15718-21446
HmHel-6	CAEZ01007937:4855-4972
HmHel-6	CAEZ01007937:58091-58189
HmHel-6	CAEZ01007937:20596-24371
HmHel-6	CAEZ01007682:12579-12726
HmHel-6	CAEZ01007682:14626-14886
HmHel-6	CAEZ01007127:276-461
HmHel-6	CAEZ01007127:822-989
HmHel-6	CAEZ01007116:1008-1098
HmHel-6	CAEZ01007116:1131-1311
HmHel-6	CAEZ01006589:13198-13895
HmHel-6	CAEZ01006589:111283-111424
HmHel-6	CAEZ01006589:39816-48976
HmHel-6	CAEZ01006499:679-834
HmHel-6	CAEZ01006489:567-692
HmHel-6	CAEZ01006489:73254-80524
HmHel-6	CAEZ01005588:11326-11487
HmHel-6	CAEZ01005389:8294-8425
HmHel-6	CAEZ01005359:11478-11585
HmHel-6	CAEZ01005359:26283-26421
HmHel-6	CAEZ01005359:4624-4745
HmHel-6	CAEZ01004972:10179-10306
HmHel-6	CAEZ01004972:25935-29605
HmHel-6	CAEZ01004972:39226-39448
HmHel-6	CAEZ01004972:8115-18843
HmHel-6	CAEZ01004798:19063-19234
HmHel-6	CAEZ01004798:6384-6577
HmHel-6	CAEZ01004008:20643-23051
HmHel-6	CAEZ01004008:59180-59307
HmHel-6	CAEZ01004008:47341-47497
HmHel-6	CAEZ01003821:2412-2575
HmHel-6	CAEZ01003524:1897-2032
HmHel-6	CAEZ01003524:6790-6996
HmHel-6	CAEZ01003442:8620-8771
HmHel-6	CAEZ01002633:19468-27791
HmHel-6	CAEZ01002633:39302-39407
HmHel-6	CAEZ01002633:20075-20242
HmHel-6	CAEZ01002631:11020-11217

HmHel-6	CAEZ01002631:24292-24492
HmHel-6	CAEZ01002631:9090-13366
HmHel-6	CAEZ01002343:1660-1894
HmHel-6	CAEZ01001880:8656-8893
HmHel-6	CAEZ01000992:4886-5012
HmHel-6	CAEZ01000992:30526-30685
HmHel-6	CAEZ01000992:11749-14208
HmHel-6	CAEZ01011009:8106-8212
HmHel-6	CAEZ01011009:24225-24457
HmHel-6	CAEZ01011009:32745-32949
HmHel-6	CAEZ01011009:18896-19100
HmHel-6	CAEZ01011009:4753-4935
HmHel-6	CAEZ01010940:6778-7049
HmHel-6	CAEZ01010940:16469-16638
HmHel-6	CAEZ01010926:27326-27473
HmHel-6	CAEZ01010926:44783-44997
HmHel-6	CAEZ01010926:97868-97992
HmHel-6	CAEZ01010926:124186-125036
HmHel-6	CAEZ01010926:87625-87776
HmHel-6	CAEZ01010912:15552-15758
HmHel-6	CAEZ01010912:988-1071
HmHel-6	CAEZ01010662:6349-6559
HmHel-6	CAEZ01010662:7511-7724
HmHel-6	CAEZ01010596:2687-2901
HmHel-6	CAEZ01010037:7490-10752
HmHel-6	CAEZ01010037:13080-13173
HmHel-6	CAEZ01010037:683-821
HmHel-6	CAEZ01009805:12153-12300
HmHel-6	CAEZ01009805:5769-5871
HmHel-6	CAEZ01009773:2697-2865
HmHel-6	CAEZ01009773:30453-30581
HmHel-6	CAEZ01009773:14537-17461
HmHel-6	CAEZ01009511:18485-18641
HmHel-6	CAEZ01009511:30831-31074
HmHel-6	CAEZ01009511:28323-28542
HmHel-6	CAEZ01008740:14721-14904
HmHel-6	CAEZ01008740:65370-65595
HmHel-6	CAEZ01008740:99159-99356
HmHel-6	CAEZ01008740:80472-81556
HmHel-6	CAEZ01008740:33908-34036
HmHel-6	CAEZ01008482:988-5279
HmHel-6	CAEZ01008482:18285-18396
HmHel-6	CAEZ01008482:5958-6076
HmHel-6	CAEZ01008372:68614-68795
HmHel-6	CAEZ01008372:98282-98434
HmHel-6	CAEZ01008372:121216-121360
HmHel-6	CAEZ01008372:195195-195388
HmHel-6	CAEZ01008372:221720-221837

HmHel-6	CAEZ01008372:271037-271297
HmHel-6	CAEZ01008372:292822-292931
HmHel-6	CAEZ01008372:316509-316762
HmHel-6	CAEZ01008372:269820-272411
HmHel-6	CAEZ01008372:249251-249499
HmHel-6	CAEZ01008372:218503-225052
HmHel-6	CAEZ01008372:180522-180755
HmHel-6	CAEZ01008372:145501-145580
HmHel-6	CAEZ01008372:127242-127530
HmHel-6	CAEZ01008372:106609-106741
HmHel-6	CAEZ01008372:55898-56099
HmHel-6	CAEZ01008372:25363-25454
HmHel-6	CAEZ01008364:7459-7609
HmHel-6	CAEZ01008302:341-6759
HmHel-6	CAEZ01008302:1279-1411
HmHel-6	CAEZ01008105:54564-58149
HmHel-6	CAEZ01008105:93152-93402
HmHel-6	CAEZ01008105:124545-129848
HmHel-6	CAEZ01008105:34508-34639
HmHel-6	CAEZ01008105:23186-23290
HmHel-6	CAEZ01008105:12550-12629
HmHel-6	CAEZ01008017:4369-4448
HmHel-6	CAEZ01008017:7676-7936
HmHel-6	CAEZ01007943:10416-10574
HmHel-6	CAEZ01007943:89-8956
HmHel-6	CAEZ01007737:27120-27262
HmHel-6	CAEZ01007737:57712-57890
HmHel-6	CAEZ01007737:57427-64901
HmHel-6	CAEZ01007737:6482-8415
HmHel-6	CAEZ01007465:17144-17266
HmHel-6	CAEZ01007465:12599-12764
HmHel-6	CAEZ01007391:6425-6539
HmHel-6	CAEZ01007391:21627-21729
HmHel-6	CAEZ01007391:17366-21777
HmHel-6	CAEZ01007391:6300-6411
HmHel-6	CAEZ01007205:10470-12648
HmHel-6	CAEZ01007205:7336-7423
HmHel-6	CAEZ01006958:2144-2349
HmHel-6	CAEZ01006958:19114-19215
HmHel-6	CAEZ01006785:6195-6337
HmHel-6	CAEZ01006785:14106-14304
HmHel-6	CAEZ01006771:1415-1629
HmHel-6	CAEZ01006771:4984-5096
HmHel-6	CAEZ01006434:19752-19831
HmHel-6	CAEZ01006434:42925-43181
HmHel-6	CAEZ01006434:70761-70887
HmHel-6	CAEZ01006434:84542-84671
HmHel-6	CAEZ01006434:100226-100418

HmHel-6	CAEZ01006434:113322-113479
HmHel-6	CAEZ01006434:112449-114775
HmHel-6	CAEZ01006434:97095-97267
HmHel-6	CAEZ01006434:60129-60329
HmHel-6	CAEZ01006434:6823-7028
HmHel-6	CAEZ01006380:2408-2502
HmHel-6	CAEZ01006380:11340-11478
HmHel-6	CAEZ01006299:9897-10111
HmHel-6	CAEZ01006299:36081-37018
HmHel-6	CAEZ01006299:67241-70166
HmHel-6	CAEZ01006299:14309-14467
HmHel-6	CAEZ01006274:11451-15247
HmHel-6	CAEZ01005972:9385-9530
HmHel-6	CAEZ01005972:35148-35271
HmHel-6	CAEZ01005972:63923-64132
HmHel-6	CAEZ01005972:38038-52364
HmHel-6	CAEZ01005938:1124-1324
HmHel-6	CAEZ01005938:16290-16414
HmHel-6	CAEZ01005798:1148-6423
HmHel-6	CAEZ01005587:1023-1109
HmHel-6	CAEZ01005587:40498-40677
HmHel-6	CAEZ01005587:12453-12593
HmHel-6	CAEZ01005097:29932-30146
HmHel-6	CAEZ01004489:15035-15216
HmHel-6	CAEZ01004451:2047-2189
HmHel-6	CAEZ01004451:5954-7414
HmHel-6	CAEZ01004088:4238-4412
HmHel-6	CAEZ01004074:1096-6061
HmHel-6	CAEZ01003775:1086-1227
HmHel-6	CAEZ01003489:8259-8458
HmHel-6	CAEZ01003489:915-1085
HmHel-6	CAEZ01003437:3335-7031
HmHel-6	CAEZ01003437:4459-4580
HmHel-6	CAEZ01002754:40715-40841
HmHel-6	CAEZ01002754:75428-75589
HmHel-6	CAEZ01002754:57874-58017
HmHel-6	CAEZ01002754:41840-41941
HmHel-6	CAEZ01002754:30198-30288
HmHel-6	CAEZ01002754:13433-13530
HmHel-6	CAEZ01002712:21797-21880
HmHel-6	CAEZ01002712:22520-22728
HmHel-6	CAEZ01002362:5442-5574
HmHel-6	CAEZ01002362:38147-45195
HmHel-6	CAEZ01002362:35179-40420
HmHel-6	CAEZ01002362:23266-23404
HmHel-6	CAEZ01001853:5500-5618
HmHel-6	CAEZ01001458:38287-38523
HmHel-6	CAEZ01001458:14362-19081

HmHel-6	CAEZ01001375:10819-10939
HmHel-6	CAEZ01001375:11451-11697
HmHel-6	CAEZ01001375:661-879
HmHel-6	CAEZ01001129:9571-9783
HmHel-6	CAEZ01001129:21499-21624
HmHel-6	CAEZ01001129:6544-6672
HmHel-6	CAEZ01001012:1299-1563
HmHel-6	CAEZ01000905:9621-9847
HmHel-6	CAEZ01000820:6179-6297
HmHel-6	CAEZ01000820:52072-52160
HmHel-6	CAEZ01000820:61395-61599
HmHel-6	CAEZ01000820:48963-49097
HmHel-6	CAEZ01000820:2148-2330
HmHel-6	CAEZ01000654:471-589
HmHel-6	CAEZ01011266:3647-5159
HmHel-6	CAEZ01010743:2850-3007
HmHel-6	CAEZ01010182:10661-16273
HmHel-6	CAEZ01010182:5926-6120
HmHel-6	CAEZ01009917:2657-2853
HmHel-6	CAEZ01009830:8677-8947
HmHel-6	CAEZ01009708:12760-32067
HmHel-6	CAEZ01009708:49983-50207
HmHel-6	CAEZ01009708:29165-29286
HmHel-6	CAEZ01009708:4449-4548
HmHel-6	CAEZ01009430:2076-8127
HmHel-6	CAEZ01009430:21678-21831
HmHel-6	CAEZ01009430:25833-25999
HmHel-6	CAEZ01009066:8168-8293
HmHel-6	CAEZ01009018:3894-4154
HmHel-6	CAEZ01009018:5610-7464
HmHel-6	CAEZ01008835:793-905
HmHel-6	CAEZ01008835:8686-8875
HmHel-6	CAEZ01008022:16209-17991
HmHel-6	CAEZ01007704:6114-6327
HmHel-6	CAEZ01007629:13749-13936
HmHel-6	CAEZ01007530:2966-3184
HmHel-6	CAEZ01007513:6727-6907
HmHel-6	CAEZ01007122:5847-12721
HmHel-6	CAEZ01007122:46818-47026
HmHel-6	CAEZ01007122:56309-56458
HmHel-6	CAEZ01007122:32605-32762
HmHel-6	CAEZ01007122:19283-20340
HmHel-6	CAEZ01006930:7754-10190
HmHel-6	CAEZ01006637:1218-1463
HmHel-6	CAEZ01006637:3606-3748
HmHel-6	CAEZ01006472:2576-2781
HmHel-6	CAEZ01006472:18284-18424
HmHel-6	CAEZ01006472:16987-17152

HmHel-6	CAEZ01006341:3894-4003
HmHel-6	CAEZ01006249:21239-21343
HmHel-6	CAEZ01006249:58751-58952
HmHel-6	CAEZ01006249:45392-46897
HmHel-6	CAEZ01006249:2883-11835
HmHel-6	CAEZ01006042:241-420
HmHel-6	CAEZ01005989:3920-4057
HmHel-6	CAEZ01005293:5837-6014
HmHel-6	CAEZ01005282:4908-10324
HmHel-6	CAEZ01005243:12974-13211
HmHel-6	CAEZ01004884:4200-4325
HmHel-6	CAEZ01004884:4770-4978
HmHel-6	CAEZ01004678:19081-19278
HmHel-6	CAEZ01004678:19891-19996
HmHel-6	CAEZ01003510:65626-65718
HmHel-6	CAEZ01003510:68664-68904
HmHel-6	CAEZ01003510:32291-32384
HmHel-6	CAEZ01003510:12734-12869
HmHel-6	CAEZ01003464:2770-4583
HmHel-6	CAEZ01003464:19371-19453
HmHel-6	CAEZ01003464:16850-16962
HmHel-6	CAEZ01003464:1624-1837
HmHel-6	CAEZ01003272:495-652
HmHel-6	CAEZ01003272:13261-13370
HmHel-6	CAEZ01003238:1370-3258
HmHel-6	CAEZ01003175:5711-5876
HmHel-6	CAEZ01003175:22063-22211
HmHel-6	CAEZ01003175:14196-15283
HmHel-6	CAEZ01002170:6808-6951
HmHel-6	CAEZ01002170:6239-6460
HmHel-6	CAEZ01000876:9683-9919
HmHel-6	CAEZ01000876:31024-31156
HmHel-6	CAEZ01000876:50806-51020
HmHel-6	CAEZ01000876:19765-19850
HmHel-6	CAEZ01011639:18204-18421
HmHel-6	CAEZ01011639:20071-20179
HmHel-6	CAEZ01011057:23883-24102
HmHel-6	CAEZ01011057:5217-8855
HmHel-6	CAEZ01010894:9652-9825
HmHel-6	CAEZ01010894:91185-91386
HmHel-6	CAEZ01010894:104058-104175
HmHel-6	CAEZ01010894:141294-141516
HmHel-6	CAEZ01010894:170058-170186
HmHel-6	CAEZ01010894:222741-222881
HmHel-6	CAEZ01010894:236219-236397
HmHel-6	CAEZ01010894:159277-159377
HmHel-6	CAEZ01010894:127162-127282
HmHel-6	CAEZ01010894:115057-115252

HmHel-6	CAEZ01010894:87643-87800
HmHel-6	CAEZ01010810:3603-3973
HmHel-6	CAEZ01010810:24216-24365
HmHel-6	CAEZ01010810:23598-23683
HmHel-6	CAEZ01010667:5589-8577
HmHel-6	CAEZ01010667:41565-41788
HmHel-6	CAEZ01010667:49472-49699
HmHel-6	CAEZ01010667:18594-18700
HmHel-6	CAEZ01010608:3954-4094
HmHel-6	CAEZ01010602:13611-13842
HmHel-6	CAEZ01010602:30979-31189
HmHel-6	CAEZ01010591:10572-10690
HmHel-6	CAEZ01010591:33996-34148
HmHel-6	CAEZ01010591:20360-20507
HmHel-6	CAEZ01010591:5212-5356
HmHel-6	CAEZ01010428:14475-14607
HmHel-6	CAEZ01010428:97386-99073
HmHel-6	CAEZ01010428:117860-118010
HmHel-6	CAEZ01010428:96770-96981
HmHel-6	CAEZ01010428:85621-85757
HmHel-6	CAEZ01010428:53800-60323
HmHel-6	CAEZ01010428:15607-15755
HmHel-6	CAEZ01010375:17769-17892
HmHel-6	CAEZ01010375:6609-6732
HmHel-6	CAEZ01010174:8817-8928
HmHel-6	CAEZ01010174:26652-26875
HmHel-6	CAEZ01010174:21809-30371
HmHel-6	CAEZ01010003:1344-1468
HmHel-6	CAEZ01010003:11555-11637
HmHel-6	CAEZ01009451:12544-12761
HmHel-6	CAEZ01009451:4740-10335
HmHel-6	CAEZ01009300:37010-37095
HmHel-6	CAEZ01009300:50083-56399
HmHel-6	CAEZ01009300:53479-59116
HmHel-6	CAEZ01009300:34637-34832
HmHel-6	CAEZ01009300:6567-6667
HmHel-6	CAEZ01008661:2959-3107
HmHel-6	CAEZ01008661:15177-15313
HmHel-6	CAEZ01008661:1423-1535
HmHel-6	CAEZ01008592:1960-2180
HmHel-6	CAEZ01008402:6080-6205
HmHel-6	CAEZ01008402:5030-5162
HmHel-6	CAEZ01008370:29967-30148
HmHel-6	CAEZ01008370:3922-6633
HmHel-6	CAEZ01008296:9669-9864
HmHel-6	CAEZ01008296:1612-5533
HmHel-6	CAEZ01008287:20275-20460
HmHel-6	CAEZ01008084:2154-2273

HmHel-6	CAEZ01008084:10279-10493
HmHel-6	CAEZ01007259:13563-13767
HmHel-6	CAEZ01007259:38285-38445
HmHel-6	CAEZ01007259:88314-88429
HmHel-6	CAEZ01007259:80507-80713
HmHel-6	CAEZ01007259:50892-50991
HmHel-6	CAEZ01007259:14593-14796
HmHel-6	CAEZ01006643:5445-5600
HmHel-6	CAEZ01006643:13987-14152
HmHel-6	CAEZ01006167:5398-5612
HmHel-6	CAEZ01006167:602-2921
HmHel-6	CAEZ01006139:2973-3209
HmHel-6	CAEZ01005846:6034-8478
HmHel-6	CAEZ01005846:3639-3874
HmHel-6	CAEZ01005474:31239-35104
HmHel-6	CAEZ01005474:42657-42871
HmHel-6	CAEZ01005448:464-685
HmHel-6	CAEZ01005226:9480-9644
HmHel-6	CAEZ01005226:20384-20465
HmHel-6	CAEZ01005162:22342-22498
HmHel-6	CAEZ01005162:78570-78653
HmHel-6	CAEZ01005162:52252-52353
HmHel-6	CAEZ01005162:21763-28288
HmHel-6	CAEZ01004976:1431-1622
HmHel-6	CAEZ01004958:44192-44416
HmHel-6	CAEZ01004958:38597-38778
HmHel-6	CAEZ01004958:11327-13667
HmHel-6	CAEZ01004699:3217-3315
HmHel-6	CAEZ01004626:4511-4707
HmHel-6	CAEZ01004626:2838-2972
HmHel-6	CAEZ01004295:796-899
HmHel-6	CAEZ01004295:8323-8556
HmHel-6	CAEZ01004232:7488-7594
HmHel-6	CAEZ01004232:6728-6858
HmHel-6	CAEZ01003840:6207-6415
HmHel-6	CAEZ01003507:23299-23420
HmHel-6	CAEZ01003507:5718-5845
HmHel-6	CAEZ01003336:2147-2409
HmHel-6	CAEZ01003323:11214-11346
HmHel-6	CAEZ01002928:12960-13191
HmHel-6	CAEZ01002928:8674-8838
HmHel-6	CAEZ01002431:12345-12485
HmHel-6	CAEZ01002431:24013-24246
HmHel-6	CAEZ01002431:58524-61654
HmHel-6	CAEZ01002431:56388-56487
HmHel-6	CAEZ01002431:22439-22607
HmHel-6	CAEZ01002153:7986-8102
HmHel-6	CAEZ01002100:18502-18667

HmHel-6	CAEZ01002100:8050-8203
HmHel-6	CAEZ01002066:21726-21856
HmHel-6	CAEZ01002066:18436-18584
HmHel-6	CAEZ01002066:4209-4331
HmHel-6	CAEZ01001822:16077-16261
HmHel-6	CAEZ01001822:20141-20342
HmHel-6	CAEZ01000832:3734-3990
HmHel-6	CAEZ01000801:5675-5867
HmHel-6	CAEZ01000482:660-812
HmHel-6	CAEZ01011583:2080-2202
HmHel-6	CAEZ01011558:3263-10905
HmHel-6	CAEZ01011502:12566-12712
HmHel-6	CAEZ01011502:244-457
HmHel-6	CAEZ01011436:3160-3288
HmHel-6	CAEZ01011436:5679-5792
HmHel-6	CAEZ01011376:1460-1701
HmHel-6	CAEZ01011376:922-1121
HmHel-6	CAEZ01011355:3500-3651
HmHel-6	CAEZ01011355:728-2282
HmHel-6	CAEZ01011186:3818-4161
HmHel-6	CAEZ01011060:8856-8979
HmHel-6	CAEZ01010997:8360-8515
HmHel-6	CAEZ01010997:17699-17803
HmHel-6	CAEZ01010946:7451-10117
HmHel-6	CAEZ01010909:13436-17977
HmHel-6	CAEZ01010909:1481-1735
HmHel-6	CAEZ01010417:6078-6310
HmHel-6	CAEZ01009759:10268-15321
HmHel-6	CAEZ01009759:4179-4261
HmHel-6	CAEZ01009757:3608-3799
HmHel-6	CAEZ01009700:5505-8828
HmHel-6	CAEZ01009700:100-289
HmHel-6	CAEZ01009520:1209-1415
HmHel-6	CAEZ01009520:3070-3229
HmHel-6	CAEZ01009374:23339-23565
HmHel-6	CAEZ01009374:45220-45332
HmHel-6	CAEZ01009374:74195-74446
HmHel-6	CAEZ01009374:103398-106886
HmHel-6	CAEZ01009374:87885-88099
HmHel-6	CAEZ01009374:53575-53662
HmHel-6	CAEZ01009374:29568-29657
HmHel-6	CAEZ01009229:3478-3696
HmHel-6	CAEZ01009229:45797-48228
HmHel-6	CAEZ01009229:4400-12336
HmHel-6	CAEZ01008785:7989-8199
HmHel-6	CAEZ01008785:38662-44161
HmHel-6	CAEZ01008785:3533-10170
HmHel-6	CAEZ01008424:4489-4697

HmHel-6	CAEZ01008424:22228-22393
HmHel-6	CAEZ01008424:19510-21644
HmHel-6	CAEZ01008397:3333-3559
HmHel-6	CAEZ01008148:3156-3355
HmHel-6	CAEZ01008148:22714-22837
HmHel-6	CAEZ01007999:1216-1470
HmHel-6	CAEZ01007999:17583-17669
HmHel-6	CAEZ01007999:6073-6204
HmHel-6	CAEZ01007155:140-306
HmHel-6	CAEZ01007081:2000-2174
HmHel-6	CAEZ01007081:7039-7166
HmHel-6	CAEZ01006820:8034-9893
HmHel-6	CAEZ01006820:30136-30350
HmHel-6	CAEZ01006596:33642-33723
HmHel-6	CAEZ01006596:49872-49979
HmHel-6	CAEZ01006596:50770-50956
HmHel-6	CAEZ01006376:15737-15964
HmHel-6	CAEZ01006376:26265-26430
HmHel-6	CAEZ01006376:15266-15364
HmHel-6	CAEZ01006371:5207-6464
HmHel-6	CAEZ01006371:472-4248
HmHel-6	CAEZ01006273:2776-2867
HmHel-6	CAEZ01006273:6075-6206
HmHel-6	CAEZ01005922:1210-1292
HmHel-6	CAEZ01005922:11368-11563
HmHel-6	CAEZ01005693:5672-5759
HmHel-6	CAEZ01005693:27558-27666
HmHel-6	CAEZ01005693:1254-9582
HmHel-6	CAEZ01005210:330-430
HmHel-6	CAEZ01005210:14186-14269
HmHel-6	CAEZ01005210:25730-25827
HmHel-6	CAEZ01005210:49602-49701
HmHel-6	CAEZ01005210:91002-91116
HmHel-6	CAEZ01005210:9033-9158
HmHel-6	CAEZ01004125:28567-32078
HmHel-6	CAEZ01004125:20382-20502
HmHel-6	CAEZ01003848:13591-13722
HmHel-6	CAEZ01003822:5369-5568
HmHel-6	CAEZ01003503:4851-8370
HmHel-6	CAEZ01003405:10766-10957
HmHel-6	CAEZ01003405:5362-9046
HmHel-6	CAEZ01003129:1685-1887
HmHel-6	CAEZ01002681:7964-8171
HmHel-6	CAEZ01002681:559-6884
HmHel-6	CAEZ01001367:1993-2156
HmHel-6	CAEZ01001367:15960-16087
HmHel-6	CAEZ01001080:9015-9238
HmHel-6	CAEZ01001080:11630-11790

HmHel-6	CAEZ01000877:1007-1175
HmHel-6	CAEZ01000826:1587-1794
HmHel-6	CAEZ01000208:4-131
HmHel-6	CAEZ01011518:3176-3302
HmHel-6	CAEZ01010869:1047-10037
HmHel-6	CAEZ01010869:73565-73687
HmHel-6	CAEZ01010869:83072-83245
HmHel-6	CAEZ01010869:34363-43336
HmHel-6	CAEZ01010746:2043-12140
HmHel-6	CAEZ01010746:44539-44657
HmHel-6	CAEZ01010287:53029-53139
HmHel-6	CAEZ01010287:72870-73106
HmHel-6	CAEZ01010287:37244-37398
HmHel-6	CAEZ01010287:21111-21228
HmHel-6	CAEZ01010287:1601-7827
HmHel-6	CAEZ01010219:23959-24109
HmHel-6	CAEZ01010219:43078-43263
HmHel-6	CAEZ01010219:67721-67885
HmHel-6	CAEZ01010219:105818-105901
HmHel-6	CAEZ01010219:120838-120984
HmHel-6	CAEZ01010219:163528-163714
HmHel-6	CAEZ01010219:93753-93914
HmHel-6	CAEZ01010219:54670-54773
HmHel-6	CAEZ01010219:24523-24682
HmHel-6	CAEZ01010206:3636-9303
HmHel-6	CAEZ01009226:2555-10915
HmHel-6	CAEZ01009226:22882-23036
HmHel-6	CAEZ01009063:13879-14061
HmHel-6	CAEZ01009063:4850-4963
HmHel-6	CAEZ01008942:3625-3787
HmHel-6	CAEZ01008942:9893-10011
HmHel-6	CAEZ01008853:10188-10364
HmHel-6	CAEZ01008853:14316-14464
HmHel-6	CAEZ01008765:4491-4600
HmHel-6	CAEZ01008765:74886-74972
HmHel-6	CAEZ01008765:86260-86437
HmHel-6	CAEZ01008765:78669-78806
HmHel-6	CAEZ01008765:66374-66606
HmHel-6	CAEZ01008765:30072-30220
HmHel-6	CAEZ01008765:3263-9333
HmHel-6	CAEZ01008709:14623-14792
HmHel-6	CAEZ01008709:21771-21995
HmHel-6	CAEZ01008666:7206-7400
HmHel-6	CAEZ01008653:4988-5222
HmHel-6	CAEZ01008653:4097-4281
HmHel-6	CAEZ01008477:11081-11231
HmHel-6	CAEZ01008477:25026-25168
HmHel-6	CAEZ01008477:21841-21948

HmHel-6	CAEZ01007836:9352-13208
HmHel-6	CAEZ01007836:10404-10612
HmHel-6	CAEZ01007592:12816-12960
HmHel-6	CAEZ01007592:23906-24026
HmHel-6	CAEZ01007252:150640-150770
HmHel-6	CAEZ01007252:200253-200463
HmHel-6	CAEZ01007252:219169-224365
HmHel-6	CAEZ01007252:245888-247888
HmHel-6	CAEZ01007252:209254-209381
HmHel-6	CAEZ01007252:110601-110711
HmHel-6	CAEZ01007252:79412-89458
HmHel-6	CAEZ01007252:35426-41041
HmHel-6	CAEZ01007192:12058-12287
HmHel-6	CAEZ01007135:1095-1194
HmHel-6	CAEZ01007135:27516-27619
HmHel-6	CAEZ01007135:32507-32698
HmHel-6	CAEZ01007135:17802-17912
HmHel-6	CAEZ01007050:76-290
HmHel-6	CAEZ01007020:6712-13186
HmHel-6	CAEZ01007020:16287-16445
HmHel-6	CAEZ01007004:6747-6935
HmHel-6	CAEZ01006927:3224-3555
HmHel-6	CAEZ01006927:2687-2825
HmHel-6	CAEZ01006539:2486-3809
HmHel-6	CAEZ01005848:2055-3550
HmHel-6	CAEZ01005608:6776-6927
HmHel-6	CAEZ01005403:10456-14476
HmHel-6	CAEZ01005403:38004-38536
HmHel-6	CAEZ01005403:58604-58813
HmHel-6	CAEZ01005403:55711-55880
HmHel-6	CAEZ01005403:14696-14824
HmHel-6	CAEZ01005329:11692-11854
HmHel-6	CAEZ01005212:5791-5929
HmHel-6	CAEZ01005212:7911-8125
HmHel-6	CAEZ01005039:3037-3227
HmHel-6	CAEZ01004822:14659-17020
HmHel-6	CAEZ01004822:46647-46813
HmHel-6	CAEZ01004822:71376-71583
HmHel-6	CAEZ01004822:47777-51297
HmHel-6	CAEZ01004822:25294-25390
HmHel-6	CAEZ01004822:14306-14491
HmHel-6	CAEZ01004638:1472-1665
HmHel-6	CAEZ01004528:11911-12145
HmHel-6	CAEZ01004316:9361-9540
HmHel-6	CAEZ01004112:5228-5429
HmHel-6	CAEZ01004112:16476-16574
HmHel-6	CAEZ01003650:10087-10195
HmHel-6	CAEZ01003650:20732-20931

HmHel-6	CAEZ01003328:12302-12542
HmHel-6	CAEZ01003328:46178-46332
HmHel-6	CAEZ01003328:70941-71082
HmHel-6	CAEZ01003328:142389-142502
HmHel-6	CAEZ01003328:122082-123421
HmHel-6	CAEZ01003328:99524-99654
HmHel-6	CAEZ01003328:27264-27370
HmHel-6	CAEZ01003300:1894-2020
HmHel-6	CAEZ01003300:22842-23054
HmHel-6	CAEZ01003300:7133-7221
HmHel-6	CAEZ01003067:59361-59448
HmHel-6	CAEZ01003067:73079-73241
HmHel-6	CAEZ01003067:97618-97713
HmHel-6	CAEZ01003067:117310-117404
HmHel-6	CAEZ01003067:46307-46448
HmHel-6	CAEZ01002264:1538-1730
HmHel-6	CAEZ01001312:3419-3540
HmHel-6	CAEZ01001312:11564-11708
HmHel-6	CAEZ01011426:589-794
HmHel-6	CAEZ01011426:2987-3076
HmHel-6	CAEZ01011093:19619-19773
HmHel-6	CAEZ01011093:36012-36098
HmHel-6	CAEZ01011093:121784-121899
HmHel-6	CAEZ01011093:142505-142591
HmHel-6	CAEZ01011093:95064-95263
HmHel-6	CAEZ01011093:53637-54989
HmHel-6	CAEZ01011093:33514-42371
HmHel-6	CAEZ01010756:9864-10052
HmHel-6	CAEZ01010756:1640-1713
HmHel-6	CAEZ01010585:85541-85625
HmHel-6	CAEZ01010585:110457-110598
HmHel-6	CAEZ01010585:165767-165981
HmHel-6	CAEZ01010585:192814-199895
HmHel-6	CAEZ01010585:180485-180607
HmHel-6	CAEZ01010585:115108-115294
HmHel-6	CAEZ01010585:45158-45246
HmHel-6	CAEZ01009947:3989-4149
HmHel-6	CAEZ01009923:6322-6507
HmHel-6	CAEZ01009580:11914-12025
HmHel-6	CAEZ01009580:26284-26432
HmHel-6	CAEZ01009287:462-626
HmHel-6	CAEZ01009287:20563-20660
HmHel-6	CAEZ01009287:34520-34712
HmHel-6	CAEZ01008962:2866-11656
HmHel-6	CAEZ01008962:7963-8080
HmHel-6	CAEZ01008891:6968-7152
HmHel-6	CAEZ01008891:16126-16203
HmHel-6	CAEZ01008804:3096-9908

HmHel-6	CAEZ01008804:20837-25393
HmHel-6	CAEZ01008804:23351-23525
HmHel-6	CAEZ01008606:3447-12710
HmHel-6	CAEZ01008606:55530-55633
HmHel-6	CAEZ01008606:79585-79784
HmHel-6	CAEZ01008606:85285-85389
HmHel-6	CAEZ01008606:61556-69818
HmHel-6	CAEZ01008606:32475-32656
HmHel-6	CAEZ01008606:10486-10635
HmHel-6	CAEZ01008253:2291-2489
HmHel-6	CAEZ01008102:9267-9482
HmHel-6	CAEZ01007570:6883-7102
HmHel-6	CAEZ01007570:5587-5789
HmHel-6	CAEZ01007493:13540-13806
HmHel-6	CAEZ01007493:9896-10014
HmHel-6	CAEZ01007224:1479-1707
HmHel-6	CAEZ01007224:37758-41401
HmHel-6	CAEZ01007224:43051-43158
HmHel-6	CAEZ01007224:12972-13149
HmHel-6	CAEZ01006850:13480-13701
HmHel-6	CAEZ01006838:3651-3836
HmHel-6	CAEZ01006764:10024-10180
HmHel-6	CAEZ01006764:8936-9156
HmHel-6	CAEZ01006745:4063-4237
HmHel-6	CAEZ01006745:29972-30072
HmHel-6	CAEZ01006745:6511-13195
HmHel-6	CAEZ01006686:68184-68282
HmHel-6	CAEZ01006686:76365-76452
HmHel-6	CAEZ01006686:64451-64636
HmHel-6	CAEZ01006336:7241-7377
HmHel-6	CAEZ01006336:27171-28045
HmHel-6	CAEZ01006190:434-544
HmHel-6	CAEZ01006190:6519-6640
HmHel-6	CAEZ01005970:10822-12133
HmHel-6	CAEZ01005970:27208-27409
HmHel-6	CAEZ01005970:5830-11268
HmHel-6	CAEZ01005631:1810-1973
HmHel-6	CAEZ01005322:3584-3755
HmHel-6	CAEZ01005322:5022-5128
HmHel-6	CAEZ01005314:2131-2251
HmHel-6	CAEZ01005314:5913-6023
HmHel-6	CAEZ01005177:64419-67043
HmHel-6	CAEZ01005177:88898-88979
HmHel-6	CAEZ01005177:59962-65451
HmHel-6	CAEZ01005177:23378-23543
HmHel-6	CAEZ01005173:84778-93428
HmHel-6	CAEZ01005173:141640-141765
HmHel-6	CAEZ01005173:115940-116092

HmHel-6	CAEZ01005173:75755-75951
HmHel-6	CAEZ01005173:18782-18863
HmHel-6	CAEZ01005150:20320-20402
HmHel-6	CAEZ01005150:20969-25689
HmHel-6	CAEZ01004893:983-1080
HmHel-6	CAEZ01004670:1170-1335
HmHel-6	CAEZ01004670:6666-6903
HmHel-6	CAEZ01004139:9720-9837
HmHel-6	CAEZ01004139:3868-4109
HmHel-6	CAEZ01003850:32776-38059
HmHel-6	CAEZ01003838:5360-5482
HmHel-6	CAEZ01003337:11154-11271
HmHel-6	CAEZ01003337:798-1042
HmHel-6	CAEZ01003286:8725-18859
HmHel-6	CAEZ01003231:23103-23273
HmHel-6	CAEZ01003115:8191-8430
HmHel-6	CAEZ01003115:8852-8939
HmHel-6	CAEZ01002598:2919-3133
HmHel-6	CAEZ01002439:2315-2514
HmHel-6	CAEZ01002220:8468-8681
HmHel-6	CAEZ01002141:7434-7666
HmHel-6	CAEZ01002022:27338-27503
HmHel-6	CAEZ01002022:4998-5137
HmHel-6	CAEZ01001889:8808-8989
HmHel-6	CAEZ01001889:6207-6327
HmHel-6	CAEZ01001831:6033-11328
HmHel-6	CAEZ01001831:40136-47849
HmHel-6	CAEZ01001831:59717-59831
HmHel-6	CAEZ01001831:32480-32597
HmHel-6	CAEZ01001496:10581-10728
HmHel-6	CAEZ01001075:6201-6406
HmHel-6	CAEZ01001075:7727-7868
HmHel-6	CAEZ01000193:213-335
HmHel-6	CAEZ01000051:16483-16608
HmHel-6	CAEZ01000051:41879-42064
HmHel-6	CAEZ01000051:6105-6215
HmHel-6	CAEZ01011599:8267-8391
HmHel-6	CAEZ01011599:23254-23377
HmHel-6	CAEZ01011245:78969-79209
HmHel-6	CAEZ01011245:96078-96886
HmHel-6	CAEZ01011245:69376-69509
HmHel-6	CAEZ01011245:47518-47609
HmHel-6	CAEZ01011245:13622-13714
HmHel-6	CAEZ01010784:23180-23300
HmHel-6	CAEZ01010784:8124-8311
HmHel-6	CAEZ01010127:36762-36889
HmHel-6	CAEZ01010127:63487-64273
HmHel-6	CAEZ01010127:115416-115572

HmHel-6	CAEZ01010127:101651-101745
HmHel-6	CAEZ01010127:82307-82487
HmHel-6	CAEZ01010127:35762-35847
HmHel-6	CAEZ01009898:72043-72249
HmHel-6	CAEZ01009898:62054-62148
HmHel-6	CAEZ01009898:13412-20266
HmHel-6	CAEZ01009475:6224-6433
HmHel-6	CAEZ01009475:21815-21923
HmHel-6	CAEZ01009457:23701-23861
HmHel-6	CAEZ01009457:11959-12086
HmHel-6	CAEZ01009297:9232-9412
HmHel-6	CAEZ01009297:7112-7220
HmHel-6	CAEZ01009002:1233-1369
HmHel-6	CAEZ01009002:27007-30459
HmHel-6	CAEZ01009002:25082-26550
HmHel-6	CAEZ01009002:3250-3447
HmHel-6	CAEZ01008464:442-594
HmHel-6	CAEZ01008400:63473-72220
HmHel-6	CAEZ01008400:89806-90515
HmHel-6	CAEZ01008400:53933-54077
HmHel-6	CAEZ01008400:8613-8768
HmHel-6	CAEZ01008393:22999-32361
HmHel-6	CAEZ01008393:54860-54941
HmHel-6	CAEZ01008393:76775-86076
HmHel-6	CAEZ01008393:97242-97374
HmHel-6	CAEZ01008393:123214-123442
HmHel-6	CAEZ01008393:134748-134932
HmHel-6	CAEZ01008393:121815-121927
HmHel-6	CAEZ01008393:33635-35083
HmHel-6	CAEZ01008393:18097-19225
HmHel-6	CAEZ01008357:5852-6081
HmHel-6	CAEZ01008272:24615-24791
HmHel-6	CAEZ01008272:26570-26660
HmHel-6	CAEZ01008272:8410-8521
HmHel-6	CAEZ01007799:4006-4174
HmHel-6	CAEZ01007799:19895-22129
HmHel-6	CAEZ01007495:2837-2969
HmHel-6	CAEZ01007495:37458-37577
HmHel-6	CAEZ01007227:5442-5622
HmHel-6	CAEZ01007114:10133-10345
HmHel-6	CAEZ01007023:4298-4494
HmHel-6	CAEZ01006983:13482-13717
HmHel-6	CAEZ01006871:7882-8472
HmHel-6	CAEZ01006871:15297-15482
HmHel-6	CAEZ01006811:7510-7677
HmHel-6	CAEZ01006730:2274-2406
HmHel-6	CAEZ01006350:15643-16070
HmHel-6	CAEZ01006350:36845-37029

HmHel-6	CAEZ01006350:20913-21145
HmHel-6	CAEZ01006103:5300-5513
HmHel-6	CAEZ01006039:7721-10987
HmHel-6	CAEZ01006039:9771-9927
HmHel-6	CAEZ01005502:5820-5982
HmHel-6	CAEZ01005225:36273-36395
HmHel-6	CAEZ01005225:26633-26861
HmHel-6	CAEZ01005137:5816-6016
HmHel-6	CAEZ01005131:3608-3818
HmHel-6	CAEZ01005131:14873-15001
HmHel-6	CAEZ01004890:2901-3066
HmHel-6	CAEZ01004890:1681-1773
HmHel-6	CAEZ01004846:20057-20225
HmHel-6	CAEZ01004772:3874-4026
HmHel-6	CAEZ01003828:48985-49109
HmHel-6	CAEZ01003828:76965-77088
HmHel-6	CAEZ01003828:91026-91186
HmHel-6	CAEZ01003828:64896-65136
HmHel-6	CAEZ01003828:42482-42655
HmHel-6	CAEZ01003828:21611-21789
HmHel-6	CAEZ01003765:3136-11637
HmHel-6	CAEZ01003765:31602-31762
HmHel-6	CAEZ01003765:28548-29949
HmHel-6	CAEZ01003530:3736-3881
HmHel-6	CAEZ01003530:4946-5051
HmHel-6	CAEZ01003392:16621-16832
HmHel-6	CAEZ01003392:8607-8731
HmHel-6	CAEZ01002927:1550-1678
HmHel-6	CAEZ01002927:9365-9554
HmHel-6	CAEZ01002844:3736-3868
HmHel-6	CAEZ01002825:6521-6625
HmHel-6	CAEZ01002825:19747-21984
HmHel-6	CAEZ01002762:25791-25924
HmHel-6	CAEZ01002762:11303-11412
HmHel-6	CAEZ01002321:11129-11318
HmHel-6	CAEZ01002321:38053-38165
HmHel-6	CAEZ01002321:58215-60852
HmHel-6	CAEZ01001904:1212-1340
HmHel-6	CAEZ01001904:7925-11659
HmHel-6	CAEZ01001777:14286-14418
HmHel-6	CAEZ01001648:1331-1479
HmHel-6	CAEZ01000036:7100-7272
HmHel-6	CAEZ01000036:20841-20997
HmHel-6	CAEZ01011627:68368-68475
HmHel-6	CAEZ01011627:91141-91265
HmHel-6	CAEZ01011461:9305-9398
HmHel-6	CAEZ01011461:25316-25475
HmHel-6	CAEZ01011461:33373-33459

HmHel-6	CAEZ01011461:2145-9526
HmHel-6	CAEZ01011274:10145-13705
HmHel-6	CAEZ01011036:830-10117
HmHel-6	CAEZ01011036:22581-27927
HmHel-6	CAEZ01011036:30785-30953
HmHel-6	CAEZ01011036:14973-18662
HmHel-6	CAEZ01010672:438-652
HmHel-6	CAEZ01009934:6335-6495
HmHel-6	CAEZ01009869:10784-10976
HmHel-6	CAEZ01009869:8440-9041
HmHel-6	CAEZ01009746:10468-10634
HmHel-6	CAEZ01009746:16623-16807
HmHel-6	CAEZ01009746:677-823
HmHel-6	CAEZ01008854:9528-9693
HmHel-6	CAEZ01008854:2241-2355
HmHel-6	CAEZ01008450:35349-36134
HmHel-6	CAEZ01008450:54038-54189
HmHel-6	CAEZ01008450:78569-79515
HmHel-6	CAEZ01008450:34361-34479
HmHel-6	CAEZ01008450:18582-18738
HmHel-6	CAEZ01008182:10414-18403
HmHel-6	CAEZ01008182:40674-40844
HmHel-6	CAEZ01008182:66520-66641
HmHel-6	CAEZ01008182:115528-119850
HmHel-6	CAEZ01008182:64829-65022
HmHel-6	CAEZ01008182:6424-6590
HmHel-6	CAEZ01007721:10167-10307
HmHel-6	CAEZ01007721:20948-21171
HmHel-6	CAEZ01007721:11080-11312
HmHel-6	CAEZ01007556:5532-6277
HmHel-6	CAEZ01007556:12061-12216
HmHel-6	CAEZ01007502:3777-3873
HmHel-6	CAEZ01007502:16111-16322
HmHel-6	CAEZ01007419:4339-4549
HmHel-6	CAEZ01007419:10745-10883
HmHel-6	CAEZ01007327:7301-10595
HmHel-6	CAEZ01007327:22612-22735
HmHel-6	CAEZ01007215:1609-4718
HmHel-6	CAEZ01007128:4548-4715
HmHel-6	CAEZ01006923:9845-12999
HmHel-6	CAEZ01006923:4488-4717
HmHel-6	CAEZ01006760:2083-2295
HmHel-6	CAEZ01006760:81282-81451
HmHel-6	CAEZ01006760:99896-100033
HmHel-6	CAEZ01006760:43620-43716
HmHel-6	CAEZ01006760:10427-10607
HmHel-6	CAEZ01006625:2345-2495
HmHel-6	CAEZ01006625:2889-2981

HmHel-6	CAEZ01006617:10185-10375
HmHel-6	CAEZ01006617:42837-43024
HmHel-6	CAEZ01006617:56046-56135
HmHel-6	CAEZ01006602:23417-23503
HmHel-6	CAEZ01006602:12964-16678
HmHel-6	CAEZ01006535:34945-35083
HmHel-6	CAEZ01006535:24595-24682
HmHel-6	CAEZ01006535:4030-4280
HmHel-6	CAEZ01006500:8427-8566
HmHel-6	CAEZ01006442:20156-20340
HmHel-6	CAEZ01006442:35485-48547
HmHel-6	CAEZ01006442:57611-57848
HmHel-6	CAEZ01006442:973-1052
HmHel-6	CAEZ01005933:11217-11324
HmHel-6	CAEZ01005933:37878-37985
HmHel-6	CAEZ01005933:7045-7228
HmHel-6	CAEZ01005810:14824-15040
HmHel-6	CAEZ01005810:3964-4196
HmHel-6	CAEZ01005790:3054-3161
HmHel-6	CAEZ01005790:6655-9231
HmHel-6	CAEZ01005556:6657-6858
HmHel-6	CAEZ01005509:15657-15741
HmHel-6	CAEZ01005509:44205-44345
HmHel-6	CAEZ01005509:64221-64332
HmHel-6	CAEZ01005509:22870-23102
HmHel-6	CAEZ01005509:10436-10563
HmHel-6	CAEZ01005139:3466-3665
HmHel-6	CAEZ01004755:12789-12976
HmHel-6	CAEZ01004620:3112-3243
HmHel-6	CAEZ01004564:2734-2915
HmHel-6	CAEZ01004564:30527-30690
HmHel-6	CAEZ01004564:22153-25650
HmHel-6	CAEZ01004356:3582-3816
HmHel-6	CAEZ01004356:7946-8097
HmHel-6	CAEZ01004153:9658-9866
HmHel-6	CAEZ01003933:13335-13544
HmHel-6	CAEZ01003933:40398-40559
HmHel-6	CAEZ01003771:10751-14286
HmHel-6	CAEZ01003771:25593-25811
HmHel-6	CAEZ01003713:2060-2191
HmHel-6	CAEZ01003519:5405-5540
HmHel-6	CAEZ01003420:7465-7665
HmHel-6	CAEZ01003420:27622-27705
HmHel-6	CAEZ01003420:14196-21555
HmHel-6	CAEZ01003416:49288-49374
HmHel-6	CAEZ01003416:93250-93413
HmHel-6	CAEZ01003416:99337-99417
HmHel-6	CAEZ01003416:63916-64070

HmHel-6	CAEZ01003416:21012-25521
HmHel-6	CAEZ01003062:9687-9803
HmHel-6	CAEZ01003062:7790-7883
HmHel-6	CAEZ01002083:3067-3194
HmHel-6	CAEZ01001936:3255-6510
HmHel-6	CAEZ01001936:4326-6039
HmHel-6	CAEZ01001877:4942-5126
HmHel-6	CAEZ01001877:2804-7376
HmHel-6	CAEZ01001752:155-304
HmHel-6	CAEZ01001752:4763-4864
HmHel-6	CAEZ01001121:377-574
HmHel-6	CAEZ01000637:508-715
HmHel-6	CAEZ01011135:31325-31443
HmHel-6	CAEZ01011135:39207-39422
HmHel-6	CAEZ01011084:857-991
HmHel-6	CAEZ01010761:2163-2269
HmHel-6	CAEZ01010734:12100-12179
HmHel-6	CAEZ01010734:22316-31123
HmHel-6	CAEZ01010734:93258-93356
HmHel-6	CAEZ01010734:126221-126303
HmHel-6	CAEZ01010734:108958-109081
HmHel-6	CAEZ01010734:78665-78782
HmHel-6	CAEZ01010734:59957-67115
HmHel-6	CAEZ01010734:20102-20321
HmHel-6	CAEZ01010070:2938-3031
HmHel-6	CAEZ01010070:991-1185
HmHel-6	CAEZ01009855:232-386
HmHel-6	CAEZ01009855:15079-15207
HmHel-6	CAEZ01009855:3411-8003
HmHel-6	CAEZ01009628:11339-11500
HmHel-6	CAEZ01009443:5909-6005
HmHel-6	CAEZ01009443:23903-36578
HmHel-6	CAEZ01009443:57912-61884
HmHel-6	CAEZ01009443:34475-34609
HmHel-6	CAEZ01009443:19511-19628
HmHel-6	CAEZ01009417:19414-22387
HmHel-6	CAEZ01009417:13135-13308
HmHel-6	CAEZ01009153:9756-9961
HmHel-6	CAEZ01009153:7118-7332
HmHel-6	CAEZ01008847:5442-5656
HmHel-6	CAEZ01008847:4841-16086
HmHel-6	CAEZ01008669:6078-17218
HmHel-6	CAEZ01008669:2658-2740
HmHel-6	CAEZ01008260:16229-16321
HmHel-6	CAEZ01008260:5310-5456
HmHel-6	CAEZ01008178:1242-1440
HmHel-6	CAEZ01007950:8069-8283
HmHel-6	CAEZ01007950:17913-17994

HmHel-6	CAEZ01007950:1823-2053
HmHel-6	CAEZ01007522:1877-2107
HmHel-6	CAEZ01007522:4138-4226
HmHel-6	CAEZ01006901:11469-11683
HmHel-6	CAEZ01006901:1066-5648
HmHel-6	CAEZ01006799:19981-20118
HmHel-6	CAEZ01006799:39804-39933
HmHel-6	CAEZ01006799:105742-105846
HmHel-6	CAEZ01006799:133714-137450
HmHel-6	CAEZ01006799:115575-115657
HmHel-6	CAEZ01006799:86227-86392
HmHel-6	CAEZ01006473:14070-14225
HmHel-6	CAEZ01006473:20548-23951
HmHel-6	CAEZ01006416:7873-15526
HmHel-6	CAEZ01006387:31031-31350
HmHel-6	CAEZ01006387:29248-29430
HmHel-6	CAEZ01006302:4207-4337
HmHel-6	CAEZ01006302:3758-3852
HmHel-6	CAEZ01005842:1225-1304
HmHel-6	CAEZ01005842:4049-4204
HmHel-6	CAEZ01005382:36968-37178
HmHel-6	CAEZ01005382:12903-13068
HmHel-6	CAEZ01005142:1-209
HmHel-6	CAEZ01004901:7135-7266
HmHel-6	CAEZ01004901:22797-22923
HmHel-6	CAEZ01004667:634-1023
HmHel-6	CAEZ01004667:4139-4248
HmHel-6	CAEZ01004538:4363-4504
HmHel-6	CAEZ01004538:4242-4360
HmHel-6	CAEZ01004365:29-164
HmHel-6	CAEZ01004365:23196-23378
HmHel-6	CAEZ01003891:11377-11637
HmHel-6	CAEZ01003891:3670-3852
HmHel-6	CAEZ01003651:19894-25242
HmHel-6	CAEZ01003651:23450-23604
HmHel-6	CAEZ01003541:345-523
HmHel-6	CAEZ01003541:20040-20122
HmHel-6	CAEZ01003541:16165-16289
HmHel-6	CAEZ01003435:3386-3558
HmHel-6	CAEZ01003435:4952-5575
HmHel-6	CAEZ01003376:1674-1893
HmHel-6	CAEZ01002650:8981-9139
HmHel-6	CAEZ01002650:46226-46406
HmHel-6	CAEZ01002650:8332-8490
HmHel-6	CAEZ01002015:12756-16821
HmHel-6	CAEZ01002015:22544-22692
HmHel-6	CAEZ01002015:4057-4181
HmHel-6	CAEZ01001924:1346-1556

HmHel-6	CAEZ01001924:406-502
HmHel-6	CAEZ01001741:7902-8064
HmHel-6	CAEZ01001741:932-1076
HmHel-6	CAEZ01000062:7738-15029
HmHel-6	CAEZ01000062:41086-41289
HmHel-6	CAEZ01000062:4204-4311
HmHel-6	CAEZ01011491:3725-3956
HmHel-6	CAEZ01011306:7519-7656
HmHel-6	CAEZ01010748:13322-13538
HmHel-6	CAEZ01010748:4208-5355
HmHel-6	CAEZ01010097:99-5846
HmHel-6	CAEZ01010097:1661-1754
HmHel-6	CAEZ01010045:5926-6103
HmHel-6	CAEZ01009885:11796-11957
HmHel-6	CAEZ01009885:31578-31706
HmHel-6	CAEZ01009885:61393-61485
HmHel-6	CAEZ01009885:28986-29115
HmHel-6	CAEZ01009806:5315-5497
HmHel-6	CAEZ01009734:530-1431
HmHel-6	CAEZ01009734:11737-11928
HmHel-6	CAEZ01009734:13719-13812
HmHel-6	CAEZ01009711:4906-5035
HmHel-6	CAEZ01009484:10082-10196
HmHel-6	CAEZ01009484:28208-28301
HmHel-6	CAEZ01009484:59315-59517
HmHel-6	CAEZ01009484:72709-72914
HmHel-6	CAEZ01009484:120471-120624
HmHel-6	CAEZ01009484:125121-125228
HmHel-6	CAEZ01009484:86878-86958
HmHel-6	CAEZ01009484:45415-45550
HmHel-6	CAEZ01009484:5743-5956
HmHel-6	CAEZ01008331:1238-1379
HmHel-6	CAEZ01008331:8047-9771
HmHel-6	CAEZ01008204:29812-29986
HmHel-6	CAEZ01008204:5721-5846
HmHel-6	CAEZ01008198:1941-2034
HmHel-6	CAEZ01008198:3504-3583
HmHel-6	CAEZ01007940:26084-26302
HmHel-6	CAEZ01007940:44922-45059
HmHel-6	CAEZ01007940:13884-21689
HmHel-6	CAEZ01007627:3063-3191
HmHel-6	CAEZ01007627:4166-9082
HmHel-6	CAEZ01007525:15835-15968
HmHel-6	CAEZ01007525:2150-2295
HmHel-6	CAEZ01007394:11401-11578
HmHel-6	CAEZ01006736:6999-7100
HmHel-6	CAEZ01006736:17194-21928
HmHel-6	CAEZ01006736:2789-8369

HmHel-6	CAEZ01006731:8197-8386
HmHel-6	CAEZ01006731:18300-19560
HmHel-6	CAEZ01006094:18093-18274
HmHel-6	CAEZ01006094:54676-60787
HmHel-6	CAEZ01006094:15820-16034
HmHel-6	CAEZ01005797:33234-33432
HmHel-6	CAEZ01005797:13263-13364
HmHel-6	CAEZ01005724:9666-9900
HmHel-6	CAEZ01005724:7208-7324
HmHel-6	CAEZ01005679:6596-6741
HmHel-6	CAEZ01005679:29118-29239
HmHel-6	CAEZ01005429:3886-3991
HmHel-6	CAEZ01005429:16850-17082
HmHel-6	CAEZ01005429:66602-66794
HmHel-6	CAEZ01005429:15569-15689
HmHel-6	CAEZ01005145:46772-48762
HmHel-6	CAEZ01005145:40779-40866
HmHel-6	CAEZ01005145:23316-23508
HmHel-6	CAEZ01005073:5616-5813
HmHel-6	CAEZ01004941:15060-15223
HmHel-6	CAEZ01004941:46779-50841
HmHel-6	CAEZ01004941:76819-76930
HmHel-6	CAEZ01004941:117321-117463
HmHel-6	CAEZ01004941:92457-92559
HmHel-6	CAEZ01004941:41805-41948
HmHel-6	CAEZ01004906:5335-5480
HmHel-6	CAEZ01004869:25571-25734
HmHel-6	CAEZ01004869:50109-50262
HmHel-6	CAEZ01004869:45973-46935
HmHel-6	CAEZ01004869:9663-17554
HmHel-6	CAEZ01004757:23317-23509
HmHel-6	CAEZ01004680:20394-20602
HmHel-6	CAEZ01004680:39171-49338
HmHel-6	CAEZ01004601:5230-5384
HmHel-6	CAEZ01004566:4406-10299
HmHel-6	CAEZ01004566:55112-55244
HmHel-6	CAEZ01004566:25284-30530
HmHel-6	CAEZ01004256:7266-7480
HmHel-6	CAEZ01004224:7431-7568
HmHel-6	CAEZ01003964:1237-1446
HmHel-6	CAEZ01003894:1565-1699
HmHel-6	CAEZ01003894:22452-22685
HmHel-6	CAEZ01003894:11007-11142
HmHel-6	CAEZ01003465:3568-13267
HmHel-6	CAEZ01003465:3153-3356
HmHel-6	CAEZ01003144:22756-22869
HmHel-6	CAEZ01003144:1128-1395
HmHel-6	CAEZ01002834:10872-11058

HmHel-6	CAEZ01002834:1404-5488
HmHel-6	CAEZ01002808:19170-19266
HmHel-6	CAEZ01002808:158578-158662
HmHel-6	CAEZ01002808:179965-180129
HmHel-6	CAEZ01002808:166412-166540
HmHel-6	CAEZ01002808:148365-148449
HmHel-6	CAEZ01002808:102932-111222
HmHel-6	CAEZ01002808:64101-72000
HmHel-6	CAEZ01002808:4192-4311
HmHel-6	CAEZ01002632:3189-3321
HmHel-6	CAEZ01002632:3723-3940
HmHel-6	CAEZ01002407:3656-3820
HmHel-6	CAEZ01002371:13810-19929
HmHel-6	CAEZ01002371:118686-118805
HmHel-6	CAEZ01002371:67543-76178
HmHel-6	CAEZ01002371:56835-57024
HmHel-6	CAEZ01002371:23461-23656
HmHel-6	CAEZ01002130:6684-6954
HmHel-6	CAEZ01002130:5098-5199
HmHel-6	CAEZ01002072:149-274
HmHel-6	CAEZ01002072:4028-4112
HmHel-6	CAEZ01001984:6771-12448
HmHel-6	CAEZ01001984:30496-37345
HmHel-6	CAEZ01001984:20775-20932
HmHel-6	CAEZ01001888:4427-4534
HmHel-6	CAEZ01001888:33855-33954
HmHel-6	CAEZ01001888:28179-34500
HmHel-6	CAEZ01001634:648-873
HmHel-6	CAEZ01001517:1013-11102
HmHel-6	CAEZ01001517:1707-1811
HmHel-6	CAEZ01001439:660-872
HmHel-6	CAEZ01000893:13391-13564
HmHel-6	CAEZ01000567:2518-2689
HmHel-6	CAEZ01011462:750-964
HmHel-6	CAEZ01011462:6065-6189
HmHel-6	CAEZ01011275:2406-2569
HmHel-6	CAEZ01010961:16797-16931
HmHel-6	CAEZ01010961:4448-4664
HmHel-6	CAEZ01010903:18851-18959
HmHel-6	CAEZ01010897:20966-21047
HmHel-6	CAEZ01010897:41757-41944
HmHel-6	CAEZ01010897:42939-43080
HmHel-6	CAEZ01010897:19158-28169
HmHel-6	CAEZ01010897:7313-7405
HmHel-6	CAEZ01010534:32742-32955
HmHel-6	CAEZ01010534:44705-44824
HmHel-6	CAEZ01010534:90453-90597
HmHel-6	CAEZ01010534:58707-61011

HmHel-6	CAEZ01010534:31860-31943
HmHel-6	CAEZ01010534:4505-4741
HmHel-6	CAEZ01010060:20-140
HmHel-6	CAEZ01010001:4755-10134
HmHel-6	CAEZ01010001:29715-29847
HmHel-6	CAEZ01010001:13084-13294
HmHel-6	CAEZ01009647:8128-8924
HmHel-6	CAEZ01009647:12054-12152
HmHel-6	CAEZ01009398:9745-9933
HmHel-6	CAEZ01009398:13398-13589
HmHel-6	CAEZ01009393:555-635
HmHel-6	CAEZ01009393:8876-9096
HmHel-6	CAEZ01009178:4388-4547
HmHel-6	CAEZ01009138:14811-14927
HmHel-6	CAEZ01009138:32826-33036
HmHel-6	CAEZ01009138:51952-52068
HmHel-6	CAEZ01009138:20343-20426
HmHel-6	CAEZ01009040:5910-6126
HmHel-6	CAEZ01009040:11277-11441
HmHel-6	CAEZ01008670:6391-6560
HmHel-6	CAEZ01008670:34865-35088
HmHel-6	CAEZ01008310:891-1128
HmHel-6	CAEZ01008221:17570-17768
HmHel-6	CAEZ01008221:22548-22698
HmHel-6	CAEZ01007888:5787-5911
HmHel-6	CAEZ01007794:3008-3159
HmHel-6	CAEZ01007794:5551-5722
HmHel-6	CAEZ01007766:5244-5417
HmHel-6	CAEZ01007764:7347-7479
HmHel-6	CAEZ01007764:26056-26139
HmHel-6	CAEZ01007764:14833-14964
HmHel-6	CAEZ01007711:27102-27276
HmHel-6	CAEZ01007642:9605-9684
HmHel-6	CAEZ01007642:30804-31012
HmHel-6	CAEZ01007642:13089-13214
HmHel-6	CAEZ01007403:34680-34868
HmHel-6	CAEZ01007403:13000-13177
HmHel-6	CAEZ01007292:18583-18714
HmHel-6	CAEZ01007079:835-3375
HmHel-6	CAEZ01007079:11129-11233
HmHel-6	CAEZ01006981:1740-1925
HmHel-6	CAEZ01006981:14919-19474
HmHel-6	CAEZ01006652:3636-3843
HmHel-6	CAEZ01006652:25887-26095
HmHel-6	CAEZ01006652:2559-2659
HmHel-6	CAEZ01006463:1890-2128
HmHel-6	CAEZ01006101:2009-2128
HmHel-6	CAEZ01006101:14667-14844

HmHel-6	CAEZ01006101:3060-3165
HmHel-6	CAEZ01005817:6101-6277
HmHel-6	CAEZ01005817:16603-16788
HmHel-6	CAEZ01005817:43589-43798
HmHel-6	CAEZ01005817:33029-33209
HmHel-6	CAEZ01005667:10703-10893
HmHel-6	CAEZ01005667:52362-52450
HmHel-6	CAEZ01005667:49934-50093
HmHel-6	CAEZ01005667:26198-26324
HmHel-6	CAEZ01005667:5520-11312
HmHel-6	CAEZ01005642:5500-10500
HmHel-6	CAEZ01005609:1630-1716
HmHel-6	CAEZ01005609:15445-16096
HmHel-6	CAEZ01005609:11577-11752
HmHel-6	CAEZ01005609:1065-1148
HmHel-6	CAEZ01005252:15178-15285
HmHel-6	CAEZ01005252:42290-42418
HmHel-6	CAEZ01005252:72240-72443
HmHel-6	CAEZ01005252:39340-39551
HmHel-6	CAEZ01005252:9066-9230
HmHel-6	CAEZ01005036:3083-3203
HmHel-6	CAEZ01005036:32218-32307
HmHel-6	CAEZ01005036:23108-23237
HmHel-6	CAEZ01005036:5474-5606
HmHel-6	CAEZ01004306:4255-4383
HmHel-6	CAEZ01004306:12964-15771
HmHel-6	CAEZ01004117:6855-6975
HmHel-6	CAEZ01004115:10821-10941
HmHel-6	CAEZ01004115:11731-11858
HmHel-6	CAEZ01003711:25029-25109
HmHel-6	CAEZ01003711:25788-25932
HmHel-6	CAEZ01003127:2636-2824
HmHel-6	CAEZ01002936:14828-14973
HmHel-6	CAEZ01002936:34050-35023
HmHel-6	CAEZ01002904:25888-26085
HmHel-6	CAEZ01002904:2217-2332
HmHel-6	CAEZ01002612:11699-11859
HmHel-6	CAEZ01002453:2566-2674
HmHel-6	CAEZ01002311:400-619
HmHel-6	CAEZ01002194:78329-78449
HmHel-6	CAEZ01002194:77475-77560
HmHel-6	CAEZ01002194:49122-49208
HmHel-6	CAEZ01002032:1568-1756
HmHel-6	CAEZ01002032:19-5227
HmHel-6	CAEZ01001450:790-910
HmHel-6	CAEZ01001147:35910-38964
HmHel-6	CAEZ01001147:32528-32706
HmHel-6	CAEZ01001147:9337-16541

HmHel-6	CAEZ01011595:9503-9627
HmHel-6	CAEZ01011595:3094-3329
HmHel-6	CAEZ01011554:6207-6399
HmHel-6	CAEZ01011554:33128-33316
HmHel-6	CAEZ01011336:2816-2955
HmHel-6	CAEZ01011336:9685-9798
HmHel-6	CAEZ01011051:5094-5184
HmHel-6	CAEZ01011051:6728-11504
HmHel-6	CAEZ01010995:27002-27227
HmHel-6	CAEZ01010995:19539-19676
HmHel-6	CAEZ01010806:368-494
HmHel-6	CAEZ01010806:13409-13643
HmHel-6	CAEZ01010806:46179-49917
HmHel-6	CAEZ01010806:66718-66882
HmHel-6	CAEZ01010806:28080-31010
HmHel-6	CAEZ01010806:8614-8732
HmHel-6	CAEZ01010707:14107-21826
HmHel-6	CAEZ01010707:41680-49433
HmHel-6	CAEZ01010707:156362-156485
HmHel-6	CAEZ01010707:175820-180425
HmHel-6	CAEZ01010707:138312-138441
HmHel-6	CAEZ01010707:113086-113316
HmHel-6	CAEZ01010374:1578-1815
HmHel-6	CAEZ01010146:8834-9057
HmHel-6	CAEZ01010146:16568-16693
HmHel-6	CAEZ01010096:14244-14359
HmHel-6	CAEZ01010041:6464-6592
HmHel-6	CAEZ01010041:29197-29438
HmHel-6	CAEZ01010041:9041-9185
HmHel-6	CAEZ01009932:79858-79944
HmHel-6	CAEZ01009932:61740-61906
HmHel-6	CAEZ01009826:2557-2641
HmHel-6	CAEZ01009826:12681-12843
HmHel-6	CAEZ01009826:2722-2853
HmHel-6	CAEZ01009587:1654-1869
HmHel-6	CAEZ01009519:6144-6363
HmHel-6	CAEZ01009519:8227-8335
HmHel-6	CAEZ01009444:1171-8836
HmHel-6	CAEZ01009444:23563-23768
HmHel-6	CAEZ01009098:2012-2189
HmHel-6	CAEZ01009098:14203-17474
HmHel-6	CAEZ01009096:3103-3283
HmHel-6	CAEZ01007947:1684-1889
HmHel-6	CAEZ01007945:7455-7621
HmHel-6	CAEZ01007560:440-567
HmHel-6	CAEZ01007551:4989-5116
HmHel-6	CAEZ01007551:13217-13296
HmHel-6	CAEZ01007551:2844-2940

HmHel-6	CAEZ01007496:21890-31122
HmHel-6	CAEZ01007496:50279-50496
HmHel-6	CAEZ01007496:22724-22841
HmHel-6	CAEZ01007372:2441-2603
HmHel-6	CAEZ01007358:10881-10980
HmHel-6	CAEZ01007358:3431-3553
HmHel-6	CAEZ01007141:14247-14374
HmHel-6	CAEZ01007141:27873-27966
HmHel-6	CAEZ01006455:5415-8329
HmHel-6	CAEZ01006246:11156-11360
HmHel-6	CAEZ01006246:24454-24633
HmHel-6	CAEZ01006246:10315-13314
HmHel-6	CAEZ01006179:6882-6985
HmHel-6	CAEZ01006020:6815-6904
HmHel-6	CAEZ01006020:33507-33675
HmHel-6	CAEZ01006020:23435-23598
HmHel-6	CAEZ01005232:828-1064
HmHel-6	CAEZ01005087:4640-4730
HmHel-6	CAEZ01005087:5616-5810
HmHel-6	CAEZ01004894:23885-24012
HmHel-6	CAEZ01004894:29691-29927
HmHel-6	CAEZ01004788:5458-5573
HmHel-6	CAEZ01004534:4079-4289
HmHel-6	CAEZ01004299:14819-14974
HmHel-6	CAEZ01003888:17346-17481
HmHel-6	CAEZ01003183:13453-13576
HmHel-6	CAEZ01003054:11544-11675
HmHel-6	CAEZ01002675:7479-7603
HmHel-6	CAEZ01002608:26259-26434
HmHel-6	CAEZ01002608:46531-46626
HmHel-6	CAEZ01002608:39180-39300
HmHel-6	CAEZ01002257:21077-21161
HmHel-6	CAEZ01002257:59126-59227
HmHel-6	CAEZ01002257:52034-52205
HmHel-6	CAEZ01002054:237-471
HmHel-6	CAEZ01002054:1666-1798
HmHel-6	CAEZ01002049:3526-3775
HmHel-6	CAEZ01002049:39492-39628
HmHel-6	CAEZ01002049:65193-65359
HmHel-6	CAEZ01002049:79223-79313
HmHel-6	CAEZ01002049:98885-98990
HmHel-6	CAEZ01002049:61170-61254
HmHel-6	CAEZ01002049:33295-33449
HmHel-6	CAEZ01002049:1004-1155
HmHel-6	CAEZ01001779:14534-14743
HmHel-6	CAEZ01001779:26176-26262
HmHel-6	CAEZ01001779:3719-3940
HmHel-6	CAEZ01001285:4568-4649

HmHel-6	CAEZ01001285:1798-2002
HmHel-6	CAEZ01000677:435-555
HmHel-6	CAEZ01000067:11634-11795
HmHel-6	CAEZ01011637:10214-10337
HmHel-6	CAEZ01011532:3277-3403
HmHel-6	CAEZ01011030:3895-4003
HmHel-6	CAEZ01011030:2142-2259
HmHel-6	CAEZ01010937:5502-5672
HmHel-6	CAEZ01010937:26257-26403
HmHel-6	CAEZ01010868:17903-18019
HmHel-6	CAEZ01010868:57052-57270
HmHel-6	CAEZ01010868:131465-131632
HmHel-6	CAEZ01010868:111059-111210
HmHel-6	CAEZ01010868:85016-85099
HmHel-6	CAEZ01010868:4244-4334
HmHel-6	CAEZ01010851:5423-10397
HmHel-6	CAEZ01010851:37962-38158
HmHel-6	CAEZ01010851:35206-35442
HmHel-6	CAEZ01010832:2277-2438
HmHel-6	CAEZ01010832:26818-26946
HmHel-6	CAEZ01010832:1610-11666
HmHel-6	CAEZ01010781:21401-21636
HmHel-6	CAEZ01010691:698-808
HmHel-6	CAEZ01010637:1370-1538
HmHel-6	CAEZ01010521:5455-5634
HmHel-6	CAEZ01010521:12489-12651
HmHel-6	CAEZ01010500:6175-6301
HmHel-6	CAEZ01010342:368-602
HmHel-6	CAEZ01009833:11275-12039
HmHel-6	CAEZ01009833:21216-26633
HmHel-6	CAEZ01009833:7046-7255
HmHel-6	CAEZ01009742:9974-10076
HmHel-6	CAEZ01009742:25571-25697
HmHel-6	CAEZ01009742:26618-26710
HmHel-6	CAEZ01009742:6319-6581
HmHel-6	CAEZ01009660:2496-2603
HmHel-6	CAEZ01009553:854-997
HmHel-6	CAEZ01009553:29560-29666
HmHel-6	CAEZ01009501:952-1051
HmHel-6	CAEZ01009501:14073-14276
HmHel-6	CAEZ01009501:99935-100061
HmHel-6	CAEZ01009501:100514-100652
HmHel-6	CAEZ01009501:1216-1430
HmHel-6	CAEZ01009331:20552-25849
HmHel-6	CAEZ01009331:78794-78896
HmHel-6	CAEZ01009331:37196-37349
HmHel-6	CAEZ01009331:17959-18054
HmHel-6	CAEZ01009310:13885-14102

HmHel-6	CAEZ01009214:16913-17072
HmHel-6	CAEZ01008819:14439-14553
HmHel-6	CAEZ01008819:5809-5961
HmHel-6	CAEZ01008815:2863-7097
HmHel-6	CAEZ01008815:2702-8150
HmHel-6	CAEZ01008729:6524-6769
HmHel-6	CAEZ01008729:37220-37348
HmHel-6	CAEZ01008729:87107-87212
HmHel-6	CAEZ01008729:108547-108628
HmHel-6	CAEZ01008729:51475-51569
HmHel-6	CAEZ01008729:40278-40475
HmHel-6	CAEZ01008630:2119-2256
HmHel-6	CAEZ01008630:5389-5571
HmHel-6	CAEZ01008622:3536-3669
HmHel-6	CAEZ01008589:2367-8465
HmHel-6	CAEZ01008589:20553-20680
HmHel-6	CAEZ01008589:25838-25993
HmHel-6	CAEZ01008589:4392-9919
HmHel-6	CAEZ01007688:10614-11872
HmHel-6	CAEZ01007346:1035-10442
HmHel-6	CAEZ01007346:2242-2377
HmHel-6	CAEZ01007146:1868-2055
HmHel-6	CAEZ01007146:2413-2658
HmHel-6	CAEZ01006962:2322-7789
HmHel-6	CAEZ01006962:20350-20561
HmHel-6	CAEZ01006819:1326-1519
HmHel-6	CAEZ01006819:4656-14653
HmHel-6	CAEZ01006427:3164-3290
HmHel-6	CAEZ01006378:3067-4661
HmHel-6	CAEZ01006378:24986-25071
HmHel-6	CAEZ01006378:11488-14900
HmHel-6	CAEZ01006157:403-617
HmHel-6	CAEZ01006157:996-1102
HmHel-6	CAEZ01005921:13170-14563
HmHel-6	CAEZ01005921:25277-31735
HmHel-6	CAEZ01005913:1440-1557
HmHel-6	CAEZ01005913:31807-31992
HmHel-6	CAEZ01005913:23816-23968
HmHel-6	CAEZ01005770:17234-22065
HmHel-6	CAEZ01005770:16761-16940
HmHel-6	CAEZ01005770:5508-5646
HmHel-6	CAEZ01005436:22141-22231
HmHel-6	CAEZ01005436:221-435
HmHel-6	CAEZ01005290:25687-25813
HmHel-6	CAEZ01005290:6820-6974
HmHel-6	CAEZ01005201:25392-25503
HmHel-6	CAEZ01005201:1255-1467
HmHel-6	CAEZ01004952:8279-8444

HmHel-6	CAEZ01004952:15686-15846
HmHel-6	CAEZ01004278:3803-4008
HmHel-6	CAEZ01004278:11850-11984
HmHel-6	CAEZ01004278:1244-1331
HmHel-6	CAEZ01004062:3118-3235
HmHel-6	CAEZ01003349:12064-12263
HmHel-6	CAEZ01003349:57645-57811
HmHel-6	CAEZ01003349:48338-53974
HmHel-6	CAEZ01003349:21283-21509
HmHel-6	CAEZ01003304:16509-23024
HmHel-6	CAEZ01003250:27358-27448
HmHel-6	CAEZ01003250:70355-70440
HmHel-6	CAEZ01003250:97950-98111
HmHel-6	CAEZ01003250:154089-154205
HmHel-6	CAEZ01003250:174223-174340
HmHel-6	CAEZ01003250:197983-198121
HmHel-6	CAEZ01003250:212959-222745
HmHel-6	CAEZ01003250:204035-206467
HmHel-6	CAEZ01003250:141366-153313
HmHel-6	CAEZ01003250:86104-86251
HmHel-6	CAEZ01002884:10907-11057
HmHel-6	CAEZ01002884:5718-5878
HmHel-6	CAEZ01002853:5224-5444
HmHel-6	CAEZ01002761:1997-2115
HmHel-6	CAEZ01002672:6872-6994
HmHel-6	CAEZ01002634:686-789
HmHel-6	CAEZ01002634:35543-35673
HmHel-6	CAEZ01002634:57438-57661
HmHel-6	CAEZ01002428:9253-9422
HmHel-6	CAEZ01002428:8020-12665
HmHel-6	CAEZ01002245:6550-6760
HmHel-6	CAEZ01002245:10493-10615
HmHel-6	CAEZ01001333:1445-1659
HmHel-6	CAEZ01001333:1826-1932
HmHel-6	CAEZ01000944:6511-6629
HmHel-6	CAEZ01011529:22044-28136
HmHel-6	CAEZ01011529:48578-48783
HmHel-6	CAEZ01011529:42908-43081
HmHel-6	CAEZ01011529:26964-27156
HmHel-6	CAEZ01011529:2479-2674
HmHel-6	CAEZ01011077:8800-8968
HmHel-6	CAEZ01010451:20625-20769
HmHel-6	CAEZ01010451:44731-44877
HmHel-6	CAEZ01010451:66459-66616
HmHel-6	CAEZ01010451:76850-76952
HmHel-6	CAEZ01010451:99485-99690
HmHel-6	CAEZ01010451:111175-111304
HmHel-6	CAEZ01010451:49434-62160

HmHel-6	CAEZ01010357:21594-23322
HmHel-6	CAEZ01010357:937-4446
HmHel-6	CAEZ01010289:6146-16057
HmHel-6	CAEZ01010289:39671-39818
HmHel-6	CAEZ01010289:98846-99004
HmHel-6	CAEZ01010289:113189-113300
HmHel-6	CAEZ01010289:142569-142667
HmHel-6	CAEZ01010289:163767-163864
HmHel-6	CAEZ01010289:135663-135864
HmHel-6	CAEZ01010289:32685-32770
HmHel-6	CAEZ01010172:4376-9467
HmHel-6	CAEZ01010086:4809-5363
HmHel-6	CAEZ01010086:22124-23878
HmHel-6	CAEZ01010086:794-7378
HmHel-6	CAEZ01010022:1972-2177
HmHel-6	CAEZ01009770:18951-19081
HmHel-6	CAEZ01009770:53950-54062
HmHel-6	CAEZ01009770:78894-79011
HmHel-6	CAEZ01009770:97325-102889
HmHel-6	CAEZ01009770:58080-59052
HmHel-6	CAEZ01009770:34516-37294
HmHel-6	CAEZ01009409:16776-16952
HmHel-6	CAEZ01009314:369-563
HmHel-6	CAEZ01009314:17044-17230
HmHel-6	CAEZ01009131:8743-8967
HmHel-6	CAEZ01009073:1365-1474
HmHel-6	CAEZ01008909:2522-2650
HmHel-6	CAEZ01008565:14767-15024
HmHel-6	CAEZ01008181:12995-13102
HmHel-6	CAEZ01008181:33538-33642
HmHel-6	CAEZ01008181:16071-18326
HmHel-6	CAEZ01008024:5988-6191
HmHel-6	CAEZ01007886:15809-15970
HmHel-6	CAEZ01007800:2059-10676
HmHel-6	CAEZ01007800:643-752
HmHel-6	CAEZ01007797:8431-8601
HmHel-6	CAEZ01007797:6100-6304
HmHel-6	CAEZ01007699:422-579
HmHel-6	CAEZ01007647:15345-15470
HmHel-6	CAEZ01007647:33507-33586
HmHel-6	CAEZ01007647:46968-47105
HmHel-6	CAEZ01007647:42080-42181
HmHel-6	CAEZ01007535:12284-12492
HmHel-6	CAEZ01007535:30502-30760
HmHel-6	CAEZ01007515:12015-12157
HmHel-6	CAEZ01007515:46224-46345
HmHel-6	CAEZ01007385:6040-6205
HmHel-6	CAEZ01007385:5187-5291

HmHel-6	CAEZ01007306:9003-9207
HmHel-6	CAEZ01007306:9346-9440
HmHel-6	CAEZ01007254:14057-14162
HmHel-6	CAEZ01007254:24299-24383
HmHel-6	CAEZ01007254:9399-9591
HmHel-6	CAEZ01007210:1672-1897
HmHel-6	CAEZ01006961:30612-30775
HmHel-6	CAEZ01006961:50699-50874
HmHel-6	CAEZ01006961:9828-9907
HmHel-6	CAEZ01006740:4539-7071
HmHel-6	CAEZ01006740:8407-8574
HmHel-6	CAEZ01006477:8993-9118
HmHel-6	CAEZ01006445:27992-36857
HmHel-6	CAEZ01006445:50126-50306
HmHel-6	CAEZ01006445:96945-97100
HmHel-6	CAEZ01006445:160255-160348
HmHel-6	CAEZ01006445:196767-196915
HmHel-6	CAEZ01006445:218620-218703
HmHel-6	CAEZ01006445:234222-243136
HmHel-6	CAEZ01006445:267541-267629
HmHel-6	CAEZ01006445:139182-139281
HmHel-6	CAEZ01006445:54919-55131
HmHel-6	CAEZ01006445:4935-5019
HmHel-6	CAEZ01006254:2804-3042
HmHel-6	CAEZ01005882:8337-10456
HmHel-6	CAEZ01005882:13569-13706
HmHel-6	CAEZ01005681:15395-15543
HmHel-6	CAEZ01005681:25730-25853
HmHel-6	CAEZ01005681:1685-1898
HmHel-6	CAEZ01005575:11543-11779
HmHel-6	CAEZ01005575:20041-20128
HmHel-6	CAEZ01005485:7779-10549
HmHel-6	CAEZ01005485:39667-39829
HmHel-6	CAEZ01005485:69583-69745
HmHel-6	CAEZ01005485:49752-49940
HmHel-6	CAEZ01005441:9127-9251
HmHel-6	CAEZ01005312:392-3662
HmHel-6	CAEZ01005312:2996-3142
HmHel-6	CAEZ01005288:35891-41589
HmHel-6	CAEZ01005288:29334-29429
HmHel-6	CAEZ01004841:15961-18561
HmHel-6	CAEZ01004841:31726-36234
HmHel-6	CAEZ01004841:40443-40582
HmHel-6	CAEZ01004841:8847-9037
HmHel-6	CAEZ01004776:1495-1614
HmHel-6	CAEZ01004593:1250-1424
HmHel-6	CAEZ01004525:763-970
HmHel-6	CAEZ01004223:6859-6996

HmHel-6	CAEZ01004223:735-877
HmHel-6	CAEZ01004148:11481-11682
HmHel-6	CAEZ01003915:13401-13491
HmHel-6	CAEZ01003915:2111-2319
HmHel-6	CAEZ01003896:309-442
HmHel-6	CAEZ01003896:18786-19028
HmHel-6	CAEZ01003402:6750-6858
HmHel-6	CAEZ01003305:3678-3806
HmHel-6	CAEZ01003305:25530-25626
HmHel-6	CAEZ01003305:18989-21500
HmHel-6	CAEZ01003305:7446-7529
HmHel-6	CAEZ01003202:4208-4290
HmHel-6	CAEZ01003202:47026-53284
HmHel-6	CAEZ01003202:98879-99072
HmHel-6	CAEZ01003202:61117-65654
HmHel-6	CAEZ01003075:35942-36088
HmHel-6	CAEZ01003075:88400-88622
HmHel-6	CAEZ01003075:73873-74034
HmHel-6	CAEZ01003075:32038-39756
HmHel-6	CAEZ01003030:1425-1610
HmHel-6	CAEZ01002666:8993-9166
HmHel-6	CAEZ01002666:32193-34120
HmHel-6	CAEZ01002666:61543-65816
HmHel-6	CAEZ01002666:76040-76247
HmHel-6	CAEZ01002666:55373-55518
HmHel-6	CAEZ01002666:26083-26267
HmHel-6	CAEZ01002147:4533-4638
HmHel-6	CAEZ01002147:13828-13977
HmHel-6	CAEZ01001773:4599-4779
HmHel-6	CAEZ01001773:34762-34850
HmHel-6	CAEZ01001773:31962-32102
HmHel-6	CAEZ01001773:2533-10837
HmHel-6	CAEZ01001201:10000-10163
HmHel-6	CAEZ01001201:12618-12759
HmHel-6	CAEZ01000864:5546-5655
HmHel-6	CAEZ01011544:3875-6467
HmHel-6	CAEZ01011544:616-842
HmHel-6	CAEZ01011506:4274-4418
HmHel-6	CAEZ01011506:30904-31027
HmHel-6	CAEZ01011506:10289-16096
HmHel-6	CAEZ01011111:6354-6478
HmHel-6	CAEZ01010446:66758-66890
HmHel-6	CAEZ01010446:10420-17694
HmHel-6	CAEZ01010293:39504-39618
HmHel-6	CAEZ01010293:10948-11113
HmHel-6	CAEZ01010247:10887-11088
HmHel-6	CAEZ01010195:10771-10955
HmHel-6	CAEZ01010118:3745-3912

HmHel-6	CAEZ01010118:22759-22844
HmHel-6	CAEZ01010118:41559-41739
HmHel-6	CAEZ01010118:24915-25030
HmHel-6	CAEZ01010067:3577-3767
HmHel-6	CAEZ01010067:9888-10073
HmHel-6	CAEZ01010056:13544-13708
HmHel-6	CAEZ01009290:4395-7255
HmHel-6	CAEZ01009290:23492-23616
HmHel-6	CAEZ01009290:14526-14721
HmHel-6	CAEZ01009290:2939-3680
HmHel-6	CAEZ01009139:14204-14324
HmHel-6	CAEZ01009139:11561-11675
HmHel-6	CAEZ01008620:25607-25695
HmHel-6	CAEZ01008620:62359-67782
HmHel-6	CAEZ01008620:89255-89439
HmHel-6	CAEZ01008620:111765-111892
HmHel-6	CAEZ01008620:156617-156773
HmHel-6	CAEZ01008620:169773-175061
HmHel-6	CAEZ01008620:158778-158905
HmHel-6	CAEZ01008620:129725-129812
HmHel-6	CAEZ01008620:90858-90937
HmHel-6	CAEZ01008620:7685-7910
HmHel-6	CAEZ01008381:17896-26768
HmHel-6	CAEZ01008265:598-845
HmHel-6	CAEZ01008265:2285-5265
HmHel-6	CAEZ01008220:14923-17119
HmHel-6	CAEZ01008220:10694-16156
HmHel-6	CAEZ01008142:4712-4844
HmHel-6	CAEZ01007923:2772-3004
HmHel-6	CAEZ01007923:17646-17871
HmHel-6	CAEZ01007923:16036-18926
HmHel-6	CAEZ01007734:7191-7392
HmHel-6	CAEZ01007681:5583-5786
HmHel-6	CAEZ01007681:670-10330
HmHel-6	CAEZ01007623:5909-6109
HmHel-6	CAEZ01007623:10001-18962
HmHel-6	CAEZ01007392:11462-11694
HmHel-6	CAEZ01007013:42783-42914
HmHel-6	CAEZ01007013:35625-35726
HmHel-6	CAEZ01006982:35-7371
HmHel-6	CAEZ01006135:5461-5635
HmHel-6	CAEZ01006078:8298-8388
HmHel-6	CAEZ01006078:16507-16622
HmHel-6	CAEZ01005965:12381-12485
HmHel-6	CAEZ01005783:12512-12624
HmHel-6	CAEZ01005783:1122-1222
HmHel-6	CAEZ01004905:16218-16359
HmHel-6	CAEZ01004905:44389-44545

HmHel-6	CAEZ01004905:8189-8294
HmHel-6	CAEZ01004627:1006-1181
HmHel-6	CAEZ01004310:1998-2242
HmHel-6	CAEZ01003917:7613-7725
HmHel-6	CAEZ01003917:28067-28167
HmHel-6	CAEZ01003917:4317-4498
HmHel-6	CAEZ01003153:23991-24085
HmHel-6	CAEZ01003153:10599-10724
HmHel-6	CAEZ01003123:2893-3057
HmHel-6	CAEZ01003123:31013-31129
HmHel-6	CAEZ01003123:17136-17277
HmHel-6	CAEZ01003060:18770-18927
HmHel-6	CAEZ01003060:32385-32525
HmHel-6	CAEZ01003060:37844-38057
HmHel-6	CAEZ01003060:14924-15008
HmHel-6	CAEZ01003059:28136-28232
HmHel-6	CAEZ01003059:32872-33053
HmHel-6	CAEZ01003034:4340-4504
HmHel-6	CAEZ01003034:11121-11331
HmHel-6	CAEZ01002921:11878-11993
HmHel-6	CAEZ01002818:8006-8158
HmHel-6	CAEZ01002415:4082-4225
HmHel-6	CAEZ01002415:77373-77500
HmHel-6	CAEZ01002415:40034-40155
HmHel-6	CAEZ01002415:9240-9392
HmHel-6	CAEZ01001736:22051-22213
HmHel-6	CAEZ01001736:67063-67288
HmHel-6	CAEZ01001736:105878-105966
HmHel-6	CAEZ01001736:102977-103124
HmHel-6	CAEZ01001736:65123-75079
HmHel-6	CAEZ01001736:49048-49180
HmHel-6	CAEZ01001579:3137-3336
HmHel-6	CAEZ01001579:62216-62420
HmHel-6	CAEZ01001579:63575-67289
HmHel-6	CAEZ01001579:51101-51282
HmHel-6	CAEZ01001509:2108-2235
HmHel-6	CAEZ01001509:2272-2374
HmHel-6	CAEZ01001351:5423-5597
HmHel-6	CAEZ01001351:17596-17740
HmHel-6	CAEZ01001351:2534-2988
HmHel-6	CAEZ01000998:6657-6780
HmHel-6	CAEZ01000998:33124-33210
HmHel-6	CAEZ01000998:51371-51587
HmHel-6	CAEZ01000998:27146-27315
HmHel-6	CAEZ01000895:6351-8652
HmHel-6	CAEZ01000895:53105-53253
HmHel-6	CAEZ01000895:80534-80650
HmHel-6	CAEZ01000895:69031-69125

HmHel-6	CAEZ01000895:18838-18958
HmHel-6	CAEZ01000688:411-625
HmHel-6	CAEZ01000645:2189-2393
HmHel-6	CAEZ01000083:11138-11238
HmHel-6	CAEZ01000083:9800-9900
HmHel-6	CAEZ01000055:3686-3843
HmHel-6	CAEZ01000055:1038-1157
HmHel-6	CAEZ01011484:1869-3573
HmHel-6	CAEZ01011484:3983-4119
HmHel-6	CAEZ01011425:391-551
HmHel-6	CAEZ01011425:8577-8657
HmHel-6	CAEZ01011043:1294-8610
HmHel-6	CAEZ01011043:30619-40738
HmHel-6	CAEZ01010862:6375-6575
HmHel-6	CAEZ01010808:17975-21729
HmHel-6	CAEZ01010808:60316-60422
HmHel-6	CAEZ01010808:81673-88325
HmHel-6	CAEZ01010808:112071-112164
HmHel-6	CAEZ01010808:125053-125161
HmHel-6	CAEZ01010808:153382-153466
HmHel-6	CAEZ01010808:169191-169303
HmHel-6	CAEZ01010808:182291-182409
HmHel-6	CAEZ01010808:216694-216784
HmHel-6	CAEZ01010808:202484-205620
HmHel-6	CAEZ01010808:181144-184846
HmHel-6	CAEZ01010808:149216-150690
HmHel-6	CAEZ01010808:112481-112601
HmHel-6	CAEZ01010808:168-303
HmHel-6	CAEZ01010606:21960-22046
HmHel-6	CAEZ01010606:14345-14505
HmHel-6	CAEZ01010535:5222-5440
HmHel-6	CAEZ01010226:79161-79329
HmHel-6	CAEZ01010226:11607-11786
HmHel-6	CAEZ01010112:14000-14201
HmHel-6	CAEZ01010112:30205-30414
HmHel-6	CAEZ01010112:3293-3500
HmHel-6	CAEZ01009479:11468-11562
HmHel-6	CAEZ01009479:36095-36228
HmHel-6	CAEZ01009479:49082-49235
HmHel-6	CAEZ01009479:56499-56623
HmHel-6	CAEZ01009479:4946-5088
HmHel-6	CAEZ01009424:36176-36308
HmHel-6	CAEZ01009211:20184-20406
HmHel-6	CAEZ01009111:5337-5599
HmHel-6	CAEZ01008905:4383-4596
HmHel-6	CAEZ01008905:17822-23541
HmHel-6	CAEZ01008905:19706-19804
HmHel-6	CAEZ01008493:3152-3305

HmHel-6	CAEZ01008139:15359-15523
HmHel-6	CAEZ01008139:38528-38736
HmHel-6	CAEZ01008139:32685-32825
HmHel-6	CAEZ01008139:14301-14515
HmHel-6	CAEZ01008079:2983-3126
HmHel-6	CAEZ01008079:5648-9451
HmHel-6	CAEZ01008055:10456-10584
HmHel-6	CAEZ01007723:3939-4045
HmHel-6	CAEZ01007723:10765-10867
HmHel-6	CAEZ01007723:479-573
HmHel-6	CAEZ01007694:4106-4289
HmHel-6	CAEZ01007694:2087-2254
HmHel-6	CAEZ01007329:751-2631
HmHel-6	CAEZ01007329:29057-29267
HmHel-6	CAEZ01007329:6926-7279
HmHel-6	CAEZ01007290:3401-3534
HmHel-6	CAEZ01007290:9067-9247
HmHel-6	CAEZ01007062:3594-3686
HmHel-6	CAEZ01007062:23166-23287
HmHel-6	CAEZ01007062:32287-32403
HmHel-6	CAEZ01007062:4121-4320
HmHel-6	CAEZ01006832:7846-13773
HmHel-6	CAEZ01006832:15143-15241
HmHel-6	CAEZ01005947:4770-11873
HmHel-6	CAEZ01005947:7285-7418
HmHel-6	CAEZ01005912:2903-3040
HmHel-6	CAEZ01005912:472-568
HmHel-6	CAEZ01005316:36168-36381
HmHel-6	CAEZ01005316:17670-27436
HmHel-6	CAEZ01005316:2923-3074
HmHel-6	CAEZ01005222:6775-6884
HmHel-6	CAEZ01005222:21842-22009
HmHel-6	CAEZ01005222:23216-23319
HmHel-6	CAEZ01005161:12741-12898
HmHel-6	CAEZ01005161:30767-30886
HmHel-6	CAEZ01005089:78000-80399
HmHel-6	CAEZ01005089:92100-92181
HmHel-6	CAEZ01005089:115394-118525
HmHel-6	CAEZ01005089:117510-117608
HmHel-6	CAEZ01005089:83094-93158
HmHel-6	CAEZ01005089:70517-70726
HmHel-6	CAEZ01005089:25218-25310
HmHel-6	CAEZ01005089:9770-9877
HmHel-6	CAEZ01004987:7285-10826
HmHel-6	CAEZ01004987:8668-8900
HmHel-6	CAEZ01004789:637-760
HmHel-6	CAEZ01004789:18105-18247
HmHel-6	CAEZ01004789:3710-9381

HmHel-6	CAEZ01004750:753-950
HmHel-6	CAEZ01004750:1112-1218
HmHel-6	CAEZ01004056:7180-7261
HmHel-6	CAEZ01004056:25664-25819
HmHel-6	CAEZ01003767:11949-12112
HmHel-6	CAEZ01003767:3082-3192
HmHel-6	CAEZ01003517:22847-22977
HmHel-6	CAEZ01003517:1878-1991
HmHel-6	CAEZ01003324:1385-1583
HmHel-6	CAEZ01003269:25149-25324
HmHel-6	CAEZ01003269:27765-28001
HmHel-6	CAEZ01003269:17204-17390
HmHel-6	CAEZ01002638:14265-14488
HmHel-6	CAEZ01002579:32-120
HmHel-6	CAEZ01002579:13521-13606
HmHel-6	CAEZ01002579:5829-6018
HmHel-6	CAEZ01002222:262-368
HmHel-6	CAEZ01002222:3989-9076
HmHel-6	CAEZ01002008:254-381
HmHel-6	CAEZ01001337:12181-12412
HmHel-6	CAEZ01001220:9587-9698
HmHel-6	CAEZ01001220:16486-16617
HmHel-6	CAEZ01000961:13103-23070
HmHel-6	CAEZ01000961:11920-12023
HmHel-6	CAEZ01000855:19471-19569
HmHel-6	CAEZ01000855:11224-19997
HmHel-6	CAEZ01011034:2471-2573
HmHel-6	CAEZ01010660:381-586
HmHel-6	CAEZ01010628:308-398
HmHel-6	CAEZ01010628:43889-44018
HmHel-6	CAEZ01010628:41662-41912
HmHel-6	CAEZ01010628:4068-13356
HmHel-6	CAEZ01010160:767-902
HmHel-6	CAEZ01009727:18878-19000
HmHel-6	CAEZ01009727:12072-12227
HmHel-6	CAEZ01009719:12955-13070
HmHel-6	CAEZ01009719:31533-31723
HmHel-6	CAEZ01009719:45672-45776
HmHel-6	CAEZ01009686:2318-2424
HmHel-6	CAEZ01009686:18479-18611
HmHel-6	CAEZ01009686:45630-45847
HmHel-6	CAEZ01009515:879-1093
HmHel-6	CAEZ01009507:8662-8857
HmHel-6	CAEZ01009507:69179-69273
HmHel-6	CAEZ01009507:88894-88979
HmHel-6	CAEZ01009507:29047-30894
HmHel-6	CAEZ01009507:7312-7415
HmHel-6	CAEZ01008215:8750-11067

HmHel-6	CAEZ01008215:19001-19114
HmHel-6	CAEZ01007957:35780-35861
HmHel-6	CAEZ01007957:57332-57488
HmHel-6	CAEZ01007957:24861-25019
HmHel-6	CAEZ01007957:1284-1364
HmHel-6	CAEZ01007615:2188-2401
HmHel-6	CAEZ01007599:11023-11116
HmHel-6	CAEZ01007599:23258-23408
HmHel-6	CAEZ01007599:37120-37211
HmHel-6	CAEZ01007599:41618-41821
HmHel-6	CAEZ01007599:27955-30364
HmHel-6	CAEZ01007599:5848-5953
HmHel-6	CAEZ01007580:3190-5046
HmHel-6	CAEZ01007538:3841-3958
HmHel-6	CAEZ01007538:6230-8044
HmHel-6	CAEZ01007405:8415-9746
HmHel-6	CAEZ01007405:5625-5804
HmHel-6	CAEZ01007147:6393-6579
HmHel-6	CAEZ01007100:20387-23839
HmHel-6	CAEZ01007100:53151-53274
HmHel-6	CAEZ01007100:70429-70512
HmHel-6	CAEZ01007100:61018-61226
HmHel-6	CAEZ01007100:34382-39066
HmHel-6	CAEZ01007028:764-866
HmHel-6	CAEZ01007021:7906-8046
HmHel-6	CAEZ01007021:14927-15827
HmHel-6	CAEZ01006954:6404-7085
HmHel-6	CAEZ01006885:4122-4334
HmHel-6	CAEZ01006885:757-879
HmHel-6	CAEZ01006536:11425-13632
HmHel-6	CAEZ01006536:27524-32538
HmHel-6	CAEZ01006536:1394-1529
HmHel-6	CAEZ01006386:6136-6298
HmHel-6	CAEZ01006287:24690-24848
HmHel-6	CAEZ01006287:52483-62012
HmHel-6	CAEZ01006287:1104-1311
HmHel-6	CAEZ01006017:10706-10916
HmHel-6	CAEZ01005915:6314-6478
HmHel-6	CAEZ01005915:7423-7573
HmHel-6	CAEZ01005887:1382-1500
HmHel-6	CAEZ01005843:6146-6305
HmHel-6	CAEZ01005843:20553-20656
HmHel-6	CAEZ01005843:6598-6720
HmHel-6	CAEZ01005659:2911-3079
HmHel-6	CAEZ01005659:15259-15422
HmHel-6	CAEZ01005586:14916-15049
HmHel-6	CAEZ01005586:27786-27964
HmHel-6	CAEZ01005258:12257-12379

HmHel-6	CAEZ01004953:11482-13339
HmHel-6	CAEZ01004953:14492-14674
HmHel-6	CAEZ01004939:1518-1617
HmHel-6	CAEZ01004939:4618-4737
HmHel-6	CAEZ01004744:14858-15013
HmHel-6	CAEZ01004744:25926-26020
HmHel-6	CAEZ01004595:5624-5750
HmHel-6	CAEZ01004595:6865-7019
HmHel-6	CAEZ01004170:8206-8291
HmHel-6	CAEZ01004044:38504-38620
HmHel-6	CAEZ01004044:31232-31389
HmHel-6	CAEZ01004022:6885-8482
HmHel-6	CAEZ01004022:9017-9175
HmHel-6	CAEZ01004017:7629-7775
HmHel-6	CAEZ01004001:1193-1331
HmHel-6	CAEZ01004001:6404-6580
HmHel-6	CAEZ01003786:1018-2148
HmHel-6	CAEZ01003786:1591-1737
HmHel-6	CAEZ01003599:8210-8462
HmHel-6	CAEZ01003599:10819-10902
HmHel-6	CAEZ01002945:1306-1428
HmHel-6	CAEZ01002725:2525-2621
HmHel-6	CAEZ01002725:1942-2060
HmHel-6	CAEZ01002674:2669-5538
HmHel-6	CAEZ01002125:2076-2194
HmHel-6	CAEZ01001907:544-670
HmHel-6	CAEZ01001764:6762-8100
HmHel-6	CAEZ01001764:13176-13276
HmHel-6	CAEZ01001102:4633-4834
HmHel-6	CAEZ01001102:28147-28235
HmHel-6	CAEZ01001102:67120-67230
HmHel-6	CAEZ01001102:29661-29873
HmHel-6	CAEZ01001101:13478-15740
HmHel-6	CAEZ01001101:12925-13065
HmHel-6	CAEZ01000940:348-501
HmHel-6	CAEZ01000732:2216-2334
HmHel-6	CAEZ01000124:2359-2497
HmHel-6	CAEZ01000046:1257-5680
HmHel-6	CAEZ01011623:3585-6856
HmHel-6	CAEZ01011292:3422-3567
HmHel-6	CAEZ01011292:20170-24167
HmHel-6	CAEZ01011201:4703-4820
HmHel-6	CAEZ01011201:3745-3841
HmHel-6	CAEZ01010817:4824-4903
HmHel-6	CAEZ01010817:16740-16846
HmHel-6	CAEZ01010817:12212-12443
HmHel-6	CAEZ01010801:12597-12725
HmHel-6	CAEZ01010801:15473-15607

HmHel-6	CAEZ01010789:2313-2450
HmHel-6	CAEZ01010343:2289-2378
HmHel-6	CAEZ01010183:10619-10745
HmHel-6	CAEZ01010183:15762-19667
HmHel-6	CAEZ01009966:5026-5136
HmHel-6	CAEZ01009966:1434-2246
HmHel-6	CAEZ01009939:5555-5670
HmHel-6	CAEZ01009939:1583-6769
HmHel-6	CAEZ01009793:3281-5320
HmHel-6	CAEZ01009566:9619-9802
HmHel-6	CAEZ01009566:4066-4240
HmHel-6	CAEZ01009539:24178-24287
HmHel-6	CAEZ01009539:6039-6179
HmHel-6	CAEZ01009527:10861-10960
HmHel-6	CAEZ01009527:12165-12359
HmHel-6	CAEZ01008809:6936-7063
HmHel-6	CAEZ01008794:363-8668
HmHel-6	CAEZ01007442:16427-16592
HmHel-6	CAEZ01007442:10945-11154
HmHel-6	CAEZ01007382:1535-1663
HmHel-6	CAEZ01007257:12617-12750
HmHel-6	CAEZ01007257:3730-3959
HmHel-6	CAEZ01007173:12019-12264
HmHel-6	CAEZ01007173:26604-26714
HmHel-6	CAEZ01007173:5198-5368
HmHel-6	CAEZ01006991:12369-15579
HmHel-6	CAEZ01006842:10611-14573
HmHel-6	CAEZ01006842:8307-8459
HmHel-6	CAEZ01006491:44545-53794
HmHel-6	CAEZ01006491:9393-9555
HmHel-6	CAEZ01006351:7150-7365
HmHel-6	CAEZ01006330:556-2332
HmHel-6	CAEZ01006091:13701-13806
HmHel-6	CAEZ01005723:7559-7763
HmHel-6	CAEZ01005404:39183-39393
HmHel-6	CAEZ01005404:27890-29964
HmHel-6	CAEZ01005404:2955-3088
HmHel-6	CAEZ01005369:5916-6123
HmHel-6	CAEZ01005285:757-1004
HmHel-6	CAEZ01005215:1577-1731
HmHel-6	CAEZ01005215:20889-22123
HmHel-6	CAEZ01005215:5502-5695
HmHel-6	CAEZ01004574:41790-41995
HmHel-6	CAEZ01004432:19362-19479
HmHel-6	CAEZ01004432:43666-43837
HmHel-6	CAEZ01004432:53250-53332
HmHel-6	CAEZ01004432:22363-22475
HmHel-6	CAEZ01004432:4438-4656

HmHel-6	CAEZ01004417:12079-12315
HmHel-6	CAEZ01004417:11360-15572
HmHel-6	CAEZ01004121:5513-11078
HmHel-6	CAEZ01003952:20306-20468
HmHel-6	CAEZ01003952:37422-37531
HmHel-6	CAEZ01003609:2043-2136
HmHel-6	CAEZ01003388:1828-2050
HmHel-6	CAEZ01002896:32550-32661
HmHel-6	CAEZ01002896:35434-35535
HmHel-6	CAEZ01002896:10759-16306
HmHel-6	CAEZ01002850:5617-5817
HmHel-6	CAEZ01001934:14659-14775
HmHel-6	CAEZ01001934:37201-37363
HmHel-6	CAEZ01001934:49242-49364
HmHel-6	CAEZ01001934:19410-19499
HmHel-6	CAEZ01001931:3374-3532
HmHel-6	CAEZ01001926:2117-2226
HmHel-6	CAEZ01001926:11068-11221
HmHel-6	CAEZ01001219:8791-8933
HmHel-6	CAEZ01001219:6635-6817
HmHel-6	CAEZ01001195:11369-11545
HmHel-6	CAEZ01001195:10563-10680
HmHel-6	CAEZ01001191:12266-12345
HmHel-6	CAEZ01001191:11538-11775
HmHel-6	CAEZ01000843:98-288
HmHel-6	CAEZ01000674:24-231
HmHel-6	CAEZ01011549:3931-4743
HmHel-6	CAEZ01011293:11812-11952
HmHel-6	CAEZ01011146:20254-20391
HmHel-6	CAEZ01011107:50174-50283
HmHel-6	CAEZ01011107:84229-84342
HmHel-6	CAEZ01011107:106973-107101
HmHel-6	CAEZ01011107:59940-60099
HmHel-6	CAEZ01011107:48677-48824
HmHel-6	CAEZ01010964:3799-4038
HmHel-6	CAEZ01010842:6595-6756
HmHel-6	CAEZ01010842:7032-7203
HmHel-6	CAEZ01010833:14686-14810
HmHel-6	CAEZ01010833:22898-23094
HmHel-6	CAEZ01010712:7661-7752
HmHel-6	CAEZ01010712:9520-12276
HmHel-6	CAEZ01010711:2173-2367
HmHel-6	CAEZ01010636:19606-19689
HmHel-6	CAEZ01010636:10376-10540
HmHel-6	CAEZ01010582:3606-3826
HmHel-6	CAEZ01010385:122-293
HmHel-6	CAEZ01010385:10938-11021
HmHel-6	CAEZ01010385:17265-17394

HmHel-6	CAEZ01010292:9446-9577
HmHel-6	CAEZ01010111:26489-26612
HmHel-6	CAEZ01010111:21047-21131
HmHel-6	CAEZ01010111:1533-1709
HmHel-6	CAEZ01009400:532-712
HmHel-6	CAEZ01009306:19066-22529
HmHel-6	CAEZ01009306:57802-60733
HmHel-6	CAEZ01009306:83774-85855
HmHel-6	CAEZ01009306:65661-76180
HmHel-6	CAEZ01009306:7009-7207
HmHel-6	CAEZ01008928:4908-5025
HmHel-6	CAEZ01008928:38057-38186
HmHel-6	CAEZ01008928:20422-20658
HmHel-6	CAEZ01008895:6547-6748
HmHel-6	CAEZ01008895:4377-7688
HmHel-6	CAEZ01008717:61057-61167
HmHel-6	CAEZ01008717:74957-75089
HmHel-6	CAEZ01008717:67883-67990
HmHel-6	CAEZ01008650:1087-1250
HmHel-6	CAEZ01008650:10819-14669
HmHel-6	CAEZ01007635:2345-2501
HmHel-6	CAEZ01007174:3444-3576
HmHel-6	CAEZ01006951:1623-1707
HmHel-6	CAEZ01006925:6682-6888
HmHel-6	CAEZ01006452:6006-13817
HmHel-6	CAEZ01006264:2807-10199
HmHel-6	CAEZ01006264:867-1081
HmHel-6	CAEZ01006253:2327-2421
HmHel-6	CAEZ01006253:6829-6948
HmHel-6	CAEZ01006107:2061-2276
HmHel-6	CAEZ01006047:16972-17056
HmHel-6	CAEZ01006047:9156-9361
HmHel-6	CAEZ01005876:9805-12928
HmHel-6	CAEZ01005876:1803-9015
HmHel-6	CAEZ01005820:3578-8416
HmHel-6	CAEZ01005820:11278-11401
HmHel-6	CAEZ01005164:3365-11223
HmHel-6	CAEZ01005164:5731-10001
HmHel-6	CAEZ01004777:19346-19470
HmHel-6	CAEZ01004777:13020-15024
HmHel-6	CAEZ01004777:787-882
HmHel-6	CAEZ01004694:4357-6727
HmHel-6	CAEZ01004694:995-1157
HmHel-6	CAEZ01004616:495-709
HmHel-6	CAEZ01004616:795-2711
HmHel-6	CAEZ01004280:504-686
HmHel-6	CAEZ01004279:2334-2456
HmHel-6	CAEZ01004279:3819-5654

HmHel-6	CAEZ01004210:3674-3758
HmHel-6	CAEZ01004067:6696-6793
HmHel-6	CAEZ01004067:32014-32220
HmHel-6	CAEZ01004067:50783-50967
HmHel-6	CAEZ01004067:7275-11191
HmHel-6	CAEZ01003810:13185-13446
HmHel-6	CAEZ01003810:14141-14233
HmHel-6	CAEZ01003686:2708-2920
HmHel-6	CAEZ01003686:7871-7979
HmHel-6	CAEZ01003674:8493-10417
HmHel-6	CAEZ01003629:3328-3442
HmHel-6	CAEZ01003629:19997-20103
HmHel-6	CAEZ01003629:5637-5809
HmHel-6	CAEZ01003410:23230-23365
HmHel-6	CAEZ01003267:1113-1280
HmHel-6	CAEZ01003130:11869-11951
HmHel-6	CAEZ01003130:18854-18951
HmHel-6	CAEZ01001981:16693-16832
HmHel-6	CAEZ01001981:27077-29427
HmHel-6	CAEZ01001981:62954-63165
HmHel-6	CAEZ01001981:59391-59510
HmHel-6	CAEZ01001981:18107-18232
HmHel-6	CAEZ01001494:1540-1696
HmHel-6	CAEZ01001494:21792-21895
HmHel-6	CAEZ01001494:17419-18782
HmHel-6	CAEZ01000701:2408-2624
HmHel-6	CAEZ01011423:4560-4708
HmHel-6	CAEZ01011249:9359-9533
HmHel-6	CAEZ01011249:7307-7391
HmHel-6	CAEZ01010700:14434-14645
HmHel-6	CAEZ01010700:58077-58265
HmHel-6	CAEZ01010700:94854-95058
HmHel-6	CAEZ01010700:43436-43541
HmHel-6	CAEZ01010700:19437-19531
HmHel-6	CAEZ01010700:7783-7970
HmHel-6	CAEZ01010645:5829-6033
HmHel-6	CAEZ01010605:12212-12335
HmHel-6	CAEZ01010523:9607-9746
HmHel-6	CAEZ01010455:37093-45127
HmHel-6	CAEZ01010455:96262-101928
HmHel-6	CAEZ01010455:104790-104908
HmHel-6	CAEZ01010455:65257-74642
HmHel-6	CAEZ01010455:6500-6580
HmHel-6	CAEZ01010284:22465-22675
HmHel-6	CAEZ01010243:54148-54275
HmHel-6	CAEZ01010243:9169-12193
HmHel-6	CAEZ01010194:11190-11441
HmHel-6	CAEZ01010194:21995-23940

HmHel-6	CAEZ01009545:3397-5780
HmHel-6	CAEZ01009545:9865-11880
HmHel-6	CAEZ01009381:10734-16443
HmHel-6	CAEZ01009381:24512-24607
HmHel-6	CAEZ01009184:938-3377
HmHel-6	CAEZ01009184:20839-20960
HmHel-6	CAEZ01009184:1249-1432
HmHel-6	CAEZ01008860:5213-5395
HmHel-6	CAEZ01008472:14220-14305
HmHel-6	CAEZ01008472:33856-33939
HmHel-6	CAEZ01008472:62668-62890
HmHel-6	CAEZ01008472:30463-33319
HmHel-6	CAEZ01008243:6863-7058
HmHel-6	CAEZ01008183:10010-10205
HmHel-6	CAEZ01008183:23344-23458
HmHel-6	CAEZ01008183:75019-75113
HmHel-6	CAEZ01008183:54896-54976
HmHel-6	CAEZ01008183:21093-21244
HmHel-6	CAEZ01008076:11195-11343
HmHel-6	CAEZ01008047:3925-4098
HmHel-6	CAEZ01008031:18583-18673
HmHel-6	CAEZ01008031:31776-31863
HmHel-6	CAEZ01008031:23334-23549
HmHel-6	CAEZ01008031:12348-12589
HmHel-6	CAEZ01007854:3456-3613
HmHel-6	CAEZ01007843:3176-3283
HmHel-6	CAEZ01007684:10883-11081
HmHel-6	CAEZ01007684:7524-7686
HmHel-6	CAEZ01007289:1068-1276
HmHel-6	CAEZ01006869:6048-6211
HmHel-6	CAEZ01006869:10511-10610
HmHel-6	CAEZ01006845:2220-2394
HmHel-6	CAEZ01006845:22245-22369
HmHel-6	CAEZ01006845:57771-57902
HmHel-6	CAEZ01006845:3725-6520
HmHel-6	CAEZ01006653:3255-3463
HmHel-6	CAEZ01006278:5478-5572
HmHel-6	CAEZ01006278:17616-17760
HmHel-6	CAEZ01006209:2068-3830
HmHel-6	CAEZ01006206:5521-5698
HmHel-6	CAEZ01006206:6812-14567
HmHel-6	CAEZ01006045:10441-10606
HmHel-6	CAEZ01006045:8684-10706
HmHel-6	CAEZ01005956:2608-2770
HmHel-6	CAEZ01005942:7436-7527
HmHel-6	CAEZ01005942:17685-24263
HmHel-6	CAEZ01005858:9442-9564
HmHel-6	CAEZ01005858:2365-2538

HmHel-6	CAEZ01005838:6258-6419
HmHel-6	CAEZ01005822:1868-1995
HmHel-6	CAEZ01005552:1413-1585
HmHel-6	CAEZ01005552:13407-13504
HmHel-6	CAEZ01005552:6934-7102
HmHel-6	CAEZ01004819:1879-2017
HmHel-6	CAEZ01004819:13982-14175
HmHel-6	CAEZ01004819:21898-23956
HmHel-6	CAEZ01004753:8303-8485
HmHel-6	CAEZ01004665:13589-13785
HmHel-6	CAEZ01004656:21026-27170
HmHel-6	CAEZ01004656:55465-55674
HmHel-6	CAEZ01004529:2700-10205
HmHel-6	CAEZ01004529:13733-13872
HmHel-6	CAEZ01004285:11919-16698
HmHel-6	CAEZ01004285:33944-34140
HmHel-6	CAEZ01004285:26011-26156
HmHel-6	CAEZ01004202:16312-25121
HmHel-6	CAEZ01004202:38712-39754
HmHel-6	CAEZ01004202:41231-41409
HmHel-6	CAEZ01004095:13730-13938
HmHel-6	CAEZ01004002:14106-14310
HmHel-6	CAEZ01003906:11448-11563
HmHel-6	CAEZ01003730:3425-3523
HmHel-6	CAEZ01003730:15858-16944
HmHel-6	CAEZ01003730:14099-14305
HmHel-6	CAEZ01003641:6785-6908
HmHel-6	CAEZ01003496:24917-24996
HmHel-6	CAEZ01003496:3667-3829
HmHel-6	CAEZ01002767:39696-39853
HmHel-6	CAEZ01002765:52122-52278
HmHel-6	CAEZ01002765:23715-27889
HmHel-6	CAEZ01001912:6384-8226
HmHel-6	CAEZ01001891:18363-18588
HmHel-6	CAEZ01001891:49299-49390
HmHel-6	CAEZ01001891:35380-35467
HmHel-6	CAEZ01001787:3799-8637
HmHel-6	CAEZ01001787:9005-9203
HmHel-6	CAEZ01001282:2761-2922
HmHel-6	CAEZ01001282:507-630
HmHel-6	CAEZ01001064:6932-10461
HmHel-6	CAEZ01000913:12698-12869
HmHel-6	CAEZ01000719:1623-1770
HmHel-6	CAEZ01000650:1729-1898
HmHel-6	CAEZ01000053:6567-6701
HmHel-6	CAEZ01011317:99-249
HmHel-6	CAEZ01011142:1186-1334
HmHel-6	CAEZ01011142:25339-33489

HmHel-6	CAEZ01011142:29212-29422
HmHel-6	CAEZ01011142:9559-9669
HmHel-6	CAEZ01010831:2355-2520
HmHel-6	CAEZ01010831:2778-2872
HmHel-6	CAEZ01010191:5238-5352
HmHel-6	CAEZ01009838:20080-20234
HmHel-6	CAEZ01009838:22542-22673
HmHel-6	CAEZ01009775:7695-16593
HmHel-6	CAEZ01009762:1304-1517
HmHel-6	CAEZ01009733:7730-7848
HmHel-6	CAEZ01009733:32426-36240
HmHel-6	CAEZ01009733:78436-78606
HmHel-6	CAEZ01009733:68454-68539
HmHel-6	CAEZ01009733:51841-51923
HmHel-6	CAEZ01009733:1685-1910
HmHel-6	CAEZ01009685:27178-27302
HmHel-6	CAEZ01009642:2706-2824
HmHel-6	CAEZ01009602:12792-17101
HmHel-6	CAEZ01009592:7250-7397
HmHel-6	CAEZ01009584:8092-8220
HmHel-6	CAEZ01009584:6620-6778
HmHel-6	CAEZ01009304:31121-31227
HmHel-6	CAEZ01009304:31394-31608
HmHel-6	CAEZ01008730:6612-6802
HmHel-6	CAEZ01008580:12488-12694
HmHel-6	CAEZ01008497:5558-5704
HmHel-6	CAEZ01008497:16190-25287
HmHel-6	CAEZ01008497:19297-25824
HmHel-6	CAEZ01008463:6281-6411
HmHel-6	CAEZ01008454:7304-7453
HmHel-6	CAEZ01008201:6267-6470
HmHel-6	CAEZ01008201:18736-21895
HmHel-6	CAEZ01008201:12131-12360
HmHel-6	CAEZ01008168:3831-9777
HmHel-6	CAEZ01008168:27230-37248
HmHel-6	CAEZ01008168:36164-36261
HmHel-6	CAEZ01008168:3438-5104
HmHel-6	CAEZ01008025:17155-17309
HmHel-6	CAEZ01008025:5611-5767
HmHel-6	CAEZ01007601:18083-18275
HmHel-6	CAEZ01007601:919-1159
HmHel-6	CAEZ01007539:6993-7223
HmHel-6	CAEZ01007539:8305-8474
HmHel-6	CAEZ01006921:5304-8481
HmHel-6	CAEZ01006921:7594-7785
HmHel-6	CAEZ01006857:22422-22612
HmHel-6	CAEZ01006857:4462-18258
HmHel-6	CAEZ01006790:14347-14461

HmHel-6	CAEZ01006790:11403-11558
HmHel-6	CAEZ01006639:6290-6491
HmHel-6	CAEZ01006331:4795-4953
HmHel-6	CAEZ01006279:5386-5573
HmHel-6	CAEZ01006279:17613-17740
HmHel-6	CAEZ01006279:7833-7999
HmHel-6	CAEZ01006081:3788-7716
HmHel-6	CAEZ01006081:34606-34715
HmHel-6	CAEZ01006081:31883-33480
HmHel-6	CAEZ01006081:9699-19157
HmHel-6	CAEZ01005973:28751-28918
HmHel-6	CAEZ01005973:68516-68628
HmHel-6	CAEZ01005973:97225-97329
HmHel-6	CAEZ01005973:29831-29940
HmHel-6	CAEZ01005816:1773-1875
HmHel-6	CAEZ01005756:8163-14858
HmHel-6	CAEZ01005756:5921-11385
HmHel-6	CAEZ01005755:3010-3186
HmHel-6	CAEZ01005755:1506-1723
HmHel-6	CAEZ01005625:4519-4707
HmHel-6	CAEZ01005550:6336-6998
HmHel-6	CAEZ01005214:3220-3400
HmHel-6	CAEZ01005083:24483-34507
HmHel-6	CAEZ01005083:73221-73387
HmHel-6	CAEZ01005083:5290-5503
HmHel-6	CAEZ01004748:11453-11650
HmHel-6	CAEZ01004703:19985-20135
HmHel-6	CAEZ01004703:6916-7043
HmHel-6	CAEZ01004662:4291-4373
HmHel-6	CAEZ01004662:17552-17696
HmHel-6	CAEZ01004662:75200-75405
HmHel-6	CAEZ01004662:47266-47372
HmHel-6	CAEZ01004662:31517-31654
HmHel-6	CAEZ01004542:1693-1772
HmHel-6	CAEZ01004542:26780-26908
HmHel-6	CAEZ01004347:4821-4939
HmHel-6	CAEZ01004135:17348-17467
HmHel-6	CAEZ01004135:14571-14757
HmHel-6	CAEZ01003968:5307-5433
HmHel-6	CAEZ01003968:9231-9355
HmHel-6	CAEZ01003936:13161-13308
HmHel-6	CAEZ01003773:1228-1472
HmHel-6	CAEZ01003560:443-549
HmHel-6	CAEZ01003560:890-1008
HmHel-6	CAEZ01003514:4860-4975
HmHel-6	CAEZ01003514:7032-7126
HmHel-6	CAEZ01003138:19328-19457
HmHel-6	CAEZ01003138:21233-21451

HmHel-6	CAEZ01002689:16394-16498
HmHel-6	CAEZ01002358:1674-2685
HmHel-6	CAEZ01002183:7354-12022
HmHel-6	CAEZ01002118:11825-12003
HmHel-6	CAEZ01001402:19005-19185
HmHel-6	CAEZ01001402:33385-33513
HmHel-6	CAEZ01001402:14727-24717
HmHel-6	CAEZ01000960:15139-16825
HmHel-6	CAEZ01000896:1598-1791
HmHel-6	CAEZ01000712:2389-2512
HmHel-6	CAEZ01000712:1094-1257
HmHel-6	CAEZ01000065:13302-13400
HmHel-6	CAEZ01000065:18896-19086
HmHel-6	CAEZ01000065:3130-7847
HmHel-6	CAEZ01011429:2900-3025
HmHel-6	CAEZ01011204:5256-5412
HmHel-6	CAEZ01011090:1828-1914
HmHel-6	CAEZ01011090:4880-5001
HmHel-6	CAEZ01011054:4446-9598
HmHel-6	CAEZ01011054:40972-41137
HmHel-6	CAEZ01011053:4015-4186
HmHel-6	CAEZ01010809:966-1144
HmHel-6	CAEZ01010809:56882-60216
HmHel-6	CAEZ01010809:29439-35784
HmHel-6	CAEZ01010809:7254-7353
HmHel-6	CAEZ01010719:197-296
HmHel-6	CAEZ01010719:20649-20814
HmHel-6	CAEZ01010719:16579-21311
HmHel-6	CAEZ01010717:544-722
HmHel-6	CAEZ01010717:3528-7488
HmHel-6	CAEZ01010600:40583-40752
HmHel-6	CAEZ01010589:2243-2340
HmHel-6	CAEZ01010162:6719-6846
HmHel-6	CAEZ01010162:5949-6050
HmHel-6	CAEZ01010012:4518-4647
HmHel-6	CAEZ01010012:9448-9626
HmHel-6	CAEZ01009766:11989-12118
HmHel-6	CAEZ01009766:29411-39540
HmHel-6	CAEZ01009766:49822-49913
HmHel-6	CAEZ01009760:17490-17607
HmHel-6	CAEZ01009755:2261-2435
HmHel-6	CAEZ01009521:7514-7668
HmHel-6	CAEZ01009521:6269-6484
HmHel-6	CAEZ01009410:5168-5353
HmHel-6	CAEZ01009366:27275-29843
HmHel-6	CAEZ01009366:58173-62956
HmHel-6	CAEZ01009366:81963-82100
HmHel-6	CAEZ01009366:113667-120189

HmHel-6	CAEZ01009366:86012-86248
HmHel-6	CAEZ01009366:67790-67905
HmHel-6	CAEZ01009366:46005-51552
HmHel-6	CAEZ01009064:11924-12085
HmHel-6	CAEZ01008931:11692-11904
HmHel-6	CAEZ01008877:1995-2104
HmHel-6	CAEZ01008803:19602-19735
HmHel-6	CAEZ01008763:17766-17875
HmHel-6	CAEZ01008763:14860-15062
HmHel-6	CAEZ01008763:4329-4450
HmHel-6	CAEZ01008478:11974-12238
HmHel-6	CAEZ01008478:5690-10184
HmHel-6	CAEZ01008335:4217-4382
HmHel-6	CAEZ01008335:14661-14811
HmHel-6	CAEZ01008318:1624-1741
HmHel-6	CAEZ01008164:14300-14446
HmHel-6	CAEZ01008164:38987-39108
HmHel-6	CAEZ01008164:30706-32857
HmHel-6	CAEZ01008164:9960-10118
HmHel-6	CAEZ01008038:85-6178
HmHel-6	CAEZ01008038:10571-10668
HmHel-6	CAEZ01007958:2191-8885
HmHel-6	CAEZ01007958:24864-25000
HmHel-6	CAEZ01007834:4406-4586
HmHel-6	CAEZ01007834:7606-7820
HmHel-6	CAEZ01007157:15264-20843
HmHel-6	CAEZ01007157:29372-29536
HmHel-6	CAEZ01005984:23776-23947
HmHel-6	CAEZ01005703:7873-7953
HmHel-6	CAEZ01005703:4782-4898
HmHel-6	CAEZ01005477:17824-18042
HmHel-6	CAEZ01005477:32164-32299
HmHel-6	CAEZ01005477:34895-39539
HmHel-6	CAEZ01005477:9510-9645
HmHel-6	CAEZ01005439:13314-13396
HmHel-6	CAEZ01005439:74131-74309
HmHel-6	CAEZ01005439:102105-102222
HmHel-6	CAEZ01005439:142741-142970
HmHel-6	CAEZ01005439:164539-164793
HmHel-6	CAEZ01005439:156032-157647
HmHel-6	CAEZ01005439:105656-106701
HmHel-6	CAEZ01005439:69474-69610
HmHel-6	CAEZ01005439:27225-36434
HmHel-6	CAEZ01005439:14360-14439
HmHel-6	CAEZ01005318:25150-26578
HmHel-6	CAEZ01005318:26947-27194
HmHel-6	CAEZ01005109:12880-13010
HmHel-6	CAEZ01005109:13828-13929

HmHel-6	CAEZ01005064:2776-2929
HmHel-6	CAEZ01005061:8733-12993
HmHel-6	CAEZ01004963:1579-1794
HmHel-6	CAEZ01004963:325-482
HmHel-6	CAEZ01004719:585-698
HmHel-6	CAEZ01004707:5392-5473
HmHel-6	CAEZ01004707:17632-17766
HmHel-6	CAEZ01004707:10973-11217
HmHel-6	CAEZ01004637:2597-11626
HmHel-6	CAEZ01004637:2026-9054
HmHel-6	CAEZ01004282:3399-11539
HmHel-6	CAEZ01004255:5078-7800
HmHel-6	CAEZ01004255:4631-4786
HmHel-6	CAEZ01004205:3997-4153
HmHel-6	CAEZ01004205:7325-7486
HmHel-6	CAEZ01004083:8611-14590
HmHel-6	CAEZ01004083:15526-15626
HmHel-6	CAEZ01004034:29229-29336
HmHel-6	CAEZ01004034:40695-40793
HmHel-6	CAEZ01004034:58680-58781
HmHel-6	CAEZ01004034:78813-78913
HmHel-6	CAEZ01004034:84024-84106
HmHel-6	CAEZ01004034:50722-50830
HmHel-6	CAEZ01004034:4318-9998
HmHel-6	CAEZ01003832:4169-8399
HmHel-6	CAEZ01003832:11137-12596
HmHel-6	CAEZ01003784:16641-16745
HmHel-6	CAEZ01003784:50932-51047
HmHel-6	CAEZ01003769:7881-8137
HmHel-6	CAEZ01003622:2255-2438
HmHel-6	CAEZ01003622:24943-34424
HmHel-6	CAEZ01003611:5922-6027
HmHel-6	CAEZ01003611:7428-7549
HmHel-6	CAEZ01003334:17323-17420
HmHel-6	CAEZ01003313:6719-6848
HmHel-6	CAEZ01002849:1470-1663
HmHel-6	CAEZ01002849:20401-20518
HmHel-6	CAEZ01002673:459-640
HmHel-6	CAEZ01002673:18863-19004
HmHel-6	CAEZ01001601:1765-1987
HmHel-6	CAEZ01001470:11790-11922
HmHel-6	CAEZ01001470:11439-13611
HmHel-6	CAEZ01001043:994-1219
HmHel-6	CAEZ01001025:7123-8207
HmHel-6	CAEZ01001025:23459-26089
HmHel-6	CAEZ01001025:12635-12721
HmHel-6	CAEZ01000862:7913-8086
HmHel-6	CAEZ01000673:1065-1225

HmHel-6	CAEZ01000673:1980-2240
HmHel-6	CAEZ01011581:3836-4007
HmHel-6	CAEZ01011139:20718-20807
HmHel-6	CAEZ01011139:99624-99724
HmHel-6	CAEZ01011139:72273-76220
HmHel-6	CAEZ01011089:8568-9495
HmHel-6	CAEZ01011089:12691-12843
HmHel-6	CAEZ01010760:10093-10221
HmHel-6	CAEZ01010760:19763-19915
HmHel-6	CAEZ01010126:8476-8666
HmHel-6	CAEZ01010126:23739-23858
HmHel-6	CAEZ01010126:53620-53832
HmHel-6	CAEZ01010115:7678-10887
HmHel-6	CAEZ01010115:13070-13164
HmHel-6	CAEZ01009827:2838-2935
HmHel-6	CAEZ01009827:13403-14856
HmHel-6	CAEZ01009670:4837-4985
HmHel-6	CAEZ01009670:18778-18949
HmHel-6	CAEZ01009340:7893-7989
HmHel-6	CAEZ01009340:2467-2601
HmHel-6	CAEZ01008947:11939-12959
HmHel-6	CAEZ01008947:9492-9621
HmHel-6	CAEZ01008901:5820-8676
HmHel-6	CAEZ01008830:7155-7323
HmHel-6	CAEZ01008624:12609-12739
HmHel-6	CAEZ01008624:14859-15040
HmHel-6	CAEZ01007935:6231-6327
HmHel-6	CAEZ01007900:40385-40581
HmHel-6	CAEZ01007798:4333-4554
HmHel-6	CAEZ01007798:2940-3161
HmHel-6	CAEZ01007640:3960-4068
HmHel-6	CAEZ01007640:10590-10746
HmHel-6	CAEZ01007286:16094-16258
HmHel-6	CAEZ01007286:20828-20921
HmHel-6	CAEZ01007087:194-361
HmHel-6	CAEZ01007087:12492-12574
HmHel-6	CAEZ01007087:6544-6692
HmHel-6	CAEZ01007061:25757-35380
HmHel-6	CAEZ01007061:41872-42078
HmHel-6	CAEZ01006795:9926-10042
HmHel-6	CAEZ01006379:17429-17529
HmHel-6	CAEZ01006171:13478-13689
HmHel-6	CAEZ01006171:27463-27676
HmHel-6	CAEZ01006171:79359-79442
HmHel-6	CAEZ01006171:104282-104381
HmHel-6	CAEZ01006171:63967-64052
HmHel-6	CAEZ01006171:36310-36395
HmHel-6	CAEZ01005773:733-923

HmHel-6	CAEZ01005743:22737-23625
HmHel-6	CAEZ01005743:43934-44033
HmHel-6	CAEZ01005743:63692-69075
HmHel-6	CAEZ01005743:64386-64561
HmHel-6	CAEZ01005743:24412-24537
HmHel-6	CAEZ01005743:3385-3519
HmHel-6	CAEZ01005507:14665-14897
HmHel-6	CAEZ01005507:9806-9955
HmHel-6	CAEZ01005417:19222-19314
HmHel-6	CAEZ01005417:15456-22958
HmHel-6	CAEZ01005363:14-178
HmHel-6	CAEZ01005319:37833-38049
HmHel-6	CAEZ01005308:12573-12728
HmHel-6	CAEZ01005308:48432-48540
HmHel-6	CAEZ01005308:17376-17522
HmHel-6	CAEZ01005255:6053-6173
HmHel-6	CAEZ01005255:6567-6708
HmHel-6	CAEZ01005186:46021-46113
HmHel-6	CAEZ01005186:19892-20709
HmHel-6	CAEZ01005062:5993-7509
HmHel-6	CAEZ01005051:11708-17469
HmHel-6	CAEZ01005051:10874-21347
HmHel-6	CAEZ01005050:519-643
HmHel-6	CAEZ01005050:1012-1142
HmHel-6	CAEZ01005014:13872-14013
HmHel-6	CAEZ01004989:29312-29478
HmHel-6	CAEZ01004989:26830-27002
HmHel-6	CAEZ01004989:3404-3500
HmHel-6	CAEZ01004928:10502-10726
HmHel-6	CAEZ01004716:6879-7059
HmHel-6	CAEZ01004633:36875-37069
HmHel-6	CAEZ01004633:46454-46541
HmHel-6	CAEZ01004633:21010-21173
HmHel-6	CAEZ01004377:6823-6955
HmHel-6	CAEZ01004377:8980-9129
HmHel-6	CAEZ01003999:13404-13492
HmHel-6	CAEZ01003863:6809-11783
HmHel-6	CAEZ01003863:19148-19277
HmHel-6	CAEZ01003863:1098-1191
HmHel-6	CAEZ01003800:3800-3908
HmHel-6	CAEZ01003762:21514-21628
HmHel-6	CAEZ01003762:18145-18321
HmHel-6	CAEZ01003571:1817-1994
HmHel-6	CAEZ01003482:5571-11080
HmHel-6	CAEZ01003214:296-476
HmHel-6	CAEZ01003173:35058-42815
HmHel-6	CAEZ01003173:55901-56025
HmHel-6	CAEZ01003173:16257-16411

HmHel-6	CAEZ01002954:3463-3629
HmHel-6	CAEZ01002925:4439-4533
HmHel-6	CAEZ01002925:39605-39822
HmHel-6	CAEZ01002925:35897-36001
HmHel-6	CAEZ01002925:713-853
HmHel-6	CAEZ01002709:3787-13409
HmHel-6	CAEZ01002709:19901-20107
HmHel-6	CAEZ01002603:4147-5935
HmHel-6	CAEZ01002335:2073-2201
HmHel-6	CAEZ01002048:12034-12134
HmHel-6	CAEZ01002048:24586-24778
HmHel-6	CAEZ01002048:19378-19615
HmHel-6	CAEZ01002048:1751-1901
HmHel-6	CAEZ01001429:678-6471
HmHel-6	CAEZ01001037:5002-5170
HmHel-6	CAEZ01000881:21795-21923
HmHel-6	CAEZ01000881:27042-27253
HmHel-6	CAEZ01000817:11135-11220
HmHel-6	CAEZ01000817:32784-32879
HmHel-6	CAEZ01000817:16773-17024
HmHel-6	CAEZ01000817:1227-2134
HmHel-6	CAEZ01000646:1925-2113
HmHel-6	CAEZ01000211:197-365
HmHel-6	CAEZ01011460:829-916
HmHel-6	CAEZ01011460:3382-3480
HmHel-6	CAEZ01011295:14641-14747
HmHel-6	CAEZ01011295:24982-33062
HmHel-6	CAEZ01011295:50764-50871
HmHel-6	CAEZ01011295:42234-42329
HmHel-6	CAEZ01011295:30552-30648
HmHel-6	CAEZ01011295:8906-14637
HmHel-6	CAEZ01011180:2463-2555
HmHel-6	CAEZ01011180:4229-4449
HmHel-6	CAEZ01011153:12583-12717
HmHel-6	CAEZ01011019:4802-7830
HmHel-6	CAEZ01011019:25607-25727
HmHel-6	CAEZ01011019:27173-27326
HmHel-6	CAEZ01011019:2648-15106
HmHel-6	CAEZ01010900:47222-49262
HmHel-6	CAEZ01010900:55531-55677
HmHel-6	CAEZ01010900:7844-7975
HmHel-6	CAEZ01010664:4583-4726
HmHel-6	CAEZ01010379:8153-8302
HmHel-6	CAEZ01010379:2757-4117
HmHel-6	CAEZ01010180:1764-1947
HmHel-6	CAEZ01010180:9966-10110
HmHel-6	CAEZ01010178:8036-8240
HmHel-6	CAEZ01010040:4182-4333

HmHel-6	CAEZ01009936:405-531
HmHel-6	CAEZ01009578:9419-15295
HmHel-6	CAEZ01009578:11858-11986
HmHel-6	CAEZ01009554:13426-13522
HmHel-6	CAEZ01009554:3601-12360
HmHel-6	CAEZ01009536:13942-22444
HmHel-6	CAEZ01009536:14899-15080
HmHel-6	CAEZ01009506:16128-16254
HmHel-6	CAEZ01009506:45628-45789
HmHel-6	CAEZ01009506:25561-38488
HmHel-6	CAEZ01009506:2263-2358
HmHel-6	CAEZ01009386:2987-3080
HmHel-6	CAEZ01009386:13534-17603
HmHel-6	CAEZ01009106:3541-3648
HmHel-6	CAEZ01009106:11393-11524
HmHel-6	CAEZ01009097:19965-20148
HmHel-6	CAEZ01008745:2058-2281
HmHel-6	CAEZ01008732:27785-27975
HmHel-6	CAEZ01008732:61625-61833
HmHel-6	CAEZ01008732:123904-124119
HmHel-6	CAEZ01008732:108836-108922
HmHel-6	CAEZ01008732:66099-80945
HmHel-6	CAEZ01008732:43900-44025
HmHel-6	CAEZ01008732:31846-31956
HmHel-6	CAEZ01008731:3673-3780
HmHel-6	CAEZ01008731:38461-41117
HmHel-6	CAEZ01008731:49202-49293
HmHel-6	CAEZ01008731:35495-35602
HmHel-6	CAEZ01008330:6992-7082
HmHel-6	CAEZ01008330:4031-4138
HmHel-6	CAEZ01008216:5683-5846
HmHel-6	CAEZ01008216:12460-12574
HmHel-6	CAEZ01008077:11156-11335
HmHel-6	CAEZ01008077:13949-19378
HmHel-6	CAEZ01007903:19863-19985
HmHel-6	CAEZ01007903:2731-2946
HmHel-6	CAEZ01007889:5740-5839
HmHel-6	CAEZ01007785:8279-8471
HmHel-6	CAEZ01007724:1570-1682
HmHel-6	CAEZ01007724:67494-76825
HmHel-6	CAEZ01007344:2675-5482
HmHel-6	CAEZ01007344:6696-6783
HmHel-6	CAEZ01006890:793-1999
HmHel-6	CAEZ01006884:11271-11456
HmHel-6	CAEZ01006884:19787-19927
HmHel-6	CAEZ01006884:6374-7880
HmHel-6	CAEZ01006880:12417-12601
HmHel-6	CAEZ01006880:18505-18698

HmHel-6	CAEZ01006741:15430-22755
HmHel-6	CAEZ01006505:11103-11250
HmHel-6	CAEZ01006315:2561-2652
HmHel-6	CAEZ01006100:4506-4670
HmHel-6	CAEZ01005883:2858-3095
HmHel-6	CAEZ01005829:26036-26118
HmHel-6	CAEZ01005829:14000-14080
HmHel-6	CAEZ01005775:7072-7255
HmHel-6	CAEZ01005678:2626-2786
HmHel-6	CAEZ01005678:6654-6794
HmHel-6	CAEZ01005551:2176-2327
HmHel-6	CAEZ01005551:15533-15705
HmHel-6	CAEZ01005256:1606-1753
HmHel-6	CAEZ01005242:2001-2106
HmHel-6	CAEZ01005242:2272-2469
HmHel-6	CAEZ01005159:7411-7557
HmHel-6	CAEZ01005128:5146-5284
HmHel-6	CAEZ01005100:9987-10155
HmHel-6	CAEZ01005100:8485-8605
HmHel-6	CAEZ01005006:3129-3248
HmHel-6	CAEZ01004592:8223-8357
HmHel-6	CAEZ01004592:15927-16073
HmHel-6	CAEZ01004408:9420-9534
HmHel-6	CAEZ01004119:8514-8633
HmHel-6	CAEZ01003778:3332-3424
HmHel-6	CAEZ01003778:27714-27924
HmHel-6	CAEZ01003671:3571-4574
HmHel-6	CAEZ01003448:1798-1937
HmHel-6	CAEZ01003447:675-814
HmHel-6	CAEZ01003343:2059-2164
HmHel-6	CAEZ01003343:2324-2521
HmHel-6	CAEZ01003335:91-286
HmHel-6	CAEZ01003210:4116-8561
HmHel-6	CAEZ01003210:30388-30597
HmHel-6	CAEZ01003210:17490-17675
HmHel-6	CAEZ01002924:41809-42013
HmHel-6	CAEZ01002924:130636-137973
HmHel-6	CAEZ01002924:163486-169403
HmHel-6	CAEZ01002924:150746-150829
HmHel-6	CAEZ01002924:74123-74204
HmHel-6	CAEZ01002924:41533-41674
HmHel-6	CAEZ01002738:1396-1590
HmHel-6	CAEZ01002738:11092-15489
HmHel-6	CAEZ01002648:4520-4755
HmHel-6	CAEZ01002648:467-9292
HmHel-6	CAEZ01002644:17016-17213
HmHel-6	CAEZ01002644:17381-17486
HmHel-6	CAEZ01002588:501-698

HmHel-6	CAEZ01002135:1684-1887
HmHel-6	CAEZ01001969:23151-23304
HmHel-6	CAEZ01001629:342-477
HmHel-6	CAEZ01001408:23948-24064
HmHel-6	CAEZ01001408:48786-48880
HmHel-6	CAEZ01001408:161202-165372
HmHel-6	CAEZ01001408:192225-195600
HmHel-6	CAEZ01001408:221739-221869
HmHel-6	CAEZ01001408:203457-203552
HmHel-6	CAEZ01001408:172113-172208
HmHel-6	CAEZ01001408:134880-135031
HmHel-6	CAEZ01001303:1503-1758
HmHel-6	CAEZ01001253:1145-1342
HmHel-6	CAEZ01001253:1512-1615
HmHel-6	CAEZ01000676:658-821
HmHel-6	CAEZ01000676:4631-4750
HmHel-6	CAEZ01000178:1722-1936
HmHel-6	CAEZ01011039:13132-13369
HmHel-6	CAEZ01011039:21759-23772
HmHel-6	CAEZ01010968:16987-19325
HmHel-6	CAEZ01010968:36204-41550
HmHel-6	CAEZ01010920:3360-3466
HmHel-6	CAEZ01010920:17021-17196
HmHel-6	CAEZ01010920:47625-47735
HmHel-6	CAEZ01010920:40770-43796
HmHel-6	CAEZ01010913:798-991
HmHel-6	CAEZ01010913:19211-19380
HmHel-6	CAEZ01010558:5932-6065
HmHel-6	CAEZ01010558:2944-5690
HmHel-6	CAEZ01010242:18985-19143
HmHel-6	CAEZ01010242:30684-30846
HmHel-6	CAEZ01010232:50545-50655
HmHel-6	CAEZ01010053:7750-7955
HmHel-6	CAEZ01010035:15673-15786
HmHel-6	CAEZ01009971:9649-9814
HmHel-6	CAEZ01009971:31760-41776
HmHel-6	CAEZ01009971:83151-83287
HmHel-6	CAEZ01009971:37617-37719
HmHel-6	CAEZ01009842:1606-1763
HmHel-6	CAEZ01009842:16719-16949
HmHel-6	CAEZ01009744:2865-3568
HmHel-6	CAEZ01009744:5635-5835
HmHel-6	CAEZ01009516:11460-11586
HmHel-6	CAEZ01009516:2978-3162
HmHel-6	CAEZ01009324:6315-7903
HmHel-6	CAEZ01009324:1920-2087
HmHel-6	CAEZ01009295:16789-16869
HmHel-6	CAEZ01009295:13689-13811

HmHel-6	CAEZ01009221:18091-18213
HmHel-6	CAEZ01009194:13569-13710
HmHel-6	CAEZ01008848:2100-2302
HmHel-6	CAEZ01008377:1704-11257
HmHel-6	CAEZ01008377:14112-14241
HmHel-6	CAEZ01008203:4145-4681
HmHel-6	CAEZ01008203:2439-8758
HmHel-6	CAEZ01007717:7329-7442
HmHel-6	CAEZ01007603:9686-9784
HmHel-6	CAEZ01007294:2749-2880
HmHel-6	CAEZ01007269:43818-44426
HmHel-6	CAEZ01007269:43386-51738
HmHel-6	CAEZ01006492:919-2339
HmHel-6	CAEZ01006492:17060-17166
HmHel-6	CAEZ01006492:3857-9558
HmHel-6	CAEZ01006421:22080-22198
HmHel-6	CAEZ01006027:547-2181
HmHel-6	CAEZ01006027:20994-21155
HmHel-6	CAEZ01006027:23834-25944
HmHel-6	CAEZ01006022:6165-6259
HmHel-6	CAEZ01005712:13354-13516
HmHel-6	CAEZ01005712:14981-15155
HmHel-6	CAEZ01005615:3378-3558
HmHel-6	CAEZ01005615:41797-41907
HmHel-6	CAEZ01005615:11101-11227
HmHel-6	CAEZ01005559:10210-10301
HmHel-6	CAEZ01005559:261-3191
HmHel-6	CAEZ01005529:1212-1456
HmHel-6	CAEZ01005528:573-696
HmHel-6	CAEZ01005528:16516-16754
HmHel-6	CAEZ01005528:38809-43177
HmHel-6	CAEZ01005415:16407-16567
HmHel-6	CAEZ01005415:39436-39542
HmHel-6	CAEZ01005119:8209-8417
HmHel-6	CAEZ01005046:7168-7413
HmHel-6	CAEZ01005045:4271-4407
HmHel-6	CAEZ01005045:34042-34701
HmHel-6	CAEZ01005045:11044-11161
HmHel-6	CAEZ01004632:29873-30017
HmHel-6	CAEZ01004632:93514-93658
HmHel-6	CAEZ01004632:112151-112295
HmHel-6	CAEZ01004632:15261-19989
HmHel-6	CAEZ01004594:2340-2541
HmHel-6	CAEZ01004572:2509-2595
HmHel-6	CAEZ01004572:13654-13760
HmHel-6	CAEZ01004431:1051-1200
HmHel-6	CAEZ01004270:1882-1962
HmHel-6	CAEZ01004270:20634-20868

HmHel-6	CAEZ01004270:3329-3459
HmHel-6	CAEZ01004023:2075-2193
HmHel-6	CAEZ01004023:12507-12594
HmHel-6	CAEZ01003957:1936-2090
HmHel-6	CAEZ01003925:4318-4483
HmHel-6	CAEZ01003747:29789-29993
HmHel-6	CAEZ01003747:12146-12269
HmHel-6	CAEZ01003621:7993-8174
HmHel-6	CAEZ01003547:1222-1376
HmHel-6	CAEZ01003525:1699-1797
HmHel-6	CAEZ01003135:1602-1805
HmHel-6	CAEZ01003135:8541-8659
HmHel-6	CAEZ01003106:5002-9992
HmHel-6	CAEZ01003106:10314-10505
HmHel-6	CAEZ01002846:11466-11569
HmHel-6	CAEZ01002846:30113-30192
HmHel-6	CAEZ01002312:3532-3704
HmHel-6	CAEZ01002312:454-9024
HmHel-6	CAEZ01002206:1155-1281
HmHel-6	CAEZ01001809:3682-4537
HmHel-6	CAEZ01001581:3126-3244
HmHel-6	CAEZ01001581:34556-34737
HmHel-6	CAEZ01001581:36095-36186
HmHel-6	CAEZ01001417:29552-29710
HmHel-6	CAEZ01001417:26361-26543
HmHel-6	CAEZ01001417:13321-13483
HmHel-6	CAEZ01001299:7-161
HmHel-6	CAEZ01001137:6715-6900
HmHel-6	CAEZ01001137:10343-10449
HmHel-6	CAEZ01001036:15738-15828
HmHel-6	CAEZ01000903:662-760
HmHel-6	CAEZ01000605:5679-5868
HmHel-6	CAEZ01011537:5781-6025
HmHel-6	CAEZ01011537:27928-28067
HmHel-6	CAEZ01011537:794-8022
HmHel-6	CAEZ01011472:7937-8125
HmHel-6	CAEZ01011472:10243-10395
HmHel-6	CAEZ01011348:5137-5282
HmHel-6	CAEZ01010689:1590-1711
HmHel-6	CAEZ01010689:10456-10591
HmHel-6	CAEZ01010211:14444-14577
HmHel-6	CAEZ01010106:1745-6886
HmHel-6	CAEZ01009938:7708-7901
HmHel-6	CAEZ01009928:22117-22211
HmHel-6	CAEZ01009928:8457-8643
HmHel-6	CAEZ01009851:15909-16928
HmHel-6	CAEZ01009851:9090-9274
HmHel-6	CAEZ01009625:440-676

HmHel-6	CAEZ01009379:19133-24163
HmHel-6	CAEZ01009379:44710-44904
HmHel-6	CAEZ01009379:55191-55360
HmHel-6	CAEZ01009379:9897-10002
HmHel-6	CAEZ01009233:3679-3848
HmHel-6	CAEZ01009233:27892-28008
HmHel-6	CAEZ01009213:8285-14717
HmHel-6	CAEZ01009189:4811-11201
HmHel-6	CAEZ01009078:19579-19795
HmHel-6	CAEZ01009078:44791-44881
HmHel-6	CAEZ01009078:64664-73783
HmHel-6	CAEZ01009078:73034-73177
HmHel-6	CAEZ01009078:35424-35520
HmHel-6	CAEZ01008694:4435-4548
HmHel-6	CAEZ01008593:5188-5306
HmHel-6	CAEZ01007953:3399-10775
HmHel-6	CAEZ01007953:10960-11188
HmHel-6	CAEZ01007670:1025-1150
HmHel-6	CAEZ01007670:7728-7891
HmHel-6	CAEZ01007508:959-1100
HmHel-6	CAEZ01007508:1905-4349
HmHel-6	CAEZ01007454:40844-40968
HmHel-6	CAEZ01007454:5284-5495
HmHel-6	CAEZ01007430:15087-15226
HmHel-6	CAEZ01007398:9237-9366
HmHel-6	CAEZ01006945:5400-6572
HmHel-6	CAEZ01006899:4248-4355
HmHel-6	CAEZ01006899:10329-10457
HmHel-6	CAEZ01006891:1532-1669
HmHel-6	CAEZ01006707:3781-3982
HmHel-6	CAEZ01006591:1134-1217
HmHel-6	CAEZ01006488:28589-28717
HmHel-6	CAEZ01006488:51102-51208
HmHel-6	CAEZ01006488:125283-132447
HmHel-6	CAEZ01006488:116628-116716
HmHel-6	CAEZ01006454:18487-18594
HmHel-6	CAEZ01006454:1295-1493
HmHel-6	CAEZ01006155:6506-6659
HmHel-6	CAEZ01006155:2218-2364
HmHel-6	CAEZ01006149:5765-5859
HmHel-6	CAEZ01006149:17797-17929
HmHel-6	CAEZ01006054:1039-1248
HmHel-6	CAEZ01006054:32892-32996
HmHel-6	CAEZ01006054:31775-31927
HmHel-6	CAEZ01006004:8839-8994
HmHel-6	CAEZ01006004:42644-42827
HmHel-6	CAEZ01006004:83440-83553
HmHel-6	CAEZ01006004:37022-37165

HmHel-6	CAEZ01006004:7343-10843
HmHel-6	CAEZ01005780:14116-16989
HmHel-6	CAEZ01005780:19225-19622
HmHel-6	CAEZ01005780:6846-6983
HmHel-6	CAEZ01005779:58553-58654
HmHel-6	CAEZ01005779:40964-43725
HmHel-6	CAEZ01005742:1697-2889
HmHel-6	CAEZ01005742:3870-3955
HmHel-6	CAEZ01005686:2102-2215
HmHel-6	CAEZ01005466:8019-8100
HmHel-6	CAEZ01005466:6922-7059
HmHel-6	CAEZ01005454:42228-52217
HmHel-6	CAEZ01005454:45562-45644
HmHel-6	CAEZ01005454:2943-12009
HmHel-6	CAEZ01005452:11494-11583
HmHel-6	CAEZ01005284:6200-6448
HmHel-6	CAEZ01005229:6857-6976
HmHel-6	CAEZ01005229:43745-46995
HmHel-6	CAEZ01005229:12800-15754
HmHel-6	CAEZ01005229:623-800
HmHel-6	CAEZ01004978:6545-6719
HmHel-6	CAEZ01004881:127-244
HmHel-6	CAEZ01004625:3312-3470
HmHel-6	CAEZ01004370:30272-30355
HmHel-6	CAEZ01004370:70619-71608
HmHel-6	CAEZ01004370:50283-55634
HmHel-6	CAEZ01004370:20930-21032
HmHel-6	CAEZ01004248:8899-9109
HmHel-6	CAEZ01004248:2352-2477
HmHel-6	CAEZ01004245:1285-1461
HmHel-6	CAEZ01003831:6399-8945
HmHel-6	CAEZ01003582:2769-3003
HmHel-6	CAEZ01003582:72950-73087
HmHel-6	CAEZ01003582:54372-59336
HmHel-6	CAEZ01003582:22339-26613
HmHel-6	CAEZ01003536:3404-3525
HmHel-6	CAEZ01003432:18216-21642
HmHel-6	CAEZ01003432:9106-9309
HmHel-6	CAEZ01003408:14585-14767
HmHel-6	CAEZ01003408:5605-5716
HmHel-6	CAEZ01002862:1783-1908
HmHel-6	CAEZ01002249:3016-3129
HmHel-6	CAEZ01002249:12530-12695
HmHel-6	CAEZ01002055:50934-51047
HmHel-6	CAEZ01002055:74291-74388
HmHel-6	CAEZ01002055:63240-63408
HmHel-6	CAEZ01001858:27733-27898
HmHel-6	CAEZ01001858:26544-26711

HmHel-6	CAEZ01001817:3699-3788
HmHel-6	CAEZ01001817:12665-12802
HmHel-6	CAEZ01001074:16065-16204
HmHel-6	CAEZ01001042:10508-10641
HmHel-6	CAEZ01001021:23322-23535
HmHel-6	CAEZ01001021:28291-28429
HmHel-6	CAEZ01001021:16733-16844
HmHel-6	CAEZ01000946:17279-17403
HmHel-6	CAEZ01000945:1560-10996
HmHel-6	CAEZ01000945:13272-13366
HmHel-6	CAEZ01000788:3657-3747
HmHel-6	CAEZ01000788:2395-2536
HmHel-6	CAEZ01011600:60-183
HmHel-6	CAEZ01011319:365-578
HmHel-6	CAEZ01011316:9256-9404
HmHel-6	CAEZ01011213:6817-6913
HmHel-6	CAEZ01011213:3442-6822
HmHel-6	CAEZ01011017:319-3496
HmHel-6	CAEZ01011017:1619-6535
HmHel-6	CAEZ01011014:8682-8805
HmHel-6	CAEZ01011014:11274-11394
HmHel-6	CAEZ01010823:19363-24420
HmHel-6	CAEZ01010823:69828-70261
HmHel-6	CAEZ01010823:84148-89069
HmHel-6	CAEZ01010823:28495-29830
HmHel-6	CAEZ01010823:7106-7236
HmHel-6	CAEZ01010726:13948-14104
HmHel-6	CAEZ01010676:4816-4934
HmHel-6	CAEZ01010676:7168-7406
HmHel-6	CAEZ01010611:1589-1693
HmHel-6	CAEZ01010611:1861-1998
HmHel-6	CAEZ01010431:18562-18730
HmHel-6	CAEZ01010431:30757-30860
HmHel-6	CAEZ01010431:6038-6117
HmHel-6	CAEZ01010390:4116-4208
HmHel-6	CAEZ01010390:400-570
HmHel-6	CAEZ01010201:973-1128
HmHel-6	CAEZ01010201:7205-7400
HmHel-6	CAEZ01009952:1757-1901
HmHel-6	CAEZ01009952:1906-2007
HmHel-6	CAEZ01009412:587-769
HmHel-6	CAEZ01009412:15587-16182
HmHel-6	CAEZ01009360:3804-3958
HmHel-6	CAEZ01009360:5171-5331
HmHel-6	CAEZ01009352:6411-6632
HmHel-6	CAEZ01009160:3585-3811
HmHel-6	CAEZ01009104:3385-3577
HmHel-6	CAEZ01008924:6304-6434

HmHel-6	CAEZ01008924:30591-31154
HmHel-6	CAEZ01008924:44549-44752
HmHel-6	CAEZ01008752:9289-9397
HmHel-6	CAEZ01008752:8299-8536
HmHel-6	CAEZ01008667:1478-1596
HmHel-6	CAEZ01008667:12321-15414
HmHel-6	CAEZ01008667:13389-16257
HmHel-6	CAEZ01008667:139-316
HmHel-6	CAEZ01008473:346-570
HmHel-6	CAEZ01008304:9365-9581
HmHel-6	CAEZ01008304:12915-13059
HmHel-6	CAEZ01008041:2170-5669
HmHel-6	CAEZ01007728:22209-22352
HmHel-6	CAEZ01007728:50640-50740
HmHel-6	CAEZ01007728:63005-63099
HmHel-6	CAEZ01007728:35135-35324
HmHel-6	CAEZ01007634:3318-3484
HmHel-6	CAEZ01007617:4490-7745
HmHel-6	CAEZ01007451:2375-2558
HmHel-6	CAEZ01007451:11230-11314
HmHel-6	CAEZ01007406:703-835
HmHel-6	CAEZ01007406:1518-3112
HmHel-6	CAEZ01007317:13903-14008
HmHel-6	CAEZ01007317:17761-17911
HmHel-6	CAEZ01007022:4219-4328
HmHel-6	CAEZ01007022:17828-18065
HmHel-6	CAEZ01006969:4972-11674
HmHel-6	CAEZ01006474:9968-15206
HmHel-6	CAEZ01006474:1230-9692
HmHel-6	CAEZ01006108:4717-13900
HmHel-6	CAEZ01006076:1696-2605
HmHel-6	CAEZ01006076:9209-9345
HmHel-6	CAEZ01005849:300-5422
HmHel-6	CAEZ01005849:2485-2733
HmHel-6	CAEZ01005626:14879-15065
HmHel-6	CAEZ01005570:2051-2255
HmHel-6	CAEZ01005506:2750-2893
HmHel-6	CAEZ01005471:7368-7525
HmHel-6	CAEZ01005471:4118-6898
HmHel-6	CAEZ01005462:14217-22690
HmHel-6	CAEZ01005462:15585-18284
HmHel-6	CAEZ01005218:2301-2381
HmHel-6	CAEZ01005218:5088-5190
HmHel-6	CAEZ01005190:68-164
HmHel-6	CAEZ01005190:1746-1868
HmHel-6	CAEZ01004830:12853-12941
HmHel-6	CAEZ01004830:2673-2829
HmHel-6	CAEZ01004481:10693-10793

HmHel-6	CAEZ01004481:21030-21788
HmHel-6	CAEZ01004481:25874-27924
HmHel-6	CAEZ01004241:2979-3115
HmHel-6	CAEZ01003721:5656-5808
HmHel-6	CAEZ01003666:9760-9896
HmHel-6	CAEZ01003645:12279-12438
HmHel-6	CAEZ01003553:4328-4456
HmHel-6	CAEZ01002906:28709-28833
HmHel-6	CAEZ01002906:24597-24806
HmHel-6	CAEZ01002906:1339-1465
HmHel-6	CAEZ01002799:2529-2633
HmHel-6	CAEZ01002799:579-707
HmHel-6	CAEZ01001206:5364-8836
HmHel-6	CAEZ01001206:11695-11866
HmHel-6	CAEZ01000682:497-952
HmHel-6	CAEZ01000194:1049-1219
HmHel-6	CAEZ01011430:454-6530
HmHel-6	CAEZ01011430:8822-8933
HmHel-6	CAEZ01011050:6194-6305
HmHel-6	CAEZ01011050:43250-45473
HmHel-6	CAEZ01011050:9914-10036
HmHel-6	CAEZ01010522:3543-3712
HmHel-6	CAEZ01010522:2803-3009
HmHel-6	CAEZ01010472:2082-6477
HmHel-6	CAEZ01010327:6176-6283
HmHel-6	CAEZ01010220:1286-1417
HmHel-6	CAEZ01010220:1599-1692
HmHel-6	CAEZ01010068:1852-1956
HmHel-6	CAEZ01010068:28795-28966
HmHel-6	CAEZ01009935:6070-6196
HmHel-6	CAEZ01009899:768-927
HmHel-6	CAEZ01009761:1974-2092
HmHel-6	CAEZ01009761:7056-7233
HmHel-6	CAEZ01009732:6935-7082
HmHel-6	CAEZ01009732:4644-4777
HmHel-6	CAEZ01009607:38214-38298
HmHel-6	CAEZ01009607:43636-43756
HmHel-6	CAEZ01009607:29281-29502
HmHel-6	CAEZ01009596:6159-6266
HmHel-6	CAEZ01009193:11393-17066
HmHel-6	CAEZ01009009:98-295
HmHel-6	CAEZ01009009:463-568
HmHel-6	CAEZ01008798:285-440
HmHel-6	CAEZ01008764:3787-3906
HmHel-6	CAEZ01008608:4491-4577
HmHel-6	CAEZ01008608:28639-28737
HmHel-6	CAEZ01008608:58697-63670
HmHel-6	CAEZ01008608:75449-80445

HmHel-6	CAEZ01008608:45479-45741
HmHel-6	CAEZ01008187:896-1028
HmHel-6	CAEZ01008176:4414-4588
HmHel-6	CAEZ01008176:26844-27026
HmHel-6	CAEZ01008176:11164-11270
HmHel-6	CAEZ01008099:10411-14274
HmHel-6	CAEZ01007763:6213-6327
HmHel-6	CAEZ01007696:11334-11435
HmHel-6	CAEZ01007696:43091-43192
HmHel-6	CAEZ01007696:26452-26581
HmHel-6	CAEZ01007696:7414-7520
HmHel-6	CAEZ01007295:1509-4737
HmHel-6	CAEZ01007295:2485-4324
HmHel-6	CAEZ01007284:1513-1622
HmHel-6	CAEZ01007188:383-553
HmHel-6	CAEZ01007068:11349-11520
HmHel-6	CAEZ01007068:10619-11620
HmHel-6	CAEZ01006907:6903-11778
HmHel-6	CAEZ01006887:10112-11991
HmHel-6	CAEZ01006848:3269-3394
HmHel-6	CAEZ01006848:13902-14008
HmHel-6	CAEZ01006362:291-457
HmHel-6	CAEZ01006311:1923-2100
HmHel-6	CAEZ01006311:574-714
HmHel-6	CAEZ01006176:4277-5005
HmHel-6	CAEZ01006176:9270-9397
HmHel-6	CAEZ01006079:1478-1601
HmHel-6	CAEZ01006079:3400-9654
HmHel-6	CAEZ01005919:13084-13294
HmHel-6	CAEZ01005919:522-633
HmHel-6	CAEZ01005684:2829-3043
HmHel-6	CAEZ01005473:23034-23128
HmHel-6	CAEZ01005473:42790-42888
HmHel-6	CAEZ01005473:38489-46074
HmHel-6	CAEZ01005473:22915-22997
HmHel-6	CAEZ01005088:18090-18297
HmHel-6	CAEZ01005088:22755-22895
HmHel-6	CAEZ01005069:7302-7445
HmHel-6	CAEZ01005056:1702-1843
HmHel-6	CAEZ01005020:1066-1253
HmHel-6	CAEZ01005020:1422-1527
HmHel-6	CAEZ01004983:4655-4750
HmHel-6	CAEZ01004983:1616-1695
HmHel-6	CAEZ01004858:258-393
HmHel-6	CAEZ01004858:18445-20934
HmHel-6	CAEZ01004858:575-668
HmHel-6	CAEZ01004700:458-4436
HmHel-6	CAEZ01004700:10589-10714

HmHel-6	CAEZ01004641:10646-10768
HmHel-6	CAEZ01004573:766-869
HmHel-6	CAEZ01004383:8628-8755
HmHel-6	CAEZ01003839:12-135
HmHel-6	CAEZ01003598:7130-7304
HmHel-6	CAEZ01003163:71226-71351
HmHel-6	CAEZ01003163:21867-22035
HmHel-6	CAEZ01003124:17749-17898
HmHel-6	CAEZ01003124:8627-8758
HmHel-6	CAEZ01002804:30899-31042
HmHel-6	CAEZ01002804:41917-45013
HmHel-6	CAEZ01002804:4707-4835
HmHel-6	CAEZ01002667:1446-1552
HmHel-6	CAEZ01002667:23960-24061
HmHel-6	CAEZ01002667:23795-29498
HmHel-6	CAEZ01002667:1719-1934
HmHel-6	CAEZ01002354:5640-5792
HmHel-6	CAEZ01002221:11515-11621
HmHel-6	CAEZ01002221:9399-10901
HmHel-6	CAEZ01002218:4462-4617
HmHel-6	CAEZ01001808:68-244
HmHel-6	CAEZ01001492:699-861
HmHel-6	CAEZ01001006:187-384
HmHel-6	CAEZ01000537:960-1058
HmHel-6	CAEZ01000079:4668-4839
HmHel-6	CAEZ01011596:1674-1813
HmHel-6	CAEZ01011476:7660-7787
HmHel-6	CAEZ01011468:4190-4392
HmHel-6	CAEZ01011198:1835-1951
HmHel-6	CAEZ01011120:31535-31689
HmHel-6	CAEZ01011120:41079-41273
HmHel-6	CAEZ01011120:18499-18675
HmHel-6	CAEZ01011097:856-1042
HmHel-6	CAEZ01010852:3021-3223
HmHel-6	CAEZ01010671:4558-14634
HmHel-6	CAEZ01010671:2690-2819
HmHel-6	CAEZ01010615:47290-47433
HmHel-6	CAEZ01010615:24019-24211
HmHel-6	CAEZ01010615:4193-4277
HmHel-6	CAEZ01010400:1386-1487
HmHel-6	CAEZ01010170:3660-3814
HmHel-6	CAEZ01010024:8554-8779
HmHel-6	CAEZ01009595:18063-18143
HmHel-6	CAEZ01009575:3288-3431
HmHel-6	CAEZ01009574:4001-4154
HmHel-6	CAEZ01009574:1411-1499
HmHel-6	CAEZ01009529:16541-16739
HmHel-6	CAEZ01009529:31926-32070

HmHel-6	CAEZ01009336:24791-24950
HmHel-6	CAEZ01009174:4520-12631
HmHel-6	CAEZ01009150:10420-16314
HmHel-6	CAEZ01009150:39454-49472
HmHel-6	CAEZ01009150:72275-72431
HmHel-6	CAEZ01009150:8883-9018
HmHel-6	CAEZ01009108:4315-4466
HmHel-6	CAEZ01008993:17714-17829
HmHel-6	CAEZ01008993:2375-2576
HmHel-6	CAEZ01008964:1286-5624
HmHel-6	CAEZ01008964:1510-9559
HmHel-6	CAEZ01008902:4635-4828
HmHel-6	CAEZ01008868:12341-12462
HmHel-6	CAEZ01008868:12794-13479
HmHel-6	CAEZ01008639:437-5134
HmHel-6	CAEZ01008639:25495-25770
HmHel-6	CAEZ01008489:839-992
HmHel-6	CAEZ01008489:13511-28004
HmHel-6	CAEZ01008489:4621-4700
HmHel-6	CAEZ01008480:16503-21495
HmHel-6	CAEZ01008480:42640-42733
HmHel-6	CAEZ01008480:24164-24275
HmHel-6	CAEZ01008480:3559-5046
HmHel-6	CAEZ01007989:436-523
HmHel-6	CAEZ01007989:19882-19972
HmHel-6	CAEZ01007585:24437-24584
HmHel-6	CAEZ01007585:47771-47882
HmHel-6	CAEZ01007585:43355-51053
HmHel-6	CAEZ01007585:3548-3752
HmHel-6	CAEZ01007075:95-193
HmHel-6	CAEZ01006758:5079-5228
HmHel-6	CAEZ01006324:28834-31838
HmHel-6	CAEZ01006324:43842-51843
HmHel-6	CAEZ01006324:76756-83481
HmHel-6	CAEZ01006324:107573-107674
HmHel-6	CAEZ01006324:71845-71958
HmHel-6	CAEZ01006324:45988-46128
HmHel-6	CAEZ01006324:2027-2155
HmHel-6	CAEZ01006064:1362-1444
HmHel-6	CAEZ01006002:10311-14002
HmHel-6	CAEZ01005955:20828-26698
HmHel-6	CAEZ01005955:33880-35207
HmHel-6	CAEZ01005924:5712-5864
HmHel-6	CAEZ01005924:32365-32446
HmHel-6	CAEZ01005910:4965-5147
HmHel-6	CAEZ01005910:3731-3821
HmHel-6	CAEZ01005868:6520-15064
HmHel-6	CAEZ01005868:11598-16065

HmHel-6	CAEZ01005677:23879-30974
HmHel-6	CAEZ01005677:23158-23261
HmHel-6	CAEZ01005410:3110-12841
HmHel-6	CAEZ01005410:87030-93589
HmHel-6	CAEZ01005410:105059-105140
HmHel-6	CAEZ01005410:156730-158505
HmHel-6	CAEZ01005410:194214-194339
HmHel-6	CAEZ01005410:170388-170471
HmHel-6	CAEZ01004995:5569-10494
HmHel-6	CAEZ01004995:15522-15674
HmHel-6	CAEZ01004981:16309-16412
HmHel-6	CAEZ01004981:28156-28255
HmHel-6	CAEZ01004981:6355-6465
HmHel-6	CAEZ01004837:1522-1657
HmHel-6	CAEZ01004749:1685-1886
HmHel-6	CAEZ01004749:21641-22914
HmHel-6	CAEZ01004547:1894-4825
HmHel-6	CAEZ01004360:6038-12243
HmHel-6	CAEZ01004349:4214-9274
HmHel-6	CAEZ01004349:20042-23870
HmHel-6	CAEZ01004237:4236-7502
HmHel-6	CAEZ01004189:11452-11598
HmHel-6	CAEZ01004189:13825-13935
HmHel-6	CAEZ01004187:6210-6380
HmHel-6	CAEZ01004123:23746-23954
HmHel-6	CAEZ01004101:3375-9475
HmHel-6	CAEZ01004101:3784-7170
HmHel-6	CAEZ01003924:5115-5213
HmHel-6	CAEZ01003882:35575-35673
HmHel-6	CAEZ01003882:23591-23710
HmHel-6	CAEZ01003799:4048-8141
HmHel-6	CAEZ01003799:11585-11769
HmHel-6	CAEZ01003770:14496-14605
HmHel-6	CAEZ01003770:444-599
HmHel-6	CAEZ01003733:1976-5997
HmHel-6	CAEZ01003634:5718-5924
HmHel-6	CAEZ01003513:2568-2703
HmHel-6	CAEZ01003513:4796-4890
HmHel-6	CAEZ01003434:4302-4524
HmHel-6	CAEZ01003414:5153-5245
HmHel-6	CAEZ01003414:26653-26809
HmHel-6	CAEZ01003342:964-1066
HmHel-6	CAEZ01003331:21836-22912
HmHel-6	CAEZ01002721:8149-8237
HmHel-6	CAEZ01002721:32172-38940
HmHel-6	CAEZ01002721:54592-54682
HmHel-6	CAEZ01002721:23396-31132
HmHel-6	CAEZ01002721:11015-11224

HmHel-6	CAEZ01002705:10740-10919
HmHel-6	CAEZ01002705:21422-21593
HmHel-6	CAEZ01002705:16843-23418
HmHel-6	CAEZ01002275:685-881
HmHel-6	CAEZ01002275:10236-10469
HmHel-6	CAEZ01002244:6526-6665
HmHel-6	CAEZ01000807:636-719
HmHel-6	CAEZ01000660:2597-3565
HmHel-6	CAEZ01011576:28357-28445
HmHel-6	CAEZ01011576:15097-15287
HmHel-6	CAEZ01011340:3729-8230
HmHel-6	CAEZ01011340:26514-26680
HmHel-6	CAEZ01011291:9480-9596
HmHel-6	CAEZ01010967:19584-19721
HmHel-6	CAEZ01010967:1764-1883
HmHel-6	CAEZ01010794:9056-9238
HmHel-6	CAEZ01010517:620-4255
HmHel-6	CAEZ01010517:42341-50659
HmHel-6	CAEZ01010517:23069-26317
HmHel-6	CAEZ01009988:22193-27809
HmHel-6	CAEZ01009988:26477-26604
HmHel-6	CAEZ01009743:639-784
HmHel-6	CAEZ01009720:1604-1756
HmHel-6	CAEZ01009535:19196-19389
HmHel-6	CAEZ01009033:10618-10762
HmHel-6	CAEZ01008663:3104-5500
HmHel-6	CAEZ01008324:4988-5194
HmHel-6	CAEZ01008324:19873-19952
HmHel-6	CAEZ01008245:8681-8830
HmHel-6	CAEZ01008245:641-790
HmHel-6	CAEZ01007921:13527-13646
HmHel-6	CAEZ01007921:961-1629
HmHel-6	CAEZ01007822:8044-8216
HmHel-6	CAEZ01007822:20457-20550
HmHel-6	CAEZ01007782:725-895
HmHel-6	CAEZ01007782:12905-13051
HmHel-6	CAEZ01007777:38059-42170
HmHel-6	CAEZ01007777:21955-22087
HmHel-6	CAEZ01007777:2698-2839
HmHel-6	CAEZ01007552:11007-11140
HmHel-6	CAEZ01007431:3085-3302
HmHel-6	CAEZ01007144:10685-10883
HmHel-6	CAEZ01007139:3943-4057
HmHel-6	CAEZ01007134:3617-5623
HmHel-6	CAEZ01007134:17559-17646
HmHel-6	CAEZ01006883:11211-11393
HmHel-6	CAEZ01006658:2905-3101
HmHel-6	CAEZ01006577:1659-8994

HmHel-6	CAEZ01006577:6661-6804
HmHel-6	CAEZ01006479:15181-15304
HmHel-6	CAEZ01006479:13720-13881
HmHel-6	CAEZ01006334:22086-22194
HmHel-6	CAEZ01006106:6874-7026
HmHel-6	CAEZ01006104:2292-2487
HmHel-6	CAEZ01005960:6866-7050
HmHel-6	CAEZ01005446:4512-4676
HmHel-6	CAEZ01005446:8692-8777
HmHel-6	CAEZ01005279:15121-15223
HmHel-6	CAEZ01005279:38552-38726
HmHel-6	CAEZ01005279:67786-67921
HmHel-6	CAEZ01005279:104206-104291
HmHel-6	CAEZ01005279:101832-101953
HmHel-6	CAEZ01005279:28545-28722
HmHel-6	CAEZ01005165:3970-4121
HmHel-6	CAEZ01004464:6824-6953
HmHel-6	CAEZ01004405:3612-3753
HmHel-6	CAEZ01004075:20258-20379
HmHel-6	CAEZ01004075:30241-30362
HmHel-6	CAEZ01004014:1647-1790
HmHel-6	CAEZ01004014:20726-20823
HmHel-6	CAEZ01004014:34710-34919
HmHel-6	CAEZ01004014:53844-53968
HmHel-6	CAEZ01004014:55821-63744
HmHel-6	CAEZ01003960:3668-3836
HmHel-6	CAEZ01003787:2618-2874
HmHel-6	CAEZ01003787:555-641
HmHel-6	CAEZ01003418:6677-6778
HmHel-6	CAEZ01003418:5790-8878
HmHel-6	CAEZ01003359:12154-12818
HmHel-6	CAEZ01003359:7367-7547
HmHel-6	CAEZ01003198:3135-3259
HmHel-6	CAEZ01003120:4862-4983
HmHel-6	CAEZ01002982:11769-16862
HmHel-6	CAEZ01002982:6982-7103
HmHel-6	CAEZ01002813:5385-9250
HmHel-6	CAEZ01002813:21496-28942
HmHel-6	CAEZ01002813:48612-49161
HmHel-6	CAEZ01002813:65449-65612
HmHel-6	CAEZ01002747:5979-6148
HmHel-6	CAEZ01002747:5044-5166
HmHel-6	CAEZ01002731:8798-9727
HmHel-6	CAEZ01002731:5918-6122
HmHel-6	CAEZ01002694:24236-24402
HmHel-6	CAEZ01001946:3891-4033
HmHel-6	CAEZ01001751:11526-11680
HmHel-6	CAEZ01001751:1088-1209

HmHel-6	CAEZ01001602:5136-5214
HmHel-6	CAEZ01001602:1527-1647
HmHel-6	CAEZ01001302:9303-9495
HmHel-6	CAEZ01001302:3025-3218
HmHel-6	CAEZ01000870:3460-3647
HmHel-6	CAEZ01000572:3268-6439
HmHel-6	CAEZ01000126:2328-2468
HmHel-6	CAEZ01011597:218-433
HmHel-6	CAEZ01011533:19479-19602
HmHel-6	CAEZ01011533:9600-9708
HmHel-6	CAEZ01011411:9017-9196
HmHel-6	CAEZ01011411:3937-4104
HmHel-6	CAEZ01011242:13772-15455
HmHel-6	CAEZ01011119:4248-4431
HmHel-6	CAEZ01010919:1172-1296
HmHel-6	CAEZ01010767:5937-6179
HmHel-6	CAEZ01010716:11433-11605
HmHel-6	CAEZ01010716:3834-4000
HmHel-6	CAEZ01010641:2832-2951
HmHel-6	CAEZ01010641:21725-21819
HmHel-6	CAEZ01010509:11534-11667
HmHel-6	CAEZ01010450:50554-50659
HmHel-6	CAEZ01010450:62112-62242
HmHel-6	CAEZ01010450:20716-20896
HmHel-6	CAEZ01010427:29716-29846
HmHel-6	CAEZ01010427:10680-10772
HmHel-6	CAEZ01010401:8216-8302
HmHel-6	CAEZ01010401:14204-14361
HmHel-6	CAEZ01010326:4715-4847
HmHel-6	CAEZ01010326:49021-49153
HmHel-6	CAEZ01010326:42313-42457
HmHel-6	CAEZ01010278:5866-6050
HmHel-6	CAEZ01010278:18384-26183
HmHel-6	CAEZ01010171:26029-26201
HmHel-6	CAEZ01010084:40278-40496
HmHel-6	CAEZ01010084:2721-8473
HmHel-6	CAEZ01009705:44152-44290
HmHel-6	CAEZ01009705:56020-56214
HmHel-6	CAEZ01009699:8690-8866
HmHel-6	CAEZ01009699:24805-27529
HmHel-6	CAEZ01009699:7115-13976
HmHel-6	CAEZ01009494:2450-2611
HmHel-6	CAEZ01009251:6303-6439
HmHel-6	CAEZ01009251:11073-12631
HmHel-6	CAEZ01009157:2398-2498
HmHel-6	CAEZ01009011:1748-1890
HmHel-6	CAEZ01008998:1640-2332
HmHel-6	CAEZ01008998:4333-4457

HmHel-6	CAEZ01008846:3247-3347
HmHel-6	CAEZ01008733:4058-4184
HmHel-6	CAEZ01008733:9336-9532
HmHel-6	CAEZ01008447:2845-2940
HmHel-6	CAEZ01008366:4698-4884
HmHel-6	CAEZ01008366:13949-19601
HmHel-6	CAEZ01008332:7960-8129
HmHel-6	CAEZ01008227:4402-4556
HmHel-6	CAEZ01008227:7844-7960
HmHel-6	CAEZ01008224:11299-20664
HmHel-6	CAEZ01008137:2278-2386
HmHel-6	CAEZ01007589:3235-3395
HmHel-6	CAEZ01007589:19978-20058
HmHel-6	CAEZ01007550:20060-20156
HmHel-6	CAEZ01007550:24493-24976
HmHel-6	CAEZ01007550:8713-8852
HmHel-6	CAEZ01007177:15028-15124
HmHel-6	CAEZ01006984:5184-5392
HmHel-6	CAEZ01006642:5772-5888
HmHel-6	CAEZ01006160:5753-5861
HmHel-6	CAEZ01006160:1716-3424
HmHel-6	CAEZ01006037:659-856
HmHel-6	CAEZ01006037:6551-6763
HmHel-6	CAEZ01005998:14461-14656
HmHel-6	CAEZ01005998:4568-4786
HmHel-6	CAEZ01005994:7770-13898
HmHel-6	CAEZ01005994:1348-1431
HmHel-6	CAEZ01005990:6066-6222
HmHel-6	CAEZ01005866:3412-3553
HmHel-6	CAEZ01005866:27718-27848
HmHel-6	CAEZ01005866:7089-12400
HmHel-6	CAEZ01005840:2572-2711
HmHel-6	CAEZ01005517:2896-2983
HmHel-6	CAEZ01005375:7366-7515
HmHel-6	CAEZ01004835:11609-11721
HmHel-6	CAEZ01004835:23716-23926
HmHel-6	CAEZ01004715:1661-1849
HmHel-6	CAEZ01004674:31-135
HmHel-6	CAEZ01004674:13157-14120
HmHel-6	CAEZ01004582:49790-49980
HmHel-6	CAEZ01004582:73717-73912
HmHel-6	CAEZ01004582:48326-48509
HmHel-6	CAEZ01004582:27406-27492
HmHel-6	CAEZ01004582:1078-1191
HmHel-6	CAEZ01004309:20457-20537
HmHel-6	CAEZ01004309:23387-23683
HmHel-6	CAEZ01004033:29180-33200
HmHel-6	CAEZ01004033:56523-63791

HmHel-6	CAEZ01004033:78399-78608
HmHel-6	CAEZ01004033:97797-97998
HmHel-6	CAEZ01004033:190368-190472
HmHel-6	CAEZ01004033:177856-177936
HmHel-6	CAEZ01004033:105896-105984
HmHel-6	CAEZ01004033:85447-92514
HmHel-6	CAEZ01004033:60081-60726
HmHel-6	CAEZ01004033:33840-33940
HmHel-6	CAEZ01003926:5548-5712
HmHel-6	CAEZ01003926:7583-7667
HmHel-6	CAEZ01003794:10874-11003
HmHel-6	CAEZ01003794:48844-48964
HmHel-6	CAEZ01003794:56900-64622
HmHel-6	CAEZ01003794:38773-38891
HmHel-6	CAEZ01003689:9008-15923
HmHel-6	CAEZ01003689:17712-17932
HmHel-6	CAEZ01003241:3810-3932
HmHel-6	CAEZ01003241:622-823
HmHel-6	CAEZ01002969:7126-7214
HmHel-6	CAEZ01002770:23814-23944
HmHel-6	CAEZ01002769:7611-7711
HmHel-6	CAEZ01002769:45963-46045
HmHel-6	CAEZ01002769:75486-75569
HmHel-6	CAEZ01002769:76815-76922
HmHel-6	CAEZ01002769:32030-32116
HmHel-6	CAEZ01002577:9123-9348
HmHel-6	CAEZ01002577:5059-6223
HmHel-6	CAEZ01002383:2827-2908
HmHel-6	CAEZ01002383:24450-28747
HmHel-6	CAEZ01002383:28002-28166
HmHel-6	CAEZ01002383:8202-14150
HmHel-6	CAEZ01002237:3238-5632
HmHel-6	CAEZ01001580:12954-13114
HmHel-6	CAEZ01001580:15581-15787
HmHel-6	CAEZ01001506:603-696
HmHel-6	CAEZ01001479:3298-3445
HmHel-6	CAEZ01000769:915-1067
HmHel-6	CAEZ01000039:9202-9302
HmHel-6	CAEZ01011613:308-431
HmHel-6	CAEZ01011613:13496-13630
HmHel-6	CAEZ01011613:15307-15471
HmHel-6	CAEZ01011183:4398-4494
HmHel-6	CAEZ01011183:1109-1228
HmHel-6	CAEZ01011103:32471-32638
HmHel-6	CAEZ01011103:6467-6584
HmHel-6	CAEZ01011078:319-599
HmHel-6	CAEZ01011048:75632-75829
HmHel-6	CAEZ01011048:52749-52833

HmHel-6	CAEZ01011048:27937-28036
HmHel-6	CAEZ01010971:25710-25817
HmHel-6	CAEZ01010971:55603-55683
HmHel-6	CAEZ01010971:41420-46029
HmHel-6	CAEZ01010706:77838-77964
HmHel-6	CAEZ01010706:95498-95637
HmHel-6	CAEZ01010706:72574-72761
HmHel-6	CAEZ01010706:40255-40379
HmHel-6	CAEZ01010706:8904-11939
HmHel-6	CAEZ01010647:1699-1786
HmHel-6	CAEZ01010646:2460-2547
HmHel-6	CAEZ01010609:11003-11181
HmHel-6	CAEZ01010599:2922-3123
HmHel-6	CAEZ01010560:348-429
HmHel-6	CAEZ01010560:11307-11635
HmHel-6	CAEZ01010504:1656-6608
HmHel-6	CAEZ01010486:338-445
HmHel-6	CAEZ01010486:7097-7305
HmHel-6	CAEZ01010432:10297-10496
HmHel-6	CAEZ01010418:4648-4790
HmHel-6	CAEZ01010193:13273-13478
HmHel-6	CAEZ01010092:2376-2556
HmHel-6	CAEZ01009682:2296-2396
HmHel-6	CAEZ01009682:21334-21441
HmHel-6	CAEZ01009682:20603-22018
HmHel-6	CAEZ01009648:2695-4734
HmHel-6	CAEZ01009263:3311-3536
HmHel-6	CAEZ01009263:25167-35027
HmHel-6	CAEZ01009188:2144-2339
HmHel-6	CAEZ01009188:643-796
HmHel-6	CAEZ01009165:3381-3579
HmHel-6	CAEZ01009165:2745-2884
HmHel-6	CAEZ01008888:6619-25221
HmHel-6	CAEZ01008888:30474-35624
HmHel-6	CAEZ01008825:22465-22623
HmHel-6	CAEZ01008825:13927-14054
HmHel-6	CAEZ01008681:1794-1885
HmHel-6	CAEZ01008604:2328-11757
HmHel-6	CAEZ01008542:16430-16582
HmHel-6	CAEZ01008491:6223-6331
HmHel-6	CAEZ01008466:6320-6453
HmHel-6	CAEZ01008466:3791-4781
HmHel-6	CAEZ01008214:3355-3536
HmHel-6	CAEZ01008212:3558-3760
HmHel-6	CAEZ01008212:15521-15671
HmHel-6	CAEZ01008212:11434-14194
HmHel-6	CAEZ01007859:1609-1716
HmHel-6	CAEZ01007859:21720-24212

HmHel-6	CAEZ01007820:1922-2100
HmHel-6	CAEZ01007218:4365-4453
HmHel-6	CAEZ01007218:12455-12598
HmHel-6	CAEZ01007195:3655-3841
HmHel-6	CAEZ01006876:6112-6324
HmHel-6	CAEZ01006876:22217-22378
HmHel-6	CAEZ01006876:9061-9162
HmHel-6	CAEZ01006794:2361-2533
HmHel-6	CAEZ01006080:7580-12030
HmHel-6	CAEZ01006080:10815-10986
HmHel-6	CAEZ01006025:336-438
HmHel-6	CAEZ01006025:1909-1996
HmHel-6	CAEZ01005824:8619-8713
HmHel-6	CAEZ01005824:3082-3205
HmHel-6	CAEZ01005806:5700-10979
HmHel-6	CAEZ01005806:23052-23242
HmHel-6	CAEZ01005806:31121-31221
HmHel-6	CAEZ01005610:2506-2649
HmHel-6	CAEZ01005610:18145-18379
HmHel-6	CAEZ01005600:9834-9997
HmHel-6	CAEZ01005566:2333-3033
HmHel-6	CAEZ01005566:14170-19655
HmHel-6	CAEZ01005530:125-224
HmHel-6	CAEZ01005270:13686-21548
HmHel-6	CAEZ01005270:29599-29824
HmHel-6	CAEZ01004897:5335-5487
HmHel-6	CAEZ01004897:3268-3359
HmHel-6	CAEZ01004211:5682-5777
HmHel-6	CAEZ01004211:28613-28740
HmHel-6	CAEZ01004211:757-9329
HmHel-6	CAEZ01004178:989-1155
HmHel-6	CAEZ01003929:10181-10368
HmHel-6	CAEZ01003902:6568-6655
HmHel-6	CAEZ01003902:21621-21720
HmHel-6	CAEZ01003902:17366-17467
HmHel-6	CAEZ01003515:3472-3600
HmHel-6	CAEZ01003322:16634-16749
HmHel-6	CAEZ01003322:8052-8180
HmHel-6	CAEZ01003270:10039-10139
HmHel-6	CAEZ01003270:14670-14774
HmHel-6	CAEZ01003254:10085-10187
HmHel-6	CAEZ01003254:20271-20397
HmHel-6	CAEZ01003254:1809-7594
HmHel-6	CAEZ01003142:16087-16198
HmHel-6	CAEZ01003142:38123-38236
HmHel-6	CAEZ01003061:16744-16903
HmHel-6	CAEZ01002940:4814-7619
HmHel-6	CAEZ01002704:24658-24791

HmHel-6	CAEZ01002609:4629-4817
HmHel-6	CAEZ01002369:4279-7485
HmHel-6	CAEZ01002369:64942-65034
HmHel-6	CAEZ01002369:41059-41162
HmHel-6	CAEZ01002369:20201-27216
HmHel-6	CAEZ01002001:11478-13718
HmHel-6	CAEZ01002001:18729-18881
HmHel-6	CAEZ01001920:5615-5730
HmHel-6	CAEZ01001788:8328-8501
HmHel-6	CAEZ01001668:25536-25637
HmHel-6	CAEZ01001668:14199-18413
HmHel-6	CAEZ01001533:24732-24822
HmHel-6	CAEZ01001533:3771-3944
HmHel-6	CAEZ01001347:8357-8568
HmHel-6	CAEZ01001347:10474-10669
HmHel-6	CAEZ01001013:1879-2026
HmHel-6	CAEZ01001013:11353-11440
HmHel-6	CAEZ01000938:2104-2289
HmHel-6	CAEZ01000926:268-422
HmHel-6	CAEZ01000448:155-246
HmHel-6	CAEZ01011629:29118-40277
HmHel-6	CAEZ01011263:7197-7393
HmHel-6	CAEZ01011172:5108-5308
HmHel-6	CAEZ01010931:951-1077
HmHel-6	CAEZ01010931:19768-19943
HmHel-6	CAEZ01010859:6693-6821
HmHel-6	CAEZ01010859:6568-6670
HmHel-6	CAEZ01010697:33237-33386
HmHel-6	CAEZ01010697:47886-47992
HmHel-6	CAEZ01010697:25964-26055
HmHel-6	CAEZ01010559:2-139
HmHel-6	CAEZ01010559:7087-7248
HmHel-6	CAEZ01010132:1596-1777
HmHel-6	CAEZ01010132:5526-5688
HmHel-6	CAEZ01010071:9659-9791
HmHel-6	CAEZ01009945:294-412
HmHel-6	CAEZ01009915:4637-4745
HmHel-6	CAEZ01009864:10707-10841
HmHel-6	CAEZ01009856:16799-17072
HmHel-6	CAEZ01009690:9555-9645
HmHel-6	CAEZ01009467:7902-8012
HmHel-6	CAEZ01009467:2192-2297
HmHel-6	CAEZ01009311:995-1094
HmHel-6	CAEZ01009311:11320-15465
HmHel-6	CAEZ01009311:38266-38442
HmHel-6	CAEZ01009311:17594-29638
HmHel-6	CAEZ01009144:4872-5074
HmHel-6	CAEZ01008698:2882-2976

HmHel-6	CAEZ01008698:1011-1142
HmHel-6	CAEZ01008521:8497-8662
HmHel-6	CAEZ01008033:6598-6799
HmHel-6	CAEZ01007691:2547-2705
HmHel-6	CAEZ01007475:58373-61598
HmHel-6	CAEZ01007475:87647-87738
HmHel-6	CAEZ01007475:88204-93233
HmHel-6	CAEZ01007475:59526-72038
HmHel-6	CAEZ01007475:35361-35542
HmHel-6	CAEZ01007165:17026-17185
HmHel-6	CAEZ01007160:968-1228
HmHel-6	CAEZ01007160:17831-17917
HmHel-6	CAEZ01007160:9388-9607
HmHel-6	CAEZ01006928:3370-6198
HmHel-6	CAEZ01006690:11575-14679
HmHel-6	CAEZ01006690:12779-12953
HmHel-6	CAEZ01006418:38190-38345
HmHel-6	CAEZ01006418:50385-50479
HmHel-6	CAEZ01006418:43349-43643
HmHel-6	CAEZ01006369:17768-21312
HmHel-6	CAEZ01006369:39454-39570
HmHel-6	CAEZ01006369:20636-20716
HmHel-6	CAEZ01006158:15828-15938
HmHel-6	CAEZ01006158:1355-1522
HmHel-6	CAEZ01005794:1907-2087
HmHel-6	CAEZ01005704:9734-9900
HmHel-6	CAEZ01005427:15115-15330
HmHel-6	CAEZ01005427:37793-43097
HmHel-6	CAEZ01005427:71814-74689
HmHel-6	CAEZ01005427:38249-38361
HmHel-6	CAEZ01005236:4878-6342
HmHel-6	CAEZ01005227:7430-7625
HmHel-6	CAEZ01004802:5231-5321
HmHel-6	CAEZ01004598:13034-13167
HmHel-6	CAEZ01004558:20596-20805
HmHel-6	CAEZ01004558:39354-39552
HmHel-6	CAEZ01004558:23389-23604
HmHel-6	CAEZ01004558:551-701
HmHel-6	CAEZ01004506:1892-7431
HmHel-6	CAEZ01004437:4762-4872
HmHel-6	CAEZ01004437:18178-18297
HmHel-6	CAEZ01004437:19421-19503
HmHel-6	CAEZ01004393:9061-9194
HmHel-6	CAEZ01004393:3834-3940
HmHel-6	CAEZ01004292:22881-23003
HmHel-6	CAEZ01004164:993-1091
HmHel-6	CAEZ01004030:14475-14688
HmHel-6	CAEZ01004030:11610-11696

HmHel-6	CAEZ01003898:16894-19801
HmHel-6	CAEZ01003898:12895-14413
HmHel-6	CAEZ01003395:2773-4136
HmHel-6	CAEZ01003282:2349-2450
HmHel-6	CAEZ01003159:5747-5985
HmHel-6	CAEZ01003159:8096-8193
HmHel-6	CAEZ01003150:10889-10979
HmHel-6	CAEZ01003150:6526-6668
HmHel-6	CAEZ01002881:9987-10148
HmHel-6	CAEZ01002881:15259-15342
HmHel-6	CAEZ01002263:1068-1222
HmHel-6	CAEZ01002200:11177-11307
HmHel-6	CAEZ01002053:8815-8972
HmHel-6	CAEZ01002053:984-1084
HmHel-6	CAEZ01000886:3880-10294
HmHel-6	CAEZ01000886:8925-9022
HmHel-6	CAEZ01000844:576-836
HmHel-6	CAEZ01000844:6207-6301
HmHel-6	CAEZ01000814:2036-3362
HmHel-6	CAEZ01011482:2317-2426
HmHel-6	CAEZ01011463:12849-17757
HmHel-6	CAEZ01011297:12116-12217
HmHel-6	CAEZ01011297:29794-29931
HmHel-6	CAEZ01011297:62220-70616
HmHel-6	CAEZ01011297:86240-86321
HmHel-6	CAEZ01011297:100279-100376
HmHel-6	CAEZ01011297:69392-69525
HmHel-6	CAEZ01011289:1512-1685
HmHel-6	CAEZ01011289:18267-18418
HmHel-6	CAEZ01011236:5545-5651
HmHel-6	CAEZ01010857:4007-6232
HmHel-6	CAEZ01010857:15187-15352
HmHel-6	CAEZ01010651:1778-1903
HmHel-6	CAEZ01010548:4997-5156
HmHel-6	CAEZ01010548:7779-7858
HmHel-6	CAEZ01010516:15400-15481
HmHel-6	CAEZ01010516:28973-29064
HmHel-6	CAEZ01010516:24772-24910
HmHel-6	CAEZ01010516:6587-6747
HmHel-6	CAEZ01010492:1373-1939
HmHel-6	CAEZ01010492:1430-1510
HmHel-6	CAEZ01010209:6347-6452
HmHel-6	CAEZ01009782:1026-1219
HmHel-6	CAEZ01009559:13-140
HmHel-6	CAEZ01009375:14205-14346
HmHel-6	CAEZ01009375:46335-46451
HmHel-6	CAEZ01009375:65685-65785
HmHel-6	CAEZ01009375:71544-74578

HmHel-6	CAEZ01009375:20028-20210
HmHel-6	CAEZ01009212:10989-11086
HmHel-6	CAEZ01009212:32497-40882
HmHel-6	CAEZ01009212:13847-13968
HmHel-6	CAEZ01009145:14249-14346
HmHel-6	CAEZ01009145:5868-5976
HmHel-6	CAEZ01008957:15146-15263
HmHel-6	CAEZ01008957:3662-3766
HmHel-6	CAEZ01008929:10962-11112
HmHel-6	CAEZ01008535:30609-30752
HmHel-6	CAEZ01008535:33520-33624
HmHel-6	CAEZ01008511:24837-24930
HmHel-6	CAEZ01008368:26093-26262
HmHel-6	CAEZ01008368:47754-47886
HmHel-6	CAEZ01008368:48615-48710
HmHel-6	CAEZ01008368:14876-15081
HmHel-6	CAEZ01007997:10654-10755
HmHel-6	CAEZ01007997:11098-11207
HmHel-6	CAEZ01007743:3600-3751
HmHel-6	CAEZ01007743:2972-3129
HmHel-6	CAEZ01007351:4059-4252
HmHel-6	CAEZ01007274:2789-2870
HmHel-6	CAEZ01007274:8954-9080
HmHel-6	CAEZ01007263:3907-4007
HmHel-6	CAEZ01007263:55910-56018
HmHel-6	CAEZ01007263:66733-66821
HmHel-6	CAEZ01007263:84820-84952
HmHel-6	CAEZ01007263:82886-82975
HmHel-6	CAEZ01007263:57526-57720
HmHel-6	CAEZ01007263:245-328
HmHel-6	CAEZ01007169:6717-6896
HmHel-6	CAEZ01007154:4817-5005
HmHel-6	CAEZ01007084:3703-3800
HmHel-6	CAEZ01006943:7385-7593
HmHel-6	CAEZ01006647:4106-4248
HmHel-6	CAEZ01006647:4748-4854
HmHel-6	CAEZ01006342:7806-13945
HmHel-6	CAEZ01006342:7347-7548
HmHel-6	CAEZ01006031:672-765
HmHel-6	CAEZ01005722:9247-9396
HmHel-6	CAEZ01005722:58662-58884
HmHel-6	CAEZ01005722:24901-25016
HmHel-6	CAEZ01005560:14090-14245
HmHel-6	CAEZ01005560:16565-16766
HmHel-6	CAEZ01005486:7592-8336
HmHel-6	CAEZ01005486:19996-20222
HmHel-6	CAEZ01005460:15079-15428
HmHel-6	CAEZ01005460:26227-26330

HmHel-6	CAEZ01005460:1444-1783
HmHel-6	CAEZ01005340:12292-12412
HmHel-6	CAEZ01005340:11596-11717
HmHel-6	CAEZ01004991:4545-4662
HmHel-6	CAEZ01004754:1929-3213
HmHel-6	CAEZ01004668:1098-1211
HmHel-6	CAEZ01004433:4269-4416
HmHel-6	CAEZ01004272:12739-21599
HmHel-6	CAEZ01004268:7352-9742
HmHel-6	CAEZ01004253:3719-3809
HmHel-6	CAEZ01004253:5168-5286
HmHel-6	CAEZ01004099:267-448
HmHel-6	CAEZ01003817:36094-36259
HmHel-6	CAEZ01003617:6546-6668
HmHel-6	CAEZ01003617:27579-27760
HmHel-6	CAEZ01003617:5042-5136
HmHel-6	CAEZ01003409:2071-2219
HmHel-6	CAEZ01003409:24068-33805
HmHel-6	CAEZ01003409:27187-27269
HmHel-6	CAEZ01003409:1202-1310
HmHel-6	CAEZ01003237:30161-30279
HmHel-6	CAEZ01003237:21959-37648
HmHel-6	CAEZ01003126:2279-2428
HmHel-6	CAEZ01002757:5546-5722
HmHel-6	CAEZ01002757:17419-17598
HmHel-6	CAEZ01002329:12065-12213
HmHel-6	CAEZ01002329:576-740
HmHel-6	CAEZ01002287:213-407
HmHel-6	CAEZ01002207:939-1176
HmHel-6	CAEZ01001521:14605-14783
HmHel-6	CAEZ01001079:5386-5563
HmHel-6	CAEZ01001079:1304-1455
HmHel-6	CAEZ01000628:2709-2862
HmHel-6	CAEZ01000106:740-972
HmHel-6	CAEZ01011569:1148-1376
HmHel-6	CAEZ01010741:12158-12274
HmHel-6	CAEZ01010587:10047-10155
HmHel-6	CAEZ01010587:7971-8073
HmHel-6	CAEZ01010490:1942-2021
HmHel-6	CAEZ01010490:1410-1510
HmHel-6	CAEZ01010419:6103-10389
HmHel-6	CAEZ01009991:28925-29138
HmHel-6	CAEZ01009991:30075-30201
HmHel-6	CAEZ01009991:10311-10475
HmHel-6	CAEZ01009777:20178-26356
HmHel-6	CAEZ01009777:56239-56423
HmHel-6	CAEZ01009777:15067-15162
HmHel-6	CAEZ01009563:6909-8134

HmHel-6	CAEZ01009563:1908-2120
HmHel-6	CAEZ01009427:13126-19734
HmHel-6	CAEZ01009364:12854-12976
HmHel-6	CAEZ01009364:41686-50525
HmHel-6	CAEZ01009364:43271-49342
HmHel-6	CAEZ01009364:23109-23955
HmHel-6	CAEZ01008818:20164-20300
HmHel-6	CAEZ01008797:6485-6639
HmHel-6	CAEZ01008458:6035-6166
HmHel-6	CAEZ01008458:16509-16696
HmHel-6	CAEZ01008225:17137-17320
HmHel-6	CAEZ01008225:3102-4493
HmHel-6	CAEZ01008069:13901-14021
HmHel-6	CAEZ01008001:26530-26653
HmHel-6	CAEZ01008001:36812-38596
HmHel-6	CAEZ01008001:20533-25332
HmHel-6	CAEZ01007949:15485-15688
HmHel-6	CAEZ01007936:170-287
HmHel-6	CAEZ01007841:7479-10021
HmHel-6	CAEZ01007841:6895-7037
HmHel-6	CAEZ01007624:81698-81786
HmHel-6	CAEZ01007597:2373-2470
HmHel-6	CAEZ01007597:19570-19701
HmHel-6	CAEZ01007558:11520-11668
HmHel-6	CAEZ01007558:16239-16910
HmHel-6	CAEZ01007356:1653-1732
HmHel-6	CAEZ01007356:2962-3135
HmHel-6	CAEZ01007226:2170-2366
HmHel-6	CAEZ01007136:9140-9284
HmHel-6	CAEZ01007049:2214-2382
HmHel-6	CAEZ01007016:4050-4138
HmHel-6	CAEZ01006957:5844-6044
HmHel-6	CAEZ01006914:13070-13188
HmHel-6	CAEZ01006867:5098-8068
HmHel-6	CAEZ01006134:9425-9620
HmHel-6	CAEZ01006134:7217-15028
HmHel-6	CAEZ01005845:7624-7712
HmHel-6	CAEZ01005845:35651-35750
HmHel-6	CAEZ01005483:32951-33158
HmHel-6	CAEZ01005483:11695-11781
HmHel-6	CAEZ01005406:10544-10648
HmHel-6	CAEZ01005406:12058-12162
HmHel-6	CAEZ01004919:57-262
HmHel-6	CAEZ01004913:7840-7976
HmHel-6	CAEZ01004817:2653-2749
HmHel-6	CAEZ01004499:30045-30165
HmHel-6	CAEZ01004499:20813-20920
HmHel-6	CAEZ01004358:1521-1611

HmHel-6	CAEZ01004358:17536-18625
HmHel-6	CAEZ01004236:1139-7756
HmHel-6	CAEZ01004236:33269-33369
HmHel-6	CAEZ01004236:21138-21332
HmHel-6	CAEZ01004039:6752-6855
HmHel-6	CAEZ01004039:25280-25464
HmHel-6	CAEZ01004039:21061-24837
HmHel-6	CAEZ01003956:7390-7568
HmHel-6	CAEZ01003903:464-560
HmHel-6	CAEZ01003685:2343-2442
HmHel-6	CAEZ01003668:6824-6936
HmHel-6	CAEZ01003424:37616-37716
HmHel-6	CAEZ01003424:6432-6544
HmHel-6	CAEZ01003132:17989-18100
HmHel-6	CAEZ01002908:3220-3321
HmHel-6	CAEZ01001954:21949-22133
HmHel-6	CAEZ01001954:39017-39174
HmHel-6	CAEZ01001954:8737-8839
HmHel-6	CAEZ01001759:3339-3530
HmHel-6	CAEZ01001759:65-3990
HmHel-6	CAEZ01001320:4617-4782
HmHel-6	CAEZ01000959:2303-2491
HmHel-6	CAEZ01000932:8065-8203
HmHel-6	CAEZ01000932:33994-34114
HmHel-6	CAEZ01000932:8764-16385
HmHel-6	CAEZ01000091:443-590
HmHel-6	CAEZ01000052:2705-2877
HmHel-6	CAEZ01000052:14595-14762
HmHel-6	CAEZ01011434:11774-11870
HmHel-6	CAEZ01011434:4688-4858
HmHel-6	CAEZ01011211:2568-3528
HmHel-6	CAEZ01011184:15678-15821
HmHel-6	CAEZ01010840:942-1055
HmHel-6	CAEZ01010607:2403-2526
HmHel-6	CAEZ01010551:1449-1659
HmHel-6	CAEZ01010551:24870-24955
HmHel-6	CAEZ01009556:6736-6891
HmHel-6	CAEZ01009556:17642-17757
HmHel-6	CAEZ01009166:1781-1880
HmHel-6	CAEZ01008391:9970-10118
HmHel-6	CAEZ01008391:16831-23640
HmHel-6	CAEZ01008283:2237-2324
HmHel-6	CAEZ01008230:8497-8631
HmHel-6	CAEZ01008040:9-172
HmHel-6	CAEZ01007965:1715-4161
HmHel-6	CAEZ01007965:3387-3489
HmHel-6	CAEZ01007890:5835-10611
HmHel-6	CAEZ01007890:14572-14731

HmHel-6	CAEZ01007774:427-7868
HmHel-6	CAEZ01007772:43703-43852
HmHel-6	CAEZ01007772:56258-56339
HmHel-6	CAEZ01007772:32260-32351
HmHel-6	CAEZ01007772:17308-17397
HmHel-6	CAEZ01007378:14191-20753
HmHel-6	CAEZ01007324:12522-12653
HmHel-6	CAEZ01007225:16403-16543
HmHel-6	CAEZ01006946:2708-2794
HmHel-6	CAEZ01006946:4691-4811
HmHel-6	CAEZ01006259:1551-1642
HmHel-6	CAEZ01006234:32920-33030
HmHel-6	CAEZ01006234:28724-28867
HmHel-6	CAEZ01006234:7137-7257
HmHel-6	CAEZ01006227:12365-12564
HmHel-6	CAEZ01006193:4527-4690
HmHel-6	CAEZ01006105:5924-6071
HmHel-6	CAEZ01006008:9698-9905
HmHel-6	CAEZ01005909:29518-32528
HmHel-6	CAEZ01005909:1356-1518
HmHel-6	CAEZ01005894:2963-3122
HmHel-6	CAEZ01005641:22936-23035
HmHel-6	CAEZ01005581:11829-11930
HmHel-6	CAEZ01005581:20115-20267
HmHel-6	CAEZ01005512:22783-22969
HmHel-6	CAEZ01005472:9288-9432
HmHel-6	CAEZ01005472:48162-48247
HmHel-6	CAEZ01005472:15193-15320
HmHel-6	CAEZ01005398:5370-12691
HmHel-6	CAEZ01005353:476-3688
HmHel-6	CAEZ01004964:5665-5817
HmHel-6	CAEZ01004944:3558-3675
HmHel-6	CAEZ01004944:10440-10606
HmHel-6	CAEZ01004873:19884-19979
HmHel-6	CAEZ01004873:15622-15725
HmHel-6	CAEZ01004806:3734-3989
HmHel-6	CAEZ01004720:13579-13714
HmHel-6	CAEZ01004685:1620-11564
HmHel-6	CAEZ01004586:7955-8134
HmHel-6	CAEZ01004496:1150-1344
HmHel-6	CAEZ01004496:61565-61798
HmHel-6	CAEZ01004496:13619-17931
HmHel-6	CAEZ01004367:4667-4746
HmHel-6	CAEZ01004355:17611-17752
HmHel-6	CAEZ01004166:3964-4063
HmHel-6	CAEZ01004163:6798-6885
HmHel-6	CAEZ01004163:12257-12461
HmHel-6	CAEZ01003887:2692-2778

HmHel-6	CAEZ01003887:17918-18105
HmHel-6	CAEZ01003887:17028-17194
HmHel-6	CAEZ01003698:16827-16921
HmHel-6	CAEZ01003649:4161-4349
HmHel-6	CAEZ01003649:11868-11948
HmHel-6	CAEZ01003623:589-716
HmHel-6	CAEZ01003623:2463-2580
HmHel-6	CAEZ01003589:3899-12229
HmHel-6	CAEZ01003589:8375-14353
HmHel-6	CAEZ01003561:4896-5093
HmHel-6	CAEZ01003377:3072-3242
HmHel-6	CAEZ01003377:8850-8999
HmHel-6	CAEZ01003329:1361-1585
HmHel-6	CAEZ01003301:23748-23956
HmHel-6	CAEZ01003301:34631-34742
HmHel-6	CAEZ01003301:20632-20789
HmHel-6	CAEZ01003182:10090-10248
HmHel-6	CAEZ01003180:1464-8588
HmHel-6	CAEZ01002959:1235-3361
HmHel-6	CAEZ01002878:4747-11213
HmHel-6	CAEZ01002878:19817-22834
HmHel-6	CAEZ01002336:8634-8761
HmHel-6	CAEZ01002285:29786-29915
HmHel-6	CAEZ01002285:27417-27540
HmHel-6	CAEZ01002285:16583-16698
HmHel-6	CAEZ01002280:7521-7640
HmHel-6	CAEZ01002122:3178-3297
HmHel-6	CAEZ01001906:5451-8378
HmHel-6	CAEZ01001906:10503-14398
HmHel-6	CAEZ01001859:16435-19708
HmHel-6	CAEZ01001606:5773-5940
HmHel-6	CAEZ01001134:747-903
HmHel-6	CAEZ01001134:2169-2323
HmHel-6	CAEZ01001133:10230-10377
HmHel-6	CAEZ01000955:1-84
HmHel-6	CAEZ01000777:1523-1658
HmHel-6	CAEZ01000098:1111-1218
HmHel-6	CAEZ01000058:2675-6481
HmHel-6	CAEZ01011574:1891-6561
HmHel-6	CAEZ01011564:4499-4582
HmHel-6	CAEZ01011564:21632-21758
HmHel-6	CAEZ01011564:38792-51912
HmHel-6	CAEZ01011564:18741-18889
HmHel-6	CAEZ01011564:7890-8050
HmHel-6	CAEZ01011391:9152-9286
HmHel-6	CAEZ01010886:1707-1842
HmHel-6	CAEZ01010703:61399-61520
HmHel-6	CAEZ01010703:85491-85661

HmHel-6	CAEZ01010703:67487-67722
HmHel-6	CAEZ01010703:9683-9780
HmHel-6	CAEZ01010674:1377-1485
HmHel-6	CAEZ01010674:51-846
HmHel-6	CAEZ01010276:19122-19231
HmHel-6	CAEZ01010276:48677-48821
HmHel-6	CAEZ01010276:8496-17020
HmHel-6	CAEZ01010114:3742-3884
HmHel-6	CAEZ01009861:7778-7904
HmHel-6	CAEZ01009684:23551-23708
HmHel-6	CAEZ01009622:11242-12820
HmHel-6	CAEZ01009450:14200-14287
HmHel-6	CAEZ01009450:18596-28514
HmHel-6	CAEZ01009357:9540-9651
HmHel-6	CAEZ01009320:7471-7587
HmHel-6	CAEZ01009308:7933-8464
HmHel-6	CAEZ01009281:7699-7835
HmHel-6	CAEZ01009281:34475-39800
HmHel-6	CAEZ01009281:12185-12399
HmHel-6	CAEZ01009070:7646-8065
HmHel-6	CAEZ01009070:29346-29475
HmHel-6	CAEZ01008897:621-763
HmHel-6	CAEZ01008775:7048-7158
HmHel-6	CAEZ01008518:22727-22851
HmHel-6	CAEZ01008518:32860-32969
HmHel-6	CAEZ01008518:22281-22383
HmHel-6	CAEZ01008336:793-875
HmHel-6	CAEZ01008199:6580-6722
HmHel-6	CAEZ01008059:4959-5061
HmHel-6	CAEZ01008046:829-1058
HmHel-6	CAEZ01008046:11517-11715
HmHel-6	CAEZ01007788:741-870
HmHel-6	CAEZ01007656:5406-5548
HmHel-6	CAEZ01007656:2663-2894
HmHel-6	CAEZ01007310:20390-20484
HmHel-6	CAEZ01007310:9274-9412
HmHel-6	CAEZ01007092:2616-2735
HmHel-6	CAEZ01007027:3334-3492
HmHel-6	CAEZ01007027:11846-17556
HmHel-6	CAEZ01006728:8187-8325
HmHel-6	CAEZ01006724:8990-9256
HmHel-6	CAEZ01006496:10452-10641
HmHel-6	CAEZ01006496:10811-10908
HmHel-6	CAEZ01006344:5386-5609
HmHel-6	CAEZ01006166:19045-19138
HmHel-6	CAEZ01005408:4144-4254
HmHel-6	CAEZ01005391:12173-12390
HmHel-6	CAEZ01005391:11116-11269

HmHel-6	CAEZ01005281:3151-3258
HmHel-6	CAEZ01005082:7937-8113
HmHel-6	CAEZ01004730:8821-8969
HmHel-6	CAEZ01004532:388-486
HmHel-6	CAEZ01004041:19333-19458
HmHel-6	CAEZ01003901:8319-8438
HmHel-6	CAEZ01003901:4065-4275
HmHel-6	CAEZ01003625:579-709
HmHel-6	CAEZ01003520:2996-3102
HmHel-6	CAEZ01003174:13965-14095
HmHel-6	CAEZ01003174:17309-17417
HmHel-6	CAEZ01003174:2319-2405
HmHel-6	CAEZ01003015:1722-1845
HmHel-6	CAEZ01003015:15462-17691
HmHel-6	CAEZ01003015:22071-22184
HmHel-6	CAEZ01003007:54369-54544
HmHel-6	CAEZ01003007:36284-38635
HmHel-6	CAEZ01003007:24743-26192
HmHel-6	CAEZ01002730:2744-2930
HmHel-6	CAEZ01002499:4908-5006
HmHel-6	CAEZ01002003:8317-8519
HmHel-6	CAEZ01001356:8858-18580
HmHel-6	CAEZ01001356:21944-22072
HmHel-6	CAEZ01000662:1290-1440
HmHel-6	CAEZ01000662:7808-7916
HmHel-6	CAEZ01000551:295-487
HmHel-6	CAEZ01000551:1960-2049
HmHel-6	CAEZ01000542:2548-2658
HmHel-6	CAEZ01000034:1639-4088
HmHel-6	CAEZ01011610:15132-15213
HmHel-6	CAEZ01011509:3415-3603
HmHel-6	CAEZ01011509:13518-14926
HmHel-6	CAEZ01011271:5362-9870
HmHel-6	CAEZ01011252:4657-4896
HmHel-6	CAEZ01011252:17689-17810
HmHel-6	CAEZ01011155:8726-8969
HmHel-6	CAEZ01011155:26907-27096
HmHel-6	CAEZ01011155:46930-47044
HmHel-6	CAEZ01011155:22340-24680
HmHel-6	CAEZ01010843:25958-26086
HmHel-6	CAEZ01010843:8560-14901
HmHel-6	CAEZ01010634:6129-6263
HmHel-6	CAEZ01010166:14613-14690
HmHel-6	CAEZ01010166:1245-1389
HmHel-6	CAEZ01009974:25669-31239
HmHel-6	CAEZ01009974:16769-16917
HmHel-6	CAEZ01009974:409-535
HmHel-6	CAEZ01009819:8955-9124

HmHel-6	CAEZ01009552:4692-4810
HmHel-6	CAEZ01009552:1372-1578
HmHel-6	CAEZ01009296:5557-5640
HmHel-6	CAEZ01009296:17475-17604
HmHel-6	CAEZ01009125:2177-2362
HmHel-6	CAEZ01009125:16945-17051
HmHel-6	CAEZ01009003:4053-4242
HmHel-6	CAEZ01008612:2652-2979
HmHel-6	CAEZ01008612:11065-11150
HmHel-6	CAEZ01008584:2010-2174
HmHel-6	CAEZ01008309:7997-8114
HmHel-6	CAEZ01008012:499-689
HmHel-6	CAEZ01008012:9149-9294
HmHel-6	CAEZ01007964:17155-20752
HmHel-6	CAEZ01007944:10287-10392
HmHel-6	CAEZ01007930:168-273
HmHel-6	CAEZ01007930:18025-18141
HmHel-6	CAEZ01007930:26445-26574
HmHel-6	CAEZ01007837:9689-9772
HmHel-6	CAEZ01007837:8096-8343
HmHel-6	CAEZ01007818:5079-5171
HmHel-6	CAEZ01007818:3489-3586
HmHel-6	CAEZ01007579:1001-1101
HmHel-6	CAEZ01007579:1638-1765
HmHel-6	CAEZ01006793:824-961
HmHel-6	CAEZ01006793:1907-1989
HmHel-6	CAEZ01006623:9505-19539
HmHel-6	CAEZ01006623:22204-22311
HmHel-6	CAEZ01006604:3023-3220
HmHel-6	CAEZ01006528:3712-3821
HmHel-6	CAEZ01006528:6537-6691
HmHel-6	CAEZ01006521:8647-8732
HmHel-6	CAEZ01006503:3122-3259
HmHel-6	CAEZ01006240:1671-4878
HmHel-6	CAEZ01005980:8973-9554
HmHel-6	CAEZ01005980:34219-34415
HmHel-6	CAEZ01005980:1295-1492
HmHel-6	CAEZ01005923:3585-3714
HmHel-6	CAEZ01005671:1930-5757
HmHel-6	CAEZ01005396:2431-2516
HmHel-6	CAEZ01005396:626-10737
HmHel-6	CAEZ01005291:3086-3203
HmHel-6	CAEZ01005273:8566-8686
HmHel-6	CAEZ01005273:17298-21899
HmHel-6	CAEZ01004726:327-443
HmHel-6	CAEZ01004726:3425-3530
HmHel-6	CAEZ01004705:2946-3074
HmHel-6	CAEZ01004276:11914-12060

HmHel-6	CAEZ01004276:27093-27215
HmHel-6	CAEZ01004276:15896-15983
HmHel-6	CAEZ01004274:1349-5988
HmHel-6	CAEZ01004262:7723-7822
HmHel-6	CAEZ01004262:1997-11730
HmHel-6	CAEZ01004082:10358-10451
HmHel-6	CAEZ01004082:20417-23787
HmHel-6	CAEZ01004054:2138-2227
HmHel-6	CAEZ01003723:14597-14773
HmHel-6	CAEZ01003259:5905-6057
HmHel-6	CAEZ01003219:13008-13229
HmHel-6	CAEZ01003219:24444-24560
HmHel-6	CAEZ01003072:1837-1982
HmHel-6	CAEZ01002933:953-2250
HmHel-6	CAEZ01002933:1515-1596
HmHel-6	CAEZ01002658:22505-28438
HmHel-6	CAEZ01002658:33488-33612
HmHel-6	CAEZ01002658:7549-7630
HmHel-6	CAEZ01001827:7383-7513
HmHel-6	CAEZ01001827:1662-1793
HmHel-6	CAEZ01001749:10700-10932
HmHel-6	CAEZ01001749:8-97
HmHel-6	CAEZ01001414:5831-5952
HmHel-6	CAEZ01001225:1862-2039
HmHel-6	CAEZ01000748:439-580
HmHel-6	CAEZ01000032:8698-18113
HmHel-6	CAEZ01000032:25519-25729
HmHel-6	CAEZ01011577:14104-14240
HmHel-6	CAEZ01011577:30577-30768
HmHel-6	CAEZ01011577:5252-5333
HmHel-6	CAEZ01011280:4053-4249
HmHel-6	CAEZ01011168:3436-3544
HmHel-6	CAEZ01010841:5125-5221
HmHel-6	CAEZ01010513:897-1051
HmHel-6	CAEZ01010502:4686-9868
HmHel-6	CAEZ01010397:11540-11668
HmHel-6	CAEZ01010317:15157-15296
HmHel-6	CAEZ01010253:4517-7766
HmHel-6	CAEZ01009907:6439-6641
HmHel-6	CAEZ01009342:18222-18424
HmHel-6	CAEZ01009342:2496-2690
HmHel-6	CAEZ01009312:4374-4486
HmHel-6	CAEZ01009312:3637-3799
HmHel-6	CAEZ01009056:16661-16789
HmHel-6	CAEZ01009056:18181-18322
HmHel-6	CAEZ01008990:13248-13408
HmHel-6	CAEZ01008958:9050-10520
HmHel-6	CAEZ01008946:3270-3381

HmHel-6	CAEZ01008946:1730-6164
HmHel-6	CAEZ01008551:1494-1657
HmHel-6	CAEZ01008363:11426-11656
HmHel-6	CAEZ01007695:3644-3736
HmHel-6	CAEZ01007695:10763-11997
HmHel-6	CAEZ01007671:1687-1771
HmHel-6	CAEZ01007660:24234-24345
HmHel-6	CAEZ01007660:11318-11523
HmHel-6	CAEZ01007631:3508-3618
HmHel-6	CAEZ01007631:52877-53084
HmHel-6	CAEZ01007631:97012-97151
HmHel-6	CAEZ01007631:56449-57931
HmHel-6	CAEZ01007631:12153-12315
HmHel-6	CAEZ01007487:10108-10236
HmHel-6	CAEZ01007302:1674-1755
HmHel-6	CAEZ01007302:18359-18474
HmHel-6	CAEZ01007302:23987-24088
HmHel-6	CAEZ01007302:5961-6061
HmHel-6	CAEZ01006766:1571-1655
HmHel-6	CAEZ01006683:387-569
HmHel-6	CAEZ01006609:2480-2627
HmHel-6	CAEZ01006538:4282-4410
HmHel-6	CAEZ01006407:25844-25991
HmHel-6	CAEZ01006407:9563-9641
HmHel-6	CAEZ01006172:10348-13201
HmHel-6	CAEZ01006172:12412-12499
HmHel-6	CAEZ01006015:8123-8203
HmHel-6	CAEZ01006015:10584-10701
HmHel-6	CAEZ01005943:4655-4751
HmHel-6	CAEZ01005916:1511-3837
HmHel-6	CAEZ01005856:10976-11181
HmHel-6	CAEZ01005856:2956-6344
HmHel-6	CAEZ01005778:5547-5628
HmHel-6	CAEZ01005778:35174-35281
HmHel-6	CAEZ01005778:15056-15164
HmHel-6	CAEZ01004721:11908-12149
HmHel-6	CAEZ01004490:1034-1121
HmHel-6	CAEZ01004414:1375-1590
HmHel-6	CAEZ01004390:2474-7391
HmHel-6	CAEZ01004390:3232-8897
HmHel-6	CAEZ01003961:14260-14342
HmHel-6	CAEZ01003939:3977-7246
HmHel-6	CAEZ01003939:19188-25081
HmHel-6	CAEZ01003939:4744-9458
HmHel-6	CAEZ01003846:6034-7093
HmHel-6	CAEZ01003846:4633-4745
HmHel-6	CAEZ01003697:83-190
HmHel-6	CAEZ01003563:6170-10852

HmHel-6	CAEZ01003368:9214-10558
HmHel-6	CAEZ01003262:4982-5102
HmHel-6	CAEZ01003262:17698-20442
HmHel-6	CAEZ01003177:7146-7302
HmHel-6	CAEZ01003177:5375-5476
HmHel-6	CAEZ01002996:1320-1483
HmHel-6	CAEZ01002909:3044-3144
HmHel-6	CAEZ01002886:951-1076
HmHel-6	CAEZ01002768:7134-7226
HmHel-6	CAEZ01002768:37537-37670
HmHel-6	CAEZ01002768:4769-10170
HmHel-6	CAEZ01002198:8757-8889
HmHel-6	CAEZ01001800:4383-4512
HmHel-6	CAEZ01001800:2007-2119
HmHel-6	CAEZ01001315:8036-8227
HmHel-6	CAEZ01001082:2768-2977
HmHel-6	CAEZ01000798:324-408
HmHel-6	CAEZ01000709:6083-6191
HmHel-6	CAEZ01000671:2185-2285
HmHel-6	CAEZ01000230:19-127
HmHel-6	CAEZ01011621:8055-8155
HmHel-6	CAEZ01011235:4493-7109
HmHel-6	CAEZ01011065:9369-9456
HmHel-6	CAEZ01011065:6730-6878
HmHel-6	CAEZ01011032:4136-8416
HmHel-6	CAEZ01010944:5991-6147
HmHel-6	CAEZ01010616:30550-30643
HmHel-6	CAEZ01010616:38666-38757
HmHel-6	CAEZ01010616:6992-7179
HmHel-6	CAEZ01010373:5639-5769
HmHel-6	CAEZ01010252:58-177
HmHel-6	CAEZ01010252:36963-40526
HmHel-6	CAEZ01009927:3012-3123
HmHel-6	CAEZ01009850:3077-3217
HmHel-6	CAEZ01009666:6639-6774
HmHel-6	CAEZ01009626:39-142
HmHel-6	CAEZ01009478:13958-20331
HmHel-6	CAEZ01009323:1210-1336
HmHel-6	CAEZ01009053:5225-5338
HmHel-6	CAEZ01009053:11217-11331
HmHel-6	CAEZ01008972:2104-3815
HmHel-6	CAEZ01008972:17792-20663
HmHel-6	CAEZ01008802:2923-3042
HmHel-6	CAEZ01008780:8260-8515
HmHel-6	CAEZ01008780:9338-9452
HmHel-6	CAEZ01008738:5889-6027
HmHel-6	CAEZ01008621:39763-49642
HmHel-6	CAEZ01008621:13105-13235

HmHel-6	CAEZ01008614:39703-39806
HmHel-6	CAEZ01008614:5208-5397
HmHel-6	CAEZ01008567:8977-9092
HmHel-6	CAEZ01008481:15741-15909
HmHel-6	CAEZ01008481:12489-12675
HmHel-6	CAEZ01008379:9003-9177
HmHel-6	CAEZ01008379:7449-8301
HmHel-6	CAEZ01008373:26982-27151
HmHel-6	CAEZ01008320:8857-9004
HmHel-6	CAEZ01007434:26815-26946
HmHel-6	CAEZ01007288:6197-6373
HmHel-6	CAEZ01007167:21375-21501
HmHel-6	CAEZ01007167:47704-47787
HmHel-6	CAEZ01006716:4316-4407
HmHel-6	CAEZ01006716:6168-6305
HmHel-6	CAEZ01006629:11653-11745
HmHel-6	CAEZ01006629:13765-13892
HmHel-6	CAEZ01006515:1287-10746
HmHel-6	CAEZ01006515:31102-31302
HmHel-6	CAEZ01006459:10934-11098
HmHel-6	CAEZ01006459:8418-8504
HmHel-6	CAEZ01006192:20637-20875
HmHel-6	CAEZ01006147:2010-2141
HmHel-6	CAEZ01006147:17158-17273
HmHel-6	CAEZ01005898:6016-10226
HmHel-6	CAEZ01005898:38716-40359
HmHel-6	CAEZ01005697:11220-11807
HmHel-6	CAEZ01005639:3582-5654
HmHel-6	CAEZ01005574:348-5477
HmHel-6	CAEZ01005445:5791-13014
HmHel-6	CAEZ01005435:661-821
HmHel-6	CAEZ01005435:6276-6415
HmHel-6	CAEZ01005362:6582-6736
HmHel-6	CAEZ01005326:9826-9918
HmHel-6	CAEZ01005326:5725-5817
HmHel-6	CAEZ01005272:19600-19767
HmHel-6	CAEZ01005272:7998-14122
HmHel-6	CAEZ01005093:13878-14258
HmHel-6	CAEZ01004996:12489-12723
HmHel-6	CAEZ01004996:32672-32756
HmHel-6	CAEZ01004996:3856-5366
HmHel-6	CAEZ01004844:894-1033
HmHel-6	CAEZ01004708:6287-6410
HmHel-6	CAEZ01004708:14181-19100
HmHel-6	CAEZ01004551:19401-21610
HmHel-6	CAEZ01004063:1873-1963
HmHel-6	CAEZ01003704:814-950
HmHel-6	CAEZ01003477:6041-6676

HmHel-6	CAEZ01003366:8294-8416
HmHel-6	CAEZ01002847:5166-5357
HmHel-6	CAEZ01002602:7937-8158
HmHel-6	CAEZ01002243:3681-3868
HmHel-6	CAEZ01002067:540-735
HmHel-6	CAEZ01002060:1054-1169
HmHel-6	CAEZ01002004:1622-1701
HmHel-6	CAEZ01001850:518-9928
HmHel-6	CAEZ01001850:44532-44720
HmHel-6	CAEZ01001850:8218-8391
HmHel-6	CAEZ01000699:5322-5473
HmHel-6	CAEZ01000063:11358-13794
HmHel-6	CAEZ01000063:36573-36664
HmHel-6	CAEZ01011636:5368-5593
HmHel-6	CAEZ01011636:131-234
HmHel-6	CAEZ01011368:5678-5820
HmHel-6	CAEZ01011368:1453-1583
HmHel-6	CAEZ01011233:2128-2234
HmHel-6	CAEZ01011212:306-441
HmHel-6	CAEZ01011212:4185-11739
HmHel-6	CAEZ01011069:21377-21590
HmHel-6	CAEZ01010945:1415-1517
HmHel-6	CAEZ01010876:16006-16203
HmHel-6	CAEZ01010876:18129-18306
HmHel-6	CAEZ01010649:1382-1501
HmHel-6	CAEZ01010649:8767-8873
HmHel-6	CAEZ01010569:4014-4150
HmHel-6	CAEZ01010568:8183-8270
HmHel-6	CAEZ01010568:9330-9527
HmHel-6	CAEZ01010519:973-4227
HmHel-6	CAEZ01010403:5908-6038
HmHel-6	CAEZ01010345:6781-6927
HmHel-6	CAEZ01010298:3592-3701
HmHel-6	CAEZ01010192:1017-1151
HmHel-6	CAEZ01009930:21330-27833
HmHel-6	CAEZ01009930:29688-29774
HmHel-6	CAEZ01009787:7049-7226
HmHel-6	CAEZ01009522:7176-7270
HmHel-6	CAEZ01008793:1108-1209
HmHel-6	CAEZ01008793:34353-34557
HmHel-6	CAEZ01008793:13541-13695
HmHel-6	CAEZ01008728:1168-1322
HmHel-6	CAEZ01008728:29333-33099
HmHel-6	CAEZ01008728:32236-32320
HmHel-6	CAEZ01008728:12240-12418
HmHel-6	CAEZ01008555:5662-5775
HmHel-6	CAEZ01008474:6605-14780
HmHel-6	CAEZ01008213:2066-2262

HmHel-6	CAEZ01008083:503-597
HmHel-6	CAEZ01007759:6130-6279
HmHel-6	CAEZ01007759:2097-2222
HmHel-6	CAEZ01007729:3649-3760
HmHel-6	CAEZ01007679:5864-5962
HmHel-6	CAEZ01007677:10623-10774
HmHel-6	CAEZ01007498:21367-21545
HmHel-6	CAEZ01007498:56525-63064
HmHel-6	CAEZ01007498:32777-32865
HmHel-6	CAEZ01007414:16204-18701
HmHel-6	CAEZ01007376:23267-23357
HmHel-6	CAEZ01007376:37011-37089
HmHel-6	CAEZ01007363:11766-11853
HmHel-6	CAEZ01007363:11068-11217
HmHel-6	CAEZ01007311:21-5545
HmHel-6	CAEZ01007311:6887-7026
HmHel-6	CAEZ01007304:716-874
HmHel-6	CAEZ01007304:2131-2315
HmHel-6	CAEZ01007303:18069-18226
HmHel-6	CAEZ01007303:16721-16948
HmHel-6	CAEZ01007303:2192-2367
HmHel-6	CAEZ01007112:9193-9300
HmHel-6	CAEZ01007018:11489-11578
HmHel-6	CAEZ01007018:10792-10900
HmHel-6	CAEZ01006822:2643-2729
HmHel-6	CAEZ01006822:11602-11684
HmHel-6	CAEZ01006816:1970-6017
HmHel-6	CAEZ01006803:22573-23526
HmHel-6	CAEZ01006803:2742-2840
HmHel-6	CAEZ01006738:14497-14603
HmHel-6	CAEZ01006641:2960-9666
HmHel-6	CAEZ01006641:4610-7559
HmHel-6	CAEZ01006368:7671-7781
HmHel-6	CAEZ01006358:325-435
HmHel-6	CAEZ01006358:7370-7528
HmHel-6	CAEZ01006335:7539-14019
HmHel-6	CAEZ01006335:7292-7484
HmHel-6	CAEZ01006140:4585-4731
HmHel-6	CAEZ01006062:4276-5324
HmHel-6	CAEZ01006062:9654-9736
HmHel-6	CAEZ01006033:20434-20683
HmHel-6	CAEZ01005999:5329-5423
HmHel-6	CAEZ01005999:17205-17328
HmHel-6	CAEZ01005823:15320-15455
HmHel-6	CAEZ01005823:27875-27957
HmHel-6	CAEZ01005563:4354-6698
HmHel-6	CAEZ01005453:12368-12463
HmHel-6	CAEZ01005453:41640-42424

HmHel-6	CAEZ01005453:10295-10459
HmHel-6	CAEZ01005155:16605-16847
HmHel-6	CAEZ01004634:535-720
HmHel-6	CAEZ01004634:9151-9338
HmHel-6	CAEZ01004440:4613-4798
HmHel-6	CAEZ01004440:3854-7632
HmHel-6	CAEZ01004287:15439-15586
HmHel-6	CAEZ01004287:9101-9251
HmHel-6	CAEZ01003969:2558-2762
HmHel-6	CAEZ01003885:21265-21381
HmHel-6	CAEZ01003885:27792-33267
HmHel-6	CAEZ01003494:5103-5213
HmHel-6	CAEZ01003312:13255-13362
HmHel-6	CAEZ01003312:10242-10340
HmHel-6	CAEZ01003264:4686-4803
HmHel-6	CAEZ01003097:11270-11400
HmHel-6	CAEZ01002790:4141-4224
HmHel-6	CAEZ01002790:11469-11591
HmHel-6	CAEZ01002771:18305-18393
HmHel-6	CAEZ01002771:30402-30503
HmHel-6	CAEZ01002771:17910-25112
HmHel-6	CAEZ01002653:9582-9715
HmHel-6	CAEZ01002653:10614-10815
HmHel-6	CAEZ01002640:3253-3422
HmHel-6	CAEZ01002640:294-1589
HmHel-6	CAEZ01002163:1756-1968
HmHel-6	CAEZ01002146:2615-11247
HmHel-6	CAEZ01002089:892-1060
HmHel-6	CAEZ01002064:24358-24441
HmHel-6	CAEZ01002064:22753-32798
HmHel-6	CAEZ01002042:2794-2899
HmHel-6	CAEZ01001846:8819-8901
HmHel-6	CAEZ01001104:1463-1565
HmHel-6	CAEZ01000800:12270-12439
HmHel-6	CAEZ01000739:2489-2575
HmHel-6	CAEZ01000739:2619-2737
HmHel-6	CAEZ01000726:1560-4134
HmHel-6	CAEZ01000726:2557-12674
HmHel-6	CAEZ01000176:1727-1848
HmHel-6	CAEZ01000176:105-203
HmHel-6	CAEZ01000054:2305-2443
HmHel-6	CAEZ01011505:9302-9465
HmHel-6	CAEZ01011181:3890-4019
HmHel-6	CAEZ01011104:14493-14585
HmHel-6	CAEZ01011104:113971-114132
HmHel-6	CAEZ01011104:41955-42048
HmHel-6	CAEZ01011104:5630-13951
HmHel-6	CAEZ01011006:5128-5244

HmHel-6	CAEZ01010957:8061-8312
HmHel-6	CAEZ01010787:11258-18515
HmHel-6	CAEZ01010202:1119-2528
HmHel-6	CAEZ01010136:7304-7401
HmHel-6	CAEZ01010062:1577-1741
HmHel-6	CAEZ01010009:797-903
HmHel-6	CAEZ01009958:7283-7363
HmHel-6	CAEZ01009958:3742-5483
HmHel-6	CAEZ01009890:799-960
HmHel-6	CAEZ01009890:6712-6798
HmHel-6	CAEZ01009637:2687-11153
HmHel-6	CAEZ01009477:2977-3137
HmHel-6	CAEZ01009265:16484-16681
HmHel-6	CAEZ01009265:32089-32311
HmHel-6	CAEZ01009238:1873-2006
HmHel-6	CAEZ01009175:8918-9107
HmHel-6	CAEZ01009175:10668-10762
HmHel-6	CAEZ01009061:6233-6338
HmHel-6	CAEZ01009017:13930-14116
HmHel-6	CAEZ01009017:55519-61277
HmHel-6	CAEZ01009017:22547-22631
HmHel-6	CAEZ01008995:5820-5961
HmHel-6	CAEZ01008807:1699-3476
HmHel-6	CAEZ01008807:10852-10965
HmHel-6	CAEZ01008762:53119-53279
HmHel-6	CAEZ01008762:64878-64988
HmHel-6	CAEZ01008762:2711-2936
HmHel-6	CAEZ01008648:14181-14271
HmHel-6	CAEZ01008648:18392-18493
HmHel-6	CAEZ01008512:50225-50312
HmHel-6	CAEZ01008512:85879-86027
HmHel-6	CAEZ01008512:82412-82589
HmHel-6	CAEZ01008512:45210-45331
HmHel-6	CAEZ01008505:4834-4955
HmHel-6	CAEZ01008388:19209-19373
HmHel-6	CAEZ01008257:9310-10501
HmHel-6	CAEZ01008257:34338-34945
HmHel-6	CAEZ01008257:12405-12500
HmHel-6	CAEZ01008044:13695-13790
HmHel-6	CAEZ01008044:6403-6520
HmHel-6	CAEZ01008006:7474-7581
HmHel-6	CAEZ01008002:634-3194
HmHel-6	CAEZ01007813:9368-9453
HmHel-6	CAEZ01007813:32962-33154
HmHel-6	CAEZ01007473:14175-14271
HmHel-6	CAEZ01007448:1933-2030
HmHel-6	CAEZ01007448:12191-12346
HmHel-6	CAEZ01007359:2660-3812

HmHel-6	CAEZ01007359:6494-13954
HmHel-6	CAEZ01007301:8657-8832
HmHel-6	CAEZ01007301:4607-4768
HmHel-6	CAEZ01007025:5046-5143
HmHel-6	CAEZ01006860:4195-4401
HmHel-6	CAEZ01006800:17648-17834
HmHel-6	CAEZ01006687:12516-13467
HmHel-6	CAEZ01006631:9724-9901
HmHel-6	CAEZ01006592:16565-16651
HmHel-6	CAEZ01006592:58458-58707
HmHel-6	CAEZ01006592:87722-90780
HmHel-6	CAEZ01006592:131390-131613
HmHel-6	CAEZ01006592:121336-126528
HmHel-6	CAEZ01006592:105104-105184
HmHel-6	CAEZ01006592:65610-65720
HmHel-6	CAEZ01006592:17084-18961
HmHel-6	CAEZ01006219:6371-6635
HmHel-6	CAEZ01006046:10400-10528
HmHel-6	CAEZ01005901:4061-4163
HmHel-6	CAEZ01005901:11242-11447
HmHel-6	CAEZ01005709:2986-3108
HmHel-6	CAEZ01005708:21896-22020
HmHel-6	CAEZ01005484:39139-39296
HmHel-6	CAEZ01005484:28603-37307
HmHel-6	CAEZ01005484:1288-1464
HmHel-6	CAEZ01005268:3517-3689
HmHel-6	CAEZ01005259:6149-8248
HmHel-6	CAEZ01005244:5122-5313
HmHel-6	CAEZ01005237:3938-4039
HmHel-6	CAEZ01005209:3864-4002
HmHel-6	CAEZ01005209:4315-7785
HmHel-6	CAEZ01005138:4216-4362
HmHel-6	CAEZ01005138:747-964
HmHel-6	CAEZ01004985:9373-9528
HmHel-6	CAEZ01004946:16120-16201
HmHel-6	CAEZ01004946:28631-28736
HmHel-6	CAEZ01004839:2794-3022
HmHel-6	CAEZ01004336:690-793
HmHel-6	CAEZ01004315:10631-10755
HmHel-6	CAEZ01004311:1918-2037
HmHel-6	CAEZ01004311:5861-5957
HmHel-6	CAEZ01004273:4255-4364
HmHel-6	CAEZ01004273:6661-6786
HmHel-6	CAEZ01004243:10687-10824
HmHel-6	CAEZ01004243:9166-9303
HmHel-6	CAEZ01003867:1496-1605
HmHel-6	CAEZ01003854:6088-6204
HmHel-6	CAEZ01003318:9888-9981

HmHel-6	CAEZ01003318:11568-11682
HmHel-6	CAEZ01002963:14393-14577
HmHel-6	CAEZ01002955:549-793
HmHel-6	CAEZ01002937:1469-1647
HmHel-6	CAEZ01002755:26286-26383
HmHel-6	CAEZ01002755:44677-44810
HmHel-6	CAEZ01002755:38979-39084
HmHel-6	CAEZ01002351:8799-8978
HmHel-6	CAEZ01002238:3825-3979
HmHel-6	CAEZ01002238:609-698
HmHel-6	CAEZ01001424:2990-3175
HmHel-6	CAEZ01001267:392-513
HmHel-6	CAEZ01000710:461-590
HmHel-6	CAEZ01000705:3471-3596
HmHel-6	CAEZ01000575:991-1092
HmHel-6	CAEZ01011129:3223-3323
HmHel-6	CAEZ01011121:392-504
HmHel-6	CAEZ01011074:184-5354
HmHel-6	CAEZ01010891:7102-15761
HmHel-6	CAEZ01010891:56076-56171
HmHel-6	CAEZ01010887:13814-13933
HmHel-6	CAEZ01010814:10649-17286
HmHel-6	CAEZ01010764:6501-6585
HmHel-6	CAEZ01010764:8685-10042
HmHel-6	CAEZ01010653:5578-5686
HmHel-6	CAEZ01010632:6046-6200
HmHel-6	CAEZ01010632:6529-6675
HmHel-6	CAEZ01010620:28060-28280
HmHel-6	CAEZ01010620:162431-162509
HmHel-6	CAEZ01010620:151710-151833
HmHel-6	CAEZ01010620:120517-129386
HmHel-6	CAEZ01010435:15205-19754
HmHel-6	CAEZ01010435:22595-22723
HmHel-6	CAEZ01010149:1764-1911
HmHel-6	CAEZ01010149:13377-13461
HmHel-6	CAEZ01010108:3411-3511
HmHel-6	CAEZ01010108:1151-1243
HmHel-6	CAEZ01009992:1542-1663
HmHel-6	CAEZ01009076:24463-28589
HmHel-6	CAEZ01009076:27874-31366
HmHel-6	CAEZ01009076:5919-6006
HmHel-6	CAEZ01008781:1190-1299
HmHel-6	CAEZ01008453:800-2518
HmHel-6	CAEZ01008421:31719-39060
HmHel-6	CAEZ01008421:49938-50804
HmHel-6	CAEZ01008421:27546-27741
HmHel-6	CAEZ01008085:7668-7828
HmHel-6	CAEZ01007973:9879-9985

HmHel-6	CAEZ01007973:24719-24804
HmHel-6	CAEZ01007973:10980-15510
HmHel-6	CAEZ01007849:30122-36216
HmHel-6	CAEZ01007849:29682-29911
HmHel-6	CAEZ01007849:16536-16627
HmHel-6	CAEZ01007762:3950-4034
HmHel-6	CAEZ01007718:7389-10873
HmHel-6	CAEZ01007718:20921-21094
HmHel-6	CAEZ01007718:6221-6350
HmHel-6	CAEZ01007438:3706-3874
HmHel-6	CAEZ01007423:1473-1670
HmHel-6	CAEZ01007053:2997-3199
HmHel-6	CAEZ01006936:525-764
HmHel-6	CAEZ01006932:12550-12760
HmHel-6	CAEZ01006932:18885-18988
HmHel-6	CAEZ01006859:6009-6208
HmHel-6	CAEZ01006123:7947-8051
HmHel-6	CAEZ01005630:6649-8411
HmHel-6	CAEZ01005628:1093-1177
HmHel-6	CAEZ01005596:9118-9233
HmHel-6	CAEZ01004992:5927-6166
HmHel-6	CAEZ01004900:5991-6151
HmHel-6	CAEZ01004857:26301-26540
HmHel-6	CAEZ01004677:3913-4003
HmHel-6	CAEZ01004677:36803-40151
HmHel-6	CAEZ01004677:80709-86948
HmHel-6	CAEZ01004677:67844-67948
HmHel-6	CAEZ01004677:55090-55210
HmHel-6	CAEZ01004581:286-392
HmHel-6	CAEZ01004493:8400-8562
HmHel-6	CAEZ01004493:7961-8083
HmHel-6	CAEZ01004404:2728-3846
HmHel-6	CAEZ01004404:8966-9077
HmHel-6	CAEZ01004361:9965-10050
HmHel-6	CAEZ01004297:21041-22710
HmHel-6	CAEZ01003972:10404-10529
HmHel-6	CAEZ01003910:6487-6634
HmHel-6	CAEZ01003889:6992-7120
HmHel-6	CAEZ01003695:1037-1145
HmHel-6	CAEZ01003642:3385-3498
HmHel-6	CAEZ01003620:3529-3613
HmHel-6	CAEZ01003590:4725-4873
HmHel-6	CAEZ01003584:1957-2237
HmHel-6	CAEZ01003523:13481-13674
HmHel-6	CAEZ01003468:2279-2391
HmHel-6	CAEZ01003273:1270-6454
HmHel-6	CAEZ01003229:3304-3391
HmHel-6	CAEZ01003164:4663-4796

HmHel-6	CAEZ01003099:4593-4696
HmHel-6	CAEZ01002916:764-887
HmHel-6	CAEZ01002873:2115-2200
HmHel-6	CAEZ01002873:19533-22603
HmHel-6	CAEZ01002873:28189-28356
HmHel-6	CAEZ01002873:12856-13080
HmHel-6	CAEZ01002835:5942-6107
HmHel-6	CAEZ01002835:9173-9265
HmHel-6	CAEZ01002783:2768-12027
HmHel-6	CAEZ01002783:5258-5429
HmHel-6	CAEZ01002756:4260-4348
HmHel-6	CAEZ01002756:5675-5823
HmHel-6	CAEZ01002746:7044-13374
HmHel-6	CAEZ01002746:7455-7622
HmHel-6	CAEZ01002155:37121-37351
HmHel-6	CAEZ01002155:18640-18728
HmHel-6	CAEZ01002058:5450-14819
HmHel-6	CAEZ01002058:4381-11368
HmHel-6	CAEZ01001878:1695-5084
HmHel-6	CAEZ01001878:1847-5056
HmHel-6	CAEZ01001866:3433-3533
HmHel-6	CAEZ01001866:2629-2724
HmHel-6	CAEZ01001739:3331-3480
HmHel-6	CAEZ01001715:4010-4248
HmHel-6	CAEZ01001449:4071-4215
HmHel-6	CAEZ01001416:3437-4919
HmHel-6	CAEZ01001120:309-412
HmHel-6	CAEZ01001097:16928-17015
HmHel-6	CAEZ01001097:23617-23772
HmHel-6	CAEZ01001097:8443-8631
HmHel-6	CAEZ01000602:215-311
HmHel-6	CAEZ01000217:3240-3489
HmHel-6	CAEZ01011514:4013-4112
HmHel-6	CAEZ01011428:4504-6929
HmHel-6	CAEZ01010892:13322-18344
HmHel-6	CAEZ01010892:12286-19626
HmHel-6	CAEZ01010828:2459-6831
HmHel-6	CAEZ01010828:33762-33873
HmHel-6	CAEZ01010798:19526-19626
HmHel-6	CAEZ01010798:23731-23815
HmHel-6	CAEZ01010798:8419-8570
HmHel-6	CAEZ01010744:193-349
HmHel-6	CAEZ01010198:23086-23248
HmHel-6	CAEZ01010198:18904-19032
HmHel-6	CAEZ01010157:22981-23092
HmHel-6	CAEZ01010105:514-595
HmHel-6	CAEZ01009998:11845-12064
HmHel-6	CAEZ01009998:11030-11127

HmHel-6	CAEZ01009990:14820-15066
HmHel-6	CAEZ01009817:5513-14188
HmHel-6	CAEZ01009817:6185-11649
HmHel-6	CAEZ01009741:2538-3178
HmHel-6	CAEZ01009413:339-489
HmHel-6	CAEZ01009346:10154-10298
HmHel-6	CAEZ01009245:1496-1597
HmHel-6	CAEZ01009013:28369-28475
HmHel-6	CAEZ01008810:4060-4200
HmHel-6	CAEZ01008623:3604-3682
HmHel-6	CAEZ01008623:17444-17614
HmHel-6	CAEZ01008623:9513-9630
HmHel-6	CAEZ01008597:6269-6368
HmHel-6	CAEZ01008597:2620-8100
HmHel-6	CAEZ01008422:12235-12350
HmHel-6	CAEZ01008422:20896-23844
HmHel-6	CAEZ01008422:10319-10494
HmHel-6	CAEZ01008340:18230-18329
HmHel-6	CAEZ01008256:10040-13794
HmHel-6	CAEZ01008256:6549-14070
HmHel-6	CAEZ01007856:4089-4222
HmHel-6	CAEZ01007698:16821-23094
HmHel-6	CAEZ01007698:21605-21756
HmHel-6	CAEZ01007470:20326-28147
HmHel-6	CAEZ01007435:18523-27027
HmHel-6	CAEZ01007435:22450-22550
HmHel-6	CAEZ01007435:8167-8369
HmHel-6	CAEZ01007412:4519-4720
HmHel-6	CAEZ01007412:21014-21111
HmHel-6	CAEZ01007364:2137-2248
HmHel-6	CAEZ01007364:837-5260
HmHel-6	CAEZ01006744:8954-9141
HmHel-6	CAEZ01006694:13619-13790
HmHel-6	CAEZ01005902:2778-2937
HmHel-6	CAEZ01005819:11717-11803
HmHel-6	CAEZ01005802:23085-23170
HmHel-6	CAEZ01005802:19175-19282
HmHel-6	CAEZ01005643:22383-31565
HmHel-6	CAEZ01005643:72676-72807
HmHel-6	CAEZ01005643:72058-72230
HmHel-6	CAEZ01005643:24476-24586
HmHel-6	CAEZ01005643:10920-11052
HmHel-6	CAEZ01005621:6398-6533
HmHel-6	CAEZ01005414:21381-21515
HmHel-6	CAEZ01004984:4590-4721
HmHel-6	CAEZ01004731:4217-4362
HmHel-6	CAEZ01004679:11621-11741
HmHel-6	CAEZ01004653:7458-7570

HmHel-6	CAEZ01004653:10696-15945
HmHel-6	CAEZ01004049:758-899
HmHel-6	CAEZ01003818:2161-2345
HmHel-6	CAEZ01003673:5408-5495
HmHel-6	CAEZ01003529:716-3718
HmHel-6	CAEZ01003398:54149-74738
HmHel-6	CAEZ01003398:65269-73904
HmHel-6	CAEZ01003398:38576-44235
HmHel-6	CAEZ01003357:6895-12052
HmHel-6	CAEZ01003193:6727-6854
HmHel-6	CAEZ01003193:31197-36565
HmHel-6	CAEZ01003193:41112-41234
HmHel-6	CAEZ01002901:3346-3439
HmHel-6	CAEZ01002901:5354-5532
HmHel-6	CAEZ01002891:11413-11516
HmHel-6	CAEZ01002714:4031-14031
HmHel-6	CAEZ01002714:22121-22208
HmHel-6	CAEZ01002714:5920-6007
HmHel-6	CAEZ01002177:441-555
HmHel-6	CAEZ01001980:13017-13137
HmHel-6	CAEZ01001892:7338-9179
HmHel-6	CAEZ01001892:35251-36435
HmHel-6	CAEZ01001892:31811-31887
HmHel-6	CAEZ01001892:13403-13587
HmHel-6	CAEZ01001053:19688-25286
HmHel-6	CAEZ01001053:19500-32530
HmHel-6	CAEZ01001033:2989-3095
HmHel-6	CAEZ01000861:2144-2360
HmHel-6	CAEZ01000830:1340-1463
HmHel-6	CAEZ01000735:5993-6105
HmHel-6	CAEZ01000735:5544-5627
HmHel-6	CAEZ01000728:5386-5571
HmHel-6	CAEZ01000728:5980-6135
HmHel-6	CAEZ01000672:1516-1615
HmHel-6	CAEZ01000672:1431-1512
HmHel-6	CAEZ01000544:3355-3540
HmHel-6	CAEZ01000457:1253-1371
HmHel-6	CAEZ01000073:5450-5536
HmHel-6	CAEZ01000073:21608-21786
HmHel-6	CAEZ01000073:1293-1402
HmHel-6	CAEZ01000057:4617-4711
HmHel-6	CAEZ01000043:8713-8796
HmHel-6	CAEZ01000041:30868-30983
HmHel-6	CAEZ01000041:19610-19723
HmHel-6	CAEZ01011589:17821-17927
HmHel-6	CAEZ01011589:1908-2118
HmHel-6	CAEZ01011419:8838-13238
HmHel-6	CAEZ01011419:9933-10045

HmHel-6	CAEZ01011300:8129-8278
HmHel-6	CAEZ01011299:13633-13761
HmHel-6	CAEZ01011284:15694-20487
HmHel-6	CAEZ01011284:29169-36353
HmHel-6	CAEZ01010996:3821-3966
HmHel-6	CAEZ01010847:1388-1479
HmHel-6	CAEZ01010753:8736-14694
HmHel-6	CAEZ01010753:12595-12731
HmHel-6	CAEZ01010299:4010-4178
HmHel-6	CAEZ01010279:15727-21272
HmHel-6	CAEZ01010080:12728-12808
HmHel-6	CAEZ01010080:4621-4795
HmHel-6	CAEZ01009904:2771-2900
HmHel-6	CAEZ01009639:5577-8821
HmHel-6	CAEZ01009433:5820-5972
HmHel-6	CAEZ01009345:2148-4210
HmHel-6	CAEZ01009328:4563-4668
HmHel-6	CAEZ01009328:4060-4166
HmHel-6	CAEZ01008845:6389-6542
HmHel-6	CAEZ01008308:1144-1342
HmHel-6	CAEZ01008307:6642-6841
HmHel-6	CAEZ01008218:24136-24286
HmHel-6	CAEZ01008218:22403-22494
HmHel-6	CAEZ01007806:12006-12090
HmHel-6	CAEZ01007784:2789-2982
HmHel-6	CAEZ01007740:20702-20846
HmHel-6	CAEZ01007740:55208-65244
HmHel-6	CAEZ01007740:77747-77852
HmHel-6	CAEZ01007740:41567-50678
HmHel-6	CAEZ01007596:2998-3088
HmHel-6	CAEZ01007594:2508-9246
HmHel-6	CAEZ01007541:4218-4329
HmHel-6	CAEZ01007158:1289-1455
HmHel-6	CAEZ01007153:4425-4555
HmHel-6	CAEZ01006780:7866-7946
HmHel-6	CAEZ01006780:9340-11066
HmHel-6	CAEZ01006255:6618-6717
HmHel-6	CAEZ01006255:494-581
HmHel-6	CAEZ01006169:9866-9971
HmHel-6	CAEZ01006169:22883-22965
HmHel-6	CAEZ01006169:23027-23151
HmHel-6	CAEZ01006099:25654-25772
HmHel-6	CAEZ01006099:20862-21541
HmHel-6	CAEZ01006028:10619-14708
HmHel-6	CAEZ01005490:5830-5924
HmHel-6	CAEZ01005374:7-101
HmHel-6	CAEZ01005247:17900-18094
HmHel-6	CAEZ01005172:25847-25969

HmHel-6	CAEZ01005052:6202-6431
HmHel-6	CAEZ01004970:3802-3920
HmHel-6	CAEZ01004833:16381-19161
HmHel-6	CAEZ01004833:17470-17564
HmHel-6	CAEZ01004725:16652-16754
HmHel-6	CAEZ01004570:4297-4392
HmHel-6	CAEZ01004570:2675-2801
HmHel-6	CAEZ01004269:5289-5378
HmHel-6	CAEZ01004269:9103-9245
HmHel-6	CAEZ01003511:2397-2480
HmHel-6	CAEZ01003483:9630-18219
HmHel-6	CAEZ01003440:4543-4633
HmHel-6	CAEZ01003253:3416-3514
HmHel-6	CAEZ01003041:5332-5462
HmHel-6	CAEZ01002735:19821-20014
HmHel-6	CAEZ01002735:5994-6132
HmHel-6	CAEZ01002376:17945-30021
HmHel-6	CAEZ01002376:3997-8309
HmHel-6	CAEZ01002274:5257-13986
HmHel-6	CAEZ01002274:8035-8152
HmHel-6	CAEZ01001407:34-140
HmHel-6	CAEZ01001407:305-410
HmHel-6	CAEZ01000839:27837-27955
HmHel-6	CAEZ01000591:3411-3493
HmHel-6	CAEZ01000547:7515-7677
HmHel-6	CAEZ01000443:338-2405
HmHel-6	CAEZ01011511:803-900
HmHel-6	CAEZ01011499:2504-2614
HmHel-6	CAEZ01011483:849-2928
HmHel-6	CAEZ01011477:20232-20325
HmHel-6	CAEZ01011477:8829-17849
HmHel-6	CAEZ01011416:17982-18115
HmHel-6	CAEZ01011416:43496-47483
HmHel-6	CAEZ01011416:91886-91985
HmHel-6	CAEZ01011416:74192-74310
HmHel-6	CAEZ01011405:1229-5351
HmHel-6	CAEZ01011342:7686-7870
HmHel-6	CAEZ01010877:13879-14099
HmHel-6	CAEZ01010877:16290-16391
HmHel-6	CAEZ01010819:4916-5065
HmHel-6	CAEZ01010750:2590-2736
HmHel-6	CAEZ01010592:481-1012
HmHel-6	CAEZ01010553:10832-10915
HmHel-6	CAEZ01010549:1481-1585
HmHel-6	CAEZ01010549:14768-14873
HmHel-6	CAEZ01010479:9660-9745
HmHel-6	CAEZ01010421:110-7039
HmHel-6	CAEZ01010415:23961-27952

HmHel-6	CAEZ01010415:6940-7730
HmHel-6	CAEZ01010004:30797-30878
HmHel-6	CAEZ01010004:8899-8987
HmHel-6	CAEZ01009957:7957-8046
HmHel-6	CAEZ01009957:5990-8868
HmHel-6	CAEZ01009789:9501-9646
HmHel-6	CAEZ01009756:1214-1362
HmHel-6	CAEZ01009728:2888-3760
HmHel-6	CAEZ01009674:8975-9217
HmHel-6	CAEZ01009674:2625-2737
HmHel-6	CAEZ01009651:3019-3212
HmHel-6	CAEZ01009651:6111-6672
HmHel-6	CAEZ01009426:3281-3374
HmHel-6	CAEZ01009407:5934-6059
HmHel-6	CAEZ01009407:9821-9965
HmHel-6	CAEZ01009315:3941-4146
HmHel-6	CAEZ01009204:21338-22712
HmHel-6	CAEZ01009185:6065-6270
HmHel-6	CAEZ01009114:15449-15562
HmHel-6	CAEZ01009001:6020-6113
HmHel-6	CAEZ01008685:618-748
HmHel-6	CAEZ01008685:9912-10052
HmHel-6	CAEZ01008540:10058-10140
HmHel-6	CAEZ01008540:26432-26569
HmHel-6	CAEZ01008485:6055-9408
HmHel-6	CAEZ01008337:16366-16456
HmHel-6	CAEZ01008337:40103-40203
HmHel-6	CAEZ01008229:3616-7214
HmHel-6	CAEZ01008229:4421-14470
HmHel-6	CAEZ01007761:8350-8535
HmHel-6	CAEZ01007761:35824-35983
HmHel-6	CAEZ01007583:4045-4167
HmHel-6	CAEZ01007469:6220-9733
HmHel-6	CAEZ01007469:70011-70167
HmHel-6	CAEZ01007373:20171-21447
HmHel-6	CAEZ01007318:3541-3654
HmHel-6	CAEZ01007240:255-360
HmHel-6	CAEZ01007132:673-766
HmHel-6	CAEZ01007132:2439-2553
HmHel-6	CAEZ01007124:57466-58831
HmHel-6	CAEZ01007124:74265-74348
HmHel-6	CAEZ01007124:111604-111698
HmHel-6	CAEZ01007124:97460-97589
HmHel-6	CAEZ01007031:12188-12345
HmHel-6	CAEZ01006894:8867-8968
HmHel-6	CAEZ01006851:1162-1251
HmHel-6	CAEZ01006680:13310-13391
HmHel-6	CAEZ01006630:3453-3704

HmHel-6	CAEZ01006621:2186-2283
HmHel-6	CAEZ01006621:10394-10551
HmHel-6	CAEZ01006495:38263-42345
HmHel-6	CAEZ01006495:18443-18530
HmHel-6	CAEZ01006482:8872-8953
HmHel-6	CAEZ01006482:9346-9533
HmHel-6	CAEZ01006373:4030-4119
HmHel-6	CAEZ01006373:14916-15021
HmHel-6	CAEZ01006189:9195-10993
HmHel-6	CAEZ01006189:5069-8154
HmHel-6	CAEZ01006120:11563-11744
HmHel-6	CAEZ01006120:41379-41497
HmHel-6	CAEZ01006120:526-631
HmHel-6	CAEZ01005518:10192-10348
HmHel-6	CAEZ01005450:5569-5677
HmHel-6	CAEZ01005386:7148-10641
HmHel-6	CAEZ01005386:22363-22449
HmHel-6	CAEZ01005386:13659-13871
HmHel-6	CAEZ01005365:6167-6305
HmHel-6	CAEZ01005187:4638-4761
HmHel-6	CAEZ01005143:5629-5764
HmHel-6	CAEZ01004895:698-829
HmHel-6	CAEZ01004888:14422-14525
HmHel-6	CAEZ01004624:9221-9325
HmHel-6	CAEZ01004503:2999-3087
HmHel-6	CAEZ01004391:5065-5188
HmHel-6	CAEZ01004305:2626-2781
HmHel-6	CAEZ01004305:5282-5370
HmHel-6	CAEZ01003940:1560-1678
HmHel-6	CAEZ01003940:13935-14088
HmHel-6	CAEZ01003764:12027-12120
HmHel-6	CAEZ01003688:1156-1245
HmHel-6	CAEZ01003586:3676-3789
HmHel-6	CAEZ01003206:17008-17118
HmHel-6	CAEZ01003206:2521-2692
HmHel-6	CAEZ01003027:1882-1975
HmHel-6	CAEZ01002890:31425-31562
HmHel-6	CAEZ01002890:11891-21677
HmHel-6	CAEZ01002652:4203-4369
HmHel-6	CAEZ01002652:12405-12529
HmHel-6	CAEZ01002647:706-851
HmHel-6	CAEZ01002580:1857-2053
HmHel-6	CAEZ01002580:2223-2328
HmHel-6	CAEZ01002242:7673-7766
HmHel-6	CAEZ01002242:1531-10381
HmHel-6	CAEZ01002005:850-955
HmHel-6	CAEZ01001821:18244-18345
HmHel-6	CAEZ01001821:25507-25623

HmHel-6	CAEZ01001821:13132-13306
HmHel-6	CAEZ01001775:19932-20062
HmHel-6	CAEZ01001649:629-726
HmHel-6	CAEZ01001513:856-961
HmHel-6	CAEZ01001188:449-554
HmHel-6	CAEZ01001115:11261-11387
HmHel-6	CAEZ01001115:3632-3744
HmHel-6	CAEZ01001018:639-770
HmHel-6	CAEZ01001018:4125-4274
HmHel-6	CAEZ01000942:4319-4408
HmHel-6	CAEZ01000687:477-570
HmHel-6	CAEZ01000476:272-369
HmHel-6	CAEZ01000147:326-408
HmHel-6	CAEZ01011632:773-853
HmHel-6	CAEZ01011328:1580-1660
HmHel-6	CAEZ01011328:2508-2592
HmHel-6	CAEZ01010765:7736-7926
HmHel-6	CAEZ01010765:4876-5093
HmHel-6	CAEZ01010758:12413-14927
HmHel-6	CAEZ01010735:7968-11555
HmHel-6	CAEZ01010735:14510-14671
HmHel-6	CAEZ01010673:2753-2973
HmHel-6	CAEZ01010550:9803-9993
HmHel-6	CAEZ01010416:14293-16462
HmHel-6	CAEZ01010155:221-309
HmHel-6	CAEZ01010082:9562-9754
HmHel-6	CAEZ01010082:4037-4121
HmHel-6	CAEZ01009879:21770-21967
HmHel-6	CAEZ01009879:2657-8547
HmHel-6	CAEZ01009597:2715-2795
HmHel-6	CAEZ01009597:7952-8084
HmHel-6	CAEZ01009546:4252-4331
HmHel-6	CAEZ01009546:21362-24049
HmHel-6	CAEZ01009460:32749-32978
HmHel-6	CAEZ01009460:5668-5825
HmHel-6	CAEZ01009389:6727-6850
HmHel-6	CAEZ01009384:16474-16627
HmHel-6	CAEZ01009176:7073-7191
HmHel-6	CAEZ01008952:1071-1263
HmHel-6	CAEZ01008952:24502-29053
HmHel-6	CAEZ01008952:31478-31603
HmHel-6	CAEZ01008937:1827-1927
HmHel-6	CAEZ01008937:10818-14523
HmHel-6	CAEZ01008749:14875-15020
HmHel-6	CAEZ01008643:29083-29214
HmHel-6	CAEZ01008643:11422-11580
HmHel-6	CAEZ01008401:3897-7694
HmHel-6	CAEZ01008401:9007-9105

HmHel-6	CAEZ01008301:11002-18434
HmHel-6	CAEZ01008301:10254-12394
HmHel-6	CAEZ01008196:18179-21047
HmHel-6	CAEZ01008196:34649-34772
HmHel-6	CAEZ01008146:2545-2637
HmHel-6	CAEZ01008118:4598-4787
HmHel-6	CAEZ01008106:2972-3079
HmHel-6	CAEZ01008106:16477-18291
HmHel-6	CAEZ01008106:3803-3916
HmHel-6	CAEZ01008087:10051-10208
HmHel-6	CAEZ01008087:26665-26830
HmHel-6	CAEZ01008087:32242-32369
HmHel-6	CAEZ01008087:12903-14852
HmHel-6	CAEZ01008050:37335-37427
HmHel-6	CAEZ01008050:82925-83083
HmHel-6	CAEZ01008050:5215-5307
HmHel-6	CAEZ01007831:2456-2540
HmHel-6	CAEZ01007459:15417-15627
HmHel-6	CAEZ01006935:5917-6093
HmHel-6	CAEZ01006824:4798-4878
HmHel-6	CAEZ01006409:27985-28121
HmHel-6	CAEZ01006409:71874-72089
HmHel-6	CAEZ01006409:92728-92837
HmHel-6	CAEZ01006409:84675-84765
HmHel-6	CAEZ01006409:35637-37624
HmHel-6	CAEZ01006202:19045-19188
HmHel-6	CAEZ01005792:1354-1534
HmHel-6	CAEZ01005645:12215-12299
HmHel-6	CAEZ01005645:36171-36257
HmHel-6	CAEZ01005645:17331-17484
HmHel-6	CAEZ01005355:9777-9870
HmHel-6	CAEZ01004816:17522-18184
HmHel-6	CAEZ01004816:63461-67648
HmHel-6	CAEZ01004816:107733-107820
HmHel-6	CAEZ01004816:88541-88706
HmHel-6	CAEZ01004816:38695-39994
HmHel-6	CAEZ01004739:6566-15310
HmHel-6	CAEZ01004739:28536-28621
HmHel-6	CAEZ01004739:24337-27244
HmHel-6	CAEZ01004739:11168-11382
HmHel-6	CAEZ01004502:381-520
HmHel-6	CAEZ01004085:8131-8215
HmHel-6	CAEZ01004053:3433-3521
HmHel-6	CAEZ01003998:21921-22671
HmHel-6	CAEZ01003868:19537-20507
HmHel-6	CAEZ01003372:2092-3601
HmHel-6	CAEZ01003042:7732-7820
HmHel-6	CAEZ01003025:2855-3035

HmHel-6	CAEZ01003025:5919-6009
HmHel-6	CAEZ01002894:6807-6914
HmHel-6	CAEZ01002788:29066-29161
HmHel-6	CAEZ01002788:3907-3991
HmHel-6	CAEZ01002713:1785-1917
HmHel-6	CAEZ01002713:20895-36503
HmHel-6	CAEZ01002713:19983-20102
HmHel-6	CAEZ01002713:9059-9161
HmHel-6	CAEZ01002663:5535-6461
HmHel-6	CAEZ01002298:1124-1220
HmHel-6	CAEZ01002047:11431-11534
HmHel-6	CAEZ01002047:21200-21288
HmHel-6	CAEZ01002047:9817-9908
HmHel-6	CAEZ01001896:431-581
HmHel-6	CAEZ01001550:11156-11279
HmHel-6	CAEZ01001276:15881-16122
HmHel-6	CAEZ01001196:41208-48773
HmHel-6	CAEZ01001196:36114-36196
HmHel-6	CAEZ01001196:6055-6139
HmHel-6	CAEZ01000917:1879-1971
HmHel-6	CAEZ01000680:5781-5925
HmHel-6	CAEZ01000441:309-417
HmHel-6	CAEZ01011625:5702-5816
HmHel-6	CAEZ01011585:1544-1644
HmHel-6	CAEZ01011479:3858-4077
HmHel-6	CAEZ01011375:10437-10605
HmHel-6	CAEZ01011367:24516-24646
HmHel-6	CAEZ01011241:9366-9461
HmHel-6	CAEZ01011177:2429-6204
HmHel-6	CAEZ01011127:4901-5059
HmHel-6	CAEZ01011062:10947-11140
HmHel-6	CAEZ01011024:14163-14262
HmHel-6	CAEZ01011003:4106-4222
HmHel-6	CAEZ01010936:15276-15513
HmHel-6	CAEZ01010936:10510-10603
HmHel-6	CAEZ01010718:7910-14469
HmHel-6	CAEZ01010630:5258-5348
HmHel-6	CAEZ01010627:9184-11543
HmHel-6	CAEZ01010627:12705-12913
HmHel-6	CAEZ01010593:6375-6475
HmHel-6	CAEZ01010547:6489-6580
HmHel-6	CAEZ01010443:4297-4388
HmHel-6	CAEZ01010355:16298-16406
HmHel-6	CAEZ01010355:26699-26853
HmHel-6	CAEZ01010355:28924-43775
HmHel-6	CAEZ01010321:7392-7571
HmHel-6	CAEZ01010239:4435-4518
HmHel-6	CAEZ01010239:29553-38929

HmHel-6	CAEZ01010019:7942-8027
HmHel-6	CAEZ01010019:9676-9841
HmHel-6	CAEZ01009978:17420-21188
HmHel-6	CAEZ01009978:43263-47960
HmHel-6	CAEZ01009978:14851-21780
HmHel-6	CAEZ01009857:333-474
HmHel-6	CAEZ01009571:11581-11676
HmHel-6	CAEZ01009497:22736-22859
HmHel-6	CAEZ01009179:5382-5481
HmHel-6	CAEZ01009171:12192-12336
HmHel-6	CAEZ01008725:17812-17929
HmHel-6	CAEZ01008725:11530-11669
HmHel-6	CAEZ01008631:47926-48122
HmHel-6	CAEZ01008631:26808-26935
HmHel-6	CAEZ01008595:1264-2441
HmHel-6	CAEZ01008595:2170-2305
HmHel-6	CAEZ01008509:12976-18282
HmHel-6	CAEZ01008509:7266-7444
HmHel-6	CAEZ01008415:24663-24780
HmHel-6	CAEZ01008415:1735-1895
HmHel-6	CAEZ01008389:1290-1380
HmHel-6	CAEZ01008389:5180-5270
HmHel-6	CAEZ01008233:13611-13766
HmHel-6	CAEZ01008233:32507-32619
HmHel-6	CAEZ01008233:76917-77026
HmHel-6	CAEZ01008233:74978-84806
HmHel-6	CAEZ01008233:2036-2115
HmHel-6	CAEZ01008115:25023-25129
HmHel-6	CAEZ01008115:27270-27365
HmHel-6	CAEZ01007685:8842-9771
HmHel-6	CAEZ01007685:75514-75686
HmHel-6	CAEZ01007685:46944-52419
HmHel-6	CAEZ01007685:16715-16870
HmHel-6	CAEZ01007576:12183-15904
HmHel-6	CAEZ01007576:39184-39273
HmHel-6	CAEZ01007443:1819-1991
HmHel-6	CAEZ01007369:4344-4465
HmHel-6	CAEZ01007171:846-962
HmHel-6	CAEZ01007126:902-989
HmHel-6	CAEZ01007085:10571-10674
HmHel-6	CAEZ01007085:99-280
HmHel-6	CAEZ01006559:18162-22274
HmHel-6	CAEZ01006514:15092-15203
HmHel-6	CAEZ01006449:15276-15399
HmHel-6	CAEZ01006449:12701-12894
HmHel-6	CAEZ01006385:8297-10451
HmHel-6	CAEZ01006321:517-599
HmHel-6	CAEZ01006224:15918-19566

HmHel-6	CAEZ01006089:8267-8380
HmHel-6	CAEZ01006089:6022-6116
HmHel-6	CAEZ01005753:369-526
HmHel-6	CAEZ01005481:26315-26396
HmHel-6	CAEZ01005481:39210-39339
HmHel-6	CAEZ01005481:63118-63232
HmHel-6	CAEZ01005481:93163-93346
HmHel-6	CAEZ01005481:12610-15145
HmHel-6	CAEZ01005315:1007-1135
HmHel-6	CAEZ01005167:3078-3256
HmHel-6	CAEZ01004997:10421-10558
HmHel-6	CAEZ01004997:4193-4312
HmHel-6	CAEZ01004933:6761-6876
HmHel-6	CAEZ01004933:3163-11063
HmHel-6	CAEZ01004878:1050-1185
HmHel-6	CAEZ01004756:6586-6769
HmHel-6	CAEZ01004724:18384-18474
HmHel-6	CAEZ01004704:3066-5127
HmHel-6	CAEZ01004693:6533-6659
HmHel-6	CAEZ01004693:1164-1310
HmHel-6	CAEZ01004622:8760-8843
HmHel-6	CAEZ01004622:7382-7513
HmHel-6	CAEZ01004345:10435-10533
HmHel-6	CAEZ01004284:15515-20684
HmHel-6	CAEZ01004196:10898-11045
HmHel-6	CAEZ01004159:5847-6036
HmHel-6	CAEZ01004040:6207-6365
HmHel-6	CAEZ01003975:10746-10878
HmHel-6	CAEZ01003866:7681-7826
HmHel-6	CAEZ01003521:3722-4370
HmHel-6	CAEZ01003521:3376-3519
HmHel-6	CAEZ01003356:1481-1568
HmHel-6	CAEZ01003136:792-923
HmHel-6	CAEZ01002827:6220-6395
HmHel-6	CAEZ01002827:5278-5375
HmHel-6	CAEZ01002807:38641-38745
HmHel-6	CAEZ01002807:4008-4145
HmHel-6	CAEZ01002758:1228-1349
HmHel-6	CAEZ01002758:35538-35617
HmHel-6	CAEZ01002758:9367-9585
HmHel-6	CAEZ01002645:2486-2667
HmHel-6	CAEZ01001973:4098-4213
HmHel-6	CAEZ01001761:683-790
HmHel-6	CAEZ01001731:9356-9519
HmHel-6	CAEZ01001160:4152-9109
HmHel-6	CAEZ01001160:14102-20988
HmHel-6	CAEZ01001159:4118-6978
HmHel-6	CAEZ01000859:5389-5526

HmHel-6	CAEZ01000664:2724-2847
HmHel-6	CAEZ01000622:1058-1239
HmHel-6	CAEZ01000564:2043-2154
HmHel-6	CAEZ01000564:5288-5379
HmHel-6	CAEZ01011301:7796-7956
HmHel-6	CAEZ01011301:11139-11234
HmHel-6	CAEZ01011286:10346-10456
HmHel-6	CAEZ01011286:8499-8595
HmHel-6	CAEZ01011207:7856-8300
HmHel-6	CAEZ01011207:7048-7165
HmHel-6	CAEZ01011049:1635-1726
HmHel-6	CAEZ01011049:43891-43983
HmHel-6	CAEZ01011049:62837-62999
HmHel-6	CAEZ01010984:222-368
HmHel-6	CAEZ01010976:3383-4567
HmHel-6	CAEZ01010976:1234-1326
HmHel-6	CAEZ01010878:4959-5049
HmHel-6	CAEZ01010710:17474-17572
HmHel-6	CAEZ01010583:12194-12297
HmHel-6	CAEZ01010583:25640-25776
HmHel-6	CAEZ01010398:29340-29456
HmHel-6	CAEZ01010398:21724-21812
HmHel-6	CAEZ01010395:948-3448
HmHel-6	CAEZ01010395:14347-14497
HmHel-6	CAEZ01010250:8427-14541
HmHel-6	CAEZ01010063:1286-1391
HmHel-6	CAEZ01009986:23113-25605
HmHel-6	CAEZ01009913:696-779
HmHel-6	CAEZ01009913:10788-10874
HmHel-6	CAEZ01009913:7005-7138
HmHel-6	CAEZ01009865:28-129
HmHel-6	CAEZ01009865:394-483
HmHel-6	CAEZ01009739:717-835
HmHel-6	CAEZ01009739:6037-6190
HmHel-6	CAEZ01009510:5603-14943
HmHel-6	CAEZ01009510:1657-1747
HmHel-6	CAEZ01009490:911-2794
HmHel-6	CAEZ01009434:2226-2409
HmHel-6	CAEZ01009434:3293-3378
HmHel-6	CAEZ01009302:32459-32553
HmHel-6	CAEZ01009218:921-1014
HmHel-6	CAEZ01009218:18301-21402
HmHel-6	CAEZ01009135:22749-30211
HmHel-6	CAEZ01009135:43795-43878
HmHel-6	CAEZ01009135:17249-17468
HmHel-6	CAEZ01009049:2385-7032
HmHel-6	CAEZ01008945:1588-1669
HmHel-6	CAEZ01008945:35649-35796

HmHel-6	CAEZ01008945:28129-28231
HmHel-6	CAEZ01008932:6299-6496
HmHel-6	CAEZ01008932:7021-7104
HmHel-6	CAEZ01008844:19605-19747
HmHel-6	CAEZ01008784:17515-17642
HmHel-6	CAEZ01008530:7989-8759
HmHel-6	CAEZ01008530:448-530
HmHel-6	CAEZ01008067:8814-8920
HmHel-6	CAEZ01007805:17337-17532
HmHel-6	CAEZ01007621:11547-11644
HmHel-6	CAEZ01007328:4850-8950
HmHel-6	CAEZ01007328:6660-6750
HmHel-6	CAEZ01007065:3657-3779
HmHel-6	CAEZ01006967:9621-9754
HmHel-6	CAEZ01006924:2227-2392
HmHel-6	CAEZ01006895:1585-1714
HmHel-6	CAEZ01006893:19802-19912
HmHel-6	CAEZ01006271:12360-12442
HmHel-6	CAEZ01006138:45676-45790
HmHel-6	CAEZ01006138:9601-9687
HmHel-6	CAEZ01006034:4417-4572
HmHel-6	CAEZ01006034:7225-15586
HmHel-6	CAEZ01006007:11571-11653
HmHel-6	CAEZ01006007:15208-15330
HmHel-6	CAEZ01005847:19548-19797
HmHel-6	CAEZ01005847:18463-18562
HmHel-6	CAEZ01005815:30625-30814
HmHel-6	CAEZ01005655:669-830
HmHel-6	CAEZ01005602:9624-9747
HmHel-6	CAEZ01005532:1293-1403
HmHel-6	CAEZ01005488:5416-5502
HmHel-6	CAEZ01005421:34346-34444
HmHel-6	CAEZ01005295:2041-2171
HmHel-6	CAEZ01005126:3243-3426
HmHel-6	CAEZ01005042:7252-7364
HmHel-6	CAEZ01005018:484-563
HmHel-6	CAEZ01005018:1701-1873
HmHel-6	CAEZ01004663:3427-3613
HmHel-6	CAEZ01004515:5493-5580
HmHel-6	CAEZ01004515:40927-41112
HmHel-6	CAEZ01004515:223-328
HmHel-6	CAEZ01004426:4426-4572
HmHel-6	CAEZ01004385:6282-6380
HmHel-6	CAEZ01004385:6914-7033
HmHel-6	CAEZ01004351:1981-2095
HmHel-6	CAEZ01004266:4908-5490
HmHel-6	CAEZ01004229:7379-7505
HmHel-6	CAEZ01004229:3538-6685

HmHel-6	CAEZ01004201:1257-1363
HmHel-6	CAEZ01004136:120-239
HmHel-6	CAEZ01003931:5035-10015
HmHel-6	CAEZ01003931:2964-3058
HmHel-6	CAEZ01003809:368-1452
HmHel-6	CAEZ01003613:8262-8360
HmHel-6	CAEZ01003613:11732-15756
HmHel-6	CAEZ01003532:6609-6699
HmHel-6	CAEZ01003505:1954-2174
HmHel-6	CAEZ01003251:499-689
HmHel-6	CAEZ01003179:4366-9596
HmHel-6	CAEZ01003179:7876-13095
HmHel-6	CAEZ01003152:25026-25147
HmHel-6	CAEZ01003145:795-7538
HmHel-6	CAEZ01003145:5228-5322
HmHel-6	CAEZ01003137:3777-3985
HmHel-6	CAEZ01003137:21094-21180
HmHel-6	CAEZ01003137:5747-5831
HmHel-6	CAEZ01003096:10941-19358
HmHel-6	CAEZ01003096:22290-22468
HmHel-6	CAEZ01003092:980-1086
HmHel-6	CAEZ01002912:404-531
HmHel-6	CAEZ01002253:2111-2246
HmHel-6	CAEZ01002253:3703-22262
HmHel-6	CAEZ01002166:1647-1755
HmHel-6	CAEZ01002166:19950-20110
HmHel-6	CAEZ01002164:10099-10273
HmHel-6	CAEZ01002164:5562-5696
HmHel-6	CAEZ01001562:10071-10190
HmHel-6	CAEZ01001426:6554-6774
HmHel-6	CAEZ01001426:1204-1314
HmHel-6	CAEZ01001289:15420-15549
HmHel-6	CAEZ01001155:480-624
HmHel-6	CAEZ01000860:15365-17499
HmHel-6	CAEZ01000860:1434-1596
HmHel-6	CAEZ01000658:2556-2682
HmHel-6	CAEZ01000548:1605-1732
HmHel-6	CAEZ01000071:13109-13219
HmHel-6	CAEZ01011570:3927-4070
HmHel-6	CAEZ01011520:1189-1286
HmHel-6	CAEZ01011498:1348-1433
HmHel-6	CAEZ01011325:1286-1403
HmHel-6	CAEZ01011244:5149-6174
HmHel-6	CAEZ01011244:3214-3318
HmHel-6	CAEZ01011238:3154-9140
HmHel-6	CAEZ01011151:24114-24201
HmHel-6	CAEZ01010846:4660-4752
HmHel-6	CAEZ01010768:8521-8683

HmHel-6	CAEZ01010768:2901-12729
HmHel-6	CAEZ01010604:3038-3191
HmHel-6	CAEZ01010578:2503-2596
HmHel-6	CAEZ01010578:13101-13338
HmHel-6	CAEZ01009834:2639-6790
HmHel-6	CAEZ01009620:1591-1720
HmHel-6	CAEZ01009620:5065-5175
HmHel-6	CAEZ01009604:4994-13449
HmHel-6	CAEZ01009491:4599-4684
HmHel-6	CAEZ01009491:1019-7533
HmHel-6	CAEZ01009418:1477-1582
HmHel-6	CAEZ01009380:5226-5361
HmHel-6	CAEZ01009380:25597-25690
HmHel-6	CAEZ01009380:50198-50298
HmHel-6	CAEZ01009380:87316-87391
HmHel-6	CAEZ01009380:73208-73434
HmHel-6	CAEZ01009380:46080-46212
HmHel-6	CAEZ01009380:11041-11188
HmHel-6	CAEZ01009080:826-987
HmHel-6	CAEZ01009074:4100-4314
HmHel-6	CAEZ01009010:23976-24057
HmHel-6	CAEZ01009010:16409-16781
HmHel-6	CAEZ01008973:4445-4610
HmHel-6	CAEZ01008973:16123-16226
HmHel-6	CAEZ01008626:6814-6899
HmHel-6	CAEZ01008432:6553-6682
HmHel-6	CAEZ01008355:7973-8057
HmHel-6	CAEZ01008355:14194-24275
HmHel-6	CAEZ01008250:316-428
HmHel-6	CAEZ01008136:4913-5022
HmHel-6	CAEZ01008131:8616-8749
HmHel-6	CAEZ01008064:16610-16707
HmHel-6	CAEZ01008013:11252-11389
HmHel-6	CAEZ01008013:34237-34398
HmHel-6	CAEZ01008013:6804-11232
HmHel-6	CAEZ01007986:2308-12487
HmHel-6	CAEZ01007986:13775-13943
HmHel-6	CAEZ01007966:58-167
HmHel-6	CAEZ01007887:11991-12084
HmHel-6	CAEZ01007604:19221-19314
HmHel-6	CAEZ01007604:14018-14134
HmHel-6	CAEZ01007587:21778-21901
HmHel-6	CAEZ01007393:6234-6347
HmHel-6	CAEZ01007366:48-145
HmHel-6	CAEZ01007161:395-476
HmHel-6	CAEZ01007149:9855-9959
HmHel-6	CAEZ01007046:12536-12630
HmHel-6	CAEZ01007046:521-626

HmHel-6	CAEZ01007032:950-1109
HmHel-6	CAEZ01007019:6119-10178
HmHel-6	CAEZ01006849:18300-18454
HmHel-6	CAEZ01006849:6601-6684
HmHel-6	CAEZ01006608:10751-10872
HmHel-6	CAEZ01006250:19233-19355
HmHel-6	CAEZ01006250:1406-1491
HmHel-6	CAEZ01006229:1515-1620
HmHel-6	CAEZ01006090:1864-3470
HmHel-6	CAEZ01006090:41145-41248
HmHel-6	CAEZ01006070:6691-6856
HmHel-6	CAEZ01005963:15872-16021
HmHel-6	CAEZ01005958:3630-3831
HmHel-6	CAEZ01005948:6252-7074
HmHel-6	CAEZ01005948:20548-20682
HmHel-6	CAEZ01005948:11024-11109
HmHel-6	CAEZ01005895:7256-7352
HmHel-6	CAEZ01005895:3140-3269
HmHel-6	CAEZ01005672:9060-10318
HmHel-6	CAEZ01005569:2537-2753
HmHel-6	CAEZ01005557:14874-14983
HmHel-6	CAEZ01005557:3965-4079
HmHel-6	CAEZ01005480:35868-35964
HmHel-6	CAEZ01005480:19675-19835
HmHel-6	CAEZ01005428:54405-56313
HmHel-6	CAEZ01005428:80323-80428
HmHel-6	CAEZ01005428:121618-121702
HmHel-6	CAEZ01005428:13983-23343
HmHel-6	CAEZ01005424:10323-20057
HmHel-6	CAEZ01005407:888-4078
HmHel-6	CAEZ01005346:2636-2745
HmHel-6	CAEZ01005346:14034-14250
HmHel-6	CAEZ01005324:896-1025
HmHel-6	CAEZ01005301:2659-2784
HmHel-6	CAEZ01005112:952-1097
HmHel-6	CAEZ01005048:33391-33523
HmHel-6	CAEZ01005048:39359-39465
HmHel-6	CAEZ01005048:13945-14035
HmHel-6	CAEZ01004863:1244-1349
HmHel-6	CAEZ01004510:6755-13628
HmHel-6	CAEZ01004505:9145-17791
HmHel-6	CAEZ01004505:3222-3343
HmHel-6	CAEZ01004353:10243-18078
HmHel-6	CAEZ01004353:11984-12166
HmHel-6	CAEZ01004265:10784-10913
HmHel-6	CAEZ01003980:5944-6037
HmHel-6	CAEZ01003748:21693-27330
HmHel-6	CAEZ01003472:13045-13256

HmHel-6	CAEZ01003462:8717-8912
HmHel-6	CAEZ01003411:2229-2338
HmHel-6	CAEZ01003407:34275-39795
HmHel-6	CAEZ01003407:59329-62114
HmHel-6	CAEZ01003407:16208-25032
HmHel-6	CAEZ01003148:6668-6800
HmHel-6	CAEZ01003133:11209-13590
HmHel-6	CAEZ01003133:1474-8746
HmHel-6	CAEZ01003108:8414-8610
HmHel-6	CAEZ01003006:3500-11256
HmHel-6	CAEZ01002899:2188-2283
HmHel-6	CAEZ01002822:9164-9294
HmHel-6	CAEZ01002781:777-898
HmHel-6	CAEZ01002703:7051-7176
HmHel-6	CAEZ01002687:3196-3277
HmHel-6	CAEZ01001794:877-982
HmHel-6	CAEZ01001518:15511-15748
HmHel-6	CAEZ01001518:21924-22060
HmHel-6	CAEZ01001427:4521-4637
HmHel-6	CAEZ01001268:225-330
HmHel-6	CAEZ01001215:546-651
HmHel-6	CAEZ01000993:4285-4460
HmHel-6	CAEZ01000865:2307-7152
HmHel-6	CAEZ01011467:4090-4190
HmHel-6	CAEZ01011277:5433-5588
HmHel-6	CAEZ01011199:8966-9101
HmHel-6	CAEZ01011199:11382-11540
HmHel-6	CAEZ01011044:13993-14079
HmHel-6	CAEZ01011044:61205-61388
HmHel-6	CAEZ01011044:93600-97214
HmHel-6	CAEZ01011044:70834-70911
HmHel-6	CAEZ01011044:37268-37470
HmHel-6	CAEZ01011044:23966-24073
HmHel-6	CAEZ01011044:4522-4642
HmHel-6	CAEZ01010557:2197-10390
HmHel-6	CAEZ01010510:1397-1507
HmHel-6	CAEZ01010330:1223-3126
HmHel-6	CAEZ01010282:14370-20447
HmHel-6	CAEZ01010282:1212-1295
HmHel-6	CAEZ01010163:5132-5312
HmHel-6	CAEZ01010101:5907-5995
HmHel-6	CAEZ01010101:5006-7480
HmHel-6	CAEZ01009877:7056-7242
HmHel-6	CAEZ01009866:5065-5319
HmHel-6	CAEZ01009795:2878-2969
HmHel-6	CAEZ01009590:1888-2008
HmHel-6	CAEZ01009453:3384-4911
HmHel-6	CAEZ01009453:54216-56752

HmHel-6	CAEZ01009453:1369-7060
HmHel-6	CAEZ01009395:4417-4605
HmHel-6	CAEZ01009350:7603-7682
HmHel-6	CAEZ01009350:9862-16389
HmHel-6	CAEZ01009182:8299-13106
HmHel-6	CAEZ01009110:1661-1807
HmHel-6	CAEZ01009086:9144-9260
HmHel-6	CAEZ01009086:14843-14963
HmHel-6	CAEZ01008951:2491-2646
HmHel-6	CAEZ01008727:7022-7106
HmHel-6	CAEZ01008692:1185-1302
HmHel-6	CAEZ01008692:9828-9928
HmHel-6	CAEZ01008645:2490-2641
HmHel-6	CAEZ01008609:1914-3441
HmHel-6	CAEZ01008582:6124-6263
HmHel-6	CAEZ01008443:331-4155
HmHel-6	CAEZ01008238:3964-4075
HmHel-6	CAEZ01008238:41365-43511
HmHel-6	CAEZ01008238:58456-58621
HmHel-6	CAEZ01008235:3861-10481
HmHel-6	CAEZ01008235:32735-33674
HmHel-6	CAEZ01008235:55384-55470
HmHel-6	CAEZ01008235:42634-43290
HmHel-6	CAEZ01008235:24639-24745
HmHel-6	CAEZ01008135:17312-17463
HmHel-6	CAEZ01007972:23982-25958
HmHel-6	CAEZ01007972:27373-27457
HmHel-6	CAEZ01007665:118530-118691
HmHel-6	CAEZ01007665:118033-118134
HmHel-6	CAEZ01007632:28585-28666
HmHel-6	CAEZ01007547:701-822
HmHel-6	CAEZ01007447:4857-11371
HmHel-6	CAEZ01007447:12713-12845
HmHel-6	CAEZ01007238:9372-9515
HmHel-6	CAEZ01007175:3350-3432
HmHel-6	CAEZ01007131:2881-3449
HmHel-6	CAEZ01007131:1679-1874
HmHel-6	CAEZ01006950:1424-1524
HmHel-6	CAEZ01006912:204-366
HmHel-6	CAEZ01006878:10194-16518
HmHel-6	CAEZ01006878:18755-18879
HmHel-6	CAEZ01006316:207-291
HmHel-6	CAEZ01006316:4044-4236
HmHel-6	CAEZ01006225:1428-1557
HmHel-6	CAEZ01006082:7121-7249
HmHel-6	CAEZ01005881:17027-17176
HmHel-6	CAEZ01005881:15325-18674
HmHel-6	CAEZ01005852:8513-8600

HmHel-6	CAEZ01005852:14054-14158
HmHel-6	CAEZ01005385:29365-29497
HmHel-6	CAEZ01005361:11759-18218
HmHel-6	CAEZ01005361:24317-27514
HmHel-6	CAEZ01005361:1876-4677
HmHel-6	CAEZ01005024:5396-5593
HmHel-6	CAEZ01004868:15081-15234
HmHel-6	CAEZ01004690:20007-20092
HmHel-6	CAEZ01004690:24834-24935
HmHel-6	CAEZ01004675:3046-6323
HmHel-6	CAEZ01004645:2359-2500
HmHel-6	CAEZ01004645:29755-29847
HmHel-6	CAEZ01004645:1773-1866
HmHel-6	CAEZ01004275:3532-3641
HmHel-6	CAEZ01004204:473-557
HmHel-6	CAEZ01004133:13073-13226
HmHel-6	CAEZ01004118:4449-4616
HmHel-6	CAEZ01004118:6662-8492
HmHel-6	CAEZ01003963:4306-4514
HmHel-6	CAEZ01003963:2860-6458
HmHel-6	CAEZ01003872:9297-9505
HmHel-6	CAEZ01003614:1692-1905
HmHel-6	CAEZ01003585:8361-8481
HmHel-6	CAEZ01003572:4677-4781
HmHel-6	CAEZ01003326:37209-46088
HmHel-6	CAEZ01003326:19451-19547
HmHel-6	CAEZ01002980:3913-4001
HmHel-6	CAEZ01002184:33113-33329
HmHel-6	CAEZ01002184:9076-9184
HmHel-6	CAEZ01002112:4719-4870
HmHel-6	CAEZ01002011:1380-3629
HmHel-6	CAEZ01001869:15300-16502
HmHel-6	CAEZ01001819:12243-12421
HmHel-6	CAEZ01001247:518-634
HmHel-6	CAEZ01000857:3326-4463
HmHel-6	CAEZ01000695:4810-4922
HmHel-6	CAEZ01000644:6245-6370
HmHel-6	CAEZ01000540:5389-5481
HmHel-6	CAEZ01000453:1374-1454
HmHel-6	CAEZ01011563:11383-11489
HmHel-6	CAEZ01011563:16864-19464
HmHel-6	CAEZ01011344:3618-3704
HmHel-6	CAEZ01011152:6547-6737
HmHel-6	CAEZ01011075:4489-4573
HmHel-6	CAEZ01011037:5249-5483
HmHel-6	CAEZ01010993:12209-12320
HmHel-6	CAEZ01010982:13944-14027
HmHel-6	CAEZ01010956:9083-9224

HmHel-6	CAEZ01010956:1-91
HmHel-6	CAEZ01010882:35784-35928
HmHel-6	CAEZ01010882:5041-10520
HmHel-6	CAEZ01010825:15837-15927
HmHel-6	CAEZ01010825:46654-46751
HmHel-6	CAEZ01010825:47576-50699
HmHel-6	CAEZ01010631:11201-11291
HmHel-6	CAEZ01010631:20498-20613
HmHel-6	CAEZ01010404:1291-1378
HmHel-6	CAEZ01010354:13853-13936
HmHel-6	CAEZ01010354:43875-44006
HmHel-6	CAEZ01010264:4994-5103
HmHel-6	CAEZ01010264:33780-37658
HmHel-6	CAEZ01010264:11176-17081
HmHel-6	CAEZ01009997:1692-1779
HmHel-6	CAEZ01009956:3955-8758
HmHel-6	CAEZ01009922:2461-2560
HmHel-6	CAEZ01009531:12786-12916
HmHel-6	CAEZ01009531:2728-4040
HmHel-6	CAEZ01009524:617-1517
HmHel-6	CAEZ01009524:4891-5002
HmHel-6	CAEZ01009192:6942-7094
HmHel-6	CAEZ01009192:9989-10070
HmHel-6	CAEZ01009112:3146-3245
HmHel-6	CAEZ01009052:6359-7087
HmHel-6	CAEZ01009052:8466-12338
HmHel-6	CAEZ01008927:8136-8248
HmHel-6	CAEZ01008927:6124-6266
HmHel-6	CAEZ01008879:106311-106417
HmHel-6	CAEZ01008879:53426-63444
HmHel-6	CAEZ01008879:11909-11996
HmHel-6	CAEZ01008873:6242-6351
HmHel-6	CAEZ01008541:5684-5800
HmHel-6	CAEZ01008507:939-1058
HmHel-6	CAEZ01008417:10952-11048
HmHel-6	CAEZ01008417:36848-36936
HmHel-6	CAEZ01008417:38532-38736
HmHel-6	CAEZ01007862:10026-10120
HmHel-6	CAEZ01007862:934-1048
HmHel-6	CAEZ01007842:2325-5201
HmHel-6	CAEZ01007842:3722-3851
HmHel-6	CAEZ01007528:3769-3992
HmHel-6	CAEZ01007424:7521-7612
HmHel-6	CAEZ01007404:2235-2370
HmHel-6	CAEZ01007404:4607-10520
HmHel-6	CAEZ01007331:20330-20432
HmHel-6	CAEZ01006841:1272-1395
HmHel-6	CAEZ01006665:3663-3770

HmHel-6	CAEZ01006626:12140-12242
HmHel-6	CAEZ01006519:8522-8606
HmHel-6	CAEZ01006519:5876-6103
HmHel-6	CAEZ01006284:1940-2062
HmHel-6	CAEZ01006238:20466-21089
HmHel-6	CAEZ01005864:31257-31359
HmHel-6	CAEZ01005832:360-9294
HmHel-6	CAEZ01005832:4706-12456
HmHel-6	CAEZ01005811:4441-4522
HmHel-6	CAEZ01005811:8123-8228
HmHel-6	CAEZ01005647:25123-25258
HmHel-6	CAEZ01005647:25823-31824
HmHel-6	CAEZ01005576:9497-9622
HmHel-6	CAEZ01005576:14306-17367
HmHel-6	CAEZ01005549:850-976
HmHel-6	CAEZ01005519:2433-2532
HmHel-6	CAEZ01005519:6554-6707
HmHel-6	CAEZ01005459:7781-7876
HmHel-6	CAEZ01004980:8251-24358
HmHel-6	CAEZ01004980:8822-9052
HmHel-6	CAEZ01004849:3909-8836
HmHel-6	CAEZ01004849:1666-4512
HmHel-6	CAEZ01004346:1263-1361
HmHel-6	CAEZ01004330:1857-1960
HmHel-6	CAEZ01004188:16761-16888
HmHel-6	CAEZ01004006:53360-61390
HmHel-6	CAEZ01004006:57848-63768
HmHel-6	CAEZ01004006:46568-46783
HmHel-6	CAEZ01003860:8983-9070
HmHel-6	CAEZ01003804:1426-1537
HmHel-6	CAEZ01003804:11494-11609
HmHel-6	CAEZ01003691:4539-4643
HmHel-6	CAEZ01003691:403-969
HmHel-6	CAEZ01003665:8191-8295
HmHel-6	CAEZ01003657:6460-6573
HmHel-6	CAEZ01003653:3641-3795
HmHel-6	CAEZ01003315:11-138
HmHel-6	CAEZ01002895:1648-1751
HmHel-6	CAEZ01002791:8090-10350
HmHel-6	CAEZ01002791:2471-10095
HmHel-6	CAEZ01002686:324-411
HmHel-6	CAEZ01002613:1388-1499
HmHel-6	CAEZ01002379:3269-3383
HmHel-6	CAEZ01002377:3153-3280
HmHel-6	CAEZ01002040:12571-12690
HmHel-6	CAEZ01002040:8264-8421
HmHel-6	CAEZ01001982:26226-28515
HmHel-6	CAEZ01001729:15924-16076

HmHel-6	CAEZ01001352:1120-1214
HmHel-6	CAEZ01001346:4952-5077
HmHel-6	CAEZ01000744:5939-6042
HmHel-6	CAEZ01000649:5228-5323
HmHel-6	CAEZ01000643:447-1983
HmHel-6	CAEZ01000643:4683-7792
HmHel-6	CAEZ01000569:2977-3100
HmHel-6	CAEZ01011622:5747-5832
HmHel-6	CAEZ01011622:4262-4348
HmHel-6	CAEZ01011609:12463-12553
HmHel-6	CAEZ01011420:7057-7167
HmHel-6	CAEZ01011420:1696-1781
HmHel-6	CAEZ01011332:1839-5861
HmHel-6	CAEZ01011100:4453-4555
HmHel-6	CAEZ01011100:12201-22169
HmHel-6	CAEZ01010529:1471-1584
HmHel-6	CAEZ01010524:10177-10338
HmHel-6	CAEZ01010524:6199-6947
HmHel-6	CAEZ01010377:3810-12097
HmHel-6	CAEZ01010336:7451-8828
HmHel-6	CAEZ01010336:22371-22472
HmHel-6	CAEZ01010335:12977-15621
HmHel-6	CAEZ01010335:823-917
HmHel-6	CAEZ01009629:2285-2374
HmHel-6	CAEZ01009629:1172-1396
HmHel-6	CAEZ01009449:726-6747
HmHel-6	CAEZ01009441:13049-13184
HmHel-6	CAEZ01009361:5649-7559
HmHel-6	CAEZ01009361:1544-10470
HmHel-6	CAEZ01009257:140-266
HmHel-6	CAEZ01009141:8585-17079
HmHel-6	CAEZ01009141:21837-21924
HmHel-6	CAEZ01008702:235-371
HmHel-6	CAEZ01008695:16493-16580
HmHel-6	CAEZ01008695:10337-10434
HmHel-6	CAEZ01008655:846-1060
HmHel-6	CAEZ01008571:12793-12947
HmHel-6	CAEZ01008571:13433-13643
HmHel-6	CAEZ01008457:12294-12388
HmHel-6	CAEZ01008344:14124-14214
HmHel-6	CAEZ01008329:6168-6250
HmHel-6	CAEZ01008329:17025-21697
HmHel-6	CAEZ01008329:7492-7641
HmHel-6	CAEZ01008286:5118-5232
HmHel-6	CAEZ01008070:4585-4710
HmHel-6	CAEZ01007869:1821-1905
HmHel-6	CAEZ01007869:19509-19663
HmHel-6	CAEZ01007869:15878-16035

HmHel-6	CAEZ01007848:30312-30534
HmHel-6	CAEZ01007833:4641-4772
HmHel-6	CAEZ01007750:3883-3989
HmHel-6	CAEZ01007750:4642-4741
HmHel-6	CAEZ01007719:4080-9430
HmHel-6	CAEZ01007719:17252-17342
HmHel-6	CAEZ01007715:3893-4010
HmHel-6	CAEZ01007458:5720-9171
HmHel-6	CAEZ01007458:19172-19314
HmHel-6	CAEZ01007251:8099-11597
HmHel-6	CAEZ01007164:9042-9157
HmHel-6	CAEZ01007117:4354-4449
HmHel-6	CAEZ01006915:1618-3327
HmHel-6	CAEZ01006896:4552-4657
HmHel-6	CAEZ01006856:2699-2781
HmHel-6	CAEZ01006587:19107-20853
HmHel-6	CAEZ01006469:5680-5770
HmHel-6	CAEZ01006307:4651-4804
HmHel-6	CAEZ01006241:26578-34290
HmHel-6	CAEZ01006241:30084-30300
HmHel-6	CAEZ01006241:1251-1389
HmHel-6	CAEZ01005939:14079-14240
HmHel-6	CAEZ01005939:2437-2751
HmHel-6	CAEZ01005657:5804-7287
HmHel-6	CAEZ01005657:2397-2565
HmHel-6	CAEZ01005565:1367-1533
HmHel-6	CAEZ01005426:4282-9683
HmHel-6	CAEZ01005426:14329-14444
HmHel-6	CAEZ01005090:1663-1788
HmHel-6	CAEZ01005086:1964-2050
HmHel-6	CAEZ01005026:12362-18515
HmHel-6	CAEZ01004902:1098-7689
HmHel-6	CAEZ01004902:4392-10603
HmHel-6	CAEZ01004879:10458-10597
HmHel-6	CAEZ01004879:11873-12023
HmHel-6	CAEZ01004786:1628-5518
HmHel-6	CAEZ01004786:35029-35181
HmHel-6	CAEZ01004786:51736-51859
HmHel-6	CAEZ01004786:70079-70202
HmHel-6	CAEZ01004786:58462-58594
HmHel-6	CAEZ01004711:1782-2610
HmHel-6	CAEZ01004642:5563-5688
HmHel-6	CAEZ01004642:1626-11254
HmHel-6	CAEZ01004607:2893-2987
HmHel-6	CAEZ01004607:10154-10285
HmHel-6	CAEZ01004342:2395-5235
HmHel-6	CAEZ01004342:3027-3219
HmHel-6	CAEZ01004168:7859-7953

HmHel-6	CAEZ01004168:4886-5119
HmHel-6	CAEZ01004152:1184-1405
HmHel-6	CAEZ01004073:7271-7369
HmHel-6	CAEZ01004073:18357-19037
HmHel-6	CAEZ01004020:19576-19662
HmHel-6	CAEZ01004020:22713-22843
HmHel-6	CAEZ01003811:19986-20137
HmHel-6	CAEZ01003763:9827-9920
HmHel-6	CAEZ01003638:4186-4272
HmHel-6	CAEZ01003628:4875-6572
HmHel-6	CAEZ01003579:4592-10363
HmHel-6	CAEZ01003574:3034-3152
HmHel-6	CAEZ01003552:10367-10535
HmHel-6	CAEZ01003545:1521-1607
HmHel-6	CAEZ01003480:507-633
HmHel-6	CAEZ01003476:2435-2521
HmHel-6	CAEZ01003066:2709-3305
HmHel-6	CAEZ01002657:7091-7179
HmHel-6	CAEZ01002657:32840-37537
HmHel-6	CAEZ01002657:16404-19105
HmHel-6	CAEZ01002248:163-257
HmHel-6	CAEZ01002143:318-424
HmHel-6	CAEZ01001410:1225-1319
HmHel-6	CAEZ01001236:547-751
HmHel-6	CAEZ01001236:1278-1393
HmHel-6	CAEZ01001157:9803-9961
HmHel-6	CAEZ01001157:4556-4713
HmHel-6	CAEZ01000958:7248-7440
HmHel-6	CAEZ01000958:8591-9021
HmHel-6	CAEZ01011517:792-877
HmHel-6	CAEZ01011504:3693-9352
HmHel-6	CAEZ01011504:6178-6321
HmHel-6	CAEZ01011413:25317-25417
HmHel-6	CAEZ01011413:50467-50606
HmHel-6	CAEZ01011413:34129-34254
HmHel-6	CAEZ01011246:1755-6559
HmHel-6	CAEZ01011221:3002-3085
HmHel-6	CAEZ01011176:802-928
HmHel-6	CAEZ01011105:19119-19204
HmHel-6	CAEZ01010992:2253-2465
HmHel-6	CAEZ01010929:432-7882
HmHel-6	CAEZ01010858:27847-36898
HmHel-6	CAEZ01010858:30087-30172
HmHel-6	CAEZ01010749:6900-7006
HmHel-6	CAEZ01010749:2756-2837
HmHel-6	CAEZ01010420:20934-21027
HmHel-6	CAEZ01010352:1212-1464
HmHel-6	CAEZ01009849:2934-3114

HmHel-6	CAEZ01009667:6241-6322
HmHel-6	CAEZ01009667:1819-1926
HmHel-6	CAEZ01009653:4582-11776
HmHel-6	CAEZ01009653:14188-14285
HmHel-6	CAEZ01009653:227-1479
HmHel-6	CAEZ01009643:5100-5205
HmHel-6	CAEZ01009542:5238-8027
HmHel-6	CAEZ01009362:3644-3757
HmHel-6	CAEZ01009362:1769-1861
HmHel-6	CAEZ01009298:2136-2251
HmHel-6	CAEZ01009177:5094-5191
HmHel-6	CAEZ01009140:828-917
HmHel-6	CAEZ01009140:13833-13964
HmHel-6	CAEZ01009140:15635-15759
HmHel-6	CAEZ01009054:7318-7404
HmHel-6	CAEZ01009054:5219-5304
HmHel-6	CAEZ01008954:10920-15428
HmHel-6	CAEZ01008925:49417-49592
HmHel-6	CAEZ01008925:21315-21446
HmHel-6	CAEZ01008917:5767-9970
HmHel-6	CAEZ01008893:2078-2290
HmHel-6	CAEZ01008562:17615-17720
HmHel-6	CAEZ01008548:16651-16846
HmHel-6	CAEZ01008442:18971-19078
HmHel-6	CAEZ01008442:15048-21158
HmHel-6	CAEZ01008431:35244-35345
HmHel-6	CAEZ01008431:3128-3209
HmHel-6	CAEZ01008419:40307-40394
HmHel-6	CAEZ01008419:30853-30966
HmHel-6	CAEZ01008411:733-838
HmHel-6	CAEZ01008205:12421-12579
HmHel-6	CAEZ01008205:13864-13948
HmHel-6	CAEZ01008132:767-900
HmHel-6	CAEZ01008080:1438-7743
HmHel-6	CAEZ01008080:13010-13091
HmHel-6	CAEZ01008075:10204-14183
HmHel-6	CAEZ01008063:5106-5243
HmHel-6	CAEZ01008011:12453-12566
HmHel-6	CAEZ01008011:7080-7169
HmHel-6	CAEZ01007977:2110-2206
HmHel-6	CAEZ01007962:37676-37774
HmHel-6	CAEZ01007962:28611-28747
HmHel-6	CAEZ01007868:5444-5556
HmHel-6	CAEZ01007838:4675-4767
HmHel-6	CAEZ01007829:12438-12531
HmHel-6	CAEZ01007775:3393-3501
HmHel-6	CAEZ01007613:8400-11566
HmHel-6	CAEZ01007490:4484-4638

HmHel-6	CAEZ01007460:10447-11775
HmHel-6	CAEZ01007460:16872-17043
HmHel-6	CAEZ01007460:3478-3699
HmHel-6	CAEZ01007386:10198-10303
HmHel-6	CAEZ01007386:11880-11998
HmHel-6	CAEZ01007221:7661-8331
HmHel-6	CAEZ01007221:15819-15963
HmHel-6	CAEZ01007211:5788-6011
HmHel-6	CAEZ01007096:9994-10075
HmHel-6	CAEZ01006892:15444-15524
HmHel-6	CAEZ01006393:1737-1842
HmHel-6	CAEZ01006314:1725-1901
HmHel-6	CAEZ01006276:9155-9237
HmHel-6	CAEZ01005940:10581-10757
HmHel-6	CAEZ01005804:2475-4509
HmHel-6	CAEZ01005765:10148-10303
HmHel-6	CAEZ01005320:7832-7937
HmHel-6	CAEZ01004979:4102-4195
HmHel-6	CAEZ01004681:17652-17747
HmHel-6	CAEZ01004681:22700-22788
HmHel-6	CAEZ01004568:2224-3828
HmHel-6	CAEZ01004555:6859-11330
HmHel-6	CAEZ01004555:8199-13899
HmHel-6	CAEZ01004511:15958-17375
HmHel-6	CAEZ01004485:400-481
HmHel-6	CAEZ01004473:13225-13334
HmHel-6	CAEZ01004352:1176-2725
HmHel-6	CAEZ01004302:6608-6751
HmHel-6	CAEZ01004291:11605-11706
HmHel-6	CAEZ01004291:10693-14057
HmHel-6	CAEZ01004106:6534-11934
HmHel-6	CAEZ01003970:10079-12356
HmHel-6	CAEZ01003970:13641-13758
HmHel-6	CAEZ01003919:374-2976
HmHel-6	CAEZ01003761:20223-20327
HmHel-6	CAEZ01003643:281-394
HmHel-6	CAEZ01003605:3544-3634
HmHel-6	CAEZ01003605:3230-3323
HmHel-6	CAEZ01003456:1245-1338
HmHel-6	CAEZ01003456:4369-4491
HmHel-6	CAEZ01003436:17842-26537
HmHel-6	CAEZ01003215:2727-2864
HmHel-6	CAEZ01002992:6703-16195
HmHel-6	CAEZ01002991:54695-54775
HmHel-6	CAEZ01002991:68990-74007
HmHel-6	CAEZ01002991:86552-86630
HmHel-6	CAEZ01002983:2522-2701
HmHel-6	CAEZ01002824:5226-7596

HmHel-6	CAEZ01002824:4136-4283
HmHel-6	CAEZ01002649:1019-1135
HmHel-6	CAEZ01002626:2653-2798
HmHel-6	CAEZ01002626:3272-4687
HmHel-6	CAEZ01002610:7923-8058
HmHel-6	CAEZ01002610:7735-7905
HmHel-6	CAEZ01002282:6276-6357
HmHel-6	CAEZ01001970:15405-15491
HmHel-6	CAEZ01001970:13079-19334
HmHel-6	CAEZ01001477:14334-14550
HmHel-6	CAEZ01001433:1628-1794
HmHel-6	CAEZ01001207:7526-9627
HmHel-6	CAEZ01000989:61-2616
HmHel-6	CAEZ01000822:2343-2496
HmHel-6	CAEZ01000809:7330-7473
HmHel-6	CAEZ01000809:3968-4049
HmHel-6	CAEZ01000804:3129-4760
HmHel-6	CAEZ01000715:15177-15257
HmHel-6	CAEZ01000715:4055-4144
HmHel-6	CAEZ01000641:3901-4010
HmHel-6	CAEZ01000467:217-400
HmHel-6	CAEZ01000135:733-897
HmHel-6	CAEZ01000070:3815-3971
HmHel-6	CAEZ01011370:112-311
HmHel-6	CAEZ01011279:11413-15815
HmHel-6	CAEZ01011279:15517-15653
HmHel-6	CAEZ01011270:1707-1787
HmHel-6	CAEZ01011270:4738-4842
HmHel-6	CAEZ01011262:4097-5776
HmHel-6	CAEZ01011262:1661-1762
HmHel-6	CAEZ01011143:7957-11389
HmHel-6	CAEZ01011113:4216-4377
HmHel-6	CAEZ01011071:10086-10174
HmHel-6	CAEZ01011038:4824-7942
HmHel-6	CAEZ01011038:9021-9114
HmHel-6	CAEZ01010963:6144-6303
HmHel-6	CAEZ01010888:17737-26727
HmHel-6	CAEZ01010757:16267-16355
HmHel-6	CAEZ01010635:15894-18307
HmHel-6	CAEZ01010635:2932-9617
HmHel-6	CAEZ01010623:17956-18091
HmHel-6	CAEZ01010412:1073-1243
HmHel-6	CAEZ01010380:2320-2412
HmHel-6	CAEZ01010207:3971-4067
HmHel-6	CAEZ01009754:7307-7419
HmHel-6	CAEZ01009738:6540-6711
HmHel-6	CAEZ01009528:19752-19924
HmHel-6	CAEZ01009280:29577-29673

HmHel-6	CAEZ01009280:19261-19340
HmHel-6	CAEZ01009280:5739-5859
HmHel-6	CAEZ01008647:1322-1434
HmHel-6	CAEZ01008605:7192-7959
HmHel-6	CAEZ01008515:52222-52350
HmHel-6	CAEZ01008515:36070-36193
HmHel-6	CAEZ01008300:17432-17512
HmHel-6	CAEZ01008300:3685-3770
HmHel-6	CAEZ01008244:24176-24259
HmHel-6	CAEZ01008244:4659-7352
HmHel-6	CAEZ01008090:2492-2630
HmHel-6	CAEZ01007971:10447-10543
HmHel-6	CAEZ01007971:11882-14663
HmHel-6	CAEZ01007919:645-733
HmHel-6	CAEZ01007807:7510-7608
HmHel-6	CAEZ01007531:4110-4192
HmHel-6	CAEZ01007531:2556-6954
HmHel-6	CAEZ01007362:9834-10635
HmHel-6	CAEZ01007214:487-570
HmHel-6	CAEZ01007138:23284-23412
HmHel-6	CAEZ01007138:21663-21755
HmHel-6	CAEZ01007055:2611-7452
HmHel-6	CAEZ01006801:2118-4561
HmHel-6	CAEZ01006628:56445-58488
HmHel-6	CAEZ01006628:34228-40298
HmHel-6	CAEZ01006551:3765-12532
HmHel-6	CAEZ01006429:3382-3471
HmHel-6	CAEZ01006306:3181-3300
HmHel-6	CAEZ01005465:4580-4692
HmHel-6	CAEZ01004717:2706-2817
HmHel-6	CAEZ01004717:7692-8276
HmHel-6	CAEZ01004498:4441-4525
HmHel-6	CAEZ01004412:7039-7122
HmHel-6	CAEZ01004364:3158-3274
HmHel-6	CAEZ01004364:8683-10157
HmHel-6	CAEZ01004341:4923-5082
HmHel-6	CAEZ01004141:1378-1478
HmHel-6	CAEZ01004141:13981-14072
HmHel-6	CAEZ01003949:7710-7842
HmHel-6	CAEZ01003875:13731-13843
HmHel-6	CAEZ01003443:9392-9528
HmHel-6	CAEZ01003429:8087-10917
HmHel-6	CAEZ01003247:3471-3556
HmHel-6	CAEZ01003247:29635-29768
HmHel-6	CAEZ01003222:6861-6965
HmHel-6	CAEZ01003222:70859-70983
HmHel-6	CAEZ01003213:2853-3373
HmHel-6	CAEZ01003131:980-1187

HmHel-6	CAEZ01002976:3043-3167
HmHel-6	CAEZ01002958:2662-7728
HmHel-6	CAEZ01002930:897-1005
HmHel-6	CAEZ01002303:245-350
HmHel-6	CAEZ01002231:26783-26915
HmHel-6	CAEZ01002231:14806-14945
HmHel-6	CAEZ01002128:1190-1310
HmHel-6	CAEZ01002126:718-828
HmHel-6	CAEZ01002126:5305-5421
HmHel-6	CAEZ01001758:6846-7492
HmHel-6	CAEZ01001750:5996-6564
HmHel-6	CAEZ01001750:8967-9054
HmHel-6	CAEZ01001589:960-1076
HmHel-6	CAEZ01001589:2244-2355
HmHel-6	CAEZ01001249:866-966
HmHel-6	CAEZ01001218:3214-3296
HmHel-6	CAEZ01001128:2715-11969
HmHel-6	CAEZ01000675:4882-5044
HmHel-6	CAEZ01000675:7346-7430
HmHel-6	CAEZ01011645:7422-7571
HmHel-6	CAEZ01011522:22334-22529
HmHel-6	CAEZ01011346:802-894
HmHel-6	CAEZ01011259:7811-7942
HmHel-6	CAEZ01011004:22367-22450
HmHel-6	CAEZ01010625:3460-3659
HmHel-6	CAEZ01010526:11436-11564
HmHel-6	CAEZ01010526:14251-14336
HmHel-6	CAEZ01010471:6649-6849
HmHel-6	CAEZ01010471:5730-5837
HmHel-6	CAEZ01010447:15551-15727
HmHel-6	CAEZ01010261:9450-9553
HmHel-6	CAEZ01009973:20012-20103
HmHel-6	CAEZ01009914:5148-5237
HmHel-6	CAEZ01009810:2529-2614
HmHel-6	CAEZ01009663:8218-8297
HmHel-6	CAEZ01009498:4110-4257
HmHel-6	CAEZ01009498:3690-3774
HmHel-6	CAEZ01009414:11607-11765
HmHel-6	CAEZ01009369:777-930
HmHel-6	CAEZ01009172:6044-6142
HmHel-6	CAEZ01009168:3422-3579
HmHel-6	CAEZ01009158:5294-5372
HmHel-6	CAEZ01009143:2627-2761
HmHel-6	CAEZ01008930:332-417
HmHel-6	CAEZ01008837:6594-6679
HmHel-6	CAEZ01008837:8352-8532
HmHel-6	CAEZ01008801:12554-12704
HmHel-6	CAEZ01008755:5586-6227

HmHel-6	CAEZ01008237:2529-3566
HmHel-6	CAEZ01008237:33712-33819
HmHel-6	CAEZ01008021:5287-5382
HmHel-6	CAEZ01008021:1424-4258
HmHel-6	CAEZ01007993:8389-9058
HmHel-6	CAEZ01007917:177-362
HmHel-6	CAEZ01007901:7273-7376
HmHel-6	CAEZ01007657:3947-4114
HmHel-6	CAEZ01007649:7724-7856
HmHel-6	CAEZ01007641:3618-9519
HmHel-6	CAEZ01007607:33595-33824
HmHel-6	CAEZ01007439:2664-2764
HmHel-6	CAEZ01007439:8141-8225
HmHel-6	CAEZ01007340:5066-5173
HmHel-6	CAEZ01007246:437-563
HmHel-6	CAEZ01007094:7553-7696
HmHel-6	CAEZ01006809:277-457
HmHel-6	CAEZ01006781:27485-27583
HmHel-6	CAEZ01006574:1121-1280
HmHel-6	CAEZ01006430:2095-2181
HmHel-6	CAEZ01006044:39147-39264
HmHel-6	CAEZ01006044:16337-16476
HmHel-6	CAEZ01006043:8762-8921
HmHel-6	CAEZ01006000:22693-22783
HmHel-6	CAEZ01005735:9503-9586
HmHel-6	CAEZ01005431:5764-5863
HmHel-6	CAEZ01005431:10254-10344
HmHel-6	CAEZ01004982:11021-11104
HmHel-6	CAEZ01004823:1483-1614
HmHel-6	CAEZ01004823:23846-23942
HmHel-6	CAEZ01004610:16625-16724
HmHel-6	CAEZ01004474:2259-2419
HmHel-6	CAEZ01004470:8028-8135
HmHel-6	CAEZ01004384:4070-4286
HmHel-6	CAEZ01004289:3904-10951
HmHel-6	CAEZ01004289:5263-13189
HmHel-6	CAEZ01004213:53782-53908
HmHel-6	CAEZ01004213:38115-48203
HmHel-6	CAEZ01004090:13957-14043
HmHel-6	CAEZ01004086:7327-7483
HmHel-6	CAEZ01004086:4193-5203
HmHel-6	CAEZ01004028:2600-2765
HmHel-6	CAEZ01003943:7627-7711
HmHel-6	CAEZ01003943:23569-23719
HmHel-6	CAEZ01003921:17897-17997
HmHel-6	CAEZ01003776:1080-1293
HmHel-6	CAEZ01003756:14557-14656
HmHel-6	CAEZ01003709:3185-3369

HmHel-6	CAEZ01003546:3347-3445
HmHel-6	CAEZ01003542:1209-1433
HmHel-6	CAEZ01003512:13893-13982
HmHel-6	CAEZ01003512:12948-13071
HmHel-6	CAEZ01003426:5233-5320
HmHel-6	CAEZ01003358:600-682
HmHel-6	CAEZ01003263:5607-5829
HmHel-6	CAEZ01002977:1015-1126
HmHel-6	CAEZ01002865:13623-13710
HmHel-6	CAEZ01002734:3545-3719
HmHel-6	CAEZ01002734:5638-5813
HmHel-6	CAEZ01002226:6605-6728
HmHel-6	CAEZ01002127:3843-5246
HmHel-6	CAEZ01001935:444-704
HmHel-6	CAEZ01001423:2072-2150
HmHel-6	CAEZ01001395:9627-9714
HmHel-6	CAEZ01001237:293-453
HmHel-6	CAEZ01000595:2293-2464
HmHel-6	CAEZ01000409:819-930
HmHel-6	CAEZ01000241:1260-1343
HmHel-6	CAEZ01000239:2698-2820
HmHel-6	CAEZ01000089:823-906
HmHel-6	CAEZ01000085:1728-1859
HmHel-6	CAEZ01011565:3831-7754
HmHel-6	CAEZ01011548:2808-2903
HmHel-6	CAEZ01011548:3406-5490
HmHel-6	CAEZ01011431:3499-3578
HmHel-6	CAEZ01011374:3-112
HmHel-6	CAEZ01011374:3478-3589
HmHel-6	CAEZ01011239:16621-16734
HmHel-6	CAEZ01010962:16178-16286
HmHel-6	CAEZ01010949:179-261
HmHel-6	CAEZ01010785:701-783
HmHel-6	CAEZ01010708:17684-17794
HmHel-6	CAEZ01010657:9415-9501
HmHel-6	CAEZ01010518:833-938
HmHel-6	CAEZ01010300:13684-13875
HmHel-6	CAEZ01010300:23302-23389
HmHel-6	CAEZ01010300:8514-8610
HmHel-6	CAEZ01009987:9094-9300
HmHel-6	CAEZ01009803:5235-5357
HmHel-6	CAEZ01009289:13432-13528
HmHel-6	CAEZ01009286:63924-64043
HmHel-6	CAEZ01009286:23766-27949
HmHel-6	CAEZ01009258:104-230
HmHel-6	CAEZ01009200:9587-17808
HmHel-6	CAEZ01009200:23635-23761
HmHel-6	CAEZ01009036:9658-9740

HmHel-6	CAEZ01009036:4425-7394
HmHel-6	CAEZ01008852:17916-23491
HmHel-6	CAEZ01008753:3844-3934
HmHel-6	CAEZ01008564:657-847
HmHel-6	CAEZ01008529:220-378
HmHel-6	CAEZ01008217:12866-12960
HmHel-6	CAEZ01008217:9739-9836
HmHel-6	CAEZ01008166:1260-1404
HmHel-6	CAEZ01008162:1910-1988
HmHel-6	CAEZ01008162:13189-13315
HmHel-6	CAEZ01007811:16573-16668
HmHel-6	CAEZ01007786:1528-1626
HmHel-6	CAEZ01007371:22624-22717
HmHel-6	CAEZ01007371:2139-2231
HmHel-6	CAEZ01007034:2439-2523
HmHel-6	CAEZ01007034:13206-13308
HmHel-6	CAEZ01007009:19249-19363
HmHel-6	CAEZ01007009:10491-10623
HmHel-6	CAEZ01006986:4804-4935
HmHel-6	CAEZ01006939:4400-4502
HmHel-6	CAEZ01006906:19646-20080
HmHel-6	CAEZ01006906:5653-5778
HmHel-6	CAEZ01006854:3664-5838
HmHel-6	CAEZ01006854:11136-11222
HmHel-6	CAEZ01006762:4512-4656
HmHel-6	CAEZ01006664:15480-15570
HmHel-6	CAEZ01006664:4950-5107
HmHel-6	CAEZ01006513:3471-3569
HmHel-6	CAEZ01006457:1389-1610
HmHel-6	CAEZ01006221:2858-3012
HmHel-6	CAEZ01005907:408-4676
HmHel-6	CAEZ01005879:565-800
HmHel-6	CAEZ01005727:1302-1466
HmHel-6	CAEZ01005725:2760-2929
HmHel-6	CAEZ01005535:2940-3024
HmHel-6	CAEZ01005467:2838-3070
HmHel-6	CAEZ01005467:5576-5660
HmHel-6	CAEZ01005248:2790-2868
HmHel-6	CAEZ01005248:1364-1615
HmHel-6	CAEZ01005094:1711-2366
HmHel-6	CAEZ01005054:1912-2014
HmHel-6	CAEZ01004866:3804-3914
HmHel-6	CAEZ01004698:14292-14374
HmHel-6	CAEZ01004587:2006-2107
HmHel-6	CAEZ01004504:7822-7904
HmHel-6	CAEZ01004480:2661-7814
HmHel-6	CAEZ01004465:397-3512
HmHel-6	CAEZ01004413:2200-2309

HmHel-6	CAEZ01003690:9222-9320
HmHel-6	CAEZ01003690:20395-20530
HmHel-6	CAEZ01003690:18686-18882
HmHel-6	CAEZ01003661:4049-6316
HmHel-6	CAEZ01003516:10303-10424
HmHel-6	CAEZ01003516:30065-35550
HmHel-6	CAEZ01003516:5413-14092
HmHel-6	CAEZ01003404:42020-42129
HmHel-6	CAEZ01003404:57129-57268
HmHel-6	CAEZ01003404:53915-54024
HmHel-6	CAEZ01003390:1563-1649
HmHel-6	CAEZ01003039:3071-3229
HmHel-6	CAEZ01003039:76-203
HmHel-6	CAEZ01002984:15701-17336
HmHel-6	CAEZ01002892:5135-5217
HmHel-6	CAEZ01002646:21672-21759
HmHel-6	CAEZ01002646:13103-13257
HmHel-6	CAEZ01002641:6188-6318
HmHel-6	CAEZ01002621:16664-16790
HmHel-6	CAEZ01002621:17622-17716
HmHel-6	CAEZ01002375:4800-4918
HmHel-6	CAEZ01002258:1940-2122
HmHel-6	CAEZ01001811:532-689
HmHel-6	CAEZ01001232:2585-2675
HmHel-6	CAEZ01000994:476-558
HmHel-6	CAEZ01000875:1810-1979
HmHel-6	CAEZ01000828:1539-5511
HmHel-6	CAEZ01000805:11776-14558
HmHel-6	CAEZ01000805:24587-24751
HmHel-6	CAEZ01000722:524-606
HmHel-6	CAEZ01000578:1160-1297
HmHel-6	CAEZ01011464:1046-2017
HmHel-6	CAEZ01011464:1518-4639
HmHel-6	CAEZ01011392:965-1054
HmHel-6	CAEZ01011392:16980-17109
HmHel-6	CAEZ01011324:13276-13393
HmHel-6	CAEZ01011302:6150-6296
HmHel-6	CAEZ01011302:348-461
HmHel-6	CAEZ01011240:1389-1490
HmHel-6	CAEZ01011205:7924-8078
HmHel-6	CAEZ01011150:1497-9178
HmHel-6	CAEZ01010678:3752-3839
HmHel-6	CAEZ01010564:8117-11768
HmHel-6	CAEZ01010344:9172-9340
HmHel-6	CAEZ01010338:7296-7537
HmHel-6	CAEZ01010173:6495-6580
HmHel-6	CAEZ01010052:6675-6804
HmHel-6	CAEZ01010052:9014-9100

HmHel-6	CAEZ01010034:64313-64444
HmHel-6	CAEZ01010027:8314-10464
HmHel-6	CAEZ01009858:4736-4856
HmHel-6	CAEZ01009695:4018-5386
HmHel-6	CAEZ01009428:1437-1518
HmHel-6	CAEZ01009402:13503-13641
HmHel-6	CAEZ01008953:422-527
HmHel-6	CAEZ01008881:10737-10861
HmHel-6	CAEZ01008760:11352-11465
HmHel-6	CAEZ01008552:27930-28020
HmHel-6	CAEZ01008284:13453-13579
HmHel-6	CAEZ01007970:3856-3935
HmHel-6	CAEZ01007970:5691-5792
HmHel-6	CAEZ01007933:8514-8715
HmHel-6	CAEZ01007910:1629-1732
HmHel-6	CAEZ01007866:2165-2285
HmHel-6	CAEZ01007683:5549-6959
HmHel-6	CAEZ01007466:3928-4065
HmHel-6	CAEZ01007200:2994-3119
HmHel-6	CAEZ01006980:8700-8796
HmHel-6	CAEZ01006882:6861-6954
HmHel-6	CAEZ01006846:18014-21852
HmHel-6	CAEZ01006531:9242-9323
HmHel-6	CAEZ01006506:13774-13879
HmHel-6	CAEZ01005740:11763-11997
HmHel-6	CAEZ01005690:16422-16602
HmHel-6	CAEZ01004801:6485-6595
HmHel-6	CAEZ01004778:1272-1396
HmHel-6	CAEZ01004327:4807-7876
HmHel-6	CAEZ01004281:7487-7628
HmHel-6	CAEZ01004132:8503-8583
HmHel-6	CAEZ01004108:5037-5155
HmHel-6	CAEZ01004108:2458-2587
HmHel-6	CAEZ01004005:751-836
HmHel-6	CAEZ01003792:15399-15529
HmHel-6	CAEZ01003785:2415-2513
HmHel-6	CAEZ01003785:28026-28131
HmHel-6	CAEZ01003785:15775-15866
HmHel-6	CAEZ01003680:6352-10901
HmHel-6	CAEZ01003604:132-217
HmHel-6	CAEZ01003309:17481-17590
HmHel-6	CAEZ01003186:12540-12704
HmHel-6	CAEZ01002739:6923-7105
HmHel-6	CAEZ01002584:1264-1428
HmHel-6	CAEZ01002140:8063-8172
HmHel-6	CAEZ01002102:5487-5684
HmHel-6	CAEZ01001854:2336-7344
HmHel-6	CAEZ01001493:9223-18418

HmHel-6	CAEZ01001398:558-703
HmHel-6	CAEZ01000962:4808-4909
HmHel-6	CAEZ01000747:333-425
HmHel-6	CAEZ01000629:2349-2459
HmHel-6	CAEZ01000629:334-420
HmHel-6	CAEZ01000586:7082-7164
HmHel-6	CAEZ01000586:6114-6224
HmHel-6	CAEZ01000064:4838-4936
HmHel-6	CAEZ01000033:14871-17583
HmHel-6	CAEZ01011642:13133-13212
HmHel-6	CAEZ01011604:9515-9626
HmHel-6	CAEZ01011440:10154-10286
HmHel-6	CAEZ01011243:5467-5695
HmHel-6	CAEZ01011085:8175-8270
HmHel-6	CAEZ01011000:8167-8270
HmHel-6	CAEZ01011000:34910-35003
HmHel-6	CAEZ01010978:9881-10001
HmHel-6	CAEZ01010960:3127-3247
HmHel-6	CAEZ01010838:11621-11705
HmHel-6	CAEZ01010263:2267-2418
HmHel-6	CAEZ01010263:4687-4772
HmHel-6	CAEZ01010099:4750-4893
HmHel-6	CAEZ01009472:5482-5566
HmHel-6	CAEZ01009390:2866-2965
HmHel-6	CAEZ01009260:12689-12817
HmHel-6	CAEZ01009250:17558-17662
HmHel-6	CAEZ01009236:5473-6077
HmHel-6	CAEZ01009219:1014-1115
HmHel-6	CAEZ01009146:1112-10287
HmHel-6	CAEZ01009146:7695-7888
HmHel-6	CAEZ01009068:4832-4916
HmHel-6	CAEZ01009012:2672-2772
HmHel-6	CAEZ01009008:6805-6926
HmHel-6	CAEZ01008974:2310-6293
HmHel-6	CAEZ01008904:2085-2173
HmHel-6	CAEZ01008496:12579-12670
HmHel-6	CAEZ01008492:1606-1691
HmHel-6	CAEZ01008384:4259-9273
HmHel-6	CAEZ01008321:757-841
HmHel-6	CAEZ01008319:7606-7726
HmHel-6	CAEZ01008045:13288-13439
HmHel-6	CAEZ01008045:11040-16458
HmHel-6	CAEZ01007609:51182-51294
HmHel-6	CAEZ01007606:3386-10771
HmHel-6	CAEZ01007280:12920-13016
HmHel-6	CAEZ01007039:565-3825
HmHel-6	CAEZ01006879:8491-9702
HmHel-6	CAEZ01006879:2379-2464

HmHel-6	CAEZ01006836:12440-12525
HmHel-6	CAEZ01006836:3926-4025
HmHel-6	CAEZ01006830:18303-18402
HmHel-6	CAEZ01006825:5746-5838
HmHel-6	CAEZ01006657:5643-5796
HmHel-6	CAEZ01006524:2458-2625
HmHel-6	CAEZ01006404:10431-10581
HmHel-6	CAEZ01006389:3435-9657
HmHel-6	CAEZ01006245:1701-1801
HmHel-6	CAEZ01006216:5889-6067
HmHel-6	CAEZ01006187:14966-15077
HmHel-6	CAEZ01006035:6000-6111
HmHel-6	CAEZ01006014:1484-1636
HmHel-6	CAEZ01005752:8487-8680
HmHel-6	CAEZ01005592:22428-22581
HmHel-6	CAEZ01005592:627-719
HmHel-6	CAEZ01005571:4435-4565
HmHel-6	CAEZ01005545:10601-19788
HmHel-6	CAEZ01005344:17469-17565
HmHel-6	CAEZ01005027:13712-21438
HmHel-6	CAEZ01005027:317-404
HmHel-6	CAEZ01004363:4132-4315
HmHel-6	CAEZ01004363:13509-16669
HmHel-6	CAEZ01004326:1211-1296
HmHel-6	CAEZ01004307:2163-10717
HmHel-6	CAEZ01004294:11193-11302
HmHel-6	CAEZ01004259:32360-40149
HmHel-6	CAEZ01004259:25229-33583
HmHel-6	CAEZ01003755:10034-13167
HmHel-6	CAEZ01003655:7693-7785
HmHel-6	CAEZ01002934:5976-6098
HmHel-6	CAEZ01002283:6336-6420
HmHel-6	CAEZ01002073:4896-4984
HmHel-6	CAEZ01002030:326-422
HmHel-6	CAEZ01001997:7609-7688
HmHel-6	CAEZ01001978:12314-12406
HmHel-6	CAEZ01001978:12888-12969
HmHel-6	CAEZ01001204:7309-7474
HmHel-6	CAEZ01000935:6389-6505
HmHel-6	CAEZ01000684:1532-1620
HmHel-6	CAEZ01000469:619-770
HmHel-6	CAEZ01000358:46-143
HmHel-6	CAEZ01000199:644-787
HmHel-6	CAEZ01000143:629-753
HmHel-6	CAEZ01000042:5017-5096
HmHel-6	CAEZ01000042:26272-26994
HmHel-6	CAEZ01000042:28438-28529
HmHel-6	CAEZ01011559:1619-9549

HmHel-6	CAEZ01011002:7967-9638
HmHel-6	CAEZ01010682:3046-3959
HmHel-6	CAEZ01010682:2331-2460
HmHel-6	CAEZ01010679:4135-4223
HmHel-6	CAEZ01010477:8125-8219
HmHel-6	CAEZ01010474:22265-22385
HmHel-6	CAEZ01010467:1466-1552
HmHel-6	CAEZ01010433:3728-3822
HmHel-6	CAEZ01010267:22062-22156
HmHel-6	CAEZ01010240:14250-17268
HmHel-6	CAEZ01010240:15278-18372
HmHel-6	CAEZ01010125:7635-7745
HmHel-6	CAEZ01010102:5958-6081
HmHel-6	CAEZ01010102:4919-5041
HmHel-6	CAEZ01009944:2562-2653
HmHel-6	CAEZ01009937:8106-8239
HmHel-6	CAEZ01009937:3284-3384
HmHel-6	CAEZ01009783:4289-4444
HmHel-6	CAEZ01009768:17730-17825
HmHel-6	CAEZ01009543:4569-4692
HmHel-6	CAEZ01008968:5322-5409
HmHel-6	CAEZ01008842:23911-23994
HmHel-6	CAEZ01008834:619-731
HmHel-6	CAEZ01008690:10791-10872
HmHel-6	CAEZ01008690:428-518
HmHel-6	CAEZ01008476:5716-6784
HmHel-6	CAEZ01008476:13832-14032
HmHel-6	CAEZ01008298:6613-6730
HmHel-6	CAEZ01007988:10089-10175
HmHel-6	CAEZ01007920:9267-9366
HmHel-6	CAEZ01007402:2837-11447
HmHel-6	CAEZ01007365:10880-11075
HmHel-6	CAEZ01007365:40560-40691
HmHel-6	CAEZ01007105:4853-5031
HmHel-6	CAEZ01007089:23147-23267
HmHel-6	CAEZ01007008:823-907
HmHel-6	CAEZ01006994:9258-9337
HmHel-6	CAEZ01006973:8663-8831
HmHel-6	CAEZ01006810:5520-15472
HmHel-6	CAEZ01006650:12604-12700
HmHel-6	CAEZ01006597:1527-1638
HmHel-6	CAEZ01006471:11818-12065
HmHel-6	CAEZ01006223:2166-2907
HmHel-6	CAEZ01006223:433-568
HmHel-6	CAEZ01005969:11802-11907
HmHel-6	CAEZ01005969:3443-3563
HmHel-6	CAEZ01005701:587-764
HmHel-6	CAEZ01005652:2696-7208

HmHel-6	CAEZ01005585:11990-12113
HmHel-6	CAEZ01005531:9482-9590
HmHel-6	CAEZ01005470:16975-17070
HmHel-6	CAEZ01005341:1466-1590
HmHel-6	CAEZ01005278:5211-5335
HmHel-6	CAEZ01005049:9105-9212
HmHel-6	CAEZ01004682:61375-67874
HmHel-6	CAEZ01004682:23769-26027
HmHel-6	CAEZ01004277:4176-4326
HmHel-6	CAEZ01004076:13825-13924
HmHel-6	CAEZ01003927:5173-11415
HmHel-6	CAEZ01003862:3534-4366
HmHel-6	CAEZ01003844:17895-17982
HmHel-6	CAEZ01003844:5861-5979
HmHel-6	CAEZ01003581:4496-4579
HmHel-6	CAEZ01003562:54395-54498
HmHel-6	CAEZ01003457:329-412
HmHel-6	CAEZ01003257:1262-1414
HmHel-6	CAEZ01003221:12860-12939
HmHel-6	CAEZ01003122:2923-3089
HmHel-6	CAEZ01003113:465-544
HmHel-6	CAEZ01003045:1449-11243
HmHel-6	CAEZ01002836:2110-11702
HmHel-6	CAEZ01002836:3595-4381
HmHel-6	CAEZ01002831:648-727
HmHel-6	CAEZ01002817:1445-1556
HmHel-6	CAEZ01002752:1665-1751
HmHel-6	CAEZ01002144:284-785
HmHel-6	CAEZ01002079:5285-5372
HmHel-6	CAEZ01001732:3306-3521
HmHel-6	CAEZ01001732:6349-6432
HmHel-6	CAEZ01001422:11821-11899
HmHel-6	CAEZ01001105:10544-10647
HmHel-6	CAEZ01001105:4852-9041
HmHel-6	CAEZ01001063:475-571
HmHel-6	CAEZ01001023:12413-12538
HmHel-6	CAEZ01000812:1766-1848
HmHel-6	CAEZ01011584:645-758
HmHel-6	CAEZ01011573:10187-10287
HmHel-6	CAEZ01011433:2293-2372
HmHel-6	CAEZ01011384:4739-4819
HmHel-6	CAEZ01011384:3071-5590
HmHel-6	CAEZ01011311:3718-3843
HmHel-6	CAEZ01011126:30344-30529
HmHel-6	CAEZ01010994:324-443
HmHel-6	CAEZ01010770:22683-22822
HmHel-6	CAEZ01010770:12259-12348
HmHel-6	CAEZ01010258:2638-2764

HmHel-6	CAEZ01010258:4242-4342
HmHel-6	CAEZ01010237:11051-11156
HmHel-6	CAEZ01010237:85248-89811
HmHel-6	CAEZ01010237:121502-121588
HmHel-6	CAEZ01010237:53112-53220
HmHel-6	CAEZ01010011:16887-17001
HmHel-6	CAEZ01009993:9381-9523
HmHel-6	CAEZ01009847:998-4874
HmHel-6	CAEZ01009618:8743-8845
HmHel-6	CAEZ01009599:639-768
HmHel-6	CAEZ01009526:2391-2488
HmHel-6	CAEZ01009493:2588-2694
HmHel-6	CAEZ01009493:3239-3329
HmHel-6	CAEZ01009142:14831-14930
HmHel-6	CAEZ01009142:3810-12031
HmHel-6	CAEZ01009000:3493-5872
HmHel-6	CAEZ01008792:2355-2470
HmHel-6	CAEZ01008269:38798-38891
HmHel-6	CAEZ01008078:2542-2684
HmHel-6	CAEZ01008030:8331-8452
HmHel-6	CAEZ01008030:5952-6054
HmHel-6	CAEZ01007990:3357-12028
HmHel-6	CAEZ01007961:758-884
HmHel-6	CAEZ01007832:1983-4051
HmHel-6	CAEZ01007605:3286-12836
HmHel-6	CAEZ01007553:423-528
HmHel-6	CAEZ01007521:7991-8089
HmHel-6	CAEZ01007517:4264-13813
HmHel-6	CAEZ01007501:566-660
HmHel-6	CAEZ01007339:2460-2709
HmHel-6	CAEZ01007339:20120-20231
HmHel-6	CAEZ01007030:7506-7643
HmHel-6	CAEZ01006868:7087-7213
HmHel-6	CAEZ01006868:10564-10657
HmHel-6	CAEZ01006674:11660-11742
HmHel-6	CAEZ01006437:21231-26797
HmHel-6	CAEZ01006191:2747-2873
HmHel-6	CAEZ01006191:2113-7520
HmHel-6	CAEZ01006188:31196-31289
HmHel-6	CAEZ01006188:29276-29518
HmHel-6	CAEZ01005981:2044-2209
HmHel-6	CAEZ01005855:6084-8435
HmHel-6	CAEZ01005754:4185-5064
HmHel-6	CAEZ01005754:21804-21888
HmHel-6	CAEZ01005023:6253-6439
HmHel-6	CAEZ01004961:11139-11253
HmHel-6	CAEZ01004930:12263-12480
HmHel-6	CAEZ01004924:4495-4581

HmHel-6	CAEZ01004924:6154-6273
HmHel-6	CAEZ01004904:6601-15931
HmHel-6	CAEZ01004904:16382-16472
HmHel-6	CAEZ01004812:1617-2339
HmHel-6	CAEZ01004799:7502-7585
HmHel-6	CAEZ01004340:3658-9468
HmHel-6	CAEZ01004340:6776-6878
HmHel-6	CAEZ01004335:11564-15228
HmHel-6	CAEZ01004335:12926-13473
HmHel-6	CAEZ01004203:3840-3922
HmHel-6	CAEZ01003907:17037-23162
HmHel-6	CAEZ01003742:4272-4504
HmHel-6	CAEZ01003417:3614-3733
HmHel-6	CAEZ01003417:19918-20064
HmHel-6	CAEZ01003417:31442-32751
HmHel-6	CAEZ01003417:45331-45427
HmHel-6	CAEZ01003327:11349-11443
HmHel-6	CAEZ01003275:26035-30662
HmHel-6	CAEZ01003156:4389-4493
HmHel-6	CAEZ01003058:450-614
HmHel-6	CAEZ01002938:6254-6617
HmHel-6	CAEZ01002240:8051-8161
HmHel-6	CAEZ01002185:533-635
HmHel-6	CAEZ01002154:3861-4006
HmHel-6	CAEZ01001963:2957-3927
HmHel-6	CAEZ01001785:5609-5758
HmHel-6	CAEZ01001428:5338-5420
HmHel-6	CAEZ01001428:2320-2421
HmHel-6	CAEZ01001305:4131-4225
HmHel-6	CAEZ01001235:8155-8245
HmHel-6	CAEZ01001153:20394-20496
HmHel-6	CAEZ01001125:2331-6938
HmHel-6	CAEZ01000947:465-603
HmHel-6	CAEZ01000773:1953-2083
HmHel-6	CAEZ01000238:138-287
HmHel-6	CAEZ01000114:79-177
HmHel-6	CAEZ01011620:6001-6126
HmHel-6	CAEZ01011582:351-500
HmHel-6	CAEZ01011422:2102-2216
HmHel-6	CAEZ01011395:6983-9671
HmHel-6	CAEZ01011158:2937-5381
HmHel-6	CAEZ01010908:15068-15189
HmHel-6	CAEZ01010658:15436-15540
HmHel-6	CAEZ01010644:2929-3022
HmHel-6	CAEZ01010638:3354-5081
HmHel-6	CAEZ01010382:5709-5865
HmHel-6	CAEZ01010382:16225-16317
HmHel-6	CAEZ01010340:1957-2051

HmHel-6	CAEZ01009929:5458-5557
HmHel-6	CAEZ01009916:3553-3649
HmHel-6	CAEZ01009852:632-759
HmHel-6	CAEZ01009740:2505-2606
HmHel-6	CAEZ01009517:4738-4931
HmHel-6	CAEZ01009463:20639-20884
HmHel-6	CAEZ01009463:3735-3963
HmHel-6	CAEZ01009354:12864-12949
HmHel-6	CAEZ01009005:10428-10529
HmHel-6	CAEZ01008985:5660-5803
HmHel-6	CAEZ01008900:6301-16926
HmHel-6	CAEZ01008900:12795-12875
HmHel-6	CAEZ01008672:19214-19340
HmHel-6	CAEZ01008625:7000-7132
HmHel-6	CAEZ01008094:13619-13820
HmHel-6	CAEZ01007978:23308-23391
HmHel-6	CAEZ01007978:7231-7340
HmHel-6	CAEZ01007906:1155-1240
HmHel-6	CAEZ01007885:4042-5051
HmHel-6	CAEZ01007767:2185-2311
HmHel-6	CAEZ01007701:5527-5727
HmHel-6	CAEZ01007024:9525-9606
HmHel-6	CAEZ01007024:8885-16072
HmHel-6	CAEZ01007003:3730-8557
HmHel-6	CAEZ01006975:6988-7085
HmHel-6	CAEZ01006922:2627-2764
HmHel-6	CAEZ01006818:5645-7575
HmHel-6	CAEZ01006476:12884-12997
HmHel-6	CAEZ01006382:4689-5822
HmHel-6	CAEZ01006207:1651-1751
HmHel-6	CAEZ01006207:2117-2218
HmHel-6	CAEZ01006011:11003-13147
HmHel-6	CAEZ01005944:9378-9507
HmHel-6	CAEZ01005888:906-995
HmHel-6	CAEZ01005640:3056-3208
HmHel-6	CAEZ01005567:1270-1358
HmHel-6	CAEZ01005464:2958-3098
HmHel-6	CAEZ01005213:12284-15808
HmHel-6	CAEZ01004937:6558-6664
HmHel-6	CAEZ01004644:4302-7371
HmHel-6	CAEZ01004563:12085-12178
HmHel-6	CAEZ01004526:740-841
HmHel-6	CAEZ01004411:1055-1152
HmHel-6	CAEZ01004379:2469-2573
HmHel-6	CAEZ01004375:11863-15153
HmHel-6	CAEZ01004231:917-1009
HmHel-6	CAEZ01004231:3025-3110
HmHel-6	CAEZ01004185:12990-13074

HmHel-6	CAEZ01004176:951-1030
HmHel-6	CAEZ01004114:7408-7489
HmHel-6	CAEZ01004042:2565-2662
HmHel-6	CAEZ01003830:18791-18876
HmHel-6	CAEZ01003644:5685-6502
HmHel-6	CAEZ01003615:4492-8145
HmHel-6	CAEZ01003557:331-472
HmHel-6	CAEZ01003508:2871-2975
HmHel-6	CAEZ01002656:15312-15403
HmHel-6	CAEZ01002250:2755-4582
HmHel-6	CAEZ01002156:1434-1566
HmHel-6	CAEZ01001763:5637-5773
HmHel-6	CAEZ01001762:2732-2924
HmHel-6	CAEZ01001505:5710-5815
HmHel-6	CAEZ01001295:119-256
HmHel-6	CAEZ01000866:968-1069
HmHel-6	CAEZ01000616:748-6380
HmHel-6	CAEZ01000558:1144-1470
HmHel-6	CAEZ01011633:24679-25030
HmHel-6	CAEZ01011335:21345-21445
HmHel-6	CAEZ01011333:2151-2243
HmHel-6	CAEZ01010861:3587-8986
HmHel-6	CAEZ01010795:14840-14972
HmHel-6	CAEZ01010654:7866-7946
HmHel-6	CAEZ01010376:177-264
HmHel-6	CAEZ01010339:83-176
HmHel-6	CAEZ01010021:23613-23717
HmHel-6	CAEZ01009960:6219-6391
HmHel-6	CAEZ01009960:15072-15152
HmHel-6	CAEZ01009942:244-403
HmHel-6	CAEZ01009924:4632-4826
HmHel-6	CAEZ01009518:2690-2882
HmHel-6	CAEZ01009514:3602-3706
HmHel-6	CAEZ01009423:2612-3156
HmHel-6	CAEZ01009313:2135-2264
HmHel-6	CAEZ01009252:5483-5568
HmHel-6	CAEZ01009216:17530-18557
HmHel-6	CAEZ01008898:20291-24433
HmHel-6	CAEZ01008644:4313-4405
HmHel-6	CAEZ01008617:3850-3959
HmHel-6	CAEZ01008513:2462-4348
HmHel-6	CAEZ01008333:7029-7114
HmHel-6	CAEZ01008267:9934-10026
HmHel-6	CAEZ01008121:13349-14978
HmHel-6	CAEZ01007907:9397-18760
HmHel-6	CAEZ01007839:4408-4590
HmHel-6	CAEZ01007614:917-1051
HmHel-6	CAEZ01007532:974-1113

HmHel-6	CAEZ01007529:176-399
HmHel-6	CAEZ01007010:12084-12165
HmHel-6	CAEZ01007010:11094-11206
HmHel-6	CAEZ01006719:7119-7264
HmHel-6	CAEZ01006691:7989-8163
HmHel-6	CAEZ01006627:5119-5204
HmHel-6	CAEZ01006610:5669-12607
HmHel-6	CAEZ01006610:14815-14903
HmHel-6	CAEZ01006599:2315-2474
HmHel-6	CAEZ01006599:7290-7412
HmHel-6	CAEZ01006485:668-3359
HmHel-6	CAEZ01006485:2511-2636
HmHel-6	CAEZ01006431:12697-12781
HmHel-6	CAEZ01006397:5993-6104
HmHel-6	CAEZ01006357:14904-14988
HmHel-6	CAEZ01006267:3651-3728
HmHel-6	CAEZ01005932:5905-6021
HmHel-6	CAEZ01005844:6636-16189
HmHel-6	CAEZ01005637:3710-4789
HmHel-6	CAEZ01005568:3167-3319
HmHel-6	CAEZ01005447:1746-2559
HmHel-6	CAEZ01005297:1694-1778
HmHel-6	CAEZ01005286:5699-5864
HmHel-6	CAEZ01005286:2090-2262
HmHel-6	CAEZ01005265:2823-2968
HmHel-6	CAEZ01005195:2558-2661
HmHel-6	CAEZ01005156:3993-4138
HmHel-6	CAEZ01004576:5266-5377
HmHel-6	CAEZ01004512:1651-1854
HmHel-6	CAEZ01004334:6116-11602
HmHel-6	CAEZ01004197:4381-4497
HmHel-6	CAEZ01004079:2544-2625
HmHel-6	CAEZ01004045:10682-14293
HmHel-6	CAEZ01003990:3661-3801
HmHel-6	CAEZ01003990:1122-1210
HmHel-6	CAEZ01003731:5603-5737
HmHel-6	CAEZ01003601:3574-3724
HmHel-6	CAEZ01003601:5345-5583
HmHel-6	CAEZ01003001:2011-2216
HmHel-6	CAEZ01002971:1210-1306
HmHel-6	CAEZ01002696:1135-1286
HmHel-6	CAEZ01002228:16627-17270
HmHel-6	CAEZ01002168:10759-10846
HmHel-6	CAEZ01001275:4355-4992
HmHel-6	CAEZ01001100:1867-2000
HmHel-6	CAEZ01001045:528-647
HmHel-6	CAEZ01000247:355-470
HmHel-6	CAEZ01000161:539-659

HmHel-6	CAEZ01000086:900-1008
HmHel-6	CAEZ01011551:15864-16007
HmHel-6	CAEZ01011508:7017-7103
HmHel-6	CAEZ01011397:7360-7615
HmHel-6	CAEZ01011386:222-7438
HmHel-6	CAEZ01010622:9914-12621
HmHel-6	CAEZ01010493:1828-1939
HmHel-6	CAEZ01010334:4327-4486
HmHel-6	CAEZ01010308:28541-28630
HmHel-6	CAEZ01010308:12096-12188
HmHel-6	CAEZ01010255:811-4189
HmHel-6	CAEZ01010179:5069-5160
HmHel-6	CAEZ01009665:6375-6486
HmHel-6	CAEZ01009492:6118-6214
HmHel-6	CAEZ01009273:1352-1430
HmHel-6	CAEZ01009273:2642-2801
HmHel-6	CAEZ01009235:3280-3407
HmHel-6	CAEZ01009007:10108-10215
HmHel-6	CAEZ01008735:40187-40282
HmHel-6	CAEZ01008475:1587-4314
HmHel-6	CAEZ01008475:35682-36628
HmHel-6	CAEZ01008433:19208-19298
HmHel-6	CAEZ01008316:15761-15848
HmHel-6	CAEZ01007825:135-261
HmHel-6	CAEZ01007815:4894-11814
HmHel-6	CAEZ01007600:3245-3439
HmHel-6	CAEZ01007598:9200-9304
HmHel-6	CAEZ01007307:7810-7897
HmHel-6	CAEZ01007163:2256-2404
HmHel-6	CAEZ01007163:10383-10524
HmHel-6	CAEZ01007104:1675-1791
HmHel-6	CAEZ01006938:2950-3028
HmHel-6	CAEZ01006606:1454-1561
HmHel-6	CAEZ01006537:3436-8249
HmHel-6	CAEZ01006508:13282-13488
HmHel-6	CAEZ01006356:9453-9548
HmHel-6	CAEZ01006009:8586-8666
HmHel-6	CAEZ01005957:10868-10971
HmHel-6	CAEZ01005874:9290-9497
HmHel-6	CAEZ01005801:2661-2788
HmHel-6	CAEZ01005541:15673-15803
HmHel-6	CAEZ01005366:19339-19466
HmHel-6	CAEZ01005332:4956-5116
HmHel-6	CAEZ01005058:12142-12326
HmHel-6	CAEZ01005005:13906-14039
HmHel-6	CAEZ01005005:4174-4297
HmHel-6	CAEZ01004536:1812-1930
HmHel-6	CAEZ01004469:10146-11433

HmHel-6	CAEZ01004209:8338-8481
HmHel-6	CAEZ01004209:758-7744
HmHel-6	CAEZ01004182:9342-9542
HmHel-6	CAEZ01004091:1329-1423
HmHel-6	CAEZ01004064:5475-11024
HmHel-6	CAEZ01004052:14671-14750
HmHel-6	CAEZ01004052:757-849
HmHel-6	CAEZ01003744:577-705
HmHel-6	CAEZ01003705:5097-8880
HmHel-6	CAEZ01003454:1038-1193
HmHel-6	CAEZ01003081:2130-2236
HmHel-6	CAEZ01002302:868-987
HmHel-6	CAEZ01002010:1804-1887
HmHel-6	CAEZ01001927:6218-6321
HmHel-6	CAEZ01001927:7058-7139
HmHel-6	CAEZ01001816:36-166
HmHel-6	CAEZ01001419:7727-7961
HmHel-6	CAEZ01000787:1931-2026
HmHel-6	CAEZ01000727:4335-4462
HmHel-6	CAEZ01000714:4238-10710
HmHel-6	CAEZ01000653:1181-1301
HmHel-6	CAEZ01000639:2106-2185
HmHel-6	CAEZ01000424:593-773
HmHel-6	CAEZ01011560:16240-16334
HmHel-6	CAEZ01011449:15102-21627
HmHel-6	CAEZ01010860:2658-2761
HmHel-6	CAEZ01010779:6332-7784
HmHel-6	CAEZ01010762:17259-17496
HmHel-6	CAEZ01010650:16902-17062
HmHel-6	CAEZ01010434:9348-9506
HmHel-6	CAEZ01010290:2783-8747
HmHel-6	CAEZ01010152:19007-19141
HmHel-6	CAEZ01010076:44-185
HmHel-6	CAEZ01009994:11390-11475
HmHel-6	CAEZ01009994:37805-37929
HmHel-6	CAEZ01009812:3785-3918
HmHel-6	CAEZ01009655:14340-14506
HmHel-6	CAEZ01009655:24790-24991
HmHel-6	CAEZ01009655:22363-22490
HmHel-6	CAEZ01009489:1797-1891
HmHel-6	CAEZ01009119:11821-11922
HmHel-6	CAEZ01009119:11273-11363
HmHel-6	CAEZ01009055:90-1858
HmHel-6	CAEZ01008906:1271-1373
HmHel-6	CAEZ01008800:17277-17363
HmHel-6	CAEZ01008697:13372-13466
HmHel-6	CAEZ01008334:6427-6509
HmHel-6	CAEZ01008248:5074-5169

HmHel-6	CAEZ01008248:34732-34869
HmHel-6	CAEZ01008248:14392-14496
HmHel-6	CAEZ01007760:750-856
HmHel-6	CAEZ01007756:19368-20860
HmHel-6	CAEZ01007690:28869-28967
HmHel-6	CAEZ01007690:8630-15966
HmHel-6	CAEZ01007338:5155-5241
HmHel-6	CAEZ01006955:265-947
HmHel-6	CAEZ01006909:539-621
HmHel-6	CAEZ01006701:2354-12199
HmHel-6	CAEZ01006396:22794-23010
HmHel-6	CAEZ01006355:601-691
HmHel-6	CAEZ01006291:10059-10186
HmHel-6	CAEZ01006215:10916-12482
HmHel-6	CAEZ01005983:9357-9503
HmHel-6	CAEZ01005818:27792-27901
HmHel-6	CAEZ01005759:14387-14473
HmHel-6	CAEZ01005741:2909-3022
HmHel-6	CAEZ01005548:3410-6242
HmHel-6	CAEZ01005543:384-10180
HmHel-6	CAEZ01005349:22472-22645
HmHel-6	CAEZ01005016:331-488
HmHel-6	CAEZ01004977:2734-2889
HmHel-6	CAEZ01004977:97-7624
HmHel-6	CAEZ01004899:12588-12681
HmHel-6	CAEZ01004862:12756-12843
HmHel-6	CAEZ01004714:5355-5476
HmHel-6	CAEZ01004664:4976-5066
HmHel-6	CAEZ01004560:28951-29049
HmHel-6	CAEZ01004560:18425-18562
HmHel-6	CAEZ01004543:1222-1307
HmHel-6	CAEZ01004416:750-844
HmHel-6	CAEZ01004416:2278-2448
HmHel-6	CAEZ01004318:6109-10808
HmHel-6	CAEZ01004247:4908-5065
HmHel-6	CAEZ01004215:3-105
HmHel-6	CAEZ01003892:2869-2948
HmHel-6	CAEZ01003880:1359-8165
HmHel-6	CAEZ01003808:9500-9627
HmHel-6	CAEZ01003715:6343-6503
HmHel-6	CAEZ01003715:6995-7092
HmHel-6	CAEZ01003636:7780-7917
HmHel-6	CAEZ01003603:1103-1200
HmHel-6	CAEZ01003244:6417-15731
HmHel-6	CAEZ01002978:449-536
HmHel-6	CAEZ01002852:3872-3986
HmHel-6	CAEZ01002816:5573-5655
HmHel-6	CAEZ01002763:401-571

HmHel-6	CAEZ01002708:5180-5278
HmHel-6	CAEZ01002436:5293-5395
HmHel-6	CAEZ01002313:4471-4585
HmHel-6	CAEZ01002290:4864-10862
HmHel-6	CAEZ01002290:5959-6083
HmHel-6	CAEZ01002148:7105-7287
HmHel-6	CAEZ01002051:3689-6944
HmHel-6	CAEZ01002051:3509-3640
HmHel-6	CAEZ01001845:2699-2808
HmHel-6	CAEZ01000081:19995-20091
HmHel-6	CAEZ01011543:10296-10377
HmHel-6	CAEZ01011308:184-292
HmHel-6	CAEZ01011040:5193-5316
HmHel-6	CAEZ01011028:477-10768
HmHel-6	CAEZ01011028:10191-10277
HmHel-6	CAEZ01010895:10974-11127
HmHel-6	CAEZ01010254:2430-2526
HmHel-6	CAEZ01010230:3877-4073
HmHel-6	CAEZ01010210:7913-8005
HmHel-6	CAEZ01010120:14795-14887
HmHel-6	CAEZ01010109:3907-5673
HmHel-6	CAEZ01010093:6582-6675
HmHel-6	CAEZ01009984:10602-10694
HmHel-6	CAEZ01009829:1459-1571
HmHel-6	CAEZ01009791:1961-2050
HmHel-6	CAEZ01009736:5104-9494
HmHel-6	CAEZ01009668:1306-7534
HmHel-6	CAEZ01009624:7392-7604
HmHel-6	CAEZ01009555:2082-2178
HmHel-6	CAEZ01009372:1188-1269
HmHel-6	CAEZ01009197:8817-15832
HmHel-6	CAEZ01009090:1191-1288
HmHel-6	CAEZ01008984:6255-6336
HmHel-6	CAEZ01008936:2616-2701
HmHel-6	CAEZ01008772:16752-17270
HmHel-6	CAEZ01008664:2536-2637
HmHel-6	CAEZ01008382:6934-7019
HmHel-6	CAEZ01008072:5685-5766
HmHel-6	CAEZ01007744:11193-11301
HmHel-6	CAEZ01006978:7155-7243
HmHel-6	CAEZ01006978:1077-1173
HmHel-6	CAEZ01006756:1908-2013
HmHel-6	CAEZ01006670:690-822
HmHel-6	CAEZ01006244:9147-10259
HmHel-6	CAEZ01005814:1221-1338
HmHel-6	CAEZ01005758:19949-20050
HmHel-6	CAEZ01005624:6589-6670
HmHel-6	CAEZ01005522:10207-10332

HmHel-6	CAEZ01005305:820-1619
HmHel-6	CAEZ01005103:5344-5437
HmHel-6	CAEZ01004974:2689-2774
HmHel-6	CAEZ01004804:9398-9486
HmHel-6	CAEZ01004804:2870-2956
HmHel-6	CAEZ01004398:9452-9595
HmHel-6	CAEZ01004172:1335-1429
HmHel-6	CAEZ01004009:1488-1593
HmHel-6	CAEZ01003851:23389-23518
HmHel-6	CAEZ01003669:22273-22382
HmHel-6	CAEZ01003595:3836-3936
HmHel-6	CAEZ01003518:4744-4826
HmHel-6	CAEZ01003320:219-364
HmHel-6	CAEZ01003185:2510-4728
HmHel-6	CAEZ01003078:1676-3005
HmHel-6	CAEZ01002841:12492-12580
HmHel-6	CAEZ01002356:102-195
HmHel-6	CAEZ01002070:4818-4919
HmHel-6	CAEZ01002070:3768-3959
HmHel-6	CAEZ01001421:1989-9421
HmHel-6	CAEZ01001421:1166-10006
HmHel-6	CAEZ01001142:2834-4691
HmHel-6	CAEZ01001085:4337-4436
HmHel-6	CAEZ01000795:3872-3970
HmHel-6	CAEZ01000670:6716-6805
HmHel-6	CAEZ01000668:1827-1920
HmHel-6	CAEZ01011388:997-1084
HmHel-6	CAEZ01011254:12725-12805
HmHel-6	CAEZ01011175:6353-6453
HmHel-6	CAEZ01011027:3094-3268
HmHel-6	CAEZ01011027:5169-10729
HmHel-6	CAEZ01010540:13765-13857
HmHel-6	CAEZ01010465:506-586
HmHel-6	CAEZ01010383:1599-5813
HmHel-6	CAEZ01010372:7435-7526
HmHel-6	CAEZ01010225:2530-2615
HmHel-6	CAEZ01010165:3313-3408
HmHel-6	CAEZ01009959:2534-2672
HmHel-6	CAEZ01009786:6222-6306
HmHel-6	CAEZ01009786:6364-8776
HmHel-6	CAEZ01009774:15984-16232
HmHel-6	CAEZ01009564:41-204
HmHel-6	CAEZ01008715:1628-1708
HmHel-6	CAEZ01008531:26451-26591
HmHel-6	CAEZ01008395:7016-7107
HmHel-6	CAEZ01008066:7258-7370
HmHel-6	CAEZ01007646:14713-14832
HmHel-6	CAEZ01007437:2323-6769

HmHel-6	CAEZ01007216:4416-5679
HmHel-6	CAEZ01007148:10177-10257
HmHel-6	CAEZ01007048:19327-23714
HmHel-6	CAEZ01006905:5787-5883
HmHel-6	CAEZ01006792:8654-9137
HmHel-6	CAEZ01006784:9879-9975
HmHel-6	CAEZ01006601:4375-6860
HmHel-6	CAEZ01006289:4173-10042
HmHel-6	CAEZ01005917:1371-4459
HmHel-6	CAEZ01005614:1051-1284
HmHel-6	CAEZ01005397:3290-11703
HmHel-6	CAEZ01004509:6816-6896
HmHel-6	CAEZ01004477:18347-18477
HmHel-6	CAEZ01004477:12650-12734
HmHel-6	CAEZ01004325:16531-16685
HmHel-6	CAEZ01004200:398-521
HmHel-6	CAEZ01004199:5036-5138
HmHel-6	CAEZ01004092:4511-4675
HmHel-6	CAEZ01003675:969-2623
HmHel-6	CAEZ01003630:7484-7575
HmHel-6	CAEZ01003506:8158-8265
HmHel-6	CAEZ01003469:15567-15650
HmHel-6	CAEZ01003445:5314-5477
HmHel-6	CAEZ01003029:11151-11384
HmHel-6	CAEZ01002259:2793-2948
HmHel-6	CAEZ01002142:2071-2495
HmHel-6	CAEZ01001887:1964-2083
HmHel-6	CAEZ01001837:811-929
HmHel-6	CAEZ01001802:12911-13014
HmHel-6	CAEZ01001221:7306-7450
HmHel-6	CAEZ01000891:68-152
HmHel-6	CAEZ01000853:767-867
HmHel-6	CAEZ01000685:317-439
HmHel-6	CAEZ01000642:394-527
HmHel-6	CAEZ01000562:943-1031
HmHel-6	CAEZ01011615:4670-6767
HmHel-6	CAEZ01011607:6218-6295
HmHel-6	CAEZ01010815:1955-2169
HmHel-6	CAEZ01010786:253-364
HmHel-6	CAEZ01010525:4481-6632
HmHel-6	CAEZ01010348:10020-10106
HmHel-6	CAEZ01010348:4313-4405
HmHel-6	CAEZ01009378:2700-2811
HmHel-6	CAEZ01009378:12711-12943
HmHel-6	CAEZ01009020:18583-18719
HmHel-6	CAEZ01009020:20534-21188
HmHel-6	CAEZ01008791:4767-4919
HmHel-6	CAEZ01008636:1489-1580

HmHel-6	CAEZ01008557:11342-11525
HmHel-6	CAEZ01008061:18888-18979
HmHel-6	CAEZ01008023:7031-7482
HmHel-6	CAEZ01007846:1413-3045
HmHel-6	CAEZ01007648:4396-4572
HmHel-6	CAEZ01007611:5123-5206
HmHel-6	CAEZ01007305:568-662
HmHel-6	CAEZ01007236:3653-3767
HmHel-6	CAEZ01007236:15-8001
HmHel-6	CAEZ01007005:5769-7025
HmHel-6	CAEZ01006952:1874-1973
HmHel-6	CAEZ01006277:1053-1147
HmHel-6	CAEZ01006236:5463-5557
HmHel-6	CAEZ01006053:3147-3234
HmHel-6	CAEZ01005889:1425-1516
HmHel-6	CAEZ01005851:21930-22016
HmHel-6	CAEZ01005656:10426-10505
HmHel-6	CAEZ01005456:10573-10655
HmHel-6	CAEZ01005146:10776-10878
HmHel-6	CAEZ01004994:2823-2906
HmHel-6	CAEZ01004934:7555-12884
HmHel-6	CAEZ01004791:3979-4058
HmHel-6	CAEZ01004508:1274-1365
HmHel-6	CAEZ01004357:6288-6399
HmHel-6	CAEZ01004328:13659-16791
HmHel-6	CAEZ01003606:1004-1078
HmHel-6	CAEZ01003535:14726-14808
HmHel-6	CAEZ01003490:1743-1852
HmHel-6	CAEZ01003064:274-877
HmHel-6	CAEZ01003050:10957-11056
HmHel-6	CAEZ01002970:1004-1238
HmHel-6	CAEZ01002920:2228-2318
HmHel-6	CAEZ01001499:1318-1452
HmHel-6	CAEZ01001497:52-176
HmHel-6	CAEZ01001497:597-773
HmHel-6	CAEZ01001073:4366-9644
HmHel-6	CAEZ01001073:19534-19753
HmHel-6	CAEZ01000656:1716-1851
HmHel-6	CAEZ01000656:6352-6466
HmHel-6	CAEZ01000596:193-361
HmHel-6	CAEZ01000302:358-453
HmHel-6	CAEZ01011603:5307-5405
HmHel-6	CAEZ01010983:2228-8883
HmHel-6	CAEZ01010911:3455-7008
HmHel-6	CAEZ01010763:4067-4197
HmHel-6	CAEZ01010656:4594-6829
HmHel-6	CAEZ01009568:9736-11910
HmHel-6	CAEZ01009568:1126-5528

HmHel-6	CAEZ01009356:10859-10941
HmHel-6	CAEZ01009093:6515-8545
HmHel-6	CAEZ01009093:2772-2882
HmHel-6	CAEZ01009058:3119-3232
HmHel-6	CAEZ01008782:2799-6756
HmHel-6	CAEZ01008297:4355-4438
HmHel-6	CAEZ01008175:12784-12877
HmHel-6	CAEZ01008109:1951-2038
HmHel-6	CAEZ01007826:10676-10774
HmHel-6	CAEZ01007826:136-217
HmHel-6	CAEZ01007697:3422-3532
HmHel-6	CAEZ01007350:639-2898
HmHel-6	CAEZ01007350:1607-1716
HmHel-6	CAEZ01007197:861-942
HmHel-6	CAEZ01006855:3875-3985
HmHel-6	CAEZ01006761:5262-5416
HmHel-6	CAEZ01006600:783-900
HmHel-6	CAEZ01006520:14214-14396
HmHel-6	CAEZ01006516:4734-4860
HmHel-6	CAEZ01006282:3900-3993
HmHel-6	CAEZ01005651:764-881
HmHel-6	CAEZ01005176:853-983
HmHel-6	CAEZ01005028:1214-1320
HmHel-6	CAEZ01005028:2635-2735
HmHel-6	CAEZ01004192:2772-6389
HmHel-6	CAEZ01004192:1886-5476
HmHel-6	CAEZ01004129:17425-17526
HmHel-6	CAEZ01003930:4549-4739
HmHel-6	CAEZ01003841:2328-3492
HmHel-6	CAEZ01003706:1453-1539
HmHel-6	CAEZ01003678:10105-10187
HmHel-6	CAEZ01003656:3207-6830
HmHel-6	CAEZ01003492:824-957
HmHel-6	CAEZ01003466:1797-1924
HmHel-6	CAEZ01003274:1389-1470
HmHel-6	CAEZ01003266:3443-3594
HmHel-6	CAEZ01002018:13129-13230
HmHel-6	CAEZ01001947:3203-3288
HmHel-6	CAEZ01001504:2260-2370
HmHel-6	CAEZ01000920:38351-38463
HmHel-6	CAEZ01000666:3352-3446
HmHel-6	CAEZ01000543:651-775
HmHel-6	CAEZ01000077:2835-2924
HmHel-6	CAEZ01011507:10992-11101
HmHel-6	CAEZ01011365:505-8751
HmHel-6	CAEZ01011257:9257-9373
HmHel-6	CAEZ01011167:2685-2824
HmHel-6	CAEZ01010745:5037-5132

HmHel-6	CAEZ01010694:2825-2922
HmHel-6	CAEZ01010508:8946-9059
HmHel-6	CAEZ01010508:3086-7796
HmHel-6	CAEZ01010494:1846-1928
HmHel-6	CAEZ01010488:5025-5211
HmHel-6	CAEZ01010473:2886-10143
HmHel-6	CAEZ01009513:1342-1570
HmHel-6	CAEZ01009246:14328-24025
HmHel-6	CAEZ01009246:8428-9174
HmHel-6	CAEZ01008865:2444-2547
HmHel-6	CAEZ01008703:722-10043
HmHel-6	CAEZ01008680:618-697
HmHel-6	CAEZ01008495:9407-9504
HmHel-6	CAEZ01008222:199-300
HmHel-6	CAEZ01007875:7651-7731
HmHel-6	CAEZ01006468:8751-8884
HmHel-6	CAEZ01006178:7550-7635
HmHel-6	CAEZ01006087:6897-6978
HmHel-6	CAEZ01006051:1966-7402
HmHel-6	CAEZ01005021:1663-2422
HmHel-6	CAEZ01004969:2361-2442
HmHel-6	CAEZ01004611:7953-8040
HmHel-6	CAEZ01004332:6603-6793
HmHel-6	CAEZ01004312:13663-19138
HmHel-6	CAEZ01004242:1016-10625
HmHel-6	CAEZ01004046:8157-8254
HmHel-6	CAEZ01003782:1219-5967
HmHel-6	CAEZ01003663:3786-3889
HmHel-6	CAEZ01003367:774-4577
HmHel-6	CAEZ01003155:4295-4447
HmHel-6	CAEZ01003076:11485-11570
HmHel-6	CAEZ01002941:21621-21753
HmHel-6	CAEZ01002796:1909-5284
HmHel-6	CAEZ01002639:565-673
HmHel-6	CAEZ01002590:900-1034
HmHel-6	CAEZ01001508:508-718
HmHel-6	CAEZ01001015:743-881
HmHel-6	CAEZ01000977:8510-8603
HmHel-6	CAEZ01000892:810-2914
HmHel-6	CAEZ01000762:2366-2475
HmHel-6	CAEZ01011444:6924-7010
HmHel-6	CAEZ01011108:1169-1291
HmHel-6	CAEZ01011007:6207-6364
HmHel-6	CAEZ01010544:27520-27600
HmHel-6	CAEZ01010154:1186-6633
HmHel-6	CAEZ01009309:16402-16537
HmHel-6	CAEZ01008081:5462-5794
HmHel-6	CAEZ01007471:1074-1161

HmHel-6	CAEZ01007468:250-337
HmHel-6	CAEZ01007182:7243-7342
HmHel-6	CAEZ01007012:4569-11332
HmHel-6	CAEZ01006942:466-714
HmHel-6	CAEZ01006806:2706-2798
HmHel-6	CAEZ01006751:1127-1215
HmHel-6	CAEZ01006504:10429-10517
HmHel-6	CAEZ01006243:15561-15725
HmHel-6	CAEZ01006211:4170-4266
HmHel-6	CAEZ01005928:6103-10377
HmHel-6	CAEZ01005813:5682-5762
HmHel-6	CAEZ01005766:7481-7575
HmHel-6	CAEZ01005766:23881-23970
HmHel-6	CAEZ01005731:5423-8581
HmHel-6	CAEZ01005516:12462-12547
HmHel-6	CAEZ01005038:11740-11952
HmHel-6	CAEZ01004790:276-359
HmHel-6	CAEZ01004524:1680-7056
HmHel-6	CAEZ01004146:884-1010
HmHel-6	CAEZ01003874:3285-3441
HmHel-6	CAEZ01003819:22684-22788
HmHel-6	CAEZ01003703:500-8147
HmHel-6	CAEZ01003364:1140-1235
HmHel-6	CAEZ01003087:6626-6714
HmHel-6	CAEZ01002898:2210-7156
HmHel-6	CAEZ01001310:1021-1145
HmHel-6	CAEZ01001217:77-160
HmHel-6	CAEZ01001108:5785-6020
HmHel-6	CAEZ01001032:1374-1462
HmHel-6	CAEZ01000808:864-948
HmHel-6	CAEZ01000755:1340-1427
HmHel-6	CAEZ01011351:2148-2246
HmHel-6	CAEZ01011112:4983-6504
HmHel-6	CAEZ01011076:1220-1463
HmHel-6	CAEZ01010642:6458-6555
HmHel-6	CAEZ01009794:893-2729
HmHel-6	CAEZ01009365:2311-4520
HmHel-6	CAEZ01008758:6-96
HmHel-6	CAEZ01008665:8321-18447
HmHel-6	CAEZ01008524:16909-21453
HmHel-6	CAEZ01008053:2990-6893
HmHel-6	CAEZ01008048:572-671
HmHel-6	CAEZ01007546:12843-13468
HmHel-6	CAEZ01007095:1612-8840
HmHel-6	CAEZ01006814:6025-6112
HmHel-6	CAEZ01005413:9397-10126
HmHel-6	CAEZ01004640:2313-2444
HmHel-6	CAEZ01004065:1677-1838

HmHel-6	CAEZ01004000:6627-6763
HmHel-6	CAEZ01003869:1904-1996
HmHel-6	CAEZ01003728:2195-2318
HmHel-6	CAEZ01002907:8247-8477
HmHel-6	CAEZ01002069:266-350
HmHel-6	CAEZ01001985:3492-12992
HmHel-6	CAEZ01001856:1452-7786
HmHel-6	CAEZ01001364:3706-3816
HmHel-6	CAEZ01001304:6383-6491
HmHel-6	CAEZ01001304:2864-3937
HmHel-6	CAEZ01001291:4504-4693
HmHel-6	CAEZ01001127:247-346
HmHel-6	CAEZ01000665:5443-5567
HmHel-6	CAEZ01000420:1164-1313
HmHel-6	CAEZ01011264:4646-4739
HmHel-6	CAEZ01011229:1149-2087
HmHel-6	CAEZ01011156:2994-4411
HmHel-6	CAEZ01011149:349-1748
HmHel-6	CAEZ01010670:1963-2494
HmHel-6	CAEZ01010670:4594-5546
HmHel-6	CAEZ01009100:5814-5899
HmHel-6	CAEZ01008724:18605-18711
HmHel-6	CAEZ01008724:31398-31490
HmHel-6	CAEZ01008412:6474-6557
HmHel-6	CAEZ01006576:515-656
HmHel-6	CAEZ01006200:1034-1844
HmHel-6	CAEZ01006110:5764-5904
HmHel-6	CAEZ01006065:2108-2310
HmHel-6	CAEZ01006055:1437-1523
HmHel-6	CAEZ01005339:2292-2387
HmHel-6	CAEZ01005134:10658-10740
HmHel-6	CAEZ01004657:528-618
HmHel-6	CAEZ01004556:909-1026
HmHel-6	CAEZ01003687:1200-1322
HmHel-6	CAEZ01003463:474-610
HmHel-6	CAEZ01003439:9763-9864
HmHel-6	CAEZ01002692:11767-11850
HmHel-6	CAEZ01002397:5655-5757
HmHel-6	CAEZ01002107:6213-7579
HmHel-6	CAEZ01001444:18714-18804
HmHel-6	CAEZ01001440:10575-14551
HmHel-6	CAEZ01001026:948-2337
HmHel-6	CAEZ01000835:6458-13504
HmHel-6	CAEZ01000191:1932-2050
HmHel-6	CAEZ01005377:2940-6816
HmHel-6	CAEZ01003953:4839-4940
HmHel-6	CAEZ01003580:5124-12459
HmHel-6	CAEZ01002902:8267-8366

HmHel-6	CAEZ01001953:10877-10962
HmHel-6	CAEZ01000690:405-505
HmHel-6	CAEZ01011086:3201-3325
HmHel-6	CAEZ01010119:6686-6766
HmHel-6	CAEZ01009908:14183-14310
HmHel-6	CAEZ01009169:5873-5957
HmHel-6	CAEZ01004520:937-1481
HmHel-6	CAEZ01004410:6250-7843
HmHel-6	CAEZ01003913:10062-10146
HmHel-6	CAEZ01002159:236-396
HmHel-6	CAEZ01001824:1378-1466
HmHel-6	CAEZ01001357:1251-1335
HmHel-6	CAEZ01000573:1788-1868
HmHel-6	CAEZ01000450:1234-1326
HmHel-6	CAEZ01011496:2638-2717
HmHel-6	CAEZ01011247:1756-1839
HmHel-6	CAEZ01009544:4970-5064
HmHel-6	CAEZ01008720:7975-8054
HmHel-6	CAEZ01007905:14606-14721
HmHel-6	CAEZ01006186:8333-8431
HmHel-6	CAEZ01003707:7194-7293
HmHel-6	CAEZ01002879:9020-9106
HmHel-6	CAEZ01000040:6088-6175
	:-
HmHel-7	CAEZ01007964:11118-14656
HmHel-7	CAEZ01005443:14141-17641
HmHel-7	CAEZ01005977:114496-117036
HmHel-7	CAEZ01007263:75733-76579
HmHel-7	CAEZ01007263:790-3779
HmHel-7	CAEZ01007751:75451-78933
HmHel-7	CAEZ01004011:26037-27411
HmHel-7	CAEZ01004011:99135-100418
HmHel-7	CAEZ01004011:154144-155360
HmHel-7	CAEZ01004011:55698-58210
HmHel-7	CAEZ01006176:5326-8748
HmHel-7	CAEZ01008248:35002-36352
HmHel-7	CAEZ01008248:45995-48376
HmHel-7	CAEZ01008248:32-972
HmHel-7	CAEZ01008294:194082-195383
HmHel-7	CAEZ01008294:152626-153749
HmHel-7	CAEZ01008294:52570-55120
HmHel-7	CAEZ01007234:1-2544
HmHel-7	CAEZ01008435:163178-164518
HmHel-7	CAEZ01008435:230911-232201
HmHel-7	CAEZ01008435:260084-262874
HmHel-7	CAEZ01008604:12461-15634
HmHel-7	CAEZ01011143:1-1921
HmHel-7	CAEZ01005416:52409-53763

HmHel-7	CAEZ01005416:47108-48077
HmHel-7	CAEZ01003818:3292-6713
HmHel-7	CAEZ01008234:1-1578
HmHel-7	CAEZ01008234:99259-100411
HmHel-7	CAEZ01008234:153858-155210
HmHel-7	CAEZ01008234:37315-38666
HmHel-7	CAEZ01008234:20145-20371
HmHel-7	CAEZ01007322:3-1553
HmHel-7	CAEZ01009969:40747-41252
HmHel-7	CAEZ01002638:1977-9287
HmHel-7	CAEZ01002821:119748-121051
HmHel-7	CAEZ01002821:8154-11613
HmHel-7	CAEZ01008159:7648-11088
HmHel-7	CAEZ01008233:42619-43907
HmHel-7	CAEZ01008233:94551-96617
HmHel-7	CAEZ01002286:16996-18530
HmHel-7	CAEZ01003510:84093-86684
HmHel-7	CAEZ01003510:4988-6319
HmHel-7	CAEZ01000983:3-1364
HmHel-7	CAEZ01007372:5270-8154
HmHel-7	CAEZ01006660:16731-20078
HmHel-7	CAEZ01011414:19044-22476
HmHel-7	CAEZ01011414:95314-95729
HmHel-7	CAEZ01004822:86926-89418
HmHel-7	CAEZ01009586:58629-59948
HmHel-7	CAEZ01009586:4086-6538
HmHel-7	CAEZ01009150:13481-14820
HmHel-7	CAEZ01009150:44299-44963
HmHel-7	CAEZ01009150:44081-44300
HmHel-7	CAEZ01004796:14480-15817
HmHel-7	CAEZ01001326:11516-12851
HmHel-7	CAEZ01001326:43344-44548
HmHel-7	CAEZ01005558:190-1302
HmHel-7	CAEZ01001184:1-2009
HmHel-7	CAEZ01007275:68762-70045
HmHel-7	CAEZ01007275:74176-75529
HmHel-7	CAEZ01009044:35984-37366
HmHel-7	CAEZ01009044:135356-137688
HmHel-7	CAEZ01002208:1-2249
HmHel-7	CAEZ01010451:89371-90672
HmHel-7	CAEZ01010451:71855-73178
HmHel-7	CAEZ01005428:126365-129677
HmHel-7	CAEZ01005428:100811-101969
HmHel-7	CAEZ01007267:15936-19214
HmHel-7	CAEZ01010583:1-2284
HmHel-7	CAEZ01005934:622-1727
HmHel-7	CAEZ01003938:5968-7179
HmHel-7	CAEZ01007262:5237-6157

HmHel-7	CAEZ01011142:35737-36682
HmHel-7	CAEZ01011142:2-449
HmHel-7	CAEZ01010960:7307-8641
HmHel-7	CAEZ01007740:18335-18458
HmHel-7	CAEZ01007740:89399-90707
HmHel-7	CAEZ01007740:3408-4719
HmHel-7	CAEZ01008691:1443-2812
HmHel-7	CAEZ01001450:56616-57927
HmHel-7	CAEZ01005044:30646-31795
HmHel-7	CAEZ01003511:1-956
HmHel-7	CAEZ01005215:6715-8064
HmHel-7	CAEZ01002062:1143-2255
HmHel-7	CAEZ01006675:62983-64333
HmHel-7	CAEZ01006675:47890-49211
HmHel-7	CAEZ01002984:19140-21664
HmHel-7	CAEZ01008091:32793-34108
HmHel-7	CAEZ01008091:84031-85319
HmHel-7	CAEZ01002974:1-1653
HmHel-7	CAEZ01008621:4-614
HmHel-7	CAEZ01008621:51804-54953
HmHel-7	CAEZ01011135:1399-2708
HmHel-7	CAEZ01011135:47448-48804
HmHel-7	CAEZ01009404:40056-41408
HmHel-7	CAEZ01010026:101921-103278
HmHel-7	CAEZ01010026:161926-163247
HmHel-7	CAEZ01003019:1-1285
HmHel-7	CAEZ01003019:78675-80030
HmHel-7	CAEZ01003019:113570-114905
HmHel-7	CAEZ01003019:24325-25662
HmHel-7	CAEZ01003595:11817-13133
HmHel-7	CAEZ01002258:15117-16473
HmHel-7	CAEZ01002620:1954-5533
HmHel-7	CAEZ01002620:15896-18046
HmHel-7	CAEZ01005949:66329-67688
HmHel-7	CAEZ01006365:11086-12440
HmHel-7	CAEZ01008800:5464-6813
HmHel-7	CAEZ01005674:218-1571
HmHel-7	CAEZ01003338:11843-13178
HmHel-7	CAEZ01004096:41896-43028
HmHel-7	CAEZ01004096:25397-26745
HmHel-7	CAEZ01001898:6574-7859
HmHel-7	CAEZ01001898:4129-5480
HmHel-7	CAEZ01006352:35322-36675
HmHel-7	CAEZ01006352:61680-63028
HmHel-7	CAEZ01010707:717-2065
HmHel-7	CAEZ01002780:10666-12019
HmHel-7	CAEZ01009975:56613-57895
HmHel-7	CAEZ01009975:111538-112243

HmHel-7	CAEZ01009975:136178-137536
HmHel-7	CAEZ01009975:113258-114590
HmHel-7	CAEZ01009975:71011-72357
HmHel-7	CAEZ01009975:4802-6149
HmHel-7	CAEZ01008946:7602-8897
HmHel-7	CAEZ01008367:28723-29857
HmHel-7	CAEZ01008367:125628-126995
HmHel-7	CAEZ01008367:149558-150913
HmHel-7	CAEZ01007061:12437-13797
HmHel-7	CAEZ01004042:3493-4418
HmHel-7	CAEZ01011134:86639-87969
HmHel-7	CAEZ01011134:126084-126989
HmHel-7	CAEZ01008405:10201-11583
HmHel-7	CAEZ01004038:27867-29207
HmHel-7	CAEZ01004038:15455-16793
HmHel-7	CAEZ01006202:34031-35381
HmHel-7	CAEZ01006202:20466-21751
HmHel-7	CAEZ01010270:67479-68828
HmHel-7	CAEZ01010270:182398-183747
HmHel-7	CAEZ01010270:290678-291251
HmHel-7	CAEZ01010270:146884-148250
HmHel-7	CAEZ01010270:83834-85114
HmHel-7	CAEZ01009381:3277-4631
HmHel-7	CAEZ01009381:10939-12108
HmHel-7	CAEZ01003760:8115-9619
HmHel-7	CAEZ01003760:25608-26920
HmHel-7	CAEZ01010496:1-1146
HmHel-7	CAEZ01008921:94117-96447
HmHel-7	CAEZ01008921:141283-142638
HmHel-7	CAEZ01005152:8464-10277
HmHel-7	CAEZ01005152:18613-19932
HmHel-7	CAEZ01002924:112555-113908
HmHel-7	CAEZ01002924:139251-140531
HmHel-7	CAEZ01002924:13339-14686
HmHel-7	CAEZ01009984:10826-12187
HmHel-7	CAEZ01008404:94513-95878
HmHel-7	CAEZ01003828:329-1631
HmHel-7	CAEZ01003828:95780-97124
HmHel-7	CAEZ01003828:26142-27490
HmHel-7	CAEZ01010915:54359-55721
HmHel-7	CAEZ01010915:17624-18962
HmHel-7	CAEZ01010915:1414-2136
HmHel-7	CAEZ01008499:28732-30082
HmHel-7	CAEZ01008499:5899-7258
HmHel-7	CAEZ01008230:8-929
HmHel-7	CAEZ01006286:39916-41276
HmHel-7	CAEZ01006286:79753-81099
HmHel-7	CAEZ01006286:65433-66727

HmHel-7	CAEZ01004752:20643-21332
HmHel-7	CAEZ01004752:117792-119075
HmHel-7	CAEZ01004752:2371-3727
HmHel-7	CAEZ01000820:33705-35047
HmHel-7	CAEZ01009736:6191-7549
HmHel-7	CAEZ01010424:163548-164823
HmHel-7	CAEZ01010424:97289-98661
HmHel-7	CAEZ01004097:60104-61467
HmHel-7	CAEZ01006400:5349-6671
HmHel-7	CAEZ01006400:22063-23379
HmHel-7	CAEZ01006400:27699-28985
HmHel-7	CAEZ01006562:226977-228314
HmHel-7	CAEZ01004656:31857-32984
HmHel-7	CAEZ01004724:15412-16692
HmHel-7	CAEZ01002108:1-985
HmHel-7	CAEZ01001510:10698-12064
HmHel-7	CAEZ01001510:32890-33925
HmHel-7	CAEZ01006300:30-948
HmHel-7	CAEZ01011050:3402-4754
HmHel-7	CAEZ01008443:16263-17613
HmHel-7	CAEZ01005746:65827-67206
HmHel-7	CAEZ01011529:35786-37143
HmHel-7	CAEZ01009359:11244-12594
HmHel-7	CAEZ01007329:48839-49561
HmHel-7	CAEZ01007329:11064-12416
HmHel-7	CAEZ01006149:25867-27219
HmHel-7	CAEZ01005475:145697-147047
HmHel-7	CAEZ01005388:1-326
HmHel-7	CAEZ01005388:558-1825
HmHel-7	CAEZ01003413:9323-10661
HmHel-7	CAEZ01002767:29995-35374
HmHel-7	CAEZ01001891:10-578
HmHel-7	CAEZ01001891:45146-46482
HmHel-7	CAEZ01010755:13929-15054
HmHel-7	CAEZ01010237:59553-60913
HmHel-7	CAEZ01010237:17123-18476
HmHel-7	CAEZ01001416:18-1069
HmHel-7	CAEZ01010507:6885-7446
HmHel-7	CAEZ01010507:5061-6393
HmHel-7	CAEZ01009762:5127-6040
HmHel-7	CAEZ01008122:11396-12751
HmHel-7	CAEZ01008122:68575-69086
HmHel-7	CAEZ01008122:1-162
HmHel-7	CAEZ01007975:45071-46429
HmHel-7	CAEZ01007975:37789-39083
HmHel-7	CAEZ01005496:7842-8722
HmHel-7	CAEZ01005496:68981-70333
HmHel-7	CAEZ01005496:110315-110734

HmHel-7	CAEZ01011495:93571-94445
HmHel-7	CAEZ01011495:74683-75964
HmHel-7	CAEZ01007118:23621-24987
HmHel-7	CAEZ01002589:19869-21230
HmHel-7	CAEZ01002589:1307-1749
HmHel-7	CAEZ01011417:2903-4259
HmHel-7	CAEZ01010725:1195-2538
HmHel-7	CAEZ01010174:12955-14305
HmHel-7	CAEZ01009982:98901-100212
HmHel-7	CAEZ01009982:69414-69957
HmHel-7	CAEZ01009982:30021-33138
HmHel-7	CAEZ01008100:75890-77226
HmHel-7	CAEZ01006406:54494-55822
HmHel-7	CAEZ01006406:83212-84575
HmHel-7	CAEZ01006406:73038-74320
HmHel-7	CAEZ01006346:11080-12212
HmHel-7	CAEZ01005669:37017-37906
HmHel-7	CAEZ01002867:282-1280
HmHel-7	CAEZ01005722:21176-22303
HmHel-7	CAEZ01005473:26564-27912
HmHel-7	CAEZ01010737:1-943
HmHel-7	CAEZ01010737:61589-62934
HmHel-7	CAEZ01010737:129150-130507
HmHel-7	CAEZ01010161:3839-5176
HmHel-7	CAEZ01007603:52744-54082
HmHel-7	CAEZ01007603:27385-28732
HmHel-7	CAEZ01003719:23822-25174
HmHel-7	CAEZ01001908:6921-8267
HmHel-7	CAEZ01009766:43512-44208
HmHel-7	CAEZ01009766:59485-60828
HmHel-7	CAEZ01009766:63342-63937
HmHel-7	CAEZ01009417:33299-34660
HmHel-7	CAEZ01008183:71973-73321
HmHel-7	CAEZ01008183:50246-51617
HmHel-7	CAEZ01005840:6080-7436
HmHel-7	CAEZ01005392:1-1377
HmHel-7	CAEZ01005392:83448-84334
HmHel-7	CAEZ01003363:712-2519
HmHel-7	CAEZ01003006:11456-12808
HmHel-7	CAEZ01002849:17355-18717
HmHel-7	CAEZ01009186:20912-22270
HmHel-7	CAEZ01009033:13419-14767
HmHel-7	CAEZ01006169:459-984
HmHel-7	CAEZ01005964:1-1048
HmHel-7	CAEZ01005964:24089-25405
HmHel-7	CAEZ01003610:17822-19183
HmHel-7	CAEZ01011169:3793-5145
HmHel-7	CAEZ01010974:14765-16033

HmHel-7	CAEZ01010974:9128-10463
HmHel-7	CAEZ01007498:64223-65601
HmHel-7	CAEZ01007498:38328-39663
HmHel-7	CAEZ01007498:17036-18170
HmHel-7	CAEZ01004734:1-1343
HmHel-7	CAEZ01010437:61535-62894
HmHel-7	CAEZ01010370:6950-8310
HmHel-7	CAEZ01003875:14672-15996
HmHel-7	CAEZ01003875:4859-6211
HmHel-7	CAEZ01003717:32162-33441
HmHel-7	CAEZ01003717:52826-54187
HmHel-7	CAEZ01003717:123379-124345
HmHel-7	CAEZ01001743:59427-60771
HmHel-7	CAEZ01000802:2630-3987
HmHel-7	CAEZ01010706:4001-5358
HmHel-7	CAEZ01010706:59468-60752
HmHel-7	CAEZ01008105:129886-130708
HmHel-7	CAEZ01008105:25732-27093
HmHel-7	CAEZ01005393:1-331
HmHel-7	CAEZ01005393:70088-71393
HmHel-7	CAEZ01005393:128480-129769
HmHel-7	CAEZ01003747:25915-27267
HmHel-7	CAEZ01004631:320627-321984
HmHel-7	CAEZ01004631:260177-261051
HmHel-7	CAEZ01004136:9463-10825
HmHel-7	CAEZ01004068:3252-4569
HmHel-7	CAEZ01002729:29839-31182
HmHel-7	CAEZ01002729:35442-36805
HmHel-7	CAEZ01010127:89458-90810
HmHel-7	CAEZ01008879:58538-61716
HmHel-7	CAEZ01005270:10761-11580
HmHel-7	CAEZ01005270:22915-24183
HmHel-7	CAEZ01003247:12142-13103
HmHel-7	CAEZ01003247:23771-25119
HmHel-7	CAEZ01003075:28223-29579
HmHel-7	CAEZ01009929:39461-40817
HmHel-7	CAEZ01009929:42301-43521
HmHel-7	CAEZ01007475:83573-84906
HmHel-7	CAEZ01010745:1727-3054
HmHel-7	CAEZ01008619:3225-4041
HmHel-7	CAEZ01008619:98193-98462
HmHel-7	CAEZ01002772:32794-34125
HmHel-7	CAEZ01009996:40130-40905
HmHel-7	CAEZ01009996:73543-75749
HmHel-7	CAEZ01009996:31836-33202
HmHel-7	CAEZ01009336:4803-6150
HmHel-7	CAEZ01008425:99244-100598
HmHel-7	CAEZ01008425:126874-127645

HmHel-7	CAEZ01007747:241002-242277
HmHel-7	CAEZ01007747:77192-78525
HmHel-7	CAEZ01008398:68257-69593
HmHel-7	CAEZ01008398:27605-28955
HmHel-7	CAEZ01007662:1-1157
HmHel-7	CAEZ01007222:208672-209812
HmHel-7	CAEZ01007222:266097-267406
HmHel-7	CAEZ01007222:230576-231891
HmHel-7	CAEZ01006749:39328-40650
HmHel-7	CAEZ01005894:9102-10147
HmHel-7	CAEZ01003345:49099-50455
HmHel-7	CAEZ01003345:25823-27162
HmHel-7	CAEZ01003192:33999-35357
HmHel-7	CAEZ01003192:121903-123255
HmHel-7	CAEZ01003192:117058-118342
HmHel-7	CAEZ01003171:3668-5019
HmHel-7	CAEZ01002238:1584-2944
HmHel-7	CAEZ01010868:50547-51863
HmHel-7	CAEZ01006617:21956-23096
HmHel-7	CAEZ01006617:51268-52575
HmHel-7	CAEZ01001598:2215-3353
HmHel-7	CAEZ01001598:779-1986
HmHel-7	CAEZ01001444:14012-15355
HmHel-7	CAEZ01001444:10531-11780
HmHel-7	CAEZ01010581:11122-12480
HmHel-7	CAEZ01008423:49470-50821
HmHel-7	CAEZ01004740:34558-35898
HmHel-7	CAEZ01003645:10832-12167
HmHel-7	CAEZ01011307:547-2333
HmHel-7	CAEZ01008784:13275-14630
HmHel-7	CAEZ01008620:44745-46105
HmHel-7	CAEZ01008620:213712-215006
HmHel-7	CAEZ01008620:95342-96695
HmHel-7	CAEZ01008620:175-1355
HmHel-7	CAEZ01008615:1397-2743
HmHel-7	CAEZ01007754:28942-30298
HmHel-7	CAEZ01006433:21882-23241
HmHel-7	CAEZ01006433:43854-45156
HmHel-7	CAEZ01005219:36996-38343
HmHel-7	CAEZ01000907:2061-3395
HmHel-7	CAEZ01008018:3195-4489
HmHel-7	CAEZ01005942:22303-23667
HmHel-7	CAEZ01005438:20061-21402
HmHel-7	CAEZ01004634:10562-11907
HmHel-7	CAEZ01002663:10057-11412
HmHel-7	CAEZ01006130:62633-63964
HmHel-7	CAEZ01006130:198537-199838
HmHel-7	CAEZ01005511:847-2187

HmHel-7	CAEZ01009894:32052-33883
HmHel-7	CAEZ01006772:17026-18363
HmHel-7	CAEZ01009631:52056-53374
HmHel-7	CAEZ01008245:5576-6933
HmHel-7	CAEZ01005154:1903-3023
HmHel-7	CAEZ01004811:19067-19977
HmHel-7	CAEZ01004811:434393-434773
HmHel-7	CAEZ01004811:165607-166966
HmHel-7	CAEZ01002736:21162-22519
HmHel-7	CAEZ01002383:21982-23328
HmHel-7	CAEZ01009456:55352-56710
HmHel-7	CAEZ01009456:152811-154137
HmHel-7	CAEZ01009145:8225-9559
HmHel-7	CAEZ01009145:18273-18485
HmHel-7	CAEZ01007018:6058-7402
HmHel-7	CAEZ01004150:1431-2779
HmHel-7	CAEZ01004150:6442-6703
HmHel-7	CAEZ01010887:18102-18893
HmHel-7	CAEZ01010865:8066-9424
HmHel-7	CAEZ01010580:4240-5526
HmHel-7	CAEZ01003881:801-2286
HmHel-7	CAEZ01003881:40247-41511
HmHel-7	CAEZ01003881:13709-15061
HmHel-7	CAEZ01003208:31503-33306
HmHel-7	CAEZ01001765:27814-29162
HmHel-7	CAEZ01001765:72491-73789
HmHel-7	CAEZ01011088:1636-2970
HmHel-7	CAEZ01009845:21322-22679
HmHel-7	CAEZ01009845:15456-16669
HmHel-7	CAEZ01008420:1-1250
HmHel-7	CAEZ01008420:162213-163548
HmHel-7	CAEZ01008327:13425-14728
HmHel-7	CAEZ01008327:65750-67071
HmHel-7	CAEZ01007499:158023-159316
HmHel-7	CAEZ01007499:132398-133641
HmHel-7	CAEZ01005458:15935-17283
HmHel-7	CAEZ01004534:8441-9429
HmHel-7	CAEZ01001403:36662-37950
HmHel-7	CAEZ01010795:26108-28043
HmHel-7	CAEZ01003761:431-1382
HmHel-7	CAEZ01009791:557-1918
HmHel-7	CAEZ01008721:205898-207221
HmHel-7	CAEZ01008721:44910-46267
HmHel-7	CAEZ01008383:8406-9759
HmHel-7	CAEZ01004810:1-1027
HmHel-7	CAEZ01001890:10862-12081
HmHel-7	CAEZ01001890:1-867
HmHel-7	CAEZ01001449:14910-16136

HmHel-7	CAEZ01010885:1246-2593
HmHel-7	CAEZ01003827:1123-2173
HmHel-7	CAEZ01003105:4705-6059
HmHel-7	CAEZ01009807:20265-20794
HmHel-7	CAEZ01009807:15494-16854
HmHel-7	CAEZ01003222:73349-75101
HmHel-7	CAEZ01006061:7397-8732
HmHel-7	CAEZ01005221:38109-38958
HmHel-7	CAEZ01005221:1-925
HmHel-7	CAEZ01004179:1-1276
HmHel-7	CAEZ01008331:3140-4487
HmHel-7	CAEZ01007982:16253-17563
HmHel-7	CAEZ01007982:4875-6225
HmHel-7	CAEZ01004259:18413-19767
HmHel-7	CAEZ01004259:57917-58962
HmHel-7	CAEZ01007373:565-1909
HmHel-7	CAEZ01002904:29-551
HmHel-7	CAEZ01002904:13235-14586
HmHel-7	CAEZ01010667:29466-30807
HmHel-7	CAEZ01010667:53843-55028
HmHel-7	CAEZ01008783:3499-4821
HmHel-7	CAEZ01006638:900-2178
HmHel-7	CAEZ01006184:34-1048
HmHel-7	CAEZ01006053:4896-6248
HmHel-7	CAEZ01002662:536-1798
HmHel-7	CAEZ01002361:16714-18065
HmHel-7	CAEZ01002361:52227-53480
HmHel-7	CAEZ01010301:62787-63502
HmHel-7	CAEZ01010301:74295-75643
HmHel-7	CAEZ01010301:13693-14976
HmHel-7	CAEZ01008576:70580-71932
HmHel-7	CAEZ01008576:4065-5425
HmHel-7	CAEZ01006249:25589-26922
HmHel-7	CAEZ01006249:64765-66024
HmHel-7	CAEZ01006249:45554-46842
HmHel-7	CAEZ01004270:24665-26008
HmHel-7	CAEZ01004226:23458-24551
HmHel-7	CAEZ01010703:56759-58099
HmHel-7	CAEZ01010703:14154-15340
HmHel-7	CAEZ01008260:29644-30970
HmHel-7	CAEZ01003005:4158-5445
HmHel-7	CAEZ01010770:30471-31832
HmHel-7	CAEZ01010691:2696-4042
HmHel-7	CAEZ01010405:1-1196
HmHel-7	CAEZ01009843:39098-40382
HmHel-7	CAEZ01009843:27022-28148
HmHel-7	CAEZ01009843:1-369
HmHel-7	CAEZ01006479:4643-6000

HmHel-7	CAEZ01006618:213108-214383
HmHel-7	CAEZ01006618:275353-276703
HmHel-7	CAEZ01006618:40045-41324
HmHel-7	CAEZ01006372:40740-43704
HmHel-7	CAEZ01006372:62714-64045
HmHel-7	CAEZ01005269:36362-37540
HmHel-7	CAEZ01005269:76684-78108
HmHel-7	CAEZ01001850:33526-34862
HmHel-7	CAEZ01011027:8007-9305
HmHel-7	CAEZ01010130:20758-21958
HmHel-7	CAEZ01010130:34455-35802
HmHel-7	CAEZ01004633:1-724
HmHel-7	CAEZ01007812:98053-99401
HmHel-7	CAEZ01007812:243218-244543
HmHel-7	CAEZ01007812:153158-154527
HmHel-7	CAEZ01010735:14996-16344
HmHel-7	CAEZ01009767:2958-4292
HmHel-7	CAEZ01004273:3-807
HmHel-7	CAEZ01009279:1631-3359
HmHel-7	CAEZ01006632:15779-17119
HmHel-7	CAEZ01006416:11729-13060
HmHel-7	CAEZ01008614:2-1004
HmHel-7	CAEZ01008614:49336-50142
HmHel-7	CAEZ01008431:26929-28291
HmHel-7	CAEZ01005239:3751-5083
HmHel-7	CAEZ01005239:6242-7563
HmHel-7	CAEZ01000935:1524-2884
HmHel-7	CAEZ01011179:27593-28934
HmHel-7	CAEZ01006616:78339-79573
HmHel-7	CAEZ01006616:240147-241500
HmHel-7	CAEZ01000850:104926-106179
HmHel-7	CAEZ01009828:28193-29548
HmHel-7	CAEZ01010278:22113-23468
HmHel-7	CAEZ01008258:100964-102184
HmHel-7	CAEZ01008258:76816-78125
HmHel-7	CAEZ01002866:82172-82969
HmHel-7	CAEZ01002866:160132-161482
HmHel-7	CAEZ01009460:34910-35615
HmHel-7	CAEZ01009460:8556-9910
HmHel-7	CAEZ01007624:18993-26409
HmHel-7	CAEZ01003081:6474-7291
HmHel-7	CAEZ01008961:50140-51493
HmHel-7	CAEZ01007631:50078-51423
HmHel-7	CAEZ01001965:26935-28286
HmHel-7	CAEZ01001965:18806-20163
HmHel-7	CAEZ01007638:3-1014
HmHel-7	CAEZ01003735:62-1339
HmHel-7	CAEZ01007530:8039-9044

HmHel-7	CAEZ01005025:2761-4051
HmHel-7	CAEZ01005025:55775-57116
HmHel-7	CAEZ01005025:109890-111245
HmHel-7	CAEZ01005025:117397-118746
HmHel-7	CAEZ01001033:4408-5678
HmHel-7	CAEZ01006369:27082-28399
HmHel-7	CAEZ01003987:1989-3349
HmHel-7	CAEZ01000858:20197-21181
HmHel-7	CAEZ01005083:29125-30455
HmHel-7	CAEZ01005083:62251-63611
HmHel-7	CAEZ01005083:94805-95390
HmHel-7	CAEZ01005083:53384-53947
HmHel-7	CAEZ01008677:18321-19639
HmHel-7	CAEZ01003250:24839-26196
HmHel-7	CAEZ01003250:159078-160424
HmHel-7	CAEZ01001287:1-1086
HmHel-7	CAEZ01007993:2958-4251
HmHel-7	CAEZ01007730:1317-2666
HmHel-7	CAEZ01007730:89414-90763
HmHel-7	CAEZ01007730:75244-76550
HmHel-7	CAEZ01010291:1147-2513
HmHel-7	CAEZ01008436:1094-2433
HmHel-7	CAEZ01006227:454-1737
HmHel-7	CAEZ01006073:63265-64620
HmHel-7	CAEZ01007463:12575-13829
HmHel-7	CAEZ01002634:17067-18388
HmHel-7	CAEZ01002634:3282-4555
HmHel-7	CAEZ01010361:8874-10193
HmHel-7	CAEZ01007177:1159-2495
HmHel-7	CAEZ01001738:46432-47783
HmHel-7	CAEZ01001738:13763-15116
HmHel-7	CAEZ01009556:8395-9742
HmHel-7	CAEZ01003879:9375-10378
HmHel-7	CAEZ01009010:13187-13593
HmHel-7	CAEZ01009010:11785-11982
HmHel-7	CAEZ01008899:4791-6101
HmHel-7	CAEZ01001457:1-10188
HmHel-7	CAEZ01010139:30800-32156
HmHel-7	CAEZ01010139:61396-62736
HmHel-7	CAEZ01010139:111185-111961
HmHel-7	CAEZ01010139:84280-85627
HmHel-7	CAEZ01005474:46324-47673
HmHel-7	CAEZ01004815:102551-103918
HmHel-7	CAEZ01004986:1756-3114
HmHel-7	CAEZ01002097:62952-64252
HmHel-7	CAEZ01002097:38756-40113
HmHel-7	CAEZ01009213:1-1302
HmHel-7	CAEZ01008418:82413-83746

HmHel-7	CAEZ01008950:5-791
HmHel-7	CAEZ01006030:8454-9264
HmHel-7	CAEZ01008857:12479-13706
HmHel-7	CAEZ01005268:9642-11005
HmHel-7	CAEZ01005268:11547-12829
HmHel-7	CAEZ01007066:25760-27022
HmHel-7	CAEZ01007066:77619-78989
HmHel-7	CAEZ01007066:64881-66015
HmHel-7	CAEZ01005425:20975-22315
HmHel-7	CAEZ01001317:4438-5654
HmHel-7	CAEZ01005498:1436-2718
HmHel-7	CAEZ01008920:11400-12755
HmHel-7	CAEZ01004076:2636-3994
HmHel-7	CAEZ01006343:998-2346
HmHel-7	CAEZ01005850:711-2076
HmHel-7	CAEZ01001775:35386-36764
HmHel-7	CAEZ01010704:44447-45770
HmHel-7	CAEZ01004033:3864-5155
HmHel-7	CAEZ01004033:201112-202379
HmHel-7	CAEZ01004033:116036-117221
HmHel-7	CAEZ01011411:1-1309
HmHel-7	CAEZ01006548:64480-65790
HmHel-7	CAEZ01006548:99536-102024
HmHel-7	CAEZ01008360:30000-31327
HmHel-7	CAEZ01008360:43124-43817
HmHel-7	CAEZ01008360:37501-38733
HmHel-7	CAEZ01010863:18181-20802
HmHel-7	CAEZ01010863:38213-39591
HmHel-7	CAEZ01000968:40791-41644
HmHel-7	CAEZ01000968:6488-7590
HmHel-7	CAEZ01008179:9210-9773
HmHel-7	CAEZ01008179:4-751
HmHel-7	CAEZ01010129:68170-69521
HmHel-7	CAEZ01002321:48296-48772
HmHel-7	CAEZ01002321:64305-64998
HmHel-7	CAEZ01010219:151318-152674
HmHel-7	CAEZ01010219:70918-72275
HmHel-7	CAEZ01010219:1897-3182
HmHel-7	CAEZ01009153:19533-20250
HmHel-7	CAEZ01009153:20366-21155
HmHel-7	CAEZ01006693:503-1772
HmHel-7	CAEZ01004449:2404-3741
HmHel-7	CAEZ01008606:132-1344
HmHel-7	CAEZ01007441:5717-6416
HmHel-7	CAEZ01006272:1318-2053
HmHel-7	CAEZ01006272:5-500
HmHel-7	CAEZ01005810:20325-21039
HmHel-7	CAEZ01005400:1-1212

HmHel-7	CAEZ01005088:26634-28163
HmHel-7	CAEZ01005088:37314-38625
HmHel-7	CAEZ01011104:52941-53930
HmHel-7	CAEZ01011104:107890-109218
HmHel-7	CAEZ01011104:10828-11667
HmHel-7	CAEZ01007660:12039-13414
HmHel-7	CAEZ01007456:481-1341
HmHel-7	CAEZ01006634:34587-42419
HmHel-7	CAEZ01009916:2-851
HmHel-7	CAEZ01006845:46835-48167
HmHel-7	CAEZ01004903:17474-19118
HmHel-7	CAEZ01003722:20547-21882
HmHel-7	CAEZ01003722:47823-49160
HmHel-7	CAEZ01011132:33-440
HmHel-7	CAEZ01011132:43255-44595
HmHel-7	CAEZ01009859:10065-11389
HmHel-7	CAEZ01008194:20157-21511
HmHel-7	CAEZ01008194:34387-35707
HmHel-7	CAEZ01006143:4804-5971
HmHel-7	CAEZ01005465:6185-7368
HmHel-7	CAEZ01007845:1-977
HmHel-7	CAEZ01007453:35351-36632
HmHel-7	CAEZ01006454:11077-12442
HmHel-7	CAEZ01001940:467-1771
HmHel-7	CAEZ01007090:7265-8537
HmHel-7	CAEZ01010920:1-550
HmHel-7	CAEZ01010920:38085-39371
HmHel-7	CAEZ01010702:10065-12111
HmHel-7	CAEZ01009274:20498-21777
HmHel-7	CAEZ01008263:14072-15346
HmHel-7	CAEZ01008247:12088-12502
HmHel-7	CAEZ01008247:10983-12089
HmHel-7	CAEZ01002459:1-1172
HmHel-7	CAEZ01009024:147-1338
HmHel-7	CAEZ01008655:9-699
HmHel-7	CAEZ01001025:16016-17194
HmHel-7	CAEZ01011139:21609-22972
HmHel-7	CAEZ01011139:87048-88430
HmHel-7	CAEZ01011139:63727-65078
HmHel-7	CAEZ01009431:16539-17883
HmHel-7	CAEZ01008427:75897-77183
HmHel-7	CAEZ01008427:62037-63373
HmHel-7	CAEZ01007785:18374-19662
HmHel-7	CAEZ01007785:1-385
HmHel-7	CAEZ01005139:6106-7369
HmHel-7	CAEZ01002184:28573-29903
HmHel-7	CAEZ01002184:1-1218
HmHel-7	CAEZ01003854:26899-28247

HmHel-7	CAEZ01002194:62602-63971
HmHel-7	CAEZ01002194:39620-40923
HmHel-7	CAEZ01010272:1-1231
HmHel-7	CAEZ01010272:90955-92237
HmHel-7	CAEZ01008884:54465-55773
HmHel-7	CAEZ01006347:10641-11923
HmHel-7	CAEZ01006347:61705-62139
HmHel-7	CAEZ01006347:235961-236307
HmHel-7	CAEZ01006347:41956-43228
HmHel-7	CAEZ01006347:25349-26708
HmHel-7	CAEZ01005163:26613-27698
HmHel-7	CAEZ01004930:17379-18724
HmHel-7	CAEZ01000070:5086-6199
HmHel-7	CAEZ01009999:55805-57002
HmHel-7	CAEZ01008771:108347-109679
HmHel-7	CAEZ01008771:19110-25402
HmHel-7	CAEZ01007765:262651-263955
HmHel-7	CAEZ01007271:17769-19070
HmHel-7	CAEZ01007271:1955-3318
HmHel-7	CAEZ01006218:44163-45011
HmHel-7	CAEZ01005886:26411-27782
HmHel-7	CAEZ01003548:32178-32924
HmHel-7	CAEZ01007960:19910-21196
HmHel-7	CAEZ01007428:7176-8313
HmHel-7	CAEZ01007100:27400-28684
HmHel-7	CAEZ01007047:5505-6786
HmHel-7	CAEZ01002825:7303-8590
HmHel-7	CAEZ01002147:5600-6928
HmHel-7	CAEZ01002147:1-467
HmHel-7	CAEZ01011278:182-1494
HmHel-7	CAEZ01009733:37334-38619
HmHel-7	CAEZ01009592:11415-12662
HmHel-7	CAEZ01007609:18382-19654
HmHel-7	CAEZ01007609:45431-47217
HmHel-7	CAEZ01007609:5086-6776
HmHel-7	CAEZ01006145:18744-19429
HmHel-7	CAEZ01004524:7455-8206
HmHel-7	CAEZ01010819:7674-8980
HmHel-7	CAEZ01009891:7982-9336
HmHel-7	CAEZ01009891:70082-71437
HmHel-7	CAEZ01008603:25239-26556
HmHel-7	CAEZ01007937:28383-29732
HmHel-7	CAEZ01005491:8949-10058
HmHel-7	CAEZ01002616:475-1762
HmHel-7	CAEZ01001007:1-719
HmHel-7	CAEZ01000967:4455-5640
HmHel-7	CAEZ01009928:42494-43828
HmHel-7	CAEZ01009806:2357-3647

HmHel-7	CAEZ01008178:12639-13251
HmHel-7	CAEZ01007731:72785-74121
HmHel-7	CAEZ01007731:92762-92889
HmHel-7	CAEZ01006228:62654-64007
HmHel-7	CAEZ01006228:12255-13460
HmHel-7	CAEZ01005320:5578-6944
HmHel-7	CAEZ01004284:4351-5725
HmHel-7	CAEZ01011007:6695-8079
HmHel-7	CAEZ01010212:22388-23683
HmHel-7	CAEZ01009716:203941-205103
HmHel-7	CAEZ01009321:9799-11082
HmHel-7	CAEZ01008112:561-1903
HmHel-7	CAEZ01008112:5805-7082
HmHel-7	CAEZ01006778:133866-135136
HmHel-7	CAEZ01003138:21976-23309
HmHel-7	CAEZ01001466:8813-10100
HmHel-7	CAEZ01001466:79483-80724
HmHel-7	CAEZ01010677:232609-234291
HmHel-7	CAEZ01010677:31437-32719
HmHel-7	CAEZ01009258:14279-15627
HmHel-7	CAEZ01002754:15834-17115
HmHel-7	CAEZ01011547:2091-3407
HmHel-7	CAEZ01011399:30051-31016
HmHel-7	CAEZ01009011:7951-9159
HmHel-7	CAEZ01008527:1-1036
HmHel-7	CAEZ01007769:14674-16013
HmHel-7	CAEZ01006529:7479-8825
HmHel-7	CAEZ01006529:1-425
HmHel-7	CAEZ01005711:163148-164498
HmHel-7	CAEZ01008437:74798-76115
HmHel-7	CAEZ01008437:53745-54780
HmHel-7	CAEZ01007269:54575-55861
HmHel-7	CAEZ01006008:1-306
HmHel-7	CAEZ01006008:10458-11795
HmHel-7	CAEZ01011631:2-979
HmHel-7	CAEZ01011437:6141-7470
HmHel-7	CAEZ01010460:46950-48237
HmHel-7	CAEZ01010460:104167-105390
HmHel-7	CAEZ01009607:25558-26849
HmHel-7	CAEZ01008246:7347-8638
HmHel-7	CAEZ01008164:43423-44708
HmHel-7	CAEZ01007413:74250-75872
HmHel-7	CAEZ01007413:14539-15832
HmHel-7	CAEZ01005981:9142-10408
HmHel-7	CAEZ01005981:20957-22201
HmHel-7	CAEZ01005911:160068-161448
HmHel-7	CAEZ01004829:40220-41507
HmHel-7	CAEZ01003962:294-1576

HmHel-7	CAEZ01003933:11187-12474
HmHel-7	CAEZ01003933:26680-28007
HmHel-7	CAEZ01003149:13422-14308
HmHel-7	CAEZ01002847:1805-3163
HmHel-7	CAEZ01002847:388-1676
HmHel-7	CAEZ01002341:15930-16608
HmHel-7	CAEZ01002341:6020-7345
HmHel-7	CAEZ01002172:4938-7192
HmHel-7	CAEZ01000994:756-1401
HmHel-7	CAEZ01010218:37354-38665
HmHel-7	CAEZ01010218:66717-67248
HmHel-7	CAEZ01010218:51077-52455
HmHel-7	CAEZ01010216:8592-9953
HmHel-7	CAEZ01009974:13317-14655
HmHel-7	CAEZ01009899:15283-16640
HmHel-7	CAEZ01009229:43172-44451
HmHel-7	CAEZ01009212:47491-48411
HmHel-7	CAEZ01009120:80385-81579
HmHel-7	CAEZ01008963:43994-45352
HmHel-7	CAEZ01008963:105215-106570
HmHel-7	CAEZ01008963:120783-123768
HmHel-7	CAEZ01008963:181687-183030
HmHel-7	CAEZ01008182:67467-68836
HmHel-7	CAEZ01007695:1-410
HmHel-7	CAEZ01007695:13770-15056
HmHel-7	CAEZ01007184:2575-5057
HmHel-7	CAEZ01007184:257295-258633
HmHel-7	CAEZ01006908:7445-8731
HmHel-7	CAEZ01005198:22666-24002
HmHel-7	CAEZ01005198:76380-77700
HmHel-7	CAEZ01010828:4779-6059
HmHel-7	CAEZ01010701:154064-155401
HmHel-7	CAEZ01009875:1-1129
HmHel-7	CAEZ01009170:11998-13266
HmHel-7	CAEZ01006495:25422-25991
HmHel-7	CAEZ01006495:1-724
HmHel-7	CAEZ01006350:37072-38002
HmHel-7	CAEZ01006350:12974-14329
HmHel-7	CAEZ01006131:9982-10642
HmHel-7	CAEZ01006131:226933-228287
HmHel-7	CAEZ01006131:40124-41483
HmHel-7	CAEZ01006007:13326-14617
HmHel-7	CAEZ01006007:42825-44117
HmHel-7	CAEZ01005451:99169-102042
HmHel-7	CAEZ01005451:114176-115495
HmHel-7	CAEZ01005373:120285-121613
HmHel-7	CAEZ01002211:47392-48653
HmHel-7	CAEZ01000823:1-1317

HmHel-7	CAEZ01010252:9050-10385
HmHel-7	CAEZ01010250:5589-6875
HmHel-7	CAEZ01009629:5-842
HmHel-7	CAEZ01003070:42874-45451
HmHel-7	CAEZ01003070:199431-200773
HmHel-7	CAEZ01002813:6-843
HmHel-7	CAEZ01011171:1130-2483
HmHel-7	CAEZ01011171:11120-12415
HmHel-7	CAEZ01010952:6535-7769
HmHel-7	CAEZ01010952:21988-23336
HmHel-7	CAEZ01010952:93228-94502
HmHel-7	CAEZ01010952:106830-107737
HmHel-7	CAEZ01010535:9-1291
HmHel-7	CAEZ01009801:44582-45920
HmHel-7	CAEZ01009801:60126-61458
HmHel-7	CAEZ01008110:19716-21349
HmHel-7	CAEZ01007978:14941-16291
HmHel-7	CAEZ01005183:9116-10403
HmHel-7	CAEZ01003385:16858-18144
HmHel-7	CAEZ01002875:51143-52435
HmHel-7	CAEZ01002875:116604-117869
HmHel-7	CAEZ01002875:87921-89234
HmHel-7	CAEZ01001349:1511-2889
HmHel-7	CAEZ01001349:71793-72155
HmHel-7	CAEZ01001349:5-746
HmHel-7	CAEZ01011292:27075-28338
HmHel-7	CAEZ01010455:100316-101575
HmHel-7	CAEZ01010094:16075-17112
HmHel-7	CAEZ01010027:12954-14317
HmHel-7	CAEZ01009435:43122-44464
HmHel-7	CAEZ01009435:65730-66980
HmHel-7	CAEZ01008926:26051-27360
HmHel-7	CAEZ01005478:24338-25645
HmHel-7	CAEZ01005478:70331-71649
HmHel-7	CAEZ01005015:168932-170290
HmHel-7	CAEZ01005015:230862-232153
HmHel-7	CAEZ01005015:157814-159118
HmHel-7	CAEZ01004632:84135-85510
HmHel-7	CAEZ01004632:109995-111275
HmHel-7	CAEZ01004632:118914-119781
HmHel-7	CAEZ01004452:22383-23693
HmHel-7	CAEZ01004452:55255-56600
HmHel-7	CAEZ01003863:12029-13376
HmHel-7	CAEZ01001463:49420-50704
HmHel-7	CAEZ01001147:25911-27248
HmHel-7	CAEZ01011020:15692-16985
HmHel-7	CAEZ01010444:6628-7963
HmHel-7	CAEZ01010039:191518-192851

HmHel-7	CAEZ01010039:101157-102491
HmHel-7	CAEZ01010039:52224-52314
HmHel-7	CAEZ01006283:46478-47371
HmHel-7	CAEZ01005489:8865-10230
HmHel-7	CAEZ01003077:60128-61152
HmHel-7	CAEZ01001239:6181-6699
HmHel-7	CAEZ01001239:1578-2865
HmHel-7	CAEZ01011107:41062-42349
HmHel-7	CAEZ01011107:75928-77266
HmHel-7	CAEZ01011093:22105-23463
HmHel-7	CAEZ01011093:193594-194970
HmHel-7	CAEZ01011093:134793-136069
HmHel-7	CAEZ01010900:53884-55236
HmHel-7	CAEZ01007385:10048-11337
HmHel-7	CAEZ01005418:257624-258908
HmHel-7	CAEZ01005418:259487-263988
HmHel-7	CAEZ01004983:1-1159
HmHel-7	CAEZ01010851:45305-46608
HmHel-7	CAEZ01009583:4400-5529
HmHel-7	CAEZ01009583:89837-90577
HmHel-7	CAEZ01009583:20974-22347
HmHel-7	CAEZ01009481:8312-9656
HmHel-7	CAEZ01009481:48115-48199
HmHel-7	CAEZ01009481:34530-35551
HmHel-7	CAEZ01007706:120626-121910
HmHel-7	CAEZ01007063:23098-24382
HmHel-7	CAEZ01003349:59607-60895
HmHel-7	CAEZ01003349:35067-36409
HmHel-7	CAEZ01003111:45249-46531
HmHel-7	CAEZ01002892:19404-20692
HmHel-7	CAEZ01010907:114833-116199
HmHel-7	CAEZ01009788:80663-81732
HmHel-7	CAEZ01009788:30486-31837
HmHel-7	CAEZ01009788:9836-11121
HmHel-7	CAEZ01009085:87474-88403
HmHel-7	CAEZ01009085:97698-98930
HmHel-7	CAEZ01009085:76779-78133
HmHel-7	CAEZ01009085:13204-14985
HmHel-7	CAEZ01008915:73041-74382
HmHel-7	CAEZ01008287:15752-17043
HmHel-7	CAEZ01007772:52260-53593
HmHel-7	CAEZ01007325:58142-59500
HmHel-7	CAEZ01004362:10894-12235
HmHel-7	CAEZ01003123:4214-5502
HmHel-7	CAEZ01002180:92309-93646
HmHel-7	CAEZ01001556:3993-5343
HmHel-7	CAEZ01001556:11679-13030
HmHel-7	CAEZ01010918:17624-18913

HmHel-7	CAEZ01010918:115263-115680
HmHel-7	CAEZ01008021:18732-20013
HmHel-7	CAEZ01007939:56822-57929
HmHel-7	CAEZ01007939:40-959
HmHel-7	CAEZ01007576:16263-17639
HmHel-7	CAEZ01007576:133-503
HmHel-7	CAEZ01006967:1-700
HmHel-7	CAEZ01004387:9-759
HmHel-7	CAEZ01003647:5016-6372
HmHel-7	CAEZ01002203:29152-30436
HmHel-7	CAEZ01002015:24218-25534
HmHel-7	CAEZ01009066:16-856
HmHel-7	CAEZ01009066:4245-5577
HmHel-7	CAEZ01005250:45552-46871
HmHel-7	CAEZ01005250:88577-89853
HmHel-7	CAEZ01005250:27221-28569
HmHel-7	CAEZ01003557:15718-16729
HmHel-7	CAEZ01002814:186230-187593
HmHel-7	CAEZ01010300:3257-4480
HmHel-7	CAEZ01009233:15013-16405
HmHel-7	CAEZ01009233:3-391
HmHel-7	CAEZ01009078:11986-14381
HmHel-7	CAEZ01009030:162-1445
HmHel-7	CAEZ01009030:100950-101530
HmHel-7	CAEZ01009030:67752-68311
HmHel-7	CAEZ01008919:83565-83799
HmHel-7	CAEZ01008919:72624-73780
HmHel-7	CAEZ01008191:1-933
HmHel-7	CAEZ01008176:12696-14016
HmHel-7	CAEZ01008106:19477-20843
HmHel-7	CAEZ01008106:1-443
HmHel-7	CAEZ01007621:7409-8747
HmHel-7	CAEZ01006799:58552-58783
HmHel-7	CAEZ01006799:96070-97175
HmHel-7	CAEZ01006799:65497-66604
HmHel-7	CAEZ01006750:8535-9802
HmHel-7	CAEZ01006750:6622-7714
HmHel-7	CAEZ01005259:6342-7702
HmHel-7	CAEZ01005097:10766-12046
HmHel-7	CAEZ01002712:18248-19536
HmHel-7	CAEZ01001888:19034-20160
HmHel-7	CAEZ01011120:44700-45986
HmHel-7	CAEZ01010827:384402-385676
HmHel-7	CAEZ01010827:8250-9627
HmHel-7	CAEZ01009844:36403-37759
HmHel-7	CAEZ01009506:51475-52681
HmHel-7	CAEZ01007728:4052-5393
HmHel-7	CAEZ01007728:11650-12991

HmHel-7	CAEZ01007721:25476-26843
HmHel-7	CAEZ01007721:17766-18733
HmHel-7	CAEZ01006434:30159-31416
HmHel-7	CAEZ01004741:30623-31905
HmHel-7	CAEZ01004662:29515-30849
HmHel-7	CAEZ01004662:48492-49811
HmHel-7	CAEZ01004662:71930-73239
HmHel-7	CAEZ01004662:810-2162
HmHel-7	CAEZ01002646:19888-21143
HmHel-7	CAEZ01002646:8375-9554
HmHel-7	CAEZ01002134:2787-4072
HmHel-7	CAEZ01006423:225539-226865
HmHel-7	CAEZ01006423:274825-276069
HmHel-7	CAEZ01006423:334027-335335
HmHel-7	CAEZ01006423:292743-294059
HmHel-7	CAEZ01003099:7343-8688
HmHel-7	CAEZ01002301:460-1743
HmHel-7	CAEZ01000839:23357-24697
HmHel-7	CAEZ01000839:10984-12318
HmHel-7	CAEZ01011651:17333-18607
HmHel-7	CAEZ01010730:32868-34152
HmHel-7	CAEZ01010257:18118-19454
HmHel-7	CAEZ01009887:4428-5792
HmHel-7	CAEZ01009887:167660-168995
HmHel-7	CAEZ01009887:225570-227731
HmHel-7	CAEZ01007784:13504-14360
HmHel-7	CAEZ01007245:1820-3134
HmHel-7	CAEZ01007232:48000-49352
HmHel-7	CAEZ01007232:24613-25836
HmHel-7	CAEZ01006148:6155-7637
HmHel-7	CAEZ01003715:1107-2458
HmHel-7	CAEZ01001879:1256-2188
HmHel-7	CAEZ01010926:126369-127703
HmHel-7	CAEZ01010232:5547-6814
HmHel-7	CAEZ01009346:19893-21209
HmHel-7	CAEZ01008602:11194-13529
HmHel-7	CAEZ01008602:6549-7804
HmHel-7	CAEZ01003635:34402-35663
HmHel-7	CAEZ01002363:9377-10737
HmHel-7	CAEZ01001779:4247-5517
HmHel-7	CAEZ01001496:24106-25423
HmHel-7	CAEZ01000849:86724-88060
HmHel-7	CAEZ01000067:3404-4740
HmHel-7	CAEZ01011137:18714-20113
HmHel-7	CAEZ01010668:15431-16572
HmHel-7	CAEZ01010668:21737-22053
HmHel-7	CAEZ01010450:6733-8011
HmHel-7	CAEZ01010450:31672-33045

HmHel-7	CAEZ01010028:68186-69295
HmHel-7	CAEZ01009246:6988-8269
HmHel-7	CAEZ01007012:908-2195
HmHel-7	CAEZ01005716:69115-70472
HmHel-7	CAEZ01003963:1101-2380
HmHel-7	CAEZ01003746:43172-44513
HmHel-7	CAEZ01003401:24697-25978
HmHel-7	CAEZ01002771:25355-26417
HmHel-7	CAEZ01002771:7729-9081
HmHel-7	CAEZ01001133:6914-8182
HmHel-7	CAEZ01001088:311-1052
HmHel-7	CAEZ01011272:4683-6057
HmHel-7	CAEZ01011230:6991-8271
HmHel-7	CAEZ01009638:19657-20997
HmHel-7	CAEZ01007370:18521-19443
HmHel-7	CAEZ01006970:9004-10323
HmHel-7	CAEZ01005876:5049-6384
HmHel-7	CAEZ01005271:1-723
HmHel-7	CAEZ01003575:8248-9407
HmHel-7	CAEZ01003346:53746-55083
HmHel-7	CAEZ01003346:44196-45534
HmHel-7	CAEZ01003305:1-740
HmHel-7	CAEZ01002713:23132-24417
HmHel-7	CAEZ01002318:1550-2828
HmHel-7	CAEZ01009730:3159-4439
HmHel-7	CAEZ01007207:36378-37723
HmHel-7	CAEZ01006299:47247-48603
HmHel-7	CAEZ01006299:27358-27826
HmHel-7	CAEZ01005696:1677-2963
HmHel-7	CAEZ01004972:47277-48565
HmHel-7	CAEZ01004771:5543-6881
HmHel-7	CAEZ01004117:8794-9116
HmHel-7	CAEZ01003137:7781-9031
HmHel-7	CAEZ01002603:6222-7572
HmHel-7	CAEZ01001805:8673-10031
HmHel-7	CAEZ01001805:88806-90142
HmHel-7	CAEZ01001214:726-1121
HmHel-7	CAEZ01009224:1526-2859
HmHel-7	CAEZ01009224:56507-56900
HmHel-7	CAEZ01009023:82791-83473
HmHel-7	CAEZ01009023:95710-96636
HmHel-7	CAEZ01008616:4846-6142
HmHel-7	CAEZ01008391:24275-25569
HmHel-7	CAEZ01007259:84107-85465
HmHel-7	CAEZ01006252:128986-130371
HmHel-7	CAEZ01006252:63098-64443
HmHel-7	CAEZ01006252:18675-19955
HmHel-7	CAEZ01005750:131-1056

HmHel-7	CAEZ01005606:47843-49134
HmHel-7	CAEZ01005606:70328-71555
HmHel-7	CAEZ01005606:86749-88058
HmHel-7	CAEZ01005606:41613-42896
HmHel-7	CAEZ01005572:13734-14181
HmHel-7	CAEZ01005410:42154-43513
HmHel-7	CAEZ01005410:28937-30224
HmHel-7	CAEZ01003416:29704-30966
HmHel-7	CAEZ01003415:67031-68391
HmHel-7	CAEZ01003415:8860-10218
HmHel-7	CAEZ01003079:4849-6116
HmHel-7	CAEZ01002105:1441-2801
HmHel-7	CAEZ01011055:721-1947
HmHel-7	CAEZ01010700:88831-90112
HmHel-7	CAEZ01009674:1-1332
HmHel-7	CAEZ01009209:11602-12185
HmHel-7	CAEZ01007121:25699-27040
HmHel-7	CAEZ01007121:44730-46087
HmHel-7	CAEZ01006577:2887-4222
HmHel-7	CAEZ01004816:47747-49026
HmHel-7	CAEZ01003929:1084-2422
HmHel-7	CAEZ01003365:1-679
HmHel-7	CAEZ01003322:4826-6219
HmHel-7	CAEZ01003235:99102-100430
HmHel-7	CAEZ01003235:87701-88945
HmHel-7	CAEZ01001233:11-418
HmHel-7	CAEZ01001233:6891-8242
HmHel-7	CAEZ01009134:2208-2751
HmHel-7	CAEZ01008099:2459-3580
HmHel-7	CAEZ01007700:3379-4727
HmHel-7	CAEZ01007647:2943-4282
HmHel-7	CAEZ01007611:1108-2447
HmHel-7	CAEZ01006230:25253-26570
HmHel-7	CAEZ01005514:1-553
HmHel-7	CAEZ01003894:4464-5786
HmHel-7	CAEZ01001330:7377-8728
HmHel-7	CAEZ01011494:35480-36745
HmHel-7	CAEZ01009770:119752-120925
HmHel-7	CAEZ01009770:103472-104795
HmHel-7	CAEZ01008372:295573-296913
HmHel-7	CAEZ01007749:64203-65354
HmHel-7	CAEZ01007297:1438-2666
HmHel-7	CAEZ01006196:4733-6545
HmHel-7	CAEZ01006196:61491-62772
HmHel-7	CAEZ01002642:22383-23727
HmHel-7	CAEZ01001665:802-1722
HmHel-7	CAEZ01001454:3291-4571
HmHel-7	CAEZ01011410:37639-38921

HmHel-7	CAEZ01011410:57034-57419
HmHel-7	CAEZ01010285:51323-52664
HmHel-7	CAEZ01010285:105519-106806
HmHel-7	CAEZ01009268:52177-53541
HmHel-7	CAEZ01009077:160014-161322
HmHel-7	CAEZ01008460:10246-11537
HmHel-7	CAEZ01008121:21729-22705
HmHel-7	CAEZ01008121:371-1462
HmHel-7	CAEZ01008104:38180-39520
HmHel-7	CAEZ01007426:4069-5431
HmHel-7	CAEZ01007395:46674-47998
HmHel-7	CAEZ01007395:14580-15752
HmHel-7	CAEZ01007264:13192-14552
HmHel-7	CAEZ01006823:6422-7707
HmHel-7	CAEZ01003260:82893-84128
HmHel-7	CAEZ01001166:2235-3518
HmHel-7	CAEZ01007941:156130-157458
HmHel-7	CAEZ01007446:1-696
HmHel-7	CAEZ01007446:27418-28696
HmHel-7	CAEZ01006129:5244-6589
HmHel-7	CAEZ01005735:31688-32694
HmHel-7	CAEZ01005319:28529-29839
HmHel-7	CAEZ01004814:8171-9452
HmHel-7	CAEZ01003718:1211-2561
HmHel-7	CAEZ01002170:1-1289
HmHel-7	CAEZ01001871:1-807
HmHel-7	CAEZ01010893:68770-70087
HmHel-7	CAEZ01010132:8881-9811
HmHel-7	CAEZ01009380:24155-25475
HmHel-7	CAEZ01007951:3185-4577
HmHel-7	CAEZ01007809:283889-285144
HmHel-7	CAEZ01007809:1-289
HmHel-7	CAEZ01005924:25510-26845
HmHel-7	CAEZ01005860:30225-31554
HmHel-7	CAEZ01005860:1-458
HmHel-7	CAEZ01005827:76236-77330
HmHel-7	CAEZ01005827:73258-74530
HmHel-7	CAEZ01005426:18620-19900
HmHel-7	CAEZ01002811:153968-155256
HmHel-7	CAEZ01002811:118077-119337
HmHel-7	CAEZ01001419:616-1994
HmHel-7	CAEZ01010287:68180-69447
HmHel-7	CAEZ01010071:10-1299
HmHel-7	CAEZ01009742:15773-17056
HmHel-7	CAEZ01008186:9163-10509
HmHel-7	CAEZ01007272:37463-38851
HmHel-7	CAEZ01007272:7968-9334
HmHel-7	CAEZ01006992:2966-4301

HmHel-7	CAEZ01006883:1726-3054
HmHel-7	CAEZ01006883:52-567
HmHel-7	CAEZ01006302:10722-12053
HmHel-7	CAEZ01005290:27394-28752
HmHel-7	CAEZ01005175:40926-42165
HmHel-7	CAEZ01005175:1180-2414
HmHel-7	CAEZ01004833:21031-22269
HmHel-7	CAEZ01003940:42-959
HmHel-7	CAEZ01003767:2289-2954
HmHel-7	CAEZ01003496:14705-16002
HmHel-7	CAEZ01003407:65244-66457
HmHel-7	CAEZ01011545:6570-7929
HmHel-7	CAEZ01010820:3-793
HmHel-7	CAEZ01010820:13668-14977
HmHel-7	CAEZ01009306:86076-87402
HmHel-7	CAEZ01009068:259-1626
HmHel-7	CAEZ01007606:1-717
HmHel-7	CAEZ01007224:3049-4333
HmHel-7	CAEZ01005741:408-1691
HmHel-7	CAEZ01005693:17285-18635
HmHel-7	CAEZ01005355:1308-2591
HmHel-7	CAEZ01005114:13611-14958
HmHel-7	CAEZ01003750:113339-114700
HmHel-7	CAEZ01003750:173546-174909
HmHel-7	CAEZ01003750:89064-90436
HmHel-7	CAEZ01002230:12700-13618
HmHel-7	CAEZ01001595:131961-132821
HmHel-7	CAEZ01001526:7934-9297
HmHel-7	CAEZ01011245:59222-60540
HmHel-7	CAEZ01011149:6175-6914
HmHel-7	CAEZ01010969:17723-20303
HmHel-7	CAEZ01010969:106713-108081
HmHel-7	CAEZ01010969:242594-243328
HmHel-7	CAEZ01010206:17079-18084
HmHel-7	CAEZ01010166:8116-9471
HmHel-7	CAEZ01008638:3649-4654
HmHel-7	CAEZ01007620:24531-25591
HmHel-7	CAEZ01003714:11429-12737
HmHel-7	CAEZ01003629:9012-10326
HmHel-7	CAEZ01003629:16345-17736
HmHel-7	CAEZ01003175:32768-34120
HmHel-7	CAEZ01003151:69026-70401
HmHel-7	CAEZ01010461:6417-7692
HmHel-7	CAEZ01010335:10176-11554
HmHel-7	CAEZ01008162:20496-21321
HmHel-7	CAEZ01006758:6595-7929
HmHel-7	CAEZ01006503:7474-8837
HmHel-7	CAEZ01006010:13160-14477

HmHel-7	CAEZ01005721:132588-132721
HmHel-7	CAEZ01005721:81220-82546
HmHel-7	CAEZ01005721:33562-34897
HmHel-7	CAEZ01005508:122481-123692
HmHel-7	CAEZ01005508:87474-88511
HmHel-7	CAEZ01003109:13692-15051
HmHel-7	CAEZ01001877:1-793
HmHel-7	CAEZ01009978:18718-20067
HmHel-7	CAEZ01009458:182923-183929
HmHel-7	CAEZ01009458:188590-189226
HmHel-7	CAEZ01009458:87041-87138
HmHel-7	CAEZ01009458:3591-4864
HmHel-7	CAEZ01009302:22138-23424
HmHel-7	CAEZ01008525:523-1803
HmHel-7	CAEZ01008490:938-1958
HmHel-7	CAEZ01006255:3950-5288
HmHel-7	CAEZ01004793:123950-125122
HmHel-7	CAEZ01004793:12094-13373
HmHel-7	CAEZ01003690:12475-13811
HmHel-7	CAEZ01009763:354-1646
HmHel-7	CAEZ01008886:10549-11907
HmHel-7	CAEZ01008886:15895-17496
HmHel-7	CAEZ01008043:8936-9682
HmHel-7	CAEZ01006747:6963-8323
HmHel-7	CAEZ01003248:70056-71405
HmHel-7	CAEZ01001070:59245-60594
HmHel-7	CAEZ01001070:37986-39333
HmHel-7	CAEZ01011259:8431-9555
HmHel-7	CAEZ01010888:9203-10257
HmHel-7	CAEZ01010888:1-587
HmHel-7	CAEZ01009945:1354-2704
HmHel-7	CAEZ01009300:4864-6184
HmHel-7	CAEZ01009300:61307-62592
HmHel-7	CAEZ01008918:2039-3407
HmHel-7	CAEZ01008751:86201-87489
HmHel-7	CAEZ01007601:6-648
HmHel-7	CAEZ01007601:21879-23228
HmHel-7	CAEZ01007601:8509-9367
HmHel-7	CAEZ01007273:16041-17235
HmHel-7	CAEZ01007273:54163-55368
HmHel-7	CAEZ01007273:108334-109566
HmHel-7	CAEZ01006062:11375-13614
HmHel-7	CAEZ01005437:1-889
HmHel-7	CAEZ01005437:22646-23923
HmHel-7	CAEZ01005437:31386-31794
HmHel-7	CAEZ01004014:52461-53773
HmHel-7	CAEZ01003245:1783-2638
HmHel-7	CAEZ01001802:5619-9343

HmHel-7	CAEZ01001435:168657-169990
HmHel-7	CAEZ01000986:2545-3880
HmHel-7	CAEZ01009842:21197-21806
HmHel-7	CAEZ01009842:1-677
HmHel-7	CAEZ01009379:7532-8812
HmHel-7	CAEZ01009017:84797-86184
HmHel-7	CAEZ01008257:1549-2868
HmHel-7	CAEZ01006491:10806-12152
HmHel-7	CAEZ01006491:26205-26343
HmHel-7	CAEZ01006491:10589-13296
HmHel-7	CAEZ01006491:4-476
HmHel-7	CAEZ01006014:2643-3995
HmHel-7	CAEZ01005038:584-948
HmHel-7	CAEZ01005038:3466-4998
HmHel-7	CAEZ01003231:10488-11818
HmHel-7	CAEZ01002742:1-563
HmHel-7	CAEZ01002742:154010-155221
HmHel-7	CAEZ01002742:219532-220850
HmHel-7	CAEZ01001557:14186-14403
HmHel-7	CAEZ01001557:31372-32654
HmHel-7	CAEZ01000046:6665-8023
HmHel-7	CAEZ01011413:1243-1662
HmHel-7	CAEZ01011413:36334-37701
HmHel-7	CAEZ01011293:5762-7063
HmHel-7	CAEZ01009627:21418-22822
HmHel-7	CAEZ01008498:604-926
HmHel-7	CAEZ01008352:121873-123219
HmHel-7	CAEZ01008116:3659-5004
HmHel-7	CAEZ01007983:6690-8035
HmHel-7	CAEZ01007983:120550-120910
HmHel-7	CAEZ01006803:44431-45763
HmHel-7	CAEZ01004213:3122-5362
HmHel-7	CAEZ01001829:376-1627
HmHel-7	CAEZ01001196:46159-47484
HmHel-7	CAEZ01001064:28046-29323
HmHel-7	CAEZ01010886:22158-23382
HmHel-7	CAEZ01010466:24208-25544
HmHel-7	CAEZ01010138:508-2699
HmHel-7	CAEZ01009989:68549-69837
HmHel-7	CAEZ01009609:56570-57850
HmHel-7	CAEZ01009293:1-633
HmHel-7	CAEZ01008713:134133-135406
HmHel-7	CAEZ01008713:17730-18969
HmHel-7	CAEZ01008597:12699-13980
HmHel-7	CAEZ01007708:62127-63446
HmHel-7	CAEZ01007708:91977-93189
HmHel-7	CAEZ01007708:52333-52901
HmHel-7	CAEZ01007708:29565-30931

HmHel-7	CAEZ01006521:11051-11985
HmHel-7	CAEZ01005396:20448-21815
HmHel-7	CAEZ01005396:6371-7732
HmHel-7	CAEZ01005153:70024-71354
HmHel-7	CAEZ01003686:10087-11345
HmHel-7	CAEZ01003682:3672-5018
HmHel-7	CAEZ01001777:12453-13815
HmHel-7	CAEZ01001579:10742-12121
HmHel-7	CAEZ01001579:55237-57126
HmHel-7	CAEZ01001579:32489-33809
HmHel-7	CAEZ01001441:34347-35709
HmHel-7	CAEZ01001234:1-899
HmHel-7	CAEZ01009495:13358-14563
HmHel-7	CAEZ01007758:5806-7168
HmHel-7	CAEZ01007168:1-1301
HmHel-7	CAEZ01006370:40366-41710
HmHel-7	CAEZ01006370:7621-8998
HmHel-7	CAEZ01006126:10553-10644
HmHel-7	CAEZ01006126:44583-45932
HmHel-7	CAEZ01006126:4058-4609
HmHel-7	CAEZ01005145:4-905
HmHel-7	CAEZ01004757:4-905
HmHel-7	CAEZ01004261:387-1796
HmHel-7	CAEZ01004044:20030-21381
HmHel-7	CAEZ01003695:20984-22337
HmHel-7	CAEZ01001501:52-787
HmHel-7	CAEZ01011489:90-1445
HmHel-7	CAEZ01010916:28-1420
HmHel-7	CAEZ01008719:54439-55793
HmHel-7	CAEZ01008323:850-2168
HmHel-7	CAEZ01005910:27686-28417
HmHel-7	CAEZ01005910:26419-27690
HmHel-7	CAEZ01004832:28999-30315
HmHel-7	CAEZ01003964:4379-5055
HmHel-7	CAEZ01003626:8125-9468
HmHel-7	CAEZ01003041:1-910
HmHel-7	CAEZ01009946:821-2197
HmHel-7	CAEZ01009223:70306-71591
HmHel-7	CAEZ01008500:128528-129866
HmHel-7	CAEZ01008500:34814-36084
HmHel-7	CAEZ01008500:1-805
HmHel-7	CAEZ01008168:14468-15639
HmHel-7	CAEZ01008168:19213-20440
HmHel-7	CAEZ01007587:27007-28373
HmHel-7	CAEZ01006926:1-822
HmHel-7	CAEZ01006020:4836-6194
HmHel-7	CAEZ01011285:48520-49872
HmHel-7	CAEZ01011283:79913-81255

HmHel-7	CAEZ01011283:77214-78577
HmHel-7	CAEZ01007464:3736-5094
HmHel-7	CAEZ01006640:7436-8775
HmHel-7	CAEZ01003513:6004-7343
HmHel-7	CAEZ01001919:3950-4887
HmHel-7	CAEZ01011554:10996-12386
HmHel-7	CAEZ01011022:4917-6214
HmHel-7	CAEZ01010242:14481-15813
HmHel-7	CAEZ01010221:94837-96115
HmHel-7	CAEZ01010018:643-1978
HmHel-7	CAEZ01008947:6149-7506
HmHel-7	CAEZ01008598:13-1369
HmHel-7	CAEZ01008515:46231-47515
HmHel-7	CAEZ01008280:778-3066
HmHel-7	CAEZ01008269:3595-4871
HmHel-7	CAEZ01008175:19690-21043
HmHel-7	CAEZ01007492:59047-60412
HmHel-7	CAEZ01006534:15692-17051
HmHel-7	CAEZ01006399:14381-15745
HmHel-7	CAEZ01006399:7690-9027
HmHel-7	CAEZ01005603:12834-14191
HmHel-7	CAEZ01005449:14850-15773
HmHel-7	CAEZ01005402:13351-13976
HmHel-7	CAEZ01005402:56873-58232
HmHel-7	CAEZ01005122:25030-25629
HmHel-7	CAEZ01004538:4758-6072
HmHel-7	CAEZ01004195:5772-7129
HmHel-7	CAEZ01003762:26291-27631
HmHel-7	CAEZ01002289:449-1570
HmHel-7	CAEZ01001518:12899-14233
HmHel-7	CAEZ01011616:35-1375
HmHel-7	CAEZ01010719:6770-8148
HmHel-7	CAEZ01005660:72004-73360
HmHel-7	CAEZ01005040:1-604
HmHel-7	CAEZ01004823:19241-20590
HmHel-7	CAEZ01004755:9-329
HmHel-7	CAEZ01004755:14142-15474
HmHel-7	CAEZ01003678:7552-8654
HmHel-7	CAEZ01002038:4584-5842
HmHel-7	CAEZ01001458:4750-5976
HmHel-7	CAEZ01000969:1-651
HmHel-7	CAEZ01000969:15274-16605
HmHel-7	CAEZ01011522:34733-36018
HmHel-7	CAEZ01011269:5714-6985
HmHel-7	CAEZ01008835:3531-4858
HmHel-7	CAEZ01007985:41937-43062
HmHel-7	CAEZ01006396:18446-19783
HmHel-7	CAEZ01006339:71672-73014

HmHel-7	CAEZ01005556:9589-10961
HmHel-7	CAEZ01005233:9692-11040
HmHel-7	CAEZ01005016:7107-8406
HmHel-7	CAEZ01004867:22-824
HmHel-7	CAEZ01004562:87380-89308
HmHel-7	CAEZ01001062:64154-65482
HmHel-7	CAEZ01011207:1149-2462
HmHel-7	CAEZ01010803:13859-15196
HmHel-7	CAEZ01010803:1463-2830
HmHel-7	CAEZ01009992:1-692
HmHel-7	CAEZ01007528:644-2015
HmHel-7	CAEZ01004941:4738-6057
HmHel-7	CAEZ01004408:10119-11481
HmHel-7	CAEZ01002792:19131-20515
HmHel-7	CAEZ01010864:137570-138805
HmHel-7	CAEZ01010318:33910-35222
HmHel-7	CAEZ01009939:12328-13673
HmHel-7	CAEZ01009823:10750-12048
HmHel-7	CAEZ01008607:8568-9872
HmHel-7	CAEZ01008361:3680-4476
HmHel-7	CAEZ01008261:114199-115692
HmHel-7	CAEZ01007225:794-2130
HmHel-7	CAEZ01006907:21031-22364
HmHel-7	CAEZ01006335:1-911
HmHel-7	CAEZ01006101:34-946
HmHel-7	CAEZ01004792:5770-7107
HmHel-7	CAEZ01004079:7965-9754
HmHel-7	CAEZ01003904:9870-10951
HmHel-7	CAEZ01000991:1-788
HmHel-7	CAEZ01010805:74095-75404
HmHel-7	CAEZ01009994:4570-5915
HmHel-7	CAEZ01009138:30953-32305
HmHel-7	CAEZ01009138:10463-11731
HmHel-7	CAEZ01008680:7353-8883
HmHel-7	CAEZ01008211:164810-166163
HmHel-7	CAEZ01007777:12850-14187
HmHel-7	CAEZ01007473:20091-20744
HmHel-7	CAEZ01007380:9416-10670
HmHel-7	CAEZ01007374:3145-4513
HmHel-7	CAEZ01007374:1735-3020
HmHel-7	CAEZ01006880:19901-20576
HmHel-7	CAEZ01005676:7940-9039
HmHel-7	CAEZ01005430:17109-17697
HmHel-7	CAEZ01004804:12357-13429
HmHel-7	CAEZ01003320:390-1445
HmHel-7	CAEZ01000864:17243-18579
HmHel-7	CAEZ01011625:9517-10851
HmHel-7	CAEZ01008968:7-347

HmHel-7	CAEZ01008069:15354-16036
HmHel-7	CAEZ01005611:13109-15098
HmHel-7	CAEZ01005274:122627-123867
HmHel-7	CAEZ01005255:1-1073
HmHel-7	CAEZ01004934:8018-9412
HmHel-7	CAEZ01004927:18852-20098
HmHel-7	CAEZ01002368:35437-36484
HmHel-7	CAEZ01000904:1-584
HmHel-7	CAEZ01010103:297-1613
HmHel-7	CAEZ01010030:53748-55150
HmHel-7	CAEZ01008838:249-1420
HmHel-7	CAEZ01008799:5-878
HmHel-7	CAEZ01007093:8891-10221
HmHel-7	CAEZ01007093:979-2288
HmHel-7	CAEZ01006974:2336-3692
HmHel-7	CAEZ01005429:2-922
HmHel-7	CAEZ01005429:42907-44176
HmHel-7	CAEZ01005407:10788-12032
HmHel-7	CAEZ01004684:15277-16634
HmHel-7	CAEZ01004515:12977-14323
HmHel-7	CAEZ01002098:16849-17867
HmHel-7	CAEZ01010584:26914-28285
HmHel-7	CAEZ01010347:3162-4407
HmHel-7	CAEZ01009484:8380-9748
HmHel-7	CAEZ01009192:15611-15972
HmHel-7	CAEZ01009192:1-890
HmHel-7	CAEZ01008353:52465-53830
HmHel-7	CAEZ01007986:2553-3715
HmHel-7	CAEZ01005093:11950-13102
HmHel-7	CAEZ01001968:933-2242
HmHel-7	CAEZ01001446:126511-127798
HmHel-7	CAEZ01001446:144450-145671
HmHel-7	CAEZ01000942:2367-3648
HmHel-7	CAEZ01010965:13082-14384
HmHel-7	CAEZ01010965:1-416
HmHel-7	CAEZ01010924:74-1141
HmHel-7	CAEZ01009056:1-1134
HmHel-7	CAEZ01008632:20145-21458
HmHel-7	CAEZ01007804:199751-201107
HmHel-7	CAEZ01002749:2254-3578
HmHel-7	CAEZ01002332:32742-34090
HmHel-7	CAEZ01000970:19973-21318
HmHel-7	CAEZ01011046:1-740
HmHel-7	CAEZ01011046:123066-124413
HmHel-7	CAEZ01011046:89414-90765
HmHel-7	CAEZ01010112:1494-2776
HmHel-7	CAEZ01009232:32424-33788
HmHel-7	CAEZ01007449:25372-26662

HmHel-7	CAEZ01006220:67839-68723
HmHel-7	CAEZ01006177:224025-225404
HmHel-7	CAEZ01005244:8846-10066
HmHel-7	CAEZ01003265:4092-5415
HmHel-7	CAEZ01001668:1816-3184
HmHel-7	CAEZ01001550:5-1315
HmHel-7	CAEZ01011640:56084-57439
HmHel-7	CAEZ01011158:8099-9382
HmHel-7	CAEZ01009465:18984-19866
HmHel-7	CAEZ01008249:474-1078
HmHel-7	CAEZ01007402:948-2312
HmHel-7	CAEZ01007140:30265-31544
HmHel-7	CAEZ01001114:3686-4973
HmHel-7	CAEZ01001114:14157-15314
HmHel-7	CAEZ01010636:7247-8582
HmHel-7	CAEZ01009025:24836-26174
HmHel-7	CAEZ01008290:99394-100724
HmHel-7	CAEZ01005504:13082-14463
HmHel-7	CAEZ01005495:35257-36448
HmHel-7	CAEZ01005086:5070-6420
HmHel-7	CAEZ01004407:1-1352
HmHel-7	CAEZ01003114:1-748
HmHel-7	CAEZ01001660:693-1027
HmHel-7	CAEZ01010666:6052-7387
HmHel-7	CAEZ01010190:44656-45984
HmHel-7	CAEZ01009746:5650-6748
HmHel-7	CAEZ01004635:1-1224
HmHel-7	CAEZ01004083:25452-26822
HmHel-7	CAEZ01003823:3783-5008
HmHel-7	CAEZ01003823:15498-15835
HmHel-7	CAEZ01001703:24237-25453
HmHel-7	CAEZ01011577:4141-5060
HmHel-7	CAEZ01011577:3008-4117
HmHel-7	CAEZ01010981:38091-39448
HmHel-7	CAEZ01010734:7283-8568
HmHel-7	CAEZ01009691:70934-72136
HmHel-7	CAEZ01009005:10742-12051
HmHel-7	CAEZ01007912:116025-117374
HmHel-7	CAEZ01007902:6982-8340
HmHel-7	CAEZ01007518:28-372
HmHel-7	CAEZ01007518:1811-3153
HmHel-7	CAEZ01007429:339-548
HmHel-7	CAEZ01007429:86914-88301
HmHel-7	CAEZ01003653:5242-6550
HmHel-7	CAEZ01003097:13591-14918
HmHel-7	CAEZ01002991:70046-71263
HmHel-7	CAEZ01011636:12908-13695
HmHel-7	CAEZ01008329:19251-20529

HmHel-7	CAEZ01008329:2131-3460
HmHel-7	CAEZ01005883:7698-9024
HmHel-7	CAEZ01004236:10648-11999
HmHel-7	CAEZ01003506:1-1147
HmHel-7	CAEZ01008707:89007-90173
HmHel-7	CAEZ01008707:85237-86545
HmHel-7	CAEZ01008707:55999-56904
HmHel-7	CAEZ01008119:9946-11266
HmHel-7	CAEZ01008119:1666-3024
HmHel-7	CAEZ01007759:3177-4526
HmHel-7	CAEZ01006417:149975-151333
HmHel-7	CAEZ01005149:20711-21926
HmHel-7	CAEZ01002255:47-568
HmHel-7	CAEZ01002255:2307-3668
HmHel-7	CAEZ01010199:337-1623
HmHel-7	CAEZ01007495:34053-35377
HmHel-7	CAEZ01006621:2552-3884
HmHel-7	CAEZ01006200:1-710
HmHel-7	CAEZ01004677:55441-56503
HmHel-7	CAEZ01004677:33343-34679
HmHel-7	CAEZ01004370:64994-66111
HmHel-7	CAEZ01002650:55919-56849
HmHel-7	CAEZ01002415:29199-30623
HmHel-7	CAEZ01002415:1744-3083
HmHel-7	CAEZ01010493:4030-5019
HmHel-7	CAEZ01007626:43796-45142
HmHel-7	CAEZ01007029:3260-4613
HmHel-7	CAEZ01004334:3588-4934
HmHel-7	CAEZ01003977:1279-2400
HmHel-7	CAEZ01003102:9416-10775
HmHel-7	CAEZ01002769:29120-30397
HmHel-7	CAEZ01002769:21760-23006
HmHel-7	CAEZ01010927:89393-90756
HmHel-7	CAEZ01010288:203925-205246
HmHel-7	CAEZ01010288:291076-292243
HmHel-7	CAEZ01008330:8111-8721
HmHel-7	CAEZ01002049:57797-59159
HmHel-7	CAEZ01001157:11885-13223
HmHel-7	CAEZ01011063:8530-9787
HmHel-7	CAEZ01010699:3758-5105
HmHel-7	CAEZ01008756:1-714
HmHel-7	CAEZ01006643:8297-9580
HmHel-7	CAEZ01006540:63507-64865
HmHel-7	CAEZ01006002:15530-16742
HmHel-7	CAEZ01005230:2015-2983
HmHel-7	CAEZ01005053:24505-25871
HmHel-7	CAEZ01004914:9037-10397
HmHel-7	CAEZ01004681:1799-3162

HmHel-7	CAEZ01004681:31743-32270
HmHel-7	CAEZ01003211:45943-47209
HmHel-7	CAEZ01003211:25243-26502
HmHel-7	CAEZ01002870:21950-23146
HmHel-7	CAEZ01001778:17-1174
HmHel-7	CAEZ01000937:19-1241
HmHel-7	CAEZ01010617:1-577
HmHel-7	CAEZ01010348:4973-6291
HmHel-7	CAEZ01009617:43263-44439
HmHel-7	CAEZ01009124:62809-63986
HmHel-7	CAEZ01009124:64014-66278
HmHel-7	CAEZ01009124:24250-25548
HmHel-7	CAEZ01009060:1-693
HmHel-7	CAEZ01003439:11032-12358
HmHel-7	CAEZ01003202:74739-76076
HmHel-7	CAEZ01010260:25406-26770
HmHel-7	CAEZ01010260:8624-9904
HmHel-7	CAEZ01010157:17531-18874
HmHel-7	CAEZ01007958:18598-19904
HmHel-7	CAEZ01007727:26894-27928
HmHel-7	CAEZ01007727:7945-9282
HmHel-7	CAEZ01005578:11296-12652
HmHel-7	CAEZ01004733:20418-21725
HmHel-7	CAEZ01004733:102211-103024
HmHel-7	CAEZ01001249:20928-21846
HmHel-7	CAEZ01001249:1-520
HmHel-7	CAEZ01011032:19014-20096
HmHel-7	CAEZ01010771:746-1587
HmHel-7	CAEZ01010771:31897-32062
HmHel-7	CAEZ01009473:16547-17942
HmHel-7	CAEZ01007458:12451-13597
HmHel-7	CAEZ01007458:1-184
HmHel-7	CAEZ01006492:5303-6620
HmHel-7	CAEZ01006303:9656-10674
HmHel-7	CAEZ01005195:8604-9913
HmHel-7	CAEZ01009003:1-601
HmHel-7	CAEZ01008957:16201-16885
HmHel-7	CAEZ01007433:414-1614
HmHel-7	CAEZ01005196:10324-11617
HmHel-7	CAEZ01004574:18543-19891
HmHel-7	CAEZ01002636:1962-3315
HmHel-7	CAEZ01001417:7361-8690
HmHel-7	CAEZ01007917:6344-7336
HmHel-7	CAEZ01003187:1209-2560
HmHel-7	CAEZ01002243:185-1550
HmHel-7	CAEZ01010453:60357-61461
HmHel-7	CAEZ01010453:124651-125981
HmHel-7	CAEZ01010453:35839-37116

HmHel-7	CAEZ01006098:21100-22448
HmHel-7	CAEZ01006098:31158-32512
HmHel-7	CAEZ01004921:10686-12005
HmHel-7	CAEZ01010431:5-3093
HmHel-7	CAEZ01010428:38529-39852
HmHel-7	CAEZ01010428:42136-43474
HmHel-7	CAEZ01008508:823-2130
HmHel-7	CAEZ01007457:196-1170
HmHel-7	CAEZ01007358:5940-7258
HmHel-7	CAEZ01006558:4918-6231
HmHel-7	CAEZ01004981:9422-10801
HmHel-7	CAEZ01010972:8744-10093
HmHel-7	CAEZ01007893:8-877
HmHel-7	CAEZ01007863:83207-84234
HmHel-7	CAEZ01007722:39260-40360
HmHel-7	CAEZ01007722:148443-149794
HmHel-7	CAEZ01007722:30465-31823
HmHel-7	CAEZ01007722:9259-10387
HmHel-7	CAEZ01007568:44618-46042
HmHel-7	CAEZ01004809:16578-17636
HmHel-7	CAEZ01003989:350-1707
HmHel-7	CAEZ01003989:3668-4354
HmHel-7	CAEZ01003641:4097-4983
HmHel-7	CAEZ01000879:181388-182619
HmHel-7	CAEZ01008356:7939-9286
HmHel-7	CAEZ01004649:9294-9859
HmHel-7	CAEZ01002635:60166-61527
HmHel-7	CAEZ01002635:63118-64445
HmHel-7	CAEZ01002635:33736-35065
HmHel-7	CAEZ01001658:1-687
HmHel-7	CAEZ01010505:2-400
HmHel-7	CAEZ01010505:14750-16117
HmHel-7	CAEZ01008416:970-2319
HmHel-7	CAEZ01008204:9443-11829
HmHel-7	CAEZ01006835:28321-29611
HmHel-7	CAEZ01004492:24483-25833
HmHel-7	CAEZ01003549:1-478
HmHel-7	CAEZ01003491:963-2017
HmHel-7	CAEZ01011195:14574-15919
HmHel-7	CAEZ01009032:2369-3747
HmHel-7	CAEZ01007336:6697-7909
HmHel-7	CAEZ01007317:20189-21495
HmHel-7	CAEZ01002732:43565-44946
HmHel-7	CAEZ01009064:6968-8177
HmHel-7	CAEZ01005134:1-555
HmHel-7	CAEZ01003517:27512-28862
HmHel-7	CAEZ01001336:1567-2932
HmHel-7	CAEZ01010411:39154-40072

HmHel-7	CAEZ01008381:1150-3091
HmHel-7	CAEZ01004693:18166-19410
HmHel-7	CAEZ01003667:6882-8228
HmHel-7	CAEZ01001673:1-1179
HmHel-7	CAEZ01001572:29406-30728
HmHel-7	CAEZ01001572:1-415
HmHel-7	CAEZ01009135:2-573
HmHel-7	CAEZ01004826:70320-71675
HmHel-7	CAEZ01001408:38603-39935
HmHel-7	CAEZ01006833:3043-4316
HmHel-7	CAEZ01004119:4392-5642
HmHel-7	CAEZ01010036:21140-22415
HmHel-7	CAEZ01009459:1-580
HmHel-7	CAEZ01010930:4554-5466
HmHel-7	CAEZ01010619:32394-33489
HmHel-7	CAEZ01002833:2015-5230
HmHel-7	CAEZ01011204:347-1681
HmHel-7	CAEZ01010799:30288-31614
HmHel-7	CAEZ01010575:2-569
HmHel-7	CAEZ01008392:18382-19730
HmHel-7	CAEZ01003868:9350-10699
HmHel-7	CAEZ01002759:4820-6137
HmHel-7	CAEZ01002674:5828-7097
HmHel-7	CAEZ01002118:15971-16549
HmHel-7	CAEZ01006824:8947-10195
HmHel-7	CAEZ01005515:32603-33946
HmHel-7	CAEZ01005515:1-397
HmHel-7	CAEZ01009872:15509-16875
HmHel-7	CAEZ01009255:4-569
HmHel-7	CAEZ01007517:19525-20155
HmHel-7	CAEZ01004745:18584-19710
HmHel-7	CAEZ01002401:1142-1878
HmHel-7	CAEZ01011394:11171-12384
HmHel-7	CAEZ01010351:90389-91738
HmHel-7	CAEZ01006318:19257-19882
HmHel-7	CAEZ01006318:3294-4473
HmHel-7	CAEZ01005623:12537-13655
HmHel-7	CAEZ01003263:1-1026
HmHel-7	CAEZ01010993:3000-4326
HmHel-7	CAEZ01010808:195014-196098
HmHel-7	CAEZ01002633:32693-33989
HmHel-7	CAEZ01011197:75309-76604
HmHel-7	CAEZ01005296:9591-10142
HmHel-7	CAEZ01000527:356-1028
HmHel-7	CAEZ01011415:1-823
HmHel-7	CAEZ01011415:13714-14299
HmHel-7	CAEZ01007348:3129-4479
HmHel-7	CAEZ01006866:20643-21969

HmHel-7	CAEZ01003701:224-1508
HmHel-7	CAEZ01009452:101539-102086
HmHel-7	CAEZ01008315:66537-67872
HmHel-7	CAEZ01008315:370564-370685
HmHel-7	CAEZ01008315:82821-84169
HmHel-7	CAEZ01008238:23270-24705
HmHel-7	CAEZ01005586:1-949
HmHel-7	CAEZ01005586:17414-18689
HmHel-7	CAEZ01005082:52965-54065
HmHel-7	CAEZ01005082:2107-3258
HmHel-7	CAEZ01004975:37517-38760
HmHel-7	CAEZ01004855:16245-17504
HmHel-7	CAEZ01003785:24872-25366
HmHel-7	CAEZ01003785:18635-19955
HmHel-7	CAEZ01002724:7581-8819
HmHel-7	CAEZ01008904:7452-8767
HmHel-7	CAEZ01006840:84-1409
HmHel-7	CAEZ01006649:11853-12490
HmHel-7	CAEZ01002919:4901-5975
HmHel-7	CAEZ01006389:4975-6327
HmHel-7	CAEZ01005624:17932-19259
HmHel-7	CAEZ01010423:48680-49859
HmHel-7	CAEZ01006901:12299-13597
HmHel-7	CAEZ01003420:6066-6995
HmHel-7	CAEZ01000953:346-1015
HmHel-7	CAEZ01008055:16606-17946
HmHel-7	CAEZ01007661:18068-19268
HmHel-7	CAEZ01004198:13-576
HmHel-7	CAEZ01000838:3523-4780
HmHel-7	CAEZ01007233:6608-7138
HmHel-7	CAEZ01005433:80012-80554
HmHel-7	CAEZ01010697:858-2196
HmHel-7	CAEZ01009271:101754-103063
HmHel-7	CAEZ01008061:1-602
HmHel-7	CAEZ01007565:1056-2350
HmHel-7	CAEZ01006661:4687-5989
HmHel-7	CAEZ01004542:23289-24624
HmHel-7	CAEZ01004542:5447-6679
HmHel-7	CAEZ01009470:1076-1836
HmHel-7	CAEZ01009470:79884-81236
HmHel-7	CAEZ01009470:118494-119853
HmHel-7	CAEZ01009470:72734-74079
HmHel-7	CAEZ01008617:1-1079
HmHel-7	CAEZ01008617:5744-7090
HmHel-7	CAEZ01006863:98615-99959
HmHel-7	CAEZ01005084:1-643
HmHel-7	CAEZ01003334:17470-18808
HmHel-7	CAEZ01009181:3532-4433

HmHel-7	CAEZ01008101:32754-33345
HmHel-7	CAEZ01004385:9890-11238
HmHel-7	CAEZ01004274:15305-15715
HmHel-7	CAEZ01004274:4603-5948
HmHel-7	CAEZ01011504:4798-6101
HmHel-7	CAEZ01011310:15-1272
HmHel-7	CAEZ01010740:76708-77298
HmHel-7	CAEZ01010740:9073-10356
HmHel-7	CAEZ01002535:1-667
HmHel-7	CAEZ01002181:21900-23192
HmHel-7	CAEZ01009444:32244-35641
HmHel-7	CAEZ01008128:1-536
HmHel-7	CAEZ01006287:22632-24049
HmHel-7	CAEZ01005321:19666-20989
HmHel-7	CAEZ01004351:1-1231
HmHel-7	CAEZ01005582:2543-3881
HmHel-7	CAEZ01004786:38645-39985
HmHel-7	CAEZ01003162:2029-3399
HmHel-7	CAEZ01009970:1-609
HmHel-7	CAEZ01008279:13458-13612
HmHel-7	CAEZ01008279:1-542
HmHel-7	CAEZ01002992:22523-23093
HmHel-7	CAEZ01001645:30445-31759
HmHel-7	CAEZ01007431:46593-46725
HmHel-7	CAEZ01007431:22818-24155
HmHel-7	CAEZ01009822:4268-5022
HmHel-7	CAEZ01009822:19761-20308
HmHel-7	CAEZ01009776:11053-12367
HmHel-7	CAEZ01008130:7860-9106
HmHel-7	CAEZ01004404:14044-14806
HmHel-7	CAEZ01002841:13973-14565
HmHel-7	CAEZ01004500:1626-2616
HmHel-7	CAEZ01002982:3624-4939
HmHel-7	CAEZ01004377:5269-6566
HmHel-7	CAEZ01002961:7646-8872
HmHel-7	CAEZ01001799:9622-10163
HmHel-7	CAEZ01006853:22376-24043
HmHel-7	CAEZ01006056:6572-7866
HmHel-7	CAEZ01005204:10427-11017
HmHel-7	CAEZ01010034:28746-29870
HmHel-7	CAEZ01008041:6384-7792
HmHel-7	CAEZ01006761:17045-17607
HmHel-7	CAEZ01006761:548-1938
HmHel-7	CAEZ01005180:15065-15645
HmHel-7	CAEZ01010643:24257-25624
HmHel-7	CAEZ01005628:4156-5479
HmHel-7	CAEZ01004697:3464-4000
HmHel-7	CAEZ01007053:642-1988

HmHel-7	CAEZ01002948:7-636
HmHel-7	CAEZ01001210:1343-2695
HmHel-7	CAEZ01000895:41810-43120
HmHel-7	CAEZ01010100:26168-26870
HmHel-7	CAEZ01008734:58994-60333
HmHel-7	CAEZ01008629:20832-21226
HmHel-7	CAEZ01008629:6079-7418
HmHel-7	CAEZ01007817:1-1201
HmHel-7	CAEZ01008678:46552-47121
HmHel-7	CAEZ01006567:66099-68535
HmHel-7	CAEZ01009197:22-1353
HmHel-7	CAEZ01009178:5196-6524
HmHel-7	CAEZ01003148:351-1655
HmHel-7	CAEZ01000020:1-551
HmHel-7	CAEZ01010164:8567-9917
HmHel-7	CAEZ01011598:1-1084
HmHel-7	CAEZ01011598:38139-39413
HmHel-7	CAEZ01008943:1082-1983
HmHel-7	CAEZ01008943:21731-23026
HmHel-7	CAEZ01008943:4-526
HmHel-7	CAEZ01005954:4468-5808
HmHel-7	CAEZ01002850:1-1338
HmHel-7	CAEZ01001278:4385-5652
HmHel-7	CAEZ01010414:16255-16845
HmHel-7	CAEZ01008532:86042-87420
HmHel-7	CAEZ01002818:16918-18247
HmHel-7	CAEZ01010814:307-1667
HmHel-7	CAEZ01007950:13103-14377
HmHel-7	CAEZ01003766:8-1224
HmHel-7	CAEZ01003507:4-1264
HmHel-7	CAEZ01011392:58-640
HmHel-7	CAEZ01009422:101325-102668
HmHel-7	CAEZ01009422:134767-135002
HmHel-7	CAEZ01008654:4257-4661
HmHel-7	CAEZ01008410:93936-94845
HmHel-7	CAEZ01006611:9463-10026
HmHel-7	CAEZ01003210:22742-24083
HmHel-7	CAEZ01002656:322-1032
HmHel-7	CAEZ01002656:6381-7670
HmHel-7	CAEZ01010541:224246-225514
HmHel-7	CAEZ01008413:21344-22849
HmHel-7	CAEZ01005846:6234-7466
HmHel-7	CAEZ01005299:60799-61386
HmHel-7	CAEZ01001933:2218-2794
HmHel-7	CAEZ01004794:11727-12257
HmHel-7	CAEZ01010831:4150-5526
HmHel-7	CAEZ01006877:3119-4451
HmHel-7	CAEZ01004468:15904-16429

HmHel-7	CAEZ01008841:1-1142
HmHel-7	CAEZ01007157:36745-38047
HmHel-7	CAEZ01002860:379-1000
HmHel-7	CAEZ01002838:11908-13265
HmHel-7	CAEZ01002711:31710-32238
HmHel-7	CAEZ01010137:1919-3206
HmHel-7	CAEZ01008477:31428-32543
HmHel-7	CAEZ01007571:45770-46994
HmHel-7	CAEZ01007571:41951-43276
HmHel-7	CAEZ01011412:13290-13776
HmHel-7	CAEZ01008140:1-515
HmHel-7	CAEZ01007633:21526-22888
HmHel-7	CAEZ01004465:1484-2752
HmHel-7	CAEZ01004663:1606-2903
HmHel-7	CAEZ01003592:808-2110
HmHel-7	CAEZ01001806:120122-121228
HmHel-7	CAEZ01009463:17312-18665
HmHel-7	CAEZ01008966:3657-4687
HmHel-7	CAEZ01007676:137641-139024
HmHel-7	CAEZ01005585:13287-14037
HmHel-7	CAEZ01003453:5070-5621
HmHel-7	CAEZ01011193:108570-109846
HmHel-7	CAEZ01008960:45156-46483
HmHel-7	CAEZ01006948:18476-19771
HmHel-7	CAEZ01003173:18406-19693
HmHel-7	CAEZ01009709:99142-100408
HmHel-7	CAEZ01004995:14387-15502
HmHel-7	CAEZ01004034:20610-23419
HmHel-7	CAEZ01001740:33068-34368
HmHel-7	CAEZ01006530:269277-270539
HmHel-7	CAEZ01005569:9989-11310
HmHel-7	CAEZ01004949:804-2166
HmHel-7	CAEZ01001571:23466-24547
HmHel-7	CAEZ01001571:30429-30837
HmHel-7	CAEZ01010111:36642-37305
HmHel-7	CAEZ01009029:1724-2252
HmHel-7	CAEZ01005210:21590-22932
HmHel-7	CAEZ01002338:11508-12412
HmHel-7	CAEZ01010029:53992-55285
HmHel-7	CAEZ01010029:75520-76230
HmHel-7	CAEZ01010029:28875-30119
HmHel-7	CAEZ01009331:14175-15479
HmHel-7	CAEZ01006322:21507-21836
HmHel-7	CAEZ01006322:1-599
HmHel-7	CAEZ01004738:1-369
HmHel-7	CAEZ01004738:63943-64023
HmHel-7	CAEZ01004738:62444-63766
HmHel-7	CAEZ01001972:1875-3143

HmHel-7	CAEZ01005486:1-529
HmHel-7	CAEZ01011371:15831-16363
HmHel-7	CAEZ01009384:7533-8868
HmHel-7	CAEZ01007716:5463-6807
HmHel-7	CAEZ01007191:2840-3779
HmHel-7	CAEZ01007163:14742-15618
HmHel-7	CAEZ01001825:13120-14456
HmHel-7	CAEZ01011219:18359-19654
HmHel-7	CAEZ01010133:1-314
HmHel-7	CAEZ01010133:22283-23676
HmHel-7	CAEZ01010113:9914-12011
HmHel-7	CAEZ01008031:24174-25519
HmHel-7	CAEZ01005307:6198-7623
HmHel-7	CAEZ01005068:1-882
HmHel-7	CAEZ01007228:1-1028
HmHel-7	CAEZ01006850:444-1708
HmHel-7	CAEZ01011048:62806-63785
HmHel-7	CAEZ01003738:13825-14442
HmHel-7	CAEZ01001789:12102-12594
HmHel-7	CAEZ01008636:19764-21087
HmHel-7	CAEZ01007008:1-528
HmHel-7	CAEZ01003325:588-3148
HmHel-7	CAEZ01003325:1-543
HmHel-7	CAEZ01001060:7-569
HmHel-7	CAEZ01002883:2-508
HmHel-7	CAEZ01004923:46362-47627
HmHel-7	CAEZ01003633:59554-60206
HmHel-7	CAEZ01002654:9932-10536
HmHel-7	CAEZ01011559:4195-5520
HmHel-7	CAEZ01010283:393-1116
HmHel-7	CAEZ01007359:21011-22349
HmHel-7	CAEZ01006279:11153-12496
HmHel-7	CAEZ01004996:6357-7656
HmHel-7	CAEZ01001868:1-586
HmHel-7	CAEZ01006490:107441-107752
HmHel-7	CAEZ01006490:111490-112190
HmHel-7	CAEZ01009931:6314-7016
HmHel-7	CAEZ01009196:10555-12502
HmHel-7	CAEZ01006125:15938-17279
HmHel-7	CAEZ01002644:1-528
HmHel-7	CAEZ01010264:1112-2421
HmHel-7	CAEZ01010001:1-1013
HmHel-7	CAEZ01008295:5322-6655
HmHel-7	CAEZ01007567:735-2039
HmHel-7	CAEZ01001811:1929-2480
HmHel-7	CAEZ01011649:10227-11537
HmHel-7	CAEZ01010185:12674-13144
HmHel-7	CAEZ01006231:1-574

HmHel-7	CAEZ01009364:32992-34132
HmHel-7	CAEZ01003189:14906-15406
HmHel-7	CAEZ01005366:29279-30255
HmHel-7	CAEZ01002300:693-1180
HmHel-7	CAEZ01001922:2-988
HmHel-7	CAEZ01010266:11085-12383
HmHel-7	CAEZ01008656:389-1718
HmHel-7	CAEZ01005098:1463-2794
HmHel-7	CAEZ01003733:1-519
HmHel-7	CAEZ01008166:2811-2940
HmHel-7	CAEZ01008166:1-572
HmHel-7	CAEZ01003741:2177-3509
HmHel-7	CAEZ01008967:20450-20970
HmHel-7	CAEZ01003321:4725-6042
HmHel-7	CAEZ01001776:1-996
HmHel-7	CAEZ01010582:4746-5221
HmHel-7	CAEZ01008063:1-491
HmHel-7	CAEZ01007205:9018-10338
HmHel-7	CAEZ01006502:10857-11357
HmHel-7	CAEZ01003071:31742-32232
HmHel-7	CAEZ01007705:37237-37991
HmHel-7	CAEZ01004020:9483-11165
HmHel-7	CAEZ01009685:1-492
HmHel-7	CAEZ01004118:1-540
HmHel-7	CAEZ01010509:15760-17015
HmHel-7	CAEZ01006776:4762-5654
HmHel-7	CAEZ01008350:62075-63635
HmHel-7	CAEZ01004754:6077-6603
HmHel-7	CAEZ01010695:13172-14249
HmHel-7	CAEZ01004495:574-1449
HmHel-7	CAEZ01010953:1-466
HmHel-7	CAEZ01009676:28130-29343
HmHel-7	CAEZ01007725:131741-132921
HmHel-7	CAEZ01007725:1-386
HmHel-7	CAEZ01002112:3742-4678
HmHel-7	CAEZ01010635:16566-17478
HmHel-7	CAEZ01008184:1-457
HmHel-7	CAEZ01005241:8204-9552
HmHel-7	CAEZ01002608:51335-51811
HmHel-7	CAEZ01007389:30441-30531
HmHel-7	CAEZ01007389:119774-121084
HmHel-7	CAEZ01006972:21097-22317
HmHel-7	CAEZ01005243:16164-18346
HmHel-7	CAEZ01004254:24977-26297
HmHel-7	CAEZ01010202:9089-10369
HmHel-7	CAEZ01009462:243-791
HmHel-7	CAEZ01009225:1-521
HmHel-7	CAEZ01008169:22249-25566

HmHel-7	CAEZ01006773:113008-114353
HmHel-7	CAEZ01009826:27-121
HmHel-7	CAEZ01009826:4724-5586
HmHel-7	CAEZ01008278:4-1134
HmHel-7	CAEZ01009374:34807-36158
HmHel-7	CAEZ01000038:505-1848
HmHel-7	CAEZ01008546:425-831
HmHel-7	CAEZ01001679:596-1094
HmHel-7	CAEZ01007704:18447-19149
HmHel-7	CAEZ01008458:2271-3656
HmHel-7	CAEZ01007238:10556-11812
HmHel-7	CAEZ01010547:6773-7207
HmHel-7	CAEZ01008190:2-612
HmHel-7	CAEZ01008190:19019-19841
HmHel-7	CAEZ01008190:1067-3336
HmHel-7	CAEZ01005573:1-518
HmHel-7	CAEZ01004496:8064-9287
HmHel-7	CAEZ01011314:23860-25017
HmHel-7	CAEZ01003155:12109-12940
HmHel-7	CAEZ01003155:1-452
HmHel-7	CAEZ01008595:13825-14271
HmHel-7	CAEZ01000999:39823-41173
HmHel-7	CAEZ01005513:2472-2929
HmHel-7	CAEZ01005513:1-466
HmHel-7	CAEZ01002886:1866-2979
HmHel-7	CAEZ01007204:24323-25638
HmHel-7	CAEZ01005334:12858-14212
HmHel-7	CAEZ01000840:1-465
HmHel-7	CAEZ01009342:7873-9277
HmHel-7	CAEZ01002675:7754-9097
HmHel-7	CAEZ01011110:498-1464
HmHel-7	CAEZ01011070:1-459
HmHel-7	CAEZ01006555:4363-6121
HmHel-7	CAEZ01005203:2013-2755
HmHel-7	CAEZ01005203:14196-14801
HmHel-7	CAEZ01002583:1283-1783
HmHel-7	CAEZ01010938:11681-12689
HmHel-7	CAEZ01009560:21227-22306
HmHel-7	CAEZ01001158:1-473
HmHel-7	CAEZ01011615:9550-10557
HmHel-7	CAEZ01010522:7870-8650
HmHel-7	CAEZ01010247:17014-17476
HmHel-7	CAEZ01009593:10968-11597
HmHel-7	CAEZ01006088:16910-19148
HmHel-7	CAEZ01002254:31002-31494
HmHel-7	CAEZ01011105:44357-45165
HmHel-7	CAEZ01010435:30998-31531
HmHel-7	CAEZ01009210:1-497

HmHel-7	CAEZ01004565:6-1325
HmHel-7	CAEZ01008170:41746-42911
HmHel-7	CAEZ01008170:24510-26997
HmHel-7	CAEZ01005382:28973-30247
HmHel-7	CAEZ01008419:40445-41549
HmHel-7	CAEZ01000061:32386-33550
HmHel-7	CAEZ01010615:43448-45713
HmHel-7	CAEZ01004723:15882-16355
HmHel-7	CAEZ01001624:1-464
HmHel-7	CAEZ01001566:712-1462
HmHel-7	CAEZ01005626:1-493
HmHel-7	CAEZ01008994:1-1059
HmHel-7	CAEZ01011296:42135-44119
HmHel-7	CAEZ01011519:64722-65941
HmHel-7	CAEZ01004958:28282-29622
HmHel-7	CAEZ01002678:13818-15077
HmHel-7	CAEZ01010449:25365-25732
HmHel-7	CAEZ01010449:1-445
HmHel-7	CAEZ01007292:1647-3514
HmHel-7	CAEZ01006652:31216-32482
HmHel-7	CAEZ01005995:4314-5656
HmHel-7	CAEZ01004389:1-496
HmHel-7	CAEZ01010944:16046-16524
HmHel-7	CAEZ01007929:10528-11844
HmHel-7	CAEZ01007362:4062-5206
HmHel-7	CAEZ01001849:153-621
HmHel-7	CAEZ01007579:10113-11190
HmHel-7	CAEZ01005935:1045-2176
HmHel-7	CAEZ01003283:609-1920
HmHel-7	CAEZ01002836:2-1128
HmHel-7	CAEZ01010413:12493-12934
HmHel-7	CAEZ01007057:1686-2126
HmHel-7	CAEZ01009936:12299-13543
HmHel-7	CAEZ01009681:50013-51354
HmHel-7	CAEZ01004904:14471-15726
HmHel-7	CAEZ01003945:1736-2970
HmHel-7	CAEZ01003227:4172-4607
HmHel-7	CAEZ01011256:11710-12182
HmHel-7	CAEZ01005806:15255-16324
HmHel-7	CAEZ01005806:34022-34771
HmHel-7	CAEZ01005806:13545-14922
HmHel-7	CAEZ01003303:1-469
HmHel-7	CAEZ01001739:9328-10627
HmHel-7	CAEZ01001681:1-1072
HmHel-7	CAEZ01001056:930-2253
HmHel-7	CAEZ01005891:1-937
HmHel-7	CAEZ01004360:22549-23894
HmHel-7	CAEZ01000964:1-911

HmHel-7	CAEZ01008254:16744-17183
HmHel-7	CAEZ01008724:3781-5057
HmHel-7	CAEZ01002040:3601-4133
HmHel-7	CAEZ01010070:3559-4958
HmHel-7	CAEZ01006071:10932-11376
HmHel-7	CAEZ01005427:1-435
HmHel-7	CAEZ01005267:16367-17669
HmHel-7	CAEZ01000926:12316-13414
HmHel-7	CAEZ01004381:19987-21314
HmHel-7	CAEZ01002615:3558-5629
HmHel-7	CAEZ01003249:2320-3602
HmHel-7	CAEZ01002625:3822-4995
HmHel-7	CAEZ01010462:100062-101382
HmHel-7	CAEZ01006003:879-1274
HmHel-7	CAEZ01005460:1-434
HmHel-7	CAEZ01003332:1924-3295
HmHel-7	CAEZ01006405:35709-37047
HmHel-7	CAEZ01004637:1-444
HmHel-7	CAEZ01005424:470-1849
HmHel-7	CAEZ01011325:6213-7415
HmHel-7	CAEZ01010614:21123-21598
HmHel-7	CAEZ01010053:12453-13759
HmHel-7	CAEZ01008671:4078-5369
HmHel-7	CAEZ01006349:22789-23876
HmHel-7	CAEZ01000995:435-1537
HmHel-7	CAEZ01010800:1-396
HmHel-7	CAEZ01005199:30058-31399
HmHel-7	CAEZ01007067:5071-6037
HmHel-7	CAEZ01007185:13807-16145
HmHel-7	CAEZ01005159:9247-10363
HmHel-7	CAEZ01006450:8011-8478
HmHel-7	CAEZ01005117:7005-7955
HmHel-7	CAEZ01002890:12064-13401
HmHel-7	CAEZ01000019:1-705
HmHel-7	CAEZ01010430:24574-25670
HmHel-7	CAEZ01001369:1-1024
HmHel-7	CAEZ01008082:10181-11217
HmHel-7	CAEZ01004737:7001-7403
HmHel-7	CAEZ01004139:19257-20617
HmHel-7	CAEZ01011312:13493-14466
HmHel-7	CAEZ01003885:1215-1945
HmHel-7	CAEZ01008355:1-417
HmHel-7	CAEZ01002262:1522-2821
HmHel-7	CAEZ01009898:24841-27315
HmHel-7	CAEZ01007724:80457-81520
HmHel-7	CAEZ01004936:23999-25172
HmHel-7	CAEZ01009386:1-1126
HmHel-7	CAEZ01009386:80-218

HmHel-7	CAEZ01006699:25799-27087
HmHel-7	CAEZ01003392:5436-6726
HmHel-7	CAEZ01010922:36468-37609
HmHel-7	CAEZ01008493:6-434
HmHel-7	CAEZ01003373:10-472
HmHel-7	CAEZ01004939:13258-13673
HmHel-7	CAEZ01003529:10990-12146
HmHel-7	CAEZ01005890:20370-20922
HmHel-7	CAEZ01001172:21485-22578
HmHel-7	CAEZ01011372:9411-9811
HmHel-7	CAEZ01010273:126472-126851
HmHel-7	CAEZ01005391:16157-16576
HmHel-7	CAEZ01005059:1-452
HmHel-7	CAEZ01008935:3388-4692
HmHel-7	CAEZ01008935:16558-17565
HmHel-7	CAEZ01005177:28-1051
HmHel-7	CAEZ01005361:5846-7211
HmHel-7	CAEZ01005361:16-271
HmHel-7	CAEZ01003562:35604-36923
HmHel-7	CAEZ01003562:29027-30040
HmHel-7	CAEZ01001764:20615-22225
HmHel-7	CAEZ01008888:614-837
HmHel-7	CAEZ01008888:1-425
HmHel-7	CAEZ01007035:1-417
HmHel-7	CAEZ01009576:43123-44201
HmHel-7	CAEZ01009511:2615-3714
HmHel-7	CAEZ01007349:4988-5430
HmHel-7	CAEZ01003936:1-445
HmHel-7	CAEZ01004644:2724-3977
HmHel-7	CAEZ01003091:1-1741
HmHel-7	CAEZ01003091:8554-9131
HmHel-7	CAEZ01005895:13-110
HmHel-7	CAEZ01005895:23300-23712
HmHel-7	CAEZ01000027:1965-2329
HmHel-7	CAEZ01011421:17366-17741
HmHel-7	CAEZ01011404:743-1110
HmHel-7	CAEZ01010764:3034-6078
HmHel-7	CAEZ01010338:10762-11392
HmHel-7	CAEZ01010338:1-224
HmHel-7	CAEZ01009959:883-2160
HmHel-7	CAEZ01000263:3-428
HmHel-7	CAEZ01005436:28344-28752
HmHel-7	CAEZ01002840:424-1286
HmHel-7	CAEZ01011528:6884-8075
HmHel-7	CAEZ01011528:51468-52267
HmHel-7	CAEZ01008492:1879-3267
HmHel-7	CAEZ01007434:24819-25952
HmHel-7	CAEZ01005360:56536-57828

HmHel-7	CAEZ01004399:44658-45033
HmHel-7	CAEZ01003035:8-853
HmHel-7	CAEZ01002041:76486-77210
HmHel-7	CAEZ01011280:4820-5184
HmHel-7	CAEZ01009679:13719-15057
HmHel-7	CAEZ01006154:1-361
HmHel-7	CAEZ01008276:600-1046
HmHel-7	CAEZ01004679:1-413
HmHel-7	CAEZ01005662:1-363
HmHel-7	CAEZ01005521:1-387
HmHel-7	CAEZ01008871:1-370
HmHel-7	CAEZ01008643:53939-55211
HmHel-7	CAEZ01004820:67090-68394
HmHel-7	CAEZ01002607:135503-141245
HmHel-7	CAEZ01009911:1-821
HmHel-7	CAEZ01008124:75839-76963
HmHel-7	CAEZ01008124:1-385
HmHel-7	CAEZ01006816:10365-11719
HmHel-7	CAEZ01010203:4-415
HmHel-7	CAEZ01009063:39822-40539
HmHel-7	CAEZ01007908:2696-3747
HmHel-7	CAEZ01001094:1067-1546
HmHel-7	CAEZ01010743:1243-2628
HmHel-7	CAEZ01010271:1-445
HmHel-7	CAEZ01005085:12831-14199
HmHel-7	CAEZ01001523:2753-3170
HmHel-7	CAEZ01010534:20493-21886
HmHel-7	CAEZ01010534:114297-115052
HmHel-7	CAEZ01009694:40753-41701
HmHel-7	CAEZ01009659:43892-46866
HmHel-7	CAEZ01009252:11462-12723
HmHel-7	CAEZ01007282:20126-21795
HmHel-7	CAEZ01003237:49905-50291
HmHel-7	CAEZ01001041:404-1512
HmHel-7	CAEZ01004795:2-771
HmHel-7	CAEZ01003640:6613-7933
HmHel-7	CAEZ01003323:1-950
HmHel-7	CAEZ01010687:5998-6450
HmHel-7	CAEZ01009824:93204-94988
HmHel-7	CAEZ01004165:1-269
HmHel-7	CAEZ01004165:16474-18132
HmHel-7	CAEZ01004165:7504-8881
HmHel-7	CAEZ01003388:4575-5907
HmHel-7	CAEZ01002250:1-368
HmHel-7	CAEZ01007366:6853-8214
HmHel-7	CAEZ01006775:5633-6504
HmHel-7	CAEZ01004636:3376-4315
HmHel-7	CAEZ01003432:13030-14348

HmHel-7	CAEZ01007038:1527-1939
HmHel-7	CAEZ01006672:12632-13869
HmHel-7	CAEZ01003661:11041-11466
HmHel-7	CAEZ01010769:1220-1596
HmHel-7	CAEZ01003196:58384-59641
HmHel-7	CAEZ01011618:896-1415
HmHel-7	CAEZ01010201:1-352
HmHel-7	CAEZ01009474:59459-60615
HmHel-7	CAEZ01009469:3443-3838
HmHel-7	CAEZ01009075:5857-6240
HmHel-7	CAEZ01004519:5-379
HmHel-7	CAEZ01001945:3-836
HmHel-7	CAEZ01007208:37644-38000
HmHel-7	CAEZ01005743:45350-45618
HmHel-7	CAEZ01005743:84325-85086
HmHel-7	CAEZ01004511:34903-35773
HmHel-7	CAEZ01007938:40908-41973
HmHel-7	CAEZ01007938:136911-137302
HmHel-7	CAEZ01002075:696-1720
HmHel-7	CAEZ01007736:52387-53409
HmHel-7	CAEZ01003731:6190-6573
HmHel-7	CAEZ01002609:17-858
HmHel-7	CAEZ01000339:1-458
HmHel-7	CAEZ01009350:17079-18434
HmHel-7	CAEZ01006812:12022-12512
HmHel-7	CAEZ01008506:9464-10451
HmHel-7	CAEZ01005026:10810-11411
HmHel-7	CAEZ01003544:1-1188
HmHel-7	CAEZ01001318:1-1066
HmHel-7	CAEZ01008648:1-354
HmHel-7	CAEZ01003577:1-367
HmHel-7	CAEZ01009573:2-935
HmHel-7	CAEZ01007811:26770-27096
HmHel-7	CAEZ01005710:1-825
HmHel-7	CAEZ01001353:1-344
HmHel-7	CAEZ01010628:14334-15381
HmHel-7	CAEZ01010325:1-860
HmHel-7	CAEZ01008202:10857-11578
HmHel-7	CAEZ01006549:14326-15732
HmHel-7	CAEZ01005173:149022-150129
HmHel-7	CAEZ01006651:28719-30290
HmHel-7	CAEZ01002843:539-904
HmHel-7	CAEZ01002187:787-1582
HmHel-7	CAEZ01008939:6664-7527
HmHel-7	CAEZ01007502:1-336
HmHel-7	CAEZ01007048:9038-9758
HmHel-7	CAEZ01011282:75254-76598
HmHel-7	CAEZ01010141:3367-4453

HmHel-7	CAEZ01007037:17-402
HmHel-7	CAEZ01009012:1173-1306
HmHel-7	CAEZ01009012:11851-13198
HmHel-7	CAEZ01002267:1786-2810
HmHel-7	CAEZ01008822:72325-72712
HmHel-7	CAEZ01008887:16711-17326
HmHel-7	CAEZ01007251:34724-35283
HmHel-7	CAEZ01002065:1-437
HmHel-7	CAEZ01011474:6504-7417
HmHel-7	CAEZ01011313:8709-10032
HmHel-7	CAEZ01008722:36108-37398
HmHel-7	CAEZ01004121:2-355
HmHel-7	CAEZ01004168:6089-7172
HmHel-7	CAEZ01002414:145874-147277
HmHel-7	CAEZ01009088:1-911
HmHel-7	CAEZ01004145:3518-4678
HmHel-7	CAEZ01010102:2036-3141
HmHel-7	CAEZ01007602:1-837
HmHel-7	CAEZ01006769:32474-33290
HmHel-7	CAEZ01003819:6934-8220
HmHel-7	CAEZ01003784:19730-21085
HmHel-7	CAEZ01001630:652-1013
HmHel-7	CAEZ01011065:2-378
HmHel-7	CAEZ01009841:109263-109687
HmHel-7	CAEZ01008630:1-919
HmHel-7	CAEZ01008177:4513-4921
HmHel-7	CAEZ01005061:1-1062
HmHel-7	CAEZ01004812:1-871
HmHel-7	CAEZ01004673:1-369
HmHel-7	CAEZ01002906:1-1017
HmHel-7	CAEZ01001087:4328-5081
HmHel-7	CAEZ01006345:1594-1933
HmHel-7	CAEZ01011595:1633-2406
HmHel-7	CAEZ01006152:148-1124
HmHel-7	CAEZ01003072:1-825
HmHel-7	CAEZ01002051:6968-8330
HmHel-7	CAEZ01001402:26530-27864
HmHel-7	CAEZ01010434:67-491
HmHel-7	CAEZ01008231:24727-25953
HmHel-7	CAEZ01008231:33215-33643
HmHel-7	CAEZ01008102:33-357
HmHel-7	CAEZ01005836:1-395
HmHel-7	CAEZ01005280:5850-6422
HmHel-7	CAEZ01003944:3822-5087
HmHel-7	CAEZ01002288:3361-3769
HmHel-7	CAEZ01011058:11785-12778
HmHel-7	CAEZ01010605:876-1657
HmHel-7	CAEZ01010506:10-417

HmHel-7	CAEZ01010420:1-900
HmHel-7	CAEZ01008987:2-829
HmHel-7	CAEZ01008255:1-508
HmHel-7	CAEZ01008161:1-717
HmHel-7	CAEZ01004682:1-937
HmHel-7	CAEZ01004412:1-733
HmHel-7	CAEZ01003700:11139-11557
HmHel-7	CAEZ01003398:46420-47526
HmHel-7	CAEZ01001971:12660-13020
HmHel-7	CAEZ01001512:10-417
HmHel-7	CAEZ01006198:2078-2908
HmHel-7	CAEZ01001026:12696-14000
HmHel-7	CAEZ01005368:7686-8750
HmHel-7	CAEZ01008167:62-876
HmHel-7	CAEZ01007768:2205-2624
HmHel-7	CAEZ01006494:50561-51061
HmHel-7	CAEZ01006009:1-500
HmHel-7	CAEZ01003853:2094-2787
HmHel-7	CAEZ01002798:27833-28607
HmHel-7	CAEZ01001867:15397-15753
HmHel-7	CAEZ01000990:39008-39401
HmHel-7	CAEZ01011488:27505-28086
HmHel-7	CAEZ01010736:10-385
HmHel-7	CAEZ01010736:57674-57960
HmHel-7	CAEZ01006903:32-355
HmHel-7	CAEZ01006762:69-473
HmHel-7	CAEZ01006762:14333-14611
HmHel-7	CAEZ01005304:937-2275
HmHel-7	CAEZ01004940:1-522
HmHel-7	CAEZ01004717:10027-10995
HmHel-7	CAEZ01002661:86-409
HmHel-7	CAEZ01001339:5789-6153
HmHel-7	CAEZ01001046:70-477
HmHel-7	CAEZ01011593:378-1598
HmHel-7	CAEZ01009912:2376-2734
HmHel-7	CAEZ01001164:1-339
HmHel-7	CAEZ01006153:83002-83834
HmHel-7	CAEZ01001760:1-777
HmHel-7	CAEZ01011630:6912-7236
HmHel-7	CAEZ01010448:3314-3965
HmHel-7	CAEZ01010440:53185-54515
HmHel-7	CAEZ01009971:23430-24772
HmHel-7	CAEZ01009803:93-512
HmHel-7	CAEZ01006956:4-380
HmHel-7	CAEZ01006151:100720-101069
HmHel-7	CAEZ01002064:40505-40867
HmHel-7	CAEZ01010289:9015-10396
HmHel-7	CAEZ01010248:1-744

HmHel-7	CAEZ01010101:1-304
HmHel-7	CAEZ01009618:3-366
HmHel-7	CAEZ01009253:9-371
HmHel-7	CAEZ01008062:10128-10525
HmHel-7	CAEZ01004618:1403-1851
HmHel-7	CAEZ01003223:1-830
HmHel-7	CAEZ01002146:12801-13318
HmHel-7	CAEZ01001245:722-1226
HmHel-7	CAEZ01011523:67318-68492
HmHel-7	CAEZ01011523:82-469
HmHel-7	CAEZ01009155:7-661
HmHel-7	CAEZ01008997:5360-6037
HmHel-7	CAEZ01008882:45361-46614
HmHel-7	CAEZ01008882:28601-28717
HmHel-7	CAEZ01008882:8183-8812
HmHel-7	CAEZ01006100:5920-6828
HmHel-7	CAEZ01003737:4347-5708
HmHel-7	CAEZ01011349:6706-7051
HmHel-7	CAEZ01011059:1-335
HmHel-7	CAEZ01009991:53668-54261
HmHel-7	CAEZ01009535:10351-11704
HmHel-7	CAEZ01008341:45135-45839
HmHel-7	CAEZ01002374:9-367
HmHel-7	CAEZ01001926:1-351
HmHel-7	CAEZ01011373:1-818
HmHel-7	CAEZ01009684:27833-28404
HmHel-7	CAEZ01009254:14643-15051
HmHel-7	CAEZ01009193:1-537
HmHel-7	CAEZ01008070:76-495
HmHel-7	CAEZ01007816:8492-14219
HmHel-7	CAEZ01007501:6594-7301
HmHel-7	CAEZ01005459:51514-52011
HmHel-7	CAEZ01005220:57655-58425
HmHel-7	CAEZ01003082:10-385
HmHel-7	CAEZ01001939:855-1448
HmHel-7	CAEZ01001892:1-353
HmHel-7	CAEZ01001892:37526-37717
HmHel-7	CAEZ01001366:7568-7975
HmHel-7	CAEZ01011163:11175-11581
HmHel-7	CAEZ01007227:5677-6067
HmHel-7	CAEZ01006221:23-446
HmHel-7	CAEZ01005855:1-371
HmHel-7	CAEZ01005137:2576-3154
HmHel-7	CAEZ01004906:10233-11241
HmHel-7	CAEZ01002717:4748-6010
HmHel-7	CAEZ01002183:16224-17179
HmHel-7	CAEZ01001189:1-371
HmHel-7	CAEZ01009156:884-1212

HmHel-7	CAEZ01005479:7264-7687
HmHel-7	CAEZ01004953:43-802
HmHel-7	CAEZ01001038:3522-3823
HmHel-7	CAEZ01011010:1-340
HmHel-7	CAEZ01006323:15-898
HmHel-7	CAEZ01001467:750-1071
HmHel-7	CAEZ01010274:29-665
HmHel-7	CAEZ01010107:5-297
HmHel-7	CAEZ01009087:2140-2449
HmHel-7	CAEZ01008798:6627-7021
HmHel-7	CAEZ01008757:1885-2292
HmHel-7	CAEZ01008647:4013-4419
HmHel-7	CAEZ01008354:71253-72214
HmHel-7	CAEZ01007445:3575-3880
HmHel-7	CAEZ01007445:3357-3576
HmHel-7	CAEZ01006072:6-381
HmHel-7	CAEZ01002658:52758-53120
HmHel-7	CAEZ01010903:1423-2013
HmHel-7	CAEZ01005661:620-942
HmHel-7	CAEZ01005641:31627-32147
HmHel-7	CAEZ01005385:1927-2971
HmHel-7	CAEZ01004501:7-380
HmHel-7	CAEZ01004405:65-471
HmHel-7	CAEZ01003324:64-483
HmHel-7	CAEZ01002990:2231-3192
HmHel-7	CAEZ01001704:25-431
HmHel-7	CAEZ01006841:6541-6934
HmHel-7	CAEZ01002366:445-747
HmHel-7	CAEZ01001857:2906-3761
HmHel-7	CAEZ01006004:3-530
HmHel-7	CAEZ01010772:1-927
HmHel-7	CAEZ01008123:57025-57810
HmHel-7	CAEZ01003602:3116-3469
HmHel-7	CAEZ01003454:47-466
HmHel-7	CAEZ01002322:11-556
HmHel-7	CAEZ01001577:7309-7940
HmHel-7	CAEZ01010548:1-441
HmHel-7	CAEZ01010356:57-648
HmHel-7	CAEZ01008328:60-478
HmHel-7	CAEZ01008328:11264-11941
HmHel-7	CAEZ01007703:32762-33714
HmHel-7	CAEZ01005902:13957-14361
HmHel-7	CAEZ01005417:1-317
HmHel-7	CAEZ01005205:3-528
HmHel-7	CAEZ01004650:12-412
HmHel-7	CAEZ01003426:1-316
HmHel-7	CAEZ01002416:87035-87441
HmHel-7	CAEZ01004446:34-458

HmHel-7	CAEZ01003167:29919-30227
HmHel-7	CAEZ01010923:3038-6255
HmHel-7	CAEZ01010923:26776-27027
HmHel-7	CAEZ01005181:1-828
HmHel-7	CAEZ01005108:116104-117435
HmHel-7	CAEZ01004692:3950-4280
HmHel-7	CAEZ01009133:33067-33600
HmHel-7	CAEZ01006045:11-668
HmHel-7	CAEZ01003623:5442-5747
HmHel-7	CAEZ01009073:62-468
HmHel-7	CAEZ01007713:55197-56335
HmHel-7	CAEZ01007713:26166-26901
HmHel-7	CAEZ01007105:1-568
HmHel-7	CAEZ01005920:751-1067
HmHel-7	CAEZ01002706:54781-55326
HmHel-7	CAEZ01006947:1-890
HmHel-7	CAEZ01000021:12-461
HmHel-7	CAEZ01008316:49883-50215
HmHel-7	CAEZ01011182:15-421
HmHel-7	CAEZ01006745:1204-2228
HmHel-7	CAEZ01006326:87177-88363
HmHel-7	CAEZ01003603:1-417
HmHel-7	CAEZ01002910:5-297
HmHel-7	CAEZ01002019:11-595
HmHel-7	CAEZ01001880:1-899
HmHel-7	CAEZ01006637:22-341
HmHel-7	CAEZ01005896:53-468
HmHel-7	CAEZ01005058:12649-13324
HmHel-7	CAEZ01002249:24826-25790
HmHel-7	CAEZ01009628:12099-12393
HmHel-7	CAEZ01003588:15-394
HmHel-7	CAEZ01003337:1-504
HmHel-7	CAEZ01008188:1-531
HmHel-7	CAEZ01008127:13445-14910
HmHel-7	CAEZ01009031:7-339
HmHel-7	CAEZ01006354:1450-2088
HmHel-7	CAEZ01004678:24322-25194
HmHel-7	CAEZ01002629:42456-43760
HmHel-7	CAEZ01001465:1-832
HmHel-7	CAEZ01001465:110106-110984
HmHel-7	CAEZ01001465:98129-98514
HmHel-7	CAEZ01009986:34-440
HmHel-7	CAEZ01009944:741-1755
HmHel-7	CAEZ01005383:13267-14316
HmHel-7	CAEZ01001782:109-526
HmHel-7	CAEZ01001363:1-350
HmHel-7	CAEZ01006744:11336-11623
HmHel-7	CAEZ01001439:39068-40536

HmHel-7	CAEZ01001085:1-339
HmHel-7	CAEZ01008623:18457-19505
HmHel-7	CAEZ01006142:1436-1788
HmHel-7	CAEZ01003274:13-592
HmHel-7	CAEZ01003106:1-282
HmHel-7	CAEZ01002386:1221-1865
HmHel-7	CAEZ01000768:1-591
HmHel-7	CAEZ01004859:1-878
HmHel-7	CAEZ01010853:2346-2656
HmHel-7	CAEZ01009531:12-670
HmHel-7	CAEZ01006946:8780-9106
HmHel-7	CAEZ01002963:3954-4257
HmHel-7	CAEZ01010690:9876-11637
HmHel-7	CAEZ01008986:20219-20520
HmHel-7	CAEZ01008497:33942-34803
HmHel-7	CAEZ01007031:23-275
HmHel-7	CAEZ01005512:23645-23964
HmHel-7	CAEZ01004339:25608-25879
HmHel-7	CAEZ01003364:4475-4882
HmHel-7	CAEZ01002812:45244-45636
HmHel-7	CAEZ01011167:3498-3805
HmHel-7	CAEZ01010852:19426-20128
HmHel-7	CAEZ01009084:311-912
HmHel-7	CAEZ01006910:31023-31314
HmHel-7	CAEZ01005737:3-285
HmHel-7	CAEZ01002354:3928-5083
HmHel-7	CAEZ01008758:3121-3874
HmHel-7	CAEZ01008212:16025-17364
HmHel-7	CAEZ01001226:1004-1595
HmHel-7	CAEZ01001131:4376-4626
HmHel-7	CAEZ01011389:16-350
HmHel-7	CAEZ01010182:1365-2154
HmHel-7	CAEZ01001858:1-306
HmHel-7	CAEZ01001443:57366-57626
HmHel-7	CAEZ01008311:1-268
HmHel-7	CAEZ01004233:1-276
HmHel-7	CAEZ01003913:1-349
HmHel-7	CAEZ01010511:40676-41249
HmHel-7	CAEZ01009365:25-275
HmHel-7	CAEZ01006113:32-367
HmHel-7	CAEZ01004672:2031-2551
HmHel-7	CAEZ01004116:5195-5567
HmHel-7	CAEZ01003943:3880-4750
HmHel-7	CAEZ01000987:632-971
HmHel-7	CAEZ01009315:1-847
HmHel-7	CAEZ01005471:1-382
HmHel-7	CAEZ01003880:1938-2833
HmHel-7	CAEZ01001925:1149-1894

HmHel-7	CAEZ01011190:29-735
HmHel-7	CAEZ01009698:5-493
HmHel-7	CAEZ01007808:114767-115192
HmHel-7	CAEZ01006578:1759-2098
HmHel-7	CAEZ01005757:5452-6373
HmHel-7	CAEZ01005136:38839-39088
HmHel-7	CAEZ01001368:15-1024
HmHel-7	CAEZ01001203:8785-9516
HmHel-7	CAEZ01008995:3131-3430
HmHel-7	CAEZ01001347:14640-15435
HmHel-7	CAEZ01001303:1-436
HmHel-7	CAEZ01001162:10182-10903
HmHel-7	CAEZ01000628:28-280
HmHel-7	CAEZ01010616:52798-53176
HmHel-7	CAEZ01007838:4846-5096
HmHel-7	CAEZ01001516:301-639
HmHel-7	CAEZ01008823:1-859
HmHel-7	CAEZ01007605:23742-24071
HmHel-7	CAEZ01007482:11348-11870
HmHel-7	CAEZ01006351:4-764
HmHel-7	CAEZ01005372:14-263
HmHel-7	CAEZ01005176:9815-10662
HmHel-7	CAEZ01002723:834-1103
HmHel-7	CAEZ01010527:32692-33132
HmHel-7	CAEZ01008165:2212-2874
HmHel-7	CAEZ01007829:1-506
HmHel-7	CAEZ01006348:1-862
HmHel-7	CAEZ01006178:11-349
HmHel-7	CAEZ01004137:22031-23659
HmHel-7	CAEZ01001117:21-358
HmHel-7	CAEZ01011150:2-253
HmHel-7	CAEZ01007918:1-253
HmHel-7	CAEZ01007277:1-697
HmHel-7	CAEZ01004836:34915-35214
HmHel-7	CAEZ01001464:34239-34494
HmHel-7	CAEZ01009034:5-528
HmHel-7	CAEZ01008545:24756-25043
HmHel-7	CAEZ01007588:222-508
HmHel-7	CAEZ01006813:3-739
HmHel-7	CAEZ01001790:1-763
HmHel-7	CAEZ01001672:593-1323
HmHel-7	CAEZ01010779:4-584
HmHel-7	CAEZ01010468:1-701
HmHel-7	CAEZ01009182:24571-25074
HmHel-7	CAEZ01005921:11-271
HmHel-7	CAEZ01004463:1-535
HmHel-7	CAEZ01003304:47655-48264
HmHel-7	CAEZ01002844:1-436

HmHel-7	CAEZ01001901:48-570
HmHel-7	CAEZ01009507:1-383
HmHel-7	CAEZ01010313:4553-5553
HmHel-7	CAEZ01009390:1-285
HmHel-7	CAEZ01004003:4-513
HmHel-7	CAEZ01002252:733-1730
HmHel-7	CAEZ01009453:1-748
HmHel-7	CAEZ01005123:2-277
HmHel-7	CAEZ01000003:1-408
HmHel-7	CAEZ01007219:4322-4620
HmHel-7	CAEZ01006859:1-230
HmHel-7	CAEZ01002697:6199-6476
HmHel-7	CAEZ01011597:2808-3167
HmHel-7	CAEZ01008044:13-538
HmHel-7	CAEZ01007092:5376-5875
HmHel-7	CAEZ01003215:1-478
HmHel-7	CAEZ01000004:1-389
HmHel-7	CAEZ01007470:21669-22264
HmHel-7	CAEZ01006921:13109-13891
HmHel-7	CAEZ01004124:3-387
HmHel-7	CAEZ01000071:372-607
HmHel-7	CAEZ01010531:1139-1888
HmHel-7	CAEZ01008782:7966-8350
HmHel-7	CAEZ01007821:9-852
HmHel-7	CAEZ01007058:1-735
HmHel-7	CAEZ01005928:20873-21143
HmHel-7	CAEZ01005420:29909-33834
HmHel-7	CAEZ01003435:6860-7097
HmHel-7	CAEZ01010095:1-832
HmHel-7	CAEZ01006219:1-493
HmHel-7	CAEZ01010355:49801-50041
HmHel-7	CAEZ01009764:1-336
HmHel-7	CAEZ01009389:13487-14265
HmHel-7	CAEZ01008936:11-263
HmHel-7	CAEZ01006882:10901-11276
HmHel-7	CAEZ01005654:1-329
HmHel-7	CAEZ01003861:16871-17172
HmHel-7	CAEZ01001817:14732-15317
HmHel-7	CAEZ01011416:23-407
HmHel-7	CAEZ01011076:1-442
HmHel-7	CAEZ01009697:3894-4436
HmHel-7	CAEZ01005655:6567-6794
HmHel-7	CAEZ01003966:1-236
HmHel-7	CAEZ01002362:15-541
HmHel-7	CAEZ01010945:1-480
HmHel-7	CAEZ01007520:4576-5142
HmHel-7	CAEZ01006784:21285-21789
HmHel-7	CAEZ01005590:6971-7747

HmHel-7	CAEZ01008905:27614-28417
HmHel-7	CAEZ01008531:845-1576
HmHel-7	CAEZ01005574:5840-6614
HmHel-7	CAEZ01001437:36228-36746
HmHel-7	CAEZ01003576:4542-4778
HmHel-7	CAEZ01001229:958-1253
HmHel-7	CAEZ01011281:1298-1770
HmHel-7	CAEZ01009510:26119-26362
HmHel-7	CAEZ01008115:37195-37429
HmHel-7	CAEZ01007762:1-335
HmHel-7	CAEZ01003302:33121-33596
HmHel-7	CAEZ01000032:22-273
HmHel-7	CAEZ01011493:15582-16695
HmHel-7	CAEZ01001180:1-712
HmHel-7	CAEZ01005614:1-479
HmHel-7	CAEZ01005399:29815-30156
HmHel-7	CAEZ01005485:79108-79741
HmHel-7	CAEZ01011627:26870-27521
HmHel-7	CAEZ01011551:1-732
HmHel-7	CAEZ01003765:1-510
HmHel-7	CAEZ01003246:1-227
HmHel-7	CAEZ01003038:18303-18823
HmHel-7	CAEZ01002920:414-660
HmHel-7	CAEZ01001251:1628-1890
HmHel-7	CAEZ01009111:1-238
HmHel-7	CAEZ01008958:1222-1567
HmHel-7	CAEZ01008870:2517-2770
HmHel-7	CAEZ01011432:441-954
HmHel-7	CAEZ01010579:7847-8990
HmHel-7	CAEZ01010579:10805-10903
HmHel-7	CAEZ01004597:21587-22060
HmHel-7	CAEZ01004597:21737-22007
HmHel-7	CAEZ01004406:14660-14900
HmHel-7	CAEZ01000821:9665-10178
HmHel-7	CAEZ01010186:1-736
HmHel-7	CAEZ01009146:381-616
HmHel-7	CAEZ01008806:38964-39476
HmHel-7	CAEZ01001766:101531-101766
HmHel-7	CAEZ01001361:9-531
HmHel-7	CAEZ01008160:1-227
HmHel-7	CAEZ01005369:1-488
HmHel-7	CAEZ01000227:1-227
HmHel-7	CAEZ01003994:22-387
HmHel-7	CAEZ01001563:8851-9557
HmHel-7	CAEZ01010982:7606-7842
HmHel-7	CAEZ01003935:1679-2188
HmHel-7	CAEZ01003732:5444-5656
HmHel-7	CAEZ01002673:26-278

HmHel-7	CAEZ01009624:1330-1553
HmHel-7	CAEZ01005231:9827-10062
HmHel-7	CAEZ01003939:30789-31047
HmHel-7	CAEZ01009249:9-538
HmHel-7	CAEZ01003624:1022-1601
HmHel-7	CAEZ01011475:1-312
HmHel-7	CAEZ01010436:2-445
HmHel-7	CAEZ01001351:23-539
HmHel-7	CAEZ01003826:20285-20541
HmHel-7	CAEZ01000846:876-1100
HmHel-7	CAEZ01008189:9-348
HmHel-7	CAEZ01007656:4718-5053
HmHel-7	CAEZ01002901:1-536
HmHel-7	CAEZ01002643:57403-57932
HmHel-7	CAEZ01008129:1-533
HmHel-7	CAEZ01005892:1-204
HmHel-7	CAEZ01000677:1-282
HmHel-7	CAEZ01000176:5094-5317
HmHel-7	CAEZ01009154:22-477
HmHel-7	CAEZ01005520:9975-10469
HmHel-7	CAEZ01006358:1-296
HmHel-7	CAEZ01002708:9263-9622
HmHel-7	CAEZ01001787:1-207
HmHel-7	CAEZ01010149:10609-10985
HmHel-7	CAEZ01011255:2383-2941
HmHel-7	CAEZ01004055:6424-6956
HmHel-7	CAEZ01004469:8-494
HmHel-7	CAEZ01001773:601-819
HmHel-7	CAEZ01002876:48244-48654
HmHel-7	CAEZ01009957:25602-25800
HmHel-7	CAEZ01008380:2-196
HmHel-7	CAEZ01002189:1056-1838
HmHel-7	CAEZ01001096:7-555
HmHel-7	CAEZ01007618:4910-5780
HmHel-7	CAEZ01005967:15230-16290
HmHel-7	CAEZ01001544:1050-1260
HmHel-7	CAEZ01003135:2047-2283
HmHel-7	CAEZ01009881:22778-23972
HmHel-7	CAEZ01007737:194-381
HmHel-7	CAEZ01000405:1-203
HmHel-7	CAEZ01005125:1-267
HmHel-7	CAEZ01006199:5631-6019
HmHel-7	CAEZ01002718:5-566
HmHel-7	CAEZ01004232:9848-10443
HmHel-7	CAEZ01011144:1-203
HmHel-7	CAEZ01006796:31611-32035
HmHel-7	CAEZ01006182:735-958
HmHel-7	CAEZ01006112:1798-1982

HmHel-7	CAEZ01004244:111877-112793
HmHel-7	CAEZ01000321:56-254
HmHel-7	CAEZ01006277:55-322
HmHel-7	CAEZ01006610:15243-15555
HmHel-7	CAEZ01011214:5277-5442
HmHel-7	CAEZ01003953:7353-7521
HmHel-7	CAEZ01011623:16187-16609
HmHel-7	CAEZ01001876:1834-1997
HmHel-7	CAEZ01010167:1-172
HmHel-7	CAEZ01005670:1-265
HmHel-7	CAEZ01007350:1-154
HmHel-7	CAEZ01006273:2-175
HmHel-7	CAEZ01000018:44-526
HmHel-7	CAEZ01011555:1-143
HmHel-7	CAEZ01010658:9542-9750
HmHel-7	CAEZ01005792:2-409
HmHel-7	CAEZ01007519:5229-5414
HmHel-7	CAEZ01004942:82114-82265
HmHel-7	CAEZ01008471:609-773
HmHel-7	CAEZ01008056:19-130
HmHel-7	CAEZ01006161:18290-18455
HmHel-7	CAEZ01008644:599-721
HmHel-7	CAEZ01004509:8318-8948
HmHel-7	CAEZ01007711:23344-23548
HmHel-7	CAEZ01010608:5712-5987
HmHel-7	CAEZ01001286:5581-5711
HmHel-7	CAEZ01010890:8042-8170
HmHel-7	CAEZ01009900:2114-2236
HmHel-7	CAEZ01005027:35-187
HmHel-7	CAEZ01004521:936-1054
HmHel-7	CAEZ01007252:96579-96729
HmHel-7	CAEZ01003912:9280-9682
HmHel-7	CAEZ01002641:1911-2041
HmHel-7	CAEZ01000773:2379-2487
HmHel-7	CAEZ01008524:22883-22985
HmHel-7	CAEZ01009420:5230-5359
HmHel-7	CAEZ01001505:3-125
HmHel-7	CAEZ01005987:22868-22970
HmHel-7	CAEZ01006559:19532-20086
HmHel-7	CAEZ01004561:1-105
HmHel-7	CAEZ01009227:7423-7561
HmHel-7	CAEZ01000394:1-92
HmHel-7	CAEZ01000387:1-85
HmHel-7	CAEZ01006422:15949-16085
HmHel-7	CAEZ01010539:9355-9481
HmHel-7	CAEZ01009074:816-925
HmHel-7	CAEZ01011051:23483-23646
HmHel-7	CAEZ01000400:4-92

HmHel-7	CAEZ01009475:23636-23808
HmHel-7	CAEZ01010727:29986-30157
HmHel-7	CAEZ01005980:10690-10867
HmHel-7	CAEZ01004848:1215-1294
HmHel-7	CAEZ01006005:14628-14716
	:-
HmHel-8	CAEZ01007114:9082-9377
HmHel-8	CAEZ01005258:11178-11473
HmHel-8	CAEZ01000908:18505-18796
HmHel-8	CAEZ01010927:136202-136493
HmHel-8	CAEZ01005402:30580-30875
HmHel-8	CAEZ01000909:1119-1414
HmHel-8	CAEZ01009221:2031-2327
HmHel-8	CAEZ01005961:653-949
HmHel-8	CAEZ01011046:177875-178170
HmHel-8	CAEZ01010270:90474-90769
HmHel-8	CAEZ01010191:948-1243
HmHel-8	CAEZ01009828:23832-25764
HmHel-8	CAEZ01009828:17083-17378
HmHel-8	CAEZ01007761:33854-34149
HmHel-8	CAEZ01009501:21004-21299
HmHel-8	CAEZ01010693:10194-10488
HmHel-8	CAEZ01010543:18266-18561
HmHel-8	CAEZ01008279:22385-22680
HmHel-8	CAEZ01007849:25847-26142
HmHel-8	CAEZ01005638:2048-2343
HmHel-8	CAEZ01003838:5840-6135
HmHel-8	CAEZ01006150:19309-19605
HmHel-8	CAEZ01002043:1825-2121
HmHel-8	CAEZ01002158:2751-3037
HmHel-8	CAEZ01007747:260144-260440
HmHel-8	CAEZ01005732:13878-14176
HmHel-8	CAEZ01000901:608-904
HmHel-8	CAEZ01010190:27853-28148
HmHel-8	CAEZ01010190:54177-54478
HmHel-8	CAEZ01006174:63496-63786
HmHel-8	CAEZ01005442:482-777
HmHel-8	CAEZ01005415:28718-29013
HmHel-8	CAEZ01005466:18947-19244
HmHel-8	CAEZ01009832:32215-32511
HmHel-8	CAEZ01009420:5664-5959
HmHel-8	CAEZ01006286:20227-20522
HmHel-8	CAEZ01005089:37730-38025
HmHel-8	CAEZ01003099:21457-21752
HmHel-8	CAEZ01009801:66309-66603
HmHel-8	CAEZ01008374:25380-25672
HmHel-8	CAEZ01001912:13550-13843
HmHel-8	CAEZ01006078:36025-36320

HmHel-8	CAEZ01005119:4012-4303
HmHel-8	CAEZ01005118:61-356
HmHel-8	CAEZ01005117:31986-32281
HmHel-8	CAEZ01003834:16620-16902
HmHel-8	CAEZ01007971:14030-14328
HmHel-8	CAEZ01002811:70394-70690
HmHel-8	CAEZ01007863:146052-146347
HmHel-8	CAEZ01004990:8907-9198
HmHel-8	CAEZ01004497:10373-10666
HmHel-8	CAEZ01005359:783-1081
HmHel-8	CAEZ01011031:1128-1423
HmHel-8	CAEZ01003365:4368-4660
HmHel-8	CAEZ01009824:79040-79334
HmHel-8	CAEZ01009824:92206-92501
HmHel-8	CAEZ01011215:126261-126553
HmHel-8	CAEZ01006131:14272-14565
HmHel-8	CAEZ01006395:7154-7414
HmHel-8	CAEZ01008576:16817-17111
HmHel-8	CAEZ01006433:10155-10443
HmHel-8	CAEZ01003673:5940-6243
HmHel-8	CAEZ01010300:6119-6491
HmHel-8	CAEZ01003748:7037-7319
HmHel-8	CAEZ01011336:11859-12148
HmHel-8	CAEZ01000427:444-570
HmHel-8	CAEZ01006769:8411-8629
HmHel-8	CAEZ01008029:12047-12165
HmHel-8	CAEZ01009032:19109-19286
HmHel-8	CAEZ01004360:31207-31502
HmHel-8	CAEZ01006320:17739-17923
HmHel-8	CAEZ01010129:82722-83006
HmHel-8	CAEZ01008571:1595-1879
HmHel-8	CAEZ01011529:43607-43894
HmHel-8	CAEZ01011519:62799-62892<br>:-
HmHel-9	CAEZ01001981:45905-46316
HmHel-9	CAEZ01007390:3282-3700
HmHel-9	CAEZ01008029:13314-13723
HmHel-9	CAEZ01004795:31605-32018
HmHel-9	CAEZ01006405:15251-15668
HmHel-9	CAEZ01005153:86354-86772
HmHel-9	CAEZ01007201:88069-88146
HmHel-9	CAEZ01007201:106684-107031
HmHel-9	CAEZ01007201:102537-102837
HmHel-9	CAEZ01007708:40954-41372
HmHel-9	CAEZ01007708:76320-76615
HmHel-9	CAEZ01001578:49945-50363
HmHel-9	CAEZ01008919:62143-62569
HmHel-9	CAEZ01004127:86972-87393

HmHel-9	CAEZ01004127:43728-43937
HmHel-9	CAEZ01004127:10684-10764
HmHel-9	CAEZ01010566:9141-9557
HmHel-9	CAEZ01008921:112237-112628
HmHel-9	CAEZ01008921:22823-23234
HmHel-9	CAEZ01010697:48486-48907
HmHel-9	CAEZ01009241:2486-2906
HmHel-9	CAEZ01008294:13433-13855
HmHel-9	CAEZ01004814:36611-37019
HmHel-9	CAEZ01001715:29270-29686
HmHel-9	CAEZ01001806:74040-74450
HmHel-9	CAEZ01006604:6467-6868
HmHel-9	CAEZ01007950:18579-18999
HmHel-9	CAEZ01006639:1826-2246
HmHel-9	CAEZ01006639:3146-3498
HmHel-9	CAEZ01006109:13717-14130
HmHel-9	CAEZ01000063:32486-32906
HmHel-9	CAEZ01008890:1535-1927
HmHel-9	CAEZ01009716:90069-90455
HmHel-9	CAEZ01009716:198371-198477
HmHel-9	CAEZ01003794:63010-63422
HmHel-9	CAEZ01002838:14999-15414
HmHel-9	CAEZ01010749:796-1202
HmHel-9	CAEZ01004641:2280-2669
HmHel-9	CAEZ01008960:226511-226927
HmHel-9	CAEZ01008522:6547-6951
HmHel-9	CAEZ01003292:99352-99755
HmHel-9	CAEZ01003292:69089-69479
HmHel-9	CAEZ01003292:39471-39801
HmHel-9	CAEZ01009670:9876-10294
HmHel-9	CAEZ01009370:154591-155016
HmHel-9	CAEZ01007046:23308-23666
HmHel-9	CAEZ01010701:190295-190711
HmHel-9	CAEZ01003634:16241-16657
HmHel-9	CAEZ01004313:2872-3293
HmHel-9	CAEZ01006659:1443-1797
HmHel-9	CAEZ01010030:241921-242226
HmHel-9	CAEZ01010030:126972-127380
HmHel-9	CAEZ01000876:58219-58636
HmHel-9	CAEZ01009443:62316-62732
HmHel-9	CAEZ01009965:21295-21651
HmHel-9	CAEZ01003435:2699-3107
HmHel-9	CAEZ01010388:1259-1672
HmHel-9	CAEZ01007802:25596-25712
HmHel-9	CAEZ01007802:20992-21408
HmHel-9	CAEZ01010728:14255-14646
HmHel-9	CAEZ01010728:57443-57726
HmHel-9	CAEZ01010728:20068-20382

HmHel-9	CAEZ01009892:104405-104737
HmHel-9	CAEZ01008034:22384-22802
HmHel-9	CAEZ01008034:5171-5452
HmHel-9	CAEZ01011222:1774-2149
HmHel-9	CAEZ01006117:6399-6804
HmHel-9	CAEZ01007803:4852-5248
HmHel-9	CAEZ01009249:15775-16189
HmHel-9	CAEZ01006299:5453-5859
HmHel-9	CAEZ01006299:22234-22399
HmHel-9	CAEZ01006299:62320-62689
HmHel-9	CAEZ01008915:312709-313050
HmHel-9	CAEZ01008478:10712-11110
HmHel-9	CAEZ01006230:8013-8435
HmHel-9	CAEZ01005607:9453-9871
HmHel-9	CAEZ01004952:20949-21354
HmHel-9	CAEZ01005772:28463-28818
HmHel-9	CAEZ01007707:18849-19237
HmHel-9	CAEZ01007707:157698-157893
HmHel-9	CAEZ01010010:4125-4512
HmHel-9	CAEZ01006778:177469-177858
HmHel-9	CAEZ01006505:1011-1391
HmHel-9	CAEZ01006470:5238-5606
HmHel-9	CAEZ01005714:40886-41251
HmHel-9	CAEZ01000909:44927-45342
HmHel-9	CAEZ01006711:65596-66000
HmHel-9	CAEZ01003163:24019-24484
HmHel-9	CAEZ01000999:52338-52741
HmHel-9	CAEZ01007861:103650-104053
HmHel-9	CAEZ01003714:13504-13930
HmHel-9	CAEZ01008281:20392-20560
HmHel-9	CAEZ01008281:8697-9082
HmHel-9	CAEZ01005210:5053-5410
HmHel-9	CAEZ01005210:73267-73384
HmHel-9	CAEZ01004918:64482-64790
HmHel-9	CAEZ01003055:7719-8141
HmHel-9	CAEZ01009701:569-988
HmHel-9	CAEZ01005442:128879-129287
HmHel-9	CAEZ01008737:128536-128835
HmHel-9	CAEZ01008737:73256-73542
HmHel-9	CAEZ01007808:23820-24148
HmHel-9	CAEZ01010049:17773-17890
HmHel-9	CAEZ01010049:3128-3525
HmHel-9	CAEZ01002155:12256-12638
HmHel-9	CAEZ01011484:2735-3135
HmHel-9	CAEZ01009883:58118-58541
HmHel-9	CAEZ01009883:40063-40290
HmHel-9	CAEZ01010427:1200-1613
HmHel-9	CAEZ01009999:19222-19536

HmHel-9	CAEZ01009403:7160-7547
HmHel-9	CAEZ01002097:30143-30560
HmHel-9	CAEZ01006787:15425-15826
HmHel-9	CAEZ01001832:49166-49612
HmHel-9	CAEZ01010787:5395-5788
HmHel-9	CAEZ01008731:39477-39770
HmHel-9	CAEZ01008138:344279-344598
HmHel-9	CAEZ01006504:1017-1310
HmHel-9	CAEZ01008278:9632-10061
HmHel-9	CAEZ01010935:6430-6882
HmHel-9	CAEZ01006164:31665-32005
HmHel-9	CAEZ01005773:1-280
HmHel-9	CAEZ01003663:10510-10933
HmHel-9	CAEZ01005474:1852-2235
HmHel-9	CAEZ01000076:1892-2255
HmHel-9	CAEZ01009421:56135-56417
HmHel-9	CAEZ01004975:31883-32275
HmHel-9	CAEZ01004796:37547-37862
HmHel-9	CAEZ01002893:697-1039
HmHel-9	CAEZ01007655:476-896
HmHel-9	CAEZ01010301:75924-76348
HmHel-9	CAEZ01002892:25802-26148
HmHel-9	CAEZ01001561:477-825
HmHel-9	CAEZ01007049:9908-10236
HmHel-9	CAEZ01006153:77869-78245
HmHel-9	CAEZ01002153:5737-6148
HmHel-9	CAEZ01010387:4126-4558
HmHel-9	CAEZ01007455:410-757
HmHel-9	CAEZ01006474:513-918
HmHel-9	CAEZ01009672:12594-12929
HmHel-9	CAEZ01002431:63567-63912
HmHel-9	CAEZ01001874:701-974
HmHel-9	CAEZ01009835:4363-4711
HmHel-9	CAEZ01006138:83604-83950
HmHel-9	CAEZ01003335:1992-2296
HmHel-9	CAEZ01009802:270977-271361
HmHel-9	CAEZ01007798:1629-1953
HmHel-9	CAEZ01006257:1138-1411
HmHel-9	CAEZ01005392:33170-33479
HmHel-9	CAEZ01000846:3695-4100
HmHel-9	CAEZ01008920:13375-13734
HmHel-9	CAEZ01008124:119820-120281
HmHel-9	CAEZ01000854:62642-62946
HmHel-9	CAEZ01007785:6983-7269
HmHel-9	CAEZ01011623:8342-8712
HmHel-9	CAEZ01010755:2426-2856
HmHel-9	CAEZ01008270:31254-31626
HmHel-9	CAEZ01006154:63924-64279

HmHel-9	CAEZ01002563:33-381
HmHel-9	CAEZ01004768:16257-16562
HmHel-9	CAEZ01008803:8771-9055
HmHel-9	CAEZ01006084:6833-7248
HmHel-9	CAEZ01009136:114282-114641
HmHel-9	CAEZ01009136:97971-98167
HmHel-9	CAEZ01007492:41370-41698
HmHel-9	CAEZ01011473:9095-9369
HmHel-9	CAEZ01009240:12728-13126
HmHel-9	CAEZ01003452:15152-15435
HmHel-9	CAEZ01007782:4833-5188
HmHel-9	CAEZ01008164:31593-32014
HmHel-9	CAEZ01003120:161-568
HmHel-9	CAEZ01000981:1313-1638
HmHel-9	CAEZ01009512:34706-35087
HmHel-9	CAEZ01003145:19499-19846
HmHel-9	CAEZ01010350:7059-7498
HmHel-9	CAEZ01003954:5334-5674
HmHel-9	CAEZ01010927:51969-52344
HmHel-9	CAEZ01005211:132983-133273
HmHel-9	CAEZ01011554:21248-21541
HmHel-9	CAEZ01004921:31621-31894
HmHel-9	CAEZ01003404:53220-53604
HmHel-9	CAEZ01010103:10238-10510
HmHel-9	CAEZ01007403:26044-26347
HmHel-9	CAEZ01006380:8207-8899
HmHel-9	CAEZ01010597:20709-20976
HmHel-9	CAEZ01009748:10250-10546
HmHel-9	CAEZ01003889:2373-2677
HmHel-9	CAEZ01007184:236645-236844
HmHel-9	CAEZ01007184:280452-280807
HmHel-9	CAEZ01007184:311564-311713
HmHel-9	CAEZ01003260:49507-49903
HmHel-9	CAEZ01004992:4207-4575
HmHel-9	CAEZ01004821:1418-1731
HmHel-9	CAEZ01011482:4209-4605
HmHel-9	CAEZ01009120:17320-17761
HmHel-9	CAEZ01009120:76911-77788
HmHel-9	CAEZ01009816:2085-2337
HmHel-9	CAEZ01007222:201322-201784
HmHel-9	CAEZ01007222:125653-125807
HmHel-9	CAEZ01003931:6881-7198
HmHel-9	CAEZ01010293:61164-61460
HmHel-9	CAEZ01009470:88476-88836
HmHel-9	CAEZ01009470:5601-5926
HmHel-9	CAEZ01003195:5853-6257
HmHel-9	CAEZ01002650:19441-19742
HmHel-9	CAEZ01009084:84634-85295

HmHel-9	CAEZ01008305:84894-85140
HmHel-9	CAEZ01003110:26538-26881
HmHel-9	CAEZ01010796:21392-21726
HmHel-9	CAEZ01010659:62186-62532
HmHel-9	CAEZ01009077:203282-203732
HmHel-9	CAEZ01008984:5549-5911
HmHel-9	CAEZ01008409:7790-8070
HmHel-9	CAEZ01008315:441161-441403
HmHel-9	CAEZ01007361:22280-22549
HmHel-9	CAEZ01005499:35184-35484
HmHel-9	CAEZ01004168:1560-1877
HmHel-9	CAEZ01010464:18805-19062
HmHel-9	CAEZ01004190:10322-10772
HmHel-9	CAEZ01010869:49660-50053
HmHel-9	CAEZ01008230:4878-5165
HmHel-9	CAEZ01000250:541-911
HmHel-9	CAEZ01009815:10538-10786
HmHel-9	CAEZ01009585:12812-13203
HmHel-9	CAEZ01007389:31114-31491
HmHel-9	CAEZ01005687:46542-46815
HmHel-9	CAEZ01011020:892-3901
HmHel-9	CAEZ01007788:5000-5293
HmHel-9	CAEZ01005716:116161-116554
HmHel-9	CAEZ01004586:11212-11585
HmHel-9	CAEZ01004158:5006-5401
HmHel-9	CAEZ01002635:45893-46272
HmHel-9	CAEZ01009788:54125-54347
HmHel-9	CAEZ01009788:24964-25270
HmHel-9	CAEZ01005350:4048-4406
HmHel-9	CAEZ01004733:73817-74215
HmHel-9	CAEZ01002611:3432-3712
HmHel-9	CAEZ01010489:36866-37271
HmHel-9	CAEZ01009193:1633-1882
HmHel-9	CAEZ01007912:100537-100958
HmHel-9	CAEZ01003898:3509-3728
HmHel-9	CAEZ01001668:23390-23640
HmHel-9	CAEZ01005121:926-1187
HmHel-9	CAEZ01010925:10411-10808
HmHel-9	CAEZ01005203:43861-44235
HmHel-9	CAEZ01004174:11008-11188
HmHel-9	CAEZ01001441:15270-15799
HmHel-9	CAEZ01005780:10270-10622
HmHel-9	CAEZ01002576:19834-20221
HmHel-9	CAEZ01005808:14493-14877
HmHel-9	CAEZ01006705:2046-2342
HmHel-9	CAEZ01006151:65570-65964
HmHel-9	CAEZ01004903:5071-5273
HmHel-9	CAEZ01006635:10600-11103

HmHel-9	CAEZ01000867:89946-90168
HmHel-9	CAEZ01009455:18748-18964
HmHel-9	CAEZ01009422:60880-60966
HmHel-9	CAEZ01009422:103747-104025
HmHel-9	CAEZ01001920:6228-6635
HmHel-9	CAEZ01011525:14895-15227
HmHel-9	CAEZ01010367:9532-9792
HmHel-9	CAEZ01006760:18777-18909
HmHel-9	CAEZ01006760:93019-93488
HmHel-9	CAEZ01005871:14391-14590
HmHel-9	CAEZ01009978:2600-2866
HmHel-9	CAEZ01000121:839-1609
HmHel-9	CAEZ01002866:139118-139334
HmHel-9	CAEZ01009357:10845-11157
HmHel-9	CAEZ01010568:1331-1543
HmHel-9	CAEZ01009631:79099-79379
HmHel-9	CAEZ01009631:64300-64626
HmHel-9	CAEZ01007448:2622-3064
HmHel-9	CAEZ01008261:26100-26637
HmHel-9	CAEZ01007737:7094-7436
HmHel-9	CAEZ01005044:5677-6024
HmHel-9	CAEZ01003478:16582-16806
HmHel-9	CAEZ01009536:19391-20135
HmHel-9	CAEZ01007705:60562-60764
HmHel-9	CAEZ01007705:83192-83380
HmHel-9	CAEZ01007705:50253-50854
HmHel-9	CAEZ01011421:1-239
HmHel-9	CAEZ01008943:10891-11307
HmHel-9	CAEZ01006675:43046-43303
HmHel-9	CAEZ01006675:68138-68561
HmHel-9	CAEZ01009433:6288-6653
HmHel-9	CAEZ01008939:19311-19619
HmHel-9	CAEZ01008732:28167-28449
HmHel-9	CAEZ01008732:139531-139628
HmHel-9	CAEZ01011445:969-1195
HmHel-9	CAEZ01011444:13386-13612
HmHel-9	CAEZ01010974:5763-6175
HmHel-9	CAEZ01001590:27-175
HmHel-9	CAEZ01008706:5422-5776
HmHel-9	CAEZ01009027:15487-15712
HmHel-9	CAEZ01004699:17800-18061
HmHel-9	CAEZ01002323:67044-67782
HmHel-9	CAEZ01002323:34686-35049
HmHel-9	CAEZ01011405:179-401
HmHel-9	CAEZ01008644:4014-4237
HmHel-9	CAEZ01006561:36587-36957
HmHel-9	CAEZ01006392:39576-39842
HmHel-9	CAEZ01006392:37787-37942

HmHel-9	CAEZ01004813:63952-64170
HmHel-9	CAEZ01004094:82192-82410
HmHel-9	CAEZ01008398:8181-8382
HmHel-9	CAEZ01005045:29260-29476
HmHel-9	CAEZ01008209:102987-103232
HmHel-9	CAEZ01006088:42127-42303
HmHel-9	CAEZ01004741:56844-57181
HmHel-9	CAEZ01004395:2890-3162
HmHel-9	CAEZ01007662:4852-5155
HmHel-9	CAEZ01009589:25737-26089
HmHel-9	CAEZ01008211:70712-70861
HmHel-9	CAEZ01008211:181388-181486
HmHel-9	CAEZ01010881:22416-22638
HmHel-9	CAEZ01003063:16864-17147
HmHel-9	CAEZ01009485:8485-8703
HmHel-9	CAEZ01004368:5447-5675
HmHel-9	CAEZ01005622:157-420
HmHel-9	CAEZ01009886:62229-62517
HmHel-9	CAEZ01006948:14944-15269
HmHel-9	CAEZ01005157:3394-3537
HmHel-9	CAEZ01004629:22400-22590
HmHel-9	CAEZ01007057:875-1192
HmHel-9	CAEZ01006691:29549-29792
HmHel-9	CAEZ01005182:1-224
HmHel-9	CAEZ01001983:3726-4122
HmHel-9	CAEZ01000807:1558-1778
HmHel-9	CAEZ01007035:601-881
HmHel-9	CAEZ01004995:1087-1251
HmHel-9	CAEZ01003582:88244-88412
HmHel-9	CAEZ01007711:2280-2625
HmHel-9	CAEZ01006775:12938-13143
HmHel-9	CAEZ01004506:2779-2965
HmHel-9	CAEZ01011522:23562-23751
HmHel-9	CAEZ01005606:27725-27934
HmHel-9	CAEZ01008639:25138-25334
HmHel-9	CAEZ01002736:16100-16481
HmHel-9	CAEZ01008874:105603-105916
HmHel-9	CAEZ01005220:38715-39028
HmHel-9	CAEZ01009353:1988-2223
HmHel-9	CAEZ01010250:9292-9422
HmHel-9	CAEZ01006908:13967-14222
HmHel-9	CAEZ01003815:12586-13989
HmHel-9	CAEZ01000879:160388-160680
HmHel-9	CAEZ01007143:24677-24952
HmHel-9	CAEZ01006690:25919-26044
HmHel-9	CAEZ01005402:31863-31993
HmHel-9	CAEZ01004353:16176-16335
HmHel-9	CAEZ01009548:8430-8607

HmHel-9	CAEZ01005071:22983-23128
HmHel-9	CAEZ01010737:51978-52288
HmHel-9	CAEZ01009228:30952-31082
HmHel-9	CAEZ01009123:3625-4075
HmHel-9	CAEZ01007365:9841-10291
HmHel-9	CAEZ01004198:30650-30820
HmHel-9	CAEZ01008879:104702-104847
HmHel-9	CAEZ01004222:63503-63654
HmHel-9	CAEZ01011046:131861-132097
HmHel-9	CAEZ01009881:41277-41388
HmHel-9	CAEZ01008979:7640-7771
HmHel-9	CAEZ01004978:3598-3758
HmHel-9	CAEZ01008396:6854-6984
HmHel-9	CAEZ01006325:10507-10654
HmHel-9	CAEZ01010384:748-953
HmHel-9	CAEZ01010023:7293-7514
HmHel-9	CAEZ01004909:29365-29791
HmHel-9	CAEZ01004869:43033-43499
HmHel-9	CAEZ01010267:7577-7738
HmHel-9	CAEZ01009223:57072-57280
HmHel-9	CAEZ01009078:34248-34436
HmHel-9	CAEZ01005970:26556-26713
HmHel-9	CAEZ01005043:180060-180156
HmHel-9	CAEZ01003849:15928-16059
HmHel-9	CAEZ01011519:158780-158922
HmHel-9	CAEZ01010376:997-1253
HmHel-9	CAEZ01009630:31316-31611
HmHel-9	CAEZ01009630:833-962
HmHel-9	CAEZ01007865:32960-33188
HmHel-9	CAEZ01003562:32155-32249
HmHel-9	CAEZ01008279:58599-58845
HmHel-9	CAEZ01006515:21517-21700
HmHel-9	CAEZ01009023:14015-14231
HmHel-9	CAEZ01008868:9226-9436
HmHel-9	CAEZ01008437:117950-118209
HmHel-9	CAEZ01006463:986-1257
HmHel-9	CAEZ01001042:7629-7773
HmHel-9	CAEZ01010969:145973-146060
HmHel-9	CAEZ01005166:8302-8527
HmHel-9	CAEZ01000061:23852-24074
HmHel-9	CAEZ01006617:45322-45495
HmHel-9	CAEZ01010797:86946-88117
HmHel-9	CAEZ01007713:34982-35081
HmHel-9	CAEZ01008103:5877-6164
HmHel-9	CAEZ01001403:31677-31837
HmHel-9	CAEZ01001765:58658-58764
HmHel-9	CAEZ01001416:6505-6771
HmHel-9	CAEZ01004034:92630-92793

HmHel-9	CAEZ01010805:7386-7603
HmHel-9	CAEZ01009732:4365-4567
HmHel-9	CAEZ01009233:2321-2400
HmHel-9	CAEZ01007047:4483-4602
HmHel-9	CAEZ01009988:5824-5922
HmHel-9	CAEZ01009765:1042-1207
HmHel-9	CAEZ01009610:37664-37837
HmHel-9	CAEZ01009374:43479-43621
HmHel-9	CAEZ01007738:77942-78126
HmHel-9	CAEZ01007539:2884-2965
HmHel-9	CAEZ01002811:153004-153114
HmHel-9	CAEZ01010078:25194-25303
HmHel-9	CAEZ01004569:7008-7179
HmHel-9	CAEZ01001625:1235-1316
HmHel-9	CAEZ01006790:13296-13497
HmHel-9	CAEZ01010776:2833-2912
HmHel-9	CAEZ01010667:19806-19961
HmHel-9	CAEZ01011313:1510-1647
HmHel-9	CAEZ01008140:1274-1404
HmHel-9	CAEZ01010516:11991-12213
HmHel-9	CAEZ01007631:64544-64661
HmHel-9	CAEZ01007259:9539-9655
HmHel-9	CAEZ01002100:8474-8664
HmHel-9	CAEZ01011054:29384-29479
HmHel-9	CAEZ01006116:2378-2517
HmHel-9	CAEZ01010193:10434-10679
HmHel-9	CAEZ01004590:1514-1621
HmHel-9	CAEZ01005269:87612-87694
HmHel-9	CAEZ01003422:62541-62771
HmHel-9	CAEZ01010753:25871-26008
HmHel-9	CAEZ01009436:1184-11118
HmHel-9	CAEZ01006365:10258-10343
HmHel-9	CAEZ01009248:58186-58396
HmHel-9	CAEZ01004905:31863-32167
HmHel-9	CAEZ01010112:2789-3023
HmHel-9	CAEZ01006947:1321-1441
HmHel-9	CAEZ01006085:38455-38690
HmHel-9	CAEZ01003209:1929-2024
HmHel-9	CAEZ01010940:2983-3085
HmHel-9	CAEZ01006618:82508-82655
HmHel-9	CAEZ01008125:119761-119931
HmHel-9	CAEZ01008120:6197-6282
HmHel-9	CAEZ01005810:20129-20272
HmHel-9	CAEZ01004828:2005-2106
HmHel-9	CAEZ01010494:2736-2835
HmHel-9	CAEZ01010032:869-976
HmHel-9	CAEZ01009686:35288-35398
HmHel-9	CAEZ01011529:49645-49751

HmHel-9	CAEZ01011491:6462-6649
HmHel-9	CAEZ01010230:5423-5539
HmHel-9	CAEZ01007263:88082-88310
HmHel-9	CAEZ01010953:5336-5545
HmHel-9	CAEZ01007006:19293-19409
HmHel-9	CAEZ01002821:74838-75011
HmHel-9	CAEZ01010709:69945-70091
HmHel-9	CAEZ01007761:17498-17624
HmHel-9	CAEZ01003888:20857-20948
HmHel-9	CAEZ01009391:16330-16459
HmHel-9	CAEZ01007469:32898-33067
HmHel-9	CAEZ01007062:22684-22796
HmHel-9	CAEZ01005765:8947-9069
HmHel-9	CAEZ01005359:8187-8355
HmHel-9	CAEZ01004582:45962-46072
HmHel-9	CAEZ01003334:34818-34904
HmHel-9	CAEZ01002208:116983-117137
HmHel-9	CAEZ01002208:15512-15775
HmHel-9	CAEZ01008220:29076-29379
HmHel-9	CAEZ01004798:9113-9241
HmHel-9	CAEZ01001064:27150-27251
HmHel-9	CAEZ01011521:10072-10192
HmHel-9	CAEZ01005162:2694-2805
HmHel-9	CAEZ01009482:8005-8256
HmHel-9	CAEZ01009320:3026-3149
HmHel-9	CAEZ01009098:4549-4664
HmHel-9	CAEZ01008196:29066-29149
HmHel-9	CAEZ01005199:71881-72030
HmHel-9	CAEZ01004738:233675-233782
HmHel-9	CAEZ01003194:29495-29590
HmHel-9	CAEZ01009665:14618-14773
HmHel-9	CAEZ01008833:15083-15245
HmHel-9	CAEZ01008688:5737-5870
HmHel-9	CAEZ01008666:2762-2868
HmHel-9	CAEZ01008592:15212-15314
HmHel-9	CAEZ01008574:61180-61298
HmHel-9	CAEZ01007178:727-839
HmHel-9	CAEZ01006162:71099-71271
HmHel-9	CAEZ01005963:22725-22871
HmHel-9	CAEZ01005911:72695-72942
HmHel-9	CAEZ01004662:34337-34530
HmHel-9	CAEZ01006525:25891-26012
HmHel-9	CAEZ01005775:5436-5525
HmHel-9	CAEZ01003201:1452-1600
HmHel-9	CAEZ01011527:19298-19413
HmHel-9	CAEZ01009895:28136-28235
HmHel-9	CAEZ01009889:19318-19434
HmHel-9	CAEZ01006551:4799-4926

HmHel-9	CAEZ01005439:69242-69346
HmHel-9	CAEZ01002964:53971-54055
HmHel-9	CAEZ01001934:31981-32301
HmHel-9	CAEZ01011494:52603-52693
HmHel-9	CAEZ01011483:1573-1691
HmHel-9	CAEZ01009885:55278-55406
HmHel-9	CAEZ01009424:21626-21777
HmHel-9	CAEZ01007188:8281-8433
HmHel-9	CAEZ01003291:119462-119552
HmHel-9	CAEZ01002815:27559-27699
HmHel-9	CAEZ01002140:11367-11470
HmHel-9	CAEZ01008769:74798-74877
HmHel-9	CAEZ01005711:170662-170752
HmHel-9	CAEZ01001386:28661-28742
HmHel-9	CAEZ01010039:113993-114098
HmHel-9	CAEZ01008353:20082-20358
HmHel-9	CAEZ01007804:70245-70398
HmHel-9	CAEZ01006286:132057-132167
HmHel-9	CAEZ01005605:93509-93634
HmHel-9	CAEZ01005430:13441-13574
HmHel-9	CAEZ01001221:25992-26120
HmHel-9	CAEZ01010972:57437-57643
HmHel-9	CAEZ01008771:3717-3805
HmHel-9	CAEZ01008771:36965-37081
HmHel-9	CAEZ01006564:24610-24693
HmHel-9	CAEZ01006044:21897-21978
HmHel-9	CAEZ01010900:24615-24714
HmHel-9	CAEZ01010713:4552-4689
HmHel-9	CAEZ01009678:91-261
HmHel-9	CAEZ01009021:6374-6517
HmHel-9	CAEZ01008182:16881-16984
HmHel-9	CAEZ01007269:44809-44904
HmHel-9	CAEZ01011617:9591-9739
HmHel-9	CAEZ01007669:24235-24365
HmHel-9	CAEZ01007219:9844-9926
HmHel-9	CAEZ01004011:121404-121502
HmHel-9	CAEZ01011526:10709-10794
HmHel-9	CAEZ01008838:19364-19472
HmHel-9	CAEZ01008345:2550-2651
HmHel-9	CAEZ01007795:21354-21515
HmHel-9	CAEZ01007745:98193-98290
HmHel-9	CAEZ01006610:9927-10016
HmHel-9	CAEZ01005187:8127-8255
HmHel-9	CAEZ01002875:108949-109054
HmHel-9	CAEZ01002046:33674-33767
HmHel-9	CAEZ01001976:557-736
HmHel-9	CAEZ01010251:1592-1692
HmHel-9	CAEZ01007893:2253-2348

HmHel-9	CAEZ01006773:221664-221792
HmHel-9	CAEZ01006534:38054-38244
HmHel-9	CAEZ01001501:62977-63076
HmHel-9	CAEZ01008740:18062-18211
HmHel-9	CAEZ01008512:75850-75942
HmHel-9	CAEZ01007894:2671-2872
HmHel-9	CAEZ01007502:11793-11897
HmHel-9	CAEZ01007040:20011-20094
HmHel-9	CAEZ01006772:124223-124317
HmHel-9	CAEZ01010332:12764-12874
HmHel-9	CAEZ01010117:27819-27988
HmHel-9	CAEZ01009896:21554-21647
HmHel-9	CAEZ01007454:4127-4213
HmHel-9	CAEZ01006378:13021-13125
HmHel-9	CAEZ01006337:73302-73393
HmHel-9	CAEZ01004541:86283-86397
HmHel-9	CAEZ01003080:156978-157060
HmHel-9	CAEZ01007941:29520-29605
HmHel-9	CAEZ01007584:51090-51371
HmHel-9	CAEZ01011560:21245-21353
HmHel-9	CAEZ01010720:500-580
HmHel-9	CAEZ01009414:7600-7684
HmHel-9	CAEZ01007353:5476-5563
HmHel-9	CAEZ01006820:8413-8497
HmHel-9	CAEZ01004070:4765-4845
HmHel-9	CAEZ01002719:26670-26755
HmHel-9	CAEZ01002144:4259-4356
HmHel-9	CAEZ01001650:38998-39093
	:-
HmHel-10	CAEZ01007872:26381-26776
HmHel-10	CAEZ01007872:87232-87912
HmHel-10	CAEZ01007872:98339-98669
HmHel-10	CAEZ01007872:83394-87839
HmHel-10	CAEZ01010193:11085-11771
HmHel-10	CAEZ01006785:4914-5590
HmHel-10	CAEZ01006785:8987-9660
HmHel-10	CAEZ01001806:82050-82586
HmHel-10	CAEZ01008978:14194-14878
HmHel-10	CAEZ01006283:2334-8934
HmHel-10	CAEZ01006283:36585-37161
HmHel-10	CAEZ01006283:20583-21141
HmHel-10	CAEZ01008635:26642-27342
HmHel-10	CAEZ01009120:79417-80121
HmHel-10	CAEZ01005780:239-898
HmHel-10	CAEZ01005780:17162-17823
HmHel-10	CAEZ01010697:48908-49554
HmHel-10	CAEZ01005783:4159-4842
HmHel-10	CAEZ01006326:3571-7659

HmHel-10	CAEZ01004990:35874-36057
HmHel-10	CAEZ01004990:76726-77095
HmHel-10	CAEZ01004990:65151-65688
HmHel-10	CAEZ01004990:49279-52664
HmHel-10	CAEZ01010979:4091-4773
HmHel-10	CAEZ01006494:10934-11326
HmHel-10	CAEZ01008912:12101-12773
HmHel-10	CAEZ01008872:19690-20271
HmHel-10	CAEZ01008872:3297-3669
HmHel-10	CAEZ01009989:37721-38361
HmHel-10	CAEZ01009420:27365-27995
HmHel-10	CAEZ01009249:18604-18776
HmHel-10	CAEZ01009249:20047-20620
HmHel-10	CAEZ01003292:95185-95686
HmHel-10	CAEZ01008963:32774-33162
HmHel-10	CAEZ01008963:88014-88710
HmHel-10	CAEZ01008963:78169-78314
HmHel-10	CAEZ01008963:41089-41761
HmHel-10	CAEZ01011169:8152-8845
HmHel-10	CAEZ01011169:27976-28648
HmHel-10	CAEZ01009816:23681-24351
HmHel-10	CAEZ01000865:738-1379
HmHel-10	CAEZ01007708:49745-49907
HmHel-10	CAEZ01007708:48043-48723
HmHel-10	CAEZ01007708:2403-2966
HmHel-10	CAEZ01006281:11129-14621
HmHel-10	CAEZ01006281:19395-19568
HmHel-10	CAEZ01003794:37216-37855
HmHel-10	CAEZ01003794:47251-47936
HmHel-10	CAEZ01003794:14238-14894
HmHel-10	CAEZ01009673:55104-55755
HmHel-10	CAEZ01005403:42549-43182
HmHel-10	CAEZ01005403:33664-33768
HmHel-10	CAEZ01006348:28785-29478
HmHel-10	CAEZ01007114:28038-28755
HmHel-10	CAEZ01003452:22949-23582
HmHel-10	CAEZ01006717:11961-12622
HmHel-10	CAEZ01004957:3781-4399
HmHel-10	CAEZ01000967:4111-4456
HmHel-10	CAEZ01010305:78232-78626
HmHel-10	CAEZ01009733:82031-82817
HmHel-10	CAEZ01009733:34665-34794
HmHel-10	CAEZ01007585:22959-23336
HmHel-10	CAEZ01010569:4306-4977
HmHel-10	CAEZ01008313:52678-53333
HmHel-10	CAEZ01005843:4112-4763
HmHel-10	CAEZ01005662:9928-10551
HmHel-10	CAEZ01006700:10997-11393

HmHel-10	CAEZ01007251:12184-12973
HmHel-10	CAEZ01007251:13966-14633
HmHel-10	CAEZ01007073:11557-11934
HmHel-10	CAEZ01003207:3752-3939
HmHel-10	CAEZ01000063:30781-31384
HmHel-10	CAEZ01000063:53847-54156
HmHel-10	CAEZ01000063:11936-12072
HmHel-10	CAEZ01010005:18741-19352
HmHel-10	CAEZ01009456:163992-164646
HmHel-10	CAEZ01007747:245884-246231
HmHel-10	CAEZ01007709:11150-11746
HmHel-10	CAEZ01007709:26870-29571
HmHel-10	CAEZ01007709:63947-64147
HmHel-10	CAEZ01007709:27675-36820
HmHel-10	CAEZ01006665:372-688
HmHel-10	CAEZ01006086:37025-37735
HmHel-10	CAEZ01003943:8544-9228
HmHel-10	CAEZ01003750:91115-91685
HmHel-10	CAEZ01008637:13379-14039
HmHel-10	CAEZ01008138:360335-361019
HmHel-10	CAEZ01006365:17487-17867
HmHel-10	CAEZ01006365:4741-5229
HmHel-10	CAEZ01005111:22377-22917
HmHel-10	CAEZ01005083:50862-51348
HmHel-10	CAEZ01005083:85805-86001
HmHel-10	CAEZ01005083:48204-48360
HmHel-10	CAEZ01005083:13727-14401
HmHel-10	CAEZ01010727:30749-31380
HmHel-10	CAEZ01010039:22268-22466
HmHel-10	CAEZ01008518:5970-6156
HmHel-10	CAEZ01006299:28739-29266
HmHel-10	CAEZ01006299:43567-50830
HmHel-10	CAEZ01005478:16953-17151
HmHel-10	CAEZ01011558:11558-12407
HmHel-10	CAEZ01008765:68442-68962
HmHel-10	CAEZ01007273:131113-131581
HmHel-10	CAEZ01009016:41261-41768
HmHel-10	CAEZ01005551:5951-6209
HmHel-10	CAEZ01010944:15541-15733
HmHel-10	CAEZ01008751:47382-47776
HmHel-10	CAEZ01003331:23598-24214
HmHel-10	CAEZ01009628:4909-5165
HmHel-10	CAEZ01008925:14565-15250
HmHel-10	CAEZ01008262:6694-7073
HmHel-10	CAEZ01008262:17090-17721
HmHel-10	CAEZ01007713:22281-22994
HmHel-10	CAEZ01007713:54776-55156
HmHel-10	CAEZ01007713:51224-51908

HmHel-10	CAEZ01007713:3014-3593
HmHel-10	CAEZ01007711:22984-23244
HmHel-10	CAEZ01007711:34451-36855
HmHel-10	CAEZ01007711:27313-27507
HmHel-10	CAEZ01005099:34140-34479
HmHel-10	CAEZ01001765:100890-101407
HmHel-10	CAEZ01007724:43221-43386
HmHel-10	CAEZ01004876:32659-33124
HmHel-10	CAEZ01009451:15203-15561
HmHel-10	CAEZ01007707:8740-9295
HmHel-10	CAEZ01007707:35331-36414
HmHel-10	CAEZ01007707:46995-47288
HmHel-10	CAEZ01007707:84350-84718
HmHel-10	CAEZ01007707:143835-144060
HmHel-10	CAEZ01007707:116141-116390
HmHel-10	CAEZ01005418:68226-68859
HmHel-10	CAEZ01005418:206112-206454
HmHel-10	CAEZ01009481:15799-16442
HmHel-10	CAEZ01009481:21871-21983
HmHel-10	CAEZ01004432:26438-26640
HmHel-10	CAEZ01004432:6449-6851
HmHel-10	CAEZ01002607:200960-201358
HmHel-10	CAEZ01001427:453-1112
HmHel-10	CAEZ01010725:23515-23708
HmHel-10	CAEZ01002635:38449-39114
HmHel-10	CAEZ01002635:52225-52856
HmHel-10	CAEZ01004751:1348-1975
HmHel-10	CAEZ01004008:57889-58683
HmHel-10	CAEZ01000970:45024-45208
HmHel-10	CAEZ01010978:10253-10428
HmHel-10	CAEZ01010978:12109-12789
HmHel-10	CAEZ01007095:7350-7766
HmHel-10	CAEZ01006708:2204-2559
HmHel-10	CAEZ01009799:911-1097
HmHel-10	CAEZ01009751:12404-12714
HmHel-10	CAEZ01006286:6996-7653
HmHel-10	CAEZ01006286:16843-17030
HmHel-10	CAEZ01001934:14876-15559
HmHel-10	CAEZ01008915:7892-8459
HmHel-10	CAEZ01008915:123762-123987
HmHel-10	CAEZ01008915:218605-218708
HmHel-10	CAEZ01008915:233897-234099
HmHel-10	CAEZ01005980:21898-22494
HmHel-10	CAEZ01005980:10443-12809
HmHel-10	CAEZ01005219:42000-42566
HmHel-10	CAEZ01004633:42340-43162
HmHel-10	CAEZ01010728:11742-12765
HmHel-10	CAEZ01010728:70510-73670

HmHel-10	CAEZ01010475:363-1043
HmHel-10	CAEZ01010117:58190-58837
HmHel-10	CAEZ01005229:35554-35729
HmHel-10	CAEZ01009769:43282-43594
HmHel-10	CAEZ01008736:6136-13846
HmHel-10	CAEZ01008736:13458-17103
HmHel-10	CAEZ01007330:6537-7029
HmHel-10	CAEZ01006153:49628-50190
HmHel-10	CAEZ01006153:72696-72843
HmHel-10	CAEZ01005241:17938-18575
HmHel-10	CAEZ01009727:10710-11000
HmHel-10	CAEZ01009221:7478-8016
HmHel-10	CAEZ01006297:5384-5930
HmHel-10	CAEZ01005452:6480-6669
HmHel-10	CAEZ01005113:10272-10952
HmHel-10	CAEZ01003222:16978-17174
HmHel-10	CAEZ01010775:788-1091
HmHel-10	CAEZ01008272:11914-12174
HmHel-10	CAEZ01004430:27096-27805
HmHel-10	CAEZ01002099:30718-31243
HmHel-10	CAEZ01002099:21969-22448
HmHel-10	CAEZ01010926:38405-38610
HmHel-10	CAEZ01010926:89671-89991
HmHel-10	CAEZ01010926:112531-113145
HmHel-10	CAEZ01010926:100565-100657
HmHel-10	CAEZ01010926:52482-52984
HmHel-10	CAEZ01009786:33162-34111
HmHel-10	CAEZ01010974:23276-23620
HmHel-10	CAEZ01007767:6870-7560
HmHel-10	CAEZ01007674:10116-10425
HmHel-10	CAEZ01002863:96620-97310
HmHel-10	CAEZ01001103:32433-32631
HmHel-10	CAEZ01010261:4761-5042
HmHel-10	CAEZ01006689:68723-69374
HmHel-10	CAEZ01005606:15129-20228
HmHel-10	CAEZ01008100:75199-75859
HmHel-10	CAEZ01004958:16000-16620
HmHel-10	CAEZ01004958:31958-32524
HmHel-10	CAEZ01004958:924-1852
HmHel-10	CAEZ01003562:50490-50806
HmHel-10	CAEZ01003562:32326-32513
HmHel-10	CAEZ01003119:11406-12068
HmHel-10	CAEZ01011519:78833-78938
HmHel-10	CAEZ01011519:97866-98198
HmHel-10	CAEZ01011296:156222-156852
HmHel-10	CAEZ01010614:28155-28674
HmHel-10	CAEZ01010614:32823-33163
HmHel-10	CAEZ01010614:7510-8130

HmHel-10	CAEZ01003495:14059-14541
HmHel-10	CAEZ01006228:60988-61204
HmHel-10	CAEZ01010318:90019-90466
HmHel-10	CAEZ01010318:48527-48696
HmHel-10	CAEZ01009271:122991-123650
HmHel-10	CAEZ01007204:46243-46398
HmHel-10	CAEZ01005930:20447-21027
HmHel-10	CAEZ01008643:14825-15426
HmHel-10	CAEZ01008643:46367-46896
HmHel-10	CAEZ01006154:104703-105404
HmHel-10	CAEZ01003774:10435-10989
HmHel-10	CAEZ01009357:1101-1604
HmHel-10	CAEZ01009233:5330-5429
HmHel-10	CAEZ01009233:23782-24351
HmHel-10	CAEZ01009233:20991-21403
HmHel-10	CAEZ01007960:75191-75371
HmHel-10	CAEZ01007682:16487-16674
HmHel-10	CAEZ01006993:1647-2046
HmHel-10	CAEZ01004477:615-1357
HmHel-10	CAEZ01009977:1889-2426
HmHel-10	CAEZ01009977:13730-14251
HmHel-10	CAEZ01007706:13199-13480
HmHel-10	CAEZ01007706:124022-124660
HmHel-10	CAEZ01007706:141970-142093
HmHel-10	CAEZ01007706:268176-268738
HmHel-10	CAEZ01007706:280665-281253
HmHel-10	CAEZ01007706:126545-126928
HmHel-10	CAEZ01005721:1598-1944
HmHel-10	CAEZ01004020:4874-5087
HmHel-10	CAEZ01010488:1143-1296
HmHel-10	CAEZ01008101:18668-19097
HmHel-10	CAEZ01008101:29704-29925
HmHel-10	CAEZ01004545:46181-46559
HmHel-10	CAEZ01003233:42599-42763
HmHel-10	CAEZ01002359:1035-1444
HmHel-10	CAEZ01011568:575-1211
HmHel-10	CAEZ01010594:64618-65218
HmHel-10	CAEZ01009633:35001-35218
HmHel-10	CAEZ01008186:4405-5085
HmHel-10	CAEZ01007870:103520-103845
HmHel-10	CAEZ01000874:1183-1429
HmHel-10	CAEZ01007928:22045-22694
HmHel-10	CAEZ01006710:7910-14755
HmHel-10	CAEZ01006490:41565-41911
HmHel-10	CAEZ01005557:2993-3318
HmHel-10	CAEZ01008882:37413-37978
HmHel-10	CAEZ01006698:34227-34939
HmHel-10	CAEZ01003335:45378-46126

HmHel-10	CAEZ01003335:5337-5915
HmHel-10	CAEZ01010260:63409-63991
HmHel-10	CAEZ01007267:909-1103
HmHel-10	CAEZ01003254:9084-9176
HmHel-10	CAEZ01003254:3063-3582
HmHel-10	CAEZ01002806:689-1047
HmHel-10	CAEZ01007500:36536-37027
HmHel-10	CAEZ01005082:31690-32181
HmHel-10	CAEZ01008979:8541-9070
HmHel-10	CAEZ01008351:30096-30713
HmHel-10	CAEZ01008351:2171-2281
HmHel-10	CAEZ01007446:12469-12877
HmHel-10	CAEZ01005659:3526-3673
HmHel-10	CAEZ01004321:12106-12339
HmHel-10	CAEZ01002636:6344-6699
HmHel-10	CAEZ01010389:9101-9554
HmHel-10	CAEZ01009841:5433-5619
HmHel-10	CAEZ01008125:118358-119138
HmHel-10	CAEZ01008035:9018-9180
HmHel-10	CAEZ01001174:6793-6939
HmHel-10	CAEZ01008743:158397-158709
HmHel-10	CAEZ01008743:158875-159066
HmHel-10	CAEZ01007249:4791-5251
HmHel-10	CAEZ01006716:2742-3759
HmHel-10	CAEZ01004415:4926-5378
HmHel-10	CAEZ01003334:33367-33740
HmHel-10	CAEZ01003334:21736-22438
HmHel-10	CAEZ01003063:18151-18439
HmHel-10	CAEZ01000966:1807-2071
HmHel-10	CAEZ01000966:1505-1648
HmHel-10	CAEZ01000723:3826-4319
HmHel-10	CAEZ01011193:110729-110942
HmHel-10	CAEZ01008922:67423-67933
HmHel-10	CAEZ01007710:5040-5603
HmHel-10	CAEZ01007067:13339-13943
HmHel-10	CAEZ01005999:33560-33871
HmHel-10	CAEZ01005433:6576-7259
HmHel-10	CAEZ01004825:14139-14325
HmHel-10	CAEZ01006664:10011-10714
HmHel-10	CAEZ01009085:4116-4380
HmHel-10	CAEZ01008916:3653-3830
HmHel-10	CAEZ01008034:23428-24001
HmHel-10	CAEZ01003120:828-1314
HmHel-10	CAEZ01008879:24566-24876
HmHel-10	CAEZ01008879:50390-50618
HmHel-10	CAEZ01007861:30158-30438
HmHel-10	CAEZ01007379:8297-8871
HmHel-10	CAEZ01011171:2536-3203

HmHel-10	CAEZ01010405:10343-10680
HmHel-10	CAEZ01007049:8974-9632
HmHel-10	CAEZ01006412:124143-124297
HmHel-10	CAEZ01000854:83407-83586
HmHel-10	CAEZ01008783:11795-13532
HmHel-10	CAEZ01008737:35977-36131
HmHel-10	CAEZ01008030:3892-4421
HmHel-10	CAEZ01005507:8033-8428
HmHel-10	CAEZ01003146:6106-6256
HmHel-10	CAEZ01003146:10447-10744
HmHel-10	CAEZ01001668:1081-4682
HmHel-10	CAEZ01011060:10694-11062
HmHel-10	CAEZ01010351:40432-40800
HmHel-10	CAEZ01007272:83705-83994
HmHel-10	CAEZ01006504:8840-9008
HmHel-10	CAEZ01003326:52749-53302
HmHel-10	CAEZ01008702:8786-9362
HmHel-10	CAEZ01006418:2995-3211
HmHel-10	CAEZ01005404:25581-26184
HmHel-10	CAEZ01005404:340-985
HmHel-10	CAEZ01002894:15495-16092
HmHel-10	CAEZ01000550:1058-1266
HmHel-10	CAEZ01007766:7669-7933
HmHel-10	CAEZ01006224:14635-14768
HmHel-10	CAEZ01006073:44369-45981
HmHel-10	CAEZ01003362:13764-13889
HmHel-10	CAEZ01008371:25507-26054
HmHel-10	CAEZ01006285:10942-12625
HmHel-10	CAEZ01006182:27309-28007
HmHel-10	CAEZ01008029:12539-12788
HmHel-10	CAEZ01005279:71105-71843
HmHel-10	CAEZ01011477:2493-2984
HmHel-10	CAEZ01010370:12163-12319
HmHel-10	CAEZ01008874:131046-131249
HmHel-10	CAEZ01008874:11422-11958
HmHel-10	CAEZ01008822:38686-38827
HmHel-10	CAEZ01008822:49380-49907
HmHel-10	CAEZ01006634:42505-42864
HmHel-10	CAEZ01006298:3416-3906
HmHel-10	CAEZ01003432:17161-17675
HmHel-10	CAEZ01003432:15551-15822
HmHel-10	CAEZ01011571:3637-4102
HmHel-10	CAEZ01008408:9555-10205
HmHel-10	CAEZ01008408:14636-14713
HmHel-10	CAEZ01002068:1065-1220
HmHel-10	CAEZ01007656:2244-2366
HmHel-10	CAEZ01006561:40113-40747
HmHel-10	CAEZ01005100:12300-12918

HmHel-10	CAEZ01004596:10272-10713
HmHel-10	CAEZ01004016:35995-38454
HmHel-10	CAEZ01001338:15786-15984
HmHel-10	CAEZ01004599:10105-10857
HmHel-10	CAEZ01001802:16380-16651
HmHel-10	CAEZ01007046:22943-23951
HmHel-10	CAEZ01000879:86221-87281
HmHel-10	CAEZ01000879:42287-42511
HmHel-10	CAEZ01011474:3608-3790
HmHel-10	CAEZ01009833:32506-32809
HmHel-10	CAEZ01007590:53735-53846
HmHel-10	CAEZ01007093:10679-11300
HmHel-10	CAEZ01004028:924-1340
HmHel-10	CAEZ01003209:1394-1828
HmHel-10	CAEZ01001431:5315-5850
HmHel-10	CAEZ01010606:14904-15226
HmHel-10	CAEZ01008753:2232-2630
HmHel-10	CAEZ01004707:1302-1503
HmHel-10	CAEZ01000061:11336-12266
HmHel-10	CAEZ01008921:90867-91953
HmHel-10	CAEZ01008921:58491-59096
HmHel-10	CAEZ01006772:24745-25007
HmHel-10	CAEZ01002211:43964-44296
HmHel-10	CAEZ01007185:1521-2197
HmHel-10	CAEZ01004733:35902-36179
HmHel-10	CAEZ01004054:4085-4689
HmHel-10	CAEZ01009248:57423-58053
HmHel-10	CAEZ01009248:34880-34985
HmHel-10	CAEZ01005998:11503-12608
HmHel-10	CAEZ01004368:48780-49152
HmHel-10	CAEZ01010613:15989-16153
HmHel-10	CAEZ01009548:557-1056
HmHel-10	CAEZ01007712:6539-7539
HmHel-10	CAEZ01003496:379-606
HmHel-10	CAEZ01009177:7851-7967
HmHel-10	CAEZ01008122:23388-23520
HmHel-10	CAEZ01003126:1720-1931
HmHel-10	CAEZ01008642:1070-1268
HmHel-10	CAEZ01008636:8929-9726
HmHel-10	CAEZ01006347:181926-182511
HmHel-10	CAEZ01005402:19376-19913
HmHel-10	CAEZ01003260:17920-18099
HmHel-10	CAEZ01002719:29669-29839
HmHel-10	CAEZ01008887:9494-9688
HmHel-10	CAEZ01008887:6924-7104
HmHel-10	CAEZ01006787:14284-14610
HmHel-10	CAEZ01006699:2205-3645
HmHel-10	CAEZ01009224:6856-7446

HmHel-10	CAEZ01005371:30819-31005
HmHel-10	CAEZ01011554:27856-28401
HmHel-10	CAEZ01009264:57681-57819
HmHel-10	CAEZ01009264:57837-58057
HmHel-10	CAEZ01005711:22196-22515
HmHel-10	CAEZ01004603:474-636
HmHel-10	CAEZ01010709:3734-4336
HmHel-10	CAEZ01011358:12933-13058
HmHel-10	CAEZ01010827:243407-243989
HmHel-10	CAEZ01010267:7464-7560
HmHel-10	CAEZ01009304:11402-14067
HmHel-10	CAEZ01008641:22463-22596
HmHel-10	CAEZ01008273:14012-14349
HmHel-10	CAEZ01005586:41181-41388
HmHel-10	CAEZ01004923:67319-72067
HmHel-10	CAEZ01004226:38780-39274
HmHel-10	CAEZ01001238:3042-3726
HmHel-10	CAEZ01009752:2008-2140
HmHel-10	CAEZ01005197:428-586
HmHel-10	CAEZ01009982:109541-110708
HmHel-10	CAEZ01009781:47766-47860
HmHel-10	CAEZ01008888:20477-20892
HmHel-10	CAEZ01008400:34089-34183
HmHel-10	CAEZ01005119:21756-21890
HmHel-10	CAEZ01004811:580-905
HmHel-10	CAEZ01010312:28699-28954
HmHel-10	CAEZ01006371:8995-9407
HmHel-10	CAEZ01005693:10110-10215
HmHel-10	CAEZ01002746:7630-7733
HmHel-10	CAEZ01002059:1467-2825
HmHel-10	CAEZ01009355:97729-99606
HmHel-10	CAEZ01008435:1100-1425
HmHel-10	CAEZ01008435:28549-29091
HmHel-10	CAEZ01008435:8604-9295
HmHel-10	CAEZ01007595:725-1035
HmHel-10	CAEZ01007595:14828-20387
HmHel-10	CAEZ01006786:7593-7773
HmHel-10	CAEZ01004992:1089-1398
HmHel-10	CAEZ01011093:14027-14458
HmHel-10	CAEZ01010732:773-971
HmHel-10	CAEZ01010726:9583-10099
HmHel-10	CAEZ01006790:14482-14792
HmHel-10	CAEZ01006248:1001-1534
HmHel-10	CAEZ01005462:6535-6658
HmHel-10	CAEZ01004808:18-603
HmHel-10	CAEZ01003230:254-412
HmHel-10	CAEZ01011564:29498-29600
HmHel-10	CAEZ01010029:68797-69089

HmHel-10	CAEZ01010010:6620-7715
HmHel-10	CAEZ01009536:20144-20280
HmHel-10	CAEZ01008839:26016-26578
HmHel-10	CAEZ01006501:17095-17404
HmHel-10	CAEZ01010729:66692-66880
HmHel-10	CAEZ01006338:41885-41979
HmHel-10	CAEZ01002811:150487-150738
HmHel-10	CAEZ01001426:10436-11042
HmHel-10	CAEZ01010737:100620-100824
HmHel-10	CAEZ01010737:139785-139895
HmHel-10	CAEZ01009124:20749-21014
HmHel-10	CAEZ01005438:991-1661
HmHel-10	CAEZ01005252:11065-11374
HmHel-10	CAEZ01004917:10344-10996
HmHel-10	CAEZ01001317:3323-3495
HmHel-10	CAEZ01011572:9749-9875
HmHel-10	CAEZ01009834:1285-1502
HmHel-10	CAEZ01007676:214214-214700
HmHel-10	CAEZ01005166:18772-18871
HmHel-10	CAEZ01003381:5084-5197
HmHel-10	CAEZ01008211:169481-169617
HmHel-10	CAEZ01007047:4793-6907
HmHel-10	CAEZ01006781:21083-21322
HmHel-10	CAEZ01005817:2230-2476
HmHel-10	CAEZ01005385:31188-31887
HmHel-10	CAEZ01005269:11506-11640
HmHel-10	CAEZ01004479:39780-39963
HmHel-10	CAEZ01008532:24704-24859
HmHel-10	CAEZ01008532:85229-85333
HmHel-10	CAEZ01008248:34125-34243
HmHel-10	CAEZ01007184:98484-98588
HmHel-10	CAEZ01008878:47659-47979
HmHel-10	CAEZ01004921:10539-10635
HmHel-10	CAEZ01010030:225869-226534
HmHel-10	CAEZ01008270:91689-91808
HmHel-10	CAEZ01008270:33270-33844
HmHel-10	CAEZ01007626:17883-17970
HmHel-10	CAEZ01004055:5990-12394
HmHel-10	CAEZ01010301:68544-68894
HmHel-10	CAEZ01010130:114179-114270
HmHel-10	CAEZ01009873:55837-56325
HmHel-10	CAEZ01005453:2248-2787
HmHel-10	CAEZ01005340:3253-3358
HmHel-10	CAEZ01007705:12490-12663
HmHel-10	CAEZ01007413:37907-37995
HmHel-10	CAEZ01007413:89661-90473
HmHel-10	CAEZ01007147:10561-10645
HmHel-10	CAEZ01004706:8852-9041

HmHel-10	CAEZ01002691:3392-3792
HmHel-10	CAEZ01000849:51294-51902
HmHel-10	CAEZ01005964:60011-60102
HmHel-10	CAEZ01004060:443-1055
HmHel-10	CAEZ01000601:1615-1753
HmHel-10	CAEZ01003127:1741-2394
HmHel-10	CAEZ01001368:1482-1596
HmHel-10	CAEZ01010682:10033-10118
HmHel-10	CAEZ01008337:11592-11730
HmHel-10	CAEZ01007689:46455-46807
HmHel-10	CAEZ01001456:1721-1934
HmHel-10	CAEZ01011612:11671-12201
HmHel-10	CAEZ01008421:39570-39674
HmHel-10	CAEZ01006548:70334-70456
HmHel-10	CAEZ01004768:24533-24614
HmHel-10	CAEZ01004256:1150-1324
HmHel-10	CAEZ01006477:2432-2542
HmHel-10	CAEZ01004786:20227-20328
HmHel-10	CAEZ01009017:75882-75970
HmHel-10	CAEZ01010816:49735-49924
HmHel-10	CAEZ01010268:26080-26293
HmHel-10	CAEZ01009458:131508-131695
HmHel-10	CAEZ01008889:26481-26715
HmHel-10	CAEZ01007987:1977-2191
HmHel-10	CAEZ01004056:2353-2452
HmHel-10	CAEZ01002875:68337-68444
HmHel-10	CAEZ01009404:67302-67396
HmHel-10	CAEZ01005772:11668-11806
HmHel-10	CAEZ01001715:33779-33908
HmHel-10	CAEZ01005437:30944-31382
HmHel-10	CAEZ01009509:161704-161804
HmHel-10	CAEZ01000878:29520-29779
HmHel-10	CAEZ01003914:18784-18946
HmHel-10	CAEZ01005477:2915-3033
HmHel-10	CAEZ01005122:19158-19359
HmHel-10	CAEZ01004601:4038-4437
HmHel-10	CAEZ01009321:9074-9284
HmHel-10	CAEZ01009230:10375-10508
HmHel-10	CAEZ01005377:1155-1290
HmHel-10	CAEZ01007397:9685-9800
HmHel-10	CAEZ01009452:5712-5876
HmHel-10	CAEZ01008210:64853-64934
HmHel-10	CAEZ01005199:9802-9932
HmHel-10	CAEZ01008801:3661-3764
HmHel-10	CAEZ01006617:53618-53700
HmHel-10	CAEZ01003517:35650-35789
HmHel-10	CAEZ01005506:758-925
HmHel-10	CAEZ01008703:8903-8983

HmHel-10	CAEZ01004936:387-517
HmHel-10	CAEZ01004991:11130-11329
HmHel-10	CAEZ01004906:8293-8554
HmHel-10	CAEZ01011495:76602-76753
HmHel-10	CAEZ01003332:6940-7271
HmHel-10	CAEZ01009242:80213-80462
HmHel-10	CAEZ01006861:20308-20403
HmHel-10	CAEZ01006207:2655-2822
HmHel-10	CAEZ01004496:9743-9913
HmHel-10	CAEZ01001395:6273-6360
HmHel-10	CAEZ01005381:6146-6413
HmHel-10	CAEZ01005682:15459-15554
HmHel-10	CAEZ01008721:30099-30180
HmHel-10	CAEZ01003598:9551-9652
